# RedundancyMiner: De-replication of redundant GO categories in microarray and proteomics analysis

**DOI:** 10.1186/1471-2105-12-52

**Published:** 2011-02-10

**Authors:** Barry R Zeeberg, Hongfang Liu, Ari B Kahn, Martin Ehler, Vinodh N Rajapakse, Robert F Bonner, Jacob D Brown, Brian P Brooks, Vladimir L Larionov, William Reinhold, John N Weinstein, Yves G Pommier

**Affiliations:** 1 Laboratory of Molecular Pharmacology, Center for Cancer Research, National Cancer Institute, NIH, Room 5068, Building 37, 37 Convent Drive, Bethesda, MD, 20892, USA; 2 Department of Biostatistics, Bioinformatics, and Biomathematics, Georgetown University Medical Center, 4000 Reservoir Road, NW, Washington, DC 20007, USA; 3 SRA International, Inc., Fairfax, VA, USA; 4 National Institutes of Health, Eunice Kennedy Shriver National Institute of Child Health and Human Development, Section on Medical Biophysics, Bethesda, MD 20892, USA; 5 University of Maryland, Department of Mathematics, College Park, MD 20742, USA; 6 National Institutes of Health, National Eye Institute, Ophthalmic Genetics and Visual Function Branch, Bethesda, MD 20892, USA; 7 Departments of Bioinformatics and Computational Biology and Systems Biology, M.D. Anderson Cancer Center, Houston, TX 77030, USA

## Abstract

**Background:**

The Gene Ontology (GO) Consortium organizes genes into hierarchical categories based on biological process, molecular function and subcellular localization. Tools such as GoMiner can leverage GO to perform ontological analysis of microarray and proteomics studies, typically generating a list of significant functional categories. Two or more of the categories are often redundant, in the sense that identical or nearly-identical sets of genes map to the categories. The redundancy might typically inflate the report of significant categories by a factor of three-fold, create an illusion of an overly long list of significant categories, and obscure the relevant biological interpretation.

**Results:**

We now introduce a new resource, RedundancyMiner, that de-replicates the redundant and nearly-redundant GO categories that had been determined by first running GoMiner. The main algorithm of RedundancyMiner, MultiClust, performs a novel form of cluster analysis in which a GO category might belong to several category clusters. Each category cluster follows a "complete linkage" paradigm. The metric is a similarity measure that captures the overlap in gene mapping between pairs of categories.

**Conclusions:**

RedundancyMiner effectively eliminated redundancies from a set of GO categories. For illustration, we have applied it to the clarification of the results arising from two current studies: (1) assessment of the gene expression profiles obtained by laser capture microdissection (LCM) of serial cryosections of the retina at the site of final optic fissure closure in the mouse embryos at specific embryonic stages, and (2) analysis of a conceptual data set obtained by examining a list of genes deemed to be "kinetochore" genes.

## Background

We previously developed GoMiner [1] and High-Throughput GoMiner [2], applications that organize lists of "interesting" genes (for example, under-and over-expressed genes from a microarray experiment) for biological interpretation in the context of the Gene Ontology [3,4]. GoMiner and related tools typically generate a list of significant functional categories. In addition to lists and tables, High-Throughput GoMiner also provides a valuable graphical output termed a "clustered image map" (CIM). The "integrative" and "individual" CIMS can depict the relationship between categories and either multiple experiments or genes, respectively.

When designing an algorithm for a program like GoMiner, a number of implementation decisions must be made. One such decision is how to handle genes mapping to a category that is a child of the category under consideration. The particular algorithm adopted by GoMiner "rolls up" genes mapping to a child category; that is, genes mapping to a child category are (recursively) assigned to the parent of that child category. Although that approach provides robust protection against variability in curation techniques, it can result in redundancy between parent and child categories.

Even in the absence of "rolling up," redundancy can be an important issue. That is, two non-parent/child categories may include identical or nearly-identical sets of genes. Overall, the redundancy can easily inflate by a factor of about three the number of categories that are considered statistically significant, create an illusion of an overly long list of significant categories, and obscure the relevant biological interpretation.

One way of addressing redundancy is exemplified by GO slims [5]: "GO slims are cut-down versions of the GO ontologies containing a subset of the terms in the whole GO. They give a broad overview of the ontology content without the detail of the specific fine grained terms. GO slims are particularly useful for giving a summary of the results of GO annotation of a genome, microarray, or cDNA collection when broad classification of gene product function is required."

However, in the context of GoMiner analysis, the GO slims approach has several drawbacks:

• It cannot deal with redundancy that might not result from "rolling up"

• It is rather inflexible, as it is pre-computed and cannot adapt to the characteristics of a particular data set

• It "throws out the baby with the bathwater:" a simplified view might be a useful first approximation, but the molecular biologist also needs to be able to "drill down" to see the full details

We propose here a solution that overcomes these limitations of GO slims. Full details are given in the Methods section. Briefly, our approach, RedundancyMiner, de-replicates the (fully- or partially-) redundant GO categories: the user selects a desired redundancy threshold, and a new reduced clustered imaged map (CIM) is created. That CIM represents those categories that were not affected by the processing, as well as composite categories that represent groups of merged categories. An additional new type of CIM is also created, which we term a "META CIM." The META CIM conveniently visualizes the pattern of grouping within the merged categories. Thus, an overview is afforded by the reduced CIM, and the details by the META CIM.

Furthermore, the redundancy computation can be based on either (a) all genes that map to a category or (b) just the genes that exhibited altered expression levels in the current experiment. The latter approach (b) will provide a META CIM that reflects redundancy and interaction between categories that is specific to the conditions of the study. This pattern may be significantly different from the static reference behavior obtained by approach (a), and it may suggest the underlying systems biology.

The META CIM does not simply discard redundancy, as might be the case for GO slims; rather, it processes the patterns of redundancy and extracts information from them. Ignoring the existence of redundancy, as GO slims does, is an oversimplification that may throw away valuable information.

A number of other earlier papers address related issues. Several of those papers address approaches to studying gene enrichment, but not specifically the redundancy problem. For example, Pehkonen [6] developed a method that clusters genes to groups with homogenous functionalities. The method uses Nonnegative Matrix Factorization (NMF) to create several clustering results with varying numbers of clusters. The clustering results are combined into a simple graphical presentation showing the functional groups over-represented in the analyzed gene list. Prufer [7] developed "FUNC," a package for detecting significant associations between gene sets and ontological annotations. Xu [8] developed "CeaGO," enriching clustered GO terms based on semantic similarity. Hermann [9] developed "SimCT," which draws a simplified representation of biological terms present in the set of objects

Several papers address approaches to address the redundancy problem within the context of studying gene enrichment, and are therefore potentially more germane. For example, Alexa [10] proposed a method "TopW" to eliminate local dependencies between GO terms; Lu [11] developed "GenGO," a generative probabilistic model which identifies a small subset of categories that, together, explain the selected gene set; and Grossmann [12] developed the "Ontologizer" that uses the parent child union (PCU) to reduce the dependencies between the individual term's measurements, and recomputes the P-value for a specific category by taking into account the immediately more general terms (the parents). That procedure can often lead to the removal of false positives, since some of the more specific categories are eliminated if their parent category is determined to be significant. The more recent global "MGSA" method of Bauer [13] outperformed the local methods of Alexa, Lu, and Grossmann. Finally, Richards [14] recently developed a novel global approach to assess the functional coherence of gene sets by taking into account both the enrichment of GO terms and their relationships among terms.

In summary, the two most promising of the previously published methods for addressing redundancy are those of Bauer and Richards. However, unlike RedundancyMiner, neither of those methods takes advantage of the redundancy patterns to infer subtle nuanced themes among groups of GO categories. RedundancyMiner's META CIM is shown here to be of great potential value in such analyses.

## Implementation

### Overview

A typical sequence of steps would be:

• prepare a list of "changed" or "interesting" genes

○ *i.e*., over-expressed genes in a microarray experiment

• prepare a list of "total" genes

○ *i.e*., all genes appearing on the microarray

• run High-Throughput GoMiner (HTGM) on the two gene lists

○ generate a mapping of "changed" genes to statistically significant GO categories

• run RedundancyMiner on the CIM representing the gene to category mapping

○ generate a reduced-redundancy CIM

○ generate a META CIM that captures the nuances of the redundancy

The first 3 steps are preparatory to running RedundancyMiner; they are not an integral part of RedundancyMiner itself.

### High-Throughput GoMiner (HTGM)

GoMiner [1] is a tool for biological interpretation of 'omic' data, including data from gene expression microarrays and state of the art sequencing technologies. It leverages the Gene Ontology (GO) to identify "biological processes," "molecular functions," and "cellular components" represented in a list of genes. High-Throughput GoMiner (HTGM) [2], which was used for many of the analyses reported here, is an enhancement of GoMiner that efficiently performs the computationally-challenging task of automated batch processing of an arbitrary number of such gene lists. In addition to generating results for each individual input gene list, HTGM also generates integrative results that relate the entire set of input files. In particular, HTGM generates an integrative CIM that shows the FDR of the significant GO categories *versus *the experiments.

A GO category is *enriched *if the number of changed genes that HTGM assigned to it is statistically significantly greater than the number expected by chance. A category is considered *significant *if its Fisher's Exact p-value and its false discovery rate (FDR) are both less than or equal to a user-selected threshold (typically 0.10). See [1,2] for detailed discussions of GoMiner and HTGM, including calculations of statistical significance.

The parameters used in all of the HTGM analyses are listed in Additional file 1.

### Clustered Image Maps

Clustered image maps (CIMs), first introduced for omic studies in the mid-1990's by members of our group [15,16], were produced here with the Genesis program [17]. In general, a CIM is a visual representation of a two-dimensional table of numerical values, in which hierarchical clustering has been performed along one or both axes. The numerical values are mapped to a pseudo-color scale. We often use CIMs to represent the mapping of genes to GO categories. One axis represents genes and the other axis represents GO categories. The numerical values in the table are 0's (yellow) and 1's (red), designating the absence (0) or presence (1) of the gene in the category. We selected the Euclidean distance metric and average linkage for hierarchal clustering. To facilitate visualization, we implemented a recently-added optional feature of GoMiner to remove very large generic categories from all CIMs.

### Overview of RedundancyMiner computation stream

There are three salient features of our practical solution to the redundancy problem:

(1) definition of a similarity metric to capture the degree of relatedness of GO categories

(2) generation of a set of non-redundant groups of GO categories clustered by clique decomposition, as implemented by the MultiClust algorithm

(3) visualization of CIMs of the non-redundant groups

We will describe our approach to those three features, and demonstrate their effectiveness in the context of analysis of gene expression microarray data.

### Definition of a similarity metric

The similarity metric is the Fisher's exact p-value. For each pair of significant categories, we compute the Fisher's exact p-value corresponding to the null hypothesis that the sets of genes mapping to the two categories are statistically independent. The 2 × 2 contingency table for the one-tail Fisher's exact test is given in Table 1. Our Java implementation of the Fisher's exact test is based on Javascript by Øyvind Langsrud [18].

**Table 1:** 2 × 2 contingency table for the one-tail Fisher's exact test

	*In first category*	*Not in first category*	*Row sums*
***In second category***	{a_i_} ∩ {b_i_}	{a_i_}' ∩ {b_i_}	{b_i_}
***Not in second category***	{a_i_} ∩ {b_i_}'	{a_i_}' ∩ {b_i_}'	{b_i_}'
***Column sums***	{a_i_}	{a_i_}'	{a_i_} ∪ {a_i_}'

{a_i_} and {b_i_} are the sets of genes mapping to the first and second categories, respectively. Prime indicates the complement of a set. {a_i_} ∪ {a_i_}' represents the set of genes mapping to the root GO category.

As suggested by Wang [19], bias in the annotation of GO can result in bias in subsequent analyses that use GO. Wang observed a correlation between a protein's annotation length (*i.e*., the number of annotation terms for the proteins) and the semantic similarity scores. Such a bias is not expected to affect the RedundancyMiner similarity metric, since

• The bias studied by Wang arises from the fact that a heavily-studied protein will be mapped to a relatively large number of GO categories, since relatively more is known about the functions of that protein. Thus, when that protein is represented as a vector of associated GO categories, that vector will be overly long. However, we are here proposing RedundancyMiner as a method to compare vectors of GO categories, not vectors of proteins. Biases in the number of studies for a given protein will not differentially bias the length (*i.e*., the number of 1's) in a GO category vector.

• The metric that we use is based upon Fisher's Exact p-value. Thus, if two GO category vectors are both highly populated with 1's, a high degree of overlap will not produce an artifactually significant (*i.e*., low p-value) score, since the computation of the p-value is explicitly based upon the probability of the observed degree of overlap relative to the proportion of 1's in the two vectors being compared. The relevant 2 × 2 contingency table, which explicitly takes the proportion of 1's into account, is shown in Table 1.

### Generation of a set of de-replicated groups by RedundancyMiner's MultiClust algorithm

We first compute a similarity matrix composed of Fisher's exact p-values. Next we select a desired p-value threshold. This selection is based on an estimate of "nominal number of merged pairs" (*i.e*., we count how many pairs of categories have a similarity score that is less than this threshold; we do not know what the actual degree of merging will be until after the merging is performed). We then apply the selected p-value threshold to the elements of the matrix to generate an undirected graph G whose vertices are GO categories. Two vertices are connected if the similarity between the two vertices is larger than the given threshold. The goal of this process is to find a clique decomposition of G and then consider each clique as a de-replicated functional group [20]. The clique decomposition is equivalent to the identification of a set of maximal cliques that cover all vertices in G. The procedure is summarized here in pseudo-code:

Variables:

• *reference *- list of n significant categories

• *similarity *- n × n matrix of pairwise similarity values

• *cluster *- list of current set of clusters

• *change *- an indication that there is a change in cluster

*change *= true

while (*change *is true){

      *change *= false

      for each *c *in *cluster*

         for each *ref *in *reference *but not in *c*

            if the similarity between *ref *and every vertex in *c *is larger than the given threshold

                  *change *= true

                  update *c *to include *ref*

            endif

         endfor

      endfor

   *cluster *= list of current set of unique clusters

endwhile

The condition "if the similarity between *ref *and every vertex in *c *is larger than the given threshold" corresponds to "complete linkage clustering," so the result is independent of the order of computing the elements of *cluster c*.

### Visualization of CIMs of the de-replicated groups

After deriving the new groups, each of which corresponds to a clique obtained in the previous step, the value displayed in the modified CIM depends on the type of input CIM, as shown in Table 2.

**Table 2:** Computation of values in the output CIM

*input type*	*output values*
***categories versus genes***	average of the values for the categories that were merged

***experiments versus genes***	minimum FDR among the categories that were merged

To summarize, the input is a (possibly redundant) CIM, and the output is a de-replicated CIM with the original redundant categories merged into a group whose name is the concatenation of names of the member categories. It is possible (in fact it is common) for any of the original categories to appear in multiple merged groups.

#### Running RedundancyMiner

##### Overview

RedundancyMiner consists of a set of perl modules (described in Additional file 2) and a java GUI. The java GUI is intended to provide optimal convenience for the molecular biologist, whereas the perl modules allow the developer to integrate RedundancyMiner's functionalities into a custom high-throughput data processing stream. The GUI can be invoked either by double-clicking the icon, or by entering "java -jar RedundancyMiner.jar" in a terminal window. The latter method is preferred, since it provides a trace of the execution. The archived java and perl modules are available as Additional file 3 and the most recent version can be downloaded from the Supplementary Materials web page. The various types of HTGM output files that may be used as input to RedundancyMiner are tabulated in Additional file 4.

##### Modes

• Default Mode: The goal is to generate a consistent set of collapsed CIMs for an entire HTGM output directory based on a common META CIM pattern derived from the integrative CIM

○ The user selects the HTGM output directory

○ RedundancyMiner generates a META CIM only for the HTGM integrative CIM, but not for each HTGM individual CIM

○ The collapsing pattern for the integrative CIM is used for collapsing each HTGM individual CIM

○ The resulting reduced CIMs are stored in the same directory as the corresponding HTGM CIM

• Custom Mode: Compared to the default mode, the user has more control over the threshold and the choice of the file to define the categories/genes structure

○ The user selects one specific CIM to process

○ The user selects a threshold for collapsing

○ The user selects a file type (.tvt., .gce., or the CIM itself) to define the categories/genes structure

○ The output (*i.e*., reduced CIM and META CIM) is directed to a special temp directory

##### User's Manual

A user's manual is provided in the form of a PowerPoint presentation (Additional file 5) and a .pdf file (Additional file 6). A simplified version of the HTGM result directory that is exemplified in the user's manual is available as Additional file 7.

##### Experimental studies

###### Retinal development

Full details are provided in [21]. Briefly, to better understand gene regulatory patterns in congenital defects of ocular development, we laser capture-microdissected tissue samples from embryonic mouse retina at the site of optic fissure closure at 8 time points (days 10.5 through 12.5). The Affymetrix MOE 430 2.0 microarray was used to analyze gene expression levels. To identify associated gene regulation patterns in 3416 genes whose expression levels varied between 4-fold and 26-fold, we applied Laplacian Eigenmaps (nonlinear dimensionality reduction) to the temporal microarray data. k-means clustering of the temporal expression profiles generated 24 coherent gene groups. We used GoMiner to facilitate identification of further gene regulatory associations in human ocular development, and to determine the biological themes that are represented by the genes in each cluster. We chose the largest cluster (cluster number 22; see "Retinal development HTGM download" in Additional file 8), containing 161 genes (of which 62 genes were recognized by GoMiner), for RedundancyMiner analysis.

###### Kinetochore genes

The list of 74 kinetochore genes (Additional file 9) was manually compiled by expert literature curation by one of us (V.L.L.). The GoMiner results are available as Additional file 10.

## Results and discussion

To highlight the characteristics and value of the RedundancyMiner approach, we next present analyses of two different types of data sets:

• the gene expression profile obtained by laser-capture microdissection (LCM) of serial cryosections of the retina at the site of final optic fissure closure in the mouse embryos at specific embryonic stages [21].

• a conceptual data set obtained by examining a list of genes deemed to be "kinetochore" genes

### Retinal development

**Figure 1:**
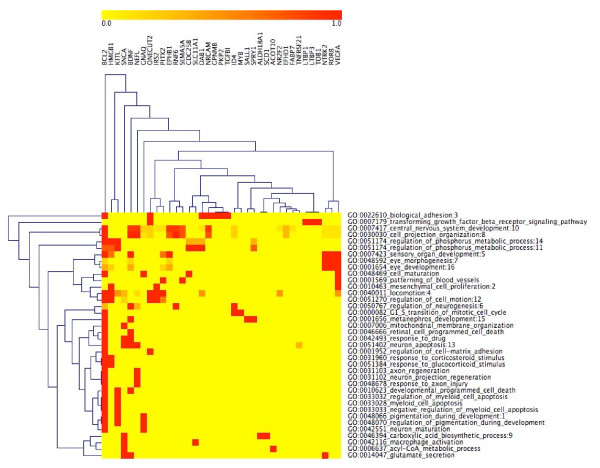
**Reduced CIM of retinal development genes versus categories**. After we ran RedundancyMiner, we found that certain GO categories in the original CIM (Additional file 11) were sufficiently redundant to be grouped together. GO categories whose names are followed by ":N" (where "N" stands for an integer) are the "representative" members of the "N^th^" group. Categories other than the "representative" ones in those groups are not shown in the reduced CIM. Instead, they are shown in the associated META CIM (Figures 2; Additional file 12).

Each row of the original CIM (Additional file 11) represents a statistically significant GO category, and each column represents an input gene that was mapped to at least one of these categories. That CIM is quite complex, containing 73 categories, many of which are redundant (*i.e*., categories that contain many genes in common) with respect to one another. To remove that complexity from the original CIM and transfer the information to the META CIM, we ran RedundancyMiner at a level of stringency corresponding to a nominal number of merged pairs = 128. The reduced CIM (Figure 1; Additional file 12) was significantly less complex, containing only 38 categories (compression ratio = 1.92). Although the experience is inherently subjective, it seems clear that the reduced CIM is more readily amenable to visual interpretation. The smaller number of categories and the reduction of redundancy make it easier to discern the major biological themes that are relevant to retinal development, *e.g*., biological adhesion, cell projection organization, eye morphogenesis, and axon regeneration.

**Figure 2:**
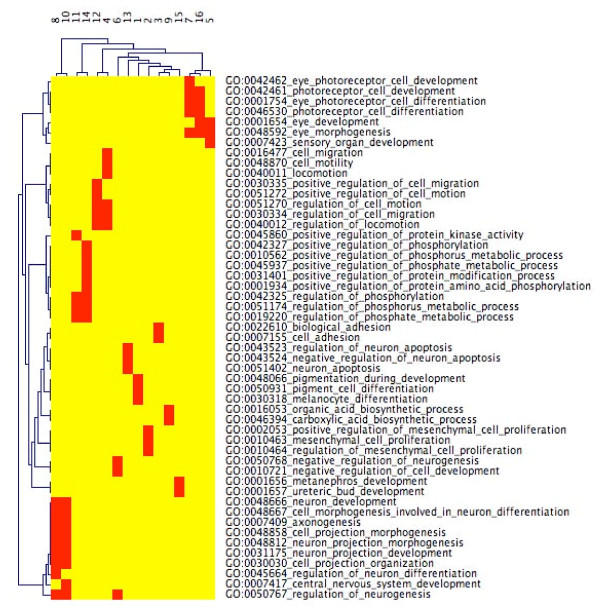
**META CIM of merged retinal development categories**. After we ran RedundancyMiner, we found that certain GO categories in the original CIM (Additional file 11) were sufficiently redundant to be grouped together. Those redundant groups are visualized here. For example, group 5 is comprised of the eye development, eye morphogenesis, and sensory organ development categories. The eye development category is also included in group 16, but in the context of a slightly different group of companion categories. Thus, groups 5 and 16 capture the eye development category in subtly different nuanced roles.

The clustering performed by RedundancyMiner is more complex than traditional clustering, since a category may appear in more than a single cluster. For instance, regulation of phosphorus metabolic process appears twice in the reduced CIM (Figure 1; Additional file 12), in RedundancyMiner clusters 11 and 14. The subtle reason for this dichotomy is revealed by the META CIM (Figure 2; Additional file 13). Cluster 11 reflects the sub-theme positive regulation of protein kinase activity, whereas cluster 14 reflects a related, but slightly different, sub-theme, positive regulation of protein amino acid phosphorylation.

All instances of this type of relationship can easily be found by examination of the META CIM. Consider the row corresponding to a particular category of interest. If there is more than a single red element in that row, then that category is involved in multiple RedundancyMiner clusters. For example, axonogenesis is a member of clusters 8 and 10. Cluster 8 reflects regulation of neuron differentation, whereas cluster 10 reflects central nervous system development. Just as genes may map to multiple categories in GO, the next higher level of organization involves the mapping of categories to different category clusters that represent subtly different functionalities.

Note that in the GO slims approach, the simplification is performed prior to the experiment, and so the pattern of redundancy reduction and information persistence is not dynamically optimized for the experiment at hand. Furthermore, GO slims would entirely miss the richness of detail that is available in the META CIM.

### Kinetochore genes

As was the case for retinal development, the original CIM (Additional file 14) is again quite complex, containing 66 categories. To reduce the complexity in the original CIM and transfer the information to the META CIM, we compared the effect of running RedundancyMiner at two levels of stringency. The more stringent level (nominal number of merged pairs = 169) yields a reduced CIM (Additional file 15) containing 27 categories (compression ratio = 2.44), and a META CIM containing 13 clusters (Additional file 16). The more permissive level (nominal number of merged pairs = 281) yielded a reduced CIM (Additional file 17) containing 20 categories (compression ratio = 3.30), and a META CIM containing 18 clusters (Additional file 18).

The two different compression ratios demonstrate the fine control that the user has in partitioning the complexity and information between the CIM and the META CIM. For instance, the more complex META CIM (Additional file 18) contains an instance of categories (nuclear transport and nucleocytoplasmic transport) that are involved in three different clusters (clusters 4, 5, and 18). Those clusters represent RNA localization, intracellular transport, and nuclear transport, respectively. As the stringency level is reduced, there is often an increase in the multiplicity of clusters containing a common category. The user can choose to explore the META CIMs generated at several different stringency levels, to find the optimal visualization of the relationship(s) of the most interest.

## Conclusions

We have presented the RedundancyMiner analysis of retinal development and kinetochore genes.

In both cases, RedundancyMiner facilitates the visual interpretation of the primary CIM image. In the original CIM images, there is a high degree of complexity arising from detailed, but relatively redundant, information. The visual complexity is significantly reduced by shifting the information in the redundant categories to the META CIM. In contrast to the original CIM, the display of the redundancy pattern in the META CIM provides valuable insight into the fine structure of the biological correlate of the gene expression pattern.

## Availability and Requirements

The most recent versions of the RedundancyMiner program package and user's manual can be downloaded from http://discover.nci.nih.gov/rm/supplementaryMaterials.html. Additional file 19 provides information for developers about the relationships of the RM and HTGM files. RedundancyMiner was developed using Perl (version 5.8) and Java (standard edition 6.0). In order to execute RedundancyMiner, Perl (with version no earlier than 5.8) and Java virtual machine (JVM 6.0) need to be installed and accessible to the operating system.

## Authors' contributions

BRZ developed the project, and wrote the manuscript. HL devised and implemented the clustering algorithm, produced the java and perl code, and constructed the PowerPoint user manual. ABK and JNW were involved in initial discussions about potential approaches to address the redundancy issue. ME, VNR, and RFB performed the clustering analysis of the retinal data sets. JDB and BPB generated retinal data. VLL and WR curated and maintained the kinetochore data set. YP provided overall scientific guidance for all of the work that was performed. All authors read and approved the final manuscript.

## Supplementary Material

Additional file 1 **Parameters used in HTGM analyses**. table of parameters used in HTGM analyses. Click here for file

Additional file 2 **Perl modules**. table of perl modules and their function. Click here for file

Additional file 3 **RedundancyMiner program package download**. the code for running RedundancyMiner on your own computer. Click here for file

Additional file 4 **Types of HTGM gene-category association files used by RedundancyMiner**. table of types and descriptions of HTGM files used by RedundancyMiner. Click here for file

Additional file 5 **PowerPoint user's manual**. Powerpoint version of the RedundancyMiner user's manual. Click here for file

Additional file 6 **PDF format of PowerPoint user's manual**. .pdf version of the RedundancyMiner user's manual. Click here for file

Additional file 7 **Simplified version of the HTGM result directory that is exemplified in the user's manual**. HTGM result directory to be used in conjunction with the examples given in the RedundancyMiner user's manual. Click here for file

Additional file 8 **Retinal development HTGM download**. compressed package of the results of running HTGM on the retinal development genes list. Click here for file

Additional file 9 **Kinetochore genes**. table listing kinetochore genes. Click here for file

Additional file 10 **Kinetochore genes HTGM download**. compressed package of the results of running HTGM on the kinetochore genes list. Click here for file

Additional file 11 **Original genes versus categories CIM for an interesting retinal development time-series cluster**. .png image of original genes versus categories CIM for an interesting retinal development time-series cluster. Click here for file

Additional file 12 **Reduced CIM of retinal development genes versus categories**. .png image of reduced CIM of retinal development genes versus categories. Click here for file

Additional file 13 **META CIM of retinal development categories *versus *META CIM group**. .png image of META CIM of retinal development categories *versus *META CIM group. Click here for file

Additional file 14 **Original kinetochore genes versus categories CIM**. .png image of original kinetochore genes versus categories CIM. Click here for file

Additional file 15 **Reduced CIM of kinetochore genes versus categories**. .png image of reduced CIM of kinetochore genes versus categories. Click here for file

Additional file 16 **META CIM of kinetochore categories *versus *META CIM group**. .png image of META CIM of kinetochore categories *versus *META CIM group. Click here for file

Additional file 17 **Reduced CIM of kinetochore genes versus categories**. .png image of reduced CIM of kinetochore genes versus categories. Click here for file

Additional file 18 **META CIM of kinetochore genes versus categories**. .png image of META CIM of kinetochore genes versus categories. Click here for file

Additional file 19 **Information for developers**. table of the relationship of RM and HTGM files. Click here for file
