# Supplementary material for: RedundancyMiner: De-replication of redundant GO categories in microarray and proteomics analysis
Source: BMC Bioinformatics. 2011 Feb 10;12:52. doi: 10.1186/1471-2105-12-52 (PMC3223614; doi:10.1186/1471-2105-12-52)
Supplement: Additional file 1 — Parameters used in HTGM analyses. table of parameters used in HTGM analyses. [file 1471-2105-12-52-S1.DOC]

Additional file 1. Parameters used in HTGM analyses

| **parameter** | **study** | |
| --- | --- | --- |
|  | **retinal development** | **kinetochore genes** |
| GO database version | Sept, 2009 | Sept, 2009 |
| GoMiner version | command line HTGM | web HTGM |
| GoMiner build | 248 | 248 |
| datasource | all | UniProtKB |
| organism | mouse | human |
| evidence code level | 3 | 1 |
| cross reference | true | false |
| synonym | true | false |
| FDR threshold | 0.15 | 0.10 |
| p value threshold | 0.15 | 0.10 |
| randomizations | 100 | 100 |
| smallest category size | 5 | 5 |
| largest category size | 300 | 1000 |
| root category | GO:0008150 biological process | GO:0008150 biological process |
