# Supplementary material for: RedundancyMiner: De-replication of redundant GO categories in microarray and proteomics analysis
Source: BMC Bioinformatics. 2011 Feb 10;12:52. doi: 10.1186/1471-2105-12-52 (PMC3223614; doi:10.1186/1471-2105-12-52)
Supplement: Additional file 2 — Perl modules. table of perl modules and their function. [file 1471-2105-12-52-S2.DOC]

Additional file 2. Perl modules

| **module** | **function** |
| --- | --- |
| prepareData.pl  prepareData_dir.pl | Calculate the similarity scores between GO categories |
| obtainStatistics.pl | Provide a summary of the distribution of the similarities and the number of pairs with similarities over the corresponding threshold. |
| multiCluster.pl  multiCluster_dir.pl | Iteratively merge pairs and generate new nodes for a given threshold. Note that one GO category can appear in several new nodes. |
| collapseCIM.pl  collapseCIM_dir.pl | Regenerate CIMs according to the new nodes. |

The modules whose names contain “_dir” are the versions that are used for the “default” mode.
