# Supplementary material for: RedundancyMiner: De-replication of redundant GO categories in microarray and proteomics analysis
Source: BMC Bioinformatics. 2011 Feb 10;12:52. doi: 10.1186/1471-2105-12-52 (PMC3223614; doi:10.1186/1471-2105-12-52)
Supplement: Additional file 4 — Types of HTGM gene-category association files used by RedundancyMiner. table of types and descriptions of HTGM files used by RedundancyMiner. [file 1471-2105-12-52-S4.DOC]

Additional file 4. Types of HTGM gene-category association files used by RedundancyMiner

| **type** | **description** | **default**  **mode** | **custom mode** |
| --- | --- | --- | --- |
| .tvt | mapping of all genes to a given category | primary | optional |
| .gce | mapping of “changed” genes to a given category | used if .tvt missing (for backward compatibility with older versions of GoMiner) |  |
| .CIM |  | not used |  |

The computation of similarity between two categories can be based on either (a) all of the genes that map to the categories or (b) the “changed” genes that map to the categories.

There is no difference between using .gce and .CIM as the gene-category association file.

The “default” mode (please see Table S2) is intended to be HTGM-specific, but the custom mode with .CIM rather than .tvt or .gce will permit the use of any CIM-format file, with one *caveat*: a collapsed category cluster is named after one of the categories in the cluster. The perl modules attempt to select the name of the most generic category. That computation is based upon the category size (*i.e.*, the number of genes), as reflected in the parameter to the argument *gcefile*. That computation is most accurate when that parameter is .tvt.
