# Supplementary material for: RedundancyMiner: De-replication of redundant GO categories in microarray and proteomics analysis
Source: BMC Bioinformatics. 2011 Feb 10;12:52. doi: 10.1186/1471-2105-12-52 (PMC3223614; doi:10.1186/1471-2105-12-52)

RedundancyMiner
User's Manual

To follow along with the presentation, please download a simplified version of the HTGM result directory that is exemplified in the user's manual.

This is available from http://discover.nci.nih.gov/rm/supplementaryMaterials.html

Custom Mode

- Computing the similarity matrix
  - Select .CIM file
  - Select .gce or .tvt file
  - Submit the job
- Clustering
  - Select P-value threshold
  - Submit the job
- File management
  - Save the working directory
  - Retrieve the working directory

Custom Mode: Select Custom Mode

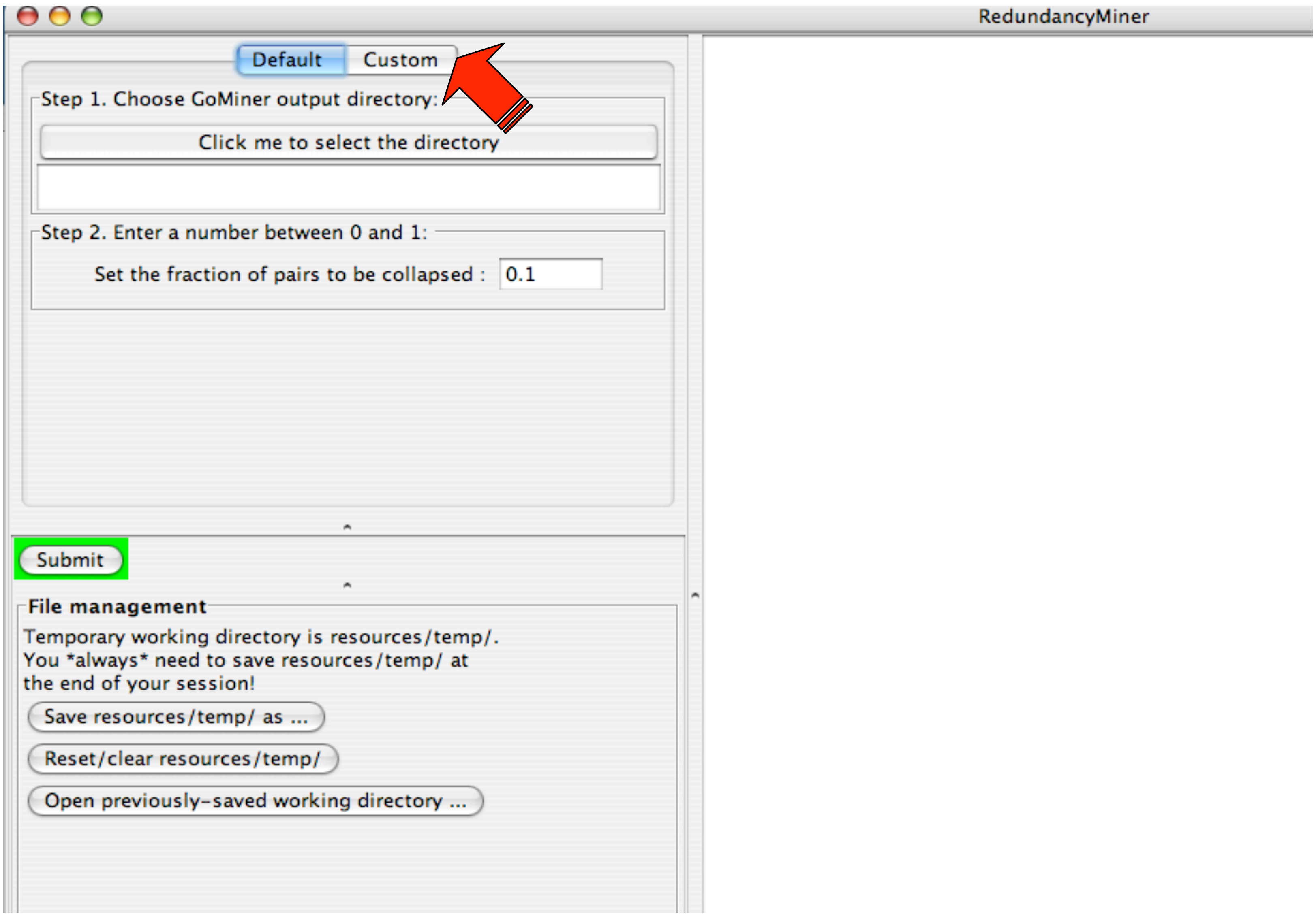

Compute the similarity matrix: Choose .CIM (Step 1)

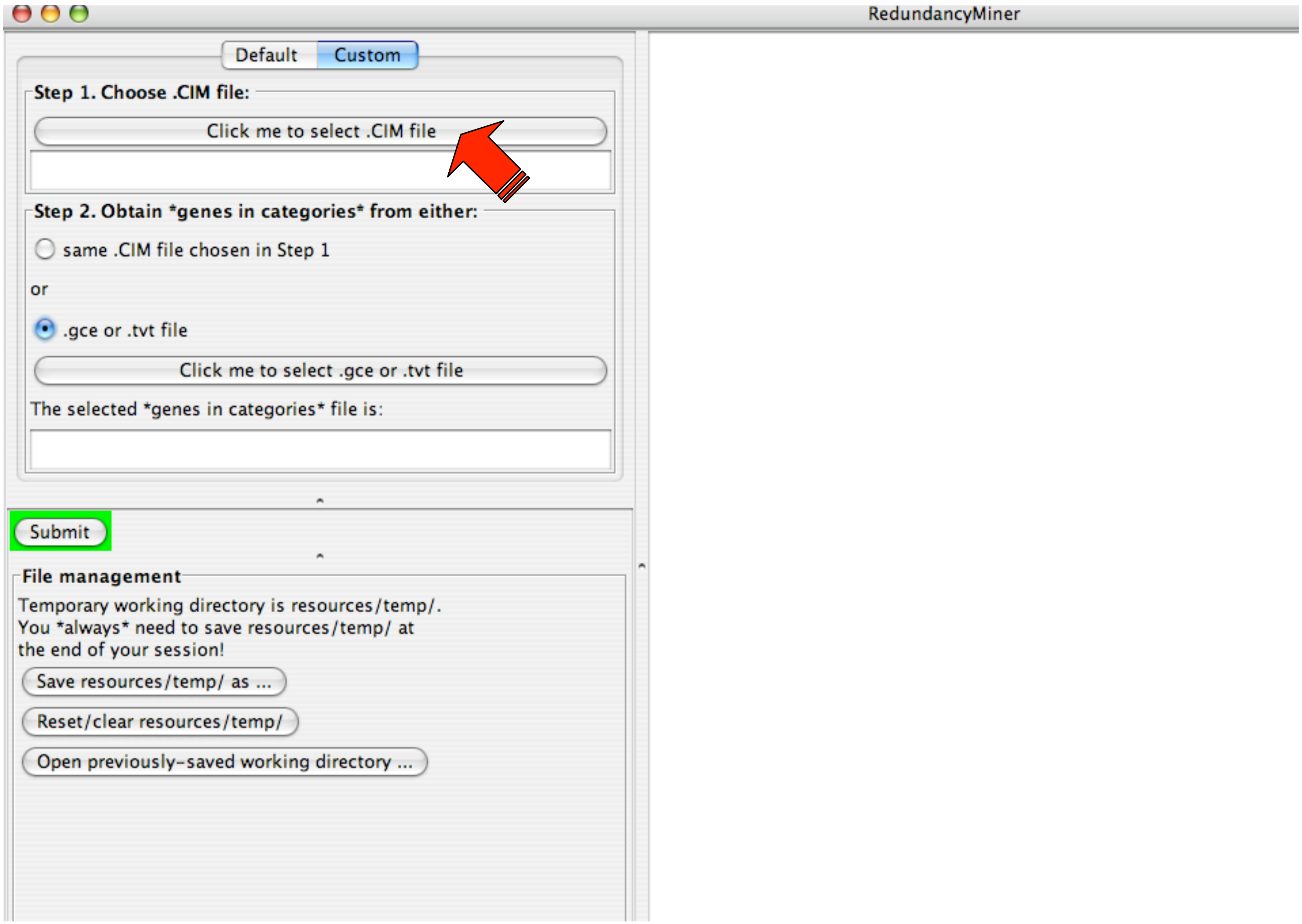

Compute the similarity matrix: Choose .CIM (Step 2)

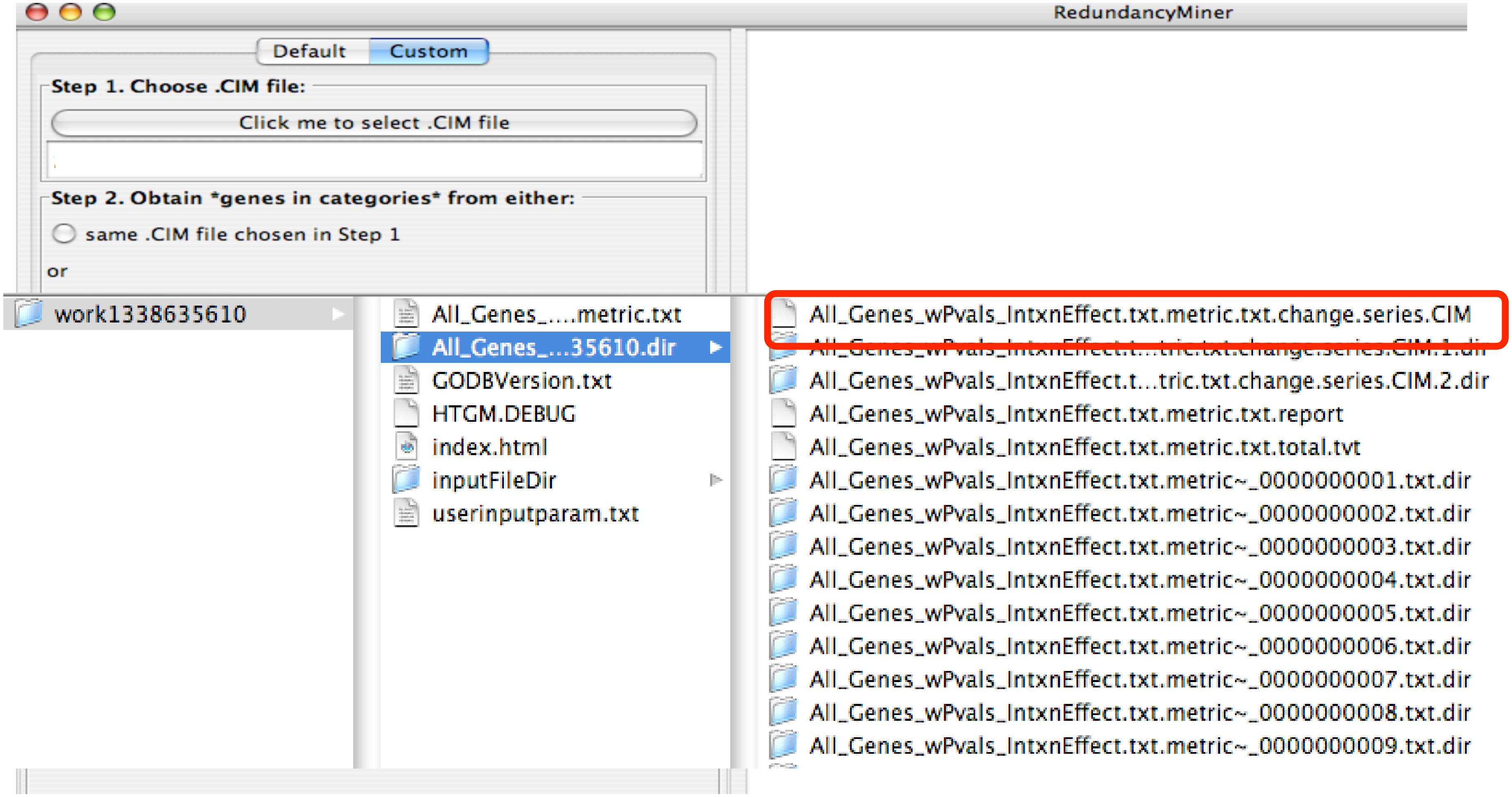

Compute the similarity matrix: Choose .gce or .tvt (Step 1)

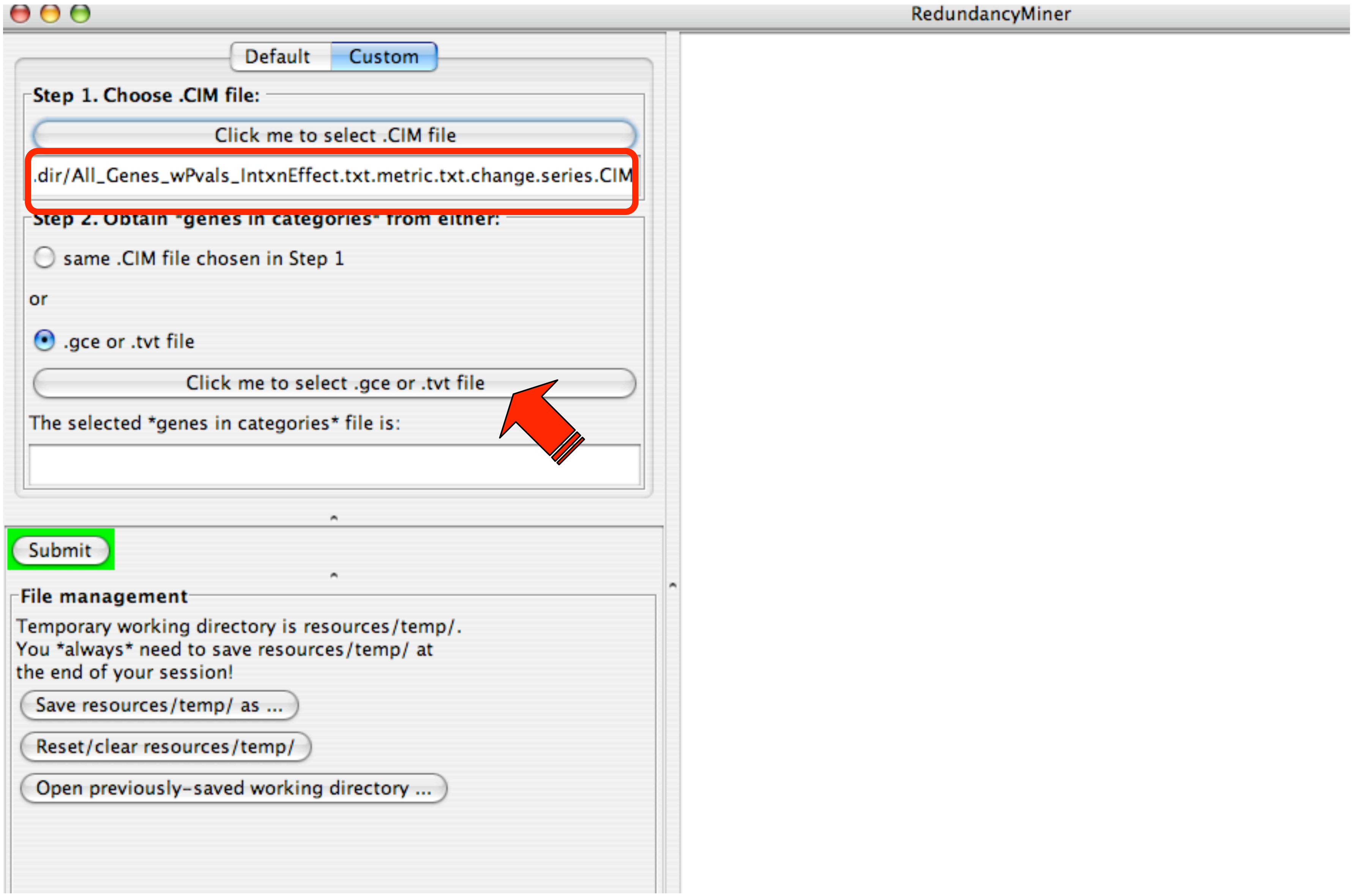

Compute the similarity matrix: Choose .gce or tvt (Step 2)

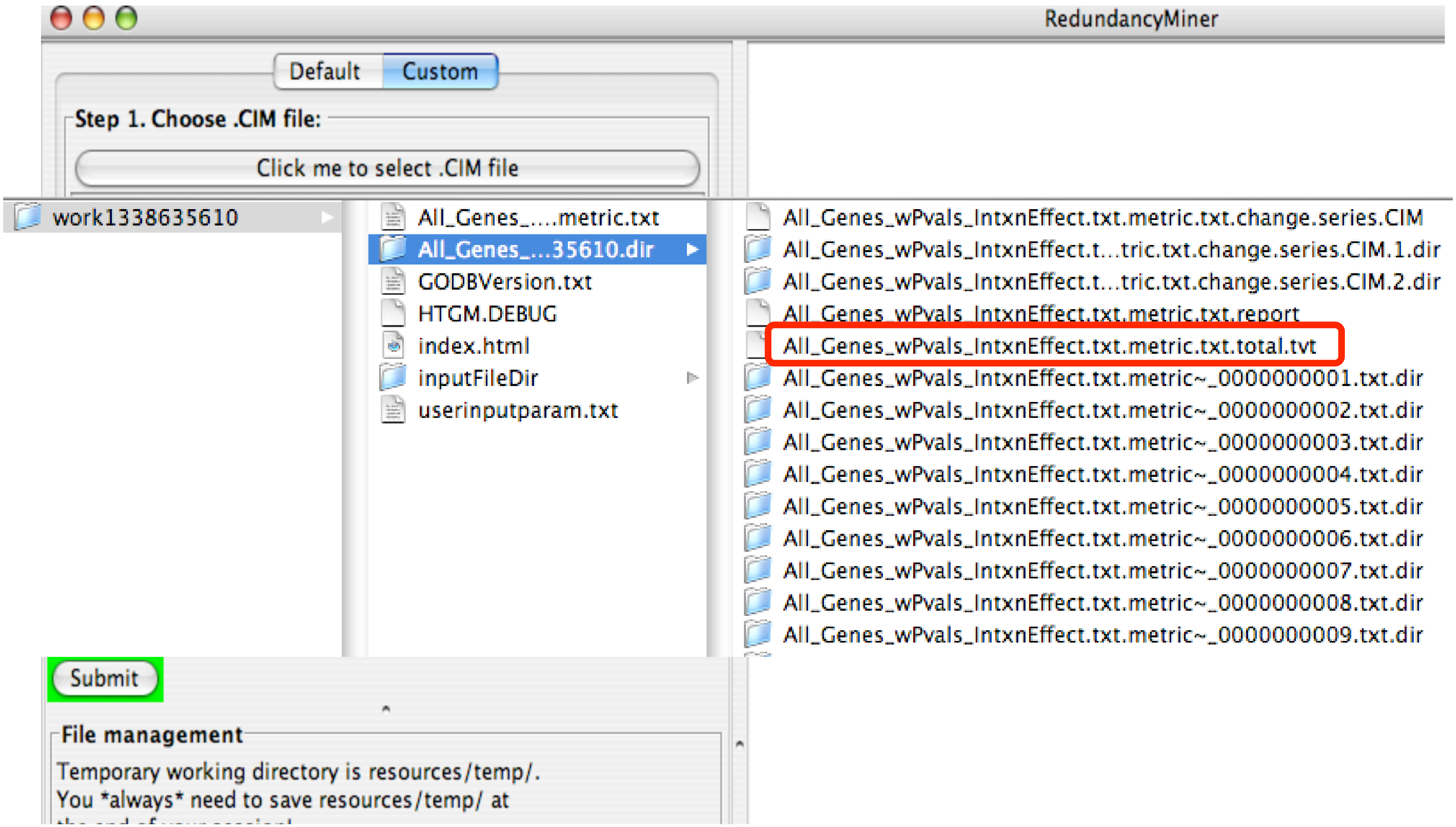

Compute the similarity matrix: Submit the job

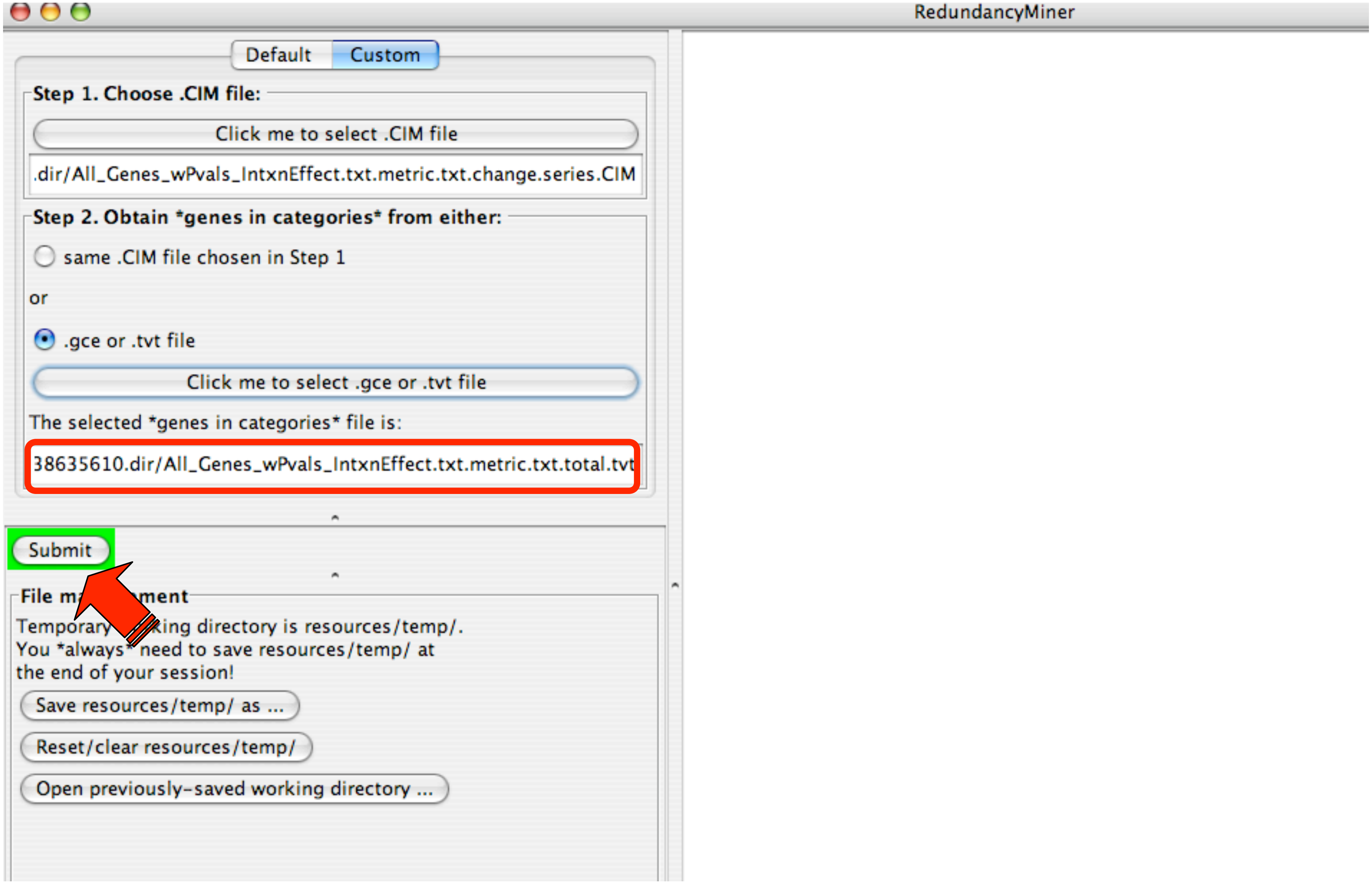

Cluster: Select P-value threshold (Step 1)

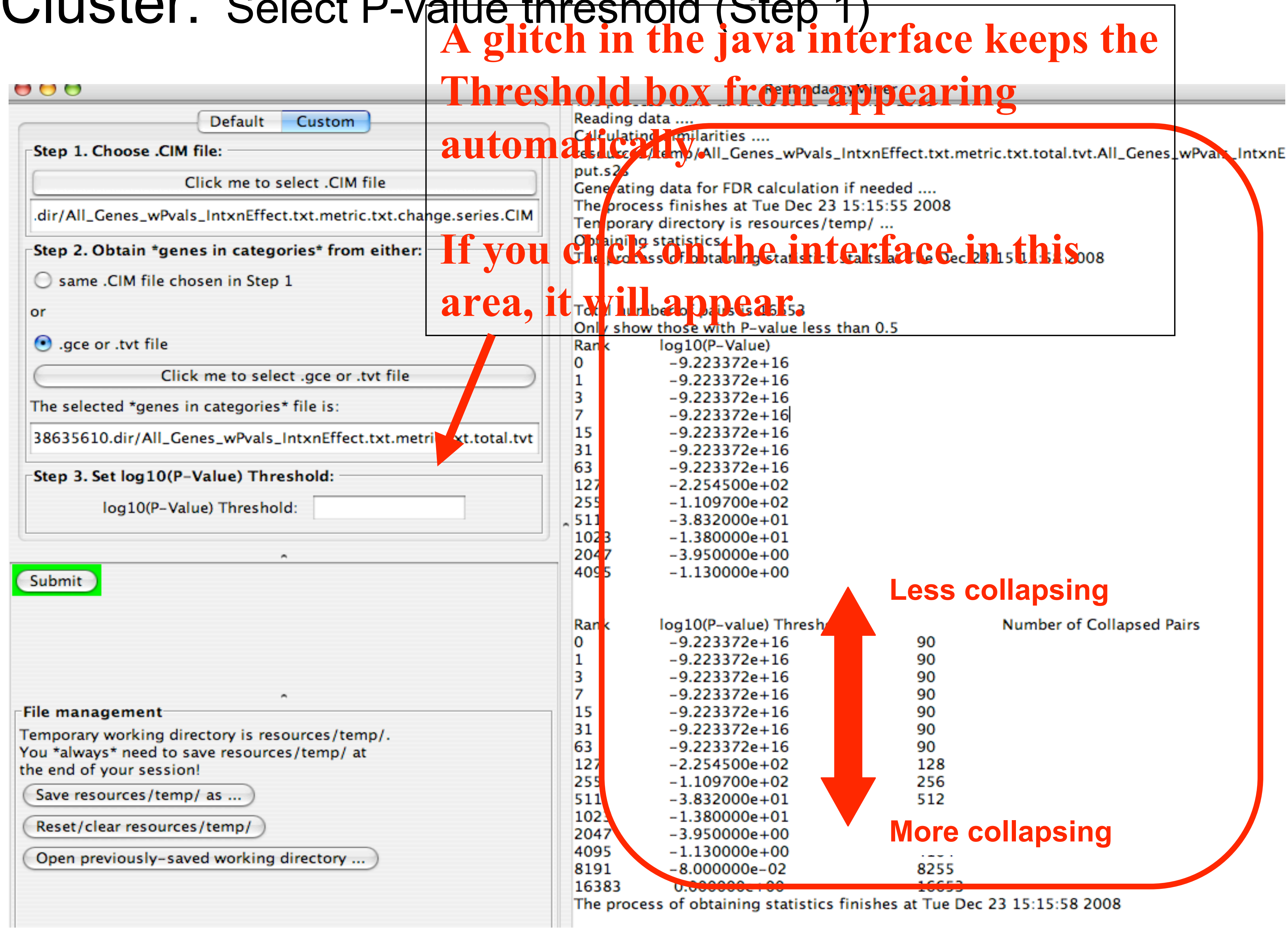

Cluster: Select P-value threshold (Step 2)

A glitch in the java interface keeps the Threshold box from appearing automatically.

If you click on the interface in this area, it will appear.

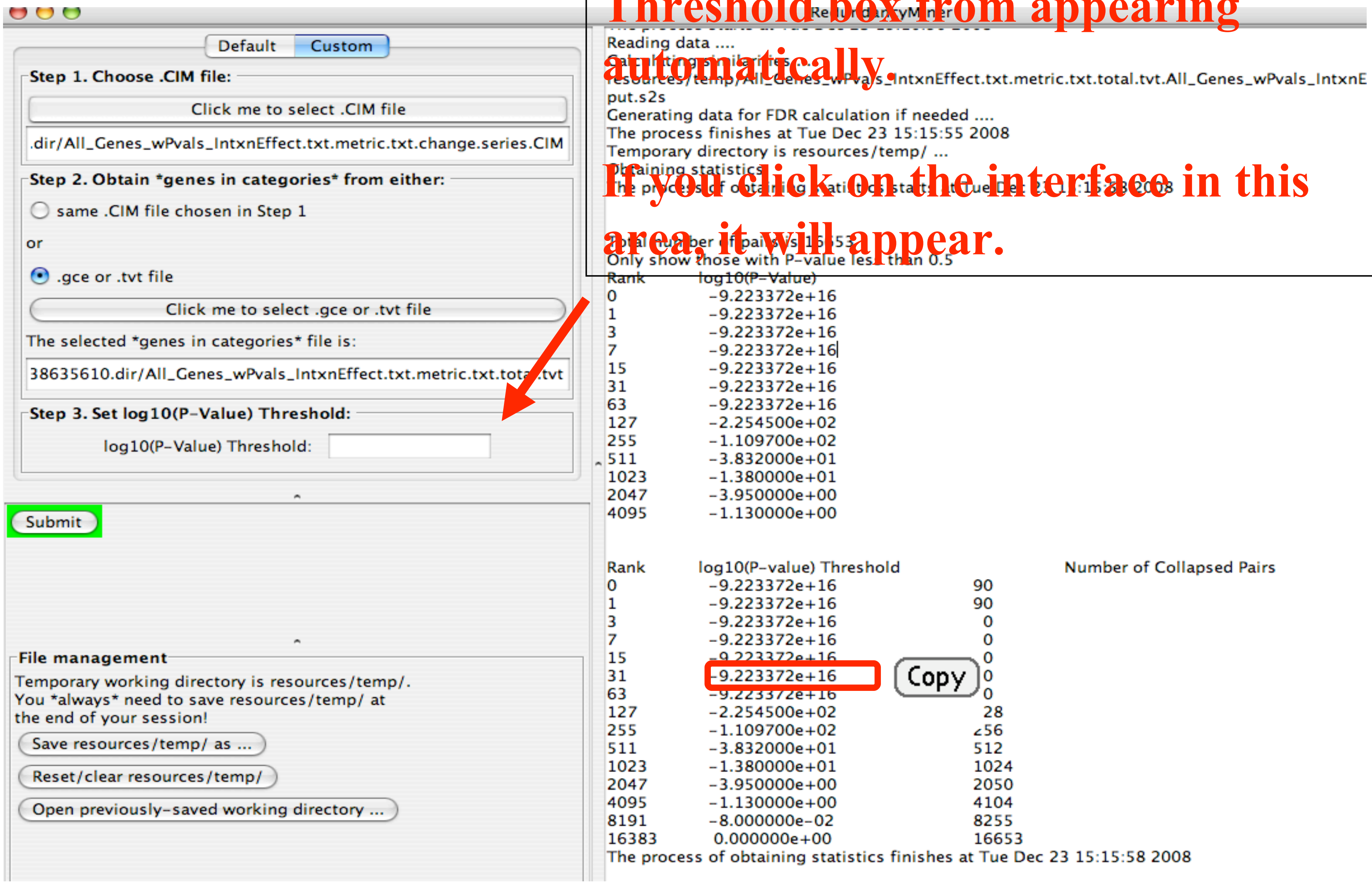

Cluster: Select P-value threshold (Step 3)

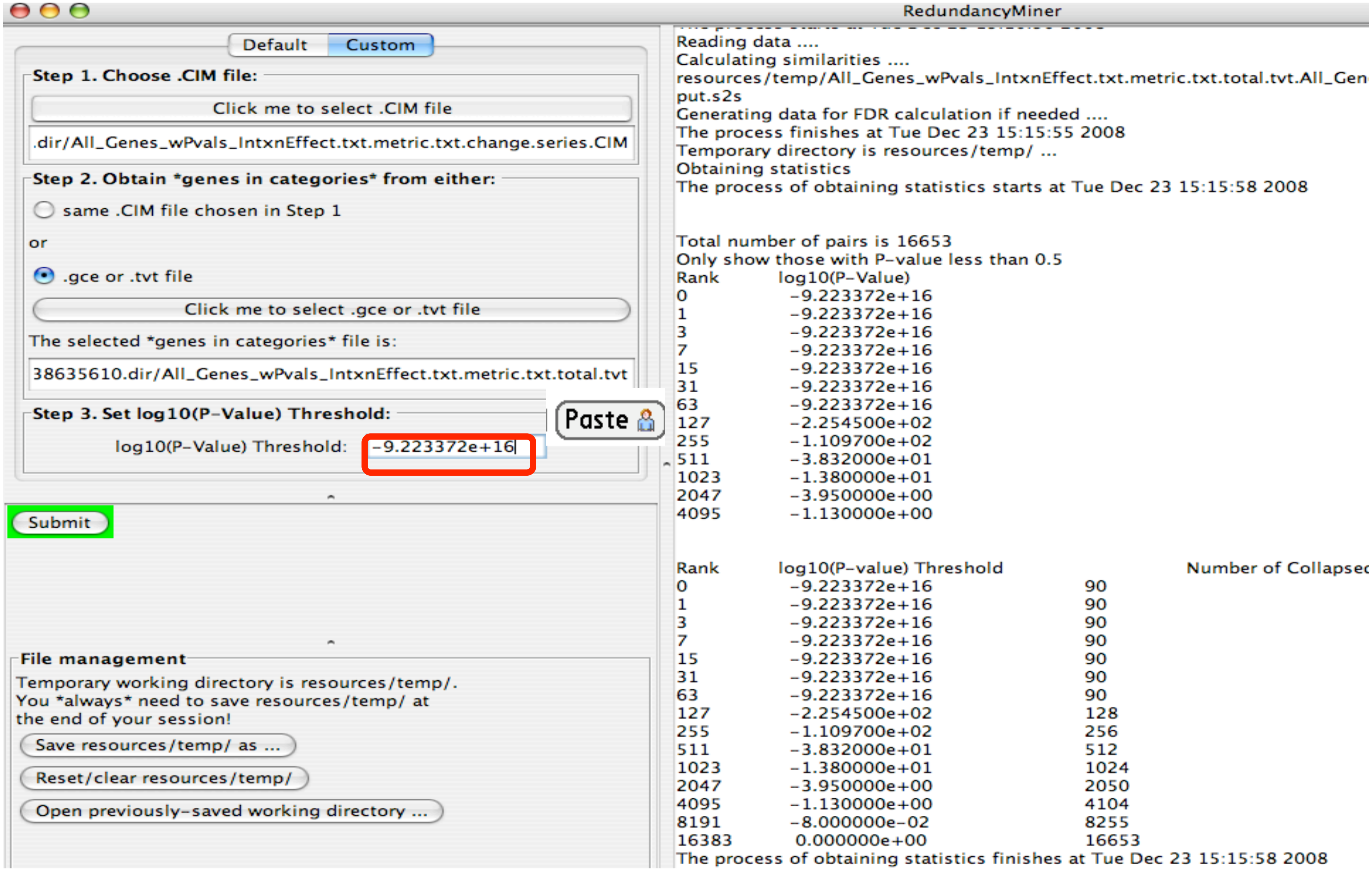

Cluster: Submit the job

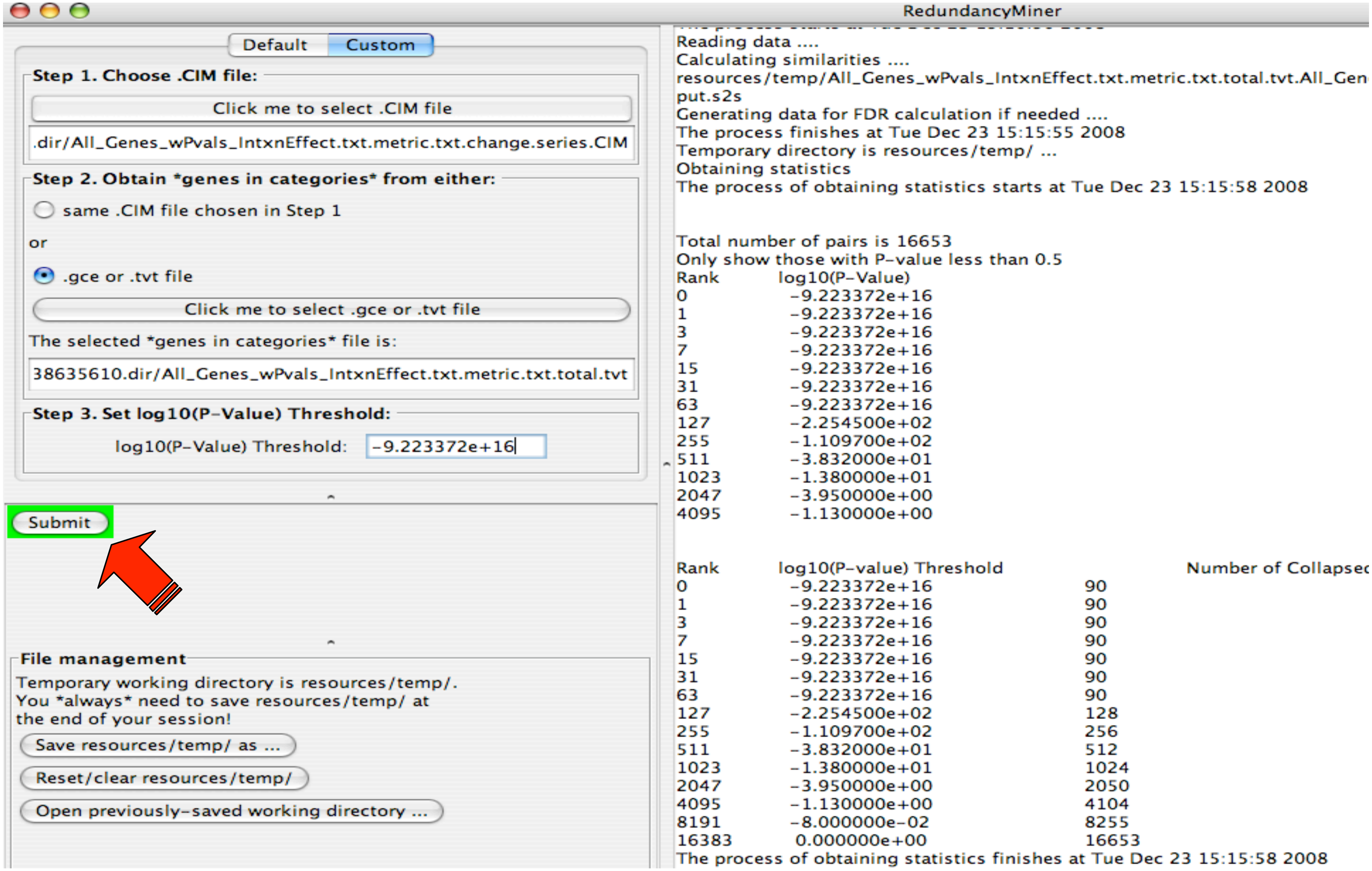

Cluster: Completed

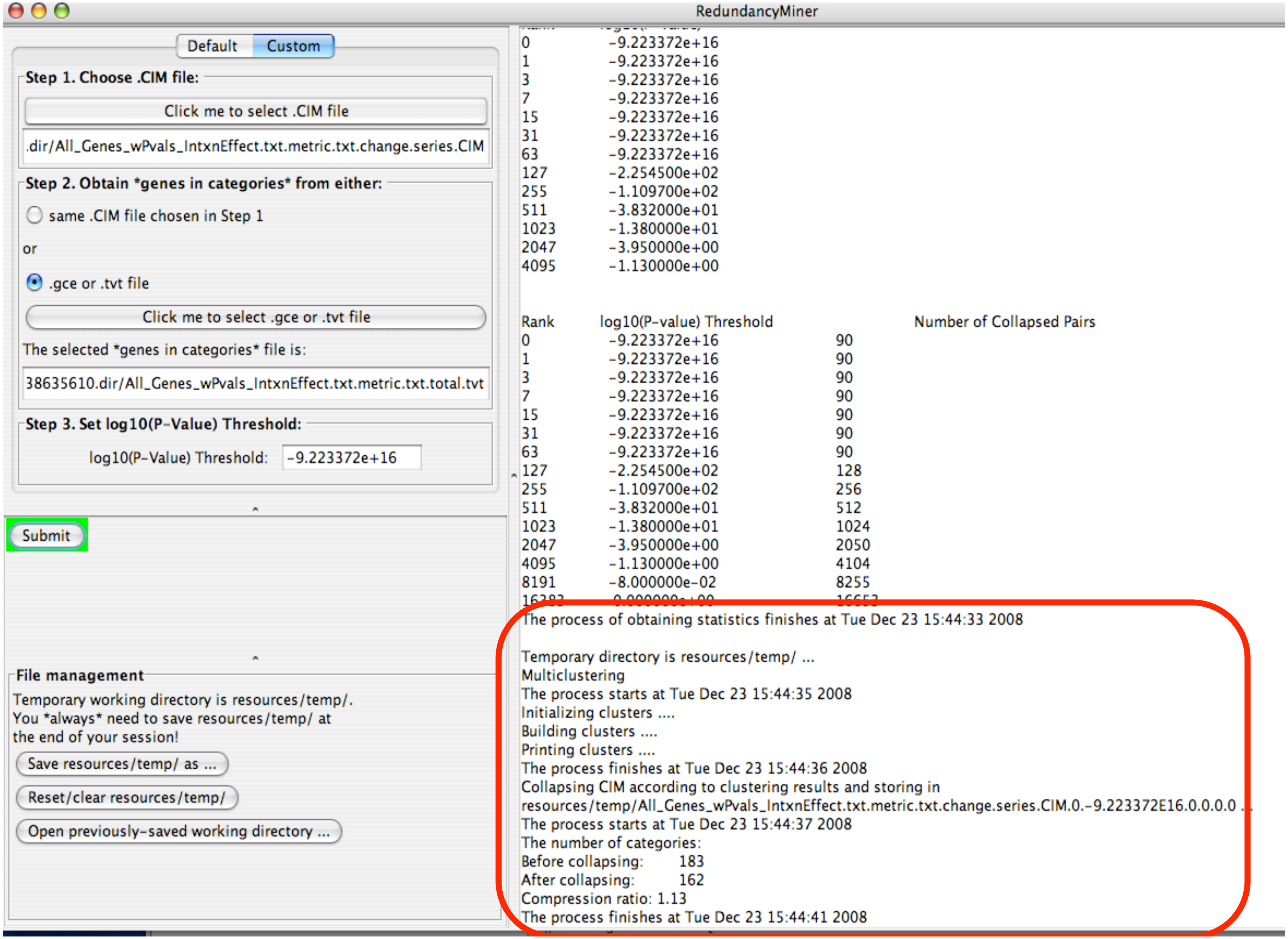

File management: Save the working directory (Step 1)

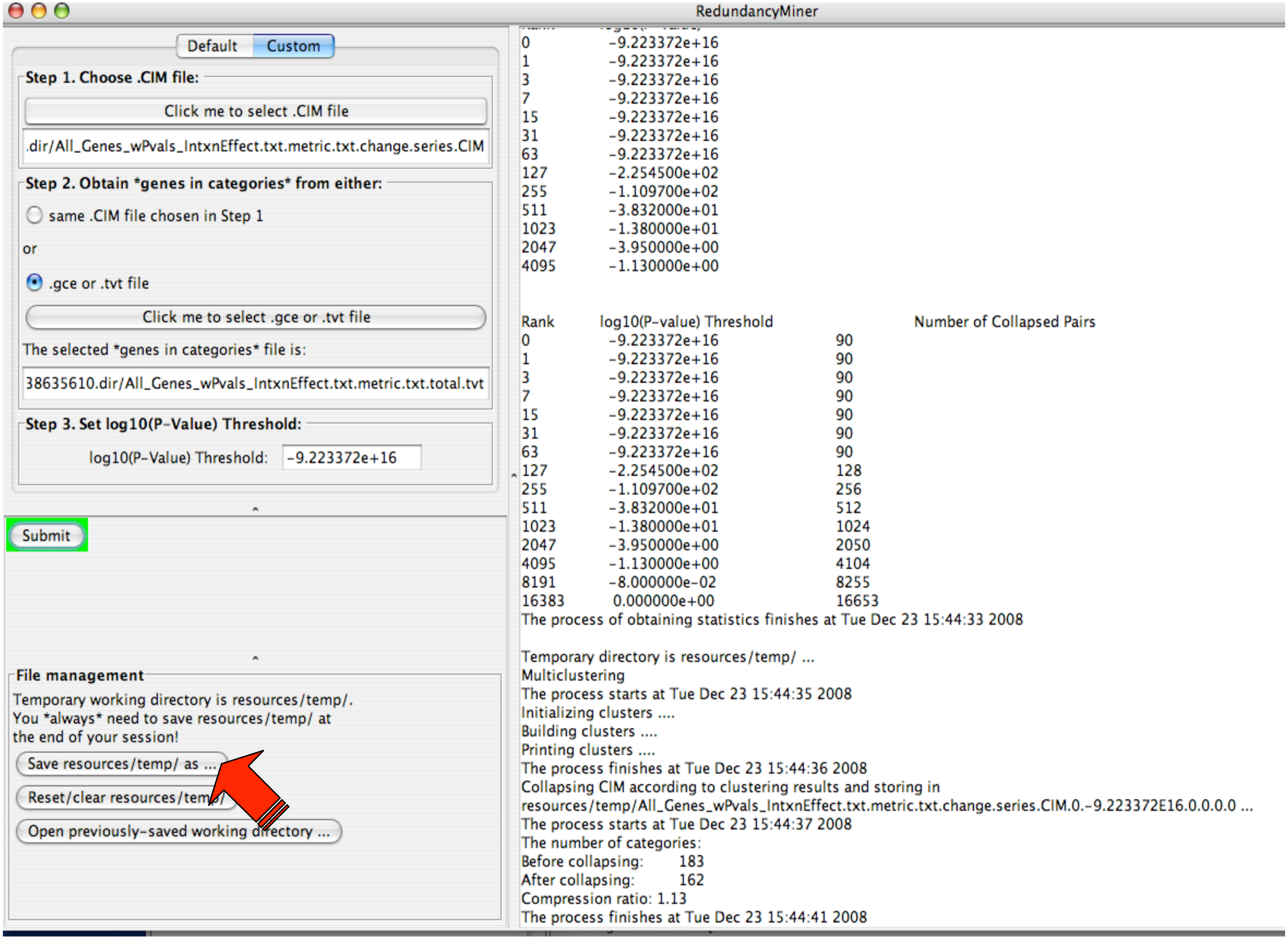

File management: Save the working directory (Step 2)

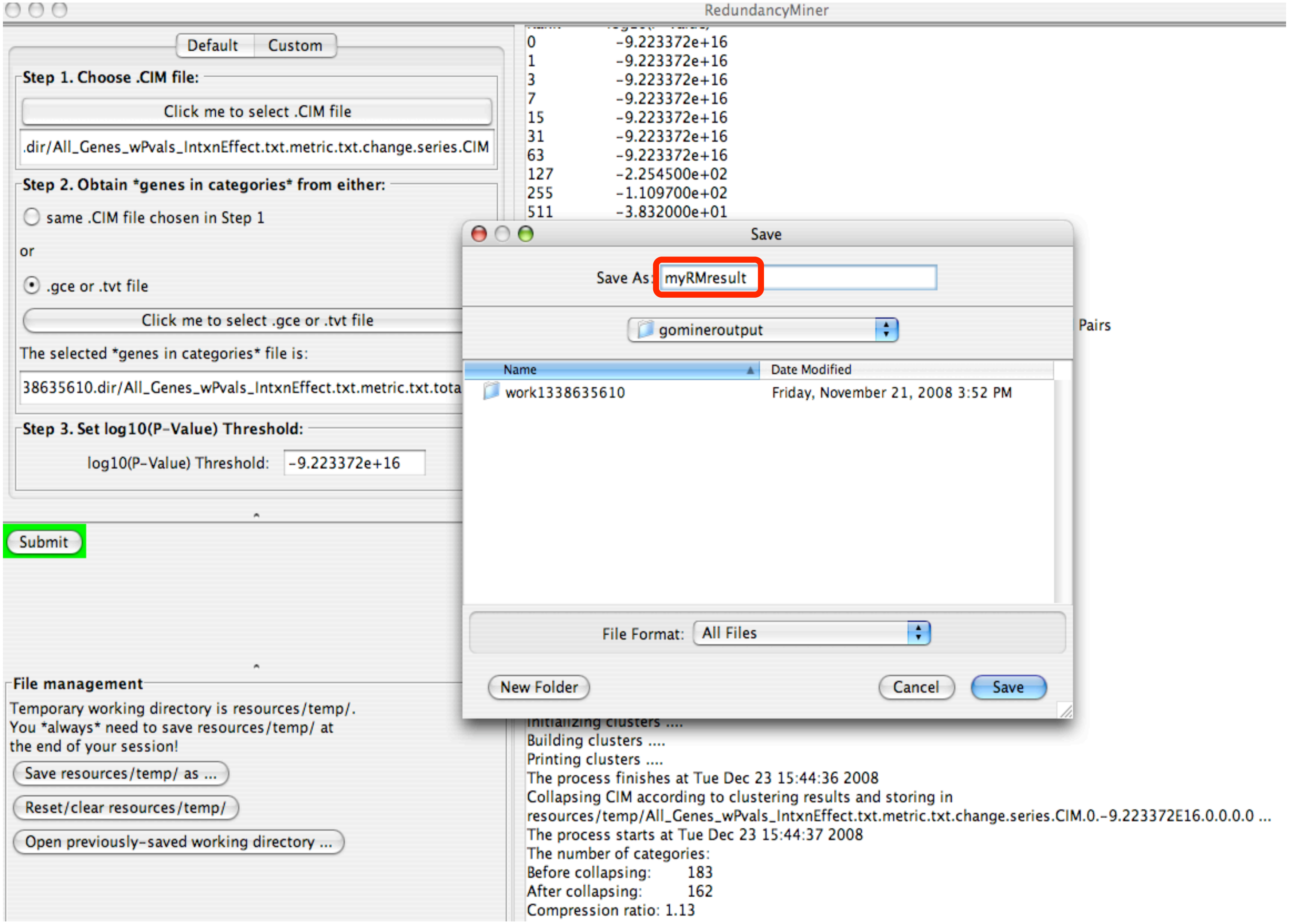

File management: Save completed

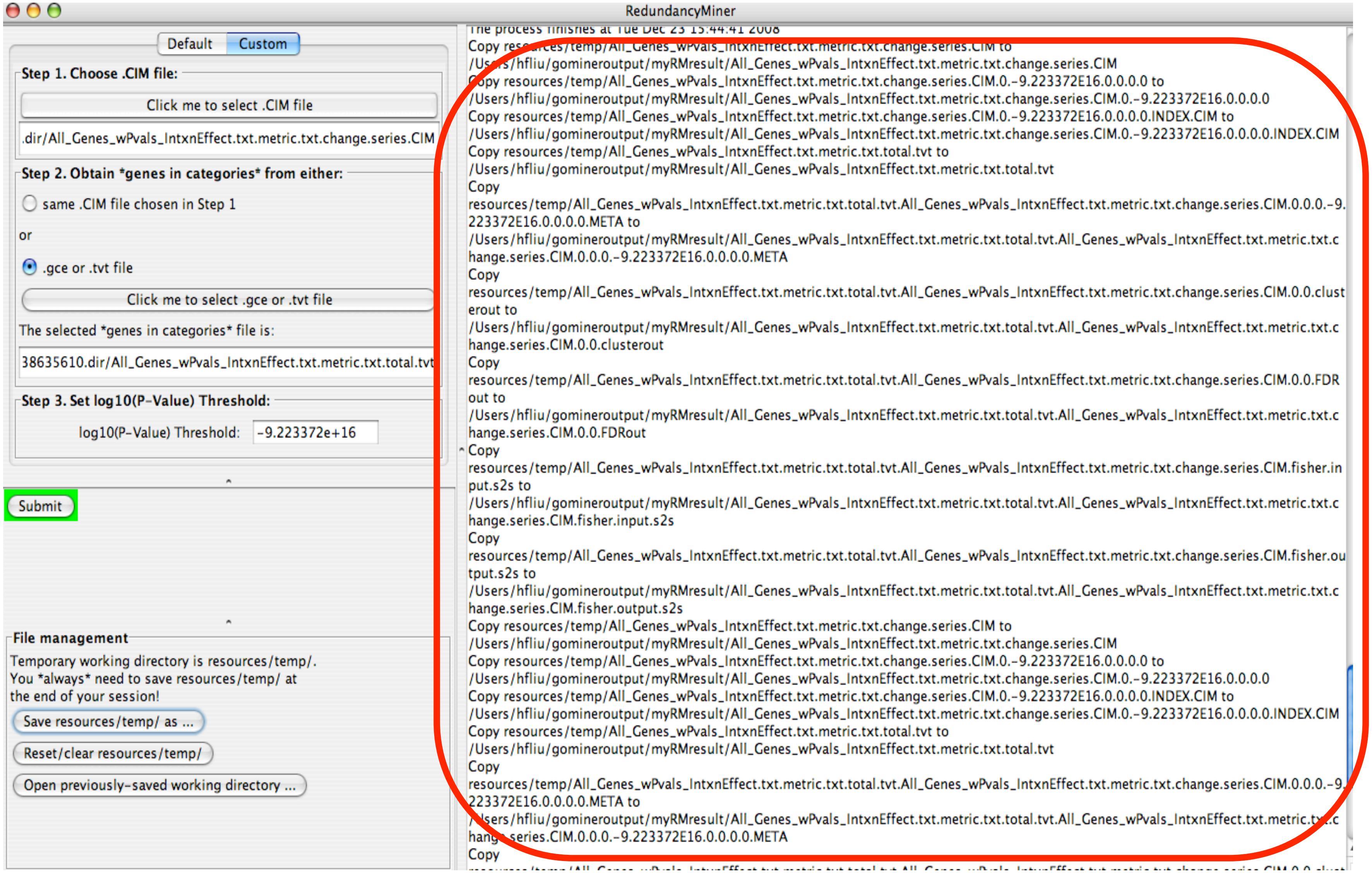

File management: Retrieve the working directory (Step 1)

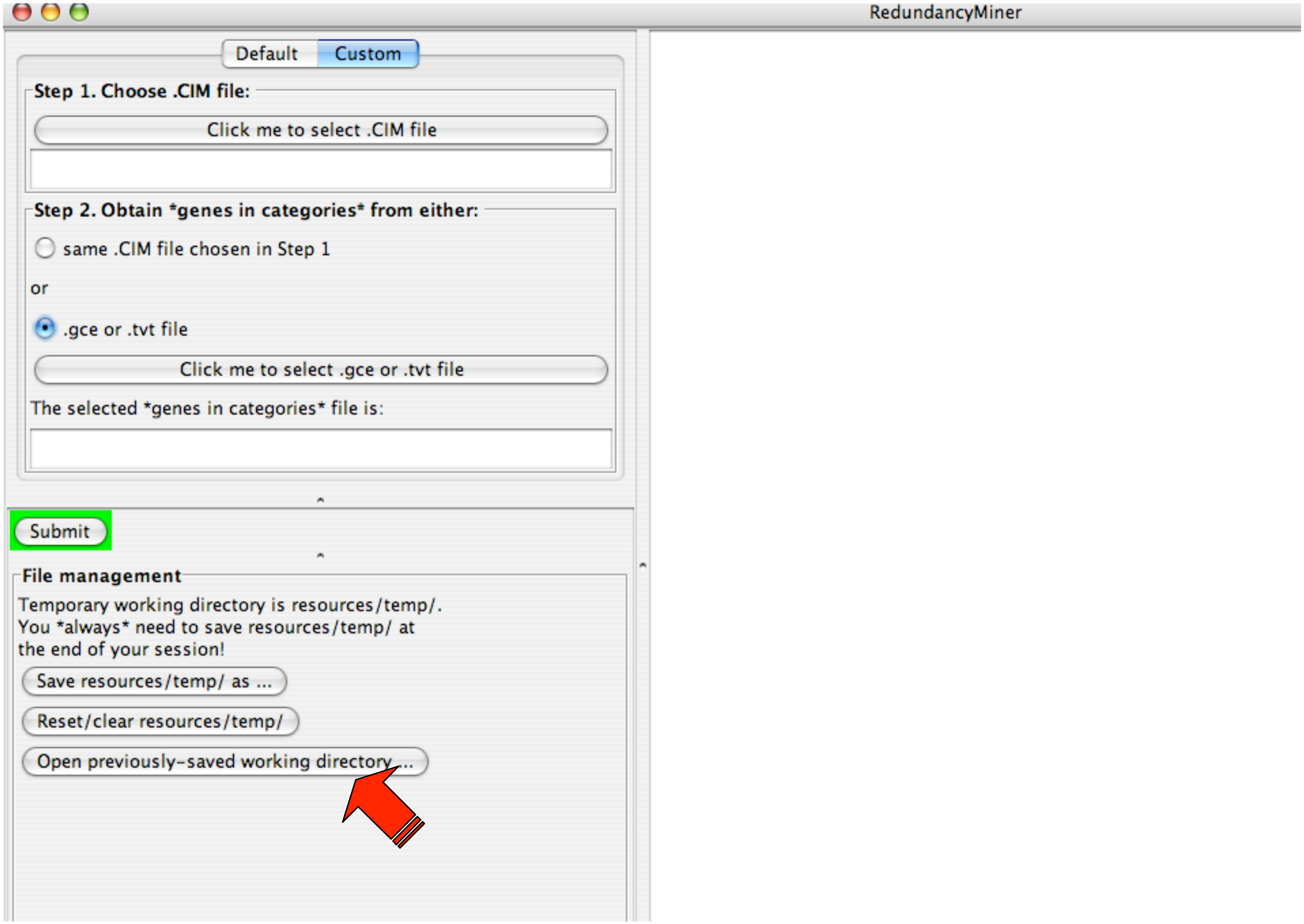

File management: Retrieve the working directory (Step 2)

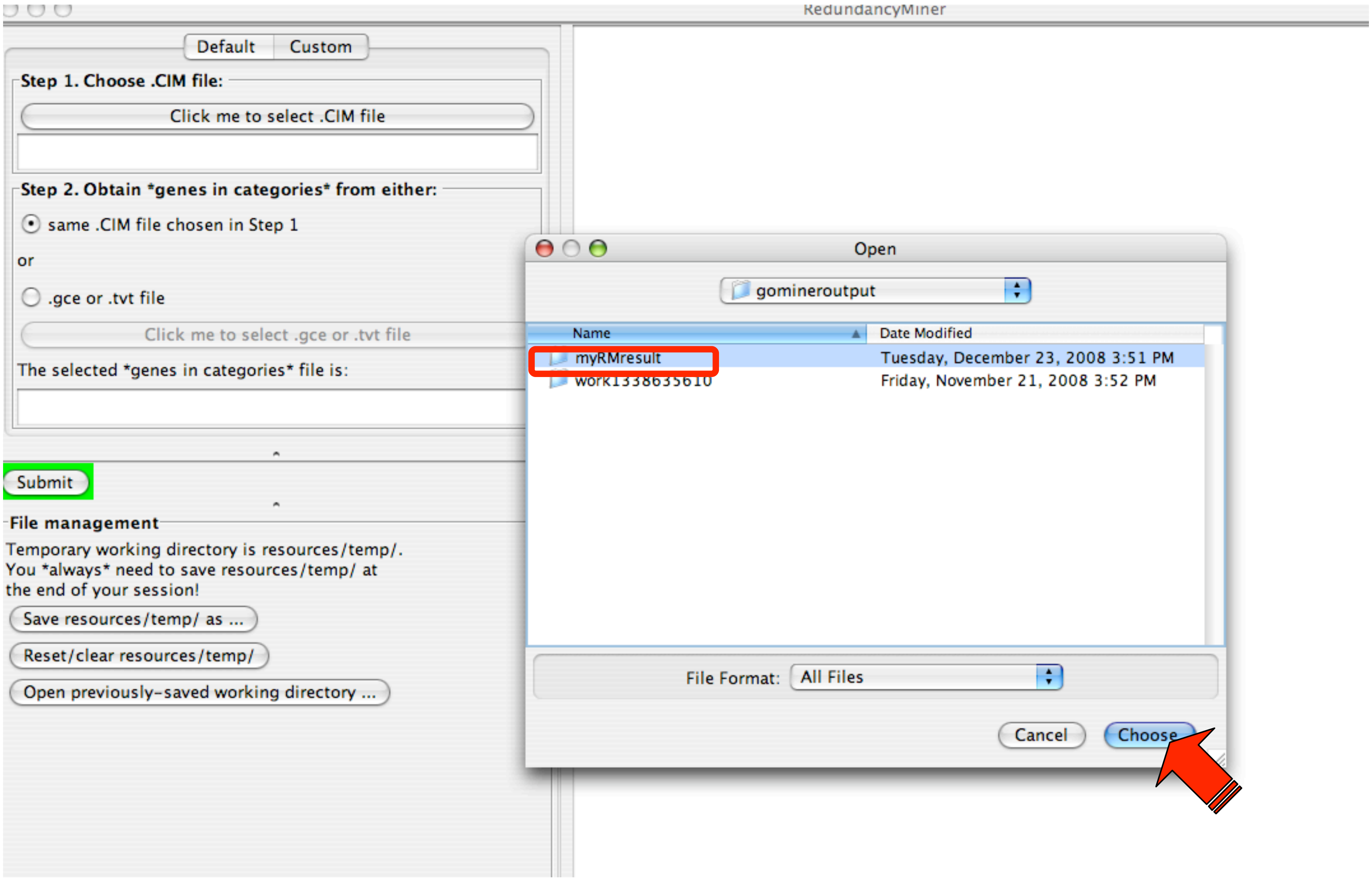

File management: Retrieval completed

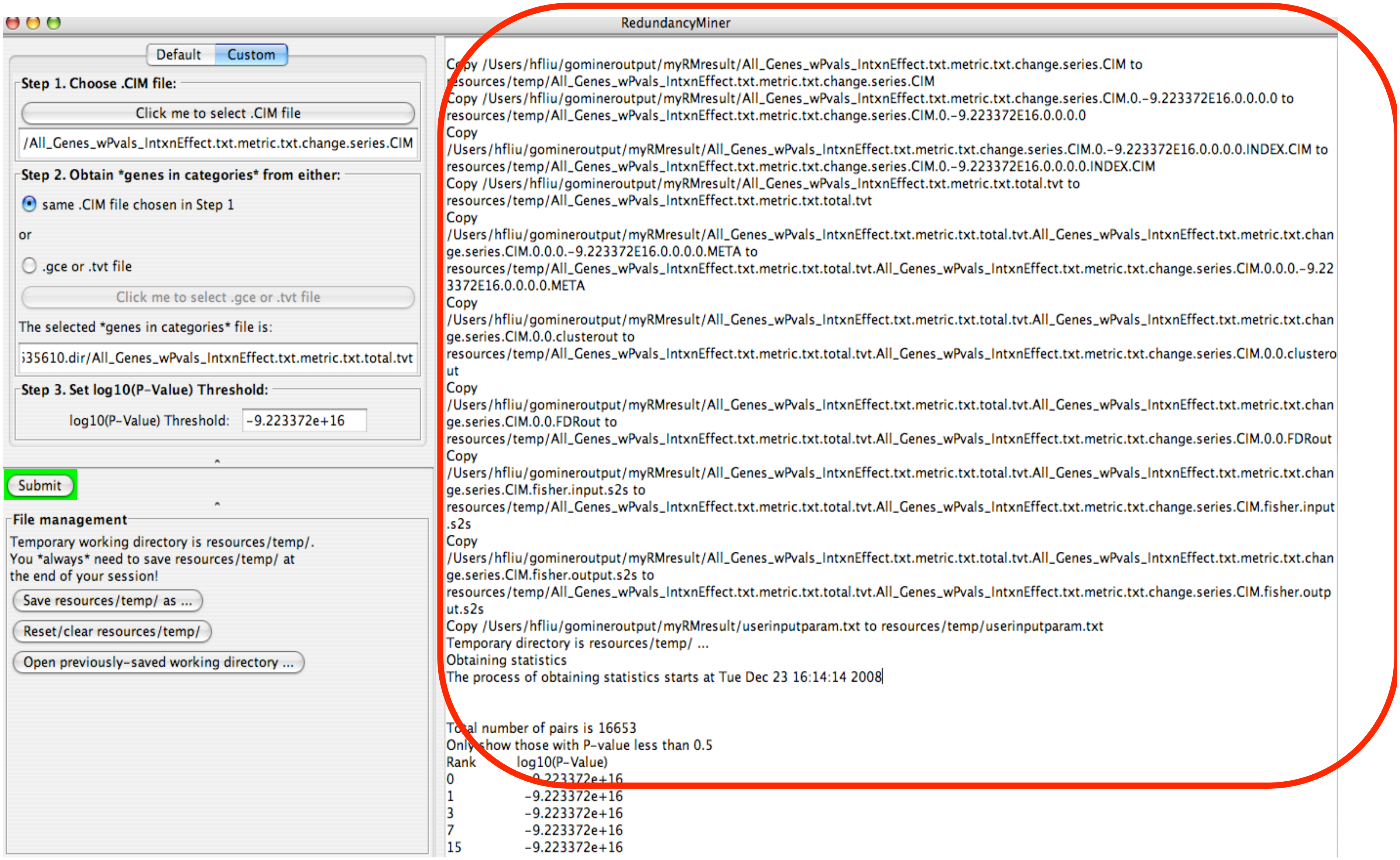

Default Mode

The default mode will generate results on the selected HTGM output directory.

- Select HTGM output directory
- Submit the job

Select Default mode

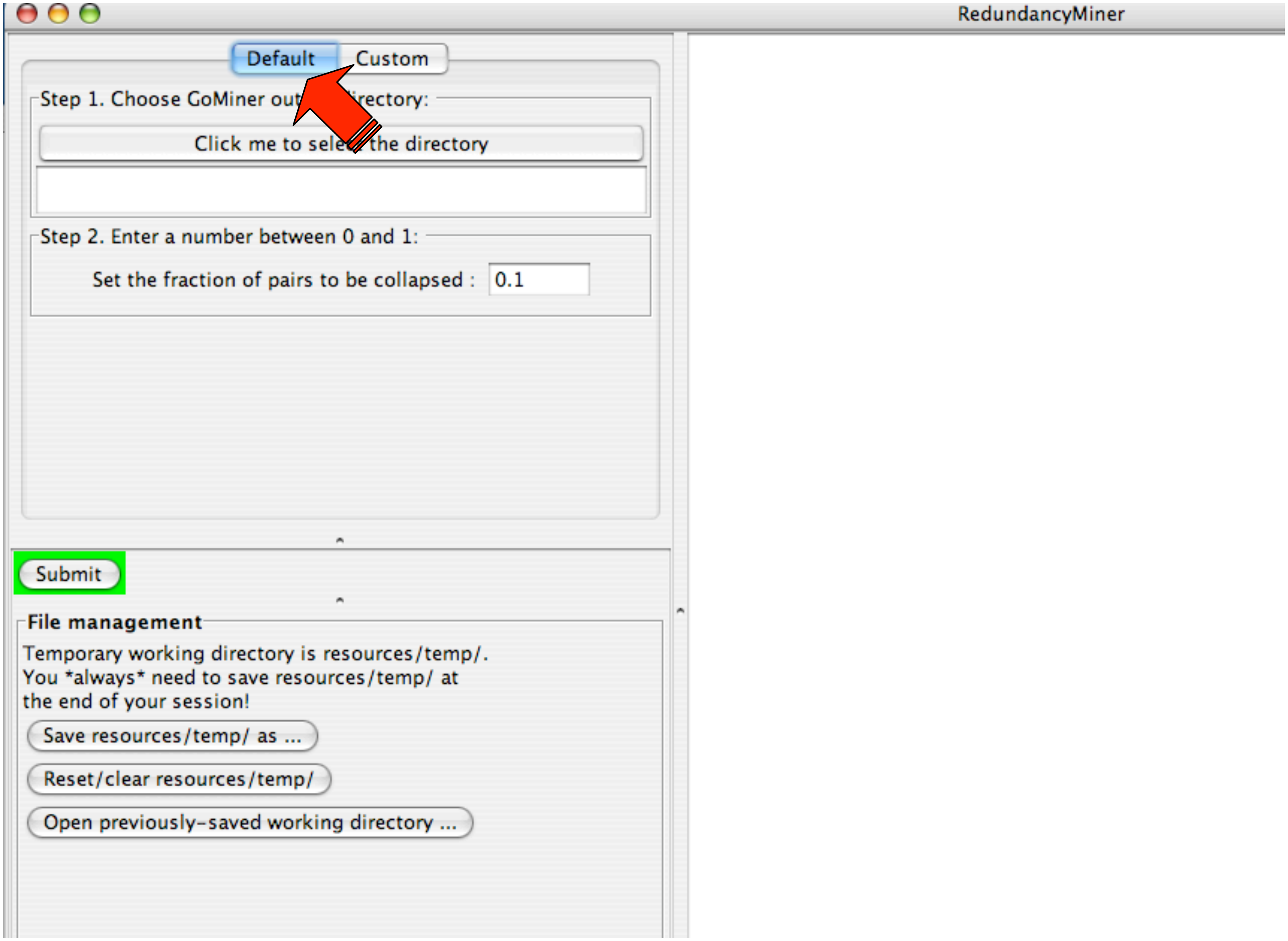

Select HTGM output directory (Step 1)

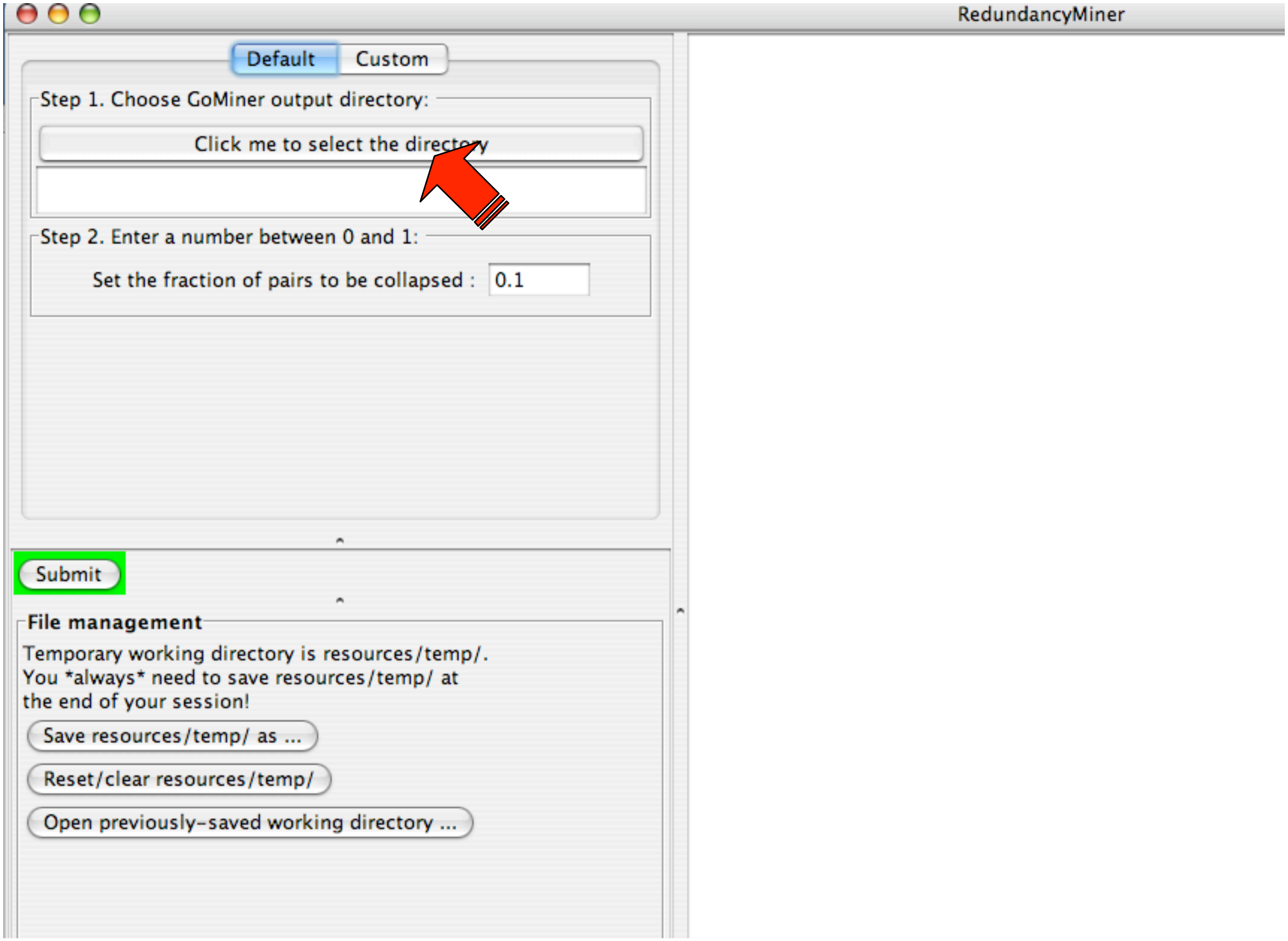

Select HTGM output directory (Step 2)

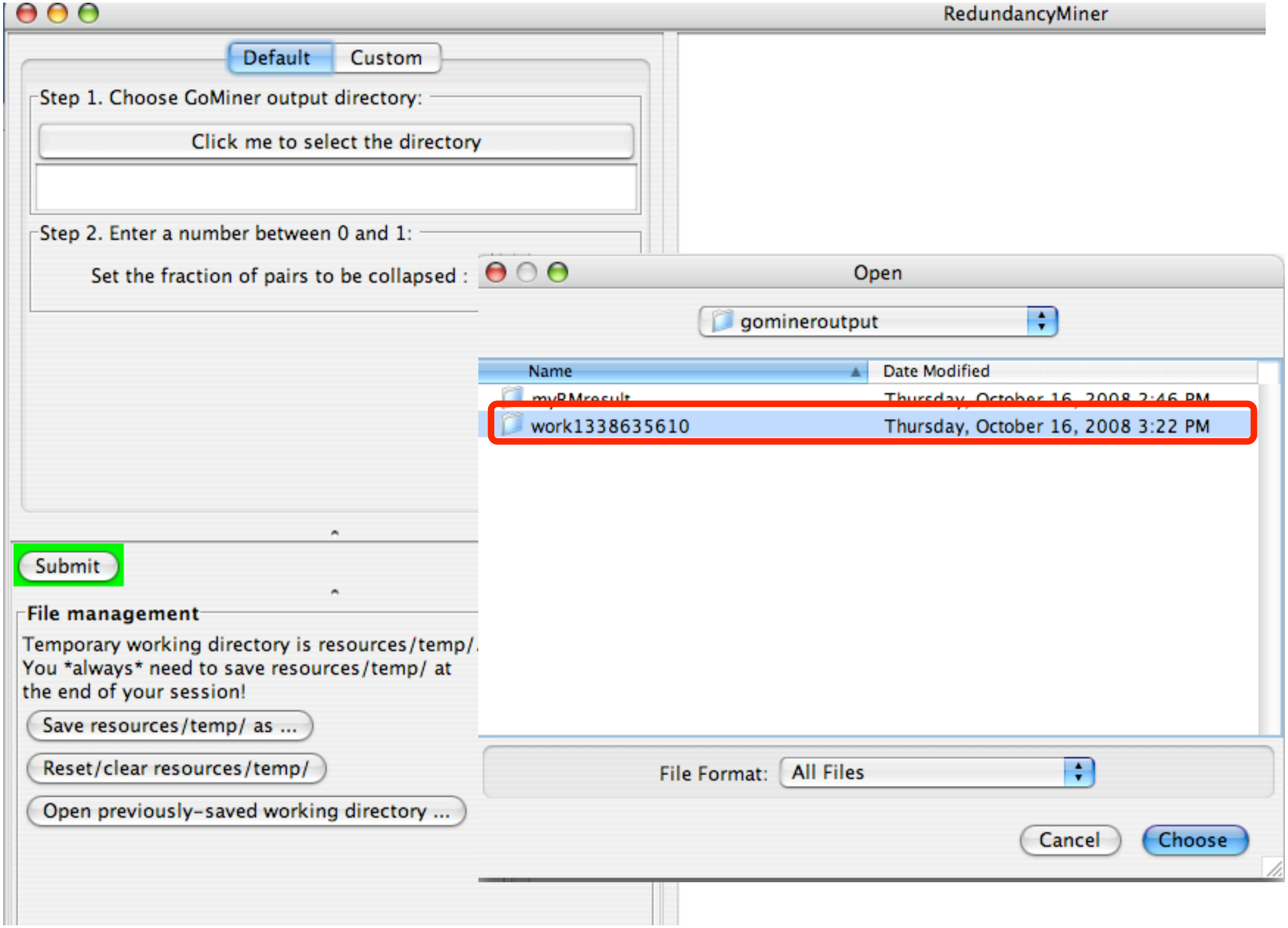

Submit the job (Step 3)

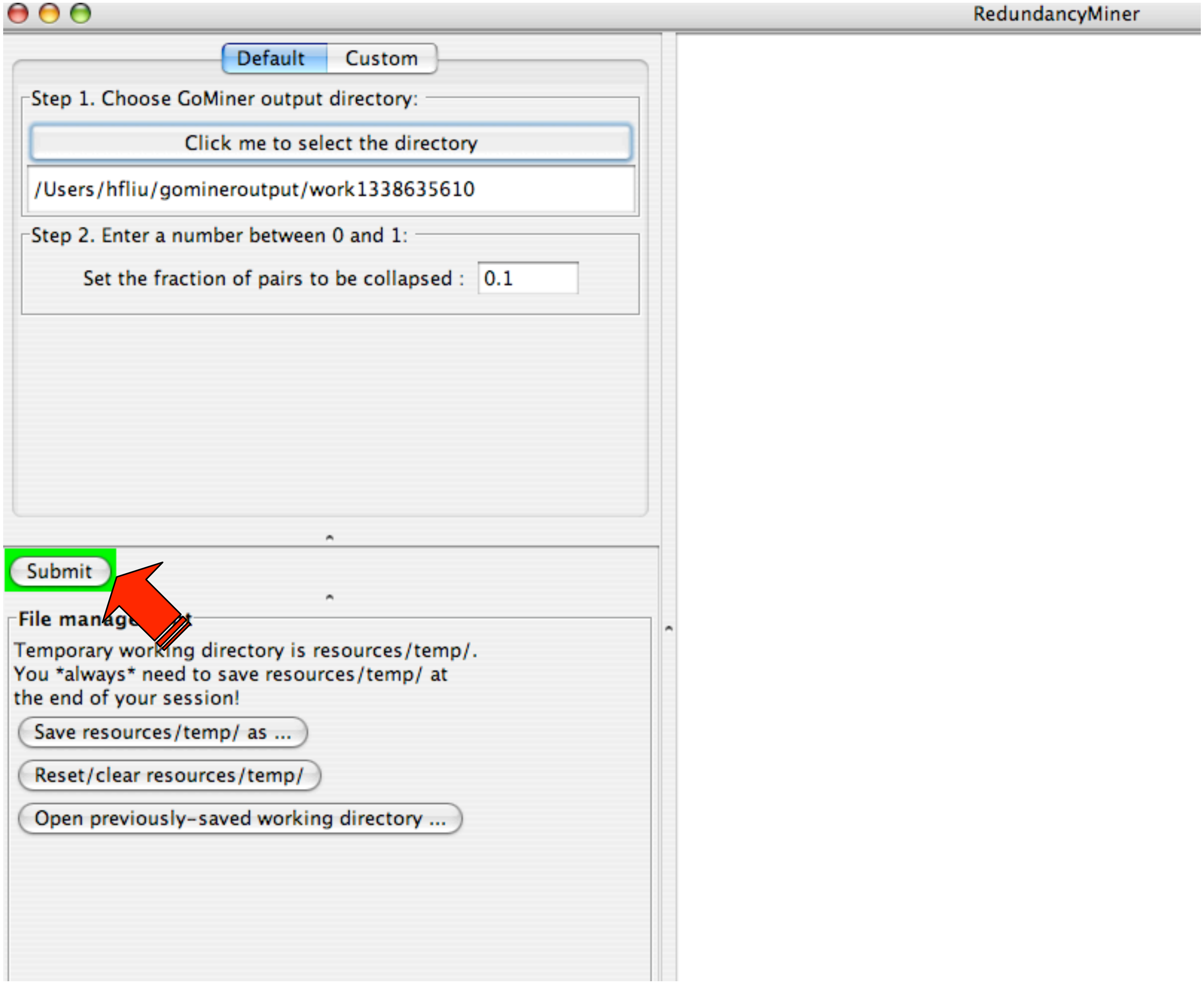

Default mode completed

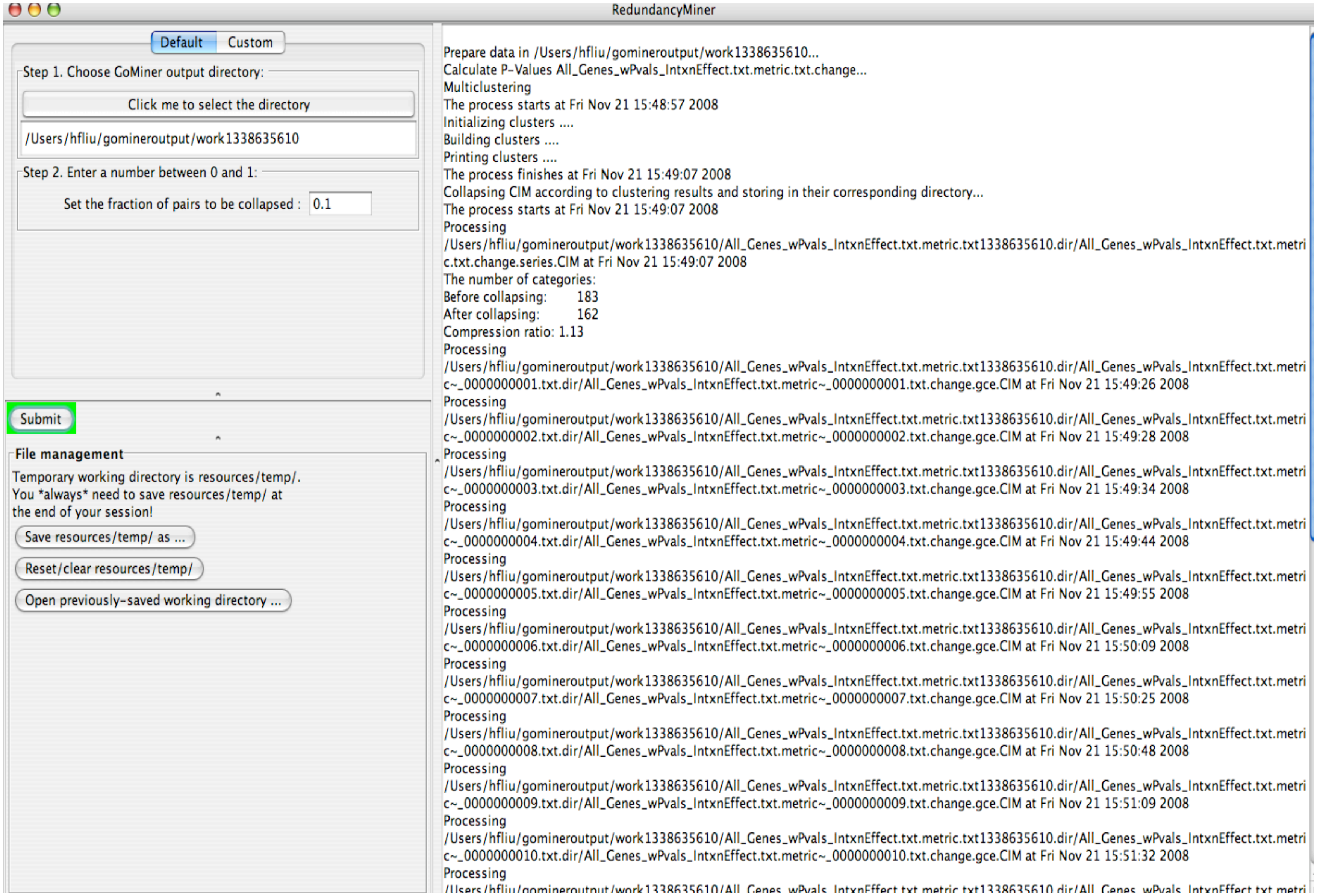

Supplement: Additional file 6 — PDF format of PowerPoint user's manual. .pdf version of the RedundancyMiner user's manual. [file 1471-2105-12-52-S6.PDF]
