# Supplementary material for: RedundancyMiner: De-replication of redundant GO categories in microarray and proteomics analysis
Source: BMC Bioinformatics. 2011 Feb 10;12:52. doi: 10.1186/1471-2105-12-52 (PMC3223614; doi:10.1186/1471-2105-12-52)
Supplement: Additional file 7 — Simplified version of the HTGM result directory that is exemplified in the user's manual. HTGM result directory to be used in conjunction with the examples given in the RedundancyMiner user's manual. [file 1471-2105-12-52-S7.GZ › work1338635610/index.html]

HTGM Job Summary

# HTGM Job Summary

| Input and Parameters | | |
| --- | --- | --- |
| Type | File | Description |
| Total File | All\_Genes\_wPvals\_IntxnEffect.txt.metric.txt | User-submitted list of total genes in the experiment or analysis |
| Changed File | All\_Genes\_wPvals\_IntxnEffect.txt.metric~\_0000000001.txt | User-submitted list of changed genes in the experiment or analysis |
| Changed File | All\_Genes\_wPvals\_IntxnEffect.txt.metric~\_0000000002.txt | User-submitted list of changed genes in the experiment or analysis |
| Changed File | All\_Genes\_wPvals\_IntxnEffect.txt.metric~\_0000000003.txt | User-submitted list of changed genes in the experiment or analysis |
| Changed File | All\_Genes\_wPvals\_IntxnEffect.txt.metric~\_0000000004.txt | User-submitted list of changed genes in the experiment or analysis |
| Changed File | All\_Genes\_wPvals\_IntxnEffect.txt.metric~\_0000000005.txt | User-submitted list of changed genes in the experiment or analysis |
| Changed File | All\_Genes\_wPvals\_IntxnEffect.txt.metric~\_0000000006.txt | User-submitted list of changed genes in the experiment or analysis |
| Changed File | All\_Genes\_wPvals\_IntxnEffect.txt.metric~\_0000000007.txt | User-submitted list of changed genes in the experiment or analysis |
| Changed File | All\_Genes\_wPvals\_IntxnEffect.txt.metric~\_0000000008.txt | User-submitted list of changed genes in the experiment or analysis |
| Changed File | All\_Genes\_wPvals\_IntxnEffect.txt.metric~\_0000000009.txt | User-submitted list of changed genes in the experiment or analysis |
| Changed File | All\_Genes\_wPvals\_IntxnEffect.txt.metric~\_0000000010.txt | User-submitted list of changed genes in the experiment or analysis |
| Changed File | All\_Genes\_wPvals\_IntxnEffect.txt.metric~\_0000000011.txt | User-submitted list of changed genes in the experiment or analysis |
| Changed File | All\_Genes\_wPvals\_IntxnEffect.txt.metric~\_0000000012.txt | User-submitted list of changed genes in the experiment or analysis |
| Changed File | All\_Genes\_wPvals\_IntxnEffect.txt.metric~\_0000000013.txt | User-submitted list of changed genes in the experiment or analysis |
| Changed File | All\_Genes\_wPvals\_IntxnEffect.txt.metric~\_0000000014.txt | User-submitted list of changed genes in the experiment or analysis |
| Changed File | All\_Genes\_wPvals\_IntxnEffect.txt.metric~\_0000000015.txt | User-submitted list of changed genes in the experiment or analysis |
| Changed File | All\_Genes\_wPvals\_IntxnEffect.txt.metric~\_0000000016.txt | User-submitted list of changed genes in the experiment or analysis |
| Changed File | All\_Genes\_wPvals\_IntxnEffect.txt.metric~\_0000000017.txt | User-submitted list of changed genes in the experiment or analysis |
| Changed File | All\_Genes\_wPvals\_IntxnEffect.txt.metric~\_0000000018.txt | User-submitted list of changed genes in the experiment or analysis |
| Changed File | All\_Genes\_wPvals\_IntxnEffect.txt.metric~\_0000000019.txt | User-submitted list of changed genes in the experiment or analysis |
| Summary of options | userinputparam.txt | A summary of the parameters selected by the user for this job |
| Database version | GODBVersion.txt | The version of the GO database used to process this job |
| --- | | ||| Results | | |
| --- | --- | --- |
| Type | File | Description |
| Archive Results (Zip) | HighThruputResult1338635610.zip | An archive of all of the result files for this job |
| Browsable Results (HTML) | Browse Results | A browsable collection of pages to navigate the result files for this job |
