# Supplementary material for: RedundancyMiner: De-replication of redundant GO categories in microarray and proteomics analysis
Source: BMC Bioinformatics. 2011 Feb 10;12:52. doi: 10.1186/1471-2105-12-52 (PMC3223614; doi:10.1186/1471-2105-12-52)
Supplement: Additional file 8 — Retinal development HTGM download. compressed package of the results of running HTGM on the retinal development genes list. [file 1471-2105-12-52-S8.ZIP › SCENARIO_2_MODIFIED/total.txt.total.txt.dir/Exp1_BestClusterMap_LEIGS_KM_24.csv.join.1.txt.dir/Exp1_BestClusterMap_LEIGS_KM_24.csv.join.1.txt.change.gce.html]

Gene Category Report for Exp1\_BestClusterMap\_LEIGS\_KM\_24.csv.join.1.txt

# Gene Category Report for Exp1\_BestClusterMap\_LEIGS\_KM\_24.csv.join.1.txt

| HYPERLINKED GO CATEGORY | HYPERLINKED GENE NAME | TOTAL GENES | CHANGED GENES | ENRICHMENT | LOG10(p) | CUMULATIVE NUMBER OF CATEGORIES | CUMULATIVE RANDOMS MEAN | FALSE DISCOVERY RATE |
| --- | --- | --- | --- | --- | --- | --- | --- | --- |
| GO:0000077\_DNA\_damage\_checkpoint | MSH2 | 14 | 2 | 25.302198 | -2.572529 | 1 | 2.94 | 2.940000 |
| GO:0000077\_DNA\_damage\_checkpoint | TIPIN | 14 | 2 | 25.302198 | -2.572529 | 1 | 2.94 | 2.940000 |
| GO:0031570\_DNA\_integrity\_checkpoint | MSH2 | 16 | 2 | 22.139423 | -2.455404 | 2 | 3.52 | 1.760000 |
| GO:0031570\_DNA\_integrity\_checkpoint | TIPIN | 16 | 2 | 22.139423 | -2.455404 | 2 | 3.52 | 1.760000 |
| GO:0043933\_macromolecular\_complex\_subunit\_organization | SPAG9 | 117 | 4 | 6.055227 | -2.415684 | 3 | 3.66 | 1.220000 |
| GO:0043933\_macromolecular\_complex\_subunit\_organization | PTBP2 | 117 | 4 | 6.055227 | -2.415684 | 3 | 3.66 | 1.220000 |
| GO:0043933\_macromolecular\_complex\_subunit\_organization | SMARCA4 | 117 | 4 | 6.055227 | -2.415684 | 3 | 3.66 | 1.220000 |
| GO:0043933\_macromolecular\_complex\_subunit\_organization | DSTN | 117 | 4 | 6.055227 | -2.415684 | 3 | 3.66 | 1.220000 |
| GO:0016458\_gene\_silencing | DNMT1 | 18 | 2 | 19.679487 | -2.352906 | 4 | 4.3 | 1.075000 |
| GO:0016458\_gene\_silencing | SMARCA4 | 18 | 2 | 19.679487 | -2.352906 | 4 | 4.3 | 1.075000 |
| GO:0006933\_negative\_regulation\_of\_cell\_adhesion\_involved\_in\_substrate-bound\_cell\_migration | ATP5B | 1 | 1 |  |  |  |  |  |  |
| GO:0033158\_regulation\_of\_protein\_import\_into\_nucleus\_\_translocation | SMAD4 | 1 | 1 |  |  |  |  |  |  |
| GO:0033160\_positive\_regulation\_of\_protein\_import\_into\_nucleus\_\_translocation | SMAD4 | 1 | 1 |  |  |  |  |  |  |
| GO:0060390\_regulation\_of\_SMAD\_protein\_nuclear\_translocation | SMAD4 | 1 | 1 |  |  |  |  |  |  |
| GO:0060391\_positive\_regulation\_of\_SMAD\_protein\_nuclear\_translocation | SMAD4 | 1 | 1 |  |  |  |  |  |  |
| GO:0000075\_cell\_cycle\_checkpoint | MSH2 | 21 | 2 | 16.868132 | -2.219893 | 5 | 5.47 | 1.094000 |
| GO:0000075\_cell\_cycle\_checkpoint | TIPIN | 21 | 2 | 16.868132 | -2.219893 | 5 | 5.47 | 1.094000 |
| GO:0034621\_cellular\_macromolecular\_complex\_subunit\_organization | PTBP2 | 76 | 3 | 6.991397 | -2.068016 | 6 | 7.24 | 1.206667 |
| GO:0034621\_cellular\_macromolecular\_complex\_subunit\_organization | DSTN | 76 | 3 | 6.991397 | -2.068016 | 6 | 7.24 | 1.206667 |
| GO:0034621\_cellular\_macromolecular\_complex\_subunit\_organization | SMARCA4 | 76 | 3 | 6.991397 | -2.068016 | 6 | 7.24 | 1.206667 |
| GO:0040029\_regulation\_of\_gene\_expression\_\_epigenetic | DNMT1 | 26 | 2 | 13.624260 | -2.037746 | 7 | 7.58 | 1.082857 |
| GO:0040029\_regulation\_of\_gene\_expression\_\_epigenetic | SMARCA4 | 26 | 2 | 13.624260 | -2.037746 | 7 | 7.58 | 1.082857 |
| GO:0032268\_regulation\_of\_cellular\_protein\_metabolic\_process | EIF4G2 | 152 | 4 | 4.660931 | -2.014135 | 8 | 7.76 | 0.970000 |
| GO:0032268\_regulation\_of\_cellular\_protein\_metabolic\_process | ACO1 | 152 | 4 | 4.660931 | -2.014135 | 8 | 7.76 | 0.970000 |
| GO:0032268\_regulation\_of\_cellular\_protein\_metabolic\_process | SMAD4 | 152 | 4 | 4.660931 | -2.014135 | 8 | 7.76 | 0.970000 |
| GO:0032268\_regulation\_of\_cellular\_protein\_metabolic\_process | DSTN | 152 | 4 | 4.660931 | -2.014135 | 8 | 7.76 | 0.970000 |
| GO:0009411\_response\_to\_UV | MSH2 | 27 | 2 | 13.119658 | -2.005824 | 9 | 7.93 | 0.881111 |
| GO:0009411\_response\_to\_UV | TIPIN | 27 | 2 | 13.119658 | -2.005824 | 9 | 7.93 | 0.881111 |
| GO:0002266\_follicular\_dendritic\_cell\_activation | NFKB2 | 2 | 1 |  |  |  |  |  |  |
| GO:0002268\_follicular\_dendritic\_cell\_differentiation | NFKB2 | 2 | 1 |  |  |  |  |  |  |
| GO:0007527\_adult\_somatic\_muscle\_development | UTRN | 2 | 1 |  |  |  |  |  |  |
| GO:0010718\_positive\_regulation\_of\_epithelial\_to\_mesenchymal\_transition | SMAD4 | 2 | 1 |  |  |  |  |  |  |
| GO:0010770\_positive\_regulation\_of\_cell\_morphogenesis\_involved\_in\_differentiation | SMAD4 | 2 | 1 |  |  |  |  |  |  |
| GO:0010862\_positive\_regulation\_of\_pathway-restricted\_SMAD\_protein\_phosphorylation | SMAD4 | 2 | 1 |  |  |  |  |  |  |
| GO:0031573\_intra-S\_DNA\_damage\_checkpoint | MSH2 | 2 | 1 |  |  |  |  |  |  |
| GO:0046950\_cellular\_ketone\_body\_metabolic\_process | OXCT1 | 2 | 1 |  |  |  |  |  |  |
| GO:0060393\_regulation\_of\_pathway-restricted\_SMAD\_protein\_phosphorylation | SMAD4 | 2 | 1 |  |  |  |  |  |  |
| GO:0006417\_regulation\_of\_translation | EIF4G2 | 29 | 2 | 12.214854 | -1.945608 | 11 | 8.85 | 0.804545 |
| GO:0006417\_regulation\_of\_translation | ACO1 | 29 | 2 | 12.214854 | -1.945608 | 11 | 8.85 | 0.804545 |
| GO:0042770\_DNA\_damage\_response\_\_signal\_transduction | MSH2 | 29 | 2 | 12.214854 | -1.945608 | 11 | 8.85 | 0.804545 |
| GO:0042770\_DNA\_damage\_response\_\_signal\_transduction | TIPIN | 29 | 2 | 12.214854 | -1.945608 | 11 | 8.85 | 0.804545 |
| GO:0051246\_regulation\_of\_protein\_metabolic\_process | EIF4G2 | 170 | 4 | 4.167421 | -1.847846 | 12 | 10.5 | 0.875000 |
| GO:0051246\_regulation\_of\_protein\_metabolic\_process | ACO1 | 170 | 4 | 4.167421 | -1.847846 | 12 | 10.5 | 0.875000 |
| GO:0051246\_regulation\_of\_protein\_metabolic\_process | SMAD4 | 170 | 4 | 4.167421 | -1.847846 | 12 | 10.5 | 0.875000 |
| GO:0051246\_regulation\_of\_protein\_metabolic\_process | DSTN | 170 | 4 | 4.167421 | -1.847846 | 12 | 10.5 | 0.875000 |
| GO:0065003\_macromolecular\_complex\_assembly | SPAG9 | 93 | 3 | 5.713400 | -1.829329 | 13 | 11.02 | 0.847692 |
| GO:0065003\_macromolecular\_complex\_assembly | PTBP2 | 93 | 3 | 5.713400 | -1.829329 | 13 | 11.02 | 0.847692 |
| GO:0065003\_macromolecular\_complex\_assembly | SMARCA4 | 93 | 3 | 5.713400 | -1.829329 | 13 | 11.02 | 0.847692 |
| GO:0051052\_regulation\_of\_DNA\_metabolic\_process | MSH2 | 34 | 2 | 10.418552 | -1.812670 | 14 | 11.42 | 0.815714 |
| GO:0051052\_regulation\_of\_DNA\_metabolic\_process | DNMT1 | 34 | 2 | 10.418552 | -1.812670 | 14 | 11.42 | 0.815714 |
| GO:0006301\_postreplication\_repair | MSH2 | 3 | 1 |  |  |  |  |  |  |
| GO:0007403\_glial\_cell\_fate\_determination | SMARCA4 | 3 | 1 |  |  |  |  |  |  |
| GO:0007525\_somatic\_muscle\_development | UTRN | 3 | 1 |  |  |  |  |  |  |
| GO:0010216\_maintenance\_of\_DNA\_methylation | DNMT1 | 3 | 1 |  |  |  |  |  |  |
| GO:0010717\_regulation\_of\_epithelial\_to\_mesenchymal\_transition | SMAD4 | 3 | 1 |  |  |  |  |  |  |
| GO:0030836\_positive\_regulation\_of\_actin\_filament\_depolymerization | DSTN | 3 | 1 |  |  |  |  |  |  |
| GO:0043243\_positive\_regulation\_of\_protein\_complex\_disassembly | DSTN | 3 | 1 |  |  |  |  |  |  |
| GO:0044030\_regulation\_of\_DNA\_methylation | DNMT1 | 3 | 1 |  |  |  |  |  |  |
| GO:0001835\_blastocyst\_hatching | SMARCA4 | 4 | 1 |  |  |  |  |  |  |
| GO:0006334\_nucleosome\_assembly | SMARCA4 | 4 | 1 |  |  |  |  |  |  |
| GO:0007184\_SMAD\_protein\_nuclear\_translocation | SMAD4 | 4 | 1 |  |  |  |  |  |  |
| GO:0010224\_response\_to\_UV-B | MSH2 | 4 | 1 |  |  |  |  |  |  |
| GO:0032715\_negative\_regulation\_of\_interleukin-6\_production | H47 | 4 | 1 |  |  |  |  |  |  |
| GO:0035188\_hatching | SMARCA4 | 4 | 1 |  |  |  |  |  |  |
| GO:0006346\_methylation-dependent\_chromatin\_silencing | SMARCA4 | 5 | 1 | 35.423077 | -1.554004 | 20 | 26.17 | 1.308500 |
| GO:0006376\_mRNA\_splice\_site\_selection | PTBP2 | 5 | 1 | 35.423077 | -1.554004 | 20 | 26.17 | 1.308500 |
| GO:0006929\_substrate-bound\_cell\_migration | ATP5B | 5 | 1 | 35.423077 | -1.554004 | 20 | 26.17 | 1.308500 |
| GO:0030042\_actin\_filament\_depolymerization | DSTN | 5 | 1 | 35.423077 | -1.554004 | 20 | 26.17 | 1.308500 |
| GO:0030834\_regulation\_of\_actin\_filament\_depolymerization | DSTN | 5 | 1 | 35.423077 | -1.554004 | 20 | 26.17 | 1.308500 |
| GO:0032720\_negative\_regulation\_of\_tumor\_necrosis\_factor\_production | H47 | 5 | 1 | 35.423077 | -1.554004 | 20 | 26.17 | 1.308500 |
| GO:0000245\_spliceosome\_assembly | PTBP2 | 6 | 1 | 29.519231 | -1.476000 | 23 | 34.76 | 1.511304 |
| GO:0060389\_pathway-restricted\_SMAD\_protein\_phosphorylation | SMAD4 | 6 | 1 | 29.519231 | -1.476000 | 23 | 34.76 | 1.511304 |
| GO:0065004\_protein-DNA\_complex\_assembly | SMARCA4 | 6 | 1 | 29.519231 | -1.476000 | 23 | 34.76 | 1.511304 |
| GO:0010608\_posttranscriptional\_regulation\_of\_gene\_expression | EIF4G2 | 52 | 2 | 6.812130 | -1.465995 | 24 | 34.96 | 1.456667 |
| GO:0010608\_posttranscriptional\_regulation\_of\_gene\_expression | ACO1 | 52 | 2 | 6.812130 | -1.465995 | 24 | 34.96 | 1.456667 |
| GO:0006412\_translation | EIF4G2 | 54 | 2 | 6.559829 | -1.435879 | 25 | 36.24 | 1.449600 |
| GO:0006412\_translation | ACO1 | 54 | 2 | 6.559829 | -1.435879 | 25 | 36.24 | 1.449600 |
| GO:0006119\_oxidative\_phosphorylation | MSH2 | 7 | 1 | 25.302198 | -1.410229 | 30 | 43.88 | 1.462667 |
| GO:0006298\_mismatch\_repair | MSH2 | 7 | 1 | 25.302198 | -1.410229 | 30 | 43.88 | 1.462667 |
| GO:0008340\_determination\_of\_adult\_lifespan | MSH2 | 7 | 1 | 25.302198 | -1.410229 | 30 | 43.88 | 1.462667 |
| GO:0022618\_ribonucleoprotein\_complex\_assembly | PTBP2 | 7 | 1 | 25.302198 | -1.410229 | 30 | 43.88 | 1.462667 |
| GO:0031497\_chromatin\_assembly | SMARCA4 | 7 | 1 | 25.302198 | -1.410229 | 30 | 43.88 | 1.462667 |
| GO:0034622\_cellular\_macromolecular\_complex\_assembly | PTBP2 | 58 | 2 | 6.107427 | -1.379201 | 31 | 45.4 | 1.464516 |
| GO:0034622\_cellular\_macromolecular\_complex\_assembly | SMARCA4 | 58 | 2 | 6.107427 | -1.379201 | 31 | 45.4 | 1.464516 |
| GO:0031323\_regulation\_of\_cellular\_metabolic\_process | SPAG9 | 1015 | 10 | 1.744979 | -1.368854 | 32 | 45.82 | 1.431875 |
| GO:0031323\_regulation\_of\_cellular\_metabolic\_process | EIF4G2 | 1015 | 10 | 1.744979 | -1.368854 | 32 | 45.82 | 1.431875 |
| GO:0031323\_regulation\_of\_cellular\_metabolic\_process | ACO1 | 1015 | 10 | 1.744979 | -1.368854 | 32 | 45.82 | 1.431875 |
| GO:0031323\_regulation\_of\_cellular\_metabolic\_process | MSH2 | 1015 | 10 | 1.744979 | -1.368854 | 32 | 45.82 | 1.431875 |
| GO:0031323\_regulation\_of\_cellular\_metabolic\_process | CREB1 | 1015 | 10 | 1.744979 | -1.368854 | 32 | 45.82 | 1.431875 |
| GO:0031323\_regulation\_of\_cellular\_metabolic\_process | SMAD4 | 1015 | 10 | 1.744979 | -1.368854 | 32 | 45.82 | 1.431875 |
| GO:0031323\_regulation\_of\_cellular\_metabolic\_process | DNMT1 | 1015 | 10 | 1.744979 | -1.368854 | 32 | 45.82 | 1.431875 |
| GO:0031323\_regulation\_of\_cellular\_metabolic\_process | TMPO | 1015 | 10 | 1.744979 | -1.368854 | 32 | 45.82 | 1.431875 |
| GO:0031323\_regulation\_of\_cellular\_metabolic\_process | DSTN | 1015 | 10 | 1.744979 | -1.368854 | 32 | 45.82 | 1.431875 |
| GO:0031323\_regulation\_of\_cellular\_metabolic\_process | SMARCA4 | 1015 | 10 | 1.744979 | -1.368854 | 32 | 45.82 | 1.431875 |
| GO:0000910\_cytokinesis | DSTN | 8 | 1 | 22.139423 | -1.353412 | 39 | 51.19 | 1.312564 |
| GO:0002566\_somatic\_diversification\_of\_immune\_receptors\_via\_somatic\_mutation | MSH2 | 8 | 1 | 22.139423 | -1.353412 | 39 | 51.19 | 1.312564 |
| GO:0016446\_somatic\_hypermutation\_of\_immunoglobulin\_genes | MSH2 | 8 | 1 | 22.139423 | -1.353412 | 39 | 51.19 | 1.312564 |
| GO:0021781\_glial\_cell\_fate\_commitment | SMARCA4 | 8 | 1 | 22.139423 | -1.353412 | 39 | 51.19 | 1.312564 |
| GO:0034728\_nucleosome\_organization | SMARCA4 | 8 | 1 | 22.139423 | -1.353412 | 39 | 51.19 | 1.312564 |
| GO:0045910\_negative\_regulation\_of\_DNA\_recombination | MSH2 | 8 | 1 | 22.139423 | -1.353412 | 39 | 51.19 | 1.312564 |
| GO:0060347\_heart\_trabecula\_formation | SMARCA4 | 8 | 1 | 22.139423 | -1.353412 | 39 | 51.19 | 1.312564 |
| GO:0035239\_tube\_morphogenesis | NUP50 | 143 | 3 | 3.715707 | -1.344417 | 40 | 51.22 | 1.280500 |
| GO:0035239\_tube\_morphogenesis | SMAD4 | 143 | 3 | 3.715707 | -1.344417 | 40 | 51.22 | 1.280500 |
| GO:0035239\_tube\_morphogenesis | MYCN | 143 | 3 | 3.715707 | -1.344417 | 40 | 51.22 | 1.280500 |
| GO:0032270\_positive\_regulation\_of\_cellular\_protein\_metabolic\_process | SMAD4 | 61 | 2 | 5.807062 | -1.339485 | 41 | 51.49 | 1.255854 |
| GO:0032270\_positive\_regulation\_of\_cellular\_protein\_metabolic\_process | DSTN | 61 | 2 | 5.807062 | -1.339485 | 41 | 51.49 | 1.255854 |
| GO:0010165\_response\_to\_X-ray | MSH2 | 9 | 1 | 19.679487 | -1.303434 | 43 | 58.56 | 1.361860 |
| GO:0032388\_positive\_regulation\_of\_intracellular\_transport | SMAD4 | 9 | 1 | 19.679487 | -1.303434 | 43 | 58.56 | 1.361860 |
| GO:0051130\_positive\_regulation\_of\_cellular\_component\_organization | SMAD4 | 66 | 2 | 5.367133 | -1.277935 | 44 | 59.97 | 1.362955 |
| GO:0051130\_positive\_regulation\_of\_cellular\_component\_organization | DSTN | 66 | 2 | 5.367133 | -1.277935 | 44 | 59.97 | 1.362955 |
| GO:0010467\_gene\_expression | EIF4G2 | 905 | 9 | 1.761368 | -1.273399 | 45 | 60.08 | 1.335111 |
| GO:0010467\_gene\_expression | ACO1 | 905 | 9 | 1.761368 | -1.273399 | 45 | 60.08 | 1.335111 |
| GO:0010467\_gene\_expression | CREB1 | 905 | 9 | 1.761368 | -1.273399 | 45 | 60.08 | 1.335111 |
| GO:0010467\_gene\_expression | SMAD4 | 905 | 9 | 1.761368 | -1.273399 | 45 | 60.08 | 1.335111 |
| GO:0010467\_gene\_expression | DNMT1 | 905 | 9 | 1.761368 | -1.273399 | 45 | 60.08 | 1.335111 |
| GO:0010467\_gene\_expression | PTBP2 | 905 | 9 | 1.761368 | -1.273399 | 45 | 60.08 | 1.335111 |
| GO:0010467\_gene\_expression | RBM39 | 905 | 9 | 1.761368 | -1.273399 | 45 | 60.08 | 1.335111 |
| GO:0010467\_gene\_expression | TMPO | 905 | 9 | 1.761368 | -1.273399 | 45 | 60.08 | 1.335111 |
| GO:0010467\_gene\_expression | SMARCA4 | 905 | 9 | 1.761368 | -1.273399 | 45 | 60.08 | 1.335111 |
| GO:0051247\_positive\_regulation\_of\_protein\_metabolic\_process | SMAD4 | 67 | 2 | 5.287026 | -1.266258 | 46 | 60.4 | 1.313043 |
| GO:0051247\_positive\_regulation\_of\_protein\_metabolic\_process | DSTN | 67 | 2 | 5.287026 | -1.266258 | 46 | 60.4 | 1.313043 |
| GO:0001832\_blastocyst\_growth | SMARCA4 | 10 | 1 | 17.711538 | -1.258850 | 52 | 66.8 | 1.284615 |
| GO:0006342\_chromatin\_silencing | SMARCA4 | 10 | 1 | 17.711538 | -1.258850 | 52 | 66.8 | 1.284615 |
| GO:0045814\_negative\_regulation\_of\_gene\_expression\_\_epigenetic | SMARCA4 | 10 | 1 | 17.711538 | -1.258850 | 52 | 66.8 | 1.284615 |
| GO:0046887\_positive\_regulation\_of\_hormone\_secretion | CREB1 | 10 | 1 | 17.711538 | -1.258850 | 52 | 66.8 | 1.284615 |
| GO:0050892\_intestinal\_absorption | ACO1 | 10 | 1 | 17.711538 | -1.258850 | 52 | 66.8 | 1.284615 |
| GO:0060343\_trabecula\_formation | SMARCA4 | 10 | 1 | 17.711538 | -1.258850 | 52 | 66.8 | 1.284615 |
| GO:0006913\_nucleocytoplasmic\_transport | NUP50 | 71 | 2 | 4.989166 | -1.221451 | 53 | 68.83 | 1.298679 |
| GO:0006913\_nucleocytoplasmic\_transport | SMAD4 | 71 | 2 | 4.989166 | -1.221451 | 53 | 68.83 | 1.298679 |
| GO:0001837\_epithelial\_to\_mesenchymal\_transition | SMAD4 | 11 | 1 | 16.101399 | -1.218630 | 58 | 74.35 | 1.281897 |
| GO:0002467\_germinal\_center\_formation | NFKB2 | 11 | 1 | 16.101399 | -1.218630 | 58 | 74.35 | 1.281897 |
| GO:0006333\_chromatin\_assembly\_or\_disassembly | SMARCA4 | 11 | 1 | 16.101399 | -1.218630 | 58 | 74.35 | 1.281897 |
| GO:0007162\_negative\_regulation\_of\_cell\_adhesion | ATP5B | 11 | 1 | 16.101399 | -1.218630 | 58 | 74.35 | 1.281897 |
| GO:0010259\_multicellular\_organismal\_aging | MSH2 | 11 | 1 | 16.101399 | -1.218630 | 58 | 74.35 | 1.281897 |
| GO:0080090\_regulation\_of\_primary\_metabolic\_process | EIF4G2 | 926 | 9 | 1.721424 | -1.218337 | 59 | 74.37 | 1.260508 |
| GO:0080090\_regulation\_of\_primary\_metabolic\_process | ACO1 | 926 | 9 | 1.721424 | -1.218337 | 59 | 74.37 | 1.260508 |
| GO:0080090\_regulation\_of\_primary\_metabolic\_process | MSH2 | 926 | 9 | 1.721424 | -1.218337 | 59 | 74.37 | 1.260508 |
| GO:0080090\_regulation\_of\_primary\_metabolic\_process | CREB1 | 926 | 9 | 1.721424 | -1.218337 | 59 | 74.37 | 1.260508 |
| GO:0080090\_regulation\_of\_primary\_metabolic\_process | SMAD4 | 926 | 9 | 1.721424 | -1.218337 | 59 | 74.37 | 1.260508 |
| GO:0080090\_regulation\_of\_primary\_metabolic\_process | DNMT1 | 926 | 9 | 1.721424 | -1.218337 | 59 | 74.37 | 1.260508 |
| GO:0080090\_regulation\_of\_primary\_metabolic\_process | TMPO | 926 | 9 | 1.721424 | -1.218337 | 59 | 74.37 | 1.260508 |
| GO:0080090\_regulation\_of\_primary\_metabolic\_process | DSTN | 926 | 9 | 1.721424 | -1.218337 | 59 | 74.37 | 1.260508 |
| GO:0080090\_regulation\_of\_primary\_metabolic\_process | SMARCA4 | 926 | 9 | 1.721424 | -1.218337 | 59 | 74.37 | 1.260508 |
| GO:0051169\_nuclear\_transport | NUP50 | 72 | 2 | 4.919872 | -1.210698 | 60 | 75.18 | 1.253000 |
| GO:0051169\_nuclear\_transport | SMAD4 | 72 | 2 | 4.919872 | -1.210698 | 60 | 75.18 | 1.253000 |
| GO:0034960\_cellular\_biopolymer\_metabolic\_process | SPAG9 | 1395 | 12 | 1.523573 | -1.194156 | 61 | 76.16 | 1.248525 |
| GO:0034960\_cellular\_biopolymer\_metabolic\_process | EIF4G2 | 1395 | 12 | 1.523573 | -1.194156 | 61 | 76.16 | 1.248525 |
| GO:0034960\_cellular\_biopolymer\_metabolic\_process | ACO1 | 1395 | 12 | 1.523573 | -1.194156 | 61 | 76.16 | 1.248525 |
| GO:0034960\_cellular\_biopolymer\_metabolic\_process | MSH2 | 1395 | 12 | 1.523573 | -1.194156 | 61 | 76.16 | 1.248525 |
| GO:0034960\_cellular\_biopolymer\_metabolic\_process | CREB1 | 1395 | 12 | 1.523573 | -1.194156 | 61 | 76.16 | 1.248525 |
| GO:0034960\_cellular\_biopolymer\_metabolic\_process | SMAD4 | 1395 | 12 | 1.523573 | -1.194156 | 61 | 76.16 | 1.248525 |
| GO:0034960\_cellular\_biopolymer\_metabolic\_process | DNMT1 | 1395 | 12 | 1.523573 | -1.194156 | 61 | 76.16 | 1.248525 |
| GO:0034960\_cellular\_biopolymer\_metabolic\_process | RBM39 | 1395 | 12 | 1.523573 | -1.194156 | 61 | 76.16 | 1.248525 |
| GO:0034960\_cellular\_biopolymer\_metabolic\_process | TMPO | 1395 | 12 | 1.523573 | -1.194156 | 61 | 76.16 | 1.248525 |
| GO:0034960\_cellular\_biopolymer\_metabolic\_process | PTBP2 | 1395 | 12 | 1.523573 | -1.194156 | 61 | 76.16 | 1.248525 |
| GO:0034960\_cellular\_biopolymer\_metabolic\_process | DSTN | 1395 | 12 | 1.523573 | -1.194156 | 61 | 76.16 | 1.248525 |
| GO:0034960\_cellular\_biopolymer\_metabolic\_process | SMARCA4 | 1395 | 12 | 1.523573 | -1.194156 | 61 | 76.16 | 1.248525 |
| GO:0060255\_regulation\_of\_macromolecule\_metabolic\_process | EIF4G2 | 936 | 9 | 1.703033 | -1.192869 | 62 | 76.19 | 1.228871 |
| GO:0060255\_regulation\_of\_macromolecule\_metabolic\_process | MSH2 | 936 | 9 | 1.703033 | -1.192869 | 62 | 76.19 | 1.228871 |
| GO:0060255\_regulation\_of\_macromolecule\_metabolic\_process | ACO1 | 936 | 9 | 1.703033 | -1.192869 | 62 | 76.19 | 1.228871 |
| GO:0060255\_regulation\_of\_macromolecule\_metabolic\_process | CREB1 | 936 | 9 | 1.703033 | -1.192869 | 62 | 76.19 | 1.228871 |
| GO:0060255\_regulation\_of\_macromolecule\_metabolic\_process | SMAD4 | 936 | 9 | 1.703033 | -1.192869 | 62 | 76.19 | 1.228871 |
| GO:0060255\_regulation\_of\_macromolecule\_metabolic\_process | DNMT1 | 936 | 9 | 1.703033 | -1.192869 | 62 | 76.19 | 1.228871 |
| GO:0060255\_regulation\_of\_macromolecule\_metabolic\_process | TMPO | 936 | 9 | 1.703033 | -1.192869 | 62 | 76.19 | 1.228871 |
| GO:0060255\_regulation\_of\_macromolecule\_metabolic\_process | DSTN | 936 | 9 | 1.703033 | -1.192869 | 62 | 76.19 | 1.228871 |
| GO:0060255\_regulation\_of\_macromolecule\_metabolic\_process | SMARCA4 | 936 | 9 | 1.703033 | -1.192869 | 62 | 76.19 | 1.228871 |
| GO:0009416\_response\_to\_light\_stimulus | MSH2 | 74 | 2 | 4.786902 | -1.189696 | 63 | 76.46 | 1.213651 |
| GO:0009416\_response\_to\_light\_stimulus | TIPIN | 74 | 2 | 4.786902 | -1.189696 | 63 | 76.46 | 1.213651 |
| GO:0019222\_regulation\_of\_metabolic\_process | SPAG9 | 1088 | 10 | 1.627899 | -1.185920 | 64 | 76.54 | 1.195938 |
| GO:0019222\_regulation\_of\_metabolic\_process | EIF4G2 | 1088 | 10 | 1.627899 | -1.185920 | 64 | 76.54 | 1.195938 |
| GO:0019222\_regulation\_of\_metabolic\_process | MSH2 | 1088 | 10 | 1.627899 | -1.185920 | 64 | 76.54 | 1.195938 |
| GO:0019222\_regulation\_of\_metabolic\_process | ACO1 | 1088 | 10 | 1.627899 | -1.185920 | 64 | 76.54 | 1.195938 |
| GO:0019222\_regulation\_of\_metabolic\_process | CREB1 | 1088 | 10 | 1.627899 | -1.185920 | 64 | 76.54 | 1.195938 |
| GO:0019222\_regulation\_of\_metabolic\_process | SMAD4 | 1088 | 10 | 1.627899 | -1.185920 | 64 | 76.54 | 1.195938 |
| GO:0019222\_regulation\_of\_metabolic\_process | DNMT1 | 1088 | 10 | 1.627899 | -1.185920 | 64 | 76.54 | 1.195938 |
| GO:0019222\_regulation\_of\_metabolic\_process | TMPO | 1088 | 10 | 1.627899 | -1.185920 | 64 | 76.54 | 1.195938 |
| GO:0019222\_regulation\_of\_metabolic\_process | DSTN | 1088 | 10 | 1.627899 | -1.185920 | 64 | 76.54 | 1.195938 |
| GO:0019222\_regulation\_of\_metabolic\_process | SMARCA4 | 1088 | 10 | 1.627899 | -1.185920 | 64 | 76.54 | 1.195938 |
| GO:0000375\_RNA\_splicing\_\_via\_transesterification\_reactions | PTBP2 | 12 | 1 | 14.759615 | -1.182013 | 72 | 82.26 | 1.142500 |
| GO:0000377\_RNA\_splicing\_\_via\_transesterification\_reactions\_with\_bulged\_adenosine\_as\_nucleophile | PTBP2 | 12 | 1 | 14.759615 | -1.182013 | 72 | 82.26 | 1.142500 |
| GO:0000398\_nuclear\_mRNA\_splicing\_\_via\_spliceosome | PTBP2 | 12 | 1 | 14.759615 | -1.182013 | 72 | 82.26 | 1.142500 |
| GO:0006413\_translational\_initiation | EIF4G2 | 12 | 1 | 14.759615 | -1.182013 | 72 | 82.26 | 1.142500 |
| GO:0006446\_regulation\_of\_translational\_initiation | EIF4G2 | 12 | 1 | 14.759615 | -1.182013 | 72 | 82.26 | 1.142500 |
| GO:0006879\_cellular\_iron\_ion\_homeostasis | ACO1 | 12 | 1 | 14.759615 | -1.182013 | 72 | 82.26 | 1.142500 |
| GO:0043624\_cellular\_protein\_complex\_disassembly | DSTN | 12 | 1 | 14.759615 | -1.182013 | 72 | 82.26 | 1.142500 |
| GO:0051261\_protein\_depolymerization | DSTN | 12 | 1 | 14.759615 | -1.182013 | 72 | 82.26 | 1.142500 |
| GO:0051050\_positive\_regulation\_of\_transport | CREB1 | 75 | 2 | 4.723077 | -1.179438 | 73 | 82.52 | 1.130411 |
| GO:0051050\_positive\_regulation\_of\_transport | SMAD4 | 75 | 2 | 4.723077 | -1.179438 | 73 | 82.52 | 1.130411 |
| GO:0034961\_cellular\_biopolymer\_biosynthetic\_process | EIF4G2 | 804 | 8 | 1.762342 | -1.159232 | 74 | 83.65 | 1.130405 |
| GO:0034961\_cellular\_biopolymer\_biosynthetic\_process | ACO1 | 804 | 8 | 1.762342 | -1.159232 | 74 | 83.65 | 1.130405 |
| GO:0034961\_cellular\_biopolymer\_biosynthetic\_process | CREB1 | 804 | 8 | 1.762342 | -1.159232 | 74 | 83.65 | 1.130405 |
| GO:0034961\_cellular\_biopolymer\_biosynthetic\_process | SMAD4 | 804 | 8 | 1.762342 | -1.159232 | 74 | 83.65 | 1.130405 |
| GO:0034961\_cellular\_biopolymer\_biosynthetic\_process | DNMT1 | 804 | 8 | 1.762342 | -1.159232 | 74 | 83.65 | 1.130405 |
| GO:0034961\_cellular\_biopolymer\_biosynthetic\_process | RBM39 | 804 | 8 | 1.762342 | -1.159232 | 74 | 83.65 | 1.130405 |
| GO:0034961\_cellular\_biopolymer\_biosynthetic\_process | TMPO | 804 | 8 | 1.762342 | -1.159232 | 74 | 83.65 | 1.130405 |
| GO:0034961\_cellular\_biopolymer\_biosynthetic\_process | SMARCA4 | 804 | 8 | 1.762342 | -1.159232 | 74 | 83.65 | 1.130405 |
| GO:0043284\_biopolymer\_biosynthetic\_process | EIF4G2 | 807 | 8 | 1.755791 | -1.151298 | 75 | 83.76 | 1.116800 |
| GO:0043284\_biopolymer\_biosynthetic\_process | ACO1 | 807 | 8 | 1.755791 | -1.151298 | 75 | 83.76 | 1.116800 |
| GO:0043284\_biopolymer\_biosynthetic\_process | CREB1 | 807 | 8 | 1.755791 | -1.151298 | 75 | 83.76 | 1.116800 |
| GO:0043284\_biopolymer\_biosynthetic\_process | SMAD4 | 807 | 8 | 1.755791 | -1.151298 | 75 | 83.76 | 1.116800 |
| GO:0043284\_biopolymer\_biosynthetic\_process | DNMT1 | 807 | 8 | 1.755791 | -1.151298 | 75 | 83.76 | 1.116800 |
| GO:0043284\_biopolymer\_biosynthetic\_process | RBM39 | 807 | 8 | 1.755791 | -1.151298 | 75 | 83.76 | 1.116800 |
| GO:0043284\_biopolymer\_biosynthetic\_process | TMPO | 807 | 8 | 1.755791 | -1.151298 | 75 | 83.76 | 1.116800 |
| GO:0043284\_biopolymer\_biosynthetic\_process | SMARCA4 | 807 | 8 | 1.755791 | -1.151298 | 75 | 83.76 | 1.116800 |
| GO:0043241\_protein\_complex\_disassembly | DSTN | 13 | 1 | 13.624260 | -1.148422 | 78 | 89.42 | 1.146410 |
| GO:0043244\_regulation\_of\_protein\_complex\_disassembly | DSTN | 13 | 1 | 13.624260 | -1.148422 | 78 | 89.42 | 1.146410 |
| GO:0051495\_positive\_regulation\_of\_cytoskeleton\_organization | DSTN | 13 | 1 | 13.624260 | -1.148422 | 78 | 89.42 | 1.146410 |
| GO:0002250\_adaptive\_immune\_response | MSH2 | 80 | 2 | 4.427885 | -1.130407 | 80 | 90.67 | 1.133375 |
| GO:0002250\_adaptive\_immune\_response | NFKB2 | 80 | 2 | 4.427885 | -1.130407 | 80 | 90.67 | 1.133375 |
| GO:0002460\_adaptive\_immune\_response\_based\_on\_somatic\_recombination\_of\_immune\_receptors\_built\_from\_immunoglobulin\_superfamily\_domains | MSH2 | 80 | 2 | 4.427885 | -1.130407 | 80 | 90.67 | 1.133375 |
| GO:0002460\_adaptive\_immune\_response\_based\_on\_somatic\_recombination\_of\_immune\_receptors\_built\_from\_immunoglobulin\_superfamily\_domains | NFKB2 | 80 | 2 | 4.427885 | -1.130407 | 80 | 90.67 | 1.133375 |
| GO:0000060\_protein\_import\_into\_nucleus\_\_translocation | SMAD4 | 14 | 1 | 12.651099 | -1.117407 | 88 | 96.42 | 1.095682 |
| GO:0006304\_DNA\_modification | DNMT1 | 14 | 1 | 12.651099 | -1.117407 | 88 | 96.42 | 1.095682 |
| GO:0006305\_DNA\_alkylation | DNMT1 | 14 | 1 | 12.651099 | -1.117407 | 88 | 96.42 | 1.095682 |
| GO:0006306\_DNA\_methylation | DNMT1 | 14 | 1 | 12.651099 | -1.117407 | 88 | 96.42 | 1.095682 |
| GO:0008064\_regulation\_of\_actin\_polymerization\_or\_depolymerization | DSTN | 14 | 1 | 12.651099 | -1.117407 | 88 | 96.42 | 1.095682 |
| GO:0030832\_regulation\_of\_actin\_filament\_length | DSTN | 14 | 1 | 12.651099 | -1.117407 | 88 | 96.42 | 1.095682 |
| GO:0034623\_cellular\_macromolecular\_complex\_disassembly | DSTN | 14 | 1 | 12.651099 | -1.117407 | 88 | 96.42 | 1.095682 |
| GO:0051053\_negative\_regulation\_of\_DNA\_metabolic\_process | MSH2 | 14 | 1 | 12.651099 | -1.117407 | 88 | 96.42 | 1.095682 |
| GO:0030198\_extracellular\_matrix\_organization | NFKB2 | 83 | 2 | 4.267841 | -1.102663 | 89 | 97.29 | 1.093146 |
| GO:0030198\_extracellular\_matrix\_organization | SMARCA4 | 83 | 2 | 4.267841 | -1.102663 | 89 | 97.29 | 1.093146 |
| GO:0022600\_digestive\_system\_process | ACO1 | 15 | 1 | 11.807692 | -1.088613 | 92 | 102.8 | 1.117391 |
| GO:0035116\_embryonic\_hindlimb\_morphogenesis | SMARCA4 | 15 | 1 | 11.807692 | -1.088613 | 92 | 102.8 | 1.117391 |
| GO:0042306\_regulation\_of\_protein\_import\_into\_nucleus | SMAD4 | 15 | 1 | 11.807692 | -1.088613 | 92 | 102.8 | 1.117391 |
| GO:0044260\_cellular\_macromolecule\_metabolic\_process | SPAG9 | 1447 | 12 | 1.468821 | -1.086454 | 93 | 102.91 | 1.106559 |
| GO:0044260\_cellular\_macromolecule\_metabolic\_process | EIF4G2 | 1447 | 12 | 1.468821 | -1.086454 | 93 | 102.91 | 1.106559 |
| GO:0044260\_cellular\_macromolecule\_metabolic\_process | MSH2 | 1447 | 12 | 1.468821 | -1.086454 | 93 | 102.91 | 1.106559 |
| GO:0044260\_cellular\_macromolecule\_metabolic\_process | ACO1 | 1447 | 12 | 1.468821 | -1.086454 | 93 | 102.91 | 1.106559 |
| GO:0044260\_cellular\_macromolecule\_metabolic\_process | CREB1 | 1447 | 12 | 1.468821 | -1.086454 | 93 | 102.91 | 1.106559 |
| GO:0044260\_cellular\_macromolecule\_metabolic\_process | SMAD4 | 1447 | 12 | 1.468821 | -1.086454 | 93 | 102.91 | 1.106559 |
| GO:0044260\_cellular\_macromolecule\_metabolic\_process | DNMT1 | 1447 | 12 | 1.468821 | -1.086454 | 93 | 102.91 | 1.106559 |
| GO:0044260\_cellular\_macromolecule\_metabolic\_process | PTBP2 | 1447 | 12 | 1.468821 | -1.086454 | 93 | 102.91 | 1.106559 |
| GO:0044260\_cellular\_macromolecule\_metabolic\_process | RBM39 | 1447 | 12 | 1.468821 | -1.086454 | 93 | 102.91 | 1.106559 |
| GO:0044260\_cellular\_macromolecule\_metabolic\_process | TMPO | 1447 | 12 | 1.468821 | -1.086454 | 93 | 102.91 | 1.106559 |
| GO:0044260\_cellular\_macromolecule\_metabolic\_process | DSTN | 1447 | 12 | 1.468821 | -1.086454 | 93 | 102.91 | 1.106559 |
| GO:0044260\_cellular\_macromolecule\_metabolic\_process | SMARCA4 | 1447 | 12 | 1.468821 | -1.086454 | 93 | 102.91 | 1.106559 |
| GO:0006605\_protein\_targeting | YWHAG | 86 | 2 | 4.118962 | -1.076063 | 94 | 103.87 | 1.105000 |
| GO:0006605\_protein\_targeting | SMAD4 | 86 | 2 | 4.118962 | -1.076063 | 94 | 103.87 | 1.105000 |
| GO:0032956\_regulation\_of\_actin\_cytoskeleton\_organization | DSTN | 16 | 1 | 11.069712 | -1.061752 | 95 | 108.69 | 1.144105 |
| GO:0048754\_branching\_morphogenesis\_of\_a\_tube | SMAD4 | 88 | 2 | 4.025350 | -1.058927 | 96 | 108.88 | 1.134167 |
| GO:0048754\_branching\_morphogenesis\_of\_a\_tube | MYCN | 88 | 2 | 4.025350 | -1.058927 | 96 | 108.88 | 1.134167 |
| GO:0006323\_DNA\_packaging | SMARCA4 | 17 | 1 | 10.418552 | -1.036590 | 101 | 114.43 | 1.132970 |
| GO:0008380\_RNA\_splicing | PTBP2 | 17 | 1 | 10.418552 | -1.036590 | 101 | 114.43 | 1.132970 |
| GO:0032535\_regulation\_of\_cellular\_component\_size | DSTN | 17 | 1 | 10.418552 | -1.036590 | 101 | 114.43 | 1.132970 |
| GO:0032970\_regulation\_of\_actin\_filament-based\_process | DSTN | 17 | 1 | 10.418552 | -1.036590 | 101 | 114.43 | 1.132970 |
| GO:0055072\_iron\_ion\_homeostasis | ACO1 | 17 | 1 | 10.418552 | -1.036590 | 101 | 114.43 | 1.132970 |
| GO:0046907\_intracellular\_transport | YWHAG | 194 | 3 | 2.738898 | -1.026275 | 102 | 114.97 | 1.127157 |
| GO:0046907\_intracellular\_transport | NUP50 | 194 | 3 | 2.738898 | -1.026275 | 102 | 114.97 | 1.127157 |
| GO:0046907\_intracellular\_transport | SMAD4 | 194 | 3 | 2.738898 | -1.026275 | 102 | 114.97 | 1.127157 |
| GO:0001818\_negative\_regulation\_of\_cytokine\_production | H47 | 18 | 1 | 9.839744 | -1.012933 | 108 | 120.5 | 1.115741 |
| GO:0032984\_macromolecular\_complex\_disassembly | DSTN | 18 | 1 | 9.839744 | -1.012933 | 108 | 120.5 | 1.115741 |
| GO:0033157\_regulation\_of\_intracellular\_protein\_transport | SMAD4 | 18 | 1 | 9.839744 | -1.012933 | 108 | 120.5 | 1.115741 |
| GO:0048535\_lymph\_node\_development | NFKB2 | 18 | 1 | 9.839744 | -1.012933 | 108 | 120.5 | 1.115741 |
| GO:0048730\_epidermis\_morphogenesis | SMARCA4 | 18 | 1 | 9.839744 | -1.012933 | 108 | 120.5 | 1.115741 |
| GO:0051222\_positive\_regulation\_of\_protein\_transport | SMAD4 | 18 | 1 | 9.839744 | -1.012933 | 108 | 120.5 | 1.115741 |
| GO:0034984\_cellular\_response\_to\_DNA\_damage\_stimulus | MSH2 | 94 | 2 | 3.768412 | -1.010151 | 109 | 121.25 | 1.112385 |
| GO:0034984\_cellular\_response\_to\_DNA\_damage\_stimulus | TIPIN | 94 | 2 | 3.768412 | -1.010151 | 109 | 121.25 | 1.112385 |
| GO:0043283\_biopolymer\_metabolic\_process | SPAG9 | 1490 | 12 | 1.426433 | -1.003163 | 110 | 121.77 | 1.107000 |
| GO:0043283\_biopolymer\_metabolic\_process | EIF4G2 | 1490 | 12 | 1.426433 | -1.003163 | 110 | 121.77 | 1.107000 |
| GO:0043283\_biopolymer\_metabolic\_process | MSH2 | 1490 | 12 | 1.426433 | -1.003163 | 110 | 121.77 | 1.107000 |
| GO:0043283\_biopolymer\_metabolic\_process | ACO1 | 1490 | 12 | 1.426433 | -1.003163 | 110 | 121.77 | 1.107000 |
| GO:0043283\_biopolymer\_metabolic\_process | CREB1 | 1490 | 12 | 1.426433 | -1.003163 | 110 | 121.77 | 1.107000 |
| GO:0043283\_biopolymer\_metabolic\_process | SMAD4 | 1490 | 12 | 1.426433 | -1.003163 | 110 | 121.77 | 1.107000 |
| GO:0043283\_biopolymer\_metabolic\_process | DNMT1 | 1490 | 12 | 1.426433 | -1.003163 | 110 | 121.77 | 1.107000 |
| GO:0043283\_biopolymer\_metabolic\_process | RBM39 | 1490 | 12 | 1.426433 | -1.003163 | 110 | 121.77 | 1.107000 |
| GO:0043283\_biopolymer\_metabolic\_process | TMPO | 1490 | 12 | 1.426433 | -1.003163 | 110 | 121.77 | 1.107000 |
| GO:0043283\_biopolymer\_metabolic\_process | PTBP2 | 1490 | 12 | 1.426433 | -1.003163 | 110 | 121.77 | 1.107000 |
| GO:0043283\_biopolymer\_metabolic\_process | SMARCA4 | 1490 | 12 | 1.426433 | -1.003163 | 110 | 121.77 | 1.107000 |
| GO:0043283\_biopolymer\_metabolic\_process | DSTN | 1490 | 12 | 1.426433 | -1.003163 | 110 | 121.77 | 1.107000 |
| GO:0007595\_lactation | CREB1 | 19 | 1 | 9.321862 | -0.990617 | 112 | 125.92 | 1.124286 |
| GO:0048536\_spleen\_development | NFKB2 | 19 | 1 | 9.321862 | -0.990617 | 112 | 125.92 | 1.124286 |
| GO:0060341\_regulation\_of\_cellular\_localization | CREB1 | 97 | 2 | 3.651864 | -0.987128 | 113 | 126.14 | 1.116283 |
| GO:0060341\_regulation\_of\_cellular\_localization | SMAD4 | 97 | 2 | 3.651864 | -0.987128 | 113 | 126.14 | 1.116283 |
| GO:0009314\_response\_to\_radiation | MSH2 | 98 | 2 | 3.614600 | -0.979643 | 114 | 126.87 | 1.112895 |
| GO:0009314\_response\_to\_radiation | TIPIN | 98 | 2 | 3.614600 | -0.979643 | 114 | 126.87 | 1.112895 |
| GO:0022607\_cellular\_component\_assembly | SPAG9 | 204 | 3 | 2.604638 | -0.976399 | 115 | 127.02 | 1.104522 |
| GO:0022607\_cellular\_component\_assembly | PTBP2 | 204 | 3 | 2.604638 | -0.976399 | 115 | 127.02 | 1.104522 |
| GO:0022607\_cellular\_component\_assembly | SMARCA4 | 204 | 3 | 2.604638 | -0.976399 | 115 | 127.02 | 1.104522 |
| GO:0060562\_epithelial\_tube\_morphogenesis | NUP50 | 99 | 2 | 3.578089 | -0.972248 | 116 | 127.46 | 1.098793 |
| GO:0060562\_epithelial\_tube\_morphogenesis | SMAD4 | 99 | 2 | 3.578089 | -0.972248 | 116 | 127.46 | 1.098793 |
| GO:0007586\_digestion | ACO1 | 20 | 1 | 8.855769 | -0.969505 | 120 | 130.67 | 1.088917 |
| GO:0032640\_tumor\_necrosis\_factor\_production | H47 | 20 | 1 | 8.855769 | -0.969505 | 120 | 130.67 | 1.088917 |
| GO:0032680\_regulation\_of\_tumor\_necrosis\_factor\_production | H47 | 20 | 1 | 8.855769 | -0.969505 | 120 | 130.67 | 1.088917 |
| GO:0046822\_regulation\_of\_nucleocytoplasmic\_transport | SMAD4 | 20 | 1 | 8.855769 | -0.969505 | 120 | 130.67 | 1.088917 |
| GO:0000018\_regulation\_of\_DNA\_recombination | MSH2 | 21 | 1 | 8.434066 | -0.949479 | 125 | 136.23 | 1.089840 |
| GO:0001702\_gastrulation\_with\_mouth\_forming\_second | SMAD4 | 21 | 1 | 8.434066 | -0.949479 | 125 | 136.23 | 1.089840 |
| GO:0001709\_cell\_fate\_determination | SMARCA4 | 21 | 1 | 8.434066 | -0.949479 | 125 | 136.23 | 1.089840 |
| GO:0008154\_actin\_polymerization\_or\_depolymerization | DSTN | 21 | 1 | 8.434066 | -0.949479 | 125 | 136.23 | 1.089840 |
| GO:0030216\_keratinocyte\_differentiation | SMARCA4 | 21 | 1 | 8.434066 | -0.949479 | 125 | 136.23 | 1.089840 |
| GO:0010556\_regulation\_of\_macromolecule\_biosynthetic\_process | EIF4G2 | 745 | 7 | 1.664171 | -0.943800 | 126 | 136.4 | 1.082540 |
| GO:0010556\_regulation\_of\_macromolecule\_biosynthetic\_process | ACO1 | 745 | 7 | 1.664171 | -0.943800 | 126 | 136.4 | 1.082540 |
| GO:0010556\_regulation\_of\_macromolecule\_biosynthetic\_process | CREB1 | 745 | 7 | 1.664171 | -0.943800 | 126 | 136.4 | 1.082540 |
| GO:0010556\_regulation\_of\_macromolecule\_biosynthetic\_process | SMAD4 | 745 | 7 | 1.664171 | -0.943800 | 126 | 136.4 | 1.082540 |
| GO:0010556\_regulation\_of\_macromolecule\_biosynthetic\_process | DNMT1 | 745 | 7 | 1.664171 | -0.943800 | 126 | 136.4 | 1.082540 |
| GO:0010556\_regulation\_of\_macromolecule\_biosynthetic\_process | TMPO | 745 | 7 | 1.664171 | -0.943800 | 126 | 136.4 | 1.082540 |
| GO:0010556\_regulation\_of\_macromolecule\_biosynthetic\_process | SMARCA4 | 745 | 7 | 1.664171 | -0.943800 | 126 | 136.4 | 1.082540 |
| GO:0035295\_tube\_development | NUP50 | 212 | 3 | 2.506350 | -0.938773 | 127 | 136.91 | 1.078031 |
| GO:0035295\_tube\_development | SMAD4 | 212 | 3 | 2.506350 | -0.938773 | 127 | 136.91 | 1.078031 |
| GO:0035295\_tube\_development | MYCN | 212 | 3 | 2.506350 | -0.938773 | 127 | 136.91 | 1.078031 |
| GO:0040018\_positive\_regulation\_of\_multicellular\_organism\_growth | CREB1 | 22 | 1 | 8.050699 | -0.930439 | 130 | 142.24 | 1.094154 |
| GO:0046883\_regulation\_of\_hormone\_secretion | CREB1 | 22 | 1 | 8.050699 | -0.930439 | 130 | 142.24 | 1.094154 |
| GO:0051260\_protein\_homooligomerization | SPAG9 | 22 | 1 | 8.050699 | -0.930439 | 130 | 142.24 | 1.094154 |
| GO:0034645\_cellular\_macromolecule\_biosynthetic\_process | EIF4G2 | 901 | 8 | 1.572612 | -0.926316 | 131 | 142.37 | 1.086794 |
| GO:0034645\_cellular\_macromolecule\_biosynthetic\_process | ACO1 | 901 | 8 | 1.572612 | -0.926316 | 131 | 142.37 | 1.086794 |
| GO:0034645\_cellular\_macromolecule\_biosynthetic\_process | CREB1 | 901 | 8 | 1.572612 | -0.926316 | 131 | 142.37 | 1.086794 |
| GO:0034645\_cellular\_macromolecule\_biosynthetic\_process | SMAD4 | 901 | 8 | 1.572612 | -0.926316 | 131 | 142.37 | 1.086794 |
| GO:0034645\_cellular\_macromolecule\_biosynthetic\_process | DNMT1 | 901 | 8 | 1.572612 | -0.926316 | 131 | 142.37 | 1.086794 |
| GO:0034645\_cellular\_macromolecule\_biosynthetic\_process | RBM39 | 901 | 8 | 1.572612 | -0.926316 | 131 | 142.37 | 1.086794 |
| GO:0034645\_cellular\_macromolecule\_biosynthetic\_process | TMPO | 901 | 8 | 1.572612 | -0.926316 | 131 | 142.37 | 1.086794 |
| GO:0034645\_cellular\_macromolecule\_biosynthetic\_process | SMARCA4 | 901 | 8 | 1.572612 | -0.926316 | 131 | 142.37 | 1.086794 |
| GO:0002204\_somatic\_recombination\_of\_immunoglobulin\_genes\_during\_immune\_response | MSH2 | 23 | 1 | 7.700669 | -0.912295 | 138 | 146.36 | 1.060580 |
| GO:0002208\_somatic\_diversification\_of\_immunoglobulins\_during\_immune\_response | MSH2 | 23 | 1 | 7.700669 | -0.912295 | 138 | 146.36 | 1.060580 |
| GO:0006397\_mRNA\_processing | PTBP2 | 23 | 1 | 7.700669 | -0.912295 | 138 | 146.36 | 1.060580 |
| GO:0022613\_ribonucleoprotein\_complex\_biogenesis | PTBP2 | 23 | 1 | 7.700669 | -0.912295 | 138 | 146.36 | 1.060580 |
| GO:0032635\_interleukin-6\_production | H47 | 23 | 1 | 7.700669 | -0.912295 | 138 | 146.36 | 1.060580 |
| GO:0032675\_regulation\_of\_interleukin-6\_production | H47 | 23 | 1 | 7.700669 | -0.912295 | 138 | 146.36 | 1.060580 |
| GO:0045190\_isotype\_switching | MSH2 | 23 | 1 | 7.700669 | -0.912295 | 138 | 146.36 | 1.060580 |
| GO:0051649\_establishment\_of\_localization\_in\_cell | YWHAG | 342 | 4 | 2.071525 | -0.911214 | 139 | 146.52 | 1.054101 |
| GO:0051649\_establishment\_of\_localization\_in\_cell | CREB1 | 342 | 4 | 2.071525 | -0.911214 | 139 | 146.52 | 1.054101 |
| GO:0051649\_establishment\_of\_localization\_in\_cell | NUP50 | 342 | 4 | 2.071525 | -0.911214 | 139 | 146.52 | 1.054101 |
| GO:0051649\_establishment\_of\_localization\_in\_cell | SMAD4 | 342 | 4 | 2.071525 | -0.911214 | 139 | 146.52 | 1.054101 |
| GO:0009059\_macromolecule\_biosynthetic\_process | EIF4G2 | 910 | 8 | 1.557058 | -0.906988 | 140 | 146.85 | 1.048929 |
| GO:0009059\_macromolecule\_biosynthetic\_process | ACO1 | 910 | 8 | 1.557058 | -0.906988 | 140 | 146.85 | 1.048929 |
| GO:0009059\_macromolecule\_biosynthetic\_process | CREB1 | 910 | 8 | 1.557058 | -0.906988 | 140 | 146.85 | 1.048929 |
| GO:0009059\_macromolecule\_biosynthetic\_process | SMAD4 | 910 | 8 | 1.557058 | -0.906988 | 140 | 146.85 | 1.048929 |
| GO:0009059\_macromolecule\_biosynthetic\_process | DNMT1 | 910 | 8 | 1.557058 | -0.906988 | 140 | 146.85 | 1.048929 |
| GO:0009059\_macromolecule\_biosynthetic\_process | RBM39 | 910 | 8 | 1.557058 | -0.906988 | 140 | 146.85 | 1.048929 |
| GO:0009059\_macromolecule\_biosynthetic\_process | TMPO | 910 | 8 | 1.557058 | -0.906988 | 140 | 146.85 | 1.048929 |
| GO:0009059\_macromolecule\_biosynthetic\_process | SMARCA4 | 910 | 8 | 1.557058 | -0.906988 | 140 | 146.85 | 1.048929 |
| GO:0002381\_immunoglobulin\_production\_during\_immune\_response | MSH2 | 24 | 1 | 7.379808 | -0.894972 | 143 | 151.33 | 1.058252 |
| GO:0007050\_cell\_cycle\_arrest | MSH2 | 24 | 1 | 7.379808 | -0.894972 | 143 | 151.33 | 1.058252 |
| GO:0032386\_regulation\_of\_intracellular\_transport | SMAD4 | 24 | 1 | 7.379808 | -0.894972 | 143 | 151.33 | 1.058252 |
| GO:0006302\_double-strand\_break\_repair | MSH2 | 25 | 1 | 7.084615 | -0.878403 | 146 | 155.19 | 1.062945 |
| GO:0021983\_pituitary\_gland\_development | CREB1 | 25 | 1 | 7.084615 | -0.878403 | 146 | 155.19 | 1.062945 |
| GO:0035137\_hindlimb\_morphogenesis | SMARCA4 | 25 | 1 | 7.084615 | -0.878403 | 146 | 155.19 | 1.062945 |
| GO:0006974\_response\_to\_DNA\_damage\_stimulus | MSH2 | 113 | 2 | 3.134786 | -0.877317 | 147 | 155.49 | 1.057755 |
| GO:0006974\_response\_to\_DNA\_damage\_stimulus | TIPIN | 113 | 2 | 3.134786 | -0.877317 | 147 | 155.49 | 1.057755 |
| GO:0010468\_regulation\_of\_gene\_expression | EIF4G2 | 778 | 7 | 1.593583 | -0.867847 | 148 | 156.14 | 1.055000 |
| GO:0010468\_regulation\_of\_gene\_expression | ACO1 | 778 | 7 | 1.593583 | -0.867847 | 148 | 156.14 | 1.055000 |
| GO:0010468\_regulation\_of\_gene\_expression | CREB1 | 778 | 7 | 1.593583 | -0.867847 | 148 | 156.14 | 1.055000 |
| GO:0010468\_regulation\_of\_gene\_expression | SMAD4 | 778 | 7 | 1.593583 | -0.867847 | 148 | 156.14 | 1.055000 |
| GO:0010468\_regulation\_of\_gene\_expression | DNMT1 | 778 | 7 | 1.593583 | -0.867847 | 148 | 156.14 | 1.055000 |
| GO:0010468\_regulation\_of\_gene\_expression | TMPO | 778 | 7 | 1.593583 | -0.867847 | 148 | 156.14 | 1.055000 |
| GO:0010468\_regulation\_of\_gene\_expression | SMARCA4 | 778 | 7 | 1.593583 | -0.867847 | 148 | 156.14 | 1.055000 |
| GO:0001658\_branching\_involved\_in\_ureteric\_bud\_morphogenesis | SMAD4 | 26 | 1 | 6.812130 | -0.862529 | 151 | 159.77 | 1.058079 |
| GO:0010212\_response\_to\_ionizing\_radiation | MSH2 | 26 | 1 | 6.812130 | -0.862529 | 151 | 159.77 | 1.058079 |
| GO:0060675\_ureteric\_bud\_morphogenesis | SMAD4 | 26 | 1 | 6.812130 | -0.862529 | 151 | 159.77 | 1.058079 |
| GO:0043170\_macromolecule\_metabolic\_process | SPAG9 | 1576 | 12 | 1.348594 | -0.851139 | 152 | 160.65 | 1.056908 |
| GO:0043170\_macromolecule\_metabolic\_process | EIF4G2 | 1576 | 12 | 1.348594 | -0.851139 | 152 | 160.65 | 1.056908 |
| GO:0043170\_macromolecule\_metabolic\_process | MSH2 | 1576 | 12 | 1.348594 | -0.851139 | 152 | 160.65 | 1.056908 |
| GO:0043170\_macromolecule\_metabolic\_process | ACO1 | 1576 | 12 | 1.348594 | -0.851139 | 152 | 160.65 | 1.056908 |
| GO:0043170\_macromolecule\_metabolic\_process | CREB1 | 1576 | 12 | 1.348594 | -0.851139 | 152 | 160.65 | 1.056908 |
| GO:0043170\_macromolecule\_metabolic\_process | SMAD4 | 1576 | 12 | 1.348594 | -0.851139 | 152 | 160.65 | 1.056908 |
| GO:0043170\_macromolecule\_metabolic\_process | DNMT1 | 1576 | 12 | 1.348594 | -0.851139 | 152 | 160.65 | 1.056908 |
| GO:0043170\_macromolecule\_metabolic\_process | PTBP2 | 1576 | 12 | 1.348594 | -0.851139 | 152 | 160.65 | 1.056908 |
| GO:0043170\_macromolecule\_metabolic\_process | RBM39 | 1576 | 12 | 1.348594 | -0.851139 | 152 | 160.65 | 1.056908 |
| GO:0043170\_macromolecule\_metabolic\_process | TMPO | 1576 | 12 | 1.348594 | -0.851139 | 152 | 160.65 | 1.056908 |
| GO:0043170\_macromolecule\_metabolic\_process | DSTN | 1576 | 12 | 1.348594 | -0.851139 | 152 | 160.65 | 1.056908 |
| GO:0043170\_macromolecule\_metabolic\_process | SMARCA4 | 1576 | 12 | 1.348594 | -0.851139 | 152 | 160.65 | 1.056908 |
| GO:0009913\_epidermal\_cell\_differentiation | SMARCA4 | 27 | 1 | 6.559829 | -0.847297 | 154 | 163.44 | 1.061299 |
| GO:0010638\_positive\_regulation\_of\_organelle\_organization | DSTN | 27 | 1 | 6.559829 | -0.847297 | 154 | 163.44 | 1.061299 |
| GO:0044085\_cellular\_component\_biogenesis | SPAG9 | 237 | 3 | 2.241967 | -0.832564 | 155 | 167.07 | 1.077871 |
| GO:0044085\_cellular\_component\_biogenesis | PTBP2 | 237 | 3 | 2.241967 | -0.832564 | 155 | 167.07 | 1.077871 |
| GO:0044085\_cellular\_component\_biogenesis | SMARCA4 | 237 | 3 | 2.241967 | -0.832564 | 155 | 167.07 | 1.077871 |
| GO:0051726\_regulation\_of\_cell\_cycle | MSH2 | 121 | 2 | 2.927527 | -0.829319 | 156 | 167.37 | 1.072885 |
| GO:0051726\_regulation\_of\_cell\_cycle | TIPIN | 121 | 2 | 2.927527 | -0.829319 | 156 | 167.37 | 1.072885 |
| GO:0006886\_intracellular\_protein\_transport | YWHAG | 122 | 2 | 2.903531 | -0.823597 | 157 | 168.5 | 1.073248 |
| GO:0006886\_intracellular\_protein\_transport | SMAD4 | 122 | 2 | 2.903531 | -0.823597 | 157 | 168.5 | 1.073248 |
| GO:0051641\_cellular\_localization | YWHAG | 370 | 4 | 1.914761 | -0.820133 | 158 | 168.69 | 1.067658 |
| GO:0051641\_cellular\_localization | CREB1 | 370 | 4 | 1.914761 | -0.820133 | 158 | 168.69 | 1.067658 |
| GO:0051641\_cellular\_localization | NUP50 | 370 | 4 | 1.914761 | -0.820133 | 158 | 168.69 | 1.067658 |
| GO:0051641\_cellular\_localization | SMAD4 | 370 | 4 | 1.914761 | -0.820133 | 158 | 168.69 | 1.067658 |
| GO:0001934\_positive\_regulation\_of\_protein\_amino\_acid\_phosphorylation | SMAD4 | 29 | 1 | 6.107427 | -0.818576 | 161 | 171.67 | 1.066273 |
| GO:0016447\_somatic\_recombination\_of\_immunoglobulin\_gene\_segments | MSH2 | 29 | 1 | 6.107427 | -0.818576 | 161 | 171.67 | 1.066273 |
| GO:0051301\_cell\_division | DSTN | 29 | 1 | 6.107427 | -0.818576 | 161 | 171.67 | 1.066273 |
| GO:0001763\_morphogenesis\_of\_a\_branching\_structure | SMAD4 | 125 | 2 | 2.833846 | -0.806778 | 163 | 172.63 | 1.059080 |
| GO:0001763\_morphogenesis\_of\_a\_branching\_structure | MYCN | 125 | 2 | 2.833846 | -0.806778 | 163 | 172.63 | 1.059080 |
| GO:0043062\_extracellular\_structure\_organization | NFKB2 | 125 | 2 | 2.833846 | -0.806778 | 163 | 172.63 | 1.059080 |
| GO:0043062\_extracellular\_structure\_organization | SMARCA4 | 125 | 2 | 2.833846 | -0.806778 | 163 | 172.63 | 1.059080 |
| GO:0000187\_activation\_of\_MAPK\_activity | SPAG9 | 30 | 1 | 5.903846 | -0.805008 | 167 | 176.17 | 1.054910 |
| GO:0016445\_somatic\_diversification\_of\_immunoglobulins | MSH2 | 30 | 1 | 5.903846 | -0.805008 | 167 | 176.17 | 1.054910 |
| GO:0022411\_cellular\_component\_disassembly | DSTN | 30 | 1 | 5.903846 | -0.805008 | 167 | 176.17 | 1.054910 |
| GO:0060021\_palate\_development | SMAD4 | 30 | 1 | 5.903846 | -0.805008 | 167 | 176.17 | 1.054910 |
| GO:0016043\_cellular\_component\_organization | SPAG9 | 964 | 8 | 1.469837 | -0.798243 | 168 | 176.43 | 1.050179 |
| GO:0016043\_cellular\_component\_organization | CREB1 | 964 | 8 | 1.469837 | -0.798243 | 168 | 176.43 | 1.050179 |
| GO:0016043\_cellular\_component\_organization | SMAD4 | 964 | 8 | 1.469837 | -0.798243 | 168 | 176.43 | 1.050179 |
| GO:0016043\_cellular\_component\_organization | RAB5A | 964 | 8 | 1.469837 | -0.798243 | 168 | 176.43 | 1.050179 |
| GO:0016043\_cellular\_component\_organization | NFKB2 | 964 | 8 | 1.469837 | -0.798243 | 168 | 176.43 | 1.050179 |
| GO:0016043\_cellular\_component\_organization | PTBP2 | 964 | 8 | 1.469837 | -0.798243 | 168 | 176.43 | 1.050179 |
| GO:0016043\_cellular\_component\_organization | DSTN | 964 | 8 | 1.469837 | -0.798243 | 168 | 176.43 | 1.050179 |
| GO:0016043\_cellular\_component\_organization | SMARCA4 | 964 | 8 | 1.469837 | -0.798243 | 168 | 176.43 | 1.050179 |
| GO:0031326\_regulation\_of\_cellular\_biosynthetic\_process | EIF4G2 | 812 | 7 | 1.526857 | -0.795475 | 169 | 176.59 | 1.044911 |
| GO:0031326\_regulation\_of\_cellular\_biosynthetic\_process | ACO1 | 812 | 7 | 1.526857 | -0.795475 | 169 | 176.59 | 1.044911 |
| GO:0031326\_regulation\_of\_cellular\_biosynthetic\_process | CREB1 | 812 | 7 | 1.526857 | -0.795475 | 169 | 176.59 | 1.044911 |
| GO:0031326\_regulation\_of\_cellular\_biosynthetic\_process | SMAD4 | 812 | 7 | 1.526857 | -0.795475 | 169 | 176.59 | 1.044911 |
| GO:0031326\_regulation\_of\_cellular\_biosynthetic\_process | DNMT1 | 812 | 7 | 1.526857 | -0.795475 | 169 | 176.59 | 1.044911 |
| GO:0031326\_regulation\_of\_cellular\_biosynthetic\_process | TMPO | 812 | 7 | 1.526857 | -0.795475 | 169 | 176.59 | 1.044911 |
| GO:0031326\_regulation\_of\_cellular\_biosynthetic\_process | SMARCA4 | 812 | 7 | 1.526857 | -0.795475 | 169 | 176.59 | 1.044911 |
| GO:0010562\_positive\_regulation\_of\_phosphorus\_metabolic\_process | SMAD4 | 31 | 1 | 5.713400 | -0.791921 | 172 | 181.68 | 1.056279 |
| GO:0042327\_positive\_regulation\_of\_phosphorylation | SMAD4 | 31 | 1 | 5.713400 | -0.791921 | 172 | 181.68 | 1.056279 |
| GO:0045937\_positive\_regulation\_of\_phosphate\_metabolic\_process | SMAD4 | 31 | 1 | 5.713400 | -0.791921 | 172 | 181.68 | 1.056279 |
| GO:0009889\_regulation\_of\_biosynthetic\_process | EIF4G2 | 815 | 7 | 1.521236 | -0.789359 | 173 | 182.27 | 1.053584 |
| GO:0009889\_regulation\_of\_biosynthetic\_process | ACO1 | 815 | 7 | 1.521236 | -0.789359 | 173 | 182.27 | 1.053584 |
| GO:0009889\_regulation\_of\_biosynthetic\_process | CREB1 | 815 | 7 | 1.521236 | -0.789359 | 173 | 182.27 | 1.053584 |
| GO:0009889\_regulation\_of\_biosynthetic\_process | SMAD4 | 815 | 7 | 1.521236 | -0.789359 | 173 | 182.27 | 1.053584 |
| GO:0009889\_regulation\_of\_biosynthetic\_process | DNMT1 | 815 | 7 | 1.521236 | -0.789359 | 173 | 182.27 | 1.053584 |
| GO:0009889\_regulation\_of\_biosynthetic\_process | TMPO | 815 | 7 | 1.521236 | -0.789359 | 173 | 182.27 | 1.053584 |
| GO:0009889\_regulation\_of\_biosynthetic\_process | SMARCA4 | 815 | 7 | 1.521236 | -0.789359 | 173 | 182.27 | 1.053584 |
| GO:0045165\_cell\_fate\_commitment | SMAD4 | 130 | 2 | 2.724852 | -0.779844 | 174 | 183.16 | 1.052644 |
| GO:0045165\_cell\_fate\_commitment | SMARCA4 | 130 | 2 | 2.724852 | -0.779844 | 174 | 183.16 | 1.052644 |
| GO:0051259\_protein\_oligomerization | SPAG9 | 32 | 1 | 5.534856 | -0.779287 | 176 | 185.4 | 1.053409 |
| GO:0051493\_regulation\_of\_cytoskeleton\_organization | DSTN | 32 | 1 | 5.534856 | -0.779287 | 176 | 185.4 | 1.053409 |
| GO:0002562\_somatic\_diversification\_of\_immune\_receptors\_via\_germline\_recombination\_within\_a\_single\_locus | MSH2 | 33 | 1 | 5.367133 | -0.767075 | 180 | 188.12 | 1.045111 |
| GO:0008584\_male\_gonad\_development | MSH2 | 33 | 1 | 5.367133 | -0.767075 | 180 | 188.12 | 1.045111 |
| GO:0016444\_somatic\_cell\_DNA\_recombination | MSH2 | 33 | 1 | 5.367133 | -0.767075 | 180 | 188.12 | 1.045111 |
| GO:0021536\_diencephalon\_development | CREB1 | 33 | 1 | 5.367133 | -0.767075 | 180 | 188.12 | 1.045111 |
| GO:0048729\_tissue\_morphogenesis | NUP50 | 255 | 3 | 2.083710 | -0.765260 | 181 | 188.51 | 1.041492 |
| GO:0048729\_tissue\_morphogenesis | SMAD4 | 255 | 3 | 2.083710 | -0.765260 | 181 | 188.51 | 1.041492 |
| GO:0048729\_tissue\_morphogenesis | SMARCA4 | 255 | 3 | 2.083710 | -0.765260 | 181 | 188.51 | 1.041492 |
| GO:0002200\_somatic\_diversification\_of\_immune\_receptors | MSH2 | 34 | 1 | 5.209276 | -0.755262 | 186 | 192.24 | 1.033548 |
| GO:0007568\_aging | MSH2 | 34 | 1 | 5.209276 | -0.755262 | 186 | 192.24 | 1.033548 |
| GO:0010720\_positive\_regulation\_of\_cell\_development | SMAD4 | 34 | 1 | 5.209276 | -0.755262 | 186 | 192.24 | 1.033548 |
| GO:0045927\_positive\_regulation\_of\_growth | CREB1 | 34 | 1 | 5.209276 | -0.755262 | 186 | 192.24 | 1.033548 |
| GO:0051047\_positive\_regulation\_of\_secretion | CREB1 | 34 | 1 | 5.209276 | -0.755262 | 186 | 192.24 | 1.033548 |
| GO:0043406\_positive\_regulation\_of\_MAP\_kinase\_activity | SPAG9 | 35 | 1 | 5.060440 | -0.743823 | 187 | 195.07 | 1.043155 |
| GO:0034613\_cellular\_protein\_localization | YWHAG | 139 | 2 | 2.548423 | -0.734532 | 188 | 195.98 | 1.042447 |
| GO:0034613\_cellular\_protein\_localization | SMAD4 | 139 | 2 | 2.548423 | -0.734532 | 188 | 195.98 | 1.042447 |
| GO:0051223\_regulation\_of\_protein\_transport | SMAD4 | 36 | 1 | 4.919872 | -0.732738 | 189 | 198.88 | 1.052275 |
| GO:0006139\_nucleobase\_\_nucleoside\_\_nucleotide\_and\_nucleic\_acid\_metabolic\_process | MSH2 | 1002 | 8 | 1.414095 | -0.728635 | 190 | 199.49 | 1.049947 |
| GO:0006139\_nucleobase\_\_nucleoside\_\_nucleotide\_and\_nucleic\_acid\_metabolic\_process | CREB1 | 1002 | 8 | 1.414095 | -0.728635 | 190 | 199.49 | 1.049947 |
| GO:0006139\_nucleobase\_\_nucleoside\_\_nucleotide\_and\_nucleic\_acid\_metabolic\_process | SMAD4 | 1002 | 8 | 1.414095 | -0.728635 | 190 | 199.49 | 1.049947 |
| GO:0006139\_nucleobase\_\_nucleoside\_\_nucleotide\_and\_nucleic\_acid\_metabolic\_process | DNMT1 | 1002 | 8 | 1.414095 | -0.728635 | 190 | 199.49 | 1.049947 |
| GO:0006139\_nucleobase\_\_nucleoside\_\_nucleotide\_and\_nucleic\_acid\_metabolic\_process | PTBP2 | 1002 | 8 | 1.414095 | -0.728635 | 190 | 199.49 | 1.049947 |
| GO:0006139\_nucleobase\_\_nucleoside\_\_nucleotide\_and\_nucleic\_acid\_metabolic\_process | RBM39 | 1002 | 8 | 1.414095 | -0.728635 | 190 | 199.49 | 1.049947 |
| GO:0006139\_nucleobase\_\_nucleoside\_\_nucleotide\_and\_nucleic\_acid\_metabolic\_process | TMPO | 1002 | 8 | 1.414095 | -0.728635 | 190 | 199.49 | 1.049947 |
| GO:0006139\_nucleobase\_\_nucleoside\_\_nucleotide\_and\_nucleic\_acid\_metabolic\_process | SMARCA4 | 1002 | 8 | 1.414095 | -0.728635 | 190 | 199.49 | 1.049947 |
| GO:0070727\_cellular\_macromolecule\_localization | YWHAG | 141 | 2 | 2.512275 | -0.724973 | 191 | 199.79 | 1.046021 |
| GO:0070727\_cellular\_macromolecule\_localization | SMAD4 | 141 | 2 | 2.512275 | -0.724973 | 191 | 199.79 | 1.046021 |
| GO:0045934\_negative\_regulation\_of\_nucleobase\_\_nucleoside\_\_nucleotide\_and\_nucleic\_acid\_metabolic\_process | MSH2 | 270 | 3 | 1.967949 | -0.714149 | 192 | 202.41 | 1.054219 |
| GO:0045934\_negative\_regulation\_of\_nucleobase\_\_nucleoside\_\_nucleotide\_and\_nucleic\_acid\_metabolic\_process | DNMT1 | 270 | 3 | 1.967949 | -0.714149 | 192 | 202.41 | 1.054219 |
| GO:0045934\_negative\_regulation\_of\_nucleobase\_\_nucleoside\_\_nucleotide\_and\_nucleic\_acid\_metabolic\_process | SMARCA4 | 270 | 3 | 1.967949 | -0.714149 | 192 | 202.41 | 1.054219 |
| GO:0006350\_transcription | CREB1 | 701 | 6 | 1.515966 | -0.713143 | 193 | 202.51 | 1.049275 |
| GO:0006350\_transcription | SMAD4 | 701 | 6 | 1.515966 | -0.713143 | 193 | 202.51 | 1.049275 |
| GO:0006350\_transcription | DNMT1 | 701 | 6 | 1.515966 | -0.713143 | 193 | 202.51 | 1.049275 |
| GO:0006350\_transcription | RBM39 | 701 | 6 | 1.515966 | -0.713143 | 193 | 202.51 | 1.049275 |
| GO:0006350\_transcription | TMPO | 701 | 6 | 1.515966 | -0.713143 | 193 | 202.51 | 1.049275 |
| GO:0006350\_transcription | SMARCA4 | 701 | 6 | 1.515966 | -0.713143 | 193 | 202.51 | 1.049275 |
| GO:0001570\_vasculogenesis | SMARCA4 | 38 | 1 | 4.660931 | -0.711554 | 200 | 206.12 | 1.030600 |
| GO:0001657\_ureteric\_bud\_development | SMAD4 | 38 | 1 | 4.660931 | -0.711554 | 200 | 206.12 | 1.030600 |
| GO:0008016\_regulation\_of\_heart\_contraction | GNAI3 | 38 | 1 | 4.660931 | -0.711554 | 200 | 206.12 | 1.030600 |
| GO:0031401\_positive\_regulation\_of\_protein\_modification\_process | SMAD4 | 38 | 1 | 4.660931 | -0.711554 | 200 | 206.12 | 1.030600 |
| GO:0032259\_methylation | DNMT1 | 38 | 1 | 4.660931 | -0.711554 | 200 | 206.12 | 1.030600 |
| GO:0042493\_response\_to\_drug | CREB1 | 38 | 1 | 4.660931 | -0.711554 | 200 | 206.12 | 1.030600 |
| GO:0043414\_biopolymer\_methylation | DNMT1 | 38 | 1 | 4.660931 | -0.711554 | 200 | 206.12 | 1.030600 |
| GO:0050789\_regulation\_of\_biological\_process | GNAI3 | 2357 | 16 | 1.202311 | -0.711000 | 201 | 206.23 | 1.026020 |
| GO:0050789\_regulation\_of\_biological\_process | ACO1 | 2357 | 16 | 1.202311 | -0.711000 | 201 | 206.23 | 1.026020 |
| GO:0050789\_regulation\_of\_biological\_process | MSH2 | 2357 | 16 | 1.202311 | -0.711000 | 201 | 206.23 | 1.026020 |
| GO:0050789\_regulation\_of\_biological\_process | CREB1 | 2357 | 16 | 1.202311 | -0.711000 | 201 | 206.23 | 1.026020 |
| GO:0050789\_regulation\_of\_biological\_process | ATP5B | 2357 | 16 | 1.202311 | -0.711000 | 201 | 206.23 | 1.026020 |
| GO:0050789\_regulation\_of\_biological\_process | TIPIN | 2357 | 16 | 1.202311 | -0.711000 | 201 | 206.23 | 1.026020 |
| GO:0050789\_regulation\_of\_biological\_process | SMAD4 | 2357 | 16 | 1.202311 | -0.711000 | 201 | 206.23 | 1.026020 |
| GO:0050789\_regulation\_of\_biological\_process | DSTN | 2357 | 16 | 1.202311 | -0.711000 | 201 | 206.23 | 1.026020 |
| GO:0050789\_regulation\_of\_biological\_process | MYCN | 2357 | 16 | 1.202311 | -0.711000 | 201 | 206.23 | 1.026020 |
| GO:0050789\_regulation\_of\_biological\_process | EIF4G2 | 2357 | 16 | 1.202311 | -0.711000 | 201 | 206.23 | 1.026020 |
| GO:0050789\_regulation\_of\_biological\_process | SPAG9 | 2357 | 16 | 1.202311 | -0.711000 | 201 | 206.23 | 1.026020 |
| GO:0050789\_regulation\_of\_biological\_process | SDCBP | 2357 | 16 | 1.202311 | -0.711000 | 201 | 206.23 | 1.026020 |
| GO:0050789\_regulation\_of\_biological\_process | DNMT1 | 2357 | 16 | 1.202311 | -0.711000 | 201 | 206.23 | 1.026020 |
| GO:0050789\_regulation\_of\_biological\_process | TMPO | 2357 | 16 | 1.202311 | -0.711000 | 201 | 206.23 | 1.026020 |
| GO:0050789\_regulation\_of\_biological\_process | H47 | 2357 | 16 | 1.202311 | -0.711000 | 201 | 206.23 | 1.026020 |
| GO:0050789\_regulation\_of\_biological\_process | SMARCA4 | 2357 | 16 | 1.202311 | -0.711000 | 201 | 206.23 | 1.026020 |
| GO:0051172\_negative\_regulation\_of\_nitrogen\_compound\_metabolic\_process | MSH2 | 271 | 3 | 1.960687 | -0.710888 | 202 | 206.49 | 1.022228 |
| GO:0051172\_negative\_regulation\_of\_nitrogen\_compound\_metabolic\_process | DNMT1 | 271 | 3 | 1.960687 | -0.710888 | 202 | 206.49 | 1.022228 |
| GO:0051172\_negative\_regulation\_of\_nitrogen\_compound\_metabolic\_process | SMARCA4 | 271 | 3 | 1.960687 | -0.710888 | 202 | 206.49 | 1.022228 |
| GO:0007242\_intracellular\_signaling\_cascade | SPAG9 | 411 | 4 | 1.723751 | -0.704317 | 203 | 207.28 | 1.021084 |
| GO:0007242\_intracellular\_signaling\_cascade | MSH2 | 411 | 4 | 1.723751 | -0.704317 | 203 | 207.28 | 1.021084 |
| GO:0007242\_intracellular\_signaling\_cascade | TIPIN | 411 | 4 | 1.723751 | -0.704317 | 203 | 207.28 | 1.021084 |
| GO:0007242\_intracellular\_signaling\_cascade | SDCBP | 411 | 4 | 1.723751 | -0.704317 | 203 | 207.28 | 1.021084 |
| GO:0030900\_forebrain\_development | CREB1 | 146 | 2 | 2.426238 | -0.701824 | 204 | 207.71 | 1.018186 |
| GO:0030900\_forebrain\_development | SMARCA4 | 146 | 2 | 2.426238 | -0.701824 | 204 | 207.71 | 1.018186 |
| GO:0006730\_one-carbon\_metabolic\_process | DNMT1 | 39 | 1 | 4.541420 | -0.701420 | 208 | 210.25 | 1.010817 |
| GO:0043524\_negative\_regulation\_of\_neuron\_apoptosis | MSH2 | 39 | 1 | 4.541420 | -0.701420 | 208 | 210.25 | 1.010817 |
| GO:0048663\_neuron\_fate\_commitment | SMAD4 | 39 | 1 | 4.541420 | -0.701420 | 208 | 210.25 | 1.010817 |
| GO:0070201\_regulation\_of\_establishment\_of\_protein\_localization | SMAD4 | 39 | 1 | 4.541420 | -0.701420 | 208 | 210.25 | 1.010817 |
| GO:0050794\_regulation\_of\_cellular\_process | GNAI3 | 2190 | 15 | 1.213119 | -0.698544 | 209 | 210.57 | 1.007512 |
| GO:0050794\_regulation\_of\_cellular\_process | ACO1 | 2190 | 15 | 1.213119 | -0.698544 | 209 | 210.57 | 1.007512 |
| GO:0050794\_regulation\_of\_cellular\_process | MSH2 | 2190 | 15 | 1.213119 | -0.698544 | 209 | 210.57 | 1.007512 |
| GO:0050794\_regulation\_of\_cellular\_process | CREB1 | 2190 | 15 | 1.213119 | -0.698544 | 209 | 210.57 | 1.007512 |
| GO:0050794\_regulation\_of\_cellular\_process | ATP5B | 2190 | 15 | 1.213119 | -0.698544 | 209 | 210.57 | 1.007512 |
| GO:0050794\_regulation\_of\_cellular\_process | TIPIN | 2190 | 15 | 1.213119 | -0.698544 | 209 | 210.57 | 1.007512 |
| GO:0050794\_regulation\_of\_cellular\_process | SMAD4 | 2190 | 15 | 1.213119 | -0.698544 | 209 | 210.57 | 1.007512 |
| GO:0050794\_regulation\_of\_cellular\_process | MYCN | 2190 | 15 | 1.213119 | -0.698544 | 209 | 210.57 | 1.007512 |
| GO:0050794\_regulation\_of\_cellular\_process | DSTN | 2190 | 15 | 1.213119 | -0.698544 | 209 | 210.57 | 1.007512 |
| GO:0050794\_regulation\_of\_cellular\_process | SPAG9 | 2190 | 15 | 1.213119 | -0.698544 | 209 | 210.57 | 1.007512 |
| GO:0050794\_regulation\_of\_cellular\_process | EIF4G2 | 2190 | 15 | 1.213119 | -0.698544 | 209 | 210.57 | 1.007512 |
| GO:0050794\_regulation\_of\_cellular\_process | SDCBP | 2190 | 15 | 1.213119 | -0.698544 | 209 | 210.57 | 1.007512 |
| GO:0050794\_regulation\_of\_cellular\_process | DNMT1 | 2190 | 15 | 1.213119 | -0.698544 | 209 | 210.57 | 1.007512 |
| GO:0050794\_regulation\_of\_cellular\_process | TMPO | 2190 | 15 | 1.213119 | -0.698544 | 209 | 210.57 | 1.007512 |
| GO:0050794\_regulation\_of\_cellular\_process | SMARCA4 | 2190 | 15 | 1.213119 | -0.698544 | 209 | 210.57 | 1.007512 |
| GO:0044267\_cellular\_protein\_metabolic\_process | SPAG9 | 559 | 5 | 1.584216 | -0.696972 | 210 | 210.89 | 1.004238 |
| GO:0044267\_cellular\_protein\_metabolic\_process | EIF4G2 | 559 | 5 | 1.584216 | -0.696972 | 210 | 210.89 | 1.004238 |
| GO:0044267\_cellular\_protein\_metabolic\_process | ACO1 | 559 | 5 | 1.584216 | -0.696972 | 210 | 210.89 | 1.004238 |
| GO:0044267\_cellular\_protein\_metabolic\_process | SMAD4 | 559 | 5 | 1.584216 | -0.696972 | 210 | 210.89 | 1.004238 |
| GO:0044267\_cellular\_protein\_metabolic\_process | DSTN | 559 | 5 | 1.584216 | -0.696972 | 210 | 210.89 | 1.004238 |
| GO:0001824\_blastocyst\_development | SMARCA4 | 40 | 1 | 4.427885 | -0.691571 | 214 | 213.72 | 0.998692 |
| GO:0014031\_mesenchymal\_cell\_development | SMAD4 | 40 | 1 | 4.427885 | -0.691571 | 214 | 213.72 | 0.998692 |
| GO:0016071\_mRNA\_metabolic\_process | PTBP2 | 40 | 1 | 4.427885 | -0.691571 | 214 | 213.72 | 0.998692 |
| GO:0017015\_regulation\_of\_transforming\_growth\_factor\_beta\_receptor\_signaling\_pathway | SMAD4 | 40 | 1 | 4.427885 | -0.691571 | 214 | 213.72 | 0.998692 |
| GO:0006810\_transport | ATP2B1 | 718 | 6 | 1.480073 | -0.679456 | 215 | 217.95 | 1.013721 |
| GO:0006810\_transport | YWHAG | 718 | 6 | 1.480073 | -0.679456 | 215 | 217.95 | 1.013721 |
| GO:0006810\_transport | CREB1 | 718 | 6 | 1.480073 | -0.679456 | 215 | 217.95 | 1.013721 |
| GO:0006810\_transport | NUP50 | 718 | 6 | 1.480073 | -0.679456 | 215 | 217.95 | 1.013721 |
| GO:0006810\_transport | SMAD4 | 718 | 6 | 1.480073 | -0.679456 | 215 | 217.95 | 1.013721 |
| GO:0006810\_transport | RAB5A | 718 | 6 | 1.480073 | -0.679456 | 215 | 217.95 | 1.013721 |
| GO:0008361\_regulation\_of\_cell\_size | CREB1 | 42 | 1 | 4.217033 | -0.672671 | 217 | 222.18 | 1.023871 |
| GO:0010769\_regulation\_of\_cell\_morphogenesis\_involved\_in\_differentiation | SMAD4 | 42 | 1 | 4.217033 | -0.672671 | 217 | 222.18 | 1.023871 |
| GO:0001841\_neural\_tube\_formation | NUP50 | 43 | 1 | 4.118962 | -0.663595 | 221 | 226.23 | 1.023665 |
| GO:0010001\_glial\_cell\_differentiation | SMARCA4 | 43 | 1 | 4.118962 | -0.663595 | 221 | 226.23 | 1.023665 |
| GO:0046879\_hormone\_secretion | CREB1 | 43 | 1 | 4.118962 | -0.663595 | 221 | 226.23 | 1.023665 |
| GO:0048762\_mesenchymal\_cell\_differentiation | SMAD4 | 43 | 1 | 4.118962 | -0.663595 | 221 | 226.23 | 1.023665 |
| GO:0051234\_establishment\_of\_localization | ATP2B1 | 729 | 6 | 1.457740 | -0.658449 | 222 | 226.87 | 1.021937 |
| GO:0051234\_establishment\_of\_localization | YWHAG | 729 | 6 | 1.457740 | -0.658449 | 222 | 226.87 | 1.021937 |
| GO:0051234\_establishment\_of\_localization | CREB1 | 729 | 6 | 1.457740 | -0.658449 | 222 | 226.87 | 1.021937 |
| GO:0051234\_establishment\_of\_localization | NUP50 | 729 | 6 | 1.457740 | -0.658449 | 222 | 226.87 | 1.021937 |
| GO:0051234\_establishment\_of\_localization | SMAD4 | 729 | 6 | 1.457740 | -0.658449 | 222 | 226.87 | 1.021937 |
| GO:0051234\_establishment\_of\_localization | RAB5A | 729 | 6 | 1.457740 | -0.658449 | 222 | 226.87 | 1.021937 |
| GO:0002377\_immunoglobulin\_production | MSH2 | 44 | 1 | 4.025350 | -0.654753 | 228 | 231.05 | 1.013377 |
| GO:0006606\_protein\_import\_into\_nucleus | SMAD4 | 44 | 1 | 4.025350 | -0.654753 | 228 | 231.05 | 1.013377 |
| GO:0009914\_hormone\_transport | CREB1 | 44 | 1 | 4.025350 | -0.654753 | 228 | 231.05 | 1.013377 |
| GO:0016064\_immunoglobulin\_mediated\_immune\_response | MSH2 | 44 | 1 | 4.025350 | -0.654753 | 228 | 231.05 | 1.013377 |
| GO:0051170\_nuclear\_import | SMAD4 | 44 | 1 | 4.025350 | -0.654753 | 228 | 231.05 | 1.013377 |
| GO:0060485\_mesenchyme\_development | SMAD4 | 44 | 1 | 4.025350 | -0.654753 | 228 | 231.05 | 1.013377 |
| GO:0001838\_embryonic\_epithelial\_tube\_formation | NUP50 | 45 | 1 | 3.935897 | -0.646134 | 230 | 232.95 | 1.012826 |
| GO:0046546\_development\_of\_primary\_male\_sexual\_characteristics | MSH2 | 45 | 1 | 3.935897 | -0.646134 | 230 | 232.95 | 1.012826 |
| GO:0051128\_regulation\_of\_cellular\_component\_organization | SMAD4 | 160 | 2 | 2.213942 | -0.642186 | 231 | 233.33 | 1.010087 |
| GO:0051128\_regulation\_of\_cellular\_component\_organization | DSTN | 160 | 2 | 2.213942 | -0.642186 | 231 | 233.33 | 1.010087 |
| GO:0019724\_B\_cell\_mediated\_immunity | MSH2 | 46 | 1 | 3.850334 | -0.637729 | 234 | 235.65 | 1.007051 |
| GO:0042063\_gliogenesis | SMARCA4 | 46 | 1 | 3.850334 | -0.637729 | 234 | 235.65 | 1.007051 |
| GO:0051098\_regulation\_of\_binding | SMAD4 | 46 | 1 | 3.850334 | -0.637729 | 234 | 235.65 | 1.007051 |
| GO:0051179\_localization | ATP2B1 | 1058 | 8 | 1.339247 | -0.635468 | 235 | 236.33 | 1.005660 |
| GO:0051179\_localization | YWHAG | 1058 | 8 | 1.339247 | -0.635468 | 235 | 236.33 | 1.005660 |
| GO:0051179\_localization | ATP5B | 1058 | 8 | 1.339247 | -0.635468 | 235 | 236.33 | 1.005660 |
| GO:0051179\_localization | CREB1 | 1058 | 8 | 1.339247 | -0.635468 | 235 | 236.33 | 1.005660 |
| GO:0051179\_localization | NUP50 | 1058 | 8 | 1.339247 | -0.635468 | 235 | 236.33 | 1.005660 |
| GO:0051179\_localization | RAB5A | 1058 | 8 | 1.339247 | -0.635468 | 235 | 236.33 | 1.005660 |
| GO:0051179\_localization | SMAD4 | 1058 | 8 | 1.339247 | -0.635468 | 235 | 236.33 | 1.005660 |
| GO:0051179\_localization | DSTN | 1058 | 8 | 1.339247 | -0.635468 | 235 | 236.33 | 1.005660 |
| GO:0009628\_response\_to\_abiotic\_stimulus | MSH2 | 162 | 2 | 2.186610 | -0.634233 | 236 | 236.66 | 1.002797 |
| GO:0009628\_response\_to\_abiotic\_stimulus | TIPIN | 162 | 2 | 2.186610 | -0.634233 | 236 | 236.66 | 1.002797 |
| GO:0006396\_RNA\_processing | PTBP2 | 47 | 1 | 3.768412 | -0.629529 | 238 | 239.7 | 1.007143 |
| GO:0030183\_B\_cell\_differentiation | MSH2 | 47 | 1 | 3.768412 | -0.629529 | 238 | 239.7 | 1.007143 |
| GO:0042325\_regulation\_of\_phosphorylation | SPAG9 | 164 | 2 | 2.159944 | -0.626411 | 239 | 240.18 | 1.004937 |
| GO:0042325\_regulation\_of\_phosphorylation | SMAD4 | 164 | 2 | 2.159944 | -0.626411 | 239 | 240.18 | 1.004937 |
| GO:0048598\_embryonic\_morphogenesis | NUP50 | 299 | 3 | 1.777077 | -0.626270 | 240 | 240.32 | 1.001333 |
| GO:0048598\_embryonic\_morphogenesis | SMAD4 | 299 | 3 | 1.777077 | -0.626270 | 240 | 240.32 | 1.001333 |
| GO:0048598\_embryonic\_morphogenesis | SMARCA4 | 299 | 3 | 1.777077 | -0.626270 | 240 | 240.32 | 1.001333 |
| GO:0006259\_DNA\_metabolic\_process | MSH2 | 165 | 2 | 2.146853 | -0.622548 | 243 | 241.27 | 0.992881 |
| GO:0006259\_DNA\_metabolic\_process | DNMT1 | 165 | 2 | 2.146853 | -0.622548 | 243 | 241.27 | 0.992881 |
| GO:0019220\_regulation\_of\_phosphate\_metabolic\_process | SPAG9 | 165 | 2 | 2.146853 | -0.622548 | 243 | 241.27 | 0.992881 |
| GO:0019220\_regulation\_of\_phosphate\_metabolic\_process | SMAD4 | 165 | 2 | 2.146853 | -0.622548 | 243 | 241.27 | 0.992881 |
| GO:0051174\_regulation\_of\_phosphorus\_metabolic\_process | SPAG9 | 165 | 2 | 2.146853 | -0.622548 | 243 | 241.27 | 0.992881 |
| GO:0051174\_regulation\_of\_phosphorus\_metabolic\_process | SMAD4 | 165 | 2 | 2.146853 | -0.622548 | 243 | 241.27 | 0.992881 |
| GO:0034504\_protein\_localization\_in\_nucleus | SMAD4 | 48 | 1 | 3.689904 | -0.621524 | 244 | 242.74 | 0.994836 |
| GO:0044238\_primary\_metabolic\_process | MSH2 | 1905 | 13 | 1.208661 | -0.617019 | 245 | 243.24 | 0.992816 |
| GO:0044238\_primary\_metabolic\_process | ACO1 | 1905 | 13 | 1.208661 | -0.617019 | 245 | 243.24 | 0.992816 |
| GO:0044238\_primary\_metabolic\_process | CREB1 | 1905 | 13 | 1.208661 | -0.617019 | 245 | 243.24 | 0.992816 |
| GO:0044238\_primary\_metabolic\_process | ATP5B | 1905 | 13 | 1.208661 | -0.617019 | 245 | 243.24 | 0.992816 |
| GO:0044238\_primary\_metabolic\_process | SMAD4 | 1905 | 13 | 1.208661 | -0.617019 | 245 | 243.24 | 0.992816 |
| GO:0044238\_primary\_metabolic\_process | DSTN | 1905 | 13 | 1.208661 | -0.617019 | 245 | 243.24 | 0.992816 |
| GO:0044238\_primary\_metabolic\_process | EIF4G2 | 1905 | 13 | 1.208661 | -0.617019 | 245 | 243.24 | 0.992816 |
| GO:0044238\_primary\_metabolic\_process | SPAG9 | 1905 | 13 | 1.208661 | -0.617019 | 245 | 243.24 | 0.992816 |
| GO:0044238\_primary\_metabolic\_process | DNMT1 | 1905 | 13 | 1.208661 | -0.617019 | 245 | 243.24 | 0.992816 |
| GO:0044238\_primary\_metabolic\_process | TMPO | 1905 | 13 | 1.208661 | -0.617019 | 245 | 243.24 | 0.992816 |
| GO:0044238\_primary\_metabolic\_process | RBM39 | 1905 | 13 | 1.208661 | -0.617019 | 245 | 243.24 | 0.992816 |
| GO:0044238\_primary\_metabolic\_process | PTBP2 | 1905 | 13 | 1.208661 | -0.617019 | 245 | 243.24 | 0.992816 |
| GO:0044238\_primary\_metabolic\_process | SMARCA4 | 1905 | 13 | 1.208661 | -0.617019 | 245 | 243.24 | 0.992816 |
| GO:0010926\_anatomical\_structure\_formation | SPAG9 | 447 | 4 | 1.584925 | -0.616939 | 246 | 243.38 | 0.989350 |
| GO:0010926\_anatomical\_structure\_formation | NUP50 | 447 | 4 | 1.584925 | -0.616939 | 246 | 243.38 | 0.989350 |
| GO:0010926\_anatomical\_structure\_formation | PTBP2 | 447 | 4 | 1.584925 | -0.616939 | 246 | 243.38 | 0.989350 |
| GO:0010926\_anatomical\_structure\_formation | SMARCA4 | 447 | 4 | 1.584925 | -0.616939 | 246 | 243.38 | 0.989350 |
| GO:0051049\_regulation\_of\_transport | CREB1 | 167 | 2 | 2.121142 | -0.614917 | 247 | 243.89 | 0.987409 |
| GO:0051049\_regulation\_of\_transport | SMAD4 | 167 | 2 | 2.121142 | -0.614917 | 247 | 243.89 | 0.987409 |
| GO:0002440\_production\_of\_molecular\_mediator\_of\_immune\_response | MSH2 | 49 | 1 | 3.614600 | -0.613707 | 251 | 246.21 | 0.980916 |
| GO:0003015\_heart\_process | GNAI3 | 49 | 1 | 3.614600 | -0.613707 | 251 | 246.21 | 0.980916 |
| GO:0046661\_male\_sex\_differentiation | MSH2 | 49 | 1 | 3.614600 | -0.613707 | 251 | 246.21 | 0.980916 |
| GO:0060047\_heart\_contraction | GNAI3 | 49 | 1 | 3.614600 | -0.613707 | 251 | 246.21 | 0.980916 |
| GO:0019219\_regulation\_of\_nucleobase\_\_nucleoside\_\_nucleotide\_and\_nucleic\_acid\_metabolic\_process | MSH2 | 757 | 6 | 1.403821 | -0.607632 | 252 | 246.68 | 0.978889 |
| GO:0019219\_regulation\_of\_nucleobase\_\_nucleoside\_\_nucleotide\_and\_nucleic\_acid\_metabolic\_process | CREB1 | 757 | 6 | 1.403821 | -0.607632 | 252 | 246.68 | 0.978889 |
| GO:0019219\_regulation\_of\_nucleobase\_\_nucleoside\_\_nucleotide\_and\_nucleic\_acid\_metabolic\_process | SMAD4 | 757 | 6 | 1.403821 | -0.607632 | 252 | 246.68 | 0.978889 |
| GO:0019219\_regulation\_of\_nucleobase\_\_nucleoside\_\_nucleotide\_and\_nucleic\_acid\_metabolic\_process | DNMT1 | 757 | 6 | 1.403821 | -0.607632 | 252 | 246.68 | 0.978889 |
| GO:0019219\_regulation\_of\_nucleobase\_\_nucleoside\_\_nucleotide\_and\_nucleic\_acid\_metabolic\_process | TMPO | 757 | 6 | 1.403821 | -0.607632 | 252 | 246.68 | 0.978889 |
| GO:0019219\_regulation\_of\_nucleobase\_\_nucleoside\_\_nucleotide\_and\_nucleic\_acid\_metabolic\_process | SMARCA4 | 757 | 6 | 1.403821 | -0.607632 | 252 | 246.68 | 0.978889 |
| GO:0001656\_metanephros\_development | SMAD4 | 50 | 1 | 3.542308 | -0.606070 | 255 | 248.61 | 0.974941 |
| GO:0007015\_actin\_filament\_organization | DSTN | 50 | 1 | 3.542308 | -0.606070 | 255 | 248.61 | 0.974941 |
| GO:0017038\_protein\_import | SMAD4 | 50 | 1 | 3.542308 | -0.606070 | 255 | 248.61 | 0.974941 |
| GO:0016310\_phosphorylation | SPAG9 | 309 | 3 | 1.719567 | -0.598898 | 256 | 249.49 | 0.974570 |
| GO:0016310\_phosphorylation | MSH2 | 309 | 3 | 1.719567 | -0.598898 | 256 | 249.49 | 0.974570 |
| GO:0016310\_phosphorylation | SMAD4 | 309 | 3 | 1.719567 | -0.598898 | 256 | 249.49 | 0.974570 |
| GO:0016569\_covalent\_chromatin\_modification | SMARCA4 | 51 | 1 | 3.472851 | -0.598606 | 258 | 251.74 | 0.975736 |
| GO:0032880\_regulation\_of\_protein\_localization | SMAD4 | 51 | 1 | 3.472851 | -0.598606 | 258 | 251.74 | 0.975736 |
| GO:0015031\_protein\_transport | YWHAG | 175 | 2 | 2.024176 | -0.585593 | 259 | 254.96 | 0.984402 |
| GO:0015031\_protein\_transport | SMAD4 | 175 | 2 | 2.024176 | -0.585593 | 259 | 254.96 | 0.984402 |
| GO:0051171\_regulation\_of\_nitrogen\_compound\_metabolic\_process | MSH2 | 771 | 6 | 1.378330 | -0.583585 | 260 | 256.69 | 0.987269 |
| GO:0051171\_regulation\_of\_nitrogen\_compound\_metabolic\_process | CREB1 | 771 | 6 | 1.378330 | -0.583585 | 260 | 256.69 | 0.987269 |
| GO:0051171\_regulation\_of\_nitrogen\_compound\_metabolic\_process | SMAD4 | 771 | 6 | 1.378330 | -0.583585 | 260 | 256.69 | 0.987269 |
| GO:0051171\_regulation\_of\_nitrogen\_compound\_metabolic\_process | DNMT1 | 771 | 6 | 1.378330 | -0.583585 | 260 | 256.69 | 0.987269 |
| GO:0051171\_regulation\_of\_nitrogen\_compound\_metabolic\_process | TMPO | 771 | 6 | 1.378330 | -0.583585 | 260 | 256.69 | 0.987269 |
| GO:0051171\_regulation\_of\_nitrogen\_compound\_metabolic\_process | SMARCA4 | 771 | 6 | 1.378330 | -0.583585 | 260 | 256.69 | 0.987269 |
| GO:0006091\_generation\_of\_precursor\_metabolites\_and\_energy | MSH2 | 54 | 1 | 3.279915 | -0.577184 | 263 | 259.51 | 0.986730 |
| GO:0007265\_Ras\_protein\_signal\_transduction | SDCBP | 54 | 1 | 3.279915 | -0.577184 | 263 | 259.51 | 0.986730 |
| GO:0043405\_regulation\_of\_MAP\_kinase\_activity | SPAG9 | 54 | 1 | 3.279915 | -0.577184 | 263 | 259.51 | 0.986730 |
| GO:0006310\_DNA\_recombination | MSH2 | 55 | 1 | 3.220280 | -0.570347 | 264 | 262.16 | 0.993030 |
| GO:0045184\_establishment\_of\_protein\_localization | YWHAG | 180 | 2 | 1.967949 | -0.568186 | 265 | 262.31 | 0.989849 |
| GO:0045184\_establishment\_of\_protein\_localization | SMAD4 | 180 | 2 | 1.967949 | -0.568186 | 265 | 262.31 | 0.989849 |
| GO:0033365\_protein\_localization\_in\_organelle | SMAD4 | 57 | 1 | 3.107287 | -0.557096 | 267 | 268.7 | 1.006367 |
| GO:0043523\_regulation\_of\_neuron\_apoptosis | MSH2 | 57 | 1 | 3.107287 | -0.557096 | 267 | 268.7 | 1.006367 |
| GO:0030902\_hindbrain\_development | SMARCA4 | 58 | 1 | 3.053714 | -0.550673 | 269 | 270.65 | 1.006134 |
| GO:0033043\_regulation\_of\_organelle\_organization | DSTN | 58 | 1 | 3.053714 | -0.550673 | 269 | 270.65 | 1.006134 |
| GO:0008152\_metabolic\_process | MSH2 | 2133 | 14 | 1.162501 | -0.549606 | 270 | 270.77 | 1.002852 |
| GO:0008152\_metabolic\_process | ACO1 | 2133 | 14 | 1.162501 | -0.549606 | 270 | 270.77 | 1.002852 |
| GO:0008152\_metabolic\_process | ATP5B | 2133 | 14 | 1.162501 | -0.549606 | 270 | 270.77 | 1.002852 |
| GO:0008152\_metabolic\_process | CREB1 | 2133 | 14 | 1.162501 | -0.549606 | 270 | 270.77 | 1.002852 |
| GO:0008152\_metabolic\_process | SMAD4 | 2133 | 14 | 1.162501 | -0.549606 | 270 | 270.77 | 1.002852 |
| GO:0008152\_metabolic\_process | DSTN | 2133 | 14 | 1.162501 | -0.549606 | 270 | 270.77 | 1.002852 |
| GO:0008152\_metabolic\_process | EIF4G2 | 2133 | 14 | 1.162501 | -0.549606 | 270 | 270.77 | 1.002852 |
| GO:0008152\_metabolic\_process | SPAG9 | 2133 | 14 | 1.162501 | -0.549606 | 270 | 270.77 | 1.002852 |
| GO:0008152\_metabolic\_process | OXCT1 | 2133 | 14 | 1.162501 | -0.549606 | 270 | 270.77 | 1.002852 |
| GO:0008152\_metabolic\_process | DNMT1 | 2133 | 14 | 1.162501 | -0.549606 | 270 | 270.77 | 1.002852 |
| GO:0008152\_metabolic\_process | TMPO | 2133 | 14 | 1.162501 | -0.549606 | 270 | 270.77 | 1.002852 |
| GO:0008152\_metabolic\_process | RBM39 | 2133 | 14 | 1.162501 | -0.549606 | 270 | 270.77 | 1.002852 |
| GO:0008152\_metabolic\_process | PTBP2 | 2133 | 14 | 1.162501 | -0.549606 | 270 | 270.77 | 1.002852 |
| GO:0008152\_metabolic\_process | SMARCA4 | 2133 | 14 | 1.162501 | -0.549606 | 270 | 270.77 | 1.002852 |
| GO:0035270\_endocrine\_system\_development | CREB1 | 59 | 1 | 3.001956 | -0.544377 | 271 | 273.37 | 1.008745 |
| GO:0010605\_negative\_regulation\_of\_macromolecule\_metabolic\_process | MSH2 | 331 | 3 | 1.605275 | -0.543276 | 272 | 273.66 | 1.006103 |
| GO:0010605\_negative\_regulation\_of\_macromolecule\_metabolic\_process | DNMT1 | 331 | 3 | 1.605275 | -0.543276 | 272 | 273.66 | 1.006103 |
| GO:0010605\_negative\_regulation\_of\_macromolecule\_metabolic\_process | SMARCA4 | 331 | 3 | 1.605275 | -0.543276 | 272 | 273.66 | 1.006103 |
| GO:0031324\_negative\_regulation\_of\_cellular\_metabolic\_process | MSH2 | 332 | 3 | 1.600440 | -0.540887 | 273 | 274.21 | 1.004432 |
| GO:0031324\_negative\_regulation\_of\_cellular\_metabolic\_process | DNMT1 | 332 | 3 | 1.600440 | -0.540887 | 273 | 274.21 | 1.004432 |
| GO:0031324\_negative\_regulation\_of\_cellular\_metabolic\_process | SMARCA4 | 332 | 3 | 1.600440 | -0.540887 | 273 | 274.21 | 1.004432 |
| GO:0044237\_cellular\_metabolic\_process | MSH2 | 1974 | 13 | 1.166413 | -0.532737 | 274 | 275.01 | 1.003686 |
| GO:0044237\_cellular\_metabolic\_process | ACO1 | 1974 | 13 | 1.166413 | -0.532737 | 274 | 275.01 | 1.003686 |
| GO:0044237\_cellular\_metabolic\_process | CREB1 | 1974 | 13 | 1.166413 | -0.532737 | 274 | 275.01 | 1.003686 |
| GO:0044237\_cellular\_metabolic\_process | SMAD4 | 1974 | 13 | 1.166413 | -0.532737 | 274 | 275.01 | 1.003686 |
| GO:0044237\_cellular\_metabolic\_process | DSTN | 1974 | 13 | 1.166413 | -0.532737 | 274 | 275.01 | 1.003686 |
| GO:0044237\_cellular\_metabolic\_process | SPAG9 | 1974 | 13 | 1.166413 | -0.532737 | 274 | 275.01 | 1.003686 |
| GO:0044237\_cellular\_metabolic\_process | EIF4G2 | 1974 | 13 | 1.166413 | -0.532737 | 274 | 275.01 | 1.003686 |
| GO:0044237\_cellular\_metabolic\_process | OXCT1 | 1974 | 13 | 1.166413 | -0.532737 | 274 | 275.01 | 1.003686 |
| GO:0044237\_cellular\_metabolic\_process | DNMT1 | 1974 | 13 | 1.166413 | -0.532737 | 274 | 275.01 | 1.003686 |
| GO:0044237\_cellular\_metabolic\_process | TMPO | 1974 | 13 | 1.166413 | -0.532737 | 274 | 275.01 | 1.003686 |
| GO:0044237\_cellular\_metabolic\_process | RBM39 | 1974 | 13 | 1.166413 | -0.532737 | 274 | 275.01 | 1.003686 |
| GO:0044237\_cellular\_metabolic\_process | PTBP2 | 1974 | 13 | 1.166413 | -0.532737 | 274 | 275.01 | 1.003686 |
| GO:0044237\_cellular\_metabolic\_process | SMARCA4 | 1974 | 13 | 1.166413 | -0.532737 | 274 | 275.01 | 1.003686 |
| GO:0022604\_regulation\_of\_cell\_morphogenesis | SMAD4 | 62 | 1 | 2.856700 | -0.526217 | 277 | 278.68 | 1.006065 |
| GO:0030155\_regulation\_of\_cell\_adhesion | ATP5B | 62 | 1 | 2.856700 | -0.526217 | 277 | 278.68 | 1.006065 |
| GO:0040014\_regulation\_of\_multicellular\_organism\_growth | CREB1 | 62 | 1 | 2.856700 | -0.526217 | 277 | 278.68 | 1.006065 |
| GO:0006793\_phosphorus\_metabolic\_process | SPAG9 | 340 | 3 | 1.562783 | -0.522188 | 279 | 279.48 | 1.001720 |
| GO:0006793\_phosphorus\_metabolic\_process | MSH2 | 340 | 3 | 1.562783 | -0.522188 | 279 | 279.48 | 1.001720 |
| GO:0006793\_phosphorus\_metabolic\_process | SMAD4 | 340 | 3 | 1.562783 | -0.522188 | 279 | 279.48 | 1.001720 |
| GO:0006796\_phosphate\_metabolic\_process | SPAG9 | 340 | 3 | 1.562783 | -0.522188 | 279 | 279.48 | 1.001720 |
| GO:0006796\_phosphate\_metabolic\_process | MSH2 | 340 | 3 | 1.562783 | -0.522188 | 279 | 279.48 | 1.001720 |
| GO:0006796\_phosphate\_metabolic\_process | SMAD4 | 340 | 3 | 1.562783 | -0.522188 | 279 | 279.48 | 1.001720 |
| GO:0007369\_gastrulation | SMAD4 | 63 | 1 | 2.811355 | -0.520393 | 280 | 280.99 | 1.003536 |
| GO:0007507\_heart\_development | VCAN | 195 | 2 | 1.816568 | -0.519783 | 281 | 281.6 | 1.002135 |
| GO:0007507\_heart\_development | SMARCA4 | 195 | 2 | 1.816568 | -0.519783 | 281 | 281.6 | 1.002135 |
| GO:0033554\_cellular\_response\_to\_stress | MSH2 | 196 | 2 | 1.807300 | -0.516744 | 282 | 282.06 | 1.000213 |
| GO:0033554\_cellular\_response\_to\_stress | TIPIN | 196 | 2 | 1.807300 | -0.516744 | 282 | 282.06 | 1.000213 |
| GO:0019538\_protein\_metabolic\_process | SPAG9 | 655 | 5 | 1.352026 | -0.512712 | 283 | 284.13 | 1.003993 |
| GO:0019538\_protein\_metabolic\_process | EIF4G2 | 655 | 5 | 1.352026 | -0.512712 | 283 | 284.13 | 1.003993 |
| GO:0019538\_protein\_metabolic\_process | ACO1 | 655 | 5 | 1.352026 | -0.512712 | 283 | 284.13 | 1.003993 |
| GO:0019538\_protein\_metabolic\_process | SMAD4 | 655 | 5 | 1.352026 | -0.512712 | 283 | 284.13 | 1.003993 |
| GO:0019538\_protein\_metabolic\_process | DSTN | 655 | 5 | 1.352026 | -0.512712 | 283 | 284.13 | 1.003993 |
| GO:0002009\_morphogenesis\_of\_an\_epithelium | NUP50 | 198 | 2 | 1.789044 | -0.510733 | 285 | 284.57 | 0.998491 |
| GO:0002009\_morphogenesis\_of\_an\_epithelium | SMAD4 | 198 | 2 | 1.789044 | -0.510733 | 285 | 284.57 | 0.998491 |
| GO:0060429\_epithelium\_development | NUP50 | 198 | 2 | 1.789044 | -0.510733 | 285 | 284.57 | 0.998491 |
| GO:0060429\_epithelium\_development | SMAD4 | 198 | 2 | 1.789044 | -0.510733 | 285 | 284.57 | 0.998491 |
| GO:0006807\_nitrogen\_compound\_metabolic\_process | MSH2 | 1147 | 8 | 1.235330 | -0.507906 | 286 | 285.66 | 0.998811 |
| GO:0006807\_nitrogen\_compound\_metabolic\_process | CREB1 | 1147 | 8 | 1.235330 | -0.507906 | 286 | 285.66 | 0.998811 |
| GO:0006807\_nitrogen\_compound\_metabolic\_process | SMAD4 | 1147 | 8 | 1.235330 | -0.507906 | 286 | 285.66 | 0.998811 |
| GO:0006807\_nitrogen\_compound\_metabolic\_process | DNMT1 | 1147 | 8 | 1.235330 | -0.507906 | 286 | 285.66 | 0.998811 |
| GO:0006807\_nitrogen\_compound\_metabolic\_process | PTBP2 | 1147 | 8 | 1.235330 | -0.507906 | 286 | 285.66 | 0.998811 |
| GO:0006807\_nitrogen\_compound\_metabolic\_process | RBM39 | 1147 | 8 | 1.235330 | -0.507906 | 286 | 285.66 | 0.998811 |
| GO:0006807\_nitrogen\_compound\_metabolic\_process | TMPO | 1147 | 8 | 1.235330 | -0.507906 | 286 | 285.66 | 0.998811 |
| GO:0006807\_nitrogen\_compound\_metabolic\_process | SMARCA4 | 1147 | 8 | 1.235330 | -0.507906 | 286 | 285.66 | 0.998811 |
| GO:0016070\_RNA\_metabolic\_process | CREB1 | 658 | 5 | 1.345862 | -0.507776 | 287 | 285.8 | 0.995819 |
| GO:0016070\_RNA\_metabolic\_process | SMAD4 | 658 | 5 | 1.345862 | -0.507776 | 287 | 285.8 | 0.995819 |
| GO:0016070\_RNA\_metabolic\_process | DNMT1 | 658 | 5 | 1.345862 | -0.507776 | 287 | 285.8 | 0.995819 |
| GO:0016070\_RNA\_metabolic\_process | PTBP2 | 658 | 5 | 1.345862 | -0.507776 | 287 | 285.8 | 0.995819 |
| GO:0016070\_RNA\_metabolic\_process | SMARCA4 | 658 | 5 | 1.345862 | -0.507776 | 287 | 285.8 | 0.995819 |
| GO:0000904\_cell\_morphogenesis\_involved\_in\_differentiation | CREB1 | 199 | 2 | 1.780054 | -0.507761 | 288 | 286.01 | 0.993090 |
| GO:0000904\_cell\_morphogenesis\_involved\_in\_differentiation | SMAD4 | 199 | 2 | 1.780054 | -0.507761 | 288 | 286.01 | 0.993090 |
| GO:0009892\_negative\_regulation\_of\_metabolic\_process | MSH2 | 348 | 3 | 1.526857 | -0.504192 | 289 | 286.13 | 0.990069 |
| GO:0009892\_negative\_regulation\_of\_metabolic\_process | DNMT1 | 348 | 3 | 1.526857 | -0.504192 | 289 | 286.13 | 0.990069 |
| GO:0009892\_negative\_regulation\_of\_metabolic\_process | SMARCA4 | 348 | 3 | 1.526857 | -0.504192 | 289 | 286.13 | 0.990069 |
| GO:0044249\_cellular\_biosynthetic\_process | EIF4G2 | 1150 | 8 | 1.232107 | -0.504006 | 290 | 286.26 | 0.987103 |
| GO:0044249\_cellular\_biosynthetic\_process | ACO1 | 1150 | 8 | 1.232107 | -0.504006 | 290 | 286.26 | 0.987103 |
| GO:0044249\_cellular\_biosynthetic\_process | CREB1 | 1150 | 8 | 1.232107 | -0.504006 | 290 | 286.26 | 0.987103 |
| GO:0044249\_cellular\_biosynthetic\_process | SMAD4 | 1150 | 8 | 1.232107 | -0.504006 | 290 | 286.26 | 0.987103 |
| GO:0044249\_cellular\_biosynthetic\_process | DNMT1 | 1150 | 8 | 1.232107 | -0.504006 | 290 | 286.26 | 0.987103 |
| GO:0044249\_cellular\_biosynthetic\_process | RBM39 | 1150 | 8 | 1.232107 | -0.504006 | 290 | 286.26 | 0.987103 |
| GO:0044249\_cellular\_biosynthetic\_process | TMPO | 1150 | 8 | 1.232107 | -0.504006 | 290 | 286.26 | 0.987103 |
| GO:0044249\_cellular\_biosynthetic\_process | SMARCA4 | 1150 | 8 | 1.232107 | -0.504006 | 290 | 286.26 | 0.987103 |
| GO:0007179\_transforming\_growth\_factor\_beta\_receptor\_signaling\_pathway | SMAD4 | 66 | 1 | 2.683566 | -0.503557 | 293 | 287.71 | 0.981945 |
| GO:0045860\_positive\_regulation\_of\_protein\_kinase\_activity | SPAG9 | 66 | 1 | 2.683566 | -0.503557 | 293 | 287.71 | 0.981945 |
| GO:0051402\_neuron\_apoptosis | MSH2 | 66 | 1 | 2.683566 | -0.503557 | 293 | 287.71 | 0.981945 |
| GO:0003007\_heart\_morphogenesis | SMARCA4 | 67 | 1 | 2.643513 | -0.498148 | 295 | 289.28 | 0.980610 |
| GO:0009791\_post-embryonic\_development | ACO1 | 67 | 1 | 2.643513 | -0.498148 | 295 | 289.28 | 0.980610 |
| GO:0006955\_immune\_response | MSH2 | 205 | 2 | 1.727955 | -0.490367 | 296 | 291.5 | 0.984797 |
| GO:0006955\_immune\_response | NFKB2 | 205 | 2 | 1.727955 | -0.490367 | 296 | 291.5 | 0.984797 |
| GO:0001932\_regulation\_of\_protein\_amino\_acid\_phosphorylation | SMAD4 | 69 | 1 | 2.566890 | -0.487612 | 298 | 292.72 | 0.982282 |
| GO:0006816\_calcium\_ion\_transport | ATP2B1 | 69 | 1 | 2.566890 | -0.487612 | 298 | 292.72 | 0.982282 |
| GO:0008406\_gonad\_development | MSH2 | 70 | 1 | 2.530220 | -0.482481 | 300 | 294.36 | 0.981200 |
| GO:0070838\_divalent\_metal\_ion\_transport | ATP2B1 | 70 | 1 | 2.530220 | -0.482481 | 300 | 294.36 | 0.981200 |
| GO:0045449\_regulation\_of\_transcription | CREB1 | 676 | 5 | 1.310025 | -0.479078 | 301 | 295.18 | 0.980664 |
| GO:0045449\_regulation\_of\_transcription | SMAD4 | 676 | 5 | 1.310025 | -0.479078 | 301 | 295.18 | 0.980664 |
| GO:0045449\_regulation\_of\_transcription | DNMT1 | 676 | 5 | 1.310025 | -0.479078 | 301 | 295.18 | 0.980664 |
| GO:0045449\_regulation\_of\_transcription | TMPO | 676 | 5 | 1.310025 | -0.479078 | 301 | 295.18 | 0.980664 |
| GO:0045449\_regulation\_of\_transcription | SMARCA4 | 676 | 5 | 1.310025 | -0.479078 | 301 | 295.18 | 0.980664 |
| GO:0006281\_DNA\_repair | MSH2 | 71 | 1 | 2.494583 | -0.477438 | 304 | 296.55 | 0.975493 |
| GO:0016331\_morphogenesis\_of\_embryonic\_epithelium | NUP50 | 71 | 1 | 2.494583 | -0.477438 | 304 | 296.55 | 0.975493 |
| GO:0033674\_positive\_regulation\_of\_kinase\_activity | SPAG9 | 71 | 1 | 2.494583 | -0.477438 | 304 | 296.55 | 0.975493 |
| GO:0007264\_small\_GTPase\_mediated\_signal\_transduction | SDCBP | 72 | 1 | 2.459936 | -0.472480 | 309 | 299.72 | 0.969968 |
| GO:0016568\_chromatin\_modification | SMARCA4 | 72 | 1 | 2.459936 | -0.472480 | 309 | 299.72 | 0.969968 |
| GO:0021915\_neural\_tube\_development | NUP50 | 72 | 1 | 2.459936 | -0.472480 | 309 | 299.72 | 0.969968 |
| GO:0030879\_mammary\_gland\_development | CREB1 | 72 | 1 | 2.459936 | -0.472480 | 309 | 299.72 | 0.969968 |
| GO:0051347\_positive\_regulation\_of\_transferase\_activity | SPAG9 | 72 | 1 | 2.459936 | -0.472480 | 309 | 299.72 | 0.969968 |
| GO:0009058\_biosynthetic\_process | EIF4G2 | 1175 | 8 | 1.205892 | -0.472441 | 310 | 299.84 | 0.967226 |
| GO:0009058\_biosynthetic\_process | ACO1 | 1175 | 8 | 1.205892 | -0.472441 | 310 | 299.84 | 0.967226 |
| GO:0009058\_biosynthetic\_process | CREB1 | 1175 | 8 | 1.205892 | -0.472441 | 310 | 299.84 | 0.967226 |
| GO:0009058\_biosynthetic\_process | SMAD4 | 1175 | 8 | 1.205892 | -0.472441 | 310 | 299.84 | 0.967226 |
| GO:0009058\_biosynthetic\_process | DNMT1 | 1175 | 8 | 1.205892 | -0.472441 | 310 | 299.84 | 0.967226 |
| GO:0009058\_biosynthetic\_process | RBM39 | 1175 | 8 | 1.205892 | -0.472441 | 310 | 299.84 | 0.967226 |
| GO:0009058\_biosynthetic\_process | TMPO | 1175 | 8 | 1.205892 | -0.472441 | 310 | 299.84 | 0.967226 |
| GO:0009058\_biosynthetic\_process | SMARCA4 | 1175 | 8 | 1.205892 | -0.472441 | 310 | 299.84 | 0.967226 |
| GO:0043009\_chordate\_embryonic\_development | MSH2 | 365 | 3 | 1.455743 | -0.468132 | 311 | 300.83 | 0.967299 |
| GO:0043009\_chordate\_embryonic\_development | NUP50 | 365 | 3 | 1.455743 | -0.468132 | 311 | 300.83 | 0.967299 |
| GO:0043009\_chordate\_embryonic\_development | SMARCA4 | 365 | 3 | 1.455743 | -0.468132 | 311 | 300.83 | 0.967299 |
| GO:0009792\_embryonic\_development\_ending\_in\_birth\_or\_egg\_hatching | MSH2 | 368 | 3 | 1.443875 | -0.462060 | 312 | 303.5 | 0.972756 |
| GO:0009792\_embryonic\_development\_ending\_in\_birth\_or\_egg\_hatching | NUP50 | 368 | 3 | 1.443875 | -0.462060 | 312 | 303.5 | 0.972756 |
| GO:0009792\_embryonic\_development\_ending\_in\_birth\_or\_egg\_hatching | SMARCA4 | 368 | 3 | 1.443875 | -0.462060 | 312 | 303.5 | 0.972756 |
| GO:0007281\_germ\_cell\_development | MSH2 | 75 | 1 | 2.361538 | -0.458095 | 314 | 304.85 | 0.970860 |
| GO:0048589\_developmental\_growth | SMARCA4 | 75 | 1 | 2.361538 | -0.458095 | 314 | 304.85 | 0.970860 |
| GO:0040007\_growth | CREB1 | 217 | 2 | 1.632400 | -0.457718 | 315 | 305.39 | 0.969492 |
| GO:0040007\_growth | SMARCA4 | 217 | 2 | 1.632400 | -0.457718 | 315 | 305.39 | 0.969492 |
| GO:0045892\_negative\_regulation\_of\_transcription\_\_DNA-dependent | DNMT1 | 218 | 2 | 1.624912 | -0.455118 | 316 | 305.85 | 0.967880 |
| GO:0045892\_negative\_regulation\_of\_transcription\_\_DNA-dependent | SMARCA4 | 218 | 2 | 1.624912 | -0.455118 | 316 | 305.85 | 0.967880 |
| GO:0048519\_negative\_regulation\_of\_biological\_process | MSH2 | 859 | 6 | 1.237127 | -0.450943 | 317 | 307.6 | 0.970347 |
| GO:0048519\_negative\_regulation\_of\_biological\_process | ATP5B | 859 | 6 | 1.237127 | -0.450943 | 317 | 307.6 | 0.970347 |
| GO:0048519\_negative\_regulation\_of\_biological\_process | SMAD4 | 859 | 6 | 1.237127 | -0.450943 | 317 | 307.6 | 0.970347 |
| GO:0048519\_negative\_regulation\_of\_biological\_process | DNMT1 | 859 | 6 | 1.237127 | -0.450943 | 317 | 307.6 | 0.970347 |
| GO:0048519\_negative\_regulation\_of\_biological\_process | H47 | 859 | 6 | 1.237127 | -0.450943 | 317 | 307.6 | 0.970347 |
| GO:0048519\_negative\_regulation\_of\_biological\_process | SMARCA4 | 859 | 6 | 1.237127 | -0.450943 | 317 | 307.6 | 0.970347 |
| GO:0051253\_negative\_regulation\_of\_RNA\_metabolic\_process | DNMT1 | 220 | 2 | 1.610140 | -0.449971 | 318 | 307.87 | 0.968145 |
| GO:0051253\_negative\_regulation\_of\_RNA\_metabolic\_process | SMARCA4 | 220 | 2 | 1.610140 | -0.449971 | 318 | 307.87 | 0.968145 |
| GO:0051241\_negative\_regulation\_of\_multicellular\_organismal\_process | H47 | 77 | 1 | 2.300200 | -0.448889 | 319 | 308.52 | 0.967147 |
| GO:0001701\_in\_utero\_embryonic\_development | MSH2 | 221 | 2 | 1.602854 | -0.447424 | 320 | 308.69 | 0.964656 |
| GO:0001701\_in\_utero\_embryonic\_development | SMARCA4 | 221 | 2 | 1.602854 | -0.447424 | 320 | 308.69 | 0.964656 |
| GO:0048513\_organ\_development | MSH2 | 1365 | 9 | 1.167794 | -0.447316 | 321 | 308.81 | 0.962025 |
| GO:0048513\_organ\_development | CREB1 | 1365 | 9 | 1.167794 | -0.447316 | 321 | 308.81 | 0.962025 |
| GO:0048513\_organ\_development | UTRN | 1365 | 9 | 1.167794 | -0.447316 | 321 | 308.81 | 0.962025 |
| GO:0048513\_organ\_development | NUP50 | 1365 | 9 | 1.167794 | -0.447316 | 321 | 308.81 | 0.962025 |
| GO:0048513\_organ\_development | SMAD4 | 1365 | 9 | 1.167794 | -0.447316 | 321 | 308.81 | 0.962025 |
| GO:0048513\_organ\_development | VCAN | 1365 | 9 | 1.167794 | -0.447316 | 321 | 308.81 | 0.962025 |
| GO:0048513\_organ\_development | NFKB2 | 1365 | 9 | 1.167794 | -0.447316 | 321 | 308.81 | 0.962025 |
| GO:0048513\_organ\_development | MYCN | 1365 | 9 | 1.167794 | -0.447316 | 321 | 308.81 | 0.962025 |
| GO:0048513\_organ\_development | SMARCA4 | 1365 | 9 | 1.167794 | -0.447316 | 321 | 308.81 | 0.962025 |
| GO:0006461\_protein\_complex\_assembly | SPAG9 | 78 | 1 | 2.270710 | -0.444396 | 325 | 311.12 | 0.957292 |
| GO:0030326\_embryonic\_limb\_morphogenesis | SMARCA4 | 78 | 1 | 2.270710 | -0.444396 | 325 | 311.12 | 0.957292 |
| GO:0035113\_embryonic\_appendage\_morphogenesis | SMARCA4 | 78 | 1 | 2.270710 | -0.444396 | 325 | 311.12 | 0.957292 |
| GO:0070271\_protein\_complex\_biogenesis | SPAG9 | 78 | 1 | 2.270710 | -0.444396 | 325 | 311.12 | 0.957292 |
| GO:0015674\_di-\_\_tri-valent\_inorganic\_cation\_transport | ATP2B1 | 79 | 1 | 2.241967 | -0.439973 | 327 | 312.06 | 0.954312 |
| GO:0051046\_regulation\_of\_secretion | CREB1 | 79 | 1 | 2.241967 | -0.439973 | 327 | 312.06 | 0.954312 |
| GO:0065007\_biological\_regulation | GNAI3 | 2593 | 16 | 1.092883 | -0.432067 | 328 | 313.93 | 0.957104 |
| GO:0065007\_biological\_regulation | ACO1 | 2593 | 16 | 1.092883 | -0.432067 | 328 | 313.93 | 0.957104 |
| GO:0065007\_biological\_regulation | MSH2 | 2593 | 16 | 1.092883 | -0.432067 | 328 | 313.93 | 0.957104 |
| GO:0065007\_biological\_regulation | CREB1 | 2593 | 16 | 1.092883 | -0.432067 | 328 | 313.93 | 0.957104 |
| GO:0065007\_biological\_regulation | ATP5B | 2593 | 16 | 1.092883 | -0.432067 | 328 | 313.93 | 0.957104 |
| GO:0065007\_biological\_regulation | TIPIN | 2593 | 16 | 1.092883 | -0.432067 | 328 | 313.93 | 0.957104 |
| GO:0065007\_biological\_regulation | SMAD4 | 2593 | 16 | 1.092883 | -0.432067 | 328 | 313.93 | 0.957104 |
| GO:0065007\_biological\_regulation | DSTN | 2593 | 16 | 1.092883 | -0.432067 | 328 | 313.93 | 0.957104 |
| GO:0065007\_biological\_regulation | MYCN | 2593 | 16 | 1.092883 | -0.432067 | 328 | 313.93 | 0.957104 |
| GO:0065007\_biological\_regulation | EIF4G2 | 2593 | 16 | 1.092883 | -0.432067 | 328 | 313.93 | 0.957104 |
| GO:0065007\_biological\_regulation | SPAG9 | 2593 | 16 | 1.092883 | -0.432067 | 328 | 313.93 | 0.957104 |
| GO:0065007\_biological\_regulation | SDCBP | 2593 | 16 | 1.092883 | -0.432067 | 328 | 313.93 | 0.957104 |
| GO:0065007\_biological\_regulation | DNMT1 | 2593 | 16 | 1.092883 | -0.432067 | 328 | 313.93 | 0.957104 |
| GO:0065007\_biological\_regulation | TMPO | 2593 | 16 | 1.092883 | -0.432067 | 328 | 313.93 | 0.957104 |
| GO:0065007\_biological\_regulation | H47 | 2593 | 16 | 1.092883 | -0.432067 | 328 | 313.93 | 0.957104 |
| GO:0065007\_biological\_regulation | SMARCA4 | 2593 | 16 | 1.092883 | -0.432067 | 328 | 313.93 | 0.957104 |
| GO:0006325\_chromatin\_organization | SMARCA4 | 83 | 1 | 2.133920 | -0.422951 | 329 | 319.43 | 0.970912 |
| GO:0007420\_brain\_development | CREB1 | 231 | 2 | 1.533467 | -0.422876 | 330 | 319.66 | 0.968667 |
| GO:0007420\_brain\_development | SMARCA4 | 231 | 2 | 1.533467 | -0.422876 | 330 | 319.66 | 0.968667 |
| GO:0030005\_cellular\_di-\_\_tri-valent\_inorganic\_cation\_homeostasis | ACO1 | 84 | 1 | 2.108516 | -0.418855 | 332 | 320.58 | 0.965602 |
| GO:0045137\_development\_of\_primary\_sexual\_characteristics | MSH2 | 84 | 1 | 2.108516 | -0.418855 | 332 | 320.58 | 0.965602 |
| GO:0002449\_lymphocyte\_mediated\_immunity | MSH2 | 85 | 1 | 2.083710 | -0.414819 | 333 | 322.55 | 0.968619 |
| GO:0042127\_regulation\_of\_cell\_proliferation | SMAD4 | 393 | 3 | 1.352026 | -0.414581 | 334 | 322.73 | 0.966257 |
| GO:0042127\_regulation\_of\_cell\_proliferation | DNMT1 | 393 | 3 | 1.352026 | -0.414581 | 334 | 322.73 | 0.966257 |
| GO:0042127\_regulation\_of\_cell\_proliferation | MYCN | 393 | 3 | 1.352026 | -0.414581 | 334 | 322.73 | 0.966257 |
| GO:0006897\_endocytosis | RAB5A | 86 | 1 | 2.059481 | -0.410843 | 338 | 325.08 | 0.961775 |
| GO:0010324\_membrane\_invagination | RAB5A | 86 | 1 | 2.059481 | -0.410843 | 338 | 325.08 | 0.961775 |
| GO:0032504\_multicellular\_organism\_reproduction | CREB1 | 86 | 1 | 2.059481 | -0.410843 | 338 | 325.08 | 0.961775 |
| GO:0048609\_reproductive\_process\_in\_a\_multicellular\_organism | CREB1 | 86 | 1 | 2.059481 | -0.410843 | 338 | 325.08 | 0.961775 |
| GO:0006468\_protein\_amino\_acid\_phosphorylation | SPAG9 | 237 | 2 | 1.494645 | -0.408912 | 339 | 326.21 | 0.962271 |
| GO:0006468\_protein\_amino\_acid\_phosphorylation | SMAD4 | 237 | 2 | 1.494645 | -0.408912 | 339 | 326.21 | 0.962271 |
| GO:0001822\_kidney\_development | SMAD4 | 87 | 1 | 2.035809 | -0.406924 | 342 | 328.39 | 0.960205 |
| GO:0003001\_generation\_of\_a\_signal\_involved\_in\_cell-cell\_signaling | CREB1 | 87 | 1 | 2.035809 | -0.406924 | 342 | 328.39 | 0.960205 |
| GO:0007178\_transmembrane\_receptor\_protein\_serine\_threonine\_kinase\_signaling\_pathway | SMAD4 | 87 | 1 | 2.035809 | -0.406924 | 342 | 328.39 | 0.960205 |
| GO:0007049\_cell\_cycle | MSH2 | 238 | 2 | 1.488365 | -0.406637 | 343 | 328.6 | 0.958017 |
| GO:0007049\_cell\_cycle | TIPIN | 238 | 2 | 1.488365 | -0.406637 | 343 | 328.6 | 0.958017 |
| GO:0009790\_embryonic\_development | MSH2 | 567 | 4 | 1.249491 | -0.397329 | 344 | 330.26 | 0.960058 |
| GO:0009790\_embryonic\_development | NUP50 | 567 | 4 | 1.249491 | -0.397329 | 344 | 330.26 | 0.960058 |
| GO:0009790\_embryonic\_development | SMAD4 | 567 | 4 | 1.249491 | -0.397329 | 344 | 330.26 | 0.960058 |
| GO:0009790\_embryonic\_development | SMARCA4 | 567 | 4 | 1.249491 | -0.397329 | 344 | 330.26 | 0.960058 |
| GO:0030003\_cellular\_cation\_homeostasis | ACO1 | 90 | 1 | 1.967949 | -0.395501 | 348 | 331.41 | 0.952328 |
| GO:0030324\_lung\_development | MYCN | 90 | 1 | 1.967949 | -0.395501 | 348 | 331.41 | 0.952328 |
| GO:0035264\_multicellular\_organism\_growth | CREB1 | 90 | 1 | 1.967949 | -0.395501 | 348 | 331.41 | 0.952328 |
| GO:0042113\_B\_cell\_activation | MSH2 | 90 | 1 | 1.967949 | -0.395501 | 348 | 331.41 | 0.952328 |
| GO:0002443\_leukocyte\_mediated\_immunity | MSH2 | 91 | 1 | 1.946323 | -0.391801 | 351 | 332.76 | 0.948034 |
| GO:0008544\_epidermis\_development | SMARCA4 | 91 | 1 | 1.946323 | -0.391801 | 351 | 332.76 | 0.948034 |
| GO:0031399\_regulation\_of\_protein\_modification\_process | SMAD4 | 91 | 1 | 1.946323 | -0.391801 | 351 | 332.76 | 0.948034 |
| GO:0030323\_respiratory\_tube\_development | MYCN | 92 | 1 | 1.925167 | -0.388152 | 352 | 333.56 | 0.947614 |
| GO:0006355\_regulation\_of\_transcription\_\_DNA-dependent | CREB1 | 575 | 4 | 1.232107 | -0.385784 | 353 | 333.99 | 0.946147 |
| GO:0006355\_regulation\_of\_transcription\_\_DNA-dependent | SMAD4 | 575 | 4 | 1.232107 | -0.385784 | 353 | 333.99 | 0.946147 |
| GO:0006355\_regulation\_of\_transcription\_\_DNA-dependent | DNMT1 | 575 | 4 | 1.232107 | -0.385784 | 353 | 333.99 | 0.946147 |
| GO:0006355\_regulation\_of\_transcription\_\_DNA-dependent | SMARCA4 | 575 | 4 | 1.232107 | -0.385784 | 353 | 333.99 | 0.946147 |
| GO:0032879\_regulation\_of\_localization | CREB1 | 248 | 2 | 1.428350 | -0.384686 | 354 | 334.44 | 0.944746 |
| GO:0032879\_regulation\_of\_localization | SMAD4 | 248 | 2 | 1.428350 | -0.384686 | 354 | 334.44 | 0.944746 |
| GO:0035107\_appendage\_morphogenesis | SMARCA4 | 93 | 1 | 1.904467 | -0.384553 | 357 | 335.74 | 0.940448 |
| GO:0035108\_limb\_morphogenesis | SMARCA4 | 93 | 1 | 1.904467 | -0.384553 | 357 | 335.74 | 0.940448 |
| GO:0055066\_di-\_\_tri-valent\_inorganic\_cation\_homeostasis | ACO1 | 93 | 1 | 1.904467 | -0.384553 | 357 | 335.74 | 0.940448 |
| GO:0007165\_signal\_transduction | SPAG9 | 915 | 6 | 1.161412 | -0.381058 | 358 | 336.41 | 0.939693 |
| GO:0007165\_signal\_transduction | GNAI3 | 915 | 6 | 1.161412 | -0.381058 | 358 | 336.41 | 0.939693 |
| GO:0007165\_signal\_transduction | MSH2 | 915 | 6 | 1.161412 | -0.381058 | 358 | 336.41 | 0.939693 |
| GO:0007165\_signal\_transduction | TIPIN | 915 | 6 | 1.161412 | -0.381058 | 358 | 336.41 | 0.939693 |
| GO:0007165\_signal\_transduction | SMAD4 | 915 | 6 | 1.161412 | -0.381058 | 358 | 336.41 | 0.939693 |
| GO:0007165\_signal\_transduction | SDCBP | 915 | 6 | 1.161412 | -0.381058 | 358 | 336.41 | 0.939693 |
| GO:0008104\_protein\_localization | YWHAG | 251 | 2 | 1.411278 | -0.378370 | 359 | 338.68 | 0.943398 |
| GO:0008104\_protein\_localization | SMAD4 | 251 | 2 | 1.411278 | -0.378370 | 359 | 338.68 | 0.943398 |
| GO:0016481\_negative\_regulation\_of\_transcription | DNMT1 | 253 | 2 | 1.400122 | -0.374226 | 360 | 340.12 | 0.944778 |
| GO:0016481\_negative\_regulation\_of\_transcription | SMARCA4 | 253 | 2 | 1.400122 | -0.374226 | 360 | 340.12 | 0.944778 |
| GO:0006954\_inflammatory\_response | H47 | 96 | 1 | 1.844952 | -0.374048 | 363 | 341.86 | 0.941763 |
| GO:0048736\_appendage\_development | SMARCA4 | 96 | 1 | 1.844952 | -0.374048 | 363 | 341.86 | 0.941763 |
| GO:0060173\_limb\_development | SMARCA4 | 96 | 1 | 1.844952 | -0.374048 | 363 | 341.86 | 0.941763 |
| GO:0007154\_cell\_communication | SPAG9 | 1096 | 7 | 1.131211 | -0.369293 | 364 | 343.48 | 0.943626 |
| GO:0007154\_cell\_communication | GNAI3 | 1096 | 7 | 1.131211 | -0.369293 | 364 | 343.48 | 0.943626 |
| GO:0007154\_cell\_communication | MSH2 | 1096 | 7 | 1.131211 | -0.369293 | 364 | 343.48 | 0.943626 |
| GO:0007154\_cell\_communication | CREB1 | 1096 | 7 | 1.131211 | -0.369293 | 364 | 343.48 | 0.943626 |
| GO:0007154\_cell\_communication | TIPIN | 1096 | 7 | 1.131211 | -0.369293 | 364 | 343.48 | 0.943626 |
| GO:0007154\_cell\_communication | SMAD4 | 1096 | 7 | 1.131211 | -0.369293 | 364 | 343.48 | 0.943626 |
| GO:0007154\_cell\_communication | SDCBP | 1096 | 7 | 1.131211 | -0.369293 | 364 | 343.48 | 0.943626 |
| GO:0051239\_regulation\_of\_multicellular\_organismal\_process | GNAI3 | 587 | 4 | 1.206919 | -0.369063 | 365 | 343.69 | 0.941616 |
| GO:0051239\_regulation\_of\_multicellular\_organismal\_process | CREB1 | 587 | 4 | 1.206919 | -0.369063 | 365 | 343.69 | 0.941616 |
| GO:0051239\_regulation\_of\_multicellular\_organismal\_process | SMAD4 | 587 | 4 | 1.206919 | -0.369063 | 365 | 343.69 | 0.941616 |
| GO:0051239\_regulation\_of\_multicellular\_organismal\_process | H47 | 587 | 4 | 1.206919 | -0.369063 | 365 | 343.69 | 0.941616 |
| GO:0007548\_sex\_differentiation | MSH2 | 98 | 1 | 1.807300 | -0.367278 | 367 | 345.53 | 0.941499 |
| GO:0060541\_respiratory\_system\_development | MYCN | 98 | 1 | 1.807300 | -0.367278 | 367 | 345.53 | 0.941499 |
| GO:0051252\_regulation\_of\_RNA\_metabolic\_process | CREB1 | 590 | 4 | 1.200782 | -0.364992 | 368 | 345.95 | 0.940082 |
| GO:0051252\_regulation\_of\_RNA\_metabolic\_process | SMAD4 | 590 | 4 | 1.200782 | -0.364992 | 368 | 345.95 | 0.940082 |
| GO:0051252\_regulation\_of\_RNA\_metabolic\_process | DNMT1 | 590 | 4 | 1.200782 | -0.364992 | 368 | 345.95 | 0.940082 |
| GO:0051252\_regulation\_of\_RNA\_metabolic\_process | SMARCA4 | 590 | 4 | 1.200782 | -0.364992 | 368 | 345.95 | 0.940082 |
| GO:0022008\_neurogenesis | CREB1 | 423 | 3 | 1.256137 | -0.364225 | 369 | 346.21 | 0.938238 |
| GO:0022008\_neurogenesis | SMAD4 | 423 | 3 | 1.256137 | -0.364225 | 369 | 346.21 | 0.938238 |
| GO:0022008\_neurogenesis | SMARCA4 | 423 | 3 | 1.256137 | -0.364225 | 369 | 346.21 | 0.938238 |
| GO:0001817\_regulation\_of\_cytokine\_production | H47 | 99 | 1 | 1.789044 | -0.363960 | 371 | 347.37 | 0.936307 |
| GO:0007398\_ectoderm\_development | SMARCA4 | 99 | 1 | 1.789044 | -0.363960 | 371 | 347.37 | 0.936307 |
| GO:0006351\_transcription\_\_DNA-dependent | CREB1 | 594 | 4 | 1.192696 | -0.359630 | 372 | 347.96 | 0.935376 |
| GO:0006351\_transcription\_\_DNA-dependent | SMAD4 | 594 | 4 | 1.192696 | -0.359630 | 372 | 347.96 | 0.935376 |
| GO:0006351\_transcription\_\_DNA-dependent | DNMT1 | 594 | 4 | 1.192696 | -0.359630 | 372 | 347.96 | 0.935376 |
| GO:0006351\_transcription\_\_DNA-dependent | SMARCA4 | 594 | 4 | 1.192696 | -0.359630 | 372 | 347.96 | 0.935376 |
| GO:0032774\_RNA\_biosynthetic\_process | CREB1 | 595 | 4 | 1.190692 | -0.358301 | 373 | 348.14 | 0.933351 |
| GO:0032774\_RNA\_biosynthetic\_process | SMAD4 | 595 | 4 | 1.190692 | -0.358301 | 373 | 348.14 | 0.933351 |
| GO:0032774\_RNA\_biosynthetic\_process | DNMT1 | 595 | 4 | 1.190692 | -0.358301 | 373 | 348.14 | 0.933351 |
| GO:0032774\_RNA\_biosynthetic\_process | SMARCA4 | 595 | 4 | 1.190692 | -0.358301 | 373 | 348.14 | 0.933351 |
| GO:0001775\_cell\_activation | MSH2 | 262 | 2 | 1.352026 | -0.356208 | 375 | 349.82 | 0.932853 |
| GO:0001775\_cell\_activation | NFKB2 | 262 | 2 | 1.352026 | -0.356208 | 375 | 349.82 | 0.932853 |
| GO:0010629\_negative\_regulation\_of\_gene\_expression | DNMT1 | 262 | 2 | 1.352026 | -0.356208 | 375 | 349.82 | 0.932853 |
| GO:0010629\_negative\_regulation\_of\_gene\_expression | SMARCA4 | 262 | 2 | 1.352026 | -0.356208 | 375 | 349.82 | 0.932853 |
| GO:0030036\_actin\_cytoskeleton\_organization | DSTN | 102 | 1 | 1.736425 | -0.354261 | 376 | 350.67 | 0.932633 |
| GO:0003013\_circulatory\_system\_process | GNAI3 | 103 | 1 | 1.719567 | -0.351111 | 378 | 352.15 | 0.931614 |
| GO:0008015\_blood\_circulation | GNAI3 | 103 | 1 | 1.719567 | -0.351111 | 378 | 352.15 | 0.931614 |
| GO:0048523\_negative\_regulation\_of\_cellular\_process | MSH2 | 774 | 5 | 1.144156 | -0.347244 | 379 | 353.71 | 0.933272 |
| GO:0048523\_negative\_regulation\_of\_cellular\_process | ATP5B | 774 | 5 | 1.144156 | -0.347244 | 379 | 353.71 | 0.933272 |
| GO:0048523\_negative\_regulation\_of\_cellular\_process | SMAD4 | 774 | 5 | 1.144156 | -0.347244 | 379 | 353.71 | 0.933272 |
| GO:0048523\_negative\_regulation\_of\_cellular\_process | DNMT1 | 774 | 5 | 1.144156 | -0.347244 | 379 | 353.71 | 0.933272 |
| GO:0048523\_negative\_regulation\_of\_cellular\_process | SMARCA4 | 774 | 5 | 1.144156 | -0.347244 | 379 | 353.71 | 0.933272 |
| GO:0010817\_regulation\_of\_hormone\_levels | CREB1 | 106 | 1 | 1.670900 | -0.341898 | 380 | 355.06 | 0.934368 |
| GO:0045859\_regulation\_of\_protein\_kinase\_activity | SPAG9 | 107 | 1 | 1.655284 | -0.338903 | 381 | 356.14 | 0.934751 |
| GO:0051240\_positive\_regulation\_of\_multicellular\_organismal\_process | CREB1 | 108 | 1 | 1.639957 | -0.335945 | 382 | 356.73 | 0.933848 |
| GO:0051716\_cellular\_response\_to\_stimulus | MSH2 | 273 | 2 | 1.297549 | -0.335508 | 383 | 357.28 | 0.932846 |
| GO:0051716\_cellular\_response\_to\_stimulus | TIPIN | 273 | 2 | 1.297549 | -0.335508 | 383 | 357.28 | 0.932846 |
| GO:0010558\_negative\_regulation\_of\_macromolecule\_biosynthetic\_process | DNMT1 | 274 | 2 | 1.292813 | -0.333694 | 385 | 358.03 | 0.929948 |
| GO:0010558\_negative\_regulation\_of\_macromolecule\_biosynthetic\_process | SMARCA4 | 274 | 2 | 1.292813 | -0.333694 | 385 | 358.03 | 0.929948 |
| GO:0033036\_macromolecule\_localization | YWHAG | 274 | 2 | 1.292813 | -0.333694 | 385 | 358.03 | 0.929948 |
| GO:0033036\_macromolecule\_localization | SMAD4 | 274 | 2 | 1.292813 | -0.333694 | 385 | 358.03 | 0.929948 |
| GO:0030029\_actin\_filament-based\_process | DSTN | 109 | 1 | 1.624912 | -0.333024 | 386 | 358.32 | 0.928290 |
| GO:0055080\_cation\_homeostasis | ACO1 | 110 | 1 | 1.610140 | -0.330138 | 387 | 360.23 | 0.930827 |
| GO:0048534\_hemopoietic\_or\_lymphoid\_organ\_development | MSH2 | 277 | 2 | 1.278811 | -0.328318 | 389 | 360.89 | 0.927738 |
| GO:0048534\_hemopoietic\_or\_lymphoid\_organ\_development | NFKB2 | 277 | 2 | 1.278811 | -0.328318 | 389 | 360.89 | 0.927738 |
| GO:0048646\_anatomical\_structure\_formation\_involved\_in\_morphogenesis | NUP50 | 277 | 2 | 1.278811 | -0.328318 | 389 | 360.89 | 0.927738 |
| GO:0048646\_anatomical\_structure\_formation\_involved\_in\_morphogenesis | SMARCA4 | 277 | 2 | 1.278811 | -0.328318 | 389 | 360.89 | 0.927738 |
| GO:0007399\_nervous\_system\_development | CREB1 | 621 | 4 | 1.140840 | -0.325333 | 390 | 362.01 | 0.928231 |
| GO:0007399\_nervous\_system\_development | NUP50 | 621 | 4 | 1.140840 | -0.325333 | 390 | 362.01 | 0.928231 |
| GO:0007399\_nervous\_system\_development | SMAD4 | 621 | 4 | 1.140840 | -0.325333 | 390 | 362.01 | 0.928231 |
| GO:0007399\_nervous\_system\_development | SMARCA4 | 621 | 4 | 1.140840 | -0.325333 | 390 | 362.01 | 0.928231 |
| GO:0065009\_regulation\_of\_molecular\_function | SPAG9 | 279 | 2 | 1.269644 | -0.324788 | 391 | 362.48 | 0.927059 |
| GO:0065009\_regulation\_of\_molecular\_function | SMAD4 | 279 | 2 | 1.269644 | -0.324788 | 391 | 362.48 | 0.927059 |
| GO:0043549\_regulation\_of\_kinase\_activity | SPAG9 | 112 | 1 | 1.581387 | -0.324470 | 392 | 363.04 | 0.926122 |
| GO:0040008\_regulation\_of\_growth | CREB1 | 113 | 1 | 1.567393 | -0.321687 | 393 | 364.1 | 0.926463 |
| GO:0031327\_negative\_regulation\_of\_cellular\_biosynthetic\_process | DNMT1 | 282 | 2 | 1.256137 | -0.319571 | 394 | 364.52 | 0.925178 |
| GO:0031327\_negative\_regulation\_of\_cellular\_biosynthetic\_process | SMARCA4 | 282 | 2 | 1.256137 | -0.319571 | 394 | 364.52 | 0.925178 |
| GO:0000165\_MAPKKK\_cascade | SPAG9 | 114 | 1 | 1.553644 | -0.318937 | 395 | 365.28 | 0.924759 |
| GO:0000902\_cell\_morphogenesis | CREB1 | 283 | 2 | 1.251699 | -0.317853 | 396 | 365.66 | 0.923384 |
| GO:0000902\_cell\_morphogenesis | SMAD4 | 283 | 2 | 1.251699 | -0.317853 | 396 | 365.66 | 0.923384 |
| GO:0051338\_regulation\_of\_transferase\_activity | SPAG9 | 115 | 1 | 1.540134 | -0.316220 | 397 | 366.28 | 0.922620 |
| GO:0009890\_negative\_regulation\_of\_biosynthetic\_process | DNMT1 | 284 | 2 | 1.247291 | -0.316145 | 398 | 366.57 | 0.921030 |
| GO:0009890\_negative\_regulation\_of\_biosynthetic\_process | SMARCA4 | 284 | 2 | 1.247291 | -0.316145 | 398 | 366.57 | 0.921030 |
| GO:0048608\_reproductive\_structure\_development | MSH2 | 116 | 1 | 1.526857 | -0.313534 | 399 | 368.12 | 0.922607 |
| GO:0043412\_biopolymer\_modification | SPAG9 | 458 | 3 | 1.160144 | -0.313271 | 400 | 368.6 | 0.921500 |
| GO:0043412\_biopolymer\_modification | SMAD4 | 458 | 3 | 1.160144 | -0.313271 | 400 | 368.6 | 0.921500 |
| GO:0043412\_biopolymer\_modification | DNMT1 | 458 | 3 | 1.160144 | -0.313271 | 400 | 368.6 | 0.921500 |
| GO:0007417\_central\_nervous\_system\_development | CREB1 | 287 | 2 | 1.234254 | -0.311082 | 401 | 368.88 | 0.919900 |
| GO:0007417\_central\_nervous\_system\_development | SMARCA4 | 287 | 2 | 1.234254 | -0.311082 | 401 | 368.88 | 0.919900 |
| GO:0001816\_cytokine\_production | H47 | 122 | 1 | 1.451765 | -0.298060 | 405 | 375.15 | 0.926296 |
| GO:0002252\_immune\_effector\_process | MSH2 | 122 | 1 | 1.451765 | -0.298060 | 405 | 375.15 | 0.926296 |
| GO:0030001\_metal\_ion\_transport | ATP2B1 | 122 | 1 | 1.451765 | -0.298060 | 405 | 375.15 | 0.926296 |
| GO:0060284\_regulation\_of\_cell\_development | SMAD4 | 122 | 1 | 1.451765 | -0.298060 | 405 | 375.15 | 0.926296 |
| GO:0002520\_immune\_system\_development | MSH2 | 295 | 2 | 1.200782 | -0.298012 | 406 | 375.91 | 0.925887 |
| GO:0002520\_immune\_system\_development | NFKB2 | 295 | 2 | 1.200782 | -0.298012 | 406 | 375.91 | 0.925887 |
| GO:0030098\_lymphocyte\_differentiation | MSH2 | 124 | 1 | 1.428350 | -0.293133 | 407 | 376.67 | 0.925479 |
| GO:0048468\_cell\_development | MSH2 | 654 | 4 | 1.083275 | -0.287568 | 408 | 378.12 | 0.926765 |
| GO:0048468\_cell\_development | CREB1 | 654 | 4 | 1.083275 | -0.287568 | 408 | 378.12 | 0.926765 |
| GO:0048468\_cell\_development | SMAD4 | 654 | 4 | 1.083275 | -0.287568 | 408 | 378.12 | 0.926765 |
| GO:0048468\_cell\_development | SMARCA4 | 654 | 4 | 1.083275 | -0.287568 | 408 | 378.12 | 0.926765 |
| GO:0001655\_urogenital\_system\_development | SMAD4 | 128 | 1 | 1.383714 | -0.283603 | 410 | 379.58 | 0.925805 |
| GO:0045597\_positive\_regulation\_of\_cell\_differentiation | SMAD4 | 128 | 1 | 1.383714 | -0.283603 | 410 | 379.58 | 0.925805 |
| GO:0051276\_chromosome\_organization | SMARCA4 | 129 | 1 | 1.372987 | -0.281285 | 411 | 380.53 | 0.925864 |
| GO:0032989\_cellular\_component\_morphogenesis | CREB1 | 307 | 2 | 1.153846 | -0.279520 | 412 | 381.38 | 0.925680 |
| GO:0032989\_cellular\_component\_morphogenesis | SMAD4 | 307 | 2 | 1.153846 | -0.279520 | 412 | 381.38 | 0.925680 |
| GO:0009952\_anterior\_posterior\_pattern\_formation | SMAD4 | 133 | 1 | 1.331695 | -0.272263 | 414 | 384.02 | 0.927585 |
| GO:0044057\_regulation\_of\_system\_process | GNAI3 | 133 | 1 | 1.331695 | -0.272263 | 414 | 384.02 | 0.927585 |
| GO:0016044\_membrane\_organization | RAB5A | 140 | 1 | 1.265110 | -0.257368 | 415 | 387.19 | 0.932988 |
| GO:0003006\_reproductive\_developmental\_process | MSH2 | 141 | 1 | 1.256137 | -0.255328 | 416 | 388.2 | 0.933173 |
| GO:0007186\_G-protein\_coupled\_receptor\_protein\_signaling\_pathway | GNAI3 | 144 | 1 | 1.229968 | -0.249331 | 417 | 389.46 | 0.933957 |
| GO:0007275\_multicellular\_organismal\_development | ACO1 | 1760 | 10 | 1.006337 | -0.248833 | 418 | 389.58 | 0.932010 |
| GO:0007275\_multicellular\_organismal\_development | MSH2 | 1760 | 10 | 1.006337 | -0.248833 | 418 | 389.58 | 0.932010 |
| GO:0007275\_multicellular\_organismal\_development | CREB1 | 1760 | 10 | 1.006337 | -0.248833 | 418 | 389.58 | 0.932010 |
| GO:0007275\_multicellular\_organismal\_development | UTRN | 1760 | 10 | 1.006337 | -0.248833 | 418 | 389.58 | 0.932010 |
| GO:0007275\_multicellular\_organismal\_development | NUP50 | 1760 | 10 | 1.006337 | -0.248833 | 418 | 389.58 | 0.932010 |
| GO:0007275\_multicellular\_organismal\_development | SMAD4 | 1760 | 10 | 1.006337 | -0.248833 | 418 | 389.58 | 0.932010 |
| GO:0007275\_multicellular\_organismal\_development | VCAN | 1760 | 10 | 1.006337 | -0.248833 | 418 | 389.58 | 0.932010 |
| GO:0007275\_multicellular\_organismal\_development | NFKB2 | 1760 | 10 | 1.006337 | -0.248833 | 418 | 389.58 | 0.932010 |
| GO:0007275\_multicellular\_organismal\_development | MYCN | 1760 | 10 | 1.006337 | -0.248833 | 418 | 389.58 | 0.932010 |
| GO:0007275\_multicellular\_organismal\_development | SMARCA4 | 1760 | 10 | 1.006337 | -0.248833 | 418 | 389.58 | 0.932010 |
| GO:0006928\_cell\_motion | ATP5B | 330 | 2 | 1.073427 | -0.247446 | 421 | 390.75 | 0.928147 |
| GO:0006928\_cell\_motion | DSTN | 330 | 2 | 1.073427 | -0.247446 | 421 | 390.75 | 0.928147 |
| GO:0010646\_regulation\_of\_cell\_communication | CREB1 | 330 | 2 | 1.073427 | -0.247446 | 421 | 390.75 | 0.928147 |
| GO:0010646\_regulation\_of\_cell\_communication | SMAD4 | 330 | 2 | 1.073427 | -0.247446 | 421 | 390.75 | 0.928147 |
| GO:0051674\_localization\_of\_cell | ATP5B | 330 | 2 | 1.073427 | -0.247446 | 421 | 390.75 | 0.928147 |
| GO:0051674\_localization\_of\_cell | DSTN | 330 | 2 | 1.073427 | -0.247446 | 421 | 390.75 | 0.928147 |
| GO:0006812\_cation\_transport | ATP2B1 | 146 | 1 | 1.213119 | -0.245433 | 422 | 392.51 | 0.930118 |
| GO:0022603\_regulation\_of\_anatomical\_structure\_morphogenesis | SMAD4 | 147 | 1 | 1.204867 | -0.243514 | 423 | 393.46 | 0.930165 |
| GO:0043085\_positive\_regulation\_of\_catalytic\_activity | SPAG9 | 148 | 1 | 1.196726 | -0.241613 | 424 | 394.35 | 0.930071 |
| GO:0032940\_secretion\_by\_cell | CREB1 | 149 | 1 | 1.188694 | -0.239732 | 425 | 395.02 | 0.929459 |
| GO:0009888\_tissue\_development | NUP50 | 525 | 3 | 1.012088 | -0.234636 | 426 | 396.57 | 0.930915 |
| GO:0009888\_tissue\_development | SMAD4 | 525 | 3 | 1.012088 | -0.234636 | 426 | 396.57 | 0.930915 |
| GO:0009888\_tissue\_development | SMARCA4 | 525 | 3 | 1.012088 | -0.234636 | 426 | 396.57 | 0.930915 |
| GO:0007517\_muscle\_organ\_development | UTRN | 153 | 1 | 1.157617 | -0.232389 | 427 | 397.41 | 0.930703 |
| GO:0048731\_system\_development | MSH2 | 1609 | 9 | 0.990701 | -0.231157 | 428 | 397.89 | 0.929650 |
| GO:0048731\_system\_development | CREB1 | 1609 | 9 | 0.990701 | -0.231157 | 428 | 397.89 | 0.929650 |
| GO:0048731\_system\_development | UTRN | 1609 | 9 | 0.990701 | -0.231157 | 428 | 397.89 | 0.929650 |
| GO:0048731\_system\_development | NUP50 | 1609 | 9 | 0.990701 | -0.231157 | 428 | 397.89 | 0.929650 |
| GO:0048731\_system\_development | SMAD4 | 1609 | 9 | 0.990701 | -0.231157 | 428 | 397.89 | 0.929650 |
| GO:0048731\_system\_development | VCAN | 1609 | 9 | 0.990701 | -0.231157 | 428 | 397.89 | 0.929650 |
| GO:0048731\_system\_development | NFKB2 | 1609 | 9 | 0.990701 | -0.231157 | 428 | 397.89 | 0.929650 |
| GO:0048731\_system\_development | MYCN | 1609 | 9 | 0.990701 | -0.231157 | 428 | 397.89 | 0.929650 |
| GO:0048731\_system\_development | SMARCA4 | 1609 | 9 | 0.990701 | -0.231157 | 428 | 397.89 | 0.929650 |
| GO:0008285\_negative\_regulation\_of\_cell\_proliferation | SMAD4 | 155 | 1 | 1.142680 | -0.228825 | 430 | 399.2 | 0.928372 |
| GO:0022402\_cell\_cycle\_process | MSH2 | 155 | 1 | 1.142680 | -0.228825 | 430 | 399.2 | 0.928372 |
| GO:0009987\_cellular\_process | GNAI3 | 3868 | 22 | 1.007378 | -0.225478 | 431 | 399.79 | 0.927587 |
| GO:0009987\_cellular\_process | ACO1 | 3868 | 22 | 1.007378 | -0.225478 | 431 | 399.79 | 0.927587 |
| GO:0009987\_cellular\_process | MSH2 | 3868 | 22 | 1.007378 | -0.225478 | 431 | 399.79 | 0.927587 |
| GO:0009987\_cellular\_process | CREB1 | 3868 | 22 | 1.007378 | -0.225478 | 431 | 399.79 | 0.927587 |
| GO:0009987\_cellular\_process | ATP5B | 3868 | 22 | 1.007378 | -0.225478 | 431 | 399.79 | 0.927587 |
| GO:0009987\_cellular\_process | TIPIN | 3868 | 22 | 1.007378 | -0.225478 | 431 | 399.79 | 0.927587 |
| GO:0009987\_cellular\_process | SMAD4 | 3868 | 22 | 1.007378 | -0.225478 | 431 | 399.79 | 0.927587 |
| GO:0009987\_cellular\_process | NFKB2 | 3868 | 22 | 1.007378 | -0.225478 | 431 | 399.79 | 0.927587 |
| GO:0009987\_cellular\_process | MYCN | 3868 | 22 | 1.007378 | -0.225478 | 431 | 399.79 | 0.927587 |
| GO:0009987\_cellular\_process | DSTN | 3868 | 22 | 1.007378 | -0.225478 | 431 | 399.79 | 0.927587 |
| GO:0009987\_cellular\_process | EIF4G2 | 3868 | 22 | 1.007378 | -0.225478 | 431 | 399.79 | 0.927587 |
| GO:0009987\_cellular\_process | SPAG9 | 3868 | 22 | 1.007378 | -0.225478 | 431 | 399.79 | 0.927587 |
| GO:0009987\_cellular\_process | YWHAG | 3868 | 22 | 1.007378 | -0.225478 | 431 | 399.79 | 0.927587 |
| GO:0009987\_cellular\_process | OXCT1 | 3868 | 22 | 1.007378 | -0.225478 | 431 | 399.79 | 0.927587 |
| GO:0009987\_cellular\_process | NUP50 | 3868 | 22 | 1.007378 | -0.225478 | 431 | 399.79 | 0.927587 |
| GO:0009987\_cellular\_process | RAB5A | 3868 | 22 | 1.007378 | -0.225478 | 431 | 399.79 | 0.927587 |
| GO:0009987\_cellular\_process | DNMT1 | 3868 | 22 | 1.007378 | -0.225478 | 431 | 399.79 | 0.927587 |
| GO:0009987\_cellular\_process | SDCBP | 3868 | 22 | 1.007378 | -0.225478 | 431 | 399.79 | 0.927587 |
| GO:0009987\_cellular\_process | RBM39 | 3868 | 22 | 1.007378 | -0.225478 | 431 | 399.79 | 0.927587 |
| GO:0009987\_cellular\_process | TMPO | 3868 | 22 | 1.007378 | -0.225478 | 431 | 399.79 | 0.927587 |
| GO:0009987\_cellular\_process | PTBP2 | 3868 | 22 | 1.007378 | -0.225478 | 431 | 399.79 | 0.927587 |
| GO:0009987\_cellular\_process | SMARCA4 | 3868 | 22 | 1.007378 | -0.225478 | 431 | 399.79 | 0.927587 |
| GO:0007409\_axonogenesis | CREB1 | 158 | 1 | 1.120983 | -0.223605 | 433 | 402.01 | 0.928430 |
| GO:0048514\_blood\_vessel\_morphogenesis | SMARCA4 | 158 | 1 | 1.120983 | -0.223605 | 433 | 402.01 | 0.928430 |
| GO:0002521\_leukocyte\_differentiation | MSH2 | 161 | 1 | 1.100096 | -0.218534 | 434 | 403.44 | 0.929585 |
| GO:0008283\_cell\_proliferation | SMAD4 | 544 | 3 | 0.976739 | -0.216077 | 435 | 404.36 | 0.929563 |
| GO:0008283\_cell\_proliferation | DNMT1 | 544 | 3 | 0.976739 | -0.216077 | 435 | 404.36 | 0.929563 |
| GO:0008283\_cell\_proliferation | MYCN | 544 | 3 | 0.976739 | -0.216077 | 435 | 404.36 | 0.929563 |
| GO:0030182\_neuron\_differentiation | CREB1 | 356 | 2 | 0.995030 | -0.215830 | 436 | 404.58 | 0.927936 |
| GO:0030182\_neuron\_differentiation | SMAD4 | 356 | 2 | 0.995030 | -0.215830 | 436 | 404.58 | 0.927936 |
| GO:0006950\_response\_to\_stress | MSH2 | 549 | 3 | 0.967844 | -0.211432 | 437 | 407.33 | 0.932105 |
| GO:0006950\_response\_to\_stress | TIPIN | 549 | 3 | 0.967844 | -0.211432 | 437 | 407.33 | 0.932105 |
| GO:0006950\_response\_to\_stress | H47 | 549 | 3 | 0.967844 | -0.211432 | 437 | 407.33 | 0.932105 |
| GO:0048812\_neuron\_projection\_morphogenesis | CREB1 | 170 | 1 | 1.041855 | -0.204152 | 438 | 411.67 | 0.939886 |
| GO:0032501\_multicellular\_organismal\_process | GNAI3 | 2183 | 12 | 0.973607 | -0.203496 | 439 | 411.79 | 0.938018 |
| GO:0032501\_multicellular\_organismal\_process | MSH2 | 2183 | 12 | 0.973607 | -0.203496 | 439 | 411.79 | 0.938018 |
| GO:0032501\_multicellular\_organismal\_process | ACO1 | 2183 | 12 | 0.973607 | -0.203496 | 439 | 411.79 | 0.938018 |
| GO:0032501\_multicellular\_organismal\_process | CREB1 | 2183 | 12 | 0.973607 | -0.203496 | 439 | 411.79 | 0.938018 |
| GO:0032501\_multicellular\_organismal\_process | UTRN | 2183 | 12 | 0.973607 | -0.203496 | 439 | 411.79 | 0.938018 |
| GO:0032501\_multicellular\_organismal\_process | NUP50 | 2183 | 12 | 0.973607 | -0.203496 | 439 | 411.79 | 0.938018 |
| GO:0032501\_multicellular\_organismal\_process | SMAD4 | 2183 | 12 | 0.973607 | -0.203496 | 439 | 411.79 | 0.938018 |
| GO:0032501\_multicellular\_organismal\_process | VCAN | 2183 | 12 | 0.973607 | -0.203496 | 439 | 411.79 | 0.938018 |
| GO:0032501\_multicellular\_organismal\_process | NFKB2 | 2183 | 12 | 0.973607 | -0.203496 | 439 | 411.79 | 0.938018 |
| GO:0032501\_multicellular\_organismal\_process | H47 | 2183 | 12 | 0.973607 | -0.203496 | 439 | 411.79 | 0.938018 |
| GO:0032501\_multicellular\_organismal\_process | MYCN | 2183 | 12 | 0.973607 | -0.203496 | 439 | 411.79 | 0.938018 |
| GO:0032501\_multicellular\_organismal\_process | SMARCA4 | 2183 | 12 | 0.973607 | -0.203496 | 439 | 411.79 | 0.938018 |
| GO:0009611\_response\_to\_wounding | H47 | 172 | 1 | 1.029741 | -0.201115 | 440 | 413.44 | 0.939636 |
| GO:0044093\_positive\_regulation\_of\_molecular\_function | SPAG9 | 173 | 1 | 1.023788 | -0.199618 | 442 | 414.73 | 0.938303 |
| GO:0048667\_cell\_morphogenesis\_involved\_in\_neuron\_differentiation | CREB1 | 173 | 1 | 1.023788 | -0.199618 | 442 | 414.73 | 0.938303 |
| GO:0000122\_negative\_regulation\_of\_transcription\_from\_RNA\_polymerase\_II\_promoter | SMARCA4 | 175 | 1 | 1.012088 | -0.196663 | 444 | 416.41 | 0.937860 |
| GO:0046903\_secretion | CREB1 | 175 | 1 | 1.012088 | -0.196663 | 444 | 416.41 | 0.937860 |
| GO:0006873\_cellular\_ion\_homeostasis | ACO1 | 176 | 1 | 1.006337 | -0.195205 | 447 | 417.72 | 0.934497 |
| GO:0043066\_negative\_regulation\_of\_apoptosis | MSH2 | 176 | 1 | 1.006337 | -0.195205 | 447 | 417.72 | 0.934497 |
| GO:0048858\_cell\_projection\_morphogenesis | CREB1 | 176 | 1 | 1.006337 | -0.195205 | 447 | 417.72 | 0.934497 |
| GO:0022414\_reproductive\_process | MSH2 | 376 | 2 | 0.942103 | -0.194392 | 448 | 417.99 | 0.933013 |
| GO:0022414\_reproductive\_process | CREB1 | 376 | 2 | 0.942103 | -0.194392 | 448 | 417.99 | 0.933013 |
| GO:0000003\_reproduction | MSH2 | 379 | 2 | 0.934646 | -0.191371 | 449 | 418.69 | 0.932494 |
| GO:0000003\_reproduction | CREB1 | 379 | 2 | 0.934646 | -0.191371 | 449 | 418.69 | 0.932494 |
| GO:0043069\_negative\_regulation\_of\_programmed\_cell\_death | MSH2 | 179 | 1 | 0.989471 | -0.190911 | 452 | 419.98 | 0.929159 |
| GO:0048732\_gland\_development | CREB1 | 179 | 1 | 0.989471 | -0.190911 | 452 | 419.98 | 0.929159 |
| GO:0060548\_negative\_regulation\_of\_cell\_death | MSH2 | 179 | 1 | 0.989471 | -0.190911 | 452 | 419.98 | 0.929159 |
| GO:0055082\_cellular\_chemical\_homeostasis | ACO1 | 181 | 1 | 0.978538 | -0.188111 | 453 | 421.89 | 0.931325 |
| GO:0043687\_post-translational\_protein\_modification | SPAG9 | 384 | 2 | 0.922476 | -0.186443 | 454 | 422.64 | 0.930925 |
| GO:0043687\_post-translational\_protein\_modification | SMAD4 | 384 | 2 | 0.922476 | -0.186443 | 454 | 422.64 | 0.930925 |
| GO:0016192\_vesicle-mediated\_transport | RAB5A | 184 | 1 | 0.962584 | -0.184003 | 456 | 423.8 | 0.929386 |
| GO:0032990\_cell\_part\_morphogenesis | CREB1 | 184 | 1 | 0.962584 | -0.184003 | 456 | 423.8 | 0.929386 |
| GO:0009653\_anatomical\_structure\_morphogenesis | CREB1 | 958 | 5 | 0.924402 | -0.183596 | 457 | 424.18 | 0.928184 |
| GO:0009653\_anatomical\_structure\_morphogenesis | NUP50 | 958 | 5 | 0.924402 | -0.183596 | 457 | 424.18 | 0.928184 |
| GO:0009653\_anatomical\_structure\_morphogenesis | SMAD4 | 958 | 5 | 0.924402 | -0.183596 | 457 | 424.18 | 0.928184 |
| GO:0009653\_anatomical\_structure\_morphogenesis | SMARCA4 | 958 | 5 | 0.924402 | -0.183596 | 457 | 424.18 | 0.928184 |
| GO:0009653\_anatomical\_structure\_morphogenesis | MYCN | 958 | 5 | 0.924402 | -0.183596 | 457 | 424.18 | 0.928184 |
| GO:0007010\_cytoskeleton\_organization | DSTN | 185 | 1 | 0.957380 | -0.182658 | 458 | 424.7 | 0.927293 |
| GO:0048856\_anatomical\_structure\_development | MSH2 | 1688 | 9 | 0.944336 | -0.182458 | 459 | 425.13 | 0.926209 |
| GO:0048856\_anatomical\_structure\_development | CREB1 | 1688 | 9 | 0.944336 | -0.182458 | 459 | 425.13 | 0.926209 |
| GO:0048856\_anatomical\_structure\_development | UTRN | 1688 | 9 | 0.944336 | -0.182458 | 459 | 425.13 | 0.926209 |
| GO:0048856\_anatomical\_structure\_development | NUP50 | 1688 | 9 | 0.944336 | -0.182458 | 459 | 425.13 | 0.926209 |
| GO:0048856\_anatomical\_structure\_development | SMAD4 | 1688 | 9 | 0.944336 | -0.182458 | 459 | 425.13 | 0.926209 |
| GO:0048856\_anatomical\_structure\_development | VCAN | 1688 | 9 | 0.944336 | -0.182458 | 459 | 425.13 | 0.926209 |
| GO:0048856\_anatomical\_structure\_development | NFKB2 | 1688 | 9 | 0.944336 | -0.182458 | 459 | 425.13 | 0.926209 |
| GO:0048856\_anatomical\_structure\_development | MYCN | 1688 | 9 | 0.944336 | -0.182458 | 459 | 425.13 | 0.926209 |
| GO:0048856\_anatomical\_structure\_development | SMARCA4 | 1688 | 9 | 0.944336 | -0.182458 | 459 | 425.13 | 0.926209 |
| GO:0006811\_ion\_transport | ATP2B1 | 186 | 1 | 0.952233 | -0.181324 | 462 | 426.31 | 0.922749 |
| GO:0007155\_cell\_adhesion | ATP5B | 186 | 1 | 0.952233 | -0.181324 | 462 | 426.31 | 0.922749 |
| GO:0022610\_biological\_adhesion | ATP5B | 186 | 1 | 0.952233 | -0.181324 | 462 | 426.31 | 0.922749 |
| GO:0006952\_defense\_response | H47 | 187 | 1 | 0.947141 | -0.180002 | 463 | 427.0 | 0.922246 |
| GO:0007276\_gamete\_generation | MSH2 | 188 | 1 | 0.942103 | -0.178691 | 464 | 428.13 | 0.922694 |
| GO:0048699\_generation\_of\_neurons | CREB1 | 396 | 2 | 0.894522 | -0.175137 | 465 | 429.12 | 0.922839 |
| GO:0048699\_generation\_of\_neurons | SMAD4 | 396 | 2 | 0.894522 | -0.175137 | 465 | 429.12 | 0.922839 |
| GO:0003002\_regionalization | SMAD4 | 195 | 1 | 0.908284 | -0.169827 | 467 | 431.61 | 0.924218 |
| GO:0019725\_cellular\_homeostasis | ACO1 | 195 | 1 | 0.908284 | -0.169827 | 467 | 431.61 | 0.924218 |
| GO:0031175\_neuron\_projection\_development | CREB1 | 197 | 1 | 0.899063 | -0.167390 | 469 | 432.95 | 0.923134 |
| GO:0050801\_ion\_homeostasis | ACO1 | 197 | 1 | 0.899063 | -0.167390 | 469 | 432.95 | 0.923134 |
| GO:0001568\_blood\_vessel\_development | SMARCA4 | 203 | 1 | 0.872490 | -0.160322 | 470 | 435.81 | 0.927255 |
| GO:0007243\_protein\_kinase\_cascade | SPAG9 | 205 | 1 | 0.863977 | -0.158044 | 471 | 437.18 | 0.928195 |
| GO:0001944\_vasculature\_development | SMARCA4 | 208 | 1 | 0.851516 | -0.154697 | 473 | 438.73 | 0.927548 |
| GO:0008284\_positive\_regulation\_of\_cell\_proliferation | MYCN | 208 | 1 | 0.851516 | -0.154697 | 473 | 438.73 | 0.927548 |
| GO:0010604\_positive\_regulation\_of\_macromolecule\_metabolic\_process | SMAD4 | 433 | 2 | 0.818085 | -0.144453 | 474 | 442.27 | 0.933059 |
| GO:0010604\_positive\_regulation\_of\_macromolecule\_metabolic\_process | DSTN | 433 | 2 | 0.818085 | -0.144453 | 474 | 442.27 | 0.933059 |
| GO:0006357\_regulation\_of\_transcription\_from\_RNA\_polymerase\_II\_promoter | SMAD4 | 435 | 2 | 0.814324 | -0.142957 | 475 | 443.29 | 0.933242 |
| GO:0006357\_regulation\_of\_transcription\_from\_RNA\_polymerase\_II\_promoter | SMARCA4 | 435 | 2 | 0.814324 | -0.142957 | 475 | 443.29 | 0.933242 |
| GO:0009887\_organ\_morphogenesis | NUP50 | 642 | 3 | 0.827642 | -0.140471 | 476 | 444.17 | 0.933130 |
| GO:0009887\_organ\_morphogenesis | SMAD4 | 642 | 3 | 0.827642 | -0.140471 | 476 | 444.17 | 0.933130 |
| GO:0009887\_organ\_morphogenesis | SMARCA4 | 642 | 3 | 0.827642 | -0.140471 | 476 | 444.17 | 0.933130 |
| GO:0006464\_protein\_modification\_process | SPAG9 | 439 | 2 | 0.806904 | -0.140011 | 477 | 444.44 | 0.931740 |
| GO:0006464\_protein\_modification\_process | SMAD4 | 439 | 2 | 0.806904 | -0.140011 | 477 | 444.44 | 0.931740 |
| GO:0031325\_positive\_regulation\_of\_cellular\_metabolic\_process | SMAD4 | 442 | 2 | 0.801427 | -0.137842 | 478 | 444.77 | 0.930481 |
| GO:0031325\_positive\_regulation\_of\_cellular\_metabolic\_process | DSTN | 442 | 2 | 0.801427 | -0.137842 | 478 | 444.77 | 0.930481 |
| GO:0006366\_transcription\_from\_RNA\_polymerase\_II\_promoter | SMAD4 | 444 | 2 | 0.797817 | -0.136414 | 479 | 445.35 | 0.929749 |
| GO:0006366\_transcription\_from\_RNA\_polymerase\_II\_promoter | SMARCA4 | 444 | 2 | 0.797817 | -0.136414 | 479 | 445.35 | 0.929749 |
| GO:0019953\_sexual\_reproduction | MSH2 | 228 | 1 | 0.776822 | -0.134353 | 481 | 446.33 | 0.927921 |
| GO:0046649\_lymphocyte\_activation | MSH2 | 228 | 1 | 0.776822 | -0.134353 | 481 | 446.33 | 0.927921 |
| GO:0007167\_enzyme\_linked\_receptor\_protein\_signaling\_pathway | SMAD4 | 229 | 1 | 0.773430 | -0.133419 | 482 | 447.01 | 0.927407 |
| GO:0006996\_organelle\_organization | DSTN | 449 | 2 | 0.788933 | -0.132909 | 483 | 447.61 | 0.926729 |
| GO:0006996\_organelle\_organization | SMARCA4 | 449 | 2 | 0.788933 | -0.132909 | 483 | 447.61 | 0.926729 |
| GO:0050790\_regulation\_of\_catalytic\_activity | SPAG9 | 233 | 1 | 0.760152 | -0.129754 | 484 | 449.78 | 0.929298 |
| GO:0016477\_cell\_migration | ATP5B | 234 | 1 | 0.756903 | -0.128856 | 485 | 450.32 | 0.928495 |
| GO:0009893\_positive\_regulation\_of\_metabolic\_process | SMAD4 | 458 | 2 | 0.773430 | -0.126822 | 486 | 451.19 | 0.928374 |
| GO:0009893\_positive\_regulation\_of\_metabolic\_process | DSTN | 458 | 2 | 0.773430 | -0.126822 | 486 | 451.19 | 0.928374 |
| GO:0030154\_cell\_differentiation | MSH2 | 1060 | 5 | 0.835450 | -0.125809 | 487 | 452.2 | 0.928542 |
| GO:0030154\_cell\_differentiation | CREB1 | 1060 | 5 | 0.835450 | -0.125809 | 487 | 452.2 | 0.928542 |
| GO:0030154\_cell\_differentiation | SMAD4 | 1060 | 5 | 0.835450 | -0.125809 | 487 | 452.2 | 0.928542 |
| GO:0030154\_cell\_differentiation | NFKB2 | 1060 | 5 | 0.835450 | -0.125809 | 487 | 452.2 | 0.928542 |
| GO:0030154\_cell\_differentiation | SMARCA4 | 1060 | 5 | 0.835450 | -0.125809 | 487 | 452.2 | 0.928542 |
| GO:0045321\_leukocyte\_activation | MSH2 | 248 | 1 | 0.714175 | -0.116982 | 488 | 454.38 | 0.931107 |
| GO:0007389\_pattern\_specification\_process | SMAD4 | 250 | 1 | 0.708462 | -0.115387 | 489 | 454.65 | 0.929755 |
| GO:0007267\_cell-cell\_signaling | CREB1 | 252 | 1 | 0.702839 | -0.113816 | 490 | 455.52 | 0.929633 |
| GO:0030097\_hemopoiesis | MSH2 | 253 | 1 | 0.700061 | -0.113040 | 491 | 456.17 | 0.929063 |
| GO:0048878\_chemical\_homeostasis | ACO1 | 254 | 1 | 0.697305 | -0.112269 | 492 | 456.6 | 0.928049 |
| GO:0065008\_regulation\_of\_biological\_quality | ACO1 | 693 | 3 | 0.766733 | -0.111714 | 493 | 456.95 | 0.926876 |
| GO:0065008\_regulation\_of\_biological\_quality | CREB1 | 693 | 3 | 0.766733 | -0.111714 | 493 | 456.95 | 0.926876 |
| GO:0065008\_regulation\_of\_biological\_quality | DSTN | 693 | 3 | 0.766733 | -0.111714 | 493 | 456.95 | 0.926876 |
| GO:0048522\_positive\_regulation\_of\_cellular\_process | CREB1 | 895 | 4 | 0.791577 | -0.111659 | 494 | 457.1 | 0.925304 |
| GO:0048522\_positive\_regulation\_of\_cellular\_process | SMAD4 | 895 | 4 | 0.791577 | -0.111659 | 494 | 457.1 | 0.925304 |
| GO:0048522\_positive\_regulation\_of\_cellular\_process | DSTN | 895 | 4 | 0.791577 | -0.111659 | 494 | 457.1 | 0.925304 |
| GO:0048522\_positive\_regulation\_of\_cellular\_process | MYCN | 895 | 4 | 0.791577 | -0.111659 | 494 | 457.1 | 0.925304 |
| GO:0009966\_regulation\_of\_signal\_transduction | SMAD4 | 256 | 1 | 0.691857 | -0.110745 | 495 | 457.96 | 0.925172 |
| GO:0048870\_cell\_motility | ATP5B | 257 | 1 | 0.689165 | -0.109991 | 496 | 458.31 | 0.924012 |
| GO:0048666\_neuron\_development | CREB1 | 262 | 1 | 0.676013 | -0.106307 | 497 | 460.2 | 0.925956 |
| GO:0030030\_cell\_projection\_organization | CREB1 | 263 | 1 | 0.673443 | -0.105587 | 498 | 460.54 | 0.924779 |
| GO:0050896\_response\_to\_stimulus | MSH2 | 1107 | 5 | 0.799979 | -0.104956 | 499 | 460.73 | 0.923307 |
| GO:0050896\_response\_to\_stimulus | CREB1 | 1107 | 5 | 0.799979 | -0.104956 | 499 | 460.73 | 0.923307 |
| GO:0050896\_response\_to\_stimulus | TIPIN | 1107 | 5 | 0.799979 | -0.104956 | 499 | 460.73 | 0.923307 |
| GO:0050896\_response\_to\_stimulus | NFKB2 | 1107 | 5 | 0.799979 | -0.104956 | 499 | 460.73 | 0.923307 |
| GO:0050896\_response\_to\_stimulus | H47 | 1107 | 5 | 0.799979 | -0.104956 | 499 | 460.73 | 0.923307 |
| GO:0048869\_cellular\_developmental\_process | MSH2 | 1113 | 5 | 0.795667 | -0.102520 | 500 | 461.79 | 0.923580 |
| GO:0048869\_cellular\_developmental\_process | CREB1 | 1113 | 5 | 0.795667 | -0.102520 | 500 | 461.79 | 0.923580 |
| GO:0048869\_cellular\_developmental\_process | SMAD4 | 1113 | 5 | 0.795667 | -0.102520 | 500 | 461.79 | 0.923580 |
| GO:0048869\_cellular\_developmental\_process | NFKB2 | 1113 | 5 | 0.795667 | -0.102520 | 500 | 461.79 | 0.923580 |
| GO:0048869\_cellular\_developmental\_process | SMARCA4 | 1113 | 5 | 0.795667 | -0.102520 | 500 | 461.79 | 0.923580 |
| GO:0045944\_positive\_regulation\_of\_transcription\_from\_RNA\_polymerase\_II\_promoter | SMAD4 | 269 | 1 | 0.658422 | -0.101374 | 501 | 462.08 | 0.922315 |
| GO:0002376\_immune\_system\_process | MSH2 | 505 | 2 | 0.701447 | -0.099219 | 502 | 463.18 | 0.922669 |
| GO:0002376\_immune\_system\_process | NFKB2 | 505 | 2 | 0.701447 | -0.099219 | 502 | 463.18 | 0.922669 |
| GO:0032502\_developmental\_process | ACO1 | 2060 | 10 | 0.859783 | -0.097180 | 503 | 464.42 | 0.923300 |
| GO:0032502\_developmental\_process | MSH2 | 2060 | 10 | 0.859783 | -0.097180 | 503 | 464.42 | 0.923300 |
| GO:0032502\_developmental\_process | CREB1 | 2060 | 10 | 0.859783 | -0.097180 | 503 | 464.42 | 0.923300 |
| GO:0032502\_developmental\_process | UTRN | 2060 | 10 | 0.859783 | -0.097180 | 503 | 464.42 | 0.923300 |
| GO:0032502\_developmental\_process | NUP50 | 2060 | 10 | 0.859783 | -0.097180 | 503 | 464.42 | 0.923300 |
| GO:0032502\_developmental\_process | SMAD4 | 2060 | 10 | 0.859783 | -0.097180 | 503 | 464.42 | 0.923300 |
| GO:0032502\_developmental\_process | VCAN | 2060 | 10 | 0.859783 | -0.097180 | 503 | 464.42 | 0.923300 |
| GO:0032502\_developmental\_process | NFKB2 | 2060 | 10 | 0.859783 | -0.097180 | 503 | 464.42 | 0.923300 |
| GO:0032502\_developmental\_process | MYCN | 2060 | 10 | 0.859783 | -0.097180 | 503 | 464.42 | 0.923300 |
| GO:0032502\_developmental\_process | SMARCA4 | 2060 | 10 | 0.859783 | -0.097180 | 503 | 464.42 | 0.923300 |
| GO:0003008\_system\_process | GNAI3 | 516 | 2 | 0.686494 | -0.093659 | 504 | 466.43 | 0.925456 |
| GO:0003008\_system\_process | ACO1 | 516 | 2 | 0.686494 | -0.093659 | 504 | 466.43 | 0.925456 |
| GO:0006629\_lipid\_metabolic\_process | ATP5B | 285 | 1 | 0.621457 | -0.091006 | 505 | 467.76 | 0.926257 |
| GO:0040011\_locomotion | ATP5B | 295 | 1 | 0.600391 | -0.085109 | 507 | 470.19 | 0.927396 |
| GO:0045595\_regulation\_of\_cell\_differentiation | SMAD4 | 295 | 1 | 0.600391 | -0.085109 | 507 | 470.19 | 0.927396 |
| GO:0045893\_positive\_regulation\_of\_transcription\_\_DNA-dependent | SMAD4 | 306 | 1 | 0.578808 | -0.079090 | 509 | 471.98 | 0.927269 |
| GO:0051254\_positive\_regulation\_of\_RNA\_metabolic\_process | SMAD4 | 306 | 1 | 0.578808 | -0.079090 | 509 | 471.98 | 0.927269 |
| GO:0051094\_positive\_regulation\_of\_developmental\_process | SMAD4 | 308 | 1 | 0.575050 | -0.078045 | 510 | 472.91 | 0.927275 |
| GO:0048518\_positive\_regulation\_of\_biological\_process | CREB1 | 995 | 4 | 0.712022 | -0.073106 | 511 | 474.47 | 0.928513 |
| GO:0048518\_positive\_regulation\_of\_biological\_process | SMAD4 | 995 | 4 | 0.712022 | -0.073106 | 511 | 474.47 | 0.928513 |
| GO:0048518\_positive\_regulation\_of\_biological\_process | MYCN | 995 | 4 | 0.712022 | -0.073106 | 511 | 474.47 | 0.928513 |
| GO:0048518\_positive\_regulation\_of\_biological\_process | DSTN | 995 | 4 | 0.712022 | -0.073106 | 511 | 474.47 | 0.928513 |
| GO:0051093\_negative\_regulation\_of\_developmental\_process | MSH2 | 331 | 1 | 0.535092 | -0.067021 | 512 | 476.88 | 0.931406 |
| GO:0045941\_positive\_regulation\_of\_transcription | SMAD4 | 338 | 1 | 0.524010 | -0.063999 | 513 | 478.43 | 0.932612 |
| GO:0009605\_response\_to\_external\_stimulus | H47 | 339 | 1 | 0.522464 | -0.063579 | 514 | 478.69 | 0.931304 |
| GO:0007166\_cell\_surface\_receptor\_linked\_signal\_transduction | GNAI3 | 597 | 2 | 0.593351 | -0.061015 | 515 | 480.68 | 0.933359 |
| GO:0007166\_cell\_surface\_receptor\_linked\_signal\_transduction | SMAD4 | 597 | 2 | 0.593351 | -0.061015 | 515 | 480.68 | 0.933359 |
| GO:0010628\_positive\_regulation\_of\_gene\_expression | SMAD4 | 346 | 1 | 0.511894 | -0.060718 | 516 | 480.98 | 0.932132 |
| GO:0045935\_positive\_regulation\_of\_nucleobase\_\_nucleoside\_\_nucleotide\_and\_nucleic\_acid\_metabolic\_process | SMAD4 | 352 | 1 | 0.503169 | -0.058372 | 517 | 481.73 | 0.931779 |
| GO:0042981\_regulation\_of\_apoptosis | MSH2 | 360 | 1 | 0.491987 | -0.055389 | 518 | 482.25 | 0.930985 |
| GO:0051173\_positive\_regulation\_of\_nitrogen\_compound\_metabolic\_process | SMAD4 | 361 | 1 | 0.490624 | -0.055027 | 519 | 482.73 | 0.930116 |
| GO:0010941\_regulation\_of\_cell\_death | MSH2 | 365 | 1 | 0.485248 | -0.053605 | 521 | 483.74 | 0.928484 |
| GO:0043067\_regulation\_of\_programmed\_cell\_death | MSH2 | 365 | 1 | 0.485248 | -0.053605 | 521 | 483.74 | 0.928484 |
| GO:0010557\_positive\_regulation\_of\_macromolecule\_biosynthetic\_process | SMAD4 | 371 | 1 | 0.477400 | -0.051541 | 522 | 484.52 | 0.928199 |
| GO:0031328\_positive\_regulation\_of\_cellular\_biosynthetic\_process | SMAD4 | 387 | 1 | 0.457662 | -0.046427 | 523 | 486.42 | 0.930057 |
| GO:0009891\_positive\_regulation\_of\_biosynthetic\_process | SMAD4 | 388 | 1 | 0.456483 | -0.046125 | 524 | 486.67 | 0.928760 |
| GO:0042221\_response\_to\_chemical\_stimulus | CREB1 | 409 | 1 | 0.433045 | -0.040225 | 525 | 488.9 | 0.931238 |
| GO:0042592\_homeostatic\_process | ACO1 | 419 | 1 | 0.422710 | -0.037690 | 526 | 489.71 | 0.931008 |
| GO:0006915\_apoptosis | MSH2 | 427 | 1 | 0.414790 | -0.035778 | 527 | 490.72 | 0.931157 |
| GO:0012501\_programmed\_cell\_death | MSH2 | 433 | 1 | 0.409042 | -0.034408 | 528 | 491.52 | 0.930909 |
| GO:0050793\_regulation\_of\_developmental\_process | MSH2 | 703 | 2 | 0.503884 | -0.034338 | 529 | 491.65 | 0.929395 |
| GO:0050793\_regulation\_of\_developmental\_process | SMAD4 | 703 | 2 | 0.503884 | -0.034338 | 529 | 491.65 | 0.929395 |
| GO:0008219\_cell\_death | MSH2 | 444 | 1 | 0.398909 | -0.032032 | 530 | 492.87 | 0.929943 |
| GO:0016265\_death | MSH2 | 450 | 1 | 0.393590 | -0.030806 | 531 | 493.7 | 0.929755 |
| GO:0008150\_biological\_process | GNAI3 | 4605 | 26 | 1.000000 | 0.000000 | 1725 | 1718.88 | 0.996452 |
| GO:0008150\_biological\_process | ATP5B | 4605 | 26 | 1.000000 | 0.000000 | 1725 | 1718.88 | 0.996452 |
| GO:0008150\_biological\_process | UTRN | 4605 | 26 | 1.000000 | 0.000000 | 1725 | 1718.88 | 0.996452 |
| GO:0008150\_biological\_process | TIPIN | 4605 | 26 | 1.000000 | 0.000000 | 1725 | 1718.88 | 0.996452 |
| GO:0008150\_biological\_process | NFKB2 | 4605 | 26 | 1.000000 | 0.000000 | 1725 | 1718.88 | 0.996452 |
| GO:0008150\_biological\_process | DSTN | 4605 | 26 | 1.000000 | 0.000000 | 1725 | 1718.88 | 0.996452 |
| GO:0008150\_biological\_process | ATP2B1 | 4605 | 26 | 1.000000 | 0.000000 | 1725 | 1718.88 | 0.996452 |
| GO:0008150\_biological\_process | OXCT1 | 4605 | 26 | 1.000000 | 0.000000 | 1725 | 1718.88 | 0.996452 |
| GO:0008150\_biological\_process | NUP50 | 4605 | 26 | 1.000000 | 0.000000 | 1725 | 1718.88 | 0.996452 |
| GO:0008150\_biological\_process | PTBP2 | 4605 | 26 | 1.000000 | 0.000000 | 1725 | 1718.88 | 0.996452 |
| GO:0008150\_biological\_process | ACO1 | 4605 | 26 | 1.000000 | 0.000000 | 1725 | 1718.88 | 0.996452 |
| GO:0008150\_biological\_process | MSH2 | 4605 | 26 | 1.000000 | 0.000000 | 1725 | 1718.88 | 0.996452 |
| GO:0008150\_biological\_process | CREB1 | 4605 | 26 | 1.000000 | 0.000000 | 1725 | 1718.88 | 0.996452 |
| GO:0008150\_biological\_process | SMAD4 | 4605 | 26 | 1.000000 | 0.000000 | 1725 | 1718.88 | 0.996452 |
| GO:0008150\_biological\_process | MYCN | 4605 | 26 | 1.000000 | 0.000000 | 1725 | 1718.88 | 0.996452 |
| GO:0008150\_biological\_process | SPAG9 | 4605 | 26 | 1.000000 | 0.000000 | 1725 | 1718.88 | 0.996452 |
| GO:0008150\_biological\_process | EIF4G2 | 4605 | 26 | 1.000000 | 0.000000 | 1725 | 1718.88 | 0.996452 |
| GO:0008150\_biological\_process | YWHAG | 4605 | 26 | 1.000000 | 0.000000 | 1725 | 1718.88 | 0.996452 |
| GO:0008150\_biological\_process | RAB5A | 4605 | 26 | 1.000000 | 0.000000 | 1725 | 1718.88 | 0.996452 |
| GO:0008150\_biological\_process | DNMT1 | 4605 | 26 | 1.000000 | 0.000000 | 1725 | 1718.88 | 0.996452 |
| GO:0008150\_biological\_process | SDCBP | 4605 | 26 | 1.000000 | 0.000000 | 1725 | 1718.88 | 0.996452 |
| GO:0008150\_biological\_process | VCAN | 4605 | 26 | 1.000000 | 0.000000 | 1725 | 1718.88 | 0.996452 |
| GO:0008150\_biological\_process | RBM39 | 4605 | 26 | 1.000000 | 0.000000 | 1725 | 1718.88 | 0.996452 |
| GO:0008150\_biological\_process | TMPO | 4605 | 26 | 1.000000 | 0.000000 | 1725 | 1718.88 | 0.996452 |
| GO:0008150\_biological\_process | H47 | 4605 | 26 | 1.000000 | 0.000000 | 1725 | 1718.88 | 0.996452 |
| GO:0008150\_biological\_process | SMARCA4 | 4605 | 26 | 1.000000 | 0.000000 | 1725 | 1718.88 | 0.996452 |
