# Supplementary material for: RedundancyMiner: De-replication of redundant GO categories in microarray and proteomics analysis
Source: BMC Bioinformatics. 2011 Feb 10;12:52. doi: 10.1186/1471-2105-12-52 (PMC3223614; doi:10.1186/1471-2105-12-52)
Supplement: Additional file 8 — Retinal development HTGM download. compressed package of the results of running HTGM on the retinal development genes list. [file 1471-2105-12-52-S8.ZIP › SCENARIO_2_MODIFIED/total.txt.total.txt.dir/Exp1_BestClusterMap_LEIGS_KM_24.csv.join.1.txt.dir/Exp1_BestClusterMap_LEIGS_KM_24.csv.join.1.txt.change.html]

Category Summary Report for Exp1\_BestClusterMap\_LEIGS\_KM\_24.csv.join.1.txt

# Category Summary Report for Exp1\_BestClusterMap\_LEIGS\_KM\_24.csv.join.1.txt

| HYPERLINKED GO CATEGORY | TOTAL GENES | CHANGED GENES | ENRICHMENT | LOG10(p) | CUMULATIVE NUMBER OF CATEGORIES | CUMULATIVE RANDOMS LOWER BOUND | CUMULATIVE RANDOMS MEAN | CUMULATIVE RANDOMS UPPER BOUND | FALSE DISCOVERY RATE |
| --- | --- | --- | --- | --- | --- | --- | --- | --- | --- |
| GO:0000077\_DNA\_damage\_checkpoint | 14 | 2 | 25.302198 | -2.572529 | 1 | -1.610103 | 2.94 | 7.490103 | 2.940000 |
| GO:0031570\_DNA\_integrity\_checkpoint | 16 | 2 | 22.139423 | -2.455404 | 2 | -1.516192 | 3.52 | 8.556192 | 1.760000 |
| GO:0043933\_macromolecular\_complex\_subunit\_organization | 117 | 4 | 6.055227 | -2.415684 | 3 | -1.514178 | 3.66 | 8.834178 | 1.220000 |
| GO:0016458\_gene\_silencing | 18 | 2 | 19.679487 | -2.352906 | 4 | -1.490100 | 4.3 | 10.090100 | 1.075000 |
| GO:0006933\_negative\_regulation\_of\_cell\_adhesion\_involved\_in\_substrate-bound\_cell\_migration | 1 | 1 |  |  |  |  |  |  |  |  |
| GO:0033158\_regulation\_of\_protein\_import\_into\_nucleus\_\_translocation | 1 | 1 |  |  |  |  |  |  |  |  |
| GO:0033160\_positive\_regulation\_of\_protein\_import\_into\_nucleus\_\_translocation | 1 | 1 |  |  |  |  |  |  |  |  |
| GO:0060390\_regulation\_of\_SMAD\_protein\_nuclear\_translocation | 1 | 1 |  |  |  |  |  |  |  |  |
| GO:0060391\_positive\_regulation\_of\_SMAD\_protein\_nuclear\_translocation | 1 | 1 |  |  |  |  |  |  |  |  |
| GO:0000075\_cell\_cycle\_checkpoint | 21 | 2 | 16.868132 | -2.219893 | 5 | -1.534263 | 5.47 | 12.474263 | 1.094000 |
| GO:0034621\_cellular\_macromolecular\_complex\_subunit\_organization | 76 | 3 | 6.991397 | -2.068016 | 6 | -1.525313 | 7.24 | 16.005313 | 1.206667 |
| GO:0040029\_regulation\_of\_gene\_expression\_\_epigenetic | 26 | 2 | 13.624260 | -2.037746 | 7 | -1.669821 | 7.58 | 16.829821 | 1.082857 |
| GO:0032268\_regulation\_of\_cellular\_protein\_metabolic\_process | 152 | 4 | 4.660931 | -2.014135 | 8 | -1.738666 | 7.76 | 17.258666 | 0.970000 |
| GO:0009411\_response\_to\_UV | 27 | 2 | 13.119658 | -2.005824 | 9 | -1.804096 | 7.93 | 17.664096 | 0.881111 |
| GO:0002266\_follicular\_dendritic\_cell\_activation | 2 | 1 |  |  |  |  |  |  |  |  |
| GO:0002268\_follicular\_dendritic\_cell\_differentiation | 2 | 1 |  |  |  |  |  |  |  |  |
| GO:0007527\_adult\_somatic\_muscle\_development | 2 | 1 |  |  |  |  |  |  |  |  |
| GO:0010718\_positive\_regulation\_of\_epithelial\_to\_mesenchymal\_transition | 2 | 1 |  |  |  |  |  |  |  |  |
| GO:0010770\_positive\_regulation\_of\_cell\_morphogenesis\_involved\_in\_differentiation | 2 | 1 |  |  |  |  |  |  |  |  |
| GO:0010862\_positive\_regulation\_of\_pathway-restricted\_SMAD\_protein\_phosphorylation | 2 | 1 |  |  |  |  |  |  |  |  |
| GO:0031573\_intra-S\_DNA\_damage\_checkpoint | 2 | 1 |  |  |  |  |  |  |  |  |
| GO:0046950\_cellular\_ketone\_body\_metabolic\_process | 2 | 1 |  |  |  |  |  |  |  |  |
| GO:0060393\_regulation\_of\_pathway-restricted\_SMAD\_protein\_phosphorylation | 2 | 1 |  |  |  |  |  |  |  |  |
| GO:0006417\_regulation\_of\_translation | 29 | 2 | 12.214854 | -1.945608 | 11 | -2.109406 | 8.85 | 19.809406 | 0.804545 |
| GO:0042770\_DNA\_damage\_response\_\_signal\_transduction | 29 | 2 | 12.214854 | -1.945608 | 11 | -2.109406 | 8.85 | 19.809406 | 0.804545 |
| GO:0051246\_regulation\_of\_protein\_metabolic\_process | 170 | 4 | 4.167421 | -1.847846 | 12 | -1.913336 | 10.5 | 22.913336 | 0.875000 |
| GO:0065003\_macromolecular\_complex\_assembly | 93 | 3 | 5.713400 | -1.829329 | 13 | -2.101022 | 11.02 | 24.141022 | 0.847692 |
| GO:0051052\_regulation\_of\_DNA\_metabolic\_process | 34 | 2 | 10.418552 | -1.812670 | 14 | -1.920286 | 11.42 | 24.760286 | 0.815714 |
| GO:0006301\_postreplication\_repair | 3 | 1 |  |  |  |  |  |  |  |  |
| GO:0007403\_glial\_cell\_fate\_determination | 3 | 1 |  |  |  |  |  |  |  |  |
| GO:0007525\_somatic\_muscle\_development | 3 | 1 |  |  |  |  |  |  |  |  |
| GO:0010216\_maintenance\_of\_DNA\_methylation | 3 | 1 |  |  |  |  |  |  |  |  |
| GO:0010717\_regulation\_of\_epithelial\_to\_mesenchymal\_transition | 3 | 1 |  |  |  |  |  |  |  |  |
| GO:0030836\_positive\_regulation\_of\_actin\_filament\_depolymerization | 3 | 1 |  |  |  |  |  |  |  |  |
| GO:0043243\_positive\_regulation\_of\_protein\_complex\_disassembly | 3 | 1 |  |  |  |  |  |  |  |  |
| GO:0044030\_regulation\_of\_DNA\_methylation | 3 | 1 |  |  |  |  |  |  |  |  |
| GO:0001835\_blastocyst\_hatching | 4 | 1 |  |  |  |  |  |  |  |  |
| GO:0006334\_nucleosome\_assembly | 4 | 1 |  |  |  |  |  |  |  |  |
| GO:0007184\_SMAD\_protein\_nuclear\_translocation | 4 | 1 |  |  |  |  |  |  |  |  |
| GO:0010224\_response\_to\_UV-B | 4 | 1 |  |  |  |  |  |  |  |  |
| GO:0032715\_negative\_regulation\_of\_interleukin-6\_production | 4 | 1 |  |  |  |  |  |  |  |  |
| GO:0035188\_hatching | 4 | 1 |  |  |  |  |  |  |  |  |
| GO:0006346\_methylation-dependent\_chromatin\_silencing | 5 | 1 | 35.423077 | -1.554004 | 20 | 4.462554 | 26.17 | 47.877446 | 1.308500 |
| GO:0006376\_mRNA\_splice\_site\_selection | 5 | 1 | 35.423077 | -1.554004 | 20 | 4.462554 | 26.17 | 47.877446 | 1.308500 |
| GO:0006929\_substrate-bound\_cell\_migration | 5 | 1 | 35.423077 | -1.554004 | 20 | 4.462554 | 26.17 | 47.877446 | 1.308500 |
| GO:0030042\_actin\_filament\_depolymerization | 5 | 1 | 35.423077 | -1.554004 | 20 | 4.462554 | 26.17 | 47.877446 | 1.308500 |
| GO:0030834\_regulation\_of\_actin\_filament\_depolymerization | 5 | 1 | 35.423077 | -1.554004 | 20 | 4.462554 | 26.17 | 47.877446 | 1.308500 |
| GO:0032720\_negative\_regulation\_of\_tumor\_necrosis\_factor\_production | 5 | 1 | 35.423077 | -1.554004 | 20 | 4.462554 | 26.17 | 47.877446 | 1.308500 |
| GO:0000245\_spliceosome\_assembly | 6 | 1 | 29.519231 | -1.476000 | 23 | 9.634408 | 34.76 | 59.885592 | 1.511304 |
| GO:0060389\_pathway-restricted\_SMAD\_protein\_phosphorylation | 6 | 1 | 29.519231 | -1.476000 | 23 | 9.634408 | 34.76 | 59.885592 | 1.511304 |
| GO:0065004\_protein-DNA\_complex\_assembly | 6 | 1 | 29.519231 | -1.476000 | 23 | 9.634408 | 34.76 | 59.885592 | 1.511304 |
| GO:0010608\_posttranscriptional\_regulation\_of\_gene\_expression | 52 | 2 | 6.812130 | -1.465995 | 24 | 9.601591 | 34.96 | 60.318409 | 1.456667 |
| GO:0006412\_translation | 54 | 2 | 6.559829 | -1.435879 | 25 | 10.114383 | 36.24 | 62.365617 | 1.449600 |
| GO:0006119\_oxidative\_phosphorylation | 7 | 1 | 25.302198 | -1.410229 | 30 | 15.444612 | 43.88 | 72.315388 | 1.462667 |
| GO:0006298\_mismatch\_repair | 7 | 1 | 25.302198 | -1.410229 | 30 | 15.444612 | 43.88 | 72.315388 | 1.462667 |
| GO:0008340\_determination\_of\_adult\_lifespan | 7 | 1 | 25.302198 | -1.410229 | 30 | 15.444612 | 43.88 | 72.315388 | 1.462667 |
| GO:0022618\_ribonucleoprotein\_complex\_assembly | 7 | 1 | 25.302198 | -1.410229 | 30 | 15.444612 | 43.88 | 72.315388 | 1.462667 |
| GO:0031497\_chromatin\_assembly | 7 | 1 | 25.302198 | -1.410229 | 30 | 15.444612 | 43.88 | 72.315388 | 1.462667 |
| GO:0034622\_cellular\_macromolecular\_complex\_assembly | 58 | 2 | 6.107427 | -1.379201 | 31 | 16.157372 | 45.4 | 74.642628 | 1.464516 |
| GO:0031323\_regulation\_of\_cellular\_metabolic\_process | 1015 | 10 | 1.744979 | -1.368854 | 32 | 16.370718 | 45.82 | 75.269282 | 1.431875 |
| GO:0000910\_cytokinesis | 8 | 1 | 22.139423 | -1.353412 | 39 | 19.812953 | 51.19 | 82.567047 | 1.312564 |
| GO:0002566\_somatic\_diversification\_of\_immune\_receptors\_via\_somatic\_mutation | 8 | 1 | 22.139423 | -1.353412 | 39 | 19.812953 | 51.19 | 82.567047 | 1.312564 |
| GO:0016446\_somatic\_hypermutation\_of\_immunoglobulin\_genes | 8 | 1 | 22.139423 | -1.353412 | 39 | 19.812953 | 51.19 | 82.567047 | 1.312564 |
| GO:0021781\_glial\_cell\_fate\_commitment | 8 | 1 | 22.139423 | -1.353412 | 39 | 19.812953 | 51.19 | 82.567047 | 1.312564 |
| GO:0034728\_nucleosome\_organization | 8 | 1 | 22.139423 | -1.353412 | 39 | 19.812953 | 51.19 | 82.567047 | 1.312564 |
| GO:0045910\_negative\_regulation\_of\_DNA\_recombination | 8 | 1 | 22.139423 | -1.353412 | 39 | 19.812953 | 51.19 | 82.567047 | 1.312564 |
| GO:0060347\_heart\_trabecula\_formation | 8 | 1 | 22.139423 | -1.353412 | 39 | 19.812953 | 51.19 | 82.567047 | 1.312564 |
| GO:0035239\_tube\_morphogenesis | 143 | 3 | 3.715707 | -1.344417 | 40 | 19.835265 | 51.22 | 82.604735 | 1.280500 |
| GO:0032270\_positive\_regulation\_of\_cellular\_protein\_metabolic\_process | 61 | 2 | 5.807062 | -1.339485 | 41 | 19.987717 | 51.49 | 82.992283 | 1.255854 |
| GO:0010165\_response\_to\_X-ray | 9 | 1 | 19.679487 | -1.303434 | 43 | 24.230544 | 58.56 | 92.889456 | 1.361860 |
| GO:0032388\_positive\_regulation\_of\_intracellular\_transport | 9 | 1 | 19.679487 | -1.303434 | 43 | 24.230544 | 58.56 | 92.889456 | 1.361860 |
| GO:0051130\_positive\_regulation\_of\_cellular\_component\_organization | 66 | 2 | 5.367133 | -1.277935 | 44 | 24.772016 | 59.97 | 95.167984 | 1.362955 |
| GO:0010467\_gene\_expression | 905 | 9 | 1.761368 | -1.273399 | 45 | 24.832339 | 60.08 | 95.327661 | 1.335111 |
| GO:0051247\_positive\_regulation\_of\_protein\_metabolic\_process | 67 | 2 | 5.287026 | -1.266258 | 46 | 24.998408 | 60.4 | 95.801592 | 1.313043 |
| GO:0001832\_blastocyst\_growth | 10 | 1 | 17.711538 | -1.258850 | 52 | 29.034910 | 66.8 | 104.565090 | 1.284615 |
| GO:0006342\_chromatin\_silencing | 10 | 1 | 17.711538 | -1.258850 | 52 | 29.034910 | 66.8 | 104.565090 | 1.284615 |
| GO:0045814\_negative\_regulation\_of\_gene\_expression\_\_epigenetic | 10 | 1 | 17.711538 | -1.258850 | 52 | 29.034910 | 66.8 | 104.565090 | 1.284615 |
| GO:0046887\_positive\_regulation\_of\_hormone\_secretion | 10 | 1 | 17.711538 | -1.258850 | 52 | 29.034910 | 66.8 | 104.565090 | 1.284615 |
| GO:0050892\_intestinal\_absorption | 10 | 1 | 17.711538 | -1.258850 | 52 | 29.034910 | 66.8 | 104.565090 | 1.284615 |
| GO:0060343\_trabecula\_formation | 10 | 1 | 17.711538 | -1.258850 | 52 | 29.034910 | 66.8 | 104.565090 | 1.284615 |
| GO:0006913\_nucleocytoplasmic\_transport | 71 | 2 | 4.989166 | -1.221451 | 53 | 29.931776 | 68.83 | 107.728224 | 1.298679 |
| GO:0001837\_epithelial\_to\_mesenchymal\_transition | 11 | 1 | 16.101399 | -1.218630 | 58 | 33.596150 | 74.35 | 115.103850 | 1.281897 |
| GO:0002467\_germinal\_center\_formation | 11 | 1 | 16.101399 | -1.218630 | 58 | 33.596150 | 74.35 | 115.103850 | 1.281897 |
| GO:0006333\_chromatin\_assembly\_or\_disassembly | 11 | 1 | 16.101399 | -1.218630 | 58 | 33.596150 | 74.35 | 115.103850 | 1.281897 |
| GO:0007162\_negative\_regulation\_of\_cell\_adhesion | 11 | 1 | 16.101399 | -1.218630 | 58 | 33.596150 | 74.35 | 115.103850 | 1.281897 |
| GO:0010259\_multicellular\_organismal\_aging | 11 | 1 | 16.101399 | -1.218630 | 58 | 33.596150 | 74.35 | 115.103850 | 1.281897 |
| GO:0080090\_regulation\_of\_primary\_metabolic\_process | 926 | 9 | 1.721424 | -1.218337 | 59 | 33.590808 | 74.37 | 115.149192 | 1.260508 |
| GO:0051169\_nuclear\_transport | 72 | 2 | 4.919872 | -1.210698 | 60 | 33.908693 | 75.18 | 116.451307 | 1.253000 |
| GO:0034960\_cellular\_biopolymer\_metabolic\_process | 1395 | 12 | 1.523573 | -1.194156 | 61 | 34.190381 | 76.16 | 118.129619 | 1.248525 |
| GO:0060255\_regulation\_of\_macromolecule\_metabolic\_process | 936 | 9 | 1.703033 | -1.192869 | 62 | 34.187187 | 76.19 | 118.192813 | 1.228871 |
| GO:0009416\_response\_to\_light\_stimulus | 74 | 2 | 4.786902 | -1.189696 | 63 | 34.443789 | 76.46 | 118.476211 | 1.213651 |
| GO:0019222\_regulation\_of\_metabolic\_process | 1088 | 10 | 1.627899 | -1.185920 | 64 | 34.501677 | 76.54 | 118.578323 | 1.195938 |
| GO:0000375\_RNA\_splicing\_\_via\_transesterification\_reactions | 12 | 1 | 14.759615 | -1.182013 | 72 | 38.294996 | 82.26 | 126.225004 | 1.142500 |
| GO:0000377\_RNA\_splicing\_\_via\_transesterification\_reactions\_with\_bulged\_adenosine\_as\_nucleophile | 12 | 1 | 14.759615 | -1.182013 | 72 | 38.294996 | 82.26 | 126.225004 | 1.142500 |
| GO:0000398\_nuclear\_mRNA\_splicing\_\_via\_spliceosome | 12 | 1 | 14.759615 | -1.182013 | 72 | 38.294996 | 82.26 | 126.225004 | 1.142500 |
| GO:0006413\_translational\_initiation | 12 | 1 | 14.759615 | -1.182013 | 72 | 38.294996 | 82.26 | 126.225004 | 1.142500 |
| GO:0006446\_regulation\_of\_translational\_initiation | 12 | 1 | 14.759615 | -1.182013 | 72 | 38.294996 | 82.26 | 126.225004 | 1.142500 |
| GO:0006879\_cellular\_iron\_ion\_homeostasis | 12 | 1 | 14.759615 | -1.182013 | 72 | 38.294996 | 82.26 | 126.225004 | 1.142500 |
| GO:0043624\_cellular\_protein\_complex\_disassembly | 12 | 1 | 14.759615 | -1.182013 | 72 | 38.294996 | 82.26 | 126.225004 | 1.142500 |
| GO:0051261\_protein\_depolymerization | 12 | 1 | 14.759615 | -1.182013 | 72 | 38.294996 | 82.26 | 126.225004 | 1.142500 |
| GO:0051050\_positive\_regulation\_of\_transport | 75 | 2 | 4.723077 | -1.179438 | 73 | 38.434115 | 82.52 | 126.605885 | 1.130411 |
| GO:0034961\_cellular\_biopolymer\_biosynthetic\_process | 804 | 8 | 1.762342 | -1.159232 | 74 | 39.006547 | 83.65 | 128.293453 | 1.130405 |
| GO:0043284\_biopolymer\_biosynthetic\_process | 807 | 8 | 1.755791 | -1.151298 | 75 | 39.030709 | 83.76 | 128.489291 | 1.116800 |
| GO:0043241\_protein\_complex\_disassembly | 13 | 1 | 13.624260 | -1.148422 | 78 | 42.788372 | 89.42 | 136.051628 | 1.146410 |
| GO:0043244\_regulation\_of\_protein\_complex\_disassembly | 13 | 1 | 13.624260 | -1.148422 | 78 | 42.788372 | 89.42 | 136.051628 | 1.146410 |
| GO:0051495\_positive\_regulation\_of\_cytoskeleton\_organization | 13 | 1 | 13.624260 | -1.148422 | 78 | 42.788372 | 89.42 | 136.051628 | 1.146410 |
| GO:0002250\_adaptive\_immune\_response | 80 | 2 | 4.427885 | -1.130407 | 80 | 43.416748 | 90.67 | 137.923252 | 1.133375 |
| GO:0002460\_adaptive\_immune\_response\_based\_on\_somatic\_recombination\_of\_immune\_receptors\_built\_from\_immunoglobulin\_superfamily\_domains | 80 | 2 | 4.427885 | -1.130407 | 80 | 43.416748 | 90.67 | 137.923252 | 1.133375 |
| GO:0000060\_protein\_import\_into\_nucleus\_\_translocation | 14 | 1 | 12.651099 | -1.117407 | 88 | 47.117557 | 96.42 | 145.722443 | 1.095682 |
| GO:0006304\_DNA\_modification | 14 | 1 | 12.651099 | -1.117407 | 88 | 47.117557 | 96.42 | 145.722443 | 1.095682 |
| GO:0006305\_DNA\_alkylation | 14 | 1 | 12.651099 | -1.117407 | 88 | 47.117557 | 96.42 | 145.722443 | 1.095682 |
| GO:0006306\_DNA\_methylation | 14 | 1 | 12.651099 | -1.117407 | 88 | 47.117557 | 96.42 | 145.722443 | 1.095682 |
| GO:0008064\_regulation\_of\_actin\_polymerization\_or\_depolymerization | 14 | 1 | 12.651099 | -1.117407 | 88 | 47.117557 | 96.42 | 145.722443 | 1.095682 |
| GO:0030832\_regulation\_of\_actin\_filament\_length | 14 | 1 | 12.651099 | -1.117407 | 88 | 47.117557 | 96.42 | 145.722443 | 1.095682 |
| GO:0034623\_cellular\_macromolecular\_complex\_disassembly | 14 | 1 | 12.651099 | -1.117407 | 88 | 47.117557 | 96.42 | 145.722443 | 1.095682 |
| GO:0051053\_negative\_regulation\_of\_DNA\_metabolic\_process | 14 | 1 | 12.651099 | -1.117407 | 88 | 47.117557 | 96.42 | 145.722443 | 1.095682 |
| GO:0030198\_extracellular\_matrix\_organization | 83 | 2 | 4.267841 | -1.102663 | 89 | 47.646362 | 97.29 | 146.933638 | 1.093146 |
| GO:0022600\_digestive\_system\_process | 15 | 1 | 11.807692 | -1.088613 | 92 | 51.715400 | 102.8 | 153.884600 | 1.117391 |
| GO:0035116\_embryonic\_hindlimb\_morphogenesis | 15 | 1 | 11.807692 | -1.088613 | 92 | 51.715400 | 102.8 | 153.884600 | 1.117391 |
| GO:0042306\_regulation\_of\_protein\_import\_into\_nucleus | 15 | 1 | 11.807692 | -1.088613 | 92 | 51.715400 | 102.8 | 153.884600 | 1.117391 |
| GO:0044260\_cellular\_macromolecule\_metabolic\_process | 1447 | 12 | 1.468821 | -1.086454 | 93 | 51.694256 | 102.91 | 154.125744 | 1.106559 |
| GO:0006605\_protein\_targeting | 86 | 2 | 4.118962 | -1.076063 | 94 | 52.431372 | 103.87 | 155.308628 | 1.105000 |
| GO:0032956\_regulation\_of\_actin\_cytoskeleton\_organization | 16 | 1 | 11.069712 | -1.061752 | 95 | 55.434812 | 108.69 | 161.945188 | 1.144105 |
| GO:0048754\_branching\_morphogenesis\_of\_a\_tube | 88 | 2 | 4.025350 | -1.058927 | 96 | 55.497772 | 108.88 | 162.262228 | 1.134167 |
| GO:0006323\_DNA\_packaging | 17 | 1 | 10.418552 | -1.036590 | 101 | 59.619905 | 114.43 | 169.240095 | 1.132970 |
| GO:0008380\_RNA\_splicing | 17 | 1 | 10.418552 | -1.036590 | 101 | 59.619905 | 114.43 | 169.240095 | 1.132970 |
| GO:0032535\_regulation\_of\_cellular\_component\_size | 17 | 1 | 10.418552 | -1.036590 | 101 | 59.619905 | 114.43 | 169.240095 | 1.132970 |
| GO:0032970\_regulation\_of\_actin\_filament-based\_process | 17 | 1 | 10.418552 | -1.036590 | 101 | 59.619905 | 114.43 | 169.240095 | 1.132970 |
| GO:0055072\_iron\_ion\_homeostasis | 17 | 1 | 10.418552 | -1.036590 | 101 | 59.619905 | 114.43 | 169.240095 | 1.132970 |
| GO:0046907\_intracellular\_transport | 194 | 3 | 2.738898 | -1.026275 | 102 | 59.882291 | 114.97 | 170.057709 | 1.127157 |
| GO:0001818\_negative\_regulation\_of\_cytokine\_production | 18 | 1 | 9.839744 | -1.012933 | 108 | 63.739205 | 120.5 | 177.260795 | 1.115741 |
| GO:0032984\_macromolecular\_complex\_disassembly | 18 | 1 | 9.839744 | -1.012933 | 108 | 63.739205 | 120.5 | 177.260795 | 1.115741 |
| GO:0033157\_regulation\_of\_intracellular\_protein\_transport | 18 | 1 | 9.839744 | -1.012933 | 108 | 63.739205 | 120.5 | 177.260795 | 1.115741 |
| GO:0048535\_lymph\_node\_development | 18 | 1 | 9.839744 | -1.012933 | 108 | 63.739205 | 120.5 | 177.260795 | 1.115741 |
| GO:0048730\_epidermis\_morphogenesis | 18 | 1 | 9.839744 | -1.012933 | 108 | 63.739205 | 120.5 | 177.260795 | 1.115741 |
| GO:0051222\_positive\_regulation\_of\_protein\_transport | 18 | 1 | 9.839744 | -1.012933 | 108 | 63.739205 | 120.5 | 177.260795 | 1.115741 |
| GO:0034984\_cellular\_response\_to\_DNA\_damage\_stimulus | 94 | 2 | 3.768412 | -1.010151 | 109 | 64.154055 | 121.25 | 178.345945 | 1.112385 |
| GO:0043283\_biopolymer\_metabolic\_process | 1490 | 12 | 1.426433 | -1.003163 | 110 | 64.428060 | 121.77 | 179.111940 | 1.107000 |
| GO:0007595\_lactation | 19 | 1 | 9.321862 | -0.990617 | 112 | 67.138831 | 125.92 | 184.701169 | 1.124286 |
| GO:0048536\_spleen\_development | 19 | 1 | 9.321862 | -0.990617 | 112 | 67.138831 | 125.92 | 184.701169 | 1.124286 |
| GO:0060341\_regulation\_of\_cellular\_localization | 97 | 2 | 3.651864 | -0.987128 | 113 | 67.186668 | 126.14 | 185.093332 | 1.116283 |
| GO:0009314\_response\_to\_radiation | 98 | 2 | 3.614600 | -0.979643 | 114 | 67.588842 | 126.87 | 186.151158 | 1.112895 |
| GO:0022607\_cellular\_component\_assembly | 204 | 3 | 2.604638 | -0.976399 | 115 | 67.763669 | 127.02 | 186.276331 | 1.104522 |
| GO:0060562\_epithelial\_tube\_morphogenesis | 99 | 2 | 3.578089 | -0.972248 | 116 | 68.082009 | 127.46 | 186.837991 | 1.098793 |
| GO:0007586\_digestion | 20 | 1 | 8.855769 | -0.969505 | 120 | 70.299418 | 130.67 | 191.040582 | 1.088917 |
| GO:0032640\_tumor\_necrosis\_factor\_production | 20 | 1 | 8.855769 | -0.969505 | 120 | 70.299418 | 130.67 | 191.040582 | 1.088917 |
| GO:0032680\_regulation\_of\_tumor\_necrosis\_factor\_production | 20 | 1 | 8.855769 | -0.969505 | 120 | 70.299418 | 130.67 | 191.040582 | 1.088917 |
| GO:0046822\_regulation\_of\_nucleocytoplasmic\_transport | 20 | 1 | 8.855769 | -0.969505 | 120 | 70.299418 | 130.67 | 191.040582 | 1.088917 |
| GO:0000018\_regulation\_of\_DNA\_recombination | 21 | 1 | 8.434066 | -0.949479 | 125 | 73.942161 | 136.23 | 198.517839 | 1.089840 |
| GO:0001702\_gastrulation\_with\_mouth\_forming\_second | 21 | 1 | 8.434066 | -0.949479 | 125 | 73.942161 | 136.23 | 198.517839 | 1.089840 |
| GO:0001709\_cell\_fate\_determination | 21 | 1 | 8.434066 | -0.949479 | 125 | 73.942161 | 136.23 | 198.517839 | 1.089840 |
| GO:0008154\_actin\_polymerization\_or\_depolymerization | 21 | 1 | 8.434066 | -0.949479 | 125 | 73.942161 | 136.23 | 198.517839 | 1.089840 |
| GO:0030216\_keratinocyte\_differentiation | 21 | 1 | 8.434066 | -0.949479 | 125 | 73.942161 | 136.23 | 198.517839 | 1.089840 |
| GO:0010556\_regulation\_of\_macromolecule\_biosynthetic\_process | 745 | 7 | 1.664171 | -0.943800 | 126 | 74.050545 | 136.4 | 198.749455 | 1.082540 |
| GO:0035295\_tube\_development | 212 | 3 | 2.506350 | -0.938773 | 127 | 74.407621 | 136.91 | 199.412379 | 1.078031 |
| GO:0040018\_positive\_regulation\_of\_multicellular\_organism\_growth | 22 | 1 | 8.050699 | -0.930439 | 130 | 78.468451 | 142.24 | 206.011549 | 1.094154 |
| GO:0046883\_regulation\_of\_hormone\_secretion | 22 | 1 | 8.050699 | -0.930439 | 130 | 78.468451 | 142.24 | 206.011549 | 1.094154 |
| GO:0051260\_protein\_homooligomerization | 22 | 1 | 8.050699 | -0.930439 | 130 | 78.468451 | 142.24 | 206.011549 | 1.094154 |
| GO:0034645\_cellular\_macromolecule\_biosynthetic\_process | 901 | 8 | 1.572612 | -0.926316 | 131 | 78.533299 | 142.37 | 206.206701 | 1.086794 |
| GO:0002204\_somatic\_recombination\_of\_immunoglobulin\_genes\_during\_immune\_response | 23 | 1 | 7.700669 | -0.912295 | 138 | 81.643599 | 146.36 | 211.076401 | 1.060580 |
| GO:0002208\_somatic\_diversification\_of\_immunoglobulins\_during\_immune\_response | 23 | 1 | 7.700669 | -0.912295 | 138 | 81.643599 | 146.36 | 211.076401 | 1.060580 |
| GO:0006397\_mRNA\_processing | 23 | 1 | 7.700669 | -0.912295 | 138 | 81.643599 | 146.36 | 211.076401 | 1.060580 |
| GO:0022613\_ribonucleoprotein\_complex\_biogenesis | 23 | 1 | 7.700669 | -0.912295 | 138 | 81.643599 | 146.36 | 211.076401 | 1.060580 |
| GO:0032635\_interleukin-6\_production | 23 | 1 | 7.700669 | -0.912295 | 138 | 81.643599 | 146.36 | 211.076401 | 1.060580 |
| GO:0032675\_regulation\_of\_interleukin-6\_production | 23 | 1 | 7.700669 | -0.912295 | 138 | 81.643599 | 146.36 | 211.076401 | 1.060580 |
| GO:0045190\_isotype\_switching | 23 | 1 | 7.700669 | -0.912295 | 138 | 81.643599 | 146.36 | 211.076401 | 1.060580 |
| GO:0051649\_establishment\_of\_localization\_in\_cell | 342 | 4 | 2.071525 | -0.911214 | 139 | 81.723585 | 146.52 | 211.316415 | 1.054101 |
| GO:0009059\_macromolecule\_biosynthetic\_process | 910 | 8 | 1.557058 | -0.906988 | 140 | 81.905832 | 146.85 | 211.794168 | 1.048929 |
| GO:0002381\_immunoglobulin\_production\_during\_immune\_response | 24 | 1 | 7.379808 | -0.894972 | 143 | 85.123550 | 151.33 | 217.536450 | 1.058252 |
| GO:0007050\_cell\_cycle\_arrest | 24 | 1 | 7.379808 | -0.894972 | 143 | 85.123550 | 151.33 | 217.536450 | 1.058252 |
| GO:0032386\_regulation\_of\_intracellular\_transport | 24 | 1 | 7.379808 | -0.894972 | 143 | 85.123550 | 151.33 | 217.536450 | 1.058252 |
| GO:0006302\_double-strand\_break\_repair | 25 | 1 | 7.084615 | -0.878403 | 146 | 87.603363 | 155.19 | 222.776637 | 1.062945 |
| GO:0021983\_pituitary\_gland\_development | 25 | 1 | 7.084615 | -0.878403 | 146 | 87.603363 | 155.19 | 222.776637 | 1.062945 |
| GO:0035137\_hindlimb\_morphogenesis | 25 | 1 | 7.084615 | -0.878403 | 146 | 87.603363 | 155.19 | 222.776637 | 1.062945 |
| GO:0006974\_response\_to\_DNA\_damage\_stimulus | 113 | 2 | 3.134786 | -0.877317 | 147 | 87.779014 | 155.49 | 223.200986 | 1.057755 |
| GO:0010468\_regulation\_of\_gene\_expression | 778 | 7 | 1.593583 | -0.867847 | 148 | 88.179224 | 156.14 | 224.100776 | 1.055000 |
| GO:0001658\_branching\_involved\_in\_ureteric\_bud\_morphogenesis | 26 | 1 | 6.812130 | -0.862529 | 151 | 90.808020 | 159.77 | 228.731980 | 1.058079 |
| GO:0010212\_response\_to\_ionizing\_radiation | 26 | 1 | 6.812130 | -0.862529 | 151 | 90.808020 | 159.77 | 228.731980 | 1.058079 |
| GO:0060675\_ureteric\_bud\_morphogenesis | 26 | 1 | 6.812130 | -0.862529 | 151 | 90.808020 | 159.77 | 228.731980 | 1.058079 |
| GO:0043170\_macromolecule\_metabolic\_process | 1576 | 12 | 1.348594 | -0.851139 | 152 | 91.430488 | 160.65 | 229.869512 | 1.056908 |
| GO:0009913\_epidermal\_cell\_differentiation | 27 | 1 | 6.559829 | -0.847297 | 154 | 93.477626 | 163.44 | 233.402374 | 1.061299 |
| GO:0010638\_positive\_regulation\_of\_organelle\_organization | 27 | 1 | 6.559829 | -0.847297 | 154 | 93.477626 | 163.44 | 233.402374 | 1.061299 |
| GO:0044085\_cellular\_component\_biogenesis | 237 | 3 | 2.241967 | -0.832564 | 155 | 95.835538 | 167.07 | 238.304462 | 1.077871 |
| GO:0051726\_regulation\_of\_cell\_cycle | 121 | 2 | 2.927527 | -0.829319 | 156 | 96.091962 | 167.37 | 238.648038 | 1.072885 |
| GO:0006886\_intracellular\_protein\_transport | 122 | 2 | 2.903531 | -0.823597 | 157 | 96.914422 | 168.5 | 240.085578 | 1.073248 |
| GO:0051641\_cellular\_localization | 370 | 4 | 1.914761 | -0.820133 | 158 | 96.998081 | 168.69 | 240.381919 | 1.067658 |
| GO:0001934\_positive\_regulation\_of\_protein\_amino\_acid\_phosphorylation | 29 | 1 | 6.107427 | -0.818576 | 161 | 99.473991 | 171.67 | 243.866009 | 1.066273 |
| GO:0016447\_somatic\_recombination\_of\_immunoglobulin\_gene\_segments | 29 | 1 | 6.107427 | -0.818576 | 161 | 99.473991 | 171.67 | 243.866009 | 1.066273 |
| GO:0051301\_cell\_division | 29 | 1 | 6.107427 | -0.818576 | 161 | 99.473991 | 171.67 | 243.866009 | 1.066273 |
| GO:0001763\_morphogenesis\_of\_a\_branching\_structure | 125 | 2 | 2.833846 | -0.806778 | 163 | 100.074531 | 172.63 | 245.185469 | 1.059080 |
| GO:0043062\_extracellular\_structure\_organization | 125 | 2 | 2.833846 | -0.806778 | 163 | 100.074531 | 172.63 | 245.185469 | 1.059080 |
| GO:0000187\_activation\_of\_MAPK\_activity | 30 | 1 | 5.903846 | -0.805008 | 167 | 102.607815 | 176.17 | 249.732185 | 1.054910 |
| GO:0016445\_somatic\_diversification\_of\_immunoglobulins | 30 | 1 | 5.903846 | -0.805008 | 167 | 102.607815 | 176.17 | 249.732185 | 1.054910 |
| GO:0022411\_cellular\_component\_disassembly | 30 | 1 | 5.903846 | -0.805008 | 167 | 102.607815 | 176.17 | 249.732185 | 1.054910 |
| GO:0060021\_palate\_development | 30 | 1 | 5.903846 | -0.805008 | 167 | 102.607815 | 176.17 | 249.732185 | 1.054910 |
| GO:0016043\_cellular\_component\_organization | 964 | 8 | 1.469837 | -0.798243 | 168 | 102.803553 | 176.43 | 250.056447 | 1.050179 |
| GO:0031326\_regulation\_of\_cellular\_biosynthetic\_process | 812 | 7 | 1.526857 | -0.795475 | 169 | 102.917630 | 176.59 | 250.262370 | 1.044911 |
| GO:0010562\_positive\_regulation\_of\_phosphorus\_metabolic\_process | 31 | 1 | 5.713400 | -0.791921 | 172 | 106.454564 | 181.68 | 256.905436 | 1.056279 |
| GO:0042327\_positive\_regulation\_of\_phosphorylation | 31 | 1 | 5.713400 | -0.791921 | 172 | 106.454564 | 181.68 | 256.905436 | 1.056279 |
| GO:0045937\_positive\_regulation\_of\_phosphate\_metabolic\_process | 31 | 1 | 5.713400 | -0.791921 | 172 | 106.454564 | 181.68 | 256.905436 | 1.056279 |
| GO:0009889\_regulation\_of\_biosynthetic\_process | 815 | 7 | 1.521236 | -0.789359 | 173 | 106.914700 | 182.27 | 257.625300 | 1.053584 |
| GO:0045165\_cell\_fate\_commitment | 130 | 2 | 2.724852 | -0.779844 | 174 | 107.563554 | 183.16 | 258.756446 | 1.052644 |
| GO:0051259\_protein\_oligomerization | 32 | 1 | 5.534856 | -0.779287 | 176 | 109.407576 | 185.4 | 261.392424 | 1.053409 |
| GO:0051493\_regulation\_of\_cytoskeleton\_organization | 32 | 1 | 5.534856 | -0.779287 | 176 | 109.407576 | 185.4 | 261.392424 | 1.053409 |
| GO:0002562\_somatic\_diversification\_of\_immune\_receptors\_via\_germline\_recombination\_within\_a\_single\_locus | 33 | 1 | 5.367133 | -0.767075 | 180 | 111.151913 | 188.12 | 265.088087 | 1.045111 |
| GO:0008584\_male\_gonad\_development | 33 | 1 | 5.367133 | -0.767075 | 180 | 111.151913 | 188.12 | 265.088087 | 1.045111 |
| GO:0016444\_somatic\_cell\_DNA\_recombination | 33 | 1 | 5.367133 | -0.767075 | 180 | 111.151913 | 188.12 | 265.088087 | 1.045111 |
| GO:0021536\_diencephalon\_development | 33 | 1 | 5.367133 | -0.767075 | 180 | 111.151913 | 188.12 | 265.088087 | 1.045111 |
| GO:0048729\_tissue\_morphogenesis | 255 | 3 | 2.083710 | -0.765260 | 181 | 111.418883 | 188.51 | 265.601117 | 1.041492 |
| GO:0002200\_somatic\_diversification\_of\_immune\_receptors | 34 | 1 | 5.209276 | -0.755262 | 186 | 114.171250 | 192.24 | 270.308750 | 1.033548 |
| GO:0007568\_aging | 34 | 1 | 5.209276 | -0.755262 | 186 | 114.171250 | 192.24 | 270.308750 | 1.033548 |
| GO:0010720\_positive\_regulation\_of\_cell\_development | 34 | 1 | 5.209276 | -0.755262 | 186 | 114.171250 | 192.24 | 270.308750 | 1.033548 |
| GO:0045927\_positive\_regulation\_of\_growth | 34 | 1 | 5.209276 | -0.755262 | 186 | 114.171250 | 192.24 | 270.308750 | 1.033548 |
| GO:0051047\_positive\_regulation\_of\_secretion | 34 | 1 | 5.209276 | -0.755262 | 186 | 114.171250 | 192.24 | 270.308750 | 1.033548 |
| GO:0043406\_positive\_regulation\_of\_MAP\_kinase\_activity | 35 | 1 | 5.060440 | -0.743823 | 187 | 116.256160 | 195.07 | 273.883840 | 1.043155 |
| GO:0034613\_cellular\_protein\_localization | 139 | 2 | 2.548423 | -0.734532 | 188 | 116.891764 | 195.98 | 275.068236 | 1.042447 |
| GO:0051223\_regulation\_of\_protein\_transport | 36 | 1 | 4.919872 | -0.732738 | 189 | 119.006706 | 198.88 | 278.753294 | 1.052275 |
| GO:0006139\_nucleobase\_\_nucleoside\_\_nucleotide\_and\_nucleic\_acid\_metabolic\_process | 1002 | 8 | 1.414095 | -0.728635 | 190 | 119.536542 | 199.49 | 279.443458 | 1.049947 |
| GO:0070727\_cellular\_macromolecule\_localization | 141 | 2 | 2.512275 | -0.724973 | 191 | 119.812188 | 199.79 | 279.767812 | 1.046021 |
| GO:0045934\_negative\_regulation\_of\_nucleobase\_\_nucleoside\_\_nucleotide\_and\_nucleic\_acid\_metabolic\_process | 270 | 3 | 1.967949 | -0.714149 | 192 | 121.591198 | 202.41 | 283.228802 | 1.054219 |
| GO:0006350\_transcription | 701 | 6 | 1.515966 | -0.713143 | 193 | 121.661783 | 202.51 | 283.358217 | 1.049275 |
| GO:0001570\_vasculogenesis | 38 | 1 | 4.660931 | -0.711554 | 200 | 124.786259 | 206.12 | 287.453741 | 1.030600 |
| GO:0001657\_ureteric\_bud\_development | 38 | 1 | 4.660931 | -0.711554 | 200 | 124.786259 | 206.12 | 287.453741 | 1.030600 |
| GO:0008016\_regulation\_of\_heart\_contraction | 38 | 1 | 4.660931 | -0.711554 | 200 | 124.786259 | 206.12 | 287.453741 | 1.030600 |
| GO:0031401\_positive\_regulation\_of\_protein\_modification\_process | 38 | 1 | 4.660931 | -0.711554 | 200 | 124.786259 | 206.12 | 287.453741 | 1.030600 |
| GO:0032259\_methylation | 38 | 1 | 4.660931 | -0.711554 | 200 | 124.786259 | 206.12 | 287.453741 | 1.030600 |
| GO:0042493\_response\_to\_drug | 38 | 1 | 4.660931 | -0.711554 | 200 | 124.786259 | 206.12 | 287.453741 | 1.030600 |
| GO:0043414\_biopolymer\_methylation | 38 | 1 | 4.660931 | -0.711554 | 200 | 124.786259 | 206.12 | 287.453741 | 1.030600 |
| GO:0050789\_regulation\_of\_biological\_process | 2357 | 16 | 1.202311 | -0.711000 | 201 | 124.909726 | 206.23 | 287.550274 | 1.026020 |
| GO:0051172\_negative\_regulation\_of\_nitrogen\_compound\_metabolic\_process | 271 | 3 | 1.960687 | -0.710888 | 202 | 125.131274 | 206.49 | 287.848726 | 1.022228 |
| GO:0007242\_intracellular\_signaling\_cascade | 411 | 4 | 1.723751 | -0.704317 | 203 | 125.533029 | 207.28 | 289.026971 | 1.021084 |
| GO:0030900\_forebrain\_development | 146 | 2 | 2.426238 | -0.701824 | 204 | 125.910751 | 207.71 | 289.509249 | 1.018186 |
| GO:0006730\_one-carbon\_metabolic\_process | 39 | 1 | 4.541420 | -0.701420 | 208 | 128.052114 | 210.25 | 292.447886 | 1.010817 |
| GO:0043524\_negative\_regulation\_of\_neuron\_apoptosis | 39 | 1 | 4.541420 | -0.701420 | 208 | 128.052114 | 210.25 | 292.447886 | 1.010817 |
| GO:0048663\_neuron\_fate\_commitment | 39 | 1 | 4.541420 | -0.701420 | 208 | 128.052114 | 210.25 | 292.447886 | 1.010817 |
| GO:0070201\_regulation\_of\_establishment\_of\_protein\_localization | 39 | 1 | 4.541420 | -0.701420 | 208 | 128.052114 | 210.25 | 292.447886 | 1.010817 |
| GO:0050794\_regulation\_of\_cellular\_process | 2190 | 15 | 1.213119 | -0.698544 | 209 | 128.378642 | 210.57 | 292.761358 | 1.007512 |
| GO:0044267\_cellular\_protein\_metabolic\_process | 559 | 5 | 1.584216 | -0.696972 | 210 | 128.525462 | 210.89 | 293.254538 | 1.004238 |
| GO:0001824\_blastocyst\_development | 40 | 1 | 4.427885 | -0.691571 | 214 | 130.665669 | 213.72 | 296.774331 | 0.998692 |
| GO:0014031\_mesenchymal\_cell\_development | 40 | 1 | 4.427885 | -0.691571 | 214 | 130.665669 | 213.72 | 296.774331 | 0.998692 |
| GO:0016071\_mRNA\_metabolic\_process | 40 | 1 | 4.427885 | -0.691571 | 214 | 130.665669 | 213.72 | 296.774331 | 0.998692 |
| GO:0017015\_regulation\_of\_transforming\_growth\_factor\_beta\_receptor\_signaling\_pathway | 40 | 1 | 4.427885 | -0.691571 | 214 | 130.665669 | 213.72 | 296.774331 | 0.998692 |
| GO:0006810\_transport | 718 | 6 | 1.480073 | -0.679456 | 215 | 133.471520 | 217.95 | 302.428480 | 1.013721 |
| GO:0008361\_regulation\_of\_cell\_size | 42 | 1 | 4.217033 | -0.672671 | 217 | 136.664643 | 222.18 | 307.695357 | 1.023871 |
| GO:0010769\_regulation\_of\_cell\_morphogenesis\_involved\_in\_differentiation | 42 | 1 | 4.217033 | -0.672671 | 217 | 136.664643 | 222.18 | 307.695357 | 1.023871 |
| GO:0001841\_neural\_tube\_formation | 43 | 1 | 4.118962 | -0.663595 | 221 | 139.766736 | 226.23 | 312.693264 | 1.023665 |
| GO:0010001\_glial\_cell\_differentiation | 43 | 1 | 4.118962 | -0.663595 | 221 | 139.766736 | 226.23 | 312.693264 | 1.023665 |
| GO:0046879\_hormone\_secretion | 43 | 1 | 4.118962 | -0.663595 | 221 | 139.766736 | 226.23 | 312.693264 | 1.023665 |
| GO:0048762\_mesenchymal\_cell\_differentiation | 43 | 1 | 4.118962 | -0.663595 | 221 | 139.766736 | 226.23 | 312.693264 | 1.023665 |
| GO:0051234\_establishment\_of\_localization | 729 | 6 | 1.457740 | -0.658449 | 222 | 140.247207 | 226.87 | 313.492793 | 1.021937 |
| GO:0002377\_immunoglobulin\_production | 44 | 1 | 4.025350 | -0.654753 | 228 | 143.270504 | 231.05 | 318.829496 | 1.013377 |
| GO:0006606\_protein\_import\_into\_nucleus | 44 | 1 | 4.025350 | -0.654753 | 228 | 143.270504 | 231.05 | 318.829496 | 1.013377 |
| GO:0009914\_hormone\_transport | 44 | 1 | 4.025350 | -0.654753 | 228 | 143.270504 | 231.05 | 318.829496 | 1.013377 |
| GO:0016064\_immunoglobulin\_mediated\_immune\_response | 44 | 1 | 4.025350 | -0.654753 | 228 | 143.270504 | 231.05 | 318.829496 | 1.013377 |
| GO:0051170\_nuclear\_import | 44 | 1 | 4.025350 | -0.654753 | 228 | 143.270504 | 231.05 | 318.829496 | 1.013377 |
| GO:0060485\_mesenchyme\_development | 44 | 1 | 4.025350 | -0.654753 | 228 | 143.270504 | 231.05 | 318.829496 | 1.013377 |
| GO:0001838\_embryonic\_epithelial\_tube\_formation | 45 | 1 | 3.935897 | -0.646134 | 230 | 144.571981 | 232.95 | 321.328019 | 1.012826 |
| GO:0046546\_development\_of\_primary\_male\_sexual\_characteristics | 45 | 1 | 3.935897 | -0.646134 | 230 | 144.571981 | 232.95 | 321.328019 | 1.012826 |
| GO:0051128\_regulation\_of\_cellular\_component\_organization | 160 | 2 | 2.213942 | -0.642186 | 231 | 144.844305 | 233.33 | 321.815695 | 1.010087 |
| GO:0019724\_B\_cell\_mediated\_immunity | 46 | 1 | 3.850334 | -0.637729 | 234 | 146.431352 | 235.65 | 324.868648 | 1.007051 |
| GO:0042063\_gliogenesis | 46 | 1 | 3.850334 | -0.637729 | 234 | 146.431352 | 235.65 | 324.868648 | 1.007051 |
| GO:0051098\_regulation\_of\_binding | 46 | 1 | 3.850334 | -0.637729 | 234 | 146.431352 | 235.65 | 324.868648 | 1.007051 |
| GO:0051179\_localization | 1058 | 8 | 1.339247 | -0.635468 | 235 | 146.791897 | 236.33 | 325.868103 | 1.005660 |
| GO:0009628\_response\_to\_abiotic\_stimulus | 162 | 2 | 2.186610 | -0.634233 | 236 | 147.138577 | 236.66 | 326.181423 | 1.002797 |
| GO:0006396\_RNA\_processing | 47 | 1 | 3.768412 | -0.629529 | 238 | 149.546763 | 239.7 | 329.853237 | 1.007143 |
| GO:0030183\_B\_cell\_differentiation | 47 | 1 | 3.768412 | -0.629529 | 238 | 149.546763 | 239.7 | 329.853237 | 1.007143 |
| GO:0042325\_regulation\_of\_phosphorylation | 164 | 2 | 2.159944 | -0.626411 | 239 | 149.909992 | 240.18 | 330.450008 | 1.004937 |
| GO:0048598\_embryonic\_morphogenesis | 299 | 3 | 1.777077 | -0.626270 | 240 | 150.053964 | 240.32 | 330.586036 | 1.001333 |
| GO:0006259\_DNA\_metabolic\_process | 165 | 2 | 2.146853 | -0.622548 | 243 | 150.742828 | 241.27 | 331.797172 | 0.992881 |
| GO:0019220\_regulation\_of\_phosphate\_metabolic\_process | 165 | 2 | 2.146853 | -0.622548 | 243 | 150.742828 | 241.27 | 331.797172 | 0.992881 |
| GO:0051174\_regulation\_of\_phosphorus\_metabolic\_process | 165 | 2 | 2.146853 | -0.622548 | 243 | 150.742828 | 241.27 | 331.797172 | 0.992881 |
| GO:0034504\_protein\_localization\_in\_nucleus | 48 | 1 | 3.689904 | -0.621524 | 244 | 151.815387 | 242.74 | 333.664613 | 0.994836 |
| GO:0044238\_primary\_metabolic\_process | 1905 | 13 | 1.208661 | -0.617019 | 245 | 152.182340 | 243.24 | 334.297660 | 0.992816 |
| GO:0010926\_anatomical\_structure\_formation | 447 | 4 | 1.584925 | -0.616939 | 246 | 152.358105 | 243.38 | 334.401895 | 0.989350 |
| GO:0051049\_regulation\_of\_transport | 167 | 2 | 2.121142 | -0.614917 | 247 | 152.586876 | 243.89 | 335.193124 | 0.987409 |
| GO:0002440\_production\_of\_molecular\_mediator\_of\_immune\_response | 49 | 1 | 3.614600 | -0.613707 | 251 | 154.712769 | 246.21 | 337.707231 | 0.980916 |
| GO:0003015\_heart\_process | 49 | 1 | 3.614600 | -0.613707 | 251 | 154.712769 | 246.21 | 337.707231 | 0.980916 |
| GO:0046661\_male\_sex\_differentiation | 49 | 1 | 3.614600 | -0.613707 | 251 | 154.712769 | 246.21 | 337.707231 | 0.980916 |
| GO:0060047\_heart\_contraction | 49 | 1 | 3.614600 | -0.613707 | 251 | 154.712769 | 246.21 | 337.707231 | 0.980916 |
| GO:0019219\_regulation\_of\_nucleobase\_\_nucleoside\_\_nucleotide\_and\_nucleic\_acid\_metabolic\_process | 757 | 6 | 1.403821 | -0.607632 | 252 | 154.962617 | 246.68 | 338.397383 | 0.978889 |
| GO:0001656\_metanephros\_development | 50 | 1 | 3.542308 | -0.606070 | 255 | 156.459936 | 248.61 | 340.760064 | 0.974941 |
| GO:0007015\_actin\_filament\_organization | 50 | 1 | 3.542308 | -0.606070 | 255 | 156.459936 | 248.61 | 340.760064 | 0.974941 |
| GO:0017038\_protein\_import | 50 | 1 | 3.542308 | -0.606070 | 255 | 156.459936 | 248.61 | 340.760064 | 0.974941 |
| GO:0016310\_phosphorylation | 309 | 3 | 1.719567 | -0.598898 | 256 | 156.964001 | 249.49 | 342.015999 | 0.974570 |
| GO:0016569\_covalent\_chromatin\_modification | 51 | 1 | 3.472851 | -0.598606 | 258 | 158.627206 | 251.74 | 344.852794 | 0.975736 |
| GO:0032880\_regulation\_of\_protein\_localization | 51 | 1 | 3.472851 | -0.598606 | 258 | 158.627206 | 251.74 | 344.852794 | 0.975736 |
| GO:0015031\_protein\_transport | 175 | 2 | 2.024176 | -0.585593 | 259 | 160.872901 | 254.96 | 349.047099 | 0.984402 |
| GO:0051171\_regulation\_of\_nitrogen\_compound\_metabolic\_process | 771 | 6 | 1.378330 | -0.583585 | 260 | 162.481688 | 256.69 | 350.898312 | 0.987269 |
| GO:0006091\_generation\_of\_precursor\_metabolites\_and\_energy | 54 | 1 | 3.279915 | -0.577184 | 263 | 164.815436 | 259.51 | 354.204564 | 0.986730 |
| GO:0007265\_Ras\_protein\_signal\_transduction | 54 | 1 | 3.279915 | -0.577184 | 263 | 164.815436 | 259.51 | 354.204564 | 0.986730 |
| GO:0043405\_regulation\_of\_MAP\_kinase\_activity | 54 | 1 | 3.279915 | -0.577184 | 263 | 164.815436 | 259.51 | 354.204564 | 0.986730 |
| GO:0006310\_DNA\_recombination | 55 | 1 | 3.220280 | -0.570347 | 264 | 166.892411 | 262.16 | 357.427589 | 0.993030 |
| GO:0045184\_establishment\_of\_protein\_localization | 180 | 2 | 1.967949 | -0.568186 | 265 | 167.005735 | 262.31 | 357.614265 | 0.989849 |
| GO:0033365\_protein\_localization\_in\_organelle | 57 | 1 | 3.107287 | -0.557096 | 267 | 172.431069 | 268.7 | 364.968931 | 1.006367 |
| GO:0043523\_regulation\_of\_neuron\_apoptosis | 57 | 1 | 3.107287 | -0.557096 | 267 | 172.431069 | 268.7 | 364.968931 | 1.006367 |
| GO:0030902\_hindbrain\_development | 58 | 1 | 3.053714 | -0.550673 | 269 | 174.001373 | 270.65 | 367.298627 | 1.006134 |
| GO:0033043\_regulation\_of\_organelle\_organization | 58 | 1 | 3.053714 | -0.550673 | 269 | 174.001373 | 270.65 | 367.298627 | 1.006134 |
| GO:0008152\_metabolic\_process | 2133 | 14 | 1.162501 | -0.549606 | 270 | 174.080676 | 270.77 | 367.459324 | 1.002852 |
| GO:0035270\_endocrine\_system\_development | 59 | 1 | 3.001956 | -0.544377 | 271 | 176.175240 | 273.37 | 370.564760 | 1.008745 |
| GO:0010605\_negative\_regulation\_of\_macromolecule\_metabolic\_process | 331 | 3 | 1.605275 | -0.543276 | 272 | 176.416972 | 273.66 | 370.903028 | 1.006103 |
| GO:0031324\_negative\_regulation\_of\_cellular\_metabolic\_process | 332 | 3 | 1.600440 | -0.540887 | 273 | 176.906321 | 274.21 | 371.513679 | 1.004432 |
| GO:0044237\_cellular\_metabolic\_process | 1974 | 13 | 1.166413 | -0.532737 | 274 | 177.627022 | 275.01 | 372.392978 | 1.003686 |
| GO:0022604\_regulation\_of\_cell\_morphogenesis | 62 | 1 | 2.856700 | -0.526217 | 277 | 180.557125 | 278.68 | 376.802875 | 1.006065 |
| GO:0030155\_regulation\_of\_cell\_adhesion | 62 | 1 | 2.856700 | -0.526217 | 277 | 180.557125 | 278.68 | 376.802875 | 1.006065 |
| GO:0040014\_regulation\_of\_multicellular\_organism\_growth | 62 | 1 | 2.856700 | -0.526217 | 277 | 180.557125 | 278.68 | 376.802875 | 1.006065 |
| GO:0006793\_phosphorus\_metabolic\_process | 340 | 3 | 1.562783 | -0.522188 | 279 | 181.202360 | 279.48 | 377.757640 | 1.001720 |
| GO:0006796\_phosphate\_metabolic\_process | 340 | 3 | 1.562783 | -0.522188 | 279 | 181.202360 | 279.48 | 377.757640 | 1.001720 |
| GO:0007369\_gastrulation | 63 | 1 | 2.811355 | -0.520393 | 280 | 182.589818 | 280.99 | 379.390182 | 1.003536 |
| GO:0007507\_heart\_development | 195 | 2 | 1.816568 | -0.519783 | 281 | 183.019673 | 281.6 | 380.180327 | 1.002135 |
| GO:0033554\_cellular\_response\_to\_stress | 196 | 2 | 1.807300 | -0.516744 | 282 | 183.333071 | 282.06 | 380.786929 | 1.000213 |
| GO:0019538\_protein\_metabolic\_process | 655 | 5 | 1.352026 | -0.512712 | 283 | 184.728086 | 284.13 | 383.531914 | 1.003993 |
| GO:0002009\_morphogenesis\_of\_an\_epithelium | 198 | 2 | 1.789044 | -0.510733 | 285 | 185.034995 | 284.57 | 384.105005 | 0.998491 |
| GO:0060429\_epithelium\_development | 198 | 2 | 1.789044 | -0.510733 | 285 | 185.034995 | 284.57 | 384.105005 | 0.998491 |
| GO:0006807\_nitrogen\_compound\_metabolic\_process | 1147 | 8 | 1.235330 | -0.507906 | 286 | 185.856231 | 285.66 | 385.463769 | 0.998811 |
| GO:0016070\_RNA\_metabolic\_process | 658 | 5 | 1.345862 | -0.507776 | 287 | 186.012853 | 285.8 | 385.587147 | 0.995819 |
| GO:0000904\_cell\_morphogenesis\_involved\_in\_differentiation | 199 | 2 | 1.780054 | -0.507761 | 288 | 186.158343 | 286.01 | 385.861657 | 0.993090 |
| GO:0009892\_negative\_regulation\_of\_metabolic\_process | 348 | 3 | 1.526857 | -0.504192 | 289 | 186.286116 | 286.13 | 385.973884 | 0.990069 |
| GO:0044249\_cellular\_biosynthetic\_process | 1150 | 8 | 1.232107 | -0.504006 | 290 | 186.368178 | 286.26 | 386.151822 | 0.987103 |
| GO:0007179\_transforming\_growth\_factor\_beta\_receptor\_signaling\_pathway | 66 | 1 | 2.683566 | -0.503557 | 293 | 187.487390 | 287.71 | 387.932610 | 0.981945 |
| GO:0045860\_positive\_regulation\_of\_protein\_kinase\_activity | 66 | 1 | 2.683566 | -0.503557 | 293 | 187.487390 | 287.71 | 387.932610 | 0.981945 |
| GO:0051402\_neuron\_apoptosis | 66 | 1 | 2.683566 | -0.503557 | 293 | 187.487390 | 287.71 | 387.932610 | 0.981945 |
| GO:0003007\_heart\_morphogenesis | 67 | 1 | 2.643513 | -0.498148 | 295 | 188.774502 | 289.28 | 389.785498 | 0.980610 |
| GO:0009791\_post-embryonic\_development | 67 | 1 | 2.643513 | -0.498148 | 295 | 188.774502 | 289.28 | 389.785498 | 0.980610 |
| GO:0006955\_immune\_response | 205 | 2 | 1.727955 | -0.490367 | 296 | 190.595154 | 291.5 | 392.404846 | 0.984797 |
| GO:0001932\_regulation\_of\_protein\_amino\_acid\_phosphorylation | 69 | 1 | 2.566890 | -0.487612 | 298 | 191.514131 | 292.72 | 393.925869 | 0.982282 |
| GO:0006816\_calcium\_ion\_transport | 69 | 1 | 2.566890 | -0.487612 | 298 | 191.514131 | 292.72 | 393.925869 | 0.982282 |
| GO:0008406\_gonad\_development | 70 | 1 | 2.530220 | -0.482481 | 300 | 192.906570 | 294.36 | 395.813430 | 0.981200 |
| GO:0070838\_divalent\_metal\_ion\_transport | 70 | 1 | 2.530220 | -0.482481 | 300 | 192.906570 | 294.36 | 395.813430 | 0.981200 |
| GO:0045449\_regulation\_of\_transcription | 676 | 5 | 1.310025 | -0.479078 | 301 | 193.547728 | 295.18 | 396.812272 | 0.980664 |
| GO:0006281\_DNA\_repair | 71 | 1 | 2.494583 | -0.477438 | 304 | 194.638137 | 296.55 | 398.461863 | 0.975493 |
| GO:0016331\_morphogenesis\_of\_embryonic\_epithelium | 71 | 1 | 2.494583 | -0.477438 | 304 | 194.638137 | 296.55 | 398.461863 | 0.975493 |
| GO:0033674\_positive\_regulation\_of\_kinase\_activity | 71 | 1 | 2.494583 | -0.477438 | 304 | 194.638137 | 296.55 | 398.461863 | 0.975493 |
| GO:0007264\_small\_GTPase\_mediated\_signal\_transduction | 72 | 1 | 2.459936 | -0.472480 | 309 | 197.459481 | 299.72 | 401.980519 | 0.969968 |
| GO:0016568\_chromatin\_modification | 72 | 1 | 2.459936 | -0.472480 | 309 | 197.459481 | 299.72 | 401.980519 | 0.969968 |
| GO:0021915\_neural\_tube\_development | 72 | 1 | 2.459936 | -0.472480 | 309 | 197.459481 | 299.72 | 401.980519 | 0.969968 |
| GO:0030879\_mammary\_gland\_development | 72 | 1 | 2.459936 | -0.472480 | 309 | 197.459481 | 299.72 | 401.980519 | 0.969968 |
| GO:0051347\_positive\_regulation\_of\_transferase\_activity | 72 | 1 | 2.459936 | -0.472480 | 309 | 197.459481 | 299.72 | 401.980519 | 0.969968 |
| GO:0009058\_biosynthetic\_process | 1175 | 8 | 1.205892 | -0.472441 | 310 | 197.553048 | 299.84 | 402.126952 | 0.967226 |
| GO:0043009\_chordate\_embryonic\_development | 365 | 3 | 1.455743 | -0.468132 | 311 | 198.153096 | 300.83 | 403.506904 | 0.967299 |
| GO:0009792\_embryonic\_development\_ending\_in\_birth\_or\_egg\_hatching | 368 | 3 | 1.443875 | -0.462060 | 312 | 200.454703 | 303.5 | 406.545297 | 0.972756 |
| GO:0007281\_germ\_cell\_development | 75 | 1 | 2.361538 | -0.458095 | 314 | 201.472456 | 304.85 | 408.227544 | 0.970860 |
| GO:0048589\_developmental\_growth | 75 | 1 | 2.361538 | -0.458095 | 314 | 201.472456 | 304.85 | 408.227544 | 0.970860 |
| GO:0040007\_growth | 217 | 2 | 1.632400 | -0.457718 | 315 | 201.862431 | 305.39 | 408.917569 | 0.969492 |
| GO:0045892\_negative\_regulation\_of\_transcription\_\_DNA-dependent | 218 | 2 | 1.624912 | -0.455118 | 316 | 202.203420 | 305.85 | 409.496580 | 0.967880 |
| GO:0048519\_negative\_regulation\_of\_biological\_process | 859 | 6 | 1.237127 | -0.450943 | 317 | 203.763493 | 307.6 | 411.436507 | 0.970347 |
| GO:0051253\_negative\_regulation\_of\_RNA\_metabolic\_process | 220 | 2 | 1.610140 | -0.449971 | 318 | 203.975176 | 307.87 | 411.764824 | 0.968145 |
| GO:0051241\_negative\_regulation\_of\_multicellular\_organismal\_process | 77 | 1 | 2.300200 | -0.448889 | 319 | 204.555993 | 308.52 | 412.484007 | 0.967147 |
| GO:0001701\_in\_utero\_embryonic\_development | 221 | 2 | 1.602854 | -0.447424 | 320 | 204.720629 | 308.69 | 412.659371 | 0.964656 |
| GO:0048513\_organ\_development | 1365 | 9 | 1.167794 | -0.447316 | 321 | 204.794199 | 308.81 | 412.825801 | 0.962025 |
| GO:0006461\_protein\_complex\_assembly | 78 | 1 | 2.270710 | -0.444396 | 325 | 206.720875 | 311.12 | 415.519125 | 0.957292 |
| GO:0030326\_embryonic\_limb\_morphogenesis | 78 | 1 | 2.270710 | -0.444396 | 325 | 206.720875 | 311.12 | 415.519125 | 0.957292 |
| GO:0035113\_embryonic\_appendage\_morphogenesis | 78 | 1 | 2.270710 | -0.444396 | 325 | 206.720875 | 311.12 | 415.519125 | 0.957292 |
| GO:0070271\_protein\_complex\_biogenesis | 78 | 1 | 2.270710 | -0.444396 | 325 | 206.720875 | 311.12 | 415.519125 | 0.957292 |
| GO:0015674\_di-\_\_tri-valent\_inorganic\_cation\_transport | 79 | 1 | 2.241967 | -0.439973 | 327 | 207.515696 | 312.06 | 416.604304 | 0.954312 |
| GO:0051046\_regulation\_of\_secretion | 79 | 1 | 2.241967 | -0.439973 | 327 | 207.515696 | 312.06 | 416.604304 | 0.954312 |
| GO:0065007\_biological\_regulation | 2593 | 16 | 1.092883 | -0.432067 | 328 | 209.037919 | 313.93 | 418.822081 | 0.957104 |
| GO:0006325\_chromatin\_organization | 83 | 1 | 2.133920 | -0.422951 | 329 | 213.819867 | 319.43 | 425.040133 | 0.970912 |
| GO:0007420\_brain\_development | 231 | 2 | 1.533467 | -0.422876 | 330 | 214.009230 | 319.66 | 425.310770 | 0.968667 |
| GO:0030005\_cellular\_di-\_\_tri-valent\_inorganic\_cation\_homeostasis | 84 | 1 | 2.108516 | -0.418855 | 332 | 214.689721 | 320.58 | 426.470279 | 0.965602 |
| GO:0045137\_development\_of\_primary\_sexual\_characteristics | 84 | 1 | 2.108516 | -0.418855 | 332 | 214.689721 | 320.58 | 426.470279 | 0.965602 |
| GO:0002449\_lymphocyte\_mediated\_immunity | 85 | 1 | 2.083710 | -0.414819 | 333 | 216.525763 | 322.55 | 428.574237 | 0.968619 |
| GO:0042127\_regulation\_of\_cell\_proliferation | 393 | 3 | 1.352026 | -0.414581 | 334 | 216.642571 | 322.73 | 428.817429 | 0.966257 |
| GO:0006897\_endocytosis | 86 | 1 | 2.059481 | -0.410843 | 338 | 218.981068 | 325.08 | 431.178932 | 0.961775 |
| GO:0010324\_membrane\_invagination | 86 | 1 | 2.059481 | -0.410843 | 338 | 218.981068 | 325.08 | 431.178932 | 0.961775 |
| GO:0032504\_multicellular\_organism\_reproduction | 86 | 1 | 2.059481 | -0.410843 | 338 | 218.981068 | 325.08 | 431.178932 | 0.961775 |
| GO:0048609\_reproductive\_process\_in\_a\_multicellular\_organism | 86 | 1 | 2.059481 | -0.410843 | 338 | 218.981068 | 325.08 | 431.178932 | 0.961775 |
| GO:0006468\_protein\_amino\_acid\_phosphorylation | 237 | 2 | 1.494645 | -0.408912 | 339 | 219.962025 | 326.21 | 432.457975 | 0.962271 |
| GO:0001822\_kidney\_development | 87 | 1 | 2.035809 | -0.406924 | 342 | 221.627742 | 328.39 | 435.152258 | 0.960205 |
| GO:0003001\_generation\_of\_a\_signal\_involved\_in\_cell-cell\_signaling | 87 | 1 | 2.035809 | -0.406924 | 342 | 221.627742 | 328.39 | 435.152258 | 0.960205 |
| GO:0007178\_transmembrane\_receptor\_protein\_serine\_threonine\_kinase\_signaling\_pathway | 87 | 1 | 2.035809 | -0.406924 | 342 | 221.627742 | 328.39 | 435.152258 | 0.960205 |
| GO:0007049\_cell\_cycle | 238 | 2 | 1.488365 | -0.406637 | 343 | 221.769444 | 328.6 | 435.430556 | 0.958017 |
| GO:0009790\_embryonic\_development | 567 | 4 | 1.249491 | -0.397329 | 344 | 223.150821 | 330.26 | 437.369179 | 0.960058 |
| GO:0030003\_cellular\_cation\_homeostasis | 90 | 1 | 1.967949 | -0.395501 | 348 | 224.068471 | 331.41 | 438.751529 | 0.952328 |
| GO:0030324\_lung\_development | 90 | 1 | 1.967949 | -0.395501 | 348 | 224.068471 | 331.41 | 438.751529 | 0.952328 |
| GO:0035264\_multicellular\_organism\_growth | 90 | 1 | 1.967949 | -0.395501 | 348 | 224.068471 | 331.41 | 438.751529 | 0.952328 |
| GO:0042113\_B\_cell\_activation | 90 | 1 | 1.967949 | -0.395501 | 348 | 224.068471 | 331.41 | 438.751529 | 0.952328 |
| GO:0002443\_leukocyte\_mediated\_immunity | 91 | 1 | 1.946323 | -0.391801 | 351 | 225.298086 | 332.76 | 440.221914 | 0.948034 |
| GO:0008544\_epidermis\_development | 91 | 1 | 1.946323 | -0.391801 | 351 | 225.298086 | 332.76 | 440.221914 | 0.948034 |
| GO:0031399\_regulation\_of\_protein\_modification\_process | 91 | 1 | 1.946323 | -0.391801 | 351 | 225.298086 | 332.76 | 440.221914 | 0.948034 |
| GO:0030323\_respiratory\_tube\_development | 92 | 1 | 1.925167 | -0.388152 | 352 | 226.021205 | 333.56 | 441.098795 | 0.947614 |
| GO:0006355\_regulation\_of\_transcription\_\_DNA-dependent | 575 | 4 | 1.232107 | -0.385784 | 353 | 226.317893 | 333.99 | 441.662107 | 0.946147 |
| GO:0032879\_regulation\_of\_localization | 248 | 2 | 1.428350 | -0.384686 | 354 | 226.620349 | 334.44 | 442.259651 | 0.944746 |
| GO:0035107\_appendage\_morphogenesis | 93 | 1 | 1.904467 | -0.384553 | 357 | 227.754533 | 335.74 | 443.725467 | 0.940448 |
| GO:0035108\_limb\_morphogenesis | 93 | 1 | 1.904467 | -0.384553 | 357 | 227.754533 | 335.74 | 443.725467 | 0.940448 |
| GO:0055066\_di-\_\_tri-valent\_inorganic\_cation\_homeostasis | 93 | 1 | 1.904467 | -0.384553 | 357 | 227.754533 | 335.74 | 443.725467 | 0.940448 |
| GO:0007165\_signal\_transduction | 915 | 6 | 1.161412 | -0.381058 | 358 | 228.360526 | 336.41 | 444.459474 | 0.939693 |
| GO:0008104\_protein\_localization | 251 | 2 | 1.411278 | -0.378370 | 359 | 230.417610 | 338.68 | 446.942390 | 0.943398 |
| GO:0016481\_negative\_regulation\_of\_transcription | 253 | 2 | 1.400122 | -0.374226 | 360 | 231.766642 | 340.12 | 448.473358 | 0.944778 |
| GO:0006954\_inflammatory\_response | 96 | 1 | 1.844952 | -0.374048 | 363 | 233.474416 | 341.86 | 450.245584 | 0.941763 |
| GO:0048736\_appendage\_development | 96 | 1 | 1.844952 | -0.374048 | 363 | 233.474416 | 341.86 | 450.245584 | 0.941763 |
| GO:0060173\_limb\_development | 96 | 1 | 1.844952 | -0.374048 | 363 | 233.474416 | 341.86 | 450.245584 | 0.941763 |
| GO:0007154\_cell\_communication | 1096 | 7 | 1.131211 | -0.369293 | 364 | 234.899677 | 343.48 | 452.060323 | 0.943626 |
| GO:0051239\_regulation\_of\_multicellular\_organismal\_process | 587 | 4 | 1.206919 | -0.369063 | 365 | 235.055250 | 343.69 | 452.324750 | 0.941616 |
| GO:0007548\_sex\_differentiation | 98 | 1 | 1.807300 | -0.367278 | 367 | 236.688028 | 345.53 | 454.371972 | 0.941499 |
| GO:0060541\_respiratory\_system\_development | 98 | 1 | 1.807300 | -0.367278 | 367 | 236.688028 | 345.53 | 454.371972 | 0.941499 |
| GO:0051252\_regulation\_of\_RNA\_metabolic\_process | 590 | 4 | 1.200782 | -0.364992 | 368 | 237.031035 | 345.95 | 454.868965 | 0.940082 |
| GO:0022008\_neurogenesis | 423 | 3 | 1.256137 | -0.364225 | 369 | 237.248113 | 346.21 | 455.171887 | 0.938238 |
| GO:0001817\_regulation\_of\_cytokine\_production | 99 | 1 | 1.789044 | -0.363960 | 371 | 238.196831 | 347.37 | 456.543169 | 0.936307 |
| GO:0007398\_ectoderm\_development | 99 | 1 | 1.789044 | -0.363960 | 371 | 238.196831 | 347.37 | 456.543169 | 0.936307 |
| GO:0006351\_transcription\_\_DNA-dependent | 594 | 4 | 1.192696 | -0.359630 | 372 | 238.648110 | 347.96 | 457.271890 | 0.935376 |
| GO:0032774\_RNA\_biosynthetic\_process | 595 | 4 | 1.190692 | -0.358301 | 373 | 238.778675 | 348.14 | 457.501325 | 0.933351 |
| GO:0001775\_cell\_activation | 262 | 2 | 1.352026 | -0.356208 | 375 | 240.265495 | 349.82 | 459.374505 | 0.932853 |
| GO:0010629\_negative\_regulation\_of\_gene\_expression | 262 | 2 | 1.352026 | -0.356208 | 375 | 240.265495 | 349.82 | 459.374505 | 0.932853 |
| GO:0030036\_actin\_cytoskeleton\_organization | 102 | 1 | 1.736425 | -0.354261 | 376 | 241.018020 | 350.67 | 460.321980 | 0.932633 |
| GO:0003013\_circulatory\_system\_process | 103 | 1 | 1.719567 | -0.351111 | 378 | 242.374529 | 352.15 | 461.925471 | 0.931614 |
| GO:0008015\_blood\_circulation | 103 | 1 | 1.719567 | -0.351111 | 378 | 242.374529 | 352.15 | 461.925471 | 0.931614 |
| GO:0048523\_negative\_regulation\_of\_cellular\_process | 774 | 5 | 1.144156 | -0.347244 | 379 | 243.700331 | 353.71 | 463.719669 | 0.933272 |
| GO:0010817\_regulation\_of\_hormone\_levels | 106 | 1 | 1.670900 | -0.341898 | 380 | 244.719039 | 355.06 | 465.400961 | 0.934368 |
| GO:0045859\_regulation\_of\_protein\_kinase\_activity | 107 | 1 | 1.655284 | -0.338903 | 381 | 245.520165 | 356.14 | 466.759835 | 0.934751 |
| GO:0051240\_positive\_regulation\_of\_multicellular\_organismal\_process | 108 | 1 | 1.639957 | -0.335945 | 382 | 246.017445 | 356.73 | 467.442555 | 0.933848 |
| GO:0051716\_cellular\_response\_to\_stimulus | 273 | 2 | 1.297549 | -0.335508 | 383 | 246.463829 | 357.28 | 468.096171 | 0.932846 |
| GO:0010558\_negative\_regulation\_of\_macromolecule\_biosynthetic\_process | 274 | 2 | 1.292813 | -0.333694 | 385 | 247.086438 | 358.03 | 468.973562 | 0.929948 |
| GO:0033036\_macromolecule\_localization | 274 | 2 | 1.292813 | -0.333694 | 385 | 247.086438 | 358.03 | 468.973562 | 0.929948 |
| GO:0030029\_actin\_filament-based\_process | 109 | 1 | 1.624912 | -0.333024 | 386 | 247.335892 | 358.32 | 469.304108 | 0.928290 |
| GO:0055080\_cation\_homeostasis | 110 | 1 | 1.610140 | -0.330138 | 387 | 248.932069 | 360.23 | 471.527931 | 0.930827 |
| GO:0048534\_hemopoietic\_or\_lymphoid\_organ\_development | 277 | 2 | 1.278811 | -0.328318 | 389 | 249.540891 | 360.89 | 472.239109 | 0.927738 |
| GO:0048646\_anatomical\_structure\_formation\_involved\_in\_morphogenesis | 277 | 2 | 1.278811 | -0.328318 | 389 | 249.540891 | 360.89 | 472.239109 | 0.927738 |
| GO:0007399\_nervous\_system\_development | 621 | 4 | 1.140840 | -0.325333 | 390 | 250.568889 | 362.01 | 473.451111 | 0.928231 |
| GO:0065009\_regulation\_of\_molecular\_function | 279 | 2 | 1.269644 | -0.324788 | 391 | 250.925558 | 362.48 | 474.034442 | 0.927059 |
| GO:0043549\_regulation\_of\_kinase\_activity | 112 | 1 | 1.581387 | -0.324470 | 392 | 251.432379 | 363.04 | 474.647621 | 0.926122 |
| GO:0040008\_regulation\_of\_growth | 113 | 1 | 1.567393 | -0.321687 | 393 | 252.308753 | 364.1 | 475.891247 | 0.926463 |
| GO:0031327\_negative\_regulation\_of\_cellular\_biosynthetic\_process | 282 | 2 | 1.256137 | -0.319571 | 394 | 252.663898 | 364.52 | 476.376102 | 0.925178 |
| GO:0000165\_MAPKKK\_cascade | 114 | 1 | 1.553644 | -0.318937 | 395 | 253.376084 | 365.28 | 477.183916 | 0.924759 |
| GO:0000902\_cell\_morphogenesis | 283 | 2 | 1.251699 | -0.317853 | 396 | 253.749031 | 365.66 | 477.570969 | 0.923384 |
| GO:0051338\_regulation\_of\_transferase\_activity | 115 | 1 | 1.540134 | -0.316220 | 397 | 254.222468 | 366.28 | 478.337532 | 0.922620 |
| GO:0009890\_negative\_regulation\_of\_biosynthetic\_process | 284 | 2 | 1.247291 | -0.316145 | 398 | 254.465679 | 366.57 | 478.674321 | 0.921030 |
| GO:0048608\_reproductive\_structure\_development | 116 | 1 | 1.526857 | -0.313534 | 399 | 255.838582 | 368.12 | 480.401418 | 0.922607 |
| GO:0043412\_biopolymer\_modification | 458 | 3 | 1.160144 | -0.313271 | 400 | 256.262845 | 368.6 | 480.937155 | 0.921500 |
| GO:0007417\_central\_nervous\_system\_development | 287 | 2 | 1.234254 | -0.311082 | 401 | 256.554959 | 368.88 | 481.205041 | 0.919900 |
| GO:0001816\_cytokine\_production | 122 | 1 | 1.451765 | -0.298060 | 405 | 262.244804 | 375.15 | 488.055196 | 0.926296 |
| GO:0002252\_immune\_effector\_process | 122 | 1 | 1.451765 | -0.298060 | 405 | 262.244804 | 375.15 | 488.055196 | 0.926296 |
| GO:0030001\_metal\_ion\_transport | 122 | 1 | 1.451765 | -0.298060 | 405 | 262.244804 | 375.15 | 488.055196 | 0.926296 |
| GO:0060284\_regulation\_of\_cell\_development | 122 | 1 | 1.451765 | -0.298060 | 405 | 262.244804 | 375.15 | 488.055196 | 0.926296 |
| GO:0002520\_immune\_system\_development | 295 | 2 | 1.200782 | -0.298012 | 406 | 263.078393 | 375.91 | 488.741607 | 0.925887 |
| GO:0030098\_lymphocyte\_differentiation | 124 | 1 | 1.428350 | -0.293133 | 407 | 263.777806 | 376.67 | 489.562194 | 0.925479 |
| GO:0048468\_cell\_development | 654 | 4 | 1.083275 | -0.287568 | 408 | 265.037097 | 378.12 | 491.202903 | 0.926765 |
| GO:0001655\_urogenital\_system\_development | 128 | 1 | 1.383714 | -0.283603 | 410 | 266.354902 | 379.58 | 492.805098 | 0.925805 |
| GO:0045597\_positive\_regulation\_of\_cell\_differentiation | 128 | 1 | 1.383714 | -0.283603 | 410 | 266.354902 | 379.58 | 492.805098 | 0.925805 |
| GO:0051276\_chromosome\_organization | 129 | 1 | 1.372987 | -0.281285 | 411 | 267.224348 | 380.53 | 493.835652 | 0.925864 |
| GO:0032989\_cellular\_component\_morphogenesis | 307 | 2 | 1.153846 | -0.279520 | 412 | 267.986453 | 381.38 | 494.773547 | 0.925680 |
| GO:0009952\_anterior\_posterior\_pattern\_formation | 133 | 1 | 1.331695 | -0.272263 | 414 | 270.515052 | 384.02 | 497.524948 | 0.927585 |
| GO:0044057\_regulation\_of\_system\_process | 133 | 1 | 1.331695 | -0.272263 | 414 | 270.515052 | 384.02 | 497.524948 | 0.927585 |
| GO:0016044\_membrane\_organization | 140 | 1 | 1.265110 | -0.257368 | 415 | 273.535491 | 387.19 | 500.844509 | 0.932988 |
| GO:0003006\_reproductive\_developmental\_process | 141 | 1 | 1.256137 | -0.255328 | 416 | 274.393324 | 388.2 | 502.006676 | 0.933173 |
| GO:0007186\_G-protein\_coupled\_receptor\_protein\_signaling\_pathway | 144 | 1 | 1.229968 | -0.249331 | 417 | 275.467051 | 389.46 | 503.452949 | 0.933957 |
| GO:0007275\_multicellular\_organismal\_development | 1760 | 10 | 1.006337 | -0.248833 | 418 | 275.639542 | 389.58 | 503.520458 | 0.932010 |
| GO:0006928\_cell\_motion | 330 | 2 | 1.073427 | -0.247446 | 421 | 276.791885 | 390.75 | 504.708115 | 0.928147 |
| GO:0010646\_regulation\_of\_cell\_communication | 330 | 2 | 1.073427 | -0.247446 | 421 | 276.791885 | 390.75 | 504.708115 | 0.928147 |
| GO:0051674\_localization\_of\_cell | 330 | 2 | 1.073427 | -0.247446 | 421 | 276.791885 | 390.75 | 504.708115 | 0.928147 |
| GO:0006812\_cation\_transport | 146 | 1 | 1.213119 | -0.245433 | 422 | 278.249223 | 392.51 | 506.770777 | 0.930118 |
| GO:0022603\_regulation\_of\_anatomical\_structure\_morphogenesis | 147 | 1 | 1.204867 | -0.243514 | 423 | 279.151944 | 393.46 | 507.768056 | 0.930165 |
| GO:0043085\_positive\_regulation\_of\_catalytic\_activity | 148 | 1 | 1.196726 | -0.241613 | 424 | 279.988763 | 394.35 | 508.711237 | 0.930071 |
| GO:0032940\_secretion\_by\_cell | 149 | 1 | 1.188694 | -0.239732 | 425 | 280.596899 | 395.02 | 509.443101 | 0.929459 |
| GO:0009888\_tissue\_development | 525 | 3 | 1.012088 | -0.234636 | 426 | 282.120306 | 396.57 | 511.019694 | 0.930915 |
| GO:0007517\_muscle\_organ\_development | 153 | 1 | 1.157617 | -0.232389 | 427 | 282.895381 | 397.41 | 511.924619 | 0.930703 |
| GO:0048731\_system\_development | 1609 | 9 | 0.990701 | -0.231157 | 428 | 283.418364 | 397.89 | 512.361636 | 0.929650 |
| GO:0008285\_negative\_regulation\_of\_cell\_proliferation | 155 | 1 | 1.142680 | -0.228825 | 430 | 284.590606 | 399.2 | 513.809394 | 0.928372 |
| GO:0022402\_cell\_cycle\_process | 155 | 1 | 1.142680 | -0.228825 | 430 | 284.590606 | 399.2 | 513.809394 | 0.928372 |
| GO:0009987\_cellular\_process | 3868 | 22 | 1.007378 | -0.225478 | 431 | 285.161897 | 399.79 | 514.418103 | 0.927587 |
| GO:0007409\_axonogenesis | 158 | 1 | 1.120983 | -0.223605 | 433 | 287.243703 | 402.01 | 516.776297 | 0.928430 |
| GO:0048514\_blood\_vessel\_morphogenesis | 158 | 1 | 1.120983 | -0.223605 | 433 | 287.243703 | 402.01 | 516.776297 | 0.928430 |
| GO:0002521\_leukocyte\_differentiation | 161 | 1 | 1.100096 | -0.218534 | 434 | 288.676535 | 403.44 | 518.203465 | 0.929585 |
| GO:0008283\_cell\_proliferation | 544 | 3 | 0.976739 | -0.216077 | 435 | 289.647930 | 404.36 | 519.072070 | 0.929563 |
| GO:0030182\_neuron\_differentiation | 356 | 2 | 0.995030 | -0.215830 | 436 | 289.813291 | 404.58 | 519.346709 | 0.927936 |
| GO:0006950\_response\_to\_stress | 549 | 3 | 0.967844 | -0.211432 | 437 | 292.453953 | 407.33 | 522.206047 | 0.932105 |
| GO:0048812\_neuron\_projection\_morphogenesis | 170 | 1 | 1.041855 | -0.204152 | 438 | 297.002007 | 411.67 | 526.337993 | 0.939886 |
| GO:0032501\_multicellular\_organismal\_process | 2183 | 12 | 0.973607 | -0.203496 | 439 | 297.108685 | 411.79 | 526.471315 | 0.938018 |
| GO:0009611\_response\_to\_wounding | 172 | 1 | 1.029741 | -0.201115 | 440 | 298.809516 | 413.44 | 528.070484 | 0.939636 |
| GO:0044093\_positive\_regulation\_of\_molecular\_function | 173 | 1 | 1.023788 | -0.199618 | 442 | 300.130843 | 414.73 | 529.329157 | 0.938303 |
| GO:0048667\_cell\_morphogenesis\_involved\_in\_neuron\_differentiation | 173 | 1 | 1.023788 | -0.199618 | 442 | 300.130843 | 414.73 | 529.329157 | 0.938303 |
| GO:0000122\_negative\_regulation\_of\_transcription\_from\_RNA\_polymerase\_II\_promoter | 175 | 1 | 1.012088 | -0.196663 | 444 | 301.813554 | 416.41 | 531.006446 | 0.937860 |
| GO:0046903\_secretion | 175 | 1 | 1.012088 | -0.196663 | 444 | 301.813554 | 416.41 | 531.006446 | 0.937860 |
| GO:0006873\_cellular\_ion\_homeostasis | 176 | 1 | 1.006337 | -0.195205 | 447 | 303.228231 | 417.72 | 532.211769 | 0.934497 |
| GO:0043066\_negative\_regulation\_of\_apoptosis | 176 | 1 | 1.006337 | -0.195205 | 447 | 303.228231 | 417.72 | 532.211769 | 0.934497 |
| GO:0048858\_cell\_projection\_morphogenesis | 176 | 1 | 1.006337 | -0.195205 | 447 | 303.228231 | 417.72 | 532.211769 | 0.934497 |
| GO:0022414\_reproductive\_process | 376 | 2 | 0.942103 | -0.194392 | 448 | 303.526606 | 417.99 | 532.453394 | 0.933013 |
| GO:0000003\_reproduction | 379 | 2 | 0.934646 | -0.191371 | 449 | 304.337391 | 418.69 | 533.042609 | 0.932494 |
| GO:0043069\_negative\_regulation\_of\_programmed\_cell\_death | 179 | 1 | 0.989471 | -0.190911 | 452 | 305.672513 | 419.98 | 534.287487 | 0.929159 |
| GO:0048732\_gland\_development | 179 | 1 | 0.989471 | -0.190911 | 452 | 305.672513 | 419.98 | 534.287487 | 0.929159 |
| GO:0060548\_negative\_regulation\_of\_cell\_death | 179 | 1 | 0.989471 | -0.190911 | 452 | 305.672513 | 419.98 | 534.287487 | 0.929159 |
| GO:0055082\_cellular\_chemical\_homeostasis | 181 | 1 | 0.978538 | -0.188111 | 453 | 307.715059 | 421.89 | 536.064941 | 0.931325 |
| GO:0043687\_post-translational\_protein\_modification | 384 | 2 | 0.922476 | -0.186443 | 454 | 308.486239 | 422.64 | 536.793761 | 0.930925 |
| GO:0016192\_vesicle-mediated\_transport | 184 | 1 | 0.962584 | -0.184003 | 456 | 309.592233 | 423.8 | 538.007767 | 0.929386 |
| GO:0032990\_cell\_part\_morphogenesis | 184 | 1 | 0.962584 | -0.184003 | 456 | 309.592233 | 423.8 | 538.007767 | 0.929386 |
| GO:0009653\_anatomical\_structure\_morphogenesis | 958 | 5 | 0.924402 | -0.183596 | 457 | 310.046870 | 424.18 | 538.313130 | 0.928184 |
| GO:0007010\_cytoskeleton\_organization | 185 | 1 | 0.957380 | -0.182658 | 458 | 310.539250 | 424.7 | 538.860750 | 0.927293 |
| GO:0048856\_anatomical\_structure\_development | 1688 | 9 | 0.944336 | -0.182458 | 459 | 310.937123 | 425.13 | 539.322877 | 0.926209 |
| GO:0006811\_ion\_transport | 186 | 1 | 0.952233 | -0.181324 | 462 | 312.023836 | 426.31 | 540.596164 | 0.922749 |
| GO:0007155\_cell\_adhesion | 186 | 1 | 0.952233 | -0.181324 | 462 | 312.023836 | 426.31 | 540.596164 | 0.922749 |
| GO:0022610\_biological\_adhesion | 186 | 1 | 0.952233 | -0.181324 | 462 | 312.023836 | 426.31 | 540.596164 | 0.922749 |
| GO:0006952\_defense\_response | 187 | 1 | 0.947141 | -0.180002 | 463 | 312.899300 | 427.0 | 541.100700 | 0.922246 |
| GO:0007276\_gamete\_generation | 188 | 1 | 0.942103 | -0.178691 | 464 | 314.069441 | 428.13 | 542.190559 | 0.922694 |
| GO:0048699\_generation\_of\_neurons | 396 | 2 | 0.894522 | -0.175137 | 465 | 315.072315 | 429.12 | 543.167685 | 0.922839 |
| GO:0003002\_regionalization | 195 | 1 | 0.908284 | -0.169827 | 467 | 317.685792 | 431.61 | 545.534208 | 0.924218 |
| GO:0019725\_cellular\_homeostasis | 195 | 1 | 0.908284 | -0.169827 | 467 | 317.685792 | 431.61 | 545.534208 | 0.924218 |
| GO:0031175\_neuron\_projection\_development | 197 | 1 | 0.899063 | -0.167390 | 469 | 319.066631 | 432.95 | 546.833369 | 0.923134 |
| GO:0050801\_ion\_homeostasis | 197 | 1 | 0.899063 | -0.167390 | 469 | 319.066631 | 432.95 | 546.833369 | 0.923134 |
| GO:0001568\_blood\_vessel\_development | 203 | 1 | 0.872490 | -0.160322 | 470 | 322.117015 | 435.81 | 549.502985 | 0.927255 |
| GO:0007243\_protein\_kinase\_cascade | 205 | 1 | 0.863977 | -0.158044 | 471 | 323.520631 | 437.18 | 550.839369 | 0.928195 |
| GO:0001944\_vasculature\_development | 208 | 1 | 0.851516 | -0.154697 | 473 | 325.161718 | 438.73 | 552.298282 | 0.927548 |
| GO:0008284\_positive\_regulation\_of\_cell\_proliferation | 208 | 1 | 0.851516 | -0.154697 | 473 | 325.161718 | 438.73 | 552.298282 | 0.927548 |
| GO:0010604\_positive\_regulation\_of\_macromolecule\_metabolic\_process | 433 | 2 | 0.818085 | -0.144453 | 474 | 328.986156 | 442.27 | 555.553844 | 0.933059 |
| GO:0006357\_regulation\_of\_transcription\_from\_RNA\_polymerase\_II\_promoter | 435 | 2 | 0.814324 | -0.142957 | 475 | 330.085412 | 443.29 | 556.494588 | 0.933242 |
| GO:0009887\_organ\_morphogenesis | 642 | 3 | 0.827642 | -0.140471 | 476 | 331.049785 | 444.17 | 557.290215 | 0.933130 |
| GO:0006464\_protein\_modification\_process | 439 | 2 | 0.806904 | -0.140011 | 477 | 331.396045 | 444.44 | 557.483955 | 0.931740 |
| GO:0031325\_positive\_regulation\_of\_cellular\_metabolic\_process | 442 | 2 | 0.801427 | -0.137842 | 478 | 331.779176 | 444.77 | 557.760824 | 0.930481 |
| GO:0006366\_transcription\_from\_RNA\_polymerase\_II\_promoter | 444 | 2 | 0.797817 | -0.136414 | 479 | 332.479969 | 445.35 | 558.220031 | 0.929749 |
| GO:0019953\_sexual\_reproduction | 228 | 1 | 0.776822 | -0.134353 | 481 | 333.593868 | 446.33 | 559.066132 | 0.927921 |
| GO:0046649\_lymphocyte\_activation | 228 | 1 | 0.776822 | -0.134353 | 481 | 333.593868 | 446.33 | 559.066132 | 0.927921 |
| GO:0007167\_enzyme\_linked\_receptor\_protein\_signaling\_pathway | 229 | 1 | 0.773430 | -0.133419 | 482 | 334.245071 | 447.01 | 559.774929 | 0.927407 |
| GO:0006996\_organelle\_organization | 449 | 2 | 0.788933 | -0.132909 | 483 | 335.005131 | 447.61 | 560.214869 | 0.926729 |
| GO:0050790\_regulation\_of\_catalytic\_activity | 233 | 1 | 0.760152 | -0.129754 | 484 | 337.392271 | 449.78 | 562.167729 | 0.929298 |
| GO:0016477\_cell\_migration | 234 | 1 | 0.756903 | -0.128856 | 485 | 338.054992 | 450.32 | 562.585008 | 0.928495 |
| GO:0009893\_positive\_regulation\_of\_metabolic\_process | 458 | 2 | 0.773430 | -0.126822 | 486 | 339.081584 | 451.19 | 563.298416 | 0.928374 |
| GO:0030154\_cell\_differentiation | 1060 | 5 | 0.835450 | -0.125809 | 487 | 340.067593 | 452.2 | 564.332407 | 0.928542 |
| GO:0045321\_leukocyte\_activation | 248 | 1 | 0.714175 | -0.116982 | 488 | 342.576350 | 454.38 | 566.183650 | 0.931107 |
| GO:0007389\_pattern\_specification\_process | 250 | 1 | 0.708462 | -0.115387 | 489 | 342.889490 | 454.65 | 566.410510 | 0.929755 |
| GO:0007267\_cell-cell\_signaling | 252 | 1 | 0.702839 | -0.113816 | 490 | 343.849717 | 455.52 | 567.190283 | 0.929633 |
| GO:0030097\_hemopoiesis | 253 | 1 | 0.700061 | -0.113040 | 491 | 344.574948 | 456.17 | 567.765052 | 0.929063 |
| GO:0048878\_chemical\_homeostasis | 254 | 1 | 0.697305 | -0.112269 | 492 | 345.082732 | 456.6 | 568.117268 | 0.928049 |
| GO:0065008\_regulation\_of\_biological\_quality | 693 | 3 | 0.766733 | -0.111714 | 493 | 345.455707 | 456.95 | 568.444293 | 0.926876 |
| GO:0048522\_positive\_regulation\_of\_cellular\_process | 895 | 4 | 0.791577 | -0.111659 | 494 | 345.686628 | 457.1 | 568.513372 | 0.925304 |
| GO:0009966\_regulation\_of\_signal\_transduction | 256 | 1 | 0.691857 | -0.110745 | 495 | 346.679944 | 457.96 | 569.240056 | 0.925172 |
| GO:0048870\_cell\_motility | 257 | 1 | 0.689165 | -0.109991 | 496 | 347.054747 | 458.31 | 569.565253 | 0.924012 |
| GO:0048666\_neuron\_development | 262 | 1 | 0.676013 | -0.106307 | 497 | 349.259940 | 460.2 | 571.140060 | 0.925956 |
| GO:0030030\_cell\_projection\_organization | 263 | 1 | 0.673443 | -0.105587 | 498 | 349.689348 | 460.54 | 571.390652 | 0.924779 |
| GO:0050896\_response\_to\_stimulus | 1107 | 5 | 0.799979 | -0.104956 | 499 | 349.927340 | 460.73 | 571.532660 | 0.923307 |
| GO:0048869\_cellular\_developmental\_process | 1113 | 5 | 0.795667 | -0.102520 | 500 | 351.142384 | 461.79 | 572.437616 | 0.923580 |
| GO:0045944\_positive\_regulation\_of\_transcription\_from\_RNA\_polymerase\_II\_promoter | 269 | 1 | 0.658422 | -0.101374 | 501 | 351.501569 | 462.08 | 572.658431 | 0.922315 |
| GO:0002376\_immune\_system\_process | 505 | 2 | 0.701447 | -0.099219 | 502 | 352.751141 | 463.18 | 573.608859 | 0.922669 |
| GO:0032502\_developmental\_process | 2060 | 10 | 0.859783 | -0.097180 | 503 | 354.265271 | 464.42 | 574.574729 | 0.923300 |
| GO:0003008\_system\_process | 516 | 2 | 0.686494 | -0.093659 | 504 | 356.664938 | 466.43 | 576.195062 | 0.925456 |
| GO:0006629\_lipid\_metabolic\_process | 285 | 1 | 0.621457 | -0.091006 | 505 | 358.107001 | 467.76 | 577.412999 | 0.926257 |
| GO:0040011\_locomotion | 295 | 1 | 0.600391 | -0.085109 | 507 | 361.023396 | 470.19 | 579.356604 | 0.927396 |
| GO:0045595\_regulation\_of\_cell\_differentiation | 295 | 1 | 0.600391 | -0.085109 | 507 | 361.023396 | 470.19 | 579.356604 | 0.927396 |
| GO:0045893\_positive\_regulation\_of\_transcription\_\_DNA-dependent | 306 | 1 | 0.578808 | -0.079090 | 509 | 363.354615 | 471.98 | 580.605385 | 0.927269 |
| GO:0051254\_positive\_regulation\_of\_RNA\_metabolic\_process | 306 | 1 | 0.578808 | -0.079090 | 509 | 363.354615 | 471.98 | 580.605385 | 0.927269 |
| GO:0051094\_positive\_regulation\_of\_developmental\_process | 308 | 1 | 0.575050 | -0.078045 | 510 | 364.436003 | 472.91 | 581.383997 | 0.927275 |
| GO:0048518\_positive\_regulation\_of\_biological\_process | 995 | 4 | 0.712022 | -0.073106 | 511 | 366.580658 | 474.47 | 582.359342 | 0.928513 |
| GO:0051093\_negative\_regulation\_of\_developmental\_process | 331 | 1 | 0.535092 | -0.067021 | 512 | 369.534501 | 476.88 | 584.225499 | 0.931406 |
| GO:0045941\_positive\_regulation\_of\_transcription | 338 | 1 | 0.524010 | -0.063999 | 513 | 371.638193 | 478.43 | 585.221807 | 0.932612 |
| GO:0009605\_response\_to\_external\_stimulus | 339 | 1 | 0.522464 | -0.063579 | 514 | 371.952174 | 478.69 | 585.427826 | 0.931304 |
| GO:0007166\_cell\_surface\_receptor\_linked\_signal\_transduction | 597 | 2 | 0.593351 | -0.061015 | 515 | 374.402787 | 480.68 | 586.957213 | 0.933359 |
| GO:0010628\_positive\_regulation\_of\_gene\_expression | 346 | 1 | 0.511894 | -0.060718 | 516 | 374.862952 | 480.98 | 587.097048 | 0.932132 |
| GO:0045935\_positive\_regulation\_of\_nucleobase\_\_nucleoside\_\_nucleotide\_and\_nucleic\_acid\_metabolic\_process | 352 | 1 | 0.503169 | -0.058372 | 517 | 375.956671 | 481.73 | 587.503329 | 0.931779 |
| GO:0042981\_regulation\_of\_apoptosis | 360 | 1 | 0.491987 | -0.055389 | 518 | 376.626753 | 482.25 | 587.873247 | 0.930985 |
| GO:0051173\_positive\_regulation\_of\_nitrogen\_compound\_metabolic\_process | 361 | 1 | 0.490624 | -0.055027 | 519 | 377.285979 | 482.73 | 588.174021 | 0.930116 |
| GO:0010941\_regulation\_of\_cell\_death | 365 | 1 | 0.485248 | -0.053605 | 521 | 378.498419 | 483.74 | 588.981581 | 0.928484 |
| GO:0043067\_regulation\_of\_programmed\_cell\_death | 365 | 1 | 0.485248 | -0.053605 | 521 | 378.498419 | 483.74 | 588.981581 | 0.928484 |
| GO:0010557\_positive\_regulation\_of\_macromolecule\_biosynthetic\_process | 371 | 1 | 0.477400 | -0.051541 | 522 | 379.365810 | 484.52 | 589.674190 | 0.928199 |
| GO:0031328\_positive\_regulation\_of\_cellular\_biosynthetic\_process | 387 | 1 | 0.457662 | -0.046427 | 523 | 381.876434 | 486.42 | 590.963566 | 0.930057 |
| GO:0009891\_positive\_regulation\_of\_biosynthetic\_process | 388 | 1 | 0.456483 | -0.046125 | 524 | 382.243325 | 486.67 | 591.096675 | 0.928760 |
| GO:0042221\_response\_to\_chemical\_stimulus | 409 | 1 | 0.433045 | -0.040225 | 525 | 385.159892 | 488.9 | 592.640108 | 0.931238 |
| GO:0042592\_homeostatic\_process | 419 | 1 | 0.422710 | -0.037690 | 526 | 386.228650 | 489.71 | 593.191350 | 0.931008 |
| GO:0006915\_apoptosis | 427 | 1 | 0.414790 | -0.035778 | 527 | 387.592044 | 490.72 | 593.847956 | 0.931157 |
| GO:0012501\_programmed\_cell\_death | 433 | 1 | 0.409042 | -0.034408 | 528 | 388.822397 | 491.52 | 594.217603 | 0.930909 |
| GO:0050793\_regulation\_of\_developmental\_process | 703 | 2 | 0.503884 | -0.034338 | 529 | 388.968146 | 491.65 | 594.331854 | 0.929395 |
| GO:0008219\_cell\_death | 444 | 1 | 0.398909 | -0.032032 | 530 | 390.734058 | 492.87 | 595.005942 | 0.929943 |
| GO:0016265\_death | 450 | 1 | 0.393590 | -0.030806 | 531 | 391.774943 | 493.7 | 595.625057 | 0.929755 |
| GO:0006641\_triglyceride\_metabolic\_process | 29 | 0 | 0.000000 | -0.000000 | 548 | 423.384072 | 520.19 | 616.995928 | 0.949252 |
| GO:0006909\_phagocytosis | 29 | 0 | 0.000000 | -0.000000 | 548 | 423.384072 | 520.19 | 616.995928 | 0.949252 |
| GO:0007190\_activation\_of\_adenylate\_cyclase\_activity | 29 | 0 | 0.000000 | -0.000000 | 548 | 423.384072 | 520.19 | 616.995928 | 0.949252 |
| GO:0010564\_regulation\_of\_cell\_cycle\_process | 29 | 0 | 0.000000 | -0.000000 | 548 | 423.384072 | 520.19 | 616.995928 | 0.949252 |
| GO:0021761\_limbic\_system\_development | 29 | 0 | 0.000000 | -0.000000 | 548 | 423.384072 | 520.19 | 616.995928 | 0.949252 |
| GO:0042176\_regulation\_of\_protein\_catabolic\_process | 29 | 0 | 0.000000 | -0.000000 | 548 | 423.384072 | 520.19 | 616.995928 | 0.949252 |
| GO:0042490\_mechanoreceptor\_differentiation | 29 | 0 | 0.000000 | -0.000000 | 548 | 423.384072 | 520.19 | 616.995928 | 0.949252 |
| GO:0043281\_regulation\_of\_caspase\_activity | 29 | 0 | 0.000000 | -0.000000 | 548 | 423.384072 | 520.19 | 616.995928 | 0.949252 |
| GO:0044087\_regulation\_of\_cellular\_component\_biogenesis | 29 | 0 | 0.000000 | -0.000000 | 548 | 423.384072 | 520.19 | 616.995928 | 0.949252 |
| GO:0044270\_nitrogen\_compound\_catabolic\_process | 29 | 0 | 0.000000 | -0.000000 | 548 | 423.384072 | 520.19 | 616.995928 | 0.949252 |
| GO:0045621\_positive\_regulation\_of\_lymphocyte\_differentiation | 29 | 0 | 0.000000 | -0.000000 | 548 | 423.384072 | 520.19 | 616.995928 | 0.949252 |
| GO:0046634\_regulation\_of\_alpha-beta\_T\_cell\_activation | 29 | 0 | 0.000000 | -0.000000 | 548 | 423.384072 | 520.19 | 616.995928 | 0.949252 |
| GO:0048066\_pigmentation\_during\_development | 29 | 0 | 0.000000 | -0.000000 | 548 | 423.384072 | 520.19 | 616.995928 | 0.949252 |
| GO:0050769\_positive\_regulation\_of\_neurogenesis | 29 | 0 | 0.000000 | -0.000000 | 548 | 423.384072 | 520.19 | 616.995928 | 0.949252 |
| GO:0052548\_regulation\_of\_endopeptidase\_activity | 29 | 0 | 0.000000 | -0.000000 | 548 | 423.384072 | 520.19 | 616.995928 | 0.949252 |
| GO:0060041\_retina\_development\_in\_camera-type\_eye | 29 | 0 | 0.000000 | -0.000000 | 548 | 423.384072 | 520.19 | 616.995928 | 0.949252 |
| GO:0070302\_regulation\_of\_stress-activated\_protein\_kinase\_signaling\_pathway | 29 | 0 | 0.000000 | -0.000000 | 548 | 423.384072 | 520.19 | 616.995928 | 0.949252 |
| GO:0001708\_cell\_fate\_specification | 56 | 0 | 0.000000 | -0.000000 | 558 | 432.401695 | 528.58 | 624.758305 | 0.947276 |
| GO:0002683\_negative\_regulation\_of\_immune\_system\_process | 56 | 0 | 0.000000 | -0.000000 | 558 | 432.401695 | 528.58 | 624.758305 | 0.947276 |
| GO:0002703\_regulation\_of\_leukocyte\_mediated\_immunity | 56 | 0 | 0.000000 | -0.000000 | 558 | 432.401695 | 528.58 | 624.758305 | 0.947276 |
| GO:0006790\_sulfur\_metabolic\_process | 56 | 0 | 0.000000 | -0.000000 | 558 | 432.401695 | 528.58 | 624.758305 | 0.947276 |
| GO:0009187\_cyclic\_nucleotide\_metabolic\_process | 56 | 0 | 0.000000 | -0.000000 | 558 | 432.401695 | 528.58 | 624.758305 | 0.947276 |
| GO:0042089\_cytokine\_biosynthetic\_process | 56 | 0 | 0.000000 | -0.000000 | 558 | 432.401695 | 528.58 | 624.758305 | 0.947276 |
| GO:0042107\_cytokine\_metabolic\_process | 56 | 0 | 0.000000 | -0.000000 | 558 | 432.401695 | 528.58 | 624.758305 | 0.947276 |
| GO:0046486\_glycerolipid\_metabolic\_process | 56 | 0 | 0.000000 | -0.000000 | 558 | 432.401695 | 528.58 | 624.758305 | 0.947276 |
| GO:0050678\_regulation\_of\_epithelial\_cell\_proliferation | 56 | 0 | 0.000000 | -0.000000 | 558 | 432.401695 | 528.58 | 624.758305 | 0.947276 |
| GO:0051321\_meiotic\_cell\_cycle | 56 | 0 | 0.000000 | -0.000000 | 558 | 432.401695 | 528.58 | 624.758305 | 0.947276 |
| GO:0001843\_neural\_tube\_closure | 33 | 0 | 0.000000 | -0.000000 | 569 | 445.882907 | 541.44 | 636.997093 | 0.951564 |
| GO:0006643\_membrane\_lipid\_metabolic\_process | 33 | 0 | 0.000000 | -0.000000 | 569 | 445.882907 | 541.44 | 636.997093 | 0.951564 |
| GO:0007188\_G-protein\_signaling\_\_coupled\_to\_cAMP\_nucleotide\_second\_messenger | 33 | 0 | 0.000000 | -0.000000 | 569 | 445.882907 | 541.44 | 636.997093 | 0.951564 |
| GO:0007270\_nerve-nerve\_synaptic\_transmission | 33 | 0 | 0.000000 | -0.000000 | 569 | 445.882907 | 541.44 | 636.997093 | 0.951564 |
| GO:0007431\_salivary\_gland\_development | 33 | 0 | 0.000000 | -0.000000 | 569 | 445.882907 | 541.44 | 636.997093 | 0.951564 |
| GO:0007565\_female\_pregnancy | 33 | 0 | 0.000000 | -0.000000 | 569 | 445.882907 | 541.44 | 636.997093 | 0.951564 |
| GO:0008643\_carbohydrate\_transport | 33 | 0 | 0.000000 | -0.000000 | 569 | 445.882907 | 541.44 | 636.997093 | 0.951564 |
| GO:0021987\_cerebral\_cortex\_development | 33 | 0 | 0.000000 | -0.000000 | 569 | 445.882907 | 541.44 | 636.997093 | 0.951564 |
| GO:0022037\_metencephalon\_development | 33 | 0 | 0.000000 | -0.000000 | 569 | 445.882907 | 541.44 | 636.997093 | 0.951564 |
| GO:0042108\_positive\_regulation\_of\_cytokine\_biosynthetic\_process | 33 | 0 | 0.000000 | -0.000000 | 569 | 445.882907 | 541.44 | 636.997093 | 0.951564 |
| GO:0060606\_tube\_closure | 33 | 0 | 0.000000 | -0.000000 | 569 | 445.882907 | 541.44 | 636.997093 | 0.951564 |
| GO:0009968\_negative\_regulation\_of\_signal\_transduction | 103 | 0 | 0.000000 | -0.000000 | 570 | 447.729034 | 543.04 | 638.350966 | 0.952702 |
| GO:0044265\_cellular\_macromolecule\_catabolic\_process | 75 | 0 | 0.000000 | -0.000000 | 571 | 450.806190 | 545.71 | 640.613810 | 0.955709 |
| GO:0002573\_myeloid\_leukocyte\_differentiation | 50 | 0 | 0.000000 | -0.000000 | 576 | 457.527932 | 551.89 | 646.252068 | 0.958142 |
| GO:0009190\_cyclic\_nucleotide\_biosynthetic\_process | 50 | 0 | 0.000000 | -0.000000 | 576 | 457.527932 | 551.89 | 646.252068 | 0.958142 |
| GO:0042129\_regulation\_of\_T\_cell\_proliferation | 50 | 0 | 0.000000 | -0.000000 | 576 | 457.527932 | 551.89 | 646.252068 | 0.958142 |
| GO:0051606\_detection\_of\_stimulus | 50 | 0 | 0.000000 | -0.000000 | 576 | 457.527932 | 551.89 | 646.252068 | 0.958142 |
| GO:0070647\_protein\_modification\_by\_small\_protein\_conjugation\_or\_removal | 50 | 0 | 0.000000 | -0.000000 | 576 | 457.527932 | 551.89 | 646.252068 | 0.958142 |
| GO:0005975\_carbohydrate\_metabolic\_process | 146 | 0 | 0.000000 | -0.000000 | 577 | 459.211556 | 553.22 | 647.228444 | 0.958787 |
| GO:0002694\_regulation\_of\_leukocyte\_activation | 121 | 0 | 0.000000 | -0.000000 | 580 | 461.446848 | 555.3 | 649.153152 | 0.957414 |
| GO:0006917\_induction\_of\_apoptosis | 121 | 0 | 0.000000 | -0.000000 | 580 | 461.446848 | 555.3 | 649.153152 | 0.957414 |
| GO:0012502\_induction\_of\_programmed\_cell\_death | 121 | 0 | 0.000000 | -0.000000 | 580 | 461.446848 | 555.3 | 649.153152 | 0.957414 |
| GO:0060249\_anatomical\_structure\_homeostasis | 96 | 0 | 0.000000 | -0.000000 | 582 | 464.486729 | 558.1 | 651.713271 | 0.958935 |
| GO:0070661\_leukocyte\_proliferation | 96 | 0 | 0.000000 | -0.000000 | 582 | 464.486729 | 558.1 | 651.713271 | 0.958935 |
| GO:0000027\_ribosomal\_large\_subunit\_assembly | 1 | 0 |  |  |  |  |  |  |  |  |
| GO:0000042\_protein\_targeting\_to\_Golgi | 1 | 0 |  |  |  |  |  |  |  |  |
| GO:0000046\_autophagic\_vacuole\_fusion | 1 | 0 |  |  |  |  |  |  |  |  |
| GO:0000050\_urea\_cycle | 1 | 0 |  |  |  |  |  |  |  |  |
| GO:0000054\_ribosome\_export\_from\_nucleus | 1 | 0 |  |  |  |  |  |  |  |  |
| GO:0000055\_ribosomal\_large\_subunit\_export\_from\_nucleus | 1 | 0 |  |  |  |  |  |  |  |  |
| GO:0000056\_ribosomal\_small\_subunit\_export\_from\_nucleus | 1 | 0 |  |  |  |  |  |  |  |  |
| GO:0000072\_M\_phase\_specific\_microtubule\_process | 1 | 0 |  |  |  |  |  |  |  |  |
| GO:0000101\_sulfur\_amino\_acid\_transport | 1 | 0 |  |  |  |  |  |  |  |  |
| GO:0000147\_actin\_cortical\_patch\_assembly | 1 | 0 |  |  |  |  |  |  |  |  |
| GO:0000154\_rRNA\_modification | 1 | 0 |  |  |  |  |  |  |  |  |
| GO:0000183\_chromatin\_silencing\_at\_rDNA | 1 | 0 |  |  |  |  |  |  |  |  |
| GO:0000185\_activation\_of\_MAPKKK\_activity | 1 | 0 |  |  |  |  |  |  |  |  |
| GO:0000238\_zygotene | 1 | 0 |  |  |  |  |  |  |  |  |
| GO:0000255\_allantoin\_metabolic\_process | 1 | 0 |  |  |  |  |  |  |  |  |
| GO:0000266\_mitochondrial\_fission | 1 | 0 |  |  |  |  |  |  |  |  |
| GO:0000273\_lipoic\_acid\_metabolic\_process | 1 | 0 |  |  |  |  |  |  |  |  |
| GO:0000301\_retrograde\_transport\_\_vesicle\_recycling\_within\_Golgi | 1 | 0 |  |  |  |  |  |  |  |  |
| GO:0000394\_RNA\_splicing\_\_via\_endonucleolytic\_cleavage\_and\_ligation | 1 | 0 |  |  |  |  |  |  |  |  |
| GO:0000429\_regulation\_of\_transcription\_from\_RNA\_polymerase\_II\_promoter\_by\_carbon\_catabolites | 1 | 0 |  |  |  |  |  |  |  |  |
| GO:0000430\_regulation\_of\_transcription\_from\_RNA\_polymerase\_II\_promoter\_by\_glucose | 1 | 0 |  |  |  |  |  |  |  |  |
| GO:0000432\_positive\_regulation\_of\_transcription\_from\_RNA\_polymerase\_II\_promoter\_by\_glucose | 1 | 0 |  |  |  |  |  |  |  |  |
| GO:0000436\_positive\_regulation\_of\_transcription\_from\_RNA\_polymerase\_II\_promoter\_by\_carbon\_catabolites | 1 | 0 |  |  |  |  |  |  |  |  |
| GO:0000448\_cleavage\_in\_ITS2\_between\_5.8S\_rRNA\_and\_LSU-rRNA\_of\_tricistronic\_rRNA\_transcript\_(SSU-rRNA\_\_5.8S\_rRNA\_\_LSU-rRNA) | 1 | 0 |  |  |  |  |  |  |  |  |
| GO:0000460\_maturation\_of\_5.8S\_rRNA | 1 | 0 |  |  |  |  |  |  |  |  |
| GO:0000463\_maturation\_of\_LSU-rRNA\_from\_tricistronic\_rRNA\_transcript\_(SSU-rRNA\_\_5.8S\_rRNA\_\_LSU-rRNA) | 1 | 0 |  |  |  |  |  |  |  |  |
| GO:0000466\_maturation\_of\_5.8S\_rRNA\_from\_tricistronic\_rRNA\_transcript\_(SSU-rRNA\_\_5.8S\_rRNA\_\_LSU-rRNA) | 1 | 0 |  |  |  |  |  |  |  |  |
| GO:0000469\_cleavages\_during\_rRNA\_processing | 1 | 0 |  |  |  |  |  |  |  |  |
| GO:0000470\_maturation\_of\_LSU-rRNA | 1 | 0 |  |  |  |  |  |  |  |  |
| GO:0000478\_endonucleolytic\_cleavages\_during\_rRNA\_processing | 1 | 0 |  |  |  |  |  |  |  |  |
| GO:0000479\_endonucleolytic\_cleavage\_of\_tricistronic\_rRNA\_transcript\_(SSU-rRNA\_\_5.8S\_rRNA\_\_LSU-rRNA) | 1 | 0 |  |  |  |  |  |  |  |  |
| GO:0000705\_achiasmate\_meiosis\_I | 1 | 0 |  |  |  |  |  |  |  |  |
| GO:0000966\_RNA\_5'-end\_processing | 1 | 0 |  |  |  |  |  |  |  |  |
| GO:0001300\_chronological\_cell\_aging | 1 | 0 |  |  |  |  |  |  |  |  |
| GO:0001547\_antral\_ovarian\_follicle\_growth | 1 | 0 |  |  |  |  |  |  |  |  |
| GO:0001555\_oocyte\_growth | 1 | 0 |  |  |  |  |  |  |  |  |
| GO:0001560\_regulation\_of\_cell\_growth\_by\_extracellular\_stimulus | 1 | 0 |  |  |  |  |  |  |  |  |
| GO:0001660\_fever | 1 | 0 |  |  |  |  |  |  |  |  |
| GO:0001696\_gastric\_acid\_secretion | 1 | 0 |  |  |  |  |  |  |  |  |
| GO:0001712\_ectodermal\_cell\_fate\_commitment | 1 | 0 |  |  |  |  |  |  |  |  |
| GO:0001714\_endodermal\_cell\_fate\_specification | 1 | 0 |  |  |  |  |  |  |  |  |
| GO:0001762\_beta-alanine\_transport | 1 | 0 |  |  |  |  |  |  |  |  |
| GO:0001766\_membrane\_raft\_polarization | 1 | 0 |  |  |  |  |  |  |  |  |
| GO:0001811\_negative\_regulation\_of\_type\_I\_hypersensitivity | 1 | 0 |  |  |  |  |  |  |  |  |
| GO:0001821\_histamine\_secretion | 1 | 0 |  |  |  |  |  |  |  |  |
| GO:0001826\_inner\_cell\_mass\_cell\_differentiation | 1 | 0 |  |  |  |  |  |  |  |  |
| GO:0001830\_trophectodermal\_cell\_fate\_commitment | 1 | 0 |  |  |  |  |  |  |  |  |
| GO:0001834\_trophectodermal\_cell\_proliferation | 1 | 0 |  |  |  |  |  |  |  |  |
| GO:0001867\_complement\_activation\_\_lectin\_pathway | 1 | 0 |  |  |  |  |  |  |  |  |
| GO:0001880\_Mullerian\_duct\_regression | 1 | 0 |  |  |  |  |  |  |  |  |
| GO:0001887\_selenium\_metabolic\_process | 1 | 0 |  |  |  |  |  |  |  |  |
| GO:0001922\_B-1\_B\_cell\_homeostasis | 1 | 0 |  |  |  |  |  |  |  |  |
| GO:0001923\_B-1\_B\_cell\_differentiation | 1 | 0 |  |  |  |  |  |  |  |  |
| GO:0001941\_postsynaptic\_membrane\_organization | 1 | 0 |  |  |  |  |  |  |  |  |
| GO:0001946\_lymphangiogenesis | 1 | 0 |  |  |  |  |  |  |  |  |
| GO:0001956\_positive\_regulation\_of\_neurotransmitter\_secretion | 1 | 0 |  |  |  |  |  |  |  |  |
| GO:0001961\_positive\_regulation\_of\_cytokine-mediated\_signaling\_pathway | 1 | 0 |  |  |  |  |  |  |  |  |
| GO:0001979\_regulation\_of\_systemic\_arterial\_blood\_pressure\_by\_chemoreceptor\_signaling | 1 | 0 |  |  |  |  |  |  |  |  |
| GO:0001980\_regulation\_of\_systemic\_arterial\_blood\_pressure\_by\_ischemic\_conditions | 1 | 0 |  |  |  |  |  |  |  |  |
| GO:0001984\_vasodilation\_of\_artery\_during\_baroreceptor\_response\_to\_increased\_systemic\_arterial\_blood\_pressure | 1 | 0 |  |  |  |  |  |  |  |  |
| GO:0001985\_negative\_regulation\_of\_heart\_rate\_in\_baroreceptor\_response\_to\_increased\_systemic\_arterial\_blood\_pressure | 1 | 0 |  |  |  |  |  |  |  |  |
| GO:0001987\_vasoconstriction\_of\_artery\_involved\_in\_baroreceptor\_response\_to\_lowering\_of\_systemic\_arterial\_blood\_pressure | 1 | 0 |  |  |  |  |  |  |  |  |
| GO:0001988\_positive\_regulation\_of\_heart\_rate\_in\_baroreceptor\_response\_to\_decreased\_systemic\_arterial\_blood\_pressure | 1 | 0 |  |  |  |  |  |  |  |  |
| GO:0001994\_norepinephrine-epinephrine\_vasoconstriction\_involved\_in\_regulation\_of\_systemic\_arterial\_blood\_pressure | 1 | 0 |  |  |  |  |  |  |  |  |
| GO:0002001\_renin\_secretion\_into\_blood\_stream | 1 | 0 |  |  |  |  |  |  |  |  |
| GO:0002002\_regulation\_of\_angiotensin\_levels\_in\_blood | 1 | 0 |  |  |  |  |  |  |  |  |
| GO:0002003\_angiotensin\_maturation | 1 | 0 |  |  |  |  |  |  |  |  |
| GO:0002007\_detection\_of\_hypoxic\_conditions\_in\_blood\_by\_chemoreceptor\_signaling | 1 | 0 |  |  |  |  |  |  |  |  |
| GO:0002017\_regulation\_of\_blood\_volume\_by\_renal\_aldosterone | 1 | 0 |  |  |  |  |  |  |  |  |
| GO:0002023\_reduction\_of\_food\_intake\_in\_response\_to\_dietary\_excess | 1 | 0 |  |  |  |  |  |  |  |  |
| GO:0002031\_G-protein\_coupled\_receptor\_internalization | 1 | 0 |  |  |  |  |  |  |  |  |
| GO:0002036\_regulation\_of\_L-glutamate\_transport | 1 | 0 |  |  |  |  |  |  |  |  |
| GO:0002040\_sprouting\_angiogenesis | 1 | 0 |  |  |  |  |  |  |  |  |
| GO:0002041\_intussusceptive\_angiogenesis | 1 | 0 |  |  |  |  |  |  |  |  |
| GO:0002068\_glandular\_epithelial\_cell\_development | 1 | 0 |  |  |  |  |  |  |  |  |
| GO:0002069\_columnar\_cuboidal\_epithelial\_cell\_maturation | 1 | 0 |  |  |  |  |  |  |  |  |
| GO:0002071\_glandular\_epithelial\_cell\_maturation | 1 | 0 |  |  |  |  |  |  |  |  |
| GO:0002082\_regulation\_of\_oxidative\_phosphorylation | 1 | 0 |  |  |  |  |  |  |  |  |
| GO:0002084\_protein\_depalmitoylation | 1 | 0 |  |  |  |  |  |  |  |  |
| GO:0002085\_inhibition\_of\_neuroepithelial\_cell\_differentiation | 1 | 0 |  |  |  |  |  |  |  |  |
| GO:0002086\_diaphragm\_contraction | 1 | 0 |  |  |  |  |  |  |  |  |
| GO:0002118\_aggressive\_behavior | 1 | 0 |  |  |  |  |  |  |  |  |
| GO:0002121\_inter-male\_aggressive\_behavior | 1 | 0 |  |  |  |  |  |  |  |  |
| GO:0002124\_territorial\_aggressive\_behavior | 1 | 0 |  |  |  |  |  |  |  |  |
| GO:0002227\_innate\_immune\_response\_in\_mucosa | 1 | 0 |  |  |  |  |  |  |  |  |
| GO:0002232\_leukocyte\_chemotaxis\_during\_inflammatory\_response | 1 | 0 |  |  |  |  |  |  |  |  |
| GO:0002248\_connective\_tissue\_replacement\_during\_inflammatory\_response | 1 | 0 |  |  |  |  |  |  |  |  |
| GO:0002282\_microglial\_cell\_activation\_during\_immune\_response | 1 | 0 |  |  |  |  |  |  |  |  |
| GO:0002287\_alpha-beta\_T\_cell\_activation\_during\_immune\_response | 1 | 0 |  |  |  |  |  |  |  |  |
| GO:0002314\_germinal\_center\_B\_cell\_differentiation | 1 | 0 |  |  |  |  |  |  |  |  |
| GO:0002315\_marginal\_zone\_B\_cell\_differentiation | 1 | 0 |  |  |  |  |  |  |  |  |
| GO:0002316\_follicular\_B\_cell\_differentiation | 1 | 0 |  |  |  |  |  |  |  |  |
| GO:0002317\_plasma\_cell\_differentiation | 1 | 0 |  |  |  |  |  |  |  |  |
| GO:0002349\_histamine\_production\_during\_acute\_inflammatory\_response | 1 | 0 |  |  |  |  |  |  |  |  |
| GO:0002351\_serotonin\_production\_during\_acute\_inflammatory\_response | 1 | 0 |  |  |  |  |  |  |  |  |
| GO:0002355\_detection\_of\_tumor\_cell | 1 | 0 |  |  |  |  |  |  |  |  |
| GO:0002370\_natural\_killer\_cell\_cytokine\_production | 1 | 0 |  |  |  |  |  |  |  |  |
| GO:0002371\_dendritic\_cell\_cytokine\_production | 1 | 0 |  |  |  |  |  |  |  |  |
| GO:0002380\_immunoglobulin\_secretion\_during\_immune\_response | 1 | 0 |  |  |  |  |  |  |  |  |
| GO:0002396\_MHC\_protein\_complex\_assembly | 1 | 0 |  |  |  |  |  |  |  |  |
| GO:0002397\_MHC\_class\_I\_protein\_complex\_assembly | 1 | 0 |  |  |  |  |  |  |  |  |
| GO:0002420\_natural\_killer\_cell\_mediated\_cytotoxicity\_directed\_against\_tumor\_cell\_target | 1 | 0 |  |  |  |  |  |  |  |  |
| GO:0002423\_natural\_killer\_cell\_mediated\_immune\_response\_to\_tumor\_cell | 1 | 0 |  |  |  |  |  |  |  |  |
| GO:0002424\_T\_cell\_mediated\_immune\_response\_to\_tumor\_cell | 1 | 0 |  |  |  |  |  |  |  |  |
| GO:0002426\_immunoglobulin\_production\_in\_mucosal\_tissue | 1 | 0 |  |  |  |  |  |  |  |  |
| GO:0002431\_Fc\_receptor\_mediated\_stimulatory\_signaling\_pathway | 1 | 0 |  |  |  |  |  |  |  |  |
| GO:0002432\_granuloma\_formation | 1 | 0 |  |  |  |  |  |  |  |  |
| GO:0002441\_histamine\_secretion\_during\_acute\_inflammatory\_response | 1 | 0 |  |  |  |  |  |  |  |  |
| GO:0002442\_serotonin\_secretion\_during\_acute\_inflammatory\_response | 1 | 0 |  |  |  |  |  |  |  |  |
| GO:0002457\_T\_cell\_antigen\_processing\_and\_presentation | 1 | 0 |  |  |  |  |  |  |  |  |
| GO:0002458\_peripheral\_T\_cell\_tolerance\_induction | 1 | 0 |  |  |  |  |  |  |  |  |
| GO:0002461\_tolerance\_induction\_dependent\_upon\_immune\_response | 1 | 0 |  |  |  |  |  |  |  |  |
| GO:0002465\_peripheral\_tolerance\_induction | 1 | 0 |  |  |  |  |  |  |  |  |
| GO:0002468\_dendritic\_cell\_antigen\_processing\_and\_presentation | 1 | 0 |  |  |  |  |  |  |  |  |
| GO:0002476\_antigen\_processing\_and\_presentation\_of\_endogenous\_peptide\_antigen\_via\_MHC\_class\_Ib | 1 | 0 |  |  |  |  |  |  |  |  |
| GO:0002479\_antigen\_processing\_and\_presentation\_of\_exogenous\_peptide\_antigen\_via\_MHC\_class\_I\_\_TAP-dependent | 1 | 0 |  |  |  |  |  |  |  |  |
| GO:0002483\_antigen\_processing\_and\_presentation\_of\_endogenous\_peptide\_antigen | 1 | 0 |  |  |  |  |  |  |  |  |
| GO:0002501\_peptide\_antigen\_assembly\_with\_MHC\_protein\_complex | 1 | 0 |  |  |  |  |  |  |  |  |
| GO:0002502\_peptide\_antigen\_assembly\_with\_MHC\_class\_I\_protein\_complex | 1 | 0 |  |  |  |  |  |  |  |  |
| GO:0002508\_central\_tolerance\_induction | 1 | 0 |  |  |  |  |  |  |  |  |
| GO:0002510\_central\_B\_cell\_tolerance\_induction | 1 | 0 |  |  |  |  |  |  |  |  |
| GO:0002545\_chronic\_inflammatory\_response\_to\_non-antigenic\_stimulus | 1 | 0 |  |  |  |  |  |  |  |  |
| GO:0002553\_histamine\_secretion\_by\_mast\_cell | 1 | 0 |  |  |  |  |  |  |  |  |
| GO:0002554\_serotonin\_secretion\_by\_platelet | 1 | 0 |  |  |  |  |  |  |  |  |
| GO:0002572\_pro-T\_cell\_differentiation | 1 | 0 |  |  |  |  |  |  |  |  |
| GO:0002577\_regulation\_of\_antigen\_processing\_and\_presentation | 1 | 0 |  |  |  |  |  |  |  |  |
| GO:0002579\_positive\_regulation\_of\_antigen\_processing\_and\_presentation | 1 | 0 |  |  |  |  |  |  |  |  |
| GO:0002604\_regulation\_of\_dendritic\_cell\_antigen\_processing\_and\_presentation | 1 | 0 |  |  |  |  |  |  |  |  |
| GO:0002606\_positive\_regulation\_of\_dendritic\_cell\_antigen\_processing\_and\_presentation | 1 | 0 |  |  |  |  |  |  |  |  |
| GO:0002635\_negative\_regulation\_of\_germinal\_center\_formation | 1 | 0 |  |  |  |  |  |  |  |  |
| GO:0002646\_regulation\_of\_central\_tolerance\_induction | 1 | 0 |  |  |  |  |  |  |  |  |
| GO:0002648\_positive\_regulation\_of\_central\_tolerance\_induction | 1 | 0 |  |  |  |  |  |  |  |  |
| GO:0002649\_regulation\_of\_tolerance\_induction\_to\_self\_antigen | 1 | 0 |  |  |  |  |  |  |  |  |
| GO:0002651\_positive\_regulation\_of\_tolerance\_induction\_to\_self\_antigen | 1 | 0 |  |  |  |  |  |  |  |  |
| GO:0002652\_regulation\_of\_tolerance\_induction\_dependent\_upon\_immune\_response | 1 | 0 |  |  |  |  |  |  |  |  |
| GO:0002654\_positive\_regulation\_of\_tolerance\_induction\_dependent\_upon\_immune\_response | 1 | 0 |  |  |  |  |  |  |  |  |
| GO:0002658\_regulation\_of\_peripheral\_tolerance\_induction | 1 | 0 |  |  |  |  |  |  |  |  |
| GO:0002660\_positive\_regulation\_of\_peripheral\_tolerance\_induction | 1 | 0 |  |  |  |  |  |  |  |  |
| GO:0002677\_negative\_regulation\_of\_chronic\_inflammatory\_response | 1 | 0 |  |  |  |  |  |  |  |  |
| GO:0002678\_positive\_regulation\_of\_chronic\_inflammatory\_response | 1 | 0 |  |  |  |  |  |  |  |  |
| GO:0002701\_negative\_regulation\_of\_production\_of\_molecular\_mediator\_of\_immune\_response | 1 | 0 |  |  |  |  |  |  |  |  |
| GO:0002719\_negative\_regulation\_of\_cytokine\_production\_during\_immune\_response | 1 | 0 |  |  |  |  |  |  |  |  |
| GO:0002724\_regulation\_of\_T\_cell\_cytokine\_production | 1 | 0 |  |  |  |  |  |  |  |  |
| GO:0002727\_regulation\_of\_natural\_killer\_cell\_cytokine\_production | 1 | 0 |  |  |  |  |  |  |  |  |
| GO:0002729\_positive\_regulation\_of\_natural\_killer\_cell\_cytokine\_production | 1 | 0 |  |  |  |  |  |  |  |  |
| GO:0002730\_regulation\_of\_dendritic\_cell\_cytokine\_production | 1 | 0 |  |  |  |  |  |  |  |  |
| GO:0002756\_MyD88-independent\_toll-like\_receptor\_signaling\_pathway | 1 | 0 |  |  |  |  |  |  |  |  |
| GO:0002767\_immune\_response-inhibiting\_cell\_surface\_receptor\_signaling\_pathway | 1 | 0 |  |  |  |  |  |  |  |  |
| GO:0002769\_natural\_killer\_cell\_inhibitory\_signaling\_pathway | 1 | 0 |  |  |  |  |  |  |  |  |
| GO:0002840\_regulation\_of\_T\_cell\_mediated\_immune\_response\_to\_tumor\_cell | 1 | 0 |  |  |  |  |  |  |  |  |
| GO:0002842\_positive\_regulation\_of\_T\_cell\_mediated\_immune\_response\_to\_tumor\_cell | 1 | 0 |  |  |  |  |  |  |  |  |
| GO:0002849\_regulation\_of\_peripheral\_T\_cell\_tolerance\_induction | 1 | 0 |  |  |  |  |  |  |  |  |
| GO:0002851\_positive\_regulation\_of\_peripheral\_T\_cell\_tolerance\_induction | 1 | 0 |  |  |  |  |  |  |  |  |
| GO:0002855\_regulation\_of\_natural\_killer\_cell\_mediated\_immune\_response\_to\_tumor\_cell | 1 | 0 |  |  |  |  |  |  |  |  |
| GO:0002857\_positive\_regulation\_of\_natural\_killer\_cell\_mediated\_immune\_response\_to\_tumor\_cell | 1 | 0 |  |  |  |  |  |  |  |  |
| GO:0002858\_regulation\_of\_natural\_killer\_cell\_mediated\_cytotoxicity\_directed\_against\_tumor\_cell\_target | 1 | 0 |  |  |  |  |  |  |  |  |
| GO:0002860\_positive\_regulation\_of\_natural\_killer\_cell\_mediated\_cytotoxicity\_directed\_against\_tumor\_cell\_target | 1 | 0 |  |  |  |  |  |  |  |  |
| GO:0002880\_regulation\_of\_chronic\_inflammatory\_response\_to\_non-antigenic\_stimulus | 1 | 0 |  |  |  |  |  |  |  |  |
| GO:0002882\_positive\_regulation\_of\_chronic\_inflammatory\_response\_to\_non-antigenic\_stimulus | 1 | 0 |  |  |  |  |  |  |  |  |
| GO:0002895\_regulation\_of\_central\_B\_cell\_tolerance\_induction | 1 | 0 |  |  |  |  |  |  |  |  |
| GO:0002897\_positive\_regulation\_of\_central\_B\_cell\_tolerance\_induction | 1 | 0 |  |  |  |  |  |  |  |  |
| GO:0002901\_mature\_B\_cell\_apoptosis | 1 | 0 |  |  |  |  |  |  |  |  |
| GO:0002903\_negative\_regulation\_of\_B\_cell\_apoptosis | 1 | 0 |  |  |  |  |  |  |  |  |
| GO:0002905\_regulation\_of\_mature\_B\_cell\_apoptosis | 1 | 0 |  |  |  |  |  |  |  |  |
| GO:0002906\_negative\_regulation\_of\_mature\_B\_cell\_apoptosis | 1 | 0 |  |  |  |  |  |  |  |  |
| GO:0003011\_involuntary\_skeletal\_muscle\_contraction | 1 | 0 |  |  |  |  |  |  |  |  |
| GO:0003027\_regulation\_of\_systemic\_arterial\_blood\_pressure\_by\_carotid\_body\_chemoreceptor\_signaling | 1 | 0 |  |  |  |  |  |  |  |  |
| GO:0003029\_detection\_of\_hypoxic\_conditions\_in\_blood\_by\_carotid\_body\_chemoreceptor\_signaling | 1 | 0 |  |  |  |  |  |  |  |  |
| GO:0003032\_detection\_of\_oxygen | 1 | 0 |  |  |  |  |  |  |  |  |
| GO:0003056\_regulation\_of\_vascular\_smooth\_muscle\_contraction | 1 | 0 |  |  |  |  |  |  |  |  |
| GO:0003062\_regulation\_of\_heart\_rate\_by\_chemical\_signal | 1 | 0 |  |  |  |  |  |  |  |  |
| GO:0003065\_positive\_regulation\_of\_heart\_rate\_by\_epinephrine | 1 | 0 |  |  |  |  |  |  |  |  |
| GO:0003068\_regulation\_of\_systemic\_arterial\_blood\_pressure\_by\_acetylcholine | 1 | 0 |  |  |  |  |  |  |  |  |
| GO:0003069\_vasodilation\_by\_acetylcholine\_involved\_in\_regulation\_of\_systemic\_arterial\_blood\_pressure | 1 | 0 |  |  |  |  |  |  |  |  |
| GO:0003070\_regulation\_of\_systemic\_arterial\_blood\_pressure\_by\_neurotransmitter | 1 | 0 |  |  |  |  |  |  |  |  |
| GO:0003097\_renal\_water\_transport | 1 | 0 |  |  |  |  |  |  |  |  |
| GO:0005979\_regulation\_of\_glycogen\_biosynthetic\_process | 1 | 0 |  |  |  |  |  |  |  |  |
| GO:0005984\_disaccharide\_metabolic\_process | 1 | 0 |  |  |  |  |  |  |  |  |
| GO:0005988\_lactose\_metabolic\_process | 1 | 0 |  |  |  |  |  |  |  |  |
| GO:0005989\_lactose\_biosynthetic\_process | 1 | 0 |  |  |  |  |  |  |  |  |
| GO:0005997\_xylulose\_metabolic\_process | 1 | 0 |  |  |  |  |  |  |  |  |
| GO:0006000\_fructose\_metabolic\_process | 1 | 0 |  |  |  |  |  |  |  |  |
| GO:0006002\_fructose\_6-phosphate\_metabolic\_process | 1 | 0 |  |  |  |  |  |  |  |  |
| GO:0006004\_fucose\_metabolic\_process | 1 | 0 |  |  |  |  |  |  |  |  |
| GO:0006013\_mannose\_metabolic\_process | 1 | 0 |  |  |  |  |  |  |  |  |
| GO:0006060\_sorbitol\_metabolic\_process | 1 | 0 |  |  |  |  |  |  |  |  |
| GO:0006064\_glucuronate\_catabolic\_process | 1 | 0 |  |  |  |  |  |  |  |  |
| GO:0006086\_acetyl-CoA\_biosynthetic\_process\_from\_pyruvate | 1 | 0 |  |  |  |  |  |  |  |  |
| GO:0006098\_pentose-phosphate\_shunt | 1 | 0 |  |  |  |  |  |  |  |  |
| GO:0006101\_citrate\_metabolic\_process | 1 | 0 |  |  |  |  |  |  |  |  |
| GO:0006104\_succinyl-CoA\_metabolic\_process | 1 | 0 |  |  |  |  |  |  |  |  |
| GO:0006116\_NADH\_oxidation | 1 | 0 |  |  |  |  |  |  |  |  |
| GO:0006120\_mitochondrial\_electron\_transport\_\_NADH\_to\_ubiquinone | 1 | 0 |  |  |  |  |  |  |  |  |
| GO:0006154\_adenosine\_catabolic\_process | 1 | 0 |  |  |  |  |  |  |  |  |
| GO:0006157\_deoxyadenosine\_catabolic\_process | 1 | 0 |  |  |  |  |  |  |  |  |
| GO:0006167\_AMP\_biosynthetic\_process | 1 | 0 |  |  |  |  |  |  |  |  |
| GO:0006175\_dATP\_biosynthetic\_process | 1 | 0 |  |  |  |  |  |  |  |  |
| GO:0006178\_guanine\_salvage | 1 | 0 |  |  |  |  |  |  |  |  |
| GO:0006196\_AMP\_catabolic\_process | 1 | 0 |  |  |  |  |  |  |  |  |
| GO:0006203\_dGTP\_catabolic\_process | 1 | 0 |  |  |  |  |  |  |  |  |
| GO:0006208\_pyrimidine\_base\_catabolic\_process | 1 | 0 |  |  |  |  |  |  |  |  |
| GO:0006221\_pyrimidine\_nucleotide\_biosynthetic\_process | 1 | 0 |  |  |  |  |  |  |  |  |
| GO:0006235\_dTTP\_biosynthetic\_process | 1 | 0 |  |  |  |  |  |  |  |  |
| GO:0006244\_pyrimidine\_nucleotide\_catabolic\_process | 1 | 0 |  |  |  |  |  |  |  |  |
| GO:0006269\_DNA\_replication\_\_synthesis\_of\_RNA\_primer | 1 | 0 |  |  |  |  |  |  |  |  |
| GO:0006283\_transcription-coupled\_nucleotide-excision\_repair | 1 | 0 |  |  |  |  |  |  |  |  |
| GO:0006296\_nucleotide-excision\_repair\_\_DNA\_incision\_\_5'-to\_lesion | 1 | 0 |  |  |  |  |  |  |  |  |
| GO:0006307\_DNA\_dealkylation | 1 | 0 |  |  |  |  |  |  |  |  |
| GO:0006337\_nucleosome\_disassembly | 1 | 0 |  |  |  |  |  |  |  |  |
| GO:0006344\_maintenance\_of\_chromatin\_silencing | 1 | 0 |  |  |  |  |  |  |  |  |
| GO:0006356\_regulation\_of\_transcription\_from\_RNA\_polymerase\_I\_promoter | 1 | 0 |  |  |  |  |  |  |  |  |
| GO:0006388\_tRNA\_splicing\_\_via\_endonucleolytic\_cleavage\_and\_ligation | 1 | 0 |  |  |  |  |  |  |  |  |
| GO:0006407\_rRNA\_export\_from\_nucleus | 1 | 0 |  |  |  |  |  |  |  |  |
| GO:0006419\_alanyl-tRNA\_aminoacylation | 1 | 0 |  |  |  |  |  |  |  |  |
| GO:0006434\_seryl-tRNA\_aminoacylation | 1 | 0 |  |  |  |  |  |  |  |  |
| GO:0006447\_regulation\_of\_translational\_initiation\_by\_iron | 1 | 0 |  |  |  |  |  |  |  |  |
| GO:0006463\_steroid\_hormone\_receptor\_complex\_assembly | 1 | 0 |  |  |  |  |  |  |  |  |
| GO:0006467\_protein\_thiol-disulfide\_exchange | 1 | 0 |  |  |  |  |  |  |  |  |
| GO:0006474\_N-terminal\_protein\_amino\_acid\_acetylation | 1 | 0 |  |  |  |  |  |  |  |  |
| GO:0006481\_C-terminal\_protein\_amino\_acid\_methylation | 1 | 0 |  |  |  |  |  |  |  |  |
| GO:0006488\_dolichol-linked\_oligosaccharide\_biosynthetic\_process | 1 | 0 |  |  |  |  |  |  |  |  |
| GO:0006494\_protein\_amino\_acid\_terminal\_glycosylation | 1 | 0 |  |  |  |  |  |  |  |  |
| GO:0006496\_protein\_amino\_acid\_terminal\_N-glycosylation | 1 | 0 |  |  |  |  |  |  |  |  |
| GO:0006500\_N-terminal\_protein\_palmitoylation | 1 | 0 |  |  |  |  |  |  |  |  |
| GO:0006507\_GPI\_anchor\_release | 1 | 0 |  |  |  |  |  |  |  |  |
| GO:0006537\_glutamate\_biosynthetic\_process | 1 | 0 |  |  |  |  |  |  |  |  |
| GO:0006544\_glycine\_metabolic\_process | 1 | 0 |  |  |  |  |  |  |  |  |
| GO:0006549\_isoleucine\_metabolic\_process | 1 | 0 |  |  |  |  |  |  |  |  |
| GO:0006553\_lysine\_metabolic\_process | 1 | 0 |  |  |  |  |  |  |  |  |
| GO:0006554\_lysine\_catabolic\_process | 1 | 0 |  |  |  |  |  |  |  |  |
| GO:0006556\_S-adenosylmethionine\_biosynthetic\_process | 1 | 0 |  |  |  |  |  |  |  |  |
| GO:0006559\_L-phenylalanine\_catabolic\_process | 1 | 0 |  |  |  |  |  |  |  |  |
| GO:0006569\_tryptophan\_catabolic\_process | 1 | 0 |  |  |  |  |  |  |  |  |
| GO:0006572\_tyrosine\_catabolic\_process | 1 | 0 |  |  |  |  |  |  |  |  |
| GO:0006573\_valine\_metabolic\_process | 1 | 0 |  |  |  |  |  |  |  |  |
| GO:0006581\_acetylcholine\_catabolic\_process | 1 | 0 |  |  |  |  |  |  |  |  |
| GO:0006585\_dopamine\_biosynthetic\_process\_from\_tyrosine | 1 | 0 |  |  |  |  |  |  |  |  |
| GO:0006590\_thyroid\_hormone\_generation | 1 | 0 |  |  |  |  |  |  |  |  |
| GO:0006591\_ornithine\_metabolic\_process | 1 | 0 |  |  |  |  |  |  |  |  |
| GO:0006596\_polyamine\_biosynthetic\_process | 1 | 0 |  |  |  |  |  |  |  |  |
| GO:0006597\_spermine\_biosynthetic\_process | 1 | 0 |  |  |  |  |  |  |  |  |
| GO:0006601\_creatine\_biosynthetic\_process | 1 | 0 |  |  |  |  |  |  |  |  |
| GO:0006613\_cotranslational\_protein\_targeting\_to\_membrane | 1 | 0 |  |  |  |  |  |  |  |  |
| GO:0006622\_protein\_targeting\_to\_lysosome | 1 | 0 |  |  |  |  |  |  |  |  |
| GO:0006627\_mitochondrial\_protein\_processing\_during\_import | 1 | 0 |  |  |  |  |  |  |  |  |
| GO:0006653\_lecithin\_metabolic\_process | 1 | 0 |  |  |  |  |  |  |  |  |
| GO:0006654\_phosphatidic\_acid\_biosynthetic\_process | 1 | 0 |  |  |  |  |  |  |  |  |
| GO:0006658\_phosphatidylserine\_metabolic\_process | 1 | 0 |  |  |  |  |  |  |  |  |
| GO:0006659\_phosphatidylserine\_biosynthetic\_process | 1 | 0 |  |  |  |  |  |  |  |  |
| GO:0006667\_sphinganine\_metabolic\_process | 1 | 0 |  |  |  |  |  |  |  |  |
| GO:0006668\_sphinganine-1-phosphate\_metabolic\_process | 1 | 0 |  |  |  |  |  |  |  |  |
| GO:0006678\_glucosylceramide\_metabolic\_process | 1 | 0 |  |  |  |  |  |  |  |  |
| GO:0006682\_galactosylceramide\_biosynthetic\_process | 1 | 0 |  |  |  |  |  |  |  |  |
| GO:0006685\_sphingomyelin\_catabolic\_process | 1 | 0 |  |  |  |  |  |  |  |  |
| GO:0006700\_C21-steroid\_hormone\_biosynthetic\_process | 1 | 0 |  |  |  |  |  |  |  |  |
| GO:0006705\_mineralocorticoid\_biosynthetic\_process | 1 | 0 |  |  |  |  |  |  |  |  |
| GO:0006709\_progesterone\_catabolic\_process | 1 | 0 |  |  |  |  |  |  |  |  |
| GO:0006729\_tetrahydrobiopterin\_biosynthetic\_process | 1 | 0 |  |  |  |  |  |  |  |  |
| GO:0006734\_NADH\_metabolic\_process | 1 | 0 |  |  |  |  |  |  |  |  |
| GO:0006740\_NADPH\_regeneration | 1 | 0 |  |  |  |  |  |  |  |  |
| GO:0006741\_NADP\_biosynthetic\_process | 1 | 0 |  |  |  |  |  |  |  |  |
| GO:0006743\_ubiquinone\_metabolic\_process | 1 | 0 |  |  |  |  |  |  |  |  |
| GO:0006744\_ubiquinone\_biosynthetic\_process | 1 | 0 |  |  |  |  |  |  |  |  |
| GO:0006772\_thiamin\_metabolic\_process | 1 | 0 |  |  |  |  |  |  |  |  |
| GO:0006784\_heme\_a\_biosynthetic\_process | 1 | 0 |  |  |  |  |  |  |  |  |
| GO:0006797\_polyphosphate\_metabolic\_process | 1 | 0 |  |  |  |  |  |  |  |  |
| GO:0006798\_polyphosphate\_catabolic\_process | 1 | 0 |  |  |  |  |  |  |  |  |
| GO:0006824\_cobalt\_ion\_transport | 1 | 0 |  |  |  |  |  |  |  |  |
| GO:0006842\_tricarboxylic\_acid\_transport | 1 | 0 |  |  |  |  |  |  |  |  |
| GO:0006844\_acyl\_carnitine\_transport | 1 | 0 |  |  |  |  |  |  |  |  |
| GO:0006855\_multidrug\_transport | 1 | 0 |  |  |  |  |  |  |  |  |
| GO:0006863\_purine\_transport | 1 | 0 |  |  |  |  |  |  |  |  |
| GO:0006890\_retrograde\_vesicle-mediated\_transport\_\_Golgi\_to\_ER | 1 | 0 |  |  |  |  |  |  |  |  |
| GO:0006891\_intra-Golgi\_vesicle-mediated\_transport | 1 | 0 |  |  |  |  |  |  |  |  |
| GO:0006893\_Golgi\_to\_plasma\_membrane\_transport | 1 | 0 |  |  |  |  |  |  |  |  |
| GO:0006895\_Golgi\_to\_endosome\_transport | 1 | 0 |  |  |  |  |  |  |  |  |
| GO:0006896\_Golgi\_to\_vacuole\_transport | 1 | 0 |  |  |  |  |  |  |  |  |
| GO:0006900\_membrane\_budding | 1 | 0 |  |  |  |  |  |  |  |  |
| GO:0006930\_substrate-bound\_cell\_migration\_\_cell\_extension | 1 | 0 |  |  |  |  |  |  |  |  |
| GO:0006931\_substrate-bound\_cell\_migration\_\_cell\_attachment\_to\_substrate | 1 | 0 |  |  |  |  |  |  |  |  |
| GO:0006957\_complement\_activation\_\_alternative\_pathway | 1 | 0 |  |  |  |  |  |  |  |  |
| GO:0006958\_complement\_activation\_\_classical\_pathway | 1 | 0 |  |  |  |  |  |  |  |  |
| GO:0006978\_DNA\_damage\_response\_\_signal\_transduction\_by\_p53\_class\_mediator\_resulting\_in\_transcription\_of\_p21\_class\_mediator | 1 | 0 |  |  |  |  |  |  |  |  |
| GO:0007016\_cytoskeletal\_anchoring\_at\_plasma\_membrane | 1 | 0 |  |  |  |  |  |  |  |  |
| GO:0007021\_tubulin\_complex\_assembly | 1 | 0 |  |  |  |  |  |  |  |  |
| GO:0007052\_mitotic\_spindle\_organization | 1 | 0 |  |  |  |  |  |  |  |  |
| GO:0007056\_spindle\_assembly\_involved\_in\_female\_meiosis | 1 | 0 |  |  |  |  |  |  |  |  |
| GO:0007057\_spindle\_assembly\_involved\_in\_female\_meiosis\_I | 1 | 0 |  |  |  |  |  |  |  |  |
| GO:0007063\_regulation\_of\_sister\_chromatid\_cohesion | 1 | 0 |  |  |  |  |  |  |  |  |
| GO:0007065\_male\_meiosis\_sister\_chromatid\_cohesion | 1 | 0 |  |  |  |  |  |  |  |  |
| GO:0007076\_mitotic\_chromosome\_condensation | 1 | 0 |  |  |  |  |  |  |  |  |
| GO:0007095\_mitotic\_cell\_cycle\_G2\_M\_transition\_DNA\_damage\_checkpoint | 1 | 0 |  |  |  |  |  |  |  |  |
| GO:0007096\_regulation\_of\_exit\_from\_mitosis | 1 | 0 |  |  |  |  |  |  |  |  |
| GO:0007158\_neuron\_adhesion | 1 | 0 |  |  |  |  |  |  |  |  |
| GO:0007168\_receptor\_guanylyl\_cyclase\_signaling\_pathway | 1 | 0 |  |  |  |  |  |  |  |  |
| GO:0007197\_inhibition\_of\_adenylate\_cyclase\_activity\_by\_muscarinic\_acetylcholine\_receptor\_signaling\_pathway | 1 | 0 |  |  |  |  |  |  |  |  |
| GO:0007207\_activation\_of\_phospholipase\_C\_activity\_by\_muscarinic\_acetylcholine\_receptor\_signaling\_pathway | 1 | 0 |  |  |  |  |  |  |  |  |
| GO:0007208\_activation\_of\_phospholipase\_C\_activity\_by\_serotonin\_receptor\_signaling\_pathway | 1 | 0 |  |  |  |  |  |  |  |  |
| GO:0007217\_tachykinin\_receptor\_signaling\_pathway | 1 | 0 |  |  |  |  |  |  |  |  |
| GO:0007221\_positive\_regulation\_of\_transcription\_of\_Notch\_receptor\_target | 1 | 0 |  |  |  |  |  |  |  |  |
| GO:0007223\_Wnt\_receptor\_signaling\_pathway\_\_calcium\_modulating\_pathway | 1 | 0 |  |  |  |  |  |  |  |  |
| GO:0007225\_patched\_ligand\_processing | 1 | 0 |  |  |  |  |  |  |  |  |
| GO:0007227\_signal\_transduction\_downstream\_of\_smoothened | 1 | 0 |  |  |  |  |  |  |  |  |
| GO:0007228\_positive\_regulation\_of\_hh\_target\_transcription\_factor\_activity | 1 | 0 |  |  |  |  |  |  |  |  |
| GO:0007231\_osmosensory\_signaling\_pathway | 1 | 0 |  |  |  |  |  |  |  |  |
| GO:0007284\_spermatogonial\_cell\_division | 1 | 0 |  |  |  |  |  |  |  |  |
| GO:0007290\_spermatid\_nucleus\_elongation | 1 | 0 |  |  |  |  |  |  |  |  |
| GO:0007296\_vitellogenesis | 1 | 0 |  |  |  |  |  |  |  |  |
| GO:0007321\_sperm\_displacement | 1 | 0 |  |  |  |  |  |  |  |  |
| GO:0007380\_specification\_of\_segmental\_identity\_\_head | 1 | 0 |  |  |  |  |  |  |  |  |
| GO:0007382\_specification\_of\_segmental\_identity\_\_maxillary\_segment | 1 | 0 |  |  |  |  |  |  |  |  |
| GO:0007400\_neuroblast\_fate\_determination | 1 | 0 |  |  |  |  |  |  |  |  |
| GO:0007402\_ganglion\_mother\_cell\_fate\_determination | 1 | 0 |  |  |  |  |  |  |  |  |
| GO:0007495\_visceral\_mesoderm-endoderm\_interaction\_involved\_in\_midgut\_development | 1 | 0 |  |  |  |  |  |  |  |  |
| GO:0007497\_posterior\_midgut\_development | 1 | 0 |  |  |  |  |  |  |  |  |
| GO:0007499\_ectoderm\_and\_mesoderm\_interaction | 1 | 0 |  |  |  |  |  |  |  |  |
| GO:0007500\_mesodermal\_cell\_fate\_determination | 1 | 0 |  |  |  |  |  |  |  |  |
| GO:0007509\_mesoderm\_migration | 1 | 0 |  |  |  |  |  |  |  |  |
| GO:0007518\_myoblast\_cell\_fate\_determination | 1 | 0 |  |  |  |  |  |  |  |  |
| GO:0007521\_muscle\_cell\_fate\_determination | 1 | 0 |  |  |  |  |  |  |  |  |
| GO:0007522\_visceral\_muscle\_development | 1 | 0 |  |  |  |  |  |  |  |  |
| GO:0007529\_establishment\_of\_synaptic\_specificity\_at\_neuromuscular\_junction | 1 | 0 |  |  |  |  |  |  |  |  |
| GO:0007538\_primary\_sex\_determination | 1 | 0 |  |  |  |  |  |  |  |  |
| GO:0007542\_primary\_sex\_determination\_\_germ-line | 1 | 0 |  |  |  |  |  |  |  |  |
| GO:0007567\_parturition | 1 | 0 |  |  |  |  |  |  |  |  |
| GO:0007614\_short-term\_memory | 1 | 0 |  |  |  |  |  |  |  |  |
| GO:0007621\_negative\_regulation\_of\_female\_receptivity | 1 | 0 |  |  |  |  |  |  |  |  |
| GO:0008049\_male\_courtship\_behavior | 1 | 0 |  |  |  |  |  |  |  |  |
| GO:0008050\_female\_courtship\_behavior | 1 | 0 |  |  |  |  |  |  |  |  |
| GO:0008052\_sensory\_organ\_boundary\_specification | 1 | 0 |  |  |  |  |  |  |  |  |
| GO:0008054\_cyclin\_catabolic\_process | 1 | 0 |  |  |  |  |  |  |  |  |
| GO:0008057\_eye\_pigment\_granule\_organization | 1 | 0 |  |  |  |  |  |  |  |  |
| GO:0008078\_mesodermal\_cell\_migration | 1 | 0 |  |  |  |  |  |  |  |  |
| GO:0008208\_C21-steroid\_hormone\_catabolic\_process | 1 | 0 |  |  |  |  |  |  |  |  |
| GO:0008216\_spermidine\_metabolic\_process | 1 | 0 |  |  |  |  |  |  |  |  |
| GO:0008292\_acetylcholine\_biosynthetic\_process | 1 | 0 |  |  |  |  |  |  |  |  |
| GO:0008295\_spermidine\_biosynthetic\_process | 1 | 0 |  |  |  |  |  |  |  |  |
| GO:0008300\_isoprenoid\_catabolic\_process | 1 | 0 |  |  |  |  |  |  |  |  |
| GO:0008333\_endosome\_to\_lysosome\_transport | 1 | 0 |  |  |  |  |  |  |  |  |
| GO:0008355\_olfactory\_learning | 1 | 0 |  |  |  |  |  |  |  |  |
| GO:0008611\_ether\_lipid\_biosynthetic\_process | 1 | 0 |  |  |  |  |  |  |  |  |
| GO:0008626\_induction\_of\_apoptosis\_by\_granzyme | 1 | 0 |  |  |  |  |  |  |  |  |
| GO:0008633\_activation\_of\_pro-apoptotic\_gene\_products | 1 | 0 |  |  |  |  |  |  |  |  |
| GO:0008653\_lipopolysaccharide\_metabolic\_process | 1 | 0 |  |  |  |  |  |  |  |  |
| GO:0009068\_aspartate\_family\_amino\_acid\_catabolic\_process | 1 | 0 |  |  |  |  |  |  |  |  |
| GO:0009084\_glutamine\_family\_amino\_acid\_biosynthetic\_process | 1 | 0 |  |  |  |  |  |  |  |  |
| GO:0009088\_threonine\_biosynthetic\_process | 1 | 0 |  |  |  |  |  |  |  |  |
| GO:0009105\_lipoic\_acid\_biosynthetic\_process | 1 | 0 |  |  |  |  |  |  |  |  |
| GO:0009109\_coenzyme\_catabolic\_process | 1 | 0 |  |  |  |  |  |  |  |  |
| GO:0009111\_vitamin\_catabolic\_process | 1 | 0 |  |  |  |  |  |  |  |  |
| GO:0009113\_purine\_base\_biosynthetic\_process | 1 | 0 |  |  |  |  |  |  |  |  |
| GO:0009127\_purine\_nucleoside\_monophosphate\_biosynthetic\_process | 1 | 0 |  |  |  |  |  |  |  |  |
| GO:0009128\_purine\_nucleoside\_monophosphate\_catabolic\_process | 1 | 0 |  |  |  |  |  |  |  |  |
| GO:0009129\_pyrimidine\_nucleoside\_monophosphate\_metabolic\_process | 1 | 0 |  |  |  |  |  |  |  |  |
| GO:0009131\_pyrimidine\_nucleoside\_monophosphate\_catabolic\_process | 1 | 0 |  |  |  |  |  |  |  |  |
| GO:0009133\_nucleoside\_diphosphate\_biosynthetic\_process | 1 | 0 |  |  |  |  |  |  |  |  |
| GO:0009145\_purine\_nucleoside\_triphosphate\_biosynthetic\_process | 1 | 0 |  |  |  |  |  |  |  |  |
| GO:0009147\_pyrimidine\_nucleoside\_triphosphate\_metabolic\_process | 1 | 0 |  |  |  |  |  |  |  |  |
| GO:0009148\_pyrimidine\_nucleoside\_triphosphate\_biosynthetic\_process | 1 | 0 |  |  |  |  |  |  |  |  |
| GO:0009152\_purine\_ribonucleotide\_biosynthetic\_process | 1 | 0 |  |  |  |  |  |  |  |  |
| GO:0009153\_purine\_deoxyribonucleotide\_biosynthetic\_process | 1 | 0 |  |  |  |  |  |  |  |  |
| GO:0009156\_ribonucleoside\_monophosphate\_biosynthetic\_process | 1 | 0 |  |  |  |  |  |  |  |  |
| GO:0009158\_ribonucleoside\_monophosphate\_catabolic\_process | 1 | 0 |  |  |  |  |  |  |  |  |
| GO:0009159\_deoxyribonucleoside\_monophosphate\_catabolic\_process | 1 | 0 |  |  |  |  |  |  |  |  |
| GO:0009162\_deoxyribonucleoside\_monophosphate\_metabolic\_process | 1 | 0 |  |  |  |  |  |  |  |  |
| GO:0009168\_purine\_ribonucleoside\_monophosphate\_biosynthetic\_process | 1 | 0 |  |  |  |  |  |  |  |  |
| GO:0009169\_purine\_ribonucleoside\_monophosphate\_catabolic\_process | 1 | 0 |  |  |  |  |  |  |  |  |
| GO:0009176\_pyrimidine\_deoxyribonucleoside\_monophosphate\_metabolic\_process | 1 | 0 |  |  |  |  |  |  |  |  |
| GO:0009178\_pyrimidine\_deoxyribonucleoside\_monophosphate\_catabolic\_process | 1 | 0 |  |  |  |  |  |  |  |  |
| GO:0009211\_pyrimidine\_deoxyribonucleoside\_triphosphate\_metabolic\_process | 1 | 0 |  |  |  |  |  |  |  |  |
| GO:0009212\_pyrimidine\_deoxyribonucleoside\_triphosphate\_biosynthetic\_process | 1 | 0 |  |  |  |  |  |  |  |  |
| GO:0009216\_purine\_deoxyribonucleoside\_triphosphate\_biosynthetic\_process | 1 | 0 |  |  |  |  |  |  |  |  |
| GO:0009221\_pyrimidine\_deoxyribonucleotide\_biosynthetic\_process | 1 | 0 |  |  |  |  |  |  |  |  |
| GO:0009223\_pyrimidine\_deoxyribonucleotide\_catabolic\_process | 1 | 0 |  |  |  |  |  |  |  |  |
| GO:0009260\_ribonucleotide\_biosynthetic\_process | 1 | 0 |  |  |  |  |  |  |  |  |
| GO:0009405\_pathogenesis | 1 | 0 |  |  |  |  |  |  |  |  |
| GO:0009414\_response\_to\_water\_deprivation | 1 | 0 |  |  |  |  |  |  |  |  |
| GO:0009415\_response\_to\_water | 1 | 0 |  |  |  |  |  |  |  |  |
| GO:0009449\_gamma-aminobutyric\_acid\_biosynthetic\_process | 1 | 0 |  |  |  |  |  |  |  |  |
| GO:0009450\_gamma-aminobutyric\_acid\_catabolic\_process | 1 | 0 |  |  |  |  |  |  |  |  |
| GO:0009589\_detection\_of\_UV | 1 | 0 |  |  |  |  |  |  |  |  |
| GO:0009590\_detection\_of\_gravity | 1 | 0 |  |  |  |  |  |  |  |  |
| GO:0009624\_response\_to\_nematode | 1 | 0 |  |  |  |  |  |  |  |  |
| GO:0009629\_response\_to\_gravity | 1 | 0 |  |  |  |  |  |  |  |  |
| GO:0009648\_photoperiodism | 1 | 0 |  |  |  |  |  |  |  |  |
| GO:0009690\_cytokinin\_metabolic\_process | 1 | 0 |  |  |  |  |  |  |  |  |
| GO:0009691\_cytokinin\_biosynthetic\_process | 1 | 0 |  |  |  |  |  |  |  |  |
| GO:0009786\_regulation\_of\_asymmetric\_cell\_division | 1 | 0 |  |  |  |  |  |  |  |  |
| GO:0009794\_regulation\_of\_mitotic\_cell\_cycle\_\_embryonic | 1 | 0 |  |  |  |  |  |  |  |  |
| GO:0009956\_radial\_pattern\_formation | 1 | 0 |  |  |  |  |  |  |  |  |
| GO:0009957\_epidermal\_cell\_fate\_specification | 1 | 0 |  |  |  |  |  |  |  |  |
| GO:0009992\_cellular\_water\_homeostasis | 1 | 0 |  |  |  |  |  |  |  |  |
| GO:0010032\_meiotic\_chromosome\_condensation | 1 | 0 |  |  |  |  |  |  |  |  |
| GO:0010039\_response\_to\_iron\_ion | 1 | 0 |  |  |  |  |  |  |  |  |
| GO:0010042\_response\_to\_manganese\_ion | 1 | 0 |  |  |  |  |  |  |  |  |
| GO:0010045\_response\_to\_nickel\_ion | 1 | 0 |  |  |  |  |  |  |  |  |
| GO:0010046\_response\_to\_mycotoxin | 1 | 0 |  |  |  |  |  |  |  |  |
| GO:0010107\_potassium\_ion\_import | 1 | 0 |  |  |  |  |  |  |  |  |
| GO:0010155\_regulation\_of\_proton\_transport | 1 | 0 |  |  |  |  |  |  |  |  |
| GO:0010160\_formation\_of\_organ\_boundary | 1 | 0 |  |  |  |  |  |  |  |  |
| GO:0010260\_organ\_senescence | 1 | 0 |  |  |  |  |  |  |  |  |
| GO:0010310\_regulation\_of\_hydrogen\_peroxide\_metabolic\_process | 1 | 0 |  |  |  |  |  |  |  |  |
| GO:0010447\_response\_to\_acidity | 1 | 0 |  |  |  |  |  |  |  |  |
| GO:0010452\_histone\_H3-K36\_methylation | 1 | 0 |  |  |  |  |  |  |  |  |
| GO:0010455\_positive\_regulation\_of\_cell\_fate\_commitment | 1 | 0 |  |  |  |  |  |  |  |  |
| GO:0010470\_regulation\_of\_gastrulation | 1 | 0 |  |  |  |  |  |  |  |  |
| GO:0010508\_positive\_regulation\_of\_autophagy | 1 | 0 |  |  |  |  |  |  |  |  |
| GO:0010519\_negative\_regulation\_of\_phospholipase\_activity | 1 | 0 |  |  |  |  |  |  |  |  |
| GO:0010520\_regulation\_of\_reciprocal\_meiotic\_recombination | 1 | 0 |  |  |  |  |  |  |  |  |
| GO:0010523\_negative\_regulation\_of\_calcium\_ion\_transport\_into\_cytosol | 1 | 0 |  |  |  |  |  |  |  |  |
| GO:0010543\_regulation\_of\_platelet\_activation | 1 | 0 |  |  |  |  |  |  |  |  |
| GO:0010561\_negative\_regulation\_of\_glycoprotein\_biosynthetic\_process | 1 | 0 |  |  |  |  |  |  |  |  |
| GO:0010569\_regulation\_of\_double-strand\_break\_repair\_via\_homologous\_recombination | 1 | 0 |  |  |  |  |  |  |  |  |
| GO:0010572\_positive\_regulation\_of\_platelet\_activation | 1 | 0 |  |  |  |  |  |  |  |  |
| GO:0010594\_regulation\_of\_endothelial\_cell\_migration | 1 | 0 |  |  |  |  |  |  |  |  |
| GO:0010596\_negative\_regulation\_of\_endothelial\_cell\_migration | 1 | 0 |  |  |  |  |  |  |  |  |
| GO:0010611\_regulation\_of\_cardiac\_muscle\_hypertrophy | 1 | 0 |  |  |  |  |  |  |  |  |
| GO:0010612\_regulation\_of\_cardiac\_muscle\_adaptation | 1 | 0 |  |  |  |  |  |  |  |  |
| GO:0010614\_negative\_regulation\_of\_cardiac\_muscle\_hypertrophy | 1 | 0 |  |  |  |  |  |  |  |  |
| GO:0010616\_negative\_regulation\_of\_cardiac\_muscle\_adaptation | 1 | 0 |  |  |  |  |  |  |  |  |
| GO:0010634\_positive\_regulation\_of\_epithelial\_cell\_migration | 1 | 0 |  |  |  |  |  |  |  |  |
| GO:0010656\_negative\_regulation\_of\_muscle\_cell\_apoptosis | 1 | 0 |  |  |  |  |  |  |  |  |
| GO:0010657\_muscle\_cell\_apoptosis | 1 | 0 |  |  |  |  |  |  |  |  |
| GO:0010658\_striated\_muscle\_cell\_apoptosis | 1 | 0 |  |  |  |  |  |  |  |  |
| GO:0010659\_cardiac\_muscle\_cell\_apoptosis | 1 | 0 |  |  |  |  |  |  |  |  |
| GO:0010660\_regulation\_of\_muscle\_cell\_apoptosis | 1 | 0 |  |  |  |  |  |  |  |  |
| GO:0010662\_regulation\_of\_striated\_muscle\_cell\_apoptosis | 1 | 0 |  |  |  |  |  |  |  |  |
| GO:0010664\_negative\_regulation\_of\_striated\_muscle\_cell\_apoptosis | 1 | 0 |  |  |  |  |  |  |  |  |
| GO:0010665\_regulation\_of\_cardiac\_muscle\_cell\_apoptosis | 1 | 0 |  |  |  |  |  |  |  |  |
| GO:0010667\_negative\_regulation\_of\_cardiac\_muscle\_cell\_apoptosis | 1 | 0 |  |  |  |  |  |  |  |  |
| GO:0010668\_ectodermal\_cell\_differentiation | 1 | 0 |  |  |  |  |  |  |  |  |
| GO:0010671\_negative\_regulation\_of\_oxygen\_and\_reactive\_oxygen\_species\_metabolic\_process | 1 | 0 |  |  |  |  |  |  |  |  |
| GO:0010719\_negative\_regulation\_of\_epithelial\_to\_mesenchymal\_transition | 1 | 0 |  |  |  |  |  |  |  |  |
| GO:0010735\_positive\_regulation\_of\_transcription\_via\_serum\_response\_element\_binding | 1 | 0 |  |  |  |  |  |  |  |  |
| GO:0010825\_positive\_regulation\_of\_centrosome\_duplication | 1 | 0 |  |  |  |  |  |  |  |  |
| GO:0010845\_positive\_regulation\_of\_reciprocal\_meiotic\_recombination | 1 | 0 |  |  |  |  |  |  |  |  |
| GO:0010850\_chemoreceptor\_signaling\_pathway\_involved\_in\_regulation\_of\_blood\_pressure | 1 | 0 |  |  |  |  |  |  |  |  |
| GO:0010873\_positive\_regulation\_of\_cholesterol\_esterification | 1 | 0 |  |  |  |  |  |  |  |  |
| GO:0010880\_regulation\_of\_release\_of\_sequestered\_calcium\_ion\_into\_cytosol\_by\_sarcoplasmic\_reticulum | 1 | 0 |  |  |  |  |  |  |  |  |
| GO:0010881\_regulation\_of\_cardiac\_muscle\_contraction\_by\_regulation\_of\_the\_release\_of\_sequestered\_calcium\_ion | 1 | 0 |  |  |  |  |  |  |  |  |
| GO:0010882\_regulation\_of\_cardiac\_muscle\_contraction\_by\_calcium\_ion\_signaling | 1 | 0 |  |  |  |  |  |  |  |  |
| GO:0010890\_positive\_regulation\_of\_sequestering\_of\_triglyceride | 1 | 0 |  |  |  |  |  |  |  |  |
| GO:0010919\_regulation\_of\_inositol\_phosphate\_biosynthetic\_process | 1 | 0 |  |  |  |  |  |  |  |  |
| GO:0010931\_macrophage\_tolerance\_induction | 1 | 0 |  |  |  |  |  |  |  |  |
| GO:0010932\_regulation\_of\_macrophage\_tolerance\_induction | 1 | 0 |  |  |  |  |  |  |  |  |
| GO:0010933\_positive\_regulation\_of\_macrophage\_tolerance\_induction | 1 | 0 |  |  |  |  |  |  |  |  |
| GO:0010934\_macrophage\_cytokine\_production | 1 | 0 |  |  |  |  |  |  |  |  |
| GO:0010935\_regulation\_of\_macrophage\_cytokine\_production | 1 | 0 |  |  |  |  |  |  |  |  |
| GO:0010936\_negative\_regulation\_of\_macrophage\_cytokine\_production | 1 | 0 |  |  |  |  |  |  |  |  |
| GO:0010953\_regulation\_of\_protein\_maturation\_by\_peptide\_bond\_cleavage | 1 | 0 |  |  |  |  |  |  |  |  |
| GO:0010962\_regulation\_of\_glucan\_biosynthetic\_process | 1 | 0 |  |  |  |  |  |  |  |  |
| GO:0010966\_regulation\_of\_phosphate\_transport | 1 | 0 |  |  |  |  |  |  |  |  |
| GO:0014012\_axon\_regeneration\_in\_the\_peripheral\_nervous\_system | 1 | 0 |  |  |  |  |  |  |  |  |
| GO:0014016\_neuroblast\_differentiation | 1 | 0 |  |  |  |  |  |  |  |  |
| GO:0014017\_neuroblast\_fate\_commitment | 1 | 0 |  |  |  |  |  |  |  |  |
| GO:0014041\_regulation\_of\_neuron\_maturation | 1 | 0 |  |  |  |  |  |  |  |  |
| GO:0014042\_positive\_regulation\_of\_neuron\_maturation | 1 | 0 |  |  |  |  |  |  |  |  |
| GO:0014049\_positive\_regulation\_of\_glutamate\_secretion | 1 | 0 |  |  |  |  |  |  |  |  |
| GO:0014061\_regulation\_of\_norepinephrine\_secretion | 1 | 0 |  |  |  |  |  |  |  |  |
| GO:0014071\_response\_to\_cycloalkane | 1 | 0 |  |  |  |  |  |  |  |  |
| GO:0014707\_branchiomeric\_skeletal\_muscle\_development | 1 | 0 |  |  |  |  |  |  |  |  |
| GO:0014738\_regulation\_of\_muscle\_hyperplasia | 1 | 0 |  |  |  |  |  |  |  |  |
| GO:0014740\_negative\_regulation\_of\_muscle\_hyperplasia | 1 | 0 |  |  |  |  |  |  |  |  |
| GO:0014741\_negative\_regulation\_of\_muscle\_hypertrophy | 1 | 0 |  |  |  |  |  |  |  |  |
| GO:0014743\_regulation\_of\_muscle\_hypertrophy | 1 | 0 |  |  |  |  |  |  |  |  |
| GO:0014805\_smooth\_muscle\_adaptation | 1 | 0 |  |  |  |  |  |  |  |  |
| GO:0014806\_smooth\_muscle\_hyperplasia | 1 | 0 |  |  |  |  |  |  |  |  |
| GO:0014807\_regulation\_of\_somitogenesis | 1 | 0 |  |  |  |  |  |  |  |  |
| GO:0014808\_release\_of\_sequestered\_calcium\_ion\_into\_cytosol\_by\_sarcoplasmic\_reticulum | 1 | 0 |  |  |  |  |  |  |  |  |
| GO:0014813\_satellite\_cell\_commitment | 1 | 0 |  |  |  |  |  |  |  |  |
| GO:0014816\_satellite\_cell\_differentiation | 1 | 0 |  |  |  |  |  |  |  |  |
| GO:0014819\_regulation\_of\_skeletal\_muscle\_contraction | 1 | 0 |  |  |  |  |  |  |  |  |
| GO:0014852\_regulation\_of\_skeletal\_muscle\_contraction\_by\_neural\_stimulation\_via\_neuromuscular\_junction | 1 | 0 |  |  |  |  |  |  |  |  |
| GO:0014853\_regulation\_of\_excitatory\_postsynaptic\_membrane\_potential\_involved\_in\_skeletal\_muscle\_contraction | 1 | 0 |  |  |  |  |  |  |  |  |
| GO:0014856\_skeletal\_muscle\_cell\_proliferation | 1 | 0 |  |  |  |  |  |  |  |  |
| GO:0014857\_regulation\_of\_skeletal\_muscle\_cell\_proliferation | 1 | 0 |  |  |  |  |  |  |  |  |
| GO:0014858\_positive\_regulation\_of\_skeletal\_muscle\_cell\_proliferation | 1 | 0 |  |  |  |  |  |  |  |  |
| GO:0014887\_cardiac\_muscle\_adaptation | 1 | 0 |  |  |  |  |  |  |  |  |
| GO:0014889\_muscle\_atrophy | 1 | 0 |  |  |  |  |  |  |  |  |
| GO:0014896\_muscle\_hypertrophy | 1 | 0 |  |  |  |  |  |  |  |  |
| GO:0014897\_striated\_muscle\_hypertrophy | 1 | 0 |  |  |  |  |  |  |  |  |
| GO:0014898\_cardiac\_muscle\_hypertrophy | 1 | 0 |  |  |  |  |  |  |  |  |
| GO:0014900\_muscle\_hyperplasia | 1 | 0 |  |  |  |  |  |  |  |  |
| GO:0014910\_regulation\_of\_smooth\_muscle\_cell\_migration | 1 | 0 |  |  |  |  |  |  |  |  |
| GO:0014911\_positive\_regulation\_of\_smooth\_muscle\_cell\_migration | 1 | 0 |  |  |  |  |  |  |  |  |
| GO:0015014\_heparan\_sulfate\_proteoglycan\_biosynthetic\_process\_\_polysaccharide\_chain\_biosynthetic\_process | 1 | 0 |  |  |  |  |  |  |  |  |
| GO:0015074\_DNA\_integration | 1 | 0 |  |  |  |  |  |  |  |  |
| GO:0015670\_carbon\_dioxide\_transport | 1 | 0 |  |  |  |  |  |  |  |  |
| GO:0015677\_copper\_ion\_import | 1 | 0 |  |  |  |  |  |  |  |  |
| GO:0015680\_intracellular\_copper\_ion\_transport | 1 | 0 |  |  |  |  |  |  |  |  |
| GO:0015684\_ferrous\_iron\_transport | 1 | 0 |  |  |  |  |  |  |  |  |
| GO:0015707\_nitrite\_transport | 1 | 0 |  |  |  |  |  |  |  |  |
| GO:0015724\_formate\_transport | 1 | 0 |  |  |  |  |  |  |  |  |
| GO:0015734\_taurine\_transport | 1 | 0 |  |  |  |  |  |  |  |  |
| GO:0015740\_C4-dicarboxylate\_transport | 1 | 0 |  |  |  |  |  |  |  |  |
| GO:0015744\_succinate\_transport | 1 | 0 |  |  |  |  |  |  |  |  |
| GO:0015746\_citrate\_transport | 1 | 0 |  |  |  |  |  |  |  |  |
| GO:0015747\_urate\_transport | 1 | 0 |  |  |  |  |  |  |  |  |
| GO:0015791\_polyol\_transport | 1 | 0 |  |  |  |  |  |  |  |  |
| GO:0015798\_myo-inositol\_transport | 1 | 0 |  |  |  |  |  |  |  |  |
| GO:0015808\_L-alanine\_transport | 1 | 0 |  |  |  |  |  |  |  |  |
| GO:0015810\_aspartate\_transport | 1 | 0 |  |  |  |  |  |  |  |  |
| GO:0015811\_L-cystine\_transport | 1 | 0 |  |  |  |  |  |  |  |  |
| GO:0015817\_histidine\_transport | 1 | 0 |  |  |  |  |  |  |  |  |
| GO:0015822\_ornithine\_transport | 1 | 0 |  |  |  |  |  |  |  |  |
| GO:0015824\_proline\_transport | 1 | 0 |  |  |  |  |  |  |  |  |
| GO:0015851\_nucleobase\_transport | 1 | 0 |  |  |  |  |  |  |  |  |
| GO:0015864\_pyrimidine\_nucleoside\_transport | 1 | 0 |  |  |  |  |  |  |  |  |
| GO:0015874\_norepinephrine\_transport | 1 | 0 |  |  |  |  |  |  |  |  |
| GO:0015881\_creatine\_transport | 1 | 0 |  |  |  |  |  |  |  |  |
| GO:0015884\_folic\_acid\_transport | 1 | 0 |  |  |  |  |  |  |  |  |
| GO:0015886\_heme\_transport | 1 | 0 |  |  |  |  |  |  |  |  |
| GO:0015888\_thiamin\_transport | 1 | 0 |  |  |  |  |  |  |  |  |
| GO:0015938\_coenzyme\_A\_catabolic\_process | 1 | 0 |  |  |  |  |  |  |  |  |
| GO:0015939\_pantothenate\_metabolic\_process | 1 | 0 |  |  |  |  |  |  |  |  |
| GO:0016073\_snRNA\_metabolic\_process | 1 | 0 |  |  |  |  |  |  |  |  |
| GO:0016074\_snoRNA\_metabolic\_process | 1 | 0 |  |  |  |  |  |  |  |  |
| GO:0016082\_synaptic\_vesicle\_priming | 1 | 0 |  |  |  |  |  |  |  |  |
| GO:0016090\_prenol\_metabolic\_process | 1 | 0 |  |  |  |  |  |  |  |  |
| GO:0016093\_polyprenol\_metabolic\_process | 1 | 0 |  |  |  |  |  |  |  |  |
| GO:0016180\_snRNA\_processing | 1 | 0 |  |  |  |  |  |  |  |  |
| GO:0016239\_positive\_regulation\_of\_macroautophagy | 1 | 0 |  |  |  |  |  |  |  |  |
| GO:0016246\_RNA\_interference | 1 | 0 |  |  |  |  |  |  |  |  |
| GO:0016255\_attachment\_of\_GPI\_anchor\_to\_protein | 1 | 0 |  |  |  |  |  |  |  |  |
| GO:0016333\_morphogenesis\_of\_follicular\_epithelium | 1 | 0 |  |  |  |  |  |  |  |  |
| GO:0016340\_calcium-dependent\_cell-matrix\_adhesion | 1 | 0 |  |  |  |  |  |  |  |  |
| GO:0016344\_meiotic\_chromosome\_movement\_towards\_spindle\_pole | 1 | 0 |  |  |  |  |  |  |  |  |
| GO:0016482\_cytoplasmic\_transport | 1 | 0 |  |  |  |  |  |  |  |  |
| GO:0016553\_base\_conversion\_or\_substitution\_editing | 1 | 0 |  |  |  |  |  |  |  |  |
| GO:0016554\_cytidine\_to\_uridine\_editing | 1 | 0 |  |  |  |  |  |  |  |  |
| GO:0016560\_protein\_import\_into\_peroxisome\_matrix\_\_docking | 1 | 0 |  |  |  |  |  |  |  |  |
| GO:0016578\_histone\_deubiquitination | 1 | 0 |  |  |  |  |  |  |  |  |
| GO:0016598\_protein\_arginylation | 1 | 0 |  |  |  |  |  |  |  |  |
| GO:0017004\_cytochrome\_complex\_assembly | 1 | 0 |  |  |  |  |  |  |  |  |
| GO:0018022\_peptidyl-lysine\_methylation | 1 | 0 |  |  |  |  |  |  |  |  |
| GO:0018023\_peptidyl-lysine\_trimethylation | 1 | 0 |  |  |  |  |  |  |  |  |
| GO:0018120\_peptidyl-arginine\_ADP-ribosylation | 1 | 0 |  |  |  |  |  |  |  |  |
| GO:0018126\_protein\_amino\_acid\_hydroxylation | 1 | 0 |  |  |  |  |  |  |  |  |
| GO:0018146\_keratan\_sulfate\_biosynthetic\_process | 1 | 0 |  |  |  |  |  |  |  |  |
| GO:0018158\_protein\_amino\_acid\_oxidation | 1 | 0 |  |  |  |  |  |  |  |  |
| GO:0018195\_peptidyl-arginine\_modification | 1 | 0 |  |  |  |  |  |  |  |  |
| GO:0018197\_peptidyl-aspartic\_acid\_modification | 1 | 0 |  |  |  |  |  |  |  |  |
| GO:0018282\_metal\_incorporation\_into\_metallo-sulfur\_cluster | 1 | 0 |  |  |  |  |  |  |  |  |
| GO:0018283\_iron\_incorporation\_into\_metallo-sulfur\_cluster | 1 | 0 |  |  |  |  |  |  |  |  |
| GO:0018318\_protein\_amino\_acid\_palmitoylation | 1 | 0 |  |  |  |  |  |  |  |  |
| GO:0018342\_protein\_prenylation | 1 | 0 |  |  |  |  |  |  |  |  |
| GO:0018344\_protein\_geranylgeranylation | 1 | 0 |  |  |  |  |  |  |  |  |
| GO:0018410\_peptide\_or\_protein\_carboxyl-terminal\_blocking | 1 | 0 |  |  |  |  |  |  |  |  |
| GO:0018916\_nitrobenzene\_metabolic\_process | 1 | 0 |  |  |  |  |  |  |  |  |
| GO:0018931\_naphthalene\_metabolic\_process | 1 | 0 |  |  |  |  |  |  |  |  |
| GO:0018992\_germ-line\_sex\_determination | 1 | 0 |  |  |  |  |  |  |  |  |
| GO:0019042\_latent\_virus\_infection | 1 | 0 |  |  |  |  |  |  |  |  |
| GO:0019046\_reactivation\_of\_latent\_virus | 1 | 0 |  |  |  |  |  |  |  |  |
| GO:0019047\_provirus\_integration | 1 | 0 |  |  |  |  |  |  |  |  |
| GO:0019076\_release\_of\_virus\_from\_host | 1 | 0 |  |  |  |  |  |  |  |  |
| GO:0019079\_viral\_genome\_replication | 1 | 0 |  |  |  |  |  |  |  |  |
| GO:0019100\_male\_germ-line\_sex\_determination | 1 | 0 |  |  |  |  |  |  |  |  |
| GO:0019101\_female\_somatic\_sex\_determination | 1 | 0 |  |  |  |  |  |  |  |  |
| GO:0019102\_male\_somatic\_sex\_determination | 1 | 0 |  |  |  |  |  |  |  |  |
| GO:0019255\_glucose\_1-phosphate\_metabolic\_process | 1 | 0 |  |  |  |  |  |  |  |  |
| GO:0019276\_UDP-N-acetylgalactosamine\_metabolic\_process | 1 | 0 |  |  |  |  |  |  |  |  |
| GO:0019344\_cysteine\_biosynthetic\_process | 1 | 0 |  |  |  |  |  |  |  |  |
| GO:0019348\_dolichol\_metabolic\_process | 1 | 0 |  |  |  |  |  |  |  |  |
| GO:0019375\_galactolipid\_biosynthetic\_process | 1 | 0 |  |  |  |  |  |  |  |  |
| GO:0019402\_galactitol\_metabolic\_process | 1 | 0 |  |  |  |  |  |  |  |  |
| GO:0019441\_tryptophan\_catabolic\_process\_to\_kynurenine | 1 | 0 |  |  |  |  |  |  |  |  |
| GO:0019477\_L-lysine\_catabolic\_process | 1 | 0 |  |  |  |  |  |  |  |  |
| GO:0019510\_S-adenosylhomocysteine\_catabolic\_process | 1 | 0 |  |  |  |  |  |  |  |  |
| GO:0019532\_oxalate\_transport | 1 | 0 |  |  |  |  |  |  |  |  |
| GO:0019626\_short-chain\_fatty\_acid\_catabolic\_process | 1 | 0 |  |  |  |  |  |  |  |  |
| GO:0019627\_urea\_metabolic\_process | 1 | 0 |  |  |  |  |  |  |  |  |
| GO:0019676\_ammonia\_assimilation\_cycle | 1 | 0 |  |  |  |  |  |  |  |  |
| GO:0019682\_glyceraldehyde-3-phosphate\_metabolic\_process | 1 | 0 |  |  |  |  |  |  |  |  |
| GO:0019695\_choline\_metabolic\_process | 1 | 0 |  |  |  |  |  |  |  |  |
| GO:0019731\_antibacterial\_humoral\_response | 1 | 0 |  |  |  |  |  |  |  |  |
| GO:0019794\_nonprotein\_amino\_acid\_metabolic\_process | 1 | 0 |  |  |  |  |  |  |  |  |
| GO:0019858\_cytosine\_metabolic\_process | 1 | 0 |  |  |  |  |  |  |  |  |
| GO:0019883\_antigen\_processing\_and\_presentation\_of\_endogenous\_antigen | 1 | 0 |  |  |  |  |  |  |  |  |
| GO:0019889\_pteridine\_metabolic\_process | 1 | 0 |  |  |  |  |  |  |  |  |
| GO:0019896\_axon\_transport\_of\_mitochondrion | 1 | 0 |  |  |  |  |  |  |  |  |
| GO:0021508\_floor\_plate\_formation | 1 | 0 |  |  |  |  |  |  |  |  |
| GO:0021528\_commissural\_neuron\_differentiation\_in\_the\_spinal\_cord | 1 | 0 |  |  |  |  |  |  |  |  |
| GO:0021572\_rhombomere\_6\_development | 1 | 0 |  |  |  |  |  |  |  |  |
| GO:0021577\_hindbrain\_structural\_organization | 1 | 0 |  |  |  |  |  |  |  |  |
| GO:0021586\_pons\_maturation | 1 | 0 |  |  |  |  |  |  |  |  |
| GO:0021589\_cerebellum\_structural\_organization | 1 | 0 |  |  |  |  |  |  |  |  |
| GO:0021590\_cerebellum\_maturation | 1 | 0 |  |  |  |  |  |  |  |  |
| GO:0021592\_fourth\_ventricle\_development | 1 | 0 |  |  |  |  |  |  |  |  |
| GO:0021594\_rhombomere\_formation | 1 | 0 |  |  |  |  |  |  |  |  |
| GO:0021660\_rhombomere\_3\_formation | 1 | 0 |  |  |  |  |  |  |  |  |
| GO:0021664\_rhombomere\_5\_morphogenesis | 1 | 0 |  |  |  |  |  |  |  |  |
| GO:0021666\_rhombomere\_5\_formation | 1 | 0 |  |  |  |  |  |  |  |  |
| GO:0021670\_lateral\_ventricle\_development | 1 | 0 |  |  |  |  |  |  |  |  |
| GO:0021678\_third\_ventricle\_development | 1 | 0 |  |  |  |  |  |  |  |  |
| GO:0021679\_cerebellar\_molecular\_layer\_development | 1 | 0 |  |  |  |  |  |  |  |  |
| GO:0021703\_locus\_ceruleus\_development | 1 | 0 |  |  |  |  |  |  |  |  |
| GO:0021732\_midbrain-hindbrain\_boundary\_maturation | 1 | 0 |  |  |  |  |  |  |  |  |
| GO:0021747\_cochlear\_nucleus\_development | 1 | 0 |  |  |  |  |  |  |  |  |
| GO:0021750\_vestibular\_nucleus\_development | 1 | 0 |  |  |  |  |  |  |  |  |
| GO:0021759\_globus\_pallidus\_development | 1 | 0 |  |  |  |  |  |  |  |  |
| GO:0021768\_nucleus\_accumbens\_development | 1 | 0 |  |  |  |  |  |  |  |  |
| GO:0021771\_lateral\_geniculate\_nucleus\_development | 1 | 0 |  |  |  |  |  |  |  |  |
| GO:0021812\_neuronal-glial\_interaction\_involved\_in\_cerebral\_cortex\_radial\_glia\_guided\_migration | 1 | 0 |  |  |  |  |  |  |  |  |
| GO:0021813\_cell-cell\_adhesion\_involved\_in\_neuronal-glial\_interactions\_involved\_in\_cerebral\_cortex\_radial\_glia\_guided\_migration | 1 | 0 |  |  |  |  |  |  |  |  |
| GO:0021870\_Cajal-Retzius\_cell\_differentiation | 1 | 0 |  |  |  |  |  |  |  |  |
| GO:0021874\_Wnt\_receptor\_signaling\_pathway\_in\_forebrain\_neuroblast\_division | 1 | 0 |  |  |  |  |  |  |  |  |
| GO:0021896\_forebrain\_astrocyte\_differentiation | 1 | 0 |  |  |  |  |  |  |  |  |
| GO:0021897\_forebrain\_astrocyte\_development | 1 | 0 |  |  |  |  |  |  |  |  |
| GO:0021902\_commitment\_of\_a\_neuronal\_cell\_to\_a\_specific\_type\_of\_neuron\_in\_the\_forebrain | 1 | 0 |  |  |  |  |  |  |  |  |
| GO:0021905\_forebrain-midbrain\_boundary\_formation | 1 | 0 |  |  |  |  |  |  |  |  |
| GO:0021914\_negative\_regulation\_of\_smoothened\_signaling\_pathway\_involved\_in\_ventral\_spinal\_cord\_patterning | 1 | 0 |  |  |  |  |  |  |  |  |
| GO:0021917\_somatic\_motor\_neuron\_fate\_commitment | 1 | 0 |  |  |  |  |  |  |  |  |
| GO:0021918\_regulation\_of\_transcription\_from\_RNA\_polymerase\_II\_promoter\_involved\_in\_somatic\_motor\_neuron\_fate\_commitment | 1 | 0 |  |  |  |  |  |  |  |  |
| GO:0021933\_radial\_glia\_guided\_migration\_of\_granule\_cell | 1 | 0 |  |  |  |  |  |  |  |  |
| GO:0021934\_hindbrain\_tangential\_cell\_migration | 1 | 0 |  |  |  |  |  |  |  |  |
| GO:0021935\_granule\_cell\_precursor\_tangential\_migration | 1 | 0 |  |  |  |  |  |  |  |  |
| GO:0021942\_radial\_glia\_guided\_migration\_of\_Purkinje\_cell | 1 | 0 |  |  |  |  |  |  |  |  |
| GO:0021960\_anterior\_commissure\_morphogenesis | 1 | 0 |  |  |  |  |  |  |  |  |
| GO:0021997\_neural\_plate\_axis\_specification | 1 | 0 |  |  |  |  |  |  |  |  |
| GO:0021999\_neural\_plate\_anterior\_posterior\_pattern\_formation | 1 | 0 |  |  |  |  |  |  |  |  |
| GO:0022004\_midbrain-hindbrain\_boundary\_maturation\_during\_brain\_development | 1 | 0 |  |  |  |  |  |  |  |  |
| GO:0022038\_corpus\_callosum\_development | 1 | 0 |  |  |  |  |  |  |  |  |
| GO:0022605\_oogenesis\_stage | 1 | 0 |  |  |  |  |  |  |  |  |
| GO:0030011\_maintenance\_of\_cell\_polarity | 1 | 0 |  |  |  |  |  |  |  |  |
| GO:0030069\_lysogeny | 1 | 0 |  |  |  |  |  |  |  |  |
| GO:0030070\_insulin\_processing | 1 | 0 |  |  |  |  |  |  |  |  |
| GO:0030092\_regulation\_of\_flagellum\_assembly | 1 | 0 |  |  |  |  |  |  |  |  |
| GO:0030103\_vasopressin\_secretion | 1 | 0 |  |  |  |  |  |  |  |  |
| GO:0030194\_positive\_regulation\_of\_blood\_coagulation | 1 | 0 |  |  |  |  |  |  |  |  |
| GO:0030206\_chondroitin\_sulfate\_biosynthetic\_process | 1 | 0 |  |  |  |  |  |  |  |  |
| GO:0030210\_heparin\_biosynthetic\_process | 1 | 0 |  |  |  |  |  |  |  |  |
| GO:0030220\_platelet\_formation | 1 | 0 |  |  |  |  |  |  |  |  |
| GO:0030222\_eosinophil\_differentiation | 1 | 0 |  |  |  |  |  |  |  |  |
| GO:0030237\_female\_sex\_determination | 1 | 0 |  |  |  |  |  |  |  |  |
| GO:0030264\_nuclear\_fragmentation\_during\_apoptosis | 1 | 0 |  |  |  |  |  |  |  |  |
| GO:0030322\_stabilization\_of\_membrane\_potential | 1 | 0 |  |  |  |  |  |  |  |  |
| GO:0030327\_prenylated\_protein\_catabolic\_process | 1 | 0 |  |  |  |  |  |  |  |  |
| GO:0030328\_prenylcysteine\_catabolic\_process | 1 | 0 |  |  |  |  |  |  |  |  |
| GO:0030329\_prenylcysteine\_metabolic\_process | 1 | 0 |  |  |  |  |  |  |  |  |
| GO:0030382\_sperm\_mitochondrion\_organization | 1 | 0 |  |  |  |  |  |  |  |  |
| GO:0030389\_fructosamine\_metabolic\_process | 1 | 0 |  |  |  |  |  |  |  |  |
| GO:0030422\_RNA\_interference\_\_production\_of\_siRNA | 1 | 0 |  |  |  |  |  |  |  |  |
| GO:0030449\_regulation\_of\_complement\_activation | 1 | 0 |  |  |  |  |  |  |  |  |
| GO:0030497\_fatty\_acid\_elongation | 1 | 0 |  |  |  |  |  |  |  |  |
| GO:0030575\_nuclear\_body\_organization | 1 | 0 |  |  |  |  |  |  |  |  |
| GO:0030578\_PML\_body\_organization | 1 | 0 |  |  |  |  |  |  |  |  |
| GO:0030853\_negative\_regulation\_of\_granulocyte\_differentiation | 1 | 0 |  |  |  |  |  |  |  |  |
| GO:0030854\_positive\_regulation\_of\_granulocyte\_differentiation | 1 | 0 |  |  |  |  |  |  |  |  |
| GO:0030886\_negative\_regulation\_of\_myeloid\_dendritic\_cell\_activation | 1 | 0 |  |  |  |  |  |  |  |  |
| GO:0030913\_paranodal\_junction\_assembly | 1 | 0 |  |  |  |  |  |  |  |  |
| GO:0031033\_myosin\_filament\_assembly\_or\_disassembly | 1 | 0 |  |  |  |  |  |  |  |  |
| GO:0031034\_myosin\_filament\_assembly | 1 | 0 |  |  |  |  |  |  |  |  |
| GO:0031055\_chromatin\_remodeling\_at\_centromere | 1 | 0 |  |  |  |  |  |  |  |  |
| GO:0031062\_positive\_regulation\_of\_histone\_methylation | 1 | 0 |  |  |  |  |  |  |  |  |
| GO:0031115\_negative\_regulation\_of\_microtubule\_polymerization | 1 | 0 |  |  |  |  |  |  |  |  |
| GO:0031129\_inductive\_cell-cell\_signaling | 1 | 0 |  |  |  |  |  |  |  |  |
| GO:0031284\_positive\_regulation\_of\_guanylate\_cyclase\_activity | 1 | 0 |  |  |  |  |  |  |  |  |
| GO:0031498\_chromatin\_disassembly | 1 | 0 |  |  |  |  |  |  |  |  |
| GO:0031507\_heterochromatin\_formation | 1 | 0 |  |  |  |  |  |  |  |  |
| GO:0031508\_centromeric\_heterochromatin\_formation | 1 | 0 |  |  |  |  |  |  |  |  |
| GO:0031529\_ruffle\_organization | 1 | 0 |  |  |  |  |  |  |  |  |
| GO:0031536\_positive\_regulation\_of\_exit\_from\_mitosis | 1 | 0 |  |  |  |  |  |  |  |  |
| GO:0031572\_G2\_M\_transition\_DNA\_damage\_checkpoint | 1 | 0 |  |  |  |  |  |  |  |  |
| GO:0031576\_G2\_M\_transition\_checkpoint | 1 | 0 |  |  |  |  |  |  |  |  |
| GO:0031580\_membrane\_raft\_distribution | 1 | 0 |  |  |  |  |  |  |  |  |
| GO:0031583\_activation\_of\_phospholipase\_D\_activity\_by\_G-protein\_coupled\_receptor\_protein\_signaling\_pathway | 1 | 0 |  |  |  |  |  |  |  |  |
| GO:0031584\_activation\_of\_phospholipase\_D\_activity | 1 | 0 |  |  |  |  |  |  |  |  |
| GO:0031585\_regulation\_of\_inositol-1\_4\_5-triphosphate\_receptor\_activity | 1 | 0 |  |  |  |  |  |  |  |  |
| GO:0031639\_plasminogen\_activation | 1 | 0 |  |  |  |  |  |  |  |  |
| GO:0031648\_protein\_destabilization | 1 | 0 |  |  |  |  |  |  |  |  |
| GO:0031665\_negative\_regulation\_of\_lipopolysaccharide-mediated\_signaling\_pathway | 1 | 0 |  |  |  |  |  |  |  |  |
| GO:0031914\_negative\_regulation\_of\_synaptic\_plasticity | 1 | 0 |  |  |  |  |  |  |  |  |
| GO:0031944\_negative\_regulation\_of\_glucocorticoid\_metabolic\_process | 1 | 0 |  |  |  |  |  |  |  |  |
| GO:0031947\_negative\_regulation\_of\_glucocorticoid\_biosynthetic\_process | 1 | 0 |  |  |  |  |  |  |  |  |
| GO:0032025\_response\_to\_cobalt\_ion | 1 | 0 |  |  |  |  |  |  |  |  |
| GO:0032026\_response\_to\_magnesium\_ion | 1 | 0 |  |  |  |  |  |  |  |  |
| GO:0032048\_cardiolipin\_metabolic\_process | 1 | 0 |  |  |  |  |  |  |  |  |
| GO:0032066\_nucleolus\_to\_nucleoplasm\_transport | 1 | 0 |  |  |  |  |  |  |  |  |
| GO:0032091\_negative\_regulation\_of\_protein\_binding | 1 | 0 |  |  |  |  |  |  |  |  |
| GO:0032092\_positive\_regulation\_of\_protein\_binding | 1 | 0 |  |  |  |  |  |  |  |  |
| GO:0032097\_positive\_regulation\_of\_response\_to\_food | 1 | 0 |  |  |  |  |  |  |  |  |
| GO:0032100\_positive\_regulation\_of\_appetite | 1 | 0 |  |  |  |  |  |  |  |  |
| GO:0032204\_regulation\_of\_telomere\_maintenance | 1 | 0 |  |  |  |  |  |  |  |  |
| GO:0032206\_positive\_regulation\_of\_telomere\_maintenance | 1 | 0 |  |  |  |  |  |  |  |  |
| GO:0032222\_regulation\_of\_synaptic\_transmission\_\_cholinergic | 1 | 0 |  |  |  |  |  |  |  |  |
| GO:0032224\_positive\_regulation\_of\_synaptic\_transmission\_\_cholinergic | 1 | 0 |  |  |  |  |  |  |  |  |
| GO:0032229\_negative\_regulation\_of\_synaptic\_transmission\_\_GABAergic | 1 | 0 |  |  |  |  |  |  |  |  |
| GO:0032237\_activation\_of\_store-operated\_calcium\_channel\_activity | 1 | 0 |  |  |  |  |  |  |  |  |
| GO:0032239\_regulation\_of\_nucleobase\_\_nucleoside\_\_nucleotide\_and\_nucleic\_acid\_transport | 1 | 0 |  |  |  |  |  |  |  |  |
| GO:0032252\_secretory\_granule\_localization | 1 | 0 |  |  |  |  |  |  |  |  |
| GO:0032274\_gonadotropin\_secretion | 1 | 0 |  |  |  |  |  |  |  |  |
| GO:0032275\_luteinizing\_hormone\_secretion | 1 | 0 |  |  |  |  |  |  |  |  |
| GO:0032287\_myelin\_maintenance\_in\_the\_peripheral\_nervous\_system | 1 | 0 |  |  |  |  |  |  |  |  |
| GO:0032289\_myelin\_formation\_in\_the\_central\_nervous\_system | 1 | 0 |  |  |  |  |  |  |  |  |
| GO:0032303\_regulation\_of\_icosanoid\_secretion | 1 | 0 |  |  |  |  |  |  |  |  |
| GO:0032305\_positive\_regulation\_of\_icosanoid\_secretion | 1 | 0 |  |  |  |  |  |  |  |  |
| GO:0032306\_regulation\_of\_prostaglandin\_secretion | 1 | 0 |  |  |  |  |  |  |  |  |
| GO:0032308\_positive\_regulation\_of\_prostaglandin\_secretion | 1 | 0 |  |  |  |  |  |  |  |  |
| GO:0032310\_prostaglandin\_secretion | 1 | 0 |  |  |  |  |  |  |  |  |
| GO:0032313\_regulation\_of\_Rab\_GTPase\_activity | 1 | 0 |  |  |  |  |  |  |  |  |
| GO:0032314\_regulation\_of\_Rac\_GTPase\_activity | 1 | 0 |  |  |  |  |  |  |  |  |
| GO:0032317\_regulation\_of\_Rap\_GTPase\_activity | 1 | 0 |  |  |  |  |  |  |  |  |
| GO:0032324\_molybdopterin\_cofactor\_biosynthetic\_process | 1 | 0 |  |  |  |  |  |  |  |  |
| GO:0032329\_serine\_transport | 1 | 0 |  |  |  |  |  |  |  |  |
| GO:0032342\_aldosterone\_biosynthetic\_process | 1 | 0 |  |  |  |  |  |  |  |  |
| GO:0032344\_regulation\_of\_aldosterone\_metabolic\_process | 1 | 0 |  |  |  |  |  |  |  |  |
| GO:0032365\_intracellular\_lipid\_transport | 1 | 0 |  |  |  |  |  |  |  |  |
| GO:0032366\_intracellular\_sterol\_transport | 1 | 0 |  |  |  |  |  |  |  |  |
| GO:0032367\_intracellular\_cholesterol\_transport | 1 | 0 |  |  |  |  |  |  |  |  |
| GO:0032370\_positive\_regulation\_of\_lipid\_transport | 1 | 0 |  |  |  |  |  |  |  |  |
| GO:0032410\_negative\_regulation\_of\_transporter\_activity | 1 | 0 |  |  |  |  |  |  |  |  |
| GO:0032413\_negative\_regulation\_of\_ion\_transmembrane\_transporter\_activity | 1 | 0 |  |  |  |  |  |  |  |  |
| GO:0032429\_regulation\_of\_phospholipase\_A2\_activity | 1 | 0 |  |  |  |  |  |  |  |  |
| GO:0032474\_otolith\_morphogenesis | 1 | 0 |  |  |  |  |  |  |  |  |
| GO:0032482\_Rab\_protein\_signal\_transduction | 1 | 0 |  |  |  |  |  |  |  |  |
| GO:0032483\_regulation\_of\_Rab\_protein\_signal\_transduction | 1 | 0 |  |  |  |  |  |  |  |  |
| GO:0032486\_Rap\_protein\_signal\_transduction | 1 | 0 |  |  |  |  |  |  |  |  |
| GO:0032487\_regulation\_of\_Rap\_protein\_signal\_transduction | 1 | 0 |  |  |  |  |  |  |  |  |
| GO:0032594\_protein\_transport\_within\_lipid\_bilayer | 1 | 0 |  |  |  |  |  |  |  |  |
| GO:0032599\_protein\_transport\_out\_of\_membrane\_raft | 1 | 0 |  |  |  |  |  |  |  |  |
| GO:0032600\_chemokine\_receptor\_transport\_out\_of\_membrane\_raft | 1 | 0 |  |  |  |  |  |  |  |  |
| GO:0032607\_interferon-alpha\_production | 1 | 0 |  |  |  |  |  |  |  |  |
| GO:0032621\_interleukin-18\_production | 1 | 0 |  |  |  |  |  |  |  |  |
| GO:0032647\_regulation\_of\_interferon-alpha\_production | 1 | 0 |  |  |  |  |  |  |  |  |
| GO:0032656\_regulation\_of\_interleukin-13\_production | 1 | 0 |  |  |  |  |  |  |  |  |
| GO:0032682\_negative\_regulation\_of\_chemokine\_production | 1 | 0 |  |  |  |  |  |  |  |  |
| GO:0032691\_negative\_regulation\_of\_interleukin-1\_beta\_production | 1 | 0 |  |  |  |  |  |  |  |  |
| GO:0032692\_negative\_regulation\_of\_interleukin-1\_production | 1 | 0 |  |  |  |  |  |  |  |  |
| GO:0032693\_negative\_regulation\_of\_interleukin-10\_production | 1 | 0 |  |  |  |  |  |  |  |  |
| GO:0032696\_negative\_regulation\_of\_interleukin-13\_production | 1 | 0 |  |  |  |  |  |  |  |  |
| GO:0032727\_positive\_regulation\_of\_interferon-alpha\_production | 1 | 0 |  |  |  |  |  |  |  |  |
| GO:0032731\_positive\_regulation\_of\_interleukin-1\_beta\_production | 1 | 0 |  |  |  |  |  |  |  |  |
| GO:0032732\_positive\_regulation\_of\_interleukin-1\_production | 1 | 0 |  |  |  |  |  |  |  |  |
| GO:0032735\_positive\_regulation\_of\_interleukin-12\_production | 1 | 0 |  |  |  |  |  |  |  |  |
| GO:0032764\_negative\_regulation\_of\_mast\_cell\_cytokine\_production | 1 | 0 |  |  |  |  |  |  |  |  |
| GO:0032765\_positive\_regulation\_of\_mast\_cell\_cytokine\_production | 1 | 0 |  |  |  |  |  |  |  |  |
| GO:0032769\_negative\_regulation\_of\_monooxygenase\_activity | 1 | 0 |  |  |  |  |  |  |  |  |
| GO:0032781\_positive\_regulation\_of\_ATPase\_activity | 1 | 0 |  |  |  |  |  |  |  |  |
| GO:0032790\_ribosome\_disassembly | 1 | 0 |  |  |  |  |  |  |  |  |
| GO:0032799\_low-density\_lipoprotein\_receptor\_metabolic\_process | 1 | 0 |  |  |  |  |  |  |  |  |
| GO:0032802\_low-density\_lipoprotein\_receptor\_catabolic\_process | 1 | 0 |  |  |  |  |  |  |  |  |
| GO:0032803\_regulation\_of\_low-density\_lipoprotein\_receptor\_catabolic\_process | 1 | 0 |  |  |  |  |  |  |  |  |
| GO:0032817\_regulation\_of\_natural\_killer\_cell\_proliferation | 1 | 0 |  |  |  |  |  |  |  |  |
| GO:0032819\_positive\_regulation\_of\_natural\_killer\_cell\_proliferation | 1 | 0 |  |  |  |  |  |  |  |  |
| GO:0032836\_glomerular\_basement\_membrane\_development | 1 | 0 |  |  |  |  |  |  |  |  |
| GO:0032855\_positive\_regulation\_of\_Rac\_GTPase\_activity | 1 | 0 |  |  |  |  |  |  |  |  |
| GO:0032863\_activation\_of\_Rac\_GTPase\_activity | 1 | 0 |  |  |  |  |  |  |  |  |
| GO:0032864\_activation\_of\_Cdc42\_GTPase\_activity | 1 | 0 |  |  |  |  |  |  |  |  |
| GO:0032885\_regulation\_of\_polysaccharide\_biosynthetic\_process | 1 | 0 |  |  |  |  |  |  |  |  |
| GO:0032907\_transforming\_growth\_factor-beta3\_production | 1 | 0 |  |  |  |  |  |  |  |  |
| GO:0032910\_regulation\_of\_transforming\_growth\_factor-beta3\_production | 1 | 0 |  |  |  |  |  |  |  |  |
| GO:0032913\_negative\_regulation\_of\_transforming\_growth\_factor-beta3\_production | 1 | 0 |  |  |  |  |  |  |  |  |
| GO:0032924\_activin\_receptor\_signaling\_pathway | 1 | 0 |  |  |  |  |  |  |  |  |
| GO:0032925\_regulation\_of\_activin\_receptor\_signaling\_pathway | 1 | 0 |  |  |  |  |  |  |  |  |
| GO:0032960\_regulation\_of\_inositol\_trisphosphate\_biosynthetic\_process | 1 | 0 |  |  |  |  |  |  |  |  |
| GO:0032962\_positive\_regulation\_of\_inositol\_trisphosphate\_biosynthetic\_process | 1 | 0 |  |  |  |  |  |  |  |  |
| GO:0032964\_collagen\_biosynthetic\_process | 1 | 0 |  |  |  |  |  |  |  |  |
| GO:0032971\_regulation\_of\_muscle\_filament\_sliding | 1 | 0 |  |  |  |  |  |  |  |  |
| GO:0032972\_regulation\_of\_muscle\_filament\_sliding\_speed | 1 | 0 |  |  |  |  |  |  |  |  |
| GO:0032986\_protein-DNA\_complex\_disassembly | 1 | 0 |  |  |  |  |  |  |  |  |
| GO:0032988\_ribonucleoprotein\_complex\_disassembly | 1 | 0 |  |  |  |  |  |  |  |  |
| GO:0033037\_polysaccharide\_localization | 1 | 0 |  |  |  |  |  |  |  |  |
| GO:0033078\_extrathymic\_T\_cell\_differentiation | 1 | 0 |  |  |  |  |  |  |  |  |
| GO:0033085\_negative\_regulation\_of\_T\_cell\_differentiation\_in\_the\_thymus | 1 | 0 |  |  |  |  |  |  |  |  |
| GO:0033087\_negative\_regulation\_of\_immature\_T\_cell\_proliferation | 1 | 0 |  |  |  |  |  |  |  |  |
| GO:0033088\_negative\_regulation\_of\_immature\_T\_cell\_proliferation\_in\_the\_thymus | 1 | 0 |  |  |  |  |  |  |  |  |
| GO:0033108\_mitochondrial\_respiratory\_chain\_complex\_assembly | 1 | 0 |  |  |  |  |  |  |  |  |
| GO:0033127\_regulation\_of\_histone\_phosphorylation | 1 | 0 |  |  |  |  |  |  |  |  |
| GO:0033128\_negative\_regulation\_of\_histone\_phosphorylation | 1 | 0 |  |  |  |  |  |  |  |  |
| GO:0033138\_positive\_regulation\_of\_peptidyl-serine\_phosphorylation | 1 | 0 |  |  |  |  |  |  |  |  |
| GO:0033169\_histone\_H3-K9\_demethylation | 1 | 0 |  |  |  |  |  |  |  |  |
| GO:0033206\_cytokinesis\_after\_meiosis | 1 | 0 |  |  |  |  |  |  |  |  |
| GO:0033240\_positive\_regulation\_of\_cellular\_amine\_metabolic\_process | 1 | 0 |  |  |  |  |  |  |  |  |
| GO:0033313\_meiotic\_cell\_cycle\_checkpoint | 1 | 0 |  |  |  |  |  |  |  |  |
| GO:0033315\_meiotic\_cell\_cycle\_DNA\_replication\_checkpoint | 1 | 0 |  |  |  |  |  |  |  |  |
| GO:0033326\_cerebrospinal\_fluid\_secretion | 1 | 0 |  |  |  |  |  |  |  |  |
| GO:0033366\_protein\_localization\_in\_secretory\_granule | 1 | 0 |  |  |  |  |  |  |  |  |
| GO:0033367\_protein\_localization\_in\_mast\_cell\_secretory\_granule | 1 | 0 |  |  |  |  |  |  |  |  |
| GO:0033368\_protease\_localization\_in\_mast\_cell\_secretory\_granule | 1 | 0 |  |  |  |  |  |  |  |  |
| GO:0033370\_maintenance\_of\_protein\_location\_in\_mast\_cell\_secretory\_granule | 1 | 0 |  |  |  |  |  |  |  |  |
| GO:0033371\_T\_cell\_secretory\_granule\_organization | 1 | 0 |  |  |  |  |  |  |  |  |
| GO:0033373\_maintenance\_of\_protease\_location\_in\_mast\_cell\_secretory\_granule | 1 | 0 |  |  |  |  |  |  |  |  |
| GO:0033374\_protein\_localization\_in\_T\_cell\_secretory\_granule | 1 | 0 |  |  |  |  |  |  |  |  |
| GO:0033375\_protease\_localization\_in\_T\_cell\_secretory\_granule | 1 | 0 |  |  |  |  |  |  |  |  |
| GO:0033377\_maintenance\_of\_protein\_location\_in\_T\_cell\_secretory\_granule | 1 | 0 |  |  |  |  |  |  |  |  |
| GO:0033379\_maintenance\_of\_protease\_location\_in\_T\_cell\_secretory\_granule | 1 | 0 |  |  |  |  |  |  |  |  |
| GO:0033380\_granzyme\_B\_localization\_in\_T\_cell\_secretory\_granule | 1 | 0 |  |  |  |  |  |  |  |  |
| GO:0033382\_maintenance\_of\_granzyme\_B\_location\_in\_T\_cell\_secretory\_granule | 1 | 0 |  |  |  |  |  |  |  |  |
| GO:0033483\_gas\_homeostasis | 1 | 0 |  |  |  |  |  |  |  |  |
| GO:0033484\_nitric\_oxide\_homeostasis | 1 | 0 |  |  |  |  |  |  |  |  |
| GO:0033505\_floor\_plate\_morphogenesis | 1 | 0 |  |  |  |  |  |  |  |  |
| GO:0033522\_histone\_H2A\_ubiquitination | 1 | 0 |  |  |  |  |  |  |  |  |
| GO:0033523\_histone\_H2B\_ubiquitination | 1 | 0 |  |  |  |  |  |  |  |  |
| GO:0033574\_response\_to\_testosterone\_stimulus | 1 | 0 |  |  |  |  |  |  |  |  |
| GO:0033606\_chemokine\_receptor\_transport\_within\_lipid\_bilayer | 1 | 0 |  |  |  |  |  |  |  |  |
| GO:0033628\_regulation\_of\_cell\_adhesion\_mediated\_by\_integrin | 1 | 0 |  |  |  |  |  |  |  |  |
| GO:0033630\_positive\_regulation\_of\_cell\_adhesion\_mediated\_by\_integrin | 1 | 0 |  |  |  |  |  |  |  |  |
| GO:0033632\_regulation\_of\_cell-cell\_adhesion\_mediated\_by\_integrin | 1 | 0 |  |  |  |  |  |  |  |  |
| GO:0033634\_positive\_regulation\_of\_cell-cell\_adhesion\_mediated\_by\_integrin | 1 | 0 |  |  |  |  |  |  |  |  |
| GO:0033683\_nucleotide-excision\_repair\_\_DNA\_incision | 1 | 0 |  |  |  |  |  |  |  |  |
| GO:0033687\_osteoblast\_proliferation | 1 | 0 |  |  |  |  |  |  |  |  |
| GO:0033688\_regulation\_of\_osteoblast\_proliferation | 1 | 0 |  |  |  |  |  |  |  |  |
| GO:0033689\_negative\_regulation\_of\_osteoblast\_proliferation | 1 | 0 |  |  |  |  |  |  |  |  |
| GO:0033750\_ribosome\_localization | 1 | 0 |  |  |  |  |  |  |  |  |
| GO:0033753\_establishment\_of\_ribosome\_localization | 1 | 0 |  |  |  |  |  |  |  |  |
| GO:0033866\_nucleoside\_bisphosphate\_biosynthetic\_process | 1 | 0 |  |  |  |  |  |  |  |  |
| GO:0033875\_ribonucleoside\_bisphosphate\_metabolic\_process | 1 | 0 |  |  |  |  |  |  |  |  |
| GO:0034030\_ribonucleoside\_bisphosphate\_biosynthetic\_process | 1 | 0 |  |  |  |  |  |  |  |  |
| GO:0034032\_purine\_nucleoside\_bisphosphate\_metabolic\_process | 1 | 0 |  |  |  |  |  |  |  |  |
| GO:0034033\_purine\_nucleoside\_bisphosphate\_biosynthetic\_process | 1 | 0 |  |  |  |  |  |  |  |  |
| GO:0034035\_purine\_ribonucleoside\_bisphosphate\_metabolic\_process | 1 | 0 |  |  |  |  |  |  |  |  |
| GO:0034036\_purine\_ribonucleoside\_bisphosphate\_biosynthetic\_process | 1 | 0 |  |  |  |  |  |  |  |  |
| GO:0034067\_protein\_localization\_in\_Golgi\_apparatus | 1 | 0 |  |  |  |  |  |  |  |  |
| GO:0034102\_erythrocyte\_clearance | 1 | 0 |  |  |  |  |  |  |  |  |
| GO:0034106\_regulation\_of\_erythrocyte\_clearance | 1 | 0 |  |  |  |  |  |  |  |  |
| GO:0034107\_negative\_regulation\_of\_erythrocyte\_clearance | 1 | 0 |  |  |  |  |  |  |  |  |
| GO:0034110\_regulation\_of\_homotypic\_cell-cell\_adhesion | 1 | 0 |  |  |  |  |  |  |  |  |
| GO:0034111\_negative\_regulation\_of\_homotypic\_cell-cell\_adhesion | 1 | 0 |  |  |  |  |  |  |  |  |
| GO:0034113\_heterotypic\_cell-cell\_adhesion | 1 | 0 |  |  |  |  |  |  |  |  |
| GO:0034117\_erythrocyte\_aggregation | 1 | 0 |  |  |  |  |  |  |  |  |
| GO:0034118\_regulation\_of\_erythrocyte\_aggregation | 1 | 0 |  |  |  |  |  |  |  |  |
| GO:0034119\_negative\_regulation\_of\_erythrocyte\_aggregation | 1 | 0 |  |  |  |  |  |  |  |  |
| GO:0034121\_regulation\_of\_toll-like\_receptor\_signaling\_pathway | 1 | 0 |  |  |  |  |  |  |  |  |
| GO:0034122\_negative\_regulation\_of\_toll-like\_receptor\_signaling\_pathway | 1 | 0 |  |  |  |  |  |  |  |  |
| GO:0034230\_enkephalin\_processing | 1 | 0 |  |  |  |  |  |  |  |  |
| GO:0034372\_very-low-density\_lipoprotein\_particle\_remodeling | 1 | 0 |  |  |  |  |  |  |  |  |
| GO:0034379\_very-low-density\_lipoprotein\_particle\_assembly | 1 | 0 |  |  |  |  |  |  |  |  |
| GO:0034380\_high-density\_lipoprotein\_particle\_assembly | 1 | 0 |  |  |  |  |  |  |  |  |
| GO:0034394\_protein\_localization\_at\_cell\_surface | 1 | 0 |  |  |  |  |  |  |  |  |
| GO:0034405\_response\_to\_fluid\_shear\_stress | 1 | 0 |  |  |  |  |  |  |  |  |
| GO:0034472\_snRNA\_3'-end\_processing | 1 | 0 |  |  |  |  |  |  |  |  |
| GO:0034474\_U2\_snRNA\_3'-end\_processing | 1 | 0 |  |  |  |  |  |  |  |  |
| GO:0034502\_protein\_localization\_to\_chromosome | 1 | 0 |  |  |  |  |  |  |  |  |
| GO:0034505\_tooth\_mineralization | 1 | 0 |  |  |  |  |  |  |  |  |
| GO:0034508\_centromere\_complex\_assembly | 1 | 0 |  |  |  |  |  |  |  |  |
| GO:0034633\_retinol\_transport | 1 | 0 |  |  |  |  |  |  |  |  |
| GO:0034643\_mitochondrion\_localization\_\_microtubule-mediated | 1 | 0 |  |  |  |  |  |  |  |  |
| GO:0034969\_histone\_arginine\_methylation | 1 | 0 |  |  |  |  |  |  |  |  |
| GO:0034982\_mitochondrial\_protein\_processing | 1 | 0 |  |  |  |  |  |  |  |  |
| GO:0035022\_positive\_regulation\_of\_Rac\_protein\_signal\_transduction | 1 | 0 |  |  |  |  |  |  |  |  |
| GO:0035024\_negative\_regulation\_of\_Rho\_protein\_signal\_transduction | 1 | 0 |  |  |  |  |  |  |  |  |
| GO:0035026\_leading\_edge\_cell\_differentiation | 1 | 0 |  |  |  |  |  |  |  |  |
| GO:0035037\_sperm\_entry | 1 | 0 |  |  |  |  |  |  |  |  |
| GO:0035039\_male\_pronucleus\_formation | 1 | 0 |  |  |  |  |  |  |  |  |
| GO:0035066\_positive\_regulation\_of\_histone\_acetylation | 1 | 0 |  |  |  |  |  |  |  |  |
| GO:0035083\_cilium\_axoneme\_assembly | 1 | 0 |  |  |  |  |  |  |  |  |
| GO:0035090\_maintenance\_of\_apical\_basal\_cell\_polarity | 1 | 0 |  |  |  |  |  |  |  |  |
| GO:0035106\_operant\_conditioning | 1 | 0 |  |  |  |  |  |  |  |  |
| GO:0035172\_hemocyte\_proliferation | 1 | 0 |  |  |  |  |  |  |  |  |
| GO:0035227\_regulation\_of\_glutamate-cysteine\_ligase\_activity | 1 | 0 |  |  |  |  |  |  |  |  |
| GO:0035229\_positive\_regulation\_of\_glutamate-cysteine\_ligase\_activity | 1 | 0 |  |  |  |  |  |  |  |  |
| GO:0035260\_internal\_genitalia\_morphogenesis | 1 | 0 |  |  |  |  |  |  |  |  |
| GO:0035262\_gonad\_morphogenesis | 1 | 0 |  |  |  |  |  |  |  |  |
| GO:0035287\_head\_segmentation | 1 | 0 |  |  |  |  |  |  |  |  |
| GO:0035289\_posterior\_head\_segmentation | 1 | 0 |  |  |  |  |  |  |  |  |
| GO:0035303\_regulation\_of\_dephosphorylation | 1 | 0 |  |  |  |  |  |  |  |  |
| GO:0035304\_regulation\_of\_protein\_amino\_acid\_dephosphorylation | 1 | 0 |  |  |  |  |  |  |  |  |
| GO:0035305\_negative\_regulation\_of\_dephosphorylation | 1 | 0 |  |  |  |  |  |  |  |  |
| GO:0035308\_negative\_regulation\_of\_protein\_amino\_acid\_dephosphorylation | 1 | 0 |  |  |  |  |  |  |  |  |
| GO:0035313\_wound\_healing\_\_spreading\_of\_epidermal\_cells | 1 | 0 |  |  |  |  |  |  |  |  |
| GO:0040013\_negative\_regulation\_of\_locomotion | 1 | 0 |  |  |  |  |  |  |  |  |
| GO:0040019\_positive\_regulation\_of\_embryonic\_development | 1 | 0 |  |  |  |  |  |  |  |  |
| GO:0040032\_post-embryonic\_body\_morphogenesis | 1 | 0 |  |  |  |  |  |  |  |  |
| GO:0040038\_polar\_body\_extrusion\_after\_meiotic\_divisions | 1 | 0 |  |  |  |  |  |  |  |  |
| GO:0042026\_protein\_refolding | 1 | 0 |  |  |  |  |  |  |  |  |
| GO:0042048\_olfactory\_behavior | 1 | 0 |  |  |  |  |  |  |  |  |
| GO:0042059\_negative\_regulation\_of\_epidermal\_growth\_factor\_receptor\_signaling\_pathway | 1 | 0 |  |  |  |  |  |  |  |  |
| GO:0042073\_intraflagellar\_transport | 1 | 0 |  |  |  |  |  |  |  |  |
| GO:0042078\_germ-line\_stem\_cell\_division | 1 | 0 |  |  |  |  |  |  |  |  |
| GO:0042091\_interleukin-10\_biosynthetic\_process | 1 | 0 |  |  |  |  |  |  |  |  |
| GO:0042103\_positive\_regulation\_of\_T\_cell\_homeostatic\_proliferation | 1 | 0 |  |  |  |  |  |  |  |  |
| GO:0042136\_neurotransmitter\_biosynthetic\_process | 1 | 0 |  |  |  |  |  |  |  |  |
| GO:0042137\_sequestering\_of\_neurotransmitter | 1 | 0 |  |  |  |  |  |  |  |  |
| GO:0042138\_meiotic\_DNA\_double-strand\_break\_formation | 1 | 0 |  |  |  |  |  |  |  |  |
| GO:0042178\_xenobiotic\_catabolic\_process | 1 | 0 |  |  |  |  |  |  |  |  |
| GO:0042225\_interleukin-5\_biosynthetic\_process | 1 | 0 |  |  |  |  |  |  |  |  |
| GO:0042231\_interleukin-13\_biosynthetic\_process | 1 | 0 |  |  |  |  |  |  |  |  |
| GO:0042255\_ribosome\_assembly | 1 | 0 |  |  |  |  |  |  |  |  |
| GO:0042257\_ribosomal\_subunit\_assembly | 1 | 0 |  |  |  |  |  |  |  |  |
| GO:0042264\_peptidyl-aspartic\_acid\_hydroxylation | 1 | 0 |  |  |  |  |  |  |  |  |
| GO:0042276\_error-prone\_postreplication\_DNA\_repair | 1 | 0 |  |  |  |  |  |  |  |  |
| GO:0042297\_vocal\_learning | 1 | 0 |  |  |  |  |  |  |  |  |
| GO:0042309\_homoiothermy | 1 | 0 |  |  |  |  |  |  |  |  |
| GO:0042320\_regulation\_of\_circadian\_sleep\_wake\_cycle\_\_REM\_sleep | 1 | 0 |  |  |  |  |  |  |  |  |
| GO:0042339\_keratan\_sulfate\_metabolic\_process | 1 | 0 |  |  |  |  |  |  |  |  |
| GO:0042347\_negative\_regulation\_of\_NF-kappaB\_import\_into\_nucleus | 1 | 0 |  |  |  |  |  |  |  |  |
| GO:0042360\_vitamin\_E\_metabolic\_process | 1 | 0 |  |  |  |  |  |  |  |  |
| GO:0042363\_fat-soluble\_vitamin\_catabolic\_process | 1 | 0 |  |  |  |  |  |  |  |  |
| GO:0042369\_vitamin\_D\_catabolic\_process | 1 | 0 |  |  |  |  |  |  |  |  |
| GO:0042373\_vitamin\_K\_metabolic\_process | 1 | 0 |  |  |  |  |  |  |  |  |
| GO:0042404\_thyroid\_hormone\_catabolic\_process | 1 | 0 |  |  |  |  |  |  |  |  |
| GO:0042414\_epinephrine\_metabolic\_process | 1 | 0 |  |  |  |  |  |  |  |  |
| GO:0042436\_indole\_derivative\_catabolic\_process | 1 | 0 |  |  |  |  |  |  |  |  |
| GO:0042489\_negative\_regulation\_of\_odontogenesis\_of\_dentine-containing\_tooth | 1 | 0 |  |  |  |  |  |  |  |  |
| GO:0042508\_tyrosine\_phosphorylation\_of\_Stat1\_protein | 1 | 0 |  |  |  |  |  |  |  |  |
| GO:0042518\_negative\_regulation\_of\_tyrosine\_phosphorylation\_of\_Stat3\_protein | 1 | 0 |  |  |  |  |  |  |  |  |
| GO:0042524\_negative\_regulation\_of\_tyrosine\_phosphorylation\_of\_Stat5\_protein | 1 | 0 |  |  |  |  |  |  |  |  |
| GO:0042536\_negative\_regulation\_of\_tumor\_necrosis\_factor\_biosynthetic\_process | 1 | 0 |  |  |  |  |  |  |  |  |
| GO:0042538\_hyperosmotic\_salinity\_response | 1 | 0 |  |  |  |  |  |  |  |  |
| GO:0042628\_mating\_plug\_formation | 1 | 0 |  |  |  |  |  |  |  |  |
| GO:0042631\_cellular\_response\_to\_water\_deprivation | 1 | 0 |  |  |  |  |  |  |  |  |
| GO:0042637\_catagen | 1 | 0 |  |  |  |  |  |  |  |  |
| GO:0042660\_positive\_regulation\_of\_cell\_fate\_specification | 1 | 0 |  |  |  |  |  |  |  |  |
| GO:0042663\_regulation\_of\_endodermal\_cell\_fate\_specification | 1 | 0 |  |  |  |  |  |  |  |  |
| GO:0042664\_negative\_regulation\_of\_endodermal\_cell\_fate\_specification | 1 | 0 |  |  |  |  |  |  |  |  |
| GO:0042667\_auditory\_receptor\_cell\_fate\_specification | 1 | 0 |  |  |  |  |  |  |  |  |
| GO:0042694\_muscle\_cell\_fate\_specification | 1 | 0 |  |  |  |  |  |  |  |  |
| GO:0042706\_eye\_photoreceptor\_cell\_fate\_commitment | 1 | 0 |  |  |  |  |  |  |  |  |
| GO:0042713\_sperm\_ejaculation | 1 | 0 |  |  |  |  |  |  |  |  |
| GO:0042723\_thiamin\_and\_derivative\_metabolic\_process | 1 | 0 |  |  |  |  |  |  |  |  |
| GO:0042737\_drug\_catabolic\_process | 1 | 0 |  |  |  |  |  |  |  |  |
| GO:0042738\_exogenous\_drug\_catabolic\_process | 1 | 0 |  |  |  |  |  |  |  |  |
| GO:0042747\_circadian\_sleep\_wake\_cycle\_\_REM\_sleep | 1 | 0 |  |  |  |  |  |  |  |  |
| GO:0042748\_circadian\_sleep\_wake\_cycle\_\_non-REM\_sleep | 1 | 0 |  |  |  |  |  |  |  |  |
| GO:0042772\_DNA\_damage\_response\_\_signal\_transduction\_resulting\_in\_transcription | 1 | 0 |  |  |  |  |  |  |  |  |
| GO:0042790\_transcription\_of\_nuclear\_rRNA\_large\_RNA\_polymerase\_I\_transcript | 1 | 0 |  |  |  |  |  |  |  |  |
| GO:0042839\_D-glucuronate\_metabolic\_process | 1 | 0 |  |  |  |  |  |  |  |  |
| GO:0042840\_D-glucuronate\_catabolic\_process | 1 | 0 |  |  |  |  |  |  |  |  |
| GO:0042891\_antibiotic\_transport | 1 | 0 |  |  |  |  |  |  |  |  |
| GO:0042892\_chloramphenicol\_transport | 1 | 0 |  |  |  |  |  |  |  |  |
| GO:0042940\_D-amino\_acid\_transport | 1 | 0 |  |  |  |  |  |  |  |  |
| GO:0042941\_D-alanine\_transport | 1 | 0 |  |  |  |  |  |  |  |  |
| GO:0042942\_D-serine\_transport | 1 | 0 |  |  |  |  |  |  |  |  |
| GO:0042983\_amyloid\_precursor\_protein\_biosynthetic\_process | 1 | 0 |  |  |  |  |  |  |  |  |
| GO:0042984\_regulation\_of\_amyloid\_precursor\_protein\_biosynthetic\_process | 1 | 0 |  |  |  |  |  |  |  |  |
| GO:0042985\_negative\_regulation\_of\_amyloid\_precursor\_protein\_biosynthetic\_process | 1 | 0 |  |  |  |  |  |  |  |  |
| GO:0042989\_sequestering\_of\_actin\_monomers | 1 | 0 |  |  |  |  |  |  |  |  |
| GO:0043044\_ATP-dependent\_chromatin\_remodeling | 1 | 0 |  |  |  |  |  |  |  |  |
| GO:0043056\_forward\_locomotion | 1 | 0 |  |  |  |  |  |  |  |  |
| GO:0043060\_meiotic\_metaphase\_I\_plate\_congression | 1 | 0 |  |  |  |  |  |  |  |  |
| GO:0043091\_L-arginine\_import | 1 | 0 |  |  |  |  |  |  |  |  |
| GO:0043124\_negative\_regulation\_of\_I-kappaB\_kinase\_NF-kappaB\_cascade | 1 | 0 |  |  |  |  |  |  |  |  |
| GO:0043132\_NAD\_transport | 1 | 0 |  |  |  |  |  |  |  |  |
| GO:0043153\_entrainment\_of\_circadian\_clock\_by\_photoperiod | 1 | 0 |  |  |  |  |  |  |  |  |
| GO:0043171\_peptide\_catabolic\_process | 1 | 0 |  |  |  |  |  |  |  |  |
| GO:0043179\_rhythmic\_excitation | 1 | 0 |  |  |  |  |  |  |  |  |
| GO:0043206\_fibril\_organization | 1 | 0 |  |  |  |  |  |  |  |  |
| GO:0043217\_myelin\_maintenance | 1 | 0 |  |  |  |  |  |  |  |  |
| GO:0043313\_regulation\_of\_neutrophil\_degranulation | 1 | 0 |  |  |  |  |  |  |  |  |
| GO:0043316\_cytotoxic\_T\_cell\_degranulation | 1 | 0 |  |  |  |  |  |  |  |  |
| GO:0043369\_CD4-positive\_or\_CD8-positive\_\_alpha-beta\_T\_cell\_lineage\_commitment | 1 | 0 |  |  |  |  |  |  |  |  |
| GO:0043375\_CD8-positive\_\_alpha-beta\_T\_cell\_lineage\_commitment | 1 | 0 |  |  |  |  |  |  |  |  |
| GO:0043379\_memory\_T\_cell\_differentiation | 1 | 0 |  |  |  |  |  |  |  |  |
| GO:0043380\_regulation\_of\_memory\_T\_cell\_differentiation | 1 | 0 |  |  |  |  |  |  |  |  |
| GO:0043400\_cortisol\_secretion | 1 | 0 |  |  |  |  |  |  |  |  |
| GO:0043415\_positive\_regulation\_of\_skeletal\_muscle\_regeneration | 1 | 0 |  |  |  |  |  |  |  |  |
| GO:0043416\_regulation\_of\_skeletal\_muscle\_regeneration | 1 | 0 |  |  |  |  |  |  |  |  |
| GO:0043437\_butanoic\_acid\_metabolic\_process | 1 | 0 |  |  |  |  |  |  |  |  |
| GO:0043438\_acetoacetic\_acid\_metabolic\_process | 1 | 0 |  |  |  |  |  |  |  |  |
| GO:0043480\_pigment\_accumulation\_in\_tissues | 1 | 0 |  |  |  |  |  |  |  |  |
| GO:0043482\_cellular\_pigment\_accumulation | 1 | 0 |  |  |  |  |  |  |  |  |
| GO:0043486\_histone\_exchange | 1 | 0 |  |  |  |  |  |  |  |  |
| GO:0043496\_regulation\_of\_protein\_homodimerization\_activity | 1 | 0 |  |  |  |  |  |  |  |  |
| GO:0043501\_skeletal\_muscle\_adaptation | 1 | 0 |  |  |  |  |  |  |  |  |
| GO:0043508\_negative\_regulation\_of\_JUN\_kinase\_activity | 1 | 0 |  |  |  |  |  |  |  |  |
| GO:0043517\_positive\_regulation\_of\_DNA\_damage\_response\_\_signal\_transduction\_by\_p53\_class\_mediator | 1 | 0 |  |  |  |  |  |  |  |  |
| GO:0043535\_regulation\_of\_blood\_vessel\_endothelial\_cell\_migration | 1 | 0 |  |  |  |  |  |  |  |  |
| GO:0043537\_negative\_regulation\_of\_blood\_vessel\_endothelial\_cell\_migration | 1 | 0 |  |  |  |  |  |  |  |  |
| GO:0043545\_molybdopterin\_cofactor\_metabolic\_process | 1 | 0 |  |  |  |  |  |  |  |  |
| GO:0043587\_tongue\_morphogenesis | 1 | 0 |  |  |  |  |  |  |  |  |
| GO:0043604\_amide\_biosynthetic\_process | 1 | 0 |  |  |  |  |  |  |  |  |
| GO:0043628\_ncRNA\_3'-end\_processing | 1 | 0 |  |  |  |  |  |  |  |  |
| GO:0044254\_multicellular\_organismal\_protein\_catabolic\_process | 1 | 0 |  |  |  |  |  |  |  |  |
| GO:0044256\_protein\_digestion | 1 | 0 |  |  |  |  |  |  |  |  |
| GO:0044266\_multicellular\_organismal\_macromolecule\_catabolic\_process | 1 | 0 |  |  |  |  |  |  |  |  |
| GO:0045004\_DNA\_replication\_proofreading | 1 | 0 |  |  |  |  |  |  |  |  |
| GO:0045019\_negative\_regulation\_of\_nitric\_oxide\_biosynthetic\_process | 1 | 0 |  |  |  |  |  |  |  |  |
| GO:0045020\_error-prone\_DNA\_repair | 1 | 0 |  |  |  |  |  |  |  |  |
| GO:0045022\_early\_endosome\_to\_late\_endosome\_transport | 1 | 0 |  |  |  |  |  |  |  |  |
| GO:0045062\_extrathymic\_T\_cell\_selection | 1 | 0 |  |  |  |  |  |  |  |  |
| GO:0045069\_regulation\_of\_viral\_genome\_replication | 1 | 0 |  |  |  |  |  |  |  |  |
| GO:0045074\_regulation\_of\_interleukin-10\_biosynthetic\_process | 1 | 0 |  |  |  |  |  |  |  |  |
| GO:0045082\_positive\_regulation\_of\_interleukin-10\_biosynthetic\_process | 1 | 0 |  |  |  |  |  |  |  |  |
| GO:0045083\_negative\_regulation\_of\_interleukin-12\_biosynthetic\_process | 1 | 0 |  |  |  |  |  |  |  |  |
| GO:0045112\_integrin\_biosynthetic\_process | 1 | 0 |  |  |  |  |  |  |  |  |
| GO:0045113\_regulation\_of\_integrin\_biosynthetic\_process | 1 | 0 |  |  |  |  |  |  |  |  |
| GO:0045188\_regulation\_of\_circadian\_sleep\_wake\_cycle\_\_non-REM\_sleep | 1 | 0 |  |  |  |  |  |  |  |  |
| GO:0045210\_FasL\_biosynthetic\_process | 1 | 0 |  |  |  |  |  |  |  |  |
| GO:0045297\_post-mating\_behavior | 1 | 0 |  |  |  |  |  |  |  |  |
| GO:0045299\_otolith\_mineralization | 1 | 0 |  |  |  |  |  |  |  |  |
| GO:0045329\_carnitine\_biosynthetic\_process | 1 | 0 |  |  |  |  |  |  |  |  |
| GO:0045341\_MHC\_class\_I\_biosynthetic\_process | 1 | 0 |  |  |  |  |  |  |  |  |
| GO:0045343\_regulation\_of\_MHC\_class\_I\_biosynthetic\_process | 1 | 0 |  |  |  |  |  |  |  |  |
| GO:0045347\_negative\_regulation\_of\_MHC\_class\_II\_biosynthetic\_process | 1 | 0 |  |  |  |  |  |  |  |  |
| GO:0045405\_regulation\_of\_interleukin-5\_biosynthetic\_process | 1 | 0 |  |  |  |  |  |  |  |  |
| GO:0045407\_positive\_regulation\_of\_interleukin-5\_biosynthetic\_process | 1 | 0 |  |  |  |  |  |  |  |  |
| GO:0045426\_quinone\_cofactor\_biosynthetic\_process | 1 | 0 |  |  |  |  |  |  |  |  |
| GO:0045448\_mitotic\_cell\_cycle\_\_embryonic | 1 | 0 |  |  |  |  |  |  |  |  |
| GO:0045454\_cell\_redox\_homeostasis | 1 | 0 |  |  |  |  |  |  |  |  |
| GO:0045583\_regulation\_of\_cytotoxic\_T\_cell\_differentiation | 1 | 0 |  |  |  |  |  |  |  |  |
| GO:0045585\_positive\_regulation\_of\_cytotoxic\_T\_cell\_differentiation | 1 | 0 |  |  |  |  |  |  |  |  |
| GO:0045601\_regulation\_of\_endothelial\_cell\_differentiation | 1 | 0 |  |  |  |  |  |  |  |  |
| GO:0045602\_negative\_regulation\_of\_endothelial\_cell\_differentiation | 1 | 0 |  |  |  |  |  |  |  |  |
| GO:0045605\_negative\_regulation\_of\_epidermal\_cell\_differentiation | 1 | 0 |  |  |  |  |  |  |  |  |
| GO:0045606\_positive\_regulation\_of\_epidermal\_cell\_differentiation | 1 | 0 |  |  |  |  |  |  |  |  |
| GO:0045609\_positive\_regulation\_of\_auditory\_receptor\_cell\_differentiation | 1 | 0 |  |  |  |  |  |  |  |  |
| GO:0045617\_negative\_regulation\_of\_keratinocyte\_differentiation | 1 | 0 |  |  |  |  |  |  |  |  |
| GO:0045618\_positive\_regulation\_of\_keratinocyte\_differentiation | 1 | 0 |  |  |  |  |  |  |  |  |
| GO:0045626\_negative\_regulation\_of\_T-helper\_1\_cell\_differentiation | 1 | 0 |  |  |  |  |  |  |  |  |
| GO:0045633\_positive\_regulation\_of\_mechanoreceptor\_differentiation | 1 | 0 |  |  |  |  |  |  |  |  |
| GO:0045650\_negative\_regulation\_of\_macrophage\_differentiation | 1 | 0 |  |  |  |  |  |  |  |  |
| GO:0045656\_negative\_regulation\_of\_monocyte\_differentiation | 1 | 0 |  |  |  |  |  |  |  |  |
| GO:0045657\_positive\_regulation\_of\_monocyte\_differentiation | 1 | 0 |  |  |  |  |  |  |  |  |
| GO:0045659\_negative\_regulation\_of\_neutrophil\_differentiation | 1 | 0 |  |  |  |  |  |  |  |  |
| GO:0045660\_positive\_regulation\_of\_neutrophil\_differentiation | 1 | 0 |  |  |  |  |  |  |  |  |
| GO:0045721\_negative\_regulation\_of\_gluconeogenesis | 1 | 0 |  |  |  |  |  |  |  |  |
| GO:0045724\_positive\_regulation\_of\_flagellum\_assembly | 1 | 0 |  |  |  |  |  |  |  |  |
| GO:0045725\_positive\_regulation\_of\_glycogen\_biosynthetic\_process | 1 | 0 |  |  |  |  |  |  |  |  |
| GO:0045740\_positive\_regulation\_of\_DNA\_replication | 1 | 0 |  |  |  |  |  |  |  |  |
| GO:0045759\_negative\_regulation\_of\_action\_potential | 1 | 0 |  |  |  |  |  |  |  |  |
| GO:0045768\_positive\_regulation\_of\_anti-apoptosis | 1 | 0 |  |  |  |  |  |  |  |  |
| GO:0045769\_negative\_regulation\_of\_asymmetric\_cell\_division | 1 | 0 |  |  |  |  |  |  |  |  |
| GO:0045794\_negative\_regulation\_of\_cell\_volume | 1 | 0 |  |  |  |  |  |  |  |  |
| GO:0045815\_positive\_regulation\_of\_gene\_expression\_\_epigenetic | 1 | 0 |  |  |  |  |  |  |  |  |
| GO:0045818\_negative\_regulation\_of\_glycogen\_catabolic\_process | 1 | 0 |  |  |  |  |  |  |  |  |
| GO:0045842\_positive\_regulation\_of\_mitotic\_metaphase\_anaphase\_transition | 1 | 0 |  |  |  |  |  |  |  |  |
| GO:0045875\_negative\_regulation\_of\_sister\_chromatid\_cohesion | 1 | 0 |  |  |  |  |  |  |  |  |
| GO:0045898\_regulation\_of\_transcriptional\_preinitiation\_complex\_assembly | 1 | 0 |  |  |  |  |  |  |  |  |
| GO:0045899\_positive\_regulation\_of\_transcriptional\_preinitiation\_complex\_assembly | 1 | 0 |  |  |  |  |  |  |  |  |
| GO:0045906\_negative\_regulation\_of\_vasoconstriction | 1 | 0 |  |  |  |  |  |  |  |  |
| GO:0045908\_negative\_regulation\_of\_vasodilation | 1 | 0 |  |  |  |  |  |  |  |  |
| GO:0045909\_positive\_regulation\_of\_vasodilation | 1 | 0 |  |  |  |  |  |  |  |  |
| GO:0045915\_positive\_regulation\_of\_catecholamine\_metabolic\_process | 1 | 0 |  |  |  |  |  |  |  |  |
| GO:0045920\_negative\_regulation\_of\_exocytosis | 1 | 0 |  |  |  |  |  |  |  |  |
| GO:0045924\_regulation\_of\_female\_receptivity | 1 | 0 |  |  |  |  |  |  |  |  |
| GO:0045947\_negative\_regulation\_of\_translational\_initiation | 1 | 0 |  |  |  |  |  |  |  |  |
| GO:0045955\_negative\_regulation\_of\_calcium\_ion-dependent\_exocytosis | 1 | 0 |  |  |  |  |  |  |  |  |
| GO:0045956\_positive\_regulation\_of\_calcium\_ion-dependent\_exocytosis | 1 | 0 |  |  |  |  |  |  |  |  |
| GO:0045964\_positive\_regulation\_of\_dopamine\_metabolic\_process | 1 | 0 |  |  |  |  |  |  |  |  |
| GO:0045988\_negative\_regulation\_of\_striated\_muscle\_contraction | 1 | 0 |  |  |  |  |  |  |  |  |
| GO:0045989\_positive\_regulation\_of\_striated\_muscle\_contraction | 1 | 0 |  |  |  |  |  |  |  |  |
| GO:0045990\_regulation\_of\_transcription\_by\_carbon\_catabolites | 1 | 0 |  |  |  |  |  |  |  |  |
| GO:0045991\_positive\_regulation\_of\_transcription\_by\_carbon\_catabolites | 1 | 0 |  |  |  |  |  |  |  |  |
| GO:0045994\_positive\_regulation\_of\_translational\_initiation\_by\_iron | 1 | 0 |  |  |  |  |  |  |  |  |
| GO:0046007\_negative\_regulation\_of\_activated\_T\_cell\_proliferation | 1 | 0 |  |  |  |  |  |  |  |  |
| GO:0046014\_negative\_regulation\_of\_T\_cell\_homeostatic\_proliferation | 1 | 0 |  |  |  |  |  |  |  |  |
| GO:0046015\_regulation\_of\_transcription\_by\_glucose | 1 | 0 |  |  |  |  |  |  |  |  |
| GO:0046016\_positive\_regulation\_of\_transcription\_by\_glucose | 1 | 0 |  |  |  |  |  |  |  |  |
| GO:0046031\_ADP\_metabolic\_process | 1 | 0 |  |  |  |  |  |  |  |  |
| GO:0046032\_ADP\_catabolic\_process | 1 | 0 |  |  |  |  |  |  |  |  |
| GO:0046061\_dATP\_catabolic\_process | 1 | 0 |  |  |  |  |  |  |  |  |
| GO:0046075\_dTTP\_metabolic\_process | 1 | 0 |  |  |  |  |  |  |  |  |
| GO:0046078\_dUMP\_metabolic\_process | 1 | 0 |  |  |  |  |  |  |  |  |
| GO:0046079\_dUMP\_catabolic\_process | 1 | 0 |  |  |  |  |  |  |  |  |
| GO:0046086\_adenosine\_biosynthetic\_process | 1 | 0 |  |  |  |  |  |  |  |  |
| GO:0046090\_deoxyadenosine\_metabolic\_process | 1 | 0 |  |  |  |  |  |  |  |  |
| GO:0046098\_guanine\_metabolic\_process | 1 | 0 |  |  |  |  |  |  |  |  |
| GO:0046101\_hypoxanthine\_biosynthetic\_process | 1 | 0 |  |  |  |  |  |  |  |  |
| GO:0046102\_inosine\_metabolic\_process | 1 | 0 |  |  |  |  |  |  |  |  |
| GO:0046103\_inosine\_biosynthetic\_process | 1 | 0 |  |  |  |  |  |  |  |  |
| GO:0046108\_uridine\_metabolic\_process | 1 | 0 |  |  |  |  |  |  |  |  |
| GO:0046110\_xanthine\_metabolic\_process | 1 | 0 |  |  |  |  |  |  |  |  |
| GO:0046111\_xanthine\_biosynthetic\_process | 1 | 0 |  |  |  |  |  |  |  |  |
| GO:0046112\_nucleobase\_biosynthetic\_process | 1 | 0 |  |  |  |  |  |  |  |  |
| GO:0046113\_nucleobase\_catabolic\_process | 1 | 0 |  |  |  |  |  |  |  |  |
| GO:0046121\_deoxyribonucleoside\_catabolic\_process | 1 | 0 |  |  |  |  |  |  |  |  |
| GO:0046122\_purine\_deoxyribonucleoside\_metabolic\_process | 1 | 0 |  |  |  |  |  |  |  |  |
| GO:0046124\_purine\_deoxyribonucleoside\_catabolic\_process | 1 | 0 |  |  |  |  |  |  |  |  |
| GO:0046125\_pyrimidine\_deoxyribonucleoside\_metabolic\_process | 1 | 0 |  |  |  |  |  |  |  |  |
| GO:0046131\_pyrimidine\_ribonucleoside\_metabolic\_process | 1 | 0 |  |  |  |  |  |  |  |  |
| GO:0046160\_heme\_a\_metabolic\_process | 1 | 0 |  |  |  |  |  |  |  |  |
| GO:0046218\_indolalkylamine\_catabolic\_process | 1 | 0 |  |  |  |  |  |  |  |  |
| GO:0046292\_formaldehyde\_metabolic\_process | 1 | 0 |  |  |  |  |  |  |  |  |
| GO:0046294\_formaldehyde\_catabolic\_process | 1 | 0 |  |  |  |  |  |  |  |  |
| GO:0046314\_phosphocreatine\_biosynthetic\_process | 1 | 0 |  |  |  |  |  |  |  |  |
| GO:0046327\_glycerol\_biosynthetic\_process\_from\_pyruvate | 1 | 0 |  |  |  |  |  |  |  |  |
| GO:0046329\_negative\_regulation\_of\_JNK\_cascade | 1 | 0 |  |  |  |  |  |  |  |  |
| GO:0046340\_diacylglycerol\_catabolic\_process | 1 | 0 |  |  |  |  |  |  |  |  |
| GO:0046351\_disaccharide\_biosynthetic\_process | 1 | 0 |  |  |  |  |  |  |  |  |
| GO:0046356\_acetyl-CoA\_catabolic\_process | 1 | 0 |  |  |  |  |  |  |  |  |
| GO:0046358\_butyrate\_biosynthetic\_process | 1 | 0 |  |  |  |  |  |  |  |  |
| GO:0046359\_butyrate\_catabolic\_process | 1 | 0 |  |  |  |  |  |  |  |  |
| GO:0046381\_CMP-N-acetylneuraminate\_metabolic\_process | 1 | 0 |  |  |  |  |  |  |  |  |
| GO:0046415\_urate\_metabolic\_process | 1 | 0 |  |  |  |  |  |  |  |  |
| GO:0046416\_D-amino\_acid\_metabolic\_process | 1 | 0 |  |  |  |  |  |  |  |  |
| GO:0046434\_organophosphate\_catabolic\_process | 1 | 0 |  |  |  |  |  |  |  |  |
| GO:0046437\_D-amino\_acid\_biosynthetic\_process | 1 | 0 |  |  |  |  |  |  |  |  |
| GO:0046440\_L-lysine\_metabolic\_process | 1 | 0 |  |  |  |  |  |  |  |  |
| GO:0046449\_creatinine\_metabolic\_process | 1 | 0 |  |  |  |  |  |  |  |  |
| GO:0046471\_phosphatidylglycerol\_metabolic\_process | 1 | 0 |  |  |  |  |  |  |  |  |
| GO:0046473\_phosphatidic\_acid\_metabolic\_process | 1 | 0 |  |  |  |  |  |  |  |  |
| GO:0046476\_glycosylceramide\_biosynthetic\_process | 1 | 0 |  |  |  |  |  |  |  |  |
| GO:0046477\_glycosylceramide\_catabolic\_process | 1 | 0 |  |  |  |  |  |  |  |  |
| GO:0046485\_ether\_lipid\_metabolic\_process | 1 | 0 |  |  |  |  |  |  |  |  |
| GO:0046487\_glyoxylate\_metabolic\_process | 1 | 0 |  |  |  |  |  |  |  |  |
| GO:0046498\_S-adenosylhomocysteine\_metabolic\_process | 1 | 0 |  |  |  |  |  |  |  |  |
| GO:0046552\_photoreceptor\_cell\_fate\_commitment | 1 | 0 |  |  |  |  |  |  |  |  |
| GO:0046586\_regulation\_of\_calcium-dependent\_cell-cell\_adhesion | 1 | 0 |  |  |  |  |  |  |  |  |
| GO:0046587\_positive\_regulation\_of\_calcium-dependent\_cell-cell\_adhesion | 1 | 0 |  |  |  |  |  |  |  |  |
| GO:0046602\_regulation\_of\_mitotic\_centrosome\_separation | 1 | 0 |  |  |  |  |  |  |  |  |
| GO:0046604\_positive\_regulation\_of\_mitotic\_centrosome\_separation | 1 | 0 |  |  |  |  |  |  |  |  |
| GO:0046607\_positive\_regulation\_of\_centrosome\_cycle | 1 | 0 |  |  |  |  |  |  |  |  |
| GO:0046655\_folic\_acid\_metabolic\_process | 1 | 0 |  |  |  |  |  |  |  |  |
| GO:0046671\_negative\_regulation\_of\_retinal\_cell\_programmed\_cell\_death | 1 | 0 |  |  |  |  |  |  |  |  |
| GO:0046685\_response\_to\_arsenic | 1 | 0 |  |  |  |  |  |  |  |  |
| GO:0046692\_sperm\_competition | 1 | 0 |  |  |  |  |  |  |  |  |
| GO:0046707\_IDP\_metabolic\_process | 1 | 0 |  |  |  |  |  |  |  |  |
| GO:0046709\_IDP\_catabolic\_process | 1 | 0 |  |  |  |  |  |  |  |  |
| GO:0046724\_oxalic\_acid\_secretion | 1 | 0 |  |  |  |  |  |  |  |  |
| GO:0046753\_non-lytic\_viral\_release | 1 | 0 |  |  |  |  |  |  |  |  |
| GO:0046755\_non-lytic\_virus\_budding | 1 | 0 |  |  |  |  |  |  |  |  |
| GO:0046826\_negative\_regulation\_of\_protein\_export\_from\_nucleus | 1 | 0 |  |  |  |  |  |  |  |  |
| GO:0046827\_positive\_regulation\_of\_protein\_export\_from\_nucleus | 1 | 0 |  |  |  |  |  |  |  |  |
| GO:0046831\_regulation\_of\_RNA\_export\_from\_nucleus | 1 | 0 |  |  |  |  |  |  |  |  |
| GO:0046834\_lipid\_phosphorylation | 1 | 0 |  |  |  |  |  |  |  |  |
| GO:0046853\_inositol\_and\_derivative\_phosphorylation | 1 | 0 |  |  |  |  |  |  |  |  |
| GO:0046864\_isoprenoid\_transport | 1 | 0 |  |  |  |  |  |  |  |  |
| GO:0046865\_terpenoid\_transport | 1 | 0 |  |  |  |  |  |  |  |  |
| GO:0046877\_regulation\_of\_saliva\_secretion | 1 | 0 |  |  |  |  |  |  |  |  |
| GO:0046878\_positive\_regulation\_of\_saliva\_secretion | 1 | 0 |  |  |  |  |  |  |  |  |
| GO:0046884\_follicle-stimulating\_hormone\_secretion | 1 | 0 |  |  |  |  |  |  |  |  |
| GO:0046898\_response\_to\_cycloheximide | 1 | 0 |  |  |  |  |  |  |  |  |
| GO:0046929\_negative\_regulation\_of\_neurotransmitter\_secretion | 1 | 0 |  |  |  |  |  |  |  |  |
| GO:0046931\_pore\_complex\_biogenesis | 1 | 0 |  |  |  |  |  |  |  |  |
| GO:0046949\_acyl-CoA\_biosynthetic\_process | 1 | 0 |  |  |  |  |  |  |  |  |
| GO:0046958\_nonassociative\_learning | 1 | 0 |  |  |  |  |  |  |  |  |
| GO:0046960\_sensitization | 1 | 0 |  |  |  |  |  |  |  |  |
| GO:0046986\_negative\_regulation\_of\_hemoglobin\_biosynthetic\_process | 1 | 0 |  |  |  |  |  |  |  |  |
| GO:0047497\_mitochondrion\_transport\_along\_microtubule | 1 | 0 |  |  |  |  |  |  |  |  |
| GO:0048047\_mating\_behavior\_\_sex\_discrimination | 1 | 0 |  |  |  |  |  |  |  |  |
| GO:0048133\_male\_germ-line\_stem\_cell\_division | 1 | 0 |  |  |  |  |  |  |  |  |
| GO:0048137\_spermatocyte\_division | 1 | 0 |  |  |  |  |  |  |  |  |
| GO:0048143\_astrocyte\_activation | 1 | 0 |  |  |  |  |  |  |  |  |
| GO:0048170\_positive\_regulation\_of\_long-term\_neuronal\_synaptic\_plasticity | 1 | 0 |  |  |  |  |  |  |  |  |
| GO:0048199\_vesicle\_targeting\_\_to\_\_from\_or\_within\_Golgi | 1 | 0 |  |  |  |  |  |  |  |  |
| GO:0048241\_epinephrine\_transport | 1 | 0 |  |  |  |  |  |  |  |  |
| GO:0048242\_epinephrine\_secretion | 1 | 0 |  |  |  |  |  |  |  |  |
| GO:0048243\_norepinephrine\_secretion | 1 | 0 |  |  |  |  |  |  |  |  |
| GO:0048247\_lymphocyte\_chemotaxis | 1 | 0 |  |  |  |  |  |  |  |  |
| GO:0048250\_mitochondrial\_iron\_ion\_transport | 1 | 0 |  |  |  |  |  |  |  |  |
| GO:0048259\_regulation\_of\_receptor-mediated\_endocytosis | 1 | 0 |  |  |  |  |  |  |  |  |
| GO:0048260\_positive\_regulation\_of\_receptor-mediated\_endocytosis | 1 | 0 |  |  |  |  |  |  |  |  |
| GO:0048290\_isotype\_switching\_to\_IgA\_isotypes | 1 | 0 |  |  |  |  |  |  |  |  |
| GO:0048296\_regulation\_of\_isotype\_switching\_to\_IgA\_isotypes | 1 | 0 |  |  |  |  |  |  |  |  |
| GO:0048298\_positive\_regulation\_of\_isotype\_switching\_to\_IgA\_isotypes | 1 | 0 |  |  |  |  |  |  |  |  |
| GO:0048319\_axial\_mesoderm\_morphogenesis | 1 | 0 |  |  |  |  |  |  |  |  |
| GO:0048320\_axial\_mesoderm\_formation | 1 | 0 |  |  |  |  |  |  |  |  |
| GO:0048385\_regulation\_of\_retinoic\_acid\_receptor\_signaling\_pathway | 1 | 0 |  |  |  |  |  |  |  |  |
| GO:0048387\_negative\_regulation\_of\_retinoic\_acid\_receptor\_signaling\_pathway | 1 | 0 |  |  |  |  |  |  |  |  |
| GO:0048388\_endosomal\_lumen\_acidification | 1 | 0 |  |  |  |  |  |  |  |  |
| GO:0048389\_intermediate\_mesoderm\_development | 1 | 0 |  |  |  |  |  |  |  |  |
| GO:0048478\_replication\_fork\_protection | 1 | 0 |  |  |  |  |  |  |  |  |
| GO:0048496\_maintenance\_of\_organ\_identity | 1 | 0 |  |  |  |  |  |  |  |  |
| GO:0048525\_negative\_regulation\_of\_viral\_reproduction | 1 | 0 |  |  |  |  |  |  |  |  |
| GO:0048539\_bone\_marrow\_development | 1 | 0 |  |  |  |  |  |  |  |  |
| GO:0048548\_regulation\_of\_pinocytosis | 1 | 0 |  |  |  |  |  |  |  |  |
| GO:0048549\_positive\_regulation\_of\_pinocytosis | 1 | 0 |  |  |  |  |  |  |  |  |
| GO:0048553\_negative\_regulation\_of\_metalloenzyme\_activity | 1 | 0 |  |  |  |  |  |  |  |  |
| GO:0048588\_developmental\_cell\_growth | 1 | 0 |  |  |  |  |  |  |  |  |
| GO:0048601\_oocyte\_morphogenesis | 1 | 0 |  |  |  |  |  |  |  |  |
| GO:0048621\_post-embryonic\_gut\_morphogenesis | 1 | 0 |  |  |  |  |  |  |  |  |
| GO:0048640\_negative\_regulation\_of\_developmental\_growth | 1 | 0 |  |  |  |  |  |  |  |  |
| GO:0048642\_negative\_regulation\_of\_skeletal\_muscle\_tissue\_development | 1 | 0 |  |  |  |  |  |  |  |  |
| GO:0048669\_collateral\_sprouting\_in\_the\_absence\_of\_injury | 1 | 0 |  |  |  |  |  |  |  |  |
| GO:0048680\_positive\_regulation\_of\_axon\_regeneration | 1 | 0 |  |  |  |  |  |  |  |  |
| GO:0048681\_negative\_regulation\_of\_axon\_regeneration | 1 | 0 |  |  |  |  |  |  |  |  |
| GO:0048686\_regulation\_of\_sprouting\_of\_injured\_axon | 1 | 0 |  |  |  |  |  |  |  |  |
| GO:0048687\_positive\_regulation\_of\_sprouting\_of\_injured\_axon | 1 | 0 |  |  |  |  |  |  |  |  |
| GO:0048690\_regulation\_of\_axon\_extension\_involved\_in\_regeneration | 1 | 0 |  |  |  |  |  |  |  |  |
| GO:0048691\_positive\_regulation\_of\_axon\_extension\_involved\_in\_regeneration | 1 | 0 |  |  |  |  |  |  |  |  |
| GO:0048714\_positive\_regulation\_of\_oligodendrocyte\_differentiation | 1 | 0 |  |  |  |  |  |  |  |  |
| GO:0048733\_sebaceous\_gland\_development | 1 | 0 |  |  |  |  |  |  |  |  |
| GO:0048743\_positive\_regulation\_of\_skeletal\_muscle\_fiber\_development | 1 | 0 |  |  |  |  |  |  |  |  |
| GO:0048752\_semicircular\_canal\_morphogenesis | 1 | 0 |  |  |  |  |  |  |  |  |
| GO:0048773\_erythrophore\_differentiation | 1 | 0 |  |  |  |  |  |  |  |  |
| GO:0048790\_maintenance\_of\_presynaptic\_active\_zone\_structure | 1 | 0 |  |  |  |  |  |  |  |  |
| GO:0048791\_calcium\_ion-dependent\_exocytosis\_of\_neurotransmitter | 1 | 0 |  |  |  |  |  |  |  |  |
| GO:0048822\_enucleate\_erythrocyte\_development | 1 | 0 |  |  |  |  |  |  |  |  |
| GO:0048866\_stem\_cell\_fate\_specification | 1 | 0 |  |  |  |  |  |  |  |  |
| GO:0048936\_peripheral\_nervous\_system\_neuron\_axonogenesis | 1 | 0 |  |  |  |  |  |  |  |  |
| GO:0050427\_3'-phosphoadenosine\_5'-phosphosulfate\_metabolic\_process | 1 | 0 |  |  |  |  |  |  |  |  |
| GO:0050428\_3'-phosphoadenosine\_5'-phosphosulfate\_biosynthetic\_process | 1 | 0 |  |  |  |  |  |  |  |  |
| GO:0050482\_arachidonic\_acid\_secretion | 1 | 0 |  |  |  |  |  |  |  |  |
| GO:0050667\_homocysteine\_metabolic\_process | 1 | 0 |  |  |  |  |  |  |  |  |
| GO:0050674\_urothelial\_cell\_proliferation | 1 | 0 |  |  |  |  |  |  |  |  |
| GO:0050675\_regulation\_of\_urothelial\_cell\_proliferation | 1 | 0 |  |  |  |  |  |  |  |  |
| GO:0050677\_positive\_regulation\_of\_urothelial\_cell\_proliferation | 1 | 0 |  |  |  |  |  |  |  |  |
| GO:0050691\_regulation\_of\_defense\_response\_to\_virus\_by\_host | 1 | 0 |  |  |  |  |  |  |  |  |
| GO:0050748\_negative\_regulation\_of\_lipoprotein\_metabolic\_process | 1 | 0 |  |  |  |  |  |  |  |  |
| GO:0050757\_thymidylate\_synthase\_biosynthetic\_process | 1 | 0 |  |  |  |  |  |  |  |  |
| GO:0050758\_regulation\_of\_thymidylate\_synthase\_biosynthetic\_process | 1 | 0 |  |  |  |  |  |  |  |  |
| GO:0050760\_negative\_regulation\_of\_thymidylate\_synthase\_biosynthetic\_process | 1 | 0 |  |  |  |  |  |  |  |  |
| GO:0050812\_regulation\_of\_acyl-CoA\_biosynthetic\_process | 1 | 0 |  |  |  |  |  |  |  |  |
| GO:0050832\_defense\_response\_to\_fungus | 1 | 0 |  |  |  |  |  |  |  |  |
| GO:0050861\_positive\_regulation\_of\_B\_cell\_receptor\_signaling\_pathway | 1 | 0 |  |  |  |  |  |  |  |  |
| GO:0050862\_positive\_regulation\_of\_T\_cell\_receptor\_signaling\_pathway | 1 | 0 |  |  |  |  |  |  |  |  |
| GO:0050916\_sensory\_perception\_of\_sweet\_taste | 1 | 0 |  |  |  |  |  |  |  |  |
| GO:0050975\_sensory\_perception\_of\_touch | 1 | 0 |  |  |  |  |  |  |  |  |
| GO:0050995\_negative\_regulation\_of\_lipid\_catabolic\_process | 1 | 0 |  |  |  |  |  |  |  |  |
| GO:0051001\_negative\_regulation\_of\_nitric-oxide\_synthase\_activity | 1 | 0 |  |  |  |  |  |  |  |  |
| GO:0051005\_negative\_regulation\_of\_lipoprotein\_lipase\_activity | 1 | 0 |  |  |  |  |  |  |  |  |
| GO:0051006\_positive\_regulation\_of\_lipoprotein\_lipase\_activity | 1 | 0 |  |  |  |  |  |  |  |  |
| GO:0051016\_barbed-end\_actin\_filament\_capping | 1 | 0 |  |  |  |  |  |  |  |  |
| GO:0051029\_rRNA\_transport | 1 | 0 |  |  |  |  |  |  |  |  |
| GO:0051043\_regulation\_of\_membrane\_protein\_ectodomain\_proteolysis | 1 | 0 |  |  |  |  |  |  |  |  |
| GO:0051044\_positive\_regulation\_of\_membrane\_protein\_ectodomain\_proteolysis | 1 | 0 |  |  |  |  |  |  |  |  |
| GO:0051088\_PMA-inducible\_membrane\_protein\_ectodomain\_proteolysis | 1 | 0 |  |  |  |  |  |  |  |  |
| GO:0051102\_DNA\_ligation\_during\_DNA\_recombination | 1 | 0 |  |  |  |  |  |  |  |  |
| GO:0051103\_DNA\_ligation\_during\_DNA\_repair | 1 | 0 |  |  |  |  |  |  |  |  |
| GO:0051123\_transcriptional\_preinitiation\_complex\_assembly | 1 | 0 |  |  |  |  |  |  |  |  |
| GO:0051125\_regulation\_of\_actin\_nucleation | 1 | 0 |  |  |  |  |  |  |  |  |
| GO:0051127\_positive\_regulation\_of\_actin\_nucleation | 1 | 0 |  |  |  |  |  |  |  |  |
| GO:0051151\_negative\_regulation\_of\_smooth\_muscle\_cell\_differentiation | 1 | 0 |  |  |  |  |  |  |  |  |
| GO:0051154\_negative\_regulation\_of\_striated\_muscle\_cell\_differentiation | 1 | 0 |  |  |  |  |  |  |  |  |
| GO:0051155\_positive\_regulation\_of\_striated\_muscle\_cell\_differentiation | 1 | 0 |  |  |  |  |  |  |  |  |
| GO:0051156\_glucose\_6-phosphate\_metabolic\_process | 1 | 0 |  |  |  |  |  |  |  |  |
| GO:0051187\_cofactor\_catabolic\_process | 1 | 0 |  |  |  |  |  |  |  |  |
| GO:0051189\_prosthetic\_group\_metabolic\_process | 1 | 0 |  |  |  |  |  |  |  |  |
| GO:0051193\_regulation\_of\_cofactor\_metabolic\_process | 1 | 0 |  |  |  |  |  |  |  |  |
| GO:0051196\_regulation\_of\_coenzyme\_metabolic\_process | 1 | 0 |  |  |  |  |  |  |  |  |
| GO:0051255\_spindle\_midzone\_assembly | 1 | 0 |  |  |  |  |  |  |  |  |
| GO:0051257\_spindle\_midzone\_assembly\_involved\_in\_meiosis | 1 | 0 |  |  |  |  |  |  |  |  |
| GO:0051281\_positive\_regulation\_of\_release\_of\_sequestered\_calcium\_ion\_into\_cytosol | 1 | 0 |  |  |  |  |  |  |  |  |
| GO:0051290\_protein\_heterotetramerization | 1 | 0 |  |  |  |  |  |  |  |  |
| GO:0051305\_chromosome\_movement\_towards\_spindle\_pole | 1 | 0 |  |  |  |  |  |  |  |  |
| GO:0051310\_metaphase\_plate\_congression | 1 | 0 |  |  |  |  |  |  |  |  |
| GO:0051311\_meiotic\_metaphase\_plate\_congression | 1 | 0 |  |  |  |  |  |  |  |  |
| GO:0051340\_regulation\_of\_ligase\_activity | 1 | 0 |  |  |  |  |  |  |  |  |
| GO:0051351\_positive\_regulation\_of\_ligase\_activity | 1 | 0 |  |  |  |  |  |  |  |  |
| GO:0051354\_negative\_regulation\_of\_oxidoreductase\_activity | 1 | 0 |  |  |  |  |  |  |  |  |
| GO:0051355\_proprioception\_during\_equilibrioception | 1 | 0 |  |  |  |  |  |  |  |  |
| GO:0051383\_kinetochore\_organization | 1 | 0 |  |  |  |  |  |  |  |  |
| GO:0051386\_regulation\_of\_nerve\_growth\_factor\_receptor\_signaling\_pathway | 1 | 0 |  |  |  |  |  |  |  |  |
| GO:0051409\_response\_to\_nitrosative\_stress | 1 | 0 |  |  |  |  |  |  |  |  |
| GO:0051457\_maintenance\_of\_protein\_location\_in\_nucleus | 1 | 0 |  |  |  |  |  |  |  |  |
| GO:0051462\_regulation\_of\_cortisol\_secretion | 1 | 0 |  |  |  |  |  |  |  |  |
| GO:0051463\_negative\_regulation\_of\_cortisol\_secretion | 1 | 0 |  |  |  |  |  |  |  |  |
| GO:0051481\_reduction\_of\_cytosolic\_calcium\_ion\_concentration | 1 | 0 |  |  |  |  |  |  |  |  |
| GO:0051482\_elevation\_of\_cytosolic\_calcium\_ion\_concentration\_during\_G-protein\_signaling\_\_coupled\_to\_IP3\_second\_messenger\_(phospholipase\_C\_activating) | 1 | 0 |  |  |  |  |  |  |  |  |
| GO:0051542\_elastin\_biosynthetic\_process | 1 | 0 |  |  |  |  |  |  |  |  |
| GO:0051568\_histone\_H3-K4\_methylation | 1 | 0 |  |  |  |  |  |  |  |  |
| GO:0051569\_regulation\_of\_histone\_H3-K4\_methylation | 1 | 0 |  |  |  |  |  |  |  |  |
| GO:0051570\_regulation\_of\_histone\_H3-K9\_methylation | 1 | 0 |  |  |  |  |  |  |  |  |
| GO:0051573\_negative\_regulation\_of\_histone\_H3-K9\_methylation | 1 | 0 |  |  |  |  |  |  |  |  |
| GO:0051580\_regulation\_of\_neurotransmitter\_uptake | 1 | 0 |  |  |  |  |  |  |  |  |
| GO:0051582\_positive\_regulation\_of\_neurotransmitter\_uptake | 1 | 0 |  |  |  |  |  |  |  |  |
| GO:0051584\_regulation\_of\_dopamine\_uptake | 1 | 0 |  |  |  |  |  |  |  |  |
| GO:0051586\_positive\_regulation\_of\_dopamine\_uptake | 1 | 0 |  |  |  |  |  |  |  |  |
| GO:0051589\_negative\_regulation\_of\_neurotransmitter\_transport | 1 | 0 |  |  |  |  |  |  |  |  |
| GO:0051593\_response\_to\_folic\_acid | 1 | 0 |  |  |  |  |  |  |  |  |
| GO:0051615\_histamine\_uptake | 1 | 0 |  |  |  |  |  |  |  |  |
| GO:0051646\_mitochondrion\_localization | 1 | 0 |  |  |  |  |  |  |  |  |
| GO:0051654\_establishment\_of\_mitochondrion\_localization | 1 | 0 |  |  |  |  |  |  |  |  |
| GO:0051661\_maintenance\_of\_centrosome\_location | 1 | 0 |  |  |  |  |  |  |  |  |
| GO:0051665\_membrane\_raft\_localization | 1 | 0 |  |  |  |  |  |  |  |  |
| GO:0051685\_maintenance\_of\_ER\_location | 1 | 0 |  |  |  |  |  |  |  |  |
| GO:0051693\_actin\_filament\_capping | 1 | 0 |  |  |  |  |  |  |  |  |
| GO:0051701\_interaction\_with\_host | 1 | 0 |  |  |  |  |  |  |  |  |
| GO:0051754\_meiotic\_sister\_chromatid\_cohesion\_\_centromeric | 1 | 0 |  |  |  |  |  |  |  |  |
| GO:0051782\_negative\_regulation\_of\_cell\_division | 1 | 0 |  |  |  |  |  |  |  |  |
| GO:0051790\_short-chain\_fatty\_acid\_biosynthetic\_process | 1 | 0 |  |  |  |  |  |  |  |  |
| GO:0051799\_negative\_regulation\_of\_hair\_follicle\_development | 1 | 0 |  |  |  |  |  |  |  |  |
| GO:0051823\_regulation\_of\_synapse\_structural\_plasticity | 1 | 0 |  |  |  |  |  |  |  |  |
| GO:0051865\_protein\_autoubiquitination | 1 | 0 |  |  |  |  |  |  |  |  |
| GO:0051901\_positive\_regulation\_of\_mitochondrial\_depolarization | 1 | 0 |  |  |  |  |  |  |  |  |
| GO:0051917\_regulation\_of\_fibrinolysis | 1 | 0 |  |  |  |  |  |  |  |  |
| GO:0051918\_negative\_regulation\_of\_fibrinolysis | 1 | 0 |  |  |  |  |  |  |  |  |
| GO:0051929\_positive\_regulation\_of\_calcium\_ion\_transport\_via\_voltage-gated\_calcium\_channel\_activity | 1 | 0 |  |  |  |  |  |  |  |  |
| GO:0051933\_amino\_acid\_uptake\_during\_transmission\_of\_nerve\_impulse | 1 | 0 |  |  |  |  |  |  |  |  |
| GO:0051935\_glutamate\_uptake\_during\_transmission\_of\_nerve\_impulse | 1 | 0 |  |  |  |  |  |  |  |  |
| GO:0051940\_regulation\_of\_catecholamine\_uptake\_during\_transmission\_of\_nerve\_impulse | 1 | 0 |  |  |  |  |  |  |  |  |
| GO:0051944\_positive\_regulation\_of\_catecholamine\_uptake\_during\_transmission\_of\_nerve\_impulse | 1 | 0 |  |  |  |  |  |  |  |  |
| GO:0051961\_negative\_regulation\_of\_nervous\_system\_development | 1 | 0 |  |  |  |  |  |  |  |  |
| GO:0051964\_negative\_regulation\_of\_synaptogenesis | 1 | 0 |  |  |  |  |  |  |  |  |
| GO:0051968\_positive\_regulation\_of\_synaptic\_transmission\_\_glutamatergic | 1 | 0 |  |  |  |  |  |  |  |  |
| GO:0051984\_positive\_regulation\_of\_chromosome\_segregation | 1 | 0 |  |  |  |  |  |  |  |  |
| GO:0051987\_positive\_regulation\_of\_attachment\_of\_spindle\_microtubules\_to\_kinetochore | 1 | 0 |  |  |  |  |  |  |  |  |
| GO:0052173\_response\_to\_defenses\_of\_other\_organism\_during\_symbiotic\_interaction | 1 | 0 |  |  |  |  |  |  |  |  |
| GO:0052200\_response\_to\_host\_defenses | 1 | 0 |  |  |  |  |  |  |  |  |
| GO:0052551\_response\_to\_defense-related\_nitric\_oxide\_production\_by\_other\_organism\_during\_symbiotic\_interaction | 1 | 0 |  |  |  |  |  |  |  |  |
| GO:0052564\_response\_to\_immune\_response\_of\_other\_organism\_during\_symbiotic\_interaction | 1 | 0 |  |  |  |  |  |  |  |  |
| GO:0052565\_response\_to\_defense-related\_host\_nitric\_oxide\_production | 1 | 0 |  |  |  |  |  |  |  |  |
| GO:0052572\_response\_to\_host\_immune\_response | 1 | 0 |  |  |  |  |  |  |  |  |
| GO:0055005\_ventricular\_cardiac\_myofibril\_development | 1 | 0 |  |  |  |  |  |  |  |  |
| GO:0055011\_atrial\_cardiac\_muscle\_cell\_differentiation | 1 | 0 |  |  |  |  |  |  |  |  |
| GO:0055014\_atrial\_cardiac\_muscle\_cell\_development | 1 | 0 |  |  |  |  |  |  |  |  |
| GO:0055078\_sodium\_ion\_homeostasis | 1 | 0 |  |  |  |  |  |  |  |  |
| GO:0055089\_fatty\_acid\_homeostasis | 1 | 0 |  |  |  |  |  |  |  |  |
| GO:0055093\_response\_to\_hyperoxia | 1 | 0 |  |  |  |  |  |  |  |  |
| GO:0060003\_copper\_ion\_export | 1 | 0 |  |  |  |  |  |  |  |  |
| GO:0060005\_vestibular\_reflex | 1 | 0 |  |  |  |  |  |  |  |  |
| GO:0060014\_granulosa\_cell\_differentiation | 1 | 0 |  |  |  |  |  |  |  |  |
| GO:0060018\_astrocyte\_fate\_commitment | 1 | 0 |  |  |  |  |  |  |  |  |
| GO:0060020\_Bergmann\_glial\_cell\_differentiation | 1 | 0 |  |  |  |  |  |  |  |  |
| GO:0060022\_hard\_palate\_development | 1 | 0 |  |  |  |  |  |  |  |  |
| GO:0060034\_notochord\_cell\_differentiation | 1 | 0 |  |  |  |  |  |  |  |  |
| GO:0060035\_notochord\_cell\_development | 1 | 0 |  |  |  |  |  |  |  |  |
| GO:0060046\_regulation\_of\_acrosome\_reaction | 1 | 0 |  |  |  |  |  |  |  |  |
| GO:0060054\_positive\_regulation\_of\_epithelial\_cell\_proliferation\_involved\_in\_wound\_healing | 1 | 0 |  |  |  |  |  |  |  |  |
| GO:0060059\_embryonic\_retina\_morphogenesis\_in\_camera-type\_eye | 1 | 0 |  |  |  |  |  |  |  |  |
| GO:0060061\_Spemann\_organizer\_formation | 1 | 0 |  |  |  |  |  |  |  |  |
| GO:0060064\_Spemann\_organizer\_formation\_at\_the\_anterior\_end\_of\_the\_primitive\_streak | 1 | 0 |  |  |  |  |  |  |  |  |
| GO:0060071\_Wnt\_receptor\_signaling\_pathway\_\_planar\_cell\_polarity\_pathway | 1 | 0 |  |  |  |  |  |  |  |  |
| GO:0060075\_regulation\_of\_resting\_membrane\_potential | 1 | 0 |  |  |  |  |  |  |  |  |
| GO:0060082\_eye\_blink\_reflex | 1 | 0 |  |  |  |  |  |  |  |  |
| GO:0060112\_generation\_of\_ovulation\_cycle\_rhythm | 1 | 0 |  |  |  |  |  |  |  |  |
| GO:0060125\_negative\_regulation\_of\_growth\_hormone\_secretion | 1 | 0 |  |  |  |  |  |  |  |  |
| GO:0060151\_peroxisome\_localization | 1 | 0 |  |  |  |  |  |  |  |  |
| GO:0060152\_microtubule-based\_peroxisome\_localization | 1 | 0 |  |  |  |  |  |  |  |  |
| GO:0060161\_positive\_regulation\_of\_dopamine\_receptor\_signaling\_pathway | 1 | 0 |  |  |  |  |  |  |  |  |
| GO:0060163\_subpallium\_neuron\_fate\_commitment | 1 | 0 |  |  |  |  |  |  |  |  |
| GO:0060165\_regulation\_of\_timing\_of\_subpallium\_neuron\_differentiation | 1 | 0 |  |  |  |  |  |  |  |  |
| GO:0060174\_limb\_bud\_formation | 1 | 0 |  |  |  |  |  |  |  |  |
| GO:0060177\_regulation\_of\_angiotensin\_metabolic\_process | 1 | 0 |  |  |  |  |  |  |  |  |
| GO:0060197\_cloacal\_septation | 1 | 0 |  |  |  |  |  |  |  |  |
| GO:0060215\_primitive\_hemopoiesis | 1 | 0 |  |  |  |  |  |  |  |  |
| GO:0060231\_mesenchymal\_to\_epithelial\_transition | 1 | 0 |  |  |  |  |  |  |  |  |
| GO:0060254\_regulation\_of\_N-terminal\_protein\_palmitoylation | 1 | 0 |  |  |  |  |  |  |  |  |
| GO:0060261\_positive\_regulation\_of\_transcription\_initiation\_from\_RNA\_polymerase\_II\_promoter | 1 | 0 |  |  |  |  |  |  |  |  |
| GO:0060262\_negative\_regulation\_of\_N-terminal\_protein\_palmitoylation | 1 | 0 |  |  |  |  |  |  |  |  |
| GO:0060263\_regulation\_of\_respiratory\_burst | 1 | 0 |  |  |  |  |  |  |  |  |
| GO:0060264\_regulation\_of\_respiratory\_burst\_during\_acute\_inflammatory\_response | 1 | 0 |  |  |  |  |  |  |  |  |
| GO:0060265\_positive\_regulation\_of\_respiratory\_burst\_during\_acute\_inflammatory\_response | 1 | 0 |  |  |  |  |  |  |  |  |
| GO:0060267\_positive\_regulation\_of\_respiratory\_burst | 1 | 0 |  |  |  |  |  |  |  |  |
| GO:0060272\_embryonic\_skeletal\_joint\_morphogenesis | 1 | 0 |  |  |  |  |  |  |  |  |
| GO:0060297\_regulation\_of\_sarcomere\_organization | 1 | 0 |  |  |  |  |  |  |  |  |
| GO:0060298\_positive\_regulation\_of\_sarcomere\_organization | 1 | 0 |  |  |  |  |  |  |  |  |
| GO:0060315\_negative\_regulation\_of\_ryanodine-sensitive\_calcium-release\_channel\_activity | 1 | 0 |  |  |  |  |  |  |  |  |
| GO:0060319\_primitive\_erythrocyte\_differentiation | 1 | 0 |  |  |  |  |  |  |  |  |
| GO:0060371\_regulation\_of\_atrial\_cardiomyocyte\_membrane\_depolarization | 1 | 0 |  |  |  |  |  |  |  |  |
| GO:0060374\_mast\_cell\_differentiation | 1 | 0 |  |  |  |  |  |  |  |  |
| GO:0060375\_regulation\_of\_mast\_cell\_differentiation | 1 | 0 |  |  |  |  |  |  |  |  |
| GO:0060376\_positive\_regulation\_of\_mast\_cell\_differentiation | 1 | 0 |  |  |  |  |  |  |  |  |
| GO:0060398\_regulation\_of\_growth\_hormone\_receptor\_signaling\_pathway | 1 | 0 |  |  |  |  |  |  |  |  |
| GO:0060399\_positive\_regulation\_of\_growth\_hormone\_receptor\_signaling\_pathway | 1 | 0 |  |  |  |  |  |  |  |  |
| GO:0060405\_regulation\_of\_penile\_erection | 1 | 0 |  |  |  |  |  |  |  |  |
| GO:0060407\_negative\_regulation\_of\_penile\_erection | 1 | 0 |  |  |  |  |  |  |  |  |
| GO:0060413\_atrial\_septum\_morphogenesis | 1 | 0 |  |  |  |  |  |  |  |  |
| GO:0060414\_aorta\_smooth\_muscle\_tissue\_morphogenesis | 1 | 0 |  |  |  |  |  |  |  |  |
| GO:0060419\_heart\_growth | 1 | 0 |  |  |  |  |  |  |  |  |
| GO:0060420\_regulation\_of\_heart\_growth | 1 | 0 |  |  |  |  |  |  |  |  |
| GO:0060421\_positive\_regulation\_of\_heart\_growth | 1 | 0 |  |  |  |  |  |  |  |  |
| GO:0060431\_primary\_lung\_bud\_formation | 1 | 0 |  |  |  |  |  |  |  |  |
| GO:0060436\_bronchiole\_morphogenesis | 1 | 0 |  |  |  |  |  |  |  |  |
| GO:0060440\_trachea\_formation | 1 | 0 |  |  |  |  |  |  |  |  |
| GO:0060449\_bud\_elongation\_involved\_in\_lung\_branching | 1 | 0 |  |  |  |  |  |  |  |  |
| GO:0060456\_positive\_regulation\_of\_digestive\_system\_process | 1 | 0 |  |  |  |  |  |  |  |  |
| GO:0060461\_right\_lung\_morphogenesis | 1 | 0 |  |  |  |  |  |  |  |  |
| GO:0060481\_lobar\_bronchus\_epithelium\_development | 1 | 0 |  |  |  |  |  |  |  |  |
| GO:0060482\_lobar\_bronchus\_development | 1 | 0 |  |  |  |  |  |  |  |  |
| GO:0060484\_lung-associated\_mesenchyme\_development | 1 | 0 |  |  |  |  |  |  |  |  |
| GO:0060486\_Clara\_cell\_differentiation | 1 | 0 |  |  |  |  |  |  |  |  |
| GO:0060510\_Type\_II\_pneumocyte\_differentiation | 1 | 0 |  |  |  |  |  |  |  |  |
| GO:0060514\_prostate\_induction | 1 | 0 |  |  |  |  |  |  |  |  |
| GO:0060515\_prostate\_field\_specification | 1 | 0 |  |  |  |  |  |  |  |  |
| GO:0060517\_epithelial\_cell\_proliferation\_involved\_in\_prostatic\_bud\_elongation | 1 | 0 |  |  |  |  |  |  |  |  |
| GO:0060520\_activation\_of\_prostate\_induction\_by\_androgen\_receptor\_signaling\_pathway | 1 | 0 |  |  |  |  |  |  |  |  |
| GO:0060535\_trachea\_cartilage\_morphogenesis | 1 | 0 |  |  |  |  |  |  |  |  |
| GO:0060536\_cartilage\_morphogenesis | 1 | 0 |  |  |  |  |  |  |  |  |
| GO:0060563\_neuroepithelial\_cell\_differentiation | 1 | 0 |  |  |  |  |  |  |  |  |
| GO:0060577\_pulmonary\_vein\_morphogenesis | 1 | 0 |  |  |  |  |  |  |  |  |
| GO:0060578\_superior\_vena\_cava\_morphogenesis | 1 | 0 |  |  |  |  |  |  |  |  |
| GO:0060584\_regulation\_of\_prostaglandin-endoperoxide\_synthase\_activity | 1 | 0 |  |  |  |  |  |  |  |  |
| GO:0060585\_positive\_regulation\_of\_prostaglandin-endoperoxidase\_synthase\_activity | 1 | 0 |  |  |  |  |  |  |  |  |
| GO:0060598\_dichotomous\_subdivision\_of\_terminal\_units\_involved\_in\_mammary\_gland\_duct\_morphogenesis | 1 | 0 |  |  |  |  |  |  |  |  |
| GO:0060611\_mammary\_gland\_fat\_development | 1 | 0 |  |  |  |  |  |  |  |  |
| GO:0060618\_nipple\_development | 1 | 0 |  |  |  |  |  |  |  |  |
| GO:0060631\_regulation\_of\_meiosis\_I | 1 | 0 |  |  |  |  |  |  |  |  |
| GO:0060649\_mammary\_gland\_bud\_elongation | 1 | 0 |  |  |  |  |  |  |  |  |
| GO:0060658\_nipple\_morphogenesis | 1 | 0 |  |  |  |  |  |  |  |  |
| GO:0060659\_nipple\_sheath\_formation | 1 | 0 |  |  |  |  |  |  |  |  |
| GO:0060668\_regulation\_of\_branching\_involved\_in\_salivary\_gland\_morphogenesis\_by\_extracellular\_matrix-epithelial\_cell\_signaling | 1 | 0 |  |  |  |  |  |  |  |  |
| GO:0060683\_regulation\_of\_branching\_involved\_in\_salivary\_gland\_morphogenesis\_by\_epithelial-mesenchymal\_signaling | 1 | 0 |  |  |  |  |  |  |  |  |
| GO:0060691\_epithelial\_cell\_maturation\_involved\_in\_salivary\_gland\_development | 1 | 0 |  |  |  |  |  |  |  |  |
| GO:0060709\_glycogen\_cell\_development\_involved\_in\_embryonic\_placenta\_development | 1 | 0 |  |  |  |  |  |  |  |  |
| GO:0060732\_positive\_regulation\_of\_inositol\_phosphate\_biosynthetic\_process | 1 | 0 |  |  |  |  |  |  |  |  |
| GO:0060739\_mesenchymal-epithelial\_cell\_signaling\_involved\_in\_prostate\_gland\_development | 1 | 0 |  |  |  |  |  |  |  |  |
| GO:0060781\_mesenchymal\_cell\_proliferation\_involved\_in\_prostate\_gland\_development | 1 | 0 |  |  |  |  |  |  |  |  |
| GO:0060782\_regulation\_of\_mesenchymal\_cell\_proliferation\_involved\_in\_prostate\_gland\_development | 1 | 0 |  |  |  |  |  |  |  |  |
| GO:0060783\_mesenchymal\_smoothened\_signaling\_pathway\_involved\_in\_prostate\_gland\_development | 1 | 0 |  |  |  |  |  |  |  |  |
| GO:0060872\_semicircular\_canal\_development | 1 | 0 |  |  |  |  |  |  |  |  |
| GO:0060896\_neural\_plate\_pattern\_specification | 1 | 0 |  |  |  |  |  |  |  |  |
| GO:0070091\_glucagon\_secretion | 1 | 0 |  |  |  |  |  |  |  |  |
| GO:0070162\_adiponectin\_secretion | 1 | 0 |  |  |  |  |  |  |  |  |
| GO:0070163\_regulation\_of\_adiponectin\_secretion | 1 | 0 |  |  |  |  |  |  |  |  |
| GO:0070164\_negative\_regulation\_of\_adiponectin\_secretion | 1 | 0 |  |  |  |  |  |  |  |  |
| GO:0070178\_D-serine\_metabolic\_process | 1 | 0 |  |  |  |  |  |  |  |  |
| GO:0070179\_D-serine\_biosynthetic\_process | 1 | 0 |  |  |  |  |  |  |  |  |
| GO:0070296\_sarcoplasmic\_reticulum\_calcium\_ion\_transport | 1 | 0 |  |  |  |  |  |  |  |  |
| GO:0070303\_negative\_regulation\_of\_stress-activated\_protein\_kinase\_signaling\_pathway | 1 | 0 |  |  |  |  |  |  |  |  |
| GO:0070328\_triglyceride\_homeostasis | 1 | 0 |  |  |  |  |  |  |  |  |
| GO:0070365\_hepatocyte\_differentiation | 1 | 0 |  |  |  |  |  |  |  |  |
| GO:0070384\_Harderian\_gland\_development | 1 | 0 |  |  |  |  |  |  |  |  |
| GO:0070391\_response\_to\_lipoteichoic\_acid | 1 | 0 |  |  |  |  |  |  |  |  |
| GO:0070424\_regulation\_of\_nucleotide-binding\_oligomerization\_domain\_containing\_signaling\_pathway | 1 | 0 |  |  |  |  |  |  |  |  |
| GO:0070426\_positive\_regulation\_of\_nucleotide-binding\_oligomerization\_domain\_containing\_signaling\_pathway | 1 | 0 |  |  |  |  |  |  |  |  |
| GO:0070428\_regulation\_of\_nucleotide-binding\_oligomerization\_domain\_containing\_1\_signaling\_pathway | 1 | 0 |  |  |  |  |  |  |  |  |
| GO:0070430\_positive\_regulation\_of\_nucleotide-binding\_oligomerization\_domain\_containing\_1\_signaling\_pathway | 1 | 0 |  |  |  |  |  |  |  |  |
| GO:0070432\_regulation\_of\_nucleotide-binding\_oligomerization\_domain\_containing\_2\_signaling\_pathway | 1 | 0 |  |  |  |  |  |  |  |  |
| GO:0070434\_positive\_regulation\_of\_nucleotide-binding\_oligomerization\_domain\_containing\_2\_signaling\_pathway | 1 | 0 |  |  |  |  |  |  |  |  |
| GO:0070493\_thrombin\_receptor\_signaling\_pathway | 1 | 0 |  |  |  |  |  |  |  |  |
| GO:0070508\_cholesterol\_import | 1 | 0 |  |  |  |  |  |  |  |  |
| GO:0070527\_platelet\_aggregation | 1 | 0 |  |  |  |  |  |  |  |  |
| GO:0070528\_protein\_kinase\_C\_signaling\_cascade | 1 | 0 |  |  |  |  |  |  |  |  |
| GO:0070555\_response\_to\_interleukin-1 | 1 | 0 |  |  |  |  |  |  |  |  |
| GO:0070560\_protein\_secretion\_by\_platelet | 1 | 0 |  |  |  |  |  |  |  |  |
| GO:0070561\_vitamin\_D\_receptor\_signaling\_pathway | 1 | 0 |  |  |  |  |  |  |  |  |
| GO:0070562\_regulation\_of\_vitamin\_D\_receptor\_signaling\_pathway | 1 | 0 |  |  |  |  |  |  |  |  |
| GO:0070571\_negative\_regulation\_of\_neuron\_projection\_regeneration | 1 | 0 |  |  |  |  |  |  |  |  |
| GO:0070572\_positive\_regulation\_of\_neuron\_projection\_regeneration | 1 | 0 |  |  |  |  |  |  |  |  |
| GO:0070613\_regulation\_of\_protein\_processing | 1 | 0 |  |  |  |  |  |  |  |  |
| GO:0070627\_ferrous\_iron\_import | 1 | 0 |  |  |  |  |  |  |  |  |
| GO:0070669\_response\_to\_interleukin-2 | 1 | 0 |  |  |  |  |  |  |  |  |
| GO:0070670\_response\_to\_interleukin-4 | 1 | 0 |  |  |  |  |  |  |  |  |
| GO:0070671\_response\_to\_interleukin-12 | 1 | 0 |  |  |  |  |  |  |  |  |
| GO:0070672\_response\_to\_interleukin-15 | 1 | 0 |  |  |  |  |  |  |  |  |
| GO:0070673\_response\_to\_interleukin-18 | 1 | 0 |  |  |  |  |  |  |  |  |
| GO:0070828\_heterochromatin\_organization | 1 | 0 |  |  |  |  |  |  |  |  |
| GO:0070874\_negative\_regulation\_of\_glycogen\_metabolic\_process | 1 | 0 |  |  |  |  |  |  |  |  |
| GO:0075136\_response\_to\_host | 1 | 0 |  |  |  |  |  |  |  |  |
| GO:0080010\_regulation\_of\_oxygen\_and\_reactive\_oxygen\_species\_metabolic\_process | 1 | 0 |  |  |  |  |  |  |  |  |
| GO:0090032\_negative\_regulation\_of\_steroid\_hormone\_biosynthetic\_process | 1 | 0 |  |  |  |  |  |  |  |  |
| GO:0000002\_mitochondrial\_genome\_maintenance | 9 | 0 | 0.000000 | -0.000000 | 700 | 581.362235 | 672.79 | 764.217765 | 0.961129 |
| GO:0000186\_activation\_of\_MAPKK\_activity | 9 | 0 | 0.000000 | -0.000000 | 700 | 581.362235 | 672.79 | 764.217765 | 0.961129 |
| GO:0001539\_ciliary\_or\_flagellar\_motility | 9 | 0 | 0.000000 | -0.000000 | 700 | 581.362235 | 672.79 | 764.217765 | 0.961129 |
| GO:0001542\_ovulation\_from\_ovarian\_follicle | 9 | 0 | 0.000000 | -0.000000 | 700 | 581.362235 | 672.79 | 764.217765 | 0.961129 |
| GO:0001667\_ameboidal\_cell\_migration | 9 | 0 | 0.000000 | -0.000000 | 700 | 581.362235 | 672.79 | 764.217765 | 0.961129 |
| GO:0001676\_long-chain\_fatty\_acid\_metabolic\_process | 9 | 0 | 0.000000 | -0.000000 | 700 | 581.362235 | 672.79 | 764.217765 | 0.961129 |
| GO:0001935\_endothelial\_cell\_proliferation | 9 | 0 | 0.000000 | -0.000000 | 700 | 581.362235 | 672.79 | 764.217765 | 0.961129 |
| GO:0002021\_response\_to\_dietary\_excess | 9 | 0 | 0.000000 | -0.000000 | 700 | 581.362235 | 672.79 | 764.217765 | 0.961129 |
| GO:0002028\_regulation\_of\_sodium\_ion\_transport | 9 | 0 | 0.000000 | -0.000000 | 700 | 581.362235 | 672.79 | 764.217765 | 0.961129 |
| GO:0002221\_pattern\_recognition\_receptor\_signaling\_pathway | 9 | 0 | 0.000000 | -0.000000 | 700 | 581.362235 | 672.79 | 764.217765 | 0.961129 |
| GO:0002292\_T\_cell\_differentiation\_during\_immune\_response | 9 | 0 | 0.000000 | -0.000000 | 700 | 581.362235 | 672.79 | 764.217765 | 0.961129 |
| GO:0002293\_alpha-beta\_T\_cell\_differentiation\_during\_immune\_response | 9 | 0 | 0.000000 | -0.000000 | 700 | 581.362235 | 672.79 | 764.217765 | 0.961129 |
| GO:0002294\_CD4-positive\_\_alpha-beta\_T\_cell\_differentiation\_during\_immune\_response | 9 | 0 | 0.000000 | -0.000000 | 700 | 581.362235 | 672.79 | 764.217765 | 0.961129 |
| GO:0002507\_tolerance\_induction | 9 | 0 | 0.000000 | -0.000000 | 700 | 581.362235 | 672.79 | 764.217765 | 0.961129 |
| GO:0002886\_regulation\_of\_myeloid\_leukocyte\_mediated\_immunity | 9 | 0 | 0.000000 | -0.000000 | 700 | 581.362235 | 672.79 | 764.217765 | 0.961129 |
| GO:0006007\_glucose\_catabolic\_process | 9 | 0 | 0.000000 | -0.000000 | 700 | 581.362235 | 672.79 | 764.217765 | 0.961129 |
| GO:0006182\_cGMP\_biosynthetic\_process | 9 | 0 | 0.000000 | -0.000000 | 700 | 581.362235 | 672.79 | 764.217765 | 0.961129 |
| GO:0006309\_DNA\_fragmentation\_involved\_in\_apoptosis | 9 | 0 | 0.000000 | -0.000000 | 700 | 581.362235 | 672.79 | 764.217765 | 0.961129 |
| GO:0006364\_rRNA\_processing | 9 | 0 | 0.000000 | -0.000000 | 700 | 581.362235 | 672.79 | 764.217765 | 0.961129 |
| GO:0006476\_protein\_amino\_acid\_deacetylation | 9 | 0 | 0.000000 | -0.000000 | 700 | 581.362235 | 672.79 | 764.217765 | 0.961129 |
| GO:0006595\_polyamine\_metabolic\_process | 9 | 0 | 0.000000 | -0.000000 | 700 | 581.362235 | 672.79 | 764.217765 | 0.961129 |
| GO:0006611\_protein\_export\_from\_nucleus | 9 | 0 | 0.000000 | -0.000000 | 700 | 581.362235 | 672.79 | 764.217765 | 0.961129 |
| GO:0006910\_phagocytosis\_\_recognition | 9 | 0 | 0.000000 | -0.000000 | 700 | 581.362235 | 672.79 | 764.217765 | 0.961129 |
| GO:0006911\_phagocytosis\_\_engulfment | 9 | 0 | 0.000000 | -0.000000 | 700 | 581.362235 | 672.79 | 764.217765 | 0.961129 |
| GO:0007128\_meiotic\_prophase\_I | 9 | 0 | 0.000000 | -0.000000 | 700 | 581.362235 | 672.79 | 764.217765 | 0.961129 |
| GO:0007193\_inhibition\_of\_adenylate\_cyclase\_activity\_by\_G-protein\_signaling | 9 | 0 | 0.000000 | -0.000000 | 700 | 581.362235 | 672.79 | 764.217765 | 0.961129 |
| GO:0007379\_segment\_specification | 9 | 0 | 0.000000 | -0.000000 | 700 | 581.362235 | 672.79 | 764.217765 | 0.961129 |
| GO:0007617\_mating\_behavior | 9 | 0 | 0.000000 | -0.000000 | 700 | 581.362235 | 672.79 | 764.217765 | 0.961129 |
| GO:0009451\_RNA\_modification | 9 | 0 | 0.000000 | -0.000000 | 700 | 581.362235 | 672.79 | 764.217765 | 0.961129 |
| GO:0010675\_regulation\_of\_cellular\_carbohydrate\_metabolic\_process | 9 | 0 | 0.000000 | -0.000000 | 700 | 581.362235 | 672.79 | 764.217765 | 0.961129 |
| GO:0014037\_Schwann\_cell\_differentiation | 9 | 0 | 0.000000 | -0.000000 | 700 | 581.362235 | 672.79 | 764.217765 | 0.961129 |
| GO:0014073\_response\_to\_tropane | 9 | 0 | 0.000000 | -0.000000 | 700 | 581.362235 | 672.79 | 764.217765 | 0.961129 |
| GO:0015695\_organic\_cation\_transport | 9 | 0 | 0.000000 | -0.000000 | 700 | 581.362235 | 672.79 | 764.217765 | 0.961129 |
| GO:0016072\_rRNA\_metabolic\_process | 9 | 0 | 0.000000 | -0.000000 | 700 | 581.362235 | 672.79 | 764.217765 | 0.961129 |
| GO:0016601\_Rac\_protein\_signal\_transduction | 9 | 0 | 0.000000 | -0.000000 | 700 | 581.362235 | 672.79 | 764.217765 | 0.961129 |
| GO:0017145\_stem\_cell\_division | 9 | 0 | 0.000000 | -0.000000 | 700 | 581.362235 | 672.79 | 764.217765 | 0.961129 |
| GO:0019320\_hexose\_catabolic\_process | 9 | 0 | 0.000000 | -0.000000 | 700 | 581.362235 | 672.79 | 764.217765 | 0.961129 |
| GO:0021544\_subpallium\_development | 9 | 0 | 0.000000 | -0.000000 | 700 | 581.362235 | 672.79 | 764.217765 | 0.961129 |
| GO:0021936\_regulation\_of\_granule\_cell\_precursor\_proliferation | 9 | 0 | 0.000000 | -0.000000 | 700 | 581.362235 | 672.79 | 764.217765 | 0.961129 |
| GO:0021940\_positive\_regulation\_of\_granule\_cell\_precursor\_proliferation | 9 | 0 | 0.000000 | -0.000000 | 700 | 581.362235 | 672.79 | 764.217765 | 0.961129 |
| GO:0030048\_actin\_filament-based\_movement | 9 | 0 | 0.000000 | -0.000000 | 700 | 581.362235 | 672.79 | 764.217765 | 0.961129 |
| GO:0030279\_negative\_regulation\_of\_ossification | 9 | 0 | 0.000000 | -0.000000 | 700 | 581.362235 | 672.79 | 764.217765 | 0.961129 |
| GO:0030325\_adrenal\_gland\_development | 9 | 0 | 0.000000 | -0.000000 | 700 | 581.362235 | 672.79 | 764.217765 | 0.961129 |
| GO:0030728\_ovulation | 9 | 0 | 0.000000 | -0.000000 | 700 | 581.362235 | 672.79 | 764.217765 | 0.961129 |
| GO:0031023\_microtubule\_organizing\_center\_organization | 9 | 0 | 0.000000 | -0.000000 | 700 | 581.362235 | 672.79 | 764.217765 | 0.961129 |
| GO:0032606\_type\_I\_interferon\_production | 9 | 0 | 0.000000 | -0.000000 | 700 | 581.362235 | 672.79 | 764.217765 | 0.961129 |
| GO:0032814\_regulation\_of\_natural\_killer\_cell\_activation | 9 | 0 | 0.000000 | -0.000000 | 700 | 581.362235 | 672.79 | 764.217765 | 0.961129 |
| GO:0032816\_positive\_regulation\_of\_natural\_killer\_cell\_activation | 9 | 0 | 0.000000 | -0.000000 | 700 | 581.362235 | 672.79 | 764.217765 | 0.961129 |
| GO:0032963\_collagen\_metabolic\_process | 9 | 0 | 0.000000 | -0.000000 | 700 | 581.362235 | 672.79 | 764.217765 | 0.961129 |
| GO:0033028\_myeloid\_cell\_apoptosis | 9 | 0 | 0.000000 | -0.000000 | 700 | 581.362235 | 672.79 | 764.217765 | 0.961129 |
| GO:0033143\_regulation\_of\_steroid\_hormone\_receptor\_signaling\_pathway | 9 | 0 | 0.000000 | -0.000000 | 700 | 581.362235 | 672.79 | 764.217765 | 0.961129 |
| GO:0033151\_V(D)J\_recombination | 9 | 0 | 0.000000 | -0.000000 | 700 | 581.362235 | 672.79 | 764.217765 | 0.961129 |
| GO:0033344\_cholesterol\_efflux | 9 | 0 | 0.000000 | -0.000000 | 700 | 581.362235 | 672.79 | 764.217765 | 0.961129 |
| GO:0034605\_cellular\_response\_to\_heat | 9 | 0 | 0.000000 | -0.000000 | 700 | 581.362235 | 672.79 | 764.217765 | 0.961129 |
| GO:0035088\_establishment\_or\_maintenance\_of\_apical\_basal\_cell\_polarity | 9 | 0 | 0.000000 | -0.000000 | 700 | 581.362235 | 672.79 | 764.217765 | 0.961129 |
| GO:0035162\_embryonic\_hemopoiesis | 9 | 0 | 0.000000 | -0.000000 | 700 | 581.362235 | 672.79 | 764.217765 | 0.961129 |
| GO:0040020\_regulation\_of\_meiosis | 9 | 0 | 0.000000 | -0.000000 | 700 | 581.362235 | 672.79 | 764.217765 | 0.961129 |
| GO:0042058\_regulation\_of\_epidermal\_growth\_factor\_receptor\_signaling\_pathway | 9 | 0 | 0.000000 | -0.000000 | 700 | 581.362235 | 672.79 | 764.217765 | 0.961129 |
| GO:0042093\_T-helper\_cell\_differentiation | 9 | 0 | 0.000000 | -0.000000 | 700 | 581.362235 | 672.79 | 764.217765 | 0.961129 |
| GO:0042220\_response\_to\_cocaine | 9 | 0 | 0.000000 | -0.000000 | 700 | 581.362235 | 672.79 | 764.217765 | 0.961129 |
| GO:0042402\_biogenic\_amine\_catabolic\_process | 9 | 0 | 0.000000 | -0.000000 | 700 | 581.362235 | 672.79 | 764.217765 | 0.961129 |
| GO:0042509\_regulation\_of\_tyrosine\_phosphorylation\_of\_STAT\_protein | 9 | 0 | 0.000000 | -0.000000 | 700 | 581.362235 | 672.79 | 764.217765 | 0.961129 |
| GO:0042640\_anagen | 9 | 0 | 0.000000 | -0.000000 | 700 | 581.362235 | 672.79 | 764.217765 | 0.961129 |
| GO:0043242\_negative\_regulation\_of\_protein\_complex\_disassembly | 9 | 0 | 0.000000 | -0.000000 | 700 | 581.362235 | 672.79 | 764.217765 | 0.961129 |
| GO:0043299\_leukocyte\_degranulation | 9 | 0 | 0.000000 | -0.000000 | 700 | 581.362235 | 672.79 | 764.217765 | 0.961129 |
| GO:0043383\_negative\_T\_cell\_selection | 9 | 0 | 0.000000 | -0.000000 | 700 | 581.362235 | 672.79 | 764.217765 | 0.961129 |
| GO:0043409\_negative\_regulation\_of\_MAPKKK\_cascade | 9 | 0 | 0.000000 | -0.000000 | 700 | 581.362235 | 672.79 | 764.217765 | 0.961129 |
| GO:0043433\_negative\_regulation\_of\_transcription\_factor\_activity | 9 | 0 | 0.000000 | -0.000000 | 700 | 581.362235 | 672.79 | 764.217765 | 0.961129 |
| GO:0043603\_cellular\_amide\_metabolic\_process | 9 | 0 | 0.000000 | -0.000000 | 700 | 581.362235 | 672.79 | 764.217765 | 0.961129 |
| GO:0045060\_negative\_thymic\_T\_cell\_selection | 9 | 0 | 0.000000 | -0.000000 | 700 | 581.362235 | 672.79 | 764.217765 | 0.961129 |
| GO:0045109\_intermediate\_filament\_organization | 9 | 0 | 0.000000 | -0.000000 | 700 | 581.362235 | 672.79 | 764.217765 | 0.961129 |
| GO:0045136\_development\_of\_secondary\_sexual\_characteristics | 9 | 0 | 0.000000 | -0.000000 | 700 | 581.362235 | 672.79 | 764.217765 | 0.961129 |
| GO:0045185\_maintenance\_of\_protein\_location | 9 | 0 | 0.000000 | -0.000000 | 700 | 581.362235 | 672.79 | 764.217765 | 0.961129 |
| GO:0045214\_sarcomere\_organization | 9 | 0 | 0.000000 | -0.000000 | 700 | 581.362235 | 672.79 | 764.217765 | 0.961129 |
| GO:0045428\_regulation\_of\_nitric\_oxide\_biosynthetic\_process | 9 | 0 | 0.000000 | -0.000000 | 700 | 581.362235 | 672.79 | 764.217765 | 0.961129 |
| GO:0045620\_negative\_regulation\_of\_lymphocyte\_differentiation | 9 | 0 | 0.000000 | -0.000000 | 700 | 581.362235 | 672.79 | 764.217765 | 0.961129 |
| GO:0045646\_regulation\_of\_erythrocyte\_differentiation | 9 | 0 | 0.000000 | -0.000000 | 700 | 581.362235 | 672.79 | 764.217765 | 0.961129 |
| GO:0045671\_negative\_regulation\_of\_osteoclast\_differentiation | 9 | 0 | 0.000000 | -0.000000 | 700 | 581.362235 | 672.79 | 764.217765 | 0.961129 |
| GO:0045766\_positive\_regulation\_of\_angiogenesis | 9 | 0 | 0.000000 | -0.000000 | 700 | 581.362235 | 672.79 | 764.217765 | 0.961129 |
| GO:0045830\_positive\_regulation\_of\_isotype\_switching | 9 | 0 | 0.000000 | -0.000000 | 700 | 581.362235 | 672.79 | 764.217765 | 0.961129 |
| GO:0045884\_regulation\_of\_survival\_gene\_product\_expression | 9 | 0 | 0.000000 | -0.000000 | 700 | 581.362235 | 672.79 | 764.217765 | 0.961129 |
| GO:0046006\_regulation\_of\_activated\_T\_cell\_proliferation | 9 | 0 | 0.000000 | -0.000000 | 700 | 581.362235 | 672.79 | 764.217765 | 0.961129 |
| GO:0046324\_regulation\_of\_glucose\_import | 9 | 0 | 0.000000 | -0.000000 | 700 | 581.362235 | 672.79 | 764.217765 | 0.961129 |
| GO:0046365\_monosaccharide\_catabolic\_process | 9 | 0 | 0.000000 | -0.000000 | 700 | 581.362235 | 672.79 | 764.217765 | 0.961129 |
| GO:0046636\_negative\_regulation\_of\_alpha-beta\_T\_cell\_activation | 9 | 0 | 0.000000 | -0.000000 | 700 | 581.362235 | 672.79 | 764.217765 | 0.961129 |
| GO:0046641\_positive\_regulation\_of\_alpha-beta\_T\_cell\_proliferation | 9 | 0 | 0.000000 | -0.000000 | 700 | 581.362235 | 672.79 | 764.217765 | 0.961129 |
| GO:0046888\_negative\_regulation\_of\_hormone\_secretion | 9 | 0 | 0.000000 | -0.000000 | 700 | 581.362235 | 672.79 | 764.217765 | 0.961129 |
| GO:0048070\_regulation\_of\_pigmentation\_during\_development | 9 | 0 | 0.000000 | -0.000000 | 700 | 581.362235 | 672.79 | 764.217765 | 0.961129 |
| GO:0048146\_positive\_regulation\_of\_fibroblast\_proliferation | 9 | 0 | 0.000000 | -0.000000 | 700 | 581.362235 | 672.79 | 764.217765 | 0.961129 |
| GO:0048284\_organelle\_fusion | 9 | 0 | 0.000000 | -0.000000 | 700 | 581.362235 | 672.79 | 764.217765 | 0.961129 |
| GO:0048488\_synaptic\_vesicle\_endocytosis | 9 | 0 | 0.000000 | -0.000000 | 700 | 581.362235 | 672.79 | 764.217765 | 0.961129 |
| GO:0048569\_post-embryonic\_organ\_development | 9 | 0 | 0.000000 | -0.000000 | 700 | 581.362235 | 672.79 | 764.217765 | 0.961129 |
| GO:0048708\_astrocyte\_differentiation | 9 | 0 | 0.000000 | -0.000000 | 700 | 581.362235 | 672.79 | 764.217765 | 0.961129 |
| GO:0050433\_regulation\_of\_catecholamine\_secretion | 9 | 0 | 0.000000 | -0.000000 | 700 | 581.362235 | 672.79 | 764.217765 | 0.961129 |
| GO:0050856\_regulation\_of\_T\_cell\_receptor\_signaling\_pathway | 9 | 0 | 0.000000 | -0.000000 | 700 | 581.362235 | 672.79 | 764.217765 | 0.961129 |
| GO:0050884\_neuromuscular\_process\_controlling\_posture | 9 | 0 | 0.000000 | -0.000000 | 700 | 581.362235 | 672.79 | 764.217765 | 0.961129 |
| GO:0050910\_detection\_of\_mechanical\_stimulus\_involved\_in\_sensory\_perception\_of\_sound | 9 | 0 | 0.000000 | -0.000000 | 700 | 581.362235 | 672.79 | 764.217765 | 0.961129 |
| GO:0050918\_positive\_chemotaxis | 9 | 0 | 0.000000 | -0.000000 | 700 | 581.362235 | 672.79 | 764.217765 | 0.961129 |
| GO:0051023\_regulation\_of\_immunoglobulin\_secretion | 9 | 0 | 0.000000 | -0.000000 | 700 | 581.362235 | 672.79 | 764.217765 | 0.961129 |
| GO:0051297\_centrosome\_organization | 9 | 0 | 0.000000 | -0.000000 | 700 | 581.362235 | 672.79 | 764.217765 | 0.961129 |
| GO:0051324\_prophase | 9 | 0 | 0.000000 | -0.000000 | 700 | 581.362235 | 672.79 | 764.217765 | 0.961129 |
| GO:0051607\_defense\_response\_to\_virus | 9 | 0 | 0.000000 | -0.000000 | 700 | 581.362235 | 672.79 | 764.217765 | 0.961129 |
| GO:0051647\_nucleus\_localization | 9 | 0 | 0.000000 | -0.000000 | 700 | 581.362235 | 672.79 | 764.217765 | 0.961129 |
| GO:0051896\_regulation\_of\_protein\_kinase\_B\_signaling\_cascade | 9 | 0 | 0.000000 | -0.000000 | 700 | 581.362235 | 672.79 | 764.217765 | 0.961129 |
| GO:0051932\_synaptic\_transmission\_\_GABAergic | 9 | 0 | 0.000000 | -0.000000 | 700 | 581.362235 | 672.79 | 764.217765 | 0.961129 |
| GO:0051963\_regulation\_of\_synaptogenesis | 9 | 0 | 0.000000 | -0.000000 | 700 | 581.362235 | 672.79 | 764.217765 | 0.961129 |
| GO:0055012\_ventricular\_cardiac\_muscle\_cell\_differentiation | 9 | 0 | 0.000000 | -0.000000 | 700 | 581.362235 | 672.79 | 764.217765 | 0.961129 |
| GO:0055013\_cardiac\_muscle\_cell\_development | 9 | 0 | 0.000000 | -0.000000 | 700 | 581.362235 | 672.79 | 764.217765 | 0.961129 |
| GO:0060052\_neurofilament\_cytoskeleton\_organization | 9 | 0 | 0.000000 | -0.000000 | 700 | 581.362235 | 672.79 | 764.217765 | 0.961129 |
| GO:0060081\_membrane\_hyperpolarization | 9 | 0 | 0.000000 | -0.000000 | 700 | 581.362235 | 672.79 | 764.217765 | 0.961129 |
| GO:0060119\_inner\_ear\_receptor\_cell\_development | 9 | 0 | 0.000000 | -0.000000 | 700 | 581.362235 | 672.79 | 764.217765 | 0.961129 |
| GO:0060122\_inner\_ear\_receptor\_stereocilium\_organization | 9 | 0 | 0.000000 | -0.000000 | 700 | 581.362235 | 672.79 | 764.217765 | 0.961129 |
| GO:0060325\_face\_morphogenesis | 9 | 0 | 0.000000 | -0.000000 | 700 | 581.362235 | 672.79 | 764.217765 | 0.961129 |
| GO:0060513\_prostatic\_bud\_formation | 9 | 0 | 0.000000 | -0.000000 | 700 | 581.362235 | 672.79 | 764.217765 | 0.961129 |
| GO:0060602\_branch\_elongation\_of\_an\_epithelium | 9 | 0 | 0.000000 | -0.000000 | 700 | 581.362235 | 672.79 | 764.217765 | 0.961129 |
| GO:0060693\_regulation\_of\_branching\_involved\_in\_salivary\_gland\_morphogenesis | 9 | 0 | 0.000000 | -0.000000 | 700 | 581.362235 | 672.79 | 764.217765 | 0.961129 |
| GO:0070306\_lens\_fiber\_cell\_differentiation | 9 | 0 | 0.000000 | -0.000000 | 700 | 581.362235 | 672.79 | 764.217765 | 0.961129 |
| GO:0090048\_negative\_regulation\_of\_transcription\_regulator\_activity | 9 | 0 | 0.000000 | -0.000000 | 700 | 581.362235 | 672.79 | 764.217765 | 0.961129 |
| GO:0007268\_synaptic\_transmission | 154 | 0 | 0.000000 | -0.000000 | 701 | 582.013958 | 673.3 | 764.586042 | 0.960485 |
| GO:0021700\_developmental\_maturation | 81 | 0 | 0.000000 | -0.000000 | 702 | 583.380665 | 674.45 | 765.519335 | 0.960755 |
| GO:0048771\_tissue\_remodeling | 74 | 0 | 0.000000 | -0.000000 | 703 | 584.921960 | 675.78 | 766.638040 | 0.961280 |
| GO:0000086\_G2\_M\_transition\_of\_mitotic\_cell\_cycle | 4 | 0 |  |  |  |  |  |  |  |  |
| GO:0000305\_response\_to\_oxygen\_radical | 4 | 0 |  |  |  |  |  |  |  |  |
| GO:0001661\_conditioned\_taste\_aversion | 4 | 0 |  |  |  |  |  |  |  |  |
| GO:0001678\_cellular\_glucose\_homeostasis | 4 | 0 |  |  |  |  |  |  |  |  |
| GO:0001777\_T\_cell\_homeostatic\_proliferation | 4 | 0 |  |  |  |  |  |  |  |  |
| GO:0001794\_type\_IIa\_hypersensitivity | 4 | 0 |  |  |  |  |  |  |  |  |
| GO:0001796\_regulation\_of\_type\_IIa\_hypersensitivity | 4 | 0 |  |  |  |  |  |  |  |  |
| GO:0001798\_positive\_regulation\_of\_type\_IIa\_hypersensitivity | 4 | 0 |  |  |  |  |  |  |  |  |
| GO:0001810\_regulation\_of\_type\_I\_hypersensitivity | 4 | 0 |  |  |  |  |  |  |  |  |
| GO:0001820\_serotonin\_secretion | 4 | 0 |  |  |  |  |  |  |  |  |
| GO:0001842\_neural\_fold\_formation | 4 | 0 |  |  |  |  |  |  |  |  |
| GO:0001881\_receptor\_recycling | 4 | 0 |  |  |  |  |  |  |  |  |
| GO:0001938\_positive\_regulation\_of\_endothelial\_cell\_proliferation | 4 | 0 |  |  |  |  |  |  |  |  |
| GO:0001978\_regulation\_of\_systemic\_arterial\_blood\_pressure\_by\_carotid\_sinus\_baroreceptor\_feedback | 4 | 0 |  |  |  |  |  |  |  |  |
| GO:0002035\_brain\_renin-angiotensin\_system | 4 | 0 |  |  |  |  |  |  |  |  |
| GO:0002051\_osteoblast\_fate\_commitment | 4 | 0 |  |  |  |  |  |  |  |  |
| GO:0002220\_innate\_immune\_response\_activating\_cell\_surface\_receptor\_signaling\_pathway | 4 | 0 |  |  |  |  |  |  |  |  |
| GO:0002249\_lymphocyte\_anergy | 4 | 0 |  |  |  |  |  |  |  |  |
| GO:0002312\_B\_cell\_activation\_during\_immune\_response | 4 | 0 |  |  |  |  |  |  |  |  |
| GO:0002313\_mature\_B\_cell\_differentiation\_during\_immune\_response | 4 | 0 |  |  |  |  |  |  |  |  |
| GO:0002318\_myeloid\_progenitor\_cell\_differentiation | 4 | 0 |  |  |  |  |  |  |  |  |
| GO:0002326\_B\_cell\_lineage\_commitment | 4 | 0 |  |  |  |  |  |  |  |  |
| GO:0002347\_response\_to\_tumor\_cell | 4 | 0 |  |  |  |  |  |  |  |  |
| GO:0002418\_immune\_response\_to\_tumor\_cell | 4 | 0 |  |  |  |  |  |  |  |  |
| GO:0002445\_type\_II\_hypersensitivity | 4 | 0 |  |  |  |  |  |  |  |  |
| GO:0002544\_chronic\_inflammatory\_response | 4 | 0 |  |  |  |  |  |  |  |  |
| GO:0002636\_positive\_regulation\_of\_germinal\_center\_formation | 4 | 0 |  |  |  |  |  |  |  |  |
| GO:0002667\_regulation\_of\_T\_cell\_anergy | 4 | 0 |  |  |  |  |  |  |  |  |
| GO:0002669\_positive\_regulation\_of\_T\_cell\_anergy | 4 | 0 |  |  |  |  |  |  |  |  |
| GO:0002687\_positive\_regulation\_of\_leukocyte\_migration | 4 | 0 |  |  |  |  |  |  |  |  |
| GO:0002702\_positive\_regulation\_of\_production\_of\_molecular\_mediator\_of\_immune\_response | 4 | 0 |  |  |  |  |  |  |  |  |
| GO:0002718\_regulation\_of\_cytokine\_production\_during\_immune\_response | 4 | 0 |  |  |  |  |  |  |  |  |
| GO:0002829\_negative\_regulation\_of\_T-helper\_2\_type\_immune\_response | 4 | 0 |  |  |  |  |  |  |  |  |
| GO:0002833\_positive\_regulation\_of\_response\_to\_biotic\_stimulus | 4 | 0 |  |  |  |  |  |  |  |  |
| GO:0002834\_regulation\_of\_response\_to\_tumor\_cell | 4 | 0 |  |  |  |  |  |  |  |  |
| GO:0002836\_positive\_regulation\_of\_response\_to\_tumor\_cell | 4 | 0 |  |  |  |  |  |  |  |  |
| GO:0002837\_regulation\_of\_immune\_response\_to\_tumor\_cell | 4 | 0 |  |  |  |  |  |  |  |  |
| GO:0002839\_positive\_regulation\_of\_immune\_response\_to\_tumor\_cell | 4 | 0 |  |  |  |  |  |  |  |  |
| GO:0002870\_T\_cell\_anergy | 4 | 0 |  |  |  |  |  |  |  |  |
| GO:0002888\_positive\_regulation\_of\_myeloid\_leukocyte\_mediated\_immunity | 4 | 0 |  |  |  |  |  |  |  |  |
| GO:0002892\_regulation\_of\_type\_II\_hypersensitivity | 4 | 0 |  |  |  |  |  |  |  |  |
| GO:0002894\_positive\_regulation\_of\_type\_II\_hypersensitivity | 4 | 0 |  |  |  |  |  |  |  |  |
| GO:0002911\_regulation\_of\_lymphocyte\_anergy | 4 | 0 |  |  |  |  |  |  |  |  |
| GO:0002913\_positive\_regulation\_of\_lymphocyte\_anergy | 4 | 0 |  |  |  |  |  |  |  |  |
| GO:0002923\_regulation\_of\_humoral\_immune\_response\_mediated\_by\_circulating\_immunoglobulin | 4 | 0 |  |  |  |  |  |  |  |  |
| GO:0003025\_regulation\_of\_systemic\_arterial\_blood\_pressure\_by\_baroreceptor\_feedback | 4 | 0 |  |  |  |  |  |  |  |  |
| GO:0003091\_renal\_water\_homeostasis | 4 | 0 |  |  |  |  |  |  |  |  |
| GO:0005978\_glycogen\_biosynthetic\_process | 4 | 0 |  |  |  |  |  |  |  |  |
| GO:0006012\_galactose\_metabolic\_process | 4 | 0 |  |  |  |  |  |  |  |  |
| GO:0006085\_acetyl-CoA\_biosynthetic\_process | 4 | 0 |  |  |  |  |  |  |  |  |
| GO:0006111\_regulation\_of\_gluconeogenesis | 4 | 0 |  |  |  |  |  |  |  |  |
| GO:0006144\_purine\_base\_metabolic\_process | 4 | 0 |  |  |  |  |  |  |  |  |
| GO:0006290\_pyrimidine\_dimer\_repair | 4 | 0 |  |  |  |  |  |  |  |  |
| GO:0006534\_cysteine\_metabolic\_process | 4 | 0 |  |  |  |  |  |  |  |  |
| GO:0006547\_histidine\_metabolic\_process | 4 | 0 |  |  |  |  |  |  |  |  |
| GO:0006548\_histidine\_catabolic\_process | 4 | 0 |  |  |  |  |  |  |  |  |
| GO:0006555\_methionine\_metabolic\_process | 4 | 0 |  |  |  |  |  |  |  |  |
| GO:0006599\_phosphagen\_metabolic\_process | 4 | 0 |  |  |  |  |  |  |  |  |
| GO:0006623\_protein\_targeting\_to\_vacuole | 4 | 0 |  |  |  |  |  |  |  |  |
| GO:0006626\_protein\_targeting\_to\_mitochondrion | 4 | 0 |  |  |  |  |  |  |  |  |
| GO:0006684\_sphingomyelin\_metabolic\_process | 4 | 0 |  |  |  |  |  |  |  |  |
| GO:0006688\_glycosphingolipid\_biosynthetic\_process | 4 | 0 |  |  |  |  |  |  |  |  |
| GO:0006707\_cholesterol\_catabolic\_process | 4 | 0 |  |  |  |  |  |  |  |  |
| GO:0006739\_NADP\_metabolic\_process | 4 | 0 |  |  |  |  |  |  |  |  |
| GO:0006835\_dicarboxylic\_acid\_transport | 4 | 0 |  |  |  |  |  |  |  |  |
| GO:0006837\_serotonin\_transport | 4 | 0 |  |  |  |  |  |  |  |  |
| GO:0006888\_ER\_to\_Golgi\_vesicle-mediated\_transport | 4 | 0 |  |  |  |  |  |  |  |  |
| GO:0006906\_vesicle\_fusion | 4 | 0 |  |  |  |  |  |  |  |  |
| GO:0006927\_transformed\_cell\_apoptosis | 4 | 0 |  |  |  |  |  |  |  |  |
| GO:0006972\_hyperosmotic\_response | 4 | 0 |  |  |  |  |  |  |  |  |
| GO:0007028\_cytoplasm\_organization | 4 | 0 |  |  |  |  |  |  |  |  |
| GO:0007031\_peroxisome\_organization | 4 | 0 |  |  |  |  |  |  |  |  |
| GO:0007066\_female\_meiosis\_sister\_chromatid\_cohesion | 4 | 0 |  |  |  |  |  |  |  |  |
| GO:0007144\_female\_meiosis\_I | 4 | 0 |  |  |  |  |  |  |  |  |
| GO:0007216\_metabotropic\_glutamate\_receptor\_signaling\_pathway | 4 | 0 |  |  |  |  |  |  |  |  |
| GO:0007342\_fusion\_of\_sperm\_to\_egg\_plasma\_membrane | 4 | 0 |  |  |  |  |  |  |  |  |
| GO:0007386\_compartment\_specification | 4 | 0 |  |  |  |  |  |  |  |  |
| GO:0008053\_mitochondrial\_fusion | 4 | 0 |  |  |  |  |  |  |  |  |
| GO:0008207\_C21-steroid\_hormone\_metabolic\_process | 4 | 0 |  |  |  |  |  |  |  |  |
| GO:0008215\_spermine\_metabolic\_process | 4 | 0 |  |  |  |  |  |  |  |  |
| GO:0009065\_glutamine\_family\_amino\_acid\_catabolic\_process | 4 | 0 |  |  |  |  |  |  |  |  |
| GO:0009075\_histidine\_family\_amino\_acid\_metabolic\_process | 4 | 0 |  |  |  |  |  |  |  |  |
| GO:0009077\_histidine\_family\_amino\_acid\_catabolic\_process | 4 | 0 |  |  |  |  |  |  |  |  |
| GO:0009134\_nucleoside\_diphosphate\_catabolic\_process | 4 | 0 |  |  |  |  |  |  |  |  |
| GO:0009163\_nucleoside\_biosynthetic\_process | 4 | 0 |  |  |  |  |  |  |  |  |
| GO:0009225\_nucleotide-sugar\_metabolic\_process | 4 | 0 |  |  |  |  |  |  |  |  |
| GO:0009250\_glucan\_biosynthetic\_process | 4 | 0 |  |  |  |  |  |  |  |  |
| GO:0009404\_toxin\_metabolic\_process | 4 | 0 |  |  |  |  |  |  |  |  |
| GO:0009593\_detection\_of\_chemical\_stimulus | 4 | 0 |  |  |  |  |  |  |  |  |
| GO:0009595\_detection\_of\_biotic\_stimulus | 4 | 0 |  |  |  |  |  |  |  |  |
| GO:0009755\_hormone-mediated\_signaling | 4 | 0 |  |  |  |  |  |  |  |  |
| GO:0009912\_auditory\_receptor\_cell\_fate\_commitment | 4 | 0 |  |  |  |  |  |  |  |  |
| GO:0010453\_regulation\_of\_cell\_fate\_commitment | 4 | 0 |  |  |  |  |  |  |  |  |
| GO:0010506\_regulation\_of\_autophagy | 4 | 0 |  |  |  |  |  |  |  |  |
| GO:0010631\_epithelial\_cell\_migration | 4 | 0 |  |  |  |  |  |  |  |  |
| GO:0010812\_negative\_regulation\_of\_cell-substrate\_adhesion | 4 | 0 |  |  |  |  |  |  |  |  |
| GO:0010829\_negative\_regulation\_of\_glucose\_transport | 4 | 0 |  |  |  |  |  |  |  |  |
| GO:0014002\_astrocyte\_development | 4 | 0 |  |  |  |  |  |  |  |  |
| GO:0014832\_urinary\_bladder\_smooth\_muscle\_contraction | 4 | 0 |  |  |  |  |  |  |  |  |
| GO:0014848\_urinary\_tract\_smooth\_muscle\_contraction | 4 | 0 |  |  |  |  |  |  |  |  |
| GO:0015701\_bicarbonate\_transport | 4 | 0 |  |  |  |  |  |  |  |  |
| GO:0015809\_arginine\_transport | 4 | 0 |  |  |  |  |  |  |  |  |
| GO:0015850\_organic\_alcohol\_transport | 4 | 0 |  |  |  |  |  |  |  |  |
| GO:0015858\_nucleoside\_transport | 4 | 0 |  |  |  |  |  |  |  |  |
| GO:0016068\_type\_I\_hypersensitivity | 4 | 0 |  |  |  |  |  |  |  |  |
| GO:0016127\_sterol\_catabolic\_process | 4 | 0 |  |  |  |  |  |  |  |  |
| GO:0016198\_axon\_choice\_point\_recognition | 4 | 0 |  |  |  |  |  |  |  |  |
| GO:0016338\_calcium-independent\_cell-cell\_adhesion | 4 | 0 |  |  |  |  |  |  |  |  |
| GO:0018198\_peptidyl-cysteine\_modification | 4 | 0 |  |  |  |  |  |  |  |  |
| GO:0018409\_peptide\_or\_protein\_amino-terminal\_blocking | 4 | 0 |  |  |  |  |  |  |  |  |
| GO:0019377\_glycolipid\_catabolic\_process | 4 | 0 |  |  |  |  |  |  |  |  |
| GO:0019432\_triglyceride\_biosynthetic\_process | 4 | 0 |  |  |  |  |  |  |  |  |
| GO:0019530\_taurine\_metabolic\_process | 4 | 0 |  |  |  |  |  |  |  |  |
| GO:0021523\_somatic\_motor\_neuron\_differentiation | 4 | 0 |  |  |  |  |  |  |  |  |
| GO:0021535\_cell\_migration\_in\_hindbrain | 4 | 0 |  |  |  |  |  |  |  |  |
| GO:0021542\_dentate\_gyrus\_development | 4 | 0 |  |  |  |  |  |  |  |  |
| GO:0021561\_facial\_nerve\_development | 4 | 0 |  |  |  |  |  |  |  |  |
| GO:0021569\_rhombomere\_3\_development | 4 | 0 |  |  |  |  |  |  |  |  |
| GO:0021571\_rhombomere\_5\_development | 4 | 0 |  |  |  |  |  |  |  |  |
| GO:0021604\_cranial\_nerve\_structural\_organization | 4 | 0 |  |  |  |  |  |  |  |  |
| GO:0021610\_facial\_nerve\_morphogenesis | 4 | 0 |  |  |  |  |  |  |  |  |
| GO:0021612\_facial\_nerve\_structural\_organization | 4 | 0 |  |  |  |  |  |  |  |  |
| GO:0021631\_optic\_nerve\_morphogenesis | 4 | 0 |  |  |  |  |  |  |  |  |
| GO:0021681\_cerebellar\_granular\_layer\_development | 4 | 0 |  |  |  |  |  |  |  |  |
| GO:0021683\_cerebellar\_granular\_layer\_morphogenesis | 4 | 0 |  |  |  |  |  |  |  |  |
| GO:0021684\_cerebellar\_granular\_layer\_formation | 4 | 0 |  |  |  |  |  |  |  |  |
| GO:0021707\_cerebellar\_granule\_cell\_differentiation | 4 | 0 |  |  |  |  |  |  |  |  |
| GO:0021778\_oligodendrocyte\_cell\_fate\_specification | 4 | 0 |  |  |  |  |  |  |  |  |
| GO:0021779\_oligodendrocyte\_cell\_fate\_commitment | 4 | 0 |  |  |  |  |  |  |  |  |
| GO:0021780\_glial\_cell\_fate\_specification | 4 | 0 |  |  |  |  |  |  |  |  |
| GO:0021801\_cerebral\_cortex\_radial\_glia\_guided\_migration | 4 | 0 |  |  |  |  |  |  |  |  |
| GO:0021830\_interneuron\_migration\_from\_the\_subpallium\_to\_the\_cortex | 4 | 0 |  |  |  |  |  |  |  |  |
| GO:0021853\_cerebral\_cortex\_GABAergic\_interneuron\_migration | 4 | 0 |  |  |  |  |  |  |  |  |
| GO:0021877\_forebrain\_neuron\_fate\_commitment | 4 | 0 |  |  |  |  |  |  |  |  |
| GO:0021894\_cerebral\_cortex\_GABAergic\_interneuron\_development | 4 | 0 |  |  |  |  |  |  |  |  |
| GO:0021910\_smoothened\_signaling\_pathway\_involved\_in\_ventral\_spinal\_cord\_patterning | 4 | 0 |  |  |  |  |  |  |  |  |
| GO:0021913\_regulation\_of\_transcription\_from\_RNA\_polymerase\_II\_promoter\_involved\_in\_ventral\_spinal\_cord\_interneuron\_specification | 4 | 0 |  |  |  |  |  |  |  |  |
| GO:0021938\_smoothened\_signaling\_pathway\_involved\_in\_regulation\_of\_granule\_cell\_precursor\_cell\_proliferation | 4 | 0 |  |  |  |  |  |  |  |  |
| GO:0021978\_telencephalon\_regionalization | 4 | 0 |  |  |  |  |  |  |  |  |
| GO:0022011\_myelination\_in\_the\_peripheral\_nervous\_system | 4 | 0 |  |  |  |  |  |  |  |  |
| GO:0030146\_diuresis | 4 | 0 |  |  |  |  |  |  |  |  |
| GO:0030300\_regulation\_of\_intestinal\_cholesterol\_absorption | 4 | 0 |  |  |  |  |  |  |  |  |
| GO:0030800\_negative\_regulation\_of\_cyclic\_nucleotide\_metabolic\_process | 4 | 0 |  |  |  |  |  |  |  |  |
| GO:0030803\_negative\_regulation\_of\_cyclic\_nucleotide\_biosynthetic\_process | 4 | 0 |  |  |  |  |  |  |  |  |
| GO:0030809\_negative\_regulation\_of\_nucleotide\_biosynthetic\_process | 4 | 0 |  |  |  |  |  |  |  |  |
| GO:0030815\_negative\_regulation\_of\_cAMP\_metabolic\_process | 4 | 0 |  |  |  |  |  |  |  |  |
| GO:0030816\_positive\_regulation\_of\_cAMP\_metabolic\_process | 4 | 0 |  |  |  |  |  |  |  |  |
| GO:0030818\_negative\_regulation\_of\_cAMP\_biosynthetic\_process | 4 | 0 |  |  |  |  |  |  |  |  |
| GO:0030819\_positive\_regulation\_of\_cAMP\_biosynthetic\_process | 4 | 0 |  |  |  |  |  |  |  |  |
| GO:0030826\_regulation\_of\_cGMP\_biosynthetic\_process | 4 | 0 |  |  |  |  |  |  |  |  |
| GO:0030858\_positive\_regulation\_of\_epithelial\_cell\_differentiation | 4 | 0 |  |  |  |  |  |  |  |  |
| GO:0030859\_polarized\_epithelial\_cell\_differentiation | 4 | 0 |  |  |  |  |  |  |  |  |
| GO:0030949\_positive\_regulation\_of\_vascular\_endothelial\_growth\_factor\_receptor\_signaling\_pathway | 4 | 0 |  |  |  |  |  |  |  |  |
| GO:0031113\_regulation\_of\_microtubule\_polymerization | 4 | 0 |  |  |  |  |  |  |  |  |
| GO:0031365\_N-terminal\_protein\_amino\_acid\_modification | 4 | 0 |  |  |  |  |  |  |  |  |
| GO:0031424\_keratinization | 4 | 0 |  |  |  |  |  |  |  |  |
| GO:0031557\_induction\_of\_programmed\_cell\_death\_in\_response\_to\_chemical\_stimulus | 4 | 0 |  |  |  |  |  |  |  |  |
| GO:0031558\_induction\_of\_apoptosis\_in\_response\_to\_chemical\_stimulus | 4 | 0 |  |  |  |  |  |  |  |  |
| GO:0031623\_receptor\_internalization | 4 | 0 |  |  |  |  |  |  |  |  |
| GO:0032088\_negative\_regulation\_of\_NF-kappaB\_transcription\_factor\_activity | 4 | 0 |  |  |  |  |  |  |  |  |
| GO:0032098\_regulation\_of\_appetite | 4 | 0 |  |  |  |  |  |  |  |  |
| GO:0032105\_negative\_regulation\_of\_response\_to\_extracellular\_stimulus | 4 | 0 |  |  |  |  |  |  |  |  |
| GO:0032108\_negative\_regulation\_of\_response\_to\_nutrient\_levels | 4 | 0 |  |  |  |  |  |  |  |  |
| GO:0032225\_regulation\_of\_synaptic\_transmission\_\_dopaminergic | 4 | 0 |  |  |  |  |  |  |  |  |
| GO:0032292\_ensheathment\_of\_axons\_in\_the\_peripheral\_nervous\_system | 4 | 0 |  |  |  |  |  |  |  |  |
| GO:0032321\_positive\_regulation\_of\_Rho\_GTPase\_activity | 4 | 0 |  |  |  |  |  |  |  |  |
| GO:0032371\_regulation\_of\_sterol\_transport | 4 | 0 |  |  |  |  |  |  |  |  |
| GO:0032374\_regulation\_of\_cholesterol\_transport | 4 | 0 |  |  |  |  |  |  |  |  |
| GO:0032401\_establishment\_of\_melanosome\_localization | 4 | 0 |  |  |  |  |  |  |  |  |
| GO:0032608\_interferon-beta\_production | 4 | 0 |  |  |  |  |  |  |  |  |
| GO:0032611\_interleukin-1\_beta\_production | 4 | 0 |  |  |  |  |  |  |  |  |
| GO:0032612\_interleukin-1\_production | 4 | 0 |  |  |  |  |  |  |  |  |
| GO:0032648\_regulation\_of\_interferon-beta\_production | 4 | 0 |  |  |  |  |  |  |  |  |
| GO:0032651\_regulation\_of\_interleukin-1\_beta\_production | 4 | 0 |  |  |  |  |  |  |  |  |
| GO:0032652\_regulation\_of\_interleukin-1\_production | 4 | 0 |  |  |  |  |  |  |  |  |
| GO:0032689\_negative\_regulation\_of\_interferon-gamma\_production | 4 | 0 |  |  |  |  |  |  |  |  |
| GO:0032713\_negative\_regulation\_of\_interleukin-4\_production | 4 | 0 |  |  |  |  |  |  |  |  |
| GO:0032733\_positive\_regulation\_of\_interleukin-10\_production | 4 | 0 |  |  |  |  |  |  |  |  |
| GO:0032808\_lacrimal\_gland\_development | 4 | 0 |  |  |  |  |  |  |  |  |
| GO:0032835\_glomerulus\_development | 4 | 0 |  |  |  |  |  |  |  |  |
| GO:0032872\_regulation\_of\_stress-activated\_MAPK\_cascade | 4 | 0 |  |  |  |  |  |  |  |  |
| GO:0032922\_circadian\_regulation\_of\_gene\_expression | 4 | 0 |  |  |  |  |  |  |  |  |
| GO:0033026\_negative\_regulation\_of\_mast\_cell\_apoptosis | 4 | 0 |  |  |  |  |  |  |  |  |
| GO:0033079\_immature\_T\_cell\_proliferation | 4 | 0 |  |  |  |  |  |  |  |  |
| GO:0033083\_regulation\_of\_immature\_T\_cell\_proliferation | 4 | 0 |  |  |  |  |  |  |  |  |
| GO:0033089\_positive\_regulation\_of\_T\_cell\_differentiation\_in\_the\_thymus | 4 | 0 |  |  |  |  |  |  |  |  |
| GO:0033135\_regulation\_of\_peptidyl-serine\_phosphorylation | 4 | 0 |  |  |  |  |  |  |  |  |
| GO:0033299\_secretion\_of\_lysosomal\_enzymes | 4 | 0 |  |  |  |  |  |  |  |  |
| GO:0033327\_Leydig\_cell\_differentiation | 4 | 0 |  |  |  |  |  |  |  |  |
| GO:0033363\_secretory\_granule\_organization | 4 | 0 |  |  |  |  |  |  |  |  |
| GO:0033599\_regulation\_of\_mammary\_gland\_epithelial\_cell\_proliferation | 4 | 0 |  |  |  |  |  |  |  |  |
| GO:0033865\_nucleoside\_bisphosphate\_metabolic\_process | 4 | 0 |  |  |  |  |  |  |  |  |
| GO:0034204\_lipid\_translocation | 4 | 0 |  |  |  |  |  |  |  |  |
| GO:0034404\_nucleobase\_\_nucleoside\_and\_nucleotide\_biosynthetic\_process | 4 | 0 |  |  |  |  |  |  |  |  |
| GO:0034587\_piRNA\_metabolic\_process | 4 | 0 |  |  |  |  |  |  |  |  |
| GO:0034614\_cellular\_response\_to\_reactive\_oxygen\_species | 4 | 0 |  |  |  |  |  |  |  |  |
| GO:0034654\_nucleobase\_\_nucleoside\_\_nucleotide\_and\_nucleic\_acid\_biosynthetic\_process | 4 | 0 |  |  |  |  |  |  |  |  |
| GO:0035020\_regulation\_of\_Rac\_protein\_signal\_transduction | 4 | 0 |  |  |  |  |  |  |  |  |
| GO:0035082\_axoneme\_assembly | 4 | 0 |  |  |  |  |  |  |  |  |
| GO:0035235\_ionotropic\_glutamate\_receptor\_signaling\_pathway | 4 | 0 |  |  |  |  |  |  |  |  |
| GO:0042345\_regulation\_of\_NF-kappaB\_import\_into\_nucleus | 4 | 0 |  |  |  |  |  |  |  |  |
| GO:0042348\_NF-kappaB\_import\_into\_nucleus | 4 | 0 |  |  |  |  |  |  |  |  |
| GO:0042359\_vitamin\_D\_metabolic\_process | 4 | 0 |  |  |  |  |  |  |  |  |
| GO:0042428\_serotonin\_metabolic\_process | 4 | 0 |  |  |  |  |  |  |  |  |
| GO:0042451\_purine\_nucleoside\_biosynthetic\_process | 4 | 0 |  |  |  |  |  |  |  |  |
| GO:0042455\_ribonucleoside\_biosynthetic\_process | 4 | 0 |  |  |  |  |  |  |  |  |
| GO:0042473\_outer\_ear\_morphogenesis | 4 | 0 |  |  |  |  |  |  |  |  |
| GO:0042522\_regulation\_of\_tyrosine\_phosphorylation\_of\_Stat5\_protein | 4 | 0 |  |  |  |  |  |  |  |  |
| GO:0042535\_positive\_regulation\_of\_tumor\_necrosis\_factor\_biosynthetic\_process | 4 | 0 |  |  |  |  |  |  |  |  |
| GO:0042541\_hemoglobin\_biosynthetic\_process | 4 | 0 |  |  |  |  |  |  |  |  |
| GO:0042558\_pteridine\_and\_derivative\_metabolic\_process | 4 | 0 |  |  |  |  |  |  |  |  |
| GO:0042634\_regulation\_of\_hair\_cycle | 4 | 0 |  |  |  |  |  |  |  |  |
| GO:0042744\_hydrogen\_peroxide\_catabolic\_process | 4 | 0 |  |  |  |  |  |  |  |  |
| GO:0042773\_ATP\_synthesis\_coupled\_electron\_transport | 4 | 0 |  |  |  |  |  |  |  |  |
| GO:0042775\_mitochondrial\_ATP\_synthesis\_coupled\_electron\_transport | 4 | 0 |  |  |  |  |  |  |  |  |
| GO:0042832\_defense\_response\_to\_protozoan | 4 | 0 |  |  |  |  |  |  |  |  |
| GO:0042982\_amyloid\_precursor\_protein\_metabolic\_process | 4 | 0 |  |  |  |  |  |  |  |  |
| GO:0042992\_negative\_regulation\_of\_transcription\_factor\_import\_into\_nucleus | 4 | 0 |  |  |  |  |  |  |  |  |
| GO:0043043\_peptide\_biosynthetic\_process | 4 | 0 |  |  |  |  |  |  |  |  |
| GO:0043129\_surfactant\_homeostasis | 4 | 0 |  |  |  |  |  |  |  |  |
| GO:0043374\_CD8-positive\_\_alpha-beta\_T\_cell\_differentiation | 4 | 0 |  |  |  |  |  |  |  |  |
| GO:0043470\_regulation\_of\_carbohydrate\_catabolic\_process | 4 | 0 |  |  |  |  |  |  |  |  |
| GO:0043471\_regulation\_of\_cellular\_carbohydrate\_catabolic\_process | 4 | 0 |  |  |  |  |  |  |  |  |
| GO:0043484\_regulation\_of\_RNA\_splicing | 4 | 0 |  |  |  |  |  |  |  |  |
| GO:0043500\_muscle\_adaptation | 4 | 0 |  |  |  |  |  |  |  |  |
| GO:0043534\_blood\_vessel\_endothelial\_cell\_migration | 4 | 0 |  |  |  |  |  |  |  |  |
| GO:0043691\_reverse\_cholesterol\_transport | 4 | 0 |  |  |  |  |  |  |  |  |
| GO:0044243\_multicellular\_organismal\_catabolic\_process | 4 | 0 |  |  |  |  |  |  |  |  |
| GO:0044403\_symbiosis\_\_encompassing\_mutualism\_through\_parasitism | 4 | 0 |  |  |  |  |  |  |  |  |
| GO:0044419\_interspecies\_interaction\_between\_organisms | 4 | 0 |  |  |  |  |  |  |  |  |
| GO:0045066\_regulatory\_T\_cell\_differentiation | 4 | 0 |  |  |  |  |  |  |  |  |
| GO:0045078\_positive\_regulation\_of\_interferon-gamma\_biosynthetic\_process | 4 | 0 |  |  |  |  |  |  |  |  |
| GO:0045332\_phospholipid\_translocation | 4 | 0 |  |  |  |  |  |  |  |  |
| GO:0045346\_regulation\_of\_MHC\_class\_II\_biosynthetic\_process | 4 | 0 |  |  |  |  |  |  |  |  |
| GO:0045350\_interferon-beta\_biosynthetic\_process | 4 | 0 |  |  |  |  |  |  |  |  |
| GO:0045357\_regulation\_of\_interferon-beta\_biosynthetic\_process | 4 | 0 |  |  |  |  |  |  |  |  |
| GO:0045359\_positive\_regulation\_of\_interferon-beta\_biosynthetic\_process | 4 | 0 |  |  |  |  |  |  |  |  |
| GO:0045600\_positive\_regulation\_of\_fat\_cell\_differentiation | 4 | 0 |  |  |  |  |  |  |  |  |
| GO:0045616\_regulation\_of\_keratinocyte\_differentiation | 4 | 0 |  |  |  |  |  |  |  |  |
| GO:0045624\_positive\_regulation\_of\_T-helper\_cell\_differentiation | 4 | 0 |  |  |  |  |  |  |  |  |
| GO:0045628\_regulation\_of\_T-helper\_2\_cell\_differentiation | 4 | 0 |  |  |  |  |  |  |  |  |
| GO:0045634\_regulation\_of\_melanocyte\_differentiation | 4 | 0 |  |  |  |  |  |  |  |  |
| GO:0045647\_negative\_regulation\_of\_erythrocyte\_differentiation | 4 | 0 |  |  |  |  |  |  |  |  |
| GO:0045672\_positive\_regulation\_of\_osteoclast\_differentiation | 4 | 0 |  |  |  |  |  |  |  |  |
| GO:0045684\_positive\_regulation\_of\_epidermis\_development | 4 | 0 |  |  |  |  |  |  |  |  |
| GO:0045736\_negative\_regulation\_of\_cyclin-dependent\_protein\_kinase\_activity | 4 | 0 |  |  |  |  |  |  |  |  |
| GO:0045742\_positive\_regulation\_of\_epidermal\_growth\_factor\_receptor\_signaling\_pathway | 4 | 0 |  |  |  |  |  |  |  |  |
| GO:0045747\_positive\_regulation\_of\_Notch\_signaling\_pathway | 4 | 0 |  |  |  |  |  |  |  |  |
| GO:0045767\_regulation\_of\_anti-apoptosis | 4 | 0 |  |  |  |  |  |  |  |  |
| GO:0045779\_negative\_regulation\_of\_bone\_resorption | 4 | 0 |  |  |  |  |  |  |  |  |
| GO:0045923\_positive\_regulation\_of\_fatty\_acid\_metabolic\_process | 4 | 0 |  |  |  |  |  |  |  |  |
| GO:0045930\_negative\_regulation\_of\_mitotic\_cell\_cycle | 4 | 0 |  |  |  |  |  |  |  |  |
| GO:0045940\_positive\_regulation\_of\_steroid\_metabolic\_process | 4 | 0 |  |  |  |  |  |  |  |  |
| GO:0045980\_negative\_regulation\_of\_nucleotide\_metabolic\_process | 4 | 0 |  |  |  |  |  |  |  |  |
| GO:0046129\_purine\_ribonucleoside\_biosynthetic\_process | 4 | 0 |  |  |  |  |  |  |  |  |
| GO:0046173\_polyol\_biosynthetic\_process | 4 | 0 |  |  |  |  |  |  |  |  |
| GO:0046541\_saliva\_secretion | 4 | 0 |  |  |  |  |  |  |  |  |
| GO:0046548\_retinal\_rod\_cell\_development | 4 | 0 |  |  |  |  |  |  |  |  |
| GO:0046579\_positive\_regulation\_of\_Ras\_protein\_signal\_transduction | 4 | 0 |  |  |  |  |  |  |  |  |
| GO:0046639\_negative\_regulation\_of\_alpha-beta\_T\_cell\_differentiation | 4 | 0 |  |  |  |  |  |  |  |  |
| GO:0046642\_negative\_regulation\_of\_alpha-beta\_T\_cell\_proliferation | 4 | 0 |  |  |  |  |  |  |  |  |
| GO:0046668\_regulation\_of\_retinal\_cell\_programmed\_cell\_death | 4 | 0 |  |  |  |  |  |  |  |  |
| GO:0046686\_response\_to\_cadmium\_ion | 4 | 0 |  |  |  |  |  |  |  |  |
| GO:0046835\_carbohydrate\_phosphorylation | 4 | 0 |  |  |  |  |  |  |  |  |
| GO:0046902\_regulation\_of\_mitochondrial\_membrane\_permeability | 4 | 0 |  |  |  |  |  |  |  |  |
| GO:0047496\_vesicle\_transport\_along\_microtubule | 4 | 0 |  |  |  |  |  |  |  |  |
| GO:0048011\_nerve\_growth\_factor\_receptor\_signaling\_pathway | 4 | 0 |  |  |  |  |  |  |  |  |
| GO:0048024\_regulation\_of\_nuclear\_mRNA\_splicing\_\_via\_spliceosome | 4 | 0 |  |  |  |  |  |  |  |  |
| GO:0048240\_sperm\_capacitation | 4 | 0 |  |  |  |  |  |  |  |  |
| GO:0048341\_paraxial\_mesoderm\_formation | 4 | 0 |  |  |  |  |  |  |  |  |
| GO:0048484\_enteric\_nervous\_system\_development | 4 | 0 |  |  |  |  |  |  |  |  |
| GO:0048512\_circadian\_behavior | 4 | 0 |  |  |  |  |  |  |  |  |
| GO:0048558\_embryonic\_gut\_morphogenesis | 4 | 0 |  |  |  |  |  |  |  |  |
| GO:0048639\_positive\_regulation\_of\_developmental\_growth | 4 | 0 |  |  |  |  |  |  |  |  |
| GO:0048710\_regulation\_of\_astrocyte\_differentiation | 4 | 0 |  |  |  |  |  |  |  |  |
| GO:0048841\_regulation\_of\_axon\_extension\_involved\_in\_axon\_guidance | 4 | 0 |  |  |  |  |  |  |  |  |
| GO:0048843\_negative\_regulation\_of\_axon\_extension\_involved\_in\_axon\_guidance | 4 | 0 |  |  |  |  |  |  |  |  |
| GO:0048846\_axon\_extension\_involved\_in\_axon\_guidance | 4 | 0 |  |  |  |  |  |  |  |  |
| GO:0048875\_chemical\_homeostasis\_within\_a\_tissue | 4 | 0 |  |  |  |  |  |  |  |  |
| GO:0048935\_peripheral\_nervous\_system\_neuron\_development | 4 | 0 |  |  |  |  |  |  |  |  |
| GO:0050702\_interleukin-1\_beta\_secretion | 4 | 0 |  |  |  |  |  |  |  |  |
| GO:0050704\_regulation\_of\_interleukin-1\_secretion | 4 | 0 |  |  |  |  |  |  |  |  |
| GO:0050706\_regulation\_of\_interleukin-1\_beta\_secretion | 4 | 0 |  |  |  |  |  |  |  |  |
| GO:0050716\_positive\_regulation\_of\_interleukin-1\_secretion | 4 | 0 |  |  |  |  |  |  |  |  |
| GO:0050718\_positive\_regulation\_of\_interleukin-1\_beta\_secretion | 4 | 0 |  |  |  |  |  |  |  |  |
| GO:0050820\_positive\_regulation\_of\_coagulation | 4 | 0 |  |  |  |  |  |  |  |  |
| GO:0050891\_multicellular\_organismal\_water\_homeostasis | 4 | 0 |  |  |  |  |  |  |  |  |
| GO:0050919\_negative\_chemotaxis | 4 | 0 |  |  |  |  |  |  |  |  |
| GO:0050932\_regulation\_of\_pigment\_cell\_differentiation | 4 | 0 |  |  |  |  |  |  |  |  |
| GO:0050961\_detection\_of\_temperature\_stimulus\_involved\_in\_sensory\_perception | 4 | 0 |  |  |  |  |  |  |  |  |
| GO:0050965\_detection\_of\_temperature\_stimulus\_involved\_in\_sensory\_perception\_of\_pain | 4 | 0 |  |  |  |  |  |  |  |  |
| GO:0050994\_regulation\_of\_lipid\_catabolic\_process | 4 | 0 |  |  |  |  |  |  |  |  |
| GO:0051024\_positive\_regulation\_of\_immunoglobulin\_secretion | 4 | 0 |  |  |  |  |  |  |  |  |
| GO:0051055\_negative\_regulation\_of\_lipid\_biosynthetic\_process | 4 | 0 |  |  |  |  |  |  |  |  |
| GO:0051124\_synaptic\_growth\_at\_neuromuscular\_junction | 4 | 0 |  |  |  |  |  |  |  |  |
| GO:0051148\_negative\_regulation\_of\_muscle\_cell\_differentiation | 4 | 0 |  |  |  |  |  |  |  |  |
| GO:0051205\_protein\_insertion\_into\_membrane | 4 | 0 |  |  |  |  |  |  |  |  |
| GO:0051225\_spindle\_assembly | 4 | 0 |  |  |  |  |  |  |  |  |
| GO:0051341\_regulation\_of\_oxidoreductase\_activity | 4 | 0 |  |  |  |  |  |  |  |  |
| GO:0051452\_intracellular\_pH\_reduction | 4 | 0 |  |  |  |  |  |  |  |  |
| GO:0051567\_histone\_H3-K9\_methylation | 4 | 0 |  |  |  |  |  |  |  |  |
| GO:0051642\_centrosome\_localization | 4 | 0 |  |  |  |  |  |  |  |  |
| GO:0051797\_regulation\_of\_hair\_follicle\_development | 4 | 0 |  |  |  |  |  |  |  |  |
| GO:0051897\_positive\_regulation\_of\_protein\_kinase\_B\_signaling\_cascade | 4 | 0 |  |  |  |  |  |  |  |  |
| GO:0051904\_pigment\_granule\_transport | 4 | 0 |  |  |  |  |  |  |  |  |
| GO:0055009\_atrial\_cardiac\_muscle\_morphogenesis | 4 | 0 |  |  |  |  |  |  |  |  |
| GO:0060008\_Sertoli\_cell\_differentiation | 4 | 0 |  |  |  |  |  |  |  |  |
| GO:0060011\_Sertoli\_cell\_proliferation | 4 | 0 |  |  |  |  |  |  |  |  |
| GO:0060057\_apoptosis\_involved\_in\_mammary\_gland\_involution | 4 | 0 |  |  |  |  |  |  |  |  |
| GO:0060058\_positive\_regulation\_of\_apoptosis\_involved\_in\_mammary\_gland\_involution | 4 | 0 |  |  |  |  |  |  |  |  |
| GO:0060065\_uterus\_development | 4 | 0 |  |  |  |  |  |  |  |  |
| GO:0060087\_relaxation\_of\_vascular\_smooth\_muscle | 4 | 0 |  |  |  |  |  |  |  |  |
| GO:0060120\_inner\_ear\_receptor\_cell\_fate\_commitment | 4 | 0 |  |  |  |  |  |  |  |  |
| GO:0060157\_urinary\_bladder\_development | 4 | 0 |  |  |  |  |  |  |  |  |
| GO:0060158\_activation\_of\_phospholipase\_C\_activity\_by\_dopamine\_receptor\_signaling\_pathway | 4 | 0 |  |  |  |  |  |  |  |  |
| GO:0060164\_regulation\_of\_timing\_of\_neuron\_differentiation | 4 | 0 |  |  |  |  |  |  |  |  |
| GO:0060235\_lens\_induction\_in\_camera-type\_eye | 4 | 0 |  |  |  |  |  |  |  |  |
| GO:0060291\_long-term\_synaptic\_potentiation | 4 | 0 |  |  |  |  |  |  |  |  |
| GO:0060412\_ventricular\_septum\_morphogenesis | 4 | 0 |  |  |  |  |  |  |  |  |
| GO:0060459\_left\_lung\_development | 4 | 0 |  |  |  |  |  |  |  |  |
| GO:0060528\_secretory\_columnal\_luminar\_epithelial\_cell\_differentiation\_involved\_in\_prostate\_glandular\_acinus\_development | 4 | 0 |  |  |  |  |  |  |  |  |
| GO:0060561\_apoptosis\_involved\_in\_morphogenesis | 4 | 0 |  |  |  |  |  |  |  |  |
| GO:0060592\_mammary\_gland\_formation | 4 | 0 |  |  |  |  |  |  |  |  |
| GO:0060644\_mammary\_gland\_epithelial\_cell\_differentiation | 4 | 0 |  |  |  |  |  |  |  |  |
| GO:0060666\_dichotomous\_subdivision\_of\_terminal\_units\_involved\_in\_salivary\_gland\_branching | 4 | 0 |  |  |  |  |  |  |  |  |
| GO:0060737\_prostate\_gland\_morphogenetic\_growth | 4 | 0 |  |  |  |  |  |  |  |  |
| GO:0060743\_epithelial\_cell\_maturation\_involved\_in\_prostate\_gland\_development | 4 | 0 |  |  |  |  |  |  |  |  |
| GO:0060751\_mammary\_gland\_duct\_branch\_elongation | 4 | 0 |  |  |  |  |  |  |  |  |
| GO:0060900\_embryonic\_camera-type\_eye\_formation | 4 | 0 |  |  |  |  |  |  |  |  |
| GO:0070059\_apoptosis\_in\_response\_to\_endoplasmic\_reticulum\_stress | 4 | 0 |  |  |  |  |  |  |  |  |
| GO:0070254\_mucus\_secretion | 4 | 0 |  |  |  |  |  |  |  |  |
| GO:0070255\_regulation\_of\_mucus\_secretion | 4 | 0 |  |  |  |  |  |  |  |  |
| GO:0070301\_cellular\_response\_to\_hydrogen\_peroxide | 4 | 0 |  |  |  |  |  |  |  |  |
| GO:0070585\_protein\_localization\_in\_mitochondrion | 4 | 0 |  |  |  |  |  |  |  |  |
| GO:0002237\_response\_to\_molecule\_of\_bacterial\_origin | 34 | 0 | 0.000000 | -0.000000 | 715 | 600.635623 | 690.85 | 781.064377 | 0.966224 |
| GO:0002699\_positive\_regulation\_of\_immune\_effector\_process | 34 | 0 | 0.000000 | -0.000000 | 715 | 600.635623 | 690.85 | 781.064377 | 0.966224 |
| GO:0007269\_neurotransmitter\_secretion | 34 | 0 | 0.000000 | -0.000000 | 715 | 600.635623 | 690.85 | 781.064377 | 0.966224 |
| GO:0007338\_single\_fertilization | 34 | 0 | 0.000000 | -0.000000 | 715 | 600.635623 | 690.85 | 781.064377 | 0.966224 |
| GO:0010721\_negative\_regulation\_of\_cell\_development | 34 | 0 | 0.000000 | -0.000000 | 715 | 600.635623 | 690.85 | 781.064377 | 0.966224 |
| GO:0016054\_organic\_acid\_catabolic\_process | 34 | 0 | 0.000000 | -0.000000 | 715 | 600.635623 | 690.85 | 781.064377 | 0.966224 |
| GO:0019882\_antigen\_processing\_and\_presentation | 34 | 0 | 0.000000 | -0.000000 | 715 | 600.635623 | 690.85 | 781.064377 | 0.966224 |
| GO:0030509\_BMP\_signaling\_pathway | 34 | 0 | 0.000000 | -0.000000 | 715 | 600.635623 | 690.85 | 781.064377 | 0.966224 |
| GO:0046395\_carboxylic\_acid\_catabolic\_process | 34 | 0 | 0.000000 | -0.000000 | 715 | 600.635623 | 690.85 | 781.064377 | 0.966224 |
| GO:0050730\_regulation\_of\_peptidyl-tyrosine\_phosphorylation | 34 | 0 | 0.000000 | -0.000000 | 715 | 600.635623 | 690.85 | 781.064377 | 0.966224 |
| GO:0060443\_mammary\_gland\_morphogenesis | 34 | 0 | 0.000000 | -0.000000 | 715 | 600.635623 | 690.85 | 781.064377 | 0.966224 |
| GO:0060711\_labyrinthine\_layer\_development | 34 | 0 | 0.000000 | -0.000000 | 715 | 600.635623 | 690.85 | 781.064377 | 0.966224 |
| GO:0002764\_immune\_response-regulating\_signal\_transduction | 51 | 0 | 0.000000 | -0.000000 | 723 | 608.924997 | 698.38 | 787.835003 | 0.965947 |
| GO:0006520\_cellular\_amino\_acid\_metabolic\_process | 51 | 0 | 0.000000 | -0.000000 | 723 | 608.924997 | 698.38 | 787.835003 | 0.965947 |
| GO:0006887\_exocytosis | 51 | 0 | 0.000000 | -0.000000 | 723 | 608.924997 | 698.38 | 787.835003 | 0.965947 |
| GO:0007601\_visual\_perception | 51 | 0 | 0.000000 | -0.000000 | 723 | 608.924997 | 698.38 | 787.835003 | 0.965947 |
| GO:0032583\_regulation\_of\_gene-specific\_transcription | 51 | 0 | 0.000000 | -0.000000 | 723 | 608.924997 | 698.38 | 787.835003 | 0.965947 |
| GO:0043408\_regulation\_of\_MAPKKK\_cascade | 51 | 0 | 0.000000 | -0.000000 | 723 | 608.924997 | 698.38 | 787.835003 | 0.965947 |
| GO:0044106\_cellular\_amine\_metabolic\_process | 51 | 0 | 0.000000 | -0.000000 | 723 | 608.924997 | 698.38 | 787.835003 | 0.965947 |
| GO:0048747\_muscle\_fiber\_development | 51 | 0 | 0.000000 | -0.000000 | 723 | 608.924997 | 698.38 | 787.835003 | 0.965947 |
| GO:0005996\_monosaccharide\_metabolic\_process | 69 | 0 | 0.000000 | -0.000000 | 726 | 612.926024 | 701.96 | 790.993976 | 0.966887 |
| GO:0032101\_regulation\_of\_response\_to\_external\_stimulus | 69 | 0 | 0.000000 | -0.000000 | 726 | 612.926024 | 701.96 | 790.993976 | 0.966887 |
| GO:0055065\_metal\_ion\_homeostasis | 69 | 0 | 0.000000 | -0.000000 | 726 | 612.926024 | 701.96 | 790.993976 | 0.966887 |
| GO:0048584\_positive\_regulation\_of\_response\_to\_stimulus | 115 | 0 | 0.000000 | -0.000000 | 727 | 614.287357 | 703.06 | 791.832643 | 0.967070 |
| GO:0001776\_leukocyte\_homeostasis | 41 | 0 | 0.000000 | -0.000000 | 747 | 631.776441 | 719.1 | 806.423559 | 0.962651 |
| GO:0002429\_immune\_response-activating\_cell\_surface\_receptor\_signaling\_pathway | 41 | 0 | 0.000000 | -0.000000 | 747 | 631.776441 | 719.1 | 806.423559 | 0.962651 |
| GO:0006260\_DNA\_replication | 41 | 0 | 0.000000 | -0.000000 | 747 | 631.776441 | 719.1 | 806.423559 | 0.962651 |
| GO:0006836\_neurotransmitter\_transport | 41 | 0 | 0.000000 | -0.000000 | 747 | 631.776441 | 719.1 | 806.423559 | 0.962651 |
| GO:0006865\_amino\_acid\_transport | 41 | 0 | 0.000000 | -0.000000 | 747 | 631.776441 | 719.1 | 806.423559 | 0.962651 |
| GO:0006979\_response\_to\_oxidative\_stress | 41 | 0 | 0.000000 | -0.000000 | 747 | 631.776441 | 719.1 | 806.423559 | 0.962651 |
| GO:0007254\_JNK\_cascade | 41 | 0 | 0.000000 | -0.000000 | 747 | 631.776441 | 719.1 | 806.423559 | 0.962651 |
| GO:0008585\_female\_gonad\_development | 41 | 0 | 0.000000 | -0.000000 | 747 | 631.776441 | 719.1 | 806.423559 | 0.962651 |
| GO:0009894\_regulation\_of\_catabolic\_process | 41 | 0 | 0.000000 | -0.000000 | 747 | 631.776441 | 719.1 | 806.423559 | 0.962651 |
| GO:0010551\_regulation\_of\_specific\_transcription\_from\_RNA\_polymerase\_II\_promoter | 41 | 0 | 0.000000 | -0.000000 | 747 | 631.776441 | 719.1 | 806.423559 | 0.962651 |
| GO:0015833\_peptide\_transport | 41 | 0 | 0.000000 | -0.000000 | 747 | 631.776441 | 719.1 | 806.423559 | 0.962651 |
| GO:0015980\_energy\_derivation\_by\_oxidation\_of\_organic\_compounds | 41 | 0 | 0.000000 | -0.000000 | 747 | 631.776441 | 719.1 | 806.423559 | 0.962651 |
| GO:0019216\_regulation\_of\_lipid\_metabolic\_process | 41 | 0 | 0.000000 | -0.000000 | 747 | 631.776441 | 719.1 | 806.423559 | 0.962651 |
| GO:0019748\_secondary\_metabolic\_process | 41 | 0 | 0.000000 | -0.000000 | 747 | 631.776441 | 719.1 | 806.423559 | 0.962651 |
| GO:0030817\_regulation\_of\_cAMP\_biosynthetic\_process | 41 | 0 | 0.000000 | -0.000000 | 747 | 631.776441 | 719.1 | 806.423559 | 0.962651 |
| GO:0031344\_regulation\_of\_cell\_projection\_organization | 41 | 0 | 0.000000 | -0.000000 | 747 | 631.776441 | 719.1 | 806.423559 | 0.962651 |
| GO:0032569\_specific\_transcription\_from\_RNA\_polymerase\_II\_promoter | 41 | 0 | 0.000000 | -0.000000 | 747 | 631.776441 | 719.1 | 806.423559 | 0.962651 |
| GO:0032844\_regulation\_of\_homeostatic\_process | 41 | 0 | 0.000000 | -0.000000 | 747 | 631.776441 | 719.1 | 806.423559 | 0.962651 |
| GO:0033077\_T\_cell\_differentiation\_in\_the\_thymus | 41 | 0 | 0.000000 | -0.000000 | 747 | 631.776441 | 719.1 | 806.423559 | 0.962651 |
| GO:0050864\_regulation\_of\_B\_cell\_activation | 41 | 0 | 0.000000 | -0.000000 | 747 | 631.776441 | 719.1 | 806.423559 | 0.962651 |
| GO:0007346\_regulation\_of\_mitotic\_cell\_cycle | 40 | 0 | 0.000000 | -0.000000 | 756 | 642.798935 | 729.41 | 816.021065 | 0.964828 |
| GO:0007599\_hemostasis | 40 | 0 | 0.000000 | -0.000000 | 756 | 642.798935 | 729.41 | 816.021065 | 0.964828 |
| GO:0008203\_cholesterol\_metabolic\_process | 40 | 0 | 0.000000 | -0.000000 | 756 | 642.798935 | 729.41 | 816.021065 | 0.964828 |
| GO:0016358\_dendrite\_development | 40 | 0 | 0.000000 | -0.000000 | 756 | 642.798935 | 729.41 | 816.021065 | 0.964828 |
| GO:0016485\_protein\_processing | 40 | 0 | 0.000000 | -0.000000 | 756 | 642.798935 | 729.41 | 816.021065 | 0.964828 |
| GO:0019935\_cyclic-nucleotide-mediated\_signaling | 40 | 0 | 0.000000 | -0.000000 | 756 | 642.798935 | 729.41 | 816.021065 | 0.964828 |
| GO:0035272\_exocrine\_system\_development | 40 | 0 | 0.000000 | -0.000000 | 756 | 642.798935 | 729.41 | 816.021065 | 0.964828 |
| GO:0046850\_regulation\_of\_bone\_remodeling | 40 | 0 | 0.000000 | -0.000000 | 756 | 642.798935 | 729.41 | 816.021065 | 0.964828 |
| GO:0051129\_negative\_regulation\_of\_cellular\_component\_organization | 40 | 0 | 0.000000 | -0.000000 | 756 | 642.798935 | 729.41 | 816.021065 | 0.964828 |
| GO:0014706\_striated\_muscle\_tissue\_development | 120 | 0 | 0.000000 | -0.000000 | 757 | 643.467284 | 729.95 | 816.432716 | 0.964267 |
| GO:0003018\_vascular\_process\_in\_circulatory\_system | 31 | 0 | 0.000000 | -0.000000 | 786 | 672.216720 | 756.59 | 840.963280 | 0.962583 |
| GO:0006486\_protein\_amino\_acid\_glycosylation | 31 | 0 | 0.000000 | -0.000000 | 786 | 672.216720 | 756.59 | 840.963280 | 0.962583 |
| GO:0006639\_acylglycerol\_metabolic\_process | 31 | 0 | 0.000000 | -0.000000 | 786 | 672.216720 | 756.59 | 840.963280 | 0.962583 |
| GO:0006665\_sphingolipid\_metabolic\_process | 31 | 0 | 0.000000 | -0.000000 | 786 | 672.216720 | 756.59 | 840.963280 | 0.962583 |
| GO:0006694\_steroid\_biosynthetic\_process | 31 | 0 | 0.000000 | -0.000000 | 786 | 672.216720 | 756.59 | 840.963280 | 0.962583 |
| GO:0006939\_smooth\_muscle\_contraction | 31 | 0 | 0.000000 | -0.000000 | 786 | 672.216720 | 756.59 | 840.963280 | 0.962583 |
| GO:0008645\_hexose\_transport | 31 | 0 | 0.000000 | -0.000000 | 786 | 672.216720 | 756.59 | 840.963280 | 0.962583 |
| GO:0009306\_protein\_secretion | 31 | 0 | 0.000000 | -0.000000 | 786 | 672.216720 | 756.59 | 840.963280 | 0.962583 |
| GO:0015749\_monosaccharide\_transport | 31 | 0 | 0.000000 | -0.000000 | 786 | 672.216720 | 756.59 | 840.963280 | 0.962583 |
| GO:0015758\_glucose\_transport | 31 | 0 | 0.000000 | -0.000000 | 786 | 672.216720 | 756.59 | 840.963280 | 0.962583 |
| GO:0016049\_cell\_growth | 31 | 0 | 0.000000 | -0.000000 | 786 | 672.216720 | 756.59 | 840.963280 | 0.962583 |
| GO:0016311\_dephosphorylation | 31 | 0 | 0.000000 | -0.000000 | 786 | 672.216720 | 756.59 | 840.963280 | 0.962583 |
| GO:0021954\_central\_nervous\_system\_neuron\_development | 31 | 0 | 0.000000 | -0.000000 | 786 | 672.216720 | 756.59 | 840.963280 | 0.962583 |
| GO:0033555\_multicellular\_organismal\_response\_to\_stress | 31 | 0 | 0.000000 | -0.000000 | 786 | 672.216720 | 756.59 | 840.963280 | 0.962583 |
| GO:0035150\_regulation\_of\_tube\_size | 31 | 0 | 0.000000 | -0.000000 | 786 | 672.216720 | 756.59 | 840.963280 | 0.962583 |
| GO:0042157\_lipoprotein\_metabolic\_process | 31 | 0 | 0.000000 | -0.000000 | 786 | 672.216720 | 756.59 | 840.963280 | 0.962583 |
| GO:0043269\_regulation\_of\_ion\_transport | 31 | 0 | 0.000000 | -0.000000 | 786 | 672.216720 | 756.59 | 840.963280 | 0.962583 |
| GO:0043413\_biopolymer\_glycosylation | 31 | 0 | 0.000000 | -0.000000 | 786 | 672.216720 | 756.59 | 840.963280 | 0.962583 |
| GO:0045088\_regulation\_of\_innate\_immune\_response | 31 | 0 | 0.000000 | -0.000000 | 786 | 672.216720 | 756.59 | 840.963280 | 0.962583 |
| GO:0046632\_alpha-beta\_T\_cell\_differentiation | 31 | 0 | 0.000000 | -0.000000 | 786 | 672.216720 | 756.59 | 840.963280 | 0.962583 |
| GO:0048167\_regulation\_of\_synaptic\_plasticity | 31 | 0 | 0.000000 | -0.000000 | 786 | 672.216720 | 756.59 | 840.963280 | 0.962583 |
| GO:0048562\_embryonic\_organ\_morphogenesis | 31 | 0 | 0.000000 | -0.000000 | 786 | 672.216720 | 756.59 | 840.963280 | 0.962583 |
| GO:0050868\_negative\_regulation\_of\_T\_cell\_activation | 31 | 0 | 0.000000 | -0.000000 | 786 | 672.216720 | 756.59 | 840.963280 | 0.962583 |
| GO:0050880\_regulation\_of\_blood\_vessel\_size | 31 | 0 | 0.000000 | -0.000000 | 786 | 672.216720 | 756.59 | 840.963280 | 0.962583 |
| GO:0051640\_organelle\_localization | 31 | 0 | 0.000000 | -0.000000 | 786 | 672.216720 | 756.59 | 840.963280 | 0.962583 |
| GO:0051899\_membrane\_depolarization | 31 | 0 | 0.000000 | -0.000000 | 786 | 672.216720 | 756.59 | 840.963280 | 0.962583 |
| GO:0055088\_lipid\_homeostasis | 31 | 0 | 0.000000 | -0.000000 | 786 | 672.216720 | 756.59 | 840.963280 | 0.962583 |
| GO:0060512\_prostate\_gland\_morphogenesis | 31 | 0 | 0.000000 | -0.000000 | 786 | 672.216720 | 756.59 | 840.963280 | 0.962583 |
| GO:0070085\_glycosylation | 31 | 0 | 0.000000 | -0.000000 | 786 | 672.216720 | 756.59 | 840.963280 | 0.962583 |
| GO:0000038\_very-long-chain\_fatty\_acid\_metabolic\_process | 6 | 0 | 0.000000 | -0.000000 | 970 | 855.015794 | 937.37 | 1019.724206 | 0.966361 |
| GO:0000768\_syncytium\_formation\_by\_plasma\_membrane\_fusion | 6 | 0 | 0.000000 | -0.000000 | 970 | 855.015794 | 937.37 | 1019.724206 | 0.966361 |
| GO:0001710\_mesodermal\_cell\_fate\_commitment | 6 | 0 | 0.000000 | -0.000000 | 970 | 855.015794 | 937.37 | 1019.724206 | 0.966361 |
| GO:0001779\_natural\_killer\_cell\_differentiation | 6 | 0 | 0.000000 | -0.000000 | 970 | 855.015794 | 937.37 | 1019.724206 | 0.966361 |
| GO:0001885\_endothelial\_cell\_development | 6 | 0 | 0.000000 | -0.000000 | 970 | 855.015794 | 937.37 | 1019.724206 | 0.966361 |
| GO:0002016\_regulation\_of\_blood\_volume\_by\_renin-angiotensin | 6 | 0 | 0.000000 | -0.000000 | 970 | 855.015794 | 937.37 | 1019.724206 | 0.966361 |
| GO:0002335\_mature\_B\_cell\_differentiation | 6 | 0 | 0.000000 | -0.000000 | 970 | 855.015794 | 937.37 | 1019.724206 | 0.966361 |
| GO:0002360\_T\_cell\_lineage\_commitment | 6 | 0 | 0.000000 | -0.000000 | 970 | 855.015794 | 937.37 | 1019.724206 | 0.966361 |
| GO:0002367\_cytokine\_production\_during\_immune\_response | 6 | 0 | 0.000000 | -0.000000 | 970 | 855.015794 | 937.37 | 1019.724206 | 0.966361 |
| GO:0002474\_antigen\_processing\_and\_presentation\_of\_peptide\_antigen\_via\_MHC\_class\_I | 6 | 0 | 0.000000 | -0.000000 | 970 | 855.015794 | 937.37 | 1019.724206 | 0.966361 |
| GO:0002475\_antigen\_processing\_and\_presentation\_via\_MHC\_class\_Ib | 6 | 0 | 0.000000 | -0.000000 | 970 | 855.015794 | 937.37 | 1019.724206 | 0.966361 |
| GO:0002532\_production\_of\_molecular\_mediator\_of\_acute\_inflammatory\_response | 6 | 0 | 0.000000 | -0.000000 | 970 | 855.015794 | 937.37 | 1019.724206 | 0.966361 |
| GO:0002541\_activation\_of\_plasma\_proteins\_involved\_in\_acute\_inflammatory\_response | 6 | 0 | 0.000000 | -0.000000 | 970 | 855.015794 | 937.37 | 1019.724206 | 0.966361 |
| GO:0002675\_positive\_regulation\_of\_acute\_inflammatory\_response | 6 | 0 | 0.000000 | -0.000000 | 970 | 855.015794 | 937.37 | 1019.724206 | 0.966361 |
| GO:0002685\_regulation\_of\_leukocyte\_migration | 6 | 0 | 0.000000 | -0.000000 | 970 | 855.015794 | 937.37 | 1019.724206 | 0.966361 |
| GO:0002831\_regulation\_of\_response\_to\_biotic\_stimulus | 6 | 0 | 0.000000 | -0.000000 | 970 | 855.015794 | 937.37 | 1019.724206 | 0.966361 |
| GO:0002920\_regulation\_of\_humoral\_immune\_response | 6 | 0 | 0.000000 | -0.000000 | 970 | 855.015794 | 937.37 | 1019.724206 | 0.966361 |
| GO:0006071\_glycerol\_metabolic\_process | 6 | 0 | 0.000000 | -0.000000 | 970 | 855.015794 | 937.37 | 1019.724206 | 0.966361 |
| GO:0006084\_acetyl-CoA\_metabolic\_process | 6 | 0 | 0.000000 | -0.000000 | 970 | 855.015794 | 937.37 | 1019.724206 | 0.966361 |
| GO:0006264\_mitochondrial\_DNA\_replication | 6 | 0 | 0.000000 | -0.000000 | 970 | 855.015794 | 937.37 | 1019.724206 | 0.966361 |
| GO:0006402\_mRNA\_catabolic\_process | 6 | 0 | 0.000000 | -0.000000 | 970 | 855.015794 | 937.37 | 1019.724206 | 0.966361 |
| GO:0006471\_protein\_amino\_acid\_ADP-ribosylation | 6 | 0 | 0.000000 | -0.000000 | 970 | 855.015794 | 937.37 | 1019.724206 | 0.966361 |
| GO:0006536\_glutamate\_metabolic\_process | 6 | 0 | 0.000000 | -0.000000 | 970 | 855.015794 | 937.37 | 1019.724206 | 0.966361 |
| GO:0006656\_phosphatidylcholine\_biosynthetic\_process | 6 | 0 | 0.000000 | -0.000000 | 970 | 855.015794 | 937.37 | 1019.724206 | 0.966361 |
| GO:0006692\_prostanoid\_metabolic\_process | 6 | 0 | 0.000000 | -0.000000 | 970 | 855.015794 | 937.37 | 1019.724206 | 0.966361 |
| GO:0006693\_prostaglandin\_metabolic\_process | 6 | 0 | 0.000000 | -0.000000 | 970 | 855.015794 | 937.37 | 1019.724206 | 0.966361 |
| GO:0006706\_steroid\_catabolic\_process | 6 | 0 | 0.000000 | -0.000000 | 970 | 855.015794 | 937.37 | 1019.724206 | 0.966361 |
| GO:0006752\_group\_transfer\_coenzyme\_metabolic\_process | 6 | 0 | 0.000000 | -0.000000 | 970 | 855.015794 | 937.37 | 1019.724206 | 0.966361 |
| GO:0006882\_cellular\_zinc\_ion\_homeostasis | 6 | 0 | 0.000000 | -0.000000 | 970 | 855.015794 | 937.37 | 1019.724206 | 0.966361 |
| GO:0006942\_regulation\_of\_striated\_muscle\_contraction | 6 | 0 | 0.000000 | -0.000000 | 970 | 855.015794 | 937.37 | 1019.724206 | 0.966361 |
| GO:0006956\_complement\_activation | 6 | 0 | 0.000000 | -0.000000 | 970 | 855.015794 | 937.37 | 1019.724206 | 0.966361 |
| GO:0006998\_nuclear\_envelope\_organization | 6 | 0 | 0.000000 | -0.000000 | 970 | 855.015794 | 937.37 | 1019.724206 | 0.966361 |
| GO:0007032\_endosome\_organization | 6 | 0 | 0.000000 | -0.000000 | 970 | 855.015794 | 937.37 | 1019.724206 | 0.966361 |
| GO:0007176\_regulation\_of\_epidermal\_growth\_factor\_receptor\_activity | 6 | 0 | 0.000000 | -0.000000 | 970 | 855.015794 | 937.37 | 1019.724206 | 0.966361 |
| GO:0007214\_gamma-aminobutyric\_acid\_signaling\_pathway | 6 | 0 | 0.000000 | -0.000000 | 970 | 855.015794 | 937.37 | 1019.724206 | 0.966361 |
| GO:0007257\_activation\_of\_JUN\_kinase\_activity | 6 | 0 | 0.000000 | -0.000000 | 970 | 855.015794 | 937.37 | 1019.724206 | 0.966361 |
| GO:0007341\_penetration\_of\_zona\_pellucida | 6 | 0 | 0.000000 | -0.000000 | 970 | 855.015794 | 937.37 | 1019.724206 | 0.966361 |
| GO:0007406\_negative\_regulation\_of\_neuroblast\_proliferation | 6 | 0 | 0.000000 | -0.000000 | 970 | 855.015794 | 937.37 | 1019.724206 | 0.966361 |
| GO:0007442\_hindgut\_morphogenesis | 6 | 0 | 0.000000 | -0.000000 | 970 | 855.015794 | 937.37 | 1019.724206 | 0.966361 |
| GO:0007520\_myoblast\_fusion | 6 | 0 | 0.000000 | -0.000000 | 970 | 855.015794 | 937.37 | 1019.724206 | 0.966361 |
| GO:0007620\_copulation | 6 | 0 | 0.000000 | -0.000000 | 970 | 855.015794 | 937.37 | 1019.724206 | 0.966361 |
| GO:0008156\_negative\_regulation\_of\_DNA\_replication | 6 | 0 | 0.000000 | -0.000000 | 970 | 855.015794 | 937.37 | 1019.724206 | 0.966361 |
| GO:0008209\_androgen\_metabolic\_process | 6 | 0 | 0.000000 | -0.000000 | 970 | 855.015794 | 937.37 | 1019.724206 | 0.966361 |
| GO:0008625\_induction\_of\_apoptosis\_via\_death\_domain\_receptors | 6 | 0 | 0.000000 | -0.000000 | 970 | 855.015794 | 937.37 | 1019.724206 | 0.966361 |
| GO:0009067\_aspartate\_family\_amino\_acid\_biosynthetic\_process | 6 | 0 | 0.000000 | -0.000000 | 970 | 855.015794 | 937.37 | 1019.724206 | 0.966361 |
| GO:0009069\_serine\_family\_amino\_acid\_metabolic\_process | 6 | 0 | 0.000000 | -0.000000 | 970 | 855.015794 | 937.37 | 1019.724206 | 0.966361 |
| GO:0009112\_nucleobase\_metabolic\_process | 6 | 0 | 0.000000 | -0.000000 | 970 | 855.015794 | 937.37 | 1019.724206 | 0.966361 |
| GO:0009143\_nucleoside\_triphosphate\_catabolic\_process | 6 | 0 | 0.000000 | -0.000000 | 970 | 855.015794 | 937.37 | 1019.724206 | 0.966361 |
| GO:0009247\_glycolipid\_biosynthetic\_process | 6 | 0 | 0.000000 | -0.000000 | 970 | 855.015794 | 937.37 | 1019.724206 | 0.966361 |
| GO:0009650\_UV\_protection | 6 | 0 | 0.000000 | -0.000000 | 970 | 855.015794 | 937.37 | 1019.724206 | 0.966361 |
| GO:0009651\_response\_to\_salt\_stress | 6 | 0 | 0.000000 | -0.000000 | 970 | 855.015794 | 937.37 | 1019.724206 | 0.966361 |
| GO:0010466\_negative\_regulation\_of\_peptidase\_activity | 6 | 0 | 0.000000 | -0.000000 | 970 | 855.015794 | 937.37 | 1019.724206 | 0.966361 |
| GO:0010883\_regulation\_of\_lipid\_storage | 6 | 0 | 0.000000 | -0.000000 | 970 | 855.015794 | 937.37 | 1019.724206 | 0.966361 |
| GO:0010906\_regulation\_of\_glucose\_metabolic\_process | 6 | 0 | 0.000000 | -0.000000 | 970 | 855.015794 | 937.37 | 1019.724206 | 0.966361 |
| GO:0014003\_oligodendrocyte\_development | 6 | 0 | 0.000000 | -0.000000 | 970 | 855.015794 | 937.37 | 1019.724206 | 0.966361 |
| GO:0014051\_gamma-aminobutyric\_acid\_secretion | 6 | 0 | 0.000000 | -0.000000 | 970 | 855.015794 | 937.37 | 1019.724206 | 0.966361 |
| GO:0014072\_response\_to\_isoquinoline\_alkaloid | 6 | 0 | 0.000000 | -0.000000 | 970 | 855.015794 | 937.37 | 1019.724206 | 0.966361 |
| GO:0014812\_muscle\_cell\_migration | 6 | 0 | 0.000000 | -0.000000 | 970 | 855.015794 | 937.37 | 1019.724206 | 0.966361 |
| GO:0014823\_response\_to\_activity | 6 | 0 | 0.000000 | -0.000000 | 970 | 855.015794 | 937.37 | 1019.724206 | 0.966361 |
| GO:0015012\_heparan\_sulfate\_proteoglycan\_biosynthetic\_process | 6 | 0 | 0.000000 | -0.000000 | 970 | 855.015794 | 937.37 | 1019.724206 | 0.966361 |
| GO:0015812\_gamma-aminobutyric\_acid\_transport | 6 | 0 | 0.000000 | -0.000000 | 970 | 855.015794 | 937.37 | 1019.724206 | 0.966361 |
| GO:0016032\_viral\_reproduction | 6 | 0 | 0.000000 | -0.000000 | 970 | 855.015794 | 937.37 | 1019.724206 | 0.966361 |
| GO:0016574\_histone\_ubiquitination | 6 | 0 | 0.000000 | -0.000000 | 970 | 855.015794 | 937.37 | 1019.724206 | 0.966361 |
| GO:0016925\_protein\_sumoylation | 6 | 0 | 0.000000 | -0.000000 | 970 | 855.015794 | 937.37 | 1019.724206 | 0.966361 |
| GO:0019433\_triglyceride\_catabolic\_process | 6 | 0 | 0.000000 | -0.000000 | 970 | 855.015794 | 937.37 | 1019.724206 | 0.966361 |
| GO:0019835\_cytolysis | 6 | 0 | 0.000000 | -0.000000 | 970 | 855.015794 | 937.37 | 1019.724206 | 0.966361 |
| GO:0021548\_pons\_development | 6 | 0 | 0.000000 | -0.000000 | 970 | 855.015794 | 937.37 | 1019.724206 | 0.966361 |
| GO:0021783\_preganglionic\_parasympathetic\_nervous\_system\_development | 6 | 0 | 0.000000 | -0.000000 | 970 | 855.015794 | 937.37 | 1019.724206 | 0.966361 |
| GO:0021892\_cerebral\_cortex\_GABAergic\_interneuron\_differentiation | 6 | 0 | 0.000000 | -0.000000 | 970 | 855.015794 | 937.37 | 1019.724206 | 0.966361 |
| GO:0021937\_Purkinje\_cell-granule\_cell\_precursor\_cell\_signaling\_involved\_in\_regulation\_of\_granule\_cell\_precursor\_cell\_proliferation | 6 | 0 | 0.000000 | -0.000000 | 970 | 855.015794 | 937.37 | 1019.724206 | 0.966361 |
| GO:0022409\_positive\_regulation\_of\_cell-cell\_adhesion | 6 | 0 | 0.000000 | -0.000000 | 970 | 855.015794 | 937.37 | 1019.724206 | 0.966361 |
| GO:0030002\_cellular\_anion\_homeostasis | 6 | 0 | 0.000000 | -0.000000 | 970 | 855.015794 | 937.37 | 1019.724206 | 0.966361 |
| GO:0030149\_sphingolipid\_catabolic\_process | 6 | 0 | 0.000000 | -0.000000 | 970 | 855.015794 | 937.37 | 1019.724206 | 0.966361 |
| GO:0030252\_growth\_hormone\_secretion | 6 | 0 | 0.000000 | -0.000000 | 970 | 855.015794 | 937.37 | 1019.724206 | 0.966361 |
| GO:0030865\_cortical\_cytoskeleton\_organization | 6 | 0 | 0.000000 | -0.000000 | 970 | 855.015794 | 937.37 | 1019.724206 | 0.966361 |
| GO:0030947\_regulation\_of\_vascular\_endothelial\_growth\_factor\_receptor\_signaling\_pathway | 6 | 0 | 0.000000 | -0.000000 | 970 | 855.015794 | 937.37 | 1019.724206 | 0.966361 |
| GO:0031077\_post-embryonic\_camera-type\_eye\_development | 6 | 0 | 0.000000 | -0.000000 | 970 | 855.015794 | 937.37 | 1019.724206 | 0.966361 |
| GO:0031330\_negative\_regulation\_of\_cellular\_catabolic\_process | 6 | 0 | 0.000000 | -0.000000 | 970 | 855.015794 | 937.37 | 1019.724206 | 0.966361 |
| GO:0031575\_G1\_S\_transition\_checkpoint | 6 | 0 | 0.000000 | -0.000000 | 970 | 855.015794 | 937.37 | 1019.724206 | 0.966361 |
| GO:0031960\_response\_to\_corticosteroid\_stimulus | 6 | 0 | 0.000000 | -0.000000 | 970 | 855.015794 | 937.37 | 1019.724206 | 0.966361 |
| GO:0032042\_mitochondrial\_DNA\_metabolic\_process | 6 | 0 | 0.000000 | -0.000000 | 970 | 855.015794 | 937.37 | 1019.724206 | 0.966361 |
| GO:0032331\_negative\_regulation\_of\_chondrocyte\_differentiation | 6 | 0 | 0.000000 | -0.000000 | 970 | 855.015794 | 937.37 | 1019.724206 | 0.966361 |
| GO:0032392\_DNA\_geometric\_change | 6 | 0 | 0.000000 | -0.000000 | 970 | 855.015794 | 937.37 | 1019.724206 | 0.966361 |
| GO:0032438\_melanosome\_organization | 6 | 0 | 0.000000 | -0.000000 | 970 | 855.015794 | 937.37 | 1019.724206 | 0.966361 |
| GO:0032469\_endoplasmic\_reticulum\_calcium\_ion\_homeostasis | 6 | 0 | 0.000000 | -0.000000 | 970 | 855.015794 | 937.37 | 1019.724206 | 0.966361 |
| GO:0032653\_regulation\_of\_interleukin-10\_production | 6 | 0 | 0.000000 | -0.000000 | 970 | 855.015794 | 937.37 | 1019.724206 | 0.966361 |
| GO:0033238\_regulation\_of\_cellular\_amine\_metabolic\_process | 6 | 0 | 0.000000 | -0.000000 | 970 | 855.015794 | 937.37 | 1019.724206 | 0.966361 |
| GO:0034968\_histone\_lysine\_methylation | 6 | 0 | 0.000000 | -0.000000 | 970 | 855.015794 | 937.37 | 1019.724206 | 0.966361 |
| GO:0035019\_somatic\_stem\_cell\_maintenance | 6 | 0 | 0.000000 | -0.000000 | 970 | 855.015794 | 937.37 | 1019.724206 | 0.966361 |
| GO:0035094\_response\_to\_nicotine | 6 | 0 | 0.000000 | -0.000000 | 970 | 855.015794 | 937.37 | 1019.724206 | 0.966361 |
| GO:0035121\_tail\_morphogenesis | 6 | 0 | 0.000000 | -0.000000 | 970 | 855.015794 | 937.37 | 1019.724206 | 0.966361 |
| GO:0040016\_embryonic\_cleavage | 6 | 0 | 0.000000 | -0.000000 | 970 | 855.015794 | 937.37 | 1019.724206 | 0.966361 |
| GO:0040023\_establishment\_of\_nucleus\_localization | 6 | 0 | 0.000000 | -0.000000 | 970 | 855.015794 | 937.37 | 1019.724206 | 0.966361 |
| GO:0040036\_regulation\_of\_fibroblast\_growth\_factor\_receptor\_signaling\_pathway | 6 | 0 | 0.000000 | -0.000000 | 970 | 855.015794 | 937.37 | 1019.724206 | 0.966361 |
| GO:0042053\_regulation\_of\_dopamine\_metabolic\_process | 6 | 0 | 0.000000 | -0.000000 | 970 | 855.015794 | 937.37 | 1019.724206 | 0.966361 |
| GO:0042069\_regulation\_of\_catecholamine\_metabolic\_process | 6 | 0 | 0.000000 | -0.000000 | 970 | 855.015794 | 937.37 | 1019.724206 | 0.966361 |
| GO:0042246\_tissue\_regeneration | 6 | 0 | 0.000000 | -0.000000 | 970 | 855.015794 | 937.37 | 1019.724206 | 0.966361 |
| GO:0042307\_positive\_regulation\_of\_protein\_import\_into\_nucleus | 6 | 0 | 0.000000 | -0.000000 | 970 | 855.015794 | 937.37 | 1019.724206 | 0.966361 |
| GO:0042308\_negative\_regulation\_of\_protein\_import\_into\_nucleus | 6 | 0 | 0.000000 | -0.000000 | 970 | 855.015794 | 937.37 | 1019.724206 | 0.966361 |
| GO:0042403\_thyroid\_hormone\_metabolic\_process | 6 | 0 | 0.000000 | -0.000000 | 970 | 855.015794 | 937.37 | 1019.724206 | 0.966361 |
| GO:0042481\_regulation\_of\_odontogenesis | 6 | 0 | 0.000000 | -0.000000 | 970 | 855.015794 | 937.37 | 1019.724206 | 0.966361 |
| GO:0042492\_gamma-delta\_T\_cell\_differentiation | 6 | 0 | 0.000000 | -0.000000 | 970 | 855.015794 | 937.37 | 1019.724206 | 0.966361 |
| GO:0042953\_lipoprotein\_transport | 6 | 0 | 0.000000 | -0.000000 | 970 | 855.015794 | 937.37 | 1019.724206 | 0.966361 |
| GO:0043064\_flagellum\_organization | 6 | 0 | 0.000000 | -0.000000 | 970 | 855.015794 | 937.37 | 1019.724206 | 0.966361 |
| GO:0043154\_negative\_regulation\_of\_caspase\_activity | 6 | 0 | 0.000000 | -0.000000 | 970 | 855.015794 | 937.37 | 1019.724206 | 0.966361 |
| GO:0043255\_regulation\_of\_carbohydrate\_biosynthetic\_process | 6 | 0 | 0.000000 | -0.000000 | 970 | 855.015794 | 937.37 | 1019.724206 | 0.966361 |
| GO:0043271\_negative\_regulation\_of\_ion\_transport | 6 | 0 | 0.000000 | -0.000000 | 970 | 855.015794 | 937.37 | 1019.724206 | 0.966361 |
| GO:0043278\_response\_to\_morphine | 6 | 0 | 0.000000 | -0.000000 | 970 | 855.015794 | 937.37 | 1019.724206 | 0.966361 |
| GO:0043300\_regulation\_of\_leukocyte\_degranulation | 6 | 0 | 0.000000 | -0.000000 | 970 | 855.015794 | 937.37 | 1019.724206 | 0.966361 |
| GO:0043467\_regulation\_of\_generation\_of\_precursor\_metabolites\_and\_energy | 6 | 0 | 0.000000 | -0.000000 | 970 | 855.015794 | 937.37 | 1019.724206 | 0.966361 |
| GO:0043547\_positive\_regulation\_of\_GTPase\_activity | 6 | 0 | 0.000000 | -0.000000 | 970 | 855.015794 | 937.37 | 1019.724206 | 0.966361 |
| GO:0043627\_response\_to\_estrogen\_stimulus | 6 | 0 | 0.000000 | -0.000000 | 970 | 855.015794 | 937.37 | 1019.724206 | 0.966361 |
| GO:0044269\_glycerol\_ether\_catabolic\_process | 6 | 0 | 0.000000 | -0.000000 | 970 | 855.015794 | 937.37 | 1019.724206 | 0.966361 |
| GO:0045072\_regulation\_of\_interferon-gamma\_biosynthetic\_process | 6 | 0 | 0.000000 | -0.000000 | 970 | 855.015794 | 937.37 | 1019.724206 | 0.966361 |
| GO:0045084\_positive\_regulation\_of\_interleukin-12\_biosynthetic\_process | 6 | 0 | 0.000000 | -0.000000 | 970 | 855.015794 | 937.37 | 1019.724206 | 0.966361 |
| GO:0045124\_regulation\_of\_bone\_resorption | 6 | 0 | 0.000000 | -0.000000 | 970 | 855.015794 | 937.37 | 1019.724206 | 0.966361 |
| GO:0045176\_apical\_protein\_localization | 6 | 0 | 0.000000 | -0.000000 | 970 | 855.015794 | 937.37 | 1019.724206 | 0.966361 |
| GO:0045540\_regulation\_of\_cholesterol\_biosynthetic\_process | 6 | 0 | 0.000000 | -0.000000 | 970 | 855.015794 | 937.37 | 1019.724206 | 0.966361 |
| GO:0045579\_positive\_regulation\_of\_B\_cell\_differentiation | 6 | 0 | 0.000000 | -0.000000 | 970 | 855.015794 | 937.37 | 1019.724206 | 0.966361 |
| GO:0045649\_regulation\_of\_macrophage\_differentiation | 6 | 0 | 0.000000 | -0.000000 | 970 | 855.015794 | 937.37 | 1019.724206 | 0.966361 |
| GO:0045727\_positive\_regulation\_of\_translation | 6 | 0 | 0.000000 | -0.000000 | 970 | 855.015794 | 937.37 | 1019.724206 | 0.966361 |
| GO:0045778\_positive\_regulation\_of\_ossification | 6 | 0 | 0.000000 | -0.000000 | 970 | 855.015794 | 937.37 | 1019.724206 | 0.966361 |
| GO:0045822\_negative\_regulation\_of\_heart\_contraction | 6 | 0 | 0.000000 | -0.000000 | 970 | 855.015794 | 937.37 | 1019.724206 | 0.966361 |
| GO:0045824\_negative\_regulation\_of\_innate\_immune\_response | 6 | 0 | 0.000000 | -0.000000 | 970 | 855.015794 | 937.37 | 1019.724206 | 0.966361 |
| GO:0045833\_negative\_regulation\_of\_lipid\_metabolic\_process | 6 | 0 | 0.000000 | -0.000000 | 970 | 855.015794 | 937.37 | 1019.724206 | 0.966361 |
| GO:0045843\_negative\_regulation\_of\_striated\_muscle\_development | 6 | 0 | 0.000000 | -0.000000 | 970 | 855.015794 | 937.37 | 1019.724206 | 0.966361 |
| GO:0045861\_negative\_regulation\_of\_proteolysis | 6 | 0 | 0.000000 | -0.000000 | 970 | 855.015794 | 937.37 | 1019.724206 | 0.966361 |
| GO:0045913\_positive\_regulation\_of\_carbohydrate\_metabolic\_process | 6 | 0 | 0.000000 | -0.000000 | 970 | 855.015794 | 937.37 | 1019.724206 | 0.966361 |
| GO:0045931\_positive\_regulation\_of\_mitotic\_cell\_cycle | 6 | 0 | 0.000000 | -0.000000 | 970 | 855.015794 | 937.37 | 1019.724206 | 0.966361 |
| GO:0045933\_positive\_regulation\_of\_muscle\_contraction | 6 | 0 | 0.000000 | -0.000000 | 970 | 855.015794 | 937.37 | 1019.724206 | 0.966361 |
| GO:0046427\_positive\_regulation\_of\_JAK-STAT\_cascade | 6 | 0 | 0.000000 | -0.000000 | 970 | 855.015794 | 937.37 | 1019.724206 | 0.966361 |
| GO:0046460\_neutral\_lipid\_biosynthetic\_process | 6 | 0 | 0.000000 | -0.000000 | 970 | 855.015794 | 937.37 | 1019.724206 | 0.966361 |
| GO:0046461\_neutral\_lipid\_catabolic\_process | 6 | 0 | 0.000000 | -0.000000 | 970 | 855.015794 | 937.37 | 1019.724206 | 0.966361 |
| GO:0046463\_acylglycerol\_biosynthetic\_process | 6 | 0 | 0.000000 | -0.000000 | 970 | 855.015794 | 937.37 | 1019.724206 | 0.966361 |
| GO:0046464\_acylglycerol\_catabolic\_process | 6 | 0 | 0.000000 | -0.000000 | 970 | 855.015794 | 937.37 | 1019.724206 | 0.966361 |
| GO:0046466\_membrane\_lipid\_catabolic\_process | 6 | 0 | 0.000000 | -0.000000 | 970 | 855.015794 | 937.37 | 1019.724206 | 0.966361 |
| GO:0046503\_glycerolipid\_catabolic\_process | 6 | 0 | 0.000000 | -0.000000 | 970 | 855.015794 | 937.37 | 1019.724206 | 0.966361 |
| GO:0046580\_negative\_regulation\_of\_Ras\_protein\_signal\_transduction | 6 | 0 | 0.000000 | -0.000000 | 970 | 855.015794 | 937.37 | 1019.724206 | 0.966361 |
| GO:0046627\_negative\_regulation\_of\_insulin\_receptor\_signaling\_pathway | 6 | 0 | 0.000000 | -0.000000 | 970 | 855.015794 | 937.37 | 1019.724206 | 0.966361 |
| GO:0046629\_gamma-delta\_T\_cell\_activation | 6 | 0 | 0.000000 | -0.000000 | 970 | 855.015794 | 937.37 | 1019.724206 | 0.966361 |
| GO:0046666\_retinal\_cell\_programmed\_cell\_death | 6 | 0 | 0.000000 | -0.000000 | 970 | 855.015794 | 937.37 | 1019.724206 | 0.966361 |
| GO:0046852\_positive\_regulation\_of\_bone\_remodeling | 6 | 0 | 0.000000 | -0.000000 | 970 | 855.015794 | 937.37 | 1019.724206 | 0.966361 |
| GO:0046889\_positive\_regulation\_of\_lipid\_biosynthetic\_process | 6 | 0 | 0.000000 | -0.000000 | 970 | 855.015794 | 937.37 | 1019.724206 | 0.966361 |
| GO:0048041\_focal\_adhesion\_formation | 6 | 0 | 0.000000 | -0.000000 | 970 | 855.015794 | 937.37 | 1019.724206 | 0.966361 |
| GO:0048103\_somatic\_stem\_cell\_division | 6 | 0 | 0.000000 | -0.000000 | 970 | 855.015794 | 937.37 | 1019.724206 | 0.966361 |
| GO:0048147\_negative\_regulation\_of\_fibroblast\_proliferation | 6 | 0 | 0.000000 | -0.000000 | 970 | 855.015794 | 937.37 | 1019.724206 | 0.966361 |
| GO:0048333\_mesodermal\_cell\_differentiation | 6 | 0 | 0.000000 | -0.000000 | 970 | 855.015794 | 937.37 | 1019.724206 | 0.966361 |
| GO:0048340\_paraxial\_mesoderm\_morphogenesis | 6 | 0 | 0.000000 | -0.000000 | 970 | 855.015794 | 937.37 | 1019.724206 | 0.966361 |
| GO:0048541\_Peyer's\_patch\_development | 6 | 0 | 0.000000 | -0.000000 | 970 | 855.015794 | 937.37 | 1019.724206 | 0.966361 |
| GO:0048563\_post-embryonic\_organ\_morphogenesis | 6 | 0 | 0.000000 | -0.000000 | 970 | 855.015794 | 937.37 | 1019.724206 | 0.966361 |
| GO:0048617\_embryonic\_foregut\_morphogenesis | 6 | 0 | 0.000000 | -0.000000 | 970 | 855.015794 | 937.37 | 1019.724206 | 0.966361 |
| GO:0048635\_negative\_regulation\_of\_muscle\_development | 6 | 0 | 0.000000 | -0.000000 | 970 | 855.015794 | 937.37 | 1019.724206 | 0.966361 |
| GO:0048644\_muscle\_organ\_morphogenesis | 6 | 0 | 0.000000 | -0.000000 | 970 | 855.015794 | 937.37 | 1019.724206 | 0.966361 |
| GO:0048703\_embryonic\_viscerocranium\_morphogenesis | 6 | 0 | 0.000000 | -0.000000 | 970 | 855.015794 | 937.37 | 1019.724206 | 0.966361 |
| GO:0048713\_regulation\_of\_oligodendrocyte\_differentiation | 6 | 0 | 0.000000 | -0.000000 | 970 | 855.015794 | 937.37 | 1019.724206 | 0.966361 |
| GO:0048853\_forebrain\_morphogenesis | 6 | 0 | 0.000000 | -0.000000 | 970 | 855.015794 | 937.37 | 1019.724206 | 0.966361 |
| GO:0050684\_regulation\_of\_mRNA\_processing | 6 | 0 | 0.000000 | -0.000000 | 970 | 855.015794 | 937.37 | 1019.724206 | 0.966361 |
| GO:0050732\_negative\_regulation\_of\_peptidyl-tyrosine\_phosphorylation | 6 | 0 | 0.000000 | -0.000000 | 970 | 855.015794 | 937.37 | 1019.724206 | 0.966361 |
| GO:0050805\_negative\_regulation\_of\_synaptic\_transmission | 6 | 0 | 0.000000 | -0.000000 | 970 | 855.015794 | 937.37 | 1019.724206 | 0.966361 |
| GO:0050821\_protein\_stabilization | 6 | 0 | 0.000000 | -0.000000 | 970 | 855.015794 | 937.37 | 1019.724206 | 0.966361 |
| GO:0050829\_defense\_response\_to\_Gram-negative\_bacterium | 6 | 0 | 0.000000 | -0.000000 | 970 | 855.015794 | 937.37 | 1019.724206 | 0.966361 |
| GO:0050872\_white\_fat\_cell\_differentiation | 6 | 0 | 0.000000 | -0.000000 | 970 | 855.015794 | 937.37 | 1019.724206 | 0.966361 |
| GO:0050951\_sensory\_perception\_of\_temperature\_stimulus | 6 | 0 | 0.000000 | -0.000000 | 970 | 855.015794 | 937.37 | 1019.724206 | 0.966361 |
| GO:0050966\_detection\_of\_mechanical\_stimulus\_involved\_in\_sensory\_perception\_of\_pain | 6 | 0 | 0.000000 | -0.000000 | 970 | 855.015794 | 937.37 | 1019.724206 | 0.966361 |
| GO:0051058\_negative\_regulation\_of\_small\_GTPase\_mediated\_signal\_transduction | 6 | 0 | 0.000000 | -0.000000 | 970 | 855.015794 | 937.37 | 1019.724206 | 0.966361 |
| GO:0051085\_chaperone\_mediated\_protein\_folding\_requiring\_cofactor | 6 | 0 | 0.000000 | -0.000000 | 970 | 855.015794 | 937.37 | 1019.724206 | 0.966361 |
| GO:0051180\_vitamin\_transport | 6 | 0 | 0.000000 | -0.000000 | 970 | 855.015794 | 937.37 | 1019.724206 | 0.966361 |
| GO:0051384\_response\_to\_glucocorticoid\_stimulus | 6 | 0 | 0.000000 | -0.000000 | 970 | 855.015794 | 937.37 | 1019.724206 | 0.966361 |
| GO:0051592\_response\_to\_calcium\_ion | 6 | 0 | 0.000000 | -0.000000 | 970 | 855.015794 | 937.37 | 1019.724206 | 0.966361 |
| GO:0051875\_pigment\_granule\_localization | 6 | 0 | 0.000000 | -0.000000 | 970 | 855.015794 | 937.37 | 1019.724206 | 0.966361 |
| GO:0051881\_regulation\_of\_mitochondrial\_membrane\_potential | 6 | 0 | 0.000000 | -0.000000 | 970 | 855.015794 | 937.37 | 1019.724206 | 0.966361 |
| GO:0051970\_negative\_regulation\_of\_transmission\_of\_nerve\_impulse | 6 | 0 | 0.000000 | -0.000000 | 970 | 855.015794 | 937.37 | 1019.724206 | 0.966361 |
| GO:0055081\_anion\_homeostasis | 6 | 0 | 0.000000 | -0.000000 | 970 | 855.015794 | 937.37 | 1019.724206 | 0.966361 |
| GO:0060013\_righting\_reflex | 6 | 0 | 0.000000 | -0.000000 | 970 | 855.015794 | 937.37 | 1019.724206 | 0.966361 |
| GO:0060017\_parathyroid\_gland\_development | 6 | 0 | 0.000000 | -0.000000 | 970 | 855.015794 | 937.37 | 1019.724206 | 0.966361 |
| GO:0060056\_mammary\_gland\_involution | 6 | 0 | 0.000000 | -0.000000 | 970 | 855.015794 | 937.37 | 1019.724206 | 0.966361 |
| GO:0060068\_vagina\_development | 6 | 0 | 0.000000 | -0.000000 | 970 | 855.015794 | 937.37 | 1019.724206 | 0.966361 |
| GO:0060134\_prepulse\_inhibition | 6 | 0 | 0.000000 | -0.000000 | 970 | 855.015794 | 937.37 | 1019.724206 | 0.966361 |
| GO:0060136\_embryonic\_process\_involved\_in\_female\_pregnancy | 6 | 0 | 0.000000 | -0.000000 | 970 | 855.015794 | 937.37 | 1019.724206 | 0.966361 |
| GO:0060271\_cilium\_morphogenesis | 6 | 0 | 0.000000 | -0.000000 | 970 | 855.015794 | 937.37 | 1019.724206 | 0.966361 |
| GO:0060411\_heart\_septum\_morphogenesis | 6 | 0 | 0.000000 | -0.000000 | 970 | 855.015794 | 937.37 | 1019.724206 | 0.966361 |
| GO:0060638\_mesenchymal-epithelial\_cell\_signaling | 6 | 0 | 0.000000 | -0.000000 | 970 | 855.015794 | 937.37 | 1019.724206 | 0.966361 |
| GO:0060685\_regulation\_of\_prostatic\_bud\_formation | 6 | 0 | 0.000000 | -0.000000 | 970 | 855.015794 | 937.37 | 1019.724206 | 0.966361 |
| GO:0060710\_chorio-allantoic\_fusion | 6 | 0 | 0.000000 | -0.000000 | 970 | 855.015794 | 937.37 | 1019.724206 | 0.966361 |
| GO:0007626\_locomotory\_behavior | 163 | 0 | 0.000000 | -0.000000 | 972 | 857.595051 | 939.55 | 1021.504949 | 0.966615 |
| GO:0042110\_T\_cell\_activation | 163 | 0 | 0.000000 | -0.000000 | 972 | 857.595051 | 939.55 | 1021.504949 | 0.966615 |
| GO:0043065\_positive\_regulation\_of\_apoptosis | 166 | 0 | 0.000000 | -0.000000 | 973 | 858.045102 | 939.93 | 1021.814898 | 0.966012 |
| GO:0000302\_response\_to\_reactive\_oxygen\_species | 16 | 0 | 0.000000 | -0.000000 | 1019 | 903.606254 | 983.98 | 1064.353746 | 0.965633 |
| GO:0001933\_negative\_regulation\_of\_protein\_amino\_acid\_phosphorylation | 16 | 0 | 0.000000 | -0.000000 | 1019 | 903.606254 | 983.98 | 1064.353746 | 0.965633 |
| GO:0003044\_regulation\_of\_systemic\_arterial\_blood\_pressure\_mediated\_by\_a\_chemical\_signal | 16 | 0 | 0.000000 | -0.000000 | 1019 | 903.606254 | 983.98 | 1064.353746 | 0.965633 |
| GO:0006664\_glycolipid\_metabolic\_process | 16 | 0 | 0.000000 | -0.000000 | 1019 | 903.606254 | 983.98 | 1064.353746 | 0.965633 |
| GO:0006821\_chloride\_transport | 16 | 0 | 0.000000 | -0.000000 | 1019 | 903.606254 | 983.98 | 1064.353746 | 0.965633 |
| GO:0007033\_vacuole\_organization | 16 | 0 | 0.000000 | -0.000000 | 1019 | 903.606254 | 983.98 | 1064.353746 | 0.965633 |
| GO:0007156\_homophilic\_cell\_adhesion | 16 | 0 | 0.000000 | -0.000000 | 1019 | 903.606254 | 983.98 | 1064.353746 | 0.965633 |
| GO:0007602\_phototransduction | 16 | 0 | 0.000000 | -0.000000 | 1019 | 903.606254 | 983.98 | 1064.353746 | 0.965633 |
| GO:0008654\_phospholipid\_biosynthetic\_process | 16 | 0 | 0.000000 | -0.000000 | 1019 | 903.606254 | 983.98 | 1064.353746 | 0.965633 |
| GO:0009988\_cell-cell\_recognition | 16 | 0 | 0.000000 | -0.000000 | 1019 | 903.606254 | 983.98 | 1064.353746 | 0.965633 |
| GO:0010038\_response\_to\_metal\_ion | 16 | 0 | 0.000000 | -0.000000 | 1019 | 903.606254 | 983.98 | 1064.353746 | 0.965633 |
| GO:0010243\_response\_to\_organic\_nitrogen | 16 | 0 | 0.000000 | -0.000000 | 1019 | 903.606254 | 983.98 | 1064.353746 | 0.965633 |
| GO:0010876\_lipid\_localization | 16 | 0 | 0.000000 | -0.000000 | 1019 | 903.606254 | 983.98 | 1064.353746 | 0.965633 |
| GO:0014075\_response\_to\_amine\_stimulus | 16 | 0 | 0.000000 | -0.000000 | 1019 | 903.606254 | 983.98 | 1064.353746 | 0.965633 |
| GO:0016126\_sterol\_biosynthetic\_process | 16 | 0 | 0.000000 | -0.000000 | 1019 | 903.606254 | 983.98 | 1064.353746 | 0.965633 |
| GO:0019722\_calcium-mediated\_signaling | 16 | 0 | 0.000000 | -0.000000 | 1019 | 903.606254 | 983.98 | 1064.353746 | 0.965633 |
| GO:0019751\_polyol\_metabolic\_process | 16 | 0 | 0.000000 | -0.000000 | 1019 | 903.606254 | 983.98 | 1064.353746 | 0.965633 |
| GO:0019915\_lipid\_storage | 16 | 0 | 0.000000 | -0.000000 | 1019 | 903.606254 | 983.98 | 1064.353746 | 0.965633 |
| GO:0021522\_spinal\_cord\_motor\_neuron\_differentiation | 16 | 0 | 0.000000 | -0.000000 | 1019 | 903.606254 | 983.98 | 1064.353746 | 0.965633 |
| GO:0021696\_cerebellar\_cortex\_morphogenesis | 16 | 0 | 0.000000 | -0.000000 | 1019 | 903.606254 | 983.98 | 1064.353746 | 0.965633 |
| GO:0030890\_positive\_regulation\_of\_B\_cell\_proliferation | 16 | 0 | 0.000000 | -0.000000 | 1019 | 903.606254 | 983.98 | 1064.353746 | 0.965633 |
| GO:0031345\_negative\_regulation\_of\_cell\_projection\_organization | 16 | 0 | 0.000000 | -0.000000 | 1019 | 903.606254 | 983.98 | 1064.353746 | 0.965633 |
| GO:0031669\_cellular\_response\_to\_nutrient\_levels | 16 | 0 | 0.000000 | -0.000000 | 1019 | 903.606254 | 983.98 | 1064.353746 | 0.965633 |
| GO:0032663\_regulation\_of\_interleukin-2\_production | 16 | 0 | 0.000000 | -0.000000 | 1019 | 903.606254 | 983.98 | 1064.353746 | 0.965633 |
| GO:0034976\_response\_to\_endoplasmic\_reticulum\_stress | 16 | 0 | 0.000000 | -0.000000 | 1019 | 903.606254 | 983.98 | 1064.353746 | 0.965633 |
| GO:0042311\_vasodilation | 16 | 0 | 0.000000 | -0.000000 | 1019 | 903.606254 | 983.98 | 1064.353746 | 0.965633 |
| GO:0042594\_response\_to\_starvation | 16 | 0 | 0.000000 | -0.000000 | 1019 | 903.606254 | 983.98 | 1064.353746 | 0.965633 |
| GO:0042596\_fear\_response | 16 | 0 | 0.000000 | -0.000000 | 1019 | 903.606254 | 983.98 | 1064.353746 | 0.965633 |
| GO:0043087\_regulation\_of\_GTPase\_activity | 16 | 0 | 0.000000 | -0.000000 | 1019 | 903.606254 | 983.98 | 1064.353746 | 0.965633 |
| GO:0043122\_regulation\_of\_I-kappaB\_kinase\_NF-kappaB\_cascade | 16 | 0 | 0.000000 | -0.000000 | 1019 | 903.606254 | 983.98 | 1064.353746 | 0.965633 |
| GO:0043367\_CD4-positive\_\_alpha\_beta\_T\_cell\_differentiation | 16 | 0 | 0.000000 | -0.000000 | 1019 | 903.606254 | 983.98 | 1064.353746 | 0.965633 |
| GO:0045104\_intermediate\_filament\_cytoskeleton\_organization | 16 | 0 | 0.000000 | -0.000000 | 1019 | 903.606254 | 983.98 | 1064.353746 | 0.965633 |
| GO:0046148\_pigment\_biosynthetic\_process | 16 | 0 | 0.000000 | -0.000000 | 1019 | 903.606254 | 983.98 | 1064.353746 | 0.965633 |
| GO:0046364\_monosaccharide\_biosynthetic\_process | 16 | 0 | 0.000000 | -0.000000 | 1019 | 903.606254 | 983.98 | 1064.353746 | 0.965633 |
| GO:0046467\_membrane\_lipid\_biosynthetic\_process | 16 | 0 | 0.000000 | -0.000000 | 1019 | 903.606254 | 983.98 | 1064.353746 | 0.965633 |
| GO:0046633\_alpha-beta\_T\_cell\_proliferation | 16 | 0 | 0.000000 | -0.000000 | 1019 | 903.606254 | 983.98 | 1064.353746 | 0.965633 |
| GO:0046700\_heterocycle\_catabolic\_process | 16 | 0 | 0.000000 | -0.000000 | 1019 | 903.606254 | 983.98 | 1064.353746 | 0.965633 |
| GO:0048015\_phosphoinositide-mediated\_signaling | 16 | 0 | 0.000000 | -0.000000 | 1019 | 903.606254 | 983.98 | 1064.353746 | 0.965633 |
| GO:0048286\_lung\_alveolus\_development | 16 | 0 | 0.000000 | -0.000000 | 1019 | 903.606254 | 983.98 | 1064.353746 | 0.965633 |
| GO:0048483\_autonomic\_nervous\_system\_development | 16 | 0 | 0.000000 | -0.000000 | 1019 | 903.606254 | 983.98 | 1064.353746 | 0.965633 |
| GO:0050974\_detection\_of\_mechanical\_stimulus\_involved\_in\_sensory\_perception | 16 | 0 | 0.000000 | -0.000000 | 1019 | 903.606254 | 983.98 | 1064.353746 | 0.965633 |
| GO:0051048\_negative\_regulation\_of\_secretion | 16 | 0 | 0.000000 | -0.000000 | 1019 | 903.606254 | 983.98 | 1064.353746 | 0.965633 |
| GO:0051937\_catecholamine\_transport | 16 | 0 | 0.000000 | -0.000000 | 1019 | 903.606254 | 983.98 | 1064.353746 | 0.965633 |
| GO:0055007\_cardiac\_muscle\_cell\_differentiation | 16 | 0 | 0.000000 | -0.000000 | 1019 | 903.606254 | 983.98 | 1064.353746 | 0.965633 |
| GO:0060193\_positive\_regulation\_of\_lipase\_activity | 16 | 0 | 0.000000 | -0.000000 | 1019 | 903.606254 | 983.98 | 1064.353746 | 0.965633 |
| GO:0060713\_labyrinthine\_layer\_morphogenesis | 16 | 0 | 0.000000 | -0.000000 | 1019 | 903.606254 | 983.98 | 1064.353746 | 0.965633 |
| GO:0005976\_polysaccharide\_metabolic\_process | 39 | 0 | 0.000000 | -0.000000 | 1030 | 917.987578 | 997.41 | 1076.832422 | 0.968359 |
| GO:0006511\_ubiquitin-dependent\_protein\_catabolic\_process | 39 | 0 | 0.000000 | -0.000000 | 1030 | 917.987578 | 997.41 | 1076.832422 | 0.968359 |
| GO:0006644\_phospholipid\_metabolic\_process | 39 | 0 | 0.000000 | -0.000000 | 1030 | 917.987578 | 997.41 | 1076.832422 | 0.968359 |
| GO:0007160\_cell-matrix\_adhesion | 39 | 0 | 0.000000 | -0.000000 | 1030 | 917.987578 | 997.41 | 1076.832422 | 0.968359 |
| GO:0007286\_spermatid\_development | 39 | 0 | 0.000000 | -0.000000 | 1030 | 917.987578 | 997.41 | 1076.832422 | 0.968359 |
| GO:0008037\_cell\_recognition | 39 | 0 | 0.000000 | -0.000000 | 1030 | 917.987578 | 997.41 | 1076.832422 | 0.968359 |
| GO:0021953\_central\_nervous\_system\_neuron\_differentiation | 39 | 0 | 0.000000 | -0.000000 | 1030 | 917.987578 | 997.41 | 1076.832422 | 0.968359 |
| GO:0031279\_regulation\_of\_cyclase\_activity | 39 | 0 | 0.000000 | -0.000000 | 1030 | 917.987578 | 997.41 | 1076.832422 | 0.968359 |
| GO:0035148\_tube\_lumen\_formation | 39 | 0 | 0.000000 | -0.000000 | 1030 | 917.987578 | 997.41 | 1076.832422 | 0.968359 |
| GO:0042475\_odontogenesis\_of\_dentine-containing\_tooth | 39 | 0 | 0.000000 | -0.000000 | 1030 | 917.987578 | 997.41 | 1076.832422 | 0.968359 |
| GO:0051339\_regulation\_of\_lyase\_activity | 39 | 0 | 0.000000 | -0.000000 | 1030 | 917.987578 | 997.41 | 1076.832422 | 0.968359 |
| GO:0002696\_positive\_regulation\_of\_leukocyte\_activation | 82 | 0 | 0.000000 | -0.000000 | 1035 | 921.335648 | 1000.4 | 1079.464352 | 0.966570 |
| GO:0007411\_axon\_guidance | 82 | 0 | 0.000000 | -0.000000 | 1035 | 921.335648 | 1000.4 | 1079.464352 | 0.966570 |
| GO:0008202\_steroid\_metabolic\_process | 82 | 0 | 0.000000 | -0.000000 | 1035 | 921.335648 | 1000.4 | 1079.464352 | 0.966570 |
| GO:0010627\_regulation\_of\_protein\_kinase\_cascade | 82 | 0 | 0.000000 | -0.000000 | 1035 | 921.335648 | 1000.4 | 1079.464352 | 0.966570 |
| GO:0045664\_regulation\_of\_neuron\_differentiation | 82 | 0 | 0.000000 | -0.000000 | 1035 | 921.335648 | 1000.4 | 1079.464352 | 0.966570 |
| GO:0001825\_blastocyst\_formation | 18 | 0 | 0.000000 | -0.000000 | 1081 | 971.302904 | 1048.65 | 1125.997096 | 0.970074 |
| GO:0001974\_blood\_vessel\_remodeling | 18 | 0 | 0.000000 | -0.000000 | 1081 | 971.302904 | 1048.65 | 1125.997096 | 0.970074 |
| GO:0002064\_epithelial\_cell\_development | 18 | 0 | 0.000000 | -0.000000 | 1081 | 971.302904 | 1048.65 | 1125.997096 | 0.970074 |
| GO:0002285\_lymphocyte\_activation\_during\_immune\_response | 18 | 0 | 0.000000 | -0.000000 | 1081 | 971.302904 | 1048.65 | 1125.997096 | 0.970074 |
| GO:0002715\_regulation\_of\_natural\_killer\_cell\_mediated\_immunity | 18 | 0 | 0.000000 | -0.000000 | 1081 | 971.302904 | 1048.65 | 1125.997096 | 0.970074 |
| GO:0003014\_renal\_system\_process | 18 | 0 | 0.000000 | -0.000000 | 1081 | 971.302904 | 1048.65 | 1125.997096 | 0.970074 |
| GO:0006022\_aminoglycan\_metabolic\_process | 18 | 0 | 0.000000 | -0.000000 | 1081 | 971.302904 | 1048.65 | 1125.997096 | 0.970074 |
| GO:0006457\_protein\_folding | 18 | 0 | 0.000000 | -0.000000 | 1081 | 971.302904 | 1048.65 | 1125.997096 | 0.970074 |
| GO:0006940\_regulation\_of\_smooth\_muscle\_contraction | 18 | 0 | 0.000000 | -0.000000 | 1081 | 971.302904 | 1048.65 | 1125.997096 | 0.970074 |
| GO:0007140\_male\_meiosis | 18 | 0 | 0.000000 | -0.000000 | 1081 | 971.302904 | 1048.65 | 1125.997096 | 0.970074 |
| GO:0007608\_sensory\_perception\_of\_smell | 18 | 0 | 0.000000 | -0.000000 | 1081 | 971.302904 | 1048.65 | 1125.997096 | 0.970074 |
| GO:0008589\_regulation\_of\_smoothened\_signaling\_pathway | 18 | 0 | 0.000000 | -0.000000 | 1081 | 971.302904 | 1048.65 | 1125.997096 | 0.970074 |
| GO:0009063\_cellular\_amino\_acid\_catabolic\_process | 18 | 0 | 0.000000 | -0.000000 | 1081 | 971.302904 | 1048.65 | 1125.997096 | 0.970074 |
| GO:0010498\_proteasomal\_protein\_catabolic\_process | 18 | 0 | 0.000000 | -0.000000 | 1081 | 971.302904 | 1048.65 | 1125.997096 | 0.970074 |
| GO:0010553\_negative\_regulation\_of\_specific\_transcription\_from\_RNA\_polymerase\_II\_promoter | 18 | 0 | 0.000000 | -0.000000 | 1081 | 971.302904 | 1048.65 | 1125.997096 | 0.970074 |
| GO:0015711\_organic\_anion\_transport | 18 | 0 | 0.000000 | -0.000000 | 1081 | 971.302904 | 1048.65 | 1125.997096 | 0.970074 |
| GO:0021517\_ventral\_spinal\_cord\_development | 18 | 0 | 0.000000 | -0.000000 | 1081 | 971.302904 | 1048.65 | 1125.997096 | 0.970074 |
| GO:0021885\_forebrain\_cell\_migration | 18 | 0 | 0.000000 | -0.000000 | 1081 | 971.302904 | 1048.65 | 1125.997096 | 0.970074 |
| GO:0030178\_negative\_regulation\_of\_Wnt\_receptor\_signaling\_pathway | 18 | 0 | 0.000000 | -0.000000 | 1081 | 971.302904 | 1048.65 | 1125.997096 | 0.970074 |
| GO:0030203\_glycosaminoglycan\_metabolic\_process | 18 | 0 | 0.000000 | -0.000000 | 1081 | 971.302904 | 1048.65 | 1125.997096 | 0.970074 |
| GO:0030282\_bone\_mineralization | 18 | 0 | 0.000000 | -0.000000 | 1081 | 971.302904 | 1048.65 | 1125.997096 | 0.970074 |
| GO:0030318\_melanocyte\_differentiation | 18 | 0 | 0.000000 | -0.000000 | 1081 | 971.302904 | 1048.65 | 1125.997096 | 0.970074 |
| GO:0030336\_negative\_regulation\_of\_cell\_migration | 18 | 0 | 0.000000 | -0.000000 | 1081 | 971.302904 | 1048.65 | 1125.997096 | 0.970074 |
| GO:0030510\_regulation\_of\_BMP\_signaling\_pathway | 18 | 0 | 0.000000 | -0.000000 | 1081 | 971.302904 | 1048.65 | 1125.997096 | 0.970074 |
| GO:0030901\_midbrain\_development | 18 | 0 | 0.000000 | -0.000000 | 1081 | 971.302904 | 1048.65 | 1125.997096 | 0.970074 |
| GO:0032623\_interleukin-2\_production | 18 | 0 | 0.000000 | -0.000000 | 1081 | 971.302904 | 1048.65 | 1125.997096 | 0.970074 |
| GO:0035051\_cardiac\_cell\_differentiation | 18 | 0 | 0.000000 | -0.000000 | 1081 | 971.302904 | 1048.65 | 1125.997096 | 0.970074 |
| GO:0042269\_regulation\_of\_natural\_killer\_cell\_mediated\_cytotoxicity | 18 | 0 | 0.000000 | -0.000000 | 1081 | 971.302904 | 1048.65 | 1125.997096 | 0.970074 |
| GO:0043029\_T\_cell\_homeostasis | 18 | 0 | 0.000000 | -0.000000 | 1081 | 971.302904 | 1048.65 | 1125.997096 | 0.970074 |
| GO:0043161\_proteasomal\_ubiquitin-dependent\_protein\_catabolic\_process | 18 | 0 | 0.000000 | -0.000000 | 1081 | 971.302904 | 1048.65 | 1125.997096 | 0.970074 |
| GO:0044272\_sulfur\_compound\_biosynthetic\_process | 18 | 0 | 0.000000 | -0.000000 | 1081 | 971.302904 | 1048.65 | 1125.997096 | 0.970074 |
| GO:0045058\_T\_cell\_selection | 18 | 0 | 0.000000 | -0.000000 | 1081 | 971.302904 | 1048.65 | 1125.997096 | 0.970074 |
| GO:0045103\_intermediate\_filament-based\_process | 18 | 0 | 0.000000 | -0.000000 | 1081 | 971.302904 | 1048.65 | 1125.997096 | 0.970074 |
| GO:0045638\_negative\_regulation\_of\_myeloid\_cell\_differentiation | 18 | 0 | 0.000000 | -0.000000 | 1081 | 971.302904 | 1048.65 | 1125.997096 | 0.970074 |
| GO:0045807\_positive\_regulation\_of\_endocytosis | 18 | 0 | 0.000000 | -0.000000 | 1081 | 971.302904 | 1048.65 | 1125.997096 | 0.970074 |
| GO:0046578\_regulation\_of\_Ras\_protein\_signal\_transduction | 18 | 0 | 0.000000 | -0.000000 | 1081 | 971.302904 | 1048.65 | 1125.997096 | 0.970074 |
| GO:0046620\_regulation\_of\_organ\_growth | 18 | 0 | 0.000000 | -0.000000 | 1081 | 971.302904 | 1048.65 | 1125.997096 | 0.970074 |
| GO:0048813\_dendrite\_morphogenesis | 18 | 0 | 0.000000 | -0.000000 | 1081 | 971.302904 | 1048.65 | 1125.997096 | 0.970074 |
| GO:0050731\_positive\_regulation\_of\_peptidyl-tyrosine\_phosphorylation | 18 | 0 | 0.000000 | -0.000000 | 1081 | 971.302904 | 1048.65 | 1125.997096 | 0.970074 |
| GO:0050982\_detection\_of\_mechanical\_stimulus | 18 | 0 | 0.000000 | -0.000000 | 1081 | 971.302904 | 1048.65 | 1125.997096 | 0.970074 |
| GO:0051168\_nuclear\_export | 18 | 0 | 0.000000 | -0.000000 | 1081 | 971.302904 | 1048.65 | 1125.997096 | 0.970074 |
| GO:0051924\_regulation\_of\_calcium\_ion\_transport | 18 | 0 | 0.000000 | -0.000000 | 1081 | 971.302904 | 1048.65 | 1125.997096 | 0.970074 |
| GO:0055008\_cardiac\_muscle\_tissue\_morphogenesis | 18 | 0 | 0.000000 | -0.000000 | 1081 | 971.302904 | 1048.65 | 1125.997096 | 0.970074 |
| GO:0060415\_muscle\_tissue\_morphogenesis | 18 | 0 | 0.000000 | -0.000000 | 1081 | 971.302904 | 1048.65 | 1125.997096 | 0.970074 |
| GO:0060571\_morphogenesis\_of\_an\_epithelial\_fold | 18 | 0 | 0.000000 | -0.000000 | 1081 | 971.302904 | 1048.65 | 1125.997096 | 0.970074 |
| GO:0060674\_placenta\_blood\_vessel\_development | 18 | 0 | 0.000000 | -0.000000 | 1081 | 971.302904 | 1048.65 | 1125.997096 | 0.970074 |
| GO:0040012\_regulation\_of\_locomotion | 72 | 0 | 0.000000 | -0.000000 | 1086 | 979.247694 | 1055.97 | 1132.692306 | 0.972348 |
| GO:0042098\_T\_cell\_proliferation | 72 | 0 | 0.000000 | -0.000000 | 1086 | 979.247694 | 1055.97 | 1132.692306 | 0.972348 |
| GO:0044262\_cellular\_carbohydrate\_metabolic\_process | 72 | 0 | 0.000000 | -0.000000 | 1086 | 979.247694 | 1055.97 | 1132.692306 | 0.972348 |
| GO:0048839\_inner\_ear\_development | 72 | 0 | 0.000000 | -0.000000 | 1086 | 979.247694 | 1055.97 | 1132.692306 | 0.972348 |
| GO:0050673\_epithelial\_cell\_proliferation | 72 | 0 | 0.000000 | -0.000000 | 1086 | 979.247694 | 1055.97 | 1132.692306 | 0.972348 |
| GO:0018193\_peptidyl-amino\_acid\_modification | 97 | 0 | 0.000000 | -0.000000 | 1087 | 980.667443 | 1057.2 | 1133.732557 | 0.972585 |
| GO:0001654\_eye\_development | 136 | 0 | 0.000000 | -0.000000 | 1088 | 981.127891 | 1057.61 | 1134.092109 | 0.972068 |
| GO:0031347\_regulation\_of\_defense\_response | 67 | 0 | 0.000000 | -0.000000 | 1090 | 984.837454 | 1060.91 | 1136.982546 | 0.973312 |
| GO:0042445\_hormone\_metabolic\_process | 67 | 0 | 0.000000 | -0.000000 | 1090 | 984.837454 | 1060.91 | 1136.982546 | 0.973312 |
| GO:0060348\_bone\_development | 99 | 0 | 0.000000 | -0.000000 | 1091 | 987.584165 | 1063.32 | 1139.055835 | 0.974629 |
| GO:0010647\_positive\_regulation\_of\_cell\_communication | 110 | 0 | 0.000000 | -0.000000 | 1094 | 990.032285 | 1065.32 | 1140.607715 | 0.973784 |
| GO:0010648\_negative\_regulation\_of\_cell\_communication | 110 | 0 | 0.000000 | -0.000000 | 1094 | 990.032285 | 1065.32 | 1140.607715 | 0.973784 |
| GO:0043010\_camera-type\_eye\_development | 110 | 0 | 0.000000 | -0.000000 | 1094 | 990.032285 | 1065.32 | 1140.607715 | 0.973784 |
| GO:0006338\_chromatin\_remodeling | 19 | 0 | 0.000000 | -0.000000 | 1126 | 1021.642623 | 1095.85 | 1170.057377 | 0.973224 |
| GO:0006497\_protein\_amino\_acid\_lipidation | 19 | 0 | 0.000000 | -0.000000 | 1126 | 1021.642623 | 1095.85 | 1170.057377 | 0.973224 |
| GO:0006672\_ceramide\_metabolic\_process | 19 | 0 | 0.000000 | -0.000000 | 1126 | 1021.642623 | 1095.85 | 1170.057377 | 0.973224 |
| GO:0006776\_vitamin\_A\_metabolic\_process | 19 | 0 | 0.000000 | -0.000000 | 1126 | 1021.642623 | 1095.85 | 1170.057377 | 0.973224 |
| GO:0007569\_cell\_aging | 19 | 0 | 0.000000 | -0.000000 | 1126 | 1021.642623 | 1095.85 | 1170.057377 | 0.973224 |
| GO:0009584\_detection\_of\_visible\_light | 19 | 0 | 0.000000 | -0.000000 | 1126 | 1021.642623 | 1095.85 | 1170.057377 | 0.973224 |
| GO:0009798\_axis\_specification | 19 | 0 | 0.000000 | -0.000000 | 1126 | 1021.642623 | 1095.85 | 1170.057377 | 0.973224 |
| GO:0010639\_negative\_regulation\_of\_organelle\_organization | 19 | 0 | 0.000000 | -0.000000 | 1126 | 1021.642623 | 1095.85 | 1170.057377 | 0.973224 |
| GO:0010952\_positive\_regulation\_of\_peptidase\_activity | 19 | 0 | 0.000000 | -0.000000 | 1126 | 1021.642623 | 1095.85 | 1170.057377 | 0.973224 |
| GO:0019218\_regulation\_of\_steroid\_metabolic\_process | 19 | 0 | 0.000000 | -0.000000 | 1126 | 1021.642623 | 1095.85 | 1170.057377 | 0.973224 |
| GO:0021587\_cerebellum\_morphogenesis | 19 | 0 | 0.000000 | -0.000000 | 1126 | 1021.642623 | 1095.85 | 1170.057377 | 0.973224 |
| GO:0030199\_collagen\_fibril\_organization | 19 | 0 | 0.000000 | -0.000000 | 1126 | 1021.642623 | 1095.85 | 1170.057377 | 0.973224 |
| GO:0030518\_steroid\_hormone\_receptor\_signaling\_pathway | 19 | 0 | 0.000000 | -0.000000 | 1126 | 1021.642623 | 1095.85 | 1170.057377 | 0.973224 |
| GO:0030595\_leukocyte\_chemotaxis | 19 | 0 | 0.000000 | -0.000000 | 1126 | 1021.642623 | 1095.85 | 1170.057377 | 0.973224 |
| GO:0032526\_response\_to\_retinoic\_acid | 19 | 0 | 0.000000 | -0.000000 | 1126 | 1021.642623 | 1095.85 | 1170.057377 | 0.973224 |
| GO:0033002\_muscle\_cell\_proliferation | 19 | 0 | 0.000000 | -0.000000 | 1126 | 1021.642623 | 1095.85 | 1170.057377 | 0.973224 |
| GO:0033189\_response\_to\_vitamin\_A | 19 | 0 | 0.000000 | -0.000000 | 1126 | 1021.642623 | 1095.85 | 1170.057377 | 0.973224 |
| GO:0042462\_eye\_photoreceptor\_cell\_development | 19 | 0 | 0.000000 | -0.000000 | 1126 | 1021.642623 | 1095.85 | 1170.057377 | 0.973224 |
| GO:0042491\_auditory\_receptor\_cell\_differentiation | 19 | 0 | 0.000000 | -0.000000 | 1126 | 1021.642623 | 1095.85 | 1170.057377 | 0.973224 |
| GO:0043280\_positive\_regulation\_of\_caspase\_activity | 19 | 0 | 0.000000 | -0.000000 | 1126 | 1021.642623 | 1095.85 | 1170.057377 | 0.973224 |
| GO:0046165\_alcohol\_biosynthetic\_process | 19 | 0 | 0.000000 | -0.000000 | 1126 | 1021.642623 | 1095.85 | 1170.057377 | 0.973224 |
| GO:0046890\_regulation\_of\_lipid\_biosynthetic\_process | 19 | 0 | 0.000000 | -0.000000 | 1126 | 1021.642623 | 1095.85 | 1170.057377 | 0.973224 |
| GO:0048547\_gut\_morphogenesis | 19 | 0 | 0.000000 | -0.000000 | 1126 | 1021.642623 | 1095.85 | 1170.057377 | 0.973224 |
| GO:0048701\_embryonic\_cranial\_skeleton\_morphogenesis | 19 | 0 | 0.000000 | -0.000000 | 1126 | 1021.642623 | 1095.85 | 1170.057377 | 0.973224 |
| GO:0050728\_negative\_regulation\_of\_inflammatory\_response | 19 | 0 | 0.000000 | -0.000000 | 1126 | 1021.642623 | 1095.85 | 1170.057377 | 0.973224 |
| GO:0050908\_detection\_of\_light\_stimulus\_involved\_in\_visual\_perception | 19 | 0 | 0.000000 | -0.000000 | 1126 | 1021.642623 | 1095.85 | 1170.057377 | 0.973224 |
| GO:0050931\_pigment\_cell\_differentiation | 19 | 0 | 0.000000 | -0.000000 | 1126 | 1021.642623 | 1095.85 | 1170.057377 | 0.973224 |
| GO:0050962\_detection\_of\_light\_stimulus\_involved\_in\_sensory\_perception | 19 | 0 | 0.000000 | -0.000000 | 1126 | 1021.642623 | 1095.85 | 1170.057377 | 0.973224 |
| GO:0051056\_regulation\_of\_small\_GTPase\_mediated\_signal\_transduction | 19 | 0 | 0.000000 | -0.000000 | 1126 | 1021.642623 | 1095.85 | 1170.057377 | 0.973224 |
| GO:0060079\_regulation\_of\_excitatory\_postsynaptic\_membrane\_potential | 19 | 0 | 0.000000 | -0.000000 | 1126 | 1021.642623 | 1095.85 | 1170.057377 | 0.973224 |
| GO:0060326\_cell\_chemotaxis | 19 | 0 | 0.000000 | -0.000000 | 1126 | 1021.642623 | 1095.85 | 1170.057377 | 0.973224 |
| GO:0060444\_branching\_involved\_in\_mammary\_gland\_duct\_morphogenesis | 19 | 0 | 0.000000 | -0.000000 | 1126 | 1021.642623 | 1095.85 | 1170.057377 | 0.973224 |
| GO:0000280\_nuclear\_division | 24 | 0 | 0.000000 | -0.000000 | 1152 | 1048.060962 | 1121.14 | 1194.219038 | 0.973212 |
| GO:0001541\_ovarian\_follicle\_development | 24 | 0 | 0.000000 | -0.000000 | 1152 | 1048.060962 | 1121.14 | 1194.219038 | 0.973212 |
| GO:0006650\_glycerophospholipid\_metabolic\_process | 24 | 0 | 0.000000 | -0.000000 | 1152 | 1048.060962 | 1121.14 | 1194.219038 | 0.973212 |
| GO:0006941\_striated\_muscle\_contraction | 24 | 0 | 0.000000 | -0.000000 | 1152 | 1048.060962 | 1121.14 | 1194.219038 | 0.973212 |
| GO:0006959\_humoral\_immune\_response | 24 | 0 | 0.000000 | -0.000000 | 1152 | 1048.060962 | 1121.14 | 1194.219038 | 0.973212 |
| GO:0007067\_mitosis | 24 | 0 | 0.000000 | -0.000000 | 1152 | 1048.060962 | 1121.14 | 1194.219038 | 0.973212 |
| GO:0007204\_elevation\_of\_cytosolic\_calcium\_ion\_concentration | 24 | 0 | 0.000000 | -0.000000 | 1152 | 1048.060962 | 1121.14 | 1194.219038 | 0.973212 |
| GO:0007259\_JAK-STAT\_cascade | 24 | 0 | 0.000000 | -0.000000 | 1152 | 1048.060962 | 1121.14 | 1194.219038 | 0.973212 |
| GO:0007266\_Rho\_protein\_signal\_transduction | 24 | 0 | 0.000000 | -0.000000 | 1152 | 1048.060962 | 1121.14 | 1194.219038 | 0.973212 |
| GO:0007632\_visual\_behavior | 24 | 0 | 0.000000 | -0.000000 | 1152 | 1048.060962 | 1121.14 | 1194.219038 | 0.973212 |
| GO:0008629\_induction\_of\_apoptosis\_by\_intracellular\_signals | 24 | 0 | 0.000000 | -0.000000 | 1152 | 1048.060962 | 1121.14 | 1194.219038 | 0.973212 |
| GO:0009612\_response\_to\_mechanical\_stimulus | 24 | 0 | 0.000000 | -0.000000 | 1152 | 1048.060962 | 1121.14 | 1194.219038 | 0.973212 |
| GO:0014070\_response\_to\_organic\_cyclic\_substance | 24 | 0 | 0.000000 | -0.000000 | 1152 | 1048.060962 | 1121.14 | 1194.219038 | 0.973212 |
| GO:0021515\_cell\_differentiation\_in\_spinal\_cord | 24 | 0 | 0.000000 | -0.000000 | 1152 | 1048.060962 | 1121.14 | 1194.219038 | 0.973212 |
| GO:0042158\_lipoprotein\_biosynthetic\_process | 24 | 0 | 0.000000 | -0.000000 | 1152 | 1048.060962 | 1121.14 | 1194.219038 | 0.973212 |
| GO:0042632\_cholesterol\_homeostasis | 24 | 0 | 0.000000 | -0.000000 | 1152 | 1048.060962 | 1121.14 | 1194.219038 | 0.973212 |
| GO:0043410\_positive\_regulation\_of\_MAPKKK\_cascade | 24 | 0 | 0.000000 | -0.000000 | 1152 | 1048.060962 | 1121.14 | 1194.219038 | 0.973212 |
| GO:0043588\_skin\_development | 24 | 0 | 0.000000 | -0.000000 | 1152 | 1048.060962 | 1121.14 | 1194.219038 | 0.973212 |
| GO:0048002\_antigen\_processing\_and\_presentation\_of\_peptide\_antigen | 24 | 0 | 0.000000 | -0.000000 | 1152 | 1048.060962 | 1121.14 | 1194.219038 | 0.973212 |
| GO:0048546\_digestive\_tract\_morphogenesis | 24 | 0 | 0.000000 | -0.000000 | 1152 | 1048.060962 | 1121.14 | 1194.219038 | 0.973212 |
| GO:0050679\_positive\_regulation\_of\_epithelial\_cell\_proliferation | 24 | 0 | 0.000000 | -0.000000 | 1152 | 1048.060962 | 1121.14 | 1194.219038 | 0.973212 |
| GO:0051099\_positive\_regulation\_of\_binding | 24 | 0 | 0.000000 | -0.000000 | 1152 | 1048.060962 | 1121.14 | 1194.219038 | 0.973212 |
| GO:0055092\_sterol\_homeostasis | 24 | 0 | 0.000000 | -0.000000 | 1152 | 1048.060962 | 1121.14 | 1194.219038 | 0.973212 |
| GO:0060078\_regulation\_of\_postsynaptic\_membrane\_potential | 24 | 0 | 0.000000 | -0.000000 | 1152 | 1048.060962 | 1121.14 | 1194.219038 | 0.973212 |
| GO:0060113\_inner\_ear\_receptor\_cell\_differentiation | 24 | 0 | 0.000000 | -0.000000 | 1152 | 1048.060962 | 1121.14 | 1194.219038 | 0.973212 |
| GO:0070667\_negative\_regulation\_of\_mast\_cell\_proliferation | 24 | 0 | 0.000000 | -0.000000 | 1152 | 1048.060962 | 1121.14 | 1194.219038 | 0.973212 |
| GO:0001704\_formation\_of\_primary\_germ\_layer | 36 | 0 | 0.000000 | -0.000000 | 1169 | 1064.026148 | 1136.03 | 1208.033852 | 0.971796 |
| GO:0001819\_positive\_regulation\_of\_cytokine\_production | 36 | 0 | 0.000000 | -0.000000 | 1169 | 1064.026148 | 1136.03 | 1208.033852 | 0.971796 |
| GO:0001889\_liver\_development | 36 | 0 | 0.000000 | -0.000000 | 1169 | 1064.026148 | 1136.03 | 1208.033852 | 0.971796 |
| GO:0006469\_negative\_regulation\_of\_protein\_kinase\_activity | 36 | 0 | 0.000000 | -0.000000 | 1169 | 1064.026148 | 1136.03 | 1208.033852 | 0.971796 |
| GO:0007187\_G-protein\_signaling\_\_coupled\_to\_cyclic\_nucleotide\_second\_messenger | 36 | 0 | 0.000000 | -0.000000 | 1169 | 1064.026148 | 1136.03 | 1208.033852 | 0.971796 |
| GO:0007368\_determination\_of\_left\_right\_symmetry | 36 | 0 | 0.000000 | -0.000000 | 1169 | 1064.026148 | 1136.03 | 1208.033852 | 0.971796 |
| GO:0007631\_feeding\_behavior | 36 | 0 | 0.000000 | -0.000000 | 1169 | 1064.026148 | 1136.03 | 1208.033852 | 0.971796 |
| GO:0014020\_primary\_neural\_tube\_formation | 36 | 0 | 0.000000 | -0.000000 | 1169 | 1064.026148 | 1136.03 | 1208.033852 | 0.971796 |
| GO:0019228\_regulation\_of\_action\_potential\_in\_neuron | 36 | 0 | 0.000000 | -0.000000 | 1169 | 1064.026148 | 1136.03 | 1208.033852 | 0.971796 |
| GO:0021510\_spinal\_cord\_development | 36 | 0 | 0.000000 | -0.000000 | 1169 | 1064.026148 | 1136.03 | 1208.033852 | 0.971796 |
| GO:0022602\_ovulation\_cycle\_process | 36 | 0 | 0.000000 | -0.000000 | 1169 | 1064.026148 | 1136.03 | 1208.033852 | 0.971796 |
| GO:0030072\_peptide\_hormone\_secretion | 36 | 0 | 0.000000 | -0.000000 | 1169 | 1064.026148 | 1136.03 | 1208.033852 | 0.971796 |
| GO:0030278\_regulation\_of\_ossification | 36 | 0 | 0.000000 | -0.000000 | 1169 | 1064.026148 | 1136.03 | 1208.033852 | 0.971796 |
| GO:0033673\_negative\_regulation\_of\_kinase\_activity | 36 | 0 | 0.000000 | -0.000000 | 1169 | 1064.026148 | 1136.03 | 1208.033852 | 0.971796 |
| GO:0042742\_defense\_response\_to\_bacterium | 36 | 0 | 0.000000 | -0.000000 | 1169 | 1064.026148 | 1136.03 | 1208.033852 | 0.971796 |
| GO:0050851\_antigen\_receptor-mediated\_signaling\_pathway | 36 | 0 | 0.000000 | -0.000000 | 1169 | 1064.026148 | 1136.03 | 1208.033852 | 0.971796 |
| GO:0050900\_leukocyte\_migration | 36 | 0 | 0.000000 | -0.000000 | 1169 | 1064.026148 | 1136.03 | 1208.033852 | 0.971796 |
| GO:0006163\_purine\_nucleotide\_metabolic\_process | 73 | 0 | 0.000000 | -0.000000 | 1174 | 1067.743744 | 1139.28 | 1210.816256 | 0.970426 |
| GO:0006936\_muscle\_contraction | 73 | 0 | 0.000000 | -0.000000 | 1174 | 1067.743744 | 1139.28 | 1210.816256 | 0.970426 |
| GO:0048706\_embryonic\_skeletal\_system\_development | 73 | 0 | 0.000000 | -0.000000 | 1174 | 1067.743744 | 1139.28 | 1210.816256 | 0.970426 |
| GO:0051270\_regulation\_of\_cell\_motion | 73 | 0 | 0.000000 | -0.000000 | 1174 | 1067.743744 | 1139.28 | 1210.816256 | 0.970426 |
| GO:0051336\_regulation\_of\_hydrolase\_activity | 73 | 0 | 0.000000 | -0.000000 | 1174 | 1067.743744 | 1139.28 | 1210.816256 | 0.970426 |
| GO:0000012\_single\_strand\_break\_repair | 2 | 0 |  |  |  |  |  |  |  |  |
| GO:0000019\_regulation\_of\_mitotic\_recombination | 2 | 0 |  |  |  |  |  |  |  |  |
| GO:0000076\_DNA\_replication\_checkpoint | 2 | 0 |  |  |  |  |  |  |  |  |
| GO:0000080\_G1\_phase\_of\_mitotic\_cell\_cycle | 2 | 0 |  |  |  |  |  |  |  |  |
| GO:0000083\_regulation\_of\_transcription\_of\_G1\_S-phase\_of\_mitotic\_cell\_cycle | 2 | 0 |  |  |  |  |  |  |  |  |
| GO:0000085\_G2\_phase\_of\_mitotic\_cell\_cycle | 2 | 0 |  |  |  |  |  |  |  |  |
| GO:0000289\_nuclear-transcribed\_mRNA\_poly(A)\_tail\_shortening | 2 | 0 |  |  |  |  |  |  |  |  |
| GO:0000381\_regulation\_of\_alternative\_nuclear\_mRNA\_splicing\_\_via\_spliceosome | 2 | 0 |  |  |  |  |  |  |  |  |
| GO:0000712\_resolution\_of\_meiotic\_joint\_molecules\_as\_recombinants | 2 | 0 |  |  |  |  |  |  |  |  |
| GO:0000720\_pyrimidine\_dimer\_repair\_by\_nucleotide-excision\_repair | 2 | 0 |  |  |  |  |  |  |  |  |
| GO:0001302\_replicative\_cell\_aging | 2 | 0 |  |  |  |  |  |  |  |  |
| GO:0001306\_age-dependent\_response\_to\_oxidative\_stress | 2 | 0 |  |  |  |  |  |  |  |  |
| GO:0001514\_selenocysteine\_incorporation | 2 | 0 |  |  |  |  |  |  |  |  |
| GO:0001522\_pseudouridine\_synthesis | 2 | 0 |  |  |  |  |  |  |  |  |
| GO:0001543\_ovarian\_follicle\_rupture | 2 | 0 |  |  |  |  |  |  |  |  |
| GO:0001561\_fatty\_acid\_alpha-oxidation | 2 | 0 |  |  |  |  |  |  |  |  |
| GO:0001675\_acrosome\_assembly | 2 | 0 |  |  |  |  |  |  |  |  |
| GO:0001743\_optic\_placode\_formation | 2 | 0 |  |  |  |  |  |  |  |  |
| GO:0001767\_establishment\_of\_lymphocyte\_polarity | 2 | 0 |  |  |  |  |  |  |  |  |
| GO:0001768\_establishment\_of\_T\_cell\_polarity | 2 | 0 |  |  |  |  |  |  |  |  |
| GO:0001771\_formation\_of\_immunological\_synapse | 2 | 0 |  |  |  |  |  |  |  |  |
| GO:0001774\_microglial\_cell\_activation | 2 | 0 |  |  |  |  |  |  |  |  |
| GO:0001781\_neutrophil\_apoptosis | 2 | 0 |  |  |  |  |  |  |  |  |
| GO:0001787\_natural\_killer\_cell\_proliferation | 2 | 0 |  |  |  |  |  |  |  |  |
| GO:0001788\_antibody-dependent\_cellular\_cytotoxicity | 2 | 0 |  |  |  |  |  |  |  |  |
| GO:0001806\_type\_IV\_hypersensitivity | 2 | 0 |  |  |  |  |  |  |  |  |
| GO:0001807\_regulation\_of\_type\_IV\_hypersensitivity | 2 | 0 |  |  |  |  |  |  |  |  |
| GO:0001808\_negative\_regulation\_of\_type\_IV\_hypersensitivity | 2 | 0 |  |  |  |  |  |  |  |  |
| GO:0001823\_mesonephros\_development | 2 | 0 |  |  |  |  |  |  |  |  |
| GO:0001845\_phagolysosome\_formation | 2 | 0 |  |  |  |  |  |  |  |  |
| GO:0001866\_NK\_T\_cell\_proliferation | 2 | 0 |  |  |  |  |  |  |  |  |
| GO:0001879\_detection\_of\_yeast | 2 | 0 |  |  |  |  |  |  |  |  |
| GO:0001886\_endothelial\_cell\_morphogenesis | 2 | 0 |  |  |  |  |  |  |  |  |
| GO:0001919\_regulation\_of\_receptor\_recycling | 2 | 0 |  |  |  |  |  |  |  |  |
| GO:0001954\_positive\_regulation\_of\_cell-matrix\_adhesion | 2 | 0 |  |  |  |  |  |  |  |  |
| GO:0001977\_renal\_system\_process\_involved\_in\_regulation\_of\_blood\_volume | 2 | 0 |  |  |  |  |  |  |  |  |
| GO:0001982\_baroreceptor\_response\_to\_decreased\_systemic\_arterial\_blood\_pressure | 2 | 0 |  |  |  |  |  |  |  |  |
| GO:0001983\_baroreceptor\_response\_to\_increased\_systemic\_arterial\_blood\_pressure | 2 | 0 |  |  |  |  |  |  |  |  |
| GO:0001992\_regulation\_of\_systemic\_arterial\_blood\_pressure\_by\_vasopressin | 2 | 0 |  |  |  |  |  |  |  |  |
| GO:0001997\_positive\_regulation\_of\_the\_force\_of\_heart\_contraction\_by\_epinephrine-norepinephrine | 2 | 0 |  |  |  |  |  |  |  |  |
| GO:0001998\_angiotensin\_mediated\_vasoconstriction\_involved\_in\_regulation\_of\_systemic\_arterial\_blood\_pressure | 2 | 0 |  |  |  |  |  |  |  |  |
| GO:0001999\_renal\_response\_to\_blood\_flow\_during\_renin-angiotensin\_regulation\_of\_systemic\_arterial\_blood\_pressure | 2 | 0 |  |  |  |  |  |  |  |  |
| GO:0002018\_renin-angiotensin\_regulation\_of\_aldosterone\_production | 2 | 0 |  |  |  |  |  |  |  |  |
| GO:0002019\_regulation\_of\_renal\_output\_by\_angiotensin | 2 | 0 |  |  |  |  |  |  |  |  |
| GO:0002024\_diet\_induced\_thermogenesis | 2 | 0 |  |  |  |  |  |  |  |  |
| GO:0002025\_vasodilation\_by\_norepinephrine-epinephrine\_involved\_in\_regulation\_of\_systemic\_arterial\_blood\_pressure | 2 | 0 |  |  |  |  |  |  |  |  |
| GO:0002029\_desensitization\_of\_G-protein\_coupled\_receptor\_protein\_signaling\_pathway | 2 | 0 |  |  |  |  |  |  |  |  |
| GO:0002033\_vasodilation\_by\_angiotensin\_involved\_in\_regulation\_of\_systemic\_arterial\_blood\_pressure | 2 | 0 |  |  |  |  |  |  |  |  |
| GO:0002066\_columnar\_cuboidal\_epithelial\_cell\_development | 2 | 0 |  |  |  |  |  |  |  |  |
| GO:0002072\_optic\_cup\_morphogenesis\_involved\_in\_camera-type\_eye\_development | 2 | 0 |  |  |  |  |  |  |  |  |
| GO:0002074\_extraocular\_skeletal\_muscle\_development | 2 | 0 |  |  |  |  |  |  |  |  |
| GO:0002138\_retinoic\_acid\_biosynthetic\_process | 2 | 0 |  |  |  |  |  |  |  |  |
| GO:0002223\_stimulatory\_C-type\_lectin\_receptor\_signaling\_pathway | 2 | 0 |  |  |  |  |  |  |  |  |
| GO:0002246\_healing\_during\_inflammatory\_response | 2 | 0 |  |  |  |  |  |  |  |  |
| GO:0002251\_organ\_or\_tissue\_specific\_immune\_response | 2 | 0 |  |  |  |  |  |  |  |  |
| GO:0002327\_immature\_B\_cell\_differentiation | 2 | 0 |  |  |  |  |  |  |  |  |
| GO:0002329\_pre-B\_cell\_differentiation | 2 | 0 |  |  |  |  |  |  |  |  |
| GO:0002339\_B\_cell\_selection | 2 | 0 |  |  |  |  |  |  |  |  |
| GO:0002352\_B\_cell\_negative\_selection | 2 | 0 |  |  |  |  |  |  |  |  |
| GO:0002358\_B\_cell\_homeostatic\_proliferation | 2 | 0 |  |  |  |  |  |  |  |  |
| GO:0002385\_mucosal\_immune\_response | 2 | 0 |  |  |  |  |  |  |  |  |
| GO:0002514\_B\_cell\_tolerance\_induction | 2 | 0 |  |  |  |  |  |  |  |  |
| GO:0002523\_leukocyte\_migration\_during\_inflammatory\_response | 2 | 0 |  |  |  |  |  |  |  |  |
| GO:0002536\_respiratory\_burst\_during\_acute\_inflammatory\_response | 2 | 0 |  |  |  |  |  |  |  |  |
| GO:0002537\_production\_of\_nitric\_oxide\_during\_acute\_inflammatory\_response | 2 | 0 |  |  |  |  |  |  |  |  |
| GO:0002576\_platelet\_degranulation | 2 | 0 |  |  |  |  |  |  |  |  |
| GO:0002639\_positive\_regulation\_of\_immunoglobulin\_production | 2 | 0 |  |  |  |  |  |  |  |  |
| GO:0002661\_regulation\_of\_B\_cell\_tolerance\_induction | 2 | 0 |  |  |  |  |  |  |  |  |
| GO:0002663\_positive\_regulation\_of\_B\_cell\_tolerance\_induction | 2 | 0 |  |  |  |  |  |  |  |  |
| GO:0002676\_regulation\_of\_chronic\_inflammatory\_response | 2 | 0 |  |  |  |  |  |  |  |  |
| GO:0002679\_respiratory\_burst\_during\_defense\_response | 2 | 0 |  |  |  |  |  |  |  |  |
| GO:0002686\_negative\_regulation\_of\_leukocyte\_migration | 2 | 0 |  |  |  |  |  |  |  |  |
| GO:0002720\_positive\_regulation\_of\_cytokine\_production\_during\_immune\_response | 2 | 0 |  |  |  |  |  |  |  |  |
| GO:0002752\_cell\_surface\_pattern\_recognition\_receptor\_signaling\_pathway | 2 | 0 |  |  |  |  |  |  |  |  |
| GO:0002755\_MyD88-dependent\_toll-like\_receptor\_signaling\_pathway | 2 | 0 |  |  |  |  |  |  |  |  |
| GO:0002765\_immune\_response-inhibiting\_signal\_transduction | 2 | 0 |  |  |  |  |  |  |  |  |
| GO:0002921\_negative\_regulation\_of\_humoral\_immune\_response | 2 | 0 |  |  |  |  |  |  |  |  |
| GO:0002922\_positive\_regulation\_of\_humoral\_immune\_response | 2 | 0 |  |  |  |  |  |  |  |  |
| GO:0002924\_negative\_regulation\_of\_humoral\_immune\_response\_mediated\_by\_circulating\_immunoglobulin | 2 | 0 |  |  |  |  |  |  |  |  |
| GO:0002925\_positive\_regulation\_of\_humoral\_immune\_response\_mediated\_by\_circulating\_immunoglobulin | 2 | 0 |  |  |  |  |  |  |  |  |
| GO:0003057\_regulation\_of\_the\_force\_of\_heart\_contraction\_by\_chemical\_signal | 2 | 0 |  |  |  |  |  |  |  |  |
| GO:0003099\_positive\_regulation\_of\_the\_force\_of\_heart\_contraction\_by\_chemical\_signal | 2 | 0 |  |  |  |  |  |  |  |  |
| GO:0005981\_regulation\_of\_glycogen\_catabolic\_process | 2 | 0 |  |  |  |  |  |  |  |  |
| GO:0006021\_inositol\_biosynthetic\_process | 2 | 0 |  |  |  |  |  |  |  |  |
| GO:0006042\_glucosamine\_biosynthetic\_process | 2 | 0 |  |  |  |  |  |  |  |  |
| GO:0006045\_N-acetylglucosamine\_biosynthetic\_process | 2 | 0 |  |  |  |  |  |  |  |  |
| GO:0006048\_UDP-N-acetylglucosamine\_biosynthetic\_process | 2 | 0 |  |  |  |  |  |  |  |  |
| GO:0006054\_N-acetylneuraminate\_metabolic\_process | 2 | 0 |  |  |  |  |  |  |  |  |
| GO:0006059\_hexitol\_metabolic\_process | 2 | 0 |  |  |  |  |  |  |  |  |
| GO:0006063\_uronic\_acid\_metabolic\_process | 2 | 0 |  |  |  |  |  |  |  |  |
| GO:0006068\_ethanol\_catabolic\_process | 2 | 0 |  |  |  |  |  |  |  |  |
| GO:0006083\_acetate\_metabolic\_process | 2 | 0 |  |  |  |  |  |  |  |  |
| GO:0006089\_lactate\_metabolic\_process | 2 | 0 |  |  |  |  |  |  |  |  |
| GO:0006105\_succinate\_metabolic\_process | 2 | 0 |  |  |  |  |  |  |  |  |
| GO:0006106\_fumarate\_metabolic\_process | 2 | 0 |  |  |  |  |  |  |  |  |
| GO:0006110\_regulation\_of\_glycolysis | 2 | 0 |  |  |  |  |  |  |  |  |
| GO:0006113\_fermentation | 2 | 0 |  |  |  |  |  |  |  |  |
| GO:0006114\_glycerol\_biosynthetic\_process | 2 | 0 |  |  |  |  |  |  |  |  |
| GO:0006122\_mitochondrial\_electron\_transport\_\_ubiquinol\_to\_cytochrome\_c | 2 | 0 |  |  |  |  |  |  |  |  |
| GO:0006152\_purine\_nucleoside\_catabolic\_process | 2 | 0 |  |  |  |  |  |  |  |  |
| GO:0006168\_adenine\_salvage | 2 | 0 |  |  |  |  |  |  |  |  |
| GO:0006200\_ATP\_catabolic\_process | 2 | 0 |  |  |  |  |  |  |  |  |
| GO:0006206\_pyrimidine\_base\_metabolic\_process | 2 | 0 |  |  |  |  |  |  |  |  |
| GO:0006213\_pyrimidine\_nucleoside\_metabolic\_process | 2 | 0 |  |  |  |  |  |  |  |  |
| GO:0006265\_DNA\_topological\_change | 2 | 0 |  |  |  |  |  |  |  |  |
| GO:0006278\_RNA-dependent\_DNA\_replication | 2 | 0 |  |  |  |  |  |  |  |  |
| GO:0006312\_mitotic\_recombination | 2 | 0 |  |  |  |  |  |  |  |  |
| GO:0006398\_histone\_mRNA\_3'-end\_processing | 2 | 0 |  |  |  |  |  |  |  |  |
| GO:0006418\_tRNA\_aminoacylation\_for\_protein\_translation | 2 | 0 |  |  |  |  |  |  |  |  |
| GO:0006451\_translational\_readthrough | 2 | 0 |  |  |  |  |  |  |  |  |
| GO:0006477\_protein\_amino\_acid\_sulfation | 2 | 0 |  |  |  |  |  |  |  |  |
| GO:0006482\_protein\_amino\_acid\_demethylation | 2 | 0 |  |  |  |  |  |  |  |  |
| GO:0006499\_N-terminal\_protein\_myristoylation | 2 | 0 |  |  |  |  |  |  |  |  |
| GO:0006525\_arginine\_metabolic\_process | 2 | 0 |  |  |  |  |  |  |  |  |
| GO:0006527\_arginine\_catabolic\_process | 2 | 0 |  |  |  |  |  |  |  |  |
| GO:0006532\_aspartate\_biosynthetic\_process | 2 | 0 |  |  |  |  |  |  |  |  |
| GO:0006538\_glutamate\_catabolic\_process | 2 | 0 |  |  |  |  |  |  |  |  |
| GO:0006558\_L-phenylalanine\_metabolic\_process | 2 | 0 |  |  |  |  |  |  |  |  |
| GO:0006563\_L-serine\_metabolic\_process | 2 | 0 |  |  |  |  |  |  |  |  |
| GO:0006566\_threonine\_metabolic\_process | 2 | 0 |  |  |  |  |  |  |  |  |
| GO:0006568\_tryptophan\_metabolic\_process | 2 | 0 |  |  |  |  |  |  |  |  |
| GO:0006583\_melanin\_biosynthetic\_process\_from\_tyrosine | 2 | 0 |  |  |  |  |  |  |  |  |
| GO:0006600\_creatine\_metabolic\_process | 2 | 0 |  |  |  |  |  |  |  |  |
| GO:0006603\_phosphocreatine\_metabolic\_process | 2 | 0 |  |  |  |  |  |  |  |  |
| GO:0006610\_ribosomal\_protein\_import\_into\_nucleus | 2 | 0 |  |  |  |  |  |  |  |  |
| GO:0006642\_triglyceride\_mobilization | 2 | 0 |  |  |  |  |  |  |  |  |
| GO:0006649\_phospholipid\_transfer\_to\_membrane | 2 | 0 |  |  |  |  |  |  |  |  |
| GO:0006681\_galactosylceramide\_metabolic\_process | 2 | 0 |  |  |  |  |  |  |  |  |
| GO:0006686\_sphingomyelin\_biosynthetic\_process | 2 | 0 |  |  |  |  |  |  |  |  |
| GO:0006702\_androgen\_biosynthetic\_process | 2 | 0 |  |  |  |  |  |  |  |  |
| GO:0006750\_glutathione\_biosynthetic\_process | 2 | 0 |  |  |  |  |  |  |  |  |
| GO:0006760\_folic\_acid\_and\_derivative\_metabolic\_process | 2 | 0 |  |  |  |  |  |  |  |  |
| GO:0006808\_regulation\_of\_nitrogen\_utilization | 2 | 0 |  |  |  |  |  |  |  |  |
| GO:0006868\_glutamine\_transport | 2 | 0 |  |  |  |  |  |  |  |  |
| GO:0006907\_pinocytosis | 2 | 0 |  |  |  |  |  |  |  |  |
| GO:0006925\_inflammatory\_cell\_apoptosis | 2 | 0 |  |  |  |  |  |  |  |  |
| GO:0006977\_DNA\_damage\_response\_\_signal\_transduction\_by\_p53\_class\_mediator\_resulting\_in\_cell\_cycle\_arrest | 2 | 0 |  |  |  |  |  |  |  |  |
| GO:0006991\_response\_to\_sterol\_depletion | 2 | 0 |  |  |  |  |  |  |  |  |
| GO:0007004\_telomere\_maintenance\_via\_telomerase | 2 | 0 |  |  |  |  |  |  |  |  |
| GO:0007020\_microtubule\_nucleation | 2 | 0 |  |  |  |  |  |  |  |  |
| GO:0007030\_Golgi\_organization | 2 | 0 |  |  |  |  |  |  |  |  |
| GO:0007035\_vacuolar\_acidification | 2 | 0 |  |  |  |  |  |  |  |  |
| GO:0007042\_lysosomal\_lumen\_acidification | 2 | 0 |  |  |  |  |  |  |  |  |
| GO:0007060\_male\_meiosis\_chromosome\_segregation | 2 | 0 |  |  |  |  |  |  |  |  |
| GO:0007089\_traversing\_start\_control\_point\_of\_mitotic\_cell\_cycle | 2 | 0 |  |  |  |  |  |  |  |  |
| GO:0007094\_mitotic\_cell\_cycle\_spindle\_assembly\_checkpoint | 2 | 0 |  |  |  |  |  |  |  |  |
| GO:0007097\_nuclear\_migration | 2 | 0 |  |  |  |  |  |  |  |  |
| GO:0007100\_mitotic\_centrosome\_separation | 2 | 0 |  |  |  |  |  |  |  |  |
| GO:0007132\_meiotic\_metaphase\_I | 2 | 0 |  |  |  |  |  |  |  |  |
| GO:0007171\_activation\_of\_transmembrane\_receptor\_protein\_tyrosine\_kinase\_activity | 2 | 0 |  |  |  |  |  |  |  |  |
| GO:0007182\_common-partner\_SMAD\_protein\_phosphorylation | 2 | 0 |  |  |  |  |  |  |  |  |
| GO:0007185\_transmembrane\_receptor\_protein\_tyrosine\_phosphatase\_signaling\_pathway | 2 | 0 |  |  |  |  |  |  |  |  |
| GO:0007205\_activation\_of\_protein\_kinase\_C\_activity\_by\_G-protein\_coupled\_receptor\_protein\_signaling\_pathway | 2 | 0 |  |  |  |  |  |  |  |  |
| GO:0007210\_serotonin\_receptor\_signaling\_pathway | 2 | 0 |  |  |  |  |  |  |  |  |
| GO:0007220\_Notch\_receptor\_processing | 2 | 0 |  |  |  |  |  |  |  |  |
| GO:0007256\_activation\_of\_JNKK\_activity | 2 | 0 |  |  |  |  |  |  |  |  |
| GO:0007258\_JUN\_phosphorylation | 2 | 0 |  |  |  |  |  |  |  |  |
| GO:0007263\_nitric\_oxide\_mediated\_signal\_transduction | 2 | 0 |  |  |  |  |  |  |  |  |
| GO:0007289\_spermatid\_nucleus\_differentiation | 2 | 0 |  |  |  |  |  |  |  |  |
| GO:0007343\_egg\_activation | 2 | 0 |  |  |  |  |  |  |  |  |
| GO:0007351\_tripartite\_regional\_subdivision | 2 | 0 |  |  |  |  |  |  |  |  |
| GO:0007418\_ventral\_midline\_development | 2 | 0 |  |  |  |  |  |  |  |  |
| GO:0007494\_midgut\_development | 2 | 0 |  |  |  |  |  |  |  |  |
| GO:0007549\_dosage\_compensation | 2 | 0 |  |  |  |  |  |  |  |  |
| GO:0007571\_age-dependent\_general\_metabolic\_decline | 2 | 0 |  |  |  |  |  |  |  |  |
| GO:0007603\_phototransduction\_\_visible\_light | 2 | 0 |  |  |  |  |  |  |  |  |
| GO:0007619\_courtship\_behavior | 2 | 0 |  |  |  |  |  |  |  |  |
| GO:0008065\_establishment\_of\_blood-nerve\_barrier | 2 | 0 |  |  |  |  |  |  |  |  |
| GO:0008089\_anterograde\_axon\_cargo\_transport | 2 | 0 |  |  |  |  |  |  |  |  |
| GO:0008210\_estrogen\_metabolic\_process | 2 | 0 |  |  |  |  |  |  |  |  |
| GO:0008212\_mineralocorticoid\_metabolic\_process | 2 | 0 |  |  |  |  |  |  |  |  |
| GO:0008214\_protein\_amino\_acid\_dealkylation | 2 | 0 |  |  |  |  |  |  |  |  |
| GO:0008228\_opsonization | 2 | 0 |  |  |  |  |  |  |  |  |
| GO:0008272\_sulfate\_transport | 2 | 0 |  |  |  |  |  |  |  |  |
| GO:0008291\_acetylcholine\_metabolic\_process | 2 | 0 |  |  |  |  |  |  |  |  |
| GO:0008298\_intracellular\_mRNA\_localization | 2 | 0 |  |  |  |  |  |  |  |  |
| GO:0008334\_histone\_mRNA\_metabolic\_process | 2 | 0 |  |  |  |  |  |  |  |  |
| GO:0008356\_asymmetric\_cell\_division | 2 | 0 |  |  |  |  |  |  |  |  |
| GO:0008582\_regulation\_of\_synaptic\_growth\_at\_neuromuscular\_junction | 2 | 0 |  |  |  |  |  |  |  |  |
| GO:0008594\_photoreceptor\_cell\_morphogenesis | 2 | 0 |  |  |  |  |  |  |  |  |
| GO:0008595\_determination\_of\_anterior\_posterior\_axis\_\_embryo | 2 | 0 |  |  |  |  |  |  |  |  |
| GO:0008608\_attachment\_of\_spindle\_microtubules\_to\_kinetochore | 2 | 0 |  |  |  |  |  |  |  |  |
| GO:0008616\_queuosine\_biosynthetic\_process | 2 | 0 |  |  |  |  |  |  |  |  |
| GO:0008617\_guanosine\_metabolic\_process | 2 | 0 |  |  |  |  |  |  |  |  |
| GO:0008618\_7-methylguanosine\_metabolic\_process | 2 | 0 |  |  |  |  |  |  |  |  |
| GO:0008634\_negative\_regulation\_of\_survival\_gene\_product\_expression | 2 | 0 |  |  |  |  |  |  |  |  |
| GO:0009048\_dosage\_compensation\_\_by\_inactivation\_of\_X\_chromosome | 2 | 0 |  |  |  |  |  |  |  |  |
| GO:0009070\_serine\_family\_amino\_acid\_biosynthetic\_process | 2 | 0 |  |  |  |  |  |  |  |  |
| GO:0009071\_serine\_family\_amino\_acid\_catabolic\_process | 2 | 0 |  |  |  |  |  |  |  |  |
| GO:0009074\_aromatic\_amino\_acid\_family\_catabolic\_process | 2 | 0 |  |  |  |  |  |  |  |  |
| GO:0009083\_branched\_chain\_family\_amino\_acid\_catabolic\_process | 2 | 0 |  |  |  |  |  |  |  |  |
| GO:0009093\_cysteine\_catabolic\_process | 2 | 0 |  |  |  |  |  |  |  |  |
| GO:0009120\_deoxyribonucleoside\_metabolic\_process | 2 | 0 |  |  |  |  |  |  |  |  |
| GO:0009125\_nucleoside\_monophosphate\_catabolic\_process | 2 | 0 |  |  |  |  |  |  |  |  |
| GO:0009126\_purine\_nucleoside\_monophosphate\_metabolic\_process | 2 | 0 |  |  |  |  |  |  |  |  |
| GO:0009142\_nucleoside\_triphosphate\_biosynthetic\_process | 2 | 0 |  |  |  |  |  |  |  |  |
| GO:0009161\_ribonucleoside\_monophosphate\_metabolic\_process | 2 | 0 |  |  |  |  |  |  |  |  |
| GO:0009164\_nucleoside\_catabolic\_process | 2 | 0 |  |  |  |  |  |  |  |  |
| GO:0009167\_purine\_ribonucleoside\_monophosphate\_metabolic\_process | 2 | 0 |  |  |  |  |  |  |  |  |
| GO:0009202\_deoxyribonucleoside\_triphosphate\_biosynthetic\_process | 2 | 0 |  |  |  |  |  |  |  |  |
| GO:0009203\_ribonucleoside\_triphosphate\_catabolic\_process | 2 | 0 |  |  |  |  |  |  |  |  |
| GO:0009207\_purine\_ribonucleoside\_triphosphate\_catabolic\_process | 2 | 0 |  |  |  |  |  |  |  |  |
| GO:0009219\_pyrimidine\_deoxyribonucleotide\_metabolic\_process | 2 | 0 |  |  |  |  |  |  |  |  |
| GO:0009265\_2'-deoxyribonucleotide\_biosynthetic\_process | 2 | 0 |  |  |  |  |  |  |  |  |
| GO:0009268\_response\_to\_pH | 2 | 0 |  |  |  |  |  |  |  |  |
| GO:0009313\_oligosaccharide\_catabolic\_process | 2 | 0 |  |  |  |  |  |  |  |  |
| GO:0009395\_phospholipid\_catabolic\_process | 2 | 0 |  |  |  |  |  |  |  |  |
| GO:0009435\_NAD\_biosynthetic\_process | 2 | 0 |  |  |  |  |  |  |  |  |
| GO:0009608\_response\_to\_symbiont | 2 | 0 |  |  |  |  |  |  |  |  |
| GO:0009609\_response\_to\_symbiotic\_bacterium | 2 | 0 |  |  |  |  |  |  |  |  |
| GO:0009649\_entrainment\_of\_circadian\_clock | 2 | 0 |  |  |  |  |  |  |  |  |
| GO:0009996\_negative\_regulation\_of\_cell\_fate\_specification | 2 | 0 |  |  |  |  |  |  |  |  |
| GO:0010002\_cardioblast\_differentiation | 2 | 0 |  |  |  |  |  |  |  |  |
| GO:0010149\_senescence | 2 | 0 |  |  |  |  |  |  |  |  |
| GO:0010225\_response\_to\_UV-C | 2 | 0 |  |  |  |  |  |  |  |  |
| GO:0010389\_regulation\_of\_G2\_M\_transition\_of\_mitotic\_cell\_cycle | 2 | 0 |  |  |  |  |  |  |  |  |
| GO:0010458\_exit\_from\_mitosis | 2 | 0 |  |  |  |  |  |  |  |  |
| GO:0010459\_negative\_regulation\_of\_heart\_rate | 2 | 0 |  |  |  |  |  |  |  |  |
| GO:0010559\_regulation\_of\_glycoprotein\_biosynthetic\_process | 2 | 0 |  |  |  |  |  |  |  |  |
| GO:0010633\_negative\_regulation\_of\_epithelial\_cell\_migration | 2 | 0 |  |  |  |  |  |  |  |  |
| GO:0010677\_negative\_regulation\_of\_cellular\_carbohydrate\_metabolic\_process | 2 | 0 |  |  |  |  |  |  |  |  |
| GO:0010742\_foam\_cell\_differentiation | 2 | 0 |  |  |  |  |  |  |  |  |
| GO:0010743\_regulation\_of\_foam\_cell\_differentiation | 2 | 0 |  |  |  |  |  |  |  |  |
| GO:0010744\_positive\_regulation\_of\_foam\_cell\_differentiation | 2 | 0 |  |  |  |  |  |  |  |  |
| GO:0010765\_positive\_regulation\_of\_sodium\_ion\_transport | 2 | 0 |  |  |  |  |  |  |  |  |
| GO:0010766\_negative\_regulation\_of\_sodium\_ion\_transport | 2 | 0 |  |  |  |  |  |  |  |  |
| GO:0010771\_negative\_regulation\_of\_cell\_morphogenesis\_involved\_in\_differentiation | 2 | 0 |  |  |  |  |  |  |  |  |
| GO:0010824\_regulation\_of\_centrosome\_duplication | 2 | 0 |  |  |  |  |  |  |  |  |
| GO:0010833\_telomere\_maintenance\_via\_telomere\_lengthening | 2 | 0 |  |  |  |  |  |  |  |  |
| GO:0010872\_regulation\_of\_cholesterol\_esterification | 2 | 0 |  |  |  |  |  |  |  |  |
| GO:0010878\_cholesterol\_storage | 2 | 0 |  |  |  |  |  |  |  |  |
| GO:0010885\_regulation\_of\_cholesterol\_storage | 2 | 0 |  |  |  |  |  |  |  |  |
| GO:0010886\_positive\_regulation\_of\_cholesterol\_storage | 2 | 0 |  |  |  |  |  |  |  |  |
| GO:0010891\_negative\_regulation\_of\_sequestering\_of\_triglyceride | 2 | 0 |  |  |  |  |  |  |  |  |
| GO:0010896\_regulation\_of\_triglyceride\_catabolic\_process | 2 | 0 |  |  |  |  |  |  |  |  |
| GO:0010898\_positive\_regulation\_of\_triglyceride\_catabolic\_process | 2 | 0 |  |  |  |  |  |  |  |  |
| GO:0010907\_positive\_regulation\_of\_glucose\_metabolic\_process | 2 | 0 |  |  |  |  |  |  |  |  |
| GO:0014028\_notochord\_formation | 2 | 0 |  |  |  |  |  |  |  |  |
| GO:0014048\_regulation\_of\_glutamate\_secretion | 2 | 0 |  |  |  |  |  |  |  |  |
| GO:0014052\_regulation\_of\_gamma-aminobutyric\_acid\_secretion | 2 | 0 |  |  |  |  |  |  |  |  |
| GO:0014054\_positive\_regulation\_of\_gamma-aminobutyric\_acid\_secretion | 2 | 0 |  |  |  |  |  |  |  |  |
| GO:0014055\_acetylcholine\_secretion | 2 | 0 |  |  |  |  |  |  |  |  |
| GO:0014056\_regulation\_of\_acetylcholine\_secretion | 2 | 0 |  |  |  |  |  |  |  |  |
| GO:0014067\_negative\_regulation\_of\_phosphoinositide\_3-kinase\_cascade | 2 | 0 |  |  |  |  |  |  |  |  |
| GO:0014745\_negative\_regulation\_of\_muscle\_adaptation | 2 | 0 |  |  |  |  |  |  |  |  |
| GO:0014829\_vascular\_smooth\_muscle\_contraction | 2 | 0 |  |  |  |  |  |  |  |  |
| GO:0014850\_response\_to\_muscle\_activity | 2 | 0 |  |  |  |  |  |  |  |  |
| GO:0014866\_skeletal\_myofibril\_assembly | 2 | 0 |  |  |  |  |  |  |  |  |
| GO:0014888\_striated\_muscle\_adaptation | 2 | 0 |  |  |  |  |  |  |  |  |
| GO:0014916\_regulation\_of\_lung\_blood\_pressure | 2 | 0 |  |  |  |  |  |  |  |  |
| GO:0015671\_oxygen\_transport | 2 | 0 |  |  |  |  |  |  |  |  |
| GO:0015696\_ammonium\_transport | 2 | 0 |  |  |  |  |  |  |  |  |
| GO:0015732\_prostaglandin\_transport | 2 | 0 |  |  |  |  |  |  |  |  |
| GO:0015819\_lysine\_transport | 2 | 0 |  |  |  |  |  |  |  |  |
| GO:0015840\_urea\_transport | 2 | 0 |  |  |  |  |  |  |  |  |
| GO:0015860\_purine\_nucleoside\_transport | 2 | 0 |  |  |  |  |  |  |  |  |
| GO:0015870\_acetylcholine\_transport | 2 | 0 |  |  |  |  |  |  |  |  |
| GO:0015937\_coenzyme\_A\_biosynthetic\_process | 2 | 0 |  |  |  |  |  |  |  |  |
| GO:0016045\_detection\_of\_bacterium | 2 | 0 |  |  |  |  |  |  |  |  |
| GO:0016046\_detection\_of\_fungus | 2 | 0 |  |  |  |  |  |  |  |  |
| GO:0016080\_synaptic\_vesicle\_targeting | 2 | 0 |  |  |  |  |  |  |  |  |
| GO:0016199\_axon\_midline\_choice\_point\_recognition | 2 | 0 |  |  |  |  |  |  |  |  |
| GO:0016226\_iron-sulfur\_cluster\_assembly | 2 | 0 |  |  |  |  |  |  |  |  |
| GO:0016233\_telomere\_capping | 2 | 0 |  |  |  |  |  |  |  |  |
| GO:0016242\_negative\_regulation\_of\_macroautophagy | 2 | 0 |  |  |  |  |  |  |  |  |
| GO:0016441\_posttranscriptional\_gene\_silencing | 2 | 0 |  |  |  |  |  |  |  |  |
| GO:0016540\_protein\_autoprocessing | 2 | 0 |  |  |  |  |  |  |  |  |
| GO:0016558\_protein\_import\_into\_peroxisome\_matrix | 2 | 0 |  |  |  |  |  |  |  |  |
| GO:0016572\_histone\_phosphorylation | 2 | 0 |  |  |  |  |  |  |  |  |
| GO:0016577\_histone\_demethylation | 2 | 0 |  |  |  |  |  |  |  |  |
| GO:0016584\_nucleosome\_positioning | 2 | 0 |  |  |  |  |  |  |  |  |
| GO:0016926\_protein\_desumoylation | 2 | 0 |  |  |  |  |  |  |  |  |
| GO:0017014\_protein\_amino\_acid\_nitrosylation | 2 | 0 |  |  |  |  |  |  |  |  |
| GO:0017144\_drug\_metabolic\_process | 2 | 0 |  |  |  |  |  |  |  |  |
| GO:0018094\_protein\_polyglycylation | 2 | 0 |  |  |  |  |  |  |  |  |
| GO:0018119\_peptidyl-cysteine\_S-nitrosylation | 2 | 0 |  |  |  |  |  |  |  |  |
| GO:0018125\_peptidyl-cysteine\_methylation | 2 | 0 |  |  |  |  |  |  |  |  |
| GO:0018205\_peptidyl-lysine\_modification | 2 | 0 |  |  |  |  |  |  |  |  |
| GO:0018319\_protein\_amino\_acid\_myristoylation | 2 | 0 |  |  |  |  |  |  |  |  |
| GO:0018377\_protein\_myristoylation | 2 | 0 |  |  |  |  |  |  |  |  |
| GO:0018401\_peptidyl-proline\_hydroxylation\_to\_4-hydroxy-L-proline | 2 | 0 |  |  |  |  |  |  |  |  |
| GO:0018993\_somatic\_sex\_determination | 2 | 0 |  |  |  |  |  |  |  |  |
| GO:0019067\_viral\_assembly\_\_maturation\_\_egress\_\_and\_release | 2 | 0 |  |  |  |  |  |  |  |  |
| GO:0019322\_pentose\_biosynthetic\_process | 2 | 0 |  |  |  |  |  |  |  |  |
| GO:0019370\_leukotriene\_biosynthetic\_process | 2 | 0 |  |  |  |  |  |  |  |  |
| GO:0019374\_galactolipid\_metabolic\_process | 2 | 0 |  |  |  |  |  |  |  |  |
| GO:0019401\_alditol\_biosynthetic\_process | 2 | 0 |  |  |  |  |  |  |  |  |
| GO:0019448\_L-cysteine\_catabolic\_process | 2 | 0 |  |  |  |  |  |  |  |  |
| GO:0019452\_L-cysteine\_catabolic\_process\_to\_taurine | 2 | 0 |  |  |  |  |  |  |  |  |
| GO:0019471\_4-hydroxyproline\_metabolic\_process | 2 | 0 |  |  |  |  |  |  |  |  |
| GO:0019511\_peptidyl-proline\_hydroxylation | 2 | 0 |  |  |  |  |  |  |  |  |
| GO:0019550\_glutamate\_catabolic\_process\_to\_aspartate | 2 | 0 |  |  |  |  |  |  |  |  |
| GO:0019551\_glutamate\_catabolic\_process\_to\_2-oxoglutarate | 2 | 0 |  |  |  |  |  |  |  |  |
| GO:0019585\_glucuronate\_metabolic\_process | 2 | 0 |  |  |  |  |  |  |  |  |
| GO:0019730\_antimicrobial\_humoral\_response | 2 | 0 |  |  |  |  |  |  |  |  |
| GO:0019740\_nitrogen\_utilization | 2 | 0 |  |  |  |  |  |  |  |  |
| GO:0019853\_L-ascorbic\_acid\_biosynthetic\_process | 2 | 0 |  |  |  |  |  |  |  |  |
| GO:0021506\_anterior\_neuropore\_closure | 2 | 0 |  |  |  |  |  |  |  |  |
| GO:0021524\_visceral\_motor\_neuron\_differentiation | 2 | 0 |  |  |  |  |  |  |  |  |
| GO:0021526\_medial\_motor\_column\_neuron\_differentiation | 2 | 0 |  |  |  |  |  |  |  |  |
| GO:0021557\_oculomotor\_nerve\_development | 2 | 0 |  |  |  |  |  |  |  |  |
| GO:0021558\_trochlear\_nerve\_development | 2 | 0 |  |  |  |  |  |  |  |  |
| GO:0021562\_vestibulocochlear\_nerve\_development | 2 | 0 |  |  |  |  |  |  |  |  |
| GO:0021568\_rhombomere\_2\_development | 2 | 0 |  |  |  |  |  |  |  |  |
| GO:0021578\_hindbrain\_maturation | 2 | 0 |  |  |  |  |  |  |  |  |
| GO:0021593\_rhombomere\_morphogenesis | 2 | 0 |  |  |  |  |  |  |  |  |
| GO:0021626\_central\_nervous\_system\_maturation | 2 | 0 |  |  |  |  |  |  |  |  |
| GO:0021658\_rhombomere\_3\_morphogenesis | 2 | 0 |  |  |  |  |  |  |  |  |
| GO:0021754\_facial\_nucleus\_development | 2 | 0 |  |  |  |  |  |  |  |  |
| GO:0021775\_smoothened\_signaling\_pathway\_involved\_in\_ventral\_spinal\_cord\_interneuron\_specification | 2 | 0 |  |  |  |  |  |  |  |  |
| GO:0021776\_smoothened\_signaling\_pathway\_involved\_in\_spinal\_cord\_motor\_neuron\_cell\_fate\_specification | 2 | 0 |  |  |  |  |  |  |  |  |
| GO:0021796\_cerebral\_cortex\_regionalization | 2 | 0 |  |  |  |  |  |  |  |  |
| GO:0021831\_embryonic\_olfactory\_bulb\_interneuron\_precursor\_migration | 2 | 0 |  |  |  |  |  |  |  |  |
| GO:0021869\_forebrain\_ventricular\_zone\_progenitor\_cell\_division | 2 | 0 |  |  |  |  |  |  |  |  |
| GO:0021873\_forebrain\_neuroblast\_division | 2 | 0 |  |  |  |  |  |  |  |  |
| GO:0021882\_regulation\_of\_transcription\_from\_RNA\_polymerase\_II\_promoter\_involved\_in\_forebrain\_neuron\_fate\_commitment | 2 | 0 |  |  |  |  |  |  |  |  |
| GO:0021893\_cerebral\_cortex\_GABAergic\_interneuron\_fate\_commitment | 2 | 0 |  |  |  |  |  |  |  |  |
| GO:0021898\_commitment\_of\_multipotent\_stem\_cells\_to\_the\_neuronal\_lineage\_in\_the\_forebrain | 2 | 0 |  |  |  |  |  |  |  |  |
| GO:0021932\_hindbrain\_radial\_glia\_guided\_cell\_migration | 2 | 0 |  |  |  |  |  |  |  |  |
| GO:0021965\_spinal\_cord\_ventral\_commissure\_morphogenesis | 2 | 0 |  |  |  |  |  |  |  |  |
| GO:0021985\_neurohypophysis\_development | 2 | 0 |  |  |  |  |  |  |  |  |
| GO:0021990\_neural\_plate\_formation | 2 | 0 |  |  |  |  |  |  |  |  |
| GO:0021995\_neuropore\_closure | 2 | 0 |  |  |  |  |  |  |  |  |
| GO:0022028\_tangential\_migration\_from\_the\_subventricular\_zone\_to\_the\_olfactory\_bulb | 2 | 0 |  |  |  |  |  |  |  |  |
| GO:0022401\_adaptation\_of\_signaling\_pathway | 2 | 0 |  |  |  |  |  |  |  |  |
| GO:0022408\_negative\_regulation\_of\_cell-cell\_adhesion | 2 | 0 |  |  |  |  |  |  |  |  |
| GO:0022410\_circadian\_sleep\_wake\_cycle\_process | 2 | 0 |  |  |  |  |  |  |  |  |
| GO:0030046\_parallel\_actin\_filament\_bundle\_formation | 2 | 0 |  |  |  |  |  |  |  |  |
| GO:0030049\_muscle\_filament\_sliding | 2 | 0 |  |  |  |  |  |  |  |  |
| GO:0030050\_vesicle\_transport\_along\_actin\_filament | 2 | 0 |  |  |  |  |  |  |  |  |
| GO:0030071\_regulation\_of\_mitotic\_metaphase\_anaphase\_transition | 2 | 0 |  |  |  |  |  |  |  |  |
| GO:0030147\_natriuresis | 2 | 0 |  |  |  |  |  |  |  |  |
| GO:0030174\_regulation\_of\_DNA\_replication\_initiation | 2 | 0 |  |  |  |  |  |  |  |  |
| GO:0030202\_heparin\_metabolic\_process | 2 | 0 |  |  |  |  |  |  |  |  |
| GO:0030219\_megakaryocyte\_differentiation | 2 | 0 |  |  |  |  |  |  |  |  |
| GO:0030223\_neutrophil\_differentiation | 2 | 0 |  |  |  |  |  |  |  |  |
| GO:0030240\_muscle\_thin\_filament\_assembly | 2 | 0 |  |  |  |  |  |  |  |  |
| GO:0030259\_lipid\_glycosylation | 2 | 0 |  |  |  |  |  |  |  |  |
| GO:0030397\_membrane\_disassembly | 2 | 0 |  |  |  |  |  |  |  |  |
| GO:0030502\_negative\_regulation\_of\_bone\_mineralization | 2 | 0 |  |  |  |  |  |  |  |  |
| GO:0030644\_cellular\_chloride\_ion\_homeostasis | 2 | 0 |  |  |  |  |  |  |  |  |
| GO:0030825\_positive\_regulation\_of\_cGMP\_metabolic\_process | 2 | 0 |  |  |  |  |  |  |  |  |
| GO:0030828\_positive\_regulation\_of\_cGMP\_biosynthetic\_process | 2 | 0 |  |  |  |  |  |  |  |  |
| GO:0030835\_negative\_regulation\_of\_actin\_filament\_depolymerization | 2 | 0 |  |  |  |  |  |  |  |  |
| GO:0030837\_negative\_regulation\_of\_actin\_filament\_polymerization | 2 | 0 |  |  |  |  |  |  |  |  |
| GO:0030852\_regulation\_of\_granulocyte\_differentiation | 2 | 0 |  |  |  |  |  |  |  |  |
| GO:0030885\_regulation\_of\_myeloid\_dendritic\_cell\_activation | 2 | 0 |  |  |  |  |  |  |  |  |
| GO:0030910\_olfactory\_placode\_formation | 2 | 0 |  |  |  |  |  |  |  |  |
| GO:0030948\_negative\_regulation\_of\_vascular\_endothelial\_growth\_factor\_receptor\_signaling\_pathway | 2 | 0 |  |  |  |  |  |  |  |  |
| GO:0030953\_spindle\_astral\_microtubule\_organization | 2 | 0 |  |  |  |  |  |  |  |  |
| GO:0031050\_dsRNA\_fragmentation | 2 | 0 |  |  |  |  |  |  |  |  |
| GO:0031061\_negative\_regulation\_of\_histone\_methylation | 2 | 0 |  |  |  |  |  |  |  |  |
| GO:0031119\_tRNA\_pseudouridine\_synthesis | 2 | 0 |  |  |  |  |  |  |  |  |
| GO:0031163\_metallo-sulfur\_cluster\_assembly | 2 | 0 |  |  |  |  |  |  |  |  |
| GO:0031223\_auditory\_behavior | 2 | 0 |  |  |  |  |  |  |  |  |
| GO:0031296\_B\_cell\_costimulation | 2 | 0 |  |  |  |  |  |  |  |  |
| GO:0031338\_regulation\_of\_vesicle\_fusion | 2 | 0 |  |  |  |  |  |  |  |  |
| GO:0031577\_spindle\_checkpoint | 2 | 0 |  |  |  |  |  |  |  |  |
| GO:0031629\_synaptic\_vesicle\_fusion\_to\_presynaptic\_membrane | 2 | 0 |  |  |  |  |  |  |  |  |
| GO:0031630\_regulation\_of\_synaptic\_vesicle\_fusion\_to\_presynaptic\_membrane | 2 | 0 |  |  |  |  |  |  |  |  |
| GO:0031664\_regulation\_of\_lipopolysaccharide-mediated\_signaling\_pathway | 2 | 0 |  |  |  |  |  |  |  |  |
| GO:0031670\_cellular\_response\_to\_nutrient | 2 | 0 |  |  |  |  |  |  |  |  |
| GO:0031848\_protection\_from\_non-homologous\_end\_joining\_at\_telomere | 2 | 0 |  |  |  |  |  |  |  |  |
| GO:0031946\_regulation\_of\_glucocorticoid\_biosynthetic\_process | 2 | 0 |  |  |  |  |  |  |  |  |
| GO:0031952\_regulation\_of\_protein\_amino\_acid\_autophosphorylation | 2 | 0 |  |  |  |  |  |  |  |  |
| GO:0031953\_negative\_regulation\_of\_protein\_amino\_acid\_autophosphorylation | 2 | 0 |  |  |  |  |  |  |  |  |
| GO:0031958\_corticosteroid\_receptor\_signaling\_pathway | 2 | 0 |  |  |  |  |  |  |  |  |
| GO:0031987\_locomotion\_involved\_in\_locomotory\_behavior | 2 | 0 |  |  |  |  |  |  |  |  |
| GO:0032096\_negative\_regulation\_of\_response\_to\_food | 2 | 0 |  |  |  |  |  |  |  |  |
| GO:0032099\_negative\_regulation\_of\_appetite | 2 | 0 |  |  |  |  |  |  |  |  |
| GO:0032106\_positive\_regulation\_of\_response\_to\_extracellular\_stimulus | 2 | 0 |  |  |  |  |  |  |  |  |
| GO:0032109\_positive\_regulation\_of\_response\_to\_nutrient\_levels | 2 | 0 |  |  |  |  |  |  |  |  |
| GO:0032226\_positive\_regulation\_of\_synaptic\_transmission\_\_dopaminergic | 2 | 0 |  |  |  |  |  |  |  |  |
| GO:0032230\_positive\_regulation\_of\_synaptic\_transmission\_\_GABAergic | 2 | 0 |  |  |  |  |  |  |  |  |
| GO:0032234\_regulation\_of\_calcium\_ion\_transport\_via\_store-operated\_calcium\_channel\_activity | 2 | 0 |  |  |  |  |  |  |  |  |
| GO:0032236\_positive\_regulation\_of\_calcium\_ion\_transport\_via\_store-operated\_calcium\_channel\_activity | 2 | 0 |  |  |  |  |  |  |  |  |
| GO:0032297\_negative\_regulation\_of\_DNA\_replication\_initiation | 2 | 0 |  |  |  |  |  |  |  |  |
| GO:0032309\_icosanoid\_secretion | 2 | 0 |  |  |  |  |  |  |  |  |
| GO:0032328\_alanine\_transport | 2 | 0 |  |  |  |  |  |  |  |  |
| GO:0032341\_aldosterone\_metabolic\_process | 2 | 0 |  |  |  |  |  |  |  |  |
| GO:0032351\_negative\_regulation\_of\_hormone\_metabolic\_process | 2 | 0 |  |  |  |  |  |  |  |  |
| GO:0032353\_negative\_regulation\_of\_hormone\_biosynthetic\_process | 2 | 0 |  |  |  |  |  |  |  |  |
| GO:0032435\_negative\_regulation\_of\_proteasomal\_ubiquitin-dependent\_protein\_catabolic\_process | 2 | 0 |  |  |  |  |  |  |  |  |
| GO:0032471\_reduction\_of\_endoplasmic\_reticulum\_calcium\_ion\_concentration | 2 | 0 |  |  |  |  |  |  |  |  |
| GO:0032481\_positive\_regulation\_of\_type\_I\_interferon\_production | 2 | 0 |  |  |  |  |  |  |  |  |
| GO:0032488\_Cdc42\_protein\_signal\_transduction | 2 | 0 |  |  |  |  |  |  |  |  |
| GO:0032489\_regulation\_of\_Cdc42\_protein\_signal\_transduction | 2 | 0 |  |  |  |  |  |  |  |  |
| GO:0032495\_response\_to\_muramyl\_dipeptide | 2 | 0 |  |  |  |  |  |  |  |  |
| GO:0032604\_granulocyte\_macrophage\_colony-stimulating\_factor\_production | 2 | 0 |  |  |  |  |  |  |  |  |
| GO:0032616\_interleukin-13\_production | 2 | 0 |  |  |  |  |  |  |  |  |
| GO:0032645\_regulation\_of\_granulocyte\_macrophage\_colony-stimulating\_factor\_production | 2 | 0 |  |  |  |  |  |  |  |  |
| GO:0032672\_regulation\_of\_interleukin-3\_production | 2 | 0 |  |  |  |  |  |  |  |  |
| GO:0032695\_negative\_regulation\_of\_interleukin-12\_production | 2 | 0 |  |  |  |  |  |  |  |  |
| GO:0032714\_negative\_regulation\_of\_interleukin-5\_production | 2 | 0 |  |  |  |  |  |  |  |  |
| GO:0032722\_positive\_regulation\_of\_chemokine\_production | 2 | 0 |  |  |  |  |  |  |  |  |
| GO:0032743\_positive\_regulation\_of\_interleukin-2\_production | 2 | 0 |  |  |  |  |  |  |  |  |
| GO:0032762\_mast\_cell\_cytokine\_production | 2 | 0 |  |  |  |  |  |  |  |  |
| GO:0032763\_regulation\_of\_mast\_cell\_cytokine\_production | 2 | 0 |  |  |  |  |  |  |  |  |
| GO:0032768\_regulation\_of\_monooxygenase\_activity | 2 | 0 |  |  |  |  |  |  |  |  |
| GO:0032788\_saturated\_monocarboxylic\_acid\_metabolic\_process | 2 | 0 |  |  |  |  |  |  |  |  |
| GO:0032789\_unsaturated\_monocarboxylic\_acid\_metabolic\_process | 2 | 0 |  |  |  |  |  |  |  |  |
| GO:0032796\_uropod\_organization | 2 | 0 |  |  |  |  |  |  |  |  |
| GO:0032800\_receptor\_biosynthetic\_process | 2 | 0 |  |  |  |  |  |  |  |  |
| GO:0032801\_receptor\_catabolic\_process | 2 | 0 |  |  |  |  |  |  |  |  |
| GO:0032829\_regulation\_of\_CD4-positive\_\_CD25-positive\_\_alpha-beta\_regulatory\_T\_cell\_differentiation | 2 | 0 |  |  |  |  |  |  |  |  |
| GO:0032831\_positive\_regulation\_of\_CD4-positive\_\_CD25-positive\_\_alpha-beta\_regulatory\_T\_cell\_differentiation | 2 | 0 |  |  |  |  |  |  |  |  |
| GO:0032892\_positive\_regulation\_of\_organic\_acid\_transport | 2 | 0 |  |  |  |  |  |  |  |  |
| GO:0032905\_transforming\_growth\_factor-beta1\_production | 2 | 0 |  |  |  |  |  |  |  |  |
| GO:0032908\_regulation\_of\_transforming\_growth\_factor-beta1\_production | 2 | 0 |  |  |  |  |  |  |  |  |
| GO:0032914\_positive\_regulation\_of\_transforming\_growth\_factor-beta1\_production | 2 | 0 |  |  |  |  |  |  |  |  |
| GO:0032933\_SREBP-mediated\_signaling\_pathway | 2 | 0 |  |  |  |  |  |  |  |  |
| GO:0032957\_inositol\_trisphosphate\_metabolic\_process | 2 | 0 |  |  |  |  |  |  |  |  |
| GO:0032958\_inositol\_phosphate\_biosynthetic\_process | 2 | 0 |  |  |  |  |  |  |  |  |
| GO:0032959\_inositol\_trisphosphate\_biosynthetic\_process | 2 | 0 |  |  |  |  |  |  |  |  |
| GO:0033092\_positive\_regulation\_of\_immature\_T\_cell\_proliferation\_in\_the\_thymus | 2 | 0 |  |  |  |  |  |  |  |  |
| GO:0033119\_negative\_regulation\_of\_RNA\_splicing | 2 | 0 |  |  |  |  |  |  |  |  |
| GO:0033136\_serine\_phosphorylation\_of\_STAT3\_protein | 2 | 0 |  |  |  |  |  |  |  |  |
| GO:0033145\_positive\_regulation\_of\_steroid\_hormone\_receptor\_signaling\_pathway | 2 | 0 |  |  |  |  |  |  |  |  |
| GO:0033147\_negative\_regulation\_of\_estrogen\_receptor\_signaling\_pathway | 2 | 0 |  |  |  |  |  |  |  |  |
| GO:0033148\_positive\_regulation\_of\_estrogen\_receptor\_signaling\_pathway | 2 | 0 |  |  |  |  |  |  |  |  |
| GO:0033194\_response\_to\_hydroperoxide | 2 | 0 |  |  |  |  |  |  |  |  |
| GO:0033275\_actin-myosin\_filament\_sliding | 2 | 0 |  |  |  |  |  |  |  |  |
| GO:0033280\_response\_to\_vitamin\_D | 2 | 0 |  |  |  |  |  |  |  |  |
| GO:0033364\_mast\_cell\_secretory\_granule\_organization | 2 | 0 |  |  |  |  |  |  |  |  |
| GO:0033504\_floor\_plate\_development | 2 | 0 |  |  |  |  |  |  |  |  |
| GO:0033603\_positive\_regulation\_of\_dopamine\_secretion | 2 | 0 |  |  |  |  |  |  |  |  |
| GO:0033605\_positive\_regulation\_of\_catecholamine\_secretion | 2 | 0 |  |  |  |  |  |  |  |  |
| GO:0033622\_integrin\_activation | 2 | 0 |  |  |  |  |  |  |  |  |
| GO:0033623\_regulation\_of\_integrin\_activation | 2 | 0 |  |  |  |  |  |  |  |  |
| GO:0033625\_positive\_regulation\_of\_integrin\_activation | 2 | 0 |  |  |  |  |  |  |  |  |
| GO:0033700\_phospholipid\_efflux | 2 | 0 |  |  |  |  |  |  |  |  |
| GO:0034142\_toll-like\_receptor\_4\_signaling\_pathway | 2 | 0 |  |  |  |  |  |  |  |  |
| GO:0034310\_monohydric\_alcohol\_catabolic\_process | 2 | 0 |  |  |  |  |  |  |  |  |
| GO:0034341\_response\_to\_interferon-gamma | 2 | 0 |  |  |  |  |  |  |  |  |
| GO:0034370\_triglyceride-rich\_lipoprotein\_particle\_remodeling | 2 | 0 |  |  |  |  |  |  |  |  |
| GO:0034374\_low-density\_lipoprotein\_particle\_remodeling | 2 | 0 |  |  |  |  |  |  |  |  |
| GO:0034377\_plasma\_lipoprotein\_particle\_assembly | 2 | 0 |  |  |  |  |  |  |  |  |
| GO:0034384\_high-density\_lipoprotein\_particle\_clearance | 2 | 0 |  |  |  |  |  |  |  |  |
| GO:0034433\_steroid\_esterification | 2 | 0 |  |  |  |  |  |  |  |  |
| GO:0034434\_sterol\_esterification | 2 | 0 |  |  |  |  |  |  |  |  |
| GO:0034435\_cholesterol\_esterification | 2 | 0 |  |  |  |  |  |  |  |  |
| GO:0034453\_microtubule\_anchoring | 2 | 0 |  |  |  |  |  |  |  |  |
| GO:0034644\_cellular\_response\_to\_UV | 2 | 0 |  |  |  |  |  |  |  |  |
| GO:0034755\_iron\_ion\_transmembrane\_transport | 2 | 0 |  |  |  |  |  |  |  |  |
| GO:0034764\_positive\_regulation\_of\_transmembrane\_transport | 2 | 0 |  |  |  |  |  |  |  |  |
| GO:0035021\_negative\_regulation\_of\_Rac\_protein\_signal\_transduction | 2 | 0 |  |  |  |  |  |  |  |  |
| GO:0035054\_embryonic\_heart\_tube\_anterior\_posterior\_pattern\_formation | 2 | 0 |  |  |  |  |  |  |  |  |
| GO:0035092\_sperm\_chromatin\_condensation | 2 | 0 |  |  |  |  |  |  |  |  |
| GO:0035110\_leg\_morphogenesis | 2 | 0 |  |  |  |  |  |  |  |  |
| GO:0035117\_embryonic\_arm\_morphogenesis | 2 | 0 |  |  |  |  |  |  |  |  |
| GO:0035120\_post-embryonic\_appendage\_morphogenesis | 2 | 0 |  |  |  |  |  |  |  |  |
| GO:0035127\_post-embryonic\_limb\_morphogenesis | 2 | 0 |  |  |  |  |  |  |  |  |
| GO:0035129\_post-embryonic\_hindlimb\_morphogenesis | 2 | 0 |  |  |  |  |  |  |  |  |
| GO:0035140\_arm\_morphogenesis | 2 | 0 |  |  |  |  |  |  |  |  |
| GO:0035194\_posttranscriptional\_gene\_silencing\_by\_RNA | 2 | 0 |  |  |  |  |  |  |  |  |
| GO:0035195\_gene\_silencing\_by\_miRNA | 2 | 0 |  |  |  |  |  |  |  |  |
| GO:0035196\_gene\_silencing\_by\_miRNA\_\_production\_of\_miRNAs | 2 | 0 |  |  |  |  |  |  |  |  |
| GO:0035315\_hair\_cell\_differentiation | 2 | 0 |  |  |  |  |  |  |  |  |
| GO:0040009\_regulation\_of\_growth\_rate | 2 | 0 |  |  |  |  |  |  |  |  |
| GO:0040037\_negative\_regulation\_of\_fibroblast\_growth\_factor\_receptor\_signaling\_pathway | 2 | 0 |  |  |  |  |  |  |  |  |
| GO:0042119\_neutrophil\_activation | 2 | 0 |  |  |  |  |  |  |  |  |
| GO:0042147\_retrograde\_transport\_\_endosome\_to\_Golgi | 2 | 0 |  |  |  |  |  |  |  |  |
| GO:0042223\_interleukin-3\_biosynthetic\_process | 2 | 0 |  |  |  |  |  |  |  |  |
| GO:0042249\_establishment\_of\_polarity\_of\_embryonic\_epithelium | 2 | 0 |  |  |  |  |  |  |  |  |
| GO:0042253\_granulocyte\_macrophage\_colony-stimulating\_factor\_biosynthetic\_process | 2 | 0 |  |  |  |  |  |  |  |  |
| GO:0042270\_protection\_from\_natural\_killer\_cell\_mediated\_cytotoxicity | 2 | 0 |  |  |  |  |  |  |  |  |
| GO:0042274\_ribosomal\_small\_subunit\_biogenesis | 2 | 0 |  |  |  |  |  |  |  |  |
| GO:0042312\_regulation\_of\_vasodilation | 2 | 0 |  |  |  |  |  |  |  |  |
| GO:0042346\_positive\_regulation\_of\_NF-kappaB\_import\_into\_nucleus | 2 | 0 |  |  |  |  |  |  |  |  |
| GO:0042396\_phosphagen\_biosynthetic\_process | 2 | 0 |  |  |  |  |  |  |  |  |
| GO:0042454\_ribonucleoside\_catabolic\_process | 2 | 0 |  |  |  |  |  |  |  |  |
| GO:0042482\_positive\_regulation\_of\_odontogenesis | 2 | 0 |  |  |  |  |  |  |  |  |
| GO:0042483\_negative\_regulation\_of\_odontogenesis | 2 | 0 |  |  |  |  |  |  |  |  |
| GO:0042488\_positive\_regulation\_of\_odontogenesis\_of\_dentine-containing\_tooth | 2 | 0 |  |  |  |  |  |  |  |  |
| GO:0042501\_serine\_phosphorylation\_of\_STAT\_protein | 2 | 0 |  |  |  |  |  |  |  |  |
| GO:0042517\_positive\_regulation\_of\_tyrosine\_phosphorylation\_of\_Stat3\_protein | 2 | 0 |  |  |  |  |  |  |  |  |
| GO:0042532\_negative\_regulation\_of\_tyrosine\_phosphorylation\_of\_STAT\_protein | 2 | 0 |  |  |  |  |  |  |  |  |
| GO:0042559\_pteridine\_and\_derivative\_biosynthetic\_process | 2 | 0 |  |  |  |  |  |  |  |  |
| GO:0042730\_fibrinolysis | 2 | 0 |  |  |  |  |  |  |  |  |
| GO:0042749\_regulation\_of\_circadian\_sleep\_wake\_cycle | 2 | 0 |  |  |  |  |  |  |  |  |
| GO:0042886\_amide\_transport | 2 | 0 |  |  |  |  |  |  |  |  |
| GO:0042921\_glucocorticoid\_receptor\_signaling\_pathway | 2 | 0 |  |  |  |  |  |  |  |  |
| GO:0042987\_amyloid\_precursor\_protein\_catabolic\_process | 2 | 0 |  |  |  |  |  |  |  |  |
| GO:0042993\_positive\_regulation\_of\_transcription\_factor\_import\_into\_nucleus | 2 | 0 |  |  |  |  |  |  |  |  |
| GO:0042994\_cytoplasmic\_sequestering\_of\_transcription\_factor | 2 | 0 |  |  |  |  |  |  |  |  |
| GO:0043032\_positive\_regulation\_of\_macrophage\_activation | 2 | 0 |  |  |  |  |  |  |  |  |
| GO:0043038\_amino\_acid\_activation | 2 | 0 |  |  |  |  |  |  |  |  |
| GO:0043039\_tRNA\_aminoacylation | 2 | 0 |  |  |  |  |  |  |  |  |
| GO:0043084\_penile\_erection | 2 | 0 |  |  |  |  |  |  |  |  |
| GO:0043088\_regulation\_of\_Cdc42\_GTPase\_activity | 2 | 0 |  |  |  |  |  |  |  |  |
| GO:0043089\_positive\_regulation\_of\_Cdc42\_GTPase\_activity | 2 | 0 |  |  |  |  |  |  |  |  |
| GO:0043096\_purine\_base\_salvage | 2 | 0 |  |  |  |  |  |  |  |  |
| GO:0043247\_telomere\_maintenance\_in\_response\_to\_DNA\_damage | 2 | 0 |  |  |  |  |  |  |  |  |
| GO:0043297\_apical\_junction\_assembly | 2 | 0 |  |  |  |  |  |  |  |  |
| GO:0043312\_neutrophil\_degranulation | 2 | 0 |  |  |  |  |  |  |  |  |
| GO:0043320\_natural\_killer\_cell\_degranulation | 2 | 0 |  |  |  |  |  |  |  |  |
| GO:0043366\_beta\_selection | 2 | 0 |  |  |  |  |  |  |  |  |
| GO:0043450\_alkene\_biosynthetic\_process | 2 | 0 |  |  |  |  |  |  |  |  |
| GO:0043476\_pigment\_accumulation | 2 | 0 |  |  |  |  |  |  |  |  |
| GO:0043490\_malate-aspartate\_shuttle | 2 | 0 |  |  |  |  |  |  |  |  |
| GO:0043502\_regulation\_of\_muscle\_adaptation | 2 | 0 |  |  |  |  |  |  |  |  |
| GO:0043516\_regulation\_of\_DNA\_damage\_response\_\_signal\_transduction\_by\_p53\_class\_mediator | 2 | 0 |  |  |  |  |  |  |  |  |
| GO:0043568\_positive\_regulation\_of\_insulin-like\_growth\_factor\_receptor\_signaling\_pathway | 2 | 0 |  |  |  |  |  |  |  |  |
| GO:0043589\_skin\_morphogenesis | 2 | 0 |  |  |  |  |  |  |  |  |
| GO:0043618\_regulation\_of\_transcription\_from\_RNA\_polymerase\_II\_promoter\_in\_response\_to\_stress | 2 | 0 |  |  |  |  |  |  |  |  |
| GO:0043619\_regulation\_of\_transcription\_from\_RNA\_polymerase\_II\_promoter\_in\_response\_to\_oxidative\_stress | 2 | 0 |  |  |  |  |  |  |  |  |
| GO:0043620\_regulation\_of\_transcription\_in\_response\_to\_stress | 2 | 0 |  |  |  |  |  |  |  |  |
| GO:0043647\_inositol\_phosphate\_metabolic\_process | 2 | 0 |  |  |  |  |  |  |  |  |
| GO:0043654\_recognition\_of\_apoptotic\_cell | 2 | 0 |  |  |  |  |  |  |  |  |
| GO:0043966\_histone\_H3\_acetylation | 2 | 0 |  |  |  |  |  |  |  |  |
| GO:0043967\_histone\_H4\_acetylation | 2 | 0 |  |  |  |  |  |  |  |  |
| GO:0044070\_regulation\_of\_anion\_transport | 2 | 0 |  |  |  |  |  |  |  |  |
| GO:0044246\_regulation\_of\_multicellular\_organismal\_metabolic\_process | 2 | 0 |  |  |  |  |  |  |  |  |
| GO:0044253\_positive\_regulation\_of\_multicellular\_organismal\_metabolic\_process | 2 | 0 |  |  |  |  |  |  |  |  |
| GO:0044268\_multicellular\_organismal\_protein\_metabolic\_process | 2 | 0 |  |  |  |  |  |  |  |  |
| GO:0045005\_maintenance\_of\_fidelity\_during\_DNA-dependent\_DNA\_replication | 2 | 0 |  |  |  |  |  |  |  |  |
| GO:0045010\_actin\_nucleation | 2 | 0 |  |  |  |  |  |  |  |  |
| GO:0045065\_cytotoxic\_T\_cell\_differentiation | 2 | 0 |  |  |  |  |  |  |  |  |
| GO:0045077\_negative\_regulation\_of\_interferon-gamma\_biosynthetic\_process | 2 | 0 |  |  |  |  |  |  |  |  |
| GO:0045079\_negative\_regulation\_of\_chemokine\_biosynthetic\_process | 2 | 0 |  |  |  |  |  |  |  |  |
| GO:0045116\_protein\_neddylation | 2 | 0 |  |  |  |  |  |  |  |  |
| GO:0045187\_regulation\_of\_circadian\_sleep\_wake\_cycle\_\_sleep | 2 | 0 |  |  |  |  |  |  |  |  |
| GO:0045212\_neurotransmitter\_receptor\_biosynthetic\_process | 2 | 0 |  |  |  |  |  |  |  |  |
| GO:0045399\_regulation\_of\_interleukin-3\_biosynthetic\_process | 2 | 0 |  |  |  |  |  |  |  |  |
| GO:0045401\_positive\_regulation\_of\_interleukin-3\_biosynthetic\_process | 2 | 0 |  |  |  |  |  |  |  |  |
| GO:0045409\_negative\_regulation\_of\_interleukin-6\_biosynthetic\_process | 2 | 0 |  |  |  |  |  |  |  |  |
| GO:0045423\_regulation\_of\_granulocyte\_macrophage\_colony-stimulating\_factor\_biosynthetic\_process | 2 | 0 |  |  |  |  |  |  |  |  |
| GO:0045425\_positive\_regulation\_of\_granulocyte\_macrophage\_colony-stimulating\_factor\_biosynthetic\_process | 2 | 0 |  |  |  |  |  |  |  |  |
| GO:0045475\_locomotor\_rhythm | 2 | 0 |  |  |  |  |  |  |  |  |
| GO:0045578\_negative\_regulation\_of\_B\_cell\_differentiation | 2 | 0 |  |  |  |  |  |  |  |  |
| GO:0045589\_regulation\_of\_regulatory\_T\_cell\_differentiation | 2 | 0 |  |  |  |  |  |  |  |  |
| GO:0045591\_positive\_regulation\_of\_regulatory\_T\_cell\_differentiation | 2 | 0 |  |  |  |  |  |  |  |  |
| GO:0045608\_negative\_regulation\_of\_auditory\_receptor\_cell\_differentiation | 2 | 0 |  |  |  |  |  |  |  |  |
| GO:0045627\_positive\_regulation\_of\_T-helper\_1\_cell\_differentiation | 2 | 0 |  |  |  |  |  |  |  |  |
| GO:0045629\_negative\_regulation\_of\_T-helper\_2\_cell\_differentiation | 2 | 0 |  |  |  |  |  |  |  |  |
| GO:0045630\_positive\_regulation\_of\_T-helper\_2\_cell\_differentiation | 2 | 0 |  |  |  |  |  |  |  |  |
| GO:0045632\_negative\_regulation\_of\_mechanoreceptor\_differentiation | 2 | 0 |  |  |  |  |  |  |  |  |
| GO:0045636\_positive\_regulation\_of\_melanocyte\_differentiation | 2 | 0 |  |  |  |  |  |  |  |  |
| GO:0045655\_regulation\_of\_monocyte\_differentiation | 2 | 0 |  |  |  |  |  |  |  |  |
| GO:0045658\_regulation\_of\_neutrophil\_differentiation | 2 | 0 |  |  |  |  |  |  |  |  |
| GO:0045662\_negative\_regulation\_of\_myoblast\_differentiation | 2 | 0 |  |  |  |  |  |  |  |  |
| GO:0045663\_positive\_regulation\_of\_myoblast\_differentiation | 2 | 0 |  |  |  |  |  |  |  |  |
| GO:0045683\_negative\_regulation\_of\_epidermis\_development | 2 | 0 |  |  |  |  |  |  |  |  |
| GO:0045737\_positive\_regulation\_of\_cyclin-dependent\_protein\_kinase\_activity | 2 | 0 |  |  |  |  |  |  |  |  |
| GO:0045739\_positive\_regulation\_of\_DNA\_repair | 2 | 0 |  |  |  |  |  |  |  |  |
| GO:0045741\_positive\_regulation\_of\_epidermal\_growth\_factor\_receptor\_activity | 2 | 0 |  |  |  |  |  |  |  |  |
| GO:0045743\_positive\_regulation\_of\_fibroblast\_growth\_factor\_receptor\_signaling\_pathway | 2 | 0 |  |  |  |  |  |  |  |  |
| GO:0045749\_negative\_regulation\_of\_S\_phase\_of\_mitotic\_cell\_cycle | 2 | 0 |  |  |  |  |  |  |  |  |
| GO:0045819\_positive\_regulation\_of\_glycogen\_catabolic\_process | 2 | 0 |  |  |  |  |  |  |  |  |
| GO:0045821\_positive\_regulation\_of\_glycolysis | 2 | 0 |  |  |  |  |  |  |  |  |
| GO:0045835\_negative\_regulation\_of\_meiosis | 2 | 0 |  |  |  |  |  |  |  |  |
| GO:0045836\_positive\_regulation\_of\_meiosis | 2 | 0 |  |  |  |  |  |  |  |  |
| GO:0045839\_negative\_regulation\_of\_mitosis | 2 | 0 |  |  |  |  |  |  |  |  |
| GO:0045841\_negative\_regulation\_of\_mitotic\_metaphase\_anaphase\_transition | 2 | 0 |  |  |  |  |  |  |  |  |
| GO:0045872\_positive\_regulation\_of\_rhodopsin\_gene\_expression | 2 | 0 |  |  |  |  |  |  |  |  |
| GO:0045912\_negative\_regulation\_of\_carbohydrate\_metabolic\_process | 2 | 0 |  |  |  |  |  |  |  |  |
| GO:0045948\_positive\_regulation\_of\_translational\_initiation | 2 | 0 |  |  |  |  |  |  |  |  |
| GO:0045950\_negative\_regulation\_of\_mitotic\_recombination | 2 | 0 |  |  |  |  |  |  |  |  |
| GO:0046033\_AMP\_metabolic\_process | 2 | 0 |  |  |  |  |  |  |  |  |
| GO:0046060\_dATP\_metabolic\_process | 2 | 0 |  |  |  |  |  |  |  |  |
| GO:0046070\_dGTP\_metabolic\_process | 2 | 0 |  |  |  |  |  |  |  |  |
| GO:0046083\_adenine\_metabolic\_process | 2 | 0 |  |  |  |  |  |  |  |  |
| GO:0046085\_adenosine\_metabolic\_process | 2 | 0 |  |  |  |  |  |  |  |  |
| GO:0046100\_hypoxanthine\_metabolic\_process | 2 | 0 |  |  |  |  |  |  |  |  |
| GO:0046114\_guanosine\_biosynthetic\_process | 2 | 0 |  |  |  |  |  |  |  |  |
| GO:0046116\_queuosine\_metabolic\_process | 2 | 0 |  |  |  |  |  |  |  |  |
| GO:0046118\_7-methylguanosine\_biosynthetic\_process | 2 | 0 |  |  |  |  |  |  |  |  |
| GO:0046130\_purine\_ribonucleoside\_catabolic\_process | 2 | 0 |  |  |  |  |  |  |  |  |
| GO:0046146\_tetrahydrobiopterin\_metabolic\_process | 2 | 0 |  |  |  |  |  |  |  |  |
| GO:0046185\_aldehyde\_catabolic\_process | 2 | 0 |  |  |  |  |  |  |  |  |
| GO:0046208\_spermine\_catabolic\_process | 2 | 0 |  |  |  |  |  |  |  |  |
| GO:0046349\_amino\_sugar\_biosynthetic\_process | 2 | 0 |  |  |  |  |  |  |  |  |
| GO:0046439\_L-cysteine\_metabolic\_process | 2 | 0 |  |  |  |  |  |  |  |  |
| GO:0046500\_S-adenosylmethionine\_metabolic\_process | 2 | 0 |  |  |  |  |  |  |  |  |
| GO:0046501\_protoporphyrinogen\_IX\_metabolic\_process | 2 | 0 |  |  |  |  |  |  |  |  |
| GO:0046514\_ceramide\_catabolic\_process | 2 | 0 |  |  |  |  |  |  |  |  |
| GO:0046521\_sphingoid\_catabolic\_process | 2 | 0 |  |  |  |  |  |  |  |  |
| GO:0046532\_regulation\_of\_photoreceptor\_cell\_differentiation | 2 | 0 |  |  |  |  |  |  |  |  |
| GO:0046533\_negative\_regulation\_of\_photoreceptor\_cell\_differentiation | 2 | 0 |  |  |  |  |  |  |  |  |
| GO:0046544\_development\_of\_secondary\_male\_sexual\_characteristics | 2 | 0 |  |  |  |  |  |  |  |  |
| GO:0046619\_optic\_placode\_formation\_involved\_in\_camera-type\_eye | 2 | 0 |  |  |  |  |  |  |  |  |
| GO:0046984\_regulation\_of\_hemoglobin\_biosynthetic\_process | 2 | 0 |  |  |  |  |  |  |  |  |
| GO:0047484\_regulation\_of\_response\_to\_osmotic\_stress | 2 | 0 |  |  |  |  |  |  |  |  |
| GO:0048025\_negative\_regulation\_of\_nuclear\_mRNA\_splicing\_\_via\_spliceosome | 2 | 0 |  |  |  |  |  |  |  |  |
| GO:0048134\_germ-line\_cyst\_formation | 2 | 0 |  |  |  |  |  |  |  |  |
| GO:0048136\_male\_germ-line\_cyst\_formation | 2 | 0 |  |  |  |  |  |  |  |  |
| GO:0048172\_regulation\_of\_short-term\_neuronal\_synaptic\_plasticity | 2 | 0 |  |  |  |  |  |  |  |  |
| GO:0048295\_positive\_regulation\_of\_isotype\_switching\_to\_IgE\_isotypes | 2 | 0 |  |  |  |  |  |  |  |  |
| GO:0048342\_paraxial\_mesodermal\_cell\_differentiation | 2 | 0 |  |  |  |  |  |  |  |  |
| GO:0048343\_paraxial\_mesodermal\_cell\_fate\_commitment | 2 | 0 |  |  |  |  |  |  |  |  |
| GO:0048382\_mesendoderm\_development | 2 | 0 |  |  |  |  |  |  |  |  |
| GO:0048552\_regulation\_of\_metalloenzyme\_activity | 2 | 0 |  |  |  |  |  |  |  |  |
| GO:0048554\_positive\_regulation\_of\_metalloenzyme\_activity | 2 | 0 |  |  |  |  |  |  |  |  |
| GO:0048619\_embryonic\_hindgut\_morphogenesis | 2 | 0 |  |  |  |  |  |  |  |  |
| GO:0048625\_myoblast\_cell\_fate\_commitment | 2 | 0 |  |  |  |  |  |  |  |  |
| GO:0048627\_myoblast\_development | 2 | 0 |  |  |  |  |  |  |  |  |
| GO:0048643\_positive\_regulation\_of\_skeletal\_muscle\_tissue\_development | 2 | 0 |  |  |  |  |  |  |  |  |
| GO:0048661\_positive\_regulation\_of\_smooth\_muscle\_cell\_proliferation | 2 | 0 |  |  |  |  |  |  |  |  |
| GO:0048670\_regulation\_of\_collateral\_sprouting | 2 | 0 |  |  |  |  |  |  |  |  |
| GO:0048671\_negative\_regulation\_of\_collateral\_sprouting | 2 | 0 |  |  |  |  |  |  |  |  |
| GO:0048677\_axon\_extension\_involved\_in\_regeneration | 2 | 0 |  |  |  |  |  |  |  |  |
| GO:0048679\_regulation\_of\_axon\_regeneration | 2 | 0 |  |  |  |  |  |  |  |  |
| GO:0048682\_sprouting\_of\_injured\_axon | 2 | 0 |  |  |  |  |  |  |  |  |
| GO:0048702\_embryonic\_neurocranium\_morphogenesis | 2 | 0 |  |  |  |  |  |  |  |  |
| GO:0048711\_positive\_regulation\_of\_astrocyte\_differentiation | 2 | 0 |  |  |  |  |  |  |  |  |
| GO:0048712\_negative\_regulation\_of\_astrocyte\_differentiation | 2 | 0 |  |  |  |  |  |  |  |  |
| GO:0048739\_cardiac\_muscle\_fiber\_development | 2 | 0 |  |  |  |  |  |  |  |  |
| GO:0048807\_female\_genitalia\_morphogenesis | 2 | 0 |  |  |  |  |  |  |  |  |
| GO:0048808\_male\_genitalia\_morphogenesis | 2 | 0 |  |  |  |  |  |  |  |  |
| GO:0048840\_otolith\_development | 2 | 0 |  |  |  |  |  |  |  |  |
| GO:0048850\_hypophysis\_morphogenesis | 2 | 0 |  |  |  |  |  |  |  |  |
| GO:0048867\_stem\_cell\_fate\_determination | 2 | 0 |  |  |  |  |  |  |  |  |
| GO:0050000\_chromosome\_localization | 2 | 0 |  |  |  |  |  |  |  |  |
| GO:0050686\_negative\_regulation\_of\_mRNA\_processing | 2 | 0 |  |  |  |  |  |  |  |  |
| GO:0050688\_regulation\_of\_defense\_response\_to\_virus | 2 | 0 |  |  |  |  |  |  |  |  |
| GO:0050746\_regulation\_of\_lipoprotein\_metabolic\_process | 2 | 0 |  |  |  |  |  |  |  |  |
| GO:0050779\_RNA\_destabilization | 2 | 0 |  |  |  |  |  |  |  |  |
| GO:0050792\_regulation\_of\_viral\_reproduction | 2 | 0 |  |  |  |  |  |  |  |  |
| GO:0050802\_circadian\_sleep\_wake\_cycle\_\_sleep | 2 | 0 |  |  |  |  |  |  |  |  |
| GO:0050847\_progesterone\_receptor\_signaling\_pathway | 2 | 0 |  |  |  |  |  |  |  |  |
| GO:0050855\_regulation\_of\_B\_cell\_receptor\_signaling\_pathway | 2 | 0 |  |  |  |  |  |  |  |  |
| GO:0050883\_musculoskeletal\_movement\_\_spinal\_reflex\_action | 2 | 0 |  |  |  |  |  |  |  |  |
| GO:0050901\_leukocyte\_tethering\_or\_rolling | 2 | 0 |  |  |  |  |  |  |  |  |
| GO:0050907\_detection\_of\_chemical\_stimulus\_involved\_in\_sensory\_perception | 2 | 0 |  |  |  |  |  |  |  |  |
| GO:0050917\_sensory\_perception\_of\_umami\_taste | 2 | 0 |  |  |  |  |  |  |  |  |
| GO:0050942\_positive\_regulation\_of\_pigment\_cell\_differentiation | 2 | 0 |  |  |  |  |  |  |  |  |
| GO:0050955\_thermoception | 2 | 0 |  |  |  |  |  |  |  |  |
| GO:0050968\_detection\_of\_chemical\_stimulus\_involved\_in\_sensory\_perception\_of\_pain | 2 | 0 |  |  |  |  |  |  |  |  |
| GO:0050973\_detection\_of\_mechanical\_stimulus\_involved\_in\_equilibrioception | 2 | 0 |  |  |  |  |  |  |  |  |
| GO:0050999\_regulation\_of\_nitric-oxide\_synthase\_activity | 2 | 0 |  |  |  |  |  |  |  |  |
| GO:0051004\_regulation\_of\_lipoprotein\_lipase\_activity | 2 | 0 |  |  |  |  |  |  |  |  |
| GO:0051014\_actin\_filament\_severing | 2 | 0 |  |  |  |  |  |  |  |  |
| GO:0051026\_chiasma\_formation | 2 | 0 |  |  |  |  |  |  |  |  |
| GO:0051081\_nuclear\_envelope\_disassembly | 2 | 0 |  |  |  |  |  |  |  |  |
| GO:0051132\_NK\_T\_cell\_activation | 2 | 0 |  |  |  |  |  |  |  |  |
| GO:0051133\_regulation\_of\_NK\_T\_cell\_activation | 2 | 0 |  |  |  |  |  |  |  |  |
| GO:0051135\_positive\_regulation\_of\_NK\_T\_cell\_activation | 2 | 0 |  |  |  |  |  |  |  |  |
| GO:0051150\_regulation\_of\_smooth\_muscle\_cell\_differentiation | 2 | 0 |  |  |  |  |  |  |  |  |
| GO:0051220\_cytoplasmic\_sequestering\_of\_protein | 2 | 0 |  |  |  |  |  |  |  |  |
| GO:0051279\_regulation\_of\_release\_of\_sequestered\_calcium\_ion\_into\_cytosol | 2 | 0 |  |  |  |  |  |  |  |  |
| GO:0051293\_establishment\_of\_spindle\_localization | 2 | 0 |  |  |  |  |  |  |  |  |
| GO:0051295\_establishment\_of\_meiotic\_spindle\_localization | 2 | 0 |  |  |  |  |  |  |  |  |
| GO:0051299\_centrosome\_separation | 2 | 0 |  |  |  |  |  |  |  |  |
| GO:0051303\_establishment\_of\_chromosome\_localization | 2 | 0 |  |  |  |  |  |  |  |  |
| GO:0051304\_chromosome\_separation | 2 | 0 |  |  |  |  |  |  |  |  |
| GO:0051307\_meiotic\_chromosome\_separation | 2 | 0 |  |  |  |  |  |  |  |  |
| GO:0051313\_attachment\_of\_spindle\_microtubules\_to\_chromosome | 2 | 0 |  |  |  |  |  |  |  |  |
| GO:0051318\_G1\_phase | 2 | 0 |  |  |  |  |  |  |  |  |
| GO:0051319\_G2\_phase | 2 | 0 |  |  |  |  |  |  |  |  |
| GO:0051353\_positive\_regulation\_of\_oxidoreductase\_activity | 2 | 0 |  |  |  |  |  |  |  |  |
| GO:0051451\_myoblast\_migration | 2 | 0 |  |  |  |  |  |  |  |  |
| GO:0051489\_regulation\_of\_filopodium\_assembly | 2 | 0 |  |  |  |  |  |  |  |  |
| GO:0051491\_positive\_regulation\_of\_filopodium\_assembly | 2 | 0 |  |  |  |  |  |  |  |  |
| GO:0051541\_elastin\_metabolic\_process | 2 | 0 |  |  |  |  |  |  |  |  |
| GO:0051546\_keratinocyte\_migration | 2 | 0 |  |  |  |  |  |  |  |  |
| GO:0051563\_smooth\_endoplasmic\_reticulum\_calcium\_ion\_homeostasis | 2 | 0 |  |  |  |  |  |  |  |  |
| GO:0051590\_positive\_regulation\_of\_neurotransmitter\_transport | 2 | 0 |  |  |  |  |  |  |  |  |
| GO:0051602\_response\_to\_electrical\_stimulus | 2 | 0 |  |  |  |  |  |  |  |  |
| GO:0051608\_histamine\_transport | 2 | 0 |  |  |  |  |  |  |  |  |
| GO:0051643\_ER\_localization | 2 | 0 |  |  |  |  |  |  |  |  |
| GO:0051653\_spindle\_localization | 2 | 0 |  |  |  |  |  |  |  |  |
| GO:0051657\_maintenance\_of\_organelle\_location | 2 | 0 |  |  |  |  |  |  |  |  |
| GO:0051702\_interaction\_with\_symbiont | 2 | 0 |  |  |  |  |  |  |  |  |
| GO:0051781\_positive\_regulation\_of\_cell\_division | 2 | 0 |  |  |  |  |  |  |  |  |
| GO:0051784\_negative\_regulation\_of\_nuclear\_division | 2 | 0 |  |  |  |  |  |  |  |  |
| GO:0051890\_regulation\_of\_cardioblast\_differentiation | 2 | 0 |  |  |  |  |  |  |  |  |
| GO:0051891\_positive\_regulation\_of\_cardioblast\_differentiation | 2 | 0 |  |  |  |  |  |  |  |  |
| GO:0051923\_sulfation | 2 | 0 |  |  |  |  |  |  |  |  |
| GO:0051938\_L-glutamate\_import | 2 | 0 |  |  |  |  |  |  |  |  |
| GO:0051957\_positive\_regulation\_of\_amino\_acid\_transport | 2 | 0 |  |  |  |  |  |  |  |  |
| GO:0051988\_regulation\_of\_attachment\_of\_spindle\_microtubules\_to\_kinetochore | 2 | 0 |  |  |  |  |  |  |  |  |
| GO:0055057\_neuroblast\_division | 2 | 0 |  |  |  |  |  |  |  |  |
| GO:0055064\_chloride\_ion\_homeostasis | 2 | 0 |  |  |  |  |  |  |  |  |
| GO:0055075\_potassium\_ion\_homeostasis | 2 | 0 |  |  |  |  |  |  |  |  |
| GO:0055090\_acylglycerol\_homeostasis | 2 | 0 |  |  |  |  |  |  |  |  |
| GO:0055091\_phospholipid\_homeostasis | 2 | 0 |  |  |  |  |  |  |  |  |
| GO:0060012\_synaptic\_transmission\_\_glycinergic | 2 | 0 |  |  |  |  |  |  |  |  |
| GO:0060023\_soft\_palate\_development | 2 | 0 |  |  |  |  |  |  |  |  |
| GO:0060032\_notochord\_regression | 2 | 0 |  |  |  |  |  |  |  |  |
| GO:0060039\_pericardium\_development | 2 | 0 |  |  |  |  |  |  |  |  |
| GO:0060044\_negative\_regulation\_of\_cardiac\_muscle\_cell\_proliferation | 2 | 0 |  |  |  |  |  |  |  |  |
| GO:0060060\_post-embryonic\_retina\_morphogenesis\_in\_camera-type\_eye | 2 | 0 |  |  |  |  |  |  |  |  |
| GO:0060083\_smooth\_muscle\_contraction\_involved\_in\_micturition | 2 | 0 |  |  |  |  |  |  |  |  |
| GO:0060124\_positive\_regulation\_of\_growth\_hormone\_secretion | 2 | 0 |  |  |  |  |  |  |  |  |
| GO:0060133\_somatotropin\_secreting\_cell\_development | 2 | 0 |  |  |  |  |  |  |  |  |
| GO:0060155\_platelet\_dense\_granule\_organization | 2 | 0 |  |  |  |  |  |  |  |  |
| GO:0060159\_regulation\_of\_dopamine\_receptor\_signaling\_pathway | 2 | 0 |  |  |  |  |  |  |  |  |
| GO:0060160\_negative\_regulation\_of\_dopamine\_receptor\_signaling\_pathway | 2 | 0 |  |  |  |  |  |  |  |  |
| GO:0060166\_olfactory\_pit\_development | 2 | 0 |  |  |  |  |  |  |  |  |
| GO:0060179\_male\_mating\_behavior | 2 | 0 |  |  |  |  |  |  |  |  |
| GO:0060180\_female\_mating\_behavior | 2 | 0 |  |  |  |  |  |  |  |  |
| GO:0060214\_endocardium\_formation | 2 | 0 |  |  |  |  |  |  |  |  |
| GO:0060218\_hemopoietic\_stem\_cell\_differentiation | 2 | 0 |  |  |  |  |  |  |  |  |
| GO:0060259\_regulation\_of\_feeding\_behavior | 2 | 0 |  |  |  |  |  |  |  |  |
| GO:0060260\_regulation\_of\_transcription\_initiation\_from\_RNA\_polymerase\_II\_promoter | 2 | 0 |  |  |  |  |  |  |  |  |
| GO:0060292\_long\_term\_synaptic\_depression | 2 | 0 |  |  |  |  |  |  |  |  |
| GO:0060318\_definitive\_erythrocyte\_differentiation | 2 | 0 |  |  |  |  |  |  |  |  |
| GO:0060346\_bone\_trabecula\_formation | 2 | 0 |  |  |  |  |  |  |  |  |
| GO:0060363\_cranial\_suture\_morphogenesis | 2 | 0 |  |  |  |  |  |  |  |  |
| GO:0060397\_JAK-STAT\_cascade\_involved\_in\_growth\_hormone\_signaling\_pathway | 2 | 0 |  |  |  |  |  |  |  |  |
| GO:0060426\_lung\_vasculature\_development | 2 | 0 |  |  |  |  |  |  |  |  |
| GO:0060430\_lung\_saccule\_development | 2 | 0 |  |  |  |  |  |  |  |  |
| GO:0060434\_bronchus\_morphogenesis | 2 | 0 |  |  |  |  |  |  |  |  |
| GO:0060439\_trachea\_morphogenesis | 2 | 0 |  |  |  |  |  |  |  |  |
| GO:0060458\_right\_lung\_development | 2 | 0 |  |  |  |  |  |  |  |  |
| GO:0060462\_lung\_lobe\_development | 2 | 0 |  |  |  |  |  |  |  |  |
| GO:0060463\_lung\_lobe\_morphogenesis | 2 | 0 |  |  |  |  |  |  |  |  |
| GO:0060479\_lung\_cell\_differentiation | 2 | 0 |  |  |  |  |  |  |  |  |
| GO:0060487\_lung\_epithelial\_cell\_differentiation | 2 | 0 |  |  |  |  |  |  |  |  |
| GO:0060516\_primary\_prostatic\_bud\_elongation | 2 | 0 |  |  |  |  |  |  |  |  |
| GO:0060529\_squamous\_basal\_epithelial\_stem\_cell\_differentiation\_involved\_in\_prostate\_gland\_acinus\_development | 2 | 0 |  |  |  |  |  |  |  |  |
| GO:0060534\_trachea\_cartilage\_development | 2 | 0 |  |  |  |  |  |  |  |  |
| GO:0060599\_lateral\_sprouting\_involved\_in\_mammary\_gland\_duct\_morphogenesis | 2 | 0 |  |  |  |  |  |  |  |  |
| GO:0060612\_adipose\_tissue\_development | 2 | 0 |  |  |  |  |  |  |  |  |
| GO:0060615\_mammary\_gland\_bud\_formation | 2 | 0 |  |  |  |  |  |  |  |  |
| GO:0060667\_branch\_elongation\_involved\_in\_salivary\_gland\_morphogenesis | 2 | 0 |  |  |  |  |  |  |  |  |
| GO:0060690\_epithelial\_cell\_differentiation\_involved\_in\_salivary\_gland\_development | 2 | 0 |  |  |  |  |  |  |  |  |
| GO:0060738\_epithelial-mesenchymal\_signaling\_involved\_in\_prostate\_gland\_development | 2 | 0 |  |  |  |  |  |  |  |  |
| GO:0060741\_prostate\_gland\_stromal\_morphogenesis | 2 | 0 |  |  |  |  |  |  |  |  |
| GO:0060763\_mammary\_duct\_terminal\_end\_bud\_growth | 2 | 0 |  |  |  |  |  |  |  |  |
| GO:0060765\_regulation\_of\_androgen\_receptor\_signaling\_pathway | 2 | 0 |  |  |  |  |  |  |  |  |
| GO:0060766\_negative\_regulation\_of\_androgen\_receptor\_signaling\_pathway | 2 | 0 |  |  |  |  |  |  |  |  |
| GO:0060769\_positive\_regulation\_of\_epithelial\_cell\_proliferation\_involved\_in\_prostate\_gland\_development | 2 | 0 |  |  |  |  |  |  |  |  |
| GO:0065005\_protein-lipid\_complex\_assembly | 2 | 0 |  |  |  |  |  |  |  |  |
| GO:0070076\_histone\_lysine\_demethylation | 2 | 0 |  |  |  |  |  |  |  |  |
| GO:0070168\_negative\_regulation\_of\_biomineral\_formation | 2 | 0 |  |  |  |  |  |  |  |  |
| GO:0070252\_actin-mediated\_cell\_contraction | 2 | 0 |  |  |  |  |  |  |  |  |
| GO:0070256\_negative\_regulation\_of\_mucus\_secretion | 2 | 0 |  |  |  |  |  |  |  |  |
| GO:0070257\_positive\_regulation\_of\_mucus\_secretion | 2 | 0 |  |  |  |  |  |  |  |  |
| GO:0070570\_regulation\_of\_neuron\_projection\_regeneration | 2 | 0 |  |  |  |  |  |  |  |  |
| GO:0070723\_response\_to\_cholesterol | 2 | 0 |  |  |  |  |  |  |  |  |
| GO:0090030\_regulation\_of\_steroid\_hormone\_biosynthetic\_process | 2 | 0 |  |  |  |  |  |  |  |  |
| GO:0050865\_regulation\_of\_cell\_activation | 122 | 0 | 0.000000 | -0.000000 | 1175 | 1071.218290 | 1142.24 | 1213.261710 | 0.972119 |
| GO:0001502\_cartilage\_condensation | 14 | 0 | 0.000000 | -0.000000 | 1231 | 1132.692791 | 1202.07 | 1271.447209 | 0.976499 |
| GO:0001829\_trophectodermal\_cell\_differentiation | 14 | 0 | 0.000000 | -0.000000 | 1231 | 1132.692791 | 1202.07 | 1271.447209 | 0.976499 |
| GO:0002027\_regulation\_of\_heart\_rate | 14 | 0 | 0.000000 | -0.000000 | 1231 | 1132.692791 | 1202.07 | 1271.447209 | 0.976499 |
| GO:0002262\_myeloid\_cell\_homeostasis | 14 | 0 | 0.000000 | -0.000000 | 1231 | 1132.692791 | 1202.07 | 1271.447209 | 0.976499 |
| GO:0002698\_negative\_regulation\_of\_immune\_effector\_process | 14 | 0 | 0.000000 | -0.000000 | 1231 | 1132.692791 | 1202.07 | 1271.447209 | 0.976499 |
| GO:0006695\_cholesterol\_biosynthetic\_process | 14 | 0 | 0.000000 | -0.000000 | 1231 | 1132.692791 | 1202.07 | 1271.447209 | 0.976499 |
| GO:0006809\_nitric\_oxide\_biosynthetic\_process | 14 | 0 | 0.000000 | -0.000000 | 1231 | 1132.692791 | 1202.07 | 1271.447209 | 0.976499 |
| GO:0006914\_autophagy | 14 | 0 | 0.000000 | -0.000000 | 1231 | 1132.692791 | 1202.07 | 1271.447209 | 0.976499 |
| GO:0006970\_response\_to\_osmotic\_stress | 14 | 0 | 0.000000 | -0.000000 | 1231 | 1132.692791 | 1202.07 | 1271.447209 | 0.976499 |
| GO:0007157\_heterophilic\_cell\_adhesion | 14 | 0 | 0.000000 | -0.000000 | 1231 | 1132.692791 | 1202.07 | 1271.447209 | 0.976499 |
| GO:0007530\_sex\_determination | 14 | 0 | 0.000000 | -0.000000 | 1231 | 1132.692791 | 1202.07 | 1271.447209 | 0.976499 |
| GO:0007589\_body\_fluid\_secretion | 14 | 0 | 0.000000 | -0.000000 | 1231 | 1132.692791 | 1202.07 | 1271.447209 | 0.976499 |
| GO:0008306\_associative\_learning | 14 | 0 | 0.000000 | -0.000000 | 1231 | 1132.692791 | 1202.07 | 1271.447209 | 0.976499 |
| GO:0008630\_DNA\_damage\_response\_\_signal\_transduction\_resulting\_in\_induction\_of\_apoptosis | 14 | 0 | 0.000000 | -0.000000 | 1231 | 1132.692791 | 1202.07 | 1271.447209 | 0.976499 |
| GO:0009108\_coenzyme\_biosynthetic\_process | 14 | 0 | 0.000000 | -0.000000 | 1231 | 1132.692791 | 1202.07 | 1271.447209 | 0.976499 |
| GO:0009267\_cellular\_response\_to\_starvation | 14 | 0 | 0.000000 | -0.000000 | 1231 | 1132.692791 | 1202.07 | 1271.447209 | 0.976499 |
| GO:0009895\_negative\_regulation\_of\_catabolic\_process | 14 | 0 | 0.000000 | -0.000000 | 1231 | 1132.692791 | 1202.07 | 1271.447209 | 0.976499 |
| GO:0010332\_response\_to\_gamma\_radiation | 14 | 0 | 0.000000 | -0.000000 | 1231 | 1132.692791 | 1202.07 | 1271.447209 | 0.976499 |
| GO:0014855\_striated\_muscle\_cell\_proliferation | 14 | 0 | 0.000000 | -0.000000 | 1231 | 1132.692791 | 1202.07 | 1271.447209 | 0.976499 |
| GO:0016573\_histone\_acetylation | 14 | 0 | 0.000000 | -0.000000 | 1231 | 1132.692791 | 1202.07 | 1271.447209 | 0.976499 |
| GO:0018130\_heterocycle\_biosynthetic\_process | 14 | 0 | 0.000000 | -0.000000 | 1231 | 1132.692791 | 1202.07 | 1271.447209 | 0.976499 |
| GO:0019217\_regulation\_of\_fatty\_acid\_metabolic\_process | 14 | 0 | 0.000000 | -0.000000 | 1231 | 1132.692791 | 1202.07 | 1271.447209 | 0.976499 |
| GO:0021782\_glial\_cell\_development | 14 | 0 | 0.000000 | -0.000000 | 1231 | 1132.692791 | 1202.07 | 1271.447209 | 0.976499 |
| GO:0021904\_dorsal\_ventral\_neural\_tube\_patterning | 14 | 0 | 0.000000 | -0.000000 | 1231 | 1132.692791 | 1202.07 | 1271.447209 | 0.976499 |
| GO:0030032\_lamellipodium\_assembly | 14 | 0 | 0.000000 | -0.000000 | 1231 | 1132.692791 | 1202.07 | 1271.447209 | 0.976499 |
| GO:0030148\_sphingolipid\_biosynthetic\_process | 14 | 0 | 0.000000 | -0.000000 | 1231 | 1132.692791 | 1202.07 | 1271.447209 | 0.976499 |
| GO:0030162\_regulation\_of\_proteolysis | 14 | 0 | 0.000000 | -0.000000 | 1231 | 1132.692791 | 1202.07 | 1271.447209 | 0.976499 |
| GO:0031099\_regeneration | 14 | 0 | 0.000000 | -0.000000 | 1231 | 1132.692791 | 1202.07 | 1271.447209 | 0.976499 |
| GO:0031346\_positive\_regulation\_of\_cell\_projection\_organization | 14 | 0 | 0.000000 | -0.000000 | 1231 | 1132.692791 | 1202.07 | 1271.447209 | 0.976499 |
| GO:0031663\_lipopolysaccharide-mediated\_signaling\_pathway | 14 | 0 | 0.000000 | -0.000000 | 1231 | 1132.692791 | 1202.07 | 1271.447209 | 0.976499 |
| GO:0032271\_regulation\_of\_protein\_polymerization | 14 | 0 | 0.000000 | -0.000000 | 1231 | 1132.692791 | 1202.07 | 1271.447209 | 0.976499 |
| GO:0033044\_regulation\_of\_chromosome\_organization | 14 | 0 | 0.000000 | -0.000000 | 1231 | 1132.692791 | 1202.07 | 1271.447209 | 0.976499 |
| GO:0034104\_negative\_regulation\_of\_tissue\_remodeling | 14 | 0 | 0.000000 | -0.000000 | 1231 | 1132.692791 | 1202.07 | 1271.447209 | 0.976499 |
| GO:0035036\_sperm-egg\_recognition | 14 | 0 | 0.000000 | -0.000000 | 1231 | 1132.692791 | 1202.07 | 1271.447209 | 0.976499 |
| GO:0042310\_vasoconstriction | 14 | 0 | 0.000000 | -0.000000 | 1231 | 1132.692791 | 1202.07 | 1271.447209 | 0.976499 |
| GO:0042573\_retinoic\_acid\_metabolic\_process | 14 | 0 | 0.000000 | -0.000000 | 1231 | 1132.692791 | 1202.07 | 1271.447209 | 0.976499 |
| GO:0043123\_positive\_regulation\_of\_I-kappaB\_kinase\_NF-kappaB\_cascade | 14 | 0 | 0.000000 | -0.000000 | 1231 | 1132.692791 | 1202.07 | 1271.447209 | 0.976499 |
| GO:0043254\_regulation\_of\_protein\_complex\_assembly | 14 | 0 | 0.000000 | -0.000000 | 1231 | 1132.692791 | 1202.07 | 1271.447209 | 0.976499 |
| GO:0043491\_protein\_kinase\_B\_signaling\_cascade | 14 | 0 | 0.000000 | -0.000000 | 1231 | 1132.692791 | 1202.07 | 1271.447209 | 0.976499 |
| GO:0044236\_multicellular\_organismal\_metabolic\_process | 14 | 0 | 0.000000 | -0.000000 | 1231 | 1132.692791 | 1202.07 | 1271.447209 | 0.976499 |
| GO:0045061\_thymic\_T\_cell\_selection | 14 | 0 | 0.000000 | -0.000000 | 1231 | 1132.692791 | 1202.07 | 1271.447209 | 0.976499 |
| GO:0045453\_bone\_resorption | 14 | 0 | 0.000000 | -0.000000 | 1231 | 1132.692791 | 1202.07 | 1271.447209 | 0.976499 |
| GO:0045598\_regulation\_of\_fat\_cell\_differentiation | 14 | 0 | 0.000000 | -0.000000 | 1231 | 1132.692791 | 1202.07 | 1271.447209 | 0.976499 |
| GO:0045732\_positive\_regulation\_of\_protein\_catabolic\_process | 14 | 0 | 0.000000 | -0.000000 | 1231 | 1132.692791 | 1202.07 | 1271.447209 | 0.976499 |
| GO:0046209\_nitric\_oxide\_metabolic\_process | 14 | 0 | 0.000000 | -0.000000 | 1231 | 1132.692791 | 1202.07 | 1271.447209 | 0.976499 |
| GO:0048048\_embryonic\_eye\_morphogenesis | 14 | 0 | 0.000000 | -0.000000 | 1231 | 1132.692791 | 1202.07 | 1271.447209 | 0.976499 |
| GO:0048545\_response\_to\_steroid\_hormone\_stimulus | 14 | 0 | 0.000000 | -0.000000 | 1231 | 1132.692791 | 1202.07 | 1271.447209 | 0.976499 |
| GO:0048665\_neuron\_fate\_specification | 14 | 0 | 0.000000 | -0.000000 | 1231 | 1132.692791 | 1202.07 | 1271.447209 | 0.976499 |
| GO:0048844\_artery\_morphogenesis | 14 | 0 | 0.000000 | -0.000000 | 1231 | 1132.692791 | 1202.07 | 1271.447209 | 0.976499 |
| GO:0050810\_regulation\_of\_steroid\_biosynthetic\_process | 14 | 0 | 0.000000 | -0.000000 | 1231 | 1132.692791 | 1202.07 | 1271.447209 | 0.976499 |
| GO:0051017\_actin\_filament\_bundle\_formation | 14 | 0 | 0.000000 | -0.000000 | 1231 | 1132.692791 | 1202.07 | 1271.447209 | 0.976499 |
| GO:0051054\_positive\_regulation\_of\_DNA\_metabolic\_process | 14 | 0 | 0.000000 | -0.000000 | 1231 | 1132.692791 | 1202.07 | 1271.447209 | 0.976499 |
| GO:0051100\_negative\_regulation\_of\_binding | 14 | 0 | 0.000000 | -0.000000 | 1231 | 1132.692791 | 1202.07 | 1271.447209 | 0.976499 |
| GO:0051952\_regulation\_of\_amine\_transport | 14 | 0 | 0.000000 | -0.000000 | 1231 | 1132.692791 | 1202.07 | 1271.447209 | 0.976499 |
| GO:0060716\_labyrinthine\_layer\_blood\_vessel\_development | 14 | 0 | 0.000000 | -0.000000 | 1231 | 1132.692791 | 1202.07 | 1271.447209 | 0.976499 |
| GO:0060840\_artery\_development | 14 | 0 | 0.000000 | -0.000000 | 1231 | 1132.692791 | 1202.07 | 1271.447209 | 0.976499 |
| GO:0002757\_immune\_response-activating\_signal\_transduction | 47 | 0 | 0.000000 | -0.000000 | 1241 | 1142.835519 | 1211.45 | 1280.064481 | 0.976189 |
| GO:0006140\_regulation\_of\_nucleotide\_metabolic\_process | 47 | 0 | 0.000000 | -0.000000 | 1241 | 1142.835519 | 1211.45 | 1280.064481 | 0.976189 |
| GO:0016570\_histone\_modification | 47 | 0 | 0.000000 | -0.000000 | 1241 | 1142.835519 | 1211.45 | 1280.064481 | 0.976189 |
| GO:0030799\_regulation\_of\_cyclic\_nucleotide\_metabolic\_process | 47 | 0 | 0.000000 | -0.000000 | 1241 | 1142.835519 | 1211.45 | 1280.064481 | 0.976189 |
| GO:0031667\_response\_to\_nutrient\_levels | 47 | 0 | 0.000000 | -0.000000 | 1241 | 1142.835519 | 1211.45 | 1280.064481 | 0.976189 |
| GO:0034754\_cellular\_hormone\_metabolic\_process | 47 | 0 | 0.000000 | -0.000000 | 1241 | 1142.835519 | 1211.45 | 1280.064481 | 0.976189 |
| GO:0045087\_innate\_immune\_response | 47 | 0 | 0.000000 | -0.000000 | 1241 | 1142.835519 | 1211.45 | 1280.064481 | 0.976189 |
| GO:0045619\_regulation\_of\_lymphocyte\_differentiation | 47 | 0 | 0.000000 | -0.000000 | 1241 | 1142.835519 | 1211.45 | 1280.064481 | 0.976189 |
| GO:0048871\_multicellular\_organismal\_homeostasis | 47 | 0 | 0.000000 | -0.000000 | 1241 | 1142.835519 | 1211.45 | 1280.064481 | 0.976189 |
| GO:0060627\_regulation\_of\_vesicle-mediated\_transport | 47 | 0 | 0.000000 | -0.000000 | 1241 | 1142.835519 | 1211.45 | 1280.064481 | 0.976189 |
| GO:0000096\_sulfur\_amino\_acid\_metabolic\_process | 11 | 0 | 0.000000 | -0.000000 | 1320 | 1223.359808 | 1289.82 | 1356.280192 | 0.977136 |
| GO:0000271\_polysaccharide\_biosynthetic\_process | 11 | 0 | 0.000000 | -0.000000 | 1320 | 1223.359808 | 1289.82 | 1356.280192 | 0.977136 |
| GO:0000737\_DNA\_catabolic\_process\_\_endonucleolytic | 11 | 0 | 0.000000 | -0.000000 | 1320 | 1223.359808 | 1289.82 | 1356.280192 | 0.977136 |
[truncated: 255,860 more chars]
