# Supplementary material for: RedundancyMiner: De-replication of redundant GO categories in microarray and proteomics analysis
Source: BMC Bioinformatics. 2011 Feb 10;12:52. doi: 10.1186/1471-2105-12-52 (PMC3223614; doi:10.1186/1471-2105-12-52)
Supplement: Additional file 8 — Retinal development HTGM download. compressed package of the results of running HTGM on the retinal development genes list. [file 1471-2105-12-52-S8.ZIP › SCENARIO_2_MODIFIED/total.txt.total.txt.dir/Exp1_BestClusterMap_LEIGS_KM_24.csv.join.10.txt.dir/Exp1_BestClusterMap_LEIGS_KM_24.csv.join.10.txt.change.gce.html]

Gene Category Report for Exp1\_BestClusterMap\_LEIGS\_KM\_24.csv.join.10.txt

# Gene Category Report for Exp1\_BestClusterMap\_LEIGS\_KM\_24.csv.join.10.txt

| HYPERLINKED GO CATEGORY | HYPERLINKED GENE NAME | TOTAL GENES | CHANGED GENES | ENRICHMENT | LOG10(p) | CUMULATIVE NUMBER OF CATEGORIES | CUMULATIVE RANDOMS MEAN | FALSE DISCOVERY RATE |
| --- | --- | --- | --- | --- | --- | --- | --- | --- |
| GO:0042438\_melanin\_biosynthetic\_process | DCT | 7 | 2 | 62.653061 | -3.386878 | 1 | 0.4 | 0.400000 |
| GO:0042438\_melanin\_biosynthetic\_process | SI | 7 | 2 | 62.653061 | -3.386878 | 1 | 0.4 | 0.400000 |
| GO:0006582\_melanin\_metabolic\_process | DCT | 8 | 2 | 54.821429 | -3.263135 | 2 | 0.5 | 0.250000 |
| GO:0006582\_melanin\_metabolic\_process | SI | 8 | 2 | 54.821429 | -3.263135 | 2 | 0.5 | 0.250000 |
| GO:0046148\_pigment\_biosynthetic\_process | DCT | 16 | 2 | 27.410714 | -2.640669 | 3 | 2.22 | 0.740000 |
| GO:0046148\_pigment\_biosynthetic\_process | SI | 16 | 2 | 27.410714 | -2.640669 | 3 | 2.22 | 0.740000 |
| GO:0010033\_response\_to\_organic\_substance | ID2 | 216 | 5 | 5.076058 | -2.623394 | 4 | 2.23 | 0.557500 |
| GO:0010033\_response\_to\_organic\_substance | PTPN2 | 216 | 5 | 5.076058 | -2.623394 | 4 | 2.23 | 0.557500 |
| GO:0010033\_response\_to\_organic\_substance | MAPK14 | 216 | 5 | 5.076058 | -2.623394 | 4 | 2.23 | 0.557500 |
| GO:0010033\_response\_to\_organic\_substance | AARS | 216 | 5 | 5.076058 | -2.623394 | 4 | 2.23 | 0.557500 |
| GO:0010033\_response\_to\_organic\_substance | SOCS7 | 216 | 5 | 5.076058 | -2.623394 | 4 | 2.23 | 0.557500 |
| GO:0042440\_pigment\_metabolic\_process | DCT | 17 | 2 | 25.798319 | -2.587505 | 5 | 2.47 | 0.494000 |
| GO:0042440\_pigment\_metabolic\_process | SI | 17 | 2 | 25.798319 | -2.587505 | 5 | 2.47 | 0.494000 |
| GO:0045639\_positive\_regulation\_of\_myeloid\_cell\_differentiation | ID2 | 20 | 2 | 21.928571 | -2.445870 | 6 | 3.22 | 0.536667 |
| GO:0045639\_positive\_regulation\_of\_myeloid\_cell\_differentiation | MAPK14 | 20 | 2 | 21.928571 | -2.445870 | 6 | 3.22 | 0.536667 |
| GO:0006419\_alanyl-tRNA\_aminoacylation | AARS | 1 | 1 |  |  |  |  |  |  |
| GO:0030327\_prenylated\_protein\_catabolic\_process | ZMPSTE24 | 1 | 1 |  |  |  |  |  |  |
| GO:0070887\_cellular\_response\_to\_chemical\_stimulus | PTPN2 | 85 | 3 | 7.739496 | -2.197277 | 7 | 4.99 | 0.712857 |
| GO:0070887\_cellular\_response\_to\_chemical\_stimulus | AARS | 85 | 3 | 7.739496 | -2.197277 | 7 | 4.99 | 0.712857 |
| GO:0070887\_cellular\_response\_to\_chemical\_stimulus | SOCS7 | 85 | 3 | 7.739496 | -2.197277 | 7 | 4.99 | 0.712857 |
| GO:0008286\_insulin\_receptor\_signaling\_pathway | PTPN2 | 27 | 2 | 16.243386 | -2.187661 | 8 | 5.07 | 0.633750 |
| GO:0008286\_insulin\_receptor\_signaling\_pathway | SOCS7 | 27 | 2 | 16.243386 | -2.187661 | 8 | 5.07 | 0.633750 |
| GO:0009719\_response\_to\_endogenous\_stimulus | PTPN2 | 92 | 3 | 7.150621 | -2.101888 | 9 | 5.87 | 0.652222 |
| GO:0009719\_response\_to\_endogenous\_stimulus | AARS | 92 | 3 | 7.150621 | -2.101888 | 9 | 5.87 | 0.652222 |
| GO:0009719\_response\_to\_endogenous\_stimulus | SOCS7 | 92 | 3 | 7.150621 | -2.101888 | 9 | 5.87 | 0.652222 |
| GO:0050885\_neuromuscular\_process\_controlling\_balance | HEXA | 32 | 2 | 13.705357 | -2.043440 | 10 | 7.03 | 0.703000 |
| GO:0050885\_neuromuscular\_process\_controlling\_balance | AARS | 32 | 2 | 13.705357 | -2.043440 | 10 | 7.03 | 0.703000 |
| GO:0006418\_tRNA\_aminoacylation\_for\_protein\_translation | AARS | 2 | 1 |  |  |  |  |  |  |
| GO:0006583\_melanin\_biosynthetic\_process\_from\_tyrosine | DCT | 2 | 1 |  |  |  |  |  |  |
| GO:0032495\_response\_to\_muramyl\_dipeptide | MAPK14 | 2 | 1 |  |  |  |  |  |  |
| GO:0043038\_amino\_acid\_activation | AARS | 2 | 1 |  |  |  |  |  |  |
| GO:0043039\_tRNA\_aminoacylation | AARS | 2 | 1 |  |  |  |  |  |  |
| GO:0032869\_cellular\_response\_to\_insulin\_stimulus | PTPN2 | 37 | 2 | 11.853282 | -1.921395 | 11 | 8.68 | 0.789091 |
| GO:0032869\_cellular\_response\_to\_insulin\_stimulus | SOCS7 | 37 | 2 | 11.853282 | -1.921395 | 11 | 8.68 | 0.789091 |
| GO:0006689\_ganglioside\_catabolic\_process | HEXA | 3 | 1 |  |  |  |  |  |  |
| GO:0043200\_response\_to\_amino\_acid\_stimulus | AARS | 3 | 1 |  |  |  |  |  |  |
| GO:0046479\_glycosphingolipid\_catabolic\_process | HEXA | 3 | 1 |  |  |  |  |  |  |
| GO:0019748\_secondary\_metabolic\_process | DCT | 41 | 2 | 10.696864 | -1.835809 | 12 | 10.51 | 0.875833 |
| GO:0019748\_secondary\_metabolic\_process | SI | 41 | 2 | 10.696864 | -1.835809 | 12 | 10.51 | 0.875833 |
| GO:0045637\_regulation\_of\_myeloid\_cell\_differentiation | ID2 | 42 | 2 | 10.442177 | -1.815807 | 13 | 11.02 | 0.847692 |
| GO:0045637\_regulation\_of\_myeloid\_cell\_differentiation | MAPK14 | 42 | 2 | 10.442177 | -1.815807 | 13 | 11.02 | 0.847692 |
| GO:0032868\_response\_to\_insulin\_stimulus | PTPN2 | 43 | 2 | 10.199336 | -1.796311 | 15 | 11.55 | 0.770000 |
| GO:0032868\_response\_to\_insulin\_stimulus | SOCS7 | 43 | 2 | 10.199336 | -1.796311 | 15 | 11.55 | 0.770000 |
| GO:0051789\_response\_to\_protein\_stimulus | ID2 | 43 | 2 | 10.199336 | -1.796311 | 15 | 11.55 | 0.770000 |
| GO:0051789\_response\_to\_protein\_stimulus | AARS | 43 | 2 | 10.199336 | -1.796311 | 15 | 11.55 | 0.770000 |
| GO:0043283\_biopolymer\_metabolic\_process | SMARCAD1 | 1490 | 12 | 1.766059 | -1.785537 | 16 | 11.65 | 0.728125 |
| GO:0043283\_biopolymer\_metabolic\_process | CSRP2BP | 1490 | 12 | 1.766059 | -1.785537 | 16 | 11.65 | 0.728125 |
| GO:0043283\_biopolymer\_metabolic\_process | ID2 | 1490 | 12 | 1.766059 | -1.785537 | 16 | 11.65 | 0.728125 |
| GO:0043283\_biopolymer\_metabolic\_process | PTPN2 | 1490 | 12 | 1.766059 | -1.785537 | 16 | 11.65 | 0.728125 |
| GO:0043283\_biopolymer\_metabolic\_process | MAPK14 | 1490 | 12 | 1.766059 | -1.785537 | 16 | 11.65 | 0.728125 |
| GO:0043283\_biopolymer\_metabolic\_process | HEXA | 1490 | 12 | 1.766059 | -1.785537 | 16 | 11.65 | 0.728125 |
| GO:0043283\_biopolymer\_metabolic\_process | WDR77 | 1490 | 12 | 1.766059 | -1.785537 | 16 | 11.65 | 0.728125 |
| GO:0043283\_biopolymer\_metabolic\_process | RBL1 | 1490 | 12 | 1.766059 | -1.785537 | 16 | 11.65 | 0.728125 |
| GO:0043283\_biopolymer\_metabolic\_process | AARS | 1490 | 12 | 1.766059 | -1.785537 | 16 | 11.65 | 0.728125 |
| GO:0043283\_biopolymer\_metabolic\_process | SOCS7 | 1490 | 12 | 1.766059 | -1.785537 | 16 | 11.65 | 0.728125 |
| GO:0043283\_biopolymer\_metabolic\_process | TAF9 | 1490 | 12 | 1.766059 | -1.785537 | 16 | 11.65 | 0.728125 |
| GO:0043283\_biopolymer\_metabolic\_process | ZMPSTE24 | 1490 | 12 | 1.766059 | -1.785537 | 16 | 11.65 | 0.728125 |
| GO:0032870\_cellular\_response\_to\_hormone\_stimulus | PTPN2 | 45 | 2 | 9.746032 | -1.758738 | 17 | 12.49 | 0.734706 |
| GO:0032870\_cellular\_response\_to\_hormone\_stimulus | SOCS7 | 45 | 2 | 9.746032 | -1.758738 | 17 | 12.49 | 0.734706 |
| GO:0009308\_amine\_metabolic\_process | DCT | 124 | 3 | 5.305300 | -1.749951 | 18 | 12.57 | 0.698333 |
| GO:0009308\_amine\_metabolic\_process | HEXA | 124 | 3 | 5.305300 | -1.749951 | 18 | 12.57 | 0.698333 |
| GO:0009308\_amine\_metabolic\_process | AARS | 124 | 3 | 5.305300 | -1.749951 | 18 | 12.57 | 0.698333 |
| GO:0019377\_glycolipid\_catabolic\_process | HEXA | 4 | 1 |  |  |  |  |  |  |
| GO:0060528\_secretory\_columnal\_luminar\_epithelial\_cell\_differentiation\_involved\_in\_prostate\_glandular\_acinus\_development | WDR77 | 4 | 1 |  |  |  |  |  |  |
| GO:0030218\_erythrocyte\_differentiation | ID2 | 46 | 2 | 9.534161 | -1.740619 | 19 | 12.79 | 0.673158 |
| GO:0030218\_erythrocyte\_differentiation | MAPK14 | 46 | 2 | 9.534161 | -1.740619 | 19 | 12.79 | 0.673158 |
| GO:0045597\_positive\_regulation\_of\_cell\_differentiation | METRN | 128 | 3 | 5.139509 | -1.713333 | 20 | 13.15 | 0.657500 |
| GO:0045597\_positive\_regulation\_of\_cell\_differentiation | ID2 | 128 | 3 | 5.139509 | -1.713333 | 20 | 13.15 | 0.657500 |
| GO:0045597\_positive\_regulation\_of\_cell\_differentiation | MAPK14 | 128 | 3 | 5.139509 | -1.713333 | 20 | 13.15 | 0.657500 |
| GO:0034101\_erythrocyte\_homeostasis | ID2 | 49 | 2 | 8.950437 | -1.688712 | 21 | 13.93 | 0.663333 |
| GO:0034101\_erythrocyte\_homeostasis | MAPK14 | 49 | 2 | 8.950437 | -1.688712 | 21 | 13.93 | 0.663333 |
| GO:0006520\_cellular\_amino\_acid\_metabolic\_process | DCT | 51 | 2 | 8.599440 | -1.655981 | 23 | 14.59 | 0.634348 |
| GO:0006520\_cellular\_amino\_acid\_metabolic\_process | AARS | 51 | 2 | 8.599440 | -1.655981 | 23 | 14.59 | 0.634348 |
| GO:0044106\_cellular\_amine\_metabolic\_process | DCT | 51 | 2 | 8.599440 | -1.655981 | 23 | 14.59 | 0.634348 |
| GO:0044106\_cellular\_amine\_metabolic\_process | AARS | 51 | 2 | 8.599440 | -1.655981 | 23 | 14.59 | 0.634348 |
| GO:0006400\_tRNA\_modification | AARS | 5 | 1 | 43.857143 | -1.645814 | 28 | 21.66 | 0.773571 |
| GO:0006570\_tyrosine\_metabolic\_process | DCT | 5 | 1 | 43.857143 | -1.645814 | 28 | 21.66 | 0.773571 |
| GO:0032494\_response\_to\_peptidoglycan | MAPK14 | 5 | 1 | 43.857143 | -1.645814 | 28 | 21.66 | 0.773571 |
| GO:0045648\_positive\_regulation\_of\_erythrocyte\_differentiation | MAPK14 | 5 | 1 | 43.857143 | -1.645814 | 28 | 21.66 | 0.773571 |
| GO:0045651\_positive\_regulation\_of\_macrophage\_differentiation | ID2 | 5 | 1 | 43.857143 | -1.645814 | 28 | 21.66 | 0.773571 |
| GO:0050905\_neuromuscular\_process | HEXA | 53 | 2 | 8.274933 | -1.624612 | 29 | 22.15 | 0.763793 |
| GO:0050905\_neuromuscular\_process | AARS | 53 | 2 | 8.274933 | -1.624612 | 29 | 22.15 | 0.763793 |
| GO:0043434\_response\_to\_peptide\_hormone\_stimulus | PTPN2 | 55 | 2 | 7.974026 | -1.594505 | 30 | 22.89 | 0.763000 |
| GO:0043434\_response\_to\_peptide\_hormone\_stimulus | SOCS7 | 55 | 2 | 7.974026 | -1.594505 | 30 | 22.89 | 0.763000 |
| GO:0043170\_macromolecule\_metabolic\_process | SMARCAD1 | 1576 | 12 | 1.669688 | -1.585938 | 31 | 23.03 | 0.742903 |
| GO:0043170\_macromolecule\_metabolic\_process | CSRP2BP | 1576 | 12 | 1.669688 | -1.585938 | 31 | 23.03 | 0.742903 |
| GO:0043170\_macromolecule\_metabolic\_process | ID2 | 1576 | 12 | 1.669688 | -1.585938 | 31 | 23.03 | 0.742903 |
| GO:0043170\_macromolecule\_metabolic\_process | PTPN2 | 1576 | 12 | 1.669688 | -1.585938 | 31 | 23.03 | 0.742903 |
| GO:0043170\_macromolecule\_metabolic\_process | HEXA | 1576 | 12 | 1.669688 | -1.585938 | 31 | 23.03 | 0.742903 |
| GO:0043170\_macromolecule\_metabolic\_process | MAPK14 | 1576 | 12 | 1.669688 | -1.585938 | 31 | 23.03 | 0.742903 |
| GO:0043170\_macromolecule\_metabolic\_process | AARS | 1576 | 12 | 1.669688 | -1.585938 | 31 | 23.03 | 0.742903 |
| GO:0043170\_macromolecule\_metabolic\_process | WDR77 | 1576 | 12 | 1.669688 | -1.585938 | 31 | 23.03 | 0.742903 |
| GO:0043170\_macromolecule\_metabolic\_process | RBL1 | 1576 | 12 | 1.669688 | -1.585938 | 31 | 23.03 | 0.742903 |
| GO:0043170\_macromolecule\_metabolic\_process | SOCS7 | 1576 | 12 | 1.669688 | -1.585938 | 31 | 23.03 | 0.742903 |
| GO:0043170\_macromolecule\_metabolic\_process | TAF9 | 1576 | 12 | 1.669688 | -1.585938 | 31 | 23.03 | 0.742903 |
| GO:0043170\_macromolecule\_metabolic\_process | ZMPSTE24 | 1576 | 12 | 1.669688 | -1.585938 | 31 | 23.03 | 0.742903 |
| GO:0006998\_nuclear\_envelope\_organization | ZMPSTE24 | 6 | 1 | 36.547619 | -1.567575 | 35 | 28.72 | 0.820571 |
| GO:0030149\_sphingolipid\_catabolic\_process | HEXA | 6 | 1 | 36.547619 | -1.567575 | 35 | 28.72 | 0.820571 |
| GO:0045649\_regulation\_of\_macrophage\_differentiation | ID2 | 6 | 1 | 36.547619 | -1.567575 | 35 | 28.72 | 0.820571 |
| GO:0046466\_membrane\_lipid\_catabolic\_process | HEXA | 6 | 1 | 36.547619 | -1.567575 | 35 | 28.72 | 0.820571 |
| GO:0034960\_cellular\_biopolymer\_metabolic\_process | SMARCAD1 | 1395 | 11 | 1.729135 | -1.552095 | 36 | 29.14 | 0.809444 |
| GO:0034960\_cellular\_biopolymer\_metabolic\_process | CSRP2BP | 1395 | 11 | 1.729135 | -1.552095 | 36 | 29.14 | 0.809444 |
| GO:0034960\_cellular\_biopolymer\_metabolic\_process | ID2 | 1395 | 11 | 1.729135 | -1.552095 | 36 | 29.14 | 0.809444 |
| GO:0034960\_cellular\_biopolymer\_metabolic\_process | PTPN2 | 1395 | 11 | 1.729135 | -1.552095 | 36 | 29.14 | 0.809444 |
| GO:0034960\_cellular\_biopolymer\_metabolic\_process | MAPK14 | 1395 | 11 | 1.729135 | -1.552095 | 36 | 29.14 | 0.809444 |
| GO:0034960\_cellular\_biopolymer\_metabolic\_process | WDR77 | 1395 | 11 | 1.729135 | -1.552095 | 36 | 29.14 | 0.809444 |
| GO:0034960\_cellular\_biopolymer\_metabolic\_process | RBL1 | 1395 | 11 | 1.729135 | -1.552095 | 36 | 29.14 | 0.809444 |
| GO:0034960\_cellular\_biopolymer\_metabolic\_process | AARS | 1395 | 11 | 1.729135 | -1.552095 | 36 | 29.14 | 0.809444 |
| GO:0034960\_cellular\_biopolymer\_metabolic\_process | SOCS7 | 1395 | 11 | 1.729135 | -1.552095 | 36 | 29.14 | 0.809444 |
| GO:0034960\_cellular\_biopolymer\_metabolic\_process | ZMPSTE24 | 1395 | 11 | 1.729135 | -1.552095 | 36 | 29.14 | 0.809444 |
| GO:0034960\_cellular\_biopolymer\_metabolic\_process | TAF9 | 1395 | 11 | 1.729135 | -1.552095 | 36 | 29.14 | 0.809444 |
| GO:0001573\_ganglioside\_metabolic\_process | HEXA | 7 | 1 | 31.326531 | -1.501570 | 41 | 36.59 | 0.892439 |
| GO:0002067\_glandular\_epithelial\_cell\_differentiation | WDR77 | 7 | 1 | 31.326531 | -1.501570 | 41 | 36.59 | 0.892439 |
| GO:0008033\_tRNA\_processing | AARS | 7 | 1 | 31.326531 | -1.501570 | 41 | 36.59 | 0.892439 |
| GO:0043353\_enucleate\_erythrocyte\_differentiation | ID2 | 7 | 1 | 31.326531 | -1.501570 | 41 | 36.59 | 0.892439 |
| GO:0060770\_negative\_regulation\_of\_epithelial\_cell\_proliferation\_involved\_in\_prostate\_gland\_development | WDR77 | 7 | 1 | 31.326531 | -1.501570 | 41 | 36.59 | 0.892439 |
| GO:0051716\_cellular\_response\_to\_stimulus | PTPN2 | 273 | 4 | 3.212977 | -1.488138 | 42 | 37.07 | 0.882619 |
| GO:0051716\_cellular\_response\_to\_stimulus | MAPK14 | 273 | 4 | 3.212977 | -1.488138 | 42 | 37.07 | 0.882619 |
| GO:0051716\_cellular\_response\_to\_stimulus | AARS | 273 | 4 | 3.212977 | -1.488138 | 42 | 37.07 | 0.882619 |
| GO:0051716\_cellular\_response\_to\_stimulus | SOCS7 | 273 | 4 | 3.212977 | -1.488138 | 42 | 37.07 | 0.882619 |
| GO:0042221\_response\_to\_chemical\_stimulus | ID2 | 409 | 5 | 2.680754 | -1.477576 | 43 | 37.42 | 0.870233 |
| GO:0042221\_response\_to\_chemical\_stimulus | PTPN2 | 409 | 5 | 2.680754 | -1.477576 | 43 | 37.42 | 0.870233 |
| GO:0042221\_response\_to\_chemical\_stimulus | MAPK14 | 409 | 5 | 2.680754 | -1.477576 | 43 | 37.42 | 0.870233 |
| GO:0042221\_response\_to\_chemical\_stimulus | AARS | 409 | 5 | 2.680754 | -1.477576 | 43 | 37.42 | 0.870233 |
| GO:0042221\_response\_to\_chemical\_stimulus | SOCS7 | 409 | 5 | 2.680754 | -1.477576 | 43 | 37.42 | 0.870233 |
| GO:0044267\_cellular\_protein\_metabolic\_process | CSRP2BP | 559 | 6 | 2.353693 | -1.466462 | 44 | 37.81 | 0.859318 |
| GO:0044267\_cellular\_protein\_metabolic\_process | PTPN2 | 559 | 6 | 2.353693 | -1.466462 | 44 | 37.81 | 0.859318 |
| GO:0044267\_cellular\_protein\_metabolic\_process | MAPK14 | 559 | 6 | 2.353693 | -1.466462 | 44 | 37.81 | 0.859318 |
| GO:0044267\_cellular\_protein\_metabolic\_process | AARS | 559 | 6 | 2.353693 | -1.466462 | 44 | 37.81 | 0.859318 |
| GO:0044267\_cellular\_protein\_metabolic\_process | SOCS7 | 559 | 6 | 2.353693 | -1.466462 | 44 | 37.81 | 0.859318 |
| GO:0044267\_cellular\_protein\_metabolic\_process | ZMPSTE24 | 559 | 6 | 2.353693 | -1.466462 | 44 | 37.81 | 0.859318 |
| GO:0002065\_columnar\_cuboidal\_epithelial\_cell\_differentiation | WDR77 | 8 | 1 | 27.410714 | -1.444519 | 47 | 43.79 | 0.931702 |
| GO:0006399\_tRNA\_metabolic\_process | AARS | 8 | 1 | 27.410714 | -1.444519 | 47 | 43.79 | 0.931702 |
| GO:0009072\_aromatic\_amino\_acid\_family\_metabolic\_process | DCT | 8 | 1 | 27.410714 | -1.444519 | 47 | 43.79 | 0.931702 |
| GO:0044260\_cellular\_macromolecule\_metabolic\_process | SMARCAD1 | 1447 | 11 | 1.666996 | -1.436809 | 48 | 44.07 | 0.918125 |
| GO:0044260\_cellular\_macromolecule\_metabolic\_process | CSRP2BP | 1447 | 11 | 1.666996 | -1.436809 | 48 | 44.07 | 0.918125 |
| GO:0044260\_cellular\_macromolecule\_metabolic\_process | ID2 | 1447 | 11 | 1.666996 | -1.436809 | 48 | 44.07 | 0.918125 |
| GO:0044260\_cellular\_macromolecule\_metabolic\_process | PTPN2 | 1447 | 11 | 1.666996 | -1.436809 | 48 | 44.07 | 0.918125 |
| GO:0044260\_cellular\_macromolecule\_metabolic\_process | MAPK14 | 1447 | 11 | 1.666996 | -1.436809 | 48 | 44.07 | 0.918125 |
| GO:0044260\_cellular\_macromolecule\_metabolic\_process | AARS | 1447 | 11 | 1.666996 | -1.436809 | 48 | 44.07 | 0.918125 |
| GO:0044260\_cellular\_macromolecule\_metabolic\_process | WDR77 | 1447 | 11 | 1.666996 | -1.436809 | 48 | 44.07 | 0.918125 |
| GO:0044260\_cellular\_macromolecule\_metabolic\_process | RBL1 | 1447 | 11 | 1.666996 | -1.436809 | 48 | 44.07 | 0.918125 |
| GO:0044260\_cellular\_macromolecule\_metabolic\_process | SOCS7 | 1447 | 11 | 1.666996 | -1.436809 | 48 | 44.07 | 0.918125 |
| GO:0044260\_cellular\_macromolecule\_metabolic\_process | ZMPSTE24 | 1447 | 11 | 1.666996 | -1.436809 | 48 | 44.07 | 0.918125 |
| GO:0044260\_cellular\_macromolecule\_metabolic\_process | TAF9 | 1447 | 11 | 1.666996 | -1.436809 | 48 | 44.07 | 0.918125 |
| GO:0009451\_RNA\_modification | AARS | 9 | 1 | 24.365079 | -1.394307 | 51 | 50.83 | 0.996667 |
| GO:0045646\_regulation\_of\_erythrocyte\_differentiation | MAPK14 | 9 | 1 | 24.365079 | -1.394307 | 51 | 50.83 | 0.996667 |
| GO:0050884\_neuromuscular\_process\_controlling\_posture | HEXA | 9 | 1 | 24.365079 | -1.394307 | 51 | 50.83 | 0.996667 |
| GO:0006357\_regulation\_of\_transcription\_from\_RNA\_polymerase\_II\_promoter | ID2 | 435 | 5 | 2.520525 | -1.377061 | 52 | 51.85 | 0.997115 |
| GO:0006357\_regulation\_of\_transcription\_from\_RNA\_polymerase\_II\_promoter | MAPK14 | 435 | 5 | 2.520525 | -1.377061 | 52 | 51.85 | 0.997115 |
| GO:0006357\_regulation\_of\_transcription\_from\_RNA\_polymerase\_II\_promoter | WDR77 | 435 | 5 | 2.520525 | -1.377061 | 52 | 51.85 | 0.997115 |
| GO:0006357\_regulation\_of\_transcription\_from\_RNA\_polymerase\_II\_promoter | RBL1 | 435 | 5 | 2.520525 | -1.377061 | 52 | 51.85 | 0.997115 |
| GO:0006357\_regulation\_of\_transcription\_from\_RNA\_polymerase\_II\_promoter | TAF9 | 435 | 5 | 2.520525 | -1.377061 | 52 | 51.85 | 0.997115 |
| GO:0060768\_regulation\_of\_epithelial\_cell\_proliferation\_involved\_in\_prostate\_gland\_development | WDR77 | 10 | 1 | 21.928571 | -1.349489 | 53 | 58.4 | 1.101887 |
| GO:0006366\_transcription\_from\_RNA\_polymerase\_II\_promoter | ID2 | 444 | 5 | 2.469434 | -1.344155 | 54 | 58.69 | 1.086852 |
| GO:0006366\_transcription\_from\_RNA\_polymerase\_II\_promoter | MAPK14 | 444 | 5 | 2.469434 | -1.344155 | 54 | 58.69 | 1.086852 |
| GO:0006366\_transcription\_from\_RNA\_polymerase\_II\_promoter | WDR77 | 444 | 5 | 2.469434 | -1.344155 | 54 | 58.69 | 1.086852 |
| GO:0006366\_transcription\_from\_RNA\_polymerase\_II\_promoter | RBL1 | 444 | 5 | 2.469434 | -1.344155 | 54 | 58.69 | 1.086852 |
| GO:0006366\_transcription\_from\_RNA\_polymerase\_II\_promoter | TAF9 | 444 | 5 | 2.469434 | -1.344155 | 54 | 58.69 | 1.086852 |
| GO:0009725\_response\_to\_hormone\_stimulus | PTPN2 | 76 | 2 | 5.770677 | -1.336209 | 55 | 59.09 | 1.074364 |
| GO:0009725\_response\_to\_hormone\_stimulus | SOCS7 | 76 | 2 | 5.770677 | -1.336209 | 55 | 59.09 | 1.074364 |
| GO:0044238\_primary\_metabolic\_process | SMARCAD1 | 1905 | 13 | 1.496438 | -1.334694 | 56 | 59.13 | 1.055893 |
| GO:0044238\_primary\_metabolic\_process | CSRP2BP | 1905 | 13 | 1.496438 | -1.334694 | 56 | 59.13 | 1.055893 |
| GO:0044238\_primary\_metabolic\_process | PTPN2 | 1905 | 13 | 1.496438 | -1.334694 | 56 | 59.13 | 1.055893 |
| GO:0044238\_primary\_metabolic\_process | HEXA | 1905 | 13 | 1.496438 | -1.334694 | 56 | 59.13 | 1.055893 |
| GO:0044238\_primary\_metabolic\_process | RBL1 | 1905 | 13 | 1.496438 | -1.334694 | 56 | 59.13 | 1.055893 |
| GO:0044238\_primary\_metabolic\_process | AARS | 1905 | 13 | 1.496438 | -1.334694 | 56 | 59.13 | 1.055893 |
| GO:0044238\_primary\_metabolic\_process | SOCS7 | 1905 | 13 | 1.496438 | -1.334694 | 56 | 59.13 | 1.055893 |
| GO:0044238\_primary\_metabolic\_process | DCT | 1905 | 13 | 1.496438 | -1.334694 | 56 | 59.13 | 1.055893 |
| GO:0044238\_primary\_metabolic\_process | ID2 | 1905 | 13 | 1.496438 | -1.334694 | 56 | 59.13 | 1.055893 |
| GO:0044238\_primary\_metabolic\_process | MAPK14 | 1905 | 13 | 1.496438 | -1.334694 | 56 | 59.13 | 1.055893 |
| GO:0044238\_primary\_metabolic\_process | WDR77 | 1905 | 13 | 1.496438 | -1.334694 | 56 | 59.13 | 1.055893 |
| GO:0044238\_primary\_metabolic\_process | TAF9 | 1905 | 13 | 1.496438 | -1.334694 | 56 | 59.13 | 1.055893 |
| GO:0044238\_primary\_metabolic\_process | ZMPSTE24 | 1905 | 13 | 1.496438 | -1.334694 | 56 | 59.13 | 1.055893 |
| GO:0006996\_organelle\_organization | CSRP2BP | 449 | 5 | 2.441934 | -1.326267 | 57 | 59.45 | 1.042982 |
| GO:0006996\_organelle\_organization | HEXA | 449 | 5 | 2.441934 | -1.326267 | 57 | 59.45 | 1.042982 |
| GO:0006996\_organelle\_organization | FAT1 | 449 | 5 | 2.441934 | -1.326267 | 57 | 59.45 | 1.042982 |
| GO:0006996\_organelle\_organization | ZMPSTE24 | 449 | 5 | 2.441934 | -1.326267 | 57 | 59.45 | 1.042982 |
| GO:0006996\_organelle\_organization | ARID1A | 449 | 5 | 2.441934 | -1.326267 | 57 | 59.45 | 1.042982 |
| GO:0019752\_carboxylic\_acid\_metabolic\_process | DCT | 181 | 3 | 3.634570 | -1.326111 | 59 | 59.52 | 1.008814 |
| GO:0019752\_carboxylic\_acid\_metabolic\_process | MAPK14 | 181 | 3 | 3.634570 | -1.326111 | 59 | 59.52 | 1.008814 |
| GO:0019752\_carboxylic\_acid\_metabolic\_process | AARS | 181 | 3 | 3.634570 | -1.326111 | 59 | 59.52 | 1.008814 |
| GO:0043436\_oxoacid\_metabolic\_process | DCT | 181 | 3 | 3.634570 | -1.326111 | 59 | 59.52 | 1.008814 |
| GO:0043436\_oxoacid\_metabolic\_process | MAPK14 | 181 | 3 | 3.634570 | -1.326111 | 59 | 59.52 | 1.008814 |
| GO:0043436\_oxoacid\_metabolic\_process | AARS | 181 | 3 | 3.634570 | -1.326111 | 59 | 59.52 | 1.008814 |
| GO:0006082\_organic\_acid\_metabolic\_process | DCT | 182 | 3 | 3.614600 | -1.320158 | 60 | 59.69 | 0.994833 |
| GO:0006082\_organic\_acid\_metabolic\_process | MAPK14 | 182 | 3 | 3.614600 | -1.320158 | 60 | 59.69 | 0.994833 |
| GO:0006082\_organic\_acid\_metabolic\_process | AARS | 182 | 3 | 3.614600 | -1.320158 | 60 | 59.69 | 0.994833 |
| GO:0042180\_cellular\_ketone\_metabolic\_process | DCT | 183 | 3 | 3.594848 | -1.314245 | 61 | 60.15 | 0.986066 |
| GO:0042180\_cellular\_ketone\_metabolic\_process | MAPK14 | 183 | 3 | 3.594848 | -1.314245 | 61 | 60.15 | 0.986066 |
| GO:0042180\_cellular\_ketone\_metabolic\_process | AARS | 183 | 3 | 3.594848 | -1.314245 | 61 | 60.15 | 0.986066 |
| GO:0008152\_metabolic\_process | SMARCAD1 | 2133 | 14 | 1.439287 | -1.311690 | 62 | 60.21 | 0.971129 |
| GO:0008152\_metabolic\_process | CSRP2BP | 2133 | 14 | 1.439287 | -1.311690 | 62 | 60.21 | 0.971129 |
| GO:0008152\_metabolic\_process | PTPN2 | 2133 | 14 | 1.439287 | -1.311690 | 62 | 60.21 | 0.971129 |
| GO:0008152\_metabolic\_process | HEXA | 2133 | 14 | 1.439287 | -1.311690 | 62 | 60.21 | 0.971129 |
| GO:0008152\_metabolic\_process | RBL1 | 2133 | 14 | 1.439287 | -1.311690 | 62 | 60.21 | 0.971129 |
| GO:0008152\_metabolic\_process | AARS | 2133 | 14 | 1.439287 | -1.311690 | 62 | 60.21 | 0.971129 |
| GO:0008152\_metabolic\_process | SI | 2133 | 14 | 1.439287 | -1.311690 | 62 | 60.21 | 0.971129 |
| GO:0008152\_metabolic\_process | SOCS7 | 2133 | 14 | 1.439287 | -1.311690 | 62 | 60.21 | 0.971129 |
| GO:0008152\_metabolic\_process | DCT | 2133 | 14 | 1.439287 | -1.311690 | 62 | 60.21 | 0.971129 |
| GO:0008152\_metabolic\_process | ID2 | 2133 | 14 | 1.439287 | -1.311690 | 62 | 60.21 | 0.971129 |
| GO:0008152\_metabolic\_process | MAPK14 | 2133 | 14 | 1.439287 | -1.311690 | 62 | 60.21 | 0.971129 |
| GO:0008152\_metabolic\_process | WDR77 | 2133 | 14 | 1.439287 | -1.311690 | 62 | 60.21 | 0.971129 |
| GO:0008152\_metabolic\_process | TAF9 | 2133 | 14 | 1.439287 | -1.311690 | 62 | 60.21 | 0.971129 |
| GO:0008152\_metabolic\_process | ZMPSTE24 | 2133 | 14 | 1.439287 | -1.311690 | 62 | 60.21 | 0.971129 |
| GO:0001101\_response\_to\_acid | AARS | 11 | 1 | 19.935065 | -1.309036 | 67 | 64.8 | 0.967164 |
| GO:0030968\_endoplasmic\_reticulum\_unfolded\_protein\_response | AARS | 11 | 1 | 19.935065 | -1.309036 | 67 | 64.8 | 0.967164 |
| GO:0034620\_cellular\_response\_to\_unfolded\_protein | AARS | 11 | 1 | 19.935065 | -1.309036 | 67 | 64.8 | 0.967164 |
| GO:0050772\_positive\_regulation\_of\_axonogenesis | METRN | 11 | 1 | 19.935065 | -1.309036 | 67 | 64.8 | 0.967164 |
| GO:0060767\_epithelial\_cell\_proliferation\_involved\_in\_prostate\_gland\_development | WDR77 | 11 | 1 | 19.935065 | -1.309036 | 67 | 64.8 | 0.967164 |
| GO:0043412\_biopolymer\_modification | CSRP2BP | 458 | 5 | 2.393949 | -1.294756 | 68 | 65.74 | 0.966765 |
| GO:0043412\_biopolymer\_modification | PTPN2 | 458 | 5 | 2.393949 | -1.294756 | 68 | 65.74 | 0.966765 |
| GO:0043412\_biopolymer\_modification | MAPK14 | 458 | 5 | 2.393949 | -1.294756 | 68 | 65.74 | 0.966765 |
| GO:0043412\_biopolymer\_modification | AARS | 458 | 5 | 2.393949 | -1.294756 | 68 | 65.74 | 0.966765 |
| GO:0043412\_biopolymer\_modification | SOCS7 | 458 | 5 | 2.393949 | -1.294756 | 68 | 65.74 | 0.966765 |
| GO:0002763\_positive\_regulation\_of\_myeloid\_leukocyte\_differentiation | ID2 | 12 | 1 | 18.273810 | -1.272186 | 72 | 70.67 | 0.981528 |
| GO:0021680\_cerebellar\_Purkinje\_cell\_layer\_development | AARS | 12 | 1 | 18.273810 | -1.272186 | 72 | 70.67 | 0.981528 |
| GO:0030225\_macrophage\_differentiation | ID2 | 12 | 1 | 18.273810 | -1.272186 | 72 | 70.67 | 0.981528 |
| GO:0060525\_prostate\_glandular\_acinus\_development | WDR77 | 12 | 1 | 18.273810 | -1.272186 | 72 | 70.67 | 0.981528 |
| GO:0006325\_chromatin\_organization | CSRP2BP | 83 | 2 | 5.283993 | -1.267442 | 73 | 71.02 | 0.972877 |
| GO:0006325\_chromatin\_organization | ARID1A | 83 | 2 | 5.283993 | -1.267442 | 73 | 71.02 | 0.972877 |
| GO:0006807\_nitrogen\_compound\_metabolic\_process | DCT | 1147 | 9 | 1.720638 | -1.263108 | 74 | 71.11 | 0.960946 |
| GO:0006807\_nitrogen\_compound\_metabolic\_process | SMARCAD1 | 1147 | 9 | 1.720638 | -1.263108 | 74 | 71.11 | 0.960946 |
| GO:0006807\_nitrogen\_compound\_metabolic\_process | ID2 | 1147 | 9 | 1.720638 | -1.263108 | 74 | 71.11 | 0.960946 |
| GO:0006807\_nitrogen\_compound\_metabolic\_process | HEXA | 1147 | 9 | 1.720638 | -1.263108 | 74 | 71.11 | 0.960946 |
| GO:0006807\_nitrogen\_compound\_metabolic\_process | MAPK14 | 1147 | 9 | 1.720638 | -1.263108 | 74 | 71.11 | 0.960946 |
| GO:0006807\_nitrogen\_compound\_metabolic\_process | AARS | 1147 | 9 | 1.720638 | -1.263108 | 74 | 71.11 | 0.960946 |
| GO:0006807\_nitrogen\_compound\_metabolic\_process | WDR77 | 1147 | 9 | 1.720638 | -1.263108 | 74 | 71.11 | 0.960946 |
| GO:0006807\_nitrogen\_compound\_metabolic\_process | RBL1 | 1147 | 9 | 1.720638 | -1.263108 | 74 | 71.11 | 0.960946 |
| GO:0006807\_nitrogen\_compound\_metabolic\_process | TAF9 | 1147 | 9 | 1.720638 | -1.263108 | 74 | 71.11 | 0.960946 |
| GO:0006687\_glycosphingolipid\_metabolic\_process | HEXA | 13 | 1 | 16.868132 | -1.238363 | 77 | 76.85 | 0.998052 |
| GO:0006986\_response\_to\_unfolded\_protein | AARS | 13 | 1 | 16.868132 | -1.238363 | 77 | 76.85 | 0.998052 |
| GO:0060742\_epithelial\_cell\_differentiation\_involved\_in\_prostate\_gland\_development | WDR77 | 13 | 1 | 16.868132 | -1.238363 | 77 | 76.85 | 0.998052 |
| GO:0044237\_cellular\_metabolic\_process | SMARCAD1 | 1974 | 13 | 1.444131 | -1.209053 | 78 | 78.45 | 1.005769 |
| GO:0044237\_cellular\_metabolic\_process | CSRP2BP | 1974 | 13 | 1.444131 | -1.209053 | 78 | 78.45 | 1.005769 |
| GO:0044237\_cellular\_metabolic\_process | PTPN2 | 1974 | 13 | 1.444131 | -1.209053 | 78 | 78.45 | 1.005769 |
| GO:0044237\_cellular\_metabolic\_process | HEXA | 1974 | 13 | 1.444131 | -1.209053 | 78 | 78.45 | 1.005769 |
| GO:0044237\_cellular\_metabolic\_process | AARS | 1974 | 13 | 1.444131 | -1.209053 | 78 | 78.45 | 1.005769 |
| GO:0044237\_cellular\_metabolic\_process | RBL1 | 1974 | 13 | 1.444131 | -1.209053 | 78 | 78.45 | 1.005769 |
| GO:0044237\_cellular\_metabolic\_process | SOCS7 | 1974 | 13 | 1.444131 | -1.209053 | 78 | 78.45 | 1.005769 |
| GO:0044237\_cellular\_metabolic\_process | DCT | 1974 | 13 | 1.444131 | -1.209053 | 78 | 78.45 | 1.005769 |
| GO:0044237\_cellular\_metabolic\_process | ID2 | 1974 | 13 | 1.444131 | -1.209053 | 78 | 78.45 | 1.005769 |
| GO:0044237\_cellular\_metabolic\_process | MAPK14 | 1974 | 13 | 1.444131 | -1.209053 | 78 | 78.45 | 1.005769 |
| GO:0044237\_cellular\_metabolic\_process | WDR77 | 1974 | 13 | 1.444131 | -1.209053 | 78 | 78.45 | 1.005769 |
| GO:0044237\_cellular\_metabolic\_process | TAF9 | 1974 | 13 | 1.444131 | -1.209053 | 78 | 78.45 | 1.005769 |
| GO:0044237\_cellular\_metabolic\_process | ZMPSTE24 | 1974 | 13 | 1.444131 | -1.209053 | 78 | 78.45 | 1.005769 |
| GO:0000077\_DNA\_damage\_checkpoint | MAPK14 | 14 | 1 | 15.663265 | -1.207116 | 82 | 82.59 | 1.007195 |
| GO:0016573\_histone\_acetylation | CSRP2BP | 14 | 1 | 15.663265 | -1.207116 | 82 | 82.59 | 1.007195 |
| GO:0031346\_positive\_regulation\_of\_cell\_projection\_organization | METRN | 14 | 1 | 15.663265 | -1.207116 | 82 | 82.59 | 1.007195 |
| GO:0031663\_lipopolysaccharide-mediated\_signaling\_pathway | MAPK14 | 14 | 1 | 15.663265 | -1.207116 | 82 | 82.59 | 1.007195 |
| GO:0006473\_protein\_amino\_acid\_acetylation | CSRP2BP | 15 | 1 | 14.619048 | -1.178089 | 84 | 88.19 | 1.049881 |
| GO:0007040\_lysosome\_organization | HEXA | 15 | 1 | 14.619048 | -1.178089 | 84 | 88.19 | 1.049881 |
| GO:0019538\_protein\_metabolic\_process | CSRP2BP | 655 | 6 | 2.008724 | -1.176905 | 85 | 88.21 | 1.037765 |
| GO:0019538\_protein\_metabolic\_process | PTPN2 | 655 | 6 | 2.008724 | -1.176905 | 85 | 88.21 | 1.037765 |
| GO:0019538\_protein\_metabolic\_process | MAPK14 | 655 | 6 | 2.008724 | -1.176905 | 85 | 88.21 | 1.037765 |
| GO:0019538\_protein\_metabolic\_process | AARS | 655 | 6 | 2.008724 | -1.176905 | 85 | 88.21 | 1.037765 |
| GO:0019538\_protein\_metabolic\_process | SOCS7 | 655 | 6 | 2.008724 | -1.176905 | 85 | 88.21 | 1.037765 |
| GO:0019538\_protein\_metabolic\_process | ZMPSTE24 | 655 | 6 | 2.008724 | -1.176905 | 85 | 88.21 | 1.037765 |
| GO:0016070\_RNA\_metabolic\_process | ID2 | 658 | 6 | 1.999566 | -1.168880 | 86 | 88.69 | 1.031279 |
| GO:0016070\_RNA\_metabolic\_process | MAPK14 | 658 | 6 | 1.999566 | -1.168880 | 86 | 88.69 | 1.031279 |
| GO:0016070\_RNA\_metabolic\_process | WDR77 | 658 | 6 | 1.999566 | -1.168880 | 86 | 88.69 | 1.031279 |
| GO:0016070\_RNA\_metabolic\_process | RBL1 | 658 | 6 | 1.999566 | -1.168880 | 86 | 88.69 | 1.031279 |
| GO:0016070\_RNA\_metabolic\_process | AARS | 658 | 6 | 1.999566 | -1.168880 | 86 | 88.69 | 1.031279 |
| GO:0016070\_RNA\_metabolic\_process | TAF9 | 658 | 6 | 1.999566 | -1.168880 | 86 | 88.69 | 1.031279 |
| GO:0006664\_glycolipid\_metabolic\_process | HEXA | 16 | 1 | 13.705357 | -1.150997 | 94 | 92.9 | 0.988298 |
| GO:0007033\_vacuole\_organization | HEXA | 16 | 1 | 13.705357 | -1.150997 | 94 | 92.9 | 0.988298 |
| GO:0010243\_response\_to\_organic\_nitrogen | AARS | 16 | 1 | 13.705357 | -1.150997 | 94 | 92.9 | 0.988298 |
| GO:0010876\_lipid\_localization | HEXA | 16 | 1 | 13.705357 | -1.150997 | 94 | 92.9 | 0.988298 |
| GO:0014075\_response\_to\_amine\_stimulus | AARS | 16 | 1 | 13.705357 | -1.150997 | 94 | 92.9 | 0.988298 |
| GO:0019915\_lipid\_storage | HEXA | 16 | 1 | 13.705357 | -1.150997 | 94 | 92.9 | 0.988298 |
| GO:0031570\_DNA\_integrity\_checkpoint | MAPK14 | 16 | 1 | 13.705357 | -1.150997 | 94 | 92.9 | 0.988298 |
| GO:0034976\_response\_to\_endoplasmic\_reticulum\_stress | AARS | 16 | 1 | 13.705357 | -1.150997 | 94 | 92.9 | 0.988298 |
| GO:0006984\_ER-nuclear\_signaling\_pathway | AARS | 17 | 1 | 12.899160 | -1.125604 | 98 | 97.89 | 0.998878 |
| GO:0019395\_fatty\_acid\_oxidation | MAPK14 | 17 | 1 | 12.899160 | -1.125604 | 98 | 97.89 | 0.998878 |
| GO:0034440\_lipid\_oxidation | MAPK14 | 17 | 1 | 12.899160 | -1.125604 | 98 | 97.89 | 0.998878 |
| GO:0034470\_ncRNA\_processing | AARS | 17 | 1 | 12.899160 | -1.125604 | 98 | 97.89 | 0.998878 |
| GO:0006022\_aminoglycan\_metabolic\_process | HEXA | 18 | 1 | 12.182540 | -1.101716 | 102 | 103.36 | 1.013333 |
| GO:0006457\_protein\_folding | AARS | 18 | 1 | 12.182540 | -1.101716 | 102 | 103.36 | 1.013333 |
| GO:0030203\_glycosaminoglycan\_metabolic\_process | HEXA | 18 | 1 | 12.182540 | -1.101716 | 102 | 103.36 | 1.013333 |
| GO:0048535\_lymph\_node\_development | ID2 | 18 | 1 | 12.182540 | -1.101716 | 102 | 103.36 | 1.013333 |
| GO:0048872\_homeostasis\_of\_number\_of\_cells | ID2 | 105 | 2 | 4.176871 | -1.087955 | 103 | 103.84 | 1.008155 |
| GO:0048872\_homeostasis\_of\_number\_of\_cells | MAPK14 | 105 | 2 | 4.176871 | -1.087955 | 103 | 103.84 | 1.008155 |
| GO:0006672\_ceramide\_metabolic\_process | HEXA | 19 | 1 | 11.541353 | -1.079170 | 104 | 107.17 | 1.030481 |
| GO:0030099\_myeloid\_cell\_differentiation | ID2 | 108 | 2 | 4.060847 | -1.066885 | 105 | 107.65 | 1.025238 |
| GO:0030099\_myeloid\_cell\_differentiation | MAPK14 | 108 | 2 | 4.060847 | -1.066885 | 105 | 107.65 | 1.025238 |
| GO:0021695\_cerebellar\_cortex\_development | AARS | 20 | 1 | 10.964286 | -1.057828 | 106 | 110.79 | 1.045189 |
| GO:0007049\_cell\_cycle | CSRP2BP | 238 | 3 | 2.764106 | -1.039668 | 107 | 111.89 | 1.045701 |
| GO:0007049\_cell\_cycle | MAPK14 | 238 | 3 | 2.764106 | -1.039668 | 107 | 111.89 | 1.045701 |
| GO:0007049\_cell\_cycle | CCNG2 | 238 | 3 | 2.764106 | -1.039668 | 107 | 111.89 | 1.045701 |
| GO:0000018\_regulation\_of\_DNA\_recombination | SMARCAD1 | 21 | 1 | 10.442177 | -1.037573 | 111 | 116.0 | 1.045045 |
| GO:0000075\_cell\_cycle\_checkpoint | MAPK14 | 21 | 1 | 10.442177 | -1.037573 | 111 | 116.0 | 1.045045 |
| GO:0010552\_positive\_regulation\_of\_specific\_transcription\_from\_RNA\_polymerase\_II\_promoter | TAF9 | 21 | 1 | 10.442177 | -1.037573 | 111 | 116.0 | 1.045045 |
| GO:0046519\_sphingoid\_metabolic\_process | HEXA | 21 | 1 | 10.442177 | -1.037573 | 111 | 116.0 | 1.045045 |
| GO:0043687\_post-translational\_protein\_modification | CSRP2BP | 384 | 4 | 2.284226 | -1.036895 | 112 | 116.08 | 1.036429 |
| GO:0043687\_post-translational\_protein\_modification | PTPN2 | 384 | 4 | 2.284226 | -1.036895 | 112 | 116.08 | 1.036429 |
| GO:0043687\_post-translational\_protein\_modification | MAPK14 | 384 | 4 | 2.284226 | -1.036895 | 112 | 116.08 | 1.036429 |
| GO:0043687\_post-translational\_protein\_modification | SOCS7 | 384 | 4 | 2.284226 | -1.036895 | 112 | 116.08 | 1.036429 |
| GO:0009607\_response\_to\_biotic\_stimulus | MAPK14 | 114 | 2 | 3.847118 | -1.026730 | 113 | 116.48 | 1.030796 |
| GO:0009607\_response\_to\_biotic\_stimulus | AARS | 114 | 2 | 3.847118 | -1.026730 | 113 | 116.48 | 1.030796 |
| GO:0030258\_lipid\_modification | MAPK14 | 22 | 1 | 9.967532 | -1.018303 | 115 | 121.47 | 1.056261 |
| GO:0034660\_ncRNA\_metabolic\_process | AARS | 22 | 1 | 9.967532 | -1.018303 | 115 | 121.47 | 1.056261 |
| GO:0006519\_cellular\_amino\_acid\_and\_derivative\_metabolic\_process | DCT | 118 | 2 | 3.716707 | -1.001321 | 116 | 122.27 | 1.054052 |
| GO:0006519\_cellular\_amino\_acid\_and\_derivative\_metabolic\_process | AARS | 118 | 2 | 3.716707 | -1.001321 | 116 | 122.27 | 1.054052 |
| GO:0007163\_establishment\_or\_maintenance\_of\_cell\_polarity | FAT1 | 23 | 1 | 9.534161 | -0.999930 | 117 | 124.42 | 1.063419 |
| GO:0051726\_regulation\_of\_cell\_cycle | MAPK14 | 121 | 2 | 3.624557 | -0.982926 | 118 | 126.13 | 1.068898 |
| GO:0051726\_regulation\_of\_cell\_cycle | CCNG2 | 121 | 2 | 3.624557 | -0.982926 | 118 | 126.13 | 1.068898 |
| GO:0043588\_skin\_development | AARS | 24 | 1 | 9.136905 | -0.982379 | 119 | 129.4 | 1.087395 |
| GO:0007628\_adult\_walking\_behavior | HEXA | 25 | 1 | 8.771429 | -0.965582 | 121 | 133.37 | 1.102231 |
| GO:0043543\_protein\_amino\_acid\_acylation | CSRP2BP | 25 | 1 | 8.771429 | -0.965582 | 121 | 133.37 | 1.102231 |
| GO:0006355\_regulation\_of\_transcription\_\_DNA-dependent | ID2 | 575 | 5 | 1.906832 | -0.952369 | 122 | 134.38 | 1.101475 |
| GO:0006355\_regulation\_of\_transcription\_\_DNA-dependent | MAPK14 | 575 | 5 | 1.906832 | -0.952369 | 122 | 134.38 | 1.101475 |
| GO:0006355\_regulation\_of\_transcription\_\_DNA-dependent | WDR77 | 575 | 5 | 1.906832 | -0.952369 | 122 | 134.38 | 1.101475 |
| GO:0006355\_regulation\_of\_transcription\_\_DNA-dependent | RBL1 | 575 | 5 | 1.906832 | -0.952369 | 122 | 134.38 | 1.101475 |
| GO:0006355\_regulation\_of\_transcription\_\_DNA-dependent | TAF9 | 575 | 5 | 1.906832 | -0.952369 | 122 | 134.38 | 1.101475 |
| GO:0050680\_negative\_regulation\_of\_epithelial\_cell\_proliferation | WDR77 | 26 | 1 | 8.434066 | -0.949479 | 123 | 137.19 | 1.115366 |
| GO:0051276\_chromosome\_organization | CSRP2BP | 129 | 2 | 3.399779 | -0.936424 | 124 | 138.51 | 1.117016 |
| GO:0051276\_chromosome\_organization | ARID1A | 129 | 2 | 3.399779 | -0.936424 | 124 | 138.51 | 1.117016 |
| GO:0002761\_regulation\_of\_myeloid\_leukocyte\_differentiation | ID2 | 27 | 1 | 8.121693 | -0.934019 | 126 | 140.63 | 1.116111 |
| GO:0032496\_response\_to\_lipopolysaccharide | MAPK14 | 27 | 1 | 8.121693 | -0.934019 | 126 | 140.63 | 1.116111 |
| GO:0019219\_regulation\_of\_nucleobase\_\_nucleoside\_\_nucleotide\_and\_nucleic\_acid\_metabolic\_process | SMARCAD1 | 757 | 6 | 1.738064 | -0.932540 | 127 | 140.72 | 1.108031 |
| GO:0019219\_regulation\_of\_nucleobase\_\_nucleoside\_\_nucleotide\_and\_nucleic\_acid\_metabolic\_process | ID2 | 757 | 6 | 1.738064 | -0.932540 | 127 | 140.72 | 1.108031 |
| GO:0019219\_regulation\_of\_nucleobase\_\_nucleoside\_\_nucleotide\_and\_nucleic\_acid\_metabolic\_process | MAPK14 | 757 | 6 | 1.738064 | -0.932540 | 127 | 140.72 | 1.108031 |
| GO:0019219\_regulation\_of\_nucleobase\_\_nucleoside\_\_nucleotide\_and\_nucleic\_acid\_metabolic\_process | WDR77 | 757 | 6 | 1.738064 | -0.932540 | 127 | 140.72 | 1.108031 |
| GO:0019219\_regulation\_of\_nucleobase\_\_nucleoside\_\_nucleotide\_and\_nucleic\_acid\_metabolic\_process | RBL1 | 757 | 6 | 1.738064 | -0.932540 | 127 | 140.72 | 1.108031 |
| GO:0019219\_regulation\_of\_nucleobase\_\_nucleoside\_\_nucleotide\_and\_nucleic\_acid\_metabolic\_process | TAF9 | 757 | 6 | 1.738064 | -0.932540 | 127 | 140.72 | 1.108031 |
| GO:0002062\_chondrocyte\_differentiation | MAPK14 | 28 | 1 | 7.831633 | -0.919155 | 132 | 143.4 | 1.086364 |
| GO:0006470\_protein\_amino\_acid\_dephosphorylation | PTPN2 | 28 | 1 | 7.831633 | -0.919155 | 132 | 143.4 | 1.086364 |
| GO:0006997\_nucleus\_organization | ZMPSTE24 | 28 | 1 | 7.831633 | -0.919155 | 132 | 143.4 | 1.086364 |
| GO:0021549\_cerebellum\_development | AARS | 28 | 1 | 7.831633 | -0.919155 | 132 | 143.4 | 1.086364 |
| GO:0043193\_positive\_regulation\_of\_gene-specific\_transcription | TAF9 | 28 | 1 | 7.831633 | -0.919155 | 132 | 143.4 | 1.086364 |
| GO:0051252\_regulation\_of\_RNA\_metabolic\_process | ID2 | 590 | 5 | 1.858354 | -0.915999 | 133 | 143.7 | 1.080451 |
| GO:0051252\_regulation\_of\_RNA\_metabolic\_process | MAPK14 | 590 | 5 | 1.858354 | -0.915999 | 133 | 143.7 | 1.080451 |
| GO:0051252\_regulation\_of\_RNA\_metabolic\_process | WDR77 | 590 | 5 | 1.858354 | -0.915999 | 133 | 143.7 | 1.080451 |
| GO:0051252\_regulation\_of\_RNA\_metabolic\_process | RBL1 | 590 | 5 | 1.858354 | -0.915999 | 133 | 143.7 | 1.080451 |
| GO:0051252\_regulation\_of\_RNA\_metabolic\_process | TAF9 | 590 | 5 | 1.858354 | -0.915999 | 133 | 143.7 | 1.080451 |
| GO:0006351\_transcription\_\_DNA-dependent | ID2 | 594 | 5 | 1.845839 | -0.906543 | 134 | 144.57 | 1.078881 |
| GO:0006351\_transcription\_\_DNA-dependent | MAPK14 | 594 | 5 | 1.845839 | -0.906543 | 134 | 144.57 | 1.078881 |
| GO:0006351\_transcription\_\_DNA-dependent | WDR77 | 594 | 5 | 1.845839 | -0.906543 | 134 | 144.57 | 1.078881 |
| GO:0006351\_transcription\_\_DNA-dependent | RBL1 | 594 | 5 | 1.845839 | -0.906543 | 134 | 144.57 | 1.078881 |
| GO:0006351\_transcription\_\_DNA-dependent | TAF9 | 594 | 5 | 1.845839 | -0.906543 | 134 | 144.57 | 1.078881 |
| GO:0042770\_DNA\_damage\_response\_\_signal\_transduction | MAPK14 | 29 | 1 | 7.561576 | -0.904844 | 137 | 147.22 | 1.074599 |
| GO:0048066\_pigmentation\_during\_development | DCT | 29 | 1 | 7.561576 | -0.904844 | 137 | 147.22 | 1.074599 |
| GO:0050769\_positive\_regulation\_of\_neurogenesis | METRN | 29 | 1 | 7.561576 | -0.904844 | 137 | 147.22 | 1.074599 |
| GO:0032774\_RNA\_biosynthetic\_process | ID2 | 595 | 5 | 1.842737 | -0.904195 | 138 | 147.39 | 1.068043 |
| GO:0032774\_RNA\_biosynthetic\_process | MAPK14 | 595 | 5 | 1.842737 | -0.904195 | 138 | 147.39 | 1.068043 |
| GO:0032774\_RNA\_biosynthetic\_process | WDR77 | 595 | 5 | 1.842737 | -0.904195 | 138 | 147.39 | 1.068043 |
| GO:0032774\_RNA\_biosynthetic\_process | RBL1 | 595 | 5 | 1.842737 | -0.904195 | 138 | 147.39 | 1.068043 |
| GO:0032774\_RNA\_biosynthetic\_process | TAF9 | 595 | 5 | 1.842737 | -0.904195 | 138 | 147.39 | 1.068043 |
| GO:0051171\_regulation\_of\_nitrogen\_compound\_metabolic\_process | SMARCAD1 | 771 | 6 | 1.706504 | -0.903123 | 139 | 147.5 | 1.061151 |
| GO:0051171\_regulation\_of\_nitrogen\_compound\_metabolic\_process | ID2 | 771 | 6 | 1.706504 | -0.903123 | 139 | 147.5 | 1.061151 |
| GO:0051171\_regulation\_of\_nitrogen\_compound\_metabolic\_process | MAPK14 | 771 | 6 | 1.706504 | -0.903123 | 139 | 147.5 | 1.061151 |
| GO:0051171\_regulation\_of\_nitrogen\_compound\_metabolic\_process | WDR77 | 771 | 6 | 1.706504 | -0.903123 | 139 | 147.5 | 1.061151 |
| GO:0051171\_regulation\_of\_nitrogen\_compound\_metabolic\_process | RBL1 | 771 | 6 | 1.706504 | -0.903123 | 139 | 147.5 | 1.061151 |
| GO:0051171\_regulation\_of\_nitrogen\_compound\_metabolic\_process | TAF9 | 771 | 6 | 1.706504 | -0.903123 | 139 | 147.5 | 1.061151 |
| GO:0042552\_myelination | HEXA | 30 | 1 | 7.309524 | -0.891050 | 140 | 151.49 | 1.082071 |
| GO:0016043\_cellular\_component\_organization | METRN | 964 | 7 | 1.592324 | -0.883914 | 141 | 152.02 | 1.078156 |
| GO:0016043\_cellular\_component\_organization | CSRP2BP | 964 | 7 | 1.592324 | -0.883914 | 141 | 152.02 | 1.078156 |
| GO:0016043\_cellular\_component\_organization | MAPK14 | 964 | 7 | 1.592324 | -0.883914 | 141 | 152.02 | 1.078156 |
| GO:0016043\_cellular\_component\_organization | HEXA | 964 | 7 | 1.592324 | -0.883914 | 141 | 152.02 | 1.078156 |
| GO:0016043\_cellular\_component\_organization | FAT1 | 964 | 7 | 1.592324 | -0.883914 | 141 | 152.02 | 1.078156 |
| GO:0016043\_cellular\_component\_organization | ZMPSTE24 | 964 | 7 | 1.592324 | -0.883914 | 141 | 152.02 | 1.078156 |
| GO:0016043\_cellular\_component\_organization | ARID1A | 964 | 7 | 1.592324 | -0.883914 | 141 | 152.02 | 1.078156 |
| GO:0007169\_transmembrane\_receptor\_protein\_tyrosine\_kinase\_signaling\_pathway | PTPN2 | 139 | 2 | 3.155190 | -0.882968 | 142 | 152.44 | 1.073521 |
| GO:0007169\_transmembrane\_receptor\_protein\_tyrosine\_kinase\_signaling\_pathway | SOCS7 | 139 | 2 | 3.155190 | -0.882968 | 142 | 152.44 | 1.073521 |
| GO:0006665\_sphingolipid\_metabolic\_process | HEXA | 31 | 1 | 7.073733 | -0.877738 | 144 | 156.69 | 1.088125 |
| GO:0016311\_dephosphorylation | PTPN2 | 31 | 1 | 7.073733 | -0.877738 | 144 | 156.69 | 1.088125 |
| GO:0006464\_protein\_modification\_process | CSRP2BP | 439 | 4 | 1.998048 | -0.874937 | 145 | 156.92 | 1.082207 |
| GO:0006464\_protein\_modification\_process | PTPN2 | 439 | 4 | 1.998048 | -0.874937 | 145 | 156.92 | 1.082207 |
| GO:0006464\_protein\_modification\_process | MAPK14 | 439 | 4 | 1.998048 | -0.874937 | 145 | 156.92 | 1.082207 |
| GO:0006464\_protein\_modification\_process | SOCS7 | 439 | 4 | 1.998048 | -0.874937 | 145 | 156.92 | 1.082207 |
| GO:0000902\_cell\_morphogenesis | METRN | 283 | 3 | 2.324584 | -0.869751 | 146 | 157.3 | 1.077397 |
| GO:0000902\_cell\_morphogenesis | MAPK14 | 283 | 3 | 2.324584 | -0.869751 | 146 | 157.3 | 1.077397 |
| GO:0000902\_cell\_morphogenesis | FAT1 | 283 | 3 | 2.324584 | -0.869751 | 146 | 157.3 | 1.077397 |
| GO:0007272\_ensheathment\_of\_neurons | HEXA | 32 | 1 | 6.852679 | -0.864877 | 149 | 159.42 | 1.069933 |
| GO:0008366\_axon\_ensheathment | HEXA | 32 | 1 | 6.852679 | -0.864877 | 149 | 159.42 | 1.069933 |
| GO:0050770\_regulation\_of\_axonogenesis | METRN | 32 | 1 | 6.852679 | -0.864877 | 149 | 159.42 | 1.069933 |
| GO:0006643\_membrane\_lipid\_metabolic\_process | HEXA | 33 | 1 | 6.645022 | -0.852440 | 151 | 162.65 | 1.077152 |
| GO:0022037\_metencephalon\_development | AARS | 33 | 1 | 6.645022 | -0.852440 | 151 | 162.65 | 1.077152 |
| GO:0005975\_carbohydrate\_metabolic\_process | MAPK14 | 146 | 2 | 3.003914 | -0.848271 | 152 | 163.17 | 1.073487 |
| GO:0005975\_carbohydrate\_metabolic\_process | HEXA | 146 | 2 | 3.003914 | -0.848271 | 152 | 163.17 | 1.073487 |
| GO:0009058\_biosynthetic\_process | DCT | 1175 | 8 | 1.493009 | -0.847996 | 153 | 163.24 | 1.066928 |
| GO:0009058\_biosynthetic\_process | ID2 | 1175 | 8 | 1.493009 | -0.847996 | 153 | 163.24 | 1.066928 |
| GO:0009058\_biosynthetic\_process | MAPK14 | 1175 | 8 | 1.493009 | -0.847996 | 153 | 163.24 | 1.066928 |
| GO:0009058\_biosynthetic\_process | SI | 1175 | 8 | 1.493009 | -0.847996 | 153 | 163.24 | 1.066928 |
| GO:0009058\_biosynthetic\_process | AARS | 1175 | 8 | 1.493009 | -0.847996 | 153 | 163.24 | 1.066928 |
| GO:0009058\_biosynthetic\_process | WDR77 | 1175 | 8 | 1.493009 | -0.847996 | 153 | 163.24 | 1.066928 |
| GO:0009058\_biosynthetic\_process | RBL1 | 1175 | 8 | 1.493009 | -0.847996 | 153 | 163.24 | 1.066928 |
| GO:0009058\_biosynthetic\_process | TAF9 | 1175 | 8 | 1.493009 | -0.847996 | 153 | 163.24 | 1.066928 |
| GO:0002237\_response\_to\_molecule\_of\_bacterial\_origin | MAPK14 | 34 | 1 | 6.449580 | -0.840402 | 156 | 166.26 | 1.065769 |
| GO:0010720\_positive\_regulation\_of\_cell\_development | METRN | 34 | 1 | 6.449580 | -0.840402 | 156 | 166.26 | 1.065769 |
| GO:0051052\_regulation\_of\_DNA\_metabolic\_process | SMARCAD1 | 34 | 1 | 6.449580 | -0.840402 | 156 | 166.26 | 1.065769 |
| GO:0034961\_cellular\_biopolymer\_biosynthetic\_process | ID2 | 804 | 6 | 1.636461 | -0.837223 | 157 | 166.63 | 1.061338 |
| GO:0034961\_cellular\_biopolymer\_biosynthetic\_process | MAPK14 | 804 | 6 | 1.636461 | -0.837223 | 157 | 166.63 | 1.061338 |
| GO:0034961\_cellular\_biopolymer\_biosynthetic\_process | AARS | 804 | 6 | 1.636461 | -0.837223 | 157 | 166.63 | 1.061338 |
| GO:0034961\_cellular\_biopolymer\_biosynthetic\_process | WDR77 | 804 | 6 | 1.636461 | -0.837223 | 157 | 166.63 | 1.061338 |
| GO:0034961\_cellular\_biopolymer\_biosynthetic\_process | RBL1 | 804 | 6 | 1.636461 | -0.837223 | 157 | 166.63 | 1.061338 |
| GO:0034961\_cellular\_biopolymer\_biosynthetic\_process | TAF9 | 804 | 6 | 1.636461 | -0.837223 | 157 | 166.63 | 1.061338 |
| GO:0043284\_biopolymer\_biosynthetic\_process | ID2 | 807 | 6 | 1.630377 | -0.831462 | 158 | 166.95 | 1.056646 |
| GO:0043284\_biopolymer\_biosynthetic\_process | MAPK14 | 807 | 6 | 1.630377 | -0.831462 | 158 | 166.95 | 1.056646 |
| GO:0043284\_biopolymer\_biosynthetic\_process | AARS | 807 | 6 | 1.630377 | -0.831462 | 158 | 166.95 | 1.056646 |
| GO:0043284\_biopolymer\_biosynthetic\_process | WDR77 | 807 | 6 | 1.630377 | -0.831462 | 158 | 166.95 | 1.056646 |
| GO:0043284\_biopolymer\_biosynthetic\_process | RBL1 | 807 | 6 | 1.630377 | -0.831462 | 158 | 166.95 | 1.056646 |
| GO:0043284\_biopolymer\_biosynthetic\_process | TAF9 | 807 | 6 | 1.630377 | -0.831462 | 158 | 166.95 | 1.056646 |
| GO:0045595\_regulation\_of\_cell\_differentiation | METRN | 295 | 3 | 2.230024 | -0.830503 | 159 | 167.36 | 1.052579 |
| GO:0045595\_regulation\_of\_cell\_differentiation | ID2 | 295 | 3 | 2.230024 | -0.830503 | 159 | 167.36 | 1.052579 |
| GO:0045595\_regulation\_of\_cell\_differentiation | MAPK14 | 295 | 3 | 2.230024 | -0.830503 | 159 | 167.36 | 1.052579 |
| GO:0016567\_protein\_ubiquitination | SOCS7 | 35 | 1 | 6.265306 | -0.828739 | 161 | 169.37 | 1.051988 |
| GO:0044242\_cellular\_lipid\_catabolic\_process | HEXA | 35 | 1 | 6.265306 | -0.828739 | 161 | 169.37 | 1.051988 |
| GO:0019228\_regulation\_of\_action\_potential\_in\_neuron | HEXA | 36 | 1 | 6.091270 | -0.817430 | 162 | 172.76 | 1.066420 |
| GO:0006139\_nucleobase\_\_nucleoside\_\_nucleotide\_and\_nucleic\_acid\_metabolic\_process | SMARCAD1 | 1002 | 7 | 1.531936 | -0.816211 | 163 | 173.04 | 1.061595 |
| GO:0006139\_nucleobase\_\_nucleoside\_\_nucleotide\_and\_nucleic\_acid\_metabolic\_process | ID2 | 1002 | 7 | 1.531936 | -0.816211 | 163 | 173.04 | 1.061595 |
| GO:0006139\_nucleobase\_\_nucleoside\_\_nucleotide\_and\_nucleic\_acid\_metabolic\_process | MAPK14 | 1002 | 7 | 1.531936 | -0.816211 | 163 | 173.04 | 1.061595 |
| GO:0006139\_nucleobase\_\_nucleoside\_\_nucleotide\_and\_nucleic\_acid\_metabolic\_process | AARS | 1002 | 7 | 1.531936 | -0.816211 | 163 | 173.04 | 1.061595 |
| GO:0006139\_nucleobase\_\_nucleoside\_\_nucleotide\_and\_nucleic\_acid\_metabolic\_process | WDR77 | 1002 | 7 | 1.531936 | -0.816211 | 163 | 173.04 | 1.061595 |
| GO:0006139\_nucleobase\_\_nucleoside\_\_nucleotide\_and\_nucleic\_acid\_metabolic\_process | RBL1 | 1002 | 7 | 1.531936 | -0.816211 | 163 | 173.04 | 1.061595 |
| GO:0006139\_nucleobase\_\_nucleoside\_\_nucleotide\_and\_nucleic\_acid\_metabolic\_process | TAF9 | 1002 | 7 | 1.531936 | -0.816211 | 163 | 173.04 | 1.061595 |
| GO:0010975\_regulation\_of\_neuron\_projection\_development | METRN | 38 | 1 | 5.770677 | -0.795798 | 165 | 179.03 | 1.085030 |
| GO:0016042\_lipid\_catabolic\_process | HEXA | 38 | 1 | 5.770677 | -0.795798 | 165 | 179.03 | 1.085030 |
| GO:0032989\_cellular\_component\_morphogenesis | METRN | 307 | 3 | 2.142857 | -0.793409 | 166 | 179.43 | 1.080904 |
| GO:0032989\_cellular\_component\_morphogenesis | MAPK14 | 307 | 3 | 2.142857 | -0.793409 | 166 | 179.43 | 1.080904 |
| GO:0032989\_cellular\_component\_morphogenesis | FAT1 | 307 | 3 | 2.142857 | -0.793409 | 166 | 179.43 | 1.080904 |
| GO:0051094\_positive\_regulation\_of\_developmental\_process | METRN | 308 | 3 | 2.135900 | -0.790410 | 167 | 179.86 | 1.077006 |
| GO:0051094\_positive\_regulation\_of\_developmental\_process | ID2 | 308 | 3 | 2.135900 | -0.790410 | 167 | 179.86 | 1.077006 |
| GO:0051094\_positive\_regulation\_of\_developmental\_process | MAPK14 | 308 | 3 | 2.135900 | -0.790410 | 167 | 179.86 | 1.077006 |
| GO:0005976\_polysaccharide\_metabolic\_process | HEXA | 39 | 1 | 5.622711 | -0.785441 | 169 | 182.37 | 1.079112 |
| GO:0043524\_negative\_regulation\_of\_neuron\_apoptosis | AARS | 39 | 1 | 5.622711 | -0.785441 | 169 | 182.37 | 1.079112 |
| GO:0010551\_regulation\_of\_specific\_transcription\_from\_RNA\_polymerase\_II\_promoter | TAF9 | 41 | 1 | 5.348432 | -0.765567 | 173 | 189.46 | 1.095145 |
| GO:0031344\_regulation\_of\_cell\_projection\_organization | METRN | 41 | 1 | 5.348432 | -0.765567 | 173 | 189.46 | 1.095145 |
| GO:0032569\_specific\_transcription\_from\_RNA\_polymerase\_II\_promoter | TAF9 | 41 | 1 | 5.348432 | -0.765567 | 173 | 189.46 | 1.095145 |
| GO:0032844\_regulation\_of\_homeostatic\_process | MAPK14 | 41 | 1 | 5.348432 | -0.765567 | 173 | 189.46 | 1.095145 |
| GO:0006006\_glucose\_metabolic\_process | MAPK14 | 42 | 1 | 5.221088 | -0.756024 | 178 | 193.28 | 1.085843 |
| GO:0010769\_regulation\_of\_cell\_morphogenesis\_involved\_in\_differentiation | METRN | 42 | 1 | 5.221088 | -0.756024 | 178 | 193.28 | 1.085843 |
| GO:0019941\_modification-dependent\_protein\_catabolic\_process | ZMPSTE24 | 42 | 1 | 5.221088 | -0.756024 | 178 | 193.28 | 1.085843 |
| GO:0043632\_modification-dependent\_macromolecule\_catabolic\_process | ZMPSTE24 | 42 | 1 | 5.221088 | -0.756024 | 178 | 193.28 | 1.085843 |
| GO:0051603\_proteolysis\_involved\_in\_cellular\_protein\_catabolic\_process | ZMPSTE24 | 42 | 1 | 5.221088 | -0.756024 | 178 | 193.28 | 1.085843 |
| GO:0032502\_developmental\_process | DCT | 2060 | 12 | 1.277393 | -0.752010 | 179 | 193.86 | 1.083017 |
| GO:0032502\_developmental\_process | METRN | 2060 | 12 | 1.277393 | -0.752010 | 179 | 193.86 | 1.083017 |
| GO:0032502\_developmental\_process | CSRP2BP | 2060 | 12 | 1.277393 | -0.752010 | 179 | 193.86 | 1.083017 |
| GO:0032502\_developmental\_process | ID2 | 2060 | 12 | 1.277393 | -0.752010 | 179 | 193.86 | 1.083017 |
| GO:0032502\_developmental\_process | FAT1 | 2060 | 12 | 1.277393 | -0.752010 | 179 | 193.86 | 1.083017 |
| GO:0032502\_developmental\_process | HEXA | 2060 | 12 | 1.277393 | -0.752010 | 179 | 193.86 | 1.083017 |
| GO:0032502\_developmental\_process | MAPK14 | 2060 | 12 | 1.277393 | -0.752010 | 179 | 193.86 | 1.083017 |
| GO:0032502\_developmental\_process | FMR1 | 2060 | 12 | 1.277393 | -0.752010 | 179 | 193.86 | 1.083017 |
| GO:0032502\_developmental\_process | AARS | 2060 | 12 | 1.277393 | -0.752010 | 179 | 193.86 | 1.083017 |
| GO:0032502\_developmental\_process | WDR77 | 2060 | 12 | 1.277393 | -0.752010 | 179 | 193.86 | 1.083017 |
| GO:0032502\_developmental\_process | SOCS7 | 2060 | 12 | 1.277393 | -0.752010 | 179 | 193.86 | 1.083017 |
| GO:0032502\_developmental\_process | PBRM1 | 2060 | 12 | 1.277393 | -0.752010 | 179 | 193.86 | 1.083017 |
| GO:0001508\_regulation\_of\_action\_potential | HEXA | 43 | 1 | 5.099668 | -0.746726 | 182 | 197.05 | 1.082692 |
| GO:0010001\_glial\_cell\_differentiation | METRN | 43 | 1 | 5.099668 | -0.746726 | 182 | 197.05 | 1.082692 |
| GO:0032446\_protein\_modification\_by\_small\_protein\_conjugation | SOCS7 | 43 | 1 | 5.099668 | -0.746726 | 182 | 197.05 | 1.082692 |
| GO:0001942\_hair\_follicle\_development | AARS | 44 | 1 | 4.983766 | -0.737663 | 190 | 201.05 | 1.058158 |
| GO:0006606\_protein\_import\_into\_nucleus | RAN | 44 | 1 | 4.983766 | -0.737663 | 190 | 201.05 | 1.058158 |
| GO:0022404\_molting\_cycle\_process | AARS | 44 | 1 | 4.983766 | -0.737663 | 190 | 201.05 | 1.058158 |
| GO:0022405\_hair\_cycle\_process | AARS | 44 | 1 | 4.983766 | -0.737663 | 190 | 201.05 | 1.058158 |
| GO:0042303\_molting\_cycle | AARS | 44 | 1 | 4.983766 | -0.737663 | 190 | 201.05 | 1.058158 |
| GO:0042633\_hair\_cycle | AARS | 44 | 1 | 4.983766 | -0.737663 | 190 | 201.05 | 1.058158 |
| GO:0044257\_cellular\_protein\_catabolic\_process | ZMPSTE24 | 44 | 1 | 4.983766 | -0.737663 | 190 | 201.05 | 1.058158 |
| GO:0051170\_nuclear\_import | RAN | 44 | 1 | 4.983766 | -0.737663 | 190 | 201.05 | 1.058158 |
| GO:0045449\_regulation\_of\_transcription | ID2 | 676 | 5 | 1.621936 | -0.732955 | 191 | 201.66 | 1.055812 |
| GO:0045449\_regulation\_of\_transcription | MAPK14 | 676 | 5 | 1.621936 | -0.732955 | 191 | 201.66 | 1.055812 |
| GO:0045449\_regulation\_of\_transcription | WDR77 | 676 | 5 | 1.621936 | -0.732955 | 191 | 201.66 | 1.055812 |
| GO:0045449\_regulation\_of\_transcription | RBL1 | 676 | 5 | 1.621936 | -0.732955 | 191 | 201.66 | 1.055812 |
| GO:0045449\_regulation\_of\_transcription | TAF9 | 676 | 5 | 1.621936 | -0.732955 | 191 | 201.66 | 1.055812 |
| GO:0044248\_cellular\_catabolic\_process | HEXA | 173 | 2 | 2.535095 | -0.731641 | 192 | 202.18 | 1.053021 |
| GO:0044248\_cellular\_catabolic\_process | ZMPSTE24 | 173 | 2 | 2.535095 | -0.731641 | 192 | 202.18 | 1.053021 |
| GO:0000122\_negative\_regulation\_of\_transcription\_from\_RNA\_polymerase\_II\_promoter | ID2 | 175 | 2 | 2.506122 | -0.723932 | 193 | 204.77 | 1.060984 |
| GO:0000122\_negative\_regulation\_of\_transcription\_from\_RNA\_polymerase\_II\_promoter | RBL1 | 175 | 2 | 2.506122 | -0.723932 | 193 | 204.77 | 1.060984 |
| GO:0030850\_prostate\_gland\_development | WDR77 | 46 | 1 | 4.767081 | -0.720198 | 195 | 206.72 | 1.060103 |
| GO:0042063\_gliogenesis | METRN | 46 | 1 | 4.767081 | -0.720198 | 195 | 206.72 | 1.060103 |
| GO:0006396\_RNA\_processing | AARS | 47 | 1 | 4.665653 | -0.711777 | 197 | 209.58 | 1.063858 |
| GO:0016570\_histone\_modification | CSRP2BP | 47 | 1 | 4.665653 | -0.711777 | 197 | 209.58 | 1.063858 |
| GO:0019318\_hexose\_metabolic\_process | MAPK14 | 48 | 1 | 4.568452 | -0.703552 | 199 | 211.71 | 1.063869 |
| GO:0034504\_protein\_localization\_in\_nucleus | RAN | 48 | 1 | 4.568452 | -0.703552 | 199 | 211.71 | 1.063869 |
| GO:0006725\_cellular\_aromatic\_compound\_metabolic\_process | DCT | 49 | 1 | 4.475219 | -0.695516 | 201 | 214.94 | 1.069353 |
| GO:0043473\_pigmentation | DCT | 49 | 1 | 4.475219 | -0.695516 | 201 | 214.94 | 1.069353 |
| GO:0002573\_myeloid\_leukocyte\_differentiation | ID2 | 50 | 1 | 4.385714 | -0.687659 | 205 | 216.98 | 1.058439 |
| GO:0007015\_actin\_filament\_organization | FAT1 | 50 | 1 | 4.385714 | -0.687659 | 205 | 216.98 | 1.058439 |
| GO:0017038\_protein\_import | RAN | 50 | 1 | 4.385714 | -0.687659 | 205 | 216.98 | 1.058439 |
| GO:0070647\_protein\_modification\_by\_small\_protein\_conjugation\_or\_removal | SOCS7 | 50 | 1 | 4.385714 | -0.687659 | 205 | 216.98 | 1.058439 |
| GO:0006350\_transcription | ID2 | 701 | 5 | 1.564092 | -0.686866 | 206 | 217.23 | 1.054515 |
| GO:0006350\_transcription | MAPK14 | 701 | 5 | 1.564092 | -0.686866 | 206 | 217.23 | 1.054515 |
| GO:0006350\_transcription | WDR77 | 701 | 5 | 1.564092 | -0.686866 | 206 | 217.23 | 1.054515 |
| GO:0006350\_transcription | RBL1 | 701 | 5 | 1.564092 | -0.686866 | 206 | 217.23 | 1.054515 |
| GO:0006350\_transcription | TAF9 | 701 | 5 | 1.564092 | -0.686866 | 206 | 217.23 | 1.054515 |
| GO:0050793\_regulation\_of\_developmental\_process | METRN | 703 | 5 | 1.559642 | -0.683302 | 207 | 217.87 | 1.052512 |
| GO:0050793\_regulation\_of\_developmental\_process | ID2 | 703 | 5 | 1.559642 | -0.683302 | 207 | 217.87 | 1.052512 |
| GO:0050793\_regulation\_of\_developmental\_process | MAPK14 | 703 | 5 | 1.559642 | -0.683302 | 207 | 217.87 | 1.052512 |
| GO:0050793\_regulation\_of\_developmental\_process | WDR77 | 703 | 5 | 1.559642 | -0.683302 | 207 | 217.87 | 1.052512 |
| GO:0050793\_regulation\_of\_developmental\_process | AARS | 703 | 5 | 1.559642 | -0.683302 | 207 | 217.87 | 1.052512 |
| GO:0016569\_covalent\_chromatin\_modification | CSRP2BP | 51 | 1 | 4.299720 | -0.679976 | 209 | 220.01 | 1.052679 |
| GO:0032583\_regulation\_of\_gene-specific\_transcription | TAF9 | 51 | 1 | 4.299720 | -0.679976 | 209 | 220.01 | 1.052679 |
| GO:0034645\_cellular\_macromolecule\_biosynthetic\_process | ID2 | 901 | 6 | 1.460282 | -0.668405 | 210 | 222.4 | 1.059048 |
| GO:0034645\_cellular\_macromolecule\_biosynthetic\_process | MAPK14 | 901 | 6 | 1.460282 | -0.668405 | 210 | 222.4 | 1.059048 |
| GO:0034645\_cellular\_macromolecule\_biosynthetic\_process | AARS | 901 | 6 | 1.460282 | -0.668405 | 210 | 222.4 | 1.059048 |
| GO:0034645\_cellular\_macromolecule\_biosynthetic\_process | WDR77 | 901 | 6 | 1.460282 | -0.668405 | 210 | 222.4 | 1.059048 |
| GO:0034645\_cellular\_macromolecule\_biosynthetic\_process | RBL1 | 901 | 6 | 1.460282 | -0.668405 | 210 | 222.4 | 1.059048 |
| GO:0034645\_cellular\_macromolecule\_biosynthetic\_process | TAF9 | 901 | 6 | 1.460282 | -0.668405 | 210 | 222.4 | 1.059048 |
| GO:0010467\_gene\_expression | ID2 | 905 | 6 | 1.453828 | -0.662160 | 211 | 224.33 | 1.063175 |
| GO:0010467\_gene\_expression | MAPK14 | 905 | 6 | 1.453828 | -0.662160 | 211 | 224.33 | 1.063175 |
| GO:0010467\_gene\_expression | AARS | 905 | 6 | 1.453828 | -0.662160 | 211 | 224.33 | 1.063175 |
| GO:0010467\_gene\_expression | WDR77 | 905 | 6 | 1.453828 | -0.662160 | 211 | 224.33 | 1.063175 |
| GO:0010467\_gene\_expression | RBL1 | 905 | 6 | 1.453828 | -0.662160 | 211 | 224.33 | 1.063175 |
| GO:0010467\_gene\_expression | TAF9 | 905 | 6 | 1.453828 | -0.662160 | 211 | 224.33 | 1.063175 |
| GO:0006412\_translation | AARS | 54 | 1 | 4.060847 | -0.657900 | 212 | 226.57 | 1.068726 |
| GO:0009059\_macromolecule\_biosynthetic\_process | ID2 | 910 | 6 | 1.445840 | -0.654427 | 213 | 226.97 | 1.065587 |
| GO:0009059\_macromolecule\_biosynthetic\_process | MAPK14 | 910 | 6 | 1.445840 | -0.654427 | 213 | 226.97 | 1.065587 |
| GO:0009059\_macromolecule\_biosynthetic\_process | AARS | 910 | 6 | 1.445840 | -0.654427 | 213 | 226.97 | 1.065587 |
| GO:0009059\_macromolecule\_biosynthetic\_process | WDR77 | 910 | 6 | 1.445840 | -0.654427 | 213 | 226.97 | 1.065587 |
| GO:0009059\_macromolecule\_biosynthetic\_process | RBL1 | 910 | 6 | 1.445840 | -0.654427 | 213 | 226.97 | 1.065587 |
| GO:0009059\_macromolecule\_biosynthetic\_process | TAF9 | 910 | 6 | 1.445840 | -0.654427 | 213 | 226.97 | 1.065587 |
| GO:0007507\_heart\_development | ID2 | 195 | 2 | 2.249084 | -0.652658 | 214 | 227.81 | 1.064533 |
| GO:0007507\_heart\_development | PBRM1 | 195 | 2 | 2.249084 | -0.652658 | 214 | 227.81 | 1.064533 |
| GO:0006310\_DNA\_recombination | SMARCAD1 | 55 | 1 | 3.987013 | -0.650846 | 216 | 229.41 | 1.062083 |
| GO:0007605\_sensory\_perception\_of\_sound | HEXA | 55 | 1 | 3.987013 | -0.650846 | 216 | 229.41 | 1.062083 |
| GO:0033554\_cellular\_response\_to\_stress | MAPK14 | 196 | 2 | 2.237609 | -0.649349 | 217 | 229.51 | 1.057650 |
| GO:0033554\_cellular\_response\_to\_stress | AARS | 196 | 2 | 2.237609 | -0.649349 | 217 | 229.51 | 1.057650 |
| GO:0050678\_regulation\_of\_epithelial\_cell\_proliferation | WDR77 | 56 | 1 | 3.915816 | -0.643934 | 218 | 231.99 | 1.064174 |
| GO:0048869\_cellular\_developmental\_process | DCT | 1113 | 7 | 1.379155 | -0.643566 | 219 | 232.14 | 1.060000 |
| GO:0048869\_cellular\_developmental\_process | METRN | 1113 | 7 | 1.379155 | -0.643566 | 219 | 232.14 | 1.060000 |
| GO:0048869\_cellular\_developmental\_process | ID2 | 1113 | 7 | 1.379155 | -0.643566 | 219 | 232.14 | 1.060000 |
| GO:0048869\_cellular\_developmental\_process | FAT1 | 1113 | 7 | 1.379155 | -0.643566 | 219 | 232.14 | 1.060000 |
| GO:0048869\_cellular\_developmental\_process | MAPK14 | 1113 | 7 | 1.379155 | -0.643566 | 219 | 232.14 | 1.060000 |
| GO:0048869\_cellular\_developmental\_process | WDR77 | 1113 | 7 | 1.379155 | -0.643566 | 219 | 232.14 | 1.060000 |
| GO:0048869\_cellular\_developmental\_process | SOCS7 | 1113 | 7 | 1.379155 | -0.643566 | 219 | 232.14 | 1.060000 |
| GO:0008344\_adult\_locomotory\_behavior | HEXA | 57 | 1 | 3.847118 | -0.637161 | 223 | 236.13 | 1.058879 |
| GO:0033365\_protein\_localization\_in\_organelle | RAN | 57 | 1 | 3.847118 | -0.637161 | 223 | 236.13 | 1.058879 |
| GO:0043523\_regulation\_of\_neuron\_apoptosis | AARS | 57 | 1 | 3.847118 | -0.637161 | 223 | 236.13 | 1.058879 |
| GO:0045444\_fat\_cell\_differentiation | SOCS7 | 57 | 1 | 3.847118 | -0.637161 | 223 | 236.13 | 1.058879 |
| GO:0030902\_hindbrain\_development | AARS | 58 | 1 | 3.780788 | -0.630521 | 224 | 237.43 | 1.059955 |
| GO:0080090\_regulation\_of\_primary\_metabolic\_process | SMARCAD1 | 926 | 6 | 1.420858 | -0.630220 | 225 | 237.6 | 1.056000 |
| GO:0080090\_regulation\_of\_primary\_metabolic\_process | ID2 | 926 | 6 | 1.420858 | -0.630220 | 225 | 237.6 | 1.056000 |
| GO:0080090\_regulation\_of\_primary\_metabolic\_process | MAPK14 | 926 | 6 | 1.420858 | -0.630220 | 225 | 237.6 | 1.056000 |
| GO:0080090\_regulation\_of\_primary\_metabolic\_process | WDR77 | 926 | 6 | 1.420858 | -0.630220 | 225 | 237.6 | 1.056000 |
| GO:0080090\_regulation\_of\_primary\_metabolic\_process | RBL1 | 926 | 6 | 1.420858 | -0.630220 | 225 | 237.6 | 1.056000 |
| GO:0080090\_regulation\_of\_primary\_metabolic\_process | TAF9 | 926 | 6 | 1.420858 | -0.630220 | 225 | 237.6 | 1.056000 |
| GO:0060255\_regulation\_of\_macromolecule\_metabolic\_process | SMARCAD1 | 936 | 6 | 1.405678 | -0.615498 | 226 | 240.32 | 1.063363 |
| GO:0060255\_regulation\_of\_macromolecule\_metabolic\_process | ID2 | 936 | 6 | 1.405678 | -0.615498 | 226 | 240.32 | 1.063363 |
| GO:0060255\_regulation\_of\_macromolecule\_metabolic\_process | MAPK14 | 936 | 6 | 1.405678 | -0.615498 | 226 | 240.32 | 1.063363 |
| GO:0060255\_regulation\_of\_macromolecule\_metabolic\_process | WDR77 | 936 | 6 | 1.405678 | -0.615498 | 226 | 240.32 | 1.063363 |
| GO:0060255\_regulation\_of\_macromolecule\_metabolic\_process | RBL1 | 936 | 6 | 1.405678 | -0.615498 | 226 | 240.32 | 1.063363 |
| GO:0060255\_regulation\_of\_macromolecule\_metabolic\_process | TAF9 | 936 | 6 | 1.405678 | -0.615498 | 226 | 240.32 | 1.063363 |
| GO:0010556\_regulation\_of\_macromolecule\_biosynthetic\_process | ID2 | 745 | 5 | 1.471716 | -0.612407 | 227 | 240.61 | 1.059956 |
| GO:0010556\_regulation\_of\_macromolecule\_biosynthetic\_process | MAPK14 | 745 | 5 | 1.471716 | -0.612407 | 227 | 240.61 | 1.059956 |
| GO:0010556\_regulation\_of\_macromolecule\_biosynthetic\_process | WDR77 | 745 | 5 | 1.471716 | -0.612407 | 227 | 240.61 | 1.059956 |
| GO:0010556\_regulation\_of\_macromolecule\_biosynthetic\_process | RBL1 | 745 | 5 | 1.471716 | -0.612407 | 227 | 240.61 | 1.059956 |
| GO:0010556\_regulation\_of\_macromolecule\_biosynthetic\_process | TAF9 | 745 | 5 | 1.471716 | -0.612407 | 227 | 240.61 | 1.059956 |
| GO:0022604\_regulation\_of\_cell\_morphogenesis | METRN | 62 | 1 | 3.536866 | -0.605203 | 230 | 244.23 | 1.061870 |
| GO:0030855\_epithelial\_cell\_differentiation | WDR77 | 62 | 1 | 3.536866 | -0.605203 | 230 | 244.23 | 1.061870 |
| GO:0050954\_sensory\_perception\_of\_mechanical\_stimulus | HEXA | 62 | 1 | 3.536866 | -0.605203 | 230 | 244.23 | 1.061870 |
| GO:0051216\_cartilage\_development | MAPK14 | 63 | 1 | 3.480726 | -0.599165 | 231 | 245.93 | 1.064632 |
| GO:0044249\_cellular\_biosynthetic\_process | DCT | 1150 | 7 | 1.334783 | -0.593421 | 232 | 246.94 | 1.064397 |
| GO:0044249\_cellular\_biosynthetic\_process | ID2 | 1150 | 7 | 1.334783 | -0.593421 | 232 | 246.94 | 1.064397 |
| GO:0044249\_cellular\_biosynthetic\_process | MAPK14 | 1150 | 7 | 1.334783 | -0.593421 | 232 | 246.94 | 1.064397 |
| GO:0044249\_cellular\_biosynthetic\_process | AARS | 1150 | 7 | 1.334783 | -0.593421 | 232 | 246.94 | 1.064397 |
| GO:0044249\_cellular\_biosynthetic\_process | WDR77 | 1150 | 7 | 1.334783 | -0.593421 | 232 | 246.94 | 1.064397 |
| GO:0044249\_cellular\_biosynthetic\_process | RBL1 | 1150 | 7 | 1.334783 | -0.593421 | 232 | 246.94 | 1.064397 |
| GO:0044249\_cellular\_biosynthetic\_process | TAF9 | 1150 | 7 | 1.334783 | -0.593421 | 232 | 246.94 | 1.064397 |
| GO:0045892\_negative\_regulation\_of\_transcription\_\_DNA-dependent | ID2 | 218 | 2 | 2.011796 | -0.581844 | 233 | 249.99 | 1.072918 |
| GO:0045892\_negative\_regulation\_of\_transcription\_\_DNA-dependent | RBL1 | 218 | 2 | 2.011796 | -0.581844 | 233 | 249.99 | 1.072918 |
| GO:0051130\_positive\_regulation\_of\_cellular\_component\_organization | METRN | 66 | 1 | 3.322511 | -0.581688 | 235 | 251.51 | 1.070255 |
| GO:0051402\_neuron\_apoptosis | AARS | 66 | 1 | 3.322511 | -0.581688 | 235 | 251.51 | 1.070255 |
| GO:0051253\_negative\_regulation\_of\_RNA\_metabolic\_process | ID2 | 220 | 2 | 1.993506 | -0.576170 | 236 | 252.41 | 1.069534 |
| GO:0051253\_negative\_regulation\_of\_RNA\_metabolic\_process | RBL1 | 220 | 2 | 1.993506 | -0.576170 | 236 | 252.41 | 1.069534 |
| GO:0034962\_cellular\_biopolymer\_catabolic\_process | ZMPSTE24 | 68 | 1 | 3.224790 | -0.570538 | 237 | 255.57 | 1.078354 |
| GO:0005996\_monosaccharide\_metabolic\_process | MAPK14 | 69 | 1 | 3.178054 | -0.565105 | 238 | 256.9 | 1.079412 |
| GO:0010468\_regulation\_of\_gene\_expression | ID2 | 778 | 5 | 1.409291 | -0.561624 | 239 | 257.09 | 1.075690 |
| GO:0010468\_regulation\_of\_gene\_expression | MAPK14 | 778 | 5 | 1.409291 | -0.561624 | 239 | 257.09 | 1.075690 |
| GO:0010468\_regulation\_of\_gene\_expression | WDR77 | 778 | 5 | 1.409291 | -0.561624 | 239 | 257.09 | 1.075690 |
| GO:0010468\_regulation\_of\_gene\_expression | RBL1 | 778 | 5 | 1.409291 | -0.561624 | 239 | 257.09 | 1.075690 |
| GO:0010468\_regulation\_of\_gene\_expression | TAF9 | 778 | 5 | 1.409291 | -0.561624 | 239 | 257.09 | 1.075690 |
| GO:0051239\_regulation\_of\_multicellular\_organismal\_process | METRN | 587 | 4 | 1.494281 | -0.560386 | 240 | 257.44 | 1.072667 |
| GO:0051239\_regulation\_of\_multicellular\_organismal\_process | ID2 | 587 | 4 | 1.494281 | -0.560386 | 240 | 257.44 | 1.072667 |
| GO:0051239\_regulation\_of\_multicellular\_organismal\_process | MAPK14 | 587 | 4 | 1.494281 | -0.560386 | 240 | 257.44 | 1.072667 |
| GO:0051239\_regulation\_of\_multicellular\_organismal\_process | WDR77 | 587 | 4 | 1.494281 | -0.560386 | 240 | 257.44 | 1.072667 |
| GO:0009617\_response\_to\_bacterium | MAPK14 | 70 | 1 | 3.132653 | -0.559762 | 241 | 258.64 | 1.073195 |
| GO:0006913\_nucleocytoplasmic\_transport | RAN | 71 | 1 | 3.088531 | -0.554507 | 242 | 260.17 | 1.075083 |
| GO:0002682\_regulation\_of\_immune\_system\_process | ID2 | 228 | 2 | 1.923559 | -0.554166 | 243 | 260.78 | 1.073169 |
| GO:0002682\_regulation\_of\_immune\_system\_process | MAPK14 | 228 | 2 | 1.923559 | -0.554166 | 243 | 260.78 | 1.073169 |
| GO:0007167\_enzyme\_linked\_receptor\_protein\_signaling\_pathway | PTPN2 | 229 | 2 | 1.915159 | -0.551492 | 244 | 261.0 | 1.069672 |
| GO:0007167\_enzyme\_linked\_receptor\_protein\_signaling\_pathway | SOCS7 | 229 | 2 | 1.915159 | -0.551492 | 244 | 261.0 | 1.069672 |
| GO:0016568\_chromatin\_modification | CSRP2BP | 72 | 1 | 3.045635 | -0.549338 | 247 | 263.58 | 1.067126 |
| GO:0050673\_epithelial\_cell\_proliferation | WDR77 | 72 | 1 | 3.045635 | -0.549338 | 247 | 263.58 | 1.067126 |
| GO:0051169\_nuclear\_transport | RAN | 72 | 1 | 3.045635 | -0.549338 | 247 | 263.58 | 1.067126 |
| GO:0044265\_cellular\_macromolecule\_catabolic\_process | ZMPSTE24 | 75 | 1 | 2.923810 | -0.534321 | 248 | 268.4 | 1.082258 |
| GO:0001501\_skeletal\_system\_development | HEXA | 236 | 2 | 1.858354 | -0.533217 | 249 | 268.61 | 1.078755 |
| GO:0001501\_skeletal\_system\_development | MAPK14 | 236 | 2 | 1.858354 | -0.533217 | 249 | 268.61 | 1.078755 |
| GO:0006508\_proteolysis | ZMPSTE24 | 76 | 1 | 2.885338 | -0.529471 | 250 | 270.18 | 1.080720 |
| GO:0042592\_homeostatic\_process | ID2 | 419 | 3 | 1.570065 | -0.526261 | 251 | 270.58 | 1.078008 |
| GO:0042592\_homeostatic\_process | MAPK14 | 419 | 3 | 1.570065 | -0.526261 | 251 | 270.58 | 1.078008 |
| GO:0042592\_homeostatic\_process | HEXA | 419 | 3 | 1.570065 | -0.526261 | 251 | 270.58 | 1.078008 |
| GO:0001890\_placenta\_development | PBRM1 | 77 | 1 | 2.847866 | -0.524696 | 252 | 271.3 | 1.076587 |
| GO:0007519\_skeletal\_muscle\_tissue\_development | MAPK14 | 78 | 1 | 2.811355 | -0.519994 | 254 | 273.29 | 1.075945 |
| GO:0060538\_skeletal\_muscle\_organ\_development | MAPK14 | 78 | 1 | 2.811355 | -0.519994 | 254 | 273.29 | 1.075945 |
| GO:0009056\_catabolic\_process | HEXA | 243 | 2 | 1.804821 | -0.515694 | 255 | 273.8 | 1.073725 |
| GO:0009056\_catabolic\_process | ZMPSTE24 | 243 | 2 | 1.804821 | -0.515694 | 255 | 273.8 | 1.073725 |
| GO:0031326\_regulation\_of\_cellular\_biosynthetic\_process | ID2 | 812 | 5 | 1.350281 | -0.513405 | 256 | 274.6 | 1.072656 |
| GO:0031326\_regulation\_of\_cellular\_biosynthetic\_process | MAPK14 | 812 | 5 | 1.350281 | -0.513405 | 256 | 274.6 | 1.072656 |
| GO:0031326\_regulation\_of\_cellular\_biosynthetic\_process | WDR77 | 812 | 5 | 1.350281 | -0.513405 | 256 | 274.6 | 1.072656 |
| GO:0031326\_regulation\_of\_cellular\_biosynthetic\_process | RBL1 | 812 | 5 | 1.350281 | -0.513405 | 256 | 274.6 | 1.072656 |
| GO:0031326\_regulation\_of\_cellular\_biosynthetic\_process | TAF9 | 812 | 5 | 1.350281 | -0.513405 | 256 | 274.6 | 1.072656 |
| GO:0006631\_fatty\_acid\_metabolic\_process | MAPK14 | 80 | 1 | 2.741071 | -0.510800 | 257 | 276.18 | 1.074630 |
| GO:0031323\_regulation\_of\_cellular\_metabolic\_process | SMARCAD1 | 1015 | 6 | 1.296270 | -0.509417 | 258 | 276.36 | 1.071163 |
| GO:0031323\_regulation\_of\_cellular\_metabolic\_process | ID2 | 1015 | 6 | 1.296270 | -0.509417 | 258 | 276.36 | 1.071163 |
| GO:0031323\_regulation\_of\_cellular\_metabolic\_process | MAPK14 | 1015 | 6 | 1.296270 | -0.509417 | 258 | 276.36 | 1.071163 |
| GO:0031323\_regulation\_of\_cellular\_metabolic\_process | WDR77 | 1015 | 6 | 1.296270 | -0.509417 | 258 | 276.36 | 1.071163 |
| GO:0031323\_regulation\_of\_cellular\_metabolic\_process | RBL1 | 1015 | 6 | 1.296270 | -0.509417 | 258 | 276.36 | 1.071163 |
| GO:0031323\_regulation\_of\_cellular\_metabolic\_process | TAF9 | 1015 | 6 | 1.296270 | -0.509417 | 258 | 276.36 | 1.071163 |
| GO:0009889\_regulation\_of\_biosynthetic\_process | ID2 | 815 | 5 | 1.345311 | -0.509339 | 259 | 276.55 | 1.067761 |
| GO:0009889\_regulation\_of\_biosynthetic\_process | MAPK14 | 815 | 5 | 1.345311 | -0.509339 | 259 | 276.55 | 1.067761 |
| GO:0009889\_regulation\_of\_biosynthetic\_process | WDR77 | 815 | 5 | 1.345311 | -0.509339 | 259 | 276.55 | 1.067761 |
| GO:0009889\_regulation\_of\_biosynthetic\_process | RBL1 | 815 | 5 | 1.345311 | -0.509339 | 259 | 276.55 | 1.067761 |
| GO:0009889\_regulation\_of\_biosynthetic\_process | TAF9 | 815 | 5 | 1.345311 | -0.509339 | 259 | 276.55 | 1.067761 |
| GO:0007399\_nervous\_system\_development | METRN | 621 | 4 | 1.412468 | -0.506157 | 260 | 277.26 | 1.066385 |
| GO:0007399\_nervous\_system\_development | HEXA | 621 | 4 | 1.412468 | -0.506157 | 260 | 277.26 | 1.066385 |
| GO:0007399\_nervous\_system\_development | FMR1 | 621 | 4 | 1.412468 | -0.506157 | 260 | 277.26 | 1.066385 |
| GO:0007399\_nervous\_system\_development | AARS | 621 | 4 | 1.412468 | -0.506157 | 260 | 277.26 | 1.066385 |
| GO:0045664\_regulation\_of\_neuron\_differentiation | METRN | 82 | 1 | 2.674216 | -0.501875 | 261 | 278.96 | 1.068812 |
| GO:0030534\_adult\_behavior | HEXA | 83 | 1 | 2.641997 | -0.497509 | 262 | 281.16 | 1.073130 |
| GO:0016481\_negative\_regulation\_of\_transcription | ID2 | 253 | 2 | 1.733484 | -0.491878 | 264 | 282.85 | 1.071402 |
| GO:0016481\_negative\_regulation\_of\_transcription | RBL1 | 253 | 2 | 1.733484 | -0.491878 | 264 | 282.85 | 1.071402 |
| GO:0030097\_hemopoiesis | ID2 | 253 | 2 | 1.733484 | -0.491878 | 264 | 282.85 | 1.071402 |
| GO:0030097\_hemopoiesis | MAPK14 | 253 | 2 | 1.733484 | -0.491878 | 264 | 282.85 | 1.071402 |
| GO:0006605\_protein\_targeting | RAN | 86 | 1 | 2.549834 | -0.484782 | 265 | 286.62 | 1.081585 |
| GO:0016337\_cell-cell\_adhesion | FAT1 | 87 | 1 | 2.520525 | -0.480657 | 266 | 289.31 | 1.087632 |
| GO:0010629\_negative\_regulation\_of\_gene\_expression | ID2 | 262 | 2 | 1.673937 | -0.471583 | 267 | 291.32 | 1.091086 |
| GO:0010629\_negative\_regulation\_of\_gene\_expression | RBL1 | 262 | 2 | 1.673937 | -0.471583 | 267 | 291.32 | 1.091086 |
| GO:0044255\_cellular\_lipid\_metabolic\_process | HEXA | 264 | 2 | 1.661255 | -0.467212 | 268 | 293.26 | 1.094254 |
| GO:0044255\_cellular\_lipid\_metabolic\_process | MAPK14 | 264 | 2 | 1.661255 | -0.467212 | 268 | 293.26 | 1.094254 |
| GO:0008544\_epidermis\_development | AARS | 91 | 1 | 2.409733 | -0.464714 | 269 | 294.08 | 1.093234 |
| GO:0045944\_positive\_regulation\_of\_transcription\_from\_RNA\_polymerase\_II\_promoter | MAPK14 | 269 | 2 | 1.630377 | -0.456494 | 270 | 297.52 | 1.101926 |
| GO:0045944\_positive\_regulation\_of\_transcription\_from\_RNA\_polymerase\_II\_promoter | TAF9 | 269 | 2 | 1.630377 | -0.456494 | 270 | 297.52 | 1.101926 |
| GO:0030154\_cell\_differentiation | DCT | 1060 | 6 | 1.241240 | -0.456384 | 271 | 297.7 | 1.098524 |
| GO:0030154\_cell\_differentiation | METRN | 1060 | 6 | 1.241240 | -0.456384 | 271 | 297.7 | 1.098524 |
| GO:0030154\_cell\_differentiation | ID2 | 1060 | 6 | 1.241240 | -0.456384 | 271 | 297.7 | 1.098524 |
| GO:0030154\_cell\_differentiation | MAPK14 | 1060 | 6 | 1.241240 | -0.456384 | 271 | 297.7 | 1.098524 |
| GO:0030154\_cell\_differentiation | WDR77 | 1060 | 6 | 1.241240 | -0.456384 | 271 | 297.7 | 1.098524 |
| GO:0030154\_cell\_differentiation | SOCS7 | 1060 | 6 | 1.241240 | -0.456384 | 271 | 297.7 | 1.098524 |
| GO:0045934\_negative\_regulation\_of\_nucleobase\_\_nucleoside\_\_nucleotide\_and\_nucleic\_acid\_metabolic\_process | ID2 | 270 | 2 | 1.624339 | -0.454386 | 272 | 297.92 | 1.095294 |
| GO:0045934\_negative\_regulation\_of\_nucleobase\_\_nucleoside\_\_nucleotide\_and\_nucleic\_acid\_metabolic\_process | RBL1 | 270 | 2 | 1.624339 | -0.454386 | 272 | 297.92 | 1.095294 |
| GO:0048856\_anatomical\_structure\_development | METRN | 1688 | 9 | 1.169177 | -0.453728 | 273 | 298.04 | 1.091722 |
| GO:0048856\_anatomical\_structure\_development | ID2 | 1688 | 9 | 1.169177 | -0.453728 | 273 | 298.04 | 1.091722 |
| GO:0048856\_anatomical\_structure\_development | HEXA | 1688 | 9 | 1.169177 | -0.453728 | 273 | 298.04 | 1.091722 |
| GO:0048856\_anatomical\_structure\_development | FAT1 | 1688 | 9 | 1.169177 | -0.453728 | 273 | 298.04 | 1.091722 |
| GO:0048856\_anatomical\_structure\_development | MAPK14 | 1688 | 9 | 1.169177 | -0.453728 | 273 | 298.04 | 1.091722 |
| GO:0048856\_anatomical\_structure\_development | FMR1 | 1688 | 9 | 1.169177 | -0.453728 | 273 | 298.04 | 1.091722 |
| GO:0048856\_anatomical\_structure\_development | AARS | 1688 | 9 | 1.169177 | -0.453728 | 273 | 298.04 | 1.091722 |
| GO:0048856\_anatomical\_structure\_development | WDR77 | 1688 | 9 | 1.169177 | -0.453728 | 273 | 298.04 | 1.091722 |
| GO:0048856\_anatomical\_structure\_development | PBRM1 | 1688 | 9 | 1.169177 | -0.453728 | 273 | 298.04 | 1.091722 |
| GO:0034984\_cellular\_response\_to\_DNA\_damage\_stimulus | MAPK14 | 94 | 1 | 2.332827 | -0.453307 | 274 | 299.55 | 1.093248 |
| GO:0051172\_negative\_regulation\_of\_nitrogen\_compound\_metabolic\_process | ID2 | 271 | 2 | 1.618345 | -0.452290 | 275 | 300.12 | 1.091345 |
| GO:0051172\_negative\_regulation\_of\_nitrogen\_compound\_metabolic\_process | RBL1 | 271 | 2 | 1.618345 | -0.452290 | 275 | 300.12 | 1.091345 |
| GO:0042391\_regulation\_of\_membrane\_potential | HEXA | 95 | 1 | 2.308271 | -0.449603 | 277 | 300.78 | 1.085848 |
| GO:0051707\_response\_to\_other\_organism | MAPK14 | 95 | 1 | 2.308271 | -0.449603 | 277 | 300.78 | 1.085848 |
| GO:0010558\_negative\_regulation\_of\_macromolecule\_biosynthetic\_process | ID2 | 274 | 2 | 1.600626 | -0.446069 | 279 | 301.68 | 1.081290 |
| GO:0010558\_negative\_regulation\_of\_macromolecule\_biosynthetic\_process | RBL1 | 274 | 2 | 1.600626 | -0.446069 | 279 | 301.68 | 1.081290 |
| GO:0033036\_macromolecule\_localization | RAN | 274 | 2 | 1.600626 | -0.446069 | 279 | 301.68 | 1.081290 |
| GO:0033036\_macromolecule\_localization | HEXA | 274 | 2 | 1.600626 | -0.446069 | 279 | 301.68 | 1.081290 |
| GO:0048534\_hemopoietic\_or\_lymphoid\_organ\_development | ID2 | 277 | 2 | 1.583290 | -0.439950 | 280 | 304.4 | 1.087143 |
| GO:0048534\_hemopoietic\_or\_lymphoid\_organ\_development | MAPK14 | 277 | 2 | 1.583290 | -0.439950 | 280 | 304.4 | 1.087143 |
| GO:0007398\_ectoderm\_development | AARS | 99 | 1 | 2.215007 | -0.435252 | 281 | 307.63 | 1.094769 |
| GO:0001525\_angiogenesis | MAPK14 | 100 | 1 | 2.192857 | -0.431776 | 282 | 307.95 | 1.092021 |
| GO:0031327\_negative\_regulation\_of\_cellular\_biosynthetic\_process | ID2 | 282 | 2 | 1.555218 | -0.429971 | 283 | 308.22 | 1.089117 |
| GO:0031327\_negative\_regulation\_of\_cellular\_biosynthetic\_process | RBL1 | 282 | 2 | 1.555218 | -0.429971 | 283 | 308.22 | 1.089117 |
| GO:0030163\_protein\_catabolic\_process | ZMPSTE24 | 101 | 1 | 2.171146 | -0.428343 | 284 | 308.93 | 1.087782 |
| GO:0009890\_negative\_regulation\_of\_biosynthetic\_process | ID2 | 284 | 2 | 1.544266 | -0.426054 | 285 | 309.44 | 1.085754 |
| GO:0009890\_negative\_regulation\_of\_biosynthetic\_process | RBL1 | 284 | 2 | 1.544266 | -0.426054 | 285 | 309.44 | 1.085754 |
| GO:0019222\_regulation\_of\_metabolic\_process | SMARCAD1 | 1088 | 6 | 1.209296 | -0.425826 | 286 | 309.63 | 1.082622 |
| GO:0019222\_regulation\_of\_metabolic\_process | ID2 | 1088 | 6 | 1.209296 | -0.425826 | 286 | 309.63 | 1.082622 |
| GO:0019222\_regulation\_of\_metabolic\_process | MAPK14 | 1088 | 6 | 1.209296 | -0.425826 | 286 | 309.63 | 1.082622 |
| GO:0019222\_regulation\_of\_metabolic\_process | WDR77 | 1088 | 6 | 1.209296 | -0.425826 | 286 | 309.63 | 1.082622 |
| GO:0019222\_regulation\_of\_metabolic\_process | RBL1 | 1088 | 6 | 1.209296 | -0.425826 | 286 | 309.63 | 1.082622 |
| GO:0019222\_regulation\_of\_metabolic\_process | TAF9 | 1088 | 6 | 1.209296 | -0.425826 | 286 | 309.63 | 1.082622 |
| GO:0030036\_actin\_cytoskeleton\_organization | FAT1 | 102 | 1 | 2.149860 | -0.424952 | 287 | 309.95 | 1.079965 |
| GO:0006629\_lipid\_metabolic\_process | MAPK14 | 285 | 2 | 1.538847 | -0.424111 | 288 | 310.17 | 1.076979 |
| GO:0006629\_lipid\_metabolic\_process | HEXA | 285 | 2 | 1.538847 | -0.424111 | 288 | 310.17 | 1.076979 |
| GO:0007417\_central\_nervous\_system\_development | FMR1 | 287 | 2 | 1.528123 | -0.420257 | 289 | 311.25 | 1.076990 |
| GO:0007417\_central\_nervous\_system\_development | AARS | 287 | 2 | 1.528123 | -0.420257 | 289 | 311.25 | 1.076990 |
| GO:0050767\_regulation\_of\_neurogenesis | METRN | 104 | 1 | 2.108516 | -0.418292 | 290 | 311.85 | 1.075345 |
| GO:0048522\_positive\_regulation\_of\_cellular\_process | METRN | 895 | 5 | 1.225060 | -0.411077 | 291 | 312.94 | 1.075395 |
| GO:0048522\_positive\_regulation\_of\_cellular\_process | ID2 | 895 | 5 | 1.225060 | -0.411077 | 291 | 312.94 | 1.075395 |
| GO:0048522\_positive\_regulation\_of\_cellular\_process | MAPK14 | 895 | 5 | 1.225060 | -0.411077 | 291 | 312.94 | 1.075395 |
| GO:0048522\_positive\_regulation\_of\_cellular\_process | WDR77 | 895 | 5 | 1.225060 | -0.411077 | 291 | 312.94 | 1.075395 |
| GO:0048522\_positive\_regulation\_of\_cellular\_process | TAF9 | 895 | 5 | 1.225060 | -0.411077 | 291 | 312.94 | 1.075395 |
| GO:0050896\_response\_to\_stimulus | ID2 | 1107 | 6 | 1.188540 | -0.406093 | 292 | 313.62 | 1.074041 |
| GO:0050896\_response\_to\_stimulus | PTPN2 | 1107 | 6 | 1.188540 | -0.406093 | 292 | 313.62 | 1.074041 |
| GO:0050896\_response\_to\_stimulus | HEXA | 1107 | 6 | 1.188540 | -0.406093 | 292 | 313.62 | 1.074041 |
| GO:0050896\_response\_to\_stimulus | MAPK14 | 1107 | 6 | 1.188540 | -0.406093 | 292 | 313.62 | 1.074041 |
| GO:0050896\_response\_to\_stimulus | AARS | 1107 | 6 | 1.188540 | -0.406093 | 292 | 313.62 | 1.074041 |
| GO:0050896\_response\_to\_stimulus | SOCS7 | 1107 | 6 | 1.188540 | -0.406093 | 292 | 313.62 | 1.074041 |
| GO:0002520\_immune\_system\_development | ID2 | 295 | 2 | 1.486683 | -0.405242 | 293 | 314.85 | 1.074573 |
| GO:0002520\_immune\_system\_development | MAPK14 | 295 | 2 | 1.486683 | -0.405242 | 293 | 314.85 | 1.074573 |
| GO:0030029\_actin\_filament-based\_process | FAT1 | 109 | 1 | 2.011796 | -0.402322 | 294 | 315.33 | 1.072551 |
| GO:0006974\_response\_to\_DNA\_damage\_stimulus | MAPK14 | 113 | 1 | 1.940582 | -0.390199 | 295 | 319.58 | 1.083322 |
| GO:0007275\_multicellular\_organismal\_development | METRN | 1760 | 9 | 1.121347 | -0.388025 | 296 | 319.91 | 1.080777 |
| GO:0007275\_multicellular\_organismal\_development | CSRP2BP | 1760 | 9 | 1.121347 | -0.388025 | 296 | 319.91 | 1.080777 |
| GO:0007275\_multicellular\_organismal\_development | ID2 | 1760 | 9 | 1.121347 | -0.388025 | 296 | 319.91 | 1.080777 |
| GO:0007275\_multicellular\_organismal\_development | HEXA | 1760 | 9 | 1.121347 | -0.388025 | 296 | 319.91 | 1.080777 |
| GO:0007275\_multicellular\_organismal\_development | MAPK14 | 1760 | 9 | 1.121347 | -0.388025 | 296 | 319.91 | 1.080777 |
| GO:0007275\_multicellular\_organismal\_development | FMR1 | 1760 | 9 | 1.121347 | -0.388025 | 296 | 319.91 | 1.080777 |
| GO:0007275\_multicellular\_organismal\_development | AARS | 1760 | 9 | 1.121347 | -0.388025 | 296 | 319.91 | 1.080777 |
| GO:0007275\_multicellular\_organismal\_development | WDR77 | 1760 | 9 | 1.121347 | -0.388025 | 296 | 319.91 | 1.080777 |
| GO:0007275\_multicellular\_organismal\_development | PBRM1 | 1760 | 9 | 1.121347 | -0.388025 | 296 | 319.91 | 1.080777 |
| GO:0050794\_regulation\_of\_cellular\_process | SMARCAD1 | 2190 | 11 | 1.101435 | -0.387080 | 297 | 321.1 | 1.081145 |
| GO:0050794\_regulation\_of\_cellular\_process | METRN | 2190 | 11 | 1.101435 | -0.387080 | 297 | 321.1 | 1.081145 |
| GO:0050794\_regulation\_of\_cellular\_process | ID2 | 2190 | 11 | 1.101435 | -0.387080 | 297 | 321.1 | 1.081145 |
| GO:0050794\_regulation\_of\_cellular\_process | PTPN2 | 2190 | 11 | 1.101435 | -0.387080 | 297 | 321.1 | 1.081145 |
| GO:0050794\_regulation\_of\_cellular\_process | MAPK14 | 2190 | 11 | 1.101435 | -0.387080 | 297 | 321.1 | 1.081145 |
| GO:0050794\_regulation\_of\_cellular\_process | WDR77 | 2190 | 11 | 1.101435 | -0.387080 | 297 | 321.1 | 1.081145 |
| GO:0050794\_regulation\_of\_cellular\_process | RBL1 | 2190 | 11 | 1.101435 | -0.387080 | 297 | 321.1 | 1.081145 |
| GO:0050794\_regulation\_of\_cellular\_process | AARS | 2190 | 11 | 1.101435 | -0.387080 | 297 | 321.1 | 1.081145 |
| GO:0050794\_regulation\_of\_cellular\_process | SOCS7 | 2190 | 11 | 1.101435 | -0.387080 | 297 | 321.1 | 1.081145 |
| GO:0050794\_regulation\_of\_cellular\_process | TAF9 | 2190 | 11 | 1.101435 | -0.387080 | 297 | 321.1 | 1.081145 |
| GO:0050794\_regulation\_of\_cellular\_process | CCNG2 | 2190 | 11 | 1.101435 | -0.387080 | 297 | 321.1 | 1.081145 |
| GO:0045893\_positive\_regulation\_of\_transcription\_\_DNA-dependent | MAPK14 | 306 | 2 | 1.433240 | -0.385599 | 299 | 321.56 | 1.075452 |
| GO:0045893\_positive\_regulation\_of\_transcription\_\_DNA-dependent | TAF9 | 306 | 2 | 1.433240 | -0.385599 | 299 | 321.56 | 1.075452 |
| GO:0051254\_positive\_regulation\_of\_RNA\_metabolic\_process | MAPK14 | 306 | 2 | 1.433240 | -0.385599 | 299 | 321.56 | 1.075452 |
| GO:0051254\_positive\_regulation\_of\_RNA\_metabolic\_process | TAF9 | 306 | 2 | 1.433240 | -0.385599 | 299 | 321.56 | 1.075452 |
| GO:0048608\_reproductive\_structure\_development | WDR77 | 116 | 1 | 1.890394 | -0.381459 | 300 | 323.6 | 1.078667 |
| GO:0051960\_regulation\_of\_nervous\_system\_development | METRN | 118 | 1 | 1.858354 | -0.375792 | 301 | 325.21 | 1.080432 |
| GO:0014706\_striated\_muscle\_tissue\_development | MAPK14 | 120 | 1 | 1.827381 | -0.370248 | 302 | 326.07 | 1.079702 |
| GO:0006886\_intracellular\_protein\_transport | RAN | 122 | 1 | 1.797424 | -0.364822 | 304 | 329.93 | 1.085296 |
| GO:0060284\_regulation\_of\_cell\_development | METRN | 122 | 1 | 1.797424 | -0.364822 | 304 | 329.93 | 1.085296 |
| GO:0009888\_tissue\_development | MAPK14 | 525 | 3 | 1.253061 | -0.361430 | 305 | 330.55 | 1.083770 |
| GO:0009888\_tissue\_development | WDR77 | 525 | 3 | 1.253061 | -0.361430 | 305 | 330.55 | 1.083770 |
| GO:0009888\_tissue\_development | AARS | 525 | 3 | 1.253061 | -0.361430 | 305 | 330.55 | 1.083770 |
| GO:0001655\_urogenital\_system\_development | WDR77 | 128 | 1 | 1.713170 | -0.349215 | 307 | 332.82 | 1.084104 |
| GO:0060537\_muscle\_tissue\_development | MAPK14 | 128 | 1 | 1.713170 | -0.349215 | 307 | 332.82 | 1.084104 |
| GO:0043285\_biopolymer\_catabolic\_process | ZMPSTE24 | 129 | 1 | 1.699889 | -0.346707 | 308 | 333.66 | 1.083312 |
| GO:0010605\_negative\_regulation\_of\_macromolecule\_metabolic\_process | ID2 | 331 | 2 | 1.324989 | -0.344859 | 309 | 335.12 | 1.084531 |
| GO:0010605\_negative\_regulation\_of\_macromolecule\_metabolic\_process | RBL1 | 331 | 2 | 1.324989 | -0.344859 | 309 | 335.12 | 1.084531 |
| GO:0032787\_monocarboxylic\_acid\_metabolic\_process | MAPK14 | 130 | 1 | 1.686813 | -0.344225 | 310 | 336.13 | 1.084290 |
| GO:0031324\_negative\_regulation\_of\_cellular\_metabolic\_process | ID2 | 332 | 2 | 1.320998 | -0.343333 | 311 | 336.38 | 1.081608 |
| GO:0031324\_negative\_regulation\_of\_cellular\_metabolic\_process | RBL1 | 332 | 2 | 1.320998 | -0.343333 | 311 | 336.38 | 1.081608 |
| GO:0048731\_system\_development | METRN | 1609 | 8 | 1.090296 | -0.336114 | 312 | 337.93 | 1.083109 |
| GO:0048731\_system\_development | ID2 | 1609 | 8 | 1.090296 | -0.336114 | 312 | 337.93 | 1.083109 |
| GO:0048731\_system\_development | HEXA | 1609 | 8 | 1.090296 | -0.336114 | 312 | 337.93 | 1.083109 |
| GO:0048731\_system\_development | MAPK14 | 1609 | 8 | 1.090296 | -0.336114 | 312 | 337.93 | 1.083109 |
| GO:0048731\_system\_development | FMR1 | 1609 | 8 | 1.090296 | -0.336114 | 312 | 337.93 | 1.083109 |
| GO:0048731\_system\_development | AARS | 1609 | 8 | 1.090296 | -0.336114 | 312 | 337.93 | 1.083109 |
| GO:0048731\_system\_development | WDR77 | 1609 | 8 | 1.090296 | -0.336114 | 312 | 337.93 | 1.083109 |
| GO:0048731\_system\_development | PBRM1 | 1609 | 8 | 1.090296 | -0.336114 | 312 | 337.93 | 1.083109 |
| GO:0045941\_positive\_regulation\_of\_transcription | MAPK14 | 338 | 2 | 1.297549 | -0.334335 | 313 | 339.34 | 1.084153 |
| GO:0045941\_positive\_regulation\_of\_transcription | TAF9 | 338 | 2 | 1.297549 | -0.334335 | 313 | 339.34 | 1.084153 |
| GO:0006793\_phosphorus\_metabolic\_process | PTPN2 | 340 | 2 | 1.289916 | -0.331394 | 315 | 340.32 | 1.080381 |
| GO:0006793\_phosphorus\_metabolic\_process | MAPK14 | 340 | 2 | 1.289916 | -0.331394 | 315 | 340.32 | 1.080381 |
| GO:0006796\_phosphate\_metabolic\_process | PTPN2 | 340 | 2 | 1.289916 | -0.331394 | 315 | 340.32 | 1.080381 |
| GO:0006796\_phosphate\_metabolic\_process | MAPK14 | 340 | 2 | 1.289916 | -0.331394 | 315 | 340.32 | 1.080381 |
| GO:0009057\_macromolecule\_catabolic\_process | ZMPSTE24 | 137 | 1 | 1.600626 | -0.327529 | 316 | 341.26 | 1.079937 |
| GO:0034613\_cellular\_protein\_localization | RAN | 139 | 1 | 1.577595 | -0.322968 | 317 | 341.9 | 1.078549 |
| GO:0010628\_positive\_regulation\_of\_gene\_expression | MAPK14 | 346 | 2 | 1.267547 | -0.322741 | 318 | 342.19 | 1.076069 |
| GO:0010628\_positive\_regulation\_of\_gene\_expression | TAF9 | 346 | 2 | 1.267547 | -0.322741 | 318 | 342.19 | 1.076069 |
| GO:0016044\_membrane\_organization | ZMPSTE24 | 140 | 1 | 1.566327 | -0.320720 | 319 | 342.73 | 1.074389 |
| GO:0009892\_negative\_regulation\_of\_metabolic\_process | ID2 | 348 | 2 | 1.260263 | -0.319913 | 320 | 343.41 | 1.073156 |
| GO:0009892\_negative\_regulation\_of\_metabolic\_process | RBL1 | 348 | 2 | 1.260263 | -0.319913 | 320 | 343.41 | 1.073156 |
| GO:0003006\_reproductive\_developmental\_process | WDR77 | 141 | 1 | 1.555218 | -0.318493 | 322 | 344.07 | 1.068540 |
| GO:0070727\_cellular\_macromolecule\_localization | RAN | 141 | 1 | 1.555218 | -0.318493 | 322 | 344.07 | 1.068540 |
| GO:0048523\_negative\_regulation\_of\_cellular\_process | ID2 | 774 | 4 | 1.133260 | -0.318454 | 323 | 344.32 | 1.066006 |
| GO:0048523\_negative\_regulation\_of\_cellular\_process | AARS | 774 | 4 | 1.133260 | -0.318454 | 323 | 344.32 | 1.066006 |
| GO:0048523\_negative\_regulation\_of\_cellular\_process | RBL1 | 774 | 4 | 1.133260 | -0.318454 | 323 | 344.32 | 1.066006 |
| GO:0048523\_negative\_regulation\_of\_cellular\_process | WDR77 | 774 | 4 | 1.133260 | -0.318454 | 323 | 344.32 | 1.066006 |
| GO:0045935\_positive\_regulation\_of\_nucleobase\_\_nucleoside\_\_nucleotide\_and\_nucleic\_acid\_metabolic\_process | MAPK14 | 352 | 2 | 1.245942 | -0.314337 | 324 | 344.83 | 1.064290 |
| GO:0045935\_positive\_regulation\_of\_nucleobase\_\_nucleoside\_\_nucleotide\_and\_nucleic\_acid\_metabolic\_process | TAF9 | 352 | 2 | 1.245942 | -0.314337 | 324 | 344.83 | 1.064290 |
| GO:0048518\_positive\_regulation\_of\_biological\_process | METRN | 995 | 5 | 1.101938 | -0.312115 | 325 | 345.4 | 1.062769 |
| GO:0048518\_positive\_regulation\_of\_biological\_process | ID2 | 995 | 5 | 1.101938 | -0.312115 | 325 | 345.4 | 1.062769 |
| GO:0048518\_positive\_regulation\_of\_biological\_process | MAPK14 | 995 | 5 | 1.101938 | -0.312115 | 325 | 345.4 | 1.062769 |
| GO:0048518\_positive\_regulation\_of\_biological\_process | WDR77 | 995 | 5 | 1.101938 | -0.312115 | 325 | 345.4 | 1.062769 |
| GO:0048518\_positive\_regulation\_of\_biological\_process | TAF9 | 995 | 5 | 1.101938 | -0.312115 | 325 | 345.4 | 1.062769 |
| GO:0022603\_regulation\_of\_anatomical\_structure\_morphogenesis | METRN | 147 | 1 | 1.491740 | -0.305571 | 326 | 348.16 | 1.067975 |
| GO:0051173\_positive\_regulation\_of\_nitrogen\_compound\_metabolic\_process | MAPK14 | 361 | 2 | 1.214879 | -0.302176 | 327 | 349.74 | 1.069541 |
| GO:0051173\_positive\_regulation\_of\_nitrogen\_compound\_metabolic\_process | TAF9 | 361 | 2 | 1.214879 | -0.302176 | 327 | 349.74 | 1.069541 |
| GO:0007517\_muscle\_organ\_development | MAPK14 | 153 | 1 | 1.433240 | -0.293351 | 328 | 352.08 | 1.073415 |
| GO:0008285\_negative\_regulation\_of\_cell\_proliferation | WDR77 | 155 | 1 | 1.414747 | -0.289425 | 330 | 354.19 | 1.073303 |
| GO:0022402\_cell\_cycle\_process | CSRP2BP | 155 | 1 | 1.414747 | -0.289425 | 330 | 354.19 | 1.073303 |
| GO:0010557\_positive\_regulation\_of\_macromolecule\_biosynthetic\_process | MAPK14 | 371 | 2 | 1.182133 | -0.289260 | 331 | 354.53 | 1.071088 |
| GO:0010557\_positive\_regulation\_of\_macromolecule\_biosynthetic\_process | TAF9 | 371 | 2 | 1.182133 | -0.289260 | 331 | 354.53 | 1.071088 |
| GO:0051704\_multi-organism\_process | MAPK14 | 157 | 1 | 1.396724 | -0.285568 | 332 | 355.35 | 1.070331 |
| GO:0006066\_alcohol\_metabolic\_process | MAPK14 | 158 | 1 | 1.387884 | -0.283665 | 335 | 356.49 | 1.064149 |
| GO:0007409\_axonogenesis | METRN | 158 | 1 | 1.387884 | -0.283665 | 335 | 356.49 | 1.064149 |
| GO:0048514\_blood\_vessel\_morphogenesis | MAPK14 | 158 | 1 | 1.387884 | -0.283665 | 335 | 356.49 | 1.064149 |
| GO:0007166\_cell\_surface\_receptor\_linked\_signal\_transduction | PTPN2 | 597 | 3 | 1.101938 | -0.280287 | 336 | 357.87 | 1.065089 |
| GO:0007166\_cell\_surface\_receptor\_linked\_signal\_transduction | MAPK14 | 597 | 3 | 1.101938 | -0.280287 | 336 | 357.87 | 1.065089 |
| GO:0007166\_cell\_surface\_receptor\_linked\_signal\_transduction | SOCS7 | 597 | 3 | 1.101938 | -0.280287 | 336 | 357.87 | 1.065089 |
| GO:0051128\_regulation\_of\_cellular\_component\_organization | METRN | 160 | 1 | 1.370536 | -0.279910 | 337 | 358.25 | 1.063056 |
| GO:0000003\_reproduction | HEXA | 379 | 2 | 1.157181 | -0.279357 | 338 | 358.51 | 1.060680 |
| GO:0000003\_reproduction | WDR77 | 379 | 2 | 1.157181 | -0.279357 | 338 | 358.51 | 1.060680 |
| GO:0002521\_leukocyte\_differentiation | ID2 | 161 | 1 | 1.362023 | -0.278057 | 339 | 358.83 | 1.058496 |
| GO:0007626\_locomotory\_behavior | HEXA | 163 | 1 | 1.345311 | -0.274399 | 340 | 360.16 | 1.059294 |
| GO:0006259\_DNA\_metabolic\_process | SMARCAD1 | 165 | 1 | 1.329004 | -0.270804 | 341 | 361.97 | 1.061496 |
| GO:0031328\_positive\_regulation\_of\_cellular\_biosynthetic\_process | MAPK14 | 387 | 2 | 1.133260 | -0.269816 | 342 | 362.28 | 1.059298 |
| GO:0031328\_positive\_regulation\_of\_cellular\_biosynthetic\_process | TAF9 | 387 | 2 | 1.133260 | -0.269816 | 342 | 362.28 | 1.059298 |
| GO:0009891\_positive\_regulation\_of\_biosynthetic\_process | MAPK14 | 388 | 2 | 1.130339 | -0.268648 | 343 | 363.06 | 1.058484 |
| GO:0009891\_positive\_regulation\_of\_biosynthetic\_process | TAF9 | 388 | 2 | 1.130339 | -0.268648 | 343 | 363.06 | 1.058484 |
| GO:0050877\_neurological\_system\_process | HEXA | 390 | 2 | 1.124542 | -0.266328 | 344 | 364.29 | 1.058983 |
| GO:0050877\_neurological\_system\_process | AARS | 390 | 2 | 1.124542 | -0.266328 | 344 | 364.29 | 1.058983 |
| GO:0050789\_regulation\_of\_biological\_process | SMARCAD1 | 2357 | 11 | 1.023395 | -0.264557 | 345 | 364.69 | 1.057072 |
| GO:0050789\_regulation\_of\_biological\_process | METRN | 2357 | 11 | 1.023395 | -0.264557 | 345 | 364.69 | 1.057072 |
| GO:0050789\_regulation\_of\_biological\_process | ID2 | 2357 | 11 | 1.023395 | -0.264557 | 345 | 364.69 | 1.057072 |
| GO:0050789\_regulation\_of\_biological\_process | PTPN2 | 2357 | 11 | 1.023395 | -0.264557 | 345 | 364.69 | 1.057072 |
| GO:0050789\_regulation\_of\_biological\_process | MAPK14 | 2357 | 11 | 1.023395 | -0.264557 | 345 | 364.69 | 1.057072 |
| GO:0050789\_regulation\_of\_biological\_process | AARS | 2357 | 11 | 1.023395 | -0.264557 | 345 | 364.69 | 1.057072 |
| GO:0050789\_regulation\_of\_biological\_process | WDR77 | 2357 | 11 | 1.023395 | -0.264557 | 345 | 364.69 | 1.057072 |
| GO:0050789\_regulation\_of\_biological\_process | RBL1 | 2357 | 11 | 1.023395 | -0.264557 | 345 | 364.69 | 1.057072 |
| GO:0050789\_regulation\_of\_biological\_process | SOCS7 | 2357 | 11 | 1.023395 | -0.264557 | 345 | 364.69 | 1.057072 |
| GO:0050789\_regulation\_of\_biological\_process | TAF9 | 2357 | 11 | 1.023395 | -0.264557 | 345 | 364.69 | 1.057072 |
| GO:0050789\_regulation\_of\_biological\_process | CCNG2 | 2357 | 11 | 1.023395 | -0.264557 | 345 | 364.69 | 1.057072 |
| GO:0048812\_neuron\_projection\_morphogenesis | METRN | 170 | 1 | 1.289916 | -0.262082 | 346 | 365.7 | 1.056936 |
| GO:0007600\_sensory\_perception | HEXA | 172 | 1 | 1.274917 | -0.258696 | 347 | 367.09 | 1.057896 |
| GO:0048667\_cell\_morphogenesis\_involved\_in\_neuron\_differentiation | METRN | 173 | 1 | 1.267547 | -0.257024 | 348 | 368.52 | 1.058966 |
| GO:0015031\_protein\_transport | RAN | 175 | 1 | 1.253061 | -0.253722 | 349 | 369.64 | 1.059140 |
| GO:0006873\_cellular\_ion\_homeostasis | HEXA | 176 | 1 | 1.245942 | -0.252091 | 352 | 370.76 | 1.053295 |
| GO:0043066\_negative\_regulation\_of\_apoptosis | AARS | 176 | 1 | 1.245942 | -0.252091 | 352 | 370.76 | 1.053295 |
| GO:0048858\_cell\_projection\_morphogenesis | METRN | 176 | 1 | 1.245942 | -0.252091 | 352 | 370.76 | 1.053295 |
| GO:0009987\_cellular\_process | SMARCAD1 | 3868 | 18 | 1.020461 | -0.251730 | 353 | 370.98 | 1.050935 |
| GO:0009987\_cellular\_process | CSRP2BP | 3868 | 18 | 1.020461 | -0.251730 | 353 | 370.98 | 1.050935 |
| GO:0009987\_cellular\_process | RAN | 3868 | 18 | 1.020461 | -0.251730 | 353 | 370.98 | 1.050935 |
| GO:0009987\_cellular\_process | PTPN2 | 3868 | 18 | 1.020461 | -0.251730 | 353 | 370.98 | 1.050935 |
| GO:0009987\_cellular\_process | HEXA | 3868 | 18 | 1.020461 | -0.251730 | 353 | 370.98 | 1.050935 |
| GO:0009987\_cellular\_process | RBL1 | 3868 | 18 | 1.020461 | -0.251730 | 353 | 370.98 | 1.050935 |
| GO:0009987\_cellular\_process | AARS | 3868 | 18 | 1.020461 | -0.251730 | 353 | 370.98 | 1.050935 |
| GO:0009987\_cellular\_process | SOCS7 | 3868 | 18 | 1.020461 | -0.251730 | 353 | 370.98 | 1.050935 |
| GO:0009987\_cellular\_process | ARID1A | 3868 | 18 | 1.020461 | -0.251730 | 353 | 370.98 | 1.050935 |
| GO:0009987\_cellular\_process | CCNG2 | 3868 | 18 | 1.020461 | -0.251730 | 353 | 370.98 | 1.050935 |
| GO:0009987\_cellular\_process | DCT | 3868 | 18 | 1.020461 | -0.251730 | 353 | 370.98 | 1.050935 |
| GO:0009987\_cellular\_process | METRN | 3868 | 18 | 1.020461 | -0.251730 | 353 | 370.98 | 1.050935 |
| GO:0009987\_cellular\_process | ID2 | 3868 | 18 | 1.020461 | -0.251730 | 353 | 370.98 | 1.050935 |
| GO:0009987\_cellular\_process | FAT1 | 3868 | 18 | 1.020461 | -0.251730 | 353 | 370.98 | 1.050935 |
| GO:0009987\_cellular\_process | MAPK14 | 3868 | 18 | 1.020461 | -0.251730 | 353 | 370.98 | 1.050935 |
| GO:0009987\_cellular\_process | WDR77 | 3868 | 18 | 1.020461 | -0.251730 | 353 | 370.98 | 1.050935 |
| GO:0009987\_cellular\_process | TAF9 | 3868 | 18 | 1.020461 | -0.251730 | 353 | 370.98 | 1.050935 |
| GO:0009987\_cellular\_process | ZMPSTE24 | 3868 | 18 | 1.020461 | -0.251730 | 353 | 370.98 | 1.050935 |
| GO:0065007\_biological\_regulation | SMARCAD1 | 2593 | 12 | 1.014820 | -0.251559 | 354 | 371.17 | 1.048503 |
| GO:0065007\_biological\_regulation | METRN | 2593 | 12 | 1.014820 | -0.251559 | 354 | 371.17 | 1.048503 |
| GO:0065007\_biological\_regulation | ID2 | 2593 | 12 | 1.014820 | -0.251559 | 354 | 371.17 | 1.048503 |
| GO:0065007\_biological\_regulation | PTPN2 | 2593 | 12 | 1.014820 | -0.251559 | 354 | 371.17 | 1.048503 |
| GO:0065007\_biological\_regulation | HEXA | 2593 | 12 | 1.014820 | -0.251559 | 354 | 371.17 | 1.048503 |
| GO:0065007\_biological\_regulation | MAPK14 | 2593 | 12 | 1.014820 | -0.251559 | 354 | 371.17 | 1.048503 |
| GO:0065007\_biological\_regulation | AARS | 2593 | 12 | 1.014820 | -0.251559 | 354 | 371.17 | 1.048503 |
| GO:0065007\_biological\_regulation | WDR77 | 2593 | 12 | 1.014820 | -0.251559 | 354 | 371.17 | 1.048503 |
| GO:0065007\_biological\_regulation | RBL1 | 2593 | 12 | 1.014820 | -0.251559 | 354 | 371.17 | 1.048503 |
| GO:0065007\_biological\_regulation | SOCS7 | 2593 | 12 | 1.014820 | -0.251559 | 354 | 371.17 | 1.048503 |
| GO:0065007\_biological\_regulation | TAF9 | 2593 | 12 | 1.014820 | -0.251559 | 354 | 371.17 | 1.048503 |
| GO:0065007\_biological\_regulation | CCNG2 | 2593 | 12 | 1.014820 | -0.251559 | 354 | 371.17 | 1.048503 |
| GO:0043069\_negative\_regulation\_of\_programmed\_cell\_death | AARS | 179 | 1 | 1.225060 | -0.247279 | 357 | 372.33 | 1.042941 |
| GO:0048732\_gland\_development | WDR77 | 179 | 1 | 1.225060 | -0.247279 | 357 | 372.33 | 1.042941 |
| GO:0060548\_negative\_regulation\_of\_cell\_death | AARS | 179 | 1 | 1.225060 | -0.247279 | 357 | 372.33 | 1.042941 |
| GO:0045184\_establishment\_of\_protein\_localization | RAN | 180 | 1 | 1.218254 | -0.245701 | 358 | 372.71 | 1.041089 |
| GO:0048519\_negative\_regulation\_of\_biological\_process | ID2 | 859 | 4 | 1.021121 | -0.244422 | 359 | 373.32 | 1.039889 |
| GO:0048519\_negative\_regulation\_of\_biological\_process | AARS | 859 | 4 | 1.021121 | -0.244422 | 359 | 373.32 | 1.039889 |
| GO:0048519\_negative\_regulation\_of\_biological\_process | WDR77 | 859 | 4 | 1.021121 | -0.244422 | 359 | 373.32 | 1.039889 |
| GO:0048519\_negative\_regulation\_of\_biological\_process | RBL1 | 859 | 4 | 1.021121 | -0.244422 | 359 | 373.32 | 1.039889 |
| GO:0055082\_cellular\_chemical\_homeostasis | HEXA | 181 | 1 | 1.211523 | -0.244136 | 360 | 374.34 | 1.039833 |
| GO:0007242\_intracellular\_signaling\_cascade | MAPK14 | 411 | 2 | 1.067084 | -0.243212 | 361 | 374.66 | 1.037839 |
| GO:0007242\_intracellular\_signaling\_cascade | AARS | 411 | 2 | 1.067084 | -0.243212 | 361 | 374.66 | 1.037839 |
| GO:0032990\_cell\_part\_morphogenesis | METRN | 184 | 1 | 1.191770 | -0.239516 | 362 | 375.99 | 1.038646 |
| GO:0007010\_cytoskeleton\_organization | FAT1 | 185 | 1 | 1.185328 | -0.238000 | 363 | 377.07 | 1.038760 |
| GO:0007155\_cell\_adhesion | FAT1 | 186 | 1 | 1.178955 | -0.236497 | 365 | 378.16 | 1.036055 |
| GO:0022610\_biological\_adhesion | FAT1 | 186 | 1 | 1.178955 | -0.236497 | 365 | 378.16 | 1.036055 |
| GO:0019226\_transmission\_of\_nerve\_impulse | HEXA | 189 | 1 | 1.160242 | -0.232058 | 366 | 380.21 | 1.038825 |
| GO:0046907\_intracellular\_transport | RAN | 194 | 1 | 1.130339 | -0.224890 | 367 | 382.32 | 1.041744 |
| GO:0019725\_cellular\_homeostasis | HEXA | 195 | 1 | 1.124542 | -0.223490 | 368 | 383.37 | 1.041766 |
| GO:0010604\_positive\_regulation\_of\_macromolecule\_metabolic\_process | MAPK14 | 433 | 2 | 1.012867 | -0.221237 | 369 | 384.68 | 1.042493 |
| GO:0010604\_positive\_regulation\_of\_macromolecule\_metabolic\_process | TAF9 | 433 | 2 | 1.012867 | -0.221237 | 369 | 384.68 | 1.042493 |
| GO:0031175\_neuron\_projection\_development | METRN | 197 | 1 | 1.113125 | -0.220721 | 371 | 385.48 | 1.039030 |
| GO:0050801\_ion\_homeostasis | HEXA | 197 | 1 | 1.113125 | -0.220721 | 371 | 385.48 | 1.039030 |
| GO:0002009\_morphogenesis\_of\_an\_epithelium | WDR77 | 198 | 1 | 1.107504 | -0.219353 | 373 | 386.2 | 1.035389 |
| GO:0060429\_epithelium\_development | WDR77 | 198 | 1 | 1.107504 | -0.219353 | 373 | 386.2 | 1.035389 |
| GO:0000904\_cell\_morphogenesis\_involved\_in\_differentiation | METRN | 199 | 1 | 1.101938 | -0.217995 | 374 | 387.05 | 1.034893 |
| GO:0031325\_positive\_regulation\_of\_cellular\_metabolic\_process | MAPK14 | 442 | 2 | 0.992243 | -0.212850 | 375 | 387.98 | 1.034613 |
| GO:0031325\_positive\_regulation\_of\_cellular\_metabolic\_process | TAF9 | 442 | 2 | 0.992243 | -0.212850 | 375 | 387.98 | 1.034613 |
| GO:0001568\_blood\_vessel\_development | MAPK14 | 203 | 1 | 1.080225 | -0.212667 | 376 | 388.39 | 1.032952 |
| GO:0007243\_protein\_kinase\_cascade | MAPK14 | 205 | 1 | 1.069686 | -0.210063 | 377 | 390.75 | 1.036472 |
| GO:0001944\_vasculature\_development | MAPK14 | 208 | 1 | 1.054258 | -0.206229 | 379 | 392.37 | 1.035277 |
| GO:0008284\_positive\_regulation\_of\_cell\_proliferation | WDR77 | 208 | 1 | 1.054258 | -0.206229 | 379 | 392.37 | 1.035277 |
| GO:0007165\_signal\_transduction | PTPN2 | 915 | 4 | 0.958626 | -0.204507 | 380 | 392.98 | 1.034158 |
| GO:0007165\_signal\_transduction | MAPK14 | 915 | 4 | 0.958626 | -0.204507 | 380 | 392.98 | 1.034158 |
| GO:0007165\_signal\_transduction | AARS | 915 | 4 | 0.958626 | -0.204507 | 380 | 392.98 | 1.034158 |
| GO:0007165\_signal\_transduction | SOCS7 | 915 | 4 | 0.958626 | -0.204507 | 380 | 392.98 | 1.034158 |
| GO:0065008\_regulation\_of\_biological\_quality | ID2 | 693 | 3 | 0.949289 | -0.199022 | 381 | 393.94 | 1.033963 |
| GO:0065008\_regulation\_of\_biological\_quality | MAPK14 | 693 | 3 | 0.949289 | -0.199022 | 381 | 393.94 | 1.033963 |
| GO:0065008\_regulation\_of\_biological\_quality | HEXA | 693 | 3 | 0.949289 | -0.199022 | 381 | 393.94 | 1.033963 |
| GO:0009893\_positive\_regulation\_of\_metabolic\_process | MAPK14 | 458 | 2 | 0.957580 | -0.198737 | 382 | 394.59 | 1.032958 |
| GO:0009893\_positive\_regulation\_of\_metabolic\_process | TAF9 | 458 | 2 | 0.957580 | -0.198737 | 382 | 394.59 | 1.032958 |
| GO:0019953\_sexual\_reproduction | HEXA | 228 | 1 | 0.961779 | -0.182725 | 383 | 400.02 | 1.044439 |
| GO:0007420\_brain\_development | AARS | 231 | 1 | 0.949289 | -0.179483 | 384 | 400.98 | 1.044219 |
| GO:0009653\_anatomical\_structure\_morphogenesis | METRN | 958 | 4 | 0.915598 | -0.177897 | 385 | 401.19 | 1.042052 |
| GO:0009653\_anatomical\_structure\_morphogenesis | FAT1 | 958 | 4 | 0.915598 | -0.177897 | 385 | 401.19 | 1.042052 |
| GO:0009653\_anatomical\_structure\_morphogenesis | MAPK14 | 958 | 4 | 0.915598 | -0.177897 | 385 | 401.19 | 1.042052 |
| GO:0009653\_anatomical\_structure\_morphogenesis | WDR77 | 958 | 4 | 0.915598 | -0.177897 | 385 | 401.19 | 1.042052 |
| GO:0050890\_cognition | HEXA | 233 | 1 | 0.941140 | -0.177358 | 386 | 401.96 | 1.041347 |
| GO:0006468\_protein\_amino\_acid\_phosphorylation | MAPK14 | 237 | 1 | 0.925256 | -0.173199 | 387 | 403.81 | 1.043437 |
| GO:0002376\_immune\_system\_process | ID2 | 505 | 2 | 0.868458 | -0.162525 | 388 | 405.16 | 1.044227 |
| GO:0002376\_immune\_system\_process | MAPK14 | 505 | 2 | 0.868458 | -0.162525 | 388 | 405.16 | 1.044227 |
| GO:0008104\_protein\_localization | RAN | 251 | 1 | 0.873648 | -0.159511 | 389 | 406.54 | 1.045090 |
| GO:0048878\_chemical\_homeostasis | HEXA | 254 | 1 | 0.863330 | -0.156744 | 390 | 408.44 | 1.047282 |
| GO:0048729\_tissue\_morphogenesis | WDR77 | 255 | 1 | 0.859944 | -0.155834 | 391 | 409.06 | 1.046189 |
| GO:0003008\_system\_process | HEXA | 516 | 2 | 0.849945 | -0.155053 | 392 | 409.39 | 1.044362 |
| GO:0003008\_system\_process | AARS | 516 | 2 | 0.849945 | -0.155053 | 392 | 409.39 | 1.044362 |
| GO:0048666\_neuron\_development | METRN | 262 | 1 | 0.836968 | -0.149630 | 393 | 412.04 | 1.048448 |
| GO:0030030\_cell\_projection\_organization | METRN | 263 | 1 | 0.833786 | -0.148767 | 394 | 412.66 | 1.047360 |
| GO:0048646\_anatomical\_structure\_formation\_involved\_in\_morphogenesis | MAPK14 | 277 | 1 | 0.791645 | -0.137261 | 395 | 416.92 | 1.055494 |
| GO:0007610\_behavior | HEXA | 279 | 1 | 0.785970 | -0.135701 | 396 | 417.71 | 1.054823 |
| GO:0006950\_response\_to\_stress | MAPK14 | 549 | 2 | 0.798855 | -0.134617 | 397 | 417.91 | 1.052670 |
| GO:0006950\_response\_to\_stress | AARS | 549 | 2 | 0.798855 | -0.134617 | 397 | 417.91 | 1.052670 |
| GO:0032501\_multicellular\_organismal\_process | METRN | 2183 | 9 | 0.904064 | -0.132678 | 398 | 418.57 | 1.051683 |
| GO:0032501\_multicellular\_organismal\_process | CSRP2BP | 2183 | 9 | 0.904064 | -0.132678 | 398 | 418.57 | 1.051683 |
| GO:0032501\_multicellular\_organismal\_process | ID2 | 2183 | 9 | 0.904064 | -0.132678 | 398 | 418.57 | 1.051683 |
| GO:0032501\_multicellular\_organismal\_process | MAPK14 | 2183 | 9 | 0.904064 | -0.132678 | 398 | 418.57 | 1.051683 |
| GO:0032501\_multicellular\_organismal\_process | HEXA | 2183 | 9 | 0.904064 | -0.132678 | 398 | 418.57 | 1.051683 |
| GO:0032501\_multicellular\_organismal\_process | FMR1 | 2183 | 9 | 0.904064 | -0.132678 | 398 | 418.57 | 1.051683 |
| GO:0032501\_multicellular\_organismal\_process | WDR77 | 2183 | 9 | 0.904064 | -0.132678 | 398 | 418.57 | 1.051683 |
| GO:0032501\_multicellular\_organismal\_process | AARS | 2183 | 9 | 0.904064 | -0.132678 | 398 | 418.57 | 1.051683 |
| GO:0032501\_multicellular\_organismal\_process | PBRM1 | 2183 | 9 | 0.904064 | -0.132678 | 398 | 418.57 | 1.051683 |
| GO:0016310\_phosphorylation | MAPK14 | 309 | 1 | 0.709663 | -0.114521 | 399 | 425.75 | 1.067043 |
| GO:0007154\_cell\_communication | PTPN2 | 1096 | 4 | 0.800313 | -0.111816 | 400 | 426.41 | 1.066025 |
| GO:0007154\_cell\_communication | MAPK14 | 1096 | 4 | 0.800313 | -0.111816 | 400 | 426.41 | 1.066025 |
| GO:0007154\_cell\_communication | AARS | 1096 | 4 | 0.800313 | -0.111816 | 400 | 426.41 | 1.066025 |
| GO:0007154\_cell\_communication | SOCS7 | 1096 | 4 | 0.800313 | -0.111816 | 400 | 426.41 | 1.066025 |
| GO:0051093\_negative\_regulation\_of\_developmental\_process | AARS | 331 | 1 | 0.662495 | -0.101301 | 401 | 429.74 | 1.071671 |
| GO:0048513\_organ\_development | ID2 | 1365 | 5 | 0.803244 | -0.101148 | 402 | 429.91 | 1.069428 |
| GO:0048513\_organ\_development | MAPK14 | 1365 | 5 | 0.803244 | -0.101148 | 402 | 429.91 | 1.069428 |
| GO:0048513\_organ\_development | AARS | 1365 | 5 | 0.803244 | -0.101148 | 402 | 429.91 | 1.069428 |
| GO:0048513\_organ\_development | WDR77 | 1365 | 5 | 0.803244 | -0.101148 | 402 | 429.91 | 1.069428 |
| GO:0048513\_organ\_development | PBRM1 | 1365 | 5 | 0.803244 | -0.101148 | 402 | 429.91 | 1.069428 |
| GO:0051649\_establishment\_of\_localization\_in\_cell | RAN | 342 | 1 | 0.641186 | -0.095319 | 403 | 432.51 | 1.073226 |
| GO:0009887\_organ\_morphogenesis | MAPK14 | 642 | 2 | 0.683133 | -0.090133 | 404 | 434.87 | 1.076411 |
| GO:0009887\_organ\_morphogenesis | WDR77 | 642 | 2 | 0.683133 | -0.090133 | 404 | 434.87 | 1.076411 |
| GO:0030182\_neuron\_differentiation | METRN | 356 | 1 | 0.615971 | -0.088246 | 405 | 435.28 | 1.074765 |
| GO:0042981\_regulation\_of\_apoptosis | AARS | 360 | 1 | 0.609127 | -0.086329 | 406 | 435.77 | 1.073325 |
| GO:0048468\_cell\_development | DCT | 654 | 2 | 0.670599 | -0.085547 | 407 | 436.26 | 1.071892 |
| GO:0048468\_cell\_development | METRN | 654 | 2 | 0.670599 | -0.085547 | 407 | 436.26 | 1.071892 |
| GO:0010941\_regulation\_of\_cell\_death | AARS | 365 | 1 | 0.600783 | -0.083995 | 409 | 437.62 | 1.069976 |
| GO:0043067\_regulation\_of\_programmed\_cell\_death | AARS | 365 | 1 | 0.600783 | -0.083995 | 409 | 437.62 | 1.069976 |
| GO:0051641\_cellular\_localization | RAN | 370 | 1 | 0.592664 | -0.081728 | 410 | 438.44 | 1.069366 |
| GO:0022414\_reproductive\_process | WDR77 | 376 | 1 | 0.583207 | -0.079092 | 411 | 439.28 | 1.068808 |
| GO:0042127\_regulation\_of\_cell\_proliferation | WDR77 | 393 | 1 | 0.557979 | -0.072096 | 412 | 442.77 | 1.074684 |
| GO:0048699\_generation\_of\_neurons | METRN | 396 | 1 | 0.553752 | -0.070930 | 413 | 443.32 | 1.073414 |
| GO:0022008\_neurogenesis | METRN | 423 | 1 | 0.518406 | -0.061277 | 414 | 446.15 | 1.077657 |
| GO:0006915\_apoptosis | AARS | 427 | 1 | 0.513550 | -0.059966 | 415 | 446.35 | 1.075542 |
| GO:0012501\_programmed\_cell\_death | AARS | 433 | 1 | 0.506434 | -0.058054 | 416 | 446.99 | 1.074495 |
| GO:0008219\_cell\_death | AARS | 444 | 1 | 0.493887 | -0.054709 | 417 | 448.22 | 1.074868 |
| GO:0010926\_anatomical\_structure\_formation | MAPK14 | 447 | 1 | 0.490572 | -0.053832 | 418 | 448.76 | 1.073589 |
| GO:0016265\_death | AARS | 450 | 1 | 0.487302 | -0.052968 | 419 | 449.19 | 1.072053 |
| GO:0008283\_cell\_proliferation | WDR77 | 544 | 1 | 0.403099 | -0.031950 | 420 | 453.5 | 1.079762 |
| GO:0009790\_embryonic\_development | CSRP2BP | 567 | 1 | 0.386747 | -0.028229 | 421 | 454.46 | 1.079477 |
| GO:0051179\_localization | RAN | 1058 | 2 | 0.414529 | -0.013213 | 422 | 457.97 | 1.085237 |
| GO:0051179\_localization | HEXA | 1058 | 2 | 0.414529 | -0.013213 | 422 | 457.97 | 1.085237 |
| GO:0006810\_transport | RAN | 718 | 1 | 0.305412 | -0.012428 | 423 | 458.26 | 1.083357 |
| GO:0051234\_establishment\_of\_localization | RAN | 729 | 1 | 0.300803 | -0.011698 | 424 | 458.39 | 1.081108 |
| GO:0008150\_biological\_process | SMARCAD1 | 4605 | 21 | 1.000000 | 0.000000 | 1742 | 1760.18 | 1.010436 |
| GO:0008150\_biological\_process | CSRP2BP | 4605 | 21 | 1.000000 | 0.000000 | 1742 | 1760.18 | 1.010436 |
| GO:0008150\_biological\_process | RAN | 4605 | 21 | 1.000000 | 0.000000 | 1742 | 1760.18 | 1.010436 |
| GO:0008150\_biological\_process | PTPN2 | 4605 | 21 | 1.000000 | 0.000000 | 1742 | 1760.18 | 1.010436 |
| GO:0008150\_biological\_process | HEXA | 4605 | 21 | 1.000000 | 0.000000 | 1742 | 1760.18 | 1.010436 |
| GO:0008150\_biological\_process | FMR1 | 4605 | 21 | 1.000000 | 0.000000 | 1742 | 1760.18 | 1.010436 |
| GO:0008150\_biological\_process | RBL1 | 4605 | 21 | 1.000000 | 0.000000 | 1742 | 1760.18 | 1.010436 |
| GO:0008150\_biological\_process | AARS | 4605 | 21 | 1.000000 | 0.000000 | 1742 | 1760.18 | 1.010436 |
| GO:0008150\_biological\_process | SI | 4605 | 21 | 1.000000 | 0.000000 | 1742 | 1760.18 | 1.010436 |
| GO:0008150\_biological\_process | SOCS7 | 4605 | 21 | 1.000000 | 0.000000 | 1742 | 1760.18 | 1.010436 |
| GO:0008150\_biological\_process | ARID1A | 4605 | 21 | 1.000000 | 0.000000 | 1742 | 1760.18 | 1.010436 |
| GO:0008150\_biological\_process | CCNG2 | 4605 | 21 | 1.000000 | 0.000000 | 1742 | 1760.18 | 1.010436 |
| GO:0008150\_biological\_process | DCT | 4605 | 21 | 1.000000 | 0.000000 | 1742 | 1760.18 | 1.010436 |
| GO:0008150\_biological\_process | METRN | 4605 | 21 | 1.000000 | 0.000000 | 1742 | 1760.18 | 1.010436 |
| GO:0008150\_biological\_process | ID2 | 4605 | 21 | 1.000000 | 0.000000 | 1742 | 1760.18 | 1.010436 |
| GO:0008150\_biological\_process | FAT1 | 4605 | 21 | 1.000000 | 0.000000 | 1742 | 1760.18 | 1.010436 |
| GO:0008150\_biological\_process | MAPK14 | 4605 | 21 | 1.000000 | 0.000000 | 1742 | 1760.18 | 1.010436 |
| GO:0008150\_biological\_process | WDR77 | 4605 | 21 | 1.000000 | 0.000000 | 1742 | 1760.18 | 1.010436 |
| GO:0008150\_biological\_process | PBRM1 | 4605 | 21 | 1.000000 | 0.000000 | 1742 | 1760.18 | 1.010436 |
| GO:0008150\_biological\_process | TAF9 | 4605 | 21 | 1.000000 | 0.000000 | 1742 | 1760.18 | 1.010436 |
| GO:0008150\_biological\_process | ZMPSTE24 | 4605 | 21 | 1.000000 | 0.000000 | 1742 | 1760.18 | 1.010436 |
