# Supplementary material for: RedundancyMiner: De-replication of redundant GO categories in microarray and proteomics analysis
Source: BMC Bioinformatics. 2011 Feb 10;12:52. doi: 10.1186/1471-2105-12-52 (PMC3223614; doi:10.1186/1471-2105-12-52)
Supplement: Additional file 8 — Retinal development HTGM download. compressed package of the results of running HTGM on the retinal development genes list. [file 1471-2105-12-52-S8.ZIP › SCENARIO_2_MODIFIED/total.txt.total.txt.dir/Exp1_BestClusterMap_LEIGS_KM_24.csv.join.10.txt.dir/Exp1_BestClusterMap_LEIGS_KM_24.csv.join.10.txt.change.html]

Category Summary Report for Exp1\_BestClusterMap\_LEIGS\_KM\_24.csv.join.10.txt

# Category Summary Report for Exp1\_BestClusterMap\_LEIGS\_KM\_24.csv.join.10.txt

| HYPERLINKED GO CATEGORY | TOTAL GENES | CHANGED GENES | ENRICHMENT | LOG10(p) | CUMULATIVE NUMBER OF CATEGORIES | CUMULATIVE RANDOMS LOWER BOUND | CUMULATIVE RANDOMS MEAN | CUMULATIVE RANDOMS UPPER BOUND | FALSE DISCOVERY RATE |
| --- | --- | --- | --- | --- | --- | --- | --- | --- | --- |
| GO:0042438\_melanin\_biosynthetic\_process | 7 | 2 | 62.653061 | -3.386878 | 1 | -0.605038 | 0.4 | 1.405038 | 0.400000 |
| GO:0006582\_melanin\_metabolic\_process | 8 | 2 | 54.821429 | -3.263135 | 2 | -0.587115 | 0.5 | 1.587115 | 0.250000 |
| GO:0046148\_pigment\_biosynthetic\_process | 16 | 2 | 27.410714 | -2.640669 | 3 | -1.041592 | 2.22 | 5.481592 | 0.740000 |
| GO:0010033\_response\_to\_organic\_substance | 216 | 5 | 5.076058 | -2.623394 | 4 | -1.063259 | 2.23 | 5.523259 | 0.557500 |
| GO:0042440\_pigment\_metabolic\_process | 17 | 2 | 25.798319 | -2.587505 | 5 | -0.983749 | 2.47 | 5.923749 | 0.494000 |
| GO:0045639\_positive\_regulation\_of\_myeloid\_cell\_differentiation | 20 | 2 | 21.928571 | -2.445870 | 6 | -1.007328 | 3.22 | 7.447328 | 0.536667 |
| GO:0006419\_alanyl-tRNA\_aminoacylation | 1 | 1 |  |  |  |  |  |  |  |  |
| GO:0030327\_prenylated\_protein\_catabolic\_process | 1 | 1 |  |  |  |  |  |  |  |  |
| GO:0070887\_cellular\_response\_to\_chemical\_stimulus | 85 | 3 | 7.739496 | -2.197277 | 7 | -0.826200 | 4.99 | 10.806200 | 0.712857 |
| GO:0008286\_insulin\_receptor\_signaling\_pathway | 27 | 2 | 16.243386 | -2.187661 | 8 | -0.849075 | 5.07 | 10.989075 | 0.633750 |
| GO:0009719\_response\_to\_endogenous\_stimulus | 92 | 3 | 7.150621 | -2.101888 | 9 | -0.745951 | 5.87 | 12.485951 | 0.652222 |
| GO:0050885\_neuromuscular\_process\_controlling\_balance | 32 | 2 | 13.705357 | -2.043440 | 10 | -0.281890 | 7.03 | 14.341890 | 0.703000 |
| GO:0006418\_tRNA\_aminoacylation\_for\_protein\_translation | 2 | 1 |  |  |  |  |  |  |  |  |
| GO:0006583\_melanin\_biosynthetic\_process\_from\_tyrosine | 2 | 1 |  |  |  |  |  |  |  |  |
| GO:0032495\_response\_to\_muramyl\_dipeptide | 2 | 1 |  |  |  |  |  |  |  |  |
| GO:0043038\_amino\_acid\_activation | 2 | 1 |  |  |  |  |  |  |  |  |
| GO:0043039\_tRNA\_aminoacylation | 2 | 1 |  |  |  |  |  |  |  |  |
| GO:0032869\_cellular\_response\_to\_insulin\_stimulus | 37 | 2 | 11.853282 | -1.921395 | 11 | 0.101333 | 8.68 | 17.258667 | 0.789091 |
| GO:0006689\_ganglioside\_catabolic\_process | 3 | 1 |  |  |  |  |  |  |  |  |
| GO:0043200\_response\_to\_amino\_acid\_stimulus | 3 | 1 |  |  |  |  |  |  |  |  |
| GO:0046479\_glycosphingolipid\_catabolic\_process | 3 | 1 |  |  |  |  |  |  |  |  |
| GO:0019748\_secondary\_metabolic\_process | 41 | 2 | 10.696864 | -1.835809 | 12 | 0.758031 | 10.51 | 20.261969 | 0.875833 |
| GO:0045637\_regulation\_of\_myeloid\_cell\_differentiation | 42 | 2 | 10.442177 | -1.815807 | 13 | 1.010933 | 11.02 | 21.029067 | 0.847692 |
| GO:0032868\_response\_to\_insulin\_stimulus | 43 | 2 | 10.199336 | -1.796311 | 15 | 1.136940 | 11.55 | 21.963060 | 0.770000 |
| GO:0051789\_response\_to\_protein\_stimulus | 43 | 2 | 10.199336 | -1.796311 | 15 | 1.136940 | 11.55 | 21.963060 | 0.770000 |
| GO:0043283\_biopolymer\_metabolic\_process | 1490 | 12 | 1.766059 | -1.785537 | 16 | 1.171185 | 11.65 | 22.128815 | 0.728125 |
| GO:0032870\_cellular\_response\_to\_hormone\_stimulus | 45 | 2 | 9.746032 | -1.758738 | 17 | 1.080722 | 12.49 | 23.899278 | 0.734706 |
| GO:0009308\_amine\_metabolic\_process | 124 | 3 | 5.305300 | -1.749951 | 18 | 1.136172 | 12.57 | 24.003828 | 0.698333 |
| GO:0019377\_glycolipid\_catabolic\_process | 4 | 1 |  |  |  |  |  |  |  |  |
| GO:0060528\_secretory\_columnal\_luminar\_epithelial\_cell\_differentiation\_involved\_in\_prostate\_glandular\_acinus\_development | 4 | 1 |  |  |  |  |  |  |  |  |
| GO:0030218\_erythrocyte\_differentiation | 46 | 2 | 9.534161 | -1.740619 | 19 | 1.170359 | 12.79 | 24.409641 | 0.673158 |
| GO:0045597\_positive\_regulation\_of\_cell\_differentiation | 128 | 3 | 5.139509 | -1.713333 | 20 | 1.246877 | 13.15 | 25.053123 | 0.657500 |
| GO:0034101\_erythrocyte\_homeostasis | 49 | 2 | 8.950437 | -1.688712 | 21 | 1.387341 | 13.93 | 26.472659 | 0.663333 |
| GO:0006520\_cellular\_amino\_acid\_metabolic\_process | 51 | 2 | 8.599440 | -1.655981 | 23 | 1.520570 | 14.59 | 27.659430 | 0.634348 |
| GO:0044106\_cellular\_amine\_metabolic\_process | 51 | 2 | 8.599440 | -1.655981 | 23 | 1.520570 | 14.59 | 27.659430 | 0.634348 |
| GO:0006400\_tRNA\_modification | 5 | 1 | 43.857143 | -1.645814 | 28 | 5.931097 | 21.66 | 37.388903 | 0.773571 |
| GO:0006570\_tyrosine\_metabolic\_process | 5 | 1 | 43.857143 | -1.645814 | 28 | 5.931097 | 21.66 | 37.388903 | 0.773571 |
| GO:0032494\_response\_to\_peptidoglycan | 5 | 1 | 43.857143 | -1.645814 | 28 | 5.931097 | 21.66 | 37.388903 | 0.773571 |
| GO:0045648\_positive\_regulation\_of\_erythrocyte\_differentiation | 5 | 1 | 43.857143 | -1.645814 | 28 | 5.931097 | 21.66 | 37.388903 | 0.773571 |
| GO:0045651\_positive\_regulation\_of\_macrophage\_differentiation | 5 | 1 | 43.857143 | -1.645814 | 28 | 5.931097 | 21.66 | 37.388903 | 0.773571 |
| GO:0050905\_neuromuscular\_process | 53 | 2 | 8.274933 | -1.624612 | 29 | 6.143451 | 22.15 | 38.156549 | 0.763793 |
| GO:0043434\_response\_to\_peptide\_hormone\_stimulus | 55 | 2 | 7.974026 | -1.594505 | 30 | 6.349328 | 22.89 | 39.430672 | 0.763000 |
| GO:0043170\_macromolecule\_metabolic\_process | 1576 | 12 | 1.669688 | -1.585938 | 31 | 6.510373 | 23.03 | 39.549627 | 0.742903 |
| GO:0006998\_nuclear\_envelope\_organization | 6 | 1 | 36.547619 | -1.567575 | 35 | 9.797468 | 28.72 | 47.642532 | 0.820571 |
| GO:0030149\_sphingolipid\_catabolic\_process | 6 | 1 | 36.547619 | -1.567575 | 35 | 9.797468 | 28.72 | 47.642532 | 0.820571 |
| GO:0045649\_regulation\_of\_macrophage\_differentiation | 6 | 1 | 36.547619 | -1.567575 | 35 | 9.797468 | 28.72 | 47.642532 | 0.820571 |
| GO:0046466\_membrane\_lipid\_catabolic\_process | 6 | 1 | 36.547619 | -1.567575 | 35 | 9.797468 | 28.72 | 47.642532 | 0.820571 |
| GO:0034960\_cellular\_biopolymer\_metabolic\_process | 1395 | 11 | 1.729135 | -1.552095 | 36 | 9.949819 | 29.14 | 48.330181 | 0.809444 |
| GO:0001573\_ganglioside\_metabolic\_process | 7 | 1 | 31.326531 | -1.501570 | 41 | 14.277665 | 36.59 | 58.902335 | 0.892439 |
| GO:0002067\_glandular\_epithelial\_cell\_differentiation | 7 | 1 | 31.326531 | -1.501570 | 41 | 14.277665 | 36.59 | 58.902335 | 0.892439 |
| GO:0008033\_tRNA\_processing | 7 | 1 | 31.326531 | -1.501570 | 41 | 14.277665 | 36.59 | 58.902335 | 0.892439 |
| GO:0043353\_enucleate\_erythrocyte\_differentiation | 7 | 1 | 31.326531 | -1.501570 | 41 | 14.277665 | 36.59 | 58.902335 | 0.892439 |
| GO:0060770\_negative\_regulation\_of\_epithelial\_cell\_proliferation\_involved\_in\_prostate\_gland\_development | 7 | 1 | 31.326531 | -1.501570 | 41 | 14.277665 | 36.59 | 58.902335 | 0.892439 |
| GO:0051716\_cellular\_response\_to\_stimulus | 273 | 4 | 3.212977 | -1.488138 | 42 | 14.405002 | 37.07 | 59.734998 | 0.882619 |
| GO:0042221\_response\_to\_chemical\_stimulus | 409 | 5 | 2.680754 | -1.477576 | 43 | 14.472462 | 37.42 | 60.367538 | 0.870233 |
| GO:0044267\_cellular\_protein\_metabolic\_process | 559 | 6 | 2.353693 | -1.466462 | 44 | 14.482860 | 37.81 | 61.137140 | 0.859318 |
| GO:0002065\_columnar\_cuboidal\_epithelial\_cell\_differentiation | 8 | 1 | 27.410714 | -1.444519 | 47 | 17.632630 | 43.79 | 69.947370 | 0.931702 |
| GO:0006399\_tRNA\_metabolic\_process | 8 | 1 | 27.410714 | -1.444519 | 47 | 17.632630 | 43.79 | 69.947370 | 0.931702 |
| GO:0009072\_aromatic\_amino\_acid\_family\_metabolic\_process | 8 | 1 | 27.410714 | -1.444519 | 47 | 17.632630 | 43.79 | 69.947370 | 0.931702 |
| GO:0044260\_cellular\_macromolecule\_metabolic\_process | 1447 | 11 | 1.666996 | -1.436809 | 48 | 17.774379 | 44.07 | 70.365621 | 0.918125 |
| GO:0009451\_RNA\_modification | 9 | 1 | 24.365079 | -1.394307 | 51 | 21.360942 | 50.83 | 80.299058 | 0.996667 |
| GO:0045646\_regulation\_of\_erythrocyte\_differentiation | 9 | 1 | 24.365079 | -1.394307 | 51 | 21.360942 | 50.83 | 80.299058 | 0.996667 |
| GO:0050884\_neuromuscular\_process\_controlling\_posture | 9 | 1 | 24.365079 | -1.394307 | 51 | 21.360942 | 50.83 | 80.299058 | 0.996667 |
| GO:0006357\_regulation\_of\_transcription\_from\_RNA\_polymerase\_II\_promoter | 435 | 5 | 2.520525 | -1.377061 | 52 | 21.547360 | 51.85 | 82.152640 | 0.997115 |
| GO:0060768\_regulation\_of\_epithelial\_cell\_proliferation\_involved\_in\_prostate\_gland\_development | 10 | 1 | 21.928571 | -1.349489 | 53 | 25.187492 | 58.4 | 91.612508 | 1.101887 |
| GO:0006366\_transcription\_from\_RNA\_polymerase\_II\_promoter | 444 | 5 | 2.469434 | -1.344155 | 54 | 25.390413 | 58.69 | 91.989587 | 1.086852 |
| GO:0009725\_response\_to\_hormone\_stimulus | 76 | 2 | 5.770677 | -1.336209 | 55 | 25.696994 | 59.09 | 92.483006 | 1.074364 |
| GO:0044238\_primary\_metabolic\_process | 1905 | 13 | 1.496438 | -1.334694 | 56 | 25.728054 | 59.13 | 92.531946 | 1.055893 |
| GO:0006996\_organelle\_organization | 449 | 5 | 2.441934 | -1.326267 | 57 | 25.800777 | 59.45 | 93.099223 | 1.042982 |
| GO:0019752\_carboxylic\_acid\_metabolic\_process | 181 | 3 | 3.634570 | -1.326111 | 59 | 25.855140 | 59.52 | 93.184860 | 1.008814 |
| GO:0043436\_oxoacid\_metabolic\_process | 181 | 3 | 3.634570 | -1.326111 | 59 | 25.855140 | 59.52 | 93.184860 | 1.008814 |
| GO:0006082\_organic\_acid\_metabolic\_process | 182 | 3 | 3.614600 | -1.320158 | 60 | 25.847317 | 59.69 | 93.532683 | 0.994833 |
| GO:0042180\_cellular\_ketone\_metabolic\_process | 183 | 3 | 3.594848 | -1.314245 | 61 | 26.124052 | 60.15 | 94.175948 | 0.986066 |
| GO:0008152\_metabolic\_process | 2133 | 14 | 1.439287 | -1.311690 | 62 | 26.181998 | 60.21 | 94.238002 | 0.971129 |
| GO:0001101\_response\_to\_acid | 11 | 1 | 19.935065 | -1.309036 | 67 | 29.103468 | 64.8 | 100.496532 | 0.967164 |
| GO:0030968\_endoplasmic\_reticulum\_unfolded\_protein\_response | 11 | 1 | 19.935065 | -1.309036 | 67 | 29.103468 | 64.8 | 100.496532 | 0.967164 |
| GO:0034620\_cellular\_response\_to\_unfolded\_protein | 11 | 1 | 19.935065 | -1.309036 | 67 | 29.103468 | 64.8 | 100.496532 | 0.967164 |
| GO:0050772\_positive\_regulation\_of\_axonogenesis | 11 | 1 | 19.935065 | -1.309036 | 67 | 29.103468 | 64.8 | 100.496532 | 0.967164 |
| GO:0060767\_epithelial\_cell\_proliferation\_involved\_in\_prostate\_gland\_development | 11 | 1 | 19.935065 | -1.309036 | 67 | 29.103468 | 64.8 | 100.496532 | 0.967164 |
| GO:0043412\_biopolymer\_modification | 458 | 5 | 2.393949 | -1.294756 | 68 | 29.602043 | 65.74 | 101.877957 | 0.966765 |
| GO:0002763\_positive\_regulation\_of\_myeloid\_leukocyte\_differentiation | 12 | 1 | 18.273810 | -1.272186 | 72 | 32.300964 | 70.67 | 109.039036 | 0.981528 |
| GO:0021680\_cerebellar\_Purkinje\_cell\_layer\_development | 12 | 1 | 18.273810 | -1.272186 | 72 | 32.300964 | 70.67 | 109.039036 | 0.981528 |
| GO:0030225\_macrophage\_differentiation | 12 | 1 | 18.273810 | -1.272186 | 72 | 32.300964 | 70.67 | 109.039036 | 0.981528 |
| GO:0060525\_prostate\_glandular\_acinus\_development | 12 | 1 | 18.273810 | -1.272186 | 72 | 32.300964 | 70.67 | 109.039036 | 0.981528 |
| GO:0006325\_chromatin\_organization | 83 | 2 | 5.283993 | -1.267442 | 73 | 32.424983 | 71.02 | 109.615017 | 0.972877 |
| GO:0006807\_nitrogen\_compound\_metabolic\_process | 1147 | 9 | 1.720638 | -1.263108 | 74 | 32.474199 | 71.11 | 109.745801 | 0.960946 |
| GO:0006687\_glycosphingolipid\_metabolic\_process | 13 | 1 | 16.868132 | -1.238363 | 77 | 35.609299 | 76.85 | 118.090701 | 0.998052 |
| GO:0006986\_response\_to\_unfolded\_protein | 13 | 1 | 16.868132 | -1.238363 | 77 | 35.609299 | 76.85 | 118.090701 | 0.998052 |
| GO:0060742\_epithelial\_cell\_differentiation\_involved\_in\_prostate\_gland\_development | 13 | 1 | 16.868132 | -1.238363 | 77 | 35.609299 | 76.85 | 118.090701 | 0.998052 |
| GO:0044237\_cellular\_metabolic\_process | 1974 | 13 | 1.444131 | -1.209053 | 78 | 36.178302 | 78.45 | 120.721698 | 1.005769 |
| GO:0000077\_DNA\_damage\_checkpoint | 14 | 1 | 15.663265 | -1.207116 | 82 | 38.590667 | 82.59 | 126.589333 | 1.007195 |
| GO:0016573\_histone\_acetylation | 14 | 1 | 15.663265 | -1.207116 | 82 | 38.590667 | 82.59 | 126.589333 | 1.007195 |
| GO:0031346\_positive\_regulation\_of\_cell\_projection\_organization | 14 | 1 | 15.663265 | -1.207116 | 82 | 38.590667 | 82.59 | 126.589333 | 1.007195 |
| GO:0031663\_lipopolysaccharide-mediated\_signaling\_pathway | 14 | 1 | 15.663265 | -1.207116 | 82 | 38.590667 | 82.59 | 126.589333 | 1.007195 |
| GO:0006473\_protein\_amino\_acid\_acetylation | 15 | 1 | 14.619048 | -1.178089 | 84 | 41.717309 | 88.19 | 134.662691 | 1.049881 |
| GO:0007040\_lysosome\_organization | 15 | 1 | 14.619048 | -1.178089 | 84 | 41.717309 | 88.19 | 134.662691 | 1.049881 |
| GO:0019538\_protein\_metabolic\_process | 655 | 6 | 2.008724 | -1.176905 | 85 | 41.727181 | 88.21 | 134.692819 | 1.037765 |
| GO:0016070\_RNA\_metabolic\_process | 658 | 6 | 1.999566 | -1.168880 | 86 | 41.883781 | 88.69 | 135.496219 | 1.031279 |
| GO:0006664\_glycolipid\_metabolic\_process | 16 | 1 | 13.705357 | -1.150997 | 94 | 44.798781 | 92.9 | 141.001219 | 0.988298 |
| GO:0007033\_vacuole\_organization | 16 | 1 | 13.705357 | -1.150997 | 94 | 44.798781 | 92.9 | 141.001219 | 0.988298 |
| GO:0010243\_response\_to\_organic\_nitrogen | 16 | 1 | 13.705357 | -1.150997 | 94 | 44.798781 | 92.9 | 141.001219 | 0.988298 |
| GO:0010876\_lipid\_localization | 16 | 1 | 13.705357 | -1.150997 | 94 | 44.798781 | 92.9 | 141.001219 | 0.988298 |
| GO:0014075\_response\_to\_amine\_stimulus | 16 | 1 | 13.705357 | -1.150997 | 94 | 44.798781 | 92.9 | 141.001219 | 0.988298 |
| GO:0019915\_lipid\_storage | 16 | 1 | 13.705357 | -1.150997 | 94 | 44.798781 | 92.9 | 141.001219 | 0.988298 |
| GO:0031570\_DNA\_integrity\_checkpoint | 16 | 1 | 13.705357 | -1.150997 | 94 | 44.798781 | 92.9 | 141.001219 | 0.988298 |
| GO:0034976\_response\_to\_endoplasmic\_reticulum\_stress | 16 | 1 | 13.705357 | -1.150997 | 94 | 44.798781 | 92.9 | 141.001219 | 0.988298 |
| GO:0006984\_ER-nuclear\_signaling\_pathway | 17 | 1 | 12.899160 | -1.125604 | 98 | 47.968871 | 97.89 | 147.811129 | 0.998878 |
| GO:0019395\_fatty\_acid\_oxidation | 17 | 1 | 12.899160 | -1.125604 | 98 | 47.968871 | 97.89 | 147.811129 | 0.998878 |
| GO:0034440\_lipid\_oxidation | 17 | 1 | 12.899160 | -1.125604 | 98 | 47.968871 | 97.89 | 147.811129 | 0.998878 |
| GO:0034470\_ncRNA\_processing | 17 | 1 | 12.899160 | -1.125604 | 98 | 47.968871 | 97.89 | 147.811129 | 0.998878 |
| GO:0006022\_aminoglycan\_metabolic\_process | 18 | 1 | 12.182540 | -1.101716 | 102 | 50.769046 | 103.36 | 155.950954 | 1.013333 |
| GO:0006457\_protein\_folding | 18 | 1 | 12.182540 | -1.101716 | 102 | 50.769046 | 103.36 | 155.950954 | 1.013333 |
| GO:0030203\_glycosaminoglycan\_metabolic\_process | 18 | 1 | 12.182540 | -1.101716 | 102 | 50.769046 | 103.36 | 155.950954 | 1.013333 |
| GO:0048535\_lymph\_node\_development | 18 | 1 | 12.182540 | -1.101716 | 102 | 50.769046 | 103.36 | 155.950954 | 1.013333 |
| GO:0048872\_homeostasis\_of\_number\_of\_cells | 105 | 2 | 4.176871 | -1.087955 | 103 | 51.059394 | 103.84 | 156.620606 | 1.008155 |
| GO:0006672\_ceramide\_metabolic\_process | 19 | 1 | 11.541353 | -1.079170 | 104 | 53.800305 | 107.17 | 160.539695 | 1.030481 |
| GO:0030099\_myeloid\_cell\_differentiation | 108 | 2 | 4.060847 | -1.066885 | 105 | 53.793968 | 107.65 | 161.506032 | 1.025238 |
| GO:0021695\_cerebellar\_cortex\_development | 20 | 1 | 10.964286 | -1.057828 | 106 | 55.629024 | 110.79 | 165.950976 | 1.045189 |
| GO:0007049\_cell\_cycle | 238 | 3 | 2.764106 | -1.039668 | 107 | 55.645782 | 111.89 | 168.134218 | 1.045701 |
| GO:0000018\_regulation\_of\_DNA\_recombination | 21 | 1 | 10.442177 | -1.037573 | 111 | 58.148047 | 116.0 | 173.851953 | 1.045045 |
| GO:0000075\_cell\_cycle\_checkpoint | 21 | 1 | 10.442177 | -1.037573 | 111 | 58.148047 | 116.0 | 173.851953 | 1.045045 |
| GO:0010552\_positive\_regulation\_of\_specific\_transcription\_from\_RNA\_polymerase\_II\_promoter | 21 | 1 | 10.442177 | -1.037573 | 111 | 58.148047 | 116.0 | 173.851953 | 1.045045 |
| GO:0046519\_sphingoid\_metabolic\_process | 21 | 1 | 10.442177 | -1.037573 | 111 | 58.148047 | 116.0 | 173.851953 | 1.045045 |
| GO:0043687\_post-translational\_protein\_modification | 384 | 4 | 2.284226 | -1.036895 | 112 | 58.182026 | 116.08 | 173.977974 | 1.036429 |
| GO:0009607\_response\_to\_biotic\_stimulus | 114 | 2 | 3.847118 | -1.026730 | 113 | 58.293174 | 116.48 | 174.666826 | 1.030796 |
| GO:0030258\_lipid\_modification | 22 | 1 | 9.967532 | -1.018303 | 115 | 61.508151 | 121.47 | 181.431849 | 1.056261 |
| GO:0034660\_ncRNA\_metabolic\_process | 22 | 1 | 9.967532 | -1.018303 | 115 | 61.508151 | 121.47 | 181.431849 | 1.056261 |
| GO:0006519\_cellular\_amino\_acid\_and\_derivative\_metabolic\_process | 118 | 2 | 3.716707 | -1.001321 | 116 | 62.001769 | 122.27 | 182.538231 | 1.054052 |
| GO:0007163\_establishment\_or\_maintenance\_of\_cell\_polarity | 23 | 1 | 9.534161 | -0.999930 | 117 | 63.272690 | 124.42 | 185.567310 | 1.063419 |
| GO:0051726\_regulation\_of\_cell\_cycle | 121 | 2 | 3.624557 | -0.982926 | 118 | 64.365433 | 126.13 | 187.894567 | 1.068898 |
| GO:0043588\_skin\_development | 24 | 1 | 9.136905 | -0.982379 | 119 | 66.356805 | 129.4 | 192.443195 | 1.087395 |
| GO:0007628\_adult\_walking\_behavior | 25 | 1 | 8.771429 | -0.965582 | 121 | 69.230371 | 133.37 | 197.509629 | 1.102231 |
| GO:0043543\_protein\_amino\_acid\_acylation | 25 | 1 | 8.771429 | -0.965582 | 121 | 69.230371 | 133.37 | 197.509629 | 1.102231 |
| GO:0006355\_regulation\_of\_transcription\_\_DNA-dependent | 575 | 5 | 1.906832 | -0.952369 | 122 | 69.918159 | 134.38 | 198.841841 | 1.101475 |
| GO:0050680\_negative\_regulation\_of\_epithelial\_cell\_proliferation | 26 | 1 | 8.434066 | -0.949479 | 123 | 71.528841 | 137.19 | 202.851159 | 1.115366 |
| GO:0051276\_chromosome\_organization | 129 | 2 | 3.399779 | -0.936424 | 124 | 72.386532 | 138.51 | 204.633468 | 1.117016 |
| GO:0002761\_regulation\_of\_myeloid\_leukocyte\_differentiation | 27 | 1 | 8.121693 | -0.934019 | 126 | 73.749637 | 140.63 | 207.510363 | 1.116111 |
| GO:0032496\_response\_to\_lipopolysaccharide | 27 | 1 | 8.121693 | -0.934019 | 126 | 73.749637 | 140.63 | 207.510363 | 1.116111 |
| GO:0019219\_regulation\_of\_nucleobase\_\_nucleoside\_\_nucleotide\_and\_nucleic\_acid\_metabolic\_process | 757 | 6 | 1.738064 | -0.932540 | 127 | 73.840932 | 140.72 | 207.599068 | 1.108031 |
| GO:0002062\_chondrocyte\_differentiation | 28 | 1 | 7.831633 | -0.919155 | 132 | 75.439673 | 143.4 | 211.360327 | 1.086364 |
| GO:0006470\_protein\_amino\_acid\_dephosphorylation | 28 | 1 | 7.831633 | -0.919155 | 132 | 75.439673 | 143.4 | 211.360327 | 1.086364 |
| GO:0006997\_nucleus\_organization | 28 | 1 | 7.831633 | -0.919155 | 132 | 75.439673 | 143.4 | 211.360327 | 1.086364 |
| GO:0021549\_cerebellum\_development | 28 | 1 | 7.831633 | -0.919155 | 132 | 75.439673 | 143.4 | 211.360327 | 1.086364 |
| GO:0043193\_positive\_regulation\_of\_gene-specific\_transcription | 28 | 1 | 7.831633 | -0.919155 | 132 | 75.439673 | 143.4 | 211.360327 | 1.086364 |
| GO:0051252\_regulation\_of\_RNA\_metabolic\_process | 590 | 5 | 1.858354 | -0.915999 | 133 | 75.617682 | 143.7 | 211.782318 | 1.080451 |
| GO:0006351\_transcription\_\_DNA-dependent | 594 | 5 | 1.845839 | -0.906543 | 134 | 76.351656 | 144.57 | 212.788344 | 1.078881 |
| GO:0042770\_DNA\_damage\_response\_\_signal\_transduction | 29 | 1 | 7.561576 | -0.904844 | 137 | 78.456964 | 147.22 | 215.983036 | 1.074599 |
| GO:0048066\_pigmentation\_during\_development | 29 | 1 | 7.561576 | -0.904844 | 137 | 78.456964 | 147.22 | 215.983036 | 1.074599 |
| GO:0050769\_positive\_regulation\_of\_neurogenesis | 29 | 1 | 7.561576 | -0.904844 | 137 | 78.456964 | 147.22 | 215.983036 | 1.074599 |
| GO:0032774\_RNA\_biosynthetic\_process | 595 | 5 | 1.842737 | -0.904195 | 138 | 78.611201 | 147.39 | 216.168799 | 1.068043 |
| GO:0051171\_regulation\_of\_nitrogen\_compound\_metabolic\_process | 771 | 6 | 1.706504 | -0.903123 | 139 | 78.696004 | 147.5 | 216.303996 | 1.061151 |
| GO:0042552\_myelination | 30 | 1 | 7.309524 | -0.891050 | 140 | 81.535975 | 151.49 | 221.444025 | 1.082071 |
| GO:0016043\_cellular\_component\_organization | 964 | 7 | 1.592324 | -0.883914 | 141 | 81.884780 | 152.02 | 222.155220 | 1.078156 |
| GO:0007169\_transmembrane\_receptor\_protein\_tyrosine\_kinase\_signaling\_pathway | 139 | 2 | 3.155190 | -0.882968 | 142 | 82.089748 | 152.44 | 222.790252 | 1.073521 |
| GO:0006665\_sphingolipid\_metabolic\_process | 31 | 1 | 7.073733 | -0.877738 | 144 | 84.559549 | 156.69 | 228.820451 | 1.088125 |
| GO:0016311\_dephosphorylation | 31 | 1 | 7.073733 | -0.877738 | 144 | 84.559549 | 156.69 | 228.820451 | 1.088125 |
| GO:0006464\_protein\_modification\_process | 439 | 4 | 1.998048 | -0.874937 | 145 | 84.730340 | 156.92 | 229.109660 | 1.082207 |
| GO:0000902\_cell\_morphogenesis | 283 | 3 | 2.324584 | -0.869751 | 146 | 84.935672 | 157.3 | 229.664328 | 1.077397 |
| GO:0007272\_ensheathment\_of\_neurons | 32 | 1 | 6.852679 | -0.864877 | 149 | 86.253084 | 159.42 | 232.586916 | 1.069933 |
| GO:0008366\_axon\_ensheathment | 32 | 1 | 6.852679 | -0.864877 | 149 | 86.253084 | 159.42 | 232.586916 | 1.069933 |
| GO:0050770\_regulation\_of\_axonogenesis | 32 | 1 | 6.852679 | -0.864877 | 149 | 86.253084 | 159.42 | 232.586916 | 1.069933 |
| GO:0006643\_membrane\_lipid\_metabolic\_process | 33 | 1 | 6.645022 | -0.852440 | 151 | 88.210631 | 162.65 | 237.089369 | 1.077152 |
| GO:0022037\_metencephalon\_development | 33 | 1 | 6.645022 | -0.852440 | 151 | 88.210631 | 162.65 | 237.089369 | 1.077152 |
| GO:0005975\_carbohydrate\_metabolic\_process | 146 | 2 | 3.003914 | -0.848271 | 152 | 88.548253 | 163.17 | 237.791747 | 1.073487 |
| GO:0009058\_biosynthetic\_process | 1175 | 8 | 1.493009 | -0.847996 | 153 | 88.564660 | 163.24 | 237.915340 | 1.066928 |
| GO:0002237\_response\_to\_molecule\_of\_bacterial\_origin | 34 | 1 | 6.449580 | -0.840402 | 156 | 90.409453 | 166.26 | 242.110547 | 1.065769 |
| GO:0010720\_positive\_regulation\_of\_cell\_development | 34 | 1 | 6.449580 | -0.840402 | 156 | 90.409453 | 166.26 | 242.110547 | 1.065769 |
| GO:0051052\_regulation\_of\_DNA\_metabolic\_process | 34 | 1 | 6.449580 | -0.840402 | 156 | 90.409453 | 166.26 | 242.110547 | 1.065769 |
| GO:0034961\_cellular\_biopolymer\_biosynthetic\_process | 804 | 6 | 1.636461 | -0.837223 | 157 | 90.592575 | 166.63 | 242.667425 | 1.061338 |
| GO:0043284\_biopolymer\_biosynthetic\_process | 807 | 6 | 1.630377 | -0.831462 | 158 | 90.852458 | 166.95 | 243.047542 | 1.056646 |
| GO:0045595\_regulation\_of\_cell\_differentiation | 295 | 3 | 2.230024 | -0.830503 | 159 | 90.994118 | 167.36 | 243.725882 | 1.052579 |
| GO:0016567\_protein\_ubiquitination | 35 | 1 | 6.265306 | -0.828739 | 161 | 92.464751 | 169.37 | 246.275249 | 1.051988 |
| GO:0044242\_cellular\_lipid\_catabolic\_process | 35 | 1 | 6.265306 | -0.828739 | 161 | 92.464751 | 169.37 | 246.275249 | 1.051988 |
| GO:0019228\_regulation\_of\_action\_potential\_in\_neuron | 36 | 1 | 6.091270 | -0.817430 | 162 | 94.691380 | 172.76 | 250.828620 | 1.066420 |
| GO:0006139\_nucleobase\_\_nucleoside\_\_nucleotide\_and\_nucleic\_acid\_metabolic\_process | 1002 | 7 | 1.531936 | -0.816211 | 163 | 94.939454 | 173.04 | 251.140546 | 1.061595 |
| GO:0010975\_regulation\_of\_neuron\_projection\_development | 38 | 1 | 5.770677 | -0.795798 | 165 | 99.339633 | 179.03 | 258.720367 | 1.085030 |
| GO:0016042\_lipid\_catabolic\_process | 38 | 1 | 5.770677 | -0.795798 | 165 | 99.339633 | 179.03 | 258.720367 | 1.085030 |
| GO:0032989\_cellular\_component\_morphogenesis | 307 | 3 | 2.142857 | -0.793409 | 166 | 99.547731 | 179.43 | 259.312269 | 1.080904 |
| GO:0051094\_positive\_regulation\_of\_developmental\_process | 308 | 3 | 2.135900 | -0.790410 | 167 | 99.778221 | 179.86 | 259.941779 | 1.077006 |
| GO:0005976\_polysaccharide\_metabolic\_process | 39 | 1 | 5.622711 | -0.785441 | 169 | 101.390934 | 182.37 | 263.349066 | 1.079112 |
| GO:0043524\_negative\_regulation\_of\_neuron\_apoptosis | 39 | 1 | 5.622711 | -0.785441 | 169 | 101.390934 | 182.37 | 263.349066 | 1.079112 |
| GO:0010551\_regulation\_of\_specific\_transcription\_from\_RNA\_polymerase\_II\_promoter | 41 | 1 | 5.348432 | -0.765567 | 173 | 106.214056 | 189.46 | 272.705944 | 1.095145 |
| GO:0031344\_regulation\_of\_cell\_projection\_organization | 41 | 1 | 5.348432 | -0.765567 | 173 | 106.214056 | 189.46 | 272.705944 | 1.095145 |
| GO:0032569\_specific\_transcription\_from\_RNA\_polymerase\_II\_promoter | 41 | 1 | 5.348432 | -0.765567 | 173 | 106.214056 | 189.46 | 272.705944 | 1.095145 |
| GO:0032844\_regulation\_of\_homeostatic\_process | 41 | 1 | 5.348432 | -0.765567 | 173 | 106.214056 | 189.46 | 272.705944 | 1.095145 |
| GO:0006006\_glucose\_metabolic\_process | 42 | 1 | 5.221088 | -0.756024 | 178 | 108.541057 | 193.28 | 278.018943 | 1.085843 |
| GO:0010769\_regulation\_of\_cell\_morphogenesis\_involved\_in\_differentiation | 42 | 1 | 5.221088 | -0.756024 | 178 | 108.541057 | 193.28 | 278.018943 | 1.085843 |
| GO:0019941\_modification-dependent\_protein\_catabolic\_process | 42 | 1 | 5.221088 | -0.756024 | 178 | 108.541057 | 193.28 | 278.018943 | 1.085843 |
| GO:0043632\_modification-dependent\_macromolecule\_catabolic\_process | 42 | 1 | 5.221088 | -0.756024 | 178 | 108.541057 | 193.28 | 278.018943 | 1.085843 |
| GO:0051603\_proteolysis\_involved\_in\_cellular\_protein\_catabolic\_process | 42 | 1 | 5.221088 | -0.756024 | 178 | 108.541057 | 193.28 | 278.018943 | 1.085843 |
| GO:0032502\_developmental\_process | 2060 | 12 | 1.277393 | -0.752010 | 179 | 108.976239 | 193.86 | 278.743761 | 1.083017 |
| GO:0001508\_regulation\_of\_action\_potential | 43 | 1 | 5.099668 | -0.746726 | 182 | 111.240324 | 197.05 | 282.859676 | 1.082692 |
| GO:0010001\_glial\_cell\_differentiation | 43 | 1 | 5.099668 | -0.746726 | 182 | 111.240324 | 197.05 | 282.859676 | 1.082692 |
| GO:0032446\_protein\_modification\_by\_small\_protein\_conjugation | 43 | 1 | 5.099668 | -0.746726 | 182 | 111.240324 | 197.05 | 282.859676 | 1.082692 |
| GO:0001942\_hair\_follicle\_development | 44 | 1 | 4.983766 | -0.737663 | 190 | 113.805727 | 201.05 | 288.294273 | 1.058158 |
| GO:0006606\_protein\_import\_into\_nucleus | 44 | 1 | 4.983766 | -0.737663 | 190 | 113.805727 | 201.05 | 288.294273 | 1.058158 |
| GO:0022404\_molting\_cycle\_process | 44 | 1 | 4.983766 | -0.737663 | 190 | 113.805727 | 201.05 | 288.294273 | 1.058158 |
| GO:0022405\_hair\_cycle\_process | 44 | 1 | 4.983766 | -0.737663 | 190 | 113.805727 | 201.05 | 288.294273 | 1.058158 |
| GO:0042303\_molting\_cycle | 44 | 1 | 4.983766 | -0.737663 | 190 | 113.805727 | 201.05 | 288.294273 | 1.058158 |
| GO:0042633\_hair\_cycle | 44 | 1 | 4.983766 | -0.737663 | 190 | 113.805727 | 201.05 | 288.294273 | 1.058158 |
| GO:0044257\_cellular\_protein\_catabolic\_process | 44 | 1 | 4.983766 | -0.737663 | 190 | 113.805727 | 201.05 | 288.294273 | 1.058158 |
| GO:0051170\_nuclear\_import | 44 | 1 | 4.983766 | -0.737663 | 190 | 113.805727 | 201.05 | 288.294273 | 1.058158 |
| GO:0045449\_regulation\_of\_transcription | 676 | 5 | 1.621936 | -0.732955 | 191 | 114.309294 | 201.66 | 289.010706 | 1.055812 |
| GO:0044248\_cellular\_catabolic\_process | 173 | 2 | 2.535095 | -0.731641 | 192 | 114.515306 | 202.18 | 289.844694 | 1.053021 |
| GO:0000122\_negative\_regulation\_of\_transcription\_from\_RNA\_polymerase\_II\_promoter | 175 | 2 | 2.506122 | -0.723932 | 193 | 115.952078 | 204.77 | 293.587922 | 1.060984 |
| GO:0030850\_prostate\_gland\_development | 46 | 1 | 4.767081 | -0.720198 | 195 | 117.275352 | 206.72 | 296.164648 | 1.060103 |
| GO:0042063\_gliogenesis | 46 | 1 | 4.767081 | -0.720198 | 195 | 117.275352 | 206.72 | 296.164648 | 1.060103 |
| GO:0006396\_RNA\_processing | 47 | 1 | 4.665653 | -0.711777 | 197 | 119.046209 | 209.58 | 300.113791 | 1.063858 |
| GO:0016570\_histone\_modification | 47 | 1 | 4.665653 | -0.711777 | 197 | 119.046209 | 209.58 | 300.113791 | 1.063858 |
| GO:0019318\_hexose\_metabolic\_process | 48 | 1 | 4.568452 | -0.703552 | 199 | 120.423317 | 211.71 | 302.996683 | 1.063869 |
| GO:0034504\_protein\_localization\_in\_nucleus | 48 | 1 | 4.568452 | -0.703552 | 199 | 120.423317 | 211.71 | 302.996683 | 1.063869 |
| GO:0006725\_cellular\_aromatic\_compound\_metabolic\_process | 49 | 1 | 4.475219 | -0.695516 | 201 | 122.691125 | 214.94 | 307.188875 | 1.069353 |
| GO:0043473\_pigmentation | 49 | 1 | 4.475219 | -0.695516 | 201 | 122.691125 | 214.94 | 307.188875 | 1.069353 |
| GO:0002573\_myeloid\_leukocyte\_differentiation | 50 | 1 | 4.385714 | -0.687659 | 205 | 124.016123 | 216.98 | 309.943877 | 1.058439 |
| GO:0007015\_actin\_filament\_organization | 50 | 1 | 4.385714 | -0.687659 | 205 | 124.016123 | 216.98 | 309.943877 | 1.058439 |
| GO:0017038\_protein\_import | 50 | 1 | 4.385714 | -0.687659 | 205 | 124.016123 | 216.98 | 309.943877 | 1.058439 |
| GO:0070647\_protein\_modification\_by\_small\_protein\_conjugation\_or\_removal | 50 | 1 | 4.385714 | -0.687659 | 205 | 124.016123 | 216.98 | 309.943877 | 1.058439 |
| GO:0006350\_transcription | 701 | 5 | 1.564092 | -0.686866 | 206 | 124.193040 | 217.23 | 310.266960 | 1.054515 |
| GO:0050793\_regulation\_of\_developmental\_process | 703 | 5 | 1.559642 | -0.683302 | 207 | 124.569502 | 217.87 | 311.170498 | 1.052512 |
| GO:0016569\_covalent\_chromatin\_modification | 51 | 1 | 4.299720 | -0.679976 | 209 | 126.121231 | 220.01 | 313.898769 | 1.052679 |
| GO:0032583\_regulation\_of\_gene-specific\_transcription | 51 | 1 | 4.299720 | -0.679976 | 209 | 126.121231 | 220.01 | 313.898769 | 1.052679 |
| GO:0034645\_cellular\_macromolecule\_biosynthetic\_process | 901 | 6 | 1.460282 | -0.668405 | 210 | 127.694929 | 222.4 | 317.105071 | 1.059048 |
| GO:0010467\_gene\_expression | 905 | 6 | 1.453828 | -0.662160 | 211 | 129.129090 | 224.33 | 319.530910 | 1.063175 |
| GO:0006412\_translation | 54 | 1 | 4.060847 | -0.657900 | 212 | 130.735309 | 226.57 | 322.404691 | 1.068726 |
| GO:0009059\_macromolecule\_biosynthetic\_process | 910 | 6 | 1.445840 | -0.654427 | 213 | 131.017102 | 226.97 | 322.922898 | 1.065587 |
| GO:0007507\_heart\_development | 195 | 2 | 2.249084 | -0.652658 | 214 | 131.679334 | 227.81 | 323.940666 | 1.064533 |
| GO:0006310\_DNA\_recombination | 55 | 1 | 3.987013 | -0.650846 | 216 | 133.188759 | 229.41 | 325.631241 | 1.062083 |
| GO:0007605\_sensory\_perception\_of\_sound | 55 | 1 | 3.987013 | -0.650846 | 216 | 133.188759 | 229.41 | 325.631241 | 1.062083 |
| GO:0033554\_cellular\_response\_to\_stress | 196 | 2 | 2.237609 | -0.649349 | 217 | 133.292077 | 229.51 | 325.727923 | 1.057650 |
| GO:0050678\_regulation\_of\_epithelial\_cell\_proliferation | 56 | 1 | 3.915816 | -0.643934 | 218 | 135.127764 | 231.99 | 328.852236 | 1.064174 |
| GO:0048869\_cellular\_developmental\_process | 1113 | 7 | 1.379155 | -0.643566 | 219 | 135.166087 | 232.14 | 329.113913 | 1.060000 |
| GO:0008344\_adult\_locomotory\_behavior | 57 | 1 | 3.847118 | -0.637161 | 223 | 137.956102 | 236.13 | 334.303898 | 1.058879 |
| GO:0033365\_protein\_localization\_in\_organelle | 57 | 1 | 3.847118 | -0.637161 | 223 | 137.956102 | 236.13 | 334.303898 | 1.058879 |
| GO:0043523\_regulation\_of\_neuron\_apoptosis | 57 | 1 | 3.847118 | -0.637161 | 223 | 137.956102 | 236.13 | 334.303898 | 1.058879 |
| GO:0045444\_fat\_cell\_differentiation | 57 | 1 | 3.847118 | -0.637161 | 223 | 137.956102 | 236.13 | 334.303898 | 1.058879 |
| GO:0030902\_hindbrain\_development | 58 | 1 | 3.780788 | -0.630521 | 224 | 138.829464 | 237.43 | 336.030536 | 1.059955 |
| GO:0080090\_regulation\_of\_primary\_metabolic\_process | 926 | 6 | 1.420858 | -0.630220 | 225 | 138.951763 | 237.6 | 336.248237 | 1.056000 |
| GO:0060255\_regulation\_of\_macromolecule\_metabolic\_process | 936 | 6 | 1.405678 | -0.615498 | 226 | 140.739579 | 240.32 | 339.900421 | 1.063363 |
| GO:0010556\_regulation\_of\_macromolecule\_biosynthetic\_process | 745 | 5 | 1.471716 | -0.612407 | 227 | 141.005236 | 240.61 | 340.214764 | 1.059956 |
| GO:0022604\_regulation\_of\_cell\_morphogenesis | 62 | 1 | 3.536866 | -0.605203 | 230 | 143.334329 | 244.23 | 345.125671 | 1.061870 |
| GO:0030855\_epithelial\_cell\_differentiation | 62 | 1 | 3.536866 | -0.605203 | 230 | 143.334329 | 244.23 | 345.125671 | 1.061870 |
| GO:0050954\_sensory\_perception\_of\_mechanical\_stimulus | 62 | 1 | 3.536866 | -0.605203 | 230 | 143.334329 | 244.23 | 345.125671 | 1.061870 |
| GO:0051216\_cartilage\_development | 63 | 1 | 3.480726 | -0.599165 | 231 | 144.662253 | 245.93 | 347.197747 | 1.064632 |
| GO:0044249\_cellular\_biosynthetic\_process | 1150 | 7 | 1.334783 | -0.593421 | 232 | 145.374892 | 246.94 | 348.505108 | 1.064397 |
| GO:0045892\_negative\_regulation\_of\_transcription\_\_DNA-dependent | 218 | 2 | 2.011796 | -0.581844 | 233 | 147.490579 | 249.99 | 352.489421 | 1.072918 |
| GO:0051130\_positive\_regulation\_of\_cellular\_component\_organization | 66 | 1 | 3.322511 | -0.581688 | 235 | 148.451667 | 251.51 | 354.568333 | 1.070255 |
| GO:0051402\_neuron\_apoptosis | 66 | 1 | 3.322511 | -0.581688 | 235 | 148.451667 | 251.51 | 354.568333 | 1.070255 |
| GO:0051253\_negative\_regulation\_of\_RNA\_metabolic\_process | 220 | 2 | 1.993506 | -0.576170 | 236 | 149.152834 | 252.41 | 355.667166 | 1.069534 |
| GO:0034962\_cellular\_biopolymer\_catabolic\_process | 68 | 1 | 3.224790 | -0.570538 | 237 | 151.466977 | 255.57 | 359.673023 | 1.078354 |
| GO:0005996\_monosaccharide\_metabolic\_process | 69 | 1 | 3.178054 | -0.565105 | 238 | 152.370414 | 256.9 | 361.429586 | 1.079412 |
| GO:0010468\_regulation\_of\_gene\_expression | 778 | 5 | 1.409291 | -0.561624 | 239 | 152.590414 | 257.09 | 361.589586 | 1.075690 |
| GO:0051239\_regulation\_of\_multicellular\_organismal\_process | 587 | 4 | 1.494281 | -0.560386 | 240 | 152.769925 | 257.44 | 362.110075 | 1.072667 |
| GO:0009617\_response\_to\_bacterium | 70 | 1 | 3.132653 | -0.559762 | 241 | 153.700622 | 258.64 | 363.579378 | 1.073195 |
| GO:0006913\_nucleocytoplasmic\_transport | 71 | 1 | 3.088531 | -0.554507 | 242 | 155.028916 | 260.17 | 365.311084 | 1.075083 |
| GO:0002682\_regulation\_of\_immune\_system\_process | 228 | 2 | 1.923559 | -0.554166 | 243 | 155.422640 | 260.78 | 366.137360 | 1.073169 |
| GO:0007167\_enzyme\_linked\_receptor\_protein\_signaling\_pathway | 229 | 2 | 1.915159 | -0.551492 | 244 | 155.622276 | 261.0 | 366.377724 | 1.069672 |
| GO:0016568\_chromatin\_modification | 72 | 1 | 3.045635 | -0.549338 | 247 | 157.537015 | 263.58 | 369.622985 | 1.067126 |
| GO:0050673\_epithelial\_cell\_proliferation | 72 | 1 | 3.045635 | -0.549338 | 247 | 157.537015 | 263.58 | 369.622985 | 1.067126 |
| GO:0051169\_nuclear\_transport | 72 | 1 | 3.045635 | -0.549338 | 247 | 157.537015 | 263.58 | 369.622985 | 1.067126 |
| GO:0044265\_cellular\_macromolecule\_catabolic\_process | 75 | 1 | 2.923810 | -0.534321 | 248 | 161.157614 | 268.4 | 375.642386 | 1.082258 |
| GO:0001501\_skeletal\_system\_development | 236 | 2 | 1.858354 | -0.533217 | 249 | 161.302089 | 268.61 | 375.917911 | 1.078755 |
| GO:0006508\_proteolysis | 76 | 1 | 2.885338 | -0.529471 | 250 | 162.449286 | 270.18 | 377.910714 | 1.080720 |
| GO:0042592\_homeostatic\_process | 419 | 3 | 1.570065 | -0.526261 | 251 | 162.724581 | 270.58 | 378.435419 | 1.078008 |
| GO:0001890\_placenta\_development | 77 | 1 | 2.847866 | -0.524696 | 252 | 163.225437 | 271.3 | 379.374563 | 1.076587 |
| GO:0007519\_skeletal\_muscle\_tissue\_development | 78 | 1 | 2.811355 | -0.519994 | 254 | 164.748494 | 273.29 | 381.831506 | 1.075945 |
| GO:0060538\_skeletal\_muscle\_organ\_development | 78 | 1 | 2.811355 | -0.519994 | 254 | 164.748494 | 273.29 | 381.831506 | 1.075945 |
| GO:0009056\_catabolic\_process | 243 | 2 | 1.804821 | -0.515694 | 255 | 165.133147 | 273.8 | 382.466853 | 1.073725 |
| GO:0031326\_regulation\_of\_cellular\_biosynthetic\_process | 812 | 5 | 1.350281 | -0.513405 | 256 | 165.683388 | 274.6 | 383.516612 | 1.072656 |
| GO:0006631\_fatty\_acid\_metabolic\_process | 80 | 1 | 2.741071 | -0.510800 | 257 | 166.985670 | 276.18 | 385.374330 | 1.074630 |
| GO:0031323\_regulation\_of\_cellular\_metabolic\_process | 1015 | 6 | 1.296270 | -0.509417 | 258 | 167.091124 | 276.36 | 385.628876 | 1.071163 |
| GO:0009889\_regulation\_of\_biosynthetic\_process | 815 | 5 | 1.345311 | -0.509339 | 259 | 167.226056 | 276.55 | 385.873944 | 1.067761 |
| GO:0007399\_nervous\_system\_development | 621 | 4 | 1.412468 | -0.506157 | 260 | 167.907579 | 277.26 | 386.612421 | 1.066385 |
| GO:0045664\_regulation\_of\_neuron\_differentiation | 82 | 1 | 2.674216 | -0.501875 | 261 | 169.058950 | 278.96 | 388.861050 | 1.068812 |
| GO:0030534\_adult\_behavior | 83 | 1 | 2.641997 | -0.497509 | 262 | 170.782345 | 281.16 | 391.537655 | 1.073130 |
| GO:0016481\_negative\_regulation\_of\_transcription | 253 | 2 | 1.733484 | -0.491878 | 264 | 171.981584 | 282.85 | 393.718416 | 1.071402 |
| GO:0030097\_hemopoiesis | 253 | 2 | 1.733484 | -0.491878 | 264 | 171.981584 | 282.85 | 393.718416 | 1.071402 |
| GO:0006605\_protein\_targeting | 86 | 1 | 2.549834 | -0.484782 | 265 | 174.801173 | 286.62 | 398.438827 | 1.081585 |
| GO:0016337\_cell-cell\_adhesion | 87 | 1 | 2.520525 | -0.480657 | 266 | 176.700485 | 289.31 | 401.919515 | 1.087632 |
| GO:0010629\_negative\_regulation\_of\_gene\_expression | 262 | 2 | 1.673937 | -0.471583 | 267 | 178.254999 | 291.32 | 404.385001 | 1.091086 |
| GO:0044255\_cellular\_lipid\_metabolic\_process | 264 | 2 | 1.661255 | -0.467212 | 268 | 179.898095 | 293.26 | 406.621905 | 1.094254 |
| GO:0008544\_epidermis\_development | 91 | 1 | 2.409733 | -0.464714 | 269 | 180.405142 | 294.08 | 407.754858 | 1.093234 |
| GO:0045944\_positive\_regulation\_of\_transcription\_from\_RNA\_polymerase\_II\_promoter | 269 | 2 | 1.630377 | -0.456494 | 270 | 183.102416 | 297.52 | 411.937584 | 1.101926 |
| GO:0030154\_cell\_differentiation | 1060 | 6 | 1.241240 | -0.456384 | 271 | 183.182699 | 297.7 | 412.217301 | 1.098524 |
| GO:0045934\_negative\_regulation\_of\_nucleobase\_\_nucleoside\_\_nucleotide\_and\_nucleic\_acid\_metabolic\_process | 270 | 2 | 1.624339 | -0.454386 | 272 | 183.393952 | 297.92 | 412.446048 | 1.095294 |
| GO:0048856\_anatomical\_structure\_development | 1688 | 9 | 1.169177 | -0.453728 | 273 | 183.524162 | 298.04 | 412.555838 | 1.091722 |
| GO:0034984\_cellular\_response\_to\_DNA\_damage\_stimulus | 94 | 1 | 2.332827 | -0.453307 | 274 | 184.958995 | 299.55 | 414.141005 | 1.093248 |
| GO:0051172\_negative\_regulation\_of\_nitrogen\_compound\_metabolic\_process | 271 | 2 | 1.618345 | -0.452290 | 275 | 185.506704 | 300.12 | 414.733296 | 1.091345 |
| GO:0042391\_regulation\_of\_membrane\_potential | 95 | 1 | 2.308271 | -0.449603 | 277 | 186.012815 | 300.78 | 415.547185 | 1.085848 |
| GO:0051707\_response\_to\_other\_organism | 95 | 1 | 2.308271 | -0.449603 | 277 | 186.012815 | 300.78 | 415.547185 | 1.085848 |
| GO:0010558\_negative\_regulation\_of\_macromolecule\_biosynthetic\_process | 274 | 2 | 1.600626 | -0.446069 | 279 | 186.740149 | 301.68 | 416.619851 | 1.081290 |
| GO:0033036\_macromolecule\_localization | 274 | 2 | 1.600626 | -0.446069 | 279 | 186.740149 | 301.68 | 416.619851 | 1.081290 |
| GO:0048534\_hemopoietic\_or\_lymphoid\_organ\_development | 277 | 2 | 1.583290 | -0.439950 | 280 | 188.588328 | 304.4 | 420.211672 | 1.087143 |
| GO:0007398\_ectoderm\_development | 99 | 1 | 2.215007 | -0.435252 | 281 | 191.054394 | 307.63 | 424.205606 | 1.094769 |
| GO:0001525\_angiogenesis | 100 | 1 | 2.192857 | -0.431776 | 282 | 191.357697 | 307.95 | 424.542303 | 1.092021 |
| GO:0031327\_negative\_regulation\_of\_cellular\_biosynthetic\_process | 282 | 2 | 1.555218 | -0.429971 | 283 | 191.573718 | 308.22 | 424.866282 | 1.089117 |
| GO:0030163\_protein\_catabolic\_process | 101 | 1 | 2.171146 | -0.428343 | 284 | 192.075065 | 308.93 | 425.784935 | 1.087782 |
| GO:0009890\_negative\_regulation\_of\_biosynthetic\_process | 284 | 2 | 1.544266 | -0.426054 | 285 | 192.474381 | 309.44 | 426.405619 | 1.085754 |
| GO:0019222\_regulation\_of\_metabolic\_process | 1088 | 6 | 1.209296 | -0.425826 | 286 | 192.588295 | 309.63 | 426.671705 | 1.082622 |
| GO:0030036\_actin\_cytoskeleton\_organization | 102 | 1 | 2.149860 | -0.424952 | 287 | 192.858447 | 309.95 | 427.041553 | 1.079965 |
| GO:0006629\_lipid\_metabolic\_process | 285 | 2 | 1.538847 | -0.424111 | 288 | 193.057686 | 310.17 | 427.282314 | 1.076979 |
| GO:0007417\_central\_nervous\_system\_development | 287 | 2 | 1.528123 | -0.420257 | 289 | 193.997844 | 311.25 | 428.502156 | 1.076990 |
| GO:0050767\_regulation\_of\_neurogenesis | 104 | 1 | 2.108516 | -0.418292 | 290 | 194.437202 | 311.85 | 429.262798 | 1.075345 |
| GO:0048522\_positive\_regulation\_of\_cellular\_process | 895 | 5 | 1.225060 | -0.411077 | 291 | 195.332897 | 312.94 | 430.547103 | 1.075395 |
| GO:0050896\_response\_to\_stimulus | 1107 | 6 | 1.188540 | -0.406093 | 292 | 195.834561 | 313.62 | 431.405439 | 1.074041 |
| GO:0002520\_immune\_system\_development | 295 | 2 | 1.486683 | -0.405242 | 293 | 196.720438 | 314.85 | 432.979562 | 1.074573 |
| GO:0030029\_actin\_filament-based\_process | 109 | 1 | 2.011796 | -0.402322 | 294 | 197.194993 | 315.33 | 433.465007 | 1.072551 |
| GO:0006974\_response\_to\_DNA\_damage\_stimulus | 113 | 1 | 1.940582 | -0.390199 | 295 | 201.022919 | 319.58 | 438.137081 | 1.083322 |
| GO:0007275\_multicellular\_organismal\_development | 1760 | 9 | 1.121347 | -0.388025 | 296 | 201.382153 | 319.91 | 438.437847 | 1.080777 |
| GO:0050794\_regulation\_of\_cellular\_process | 2190 | 11 | 1.101435 | -0.387080 | 297 | 202.219914 | 321.1 | 439.980086 | 1.081145 |
| GO:0045893\_positive\_regulation\_of\_transcription\_\_DNA-dependent | 306 | 2 | 1.433240 | -0.385599 | 299 | 202.730496 | 321.56 | 440.389504 | 1.075452 |
| GO:0051254\_positive\_regulation\_of\_RNA\_metabolic\_process | 306 | 2 | 1.433240 | -0.385599 | 299 | 202.730496 | 321.56 | 440.389504 | 1.075452 |
| GO:0048608\_reproductive\_structure\_development | 116 | 1 | 1.890394 | -0.381459 | 300 | 204.153719 | 323.6 | 443.046281 | 1.078667 |
| GO:0051960\_regulation\_of\_nervous\_system\_development | 118 | 1 | 1.858354 | -0.375792 | 301 | 205.590207 | 325.21 | 444.829793 | 1.080432 |
| GO:0014706\_striated\_muscle\_tissue\_development | 120 | 1 | 1.827381 | -0.370248 | 302 | 206.430453 | 326.07 | 445.709547 | 1.079702 |
| GO:0006886\_intracellular\_protein\_transport | 122 | 1 | 1.797424 | -0.364822 | 304 | 209.889581 | 329.93 | 449.970419 | 1.085296 |
| GO:0060284\_regulation\_of\_cell\_development | 122 | 1 | 1.797424 | -0.364822 | 304 | 209.889581 | 329.93 | 449.970419 | 1.085296 |
| GO:0009888\_tissue\_development | 525 | 3 | 1.253061 | -0.361430 | 305 | 210.578339 | 330.55 | 450.521661 | 1.083770 |
| GO:0001655\_urogenital\_system\_development | 128 | 1 | 1.713170 | -0.349215 | 307 | 212.400499 | 332.82 | 453.239501 | 1.084104 |
| GO:0060537\_muscle\_tissue\_development | 128 | 1 | 1.713170 | -0.349215 | 307 | 212.400499 | 332.82 | 453.239501 | 1.084104 |
| GO:0043285\_biopolymer\_catabolic\_process | 129 | 1 | 1.699889 | -0.346707 | 308 | 213.253263 | 333.66 | 454.066737 | 1.083312 |
| GO:0010605\_negative\_regulation\_of\_macromolecule\_metabolic\_process | 331 | 2 | 1.324989 | -0.344859 | 309 | 214.582795 | 335.12 | 455.657205 | 1.084531 |
| GO:0032787\_monocarboxylic\_acid\_metabolic\_process | 130 | 1 | 1.686813 | -0.344225 | 310 | 215.411724 | 336.13 | 456.848276 | 1.084290 |
| GO:0031324\_negative\_regulation\_of\_cellular\_metabolic\_process | 332 | 2 | 1.320998 | -0.343333 | 311 | 215.606752 | 336.38 | 457.153248 | 1.081608 |
| GO:0048731\_system\_development | 1609 | 8 | 1.090296 | -0.336114 | 312 | 217.096342 | 337.93 | 458.763658 | 1.083109 |
| GO:0045941\_positive\_regulation\_of\_transcription | 338 | 2 | 1.297549 | -0.334335 | 313 | 218.487287 | 339.34 | 460.192713 | 1.084153 |
| GO:0006793\_phosphorus\_metabolic\_process | 340 | 2 | 1.289916 | -0.331394 | 315 | 219.388441 | 340.32 | 461.251559 | 1.080381 |
| GO:0006796\_phosphate\_metabolic\_process | 340 | 2 | 1.289916 | -0.331394 | 315 | 219.388441 | 340.32 | 461.251559 | 1.080381 |
| GO:0009057\_macromolecule\_catabolic\_process | 137 | 1 | 1.600626 | -0.327529 | 316 | 220.335228 | 341.26 | 462.184772 | 1.079937 |
| GO:0034613\_cellular\_protein\_localization | 139 | 1 | 1.577595 | -0.322968 | 317 | 220.869117 | 341.9 | 462.930883 | 1.078549 |
| GO:0010628\_positive\_regulation\_of\_gene\_expression | 346 | 2 | 1.267547 | -0.322741 | 318 | 221.172204 | 342.19 | 463.207796 | 1.076069 |
| GO:0016044\_membrane\_organization | 140 | 1 | 1.566327 | -0.320720 | 319 | 221.512952 | 342.73 | 463.947048 | 1.074389 |
| GO:0009892\_negative\_regulation\_of\_metabolic\_process | 348 | 2 | 1.260263 | -0.319913 | 320 | 222.047109 | 343.41 | 464.772891 | 1.073156 |
| GO:0003006\_reproductive\_developmental\_process | 141 | 1 | 1.555218 | -0.318493 | 322 | 222.593872 | 344.07 | 465.546128 | 1.068540 |
| GO:0070727\_cellular\_macromolecule\_localization | 141 | 1 | 1.555218 | -0.318493 | 322 | 222.593872 | 344.07 | 465.546128 | 1.068540 |
| GO:0048523\_negative\_regulation\_of\_cellular\_process | 774 | 4 | 1.133260 | -0.318454 | 323 | 222.897632 | 344.32 | 465.742368 | 1.066006 |
| GO:0045935\_positive\_regulation\_of\_nucleobase\_\_nucleoside\_\_nucleotide\_and\_nucleic\_acid\_metabolic\_process | 352 | 2 | 1.245942 | -0.314337 | 324 | 223.297774 | 344.83 | 466.362226 | 1.064290 |
| GO:0048518\_positive\_regulation\_of\_biological\_process | 995 | 5 | 1.101938 | -0.312115 | 325 | 223.829719 | 345.4 | 466.970281 | 1.062769 |
| GO:0022603\_regulation\_of\_anatomical\_structure\_morphogenesis | 147 | 1 | 1.491740 | -0.305571 | 326 | 226.558837 | 348.16 | 469.761163 | 1.067975 |
| GO:0051173\_positive\_regulation\_of\_nitrogen\_compound\_metabolic\_process | 361 | 2 | 1.214879 | -0.302176 | 327 | 227.878875 | 349.74 | 471.601125 | 1.069541 |
| GO:0007517\_muscle\_organ\_development | 153 | 1 | 1.433240 | -0.293351 | 328 | 229.978810 | 352.08 | 474.181190 | 1.073415 |
| GO:0008285\_negative\_regulation\_of\_cell\_proliferation | 155 | 1 | 1.414747 | -0.289425 | 330 | 232.117684 | 354.19 | 476.262316 | 1.073303 |
| GO:0022402\_cell\_cycle\_process | 155 | 1 | 1.414747 | -0.289425 | 330 | 232.117684 | 354.19 | 476.262316 | 1.073303 |
| GO:0010557\_positive\_regulation\_of\_macromolecule\_biosynthetic\_process | 371 | 2 | 1.182133 | -0.289260 | 331 | 232.459441 | 354.53 | 476.600559 | 1.071088 |
| GO:0051704\_multi-organism\_process | 157 | 1 | 1.396724 | -0.285568 | 332 | 233.186889 | 355.35 | 477.513111 | 1.070331 |
| GO:0006066\_alcohol\_metabolic\_process | 158 | 1 | 1.387884 | -0.283665 | 335 | 234.337711 | 356.49 | 478.642289 | 1.064149 |
| GO:0007409\_axonogenesis | 158 | 1 | 1.387884 | -0.283665 | 335 | 234.337711 | 356.49 | 478.642289 | 1.064149 |
| GO:0048514\_blood\_vessel\_morphogenesis | 158 | 1 | 1.387884 | -0.283665 | 335 | 234.337711 | 356.49 | 478.642289 | 1.064149 |
| GO:0007166\_cell\_surface\_receptor\_linked\_signal\_transduction | 597 | 3 | 1.101938 | -0.280287 | 336 | 235.647926 | 357.87 | 480.092074 | 1.065089 |
| GO:0051128\_regulation\_of\_cellular\_component\_organization | 160 | 1 | 1.370536 | -0.279910 | 337 | 235.973168 | 358.25 | 480.526832 | 1.063056 |
| GO:0000003\_reproduction | 379 | 2 | 1.157181 | -0.279357 | 338 | 236.278683 | 358.51 | 480.741317 | 1.060680 |
| GO:0002521\_leukocyte\_differentiation | 161 | 1 | 1.362023 | -0.278057 | 339 | 236.544355 | 358.83 | 481.115645 | 1.058496 |
| GO:0007626\_locomotory\_behavior | 163 | 1 | 1.345311 | -0.274399 | 340 | 237.714983 | 360.16 | 482.605017 | 1.059294 |
| GO:0006259\_DNA\_metabolic\_process | 165 | 1 | 1.329004 | -0.270804 | 341 | 239.611242 | 361.97 | 484.328758 | 1.061496 |
| GO:0031328\_positive\_regulation\_of\_cellular\_biosynthetic\_process | 387 | 2 | 1.133260 | -0.269816 | 342 | 239.943481 | 362.28 | 484.616519 | 1.059298 |
| GO:0009891\_positive\_regulation\_of\_biosynthetic\_process | 388 | 2 | 1.130339 | -0.268648 | 343 | 240.725402 | 363.06 | 485.394598 | 1.058484 |
| GO:0050877\_neurological\_system\_process | 390 | 2 | 1.124542 | -0.266328 | 344 | 241.909628 | 364.29 | 486.670372 | 1.058983 |
| GO:0050789\_regulation\_of\_biological\_process | 2357 | 11 | 1.023395 | -0.264557 | 345 | 242.239375 | 364.69 | 487.140625 | 1.057072 |
| GO:0048812\_neuron\_projection\_morphogenesis | 170 | 1 | 1.289916 | -0.262082 | 346 | 243.201309 | 365.7 | 488.198691 | 1.056936 |
| GO:0007600\_sensory\_perception | 172 | 1 | 1.274917 | -0.258696 | 347 | 244.622103 | 367.09 | 489.557897 | 1.057896 |
| GO:0048667\_cell\_morphogenesis\_involved\_in\_neuron\_differentiation | 173 | 1 | 1.267547 | -0.257024 | 348 | 246.068568 | 368.52 | 490.971432 | 1.058966 |
| GO:0015031\_protein\_transport | 175 | 1 | 1.253061 | -0.253722 | 349 | 247.049648 | 369.64 | 492.230352 | 1.059140 |
| GO:0006873\_cellular\_ion\_homeostasis | 176 | 1 | 1.245942 | -0.252091 | 352 | 248.147683 | 370.76 | 493.372317 | 1.053295 |
| GO:0043066\_negative\_regulation\_of\_apoptosis | 176 | 1 | 1.245942 | -0.252091 | 352 | 248.147683 | 370.76 | 493.372317 | 1.053295 |
| GO:0048858\_cell\_projection\_morphogenesis | 176 | 1 | 1.245942 | -0.252091 | 352 | 248.147683 | 370.76 | 493.372317 | 1.053295 |
| GO:0009987\_cellular\_process | 3868 | 18 | 1.020461 | -0.251730 | 353 | 248.308230 | 370.98 | 493.651770 | 1.050935 |
| GO:0065007\_biological\_regulation | 2593 | 12 | 1.014820 | -0.251559 | 354 | 248.458953 | 371.17 | 493.881047 | 1.048503 |
| GO:0043069\_negative\_regulation\_of\_programmed\_cell\_death | 179 | 1 | 1.225060 | -0.247279 | 357 | 249.518651 | 372.33 | 495.141349 | 1.042941 |
| GO:0048732\_gland\_development | 179 | 1 | 1.225060 | -0.247279 | 357 | 249.518651 | 372.33 | 495.141349 | 1.042941 |
| GO:0060548\_negative\_regulation\_of\_cell\_death | 179 | 1 | 1.225060 | -0.247279 | 357 | 249.518651 | 372.33 | 495.141349 | 1.042941 |
| GO:0045184\_establishment\_of\_protein\_localization | 180 | 1 | 1.218254 | -0.245701 | 358 | 249.854637 | 372.71 | 495.565363 | 1.041089 |
| GO:0048519\_negative\_regulation\_of\_biological\_process | 859 | 4 | 1.021121 | -0.244422 | 359 | 250.613083 | 373.32 | 496.026917 | 1.039889 |
| GO:0055082\_cellular\_chemical\_homeostasis | 181 | 1 | 1.211523 | -0.244136 | 360 | 251.422501 | 374.34 | 497.257499 | 1.039833 |
| GO:0007242\_intracellular\_signaling\_cascade | 411 | 2 | 1.067084 | -0.243212 | 361 | 251.668810 | 374.66 | 497.651190 | 1.037839 |
| GO:0032990\_cell\_part\_morphogenesis | 184 | 1 | 1.191770 | -0.239516 | 362 | 252.863679 | 375.99 | 499.116321 | 1.038646 |
| GO:0007010\_cytoskeleton\_organization | 185 | 1 | 1.185328 | -0.238000 | 363 | 253.996132 | 377.07 | 500.143868 | 1.038760 |
| GO:0007155\_cell\_adhesion | 186 | 1 | 1.178955 | -0.236497 | 365 | 254.992321 | 378.16 | 501.327679 | 1.036055 |
| GO:0022610\_biological\_adhesion | 186 | 1 | 1.178955 | -0.236497 | 365 | 254.992321 | 378.16 | 501.327679 | 1.036055 |
| GO:0019226\_transmission\_of\_nerve\_impulse | 189 | 1 | 1.160242 | -0.232058 | 366 | 257.025053 | 380.21 | 503.394947 | 1.038825 |
| GO:0046907\_intracellular\_transport | 194 | 1 | 1.130339 | -0.224890 | 367 | 259.081471 | 382.32 | 505.558529 | 1.041744 |
| GO:0019725\_cellular\_homeostasis | 195 | 1 | 1.124542 | -0.223490 | 368 | 260.156901 | 383.37 | 506.583099 | 1.041766 |
| GO:0010604\_positive\_regulation\_of\_macromolecule\_metabolic\_process | 433 | 2 | 1.012867 | -0.221237 | 369 | 261.407134 | 384.68 | 507.952866 | 1.042493 |
| GO:0031175\_neuron\_projection\_development | 197 | 1 | 1.113125 | -0.220721 | 371 | 262.232570 | 385.48 | 508.727430 | 1.039030 |
| GO:0050801\_ion\_homeostasis | 197 | 1 | 1.113125 | -0.220721 | 371 | 262.232570 | 385.48 | 508.727430 | 1.039030 |
| GO:0002009\_morphogenesis\_of\_an\_epithelium | 198 | 1 | 1.107504 | -0.219353 | 373 | 262.943923 | 386.2 | 509.456077 | 1.035389 |
| GO:0060429\_epithelium\_development | 198 | 1 | 1.107504 | -0.219353 | 373 | 262.943923 | 386.2 | 509.456077 | 1.035389 |
| GO:0000904\_cell\_morphogenesis\_involved\_in\_differentiation | 199 | 1 | 1.101938 | -0.217995 | 374 | 263.888500 | 387.05 | 510.211500 | 1.034893 |
| GO:0031325\_positive\_regulation\_of\_cellular\_metabolic\_process | 442 | 2 | 0.992243 | -0.212850 | 375 | 264.801967 | 387.98 | 511.158033 | 1.034613 |
| GO:0001568\_blood\_vessel\_development | 203 | 1 | 1.080225 | -0.212667 | 376 | 265.247076 | 388.39 | 511.532924 | 1.032952 |
| GO:0007243\_protein\_kinase\_cascade | 205 | 1 | 1.069686 | -0.210063 | 377 | 267.504956 | 390.75 | 513.995044 | 1.036472 |
| GO:0001944\_vasculature\_development | 208 | 1 | 1.054258 | -0.206229 | 379 | 269.120179 | 392.37 | 515.619821 | 1.035277 |
| GO:0008284\_positive\_regulation\_of\_cell\_proliferation | 208 | 1 | 1.054258 | -0.206229 | 379 | 269.120179 | 392.37 | 515.619821 | 1.035277 |
| GO:0007165\_signal\_transduction | 915 | 4 | 0.958626 | -0.204507 | 380 | 269.658299 | 392.98 | 516.301701 | 1.034158 |
| GO:0065008\_regulation\_of\_biological\_quality | 693 | 3 | 0.949289 | -0.199022 | 381 | 270.645262 | 393.94 | 517.234738 | 1.033963 |
| GO:0009893\_positive\_regulation\_of\_metabolic\_process | 458 | 2 | 0.957580 | -0.198737 | 382 | 271.377603 | 394.59 | 517.802397 | 1.032958 |
| GO:0019953\_sexual\_reproduction | 228 | 1 | 0.961779 | -0.182725 | 383 | 277.056100 | 400.02 | 522.983900 | 1.044439 |
| GO:0007420\_brain\_development | 231 | 1 | 0.949289 | -0.179483 | 384 | 278.071068 | 400.98 | 523.888932 | 1.044219 |
| GO:0009653\_anatomical\_structure\_morphogenesis | 958 | 4 | 0.915598 | -0.177897 | 385 | 278.292926 | 401.19 | 524.087074 | 1.042052 |
| GO:0050890\_cognition | 233 | 1 | 0.941140 | -0.177358 | 386 | 279.129089 | 401.96 | 524.790911 | 1.041347 |
| GO:0006468\_protein\_amino\_acid\_phosphorylation | 237 | 1 | 0.925256 | -0.173199 | 387 | 281.285651 | 403.81 | 526.334349 | 1.043437 |
| GO:0002376\_immune\_system\_process | 505 | 2 | 0.868458 | -0.162525 | 388 | 282.741301 | 405.16 | 527.578699 | 1.044227 |
| GO:0008104\_protein\_localization | 251 | 1 | 0.873648 | -0.159511 | 389 | 284.294228 | 406.54 | 528.785772 | 1.045090 |
| GO:0048878\_chemical\_homeostasis | 254 | 1 | 0.863330 | -0.156744 | 390 | 286.241261 | 408.44 | 530.638739 | 1.047282 |
| GO:0048729\_tissue\_morphogenesis | 255 | 1 | 0.859944 | -0.155834 | 391 | 286.897927 | 409.06 | 531.222073 | 1.046189 |
| GO:0003008\_system\_process | 516 | 2 | 0.849945 | -0.155053 | 392 | 287.260503 | 409.39 | 531.519497 | 1.044362 |
| GO:0048666\_neuron\_development | 262 | 1 | 0.836968 | -0.149630 | 393 | 290.328143 | 412.04 | 533.751857 | 1.048448 |
| GO:0030030\_cell\_projection\_organization | 263 | 1 | 0.833786 | -0.148767 | 394 | 291.017518 | 412.66 | 534.302482 | 1.047360 |
| GO:0048646\_anatomical\_structure\_formation\_involved\_in\_morphogenesis | 277 | 1 | 0.791645 | -0.137261 | 395 | 295.691717 | 416.92 | 538.148283 | 1.055494 |
| GO:0007610\_behavior | 279 | 1 | 0.785970 | -0.135701 | 396 | 296.533003 | 417.71 | 538.886997 | 1.054823 |
| GO:0006950\_response\_to\_stress | 549 | 2 | 0.798855 | -0.134617 | 397 | 296.793219 | 417.91 | 539.026781 | 1.052670 |
| GO:0032501\_multicellular\_organismal\_process | 2183 | 9 | 0.904064 | -0.132678 | 398 | 297.524533 | 418.57 | 539.615467 | 1.051683 |
| GO:0016310\_phosphorylation | 309 | 1 | 0.709663 | -0.114521 | 399 | 305.896354 | 425.75 | 545.603646 | 1.067043 |
| GO:0007154\_cell\_communication | 1096 | 4 | 0.800313 | -0.111816 | 400 | 306.759069 | 426.41 | 546.060931 | 1.066025 |
| GO:0051093\_negative\_regulation\_of\_developmental\_process | 331 | 1 | 0.662495 | -0.101301 | 401 | 310.760448 | 429.74 | 548.719552 | 1.071671 |
| GO:0048513\_organ\_development | 1365 | 5 | 0.803244 | -0.101148 | 402 | 310.994013 | 429.91 | 548.825987 | 1.069428 |
| GO:0051649\_establishment\_of\_localization\_in\_cell | 342 | 1 | 0.641186 | -0.095319 | 403 | 314.295720 | 432.51 | 550.724280 | 1.073226 |
| GO:0009887\_organ\_morphogenesis | 642 | 2 | 0.683133 | -0.090133 | 404 | 316.914465 | 434.87 | 552.825535 | 1.076411 |
| GO:0030182\_neuron\_differentiation | 356 | 1 | 0.615971 | -0.088246 | 405 | 317.375049 | 435.28 | 553.184951 | 1.074765 |
| GO:0042981\_regulation\_of\_apoptosis | 360 | 1 | 0.609127 | -0.086329 | 406 | 317.949570 | 435.77 | 553.590430 | 1.073325 |
| GO:0048468\_cell\_development | 654 | 2 | 0.670599 | -0.085547 | 407 | 318.571864 | 436.26 | 553.948136 | 1.071892 |
| GO:0010941\_regulation\_of\_cell\_death | 365 | 1 | 0.600783 | -0.083995 | 409 | 320.202436 | 437.62 | 555.037564 | 1.069976 |
| GO:0043067\_regulation\_of\_programmed\_cell\_death | 365 | 1 | 0.600783 | -0.083995 | 409 | 320.202436 | 437.62 | 555.037564 | 1.069976 |
| GO:0051641\_cellular\_localization | 370 | 1 | 0.592664 | -0.081728 | 410 | 321.208698 | 438.44 | 555.671302 | 1.069366 |
| GO:0022414\_reproductive\_process | 376 | 1 | 0.583207 | -0.079092 | 411 | 322.089395 | 439.28 | 556.470605 | 1.068808 |
| GO:0042127\_regulation\_of\_cell\_proliferation | 393 | 1 | 0.557979 | -0.072096 | 412 | 326.153573 | 442.77 | 559.386427 | 1.074684 |
| GO:0048699\_generation\_of\_neurons | 396 | 1 | 0.553752 | -0.070930 | 413 | 326.846577 | 443.32 | 559.793423 | 1.073414 |
| GO:0022008\_neurogenesis | 423 | 1 | 0.518406 | -0.061277 | 414 | 330.447458 | 446.15 | 561.852542 | 1.077657 |
| GO:0006915\_apoptosis | 427 | 1 | 0.513550 | -0.059966 | 415 | 330.730599 | 446.35 | 561.969401 | 1.075542 |
| GO:0012501\_programmed\_cell\_death | 433 | 1 | 0.506434 | -0.058054 | 416 | 331.659601 | 446.99 | 562.320399 | 1.074495 |
| GO:0008219\_cell\_death | 444 | 1 | 0.493887 | -0.054709 | 417 | 333.253024 | 448.22 | 563.186976 | 1.074868 |
| GO:0010926\_anatomical\_structure\_formation | 447 | 1 | 0.490572 | -0.053832 | 418 | 334.014509 | 448.76 | 563.505491 | 1.073589 |
| GO:0016265\_death | 450 | 1 | 0.487302 | -0.052968 | 419 | 334.466292 | 449.19 | 563.913708 | 1.072053 |
| GO:0008283\_cell\_proliferation | 544 | 1 | 0.403099 | -0.031950 | 420 | 340.309493 | 453.5 | 566.690507 | 1.079762 |
| GO:0009790\_embryonic\_development | 567 | 1 | 0.386747 | -0.028229 | 421 | 341.551937 | 454.46 | 567.368063 | 1.079477 |
| GO:0051179\_localization | 1058 | 2 | 0.414529 | -0.013213 | 422 | 346.474341 | 457.97 | 569.465659 | 1.085237 |
| GO:0006810\_transport | 718 | 1 | 0.305412 | -0.012428 | 423 | 346.902117 | 458.26 | 569.617883 | 1.083357 |
| GO:0051234\_establishment\_of\_localization | 729 | 1 | 0.300803 | -0.011698 | 424 | 347.079723 | 458.39 | 569.700277 | 1.081108 |
| GO:0002200\_somatic\_diversification\_of\_immune\_receptors | 34 | 0 | 0.000000 | -0.000000 | 439 | 366.364877 | 475.58 | 584.795123 | 1.083326 |
| GO:0002699\_positive\_regulation\_of\_immune\_effector\_process | 34 | 0 | 0.000000 | -0.000000 | 439 | 366.364877 | 475.58 | 584.795123 | 1.083326 |
| GO:0007269\_neurotransmitter\_secretion | 34 | 0 | 0.000000 | -0.000000 | 439 | 366.364877 | 475.58 | 584.795123 | 1.083326 |
| GO:0007338\_single\_fertilization | 34 | 0 | 0.000000 | -0.000000 | 439 | 366.364877 | 475.58 | 584.795123 | 1.083326 |
| GO:0007568\_aging | 34 | 0 | 0.000000 | -0.000000 | 439 | 366.364877 | 475.58 | 584.795123 | 1.083326 |
| GO:0010721\_negative\_regulation\_of\_cell\_development | 34 | 0 | 0.000000 | -0.000000 | 439 | 366.364877 | 475.58 | 584.795123 | 1.083326 |
| GO:0016054\_organic\_acid\_catabolic\_process | 34 | 0 | 0.000000 | -0.000000 | 439 | 366.364877 | 475.58 | 584.795123 | 1.083326 |
| GO:0019882\_antigen\_processing\_and\_presentation | 34 | 0 | 0.000000 | -0.000000 | 439 | 366.364877 | 475.58 | 584.795123 | 1.083326 |
| GO:0030509\_BMP\_signaling\_pathway | 34 | 0 | 0.000000 | -0.000000 | 439 | 366.364877 | 475.58 | 584.795123 | 1.083326 |
| GO:0045927\_positive\_regulation\_of\_growth | 34 | 0 | 0.000000 | -0.000000 | 439 | 366.364877 | 475.58 | 584.795123 | 1.083326 |
| GO:0046395\_carboxylic\_acid\_catabolic\_process | 34 | 0 | 0.000000 | -0.000000 | 439 | 366.364877 | 475.58 | 584.795123 | 1.083326 |
| GO:0050730\_regulation\_of\_peptidyl-tyrosine\_phosphorylation | 34 | 0 | 0.000000 | -0.000000 | 439 | 366.364877 | 475.58 | 584.795123 | 1.083326 |
| GO:0051047\_positive\_regulation\_of\_secretion | 34 | 0 | 0.000000 | -0.000000 | 439 | 366.364877 | 475.58 | 584.795123 | 1.083326 |
| GO:0060443\_mammary\_gland\_morphogenesis | 34 | 0 | 0.000000 | -0.000000 | 439 | 366.364877 | 475.58 | 584.795123 | 1.083326 |
| GO:0060711\_labyrinthine\_layer\_development | 34 | 0 | 0.000000 | -0.000000 | 439 | 366.364877 | 475.58 | 584.795123 | 1.083326 |
| GO:0001776\_leukocyte\_homeostasis | 41 | 0 | 0.000000 | -0.000000 | 454 | 384.151571 | 491.81 | 599.468429 | 1.083282 |
| GO:0002429\_immune\_response-activating\_cell\_surface\_receptor\_signaling\_pathway | 41 | 0 | 0.000000 | -0.000000 | 454 | 384.151571 | 491.81 | 599.468429 | 1.083282 |
| GO:0006260\_DNA\_replication | 41 | 0 | 0.000000 | -0.000000 | 454 | 384.151571 | 491.81 | 599.468429 | 1.083282 |
| GO:0006836\_neurotransmitter\_transport | 41 | 0 | 0.000000 | -0.000000 | 454 | 384.151571 | 491.81 | 599.468429 | 1.083282 |
| GO:0006865\_amino\_acid\_transport | 41 | 0 | 0.000000 | -0.000000 | 454 | 384.151571 | 491.81 | 599.468429 | 1.083282 |
| GO:0006979\_response\_to\_oxidative\_stress | 41 | 0 | 0.000000 | -0.000000 | 454 | 384.151571 | 491.81 | 599.468429 | 1.083282 |
| GO:0007254\_JNK\_cascade | 41 | 0 | 0.000000 | -0.000000 | 454 | 384.151571 | 491.81 | 599.468429 | 1.083282 |
| GO:0008585\_female\_gonad\_development | 41 | 0 | 0.000000 | -0.000000 | 454 | 384.151571 | 491.81 | 599.468429 | 1.083282 |
| GO:0009894\_regulation\_of\_catabolic\_process | 41 | 0 | 0.000000 | -0.000000 | 454 | 384.151571 | 491.81 | 599.468429 | 1.083282 |
| GO:0015833\_peptide\_transport | 41 | 0 | 0.000000 | -0.000000 | 454 | 384.151571 | 491.81 | 599.468429 | 1.083282 |
| GO:0015980\_energy\_derivation\_by\_oxidation\_of\_organic\_compounds | 41 | 0 | 0.000000 | -0.000000 | 454 | 384.151571 | 491.81 | 599.468429 | 1.083282 |
| GO:0019216\_regulation\_of\_lipid\_metabolic\_process | 41 | 0 | 0.000000 | -0.000000 | 454 | 384.151571 | 491.81 | 599.468429 | 1.083282 |
| GO:0030817\_regulation\_of\_cAMP\_biosynthetic\_process | 41 | 0 | 0.000000 | -0.000000 | 454 | 384.151571 | 491.81 | 599.468429 | 1.083282 |
| GO:0033077\_T\_cell\_differentiation\_in\_the\_thymus | 41 | 0 | 0.000000 | -0.000000 | 454 | 384.151571 | 491.81 | 599.468429 | 1.083282 |
| GO:0050864\_regulation\_of\_B\_cell\_activation | 41 | 0 | 0.000000 | -0.000000 | 454 | 384.151571 | 491.81 | 599.468429 | 1.083282 |
| GO:0000060\_protein\_import\_into\_nucleus\_\_translocation | 14 | 0 | 0.000000 | -0.000000 | 515 | 446.758044 | 552.58 | 658.401956 | 1.072971 |
| GO:0001502\_cartilage\_condensation | 14 | 0 | 0.000000 | -0.000000 | 515 | 446.758044 | 552.58 | 658.401956 | 1.072971 |
| GO:0001829\_trophectodermal\_cell\_differentiation | 14 | 0 | 0.000000 | -0.000000 | 515 | 446.758044 | 552.58 | 658.401956 | 1.072971 |
| GO:0002027\_regulation\_of\_heart\_rate | 14 | 0 | 0.000000 | -0.000000 | 515 | 446.758044 | 552.58 | 658.401956 | 1.072971 |
| GO:0002262\_myeloid\_cell\_homeostasis | 14 | 0 | 0.000000 | -0.000000 | 515 | 446.758044 | 552.58 | 658.401956 | 1.072971 |
| GO:0002698\_negative\_regulation\_of\_immune\_effector\_process | 14 | 0 | 0.000000 | -0.000000 | 515 | 446.758044 | 552.58 | 658.401956 | 1.072971 |
| GO:0006304\_DNA\_modification | 14 | 0 | 0.000000 | -0.000000 | 515 | 446.758044 | 552.58 | 658.401956 | 1.072971 |
| GO:0006305\_DNA\_alkylation | 14 | 0 | 0.000000 | -0.000000 | 515 | 446.758044 | 552.58 | 658.401956 | 1.072971 |
| GO:0006306\_DNA\_methylation | 14 | 0 | 0.000000 | -0.000000 | 515 | 446.758044 | 552.58 | 658.401956 | 1.072971 |
| GO:0006695\_cholesterol\_biosynthetic\_process | 14 | 0 | 0.000000 | -0.000000 | 515 | 446.758044 | 552.58 | 658.401956 | 1.072971 |
| GO:0006809\_nitric\_oxide\_biosynthetic\_process | 14 | 0 | 0.000000 | -0.000000 | 515 | 446.758044 | 552.58 | 658.401956 | 1.072971 |
| GO:0006914\_autophagy | 14 | 0 | 0.000000 | -0.000000 | 515 | 446.758044 | 552.58 | 658.401956 | 1.072971 |
| GO:0006970\_response\_to\_osmotic\_stress | 14 | 0 | 0.000000 | -0.000000 | 515 | 446.758044 | 552.58 | 658.401956 | 1.072971 |
| GO:0007157\_heterophilic\_cell\_adhesion | 14 | 0 | 0.000000 | -0.000000 | 515 | 446.758044 | 552.58 | 658.401956 | 1.072971 |
| GO:0007530\_sex\_determination | 14 | 0 | 0.000000 | -0.000000 | 515 | 446.758044 | 552.58 | 658.401956 | 1.072971 |
| GO:0007589\_body\_fluid\_secretion | 14 | 0 | 0.000000 | -0.000000 | 515 | 446.758044 | 552.58 | 658.401956 | 1.072971 |
| GO:0008064\_regulation\_of\_actin\_polymerization\_or\_depolymerization | 14 | 0 | 0.000000 | -0.000000 | 515 | 446.758044 | 552.58 | 658.401956 | 1.072971 |
| GO:0008306\_associative\_learning | 14 | 0 | 0.000000 | -0.000000 | 515 | 446.758044 | 552.58 | 658.401956 | 1.072971 |
| GO:0008630\_DNA\_damage\_response\_\_signal\_transduction\_resulting\_in\_induction\_of\_apoptosis | 14 | 0 | 0.000000 | -0.000000 | 515 | 446.758044 | 552.58 | 658.401956 | 1.072971 |
| GO:0009108\_coenzyme\_biosynthetic\_process | 14 | 0 | 0.000000 | -0.000000 | 515 | 446.758044 | 552.58 | 658.401956 | 1.072971 |
| GO:0009267\_cellular\_response\_to\_starvation | 14 | 0 | 0.000000 | -0.000000 | 515 | 446.758044 | 552.58 | 658.401956 | 1.072971 |
| GO:0009895\_negative\_regulation\_of\_catabolic\_process | 14 | 0 | 0.000000 | -0.000000 | 515 | 446.758044 | 552.58 | 658.401956 | 1.072971 |
| GO:0010332\_response\_to\_gamma\_radiation | 14 | 0 | 0.000000 | -0.000000 | 515 | 446.758044 | 552.58 | 658.401956 | 1.072971 |
| GO:0014855\_striated\_muscle\_cell\_proliferation | 14 | 0 | 0.000000 | -0.000000 | 515 | 446.758044 | 552.58 | 658.401956 | 1.072971 |
| GO:0018130\_heterocycle\_biosynthetic\_process | 14 | 0 | 0.000000 | -0.000000 | 515 | 446.758044 | 552.58 | 658.401956 | 1.072971 |
| GO:0019217\_regulation\_of\_fatty\_acid\_metabolic\_process | 14 | 0 | 0.000000 | -0.000000 | 515 | 446.758044 | 552.58 | 658.401956 | 1.072971 |
| GO:0021782\_glial\_cell\_development | 14 | 0 | 0.000000 | -0.000000 | 515 | 446.758044 | 552.58 | 658.401956 | 1.072971 |
| GO:0021904\_dorsal\_ventral\_neural\_tube\_patterning | 14 | 0 | 0.000000 | -0.000000 | 515 | 446.758044 | 552.58 | 658.401956 | 1.072971 |
| GO:0030032\_lamellipodium\_assembly | 14 | 0 | 0.000000 | -0.000000 | 515 | 446.758044 | 552.58 | 658.401956 | 1.072971 |
| GO:0030148\_sphingolipid\_biosynthetic\_process | 14 | 0 | 0.000000 | -0.000000 | 515 | 446.758044 | 552.58 | 658.401956 | 1.072971 |
| GO:0030162\_regulation\_of\_proteolysis | 14 | 0 | 0.000000 | -0.000000 | 515 | 446.758044 | 552.58 | 658.401956 | 1.072971 |
| GO:0030832\_regulation\_of\_actin\_filament\_length | 14 | 0 | 0.000000 | -0.000000 | 515 | 446.758044 | 552.58 | 658.401956 | 1.072971 |
| GO:0031099\_regeneration | 14 | 0 | 0.000000 | -0.000000 | 515 | 446.758044 | 552.58 | 658.401956 | 1.072971 |
| GO:0032271\_regulation\_of\_protein\_polymerization | 14 | 0 | 0.000000 | -0.000000 | 515 | 446.758044 | 552.58 | 658.401956 | 1.072971 |
| GO:0033044\_regulation\_of\_chromosome\_organization | 14 | 0 | 0.000000 | -0.000000 | 515 | 446.758044 | 552.58 | 658.401956 | 1.072971 |
| GO:0034104\_negative\_regulation\_of\_tissue\_remodeling | 14 | 0 | 0.000000 | -0.000000 | 515 | 446.758044 | 552.58 | 658.401956 | 1.072971 |
| GO:0034623\_cellular\_macromolecular\_complex\_disassembly | 14 | 0 | 0.000000 | -0.000000 | 515 | 446.758044 | 552.58 | 658.401956 | 1.072971 |
| GO:0035036\_sperm-egg\_recognition | 14 | 0 | 0.000000 | -0.000000 | 515 | 446.758044 | 552.58 | 658.401956 | 1.072971 |
| GO:0042310\_vasoconstriction | 14 | 0 | 0.000000 | -0.000000 | 515 | 446.758044 | 552.58 | 658.401956 | 1.072971 |
| GO:0042573\_retinoic\_acid\_metabolic\_process | 14 | 0 | 0.000000 | -0.000000 | 515 | 446.758044 | 552.58 | 658.401956 | 1.072971 |
| GO:0043123\_positive\_regulation\_of\_I-kappaB\_kinase\_NF-kappaB\_cascade | 14 | 0 | 0.000000 | -0.000000 | 515 | 446.758044 | 552.58 | 658.401956 | 1.072971 |
| GO:0043254\_regulation\_of\_protein\_complex\_assembly | 14 | 0 | 0.000000 | -0.000000 | 515 | 446.758044 | 552.58 | 658.401956 | 1.072971 |
| GO:0043491\_protein\_kinase\_B\_signaling\_cascade | 14 | 0 | 0.000000 | -0.000000 | 515 | 446.758044 | 552.58 | 658.401956 | 1.072971 |
| GO:0044236\_multicellular\_organismal\_metabolic\_process | 14 | 0 | 0.000000 | -0.000000 | 515 | 446.758044 | 552.58 | 658.401956 | 1.072971 |
| GO:0045061\_thymic\_T\_cell\_selection | 14 | 0 | 0.000000 | -0.000000 | 515 | 446.758044 | 552.58 | 658.401956 | 1.072971 |
| GO:0045453\_bone\_resorption | 14 | 0 | 0.000000 | -0.000000 | 515 | 446.758044 | 552.58 | 658.401956 | 1.072971 |
| GO:0045598\_regulation\_of\_fat\_cell\_differentiation | 14 | 0 | 0.000000 | -0.000000 | 515 | 446.758044 | 552.58 | 658.401956 | 1.072971 |
| GO:0045732\_positive\_regulation\_of\_protein\_catabolic\_process | 14 | 0 | 0.000000 | -0.000000 | 515 | 446.758044 | 552.58 | 658.401956 | 1.072971 |
| GO:0046209\_nitric\_oxide\_metabolic\_process | 14 | 0 | 0.000000 | -0.000000 | 515 | 446.758044 | 552.58 | 658.401956 | 1.072971 |
| GO:0048048\_embryonic\_eye\_morphogenesis | 14 | 0 | 0.000000 | -0.000000 | 515 | 446.758044 | 552.58 | 658.401956 | 1.072971 |
| GO:0048545\_response\_to\_steroid\_hormone\_stimulus | 14 | 0 | 0.000000 | -0.000000 | 515 | 446.758044 | 552.58 | 658.401956 | 1.072971 |
| GO:0048665\_neuron\_fate\_specification | 14 | 0 | 0.000000 | -0.000000 | 515 | 446.758044 | 552.58 | 658.401956 | 1.072971 |
| GO:0048844\_artery\_morphogenesis | 14 | 0 | 0.000000 | -0.000000 | 515 | 446.758044 | 552.58 | 658.401956 | 1.072971 |
| GO:0050810\_regulation\_of\_steroid\_biosynthetic\_process | 14 | 0 | 0.000000 | -0.000000 | 515 | 446.758044 | 552.58 | 658.401956 | 1.072971 |
| GO:0051017\_actin\_filament\_bundle\_formation | 14 | 0 | 0.000000 | -0.000000 | 515 | 446.758044 | 552.58 | 658.401956 | 1.072971 |
| GO:0051053\_negative\_regulation\_of\_DNA\_metabolic\_process | 14 | 0 | 0.000000 | -0.000000 | 515 | 446.758044 | 552.58 | 658.401956 | 1.072971 |
| GO:0051054\_positive\_regulation\_of\_DNA\_metabolic\_process | 14 | 0 | 0.000000 | -0.000000 | 515 | 446.758044 | 552.58 | 658.401956 | 1.072971 |
| GO:0051100\_negative\_regulation\_of\_binding | 14 | 0 | 0.000000 | -0.000000 | 515 | 446.758044 | 552.58 | 658.401956 | 1.072971 |
| GO:0051952\_regulation\_of\_amine\_transport | 14 | 0 | 0.000000 | -0.000000 | 515 | 446.758044 | 552.58 | 658.401956 | 1.072971 |
| GO:0060716\_labyrinthine\_layer\_blood\_vessel\_development | 14 | 0 | 0.000000 | -0.000000 | 515 | 446.758044 | 552.58 | 658.401956 | 1.072971 |
| GO:0060840\_artery\_development | 14 | 0 | 0.000000 | -0.000000 | 515 | 446.758044 | 552.58 | 658.401956 | 1.072971 |
| GO:0000280\_nuclear\_division | 24 | 0 | 0.000000 | -0.000000 | 543 | 473.720808 | 578.19 | 682.659192 | 1.064807 |
| GO:0001541\_ovarian\_follicle\_development | 24 | 0 | 0.000000 | -0.000000 | 543 | 473.720808 | 578.19 | 682.659192 | 1.064807 |
| GO:0002381\_immunoglobulin\_production\_during\_immune\_response | 24 | 0 | 0.000000 | -0.000000 | 543 | 473.720808 | 578.19 | 682.659192 | 1.064807 |
| GO:0006650\_glycerophospholipid\_metabolic\_process | 24 | 0 | 0.000000 | -0.000000 | 543 | 473.720808 | 578.19 | 682.659192 | 1.064807 |
| GO:0006941\_striated\_muscle\_contraction | 24 | 0 | 0.000000 | -0.000000 | 543 | 473.720808 | 578.19 | 682.659192 | 1.064807 |
| GO:0006959\_humoral\_immune\_response | 24 | 0 | 0.000000 | -0.000000 | 543 | 473.720808 | 578.19 | 682.659192 | 1.064807 |
| GO:0007050\_cell\_cycle\_arrest | 24 | 0 | 0.000000 | -0.000000 | 543 | 473.720808 | 578.19 | 682.659192 | 1.064807 |
| GO:0007067\_mitosis | 24 | 0 | 0.000000 | -0.000000 | 543 | 473.720808 | 578.19 | 682.659192 | 1.064807 |
| GO:0007204\_elevation\_of\_cytosolic\_calcium\_ion\_concentration | 24 | 0 | 0.000000 | -0.000000 | 543 | 473.720808 | 578.19 | 682.659192 | 1.064807 |
| GO:0007259\_JAK-STAT\_cascade | 24 | 0 | 0.000000 | -0.000000 | 543 | 473.720808 | 578.19 | 682.659192 | 1.064807 |
| GO:0007266\_Rho\_protein\_signal\_transduction | 24 | 0 | 0.000000 | -0.000000 | 543 | 473.720808 | 578.19 | 682.659192 | 1.064807 |
| GO:0007632\_visual\_behavior | 24 | 0 | 0.000000 | -0.000000 | 543 | 473.720808 | 578.19 | 682.659192 | 1.064807 |
| GO:0008629\_induction\_of\_apoptosis\_by\_intracellular\_signals | 24 | 0 | 0.000000 | -0.000000 | 543 | 473.720808 | 578.19 | 682.659192 | 1.064807 |
| GO:0009612\_response\_to\_mechanical\_stimulus | 24 | 0 | 0.000000 | -0.000000 | 543 | 473.720808 | 578.19 | 682.659192 | 1.064807 |
| GO:0014070\_response\_to\_organic\_cyclic\_substance | 24 | 0 | 0.000000 | -0.000000 | 543 | 473.720808 | 578.19 | 682.659192 | 1.064807 |
| GO:0021515\_cell\_differentiation\_in\_spinal\_cord | 24 | 0 | 0.000000 | -0.000000 | 543 | 473.720808 | 578.19 | 682.659192 | 1.064807 |
| GO:0032386\_regulation\_of\_intracellular\_transport | 24 | 0 | 0.000000 | -0.000000 | 543 | 473.720808 | 578.19 | 682.659192 | 1.064807 |
| GO:0042158\_lipoprotein\_biosynthetic\_process | 24 | 0 | 0.000000 | -0.000000 | 543 | 473.720808 | 578.19 | 682.659192 | 1.064807 |
| GO:0042632\_cholesterol\_homeostasis | 24 | 0 | 0.000000 | -0.000000 | 543 | 473.720808 | 578.19 | 682.659192 | 1.064807 |
| GO:0043410\_positive\_regulation\_of\_MAPKKK\_cascade | 24 | 0 | 0.000000 | -0.000000 | 543 | 473.720808 | 578.19 | 682.659192 | 1.064807 |
| GO:0048002\_antigen\_processing\_and\_presentation\_of\_peptide\_antigen | 24 | 0 | 0.000000 | -0.000000 | 543 | 473.720808 | 578.19 | 682.659192 | 1.064807 |
| GO:0048546\_digestive\_tract\_morphogenesis | 24 | 0 | 0.000000 | -0.000000 | 543 | 473.720808 | 578.19 | 682.659192 | 1.064807 |
| GO:0050679\_positive\_regulation\_of\_epithelial\_cell\_proliferation | 24 | 0 | 0.000000 | -0.000000 | 543 | 473.720808 | 578.19 | 682.659192 | 1.064807 |
| GO:0051099\_positive\_regulation\_of\_binding | 24 | 0 | 0.000000 | -0.000000 | 543 | 473.720808 | 578.19 | 682.659192 | 1.064807 |
| GO:0055092\_sterol\_homeostasis | 24 | 0 | 0.000000 | -0.000000 | 543 | 473.720808 | 578.19 | 682.659192 | 1.064807 |
| GO:0060078\_regulation\_of\_postsynaptic\_membrane\_potential | 24 | 0 | 0.000000 | -0.000000 | 543 | 473.720808 | 578.19 | 682.659192 | 1.064807 |
| GO:0060113\_inner\_ear\_receptor\_cell\_differentiation | 24 | 0 | 0.000000 | -0.000000 | 543 | 473.720808 | 578.19 | 682.659192 | 1.064807 |
| GO:0070667\_negative\_regulation\_of\_mast\_cell\_proliferation | 24 | 0 | 0.000000 | -0.000000 | 543 | 473.720808 | 578.19 | 682.659192 | 1.064807 |
| GO:0001656\_metanephros\_development | 50 | 0 | 0.000000 | -0.000000 | 547 | 480.710441 | 584.55 | 688.389559 | 1.068647 |
| GO:0009190\_cyclic\_nucleotide\_biosynthetic\_process | 50 | 0 | 0.000000 | -0.000000 | 547 | 480.710441 | 584.55 | 688.389559 | 1.068647 |
| GO:0042129\_regulation\_of\_T\_cell\_proliferation | 50 | 0 | 0.000000 | -0.000000 | 547 | 480.710441 | 584.55 | 688.389559 | 1.068647 |
| GO:0051606\_detection\_of\_stimulus | 50 | 0 | 0.000000 | -0.000000 | 547 | 480.710441 | 584.55 | 688.389559 | 1.068647 |
| GO:0006954\_inflammatory\_response | 96 | 0 | 0.000000 | -0.000000 | 552 | 484.557915 | 587.67 | 690.782085 | 1.064620 |
| GO:0048736\_appendage\_development | 96 | 0 | 0.000000 | -0.000000 | 552 | 484.557915 | 587.67 | 690.782085 | 1.064620 |
| GO:0060173\_limb\_development | 96 | 0 | 0.000000 | -0.000000 | 552 | 484.557915 | 587.67 | 690.782085 | 1.064620 |
| GO:0060249\_anatomical\_structure\_homeostasis | 96 | 0 | 0.000000 | -0.000000 | 552 | 484.557915 | 587.67 | 690.782085 | 1.064620 |
| GO:0070661\_leukocyte\_proliferation | 96 | 0 | 0.000000 | -0.000000 | 552 | 484.557915 | 587.67 | 690.782085 | 1.064620 |
| GO:0002764\_immune\_response-regulating\_signal\_transduction | 51 | 0 | 0.000000 | -0.000000 | 558 | 494.588127 | 596.61 | 698.631873 | 1.069194 |
| GO:0006887\_exocytosis | 51 | 0 | 0.000000 | -0.000000 | 558 | 494.588127 | 596.61 | 698.631873 | 1.069194 |
| GO:0007601\_visual\_perception | 51 | 0 | 0.000000 | -0.000000 | 558 | 494.588127 | 596.61 | 698.631873 | 1.069194 |
| GO:0032880\_regulation\_of\_protein\_localization | 51 | 0 | 0.000000 | -0.000000 | 558 | 494.588127 | 596.61 | 698.631873 | 1.069194 |
| GO:0043408\_regulation\_of\_MAPKKK\_cascade | 51 | 0 | 0.000000 | -0.000000 | 558 | 494.588127 | 596.61 | 698.631873 | 1.069194 |
| GO:0048747\_muscle\_fiber\_development | 51 | 0 | 0.000000 | -0.000000 | 558 | 494.588127 | 596.61 | 698.631873 | 1.069194 |
| GO:0000027\_ribosomal\_large\_subunit\_assembly | 1 | 0 |  |  |  |  |  |  |  |  |
| GO:0000042\_protein\_targeting\_to\_Golgi | 1 | 0 |  |  |  |  |  |  |  |  |
| GO:0000046\_autophagic\_vacuole\_fusion | 1 | 0 |  |  |  |  |  |  |  |  |
| GO:0000050\_urea\_cycle | 1 | 0 |  |  |  |  |  |  |  |  |
| GO:0000054\_ribosome\_export\_from\_nucleus | 1 | 0 |  |  |  |  |  |  |  |  |
| GO:0000055\_ribosomal\_large\_subunit\_export\_from\_nucleus | 1 | 0 |  |  |  |  |  |  |  |  |
| GO:0000056\_ribosomal\_small\_subunit\_export\_from\_nucleus | 1 | 0 |  |  |  |  |  |  |  |  |
| GO:0000072\_M\_phase\_specific\_microtubule\_process | 1 | 0 |  |  |  |  |  |  |  |  |
| GO:0000101\_sulfur\_amino\_acid\_transport | 1 | 0 |  |  |  |  |  |  |  |  |
| GO:0000147\_actin\_cortical\_patch\_assembly | 1 | 0 |  |  |  |  |  |  |  |  |
| GO:0000154\_rRNA\_modification | 1 | 0 |  |  |  |  |  |  |  |  |
| GO:0000183\_chromatin\_silencing\_at\_rDNA | 1 | 0 |  |  |  |  |  |  |  |  |
| GO:0000185\_activation\_of\_MAPKKK\_activity | 1 | 0 |  |  |  |  |  |  |  |  |
| GO:0000238\_zygotene | 1 | 0 |  |  |  |  |  |  |  |  |
| GO:0000255\_allantoin\_metabolic\_process | 1 | 0 |  |  |  |  |  |  |  |  |
| GO:0000266\_mitochondrial\_fission | 1 | 0 |  |  |  |  |  |  |  |  |
| GO:0000273\_lipoic\_acid\_metabolic\_process | 1 | 0 |  |  |  |  |  |  |  |  |
| GO:0000301\_retrograde\_transport\_\_vesicle\_recycling\_within\_Golgi | 1 | 0 |  |  |  |  |  |  |  |  |
| GO:0000394\_RNA\_splicing\_\_via\_endonucleolytic\_cleavage\_and\_ligation | 1 | 0 |  |  |  |  |  |  |  |  |
| GO:0000429\_regulation\_of\_transcription\_from\_RNA\_polymerase\_II\_promoter\_by\_carbon\_catabolites | 1 | 0 |  |  |  |  |  |  |  |  |
| GO:0000430\_regulation\_of\_transcription\_from\_RNA\_polymerase\_II\_promoter\_by\_glucose | 1 | 0 |  |  |  |  |  |  |  |  |
| GO:0000432\_positive\_regulation\_of\_transcription\_from\_RNA\_polymerase\_II\_promoter\_by\_glucose | 1 | 0 |  |  |  |  |  |  |  |  |
| GO:0000436\_positive\_regulation\_of\_transcription\_from\_RNA\_polymerase\_II\_promoter\_by\_carbon\_catabolites | 1 | 0 |  |  |  |  |  |  |  |  |
| GO:0000448\_cleavage\_in\_ITS2\_between\_5.8S\_rRNA\_and\_LSU-rRNA\_of\_tricistronic\_rRNA\_transcript\_(SSU-rRNA\_\_5.8S\_rRNA\_\_LSU-rRNA) | 1 | 0 |  |  |  |  |  |  |  |  |
| GO:0000460\_maturation\_of\_5.8S\_rRNA | 1 | 0 |  |  |  |  |  |  |  |  |
| GO:0000463\_maturation\_of\_LSU-rRNA\_from\_tricistronic\_rRNA\_transcript\_(SSU-rRNA\_\_5.8S\_rRNA\_\_LSU-rRNA) | 1 | 0 |  |  |  |  |  |  |  |  |
| GO:0000466\_maturation\_of\_5.8S\_rRNA\_from\_tricistronic\_rRNA\_transcript\_(SSU-rRNA\_\_5.8S\_rRNA\_\_LSU-rRNA) | 1 | 0 |  |  |  |  |  |  |  |  |
| GO:0000469\_cleavages\_during\_rRNA\_processing | 1 | 0 |  |  |  |  |  |  |  |  |
| GO:0000470\_maturation\_of\_LSU-rRNA | 1 | 0 |  |  |  |  |  |  |  |  |
| GO:0000478\_endonucleolytic\_cleavages\_during\_rRNA\_processing | 1 | 0 |  |  |  |  |  |  |  |  |
| GO:0000479\_endonucleolytic\_cleavage\_of\_tricistronic\_rRNA\_transcript\_(SSU-rRNA\_\_5.8S\_rRNA\_\_LSU-rRNA) | 1 | 0 |  |  |  |  |  |  |  |  |
| GO:0000705\_achiasmate\_meiosis\_I | 1 | 0 |  |  |  |  |  |  |  |  |
| GO:0000966\_RNA\_5'-end\_processing | 1 | 0 |  |  |  |  |  |  |  |  |
| GO:0001300\_chronological\_cell\_aging | 1 | 0 |  |  |  |  |  |  |  |  |
| GO:0001547\_antral\_ovarian\_follicle\_growth | 1 | 0 |  |  |  |  |  |  |  |  |
| GO:0001555\_oocyte\_growth | 1 | 0 |  |  |  |  |  |  |  |  |
| GO:0001560\_regulation\_of\_cell\_growth\_by\_extracellular\_stimulus | 1 | 0 |  |  |  |  |  |  |  |  |
| GO:0001660\_fever | 1 | 0 |  |  |  |  |  |  |  |  |
| GO:0001696\_gastric\_acid\_secretion | 1 | 0 |  |  |  |  |  |  |  |  |
| GO:0001712\_ectodermal\_cell\_fate\_commitment | 1 | 0 |  |  |  |  |  |  |  |  |
| GO:0001714\_endodermal\_cell\_fate\_specification | 1 | 0 |  |  |  |  |  |  |  |  |
| GO:0001762\_beta-alanine\_transport | 1 | 0 |  |  |  |  |  |  |  |  |
| GO:0001766\_membrane\_raft\_polarization | 1 | 0 |  |  |  |  |  |  |  |  |
| GO:0001811\_negative\_regulation\_of\_type\_I\_hypersensitivity | 1 | 0 |  |  |  |  |  |  |  |  |
| GO:0001821\_histamine\_secretion | 1 | 0 |  |  |  |  |  |  |  |  |
| GO:0001826\_inner\_cell\_mass\_cell\_differentiation | 1 | 0 |  |  |  |  |  |  |  |  |
| GO:0001830\_trophectodermal\_cell\_fate\_commitment | 1 | 0 |  |  |  |  |  |  |  |  |
| GO:0001834\_trophectodermal\_cell\_proliferation | 1 | 0 |  |  |  |  |  |  |  |  |
| GO:0001867\_complement\_activation\_\_lectin\_pathway | 1 | 0 |  |  |  |  |  |  |  |  |
| GO:0001880\_Mullerian\_duct\_regression | 1 | 0 |  |  |  |  |  |  |  |  |
| GO:0001887\_selenium\_metabolic\_process | 1 | 0 |  |  |  |  |  |  |  |  |
| GO:0001922\_B-1\_B\_cell\_homeostasis | 1 | 0 |  |  |  |  |  |  |  |  |
| GO:0001923\_B-1\_B\_cell\_differentiation | 1 | 0 |  |  |  |  |  |  |  |  |
| GO:0001941\_postsynaptic\_membrane\_organization | 1 | 0 |  |  |  |  |  |  |  |  |
| GO:0001946\_lymphangiogenesis | 1 | 0 |  |  |  |  |  |  |  |  |
| GO:0001956\_positive\_regulation\_of\_neurotransmitter\_secretion | 1 | 0 |  |  |  |  |  |  |  |  |
| GO:0001961\_positive\_regulation\_of\_cytokine-mediated\_signaling\_pathway | 1 | 0 |  |  |  |  |  |  |  |  |
| GO:0001979\_regulation\_of\_systemic\_arterial\_blood\_pressure\_by\_chemoreceptor\_signaling | 1 | 0 |  |  |  |  |  |  |  |  |
| GO:0001980\_regulation\_of\_systemic\_arterial\_blood\_pressure\_by\_ischemic\_conditions | 1 | 0 |  |  |  |  |  |  |  |  |
| GO:0001984\_vasodilation\_of\_artery\_during\_baroreceptor\_response\_to\_increased\_systemic\_arterial\_blood\_pressure | 1 | 0 |  |  |  |  |  |  |  |  |
| GO:0001985\_negative\_regulation\_of\_heart\_rate\_in\_baroreceptor\_response\_to\_increased\_systemic\_arterial\_blood\_pressure | 1 | 0 |  |  |  |  |  |  |  |  |
| GO:0001987\_vasoconstriction\_of\_artery\_involved\_in\_baroreceptor\_response\_to\_lowering\_of\_systemic\_arterial\_blood\_pressure | 1 | 0 |  |  |  |  |  |  |  |  |
| GO:0001988\_positive\_regulation\_of\_heart\_rate\_in\_baroreceptor\_response\_to\_decreased\_systemic\_arterial\_blood\_pressure | 1 | 0 |  |  |  |  |  |  |  |  |
| GO:0001994\_norepinephrine-epinephrine\_vasoconstriction\_involved\_in\_regulation\_of\_systemic\_arterial\_blood\_pressure | 1 | 0 |  |  |  |  |  |  |  |  |
| GO:0002001\_renin\_secretion\_into\_blood\_stream | 1 | 0 |  |  |  |  |  |  |  |  |
| GO:0002002\_regulation\_of\_angiotensin\_levels\_in\_blood | 1 | 0 |  |  |  |  |  |  |  |  |
| GO:0002003\_angiotensin\_maturation | 1 | 0 |  |  |  |  |  |  |  |  |
| GO:0002007\_detection\_of\_hypoxic\_conditions\_in\_blood\_by\_chemoreceptor\_signaling | 1 | 0 |  |  |  |  |  |  |  |  |
| GO:0002017\_regulation\_of\_blood\_volume\_by\_renal\_aldosterone | 1 | 0 |  |  |  |  |  |  |  |  |
| GO:0002023\_reduction\_of\_food\_intake\_in\_response\_to\_dietary\_excess | 1 | 0 |  |  |  |  |  |  |  |  |
| GO:0002031\_G-protein\_coupled\_receptor\_internalization | 1 | 0 |  |  |  |  |  |  |  |  |
| GO:0002036\_regulation\_of\_L-glutamate\_transport | 1 | 0 |  |  |  |  |  |  |  |  |
| GO:0002040\_sprouting\_angiogenesis | 1 | 0 |  |  |  |  |  |  |  |  |
| GO:0002041\_intussusceptive\_angiogenesis | 1 | 0 |  |  |  |  |  |  |  |  |
| GO:0002068\_glandular\_epithelial\_cell\_development | 1 | 0 |  |  |  |  |  |  |  |  |
| GO:0002069\_columnar\_cuboidal\_epithelial\_cell\_maturation | 1 | 0 |  |  |  |  |  |  |  |  |
| GO:0002071\_glandular\_epithelial\_cell\_maturation | 1 | 0 |  |  |  |  |  |  |  |  |
| GO:0002082\_regulation\_of\_oxidative\_phosphorylation | 1 | 0 |  |  |  |  |  |  |  |  |
| GO:0002084\_protein\_depalmitoylation | 1 | 0 |  |  |  |  |  |  |  |  |
| GO:0002085\_inhibition\_of\_neuroepithelial\_cell\_differentiation | 1 | 0 |  |  |  |  |  |  |  |  |
| GO:0002086\_diaphragm\_contraction | 1 | 0 |  |  |  |  |  |  |  |  |
| GO:0002118\_aggressive\_behavior | 1 | 0 |  |  |  |  |  |  |  |  |
| GO:0002121\_inter-male\_aggressive\_behavior | 1 | 0 |  |  |  |  |  |  |  |  |
| GO:0002124\_territorial\_aggressive\_behavior | 1 | 0 |  |  |  |  |  |  |  |  |
| GO:0002227\_innate\_immune\_response\_in\_mucosa | 1 | 0 |  |  |  |  |  |  |  |  |
| GO:0002232\_leukocyte\_chemotaxis\_during\_inflammatory\_response | 1 | 0 |  |  |  |  |  |  |  |  |
| GO:0002248\_connective\_tissue\_replacement\_during\_inflammatory\_response | 1 | 0 |  |  |  |  |  |  |  |  |
| GO:0002282\_microglial\_cell\_activation\_during\_immune\_response | 1 | 0 |  |  |  |  |  |  |  |  |
| GO:0002287\_alpha-beta\_T\_cell\_activation\_during\_immune\_response | 1 | 0 |  |  |  |  |  |  |  |  |
| GO:0002314\_germinal\_center\_B\_cell\_differentiation | 1 | 0 |  |  |  |  |  |  |  |  |
| GO:0002315\_marginal\_zone\_B\_cell\_differentiation | 1 | 0 |  |  |  |  |  |  |  |  |
| GO:0002316\_follicular\_B\_cell\_differentiation | 1 | 0 |  |  |  |  |  |  |  |  |
| GO:0002317\_plasma\_cell\_differentiation | 1 | 0 |  |  |  |  |  |  |  |  |
| GO:0002349\_histamine\_production\_during\_acute\_inflammatory\_response | 1 | 0 |  |  |  |  |  |  |  |  |
| GO:0002351\_serotonin\_production\_during\_acute\_inflammatory\_response | 1 | 0 |  |  |  |  |  |  |  |  |
| GO:0002355\_detection\_of\_tumor\_cell | 1 | 0 |  |  |  |  |  |  |  |  |
| GO:0002370\_natural\_killer\_cell\_cytokine\_production | 1 | 0 |  |  |  |  |  |  |  |  |
| GO:0002371\_dendritic\_cell\_cytokine\_production | 1 | 0 |  |  |  |  |  |  |  |  |
| GO:0002380\_immunoglobulin\_secretion\_during\_immune\_response | 1 | 0 |  |  |  |  |  |  |  |  |
| GO:0002396\_MHC\_protein\_complex\_assembly | 1 | 0 |  |  |  |  |  |  |  |  |
| GO:0002397\_MHC\_class\_I\_protein\_complex\_assembly | 1 | 0 |  |  |  |  |  |  |  |  |
| GO:0002420\_natural\_killer\_cell\_mediated\_cytotoxicity\_directed\_against\_tumor\_cell\_target | 1 | 0 |  |  |  |  |  |  |  |  |
| GO:0002423\_natural\_killer\_cell\_mediated\_immune\_response\_to\_tumor\_cell | 1 | 0 |  |  |  |  |  |  |  |  |
| GO:0002424\_T\_cell\_mediated\_immune\_response\_to\_tumor\_cell | 1 | 0 |  |  |  |  |  |  |  |  |
| GO:0002426\_immunoglobulin\_production\_in\_mucosal\_tissue | 1 | 0 |  |  |  |  |  |  |  |  |
| GO:0002431\_Fc\_receptor\_mediated\_stimulatory\_signaling\_pathway | 1 | 0 |  |  |  |  |  |  |  |  |
| GO:0002432\_granuloma\_formation | 1 | 0 |  |  |  |  |  |  |  |  |
| GO:0002441\_histamine\_secretion\_during\_acute\_inflammatory\_response | 1 | 0 |  |  |  |  |  |  |  |  |
| GO:0002442\_serotonin\_secretion\_during\_acute\_inflammatory\_response | 1 | 0 |  |  |  |  |  |  |  |  |
| GO:0002457\_T\_cell\_antigen\_processing\_and\_presentation | 1 | 0 |  |  |  |  |  |  |  |  |
| GO:0002458\_peripheral\_T\_cell\_tolerance\_induction | 1 | 0 |  |  |  |  |  |  |  |  |
| GO:0002461\_tolerance\_induction\_dependent\_upon\_immune\_response | 1 | 0 |  |  |  |  |  |  |  |  |
| GO:0002465\_peripheral\_tolerance\_induction | 1 | 0 |  |  |  |  |  |  |  |  |
| GO:0002468\_dendritic\_cell\_antigen\_processing\_and\_presentation | 1 | 0 |  |  |  |  |  |  |  |  |
| GO:0002476\_antigen\_processing\_and\_presentation\_of\_endogenous\_peptide\_antigen\_via\_MHC\_class\_Ib | 1 | 0 |  |  |  |  |  |  |  |  |
| GO:0002479\_antigen\_processing\_and\_presentation\_of\_exogenous\_peptide\_antigen\_via\_MHC\_class\_I\_\_TAP-dependent | 1 | 0 |  |  |  |  |  |  |  |  |
| GO:0002483\_antigen\_processing\_and\_presentation\_of\_endogenous\_peptide\_antigen | 1 | 0 |  |  |  |  |  |  |  |  |
| GO:0002501\_peptide\_antigen\_assembly\_with\_MHC\_protein\_complex | 1 | 0 |  |  |  |  |  |  |  |  |
| GO:0002502\_peptide\_antigen\_assembly\_with\_MHC\_class\_I\_protein\_complex | 1 | 0 |  |  |  |  |  |  |  |  |
| GO:0002508\_central\_tolerance\_induction | 1 | 0 |  |  |  |  |  |  |  |  |
| GO:0002510\_central\_B\_cell\_tolerance\_induction | 1 | 0 |  |  |  |  |  |  |  |  |
| GO:0002545\_chronic\_inflammatory\_response\_to\_non-antigenic\_stimulus | 1 | 0 |  |  |  |  |  |  |  |  |
| GO:0002553\_histamine\_secretion\_by\_mast\_cell | 1 | 0 |  |  |  |  |  |  |  |  |
| GO:0002554\_serotonin\_secretion\_by\_platelet | 1 | 0 |  |  |  |  |  |  |  |  |
| GO:0002572\_pro-T\_cell\_differentiation | 1 | 0 |  |  |  |  |  |  |  |  |
| GO:0002577\_regulation\_of\_antigen\_processing\_and\_presentation | 1 | 0 |  |  |  |  |  |  |  |  |
| GO:0002579\_positive\_regulation\_of\_antigen\_processing\_and\_presentation | 1 | 0 |  |  |  |  |  |  |  |  |
| GO:0002604\_regulation\_of\_dendritic\_cell\_antigen\_processing\_and\_presentation | 1 | 0 |  |  |  |  |  |  |  |  |
| GO:0002606\_positive\_regulation\_of\_dendritic\_cell\_antigen\_processing\_and\_presentation | 1 | 0 |  |  |  |  |  |  |  |  |
| GO:0002635\_negative\_regulation\_of\_germinal\_center\_formation | 1 | 0 |  |  |  |  |  |  |  |  |
| GO:0002646\_regulation\_of\_central\_tolerance\_induction | 1 | 0 |  |  |  |  |  |  |  |  |
| GO:0002648\_positive\_regulation\_of\_central\_tolerance\_induction | 1 | 0 |  |  |  |  |  |  |  |  |
| GO:0002649\_regulation\_of\_tolerance\_induction\_to\_self\_antigen | 1 | 0 |  |  |  |  |  |  |  |  |
| GO:0002651\_positive\_regulation\_of\_tolerance\_induction\_to\_self\_antigen | 1 | 0 |  |  |  |  |  |  |  |  |
| GO:0002652\_regulation\_of\_tolerance\_induction\_dependent\_upon\_immune\_response | 1 | 0 |  |  |  |  |  |  |  |  |
| GO:0002654\_positive\_regulation\_of\_tolerance\_induction\_dependent\_upon\_immune\_response | 1 | 0 |  |  |  |  |  |  |  |  |
| GO:0002658\_regulation\_of\_peripheral\_tolerance\_induction | 1 | 0 |  |  |  |  |  |  |  |  |
| GO:0002660\_positive\_regulation\_of\_peripheral\_tolerance\_induction | 1 | 0 |  |  |  |  |  |  |  |  |
| GO:0002677\_negative\_regulation\_of\_chronic\_inflammatory\_response | 1 | 0 |  |  |  |  |  |  |  |  |
| GO:0002678\_positive\_regulation\_of\_chronic\_inflammatory\_response | 1 | 0 |  |  |  |  |  |  |  |  |
| GO:0002701\_negative\_regulation\_of\_production\_of\_molecular\_mediator\_of\_immune\_response | 1 | 0 |  |  |  |  |  |  |  |  |
| GO:0002719\_negative\_regulation\_of\_cytokine\_production\_during\_immune\_response | 1 | 0 |  |  |  |  |  |  |  |  |
| GO:0002724\_regulation\_of\_T\_cell\_cytokine\_production | 1 | 0 |  |  |  |  |  |  |  |  |
| GO:0002727\_regulation\_of\_natural\_killer\_cell\_cytokine\_production | 1 | 0 |  |  |  |  |  |  |  |  |
| GO:0002729\_positive\_regulation\_of\_natural\_killer\_cell\_cytokine\_production | 1 | 0 |  |  |  |  |  |  |  |  |
| GO:0002730\_regulation\_of\_dendritic\_cell\_cytokine\_production | 1 | 0 |  |  |  |  |  |  |  |  |
| GO:0002756\_MyD88-independent\_toll-like\_receptor\_signaling\_pathway | 1 | 0 |  |  |  |  |  |  |  |  |
| GO:0002767\_immune\_response-inhibiting\_cell\_surface\_receptor\_signaling\_pathway | 1 | 0 |  |  |  |  |  |  |  |  |
| GO:0002769\_natural\_killer\_cell\_inhibitory\_signaling\_pathway | 1 | 0 |  |  |  |  |  |  |  |  |
| GO:0002840\_regulation\_of\_T\_cell\_mediated\_immune\_response\_to\_tumor\_cell | 1 | 0 |  |  |  |  |  |  |  |  |
| GO:0002842\_positive\_regulation\_of\_T\_cell\_mediated\_immune\_response\_to\_tumor\_cell | 1 | 0 |  |  |  |  |  |  |  |  |
| GO:0002849\_regulation\_of\_peripheral\_T\_cell\_tolerance\_induction | 1 | 0 |  |  |  |  |  |  |  |  |
| GO:0002851\_positive\_regulation\_of\_peripheral\_T\_cell\_tolerance\_induction | 1 | 0 |  |  |  |  |  |  |  |  |
| GO:0002855\_regulation\_of\_natural\_killer\_cell\_mediated\_immune\_response\_to\_tumor\_cell | 1 | 0 |  |  |  |  |  |  |  |  |
| GO:0002857\_positive\_regulation\_of\_natural\_killer\_cell\_mediated\_immune\_response\_to\_tumor\_cell | 1 | 0 |  |  |  |  |  |  |  |  |
| GO:0002858\_regulation\_of\_natural\_killer\_cell\_mediated\_cytotoxicity\_directed\_against\_tumor\_cell\_target | 1 | 0 |  |  |  |  |  |  |  |  |
| GO:0002860\_positive\_regulation\_of\_natural\_killer\_cell\_mediated\_cytotoxicity\_directed\_against\_tumor\_cell\_target | 1 | 0 |  |  |  |  |  |  |  |  |
| GO:0002880\_regulation\_of\_chronic\_inflammatory\_response\_to\_non-antigenic\_stimulus | 1 | 0 |  |  |  |  |  |  |  |  |
| GO:0002882\_positive\_regulation\_of\_chronic\_inflammatory\_response\_to\_non-antigenic\_stimulus | 1 | 0 |  |  |  |  |  |  |  |  |
| GO:0002895\_regulation\_of\_central\_B\_cell\_tolerance\_induction | 1 | 0 |  |  |  |  |  |  |  |  |
| GO:0002897\_positive\_regulation\_of\_central\_B\_cell\_tolerance\_induction | 1 | 0 |  |  |  |  |  |  |  |  |
| GO:0002901\_mature\_B\_cell\_apoptosis | 1 | 0 |  |  |  |  |  |  |  |  |
| GO:0002903\_negative\_regulation\_of\_B\_cell\_apoptosis | 1 | 0 |  |  |  |  |  |  |  |  |
| GO:0002905\_regulation\_of\_mature\_B\_cell\_apoptosis | 1 | 0 |  |  |  |  |  |  |  |  |
| GO:0002906\_negative\_regulation\_of\_mature\_B\_cell\_apoptosis | 1 | 0 |  |  |  |  |  |  |  |  |
| GO:0003011\_involuntary\_skeletal\_muscle\_contraction | 1 | 0 |  |  |  |  |  |  |  |  |
| GO:0003027\_regulation\_of\_systemic\_arterial\_blood\_pressure\_by\_carotid\_body\_chemoreceptor\_signaling | 1 | 0 |  |  |  |  |  |  |  |  |
| GO:0003029\_detection\_of\_hypoxic\_conditions\_in\_blood\_by\_carotid\_body\_chemoreceptor\_signaling | 1 | 0 |  |  |  |  |  |  |  |  |
| GO:0003032\_detection\_of\_oxygen | 1 | 0 |  |  |  |  |  |  |  |  |
| GO:0003056\_regulation\_of\_vascular\_smooth\_muscle\_contraction | 1 | 0 |  |  |  |  |  |  |  |  |
| GO:0003062\_regulation\_of\_heart\_rate\_by\_chemical\_signal | 1 | 0 |  |  |  |  |  |  |  |  |
| GO:0003065\_positive\_regulation\_of\_heart\_rate\_by\_epinephrine | 1 | 0 |  |  |  |  |  |  |  |  |
| GO:0003068\_regulation\_of\_systemic\_arterial\_blood\_pressure\_by\_acetylcholine | 1 | 0 |  |  |  |  |  |  |  |  |
| GO:0003069\_vasodilation\_by\_acetylcholine\_involved\_in\_regulation\_of\_systemic\_arterial\_blood\_pressure | 1 | 0 |  |  |  |  |  |  |  |  |
| GO:0003070\_regulation\_of\_systemic\_arterial\_blood\_pressure\_by\_neurotransmitter | 1 | 0 |  |  |  |  |  |  |  |  |
| GO:0003097\_renal\_water\_transport | 1 | 0 |  |  |  |  |  |  |  |  |
| GO:0005979\_regulation\_of\_glycogen\_biosynthetic\_process | 1 | 0 |  |  |  |  |  |  |  |  |
| GO:0005984\_disaccharide\_metabolic\_process | 1 | 0 |  |  |  |  |  |  |  |  |
| GO:0005988\_lactose\_metabolic\_process | 1 | 0 |  |  |  |  |  |  |  |  |
| GO:0005989\_lactose\_biosynthetic\_process | 1 | 0 |  |  |  |  |  |  |  |  |
| GO:0005997\_xylulose\_metabolic\_process | 1 | 0 |  |  |  |  |  |  |  |  |
| GO:0006000\_fructose\_metabolic\_process | 1 | 0 |  |  |  |  |  |  |  |  |
| GO:0006002\_fructose\_6-phosphate\_metabolic\_process | 1 | 0 |  |  |  |  |  |  |  |  |
| GO:0006004\_fucose\_metabolic\_process | 1 | 0 |  |  |  |  |  |  |  |  |
| GO:0006013\_mannose\_metabolic\_process | 1 | 0 |  |  |  |  |  |  |  |  |
| GO:0006060\_sorbitol\_metabolic\_process | 1 | 0 |  |  |  |  |  |  |  |  |
| GO:0006064\_glucuronate\_catabolic\_process | 1 | 0 |  |  |  |  |  |  |  |  |
| GO:0006086\_acetyl-CoA\_biosynthetic\_process\_from\_pyruvate | 1 | 0 |  |  |  |  |  |  |  |  |
| GO:0006098\_pentose-phosphate\_shunt | 1 | 0 |  |  |  |  |  |  |  |  |
| GO:0006101\_citrate\_metabolic\_process | 1 | 0 |  |  |  |  |  |  |  |  |
| GO:0006104\_succinyl-CoA\_metabolic\_process | 1 | 0 |  |  |  |  |  |  |  |  |
| GO:0006116\_NADH\_oxidation | 1 | 0 |  |  |  |  |  |  |  |  |
| GO:0006120\_mitochondrial\_electron\_transport\_\_NADH\_to\_ubiquinone | 1 | 0 |  |  |  |  |  |  |  |  |
| GO:0006154\_adenosine\_catabolic\_process | 1 | 0 |  |  |  |  |  |  |  |  |
| GO:0006157\_deoxyadenosine\_catabolic\_process | 1 | 0 |  |  |  |  |  |  |  |  |
| GO:0006167\_AMP\_biosynthetic\_process | 1 | 0 |  |  |  |  |  |  |  |  |
| GO:0006175\_dATP\_biosynthetic\_process | 1 | 0 |  |  |  |  |  |  |  |  |
| GO:0006178\_guanine\_salvage | 1 | 0 |  |  |  |  |  |  |  |  |
| GO:0006196\_AMP\_catabolic\_process | 1 | 0 |  |  |  |  |  |  |  |  |
| GO:0006203\_dGTP\_catabolic\_process | 1 | 0 |  |  |  |  |  |  |  |  |
| GO:0006208\_pyrimidine\_base\_catabolic\_process | 1 | 0 |  |  |  |  |  |  |  |  |
| GO:0006221\_pyrimidine\_nucleotide\_biosynthetic\_process | 1 | 0 |  |  |  |  |  |  |  |  |
| GO:0006235\_dTTP\_biosynthetic\_process | 1 | 0 |  |  |  |  |  |  |  |  |
| GO:0006244\_pyrimidine\_nucleotide\_catabolic\_process | 1 | 0 |  |  |  |  |  |  |  |  |
| GO:0006269\_DNA\_replication\_\_synthesis\_of\_RNA\_primer | 1 | 0 |  |  |  |  |  |  |  |  |
| GO:0006283\_transcription-coupled\_nucleotide-excision\_repair | 1 | 0 |  |  |  |  |  |  |  |  |
| GO:0006296\_nucleotide-excision\_repair\_\_DNA\_incision\_\_5'-to\_lesion | 1 | 0 |  |  |  |  |  |  |  |  |
| GO:0006307\_DNA\_dealkylation | 1 | 0 |  |  |  |  |  |  |  |  |
| GO:0006337\_nucleosome\_disassembly | 1 | 0 |  |  |  |  |  |  |  |  |
| GO:0006344\_maintenance\_of\_chromatin\_silencing | 1 | 0 |  |  |  |  |  |  |  |  |
| GO:0006356\_regulation\_of\_transcription\_from\_RNA\_polymerase\_I\_promoter | 1 | 0 |  |  |  |  |  |  |  |  |
| GO:0006388\_tRNA\_splicing\_\_via\_endonucleolytic\_cleavage\_and\_ligation | 1 | 0 |  |  |  |  |  |  |  |  |
| GO:0006407\_rRNA\_export\_from\_nucleus | 1 | 0 |  |  |  |  |  |  |  |  |
| GO:0006434\_seryl-tRNA\_aminoacylation | 1 | 0 |  |  |  |  |  |  |  |  |
| GO:0006447\_regulation\_of\_translational\_initiation\_by\_iron | 1 | 0 |  |  |  |  |  |  |  |  |
| GO:0006463\_steroid\_hormone\_receptor\_complex\_assembly | 1 | 0 |  |  |  |  |  |  |  |  |
| GO:0006467\_protein\_thiol-disulfide\_exchange | 1 | 0 |  |  |  |  |  |  |  |  |
| GO:0006474\_N-terminal\_protein\_amino\_acid\_acetylation | 1 | 0 |  |  |  |  |  |  |  |  |
| GO:0006481\_C-terminal\_protein\_amino\_acid\_methylation | 1 | 0 |  |  |  |  |  |  |  |  |
| GO:0006488\_dolichol-linked\_oligosaccharide\_biosynthetic\_process | 1 | 0 |  |  |  |  |  |  |  |  |
| GO:0006494\_protein\_amino\_acid\_terminal\_glycosylation | 1 | 0 |  |  |  |  |  |  |  |  |
| GO:0006496\_protein\_amino\_acid\_terminal\_N-glycosylation | 1 | 0 |  |  |  |  |  |  |  |  |
| GO:0006500\_N-terminal\_protein\_palmitoylation | 1 | 0 |  |  |  |  |  |  |  |  |
| GO:0006507\_GPI\_anchor\_release | 1 | 0 |  |  |  |  |  |  |  |  |
| GO:0006537\_glutamate\_biosynthetic\_process | 1 | 0 |  |  |  |  |  |  |  |  |
| GO:0006544\_glycine\_metabolic\_process | 1 | 0 |  |  |  |  |  |  |  |  |
| GO:0006549\_isoleucine\_metabolic\_process | 1 | 0 |  |  |  |  |  |  |  |  |
| GO:0006553\_lysine\_metabolic\_process | 1 | 0 |  |  |  |  |  |  |  |  |
| GO:0006554\_lysine\_catabolic\_process | 1 | 0 |  |  |  |  |  |  |  |  |
| GO:0006556\_S-adenosylmethionine\_biosynthetic\_process | 1 | 0 |  |  |  |  |  |  |  |  |
| GO:0006559\_L-phenylalanine\_catabolic\_process | 1 | 0 |  |  |  |  |  |  |  |  |
| GO:0006569\_tryptophan\_catabolic\_process | 1 | 0 |  |  |  |  |  |  |  |  |
| GO:0006572\_tyrosine\_catabolic\_process | 1 | 0 |  |  |  |  |  |  |  |  |
| GO:0006573\_valine\_metabolic\_process | 1 | 0 |  |  |  |  |  |  |  |  |
| GO:0006581\_acetylcholine\_catabolic\_process | 1 | 0 |  |  |  |  |  |  |  |  |
| GO:0006585\_dopamine\_biosynthetic\_process\_from\_tyrosine | 1 | 0 |  |  |  |  |  |  |  |  |
| GO:0006590\_thyroid\_hormone\_generation | 1 | 0 |  |  |  |  |  |  |  |  |
| GO:0006591\_ornithine\_metabolic\_process | 1 | 0 |  |  |  |  |  |  |  |  |
| GO:0006596\_polyamine\_biosynthetic\_process | 1 | 0 |  |  |  |  |  |  |  |  |
| GO:0006597\_spermine\_biosynthetic\_process | 1 | 0 |  |  |  |  |  |  |  |  |
| GO:0006601\_creatine\_biosynthetic\_process | 1 | 0 |  |  |  |  |  |  |  |  |
| GO:0006613\_cotranslational\_protein\_targeting\_to\_membrane | 1 | 0 |  |  |  |  |  |  |  |  |
| GO:0006622\_protein\_targeting\_to\_lysosome | 1 | 0 |  |  |  |  |  |  |  |  |
| GO:0006627\_mitochondrial\_protein\_processing\_during\_import | 1 | 0 |  |  |  |  |  |  |  |  |
| GO:0006653\_lecithin\_metabolic\_process | 1 | 0 |  |  |  |  |  |  |  |  |
| GO:0006654\_phosphatidic\_acid\_biosynthetic\_process | 1 | 0 |  |  |  |  |  |  |  |  |
| GO:0006658\_phosphatidylserine\_metabolic\_process | 1 | 0 |  |  |  |  |  |  |  |  |
| GO:0006659\_phosphatidylserine\_biosynthetic\_process | 1 | 0 |  |  |  |  |  |  |  |  |
| GO:0006667\_sphinganine\_metabolic\_process | 1 | 0 |  |  |  |  |  |  |  |  |
| GO:0006668\_sphinganine-1-phosphate\_metabolic\_process | 1 | 0 |  |  |  |  |  |  |  |  |
| GO:0006678\_glucosylceramide\_metabolic\_process | 1 | 0 |  |  |  |  |  |  |  |  |
| GO:0006682\_galactosylceramide\_biosynthetic\_process | 1 | 0 |  |  |  |  |  |  |  |  |
| GO:0006685\_sphingomyelin\_catabolic\_process | 1 | 0 |  |  |  |  |  |  |  |  |
| GO:0006700\_C21-steroid\_hormone\_biosynthetic\_process | 1 | 0 |  |  |  |  |  |  |  |  |
| GO:0006705\_mineralocorticoid\_biosynthetic\_process | 1 | 0 |  |  |  |  |  |  |  |  |
| GO:0006709\_progesterone\_catabolic\_process | 1 | 0 |  |  |  |  |  |  |  |  |
| GO:0006729\_tetrahydrobiopterin\_biosynthetic\_process | 1 | 0 |  |  |  |  |  |  |  |  |
| GO:0006734\_NADH\_metabolic\_process | 1 | 0 |  |  |  |  |  |  |  |  |
| GO:0006740\_NADPH\_regeneration | 1 | 0 |  |  |  |  |  |  |  |  |
| GO:0006741\_NADP\_biosynthetic\_process | 1 | 0 |  |  |  |  |  |  |  |  |
| GO:0006743\_ubiquinone\_metabolic\_process | 1 | 0 |  |  |  |  |  |  |  |  |
| GO:0006744\_ubiquinone\_biosynthetic\_process | 1 | 0 |  |  |  |  |  |  |  |  |
| GO:0006772\_thiamin\_metabolic\_process | 1 | 0 |  |  |  |  |  |  |  |  |
| GO:0006784\_heme\_a\_biosynthetic\_process | 1 | 0 |  |  |  |  |  |  |  |  |
| GO:0006797\_polyphosphate\_metabolic\_process | 1 | 0 |  |  |  |  |  |  |  |  |
| GO:0006798\_polyphosphate\_catabolic\_process | 1 | 0 |  |  |  |  |  |  |  |  |
| GO:0006824\_cobalt\_ion\_transport | 1 | 0 |  |  |  |  |  |  |  |  |
| GO:0006842\_tricarboxylic\_acid\_transport | 1 | 0 |  |  |  |  |  |  |  |  |
| GO:0006844\_acyl\_carnitine\_transport | 1 | 0 |  |  |  |  |  |  |  |  |
| GO:0006855\_multidrug\_transport | 1 | 0 |  |  |  |  |  |  |  |  |
| GO:0006863\_purine\_transport | 1 | 0 |  |  |  |  |  |  |  |  |
| GO:0006890\_retrograde\_vesicle-mediated\_transport\_\_Golgi\_to\_ER | 1 | 0 |  |  |  |  |  |  |  |  |
| GO:0006891\_intra-Golgi\_vesicle-mediated\_transport | 1 | 0 |  |  |  |  |  |  |  |  |
| GO:0006893\_Golgi\_to\_plasma\_membrane\_transport | 1 | 0 |  |  |  |  |  |  |  |  |
| GO:0006895\_Golgi\_to\_endosome\_transport | 1 | 0 |  |  |  |  |  |  |  |  |
| GO:0006896\_Golgi\_to\_vacuole\_transport | 1 | 0 |  |  |  |  |  |  |  |  |
| GO:0006900\_membrane\_budding | 1 | 0 |  |  |  |  |  |  |  |  |
| GO:0006930\_substrate-bound\_cell\_migration\_\_cell\_extension | 1 | 0 |  |  |  |  |  |  |  |  |
| GO:0006931\_substrate-bound\_cell\_migration\_\_cell\_attachment\_to\_substrate | 1 | 0 |  |  |  |  |  |  |  |  |
| GO:0006933\_negative\_regulation\_of\_cell\_adhesion\_involved\_in\_substrate-bound\_cell\_migration | 1 | 0 |  |  |  |  |  |  |  |  |
| GO:0006957\_complement\_activation\_\_alternative\_pathway | 1 | 0 |  |  |  |  |  |  |  |  |
| GO:0006958\_complement\_activation\_\_classical\_pathway | 1 | 0 |  |  |  |  |  |  |  |  |
| GO:0006978\_DNA\_damage\_response\_\_signal\_transduction\_by\_p53\_class\_mediator\_resulting\_in\_transcription\_of\_p21\_class\_mediator | 1 | 0 |  |  |  |  |  |  |  |  |
| GO:0007016\_cytoskeletal\_anchoring\_at\_plasma\_membrane | 1 | 0 |  |  |  |  |  |  |  |  |
| GO:0007021\_tubulin\_complex\_assembly | 1 | 0 |  |  |  |  |  |  |  |  |
| GO:0007052\_mitotic\_spindle\_organization | 1 | 0 |  |  |  |  |  |  |  |  |
| GO:0007056\_spindle\_assembly\_involved\_in\_female\_meiosis | 1 | 0 |  |  |  |  |  |  |  |  |
| GO:0007057\_spindle\_assembly\_involved\_in\_female\_meiosis\_I | 1 | 0 |  |  |  |  |  |  |  |  |
| GO:0007063\_regulation\_of\_sister\_chromatid\_cohesion | 1 | 0 |  |  |  |  |  |  |  |  |
| GO:0007065\_male\_meiosis\_sister\_chromatid\_cohesion | 1 | 0 |  |  |  |  |  |  |  |  |
| GO:0007076\_mitotic\_chromosome\_condensation | 1 | 0 |  |  |  |  |  |  |  |  |
| GO:0007095\_mitotic\_cell\_cycle\_G2\_M\_transition\_DNA\_damage\_checkpoint | 1 | 0 |  |  |  |  |  |  |  |  |
| GO:0007096\_regulation\_of\_exit\_from\_mitosis | 1 | 0 |  |  |  |  |  |  |  |  |
| GO:0007158\_neuron\_adhesion | 1 | 0 |  |  |  |  |  |  |  |  |
| GO:0007168\_receptor\_guanylyl\_cyclase\_signaling\_pathway | 1 | 0 |  |  |  |  |  |  |  |  |
| GO:0007197\_inhibition\_of\_adenylate\_cyclase\_activity\_by\_muscarinic\_acetylcholine\_receptor\_signaling\_pathway | 1 | 0 |  |  |  |  |  |  |  |  |
| GO:0007207\_activation\_of\_phospholipase\_C\_activity\_by\_muscarinic\_acetylcholine\_receptor\_signaling\_pathway | 1 | 0 |  |  |  |  |  |  |  |  |
| GO:0007208\_activation\_of\_phospholipase\_C\_activity\_by\_serotonin\_receptor\_signaling\_pathway | 1 | 0 |  |  |  |  |  |  |  |  |
| GO:0007217\_tachykinin\_receptor\_signaling\_pathway | 1 | 0 |  |  |  |  |  |  |  |  |
| GO:0007221\_positive\_regulation\_of\_transcription\_of\_Notch\_receptor\_target | 1 | 0 |  |  |  |  |  |  |  |  |
| GO:0007223\_Wnt\_receptor\_signaling\_pathway\_\_calcium\_modulating\_pathway | 1 | 0 |  |  |  |  |  |  |  |  |
| GO:0007225\_patched\_ligand\_processing | 1 | 0 |  |  |  |  |  |  |  |  |
| GO:0007227\_signal\_transduction\_downstream\_of\_smoothened | 1 | 0 |  |  |  |  |  |  |  |  |
| GO:0007228\_positive\_regulation\_of\_hh\_target\_transcription\_factor\_activity | 1 | 0 |  |  |  |  |  |  |  |  |
| GO:0007231\_osmosensory\_signaling\_pathway | 1 | 0 |  |  |  |  |  |  |  |  |
| GO:0007284\_spermatogonial\_cell\_division | 1 | 0 |  |  |  |  |  |  |  |  |
| GO:0007290\_spermatid\_nucleus\_elongation | 1 | 0 |  |  |  |  |  |  |  |  |
| GO:0007296\_vitellogenesis | 1 | 0 |  |  |  |  |  |  |  |  |
| GO:0007321\_sperm\_displacement | 1 | 0 |  |  |  |  |  |  |  |  |
| GO:0007380\_specification\_of\_segmental\_identity\_\_head | 1 | 0 |  |  |  |  |  |  |  |  |
| GO:0007382\_specification\_of\_segmental\_identity\_\_maxillary\_segment | 1 | 0 |  |  |  |  |  |  |  |  |
| GO:0007400\_neuroblast\_fate\_determination | 1 | 0 |  |  |  |  |  |  |  |  |
| GO:0007402\_ganglion\_mother\_cell\_fate\_determination | 1 | 0 |  |  |  |  |  |  |  |  |
| GO:0007495\_visceral\_mesoderm-endoderm\_interaction\_involved\_in\_midgut\_development | 1 | 0 |  |  |  |  |  |  |  |  |
| GO:0007497\_posterior\_midgut\_development | 1 | 0 |  |  |  |  |  |  |  |  |
| GO:0007499\_ectoderm\_and\_mesoderm\_interaction | 1 | 0 |  |  |  |  |  |  |  |  |
| GO:0007500\_mesodermal\_cell\_fate\_determination | 1 | 0 |  |  |  |  |  |  |  |  |
| GO:0007509\_mesoderm\_migration | 1 | 0 |  |  |  |  |  |  |  |  |
| GO:0007518\_myoblast\_cell\_fate\_determination | 1 | 0 |  |  |  |  |  |  |  |  |
| GO:0007521\_muscle\_cell\_fate\_determination | 1 | 0 |  |  |  |  |  |  |  |  |
| GO:0007522\_visceral\_muscle\_development | 1 | 0 |  |  |  |  |  |  |  |  |
| GO:0007529\_establishment\_of\_synaptic\_specificity\_at\_neuromuscular\_junction | 1 | 0 |  |  |  |  |  |  |  |  |
| GO:0007538\_primary\_sex\_determination | 1 | 0 |  |  |  |  |  |  |  |  |
| GO:0007542\_primary\_sex\_determination\_\_germ-line | 1 | 0 |  |  |  |  |  |  |  |  |
| GO:0007567\_parturition | 1 | 0 |  |  |  |  |  |  |  |  |
| GO:0007614\_short-term\_memory | 1 | 0 |  |  |  |  |  |  |  |  |
| GO:0007621\_negative\_regulation\_of\_female\_receptivity | 1 | 0 |  |  |  |  |  |  |  |  |
| GO:0008049\_male\_courtship\_behavior | 1 | 0 |  |  |  |  |  |  |  |  |
| GO:0008050\_female\_courtship\_behavior | 1 | 0 |  |  |  |  |  |  |  |  |
| GO:0008052\_sensory\_organ\_boundary\_specification | 1 | 0 |  |  |  |  |  |  |  |  |
| GO:0008054\_cyclin\_catabolic\_process | 1 | 0 |  |  |  |  |  |  |  |  |
| GO:0008057\_eye\_pigment\_granule\_organization | 1 | 0 |  |  |  |  |  |  |  |  |
| GO:0008078\_mesodermal\_cell\_migration | 1 | 0 |  |  |  |  |  |  |  |  |
| GO:0008208\_C21-steroid\_hormone\_catabolic\_process | 1 | 0 |  |  |  |  |  |  |  |  |
| GO:0008216\_spermidine\_metabolic\_process | 1 | 0 |  |  |  |  |  |  |  |  |
| GO:0008292\_acetylcholine\_biosynthetic\_process | 1 | 0 |  |  |  |  |  |  |  |  |
| GO:0008295\_spermidine\_biosynthetic\_process | 1 | 0 |  |  |  |  |  |  |  |  |
| GO:0008300\_isoprenoid\_catabolic\_process | 1 | 0 |  |  |  |  |  |  |  |  |
| GO:0008333\_endosome\_to\_lysosome\_transport | 1 | 0 |  |  |  |  |  |  |  |  |
| GO:0008355\_olfactory\_learning | 1 | 0 |  |  |  |  |  |  |  |  |
| GO:0008611\_ether\_lipid\_biosynthetic\_process | 1 | 0 |  |  |  |  |  |  |  |  |
| GO:0008626\_induction\_of\_apoptosis\_by\_granzyme | 1 | 0 |  |  |  |  |  |  |  |  |
| GO:0008633\_activation\_of\_pro-apoptotic\_gene\_products | 1 | 0 |  |  |  |  |  |  |  |  |
| GO:0008653\_lipopolysaccharide\_metabolic\_process | 1 | 0 |  |  |  |  |  |  |  |  |
| GO:0009068\_aspartate\_family\_amino\_acid\_catabolic\_process | 1 | 0 |  |  |  |  |  |  |  |  |
| GO:0009084\_glutamine\_family\_amino\_acid\_biosynthetic\_process | 1 | 0 |  |  |  |  |  |  |  |  |
| GO:0009088\_threonine\_biosynthetic\_process | 1 | 0 |  |  |  |  |  |  |  |  |
| GO:0009105\_lipoic\_acid\_biosynthetic\_process | 1 | 0 |  |  |  |  |  |  |  |  |
| GO:0009109\_coenzyme\_catabolic\_process | 1 | 0 |  |  |  |  |  |  |  |  |
| GO:0009111\_vitamin\_catabolic\_process | 1 | 0 |  |  |  |  |  |  |  |  |
| GO:0009113\_purine\_base\_biosynthetic\_process | 1 | 0 |  |  |  |  |  |  |  |  |
| GO:0009127\_purine\_nucleoside\_monophosphate\_biosynthetic\_process | 1 | 0 |  |  |  |  |  |  |  |  |
| GO:0009128\_purine\_nucleoside\_monophosphate\_catabolic\_process | 1 | 0 |  |  |  |  |  |  |  |  |
| GO:0009129\_pyrimidine\_nucleoside\_monophosphate\_metabolic\_process | 1 | 0 |  |  |  |  |  |  |  |  |
| GO:0009131\_pyrimidine\_nucleoside\_monophosphate\_catabolic\_process | 1 | 0 |  |  |  |  |  |  |  |  |
| GO:0009133\_nucleoside\_diphosphate\_biosynthetic\_process | 1 | 0 |  |  |  |  |  |  |  |  |
| GO:0009145\_purine\_nucleoside\_triphosphate\_biosynthetic\_process | 1 | 0 |  |  |  |  |  |  |  |  |
| GO:0009147\_pyrimidine\_nucleoside\_triphosphate\_metabolic\_process | 1 | 0 |  |  |  |  |  |  |  |  |
| GO:0009148\_pyrimidine\_nucleoside\_triphosphate\_biosynthetic\_process | 1 | 0 |  |  |  |  |  |  |  |  |
| GO:0009152\_purine\_ribonucleotide\_biosynthetic\_process | 1 | 0 |  |  |  |  |  |  |  |  |
| GO:0009153\_purine\_deoxyribonucleotide\_biosynthetic\_process | 1 | 0 |  |  |  |  |  |  |  |  |
| GO:0009156\_ribonucleoside\_monophosphate\_biosynthetic\_process | 1 | 0 |  |  |  |  |  |  |  |  |
| GO:0009158\_ribonucleoside\_monophosphate\_catabolic\_process | 1 | 0 |  |  |  |  |  |  |  |  |
| GO:0009159\_deoxyribonucleoside\_monophosphate\_catabolic\_process | 1 | 0 |  |  |  |  |  |  |  |  |
| GO:0009162\_deoxyribonucleoside\_monophosphate\_metabolic\_process | 1 | 0 |  |  |  |  |  |  |  |  |
| GO:0009168\_purine\_ribonucleoside\_monophosphate\_biosynthetic\_process | 1 | 0 |  |  |  |  |  |  |  |  |
| GO:0009169\_purine\_ribonucleoside\_monophosphate\_catabolic\_process | 1 | 0 |  |  |  |  |  |  |  |  |
| GO:0009176\_pyrimidine\_deoxyribonucleoside\_monophosphate\_metabolic\_process | 1 | 0 |  |  |  |  |  |  |  |  |
| GO:0009178\_pyrimidine\_deoxyribonucleoside\_monophosphate\_catabolic\_process | 1 | 0 |  |  |  |  |  |  |  |  |
| GO:0009211\_pyrimidine\_deoxyribonucleoside\_triphosphate\_metabolic\_process | 1 | 0 |  |  |  |  |  |  |  |  |
| GO:0009212\_pyrimidine\_deoxyribonucleoside\_triphosphate\_biosynthetic\_process | 1 | 0 |  |  |  |  |  |  |  |  |
| GO:0009216\_purine\_deoxyribonucleoside\_triphosphate\_biosynthetic\_process | 1 | 0 |  |  |  |  |  |  |  |  |
| GO:0009221\_pyrimidine\_deoxyribonucleotide\_biosynthetic\_process | 1 | 0 |  |  |  |  |  |  |  |  |
| GO:0009223\_pyrimidine\_deoxyribonucleotide\_catabolic\_process | 1 | 0 |  |  |  |  |  |  |  |  |
| GO:0009260\_ribonucleotide\_biosynthetic\_process | 1 | 0 |  |  |  |  |  |  |  |  |
| GO:0009405\_pathogenesis | 1 | 0 |  |  |  |  |  |  |  |  |
| GO:0009414\_response\_to\_water\_deprivation | 1 | 0 |  |  |  |  |  |  |  |  |
| GO:0009415\_response\_to\_water | 1 | 0 |  |  |  |  |  |  |  |  |
| GO:0009449\_gamma-aminobutyric\_acid\_biosynthetic\_process | 1 | 0 |  |  |  |  |  |  |  |  |
| GO:0009450\_gamma-aminobutyric\_acid\_catabolic\_process | 1 | 0 |  |  |  |  |  |  |  |  |
| GO:0009589\_detection\_of\_UV | 1 | 0 |  |  |  |  |  |  |  |  |
| GO:0009590\_detection\_of\_gravity | 1 | 0 |  |  |  |  |  |  |  |  |
| GO:0009624\_response\_to\_nematode | 1 | 0 |  |  |  |  |  |  |  |  |
| GO:0009629\_response\_to\_gravity | 1 | 0 |  |  |  |  |  |  |  |  |
| GO:0009648\_photoperiodism | 1 | 0 |  |  |  |  |  |  |  |  |
| GO:0009690\_cytokinin\_metabolic\_process | 1 | 0 |  |  |  |  |  |  |  |  |
| GO:0009691\_cytokinin\_biosynthetic\_process | 1 | 0 |  |  |  |  |  |  |  |  |
| GO:0009786\_regulation\_of\_asymmetric\_cell\_division | 1 | 0 |  |  |  |  |  |  |  |  |
| GO:0009794\_regulation\_of\_mitotic\_cell\_cycle\_\_embryonic | 1 | 0 |  |  |  |  |  |  |  |  |
| GO:0009956\_radial\_pattern\_formation | 1 | 0 |  |  |  |  |  |  |  |  |
| GO:0009957\_epidermal\_cell\_fate\_specification | 1 | 0 |  |  |  |  |  |  |  |  |
| GO:0009992\_cellular\_water\_homeostasis | 1 | 0 |  |  |  |  |  |  |  |  |
| GO:0010032\_meiotic\_chromosome\_condensation | 1 | 0 |  |  |  |  |  |  |  |  |
| GO:0010039\_response\_to\_iron\_ion | 1 | 0 |  |  |  |  |  |  |  |  |
| GO:0010042\_response\_to\_manganese\_ion | 1 | 0 |  |  |  |  |  |  |  |  |
| GO:0010045\_response\_to\_nickel\_ion | 1 | 0 |  |  |  |  |  |  |  |  |
| GO:0010046\_response\_to\_mycotoxin | 1 | 0 |  |  |  |  |  |  |  |  |
| GO:0010107\_potassium\_ion\_import | 1 | 0 |  |  |  |  |  |  |  |  |
| GO:0010155\_regulation\_of\_proton\_transport | 1 | 0 |  |  |  |  |  |  |  |  |
| GO:0010160\_formation\_of\_organ\_boundary | 1 | 0 |  |  |  |  |  |  |  |  |
| GO:0010260\_organ\_senescence | 1 | 0 |  |  |  |  |  |  |  |  |
| GO:0010310\_regulation\_of\_hydrogen\_peroxide\_metabolic\_process | 1 | 0 |  |  |  |  |  |  |  |  |
| GO:0010447\_response\_to\_acidity | 1 | 0 |  |  |  |  |  |  |  |  |
| GO:0010452\_histone\_H3-K36\_methylation | 1 | 0 |  |  |  |  |  |  |  |  |
| GO:0010455\_positive\_regulation\_of\_cell\_fate\_commitment | 1 | 0 |  |  |  |  |  |  |  |  |
| GO:0010470\_regulation\_of\_gastrulation | 1 | 0 |  |  |  |  |  |  |  |  |
| GO:0010508\_positive\_regulation\_of\_autophagy | 1 | 0 |  |  |  |  |  |  |  |  |
| GO:0010519\_negative\_regulation\_of\_phospholipase\_activity | 1 | 0 |  |  |  |  |  |  |  |  |
| GO:0010520\_regulation\_of\_reciprocal\_meiotic\_recombination | 1 | 0 |  |  |  |  |  |  |  |  |
| GO:0010523\_negative\_regulation\_of\_calcium\_ion\_transport\_into\_cytosol | 1 | 0 |  |  |  |  |  |  |  |  |
| GO:0010543\_regulation\_of\_platelet\_activation | 1 | 0 |  |  |  |  |  |  |  |  |
| GO:0010561\_negative\_regulation\_of\_glycoprotein\_biosynthetic\_process | 1 | 0 |  |  |  |  |  |  |  |  |
| GO:0010569\_regulation\_of\_double-strand\_break\_repair\_via\_homologous\_recombination | 1 | 0 |  |  |  |  |  |  |  |  |
| GO:0010572\_positive\_regulation\_of\_platelet\_activation | 1 | 0 |  |  |  |  |  |  |  |  |
| GO:0010594\_regulation\_of\_endothelial\_cell\_migration | 1 | 0 |  |  |  |  |  |  |  |  |
| GO:0010596\_negative\_regulation\_of\_endothelial\_cell\_migration | 1 | 0 |  |  |  |  |  |  |  |  |
| GO:0010611\_regulation\_of\_cardiac\_muscle\_hypertrophy | 1 | 0 |  |  |  |  |  |  |  |  |
| GO:0010612\_regulation\_of\_cardiac\_muscle\_adaptation | 1 | 0 |  |  |  |  |  |  |  |  |
| GO:0010614\_negative\_regulation\_of\_cardiac\_muscle\_hypertrophy | 1 | 0 |  |  |  |  |  |  |  |  |
| GO:0010616\_negative\_regulation\_of\_cardiac\_muscle\_adaptation | 1 | 0 |  |  |  |  |  |  |  |  |
| GO:0010634\_positive\_regulation\_of\_epithelial\_cell\_migration | 1 | 0 |  |  |  |  |  |  |  |  |
| GO:0010656\_negative\_regulation\_of\_muscle\_cell\_apoptosis | 1 | 0 |  |  |  |  |  |  |  |  |
| GO:0010657\_muscle\_cell\_apoptosis | 1 | 0 |  |  |  |  |  |  |  |  |
| GO:0010658\_striated\_muscle\_cell\_apoptosis | 1 | 0 |  |  |  |  |  |  |  |  |
| GO:0010659\_cardiac\_muscle\_cell\_apoptosis | 1 | 0 |  |  |  |  |  |  |  |  |
| GO:0010660\_regulation\_of\_muscle\_cell\_apoptosis | 1 | 0 |  |  |  |  |  |  |  |  |
| GO:0010662\_regulation\_of\_striated\_muscle\_cell\_apoptosis | 1 | 0 |  |  |  |  |  |  |  |  |
| GO:0010664\_negative\_regulation\_of\_striated\_muscle\_cell\_apoptosis | 1 | 0 |  |  |  |  |  |  |  |  |
| GO:0010665\_regulation\_of\_cardiac\_muscle\_cell\_apoptosis | 1 | 0 |  |  |  |  |  |  |  |  |
| GO:0010667\_negative\_regulation\_of\_cardiac\_muscle\_cell\_apoptosis | 1 | 0 |  |  |  |  |  |  |  |  |
| GO:0010668\_ectodermal\_cell\_differentiation | 1 | 0 |  |  |  |  |  |  |  |  |
| GO:0010671\_negative\_regulation\_of\_oxygen\_and\_reactive\_oxygen\_species\_metabolic\_process | 1 | 0 |  |  |  |  |  |  |  |  |
| GO:0010719\_negative\_regulation\_of\_epithelial\_to\_mesenchymal\_transition | 1 | 0 |  |  |  |  |  |  |  |  |
| GO:0010735\_positive\_regulation\_of\_transcription\_via\_serum\_response\_element\_binding | 1 | 0 |  |  |  |  |  |  |  |  |
| GO:0010825\_positive\_regulation\_of\_centrosome\_duplication | 1 | 0 |  |  |  |  |  |  |  |  |
| GO:0010845\_positive\_regulation\_of\_reciprocal\_meiotic\_recombination | 1 | 0 |  |  |  |  |  |  |  |  |
| GO:0010850\_chemoreceptor\_signaling\_pathway\_involved\_in\_regulation\_of\_blood\_pressure | 1 | 0 |  |  |  |  |  |  |  |  |
| GO:0010873\_positive\_regulation\_of\_cholesterol\_esterification | 1 | 0 |  |  |  |  |  |  |  |  |
| GO:0010880\_regulation\_of\_release\_of\_sequestered\_calcium\_ion\_into\_cytosol\_by\_sarcoplasmic\_reticulum | 1 | 0 |  |  |  |  |  |  |  |  |
| GO:0010881\_regulation\_of\_cardiac\_muscle\_contraction\_by\_regulation\_of\_the\_release\_of\_sequestered\_calcium\_ion | 1 | 0 |  |  |  |  |  |  |  |  |
| GO:0010882\_regulation\_of\_cardiac\_muscle\_contraction\_by\_calcium\_ion\_signaling | 1 | 0 |  |  |  |  |  |  |  |  |
| GO:0010890\_positive\_regulation\_of\_sequestering\_of\_triglyceride | 1 | 0 |  |  |  |  |  |  |  |  |
| GO:0010919\_regulation\_of\_inositol\_phosphate\_biosynthetic\_process | 1 | 0 |  |  |  |  |  |  |  |  |
| GO:0010931\_macrophage\_tolerance\_induction | 1 | 0 |  |  |  |  |  |  |  |  |
| GO:0010932\_regulation\_of\_macrophage\_tolerance\_induction | 1 | 0 |  |  |  |  |  |  |  |  |
| GO:0010933\_positive\_regulation\_of\_macrophage\_tolerance\_induction | 1 | 0 |  |  |  |  |  |  |  |  |
| GO:0010934\_macrophage\_cytokine\_production | 1 | 0 |  |  |  |  |  |  |  |  |
| GO:0010935\_regulation\_of\_macrophage\_cytokine\_production | 1 | 0 |  |  |  |  |  |  |  |  |
| GO:0010936\_negative\_regulation\_of\_macrophage\_cytokine\_production | 1 | 0 |  |  |  |  |  |  |  |  |
| GO:0010953\_regulation\_of\_protein\_maturation\_by\_peptide\_bond\_cleavage | 1 | 0 |  |  |  |  |  |  |  |  |
| GO:0010962\_regulation\_of\_glucan\_biosynthetic\_process | 1 | 0 |  |  |  |  |  |  |  |  |
| GO:0010966\_regulation\_of\_phosphate\_transport | 1 | 0 |  |  |  |  |  |  |  |  |
| GO:0014012\_axon\_regeneration\_in\_the\_peripheral\_nervous\_system | 1 | 0 |  |  |  |  |  |  |  |  |
| GO:0014016\_neuroblast\_differentiation | 1 | 0 |  |  |  |  |  |  |  |  |
| GO:0014017\_neuroblast\_fate\_commitment | 1 | 0 |  |  |  |  |  |  |  |  |
| GO:0014041\_regulation\_of\_neuron\_maturation | 1 | 0 |  |  |  |  |  |  |  |  |
| GO:0014042\_positive\_regulation\_of\_neuron\_maturation | 1 | 0 |  |  |  |  |  |  |  |  |
| GO:0014049\_positive\_regulation\_of\_glutamate\_secretion | 1 | 0 |  |  |  |  |  |  |  |  |
| GO:0014061\_regulation\_of\_norepinephrine\_secretion | 1 | 0 |  |  |  |  |  |  |  |  |
| GO:0014071\_response\_to\_cycloalkane | 1 | 0 |  |  |  |  |  |  |  |  |
| GO:0014707\_branchiomeric\_skeletal\_muscle\_development | 1 | 0 |  |  |  |  |  |  |  |  |
| GO:0014738\_regulation\_of\_muscle\_hyperplasia | 1 | 0 |  |  |  |  |  |  |  |  |
| GO:0014740\_negative\_regulation\_of\_muscle\_hyperplasia | 1 | 0 |  |  |  |  |  |  |  |  |
| GO:0014741\_negative\_regulation\_of\_muscle\_hypertrophy | 1 | 0 |  |  |  |  |  |  |  |  |
| GO:0014743\_regulation\_of\_muscle\_hypertrophy | 1 | 0 |  |  |  |  |  |  |  |  |
| GO:0014805\_smooth\_muscle\_adaptation | 1 | 0 |  |  |  |  |  |  |  |  |
| GO:0014806\_smooth\_muscle\_hyperplasia | 1 | 0 |  |  |  |  |  |  |  |  |
| GO:0014807\_regulation\_of\_somitogenesis | 1 | 0 |  |  |  |  |  |  |  |  |
| GO:0014808\_release\_of\_sequestered\_calcium\_ion\_into\_cytosol\_by\_sarcoplasmic\_reticulum | 1 | 0 |  |  |  |  |  |  |  |  |
| GO:0014813\_satellite\_cell\_commitment | 1 | 0 |  |  |  |  |  |  |  |  |
| GO:0014816\_satellite\_cell\_differentiation | 1 | 0 |  |  |  |  |  |  |  |  |
| GO:0014819\_regulation\_of\_skeletal\_muscle\_contraction | 1 | 0 |  |  |  |  |  |  |  |  |
| GO:0014852\_regulation\_of\_skeletal\_muscle\_contraction\_by\_neural\_stimulation\_via\_neuromuscular\_junction | 1 | 0 |  |  |  |  |  |  |  |  |
| GO:0014853\_regulation\_of\_excitatory\_postsynaptic\_membrane\_potential\_involved\_in\_skeletal\_muscle\_contraction | 1 | 0 |  |  |  |  |  |  |  |  |
| GO:0014856\_skeletal\_muscle\_cell\_proliferation | 1 | 0 |  |  |  |  |  |  |  |  |
| GO:0014857\_regulation\_of\_skeletal\_muscle\_cell\_proliferation | 1 | 0 |  |  |  |  |  |  |  |  |
| GO:0014858\_positive\_regulation\_of\_skeletal\_muscle\_cell\_proliferation | 1 | 0 |  |  |  |  |  |  |  |  |
| GO:0014887\_cardiac\_muscle\_adaptation | 1 | 0 |  |  |  |  |  |  |  |  |
| GO:0014889\_muscle\_atrophy | 1 | 0 |  |  |  |  |  |  |  |  |
| GO:0014896\_muscle\_hypertrophy | 1 | 0 |  |  |  |  |  |  |  |  |
| GO:0014897\_striated\_muscle\_hypertrophy | 1 | 0 |  |  |  |  |  |  |  |  |
| GO:0014898\_cardiac\_muscle\_hypertrophy | 1 | 0 |  |  |  |  |  |  |  |  |
| GO:0014900\_muscle\_hyperplasia | 1 | 0 |  |  |  |  |  |  |  |  |
| GO:0014910\_regulation\_of\_smooth\_muscle\_cell\_migration | 1 | 0 |  |  |  |  |  |  |  |  |
| GO:0014911\_positive\_regulation\_of\_smooth\_muscle\_cell\_migration | 1 | 0 |  |  |  |  |  |  |  |  |
| GO:0015014\_heparan\_sulfate\_proteoglycan\_biosynthetic\_process\_\_polysaccharide\_chain\_biosynthetic\_process | 1 | 0 |  |  |  |  |  |  |  |  |
| GO:0015074\_DNA\_integration | 1 | 0 |  |  |  |  |  |  |  |  |
| GO:0015670\_carbon\_dioxide\_transport | 1 | 0 |  |  |  |  |  |  |  |  |
| GO:0015677\_copper\_ion\_import | 1 | 0 |  |  |  |  |  |  |  |  |
| GO:0015680\_intracellular\_copper\_ion\_transport | 1 | 0 |  |  |  |  |  |  |  |  |
| GO:0015684\_ferrous\_iron\_transport | 1 | 0 |  |  |  |  |  |  |  |  |
| GO:0015707\_nitrite\_transport | 1 | 0 |  |  |  |  |  |  |  |  |
| GO:0015724\_formate\_transport | 1 | 0 |  |  |  |  |  |  |  |  |
| GO:0015734\_taurine\_transport | 1 | 0 |  |  |  |  |  |  |  |  |
| GO:0015740\_C4-dicarboxylate\_transport | 1 | 0 |  |  |  |  |  |  |  |  |
| GO:0015744\_succinate\_transport | 1 | 0 |  |  |  |  |  |  |  |  |
| GO:0015746\_citrate\_transport | 1 | 0 |  |  |  |  |  |  |  |  |
| GO:0015747\_urate\_transport | 1 | 0 |  |  |  |  |  |  |  |  |
| GO:0015791\_polyol\_transport | 1 | 0 |  |  |  |  |  |  |  |  |
| GO:0015798\_myo-inositol\_transport | 1 | 0 |  |  |  |  |  |  |  |  |
| GO:0015808\_L-alanine\_transport | 1 | 0 |  |  |  |  |  |  |  |  |
| GO:0015810\_aspartate\_transport | 1 | 0 |  |  |  |  |  |  |  |  |
| GO:0015811\_L-cystine\_transport | 1 | 0 |  |  |  |  |  |  |  |  |
| GO:0015817\_histidine\_transport | 1 | 0 |  |  |  |  |  |  |  |  |
| GO:0015822\_ornithine\_transport | 1 | 0 |  |  |  |  |  |  |  |  |
| GO:0015824\_proline\_transport | 1 | 0 |  |  |  |  |  |  |  |  |
| GO:0015851\_nucleobase\_transport | 1 | 0 |  |  |  |  |  |  |  |  |
| GO:0015864\_pyrimidine\_nucleoside\_transport | 1 | 0 |  |  |  |  |  |  |  |  |
| GO:0015874\_norepinephrine\_transport | 1 | 0 |  |  |  |  |  |  |  |  |
| GO:0015881\_creatine\_transport | 1 | 0 |  |  |  |  |  |  |  |  |
| GO:0015884\_folic\_acid\_transport | 1 | 0 |  |  |  |  |  |  |  |  |
| GO:0015886\_heme\_transport | 1 | 0 |  |  |  |  |  |  |  |  |
| GO:0015888\_thiamin\_transport | 1 | 0 |  |  |  |  |  |  |  |  |
| GO:0015938\_coenzyme\_A\_catabolic\_process | 1 | 0 |  |  |  |  |  |  |  |  |
| GO:0015939\_pantothenate\_metabolic\_process | 1 | 0 |  |  |  |  |  |  |  |  |
| GO:0016073\_snRNA\_metabolic\_process | 1 | 0 |  |  |  |  |  |  |  |  |
| GO:0016074\_snoRNA\_metabolic\_process | 1 | 0 |  |  |  |  |  |  |  |  |
| GO:0016082\_synaptic\_vesicle\_priming | 1 | 0 |  |  |  |  |  |  |  |  |
| GO:0016090\_prenol\_metabolic\_process | 1 | 0 |  |  |  |  |  |  |  |  |
| GO:0016093\_polyprenol\_metabolic\_process | 1 | 0 |  |  |  |  |  |  |  |  |
| GO:0016180\_snRNA\_processing | 1 | 0 |  |  |  |  |  |  |  |  |
| GO:0016239\_positive\_regulation\_of\_macroautophagy | 1 | 0 |  |  |  |  |  |  |  |  |
| GO:0016246\_RNA\_interference | 1 | 0 |  |  |  |  |  |  |  |  |
| GO:0016255\_attachment\_of\_GPI\_anchor\_to\_protein | 1 | 0 |  |  |  |  |  |  |  |  |
| GO:0016333\_morphogenesis\_of\_follicular\_epithelium | 1 | 0 |  |  |  |  |  |  |  |  |
| GO:0016340\_calcium-dependent\_cell-matrix\_adhesion | 1 | 0 |  |  |  |  |  |  |  |  |
| GO:0016344\_meiotic\_chromosome\_movement\_towards\_spindle\_pole | 1 | 0 |  |  |  |  |  |  |  |  |
| GO:0016482\_cytoplasmic\_transport | 1 | 0 |  |  |  |  |  |  |  |  |
| GO:0016553\_base\_conversion\_or\_substitution\_editing | 1 | 0 |  |  |  |  |  |  |  |  |
| GO:0016554\_cytidine\_to\_uridine\_editing | 1 | 0 |  |  |  |  |  |  |  |  |
| GO:0016560\_protein\_import\_into\_peroxisome\_matrix\_\_docking | 1 | 0 |  |  |  |  |  |  |  |  |
| GO:0016578\_histone\_deubiquitination | 1 | 0 |  |  |  |  |  |  |  |  |
| GO:0016598\_protein\_arginylation | 1 | 0 |  |  |  |  |  |  |  |  |
| GO:0017004\_cytochrome\_complex\_assembly | 1 | 0 |  |  |  |  |  |  |  |  |
| GO:0018022\_peptidyl-lysine\_methylation | 1 | 0 |  |  |  |  |  |  |  |  |
| GO:0018023\_peptidyl-lysine\_trimethylation | 1 | 0 |  |  |  |  |  |  |  |  |
| GO:0018120\_peptidyl-arginine\_ADP-ribosylation | 1 | 0 |  |  |  |  |  |  |  |  |
| GO:0018126\_protein\_amino\_acid\_hydroxylation | 1 | 0 |  |  |  |  |  |  |  |  |
| GO:0018146\_keratan\_sulfate\_biosynthetic\_process | 1 | 0 |  |  |  |  |  |  |  |  |
| GO:0018158\_protein\_amino\_acid\_oxidation | 1 | 0 |  |  |  |  |  |  |  |  |
| GO:0018195\_peptidyl-arginine\_modification | 1 | 0 |  |  |  |  |  |  |  |  |
| GO:0018197\_peptidyl-aspartic\_acid\_modification | 1 | 0 |  |  |  |  |  |  |  |  |
| GO:0018282\_metal\_incorporation\_into\_metallo-sulfur\_cluster | 1 | 0 |  |  |  |  |  |  |  |  |
| GO:0018283\_iron\_incorporation\_into\_metallo-sulfur\_cluster | 1 | 0 |  |  |  |  |  |  |  |  |
| GO:0018318\_protein\_amino\_acid\_palmitoylation | 1 | 0 |  |  |  |  |  |  |  |  |
| GO:0018342\_protein\_prenylation | 1 | 0 |  |  |  |  |  |  |  |  |
| GO:0018344\_protein\_geranylgeranylation | 1 | 0 |  |  |  |  |  |  |  |  |
| GO:0018410\_peptide\_or\_protein\_carboxyl-terminal\_blocking | 1 | 0 |  |  |  |  |  |  |  |  |
| GO:0018916\_nitrobenzene\_metabolic\_process | 1 | 0 |  |  |  |  |  |  |  |  |
| GO:0018931\_naphthalene\_metabolic\_process | 1 | 0 |  |  |  |  |  |  |  |  |
| GO:0018992\_germ-line\_sex\_determination | 1 | 0 |  |  |  |  |  |  |  |  |
| GO:0019042\_latent\_virus\_infection | 1 | 0 |  |  |  |  |  |  |  |  |
| GO:0019046\_reactivation\_of\_latent\_virus | 1 | 0 |  |  |  |  |  |  |  |  |
| GO:0019047\_provirus\_integration | 1 | 0 |  |  |  |  |  |  |  |  |
| GO:0019076\_release\_of\_virus\_from\_host | 1 | 0 |  |  |  |  |  |  |  |  |
| GO:0019079\_viral\_genome\_replication | 1 | 0 |  |  |  |  |  |  |  |  |
| GO:0019100\_male\_germ-line\_sex\_determination | 1 | 0 |  |  |  |  |  |  |  |  |
| GO:0019101\_female\_somatic\_sex\_determination | 1 | 0 |  |  |  |  |  |  |  |  |
| GO:0019102\_male\_somatic\_sex\_determination | 1 | 0 |  |  |  |  |  |  |  |  |
| GO:0019255\_glucose\_1-phosphate\_metabolic\_process | 1 | 0 |  |  |  |  |  |  |  |  |
| GO:0019276\_UDP-N-acetylgalactosamine\_metabolic\_process | 1 | 0 |  |  |  |  |  |  |  |  |
| GO:0019344\_cysteine\_biosynthetic\_process | 1 | 0 |  |  |  |  |  |  |  |  |
| GO:0019348\_dolichol\_metabolic\_process | 1 | 0 |  |  |  |  |  |  |  |  |
| GO:0019375\_galactolipid\_biosynthetic\_process | 1 | 0 |  |  |  |  |  |  |  |  |
| GO:0019402\_galactitol\_metabolic\_process | 1 | 0 |  |  |  |  |  |  |  |  |
| GO:0019441\_tryptophan\_catabolic\_process\_to\_kynurenine | 1 | 0 |  |  |  |  |  |  |  |  |
| GO:0019477\_L-lysine\_catabolic\_process | 1 | 0 |  |  |  |  |  |  |  |  |
| GO:0019510\_S-adenosylhomocysteine\_catabolic\_process | 1 | 0 |  |  |  |  |  |  |  |  |
| GO:0019532\_oxalate\_transport | 1 | 0 |  |  |  |  |  |  |  |  |
| GO:0019626\_short-chain\_fatty\_acid\_catabolic\_process | 1 | 0 |  |  |  |  |  |  |  |  |
| GO:0019627\_urea\_metabolic\_process | 1 | 0 |  |  |  |  |  |  |  |  |
| GO:0019676\_ammonia\_assimilation\_cycle | 1 | 0 |  |  |  |  |  |  |  |  |
| GO:0019682\_glyceraldehyde-3-phosphate\_metabolic\_process | 1 | 0 |  |  |  |  |  |  |  |  |
| GO:0019695\_choline\_metabolic\_process | 1 | 0 |  |  |  |  |  |  |  |  |
| GO:0019731\_antibacterial\_humoral\_response | 1 | 0 |  |  |  |  |  |  |  |  |
| GO:0019794\_nonprotein\_amino\_acid\_metabolic\_process | 1 | 0 |  |  |  |  |  |  |  |  |
| GO:0019858\_cytosine\_metabolic\_process | 1 | 0 |  |  |  |  |  |  |  |  |
| GO:0019883\_antigen\_processing\_and\_presentation\_of\_endogenous\_antigen | 1 | 0 |  |  |  |  |  |  |  |  |
| GO:0019889\_pteridine\_metabolic\_process | 1 | 0 |  |  |  |  |  |  |  |  |
| GO:0019896\_axon\_transport\_of\_mitochondrion | 1 | 0 |  |  |  |  |  |  |  |  |
| GO:0021508\_floor\_plate\_formation | 1 | 0 |  |  |  |  |  |  |  |  |
| GO:0021528\_commissural\_neuron\_differentiation\_in\_the\_spinal\_cord | 1 | 0 |  |  |  |  |  |  |  |  |
| GO:0021572\_rhombomere\_6\_development | 1 | 0 |  |  |  |  |  |  |  |  |
| GO:0021577\_hindbrain\_structural\_organization | 1 | 0 |  |  |  |  |  |  |  |  |
| GO:0021586\_pons\_maturation | 1 | 0 |  |  |  |  |  |  |  |  |
| GO:0021589\_cerebellum\_structural\_organization | 1 | 0 |  |  |  |  |  |  |  |  |
| GO:0021590\_cerebellum\_maturation | 1 | 0 |  |  |  |  |  |  |  |  |
| GO:0021592\_fourth\_ventricle\_development | 1 | 0 |  |  |  |  |  |  |  |  |
| GO:0021594\_rhombomere\_formation | 1 | 0 |  |  |  |  |  |  |  |  |
| GO:0021660\_rhombomere\_3\_formation | 1 | 0 |  |  |  |  |  |  |  |  |
| GO:0021664\_rhombomere\_5\_morphogenesis | 1 | 0 |  |  |  |  |  |  |  |  |
| GO:0021666\_rhombomere\_5\_formation | 1 | 0 |  |  |  |  |  |  |  |  |
| GO:0021670\_lateral\_ventricle\_development | 1 | 0 |  |  |  |  |  |  |  |  |
| GO:0021678\_third\_ventricle\_development | 1 | 0 |  |  |  |  |  |  |  |  |
| GO:0021679\_cerebellar\_molecular\_layer\_development | 1 | 0 |  |  |  |  |  |  |  |  |
| GO:0021703\_locus\_ceruleus\_development | 1 | 0 |  |  |  |  |  |  |  |  |
| GO:0021732\_midbrain-hindbrain\_boundary\_maturation | 1 | 0 |  |  |  |  |  |  |  |  |
| GO:0021747\_cochlear\_nucleus\_development | 1 | 0 |  |  |  |  |  |  |  |  |
| GO:0021750\_vestibular\_nucleus\_development | 1 | 0 |  |  |  |  |  |  |  |  |
| GO:0021759\_globus\_pallidus\_development | 1 | 0 |  |  |  |  |  |  |  |  |
| GO:0021768\_nucleus\_accumbens\_development | 1 | 0 |  |  |  |  |  |  |  |  |
| GO:0021771\_lateral\_geniculate\_nucleus\_development | 1 | 0 |  |  |  |  |  |  |  |  |
| GO:0021812\_neuronal-glial\_interaction\_involved\_in\_cerebral\_cortex\_radial\_glia\_guided\_migration | 1 | 0 |  |  |  |  |  |  |  |  |
| GO:0021813\_cell-cell\_adhesion\_involved\_in\_neuronal-glial\_interactions\_involved\_in\_cerebral\_cortex\_radial\_glia\_guided\_migration | 1 | 0 |  |  |  |  |  |  |  |  |
| GO:0021870\_Cajal-Retzius\_cell\_differentiation | 1 | 0 |  |  |  |  |  |  |  |  |
| GO:0021874\_Wnt\_receptor\_signaling\_pathway\_in\_forebrain\_neuroblast\_division | 1 | 0 |  |  |  |  |  |  |  |  |
| GO:0021896\_forebrain\_astrocyte\_differentiation | 1 | 0 |  |  |  |  |  |  |  |  |
| GO:0021897\_forebrain\_astrocyte\_development | 1 | 0 |  |  |  |  |  |  |  |  |
| GO:0021902\_commitment\_of\_a\_neuronal\_cell\_to\_a\_specific\_type\_of\_neuron\_in\_the\_forebrain | 1 | 0 |  |  |  |  |  |  |  |  |
| GO:0021905\_forebrain-midbrain\_boundary\_formation | 1 | 0 |  |  |  |  |  |  |  |  |
| GO:0021914\_negative\_regulation\_of\_smoothened\_signaling\_pathway\_involved\_in\_ventral\_spinal\_cord\_patterning | 1 | 0 |  |  |  |  |  |  |  |  |
| GO:0021917\_somatic\_motor\_neuron\_fate\_commitment | 1 | 0 |  |  |  |  |  |  |  |  |
| GO:0021918\_regulation\_of\_transcription\_from\_RNA\_polymerase\_II\_promoter\_involved\_in\_somatic\_motor\_neuron\_fate\_commitment | 1 | 0 |  |  |  |  |  |  |  |  |
| GO:0021933\_radial\_glia\_guided\_migration\_of\_granule\_cell | 1 | 0 |  |  |  |  |  |  |  |  |
| GO:0021934\_hindbrain\_tangential\_cell\_migration | 1 | 0 |  |  |  |  |  |  |  |  |
| GO:0021935\_granule\_cell\_precursor\_tangential\_migration | 1 | 0 |  |  |  |  |  |  |  |  |
| GO:0021942\_radial\_glia\_guided\_migration\_of\_Purkinje\_cell | 1 | 0 |  |  |  |  |  |  |  |  |
| GO:0021960\_anterior\_commissure\_morphogenesis | 1 | 0 |  |  |  |  |  |  |  |  |
| GO:0021997\_neural\_plate\_axis\_specification | 1 | 0 |  |  |  |  |  |  |  |  |
| GO:0021999\_neural\_plate\_anterior\_posterior\_pattern\_formation | 1 | 0 |  |  |  |  |  |  |  |  |
| GO:0022004\_midbrain-hindbrain\_boundary\_maturation\_during\_brain\_development | 1 | 0 |  |  |  |  |  |  |  |  |
| GO:0022038\_corpus\_callosum\_development | 1 | 0 |  |  |  |  |  |  |  |  |
| GO:0022605\_oogenesis\_stage | 1 | 0 |  |  |  |  |  |  |  |  |
| GO:0030011\_maintenance\_of\_cell\_polarity | 1 | 0 |  |  |  |  |  |  |  |  |
| GO:0030069\_lysogeny | 1 | 0 |  |  |  |  |  |  |  |  |
| GO:0030070\_insulin\_processing | 1 | 0 |  |  |  |  |  |  |  |  |
| GO:0030092\_regulation\_of\_flagellum\_assembly | 1 | 0 |  |  |  |  |  |  |  |  |
| GO:0030103\_vasopressin\_secretion | 1 | 0 |  |  |  |  |  |  |  |  |
| GO:0030194\_positive\_regulation\_of\_blood\_coagulation | 1 | 0 |  |  |  |  |  |  |  |  |
| GO:0030206\_chondroitin\_sulfate\_biosynthetic\_process | 1 | 0 |  |  |  |  |  |  |  |  |
| GO:0030210\_heparin\_biosynthetic\_process | 1 | 0 |  |  |  |  |  |  |  |  |
| GO:0030220\_platelet\_formation | 1 | 0 |  |  |  |  |  |  |  |  |
| GO:0030222\_eosinophil\_differentiation | 1 | 0 |  |  |  |  |  |  |  |  |
| GO:0030237\_female\_sex\_determination | 1 | 0 |  |  |  |  |  |  |  |  |
| GO:0030264\_nuclear\_fragmentation\_during\_apoptosis | 1 | 0 |  |  |  |  |  |  |  |  |
| GO:0030322\_stabilization\_of\_membrane\_potential | 1 | 0 |  |  |  |  |  |  |  |  |
| GO:0030328\_prenylcysteine\_catabolic\_process | 1 | 0 |  |  |  |  |  |  |  |  |
| GO:0030329\_prenylcysteine\_metabolic\_process | 1 | 0 |  |  |  |  |  |  |  |  |
| GO:0030382\_sperm\_mitochondrion\_organization | 1 | 0 |  |  |  |  |  |  |  |  |
| GO:0030389\_fructosamine\_metabolic\_process | 1 | 0 |  |  |  |  |  |  |  |  |
| GO:0030422\_RNA\_interference\_\_production\_of\_siRNA | 1 | 0 |  |  |  |  |  |  |  |  |
| GO:0030449\_regulation\_of\_complement\_activation | 1 | 0 |  |  |  |  |  |  |  |  |
| GO:0030497\_fatty\_acid\_elongation | 1 | 0 |  |  |  |  |  |  |  |  |
| GO:0030575\_nuclear\_body\_organization | 1 | 0 |  |  |  |  |  |  |  |  |
| GO:0030578\_PML\_body\_organization | 1 | 0 |  |  |  |  |  |  |  |  |
| GO:0030853\_negative\_regulation\_of\_granulocyte\_differentiation | 1 | 0 |  |  |  |  |  |  |  |  |
| GO:0030854\_positive\_regulation\_of\_granulocyte\_differentiation | 1 | 0 |  |  |  |  |  |  |  |  |
| GO:0030886\_negative\_regulation\_of\_myeloid\_dendritic\_cell\_activation | 1 | 0 |  |  |  |  |  |  |  |  |
| GO:0030913\_paranodal\_junction\_assembly | 1 | 0 |  |  |  |  |  |  |  |  |
| GO:0031033\_myosin\_filament\_assembly\_or\_disassembly | 1 | 0 |  |  |  |  |  |  |  |  |
| GO:0031034\_myosin\_filament\_assembly | 1 | 0 |  |  |  |  |  |  |  |  |
| GO:0031055\_chromatin\_remodeling\_at\_centromere | 1 | 0 |  |  |  |  |  |  |  |  |
| GO:0031062\_positive\_regulation\_of\_histone\_methylation | 1 | 0 |  |  |  |  |  |  |  |  |
| GO:0031115\_negative\_regulation\_of\_microtubule\_polymerization | 1 | 0 |  |  |  |  |  |  |  |  |
| GO:0031129\_inductive\_cell-cell\_signaling | 1 | 0 |  |  |  |  |  |  |  |  |
| GO:0031284\_positive\_regulation\_of\_guanylate\_cyclase\_activity | 1 | 0 |  |  |  |  |  |  |  |  |
| GO:0031498\_chromatin\_disassembly | 1 | 0 |  |  |  |  |  |  |  |  |
| GO:0031507\_heterochromatin\_formation | 1 | 0 |  |  |  |  |  |  |  |  |
| GO:0031508\_centromeric\_heterochromatin\_formation | 1 | 0 |  |  |  |  |  |  |  |  |
| GO:0031529\_ruffle\_organization | 1 | 0 |  |  |  |  |  |  |  |  |
| GO:0031536\_positive\_regulation\_of\_exit\_from\_mitosis | 1 | 0 |  |  |  |  |  |  |  |  |
| GO:0031572\_G2\_M\_transition\_DNA\_damage\_checkpoint | 1 | 0 |  |  |  |  |  |  |  |  |
| GO:0031576\_G2\_M\_transition\_checkpoint | 1 | 0 |  |  |  |  |  |  |  |  |
| GO:0031580\_membrane\_raft\_distribution | 1 | 0 |  |  |  |  |  |  |  |  |
| GO:0031583\_activation\_of\_phospholipase\_D\_activity\_by\_G-protein\_coupled\_receptor\_protein\_signaling\_pathway | 1 | 0 |  |  |  |  |  |  |  |  |
| GO:0031584\_activation\_of\_phospholipase\_D\_activity | 1 | 0 |  |  |  |  |  |  |  |  |
| GO:0031585\_regulation\_of\_inositol-1\_4\_5-triphosphate\_receptor\_activity | 1 | 0 |  |  |  |  |  |  |  |  |
| GO:0031639\_plasminogen\_activation | 1 | 0 |  |  |  |  |  |  |  |  |
| GO:0031648\_protein\_destabilization | 1 | 0 |  |  |  |  |  |  |  |  |
| GO:0031665\_negative\_regulation\_of\_lipopolysaccharide-mediated\_signaling\_pathway | 1 | 0 |  |  |  |  |  |  |  |  |
| GO:0031914\_negative\_regulation\_of\_synaptic\_plasticity | 1 | 0 |  |  |  |  |  |  |  |  |
| GO:0031944\_negative\_regulation\_of\_glucocorticoid\_metabolic\_process | 1 | 0 |  |  |  |  |  |  |  |  |
| GO:0031947\_negative\_regulation\_of\_glucocorticoid\_biosynthetic\_process | 1 | 0 |  |  |  |  |  |  |  |  |
| GO:0032025\_response\_to\_cobalt\_ion | 1 | 0 |  |  |  |  |  |  |  |  |
| GO:0032026\_response\_to\_magnesium\_ion | 1 | 0 |  |  |  |  |  |  |  |  |
| GO:0032048\_cardiolipin\_metabolic\_process | 1 | 0 |  |  |  |  |  |  |  |  |
| GO:0032066\_nucleolus\_to\_nucleoplasm\_transport | 1 | 0 |  |  |  |  |  |  |  |  |
| GO:0032091\_negative\_regulation\_of\_protein\_binding | 1 | 0 |  |  |  |  |  |  |  |  |
| GO:0032092\_positive\_regulation\_of\_protein\_binding | 1 | 0 |  |  |  |  |  |  |  |  |
| GO:0032097\_positive\_regulation\_of\_response\_to\_food | 1 | 0 |  |  |  |  |  |  |  |  |
| GO:0032100\_positive\_regulation\_of\_appetite | 1 | 0 |  |  |  |  |  |  |  |  |
| GO:0032204\_regulation\_of\_telomere\_maintenance | 1 | 0 |  |  |  |  |  |  |  |  |
| GO:0032206\_positive\_regulation\_of\_telomere\_maintenance | 1 | 0 |  |  |  |  |  |  |  |  |
| GO:0032222\_regulation\_of\_synaptic\_transmission\_\_cholinergic | 1 | 0 |  |  |  |  |  |  |  |  |
| GO:0032224\_positive\_regulation\_of\_synaptic\_transmission\_\_cholinergic | 1 | 0 |  |  |  |  |  |  |  |  |
| GO:0032229\_negative\_regulation\_of\_synaptic\_transmission\_\_GABAergic | 1 | 0 |  |  |  |  |  |  |  |  |
| GO:0032237\_activation\_of\_store-operated\_calcium\_channel\_activity | 1 | 0 |  |  |  |  |  |  |  |  |
| GO:0032239\_regulation\_of\_nucleobase\_\_nucleoside\_\_nucleotide\_and\_nucleic\_acid\_transport | 1 | 0 |  |  |  |  |  |  |  |  |
| GO:0032252\_secretory\_granule\_localization | 1 | 0 |  |  |  |  |  |  |  |  |
| GO:0032274\_gonadotropin\_secretion | 1 | 0 |  |  |  |  |  |  |  |  |
| GO:0032275\_luteinizing\_hormone\_secretion | 1 | 0 |  |  |  |  |  |  |  |  |
| GO:0032287\_myelin\_maintenance\_in\_the\_peripheral\_nervous\_system | 1 | 0 |  |  |  |  |  |  |  |  |
| GO:0032289\_myelin\_formation\_in\_the\_central\_nervous\_system | 1 | 0 |  |  |  |  |  |  |  |  |
| GO:0032303\_regulation\_of\_icosanoid\_secretion | 1 | 0 |  |  |  |  |  |  |  |  |
| GO:0032305\_positive\_regulation\_of\_icosanoid\_secretion | 1 | 0 |  |  |  |  |  |  |  |  |
| GO:0032306\_regulation\_of\_prostaglandin\_secretion | 1 | 0 |  |  |  |  |  |  |  |  |
| GO:0032308\_positive\_regulation\_of\_prostaglandin\_secretion | 1 | 0 |  |  |  |  |  |  |  |  |
| GO:0032310\_prostaglandin\_secretion | 1 | 0 |  |  |  |  |  |  |  |  |
| GO:0032313\_regulation\_of\_Rab\_GTPase\_activity | 1 | 0 |  |  |  |  |  |  |  |  |
| GO:0032314\_regulation\_of\_Rac\_GTPase\_activity | 1 | 0 |  |  |  |  |  |  |  |  |
| GO:0032317\_regulation\_of\_Rap\_GTPase\_activity | 1 | 0 |  |  |  |  |  |  |  |  |
| GO:0032324\_molybdopterin\_cofactor\_biosynthetic\_process | 1 | 0 |  |  |  |  |  |  |  |  |
| GO:0032329\_serine\_transport | 1 | 0 |  |  |  |  |  |  |  |  |
| GO:0032342\_aldosterone\_biosynthetic\_process | 1 | 0 |  |  |  |  |  |  |  |  |
| GO:0032344\_regulation\_of\_aldosterone\_metabolic\_process | 1 | 0 |  |  |  |  |  |  |  |  |
| GO:0032365\_intracellular\_lipid\_transport | 1 | 0 |  |  |  |  |  |  |  |  |
| GO:0032366\_intracellular\_sterol\_transport | 1 | 0 |  |  |  |  |  |  |  |  |
| GO:0032367\_intracellular\_cholesterol\_transport | 1 | 0 |  |  |  |  |  |  |  |  |
| GO:0032370\_positive\_regulation\_of\_lipid\_transport | 1 | 0 |  |  |  |  |  |  |  |  |
| GO:0032410\_negative\_regulation\_of\_transporter\_activity | 1 | 0 |  |  |  |  |  |  |  |  |
| GO:0032413\_negative\_regulation\_of\_ion\_transmembrane\_transporter\_activity | 1 | 0 |  |  |  |  |  |  |  |  |
| GO:0032429\_regulation\_of\_phospholipase\_A2\_activity | 1 | 0 |  |  |  |  |  |  |  |  |
| GO:0032474\_otolith\_morphogenesis | 1 | 0 |  |  |  |  |  |  |  |  |
| GO:0032482\_Rab\_protein\_signal\_transduction | 1 | 0 |  |  |  |  |  |  |  |  |
| GO:0032483\_regulation\_of\_Rab\_protein\_signal\_transduction | 1 | 0 |  |  |  |  |  |  |  |  |
| GO:0032486\_Rap\_protein\_signal\_transduction | 1 | 0 |  |  |  |  |  |  |  |  |
| GO:0032487\_regulation\_of\_Rap\_protein\_signal\_transduction | 1 | 0 |  |  |  |  |  |  |  |  |
| GO:0032594\_protein\_transport\_within\_lipid\_bilayer | 1 | 0 |  |  |  |  |  |  |  |  |
| GO:0032599\_protein\_transport\_out\_of\_membrane\_raft | 1 | 0 |  |  |  |  |  |  |  |  |
| GO:0032600\_chemokine\_receptor\_transport\_out\_of\_membrane\_raft | 1 | 0 |  |  |  |  |  |  |  |  |
| GO:0032607\_interferon-alpha\_production | 1 | 0 |  |  |  |  |  |  |  |  |
| GO:0032621\_interleukin-18\_production | 1 | 0 |  |  |  |  |  |  |  |  |
| GO:0032647\_regulation\_of\_interferon-alpha\_production | 1 | 0 |  |  |  |  |  |  |  |  |
| GO:0032656\_regulation\_of\_interleukin-13\_production | 1 | 0 |  |  |  |  |  |  |  |  |
| GO:0032682\_negative\_regulation\_of\_chemokine\_production | 1 | 0 |  |  |  |  |  |  |  |  |
| GO:0032691\_negative\_regulation\_of\_interleukin-1\_beta\_production | 1 | 0 |  |  |  |  |  |  |  |  |
| GO:0032692\_negative\_regulation\_of\_interleukin-1\_production | 1 | 0 |  |  |  |  |  |  |  |  |
| GO:0032693\_negative\_regulation\_of\_interleukin-10\_production | 1 | 0 |  |  |  |  |  |  |  |  |
| GO:0032696\_negative\_regulation\_of\_interleukin-13\_production | 1 | 0 |  |  |  |  |  |  |  |  |
| GO:0032727\_positive\_regulation\_of\_interferon-alpha\_production | 1 | 0 |  |  |  |  |  |  |  |  |
| GO:0032731\_positive\_regulation\_of\_interleukin-1\_beta\_production | 1 | 0 |  |  |  |  |  |  |  |  |
| GO:0032732\_positive\_regulation\_of\_interleukin-1\_production | 1 | 0 |  |  |  |  |  |  |  |  |
| GO:0032735\_positive\_regulation\_of\_interleukin-12\_production | 1 | 0 |  |  |  |  |  |  |  |  |
| GO:0032764\_negative\_regulation\_of\_mast\_cell\_cytokine\_production | 1 | 0 |  |  |  |  |  |  |  |  |
| GO:0032765\_positive\_regulation\_of\_mast\_cell\_cytokine\_production | 1 | 0 |  |  |  |  |  |  |  |  |
| GO:0032769\_negative\_regulation\_of\_monooxygenase\_activity | 1 | 0 |  |  |  |  |  |  |  |  |
| GO:0032781\_positive\_regulation\_of\_ATPase\_activity | 1 | 0 |  |  |  |  |  |  |  |  |
| GO:0032790\_ribosome\_disassembly | 1 | 0 |  |  |  |  |  |  |  |  |
| GO:0032799\_low-density\_lipoprotein\_receptor\_metabolic\_process | 1 | 0 |  |  |  |  |  |  |  |  |
| GO:0032802\_low-density\_lipoprotein\_receptor\_catabolic\_process | 1 | 0 |  |  |  |  |  |  |  |  |
| GO:0032803\_regulation\_of\_low-density\_lipoprotein\_receptor\_catabolic\_process | 1 | 0 |  |  |  |  |  |  |  |  |
| GO:0032817\_regulation\_of\_natural\_killer\_cell\_proliferation | 1 | 0 |  |  |  |  |  |  |  |  |
| GO:0032819\_positive\_regulation\_of\_natural\_killer\_cell\_proliferation | 1 | 0 |  |  |  |  |  |  |  |  |
| GO:0032836\_glomerular\_basement\_membrane\_development | 1 | 0 |  |  |  |  |  |  |  |  |
| GO:0032855\_positive\_regulation\_of\_Rac\_GTPase\_activity | 1 | 0 |  |  |  |  |  |  |  |  |
| GO:0032863\_activation\_of\_Rac\_GTPase\_activity | 1 | 0 |  |  |  |  |  |  |  |  |
| GO:0032864\_activation\_of\_Cdc42\_GTPase\_activity | 1 | 0 |  |  |  |  |  |  |  |  |
| GO:0032885\_regulation\_of\_polysaccharide\_biosynthetic\_process | 1 | 0 |  |  |  |  |  |  |  |  |
| GO:0032907\_transforming\_growth\_factor-beta3\_production | 1 | 0 |  |  |  |  |  |  |  |  |
| GO:0032910\_regulation\_of\_transforming\_growth\_factor-beta3\_production | 1 | 0 |  |  |  |  |  |  |  |  |
| GO:0032913\_negative\_regulation\_of\_transforming\_growth\_factor-beta3\_production | 1 | 0 |  |  |  |  |  |  |  |  |
| GO:0032924\_activin\_receptor\_signaling\_pathway | 1 | 0 |  |  |  |  |  |  |  |  |
| GO:0032925\_regulation\_of\_activin\_receptor\_signaling\_pathway | 1 | 0 |  |  |  |  |  |  |  |  |
| GO:0032960\_regulation\_of\_inositol\_trisphosphate\_biosynthetic\_process | 1 | 0 |  |  |  |  |  |  |  |  |
| GO:0032962\_positive\_regulation\_of\_inositol\_trisphosphate\_biosynthetic\_process | 1 | 0 |  |  |  |  |  |  |  |  |
| GO:0032964\_collagen\_biosynthetic\_process | 1 | 0 |  |  |  |  |  |  |  |  |
| GO:0032971\_regulation\_of\_muscle\_filament\_sliding | 1 | 0 |  |  |  |  |  |  |  |  |
| GO:0032972\_regulation\_of\_muscle\_filament\_sliding\_speed | 1 | 0 |  |  |  |  |  |  |  |  |
| GO:0032986\_protein-DNA\_complex\_disassembly | 1 | 0 |  |  |  |  |  |  |  |  |
| GO:0032988\_ribonucleoprotein\_complex\_disassembly | 1 | 0 |  |  |  |  |  |  |  |  |
| GO:0033037\_polysaccharide\_localization | 1 | 0 |  |  |  |  |  |  |  |  |
| GO:0033078\_extrathymic\_T\_cell\_differentiation | 1 | 0 |  |  |  |  |  |  |  |  |
| GO:0033085\_negative\_regulation\_of\_T\_cell\_differentiation\_in\_the\_thymus | 1 | 0 |  |  |  |  |  |  |  |  |
| GO:0033087\_negative\_regulation\_of\_immature\_T\_cell\_proliferation | 1 | 0 |  |  |  |  |  |  |  |  |
| GO:0033088\_negative\_regulation\_of\_immature\_T\_cell\_proliferation\_in\_the\_thymus | 1 | 0 |  |  |  |  |  |  |  |  |
| GO:0033108\_mitochondrial\_respiratory\_chain\_complex\_assembly | 1 | 0 |  |  |  |  |  |  |  |  |
| GO:0033127\_regulation\_of\_histone\_phosphorylation | 1 | 0 |  |  |  |  |  |  |  |  |
| GO:0033128\_negative\_regulation\_of\_histone\_phosphorylation | 1 | 0 |  |  |  |  |  |  |  |  |
| GO:0033138\_positive\_regulation\_of\_peptidyl-serine\_phosphorylation | 1 | 0 |  |  |  |  |  |  |  |  |
| GO:0033158\_regulation\_of\_protein\_import\_into\_nucleus\_\_translocation | 1 | 0 |  |  |  |  |  |  |  |  |
| GO:0033160\_positive\_regulation\_of\_protein\_import\_into\_nucleus\_\_translocation | 1 | 0 |  |  |  |  |  |  |  |  |
| GO:0033169\_histone\_H3-K9\_demethylation | 1 | 0 |  |  |  |  |  |  |  |  |
| GO:0033206\_cytokinesis\_after\_meiosis | 1 | 0 |  |  |  |  |  |  |  |  |
| GO:0033240\_positive\_regulation\_of\_cellular\_amine\_metabolic\_process | 1 | 0 |  |  |  |  |  |  |  |  |
| GO:0033313\_meiotic\_cell\_cycle\_checkpoint | 1 | 0 |  |  |  |  |  |  |  |  |
| GO:0033315\_meiotic\_cell\_cycle\_DNA\_replication\_checkpoint | 1 | 0 |  |  |  |  |  |  |  |  |
| GO:0033326\_cerebrospinal\_fluid\_secretion | 1 | 0 |  |  |  |  |  |  |  |  |
| GO:0033366\_protein\_localization\_in\_secretory\_granule | 1 | 0 |  |  |  |  |  |  |  |  |
| GO:0033367\_protein\_localization\_in\_mast\_cell\_secretory\_granule | 1 | 0 |  |  |  |  |  |  |  |  |
| GO:0033368\_protease\_localization\_in\_mast\_cell\_secretory\_granule | 1 | 0 |  |  |  |  |  |  |  |  |
| GO:0033370\_maintenance\_of\_protein\_location\_in\_mast\_cell\_secretory\_granule | 1 | 0 |  |  |  |  |  |  |  |  |
| GO:0033371\_T\_cell\_secretory\_granule\_organization | 1 | 0 |  |  |  |  |  |  |  |  |
| GO:0033373\_maintenance\_of\_protease\_location\_in\_mast\_cell\_secretory\_granule | 1 | 0 |  |  |  |  |  |  |  |  |
| GO:0033374\_protein\_localization\_in\_T\_cell\_secretory\_granule | 1 | 0 |  |  |  |  |  |  |  |  |
| GO:0033375\_protease\_localization\_in\_T\_cell\_secretory\_granule | 1 | 0 |  |  |  |  |  |  |  |  |
| GO:0033377\_maintenance\_of\_protein\_location\_in\_T\_cell\_secretory\_granule | 1 | 0 |  |  |  |  |  |  |  |  |
| GO:0033379\_maintenance\_of\_protease\_location\_in\_T\_cell\_secretory\_granule | 1 | 0 |  |  |  |  |  |  |  |  |
| GO:0033380\_granzyme\_B\_localization\_in\_T\_cell\_secretory\_granule | 1 | 0 |  |  |  |  |  |  |  |  |
| GO:0033382\_maintenance\_of\_granzyme\_B\_location\_in\_T\_cell\_secretory\_granule | 1 | 0 |  |  |  |  |  |  |  |  |
| GO:0033483\_gas\_homeostasis | 1 | 0 |  |  |  |  |  |  |  |  |
| GO:0033484\_nitric\_oxide\_homeostasis | 1 | 0 |  |  |  |  |  |  |  |  |
| GO:0033505\_floor\_plate\_morphogenesis | 1 | 0 |  |  |  |  |  |  |  |  |
| GO:0033522\_histone\_H2A\_ubiquitination | 1 | 0 |  |  |  |  |  |  |  |  |
| GO:0033523\_histone\_H2B\_ubiquitination | 1 | 0 |  |  |  |  |  |  |  |  |
| GO:0033574\_response\_to\_testosterone\_stimulus | 1 | 0 |  |  |  |  |  |  |  |  |
| GO:0033606\_chemokine\_receptor\_transport\_within\_lipid\_bilayer | 1 | 0 |  |  |  |  |  |  |  |  |
| GO:0033628\_regulation\_of\_cell\_adhesion\_mediated\_by\_integrin | 1 | 0 |  |  |  |  |  |  |  |  |
| GO:0033630\_positive\_regulation\_of\_cell\_adhesion\_mediated\_by\_integrin | 1 | 0 |  |  |  |  |  |  |  |  |
| GO:0033632\_regulation\_of\_cell-cell\_adhesion\_mediated\_by\_integrin | 1 | 0 |  |  |  |  |  |  |  |  |
| GO:0033634\_positive\_regulation\_of\_cell-cell\_adhesion\_mediated\_by\_integrin | 1 | 0 |  |  |  |  |  |  |  |  |
| GO:0033683\_nucleotide-excision\_repair\_\_DNA\_incision | 1 | 0 |  |  |  |  |  |  |  |  |
| GO:0033687\_osteoblast\_proliferation | 1 | 0 |  |  |  |  |  |  |  |  |
| GO:0033688\_regulation\_of\_osteoblast\_proliferation | 1 | 0 |  |  |  |  |  |  |  |  |
| GO:0033689\_negative\_regulation\_of\_osteoblast\_proliferation | 1 | 0 |  |  |  |  |  |  |  |  |
| GO:0033750\_ribosome\_localization | 1 | 0 |  |  |  |  |  |  |  |  |
| GO:0033753\_establishment\_of\_ribosome\_localization | 1 | 0 |  |  |  |  |  |  |  |  |
| GO:0033866\_nucleoside\_bisphosphate\_biosynthetic\_process | 1 | 0 |  |  |  |  |  |  |  |  |
| GO:0033875\_ribonucleoside\_bisphosphate\_metabolic\_process | 1 | 0 |  |  |  |  |  |  |  |  |
| GO:0034030\_ribonucleoside\_bisphosphate\_biosynthetic\_process | 1 | 0 |  |  |  |  |  |  |  |  |
| GO:0034032\_purine\_nucleoside\_bisphosphate\_metabolic\_process | 1 | 0 |  |  |  |  |  |  |  |  |
| GO:0034033\_purine\_nucleoside\_bisphosphate\_biosynthetic\_process | 1 | 0 |  |  |  |  |  |  |  |  |
| GO:0034035\_purine\_ribonucleoside\_bisphosphate\_metabolic\_process | 1 | 0 |  |  |  |  |  |  |  |  |
| GO:0034036\_purine\_ribonucleoside\_bisphosphate\_biosynthetic\_process | 1 | 0 |  |  |  |  |  |  |  |  |
| GO:0034067\_protein\_localization\_in\_Golgi\_apparatus | 1 | 0 |  |  |  |  |  |  |  |  |
| GO:0034102\_erythrocyte\_clearance | 1 | 0 |  |  |  |  |  |  |  |  |
| GO:0034106\_regulation\_of\_erythrocyte\_clearance | 1 | 0 |  |  |  |  |  |  |  |  |
| GO:0034107\_negative\_regulation\_of\_erythrocyte\_clearance | 1 | 0 |  |  |  |  |  |  |  |  |
| GO:0034110\_regulation\_of\_homotypic\_cell-cell\_adhesion | 1 | 0 |  |  |  |  |  |  |  |  |
| GO:0034111\_negative\_regulation\_of\_homotypic\_cell-cell\_adhesion | 1 | 0 |  |  |  |  |  |  |  |  |
| GO:0034113\_heterotypic\_cell-cell\_adhesion | 1 | 0 |  |  |  |  |  |  |  |  |
| GO:0034117\_erythrocyte\_aggregation | 1 | 0 |  |  |  |  |  |  |  |  |
| GO:0034118\_regulation\_of\_erythrocyte\_aggregation | 1 | 0 |  |  |  |  |  |  |  |  |
| GO:0034119\_negative\_regulation\_of\_erythrocyte\_aggregation | 1 | 0 |  |  |  |  |  |  |  |  |
| GO:0034121\_regulation\_of\_toll-like\_receptor\_signaling\_pathway | 1 | 0 |  |  |  |  |  |  |  |  |
| GO:0034122\_negative\_regulation\_of\_toll-like\_receptor\_signaling\_pathway | 1 | 0 |  |  |  |  |  |  |  |  |
| GO:0034230\_enkephalin\_processing | 1 | 0 |  |  |  |  |  |  |  |  |
| GO:0034372\_very-low-density\_lipoprotein\_particle\_remodeling | 1 | 0 |  |  |  |  |  |  |  |  |
| GO:0034379\_very-low-density\_lipoprotein\_particle\_assembly | 1 | 0 |  |  |  |  |  |  |  |  |
| GO:0034380\_high-density\_lipoprotein\_particle\_assembly | 1 | 0 |  |  |  |  |  |  |  |  |
| GO:0034394\_protein\_localization\_at\_cell\_surface | 1 | 0 |  |  |  |  |  |  |  |  |
| GO:0034405\_response\_to\_fluid\_shear\_stress | 1 | 0 |  |  |  |  |  |  |  |  |
| GO:0034472\_snRNA\_3'-end\_processing | 1 | 0 |  |  |  |  |  |  |  |  |
| GO:0034474\_U2\_snRNA\_3'-end\_processing | 1 | 0 |  |  |  |  |  |  |  |  |
| GO:0034502\_protein\_localization\_to\_chromosome | 1 | 0 |  |  |  |  |  |  |  |  |
| GO:0034505\_tooth\_mineralization | 1 | 0 |  |  |  |  |  |  |  |  |
| GO:0034508\_centromere\_complex\_assembly | 1 | 0 |  |  |  |  |  |  |  |  |
| GO:0034633\_retinol\_transport | 1 | 0 |  |  |  |  |  |  |  |  |
| GO:0034643\_mitochondrion\_localization\_\_microtubule-mediated | 1 | 0 |  |  |  |  |  |  |  |  |
| GO:0034969\_histone\_arginine\_methylation | 1 | 0 |  |  |  |  |  |  |  |  |
| GO:0034982\_mitochondrial\_protein\_processing | 1 | 0 |  |  |  |  |  |  |  |  |
| GO:0035022\_positive\_regulation\_of\_Rac\_protein\_signal\_transduction | 1 | 0 |  |  |  |  |  |  |  |  |
| GO:0035024\_negative\_regulation\_of\_Rho\_protein\_signal\_transduction | 1 | 0 |  |  |  |  |  |  |  |  |
| GO:0035026\_leading\_edge\_cell\_differentiation | 1 | 0 |  |  |  |  |  |  |  |  |
| GO:0035037\_sperm\_entry | 1 | 0 |  |  |  |  |  |  |  |  |
| GO:0035039\_male\_pronucleus\_formation | 1 | 0 |  |  |  |  |  |  |  |  |
| GO:0035066\_positive\_regulation\_of\_histone\_acetylation | 1 | 0 |  |  |  |  |  |  |  |  |
| GO:0035083\_cilium\_axoneme\_assembly | 1 | 0 |  |  |  |  |  |  |  |  |
| GO:0035090\_maintenance\_of\_apical\_basal\_cell\_polarity | 1 | 0 |  |  |  |  |  |  |  |  |
| GO:0035106\_operant\_conditioning | 1 | 0 |  |  |  |  |  |  |  |  |
| GO:0035172\_hemocyte\_proliferation | 1 | 0 |  |  |  |  |  |  |  |  |
| GO:0035227\_regulation\_of\_glutamate-cysteine\_ligase\_activity | 1 | 0 |  |  |  |  |  |  |  |  |
| GO:0035229\_positive\_regulation\_of\_glutamate-cysteine\_ligase\_activity | 1 | 0 |  |  |  |  |  |  |  |  |
| GO:0035260\_internal\_genitalia\_morphogenesis | 1 | 0 |  |  |  |  |  |  |  |  |
| GO:0035262\_gonad\_morphogenesis | 1 | 0 |  |  |  |  |  |  |  |  |
| GO:0035287\_head\_segmentation | 1 | 0 |  |  |  |  |  |  |  |  |
| GO:0035289\_posterior\_head\_segmentation | 1 | 0 |  |  |  |  |  |  |  |  |
| GO:0035303\_regulation\_of\_dephosphorylation | 1 | 0 |  |  |  |  |  |  |  |  |
| GO:0035304\_regulation\_of\_protein\_amino\_acid\_dephosphorylation | 1 | 0 |  |  |  |  |  |  |  |  |
| GO:0035305\_negative\_regulation\_of\_dephosphorylation | 1 | 0 |  |  |  |  |  |  |  |  |
| GO:0035308\_negative\_regulation\_of\_protein\_amino\_acid\_dephosphorylation | 1 | 0 |  |  |  |  |  |  |  |  |
| GO:0035313\_wound\_healing\_\_spreading\_of\_epidermal\_cells | 1 | 0 |  |  |  |  |  |  |  |  |
| GO:0040013\_negative\_regulation\_of\_locomotion | 1 | 0 |  |  |  |  |  |  |  |  |
| GO:0040019\_positive\_regulation\_of\_embryonic\_development | 1 | 0 |  |  |  |  |  |  |  |  |
| GO:0040032\_post-embryonic\_body\_morphogenesis | 1 | 0 |  |  |  |  |  |  |  |  |
| GO:0040038\_polar\_body\_extrusion\_after\_meiotic\_divisions | 1 | 0 |  |  |  |  |  |  |  |  |
| GO:0042026\_protein\_refolding | 1 | 0 |  |  |  |  |  |  |  |  |
| GO:0042048\_olfactory\_behavior | 1 | 0 |  |  |  |  |  |  |  |  |
| GO:0042059\_negative\_regulation\_of\_epidermal\_growth\_factor\_receptor\_signaling\_pathway | 1 | 0 |  |  |  |  |  |  |  |  |
| GO:0042073\_intraflagellar\_transport | 1 | 0 |  |  |  |  |  |  |  |  |
| GO:0042078\_germ-line\_stem\_cell\_division | 1 | 0 |  |  |  |  |  |  |  |  |
| GO:0042091\_interleukin-10\_biosynthetic\_process | 1 | 0 |  |  |  |  |  |  |  |  |
| GO:0042103\_positive\_regulation\_of\_T\_cell\_homeostatic\_proliferation | 1 | 0 |  |  |  |  |  |  |  |  |
| GO:0042136\_neurotransmitter\_biosynthetic\_process | 1 | 0 |  |  |  |  |  |  |  |  |
| GO:0042137\_sequestering\_of\_neurotransmitter | 1 | 0 |  |  |  |  |  |  |  |  |
| GO:0042138\_meiotic\_DNA\_double-strand\_break\_formation | 1 | 0 |  |  |  |  |  |  |  |  |
| GO:0042178\_xenobiotic\_catabolic\_process | 1 | 0 |  |  |  |  |  |  |  |  |
| GO:0042225\_interleukin-5\_biosynthetic\_process | 1 | 0 |  |  |  |  |  |  |  |  |
| GO:0042231\_interleukin-13\_biosynthetic\_process | 1 | 0 |  |  |  |  |  |  |  |  |
| GO:0042255\_ribosome\_assembly | 1 | 0 |  |  |  |  |  |  |  |  |
| GO:0042257\_ribosomal\_subunit\_assembly | 1 | 0 |  |  |  |  |  |  |  |  |
| GO:0042264\_peptidyl-aspartic\_acid\_hydroxylation | 1 | 0 |  |  |  |  |  |  |  |  |
| GO:0042276\_error-prone\_postreplication\_DNA\_repair | 1 | 0 |  |  |  |  |  |  |  |  |
| GO:0042297\_vocal\_learning | 1 | 0 |  |  |  |  |  |  |  |  |
| GO:0042309\_homoiothermy | 1 | 0 |  |  |  |  |  |  |  |  |
| GO:0042320\_regulation\_of\_circadian\_sleep\_wake\_cycle\_\_REM\_sleep | 1 | 0 |  |  |  |  |  |  |  |  |
| GO:0042339\_keratan\_sulfate\_metabolic\_process | 1 | 0 |  |  |  |  |  |  |  |  |
| GO:0042347\_negative\_regulation\_of\_NF-kappaB\_import\_into\_nucleus | 1 | 0 |  |  |  |  |  |  |  |  |
| GO:0042360\_vitamin\_E\_metabolic\_process | 1 | 0 |  |  |  |  |  |  |  |  |
| GO:0042363\_fat-soluble\_vitamin\_catabolic\_process | 1 | 0 |  |  |  |  |  |  |  |  |
| GO:0042369\_vitamin\_D\_catabolic\_process | 1 | 0 |  |  |  |  |  |  |  |  |
| GO:0042373\_vitamin\_K\_metabolic\_process | 1 | 0 |  |  |  |  |  |  |  |  |
| GO:0042404\_thyroid\_hormone\_catabolic\_process | 1 | 0 |  |  |  |  |  |  |  |  |
| GO:0042414\_epinephrine\_metabolic\_process | 1 | 0 |  |  |  |  |  |  |  |  |
| GO:0042436\_indole\_derivative\_catabolic\_process | 1 | 0 |  |  |  |  |  |  |  |  |
| GO:0042489\_negative\_regulation\_of\_odontogenesis\_of\_dentine-containing\_tooth | 1 | 0 |  |  |  |  |  |  |  |  |
| GO:0042508\_tyrosine\_phosphorylation\_of\_Stat1\_protein | 1 | 0 |  |  |  |  |  |  |  |  |
| GO:0042518\_negative\_regulation\_of\_tyrosine\_phosphorylation\_of\_Stat3\_protein | 1 | 0 |  |  |  |  |  |  |  |  |
| GO:0042524\_negative\_regulation\_of\_tyrosine\_phosphorylation\_of\_Stat5\_protein | 1 | 0 |  |  |  |  |  |  |  |  |
| GO:0042536\_negative\_regulation\_of\_tumor\_necrosis\_factor\_biosynthetic\_process | 1 | 0 |  |  |  |  |  |  |  |  |
| GO:0042538\_hyperosmotic\_salinity\_response | 1 | 0 |  |  |  |  |  |  |  |  |
| GO:0042628\_mating\_plug\_formation | 1 | 0 |  |  |  |  |  |  |  |  |
| GO:0042631\_cellular\_response\_to\_water\_deprivation | 1 | 0 |  |  |  |  |  |  |  |  |
| GO:0042637\_catagen | 1 | 0 |  |  |  |  |  |  |  |  |
| GO:0042660\_positive\_regulation\_of\_cell\_fate\_specification | 1 | 0 |  |  |  |  |  |  |  |  |
| GO:0042663\_regulation\_of\_endodermal\_cell\_fate\_specification | 1 | 0 |  |  |  |  |  |  |  |  |
| GO:0042664\_negative\_regulation\_of\_endodermal\_cell\_fate\_specification | 1 | 0 |  |  |  |  |  |  |  |  |
| GO:0042667\_auditory\_receptor\_cell\_fate\_specification | 1 | 0 |  |  |  |  |  |  |  |  |
| GO:0042694\_muscle\_cell\_fate\_specification | 1 | 0 |  |  |  |  |  |  |  |  |
| GO:0042706\_eye\_photoreceptor\_cell\_fate\_commitment | 1 | 0 |  |  |  |  |  |  |  |  |
| GO:0042713\_sperm\_ejaculation | 1 | 0 |  |  |  |  |  |  |  |  |
| GO:0042723\_thiamin\_and\_derivative\_metabolic\_process | 1 | 0 |  |  |  |  |  |  |  |  |
| GO:0042737\_drug\_catabolic\_process | 1 | 0 |  |  |  |  |  |  |  |  |
| GO:0042738\_exogenous\_drug\_catabolic\_process | 1 | 0 |  |  |  |  |  |  |  |  |
| GO:0042747\_circadian\_sleep\_wake\_cycle\_\_REM\_sleep | 1 | 0 |  |  |  |  |  |  |  |  |
| GO:0042748\_circadian\_sleep\_wake\_cycle\_\_non-REM\_sleep | 1 | 0 |  |  |  |  |  |  |  |  |
| GO:0042772\_DNA\_damage\_response\_\_signal\_transduction\_resulting\_in\_transcription | 1 | 0 |  |  |  |  |  |  |  |  |
| GO:0042790\_transcription\_of\_nuclear\_rRNA\_large\_RNA\_polymerase\_I\_transcript | 1 | 0 |  |  |  |  |  |  |  |  |
| GO:0042839\_D-glucuronate\_metabolic\_process | 1 | 0 |  |  |  |  |  |  |  |  |
| GO:0042840\_D-glucuronate\_catabolic\_process | 1 | 0 |  |  |  |  |  |  |  |  |
| GO:0042891\_antibiotic\_transport | 1 | 0 |  |  |  |  |  |  |  |  |
| GO:0042892\_chloramphenicol\_transport | 1 | 0 |  |  |  |  |  |  |  |  |
| GO:0042940\_D-amino\_acid\_transport | 1 | 0 |  |  |  |  |  |  |  |  |
| GO:0042941\_D-alanine\_transport | 1 | 0 |  |  |  |  |  |  |  |  |
| GO:0042942\_D-serine\_transport | 1 | 0 |  |  |  |  |  |  |  |  |
| GO:0042983\_amyloid\_precursor\_protein\_biosynthetic\_process | 1 | 0 |  |  |  |  |  |  |  |  |
| GO:0042984\_regulation\_of\_amyloid\_precursor\_protein\_biosynthetic\_process | 1 | 0 |  |  |  |  |  |  |  |  |
| GO:0042985\_negative\_regulation\_of\_amyloid\_precursor\_protein\_biosynthetic\_process | 1 | 0 |  |  |  |  |  |  |  |  |
| GO:0042989\_sequestering\_of\_actin\_monomers | 1 | 0 |  |  |  |  |  |  |  |  |
| GO:0043044\_ATP-dependent\_chromatin\_remodeling | 1 | 0 |  |  |  |  |  |  |  |  |
| GO:0043056\_forward\_locomotion | 1 | 0 |  |  |  |  |  |  |  |  |
| GO:0043060\_meiotic\_metaphase\_I\_plate\_congression | 1 | 0 |  |  |  |  |  |  |  |  |
| GO:0043091\_L-arginine\_import | 1 | 0 |  |  |  |  |  |  |  |  |
| GO:0043124\_negative\_regulation\_of\_I-kappaB\_kinase\_NF-kappaB\_cascade | 1 | 0 |  |  |  |  |  |  |  |  |
| GO:0043132\_NAD\_transport | 1 | 0 |  |  |  |  |  |  |  |  |
| GO:0043153\_entrainment\_of\_circadian\_clock\_by\_photoperiod | 1 | 0 |  |  |  |  |  |  |  |  |
| GO:0043171\_peptide\_catabolic\_process | 1 | 0 |  |  |  |  |  |  |  |  |
| GO:0043179\_rhythmic\_excitation | 1 | 0 |  |  |  |  |  |  |  |  |
| GO:0043206\_fibril\_organization | 1 | 0 |  |  |  |  |  |  |  |  |
| GO:0043217\_myelin\_maintenance | 1 | 0 |  |  |  |  |  |  |  |  |
| GO:0043313\_regulation\_of\_neutrophil\_degranulation | 1 | 0 |  |  |  |  |  |  |  |  |
| GO:0043316\_cytotoxic\_T\_cell\_degranulation | 1 | 0 |  |  |  |  |  |  |  |  |
| GO:0043369\_CD4-positive\_or\_CD8-positive\_\_alpha-beta\_T\_cell\_lineage\_commitment | 1 | 0 |  |  |  |  |  |  |  |  |
| GO:0043375\_CD8-positive\_\_alpha-beta\_T\_cell\_lineage\_commitment | 1 | 0 |  |  |  |  |  |  |  |  |
| GO:0043379\_memory\_T\_cell\_differentiation | 1 | 0 |  |  |  |  |  |  |  |  |
| GO:0043380\_regulation\_of\_memory\_T\_cell\_differentiation | 1 | 0 |  |  |  |  |  |  |  |  |
| GO:0043400\_cortisol\_secretion | 1 | 0 |  |  |  |  |  |  |  |  |
| GO:0043415\_positive\_regulation\_of\_skeletal\_muscle\_regeneration | 1 | 0 |  |  |  |  |  |  |  |  |
| GO:0043416\_regulation\_of\_skeletal\_muscle\_regeneration | 1 | 0 |  |  |  |  |  |  |  |  |
| GO:0043437\_butanoic\_acid\_metabolic\_process | 1 | 0 |  |  |  |  |  |  |  |  |
| GO:0043438\_acetoacetic\_acid\_metabolic\_process | 1 | 0 |  |  |  |  |  |  |  |  |
| GO:0043480\_pigment\_accumulation\_in\_tissues | 1 | 0 |  |  |  |  |  |  |  |  |
| GO:0043482\_cellular\_pigment\_accumulation | 1 | 0 |  |  |  |  |  |  |  |  |
| GO:0043486\_histone\_exchange | 1 | 0 |  |  |  |  |  |  |  |  |
| GO:0043496\_regulation\_of\_protein\_homodimerization\_activity | 1 | 0 |  |  |  |  |  |  |  |  |
| GO:0043501\_skeletal\_muscle\_adaptation | 1 | 0 |  |  |  |  |  |  |  |  |
| GO:0043508\_negative\_regulation\_of\_JUN\_kinase\_activity | 1 | 0 |  |  |  |  |  |  |  |  |
| GO:0043517\_positive\_regulation\_of\_DNA\_damage\_response\_\_signal\_transduction\_by\_p53\_class\_mediator | 1 | 0 |  |  |  |  |  |  |  |  |
| GO:0043535\_regulation\_of\_blood\_vessel\_endothelial\_cell\_migration | 1 | 0 |  |  |  |  |  |  |  |  |
| GO:0043537\_negative\_regulation\_of\_blood\_vessel\_endothelial\_cell\_migration | 1 | 0 |  |  |  |  |  |  |  |  |
| GO:0043545\_molybdopterin\_cofactor\_metabolic\_process | 1 | 0 |  |  |  |  |  |  |  |  |
| GO:0043587\_tongue\_morphogenesis | 1 | 0 |  |  |  |  |  |  |  |  |
| GO:0043604\_amide\_biosynthetic\_process | 1 | 0 |  |  |  |  |  |  |  |  |
| GO:0043628\_ncRNA\_3'-end\_processing | 1 | 0 |  |  |  |  |  |  |  |  |
| GO:0044254\_multicellular\_organismal\_protein\_catabolic\_process | 1 | 0 |  |  |  |  |  |  |  |  |
| GO:0044256\_protein\_digestion | 1 | 0 |  |  |  |  |  |  |  |  |
| GO:0044266\_multicellular\_organismal\_macromolecule\_catabolic\_process | 1 | 0 |  |  |  |  |  |  |  |  |
| GO:0045004\_DNA\_replication\_proofreading | 1 | 0 |  |  |  |  |  |  |  |  |
| GO:0045019\_negative\_regulation\_of\_nitric\_oxide\_biosynthetic\_process | 1 | 0 |  |  |  |  |  |  |  |  |
| GO:0045020\_error-prone\_DNA\_repair | 1 | 0 |  |  |  |  |  |  |  |  |
| GO:0045022\_early\_endosome\_to\_late\_endosome\_transport | 1 | 0 |  |  |  |  |  |  |  |  |
| GO:0045062\_extrathymic\_T\_cell\_selection | 1 | 0 |  |  |  |  |  |  |  |  |
| GO:0045069\_regulation\_of\_viral\_genome\_replication | 1 | 0 |  |  |  |  |  |  |  |  |
| GO:0045074\_regulation\_of\_interleukin-10\_biosynthetic\_process | 1 | 0 |  |  |  |  |  |  |  |  |
| GO:0045082\_positive\_regulation\_of\_interleukin-10\_biosynthetic\_process | 1 | 0 |  |  |  |  |  |  |  |  |
| GO:0045083\_negative\_regulation\_of\_interleukin-12\_biosynthetic\_process | 1 | 0 |  |  |  |  |  |  |  |  |
| GO:0045112\_integrin\_biosynthetic\_process | 1 | 0 |  |  |  |  |  |  |  |  |
| GO:0045113\_regulation\_of\_integrin\_biosynthetic\_process | 1 | 0 |  |  |  |  |  |  |  |  |
| GO:0045188\_regulation\_of\_circadian\_sleep\_wake\_cycle\_\_non-REM\_sleep | 1 | 0 |  |  |  |  |  |  |  |  |
| GO:0045210\_FasL\_biosynthetic\_process | 1 | 0 |  |  |  |  |  |  |  |  |
| GO:0045297\_post-mating\_behavior | 1 | 0 |  |  |  |  |  |  |  |  |
| GO:0045299\_otolith\_mineralization | 1 | 0 |  |  |  |  |  |  |  |  |
| GO:0045329\_carnitine\_biosynthetic\_process | 1 | 0 |  |  |  |  |  |  |  |  |
| GO:0045341\_MHC\_class\_I\_biosynthetic\_process | 1 | 0 |  |  |  |  |  |  |  |  |
| GO:0045343\_regulation\_of\_MHC\_class\_I\_biosynthetic\_process | 1 | 0 |  |  |  |  |  |  |  |  |
| GO:0045347\_negative\_regulation\_of\_MHC\_class\_II\_biosynthetic\_process | 1 | 0 |  |  |  |  |  |  |  |  |
| GO:0045405\_regulation\_of\_interleukin-5\_biosynthetic\_process | 1 | 0 |  |  |  |  |  |  |  |  |
| GO:0045407\_positive\_regulation\_of\_interleukin-5\_biosynthetic\_process | 1 | 0 |  |  |  |  |  |  |  |  |
| GO:0045426\_quinone\_cofactor\_biosynthetic\_process | 1 | 0 |  |  |  |  |  |  |  |  |
| GO:0045448\_mitotic\_cell\_cycle\_\_embryonic | 1 | 0 |  |  |  |  |  |  |  |  |
| GO:0045454\_cell\_redox\_homeostasis | 1 | 0 |  |  |  |  |  |  |  |  |
| GO:0045583\_regulation\_of\_cytotoxic\_T\_cell\_differentiation | 1 | 0 |  |  |  |  |  |  |  |  |
| GO:0045585\_positive\_regulation\_of\_cytotoxic\_T\_cell\_differentiation | 1 | 0 |  |  |  |  |  |  |  |  |
| GO:0045601\_regulation\_of\_endothelial\_cell\_differentiation | 1 | 0 |  |  |  |  |  |  |  |  |
| GO:0045602\_negative\_regulation\_of\_endothelial\_cell\_differentiation | 1 | 0 |  |  |  |  |  |  |  |  |
| GO:0045605\_negative\_regulation\_of\_epidermal\_cell\_differentiation | 1 | 0 |  |  |  |  |  |  |  |  |
| GO:0045606\_positive\_regulation\_of\_epidermal\_cell\_differentiation | 1 | 0 |  |  |  |  |  |  |  |  |
| GO:0045609\_positive\_regulation\_of\_auditory\_receptor\_cell\_differentiation | 1 | 0 |  |  |  |  |  |  |  |  |
| GO:0045617\_negative\_regulation\_of\_keratinocyte\_differentiation | 1 | 0 |  |  |  |  |  |  |  |  |
| GO:0045618\_positive\_regulation\_of\_keratinocyte\_differentiation | 1 | 0 |  |  |  |  |  |  |  |  |
| GO:0045626\_negative\_regulation\_of\_T-helper\_1\_cell\_differentiation | 1 | 0 |  |  |  |  |  |  |  |  |
| GO:0045633\_positive\_regulation\_of\_mechanoreceptor\_differentiation | 1 | 0 |  |  |  |  |  |  |  |  |
| GO:0045650\_negative\_regulation\_of\_macrophage\_differentiation | 1 | 0 |  |  |  |  |  |  |  |  |
| GO:0045656\_negative\_regulation\_of\_monocyte\_differentiation | 1 | 0 |  |  |  |  |  |  |  |  |
| GO:0045657\_positive\_regulation\_of\_monocyte\_differentiation | 1 | 0 |  |  |  |  |  |  |  |  |
| GO:0045659\_negative\_regulation\_of\_neutrophil\_differentiation | 1 | 0 |  |  |  |  |  |  |  |  |
| GO:0045660\_positive\_regulation\_of\_neutrophil\_differentiation | 1 | 0 |  |  |  |  |  |  |  |  |
| GO:0045721\_negative\_regulation\_of\_gluconeogenesis | 1 | 0 |  |  |  |  |  |  |  |  |
| GO:0045724\_positive\_regulation\_of\_flagellum\_assembly | 1 | 0 |  |  |  |  |  |  |  |  |
| GO:0045725\_positive\_regulation\_of\_glycogen\_biosynthetic\_process | 1 | 0 |  |  |  |  |  |  |  |  |
| GO:0045740\_positive\_regulation\_of\_DNA\_replication | 1 | 0 |  |  |  |  |  |  |  |  |
| GO:0045759\_negative\_regulation\_of\_action\_potential | 1 | 0 |  |  |  |  |  |  |  |  |
| GO:0045768\_positive\_regulation\_of\_anti-apoptosis | 1 | 0 |  |  |  |  |  |  |  |  |
| GO:0045769\_negative\_regulation\_of\_asymmetric\_cell\_division | 1 | 0 |  |  |  |  |  |  |  |  |
| GO:0045794\_negative\_regulation\_of\_cell\_volume | 1 | 0 |  |  |  |  |  |  |  |  |
| GO:0045815\_positive\_regulation\_of\_gene\_expression\_\_epigenetic | 1 | 0 |  |  |  |  |  |  |  |  |
| GO:0045818\_negative\_regulation\_of\_glycogen\_catabolic\_process | 1 | 0 |  |  |  |  |  |  |  |  |
| GO:0045842\_positive\_regulation\_of\_mitotic\_metaphase\_anaphase\_transition | 1 | 0 |  |  |  |  |  |  |  |  |
| GO:0045875\_negative\_regulation\_of\_sister\_chromatid\_cohesion | 1 | 0 |  |  |  |  |  |  |  |  |
| GO:0045898\_regulation\_of\_transcriptional\_preinitiation\_complex\_assembly | 1 | 0 |  |  |  |  |  |  |  |  |
| GO:0045899\_positive\_regulation\_of\_transcriptional\_preinitiation\_complex\_assembly | 1 | 0 |  |  |  |  |  |  |  |  |
| GO:0045906\_negative\_regulation\_of\_vasoconstriction | 1 | 0 |  |  |  |  |  |  |  |  |
| GO:0045908\_negative\_regulation\_of\_vasodilation | 1 | 0 |  |  |  |  |  |  |  |  |
| GO:0045909\_positive\_regulation\_of\_vasodilation | 1 | 0 |  |  |  |  |  |  |  |  |
| GO:0045915\_positive\_regulation\_of\_catecholamine\_metabolic\_process | 1 | 0 |  |  |  |  |  |  |  |  |
| GO:0045920\_negative\_regulation\_of\_exocytosis | 1 | 0 |  |  |  |  |  |  |  |  |
| GO:0045924\_regulation\_of\_female\_receptivity | 1 | 0 |  |  |  |  |  |  |  |  |
| GO:0045947\_negative\_regulation\_of\_translational\_initiation | 1 | 0 |  |  |  |  |  |  |  |  |
| GO:0045955\_negative\_regulation\_of\_calcium\_ion-dependent\_exocytosis | 1 | 0 |  |  |  |  |  |  |  |  |
| GO:0045956\_positive\_regulation\_of\_calcium\_ion-dependent\_exocytosis | 1 | 0 |  |  |  |  |  |  |  |  |
| GO:0045964\_positive\_regulation\_of\_dopamine\_metabolic\_process | 1 | 0 |  |  |  |  |  |  |  |  |
| GO:0045988\_negative\_regulation\_of\_striated\_muscle\_contraction | 1 | 0 |  |  |  |  |  |  |  |  |
| GO:0045989\_positive\_regulation\_of\_striated\_muscle\_contraction | 1 | 0 |  |  |  |  |  |  |  |  |
| GO:0045990\_regulation\_of\_transcription\_by\_carbon\_catabolites | 1 | 0 |  |  |  |  |  |  |  |  |
| GO:0045991\_positive\_regulation\_of\_transcription\_by\_carbon\_catabolites | 1 | 0 |  |  |  |  |  |  |  |  |
| GO:0045994\_positive\_regulation\_of\_translational\_initiation\_by\_iron | 1 | 0 |  |  |  |  |  |  |  |  |
| GO:0046007\_negative\_regulation\_of\_activated\_T\_cell\_proliferation | 1 | 0 |  |  |  |  |  |  |  |  |
| GO:0046014\_negative\_regulation\_of\_T\_cell\_homeostatic\_proliferation | 1 | 0 |  |  |  |  |  |  |  |  |
| GO:0046015\_regulation\_of\_transcription\_by\_glucose | 1 | 0 |  |  |  |  |  |  |  |  |
| GO:0046016\_positive\_regulation\_of\_transcription\_by\_glucose | 1 | 0 |  |  |  |  |  |  |  |  |
| GO:0046031\_ADP\_metabolic\_process | 1 | 0 |  |  |  |  |  |  |  |  |
| GO:0046032\_ADP\_catabolic\_process | 1 | 0 |  |  |  |  |  |  |  |  |
| GO:0046061\_dATP\_catabolic\_process | 1 | 0 |  |  |  |  |  |  |  |  |
| GO:0046075\_dTTP\_metabolic\_process | 1 | 0 |  |  |  |  |  |  |  |  |
| GO:0046078\_dUMP\_metabolic\_process | 1 | 0 |  |  |  |  |  |  |  |  |
| GO:0046079\_dUMP\_catabolic\_process | 1 | 0 |  |  |  |  |  |  |  |  |
| GO:0046086\_adenosine\_biosynthetic\_process | 1 | 0 |  |  |  |  |  |  |  |  |
| GO:0046090\_deoxyadenosine\_metabolic\_process | 1 | 0 |  |  |  |  |  |  |  |  |
| GO:0046098\_guanine\_metabolic\_process | 1 | 0 |  |  |  |  |  |  |  |  |
| GO:0046101\_hypoxanthine\_biosynthetic\_process | 1 | 0 |  |  |  |  |  |  |  |  |
| GO:0046102\_inosine\_metabolic\_process | 1 | 0 |  |  |  |  |  |  |  |  |
| GO:0046103\_inosine\_biosynthetic\_process | 1 | 0 |  |  |  |  |  |  |  |  |
| GO:0046108\_uridine\_metabolic\_process | 1 | 0 |  |  |  |  |  |  |  |  |
| GO:0046110\_xanthine\_metabolic\_process | 1 | 0 |  |  |  |  |  |  |  |  |
| GO:0046111\_xanthine\_biosynthetic\_process | 1 | 0 |  |  |  |  |  |  |  |  |
| GO:0046112\_nucleobase\_biosynthetic\_process | 1 | 0 |  |  |  |  |  |  |  |  |
| GO:0046113\_nucleobase\_catabolic\_process | 1 | 0 |  |  |  |  |  |  |  |  |
| GO:0046121\_deoxyribonucleoside\_catabolic\_process | 1 | 0 |  |  |  |  |  |  |  |  |
| GO:0046122\_purine\_deoxyribonucleoside\_metabolic\_process | 1 | 0 |  |  |  |  |  |  |  |  |
| GO:0046124\_purine\_deoxyribonucleoside\_catabolic\_process | 1 | 0 |  |  |  |  |  |  |  |  |
| GO:0046125\_pyrimidine\_deoxyribonucleoside\_metabolic\_process | 1 | 0 |  |  |  |  |  |  |  |  |
| GO:0046131\_pyrimidine\_ribonucleoside\_metabolic\_process | 1 | 0 |  |  |  |  |  |  |  |  |
| GO:0046160\_heme\_a\_metabolic\_process | 1 | 0 |  |  |  |  |  |  |  |  |
| GO:0046218\_indolalkylamine\_catabolic\_process | 1 | 0 |  |  |  |  |  |  |  |  |
| GO:0046292\_formaldehyde\_metabolic\_process | 1 | 0 |  |  |  |  |  |  |  |  |
| GO:0046294\_formaldehyde\_catabolic\_process | 1 | 0 |  |  |  |  |  |  |  |  |
| GO:0046314\_phosphocreatine\_biosynthetic\_process | 1 | 0 |  |  |  |  |  |  |  |  |
| GO:0046327\_glycerol\_biosynthetic\_process\_from\_pyruvate | 1 | 0 |  |  |  |  |  |  |  |  |
| GO:0046329\_negative\_regulation\_of\_JNK\_cascade | 1 | 0 |  |  |  |  |  |  |  |  |
| GO:0046340\_diacylglycerol\_catabolic\_process | 1 | 0 |  |  |  |  |  |  |  |  |
| GO:0046351\_disaccharide\_biosynthetic\_process | 1 | 0 |  |  |  |  |  |  |  |  |
| GO:0046356\_acetyl-CoA\_catabolic\_process | 1 | 0 |  |  |  |  |  |  |  |  |
| GO:0046358\_butyrate\_biosynthetic\_process | 1 | 0 |  |  |  |  |  |  |  |  |
| GO:0046359\_butyrate\_catabolic\_process | 1 | 0 |  |  |  |  |  |  |  |  |
| GO:0046381\_CMP-N-acetylneuraminate\_metabolic\_process | 1 | 0 |  |  |  |  |  |  |  |  |
| GO:0046415\_urate\_metabolic\_process | 1 | 0 |  |  |  |  |  |  |  |  |
| GO:0046416\_D-amino\_acid\_metabolic\_process | 1 | 0 |  |  |  |  |  |  |  |  |
| GO:0046434\_organophosphate\_catabolic\_process | 1 | 0 |  |  |  |  |  |  |  |  |
| GO:0046437\_D-amino\_acid\_biosynthetic\_process | 1 | 0 |  |  |  |  |  |  |  |  |
| GO:0046440\_L-lysine\_metabolic\_process | 1 | 0 |  |  |  |  |  |  |  |  |
| GO:0046449\_creatinine\_metabolic\_process | 1 | 0 |  |  |  |  |  |  |  |  |
| GO:0046471\_phosphatidylglycerol\_metabolic\_process | 1 | 0 |  |  |  |  |  |  |  |  |
| GO:0046473\_phosphatidic\_acid\_metabolic\_process | 1 | 0 |  |  |  |  |  |  |  |  |
| GO:0046476\_glycosylceramide\_biosynthetic\_process | 1 | 0 |  |  |  |  |  |  |  |  |
| GO:0046477\_glycosylceramide\_catabolic\_process | 1 | 0 |  |  |  |  |  |  |  |  |
| GO:0046485\_ether\_lipid\_metabolic\_process | 1 | 0 |  |  |  |  |  |  |  |  |
| GO:0046487\_glyoxylate\_metabolic\_process | 1 | 0 |  |  |  |  |  |  |  |  |
| GO:0046498\_S-adenosylhomocysteine\_metabolic\_process | 1 | 0 |  |  |  |  |  |  |  |  |
| GO:0046552\_photoreceptor\_cell\_fate\_commitment | 1 | 0 |  |  |  |  |  |  |  |  |
| GO:0046586\_regulation\_of\_calcium-dependent\_cell-cell\_adhesion | 1 | 0 |  |  |  |  |  |  |  |  |
| GO:0046587\_positive\_regulation\_of\_calcium-dependent\_cell-cell\_adhesion | 1 | 0 |  |  |  |  |  |  |  |  |
| GO:0046602\_regulation\_of\_mitotic\_centrosome\_separation | 1 | 0 |  |  |  |  |  |  |  |  |
| GO:0046604\_positive\_regulation\_of\_mitotic\_centrosome\_separation | 1 | 0 |  |  |  |  |  |  |  |  |
| GO:0046607\_positive\_regulation\_of\_centrosome\_cycle | 1 | 0 |  |  |  |  |  |  |  |  |
| GO:0046655\_folic\_acid\_metabolic\_process | 1 | 0 |  |  |  |  |  |  |  |  |
| GO:0046671\_negative\_regulation\_of\_retinal\_cell\_programmed\_cell\_death | 1 | 0 |  |  |  |  |  |  |  |  |
| GO:0046685\_response\_to\_arsenic | 1 | 0 |  |  |  |  |  |  |  |  |
| GO:0046692\_sperm\_competition | 1 | 0 |  |  |  |  |  |  |  |  |
| GO:0046707\_IDP\_metabolic\_process | 1 | 0 |  |  |  |  |  |  |  |  |
| GO:0046709\_IDP\_catabolic\_process | 1 | 0 |  |  |  |  |  |  |  |  |
| GO:0046724\_oxalic\_acid\_secretion | 1 | 0 |  |  |  |  |  |  |  |  |
| GO:0046753\_non-lytic\_viral\_release | 1 | 0 |  |  |  |  |  |  |  |  |
| GO:0046755\_non-lytic\_virus\_budding | 1 | 0 |  |  |  |  |  |  |  |  |
| GO:0046826\_negative\_regulation\_of\_protein\_export\_from\_nucleus | 1 | 0 |  |  |  |  |  |  |  |  |
| GO:0046827\_positive\_regulation\_of\_protein\_export\_from\_nucleus | 1 | 0 |  |  |  |  |  |  |  |  |
| GO:0046831\_regulation\_of\_RNA\_export\_from\_nucleus | 1 | 0 |  |  |  |  |  |  |  |  |
| GO:0046834\_lipid\_phosphorylation | 1 | 0 |  |  |  |  |  |  |  |  |
| GO:0046853\_inositol\_and\_derivative\_phosphorylation | 1 | 0 |  |  |  |  |  |  |  |  |
| GO:0046864\_isoprenoid\_transport | 1 | 0 |  |  |  |  |  |  |  |  |
| GO:0046865\_terpenoid\_transport | 1 | 0 |  |  |  |  |  |  |  |  |
| GO:0046877\_regulation\_of\_saliva\_secretion | 1 | 0 |  |  |  |  |  |  |  |  |
| GO:0046878\_positive\_regulation\_of\_saliva\_secretion | 1 | 0 |  |  |  |  |  |  |  |  |
| GO:0046884\_follicle-stimulating\_hormone\_secretion | 1 | 0 |  |  |  |  |  |  |  |  |
| GO:0046898\_response\_to\_cycloheximide | 1 | 0 |  |  |  |  |  |  |  |  |
| GO:0046929\_negative\_regulation\_of\_neurotransmitter\_secretion | 1 | 0 |  |  |  |  |  |  |  |  |
| GO:0046931\_pore\_complex\_biogenesis | 1 | 0 |  |  |  |  |  |  |  |  |
| GO:0046949\_acyl-CoA\_biosynthetic\_process | 1 | 0 |  |  |  |  |  |  |  |  |
| GO:0046958\_nonassociative\_learning | 1 | 0 |  |  |  |  |  |  |  |  |
| GO:0046960\_sensitization | 1 | 0 |  |  |  |  |  |  |  |  |
| GO:0046986\_negative\_regulation\_of\_hemoglobin\_biosynthetic\_process | 1 | 0 |  |  |  |  |  |  |  |  |
| GO:0047497\_mitochondrion\_transport\_along\_microtubule | 1 | 0 |  |  |  |  |  |  |  |  |
| GO:0048047\_mating\_behavior\_\_sex\_discrimination | 1 | 0 |  |  |  |  |  |  |  |  |
| GO:0048133\_male\_germ-line\_stem\_cell\_division | 1 | 0 |  |  |  |  |  |  |  |  |
| GO:0048137\_spermatocyte\_division | 1 | 0 |  |  |  |  |  |  |  |  |
| GO:0048143\_astrocyte\_activation | 1 | 0 |  |  |  |  |  |  |  |  |
| GO:0048170\_positive\_regulation\_of\_long-term\_neuronal\_synaptic\_plasticity | 1 | 0 |  |  |  |  |  |  |  |  |
| GO:0048199\_vesicle\_targeting\_\_to\_\_from\_or\_within\_Golgi | 1 | 0 |  |  |  |  |  |  |  |  |
| GO:0048241\_epinephrine\_transport | 1 | 0 |  |  |  |  |  |  |  |  |
| GO:0048242\_epinephrine\_secretion | 1 | 0 |  |  |  |  |  |  |  |  |
| GO:0048243\_norepinephrine\_secretion | 1 | 0 |  |  |  |  |  |  |  |  |
| GO:0048247\_lymphocyte\_chemotaxis | 1 | 0 |  |  |  |  |  |  |  |  |
| GO:0048250\_mitochondrial\_iron\_ion\_transport | 1 | 0 |  |  |  |  |  |  |  |  |
| GO:0048259\_regulation\_of\_receptor-mediated\_endocytosis | 1 | 0 |  |  |  |  |  |  |  |  |
| GO:0048260\_positive\_regulation\_of\_receptor-mediated\_endocytosis | 1 | 0 |  |  |  |  |  |  |  |  |
| GO:0048290\_isotype\_switching\_to\_IgA\_isotypes | 1 | 0 |  |  |  |  |  |  |  |  |
| GO:0048296\_regulation\_of\_isotype\_switching\_to\_IgA\_isotypes | 1 | 0 |  |  |  |  |  |  |  |  |
| GO:0048298\_positive\_regulation\_of\_isotype\_switching\_to\_IgA\_isotypes | 1 | 0 |  |  |  |  |  |  |  |  |
| GO:0048319\_axial\_mesoderm\_morphogenesis | 1 | 0 |  |  |  |  |  |  |  |  |
| GO:0048320\_axial\_mesoderm\_formation | 1 | 0 |  |  |  |  |  |  |  |  |
| GO:0048385\_regulation\_of\_retinoic\_acid\_receptor\_signaling\_pathway | 1 | 0 |  |  |  |  |  |  |  |  |
| GO:0048387\_negative\_regulation\_of\_retinoic\_acid\_receptor\_signaling\_pathway | 1 | 0 |  |  |  |  |  |  |  |  |
| GO:0048388\_endosomal\_lumen\_acidification | 1 | 0 |  |  |  |  |  |  |  |  |
| GO:0048389\_intermediate\_mesoderm\_development | 1 | 0 |  |  |  |  |  |  |  |  |
| GO:0048478\_replication\_fork\_protection | 1 | 0 |  |  |  |  |  |  |  |  |
| GO:0048496\_maintenance\_of\_organ\_identity | 1 | 0 |  |  |  |  |  |  |  |  |
| GO:0048525\_negative\_regulation\_of\_viral\_reproduction | 1 | 0 |  |  |  |  |  |  |  |  |
| GO:0048539\_bone\_marrow\_development | 1 | 0 |  |  |  |  |  |  |  |  |
| GO:0048548\_regulation\_of\_pinocytosis | 1 | 0 |  |  |  |  |  |  |  |  |
| GO:0048549\_positive\_regulation\_of\_pinocytosis | 1 | 0 |  |  |  |  |  |  |  |  |
| GO:0048553\_negative\_regulation\_of\_metalloenzyme\_activity | 1 | 0 |  |  |  |  |  |  |  |  |
| GO:0048588\_developmental\_cell\_growth | 1 | 0 |  |  |  |  |  |  |  |  |
| GO:0048601\_oocyte\_morphogenesis | 1 | 0 |  |  |  |  |  |  |  |  |
| GO:0048621\_post-embryonic\_gut\_morphogenesis | 1 | 0 |  |  |  |  |  |  |  |  |
| GO:0048640\_negative\_regulation\_of\_developmental\_growth | 1 | 0 |  |  |  |  |  |  |  |  |
| GO:0048642\_negative\_regulation\_of\_skeletal\_muscle\_tissue\_development | 1 | 0 |  |  |  |  |  |  |  |  |
| GO:0048669\_collateral\_sprouting\_in\_the\_absence\_of\_injury | 1 | 0 |  |  |  |  |  |  |  |  |
| GO:0048680\_positive\_regulation\_of\_axon\_regeneration | 1 | 0 |  |  |  |  |  |  |  |  |
| GO:0048681\_negative\_regulation\_of\_axon\_regeneration | 1 | 0 |  |  |  |  |  |  |  |  |
| GO:0048686\_regulation\_of\_sprouting\_of\_injured\_axon | 1 | 0 |  |  |  |  |  |  |  |  |
| GO:0048687\_positive\_regulation\_of\_sprouting\_of\_injured\_axon | 1 | 0 |  |  |  |  |  |  |  |  |
| GO:0048690\_regulation\_of\_axon\_extension\_involved\_in\_regeneration | 1 | 0 |  |  |  |  |  |  |  |  |
| GO:0048691\_positive\_regulation\_of\_axon\_extension\_involved\_in\_regeneration | 1 | 0 |  |  |  |  |  |  |  |  |
| GO:0048714\_positive\_regulation\_of\_oligodendrocyte\_differentiation | 1 | 0 |  |  |  |  |  |  |  |  |
| GO:0048733\_sebaceous\_gland\_development | 1 | 0 |  |  |  |  |  |  |  |  |
| GO:0048743\_positive\_regulation\_of\_skeletal\_muscle\_fiber\_development | 1 | 0 |  |  |  |  |  |  |  |  |
| GO:0048752\_semicircular\_canal\_morphogenesis | 1 | 0 |  |  |  |  |  |  |  |  |
| GO:0048773\_erythrophore\_differentiation | 1 | 0 |  |  |  |  |  |  |  |  |
| GO:0048790\_maintenance\_of\_presynaptic\_active\_zone\_structure | 1 | 0 |  |  |  |  |  |  |  |  |
| GO:0048791\_calcium\_ion-dependent\_exocytosis\_of\_neurotransmitter | 1 | 0 |  |  |  |  |  |  |  |  |
| GO:0048822\_enucleate\_erythrocyte\_development | 1 | 0 |  |  |  |  |  |  |  |  |
| GO:0048866\_stem\_cell\_fate\_specification | 1 | 0 |  |  |  |  |  |  |  |  |
| GO:0048936\_peripheral\_nervous\_system\_neuron\_axonogenesis | 1 | 0 |  |  |  |  |  |  |  |  |
| GO:0050427\_3'-phosphoadenosine\_5'-phosphosulfate\_metabolic\_process | 1 | 0 |  |  |  |  |  |  |  |  |
| GO:0050428\_3'-phosphoadenosine\_5'-phosphosulfate\_biosynthetic\_process | 1 | 0 |  |  |  |  |  |  |  |  |
| GO:0050482\_arachidonic\_acid\_secretion | 1 | 0 |  |  |  |  |  |  |  |  |
| GO:0050667\_homocysteine\_metabolic\_process | 1 | 0 |  |  |  |  |  |  |  |  |
| GO:0050674\_urothelial\_cell\_proliferation | 1 | 0 |  |  |  |  |  |  |  |  |
| GO:0050675\_regulation\_of\_urothelial\_cell\_proliferation | 1 | 0 |  |  |  |  |  |  |  |  |
| GO:0050677\_positive\_regulation\_of\_urothelial\_cell\_proliferation | 1 | 0 |  |  |  |  |  |  |  |  |
| GO:0050691\_regulation\_of\_defense\_response\_to\_virus\_by\_host | 1 | 0 |  |  |  |  |  |  |  |  |
| GO:0050748\_negative\_regulation\_of\_lipoprotein\_metabolic\_process | 1 | 0 |  |  |  |  |  |  |  |  |
| GO:0050757\_thymidylate\_synthase\_biosynthetic\_process | 1 | 0 |  |  |  |  |  |  |  |  |
| GO:0050758\_regulation\_of\_thymidylate\_synthase\_biosynthetic\_process | 1 | 0 |  |  |  |  |  |  |  |  |
| GO:0050760\_negative\_regulation\_of\_thymidylate\_synthase\_biosynthetic\_process | 1 | 0 |  |  |  |  |  |  |  |  |
| GO:0050812\_regulation\_of\_acyl-CoA\_biosynthetic\_process | 1 | 0 |  |  |  |  |  |  |  |  |
| GO:0050832\_defense\_response\_to\_fungus | 1 | 0 |  |  |  |  |  |  |  |  |
| GO:0050861\_positive\_regulation\_of\_B\_cell\_receptor\_signaling\_pathway | 1 | 0 |  |  |  |  |  |  |  |  |
| GO:0050862\_positive\_regulation\_of\_T\_cell\_receptor\_signaling\_pathway | 1 | 0 |  |  |  |  |  |  |  |  |
| GO:0050916\_sensory\_perception\_of\_sweet\_taste | 1 | 0 |  |  |  |  |  |  |  |  |
| GO:0050975\_sensory\_perception\_of\_touch | 1 | 0 |  |  |  |  |  |  |  |  |
| GO:0050995\_negative\_regulation\_of\_lipid\_catabolic\_process | 1 | 0 |  |  |  |  |  |  |  |  |
| GO:0051001\_negative\_regulation\_of\_nitric-oxide\_synthase\_activity | 1 | 0 |  |  |  |  |  |  |  |  |
| GO:0051005\_negative\_regulation\_of\_lipoprotein\_lipase\_activity | 1 | 0 |  |  |  |  |  |  |  |  |
| GO:0051006\_positive\_regulation\_of\_lipoprotein\_lipase\_activity | 1 | 0 |  |  |  |  |  |  |  |  |
| GO:0051016\_barbed-end\_actin\_filament\_capping | 1 | 0 |  |  |  |  |  |  |  |  |
| GO:0051029\_rRNA\_transport | 1 | 0 |  |  |  |  |  |  |  |  |
| GO:0051043\_regulation\_of\_membrane\_protein\_ectodomain\_proteolysis | 1 | 0 |  |  |  |  |  |  |  |  |
| GO:0051044\_positive\_regulation\_of\_membrane\_protein\_ectodomain\_proteolysis | 1 | 0 |  |  |  |  |  |  |  |  |
| GO:0051088\_PMA-inducible\_membrane\_protein\_ectodomain\_proteolysis | 1 | 0 |  |  |  |  |  |  |  |  |
| GO:0051102\_DNA\_ligation\_during\_DNA\_recombination | 1 | 0 |  |  |  |  |  |  |  |  |
| GO:0051103\_DNA\_ligation\_during\_DNA\_repair | 1 | 0 |  |  |  |  |  |  |  |  |
| GO:0051123\_transcriptional\_preinitiation\_complex\_assembly | 1 | 0 |  |  |  |  |  |  |  |  |
| GO:0051125\_regulation\_of\_actin\_nucleation | 1 | 0 |  |  |  |  |  |  |  |  |
| GO:0051127\_positive\_regulation\_of\_actin\_nucleation | 1 | 0 |  |  |  |  |  |  |  |  |
| GO:0051151\_negative\_regulation\_of\_smooth\_muscle\_cell\_differentiation | 1 | 0 |  |  |  |  |  |  |  |  |
| GO:0051154\_negative\_regulation\_of\_striated\_muscle\_cell\_differentiation | 1 | 0 |  |  |  |  |  |  |  |  |
| GO:0051155\_positive\_regulation\_of\_striated\_muscle\_cell\_differentiation | 1 | 0 |  |  |  |  |  |  |  |  |
| GO:0051156\_glucose\_6-phosphate\_metabolic\_process | 1 | 0 |  |  |  |  |  |  |  |  |
| GO:0051187\_cofactor\_catabolic\_process | 1 | 0 |  |  |  |  |  |  |  |  |
| GO:0051189\_prosthetic\_group\_metabolic\_process | 1 | 0 |  |  |  |  |  |  |  |  |
| GO:0051193\_regulation\_of\_cofactor\_metabolic\_process | 1 | 0 |  |  |  |  |  |  |  |  |
| GO:0051196\_regulation\_of\_coenzyme\_metabolic\_process | 1 | 0 |  |  |  |  |  |  |  |  |
| GO:0051255\_spindle\_midzone\_assembly | 1 | 0 |  |  |  |  |  |  |  |  |
| GO:0051257\_spindle\_midzone\_assembly\_involved\_in\_meiosis | 1 | 0 |  |  |  |  |  |  |  |  |
| GO:0051281\_positive\_regulation\_of\_release\_of\_sequestered\_calcium\_ion\_into\_cytosol | 1 | 0 |  |  |  |  |  |  |  |  |
| GO:0051290\_protein\_heterotetramerization | 1 | 0 |  |  |  |  |  |  |  |  |
| GO:0051305\_chromosome\_movement\_towards\_spindle\_pole | 1 | 0 |  |  |  |  |  |  |  |  |
| GO:0051310\_metaphase\_plate\_congression | 1 | 0 |  |  |  |  |  |  |  |  |
| GO:0051311\_meiotic\_metaphase\_plate\_congression | 1 | 0 |  |  |  |  |  |  |  |  |
| GO:0051340\_regulation\_of\_ligase\_activity | 1 | 0 |  |  |  |  |  |  |  |  |
| GO:0051351\_positive\_regulation\_of\_ligase\_activity | 1 | 0 |  |  |  |  |  |  |  |  |
| GO:0051354\_negative\_regulation\_of\_oxidoreductase\_activity | 1 | 0 |  |  |  |  |  |  |  |  |
| GO:0051355\_proprioception\_during\_equilibrioception | 1 | 0 |  |  |  |  |  |  |  |  |
| GO:0051383\_kinetochore\_organization | 1 | 0 |  |  |  |  |  |  |  |  |
| GO:0051386\_regulation\_of\_nerve\_growth\_factor\_receptor\_signaling\_pathway | 1 | 0 |  |  |  |  |  |  |  |  |
| GO:0051409\_response\_to\_nitrosative\_stress | 1 | 0 |  |  |  |  |  |  |  |  |
| GO:0051457\_maintenance\_of\_protein\_location\_in\_nucleus | 1 | 0 |  |  |  |  |  |  |  |  |
| GO:0051462\_regulation\_of\_cortisol\_secretion | 1 | 0 |  |  |  |  |  |  |  |  |
| GO:0051463\_negative\_regulation\_of\_cortisol\_secretion | 1 | 0 |  |  |  |  |  |  |  |  |
| GO:0051481\_reduction\_of\_cytosolic\_calcium\_ion\_concentration | 1 | 0 |  |  |  |  |  |  |  |  |
| GO:0051482\_elevation\_of\_cytosolic\_calcium\_ion\_concentration\_during\_G-protein\_signaling\_\_coupled\_to\_IP3\_second\_messenger\_(phospholipase\_C\_activating) | 1 | 0 |  |  |  |  |  |  |  |  |
| GO:0051542\_elastin\_biosynthetic\_process | 1 | 0 |  |  |  |  |  |  |  |  |
| GO:0051568\_histone\_H3-K4\_methylation | 1 | 0 |  |  |  |  |  |  |  |  |
| GO:0051569\_regulation\_of\_histone\_H3-K4\_methylation | 1 | 0 |  |  |  |  |  |  |  |  |
| GO:0051570\_regulation\_of\_histone\_H3-K9\_methylation | 1 | 0 |  |  |  |  |  |  |  |  |
| GO:0051573\_negative\_regulation\_of\_histone\_H3-K9\_methylation | 1 | 0 |  |  |  |  |  |  |  |  |
| GO:0051580\_regulation\_of\_neurotransmitter\_uptake | 1 | 0 |  |  |  |  |  |  |  |  |
| GO:0051582\_positive\_regulation\_of\_neurotransmitter\_uptake | 1 | 0 |  |  |  |  |  |  |  |  |
| GO:0051584\_regulation\_of\_dopamine\_uptake | 1 | 0 |  |  |  |  |  |  |  |  |
| GO:0051586\_positive\_regulation\_of\_dopamine\_uptake | 1 | 0 |  |  |  |  |  |  |  |  |
| GO:0051589\_negative\_regulation\_of\_neurotransmitter\_transport | 1 | 0 |  |  |  |  |  |  |  |  |
| GO:0051593\_response\_to\_folic\_acid | 1 | 0 |  |  |  |  |  |  |  |  |
| GO:0051615\_histamine\_uptake | 1 | 0 |  |  |  |  |  |  |  |  |
| GO:0051646\_mitochondrion\_localization | 1 | 0 |  |  |  |  |  |  |  |  |
| GO:0051654\_establishment\_of\_mitochondrion\_localization | 1 | 0 |  |  |  |  |  |  |  |  |
| GO:0051661\_maintenance\_of\_centrosome\_location | 1 | 0 |  |  |  |  |  |  |  |  |
| GO:0051665\_membrane\_raft\_localization | 1 | 0 |  |  |  |  |  |  |  |  |
| GO:0051685\_maintenance\_of\_ER\_location | 1 | 0 |  |  |  |  |  |  |  |  |
| GO:0051693\_actin\_filament\_capping | 1 | 0 |  |  |  |  |  |  |  |  |
| GO:0051701\_interaction\_with\_host | 1 | 0 |  |  |  |  |  |  |  |  |
| GO:0051754\_meiotic\_sister\_chromatid\_cohesion\_\_centromeric | 1 | 0 |  |  |  |  |  |  |  |  |
| GO:0051782\_negative\_regulation\_of\_cell\_division | 1 | 0 |  |  |  |  |  |  |  |  |
| GO:0051790\_short-chain\_fatty\_acid\_biosynthetic\_process | 1 | 0 |  |  |  |  |  |  |  |  |
| GO:0051799\_negative\_regulation\_of\_hair\_follicle\_development | 1 | 0 |  |  |  |  |  |  |  |  |
| GO:0051823\_regulation\_of\_synapse\_structural\_plasticity | 1 | 0 |  |  |  |  |  |  |  |  |
| GO:0051865\_protein\_autoubiquitination | 1 | 0 |  |  |  |  |  |  |  |  |
| GO:0051901\_positive\_regulation\_of\_mitochondrial\_depolarization | 1 | 0 |  |  |  |  |  |  |  |  |
| GO:0051917\_regulation\_of\_fibrinolysis | 1 | 0 |  |  |  |  |  |  |  |  |
| GO:0051918\_negative\_regulation\_of\_fibrinolysis | 1 | 0 |  |  |  |  |  |  |  |  |
| GO:0051929\_positive\_regulation\_of\_calcium\_ion\_transport\_via\_voltage-gated\_calcium\_channel\_activity | 1 | 0 |  |  |  |  |  |  |  |  |
| GO:0051933\_amino\_acid\_uptake\_during\_transmission\_of\_nerve\_impulse | 1 | 0 |  |  |  |  |  |  |  |  |
| GO:0051935\_glutamate\_uptake\_during\_transmission\_of\_nerve\_impulse | 1 | 0 |  |  |  |  |  |  |  |  |
| GO:0051940\_regulation\_of\_catecholamine\_uptake\_during\_transmission\_of\_nerve\_impulse | 1 | 0 |  |  |  |  |  |  |  |  |
| GO:0051944\_positive\_regulation\_of\_catecholamine\_uptake\_during\_transmission\_of\_nerve\_impulse | 1 | 0 |  |  |  |  |  |  |  |  |
| GO:0051961\_negative\_regulation\_of\_nervous\_system\_development | 1 | 0 |  |  |  |  |  |  |  |  |
| GO:0051964\_negative\_regulation\_of\_synaptogenesis | 1 | 0 |  |  |  |  |  |  |  |  |
| GO:0051968\_positive\_regulation\_of\_synaptic\_transmission\_\_glutamatergic | 1 | 0 |  |  |  |  |  |  |  |  |
| GO:0051984\_positive\_regulation\_of\_chromosome\_segregation | 1 | 0 |  |  |  |  |  |  |  |  |
| GO:0051987\_positive\_regulation\_of\_attachment\_of\_spindle\_microtubules\_to\_kinetochore | 1 | 0 |  |  |  |  |  |  |  |  |
| GO:0052173\_response\_to\_defenses\_of\_other\_organism\_during\_symbiotic\_interaction | 1 | 0 |  |  |  |  |  |  |  |  |
| GO:0052200\_response\_to\_host\_defenses | 1 | 0 |  |  |  |  |  |  |  |  |
| GO:0052551\_response\_to\_defense-related\_nitric\_oxide\_production\_by\_other\_organism\_during\_symbiotic\_interaction | 1 | 0 |  |  |  |  |  |  |  |  |
| GO:0052564\_response\_to\_immune\_response\_of\_other\_organism\_during\_symbiotic\_interaction | 1 | 0 |  |  |  |  |  |  |  |  |
| GO:0052565\_response\_to\_defense-related\_host\_nitric\_oxide\_production | 1 | 0 |  |  |  |  |  |  |  |  |
| GO:0052572\_response\_to\_host\_immune\_response | 1 | 0 |  |  |  |  |  |  |  |  |
| GO:0055005\_ventricular\_cardiac\_myofibril\_development | 1 | 0 |  |  |  |  |  |  |  |  |
| GO:0055011\_atrial\_cardiac\_muscle\_cell\_differentiation | 1 | 0 |  |  |  |  |  |  |  |  |
| GO:0055014\_atrial\_cardiac\_muscle\_cell\_development | 1 | 0 |  |  |  |  |  |  |  |  |
| GO:0055078\_sodium\_ion\_homeostasis | 1 | 0 |  |  |  |  |  |  |  |  |
| GO:0055089\_fatty\_acid\_homeostasis | 1 | 0 |  |  |  |  |  |  |  |  |
| GO:0055093\_response\_to\_hyperoxia | 1 | 0 |  |  |  |  |  |  |  |  |
| GO:0060003\_copper\_ion\_export | 1 | 0 |  |  |  |  |  |  |  |  |
| GO:0060005\_vestibular\_reflex | 1 | 0 |  |  |  |  |  |  |  |  |
| GO:0060014\_granulosa\_cell\_differentiation | 1 | 0 |  |  |  |  |  |  |  |  |
| GO:0060018\_astrocyte\_fate\_commitment | 1 | 0 |  |  |  |  |  |  |  |  |
| GO:0060020\_Bergmann\_glial\_cell\_differentiation | 1 | 0 |  |  |  |  |  |  |  |  |
| GO:0060022\_hard\_palate\_development | 1 | 0 |  |  |  |  |  |  |  |  |
| GO:0060034\_notochord\_cell\_differentiation | 1 | 0 |  |  |  |  |  |  |  |  |
| GO:0060035\_notochord\_cell\_development | 1 | 0 |  |  |  |  |  |  |  |  |
| GO:0060046\_regulation\_of\_acrosome\_reaction | 1 | 0 |  |  |  |  |  |  |  |  |
| GO:0060054\_positive\_regulation\_of\_epithelial\_cell\_proliferation\_involved\_in\_wound\_healing | 1 | 0 |  |  |  |  |  |  |  |  |
| GO:0060059\_embryonic\_retina\_morphogenesis\_in\_camera-type\_eye | 1 | 0 |  |  |  |  |  |  |  |  |
| GO:0060061\_Spemann\_organizer\_formation | 1 | 0 |  |  |  |  |  |  |  |  |
| GO:0060064\_Spemann\_organizer\_formation\_at\_the\_anterior\_end\_of\_the\_primitive\_streak | 1 | 0 |  |  |  |  |  |  |  |  |
| GO:0060071\_Wnt\_receptor\_signaling\_pathway\_\_planar\_cell\_polarity\_pathway | 1 | 0 |  |  |  |  |  |  |  |  |
| GO:0060075\_regulation\_of\_resting\_membrane\_potential | 1 | 0 |  |  |  |  |  |  |  |  |
| GO:0060082\_eye\_blink\_reflex | 1 | 0 |  |  |  |  |  |  |  |  |
| GO:0060112\_generation\_of\_ovulation\_cycle\_rhythm | 1 | 0 |  |  |  |  |  |  |  |  |
| GO:0060125\_negative\_regulation\_of\_growth\_hormone\_secretion | 1 | 0 |  |  |  |  |  |  |  |  |
| GO:0060151\_peroxisome\_localization | 1 | 0 |  |  |  |  |  |  |  |  |
| GO:0060152\_microtubule-based\_peroxisome\_localization | 1 | 0 |  |  |  |  |  |  |  |  |
| GO:0060161\_positive\_regulation\_of\_dopamine\_receptor\_signaling\_pathway | 1 | 0 |  |  |  |  |  |  |  |  |
| GO:0060163\_subpallium\_neuron\_fate\_commitment | 1 | 0 |  |  |  |  |  |  |  |  |
| GO:0060165\_regulation\_of\_timing\_of\_subpallium\_neuron\_differentiation | 1 | 0 |  |  |  |  |  |  |  |  |
| GO:0060174\_limb\_bud\_formation | 1 | 0 |  |  |  |  |  |  |  |  |
| GO:0060177\_regulation\_of\_angiotensin\_metabolic\_process | 1 | 0 |  |  |  |  |  |  |  |  |
| GO:0060197\_cloacal\_septation | 1 | 0 |  |  |  |  |  |  |  |  |
| GO:0060215\_primitive\_hemopoiesis | 1 | 0 |  |  |  |  |  |  |  |  |
| GO:0060231\_mesenchymal\_to\_epithelial\_transition | 1 | 0 |  |  |  |  |  |  |  |  |
| GO:0060254\_regulation\_of\_N-terminal\_protein\_palmitoylation | 1 | 0 |  |  |  |  |  |  |  |  |
| GO:0060261\_positive\_regulation\_of\_transcription\_initiation\_from\_RNA\_polymerase\_II\_promoter | 1 | 0 |  |  |  |  |  |  |  |  |
| GO:0060262\_negative\_regulation\_of\_N-terminal\_protein\_palmitoylation | 1 | 0 |  |  |  |  |  |  |  |  |
| GO:0060263\_regulation\_of\_respiratory\_burst | 1 | 0 |  |  |  |  |  |  |  |  |
| GO:0060264\_regulation\_of\_respiratory\_burst\_during\_acute\_inflammatory\_response | 1 | 0 |  |  |  |  |  |  |  |  |
| GO:0060265\_positive\_regulation\_of\_respiratory\_burst\_during\_acute\_inflammatory\_response | 1 | 0 |  |  |  |  |  |  |  |  |
| GO:0060267\_positive\_regulation\_of\_respiratory\_burst | 1 | 0 |  |  |  |  |  |  |  |  |
| GO:0060272\_embryonic\_skeletal\_joint\_morphogenesis | 1 | 0 |  |  |  |  |  |  |  |  |
| GO:0060297\_regulation\_of\_sarcomere\_organization | 1 | 0 |  |  |  |  |  |  |  |  |
| GO:0060298\_positive\_regulation\_of\_sarcomere\_organization | 1 | 0 |  |  |  |  |  |  |  |  |
| GO:0060315\_negative\_regulation\_of\_ryanodine-sensitive\_calcium-release\_channel\_activity | 1 | 0 |  |  |  |  |  |  |  |  |
| GO:0060319\_primitive\_erythrocyte\_differentiation | 1 | 0 |  |  |  |  |  |  |  |  |
| GO:0060371\_regulation\_of\_atrial\_cardiomyocyte\_membrane\_depolarization | 1 | 0 |  |  |  |  |  |  |  |  |
| GO:0060374\_mast\_cell\_differentiation | 1 | 0 |  |  |  |  |  |  |  |  |
| GO:0060375\_regulation\_of\_mast\_cell\_differentiation | 1 | 0 |  |  |  |  |  |  |  |  |
| GO:0060376\_positive\_regulation\_of\_mast\_cell\_differentiation | 1 | 0 |  |  |  |  |  |  |  |  |
| GO:0060390\_regulation\_of\_SMAD\_protein\_nuclear\_translocation | 1 | 0 |  |  |  |  |  |  |  |  |
| GO:0060391\_positive\_regulation\_of\_SMAD\_protein\_nuclear\_translocation | 1 | 0 |  |  |  |  |  |  |  |  |
| GO:0060398\_regulation\_of\_growth\_hormone\_receptor\_signaling\_pathway | 1 | 0 |  |  |  |  |  |  |  |  |
| GO:0060399\_positive\_regulation\_of\_growth\_hormone\_receptor\_signaling\_pathway | 1 | 0 |  |  |  |  |  |  |  |  |
| GO:0060405\_regulation\_of\_penile\_erection | 1 | 0 |  |  |  |  |  |  |  |  |
| GO:0060407\_negative\_regulation\_of\_penile\_erection | 1 | 0 |  |  |  |  |  |  |  |  |
| GO:0060413\_atrial\_septum\_morphogenesis | 1 | 0 |  |  |  |  |  |  |  |  |
| GO:0060414\_aorta\_smooth\_muscle\_tissue\_morphogenesis | 1 | 0 |  |  |  |  |  |  |  |  |
| GO:0060419\_heart\_growth | 1 | 0 |  |  |  |  |  |  |  |  |
| GO:0060420\_regulation\_of\_heart\_growth | 1 | 0 |  |  |  |  |  |  |  |  |
| GO:0060421\_positive\_regulation\_of\_heart\_growth | 1 | 0 |  |  |  |  |  |  |  |  |
| GO:0060431\_primary\_lung\_bud\_formation | 1 | 0 |  |  |  |  |  |  |  |  |
| GO:0060436\_bronchiole\_morphogenesis | 1 | 0 |  |  |  |  |  |  |  |  |
| GO:0060440\_trachea\_formation | 1 | 0 |  |  |  |  |  |  |  |  |
| GO:0060449\_bud\_elongation\_involved\_in\_lung\_branching | 1 | 0 |  |  |  |  |  |  |  |  |
| GO:0060456\_positive\_regulation\_of\_digestive\_system\_process | 1 | 0 |  |  |  |  |  |  |  |  |
| GO:0060461\_right\_lung\_morphogenesis | 1 | 0 |  |  |  |  |  |  |  |  |
| GO:0060481\_lobar\_bronchus\_epithelium\_development | 1 | 0 |  |  |  |  |  |  |  |  |
| GO:0060482\_lobar\_bronchus\_development | 1 | 0 |  |  |  |  |  |  |  |  |
| GO:0060484\_lung-associated\_mesenchyme\_development | 1 | 0 |  |  |  |  |  |  |  |  |
| GO:0060486\_Clara\_cell\_differentiation | 1 | 0 |  |  |  |  |  |  |  |  |
| GO:0060510\_Type\_II\_pneumocyte\_differentiation | 1 | 0 |  |  |  |  |  |  |  |  |
| GO:0060514\_prostate\_induction | 1 | 0 |  |  |  |  |  |  |  |  |
| GO:0060515\_prostate\_field\_specification | 1 | 0 |  |  |  |  |  |  |  |  |
| GO:0060517\_epithelial\_cell\_proliferation\_involved\_in\_prostatic\_bud\_elongation | 1 | 0 |  |  |  |  |  |  |  |  |
| GO:0060520\_activation\_of\_prostate\_induction\_by\_androgen\_receptor\_signaling\_pathway | 1 | 0 |  |  |  |  |  |  |  |  |
| GO:0060535\_trachea\_cartilage\_morphogenesis | 1 | 0 |  |  |  |  |  |  |  |  |
| GO:0060536\_cartilage\_morphogenesis | 1 | 0 |  |  |  |  |  |  |  |  |
| GO:0060563\_neuroepithelial\_cell\_differentiation | 1 | 0 |  |  |  |  |  |  |  |  |
| GO:0060577\_pulmonary\_vein\_morphogenesis | 1 | 0 |  |  |  |  |  |  |  |  |
| GO:0060578\_superior\_vena\_cava\_morphogenesis | 1 | 0 |  |  |  |  |  |  |  |  |
| GO:0060584\_regulation\_of\_prostaglandin-endoperoxide\_synthase\_activity | 1 | 0 |  |  |  |  |  |  |  |  |
| GO:0060585\_positive\_regulation\_of\_prostaglandin-endoperoxidase\_synthase\_activity | 1 | 0 |  |  |  |  |  |  |  |  |
| GO:0060598\_dichotomous\_subdivision\_of\_terminal\_units\_involved\_in\_mammary\_gland\_duct\_morphogenesis | 1 | 0 |  |  |  |  |  |  |  |  |
| GO:0060611\_mammary\_gland\_fat\_development | 1 | 0 |  |  |  |  |  |  |  |  |
| GO:0060618\_nipple\_development | 1 | 0 |  |  |  |  |  |  |  |  |
| GO:0060631\_regulation\_of\_meiosis\_I | 1 | 0 |  |  |  |  |  |  |  |  |
| GO:0060649\_mammary\_gland\_bud\_elongation | 1 | 0 |  |  |  |  |  |  |  |  |
| GO:0060658\_nipple\_morphogenesis | 1 | 0 |  |  |  |  |  |  |  |  |
| GO:0060659\_nipple\_sheath\_formation | 1 | 0 |  |  |  |  |  |  |  |  |
| GO:0060668\_regulation\_of\_branching\_involved\_in\_salivary\_gland\_morphogenesis\_by\_extracellular\_matrix-epithelial\_cell\_signaling | 1 | 0 |  |  |  |  |  |  |  |  |
| GO:0060683\_regulation\_of\_branching\_involved\_in\_salivary\_gland\_morphogenesis\_by\_epithelial-mesenchymal\_signaling | 1 | 0 |  |  |  |  |  |  |  |  |
| GO:0060691\_epithelial\_cell\_maturation\_involved\_in\_salivary\_gland\_development | 1 | 0 |  |  |  |  |  |  |  |  |
| GO:0060709\_glycogen\_cell\_development\_involved\_in\_embryonic\_placenta\_development | 1 | 0 |  |  |  |  |  |  |  |  |
| GO:0060732\_positive\_regulation\_of\_inositol\_phosphate\_biosynthetic\_process | 1 | 0 |  |  |  |  |  |  |  |  |
| GO:0060739\_mesenchymal-epithelial\_cell\_signaling\_involved\_in\_prostate\_gland\_development | 1 | 0 |  |  |  |  |  |  |  |  |
| GO:0060781\_mesenchymal\_cell\_proliferation\_involved\_in\_prostate\_gland\_development | 1 | 0 |  |  |  |  |  |  |  |  |
| GO:0060782\_regulation\_of\_mesenchymal\_cell\_proliferation\_involved\_in\_prostate\_gland\_development | 1 | 0 |  |  |  |  |  |  |  |  |
| GO:0060783\_mesenchymal\_smoothened\_signaling\_pathway\_involved\_in\_prostate\_gland\_development | 1 | 0 |  |  |  |  |  |  |  |  |
| GO:0060872\_semicircular\_canal\_development | 1 | 0 |  |  |  |  |  |  |  |  |
| GO:0060896\_neural\_plate\_pattern\_specification | 1 | 0 |  |  |  |  |  |  |  |  |
| GO:0070091\_glucagon\_secretion | 1 | 0 |  |  |  |  |  |  |  |  |
| GO:0070162\_adiponectin\_secretion | 1 | 0 |  |  |  |  |  |  |  |  |
| GO:0070163\_regulation\_of\_adiponectin\_secretion | 1 | 0 |  |  |  |  |  |  |  |  |
| GO:0070164\_negative\_regulation\_of\_adiponectin\_secretion | 1 | 0 |  |  |  |  |  |  |  |  |
| GO:0070178\_D-serine\_metabolic\_process | 1 | 0 |  |  |  |  |  |  |  |  |
| GO:0070179\_D-serine\_biosynthetic\_process | 1 | 0 |  |  |  |  |  |  |  |  |
| GO:0070296\_sarcoplasmic\_reticulum\_calcium\_ion\_transport | 1 | 0 |  |  |  |  |  |  |  |  |
| GO:0070303\_negative\_regulation\_of\_stress-activated\_protein\_kinase\_signaling\_pathway | 1 | 0 |  |  |  |  |  |  |  |  |
| GO:0070328\_triglyceride\_homeostasis | 1 | 0 |  |  |  |  |  |  |  |  |
| GO:0070365\_hepatocyte\_differentiation | 1 | 0 |  |  |  |  |  |  |  |  |
| GO:0070384\_Harderian\_gland\_development | 1 | 0 |  |  |  |  |  |  |  |  |
| GO:0070391\_response\_to\_lipoteichoic\_acid | 1 | 0 |  |  |  |  |  |  |  |  |
| GO:0070424\_regulation\_of\_nucleotide-binding\_oligomerization\_domain\_containing\_signaling\_pathway | 1 | 0 |  |  |  |  |  |  |  |  |
| GO:0070426\_positive\_regulation\_of\_nucleotide-binding\_oligomerization\_domain\_containing\_signaling\_pathway | 1 | 0 |  |  |  |  |  |  |  |  |
| GO:0070428\_regulation\_of\_nucleotide-binding\_oligomerization\_domain\_containing\_1\_signaling\_pathway | 1 | 0 |  |  |  |  |  |  |  |  |
| GO:0070430\_positive\_regulation\_of\_nucleotide-binding\_oligomerization\_domain\_containing\_1\_signaling\_pathway | 1 | 0 |  |  |  |  |  |  |  |  |
| GO:0070432\_regulation\_of\_nucleotide-binding\_oligomerization\_domain\_containing\_2\_signaling\_pathway | 1 | 0 |  |  |  |  |  |  |  |  |
| GO:0070434\_positive\_regulation\_of\_nucleotide-binding\_oligomerization\_domain\_containing\_2\_signaling\_pathway | 1 | 0 |  |  |  |  |  |  |  |  |
| GO:0070493\_thrombin\_receptor\_signaling\_pathway | 1 | 0 |  |  |  |  |  |  |  |  |
| GO:0070508\_cholesterol\_import | 1 | 0 |  |  |  |  |  |  |  |  |
| GO:0070527\_platelet\_aggregation | 1 | 0 |  |  |  |  |  |  |  |  |
| GO:0070528\_protein\_kinase\_C\_signaling\_cascade | 1 | 0 |  |  |  |  |  |  |  |  |
| GO:0070555\_response\_to\_interleukin-1 | 1 | 0 |  |  |  |  |  |  |  |  |
| GO:0070560\_protein\_secretion\_by\_platelet | 1 | 0 |  |  |  |  |  |  |  |  |
| GO:0070561\_vitamin\_D\_receptor\_signaling\_pathway | 1 | 0 |  |  |  |  |  |  |  |  |
| GO:0070562\_regulation\_of\_vitamin\_D\_receptor\_signaling\_pathway | 1 | 0 |  |  |  |  |  |  |  |  |
| GO:0070571\_negative\_regulation\_of\_neuron\_projection\_regeneration | 1 | 0 |  |  |  |  |  |  |  |  |
| GO:0070572\_positive\_regulation\_of\_neuron\_projection\_regeneration | 1 | 0 |  |  |  |  |  |  |  |  |
| GO:0070613\_regulation\_of\_protein\_processing | 1 | 0 |  |  |  |  |  |  |  |  |
| GO:0070627\_ferrous\_iron\_import | 1 | 0 |  |  |  |  |  |  |  |  |
| GO:0070669\_response\_to\_interleukin-2 | 1 | 0 |  |  |  |  |  |  |  |  |
| GO:0070670\_response\_to\_interleukin-4 | 1 | 0 |  |  |  |  |  |  |  |  |
| GO:0070671\_response\_to\_interleukin-12 | 1 | 0 |  |  |  |  |  |  |  |  |
| GO:0070672\_response\_to\_interleukin-15 | 1 | 0 |  |  |  |  |  |  |  |  |
| GO:0070673\_response\_to\_interleukin-18 | 1 | 0 |  |  |  |  |  |  |  |  |
| GO:0070828\_heterochromatin\_organization | 1 | 0 |  |  |  |  |  |  |  |  |
| GO:0070874\_negative\_regulation\_of\_glycogen\_metabolic\_process | 1 | 0 |  |  |  |  |  |  |  |  |
| GO:0075136\_response\_to\_host | 1 | 0 |  |  |  |  |  |  |  |  |
| GO:0080010\_regulation\_of\_oxygen\_and\_reactive\_oxygen\_species\_metabolic\_process | 1 | 0 |  |  |  |  |  |  |  |  |
| GO:0090032\_negative\_regulation\_of\_steroid\_hormone\_biosynthetic\_process | 1 | 0 |  |  |  |  |  |  |  |  |
| GO:0001708\_cell\_fate\_specification | 56 | 0 | 0.000000 | -0.000000 | 567 | 503.102100 | 604.42 | 705.737900 | 1.065996 |
| GO:0002683\_negative\_regulation\_of\_immune\_system\_process | 56 | 0 | 0.000000 | -0.000000 | 567 | 503.102100 | 604.42 | 705.737900 | 1.065996 |
| GO:0002703\_regulation\_of\_leukocyte\_mediated\_immunity | 56 | 0 | 0.000000 | -0.000000 | 567 | 503.102100 | 604.42 | 705.737900 | 1.065996 |
| GO:0006790\_sulfur\_metabolic\_process | 56 | 0 | 0.000000 | -0.000000 | 567 | 503.102100 | 604.42 | 705.737900 | 1.065996 |
| GO:0009187\_cyclic\_nucleotide\_metabolic\_process | 56 | 0 | 0.000000 | -0.000000 | 567 | 503.102100 | 604.42 | 705.737900 | 1.065996 |
| GO:0042089\_cytokine\_biosynthetic\_process | 56 | 0 | 0.000000 | -0.000000 | 567 | 503.102100 | 604.42 | 705.737900 | 1.065996 |
| GO:0042107\_cytokine\_metabolic\_process | 56 | 0 | 0.000000 | -0.000000 | 567 | 503.102100 | 604.42 | 705.737900 | 1.065996 |
| GO:0046486\_glycerolipid\_metabolic\_process | 56 | 0 | 0.000000 | -0.000000 | 567 | 503.102100 | 604.42 | 705.737900 | 1.065996 |
| GO:0051321\_meiotic\_cell\_cycle | 56 | 0 | 0.000000 | -0.000000 | 567 | 503.102100 | 604.42 | 705.737900 | 1.065996 |
| GO:0003013\_circulatory\_system\_process | 103 | 0 | 0.000000 | -0.000000 | 570 | 505.672024 | 606.71 | 707.747976 | 1.064404 |
| GO:0008015\_blood\_circulation | 103 | 0 | 0.000000 | -0.000000 | 570 | 505.672024 | 606.71 | 707.747976 | 1.064404 |
| GO:0009968\_negative\_regulation\_of\_signal\_transduction | 103 | 0 | 0.000000 | -0.000000 | 570 | 505.672024 | 606.71 | 707.747976 | 1.064404 |
| GO:0000302\_response\_to\_reactive\_oxygen\_species | 16 | 0 | 0.000000 | -0.000000 | 609 | 550.790583 | 651.06 | 751.329417 | 1.069064 |
| GO:0001933\_negative\_regulation\_of\_protein\_amino\_acid\_phosphorylation | 16 | 0 | 0.000000 | -0.000000 | 609 | 550.790583 | 651.06 | 751.329417 | 1.069064 |
| GO:0003044\_regulation\_of\_systemic\_arterial\_blood\_pressure\_mediated\_by\_a\_chemical\_signal | 16 | 0 | 0.000000 | -0.000000 | 609 | 550.790583 | 651.06 | 751.329417 | 1.069064 |
| GO:0006821\_chloride\_transport | 16 | 0 | 0.000000 | -0.000000 | 609 | 550.790583 | 651.06 | 751.329417 | 1.069064 |
| GO:0007156\_homophilic\_cell\_adhesion | 16 | 0 | 0.000000 | -0.000000 | 609 | 550.790583 | 651.06 | 751.329417 | 1.069064 |
| GO:0007602\_phototransduction | 16 | 0 | 0.000000 | -0.000000 | 609 | 550.790583 | 651.06 | 751.329417 | 1.069064 |
| GO:0008654\_phospholipid\_biosynthetic\_process | 16 | 0 | 0.000000 | -0.000000 | 609 | 550.790583 | 651.06 | 751.329417 | 1.069064 |
| GO:0009988\_cell-cell\_recognition | 16 | 0 | 0.000000 | -0.000000 | 609 | 550.790583 | 651.06 | 751.329417 | 1.069064 |
| GO:0010038\_response\_to\_metal\_ion | 16 | 0 | 0.000000 | -0.000000 | 609 | 550.790583 | 651.06 | 751.329417 | 1.069064 |
| GO:0016126\_sterol\_biosynthetic\_process | 16 | 0 | 0.000000 | -0.000000 | 609 | 550.790583 | 651.06 | 751.329417 | 1.069064 |
| GO:0019722\_calcium-mediated\_signaling | 16 | 0 | 0.000000 | -0.000000 | 609 | 550.790583 | 651.06 | 751.329417 | 1.069064 |
| GO:0019751\_polyol\_metabolic\_process | 16 | 0 | 0.000000 | -0.000000 | 609 | 550.790583 | 651.06 | 751.329417 | 1.069064 |
| GO:0021522\_spinal\_cord\_motor\_neuron\_differentiation | 16 | 0 | 0.000000 | -0.000000 | 609 | 550.790583 | 651.06 | 751.329417 | 1.069064 |
| GO:0021696\_cerebellar\_cortex\_morphogenesis | 16 | 0 | 0.000000 | -0.000000 | 609 | 550.790583 | 651.06 | 751.329417 | 1.069064 |
| GO:0030890\_positive\_regulation\_of\_B\_cell\_proliferation | 16 | 0 | 0.000000 | -0.000000 | 609 | 550.790583 | 651.06 | 751.329417 | 1.069064 |
| GO:0031345\_negative\_regulation\_of\_cell\_projection\_organization | 16 | 0 | 0.000000 | -0.000000 | 609 | 550.790583 | 651.06 | 751.329417 | 1.069064 |
| GO:0031669\_cellular\_response\_to\_nutrient\_levels | 16 | 0 | 0.000000 | -0.000000 | 609 | 550.790583 | 651.06 | 751.329417 | 1.069064 |
| GO:0032663\_regulation\_of\_interleukin-2\_production | 16 | 0 | 0.000000 | -0.000000 | 609 | 550.790583 | 651.06 | 751.329417 | 1.069064 |
| GO:0032956\_regulation\_of\_actin\_cytoskeleton\_organization | 16 | 0 | 0.000000 | -0.000000 | 609 | 550.790583 | 651.06 | 751.329417 | 1.069064 |
| GO:0042311\_vasodilation | 16 | 0 | 0.000000 | -0.000000 | 609 | 550.790583 | 651.06 | 751.329417 | 1.069064 |
| GO:0042594\_response\_to\_starvation | 16 | 0 | 0.000000 | -0.000000 | 609 | 550.790583 | 651.06 | 751.329417 | 1.069064 |
| GO:0042596\_fear\_response | 16 | 0 | 0.000000 | -0.000000 | 609 | 550.790583 | 651.06 | 751.329417 | 1.069064 |
| GO:0043087\_regulation\_of\_GTPase\_activity | 16 | 0 | 0.000000 | -0.000000 | 609 | 550.790583 | 651.06 | 751.329417 | 1.069064 |
| GO:0043122\_regulation\_of\_I-kappaB\_kinase\_NF-kappaB\_cascade | 16 | 0 | 0.000000 | -0.000000 | 609 | 550.790583 | 651.06 | 751.329417 | 1.069064 |
| GO:0043367\_CD4-positive\_\_alpha\_beta\_T\_cell\_differentiation | 16 | 0 | 0.000000 | -0.000000 | 609 | 550.790583 | 651.06 | 751.329417 | 1.069064 |
| GO:0045104\_intermediate\_filament\_cytoskeleton\_organization | 16 | 0 | 0.000000 | -0.000000 | 609 | 550.790583 | 651.06 | 751.329417 | 1.069064 |
| GO:0046364\_monosaccharide\_biosynthetic\_process | 16 | 0 | 0.000000 | -0.000000 | 609 | 550.790583 | 651.06 | 751.329417 | 1.069064 |
| GO:0046467\_membrane\_lipid\_biosynthetic\_process | 16 | 0 | 0.000000 | -0.000000 | 609 | 550.790583 | 651.06 | 751.329417 | 1.069064 |
| GO:0046633\_alpha-beta\_T\_cell\_proliferation | 16 | 0 | 0.000000 | -0.000000 | 609 | 550.790583 | 651.06 | 751.329417 | 1.069064 |
| GO:0046700\_heterocycle\_catabolic\_process | 16 | 0 | 0.000000 | -0.000000 | 609 | 550.790583 | 651.06 | 751.329417 | 1.069064 |
| GO:0048015\_phosphoinositide-mediated\_signaling | 16 | 0 | 0.000000 | -0.000000 | 609 | 550.790583 | 651.06 | 751.329417 | 1.069064 |
| GO:0048286\_lung\_alveolus\_development | 16 | 0 | 0.000000 | -0.000000 | 609 | 550.790583 | 651.06 | 751.329417 | 1.069064 |
| GO:0048483\_autonomic\_nervous\_system\_development | 16 | 0 | 0.000000 | -0.000000 | 609 | 550.790583 | 651.06 | 751.329417 | 1.069064 |
| GO:0050974\_detection\_of\_mechanical\_stimulus\_involved\_in\_sensory\_perception | 16 | 0 | 0.000000 | -0.000000 | 609 | 550.790583 | 651.06 | 751.329417 | 1.069064 |
| GO:0051048\_negative\_regulation\_of\_secretion | 16 | 0 | 0.000000 | -0.000000 | 609 | 550.790583 | 651.06 | 751.329417 | 1.069064 |
| GO:0051937\_catecholamine\_transport | 16 | 0 | 0.000000 | -0.000000 | 609 | 550.790583 | 651.06 | 751.329417 | 1.069064 |
| GO:0055007\_cardiac\_muscle\_cell\_differentiation | 16 | 0 | 0.000000 | -0.000000 | 609 | 550.790583 | 651.06 | 751.329417 | 1.069064 |
| GO:0060193\_positive\_regulation\_of\_lipase\_activity | 16 | 0 | 0.000000 | -0.000000 | 609 | 550.790583 | 651.06 | 751.329417 | 1.069064 |
| GO:0060713\_labyrinthine\_layer\_morphogenesis | 16 | 0 | 0.000000 | -0.000000 | 609 | 550.790583 | 651.06 | 751.329417 | 1.069064 |
| GO:0000082\_G1\_S\_transition\_of\_mitotic\_cell\_cycle | 23 | 0 | 0.000000 | -0.000000 | 632 | 573.510809 | 672.8 | 772.089191 | 1.064557 |
| GO:0002204\_somatic\_recombination\_of\_immunoglobulin\_genes\_during\_immune\_response | 23 | 0 | 0.000000 | -0.000000 | 632 | 573.510809 | 672.8 | 772.089191 | 1.064557 |
| GO:0002208\_somatic\_diversification\_of\_immunoglobulins\_during\_immune\_response | 23 | 0 | 0.000000 | -0.000000 | 632 | 573.510809 | 672.8 | 772.089191 | 1.064557 |
| GO:0002228\_natural\_killer\_cell\_mediated\_immunity | 23 | 0 | 0.000000 | -0.000000 | 632 | 573.510809 | 672.8 | 772.089191 | 1.064557 |
| GO:0002821\_positive\_regulation\_of\_adaptive\_immune\_response | 23 | 0 | 0.000000 | -0.000000 | 632 | 573.510809 | 672.8 | 772.089191 | 1.064557 |
| GO:0002824\_positive\_regulation\_of\_adaptive\_immune\_response\_based\_on\_somatic\_recombination\_of\_immune\_receptors\_built\_from\_immunoglobulin\_superfamily\_domains | 23 | 0 | 0.000000 | -0.000000 | 632 | 573.510809 | 672.8 | 772.089191 | 1.064557 |
| GO:0003073\_regulation\_of\_systemic\_arterial\_blood\_pressure | 23 | 0 | 0.000000 | -0.000000 | 632 | 573.510809 | 672.8 | 772.089191 | 1.064557 |
| GO:0006397\_mRNA\_processing | 23 | 0 | 0.000000 | -0.000000 | 632 | 573.510809 | 672.8 | 772.089191 | 1.064557 |
| GO:0007018\_microtubule-based\_movement | 23 | 0 | 0.000000 | -0.000000 | 632 | 573.510809 | 672.8 | 772.089191 | 1.064557 |
| GO:0007584\_response\_to\_nutrient | 23 | 0 | 0.000000 | -0.000000 | 632 | 573.510809 | 672.8 | 772.089191 | 1.064557 |
| GO:0008542\_visual\_learning | 23 | 0 | 0.000000 | -0.000000 | 632 | 573.510809 | 672.8 | 772.089191 | 1.064557 |
| GO:0009954\_proximal\_distal\_pattern\_formation | 23 | 0 | 0.000000 | -0.000000 | 632 | 573.510809 | 672.8 | 772.089191 | 1.064557 |
| GO:0015698\_inorganic\_anion\_transport | 23 | 0 | 0.000000 | -0.000000 | 632 | 573.510809 | 672.8 | 772.089191 | 1.064557 |
| GO:0022613\_ribonucleoprotein\_complex\_biogenesis | 23 | 0 | 0.000000 | -0.000000 | 632 | 573.510809 | 672.8 | 772.089191 | 1.064557 |
| GO:0030512\_negative\_regulation\_of\_transforming\_growth\_factor\_beta\_receptor\_signaling\_pathway | 23 | 0 | 0.000000 | -0.000000 | 632 | 573.510809 | 672.8 | 772.089191 | 1.064557 |
| GO:0032635\_interleukin-6\_production | 23 | 0 | 0.000000 | -0.000000 | 632 | 573.510809 | 672.8 | 772.089191 | 1.064557 |
| GO:0032675\_regulation\_of\_interleukin-6\_production | 23 | 0 | 0.000000 | -0.000000 | 632 | 573.510809 | 672.8 | 772.089191 | 1.064557 |
| GO:0042267\_natural\_killer\_cell\_mediated\_cytotoxicity | 23 | 0 | 0.000000 | -0.000000 | 632 | 573.510809 | 672.8 | 772.089191 | 1.064557 |
| GO:0043388\_positive\_regulation\_of\_DNA\_binding | 23 | 0 | 0.000000 | -0.000000 | 632 | 573.510809 | 672.8 | 772.089191 | 1.064557 |
| GO:0045190\_isotype\_switching | 23 | 0 | 0.000000 | -0.000000 | 632 | 573.510809 | 672.8 | 772.089191 | 1.064557 |
| GO:0051705\_behavioral\_interaction\_between\_organisms | 23 | 0 | 0.000000 | -0.000000 | 632 | 573.510809 | 672.8 | 772.089191 | 1.064557 |
| GO:0060349\_bone\_morphogenesis | 23 | 0 | 0.000000 | -0.000000 | 632 | 573.510809 | 672.8 | 772.089191 | 1.064557 |
| GO:0060445\_branching\_involved\_in\_salivary\_gland\_morphogenesis | 23 | 0 | 0.000000 | -0.000000 | 632 | 573.510809 | 672.8 | 772.089191 | 1.064557 |
| GO:0006812\_cation\_transport | 146 | 0 | 0.000000 | -0.000000 | 634 | 575.746366 | 674.62 | 773.493634 | 1.064069 |
| GO:0030900\_forebrain\_development | 146 | 0 | 0.000000 | -0.000000 | 634 | 575.746366 | 674.62 | 773.493634 | 1.064069 |
| GO:0000002\_mitochondrial\_genome\_maintenance | 9 | 0 | 0.000000 | -0.000000 | 751 | 692.650011 | 789.34 | 886.029989 | 1.051052 |
| GO:0000186\_activation\_of\_MAPKK\_activity | 9 | 0 | 0.000000 | -0.000000 | 751 | 692.650011 | 789.34 | 886.029989 | 1.051052 |
| GO:0001539\_ciliary\_or\_flagellar\_motility | 9 | 0 | 0.000000 | -0.000000 | 751 | 692.650011 | 789.34 | 886.029989 | 1.051052 |
| GO:0001542\_ovulation\_from\_ovarian\_follicle | 9 | 0 | 0.000000 | -0.000000 | 751 | 692.650011 | 789.34 | 886.029989 | 1.051052 |
| GO:0001667\_ameboidal\_cell\_migration | 9 | 0 | 0.000000 | -0.000000 | 751 | 692.650011 | 789.34 | 886.029989 | 1.051052 |
| GO:0001676\_long-chain\_fatty\_acid\_metabolic\_process | 9 | 0 | 0.000000 | -0.000000 | 751 | 692.650011 | 789.34 | 886.029989 | 1.051052 |
| GO:0001935\_endothelial\_cell\_proliferation | 9 | 0 | 0.000000 | -0.000000 | 751 | 692.650011 | 789.34 | 886.029989 | 1.051052 |
| GO:0002021\_response\_to\_dietary\_excess | 9 | 0 | 0.000000 | -0.000000 | 751 | 692.650011 | 789.34 | 886.029989 | 1.051052 |
| GO:0002028\_regulation\_of\_sodium\_ion\_transport | 9 | 0 | 0.000000 | -0.000000 | 751 | 692.650011 | 789.34 | 886.029989 | 1.051052 |
| GO:0002221\_pattern\_recognition\_receptor\_signaling\_pathway | 9 | 0 | 0.000000 | -0.000000 | 751 | 692.650011 | 789.34 | 886.029989 | 1.051052 |
| GO:0002292\_T\_cell\_differentiation\_during\_immune\_response | 9 | 0 | 0.000000 | -0.000000 | 751 | 692.650011 | 789.34 | 886.029989 | 1.051052 |
| GO:0002293\_alpha-beta\_T\_cell\_differentiation\_during\_immune\_response | 9 | 0 | 0.000000 | -0.000000 | 751 | 692.650011 | 789.34 | 886.029989 | 1.051052 |
| GO:0002294\_CD4-positive\_\_alpha-beta\_T\_cell\_differentiation\_during\_immune\_response | 9 | 0 | 0.000000 | -0.000000 | 751 | 692.650011 | 789.34 | 886.029989 | 1.051052 |
| GO:0002507\_tolerance\_induction | 9 | 0 | 0.000000 | -0.000000 | 751 | 692.650011 | 789.34 | 886.029989 | 1.051052 |
| GO:0002886\_regulation\_of\_myeloid\_leukocyte\_mediated\_immunity | 9 | 0 | 0.000000 | -0.000000 | 751 | 692.650011 | 789.34 | 886.029989 | 1.051052 |
| GO:0006007\_glucose\_catabolic\_process | 9 | 0 | 0.000000 | -0.000000 | 751 | 692.650011 | 789.34 | 886.029989 | 1.051052 |
| GO:0006182\_cGMP\_biosynthetic\_process | 9 | 0 | 0.000000 | -0.000000 | 751 | 692.650011 | 789.34 | 886.029989 | 1.051052 |
| GO:0006309\_DNA\_fragmentation\_involved\_in\_apoptosis | 9 | 0 | 0.000000 | -0.000000 | 751 | 692.650011 | 789.34 | 886.029989 | 1.051052 |
| GO:0006364\_rRNA\_processing | 9 | 0 | 0.000000 | -0.000000 | 751 | 692.650011 | 789.34 | 886.029989 | 1.051052 |
| GO:0006476\_protein\_amino\_acid\_deacetylation | 9 | 0 | 0.000000 | -0.000000 | 751 | 692.650011 | 789.34 | 886.029989 | 1.051052 |
| GO:0006595\_polyamine\_metabolic\_process | 9 | 0 | 0.000000 | -0.000000 | 751 | 692.650011 | 789.34 | 886.029989 | 1.051052 |
| GO:0006611\_protein\_export\_from\_nucleus | 9 | 0 | 0.000000 | -0.000000 | 751 | 692.650011 | 789.34 | 886.029989 | 1.051052 |
| GO:0006910\_phagocytosis\_\_recognition | 9 | 0 | 0.000000 | -0.000000 | 751 | 692.650011 | 789.34 | 886.029989 | 1.051052 |
| GO:0006911\_phagocytosis\_\_engulfment | 9 | 0 | 0.000000 | -0.000000 | 751 | 692.650011 | 789.34 | 886.029989 | 1.051052 |
| GO:0007128\_meiotic\_prophase\_I | 9 | 0 | 0.000000 | -0.000000 | 751 | 692.650011 | 789.34 | 886.029989 | 1.051052 |
| GO:0007193\_inhibition\_of\_adenylate\_cyclase\_activity\_by\_G-protein\_signaling | 9 | 0 | 0.000000 | -0.000000 | 751 | 692.650011 | 789.34 | 886.029989 | 1.051052 |
| GO:0007379\_segment\_specification | 9 | 0 | 0.000000 | -0.000000 | 751 | 692.650011 | 789.34 | 886.029989 | 1.051052 |
| GO:0007617\_mating\_behavior | 9 | 0 | 0.000000 | -0.000000 | 751 | 692.650011 | 789.34 | 886.029989 | 1.051052 |
| GO:0010165\_response\_to\_X-ray | 9 | 0 | 0.000000 | -0.000000 | 751 | 692.650011 | 789.34 | 886.029989 | 1.051052 |
| GO:0010675\_regulation\_of\_cellular\_carbohydrate\_metabolic\_process | 9 | 0 | 0.000000 | -0.000000 | 751 | 692.650011 | 789.34 | 886.029989 | 1.051052 |
| GO:0014037\_Schwann\_cell\_differentiation | 9 | 0 | 0.000000 | -0.000000 | 751 | 692.650011 | 789.34 | 886.029989 | 1.051052 |
| GO:0014073\_response\_to\_tropane | 9 | 0 | 0.000000 | -0.000000 | 751 | 692.650011 | 789.34 | 886.029989 | 1.051052 |
| GO:0015695\_organic\_cation\_transport | 9 | 0 | 0.000000 | -0.000000 | 751 | 692.650011 | 789.34 | 886.029989 | 1.051052 |
| GO:0016072\_rRNA\_metabolic\_process | 9 | 0 | 0.000000 | -0.000000 | 751 | 692.650011 | 789.34 | 886.029989 | 1.051052 |
| GO:0016601\_Rac\_protein\_signal\_transduction | 9 | 0 | 0.000000 | -0.000000 | 751 | 692.650011 | 789.34 | 886.029989 | 1.051052 |
| GO:0017145\_stem\_cell\_division | 9 | 0 | 0.000000 | -0.000000 | 751 | 692.650011 | 789.34 | 886.029989 | 1.051052 |
| GO:0019320\_hexose\_catabolic\_process | 9 | 0 | 0.000000 | -0.000000 | 751 | 692.650011 | 789.34 | 886.029989 | 1.051052 |
| GO:0021544\_subpallium\_development | 9 | 0 | 0.000000 | -0.000000 | 751 | 692.650011 | 789.34 | 886.029989 | 1.051052 |
| GO:0021936\_regulation\_of\_granule\_cell\_precursor\_proliferation | 9 | 0 | 0.000000 | -0.000000 | 751 | 692.650011 | 789.34 | 886.029989 | 1.051052 |
| GO:0021940\_positive\_regulation\_of\_granule\_cell\_precursor\_proliferation | 9 | 0 | 0.000000 | -0.000000 | 751 | 692.650011 | 789.34 | 886.029989 | 1.051052 |
| GO:0030048\_actin\_filament-based\_movement | 9 | 0 | 0.000000 | -0.000000 | 751 | 692.650011 | 789.34 | 886.029989 | 1.051052 |
| GO:0030279\_negative\_regulation\_of\_ossification | 9 | 0 | 0.000000 | -0.000000 | 751 | 692.650011 | 789.34 | 886.029989 | 1.051052 |
| GO:0030325\_adrenal\_gland\_development | 9 | 0 | 0.000000 | -0.000000 | 751 | 692.650011 | 789.34 | 886.029989 | 1.051052 |
| GO:0030728\_ovulation | 9 | 0 | 0.000000 | -0.000000 | 751 | 692.650011 | 789.34 | 886.029989 | 1.051052 |
| GO:0031023\_microtubule\_organizing\_center\_organization | 9 | 0 | 0.000000 | -0.000000 | 751 | 692.650011 | 789.34 | 886.029989 | 1.051052 |
| GO:0032388\_positive\_regulation\_of\_intracellular\_transport | 9 | 0 | 0.000000 | -0.000000 | 751 | 692.650011 | 789.34 | 886.029989 | 1.051052 |
| GO:0032606\_type\_I\_interferon\_production | 9 | 0 | 0.000000 | -0.000000 | 751 | 692.650011 | 789.34 | 886.029989 | 1.051052 |
| GO:0032814\_regulation\_of\_natural\_killer\_cell\_activation | 9 | 0 | 0.000000 | -0.000000 | 751 | 692.650011 | 789.34 | 886.029989 | 1.051052 |
| GO:0032816\_positive\_regulation\_of\_natural\_killer\_cell\_activation | 9 | 0 | 0.000000 | -0.000000 | 751 | 692.650011 | 789.34 | 886.029989 | 1.051052 |
| GO:0032963\_collagen\_metabolic\_process | 9 | 0 | 0.000000 | -0.000000 | 751 | 692.650011 | 789.34 | 886.029989 | 1.051052 |
| GO:0033028\_myeloid\_cell\_apoptosis | 9 | 0 | 0.000000 | -0.000000 | 751 | 692.650011 | 789.34 | 886.029989 | 1.051052 |
| GO:0033143\_regulation\_of\_steroid\_hormone\_receptor\_signaling\_pathway | 9 | 0 | 0.000000 | -0.000000 | 751 | 692.650011 | 789.34 | 886.029989 | 1.051052 |
| GO:0033151\_V(D)J\_recombination | 9 | 0 | 0.000000 | -0.000000 | 751 | 692.650011 | 789.34 | 886.029989 | 1.051052 |
| GO:0033344\_cholesterol\_efflux | 9 | 0 | 0.000000 | -0.000000 | 751 | 692.650011 | 789.34 | 886.029989 | 1.051052 |
| GO:0034605\_cellular\_response\_to\_heat | 9 | 0 | 0.000000 | -0.000000 | 751 | 692.650011 | 789.34 | 886.029989 | 1.051052 |
| GO:0035088\_establishment\_or\_maintenance\_of\_apical\_basal\_cell\_polarity | 9 | 0 | 0.000000 | -0.000000 | 751 | 692.650011 | 789.34 | 886.029989 | 1.051052 |
| GO:0035162\_embryonic\_hemopoiesis | 9 | 0 | 0.000000 | -0.000000 | 751 | 692.650011 | 789.34 | 886.029989 | 1.051052 |
| GO:0040020\_regulation\_of\_meiosis | 9 | 0 | 0.000000 | -0.000000 | 751 | 692.650011 | 789.34 | 886.029989 | 1.051052 |
| GO:0042058\_regulation\_of\_epidermal\_growth\_factor\_receptor\_signaling\_pathway | 9 | 0 | 0.000000 | -0.000000 | 751 | 692.650011 | 789.34 | 886.029989 | 1.051052 |
| GO:0042093\_T-helper\_cell\_differentiation | 9 | 0 | 0.000000 | -0.000000 | 751 | 692.650011 | 789.34 | 886.029989 | 1.051052 |
| GO:0042220\_response\_to\_cocaine | 9 | 0 | 0.000000 | -0.000000 | 751 | 692.650011 | 789.34 | 886.029989 | 1.051052 |
| GO:0042402\_biogenic\_amine\_catabolic\_process | 9 | 0 | 0.000000 | -0.000000 | 751 | 692.650011 | 789.34 | 886.029989 | 1.051052 |
| GO:0042509\_regulation\_of\_tyrosine\_phosphorylation\_of\_STAT\_protein | 9 | 0 | 0.000000 | -0.000000 | 751 | 692.650011 | 789.34 | 886.029989 | 1.051052 |
| GO:0042640\_anagen | 9 | 0 | 0.000000 | -0.000000 | 751 | 692.650011 | 789.34 | 886.029989 | 1.051052 |
| GO:0043242\_negative\_regulation\_of\_protein\_complex\_disassembly | 9 | 0 | 0.000000 | -0.000000 | 751 | 692.650011 | 789.34 | 886.029989 | 1.051052 |
| GO:0043299\_leukocyte\_degranulation | 9 | 0 | 0.000000 | -0.000000 | 751 | 692.650011 | 789.34 | 886.029989 | 1.051052 |
| GO:0043383\_negative\_T\_cell\_selection | 9 | 0 | 0.000000 | -0.000000 | 751 | 692.650011 | 789.34 | 886.029989 | 1.051052 |
| GO:0043409\_negative\_regulation\_of\_MAPKKK\_cascade | 9 | 0 | 0.000000 | -0.000000 | 751 | 692.650011 | 789.34 | 886.029989 | 1.051052 |
| GO:0043433\_negative\_regulation\_of\_transcription\_factor\_activity | 9 | 0 | 0.000000 | -0.000000 | 751 | 692.650011 | 789.34 | 886.029989 | 1.051052 |
| GO:0043603\_cellular\_amide\_metabolic\_process | 9 | 0 | 0.000000 | -0.000000 | 751 | 692.650011 | 789.34 | 886.029989 | 1.051052 |
| GO:0045060\_negative\_thymic\_T\_cell\_selection | 9 | 0 | 0.000000 | -0.000000 | 751 | 692.650011 | 789.34 | 886.029989 | 1.051052 |
| GO:0045109\_intermediate\_filament\_organization | 9 | 0 | 0.000000 | -0.000000 | 751 | 692.650011 | 789.34 | 886.029989 | 1.051052 |
| GO:0045136\_development\_of\_secondary\_sexual\_characteristics | 9 | 0 | 0.000000 | -0.000000 | 751 | 692.650011 | 789.34 | 886.029989 | 1.051052 |
| GO:0045185\_maintenance\_of\_protein\_location | 9 | 0 | 0.000000 | -0.000000 | 751 | 692.650011 | 789.34 | 886.029989 | 1.051052 |
| GO:0045214\_sarcomere\_organization | 9 | 0 | 0.000000 | -0.000000 | 751 | 692.650011 | 789.34 | 886.029989 | 1.051052 |
| GO:0045428\_regulation\_of\_nitric\_oxide\_biosynthetic\_process | 9 | 0 | 0.000000 | -0.000000 | 751 | 692.650011 | 789.34 | 886.029989 | 1.051052 |
| GO:0045620\_negative\_regulation\_of\_lymphocyte\_differentiation | 9 | 0 | 0.000000 | -0.000000 | 751 | 692.650011 | 789.34 | 886.029989 | 1.051052 |
| GO:0045671\_negative\_regulation\_of\_osteoclast\_differentiation | 9 | 0 | 0.000000 | -0.000000 | 751 | 692.650011 | 789.34 | 886.029989 | 1.051052 |
| GO:0045766\_positive\_regulation\_of\_angiogenesis | 9 | 0 | 0.000000 | -0.000000 | 751 | 692.650011 | 789.34 | 886.029989 | 1.051052 |
| GO:0045830\_positive\_regulation\_of\_isotype\_switching | 9 | 0 | 0.000000 | -0.000000 | 751 | 692.650011 | 789.34 | 886.029989 | 1.051052 |
| GO:0045884\_regulation\_of\_survival\_gene\_product\_expression | 9 | 0 | 0.000000 | -0.000000 | 751 | 692.650011 | 789.34 | 886.029989 | 1.051052 |
| GO:0046006\_regulation\_of\_activated\_T\_cell\_proliferation | 9 | 0 | 0.000000 | -0.000000 | 751 | 692.650011 | 789.34 | 886.029989 | 1.051052 |
| GO:0046324\_regulation\_of\_glucose\_import | 9 | 0 | 0.000000 | -0.000000 | 751 | 692.650011 | 789.34 | 886.029989 | 1.051052 |
| GO:0046365\_monosaccharide\_catabolic\_process | 9 | 0 | 0.000000 | -0.000000 | 751 | 692.650011 | 789.34 | 886.029989 | 1.051052 |
| GO:0046636\_negative\_regulation\_of\_alpha-beta\_T\_cell\_activation | 9 | 0 | 0.000000 | -0.000000 | 751 | 692.650011 | 789.34 | 886.029989 | 1.051052 |
| GO:0046641\_positive\_regulation\_of\_alpha-beta\_T\_cell\_proliferation | 9 | 0 | 0.000000 | -0.000000 | 751 | 692.650011 | 789.34 | 886.029989 | 1.051052 |
| GO:0046888\_negative\_regulation\_of\_hormone\_secretion | 9 | 0 | 0.000000 | -0.000000 | 751 | 692.650011 | 789.34 | 886.029989 | 1.051052 |
| GO:0048070\_regulation\_of\_pigmentation\_during\_development | 9 | 0 | 0.000000 | -0.000000 | 751 | 692.650011 | 789.34 | 886.029989 | 1.051052 |
| GO:0048146\_positive\_regulation\_of\_fibroblast\_proliferation | 9 | 0 | 0.000000 | -0.000000 | 751 | 692.650011 | 789.34 | 886.029989 | 1.051052 |
| GO:0048284\_organelle\_fusion | 9 | 0 | 0.000000 | -0.000000 | 751 | 692.650011 | 789.34 | 886.029989 | 1.051052 |
| GO:0048488\_synaptic\_vesicle\_endocytosis | 9 | 0 | 0.000000 | -0.000000 | 751 | 692.650011 | 789.34 | 886.029989 | 1.051052 |
| GO:0048569\_post-embryonic\_organ\_development | 9 | 0 | 0.000000 | -0.000000 | 751 | 692.650011 | 789.34 | 886.029989 | 1.051052 |
| GO:0048708\_astrocyte\_differentiation | 9 | 0 | 0.000000 | -0.000000 | 751 | 692.650011 | 789.34 | 886.029989 | 1.051052 |
| GO:0050433\_regulation\_of\_catecholamine\_secretion | 9 | 0 | 0.000000 | -0.000000 | 751 | 692.650011 | 789.34 | 886.029989 | 1.051052 |
| GO:0050856\_regulation\_of\_T\_cell\_receptor\_signaling\_pathway | 9 | 0 | 0.000000 | -0.000000 | 751 | 692.650011 | 789.34 | 886.029989 | 1.051052 |
| GO:0050910\_detection\_of\_mechanical\_stimulus\_involved\_in\_sensory\_perception\_of\_sound | 9 | 0 | 0.000000 | -0.000000 | 751 | 692.650011 | 789.34 | 886.029989 | 1.051052 |
| GO:0050918\_positive\_chemotaxis | 9 | 0 | 0.000000 | -0.000000 | 751 | 692.650011 | 789.34 | 886.029989 | 1.051052 |
| GO:0051023\_regulation\_of\_immunoglobulin\_secretion | 9 | 0 | 0.000000 | -0.000000 | 751 | 692.650011 | 789.34 | 886.029989 | 1.051052 |
| GO:0051297\_centrosome\_organization | 9 | 0 | 0.000000 | -0.000000 | 751 | 692.650011 | 789.34 | 886.029989 | 1.051052 |
| GO:0051324\_prophase | 9 | 0 | 0.000000 | -0.000000 | 751 | 692.650011 | 789.34 | 886.029989 | 1.051052 |
| GO:0051607\_defense\_response\_to\_virus | 9 | 0 | 0.000000 | -0.000000 | 751 | 692.650011 | 789.34 | 886.029989 | 1.051052 |
| GO:0051647\_nucleus\_localization | 9 | 0 | 0.000000 | -0.000000 | 751 | 692.650011 | 789.34 | 886.029989 | 1.051052 |
| GO:0051896\_regulation\_of\_protein\_kinase\_B\_signaling\_cascade | 9 | 0 | 0.000000 | -0.000000 | 751 | 692.650011 | 789.34 | 886.029989 | 1.051052 |
| GO:0051932\_synaptic\_transmission\_\_GABAergic | 9 | 0 | 0.000000 | -0.000000 | 751 | 692.650011 | 789.34 | 886.029989 | 1.051052 |
| GO:0051963\_regulation\_of\_synaptogenesis | 9 | 0 | 0.000000 | -0.000000 | 751 | 692.650011 | 789.34 | 886.029989 | 1.051052 |
| GO:0055012\_ventricular\_cardiac\_muscle\_cell\_differentiation | 9 | 0 | 0.000000 | -0.000000 | 751 | 692.650011 | 789.34 | 886.029989 | 1.051052 |
| GO:0055013\_cardiac\_muscle\_cell\_development | 9 | 0 | 0.000000 | -0.000000 | 751 | 692.650011 | 789.34 | 886.029989 | 1.051052 |
| GO:0060052\_neurofilament\_cytoskeleton\_organization | 9 | 0 | 0.000000 | -0.000000 | 751 | 692.650011 | 789.34 | 886.029989 | 1.051052 |
| GO:0060081\_membrane\_hyperpolarization | 9 | 0 | 0.000000 | -0.000000 | 751 | 692.650011 | 789.34 | 886.029989 | 1.051052 |
| GO:0060119\_inner\_ear\_receptor\_cell\_development | 9 | 0 | 0.000000 | -0.000000 | 751 | 692.650011 | 789.34 | 886.029989 | 1.051052 |
| GO:0060122\_inner\_ear\_receptor\_stereocilium\_organization | 9 | 0 | 0.000000 | -0.000000 | 751 | 692.650011 | 789.34 | 886.029989 | 1.051052 |
| GO:0060325\_face\_morphogenesis | 9 | 0 | 0.000000 | -0.000000 | 751 | 692.650011 | 789.34 | 886.029989 | 1.051052 |
| GO:0060513\_prostatic\_bud\_formation | 9 | 0 | 0.000000 | -0.000000 | 751 | 692.650011 | 789.34 | 886.029989 | 1.051052 |
| GO:0060602\_branch\_elongation\_of\_an\_epithelium | 9 | 0 | 0.000000 | -0.000000 | 751 | 692.650011 | 789.34 | 886.029989 | 1.051052 |
| GO:0060693\_regulation\_of\_branching\_involved\_in\_salivary\_gland\_morphogenesis | 9 | 0 | 0.000000 | -0.000000 | 751 | 692.650011 | 789.34 | 886.029989 | 1.051052 |
| GO:0070306\_lens\_fiber\_cell\_differentiation | 9 | 0 | 0.000000 | -0.000000 | 751 | 692.650011 | 789.34 | 886.029989 | 1.051052 |
| GO:0090048\_negative\_regulation\_of\_transcription\_regulator\_activity | 9 | 0 | 0.000000 | -0.000000 | 751 | 692.650011 | 789.34 | 886.029989 | 1.051052 |
| GO:0001704\_formation\_of\_primary\_germ\_layer | 36 | 0 | 0.000000 | -0.000000 | 768 | 708.815414 | 804.26 | 899.704586 | 1.047214 |
| GO:0001819\_positive\_regulation\_of\_cytokine\_production | 36 | 0 | 0.000000 | -0.000000 | 768 | 708.815414 | 804.26 | 899.704586 | 1.047214 |
| GO:0001889\_liver\_development | 36 | 0 | 0.000000 | -0.000000 | 768 | 708.815414 | 804.26 | 899.704586 | 1.047214 |
| GO:0006469\_negative\_regulation\_of\_protein\_kinase\_activity | 36 | 0 | 0.000000 | -0.000000 | 768 | 708.815414 | 804.26 | 899.704586 | 1.047214 |
| GO:0007187\_G-protein\_signaling\_\_coupled\_to\_cyclic\_nucleotide\_second\_messenger | 36 | 0 | 0.000000 | -0.000000 | 768 | 708.815414 | 804.26 | 899.704586 | 1.047214 |
| GO:0007368\_determination\_of\_left\_right\_symmetry | 36 | 0 | 0.000000 | -0.000000 | 768 | 708.815414 | 804.26 | 899.704586 | 1.047214 |
| GO:0007631\_feeding\_behavior | 36 | 0 | 0.000000 | -0.000000 | 768 | 708.815414 | 804.26 | 899.704586 | 1.047214 |
| GO:0014020\_primary\_neural\_tube\_formation | 36 | 0 | 0.000000 | -0.000000 | 768 | 708.815414 | 804.26 | 899.704586 | 1.047214 |
| GO:0021510\_spinal\_cord\_development | 36 | 0 | 0.000000 | -0.000000 | 768 | 708.815414 | 804.26 | 899.704586 | 1.047214 |
| GO:0022602\_ovulation\_cycle\_process | 36 | 0 | 0.000000 | -0.000000 | 768 | 708.815414 | 804.26 | 899.704586 | 1.047214 |
| GO:0030072\_peptide\_hormone\_secretion | 36 | 0 | 0.000000 | -0.000000 | 768 | 708.815414 | 804.26 | 899.704586 | 1.047214 |
| GO:0030278\_regulation\_of\_ossification | 36 | 0 | 0.000000 | -0.000000 | 768 | 708.815414 | 804.26 | 899.704586 | 1.047214 |
| GO:0033673\_negative\_regulation\_of\_kinase\_activity | 36 | 0 | 0.000000 | -0.000000 | 768 | 708.815414 | 804.26 | 899.704586 | 1.047214 |
| GO:0042742\_defense\_response\_to\_bacterium | 36 | 0 | 0.000000 | -0.000000 | 768 | 708.815414 | 804.26 | 899.704586 | 1.047214 |
| GO:0050851\_antigen\_receptor-mediated\_signaling\_pathway | 36 | 0 | 0.000000 | -0.000000 | 768 | 708.815414 | 804.26 | 899.704586 | 1.047214 |
| GO:0050900\_leukocyte\_migration | 36 | 0 | 0.000000 | -0.000000 | 768 | 708.815414 | 804.26 | 899.704586 | 1.047214 |
| GO:0051223\_regulation\_of\_protein\_transport | 36 | 0 | 0.000000 | -0.000000 | 768 | 708.815414 | 804.26 | 899.704586 | 1.047214 |
| GO:0000086\_G2\_M\_transition\_of\_mitotic\_cell\_cycle | 4 | 0 |  |  |  |  |  |  |  |  |
| GO:0000305\_response\_to\_oxygen\_radical | 4 | 0 |  |  |  |  |  |  |  |  |
| GO:0001661\_conditioned\_taste\_aversion | 4 | 0 |  |  |  |  |  |  |  |  |
| GO:0001678\_cellular\_glucose\_homeostasis | 4 | 0 |  |  |  |  |  |  |  |  |
| GO:0001777\_T\_cell\_homeostatic\_proliferation | 4 | 0 |  |  |  |  |  |  |  |  |
| GO:0001794\_type\_IIa\_hypersensitivity | 4 | 0 |  |  |  |  |  |  |  |  |
| GO:0001796\_regulation\_of\_type\_IIa\_hypersensitivity | 4 | 0 |  |  |  |  |  |  |  |  |
| GO:0001798\_positive\_regulation\_of\_type\_IIa\_hypersensitivity | 4 | 0 |  |  |  |  |  |  |  |  |
| GO:0001810\_regulation\_of\_type\_I\_hypersensitivity | 4 | 0 |  |  |  |  |  |  |  |  |
| GO:0001820\_serotonin\_secretion | 4 | 0 |  |  |  |  |  |  |  |  |
| GO:0001835\_blastocyst\_hatching | 4 | 0 |  |  |  |  |  |  |  |  |
| GO:0001842\_neural\_fold\_formation | 4 | 0 |  |  |  |  |  |  |  |  |
| GO:0001881\_receptor\_recycling | 4 | 0 |  |  |  |  |  |  |  |  |
| GO:0001938\_positive\_regulation\_of\_endothelial\_cell\_proliferation | 4 | 0 |  |  |  |  |  |  |  |  |
| GO:0001978\_regulation\_of\_systemic\_arterial\_blood\_pressure\_by\_carotid\_sinus\_baroreceptor\_feedback | 4 | 0 |  |  |  |  |  |  |  |  |
| GO:0002035\_brain\_renin-angiotensin\_system | 4 | 0 |  |  |  |  |  |  |  |  |
| GO:0002051\_osteoblast\_fate\_commitment | 4 | 0 |  |  |  |  |  |  |  |  |
| GO:0002220\_innate\_immune\_response\_activating\_cell\_surface\_receptor\_signaling\_pathway | 4 | 0 |  |  |  |  |  |  |  |  |
| GO:0002249\_lymphocyte\_anergy | 4 | 0 |  |  |  |  |  |  |  |  |
| GO:0002312\_B\_cell\_activation\_during\_immune\_response | 4 | 0 |  |  |  |  |  |  |  |  |
| GO:0002313\_mature\_B\_cell\_differentiation\_during\_immune\_response | 4 | 0 |  |  |  |  |  |  |  |  |
| GO:0002318\_myeloid\_progenitor\_cell\_differentiation | 4 | 0 |  |  |  |  |  |  |  |  |
| GO:0002326\_B\_cell\_lineage\_commitment | 4 | 0 |  |  |  |  |  |  |  |  |
| GO:0002347\_response\_to\_tumor\_cell | 4 | 0 |  |  |  |  |  |  |  |  |
| GO:0002418\_immune\_response\_to\_tumor\_cell | 4 | 0 |  |  |  |  |  |  |  |  |
| GO:0002445\_type\_II\_hypersensitivity | 4 | 0 |  |  |  |  |  |  |  |  |
| GO:0002544\_chronic\_inflammatory\_response | 4 | 0 |  |  |  |  |  |  |  |  |
| GO:0002636\_positive\_regulation\_of\_germinal\_center\_formation | 4 | 0 |  |  |  |  |  |  |  |  |
| GO:0002667\_regulation\_of\_T\_cell\_anergy | 4 | 0 |  |  |  |  |  |  |  |  |
| GO:0002669\_positive\_regulation\_of\_T\_cell\_anergy | 4 | 0 |  |  |  |  |  |  |  |  |
| GO:0002687\_positive\_regulation\_of\_leukocyte\_migration | 4 | 0 |  |  |  |  |  |  |  |  |
| GO:0002702\_positive\_regulation\_of\_production\_of\_molecular\_mediator\_of\_immune\_response | 4 | 0 |  |  |  |  |  |  |  |  |
| GO:0002718\_regulation\_of\_cytokine\_production\_during\_immune\_response | 4 | 0 |  |  |  |  |  |  |  |  |
| GO:0002829\_negative\_regulation\_of\_T-helper\_2\_type\_immune\_response | 4 | 0 |  |  |  |  |  |  |  |  |
| GO:0002833\_positive\_regulation\_of\_response\_to\_biotic\_stimulus | 4 | 0 |  |  |  |  |  |  |  |  |
| GO:0002834\_regulation\_of\_response\_to\_tumor\_cell | 4 | 0 |  |  |  |  |  |  |  |  |
| GO:0002836\_positive\_regulation\_of\_response\_to\_tumor\_cell | 4 | 0 |  |  |  |  |  |  |  |  |
| GO:0002837\_regulation\_of\_immune\_response\_to\_tumor\_cell | 4 | 0 |  |  |  |  |  |  |  |  |
| GO:0002839\_positive\_regulation\_of\_immune\_response\_to\_tumor\_cell | 4 | 0 |  |  |  |  |  |  |  |  |
| GO:0002870\_T\_cell\_anergy | 4 | 0 |  |  |  |  |  |  |  |  |
| GO:0002888\_positive\_regulation\_of\_myeloid\_leukocyte\_mediated\_immunity | 4 | 0 |  |  |  |  |  |  |  |  |
| GO:0002892\_regulation\_of\_type\_II\_hypersensitivity | 4 | 0 |  |  |  |  |  |  |  |  |
| GO:0002894\_positive\_regulation\_of\_type\_II\_hypersensitivity | 4 | 0 |  |  |  |  |  |  |  |  |
| GO:0002911\_regulation\_of\_lymphocyte\_anergy | 4 | 0 |  |  |  |  |  |  |  |  |
| GO:0002913\_positive\_regulation\_of\_lymphocyte\_anergy | 4 | 0 |  |  |  |  |  |  |  |  |
| GO:0002923\_regulation\_of\_humoral\_immune\_response\_mediated\_by\_circulating\_immunoglobulin | 4 | 0 |  |  |  |  |  |  |  |  |
| GO:0003025\_regulation\_of\_systemic\_arterial\_blood\_pressure\_by\_baroreceptor\_feedback | 4 | 0 |  |  |  |  |  |  |  |  |
| GO:0003091\_renal\_water\_homeostasis | 4 | 0 |  |  |  |  |  |  |  |  |
| GO:0005978\_glycogen\_biosynthetic\_process | 4 | 0 |  |  |  |  |  |  |  |  |
| GO:0006012\_galactose\_metabolic\_process | 4 | 0 |  |  |  |  |  |  |  |  |
| GO:0006085\_acetyl-CoA\_biosynthetic\_process | 4 | 0 |  |  |  |  |  |  |  |  |
| GO:0006111\_regulation\_of\_gluconeogenesis | 4 | 0 |  |  |  |  |  |  |  |  |
| GO:0006144\_purine\_base\_metabolic\_process | 4 | 0 |  |  |  |  |  |  |  |  |
| GO:0006290\_pyrimidine\_dimer\_repair | 4 | 0 |  |  |  |  |  |  |  |  |
| GO:0006334\_nucleosome\_assembly | 4 | 0 |  |  |  |  |  |  |  |  |
| GO:0006534\_cysteine\_metabolic\_process | 4 | 0 |  |  |  |  |  |  |  |  |
| GO:0006547\_histidine\_metabolic\_process | 4 | 0 |  |  |  |  |  |  |  |  |
| GO:0006548\_histidine\_catabolic\_process | 4 | 0 |  |  |  |  |  |  |  |  |
| GO:0006555\_methionine\_metabolic\_process | 4 | 0 |  |  |  |  |  |  |  |  |
| GO:0006599\_phosphagen\_metabolic\_process | 4 | 0 |  |  |  |  |  |  |  |  |
| GO:0006623\_protein\_targeting\_to\_vacuole | 4 | 0 |  |  |  |  |  |  |  |  |
| GO:0006626\_protein\_targeting\_to\_mitochondrion | 4 | 0 |  |  |  |  |  |  |  |  |
| GO:0006684\_sphingomyelin\_metabolic\_process | 4 | 0 |  |  |  |  |  |  |  |  |
| GO:0006688\_glycosphingolipid\_biosynthetic\_process | 4 | 0 |  |  |  |  |  |  |  |  |
| GO:0006707\_cholesterol\_catabolic\_process | 4 | 0 |  |  |  |  |  |  |  |  |
| GO:0006739\_NADP\_metabolic\_process | 4 | 0 |  |  |  |  |  |  |  |  |
| GO:0006835\_dicarboxylic\_acid\_transport | 4 | 0 |  |  |  |  |  |  |  |  |
| GO:0006837\_serotonin\_transport | 4 | 0 |  |  |  |  |  |  |  |  |
| GO:0006888\_ER\_to\_Golgi\_vesicle-mediated\_transport | 4 | 0 |  |  |  |  |  |  |  |  |
| GO:0006906\_vesicle\_fusion | 4 | 0 |  |  |  |  |  |  |  |  |
| GO:0006927\_transformed\_cell\_apoptosis | 4 | 0 |  |  |  |  |  |  |  |  |
| GO:0006972\_hyperosmotic\_response | 4 | 0 |  |  |  |  |  |  |  |  |
| GO:0007028\_cytoplasm\_organization | 4 | 0 |  |  |  |  |  |  |  |  |
| GO:0007031\_peroxisome\_organization | 4 | 0 |  |  |  |  |  |  |  |  |
| GO:0007066\_female\_meiosis\_sister\_chromatid\_cohesion | 4 | 0 |  |  |  |  |  |  |  |  |
| GO:0007144\_female\_meiosis\_I | 4 | 0 |  |  |  |  |  |  |  |  |
| GO:0007184\_SMAD\_protein\_nuclear\_translocation | 4 | 0 |  |  |  |  |  |  |  |  |
| GO:0007216\_metabotropic\_glutamate\_receptor\_signaling\_pathway | 4 | 0 |  |  |  |  |  |  |  |  |
| GO:0007342\_fusion\_of\_sperm\_to\_egg\_plasma\_membrane | 4 | 0 |  |  |  |  |  |  |  |  |
| GO:0007386\_compartment\_specification | 4 | 0 |  |  |  |  |  |  |  |  |
| GO:0008053\_mitochondrial\_fusion | 4 | 0 |  |  |  |  |  |  |  |  |
| GO:0008207\_C21-steroid\_hormone\_metabolic\_process | 4 | 0 |  |  |  |  |  |  |  |  |
| GO:0008215\_spermine\_metabolic\_process | 4 | 0 |  |  |  |  |  |  |  |  |
| GO:0009065\_glutamine\_family\_amino\_acid\_catabolic\_process | 4 | 0 |  |  |  |  |  |  |  |  |
| GO:0009075\_histidine\_family\_amino\_acid\_metabolic\_process | 4 | 0 |  |  |  |  |  |  |  |  |
| GO:0009077\_histidine\_family\_amino\_acid\_catabolic\_process | 4 | 0 |  |  |  |  |  |  |  |  |
| GO:0009134\_nucleoside\_diphosphate\_catabolic\_process | 4 | 0 |  |  |  |  |  |  |  |  |
| GO:0009163\_nucleoside\_biosynthetic\_process | 4 | 0 |  |  |  |  |  |  |  |  |
| GO:0009225\_nucleotide-sugar\_metabolic\_process | 4 | 0 |  |  |  |  |  |  |  |  |
| GO:0009250\_glucan\_biosynthetic\_process | 4 | 0 |  |  |  |  |  |  |  |  |
| GO:0009404\_toxin\_metabolic\_process | 4 | 0 |  |  |  |  |  |  |  |  |
| GO:0009593\_detection\_of\_chemical\_stimulus | 4 | 0 |  |  |  |  |  |  |  |  |
| GO:0009595\_detection\_of\_biotic\_stimulus | 4 | 0 |  |  |  |  |  |  |  |  |
| GO:0009755\_hormone-mediated\_signaling | 4 | 0 |  |  |  |  |  |  |  |  |
| GO:0009912\_auditory\_receptor\_cell\_fate\_commitment | 4 | 0 |  |  |  |  |  |  |  |  |
| GO:0010224\_response\_to\_UV-B | 4 | 0 |  |  |  |  |  |  |  |  |
| GO:0010453\_regulation\_of\_cell\_fate\_commitment | 4 | 0 |  |  |  |  |  |  |  |  |
| GO:0010506\_regulation\_of\_autophagy | 4 | 0 |  |  |  |  |  |  |  |  |
| GO:0010631\_epithelial\_cell\_migration | 4 | 0 |  |  |  |  |  |  |  |  |
| GO:0010812\_negative\_regulation\_of\_cell-substrate\_adhesion | 4 | 0 |  |  |  |  |  |  |  |  |
| GO:0010829\_negative\_regulation\_of\_glucose\_transport | 4 | 0 |  |  |  |  |  |  |  |  |
| GO:0014002\_astrocyte\_development | 4 | 0 |  |  |  |  |  |  |  |  |
| GO:0014832\_urinary\_bladder\_smooth\_muscle\_contraction | 4 | 0 |  |  |  |  |  |  |  |  |
| GO:0014848\_urinary\_tract\_smooth\_muscle\_contraction | 4 | 0 |  |  |  |  |  |  |  |  |
| GO:0015701\_bicarbonate\_transport | 4 | 0 |  |  |  |  |  |  |  |  |
| GO:0015809\_arginine\_transport | 4 | 0 |  |  |  |  |  |  |  |  |
| GO:0015850\_organic\_alcohol\_transport | 4 | 0 |  |  |  |  |  |  |  |  |
| GO:0015858\_nucleoside\_transport | 4 | 0 |  |  |  |  |  |  |  |  |
| GO:0016068\_type\_I\_hypersensitivity | 4 | 0 |  |  |  |  |  |  |  |  |
| GO:0016127\_sterol\_catabolic\_process | 4 | 0 |  |  |  |  |  |  |  |  |
| GO:0016198\_axon\_choice\_point\_recognition | 4 | 0 |  |  |  |  |  |  |  |  |
| GO:0016338\_calcium-independent\_cell-cell\_adhesion | 4 | 0 |  |  |  |  |  |  |  |  |
| GO:0018198\_peptidyl-cysteine\_modification | 4 | 0 |  |  |  |  |  |  |  |  |
| GO:0018409\_peptide\_or\_protein\_amino-terminal\_blocking | 4 | 0 |  |  |  |  |  |  |  |  |
| GO:0019432\_triglyceride\_biosynthetic\_process | 4 | 0 |  |  |  |  |  |  |  |  |
| GO:0019530\_taurine\_metabolic\_process | 4 | 0 |  |  |  |  |  |  |  |  |
| GO:0021523\_somatic\_motor\_neuron\_differentiation | 4 | 0 |  |  |  |  |  |  |  |  |
| GO:0021535\_cell\_migration\_in\_hindbrain | 4 | 0 |  |  |  |  |  |  |  |  |
| GO:0021542\_dentate\_gyrus\_development | 4 | 0 |  |  |  |  |  |  |  |  |
| GO:0021561\_facial\_nerve\_development | 4 | 0 |  |  |  |  |  |  |  |  |
| GO:0021569\_rhombomere\_3\_development | 4 | 0 |  |  |  |  |  |  |  |  |
| GO:0021571\_rhombomere\_5\_development | 4 | 0 |  |  |  |  |  |  |  |  |
| GO:0021604\_cranial\_nerve\_structural\_organization | 4 | 0 |  |  |  |  |  |  |  |  |
| GO:0021610\_facial\_nerve\_morphogenesis | 4 | 0 |  |  |  |  |  |  |  |  |
| GO:0021612\_facial\_nerve\_structural\_organization | 4 | 0 |  |  |  |  |  |  |  |  |
| GO:0021631\_optic\_nerve\_morphogenesis | 4 | 0 |  |  |  |  |  |  |  |  |
| GO:0021681\_cerebellar\_granular\_layer\_development | 4 | 0 |  |  |  |  |  |  |  |  |
| GO:0021683\_cerebellar\_granular\_layer\_morphogenesis | 4 | 0 |  |  |  |  |  |  |  |  |
| GO:0021684\_cerebellar\_granular\_layer\_formation | 4 | 0 |  |  |  |  |  |  |  |  |
| GO:0021707\_cerebellar\_granule\_cell\_differentiation | 4 | 0 |  |  |  |  |  |  |  |  |
| GO:0021778\_oligodendrocyte\_cell\_fate\_specification | 4 | 0 |  |  |  |  |  |  |  |  |
| GO:0021779\_oligodendrocyte\_cell\_fate\_commitment | 4 | 0 |  |  |  |  |  |  |  |  |
| GO:0021780\_glial\_cell\_fate\_specification | 4 | 0 |  |  |  |  |  |  |  |  |
| GO:0021801\_cerebral\_cortex\_radial\_glia\_guided\_migration | 4 | 0 |  |  |  |  |  |  |  |  |
| GO:0021830\_interneuron\_migration\_from\_the\_subpallium\_to\_the\_cortex | 4 | 0 |  |  |  |  |  |  |  |  |
| GO:0021853\_cerebral\_cortex\_GABAergic\_interneuron\_migration | 4 | 0 |  |  |  |  |  |  |  |  |
| GO:0021877\_forebrain\_neuron\_fate\_commitment | 4 | 0 |  |  |  |  |  |  |  |  |
| GO:0021894\_cerebral\_cortex\_GABAergic\_interneuron\_development | 4 | 0 |  |  |  |  |  |  |  |  |
| GO:0021910\_smoothened\_signaling\_pathway\_involved\_in\_ventral\_spinal\_cord\_patterning | 4 | 0 |  |  |  |  |  |  |  |  |
| GO:0021913\_regulation\_of\_transcription\_from\_RNA\_polymerase\_II\_promoter\_involved\_in\_ventral\_spinal\_cord\_interneuron\_specification | 4 | 0 |  |  |  |  |  |  |  |  |
| GO:0021938\_smoothened\_signaling\_pathway\_involved\_in\_regulation\_of\_granule\_cell\_precursor\_cell\_proliferation | 4 | 0 |  |  |  |  |  |  |  |  |
| GO:0021978\_telencephalon\_regionalization | 4 | 0 |  |  |  |  |  |  |  |  |
| GO:0022011\_myelination\_in\_the\_peripheral\_nervous\_system | 4 | 0 |  |  |  |  |  |  |  |  |
| GO:0030146\_diuresis | 4 | 0 |  |  |  |  |  |  |  |  |
| GO:0030300\_regulation\_of\_intestinal\_cholesterol\_absorption | 4 | 0 |  |  |  |  |  |  |  |  |
| GO:0030800\_negative\_regulation\_of\_cyclic\_nucleotide\_metabolic\_process | 4 | 0 |  |  |  |  |  |  |  |  |
| GO:0030803\_negative\_regulation\_of\_cyclic\_nucleotide\_biosynthetic\_process | 4 | 0 |  |  |  |  |  |  |  |  |
| GO:0030809\_negative\_regulation\_of\_nucleotide\_biosynthetic\_process | 4 | 0 |  |  |  |  |  |  |  |  |
| GO:0030815\_negative\_regulation\_of\_cAMP\_metabolic\_process | 4 | 0 |  |  |  |  |  |  |  |  |
| GO:0030816\_positive\_regulation\_of\_cAMP\_metabolic\_process | 4 | 0 |  |  |  |  |  |  |  |  |
| GO:0030818\_negative\_regulation\_of\_cAMP\_biosynthetic\_process | 4 | 0 |  |  |  |  |  |  |  |  |
| GO:0030819\_positive\_regulation\_of\_cAMP\_biosynthetic\_process | 4 | 0 |  |  |  |  |  |  |  |  |
| GO:0030826\_regulation\_of\_cGMP\_biosynthetic\_process | 4 | 0 |  |  |  |  |  |  |  |  |
| GO:0030858\_positive\_regulation\_of\_epithelial\_cell\_differentiation | 4 | 0 |  |  |  |  |  |  |  |  |
| GO:0030859\_polarized\_epithelial\_cell\_differentiation | 4 | 0 |  |  |  |  |  |  |  |  |
| GO:0030949\_positive\_regulation\_of\_vascular\_endothelial\_growth\_factor\_receptor\_signaling\_pathway | 4 | 0 |  |  |  |  |  |  |  |  |
| GO:0031113\_regulation\_of\_microtubule\_polymerization | 4 | 0 |  |  |  |  |  |  |  |  |
| GO:0031365\_N-terminal\_protein\_amino\_acid\_modification | 4 | 0 |  |  |  |  |  |  |  |  |
| GO:0031424\_keratinization | 4 | 0 |  |  |  |  |  |  |  |  |
| GO:0031557\_induction\_of\_programmed\_cell\_death\_in\_response\_to\_chemical\_stimulus | 4 | 0 |  |  |  |  |  |  |  |  |
| GO:0031558\_induction\_of\_apoptosis\_in\_response\_to\_chemical\_stimulus | 4 | 0 |  |  |  |  |  |  |  |  |
| GO:0031623\_receptor\_internalization | 4 | 0 |  |  |  |  |  |  |  |  |
| GO:0032088\_negative\_regulation\_of\_NF-kappaB\_transcription\_factor\_activity | 4 | 0 |  |  |  |  |  |  |  |  |
| GO:0032098\_regulation\_of\_appetite | 4 | 0 |  |  |  |  |  |  |  |  |
| GO:0032105\_negative\_regulation\_of\_response\_to\_extracellular\_stimulus | 4 | 0 |  |  |  |  |  |  |  |  |
| GO:0032108\_negative\_regulation\_of\_response\_to\_nutrient\_levels | 4 | 0 |  |  |  |  |  |  |  |  |
| GO:0032225\_regulation\_of\_synaptic\_transmission\_\_dopaminergic | 4 | 0 |  |  |  |  |  |  |  |  |
| GO:0032292\_ensheathment\_of\_axons\_in\_the\_peripheral\_nervous\_system | 4 | 0 |  |  |  |  |  |  |  |  |
| GO:0032321\_positive\_regulation\_of\_Rho\_GTPase\_activity | 4 | 0 |  |  |  |  |  |  |  |  |
| GO:0032371\_regulation\_of\_sterol\_transport | 4 | 0 |  |  |  |  |  |  |  |  |
| GO:0032374\_regulation\_of\_cholesterol\_transport | 4 | 0 |  |  |  |  |  |  |  |  |
| GO:0032401\_establishment\_of\_melanosome\_localization | 4 | 0 |  |  |  |  |  |  |  |  |
| GO:0032608\_interferon-beta\_production | 4 | 0 |  |  |  |  |  |  |  |  |
| GO:0032611\_interleukin-1\_beta\_production | 4 | 0 |  |  |  |  |  |  |  |  |
| GO:0032612\_interleukin-1\_production | 4 | 0 |  |  |  |  |  |  |  |  |
| GO:0032648\_regulation\_of\_interferon-beta\_production | 4 | 0 |  |  |  |  |  |  |  |  |
| GO:0032651\_regulation\_of\_interleukin-1\_beta\_production | 4 | 0 |  |  |  |  |  |  |  |  |
| GO:0032652\_regulation\_of\_interleukin-1\_production | 4 | 0 |  |  |  |  |  |  |  |  |
| GO:0032689\_negative\_regulation\_of\_interferon-gamma\_production | 4 | 0 |  |  |  |  |  |  |  |  |
| GO:0032713\_negative\_regulation\_of\_interleukin-4\_production | 4 | 0 |  |  |  |  |  |  |  |  |
| GO:0032715\_negative\_regulation\_of\_interleukin-6\_production | 4 | 0 |  |  |  |  |  |  |  |  |
| GO:0032733\_positive\_regulation\_of\_interleukin-10\_production | 4 | 0 |  |  |  |  |  |  |  |  |
| GO:0032808\_lacrimal\_gland\_development | 4 | 0 |  |  |  |  |  |  |  |  |
| GO:0032835\_glomerulus\_development | 4 | 0 |  |  |  |  |  |  |  |  |
| GO:0032872\_regulation\_of\_stress-activated\_MAPK\_cascade | 4 | 0 |  |  |  |  |  |  |  |  |
| GO:0032922\_circadian\_regulation\_of\_gene\_expression | 4 | 0 |  |  |  |  |  |  |  |  |
| GO:0033026\_negative\_regulation\_of\_mast\_cell\_apoptosis | 4 | 0 |  |  |  |  |  |  |  |  |
| GO:0033079\_immature\_T\_cell\_proliferation | 4 | 0 |  |  |  |  |  |  |  |  |
| GO:0033083\_regulation\_of\_immature\_T\_cell\_proliferation | 4 | 0 |  |  |  |  |  |  |  |  |
| GO:0033089\_positive\_regulation\_of\_T\_cell\_differentiation\_in\_the\_thymus | 4 | 0 |  |  |  |  |  |  |  |  |
| GO:0033135\_regulation\_of\_peptidyl-serine\_phosphorylation | 4 | 0 |  |  |  |  |  |  |  |  |
| GO:0033299\_secretion\_of\_lysosomal\_enzymes | 4 | 0 |  |  |  |  |  |  |  |  |
| GO:0033327\_Leydig\_cell\_differentiation | 4 | 0 |  |  |  |  |  |  |  |  |
| GO:0033363\_secretory\_granule\_organization | 4 | 0 |  |  |  |  |  |  |  |  |
| GO:0033599\_regulation\_of\_mammary\_gland\_epithelial\_cell\_proliferation | 4 | 0 |  |  |  |  |  |  |  |  |
| GO:0033865\_nucleoside\_bisphosphate\_metabolic\_process | 4 | 0 |  |  |  |  |  |  |  |  |
| GO:0034204\_lipid\_translocation | 4 | 0 |  |  |  |  |  |  |  |  |
| GO:0034404\_nucleobase\_\_nucleoside\_and\_nucleotide\_biosynthetic\_process | 4 | 0 |  |  |  |  |  |  |  |  |
| GO:0034587\_piRNA\_metabolic\_process | 4 | 0 |  |  |  |  |  |  |  |  |
| GO:0034614\_cellular\_response\_to\_reactive\_oxygen\_species | 4 | 0 |  |  |  |  |  |  |  |  |
| GO:0034654\_nucleobase\_\_nucleoside\_\_nucleotide\_and\_nucleic\_acid\_biosynthetic\_process | 4 | 0 |  |  |  |  |  |  |  |  |
| GO:0035020\_regulation\_of\_Rac\_protein\_signal\_transduction | 4 | 0 |  |  |  |  |  |  |  |  |
| GO:0035082\_axoneme\_assembly | 4 | 0 |  |  |  |  |  |  |  |  |
| GO:0035188\_hatching | 4 | 0 |  |  |  |  |  |  |  |  |
| GO:0035235\_ionotropic\_glutamate\_receptor\_signaling\_pathway | 4 | 0 |  |  |  |  |  |  |  |  |
| GO:0042345\_regulation\_of\_NF-kappaB\_import\_into\_nucleus | 4 | 0 |  |  |  |  |  |  |  |  |
| GO:0042348\_NF-kappaB\_import\_into\_nucleus | 4 | 0 |  |  |  |  |  |  |  |  |
| GO:0042359\_vitamin\_D\_metabolic\_process | 4 | 0 |  |  |  |  |  |  |  |  |
| GO:0042428\_serotonin\_metabolic\_process | 4 | 0 |  |  |  |  |  |  |  |  |
| GO:0042451\_purine\_nucleoside\_biosynthetic\_process | 4 | 0 |  |  |  |  |  |  |  |  |
| GO:0042455\_ribonucleoside\_biosynthetic\_process | 4 | 0 |  |  |  |  |  |  |  |  |
| GO:0042473\_outer\_ear\_morphogenesis | 4 | 0 |  |  |  |  |  |  |  |  |
| GO:0042522\_regulation\_of\_tyrosine\_phosphorylation\_of\_Stat5\_protein | 4 | 0 |  |  |  |  |  |  |  |  |
| GO:0042535\_positive\_regulation\_of\_tumor\_necrosis\_factor\_biosynthetic\_process | 4 | 0 |  |  |  |  |  |  |  |  |
| GO:0042541\_hemoglobin\_biosynthetic\_process | 4 | 0 |  |  |  |  |  |  |  |  |
| GO:0042558\_pteridine\_and\_derivative\_metabolic\_process | 4 | 0 |  |  |  |  |  |  |  |  |
| GO:0042634\_regulation\_of\_hair\_cycle | 4 | 0 |  |  |  |  |  |  |  |  |
| GO:0042744\_hydrogen\_peroxide\_catabolic\_process | 4 | 0 |  |  |  |  |  |  |  |  |
| GO:0042773\_ATP\_synthesis\_coupled\_electron\_transport | 4 | 0 |  |  |  |  |  |  |  |  |
| GO:0042775\_mitochondrial\_ATP\_synthesis\_coupled\_electron\_transport | 4 | 0 |  |  |  |  |  |  |  |  |
| GO:0042832\_defense\_response\_to\_protozoan | 4 | 0 |  |  |  |  |  |  |  |  |
| GO:0042982\_amyloid\_precursor\_protein\_metabolic\_process | 4 | 0 |  |  |  |  |  |  |  |  |
| GO:0042992\_negative\_regulation\_of\_transcription\_factor\_import\_into\_nucleus | 4 | 0 |  |  |  |  |  |  |  |  |
| GO:0043043\_peptide\_biosynthetic\_process | 4 | 0 |  |  |  |  |  |  |  |  |
| GO:0043129\_surfactant\_homeostasis | 4 | 0 |  |  |  |  |  |  |  |  |
| GO:0043374\_CD8-positive\_\_alpha-beta\_T\_cell\_differentiation | 4 | 0 |  |  |  |  |  |  |  |  |
| GO:0043470\_regulation\_of\_carbohydrate\_catabolic\_process | 4 | 0 |  |  |  |  |  |  |  |  |
| GO:0043471\_regulation\_of\_cellular\_carbohydrate\_catabolic\_process | 4 | 0 |  |  |  |  |  |  |  |  |
| GO:0043484\_regulation\_of\_RNA\_splicing | 4 | 0 |  |  |  |  |  |  |  |  |
| GO:0043500\_muscle\_adaptation | 4 | 0 |  |  |  |  |  |  |  |  |
| GO:0043534\_blood\_vessel\_endothelial\_cell\_migration | 4 | 0 |  |  |  |  |  |  |  |  |
| GO:0043691\_reverse\_cholesterol\_transport | 4 | 0 |  |  |  |  |  |  |  |  |
| GO:0044243\_multicellular\_organismal\_catabolic\_process | 4 | 0 |  |  |  |  |  |  |  |  |
| GO:0044403\_symbiosis\_\_encompassing\_mutualism\_through\_parasitism | 4 | 0 |  |  |  |  |  |  |  |  |
| GO:0044419\_interspecies\_interaction\_between\_organisms | 4 | 0 |  |  |  |  |  |  |  |  |
| GO:0045066\_regulatory\_T\_cell\_differentiation | 4 | 0 |  |  |  |  |  |  |  |  |
| GO:0045078\_positive\_regulation\_of\_interferon-gamma\_biosynthetic\_process | 4 | 0 |  |  |  |  |  |  |  |  |
| GO:0045332\_phospholipid\_translocation | 4 | 0 |  |  |  |  |  |  |  |  |
| GO:0045346\_regulation\_of\_MHC\_class\_II\_biosynthetic\_process | 4 | 0 |  |  |  |  |  |  |  |  |
| GO:0045350\_interferon-beta\_biosynthetic\_process | 4 | 0 |  |  |  |  |  |  |  |  |
| GO:0045357\_regulation\_of\_interferon-beta\_biosynthetic\_process | 4 | 0 |  |  |  |  |  |  |  |  |
| GO:0045359\_positive\_regulation\_of\_interferon-beta\_biosynthetic\_process | 4 | 0 |  |  |  |  |  |  |  |  |
| GO:0045600\_positive\_regulation\_of\_fat\_cell\_differentiation | 4 | 0 |  |  |  |  |  |  |  |  |
| GO:0045616\_regulation\_of\_keratinocyte\_differentiation | 4 | 0 |  |  |  |  |  |  |  |  |
| GO:0045624\_positive\_regulation\_of\_T-helper\_cell\_differentiation | 4 | 0 |  |  |  |  |  |  |  |  |
| GO:0045628\_regulation\_of\_T-helper\_2\_cell\_differentiation | 4 | 0 |  |  |  |  |  |  |  |  |
| GO:0045634\_regulation\_of\_melanocyte\_differentiation | 4 | 0 |  |  |  |  |  |  |  |  |
| GO:0045647\_negative\_regulation\_of\_erythrocyte\_differentiation | 4 | 0 |  |  |  |  |  |  |  |  |
| GO:0045672\_positive\_regulation\_of\_osteoclast\_differentiation | 4 | 0 |  |  |  |  |  |  |  |  |
| GO:0045684\_positive\_regulation\_of\_epidermis\_development | 4 | 0 |  |  |  |  |  |  |  |  |
| GO:0045736\_negative\_regulation\_of\_cyclin-dependent\_protein\_kinase\_activity | 4 | 0 |  |  |  |  |  |  |  |  |
| GO:0045742\_positive\_regulation\_of\_epidermal\_growth\_factor\_receptor\_signaling\_pathway | 4 | 0 |  |  |  |  |  |  |  |  |
| GO:0045747\_positive\_regulation\_of\_Notch\_signaling\_pathway | 4 | 0 |  |  |  |  |  |  |  |  |
| GO:0045767\_regulation\_of\_anti-apoptosis | 4 | 0 |  |  |  |  |  |  |  |  |
| GO:0045779\_negative\_regulation\_of\_bone\_resorption | 4 | 0 |  |  |  |  |  |  |  |  |
| GO:0045923\_positive\_regulation\_of\_fatty\_acid\_metabolic\_process | 4 | 0 |  |  |  |  |  |  |  |  |
| GO:0045930\_negative\_regulation\_of\_mitotic\_cell\_cycle | 4 | 0 |  |  |  |  |  |  |  |  |
| GO:0045940\_positive\_regulation\_of\_steroid\_metabolic\_process | 4 | 0 |  |  |  |  |  |  |  |  |
| GO:0045980\_negative\_regulation\_of\_nucleotide\_metabolic\_process | 4 | 0 |  |  |  |  |  |  |  |  |
| GO:0046129\_purine\_ribonucleoside\_biosynthetic\_process | 4 | 0 |  |  |  |  |  |  |  |  |
| GO:0046173\_polyol\_biosynthetic\_process | 4 | 0 |  |  |  |  |  |  |  |  |
| GO:0046541\_saliva\_secretion | 4 | 0 |  |  |  |  |  |  |  |  |
| GO:0046548\_retinal\_rod\_cell\_development | 4 | 0 |  |  |  |  |  |  |  |  |
| GO:0046579\_positive\_regulation\_of\_Ras\_protein\_signal\_transduction | 4 | 0 |  |  |  |  |  |  |  |  |
| GO:0046639\_negative\_regulation\_of\_alpha-beta\_T\_cell\_differentiation | 4 | 0 |  |  |  |  |  |  |  |  |
| GO:0046642\_negative\_regulation\_of\_alpha-beta\_T\_cell\_proliferation | 4 | 0 |  |  |  |  |  |  |  |  |
| GO:0046668\_regulation\_of\_retinal\_cell\_programmed\_cell\_death | 4 | 0 |  |  |  |  |  |  |  |  |
| GO:0046686\_response\_to\_cadmium\_ion | 4 | 0 |  |  |  |  |  |  |  |  |
| GO:0046835\_carbohydrate\_phosphorylation | 4 | 0 |  |  |  |  |  |  |  |  |
| GO:0046902\_regulation\_of\_mitochondrial\_membrane\_permeability | 4 | 0 |  |  |  |  |  |  |  |  |
| GO:0047496\_vesicle\_transport\_along\_microtubule | 4 | 0 |  |  |  |  |  |  |  |  |
| GO:0048011\_nerve\_growth\_factor\_receptor\_signaling\_pathway | 4 | 0 |  |  |  |  |  |  |  |  |
| GO:0048024\_regulation\_of\_nuclear\_mRNA\_splicing\_\_via\_spliceosome | 4 | 0 |  |  |  |  |  |  |  |  |
| GO:0048240\_sperm\_capacitation | 4 | 0 |  |  |  |  |  |  |  |  |
| GO:0048341\_paraxial\_mesoderm\_formation | 4 | 0 |  |  |  |  |  |  |  |  |
| GO:0048484\_enteric\_nervous\_system\_development | 4 | 0 |  |  |  |  |  |  |  |  |
| GO:0048512\_circadian\_behavior | 4 | 0 |  |  |  |  |  |  |  |  |
| GO:0048558\_embryonic\_gut\_morphogenesis | 4 | 0 |  |  |  |  |  |  |  |  |
| GO:0048639\_positive\_regulation\_of\_developmental\_growth | 4 | 0 |  |  |  |  |  |  |  |  |
| GO:0048710\_regulation\_of\_astrocyte\_differentiation | 4 | 0 |  |  |  |  |  |  |  |  |
| GO:0048841\_regulation\_of\_axon\_extension\_involved\_in\_axon\_guidance | 4 | 0 |  |  |  |  |  |  |  |  |
| GO:0048843\_negative\_regulation\_of\_axon\_extension\_involved\_in\_axon\_guidance | 4 | 0 |  |  |  |  |  |  |  |  |
| GO:0048846\_axon\_extension\_involved\_in\_axon\_guidance | 4 | 0 |  |  |  |  |  |  |  |  |
| GO:0048875\_chemical\_homeostasis\_within\_a\_tissue | 4 | 0 |  |  |  |  |  |  |  |  |
| GO:0048935\_peripheral\_nervous\_system\_neuron\_development | 4 | 0 |  |  |  |  |  |  |  |  |
| GO:0050702\_interleukin-1\_beta\_secretion | 4 | 0 |  |  |  |  |  |  |  |  |
| GO:0050704\_regulation\_of\_interleukin-1\_secretion | 4 | 0 |  |  |  |  |  |  |  |  |
| GO:0050706\_regulation\_of\_interleukin-1\_beta\_secretion | 4 | 0 |  |  |  |  |  |  |  |  |
| GO:0050716\_positive\_regulation\_of\_interleukin-1\_secretion | 4 | 0 |  |  |  |  |  |  |  |  |
| GO:0050718\_positive\_regulation\_of\_interleukin-1\_beta\_secretion | 4 | 0 |  |  |  |  |  |  |  |  |
| GO:0050820\_positive\_regulation\_of\_coagulation | 4 | 0 |  |  |  |  |  |  |  |  |
| GO:0050891\_multicellular\_organismal\_water\_homeostasis | 4 | 0 |  |  |  |  |  |  |  |  |
| GO:0050919\_negative\_chemotaxis | 4 | 0 |  |  |  |  |  |  |  |  |
| GO:0050932\_regulation\_of\_pigment\_cell\_differentiation | 4 | 0 |  |  |  |  |  |  |  |  |
| GO:0050961\_detection\_of\_temperature\_stimulus\_involved\_in\_sensory\_perception | 4 | 0 |  |  |  |  |  |  |  |  |
| GO:0050965\_detection\_of\_temperature\_stimulus\_involved\_in\_sensory\_perception\_of\_pain | 4 | 0 |  |  |  |  |  |  |  |  |
| GO:0050994\_regulation\_of\_lipid\_catabolic\_process | 4 | 0 |  |  |  |  |  |  |  |  |
| GO:0051024\_positive\_regulation\_of\_immunoglobulin\_secretion | 4 | 0 |  |  |  |  |  |  |  |  |
| GO:0051055\_negative\_regulation\_of\_lipid\_biosynthetic\_process | 4 | 0 |  |  |  |  |  |  |  |  |
| GO:0051124\_synaptic\_growth\_at\_neuromuscular\_junction | 4 | 0 |  |  |  |  |  |  |  |  |
| GO:0051148\_negative\_regulation\_of\_muscle\_cell\_differentiation | 4 | 0 |  |  |  |  |  |  |  |  |
| GO:0051205\_protein\_insertion\_into\_membrane | 4 | 0 |  |  |  |  |  |  |  |  |
| GO:0051225\_spindle\_assembly | 4 | 0 |  |  |  |  |  |  |  |  |
| GO:0051341\_regulation\_of\_oxidoreductase\_activity | 4 | 0 |  |  |  |  |  |  |  |  |
| GO:0051452\_intracellular\_pH\_reduction | 4 | 0 |  |  |  |  |  |  |  |  |
| GO:0051567\_histone\_H3-K9\_methylation | 4 | 0 |  |  |  |  |  |  |  |  |
| GO:0051642\_centrosome\_localization | 4 | 0 |  |  |  |  |  |  |  |  |
| GO:0051797\_regulation\_of\_hair\_follicle\_development | 4 | 0 |  |  |  |  |  |  |  |  |
| GO:0051897\_positive\_regulation\_of\_protein\_kinase\_B\_signaling\_cascade | 4 | 0 |  |  |  |  |  |  |  |  |
| GO:0051904\_pigment\_granule\_transport | 4 | 0 |  |  |  |  |  |  |  |  |
| GO:0055009\_atrial\_cardiac\_muscle\_morphogenesis | 4 | 0 |  |  |  |  |  |  |  |  |
| GO:0060008\_Sertoli\_cell\_differentiation | 4 | 0 |  |  |  |  |  |  |  |  |
| GO:0060011\_Sertoli\_cell\_proliferation | 4 | 0 |  |  |  |  |  |  |  |  |
| GO:0060057\_apoptosis\_involved\_in\_mammary\_gland\_involution | 4 | 0 |  |  |  |  |  |  |  |  |
| GO:0060058\_positive\_regulation\_of\_apoptosis\_involved\_in\_mammary\_gland\_involution | 4 | 0 |  |  |  |  |  |  |  |  |
| GO:0060065\_uterus\_development | 4 | 0 |  |  |  |  |  |  |  |  |
| GO:0060087\_relaxation\_of\_vascular\_smooth\_muscle | 4 | 0 |  |  |  |  |  |  |  |  |
| GO:0060120\_inner\_ear\_receptor\_cell\_fate\_commitment | 4 | 0 |  |  |  |  |  |  |  |  |
| GO:0060157\_urinary\_bladder\_development | 4 | 0 |  |  |  |  |  |  |  |  |
| GO:0060158\_activation\_of\_phospholipase\_C\_activity\_by\_dopamine\_receptor\_signaling\_pathway | 4 | 0 |  |  |  |  |  |  |  |  |
| GO:0060164\_regulation\_of\_timing\_of\_neuron\_differentiation | 4 | 0 |  |  |  |  |  |  |  |  |
| GO:0060235\_lens\_induction\_in\_camera-type\_eye | 4 | 0 |  |  |  |  |  |  |  |  |
| GO:0060291\_long-term\_synaptic\_potentiation | 4 | 0 |  |  |  |  |  |  |  |  |
| GO:0060412\_ventricular\_septum\_morphogenesis | 4 | 0 |  |  |  |  |  |  |  |  |
| GO:0060459\_left\_lung\_development | 4 | 0 |  |  |  |  |  |  |  |  |
| GO:0060561\_apoptosis\_involved\_in\_morphogenesis | 4 | 0 |  |  |  |  |  |  |  |  |
| GO:0060592\_mammary\_gland\_formation | 4 | 0 |  |  |  |  |  |  |  |  |
| GO:0060644\_mammary\_gland\_epithelial\_cell\_differentiation | 4 | 0 |  |  |  |  |  |  |  |  |
| GO:0060666\_dichotomous\_subdivision\_of\_terminal\_units\_involved\_in\_salivary\_gland\_branching | 4 | 0 |  |  |  |  |  |  |  |  |
| GO:0060737\_prostate\_gland\_morphogenetic\_growth | 4 | 0 |  |  |  |  |  |  |  |  |
| GO:0060743\_epithelial\_cell\_maturation\_involved\_in\_prostate\_gland\_development | 4 | 0 |  |  |  |  |  |  |  |  |
| GO:0060751\_mammary\_gland\_duct\_branch\_elongation | 4 | 0 |  |  |  |  |  |  |  |  |
| GO:0060900\_embryonic\_camera-type\_eye\_formation | 4 | 0 |  |  |  |  |  |  |  |  |
| GO:0070059\_apoptosis\_in\_response\_to\_endoplasmic\_reticulum\_stress | 4 | 0 |  |  |  |  |  |  |  |  |
| GO:0070254\_mucus\_secretion | 4 | 0 |  |  |  |  |  |  |  |  |
| GO:0070255\_regulation\_of\_mucus\_secretion | 4 | 0 |  |  |  |  |  |  |  |  |
| GO:0070301\_cellular\_response\_to\_hydrogen\_peroxide | 4 | 0 |  |  |  |  |  |  |  |  |
| GO:0070585\_protein\_localization\_in\_mitochondrion | 4 | 0 |  |  |  |  |  |  |  |  |
| GO:0006811\_ion\_transport | 186 | 0 | 0.000000 | -0.000000 | 769 | 710.322790 | 805.49 | 900.657210 | 1.047451 |
| GO:0042110\_T\_cell\_activation | 163 | 0 | 0.000000 | -0.000000 | 770 | 711.556317 | 806.38 | 901.203683 | 1.047247 |
| GO:0009416\_response\_to\_light\_stimulus | 74 | 0 | 0.000000 | -0.000000 | 772 | 713.241449 | 807.75 | 902.258551 | 1.046308 |
| GO:0048771\_tissue\_remodeling | 74 | 0 | 0.000000 | -0.000000 | 772 | 713.241449 | 807.75 | 902.258551 | 1.046308 |
| GO:0043065\_positive\_regulation\_of\_apoptosis | 166 | 0 | 0.000000 | -0.000000 | 773 | 713.795738 | 808.19 | 902.584262 | 1.045524 |
| GO:0015674\_di-\_\_tri-valent\_inorganic\_cation\_transport | 79 | 0 | 0.000000 | -0.000000 | 775 | 715.326909 | 809.46 | 903.593091 | 1.044465 |
| GO:0051046\_regulation\_of\_secretion | 79 | 0 | 0.000000 | -0.000000 | 775 | 715.326909 | 809.46 | 903.593091 | 1.044465 |
| GO:0007283\_spermatogenesis | 134 | 0 | 0.000000 | -0.000000 | 777 | 716.185118 | 810.48 | 904.774882 | 1.043089 |
| GO:0048232\_male\_gamete\_generation | 134 | 0 | 0.000000 | -0.000000 | 777 | 716.185118 | 810.48 | 904.774882 | 1.043089 |
| GO:0002694\_regulation\_of\_leukocyte\_activation | 121 | 0 | 0.000000 | -0.000000 | 780 | 718.742190 | 812.74 | 906.737810 | 1.041974 |
| GO:0006917\_induction\_of\_apoptosis | 121 | 0 | 0.000000 | -0.000000 | 780 | 718.742190 | 812.74 | 906.737810 | 1.041974 |
| GO:0012502\_induction\_of\_programmed\_cell\_death | 121 | 0 | 0.000000 | -0.000000 | 780 | 718.742190 | 812.74 | 906.737810 | 1.041974 |
| GO:0001822\_kidney\_development | 87 | 0 | 0.000000 | -0.000000 | 786 | 724.198979 | 817.38 | 910.561021 | 1.039924 |
| GO:0003001\_generation\_of\_a\_signal\_involved\_in\_cell-cell\_signaling | 87 | 0 | 0.000000 | -0.000000 | 786 | 724.198979 | 817.38 | 910.561021 | 1.039924 |
| GO:0007178\_transmembrane\_receptor\_protein\_serine\_threonine\_kinase\_signaling\_pathway | 87 | 0 | 0.000000 | -0.000000 | 786 | 724.198979 | 817.38 | 910.561021 | 1.039924 |
| GO:0022612\_gland\_morphogenesis | 87 | 0 | 0.000000 | -0.000000 | 786 | 724.198979 | 817.38 | 910.561021 | 1.039924 |
| GO:0043583\_ear\_development | 87 | 0 | 0.000000 | -0.000000 | 786 | 724.198979 | 817.38 | 910.561021 | 1.039924 |
| GO:0050778\_positive\_regulation\_of\_immune\_response | 87 | 0 | 0.000000 | -0.000000 | 786 | 724.198979 | 817.38 | 910.561021 | 1.039924 |
| GO:0001843\_neural\_tube\_closure | 33 | 0 | 0.000000 | -0.000000 | 799 | 737.768971 | 830.07 | 922.371029 | 1.038886 |
| GO:0002562\_somatic\_diversification\_of\_immune\_receptors\_via\_germline\_recombination\_within\_a\_single\_locus | 33 | 0 | 0.000000 | -0.000000 | 799 | 737.768971 | 830.07 | 922.371029 | 1.038886 |
| GO:0007188\_G-protein\_signaling\_\_coupled\_to\_cAMP\_nucleotide\_second\_messenger | 33 | 0 | 0.000000 | -0.000000 | 799 | 737.768971 | 830.07 | 922.371029 | 1.038886 |
| GO:0007270\_nerve-nerve\_synaptic\_transmission | 33 | 0 | 0.000000 | -0.000000 | 799 | 737.768971 | 830.07 | 922.371029 | 1.038886 |
| GO:0007431\_salivary\_gland\_development | 33 | 0 | 0.000000 | -0.000000 | 799 | 737.768971 | 830.07 | 922.371029 | 1.038886 |
| GO:0007565\_female\_pregnancy | 33 | 0 | 0.000000 | -0.000000 | 799 | 737.768971 | 830.07 | 922.371029 | 1.038886 |
| GO:0008584\_male\_gonad\_development | 33 | 0 | 0.000000 | -0.000000 | 799 | 737.768971 | 830.07 | 922.371029 | 1.038886 |
| GO:0008643\_carbohydrate\_transport | 33 | 0 | 0.000000 | -0.000000 | 799 | 737.768971 | 830.07 | 922.371029 | 1.038886 |
| GO:0016444\_somatic\_cell\_DNA\_recombination | 33 | 0 | 0.000000 | -0.000000 | 799 | 737.768971 | 830.07 | 922.371029 | 1.038886 |
| GO:0021536\_diencephalon\_development | 33 | 0 | 0.000000 | -0.000000 | 799 | 737.768971 | 830.07 | 922.371029 | 1.038886 |
| GO:0021987\_cerebral\_cortex\_development | 33 | 0 | 0.000000 | -0.000000 | 799 | 737.768971 | 830.07 | 922.371029 | 1.038886 |
| GO:0042108\_positive\_regulation\_of\_cytokine\_biosynthetic\_process | 33 | 0 | 0.000000 | -0.000000 | 799 | 737.768971 | 830.07 | 922.371029 | 1.038886 |
| GO:0060606\_tube\_closure | 33 | 0 | 0.000000 | -0.000000 | 799 | 737.768971 | 830.07 | 922.371029 | 1.038886 |
| GO:0021700\_developmental\_maturation | 81 | 0 | 0.000000 | -0.000000 | 800 | 738.391655 | 830.63 | 922.868345 | 1.038288 |
| GO:0001932\_regulation\_of\_protein\_amino\_acid\_phosphorylation | 69 | 0 | 0.000000 | -0.000000 | 804 | 742.553047 | 834.23 | 925.906953 | 1.037600 |
| GO:0006816\_calcium\_ion\_transport | 69 | 0 | 0.000000 | -0.000000 | 804 | 742.553047 | 834.23 | 925.906953 | 1.037600 |
| GO:0032101\_regulation\_of\_response\_to\_external\_stimulus | 69 | 0 | 0.000000 | -0.000000 | 804 | 742.553047 | 834.23 | 925.906953 | 1.037600 |
| GO:0055065\_metal\_ion\_homeostasis | 69 | 0 | 0.000000 | -0.000000 | 804 | 742.553047 | 834.23 | 925.906953 | 1.037600 |
| GO:0001841\_neural\_tube\_formation | 43 | 0 | 0.000000 | -0.000000 | 817 | 759.707895 | 849.99 | 940.272105 | 1.040379 |
| GO:0001894\_tissue\_homeostasis | 43 | 0 | 0.000000 | -0.000000 | 817 | 759.707895 | 849.99 | 940.272105 | 1.040379 |
| GO:0002819\_regulation\_of\_adaptive\_immune\_response | 43 | 0 | 0.000000 | -0.000000 | 817 | 759.707895 | 849.99 | 940.272105 | 1.040379 |
| GO:0002822\_regulation\_of\_adaptive\_immune\_response\_based\_on\_somatic\_recombination\_of\_immune\_receptors\_built\_from\_immunoglobulin\_superfamily\_domains | 43 | 0 | 0.000000 | -0.000000 | 817 | 759.707895 | 849.99 | 940.272105 | 1.040379 |
| GO:0006766\_vitamin\_metabolic\_process | 43 | 0 | 0.000000 | -0.000000 | 817 | 759.707895 | 849.99 | 940.272105 | 1.040379 |
| GO:0007224\_smoothened\_signaling\_pathway | 43 | 0 | 0.000000 | -0.000000 | 817 | 759.707895 | 849.99 | 940.272105 | 1.040379 |
| GO:0009582\_detection\_of\_abiotic\_stimulus | 43 | 0 | 0.000000 | -0.000000 | 817 | 759.707895 | 849.99 | 940.272105 | 1.040379 |
| GO:0019637\_organophosphate\_metabolic\_process | 43 | 0 | 0.000000 | -0.000000 | 817 | 759.707895 | 849.99 | 940.272105 | 1.040379 |
| GO:0030814\_regulation\_of\_cAMP\_metabolic\_process | 43 | 0 | 0.000000 | -0.000000 | 817 | 759.707895 | 849.99 | 940.272105 | 1.040379 |
| GO:0031098\_stress-activated\_protein\_kinase\_signaling\_pathway | 43 | 0 | 0.000000 | -0.000000 | 817 | 759.707895 | 849.99 | 940.272105 | 1.040379 |
| GO:0046879\_hormone\_secretion | 43 | 0 | 0.000000 | -0.000000 | 817 | 759.707895 | 849.99 | 940.272105 | 1.040379 |
| GO:0048762\_mesenchymal\_cell\_differentiation | 43 | 0 | 0.000000 | -0.000000 | 817 | 759.707895 | 849.99 | 940.272105 | 1.040379 |
| GO:0051604\_protein\_maturation | 43 | 0 | 0.000000 | -0.000000 | 817 | 759.707895 | 849.99 | 940.272105 | 1.040379 |
| GO:0000079\_regulation\_of\_cyclin-dependent\_protein\_kinase\_activity | 7 | 0 | 0.000000 | -0.000000 | 993 | 937.468949 | 1025.68 | 1113.891051 | 1.032910 |
| GO:0000188\_inactivation\_of\_MAPK\_activity | 7 | 0 | 0.000000 | -0.000000 | 993 | 937.468949 | 1025.68 | 1113.891051 | 1.032910 |
| GO:0001504\_neurotransmitter\_uptake | 7 | 0 | 0.000000 | -0.000000 | 993 | 937.468949 | 1025.68 | 1113.891051 | 1.032910 |
| GO:0001556\_oocyte\_maturation | 7 | 0 | 0.000000 | -0.000000 | 993 | 937.468949 | 1025.68 | 1113.891051 | 1.032910 |
| GO:0001736\_establishment\_of\_planar\_polarity | 7 | 0 | 0.000000 | -0.000000 | 993 | 937.468949 | 1025.68 | 1113.891051 | 1.032910 |
| GO:0001839\_neural\_plate\_morphogenesis | 7 | 0 | 0.000000 | -0.000000 | 993 | 937.468949 | 1025.68 | 1113.891051 | 1.032910 |
| GO:0001936\_regulation\_of\_endothelial\_cell\_proliferation | 7 | 0 | 0.000000 | -0.000000 | 993 | 937.468949 | 1025.68 | 1113.891051 | 1.032910 |
| GO:0001967\_suckling\_behavior | 7 | 0 | 0.000000 | -0.000000 | 993 | 937.468949 | 1025.68 | 1113.891051 | 1.032910 |
| GO:0002011\_morphogenesis\_of\_an\_epithelial\_sheet | 7 | 0 | 0.000000 | -0.000000 | 993 | 937.468949 | 1025.68 | 1113.891051 | 1.032910 |
| GO:0002052\_positive\_regulation\_of\_neuroblast\_proliferation | 7 | 0 | 0.000000 | -0.000000 | 993 | 937.468949 | 1025.68 | 1113.891051 | 1.032910 |
| GO:0002063\_chondrocyte\_development | 7 | 0 | 0.000000 | -0.000000 | 993 | 937.468949 | 1025.68 | 1113.891051 | 1.032910 |
| GO:0002076\_osteoblast\_development | 7 | 0 | 0.000000 | -0.000000 | 993 | 937.468949 | 1025.68 | 1113.891051 | 1.032910 |
| GO:0002087\_regulation\_of\_respiratory\_gaseous\_exchange\_by\_neurological\_system\_process | 7 | 0 | 0.000000 | -0.000000 | 993 | 937.468949 | 1025.68 | 1113.891051 | 1.032910 |
| GO:0002093\_auditory\_receptor\_cell\_morphogenesis | 7 | 0 | 0.000000 | -0.000000 | 993 | 937.468949 | 1025.68 | 1113.891051 | 1.032910 |
| GO:0002224\_toll-like\_receptor\_signaling\_pathway | 7 | 0 | 0.000000 | -0.000000 | 993 | 937.468949 | 1025.68 | 1113.891051 | 1.032910 |
| GO:0002455\_humoral\_immune\_response\_mediated\_by\_circulating\_immunoglobulin | 7 | 0 | 0.000000 | -0.000000 | 993 | 937.468949 | 1025.68 | 1113.891051 | 1.032910 |
| GO:0002643\_regulation\_of\_tolerance\_induction | 7 | 0 | 0.000000 | -0.000000 | 993 | 937.468949 | 1025.68 | 1113.891051 | 1.032910 |
| GO:0002645\_positive\_regulation\_of\_tolerance\_induction | 7 | 0 | 0.000000 | -0.000000 | 993 | 937.468949 | 1025.68 | 1113.891051 | 1.032910 |
| GO:0002714\_positive\_regulation\_of\_B\_cell\_mediated\_immunity | 7 | 0 | 0.000000 | -0.000000 | 993 | 937.468949 | 1025.68 | 1113.891051 | 1.032910 |
| GO:0002792\_negative\_regulation\_of\_peptide\_secretion | 7 | 0 | 0.000000 | -0.000000 | 993 | 937.468949 | 1025.68 | 1113.891051 | 1.032910 |
| GO:0002793\_positive\_regulation\_of\_peptide\_secretion | 7 | 0 | 0.000000 | -0.000000 | 993 | 937.468949 | 1025.68 | 1113.891051 | 1.032910 |
| GO:0002828\_regulation\_of\_T-helper\_2\_type\_immune\_response | 7 | 0 | 0.000000 | -0.000000 | 993 | 937.468949 | 1025.68 | 1113.891051 | 1.032910 |
| GO:0002863\_positive\_regulation\_of\_inflammatory\_response\_to\_antigenic\_stimulus | 7 | 0 | 0.000000 | -0.000000 | 993 | 937.468949 | 1025.68 | 1113.891051 | 1.032910 |
| GO:0002891\_positive\_regulation\_of\_immunoglobulin\_mediated\_immune\_response | 7 | 0 | 0.000000 | -0.000000 | 993 | 937.468949 | 1025.68 | 1113.891051 | 1.032910 |
| GO:0003084\_positive\_regulation\_of\_systemic\_arterial\_blood\_pressure | 7 | 0 | 0.000000 | -0.000000 | 993 | 937.468949 | 1025.68 | 1113.891051 | 1.032910 |
| GO:0003085\_negative\_regulation\_of\_systemic\_arterial\_blood\_pressure | 7 | 0 | 0.000000 | -0.000000 | 993 | 937.468949 | 1025.68 | 1113.891051 | 1.032910 |
| GO:0006014\_D-ribose\_metabolic\_process | 7 | 0 | 0.000000 | -0.000000 | 993 | 937.468949 | 1025.68 | 1113.891051 | 1.032910 |
| GO:0006041\_glucosamine\_metabolic\_process | 7 | 0 | 0.000000 | -0.000000 | 993 | 937.468949 | 1025.68 | 1113.891051 | 1.032910 |
| GO:0006044\_N-acetylglucosamine\_metabolic\_process | 7 | 0 | 0.000000 | -0.000000 | 993 | 937.468949 | 1025.68 | 1113.891051 | 1.032910 |
| GO:0006096\_glycolysis | 7 | 0 | 0.000000 | -0.000000 | 993 | 937.468949 | 1025.68 | 1113.891051 | 1.032910 |
| GO:0006119\_oxidative\_phosphorylation | 7 | 0 | 0.000000 | -0.000000 | 993 | 937.468949 | 1025.68 | 1113.891051 | 1.032910 |
| GO:0006275\_regulation\_of\_DNA\_replication | 7 | 0 | 0.000000 | -0.000000 | 993 | 937.468949 | 1025.68 | 1113.891051 | 1.032910 |
| GO:0006298\_mismatch\_repair | 7 | 0 | 0.000000 | -0.000000 | 993 | 937.468949 | 1025.68 | 1113.891051 | 1.032910 |
| GO:0006352\_transcription\_initiation | 7 | 0 | 0.000000 | -0.000000 | 993 | 937.468949 | 1025.68 | 1113.891051 | 1.032910 |
| GO:0006401\_RNA\_catabolic\_process | 7 | 0 | 0.000000 | -0.000000 | 993 | 937.468949 | 1025.68 | 1113.891051 | 1.032910 |
| GO:0006406\_mRNA\_export\_from\_nucleus | 7 | 0 | 0.000000 | -0.000000 | 993 | 937.468949 | 1025.68 | 1113.891051 | 1.032910 |
| GO:0006505\_GPI\_anchor\_metabolic\_process | 7 | 0 | 0.000000 | -0.000000 | 993 | 937.468949 | 1025.68 | 1113.891051 | 1.032910 |
| GO:0006516\_glycoprotein\_catabolic\_process | 7 | 0 | 0.000000 | -0.000000 | 993 | 937.468949 | 1025.68 | 1113.891051 | 1.032910 |
| GO:0006612\_protein\_targeting\_to\_membrane | 7 | 0 | 0.000000 | -0.000000 | 993 | 937.468949 | 1025.68 | 1113.891051 | 1.032910 |
| GO:0006769\_nicotinamide\_metabolic\_process | 7 | 0 | 0.000000 | -0.000000 | 993 | 937.468949 | 1025.68 | 1113.891051 | 1.032910 |
| GO:0006783\_heme\_biosynthetic\_process | 7 | 0 | 0.000000 | -0.000000 | 993 | 937.468949 | 1025.68 | 1113.891051 | 1.032910 |
| GO:0006818\_hydrogen\_transport | 7 | 0 | 0.000000 | -0.000000 | 993 | 937.468949 | 1025.68 | 1113.891051 | 1.032910 |
| GO:0006878\_cellular\_copper\_ion\_homeostasis | 7 | 0 | 0.000000 | -0.000000 | 993 | 937.468949 | 1025.68 | 1113.891051 | 1.032910 |
| GO:0006884\_cell\_volume\_homeostasis | 7 | 0 | 0.000000 | -0.000000 | 993 | 937.468949 | 1025.68 | 1113.891051 | 1.032910 |
| GO:0006949\_syncytium\_formation | 7 | 0 | 0.000000 | -0.000000 | 993 | 937.468949 | 1025.68 | 1113.891051 | 1.032910 |
| GO:0007019\_microtubule\_depolymerization | 7 | 0 | 0.000000 | -0.000000 | 993 | 937.468949 | 1025.68 | 1113.891051 | 1.032910 |
| GO:0007026\_negative\_regulation\_of\_microtubule\_depolymerization | 7 | 0 | 0.000000 | -0.000000 | 993 | 937.468949 | 1025.68 | 1113.891051 | 1.032910 |
| GO:0007034\_vacuolar\_transport | 7 | 0 | 0.000000 | -0.000000 | 993 | 937.468949 | 1025.68 | 1113.891051 | 1.032910 |
| GO:0007062\_sister\_chromatid\_cohesion | 7 | 0 | 0.000000 | -0.000000 | 993 | 937.468949 | 1025.68 | 1113.891051 | 1.032910 |
| GO:0007130\_synaptonemal\_complex\_assembly | 7 | 0 | 0.000000 | -0.000000 | 993 | 937.468949 | 1025.68 | 1113.891051 | 1.032910 |
| GO:0007164\_establishment\_of\_tissue\_polarity | 7 | 0 | 0.000000 | -0.000000 | 993 | 937.468949 | 1025.68 | 1113.891051 | 1.032910 |
| GO:0007191\_activation\_of\_adenylate\_cyclase\_activity\_by\_dopamine\_receptor\_signaling\_pathway | 7 | 0 | 0.000000 | -0.000000 | 993 | 937.468949 | 1025.68 | 1113.891051 | 1.032910 |
| GO:0007271\_synaptic\_transmission\_\_cholinergic | 7 | 0 | 0.000000 | -0.000000 | 993 | 937.468949 | 1025.68 | 1113.891051 | 1.032910 |
| GO:0007413\_axonal\_fasciculation | 7 | 0 | 0.000000 | -0.000000 | 993 | 937.468949 | 1025.68 | 1113.891051 | 1.032910 |
| GO:0007440\_foregut\_morphogenesis | 7 | 0 | 0.000000 | -0.000000 | 993 | 937.468949 | 1025.68 | 1113.891051 | 1.032910 |
| GO:0007616\_long-term\_memory | 7 | 0 | 0.000000 | -0.000000 | 993 | 937.468949 | 1025.68 | 1113.891051 | 1.032910 |
| GO:0008299\_isoprenoid\_biosynthetic\_process | 7 | 0 | 0.000000 | -0.000000 | 993 | 937.468949 | 1025.68 | 1113.891051 | 1.032910 |
| GO:0008340\_determination\_of\_adult\_lifespan | 7 | 0 | 0.000000 | -0.000000 | 993 | 937.468949 | 1025.68 | 1113.891051 | 1.032910 |
| GO:0009150\_purine\_ribonucleotide\_metabolic\_process | 7 | 0 | 0.000000 | -0.000000 | 993 | 937.468949 | 1025.68 | 1113.891051 | 1.032910 |
| GO:0009200\_deoxyribonucleoside\_triphosphate\_metabolic\_process | 7 | 0 | 0.000000 | -0.000000 | 993 | 937.468949 | 1025.68 | 1113.891051 | 1.032910 |
| GO:0009259\_ribonucleotide\_metabolic\_process | 7 | 0 | 0.000000 | -0.000000 | 993 | 937.468949 | 1025.68 | 1113.891051 | 1.032910 |
| GO:0009311\_oligosaccharide\_metabolic\_process | 7 | 0 | 0.000000 | -0.000000 | 993 | 937.468949 | 1025.68 | 1113.891051 | 1.032910 |
| GO:0009394\_2'-deoxyribonucleotide\_metabolic\_process | 7 | 0 | 0.000000 | -0.000000 | 993 | 937.468949 | 1025.68 | 1113.891051 | 1.032910 |
| GO:0009820\_alkaloid\_metabolic\_process | 7 | 0 | 0.000000 | -0.000000 | 993 | 937.468949 | 1025.68 | 1113.891051 | 1.032910 |
| GO:0010469\_regulation\_of\_receptor\_activity | 7 | 0 | 0.000000 | -0.000000 | 993 | 937.468949 | 1025.68 | 1113.891051 | 1.032910 |
| GO:0010948\_negative\_regulation\_of\_cell\_cycle\_process | 7 | 0 | 0.000000 | -0.000000 | 993 | 937.468949 | 1025.68 | 1113.891051 | 1.032910 |
| GO:0014047\_glutamate\_secretion | 7 | 0 | 0.000000 | -0.000000 | 993 | 937.468949 | 1025.68 | 1113.891051 | 1.032910 |
| GO:0014066\_regulation\_of\_phosphoinositide\_3-kinase\_cascade | 7 | 0 | 0.000000 | -0.000000 | 993 | 937.468949 | 1025.68 | 1113.891051 | 1.032910 |
| GO:0014821\_phasic\_smooth\_muscle\_contraction | 7 | 0 | 0.000000 | -0.000000 | 993 | 937.468949 | 1025.68 | 1113.891051 | 1.032910 |
| GO:0015697\_quaternary\_ammonium\_group\_transport | 7 | 0 | 0.000000 | -0.000000 | 993 | 937.468949 | 1025.68 | 1113.891051 | 1.032910 |
| GO:0015813\_L-glutamate\_transport | 7 | 0 | 0.000000 | -0.000000 | 993 | 937.468949 | 1025.68 | 1113.891051 | 1.032910 |
| GO:0015908\_fatty\_acid\_transport | 7 | 0 | 0.000000 | -0.000000 | 993 | 937.468949 | 1025.68 | 1113.891051 | 1.032910 |
| GO:0015914\_phospholipid\_transport | 7 | 0 | 0.000000 | -0.000000 | 993 | 937.468949 | 1025.68 | 1113.891051 | 1.032910 |
| GO:0015992\_proton\_transport | 7 | 0 | 0.000000 | -0.000000 | 993 | 937.468949 | 1025.68 | 1113.891051 | 1.032910 |
| GO:0016339\_calcium-dependent\_cell-cell\_adhesion | 7 | 0 | 0.000000 | -0.000000 | 993 | 937.468949 | 1025.68 | 1113.891051 | 1.032910 |
| GO:0016575\_histone\_deacetylation | 7 | 0 | 0.000000 | -0.000000 | 993 | 937.468949 | 1025.68 | 1113.891051 | 1.032910 |
| GO:0019362\_pyridine\_nucleotide\_metabolic\_process | 7 | 0 | 0.000000 | -0.000000 | 993 | 937.468949 | 1025.68 | 1113.891051 | 1.032910 |
| GO:0019692\_deoxyribose\_phosphate\_metabolic\_process | 7 | 0 | 0.000000 | -0.000000 | 993 | 937.468949 | 1025.68 | 1113.891051 | 1.032910 |
| GO:0019800\_peptide\_cross-linking\_via\_chondroitin\_4-sulfate\_glycosaminoglycan | 7 | 0 | 0.000000 | -0.000000 | 993 | 937.468949 | 1025.68 | 1113.891051 | 1.032910 |
| GO:0020027\_hemoglobin\_metabolic\_process | 7 | 0 | 0.000000 | -0.000000 | 993 | 937.468949 | 1025.68 | 1113.891051 | 1.032910 |
| GO:0021514\_ventral\_spinal\_cord\_interneuron\_differentiation | 7 | 0 | 0.000000 | -0.000000 | 993 | 937.468949 | 1025.68 | 1113.891051 | 1.032910 |
| GO:0021516\_dorsal\_spinal\_cord\_development | 7 | 0 | 0.000000 | -0.000000 | 993 | 937.468949 | 1025.68 | 1113.891051 | 1.032910 |
| GO:0021520\_spinal\_cord\_motor\_neuron\_cell\_fate\_specification | 7 | 0 | 0.000000 | -0.000000 | 993 | 937.468949 | 1025.68 | 1113.891051 | 1.032910 |
| GO:0021521\_ventral\_spinal\_cord\_interneuron\_specification | 7 | 0 | 0.000000 | -0.000000 | 993 | 937.468949 | 1025.68 | 1113.891051 | 1.032910 |
| GO:0021546\_rhombomere\_development | 7 | 0 | 0.000000 | -0.000000 | 993 | 937.468949 | 1025.68 | 1113.891051 | 1.032910 |
| GO:0021756\_striatum\_development | 7 | 0 | 0.000000 | -0.000000 | 993 | 937.468949 | 1025.68 | 1113.891051 | 1.032910 |
| GO:0021884\_forebrain\_neuron\_development | 7 | 0 | 0.000000 | -0.000000 | 993 | 937.468949 | 1025.68 | 1113.891051 | 1.032910 |
| GO:0021903\_rostrocaudal\_neural\_tube\_patterning | 7 | 0 | 0.000000 | -0.000000 | 993 | 937.468949 | 1025.68 | 1113.891051 | 1.032910 |
| GO:0021984\_adenohypophysis\_development | 7 | 0 | 0.000000 | -0.000000 | 993 | 937.468949 | 1025.68 | 1113.891051 | 1.032910 |
| GO:0022407\_regulation\_of\_cell-cell\_adhesion | 7 | 0 | 0.000000 | -0.000000 | 993 | 937.468949 | 1025.68 | 1113.891051 | 1.032910 |
| GO:0022618\_ribonucleoprotein\_complex\_assembly | 7 | 0 | 0.000000 | -0.000000 | 993 | 937.468949 | 1025.68 | 1113.891051 | 1.032910 |
| GO:0030104\_water\_homeostasis | 7 | 0 | 0.000000 | -0.000000 | 993 | 937.468949 | 1025.68 | 1113.891051 | 1.032910 |
| GO:0030201\_heparan\_sulfate\_proteoglycan\_metabolic\_process | 7 | 0 | 0.000000 | -0.000000 | 993 | 937.468949 | 1025.68 | 1113.891051 | 1.032910 |
| GO:0030432\_peristalsis | 7 | 0 | 0.000000 | -0.000000 | 993 | 937.468949 | 1025.68 | 1113.891051 | 1.032910 |
| GO:0030517\_negative\_regulation\_of\_axon\_extension | 7 | 0 | 0.000000 | -0.000000 | 993 | 937.468949 | 1025.68 | 1113.891051 | 1.032910 |
| GO:0030520\_estrogen\_receptor\_signaling\_pathway | 7 | 0 | 0.000000 | -0.000000 | 993 | 937.468949 | 1025.68 | 1113.891051 | 1.032910 |
| GO:0030521\_androgen\_receptor\_signaling\_pathway | 7 | 0 | 0.000000 | -0.000000 | 993 | 937.468949 | 1025.68 | 1113.891051 | 1.032910 |
| GO:0030903\_notochord\_development | 7 | 0 | 0.000000 | -0.000000 | 993 | 937.468949 | 1025.68 | 1113.891051 | 1.032910 |
| GO:0031017\_exocrine\_pancreas\_development | 7 | 0 | 0.000000 | -0.000000 | 993 | 937.468949 | 1025.68 | 1113.891051 | 1.032910 |
| GO:0031114\_regulation\_of\_microtubule\_depolymerization | 7 | 0 | 0.000000 | -0.000000 | 993 | 937.468949 | 1025.68 | 1113.891051 | 1.032910 |
| GO:0031124\_mRNA\_3'-end\_processing | 7 | 0 | 0.000000 | -0.000000 | 993 | 937.468949 | 1025.68 | 1113.891051 | 1.032910 |
| GO:0031497\_chromatin\_assembly | 7 | 0 | 0.000000 | -0.000000 | 993 | 937.468949 | 1025.68 | 1113.891051 | 1.032910 |
| GO:0032104\_regulation\_of\_response\_to\_extracellular\_stimulus | 7 | 0 | 0.000000 | -0.000000 | 993 | 937.468949 | 1025.68 | 1113.891051 | 1.032910 |
| GO:0032107\_regulation\_of\_response\_to\_nutrient\_levels | 7 | 0 | 0.000000 | -0.000000 | 993 | 937.468949 | 1025.68 | 1113.891051 | 1.032910 |
| GO:0032228\_regulation\_of\_synaptic\_transmission\_\_GABAergic | 7 | 0 | 0.000000 | -0.000000 | 993 | 937.468949 | 1025.68 | 1113.891051 | 1.032910 |
| GO:0032319\_regulation\_of\_Rho\_GTPase\_activity | 7 | 0 | 0.000000 | -0.000000 | 993 | 937.468949 | 1025.68 | 1113.891051 | 1.032910 |
| GO:0032387\_negative\_regulation\_of\_intracellular\_transport | 7 | 0 | 0.000000 | -0.000000 | 993 | 937.468949 | 1025.68 | 1113.891051 | 1.032910 |
| GO:0032507\_maintenance\_of\_protein\_location\_in\_cell | 7 | 0 | 0.000000 | -0.000000 | 993 | 937.468949 | 1025.68 | 1113.891051 | 1.032910 |
| GO:0033032\_regulation\_of\_myeloid\_cell\_apoptosis | 7 | 0 | 0.000000 | -0.000000 | 993 | 937.468949 | 1025.68 | 1113.891051 | 1.032910 |
| GO:0033057\_reproductive\_behavior\_in\_a\_multicellular\_organism | 7 | 0 | 0.000000 | -0.000000 | 993 | 937.468949 | 1025.68 | 1113.891051 | 1.032910 |
| GO:0034599\_cellular\_response\_to\_oxidative\_stress | 7 | 0 | 0.000000 | -0.000000 | 993 | 937.468949 | 1025.68 | 1113.891051 | 1.032910 |
| GO:0042033\_chemokine\_biosynthetic\_process | 7 | 0 | 0.000000 | -0.000000 | 993 | 937.468949 | 1025.68 | 1113.891051 | 1.032910 |
| GO:0042133\_neurotransmitter\_metabolic\_process | 7 | 0 | 0.000000 | -0.000000 | 993 | 937.468949 | 1025.68 | 1113.891051 | 1.032910 |
| GO:0042168\_heme\_metabolic\_process | 7 | 0 | 0.000000 | -0.000000 | 993 | 937.468949 | 1025.68 | 1113.891051 | 1.032910 |
| GO:0042415\_norepinephrine\_metabolic\_process | 7 | 0 | 0.000000 | -0.000000 | 993 | 937.468949 | 1025.68 | 1113.891051 | 1.032910 |
| GO:0042503\_tyrosine\_phosphorylation\_of\_Stat3\_protein | 7 | 0 | 0.000000 | -0.000000 | 993 | 937.468949 | 1025.68 | 1113.891051 | 1.032910 |
| GO:0042572\_retinol\_metabolic\_process | 7 | 0 | 0.000000 | -0.000000 | 993 | 937.468949 | 1025.68 | 1113.891051 | 1.032910 |
| GO:0043372\_positive\_regulation\_of\_CD4-positive\_\_alpha\_beta\_T\_cell\_differentiation | 7 | 0 | 0.000000 | -0.000000 | 993 | 937.468949 | 1025.68 | 1113.891051 | 1.032910 |
| GO:0043449\_cellular\_alkene\_metabolic\_process | 7 | 0 | 0.000000 | -0.000000 | 993 | 937.468949 | 1025.68 | 1113.891051 | 1.032910 |
| GO:0043507\_positive\_regulation\_of\_JUN\_kinase\_activity | 7 | 0 | 0.000000 | -0.000000 | 993 | 937.468949 | 1025.68 | 1113.891051 | 1.032910 |
| GO:0043567\_regulation\_of\_insulin-like\_growth\_factor\_receptor\_signaling\_pathway | 7 | 0 | 0.000000 | -0.000000 | 993 | 937.468949 | 1025.68 | 1113.891051 | 1.032910 |
| GO:0043584\_nose\_development | 7 | 0 | 0.000000 | -0.000000 | 993 | 937.468949 | 1025.68 | 1113.891051 | 1.032910 |
| GO:0044065\_regulation\_of\_respiratory\_system\_process | 7 | 0 | 0.000000 | -0.000000 | 993 | 937.468949 | 1025.68 | 1113.891051 | 1.032910 |
| GO:0044275\_cellular\_carbohydrate\_catabolic\_process | 7 | 0 | 0.000000 | -0.000000 | 993 | 937.468949 | 1025.68 | 1113.891051 | 1.032910 |
| GO:0045059\_positive\_thymic\_T\_cell\_selection | 7 | 0 | 0.000000 | -0.000000 | 993 | 937.468949 | 1025.68 | 1113.891051 | 1.032910 |
| GO:0045073\_regulation\_of\_chemokine\_biosynthetic\_process | 7 | 0 | 0.000000 | -0.000000 | 993 | 937.468949 | 1025.68 | 1113.891051 | 1.032910 |
| GO:0045581\_negative\_regulation\_of\_T\_cell\_differentiation | 7 | 0 | 0.000000 | -0.000000 | 993 | 937.468949 | 1025.68 | 1113.891051 | 1.032910 |
| GO:0045599\_negative\_regulation\_of\_fat\_cell\_differentiation | 7 | 0 | 0.000000 | -0.000000 | 993 | 937.468949 | 1025.68 | 1113.891051 | 1.032910 |
| GO:0045604\_regulation\_of\_epidermal\_cell\_differentiation | 7 | 0 | 0.000000 | -0.000000 | 993 | 937.468949 | 1025.68 | 1113.891051 | 1.032910 |
| GO:0045668\_negative\_regulation\_of\_osteoblast\_differentiation | 7 | 0 | 0.000000 | -0.000000 | 993 | 937.468949 | 1025.68 | 1113.891051 | 1.032910 |
| GO:0045823\_positive\_regulation\_of\_heart\_contraction | 7 | 0 | 0.000000 | -0.000000 | 993 | 937.468949 | 1025.68 | 1113.891051 | 1.032910 |
| GO:0045840\_positive\_regulation\_of\_mitosis | 7 | 0 | 0.000000 | -0.000000 | 993 | 937.468949 | 1025.68 | 1113.891051 | 1.032910 |
| GO:0045862\_positive\_regulation\_of\_proteolysis | 7 | 0 | 0.000000 | -0.000000 | 993 | 937.468949 | 1025.68 | 1113.891051 | 1.032910 |
| GO:0045879\_negative\_regulation\_of\_smoothened\_signaling\_pathway | 7 | 0 | 0.000000 | -0.000000 | 993 | 937.468949 | 1025.68 | 1113.891051 | 1.032910 |
| GO:0045880\_positive\_regulation\_of\_smoothened\_signaling\_pathway | 7 | 0 | 0.000000 | -0.000000 | 993 | 937.468949 | 1025.68 | 1113.891051 | 1.032910 |
| GO:0045986\_negative\_regulation\_of\_smooth\_muscle\_contraction | 7 | 0 | 0.000000 | -0.000000 | 993 | 937.468949 | 1025.68 | 1113.891051 | 1.032910 |
| GO:0046496\_nicotinamide\_nucleotide\_metabolic\_process | 7 | 0 | 0.000000 | -0.000000 | 993 | 937.468949 | 1025.68 | 1113.891051 | 1.032910 |
| GO:0046504\_glycerol\_ether\_biosynthetic\_process | 7 | 0 | 0.000000 | -0.000000 | 993 | 937.468949 | 1025.68 | 1113.891051 | 1.032910 |
| GO:0046513\_ceramide\_biosynthetic\_process | 7 | 0 | 0.000000 | -0.000000 | 993 | 937.468949 | 1025.68 | 1113.891051 | 1.032910 |
| GO:0046520\_sphingoid\_biosynthetic\_process | 7 | 0 | 0.000000 | -0.000000 | 993 | 937.468949 | 1025.68 | 1113.891051 | 1.032910 |
| GO:0046543\_development\_of\_secondary\_female\_sexual\_characteristics | 7 | 0 | 0.000000 | -0.000000 | 993 | 937.468949 | 1025.68 | 1113.891051 | 1.032910 |
| GO:0046622\_positive\_regulation\_of\_organ\_growth | 7 | 0 | 0.000000 | -0.000000 | 993 | 937.468949 | 1025.68 | 1113.891051 | 1.032910 |
| GO:0046626\_regulation\_of\_insulin\_receptor\_signaling\_pathway | 7 | 0 | 0.000000 | -0.000000 | 993 | 937.468949 | 1025.68 | 1113.891051 | 1.032910 |
| GO:0046676\_negative\_regulation\_of\_insulin\_secretion | 7 | 0 | 0.000000 | -0.000000 | 993 | 937.468949 | 1025.68 | 1113.891051 | 1.032910 |
| GO:0046677\_response\_to\_antibiotic | 7 | 0 | 0.000000 | -0.000000 | 993 | 937.468949 | 1025.68 | 1113.891051 | 1.032910 |
| GO:0046823\_negative\_regulation\_of\_nucleocytoplasmic\_transport | 7 | 0 | 0.000000 | -0.000000 | 993 | 937.468949 | 1025.68 | 1113.891051 | 1.032910 |
| GO:0046824\_positive\_regulation\_of\_nucleocytoplasmic\_transport | 7 | 0 | 0.000000 | -0.000000 | 993 | 937.468949 | 1025.68 | 1113.891051 | 1.032910 |
| GO:0046847\_filopodium\_assembly | 7 | 0 | 0.000000 | -0.000000 | 993 | 937.468949 | 1025.68 | 1113.891051 | 1.032910 |
| GO:0048148\_behavioral\_response\_to\_cocaine | 7 | 0 | 0.000000 | -0.000000 | 993 | 937.468949 | 1025.68 | 1113.891051 | 1.032910 |
| GO:0048304\_positive\_regulation\_of\_isotype\_switching\_to\_IgG\_isotypes | 7 | 0 | 0.000000 | -0.000000 | 993 | 937.468949 | 1025.68 | 1113.891051 | 1.032910 |
| GO:0048486\_parasympathetic\_nervous\_system\_development | 7 | 0 | 0.000000 | -0.000000 | 993 | 937.468949 | 1025.68 | 1113.891051 | 1.032910 |
| GO:0048537\_mucosal-associated\_lymphoid\_tissue\_development | 7 | 0 | 0.000000 | -0.000000 | 993 | 937.468949 | 1025.68 | 1113.891051 | 1.032910 |
| GO:0048753\_pigment\_granule\_organization | 7 | 0 | 0.000000 | -0.000000 | 993 | 937.468949 | 1025.68 | 1113.891051 | 1.032910 |
| GO:0048814\_regulation\_of\_dendrite\_morphogenesis | 7 | 0 | 0.000000 | -0.000000 | 993 | 937.468949 | 1025.68 | 1113.891051 | 1.032910 |
| GO:0048857\_neural\_nucleus\_development | 7 | 0 | 0.000000 | -0.000000 | 993 | 937.468949 | 1025.68 | 1113.891051 | 1.032910 |
| GO:0050755\_chemokine\_metabolic\_process | 7 | 0 | 0.000000 | -0.000000 | 993 | 937.468949 | 1025.68 | 1113.891051 | 1.032910 |
| GO:0050773\_regulation\_of\_dendrite\_development | 7 | 0 | 0.000000 | -0.000000 | 993 | 937.468949 | 1025.68 | 1113.891051 | 1.032910 |
| GO:0051028\_mRNA\_transport | 7 | 0 | 0.000000 | -0.000000 | 993 | 937.468949 | 1025.68 | 1113.891051 | 1.032910 |
| GO:0051785\_positive\_regulation\_of\_nuclear\_division | 7 | 0 | 0.000000 | -0.000000 | 993 | 937.468949 | 1025.68 | 1113.891051 | 1.032910 |
| GO:0051928\_positive\_regulation\_of\_calcium\_ion\_transport | 7 | 0 | 0.000000 | -0.000000 | 993 | 937.468949 | 1025.68 | 1113.891051 | 1.032910 |
| GO:0055069\_zinc\_ion\_homeostasis | 7 | 0 | 0.000000 | -0.000000 | 993 | 937.468949 | 1025.68 | 1113.891051 | 1.032910 |
| GO:0055070\_copper\_ion\_homeostasis | 7 | 0 | 0.000000 | -0.000000 | 993 | 937.468949 | 1025.68 | 1113.891051 | 1.032910 |
| GO:0060037\_pharyngeal\_system\_development | 7 | 0 | 0.000000 | -0.000000 | 993 | 937.468949 | 1025.68 | 1113.891051 | 1.032910 |
| GO:0060080\_regulation\_of\_inhibitory\_postsynaptic\_membrane\_potential | 7 | 0 | 0.000000 | -0.000000 | 993 | 937.468949 | 1025.68 | 1113.891051 | 1.032910 |
| GO:0060088\_auditory\_receptor\_cell\_stereocilium\_organization | 7 | 0 | 0.000000 | -0.000000 | 993 | 937.468949 | 1025.68 | 1113.891051 | 1.032910 |
| GO:0060117\_auditory\_receptor\_cell\_development | 7 | 0 | 0.000000 | -0.000000 | 993 | 937.468949 | 1025.68 | 1113.891051 | 1.032910 |
| GO:0060441\_branching\_involved\_in\_lung\_morphogenesis | 7 | 0 | 0.000000 | -0.000000 | 993 | 937.468949 | 1025.68 | 1113.891051 | 1.032910 |
| GO:0060526\_prostate\_glandular\_acinus\_morphogenesis | 7 | 0 | 0.000000 | -0.000000 | 993 | 937.468949 | 1025.68 | 1113.891051 | 1.032910 |
| GO:0060527\_prostate\_epithelial\_cord\_arborization\_involved\_in\_prostate\_glandular\_acinus\_morphogenesis | 7 | 0 | 0.000000 | -0.000000 | 993 | 937.468949 | 1025.68 | 1113.891051 | 1.032910 |
| GO:0060579\_ventral\_spinal\_cord\_interneuron\_fate\_commitment | 7 | 0 | 0.000000 | -0.000000 | 993 | 937.468949 | 1025.68 | 1113.891051 | 1.032910 |
| GO:0060664\_epithelial\_cell\_proliferation\_involved\_in\_salivary\_gland\_morphogenesis | 7 | 0 | 0.000000 | -0.000000 | 993 | 937.468949 | 1025.68 | 1113.891051 | 1.032910 |
| GO:0060687\_regulation\_of\_branching\_involved\_in\_prostate\_gland\_morphogenesis | 7 | 0 | 0.000000 | -0.000000 | 993 | 937.468949 | 1025.68 | 1113.891051 | 1.032910 |
| GO:0060788\_ectodermal\_placode\_formation | 7 | 0 | 0.000000 | -0.000000 | 993 | 937.468949 | 1025.68 | 1113.891051 | 1.032910 |
| GO:0060795\_cell\_fate\_commitment\_involved\_in\_the\_formation\_of\_primary\_germ\_layers | 7 | 0 | 0.000000 | -0.000000 | 993 | 937.468949 | 1025.68 | 1113.891051 | 1.032910 |
| GO:0070228\_regulation\_of\_lymphocyte\_apoptosis | 7 | 0 | 0.000000 | -0.000000 | 993 | 937.468949 | 1025.68 | 1113.891051 | 1.032910 |
| GO:0070646\_protein\_modification\_by\_small\_protein\_removal | 7 | 0 | 0.000000 | -0.000000 | 993 | 937.468949 | 1025.68 | 1113.891051 | 1.032910 |
| GO:0032940\_secretion\_by\_cell | 149 | 0 | 0.000000 | -0.000000 | 994 | 938.046267 | 1026.09 | 1114.133733 | 1.032284 |
| GO:0006753\_nucleoside\_phosphate\_metabolic\_process | 94 | 0 | 0.000000 | -0.000000 | 999 | 943.098790 | 1030.72 | 1118.341210 | 1.031752 |
| GO:0008610\_lipid\_biosynthetic\_process | 94 | 0 | 0.000000 | -0.000000 | 999 | 943.098790 | 1030.72 | 1118.341210 | 1.031752 |
| GO:0009117\_nucleotide\_metabolic\_process | 94 | 0 | 0.000000 | -0.000000 | 999 | 943.098790 | 1030.72 | 1118.341210 | 1.031752 |
| GO:0032943\_mononuclear\_cell\_proliferation | 94 | 0 | 0.000000 | -0.000000 | 999 | 943.098790 | 1030.72 | 1118.341210 | 1.031752 |
| GO:0046651\_lymphocyte\_proliferation | 94 | 0 | 0.000000 | -0.000000 | 999 | 943.098790 | 1030.72 | 1118.341210 | 1.031752 |
| GO:0000278\_mitotic\_cell\_cycle | 80 | 0 | 0.000000 | -0.000000 | 1003 | 946.921079 | 1034.07 | 1121.218921 | 1.030977 |
| GO:0002250\_adaptive\_immune\_response | 80 | 0 | 0.000000 | -0.000000 | 1003 | 946.921079 | 1034.07 | 1121.218921 | 1.030977 |
| GO:0002460\_adaptive\_immune\_response\_based\_on\_somatic\_recombination\_of\_immune\_receptors\_built\_from\_immunoglobulin\_superfamily\_domains | 80 | 0 | 0.000000 | -0.000000 | 1003 | 946.921079 | 1034.07 | 1121.218921 | 1.030977 |
| GO:0044092\_negative\_regulation\_of\_molecular\_function | 80 | 0 | 0.000000 | -0.000000 | 1003 | 946.921079 | 1034.07 | 1121.218921 | 1.030977 |
| GO:0001824\_blastocyst\_development | 40 | 0 | 0.000000 | -0.000000 | 1016 | 958.076198 | 1044.61 | 1131.143802 | 1.028159 |
| GO:0007346\_regulation\_of\_mitotic\_cell\_cycle | 40 | 0 | 0.000000 | -0.000000 | 1016 | 958.076198 | 1044.61 | 1131.143802 | 1.028159 |
| GO:0007599\_hemostasis | 40 | 0 | 0.000000 | -0.000000 | 1016 | 958.076198 | 1044.61 | 1131.143802 | 1.028159 |
| GO:0008203\_cholesterol\_metabolic\_process | 40 | 0 | 0.000000 | -0.000000 | 1016 | 958.076198 | 1044.61 | 1131.143802 | 1.028159 |
| GO:0014031\_mesenchymal\_cell\_development | 40 | 0 | 0.000000 | -0.000000 | 1016 | 958.076198 | 1044.61 | 1131.143802 | 1.028159 |
| GO:0016071\_mRNA\_metabolic\_process | 40 | 0 | 0.000000 | -0.000000 | 1016 | 958.076198 | 1044.61 | 1131.143802 | 1.028159 |
| GO:0016358\_dendrite\_development | 40 | 0 | 0.000000 | -0.000000 | 1016 | 958.076198 | 1044.61 | 1131.143802 | 1.028159 |
| GO:0016485\_protein\_processing | 40 | 0 | 0.000000 | -0.000000 | 1016 | 958.076198 | 1044.61 | 1131.143802 | 1.028159 |
| GO:0017015\_regulation\_of\_transforming\_growth\_factor\_beta\_receptor\_signaling\_pathway | 40 | 0 | 0.000000 | -0.000000 | 1016 | 958.076198 | 1044.61 | 1131.143802 | 1.028159 |
| GO:0019935\_cyclic-nucleotide-mediated\_signaling | 40 | 0 | 0.000000 | -0.000000 | 1016 | 958.076198 | 1044.61 | 1131.143802 | 1.028159 |
| GO:0035272\_exocrine\_system\_development | 40 | 0 | 0.000000 | -0.000000 | 1016 | 958.076198 | 1044.61 | 1131.143802 | 1.028159 |
| GO:0046850\_regulation\_of\_bone\_remodeling | 40 | 0 | 0.000000 | -0.000000 | 1016 | 958.076198 | 1044.61 | 1131.143802 | 1.028159 |
| GO:0051129\_negative\_regulation\_of\_cellular\_component\_organization | 40 | 0 | 0.000000 | -0.000000 | 1016 | 958.076198 | 1044.61 | 1131.143802 | 1.028159 |
| GO:0007268\_synaptic\_transmission | 154 | 0 | 0.000000 | -0.000000 | 1017 | 958.699157 | 1045.08 | 1131.460843 | 1.027611 |
| GO:0051241\_negative\_regulation\_of\_multicellular\_organismal\_process | 77 | 0 | 0.000000 | -0.000000 | 1018 | 963.089134 | 1048.83 | 1134.570866 | 1.030285 |
| GO:0001759\_induction\_of\_an\_organ | 15 | 0 | 0.000000 | -0.000000 | 1072 | 1016.622650 | 1101.08 | 1185.537350 | 1.027127 |
| GO:0001782\_B\_cell\_homeostasis | 15 | 0 | 0.000000 | -0.000000 | 1072 | 1016.622650 | 1101.08 | 1185.537350 | 1.027127 |
| GO:0001964\_startle\_response | 15 | 0 | 0.000000 | -0.000000 | 1072 | 1016.622650 | 1101.08 | 1185.537350 | 1.027127 |
| GO:0002286\_T\_cell\_activation\_during\_immune\_response | 15 | 0 | 0.000000 | -0.000000 | 1072 | 1016.622650 | 1101.08 | 1185.537350 | 1.027127 |
| GO:0002495\_antigen\_processing\_and\_presentation\_of\_peptide\_antigen\_via\_MHC\_class\_II | 15 | 0 | 0.000000 | -0.000000 | 1072 | 1016.622650 | 1101.08 | 1185.537350 | 1.027127 |
| GO:0002504\_antigen\_processing\_and\_presentation\_of\_peptide\_or\_polysaccharide\_antigen\_via\_MHC\_class\_II | 15 | 0 | 0.000000 | -0.000000 | 1072 | 1016.622650 | 1101.08 | 1185.537350 | 1.027127 |
| GO:0002709\_regulation\_of\_T\_cell\_mediated\_immunity | 15 | 0 | 0.000000 | -0.000000 | 1072 | 1016.622650 | 1101.08 | 1185.537350 | 1.027127 |
| GO:0006487\_protein\_amino\_acid\_N-linked\_glycosylation | 15 | 0 | 0.000000 | -0.000000 | 1072 | 1016.622650 | 1101.08 | 1185.537350 | 1.027127 |
| GO:0006749\_glutathione\_metabolic\_process | 15 | 0 | 0.000000 | -0.000000 | 1072 | 1016.622650 | 1101.08 | 1185.537350 | 1.027127 |
| GO:0006885\_regulation\_of\_pH | 15 | 0 | 0.000000 | -0.000000 | 1072 | 1016.622650 | 1101.08 | 1185.537350 | 1.027127 |
| GO:0007173\_epidermal\_growth\_factor\_receptor\_signaling\_pathway | 15 | 0 | 0.000000 | -0.000000 | 1072 | 1016.622650 | 1101.08 | 1185.537350 | 1.027127 |
| GO:0007200\_activation\_of\_phospholipase\_C\_activity\_by\_G-protein\_coupled\_receptor\_protein\_signaling\_pathway\_coupled\_to\_IP3\_second\_messenger | 15 | 0 | 0.000000 | -0.000000 | 1072 | 1016.622650 | 1101.08 | 1185.537350 | 1.027127 |
| GO:0007202\_activation\_of\_phospholipase\_C\_activity | 15 | 0 | 0.000000 | -0.000000 | 1072 | 1016.622650 | 1101.08 | 1185.537350 | 1.027127 |
| GO:0007218\_neuropeptide\_signaling\_pathway | 15 | 0 | 0.000000 | -0.000000 | 1072 | 1016.622650 | 1101.08 | 1185.537350 | 1.027127 |
| GO:0007588\_excretion | 15 | 0 | 0.000000 | -0.000000 | 1072 | 1016.622650 | 1101.08 | 1185.537350 | 1.027127 |
| GO:0007618\_mating | 15 | 0 | 0.000000 | -0.000000 | 1072 | 1016.622650 | 1101.08 | 1185.537350 | 1.027127 |
| GO:0008543\_fibroblast\_growth\_factor\_receptor\_signaling\_pathway | 15 | 0 | 0.000000 | -0.000000 | 1072 | 1016.622650 | 1101.08 | 1185.537350 | 1.027127 |
| GO:0009062\_fatty\_acid\_catabolic\_process | 15 | 0 | 0.000000 | -0.000000 | 1072 | 1016.622650 | 1101.08 | 1185.537350 | 1.027127 |
| GO:0009116\_nucleoside\_metabolic\_process | 15 | 0 | 0.000000 | -0.000000 | 1072 | 1016.622650 | 1101.08 | 1185.537350 | 1.027127 |
| GO:0010092\_specification\_of\_organ\_identity | 15 | 0 | 0.000000 | -0.000000 | 1072 | 1016.622650 | 1101.08 | 1185.537350 | 1.027127 |
| GO:0010171\_body\_morphogenesis | 15 | 0 | 0.000000 | -0.000000 | 1072 | 1016.622650 | 1101.08 | 1185.537350 | 1.027127 |
| GO:0010518\_positive\_regulation\_of\_phospholipase\_activity | 15 | 0 | 0.000000 | -0.000000 | 1072 | 1016.622650 | 1101.08 | 1185.537350 | 1.027127 |
| GO:0010863\_positive\_regulation\_of\_phospholipase\_C\_activity | 15 | 0 | 0.000000 | -0.000000 | 1072 | 1016.622650 | 1101.08 | 1185.537350 | 1.027127 |
| GO:0015931\_nucleobase\_\_nucleoside\_\_nucleotide\_and\_nucleic\_acid\_transport | 15 | 0 | 0.000000 | -0.000000 | 1072 | 1016.622650 | 1101.08 | 1185.537350 | 1.027127 |
| GO:0019886\_antigen\_processing\_and\_presentation\_of\_exogenous\_peptide\_antigen\_via\_MHC\_class\_II | 15 | 0 | 0.000000 | -0.000000 | 1072 | 1016.622650 | 1101.08 | 1185.537350 | 1.027127 |
| GO:0021795\_cerebral\_cortex\_cell\_migration | 15 | 0 | 0.000000 | -0.000000 | 1072 | 1016.622650 | 1101.08 | 1185.537350 | 1.027127 |
| GO:0021872\_generation\_of\_neurons\_in\_the\_forebrain | 15 | 0 | 0.000000 | -0.000000 | 1072 | 1016.622650 | 1101.08 | 1185.537350 | 1.027127 |
| GO:0022600\_digestive\_system\_process | 15 | 0 | 0.000000 | -0.000000 | 1072 | 1016.622650 | 1101.08 | 1185.537350 | 1.027127 |
| GO:0030041\_actin\_filament\_polymerization | 15 | 0 | 0.000000 | -0.000000 | 1072 | 1016.622650 | 1101.08 | 1185.537350 | 1.027127 |
| GO:0031069\_hair\_follicle\_morphogenesis | 15 | 0 | 0.000000 | -0.000000 | 1072 | 1016.622650 | 1101.08 | 1185.537350 | 1.027127 |
| GO:0031076\_embryonic\_camera-type\_eye\_development | 15 | 0 | 0.000000 | -0.000000 | 1072 | 1016.622650 | 1101.08 | 1185.537350 | 1.027127 |
| GO:0031329\_regulation\_of\_cellular\_catabolic\_process | 15 | 0 | 0.000000 | -0.000000 | 1072 | 1016.622650 | 1101.08 | 1185.537350 | 1.027127 |
| GO:0035116\_embryonic\_hindlimb\_morphogenesis | 15 | 0 | 0.000000 | -0.000000 | 1072 | 1016.622650 | 1101.08 | 1185.537350 | 1.027127 |
| GO:0035249\_synaptic\_transmission\_\_glutamatergic | 15 | 0 | 0.000000 | -0.000000 | 1072 | 1016.622650 | 1101.08 | 1185.537350 | 1.027127 |
| GO:0042306\_regulation\_of\_protein\_import\_into\_nucleus | 15 | 0 | 0.000000 | -0.000000 | 1072 | 1016.622650 | 1101.08 | 1185.537350 | 1.027127 |
| GO:0045666\_positive\_regulation\_of\_neuron\_differentiation | 15 | 0 | 0.000000 | -0.000000 | 1072 | 1016.622650 | 1101.08 | 1185.537350 | 1.027127 |
| GO:0046164\_alcohol\_catabolic\_process | 15 | 0 | 0.000000 | -0.000000 | 1072 | 1016.622650 | 1101.08 | 1185.537350 | 1.027127 |
| GO:0046638\_positive\_regulation\_of\_alpha-beta\_T\_cell\_differentiation | 15 | 0 | 0.000000 | -0.000000 | 1072 | 1016.622650 | 1101.08 | 1185.537350 | 1.027127 |
| GO:0048008\_platelet-derived\_growth\_factor\_receptor\_signaling\_pathway | 15 | 0 | 0.000000 | -0.000000 | 1072 | 1016.622650 | 1101.08 | 1185.537350 | 1.027127 |
| GO:0048010\_vascular\_endothelial\_growth\_factor\_receptor\_signaling\_pathway | 15 | 0 | 0.000000 | -0.000000 | 1072 | 1016.622650 | 1101.08 | 1185.537350 | 1.027127 |
| GO:0048144\_fibroblast\_proliferation | 15 | 0 | 0.000000 | -0.000000 | 1072 | 1016.622650 | 1101.08 | 1185.537350 | 1.027127 |
| GO:0048145\_regulation\_of\_fibroblast\_proliferation | 15 | 0 | 0.000000 | -0.000000 | 1072 | 1016.622650 | 1101.08 | 1185.537350 | 1.027127 |
| GO:0048610\_reproductive\_cellular\_process | 15 | 0 | 0.000000 | -0.000000 | 1072 | 1016.622650 | 1101.08 | 1185.537350 | 1.027127 |
| GO:0048709\_oligodendrocyte\_differentiation | 15 | 0 | 0.000000 | -0.000000 | 1072 | 1016.622650 | 1101.08 | 1185.537350 | 1.027127 |
| GO:0050729\_positive\_regulation\_of\_inflammatory\_response | 15 | 0 | 0.000000 | -0.000000 | 1072 | 1016.622650 | 1101.08 | 1185.537350 | 1.027127 |
| GO:0050796\_regulation\_of\_insulin\_secretion | 15 | 0 | 0.000000 | -0.000000 | 1072 | 1016.622650 | 1101.08 | 1185.537350 | 1.027127 |
| GO:0050798\_activated\_T\_cell\_proliferation | 15 | 0 | 0.000000 | -0.000000 | 1072 | 1016.622650 | 1101.08 | 1185.537350 | 1.027127 |
| GO:0055010\_ventricular\_cardiac\_muscle\_morphogenesis | 15 | 0 | 0.000000 | -0.000000 | 1072 | 1016.622650 | 1101.08 | 1185.537350 | 1.027127 |
| GO:0060322\_head\_development | 15 | 0 | 0.000000 | -0.000000 | 1072 | 1016.622650 | 1101.08 | 1185.537350 | 1.027127 |
| GO:0060425\_lung\_morphogenesis | 15 | 0 | 0.000000 | -0.000000 | 1072 | 1016.622650 | 1101.08 | 1185.537350 | 1.027127 |
| GO:0060442\_branching\_involved\_in\_prostate\_gland\_morphogenesis | 15 | 0 | 0.000000 | -0.000000 | 1072 | 1016.622650 | 1101.08 | 1185.537350 | 1.027127 |
| GO:0060749\_mammary\_gland\_alveolus\_development | 15 | 0 | 0.000000 | -0.000000 | 1072 | 1016.622650 | 1101.08 | 1185.537350 | 1.027127 |
| GO:0070227\_lymphocyte\_apoptosis | 15 | 0 | 0.000000 | -0.000000 | 1072 | 1016.622650 | 1101.08 | 1185.537350 | 1.027127 |
| GO:0070507\_regulation\_of\_microtubule\_cytoskeleton\_organization | 15 | 0 | 0.000000 | -0.000000 | 1072 | 1016.622650 | 1101.08 | 1185.537350 | 1.027127 |
| GO:0048584\_positive\_regulation\_of\_response\_to\_stimulus | 115 | 0 | 0.000000 | -0.000000 | 1074 | 1018.182205 | 1102.2 | 1186.217795 | 1.026257 |
| GO:0051338\_regulation\_of\_transferase\_activity | 115 | 0 | 0.000000 | -0.000000 | 1074 | 1018.182205 | 1102.2 | 1186.217795 | 1.026257 |
| GO:0007186\_G-protein\_coupled\_receptor\_protein\_signaling\_pathway | 144 | 0 | 0.000000 | -0.000000 | 1076 | 1019.430270 | 1103.22 | 1187.009730 | 1.025297 |
| GO:0045596\_negative\_regulation\_of\_cell\_differentiation | 144 | 0 | 0.000000 | -0.000000 | 1076 | 1019.430270 | 1103.22 | 1187.009730 | 1.025297 |
| GO:0018193\_peptidyl-amino\_acid\_modification | 97 | 0 | 0.000000 | -0.000000 | 1078 | 1021.004872 | 1104.44 | 1187.875128 | 1.024527 |
| GO:0060341\_regulation\_of\_cellular\_localization | 97 | 0 | 0.000000 | -0.000000 | 1078 | 1021.004872 | 1104.44 | 1187.875128 | 1.024527 |
| GO:0007264\_small\_GTPase\_mediated\_signal\_transduction | 72 | 0 | 0.000000 | -0.000000 | 1086 | 1029.994931 | 1112.39 | 1194.785069 | 1.024300 |
| GO:0021915\_neural\_tube\_development | 72 | 0 | 0.000000 | -0.000000 | 1086 | 1029.994931 | 1112.39 | 1194.785069 | 1.024300 |
| GO:0030879\_mammary\_gland\_development | 72 | 0 | 0.000000 | -0.000000 | 1086 | 1029.994931 | 1112.39 | 1194.785069 | 1.024300 |
| GO:0040012\_regulation\_of\_locomotion | 72 | 0 | 0.000000 | -0.000000 | 1086 | 1029.994931 | 1112.39 | 1194.785069 | 1.024300 |
| GO:0042098\_T\_cell\_proliferation | 72 | 0 | 0.000000 | -0.000000 | 1086 | 1029.994931 | 1112.39 | 1194.785069 | 1.024300 |
| GO:0044262\_cellular\_carbohydrate\_metabolic\_process | 72 | 0 | 0.000000 | -0.000000 | 1086 | 1029.994931 | 1112.39 | 1194.785069 | 1.024300 |
| GO:0048839\_inner\_ear\_development | 72 | 0 | 0.000000 | -0.000000 | 1086 | 1029.994931 | 1112.39 | 1194.785069 | 1.024300 |
| GO:0051347\_positive\_regulation\_of\_transferase\_activity | 72 | 0 | 0.000000 | -0.000000 | 1086 | 1029.994931 | 1112.39 | 1194.785069 | 1.024300 |
| GO:0007611\_learning\_or\_memory | 70 | 0 | 0.000000 | -0.000000 | 1090 | 1034.042386 | 1116.0 | 1197.957614 | 1.023853 |
| GO:0008406\_gonad\_development | 70 | 0 | 0.000000 | -0.000000 | 1090 | 1034.042386 | 1116.0 | 1197.957614 | 1.023853 |
| GO:0048592\_eye\_morphogenesis | 70 | 0 | 0.000000 | -0.000000 | 1090 | 1034.042386 | 1116.0 | 1197.957614 | 1.023853 |
| GO:0070838\_divalent\_metal\_ion\_transport | 70 | 0 | 0.000000 | -0.000000 | 1090 | 1034.042386 | 1116.0 | 1197.957614 | 1.023853 |
| GO:0040008\_regulation\_of\_growth | 113 | 0 | 0.000000 | -0.000000 | 1091 | 1035.304162 | 1117.16 | 1199.015838 | 1.023978 |
| GO:0006952\_defense\_response | 187 | 0 | 0.000000 | -0.000000 | 1092 | 1035.817256 | 1117.5 | 1199.182744 | 1.023352 |
| GO:0001654\_eye\_development | 136 | 0 | 0.000000 | -0.000000 | 1093 | 1036.367580 | 1118.01 | 1199.652420 | 1.022882 |
| GO:0045165\_cell\_fate\_commitment | 130 | 0 | 0.000000 | -0.000000 | 1095 | 1038.303705 | 1119.65 | 1200.996295 | 1.022511 |
| GO:0050776\_regulation\_of\_immune\_response | 130 | 0 | 0.000000 | -0.000000 | 1095 | 1038.303705 | 1119.65 | 1200.996295 | 1.022511 |
| GO:0035239\_tube\_morphogenesis | 143 | 0 | 0.000000 | -0.000000 | 1096 | 1039.434526 | 1120.49 | 1201.545474 | 1.022345 |
| GO:0002440\_production\_of\_molecular\_mediator\_of\_immune\_response | 49 | 0 | 0.000000 | -0.000000 | 1105 | 1049.821019 | 1130.01 | 1210.198981 | 1.022633 |
| GO:0003015\_heart\_process | 49 | 0 | 0.000000 | -0.000000 | 1105 | 1049.821019 | 1130.01 | 1210.198981 | 1.022633 |
| GO:0007606\_sensory\_perception\_of\_chemical\_stimulus | 49 | 0 | 0.000000 | -0.000000 | 1105 | 1049.821019 | 1130.01 | 1210.198981 | 1.022633 |
| GO:0021543\_pallium\_development | 49 | 0 | 0.000000 | -0.000000 | 1105 | 1049.821019 | 1130.01 | 1210.198981 | 1.022633 |
| GO:0042035\_regulation\_of\_cytokine\_biosynthetic\_process | 49 | 0 | 0.000000 | -0.000000 | 1105 | 1049.821019 | 1130.01 | 1210.198981 | 1.022633 |
| GO:0046660\_female\_sex\_differentiation | 49 | 0 | 0.000000 | -0.000000 | 1105 | 1049.821019 | 1130.01 | 1210.198981 | 1.022633 |
| GO:0046661\_male\_sex\_differentiation | 49 | 0 | 0.000000 | -0.000000 | 1105 | 1049.821019 | 1130.01 | 1210.198981 | 1.022633 |
| GO:0048741\_skeletal\_muscle\_fiber\_development | 49 | 0 | 0.000000 | -0.000000 | 1105 | 1049.821019 | 1130.01 | 1210.198981 | 1.022633 |
| GO:0060047\_heart\_contraction | 49 | 0 | 0.000000 | -0.000000 | 1105 | 1049.821019 | 1130.01 | 1210.198981 | 1.022633 |
| GO:0006576\_biogenic\_amine\_metabolic\_process | 53 | 0 | 0.000000 | -0.000000 | 1112 | 1056.482471 | 1136.11 | 1215.737529 | 1.021682 |
| GO:0006935\_chemotaxis | 53 | 0 | 0.000000 | -0.000000 | 1112 | 1056.482471 | 1136.11 | 1215.737529 | 1.021682 |
| GO:0030031\_cell\_projection\_assembly | 53 | 0 | 0.000000 | -0.000000 | 1112 | 1056.482471 | 1136.11 | 1215.737529 | 1.021682 |
| GO:0042330\_taxis | 53 | 0 | 0.000000 | -0.000000 | 1112 | 1056.482471 | 1136.11 | 1215.737529 | 1.021682 |
| GO:0046942\_carboxylic\_acid\_transport | 53 | 0 | 0.000000 | -0.000000 | 1112 | 1056.482471 | 1136.11 | 1215.737529 | 1.021682 |
| GO:0051248\_negative\_regulation\_of\_protein\_metabolic\_process | 53 | 0 | 0.000000 | -0.000000 | 1112 | 1056.482471 | 1136.11 | 1215.737529 | 1.021682 |
| GO:0055085\_transmembrane\_transport | 53 | 0 | 0.000000 | -0.000000 | 1112 | 1056.482471 | 1136.11 | 1215.737529 | 1.021682 |
| GO:0000038\_very-long-chain\_fatty\_acid\_metabolic\_process | 6 | 0 | 0.000000 | -0.000000 | 1295 | 1240.770299 | 1317.85 | 1394.929701 | 1.017645 |
| GO:0000245\_spliceosome\_assembly | 6 | 0 | 0.000000 | -0.000000 | 1295 | 1240.770299 | 1317.85 | 1394.929701 | 1.017645 |
| GO:0000768\_syncytium\_formation\_by\_plasma\_membrane\_fusion | 6 | 0 | 0.000000 | -0.000000 | 1295 | 1240.770299 | 1317.85 | 1394.929701 | 1.017645 |
| GO:0001710\_mesodermal\_cell\_fate\_commitment | 6 | 0 | 0.000000 | -0.000000 | 1295 | 1240.770299 | 1317.85 | 1394.929701 | 1.017645 |
| GO:0001779\_natural\_killer\_cell\_differentiation | 6 | 0 | 0.000000 | -0.000000 | 1295 | 1240.770299 | 1317.85 | 1394.929701 | 1.017645 |
| GO:0001885\_endothelial\_cell\_development | 6 | 0 | 0.000000 | -0.000000 | 1295 | 1240.770299 | 1317.85 | 1394.929701 | 1.017645 |
| GO:0002016\_regulation\_of\_blood\_volume\_by\_renin-angiotensin | 6 | 0 | 0.000000 | -0.000000 | 1295 | 1240.770299 | 1317.85 | 1394.929701 | 1.017645 |
| GO:0002335\_mature\_B\_cell\_differentiation | 6 | 0 | 0.000000 | -0.000000 | 1295 | 1240.770299 | 1317.85 | 1394.929701 | 1.017645 |
| GO:0002360\_T\_cell\_lineage\_commitment | 6 | 0 | 0.000000 | -0.000000 | 1295 | 1240.770299 | 1317.85 | 1394.929701 | 1.017645 |
| GO:0002367\_cytokine\_production\_during\_immune\_response | 6 | 0 | 0.000000 | -0.000000 | 1295 | 1240.770299 | 1317.85 | 1394.929701 | 1.017645 |
| GO:0002474\_antigen\_processing\_and\_presentation\_of\_peptide\_antigen\_via\_MHC\_class\_I | 6 | 0 | 0.000000 | -0.000000 | 1295 | 1240.770299 | 1317.85 | 1394.929701 | 1.017645 |
| GO:0002475\_antigen\_processing\_and\_presentation\_via\_MHC\_class\_Ib | 6 | 0 | 0.000000 | -0.000000 | 1295 | 1240.770299 | 1317.85 | 1394.929701 | 1.017645 |
| GO:0002532\_production\_of\_molecular\_mediator\_of\_acute\_inflammatory\_response | 6 | 0 | 0.000000 | -0.000000 | 1295 | 1240.770299 | 1317.85 | 1394.929701 | 1.017645 |
| GO:0002541\_activation\_of\_plasma\_proteins\_involved\_in\_acute\_inflammatory\_response | 6 | 0 | 0.000000 | -0.000000 | 1295 | 1240.770299 | 1317.85 | 1394.929701 | 1.017645 |
| GO:0002675\_positive\_regulation\_of\_acute\_inflammatory\_response | 6 | 0 | 0.000000 | -0.000000 | 1295 | 1240.770299 | 1317.85 | 1394.929701 | 1.017645 |
| GO:0002685\_regulation\_of\_leukocyte\_migration | 6 | 0 | 0.000000 | -0.000000 | 1295 | 1240.770299 | 1317.85 | 1394.929701 | 1.017645 |
| GO:0002831\_regulation\_of\_response\_to\_biotic\_stimulus | 6 | 0 | 0.000000 | -0.000000 | 1295 | 1240.770299 | 1317.85 | 1394.929701 | 1.017645 |
| GO:0002920\_regulation\_of\_humoral\_immune\_response | 6 | 0 | 0.000000 | -0.000000 | 1295 | 1240.770299 | 1317.85 | 1394.929701 | 1.017645 |
| GO:0006071\_glycerol\_metabolic\_process | 6 | 0 | 0.000000 | -0.000000 | 1295 | 1240.770299 | 1317.85 | 1394.929701 | 1.017645 |
| GO:0006084\_acetyl-CoA\_metabolic\_process | 6 | 0 | 0.000000 | -0.000000 | 1295 | 1240.770299 | 1317.85 | 1394.929701 | 1.017645 |
| GO:0006264\_mitochondrial\_DNA\_replication | 6 | 0 | 0.000000 | -0.000000 | 1295 | 1240.770299 | 1317.85 | 1394.929701 | 1.017645 |
| GO:0006402\_mRNA\_catabolic\_process | 6 | 0 | 0.000000 | -0.000000 | 1295 | 1240.770299 | 1317.85 | 1394.929701 | 1.017645 |
| GO:0006471\_protein\_amino\_acid\_ADP-ribosylation | 6 | 0 | 0.000000 | -0.000000 | 1295 | 1240.770299 | 1317.85 | 1394.929701 | 1.017645 |
| GO:0006536\_glutamate\_metabolic\_process | 6 | 0 | 0.000000 | -0.000000 | 1295 | 1240.770299 | 1317.85 | 1394.929701 | 1.017645 |
| GO:0006656\_phosphatidylcholine\_biosynthetic\_process | 6 | 0 | 0.000000 | -0.000000 | 1295 | 1240.770299 | 1317.85 | 1394.929701 | 1.017645 |
| GO:0006692\_prostanoid\_metabolic\_process | 6 | 0 | 0.000000 | -0.000000 | 1295 | 1240.770299 | 1317.85 | 1394.929701 | 1.017645 |
| GO:0006693\_prostaglandin\_metabolic\_process | 6 | 0 | 0.000000 | -0.000000 | 1295 | 1240.770299 | 1317.85 | 1394.929701 | 1.017645 |
| GO:0006706\_steroid\_catabolic\_process | 6 | 0 | 0.000000 | -0.000000 | 1295 | 1240.770299 | 1317.85 | 1394.929701 | 1.017645 |
| GO:0006752\_group\_transfer\_coenzyme\_metabolic\_process | 6 | 0 | 0.000000 | -0.000000 | 1295 | 1240.770299 | 1317.85 | 1394.929701 | 1.017645 |
| GO:0006882\_cellular\_zinc\_ion\_homeostasis | 6 | 0 | 0.000000 | -0.000000 | 1295 | 1240.770299 | 1317.85 | 1394.929701 | 1.017645 |
| GO:0006942\_regulation\_of\_striated\_muscle\_contraction | 6 | 0 | 0.000000 | -0.000000 | 1295 | 1240.770299 | 1317.85 | 1394.929701 | 1.017645 |
| GO:0006956\_complement\_activation | 6 | 0 | 0.000000 | -0.000000 | 1295 | 1240.770299 | 1317.85 | 1394.929701 | 1.017645 |
| GO:0007032\_endosome\_organization | 6 | 0 | 0.000000 | -0.000000 | 1295 | 1240.770299 | 1317.85 | 1394.929701 | 1.017645 |
| GO:0007176\_regulation\_of\_epidermal\_growth\_factor\_receptor\_activity | 6 | 0 | 0.000000 | -0.000000 | 1295 | 1240.770299 | 1317.85 | 1394.929701 | 1.017645 |
| GO:0007214\_gamma-aminobutyric\_acid\_signaling\_pathway | 6 | 0 | 0.000000 | -0.000000 | 1295 | 1240.770299 | 1317.85 | 1394.929701 | 1.017645 |
| GO:0007257\_activation\_of\_JUN\_kinase\_activity | 6 | 0 | 0.000000 | -0.000000 | 1295 | 1240.770299 | 1317.85 | 1394.929701 | 1.017645 |
| GO:0007341\_penetration\_of\_zona\_pellucida | 6 | 0 | 0.000000 | -0.000000 | 1295 | 1240.770299 | 1317.85 | 1394.929701 | 1.017645 |
| GO:0007406\_negative\_regulation\_of\_neuroblast\_proliferation | 6 | 0 | 0.000000 | -0.000000 | 1295 | 1240.770299 | 1317.85 | 1394.929701 | 1.017645 |
| GO:0007442\_hindgut\_morphogenesis | 6 | 0 | 0.000000 | -0.000000 | 1295 | 1240.770299 | 1317.85 | 1394.929701 | 1.017645 |
| GO:0007520\_myoblast\_fusion | 6 | 0 | 0.000000 | -0.000000 | 1295 | 1240.770299 | 1317.85 | 1394.929701 | 1.017645 |
| GO:0007620\_copulation | 6 | 0 | 0.000000 | -0.000000 | 1295 | 1240.770299 | 1317.85 | 1394.929701 | 1.017645 |
| GO:0008156\_negative\_regulation\_of\_DNA\_replication | 6 | 0 | 0.000000 | -0.000000 | 1295 | 1240.770299 | 1317.85 | 1394.929701 | 1.017645 |
| GO:0008209\_androgen\_metabolic\_process | 6 | 0 | 0.000000 | -0.000000 | 1295 | 1240.770299 | 1317.85 | 1394.929701 | 1.017645 |
| GO:0008625\_induction\_of\_apoptosis\_via\_death\_domain\_receptors | 6 | 0 | 0.000000 | -0.000000 | 1295 | 1240.770299 | 1317.85 | 1394.929701 | 1.017645 |
| GO:0009067\_aspartate\_family\_amino\_acid\_biosynthetic\_process | 6 | 0 | 0.000000 | -0.000000 | 1295 | 1240.770299 | 1317.85 | 1394.929701 | 1.017645 |
| GO:0009069\_serine\_family\_amino\_acid\_metabolic\_process | 6 | 0 | 0.000000 | -0.000000 | 1295 | 1240.770299 | 1317.85 | 1394.929701 | 1.017645 |
| GO:0009112\_nucleobase\_metabolic\_process | 6 | 0 | 0.000000 | -0.000000 | 1295 | 1240.770299 | 1317.85 | 1394.929701 | 1.017645 |
| GO:0009143\_nucleoside\_triphosphate\_catabolic\_process | 6 | 0 | 0.000000 | -0.000000 | 1295 | 1240.770299 | 1317.85 | 1394.929701 | 1.017645 |
| GO:0009247\_glycolipid\_biosynthetic\_process | 6 | 0 | 0.000000 | -0.000000 | 1295 | 1240.770299 | 1317.85 | 1394.929701 | 1.017645 |
| GO:0009650\_UV\_protection | 6 | 0 | 0.000000 | -0.000000 | 1295 | 1240.770299 | 1317.85 | 1394.929701 | 1.017645 |
| GO:0009651\_response\_to\_salt\_stress | 6 | 0 | 0.000000 | -0.000000 | 1295 | 1240.770299 | 1317.85 | 1394.929701 | 1.017645 |
| GO:0010466\_negative\_regulation\_of\_peptidase\_activity | 6 | 0 | 0.000000 | -0.000000 | 1295 | 1240.770299 | 1317.85 | 1394.929701 | 1.017645 |
| GO:0010883\_regulation\_of\_lipid\_storage | 6 | 0 | 0.000000 | -0.000000 | 1295 | 1240.770299 | 1317.85 | 1394.929701 | 1.017645 |
| GO:0010906\_regulation\_of\_glucose\_metabolic\_process | 6 | 0 | 0.000000 | -0.000000 | 1295 | 1240.770299 | 1317.85 | 1394.929701 | 1.017645 |
| GO:0014003\_oligodendrocyte\_development | 6 | 0 | 0.000000 | -0.000000 | 1295 | 1240.770299 | 1317.85 | 1394.929701 | 1.017645 |
| GO:0014051\_gamma-aminobutyric\_acid\_secretion | 6 | 0 | 0.000000 | -0.000000 | 1295 | 1240.770299 | 1317.85 | 1394.929701 | 1.017645 |
| GO:0014072\_response\_to\_isoquinoline\_alkaloid | 6 | 0 | 0.000000 | -0.000000 | 1295 | 1240.770299 | 1317.85 | 1394.929701 | 1.017645 |
| GO:0014812\_muscle\_cell\_migration | 6 | 0 | 0.000000 | -0.000000 | 1295 | 1240.770299 | 1317.85 | 1394.929701 | 1.017645 |
| GO:0014823\_response\_to\_activity | 6 | 0 | 0.000000 | -0.000000 | 1295 | 1240.770299 | 1317.85 | 1394.929701 | 1.017645 |
| GO:0015012\_heparan\_sulfate\_proteoglycan\_biosynthetic\_process | 6 | 0 | 0.000000 | -0.000000 | 1295 | 1240.770299 | 1317.85 | 1394.929701 | 1.017645 |
| GO:0015812\_gamma-aminobutyric\_acid\_transport | 6 | 0 | 0.000000 | -0.000000 | 1295 | 1240.770299 | 1317.85 | 1394.929701 | 1.017645 |
| GO:0016032\_viral\_reproduction | 6 | 0 | 0.000000 | -0.000000 | 1295 | 1240.770299 | 1317.85 | 1394.929701 | 1.017645 |
| GO:0016574\_histone\_ubiquitination | 6 | 0 | 0.000000 | -0.000000 | 1295 | 1240.770299 | 1317.85 | 1394.929701 | 1.017645 |
| GO:0016925\_protein\_sumoylation | 6 | 0 | 0.000000 | -0.000000 | 1295 | 1240.770299 | 1317.85 | 1394.929701 | 1.017645 |
| GO:0019433\_triglyceride\_catabolic\_process | 6 | 0 | 0.000000 | -0.000000 | 1295 | 1240.770299 | 1317.85 | 1394.929701 | 1.017645 |
| GO:0019835\_cytolysis | 6 | 0 | 0.000000 | -0.000000 | 1295 | 1240.770299 | 1317.85 | 1394.929701 | 1.017645 |
| GO:0021548\_pons\_development | 6 | 0 | 0.000000 | -0.000000 | 1295 | 1240.770299 | 1317.85 | 1394.929701 | 1.017645 |
| GO:0021783\_preganglionic\_parasympathetic\_nervous\_system\_development | 6 | 0 | 0.000000 | -0.000000 | 1295 | 1240.770299 | 1317.85 | 1394.929701 | 1.017645 |
| GO:0021892\_cerebral\_cortex\_GABAergic\_interneuron\_differentiation | 6 | 0 | 0.000000 | -0.000000 | 1295 | 1240.770299 | 1317.85 | 1394.929701 | 1.017645 |
| GO:0021937\_Purkinje\_cell-granule\_cell\_precursor\_cell\_signaling\_involved\_in\_regulation\_of\_granule\_cell\_precursor\_cell\_proliferation | 6 | 0 | 0.000000 | -0.000000 | 1295 | 1240.770299 | 1317.85 | 1394.929701 | 1.017645 |
| GO:0022409\_positive\_regulation\_of\_cell-cell\_adhesion | 6 | 0 | 0.000000 | -0.000000 | 1295 | 1240.770299 | 1317.85 | 1394.929701 | 1.017645 |
| GO:0030002\_cellular\_anion\_homeostasis | 6 | 0 | 0.000000 | -0.000000 | 1295 | 1240.770299 | 1317.85 | 1394.929701 | 1.017645 |
| GO:0030252\_growth\_hormone\_secretion | 6 | 0 | 0.000000 | -0.000000 | 1295 | 1240.770299 | 1317.85 | 1394.929701 | 1.017645 |
| GO:0030865\_cortical\_cytoskeleton\_organization | 6 | 0 | 0.000000 | -0.000000 | 1295 | 1240.770299 | 1317.85 | 1394.929701 | 1.017645 |
| GO:0030947\_regulation\_of\_vascular\_endothelial\_growth\_factor\_receptor\_signaling\_pathway | 6 | 0 | 0.000000 | -0.000000 | 1295 | 1240.770299 | 1317.85 | 1394.929701 | 1.017645 |
| GO:0031077\_post-embryonic\_camera-type\_eye\_development | 6 | 0 | 0.000000 | -0.000000 | 1295 | 1240.770299 | 1317.85 | 1394.929701 | 1.017645 |
| GO:0031330\_negative\_regulation\_of\_cellular\_catabolic\_process | 6 | 0 | 0.000000 | -0.000000 | 1295 | 1240.770299 | 1317.85 | 1394.929701 | 1.017645 |
| GO:0031575\_G1\_S\_transition\_checkpoint | 6 | 0 | 0.000000 | -0.000000 | 1295 | 1240.770299 | 1317.85 | 1394.929701 | 1.017645 |
| GO:0031960\_response\_to\_corticosteroid\_stimulus | 6 | 0 | 0.000000 | -0.000000 | 1295 | 1240.770299 | 1317.85 | 1394.929701 | 1.017645 |
| GO:0032042\_mitochondrial\_DNA\_metabolic\_process | 6 | 0 | 0.000000 | -0.000000 | 1295 | 1240.770299 | 1317.85 | 1394.929701 | 1.017645 |
| GO:0032331\_negative\_regulation\_of\_chondrocyte\_differentiation | 6 | 0 | 0.000000 | -0.000000 | 1295 | 1240.770299 | 1317.85 | 1394.929701 | 1.017645 |
| GO:0032392\_DNA\_geometric\_change | 6 | 0 | 0.000000 | -0.000000 | 1295 | 1240.770299 | 1317.85 | 1394.929701 | 1.017645 |
| GO:0032438\_melanosome\_organization | 6 | 0 | 0.000000 | -0.000000 | 1295 | 1240.770299 | 1317.85 | 1394.929701 | 1.017645 |
| GO:0032469\_endoplasmic\_reticulum\_calcium\_ion\_homeostasis | 6 | 0 | 0.000000 | -0.000000 | 1295 | 1240.770299 | 1317.85 | 1394.929701 | 1.017645 |
| GO:0032653\_regulation\_of\_interleukin-10\_production | 6 | 0 | 0.000000 | -0.000000 | 1295 | 1240.770299 | 1317.85 | 1394.929701 | 1.017645 |
| GO:0033238\_regulation\_of\_cellular\_amine\_metabolic\_process | 6 | 0 | 0.000000 | -0.000000 | 1295 | 1240.770299 | 1317.85 | 1394.929701 | 1.017645 |
| GO:0034968\_histone\_lysine\_methylation | 6 | 0 | 0.000000 | -0.000000 | 1295 | 1240.770299 | 1317.85 | 1394.929701 | 1.017645 |
| GO:0035019\_somatic\_stem\_cell\_maintenance | 6 | 0 | 0.000000 | -0.000000 | 1295 | 1240.770299 | 1317.85 | 1394.929701 | 1.017645 |
| GO:0035094\_response\_to\_nicotine | 6 | 0 | 0.000000 | -0.000000 | 1295 | 1240.770299 | 1317.85 | 1394.929701 | 1.017645 |
| GO:0035121\_tail\_morphogenesis | 6 | 0 | 0.000000 | -0.000000 | 1295 | 1240.770299 | 1317.85 | 1394.929701 | 1.017645 |
| GO:0040016\_embryonic\_cleavage | 6 | 0 | 0.000000 | -0.000000 | 1295 | 1240.770299 | 1317.85 | 1394.929701 | 1.017645 |
| GO:0040023\_establishment\_of\_nucleus\_localization | 6 | 0 | 0.000000 | -0.000000 | 1295 | 1240.770299 | 1317.85 | 1394.929701 | 1.017645 |
| GO:0040036\_regulation\_of\_fibroblast\_growth\_factor\_receptor\_signaling\_pathway | 6 | 0 | 0.000000 | -0.000000 | 1295 | 1240.770299 | 1317.85 | 1394.929701 | 1.017645 |
| GO:0042053\_regulation\_of\_dopamine\_metabolic\_process | 6 | 0 | 0.000000 | -0.000000 | 1295 | 1240.770299 | 1317.85 | 1394.929701 | 1.017645 |
| GO:0042069\_regulation\_of\_catecholamine\_metabolic\_process | 6 | 0 | 0.000000 | -0.000000 | 1295 | 1240.770299 | 1317.85 | 1394.929701 | 1.017645 |
| GO:0042246\_tissue\_regeneration | 6 | 0 | 0.000000 | -0.000000 | 1295 | 1240.770299 | 1317.85 | 1394.929701 | 1.017645 |
| GO:0042307\_positive\_regulation\_of\_protein\_import\_into\_nucleus | 6 | 0 | 0.000000 | -0.000000 | 1295 | 1240.770299 | 1317.85 | 1394.929701 | 1.017645 |
| GO:0042308\_negative\_regulation\_of\_protein\_import\_into\_nucleus | 6 | 0 | 0.000000 | -0.000000 | 1295 | 1240.770299 | 1317.85 | 1394.929701 | 1.017645 |
| GO:0042403\_thyroid\_hormone\_metabolic\_process | 6 | 0 | 0.000000 | -0.000000 | 1295 | 1240.770299 | 1317.85 | 1394.929701 | 1.017645 |
| GO:0042481\_regulation\_of\_odontogenesis | 6 | 0 | 0.000000 | -0.000000 | 1295 | 1240.770299 | 1317.85 | 1394.929701 | 1.017645 |
| GO:0042492\_gamma-delta\_T\_cell\_differentiation | 6 | 0 | 0.000000 | -0.000000 | 1295 | 1240.770299 | 1317.85 | 1394.929701 | 1.017645 |
| GO:0042953\_lipoprotein\_transport | 6 | 0 | 0.000000 | -0.000000 | 1295 | 1240.770299 | 1317.85 | 1394.929701 | 1.017645 |
| GO:0043064\_flagellum\_organization | 6 | 0 | 0.000000 | -0.000000 | 1295 | 1240.770299 | 1317.85 | 1394.929701 | 1.017645 |
| GO:0043154\_negative\_regulation\_of\_caspase\_activity | 6 | 0 | 0.000000 | -0.000000 | 1295 | 1240.770299 | 1317.85 | 1394.929701 | 1.017645 |
| GO:0043255\_regulation\_of\_carbohydrate\_biosynthetic\_process | 6 | 0 | 0.000000 | -0.000000 | 1295 | 1240.770299 | 1317.85 | 1394.929701 | 1.017645 |
| GO:0043271\_negative\_regulation\_of\_ion\_transport | 6 | 0 | 0.000000 | -0.000000 | 1295 | 1240.770299 | 1317.85 | 1394.929701 | 1.017645 |
| GO:0043278\_response\_to\_morphine | 6 | 0 | 0.000000 | -0.000000 | 1295 | 1240.770299 | 1317.85 | 1394.929701 | 1.017645 |
| GO:0043300\_regulation\_of\_leukocyte\_degranulation | 6 | 0 | 0.000000 | -0.000000 | 1295 | 1240.770299 | 1317.85 | 1394.929701 | 1.017645 |
| GO:0043467\_regulation\_of\_generation\_of\_precursor\_metabolites\_and\_energy | 6 | 0 | 0.000000 | -0.000000 | 1295 | 1240.770299 | 1317.85 | 1394.929701 | 1.017645 |
| GO:0043547\_positive\_regulation\_of\_GTPase\_activity | 6 | 0 | 0.000000 | -0.000000 | 1295 | 1240.770299 | 1317.85 | 1394.929701 | 1.017645 |
| GO:0043627\_response\_to\_estrogen\_stimulus | 6 | 0 | 0.000000 | -0.000000 | 1295 | 1240.770299 | 1317.85 | 1394.929701 | 1.017645 |
| GO:0044269\_glycerol\_ether\_catabolic\_process | 6 | 0 | 0.000000 | -0.000000 | 1295 | 1240.770299 | 1317.85 | 1394.929701 | 1.017645 |
| GO:0045072\_regulation\_of\_interferon-gamma\_biosynthetic\_process | 6 | 0 | 0.000000 | -0.000000 | 1295 | 1240.770299 | 1317.85 | 1394.929701 | 1.017645 |
| GO:0045084\_positive\_regulation\_of\_interleukin-12\_biosynthetic\_process | 6 | 0 | 0.000000 | -0.000000 | 1295 | 1240.770299 | 1317.85 | 1394.929701 | 1.017645 |
| GO:0045124\_regulation\_of\_bone\_resorption | 6 | 0 | 0.000000 | -0.000000 | 1295 | 1240.770299 | 1317.85 | 1394.929701 | 1.017645 |
| GO:0045176\_apical\_protein\_localization | 6 | 0 | 0.000000 | -0.000000 | 1295 | 1240.770299 | 1317.85 | 1394.929701 | 1.017645 |
| GO:0045540\_regulation\_of\_cholesterol\_biosynthetic\_process | 6 | 0 | 0.000000 | -0.000000 | 1295 | 1240.770299 | 1317.85 | 1394.929701 | 1.017645 |
| GO:0045579\_positive\_regulation\_of\_B\_cell\_differentiation | 6 | 0 | 0.000000 | -0.000000 | 1295 | 1240.770299 | 1317.85 | 1394.929701 | 1.017645 |
| GO:0045727\_positive\_regulation\_of\_translation | 6 | 0 | 0.000000 | -0.000000 | 1295 | 1240.770299 | 1317.85 | 1394.929701 | 1.017645 |
| GO:0045778\_positive\_regulation\_of\_ossification | 6 | 0 | 0.000000 | -0.000000 | 1295 | 1240.770299 | 1317.85 | 1394.929701 | 1.017645 |
| GO:0045822\_negative\_regulation\_of\_heart\_contraction | 6 | 0 | 0.000000 | -0.000000 | 1295 | 1240.770299 | 1317.85 | 1394.929701 | 1.017645 |
| GO:0045824\_negative\_regulation\_of\_innate\_immune\_response | 6 | 0 | 0.000000 | -0.000000 | 1295 | 1240.770299 | 1317.85 | 1394.929701 | 1.017645 |
| GO:0045833\_negative\_regulation\_of\_lipid\_metabolic\_process | 6 | 0 | 0.000000 | -0.000000 | 1295 | 1240.770299 | 1317.85 | 1394.929701 | 1.017645 |
| GO:0045843\_negative\_regulation\_of\_striated\_muscle\_development | 6 | 0 | 0.000000 | -0.000000 | 1295 | 1240.770299 | 1317.85 | 1394.929701 | 1.017645 |
| GO:0045861\_negative\_regulation\_of\_proteolysis | 6 | 0 | 0.000000 | -0.000000 | 1295 | 1240.770299 | 1317.85 | 1394.929701 | 1.017645 |
| GO:0045913\_positive\_regulation\_of\_carbohydrate\_metabolic\_process | 6 | 0 | 0.000000 | -0.000000 | 1295 | 1240.770299 | 1317.85 | 1394.929701 | 1.017645 |
| GO:0045931\_positive\_regulation\_of\_mitotic\_cell\_cycle | 6 | 0 | 0.000000 | -0.000000 | 1295 | 1240.770299 | 1317.85 | 1394.929701 | 1.017645 |
| GO:0045933\_positive\_regulation\_of\_muscle\_contraction | 6 | 0 | 0.000000 | -0.000000 | 1295 | 1240.770299 | 1317.85 | 1394.929701 | 1.017645 |
| GO:0046427\_positive\_regulation\_of\_JAK-STAT\_cascade | 6 | 0 | 0.000000 | -0.000000 | 1295 | 1240.770299 | 1317.85 | 1394.929701 | 1.017645 |
| GO:0046460\_neutral\_lipid\_biosynthetic\_process | 6 | 0 | 0.000000 | -0.000000 | 1295 | 1240.770299 | 1317.85 | 1394.929701 | 1.017645 |
| GO:0046461\_neutral\_lipid\_catabolic\_process | 6 | 0 | 0.000000 | -0.000000 | 1295 | 1240.770299 | 1317.85 | 1394.929701 | 1.017645 |
| GO:0046463\_acylglycerol\_biosynthetic\_process | 6 | 0 | 0.000000 | -0.000000 | 1295 | 1240.770299 | 1317.85 | 1394.929701 | 1.017645 |
| GO:0046464\_acylglycerol\_catabolic\_process | 6 | 0 | 0.000000 | -0.000000 | 1295 | 1240.770299 | 1317.85 | 1394.929701 | 1.017645 |
| GO:0046503\_glycerolipid\_catabolic\_process | 6 | 0 | 0.000000 | -0.000000 | 1295 | 1240.770299 | 1317.85 | 1394.929701 | 1.017645 |
| GO:0046580\_negative\_regulation\_of\_Ras\_protein\_signal\_transduction | 6 | 0 | 0.000000 | -0.000000 | 1295 | 1240.770299 | 1317.85 | 1394.929701 | 1.017645 |
| GO:0046627\_negative\_regulation\_of\_insulin\_receptor\_signaling\_pathway | 6 | 0 | 0.000000 | -0.000000 | 1295 | 1240.770299 | 1317.85 | 1394.929701 | 1.017645 |
| GO:0046629\_gamma-delta\_T\_cell\_activation | 6 | 0 | 0.000000 | -0.000000 | 1295 | 1240.770299 | 1317.85 | 1394.929701 | 1.017645 |
| GO:0046666\_retinal\_cell\_programmed\_cell\_death | 6 | 0 | 0.000000 | -0.000000 | 1295 | 1240.770299 | 1317.85 | 1394.929701 | 1.017645 |
| GO:0046852\_positive\_regulation\_of\_bone\_remodeling | 6 | 0 | 0.000000 | -0.000000 | 1295 | 1240.770299 | 1317.85 | 1394.929701 | 1.017645 |
| GO:0046889\_positive\_regulation\_of\_lipid\_biosynthetic\_process | 6 | 0 | 0.000000 | -0.000000 | 1295 | 1240.770299 | 1317.85 | 1394.929701 | 1.017645 |
| GO:0048041\_focal\_adhesion\_formation | 6 | 0 | 0.000000 | -0.000000 | 1295 | 1240.770299 | 1317.85 | 1394.929701 | 1.017645 |
| GO:0048103\_somatic\_stem\_cell\_division | 6 | 0 | 0.000000 | -0.000000 | 1295 | 1240.770299 | 1317.85 | 1394.929701 | 1.017645 |
| GO:0048147\_negative\_regulation\_of\_fibroblast\_proliferation | 6 | 0 | 0.000000 | -0.000000 | 1295 | 1240.770299 | 1317.85 | 1394.929701 | 1.017645 |
| GO:0048333\_mesodermal\_cell\_differentiation | 6 | 0 | 0.000000 | -0.000000 | 1295 | 1240.770299 | 1317.85 | 1394.929701 | 1.017645 |
| GO:0048340\_paraxial\_mesoderm\_morphogenesis | 6 | 0 | 0.000000 | -0.000000 | 1295 | 1240.770299 | 1317.85 | 1394.929701 | 1.017645 |
| GO:0048541\_Peyer's\_patch\_development | 6 | 0 | 0.000000 | -0.000000 | 1295 | 1240.770299 | 1317.85 | 1394.929701 | 1.017645 |
| GO:0048563\_post-embryonic\_organ\_morphogenesis | 6 | 0 | 0.000000 | -0.000000 | 1295 | 1240.770299 | 1317.85 | 1394.929701 | 1.017645 |
| GO:0048617\_embryonic\_foregut\_morphogenesis | 6 | 0 | 0.000000 | -0.000000 | 1295 | 1240.770299 | 1317.85 | 1394.929701 | 1.017645 |
| GO:0048635\_negative\_regulation\_of\_muscle\_development | 6 | 0 | 0.000000 | -0.000000 | 1295 | 1240.770299 | 1317.85 | 1394.929701 | 1.017645 |
| GO:0048644\_muscle\_organ\_morphogenesis | 6 | 0 | 0.000000 | -0.000000 | 1295 | 1240.770299 | 1317.85 | 1394.929701 | 1.017645 |
| GO:0048703\_embryonic\_viscerocranium\_morphogenesis | 6 | 0 | 0.000000 | -0.000000 | 1295 | 1240.770299 | 1317.85 | 1394.929701 | 1.017645 |
| GO:0048713\_regulation\_of\_oligodendrocyte\_differentiation | 6 | 0 | 0.000000 | -0.000000 | 1295 | 1240.770299 | 1317.85 | 1394.929701 | 1.017645 |
| GO:0048853\_forebrain\_morphogenesis | 6 | 0 | 0.000000 | -0.000000 | 1295 | 1240.770299 | 1317.85 | 1394.929701 | 1.017645 |
| GO:0050684\_regulation\_of\_mRNA\_processing | 6 | 0 | 0.000000 | -0.000000 | 1295 | 1240.770299 | 1317.85 | 1394.929701 | 1.017645 |
| GO:0050732\_negative\_regulation\_of\_peptidyl-tyrosine\_phosphorylation | 6 | 0 | 0.000000 | -0.000000 | 1295 | 1240.770299 | 1317.85 | 1394.929701 | 1.017645 |
| GO:0050805\_negative\_regulation\_of\_synaptic\_transmission | 6 | 0 | 0.000000 | -0.000000 | 1295 | 1240.770299 | 1317.85 | 1394.929701 | 1.017645 |
| GO:0050821\_protein\_stabilization | 6 | 0 | 0.000000 | -0.000000 | 1295 | 1240.770299 | 1317.85 | 1394.929701 | 1.017645 |
| GO:0050829\_defense\_response\_to\_Gram-negative\_bacterium | 6 | 0 | 0.000000 | -0.000000 | 1295 | 1240.770299 | 1317.85 | 1394.929701 | 1.017645 |
| GO:0050872\_white\_fat\_cell\_differentiation | 6 | 0 | 0.000000 | -0.000000 | 1295 | 1240.770299 | 1317.85 | 1394.929701 | 1.017645 |
| GO:0050951\_sensory\_perception\_of\_temperature\_stimulus | 6 | 0 | 0.000000 | -0.000000 | 1295 | 1240.770299 | 1317.85 | 1394.929701 | 1.017645 |
| GO:0050966\_detection\_of\_mechanical\_stimulus\_involved\_in\_sensory\_perception\_of\_pain | 6 | 0 | 0.000000 | -0.000000 | 1295 | 1240.770299 | 1317.85 | 1394.929701 | 1.017645 |
| GO:0051058\_negative\_regulation\_of\_small\_GTPase\_mediated\_signal\_transduction | 6 | 0 | 0.000000 | -0.000000 | 1295 | 1240.770299 | 1317.85 | 1394.929701 | 1.017645 |
| GO:0051085\_chaperone\_mediated\_protein\_folding\_requiring\_cofactor | 6 | 0 | 0.000000 | -0.000000 | 1295 | 1240.770299 | 1317.85 | 1394.929701 | 1.017645 |
| GO:0051180\_vitamin\_transport | 6 | 0 | 0.000000 | -0.000000 | 1295 | 1240.770299 | 1317.85 | 1394.929701 | 1.017645 |
| GO:0051384\_response\_to\_glucocorticoid\_stimulus | 6 | 0 | 0.000000 | -0.000000 | 1295 | 1240.770299 | 1317.85 | 1394.929701 | 1.017645 |
| GO:0051592\_response\_to\_calcium\_ion | 6 | 0 | 0.000000 | -0.000000 | 1295 | 1240.770299 | 1317.85 | 1394.929701 | 1.017645 |
| GO:0051875\_pigment\_granule\_localization | 6 | 0 | 0.000000 | -0.000000 | 1295 | 1240.770299 | 1317.85 | 1394.929701 | 1.017645 |
| GO:0051881\_regulation\_of\_mitochondrial\_membrane\_potential | 6 | 0 | 0.000000 | -0.000000 | 1295 | 1240.770299 | 1317.85 | 1394.929701 | 1.017645 |
| GO:0051970\_negative\_regulation\_of\_transmission\_of\_nerve\_impulse | 6 | 0 | 0.000000 | -0.000000 | 1295 | 1240.770299 | 1317.85 | 1394.929701 | 1.017645 |
| GO:0055081\_anion\_homeostasis | 6 | 0 | 0.000000 | -0.000000 | 1295 | 1240.770299 | 1317.85 | 1394.929701 | 1.017645 |
| GO:0060013\_righting\_reflex | 6 | 0 | 0.000000 | -0.000000 | 1295 | 1240.770299 | 1317.85 | 1394.929701 | 1.017645 |
| GO:0060017\_parathyroid\_gland\_development | 6 | 0 | 0.000000 | -0.000000 | 1295 | 1240.770299 | 1317.85 | 1394.929701 | 1.017645 |
| GO:0060056\_mammary\_gland\_involution | 6 | 0 | 0.000000 | -0.000000 | 1295 | 1240.770299 | 1317.85 | 1394.929701 | 1.017645 |
| GO:0060068\_vagina\_development | 6 | 0 | 0.000000 | -0.000000 | 1295 | 1240.770299 | 1317.85 | 1394.929701 | 1.017645 |
| GO:0060134\_prepulse\_inhibition | 6 | 0 | 0.000000 | -0.000000 | 1295 | 1240.770299 | 1317.85 | 1394.929701 | 1.017645 |
| GO:0060136\_embryonic\_process\_involved\_in\_female\_pregnancy | 6 | 0 | 0.000000 | -0.000000 | 1295 | 1240.770299 | 1317.85 | 1394.929701 | 1.017645 |
| GO:0060271\_cilium\_morphogenesis | 6 | 0 | 0.000000 | -0.000000 | 1295 | 1240.770299 | 1317.85 | 1394.929701 | 1.017645 |
| GO:0060389\_pathway-restricted\_SMAD\_protein\_phosphorylation | 6 | 0 | 0.000000 | -0.000000 | 1295 | 1240.770299 | 1317.85 | 1394.929701 | 1.017645 |
| GO:0060411\_heart\_septum\_morphogenesis | 6 | 0 | 0.000000 | -0.000000 | 1295 | 1240.770299 | 1317.85 | 1394.929701 | 1.017645 |
| GO:0060638\_mesenchymal-epithelial\_cell\_signaling | 6 | 0 | 0.000000 | -0.000000 | 1295 | 1240.770299 | 1317.85 | 1394.929701 | 1.017645 |
| GO:0060685\_regulation\_of\_prostatic\_bud\_formation | 6 | 0 | 0.000000 | -0.000000 | 1295 | 1240.770299 | 1317.85 | 1394.929701 | 1.017645 |
| GO:0060710\_chorio-allantoic\_fusion | 6 | 0 | 0.000000 | -0.000000 | 1295 | 1240.770299 | 1317.85 | 1394.929701 | 1.017645 |
| GO:0065004\_protein-DNA\_complex\_assembly | 6 | 0 | 0.000000 | -0.000000 | 1295 | 1240.770299 | 1317.85 | 1394.929701 | 1.017645 |
| GO:0002697\_regulation\_of\_immune\_effector\_process | 68 | 0 | 0.000000 | -0.000000 | 1298 | 1243.836162 | 1320.6 | 1397.363838 | 1.017411 |
| GO:0019932\_second-messenger-mediated\_signaling | 68 | 0 | 0.000000 | -0.000000 | 1298 | 1243.836162 | 1320.6 | 1397.363838 | 1.017411 |
| GO:0042692\_muscle\_cell\_differentiation | 68 | 0 | 0.000000 | -0.000000 | 1298 | 1243.836162 | 1320.6 | 1397.363838 | 1.017411 |
| GO:0043933\_macromolecular\_complex\_subunit\_organization | 117 | 0 | 0.000000 | -0.000000 | 1299 | 1244.479783 | 1321.12 | 1397.760217 | 1.017028 |
| GO:0001570\_vasculogenesis | 38 | 0 | 0.000000 | -0.000000 | 1317 | 1261.749403 | 1337.6 | 1413.450597 | 1.015642 |
| GO:0001649\_osteoblast\_differentiation | 38 | 0 | 0.000000 | -0.000000 | 1317 | 1261.749403 | 1337.6 | 1413.450597 | 1.015642 |
| GO:0001657\_ureteric\_bud\_development | 38 | 0 | 0.000000 | -0.000000 | 1317 | 1261.749403 | 1337.6 | 1413.450597 | 1.015642 |
| GO:0002695\_negative\_regulation\_of\_leukocyte\_activation | 38 | 0 | 0.000000 | -0.000000 | 1317 | 1261.749403 | 1337.6 | 1413.450597 | 1.015642 |
| GO:0006820\_anion\_transport | 38 | 0 | 0.000000 | -0.000000 | 1317 | 1261.749403 | 1337.6 | 1413.450597 | 1.015642 |
| GO:0007596\_blood\_coagulation | 38 | 0 | 0.000000 | -0.000000 | 1317 | 1261.749403 | 1337.6 | 1413.450597 | 1.015642 |
| GO:0008016\_regulation\_of\_heart\_contraction | 38 | 0 | 0.000000 | -0.000000 | 1317 | 1261.749403 | 1337.6 | 1413.450597 | 1.015642 |
| GO:0016053\_organic\_acid\_biosynthetic\_process | 38 | 0 | 0.000000 | -0.000000 | 1317 | 1261.749403 | 1337.6 | 1413.450597 | 1.015642 |
| GO:0031401\_positive\_regulation\_of\_protein\_modification\_process | 38 | 0 | 0.000000 | -0.000000 | 1317 | 1261.749403 | 1337.6 | 1413.450597 | 1.015642 |
| GO:0032259\_methylation | 38 | 0 | 0.000000 | -0.000000 | 1317 | 1261.749403 | 1337.6 | 1413.450597 | 1.015642 |
| GO:0042493\_response\_to\_drug | 38 | 0 | 0.000000 | -0.000000 | 1317 | 1261.749403 | 1337.6 | 1413.450597 | 1.015642 |
| GO:0043414\_biopolymer\_methylation | 38 | 0 | 0.000000 | -0.000000 | 1317 | 1261.749403 | 1337.6 | 1413.450597 | 1.015642 |
| GO:0045580\_regulation\_of\_T\_cell\_differentiation | 38 | 0 | 0.000000 | -0.000000 | 1317 | 1261.749403 | 1337.6 | 1413.450597 | 1.015642 |
| GO:0046394\_carboxylic\_acid\_biosynthetic\_process | 38 | 0 | 0.000000 | -0.000000 | 1317 | 1261.749403 | 1337.6 | 1413.450597 | 1.015642 |
| GO:0046777\_protein\_amino\_acid\_autophosphorylation | 38 | 0 | 0.000000 | -0.000000 | 1317 | 1261.749403 | 1337.6 | 1413.450597 | 1.015642 |
| GO:0050727\_regulation\_of\_inflammatory\_response | 38 | 0 | 0.000000 | -0.000000 | 1317 | 1261.749403 | 1337.6 | 1413.450597 | 1.015642 |
| GO:0050866\_negative\_regulation\_of\_cell\_activation | 38 | 0 | 0.000000 | -0.000000 | 1317 | 1261.749403 | 1337.6 | 1413.450597 | 1.015642 |
| GO:0051348\_negative\_regulation\_of\_transferase\_activity | 38 | 0 | 0.000000 | -0.000000 | 1317 | 1261.749403 | 1337.6 | 1413.450597 | 1.015642 |
| GO:0006897\_endocytosis | 86 | 0 | 0.000000 | -0.000000 | 1322 | 1266.594132 | 1341.74 | 1416.885868 | 1.014932 |
| GO:0010324\_membrane\_invagination | 86 | 0 | 0.000000 | -0.000000 | 1322 | 1266.594132 | 1341.74 | 1416.885868 | 1.014932 |
| GO:0032504\_multicellular\_organism\_reproduction | 86 | 0 | 0.000000 | -0.000000 | 1322 | 1266.594132 | 1341.74 | 1416.885868 | 1.014932 |
| GO:0034641\_cellular\_nitrogen\_compound\_metabolic\_process | 86 | 0 | 0.000000 | -0.000000 | 1322 | 1266.594132 | 1341.74 | 1416.885868 | 1.014932 |
| GO:0048609\_reproductive\_process\_in\_a\_multicellular\_organism | 86 | 0 | 0.000000 | -0.000000 | 1322 | 1266.594132 | 1341.74 | 1416.885868 | 1.014932 |
| GO:0000209\_protein\_polyubiquitination | 10 | 0 | 0.000000 | -0.000000 | 1445 | 1387.002114 | 1460.08 | 1533.157886 | 1.010436 |
| GO:0000724\_double-strand\_break\_repair\_via\_homologous\_recombination | 10 | 0 | 0.000000 | -0.000000 | 1445 | 1387.002114 | 1460.08 | 1533.157886 | 1.010436 |
| GO:0000725\_recombinational\_repair | 10 | 0 | 0.000000 | -0.000000 | 1445 | 1387.002114 | 1460.08 | 1533.157886 | 1.010436 |
| GO:0001578\_microtubule\_bundle\_formation | 10 | 0 | 0.000000 | -0.000000 | 1445 | 1387.002114 | 1460.08 | 1533.157886 | 1.010436 |
| GO:0001659\_temperature\_homeostasis | 10 | 0 | 0.000000 | -0.000000 | 1445 | 1387.002114 | 1460.08 | 1533.157886 | 1.010436 |
| GO:0001773\_myeloid\_dendritic\_cell\_activation | 10 | 0 | 0.000000 | -0.000000 | 1445 | 1387.002114 | 1460.08 | 1533.157886 | 1.010436 |
| GO:0001832\_blastocyst\_growth | 10 | 0 | 0.000000 | -0.000000 | 1445 | 1387.002114 | 1460.08 | 1533.157886 | 1.010436 |
| GO:0001914\_regulation\_of\_T\_cell\_mediated\_cytotoxicity | 10 | 0 | 0.000000 | -0.000000 | 1445 | 1387.002114 | 1460.08 | 1533.157886 | 1.010436 |
| GO:0001990\_regulation\_of\_systemic\_arterial\_blood\_pressure\_by\_hormone | 10 | 0 | 0.000000 | -0.000000 | 1445 | 1387.002114 | 1460.08 | 1533.157886 | 1.010436 |
| GO:0002070\_epithelial\_cell\_maturation | 10 | 0 | 0.000000 | -0.000000 | 1445 | 1387.002114 | 1460.08 | 1533.157886 | 1.010436 |
| GO:0002673\_regulation\_of\_acute\_inflammatory\_response | 10 | 0 | 0.000000 | -0.000000 | 1445 | 1387.002114 | 1460.08 | 1533.157886 | 1.010436 |
| GO:0002711\_positive\_regulation\_of\_T\_cell\_mediated\_immunity | 10 | 0 | 0.000000 | -0.000000 | 1445 | 1387.002114 | 1460.08 | 1533.157886 | 1.010436 |
| GO:0002762\_negative\_regulation\_of\_myeloid\_leukocyte\_differentiation | 10 | 0 | 0.000000 | -0.000000 | 1445 | 1387.002114 | 1460.08 | 1533.157886 | 1.010436 |
| GO:0006040\_amino\_sugar\_metabolic\_process | 10 | 0 | 0.000000 | -0.000000 | 1445 | 1387.002114 | 1460.08 | 1533.157886 | 1.010436 |
| GO:0006081\_cellular\_aldehyde\_metabolic\_process | 10 | 0 | 0.000000 | -0.000000 | 1445 | 1387.002114 | 1460.08 | 1533.157886 | 1.010436 |
| GO:0006109\_regulation\_of\_carbohydrate\_metabolic\_process | 10 | 0 | 0.000000 | -0.000000 | 1445 | 1387.002114 | 1460.08 | 1533.157886 | 1.010436 |
| GO:0006289\_nucleotide-excision\_repair | 10 | 0 | 0.000000 | -0.000000 | 1445 | 1387.002114 | 1460.08 | 1533.157886 | 1.010436 |
| GO:0006342\_chromatin\_silencing | 10 | 0 | 0.000000 | -0.000000 | 1445 | 1387.002114 | 1460.08 | 1533.157886 | 1.010436 |
| GO:0006405\_RNA\_export\_from\_nucleus | 10 | 0 | 0.000000 | -0.000000 | 1445 | 1387.002114 | 1460.08 | 1533.157886 | 1.010436 |
| GO:0006801\_superoxide\_metabolic\_process | 10 | 0 | 0.000000 | -0.000000 | 1445 | 1387.002114 | 1460.08 | 1533.157886 | 1.010436 |
| GO:0006805\_xenobiotic\_metabolic\_process | 10 | 0 | 0.000000 | -0.000000 | 1445 | 1387.002114 | 1460.08 | 1533.157886 | 1.010436 |
| GO:0006826\_iron\_ion\_transport | 10 | 0 | 0.000000 | -0.000000 | 1445 | 1387.002114 | 1460.08 | 1533.157886 | 1.010436 |
| GO:0006921\_cell\_structure\_disassembly\_during\_apoptosis | 10 | 0 | 0.000000 | -0.000000 | 1445 | 1387.002114 | 1460.08 | 1533.157886 | 1.010436 |
| GO:0006968\_cellular\_defense\_response | 10 | 0 | 0.000000 | -0.000000 | 1445 | 1387.002114 | 1460.08 | 1533.157886 | 1.010436 |
| GO:0007006\_mitochondrial\_membrane\_organization | 10 | 0 | 0.000000 | -0.000000 | 1445 | 1387.002114 | 1460.08 | 1533.157886 | 1.010436 |
| GO:0007044\_cell-substrate\_junction\_assembly | 10 | 0 | 0.000000 | -0.000000 | 1445 | 1387.002114 | 1460.08 | 1533.157886 | 1.010436 |
| GO:0007093\_mitotic\_cell\_cycle\_checkpoint | 10 | 0 | 0.000000 | -0.000000 | 1445 | 1387.002114 | 1460.08 | 1533.157886 | 1.010436 |
| GO:0007172\_signal\_complex\_assembly | 10 | 0 | 0.000000 | -0.000000 | 1445 | 1387.002114 | 1460.08 | 1533.157886 | 1.010436 |
| GO:0007194\_negative\_regulation\_of\_adenylate\_cyclase\_activity | 10 | 0 | 0.000000 | -0.000000 | 1445 | 1387.002114 | 1460.08 | 1533.157886 | 1.010436 |
| GO:0008088\_axon\_cargo\_transport | 10 | 0 | 0.000000 | -0.000000 | 1445 | 1387.002114 | 1460.08 | 1533.157886 | 1.010436 |
| GO:0008206\_bile\_acid\_metabolic\_process | 10 | 0 | 0.000000 | -0.000000 | 1445 | 1387.002114 | 1460.08 | 1533.157886 | 1.010436 |
| GO:0008211\_glucocorticoid\_metabolic\_process | 10 | 0 | 0.000000 | -0.000000 | 1445 | 1387.002114 | 1460.08 | 1533.157886 | 1.010436 |
| GO:0009066\_aspartate\_family\_amino\_acid\_metabolic\_process | 10 | 0 | 0.000000 | -0.000000 | 1445 | 1387.002114 | 1460.08 | 1533.157886 | 1.010436 |
| GO:0009110\_vitamin\_biosynthetic\_process | 10 | 0 | 0.000000 | -0.000000 | 1445 | 1387.002114 | 1460.08 | 1533.157886 | 1.010436 |
| GO:0009620\_response\_to\_fungus | 10 | 0 | 0.000000 | -0.000000 | 1445 | 1387.002114 | 1460.08 | 1533.157886 | 1.010436 |
| GO:0009743\_response\_to\_carbohydrate\_stimulus | 10 | 0 | 0.000000 | -0.000000 | 1445 | 1387.002114 | 1460.08 | 1533.157886 | 1.010436 |
| GO:0009948\_anterior\_posterior\_axis\_specification | 10 | 0 | 0.000000 | -0.000000 | 1445 | 1387.002114 | 1460.08 | 1533.157886 | 1.010436 |
| GO:0010827\_regulation\_of\_glucose\_transport | 10 | 0 | 0.000000 | -0.000000 | 1445 | 1387.002114 | 1460.08 | 1533.157886 | 1.010436 |
| GO:0015718\_monocarboxylic\_acid\_transport | 10 | 0 | 0.000000 | -0.000000 | 1445 | 1387.002114 | 1460.08 | 1533.157886 | 1.010436 |
| GO:0016197\_endosome\_transport | 10 | 0 | 0.000000 | -0.000000 | 1445 | 1387.002114 | 1460.08 | 1533.157886 | 1.010436 |
| GO:0016486\_peptide\_hormone\_processing | 10 | 0 | 0.000000 | -0.000000 | 1445 | 1387.002114 | 1460.08 | 1533.157886 | 1.010436 |
| GO:0017156\_calcium\_ion-dependent\_exocytosis | 10 | 0 | 0.000000 | -0.000000 | 1445 | 1387.002114 | 1460.08 | 1533.157886 | 1.010436 |
| GO:0018149\_peptide\_cross-linking | 10 | 0 | 0.000000 | -0.000000 | 1445 | 1387.002114 | 1460.08 | 1533.157886 | 1.010436 |
| GO:0019321\_pentose\_metabolic\_process | 10 | 0 | 0.000000 | -0.000000 | 1445 | 1387.002114 | 1460.08 | 1533.157886 | 1.010436 |
| GO:0021534\_cell\_proliferation\_in\_hindbrain | 10 | 0 | 0.000000 | -0.000000 | 1445 | 1387.002114 | 1460.08 | 1533.157886 | 1.010436 |
| GO:0021871\_forebrain\_regionalization | 10 | 0 | 0.000000 | -0.000000 | 1445 | 1387.002114 | 1460.08 | 1533.157886 | 1.010436 |
| GO:0021895\_cerebral\_cortex\_neuron\_differentiation | 10 | 0 | 0.000000 | -0.000000 | 1445 | 1387.002114 | 1460.08 | 1533.157886 | 1.010436 |
| GO:0021924\_cell\_proliferation\_in\_the\_external\_granule\_layer | 10 | 0 | 0.000000 | -0.000000 | 1445 | 1387.002114 | 1460.08 | 1533.157886 | 1.010436 |
| GO:0021930\_granule\_cell\_precursor\_proliferation | 10 | 0 | 0.000000 | -0.000000 | 1445 | 1387.002114 | 1460.08 | 1533.157886 | 1.010436 |
| GO:0021952\_central\_nervous\_system\_projection\_neuron\_axonogenesis | 10 | 0 | 0.000000 | -0.000000 | 1445 | 1387.002114 | 1460.08 | 1533.157886 | 1.010436 |
| GO:0022900\_electron\_transport\_chain | 10 | 0 | 0.000000 | -0.000000 | 1445 | 1387.002114 | 1460.08 | 1533.157886 | 1.010436 |
| GO:0022904\_respiratory\_electron\_transport\_chain | 10 | 0 | 0.000000 | -0.000000 | 1445 | 1387.002114 | 1460.08 | 1533.157886 | 1.010436 |
| GO:0030168\_platelet\_activation | 10 | 0 | 0.000000 | -0.000000 | 1445 | 1387.002114 | 1460.08 | 1533.157886 | 1.010436 |
| GO:0030833\_regulation\_of\_actin\_filament\_polymerization | 10 | 0 | 0.000000 | -0.000000 | 1445 | 1387.002114 | 1460.08 | 1533.157886 | 1.010436 |
| GO:0031018\_endocrine\_pancreas\_development | 10 | 0 | 0.000000 | -0.000000 | 1445 | 1387.002114 | 1460.08 | 1533.157886 | 1.010436 |
| GO:0031280\_negative\_regulation\_of\_cyclase\_activity | 10 | 0 | 0.000000 | -0.000000 | 1445 | 1387.002114 | 1460.08 | 1533.157886 | 1.010436 |
| GO:0031331\_positive\_regulation\_of\_cellular\_catabolic\_process | 10 | 0 | 0.000000 | -0.000000 | 1445 | 1387.002114 | 1460.08 | 1533.157886 | 1.010436 |
| GO:0031645\_negative\_regulation\_of\_neurological\_system\_process | 10 | 0 | 0.000000 | -0.000000 | 1445 | 1387.002114 | 1460.08 | 1533.157886 | 1.010436 |
| GO:0032318\_regulation\_of\_Ras\_GTPase\_activity | 10 | 0 | 0.000000 | -0.000000 | 1445 | 1387.002114 | 1460.08 | 1533.157886 | 1.010436 |
| GO:0032602\_chemokine\_production | 10 | 0 | 0.000000 | -0.000000 | 1445 | 1387.002114 | 1460.08 | 1533.157886 | 1.010436 |
| GO:0032633\_interleukin-4\_production | 10 | 0 | 0.000000 | -0.000000 | 1445 | 1387.002114 | 1460.08 | 1533.157886 | 1.010436 |
| GO:0032642\_regulation\_of\_chemokine\_production | 10 | 0 | 0.000000 | -0.000000 | 1445 | 1387.002114 | 1460.08 | 1533.157886 | 1.010436 |
| GO:0032673\_regulation\_of\_interleukin-4\_production | 10 | 0 | 0.000000 | -0.000000 | 1445 | 1387.002114 | 1460.08 | 1533.157886 | 1.010436 |
| GO:0032760\_positive\_regulation\_of\_tumor\_necrosis\_factor\_production | 10 | 0 | 0.000000 | -0.000000 | 1445 | 1387.002114 | 1460.08 | 1533.157886 | 1.010436 |
| GO:0033081\_regulation\_of\_T\_cell\_differentiation\_in\_the\_thymus | 10 | 0 | 0.000000 | -0.000000 | 1445 | 1387.002114 | 1460.08 | 1533.157886 | 1.010436 |
| GO:0034105\_positive\_regulation\_of\_tissue\_remodeling | 10 | 0 | 0.000000 | -0.000000 | 1445 | 1387.002114 | 1460.08 | 1533.157886 | 1.010436 |
| GO:0034637\_cellular\_carbohydrate\_biosynthetic\_process | 10 | 0 | 0.000000 | -0.000000 | 1445 | 1387.002114 | 1460.08 | 1533.157886 | 1.010436 |
| GO:0040015\_negative\_regulation\_of\_multicellular\_organism\_growth | 10 | 0 | 0.000000 | -0.000000 | 1445 | 1387.002114 | 1460.08 | 1533.157886 | 1.010436 |
| GO:0042088\_T-helper\_1\_type\_immune\_response | 10 | 0 | 0.000000 | -0.000000 | 1445 | 1387.002114 | 1460.08 | 1533.157886 | 1.010436 |
| GO:0042116\_macrophage\_activation | 10 | 0 | 0.000000 | -0.000000 | 1445 | 1387.002114 | 1460.08 | 1533.157886 | 1.010436 |
| GO:0042177\_negative\_regulation\_of\_protein\_catabolic\_process | 10 | 0 | 0.000000 | -0.000000 | 1445 | 1387.002114 | 1460.08 | 1533.157886 | 1.010436 |
| GO:0042755\_eating\_behavior | 10 | 0 | 0.000000 | -0.000000 | 1445 | 1387.002114 | 1460.08 | 1533.157886 | 1.010436 |
| GO:0043113\_receptor\_clustering | 10 | 0 | 0.000000 | -0.000000 | 1445 | 1387.002114 | 1460.08 | 1533.157886 | 1.010436 |
| GO:0043330\_response\_to\_exogenous\_dsRNA | 10 | 0 | 0.000000 | -0.000000 | 1445 | 1387.002114 | 1460.08 | 1533.157886 | 1.010436 |
| GO:0043488\_regulation\_of\_mRNA\_stability | 10 | 0 | 0.000000 | -0.000000 | 1445 | 1387.002114 | 1460.08 | 1533.157886 | 1.010436 |
| GO:0043506\_regulation\_of\_JUN\_kinase\_activity | 10 | 0 | 0.000000 | -0.000000 | 1445 | 1387.002114 | 1460.08 | 1533.157886 | 1.010436 |
| GO:0043525\_positive\_regulation\_of\_neuron\_apoptosis | 10 | 0 | 0.000000 | -0.000000 | 1445 | 1387.002114 | 1460.08 | 1533.157886 | 1.010436 |
| GO:0044259\_multicellular\_organismal\_macromolecule\_metabolic\_process | 10 | 0 | 0.000000 | -0.000000 | 1445 | 1387.002114 | 1460.08 | 1533.157886 | 1.010436 |
| GO:0045132\_meiotic\_chromosome\_segregation | 10 | 0 | 0.000000 | -0.000000 | 1445 | 1387.002114 | 1460.08 | 1533.157886 | 1.010436 |
| GO:0045446\_endothelial\_cell\_differentiation | 10 | 0 | 0.000000 | -0.000000 | 1445 | 1387.002114 | 1460.08 | 1533.157886 | 1.010436 |
| GO:0045576\_mast\_cell\_activation | 10 | 0 | 0.000000 | -0.000000 | 1445 | 1387.002114 | 1460.08 | 1533.157886 | 1.010436 |
| GO:0045669\_positive\_regulation\_of\_osteoblast\_differentiation | 10 | 0 | 0.000000 | -0.000000 | 1445 | 1387.002114 | 1460.08 | 1533.157886 | 1.010436 |
| GO:0045776\_negative\_regulation\_of\_blood\_pressure | 10 | 0 | 0.000000 | -0.000000 | 1445 | 1387.002114 | 1460.08 | 1533.157886 | 1.010436 |
| GO:0045777\_positive\_regulation\_of\_blood\_pressure | 10 | 0 | 0.000000 | -0.000000 | 1445 | 1387.002114 | 1460.08 | 1533.157886 | 1.010436 |
| GO:0045814\_negative\_regulation\_of\_gene\_expression\_\_epigenetic | 10 | 0 | 0.000000 | -0.000000 | 1445 | 1387.002114 | 1460.08 | 1533.157886 | 1.010436 |
| GO:0045911\_positive\_regulation\_of\_DNA\_recombination | 10 | 0 | 0.000000 | -0.000000 | 1445 | 1387.002114 | 1460.08 | 1533.157886 | 1.010436 |
| GO:0046887\_positive\_regulation\_of\_hormone\_secretion | 10 | 0 | 0.000000 | -0.000000 | 1445 | 1387.002114 | 1460.08 | 1533.157886 | 1.010436 |
| GO:0048291\_isotype\_switching\_to\_IgG\_isotypes | 10 | 0 | 0.000000 | -0.000000 | 1445 | 1387.002114 | 1460.08 | 1533.157886 | 1.010436 |
| GO:0048302\_regulation\_of\_isotype\_switching\_to\_IgG\_isotypes | 10 | 0 | 0.000000 | -0.000000 | 1445 | 1387.002114 | 1460.08 | 1533.157886 | 1.010436 |
| GO:0048339\_paraxial\_mesoderm\_development | 10 | 0 | 0.000000 | -0.000000 | 1445 | 1387.002114 | 1460.08 | 1533.157886 | 1.010436 |
| GO:0048384\_retinoic\_acid\_receptor\_signaling\_pathway | 10 | 0 | 0.000000 | -0.000000 | 1445 | 1387.002114 | 1460.08 | 1533.157886 | 1.010436 |
| GO:0048596\_embryonic\_camera-type\_eye\_morphogenesis | 10 | 0 | 0.000000 | -0.000000 | 1445 | 1387.002114 | 1460.08 | 1533.157886 | 1.010436 |
| GO:0048641\_regulation\_of\_skeletal\_muscle\_tissue\_development | 10 | 0 | 0.000000 | -0.000000 | 1445 | 1387.002114 | 1460.08 | 1533.157886 | 1.010436 |
| GO:0048738\_cardiac\_muscle\_tissue\_development | 10 | 0 | 0.000000 | -0.000000 | 1445 | 1387.002114 | 1460.08 | 1533.157886 | 1.010436 |
| GO:0050654\_chondroitin\_sulfate\_proteoglycan\_metabolic\_process | 10 | 0 | 0.000000 | -0.000000 | 1445 | 1387.002114 | 1460.08 | 1533.157886 | 1.010436 |
| GO:0050657\_nucleic\_acid\_transport | 10 | 0 | 0.000000 | -0.000000 | 1445 | 1387.002114 | 1460.08 | 1533.157886 | 1.010436 |
| GO:0050658\_RNA\_transport | 10 | 0 | 0.000000 | -0.000000 | 1445 | 1387.002114 | 1460.08 | 1533.157886 | 1.010436 |
| GO:0050663\_cytokine\_secretion | 10 | 0 | 0.000000 | -0.000000 | 1445 | 1387.002114 | 1460.08 | 1533.157886 | 1.010436 |
| GO:0050714\_positive\_regulation\_of\_protein\_secretion | 10 | 0 | 0.000000 | -0.000000 | 1445 | 1387.002114 | 1460.08 | 1533.157886 | 1.010436 |
| GO:0050879\_multicellular\_organismal\_movement | 10 | 0 | 0.000000 | -0.000000 | 1445 | 1387.002114 | 1460.08 | 1533.157886 | 1.010436 |
| GO:0050881\_musculoskeletal\_movement | 10 | 0 | 0.000000 | -0.000000 | 1445 | 1387.002114 | 1460.08 | 1533.157886 | 1.010436 |
| GO:0050886\_endocrine\_process | 10 | 0 | 0.000000 | -0.000000 | 1445 | 1387.002114 | 1460.08 | 1533.157886 | 1.010436 |
| GO:0050892\_intestinal\_absorption | 10 | 0 | 0.000000 | -0.000000 | 1445 | 1387.002114 | 1460.08 | 1533.157886 | 1.010436 |
| GO:0051147\_regulation\_of\_muscle\_cell\_differentiation | 10 | 0 | 0.000000 | -0.000000 | 1445 | 1387.002114 | 1460.08 | 1533.157886 | 1.010436 |
| GO:0051208\_sequestering\_of\_calcium\_ion | 10 | 0 | 0.000000 | -0.000000 | 1445 | 1387.002114 | 1460.08 | 1533.157886 | 1.010436 |
| GO:0051209\_release\_of\_sequestered\_calcium\_ion\_into\_cytosol | 10 | 0 | 0.000000 | -0.000000 | 1445 | 1387.002114 | 1460.08 | 1533.157886 | 1.010436 |
| GO:0051224\_negative\_regulation\_of\_protein\_transport | 10 | 0 | 0.000000 | -0.000000 | 1445 | 1387.002114 | 1460.08 | 1533.157886 | 1.010436 |
| GO:0051236\_establishment\_of\_RNA\_localization | 10 | 0 | 0.000000 | -0.000000 | 1445 | 1387.002114 | 1460.08 | 1533.157886 | 1.010436 |
| GO:0051238\_sequestering\_of\_metal\_ion | 10 | 0 | 0.000000 | -0.000000 | 1445 | 1387.002114 | 1460.08 | 1533.157886 | 1.010436 |
| GO:0051262\_protein\_tetramerization | 10 | 0 | 0.000000 | -0.000000 | 1445 | 1387.002114 | 1460.08 | 1533.157886 | 1.010436 |
| GO:0051282\_regulation\_of\_sequestering\_of\_calcium\_ion | 10 | 0 | 0.000000 | -0.000000 | 1445 | 1387.002114 | 1460.08 | 1533.157886 | 1.010436 |
| GO:0051283\_negative\_regulation\_of\_sequestering\_of\_calcium\_ion | 10 | 0 | 0.000000 | -0.000000 | 1445 | 1387.002114 | 1460.08 | 1533.157886 | 1.010436 |
| GO:0051350\_negative\_regulation\_of\_lyase\_activity | 10 | 0 | 0.000000 | -0.000000 | 1445 | 1387.002114 | 1460.08 | 1533.157886 | 1.010436 |
| GO:0051445\_regulation\_of\_meiotic\_cell\_cycle | 10 | 0 | 0.000000 | -0.000000 | 1445 | 1387.002114 | 1460.08 | 1533.157886 | 1.010436 |
| GO:0051650\_establishment\_of\_vesicle\_localization | 10 | 0 | 0.000000 | -0.000000 | 1445 | 1387.002114 | 1460.08 | 1533.157886 | 1.010436 |
| GO:0051651\_maintenance\_of\_location\_in\_cell | 10 | 0 | 0.000000 | -0.000000 | 1445 | 1387.002114 | 1460.08 | 1533.157886 | 1.010436 |
| GO:0060135\_maternal\_process\_involved\_in\_female\_pregnancy | 10 | 0 | 0.000000 | -0.000000 | 1445 | 1387.002114 | 1460.08 | 1533.157886 | 1.010436 |
| GO:0060216\_definitive\_hemopoiesis | 10 | 0 | 0.000000 | -0.000000 | 1445 | 1387.002114 | 1460.08 | 1533.157886 | 1.010436 |
| GO:0060323\_head\_morphogenesis | 10 | 0 | 0.000000 | -0.000000 | 1445 | 1387.002114 | 1460.08 | 1533.157886 | 1.010436 |
| GO:0060343\_trabecula\_formation | 10 | 0 | 0.000000 | -0.000000 | 1445 | 1387.002114 | 1460.08 | 1533.157886 | 1.010436 |
| GO:0060601\_lateral\_sprouting\_from\_an\_epithelium | 10 | 0 | 0.000000 | -0.000000 | 1445 | 1387.002114 | 1460.08 | 1533.157886 | 1.010436 |
| GO:0060669\_embryonic\_placenta\_morphogenesis | 10 | 0 | 0.000000 | -0.000000 | 1445 | 1387.002114 | 1460.08 | 1533.157886 | 1.010436 |
| GO:0060706\_cell\_differentiation\_involved\_in\_embryonic\_placenta\_development | 10 | 0 | 0.000000 | -0.000000 | 1445 | 1387.002114 | 1460.08 | 1533.157886 | 1.010436 |
| GO:0000165\_MAPKKK\_cascade | 114 | 0 | 0.000000 | -0.000000 | 1446 | 1388.467852 | 1461.21 | 1533.952148 | 1.010519 |
| GO:0046483\_heterocycle\_metabolic\_process | 116 | 0 | 0.000000 | -0.000000 | 1448 | 1390.671845 | 1463.04 | 1535.408155 | 1.010387 |
| GO:0080134\_regulation\_of\_response\_to\_stress | 116 | 0 | 0.000000 | -0.000000 | 1448 | 1390.671845 | 1463.04 | 1535.408155 | 1.010387 |
| GO:0010647\_positive\_regulation\_of\_cell\_communication | 110 | 0 | 0.000000 | -0.000000 | 1452 | 1394.754526 | 1466.41 | 1538.065474 | 1.009924 |
| GO:0010648\_negative\_regulation\_of\_cell\_communication | 110 | 0 | 0.000000 | -0.000000 | 1452 | 1394.754526 | 1466.41 | 1538.065474 | 1.009924 |
| GO:0043010\_camera-type\_eye\_development | 110 | 0 | 0.000000 | -0.000000 | 1452 | 1394.754526 | 1466.41 | 1538.065474 | 1.009924 |
| GO:0055080\_cation\_homeostasis | 110 | 0 | 0.000000 | -0.000000 | 1452 | 1394.754526 | 1466.41 | 1538.065474 | 1.009924 |
| GO:0000910\_cytokinesis | 8 | 0 | 0.000000 | -0.000000 | 1580 | 1523.903614 | 1593.14 | 1662.376386 | 1.008316 |
| GO:0001783\_B\_cell\_apoptosis | 8 | 0 | 0.000000 | -0.000000 | 1580 | 1523.903614 | 1593.14 | 1662.376386 | 1.008316 |
| GO:0001833\_inner\_cell\_mass\_cell\_proliferation | 8 | 0 | 0.000000 | -0.000000 | 1580 | 1523.903614 | 1593.14 | 1662.376386 | 1.008316 |
| GO:0001840\_neural\_plate\_development | 8 | 0 | 0.000000 | -0.000000 | 1580 | 1523.903614 | 1593.14 | 1662.376386 | 1.008316 |
| GO:0001893\_maternal\_placenta\_development | 8 | 0 | 0.000000 | -0.000000 | 1580 | 1523.903614 | 1593.14 | 1662.376386 | 1.008316 |
| GO:0001911\_negative\_regulation\_of\_leukocyte\_mediated\_cytotoxicity | 8 | 0 | 0.000000 | -0.000000 | 1580 | 1523.903614 | 1593.14 | 1662.376386 | 1.008316 |
| GO:0001916\_positive\_regulation\_of\_T\_cell\_mediated\_cytotoxicity | 8 | 0 | 0.000000 | -0.000000 | 1580 | 1523.903614 | 1593.14 | 1662.376386 | 1.008316 |
| GO:0002320\_lymphoid\_progenitor\_cell\_differentiation | 8 | 0 | 0.000000 | -0.000000 | 1580 | 1523.903614 | 1593.14 | 1662.376386 | 1.008316 |
| GO:0002438\_acute\_inflammatory\_response\_to\_antigenic\_stimulus | 8 | 0 | 0.000000 | -0.000000 | 1580 | 1523.903614 | 1593.14 | 1662.376386 | 1.008316 |
| GO:0002524\_hypersensitivity | 8 | 0 | 0.000000 | -0.000000 | 1580 | 1523.903614 | 1593.14 | 1662.376386 | 1.008316 |
| GO:0002566\_somatic\_diversification\_of\_immune\_receptors\_via\_somatic\_mutation | 8 | 0 | 0.000000 | -0.000000 | 1580 | 1523.903614 | 1593.14 | 1662.376386 | 1.008316 |
| GO:0002864\_regulation\_of\_acute\_inflammatory\_response\_to\_antigenic\_stimulus | 8 | 0 | 0.000000 | -0.000000 | 1580 | 1523.903614 | 1593.14 | 1662.376386 | 1.008316 |
| GO:0002883\_regulation\_of\_hypersensitivity | 8 | 0 | 0.000000 | -0.000000 | 1580 | 1523.903614 | 1593.14 | 1662.376386 | 1.008316 |
| GO:0003081\_regulation\_of\_systemic\_arterial\_blood\_pressure\_by\_renin-angiotensin | 8 | 0 | 0.000000 | -0.000000 | 1580 | 1523.903614 | 1593.14 | 1662.376386 | 1.008316 |
| GO:0006020\_inositol\_metabolic\_process | 8 | 0 | 0.000000 | -0.000000 | 1580 | 1523.903614 | 1593.14 | 1662.376386 | 1.008316 |
| GO:0006195\_purine\_nucleotide\_catabolic\_process | 8 | 0 | 0.000000 | -0.000000 | 1580 | 1523.903614 | 1593.14 | 1662.376386 | 1.008316 |
| GO:0006284\_base-excision\_repair | 8 | 0 | 0.000000 | -0.000000 | 1580 | 1523.903614 | 1593.14 | 1662.376386 | 1.008316 |
| GO:0006349\_genetic\_imprinting | 8 | 0 | 0.000000 | -0.000000 | 1580 | 1523.903614 | 1593.14 | 1662.376386 | 1.008316 |
| GO:0006360\_transcription\_from\_RNA\_polymerase\_I\_promoter | 8 | 0 | 0.000000 | -0.000000 | 1580 | 1523.903614 | 1593.14 | 1662.376386 | 1.008316 |
| GO:0006458\_'de\_novo'\_protein\_folding | 8 | 0 | 0.000000 | -0.000000 | 1580 | 1523.903614 | 1593.14 | 1662.376386 | 1.008316 |
| GO:0006493\_protein\_amino\_acid\_O-linked\_glycosylation | 8 | 0 | 0.000000 | -0.000000 | 1580 | 1523.903614 | 1593.14 | 1662.376386 | 1.008316 |
| GO:0006733\_oxidoreduction\_coenzyme\_metabolic\_process | 8 | 0 | 0.000000 | -0.000000 | 1580 | 1523.903614 | 1593.14 | 1662.376386 | 1.008316 |
| GO:0006829\_zinc\_ion\_transport | 8 | 0 | 0.000000 | -0.000000 | 1580 | 1523.903614 | 1593.14 | 1662.376386 | 1.008316 |
| GO:0007009\_plasma\_membrane\_organization | 8 | 0 | 0.000000 | -0.000000 | 1580 | 1523.903614 | 1593.14 | 1662.376386 | 1.008316 |
| GO:0007098\_centrosome\_cycle | 8 | 0 | 0.000000 | -0.000000 | 1580 | 1523.903614 | 1593.14 | 1662.376386 | 1.008316 |
| GO:0007131\_reciprocal\_meiotic\_recombination | 8 | 0 | 0.000000 | -0.000000 | 1580 | 1523.903614 | 1593.14 | 1662.376386 | 1.008316 |
| GO:0007141\_male\_meiosis\_I | 8 | 0 | 0.000000 | -0.000000 | 1580 | 1523.903614 | 1593.14 | 1662.376386 | 1.008316 |
| GO:0007625\_grooming\_behavior | 8 | 0 | 0.000000 | -0.000000 | 1580 | 1523.903614 | 1593.14 | 1662.376386 | 1.008316 |
| GO:0008105\_asymmetric\_protein\_localization | 8 | 0 | 0.000000 | -0.000000 | 1580 | 1523.903614 | 1593.14 | 1662.376386 | 1.008316 |
| GO:0008593\_regulation\_of\_Notch\_signaling\_pathway | 8 | 0 | 0.000000 | -0.000000 | 1580 | 1523.903614 | 1593.14 | 1662.376386 | 1.008316 |
| GO:0009144\_purine\_nucleoside\_triphosphate\_metabolic\_process | 8 | 0 | 0.000000 | -0.000000 | 1580 | 1523.903614 | 1593.14 | 1662.376386 | 1.008316 |
| GO:0009746\_response\_to\_hexose\_stimulus | 8 | 0 | 0.000000 | -0.000000 | 1580 | 1523.903614 | 1593.14 | 1662.376386 | 1.008316 |
| GO:0009749\_response\_to\_glucose\_stimulus | 8 | 0 | 0.000000 | -0.000000 | 1580 | 1523.903614 | 1593.14 | 1662.376386 | 1.008316 |
| GO:0014014\_negative\_regulation\_of\_gliogenesis | 8 | 0 | 0.000000 | -0.000000 | 1580 | 1523.903614 | 1593.14 | 1662.376386 | 1.008316 |
| GO:0014046\_dopamine\_secretion | 8 | 0 | 0.000000 | -0.000000 | 1580 | 1523.903614 | 1593.14 | 1662.376386 | 1.008316 |
| GO:0014059\_regulation\_of\_dopamine\_secretion | 8 | 0 | 0.000000 | -0.000000 | 1580 | 1523.903614 | 1593.14 | 1662.376386 | 1.008316 |
| GO:0014065\_phosphoinositide\_3-kinase\_cascade | 8 | 0 | 0.000000 | -0.000000 | 1580 | 1523.903614 | 1593.14 | 1662.376386 | 1.008316 |
| GO:0015800\_acidic\_amino\_acid\_transport | 8 | 0 | 0.000000 | -0.000000 | 1580 | 1523.903614 | 1593.14 | 1662.376386 | 1.008316 |
| GO:0015804\_neutral\_amino\_acid\_transport | 8 | 0 | 0.000000 | -0.000000 | 1580 | 1523.903614 | 1593.14 | 1662.376386 | 1.008316 |
| GO:0016236\_macroautophagy | 8 | 0 | 0.000000 | -0.000000 | 1580 | 1523.903614 | 1593.14 | 1662.376386 | 1.008316 |
| GO:0016446\_somatic\_hypermutation\_of\_immunoglobulin\_genes | 8 | 0 | 0.000000 | -0.000000 | 1580 | 1523.903614 | 1593.14 | 1662.376386 | 1.008316 |
| GO:0018107\_peptidyl-threonine\_phosphorylation | 8 | 0 | 0.000000 | -0.000000 | 1580 | 1523.903614 | 1593.14 | 1662.376386 | 1.008316 |
| GO:0018210\_peptidyl-threonine\_modification | 8 | 0 | 0.000000 | -0.000000 | 1580 | 1523.903614 | 1593.14 | 1662.376386 | 1.008316 |
| GO:0018345\_protein\_palmitoylation | 8 | 0 | 0.000000 | -0.000000 | 1580 | 1523.903614 | 1593.14 | 1662.376386 | 1.008316 |
| GO:0019229\_regulation\_of\_vasoconstriction | 8 | 0 | 0.000000 | -0.000000 | 1580 | 1523.903614 | 1593.14 | 1662.376386 | 1.008316 |
| GO:0019400\_alditol\_metabolic\_process | 8 | 0 | 0.000000 | -0.000000 | 1580 | 1523.903614 | 1593.14 | 1662.376386 | 1.008316 |
| GO:0021692\_cerebellar\_Purkinje\_cell\_layer\_morphogenesis | 8 | 0 | 0.000000 | -0.000000 | 1580 | 1523.903614 | 1593.14 | 1662.376386 | 1.008316 |
| GO:0021694\_cerebellar\_Purkinje\_cell\_layer\_formation | 8 | 0 | 0.000000 | -0.000000 | 1580 | 1523.903614 | 1593.14 | 1662.376386 | 1.008316 |
| GO:0021702\_cerebellar\_Purkinje\_cell\_differentiation | 8 | 0 | 0.000000 | -0.000000 | 1580 | 1523.903614 | 1593.14 | 1662.376386 | 1.008316 |
| GO:0021781\_glial\_cell\_fate\_commitment | 8 | 0 | 0.000000 | -0.000000 | 1580 | 1523.903614 | 1593.14 | 1662.376386 | 1.008316 |
| GO:0021799\_cerebral\_cortex\_radially\_oriented\_cell\_migration | 8 | 0 | 0.000000 | -0.000000 | 1580 | 1523.903614 | 1593.14 | 1662.376386 | 1.008316 |
| GO:0022898\_regulation\_of\_transmembrane\_transporter\_activity | 8 | 0 | 0.000000 | -0.000000 | 1580 | 1523.903614 | 1593.14 | 1662.376386 | 1.008316 |
| GO:0030035\_microspike\_assembly | 8 | 0 | 0.000000 | -0.000000 | 1580 | 1523.903614 | 1593.14 | 1662.376386 | 1.008316 |
| GO:0030193\_regulation\_of\_blood\_coagulation | 8 | 0 | 0.000000 | -0.000000 | 1580 | 1523.903614 | 1593.14 | 1662.376386 | 1.008316 |
| GO:0030204\_chondroitin\_sulfate\_metabolic\_process | 8 | 0 | 0.000000 | -0.000000 | 1580 | 1523.903614 | 1593.14 | 1662.376386 | 1.008316 |
| GO:0030500\_regulation\_of\_bone\_mineralization | 8 | 0 | 0.000000 | -0.000000 | 1580 | 1523.903614 | 1593.14 | 1662.376386 | 1.008316 |
| GO:0030511\_positive\_regulation\_of\_transforming\_growth\_factor\_beta\_receptor\_signaling\_pathway | 8 | 0 | 0.000000 | -0.000000 | 1580 | 1523.903614 | 1593.14 | 1662.376386 | 1.008316 |
| GO:0031102\_neuron\_projection\_regeneration | 8 | 0 | 0.000000 | -0.000000 | 1580 | 1523.903614 | 1593.14 | 1662.376386 | 1.008316 |
| GO:0031103\_axon\_regeneration | 8 | 0 | 0.000000 | -0.000000 | 1580 | 1523.903614 | 1593.14 | 1662.376386 | 1.008316 |
| GO:0031111\_negative\_regulation\_of\_microtubule\_polymerization\_or\_depolymerization | 8 | 0 | 0.000000 | -0.000000 | 1580 | 1523.903614 | 1593.14 | 1662.376386 | 1.008316 |
| GO:0031123\_RNA\_3'-end\_processing | 8 | 0 | 0.000000 | -0.000000 | 1580 | 1523.903614 | 1593.14 | 1662.376386 | 1.008316 |
| GO:0031294\_lymphocyte\_costimulation | 8 | 0 | 0.000000 | -0.000000 | 1580 | 1523.903614 | 1593.14 | 1662.376386 | 1.008316 |
| GO:0031295\_T\_cell\_costimulation | 8 | 0 | 0.000000 | -0.000000 | 1580 | 1523.903614 | 1593.14 | 1662.376386 | 1.008316 |
| GO:0031334\_positive\_regulation\_of\_protein\_complex\_assembly | 8 | 0 | 0.000000 | -0.000000 | 1580 | 1523.903614 | 1593.14 | 1662.376386 | 1.008316 |
| GO:0031342\_negative\_regulation\_of\_cell\_killing | 8 | 0 | 0.000000 | -0.000000 | 1580 | 1523.903614 | 1593.14 | 1662.376386 | 1.008316 |
| GO:0031396\_regulation\_of\_protein\_ubiquitination | 8 | 0 | 0.000000 | -0.000000 | 1580 | 1523.903614 | 1593.14 | 1662.376386 | 1.008316 |
| GO:0032094\_response\_to\_food | 8 | 0 | 0.000000 | -0.000000 | 1580 | 1523.903614 | 1593.14 | 1662.376386 | 1.008316 |
| GO:0032273\_positive\_regulation\_of\_protein\_polymerization | 8 | 0 | 0.000000 | -0.000000 | 1580 | 1523.903614 | 1593.14 | 1662.376386 | 1.008316 |
| GO:0032409\_regulation\_of\_transporter\_activity | 8 | 0 | 0.000000 | -0.000000 | 1580 | 1523.903614 | 1593.14 | 1662.376386 | 1.008316 |
| GO:0032412\_regulation\_of\_ion\_transmembrane\_transporter\_activity | 8 | 0 | 0.000000 | -0.000000 | 1580 | 1523.903614 | 1593.14 | 1662.376386 | 1.008316 |
| GO:0032613\_interleukin-10\_production | 8 | 0 | 0.000000 | -0.000000 | 1580 | 1523.903614 | 1593.14 | 1662.376386 | 1.008316 |
| GO:0033198\_response\_to\_ATP | 8 | 0 | 0.000000 | -0.000000 | 1580 | 1523.903614 | 1593.14 | 1662.376386 | 1.008316 |
| GO:0034284\_response\_to\_monosaccharide\_stimulus | 8 | 0 | 0.000000 | -0.000000 | 1580 | 1523.903614 | 1593.14 | 1662.376386 | 1.008316 |
| GO:0034728\_nucleosome\_organization | 8 | 0 | 0.000000 | -0.000000 | 1580 | 1523.903614 | 1593.14 | 1662.376386 | 1.008316 |
| GO:0035023\_regulation\_of\_Rho\_protein\_signal\_transduction | 8 | 0 | 0.000000 | -0.000000 | 1580 | 1523.903614 | 1593.14 | 1662.376386 | 1.008316 |
| GO:0035112\_genitalia\_morphogenesis | 8 | 0 | 0.000000 | -0.000000 | 1580 | 1523.903614 | 1593.14 | 1662.376386 | 1.008316 |
| GO:0040017\_positive\_regulation\_of\_locomotion | 8 | 0 | 0.000000 | -0.000000 | 1580 | 1523.903614 | 1593.14 | 1662.376386 | 1.008316 |
| GO:0040034\_regulation\_of\_development\_\_heterochronic | 8 | 0 | 0.000000 | -0.000000 | 1580 | 1523.903614 | 1593.14 | 1662.376386 | 1.008316 |
| GO:0042074\_cell\_migration\_involved\_in\_gastrulation | 8 | 0 | 0.000000 | -0.000000 | 1580 | 1523.903614 | 1593.14 | 1662.376386 | 1.008316 |
| GO:0042090\_interleukin-12\_biosynthetic\_process | 8 | 0 | 0.000000 | -0.000000 | 1580 | 1523.903614 | 1593.14 | 1662.376386 | 1.008316 |
| GO:0042092\_T-helper\_2\_type\_immune\_response | 8 | 0 | 0.000000 | -0.000000 | 1580 | 1523.903614 | 1593.14 | 1662.376386 | 1.008316 |
| GO:0042095\_interferon-gamma\_biosynthetic\_process | 8 | 0 | 0.000000 | -0.000000 | 1580 | 1523.903614 | 1593.14 | 1662.376386 | 1.008316 |
| GO:0042104\_positive\_regulation\_of\_activated\_T\_cell\_proliferation | 8 | 0 | 0.000000 | -0.000000 | 1580 | 1523.903614 | 1593.14 | 1662.376386 | 1.008316 |
| GO:0042226\_interleukin-6\_biosynthetic\_process | 8 | 0 | 0.000000 | -0.000000 | 1580 | 1523.903614 | 1593.14 | 1662.376386 | 1.008316 |
| GO:0042304\_regulation\_of\_fatty\_acid\_biosynthetic\_process | 8 | 0 | 0.000000 | -0.000000 | 1580 | 1523.903614 | 1593.14 | 1662.376386 | 1.008316 |
| GO:0042423\_catecholamine\_biosynthetic\_process | 8 | 0 | 0.000000 | -0.000000 | 1580 | 1523.903614 | 1593.14 | 1662.376386 | 1.008316 |
| GO:0042771\_DNA\_damage\_response\_\_signal\_transduction\_by\_p53\_class\_mediator\_resulting\_in\_induction\_of\_apoptosis | 8 | 0 | 0.000000 | -0.000000 | 1580 | 1523.903614 | 1593.14 | 1662.376386 | 1.008316 |
| GO:0042990\_regulation\_of\_transcription\_factor\_import\_into\_nucleus | 8 | 0 | 0.000000 | -0.000000 | 1580 | 1523.903614 | 1593.14 | 1662.376386 | 1.008316 |
| GO:0042991\_transcription\_factor\_import\_into\_nucleus | 8 | 0 | 0.000000 | -0.000000 | 1580 | 1523.903614 | 1593.14 | 1662.376386 | 1.008316 |
| GO:0043011\_myeloid\_dendritic\_cell\_differentiation | 8 | 0 | 0.000000 | -0.000000 | 1580 | 1523.903614 | 1593.14 | 1662.376386 | 1.008316 |
| GO:0043368\_positive\_T\_cell\_selection | 8 | 0 | 0.000000 | -0.000000 | 1580 | 1523.903614 | 1593.14 | 1662.376386 | 1.008316 |
| GO:0043370\_regulation\_of\_CD4-positive\_\_alpha\_beta\_T\_cell\_differentiation | 8 | 0 | 0.000000 | -0.000000 | 1580 | 1523.903614 | 1593.14 | 1662.376386 | 1.008316 |
| GO:0043542\_endothelial\_cell\_migration | 8 | 0 | 0.000000 | -0.000000 | 1580 | 1523.903614 | 1593.14 | 1662.376386 | 1.008316 |
| GO:0043616\_keratinocyte\_proliferation | 8 | 0 | 0.000000 | -0.000000 | 1580 | 1523.903614 | 1593.14 | 1662.376386 | 1.008316 |
| GO:0045075\_regulation\_of\_interleukin-12\_biosynthetic\_process | 8 | 0 | 0.000000 | -0.000000 | 1580 | 1523.903614 | 1593.14 | 1662.376386 | 1.008316 |
| GO:0045086\_positive\_regulation\_of\_interleukin-2\_biosynthetic\_process | 8 | 0 | 0.000000 | -0.000000 | 1580 | 1523.903614 | 1593.14 | 1662.376386 | 1.008316 |
| GO:0045351\_type\_I\_interferon\_biosynthetic\_process | 8 | 0 | 0.000000 | -0.000000 | 1580 | 1523.903614 | 1593.14 | 1662.376386 | 1.008316 |
| GO:0045408\_regulation\_of\_interleukin-6\_biosynthetic\_process | 8 | 0 | 0.000000 | -0.000000 | 1580 | 1523.903614 | 1593.14 | 1662.376386 | 1.008316 |
| GO:0045429\_positive\_regulation\_of\_nitric\_oxide\_biosynthetic\_process | 8 | 0 | 0.000000 | -0.000000 | 1580 | 1523.903614 | 1593.14 | 1662.376386 | 1.008316 |
| GO:0045494\_photoreceptor\_cell\_maintenance | 8 | 0 | 0.000000 | -0.000000 | 1580 | 1523.903614 | 1593.14 | 1662.376386 | 1.008316 |
| GO:0045686\_negative\_regulation\_of\_glial\_cell\_differentiation | 8 | 0 | 0.000000 | -0.000000 | 1580 | 1523.903614 | 1593.14 | 1662.376386 | 1.008316 |
| GO:0045910\_negative\_regulation\_of\_DNA\_recombination | 8 | 0 | 0.000000 | -0.000000 | 1580 | 1523.903614 | 1593.14 | 1662.376386 | 1.008316 |
| GO:0045921\_positive\_regulation\_of\_exocytosis | 8 | 0 | 0.000000 | -0.000000 | 1580 | 1523.903614 | 1593.14 | 1662.376386 | 1.008316 |
| GO:0045932\_negative\_regulation\_of\_muscle\_contraction | 8 | 0 | 0.000000 | -0.000000 | 1580 | 1523.903614 | 1593.14 | 1662.376386 | 1.008316 |
| GO:0046470\_phosphatidylcholine\_metabolic\_process | 8 | 0 | 0.000000 | -0.000000 | 1580 | 1523.903614 | 1593.14 | 1662.376386 | 1.008316 |
| GO:0048266\_behavioral\_response\_to\_pain | 8 | 0 | 0.000000 | -0.000000 | 1580 | 1523.903614 | 1593.14 | 1662.376386 | 1.008316 |
| GO:0048505\_regulation\_of\_timing\_of\_cell\_differentiation | 8 | 0 | 0.000000 | -0.000000 | 1580 | 1523.903614 | 1593.14 | 1662.376386 | 1.008316 |
| GO:0048520\_positive\_regulation\_of\_behavior | 8 | 0 | 0.000000 | -0.000000 | 1580 | 1523.903614 | 1593.14 | 1662.376386 | 1.008316 |
| GO:0048557\_embryonic\_digestive\_tract\_morphogenesis | 8 | 0 | 0.000000 | -0.000000 | 1580 | 1523.903614 | 1593.14 | 1662.376386 | 1.008316 |
| GO:0048638\_regulation\_of\_developmental\_growth | 8 | 0 | 0.000000 | -0.000000 | 1580 | 1523.903614 | 1593.14 | 1662.376386 | 1.008316 |
| GO:0048742\_regulation\_of\_skeletal\_muscle\_fiber\_development | 8 | 0 | 0.000000 | -0.000000 | 1580 | 1523.903614 | 1593.14 | 1662.376386 | 1.008316 |
| GO:0050707\_regulation\_of\_cytokine\_secretion | 8 | 0 | 0.000000 | -0.000000 | 1580 | 1523.903614 | 1593.14 | 1662.376386 | 1.008316 |
| GO:0050909\_sensory\_perception\_of\_taste | 8 | 0 | 0.000000 | -0.000000 | 1580 | 1523.903614 | 1593.14 | 1662.376386 | 1.008316 |
| GO:0050920\_regulation\_of\_chemotaxis | 8 | 0 | 0.000000 | -0.000000 | 1580 | 1523.903614 | 1593.14 | 1662.376386 | 1.008316 |
| GO:0050921\_positive\_regulation\_of\_chemotaxis | 8 | 0 | 0.000000 | -0.000000 | 1580 | 1523.903614 | 1593.14 | 1662.376386 | 1.008316 |
| GO:0050926\_regulation\_of\_positive\_chemotaxis | 8 | 0 | 0.000000 | -0.000000 | 1580 | 1523.903614 | 1593.14 | 1662.376386 | 1.008316 |
| GO:0050927\_positive\_regulation\_of\_positive\_chemotaxis | 8 | 0 | 0.000000 | -0.000000 | 1580 | 1523.903614 | 1593.14 | 1662.376386 | 1.008316 |
| GO:0050930\_induction\_of\_positive\_chemotaxis | 8 | 0 | 0.000000 | -0.000000 | 1580 | 1523.903614 | 1593.14 | 1662.376386 | 1.008316 |
| GO:0051084\_'de\_novo'\_posttranslational\_protein\_folding | 8 | 0 | 0.000000 | -0.000000 | 1580 | 1523.903614 | 1593.14 | 1662.376386 | 1.008316 |
| GO:0051181\_cofactor\_transport | 8 | 0 | 0.000000 | -0.000000 | 1580 | 1523.903614 | 1593.14 | 1662.376386 | 1.008316 |
| GO:0060043\_regulation\_of\_cardiac\_muscle\_cell\_proliferation | 8 | 0 | 0.000000 | -0.000000 | 1580 | 1523.903614 | 1593.14 | 1662.376386 | 1.008316 |
| GO:0060347\_heart\_trabecula\_formation | 8 | 0 | 0.000000 | -0.000000 | 1580 | 1523.903614 | 1593.14 | 1662.376386 | 1.008316 |
| GO:0060670\_branching\_involved\_in\_embryonic\_placenta\_morphogenesis | 8 | 0 | 0.000000 | -0.000000 | 1580 | 1523.903614 | 1593.14 | 1662.376386 | 1.008316 |
| GO:0060712\_spongiotrophoblast\_layer\_development | 8 | 0 | 0.000000 | -0.000000 | 1580 | 1523.903614 | 1593.14 | 1662.376386 | 1.008316 |
| GO:0070167\_regulation\_of\_biomineral\_formation | 8 | 0 | 0.000000 | -0.000000 | 1580 | 1523.903614 | 1593.14 | 1662.376386 | 1.008316 |
| GO:0070193\_synaptonemal\_complex\_organization | 8 | 0 | 0.000000 | -0.000000 | 1580 | 1523.903614 | 1593.14 | 1662.376386 | 1.008316 |
| GO:0070231\_T\_cell\_apoptosis | 8 | 0 | 0.000000 | -0.000000 | 1580 | 1523.903614 | 1593.14 | 1662.376386 | 1.008316 |
| GO:0070584\_mitochondrion\_morphogenesis | 8 | 0 | 0.000000 | -0.000000 | 1580 | 1523.903614 | 1593.14 | 1662.376386 | 1.008316 |
| GO:0000096\_sulfur\_amino\_acid\_metabolic\_process | 11 | 0 | 0.000000 | -0.000000 | 1659 | 1604.974777 | 1672.43 | 1739.885223 | 1.008095 |
| GO:0000271\_polysaccharide\_biosynthetic\_process | 11 | 0 | 0.000000 | -0.000000 | 1659 | 1604.974777 | 1672.43 | 1739.885223 | 1.008095 |
| GO:0000737\_DNA\_catabolic\_process\_\_endonucleolytic | 11 | 0 | 0.000000 | -0.000000 | 1659 | 1604.974777 | 1672.43 | 1739.885223 | 1.008095 |
| GO:0001837\_epithelial\_to\_mesenchymal\_transition | 11 | 0 | 0.000000 | -0.000000 | 1659 | 1604.974777 | 1672.43 | 1739.885223 | 1.008095 |
| GO:0001913\_T\_cell\_mediated\_cytotoxicity | 11 | 0 | 0.000000 | -0.000000 | 1659 | 1604.974777 | 1672.43 | 1739.885223 | 1.008095 |
| GO:0001952\_regulation\_of\_cell-matrix\_adhesion | 11 | 0 | 0.000000 | -0.000000 | 1659 | 1604.974777 | 1672.43 | 1739.885223 | 1.008095 |
| GO:0001963\_synaptic\_transmission\_\_dopaminergic | 11 | 0 | 0.000000 | -0.000000 | 1659 | 1604.974777 | 1672.43 | 1739.885223 | 1.008095 |
| GO:0002444\_myeloid\_leukocyte\_mediated\_immunity | 11 | 0 | 0.000000 | -0.000000 | 1659 | 1604.974777 | 1672.43 | 1739.885223 | 1.008095 |
| GO:0002467\_germinal\_center\_formation | 11 | 0 | 0.000000 | -0.000000 | 1659 | 1604.974777 | 1672.43 | 1739.885223 | 1.008095 |
| GO:0002758\_innate\_immune\_response-activating\_signal\_transduction | 11 | 0 | 0.000000 | -0.000000 | 1659 | 1604.974777 | 1672.43 | 1739.885223 | 1.008095 |
| GO:0006333\_chromatin\_assembly\_or\_disassembly | 11 | 0 | 0.000000 | -0.000000 | 1659 | 1604.974777 | 1672.43 | 1739.885223 | 1.008095 |
| GO:0006635\_fatty\_acid\_beta-oxidation | 11 | 0 | 0.000000 | -0.000000 | 1659 | 1604.974777 | 1672.43 | 1739.885223 | 1.008095 |
| GO:0006637\_acyl-CoA\_metabolic\_process | 11 | 0 | 0.000000 | -0.000000 | 1659 | 1604.974777 | 1672.43 | 1739.885223 | 1.008095 |
| GO:0006690\_icosanoid\_metabolic\_process | 11 | 0 | 0.000000 | -0.000000 | 1659 | 1604.974777 | 1672.43 | 1739.885223 | 1.008095 |
| GO:0006779\_porphyrin\_biosynthetic\_process | 11 | 0 | 0.000000 | -0.000000 | 1659 | 1604.974777 | 1672.43 | 1739.885223 | 1.008095 |
| GO:0007051\_spindle\_organization | 11 | 0 | 0.000000 | -0.000000 | 1659 | 1604.974777 | 1672.43 | 1739.885223 | 1.008095 |
| GO:0007088\_regulation\_of\_mitosis | 11 | 0 | 0.000000 | -0.000000 | 1659 | 1604.974777 | 1672.43 | 1739.885223 | 1.008095 |
| GO:0007159\_leukocyte\_adhesion | 11 | 0 | 0.000000 | -0.000000 | 1659 | 1604.974777 | 1672.43 | 1739.885223 | 1.008095 |
| GO:0007162\_negative\_regulation\_of\_cell\_adhesion | 11 | 0 | 0.000000 | -0.000000 | 1659 | 1604.974777 | 1672.43 | 1739.885223 | 1.008095 |
| GO:0007215\_glutamate\_signaling\_pathway | 11 | 0 | 0.000000 | -0.000000 | 1659 | 1604.974777 | 1672.43 | 1739.885223 | 1.008095 |
| GO:0007229\_integrin-mediated\_signaling\_pathway | 11 | 0 | 0.000000 | -0.000000 | 1659 | 1604.974777 | 1672.43 | 1739.885223 | 1.008095 |
| GO:0007260\_tyrosine\_phosphorylation\_of\_STAT\_protein | 11 | 0 | 0.000000 | -0.000000 | 1659 | 1604.974777 | 1672.43 | 1739.885223 | 1.008095 |
| GO:0008354\_germ\_cell\_migration | 11 | 0 | 0.000000 | -0.000000 | 1659 | 1604.974777 | 1672.43 | 1739.885223 | 1.008095 |
| GO:0008652\_cellular\_amino\_acid\_biosynthetic\_process | 11 | 0 | 0.000000 | -0.000000 | 1659 | 1604.974777 | 1672.43 | 1739.885223 | 1.008095 |
| GO:0009064\_glutamine\_family\_amino\_acid\_metabolic\_process | 11 | 0 | 0.000000 | -0.000000 | 1659 | 1604.974777 | 1672.43 | 1739.885223 | 1.008095 |
| GO:0009141\_nucleoside\_triphosphate\_metabolic\_process | 11 | 0 | 0.000000 | -0.000000 | 1659 | 1604.974777 | 1672.43 | 1739.885223 | 1.008095 |
| GO:0009166\_nucleotide\_catabolic\_process | 11 | 0 | 0.000000 | -0.000000 | 1659 | 1604.974777 | 1672.43 | 1739.885223 | 1.008095 |
| GO:0009409\_response\_to\_cold | 11 | 0 | 0.000000 | -0.000000 | 1659 | 1604.974777 | 1672.43 | 1739.885223 | 1.008095 |
| GO:0010259\_multicellular\_organismal\_aging | 11 | 0 | 0.000000 | -0.000000 | 1659 | 1604.974777 | 1672.43 | 1739.885223 | 1.008095 |
| GO:0014013\_regulation\_of\_gliogenesis | 11 | 0 | 0.000000 | -0.000000 | 1659 | 1604.974777 | 1672.43 | 1739.885223 | 1.008095 |
| GO:0014902\_myotube\_differentiation | 11 | 0 | 0.000000 | -0.000000 | 1659 | 1604.974777 | 1672.43 | 1739.885223 | 1.008095 |
| GO:0016079\_synaptic\_vesicle\_exocytosis | 11 | 0 | 0.000000 | -0.000000 | 1659 | 1604.974777 | 1672.43 | 1739.885223 | 1.008095 |
| GO:0021602\_cranial\_nerve\_morphogenesis | 11 | 0 | 0.000000 | -0.000000 | 1659 | 1604.974777 | 1672.43 | 1739.885223 | 1.008095 |
| GO:0021846\_cell\_proliferation\_in\_forebrain | 11 | 0 | 0.000000 | -0.000000 | 1659 | 1604.974777 | 1672.43 | 1739.885223 | 1.008095 |
| GO:0030238\_male\_sex\_determination | 11 | 0 | 0.000000 | -0.000000 | 1659 | 1604.974777 | 1672.43 | 1739.885223 | 1.008095 |
| GO:0030308\_negative\_regulation\_of\_cell\_growth | 11 | 0 | 0.000000 | -0.000000 | 1659 | 1604.974777 | 1672.43 | 1739.885223 | 1.008095 |
| GO:0030593\_neutrophil\_chemotaxis | 11 | 0 | 0.000000 | -0.000000 | 1659 | 1604.974777 | 1672.43 | 1739.885223 | 1.008095 |
| GO:0030856\_regulation\_of\_epithelial\_cell\_differentiation | 11 | 0 | 0.000000 | -0.000000 | 1659 | 1604.974777 | 1672.43 | 1739.885223 | 1.008095 |
| GO:0030878\_thyroid\_gland\_development | 11 | 0 | 0.000000 | -0.000000 | 1659 | 1604.974777 | 1672.43 | 1739.885223 | 1.008095 |
| GO:0031110\_regulation\_of\_microtubule\_polymerization\_or\_depolymerization | 11 | 0 | 0.000000 | -0.000000 | 1659 | 1604.974777 | 1672.43 | 1739.885223 | 1.008095 |
| GO:0031646\_positive\_regulation\_of\_neurological\_system\_process | 11 | 0 | 0.000000 | -0.000000 | 1659 | 1604.974777 | 1672.43 | 1739.885223 | 1.008095 |
| GO:0031647\_regulation\_of\_protein\_stability | 11 | 0 | 0.000000 | -0.000000 | 1659 | 1604.974777 | 1672.43 | 1739.885223 | 1.008095 |
| GO:0032655\_regulation\_of\_interleukin-12\_production | 11 | 0 | 0.000000 | -0.000000 | 1659 | 1604.974777 | 1672.43 | 1739.885223 | 1.008095 |
| GO:0033014\_tetrapyrrole\_biosynthetic\_process | 11 | 0 | 0.000000 | -0.000000 | 1659 | 1604.974777 | 1672.43 | 1739.885223 | 1.008095 |
| GO:0033059\_cellular\_pigmentation | 11 | 0 | 0.000000 | -0.000000 | 1659 | 1604.974777 | 1672.43 | 1739.885223 | 1.008095 |
| GO:0033559\_unsaturated\_fatty\_acid\_metabolic\_process | 11 | 0 | 0.000000 | -0.000000 | 1659 | 1604.974777 | 1672.43 | 1739.885223 | 1.008095 |
| GO:0034762\_regulation\_of\_transmembrane\_transport | 11 | 0 | 0.000000 | -0.000000 | 1659 | 1604.974777 | 1672.43 | 1739.885223 | 1.008095 |
| GO:0035176\_social\_behavior | 11 | 0 | 0.000000 | -0.000000 | 1659 | 1604.974777 | 1672.43 | 1739.885223 | 1.008095 |
| GO:0042036\_negative\_regulation\_of\_cytokine\_biosynthetic\_process | 11 | 0 | 0.000000 | -0.000000 | 1659 | 1604.974777 | 1672.43 | 1739.885223 | 1.008095 |
| GO:0042219\_cellular\_amino\_acid\_derivative\_catabolic\_process | 11 | 0 | 0.000000 | -0.000000 | 1659 | 1604.974777 | 1672.43 | 1739.885223 | 1.008095 |
| GO:0042401\_biogenic\_amine\_biosynthetic\_process | 11 | 0 | 0.000000 | -0.000000 | 1659 | 1604.974777 | 1672.43 | 1739.885223 | 1.008095 |
| GO:0042439\_ethanolamine\_and\_derivative\_metabolic\_process | 11 | 0 | 0.000000 | -0.000000 | 1659 | 1604.974777 | 1672.43 | 1739.885223 | 1.008095 |
| GO:0042542\_response\_to\_hydrogen\_peroxide | 11 | 0 | 0.000000 | -0.000000 | 1659 | 1604.974777 | 1672.43 | 1739.885223 | 1.008095 |
| GO:0042551\_neuron\_maturation | 11 | 0 | 0.000000 | -0.000000 | 1659 | 1604.974777 | 1672.43 | 1739.885223 | 1.008095 |
| GO:0043270\_positive\_regulation\_of\_ion\_transport | 11 | 0 | 0.000000 | -0.000000 | 1659 | 1604.974777 | 1672.43 | 1739.885223 | 1.008095 |
| GO:0043576\_regulation\_of\_respiratory\_gaseous\_exchange | 11 | 0 | 0.000000 | -0.000000 | 1659 | 1604.974777 | 1672.43 | 1739.885223 | 1.008095 |
| GO:0045026\_plasma\_membrane\_fusion | 11 | 0 | 0.000000 | -0.000000 | 1659 | 1604.974777 | 1672.43 | 1739.885223 | 1.008095 |
| GO:0045055\_regulated\_secretory\_pathway | 11 | 0 | 0.000000 | -0.000000 | 1659 | 1604.974777 | 1672.43 | 1739.885223 | 1.008095 |
| GO:0045216\_cell-cell\_junction\_organization | 11 | 0 | 0.000000 | -0.000000 | 1659 | 1604.974777 | 1672.43 | 1739.885223 | 1.008095 |
| GO:0045685\_regulation\_of\_glial\_cell\_differentiation | 11 | 0 | 0.000000 | -0.000000 | 1659 | 1604.974777 | 1672.43 | 1739.885223 | 1.008095 |
| GO:0045834\_positive\_regulation\_of\_lipid\_metabolic\_process | 11 | 0 | 0.000000 | -0.000000 | 1659 | 1604.974777 | 1672.43 | 1739.885223 | 1.008095 |
| GO:0046323\_glucose\_import | 11 | 0 | 0.000000 | -0.000000 | 1659 | 1604.974777 | 1672.43 | 1739.885223 | 1.008095 |
| GO:0046425\_regulation\_of\_JAK-STAT\_cascade | 11 | 0 | 0.000000 | -0.000000 | 1659 | 1604.974777 | 1672.43 | 1739.885223 | 1.008095 |
| GO:0046716\_muscle\_maintenance | 11 | 0 | 0.000000 | -0.000000 | 1659 | 1604.974777 | 1672.43 | 1739.885223 | 1.008095 |
| GO:0046928\_regulation\_of\_neurotransmitter\_secretion | 11 | 0 | 0.000000 | -0.000000 | 1659 | 1604.974777 | 1672.43 | 1739.885223 | 1.008095 |
| GO:0048009\_insulin-like\_growth\_factor\_receptor\_signaling\_pathway | 11 | 0 | 0.000000 | -0.000000 | 1659 | 1604.974777 | 1672.43 | 1739.885223 | 1.008095 |
| GO:0048193\_Golgi\_vesicle\_transport | 11 | 0 | 0.000000 | -0.000000 | 1659 | 1604.974777 | 1672.43 | 1739.885223 | 1.008095 |
| GO:0048678\_response\_to\_axon\_injury | 11 | 0 | 0.000000 | -0.000000 | 1659 | 1604.974777 | 1672.43 | 1739.885223 | 1.008095 |
| GO:0048745\_smooth\_muscle\_tissue\_development | 11 | 0 | 0.000000 | -0.000000 | 1659 | 1604.974777 | 1672.43 | 1739.885223 | 1.008095 |
| GO:0050806\_positive\_regulation\_of\_synaptic\_transmission | 11 | 0 | 0.000000 | -0.000000 | 1659 | 1604.974777 | 1672.43 | 1739.885223 | 1.008095 |
| GO:0050807\_regulation\_of\_synapse\_organization | 11 | 0 | 0.000000 | -0.000000 | 1659 | 1604.974777 | 1672.43 | 1739.885223 | 1.008095 |
| GO:0050830\_defense\_response\_to\_Gram-positive\_bacterium | 11 | 0 | 0.000000 | -0.000000 | 1659 | 1604.974777 | 1672.43 | 1739.885223 | 1.008095 |
| GO:0050854\_regulation\_of\_antigen\_receptor-mediated\_signaling\_pathway | 11 | 0 | 0.000000 | -0.000000 | 1659 | 1604.974777 | 1672.43 | 1739.885223 | 1.008095 |
| GO:0051092\_positive\_regulation\_of\_NF-kappaB\_transcription\_factor\_activity | 11 | 0 | 0.000000 | -0.000000 | 1659 | 1604.974777 | 1672.43 | 1739.885223 | 1.008095 |
| GO:0051494\_negative\_regulation\_of\_cytoskeleton\_organization | 11 | 0 | 0.000000 | -0.000000 | 1659 | 1604.974777 | 1672.43 | 1739.885223 | 1.008095 |
| GO:0051648\_vesicle\_localization | 11 | 0 | 0.000000 | -0.000000 | 1659 | 1604.974777 | 1672.43 | 1739.885223 | 1.008095 |
| GO:0051783\_regulation\_of\_nuclear\_division | 11 | 0 | 0.000000 | -0.000000 | 1659 | 1604.974777 | 1672.43 | 1739.885223 | 1.008095 |
| GO:0051971\_positive\_regulation\_of\_transmission\_of\_nerve\_impulse | 11 | 0 | 0.000000 | -0.000000 | 1659 | 1604.974777 | 1672.43 | 1739.885223 | 1.008095 |
| GO:0060004\_reflex | 11 | 0 | 0.000000 | -0.000000 | 1659 | 1604.974777 | 1672.43 | 1739.885223 | 1.008095 |
| GO:0007369\_gastrulation | 63 | 0 | 0.000000 | -0.000000 | 1664 | 1611.909262 | 1678.66 | 1745.410738 | 1.008810 |
| GO:0009165\_nucleotide\_biosynthetic\_process | 63 | 0 | 0.000000 | -0.000000 | 1664 | 1611.909262 | 1678.66 | 1745.410738 | 1.008810 |
| GO:0051186\_cofactor\_metabolic\_process | 63 | 0 | 0.000000 | -0.000000 | 1664 | 1611.909262 | 1678.66 | 1745.410738 | 1.008810 |
| GO:0070662\_mast\_cell\_proliferation | 63 | 0 | 0.000000 | -0.000000 | 1664 | 1611.909262 | 1678.66 | 1745.410738 | 1.008810 |
| GO:0070666\_regulation\_of\_mast\_cell\_proliferation | 63 | 0 | 0.000000 | -0.000000 | 1664 | 1611.909262 | 1678.66 | 1745.410738 | 1.008810 |
| GO:0002757\_immune\_response-activating\_signal\_transduction | 47 | 0 | 0.000000 | -0.000000 | 1674 | 1623.740039 | 1689.24 | 1754.739961 | 1.009104 |
| GO:0006140\_regulation\_of\_nucleotide\_metabolic\_process | 47 | 0 | 0.000000 | -0.000000 | 1674 | 1623.740039 | 1689.24 | 1754.739961 | 1.009104 |
| GO:0030183\_B\_cell\_differentiation | 47 | 0 | 0.000000 | -0.000000 | 1674 | 1623.740039 | 1689.24 | 1754.739961 | 1.009104 |
| GO:0030799\_regulation\_of\_cyclic\_nucleotide\_metabolic\_process | 47 | 0 | 0.000000 | -0.000000 | 1674 | 1623.740039 | 1689.24 | 1754.739961 | 1.009104 |
| GO:0031667\_response\_to\_nutrient\_levels | 47 | 0 | 0.000000 | -0.000000 | 1674 | 1623.740039 | 1689.24 | 1754.739961 | 1.009104 |
| GO:0034754\_cellular\_hormone\_metabolic\_process | 47 | 0 | 0.000000 | -0.000000 | 1674 | 1623.740039 | 1689.24 | 1754.739961 | 1.009104 |
| GO:0045087\_innate\_immune\_response | 47 | 0 | 0.000000 | -0.000000 | 1674 | 1623.740039 | 1689.24 | 1754.739961 | 1.009104 |
| GO:0045619\_regulation\_of\_lymphocyte\_differentiation | 47 | 0 | 0.000000 | -0.000000 | 1674 | 1623.740039 | 1689.24 | 1754.739961 | 1.009104 |
| GO:0048871\_multicellular\_organismal\_homeostasis | 47 | 0 | 0.000000 | -0.000000 | 1674 | 1623.740039 | 1689.24 | 1754.739961 | 1.009104 |
| GO:0060627\_regulation\_of\_vesicle-mediated\_transport | 47 | 0 | 0.000000 | -0.000000 | 1674 | 1623.740039 | 1689.24 | 1754.739961 | 1.009104 |
| GO:0006461\_protein\_complex\_assembly | 78 | 0 | 0.000000 | -0.000000 | 1679 | 1629.343552 | 1694.06 | 1758.776448 | 1.008970 |
| GO:0030326\_embryonic\_limb\_morphogenesis | 78 | 0 | 0.000000 | -0.000000 | 1679 | 1629.343552 | 1694.06 | 1758.776448 | 1.008970 |
| GO:0035113\_embryonic\_appendage\_morphogenesis | 78 | 0 | 0.000000 | -0.000000 | 1679 | 1629.343552 | 1694.06 | 1758.776448 | 1.008970 |
| GO:0051251\_positive\_regulation\_of\_lymphocyte\_activation | 78 | 0 | 0.000000 | -0.000000 | 1679 | 1629.343552 | 1694.06 | 1758.776448 | 1.008970 |
| GO:0070271\_protein\_complex\_biogenesis | 78 | 0 | 0.000000 | -0.000000 | 1679 | 1629.343552 | 1694.06 | 1758.776448 | 1.008970 |
| GO:0007281\_germ\_cell\_development | 75 | 0 | 0.000000 | -0.000000 | 1682 | 1632.457350 | 1696.84 | 1761.222650 | 1.008823 |
| GO:0048589\_developmental\_growth | 75 | 0 | 0.000000 | -0.000000 | 1682 | 1632.457350 | 1696.84 | 1761.222650 | 1.008823 |
| GO:0051050\_positive\_regulation\_of\_transport | 75 | 0 | 0.000000 | -0.000000 | 1682 | 1632.457350 | 1696.84 | 1761.222650 | 1.008823 |
| GO:0003007\_heart\_morphogenesis | 67 | 0 | 0.000000 | -0.000000 | 1687 | 1636.633063 | 1700.4 | 1764.166937 | 1.007943 |
| GO:0009791\_post-embryonic\_development | 67 | 0 | 0.000000 | -0.000000 | 1687 | 1636.633063 | 1700.4 | 1764.166937 | 1.007943 |
| GO:0031347\_regulation\_of\_defense\_response | 67 | 0 | 0.000000 | -0.000000 | 1687 | 1636.633063 | 1700.4 | 1764.166937 | 1.007943 |
| GO:0042445\_hormone\_metabolic\_process | 67 | 0 | 0.000000 | -0.000000 | 1687 | 1636.633063 | 1700.4 | 1764.166937 | 1.007943 |
| GO:0051247\_positive\_regulation\_of\_protein\_metabolic\_process | 67 | 0 | 0.000000 | -0.000000 | 1687 | 1636.633063 | 1700.4 | 1764.166937 | 1.007943 |
| GO:0044093\_positive\_regulation\_of\_molecular\_function | 173 | 0 | 0.000000 | -0.000000 | 1688 | 1638.183400 | 1701.52 | 1764.856600 | 1.008009 |
| GO:0046903\_secretion | 175 | 0 | 0.000000 | -0.000000 | 1689 | 1639.824049 | 1702.72 | 1765.615951 | 1.008123 |
| GO:0006916\_anti-apoptosis | 62 | 0 | 0.000000 | -0.000000 | 1695 | 1650.217666 | 1711.8 | 1773.382334 | 1.009912 |
| GO:0021537\_telencephalon\_development | 62 | 0 | 0.000000 | -0.000000 | 1695 | 1650.217666 | 1711.8 | 1773.382334 | 1.009912 |
| GO:0030155\_regulation\_of\_cell\_adhesion | 62 | 0 | 0.000000 | -0.000000 | 1695 | 1650.217666 | 1711.8 | 1773.382334 | 1.009912 |
| GO:0032944\_regulation\_of\_mononuclear\_cell\_proliferation | 62 | 0 | 0.000000 | -0.000000 | 1695 | 1650.217666 | 1711.8 | 1773.382334 | 1.009912 |
| GO:0040014\_regulation\_of\_multicellular\_organism\_growth | 62 | 0 | 0.000000 | -0.000000 | 1695 | 1650.217666 | 1711.8 | 1773.382334 | 1.009912 |
| GO:0050670\_regulation\_of\_lymphocyte\_proliferation | 62 | 0 | 0.000000 | -0.000000 | 1695 | 1650.217666 | 1711.8 | 1773.382334 | 1.009912 |
| GO:0001934\_positive\_regulation\_of\_protein\_amino\_acid\_phosphorylation | 29 | 0 | 0.000000 | -0.000000 | 1714 | 1670.231586 | 1730.94 | 1791.648414 | 1.009883 |
| GO:0006417\_regulation\_of\_translation | 29 | 0 | 0.000000 | -0.000000 | 1714 | 1670.231586 | 1730.94 | 1791.648414 | 1.009883 |
| GO:0006641\_triglyceride\_metabolic\_process | 29 | 0 | 0.000000 | -0.000000 | 1714 | 1670.231586 | 1730.94 | 1791.648414 | 1.009883 |
| GO:0006909\_phagocytosis | 29 | 0 | 0.000000 | -0.000000 | 1714 | 1670.231586 | 1730.94 | 1791.648414 | 1.009883 |
| GO:0007190\_activation\_of\_adenylate\_cyclase\_activity | 29 | 0 | 0.000000 | -0.000000 | 1714 | 1670.231586 | 1730.94 | 1791.648414 | 1.009883 |
| GO:0010564\_regulation\_of\_cell\_cycle\_process | 29 | 0 | 0.000000 | -0.000000 | 1714 | 1670.231586 | 1730.94 | 1791.648414 | 1.009883 |
| GO:0016447\_somatic\_recombination\_of\_immunoglobulin\_gene\_segments | 29 | 0 | 0.000000 | -0.000000 | 1714 | 1670.231586 | 1730.94 | 1791.648414 | 1.009883 |
| GO:0021761\_limbic\_system\_development | 29 | 0 | 0.000000 | -0.000000 | 1714 | 1670.231586 | 1730.94 | 1791.648414 | 1.009883 |
| GO:0042176\_regulation\_of\_protein\_catabolic\_process | 29 | 0 | 0.000000 | -0.000000 | 1714 | 1670.231586 | 1730.94 | 1791.648414 | 1.009883 |
| GO:0042490\_mechanoreceptor\_differentiation | 29 | 0 | 0.000000 | -0.000000 | 1714 | 1670.231586 | 1730.94 | 1791.648414 | 1.009883 |
| GO:0043281\_regulation\_of\_caspase\_activity | 29 | 0 | 0.000000 | -0.000000 | 1714 | 1670.231586 | 1730.94 | 1791.648414 | 1.009883 |
| GO:0044087\_regulation\_of\_cellular\_component\_biogenesis | 29 | 0 | 0.000000 | -0.000000 | 1714 | 1670.231586 | 1730.94 | 1791.648414 | 1.009883 |
| GO:0044270\_nitrogen\_compound\_catabolic\_process | 29 | 0 | 0.000000 | -0.000000 | 1714 | 1670.231586 | 1730.94 | 1791.648414 | 1.009883 |
| GO:0045621\_positive\_regulation\_of\_lymphocyte\_differentiation | 29 | 0 | 0.000000 | -0.000000 | 1714 | 1670.231586 | 1730.94 | 1791.648414 | 1.009883 |
| GO:0046634\_regulation\_of\_alpha-beta\_T\_cell\_activation | 29 | 0 | 0.000000 | -0.000000 | 1714 | 1670.231586 | 1730.94 | 1791.648414 | 1.009883 |
| GO:0051301\_cell\_division | 29 | 0 | 0.000000 | -0.000000 | 1714 | 1670.231586 | 1730.94 | 1791.648414 | 1.009883 |
[truncated: 256,072 more chars]
