# Supplementary material for: RedundancyMiner: De-replication of redundant GO categories in microarray and proteomics analysis
Source: BMC Bioinformatics. 2011 Feb 10;12:52. doi: 10.1186/1471-2105-12-52 (PMC3223614; doi:10.1186/1471-2105-12-52)
Supplement: Additional file 8 — Retinal development HTGM download. compressed package of the results of running HTGM on the retinal development genes list. [file 1471-2105-12-52-S8.ZIP › SCENARIO_2_MODIFIED/total.txt.total.txt.dir/Exp1_BestClusterMap_LEIGS_KM_24.csv.join.11.txt.dir/Exp1_BestClusterMap_LEIGS_KM_24.csv.join.11.txt.change.gce.html]

Gene Category Report for Exp1\_BestClusterMap\_LEIGS\_KM\_24.csv.join.11.txt

# Gene Category Report for Exp1\_BestClusterMap\_LEIGS\_KM\_24.csv.join.11.txt

| HYPERLINKED GO CATEGORY | HYPERLINKED GENE NAME | TOTAL GENES | CHANGED GENES | ENRICHMENT | LOG10(p) | CUMULATIVE NUMBER OF CATEGORIES | CUMULATIVE RANDOMS MEAN | FALSE DISCOVERY RATE |
| --- | --- | --- | --- | --- | --- | --- | --- | --- |
| GO:0006346\_methylation-dependent\_chromatin\_silencing | DNMT3B | 5 | 2 | 70.846154 | -3.517987 | 1 | 0.19 | 0.190000 |
| GO:0006346\_methylation-dependent\_chromatin\_silencing | HELLS | 5 | 2 | 70.846154 | -3.517987 | 1 | 0.19 | 0.190000 |
| GO:0006342\_chromatin\_silencing | DNMT3B | 10 | 2 | 35.423077 | -2.872325 | 3 | 0.82 | 0.273333 |
| GO:0006342\_chromatin\_silencing | HELLS | 10 | 2 | 35.423077 | -2.872325 | 3 | 0.82 | 0.273333 |
| GO:0045814\_negative\_regulation\_of\_gene\_expression\_\_epigenetic | DNMT3B | 10 | 2 | 35.423077 | -2.872325 | 3 | 0.82 | 0.273333 |
| GO:0045814\_negative\_regulation\_of\_gene\_expression\_\_epigenetic | HELLS | 10 | 2 | 35.423077 | -2.872325 | 3 | 0.82 | 0.273333 |
| GO:0006304\_DNA\_modification | DNMT3B | 14 | 2 | 25.302198 | -2.572529 | 6 | 1.53 | 0.255000 |
| GO:0006304\_DNA\_modification | HELLS | 14 | 2 | 25.302198 | -2.572529 | 6 | 1.53 | 0.255000 |
| GO:0006305\_DNA\_alkylation | DNMT3B | 14 | 2 | 25.302198 | -2.572529 | 6 | 1.53 | 0.255000 |
| GO:0006305\_DNA\_alkylation | HELLS | 14 | 2 | 25.302198 | -2.572529 | 6 | 1.53 | 0.255000 |
| GO:0006306\_DNA\_methylation | DNMT3B | 14 | 2 | 25.302198 | -2.572529 | 6 | 1.53 | 0.255000 |
| GO:0006306\_DNA\_methylation | HELLS | 14 | 2 | 25.302198 | -2.572529 | 6 | 1.53 | 0.255000 |
| GO:0043412\_biopolymer\_modification | DKC1 | 458 | 8 | 3.093719 | -2.553849 | 7 | 1.55 | 0.221429 |
| GO:0043412\_biopolymer\_modification | NLK | 458 | 8 | 3.093719 | -2.553849 | 7 | 1.55 | 0.221429 |
| GO:0043412\_biopolymer\_modification | MKNK2 | 458 | 8 | 3.093719 | -2.553849 | 7 | 1.55 | 0.221429 |
| GO:0043412\_biopolymer\_modification | SKP2 | 458 | 8 | 3.093719 | -2.553849 | 7 | 1.55 | 0.221429 |
| GO:0043412\_biopolymer\_modification | DNMT3B | 458 | 8 | 3.093719 | -2.553849 | 7 | 1.55 | 0.221429 |
| GO:0043412\_biopolymer\_modification | PTPN12 | 458 | 8 | 3.093719 | -2.553849 | 7 | 1.55 | 0.221429 |
| GO:0043412\_biopolymer\_modification | HELLS | 458 | 8 | 3.093719 | -2.553849 | 7 | 1.55 | 0.221429 |
| GO:0043412\_biopolymer\_modification | PTPN11 | 458 | 8 | 3.093719 | -2.553849 | 7 | 1.55 | 0.221429 |
| GO:0044238\_primary\_metabolic\_process | RAD51AP1 | 1905 | 18 | 1.673531 | -2.428729 | 8 | 2.03 | 0.253750 |
| GO:0044238\_primary\_metabolic\_process | STRAP | 1905 | 18 | 1.673531 | -2.428729 | 8 | 2.03 | 0.253750 |
| GO:0044238\_primary\_metabolic\_process | GPBP1 | 1905 | 18 | 1.673531 | -2.428729 | 8 | 2.03 | 0.253750 |
| GO:0044238\_primary\_metabolic\_process | NLK | 1905 | 18 | 1.673531 | -2.428729 | 8 | 2.03 | 0.253750 |
| GO:0044238\_primary\_metabolic\_process | OTX2 | 1905 | 18 | 1.673531 | -2.428729 | 8 | 2.03 | 0.253750 |
| GO:0044238\_primary\_metabolic\_process | MKNK2 | 1905 | 18 | 1.673531 | -2.428729 | 8 | 2.03 | 0.253750 |
| GO:0044238\_primary\_metabolic\_process | SKP2 | 1905 | 18 | 1.673531 | -2.428729 | 8 | 2.03 | 0.253750 |
| GO:0044238\_primary\_metabolic\_process | AZIN1 | 1905 | 18 | 1.673531 | -2.428729 | 8 | 2.03 | 0.253750 |
| GO:0044238\_primary\_metabolic\_process | PTPN12 | 1905 | 18 | 1.673531 | -2.428729 | 8 | 2.03 | 0.253750 |
| GO:0044238\_primary\_metabolic\_process | PTPN11 | 1905 | 18 | 1.673531 | -2.428729 | 8 | 2.03 | 0.253750 |
| GO:0044238\_primary\_metabolic\_process | QK | 1905 | 18 | 1.673531 | -2.428729 | 8 | 2.03 | 0.253750 |
| GO:0044238\_primary\_metabolic\_process | ACSM3 | 1905 | 18 | 1.673531 | -2.428729 | 8 | 2.03 | 0.253750 |
| GO:0044238\_primary\_metabolic\_process | DKC1 | 1905 | 18 | 1.673531 | -2.428729 | 8 | 2.03 | 0.253750 |
| GO:0044238\_primary\_metabolic\_process | ALDH1A7 | 1905 | 18 | 1.673531 | -2.428729 | 8 | 2.03 | 0.253750 |
| GO:0044238\_primary\_metabolic\_process | DNMT3B | 1905 | 18 | 1.673531 | -2.428729 | 8 | 2.03 | 0.253750 |
| GO:0044238\_primary\_metabolic\_process | FOXD1 | 1905 | 18 | 1.673531 | -2.428729 | 8 | 2.03 | 0.253750 |
| GO:0044238\_primary\_metabolic\_process | HELLS | 1905 | 18 | 1.673531 | -2.428729 | 8 | 2.03 | 0.253750 |
| GO:0044238\_primary\_metabolic\_process | RBM17 | 1905 | 18 | 1.673531 | -2.428729 | 8 | 2.03 | 0.253750 |
| GO:0016458\_gene\_silencing | DNMT3B | 18 | 2 | 19.679487 | -2.352906 | 9 | 2.5 | 0.277778 |
| GO:0016458\_gene\_silencing | HELLS | 18 | 2 | 19.679487 | -2.352906 | 9 | 2.5 | 0.277778 |
| GO:0006916\_anti-apoptosis | SKP2 | 62 | 3 | 8.570099 | -2.314551 | 10 | 2.59 | 0.259000 |
| GO:0006916\_anti-apoptosis | API5 | 62 | 3 | 8.570099 | -2.314551 | 10 | 2.59 | 0.259000 |
| GO:0006916\_anti-apoptosis | HELLS | 62 | 3 | 8.570099 | -2.314551 | 10 | 2.59 | 0.259000 |
| GO:0008152\_metabolic\_process | RAD51AP1 | 2133 | 19 | 1.577680 | -2.288565 | 11 | 2.83 | 0.257273 |
| GO:0008152\_metabolic\_process | STRAP | 2133 | 19 | 1.577680 | -2.288565 | 11 | 2.83 | 0.257273 |
| GO:0008152\_metabolic\_process | GPBP1 | 2133 | 19 | 1.577680 | -2.288565 | 11 | 2.83 | 0.257273 |
| GO:0008152\_metabolic\_process | NLK | 2133 | 19 | 1.577680 | -2.288565 | 11 | 2.83 | 0.257273 |
| GO:0008152\_metabolic\_process | OTX2 | 2133 | 19 | 1.577680 | -2.288565 | 11 | 2.83 | 0.257273 |
| GO:0008152\_metabolic\_process | MKNK2 | 2133 | 19 | 1.577680 | -2.288565 | 11 | 2.83 | 0.257273 |
| GO:0008152\_metabolic\_process | SKP2 | 2133 | 19 | 1.577680 | -2.288565 | 11 | 2.83 | 0.257273 |
| GO:0008152\_metabolic\_process | AZIN1 | 2133 | 19 | 1.577680 | -2.288565 | 11 | 2.83 | 0.257273 |
| GO:0008152\_metabolic\_process | PTPN12 | 2133 | 19 | 1.577680 | -2.288565 | 11 | 2.83 | 0.257273 |
| GO:0008152\_metabolic\_process | PTPN11 | 2133 | 19 | 1.577680 | -2.288565 | 11 | 2.83 | 0.257273 |
| GO:0008152\_metabolic\_process | QK | 2133 | 19 | 1.577680 | -2.288565 | 11 | 2.83 | 0.257273 |
| GO:0008152\_metabolic\_process | ACSM3 | 2133 | 19 | 1.577680 | -2.288565 | 11 | 2.83 | 0.257273 |
| GO:0008152\_metabolic\_process | TAF10 | 2133 | 19 | 1.577680 | -2.288565 | 11 | 2.83 | 0.257273 |
| GO:0008152\_metabolic\_process | DKC1 | 2133 | 19 | 1.577680 | -2.288565 | 11 | 2.83 | 0.257273 |
| GO:0008152\_metabolic\_process | ALDH1A7 | 2133 | 19 | 1.577680 | -2.288565 | 11 | 2.83 | 0.257273 |
| GO:0008152\_metabolic\_process | DNMT3B | 2133 | 19 | 1.577680 | -2.288565 | 11 | 2.83 | 0.257273 |
| GO:0008152\_metabolic\_process | FOXD1 | 2133 | 19 | 1.577680 | -2.288565 | 11 | 2.83 | 0.257273 |
| GO:0008152\_metabolic\_process | HELLS | 2133 | 19 | 1.577680 | -2.288565 | 11 | 2.83 | 0.257273 |
| GO:0008152\_metabolic\_process | RBM17 | 2133 | 19 | 1.577680 | -2.288565 | 11 | 2.83 | 0.257273 |
| GO:0000154\_rRNA\_modification | DKC1 | 1 | 1 |  |  |  |  |  |  |
| GO:0031055\_chromatin\_remodeling\_at\_centromere | HELLS | 1 | 1 |  |  |  |  |  |  |
| GO:0031507\_heterochromatin\_formation | HELLS | 1 | 1 |  |  |  |  |  |  |
| GO:0031508\_centromeric\_heterochromatin\_formation | HELLS | 1 | 1 |  |  |  |  |  |  |
| GO:0034508\_centromere\_complex\_assembly | HELLS | 1 | 1 |  |  |  |  |  |  |
| GO:0042706\_eye\_photoreceptor\_cell\_fate\_commitment | OTX2 | 1 | 1 |  |  |  |  |  |  |
| GO:0043400\_cortisol\_secretion | PTPN11 | 1 | 1 |  |  |  |  |  |  |
| GO:0046552\_photoreceptor\_cell\_fate\_commitment | OTX2 | 1 | 1 |  |  |  |  |  |  |
| GO:0051462\_regulation\_of\_cortisol\_secretion | PTPN11 | 1 | 1 |  |  |  |  |  |  |
| GO:0051463\_negative\_regulation\_of\_cortisol\_secretion | PTPN11 | 1 | 1 |  |  |  |  |  |  |
| GO:0060125\_negative\_regulation\_of\_growth\_hormone\_secretion | PTPN11 | 1 | 1 |  |  |  |  |  |  |
| GO:0070365\_hepatocyte\_differentiation | TAF10 | 1 | 1 |  |  |  |  |  |  |
| GO:0070828\_heterochromatin\_organization | HELLS | 1 | 1 |  |  |  |  |  |  |
| GO:0044237\_cellular\_metabolic\_process | RAD51AP1 | 1974 | 18 | 1.615034 | -2.229483 | 12 | 3.1 | 0.258333 |
| GO:0044237\_cellular\_metabolic\_process | STRAP | 1974 | 18 | 1.615034 | -2.229483 | 12 | 3.1 | 0.258333 |
| GO:0044237\_cellular\_metabolic\_process | GPBP1 | 1974 | 18 | 1.615034 | -2.229483 | 12 | 3.1 | 0.258333 |
| GO:0044237\_cellular\_metabolic\_process | NLK | 1974 | 18 | 1.615034 | -2.229483 | 12 | 3.1 | 0.258333 |
| GO:0044237\_cellular\_metabolic\_process | OTX2 | 1974 | 18 | 1.615034 | -2.229483 | 12 | 3.1 | 0.258333 |
| GO:0044237\_cellular\_metabolic\_process | SKP2 | 1974 | 18 | 1.615034 | -2.229483 | 12 | 3.1 | 0.258333 |
| GO:0044237\_cellular\_metabolic\_process | MKNK2 | 1974 | 18 | 1.615034 | -2.229483 | 12 | 3.1 | 0.258333 |
| GO:0044237\_cellular\_metabolic\_process | AZIN1 | 1974 | 18 | 1.615034 | -2.229483 | 12 | 3.1 | 0.258333 |
| GO:0044237\_cellular\_metabolic\_process | PTPN12 | 1974 | 18 | 1.615034 | -2.229483 | 12 | 3.1 | 0.258333 |
| GO:0044237\_cellular\_metabolic\_process | PTPN11 | 1974 | 18 | 1.615034 | -2.229483 | 12 | 3.1 | 0.258333 |
| GO:0044237\_cellular\_metabolic\_process | QK | 1974 | 18 | 1.615034 | -2.229483 | 12 | 3.1 | 0.258333 |
| GO:0044237\_cellular\_metabolic\_process | ACSM3 | 1974 | 18 | 1.615034 | -2.229483 | 12 | 3.1 | 0.258333 |
| GO:0044237\_cellular\_metabolic\_process | DKC1 | 1974 | 18 | 1.615034 | -2.229483 | 12 | 3.1 | 0.258333 |
| GO:0044237\_cellular\_metabolic\_process | ALDH1A7 | 1974 | 18 | 1.615034 | -2.229483 | 12 | 3.1 | 0.258333 |
| GO:0044237\_cellular\_metabolic\_process | DNMT3B | 1974 | 18 | 1.615034 | -2.229483 | 12 | 3.1 | 0.258333 |
| GO:0044237\_cellular\_metabolic\_process | FOXD1 | 1974 | 18 | 1.615034 | -2.229483 | 12 | 3.1 | 0.258333 |
| GO:0044237\_cellular\_metabolic\_process | HELLS | 1974 | 18 | 1.615034 | -2.229483 | 12 | 3.1 | 0.258333 |
| GO:0044237\_cellular\_metabolic\_process | RBM17 | 1974 | 18 | 1.615034 | -2.229483 | 12 | 3.1 | 0.258333 |
| GO:0006633\_fatty\_acid\_biosynthetic\_process | ACSM3 | 21 | 2 | 16.868132 | -2.219893 | 13 | 3.34 | 0.256923 |
| GO:0006633\_fatty\_acid\_biosynthetic\_process | QK | 21 | 2 | 16.868132 | -2.219893 | 13 | 3.34 | 0.256923 |
| GO:0010467\_gene\_expression | TAF10 | 905 | 11 | 2.152784 | -2.170577 | 14 | 3.71 | 0.265000 |
| GO:0010467\_gene\_expression | DKC1 | 905 | 11 | 2.152784 | -2.170577 | 14 | 3.71 | 0.265000 |
| GO:0010467\_gene\_expression | GPBP1 | 905 | 11 | 2.152784 | -2.170577 | 14 | 3.71 | 0.265000 |
| GO:0010467\_gene\_expression | STRAP | 905 | 11 | 2.152784 | -2.170577 | 14 | 3.71 | 0.265000 |
| GO:0010467\_gene\_expression | NLK | 905 | 11 | 2.152784 | -2.170577 | 14 | 3.71 | 0.265000 |
| GO:0010467\_gene\_expression | OTX2 | 905 | 11 | 2.152784 | -2.170577 | 14 | 3.71 | 0.265000 |
| GO:0010467\_gene\_expression | SKP2 | 905 | 11 | 2.152784 | -2.170577 | 14 | 3.71 | 0.265000 |
| GO:0010467\_gene\_expression | DNMT3B | 905 | 11 | 2.152784 | -2.170577 | 14 | 3.71 | 0.265000 |
| GO:0010467\_gene\_expression | FOXD1 | 905 | 11 | 2.152784 | -2.170577 | 14 | 3.71 | 0.265000 |
| GO:0010467\_gene\_expression | HELLS | 905 | 11 | 2.152784 | -2.170577 | 14 | 3.71 | 0.265000 |
| GO:0010467\_gene\_expression | RBM17 | 905 | 11 | 2.152784 | -2.170577 | 14 | 3.71 | 0.265000 |
| GO:0000082\_G1\_S\_transition\_of\_mitotic\_cell\_cycle | TAF10 | 23 | 2 | 15.401338 | -2.142000 | 15 | 3.98 | 0.265333 |
| GO:0000082\_G1\_S\_transition\_of\_mitotic\_cell\_cycle | SKP2 | 23 | 2 | 15.401338 | -2.142000 | 15 | 3.98 | 0.265333 |
| GO:0040029\_regulation\_of\_gene\_expression\_\_epigenetic | DNMT3B | 26 | 2 | 13.624260 | -2.037746 | 16 | 5.02 | 0.313750 |
| GO:0040029\_regulation\_of\_gene\_expression\_\_epigenetic | HELLS | 26 | 2 | 13.624260 | -2.037746 | 16 | 5.02 | 0.313750 |
| GO:0034960\_cellular\_biopolymer\_metabolic\_process | RAD51AP1 | 1395 | 14 | 1.777502 | -1.993254 | 17 | 5.4 | 0.317647 |
| GO:0034960\_cellular\_biopolymer\_metabolic\_process | STRAP | 1395 | 14 | 1.777502 | -1.993254 | 17 | 5.4 | 0.317647 |
| GO:0034960\_cellular\_biopolymer\_metabolic\_process | GPBP1 | 1395 | 14 | 1.777502 | -1.993254 | 17 | 5.4 | 0.317647 |
| GO:0034960\_cellular\_biopolymer\_metabolic\_process | NLK | 1395 | 14 | 1.777502 | -1.993254 | 17 | 5.4 | 0.317647 |
| GO:0034960\_cellular\_biopolymer\_metabolic\_process | OTX2 | 1395 | 14 | 1.777502 | -1.993254 | 17 | 5.4 | 0.317647 |
| GO:0034960\_cellular\_biopolymer\_metabolic\_process | SKP2 | 1395 | 14 | 1.777502 | -1.993254 | 17 | 5.4 | 0.317647 |
| GO:0034960\_cellular\_biopolymer\_metabolic\_process | MKNK2 | 1395 | 14 | 1.777502 | -1.993254 | 17 | 5.4 | 0.317647 |
| GO:0034960\_cellular\_biopolymer\_metabolic\_process | PTPN12 | 1395 | 14 | 1.777502 | -1.993254 | 17 | 5.4 | 0.317647 |
| GO:0034960\_cellular\_biopolymer\_metabolic\_process | PTPN11 | 1395 | 14 | 1.777502 | -1.993254 | 17 | 5.4 | 0.317647 |
| GO:0034960\_cellular\_biopolymer\_metabolic\_process | DKC1 | 1395 | 14 | 1.777502 | -1.993254 | 17 | 5.4 | 0.317647 |
| GO:0034960\_cellular\_biopolymer\_metabolic\_process | DNMT3B | 1395 | 14 | 1.777502 | -1.993254 | 17 | 5.4 | 0.317647 |
| GO:0034960\_cellular\_biopolymer\_metabolic\_process | FOXD1 | 1395 | 14 | 1.777502 | -1.993254 | 17 | 5.4 | 0.317647 |
| GO:0034960\_cellular\_biopolymer\_metabolic\_process | HELLS | 1395 | 14 | 1.777502 | -1.993254 | 17 | 5.4 | 0.317647 |
| GO:0034960\_cellular\_biopolymer\_metabolic\_process | RBM17 | 1395 | 14 | 1.777502 | -1.993254 | 17 | 5.4 | 0.317647 |
| GO:0009987\_cellular\_process | STRAP | 3868 | 26 | 1.190538 | -1.975186 | 18 | 5.51 | 0.306111 |
| GO:0009987\_cellular\_process | GPBP1 | 3868 | 26 | 1.190538 | -1.975186 | 18 | 5.51 | 0.306111 |
| GO:0009987\_cellular\_process | MKNK2 | 3868 | 26 | 1.190538 | -1.975186 | 18 | 5.51 | 0.306111 |
| GO:0009987\_cellular\_process | AZIN1 | 3868 | 26 | 1.190538 | -1.975186 | 18 | 5.51 | 0.306111 |
| GO:0009987\_cellular\_process | ITM2B | 3868 | 26 | 1.190538 | -1.975186 | 18 | 5.51 | 0.306111 |
| GO:0009987\_cellular\_process | DKC1 | 3868 | 26 | 1.190538 | -1.975186 | 18 | 5.51 | 0.306111 |
| GO:0009987\_cellular\_process | ALDH1A7 | 3868 | 26 | 1.190538 | -1.975186 | 18 | 5.51 | 0.306111 |
| GO:0009987\_cellular\_process | DNMT3B | 3868 | 26 | 1.190538 | -1.975186 | 18 | 5.51 | 0.306111 |
| GO:0009987\_cellular\_process | FOXD1 | 3868 | 26 | 1.190538 | -1.975186 | 18 | 5.51 | 0.306111 |
| GO:0009987\_cellular\_process | HELLS | 3868 | 26 | 1.190538 | -1.975186 | 18 | 5.51 | 0.306111 |
| GO:0009987\_cellular\_process | API5 | 3868 | 26 | 1.190538 | -1.975186 | 18 | 5.51 | 0.306111 |
| GO:0009987\_cellular\_process | RAD51AP1 | 3868 | 26 | 1.190538 | -1.975186 | 18 | 5.51 | 0.306111 |
| GO:0009987\_cellular\_process | SSBP1 | 3868 | 26 | 1.190538 | -1.975186 | 18 | 5.51 | 0.306111 |
| GO:0009987\_cellular\_process | NLK | 3868 | 26 | 1.190538 | -1.975186 | 18 | 5.51 | 0.306111 |
| GO:0009987\_cellular\_process | OTX2 | 3868 | 26 | 1.190538 | -1.975186 | 18 | 5.51 | 0.306111 |
| GO:0009987\_cellular\_process | SKP2 | 3868 | 26 | 1.190538 | -1.975186 | 18 | 5.51 | 0.306111 |
| GO:0009987\_cellular\_process | GARS | 3868 | 26 | 1.190538 | -1.975186 | 18 | 5.51 | 0.306111 |
| GO:0009987\_cellular\_process | PTPN12 | 3868 | 26 | 1.190538 | -1.975186 | 18 | 5.51 | 0.306111 |
| GO:0009987\_cellular\_process | PTPN11 | 3868 | 26 | 1.190538 | -1.975186 | 18 | 5.51 | 0.306111 |
| GO:0009987\_cellular\_process | QK | 3868 | 26 | 1.190538 | -1.975186 | 18 | 5.51 | 0.306111 |
| GO:0009987\_cellular\_process | ACSM3 | 3868 | 26 | 1.190538 | -1.975186 | 18 | 5.51 | 0.306111 |
| GO:0009987\_cellular\_process | TAF10 | 3868 | 26 | 1.190538 | -1.975186 | 18 | 5.51 | 0.306111 |
| GO:0009987\_cellular\_process | RGS2 | 3868 | 26 | 1.190538 | -1.975186 | 18 | 5.51 | 0.306111 |
| GO:0009987\_cellular\_process | VCP | 3868 | 26 | 1.190538 | -1.975186 | 18 | 5.51 | 0.306111 |
| GO:0009987\_cellular\_process | RAP1A | 3868 | 26 | 1.190538 | -1.975186 | 18 | 5.51 | 0.306111 |
| GO:0009987\_cellular\_process | RBM17 | 3868 | 26 | 1.190538 | -1.975186 | 18 | 5.51 | 0.306111 |
| GO:0008634\_negative\_regulation\_of\_survival\_gene\_product\_expression | SKP2 | 2 | 1 |  |  |  |  |  |  |
| GO:0033136\_serine\_phosphorylation\_of\_STAT3\_protein | NLK | 2 | 1 |  |  |  |  |  |  |
| GO:0033145\_positive\_regulation\_of\_steroid\_hormone\_receptor\_signaling\_pathway | SKP2 | 2 | 1 |  |  |  |  |  |  |
| GO:0033148\_positive\_regulation\_of\_estrogen\_receptor\_signaling\_pathway | SKP2 | 2 | 1 |  |  |  |  |  |  |
| GO:0042501\_serine\_phosphorylation\_of\_STAT\_protein | NLK | 2 | 1 |  |  |  |  |  |  |
| GO:0043170\_macromolecule\_metabolic\_process | RAD51AP1 | 1576 | 15 | 1.685743 | -1.928873 | 19 | 6.27 | 0.330000 |
| GO:0043170\_macromolecule\_metabolic\_process | GPBP1 | 1576 | 15 | 1.685743 | -1.928873 | 19 | 6.27 | 0.330000 |
| GO:0043170\_macromolecule\_metabolic\_process | STRAP | 1576 | 15 | 1.685743 | -1.928873 | 19 | 6.27 | 0.330000 |
| GO:0043170\_macromolecule\_metabolic\_process | NLK | 1576 | 15 | 1.685743 | -1.928873 | 19 | 6.27 | 0.330000 |
| GO:0043170\_macromolecule\_metabolic\_process | OTX2 | 1576 | 15 | 1.685743 | -1.928873 | 19 | 6.27 | 0.330000 |
| GO:0043170\_macromolecule\_metabolic\_process | SKP2 | 1576 | 15 | 1.685743 | -1.928873 | 19 | 6.27 | 0.330000 |
| GO:0043170\_macromolecule\_metabolic\_process | MKNK2 | 1576 | 15 | 1.685743 | -1.928873 | 19 | 6.27 | 0.330000 |
| GO:0043170\_macromolecule\_metabolic\_process | PTPN12 | 1576 | 15 | 1.685743 | -1.928873 | 19 | 6.27 | 0.330000 |
| GO:0043170\_macromolecule\_metabolic\_process | PTPN11 | 1576 | 15 | 1.685743 | -1.928873 | 19 | 6.27 | 0.330000 |
| GO:0043170\_macromolecule\_metabolic\_process | TAF10 | 1576 | 15 | 1.685743 | -1.928873 | 19 | 6.27 | 0.330000 |
| GO:0043170\_macromolecule\_metabolic\_process | DKC1 | 1576 | 15 | 1.685743 | -1.928873 | 19 | 6.27 | 0.330000 |
| GO:0043170\_macromolecule\_metabolic\_process | FOXD1 | 1576 | 15 | 1.685743 | -1.928873 | 19 | 6.27 | 0.330000 |
| GO:0043170\_macromolecule\_metabolic\_process | DNMT3B | 1576 | 15 | 1.685743 | -1.928873 | 19 | 6.27 | 0.330000 |
| GO:0043170\_macromolecule\_metabolic\_process | HELLS | 1576 | 15 | 1.685743 | -1.928873 | 19 | 6.27 | 0.330000 |
| GO:0043170\_macromolecule\_metabolic\_process | RBM17 | 1576 | 15 | 1.685743 | -1.928873 | 19 | 6.27 | 0.330000 |
| GO:0044260\_cellular\_macromolecule\_metabolic\_process | RAD51AP1 | 1447 | 14 | 1.713625 | -1.844346 | 20 | 7.54 | 0.377000 |
| GO:0044260\_cellular\_macromolecule\_metabolic\_process | STRAP | 1447 | 14 | 1.713625 | -1.844346 | 20 | 7.54 | 0.377000 |
| GO:0044260\_cellular\_macromolecule\_metabolic\_process | GPBP1 | 1447 | 14 | 1.713625 | -1.844346 | 20 | 7.54 | 0.377000 |
| GO:0044260\_cellular\_macromolecule\_metabolic\_process | NLK | 1447 | 14 | 1.713625 | -1.844346 | 20 | 7.54 | 0.377000 |
| GO:0044260\_cellular\_macromolecule\_metabolic\_process | OTX2 | 1447 | 14 | 1.713625 | -1.844346 | 20 | 7.54 | 0.377000 |
| GO:0044260\_cellular\_macromolecule\_metabolic\_process | SKP2 | 1447 | 14 | 1.713625 | -1.844346 | 20 | 7.54 | 0.377000 |
| GO:0044260\_cellular\_macromolecule\_metabolic\_process | MKNK2 | 1447 | 14 | 1.713625 | -1.844346 | 20 | 7.54 | 0.377000 |
| GO:0044260\_cellular\_macromolecule\_metabolic\_process | PTPN12 | 1447 | 14 | 1.713625 | -1.844346 | 20 | 7.54 | 0.377000 |
| GO:0044260\_cellular\_macromolecule\_metabolic\_process | PTPN11 | 1447 | 14 | 1.713625 | -1.844346 | 20 | 7.54 | 0.377000 |
| GO:0044260\_cellular\_macromolecule\_metabolic\_process | DKC1 | 1447 | 14 | 1.713625 | -1.844346 | 20 | 7.54 | 0.377000 |
| GO:0044260\_cellular\_macromolecule\_metabolic\_process | FOXD1 | 1447 | 14 | 1.713625 | -1.844346 | 20 | 7.54 | 0.377000 |
| GO:0044260\_cellular\_macromolecule\_metabolic\_process | DNMT3B | 1447 | 14 | 1.713625 | -1.844346 | 20 | 7.54 | 0.377000 |
| GO:0044260\_cellular\_macromolecule\_metabolic\_process | HELLS | 1447 | 14 | 1.713625 | -1.844346 | 20 | 7.54 | 0.377000 |
| GO:0044260\_cellular\_macromolecule\_metabolic\_process | RBM17 | 1447 | 14 | 1.713625 | -1.844346 | 20 | 7.54 | 0.377000 |
| GO:0051325\_interphase | TAF10 | 35 | 2 | 10.120879 | -1.788615 | 22 | 8.45 | 0.384091 |
| GO:0051325\_interphase | SKP2 | 35 | 2 | 10.120879 | -1.788615 | 22 | 8.45 | 0.384091 |
| GO:0051329\_interphase\_of\_mitotic\_cell\_cycle | TAF10 | 35 | 2 | 10.120879 | -1.788615 | 22 | 8.45 | 0.384091 |
| GO:0051329\_interphase\_of\_mitotic\_cell\_cycle | SKP2 | 35 | 2 | 10.120879 | -1.788615 | 22 | 8.45 | 0.384091 |
| GO:0010216\_maintenance\_of\_DNA\_methylation | HELLS | 3 | 1 |  |  |  |  |  |  |
| GO:0031503\_protein\_complex\_localization | DNMT3B | 3 | 1 |  |  |  |  |  |  |
| GO:0042759\_long-chain\_fatty\_acid\_biosynthetic\_process | QK | 3 | 1 |  |  |  |  |  |  |
| GO:0043045\_DNA\_methylation\_during\_embryonic\_development | DNMT3B | 3 | 1 |  |  |  |  |  |  |
| GO:0044030\_regulation\_of\_DNA\_methylation | HELLS | 3 | 1 |  |  |  |  |  |  |
| GO:0046825\_regulation\_of\_protein\_export\_from\_nucleus | PTPN11 | 3 | 1 |  |  |  |  |  |  |
| GO:0060123\_regulation\_of\_growth\_hormone\_secretion | PTPN11 | 3 | 1 |  |  |  |  |  |  |
| GO:0043283\_biopolymer\_metabolic\_process | RAD51AP1 | 1490 | 14 | 1.664171 | -1.727909 | 23 | 9.19 | 0.399565 |
| GO:0043283\_biopolymer\_metabolic\_process | GPBP1 | 1490 | 14 | 1.664171 | -1.727909 | 23 | 9.19 | 0.399565 |
| GO:0043283\_biopolymer\_metabolic\_process | STRAP | 1490 | 14 | 1.664171 | -1.727909 | 23 | 9.19 | 0.399565 |
| GO:0043283\_biopolymer\_metabolic\_process | NLK | 1490 | 14 | 1.664171 | -1.727909 | 23 | 9.19 | 0.399565 |
| GO:0043283\_biopolymer\_metabolic\_process | OTX2 | 1490 | 14 | 1.664171 | -1.727909 | 23 | 9.19 | 0.399565 |
| GO:0043283\_biopolymer\_metabolic\_process | SKP2 | 1490 | 14 | 1.664171 | -1.727909 | 23 | 9.19 | 0.399565 |
| GO:0043283\_biopolymer\_metabolic\_process | MKNK2 | 1490 | 14 | 1.664171 | -1.727909 | 23 | 9.19 | 0.399565 |
| GO:0043283\_biopolymer\_metabolic\_process | PTPN12 | 1490 | 14 | 1.664171 | -1.727909 | 23 | 9.19 | 0.399565 |
| GO:0043283\_biopolymer\_metabolic\_process | PTPN11 | 1490 | 14 | 1.664171 | -1.727909 | 23 | 9.19 | 0.399565 |
| GO:0043283\_biopolymer\_metabolic\_process | DKC1 | 1490 | 14 | 1.664171 | -1.727909 | 23 | 9.19 | 0.399565 |
| GO:0043283\_biopolymer\_metabolic\_process | DNMT3B | 1490 | 14 | 1.664171 | -1.727909 | 23 | 9.19 | 0.399565 |
| GO:0043283\_biopolymer\_metabolic\_process | FOXD1 | 1490 | 14 | 1.664171 | -1.727909 | 23 | 9.19 | 0.399565 |
| GO:0043283\_biopolymer\_metabolic\_process | HELLS | 1490 | 14 | 1.664171 | -1.727909 | 23 | 9.19 | 0.399565 |
| GO:0043283\_biopolymer\_metabolic\_process | RBM17 | 1490 | 14 | 1.664171 | -1.727909 | 23 | 9.19 | 0.399565 |
| GO:0016053\_organic\_acid\_biosynthetic\_process | ACSM3 | 38 | 2 | 9.321862 | -1.720669 | 27 | 9.61 | 0.355926 |
| GO:0016053\_organic\_acid\_biosynthetic\_process | QK | 38 | 2 | 9.321862 | -1.720669 | 27 | 9.61 | 0.355926 |
| GO:0032259\_methylation | DNMT3B | 38 | 2 | 9.321862 | -1.720669 | 27 | 9.61 | 0.355926 |
| GO:0032259\_methylation | HELLS | 38 | 2 | 9.321862 | -1.720669 | 27 | 9.61 | 0.355926 |
| GO:0043414\_biopolymer\_methylation | DNMT3B | 38 | 2 | 9.321862 | -1.720669 | 27 | 9.61 | 0.355926 |
| GO:0043414\_biopolymer\_methylation | HELLS | 38 | 2 | 9.321862 | -1.720669 | 27 | 9.61 | 0.355926 |
| GO:0046394\_carboxylic\_acid\_biosynthetic\_process | ACSM3 | 38 | 2 | 9.321862 | -1.720669 | 27 | 9.61 | 0.355926 |
| GO:0046394\_carboxylic\_acid\_biosynthetic\_process | QK | 38 | 2 | 9.321862 | -1.720669 | 27 | 9.61 | 0.355926 |
| GO:0006730\_one-carbon\_metabolic\_process | DNMT3B | 39 | 2 | 9.082840 | -1.699303 | 28 | 10.08 | 0.360000 |
| GO:0006730\_one-carbon\_metabolic\_process | HELLS | 39 | 2 | 9.082840 | -1.699303 | 28 | 10.08 | 0.360000 |
| GO:0010468\_regulation\_of\_gene\_expression | TAF10 | 778 | 9 | 2.048893 | -1.657749 | 29 | 10.81 | 0.372759 |
| GO:0010468\_regulation\_of\_gene\_expression | STRAP | 778 | 9 | 2.048893 | -1.657749 | 29 | 10.81 | 0.372759 |
| GO:0010468\_regulation\_of\_gene\_expression | GPBP1 | 778 | 9 | 2.048893 | -1.657749 | 29 | 10.81 | 0.372759 |
| GO:0010468\_regulation\_of\_gene\_expression | NLK | 778 | 9 | 2.048893 | -1.657749 | 29 | 10.81 | 0.372759 |
| GO:0010468\_regulation\_of\_gene\_expression | OTX2 | 778 | 9 | 2.048893 | -1.657749 | 29 | 10.81 | 0.372759 |
| GO:0010468\_regulation\_of\_gene\_expression | SKP2 | 778 | 9 | 2.048893 | -1.657749 | 29 | 10.81 | 0.372759 |
| GO:0010468\_regulation\_of\_gene\_expression | DNMT3B | 778 | 9 | 2.048893 | -1.657749 | 29 | 10.81 | 0.372759 |
| GO:0010468\_regulation\_of\_gene\_expression | FOXD1 | 778 | 9 | 2.048893 | -1.657749 | 29 | 10.81 | 0.372759 |
| GO:0010468\_regulation\_of\_gene\_expression | HELLS | 778 | 9 | 2.048893 | -1.657749 | 29 | 10.81 | 0.372759 |
| GO:0009755\_hormone-mediated\_signaling | PTPN11 | 4 | 1 |  |  |  |  |  |  |
| GO:0048011\_nerve\_growth\_factor\_receptor\_signaling\_pathway | PTPN11 | 4 | 1 |  |  |  |  |  |  |
| GO:0016070\_RNA\_metabolic\_process | DKC1 | 658 | 8 | 2.153379 | -1.614511 | 30 | 11.99 | 0.399667 |
| GO:0016070\_RNA\_metabolic\_process | GPBP1 | 658 | 8 | 2.153379 | -1.614511 | 30 | 11.99 | 0.399667 |
| GO:0016070\_RNA\_metabolic\_process | STRAP | 658 | 8 | 2.153379 | -1.614511 | 30 | 11.99 | 0.399667 |
| GO:0016070\_RNA\_metabolic\_process | OTX2 | 658 | 8 | 2.153379 | -1.614511 | 30 | 11.99 | 0.399667 |
| GO:0016070\_RNA\_metabolic\_process | DNMT3B | 658 | 8 | 2.153379 | -1.614511 | 30 | 11.99 | 0.399667 |
| GO:0016070\_RNA\_metabolic\_process | FOXD1 | 658 | 8 | 2.153379 | -1.614511 | 30 | 11.99 | 0.399667 |
| GO:0016070\_RNA\_metabolic\_process | HELLS | 658 | 8 | 2.153379 | -1.614511 | 30 | 11.99 | 0.399667 |
| GO:0016070\_RNA\_metabolic\_process | RBM17 | 658 | 8 | 2.153379 | -1.614511 | 30 | 11.99 | 0.399667 |
| GO:0033146\_regulation\_of\_estrogen\_receptor\_signaling\_pathway | SKP2 | 5 | 1 | 35.423077 | -1.554004 | 33 | 20.65 | 0.625758 |
| GO:0042574\_retinal\_metabolic\_process | ALDH1A7 | 5 | 1 | 35.423077 | -1.554004 | 33 | 20.65 | 0.625758 |
| GO:0043288\_apocarotenoid\_metabolic\_process | ALDH1A7 | 5 | 1 | 35.423077 | -1.554004 | 33 | 20.65 | 0.625758 |
| GO:0006396\_RNA\_processing | DKC1 | 47 | 2 | 7.536825 | -1.547254 | 34 | 20.98 | 0.617059 |
| GO:0006396\_RNA\_processing | RBM17 | 47 | 2 | 7.536825 | -1.547254 | 34 | 20.98 | 0.617059 |
| GO:0006915\_apoptosis | TAF10 | 427 | 6 | 2.488741 | -1.545866 | 35 | 21.03 | 0.600857 |
| GO:0006915\_apoptosis | VCP | 427 | 6 | 2.488741 | -1.545866 | 35 | 21.03 | 0.600857 |
| GO:0006915\_apoptosis | SKP2 | 427 | 6 | 2.488741 | -1.545866 | 35 | 21.03 | 0.600857 |
| GO:0006915\_apoptosis | ITM2B | 427 | 6 | 2.488741 | -1.545866 | 35 | 21.03 | 0.600857 |
| GO:0006915\_apoptosis | API5 | 427 | 6 | 2.488741 | -1.545866 | 35 | 21.03 | 0.600857 |
| GO:0006915\_apoptosis | HELLS | 427 | 6 | 2.488741 | -1.545866 | 35 | 21.03 | 0.600857 |
| GO:0012501\_programmed\_cell\_death | TAF10 | 433 | 6 | 2.454255 | -1.519410 | 36 | 21.46 | 0.596111 |
| GO:0012501\_programmed\_cell\_death | VCP | 433 | 6 | 2.454255 | -1.519410 | 36 | 21.46 | 0.596111 |
| GO:0012501\_programmed\_cell\_death | SKP2 | 433 | 6 | 2.454255 | -1.519410 | 36 | 21.46 | 0.596111 |
| GO:0012501\_programmed\_cell\_death | ITM2B | 433 | 6 | 2.454255 | -1.519410 | 36 | 21.46 | 0.596111 |
| GO:0012501\_programmed\_cell\_death | API5 | 433 | 6 | 2.454255 | -1.519410 | 36 | 21.46 | 0.596111 |
| GO:0012501\_programmed\_cell\_death | HELLS | 433 | 6 | 2.454255 | -1.519410 | 36 | 21.46 | 0.596111 |
| GO:0016569\_covalent\_chromatin\_modification | DNMT3B | 51 | 2 | 6.945701 | -1.481538 | 37 | 22.94 | 0.620000 |
| GO:0016569\_covalent\_chromatin\_modification | HELLS | 51 | 2 | 6.945701 | -1.481538 | 37 | 22.94 | 0.620000 |
| GO:0030252\_growth\_hormone\_secretion | PTPN11 | 6 | 1 | 29.519231 | -1.476000 | 39 | 28.66 | 0.734872 |
| GO:0065004\_protein-DNA\_complex\_assembly | HELLS | 6 | 1 | 29.519231 | -1.476000 | 39 | 28.66 | 0.734872 |
| GO:0008219\_cell\_death | TAF10 | 444 | 6 | 2.393451 | -1.472222 | 40 | 28.72 | 0.718000 |
| GO:0008219\_cell\_death | VCP | 444 | 6 | 2.393451 | -1.472222 | 40 | 28.72 | 0.718000 |
| GO:0008219\_cell\_death | SKP2 | 444 | 6 | 2.393451 | -1.472222 | 40 | 28.72 | 0.718000 |
| GO:0008219\_cell\_death | ITM2B | 444 | 6 | 2.393451 | -1.472222 | 40 | 28.72 | 0.718000 |
| GO:0008219\_cell\_death | API5 | 444 | 6 | 2.393451 | -1.472222 | 40 | 28.72 | 0.718000 |
| GO:0008219\_cell\_death | HELLS | 444 | 6 | 2.393451 | -1.472222 | 40 | 28.72 | 0.718000 |
| GO:0032787\_monocarboxylic\_acid\_metabolic\_process | ACSM3 | 130 | 3 | 4.087278 | -1.448605 | 41 | 29.53 | 0.720244 |
| GO:0032787\_monocarboxylic\_acid\_metabolic\_process | ALDH1A7 | 130 | 3 | 4.087278 | -1.448605 | 41 | 29.53 | 0.720244 |
| GO:0032787\_monocarboxylic\_acid\_metabolic\_process | QK | 130 | 3 | 4.087278 | -1.448605 | 41 | 29.53 | 0.720244 |
| GO:0016265\_death | TAF10 | 450 | 6 | 2.361538 | -1.447175 | 42 | 29.56 | 0.703810 |
| GO:0016265\_death | VCP | 450 | 6 | 2.361538 | -1.447175 | 42 | 29.56 | 0.703810 |
| GO:0016265\_death | SKP2 | 450 | 6 | 2.361538 | -1.447175 | 42 | 29.56 | 0.703810 |
| GO:0016265\_death | ITM2B | 450 | 6 | 2.361538 | -1.447175 | 42 | 29.56 | 0.703810 |
| GO:0016265\_death | API5 | 450 | 6 | 2.361538 | -1.447175 | 42 | 29.56 | 0.703810 |
| GO:0016265\_death | HELLS | 450 | 6 | 2.361538 | -1.447175 | 42 | 29.56 | 0.703810 |
| GO:0006807\_nitrogen\_compound\_metabolic\_process | RAD51AP1 | 1147 | 11 | 1.698578 | -1.412757 | 43 | 30.53 | 0.710000 |
| GO:0006807\_nitrogen\_compound\_metabolic\_process | DKC1 | 1147 | 11 | 1.698578 | -1.412757 | 43 | 30.53 | 0.710000 |
| GO:0006807\_nitrogen\_compound\_metabolic\_process | GPBP1 | 1147 | 11 | 1.698578 | -1.412757 | 43 | 30.53 | 0.710000 |
| GO:0006807\_nitrogen\_compound\_metabolic\_process | STRAP | 1147 | 11 | 1.698578 | -1.412757 | 43 | 30.53 | 0.710000 |
| GO:0006807\_nitrogen\_compound\_metabolic\_process | NLK | 1147 | 11 | 1.698578 | -1.412757 | 43 | 30.53 | 0.710000 |
| GO:0006807\_nitrogen\_compound\_metabolic\_process | OTX2 | 1147 | 11 | 1.698578 | -1.412757 | 43 | 30.53 | 0.710000 |
| GO:0006807\_nitrogen\_compound\_metabolic\_process | AZIN1 | 1147 | 11 | 1.698578 | -1.412757 | 43 | 30.53 | 0.710000 |
| GO:0006807\_nitrogen\_compound\_metabolic\_process | FOXD1 | 1147 | 11 | 1.698578 | -1.412757 | 43 | 30.53 | 0.710000 |
| GO:0006807\_nitrogen\_compound\_metabolic\_process | DNMT3B | 1147 | 11 | 1.698578 | -1.412757 | 43 | 30.53 | 0.710000 |
| GO:0006807\_nitrogen\_compound\_metabolic\_process | HELLS | 1147 | 11 | 1.698578 | -1.412757 | 43 | 30.53 | 0.710000 |
| GO:0006807\_nitrogen\_compound\_metabolic\_process | RBM17 | 1147 | 11 | 1.698578 | -1.412757 | 43 | 30.53 | 0.710000 |
| GO:0002792\_negative\_regulation\_of\_peptide\_secretion | PTPN11 | 7 | 1 | 25.302198 | -1.410229 | 47 | 37.09 | 0.789149 |
| GO:0030520\_estrogen\_receptor\_signaling\_pathway | SKP2 | 7 | 1 | 25.302198 | -1.410229 | 47 | 37.09 | 0.789149 |
| GO:0031497\_chromatin\_assembly | HELLS | 7 | 1 | 25.302198 | -1.410229 | 47 | 37.09 | 0.789149 |
| GO:0046676\_negative\_regulation\_of\_insulin\_secretion | PTPN11 | 7 | 1 | 25.302198 | -1.410229 | 47 | 37.09 | 0.789149 |
| GO:0006139\_nucleobase\_\_nucleoside\_\_nucleotide\_and\_nucleic\_acid\_metabolic\_process | RAD51AP1 | 1002 | 10 | 1.767619 | -1.403861 | 48 | 37.41 | 0.779375 |
| GO:0006139\_nucleobase\_\_nucleoside\_\_nucleotide\_and\_nucleic\_acid\_metabolic\_process | DKC1 | 1002 | 10 | 1.767619 | -1.403861 | 48 | 37.41 | 0.779375 |
| GO:0006139\_nucleobase\_\_nucleoside\_\_nucleotide\_and\_nucleic\_acid\_metabolic\_process | GPBP1 | 1002 | 10 | 1.767619 | -1.403861 | 48 | 37.41 | 0.779375 |
| GO:0006139\_nucleobase\_\_nucleoside\_\_nucleotide\_and\_nucleic\_acid\_metabolic\_process | STRAP | 1002 | 10 | 1.767619 | -1.403861 | 48 | 37.41 | 0.779375 |
| GO:0006139\_nucleobase\_\_nucleoside\_\_nucleotide\_and\_nucleic\_acid\_metabolic\_process | NLK | 1002 | 10 | 1.767619 | -1.403861 | 48 | 37.41 | 0.779375 |
| GO:0006139\_nucleobase\_\_nucleoside\_\_nucleotide\_and\_nucleic\_acid\_metabolic\_process | OTX2 | 1002 | 10 | 1.767619 | -1.403861 | 48 | 37.41 | 0.779375 |
| GO:0006139\_nucleobase\_\_nucleoside\_\_nucleotide\_and\_nucleic\_acid\_metabolic\_process | FOXD1 | 1002 | 10 | 1.767619 | -1.403861 | 48 | 37.41 | 0.779375 |
| GO:0006139\_nucleobase\_\_nucleoside\_\_nucleotide\_and\_nucleic\_acid\_metabolic\_process | DNMT3B | 1002 | 10 | 1.767619 | -1.403861 | 48 | 37.41 | 0.779375 |
| GO:0006139\_nucleobase\_\_nucleoside\_\_nucleotide\_and\_nucleic\_acid\_metabolic\_process | HELLS | 1002 | 10 | 1.767619 | -1.403861 | 48 | 37.41 | 0.779375 |
| GO:0006139\_nucleobase\_\_nucleoside\_\_nucleotide\_and\_nucleic\_acid\_metabolic\_process | RBM17 | 1002 | 10 | 1.767619 | -1.403861 | 48 | 37.41 | 0.779375 |
| GO:0034622\_cellular\_macromolecular\_complex\_assembly | TAF10 | 58 | 2 | 6.107427 | -1.379201 | 49 | 38.22 | 0.780000 |
| GO:0034622\_cellular\_macromolecular\_complex\_assembly | HELLS | 58 | 2 | 6.107427 | -1.379201 | 49 | 38.22 | 0.780000 |
| GO:0006349\_genetic\_imprinting | DNMT3B | 8 | 1 | 22.139423 | -1.353412 | 53 | 44.12 | 0.832453 |
| GO:0018107\_peptidyl-threonine\_phosphorylation | NLK | 8 | 1 | 22.139423 | -1.353412 | 53 | 44.12 | 0.832453 |
| GO:0018210\_peptidyl-threonine\_modification | NLK | 8 | 1 | 22.139423 | -1.353412 | 53 | 44.12 | 0.832453 |
| GO:0070584\_mitochondrion\_morphogenesis | SSBP1 | 8 | 1 | 22.139423 | -1.353412 | 53 | 44.12 | 0.832453 |
| GO:0042981\_regulation\_of\_apoptosis | VCP | 360 | 5 | 2.459936 | -1.321626 | 54 | 45.01 | 0.833519 |
| GO:0042981\_regulation\_of\_apoptosis | SKP2 | 360 | 5 | 2.459936 | -1.321626 | 54 | 45.01 | 0.833519 |
| GO:0042981\_regulation\_of\_apoptosis | ITM2B | 360 | 5 | 2.459936 | -1.321626 | 54 | 45.01 | 0.833519 |
| GO:0042981\_regulation\_of\_apoptosis | API5 | 360 | 5 | 2.459936 | -1.321626 | 54 | 45.01 | 0.833519 |
| GO:0042981\_regulation\_of\_apoptosis | HELLS | 360 | 5 | 2.459936 | -1.321626 | 54 | 45.01 | 0.833519 |
| GO:0001676\_long-chain\_fatty\_acid\_metabolic\_process | QK | 9 | 1 | 19.679487 | -1.303434 | 63 | 50.7 | 0.804762 |
| GO:0006364\_rRNA\_processing | DKC1 | 9 | 1 | 19.679487 | -1.303434 | 63 | 50.7 | 0.804762 |
| GO:0006595\_polyamine\_metabolic\_process | AZIN1 | 9 | 1 | 19.679487 | -1.303434 | 63 | 50.7 | 0.804762 |
| GO:0006611\_protein\_export\_from\_nucleus | PTPN11 | 9 | 1 | 19.679487 | -1.303434 | 63 | 50.7 | 0.804762 |
| GO:0009451\_RNA\_modification | DKC1 | 9 | 1 | 19.679487 | -1.303434 | 63 | 50.7 | 0.804762 |
| GO:0016072\_rRNA\_metabolic\_process | DKC1 | 9 | 1 | 19.679487 | -1.303434 | 63 | 50.7 | 0.804762 |
| GO:0033143\_regulation\_of\_steroid\_hormone\_receptor\_signaling\_pathway | SKP2 | 9 | 1 | 19.679487 | -1.303434 | 63 | 50.7 | 0.804762 |
| GO:0045884\_regulation\_of\_survival\_gene\_product\_expression | SKP2 | 9 | 1 | 19.679487 | -1.303434 | 63 | 50.7 | 0.804762 |
| GO:0046888\_negative\_regulation\_of\_hormone\_secretion | PTPN11 | 9 | 1 | 19.679487 | -1.303434 | 63 | 50.7 | 0.804762 |
| GO:0010941\_regulation\_of\_cell\_death | VCP | 365 | 5 | 2.426238 | -1.299932 | 65 | 51.14 | 0.786769 |
| GO:0010941\_regulation\_of\_cell\_death | SKP2 | 365 | 5 | 2.426238 | -1.299932 | 65 | 51.14 | 0.786769 |
| GO:0010941\_regulation\_of\_cell\_death | ITM2B | 365 | 5 | 2.426238 | -1.299932 | 65 | 51.14 | 0.786769 |
| GO:0010941\_regulation\_of\_cell\_death | API5 | 365 | 5 | 2.426238 | -1.299932 | 65 | 51.14 | 0.786769 |
| GO:0010941\_regulation\_of\_cell\_death | HELLS | 365 | 5 | 2.426238 | -1.299932 | 65 | 51.14 | 0.786769 |
| GO:0043067\_regulation\_of\_programmed\_cell\_death | VCP | 365 | 5 | 2.426238 | -1.299932 | 65 | 51.14 | 0.786769 |
| GO:0043067\_regulation\_of\_programmed\_cell\_death | SKP2 | 365 | 5 | 2.426238 | -1.299932 | 65 | 51.14 | 0.786769 |
| GO:0043067\_regulation\_of\_programmed\_cell\_death | ITM2B | 365 | 5 | 2.426238 | -1.299932 | 65 | 51.14 | 0.786769 |
| GO:0043067\_regulation\_of\_programmed\_cell\_death | API5 | 365 | 5 | 2.426238 | -1.299932 | 65 | 51.14 | 0.786769 |
| GO:0043067\_regulation\_of\_programmed\_cell\_death | HELLS | 365 | 5 | 2.426238 | -1.299932 | 65 | 51.14 | 0.786769 |
| GO:0009966\_regulation\_of\_signal\_transduction | STRAP | 256 | 4 | 2.767428 | -1.274486 | 66 | 51.94 | 0.786970 |
| GO:0009966\_regulation\_of\_signal\_transduction | NLK | 256 | 4 | 2.767428 | -1.274486 | 66 | 51.94 | 0.786970 |
| GO:0009966\_regulation\_of\_signal\_transduction | SKP2 | 256 | 4 | 2.767428 | -1.274486 | 66 | 51.94 | 0.786970 |
| GO:0009966\_regulation\_of\_signal\_transduction | PTPN11 | 256 | 4 | 2.767428 | -1.274486 | 66 | 51.94 | 0.786970 |
| GO:0042445\_hormone\_metabolic\_process | ALDH1A7 | 67 | 2 | 5.287026 | -1.266258 | 67 | 52.33 | 0.781045 |
| GO:0042445\_hormone\_metabolic\_process | PTPN11 | 67 | 2 | 5.287026 | -1.266258 | 67 | 52.33 | 0.781045 |
| GO:0000209\_protein\_polyubiquitination | SKP2 | 10 | 1 | 17.711538 | -1.258850 | 70 | 58.51 | 0.835857 |
| GO:0006081\_cellular\_aldehyde\_metabolic\_process | ALDH1A7 | 10 | 1 | 17.711538 | -1.258850 | 70 | 58.51 | 0.835857 |
| GO:0046887\_positive\_regulation\_of\_hormone\_secretion | PTPN11 | 10 | 1 | 17.711538 | -1.258850 | 70 | 58.51 | 0.835857 |
| GO:0010629\_negative\_regulation\_of\_gene\_expression | STRAP | 262 | 4 | 2.704052 | -1.243966 | 71 | 59.17 | 0.833380 |
| GO:0010629\_negative\_regulation\_of\_gene\_expression | SKP2 | 262 | 4 | 2.704052 | -1.243966 | 71 | 59.17 | 0.833380 |
| GO:0010629\_negative\_regulation\_of\_gene\_expression | DNMT3B | 262 | 4 | 2.704052 | -1.243966 | 71 | 59.17 | 0.833380 |
| GO:0010629\_negative\_regulation\_of\_gene\_expression | HELLS | 262 | 4 | 2.704052 | -1.243966 | 71 | 59.17 | 0.833380 |
| GO:0044255\_cellular\_lipid\_metabolic\_process | ACSM3 | 264 | 4 | 2.683566 | -1.233999 | 72 | 59.72 | 0.829444 |
| GO:0044255\_cellular\_lipid\_metabolic\_process | ALDH1A7 | 264 | 4 | 2.683566 | -1.233999 | 72 | 59.72 | 0.829444 |
| GO:0044255\_cellular\_lipid\_metabolic\_process | PTPN11 | 264 | 4 | 2.683566 | -1.233999 | 72 | 59.72 | 0.829444 |
| GO:0044255\_cellular\_lipid\_metabolic\_process | QK | 264 | 4 | 2.683566 | -1.233999 | 72 | 59.72 | 0.829444 |
| GO:0043687\_post-translational\_protein\_modification | NLK | 384 | 5 | 2.306190 | -1.221162 | 73 | 60.4 | 0.827397 |
| GO:0043687\_post-translational\_protein\_modification | MKNK2 | 384 | 5 | 2.306190 | -1.221162 | 73 | 60.4 | 0.827397 |
| GO:0043687\_post-translational\_protein\_modification | SKP2 | 384 | 5 | 2.306190 | -1.221162 | 73 | 60.4 | 0.827397 |
| GO:0043687\_post-translational\_protein\_modification | PTPN12 | 384 | 5 | 2.306190 | -1.221162 | 73 | 60.4 | 0.827397 |
| GO:0043687\_post-translational\_protein\_modification | PTPN11 | 384 | 5 | 2.306190 | -1.221162 | 73 | 60.4 | 0.827397 |
| GO:0006333\_chromatin\_assembly\_or\_disassembly | HELLS | 11 | 1 | 16.101399 | -1.218630 | 75 | 65.24 | 0.869867 |
| GO:0045055\_regulated\_secretory\_pathway | GARS | 11 | 1 | 16.101399 | -1.218630 | 75 | 65.24 | 0.869867 |
| GO:0016568\_chromatin\_modification | DNMT3B | 72 | 2 | 4.919872 | -1.210698 | 76 | 66.05 | 0.869079 |
| GO:0016568\_chromatin\_modification | HELLS | 72 | 2 | 4.919872 | -1.210698 | 76 | 66.05 | 0.869079 |
| GO:0060255\_regulation\_of\_macromolecule\_metabolic\_process | TAF10 | 936 | 9 | 1.703033 | -1.192869 | 77 | 66.9 | 0.868831 |
| GO:0060255\_regulation\_of\_macromolecule\_metabolic\_process | STRAP | 936 | 9 | 1.703033 | -1.192869 | 77 | 66.9 | 0.868831 |
| GO:0060255\_regulation\_of\_macromolecule\_metabolic\_process | GPBP1 | 936 | 9 | 1.703033 | -1.192869 | 77 | 66.9 | 0.868831 |
| GO:0060255\_regulation\_of\_macromolecule\_metabolic\_process | NLK | 936 | 9 | 1.703033 | -1.192869 | 77 | 66.9 | 0.868831 |
| GO:0060255\_regulation\_of\_macromolecule\_metabolic\_process | OTX2 | 936 | 9 | 1.703033 | -1.192869 | 77 | 66.9 | 0.868831 |
| GO:0060255\_regulation\_of\_macromolecule\_metabolic\_process | SKP2 | 936 | 9 | 1.703033 | -1.192869 | 77 | 66.9 | 0.868831 |
| GO:0060255\_regulation\_of\_macromolecule\_metabolic\_process | DNMT3B | 936 | 9 | 1.703033 | -1.192869 | 77 | 66.9 | 0.868831 |
| GO:0060255\_regulation\_of\_macromolecule\_metabolic\_process | FOXD1 | 936 | 9 | 1.703033 | -1.192869 | 77 | 66.9 | 0.868831 |
| GO:0060255\_regulation\_of\_macromolecule\_metabolic\_process | HELLS | 936 | 9 | 1.703033 | -1.192869 | 77 | 66.9 | 0.868831 |
| GO:0006259\_DNA\_metabolic\_process | RAD51AP1 | 165 | 3 | 3.220280 | -1.192052 | 78 | 66.99 | 0.858846 |
| GO:0006259\_DNA\_metabolic\_process | DNMT3B | 165 | 3 | 3.220280 | -1.192052 | 78 | 66.99 | 0.858846 |
| GO:0006259\_DNA\_metabolic\_process | HELLS | 165 | 3 | 3.220280 | -1.192052 | 78 | 66.99 | 0.858846 |
| GO:0019222\_regulation\_of\_metabolic\_process | TAF10 | 1088 | 10 | 1.627899 | -1.185920 | 79 | 67.27 | 0.851519 |
| GO:0019222\_regulation\_of\_metabolic\_process | GPBP1 | 1088 | 10 | 1.627899 | -1.185920 | 79 | 67.27 | 0.851519 |
| GO:0019222\_regulation\_of\_metabolic\_process | STRAP | 1088 | 10 | 1.627899 | -1.185920 | 79 | 67.27 | 0.851519 |
| GO:0019222\_regulation\_of\_metabolic\_process | NLK | 1088 | 10 | 1.627899 | -1.185920 | 79 | 67.27 | 0.851519 |
| GO:0019222\_regulation\_of\_metabolic\_process | OTX2 | 1088 | 10 | 1.627899 | -1.185920 | 79 | 67.27 | 0.851519 |
| GO:0019222\_regulation\_of\_metabolic\_process | SKP2 | 1088 | 10 | 1.627899 | -1.185920 | 79 | 67.27 | 0.851519 |
| GO:0019222\_regulation\_of\_metabolic\_process | FOXD1 | 1088 | 10 | 1.627899 | -1.185920 | 79 | 67.27 | 0.851519 |
| GO:0019222\_regulation\_of\_metabolic\_process | DNMT3B | 1088 | 10 | 1.627899 | -1.185920 | 79 | 67.27 | 0.851519 |
| GO:0019222\_regulation\_of\_metabolic\_process | HELLS | 1088 | 10 | 1.627899 | -1.185920 | 79 | 67.27 | 0.851519 |
| GO:0019222\_regulation\_of\_metabolic\_process | PTPN11 | 1088 | 10 | 1.627899 | -1.185920 | 79 | 67.27 | 0.851519 |
| GO:0034621\_cellular\_macromolecular\_complex\_subunit\_organization | TAF10 | 76 | 2 | 4.660931 | -1.169336 | 80 | 73.22 | 0.915250 |
| GO:0034621\_cellular\_macromolecular\_complex\_subunit\_organization | HELLS | 76 | 2 | 4.660931 | -1.169336 | 80 | 73.22 | 0.915250 |
| GO:0018105\_peptidyl-serine\_phosphorylation | NLK | 13 | 1 | 13.624260 | -1.148422 | 81 | 79.04 | 0.975802 |
| GO:0006629\_lipid\_metabolic\_process | ACSM3 | 285 | 4 | 2.485830 | -1.135137 | 82 | 79.56 | 0.970244 |
| GO:0006629\_lipid\_metabolic\_process | ALDH1A7 | 285 | 4 | 2.485830 | -1.135137 | 82 | 79.56 | 0.970244 |
| GO:0006629\_lipid\_metabolic\_process | PTPN11 | 285 | 4 | 2.485830 | -1.135137 | 82 | 79.56 | 0.970244 |
| GO:0006629\_lipid\_metabolic\_process | QK | 285 | 4 | 2.485830 | -1.135137 | 82 | 79.56 | 0.970244 |
| GO:0000278\_mitotic\_cell\_cycle | TAF10 | 80 | 2 | 4.427885 | -1.130407 | 84 | 80.0 | 0.952381 |
| GO:0000278\_mitotic\_cell\_cycle | SKP2 | 80 | 2 | 4.427885 | -1.130407 | 84 | 80.0 | 0.952381 |
| GO:0006631\_fatty\_acid\_metabolic\_process | ACSM3 | 80 | 2 | 4.427885 | -1.130407 | 84 | 80.0 | 0.952381 |
| GO:0006631\_fatty\_acid\_metabolic\_process | QK | 80 | 2 | 4.427885 | -1.130407 | 84 | 80.0 | 0.952381 |
| GO:0043066\_negative\_regulation\_of\_apoptosis | SKP2 | 176 | 3 | 3.019012 | -1.125079 | 85 | 80.51 | 0.947176 |
| GO:0043066\_negative\_regulation\_of\_apoptosis | API5 | 176 | 3 | 3.019012 | -1.125079 | 85 | 80.51 | 0.947176 |
| GO:0043066\_negative\_regulation\_of\_apoptosis | HELLS | 176 | 3 | 3.019012 | -1.125079 | 85 | 80.51 | 0.947176 |
| GO:0045449\_regulation\_of\_transcription | GPBP1 | 676 | 7 | 1.834035 | -1.123148 | 86 | 80.59 | 0.937093 |
| GO:0045449\_regulation\_of\_transcription | STRAP | 676 | 7 | 1.834035 | -1.123148 | 86 | 80.59 | 0.937093 |
| GO:0045449\_regulation\_of\_transcription | NLK | 676 | 7 | 1.834035 | -1.123148 | 86 | 80.59 | 0.937093 |
| GO:0045449\_regulation\_of\_transcription | OTX2 | 676 | 7 | 1.834035 | -1.123148 | 86 | 80.59 | 0.937093 |
| GO:0045449\_regulation\_of\_transcription | DNMT3B | 676 | 7 | 1.834035 | -1.123148 | 86 | 80.59 | 0.937093 |
| GO:0045449\_regulation\_of\_transcription | FOXD1 | 676 | 7 | 1.834035 | -1.123148 | 86 | 80.59 | 0.937093 |
| GO:0045449\_regulation\_of\_transcription | HELLS | 676 | 7 | 1.834035 | -1.123148 | 86 | 80.59 | 0.937093 |
| GO:0007242\_intracellular\_signaling\_cascade | NLK | 411 | 5 | 2.154688 | -1.118365 | 87 | 80.71 | 0.927701 |
| GO:0007242\_intracellular\_signaling\_cascade | SKP2 | 411 | 5 | 2.154688 | -1.118365 | 87 | 80.71 | 0.927701 |
| GO:0007242\_intracellular\_signaling\_cascade | MKNK2 | 411 | 5 | 2.154688 | -1.118365 | 87 | 80.71 | 0.927701 |
| GO:0007242\_intracellular\_signaling\_cascade | RAP1A | 411 | 5 | 2.154688 | -1.118365 | 87 | 80.71 | 0.927701 |
| GO:0007242\_intracellular\_signaling\_cascade | PTPN11 | 411 | 5 | 2.154688 | -1.118365 | 87 | 80.71 | 0.927701 |
| GO:0000077\_DNA\_damage\_checkpoint | PTPN11 | 14 | 1 | 12.651099 | -1.117407 | 89 | 85.27 | 0.958090 |
| GO:0042573\_retinoic\_acid\_metabolic\_process | ALDH1A7 | 14 | 1 | 12.651099 | -1.117407 | 89 | 85.27 | 0.958090 |
| GO:0043069\_negative\_regulation\_of\_programmed\_cell\_death | SKP2 | 179 | 3 | 2.968414 | -1.107731 | 91 | 85.75 | 0.942308 |
| GO:0043069\_negative\_regulation\_of\_programmed\_cell\_death | API5 | 179 | 3 | 2.968414 | -1.107731 | 91 | 85.75 | 0.942308 |
| GO:0043069\_negative\_regulation\_of\_programmed\_cell\_death | HELLS | 179 | 3 | 2.968414 | -1.107731 | 91 | 85.75 | 0.942308 |
| GO:0060548\_negative\_regulation\_of\_cell\_death | SKP2 | 179 | 3 | 2.968414 | -1.107731 | 91 | 85.75 | 0.942308 |
| GO:0060548\_negative\_regulation\_of\_cell\_death | API5 | 179 | 3 | 2.968414 | -1.107731 | 91 | 85.75 | 0.942308 |
| GO:0060548\_negative\_regulation\_of\_cell\_death | HELLS | 179 | 3 | 2.968414 | -1.107731 | 91 | 85.75 | 0.942308 |
| GO:0006325\_chromatin\_organization | DNMT3B | 83 | 2 | 4.267841 | -1.102663 | 92 | 86.12 | 0.936087 |
| GO:0006325\_chromatin\_organization | HELLS | 83 | 2 | 4.267841 | -1.102663 | 92 | 86.12 | 0.936087 |
| GO:0019752\_carboxylic\_acid\_metabolic\_process | ACSM3 | 181 | 3 | 2.935614 | -1.096371 | 94 | 86.36 | 0.918723 |
| GO:0019752\_carboxylic\_acid\_metabolic\_process | ALDH1A7 | 181 | 3 | 2.935614 | -1.096371 | 94 | 86.36 | 0.918723 |
| GO:0019752\_carboxylic\_acid\_metabolic\_process | QK | 181 | 3 | 2.935614 | -1.096371 | 94 | 86.36 | 0.918723 |
| GO:0043436\_oxoacid\_metabolic\_process | ACSM3 | 181 | 3 | 2.935614 | -1.096371 | 94 | 86.36 | 0.918723 |
| GO:0043436\_oxoacid\_metabolic\_process | ALDH1A7 | 181 | 3 | 2.935614 | -1.096371 | 94 | 86.36 | 0.918723 |
| GO:0043436\_oxoacid\_metabolic\_process | QK | 181 | 3 | 2.935614 | -1.096371 | 94 | 86.36 | 0.918723 |
| GO:0006082\_organic\_acid\_metabolic\_process | ACSM3 | 182 | 3 | 2.919484 | -1.090751 | 95 | 86.63 | 0.911895 |
| GO:0006082\_organic\_acid\_metabolic\_process | ALDH1A7 | 182 | 3 | 2.919484 | -1.090751 | 95 | 86.63 | 0.911895 |
| GO:0006082\_organic\_acid\_metabolic\_process | QK | 182 | 3 | 2.919484 | -1.090751 | 95 | 86.63 | 0.911895 |
| GO:0050796\_regulation\_of\_insulin\_secretion | PTPN11 | 15 | 1 | 11.807692 | -1.088613 | 96 | 91.01 | 0.948021 |
| GO:0042180\_cellular\_ketone\_metabolic\_process | ACSM3 | 183 | 3 | 2.903531 | -1.085171 | 97 | 91.12 | 0.939381 |
| GO:0042180\_cellular\_ketone\_metabolic\_process | ALDH1A7 | 183 | 3 | 2.903531 | -1.085171 | 97 | 91.12 | 0.939381 |
| GO:0042180\_cellular\_ketone\_metabolic\_process | QK | 183 | 3 | 2.903531 | -1.085171 | 97 | 91.12 | 0.939381 |
| GO:0032990\_cell\_part\_morphogenesis | SSBP1 | 184 | 3 | 2.887751 | -1.079629 | 98 | 91.51 | 0.933776 |
| GO:0032990\_cell\_part\_morphogenesis | FOXD1 | 184 | 3 | 2.887751 | -1.079629 | 98 | 91.51 | 0.933776 |
| GO:0032990\_cell\_part\_morphogenesis | PTPN11 | 184 | 3 | 2.887751 | -1.079629 | 98 | 91.51 | 0.933776 |
| GO:0031570\_DNA\_integrity\_checkpoint | PTPN11 | 16 | 1 | 11.069712 | -1.061752 | 100 | 96.88 | 0.968800 |
| GO:0051048\_negative\_regulation\_of\_secretion | PTPN11 | 16 | 1 | 11.069712 | -1.061752 | 100 | 96.88 | 0.968800 |
| GO:0006350\_transcription | GPBP1 | 701 | 7 | 1.768627 | -1.054725 | 101 | 97.2 | 0.962376 |
| GO:0006350\_transcription | STRAP | 701 | 7 | 1.768627 | -1.054725 | 101 | 97.2 | 0.962376 |
| GO:0006350\_transcription | NLK | 701 | 7 | 1.768627 | -1.054725 | 101 | 97.2 | 0.962376 |
| GO:0006350\_transcription | OTX2 | 701 | 7 | 1.768627 | -1.054725 | 101 | 97.2 | 0.962376 |
| GO:0006350\_transcription | DNMT3B | 701 | 7 | 1.768627 | -1.054725 | 101 | 97.2 | 0.962376 |
| GO:0006350\_transcription | FOXD1 | 701 | 7 | 1.768627 | -1.054725 | 101 | 97.2 | 0.962376 |
| GO:0006350\_transcription | HELLS | 701 | 7 | 1.768627 | -1.054725 | 101 | 97.2 | 0.962376 |
| GO:0002791\_regulation\_of\_peptide\_secretion | PTPN11 | 17 | 1 | 10.418552 | -1.036590 | 107 | 102.17 | 0.954860 |
| GO:0006323\_DNA\_packaging | HELLS | 17 | 1 | 10.418552 | -1.036590 | 107 | 102.17 | 0.954860 |
| GO:0006919\_activation\_of\_caspase\_activity | VCP | 17 | 1 | 10.418552 | -1.036590 | 107 | 102.17 | 0.954860 |
| GO:0008380\_RNA\_splicing | RBM17 | 17 | 1 | 10.418552 | -1.036590 | 107 | 102.17 | 0.954860 |
| GO:0034470\_ncRNA\_processing | DKC1 | 17 | 1 | 10.418552 | -1.036590 | 107 | 102.17 | 0.954860 |
| GO:0042254\_ribosome\_biogenesis | DKC1 | 17 | 1 | 10.418552 | -1.036590 | 107 | 102.17 | 0.954860 |
| GO:0006464\_protein\_modification\_process | NLK | 439 | 5 | 2.017260 | -1.021768 | 108 | 102.95 | 0.953241 |
| GO:0006464\_protein\_modification\_process | MKNK2 | 439 | 5 | 2.017260 | -1.021768 | 108 | 102.95 | 0.953241 |
| GO:0006464\_protein\_modification\_process | SKP2 | 439 | 5 | 2.017260 | -1.021768 | 108 | 102.95 | 0.953241 |
| GO:0006464\_protein\_modification\_process | PTPN12 | 439 | 5 | 2.017260 | -1.021768 | 108 | 102.95 | 0.953241 |
| GO:0006464\_protein\_modification\_process | PTPN11 | 439 | 5 | 2.017260 | -1.021768 | 108 | 102.95 | 0.953241 |
| GO:0065003\_macromolecular\_complex\_assembly | TAF10 | 93 | 2 | 3.808933 | -1.018020 | 109 | 103.51 | 0.949633 |
| GO:0065003\_macromolecular\_complex\_assembly | HELLS | 93 | 2 | 3.808933 | -1.018020 | 109 | 103.51 | 0.949633 |
| GO:0006355\_regulation\_of\_transcription\_\_DNA-dependent | GPBP1 | 575 | 6 | 1.848161 | -1.017029 | 110 | 103.58 | 0.941636 |
| GO:0006355\_regulation\_of\_transcription\_\_DNA-dependent | STRAP | 575 | 6 | 1.848161 | -1.017029 | 110 | 103.58 | 0.941636 |
| GO:0006355\_regulation\_of\_transcription\_\_DNA-dependent | OTX2 | 575 | 6 | 1.848161 | -1.017029 | 110 | 103.58 | 0.941636 |
| GO:0006355\_regulation\_of\_transcription\_\_DNA-dependent | DNMT3B | 575 | 6 | 1.848161 | -1.017029 | 110 | 103.58 | 0.941636 |
| GO:0006355\_regulation\_of\_transcription\_\_DNA-dependent | FOXD1 | 575 | 6 | 1.848161 | -1.017029 | 110 | 103.58 | 0.941636 |
| GO:0006355\_regulation\_of\_transcription\_\_DNA-dependent | HELLS | 575 | 6 | 1.848161 | -1.017029 | 110 | 103.58 | 0.941636 |
| GO:0030178\_negative\_regulation\_of\_Wnt\_receptor\_signaling\_pathway | NLK | 18 | 1 | 9.839744 | -1.012933 | 114 | 108.34 | 0.950351 |
| GO:0030901\_midbrain\_development | OTX2 | 18 | 1 | 9.839744 | -1.012933 | 114 | 108.34 | 0.950351 |
| GO:0033157\_regulation\_of\_intracellular\_protein\_transport | PTPN11 | 18 | 1 | 9.839744 | -1.012933 | 114 | 108.34 | 0.950351 |
| GO:0051168\_nuclear\_export | PTPN11 | 18 | 1 | 9.839744 | -1.012933 | 114 | 108.34 | 0.950351 |
| GO:0008610\_lipid\_biosynthetic\_process | ACSM3 | 94 | 2 | 3.768412 | -1.010151 | 115 | 109.18 | 0.949391 |
| GO:0008610\_lipid\_biosynthetic\_process | QK | 94 | 2 | 3.768412 | -1.010151 | 115 | 109.18 | 0.949391 |
| GO:0006338\_chromatin\_remodeling | HELLS | 19 | 1 | 9.321862 | -0.990617 | 120 | 113.77 | 0.948083 |
| GO:0006776\_vitamin\_A\_metabolic\_process | ALDH1A7 | 19 | 1 | 9.321862 | -0.990617 | 120 | 113.77 | 0.948083 |
| GO:0010952\_positive\_regulation\_of\_peptidase\_activity | VCP | 19 | 1 | 9.321862 | -0.990617 | 120 | 113.77 | 0.948083 |
| GO:0030518\_steroid\_hormone\_receptor\_signaling\_pathway | SKP2 | 19 | 1 | 9.321862 | -0.990617 | 120 | 113.77 | 0.948083 |
| GO:0043280\_positive\_regulation\_of\_caspase\_activity | VCP | 19 | 1 | 9.321862 | -0.990617 | 120 | 113.77 | 0.948083 |
| GO:0009967\_positive\_regulation\_of\_signal\_transduction | SKP2 | 98 | 2 | 3.614600 | -0.979643 | 121 | 114.59 | 0.947025 |
| GO:0009967\_positive\_regulation\_of\_signal\_transduction | PTPN11 | 98 | 2 | 3.614600 | -0.979643 | 121 | 114.59 | 0.947025 |
| GO:0051252\_regulation\_of\_RNA\_metabolic\_process | GPBP1 | 590 | 6 | 1.801173 | -0.975125 | 122 | 114.82 | 0.941148 |
| GO:0051252\_regulation\_of\_RNA\_metabolic\_process | STRAP | 590 | 6 | 1.801173 | -0.975125 | 122 | 114.82 | 0.941148 |
| GO:0051252\_regulation\_of\_RNA\_metabolic\_process | OTX2 | 590 | 6 | 1.801173 | -0.975125 | 122 | 114.82 | 0.941148 |
| GO:0051252\_regulation\_of\_RNA\_metabolic\_process | DNMT3B | 590 | 6 | 1.801173 | -0.975125 | 122 | 114.82 | 0.941148 |
| GO:0051252\_regulation\_of\_RNA\_metabolic\_process | FOXD1 | 590 | 6 | 1.801173 | -0.975125 | 122 | 114.82 | 0.941148 |
| GO:0051252\_regulation\_of\_RNA\_metabolic\_process | HELLS | 590 | 6 | 1.801173 | -0.975125 | 122 | 114.82 | 0.941148 |
| GO:0007243\_protein\_kinase\_cascade | NLK | 205 | 3 | 2.591932 | -0.971589 | 123 | 115.44 | 0.938537 |
| GO:0007243\_protein\_kinase\_cascade | MKNK2 | 205 | 3 | 2.591932 | -0.971589 | 123 | 115.44 | 0.938537 |
| GO:0007243\_protein\_kinase\_cascade | PTPN11 | 205 | 3 | 2.591932 | -0.971589 | 123 | 115.44 | 0.938537 |
| GO:0018209\_peptidyl-serine\_modification | NLK | 20 | 1 | 8.855769 | -0.969505 | 125 | 118.63 | 0.949040 |
| GO:0046822\_regulation\_of\_nucleocytoplasmic\_transport | PTPN11 | 20 | 1 | 8.855769 | -0.969505 | 125 | 118.63 | 0.949040 |
| GO:0006351\_transcription\_\_DNA-dependent | GPBP1 | 594 | 6 | 1.789044 | -0.964244 | 126 | 118.85 | 0.943254 |
| GO:0006351\_transcription\_\_DNA-dependent | STRAP | 594 | 6 | 1.789044 | -0.964244 | 126 | 118.85 | 0.943254 |
| GO:0006351\_transcription\_\_DNA-dependent | OTX2 | 594 | 6 | 1.789044 | -0.964244 | 126 | 118.85 | 0.943254 |
| GO:0006351\_transcription\_\_DNA-dependent | DNMT3B | 594 | 6 | 1.789044 | -0.964244 | 126 | 118.85 | 0.943254 |
| GO:0006351\_transcription\_\_DNA-dependent | FOXD1 | 594 | 6 | 1.789044 | -0.964244 | 126 | 118.85 | 0.943254 |
| GO:0006351\_transcription\_\_DNA-dependent | HELLS | 594 | 6 | 1.789044 | -0.964244 | 126 | 118.85 | 0.943254 |
| GO:0032774\_RNA\_biosynthetic\_process | GPBP1 | 595 | 6 | 1.786037 | -0.961542 | 127 | 118.92 | 0.936378 |
| GO:0032774\_RNA\_biosynthetic\_process | STRAP | 595 | 6 | 1.786037 | -0.961542 | 127 | 118.92 | 0.936378 |
| GO:0032774\_RNA\_biosynthetic\_process | OTX2 | 595 | 6 | 1.786037 | -0.961542 | 127 | 118.92 | 0.936378 |
| GO:0032774\_RNA\_biosynthetic\_process | DNMT3B | 595 | 6 | 1.786037 | -0.961542 | 127 | 118.92 | 0.936378 |
| GO:0032774\_RNA\_biosynthetic\_process | FOXD1 | 595 | 6 | 1.786037 | -0.961542 | 127 | 118.92 | 0.936378 |
| GO:0032774\_RNA\_biosynthetic\_process | HELLS | 595 | 6 | 1.786037 | -0.961542 | 127 | 118.92 | 0.936378 |
| GO:0010646\_regulation\_of\_cell\_communication | STRAP | 330 | 4 | 2.146853 | -0.953711 | 128 | 119.49 | 0.933516 |
| GO:0010646\_regulation\_of\_cell\_communication | NLK | 330 | 4 | 2.146853 | -0.953711 | 128 | 119.49 | 0.933516 |
| GO:0010646\_regulation\_of\_cell\_communication | SKP2 | 330 | 4 | 2.146853 | -0.953711 | 128 | 119.49 | 0.933516 |
| GO:0010646\_regulation\_of\_cell\_communication | PTPN11 | 330 | 4 | 2.146853 | -0.953711 | 128 | 119.49 | 0.933516 |
| GO:0010605\_negative\_regulation\_of\_macromolecule\_metabolic\_process | STRAP | 331 | 4 | 2.140367 | -0.950084 | 129 | 119.75 | 0.928295 |
| GO:0010605\_negative\_regulation\_of\_macromolecule\_metabolic\_process | SKP2 | 331 | 4 | 2.140367 | -0.950084 | 129 | 119.75 | 0.928295 |
| GO:0010605\_negative\_regulation\_of\_macromolecule\_metabolic\_process | DNMT3B | 331 | 4 | 2.140367 | -0.950084 | 129 | 119.75 | 0.928295 |
| GO:0010605\_negative\_regulation\_of\_macromolecule\_metabolic\_process | HELLS | 331 | 4 | 2.140367 | -0.950084 | 129 | 119.75 | 0.928295 |
| GO:0000075\_cell\_cycle\_checkpoint | PTPN11 | 21 | 1 | 8.434066 | -0.949479 | 131 | 124.21 | 0.948168 |
| GO:0001754\_eye\_photoreceptor\_cell\_differentiation | OTX2 | 21 | 1 | 8.434066 | -0.949479 | 131 | 124.21 | 0.948168 |
| GO:0010556\_regulation\_of\_macromolecule\_biosynthetic\_process | STRAP | 745 | 7 | 1.664171 | -0.943800 | 132 | 124.33 | 0.941894 |
| GO:0010556\_regulation\_of\_macromolecule\_biosynthetic\_process | GPBP1 | 745 | 7 | 1.664171 | -0.943800 | 132 | 124.33 | 0.941894 |
| GO:0010556\_regulation\_of\_macromolecule\_biosynthetic\_process | NLK | 745 | 7 | 1.664171 | -0.943800 | 132 | 124.33 | 0.941894 |
| GO:0010556\_regulation\_of\_macromolecule\_biosynthetic\_process | OTX2 | 745 | 7 | 1.664171 | -0.943800 | 132 | 124.33 | 0.941894 |
| GO:0010556\_regulation\_of\_macromolecule\_biosynthetic\_process | DNMT3B | 745 | 7 | 1.664171 | -0.943800 | 132 | 124.33 | 0.941894 |
| GO:0010556\_regulation\_of\_macromolecule\_biosynthetic\_process | FOXD1 | 745 | 7 | 1.664171 | -0.943800 | 132 | 124.33 | 0.941894 |
| GO:0010556\_regulation\_of\_macromolecule\_biosynthetic\_process | HELLS | 745 | 7 | 1.664171 | -0.943800 | 132 | 124.33 | 0.941894 |
| GO:0009968\_negative\_regulation\_of\_signal\_transduction | STRAP | 103 | 2 | 3.439134 | -0.943540 | 133 | 124.64 | 0.937143 |
| GO:0009968\_negative\_regulation\_of\_signal\_transduction | NLK | 103 | 2 | 3.439134 | -0.943540 | 133 | 124.64 | 0.937143 |
| GO:0001523\_retinoid\_metabolic\_process | ALDH1A7 | 22 | 1 | 8.050699 | -0.930439 | 138 | 130.18 | 0.943333 |
| GO:0006721\_terpenoid\_metabolic\_process | ALDH1A7 | 22 | 1 | 8.050699 | -0.930439 | 138 | 130.18 | 0.943333 |
| GO:0016101\_diterpenoid\_metabolic\_process | ALDH1A7 | 22 | 1 | 8.050699 | -0.930439 | 138 | 130.18 | 0.943333 |
| GO:0034660\_ncRNA\_metabolic\_process | DKC1 | 22 | 1 | 8.050699 | -0.930439 | 138 | 130.18 | 0.943333 |
| GO:0046883\_regulation\_of\_hormone\_secretion | PTPN11 | 22 | 1 | 8.050699 | -0.930439 | 138 | 130.18 | 0.943333 |
| GO:0010817\_regulation\_of\_hormone\_levels | ALDH1A7 | 106 | 2 | 3.341800 | -0.922878 | 139 | 130.53 | 0.939065 |
| GO:0010817\_regulation\_of\_hormone\_levels | PTPN11 | 106 | 2 | 3.341800 | -0.922878 | 139 | 130.53 | 0.939065 |
| GO:0006793\_phosphorus\_metabolic\_process | NLK | 340 | 4 | 2.083710 | -0.918144 | 141 | 130.96 | 0.928794 |
| GO:0006793\_phosphorus\_metabolic\_process | MKNK2 | 340 | 4 | 2.083710 | -0.918144 | 141 | 130.96 | 0.928794 |
| GO:0006793\_phosphorus\_metabolic\_process | PTPN12 | 340 | 4 | 2.083710 | -0.918144 | 141 | 130.96 | 0.928794 |
| GO:0006793\_phosphorus\_metabolic\_process | PTPN11 | 340 | 4 | 2.083710 | -0.918144 | 141 | 130.96 | 0.928794 |
| GO:0006796\_phosphate\_metabolic\_process | NLK | 340 | 4 | 2.083710 | -0.918144 | 141 | 130.96 | 0.928794 |
| GO:0006796\_phosphate\_metabolic\_process | MKNK2 | 340 | 4 | 2.083710 | -0.918144 | 141 | 130.96 | 0.928794 |
| GO:0006796\_phosphate\_metabolic\_process | PTPN12 | 340 | 4 | 2.083710 | -0.918144 | 141 | 130.96 | 0.928794 |
| GO:0006796\_phosphate\_metabolic\_process | PTPN11 | 340 | 4 | 2.083710 | -0.918144 | 141 | 130.96 | 0.928794 |
| GO:0019219\_regulation\_of\_nucleobase\_\_nucleoside\_\_nucleotide\_and\_nucleic\_acid\_metabolic\_process | STRAP | 757 | 7 | 1.637791 | -0.915499 | 142 | 131.34 | 0.924930 |
| GO:0019219\_regulation\_of\_nucleobase\_\_nucleoside\_\_nucleotide\_and\_nucleic\_acid\_metabolic\_process | GPBP1 | 757 | 7 | 1.637791 | -0.915499 | 142 | 131.34 | 0.924930 |
| GO:0019219\_regulation\_of\_nucleobase\_\_nucleoside\_\_nucleotide\_and\_nucleic\_acid\_metabolic\_process | NLK | 757 | 7 | 1.637791 | -0.915499 | 142 | 131.34 | 0.924930 |
| GO:0019219\_regulation\_of\_nucleobase\_\_nucleoside\_\_nucleotide\_and\_nucleic\_acid\_metabolic\_process | OTX2 | 757 | 7 | 1.637791 | -0.915499 | 142 | 131.34 | 0.924930 |
| GO:0019219\_regulation\_of\_nucleobase\_\_nucleoside\_\_nucleotide\_and\_nucleic\_acid\_metabolic\_process | DNMT3B | 757 | 7 | 1.637791 | -0.915499 | 142 | 131.34 | 0.924930 |
| GO:0019219\_regulation\_of\_nucleobase\_\_nucleoside\_\_nucleotide\_and\_nucleic\_acid\_metabolic\_process | FOXD1 | 757 | 7 | 1.637791 | -0.915499 | 142 | 131.34 | 0.924930 |
| GO:0019219\_regulation\_of\_nucleobase\_\_nucleoside\_\_nucleotide\_and\_nucleic\_acid\_metabolic\_process | HELLS | 757 | 7 | 1.637791 | -0.915499 | 142 | 131.34 | 0.924930 |
| GO:0006397\_mRNA\_processing | RBM17 | 23 | 1 | 7.700669 | -0.912295 | 145 | 134.15 | 0.925172 |
| GO:0022613\_ribonucleoprotein\_complex\_biogenesis | DKC1 | 23 | 1 | 7.700669 | -0.912295 | 145 | 134.15 | 0.925172 |
| GO:0030512\_negative\_regulation\_of\_transforming\_growth\_factor\_beta\_receptor\_signaling\_pathway | STRAP | 23 | 1 | 7.700669 | -0.912295 | 145 | 134.15 | 0.925172 |
| GO:0045892\_negative\_regulation\_of\_transcription\_\_DNA-dependent | STRAP | 218 | 3 | 2.437368 | -0.911779 | 146 | 134.21 | 0.919247 |
| GO:0045892\_negative\_regulation\_of\_transcription\_\_DNA-dependent | DNMT3B | 218 | 3 | 2.437368 | -0.911779 | 146 | 134.21 | 0.919247 |
| GO:0045892\_negative\_regulation\_of\_transcription\_\_DNA-dependent | HELLS | 218 | 3 | 2.437368 | -0.911779 | 146 | 134.21 | 0.919247 |
| GO:0051253\_negative\_regulation\_of\_RNA\_metabolic\_process | STRAP | 220 | 3 | 2.415210 | -0.903004 | 147 | 134.74 | 0.916599 |
| GO:0051253\_negative\_regulation\_of\_RNA\_metabolic\_process | DNMT3B | 220 | 3 | 2.415210 | -0.903004 | 147 | 134.74 | 0.916599 |
| GO:0051253\_negative\_regulation\_of\_RNA\_metabolic\_process | HELLS | 220 | 3 | 2.415210 | -0.903004 | 147 | 134.74 | 0.916599 |
| GO:0010647\_positive\_regulation\_of\_cell\_communication | SKP2 | 110 | 2 | 3.220280 | -0.896409 | 149 | 135.39 | 0.908658 |
| GO:0010647\_positive\_regulation\_of\_cell\_communication | PTPN11 | 110 | 2 | 3.220280 | -0.896409 | 149 | 135.39 | 0.908658 |
| GO:0010648\_negative\_regulation\_of\_cell\_communication | STRAP | 110 | 2 | 3.220280 | -0.896409 | 149 | 135.39 | 0.908658 |
| GO:0010648\_negative\_regulation\_of\_cell\_communication | NLK | 110 | 2 | 3.220280 | -0.896409 | 149 | 135.39 | 0.908658 |
| GO:0007259\_JAK-STAT\_cascade | NLK | 24 | 1 | 7.379808 | -0.894972 | 151 | 138.78 | 0.919073 |
| GO:0032386\_regulation\_of\_intracellular\_transport | PTPN11 | 24 | 1 | 7.379808 | -0.894972 | 151 | 138.78 | 0.919073 |
| GO:0065007\_biological\_regulation | STRAP | 2593 | 18 | 1.229494 | -0.893822 | 152 | 138.97 | 0.914276 |
| GO:0065007\_biological\_regulation | GPBP1 | 2593 | 18 | 1.229494 | -0.893822 | 152 | 138.97 | 0.914276 |
| GO:0065007\_biological\_regulation | NLK | 2593 | 18 | 1.229494 | -0.893822 | 152 | 138.97 | 0.914276 |
| GO:0065007\_biological\_regulation | OTX2 | 2593 | 18 | 1.229494 | -0.893822 | 152 | 138.97 | 0.914276 |
| GO:0065007\_biological\_regulation | MKNK2 | 2593 | 18 | 1.229494 | -0.893822 | 152 | 138.97 | 0.914276 |
| GO:0065007\_biological\_regulation | SKP2 | 2593 | 18 | 1.229494 | -0.893822 | 152 | 138.97 | 0.914276 |
| GO:0065007\_biological\_regulation | ITM2B | 2593 | 18 | 1.229494 | -0.893822 | 152 | 138.97 | 0.914276 |
| GO:0065007\_biological\_regulation | QK | 2593 | 18 | 1.229494 | -0.893822 | 152 | 138.97 | 0.914276 |
| GO:0065007\_biological\_regulation | PTPN11 | 2593 | 18 | 1.229494 | -0.893822 | 152 | 138.97 | 0.914276 |
| GO:0065007\_biological\_regulation | TAF10 | 2593 | 18 | 1.229494 | -0.893822 | 152 | 138.97 | 0.914276 |
| GO:0065007\_biological\_regulation | RGS2 | 2593 | 18 | 1.229494 | -0.893822 | 152 | 138.97 | 0.914276 |
| GO:0065007\_biological\_regulation | VCP | 2593 | 18 | 1.229494 | -0.893822 | 152 | 138.97 | 0.914276 |
| GO:0065007\_biological\_regulation | RAP1A | 2593 | 18 | 1.229494 | -0.893822 | 152 | 138.97 | 0.914276 |
| GO:0065007\_biological\_regulation | ALDH1A7 | 2593 | 18 | 1.229494 | -0.893822 | 152 | 138.97 | 0.914276 |
| GO:0065007\_biological\_regulation | DNMT3B | 2593 | 18 | 1.229494 | -0.893822 | 152 | 138.97 | 0.914276 |
| GO:0065007\_biological\_regulation | FOXD1 | 2593 | 18 | 1.229494 | -0.893822 | 152 | 138.97 | 0.914276 |
| GO:0065007\_biological\_regulation | HELLS | 2593 | 18 | 1.229494 | -0.893822 | 152 | 138.97 | 0.914276 |
| GO:0065007\_biological\_regulation | API5 | 2593 | 18 | 1.229494 | -0.893822 | 152 | 138.97 | 0.914276 |
| GO:0009892\_negative\_regulation\_of\_metabolic\_process | STRAP | 348 | 4 | 2.035809 | -0.890778 | 153 | 139.15 | 0.909477 |
| GO:0009892\_negative\_regulation\_of\_metabolic\_process | SKP2 | 348 | 4 | 2.035809 | -0.890778 | 153 | 139.15 | 0.909477 |
| GO:0009892\_negative\_regulation\_of\_metabolic\_process | DNMT3B | 348 | 4 | 2.035809 | -0.890778 | 153 | 139.15 | 0.909477 |
| GO:0009892\_negative\_regulation\_of\_metabolic\_process | HELLS | 348 | 4 | 2.035809 | -0.890778 | 153 | 139.15 | 0.909477 |
| GO:0051171\_regulation\_of\_nitrogen\_compound\_metabolic\_process | STRAP | 771 | 7 | 1.608051 | -0.883472 | 154 | 139.43 | 0.905390 |
| GO:0051171\_regulation\_of\_nitrogen\_compound\_metabolic\_process | GPBP1 | 771 | 7 | 1.608051 | -0.883472 | 154 | 139.43 | 0.905390 |
| GO:0051171\_regulation\_of\_nitrogen\_compound\_metabolic\_process | NLK | 771 | 7 | 1.608051 | -0.883472 | 154 | 139.43 | 0.905390 |
| GO:0051171\_regulation\_of\_nitrogen\_compound\_metabolic\_process | OTX2 | 771 | 7 | 1.608051 | -0.883472 | 154 | 139.43 | 0.905390 |
| GO:0051171\_regulation\_of\_nitrogen\_compound\_metabolic\_process | DNMT3B | 771 | 7 | 1.608051 | -0.883472 | 154 | 139.43 | 0.905390 |
| GO:0051171\_regulation\_of\_nitrogen\_compound\_metabolic\_process | FOXD1 | 771 | 7 | 1.608051 | -0.883472 | 154 | 139.43 | 0.905390 |
| GO:0051171\_regulation\_of\_nitrogen\_compound\_metabolic\_process | HELLS | 771 | 7 | 1.608051 | -0.883472 | 154 | 139.43 | 0.905390 |
| GO:0006775\_fat-soluble\_vitamin\_metabolic\_process | ALDH1A7 | 25 | 1 | 7.084615 | -0.878403 | 156 | 142.64 | 0.914359 |
| GO:0007492\_endoderm\_development | OTX2 | 25 | 1 | 7.084615 | -0.878403 | 156 | 142.64 | 0.914359 |
| GO:0048523\_negative\_regulation\_of\_cellular\_process | STRAP | 774 | 7 | 1.601819 | -0.876744 | 157 | 143.09 | 0.911401 |
| GO:0048523\_negative\_regulation\_of\_cellular\_process | NLK | 774 | 7 | 1.601819 | -0.876744 | 157 | 143.09 | 0.911401 |
| GO:0048523\_negative\_regulation\_of\_cellular\_process | SKP2 | 774 | 7 | 1.601819 | -0.876744 | 157 | 143.09 | 0.911401 |
| GO:0048523\_negative\_regulation\_of\_cellular\_process | DNMT3B | 774 | 7 | 1.601819 | -0.876744 | 157 | 143.09 | 0.911401 |
| GO:0048523\_negative\_regulation\_of\_cellular\_process | API5 | 774 | 7 | 1.601819 | -0.876744 | 157 | 143.09 | 0.911401 |
| GO:0048523\_negative\_regulation\_of\_cellular\_process | HELLS | 774 | 7 | 1.601819 | -0.876744 | 157 | 143.09 | 0.911401 |
| GO:0048523\_negative\_regulation\_of\_cellular\_process | PTPN11 | 774 | 7 | 1.601819 | -0.876744 | 157 | 143.09 | 0.911401 |
| GO:0001658\_branching\_involved\_in\_ureteric\_bud\_morphogenesis | FOXD1 | 26 | 1 | 6.812130 | -0.862529 | 162 | 147.35 | 0.909568 |
| GO:0006720\_isoprenoid\_metabolic\_process | ALDH1A7 | 26 | 1 | 6.812130 | -0.862529 | 162 | 147.35 | 0.909568 |
| GO:0046530\_photoreceptor\_cell\_differentiation | OTX2 | 26 | 1 | 6.812130 | -0.862529 | 162 | 147.35 | 0.909568 |
| GO:0050873\_brown\_fat\_cell\_differentiation | RGS2 | 26 | 1 | 6.812130 | -0.862529 | 162 | 147.35 | 0.909568 |
| GO:0060675\_ureteric\_bud\_morphogenesis | FOXD1 | 26 | 1 | 6.812130 | -0.862529 | 162 | 147.35 | 0.909568 |
| GO:0043933\_macromolecular\_complex\_subunit\_organization | TAF10 | 117 | 2 | 3.027613 | -0.852810 | 163 | 148.02 | 0.908098 |
| GO:0043933\_macromolecular\_complex\_subunit\_organization | HELLS | 117 | 2 | 3.027613 | -0.852810 | 163 | 148.02 | 0.908098 |
| GO:0022403\_cell\_cycle\_phase | TAF10 | 119 | 2 | 2.976729 | -0.840942 | 164 | 151.44 | 0.923415 |
| GO:0022403\_cell\_cycle\_phase | SKP2 | 119 | 2 | 2.976729 | -0.840942 | 164 | 151.44 | 0.923415 |
| GO:0006470\_protein\_amino\_acid\_dephosphorylation | PTPN12 | 28 | 1 | 6.325549 | -0.832659 | 167 | 153.99 | 0.922096 |
| GO:0030073\_insulin\_secretion | PTPN11 | 28 | 1 | 6.325549 | -0.832659 | 167 | 153.99 | 0.922096 |
| GO:0030111\_regulation\_of\_Wnt\_receptor\_signaling\_pathway | NLK | 28 | 1 | 6.325549 | -0.832659 | 167 | 153.99 | 0.922096 |
| GO:0006468\_protein\_amino\_acid\_phosphorylation | NLK | 237 | 3 | 2.241967 | -0.832564 | 169 | 154.2 | 0.912426 |
| GO:0006468\_protein\_amino\_acid\_phosphorylation | MKNK2 | 237 | 3 | 2.241967 | -0.832564 | 169 | 154.2 | 0.912426 |
| GO:0006468\_protein\_amino\_acid\_phosphorylation | PTPN11 | 237 | 3 | 2.241967 | -0.832564 | 169 | 154.2 | 0.912426 |
| GO:0044085\_cellular\_component\_biogenesis | TAF10 | 237 | 3 | 2.241967 | -0.832564 | 169 | 154.2 | 0.912426 |
| GO:0044085\_cellular\_component\_biogenesis | DKC1 | 237 | 3 | 2.241967 | -0.832564 | 169 | 154.2 | 0.912426 |
| GO:0044085\_cellular\_component\_biogenesis | HELLS | 237 | 3 | 2.241967 | -0.832564 | 169 | 154.2 | 0.912426 |
| GO:0051726\_regulation\_of\_cell\_cycle | SKP2 | 121 | 2 | 2.927527 | -0.829319 | 170 | 154.56 | 0.909176 |
| GO:0051726\_regulation\_of\_cell\_cycle | PTPN11 | 121 | 2 | 2.927527 | -0.829319 | 170 | 154.56 | 0.909176 |
| GO:0007049\_cell\_cycle | TAF10 | 238 | 3 | 2.232547 | -0.828639 | 171 | 154.65 | 0.904386 |
| GO:0007049\_cell\_cycle | SKP2 | 238 | 3 | 2.232547 | -0.828639 | 171 | 154.65 | 0.904386 |
| GO:0007049\_cell\_cycle | PTPN11 | 238 | 3 | 2.232547 | -0.828639 | 171 | 154.65 | 0.904386 |
| GO:0006641\_triglyceride\_metabolic\_process | PTPN11 | 29 | 1 | 6.107427 | -0.818576 | 175 | 158.58 | 0.906171 |
| GO:0042770\_DNA\_damage\_response\_\_signal\_transduction | PTPN11 | 29 | 1 | 6.107427 | -0.818576 | 175 | 158.58 | 0.906171 |
| GO:0043281\_regulation\_of\_caspase\_activity | VCP | 29 | 1 | 6.107427 | -0.818576 | 175 | 158.58 | 0.906171 |
| GO:0052548\_regulation\_of\_endopeptidase\_activity | VCP | 29 | 1 | 6.107427 | -0.818576 | 175 | 158.58 | 0.906171 |
| GO:0034961\_cellular\_biopolymer\_biosynthetic\_process | GPBP1 | 804 | 7 | 1.542049 | -0.811993 | 176 | 159.18 | 0.904432 |
| GO:0034961\_cellular\_biopolymer\_biosynthetic\_process | STRAP | 804 | 7 | 1.542049 | -0.811993 | 176 | 159.18 | 0.904432 |
| GO:0034961\_cellular\_biopolymer\_biosynthetic\_process | NLK | 804 | 7 | 1.542049 | -0.811993 | 176 | 159.18 | 0.904432 |
| GO:0034961\_cellular\_biopolymer\_biosynthetic\_process | OTX2 | 804 | 7 | 1.542049 | -0.811993 | 176 | 159.18 | 0.904432 |
| GO:0034961\_cellular\_biopolymer\_biosynthetic\_process | DNMT3B | 804 | 7 | 1.542049 | -0.811993 | 176 | 159.18 | 0.904432 |
| GO:0034961\_cellular\_biopolymer\_biosynthetic\_process | FOXD1 | 804 | 7 | 1.542049 | -0.811993 | 176 | 159.18 | 0.904432 |
| GO:0034961\_cellular\_biopolymer\_biosynthetic\_process | HELLS | 804 | 7 | 1.542049 | -0.811993 | 176 | 159.18 | 0.904432 |
| GO:0043284\_biopolymer\_biosynthetic\_process | GPBP1 | 807 | 7 | 1.536317 | -0.805763 | 177 | 159.58 | 0.901582 |
| GO:0043284\_biopolymer\_biosynthetic\_process | STRAP | 807 | 7 | 1.536317 | -0.805763 | 177 | 159.58 | 0.901582 |
| GO:0043284\_biopolymer\_biosynthetic\_process | NLK | 807 | 7 | 1.536317 | -0.805763 | 177 | 159.58 | 0.901582 |
| GO:0043284\_biopolymer\_biosynthetic\_process | OTX2 | 807 | 7 | 1.536317 | -0.805763 | 177 | 159.58 | 0.901582 |
| GO:0043284\_biopolymer\_biosynthetic\_process | DNMT3B | 807 | 7 | 1.536317 | -0.805763 | 177 | 159.58 | 0.901582 |
| GO:0043284\_biopolymer\_biosynthetic\_process | FOXD1 | 807 | 7 | 1.536317 | -0.805763 | 177 | 159.58 | 0.901582 |
| GO:0043284\_biopolymer\_biosynthetic\_process | HELLS | 807 | 7 | 1.536317 | -0.805763 | 177 | 159.58 | 0.901582 |
| GO:0000187\_activation\_of\_MAPK\_activity | PTPN11 | 30 | 1 | 5.903846 | -0.805008 | 184 | 162.79 | 0.884728 |
| GO:0030522\_intracellular\_receptor-mediated\_signaling\_pathway | SKP2 | 30 | 1 | 5.903846 | -0.805008 | 184 | 162.79 | 0.884728 |
| GO:0033500\_carbohydrate\_homeostasis | PTPN11 | 30 | 1 | 5.903846 | -0.805008 | 184 | 162.79 | 0.884728 |
| GO:0035265\_organ\_growth | PTPN11 | 30 | 1 | 5.903846 | -0.805008 | 184 | 162.79 | 0.884728 |
| GO:0042552\_myelination | QK | 30 | 1 | 5.903846 | -0.805008 | 184 | 162.79 | 0.884728 |
| GO:0042593\_glucose\_homeostasis | PTPN11 | 30 | 1 | 5.903846 | -0.805008 | 184 | 162.79 | 0.884728 |
| GO:0052547\_regulation\_of\_peptidase\_activity | VCP | 30 | 1 | 5.903846 | -0.805008 | 184 | 162.79 | 0.884728 |
| GO:0031326\_regulation\_of\_cellular\_biosynthetic\_process | GPBP1 | 812 | 7 | 1.526857 | -0.795475 | 185 | 163.12 | 0.881730 |
| GO:0031326\_regulation\_of\_cellular\_biosynthetic\_process | STRAP | 812 | 7 | 1.526857 | -0.795475 | 185 | 163.12 | 0.881730 |
| GO:0031326\_regulation\_of\_cellular\_biosynthetic\_process | NLK | 812 | 7 | 1.526857 | -0.795475 | 185 | 163.12 | 0.881730 |
| GO:0031326\_regulation\_of\_cellular\_biosynthetic\_process | OTX2 | 812 | 7 | 1.526857 | -0.795475 | 185 | 163.12 | 0.881730 |
| GO:0031326\_regulation\_of\_cellular\_biosynthetic\_process | FOXD1 | 812 | 7 | 1.526857 | -0.795475 | 185 | 163.12 | 0.881730 |
| GO:0031326\_regulation\_of\_cellular\_biosynthetic\_process | DNMT3B | 812 | 7 | 1.526857 | -0.795475 | 185 | 163.12 | 0.881730 |
| GO:0031326\_regulation\_of\_cellular\_biosynthetic\_process | HELLS | 812 | 7 | 1.526857 | -0.795475 | 185 | 163.12 | 0.881730 |
| GO:0006639\_acylglycerol\_metabolic\_process | PTPN11 | 31 | 1 | 5.713400 | -0.791921 | 187 | 167.43 | 0.895348 |
| GO:0016311\_dephosphorylation | PTPN12 | 31 | 1 | 5.713400 | -0.791921 | 187 | 167.43 | 0.895348 |
| GO:0001655\_urogenital\_system\_development | FOXD1 | 128 | 2 | 2.767428 | -0.790458 | 188 | 168.01 | 0.893670 |
| GO:0001655\_urogenital\_system\_development | HELLS | 128 | 2 | 2.767428 | -0.790458 | 188 | 168.01 | 0.893670 |
| GO:0009889\_regulation\_of\_biosynthetic\_process | GPBP1 | 815 | 7 | 1.521236 | -0.789359 | 189 | 168.1 | 0.889418 |
| GO:0009889\_regulation\_of\_biosynthetic\_process | STRAP | 815 | 7 | 1.521236 | -0.789359 | 189 | 168.1 | 0.889418 |
| GO:0009889\_regulation\_of\_biosynthetic\_process | NLK | 815 | 7 | 1.521236 | -0.789359 | 189 | 168.1 | 0.889418 |
| GO:0009889\_regulation\_of\_biosynthetic\_process | OTX2 | 815 | 7 | 1.521236 | -0.789359 | 189 | 168.1 | 0.889418 |
| GO:0009889\_regulation\_of\_biosynthetic\_process | FOXD1 | 815 | 7 | 1.521236 | -0.789359 | 189 | 168.1 | 0.889418 |
| GO:0009889\_regulation\_of\_biosynthetic\_process | DNMT3B | 815 | 7 | 1.521236 | -0.789359 | 189 | 168.1 | 0.889418 |
| GO:0009889\_regulation\_of\_biosynthetic\_process | HELLS | 815 | 7 | 1.521236 | -0.789359 | 189 | 168.1 | 0.889418 |
| GO:0051276\_chromosome\_organization | DNMT3B | 129 | 2 | 2.745975 | -0.785125 | 190 | 168.3 | 0.885789 |
| GO:0051276\_chromosome\_organization | HELLS | 129 | 2 | 2.745975 | -0.785125 | 190 | 168.3 | 0.885789 |
| GO:0006638\_neutral\_lipid\_metabolic\_process | PTPN11 | 32 | 1 | 5.534856 | -0.779287 | 194 | 170.96 | 0.881237 |
| GO:0006662\_glycerol\_ether\_metabolic\_process | PTPN11 | 32 | 1 | 5.534856 | -0.779287 | 194 | 170.96 | 0.881237 |
| GO:0007272\_ensheathment\_of\_neurons | QK | 32 | 1 | 5.534856 | -0.779287 | 194 | 170.96 | 0.881237 |
| GO:0008366\_axon\_ensheathment | QK | 32 | 1 | 5.534856 | -0.779287 | 194 | 170.96 | 0.881237 |
| GO:0016481\_negative\_regulation\_of\_transcription | STRAP | 253 | 3 | 2.100182 | -0.772402 | 195 | 171.52 | 0.879590 |
| GO:0016481\_negative\_regulation\_of\_transcription | DNMT3B | 253 | 3 | 2.100182 | -0.772402 | 195 | 171.52 | 0.879590 |
| GO:0016481\_negative\_regulation\_of\_transcription | HELLS | 253 | 3 | 2.100182 | -0.772402 | 195 | 171.52 | 0.879590 |
| GO:0007283\_spermatogenesis | RGS2 | 134 | 2 | 2.643513 | -0.759225 | 197 | 175.07 | 0.888680 |
| GO:0007283\_spermatogenesis | QK | 134 | 2 | 2.643513 | -0.759225 | 197 | 175.07 | 0.888680 |
| GO:0048232\_male\_gamete\_generation | RGS2 | 134 | 2 | 2.643513 | -0.759225 | 197 | 175.07 | 0.888680 |
| GO:0048232\_male\_gamete\_generation | QK | 134 | 2 | 2.643513 | -0.759225 | 197 | 175.07 | 0.888680 |
| GO:0051047\_positive\_regulation\_of\_secretion | PTPN11 | 34 | 1 | 5.209276 | -0.755262 | 199 | 178.06 | 0.894774 |
| GO:0051052\_regulation\_of\_DNA\_metabolic\_process | HELLS | 34 | 1 | 5.209276 | -0.755262 | 199 | 178.06 | 0.894774 |
| GO:0044249\_cellular\_biosynthetic\_process | ACSM3 | 1150 | 9 | 1.386120 | -0.747241 | 200 | 178.47 | 0.892350 |
| GO:0044249\_cellular\_biosynthetic\_process | GPBP1 | 1150 | 9 | 1.386120 | -0.747241 | 200 | 178.47 | 0.892350 |
| GO:0044249\_cellular\_biosynthetic\_process | STRAP | 1150 | 9 | 1.386120 | -0.747241 | 200 | 178.47 | 0.892350 |
| GO:0044249\_cellular\_biosynthetic\_process | NLK | 1150 | 9 | 1.386120 | -0.747241 | 200 | 178.47 | 0.892350 |
| GO:0044249\_cellular\_biosynthetic\_process | OTX2 | 1150 | 9 | 1.386120 | -0.747241 | 200 | 178.47 | 0.892350 |
| GO:0044249\_cellular\_biosynthetic\_process | FOXD1 | 1150 | 9 | 1.386120 | -0.747241 | 200 | 178.47 | 0.892350 |
| GO:0044249\_cellular\_biosynthetic\_process | DNMT3B | 1150 | 9 | 1.386120 | -0.747241 | 200 | 178.47 | 0.892350 |
| GO:0044249\_cellular\_biosynthetic\_process | HELLS | 1150 | 9 | 1.386120 | -0.747241 | 200 | 178.47 | 0.892350 |
| GO:0044249\_cellular\_biosynthetic\_process | QK | 1150 | 9 | 1.386120 | -0.747241 | 200 | 178.47 | 0.892350 |
| GO:0016567\_protein\_ubiquitination | SKP2 | 35 | 1 | 5.060440 | -0.743823 | 204 | 180.74 | 0.885980 |
| GO:0018904\_organic\_ether\_metabolic\_process | PTPN11 | 35 | 1 | 5.060440 | -0.743823 | 204 | 180.74 | 0.885980 |
| GO:0043406\_positive\_regulation\_of\_MAP\_kinase\_activity | PTPN11 | 35 | 1 | 5.060440 | -0.743823 | 204 | 180.74 | 0.885980 |
| GO:0051051\_negative\_regulation\_of\_transport | PTPN11 | 35 | 1 | 5.060440 | -0.743823 | 204 | 180.74 | 0.885980 |
| GO:0001889\_liver\_development | TAF10 | 36 | 1 | 4.919872 | -0.732738 | 208 | 184.83 | 0.888606 |
| GO:0019228\_regulation\_of\_action\_potential\_in\_neuron | QK | 36 | 1 | 4.919872 | -0.732738 | 208 | 184.83 | 0.888606 |
| GO:0030072\_peptide\_hormone\_secretion | PTPN11 | 36 | 1 | 4.919872 | -0.732738 | 208 | 184.83 | 0.888606 |
| GO:0051223\_regulation\_of\_protein\_transport | PTPN11 | 36 | 1 | 4.919872 | -0.732738 | 208 | 184.83 | 0.888606 |
| GO:0002790\_peptide\_secretion | PTPN11 | 37 | 1 | 4.786902 | -0.721988 | 210 | 187.58 | 0.893238 |
| GO:0051101\_regulation\_of\_DNA\_binding | TAF10 | 37 | 1 | 4.786902 | -0.721988 | 210 | 187.58 | 0.893238 |
| GO:0045934\_negative\_regulation\_of\_nucleobase\_\_nucleoside\_\_nucleotide\_and\_nucleic\_acid\_metabolic\_process | STRAP | 270 | 3 | 1.967949 | -0.714149 | 211 | 188.26 | 0.892227 |
| GO:0045934\_negative\_regulation\_of\_nucleobase\_\_nucleoside\_\_nucleotide\_and\_nucleic\_acid\_metabolic\_process | DNMT3B | 270 | 3 | 1.967949 | -0.714149 | 211 | 188.26 | 0.892227 |
| GO:0045934\_negative\_regulation\_of\_nucleobase\_\_nucleoside\_\_nucleotide\_and\_nucleic\_acid\_metabolic\_process | HELLS | 270 | 3 | 1.967949 | -0.714149 | 211 | 188.26 | 0.892227 |
| GO:0001570\_vasculogenesis | QK | 38 | 1 | 4.660931 | -0.711554 | 214 | 191.92 | 0.896822 |
| GO:0001657\_ureteric\_bud\_development | FOXD1 | 38 | 1 | 4.660931 | -0.711554 | 214 | 191.92 | 0.896822 |
| GO:0046777\_protein\_amino\_acid\_autophosphorylation | NLK | 38 | 1 | 4.660931 | -0.711554 | 214 | 191.92 | 0.896822 |
| GO:0050789\_regulation\_of\_biological\_process | STRAP | 2357 | 16 | 1.202311 | -0.711000 | 215 | 192.07 | 0.893349 |
| GO:0050789\_regulation\_of\_biological\_process | GPBP1 | 2357 | 16 | 1.202311 | -0.711000 | 215 | 192.07 | 0.893349 |
| GO:0050789\_regulation\_of\_biological\_process | NLK | 2357 | 16 | 1.202311 | -0.711000 | 215 | 192.07 | 0.893349 |
| GO:0050789\_regulation\_of\_biological\_process | OTX2 | 2357 | 16 | 1.202311 | -0.711000 | 215 | 192.07 | 0.893349 |
| GO:0050789\_regulation\_of\_biological\_process | MKNK2 | 2357 | 16 | 1.202311 | -0.711000 | 215 | 192.07 | 0.893349 |
| GO:0050789\_regulation\_of\_biological\_process | SKP2 | 2357 | 16 | 1.202311 | -0.711000 | 215 | 192.07 | 0.893349 |
| GO:0050789\_regulation\_of\_biological\_process | ITM2B | 2357 | 16 | 1.202311 | -0.711000 | 215 | 192.07 | 0.893349 |
| GO:0050789\_regulation\_of\_biological\_process | PTPN11 | 2357 | 16 | 1.202311 | -0.711000 | 215 | 192.07 | 0.893349 |
| GO:0050789\_regulation\_of\_biological\_process | TAF10 | 2357 | 16 | 1.202311 | -0.711000 | 215 | 192.07 | 0.893349 |
| GO:0050789\_regulation\_of\_biological\_process | RGS2 | 2357 | 16 | 1.202311 | -0.711000 | 215 | 192.07 | 0.893349 |
| GO:0050789\_regulation\_of\_biological\_process | VCP | 2357 | 16 | 1.202311 | -0.711000 | 215 | 192.07 | 0.893349 |
| GO:0050789\_regulation\_of\_biological\_process | RAP1A | 2357 | 16 | 1.202311 | -0.711000 | 215 | 192.07 | 0.893349 |
| GO:0050789\_regulation\_of\_biological\_process | DNMT3B | 2357 | 16 | 1.202311 | -0.711000 | 215 | 192.07 | 0.893349 |
| GO:0050789\_regulation\_of\_biological\_process | FOXD1 | 2357 | 16 | 1.202311 | -0.711000 | 215 | 192.07 | 0.893349 |
| GO:0050789\_regulation\_of\_biological\_process | HELLS | 2357 | 16 | 1.202311 | -0.711000 | 215 | 192.07 | 0.893349 |
| GO:0050789\_regulation\_of\_biological\_process | API5 | 2357 | 16 | 1.202311 | -0.711000 | 215 | 192.07 | 0.893349 |
| GO:0051172\_negative\_regulation\_of\_nitrogen\_compound\_metabolic\_process | STRAP | 271 | 3 | 1.960687 | -0.710888 | 216 | 192.52 | 0.891296 |
| GO:0051172\_negative\_regulation\_of\_nitrogen\_compound\_metabolic\_process | DNMT3B | 271 | 3 | 1.960687 | -0.710888 | 216 | 192.52 | 0.891296 |
| GO:0051172\_negative\_regulation\_of\_nitrogen\_compound\_metabolic\_process | HELLS | 271 | 3 | 1.960687 | -0.710888 | 216 | 192.52 | 0.891296 |
| GO:0031323\_regulation\_of\_cellular\_metabolic\_process | STRAP | 1015 | 8 | 1.395983 | -0.706041 | 217 | 192.97 | 0.889263 |
| GO:0031323\_regulation\_of\_cellular\_metabolic\_process | GPBP1 | 1015 | 8 | 1.395983 | -0.706041 | 217 | 192.97 | 0.889263 |
| GO:0031323\_regulation\_of\_cellular\_metabolic\_process | NLK | 1015 | 8 | 1.395983 | -0.706041 | 217 | 192.97 | 0.889263 |
| GO:0031323\_regulation\_of\_cellular\_metabolic\_process | OTX2 | 1015 | 8 | 1.395983 | -0.706041 | 217 | 192.97 | 0.889263 |
| GO:0031323\_regulation\_of\_cellular\_metabolic\_process | DNMT3B | 1015 | 8 | 1.395983 | -0.706041 | 217 | 192.97 | 0.889263 |
| GO:0031323\_regulation\_of\_cellular\_metabolic\_process | FOXD1 | 1015 | 8 | 1.395983 | -0.706041 | 217 | 192.97 | 0.889263 |
| GO:0031323\_regulation\_of\_cellular\_metabolic\_process | HELLS | 1015 | 8 | 1.395983 | -0.706041 | 217 | 192.97 | 0.889263 |
| GO:0031323\_regulation\_of\_cellular\_metabolic\_process | PTPN11 | 1015 | 8 | 1.395983 | -0.706041 | 217 | 192.97 | 0.889263 |
| GO:0009058\_biosynthetic\_process | ACSM3 | 1175 | 9 | 1.356628 | -0.705788 | 218 | 193.05 | 0.885550 |
| GO:0009058\_biosynthetic\_process | STRAP | 1175 | 9 | 1.356628 | -0.705788 | 218 | 193.05 | 0.885550 |
| GO:0009058\_biosynthetic\_process | GPBP1 | 1175 | 9 | 1.356628 | -0.705788 | 218 | 193.05 | 0.885550 |
| GO:0009058\_biosynthetic\_process | NLK | 1175 | 9 | 1.356628 | -0.705788 | 218 | 193.05 | 0.885550 |
| GO:0009058\_biosynthetic\_process | OTX2 | 1175 | 9 | 1.356628 | -0.705788 | 218 | 193.05 | 0.885550 |
| GO:0009058\_biosynthetic\_process | DNMT3B | 1175 | 9 | 1.356628 | -0.705788 | 218 | 193.05 | 0.885550 |
| GO:0009058\_biosynthetic\_process | FOXD1 | 1175 | 9 | 1.356628 | -0.705788 | 218 | 193.05 | 0.885550 |
| GO:0009058\_biosynthetic\_process | HELLS | 1175 | 9 | 1.356628 | -0.705788 | 218 | 193.05 | 0.885550 |
| GO:0009058\_biosynthetic\_process | QK | 1175 | 9 | 1.356628 | -0.705788 | 218 | 193.05 | 0.885550 |
| GO:0048519\_negative\_regulation\_of\_biological\_process | STRAP | 859 | 7 | 1.443315 | -0.704362 | 219 | 193.33 | 0.882785 |
| GO:0048519\_negative\_regulation\_of\_biological\_process | NLK | 859 | 7 | 1.443315 | -0.704362 | 219 | 193.33 | 0.882785 |
| GO:0048519\_negative\_regulation\_of\_biological\_process | SKP2 | 859 | 7 | 1.443315 | -0.704362 | 219 | 193.33 | 0.882785 |
| GO:0048519\_negative\_regulation\_of\_biological\_process | DNMT3B | 859 | 7 | 1.443315 | -0.704362 | 219 | 193.33 | 0.882785 |
| GO:0048519\_negative\_regulation\_of\_biological\_process | API5 | 859 | 7 | 1.443315 | -0.704362 | 219 | 193.33 | 0.882785 |
| GO:0048519\_negative\_regulation\_of\_biological\_process | HELLS | 859 | 7 | 1.443315 | -0.704362 | 219 | 193.33 | 0.882785 |
| GO:0048519\_negative\_regulation\_of\_biological\_process | PTPN11 | 859 | 7 | 1.443315 | -0.704362 | 219 | 193.33 | 0.882785 |
| GO:0006511\_ubiquitin-dependent\_protein\_catabolic\_process | SKP2 | 39 | 1 | 4.541420 | -0.701420 | 222 | 196.38 | 0.884595 |
| GO:0048663\_neuron\_fate\_commitment | OTX2 | 39 | 1 | 4.541420 | -0.701420 | 222 | 196.38 | 0.884595 |
| GO:0070201\_regulation\_of\_establishment\_of\_protein\_localization | PTPN11 | 39 | 1 | 4.541420 | -0.701420 | 222 | 196.38 | 0.884595 |
| GO:0010558\_negative\_regulation\_of\_macromolecule\_biosynthetic\_process | STRAP | 274 | 3 | 1.939220 | -0.701211 | 223 | 196.62 | 0.881704 |
| GO:0010558\_negative\_regulation\_of\_macromolecule\_biosynthetic\_process | DNMT3B | 274 | 3 | 1.939220 | -0.701211 | 223 | 196.62 | 0.881704 |
| GO:0010558\_negative\_regulation\_of\_macromolecule\_biosynthetic\_process | HELLS | 274 | 3 | 1.939220 | -0.701211 | 223 | 196.62 | 0.881704 |
| GO:0050794\_regulation\_of\_cellular\_process | GPBP1 | 2190 | 15 | 1.213119 | -0.698544 | 224 | 196.74 | 0.878304 |
| GO:0050794\_regulation\_of\_cellular\_process | STRAP | 2190 | 15 | 1.213119 | -0.698544 | 224 | 196.74 | 0.878304 |
| GO:0050794\_regulation\_of\_cellular\_process | NLK | 2190 | 15 | 1.213119 | -0.698544 | 224 | 196.74 | 0.878304 |
| GO:0050794\_regulation\_of\_cellular\_process | OTX2 | 2190 | 15 | 1.213119 | -0.698544 | 224 | 196.74 | 0.878304 |
| GO:0050794\_regulation\_of\_cellular\_process | SKP2 | 2190 | 15 | 1.213119 | -0.698544 | 224 | 196.74 | 0.878304 |
| GO:0050794\_regulation\_of\_cellular\_process | MKNK2 | 2190 | 15 | 1.213119 | -0.698544 | 224 | 196.74 | 0.878304 |
| GO:0050794\_regulation\_of\_cellular\_process | ITM2B | 2190 | 15 | 1.213119 | -0.698544 | 224 | 196.74 | 0.878304 |
| GO:0050794\_regulation\_of\_cellular\_process | PTPN11 | 2190 | 15 | 1.213119 | -0.698544 | 224 | 196.74 | 0.878304 |
| GO:0050794\_regulation\_of\_cellular\_process | RGS2 | 2190 | 15 | 1.213119 | -0.698544 | 224 | 196.74 | 0.878304 |
| GO:0050794\_regulation\_of\_cellular\_process | VCP | 2190 | 15 | 1.213119 | -0.698544 | 224 | 196.74 | 0.878304 |
| GO:0050794\_regulation\_of\_cellular\_process | RAP1A | 2190 | 15 | 1.213119 | -0.698544 | 224 | 196.74 | 0.878304 |
| GO:0050794\_regulation\_of\_cellular\_process | DNMT3B | 2190 | 15 | 1.213119 | -0.698544 | 224 | 196.74 | 0.878304 |
| GO:0050794\_regulation\_of\_cellular\_process | FOXD1 | 2190 | 15 | 1.213119 | -0.698544 | 224 | 196.74 | 0.878304 |
| GO:0050794\_regulation\_of\_cellular\_process | HELLS | 2190 | 15 | 1.213119 | -0.698544 | 224 | 196.74 | 0.878304 |
| GO:0050794\_regulation\_of\_cellular\_process | API5 | 2190 | 15 | 1.213119 | -0.698544 | 224 | 196.74 | 0.878304 |
| GO:0044267\_cellular\_protein\_metabolic\_process | NLK | 559 | 5 | 1.584216 | -0.696972 | 225 | 196.94 | 0.875289 |
| GO:0044267\_cellular\_protein\_metabolic\_process | MKNK2 | 559 | 5 | 1.584216 | -0.696972 | 225 | 196.94 | 0.875289 |
| GO:0044267\_cellular\_protein\_metabolic\_process | SKP2 | 559 | 5 | 1.584216 | -0.696972 | 225 | 196.94 | 0.875289 |
| GO:0044267\_cellular\_protein\_metabolic\_process | PTPN12 | 559 | 5 | 1.584216 | -0.696972 | 225 | 196.94 | 0.875289 |
| GO:0044267\_cellular\_protein\_metabolic\_process | PTPN11 | 559 | 5 | 1.584216 | -0.696972 | 225 | 196.94 | 0.875289 |
| GO:0043085\_positive\_regulation\_of\_catalytic\_activity | VCP | 148 | 2 | 2.393451 | -0.692852 | 226 | 197.17 | 0.872434 |
| GO:0043085\_positive\_regulation\_of\_catalytic\_activity | PTPN11 | 148 | 2 | 2.393451 | -0.692852 | 226 | 197.17 | 0.872434 |
| GO:0016071\_mRNA\_metabolic\_process | RBM17 | 40 | 1 | 4.427885 | -0.691571 | 228 | 199.53 | 0.875132 |
| GO:0017015\_regulation\_of\_transforming\_growth\_factor\_beta\_receptor\_signaling\_pathway | STRAP | 40 | 1 | 4.427885 | -0.691571 | 228 | 199.53 | 0.875132 |
| GO:0032940\_secretion\_by\_cell | GARS | 149 | 2 | 2.377388 | -0.688426 | 229 | 199.68 | 0.871965 |
| GO:0032940\_secretion\_by\_cell | PTPN11 | 149 | 2 | 2.377388 | -0.688426 | 229 | 199.68 | 0.871965 |
| GO:0065009\_regulation\_of\_molecular\_function | TAF10 | 279 | 3 | 1.904467 | -0.685425 | 230 | 199.94 | 0.869304 |
| GO:0065009\_regulation\_of\_molecular\_function | VCP | 279 | 3 | 1.904467 | -0.685425 | 230 | 199.94 | 0.869304 |
| GO:0065009\_regulation\_of\_molecular\_function | PTPN11 | 279 | 3 | 1.904467 | -0.685425 | 230 | 199.94 | 0.869304 |
| GO:0015833\_peptide\_transport | PTPN11 | 41 | 1 | 4.319887 | -0.681992 | 232 | 203.41 | 0.876767 |
| GO:0019748\_secondary\_metabolic\_process | ALDH1A7 | 41 | 1 | 4.319887 | -0.681992 | 232 | 203.41 | 0.876767 |
| GO:0031327\_negative\_regulation\_of\_cellular\_biosynthetic\_process | STRAP | 282 | 3 | 1.884206 | -0.676152 | 233 | 203.78 | 0.874592 |
| GO:0031327\_negative\_regulation\_of\_cellular\_biosynthetic\_process | DNMT3B | 282 | 3 | 1.884206 | -0.676152 | 233 | 203.78 | 0.874592 |
| GO:0031327\_negative\_regulation\_of\_cellular\_biosynthetic\_process | HELLS | 282 | 3 | 1.884206 | -0.676152 | 233 | 203.78 | 0.874592 |
| GO:0019941\_modification-dependent\_protein\_catabolic\_process | SKP2 | 42 | 1 | 4.217033 | -0.672671 | 237 | 207.38 | 0.875021 |
| GO:0043632\_modification-dependent\_macromolecule\_catabolic\_process | SKP2 | 42 | 1 | 4.217033 | -0.672671 | 237 | 207.38 | 0.875021 |
| GO:0051345\_positive\_regulation\_of\_hydrolase\_activity | VCP | 42 | 1 | 4.217033 | -0.672671 | 237 | 207.38 | 0.875021 |
| GO:0051603\_proteolysis\_involved\_in\_cellular\_protein\_catabolic\_process | SKP2 | 42 | 1 | 4.217033 | -0.672671 | 237 | 207.38 | 0.875021 |
| GO:0009890\_negative\_regulation\_of\_biosynthetic\_process | STRAP | 284 | 3 | 1.870937 | -0.670052 | 238 | 207.63 | 0.872395 |
| GO:0009890\_negative\_regulation\_of\_biosynthetic\_process | DNMT3B | 284 | 3 | 1.870937 | -0.670052 | 238 | 207.63 | 0.872395 |
| GO:0009890\_negative\_regulation\_of\_biosynthetic\_process | HELLS | 284 | 3 | 1.870937 | -0.670052 | 238 | 207.63 | 0.872395 |
| GO:0001508\_regulation\_of\_action\_potential | QK | 43 | 1 | 4.118962 | -0.663595 | 242 | 211.55 | 0.874174 |
| GO:0006766\_vitamin\_metabolic\_process | ALDH1A7 | 43 | 1 | 4.118962 | -0.663595 | 242 | 211.55 | 0.874174 |
| GO:0032446\_protein\_modification\_by\_small\_protein\_conjugation | SKP2 | 43 | 1 | 4.118962 | -0.663595 | 242 | 211.55 | 0.874174 |
| GO:0046879\_hormone\_secretion | PTPN11 | 43 | 1 | 4.118962 | -0.663595 | 242 | 211.55 | 0.874174 |
| GO:0022402\_cell\_cycle\_process | TAF10 | 155 | 2 | 2.285360 | -0.662664 | 243 | 211.84 | 0.871770 |
| GO:0022402\_cell\_cycle\_process | SKP2 | 155 | 2 | 2.285360 | -0.662664 | 243 | 211.84 | 0.871770 |
| GO:0009914\_hormone\_transport | PTPN11 | 44 | 1 | 4.025350 | -0.654753 | 245 | 216.13 | 0.882163 |
| GO:0044257\_cellular\_protein\_catabolic\_process | SKP2 | 44 | 1 | 4.025350 | -0.654753 | 245 | 216.13 | 0.882163 |
| GO:0007409\_axonogenesis | FOXD1 | 158 | 2 | 2.241967 | -0.650273 | 246 | 216.73 | 0.881016 |
| GO:0007409\_axonogenesis | PTPN11 | 158 | 2 | 2.241967 | -0.650273 | 246 | 216.73 | 0.881016 |
| GO:0032870\_cellular\_response\_to\_hormone\_stimulus | PTPN11 | 45 | 1 | 3.935897 | -0.646134 | 248 | 218.13 | 0.879556 |
| GO:0043623\_cellular\_protein\_complex\_assembly | TAF10 | 45 | 1 | 3.935897 | -0.646134 | 248 | 218.13 | 0.879556 |
| GO:0051098\_regulation\_of\_binding | TAF10 | 46 | 1 | 3.850334 | -0.637729 | 249 | 220.6 | 0.885944 |
| GO:0034645\_cellular\_macromolecule\_biosynthetic\_process | GPBP1 | 901 | 7 | 1.376035 | -0.630880 | 250 | 221.87 | 0.887480 |
| GO:0034645\_cellular\_macromolecule\_biosynthetic\_process | STRAP | 901 | 7 | 1.376035 | -0.630880 | 250 | 221.87 | 0.887480 |
| GO:0034645\_cellular\_macromolecule\_biosynthetic\_process | NLK | 901 | 7 | 1.376035 | -0.630880 | 250 | 221.87 | 0.887480 |
| GO:0034645\_cellular\_macromolecule\_biosynthetic\_process | OTX2 | 901 | 7 | 1.376035 | -0.630880 | 250 | 221.87 | 0.887480 |
| GO:0034645\_cellular\_macromolecule\_biosynthetic\_process | DNMT3B | 901 | 7 | 1.376035 | -0.630880 | 250 | 221.87 | 0.887480 |
| GO:0034645\_cellular\_macromolecule\_biosynthetic\_process | FOXD1 | 901 | 7 | 1.376035 | -0.630880 | 250 | 221.87 | 0.887480 |
| GO:0034645\_cellular\_macromolecule\_biosynthetic\_process | HELLS | 901 | 7 | 1.376035 | -0.630880 | 250 | 221.87 | 0.887480 |
| GO:0034754\_cellular\_hormone\_metabolic\_process | ALDH1A7 | 47 | 1 | 3.768412 | -0.629529 | 251 | 224.52 | 0.894502 |
| GO:0009059\_macromolecule\_biosynthetic\_process | STRAP | 910 | 7 | 1.362426 | -0.616036 | 252 | 228.5 | 0.906746 |
| GO:0009059\_macromolecule\_biosynthetic\_process | GPBP1 | 910 | 7 | 1.362426 | -0.616036 | 252 | 228.5 | 0.906746 |
| GO:0009059\_macromolecule\_biosynthetic\_process | NLK | 910 | 7 | 1.362426 | -0.616036 | 252 | 228.5 | 0.906746 |
| GO:0009059\_macromolecule\_biosynthetic\_process | OTX2 | 910 | 7 | 1.362426 | -0.616036 | 252 | 228.5 | 0.906746 |
| GO:0009059\_macromolecule\_biosynthetic\_process | DNMT3B | 910 | 7 | 1.362426 | -0.616036 | 252 | 228.5 | 0.906746 |
| GO:0009059\_macromolecule\_biosynthetic\_process | FOXD1 | 910 | 7 | 1.362426 | -0.616036 | 252 | 228.5 | 0.906746 |
| GO:0009059\_macromolecule\_biosynthetic\_process | HELLS | 910 | 7 | 1.362426 | -0.616036 | 252 | 228.5 | 0.906746 |
| GO:0007165\_signal\_transduction | RGS2 | 915 | 7 | 1.354981 | -0.607921 | 253 | 231.76 | 0.916047 |
| GO:0007165\_signal\_transduction | STRAP | 915 | 7 | 1.354981 | -0.607921 | 253 | 231.76 | 0.916047 |
| GO:0007165\_signal\_transduction | NLK | 915 | 7 | 1.354981 | -0.607921 | 253 | 231.76 | 0.916047 |
| GO:0007165\_signal\_transduction | SKP2 | 915 | 7 | 1.354981 | -0.607921 | 253 | 231.76 | 0.916047 |
| GO:0007165\_signal\_transduction | MKNK2 | 915 | 7 | 1.354981 | -0.607921 | 253 | 231.76 | 0.916047 |
| GO:0007165\_signal\_transduction | RAP1A | 915 | 7 | 1.354981 | -0.607921 | 253 | 231.76 | 0.916047 |
| GO:0007165\_signal\_transduction | PTPN11 | 915 | 7 | 1.354981 | -0.607921 | 253 | 231.76 | 0.916047 |
| GO:0045893\_positive\_regulation\_of\_transcription\_\_DNA-dependent | GPBP1 | 306 | 3 | 1.736425 | -0.606965 | 255 | 232.11 | 0.910235 |
| GO:0045893\_positive\_regulation\_of\_transcription\_\_DNA-dependent | OTX2 | 306 | 3 | 1.736425 | -0.606965 | 255 | 232.11 | 0.910235 |
| GO:0045893\_positive\_regulation\_of\_transcription\_\_DNA-dependent | FOXD1 | 306 | 3 | 1.736425 | -0.606965 | 255 | 232.11 | 0.910235 |
| GO:0051254\_positive\_regulation\_of\_RNA\_metabolic\_process | GPBP1 | 306 | 3 | 1.736425 | -0.606965 | 255 | 232.11 | 0.910235 |
| GO:0051254\_positive\_regulation\_of\_RNA\_metabolic\_process | OTX2 | 306 | 3 | 1.736425 | -0.606965 | 255 | 232.11 | 0.910235 |
| GO:0051254\_positive\_regulation\_of\_RNA\_metabolic\_process | FOXD1 | 306 | 3 | 1.736425 | -0.606965 | 255 | 232.11 | 0.910235 |
| GO:0001656\_metanephros\_development | FOXD1 | 50 | 1 | 3.542308 | -0.606070 | 257 | 233.95 | 0.910311 |
| GO:0070647\_protein\_modification\_by\_small\_protein\_conjugation\_or\_removal | SKP2 | 50 | 1 | 3.542308 | -0.606070 | 257 | 233.95 | 0.910311 |
| GO:0032989\_cellular\_component\_morphogenesis | SSBP1 | 307 | 3 | 1.730769 | -0.604262 | 258 | 234.07 | 0.907248 |
| GO:0032989\_cellular\_component\_morphogenesis | FOXD1 | 307 | 3 | 1.730769 | -0.604262 | 258 | 234.07 | 0.907248 |
| GO:0032989\_cellular\_component\_morphogenesis | PTPN11 | 307 | 3 | 1.730769 | -0.604262 | 258 | 234.07 | 0.907248 |
| GO:0048812\_neuron\_projection\_morphogenesis | FOXD1 | 170 | 2 | 2.083710 | -0.603700 | 259 | 234.43 | 0.905135 |
| GO:0048812\_neuron\_projection\_morphogenesis | PTPN11 | 170 | 2 | 2.083710 | -0.603700 | 259 | 234.43 | 0.905135 |
| GO:0016310\_phosphorylation | NLK | 309 | 3 | 1.719567 | -0.598898 | 260 | 234.76 | 0.902923 |
| GO:0016310\_phosphorylation | MKNK2 | 309 | 3 | 1.719567 | -0.598898 | 260 | 234.76 | 0.902923 |
| GO:0016310\_phosphorylation | PTPN11 | 309 | 3 | 1.719567 | -0.598898 | 260 | 234.76 | 0.902923 |
| GO:0006887\_exocytosis | GARS | 51 | 1 | 3.472851 | -0.598606 | 262 | 236.79 | 0.903779 |
| GO:0032880\_regulation\_of\_protein\_localization | PTPN11 | 51 | 1 | 3.472851 | -0.598606 | 262 | 236.79 | 0.903779 |
| GO:0044093\_positive\_regulation\_of\_molecular\_function | VCP | 173 | 2 | 2.047577 | -0.592750 | 264 | 237.77 | 0.900644 |
| GO:0044093\_positive\_regulation\_of\_molecular\_function | PTPN11 | 173 | 2 | 2.047577 | -0.592750 | 264 | 237.77 | 0.900644 |
| GO:0048667\_cell\_morphogenesis\_involved\_in\_neuron\_differentiation | FOXD1 | 173 | 2 | 2.047577 | -0.592750 | 264 | 237.77 | 0.900644 |
| GO:0048667\_cell\_morphogenesis\_involved\_in\_neuron\_differentiation | PTPN11 | 173 | 2 | 2.047577 | -0.592750 | 264 | 237.77 | 0.900644 |
| GO:0080090\_regulation\_of\_primary\_metabolic\_process | STRAP | 926 | 7 | 1.338885 | -0.590393 | 265 | 239.25 | 0.902830 |
| GO:0080090\_regulation\_of\_primary\_metabolic\_process | GPBP1 | 926 | 7 | 1.338885 | -0.590393 | 265 | 239.25 | 0.902830 |
| GO:0080090\_regulation\_of\_primary\_metabolic\_process | NLK | 926 | 7 | 1.338885 | -0.590393 | 265 | 239.25 | 0.902830 |
| GO:0080090\_regulation\_of\_primary\_metabolic\_process | OTX2 | 926 | 7 | 1.338885 | -0.590393 | 265 | 239.25 | 0.902830 |
| GO:0080090\_regulation\_of\_primary\_metabolic\_process | DNMT3B | 926 | 7 | 1.338885 | -0.590393 | 265 | 239.25 | 0.902830 |
| GO:0080090\_regulation\_of\_primary\_metabolic\_process | FOXD1 | 926 | 7 | 1.338885 | -0.590393 | 265 | 239.25 | 0.902830 |
| GO:0080090\_regulation\_of\_primary\_metabolic\_process | HELLS | 926 | 7 | 1.338885 | -0.590393 | 265 | 239.25 | 0.902830 |
| GO:0046903\_secretion | GARS | 175 | 2 | 2.024176 | -0.585593 | 266 | 239.94 | 0.902030 |
| GO:0046903\_secretion | PTPN11 | 175 | 2 | 2.024176 | -0.585593 | 266 | 239.94 | 0.902030 |
| GO:0006576\_biogenic\_amine\_metabolic\_process | AZIN1 | 53 | 1 | 3.341800 | -0.584169 | 267 | 241.81 | 0.905655 |
| GO:0048858\_cell\_projection\_morphogenesis | FOXD1 | 176 | 2 | 2.012675 | -0.582057 | 268 | 242.6 | 0.905224 |
| GO:0048858\_cell\_projection\_morphogenesis | PTPN11 | 176 | 2 | 2.012675 | -0.582057 | 268 | 242.6 | 0.905224 |
| GO:0043405\_regulation\_of\_MAP\_kinase\_activity | PTPN11 | 54 | 1 | 3.279915 | -0.577184 | 269 | 244.81 | 0.910074 |
| GO:0006310\_DNA\_recombination | RAD51AP1 | 55 | 1 | 3.220280 | -0.570347 | 270 | 247.67 | 0.917296 |
| GO:0001708\_cell\_fate\_specification | OTX2 | 56 | 1 | 3.162775 | -0.563653 | 272 | 250.63 | 0.921434 |
| GO:0046486\_glycerolipid\_metabolic\_process | PTPN11 | 56 | 1 | 3.162775 | -0.563653 | 272 | 250.63 | 0.921434 |
| GO:0009953\_dorsal\_ventral\_pattern\_formation | OTX2 | 57 | 1 | 3.107287 | -0.557096 | 275 | 253.96 | 0.923491 |
| GO:0042472\_inner\_ear\_morphogenesis | OTX2 | 57 | 1 | 3.107287 | -0.557096 | 275 | 253.96 | 0.923491 |
| GO:0045444\_fat\_cell\_differentiation | RGS2 | 57 | 1 | 3.107287 | -0.557096 | 275 | 253.96 | 0.923491 |
| GO:0048869\_cellular\_developmental\_process | TAF10 | 1113 | 8 | 1.273067 | -0.553872 | 276 | 254.5 | 0.922101 |
| GO:0048869\_cellular\_developmental\_process | SSBP1 | 1113 | 8 | 1.273067 | -0.553872 | 276 | 254.5 | 0.922101 |
| GO:0048869\_cellular\_developmental\_process | RGS2 | 1113 | 8 | 1.273067 | -0.553872 | 276 | 254.5 | 0.922101 |
| GO:0048869\_cellular\_developmental\_process | OTX2 | 1113 | 8 | 1.273067 | -0.553872 | 276 | 254.5 | 0.922101 |
| GO:0048869\_cellular\_developmental\_process | FOXD1 | 1113 | 8 | 1.273067 | -0.553872 | 276 | 254.5 | 0.922101 |
| GO:0048869\_cellular\_developmental\_process | HELLS | 1113 | 8 | 1.273067 | -0.553872 | 276 | 254.5 | 0.922101 |
| GO:0048869\_cellular\_developmental\_process | PTPN11 | 1113 | 8 | 1.273067 | -0.553872 | 276 | 254.5 | 0.922101 |
| GO:0048869\_cellular\_developmental\_process | QK | 1113 | 8 | 1.273067 | -0.553872 | 276 | 254.5 | 0.922101 |
| GO:0016055\_Wnt\_receptor\_signaling\_pathway | NLK | 59 | 1 | 3.001956 | -0.544377 | 277 | 258.31 | 0.932527 |
| GO:0051093\_negative\_regulation\_of\_developmental\_process | SKP2 | 331 | 3 | 1.605275 | -0.543276 | 278 | 258.69 | 0.930540 |
| GO:0051093\_negative\_regulation\_of\_developmental\_process | API5 | 331 | 3 | 1.605275 | -0.543276 | 278 | 258.69 | 0.930540 |
| GO:0051093\_negative\_regulation\_of\_developmental\_process | HELLS | 331 | 3 | 1.605275 | -0.543276 | 278 | 258.69 | 0.930540 |
| GO:0007276\_gamete\_generation | RGS2 | 188 | 2 | 1.884206 | -0.541692 | 279 | 258.98 | 0.928244 |
| GO:0007276\_gamete\_generation | QK | 188 | 2 | 1.884206 | -0.541692 | 279 | 258.98 | 0.928244 |
| GO:0031324\_negative\_regulation\_of\_cellular\_metabolic\_process | STRAP | 332 | 3 | 1.600440 | -0.540887 | 280 | 259.15 | 0.925536 |
| GO:0031324\_negative\_regulation\_of\_cellular\_metabolic\_process | DNMT3B | 332 | 3 | 1.600440 | -0.540887 | 280 | 259.15 | 0.925536 |
| GO:0031324\_negative\_regulation\_of\_cellular\_metabolic\_process | HELLS | 332 | 3 | 1.600440 | -0.540887 | 280 | 259.15 | 0.925536 |
| GO:0007005\_mitochondrion\_organization | SSBP1 | 61 | 1 | 2.903531 | -0.532153 | 281 | 261.18 | 0.929466 |
| GO:0045941\_positive\_regulation\_of\_transcription | GPBP1 | 338 | 3 | 1.572030 | -0.526796 | 282 | 261.53 | 0.927411 |
| GO:0045941\_positive\_regulation\_of\_transcription | OTX2 | 338 | 3 | 1.572030 | -0.526796 | 282 | 261.53 | 0.927411 |
| GO:0045941\_positive\_regulation\_of\_transcription | FOXD1 | 338 | 3 | 1.572030 | -0.526796 | 282 | 261.53 | 0.927411 |
| GO:0040014\_regulation\_of\_multicellular\_organism\_growth | PTPN11 | 62 | 1 | 2.856700 | -0.526217 | 283 | 263.88 | 0.932438 |
| GO:0031175\_neuron\_projection\_development | FOXD1 | 197 | 2 | 1.798126 | -0.513728 | 284 | 269.41 | 0.948627 |
| GO:0031175\_neuron\_projection\_development | PTPN11 | 197 | 2 | 1.798126 | -0.513728 | 284 | 269.41 | 0.948627 |
| GO:0019538\_protein\_metabolic\_process | NLK | 655 | 5 | 1.352026 | -0.512712 | 285 | 269.72 | 0.946386 |
| GO:0019538\_protein\_metabolic\_process | MKNK2 | 655 | 5 | 1.352026 | -0.512712 | 285 | 269.72 | 0.946386 |
| GO:0019538\_protein\_metabolic\_process | SKP2 | 655 | 5 | 1.352026 | -0.512712 | 285 | 269.72 | 0.946386 |
| GO:0019538\_protein\_metabolic\_process | PTPN12 | 655 | 5 | 1.352026 | -0.512712 | 285 | 269.72 | 0.946386 |
| GO:0019538\_protein\_metabolic\_process | PTPN11 | 655 | 5 | 1.352026 | -0.512712 | 285 | 269.72 | 0.946386 |
| GO:0042471\_ear\_morphogenesis | OTX2 | 65 | 1 | 2.724852 | -0.509066 | 286 | 271.03 | 0.947657 |
| GO:0010628\_positive\_regulation\_of\_gene\_expression | GPBP1 | 346 | 3 | 1.535683 | -0.508627 | 287 | 271.23 | 0.945052 |
| GO:0010628\_positive\_regulation\_of\_gene\_expression | OTX2 | 346 | 3 | 1.535683 | -0.508627 | 287 | 271.23 | 0.945052 |
| GO:0010628\_positive\_regulation\_of\_gene\_expression | FOXD1 | 346 | 3 | 1.535683 | -0.508627 | 287 | 271.23 | 0.945052 |
| GO:0000904\_cell\_morphogenesis\_involved\_in\_differentiation | FOXD1 | 199 | 2 | 1.780054 | -0.507761 | 288 | 271.75 | 0.943576 |
| GO:0000904\_cell\_morphogenesis\_involved\_in\_differentiation | PTPN11 | 199 | 2 | 1.780054 | -0.507761 | 288 | 271.75 | 0.943576 |
| GO:0007179\_transforming\_growth\_factor\_beta\_receptor\_signaling\_pathway | STRAP | 66 | 1 | 2.683566 | -0.503557 | 290 | 273.42 | 0.942828 |
| GO:0045860\_positive\_regulation\_of\_protein\_kinase\_activity | PTPN11 | 66 | 1 | 2.683566 | -0.503557 | 290 | 273.42 | 0.942828 |
| GO:0045935\_positive\_regulation\_of\_nucleobase\_\_nucleoside\_\_nucleotide\_and\_nucleic\_acid\_metabolic\_process | GPBP1 | 352 | 3 | 1.509506 | -0.495446 | 291 | 275.31 | 0.946082 |
| GO:0045935\_positive\_regulation\_of\_nucleobase\_\_nucleoside\_\_nucleotide\_and\_nucleic\_acid\_metabolic\_process | OTX2 | 352 | 3 | 1.509506 | -0.495446 | 291 | 275.31 | 0.946082 |
| GO:0045935\_positive\_regulation\_of\_nucleobase\_\_nucleoside\_\_nucleotide\_and\_nucleic\_acid\_metabolic\_process | FOXD1 | 352 | 3 | 1.509506 | -0.495446 | 291 | 275.31 | 0.946082 |
| GO:0022607\_cellular\_component\_assembly | TAF10 | 204 | 2 | 1.736425 | -0.493214 | 292 | 275.56 | 0.943699 |
| GO:0022607\_cellular\_component\_assembly | HELLS | 204 | 2 | 1.736425 | -0.493214 | 292 | 275.56 | 0.943699 |
| GO:0034962\_cellular\_biopolymer\_catabolic\_process | SKP2 | 68 | 1 | 2.604638 | -0.492833 | 294 | 276.58 | 0.940748 |
| GO:0042692\_muscle\_cell\_differentiation | QK | 68 | 1 | 2.604638 | -0.492833 | 294 | 276.58 | 0.940748 |
| GO:0030182\_neuron\_differentiation | OTX2 | 356 | 3 | 1.492545 | -0.486864 | 295 | 278.65 | 0.944576 |
| GO:0030182\_neuron\_differentiation | FOXD1 | 356 | 3 | 1.492545 | -0.486864 | 295 | 278.65 | 0.944576 |
| GO:0030182\_neuron\_differentiation | PTPN11 | 356 | 3 | 1.492545 | -0.486864 | 295 | 278.65 | 0.944576 |
| GO:0048592\_eye\_morphogenesis | OTX2 | 70 | 1 | 2.530220 | -0.482481 | 296 | 280.29 | 0.946926 |
| GO:0006913\_nucleocytoplasmic\_transport | PTPN11 | 71 | 1 | 2.494583 | -0.477438 | 298 | 282.57 | 0.948221 |
| GO:0033674\_positive\_regulation\_of\_kinase\_activity | PTPN11 | 71 | 1 | 2.494583 | -0.477438 | 298 | 282.57 | 0.948221 |
| GO:0051173\_positive\_regulation\_of\_nitrogen\_compound\_metabolic\_process | GPBP1 | 361 | 3 | 1.471873 | -0.476360 | 299 | 282.76 | 0.945686 |
| GO:0051173\_positive\_regulation\_of\_nitrogen\_compound\_metabolic\_process | OTX2 | 361 | 3 | 1.471873 | -0.476360 | 299 | 282.76 | 0.945686 |
| GO:0051173\_positive\_regulation\_of\_nitrogen\_compound\_metabolic\_process | FOXD1 | 361 | 3 | 1.471873 | -0.476360 | 299 | 282.76 | 0.945686 |
| GO:0007264\_small\_GTPase\_mediated\_signal\_transduction | RAP1A | 72 | 1 | 2.459936 | -0.472480 | 303 | 285.64 | 0.942706 |
| GO:0048839\_inner\_ear\_development | OTX2 | 72 | 1 | 2.459936 | -0.472480 | 303 | 285.64 | 0.942706 |
| GO:0051169\_nuclear\_transport | PTPN11 | 72 | 1 | 2.459936 | -0.472480 | 303 | 285.64 | 0.942706 |
| GO:0051347\_positive\_regulation\_of\_transferase\_activity | PTPN11 | 72 | 1 | 2.459936 | -0.472480 | 303 | 285.64 | 0.942706 |
| GO:0051336\_regulation\_of\_hydrolase\_activity | VCP | 73 | 1 | 2.426238 | -0.467605 | 304 | 287.98 | 0.947303 |
| GO:0044265\_cellular\_macromolecule\_catabolic\_process | SKP2 | 75 | 1 | 2.361538 | -0.458095 | 306 | 290.52 | 0.949412 |
| GO:0051050\_positive\_regulation\_of\_transport | PTPN11 | 75 | 1 | 2.361538 | -0.458095 | 306 | 290.52 | 0.949412 |
| GO:0010557\_positive\_regulation\_of\_macromolecule\_biosynthetic\_process | GPBP1 | 371 | 3 | 1.432200 | -0.456071 | 307 | 291.51 | 0.949544 |
| GO:0010557\_positive\_regulation\_of\_macromolecule\_biosynthetic\_process | OTX2 | 371 | 3 | 1.432200 | -0.456071 | 307 | 291.51 | 0.949544 |
| GO:0010557\_positive\_regulation\_of\_macromolecule\_biosynthetic\_process | FOXD1 | 371 | 3 | 1.432200 | -0.456071 | 307 | 291.51 | 0.949544 |
| GO:0006508\_proteolysis | SKP2 | 76 | 1 | 2.330466 | -0.453455 | 309 | 292.92 | 0.947961 |
| GO:0009725\_response\_to\_hormone\_stimulus | PTPN11 | 76 | 1 | 2.330466 | -0.453455 | 309 | 292.92 | 0.947961 |
| GO:0022414\_reproductive\_process | RGS2 | 376 | 3 | 1.413155 | -0.446273 | 310 | 294.64 | 0.950452 |
| GO:0022414\_reproductive\_process | PTPN11 | 376 | 3 | 1.413155 | -0.446273 | 310 | 294.64 | 0.950452 |
| GO:0022414\_reproductive\_process | QK | 376 | 3 | 1.413155 | -0.446273 | 310 | 294.64 | 0.950452 |
| GO:0006461\_protein\_complex\_assembly | TAF10 | 78 | 1 | 2.270710 | -0.444396 | 312 | 296.57 | 0.950545 |
| GO:0070271\_protein\_complex\_biogenesis | TAF10 | 78 | 1 | 2.270710 | -0.444396 | 312 | 296.57 | 0.950545 |
| GO:0000003\_reproduction | RGS2 | 379 | 3 | 1.401969 | -0.440500 | 313 | 296.84 | 0.948371 |
| GO:0000003\_reproduction | PTPN11 | 379 | 3 | 1.401969 | -0.440500 | 313 | 296.84 | 0.948371 |
| GO:0000003\_reproduction | QK | 379 | 3 | 1.401969 | -0.440500 | 313 | 296.84 | 0.948371 |
| GO:0051046\_regulation\_of\_secretion | PTPN11 | 79 | 1 | 2.241967 | -0.439973 | 314 | 297.47 | 0.947357 |
| GO:0032502\_developmental\_process | SSBP1 | 2060 | 13 | 1.117718 | -0.439414 | 315 | 297.52 | 0.944508 |
| GO:0032502\_developmental\_process | OTX2 | 2060 | 13 | 1.117718 | -0.439414 | 315 | 297.52 | 0.944508 |
| GO:0032502\_developmental\_process | SKP2 | 2060 | 13 | 1.117718 | -0.439414 | 315 | 297.52 | 0.944508 |
| GO:0032502\_developmental\_process | ITM2B | 2060 | 13 | 1.117718 | -0.439414 | 315 | 297.52 | 0.944508 |
| GO:0032502\_developmental\_process | QK | 2060 | 13 | 1.117718 | -0.439414 | 315 | 297.52 | 0.944508 |
| GO:0032502\_developmental\_process | PTPN11 | 2060 | 13 | 1.117718 | -0.439414 | 315 | 297.52 | 0.944508 |
| GO:0032502\_developmental\_process | TAF10 | 2060 | 13 | 1.117718 | -0.439414 | 315 | 297.52 | 0.944508 |
| GO:0032502\_developmental\_process | RGS2 | 2060 | 13 | 1.117718 | -0.439414 | 315 | 297.52 | 0.944508 |
| GO:0032502\_developmental\_process | VCP | 2060 | 13 | 1.117718 | -0.439414 | 315 | 297.52 | 0.944508 |
| GO:0032502\_developmental\_process | DNMT3B | 2060 | 13 | 1.117718 | -0.439414 | 315 | 297.52 | 0.944508 |
| GO:0032502\_developmental\_process | FOXD1 | 2060 | 13 | 1.117718 | -0.439414 | 315 | 297.52 | 0.944508 |
| GO:0032502\_developmental\_process | HELLS | 2060 | 13 | 1.117718 | -0.439414 | 315 | 297.52 | 0.944508 |
| GO:0032502\_developmental\_process | API5 | 2060 | 13 | 1.117718 | -0.439414 | 315 | 297.52 | 0.944508 |
| GO:0050793\_regulation\_of\_developmental\_process | VCP | 703 | 5 | 1.259711 | -0.438830 | 316 | 297.74 | 0.942215 |
| GO:0050793\_regulation\_of\_developmental\_process | SKP2 | 703 | 5 | 1.259711 | -0.438830 | 316 | 297.74 | 0.942215 |
| GO:0050793\_regulation\_of\_developmental\_process | ITM2B | 703 | 5 | 1.259711 | -0.438830 | 316 | 297.74 | 0.942215 |
| GO:0050793\_regulation\_of\_developmental\_process | API5 | 703 | 5 | 1.259711 | -0.438830 | 316 | 297.74 | 0.942215 |
| GO:0050793\_regulation\_of\_developmental\_process | HELLS | 703 | 5 | 1.259711 | -0.438830 | 316 | 297.74 | 0.942215 |
| GO:0019953\_sexual\_reproduction | RGS2 | 228 | 2 | 1.553644 | -0.430068 | 317 | 300.71 | 0.948612 |
| GO:0019953\_sexual\_reproduction | QK | 228 | 2 | 1.553644 | -0.430068 | 317 | 300.71 | 0.948612 |
| GO:0007167\_enzyme\_linked\_receptor\_protein\_signaling\_pathway | STRAP | 229 | 2 | 1.546859 | -0.427655 | 318 | 301.03 | 0.946635 |
| GO:0007167\_enzyme\_linked\_receptor\_protein\_signaling\_pathway | PTPN11 | 229 | 2 | 1.546859 | -0.427655 | 318 | 301.03 | 0.946635 |
| GO:0007411\_axon\_guidance | FOXD1 | 82 | 1 | 2.159944 | -0.427109 | 319 | 302.5 | 0.948276 |
| GO:0031328\_positive\_regulation\_of\_cellular\_biosynthetic\_process | GPBP1 | 387 | 3 | 1.372987 | -0.425488 | 320 | 302.7 | 0.945938 |
| GO:0031328\_positive\_regulation\_of\_cellular\_biosynthetic\_process | OTX2 | 387 | 3 | 1.372987 | -0.425488 | 320 | 302.7 | 0.945938 |
| GO:0031328\_positive\_regulation\_of\_cellular\_biosynthetic\_process | FOXD1 | 387 | 3 | 1.372987 | -0.425488 | 320 | 302.7 | 0.945938 |
| GO:0009891\_positive\_regulation\_of\_biosynthetic\_process | GPBP1 | 388 | 3 | 1.369449 | -0.423650 | 321 | 303.04 | 0.944050 |
| GO:0009891\_positive\_regulation\_of\_biosynthetic\_process | OTX2 | 388 | 3 | 1.369449 | -0.423650 | 321 | 303.04 | 0.944050 |
| GO:0009891\_positive\_regulation\_of\_biosynthetic\_process | FOXD1 | 388 | 3 | 1.369449 | -0.423650 | 321 | 303.04 | 0.944050 |
| GO:0006575\_cellular\_amino\_acid\_derivative\_metabolic\_process | AZIN1 | 83 | 1 | 2.133920 | -0.422951 | 322 | 304.85 | 0.946739 |
| GO:0050790\_regulation\_of\_catalytic\_activity | VCP | 233 | 2 | 1.520304 | -0.418160 | 323 | 306.46 | 0.948793 |
| GO:0050790\_regulation\_of\_catalytic\_activity | PTPN11 | 233 | 2 | 1.520304 | -0.418160 | 323 | 306.46 | 0.948793 |
| GO:0070887\_cellular\_response\_to\_chemical\_stimulus | PTPN11 | 85 | 1 | 2.083710 | -0.414819 | 324 | 307.92 | 0.950370 |
| GO:0006605\_protein\_targeting | PTPN11 | 86 | 1 | 2.059481 | -0.410843 | 327 | 310.4 | 0.949235 |
| GO:0032504\_multicellular\_organism\_reproduction | PTPN11 | 86 | 1 | 2.059481 | -0.410843 | 327 | 310.4 | 0.949235 |
| GO:0048609\_reproductive\_process\_in\_a\_multicellular\_organism | PTPN11 | 86 | 1 | 2.059481 | -0.410843 | 327 | 310.4 | 0.949235 |
| GO:0048699\_generation\_of\_neurons | OTX2 | 396 | 3 | 1.341783 | -0.409237 | 328 | 310.66 | 0.947134 |
| GO:0048699\_generation\_of\_neurons | FOXD1 | 396 | 3 | 1.341783 | -0.409237 | 328 | 310.66 | 0.947134 |
| GO:0048699\_generation\_of\_neurons | PTPN11 | 396 | 3 | 1.341783 | -0.409237 | 328 | 310.66 | 0.947134 |
| GO:0030154\_cell\_differentiation | TAF10 | 1060 | 7 | 1.169630 | -0.409083 | 329 | 310.95 | 0.945137 |
| GO:0030154\_cell\_differentiation | RGS2 | 1060 | 7 | 1.169630 | -0.409083 | 329 | 310.95 | 0.945137 |
| GO:0030154\_cell\_differentiation | OTX2 | 1060 | 7 | 1.169630 | -0.409083 | 329 | 310.95 | 0.945137 |
| GO:0030154\_cell\_differentiation | FOXD1 | 1060 | 7 | 1.169630 | -0.409083 | 329 | 310.95 | 0.945137 |
| GO:0030154\_cell\_differentiation | HELLS | 1060 | 7 | 1.169630 | -0.409083 | 329 | 310.95 | 0.945137 |
| GO:0030154\_cell\_differentiation | PTPN11 | 1060 | 7 | 1.169630 | -0.409083 | 329 | 310.95 | 0.945137 |
| GO:0030154\_cell\_differentiation | QK | 1060 | 7 | 1.169630 | -0.409083 | 329 | 310.95 | 0.945137 |
| GO:0001822\_kidney\_development | FOXD1 | 87 | 1 | 2.035809 | -0.406924 | 333 | 313.44 | 0.941261 |
| GO:0003001\_generation\_of\_a\_signal\_involved\_in\_cell-cell\_signaling | PTPN11 | 87 | 1 | 2.035809 | -0.406924 | 333 | 313.44 | 0.941261 |
| GO:0007178\_transmembrane\_receptor\_protein\_serine\_threonine\_kinase\_signaling\_pathway | STRAP | 87 | 1 | 2.035809 | -0.406924 | 333 | 313.44 | 0.941261 |
| GO:0043583\_ear\_development | OTX2 | 87 | 1 | 2.035809 | -0.406924 | 333 | 313.44 | 0.941261 |
| GO:0048522\_positive\_regulation\_of\_cellular\_process | GPBP1 | 895 | 6 | 1.187366 | -0.404851 | 334 | 313.83 | 0.939611 |
| GO:0048522\_positive\_regulation\_of\_cellular\_process | OTX2 | 895 | 6 | 1.187366 | -0.404851 | 334 | 313.83 | 0.939611 |
| GO:0048522\_positive\_regulation\_of\_cellular\_process | SKP2 | 895 | 6 | 1.187366 | -0.404851 | 334 | 313.83 | 0.939611 |
| GO:0048522\_positive\_regulation\_of\_cellular\_process | ITM2B | 895 | 6 | 1.187366 | -0.404851 | 334 | 313.83 | 0.939611 |
| GO:0048522\_positive\_regulation\_of\_cellular\_process | FOXD1 | 895 | 6 | 1.187366 | -0.404851 | 334 | 313.83 | 0.939611 |
| GO:0048522\_positive\_regulation\_of\_cellular\_process | PTPN11 | 895 | 6 | 1.187366 | -0.404851 | 334 | 313.83 | 0.939611 |
| GO:0048754\_branching\_morphogenesis\_of\_a\_tube | FOXD1 | 88 | 1 | 2.012675 | -0.403062 | 335 | 314.85 | 0.939851 |
| GO:0035264\_multicellular\_organism\_growth | PTPN11 | 90 | 1 | 1.967949 | -0.395501 | 336 | 316.7 | 0.942560 |
| GO:0009719\_response\_to\_endogenous\_stimulus | PTPN11 | 92 | 1 | 1.925167 | -0.388152 | 337 | 319.09 | 0.946855 |
| GO:0032943\_mononuclear\_cell\_proliferation | HELLS | 94 | 1 | 1.884206 | -0.381004 | 340 | 324.0 | 0.952941 |
| GO:0034984\_cellular\_response\_to\_DNA\_damage\_stimulus | PTPN11 | 94 | 1 | 1.884206 | -0.381004 | 340 | 324.0 | 0.952941 |
| GO:0046651\_lymphocyte\_proliferation | HELLS | 94 | 1 | 1.884206 | -0.381004 | 340 | 324.0 | 0.952941 |
| GO:0042391\_regulation\_of\_membrane\_potential | QK | 95 | 1 | 1.864372 | -0.377502 | 341 | 325.34 | 0.954076 |
| GO:0070661\_leukocyte\_proliferation | HELLS | 96 | 1 | 1.844952 | -0.374048 | 342 | 327.76 | 0.958363 |
| GO:0048878\_chemical\_homeostasis | QK | 254 | 2 | 1.394609 | -0.372174 | 343 | 328.17 | 0.956764 |
| GO:0048878\_chemical\_homeostasis | PTPN11 | 254 | 2 | 1.394609 | -0.372174 | 343 | 328.17 | 0.956764 |
| GO:0018193\_peptidyl-amino\_acid\_modification | NLK | 97 | 1 | 1.825932 | -0.370641 | 345 | 328.89 | 0.953304 |
| GO:0060341\_regulation\_of\_cellular\_localization | PTPN11 | 97 | 1 | 1.825932 | -0.370641 | 345 | 328.89 | 0.953304 |
| GO:0007154\_cell\_communication | RGS2 | 1096 | 7 | 1.131211 | -0.369293 | 346 | 329.79 | 0.953150 |
| GO:0007154\_cell\_communication | STRAP | 1096 | 7 | 1.131211 | -0.369293 | 346 | 329.79 | 0.953150 |
| GO:0007154\_cell\_communication | NLK | 1096 | 7 | 1.131211 | -0.369293 | 346 | 329.79 | 0.953150 |
| GO:0007154\_cell\_communication | SKP2 | 1096 | 7 | 1.131211 | -0.369293 | 346 | 329.79 | 0.953150 |
| GO:0007154\_cell\_communication | MKNK2 | 1096 | 7 | 1.131211 | -0.369293 | 346 | 329.79 | 0.953150 |
| GO:0007154\_cell\_communication | RAP1A | 1096 | 7 | 1.131211 | -0.369293 | 346 | 329.79 | 0.953150 |
| GO:0007154\_cell\_communication | PTPN11 | 1096 | 7 | 1.131211 | -0.369293 | 346 | 329.79 | 0.953150 |
| GO:0022008\_neurogenesis | OTX2 | 423 | 3 | 1.256137 | -0.364225 | 347 | 332.56 | 0.958386 |
| GO:0022008\_neurogenesis | FOXD1 | 423 | 3 | 1.256137 | -0.364225 | 347 | 332.56 | 0.958386 |
| GO:0022008\_neurogenesis | PTPN11 | 423 | 3 | 1.256137 | -0.364225 | 347 | 332.56 | 0.958386 |
| GO:0060562\_epithelial\_tube\_morphogenesis | FOXD1 | 99 | 1 | 1.789044 | -0.363960 | 348 | 333.83 | 0.959282 |
| GO:0030163\_protein\_catabolic\_process | SKP2 | 101 | 1 | 1.753618 | -0.357452 | 349 | 335.54 | 0.961433 |
| GO:0048666\_neuron\_development | FOXD1 | 262 | 2 | 1.352026 | -0.356208 | 350 | 336.58 | 0.961657 |
| GO:0048666\_neuron\_development | PTPN11 | 262 | 2 | 1.352026 | -0.356208 | 350 | 336.58 | 0.961657 |
| GO:0007166\_cell\_surface\_receptor\_linked\_signal\_transduction | RGS2 | 597 | 4 | 1.186703 | -0.355658 | 351 | 337.08 | 0.960342 |
| GO:0007166\_cell\_surface\_receptor\_linked\_signal\_transduction | STRAP | 597 | 4 | 1.186703 | -0.355658 | 351 | 337.08 | 0.960342 |
| GO:0007166\_cell\_surface\_receptor\_linked\_signal\_transduction | NLK | 597 | 4 | 1.186703 | -0.355658 | 351 | 337.08 | 0.960342 |
| GO:0007166\_cell\_surface\_receptor\_linked\_signal\_transduction | PTPN11 | 597 | 4 | 1.186703 | -0.355658 | 351 | 337.08 | 0.960342 |
| GO:0030030\_cell\_projection\_organization | FOXD1 | 263 | 2 | 1.346885 | -0.354268 | 352 | 337.38 | 0.958466 |
| GO:0030030\_cell\_projection\_organization | PTPN11 | 263 | 2 | 1.346885 | -0.354268 | 352 | 337.38 | 0.958466 |
| GO:0010604\_positive\_regulation\_of\_macromolecule\_metabolic\_process | GPBP1 | 433 | 3 | 1.227127 | -0.348868 | 353 | 339.84 | 0.962720 |
| GO:0010604\_positive\_regulation\_of\_macromolecule\_metabolic\_process | OTX2 | 433 | 3 | 1.227127 | -0.348868 | 353 | 339.84 | 0.962720 |
| GO:0010604\_positive\_regulation\_of\_macromolecule\_metabolic\_process | FOXD1 | 433 | 3 | 1.227127 | -0.348868 | 353 | 339.84 | 0.962720 |
| GO:0006357\_regulation\_of\_transcription\_from\_RNA\_polymerase\_II\_promoter | STRAP | 435 | 3 | 1.221485 | -0.345876 | 354 | 341.04 | 0.963390 |
| GO:0006357\_regulation\_of\_transcription\_from\_RNA\_polymerase\_II\_promoter | OTX2 | 435 | 3 | 1.221485 | -0.345876 | 354 | 341.04 | 0.963390 |
| GO:0006357\_regulation\_of\_transcription\_from\_RNA\_polymerase\_II\_promoter | FOXD1 | 435 | 3 | 1.221485 | -0.345876 | 354 | 341.04 | 0.963390 |
| GO:0045944\_positive\_regulation\_of\_transcription\_from\_RNA\_polymerase\_II\_promoter | OTX2 | 269 | 2 | 1.316843 | -0.342874 | 355 | 341.57 | 0.962169 |
| GO:0045944\_positive\_regulation\_of\_transcription\_from\_RNA\_polymerase\_II\_promoter | FOXD1 | 269 | 2 | 1.316843 | -0.342874 | 355 | 341.57 | 0.962169 |
| GO:0045859\_regulation\_of\_protein\_kinase\_activity | PTPN11 | 107 | 1 | 1.655284 | -0.338903 | 356 | 343.47 | 0.964803 |
| GO:0031325\_positive\_regulation\_of\_cellular\_metabolic\_process | GPBP1 | 442 | 3 | 1.202141 | -0.335608 | 357 | 344.28 | 0.964370 |
| GO:0031325\_positive\_regulation\_of\_cellular\_metabolic\_process | OTX2 | 442 | 3 | 1.202141 | -0.335608 | 357 | 344.28 | 0.964370 |
| GO:0031325\_positive\_regulation\_of\_cellular\_metabolic\_process | FOXD1 | 442 | 3 | 1.202141 | -0.335608 | 357 | 344.28 | 0.964370 |
| GO:0006366\_transcription\_from\_RNA\_polymerase\_II\_promoter | STRAP | 444 | 3 | 1.196726 | -0.332731 | 358 | 346.15 | 0.966899 |
| GO:0006366\_transcription\_from\_RNA\_polymerase\_II\_promoter | OTX2 | 444 | 3 | 1.196726 | -0.332731 | 358 | 346.15 | 0.966899 |
| GO:0006366\_transcription\_from\_RNA\_polymerase\_II\_promoter | FOXD1 | 444 | 3 | 1.196726 | -0.332731 | 358 | 346.15 | 0.966899 |
| GO:0016043\_cellular\_component\_organization | TAF10 | 964 | 6 | 1.102378 | -0.327787 | 359 | 348.41 | 0.970501 |
| GO:0016043\_cellular\_component\_organization | SSBP1 | 964 | 6 | 1.102378 | -0.327787 | 359 | 348.41 | 0.970501 |
| GO:0016043\_cellular\_component\_organization | FOXD1 | 964 | 6 | 1.102378 | -0.327787 | 359 | 348.41 | 0.970501 |
| GO:0016043\_cellular\_component\_organization | DNMT3B | 964 | 6 | 1.102378 | -0.327787 | 359 | 348.41 | 0.970501 |
| GO:0016043\_cellular\_component\_organization | HELLS | 964 | 6 | 1.102378 | -0.327787 | 359 | 348.41 | 0.970501 |
| GO:0016043\_cellular\_component\_organization | PTPN11 | 964 | 6 | 1.102378 | -0.327787 | 359 | 348.41 | 0.970501 |
| GO:0006996\_organelle\_organization | SSBP1 | 449 | 3 | 1.183399 | -0.325646 | 360 | 349.4 | 0.970556 |
| GO:0006996\_organelle\_organization | DNMT3B | 449 | 3 | 1.183399 | -0.325646 | 360 | 349.4 | 0.970556 |
| GO:0006996\_organelle\_organization | HELLS | 449 | 3 | 1.183399 | -0.325646 | 360 | 349.4 | 0.970556 |
| GO:0007399\_nervous\_system\_development | OTX2 | 621 | 4 | 1.140840 | -0.325333 | 361 | 349.63 | 0.968504 |
| GO:0007399\_nervous\_system\_development | FOXD1 | 621 | 4 | 1.140840 | -0.325333 | 361 | 349.63 | 0.968504 |
| GO:0007399\_nervous\_system\_development | PTPN11 | 621 | 4 | 1.140840 | -0.325333 | 361 | 349.63 | 0.968504 |
| GO:0007399\_nervous\_system\_development | QK | 621 | 4 | 1.140840 | -0.325333 | 361 | 349.63 | 0.968504 |
| GO:0043549\_regulation\_of\_kinase\_activity | PTPN11 | 112 | 1 | 1.581387 | -0.324470 | 362 | 350.92 | 0.969392 |
| GO:0006974\_response\_to\_DNA\_damage\_stimulus | PTPN11 | 113 | 1 | 1.567393 | -0.321687 | 364 | 351.91 | 0.966786 |
| GO:0040008\_regulation\_of\_growth | PTPN11 | 113 | 1 | 1.567393 | -0.321687 | 364 | 351.91 | 0.966786 |
| GO:0000165\_MAPKKK\_cascade | PTPN11 | 114 | 1 | 1.553644 | -0.318937 | 365 | 353.0 | 0.967123 |
| GO:0000902\_cell\_morphogenesis | FOXD1 | 283 | 2 | 1.251699 | -0.317853 | 366 | 353.48 | 0.965792 |
| GO:0000902\_cell\_morphogenesis | PTPN11 | 283 | 2 | 1.251699 | -0.317853 | 366 | 353.48 | 0.965792 |
| GO:0051338\_regulation\_of\_transferase\_activity | PTPN11 | 115 | 1 | 1.540134 | -0.316220 | 367 | 354.12 | 0.964905 |
| GO:0009893\_positive\_regulation\_of\_metabolic\_process | GPBP1 | 458 | 3 | 1.160144 | -0.313271 | 368 | 356.66 | 0.969185 |
| GO:0009893\_positive\_regulation\_of\_metabolic\_process | OTX2 | 458 | 3 | 1.160144 | -0.313271 | 368 | 356.66 | 0.969185 |
| GO:0009893\_positive\_regulation\_of\_metabolic\_process | FOXD1 | 458 | 3 | 1.160144 | -0.313271 | 368 | 356.66 | 0.969185 |
| GO:0006519\_cellular\_amino\_acid\_and\_derivative\_metabolic\_process | AZIN1 | 118 | 1 | 1.500978 | -0.308257 | 369 | 358.35 | 0.971138 |
| GO:0006917\_induction\_of\_apoptosis | ITM2B | 121 | 1 | 1.463764 | -0.300565 | 371 | 361.08 | 0.973261 |
| GO:0012502\_induction\_of\_programmed\_cell\_death | ITM2B | 121 | 1 | 1.463764 | -0.300565 | 371 | 361.08 | 0.973261 |
| GO:0006886\_intracellular\_protein\_transport | PTPN11 | 122 | 1 | 1.451765 | -0.298060 | 372 | 363.03 | 0.975887 |
| GO:0048518\_positive\_regulation\_of\_biological\_process | GPBP1 | 995 | 6 | 1.068032 | -0.297486 | 373 | 364.03 | 0.975952 |
| GO:0048518\_positive\_regulation\_of\_biological\_process | OTX2 | 995 | 6 | 1.068032 | -0.297486 | 373 | 364.03 | 0.975952 |
| GO:0048518\_positive\_regulation\_of\_biological\_process | SKP2 | 995 | 6 | 1.068032 | -0.297486 | 373 | 364.03 | 0.975952 |
| GO:0048518\_positive\_regulation\_of\_biological\_process | ITM2B | 995 | 6 | 1.068032 | -0.297486 | 373 | 364.03 | 0.975952 |
| GO:0048518\_positive\_regulation\_of\_biological\_process | FOXD1 | 995 | 6 | 1.068032 | -0.297486 | 373 | 364.03 | 0.975952 |
| GO:0048518\_positive\_regulation\_of\_biological\_process | PTPN11 | 995 | 6 | 1.068032 | -0.297486 | 373 | 364.03 | 0.975952 |
| GO:0009308\_amine\_metabolic\_process | AZIN1 | 124 | 1 | 1.428350 | -0.293133 | 375 | 364.78 | 0.972747 |
| GO:0030098\_lymphocyte\_differentiation | HELLS | 124 | 1 | 1.428350 | -0.293133 | 375 | 364.78 | 0.972747 |
| GO:0001763\_morphogenesis\_of\_a\_branching\_structure | FOXD1 | 125 | 1 | 1.416923 | -0.290711 | 376 | 366.24 | 0.974043 |
| GO:0043285\_biopolymer\_catabolic\_process | SKP2 | 129 | 1 | 1.372987 | -0.281285 | 377 | 368.8 | 0.978249 |
| GO:0045165\_cell\_fate\_commitment | OTX2 | 130 | 1 | 1.362426 | -0.278993 | 378 | 370.98 | 0.981429 |
| GO:0009952\_anterior\_posterior\_pattern\_formation | OTX2 | 133 | 1 | 1.331695 | -0.272263 | 379 | 372.35 | 0.982454 |
| GO:0001654\_eye\_development | OTX2 | 136 | 1 | 1.302319 | -0.265745 | 380 | 373.47 | 0.982816 |
| GO:0009057\_macromolecule\_catabolic\_process | SKP2 | 137 | 1 | 1.292813 | -0.263618 | 381 | 374.11 | 0.981916 |
| GO:0007169\_transmembrane\_receptor\_protein\_tyrosine\_kinase\_signaling\_pathway | PTPN11 | 139 | 1 | 1.274211 | -0.259430 | 383 | 374.96 | 0.979008 |
| GO:0034613\_cellular\_protein\_localization | PTPN11 | 139 | 1 | 1.274211 | -0.259430 | 383 | 374.96 | 0.979008 |
| GO:0070727\_cellular\_macromolecule\_localization | PTPN11 | 141 | 1 | 1.256137 | -0.255328 | 384 | 376.22 | 0.979740 |
| GO:0035239\_tube\_morphogenesis | FOXD1 | 143 | 1 | 1.238569 | -0.251309 | 385 | 376.74 | 0.978545 |
| GO:0007186\_G-protein\_coupled\_receptor\_protein\_signaling\_pathway | RGS2 | 144 | 1 | 1.229968 | -0.249331 | 386 | 377.43 | 0.977798 |
| GO:0030900\_forebrain\_development | OTX2 | 146 | 1 | 1.213119 | -0.245433 | 387 | 380.53 | 0.983282 |
| GO:0051649\_establishment\_of\_localization\_in\_cell | GARS | 342 | 2 | 1.035762 | -0.232288 | 388 | 386.0 | 0.994845 |
| GO:0051649\_establishment\_of\_localization\_in\_cell | PTPN11 | 342 | 2 | 1.035762 | -0.232288 | 388 | 386.0 | 0.994845 |
| GO:0048514\_blood\_vessel\_morphogenesis | QK | 158 | 1 | 1.120983 | -0.223605 | 389 | 390.25 | 1.003213 |
| GO:0002521\_leukocyte\_differentiation | HELLS | 161 | 1 | 1.100096 | -0.218534 | 390 | 391.79 | 1.004590 |
| GO:0042325\_regulation\_of\_phosphorylation | PTPN11 | 164 | 1 | 1.079972 | -0.213605 | 391 | 394.82 | 1.009770 |
| GO:0019220\_regulation\_of\_phosphate\_metabolic\_process | PTPN11 | 165 | 1 | 1.073427 | -0.211993 | 393 | 396.17 | 1.008066 |
| GO:0051174\_regulation\_of\_phosphorus\_metabolic\_process | PTPN11 | 165 | 1 | 1.073427 | -0.211993 | 393 | 396.17 | 1.008066 |
| GO:0043065\_positive\_regulation\_of\_apoptosis | ITM2B | 166 | 1 | 1.066960 | -0.210395 | 394 | 397.09 | 1.007843 |
| GO:0010942\_positive\_regulation\_of\_cell\_death | ITM2B | 167 | 1 | 1.060571 | -0.208813 | 397 | 398.52 | 1.003829 |
| GO:0043068\_positive\_regulation\_of\_programmed\_cell\_death | ITM2B | 167 | 1 | 1.060571 | -0.208813 | 397 | 398.52 | 1.003829 |
| GO:0051049\_regulation\_of\_transport | PTPN11 | 167 | 1 | 1.060571 | -0.208813 | 397 | 398.52 | 1.003829 |
| GO:0051641\_cellular\_localization | GARS | 370 | 2 | 0.957380 | -0.200582 | 398 | 402.94 | 1.012412 |
| GO:0051641\_cellular\_localization | PTPN11 | 370 | 2 | 0.957380 | -0.200582 | 398 | 402.94 | 1.012412 |
| GO:0044248\_cellular\_catabolic\_process | SKP2 | 173 | 1 | 1.023788 | -0.199618 | 399 | 404.12 | 1.012832 |
| GO:0000122\_negative\_regulation\_of\_transcription\_from\_RNA\_polymerase\_II\_promoter | STRAP | 175 | 1 | 1.012088 | -0.196663 | 401 | 405.81 | 1.011995 |
| GO:0015031\_protein\_transport | PTPN11 | 175 | 1 | 1.012088 | -0.196663 | 401 | 405.81 | 1.011995 |
| GO:0009790\_embryonic\_development | TAF10 | 567 | 3 | 0.937118 | -0.195487 | 402 | 406.06 | 1.010100 |
| GO:0009790\_embryonic\_development | OTX2 | 567 | 3 | 0.937118 | -0.195487 | 402 | 406.06 | 1.010100 |
| GO:0009790\_embryonic\_development | DNMT3B | 567 | 3 | 0.937118 | -0.195487 | 402 | 406.06 | 1.010100 |
| GO:0006873\_cellular\_ion\_homeostasis | QK | 176 | 1 | 1.006337 | -0.195205 | 403 | 407.26 | 1.010571 |
| GO:0045184\_establishment\_of\_protein\_localization | PTPN11 | 180 | 1 | 0.983974 | -0.189505 | 404 | 409.63 | 1.013936 |
| GO:0055082\_cellular\_chemical\_homeostasis | QK | 181 | 1 | 0.978538 | -0.188111 | 405 | 411.21 | 1.015333 |
| GO:0016192\_vesicle-mediated\_transport | GARS | 184 | 1 | 0.962584 | -0.184003 | 406 | 413.06 | 1.017389 |
| GO:0009653\_anatomical\_structure\_morphogenesis | SSBP1 | 958 | 5 | 0.924402 | -0.183596 | 407 | 413.48 | 1.015921 |
| GO:0009653\_anatomical\_structure\_morphogenesis | OTX2 | 958 | 5 | 0.924402 | -0.183596 | 407 | 413.48 | 1.015921 |
| GO:0009653\_anatomical\_structure\_morphogenesis | FOXD1 | 958 | 5 | 0.924402 | -0.183596 | 407 | 413.48 | 1.015921 |
| GO:0009653\_anatomical\_structure\_morphogenesis | PTPN11 | 958 | 5 | 0.924402 | -0.183596 | 407 | 413.48 | 1.015921 |
| GO:0009653\_anatomical\_structure\_morphogenesis | QK | 958 | 5 | 0.924402 | -0.183596 | 407 | 413.48 | 1.015921 |
| GO:0019226\_transmission\_of\_nerve\_impulse | QK | 189 | 1 | 0.937118 | -0.177392 | 408 | 418.86 | 1.026618 |
| GO:0046907\_intracellular\_transport | PTPN11 | 194 | 1 | 0.912966 | -0.171061 | 409 | 420.75 | 1.028729 |
| GO:0003002\_regionalization | OTX2 | 195 | 1 | 0.908284 | -0.169827 | 411 | 422.05 | 1.026886 |
| GO:0019725\_cellular\_homeostasis | QK | 195 | 1 | 0.908284 | -0.169827 | 411 | 422.05 | 1.026886 |
| GO:0033554\_cellular\_response\_to\_stress | PTPN11 | 196 | 1 | 0.903650 | -0.168603 | 412 | 422.51 | 1.025510 |
| GO:0050801\_ion\_homeostasis | QK | 197 | 1 | 0.899063 | -0.167390 | 413 | 423.32 | 1.024988 |
| GO:0002009\_morphogenesis\_of\_an\_epithelium | FOXD1 | 198 | 1 | 0.894522 | -0.166187 | 415 | 424.06 | 1.021831 |
| GO:0060429\_epithelium\_development | FOXD1 | 198 | 1 | 0.894522 | -0.166187 | 415 | 424.06 | 1.021831 |
| GO:0001568\_blood\_vessel\_development | QK | 203 | 1 | 0.872490 | -0.160322 | 416 | 426.11 | 1.024303 |
| GO:0042592\_homeostatic\_process | PTPN11 | 419 | 2 | 0.845419 | -0.155370 | 417 | 428.06 | 1.026523 |
| GO:0042592\_homeostatic\_process | QK | 419 | 2 | 0.845419 | -0.155370 | 417 | 428.06 | 1.026523 |
| GO:0001944\_vasculature\_development | QK | 208 | 1 | 0.851516 | -0.154697 | 418 | 428.9 | 1.026077 |
| GO:0035295\_tube\_development | FOXD1 | 212 | 1 | 0.835450 | -0.150360 | 419 | 430.05 | 1.026372 |
| GO:0010033\_response\_to\_organic\_substance | PTPN11 | 216 | 1 | 0.819979 | -0.146163 | 420 | 430.89 | 1.025929 |
| GO:0040007\_growth | PTPN11 | 217 | 1 | 0.816200 | -0.145135 | 421 | 431.93 | 1.025962 |
| GO:0007423\_sensory\_organ\_development | OTX2 | 219 | 1 | 0.808746 | -0.143104 | 422 | 433.21 | 1.026564 |
| GO:0009887\_organ\_morphogenesis | OTX2 | 642 | 3 | 0.827642 | -0.140471 | 423 | 434.45 | 1.027069 |
| GO:0009887\_organ\_morphogenesis | FOXD1 | 642 | 3 | 0.827642 | -0.140471 | 423 | 434.45 | 1.027069 |
| GO:0009887\_organ\_morphogenesis | QK | 642 | 3 | 0.827642 | -0.140471 | 423 | 434.45 | 1.027069 |
| GO:0046649\_lymphocyte\_activation | HELLS | 228 | 1 | 0.776822 | -0.134353 | 424 | 436.53 | 1.029552 |
| GO:0010926\_anatomical\_structure\_formation | TAF10 | 447 | 2 | 0.792463 | -0.134300 | 425 | 436.79 | 1.027741 |
| GO:0010926\_anatomical\_structure\_formation | HELLS | 447 | 2 | 0.792463 | -0.134300 | 425 | 436.79 | 1.027741 |
| GO:0048468\_cell\_development | OTX2 | 654 | 3 | 0.812456 | -0.133147 | 426 | 437.36 | 1.026667 |
| GO:0048468\_cell\_development | FOXD1 | 654 | 3 | 0.812456 | -0.133147 | 426 | 437.36 | 1.026667 |
| GO:0048468\_cell\_development | PTPN11 | 654 | 3 | 0.812456 | -0.133147 | 426 | 437.36 | 1.026667 |
| GO:0007420\_brain\_development | OTX2 | 231 | 1 | 0.766733 | -0.131572 | 427 | 438.51 | 1.026956 |
| GO:0009056\_catabolic\_process | SKP2 | 243 | 1 | 0.728870 | -0.121076 | 428 | 443.28 | 1.035701 |
| GO:0032879\_regulation\_of\_localization | PTPN11 | 248 | 1 | 0.714175 | -0.116982 | 430 | 444.43 | 1.033558 |
| GO:0045321\_leukocyte\_activation | HELLS | 248 | 1 | 0.714175 | -0.116982 | 430 | 444.43 | 1.033558 |
| GO:0007389\_pattern\_specification\_process | OTX2 | 250 | 1 | 0.708462 | -0.115387 | 431 | 444.85 | 1.032135 |
| GO:0008104\_protein\_localization | PTPN11 | 251 | 1 | 0.705639 | -0.114599 | 432 | 445.28 | 1.030741 |
| GO:0007267\_cell-cell\_signaling | PTPN11 | 252 | 1 | 0.702839 | -0.113816 | 433 | 445.72 | 1.029376 |
| GO:0030097\_hemopoiesis | HELLS | 253 | 1 | 0.700061 | -0.113040 | 434 | 446.3 | 1.028341 |
| GO:0065008\_regulation\_of\_biological\_quality | ALDH1A7 | 693 | 3 | 0.766733 | -0.111714 | 435 | 447.28 | 1.028230 |
| GO:0065008\_regulation\_of\_biological\_quality | QK | 693 | 3 | 0.766733 | -0.111714 | 435 | 447.28 | 1.028230 |
| GO:0065008\_regulation\_of\_biological\_quality | PTPN11 | 693 | 3 | 0.766733 | -0.111714 | 435 | 447.28 | 1.028230 |
| GO:0048729\_tissue\_morphogenesis | FOXD1 | 255 | 1 | 0.694570 | -0.111504 | 436 | 447.85 | 1.027179 |
| GO:0001775\_cell\_activation | HELLS | 262 | 1 | 0.676013 | -0.106307 | 437 | 450.35 | 1.030549 |
| GO:0051716\_cellular\_response\_to\_stimulus | PTPN11 | 273 | 1 | 0.648774 | -0.098668 | 438 | 453.72 | 1.035890 |
| GO:0033036\_macromolecule\_localization | PTPN11 | 274 | 1 | 0.646407 | -0.098003 | 439 | 454.47 | 1.035239 |
| GO:0048534\_hemopoietic\_or\_lymphoid\_organ\_development | HELLS | 277 | 1 | 0.639406 | -0.096039 | 440 | 455.31 | 1.034795 |
| GO:0007417\_central\_nervous\_system\_development | OTX2 | 287 | 1 | 0.617127 | -0.089793 | 441 | 458.28 | 1.039184 |
| GO:0009888\_tissue\_development | OTX2 | 525 | 2 | 0.674725 | -0.089336 | 442 | 458.63 | 1.037624 |
| GO:0009888\_tissue\_development | FOXD1 | 525 | 2 | 0.674725 | -0.089336 | 442 | 458.63 | 1.037624 |
| GO:0002520\_immune\_system\_development | HELLS | 295 | 1 | 0.600391 | -0.085109 | 443 | 460.11 | 1.038623 |
| GO:0048598\_embryonic\_morphogenesis | OTX2 | 299 | 1 | 0.592359 | -0.082866 | 444 | 460.85 | 1.037950 |
| GO:0051094\_positive\_regulation\_of\_developmental\_process | ITM2B | 308 | 1 | 0.575050 | -0.078045 | 445 | 462.89 | 1.040202 |
| GO:0006928\_cell\_motion | FOXD1 | 330 | 1 | 0.536713 | -0.067464 | 447 | 466.09 | 1.042707 |
| GO:0051674\_localization\_of\_cell | FOXD1 | 330 | 1 | 0.536713 | -0.067464 | 447 | 466.09 | 1.042707 |
| GO:0051179\_localization | GARS | 1058 | 4 | 0.669623 | -0.055350 | 448 | 471.87 | 1.053281 |
| GO:0051179\_localization | FOXD1 | 1058 | 4 | 0.669623 | -0.055350 | 448 | 471.87 | 1.053281 |
| GO:0051179\_localization | DNMT3B | 1058 | 4 | 0.669623 | -0.055350 | 448 | 471.87 | 1.053281 |
| GO:0051179\_localization | PTPN11 | 1058 | 4 | 0.669623 | -0.055350 | 448 | 471.87 | 1.053281 |
| GO:0048856\_anatomical\_structure\_development | TAF10 | 1688 | 7 | 0.734483 | -0.048516 | 449 | 475.18 | 1.058307 |
| GO:0048856\_anatomical\_structure\_development | SSBP1 | 1688 | 7 | 0.734483 | -0.048516 | 449 | 475.18 | 1.058307 |
| GO:0048856\_anatomical\_structure\_development | OTX2 | 1688 | 7 | 0.734483 | -0.048516 | 449 | 475.18 | 1.058307 |
| GO:0048856\_anatomical\_structure\_development | FOXD1 | 1688 | 7 | 0.734483 | -0.048516 | 449 | 475.18 | 1.058307 |
| GO:0048856\_anatomical\_structure\_development | HELLS | 1688 | 7 | 0.734483 | -0.048516 | 449 | 475.18 | 1.058307 |
| GO:0048856\_anatomical\_structure\_development | PTPN11 | 1688 | 7 | 0.734483 | -0.048516 | 449 | 475.18 | 1.058307 |
| GO:0048856\_anatomical\_structure\_development | QK | 1688 | 7 | 0.734483 | -0.048516 | 449 | 475.18 | 1.058307 |
| GO:0050877\_neurological\_system\_process | QK | 390 | 1 | 0.454142 | -0.045527 | 450 | 476.6 | 1.059111 |
| GO:0042221\_response\_to\_chemical\_stimulus | PTPN11 | 409 | 1 | 0.433045 | -0.040225 | 451 | 478.42 | 1.060798 |
| GO:0007275\_multicellular\_organismal\_development | TAF10 | 1760 | 7 | 0.704436 | -0.035740 | 452 | 480.42 | 1.062876 |
| GO:0007275\_multicellular\_organismal\_development | OTX2 | 1760 | 7 | 0.704436 | -0.035740 | 452 | 480.42 | 1.062876 |
| GO:0007275\_multicellular\_organismal\_development | FOXD1 | 1760 | 7 | 0.704436 | -0.035740 | 452 | 480.42 | 1.062876 |
| GO:0007275\_multicellular\_organismal\_development | DNMT3B | 1760 | 7 | 0.704436 | -0.035740 | 452 | 480.42 | 1.062876 |
| GO:0007275\_multicellular\_organismal\_development | HELLS | 1760 | 7 | 0.704436 | -0.035740 | 452 | 480.42 | 1.062876 |
| GO:0007275\_multicellular\_organismal\_development | PTPN11 | 1760 | 7 | 0.704436 | -0.035740 | 452 | 480.42 | 1.062876 |
| GO:0007275\_multicellular\_organismal\_development | QK | 1760 | 7 | 0.704436 | -0.035740 | 452 | 480.42 | 1.062876 |
| GO:0048513\_organ\_development | TAF10 | 1365 | 5 | 0.648774 | -0.035368 | 453 | 480.47 | 1.060640 |
| GO:0048513\_organ\_development | OTX2 | 1365 | 5 | 0.648774 | -0.035368 | 453 | 480.47 | 1.060640 |
| GO:0048513\_organ\_development | FOXD1 | 1365 | 5 | 0.648774 | -0.035368 | 453 | 480.47 | 1.060640 |
| GO:0048513\_organ\_development | HELLS | 1365 | 5 | 0.648774 | -0.035368 | 453 | 480.47 | 1.060640 |
| GO:0048513\_organ\_development | QK | 1365 | 5 | 0.648774 | -0.035368 | 453 | 480.47 | 1.060640 |
| GO:0006810\_transport | GARS | 718 | 2 | 0.493358 | -0.031606 | 454 | 482.57 | 1.062930 |
| GO:0006810\_transport | PTPN11 | 718 | 2 | 0.493358 | -0.031606 | 454 | 482.57 | 1.062930 |
| GO:0051234\_establishment\_of\_localization | GARS | 729 | 2 | 0.485913 | -0.029733 | 455 | 483.71 | 1.063099 |
| GO:0051234\_establishment\_of\_localization | PTPN11 | 729 | 2 | 0.485913 | -0.029733 | 455 | 483.71 | 1.063099 |
| GO:0048731\_system\_development | TAF10 | 1609 | 6 | 0.660468 | -0.029269 | 456 | 483.81 | 1.060987 |
| GO:0048731\_system\_development | OTX2 | 1609 | 6 | 0.660468 | -0.029269 | 456 | 483.81 | 1.060987 |
| GO:0048731\_system\_development | FOXD1 | 1609 | 6 | 0.660468 | -0.029269 | 456 | 483.81 | 1.060987 |
| GO:0048731\_system\_development | HELLS | 1609 | 6 | 0.660468 | -0.029269 | 456 | 483.81 | 1.060987 |
| GO:0048731\_system\_development | PTPN11 | 1609 | 6 | 0.660468 | -0.029269 | 456 | 483.81 | 1.060987 |
| GO:0048731\_system\_development | QK | 1609 | 6 | 0.660468 | -0.029269 | 456 | 483.81 | 1.060987 |
| GO:0002376\_immune\_system\_process | HELLS | 505 | 1 | 0.350724 | -0.021534 | 457 | 485.4 | 1.062144 |
| GO:0003008\_system\_process | QK | 516 | 1 | 0.343247 | -0.020043 | 458 | 485.64 | 1.060349 |
| GO:0008283\_cell\_proliferation | HELLS | 544 | 1 | 0.325580 | -0.016690 | 459 | 486.62 | 1.060174 |
| GO:0006950\_response\_to\_stress | PTPN11 | 549 | 1 | 0.322615 | -0.016153 | 460 | 486.76 | 1.058174 |
| GO:0051239\_regulation\_of\_multicellular\_organismal\_process | PTPN11 | 587 | 1 | 0.301730 | -0.012585 | 461 | 487.86 | 1.058265 |
| GO:0032501\_multicellular\_organismal\_process | TAF10 | 2183 | 7 | 0.567938 | -0.004160 | 462 | 490.51 | 1.061710 |
| GO:0032501\_multicellular\_organismal\_process | OTX2 | 2183 | 7 | 0.567938 | -0.004160 | 462 | 490.51 | 1.061710 |
| GO:0032501\_multicellular\_organismal\_process | FOXD1 | 2183 | 7 | 0.567938 | -0.004160 | 462 | 490.51 | 1.061710 |
| GO:0032501\_multicellular\_organismal\_process | DNMT3B | 2183 | 7 | 0.567938 | -0.004160 | 462 | 490.51 | 1.061710 |
| GO:0032501\_multicellular\_organismal\_process | HELLS | 2183 | 7 | 0.567938 | -0.004160 | 462 | 490.51 | 1.061710 |
| GO:0032501\_multicellular\_organismal\_process | PTPN11 | 2183 | 7 | 0.567938 | -0.004160 | 462 | 490.51 | 1.061710 |
| GO:0032501\_multicellular\_organismal\_process | QK | 2183 | 7 | 0.567938 | -0.004160 | 462 | 490.51 | 1.061710 |
| GO:0050896\_response\_to\_stimulus | PTPN11 | 1107 | 1 | 0.159996 | -0.000334 | 463 | 491.56 | 1.061685 |
| GO:0008150\_biological\_process | GPBP1 | 4605 | 26 | 1.000000 | 0.000000 | 1703 | 1715.19 | 1.007158 |
| GO:0008150\_biological\_process | STRAP | 4605 | 26 | 1.000000 | 0.000000 | 1703 | 1715.19 | 1.007158 |
| GO:0008150\_biological\_process | MKNK2 | 4605 | 26 | 1.000000 | 0.000000 | 1703 | 1715.19 | 1.007158 |
| GO:0008150\_biological\_process | AZIN1 | 4605 | 26 | 1.000000 | 0.000000 | 1703 | 1715.19 | 1.007158 |
| GO:0008150\_biological\_process | ITM2B | 4605 | 26 | 1.000000 | 0.000000 | 1703 | 1715.19 | 1.007158 |
| GO:0008150\_biological\_process | DKC1 | 4605 | 26 | 1.000000 | 0.000000 | 1703 | 1715.19 | 1.007158 |
| GO:0008150\_biological\_process | ALDH1A7 | 4605 | 26 | 1.000000 | 0.000000 | 1703 | 1715.19 | 1.007158 |
| GO:0008150\_biological\_process | FOXD1 | 4605 | 26 | 1.000000 | 0.000000 | 1703 | 1715.19 | 1.007158 |
| GO:0008150\_biological\_process | DNMT3B | 4605 | 26 | 1.000000 | 0.000000 | 1703 | 1715.19 | 1.007158 |
| GO:0008150\_biological\_process | API5 | 4605 | 26 | 1.000000 | 0.000000 | 1703 | 1715.19 | 1.007158 |
| GO:0008150\_biological\_process | HELLS | 4605 | 26 | 1.000000 | 0.000000 | 1703 | 1715.19 | 1.007158 |
| GO:0008150\_biological\_process | RAD51AP1 | 4605 | 26 | 1.000000 | 0.000000 | 1703 | 1715.19 | 1.007158 |
| GO:0008150\_biological\_process | SSBP1 | 4605 | 26 | 1.000000 | 0.000000 | 1703 | 1715.19 | 1.007158 |
| GO:0008150\_biological\_process | NLK | 4605 | 26 | 1.000000 | 0.000000 | 1703 | 1715.19 | 1.007158 |
| GO:0008150\_biological\_process | OTX2 | 4605 | 26 | 1.000000 | 0.000000 | 1703 | 1715.19 | 1.007158 |
| GO:0008150\_biological\_process | SKP2 | 4605 | 26 | 1.000000 | 0.000000 | 1703 | 1715.19 | 1.007158 |
| GO:0008150\_biological\_process | GARS | 4605 | 26 | 1.000000 | 0.000000 | 1703 | 1715.19 | 1.007158 |
| GO:0008150\_biological\_process | PTPN12 | 4605 | 26 | 1.000000 | 0.000000 | 1703 | 1715.19 | 1.007158 |
| GO:0008150\_biological\_process | PTPN11 | 4605 | 26 | 1.000000 | 0.000000 | 1703 | 1715.19 | 1.007158 |
| GO:0008150\_biological\_process | QK | 4605 | 26 | 1.000000 | 0.000000 | 1703 | 1715.19 | 1.007158 |
| GO:0008150\_biological\_process | ACSM3 | 4605 | 26 | 1.000000 | 0.000000 | 1703 | 1715.19 | 1.007158 |
| GO:0008150\_biological\_process | TAF10 | 4605 | 26 | 1.000000 | 0.000000 | 1703 | 1715.19 | 1.007158 |
| GO:0008150\_biological\_process | RGS2 | 4605 | 26 | 1.000000 | 0.000000 | 1703 | 1715.19 | 1.007158 |
| GO:0008150\_biological\_process | VCP | 4605 | 26 | 1.000000 | 0.000000 | 1703 | 1715.19 | 1.007158 |
| GO:0008150\_biological\_process | RAP1A | 4605 | 26 | 1.000000 | 0.000000 | 1703 | 1715.19 | 1.007158 |
| GO:0008150\_biological\_process | RBM17 | 4605 | 26 | 1.000000 | 0.000000 | 1703 | 1715.19 | 1.007158 |
