# Supplementary material for: RedundancyMiner: De-replication of redundant GO categories in microarray and proteomics analysis
Source: BMC Bioinformatics. 2011 Feb 10;12:52. doi: 10.1186/1471-2105-12-52 (PMC3223614; doi:10.1186/1471-2105-12-52)
Supplement: Additional file 8 — Retinal development HTGM download. compressed package of the results of running HTGM on the retinal development genes list. [file 1471-2105-12-52-S8.ZIP › SCENARIO_2_MODIFIED/total.txt.total.txt.dir/Exp1_BestClusterMap_LEIGS_KM_24.csv.join.12.txt.dir/Exp1_BestClusterMap_LEIGS_KM_24.csv.join.12.txt.change.gce.html]

Gene Category Report for Exp1\_BestClusterMap\_LEIGS\_KM\_24.csv.join.12.txt

# Gene Category Report for Exp1\_BestClusterMap\_LEIGS\_KM\_24.csv.join.12.txt

| HYPERLINKED GO CATEGORY | HYPERLINKED GENE NAME | TOTAL GENES | CHANGED GENES | ENRICHMENT | LOG10(p) | CUMULATIVE NUMBER OF CATEGORIES | CUMULATIVE RANDOMS MEAN | FALSE DISCOVERY RATE |
| --- | --- | --- | --- | --- | --- | --- | --- | --- |
| GO:0060235\_lens\_induction\_in\_camera-type\_eye | SOX2 | 4 | 2 |  |  |  |  |  |  |
| GO:0060235\_lens\_induction\_in\_camera-type\_eye | SIX3 | 4 | 2 |  |  |  |  |  |  |
| GO:0034599\_cellular\_response\_to\_oxidative\_stress | GPX1 | 7 | 2 | 45.369458 | -3.103092 | 1 | 0.98 | 0.980000 |
| GO:0034599\_cellular\_response\_to\_oxidative\_stress | HIF1A | 7 | 2 | 45.369458 | -3.103092 | 1 | 0.98 | 0.980000 |
| GO:0002089\_lens\_morphogenesis\_in\_camera-type\_eye | SOX2 | 12 | 2 | 26.465517 | -2.614256 | 2 | 2.91 | 1.455000 |
| GO:0002089\_lens\_morphogenesis\_in\_camera-type\_eye | SIX3 | 12 | 2 | 26.465517 | -2.614256 | 2 | 2.91 | 1.455000 |
| GO:0048593\_camera-type\_eye\_morphogenesis | ALDH1A1 | 44 | 3 | 10.826803 | -2.601901 | 3 | 2.96 | 0.986667 |
| GO:0048593\_camera-type\_eye\_morphogenesis | SOX2 | 44 | 3 | 10.826803 | -2.601901 | 3 | 2.96 | 0.986667 |
| GO:0048593\_camera-type\_eye\_morphogenesis | SIX3 | 44 | 3 | 10.826803 | -2.601901 | 3 | 2.96 | 0.986667 |
| GO:0048568\_embryonic\_organ\_development | ALDH1A1 | 55 | 3 | 8.661442 | -2.325212 | 4 | 4.64 | 1.160000 |
| GO:0048568\_embryonic\_organ\_development | HIF1A | 55 | 3 | 8.661442 | -2.325212 | 4 | 4.64 | 1.160000 |
| GO:0048568\_embryonic\_organ\_development | SOX2 | 55 | 3 | 8.661442 | -2.325212 | 4 | 4.64 | 1.160000 |
| GO:0006261\_DNA-dependent\_DNA\_replication | MCM7 | 17 | 2 | 18.681542 | -2.308735 | 5 | 4.9 | 0.980000 |
| GO:0006261\_DNA-dependent\_DNA\_replication | POLD1 | 17 | 2 | 18.681542 | -2.308735 | 5 | 4.9 | 0.980000 |
| GO:0030178\_negative\_regulation\_of\_Wnt\_receptor\_signaling\_pathway | SOX2 | 18 | 2 | 17.643678 | -2.259276 | 6 | 5.5 | 0.916667 |
| GO:0030178\_negative\_regulation\_of\_Wnt\_receptor\_signaling\_pathway | SIX3 | 18 | 2 | 17.643678 | -2.259276 | 6 | 5.5 | 0.916667 |
| GO:0002248\_connective\_tissue\_replacement\_during\_inflammatory\_response | HIF1A | 1 | 1 |  |  |  |  |  |  |
| GO:0006544\_glycine\_metabolic\_process | PHGDH | 1 | 1 |  |  |  |  |  |  |
| GO:0010634\_positive\_regulation\_of\_epithelial\_cell\_migration | HIF1A | 1 | 1 |  |  |  |  |  |  |
| GO:0018158\_protein\_amino\_acid\_oxidation | GPX1 | 1 | 1 |  |  |  |  |  |  |
| GO:0019682\_glyceraldehyde-3-phosphate\_metabolic\_process | TPI1 | 1 | 1 |  |  |  |  |  |  |
| GO:0032365\_intracellular\_lipid\_transport | NPC2 | 1 | 1 |  |  |  |  |  |  |
| GO:0032366\_intracellular\_sterol\_transport | NPC2 | 1 | 1 |  |  |  |  |  |  |
| GO:0032367\_intracellular\_cholesterol\_transport | NPC2 | 1 | 1 |  |  |  |  |  |  |
| GO:0045004\_DNA\_replication\_proofreading | POLD1 | 1 | 1 |  |  |  |  |  |  |
| GO:0046827\_positive\_regulation\_of\_protein\_export\_from\_nucleus | PRKACA | 1 | 1 |  |  |  |  |  |  |
| GO:0031128\_developmental\_induction | SOX2 | 20 | 2 | 15.879310 | -2.168598 | 8 | 6.38 | 0.797500 |
| GO:0031128\_developmental\_induction | SIX3 | 20 | 2 | 15.879310 | -2.168598 | 8 | 6.38 | 0.797500 |
| GO:0045168\_cell-cell\_signaling\_involved\_in\_cell\_fate\_specification | SOX2 | 20 | 2 | 15.879310 | -2.168598 | 8 | 6.38 | 0.797500 |
| GO:0045168\_cell-cell\_signaling\_involved\_in\_cell\_fate\_specification | SIX3 | 20 | 2 | 15.879310 | -2.168598 | 8 | 6.38 | 0.797500 |
| GO:0033554\_cellular\_response\_to\_stress | GPX1 | 196 | 5 | 4.050844 | -2.164771 | 9 | 6.4 | 0.711111 |
| GO:0033554\_cellular\_response\_to\_stress | HIF1A | 196 | 5 | 4.050844 | -2.164771 | 9 | 6.4 | 0.711111 |
| GO:0033554\_cellular\_response\_to\_stress | EIF2S1 | 196 | 5 | 4.050844 | -2.164771 | 9 | 6.4 | 0.711111 |
| GO:0033554\_cellular\_response\_to\_stress | POLD1 | 196 | 5 | 4.050844 | -2.164771 | 9 | 6.4 | 0.711111 |
| GO:0033554\_cellular\_response\_to\_stress | TRIP13 | 196 | 5 | 4.050844 | -2.164771 | 9 | 6.4 | 0.711111 |
| GO:0044238\_primary\_metabolic\_process | SOX2 | 1905 | 19 | 1.583763 | -2.135377 | 10 | 6.67 | 0.667000 |
| GO:0044238\_primary\_metabolic\_process | SIX3 | 1905 | 19 | 1.583763 | -2.135377 | 10 | 6.67 | 0.667000 |
| GO:0044238\_primary\_metabolic\_process | CDK4 | 1905 | 19 | 1.583763 | -2.135377 | 10 | 6.67 | 0.667000 |
| GO:0044238\_primary\_metabolic\_process | CNOT7 | 1905 | 19 | 1.583763 | -2.135377 | 10 | 6.67 | 0.667000 |
| GO:0044238\_primary\_metabolic\_process | SF3A2 | 1905 | 19 | 1.583763 | -2.135377 | 10 | 6.67 | 0.667000 |
| GO:0044238\_primary\_metabolic\_process | PPP1CB | 1905 | 19 | 1.583763 | -2.135377 | 10 | 6.67 | 0.667000 |
| GO:0044238\_primary\_metabolic\_process | MTHFD1 | 1905 | 19 | 1.583763 | -2.135377 | 10 | 6.67 | 0.667000 |
| GO:0044238\_primary\_metabolic\_process | ALDH1A1 | 1905 | 19 | 1.583763 | -2.135377 | 10 | 6.67 | 0.667000 |
| GO:0044238\_primary\_metabolic\_process | DDR1 | 1905 | 19 | 1.583763 | -2.135377 | 10 | 6.67 | 0.667000 |
| GO:0044238\_primary\_metabolic\_process | GPX1 | 1905 | 19 | 1.583763 | -2.135377 | 10 | 6.67 | 0.667000 |
| GO:0044238\_primary\_metabolic\_process | TPI1 | 1905 | 19 | 1.583763 | -2.135377 | 10 | 6.67 | 0.667000 |
| GO:0044238\_primary\_metabolic\_process | MCM7 | 1905 | 19 | 1.583763 | -2.135377 | 10 | 6.67 | 0.667000 |
| GO:0044238\_primary\_metabolic\_process | HIF1A | 1905 | 19 | 1.583763 | -2.135377 | 10 | 6.67 | 0.667000 |
| GO:0044238\_primary\_metabolic\_process | BTG1 | 1905 | 19 | 1.583763 | -2.135377 | 10 | 6.67 | 0.667000 |
| GO:0044238\_primary\_metabolic\_process | EIF2S1 | 1905 | 19 | 1.583763 | -2.135377 | 10 | 6.67 | 0.667000 |
| GO:0044238\_primary\_metabolic\_process | POLD1 | 1905 | 19 | 1.583763 | -2.135377 | 10 | 6.67 | 0.667000 |
| GO:0044238\_primary\_metabolic\_process | PHGDH | 1905 | 19 | 1.583763 | -2.135377 | 10 | 6.67 | 0.667000 |
| GO:0044238\_primary\_metabolic\_process | PRKACA | 1905 | 19 | 1.583763 | -2.135377 | 10 | 6.67 | 0.667000 |
| GO:0044238\_primary\_metabolic\_process | TRIP13 | 1905 | 19 | 1.583763 | -2.135377 | 10 | 6.67 | 0.667000 |
| GO:0043687\_post-translational\_protein\_modification | GPX1 | 384 | 7 | 2.894666 | -2.083172 | 11 | 7.51 | 0.682727 |
| GO:0043687\_post-translational\_protein\_modification | DDR1 | 384 | 7 | 2.894666 | -2.083172 | 11 | 7.51 | 0.682727 |
| GO:0043687\_post-translational\_protein\_modification | BTG1 | 384 | 7 | 2.894666 | -2.083172 | 11 | 7.51 | 0.682727 |
| GO:0043687\_post-translational\_protein\_modification | EIF2S1 | 384 | 7 | 2.894666 | -2.083172 | 11 | 7.51 | 0.682727 |
| GO:0043687\_post-translational\_protein\_modification | PRKACA | 384 | 7 | 2.894666 | -2.083172 | 11 | 7.51 | 0.682727 |
| GO:0043687\_post-translational\_protein\_modification | CDK4 | 384 | 7 | 2.894666 | -2.083172 | 11 | 7.51 | 0.682727 |
| GO:0043687\_post-translational\_protein\_modification | PPP1CB | 384 | 7 | 2.894666 | -2.083172 | 11 | 7.51 | 0.682727 |
| GO:0048592\_eye\_morphogenesis | ALDH1A1 | 70 | 3 | 6.805419 | -2.033248 | 12 | 7.94 | 0.661667 |
| GO:0048592\_eye\_morphogenesis | SOX2 | 70 | 3 | 6.805419 | -2.033248 | 12 | 7.94 | 0.661667 |
| GO:0048592\_eye\_morphogenesis | SIX3 | 70 | 3 | 6.805419 | -2.033248 | 12 | 7.94 | 0.661667 |
| GO:0000280\_nuclear\_division | BUB1B | 24 | 2 | 13.232759 | -2.013205 | 14 | 8.24 | 0.588571 |
| GO:0000280\_nuclear\_division | CDCA5 | 24 | 2 | 13.232759 | -2.013205 | 14 | 8.24 | 0.588571 |
| GO:0007067\_mitosis | BUB1B | 24 | 2 | 13.232759 | -2.013205 | 14 | 8.24 | 0.588571 |
| GO:0007067\_mitosis | CDCA5 | 24 | 2 | 13.232759 | -2.013205 | 14 | 8.24 | 0.588571 |
| GO:0000087\_M\_phase\_of\_mitotic\_cell\_cycle | BUB1B | 25 | 2 | 12.703448 | -1.978682 | 16 | 8.63 | 0.539375 |
| GO:0000087\_M\_phase\_of\_mitotic\_cell\_cycle | CDCA5 | 25 | 2 | 12.703448 | -1.978682 | 16 | 8.63 | 0.539375 |
| GO:0048285\_organelle\_fission | BUB1B | 25 | 2 | 12.703448 | -1.978682 | 16 | 8.63 | 0.539375 |
| GO:0048285\_organelle\_fission | CDCA5 | 25 | 2 | 12.703448 | -1.978682 | 16 | 8.63 | 0.539375 |
| GO:0044237\_cellular\_metabolic\_process | SOX2 | 1974 | 19 | 1.528404 | -1.940463 | 17 | 9.03 | 0.531176 |
| GO:0044237\_cellular\_metabolic\_process | SIX3 | 1974 | 19 | 1.528404 | -1.940463 | 17 | 9.03 | 0.531176 |
| GO:0044237\_cellular\_metabolic\_process | CDK4 | 1974 | 19 | 1.528404 | -1.940463 | 17 | 9.03 | 0.531176 |
| GO:0044237\_cellular\_metabolic\_process | CNOT7 | 1974 | 19 | 1.528404 | -1.940463 | 17 | 9.03 | 0.531176 |
| GO:0044237\_cellular\_metabolic\_process | SF3A2 | 1974 | 19 | 1.528404 | -1.940463 | 17 | 9.03 | 0.531176 |
| GO:0044237\_cellular\_metabolic\_process | PPP1CB | 1974 | 19 | 1.528404 | -1.940463 | 17 | 9.03 | 0.531176 |
| GO:0044237\_cellular\_metabolic\_process | MTHFD1 | 1974 | 19 | 1.528404 | -1.940463 | 17 | 9.03 | 0.531176 |
| GO:0044237\_cellular\_metabolic\_process | ALDH1A1 | 1974 | 19 | 1.528404 | -1.940463 | 17 | 9.03 | 0.531176 |
| GO:0044237\_cellular\_metabolic\_process | GPX1 | 1974 | 19 | 1.528404 | -1.940463 | 17 | 9.03 | 0.531176 |
| GO:0044237\_cellular\_metabolic\_process | DDR1 | 1974 | 19 | 1.528404 | -1.940463 | 17 | 9.03 | 0.531176 |
| GO:0044237\_cellular\_metabolic\_process | TPI1 | 1974 | 19 | 1.528404 | -1.940463 | 17 | 9.03 | 0.531176 |
| GO:0044237\_cellular\_metabolic\_process | HIF1A | 1974 | 19 | 1.528404 | -1.940463 | 17 | 9.03 | 0.531176 |
| GO:0044237\_cellular\_metabolic\_process | MCM7 | 1974 | 19 | 1.528404 | -1.940463 | 17 | 9.03 | 0.531176 |
| GO:0044237\_cellular\_metabolic\_process | BTG1 | 1974 | 19 | 1.528404 | -1.940463 | 17 | 9.03 | 0.531176 |
| GO:0044237\_cellular\_metabolic\_process | EIF2S1 | 1974 | 19 | 1.528404 | -1.940463 | 17 | 9.03 | 0.531176 |
| GO:0044237\_cellular\_metabolic\_process | POLD1 | 1974 | 19 | 1.528404 | -1.940463 | 17 | 9.03 | 0.531176 |
| GO:0044237\_cellular\_metabolic\_process | PHGDH | 1974 | 19 | 1.528404 | -1.940463 | 17 | 9.03 | 0.531176 |
| GO:0044237\_cellular\_metabolic\_process | PRKACA | 1974 | 19 | 1.528404 | -1.940463 | 17 | 9.03 | 0.531176 |
| GO:0044237\_cellular\_metabolic\_process | TRIP13 | 1974 | 19 | 1.528404 | -1.940463 | 17 | 9.03 | 0.531176 |
| GO:0002072\_optic\_cup\_morphogenesis\_involved\_in\_camera-type\_eye\_development | ALDH1A1 | 2 | 1 |  |  |  |  |  |  |
| GO:0002246\_healing\_during\_inflammatory\_response | HIF1A | 2 | 1 |  |  |  |  |  |  |
| GO:0006089\_lactate\_metabolic\_process | HIF1A | 2 | 1 |  |  |  |  |  |  |
| GO:0006563\_L-serine\_metabolic\_process | PHGDH | 2 | 1 |  |  |  |  |  |  |
| GO:0006566\_threonine\_metabolic\_process | PHGDH | 2 | 1 |  |  |  |  |  |  |
| GO:0009608\_response\_to\_symbiont | GPX1 | 2 | 1 |  |  |  |  |  |  |
| GO:0009609\_response\_to\_symbiotic\_bacterium | GPX1 | 2 | 1 |  |  |  |  |  |  |
| GO:0014850\_response\_to\_muscle\_activity | HIF1A | 2 | 1 |  |  |  |  |  |  |
| GO:0030910\_olfactory\_placode\_formation | SOX2 | 2 | 1 |  |  |  |  |  |  |
| GO:0033194\_response\_to\_hydroperoxide | GPX1 | 2 | 1 |  |  |  |  |  |  |
| GO:0043618\_regulation\_of\_transcription\_from\_RNA\_polymerase\_II\_promoter\_in\_response\_to\_stress | HIF1A | 2 | 1 |  |  |  |  |  |  |
| GO:0043619\_regulation\_of\_transcription\_from\_RNA\_polymerase\_II\_promoter\_in\_response\_to\_oxidative\_stress | HIF1A | 2 | 1 |  |  |  |  |  |  |
| GO:0043620\_regulation\_of\_transcription\_in\_response\_to\_stress | HIF1A | 2 | 1 |  |  |  |  |  |  |
| GO:0045005\_maintenance\_of\_fidelity\_during\_DNA-dependent\_DNA\_replication | POLD1 | 2 | 1 |  |  |  |  |  |  |
| GO:0050973\_detection\_of\_mechanical\_stimulus\_involved\_in\_equilibrioception | SOX2 | 2 | 1 |  |  |  |  |  |  |
| GO:0051541\_elastin\_metabolic\_process | HIF1A | 2 | 1 |  |  |  |  |  |  |
| GO:0051702\_interaction\_with\_symbiont | GPX1 | 2 | 1 |  |  |  |  |  |  |
| GO:0034960\_cellular\_biopolymer\_metabolic\_process | SOX2 | 1395 | 15 | 1.707453 | -1.900987 | 18 | 9.41 | 0.522778 |
| GO:0034960\_cellular\_biopolymer\_metabolic\_process | SIX3 | 1395 | 15 | 1.707453 | -1.900987 | 18 | 9.41 | 0.522778 |
| GO:0034960\_cellular\_biopolymer\_metabolic\_process | SF3A2 | 1395 | 15 | 1.707453 | -1.900987 | 18 | 9.41 | 0.522778 |
| GO:0034960\_cellular\_biopolymer\_metabolic\_process | CNOT7 | 1395 | 15 | 1.707453 | -1.900987 | 18 | 9.41 | 0.522778 |
| GO:0034960\_cellular\_biopolymer\_metabolic\_process | CDK4 | 1395 | 15 | 1.707453 | -1.900987 | 18 | 9.41 | 0.522778 |
| GO:0034960\_cellular\_biopolymer\_metabolic\_process | PPP1CB | 1395 | 15 | 1.707453 | -1.900987 | 18 | 9.41 | 0.522778 |
| GO:0034960\_cellular\_biopolymer\_metabolic\_process | GPX1 | 1395 | 15 | 1.707453 | -1.900987 | 18 | 9.41 | 0.522778 |
| GO:0034960\_cellular\_biopolymer\_metabolic\_process | DDR1 | 1395 | 15 | 1.707453 | -1.900987 | 18 | 9.41 | 0.522778 |
| GO:0034960\_cellular\_biopolymer\_metabolic\_process | MCM7 | 1395 | 15 | 1.707453 | -1.900987 | 18 | 9.41 | 0.522778 |
| GO:0034960\_cellular\_biopolymer\_metabolic\_process | HIF1A | 1395 | 15 | 1.707453 | -1.900987 | 18 | 9.41 | 0.522778 |
| GO:0034960\_cellular\_biopolymer\_metabolic\_process | BTG1 | 1395 | 15 | 1.707453 | -1.900987 | 18 | 9.41 | 0.522778 |
| GO:0034960\_cellular\_biopolymer\_metabolic\_process | POLD1 | 1395 | 15 | 1.707453 | -1.900987 | 18 | 9.41 | 0.522778 |
| GO:0034960\_cellular\_biopolymer\_metabolic\_process | EIF2S1 | 1395 | 15 | 1.707453 | -1.900987 | 18 | 9.41 | 0.522778 |
| GO:0034960\_cellular\_biopolymer\_metabolic\_process | PRKACA | 1395 | 15 | 1.707453 | -1.900987 | 18 | 9.41 | 0.522778 |
| GO:0034960\_cellular\_biopolymer\_metabolic\_process | TRIP13 | 1395 | 15 | 1.707453 | -1.900987 | 18 | 9.41 | 0.522778 |
| GO:0002088\_lens\_development\_in\_camera-type\_eye | SOX2 | 28 | 2 | 11.342365 | -1.883375 | 20 | 9.77 | 0.488500 |
| GO:0002088\_lens\_development\_in\_camera-type\_eye | SIX3 | 28 | 2 | 11.342365 | -1.883375 | 20 | 9.77 | 0.488500 |
| GO:0030111\_regulation\_of\_Wnt\_receptor\_signaling\_pathway | SOX2 | 28 | 2 | 11.342365 | -1.883375 | 20 | 9.77 | 0.488500 |
| GO:0030111\_regulation\_of\_Wnt\_receptor\_signaling\_pathway | SIX3 | 28 | 2 | 11.342365 | -1.883375 | 20 | 9.77 | 0.488500 |
| GO:0007049\_cell\_cycle | PHGDH | 238 | 5 | 3.335990 | -1.819383 | 21 | 10.75 | 0.511905 |
| GO:0007049\_cell\_cycle | BUB1B | 238 | 5 | 3.335990 | -1.819383 | 21 | 10.75 | 0.511905 |
| GO:0007049\_cell\_cycle | CDK4 | 238 | 5 | 3.335990 | -1.819383 | 21 | 10.75 | 0.511905 |
| GO:0007049\_cell\_cycle | CDCA5 | 238 | 5 | 3.335990 | -1.819383 | 21 | 10.75 | 0.511905 |
| GO:0007049\_cell\_cycle | TRIP13 | 238 | 5 | 3.335990 | -1.819383 | 21 | 10.75 | 0.511905 |
| GO:0022402\_cell\_cycle\_process | PHGDH | 155 | 4 | 4.097887 | -1.817636 | 22 | 10.78 | 0.490000 |
| GO:0022402\_cell\_cycle\_process | BUB1B | 155 | 4 | 4.097887 | -1.817636 | 22 | 10.78 | 0.490000 |
| GO:0022402\_cell\_cycle\_process | CDCA5 | 155 | 4 | 4.097887 | -1.817636 | 22 | 10.78 | 0.490000 |
| GO:0022402\_cell\_cycle\_process | TRIP13 | 155 | 4 | 4.097887 | -1.817636 | 22 | 10.78 | 0.490000 |
| GO:0000279\_M\_phase | BUB1B | 85 | 3 | 5.604462 | -1.804329 | 23 | 10.91 | 0.474348 |
| GO:0000279\_M\_phase | CDCA5 | 85 | 3 | 5.604462 | -1.804329 | 23 | 10.91 | 0.474348 |
| GO:0000279\_M\_phase | TRIP13 | 85 | 3 | 5.604462 | -1.804329 | 23 | 10.91 | 0.474348 |
| GO:0043170\_macromolecule\_metabolic\_process | SOX2 | 1576 | 16 | 1.612113 | -1.790052 | 24 | 11.6 | 0.483333 |
| GO:0043170\_macromolecule\_metabolic\_process | SIX3 | 1576 | 16 | 1.612113 | -1.790052 | 24 | 11.6 | 0.483333 |
| GO:0043170\_macromolecule\_metabolic\_process | CNOT7 | 1576 | 16 | 1.612113 | -1.790052 | 24 | 11.6 | 0.483333 |
| GO:0043170\_macromolecule\_metabolic\_process | SF3A2 | 1576 | 16 | 1.612113 | -1.790052 | 24 | 11.6 | 0.483333 |
| GO:0043170\_macromolecule\_metabolic\_process | CDK4 | 1576 | 16 | 1.612113 | -1.790052 | 24 | 11.6 | 0.483333 |
| GO:0043170\_macromolecule\_metabolic\_process | PPP1CB | 1576 | 16 | 1.612113 | -1.790052 | 24 | 11.6 | 0.483333 |
| GO:0043170\_macromolecule\_metabolic\_process | GPX1 | 1576 | 16 | 1.612113 | -1.790052 | 24 | 11.6 | 0.483333 |
| GO:0043170\_macromolecule\_metabolic\_process | DDR1 | 1576 | 16 | 1.612113 | -1.790052 | 24 | 11.6 | 0.483333 |
| GO:0043170\_macromolecule\_metabolic\_process | HIF1A | 1576 | 16 | 1.612113 | -1.790052 | 24 | 11.6 | 0.483333 |
| GO:0043170\_macromolecule\_metabolic\_process | MCM7 | 1576 | 16 | 1.612113 | -1.790052 | 24 | 11.6 | 0.483333 |
| GO:0043170\_macromolecule\_metabolic\_process | BTG1 | 1576 | 16 | 1.612113 | -1.790052 | 24 | 11.6 | 0.483333 |
| GO:0043170\_macromolecule\_metabolic\_process | EIF2S1 | 1576 | 16 | 1.612113 | -1.790052 | 24 | 11.6 | 0.483333 |
| GO:0043170\_macromolecule\_metabolic\_process | POLD1 | 1576 | 16 | 1.612113 | -1.790052 | 24 | 11.6 | 0.483333 |
| GO:0043170\_macromolecule\_metabolic\_process | PHGDH | 1576 | 16 | 1.612113 | -1.790052 | 24 | 11.6 | 0.483333 |
| GO:0043170\_macromolecule\_metabolic\_process | PRKACA | 1576 | 16 | 1.612113 | -1.790052 | 24 | 11.6 | 0.483333 |
| GO:0043170\_macromolecule\_metabolic\_process | TRIP13 | 1576 | 16 | 1.612113 | -1.790052 | 24 | 11.6 | 0.483333 |
| GO:0006464\_protein\_modification\_process | GPX1 | 439 | 7 | 2.532008 | -1.778019 | 25 | 11.72 | 0.468800 |
| GO:0006464\_protein\_modification\_process | DDR1 | 439 | 7 | 2.532008 | -1.778019 | 25 | 11.72 | 0.468800 |
| GO:0006464\_protein\_modification\_process | BTG1 | 439 | 7 | 2.532008 | -1.778019 | 25 | 11.72 | 0.468800 |
| GO:0006464\_protein\_modification\_process | EIF2S1 | 439 | 7 | 2.532008 | -1.778019 | 25 | 11.72 | 0.468800 |
| GO:0006464\_protein\_modification\_process | PRKACA | 439 | 7 | 2.532008 | -1.778019 | 25 | 11.72 | 0.468800 |
| GO:0006464\_protein\_modification\_process | CDK4 | 439 | 7 | 2.532008 | -1.778019 | 25 | 11.72 | 0.468800 |
| GO:0006464\_protein\_modification\_process | PPP1CB | 439 | 7 | 2.532008 | -1.778019 | 25 | 11.72 | 0.468800 |
| GO:0044260\_cellular\_macromolecule\_metabolic\_process | SOX2 | 1447 | 15 | 1.646093 | -1.748775 | 26 | 12.22 | 0.470000 |
| GO:0044260\_cellular\_macromolecule\_metabolic\_process | SIX3 | 1447 | 15 | 1.646093 | -1.748775 | 26 | 12.22 | 0.470000 |
| GO:0044260\_cellular\_macromolecule\_metabolic\_process | CNOT7 | 1447 | 15 | 1.646093 | -1.748775 | 26 | 12.22 | 0.470000 |
| GO:0044260\_cellular\_macromolecule\_metabolic\_process | SF3A2 | 1447 | 15 | 1.646093 | -1.748775 | 26 | 12.22 | 0.470000 |
| GO:0044260\_cellular\_macromolecule\_metabolic\_process | CDK4 | 1447 | 15 | 1.646093 | -1.748775 | 26 | 12.22 | 0.470000 |
| GO:0044260\_cellular\_macromolecule\_metabolic\_process | PPP1CB | 1447 | 15 | 1.646093 | -1.748775 | 26 | 12.22 | 0.470000 |
| GO:0044260\_cellular\_macromolecule\_metabolic\_process | GPX1 | 1447 | 15 | 1.646093 | -1.748775 | 26 | 12.22 | 0.470000 |
| GO:0044260\_cellular\_macromolecule\_metabolic\_process | DDR1 | 1447 | 15 | 1.646093 | -1.748775 | 26 | 12.22 | 0.470000 |
| GO:0044260\_cellular\_macromolecule\_metabolic\_process | HIF1A | 1447 | 15 | 1.646093 | -1.748775 | 26 | 12.22 | 0.470000 |
| GO:0044260\_cellular\_macromolecule\_metabolic\_process | MCM7 | 1447 | 15 | 1.646093 | -1.748775 | 26 | 12.22 | 0.470000 |
| GO:0044260\_cellular\_macromolecule\_metabolic\_process | BTG1 | 1447 | 15 | 1.646093 | -1.748775 | 26 | 12.22 | 0.470000 |
| GO:0044260\_cellular\_macromolecule\_metabolic\_process | POLD1 | 1447 | 15 | 1.646093 | -1.748775 | 26 | 12.22 | 0.470000 |
| GO:0044260\_cellular\_macromolecule\_metabolic\_process | EIF2S1 | 1447 | 15 | 1.646093 | -1.748775 | 26 | 12.22 | 0.470000 |
| GO:0044260\_cellular\_macromolecule\_metabolic\_process | PRKACA | 1447 | 15 | 1.646093 | -1.748775 | 26 | 12.22 | 0.470000 |
| GO:0044260\_cellular\_macromolecule\_metabolic\_process | TRIP13 | 1447 | 15 | 1.646093 | -1.748775 | 26 | 12.22 | 0.470000 |
| GO:0021536\_diencephalon\_development | SOX2 | 33 | 2 | 9.623824 | -1.746660 | 27 | 12.49 | 0.462593 |
| GO:0021536\_diencephalon\_development | SIX3 | 33 | 2 | 9.623824 | -1.746660 | 27 | 12.49 | 0.462593 |
| GO:0006287\_base-excision\_repair\_\_gap-filling | POLD1 | 3 | 1 |  |  |  |  |  |  |
| GO:0009448\_gamma-aminobutyric\_acid\_metabolic\_process | PHGDH | 3 | 1 |  |  |  |  |  |  |
| GO:0010632\_regulation\_of\_epithelial\_cell\_migration | HIF1A | 3 | 1 |  |  |  |  |  |  |
| GO:0019605\_butyrate\_metabolic\_process | PHGDH | 3 | 1 |  |  |  |  |  |  |
| GO:0021797\_forebrain\_anterior\_posterior\_pattern\_formation | SIX3 | 3 | 1 |  |  |  |  |  |  |
| GO:0043586\_tongue\_development | SOX2 | 3 | 1 |  |  |  |  |  |  |
| GO:0046825\_regulation\_of\_protein\_export\_from\_nucleus | PRKACA | 3 | 1 |  |  |  |  |  |  |
| GO:0048852\_diencephalon\_morphogenesis | SOX2 | 3 | 1 |  |  |  |  |  |  |
| GO:0050957\_equilibrioception | SOX2 | 3 | 1 |  |  |  |  |  |  |
| GO:0051450\_myoblast\_proliferation | GPX1 | 3 | 1 |  |  |  |  |  |  |
| GO:0060055\_angiogenesis\_involved\_in\_wound\_healing | GPX1 | 3 | 1 |  |  |  |  |  |  |
| GO:0043412\_biopolymer\_modification | GPX1 | 458 | 7 | 2.426969 | -1.684658 | 28 | 14.18 | 0.506429 |
| GO:0043412\_biopolymer\_modification | DDR1 | 458 | 7 | 2.426969 | -1.684658 | 28 | 14.18 | 0.506429 |
| GO:0043412\_biopolymer\_modification | BTG1 | 458 | 7 | 2.426969 | -1.684658 | 28 | 14.18 | 0.506429 |
| GO:0043412\_biopolymer\_modification | EIF2S1 | 458 | 7 | 2.426969 | -1.684658 | 28 | 14.18 | 0.506429 |
| GO:0043412\_biopolymer\_modification | PRKACA | 458 | 7 | 2.426969 | -1.684658 | 28 | 14.18 | 0.506429 |
| GO:0043412\_biopolymer\_modification | CDK4 | 458 | 7 | 2.426969 | -1.684658 | 28 | 14.18 | 0.506429 |
| GO:0043412\_biopolymer\_modification | PPP1CB | 458 | 7 | 2.426969 | -1.684658 | 28 | 14.18 | 0.506429 |
| GO:0021510\_spinal\_cord\_development | PHGDH | 36 | 2 | 8.821839 | -1.675003 | 29 | 14.62 | 0.504138 |
| GO:0021510\_spinal\_cord\_development | PBX3 | 36 | 2 | 8.821839 | -1.675003 | 29 | 14.62 | 0.504138 |
| GO:0060249\_anatomical\_structure\_homeostasis | HIF1A | 96 | 3 | 4.962284 | -1.664038 | 30 | 14.81 | 0.493667 |
| GO:0060249\_anatomical\_structure\_homeostasis | SOX2 | 96 | 3 | 4.962284 | -1.664038 | 30 | 14.81 | 0.493667 |
| GO:0060249\_anatomical\_structure\_homeostasis | FH1 | 96 | 3 | 4.962284 | -1.664038 | 30 | 14.81 | 0.493667 |
| GO:0009967\_positive\_regulation\_of\_signal\_transduction | GPX1 | 98 | 3 | 4.861013 | -1.640535 | 31 | 15.57 | 0.502258 |
| GO:0009967\_positive\_regulation\_of\_signal\_transduction | HIF1A | 98 | 3 | 4.861013 | -1.640535 | 31 | 15.57 | 0.502258 |
| GO:0009967\_positive\_regulation\_of\_signal\_transduction | SOX2 | 98 | 3 | 4.861013 | -1.640535 | 31 | 15.57 | 0.502258 |
| GO:0046777\_protein\_amino\_acid\_autophosphorylation | EIF2S1 | 38 | 2 | 8.357532 | -1.630751 | 32 | 16.09 | 0.502812 |
| GO:0046777\_protein\_amino\_acid\_autophosphorylation | PRKACA | 38 | 2 | 8.357532 | -1.630751 | 32 | 16.09 | 0.502812 |
| GO:0043283\_biopolymer\_metabolic\_process | SOX2 | 1490 | 15 | 1.598588 | -1.630143 | 33 | 16.12 | 0.488485 |
| GO:0043283\_biopolymer\_metabolic\_process | SIX3 | 1490 | 15 | 1.598588 | -1.630143 | 33 | 16.12 | 0.488485 |
| GO:0043283\_biopolymer\_metabolic\_process | CNOT7 | 1490 | 15 | 1.598588 | -1.630143 | 33 | 16.12 | 0.488485 |
| GO:0043283\_biopolymer\_metabolic\_process | CDK4 | 1490 | 15 | 1.598588 | -1.630143 | 33 | 16.12 | 0.488485 |
| GO:0043283\_biopolymer\_metabolic\_process | SF3A2 | 1490 | 15 | 1.598588 | -1.630143 | 33 | 16.12 | 0.488485 |
| GO:0043283\_biopolymer\_metabolic\_process | PPP1CB | 1490 | 15 | 1.598588 | -1.630143 | 33 | 16.12 | 0.488485 |
| GO:0043283\_biopolymer\_metabolic\_process | DDR1 | 1490 | 15 | 1.598588 | -1.630143 | 33 | 16.12 | 0.488485 |
| GO:0043283\_biopolymer\_metabolic\_process | GPX1 | 1490 | 15 | 1.598588 | -1.630143 | 33 | 16.12 | 0.488485 |
| GO:0043283\_biopolymer\_metabolic\_process | HIF1A | 1490 | 15 | 1.598588 | -1.630143 | 33 | 16.12 | 0.488485 |
| GO:0043283\_biopolymer\_metabolic\_process | MCM7 | 1490 | 15 | 1.598588 | -1.630143 | 33 | 16.12 | 0.488485 |
| GO:0043283\_biopolymer\_metabolic\_process | BTG1 | 1490 | 15 | 1.598588 | -1.630143 | 33 | 16.12 | 0.488485 |
| GO:0043283\_biopolymer\_metabolic\_process | EIF2S1 | 1490 | 15 | 1.598588 | -1.630143 | 33 | 16.12 | 0.488485 |
| GO:0043283\_biopolymer\_metabolic\_process | POLD1 | 1490 | 15 | 1.598588 | -1.630143 | 33 | 16.12 | 0.488485 |
| GO:0043283\_biopolymer\_metabolic\_process | PRKACA | 1490 | 15 | 1.598588 | -1.630143 | 33 | 16.12 | 0.488485 |
| GO:0043283\_biopolymer\_metabolic\_process | TRIP13 | 1490 | 15 | 1.598588 | -1.630143 | 33 | 16.12 | 0.488485 |
| GO:0006547\_histidine\_metabolic\_process | MTHFD1 | 4 | 1 |  |  |  |  |  |  |
| GO:0006548\_histidine\_catabolic\_process | MTHFD1 | 4 | 1 |  |  |  |  |  |  |
| GO:0007144\_female\_meiosis\_I | TRIP13 | 4 | 1 |  |  |  |  |  |  |
| GO:0009075\_histidine\_family\_amino\_acid\_metabolic\_process | MTHFD1 | 4 | 1 |  |  |  |  |  |  |
| GO:0009077\_histidine\_family\_amino\_acid\_catabolic\_process | MTHFD1 | 4 | 1 |  |  |  |  |  |  |
| GO:0010631\_epithelial\_cell\_migration | HIF1A | 4 | 1 |  |  |  |  |  |  |
| GO:0019530\_taurine\_metabolic\_process | PHGDH | 4 | 1 |  |  |  |  |  |  |
| GO:0030949\_positive\_regulation\_of\_vascular\_endothelial\_growth\_factor\_receptor\_signaling\_pathway | HIF1A | 4 | 1 |  |  |  |  |  |  |
| GO:0034614\_cellular\_response\_to\_reactive\_oxygen\_species | GPX1 | 4 | 1 |  |  |  |  |  |  |
| GO:0042541\_hemoglobin\_biosynthetic\_process | HIF1A | 4 | 1 |  |  |  |  |  |  |
| GO:0042744\_hydrogen\_peroxide\_catabolic\_process | GPX1 | 4 | 1 |  |  |  |  |  |  |
| GO:0043534\_blood\_vessel\_endothelial\_cell\_migration | GPX1 | 4 | 1 |  |  |  |  |  |  |
| GO:0044403\_symbiosis\_\_encompassing\_mutualism\_through\_parasitism | GPX1 | 4 | 1 |  |  |  |  |  |  |
| GO:0044419\_interspecies\_interaction\_between\_organisms | GPX1 | 4 | 1 |  |  |  |  |  |  |
| GO:0045747\_positive\_regulation\_of\_Notch\_signaling\_pathway | SOX2 | 4 | 1 |  |  |  |  |  |  |
| GO:0051897\_positive\_regulation\_of\_protein\_kinase\_B\_signaling\_cascade | GPX1 | 4 | 1 |  |  |  |  |  |  |
| GO:0060900\_embryonic\_camera-type\_eye\_formation | ALDH1A1 | 4 | 1 |  |  |  |  |  |  |
| GO:0070301\_cellular\_response\_to\_hydrogen\_peroxide | GPX1 | 4 | 1 |  |  |  |  |  |  |
| GO:0019752\_carboxylic\_acid\_metabolic\_process | ALDH1A1 | 181 | 4 | 3.509240 | -1.594785 | 35 | 16.88 | 0.482286 |
| GO:0019752\_carboxylic\_acid\_metabolic\_process | MTHFD1 | 181 | 4 | 3.509240 | -1.594785 | 35 | 16.88 | 0.482286 |
| GO:0019752\_carboxylic\_acid\_metabolic\_process | HIF1A | 181 | 4 | 3.509240 | -1.594785 | 35 | 16.88 | 0.482286 |
| GO:0019752\_carboxylic\_acid\_metabolic\_process | PHGDH | 181 | 4 | 3.509240 | -1.594785 | 35 | 16.88 | 0.482286 |
| GO:0043436\_oxoacid\_metabolic\_process | ALDH1A1 | 181 | 4 | 3.509240 | -1.594785 | 35 | 16.88 | 0.482286 |
| GO:0043436\_oxoacid\_metabolic\_process | MTHFD1 | 181 | 4 | 3.509240 | -1.594785 | 35 | 16.88 | 0.482286 |
| GO:0043436\_oxoacid\_metabolic\_process | HIF1A | 181 | 4 | 3.509240 | -1.594785 | 35 | 16.88 | 0.482286 |
| GO:0043436\_oxoacid\_metabolic\_process | PHGDH | 181 | 4 | 3.509240 | -1.594785 | 35 | 16.88 | 0.482286 |
| GO:0006082\_organic\_acid\_metabolic\_process | ALDH1A1 | 182 | 4 | 3.489958 | -1.587017 | 36 | 17.26 | 0.479444 |
| GO:0006082\_organic\_acid\_metabolic\_process | MTHFD1 | 182 | 4 | 3.489958 | -1.587017 | 36 | 17.26 | 0.479444 |
| GO:0006082\_organic\_acid\_metabolic\_process | HIF1A | 182 | 4 | 3.489958 | -1.587017 | 36 | 17.26 | 0.479444 |
| GO:0006082\_organic\_acid\_metabolic\_process | PHGDH | 182 | 4 | 3.489958 | -1.587017 | 36 | 17.26 | 0.479444 |
| GO:0051716\_cellular\_response\_to\_stimulus | GPX1 | 273 | 5 | 2.908299 | -1.585942 | 37 | 17.29 | 0.467297 |
| GO:0051716\_cellular\_response\_to\_stimulus | HIF1A | 273 | 5 | 2.908299 | -1.585942 | 37 | 17.29 | 0.467297 |
| GO:0051716\_cellular\_response\_to\_stimulus | EIF2S1 | 273 | 5 | 2.908299 | -1.585942 | 37 | 17.29 | 0.467297 |
| GO:0051716\_cellular\_response\_to\_stimulus | POLD1 | 273 | 5 | 2.908299 | -1.585942 | 37 | 17.29 | 0.467297 |
| GO:0051716\_cellular\_response\_to\_stimulus | TRIP13 | 273 | 5 | 2.908299 | -1.585942 | 37 | 17.29 | 0.467297 |
| GO:0042180\_cellular\_ketone\_metabolic\_process | ALDH1A1 | 183 | 4 | 3.470888 | -1.579301 | 38 | 17.47 | 0.459737 |
| GO:0042180\_cellular\_ketone\_metabolic\_process | MTHFD1 | 183 | 4 | 3.470888 | -1.579301 | 38 | 17.47 | 0.459737 |
| GO:0042180\_cellular\_ketone\_metabolic\_process | HIF1A | 183 | 4 | 3.470888 | -1.579301 | 38 | 17.47 | 0.459737 |
| GO:0042180\_cellular\_ketone\_metabolic\_process | PHGDH | 183 | 4 | 3.470888 | -1.579301 | 38 | 17.47 | 0.459737 |
| GO:0006260\_DNA\_replication | MCM7 | 41 | 2 | 7.746005 | -1.568933 | 40 | 18.08 | 0.452000 |
| GO:0006260\_DNA\_replication | POLD1 | 41 | 2 | 7.746005 | -1.568933 | 40 | 18.08 | 0.452000 |
| GO:0006979\_response\_to\_oxidative\_stress | GPX1 | 41 | 2 | 7.746005 | -1.568933 | 40 | 18.08 | 0.452000 |
| GO:0006979\_response\_to\_oxidative\_stress | HIF1A | 41 | 2 | 7.746005 | -1.568933 | 40 | 18.08 | 0.452000 |
| GO:0048646\_anatomical\_structure\_formation\_involved\_in\_morphogenesis | ALDH1A1 | 277 | 5 | 2.866302 | -1.561766 | 41 | 18.17 | 0.443171 |
| GO:0048646\_anatomical\_structure\_formation\_involved\_in\_morphogenesis | GPX1 | 277 | 5 | 2.866302 | -1.561766 | 41 | 18.17 | 0.443171 |
| GO:0048646\_anatomical\_structure\_formation\_involved\_in\_morphogenesis | HIF1A | 277 | 5 | 2.866302 | -1.561766 | 41 | 18.17 | 0.443171 |
| GO:0048646\_anatomical\_structure\_formation\_involved\_in\_morphogenesis | SOX2 | 277 | 5 | 2.866302 | -1.561766 | 41 | 18.17 | 0.443171 |
| GO:0048646\_anatomical\_structure\_formation\_involved\_in\_morphogenesis | PRKACA | 277 | 5 | 2.866302 | -1.561766 | 41 | 18.17 | 0.443171 |
| GO:0008152\_metabolic\_process | SOX2 | 2133 | 19 | 1.414472 | -1.537099 | 42 | 18.84 | 0.448571 |
| GO:0008152\_metabolic\_process | SIX3 | 2133 | 19 | 1.414472 | -1.537099 | 42 | 18.84 | 0.448571 |
| GO:0008152\_metabolic\_process | CDK4 | 2133 | 19 | 1.414472 | -1.537099 | 42 | 18.84 | 0.448571 |
| GO:0008152\_metabolic\_process | CNOT7 | 2133 | 19 | 1.414472 | -1.537099 | 42 | 18.84 | 0.448571 |
| GO:0008152\_metabolic\_process | SF3A2 | 2133 | 19 | 1.414472 | -1.537099 | 42 | 18.84 | 0.448571 |
| GO:0008152\_metabolic\_process | PPP1CB | 2133 | 19 | 1.414472 | -1.537099 | 42 | 18.84 | 0.448571 |
| GO:0008152\_metabolic\_process | MTHFD1 | 2133 | 19 | 1.414472 | -1.537099 | 42 | 18.84 | 0.448571 |
| GO:0008152\_metabolic\_process | ALDH1A1 | 2133 | 19 | 1.414472 | -1.537099 | 42 | 18.84 | 0.448571 |
| GO:0008152\_metabolic\_process | DDR1 | 2133 | 19 | 1.414472 | -1.537099 | 42 | 18.84 | 0.448571 |
| GO:0008152\_metabolic\_process | GPX1 | 2133 | 19 | 1.414472 | -1.537099 | 42 | 18.84 | 0.448571 |
| GO:0008152\_metabolic\_process | TPI1 | 2133 | 19 | 1.414472 | -1.537099 | 42 | 18.84 | 0.448571 |
| GO:0008152\_metabolic\_process | MCM7 | 2133 | 19 | 1.414472 | -1.537099 | 42 | 18.84 | 0.448571 |
| GO:0008152\_metabolic\_process | HIF1A | 2133 | 19 | 1.414472 | -1.537099 | 42 | 18.84 | 0.448571 |
| GO:0008152\_metabolic\_process | BTG1 | 2133 | 19 | 1.414472 | -1.537099 | 42 | 18.84 | 0.448571 |
| GO:0008152\_metabolic\_process | EIF2S1 | 2133 | 19 | 1.414472 | -1.537099 | 42 | 18.84 | 0.448571 |
| GO:0008152\_metabolic\_process | POLD1 | 2133 | 19 | 1.414472 | -1.537099 | 42 | 18.84 | 0.448571 |
| GO:0008152\_metabolic\_process | PHGDH | 2133 | 19 | 1.414472 | -1.537099 | 42 | 18.84 | 0.448571 |
| GO:0008152\_metabolic\_process | PRKACA | 2133 | 19 | 1.414472 | -1.537099 | 42 | 18.84 | 0.448571 |
| GO:0008152\_metabolic\_process | TRIP13 | 2133 | 19 | 1.414472 | -1.537099 | 42 | 18.84 | 0.448571 |
| GO:0010647\_positive\_regulation\_of\_cell\_communication | GPX1 | 110 | 3 | 4.330721 | -1.510393 | 44 | 20.34 | 0.462273 |
| GO:0010647\_positive\_regulation\_of\_cell\_communication | HIF1A | 110 | 3 | 4.330721 | -1.510393 | 44 | 20.34 | 0.462273 |
| GO:0010647\_positive\_regulation\_of\_cell\_communication | SOX2 | 110 | 3 | 4.330721 | -1.510393 | 44 | 20.34 | 0.462273 |
| GO:0043010\_camera-type\_eye\_development | ALDH1A1 | 110 | 3 | 4.330721 | -1.510393 | 44 | 20.34 | 0.462273 |
| GO:0043010\_camera-type\_eye\_development | SOX2 | 110 | 3 | 4.330721 | -1.510393 | 44 | 20.34 | 0.462273 |
| GO:0043010\_camera-type\_eye\_development | SIX3 | 110 | 3 | 4.330721 | -1.510393 | 44 | 20.34 | 0.462273 |
| GO:0000239\_pachytene | TRIP13 | 5 | 1 | 31.758621 | -1.507145 | 60 | 29.12 | 0.485333 |
| GO:0002862\_negative\_regulation\_of\_inflammatory\_response\_to\_antigenic\_stimulus | GPX1 | 5 | 1 | 31.758621 | -1.507145 | 60 | 29.12 | 0.485333 |
| GO:0006268\_DNA\_unwinding\_during\_replication | MCM7 | 5 | 1 | 31.758621 | -1.507145 | 60 | 29.12 | 0.485333 |
| GO:0006541\_glutamine\_metabolic\_process | PHGDH | 5 | 1 | 31.758621 | -1.507145 | 60 | 29.12 | 0.485333 |
| GO:0007091\_mitotic\_metaphase\_anaphase\_transition | BUB1B | 5 | 1 | 31.758621 | -1.507145 | 60 | 29.12 | 0.485333 |
| GO:0008631\_induction\_of\_apoptosis\_by\_oxidative\_stress | GPX1 | 5 | 1 | 31.758621 | -1.507145 | 60 | 29.12 | 0.485333 |
| GO:0032508\_DNA\_duplex\_unwinding | MCM7 | 5 | 1 | 31.758621 | -1.507145 | 60 | 29.12 | 0.485333 |
| GO:0035238\_vitamin\_A\_biosynthetic\_process | ALDH1A1 | 5 | 1 | 31.758621 | -1.507145 | 60 | 29.12 | 0.485333 |
| GO:0042362\_fat-soluble\_vitamin\_biosynthetic\_process | ALDH1A1 | 5 | 1 | 31.758621 | -1.507145 | 60 | 29.12 | 0.485333 |
| GO:0042904\_9-cis-retinoic\_acid\_biosynthetic\_process | ALDH1A1 | 5 | 1 | 31.758621 | -1.507145 | 60 | 29.12 | 0.485333 |
| GO:0042905\_9-cis-retinoic\_acid\_metabolic\_process | ALDH1A1 | 5 | 1 | 31.758621 | -1.507145 | 60 | 29.12 | 0.485333 |
| GO:0043403\_skeletal\_muscle\_regeneration | GPX1 | 5 | 1 | 31.758621 | -1.507145 | 60 | 29.12 | 0.485333 |
| GO:0043555\_regulation\_of\_translation\_in\_response\_to\_stress | EIF2S1 | 5 | 1 | 31.758621 | -1.507145 | 60 | 29.12 | 0.485333 |
| GO:0043558\_regulation\_of\_translational\_initiation\_in\_response\_to\_stress | EIF2S1 | 5 | 1 | 31.758621 | -1.507145 | 60 | 29.12 | 0.485333 |
| GO:0045648\_positive\_regulation\_of\_erythrocyte\_differentiation | HIF1A | 5 | 1 | 31.758621 | -1.507145 | 60 | 29.12 | 0.485333 |
| GO:0046459\_short-chain\_fatty\_acid\_metabolic\_process | PHGDH | 5 | 1 | 31.758621 | -1.507145 | 60 | 29.12 | 0.485333 |
| GO:0001885\_endothelial\_cell\_development | GPX1 | 6 | 1 | 26.465517 | -1.429282 | 67 | 37.88 | 0.565373 |
| GO:0009069\_serine\_family\_amino\_acid\_metabolic\_process | PHGDH | 6 | 1 | 26.465517 | -1.429282 | 67 | 37.88 | 0.565373 |
| GO:0014823\_response\_to\_activity | HIF1A | 6 | 1 | 26.465517 | -1.429282 | 67 | 37.88 | 0.565373 |
| GO:0030947\_regulation\_of\_vascular\_endothelial\_growth\_factor\_receptor\_signaling\_pathway | HIF1A | 6 | 1 | 26.465517 | -1.429282 | 67 | 37.88 | 0.565373 |
| GO:0032392\_DNA\_geometric\_change | MCM7 | 6 | 1 | 26.465517 | -1.429282 | 67 | 37.88 | 0.565373 |
| GO:0042246\_tissue\_regeneration | GPX1 | 6 | 1 | 26.465517 | -1.429282 | 67 | 37.88 | 0.565373 |
| GO:0048853\_forebrain\_morphogenesis | SOX2 | 6 | 1 | 26.465517 | -1.429282 | 67 | 37.88 | 0.565373 |
| GO:0022403\_cell\_cycle\_phase | BUB1B | 119 | 3 | 4.003187 | -1.423367 | 68 | 38.44 | 0.565294 |
| GO:0022403\_cell\_cycle\_phase | CDCA5 | 119 | 3 | 4.003187 | -1.423367 | 68 | 38.44 | 0.565294 |
| GO:0022403\_cell\_cycle\_phase | TRIP13 | 119 | 3 | 4.003187 | -1.423367 | 68 | 38.44 | 0.565294 |
| GO:0006520\_cellular\_amino\_acid\_metabolic\_process | MTHFD1 | 51 | 2 | 6.227181 | -1.393999 | 70 | 39.83 | 0.569000 |
| GO:0006520\_cellular\_amino\_acid\_metabolic\_process | PHGDH | 51 | 2 | 6.227181 | -1.393999 | 70 | 39.83 | 0.569000 |
| GO:0044106\_cellular\_amine\_metabolic\_process | MTHFD1 | 51 | 2 | 6.227181 | -1.393999 | 70 | 39.83 | 0.569000 |
| GO:0044106\_cellular\_amine\_metabolic\_process | PHGDH | 51 | 2 | 6.227181 | -1.393999 | 70 | 39.83 | 0.569000 |
| GO:0001556\_oocyte\_maturation | TRIP13 | 7 | 1 | 22.684729 | -1.363652 | 83 | 48.45 | 0.583735 |
| GO:0002052\_positive\_regulation\_of\_neuroblast\_proliferation | SOX2 | 7 | 1 | 22.684729 | -1.363652 | 83 | 48.45 | 0.583735 |
| GO:0002087\_regulation\_of\_respiratory\_gaseous\_exchange\_by\_neurological\_system\_process | PBX3 | 7 | 1 | 22.684729 | -1.363652 | 83 | 48.45 | 0.583735 |
| GO:0008299\_isoprenoid\_biosynthetic\_process | ALDH1A1 | 7 | 1 | 22.684729 | -1.363652 | 83 | 48.45 | 0.583735 |
| GO:0020027\_hemoglobin\_metabolic\_process | HIF1A | 7 | 1 | 22.684729 | -1.363652 | 83 | 48.45 | 0.583735 |
| GO:0021516\_dorsal\_spinal\_cord\_development | PBX3 | 7 | 1 | 22.684729 | -1.363652 | 83 | 48.45 | 0.583735 |
| GO:0021984\_adenohypophysis\_development | SOX2 | 7 | 1 | 22.684729 | -1.363652 | 83 | 48.45 | 0.583735 |
| GO:0042572\_retinol\_metabolic\_process | ALDH1A1 | 7 | 1 | 22.684729 | -1.363652 | 83 | 48.45 | 0.583735 |
| GO:0043584\_nose\_development | SOX2 | 7 | 1 | 22.684729 | -1.363652 | 83 | 48.45 | 0.583735 |
| GO:0044065\_regulation\_of\_respiratory\_system\_process | PBX3 | 7 | 1 | 22.684729 | -1.363652 | 83 | 48.45 | 0.583735 |
| GO:0045668\_negative\_regulation\_of\_osteoblast\_differentiation | SOX2 | 7 | 1 | 22.684729 | -1.363652 | 83 | 48.45 | 0.583735 |
| GO:0046824\_positive\_regulation\_of\_nucleocytoplasmic\_transport | PRKACA | 7 | 1 | 22.684729 | -1.363652 | 83 | 48.45 | 0.583735 |
| GO:0060788\_ectodermal\_placode\_formation | SOX2 | 7 | 1 | 22.684729 | -1.363652 | 83 | 48.45 | 0.583735 |
| GO:0019538\_protein\_metabolic\_process | GPX1 | 655 | 8 | 1.939458 | -1.353555 | 84 | 48.77 | 0.580595 |
| GO:0019538\_protein\_metabolic\_process | DDR1 | 655 | 8 | 1.939458 | -1.353555 | 84 | 48.77 | 0.580595 |
| GO:0019538\_protein\_metabolic\_process | HIF1A | 655 | 8 | 1.939458 | -1.353555 | 84 | 48.77 | 0.580595 |
| GO:0019538\_protein\_metabolic\_process | BTG1 | 655 | 8 | 1.939458 | -1.353555 | 84 | 48.77 | 0.580595 |
| GO:0019538\_protein\_metabolic\_process | EIF2S1 | 655 | 8 | 1.939458 | -1.353555 | 84 | 48.77 | 0.580595 |
| GO:0019538\_protein\_metabolic\_process | PRKACA | 655 | 8 | 1.939458 | -1.353555 | 84 | 48.77 | 0.580595 |
| GO:0019538\_protein\_metabolic\_process | CDK4 | 655 | 8 | 1.939458 | -1.353555 | 84 | 48.77 | 0.580595 |
| GO:0019538\_protein\_metabolic\_process | PPP1CB | 655 | 8 | 1.939458 | -1.353555 | 84 | 48.77 | 0.580595 |
| GO:0007605\_sensory\_perception\_of\_sound | GPX1 | 55 | 2 | 5.774295 | -1.334470 | 85 | 49.81 | 0.586000 |
| GO:0007605\_sensory\_perception\_of\_sound | SOX2 | 55 | 2 | 5.774295 | -1.334470 | 85 | 49.81 | 0.586000 |
| GO:0007423\_sensory\_organ\_development | ALDH1A1 | 219 | 4 | 2.900331 | -1.332657 | 86 | 49.95 | 0.580814 |
| GO:0007423\_sensory\_organ\_development | DDR1 | 219 | 4 | 2.900331 | -1.332657 | 86 | 49.95 | 0.580814 |
| GO:0007423\_sensory\_organ\_development | SOX2 | 219 | 4 | 2.900331 | -1.332657 | 86 | 49.95 | 0.580814 |
| GO:0007423\_sensory\_organ\_development | SIX3 | 219 | 4 | 2.900331 | -1.332657 | 86 | 49.95 | 0.580814 |
| GO:0032787\_monocarboxylic\_acid\_metabolic\_process | ALDH1A1 | 130 | 3 | 3.664456 | -1.327184 | 87 | 50.18 | 0.576782 |
| GO:0032787\_monocarboxylic\_acid\_metabolic\_process | HIF1A | 130 | 3 | 3.664456 | -1.327184 | 87 | 50.18 | 0.576782 |
| GO:0032787\_monocarboxylic\_acid\_metabolic\_process | PHGDH | 130 | 3 | 3.664456 | -1.327184 | 87 | 50.18 | 0.576782 |
| GO:0008283\_cell\_proliferation | GPX1 | 544 | 7 | 2.043294 | -1.323406 | 88 | 50.21 | 0.570568 |
| GO:0008283\_cell\_proliferation | DDR1 | 544 | 7 | 2.043294 | -1.323406 | 88 | 50.21 | 0.570568 |
| GO:0008283\_cell\_proliferation | MCM7 | 544 | 7 | 2.043294 | -1.323406 | 88 | 50.21 | 0.570568 |
| GO:0008283\_cell\_proliferation | BTG1 | 544 | 7 | 2.043294 | -1.323406 | 88 | 50.21 | 0.570568 |
| GO:0008283\_cell\_proliferation | MARCKSL1 | 544 | 7 | 2.043294 | -1.323406 | 88 | 50.21 | 0.570568 |
| GO:0008283\_cell\_proliferation | SOX2 | 544 | 7 | 2.043294 | -1.323406 | 88 | 50.21 | 0.570568 |
| GO:0008283\_cell\_proliferation | CDK4 | 544 | 7 | 2.043294 | -1.323406 | 88 | 50.21 | 0.570568 |
| GO:0001708\_cell\_fate\_specification | SOX2 | 56 | 2 | 5.671182 | -1.320346 | 89 | 50.68 | 0.569438 |
| GO:0001708\_cell\_fate\_specification | SIX3 | 56 | 2 | 5.671182 | -1.320346 | 89 | 50.68 | 0.569438 |
| GO:0006284\_base-excision\_repair | POLD1 | 8 | 1 | 19.849138 | -1.306976 | 94 | 57.42 | 0.610851 |
| GO:0007131\_reciprocal\_meiotic\_recombination | TRIP13 | 8 | 1 | 19.849138 | -1.306976 | 94 | 57.42 | 0.610851 |
| GO:0007141\_male\_meiosis\_I | TRIP13 | 8 | 1 | 19.849138 | -1.306976 | 94 | 57.42 | 0.610851 |
| GO:0008593\_regulation\_of\_Notch\_signaling\_pathway | SOX2 | 8 | 1 | 19.849138 | -1.306976 | 94 | 57.42 | 0.610851 |
| GO:0043542\_endothelial\_cell\_migration | GPX1 | 8 | 1 | 19.849138 | -1.306976 | 94 | 57.42 | 0.610851 |
| GO:0010646\_regulation\_of\_cell\_communication | GPX1 | 330 | 5 | 2.405956 | -1.280302 | 95 | 58.44 | 0.615158 |
| GO:0010646\_regulation\_of\_cell\_communication | HIF1A | 330 | 5 | 2.405956 | -1.280302 | 95 | 58.44 | 0.615158 |
| GO:0010646\_regulation\_of\_cell\_communication | SOX2 | 330 | 5 | 2.405956 | -1.280302 | 95 | 58.44 | 0.615158 |
| GO:0010646\_regulation\_of\_cell\_communication | SIX3 | 330 | 5 | 2.405956 | -1.280302 | 95 | 58.44 | 0.615158 |
| GO:0010646\_regulation\_of\_cell\_communication | PRKACA | 330 | 5 | 2.405956 | -1.280302 | 95 | 58.44 | 0.615158 |
| GO:0016055\_Wnt\_receptor\_signaling\_pathway | SOX2 | 59 | 2 | 5.382817 | -1.279624 | 96 | 58.68 | 0.611250 |
| GO:0016055\_Wnt\_receptor\_signaling\_pathway | SIX3 | 59 | 2 | 5.382817 | -1.279624 | 96 | 58.68 | 0.611250 |
| GO:0001654\_eye\_development | ALDH1A1 | 136 | 3 | 3.502789 | -1.278812 | 97 | 58.81 | 0.606289 |
| GO:0001654\_eye\_development | SOX2 | 136 | 3 | 3.502789 | -1.278812 | 97 | 58.81 | 0.606289 |
| GO:0001654\_eye\_development | SIX3 | 136 | 3 | 3.502789 | -1.278812 | 97 | 58.81 | 0.606289 |
| GO:0044267\_cellular\_protein\_metabolic\_process | GPX1 | 559 | 7 | 1.988465 | -1.269157 | 98 | 59.15 | 0.603571 |
| GO:0044267\_cellular\_protein\_metabolic\_process | DDR1 | 559 | 7 | 1.988465 | -1.269157 | 98 | 59.15 | 0.603571 |
| GO:0044267\_cellular\_protein\_metabolic\_process | BTG1 | 559 | 7 | 1.988465 | -1.269157 | 98 | 59.15 | 0.603571 |
| GO:0044267\_cellular\_protein\_metabolic\_process | EIF2S1 | 559 | 7 | 1.988465 | -1.269157 | 98 | 59.15 | 0.603571 |
| GO:0044267\_cellular\_protein\_metabolic\_process | PRKACA | 559 | 7 | 1.988465 | -1.269157 | 98 | 59.15 | 0.603571 |
| GO:0044267\_cellular\_protein\_metabolic\_process | CDK4 | 559 | 7 | 1.988465 | -1.269157 | 98 | 59.15 | 0.603571 |
| GO:0044267\_cellular\_protein\_metabolic\_process | PPP1CB | 559 | 7 | 1.988465 | -1.269157 | 98 | 59.15 | 0.603571 |
| GO:0006611\_protein\_export\_from\_nucleus | PRKACA | 9 | 1 | 17.643678 | -1.257138 | 107 | 65.81 | 0.615047 |
| GO:0007128\_meiotic\_prophase\_I | TRIP13 | 9 | 1 | 17.643678 | -1.257138 | 107 | 65.81 | 0.615047 |
| GO:0032388\_positive\_regulation\_of\_intracellular\_transport | PRKACA | 9 | 1 | 17.643678 | -1.257138 | 107 | 65.81 | 0.615047 |
| GO:0032963\_collagen\_metabolic\_process | HIF1A | 9 | 1 | 17.643678 | -1.257138 | 107 | 65.81 | 0.615047 |
| GO:0035162\_embryonic\_hemopoiesis | HIF1A | 9 | 1 | 17.643678 | -1.257138 | 107 | 65.81 | 0.615047 |
| GO:0045646\_regulation\_of\_erythrocyte\_differentiation | HIF1A | 9 | 1 | 17.643678 | -1.257138 | 107 | 65.81 | 0.615047 |
| GO:0050910\_detection\_of\_mechanical\_stimulus\_involved\_in\_sensory\_perception\_of\_sound | SOX2 | 9 | 1 | 17.643678 | -1.257138 | 107 | 65.81 | 0.615047 |
| GO:0051324\_prophase | TRIP13 | 9 | 1 | 17.643678 | -1.257138 | 107 | 65.81 | 0.615047 |
| GO:0051896\_regulation\_of\_protein\_kinase\_B\_signaling\_cascade | GPX1 | 9 | 1 | 17.643678 | -1.257138 | 107 | 65.81 | 0.615047 |
| GO:0021537\_telencephalon\_development | SOX2 | 62 | 2 | 5.122358 | -1.241184 | 109 | 66.93 | 0.614037 |
| GO:0021537\_telencephalon\_development | SIX3 | 62 | 2 | 5.122358 | -1.241184 | 109 | 66.93 | 0.614037 |
| GO:0050954\_sensory\_perception\_of\_mechanical\_stimulus | GPX1 | 62 | 2 | 5.122358 | -1.241184 | 109 | 66.93 | 0.614037 |
| GO:0050954\_sensory\_perception\_of\_mechanical\_stimulus | SOX2 | 62 | 2 | 5.122358 | -1.241184 | 109 | 66.93 | 0.614037 |
| GO:0006793\_phosphorus\_metabolic\_process | DDR1 | 340 | 5 | 2.335193 | -1.234186 | 111 | 67.23 | 0.605676 |
| GO:0006793\_phosphorus\_metabolic\_process | EIF2S1 | 340 | 5 | 2.335193 | -1.234186 | 111 | 67.23 | 0.605676 |
| GO:0006793\_phosphorus\_metabolic\_process | PRKACA | 340 | 5 | 2.335193 | -1.234186 | 111 | 67.23 | 0.605676 |
| GO:0006793\_phosphorus\_metabolic\_process | CDK4 | 340 | 5 | 2.335193 | -1.234186 | 111 | 67.23 | 0.605676 |
| GO:0006793\_phosphorus\_metabolic\_process | PPP1CB | 340 | 5 | 2.335193 | -1.234186 | 111 | 67.23 | 0.605676 |
| GO:0006796\_phosphate\_metabolic\_process | DDR1 | 340 | 5 | 2.335193 | -1.234186 | 111 | 67.23 | 0.605676 |
| GO:0006796\_phosphate\_metabolic\_process | EIF2S1 | 340 | 5 | 2.335193 | -1.234186 | 111 | 67.23 | 0.605676 |
| GO:0006796\_phosphate\_metabolic\_process | PRKACA | 340 | 5 | 2.335193 | -1.234186 | 111 | 67.23 | 0.605676 |
| GO:0006796\_phosphate\_metabolic\_process | CDK4 | 340 | 5 | 2.335193 | -1.234186 | 111 | 67.23 | 0.605676 |
| GO:0006796\_phosphate\_metabolic\_process | PPP1CB | 340 | 5 | 2.335193 | -1.234186 | 111 | 67.23 | 0.605676 |
| GO:0006468\_protein\_amino\_acid\_phosphorylation | DDR1 | 237 | 4 | 2.680052 | -1.228311 | 112 | 67.68 | 0.604286 |
| GO:0006468\_protein\_amino\_acid\_phosphorylation | EIF2S1 | 237 | 4 | 2.680052 | -1.228311 | 112 | 67.68 | 0.604286 |
| GO:0006468\_protein\_amino\_acid\_phosphorylation | PRKACA | 237 | 4 | 2.680052 | -1.228311 | 112 | 67.68 | 0.604286 |
| GO:0006468\_protein\_amino\_acid\_phosphorylation | CDK4 | 237 | 4 | 2.680052 | -1.228311 | 112 | 67.68 | 0.604286 |
| GO:0042060\_wound\_healing | GPX1 | 64 | 2 | 4.962284 | -1.216716 | 113 | 68.31 | 0.604513 |
| GO:0042060\_wound\_healing | HIF1A | 64 | 2 | 4.962284 | -1.216716 | 113 | 68.31 | 0.604513 |
| GO:0001659\_temperature\_homeostasis | GPX1 | 10 | 1 | 15.879310 | -1.212694 | 121 | 76.37 | 0.631157 |
| GO:0006081\_cellular\_aldehyde\_metabolic\_process | TPI1 | 10 | 1 | 15.879310 | -1.212694 | 121 | 76.37 | 0.631157 |
| GO:0009066\_aspartate\_family\_amino\_acid\_metabolic\_process | PHGDH | 10 | 1 | 15.879310 | -1.212694 | 121 | 76.37 | 0.631157 |
| GO:0009110\_vitamin\_biosynthetic\_process | ALDH1A1 | 10 | 1 | 15.879310 | -1.212694 | 121 | 76.37 | 0.631157 |
| GO:0021871\_forebrain\_regionalization | SIX3 | 10 | 1 | 15.879310 | -1.212694 | 121 | 76.37 | 0.631157 |
| GO:0044259\_multicellular\_organismal\_macromolecule\_metabolic\_process | HIF1A | 10 | 1 | 15.879310 | -1.212694 | 121 | 76.37 | 0.631157 |
| GO:0045446\_endothelial\_cell\_differentiation | GPX1 | 10 | 1 | 15.879310 | -1.212694 | 121 | 76.37 | 0.631157 |
| GO:0048596\_embryonic\_camera-type\_eye\_morphogenesis | ALDH1A1 | 10 | 1 | 15.879310 | -1.212694 | 121 | 76.37 | 0.631157 |
| GO:0001837\_epithelial\_to\_mesenchymal\_transition | HIF1A | 11 | 1 | 14.435737 | -1.172613 | 128 | 83.58 | 0.652969 |
| GO:0001952\_regulation\_of\_cell-matrix\_adhesion | DDR1 | 11 | 1 | 14.435737 | -1.172613 | 128 | 83.58 | 0.652969 |
| GO:0009064\_glutamine\_family\_amino\_acid\_metabolic\_process | PHGDH | 11 | 1 | 14.435737 | -1.172613 | 128 | 83.58 | 0.652969 |
| GO:0014902\_myotube\_differentiation | GPX1 | 11 | 1 | 14.435737 | -1.172613 | 128 | 83.58 | 0.652969 |
| GO:0042542\_response\_to\_hydrogen\_peroxide | GPX1 | 11 | 1 | 14.435737 | -1.172613 | 128 | 83.58 | 0.652969 |
| GO:0043576\_regulation\_of\_respiratory\_gaseous\_exchange | PBX3 | 11 | 1 | 14.435737 | -1.172613 | 128 | 83.58 | 0.652969 |
| GO:0046716\_muscle\_maintenance | HIF1A | 11 | 1 | 14.435737 | -1.172613 | 128 | 83.58 | 0.652969 |
| GO:0006281\_DNA\_repair | POLD1 | 71 | 2 | 4.473045 | -1.137520 | 130 | 86.09 | 0.662231 |
| GO:0006281\_DNA\_repair | TRIP13 | 71 | 2 | 4.473045 | -1.137520 | 130 | 86.09 | 0.662231 |
| GO:0006913\_nucleocytoplasmic\_transport | SIX3 | 71 | 2 | 4.473045 | -1.137520 | 130 | 86.09 | 0.662231 |
| GO:0006913\_nucleocytoplasmic\_transport | PRKACA | 71 | 2 | 4.473045 | -1.137520 | 130 | 86.09 | 0.662231 |
| GO:0000375\_RNA\_splicing\_\_via\_transesterification\_reactions | SF3A2 | 12 | 1 | 13.232759 | -1.136136 | 142 | 91.73 | 0.645986 |
| GO:0000377\_RNA\_splicing\_\_via\_transesterification\_reactions\_with\_bulged\_adenosine\_as\_nucleophile | SF3A2 | 12 | 1 | 13.232759 | -1.136136 | 142 | 91.73 | 0.645986 |
| GO:0000398\_nuclear\_mRNA\_splicing\_\_via\_spliceosome | SF3A2 | 12 | 1 | 13.232759 | -1.136136 | 142 | 91.73 | 0.645986 |
| GO:0002437\_inflammatory\_response\_to\_antigenic\_stimulus | GPX1 | 12 | 1 | 13.232759 | -1.136136 | 142 | 91.73 | 0.645986 |
| GO:0002861\_regulation\_of\_inflammatory\_response\_to\_antigenic\_stimulus | GPX1 | 12 | 1 | 13.232759 | -1.136136 | 142 | 91.73 | 0.645986 |
| GO:0006413\_translational\_initiation | EIF2S1 | 12 | 1 | 13.232759 | -1.136136 | 142 | 91.73 | 0.645986 |
| GO:0006446\_regulation\_of\_translational\_initiation | EIF2S1 | 12 | 1 | 13.232759 | -1.136136 | 142 | 91.73 | 0.645986 |
| GO:0006879\_cellular\_iron\_ion\_homeostasis | HIF1A | 12 | 1 | 13.232759 | -1.136136 | 142 | 91.73 | 0.645986 |
| GO:0007143\_female\_meiosis | TRIP13 | 12 | 1 | 13.232759 | -1.136136 | 142 | 91.73 | 0.645986 |
| GO:0042743\_hydrogen\_peroxide\_metabolic\_process | GPX1 | 12 | 1 | 13.232759 | -1.136136 | 142 | 91.73 | 0.645986 |
| GO:0048854\_brain\_morphogenesis | SOX2 | 12 | 1 | 13.232759 | -1.136136 | 142 | 91.73 | 0.645986 |
| GO:0060042\_retina\_morphogenesis\_in\_camera-type\_eye | SOX2 | 12 | 1 | 13.232759 | -1.136136 | 142 | 91.73 | 0.645986 |
| GO:0009966\_regulation\_of\_signal\_transduction | GPX1 | 256 | 4 | 2.481142 | -1.129131 | 143 | 91.85 | 0.642308 |
| GO:0009966\_regulation\_of\_signal\_transduction | HIF1A | 256 | 4 | 2.481142 | -1.129131 | 143 | 91.85 | 0.642308 |
| GO:0009966\_regulation\_of\_signal\_transduction | SOX2 | 256 | 4 | 2.481142 | -1.129131 | 143 | 91.85 | 0.642308 |
| GO:0009966\_regulation\_of\_signal\_transduction | SIX3 | 256 | 4 | 2.481142 | -1.129131 | 143 | 91.85 | 0.642308 |
| GO:0030879\_mammary\_gland\_development | DDR1 | 72 | 2 | 4.410920 | -1.126945 | 145 | 92.81 | 0.640069 |
| GO:0030879\_mammary\_gland\_development | NME1 | 72 | 2 | 4.410920 | -1.126945 | 145 | 92.81 | 0.640069 |
| GO:0051169\_nuclear\_transport | SIX3 | 72 | 2 | 4.410920 | -1.126945 | 145 | 92.81 | 0.640069 |
| GO:0051169\_nuclear\_transport | PRKACA | 72 | 2 | 4.410920 | -1.126945 | 145 | 92.81 | 0.640069 |
| GO:0048771\_tissue\_remodeling | HIF1A | 74 | 2 | 4.291705 | -1.106300 | 146 | 93.71 | 0.641849 |
| GO:0048771\_tissue\_remodeling | SOX2 | 74 | 2 | 4.291705 | -1.106300 | 146 | 93.71 | 0.641849 |
| GO:0003016\_respiratory\_system\_process | PBX3 | 13 | 1 | 12.214854 | -1.102683 | 153 | 99.61 | 0.651046 |
| GO:0007566\_embryo\_implantation | DDR1 | 13 | 1 | 12.214854 | -1.102683 | 153 | 99.61 | 0.651046 |
| GO:0009410\_response\_to\_xenobiotic\_stimulus | GPX1 | 13 | 1 | 12.214854 | -1.102683 | 153 | 99.61 | 0.651046 |
| GO:0009994\_oocyte\_differentiation | TRIP13 | 13 | 1 | 12.214854 | -1.102683 | 153 | 99.61 | 0.651046 |
| GO:0021879\_forebrain\_neuron\_differentiation | SOX2 | 13 | 1 | 12.214854 | -1.102683 | 153 | 99.61 | 0.651046 |
| GO:0030539\_male\_genitalia\_development | SOX2 | 13 | 1 | 12.214854 | -1.102683 | 153 | 99.61 | 0.651046 |
| GO:0048599\_oocyte\_development | TRIP13 | 13 | 1 | 12.214854 | -1.102683 | 153 | 99.61 | 0.651046 |
| GO:0006259\_DNA\_metabolic\_process | MCM7 | 165 | 3 | 2.887147 | -1.077611 | 154 | 100.95 | 0.655519 |
| GO:0006259\_DNA\_metabolic\_process | POLD1 | 165 | 3 | 2.887147 | -1.077611 | 154 | 100.95 | 0.655519 |
| GO:0006259\_DNA\_metabolic\_process | TRIP13 | 165 | 3 | 2.887147 | -1.077611 | 154 | 100.95 | 0.655519 |
| GO:0001890\_placenta\_development | HSP90AB1 | 77 | 2 | 4.124496 | -1.076523 | 155 | 101.17 | 0.652710 |
| GO:0001890\_placenta\_development | HIF1A | 77 | 2 | 4.124496 | -1.076523 | 155 | 101.17 | 0.652710 |
| GO:0010332\_response\_to\_gamma\_radiation | GPX1 | 14 | 1 | 11.342365 | -1.071807 | 162 | 106.01 | 0.654383 |
| GO:0021782\_glial\_cell\_development | PHGDH | 14 | 1 | 11.342365 | -1.071807 | 162 | 106.01 | 0.654383 |
| GO:0031099\_regeneration | GPX1 | 14 | 1 | 11.342365 | -1.071807 | 162 | 106.01 | 0.654383 |
| GO:0042573\_retinoic\_acid\_metabolic\_process | ALDH1A1 | 14 | 1 | 11.342365 | -1.071807 | 162 | 106.01 | 0.654383 |
| GO:0043491\_protein\_kinase\_B\_signaling\_cascade | GPX1 | 14 | 1 | 11.342365 | -1.071807 | 162 | 106.01 | 0.654383 |
| GO:0044236\_multicellular\_organismal\_metabolic\_process | HIF1A | 14 | 1 | 11.342365 | -1.071807 | 162 | 106.01 | 0.654383 |
| GO:0048048\_embryonic\_eye\_morphogenesis | ALDH1A1 | 14 | 1 | 11.342365 | -1.071807 | 162 | 106.01 | 0.654383 |
| GO:0045944\_positive\_regulation\_of\_transcription\_from\_RNA\_polymerase\_II\_promoter | HIF1A | 269 | 4 | 2.361236 | -1.066916 | 163 | 106.32 | 0.652270 |
| GO:0045944\_positive\_regulation\_of\_transcription\_from\_RNA\_polymerase\_II\_promoter | SOX2 | 269 | 4 | 2.361236 | -1.066916 | 163 | 106.32 | 0.652270 |
| GO:0045944\_positive\_regulation\_of\_transcription\_from\_RNA\_polymerase\_II\_promoter | SIX3 | 269 | 4 | 2.361236 | -1.066916 | 163 | 106.32 | 0.652270 |
| GO:0045944\_positive\_regulation\_of\_transcription\_from\_RNA\_polymerase\_II\_promoter | CNOT7 | 269 | 4 | 2.361236 | -1.066916 | 163 | 106.32 | 0.652270 |
| GO:0000278\_mitotic\_cell\_cycle | BUB1B | 80 | 2 | 3.969828 | -1.048077 | 164 | 107.76 | 0.657073 |
| GO:0000278\_mitotic\_cell\_cycle | CDCA5 | 80 | 2 | 3.969828 | -1.048077 | 164 | 107.76 | 0.657073 |
| GO:0021872\_generation\_of\_neurons\_in\_the\_forebrain | SOX2 | 15 | 1 | 10.586207 | -1.043152 | 169 | 113.31 | 0.670473 |
| GO:0031076\_embryonic\_camera-type\_eye\_development | ALDH1A1 | 15 | 1 | 10.586207 | -1.043152 | 169 | 113.31 | 0.670473 |
| GO:0045666\_positive\_regulation\_of\_neuron\_differentiation | SOX2 | 15 | 1 | 10.586207 | -1.043152 | 169 | 113.31 | 0.670473 |
| GO:0048010\_vascular\_endothelial\_growth\_factor\_receptor\_signaling\_pathway | HIF1A | 15 | 1 | 10.586207 | -1.043152 | 169 | 113.31 | 0.670473 |
| GO:0060749\_mammary\_gland\_alveolus\_development | DDR1 | 15 | 1 | 10.586207 | -1.043152 | 169 | 113.31 | 0.670473 |
| GO:0050877\_neurological\_system\_process | GPX1 | 390 | 5 | 2.035809 | -1.030052 | 170 | 113.84 | 0.669647 |
| GO:0050877\_neurological\_system\_process | SOX2 | 390 | 5 | 2.035809 | -1.030052 | 170 | 113.84 | 0.669647 |
| GO:0050877\_neurological\_system\_process | PTN | 390 | 5 | 2.035809 | -1.030052 | 170 | 113.84 | 0.669647 |
| GO:0050877\_neurological\_system\_process | PRKACA | 390 | 5 | 2.035809 | -1.030052 | 170 | 113.84 | 0.669647 |
| GO:0050877\_neurological\_system\_process | PBX3 | 390 | 5 | 2.035809 | -1.030052 | 170 | 113.84 | 0.669647 |
| GO:0042127\_regulation\_of\_cell\_proliferation | DDR1 | 393 | 5 | 2.020268 | -1.019049 | 171 | 115.13 | 0.673275 |
| GO:0042127\_regulation\_of\_cell\_proliferation | BTG1 | 393 | 5 | 2.020268 | -1.019049 | 171 | 115.13 | 0.673275 |
| GO:0042127\_regulation\_of\_cell\_proliferation | MARCKSL1 | 393 | 5 | 2.020268 | -1.019049 | 171 | 115.13 | 0.673275 |
| GO:0042127\_regulation\_of\_cell\_proliferation | SOX2 | 393 | 5 | 2.020268 | -1.019049 | 171 | 115.13 | 0.673275 |
| GO:0042127\_regulation\_of\_cell\_proliferation | CDK4 | 393 | 5 | 2.020268 | -1.019049 | 171 | 115.13 | 0.673275 |
| GO:0000302\_response\_to\_reactive\_oxygen\_species | GPX1 | 16 | 1 | 9.924569 | -1.016429 | 176 | 119.7 | 0.680114 |
| GO:0042311\_vasodilation | GPX1 | 16 | 1 | 9.924569 | -1.016429 | 176 | 119.7 | 0.680114 |
| GO:0046148\_pigment\_biosynthetic\_process | SOX2 | 16 | 1 | 9.924569 | -1.016429 | 176 | 119.7 | 0.680114 |
| GO:0046700\_heterocycle\_catabolic\_process | MTHFD1 | 16 | 1 | 9.924569 | -1.016429 | 176 | 119.7 | 0.680114 |
| GO:0050974\_detection\_of\_mechanical\_stimulus\_involved\_in\_sensory\_perception | SOX2 | 16 | 1 | 9.924569 | -1.016429 | 176 | 119.7 | 0.680114 |
| GO:0009887\_organ\_morphogenesis | ALDH1A1 | 642 | 7 | 1.731389 | -1.006531 | 177 | 120.51 | 0.680847 |
| GO:0009887\_organ\_morphogenesis | DDR1 | 642 | 7 | 1.731389 | -1.006531 | 177 | 120.51 | 0.680847 |
| GO:0009887\_organ\_morphogenesis | GPX1 | 642 | 7 | 1.731389 | -1.006531 | 177 | 120.51 | 0.680847 |
| GO:0009887\_organ\_morphogenesis | HIF1A | 642 | 7 | 1.731389 | -1.006531 | 177 | 120.51 | 0.680847 |
| GO:0009887\_organ\_morphogenesis | SOX2 | 642 | 7 | 1.731389 | -1.006531 | 177 | 120.51 | 0.680847 |
| GO:0009887\_organ\_morphogenesis | SIX3 | 642 | 7 | 1.731389 | -1.006531 | 177 | 120.51 | 0.680847 |
| GO:0009887\_organ\_morphogenesis | PRKACA | 642 | 7 | 1.731389 | -1.006531 | 177 | 120.51 | 0.680847 |
| GO:0070887\_cellular\_response\_to\_chemical\_stimulus | GPX1 | 85 | 2 | 3.736308 | -1.003361 | 178 | 120.83 | 0.678820 |
| GO:0070887\_cellular\_response\_to\_chemical\_stimulus | HIF1A | 85 | 2 | 3.736308 | -1.003361 | 178 | 120.83 | 0.678820 |
| GO:0048732\_gland\_development | DDR1 | 179 | 3 | 2.661337 | -0.996042 | 179 | 121.25 | 0.677374 |
| GO:0048732\_gland\_development | NME1 | 179 | 3 | 2.661337 | -0.996042 | 179 | 121.25 | 0.677374 |
| GO:0048732\_gland\_development | SOX2 | 179 | 3 | 2.661337 | -0.996042 | 179 | 121.25 | 0.677374 |
| GO:0006605\_protein\_targeting | SIX3 | 86 | 2 | 3.692863 | -0.994793 | 180 | 121.71 | 0.676167 |
| GO:0006605\_protein\_targeting | PRKACA | 86 | 2 | 3.692863 | -0.994793 | 180 | 121.71 | 0.676167 |
| GO:0008380\_RNA\_splicing | SF3A2 | 17 | 1 | 9.340771 | -0.991406 | 185 | 126.43 | 0.683405 |
| GO:0042440\_pigment\_metabolic\_process | SOX2 | 17 | 1 | 9.340771 | -0.991406 | 185 | 126.43 | 0.683405 |
| GO:0045667\_regulation\_of\_osteoblast\_differentiation | SOX2 | 17 | 1 | 9.340771 | -0.991406 | 185 | 126.43 | 0.683405 |
| GO:0048873\_homeostasis\_of\_number\_of\_cells\_within\_a\_tissue | FH1 | 17 | 1 | 9.340771 | -0.991406 | 185 | 126.43 | 0.683405 |
| GO:0055072\_iron\_ion\_homeostasis | HIF1A | 17 | 1 | 9.340771 | -0.991406 | 185 | 126.43 | 0.683405 |
| GO:0007417\_central\_nervous\_system\_development | SOX2 | 287 | 4 | 2.213144 | -0.987426 | 186 | 126.64 | 0.680860 |
| GO:0007417\_central\_nervous\_system\_development | PHGDH | 287 | 4 | 2.213144 | -0.987426 | 186 | 126.64 | 0.680860 |
| GO:0007417\_central\_nervous\_system\_development | SIX3 | 287 | 4 | 2.213144 | -0.987426 | 186 | 126.64 | 0.680860 |
| GO:0007417\_central\_nervous\_system\_development | PBX3 | 287 | 4 | 2.213144 | -0.987426 | 186 | 126.64 | 0.680860 |
| GO:0043583\_ear\_development | DDR1 | 87 | 2 | 3.650416 | -0.986343 | 187 | 127.36 | 0.681070 |
| GO:0043583\_ear\_development | SOX2 | 87 | 2 | 3.650416 | -0.986343 | 187 | 127.36 | 0.681070 |
| GO:0009888\_tissue\_development | ALDH1A1 | 525 | 6 | 1.814778 | -0.980844 | 188 | 127.7 | 0.679255 |
| GO:0009888\_tissue\_development | DDR1 | 525 | 6 | 1.814778 | -0.980844 | 188 | 127.7 | 0.679255 |
| GO:0009888\_tissue\_development | GPX1 | 525 | 6 | 1.814778 | -0.980844 | 188 | 127.7 | 0.679255 |
| GO:0009888\_tissue\_development | HIF1A | 525 | 6 | 1.814778 | -0.980844 | 188 | 127.7 | 0.679255 |
| GO:0009888\_tissue\_development | PTN | 525 | 6 | 1.814778 | -0.980844 | 188 | 127.7 | 0.679255 |
| GO:0009888\_tissue\_development | PRKACA | 525 | 6 | 1.814778 | -0.980844 | 188 | 127.7 | 0.679255 |
| GO:0001503\_ossification | SOX2 | 88 | 2 | 3.608934 | -0.978009 | 189 | 127.98 | 0.677143 |
| GO:0001503\_ossification | PTN | 88 | 2 | 3.608934 | -0.978009 | 189 | 127.98 | 0.677143 |
| GO:0007140\_male\_meiosis | TRIP13 | 18 | 1 | 8.821839 | -0.967886 | 196 | 134.13 | 0.684337 |
| GO:0009063\_cellular\_amino\_acid\_catabolic\_process | MTHFD1 | 18 | 1 | 8.821839 | -0.967886 | 196 | 134.13 | 0.684337 |
| GO:0030282\_bone\_mineralization | PTN | 18 | 1 | 8.821839 | -0.967886 | 196 | 134.13 | 0.684337 |
| GO:0033157\_regulation\_of\_intracellular\_protein\_transport | PRKACA | 18 | 1 | 8.821839 | -0.967886 | 196 | 134.13 | 0.684337 |
| GO:0050982\_detection\_of\_mechanical\_stimulus | SOX2 | 18 | 1 | 8.821839 | -0.967886 | 196 | 134.13 | 0.684337 |
| GO:0051168\_nuclear\_export | PRKACA | 18 | 1 | 8.821839 | -0.967886 | 196 | 134.13 | 0.684337 |
| GO:0051222\_positive\_regulation\_of\_protein\_transport | PRKACA | 18 | 1 | 8.821839 | -0.967886 | 196 | 134.13 | 0.684337 |
| GO:0042221\_response\_to\_chemical\_stimulus | ALDH1A1 | 409 | 5 | 1.941236 | -0.962473 | 197 | 134.42 | 0.682335 |
| GO:0042221\_response\_to\_chemical\_stimulus | DDR1 | 409 | 5 | 1.941236 | -0.962473 | 197 | 134.42 | 0.682335 |
| GO:0042221\_response\_to\_chemical\_stimulus | GPX1 | 409 | 5 | 1.941236 | -0.962473 | 197 | 134.42 | 0.682335 |
| GO:0042221\_response\_to\_chemical\_stimulus | HIF1A | 409 | 5 | 1.941236 | -0.962473 | 197 | 134.42 | 0.682335 |
| GO:0042221\_response\_to\_chemical\_stimulus | SOX2 | 409 | 5 | 1.941236 | -0.962473 | 197 | 134.42 | 0.682335 |
| GO:0006338\_chromatin\_remodeling | ACTL6A | 19 | 1 | 8.357532 | -0.945708 | 204 | 139.64 | 0.684510 |
| GO:0006776\_vitamin\_A\_metabolic\_process | ALDH1A1 | 19 | 1 | 8.357532 | -0.945708 | 204 | 139.64 | 0.684510 |
| GO:0007595\_lactation | NME1 | 19 | 1 | 8.357532 | -0.945708 | 204 | 139.64 | 0.684510 |
| GO:0032526\_response\_to\_retinoic\_acid | SOX2 | 19 | 1 | 8.357532 | -0.945708 | 204 | 139.64 | 0.684510 |
| GO:0033189\_response\_to\_vitamin\_A | SOX2 | 19 | 1 | 8.357532 | -0.945708 | 204 | 139.64 | 0.684510 |
| GO:0050728\_negative\_regulation\_of\_inflammatory\_response | GPX1 | 19 | 1 | 8.357532 | -0.945708 | 204 | 139.64 | 0.684510 |
| GO:0060444\_branching\_involved\_in\_mammary\_gland\_duct\_morphogenesis | DDR1 | 19 | 1 | 8.357532 | -0.945708 | 204 | 139.64 | 0.684510 |
| GO:0034984\_cellular\_response\_to\_DNA\_damage\_stimulus | POLD1 | 94 | 2 | 3.378577 | -0.930285 | 205 | 140.85 | 0.687073 |
| GO:0034984\_cellular\_response\_to\_DNA\_damage\_stimulus | TRIP13 | 94 | 2 | 3.378577 | -0.930285 | 205 | 140.85 | 0.687073 |
| GO:0042592\_homeostatic\_process | GPX1 | 419 | 5 | 1.894906 | -0.928826 | 206 | 140.94 | 0.684175 |
| GO:0042592\_homeostatic\_process | NPC2 | 419 | 5 | 1.894906 | -0.928826 | 206 | 140.94 | 0.684175 |
| GO:0042592\_homeostatic\_process | HIF1A | 419 | 5 | 1.894906 | -0.928826 | 206 | 140.94 | 0.684175 |
| GO:0042592\_homeostatic\_process | SOX2 | 419 | 5 | 1.894906 | -0.928826 | 206 | 140.94 | 0.684175 |
| GO:0042592\_homeostatic\_process | FH1 | 419 | 5 | 1.894906 | -0.928826 | 206 | 140.94 | 0.684175 |
| GO:0031214\_biomineral\_formation | PTN | 20 | 1 | 7.939655 | -0.924733 | 210 | 144.44 | 0.687810 |
| GO:0045639\_positive\_regulation\_of\_myeloid\_cell\_differentiation | HIF1A | 20 | 1 | 7.939655 | -0.924733 | 210 | 144.44 | 0.687810 |
| GO:0046822\_regulation\_of\_nucleocytoplasmic\_transport | PRKACA | 20 | 1 | 7.939655 | -0.924733 | 210 | 144.44 | 0.687810 |
| GO:0048806\_genitalia\_development | SOX2 | 20 | 1 | 7.939655 | -0.924733 | 210 | 144.44 | 0.687810 |
| GO:0046907\_intracellular\_transport | NPC2 | 194 | 3 | 2.455563 | -0.917508 | 211 | 144.88 | 0.686635 |
| GO:0046907\_intracellular\_transport | SIX3 | 194 | 3 | 2.455563 | -0.917508 | 211 | 144.88 | 0.686635 |
| GO:0046907\_intracellular\_transport | PRKACA | 194 | 3 | 2.455563 | -0.917508 | 211 | 144.88 | 0.686635 |
| GO:0006954\_inflammatory\_response | GPX1 | 96 | 2 | 3.308190 | -0.915190 | 212 | 145.24 | 0.685094 |
| GO:0006954\_inflammatory\_response | HIF1A | 96 | 2 | 3.308190 | -0.915190 | 212 | 145.24 | 0.685094 |
| GO:0045893\_positive\_regulation\_of\_transcription\_\_DNA-dependent | HIF1A | 306 | 4 | 2.075727 | -0.910945 | 214 | 145.77 | 0.681168 |
| GO:0045893\_positive\_regulation\_of\_transcription\_\_DNA-dependent | SOX2 | 306 | 4 | 2.075727 | -0.910945 | 214 | 145.77 | 0.681168 |
| GO:0045893\_positive\_regulation\_of\_transcription\_\_DNA-dependent | SIX3 | 306 | 4 | 2.075727 | -0.910945 | 214 | 145.77 | 0.681168 |
| GO:0045893\_positive\_regulation\_of\_transcription\_\_DNA-dependent | CNOT7 | 306 | 4 | 2.075727 | -0.910945 | 214 | 145.77 | 0.681168 |
| GO:0051254\_positive\_regulation\_of\_RNA\_metabolic\_process | HIF1A | 306 | 4 | 2.075727 | -0.910945 | 214 | 145.77 | 0.681168 |
| GO:0051254\_positive\_regulation\_of\_RNA\_metabolic\_process | SOX2 | 306 | 4 | 2.075727 | -0.910945 | 214 | 145.77 | 0.681168 |
| GO:0051254\_positive\_regulation\_of\_RNA\_metabolic\_process | SIX3 | 306 | 4 | 2.075727 | -0.910945 | 214 | 145.77 | 0.681168 |
| GO:0051254\_positive\_regulation\_of\_RNA\_metabolic\_process | CNOT7 | 306 | 4 | 2.075727 | -0.910945 | 214 | 145.77 | 0.681168 |
| GO:0001755\_neural\_crest\_cell\_migration | HIF1A | 21 | 1 | 7.561576 | -0.904844 | 217 | 150.8 | 0.694931 |
| GO:0019827\_stem\_cell\_maintenance | SOX2 | 21 | 1 | 7.561576 | -0.904844 | 217 | 150.8 | 0.694931 |
| GO:0050777\_negative\_regulation\_of\_immune\_response | GPX1 | 21 | 1 | 7.561576 | -0.904844 | 217 | 150.8 | 0.694931 |
| GO:0051094\_positive\_regulation\_of\_developmental\_process | ALDH1A1 | 308 | 4 | 2.062248 | -0.903299 | 218 | 151.04 | 0.692844 |
| GO:0051094\_positive\_regulation\_of\_developmental\_process | GPX1 | 308 | 4 | 2.062248 | -0.903299 | 218 | 151.04 | 0.692844 |
| GO:0051094\_positive\_regulation\_of\_developmental\_process | HIF1A | 308 | 4 | 2.062248 | -0.903299 | 218 | 151.04 | 0.692844 |
| GO:0051094\_positive\_regulation\_of\_developmental\_process | SOX2 | 308 | 4 | 2.062248 | -0.903299 | 218 | 151.04 | 0.692844 |
| GO:0016310\_phosphorylation | DDR1 | 309 | 4 | 2.055574 | -0.899504 | 219 | 151.85 | 0.693379 |
| GO:0016310\_phosphorylation | EIF2S1 | 309 | 4 | 2.055574 | -0.899504 | 219 | 151.85 | 0.693379 |
| GO:0016310\_phosphorylation | PRKACA | 309 | 4 | 2.055574 | -0.899504 | 219 | 151.85 | 0.693379 |
| GO:0016310\_phosphorylation | CDK4 | 309 | 4 | 2.055574 | -0.899504 | 219 | 151.85 | 0.693379 |
| GO:0060348\_bone\_development | SOX2 | 99 | 2 | 3.207941 | -0.893253 | 220 | 152.23 | 0.691955 |
| GO:0060348\_bone\_development | PTN | 99 | 2 | 3.207941 | -0.893253 | 220 | 152.23 | 0.691955 |
| GO:0001525\_angiogenesis | GPX1 | 100 | 2 | 3.175862 | -0.886121 | 221 | 152.49 | 0.690000 |
| GO:0001525\_angiogenesis | HIF1A | 100 | 2 | 3.175862 | -0.886121 | 221 | 152.49 | 0.690000 |
| GO:0001523\_retinoid\_metabolic\_process | ALDH1A1 | 22 | 1 | 7.217868 | -0.885940 | 232 | 158.18 | 0.681810 |
| GO:0001558\_regulation\_of\_cell\_growth | DDR1 | 22 | 1 | 7.217868 | -0.885940 | 232 | 158.18 | 0.681810 |
| GO:0001947\_heart\_looping | HIF1A | 22 | 1 | 7.217868 | -0.885940 | 232 | 158.18 | 0.681810 |
| GO:0006721\_terpenoid\_metabolic\_process | ALDH1A1 | 22 | 1 | 7.217868 | -0.885940 | 232 | 158.18 | 0.681810 |
| GO:0015918\_sterol\_transport | NPC2 | 22 | 1 | 7.217868 | -0.885940 | 232 | 158.18 | 0.681810 |
| GO:0016101\_diterpenoid\_metabolic\_process | ALDH1A1 | 22 | 1 | 7.217868 | -0.885940 | 232 | 158.18 | 0.681810 |
| GO:0030301\_cholesterol\_transport | NPC2 | 22 | 1 | 7.217868 | -0.885940 | 232 | 158.18 | 0.681810 |
| GO:0030335\_positive\_regulation\_of\_cell\_migration | HIF1A | 22 | 1 | 7.217868 | -0.885940 | 232 | 158.18 | 0.681810 |
| GO:0033273\_response\_to\_vitamin | SOX2 | 22 | 1 | 7.217868 | -0.885940 | 232 | 158.18 | 0.681810 |
| GO:0048477\_oogenesis | TRIP13 | 22 | 1 | 7.217868 | -0.885940 | 232 | 158.18 | 0.681810 |
| GO:0048864\_stem\_cell\_development | SOX2 | 22 | 1 | 7.217868 | -0.885940 | 232 | 158.18 | 0.681810 |
| GO:0065008\_regulation\_of\_biological\_quality | ALDH1A1 | 693 | 7 | 1.603971 | -0.871965 | 233 | 159.01 | 0.682446 |
| GO:0065008\_regulation\_of\_biological\_quality | GPX1 | 693 | 7 | 1.603971 | -0.871965 | 233 | 159.01 | 0.682446 |
| GO:0065008\_regulation\_of\_biological\_quality | DDR1 | 693 | 7 | 1.603971 | -0.871965 | 233 | 159.01 | 0.682446 |
| GO:0065008\_regulation\_of\_biological\_quality | NPC2 | 693 | 7 | 1.603971 | -0.871965 | 233 | 159.01 | 0.682446 |
| GO:0065008\_regulation\_of\_biological\_quality | HIF1A | 693 | 7 | 1.603971 | -0.871965 | 233 | 159.01 | 0.682446 |
| GO:0065008\_regulation\_of\_biological\_quality | SOX2 | 693 | 7 | 1.603971 | -0.871965 | 233 | 159.01 | 0.682446 |
| GO:0065008\_regulation\_of\_biological\_quality | FH1 | 693 | 7 | 1.603971 | -0.871965 | 233 | 159.01 | 0.682446 |
| GO:0006397\_mRNA\_processing | SF3A2 | 23 | 1 | 6.904048 | -0.867934 | 235 | 162.34 | 0.690809 |
| GO:0007584\_response\_to\_nutrient | SOX2 | 23 | 1 | 6.904048 | -0.867934 | 235 | 162.34 | 0.690809 |
| GO:0050896\_response\_to\_stimulus | ALDH1A1 | 1107 | 10 | 1.434445 | -0.865941 | 236 | 162.39 | 0.688093 |
| GO:0050896\_response\_to\_stimulus | DDR1 | 1107 | 10 | 1.434445 | -0.865941 | 236 | 162.39 | 0.688093 |
| GO:0050896\_response\_to\_stimulus | GPX1 | 1107 | 10 | 1.434445 | -0.865941 | 236 | 162.39 | 0.688093 |
| GO:0050896\_response\_to\_stimulus | HIF1A | 1107 | 10 | 1.434445 | -0.865941 | 236 | 162.39 | 0.688093 |
| GO:0050896\_response\_to\_stimulus | EIF2S1 | 1107 | 10 | 1.434445 | -0.865941 | 236 | 162.39 | 0.688093 |
| GO:0050896\_response\_to\_stimulus | POLD1 | 1107 | 10 | 1.434445 | -0.865941 | 236 | 162.39 | 0.688093 |
| GO:0050896\_response\_to\_stimulus | SOX2 | 1107 | 10 | 1.434445 | -0.865941 | 236 | 162.39 | 0.688093 |
| GO:0050896\_response\_to\_stimulus | PTN | 1107 | 10 | 1.434445 | -0.865941 | 236 | 162.39 | 0.688093 |
| GO:0050896\_response\_to\_stimulus | PBX3 | 1107 | 10 | 1.434445 | -0.865941 | 236 | 162.39 | 0.688093 |
| GO:0050896\_response\_to\_stimulus | TRIP13 | 1107 | 10 | 1.434445 | -0.865941 | 236 | 162.39 | 0.688093 |
| GO:0009968\_negative\_regulation\_of\_signal\_transduction | SOX2 | 103 | 2 | 3.083361 | -0.865240 | 237 | 162.73 | 0.686624 |
| GO:0009968\_negative\_regulation\_of\_signal\_transduction | SIX3 | 103 | 2 | 3.083361 | -0.865240 | 237 | 162.73 | 0.686624 |
| GO:0009790\_embryonic\_development | ALDH1A1 | 567 | 6 | 1.680350 | -0.860367 | 238 | 163.13 | 0.685420 |
| GO:0009790\_embryonic\_development | TPI1 | 567 | 6 | 1.680350 | -0.860367 | 238 | 163.13 | 0.685420 |
| GO:0009790\_embryonic\_development | HIF1A | 567 | 6 | 1.680350 | -0.860367 | 238 | 163.13 | 0.685420 |
| GO:0009790\_embryonic\_development | SOX2 | 567 | 6 | 1.680350 | -0.860367 | 238 | 163.13 | 0.685420 |
| GO:0009790\_embryonic\_development | PHGDH | 567 | 6 | 1.680350 | -0.860367 | 238 | 163.13 | 0.685420 |
| GO:0009790\_embryonic\_development | PRKACA | 567 | 6 | 1.680350 | -0.860367 | 238 | 163.13 | 0.685420 |
| GO:0048872\_homeostasis\_of\_number\_of\_cells | HIF1A | 105 | 2 | 3.024631 | -0.851732 | 239 | 163.73 | 0.685063 |
| GO:0048872\_homeostasis\_of\_number\_of\_cells | FH1 | 105 | 2 | 3.024631 | -0.851732 | 239 | 163.73 | 0.685063 |
| GO:0008629\_induction\_of\_apoptosis\_by\_intracellular\_signals | GPX1 | 24 | 1 | 6.616379 | -0.850747 | 244 | 167.91 | 0.688156 |
| GO:0009612\_response\_to\_mechanical\_stimulus | SOX2 | 24 | 1 | 6.616379 | -0.850747 | 244 | 167.91 | 0.688156 |
| GO:0032386\_regulation\_of\_intracellular\_transport | PRKACA | 24 | 1 | 6.616379 | -0.850747 | 244 | 167.91 | 0.688156 |
| GO:0042632\_cholesterol\_homeostasis | NPC2 | 24 | 1 | 6.616379 | -0.850747 | 244 | 167.91 | 0.688156 |
| GO:0055092\_sterol\_homeostasis | NPC2 | 24 | 1 | 6.616379 | -0.850747 | 244 | 167.91 | 0.688156 |
| GO:0010926\_anatomical\_structure\_formation | ALDH1A1 | 447 | 5 | 1.776209 | -0.841036 | 245 | 168.35 | 0.687143 |
| GO:0010926\_anatomical\_structure\_formation | GPX1 | 447 | 5 | 1.776209 | -0.841036 | 245 | 168.35 | 0.687143 |
| GO:0010926\_anatomical\_structure\_formation | HIF1A | 447 | 5 | 1.776209 | -0.841036 | 245 | 168.35 | 0.687143 |
| GO:0010926\_anatomical\_structure\_formation | SOX2 | 447 | 5 | 1.776209 | -0.841036 | 245 | 168.35 | 0.687143 |
| GO:0010926\_anatomical\_structure\_formation | PRKACA | 447 | 5 | 1.776209 | -0.841036 | 245 | 168.35 | 0.687143 |
| GO:0006302\_double-strand\_break\_repair | TRIP13 | 25 | 1 | 6.351724 | -0.834314 | 250 | 172.19 | 0.688760 |
| GO:0006775\_fat-soluble\_vitamin\_metabolic\_process | ALDH1A1 | 25 | 1 | 6.351724 | -0.834314 | 250 | 172.19 | 0.688760 |
| GO:0021983\_pituitary\_gland\_development | SOX2 | 25 | 1 | 6.351724 | -0.834314 | 250 | 172.19 | 0.688760 |
| GO:0031348\_negative\_regulation\_of\_defense\_response | GPX1 | 25 | 1 | 6.351724 | -0.834314 | 250 | 172.19 | 0.688760 |
| GO:0060603\_mammary\_gland\_duct\_morphogenesis | DDR1 | 25 | 1 | 6.351724 | -0.834314 | 250 | 172.19 | 0.688760 |
| GO:0010648\_negative\_regulation\_of\_cell\_communication | SOX2 | 110 | 2 | 2.887147 | -0.819317 | 251 | 173.51 | 0.691275 |
| GO:0010648\_negative\_regulation\_of\_cell\_communication | SIX3 | 110 | 2 | 2.887147 | -0.819317 | 251 | 173.51 | 0.691275 |
| GO:0001666\_response\_to\_hypoxia | HIF1A | 26 | 1 | 6.107427 | -0.818576 | 259 | 176.81 | 0.682664 |
| GO:0006720\_isoprenoid\_metabolic\_process | ALDH1A1 | 26 | 1 | 6.107427 | -0.818576 | 259 | 176.81 | 0.682664 |
| GO:0006800\_oxygen\_and\_reactive\_oxygen\_species\_metabolic\_process | GPX1 | 26 | 1 | 6.107427 | -0.818576 | 259 | 176.81 | 0.682664 |
| GO:0007405\_neuroblast\_proliferation | SOX2 | 26 | 1 | 6.107427 | -0.818576 | 259 | 176.81 | 0.682664 |
| GO:0009310\_amine\_catabolic\_process | MTHFD1 | 26 | 1 | 6.107427 | -0.818576 | 259 | 176.81 | 0.682664 |
| GO:0009636\_response\_to\_toxin | GPX1 | 26 | 1 | 6.107427 | -0.818576 | 259 | 176.81 | 0.682664 |
| GO:0010212\_response\_to\_ionizing\_radiation | GPX1 | 26 | 1 | 6.107427 | -0.818576 | 259 | 176.81 | 0.682664 |
| GO:0045665\_negative\_regulation\_of\_neuron\_differentiation | SOX2 | 26 | 1 | 6.107427 | -0.818576 | 259 | 176.81 | 0.682664 |
| GO:0040007\_growth | GPX1 | 217 | 3 | 2.195296 | -0.811866 | 260 | 177.61 | 0.683115 |
| GO:0040007\_growth | DDR1 | 217 | 3 | 2.195296 | -0.811866 | 260 | 177.61 | 0.683115 |
| GO:0040007\_growth | HIF1A | 217 | 3 | 2.195296 | -0.811866 | 260 | 177.61 | 0.683115 |
| GO:0006479\_protein\_amino\_acid\_methylation | BTG1 | 27 | 1 | 5.881226 | -0.803479 | 264 | 181.14 | 0.686136 |
| GO:0008213\_protein\_amino\_acid\_alkylation | BTG1 | 27 | 1 | 5.881226 | -0.803479 | 264 | 181.14 | 0.686136 |
| GO:0051272\_positive\_regulation\_of\_cell\_motion | HIF1A | 27 | 1 | 5.881226 | -0.803479 | 264 | 181.14 | 0.686136 |
| GO:0070482\_response\_to\_oxygen\_levels | HIF1A | 27 | 1 | 5.881226 | -0.803479 | 264 | 181.14 | 0.686136 |
| GO:0006974\_response\_to\_DNA\_damage\_stimulus | POLD1 | 113 | 2 | 2.810497 | -0.800740 | 266 | 181.54 | 0.682481 |
| GO:0006974\_response\_to\_DNA\_damage\_stimulus | TRIP13 | 113 | 2 | 2.810497 | -0.800740 | 266 | 181.54 | 0.682481 |
| GO:0040008\_regulation\_of\_growth | DDR1 | 113 | 2 | 2.810497 | -0.800740 | 266 | 181.54 | 0.682481 |
| GO:0040008\_regulation\_of\_growth | HIF1A | 113 | 2 | 2.810497 | -0.800740 | 266 | 181.54 | 0.682481 |
| GO:0045941\_positive\_regulation\_of\_transcription | HIF1A | 338 | 4 | 1.879208 | -0.796882 | 267 | 181.85 | 0.681086 |
| GO:0045941\_positive\_regulation\_of\_transcription | SOX2 | 338 | 4 | 1.879208 | -0.796882 | 267 | 181.85 | 0.681086 |
| GO:0045941\_positive\_regulation\_of\_transcription | SIX3 | 338 | 4 | 1.879208 | -0.796882 | 267 | 181.85 | 0.681086 |
| GO:0045941\_positive\_regulation\_of\_transcription | CNOT7 | 338 | 4 | 1.879208 | -0.796882 | 267 | 181.85 | 0.681086 |
| GO:0006470\_protein\_amino\_acid\_dephosphorylation | PPP1CB | 28 | 1 | 5.671182 | -0.788977 | 272 | 185.47 | 0.681875 |
| GO:0007127\_meiosis\_I | TRIP13 | 28 | 1 | 5.671182 | -0.788977 | 272 | 185.47 | 0.681875 |
| GO:0007585\_respiratory\_gaseous\_exchange | PBX3 | 28 | 1 | 5.671182 | -0.788977 | 272 | 185.47 | 0.681875 |
| GO:0045926\_negative\_regulation\_of\_growth | HIF1A | 28 | 1 | 5.671182 | -0.788977 | 272 | 185.47 | 0.681875 |
| GO:0048863\_stem\_cell\_differentiation | SOX2 | 28 | 1 | 5.671182 | -0.788977 | 272 | 185.47 | 0.681875 |
| GO:0006807\_nitrogen\_compound\_metabolic\_process | MTHFD1 | 1147 | 10 | 1.384421 | -0.788795 | 273 | 185.54 | 0.679634 |
| GO:0006807\_nitrogen\_compound\_metabolic\_process | HIF1A | 1147 | 10 | 1.384421 | -0.788795 | 273 | 185.54 | 0.679634 |
| GO:0006807\_nitrogen\_compound\_metabolic\_process | MCM7 | 1147 | 10 | 1.384421 | -0.788795 | 273 | 185.54 | 0.679634 |
| GO:0006807\_nitrogen\_compound\_metabolic\_process | POLD1 | 1147 | 10 | 1.384421 | -0.788795 | 273 | 185.54 | 0.679634 |
| GO:0006807\_nitrogen\_compound\_metabolic\_process | SOX2 | 1147 | 10 | 1.384421 | -0.788795 | 273 | 185.54 | 0.679634 |
| GO:0006807\_nitrogen\_compound\_metabolic\_process | PHGDH | 1147 | 10 | 1.384421 | -0.788795 | 273 | 185.54 | 0.679634 |
| GO:0006807\_nitrogen\_compound\_metabolic\_process | SIX3 | 1147 | 10 | 1.384421 | -0.788795 | 273 | 185.54 | 0.679634 |
| GO:0006807\_nitrogen\_compound\_metabolic\_process | SF3A2 | 1147 | 10 | 1.384421 | -0.788795 | 273 | 185.54 | 0.679634 |
| GO:0006807\_nitrogen\_compound\_metabolic\_process | CNOT7 | 1147 | 10 | 1.384421 | -0.788795 | 273 | 185.54 | 0.679634 |
| GO:0006807\_nitrogen\_compound\_metabolic\_process | TRIP13 | 1147 | 10 | 1.384421 | -0.788795 | 273 | 185.54 | 0.679634 |
| GO:0006417\_regulation\_of\_translation | EIF2S1 | 29 | 1 | 5.475624 | -0.775028 | 278 | 190.06 | 0.683669 |
| GO:0006641\_triglyceride\_metabolic\_process | GPX1 | 29 | 1 | 5.475624 | -0.775028 | 278 | 190.06 | 0.683669 |
| GO:0044270\_nitrogen\_compound\_catabolic\_process | MTHFD1 | 29 | 1 | 5.475624 | -0.775028 | 278 | 190.06 | 0.683669 |
| GO:0050769\_positive\_regulation\_of\_neurogenesis | SOX2 | 29 | 1 | 5.475624 | -0.775028 | 278 | 190.06 | 0.683669 |
| GO:0060041\_retina\_development\_in\_camera-type\_eye | SOX2 | 29 | 1 | 5.475624 | -0.775028 | 278 | 190.06 | 0.683669 |
| GO:0006519\_cellular\_amino\_acid\_and\_derivative\_metabolic\_process | MTHFD1 | 118 | 2 | 2.691409 | -0.771123 | 279 | 190.43 | 0.682545 |
| GO:0006519\_cellular\_amino\_acid\_and\_derivative\_metabolic\_process | PHGDH | 118 | 2 | 2.691409 | -0.771123 | 279 | 190.43 | 0.682545 |
| GO:0010628\_positive\_regulation\_of\_gene\_expression | HIF1A | 346 | 4 | 1.835758 | -0.770912 | 280 | 190.55 | 0.680536 |
| GO:0010628\_positive\_regulation\_of\_gene\_expression | SOX2 | 346 | 4 | 1.835758 | -0.770912 | 280 | 190.55 | 0.680536 |
| GO:0010628\_positive\_regulation\_of\_gene\_expression | SIX3 | 346 | 4 | 1.835758 | -0.770912 | 280 | 190.55 | 0.680536 |
| GO:0010628\_positive\_regulation\_of\_gene\_expression | CNOT7 | 346 | 4 | 1.835758 | -0.770912 | 280 | 190.55 | 0.680536 |
| GO:0007219\_Notch\_signaling\_pathway | SOX2 | 30 | 1 | 5.293103 | -0.761595 | 286 | 195.16 | 0.682378 |
| GO:0014032\_neural\_crest\_cell\_development | HIF1A | 30 | 1 | 5.293103 | -0.761595 | 286 | 195.16 | 0.682378 |
| GO:0014033\_neural\_crest\_cell\_differentiation | HIF1A | 30 | 1 | 5.293103 | -0.761595 | 286 | 195.16 | 0.682378 |
| GO:0032102\_negative\_regulation\_of\_response\_to\_external\_stimulus | GPX1 | 30 | 1 | 5.293103 | -0.761595 | 286 | 195.16 | 0.682378 |
| GO:0033500\_carbohydrate\_homeostasis | HIF1A | 30 | 1 | 5.293103 | -0.761595 | 286 | 195.16 | 0.682378 |
| GO:0042593\_glucose\_homeostasis | HIF1A | 30 | 1 | 5.293103 | -0.761595 | 286 | 195.16 | 0.682378 |
| GO:0045935\_positive\_regulation\_of\_nucleobase\_\_nucleoside\_\_nucleotide\_and\_nucleic\_acid\_metabolic\_process | HIF1A | 352 | 4 | 1.804467 | -0.752042 | 287 | 196.28 | 0.683902 |
| GO:0045935\_positive\_regulation\_of\_nucleobase\_\_nucleoside\_\_nucleotide\_and\_nucleic\_acid\_metabolic\_process | SOX2 | 352 | 4 | 1.804467 | -0.752042 | 287 | 196.28 | 0.683902 |
| GO:0045935\_positive\_regulation\_of\_nucleobase\_\_nucleoside\_\_nucleotide\_and\_nucleic\_acid\_metabolic\_process | SIX3 | 352 | 4 | 1.804467 | -0.752042 | 287 | 196.28 | 0.683902 |
| GO:0045935\_positive\_regulation\_of\_nucleobase\_\_nucleoside\_\_nucleotide\_and\_nucleic\_acid\_metabolic\_process | CNOT7 | 352 | 4 | 1.804467 | -0.752042 | 287 | 196.28 | 0.683902 |
| GO:0003018\_vascular\_process\_in\_circulatory\_system | GPX1 | 31 | 1 | 5.122358 | -0.748643 | 295 | 201.89 | 0.684373 |
| GO:0006639\_acylglycerol\_metabolic\_process | GPX1 | 31 | 1 | 5.122358 | -0.748643 | 295 | 201.89 | 0.684373 |
| GO:0016049\_cell\_growth | DDR1 | 31 | 1 | 5.122358 | -0.748643 | 295 | 201.89 | 0.684373 |
| GO:0016311\_dephosphorylation | PPP1CB | 31 | 1 | 5.122358 | -0.748643 | 295 | 201.89 | 0.684373 |
| GO:0035150\_regulation\_of\_tube\_size | GPX1 | 31 | 1 | 5.122358 | -0.748643 | 295 | 201.89 | 0.684373 |
| GO:0048562\_embryonic\_organ\_morphogenesis | ALDH1A1 | 31 | 1 | 5.122358 | -0.748643 | 295 | 201.89 | 0.684373 |
| GO:0050880\_regulation\_of\_blood\_vessel\_size | GPX1 | 31 | 1 | 5.122358 | -0.748643 | 295 | 201.89 | 0.684373 |
| GO:0055088\_lipid\_homeostasis | NPC2 | 31 | 1 | 5.122358 | -0.748643 | 295 | 201.89 | 0.684373 |
| GO:0006886\_intracellular\_protein\_transport | SIX3 | 122 | 2 | 2.603166 | -0.748556 | 296 | 202.77 | 0.685034 |
| GO:0006886\_intracellular\_protein\_transport | PRKACA | 122 | 2 | 2.603166 | -0.748556 | 296 | 202.77 | 0.685034 |
| GO:0050890\_cognition | GPX1 | 233 | 3 | 2.044546 | -0.747193 | 297 | 203.06 | 0.683704 |
| GO:0050890\_cognition | SOX2 | 233 | 3 | 2.044546 | -0.747193 | 297 | 203.06 | 0.683704 |
| GO:0050890\_cognition | PTN | 233 | 3 | 2.044546 | -0.747193 | 297 | 203.06 | 0.683704 |
| GO:0009308\_amine\_metabolic\_process | MTHFD1 | 124 | 2 | 2.561179 | -0.737625 | 298 | 203.69 | 0.683523 |
| GO:0009308\_amine\_metabolic\_process | PHGDH | 124 | 2 | 2.561179 | -0.737625 | 298 | 203.69 | 0.683523 |
| GO:0001707\_mesoderm\_formation | PRKACA | 32 | 1 | 4.962284 | -0.736142 | 303 | 206.13 | 0.680297 |
| GO:0006638\_neutral\_lipid\_metabolic\_process | GPX1 | 32 | 1 | 4.962284 | -0.736142 | 303 | 206.13 | 0.680297 |
| GO:0006662\_glycerol\_ether\_metabolic\_process | GPX1 | 32 | 1 | 4.962284 | -0.736142 | 303 | 206.13 | 0.680297 |
| GO:0048332\_mesoderm\_morphogenesis | PRKACA | 32 | 1 | 4.962284 | -0.736142 | 303 | 206.13 | 0.680297 |
| GO:0050885\_neuromuscular\_process\_controlling\_balance | SOX2 | 32 | 1 | 4.962284 | -0.736142 | 303 | 206.13 | 0.680297 |
| GO:0001501\_skeletal\_system\_development | HIF1A | 236 | 3 | 2.018556 | -0.735772 | 304 | 206.25 | 0.678454 |
| GO:0001501\_skeletal\_system\_development | SOX2 | 236 | 3 | 2.018556 | -0.735772 | 304 | 206.25 | 0.678454 |
| GO:0001501\_skeletal\_system\_development | PTN | 236 | 3 | 2.018556 | -0.735772 | 304 | 206.25 | 0.678454 |
| GO:0051173\_positive\_regulation\_of\_nitrogen\_compound\_metabolic\_process | HIF1A | 361 | 4 | 1.759480 | -0.724670 | 305 | 207.48 | 0.680262 |
| GO:0051173\_positive\_regulation\_of\_nitrogen\_compound\_metabolic\_process | SOX2 | 361 | 4 | 1.759480 | -0.724670 | 305 | 207.48 | 0.680262 |
| GO:0051173\_positive\_regulation\_of\_nitrogen\_compound\_metabolic\_process | SIX3 | 361 | 4 | 1.759480 | -0.724670 | 305 | 207.48 | 0.680262 |
| GO:0051173\_positive\_regulation\_of\_nitrogen\_compound\_metabolic\_process | CNOT7 | 361 | 4 | 1.759480 | -0.724670 | 305 | 207.48 | 0.680262 |
| GO:0007565\_female\_pregnancy | DDR1 | 33 | 1 | 4.811912 | -0.724065 | 307 | 210.17 | 0.684593 |
| GO:0021987\_cerebral\_cortex\_development | SOX2 | 33 | 1 | 4.811912 | -0.724065 | 307 | 210.17 | 0.684593 |
| GO:0045597\_positive\_regulation\_of\_cell\_differentiation | HIF1A | 128 | 2 | 2.481142 | -0.716432 | 308 | 210.72 | 0.684156 |
| GO:0045597\_positive\_regulation\_of\_cell\_differentiation | SOX2 | 128 | 2 | 2.481142 | -0.716432 | 308 | 210.72 | 0.684156 |
| GO:0010720\_positive\_regulation\_of\_cell\_development | SOX2 | 34 | 1 | 4.670385 | -0.712385 | 312 | 214.67 | 0.688045 |
| GO:0016054\_organic\_acid\_catabolic\_process | MTHFD1 | 34 | 1 | 4.670385 | -0.712385 | 312 | 214.67 | 0.688045 |
| GO:0046395\_carboxylic\_acid\_catabolic\_process | MTHFD1 | 34 | 1 | 4.670385 | -0.712385 | 312 | 214.67 | 0.688045 |
| GO:0060443\_mammary\_gland\_morphogenesis | DDR1 | 34 | 1 | 4.670385 | -0.712385 | 312 | 214.67 | 0.688045 |
| GO:0045165\_cell\_fate\_commitment | SOX2 | 130 | 2 | 2.442971 | -0.706155 | 313 | 215.66 | 0.689010 |
| GO:0045165\_cell\_fate\_commitment | SIX3 | 130 | 2 | 2.442971 | -0.706155 | 313 | 215.66 | 0.689010 |
| GO:0006869\_lipid\_transport | NPC2 | 35 | 1 | 4.536946 | -0.701080 | 317 | 218.19 | 0.688297 |
| GO:0007292\_female\_gamete\_generation | TRIP13 | 35 | 1 | 4.536946 | -0.701080 | 317 | 218.19 | 0.688297 |
| GO:0010810\_regulation\_of\_cell-substrate\_adhesion | DDR1 | 35 | 1 | 4.536946 | -0.701080 | 317 | 218.19 | 0.688297 |
| GO:0018904\_organic\_ether\_metabolic\_process | GPX1 | 35 | 1 | 4.536946 | -0.701080 | 317 | 218.19 | 0.688297 |
| GO:0010557\_positive\_regulation\_of\_macromolecule\_biosynthetic\_process | HIF1A | 371 | 4 | 1.712055 | -0.695509 | 318 | 218.65 | 0.687579 |
| GO:0010557\_positive\_regulation\_of\_macromolecule\_biosynthetic\_process | SOX2 | 371 | 4 | 1.712055 | -0.695509 | 318 | 218.65 | 0.687579 |
| GO:0010557\_positive\_regulation\_of\_macromolecule\_biosynthetic\_process | SIX3 | 371 | 4 | 1.712055 | -0.695509 | 318 | 218.65 | 0.687579 |
| GO:0010557\_positive\_regulation\_of\_macromolecule\_biosynthetic\_process | CNOT7 | 371 | 4 | 1.712055 | -0.695509 | 318 | 218.65 | 0.687579 |
| GO:0044057\_regulation\_of\_system\_process | PRKACA | 133 | 2 | 2.387866 | -0.691121 | 319 | 219.34 | 0.687586 |
| GO:0044057\_regulation\_of\_system\_process | PBX3 | 133 | 2 | 2.387866 | -0.691121 | 319 | 219.34 | 0.687586 |
| GO:0001704\_formation\_of\_primary\_germ\_layer | PRKACA | 36 | 1 | 4.410920 | -0.690129 | 322 | 222.87 | 0.692143 |
| GO:0030278\_regulation\_of\_ossification | SOX2 | 36 | 1 | 4.410920 | -0.690129 | 322 | 222.87 | 0.692143 |
| GO:0051223\_regulation\_of\_protein\_transport | PRKACA | 36 | 1 | 4.410920 | -0.690129 | 322 | 222.87 | 0.692143 |
| GO:0022414\_reproductive\_process | DDR1 | 376 | 4 | 1.689288 | -0.681401 | 323 | 223.71 | 0.692601 |
| GO:0022414\_reproductive\_process | NME1 | 376 | 4 | 1.689288 | -0.681401 | 323 | 223.71 | 0.692601 |
| GO:0022414\_reproductive\_process | SOX2 | 376 | 4 | 1.689288 | -0.681401 | 323 | 223.71 | 0.692601 |
| GO:0022414\_reproductive\_process | TRIP13 | 376 | 4 | 1.689288 | -0.681401 | 323 | 223.71 | 0.692601 |
| GO:0050906\_detection\_of\_stimulus\_involved\_in\_sensory\_perception | SOX2 | 37 | 1 | 4.291705 | -0.679511 | 324 | 226.09 | 0.697809 |
| GO:0007267\_cell-cell\_signaling | SOX2 | 252 | 3 | 1.890394 | -0.678257 | 325 | 226.27 | 0.696215 |
| GO:0007267\_cell-cell\_signaling | SIX3 | 252 | 3 | 1.890394 | -0.678257 | 325 | 226.27 | 0.696215 |
| GO:0007267\_cell-cell\_signaling | PRKACA | 252 | 3 | 1.890394 | -0.678257 | 325 | 226.27 | 0.696215 |
| GO:0000003\_reproduction | DDR1 | 379 | 4 | 1.675917 | -0.673082 | 326 | 226.78 | 0.695644 |
| GO:0000003\_reproduction | NME1 | 379 | 4 | 1.675917 | -0.673082 | 326 | 226.78 | 0.695644 |
| GO:0000003\_reproduction | SOX2 | 379 | 4 | 1.675917 | -0.673082 | 326 | 226.78 | 0.695644 |
| GO:0000003\_reproduction | TRIP13 | 379 | 4 | 1.675917 | -0.673082 | 326 | 226.78 | 0.695644 |
| GO:0001649\_osteoblast\_differentiation | SOX2 | 38 | 1 | 4.178766 | -0.669210 | 331 | 230.65 | 0.696828 |
| GO:0032259\_methylation | BTG1 | 38 | 1 | 4.178766 | -0.669210 | 331 | 230.65 | 0.696828 |
| GO:0042493\_response\_to\_drug | ALDH1A1 | 38 | 1 | 4.178766 | -0.669210 | 331 | 230.65 | 0.696828 |
| GO:0043414\_biopolymer\_methylation | BTG1 | 38 | 1 | 4.178766 | -0.669210 | 331 | 230.65 | 0.696828 |
| GO:0050727\_regulation\_of\_inflammatory\_response | GPX1 | 38 | 1 | 4.178766 | -0.669210 | 331 | 230.65 | 0.696828 |
| GO:0048729\_tissue\_morphogenesis | ALDH1A1 | 255 | 3 | 1.868154 | -0.668071 | 332 | 230.79 | 0.695151 |
| GO:0048729\_tissue\_morphogenesis | DDR1 | 255 | 3 | 1.868154 | -0.668071 | 332 | 230.79 | 0.695151 |
| GO:0048729\_tissue\_morphogenesis | PRKACA | 255 | 3 | 1.868154 | -0.668071 | 332 | 230.79 | 0.695151 |
| GO:0034613\_cellular\_protein\_localization | SIX3 | 139 | 2 | 2.284793 | -0.662351 | 333 | 231.5 | 0.695195 |
| GO:0034613\_cellular\_protein\_localization | PRKACA | 139 | 2 | 2.284793 | -0.662351 | 333 | 231.5 | 0.695195 |
| GO:0006730\_one-carbon\_metabolic\_process | BTG1 | 39 | 1 | 4.071618 | -0.659208 | 338 | 234.96 | 0.695148 |
| GO:0007160\_cell-matrix\_adhesion | DDR1 | 39 | 1 | 4.071618 | -0.659208 | 338 | 234.96 | 0.695148 |
| GO:0007286\_spermatid\_development | TRIP13 | 39 | 1 | 4.071618 | -0.659208 | 338 | 234.96 | 0.695148 |
| GO:0048663\_neuron\_fate\_commitment | SOX2 | 39 | 1 | 4.071618 | -0.659208 | 338 | 234.96 | 0.695148 |
| GO:0070201\_regulation\_of\_establishment\_of\_protein\_localization | PRKACA | 39 | 1 | 4.071618 | -0.659208 | 338 | 234.96 | 0.695148 |
| GO:0003008\_system\_process | GPX1 | 516 | 5 | 1.538693 | -0.659098 | 339 | 235.12 | 0.693569 |
| GO:0003008\_system\_process | SOX2 | 516 | 5 | 1.538693 | -0.659098 | 339 | 235.12 | 0.693569 |
| GO:0003008\_system\_process | PTN | 516 | 5 | 1.538693 | -0.659098 | 339 | 235.12 | 0.693569 |
| GO:0003008\_system\_process | PRKACA | 516 | 5 | 1.538693 | -0.659098 | 339 | 235.12 | 0.693569 |
| GO:0003008\_system\_process | PBX3 | 516 | 5 | 1.538693 | -0.659098 | 339 | 235.12 | 0.693569 |
| GO:0048468\_cell\_development | GPX1 | 654 | 6 | 1.456817 | -0.654585 | 340 | 235.43 | 0.692441 |
| GO:0048468\_cell\_development | HIF1A | 654 | 6 | 1.456817 | -0.654585 | 340 | 235.43 | 0.692441 |
| GO:0048468\_cell\_development | SOX2 | 654 | 6 | 1.456817 | -0.654585 | 340 | 235.43 | 0.692441 |
| GO:0048468\_cell\_development | PHGDH | 654 | 6 | 1.456817 | -0.654585 | 340 | 235.43 | 0.692441 |
| GO:0048468\_cell\_development | PBX3 | 654 | 6 | 1.456817 | -0.654585 | 340 | 235.43 | 0.692441 |
| GO:0048468\_cell\_development | TRIP13 | 654 | 6 | 1.456817 | -0.654585 | 340 | 235.43 | 0.692441 |
| GO:0070727\_cellular\_macromolecule\_localization | SIX3 | 141 | 2 | 2.252384 | -0.653124 | 341 | 235.76 | 0.691378 |
| GO:0070727\_cellular\_macromolecule\_localization | PRKACA | 141 | 2 | 2.252384 | -0.653124 | 341 | 235.76 | 0.691378 |
| GO:0031328\_positive\_regulation\_of\_cellular\_biosynthetic\_process | HIF1A | 387 | 4 | 1.641272 | -0.651418 | 342 | 236.18 | 0.690585 |
| GO:0031328\_positive\_regulation\_of\_cellular\_biosynthetic\_process | SOX2 | 387 | 4 | 1.641272 | -0.651418 | 342 | 236.18 | 0.690585 |
| GO:0031328\_positive\_regulation\_of\_cellular\_biosynthetic\_process | SIX3 | 387 | 4 | 1.641272 | -0.651418 | 342 | 236.18 | 0.690585 |
| GO:0031328\_positive\_regulation\_of\_cellular\_biosynthetic\_process | CNOT7 | 387 | 4 | 1.641272 | -0.651418 | 342 | 236.18 | 0.690585 |
| GO:0014031\_mesenchymal\_cell\_development | HIF1A | 40 | 1 | 3.969828 | -0.649491 | 345 | 238.79 | 0.692145 |
| GO:0016071\_mRNA\_metabolic\_process | SF3A2 | 40 | 1 | 3.969828 | -0.649491 | 345 | 238.79 | 0.692145 |
| GO:0046850\_regulation\_of\_bone\_remodeling | SOX2 | 40 | 1 | 3.969828 | -0.649491 | 345 | 238.79 | 0.692145 |
| GO:0009891\_positive\_regulation\_of\_biosynthetic\_process | HIF1A | 388 | 4 | 1.637042 | -0.648762 | 346 | 238.94 | 0.690578 |
| GO:0009891\_positive\_regulation\_of\_biosynthetic\_process | SOX2 | 388 | 4 | 1.637042 | -0.648762 | 346 | 238.94 | 0.690578 |
| GO:0009891\_positive\_regulation\_of\_biosynthetic\_process | SIX3 | 388 | 4 | 1.637042 | -0.648762 | 346 | 238.94 | 0.690578 |
| GO:0009891\_positive\_regulation\_of\_biosynthetic\_process | CNOT7 | 388 | 4 | 1.637042 | -0.648762 | 346 | 238.94 | 0.690578 |
| GO:0019748\_secondary\_metabolic\_process | ALDH1A1 | 41 | 1 | 3.873003 | -0.640044 | 348 | 243.76 | 0.700460 |
| GO:0032844\_regulation\_of\_homeostatic\_process | HIF1A | 41 | 1 | 3.873003 | -0.640044 | 348 | 243.76 | 0.700460 |
| GO:0044255\_cellular\_lipid\_metabolic\_process | ALDH1A1 | 264 | 3 | 1.804467 | -0.638564 | 349 | 244.39 | 0.700258 |
| GO:0044255\_cellular\_lipid\_metabolic\_process | GPX1 | 264 | 3 | 1.804467 | -0.638564 | 349 | 244.39 | 0.700258 |
| GO:0044255\_cellular\_lipid\_metabolic\_process | PHGDH | 264 | 3 | 1.804467 | -0.638564 | 349 | 244.39 | 0.700258 |
| GO:0034961\_cellular\_biopolymer\_biosynthetic\_process | HIF1A | 804 | 7 | 1.382527 | -0.634250 | 350 | 244.83 | 0.699514 |
| GO:0034961\_cellular\_biopolymer\_biosynthetic\_process | MCM7 | 804 | 7 | 1.382527 | -0.634250 | 350 | 244.83 | 0.699514 |
| GO:0034961\_cellular\_biopolymer\_biosynthetic\_process | EIF2S1 | 804 | 7 | 1.382527 | -0.634250 | 350 | 244.83 | 0.699514 |
| GO:0034961\_cellular\_biopolymer\_biosynthetic\_process | POLD1 | 804 | 7 | 1.382527 | -0.634250 | 350 | 244.83 | 0.699514 |
| GO:0034961\_cellular\_biopolymer\_biosynthetic\_process | SOX2 | 804 | 7 | 1.382527 | -0.634250 | 350 | 244.83 | 0.699514 |
| GO:0034961\_cellular\_biopolymer\_biosynthetic\_process | SIX3 | 804 | 7 | 1.382527 | -0.634250 | 350 | 244.83 | 0.699514 |
| GO:0034961\_cellular\_biopolymer\_biosynthetic\_process | CNOT7 | 804 | 7 | 1.382527 | -0.634250 | 350 | 244.83 | 0.699514 |
| GO:0006006\_glucose\_metabolic\_process | TPI1 | 42 | 1 | 3.780788 | -0.630855 | 355 | 248.87 | 0.701042 |
| GO:0008361\_regulation\_of\_cell\_size | DDR1 | 42 | 1 | 3.780788 | -0.630855 | 355 | 248.87 | 0.701042 |
| GO:0010740\_positive\_regulation\_of\_protein\_kinase\_cascade | GPX1 | 42 | 1 | 3.780788 | -0.630855 | 355 | 248.87 | 0.701042 |
| GO:0045637\_regulation\_of\_myeloid\_cell\_differentiation | HIF1A | 42 | 1 | 3.780788 | -0.630855 | 355 | 248.87 | 0.701042 |
| GO:0048515\_spermatid\_differentiation | TRIP13 | 42 | 1 | 3.780788 | -0.630855 | 355 | 248.87 | 0.701042 |
| GO:0030900\_forebrain\_development | SOX2 | 146 | 2 | 2.175248 | -0.630805 | 356 | 249.45 | 0.700702 |
| GO:0030900\_forebrain\_development | SIX3 | 146 | 2 | 2.175248 | -0.630805 | 356 | 249.45 | 0.700702 |
| GO:0043284\_biopolymer\_biosynthetic\_process | HIF1A | 807 | 7 | 1.377388 | -0.628723 | 357 | 249.57 | 0.699076 |
| GO:0043284\_biopolymer\_biosynthetic\_process | MCM7 | 807 | 7 | 1.377388 | -0.628723 | 357 | 249.57 | 0.699076 |
| GO:0043284\_biopolymer\_biosynthetic\_process | EIF2S1 | 807 | 7 | 1.377388 | -0.628723 | 357 | 249.57 | 0.699076 |
| GO:0043284\_biopolymer\_biosynthetic\_process | POLD1 | 807 | 7 | 1.377388 | -0.628723 | 357 | 249.57 | 0.699076 |
| GO:0043284\_biopolymer\_biosynthetic\_process | SOX2 | 807 | 7 | 1.377388 | -0.628723 | 357 | 249.57 | 0.699076 |
| GO:0043284\_biopolymer\_biosynthetic\_process | SIX3 | 807 | 7 | 1.377388 | -0.628723 | 357 | 249.57 | 0.699076 |
| GO:0043284\_biopolymer\_biosynthetic\_process | CNOT7 | 807 | 7 | 1.377388 | -0.628723 | 357 | 249.57 | 0.699076 |
| GO:0001894\_tissue\_homeostasis | FH1 | 43 | 1 | 3.692863 | -0.621910 | 363 | 254.13 | 0.700083 |
| GO:0006766\_vitamin\_metabolic\_process | ALDH1A1 | 43 | 1 | 3.692863 | -0.621910 | 363 | 254.13 | 0.700083 |
| GO:0009582\_detection\_of\_abiotic\_stimulus | SOX2 | 43 | 1 | 3.692863 | -0.621910 | 363 | 254.13 | 0.700083 |
| GO:0010001\_glial\_cell\_differentiation | PHGDH | 43 | 1 | 3.692863 | -0.621910 | 363 | 254.13 | 0.700083 |
| GO:0048762\_mesenchymal\_cell\_differentiation | HIF1A | 43 | 1 | 3.692863 | -0.621910 | 363 | 254.13 | 0.700083 |
| GO:0051789\_response\_to\_protein\_stimulus | DDR1 | 43 | 1 | 3.692863 | -0.621910 | 363 | 254.13 | 0.700083 |
| GO:0006606\_protein\_import\_into\_nucleus | SIX3 | 44 | 1 | 3.608934 | -0.613199 | 366 | 259.37 | 0.708661 |
| GO:0051170\_nuclear\_import | SIX3 | 44 | 1 | 3.608934 | -0.613199 | 366 | 259.37 | 0.708661 |
| GO:0060485\_mesenchyme\_development | HIF1A | 44 | 1 | 3.608934 | -0.613199 | 366 | 259.37 | 0.708661 |
| GO:0034103\_regulation\_of\_tissue\_remodeling | SOX2 | 45 | 1 | 3.528736 | -0.604712 | 368 | 261.41 | 0.710353 |
| GO:0046546\_development\_of\_primary\_male\_sexual\_characteristics | SOX2 | 45 | 1 | 3.528736 | -0.604712 | 368 | 261.41 | 0.710353 |
| GO:0007612\_learning | PTN | 46 | 1 | 3.452024 | -0.596438 | 372 | 264.56 | 0.711183 |
| GO:0009581\_detection\_of\_external\_stimulus | SOX2 | 46 | 1 | 3.452024 | -0.596438 | 372 | 264.56 | 0.711183 |
| GO:0030218\_erythrocyte\_differentiation | HIF1A | 46 | 1 | 3.452024 | -0.596438 | 372 | 264.56 | 0.711183 |
| GO:0042063\_gliogenesis | PHGDH | 46 | 1 | 3.452024 | -0.596438 | 372 | 264.56 | 0.711183 |
| GO:0008285\_negative\_regulation\_of\_cell\_proliferation | DDR1 | 155 | 2 | 2.048943 | -0.593125 | 373 | 265.15 | 0.710858 |
| GO:0008285\_negative\_regulation\_of\_cell\_proliferation | BTG1 | 155 | 2 | 2.048943 | -0.593125 | 373 | 265.15 | 0.710858 |
| GO:0006396\_RNA\_processing | SF3A2 | 47 | 1 | 3.378577 | -0.588367 | 377 | 268.5 | 0.712202 |
| GO:0031667\_response\_to\_nutrient\_levels | SOX2 | 47 | 1 | 3.378577 | -0.588367 | 377 | 268.5 | 0.712202 |
| GO:0034754\_cellular\_hormone\_metabolic\_process | ALDH1A1 | 47 | 1 | 3.378577 | -0.588367 | 377 | 268.5 | 0.712202 |
| GO:0048871\_multicellular\_organismal\_homeostasis | FH1 | 47 | 1 | 3.378577 | -0.588367 | 377 | 268.5 | 0.712202 |
| GO:0006950\_response\_to\_stress | GPX1 | 549 | 5 | 1.446203 | -0.586443 | 378 | 268.62 | 0.710635 |
| GO:0006950\_response\_to\_stress | HIF1A | 549 | 5 | 1.446203 | -0.586443 | 378 | 268.62 | 0.710635 |
| GO:0006950\_response\_to\_stress | EIF2S1 | 549 | 5 | 1.446203 | -0.586443 | 378 | 268.62 | 0.710635 |
| GO:0006950\_response\_to\_stress | POLD1 | 549 | 5 | 1.446203 | -0.586443 | 378 | 268.62 | 0.710635 |
| GO:0006950\_response\_to\_stress | TRIP13 | 549 | 5 | 1.446203 | -0.586443 | 378 | 268.62 | 0.710635 |
| GO:0051704\_multi-organism\_process | GPX1 | 157 | 2 | 2.022842 | -0.585157 | 379 | 268.86 | 0.709393 |
| GO:0051704\_multi-organism\_process | DDR1 | 157 | 2 | 2.022842 | -0.585157 | 379 | 268.86 | 0.709393 |
| GO:0048514\_blood\_vessel\_morphogenesis | GPX1 | 158 | 2 | 2.010039 | -0.581225 | 380 | 269.72 | 0.709789 |
| GO:0048514\_blood\_vessel\_morphogenesis | HIF1A | 158 | 2 | 2.010039 | -0.581225 | 380 | 269.72 | 0.709789 |
| GO:0007498\_mesoderm\_development | PRKACA | 48 | 1 | 3.308190 | -0.580493 | 384 | 271.72 | 0.707604 |
| GO:0019318\_hexose\_metabolic\_process | TPI1 | 48 | 1 | 3.308190 | -0.580493 | 384 | 271.72 | 0.707604 |
| GO:0034504\_protein\_localization\_in\_nucleus | SIX3 | 48 | 1 | 3.308190 | -0.580493 | 384 | 271.72 | 0.707604 |
| GO:0046849\_bone\_remodeling | SOX2 | 48 | 1 | 3.308190 | -0.580493 | 384 | 271.72 | 0.707604 |
| GO:0006629\_lipid\_metabolic\_process | ALDH1A1 | 285 | 3 | 1.671506 | -0.575338 | 385 | 272.34 | 0.707377 |
| GO:0006629\_lipid\_metabolic\_process | GPX1 | 285 | 3 | 1.671506 | -0.575338 | 385 | 272.34 | 0.707377 |
| GO:0006629\_lipid\_metabolic\_process | PHGDH | 285 | 3 | 1.671506 | -0.575338 | 385 | 272.34 | 0.707377 |
| GO:0003015\_heart\_process | GPX1 | 49 | 1 | 3.240676 | -0.572805 | 391 | 275.08 | 0.703529 |
| GO:0021543\_pallium\_development | SOX2 | 49 | 1 | 3.240676 | -0.572805 | 391 | 275.08 | 0.703529 |
| GO:0034101\_erythrocyte\_homeostasis | HIF1A | 49 | 1 | 3.240676 | -0.572805 | 391 | 275.08 | 0.703529 |
| GO:0046661\_male\_sex\_differentiation | SOX2 | 49 | 1 | 3.240676 | -0.572805 | 391 | 275.08 | 0.703529 |
| GO:0048741\_skeletal\_muscle\_fiber\_development | GPX1 | 49 | 1 | 3.240676 | -0.572805 | 391 | 275.08 | 0.703529 |
| GO:0060047\_heart\_contraction | GPX1 | 49 | 1 | 3.240676 | -0.572805 | 391 | 275.08 | 0.703529 |
| GO:0009628\_response\_to\_abiotic\_stimulus | GPX1 | 162 | 2 | 1.960409 | -0.565835 | 392 | 275.93 | 0.703903 |
| GO:0009628\_response\_to\_abiotic\_stimulus | SOX2 | 162 | 2 | 1.960409 | -0.565835 | 392 | 275.93 | 0.703903 |
| GO:0017038\_protein\_import | SIX3 | 50 | 1 | 3.175862 | -0.565298 | 394 | 277.73 | 0.704898 |
| GO:0051606\_detection\_of\_stimulus | SOX2 | 50 | 1 | 3.175862 | -0.565298 | 394 | 277.73 | 0.704898 |
| GO:0032880\_regulation\_of\_protein\_localization | PRKACA | 51 | 1 | 3.113590 | -0.557963 | 396 | 281.3 | 0.710354 |
| GO:0048747\_muscle\_fiber\_development | GPX1 | 51 | 1 | 3.113590 | -0.557963 | 396 | 281.3 | 0.710354 |
| GO:0048518\_positive\_regulation\_of\_biological\_process | ALDH1A1 | 995 | 8 | 1.276728 | -0.555053 | 397 | 281.41 | 0.708841 |
| GO:0048518\_positive\_regulation\_of\_biological\_process | GPX1 | 995 | 8 | 1.276728 | -0.555053 | 397 | 281.41 | 0.708841 |
| GO:0048518\_positive\_regulation\_of\_biological\_process | HIF1A | 995 | 8 | 1.276728 | -0.555053 | 397 | 281.41 | 0.708841 |
| GO:0048518\_positive\_regulation\_of\_biological\_process | MARCKSL1 | 995 | 8 | 1.276728 | -0.555053 | 397 | 281.41 | 0.708841 |
| GO:0048518\_positive\_regulation\_of\_biological\_process | SOX2 | 995 | 8 | 1.276728 | -0.555053 | 397 | 281.41 | 0.708841 |
| GO:0048518\_positive\_regulation\_of\_biological\_process | SIX3 | 995 | 8 | 1.276728 | -0.555053 | 397 | 281.41 | 0.708841 |
| GO:0048518\_positive\_regulation\_of\_biological\_process | PRKACA | 995 | 8 | 1.276728 | -0.555053 | 397 | 281.41 | 0.708841 |
| GO:0048518\_positive\_regulation\_of\_biological\_process | CNOT7 | 995 | 8 | 1.276728 | -0.555053 | 397 | 281.41 | 0.708841 |
| GO:0043065\_positive\_regulation\_of\_apoptosis | ALDH1A1 | 166 | 2 | 1.913170 | -0.550967 | 398 | 282.5 | 0.709799 |
| GO:0043065\_positive\_regulation\_of\_apoptosis | GPX1 | 166 | 2 | 1.913170 | -0.550967 | 398 | 282.5 | 0.709799 |
| GO:0010608\_posttranscriptional\_regulation\_of\_gene\_expression | EIF2S1 | 52 | 1 | 3.053714 | -0.550794 | 400 | 283.75 | 0.709375 |
| GO:0048585\_negative\_regulation\_of\_response\_to\_stimulus | GPX1 | 52 | 1 | 3.053714 | -0.550794 | 400 | 283.75 | 0.709375 |
| GO:0010942\_positive\_regulation\_of\_cell\_death | ALDH1A1 | 167 | 2 | 1.901714 | -0.547328 | 402 | 285.42 | 0.710000 |
| GO:0010942\_positive\_regulation\_of\_cell\_death | GPX1 | 167 | 2 | 1.901714 | -0.547328 | 402 | 285.42 | 0.710000 |
| GO:0043068\_positive\_regulation\_of\_programmed\_cell\_death | ALDH1A1 | 167 | 2 | 1.901714 | -0.547328 | 402 | 285.42 | 0.710000 |
| GO:0043068\_positive\_regulation\_of\_programmed\_cell\_death | GPX1 | 167 | 2 | 1.901714 | -0.547328 | 402 | 285.42 | 0.710000 |
| GO:0006139\_nucleobase\_\_nucleoside\_\_nucleotide\_and\_nucleic\_acid\_metabolic\_process | HIF1A | 1002 | 8 | 1.267809 | -0.544339 | 403 | 285.57 | 0.708610 |
| GO:0006139\_nucleobase\_\_nucleoside\_\_nucleotide\_and\_nucleic\_acid\_metabolic\_process | MCM7 | 1002 | 8 | 1.267809 | -0.544339 | 403 | 285.57 | 0.708610 |
| GO:0006139\_nucleobase\_\_nucleoside\_\_nucleotide\_and\_nucleic\_acid\_metabolic\_process | POLD1 | 1002 | 8 | 1.267809 | -0.544339 | 403 | 285.57 | 0.708610 |
| GO:0006139\_nucleobase\_\_nucleoside\_\_nucleotide\_and\_nucleic\_acid\_metabolic\_process | SOX2 | 1002 | 8 | 1.267809 | -0.544339 | 403 | 285.57 | 0.708610 |
| GO:0006139\_nucleobase\_\_nucleoside\_\_nucleotide\_and\_nucleic\_acid\_metabolic\_process | SIX3 | 1002 | 8 | 1.267809 | -0.544339 | 403 | 285.57 | 0.708610 |
| GO:0006139\_nucleobase\_\_nucleoside\_\_nucleotide\_and\_nucleic\_acid\_metabolic\_process | SF3A2 | 1002 | 8 | 1.267809 | -0.544339 | 403 | 285.57 | 0.708610 |
| GO:0006139\_nucleobase\_\_nucleoside\_\_nucleotide\_and\_nucleic\_acid\_metabolic\_process | CNOT7 | 1002 | 8 | 1.267809 | -0.544339 | 403 | 285.57 | 0.708610 |
| GO:0006139\_nucleobase\_\_nucleoside\_\_nucleotide\_and\_nucleic\_acid\_metabolic\_process | TRIP13 | 1002 | 8 | 1.267809 | -0.544339 | 403 | 285.57 | 0.708610 |
| GO:0050905\_neuromuscular\_process | SOX2 | 53 | 1 | 2.996096 | -0.543784 | 404 | 287.77 | 0.712302 |
| GO:0010604\_positive\_regulation\_of\_macromolecule\_metabolic\_process | HIF1A | 433 | 4 | 1.466911 | -0.540149 | 405 | 288.04 | 0.711210 |
| GO:0010604\_positive\_regulation\_of\_macromolecule\_metabolic\_process | SOX2 | 433 | 4 | 1.466911 | -0.540149 | 405 | 288.04 | 0.711210 |
| GO:0010604\_positive\_regulation\_of\_macromolecule\_metabolic\_process | SIX3 | 433 | 4 | 1.466911 | -0.540149 | 405 | 288.04 | 0.711210 |
| GO:0010604\_positive\_regulation\_of\_macromolecule\_metabolic\_process | CNOT7 | 433 | 4 | 1.466911 | -0.540149 | 405 | 288.04 | 0.711210 |
| GO:0007275\_multicellular\_organismal\_development | HSP90AB1 | 1760 | 13 | 1.172904 | -0.537157 | 406 | 288.3 | 0.710099 |
| GO:0007275\_multicellular\_organismal\_development | SOX2 | 1760 | 13 | 1.172904 | -0.537157 | 406 | 288.3 | 0.710099 |
| GO:0007275\_multicellular\_organismal\_development | SIX3 | 1760 | 13 | 1.172904 | -0.537157 | 406 | 288.3 | 0.710099 |
| GO:0007275\_multicellular\_organismal\_development | ALDH1A1 | 1760 | 13 | 1.172904 | -0.537157 | 406 | 288.3 | 0.710099 |
| GO:0007275\_multicellular\_organismal\_development | DDR1 | 1760 | 13 | 1.172904 | -0.537157 | 406 | 288.3 | 0.710099 |
| GO:0007275\_multicellular\_organismal\_development | GPX1 | 1760 | 13 | 1.172904 | -0.537157 | 406 | 288.3 | 0.710099 |
| GO:0007275\_multicellular\_organismal\_development | TPI1 | 1760 | 13 | 1.172904 | -0.537157 | 406 | 288.3 | 0.710099 |
| GO:0007275\_multicellular\_organismal\_development | HIF1A | 1760 | 13 | 1.172904 | -0.537157 | 406 | 288.3 | 0.710099 |
| GO:0007275\_multicellular\_organismal\_development | NME1 | 1760 | 13 | 1.172904 | -0.537157 | 406 | 288.3 | 0.710099 |
| GO:0007275\_multicellular\_organismal\_development | PHGDH | 1760 | 13 | 1.172904 | -0.537157 | 406 | 288.3 | 0.710099 |
| GO:0007275\_multicellular\_organismal\_development | PTN | 1760 | 13 | 1.172904 | -0.537157 | 406 | 288.3 | 0.710099 |
| GO:0007275\_multicellular\_organismal\_development | PRKACA | 1760 | 13 | 1.172904 | -0.537157 | 406 | 288.3 | 0.710099 |
| GO:0007275\_multicellular\_organismal\_development | PBX3 | 1760 | 13 | 1.172904 | -0.537157 | 406 | 288.3 | 0.710099 |
| GO:0048598\_embryonic\_morphogenesis | ALDH1A1 | 299 | 3 | 1.593242 | -0.537104 | 407 | 288.47 | 0.708771 |
| GO:0048598\_embryonic\_morphogenesis | SOX2 | 299 | 3 | 1.593242 | -0.537104 | 407 | 288.47 | 0.708771 |
| GO:0048598\_embryonic\_morphogenesis | PRKACA | 299 | 3 | 1.593242 | -0.537104 | 407 | 288.47 | 0.708771 |
| GO:0006412\_translation | EIF2S1 | 54 | 1 | 2.940613 | -0.536928 | 408 | 290.7 | 0.712500 |
| GO:0006357\_regulation\_of\_transcription\_from\_RNA\_polymerase\_II\_promoter | HIF1A | 435 | 4 | 1.460166 | -0.535778 | 409 | 291.24 | 0.712078 |
| GO:0006357\_regulation\_of\_transcription\_from\_RNA\_polymerase\_II\_promoter | SOX2 | 435 | 4 | 1.460166 | -0.535778 | 409 | 291.24 | 0.712078 |
| GO:0006357\_regulation\_of\_transcription\_from\_RNA\_polymerase\_II\_promoter | SIX3 | 435 | 4 | 1.460166 | -0.535778 | 409 | 291.24 | 0.712078 |
| GO:0006357\_regulation\_of\_transcription\_from\_RNA\_polymerase\_II\_promoter | CNOT7 | 435 | 4 | 1.460166 | -0.535778 | 409 | 291.24 | 0.712078 |
| GO:0048731\_system\_development | ALDH1A1 | 1609 | 12 | 1.184287 | -0.534421 | 410 | 291.45 | 0.710854 |
| GO:0048731\_system\_development | HSP90AB1 | 1609 | 12 | 1.184287 | -0.534421 | 410 | 291.45 | 0.710854 |
| GO:0048731\_system\_development | DDR1 | 1609 | 12 | 1.184287 | -0.534421 | 410 | 291.45 | 0.710854 |
| GO:0048731\_system\_development | GPX1 | 1609 | 12 | 1.184287 | -0.534421 | 410 | 291.45 | 0.710854 |
| GO:0048731\_system\_development | HIF1A | 1609 | 12 | 1.184287 | -0.534421 | 410 | 291.45 | 0.710854 |
| GO:0048731\_system\_development | NME1 | 1609 | 12 | 1.184287 | -0.534421 | 410 | 291.45 | 0.710854 |
| GO:0048731\_system\_development | SOX2 | 1609 | 12 | 1.184287 | -0.534421 | 410 | 291.45 | 0.710854 |
| GO:0048731\_system\_development | PHGDH | 1609 | 12 | 1.184287 | -0.534421 | 410 | 291.45 | 0.710854 |
| GO:0048731\_system\_development | SIX3 | 1609 | 12 | 1.184287 | -0.534421 | 410 | 291.45 | 0.710854 |
| GO:0048731\_system\_development | PTN | 1609 | 12 | 1.184287 | -0.534421 | 410 | 291.45 | 0.710854 |
| GO:0048731\_system\_development | PRKACA | 1609 | 12 | 1.184287 | -0.534421 | 410 | 291.45 | 0.710854 |
| GO:0048731\_system\_development | PBX3 | 1609 | 12 | 1.184287 | -0.534421 | 410 | 291.45 | 0.710854 |
| GO:0006310\_DNA\_recombination | TRIP13 | 55 | 1 | 2.887147 | -0.530219 | 413 | 293.55 | 0.710775 |
| GO:0007126\_meiosis | TRIP13 | 55 | 1 | 2.887147 | -0.530219 | 413 | 293.55 | 0.710775 |
| GO:0051327\_M\_phase\_of\_meiotic\_cell\_cycle | TRIP13 | 55 | 1 | 2.887147 | -0.530219 | 413 | 293.55 | 0.710775 |
| GO:0007600\_sensory\_perception | GPX1 | 172 | 2 | 1.846431 | -0.529586 | 415 | 294.16 | 0.708819 |
| GO:0007600\_sensory\_perception | SOX2 | 172 | 2 | 1.846431 | -0.529586 | 415 | 294.16 | 0.708819 |
| GO:0009611\_response\_to\_wounding | GPX1 | 172 | 2 | 1.846431 | -0.529586 | 415 | 294.16 | 0.708819 |
| GO:0009611\_response\_to\_wounding | HIF1A | 172 | 2 | 1.846431 | -0.529586 | 415 | 294.16 | 0.708819 |
| GO:0044248\_cellular\_catabolic\_process | MTHFD1 | 173 | 2 | 1.835758 | -0.526126 | 416 | 294.97 | 0.709062 |
| GO:0044248\_cellular\_catabolic\_process | GPX1 | 173 | 2 | 1.835758 | -0.526126 | 416 | 294.97 | 0.709062 |
| GO:0002683\_negative\_regulation\_of\_immune\_system\_process | GPX1 | 56 | 1 | 2.835591 | -0.523653 | 420 | 297.63 | 0.708643 |
| GO:0006790\_sulfur\_metabolic\_process | PHGDH | 56 | 1 | 2.835591 | -0.523653 | 420 | 297.63 | 0.708643 |
| GO:0046486\_glycerolipid\_metabolic\_process | GPX1 | 56 | 1 | 2.835591 | -0.523653 | 420 | 297.63 | 0.708643 |
| GO:0051321\_meiotic\_cell\_cycle | TRIP13 | 56 | 1 | 2.835591 | -0.523653 | 420 | 297.63 | 0.708643 |
| GO:0031325\_positive\_regulation\_of\_cellular\_metabolic\_process | HIF1A | 442 | 4 | 1.437042 | -0.520758 | 421 | 297.79 | 0.707340 |
| GO:0031325\_positive\_regulation\_of\_cellular\_metabolic\_process | SOX2 | 442 | 4 | 1.437042 | -0.520758 | 421 | 297.79 | 0.707340 |
| GO:0031325\_positive\_regulation\_of\_cellular\_metabolic\_process | SIX3 | 442 | 4 | 1.437042 | -0.520758 | 421 | 297.79 | 0.707340 |
| GO:0031325\_positive\_regulation\_of\_cellular\_metabolic\_process | CNOT7 | 442 | 4 | 1.437042 | -0.520758 | 421 | 297.79 | 0.707340 |
| GO:0015031\_protein\_transport | SIX3 | 175 | 2 | 1.814778 | -0.519289 | 422 | 298.33 | 0.706943 |
| GO:0015031\_protein\_transport | PRKACA | 175 | 2 | 1.814778 | -0.519289 | 422 | 298.33 | 0.706943 |
| GO:0001892\_embryonic\_placenta\_development | HIF1A | 57 | 1 | 2.785844 | -0.517224 | 430 | 301.77 | 0.701791 |
| GO:0008344\_adult\_locomotory\_behavior | PBX3 | 57 | 1 | 2.785844 | -0.517224 | 430 | 301.77 | 0.701791 |
| GO:0018108\_peptidyl-tyrosine\_phosphorylation | DDR1 | 57 | 1 | 2.785844 | -0.517224 | 430 | 301.77 | 0.701791 |
| GO:0018212\_peptidyl-tyrosine\_modification | DDR1 | 57 | 1 | 2.785844 | -0.517224 | 430 | 301.77 | 0.701791 |
| GO:0033365\_protein\_localization\_in\_organelle | SIX3 | 57 | 1 | 2.785844 | -0.517224 | 430 | 301.77 | 0.701791 |
| GO:0042472\_inner\_ear\_morphogenesis | SOX2 | 57 | 1 | 2.785844 | -0.517224 | 430 | 301.77 | 0.701791 |
| GO:0043523\_regulation\_of\_neuron\_apoptosis | GPX1 | 57 | 1 | 2.785844 | -0.517224 | 430 | 301.77 | 0.701791 |
| GO:0045444\_fat\_cell\_differentiation | GPX1 | 57 | 1 | 2.785844 | -0.517224 | 430 | 301.77 | 0.701791 |
| GO:0006366\_transcription\_from\_RNA\_polymerase\_II\_promoter | HIF1A | 444 | 4 | 1.430568 | -0.516545 | 431 | 302.02 | 0.700742 |
| GO:0006366\_transcription\_from\_RNA\_polymerase\_II\_promoter | SOX2 | 444 | 4 | 1.430568 | -0.516545 | 431 | 302.02 | 0.700742 |
| GO:0006366\_transcription\_from\_RNA\_polymerase\_II\_promoter | SIX3 | 444 | 4 | 1.430568 | -0.516545 | 431 | 302.02 | 0.700742 |
| GO:0006366\_transcription\_from\_RNA\_polymerase\_II\_promoter | CNOT7 | 444 | 4 | 1.430568 | -0.516545 | 431 | 302.02 | 0.700742 |
| GO:0050804\_regulation\_of\_synaptic\_transmission | PRKACA | 58 | 1 | 2.737812 | -0.510928 | 432 | 304.2 | 0.704167 |
| GO:0030334\_regulation\_of\_cell\_migration | HIF1A | 59 | 1 | 2.691409 | -0.504760 | 435 | 306.45 | 0.704483 |
| GO:0035270\_endocrine\_system\_development | SOX2 | 59 | 1 | 2.691409 | -0.504760 | 435 | 306.45 | 0.704483 |
| GO:0048469\_cell\_maturation | TRIP13 | 59 | 1 | 2.691409 | -0.504760 | 435 | 306.45 | 0.704483 |
| GO:0045184\_establishment\_of\_protein\_localization | SIX3 | 180 | 2 | 1.764368 | -0.502678 | 436 | 306.79 | 0.703647 |
| GO:0045184\_establishment\_of\_protein\_localization | PRKACA | 180 | 2 | 1.764368 | -0.502678 | 436 | 306.79 | 0.703647 |
| GO:0009991\_response\_to\_extracellular\_stimulus | SOX2 | 61 | 1 | 2.603166 | -0.492789 | 438 | 309.8 | 0.707306 |
| GO:0051969\_regulation\_of\_transmission\_of\_nerve\_impulse | PRKACA | 61 | 1 | 2.603166 | -0.492789 | 438 | 309.8 | 0.707306 |
| GO:0009893\_positive\_regulation\_of\_metabolic\_process | HIF1A | 458 | 4 | 1.386839 | -0.487995 | 439 | 310.95 | 0.708314 |
| GO:0009893\_positive\_regulation\_of\_metabolic\_process | SOX2 | 458 | 4 | 1.386839 | -0.487995 | 439 | 310.95 | 0.708314 |
| GO:0009893\_positive\_regulation\_of\_metabolic\_process | SIX3 | 458 | 4 | 1.386839 | -0.487995 | 439 | 310.95 | 0.708314 |
| GO:0009893\_positive\_regulation\_of\_metabolic\_process | CNOT7 | 458 | 4 | 1.386839 | -0.487995 | 439 | 310.95 | 0.708314 |
| GO:0030155\_regulation\_of\_cell\_adhesion | DDR1 | 62 | 1 | 2.561179 | -0.486980 | 440 | 313.19 | 0.711795 |
| GO:0048522\_positive\_regulation\_of\_cellular\_process | ALDH1A1 | 895 | 7 | 1.241957 | -0.484216 | 441 | 313.64 | 0.711202 |
| GO:0048522\_positive\_regulation\_of\_cellular\_process | GPX1 | 895 | 7 | 1.241957 | -0.484216 | 441 | 313.64 | 0.711202 |
| GO:0048522\_positive\_regulation\_of\_cellular\_process | HIF1A | 895 | 7 | 1.241957 | -0.484216 | 441 | 313.64 | 0.711202 |
| GO:0048522\_positive\_regulation\_of\_cellular\_process | MARCKSL1 | 895 | 7 | 1.241957 | -0.484216 | 441 | 313.64 | 0.711202 |
| GO:0048522\_positive\_regulation\_of\_cellular\_process | SOX2 | 895 | 7 | 1.241957 | -0.484216 | 441 | 313.64 | 0.711202 |
| GO:0048522\_positive\_regulation\_of\_cellular\_process | SIX3 | 895 | 7 | 1.241957 | -0.484216 | 441 | 313.64 | 0.711202 |
| GO:0048522\_positive\_regulation\_of\_cellular\_process | CNOT7 | 895 | 7 | 1.241957 | -0.484216 | 441 | 313.64 | 0.711202 |
| GO:0007369\_gastrulation | PRKACA | 63 | 1 | 2.520525 | -0.481282 | 443 | 315.96 | 0.713228 |
| GO:0051216\_cartilage\_development | HIF1A | 63 | 1 | 2.520525 | -0.481282 | 443 | 315.96 | 0.713228 |
| GO:0006952\_defense\_response | GPX1 | 187 | 2 | 1.698322 | -0.480516 | 444 | 316.17 | 0.712095 |
| GO:0006952\_defense\_response | HIF1A | 187 | 2 | 1.698322 | -0.480516 | 444 | 316.17 | 0.712095 |
| GO:0031644\_regulation\_of\_neurological\_system\_process | PRKACA | 64 | 1 | 2.481142 | -0.475692 | 445 | 317.61 | 0.713730 |
| GO:0034645\_cellular\_macromolecule\_biosynthetic\_process | HIF1A | 901 | 7 | 1.233687 | -0.475507 | 446 | 317.75 | 0.712444 |
| GO:0034645\_cellular\_macromolecule\_biosynthetic\_process | MCM7 | 901 | 7 | 1.233687 | -0.475507 | 446 | 317.75 | 0.712444 |
| GO:0034645\_cellular\_macromolecule\_biosynthetic\_process | EIF2S1 | 901 | 7 | 1.233687 | -0.475507 | 446 | 317.75 | 0.712444 |
| GO:0034645\_cellular\_macromolecule\_biosynthetic\_process | POLD1 | 901 | 7 | 1.233687 | -0.475507 | 446 | 317.75 | 0.712444 |
| GO:0034645\_cellular\_macromolecule\_biosynthetic\_process | SOX2 | 901 | 7 | 1.233687 | -0.475507 | 446 | 317.75 | 0.712444 |
| GO:0034645\_cellular\_macromolecule\_biosynthetic\_process | SIX3 | 901 | 7 | 1.233687 | -0.475507 | 446 | 317.75 | 0.712444 |
| GO:0034645\_cellular\_macromolecule\_biosynthetic\_process | CNOT7 | 901 | 7 | 1.233687 | -0.475507 | 446 | 317.75 | 0.712444 |
| GO:0042471\_ear\_morphogenesis | SOX2 | 65 | 1 | 2.442971 | -0.470207 | 447 | 318.68 | 0.712931 |
| GO:0010467\_gene\_expression | HIF1A | 905 | 7 | 1.228234 | -0.469776 | 448 | 319.04 | 0.712143 |
| GO:0010467\_gene\_expression | EIF2S1 | 905 | 7 | 1.228234 | -0.469776 | 448 | 319.04 | 0.712143 |
| GO:0010467\_gene\_expression | SOX2 | 905 | 7 | 1.228234 | -0.469776 | 448 | 319.04 | 0.712143 |
| GO:0010467\_gene\_expression | PHGDH | 905 | 7 | 1.228234 | -0.469776 | 448 | 319.04 | 0.712143 |
| GO:0010467\_gene\_expression | SIX3 | 905 | 7 | 1.228234 | -0.469776 | 448 | 319.04 | 0.712143 |
| GO:0010467\_gene\_expression | SF3A2 | 905 | 7 | 1.228234 | -0.469776 | 448 | 319.04 | 0.712143 |
| GO:0010467\_gene\_expression | CNOT7 | 905 | 7 | 1.228234 | -0.469776 | 448 | 319.04 | 0.712143 |
| GO:0031589\_cell-substrate\_adhesion | DDR1 | 66 | 1 | 2.405956 | -0.464824 | 450 | 320.67 | 0.712600 |
| GO:0051402\_neuron\_apoptosis | GPX1 | 66 | 1 | 2.405956 | -0.464824 | 450 | 320.67 | 0.712600 |
| GO:0009059\_macromolecule\_biosynthetic\_process | MCM7 | 910 | 7 | 1.221485 | -0.462695 | 451 | 321.04 | 0.711840 |
| GO:0009059\_macromolecule\_biosynthetic\_process | HIF1A | 910 | 7 | 1.221485 | -0.462695 | 451 | 321.04 | 0.711840 |
| GO:0009059\_macromolecule\_biosynthetic\_process | POLD1 | 910 | 7 | 1.221485 | -0.462695 | 451 | 321.04 | 0.711840 |
| GO:0009059\_macromolecule\_biosynthetic\_process | EIF2S1 | 910 | 7 | 1.221485 | -0.462695 | 451 | 321.04 | 0.711840 |
| GO:0009059\_macromolecule\_biosynthetic\_process | SOX2 | 910 | 7 | 1.221485 | -0.462695 | 451 | 321.04 | 0.711840 |
| GO:0009059\_macromolecule\_biosynthetic\_process | SIX3 | 910 | 7 | 1.221485 | -0.462695 | 451 | 321.04 | 0.711840 |
| GO:0009059\_macromolecule\_biosynthetic\_process | CNOT7 | 910 | 7 | 1.221485 | -0.462695 | 451 | 321.04 | 0.711840 |
| GO:0003007\_heart\_morphogenesis | HIF1A | 67 | 1 | 2.370046 | -0.459539 | 454 | 323.87 | 0.713370 |
| GO:0031347\_regulation\_of\_defense\_response | GPX1 | 67 | 1 | 2.370046 | -0.459539 | 454 | 323.87 | 0.713370 |
| GO:0042445\_hormone\_metabolic\_process | ALDH1A1 | 67 | 1 | 2.370046 | -0.459539 | 454 | 323.87 | 0.713370 |
| GO:0048513\_organ\_development | ALDH1A1 | 1365 | 10 | 1.163319 | -0.458859 | 455 | 324.05 | 0.712198 |
| GO:0048513\_organ\_development | HSP90AB1 | 1365 | 10 | 1.163319 | -0.458859 | 455 | 324.05 | 0.712198 |
| GO:0048513\_organ\_development | DDR1 | 1365 | 10 | 1.163319 | -0.458859 | 455 | 324.05 | 0.712198 |
| GO:0048513\_organ\_development | GPX1 | 1365 | 10 | 1.163319 | -0.458859 | 455 | 324.05 | 0.712198 |
| GO:0048513\_organ\_development | HIF1A | 1365 | 10 | 1.163319 | -0.458859 | 455 | 324.05 | 0.712198 |
| GO:0048513\_organ\_development | NME1 | 1365 | 10 | 1.163319 | -0.458859 | 455 | 324.05 | 0.712198 |
| GO:0048513\_organ\_development | SOX2 | 1365 | 10 | 1.163319 | -0.458859 | 455 | 324.05 | 0.712198 |
| GO:0048513\_organ\_development | SIX3 | 1365 | 10 | 1.163319 | -0.458859 | 455 | 324.05 | 0.712198 |
| GO:0048513\_organ\_development | PTN | 1365 | 10 | 1.163319 | -0.458859 | 455 | 324.05 | 0.712198 |
| GO:0048513\_organ\_development | PRKACA | 1365 | 10 | 1.163319 | -0.458859 | 455 | 324.05 | 0.712198 |
| GO:0042692\_muscle\_cell\_differentiation | GPX1 | 68 | 1 | 2.335193 | -0.454350 | 456 | 326.21 | 0.715373 |
| GO:0005996\_monosaccharide\_metabolic\_process | TPI1 | 69 | 1 | 2.301349 | -0.449253 | 458 | 328.26 | 0.716725 |
| GO:0032101\_regulation\_of\_response\_to\_external\_stimulus | GPX1 | 69 | 1 | 2.301349 | -0.449253 | 458 | 328.26 | 0.716725 |
| GO:0002009\_morphogenesis\_of\_an\_epithelium | ALDH1A1 | 198 | 2 | 1.603971 | -0.448051 | 460 | 328.88 | 0.714957 |
| GO:0002009\_morphogenesis\_of\_an\_epithelium | DDR1 | 198 | 2 | 1.603971 | -0.448051 | 460 | 328.88 | 0.714957 |
| GO:0060429\_epithelium\_development | ALDH1A1 | 198 | 2 | 1.603971 | -0.448051 | 460 | 328.88 | 0.714957 |
| GO:0060429\_epithelium\_development | DDR1 | 198 | 2 | 1.603971 | -0.448051 | 460 | 328.88 | 0.714957 |
| GO:0007611\_learning\_or\_memory | PTN | 70 | 1 | 2.268473 | -0.444247 | 462 | 330.85 | 0.716126 |
| GO:0009617\_response\_to\_bacterium | GPX1 | 70 | 1 | 2.268473 | -0.444247 | 462 | 330.85 | 0.716126 |
| GO:0009605\_response\_to\_external\_stimulus | GPX1 | 339 | 3 | 1.405249 | -0.442352 | 463 | 330.99 | 0.714881 |
| GO:0009605\_response\_to\_external\_stimulus | HIF1A | 339 | 3 | 1.405249 | -0.442352 | 463 | 330.99 | 0.714881 |
| GO:0009605\_response\_to\_external\_stimulus | SOX2 | 339 | 3 | 1.405249 | -0.442352 | 463 | 330.99 | 0.714881 |
| GO:0048856\_anatomical\_structure\_development | HSP90AB1 | 1688 | 12 | 1.128861 | -0.440176 | 464 | 331.8 | 0.715086 |
| GO:0048856\_anatomical\_structure\_development | ALDH1A1 | 1688 | 12 | 1.128861 | -0.440176 | 464 | 331.8 | 0.715086 |
| GO:0048856\_anatomical\_structure\_development | GPX1 | 1688 | 12 | 1.128861 | -0.440176 | 464 | 331.8 | 0.715086 |
| GO:0048856\_anatomical\_structure\_development | DDR1 | 1688 | 12 | 1.128861 | -0.440176 | 464 | 331.8 | 0.715086 |
| GO:0048856\_anatomical\_structure\_development | HIF1A | 1688 | 12 | 1.128861 | -0.440176 | 464 | 331.8 | 0.715086 |
| GO:0048856\_anatomical\_structure\_development | NME1 | 1688 | 12 | 1.128861 | -0.440176 | 464 | 331.8 | 0.715086 |
| GO:0048856\_anatomical\_structure\_development | SOX2 | 1688 | 12 | 1.128861 | -0.440176 | 464 | 331.8 | 0.715086 |
| GO:0048856\_anatomical\_structure\_development | PHGDH | 1688 | 12 | 1.128861 | -0.440176 | 464 | 331.8 | 0.715086 |
| GO:0048856\_anatomical\_structure\_development | SIX3 | 1688 | 12 | 1.128861 | -0.440176 | 464 | 331.8 | 0.715086 |
| GO:0048856\_anatomical\_structure\_development | PTN | 1688 | 12 | 1.128861 | -0.440176 | 464 | 331.8 | 0.715086 |
| GO:0048856\_anatomical\_structure\_development | PRKACA | 1688 | 12 | 1.128861 | -0.440176 | 464 | 331.8 | 0.715086 |
| GO:0048856\_anatomical\_structure\_development | PBX3 | 1688 | 12 | 1.128861 | -0.440176 | 464 | 331.8 | 0.715086 |
| GO:0009100\_glycoprotein\_metabolic\_process | HIF1A | 71 | 1 | 2.236523 | -0.439328 | 466 | 333.28 | 0.715193 |
| GO:0016331\_morphogenesis\_of\_embryonic\_epithelium | ALDH1A1 | 71 | 1 | 2.236523 | -0.439328 | 466 | 333.28 | 0.715193 |
| GO:0010468\_regulation\_of\_gene\_expression | HIF1A | 778 | 6 | 1.224625 | -0.438876 | 467 | 333.43 | 0.713983 |
| GO:0010468\_regulation\_of\_gene\_expression | EIF2S1 | 778 | 6 | 1.224625 | -0.438876 | 467 | 333.43 | 0.713983 |
| GO:0010468\_regulation\_of\_gene\_expression | SOX2 | 778 | 6 | 1.224625 | -0.438876 | 467 | 333.43 | 0.713983 |
| GO:0010468\_regulation\_of\_gene\_expression | PHGDH | 778 | 6 | 1.224625 | -0.438876 | 467 | 333.43 | 0.713983 |
| GO:0010468\_regulation\_of\_gene\_expression | SIX3 | 778 | 6 | 1.224625 | -0.438876 | 467 | 333.43 | 0.713983 |
| GO:0010468\_regulation\_of\_gene\_expression | CNOT7 | 778 | 6 | 1.224625 | -0.438876 | 467 | 333.43 | 0.713983 |
| GO:0051649\_establishment\_of\_localization\_in\_cell | NPC2 | 342 | 3 | 1.392922 | -0.436006 | 468 | 333.59 | 0.712799 |
| GO:0051649\_establishment\_of\_localization\_in\_cell | SIX3 | 342 | 3 | 1.392922 | -0.436006 | 468 | 333.59 | 0.712799 |
| GO:0051649\_establishment\_of\_localization\_in\_cell | PRKACA | 342 | 3 | 1.392922 | -0.436006 | 468 | 333.59 | 0.712799 |
| GO:0016568\_chromatin\_modification | ACTL6A | 72 | 1 | 2.205460 | -0.434494 | 472 | 336.9 | 0.713771 |
| GO:0021915\_neural\_tube\_development | PHGDH | 72 | 1 | 2.205460 | -0.434494 | 472 | 336.9 | 0.713771 |
| GO:0040012\_regulation\_of\_locomotion | HIF1A | 72 | 1 | 2.205460 | -0.434494 | 472 | 336.9 | 0.713771 |
| GO:0048839\_inner\_ear\_development | SOX2 | 72 | 1 | 2.205460 | -0.434494 | 472 | 336.9 | 0.713771 |
| GO:0001568\_blood\_vessel\_development | GPX1 | 203 | 2 | 1.564464 | -0.434173 | 473 | 337.12 | 0.712727 |
| GO:0001568\_blood\_vessel\_development | HIF1A | 203 | 2 | 1.564464 | -0.434173 | 473 | 337.12 | 0.712727 |
| GO:0051270\_regulation\_of\_cell\_motion | HIF1A | 73 | 1 | 2.175248 | -0.429742 | 474 | 339.01 | 0.715211 |
| GO:0001944\_vasculature\_development | GPX1 | 208 | 2 | 1.526857 | -0.420805 | 476 | 341.28 | 0.716975 |
| GO:0001944\_vasculature\_development | HIF1A | 208 | 2 | 1.526857 | -0.420805 | 476 | 341.28 | 0.716975 |
| GO:0008284\_positive\_regulation\_of\_cell\_proliferation | MARCKSL1 | 208 | 2 | 1.526857 | -0.420805 | 476 | 341.28 | 0.716975 |
| GO:0008284\_positive\_regulation\_of\_cell\_proliferation | SOX2 | 208 | 2 | 1.526857 | -0.420805 | 476 | 341.28 | 0.716975 |
| GO:0007281\_germ\_cell\_development | TRIP13 | 75 | 1 | 2.117241 | -0.420478 | 479 | 342.35 | 0.714718 |
| GO:0048589\_developmental\_growth | GPX1 | 75 | 1 | 2.117241 | -0.420478 | 479 | 342.35 | 0.714718 |
| GO:0051050\_positive\_regulation\_of\_transport | PRKACA | 75 | 1 | 2.117241 | -0.420478 | 479 | 342.35 | 0.714718 |
| GO:0030182\_neuron\_differentiation | SOX2 | 356 | 3 | 1.338144 | -0.407638 | 480 | 345.19 | 0.719146 |
| GO:0030182\_neuron\_differentiation | PHGDH | 356 | 3 | 1.338144 | -0.407638 | 480 | 345.19 | 0.719146 |
| GO:0030182\_neuron\_differentiation | PBX3 | 356 | 3 | 1.338144 | -0.407638 | 480 | 345.19 | 0.719146 |
| GO:0007519\_skeletal\_muscle\_tissue\_development | GPX1 | 78 | 1 | 2.035809 | -0.407147 | 482 | 347.62 | 0.721203 |
| GO:0060538\_skeletal\_muscle\_organ\_development | GPX1 | 78 | 1 | 2.035809 | -0.407147 | 482 | 347.62 | 0.721203 |
| GO:0042981\_regulation\_of\_apoptosis | ALDH1A1 | 360 | 3 | 1.323276 | -0.399893 | 483 | 349.58 | 0.723768 |
| GO:0042981\_regulation\_of\_apoptosis | GPX1 | 360 | 3 | 1.323276 | -0.399893 | 483 | 349.58 | 0.723768 |
| GO:0042981\_regulation\_of\_apoptosis | HIF1A | 360 | 3 | 1.323276 | -0.399893 | 483 | 349.58 | 0.723768 |
| GO:0009653\_anatomical\_structure\_morphogenesis | ALDH1A1 | 958 | 7 | 1.160284 | -0.399212 | 484 | 349.74 | 0.722603 |
| GO:0009653\_anatomical\_structure\_morphogenesis | GPX1 | 958 | 7 | 1.160284 | -0.399212 | 484 | 349.74 | 0.722603 |
| GO:0009653\_anatomical\_structure\_morphogenesis | DDR1 | 958 | 7 | 1.160284 | -0.399212 | 484 | 349.74 | 0.722603 |
| GO:0009653\_anatomical\_structure\_morphogenesis | HIF1A | 958 | 7 | 1.160284 | -0.399212 | 484 | 349.74 | 0.722603 |
| GO:0009653\_anatomical\_structure\_morphogenesis | SOX2 | 958 | 7 | 1.160284 | -0.399212 | 484 | 349.74 | 0.722603 |
| GO:0009653\_anatomical\_structure\_morphogenesis | SIX3 | 958 | 7 | 1.160284 | -0.399212 | 484 | 349.74 | 0.722603 |
| GO:0009653\_anatomical\_structure\_morphogenesis | PRKACA | 958 | 7 | 1.160284 | -0.399212 | 484 | 349.74 | 0.722603 |
| GO:0006631\_fatty\_acid\_metabolic\_process | PHGDH | 80 | 1 | 1.984914 | -0.398614 | 485 | 351.53 | 0.724804 |
| GO:0016070\_RNA\_metabolic\_process | HIF1A | 658 | 5 | 1.206635 | -0.396751 | 486 | 352.36 | 0.725021 |
| GO:0016070\_RNA\_metabolic\_process | SOX2 | 658 | 5 | 1.206635 | -0.396751 | 486 | 352.36 | 0.725021 |
| GO:0016070\_RNA\_metabolic\_process | SIX3 | 658 | 5 | 1.206635 | -0.396751 | 486 | 352.36 | 0.725021 |
| GO:0016070\_RNA\_metabolic\_process | CNOT7 | 658 | 5 | 1.206635 | -0.396751 | 486 | 352.36 | 0.725021 |
| GO:0016070\_RNA\_metabolic\_process | SF3A2 | 658 | 5 | 1.206635 | -0.396751 | 486 | 352.36 | 0.725021 |
| GO:0021700\_developmental\_maturation | TRIP13 | 81 | 1 | 1.960409 | -0.394448 | 487 | 353.07 | 0.724990 |
| GO:0010941\_regulation\_of\_cell\_death | ALDH1A1 | 365 | 3 | 1.305149 | -0.390426 | 489 | 354.73 | 0.725419 |
| GO:0010941\_regulation\_of\_cell\_death | GPX1 | 365 | 3 | 1.305149 | -0.390426 | 489 | 354.73 | 0.725419 |
| GO:0010941\_regulation\_of\_cell\_death | HIF1A | 365 | 3 | 1.305149 | -0.390426 | 489 | 354.73 | 0.725419 |
| GO:0043067\_regulation\_of\_programmed\_cell\_death | ALDH1A1 | 365 | 3 | 1.305149 | -0.390426 | 489 | 354.73 | 0.725419 |
| GO:0043067\_regulation\_of\_programmed\_cell\_death | GPX1 | 365 | 3 | 1.305149 | -0.390426 | 489 | 354.73 | 0.725419 |
| GO:0043067\_regulation\_of\_programmed\_cell\_death | HIF1A | 365 | 3 | 1.305149 | -0.390426 | 489 | 354.73 | 0.725419 |
| GO:0010627\_regulation\_of\_protein\_kinase\_cascade | GPX1 | 82 | 1 | 1.936501 | -0.390347 | 491 | 356.19 | 0.725438 |
| GO:0045664\_regulation\_of\_neuron\_differentiation | SOX2 | 82 | 1 | 1.936501 | -0.390347 | 491 | 356.19 | 0.725438 |
| GO:0006325\_chromatin\_organization | ACTL6A | 83 | 1 | 1.913170 | -0.386310 | 494 | 358.62 | 0.725951 |
| GO:0006575\_cellular\_amino\_acid\_derivative\_metabolic\_process | PHGDH | 83 | 1 | 1.913170 | -0.386310 | 494 | 358.62 | 0.725951 |
| GO:0030534\_adult\_behavior | PBX3 | 83 | 1 | 1.913170 | -0.386310 | 494 | 358.62 | 0.725951 |
| GO:0030005\_cellular\_di-\_\_tri-valent\_inorganic\_cation\_homeostasis | HIF1A | 84 | 1 | 1.890394 | -0.382334 | 496 | 359.61 | 0.725020 |
| GO:0045137\_development\_of\_primary\_sexual\_characteristics | SOX2 | 84 | 1 | 1.890394 | -0.382334 | 496 | 359.61 | 0.725020 |
| GO:0051641\_cellular\_localization | NPC2 | 370 | 3 | 1.287512 | -0.381191 | 497 | 359.8 | 0.723944 |
| GO:0051641\_cellular\_localization | SIX3 | 370 | 3 | 1.287512 | -0.381191 | 497 | 359.8 | 0.723944 |
| GO:0051641\_cellular\_localization | PRKACA | 370 | 3 | 1.287512 | -0.381191 | 497 | 359.8 | 0.723944 |
| GO:0032502\_developmental\_process | HSP90AB1 | 2060 | 14 | 1.079176 | -0.377304 | 498 | 361.17 | 0.725241 |
| GO:0032502\_developmental\_process | SOX2 | 2060 | 14 | 1.079176 | -0.377304 | 498 | 361.17 | 0.725241 |
| GO:0032502\_developmental\_process | SIX3 | 2060 | 14 | 1.079176 | -0.377304 | 498 | 361.17 | 0.725241 |
| GO:0032502\_developmental\_process | ALDH1A1 | 2060 | 14 | 1.079176 | -0.377304 | 498 | 361.17 | 0.725241 |
| GO:0032502\_developmental\_process | DDR1 | 2060 | 14 | 1.079176 | -0.377304 | 498 | 361.17 | 0.725241 |
| GO:0032502\_developmental\_process | GPX1 | 2060 | 14 | 1.079176 | -0.377304 | 498 | 361.17 | 0.725241 |
| GO:0032502\_developmental\_process | TPI1 | 2060 | 14 | 1.079176 | -0.377304 | 498 | 361.17 | 0.725241 |
| GO:0032502\_developmental\_process | HIF1A | 2060 | 14 | 1.079176 | -0.377304 | 498 | 361.17 | 0.725241 |
| GO:0032502\_developmental\_process | NME1 | 2060 | 14 | 1.079176 | -0.377304 | 498 | 361.17 | 0.725241 |
| GO:0032502\_developmental\_process | PHGDH | 2060 | 14 | 1.079176 | -0.377304 | 498 | 361.17 | 0.725241 |
| GO:0032502\_developmental\_process | PTN | 2060 | 14 | 1.079176 | -0.377304 | 498 | 361.17 | 0.725241 |
| GO:0032502\_developmental\_process | PRKACA | 2060 | 14 | 1.079176 | -0.377304 | 498 | 361.17 | 0.725241 |
| GO:0032502\_developmental\_process | PBX3 | 2060 | 14 | 1.079176 | -0.377304 | 498 | 361.17 | 0.725241 |
| GO:0032502\_developmental\_process | TRIP13 | 2060 | 14 | 1.079176 | -0.377304 | 498 | 361.17 | 0.725241 |
| GO:0032504\_multicellular\_organism\_reproduction | NME1 | 86 | 1 | 1.846431 | -0.374563 | 501 | 363.41 | 0.725369 |
| GO:0034641\_cellular\_nitrogen\_compound\_metabolic\_process | MTHFD1 | 86 | 1 | 1.846431 | -0.374563 | 501 | 363.41 | 0.725369 |
| GO:0048609\_reproductive\_process\_in\_a\_multicellular\_organism | NME1 | 86 | 1 | 1.846431 | -0.374563 | 501 | 363.41 | 0.725369 |
| GO:0002682\_regulation\_of\_immune\_system\_process | GPX1 | 228 | 2 | 1.392922 | -0.371950 | 502 | 364.1 | 0.725299 |
| GO:0002682\_regulation\_of\_immune\_system\_process | HIF1A | 228 | 2 | 1.392922 | -0.371950 | 502 | 364.1 | 0.725299 |
| GO:0022612\_gland\_morphogenesis | DDR1 | 87 | 1 | 1.825208 | -0.370764 | 503 | 366.67 | 0.728966 |
| GO:0048754\_branching\_morphogenesis\_of\_a\_tube | DDR1 | 88 | 1 | 1.804467 | -0.367022 | 504 | 368.27 | 0.730694 |
| GO:0007420\_brain\_development | SOX2 | 231 | 2 | 1.374832 | -0.365204 | 505 | 368.7 | 0.730099 |
| GO:0007420\_brain\_development | SIX3 | 231 | 2 | 1.374832 | -0.365204 | 505 | 368.7 | 0.730099 |
| GO:0030003\_cellular\_cation\_homeostasis | HIF1A | 90 | 1 | 1.764368 | -0.359700 | 506 | 370.82 | 0.732846 |
| GO:0016477\_cell\_migration | GPX1 | 234 | 2 | 1.357206 | -0.358597 | 507 | 371.07 | 0.731893 |
| GO:0016477\_cell\_migration | HIF1A | 234 | 2 | 1.357206 | -0.358597 | 507 | 371.07 | 0.731893 |
| GO:0044249\_cellular\_biosynthetic\_process | ALDH1A1 | 1150 | 8 | 1.104648 | -0.354705 | 508 | 372.81 | 0.733878 |
| GO:0044249\_cellular\_biosynthetic\_process | MCM7 | 1150 | 8 | 1.104648 | -0.354705 | 508 | 372.81 | 0.733878 |
| GO:0044249\_cellular\_biosynthetic\_process | HIF1A | 1150 | 8 | 1.104648 | -0.354705 | 508 | 372.81 | 0.733878 |
| GO:0044249\_cellular\_biosynthetic\_process | POLD1 | 1150 | 8 | 1.104648 | -0.354705 | 508 | 372.81 | 0.733878 |
| GO:0044249\_cellular\_biosynthetic\_process | EIF2S1 | 1150 | 8 | 1.104648 | -0.354705 | 508 | 372.81 | 0.733878 |
| GO:0044249\_cellular\_biosynthetic\_process | SOX2 | 1150 | 8 | 1.104648 | -0.354705 | 508 | 372.81 | 0.733878 |
| GO:0044249\_cellular\_biosynthetic\_process | SIX3 | 1150 | 8 | 1.104648 | -0.354705 | 508 | 372.81 | 0.733878 |
| GO:0044249\_cellular\_biosynthetic\_process | CNOT7 | 1150 | 8 | 1.104648 | -0.354705 | 508 | 372.81 | 0.733878 |
| GO:0055066\_di-\_\_tri-valent\_inorganic\_cation\_homeostasis | HIF1A | 93 | 1 | 1.707453 | -0.349108 | 509 | 376.81 | 0.740295 |
| GO:0008610\_lipid\_biosynthetic\_process | ALDH1A1 | 94 | 1 | 1.689288 | -0.345676 | 510 | 379.28 | 0.743686 |
| GO:0051707\_response\_to\_other\_organism | GPX1 | 95 | 1 | 1.671506 | -0.342293 | 511 | 380.12 | 0.743875 |
| GO:0009056\_catabolic\_process | MTHFD1 | 243 | 2 | 1.306939 | -0.339579 | 512 | 380.63 | 0.743418 |
| GO:0009056\_catabolic\_process | GPX1 | 243 | 2 | 1.306939 | -0.339579 | 512 | 380.63 | 0.743418 |
| GO:0048699\_generation\_of\_neurons | SOX2 | 396 | 3 | 1.202978 | -0.336659 | 513 | 383.26 | 0.747096 |
| GO:0048699\_generation\_of\_neurons | PHGDH | 396 | 3 | 1.202978 | -0.336659 | 513 | 383.26 | 0.747096 |
| GO:0048699\_generation\_of\_neurons | PBX3 | 396 | 3 | 1.202978 | -0.336659 | 513 | 383.26 | 0.747096 |
| GO:0018193\_peptidyl-amino\_acid\_modification | DDR1 | 97 | 1 | 1.637042 | -0.335666 | 515 | 384.16 | 0.745942 |
| GO:0060341\_regulation\_of\_cellular\_localization | PRKACA | 97 | 1 | 1.637042 | -0.335666 | 515 | 384.16 | 0.745942 |
| GO:0048519\_negative\_regulation\_of\_biological\_process | DDR1 | 859 | 6 | 1.109149 | -0.334645 | 516 | 384.4 | 0.744961 |
| GO:0048519\_negative\_regulation\_of\_biological\_process | GPX1 | 859 | 6 | 1.109149 | -0.334645 | 516 | 384.4 | 0.744961 |
| GO:0048519\_negative\_regulation\_of\_biological\_process | HIF1A | 859 | 6 | 1.109149 | -0.334645 | 516 | 384.4 | 0.744961 |
| GO:0048519\_negative\_regulation\_of\_biological\_process | BTG1 | 859 | 6 | 1.109149 | -0.334645 | 516 | 384.4 | 0.744961 |
| GO:0048519\_negative\_regulation\_of\_biological\_process | SOX2 | 859 | 6 | 1.109149 | -0.334645 | 516 | 384.4 | 0.744961 |
| GO:0048519\_negative\_regulation\_of\_biological\_process | SIX3 | 859 | 6 | 1.109149 | -0.334645 | 516 | 384.4 | 0.744961 |
| GO:0007548\_sex\_differentiation | SOX2 | 98 | 1 | 1.620338 | -0.332420 | 519 | 385.92 | 0.743584 |
| GO:0009314\_response\_to\_radiation | GPX1 | 98 | 1 | 1.620338 | -0.332420 | 519 | 385.92 | 0.743584 |
| GO:0060541\_respiratory\_system\_development | SOX2 | 98 | 1 | 1.620338 | -0.332420 | 519 | 385.92 | 0.743584 |
| GO:0032879\_regulation\_of\_localization | HIF1A | 248 | 2 | 1.280590 | -0.329507 | 520 | 386.47 | 0.743212 |
| GO:0032879\_regulation\_of\_localization | PRKACA | 248 | 2 | 1.280590 | -0.329507 | 520 | 386.47 | 0.743212 |
| GO:0060562\_epithelial\_tube\_morphogenesis | DDR1 | 99 | 1 | 1.603971 | -0.329219 | 521 | 387.9 | 0.744530 |
| GO:0009058\_biosynthetic\_process | ALDH1A1 | 1175 | 8 | 1.081145 | -0.328830 | 522 | 388.06 | 0.743410 |
| GO:0009058\_biosynthetic\_process | MCM7 | 1175 | 8 | 1.081145 | -0.328830 | 522 | 388.06 | 0.743410 |
| GO:0009058\_biosynthetic\_process | HIF1A | 1175 | 8 | 1.081145 | -0.328830 | 522 | 388.06 | 0.743410 |
| GO:0009058\_biosynthetic\_process | POLD1 | 1175 | 8 | 1.081145 | -0.328830 | 522 | 388.06 | 0.743410 |
| GO:0009058\_biosynthetic\_process | EIF2S1 | 1175 | 8 | 1.081145 | -0.328830 | 522 | 388.06 | 0.743410 |
| GO:0009058\_biosynthetic\_process | SOX2 | 1175 | 8 | 1.081145 | -0.328830 | 522 | 388.06 | 0.743410 |
| GO:0009058\_biosynthetic\_process | SIX3 | 1175 | 8 | 1.081145 | -0.328830 | 522 | 388.06 | 0.743410 |
| GO:0009058\_biosynthetic\_process | CNOT7 | 1175 | 8 | 1.081145 | -0.328830 | 522 | 388.06 | 0.743410 |
| GO:0008104\_protein\_localization | SIX3 | 251 | 2 | 1.265284 | -0.323624 | 523 | 389.01 | 0.743805 |
| GO:0008104\_protein\_localization | PRKACA | 251 | 2 | 1.265284 | -0.323624 | 523 | 389.01 | 0.743805 |
| GO:0016481\_negative\_regulation\_of\_transcription | SOX2 | 253 | 2 | 1.255281 | -0.319768 | 524 | 390.82 | 0.745840 |
| GO:0016481\_negative\_regulation\_of\_transcription | SIX3 | 253 | 2 | 1.255281 | -0.319768 | 524 | 390.82 | 0.745840 |
| GO:0048878\_chemical\_homeostasis | NPC2 | 254 | 2 | 1.250339 | -0.317859 | 525 | 391.21 | 0.745162 |
| GO:0048878\_chemical\_homeostasis | HIF1A | 254 | 2 | 1.250339 | -0.317859 | 525 | 391.21 | 0.745162 |
| GO:0003013\_circulatory\_system\_process | GPX1 | 103 | 1 | 1.541681 | -0.316835 | 527 | 392.32 | 0.744440 |
| GO:0008015\_blood\_circulation | GPX1 | 103 | 1 | 1.541681 | -0.316835 | 527 | 392.32 | 0.744440 |
| GO:0050767\_regulation\_of\_neurogenesis | SOX2 | 104 | 1 | 1.526857 | -0.313841 | 528 | 393.69 | 0.745625 |
| GO:0048870\_cell\_motility | GPX1 | 257 | 2 | 1.235744 | -0.312208 | 529 | 394.51 | 0.745766 |
| GO:0048870\_cell\_motility | HIF1A | 257 | 2 | 1.235744 | -0.312208 | 529 | 394.51 | 0.745766 |
| GO:0010817\_regulation\_of\_hormone\_levels | ALDH1A1 | 106 | 1 | 1.498048 | -0.307968 | 530 | 395.37 | 0.745981 |
| GO:0009987\_cellular\_process | MARCKSL1 | 3868 | 25 | 1.026326 | -0.305589 | 531 | 395.52 | 0.744859 |
| GO:0009987\_cellular\_process | SOX2 | 3868 | 25 | 1.026326 | -0.305589 | 531 | 395.52 | 0.744859 |
| GO:0009987\_cellular\_process | CNOT7 | 3868 | 25 | 1.026326 | -0.305589 | 531 | 395.52 | 0.744859 |
| GO:0009987\_cellular\_process | ALDH1A1 | 3868 | 25 | 1.026326 | -0.305589 | 531 | 395.52 | 0.744859 |
| GO:0009987\_cellular\_process | MTHFD1 | 3868 | 25 | 1.026326 | -0.305589 | 531 | 395.52 | 0.744859 |
| GO:0009987\_cellular\_process | GPX1 | 3868 | 25 | 1.026326 | -0.305589 | 531 | 395.52 | 0.744859 |
| GO:0009987\_cellular\_process | TPI1 | 3868 | 25 | 1.026326 | -0.305589 | 531 | 395.52 | 0.744859 |
| GO:0009987\_cellular\_process | MCM7 | 3868 | 25 | 1.026326 | -0.305589 | 531 | 395.52 | 0.744859 |
| GO:0009987\_cellular\_process | PRKACA | 3868 | 25 | 1.026326 | -0.305589 | 531 | 395.52 | 0.744859 |
| GO:0009987\_cellular\_process | ACTL6A | 3868 | 25 | 1.026326 | -0.305589 | 531 | 395.52 | 0.744859 |
| GO:0009987\_cellular\_process | CDCA5 | 3868 | 25 | 1.026326 | -0.305589 | 531 | 395.52 | 0.744859 |
| GO:0009987\_cellular\_process | TRIP13 | 3868 | 25 | 1.026326 | -0.305589 | 531 | 395.52 | 0.744859 |
| GO:0009987\_cellular\_process | SIX3 | 3868 | 25 | 1.026326 | -0.305589 | 531 | 395.52 | 0.744859 |
| GO:0009987\_cellular\_process | CDK4 | 3868 | 25 | 1.026326 | -0.305589 | 531 | 395.52 | 0.744859 |
| GO:0009987\_cellular\_process | SF3A2 | 3868 | 25 | 1.026326 | -0.305589 | 531 | 395.52 | 0.744859 |
| GO:0009987\_cellular\_process | PPP1CB | 3868 | 25 | 1.026326 | -0.305589 | 531 | 395.52 | 0.744859 |
| GO:0009987\_cellular\_process | DDR1 | 3868 | 25 | 1.026326 | -0.305589 | 531 | 395.52 | 0.744859 |
| GO:0009987\_cellular\_process | HIF1A | 3868 | 25 | 1.026326 | -0.305589 | 531 | 395.52 | 0.744859 |
| GO:0009987\_cellular\_process | NPC2 | 3868 | 25 | 1.026326 | -0.305589 | 531 | 395.52 | 0.744859 |
| GO:0009987\_cellular\_process | BTG1 | 3868 | 25 | 1.026326 | -0.305589 | 531 | 395.52 | 0.744859 |
| GO:0009987\_cellular\_process | EIF2S1 | 3868 | 25 | 1.026326 | -0.305589 | 531 | 395.52 | 0.744859 |
| GO:0009987\_cellular\_process | POLD1 | 3868 | 25 | 1.026326 | -0.305589 | 531 | 395.52 | 0.744859 |
| GO:0009987\_cellular\_process | PHGDH | 3868 | 25 | 1.026326 | -0.305589 | 531 | 395.52 | 0.744859 |
| GO:0009987\_cellular\_process | BUB1B | 3868 | 25 | 1.026326 | -0.305589 | 531 | 395.52 | 0.744859 |
| GO:0009987\_cellular\_process | PBX3 | 3868 | 25 | 1.026326 | -0.305589 | 531 | 395.52 | 0.744859 |
| GO:0010629\_negative\_regulation\_of\_gene\_expression | SOX2 | 262 | 2 | 1.212161 | -0.303035 | 533 | 396.81 | 0.744484 |
| GO:0010629\_negative\_regulation\_of\_gene\_expression | SIX3 | 262 | 2 | 1.212161 | -0.303035 | 533 | 396.81 | 0.744484 |
| GO:0048666\_neuron\_development | PHGDH | 262 | 2 | 1.212161 | -0.303035 | 533 | 396.81 | 0.744484 |
| GO:0048666\_neuron\_development | PBX3 | 262 | 2 | 1.212161 | -0.303035 | 533 | 396.81 | 0.744484 |
| GO:0006355\_regulation\_of\_transcription\_\_DNA-dependent | HIF1A | 575 | 4 | 1.104648 | -0.302450 | 534 | 397.05 | 0.743539 |
| GO:0006355\_regulation\_of\_transcription\_\_DNA-dependent | SOX2 | 575 | 4 | 1.104648 | -0.302450 | 534 | 397.05 | 0.743539 |
| GO:0006355\_regulation\_of\_transcription\_\_DNA-dependent | SIX3 | 575 | 4 | 1.104648 | -0.302450 | 534 | 397.05 | 0.743539 |
| GO:0006355\_regulation\_of\_transcription\_\_DNA-dependent | CNOT7 | 575 | 4 | 1.104648 | -0.302450 | 534 | 397.05 | 0.743539 |
| GO:0030099\_myeloid\_cell\_differentiation | HIF1A | 108 | 1 | 1.470307 | -0.302244 | 535 | 397.8 | 0.743551 |
| GO:0055080\_cation\_homeostasis | HIF1A | 110 | 1 | 1.443574 | -0.296665 | 536 | 400.44 | 0.747090 |
| GO:0022008\_neurogenesis | SOX2 | 423 | 3 | 1.126192 | -0.295972 | 537 | 400.66 | 0.746108 |
| GO:0022008\_neurogenesis | PHGDH | 423 | 3 | 1.126192 | -0.295972 | 537 | 400.66 | 0.746108 |
| GO:0022008\_neurogenesis | PBX3 | 423 | 3 | 1.126192 | -0.295972 | 537 | 400.66 | 0.746108 |
| GO:0006915\_apoptosis | ALDH1A1 | 427 | 3 | 1.115642 | -0.290378 | 538 | 402.44 | 0.748030 |
| GO:0006915\_apoptosis | GPX1 | 427 | 3 | 1.115642 | -0.290378 | 538 | 402.44 | 0.748030 |
| GO:0006915\_apoptosis | HIF1A | 427 | 3 | 1.115642 | -0.290378 | 538 | 402.44 | 0.748030 |
| GO:0045934\_negative\_regulation\_of\_nucleobase\_\_nucleoside\_\_nucleotide\_and\_nucleic\_acid\_metabolic\_process | SOX2 | 270 | 2 | 1.176245 | -0.288970 | 539 | 403.18 | 0.748015 |
| GO:0045934\_negative\_regulation\_of\_nucleobase\_\_nucleoside\_\_nucleotide\_and\_nucleic\_acid\_metabolic\_process | SIX3 | 270 | 2 | 1.176245 | -0.288970 | 539 | 403.18 | 0.748015 |
| GO:0030154\_cell\_differentiation | GPX1 | 1060 | 7 | 1.048634 | -0.288293 | 540 | 403.98 | 0.748111 |
| GO:0030154\_cell\_differentiation | HIF1A | 1060 | 7 | 1.048634 | -0.288293 | 540 | 403.98 | 0.748111 |
| GO:0030154\_cell\_differentiation | SOX2 | 1060 | 7 | 1.048634 | -0.288293 | 540 | 403.98 | 0.748111 |
| GO:0030154\_cell\_differentiation | SIX3 | 1060 | 7 | 1.048634 | -0.288293 | 540 | 403.98 | 0.748111 |
| GO:0030154\_cell\_differentiation | PHGDH | 1060 | 7 | 1.048634 | -0.288293 | 540 | 403.98 | 0.748111 |
| GO:0030154\_cell\_differentiation | PBX3 | 1060 | 7 | 1.048634 | -0.288293 | 540 | 403.98 | 0.748111 |
| GO:0030154\_cell\_differentiation | TRIP13 | 1060 | 7 | 1.048634 | -0.288293 | 540 | 403.98 | 0.748111 |
| GO:0051239\_regulation\_of\_multicellular\_organismal\_process | HIF1A | 587 | 4 | 1.082065 | -0.287785 | 541 | 404.17 | 0.747079 |
| GO:0051239\_regulation\_of\_multicellular\_organismal\_process | SOX2 | 587 | 4 | 1.082065 | -0.287785 | 541 | 404.17 | 0.747079 |
| GO:0051239\_regulation\_of\_multicellular\_organismal\_process | PRKACA | 587 | 4 | 1.082065 | -0.287785 | 541 | 404.17 | 0.747079 |
| GO:0051239\_regulation\_of\_multicellular\_organismal\_process | PBX3 | 587 | 4 | 1.082065 | -0.287785 | 541 | 404.17 | 0.747079 |
| GO:0010556\_regulation\_of\_macromolecule\_biosynthetic\_process | HIF1A | 745 | 5 | 1.065726 | -0.287762 | 542 | 404.36 | 0.746052 |
| GO:0010556\_regulation\_of\_macromolecule\_biosynthetic\_process | EIF2S1 | 745 | 5 | 1.065726 | -0.287762 | 542 | 404.36 | 0.746052 |
| GO:0010556\_regulation\_of\_macromolecule\_biosynthetic\_process | SOX2 | 745 | 5 | 1.065726 | -0.287762 | 542 | 404.36 | 0.746052 |
| GO:0010556\_regulation\_of\_macromolecule\_biosynthetic\_process | SIX3 | 745 | 5 | 1.065726 | -0.287762 | 542 | 404.36 | 0.746052 |
| GO:0010556\_regulation\_of\_macromolecule\_biosynthetic\_process | CNOT7 | 745 | 5 | 1.065726 | -0.287762 | 542 | 404.36 | 0.746052 |
| GO:0051172\_negative\_regulation\_of\_nitrogen\_compound\_metabolic\_process | SOX2 | 271 | 2 | 1.171905 | -0.287263 | 543 | 404.63 | 0.745175 |
| GO:0051172\_negative\_regulation\_of\_nitrogen\_compound\_metabolic\_process | SIX3 | 271 | 2 | 1.171905 | -0.287263 | 543 | 404.63 | 0.745175 |
| GO:0009607\_response\_to\_biotic\_stimulus | GPX1 | 114 | 1 | 1.392922 | -0.285918 | 544 | 405.31 | 0.745055 |
| GO:0051252\_regulation\_of\_RNA\_metabolic\_process | HIF1A | 590 | 4 | 1.076563 | -0.284223 | 545 | 405.76 | 0.744514 |
| GO:0051252\_regulation\_of\_RNA\_metabolic\_process | SOX2 | 590 | 4 | 1.076563 | -0.284223 | 545 | 405.76 | 0.744514 |
| GO:0051252\_regulation\_of\_RNA\_metabolic\_process | SIX3 | 590 | 4 | 1.076563 | -0.284223 | 545 | 405.76 | 0.744514 |
| GO:0051252\_regulation\_of\_RNA\_metabolic\_process | CNOT7 | 590 | 4 | 1.076563 | -0.284223 | 545 | 405.76 | 0.744514 |
| GO:0010558\_negative\_regulation\_of\_macromolecule\_biosynthetic\_process | SOX2 | 274 | 2 | 1.159074 | -0.282207 | 547 | 407.32 | 0.744644 |
| GO:0010558\_negative\_regulation\_of\_macromolecule\_biosynthetic\_process | SIX3 | 274 | 2 | 1.159074 | -0.282207 | 547 | 407.32 | 0.744644 |
| GO:0033036\_macromolecule\_localization | SIX3 | 274 | 2 | 1.159074 | -0.282207 | 547 | 407.32 | 0.744644 |
| GO:0033036\_macromolecule\_localization | PRKACA | 274 | 2 | 1.159074 | -0.282207 | 547 | 407.32 | 0.744644 |
| GO:0012501\_programmed\_cell\_death | ALDH1A1 | 433 | 3 | 1.100183 | -0.282183 | 548 | 407.74 | 0.744051 |
| GO:0012501\_programmed\_cell\_death | GPX1 | 433 | 3 | 1.100183 | -0.282183 | 548 | 407.74 | 0.744051 |
| GO:0012501\_programmed\_cell\_death | HIF1A | 433 | 3 | 1.100183 | -0.282183 | 548 | 407.74 | 0.744051 |
| GO:0046483\_heterocycle\_metabolic\_process | MTHFD1 | 116 | 1 | 1.368906 | -0.280740 | 550 | 408.97 | 0.743582 |
| GO:0080134\_regulation\_of\_response\_to\_stress | GPX1 | 116 | 1 | 1.368906 | -0.280740 | 550 | 408.97 | 0.743582 |
| GO:0006351\_transcription\_\_DNA-dependent | HIF1A | 594 | 4 | 1.069314 | -0.279539 | 551 | 409.38 | 0.742976 |
| GO:0006351\_transcription\_\_DNA-dependent | SOX2 | 594 | 4 | 1.069314 | -0.279539 | 551 | 409.38 | 0.742976 |
| GO:0006351\_transcription\_\_DNA-dependent | SIX3 | 594 | 4 | 1.069314 | -0.279539 | 551 | 409.38 | 0.742976 |
| GO:0006351\_transcription\_\_DNA-dependent | CNOT7 | 594 | 4 | 1.069314 | -0.279539 | 551 | 409.38 | 0.742976 |
| GO:0032774\_RNA\_biosynthetic\_process | HIF1A | 595 | 4 | 1.067517 | -0.278379 | 552 | 409.89 | 0.742554 |
| GO:0032774\_RNA\_biosynthetic\_process | SOX2 | 595 | 4 | 1.067517 | -0.278379 | 552 | 409.89 | 0.742554 |
| GO:0032774\_RNA\_biosynthetic\_process | SIX3 | 595 | 4 | 1.067517 | -0.278379 | 552 | 409.89 | 0.742554 |
| GO:0032774\_RNA\_biosynthetic\_process | CNOT7 | 595 | 4 | 1.067517 | -0.278379 | 552 | 409.89 | 0.742554 |
| GO:0051960\_regulation\_of\_nervous\_system\_development | SOX2 | 118 | 1 | 1.345704 | -0.275687 | 553 | 412.04 | 0.745099 |
| GO:0007610\_behavior | PTN | 279 | 2 | 1.138302 | -0.273995 | 554 | 413.0 | 0.745487 |
| GO:0007610\_behavior | PBX3 | 279 | 2 | 1.138302 | -0.273995 | 554 | 413.0 | 0.745487 |
| GO:0032501\_multicellular\_organismal\_process | HSP90AB1 | 2183 | 14 | 1.018371 | -0.271327 | 555 | 413.68 | 0.745369 |
| GO:0032501\_multicellular\_organismal\_process | SOX2 | 2183 | 14 | 1.018371 | -0.271327 | 555 | 413.68 | 0.745369 |
| GO:0032501\_multicellular\_organismal\_process | SIX3 | 2183 | 14 | 1.018371 | -0.271327 | 555 | 413.68 | 0.745369 |
| GO:0032501\_multicellular\_organismal\_process | ALDH1A1 | 2183 | 14 | 1.018371 | -0.271327 | 555 | 413.68 | 0.745369 |
| GO:0032501\_multicellular\_organismal\_process | GPX1 | 2183 | 14 | 1.018371 | -0.271327 | 555 | 413.68 | 0.745369 |
| GO:0032501\_multicellular\_organismal\_process | DDR1 | 2183 | 14 | 1.018371 | -0.271327 | 555 | 413.68 | 0.745369 |
| GO:0032501\_multicellular\_organismal\_process | TPI1 | 2183 | 14 | 1.018371 | -0.271327 | 555 | 413.68 | 0.745369 |
| GO:0032501\_multicellular\_organismal\_process | HIF1A | 2183 | 14 | 1.018371 | -0.271327 | 555 | 413.68 | 0.745369 |
| GO:0032501\_multicellular\_organismal\_process | NME1 | 2183 | 14 | 1.018371 | -0.271327 | 555 | 413.68 | 0.745369 |
| GO:0032501\_multicellular\_organismal\_process | PHGDH | 2183 | 14 | 1.018371 | -0.271327 | 555 | 413.68 | 0.745369 |
| GO:0032501\_multicellular\_organismal\_process | PTN | 2183 | 14 | 1.018371 | -0.271327 | 555 | 413.68 | 0.745369 |
| GO:0032501\_multicellular\_organismal\_process | PRKACA | 2183 | 14 | 1.018371 | -0.271327 | 555 | 413.68 | 0.745369 |
| GO:0032501\_multicellular\_organismal\_process | FH1 | 2183 | 14 | 1.018371 | -0.271327 | 555 | 413.68 | 0.745369 |
| GO:0032501\_multicellular\_organismal\_process | PBX3 | 2183 | 14 | 1.018371 | -0.271327 | 555 | 413.68 | 0.745369 |
| GO:0014706\_striated\_muscle\_tissue\_development | GPX1 | 120 | 1 | 1.323276 | -0.270753 | 556 | 414.09 | 0.744766 |
| GO:0031327\_negative\_regulation\_of\_cellular\_biosynthetic\_process | SOX2 | 282 | 2 | 1.126192 | -0.269192 | 557 | 414.51 | 0.744183 |
| GO:0031327\_negative\_regulation\_of\_cellular\_biosynthetic\_process | SIX3 | 282 | 2 | 1.126192 | -0.269192 | 557 | 414.51 | 0.744183 |
| GO:0006917\_induction\_of\_apoptosis | GPX1 | 121 | 1 | 1.312340 | -0.268330 | 560 | 415.96 | 0.742786 |
| GO:0012502\_induction\_of\_programmed\_cell\_death | GPX1 | 121 | 1 | 1.312340 | -0.268330 | 560 | 415.96 | 0.742786 |
| GO:0051726\_regulation\_of\_cell\_cycle | CDK4 | 121 | 1 | 1.312340 | -0.268330 | 560 | 415.96 | 0.742786 |
| GO:0008219\_cell\_death | ALDH1A1 | 444 | 3 | 1.072926 | -0.267748 | 561 | 416.46 | 0.742353 |
| GO:0008219\_cell\_death | GPX1 | 444 | 3 | 1.072926 | -0.267748 | 561 | 416.46 | 0.742353 |
| GO:0008219\_cell\_death | HIF1A | 444 | 3 | 1.072926 | -0.267748 | 561 | 416.46 | 0.742353 |
| GO:0009890\_negative\_regulation\_of\_biosynthetic\_process | SOX2 | 284 | 2 | 1.118261 | -0.266040 | 562 | 416.96 | 0.741922 |
| GO:0009890\_negative\_regulation\_of\_biosynthetic\_process | SIX3 | 284 | 2 | 1.118261 | -0.266040 | 562 | 416.96 | 0.741922 |
| GO:0060284\_regulation\_of\_cell\_development | SOX2 | 122 | 1 | 1.301583 | -0.265935 | 563 | 419.1 | 0.744405 |
| GO:0006996\_organelle\_organization | BUB1B | 449 | 3 | 1.060978 | -0.261430 | 564 | 420.22 | 0.745071 |
| GO:0006996\_organelle\_organization | ACTL6A | 449 | 3 | 1.060978 | -0.261430 | 564 | 420.22 | 0.745071 |
| GO:0006996\_organelle\_organization | CDCA5 | 449 | 3 | 1.060978 | -0.261430 | 564 | 420.22 | 0.745071 |
| GO:0016265\_death | ALDH1A1 | 450 | 3 | 1.058621 | -0.260184 | 565 | 421.52 | 0.746053 |
| GO:0016265\_death | GPX1 | 450 | 3 | 1.058621 | -0.260184 | 565 | 421.52 | 0.746053 |
| GO:0016265\_death | HIF1A | 450 | 3 | 1.058621 | -0.260184 | 565 | 421.52 | 0.746053 |
| GO:0001763\_morphogenesis\_of\_a\_branching\_structure | DDR1 | 125 | 1 | 1.270345 | -0.258917 | 566 | 422.21 | 0.745954 |
| GO:0048523\_negative\_regulation\_of\_cellular\_process | GPX1 | 774 | 5 | 1.025795 | -0.257911 | 567 | 422.56 | 0.745256 |
| GO:0048523\_negative\_regulation\_of\_cellular\_process | DDR1 | 774 | 5 | 1.025795 | -0.257911 | 567 | 422.56 | 0.745256 |
| GO:0048523\_negative\_regulation\_of\_cellular\_process | BTG1 | 774 | 5 | 1.025795 | -0.257911 | 567 | 422.56 | 0.745256 |
| GO:0048523\_negative\_regulation\_of\_cellular\_process | SOX2 | 774 | 5 | 1.025795 | -0.257911 | 567 | 422.56 | 0.745256 |
| GO:0048523\_negative\_regulation\_of\_cellular\_process | SIX3 | 774 | 5 | 1.025795 | -0.257911 | 567 | 422.56 | 0.745256 |
| GO:0060255\_regulation\_of\_macromolecule\_metabolic\_process | HIF1A | 936 | 6 | 1.017905 | -0.256160 | 568 | 422.69 | 0.744173 |
| GO:0060255\_regulation\_of\_macromolecule\_metabolic\_process | EIF2S1 | 936 | 6 | 1.017905 | -0.256160 | 568 | 422.69 | 0.744173 |
| GO:0060255\_regulation\_of\_macromolecule\_metabolic\_process | SOX2 | 936 | 6 | 1.017905 | -0.256160 | 568 | 422.69 | 0.744173 |
| GO:0060255\_regulation\_of\_macromolecule\_metabolic\_process | PHGDH | 936 | 6 | 1.017905 | -0.256160 | 568 | 422.69 | 0.744173 |
| GO:0060255\_regulation\_of\_macromolecule\_metabolic\_process | SIX3 | 936 | 6 | 1.017905 | -0.256160 | 568 | 422.69 | 0.744173 |
| GO:0060255\_regulation\_of\_macromolecule\_metabolic\_process | CNOT7 | 936 | 6 | 1.017905 | -0.256160 | 568 | 422.69 | 0.744173 |
| GO:0060537\_muscle\_tissue\_development | GPX1 | 128 | 1 | 1.240571 | -0.252138 | 569 | 424.37 | 0.745817 |
| GO:0051276\_chromosome\_organization | ACTL6A | 129 | 1 | 1.230954 | -0.249929 | 570 | 425.47 | 0.746439 |
| GO:0007399\_nervous\_system\_development | SOX2 | 621 | 4 | 1.022822 | -0.249743 | 571 | 425.71 | 0.745552 |
| GO:0007399\_nervous\_system\_development | PHGDH | 621 | 4 | 1.022822 | -0.249743 | 571 | 425.71 | 0.745552 |
| GO:0007399\_nervous\_system\_development | SIX3 | 621 | 4 | 1.022822 | -0.249743 | 571 | 425.71 | 0.745552 |
| GO:0007399\_nervous\_system\_development | PBX3 | 621 | 4 | 1.022822 | -0.249743 | 571 | 425.71 | 0.745552 |
| GO:0040011\_locomotion | GPX1 | 295 | 2 | 1.076563 | -0.249402 | 573 | 426.58 | 0.744468 |
| GO:0040011\_locomotion | HIF1A | 295 | 2 | 1.076563 | -0.249402 | 573 | 426.58 | 0.744468 |
| GO:0045595\_regulation\_of\_cell\_differentiation | HIF1A | 295 | 2 | 1.076563 | -0.249402 | 573 | 426.58 | 0.744468 |
| GO:0045595\_regulation\_of\_cell\_differentiation | SOX2 | 295 | 2 | 1.076563 | -0.249402 | 573 | 426.58 | 0.744468 |
| GO:0050776\_regulation\_of\_immune\_response | GPX1 | 130 | 1 | 1.221485 | -0.247746 | 574 | 427.66 | 0.745052 |
| GO:0048869\_cellular\_developmental\_process | GPX1 | 1113 | 7 | 0.998699 | -0.241674 | 575 | 428.53 | 0.745270 |
| GO:0048869\_cellular\_developmental\_process | HIF1A | 1113 | 7 | 0.998699 | -0.241674 | 575 | 428.53 | 0.745270 |
| GO:0048869\_cellular\_developmental\_process | SOX2 | 1113 | 7 | 0.998699 | -0.241674 | 575 | 428.53 | 0.745270 |
| GO:0048869\_cellular\_developmental\_process | PHGDH | 1113 | 7 | 0.998699 | -0.241674 | 575 | 428.53 | 0.745270 |
| GO:0048869\_cellular\_developmental\_process | SIX3 | 1113 | 7 | 0.998699 | -0.241674 | 575 | 428.53 | 0.745270 |
| GO:0048869\_cellular\_developmental\_process | PBX3 | 1113 | 7 | 0.998699 | -0.241674 | 575 | 428.53 | 0.745270 |
| GO:0048869\_cellular\_developmental\_process | TRIP13 | 1113 | 7 | 0.998699 | -0.241674 | 575 | 428.53 | 0.745270 |
| GO:0009952\_anterior\_posterior\_pattern\_formation | SIX3 | 133 | 1 | 1.193933 | -0.241340 | 576 | 429.26 | 0.745243 |
| GO:0007283\_spermatogenesis | TRIP13 | 134 | 1 | 1.185023 | -0.239252 | 578 | 429.92 | 0.743806 |
| GO:0048232\_male\_gamete\_generation | TRIP13 | 134 | 1 | 1.185023 | -0.239252 | 578 | 429.92 | 0.743806 |
| GO:0007169\_transmembrane\_receptor\_protein\_tyrosine\_kinase\_signaling\_pathway | HIF1A | 139 | 1 | 1.142396 | -0.229150 | 579 | 433.37 | 0.748480 |
| GO:0003006\_reproductive\_developmental\_process | SOX2 | 141 | 1 | 1.126192 | -0.225260 | 580 | 434.92 | 0.749862 |
| GO:0031326\_regulation\_of\_cellular\_biosynthetic\_process | HIF1A | 812 | 5 | 0.977790 | -0.222954 | 581 | 435.11 | 0.748898 |
| GO:0031326\_regulation\_of\_cellular\_biosynthetic\_process | EIF2S1 | 812 | 5 | 0.977790 | -0.222954 | 581 | 435.11 | 0.748898 |
| GO:0031326\_regulation\_of\_cellular\_biosynthetic\_process | SOX2 | 812 | 5 | 0.977790 | -0.222954 | 581 | 435.11 | 0.748898 |
| GO:0031326\_regulation\_of\_cellular\_biosynthetic\_process | SIX3 | 812 | 5 | 0.977790 | -0.222954 | 581 | 435.11 | 0.748898 |
| GO:0031326\_regulation\_of\_cellular\_biosynthetic\_process | CNOT7 | 812 | 5 | 0.977790 | -0.222954 | 581 | 435.11 | 0.748898 |
| GO:0035239\_tube\_morphogenesis | DDR1 | 143 | 1 | 1.110441 | -0.221453 | 582 | 435.5 | 0.748282 |
| GO:0009889\_regulation\_of\_biosynthetic\_process | HIF1A | 815 | 5 | 0.974191 | -0.220381 | 583 | 435.69 | 0.747324 |
| GO:0009889\_regulation\_of\_biosynthetic\_process | EIF2S1 | 815 | 5 | 0.974191 | -0.220381 | 583 | 435.69 | 0.747324 |
| GO:0009889\_regulation\_of\_biosynthetic\_process | SOX2 | 815 | 5 | 0.974191 | -0.220381 | 583 | 435.69 | 0.747324 |
| GO:0009889\_regulation\_of\_biosynthetic\_process | SIX3 | 815 | 5 | 0.974191 | -0.220381 | 583 | 435.69 | 0.747324 |
| GO:0009889\_regulation\_of\_biosynthetic\_process | CNOT7 | 815 | 5 | 0.974191 | -0.220381 | 583 | 435.69 | 0.747324 |
| GO:0045596\_negative\_regulation\_of\_cell\_differentiation | SOX2 | 144 | 1 | 1.102730 | -0.219580 | 584 | 436.42 | 0.747295 |
| GO:0005975\_carbohydrate\_metabolic\_process | TPI1 | 146 | 1 | 1.087624 | -0.215892 | 585 | 437.76 | 0.748308 |
| GO:0032268\_regulation\_of\_cellular\_protein\_metabolic\_process | EIF2S1 | 152 | 1 | 1.044691 | -0.205279 | 586 | 440.21 | 0.751212 |
| GO:0065007\_biological\_regulation | MARCKSL1 | 2593 | 16 | 0.979826 | -0.204338 | 587 | 440.34 | 0.750153 |
| GO:0065007\_biological\_regulation | SOX2 | 2593 | 16 | 0.979826 | -0.204338 | 587 | 440.34 | 0.750153 |
| GO:0065007\_biological\_regulation | SIX3 | 2593 | 16 | 0.979826 | -0.204338 | 587 | 440.34 | 0.750153 |
| GO:0065007\_biological\_regulation | CNOT7 | 2593 | 16 | 0.979826 | -0.204338 | 587 | 440.34 | 0.750153 |
| GO:0065007\_biological\_regulation | CDK4 | 2593 | 16 | 0.979826 | -0.204338 | 587 | 440.34 | 0.750153 |
| GO:0065007\_biological\_regulation | ALDH1A1 | 2593 | 16 | 0.979826 | -0.204338 | 587 | 440.34 | 0.750153 |
| GO:0065007\_biological\_regulation | GPX1 | 2593 | 16 | 0.979826 | -0.204338 | 587 | 440.34 | 0.750153 |
| GO:0065007\_biological\_regulation | DDR1 | 2593 | 16 | 0.979826 | -0.204338 | 587 | 440.34 | 0.750153 |
| GO:0065007\_biological\_regulation | NPC2 | 2593 | 16 | 0.979826 | -0.204338 | 587 | 440.34 | 0.750153 |
| GO:0065007\_biological\_regulation | HIF1A | 2593 | 16 | 0.979826 | -0.204338 | 587 | 440.34 | 0.750153 |
| GO:0065007\_biological\_regulation | BTG1 | 2593 | 16 | 0.979826 | -0.204338 | 587 | 440.34 | 0.750153 |
| GO:0065007\_biological\_regulation | EIF2S1 | 2593 | 16 | 0.979826 | -0.204338 | 587 | 440.34 | 0.750153 |
| GO:0065007\_biological\_regulation | PHGDH | 2593 | 16 | 0.979826 | -0.204338 | 587 | 440.34 | 0.750153 |
| GO:0065007\_biological\_regulation | PRKACA | 2593 | 16 | 0.979826 | -0.204338 | 587 | 440.34 | 0.750153 |
| GO:0065007\_biological\_regulation | FH1 | 2593 | 16 | 0.979826 | -0.204338 | 587 | 440.34 | 0.750153 |
| GO:0065007\_biological\_regulation | PBX3 | 2593 | 16 | 0.979826 | -0.204338 | 587 | 440.34 | 0.750153 |
| GO:0007517\_muscle\_organ\_development | GPX1 | 153 | 1 | 1.037863 | -0.203573 | 588 | 440.75 | 0.749575 |
| GO:0006928\_cell\_motion | GPX1 | 330 | 2 | 0.962382 | -0.203413 | 590 | 441.63 | 0.748525 |
| GO:0006928\_cell\_motion | HIF1A | 330 | 2 | 0.962382 | -0.203413 | 590 | 441.63 | 0.748525 |
| GO:0051674\_localization\_of\_cell | GPX1 | 330 | 2 | 0.962382 | -0.203413 | 590 | 441.63 | 0.748525 |
| GO:0051674\_localization\_of\_cell | HIF1A | 330 | 2 | 0.962382 | -0.203413 | 590 | 441.63 | 0.748525 |
| GO:0010605\_negative\_regulation\_of\_macromolecule\_metabolic\_process | SOX2 | 331 | 2 | 0.959475 | -0.202238 | 592 | 442.14 | 0.746858 |
| GO:0010605\_negative\_regulation\_of\_macromolecule\_metabolic\_process | SIX3 | 331 | 2 | 0.959475 | -0.202238 | 592 | 442.14 | 0.746858 |
| GO:0051093\_negative\_regulation\_of\_developmental\_process | GPX1 | 331 | 2 | 0.959475 | -0.202238 | 592 | 442.14 | 0.746858 |
| GO:0051093\_negative\_regulation\_of\_developmental\_process | SOX2 | 331 | 2 | 0.959475 | -0.202238 | 592 | 442.14 | 0.746858 |
| GO:0007268\_synaptic\_transmission | PRKACA | 154 | 1 | 1.031124 | -0.201885 | 593 | 442.69 | 0.746526 |
| GO:0031324\_negative\_regulation\_of\_cellular\_metabolic\_process | SOX2 | 332 | 2 | 0.956585 | -0.201070 | 594 | 443.09 | 0.745943 |
| GO:0031324\_negative\_regulation\_of\_cellular\_metabolic\_process | SIX3 | 332 | 2 | 0.956585 | -0.201070 | 594 | 443.09 | 0.745943 |
| GO:0045449\_regulation\_of\_transcription | HIF1A | 676 | 4 | 0.939604 | -0.197870 | 595 | 444.43 | 0.746941 |
| GO:0045449\_regulation\_of\_transcription | SOX2 | 676 | 4 | 0.939604 | -0.197870 | 595 | 444.43 | 0.746941 |
| GO:0045449\_regulation\_of\_transcription | SIX3 | 676 | 4 | 0.939604 | -0.197870 | 595 | 444.43 | 0.746941 |
| GO:0045449\_regulation\_of\_transcription | CNOT7 | 676 | 4 | 0.939604 | -0.197870 | 595 | 444.43 | 0.746941 |
| GO:0006066\_alcohol\_metabolic\_process | TPI1 | 158 | 1 | 1.005020 | -0.195300 | 596 | 446.09 | 0.748473 |
| GO:0007626\_locomotory\_behavior | PBX3 | 163 | 1 | 0.974191 | -0.187431 | 597 | 449.68 | 0.753233 |
| GO:0009892\_negative\_regulation\_of\_metabolic\_process | SOX2 | 348 | 2 | 0.912604 | -0.183296 | 598 | 452.02 | 0.755886 |
| GO:0009892\_negative\_regulation\_of\_metabolic\_process | SIX3 | 348 | 2 | 0.912604 | -0.183296 | 598 | 452.02 | 0.755886 |
| GO:0051049\_regulation\_of\_transport | PRKACA | 167 | 1 | 0.950857 | -0.181410 | 599 | 453.64 | 0.757329 |
| GO:0006350\_transcription | HIF1A | 701 | 4 | 0.906095 | -0.177710 | 600 | 454.38 | 0.757300 |
| GO:0006350\_transcription | SOX2 | 701 | 4 | 0.906095 | -0.177710 | 600 | 454.38 | 0.757300 |
| GO:0006350\_transcription | SIX3 | 701 | 4 | 0.906095 | -0.177710 | 600 | 454.38 | 0.757300 |
| GO:0006350\_transcription | CNOT7 | 701 | 4 | 0.906095 | -0.177710 | 600 | 454.38 | 0.757300 |
| GO:0051246\_regulation\_of\_protein\_metabolic\_process | EIF2S1 | 170 | 1 | 0.934077 | -0.177046 | 601 | 455.18 | 0.757371 |
| GO:0050793\_regulation\_of\_developmental\_process | ALDH1A1 | 703 | 4 | 0.903517 | -0.176181 | 602 | 455.4 | 0.756478 |
| GO:0050793\_regulation\_of\_developmental\_process | GPX1 | 703 | 4 | 0.903517 | -0.176181 | 602 | 455.4 | 0.756478 |
| GO:0050793\_regulation\_of\_developmental\_process | HIF1A | 703 | 4 | 0.903517 | -0.176181 | 602 | 455.4 | 0.756478 |
| GO:0050793\_regulation\_of\_developmental\_process | SOX2 | 703 | 4 | 0.903517 | -0.176181 | 602 | 455.4 | 0.756478 |
| GO:0000122\_negative\_regulation\_of\_transcription\_from\_RNA\_polymerase\_II\_promoter | SOX2 | 175 | 1 | 0.907389 | -0.170046 | 603 | 458.93 | 0.761078 |
| GO:0006873\_cellular\_ion\_homeostasis | HIF1A | 176 | 1 | 0.902234 | -0.168686 | 605 | 460.4 | 0.760992 |
| GO:0043066\_negative\_regulation\_of\_apoptosis | GPX1 | 176 | 1 | 0.902234 | -0.168686 | 605 | 460.4 | 0.760992 |
| GO:0043009\_chordate\_embryonic\_development | HIF1A | 365 | 2 | 0.870099 | -0.166168 | 606 | 461.13 | 0.760941 |
| GO:0043009\_chordate\_embryonic\_development | PHGDH | 365 | 2 | 0.870099 | -0.166168 | 606 | 461.13 | 0.760941 |
| GO:0043069\_negative\_regulation\_of\_programmed\_cell\_death | GPX1 | 179 | 1 | 0.887112 | -0.164680 | 608 | 462.73 | 0.761069 |
| GO:0060548\_negative\_regulation\_of\_cell\_death | GPX1 | 179 | 1 | 0.887112 | -0.164680 | 608 | 462.73 | 0.761069 |
| GO:0009792\_embryonic\_development\_ending\_in\_birth\_or\_egg\_hatching | HIF1A | 368 | 2 | 0.863006 | -0.163319 | 609 | 463.68 | 0.761379 |
| GO:0009792\_embryonic\_development\_ending\_in\_birth\_or\_egg\_hatching | PHGDH | 368 | 2 | 0.863006 | -0.163319 | 609 | 463.68 | 0.761379 |
| GO:0055082\_cellular\_chemical\_homeostasis | HIF1A | 181 | 1 | 0.877310 | -0.162072 | 610 | 464.88 | 0.762098 |
| GO:0050789\_regulation\_of\_biological\_process | MARCKSL1 | 2357 | 14 | 0.943192 | -0.160110 | 611 | 466.24 | 0.763077 |
| GO:0050789\_regulation\_of\_biological\_process | SOX2 | 2357 | 14 | 0.943192 | -0.160110 | 611 | 466.24 | 0.763077 |
| GO:0050789\_regulation\_of\_biological\_process | SIX3 | 2357 | 14 | 0.943192 | -0.160110 | 611 | 466.24 | 0.763077 |
| GO:0050789\_regulation\_of\_biological\_process | CNOT7 | 2357 | 14 | 0.943192 | -0.160110 | 611 | 466.24 | 0.763077 |
| GO:0050789\_regulation\_of\_biological\_process | CDK4 | 2357 | 14 | 0.943192 | -0.160110 | 611 | 466.24 | 0.763077 |
| GO:0050789\_regulation\_of\_biological\_process | ALDH1A1 | 2357 | 14 | 0.943192 | -0.160110 | 611 | 466.24 | 0.763077 |
| GO:0050789\_regulation\_of\_biological\_process | GPX1 | 2357 | 14 | 0.943192 | -0.160110 | 611 | 466.24 | 0.763077 |
| GO:0050789\_regulation\_of\_biological\_process | DDR1 | 2357 | 14 | 0.943192 | -0.160110 | 611 | 466.24 | 0.763077 |
| GO:0050789\_regulation\_of\_biological\_process | HIF1A | 2357 | 14 | 0.943192 | -0.160110 | 611 | 466.24 | 0.763077 |
| GO:0050789\_regulation\_of\_biological\_process | BTG1 | 2357 | 14 | 0.943192 | -0.160110 | 611 | 466.24 | 0.763077 |
| GO:0050789\_regulation\_of\_biological\_process | EIF2S1 | 2357 | 14 | 0.943192 | -0.160110 | 611 | 466.24 | 0.763077 |
| GO:0050789\_regulation\_of\_biological\_process | PHGDH | 2357 | 14 | 0.943192 | -0.160110 | 611 | 466.24 | 0.763077 |
| GO:0050789\_regulation\_of\_biological\_process | PRKACA | 2357 | 14 | 0.943192 | -0.160110 | 611 | 466.24 | 0.763077 |
| GO:0050789\_regulation\_of\_biological\_process | PBX3 | 2357 | 14 | 0.943192 | -0.160110 | 611 | 466.24 | 0.763077 |
| GO:0007155\_cell\_adhesion | DDR1 | 186 | 1 | 0.853726 | -0.155761 | 613 | 469.39 | 0.765726 |
| GO:0022610\_biological\_adhesion | DDR1 | 186 | 1 | 0.853726 | -0.155761 | 613 | 469.39 | 0.765726 |
| GO:0007276\_gamete\_generation | TRIP13 | 188 | 1 | 0.844644 | -0.153316 | 614 | 470.47 | 0.766238 |
| GO:0019226\_transmission\_of\_nerve\_impulse | PRKACA | 189 | 1 | 0.840175 | -0.152111 | 615 | 471.15 | 0.766098 |
| GO:0007165\_signal\_transduction | GPX1 | 915 | 5 | 0.867722 | -0.148164 | 616 | 472.1 | 0.766396 |
| GO:0007165\_signal\_transduction | HIF1A | 915 | 5 | 0.867722 | -0.148164 | 616 | 472.1 | 0.766396 |
| GO:0007165\_signal\_transduction | SOX2 | 915 | 5 | 0.867722 | -0.148164 | 616 | 472.1 | 0.766396 |
| GO:0007165\_signal\_transduction | SIX3 | 915 | 5 | 0.867722 | -0.148164 | 616 | 472.1 | 0.766396 |
| GO:0007165\_signal\_transduction | CDK4 | 915 | 5 | 0.867722 | -0.148164 | 616 | 472.1 | 0.766396 |
| GO:0019222\_regulation\_of\_metabolic\_process | HIF1A | 1088 | 6 | 0.875697 | -0.146181 | 617 | 473.29 | 0.767083 |
| GO:0019222\_regulation\_of\_metabolic\_process | EIF2S1 | 1088 | 6 | 0.875697 | -0.146181 | 617 | 473.29 | 0.767083 |
| GO:0019222\_regulation\_of\_metabolic\_process | SOX2 | 1088 | 6 | 0.875697 | -0.146181 | 617 | 473.29 | 0.767083 |
| GO:0019222\_regulation\_of\_metabolic\_process | PHGDH | 1088 | 6 | 0.875697 | -0.146181 | 617 | 473.29 | 0.767083 |
| GO:0019222\_regulation\_of\_metabolic\_process | SIX3 | 1088 | 6 | 0.875697 | -0.146181 | 617 | 473.29 | 0.767083 |
| GO:0019222\_regulation\_of\_metabolic\_process | CNOT7 | 1088 | 6 | 0.875697 | -0.146181 | 617 | 473.29 | 0.767083 |
| GO:0003002\_regionalization | SIX3 | 195 | 1 | 0.814324 | -0.145103 | 620 | 474.56 | 0.765419 |
| GO:0007507\_heart\_development | HIF1A | 195 | 1 | 0.814324 | -0.145103 | 620 | 474.56 | 0.765419 |
| GO:0019725\_cellular\_homeostasis | HIF1A | 195 | 1 | 0.814324 | -0.145103 | 620 | 474.56 | 0.765419 |
| GO:0031175\_neuron\_projection\_development | PHGDH | 197 | 1 | 0.806056 | -0.142850 | 622 | 476.02 | 0.765305 |
| GO:0050801\_ion\_homeostasis | HIF1A | 197 | 1 | 0.806056 | -0.142850 | 622 | 476.02 | 0.765305 |
| GO:0007154\_cell\_communication | GPX1 | 1096 | 6 | 0.869305 | -0.141731 | 623 | 477.17 | 0.765923 |
| GO:0007154\_cell\_communication | HIF1A | 1096 | 6 | 0.869305 | -0.141731 | 623 | 477.17 | 0.765923 |
| GO:0007154\_cell\_communication | SOX2 | 1096 | 6 | 0.869305 | -0.141731 | 623 | 477.17 | 0.765923 |
| GO:0007154\_cell\_communication | SIX3 | 1096 | 6 | 0.869305 | -0.141731 | 623 | 477.17 | 0.765923 |
| GO:0007154\_cell\_communication | PRKACA | 1096 | 6 | 0.869305 | -0.141731 | 623 | 477.17 | 0.765923 |
| GO:0007154\_cell\_communication | CDK4 | 1096 | 6 | 0.869305 | -0.141731 | 623 | 477.17 | 0.765923 |
| GO:0080090\_regulation\_of\_primary\_metabolic\_process | HIF1A | 926 | 5 | 0.857414 | -0.141651 | 624 | 477.28 | 0.764872 |
| GO:0080090\_regulation\_of\_primary\_metabolic\_process | EIF2S1 | 926 | 5 | 0.857414 | -0.141651 | 624 | 477.28 | 0.764872 |
| GO:0080090\_regulation\_of\_primary\_metabolic\_process | SOX2 | 926 | 5 | 0.857414 | -0.141651 | 624 | 477.28 | 0.764872 |
| GO:0080090\_regulation\_of\_primary\_metabolic\_process | SIX3 | 926 | 5 | 0.857414 | -0.141651 | 624 | 477.28 | 0.764872 |
| GO:0080090\_regulation\_of\_primary\_metabolic\_process | CNOT7 | 926 | 5 | 0.857414 | -0.141651 | 624 | 477.28 | 0.764872 |
| GO:0000904\_cell\_morphogenesis\_involved\_in\_differentiation | HIF1A | 199 | 1 | 0.797955 | -0.140638 | 625 | 477.97 | 0.764752 |
| GO:0019219\_regulation\_of\_nucleobase\_\_nucleoside\_\_nucleotide\_and\_nucleic\_acid\_metabolic\_process | HIF1A | 757 | 4 | 0.839065 | -0.139110 | 626 | 478.42 | 0.764249 |
| GO:0019219\_regulation\_of\_nucleobase\_\_nucleoside\_\_nucleotide\_and\_nucleic\_acid\_metabolic\_process | SOX2 | 757 | 4 | 0.839065 | -0.139110 | 626 | 478.42 | 0.764249 |
| GO:0019219\_regulation\_of\_nucleobase\_\_nucleoside\_\_nucleotide\_and\_nucleic\_acid\_metabolic\_process | SIX3 | 757 | 4 | 0.839065 | -0.139110 | 626 | 478.42 | 0.764249 |
| GO:0019219\_regulation\_of\_nucleobase\_\_nucleoside\_\_nucleotide\_and\_nucleic\_acid\_metabolic\_process | CNOT7 | 757 | 4 | 0.839065 | -0.139110 | 626 | 478.42 | 0.764249 |
| GO:0006955\_immune\_response | GPX1 | 205 | 1 | 0.774601 | -0.134230 | 628 | 480.69 | 0.765430 |
| GO:0007243\_protein\_kinase\_cascade | GPX1 | 205 | 1 | 0.774601 | -0.134230 | 628 | 480.69 | 0.765430 |
| GO:0051171\_regulation\_of\_nitrogen\_compound\_metabolic\_process | HIF1A | 771 | 4 | 0.823829 | -0.130722 | 629 | 482.31 | 0.766789 |
| GO:0051171\_regulation\_of\_nitrogen\_compound\_metabolic\_process | SOX2 | 771 | 4 | 0.823829 | -0.130722 | 629 | 482.31 | 0.766789 |
| GO:0051171\_regulation\_of\_nitrogen\_compound\_metabolic\_process | SIX3 | 771 | 4 | 0.823829 | -0.130722 | 629 | 482.31 | 0.766789 |
| GO:0051171\_regulation\_of\_nitrogen\_compound\_metabolic\_process | CNOT7 | 771 | 4 | 0.823829 | -0.130722 | 629 | 482.31 | 0.766789 |
| GO:0007166\_cell\_surface\_receptor\_linked\_signal\_transduction | HIF1A | 597 | 3 | 0.797955 | -0.127627 | 630 | 483.31 | 0.767159 |
| GO:0007166\_cell\_surface\_receptor\_linked\_signal\_transduction | SOX2 | 597 | 3 | 0.797955 | -0.127627 | 630 | 483.31 | 0.767159 |
| GO:0007166\_cell\_surface\_receptor\_linked\_signal\_transduction | SIX3 | 597 | 3 | 0.797955 | -0.127627 | 630 | 483.31 | 0.767159 |
| GO:0035295\_tube\_development | DDR1 | 212 | 1 | 0.749024 | -0.127168 | 631 | 483.93 | 0.766926 |
| GO:0010033\_response\_to\_organic\_substance | DDR1 | 216 | 1 | 0.735153 | -0.123321 | 632 | 484.64 | 0.766835 |
| GO:0048583\_regulation\_of\_response\_to\_stimulus | GPX1 | 217 | 1 | 0.731765 | -0.122380 | 633 | 485.37 | 0.766777 |
| GO:0045892\_negative\_regulation\_of\_transcription\_\_DNA-dependent | SOX2 | 218 | 1 | 0.728409 | -0.121446 | 634 | 486.16 | 0.766814 |
| GO:0016043\_cellular\_component\_organization | HIF1A | 964 | 5 | 0.823616 | -0.121021 | 635 | 486.39 | 0.765969 |
| GO:0016043\_cellular\_component\_organization | PHGDH | 964 | 5 | 0.823616 | -0.121021 | 635 | 486.39 | 0.765969 |
| GO:0016043\_cellular\_component\_organization | BUB1B | 964 | 5 | 0.823616 | -0.121021 | 635 | 486.39 | 0.765969 |
| GO:0016043\_cellular\_component\_organization | ACTL6A | 964 | 5 | 0.823616 | -0.121021 | 635 | 486.39 | 0.765969 |
| GO:0016043\_cellular\_component\_organization | CDCA5 | 964 | 5 | 0.823616 | -0.121021 | 635 | 486.39 | 0.765969 |
| GO:0051253\_negative\_regulation\_of\_RNA\_metabolic\_process | SOX2 | 220 | 1 | 0.721787 | -0.119604 | 636 | 486.99 | 0.765708 |
| GO:0001701\_in\_utero\_embryonic\_development | HIF1A | 221 | 1 | 0.718521 | -0.118694 | 637 | 487.53 | 0.765353 |
| GO:0019953\_sexual\_reproduction | TRIP13 | 228 | 1 | 0.696461 | -0.112537 | 638 | 489.81 | 0.767727 |
| GO:0007167\_enzyme\_linked\_receptor\_protein\_signaling\_pathway | HIF1A | 229 | 1 | 0.693420 | -0.111687 | 639 | 490.62 | 0.767793 |
| GO:0050790\_regulation\_of\_catalytic\_activity | HIF1A | 233 | 1 | 0.681515 | -0.108356 | 640 | 492.32 | 0.769250 |
| GO:0031323\_regulation\_of\_cellular\_metabolic\_process | HIF1A | 1015 | 5 | 0.782232 | -0.097447 | 641 | 496.73 | 0.774930 |
| GO:0031323\_regulation\_of\_cellular\_metabolic\_process | EIF2S1 | 1015 | 5 | 0.782232 | -0.097447 | 641 | 496.73 | 0.774930 |
| GO:0031323\_regulation\_of\_cellular\_metabolic\_process | SOX2 | 1015 | 5 | 0.782232 | -0.097447 | 641 | 496.73 | 0.774930 |
| GO:0031323\_regulation\_of\_cellular\_metabolic\_process | SIX3 | 1015 | 5 | 0.782232 | -0.097447 | 641 | 496.73 | 0.774930 |
| GO:0031323\_regulation\_of\_cellular\_metabolic\_process | CNOT7 | 1015 | 5 | 0.782232 | -0.097447 | 641 | 496.73 | 0.774930 |
| GO:0007389\_pattern\_specification\_process | SIX3 | 250 | 1 | 0.635172 | -0.095364 | 642 | 498.75 | 0.776869 |
| GO:0050794\_regulation\_of\_cellular\_process | ALDH1A1 | 2190 | 12 | 0.870099 | -0.095170 | 643 | 498.85 | 0.775816 |
| GO:0050794\_regulation\_of\_cellular\_process | GPX1 | 2190 | 12 | 0.870099 | -0.095170 | 643 | 498.85 | 0.775816 |
| GO:0050794\_regulation\_of\_cellular\_process | DDR1 | 2190 | 12 | 0.870099 | -0.095170 | 643 | 498.85 | 0.775816 |
| GO:0050794\_regulation\_of\_cellular\_process | HIF1A | 2190 | 12 | 0.870099 | -0.095170 | 643 | 498.85 | 0.775816 |
| GO:0050794\_regulation\_of\_cellular\_process | BTG1 | 2190 | 12 | 0.870099 | -0.095170 | 643 | 498.85 | 0.775816 |
| GO:0050794\_regulation\_of\_cellular\_process | MARCKSL1 | 2190 | 12 | 0.870099 | -0.095170 | 643 | 498.85 | 0.775816 |
| GO:0050794\_regulation\_of\_cellular\_process | EIF2S1 | 2190 | 12 | 0.870099 | -0.095170 | 643 | 498.85 | 0.775816 |
| GO:0050794\_regulation\_of\_cellular\_process | SOX2 | 2190 | 12 | 0.870099 | -0.095170 | 643 | 498.85 | 0.775816 |
| GO:0050794\_regulation\_of\_cellular\_process | SIX3 | 2190 | 12 | 0.870099 | -0.095170 | 643 | 498.85 | 0.775816 |
| GO:0050794\_regulation\_of\_cellular\_process | PRKACA | 2190 | 12 | 0.870099 | -0.095170 | 643 | 498.85 | 0.775816 |
| GO:0050794\_regulation\_of\_cellular\_process | CNOT7 | 2190 | 12 | 0.870099 | -0.095170 | 643 | 498.85 | 0.775816 |
| GO:0050794\_regulation\_of\_cellular\_process | CDK4 | 2190 | 12 | 0.870099 | -0.095170 | 643 | 498.85 | 0.775816 |
| GO:0030097\_hemopoiesis | HIF1A | 253 | 1 | 0.627641 | -0.093252 | 644 | 500.32 | 0.776894 |
| GO:0030030\_cell\_projection\_organization | PHGDH | 263 | 1 | 0.603776 | -0.086570 | 645 | 503.34 | 0.780372 |
| GO:0051179\_localization | GPX1 | 1058 | 5 | 0.750440 | -0.080767 | 646 | 505.45 | 0.782430 |
| GO:0051179\_localization | NPC2 | 1058 | 5 | 0.750440 | -0.080767 | 646 | 505.45 | 0.782430 |
| GO:0051179\_localization | HIF1A | 1058 | 5 | 0.750440 | -0.080767 | 646 | 505.45 | 0.782430 |
| GO:0051179\_localization | SIX3 | 1058 | 5 | 0.750440 | -0.080767 | 646 | 505.45 | 0.782430 |
| GO:0051179\_localization | PRKACA | 1058 | 5 | 0.750440 | -0.080767 | 646 | 505.45 | 0.782430 |
| GO:0048534\_hemopoietic\_or\_lymphoid\_organ\_development | HIF1A | 277 | 1 | 0.573260 | -0.078062 | 647 | 507.31 | 0.784096 |
| GO:0065009\_regulation\_of\_molecular\_function | HIF1A | 279 | 1 | 0.569151 | -0.076921 | 648 | 507.91 | 0.783812 |
| GO:0000902\_cell\_morphogenesis | HIF1A | 283 | 1 | 0.561106 | -0.074692 | 649 | 508.93 | 0.784176 |
| GO:0002376\_immune\_system\_process | GPX1 | 505 | 2 | 0.628884 | -0.073961 | 650 | 509.43 | 0.783738 |
| GO:0002376\_immune\_system\_process | HIF1A | 505 | 2 | 0.628884 | -0.073961 | 650 | 509.43 | 0.783738 |
| GO:0006810\_transport | NPC2 | 718 | 3 | 0.663481 | -0.069215 | 651 | 511.13 | 0.785146 |
| GO:0006810\_transport | SIX3 | 718 | 3 | 0.663481 | -0.069215 | 651 | 511.13 | 0.785146 |
| GO:0006810\_transport | PRKACA | 718 | 3 | 0.663481 | -0.069215 | 651 | 511.13 | 0.785146 |
| GO:0002520\_immune\_system\_development | HIF1A | 295 | 1 | 0.538282 | -0.068405 | 652 | 512.28 | 0.785706 |
| GO:0051234\_establishment\_of\_localization | NPC2 | 729 | 3 | 0.653470 | -0.065371 | 653 | 513.38 | 0.786187 |
| GO:0051234\_establishment\_of\_localization | SIX3 | 729 | 3 | 0.653470 | -0.065371 | 653 | 513.38 | 0.786187 |
| GO:0051234\_establishment\_of\_localization | PRKACA | 729 | 3 | 0.653470 | -0.065371 | 653 | 513.38 | 0.786187 |
| GO:0032989\_cellular\_component\_morphogenesis | HIF1A | 307 | 1 | 0.517241 | -0.062670 | 654 | 514.86 | 0.787248 |
| GO:0007242\_intracellular\_signaling\_cascade | GPX1 | 411 | 1 | 0.386358 | -0.029600 | 655 | 529.38 | 0.808214 |
| GO:0008150\_biological\_process | HSP90AB1 | 4605 | 29 | 1.000000 | 0.000000 | 2333 | 2317.39 | 0.993309 |
| GO:0008150\_biological\_process | MARCKSL1 | 4605 | 29 | 1.000000 | 0.000000 | 2333 | 2317.39 | 0.993309 |
| GO:0008150\_biological\_process | SOX2 | 4605 | 29 | 1.000000 | 0.000000 | 2333 | 2317.39 | 0.993309 |
| GO:0008150\_biological\_process | CNOT7 | 4605 | 29 | 1.000000 | 0.000000 | 2333 | 2317.39 | 0.993309 |
| GO:0008150\_biological\_process | MTHFD1 | 4605 | 29 | 1.000000 | 0.000000 | 2333 | 2317.39 | 0.993309 |
| GO:0008150\_biological\_process | ALDH1A1 | 4605 | 29 | 1.000000 | 0.000000 | 2333 | 2317.39 | 0.993309 |
| GO:0008150\_biological\_process | GPX1 | 4605 | 29 | 1.000000 | 0.000000 | 2333 | 2317.39 | 0.993309 |
| GO:0008150\_biological\_process | TPI1 | 4605 | 29 | 1.000000 | 0.000000 | 2333 | 2317.39 | 0.993309 |
| GO:0008150\_biological\_process | MCM7 | 4605 | 29 | 1.000000 | 0.000000 | 2333 | 2317.39 | 0.993309 |
| GO:0008150\_biological\_process | PTN | 4605 | 29 | 1.000000 | 0.000000 | 2333 | 2317.39 | 0.993309 |
| GO:0008150\_biological\_process | PRKACA | 4605 | 29 | 1.000000 | 0.000000 | 2333 | 2317.39 | 0.993309 |
| GO:0008150\_biological\_process | ACTL6A | 4605 | 29 | 1.000000 | 0.000000 | 2333 | 2317.39 | 0.993309 |
| GO:0008150\_biological\_process | FH1 | 4605 | 29 | 1.000000 | 0.000000 | 2333 | 2317.39 | 0.993309 |
| GO:0008150\_biological\_process | CDCA5 | 4605 | 29 | 1.000000 | 0.000000 | 2333 | 2317.39 | 0.993309 |
| GO:0008150\_biological\_process | TRIP13 | 4605 | 29 | 1.000000 | 0.000000 | 2333 | 2317.39 | 0.993309 |
| GO:0008150\_biological\_process | SIX3 | 4605 | 29 | 1.000000 | 0.000000 | 2333 | 2317.39 | 0.993309 |
| GO:0008150\_biological\_process | SF3A2 | 4605 | 29 | 1.000000 | 0.000000 | 2333 | 2317.39 | 0.993309 |
| GO:0008150\_biological\_process | CDK4 | 4605 | 29 | 1.000000 | 0.000000 | 2333 | 2317.39 | 0.993309 |
| GO:0008150\_biological\_process | PPP1CB | 4605 | 29 | 1.000000 | 0.000000 | 2333 | 2317.39 | 0.993309 |
| GO:0008150\_biological\_process | DDR1 | 4605 | 29 | 1.000000 | 0.000000 | 2333 | 2317.39 | 0.993309 |
| GO:0008150\_biological\_process | NPC2 | 4605 | 29 | 1.000000 | 0.000000 | 2333 | 2317.39 | 0.993309 |
| GO:0008150\_biological\_process | HIF1A | 4605 | 29 | 1.000000 | 0.000000 | 2333 | 2317.39 | 0.993309 |
| GO:0008150\_biological\_process | NME1 | 4605 | 29 | 1.000000 | 0.000000 | 2333 | 2317.39 | 0.993309 |
| GO:0008150\_biological\_process | BTG1 | 4605 | 29 | 1.000000 | 0.000000 | 2333 | 2317.39 | 0.993309 |
| GO:0008150\_biological\_process | POLD1 | 4605 | 29 | 1.000000 | 0.000000 | 2333 | 2317.39 | 0.993309 |
| GO:0008150\_biological\_process | EIF2S1 | 4605 | 29 | 1.000000 | 0.000000 | 2333 | 2317.39 | 0.993309 |
| GO:0008150\_biological\_process | PHGDH | 4605 | 29 | 1.000000 | 0.000000 | 2333 | 2317.39 | 0.993309 |
| GO:0008150\_biological\_process | BUB1B | 4605 | 29 | 1.000000 | 0.000000 | 2333 | 2317.39 | 0.993309 |
| GO:0008150\_biological\_process | PBX3 | 4605 | 29 | 1.000000 | 0.000000 | 2333 | 2317.39 | 0.993309 |
