# Supplementary material for: RedundancyMiner: De-replication of redundant GO categories in microarray and proteomics analysis
Source: BMC Bioinformatics. 2011 Feb 10;12:52. doi: 10.1186/1471-2105-12-52 (PMC3223614; doi:10.1186/1471-2105-12-52)
Supplement: Additional file 8 — Retinal development HTGM download. compressed package of the results of running HTGM on the retinal development genes list. [file 1471-2105-12-52-S8.ZIP › SCENARIO_2_MODIFIED/total.txt.total.txt.dir/Exp1_BestClusterMap_LEIGS_KM_24.csv.join.12.txt.dir/Exp1_BestClusterMap_LEIGS_KM_24.csv.join.12.txt.change.html]

Category Summary Report for Exp1\_BestClusterMap\_LEIGS\_KM\_24.csv.join.12.txt

# Category Summary Report for Exp1\_BestClusterMap\_LEIGS\_KM\_24.csv.join.12.txt

| HYPERLINKED GO CATEGORY | TOTAL GENES | CHANGED GENES | ENRICHMENT | LOG10(p) | CUMULATIVE NUMBER OF CATEGORIES | CUMULATIVE RANDOMS LOWER BOUND | CUMULATIVE RANDOMS MEAN | CUMULATIVE RANDOMS UPPER BOUND | FALSE DISCOVERY RATE |
| --- | --- | --- | --- | --- | --- | --- | --- | --- | --- |
| GO:0060235\_lens\_induction\_in\_camera-type\_eye | 4 | 2 |  |  |  |  |  |  |  |  |
| GO:0034599\_cellular\_response\_to\_oxidative\_stress | 7 | 2 | 45.369458 | -3.103092 | 1 | -1.415197 | 0.98 | 3.375197 | 0.980000 |
| GO:0002089\_lens\_morphogenesis\_in\_camera-type\_eye | 12 | 2 | 26.465517 | -2.614256 | 2 | -2.077044 | 2.91 | 7.897044 | 1.455000 |
| GO:0048593\_camera-type\_eye\_morphogenesis | 44 | 3 | 10.826803 | -2.601901 | 3 | -2.122988 | 2.96 | 8.042988 | 0.986667 |
| GO:0048568\_embryonic\_organ\_development | 55 | 3 | 8.661442 | -2.325212 | 4 | -2.287503 | 4.64 | 11.567503 | 1.160000 |
| GO:0006261\_DNA-dependent\_DNA\_replication | 17 | 2 | 18.681542 | -2.308735 | 5 | -2.338366 | 4.9 | 12.138366 | 0.980000 |
| GO:0030178\_negative\_regulation\_of\_Wnt\_receptor\_signaling\_pathway | 18 | 2 | 17.643678 | -2.259276 | 6 | -2.266153 | 5.5 | 13.266153 | 0.916667 |
| GO:0002248\_connective\_tissue\_replacement\_during\_inflammatory\_response | 1 | 1 |  |  |  |  |  |  |  |  |
| GO:0006544\_glycine\_metabolic\_process | 1 | 1 |  |  |  |  |  |  |  |  |
| GO:0010634\_positive\_regulation\_of\_epithelial\_cell\_migration | 1 | 1 |  |  |  |  |  |  |  |  |
| GO:0018158\_protein\_amino\_acid\_oxidation | 1 | 1 |  |  |  |  |  |  |  |  |
| GO:0019682\_glyceraldehyde-3-phosphate\_metabolic\_process | 1 | 1 |  |  |  |  |  |  |  |  |
| GO:0032365\_intracellular\_lipid\_transport | 1 | 1 |  |  |  |  |  |  |  |  |
| GO:0032366\_intracellular\_sterol\_transport | 1 | 1 |  |  |  |  |  |  |  |  |
| GO:0032367\_intracellular\_cholesterol\_transport | 1 | 1 |  |  |  |  |  |  |  |  |
| GO:0045004\_DNA\_replication\_proofreading | 1 | 1 |  |  |  |  |  |  |  |  |
| GO:0046827\_positive\_regulation\_of\_protein\_export\_from\_nucleus | 1 | 1 |  |  |  |  |  |  |  |  |
| GO:0031128\_developmental\_induction | 20 | 2 | 15.879310 | -2.168598 | 8 | -2.179689 | 6.38 | 14.939689 | 0.797500 |
| GO:0045168\_cell-cell\_signaling\_involved\_in\_cell\_fate\_specification | 20 | 2 | 15.879310 | -2.168598 | 8 | -2.179689 | 6.38 | 14.939689 | 0.797500 |
| GO:0033554\_cellular\_response\_to\_stress | 196 | 5 | 4.050844 | -2.164771 | 9 | -2.169384 | 6.4 | 14.969384 | 0.711111 |
| GO:0044238\_primary\_metabolic\_process | 1905 | 19 | 1.583763 | -2.135377 | 10 | -2.355279 | 6.67 | 15.695279 | 0.667000 |
| GO:0043687\_post-translational\_protein\_modification | 384 | 7 | 2.894666 | -2.083172 | 11 | -2.398159 | 7.51 | 17.418159 | 0.682727 |
| GO:0048592\_eye\_morphogenesis | 70 | 3 | 6.805419 | -2.033248 | 12 | -2.669525 | 7.94 | 18.549525 | 0.661667 |
| GO:0000280\_nuclear\_division | 24 | 2 | 13.232759 | -2.013205 | 14 | -2.560412 | 8.24 | 19.040412 | 0.588571 |
| GO:0007067\_mitosis | 24 | 2 | 13.232759 | -2.013205 | 14 | -2.560412 | 8.24 | 19.040412 | 0.588571 |
| GO:0000087\_M\_phase\_of\_mitotic\_cell\_cycle | 25 | 2 | 12.703448 | -1.978682 | 16 | -2.610848 | 8.63 | 19.870848 | 0.539375 |
| GO:0048285\_organelle\_fission | 25 | 2 | 12.703448 | -1.978682 | 16 | -2.610848 | 8.63 | 19.870848 | 0.539375 |
| GO:0044237\_cellular\_metabolic\_process | 1974 | 19 | 1.528404 | -1.940463 | 17 | -2.663437 | 9.03 | 20.723437 | 0.531176 |
| GO:0002072\_optic\_cup\_morphogenesis\_involved\_in\_camera-type\_eye\_development | 2 | 1 |  |  |  |  |  |  |  |  |
| GO:0002246\_healing\_during\_inflammatory\_response | 2 | 1 |  |  |  |  |  |  |  |  |
| GO:0006089\_lactate\_metabolic\_process | 2 | 1 |  |  |  |  |  |  |  |  |
| GO:0006563\_L-serine\_metabolic\_process | 2 | 1 |  |  |  |  |  |  |  |  |
| GO:0006566\_threonine\_metabolic\_process | 2 | 1 |  |  |  |  |  |  |  |  |
| GO:0009608\_response\_to\_symbiont | 2 | 1 |  |  |  |  |  |  |  |  |
| GO:0009609\_response\_to\_symbiotic\_bacterium | 2 | 1 |  |  |  |  |  |  |  |  |
| GO:0014850\_response\_to\_muscle\_activity | 2 | 1 |  |  |  |  |  |  |  |  |
| GO:0030910\_olfactory\_placode\_formation | 2 | 1 |  |  |  |  |  |  |  |  |
| GO:0033194\_response\_to\_hydroperoxide | 2 | 1 |  |  |  |  |  |  |  |  |
| GO:0043618\_regulation\_of\_transcription\_from\_RNA\_polymerase\_II\_promoter\_in\_response\_to\_stress | 2 | 1 |  |  |  |  |  |  |  |  |
| GO:0043619\_regulation\_of\_transcription\_from\_RNA\_polymerase\_II\_promoter\_in\_response\_to\_oxidative\_stress | 2 | 1 |  |  |  |  |  |  |  |  |
| GO:0043620\_regulation\_of\_transcription\_in\_response\_to\_stress | 2 | 1 |  |  |  |  |  |  |  |  |
| GO:0045005\_maintenance\_of\_fidelity\_during\_DNA-dependent\_DNA\_replication | 2 | 1 |  |  |  |  |  |  |  |  |
| GO:0050973\_detection\_of\_mechanical\_stimulus\_involved\_in\_equilibrioception | 2 | 1 |  |  |  |  |  |  |  |  |
| GO:0051541\_elastin\_metabolic\_process | 2 | 1 |  |  |  |  |  |  |  |  |
| GO:0051702\_interaction\_with\_symbiont | 2 | 1 |  |  |  |  |  |  |  |  |
| GO:0034960\_cellular\_biopolymer\_metabolic\_process | 1395 | 15 | 1.707453 | -1.900987 | 18 | -2.520011 | 9.41 | 21.340011 | 0.522778 |
| GO:0002088\_lens\_development\_in\_camera-type\_eye | 28 | 2 | 11.342365 | -1.883375 | 20 | -2.445121 | 9.77 | 21.985121 | 0.488500 |
| GO:0030111\_regulation\_of\_Wnt\_receptor\_signaling\_pathway | 28 | 2 | 11.342365 | -1.883375 | 20 | -2.445121 | 9.77 | 21.985121 | 0.488500 |
| GO:0007049\_cell\_cycle | 238 | 5 | 3.335990 | -1.819383 | 21 | -1.990118 | 10.75 | 23.490118 | 0.511905 |
| GO:0022402\_cell\_cycle\_process | 155 | 4 | 4.097887 | -1.817636 | 22 | -2.002232 | 10.78 | 23.562232 | 0.490000 |
| GO:0000279\_M\_phase | 85 | 3 | 5.604462 | -1.804329 | 23 | -1.961623 | 10.91 | 23.781623 | 0.474348 |
| GO:0043170\_macromolecule\_metabolic\_process | 1576 | 16 | 1.612113 | -1.790052 | 24 | -1.598561 | 11.6 | 24.798561 | 0.483333 |
| GO:0006464\_protein\_modification\_process | 439 | 7 | 2.532008 | -1.778019 | 25 | -1.532848 | 11.72 | 24.972848 | 0.468800 |
| GO:0044260\_cellular\_macromolecule\_metabolic\_process | 1447 | 15 | 1.646093 | -1.748775 | 26 | -1.324972 | 12.22 | 25.764972 | 0.470000 |
| GO:0021536\_diencephalon\_development | 33 | 2 | 9.623824 | -1.746660 | 27 | -1.284882 | 12.49 | 26.264882 | 0.462593 |
| GO:0006287\_base-excision\_repair\_\_gap-filling | 3 | 1 |  |  |  |  |  |  |  |  |
| GO:0009448\_gamma-aminobutyric\_acid\_metabolic\_process | 3 | 1 |  |  |  |  |  |  |  |  |
| GO:0010632\_regulation\_of\_epithelial\_cell\_migration | 3 | 1 |  |  |  |  |  |  |  |  |
| GO:0019605\_butyrate\_metabolic\_process | 3 | 1 |  |  |  |  |  |  |  |  |
| GO:0021797\_forebrain\_anterior\_posterior\_pattern\_formation | 3 | 1 |  |  |  |  |  |  |  |  |
| GO:0043586\_tongue\_development | 3 | 1 |  |  |  |  |  |  |  |  |
| GO:0046825\_regulation\_of\_protein\_export\_from\_nucleus | 3 | 1 |  |  |  |  |  |  |  |  |
| GO:0048852\_diencephalon\_morphogenesis | 3 | 1 |  |  |  |  |  |  |  |  |
| GO:0050957\_equilibrioception | 3 | 1 |  |  |  |  |  |  |  |  |
| GO:0051450\_myoblast\_proliferation | 3 | 1 |  |  |  |  |  |  |  |  |
| GO:0060055\_angiogenesis\_involved\_in\_wound\_healing | 3 | 1 |  |  |  |  |  |  |  |  |
| GO:0043412\_biopolymer\_modification | 458 | 7 | 2.426969 | -1.684658 | 28 | -0.662187 | 14.18 | 29.022187 | 0.506429 |
| GO:0021510\_spinal\_cord\_development | 36 | 2 | 8.821839 | -1.675003 | 29 | -0.413149 | 14.62 | 29.653149 | 0.504138 |
| GO:0060249\_anatomical\_structure\_homeostasis | 96 | 3 | 4.962284 | -1.664038 | 30 | -0.347553 | 14.81 | 29.967553 | 0.493667 |
| GO:0009967\_positive\_regulation\_of\_signal\_transduction | 98 | 3 | 4.861013 | -1.640535 | 31 | -0.047431 | 15.57 | 31.187431 | 0.502258 |
| GO:0046777\_protein\_amino\_acid\_autophosphorylation | 38 | 2 | 8.357532 | -1.630751 | 32 | 0.199532 | 16.09 | 31.980468 | 0.502812 |
| GO:0043283\_biopolymer\_metabolic\_process | 1490 | 15 | 1.598588 | -1.630143 | 33 | 0.192589 | 16.12 | 32.047411 | 0.488485 |
| GO:0006547\_histidine\_metabolic\_process | 4 | 1 |  |  |  |  |  |  |  |  |
| GO:0006548\_histidine\_catabolic\_process | 4 | 1 |  |  |  |  |  |  |  |  |
| GO:0007144\_female\_meiosis\_I | 4 | 1 |  |  |  |  |  |  |  |  |
| GO:0009075\_histidine\_family\_amino\_acid\_metabolic\_process | 4 | 1 |  |  |  |  |  |  |  |  |
| GO:0009077\_histidine\_family\_amino\_acid\_catabolic\_process | 4 | 1 |  |  |  |  |  |  |  |  |
| GO:0010631\_epithelial\_cell\_migration | 4 | 1 |  |  |  |  |  |  |  |  |
| GO:0019530\_taurine\_metabolic\_process | 4 | 1 |  |  |  |  |  |  |  |  |
| GO:0030949\_positive\_regulation\_of\_vascular\_endothelial\_growth\_factor\_receptor\_signaling\_pathway | 4 | 1 |  |  |  |  |  |  |  |  |
| GO:0034614\_cellular\_response\_to\_reactive\_oxygen\_species | 4 | 1 |  |  |  |  |  |  |  |  |
| GO:0042541\_hemoglobin\_biosynthetic\_process | 4 | 1 |  |  |  |  |  |  |  |  |
| GO:0042744\_hydrogen\_peroxide\_catabolic\_process | 4 | 1 |  |  |  |  |  |  |  |  |
| GO:0043534\_blood\_vessel\_endothelial\_cell\_migration | 4 | 1 |  |  |  |  |  |  |  |  |
| GO:0044403\_symbiosis\_\_encompassing\_mutualism\_through\_parasitism | 4 | 1 |  |  |  |  |  |  |  |  |
| GO:0044419\_interspecies\_interaction\_between\_organisms | 4 | 1 |  |  |  |  |  |  |  |  |
| GO:0045747\_positive\_regulation\_of\_Notch\_signaling\_pathway | 4 | 1 |  |  |  |  |  |  |  |  |
| GO:0051897\_positive\_regulation\_of\_protein\_kinase\_B\_signaling\_cascade | 4 | 1 |  |  |  |  |  |  |  |  |
| GO:0060900\_embryonic\_camera-type\_eye\_formation | 4 | 1 |  |  |  |  |  |  |  |  |
| GO:0070301\_cellular\_response\_to\_hydrogen\_peroxide | 4 | 1 |  |  |  |  |  |  |  |  |
| GO:0019752\_carboxylic\_acid\_metabolic\_process | 181 | 4 | 3.509240 | -1.594785 | 35 | 0.491854 | 16.88 | 33.268146 | 0.482286 |
| GO:0043436\_oxoacid\_metabolic\_process | 181 | 4 | 3.509240 | -1.594785 | 35 | 0.491854 | 16.88 | 33.268146 | 0.482286 |
| GO:0006082\_organic\_acid\_metabolic\_process | 182 | 4 | 3.489958 | -1.587017 | 36 | 0.632397 | 17.26 | 33.887603 | 0.479444 |
| GO:0051716\_cellular\_response\_to\_stimulus | 273 | 5 | 2.908299 | -1.585942 | 37 | 0.662595 | 17.29 | 33.917405 | 0.467297 |
| GO:0042180\_cellular\_ketone\_metabolic\_process | 183 | 4 | 3.470888 | -1.579301 | 38 | 0.832781 | 17.47 | 34.107219 | 0.459737 |
| GO:0006260\_DNA\_replication | 41 | 2 | 7.746005 | -1.568933 | 40 | 1.107247 | 18.08 | 35.052753 | 0.452000 |
| GO:0006979\_response\_to\_oxidative\_stress | 41 | 2 | 7.746005 | -1.568933 | 40 | 1.107247 | 18.08 | 35.052753 | 0.452000 |
| GO:0048646\_anatomical\_structure\_formation\_involved\_in\_morphogenesis | 277 | 5 | 2.866302 | -1.561766 | 41 | 1.109765 | 18.17 | 35.230235 | 0.443171 |
| GO:0008152\_metabolic\_process | 2133 | 19 | 1.414472 | -1.537099 | 42 | 1.496926 | 18.84 | 36.183074 | 0.448571 |
| GO:0010647\_positive\_regulation\_of\_cell\_communication | 110 | 3 | 4.330721 | -1.510393 | 44 | 1.884364 | 20.34 | 38.795636 | 0.462273 |
| GO:0043010\_camera-type\_eye\_development | 110 | 3 | 4.330721 | -1.510393 | 44 | 1.884364 | 20.34 | 38.795636 | 0.462273 |
| GO:0000239\_pachytene | 5 | 1 | 31.758621 | -1.507145 | 60 | 8.774360 | 29.12 | 49.465640 | 0.485333 |
| GO:0002862\_negative\_regulation\_of\_inflammatory\_response\_to\_antigenic\_stimulus | 5 | 1 | 31.758621 | -1.507145 | 60 | 8.774360 | 29.12 | 49.465640 | 0.485333 |
| GO:0006268\_DNA\_unwinding\_during\_replication | 5 | 1 | 31.758621 | -1.507145 | 60 | 8.774360 | 29.12 | 49.465640 | 0.485333 |
| GO:0006541\_glutamine\_metabolic\_process | 5 | 1 | 31.758621 | -1.507145 | 60 | 8.774360 | 29.12 | 49.465640 | 0.485333 |
| GO:0007091\_mitotic\_metaphase\_anaphase\_transition | 5 | 1 | 31.758621 | -1.507145 | 60 | 8.774360 | 29.12 | 49.465640 | 0.485333 |
| GO:0008631\_induction\_of\_apoptosis\_by\_oxidative\_stress | 5 | 1 | 31.758621 | -1.507145 | 60 | 8.774360 | 29.12 | 49.465640 | 0.485333 |
| GO:0032508\_DNA\_duplex\_unwinding | 5 | 1 | 31.758621 | -1.507145 | 60 | 8.774360 | 29.12 | 49.465640 | 0.485333 |
| GO:0035238\_vitamin\_A\_biosynthetic\_process | 5 | 1 | 31.758621 | -1.507145 | 60 | 8.774360 | 29.12 | 49.465640 | 0.485333 |
| GO:0042362\_fat-soluble\_vitamin\_biosynthetic\_process | 5 | 1 | 31.758621 | -1.507145 | 60 | 8.774360 | 29.12 | 49.465640 | 0.485333 |
| GO:0042904\_9-cis-retinoic\_acid\_biosynthetic\_process | 5 | 1 | 31.758621 | -1.507145 | 60 | 8.774360 | 29.12 | 49.465640 | 0.485333 |
| GO:0042905\_9-cis-retinoic\_acid\_metabolic\_process | 5 | 1 | 31.758621 | -1.507145 | 60 | 8.774360 | 29.12 | 49.465640 | 0.485333 |
| GO:0043403\_skeletal\_muscle\_regeneration | 5 | 1 | 31.758621 | -1.507145 | 60 | 8.774360 | 29.12 | 49.465640 | 0.485333 |
| GO:0043555\_regulation\_of\_translation\_in\_response\_to\_stress | 5 | 1 | 31.758621 | -1.507145 | 60 | 8.774360 | 29.12 | 49.465640 | 0.485333 |
| GO:0043558\_regulation\_of\_translational\_initiation\_in\_response\_to\_stress | 5 | 1 | 31.758621 | -1.507145 | 60 | 8.774360 | 29.12 | 49.465640 | 0.485333 |
| GO:0045648\_positive\_regulation\_of\_erythrocyte\_differentiation | 5 | 1 | 31.758621 | -1.507145 | 60 | 8.774360 | 29.12 | 49.465640 | 0.485333 |
| GO:0046459\_short-chain\_fatty\_acid\_metabolic\_process | 5 | 1 | 31.758621 | -1.507145 | 60 | 8.774360 | 29.12 | 49.465640 | 0.485333 |
| GO:0001885\_endothelial\_cell\_development | 6 | 1 | 26.465517 | -1.429282 | 67 | 14.552174 | 37.88 | 61.207826 | 0.565373 |
| GO:0009069\_serine\_family\_amino\_acid\_metabolic\_process | 6 | 1 | 26.465517 | -1.429282 | 67 | 14.552174 | 37.88 | 61.207826 | 0.565373 |
| GO:0014823\_response\_to\_activity | 6 | 1 | 26.465517 | -1.429282 | 67 | 14.552174 | 37.88 | 61.207826 | 0.565373 |
| GO:0030947\_regulation\_of\_vascular\_endothelial\_growth\_factor\_receptor\_signaling\_pathway | 6 | 1 | 26.465517 | -1.429282 | 67 | 14.552174 | 37.88 | 61.207826 | 0.565373 |
| GO:0032392\_DNA\_geometric\_change | 6 | 1 | 26.465517 | -1.429282 | 67 | 14.552174 | 37.88 | 61.207826 | 0.565373 |
| GO:0042246\_tissue\_regeneration | 6 | 1 | 26.465517 | -1.429282 | 67 | 14.552174 | 37.88 | 61.207826 | 0.565373 |
| GO:0048853\_forebrain\_morphogenesis | 6 | 1 | 26.465517 | -1.429282 | 67 | 14.552174 | 37.88 | 61.207826 | 0.565373 |
| GO:0022403\_cell\_cycle\_phase | 119 | 3 | 4.003187 | -1.423367 | 68 | 14.906521 | 38.44 | 61.973479 | 0.565294 |
| GO:0006520\_cellular\_amino\_acid\_metabolic\_process | 51 | 2 | 6.227181 | -1.393999 | 70 | 15.486476 | 39.83 | 64.173524 | 0.569000 |
| GO:0044106\_cellular\_amine\_metabolic\_process | 51 | 2 | 6.227181 | -1.393999 | 70 | 15.486476 | 39.83 | 64.173524 | 0.569000 |
| GO:0001556\_oocyte\_maturation | 7 | 1 | 22.684729 | -1.363652 | 83 | 21.193132 | 48.45 | 75.706868 | 0.583735 |
| GO:0002052\_positive\_regulation\_of\_neuroblast\_proliferation | 7 | 1 | 22.684729 | -1.363652 | 83 | 21.193132 | 48.45 | 75.706868 | 0.583735 |
| GO:0002087\_regulation\_of\_respiratory\_gaseous\_exchange\_by\_neurological\_system\_process | 7 | 1 | 22.684729 | -1.363652 | 83 | 21.193132 | 48.45 | 75.706868 | 0.583735 |
| GO:0008299\_isoprenoid\_biosynthetic\_process | 7 | 1 | 22.684729 | -1.363652 | 83 | 21.193132 | 48.45 | 75.706868 | 0.583735 |
| GO:0020027\_hemoglobin\_metabolic\_process | 7 | 1 | 22.684729 | -1.363652 | 83 | 21.193132 | 48.45 | 75.706868 | 0.583735 |
| GO:0021516\_dorsal\_spinal\_cord\_development | 7 | 1 | 22.684729 | -1.363652 | 83 | 21.193132 | 48.45 | 75.706868 | 0.583735 |
| GO:0021984\_adenohypophysis\_development | 7 | 1 | 22.684729 | -1.363652 | 83 | 21.193132 | 48.45 | 75.706868 | 0.583735 |
| GO:0042572\_retinol\_metabolic\_process | 7 | 1 | 22.684729 | -1.363652 | 83 | 21.193132 | 48.45 | 75.706868 | 0.583735 |
| GO:0043584\_nose\_development | 7 | 1 | 22.684729 | -1.363652 | 83 | 21.193132 | 48.45 | 75.706868 | 0.583735 |
| GO:0044065\_regulation\_of\_respiratory\_system\_process | 7 | 1 | 22.684729 | -1.363652 | 83 | 21.193132 | 48.45 | 75.706868 | 0.583735 |
| GO:0045668\_negative\_regulation\_of\_osteoblast\_differentiation | 7 | 1 | 22.684729 | -1.363652 | 83 | 21.193132 | 48.45 | 75.706868 | 0.583735 |
| GO:0046824\_positive\_regulation\_of\_nucleocytoplasmic\_transport | 7 | 1 | 22.684729 | -1.363652 | 83 | 21.193132 | 48.45 | 75.706868 | 0.583735 |
| GO:0060788\_ectodermal\_placode\_formation | 7 | 1 | 22.684729 | -1.363652 | 83 | 21.193132 | 48.45 | 75.706868 | 0.583735 |
| GO:0019538\_protein\_metabolic\_process | 655 | 8 | 1.939458 | -1.353555 | 84 | 21.322031 | 48.77 | 76.217969 | 0.580595 |
| GO:0007605\_sensory\_perception\_of\_sound | 55 | 2 | 5.774295 | -1.334470 | 85 | 21.845847 | 49.81 | 77.774153 | 0.586000 |
| GO:0007423\_sensory\_organ\_development | 219 | 4 | 2.900331 | -1.332657 | 86 | 21.891844 | 49.95 | 78.008156 | 0.580814 |
| GO:0032787\_monocarboxylic\_acid\_metabolic\_process | 130 | 3 | 3.664456 | -1.327184 | 87 | 21.990389 | 50.18 | 78.369611 | 0.576782 |
| GO:0008283\_cell\_proliferation | 544 | 7 | 2.043294 | -1.323406 | 88 | 21.976738 | 50.21 | 78.443262 | 0.570568 |
| GO:0001708\_cell\_fate\_specification | 56 | 2 | 5.671182 | -1.320346 | 89 | 22.140508 | 50.68 | 79.219492 | 0.569438 |
| GO:0006284\_base-excision\_repair | 8 | 1 | 19.849138 | -1.306976 | 94 | 26.536975 | 57.42 | 88.303025 | 0.610851 |
| GO:0007131\_reciprocal\_meiotic\_recombination | 8 | 1 | 19.849138 | -1.306976 | 94 | 26.536975 | 57.42 | 88.303025 | 0.610851 |
| GO:0007141\_male\_meiosis\_I | 8 | 1 | 19.849138 | -1.306976 | 94 | 26.536975 | 57.42 | 88.303025 | 0.610851 |
| GO:0008593\_regulation\_of\_Notch\_signaling\_pathway | 8 | 1 | 19.849138 | -1.306976 | 94 | 26.536975 | 57.42 | 88.303025 | 0.610851 |
| GO:0043542\_endothelial\_cell\_migration | 8 | 1 | 19.849138 | -1.306976 | 94 | 26.536975 | 57.42 | 88.303025 | 0.610851 |
| GO:0010646\_regulation\_of\_cell\_communication | 330 | 5 | 2.405956 | -1.280302 | 95 | 27.140431 | 58.44 | 89.739569 | 0.615158 |
| GO:0016055\_Wnt\_receptor\_signaling\_pathway | 59 | 2 | 5.382817 | -1.279624 | 96 | 27.270074 | 58.68 | 90.089926 | 0.611250 |
| GO:0001654\_eye\_development | 136 | 3 | 3.502789 | -1.278812 | 97 | 27.376024 | 58.81 | 90.243976 | 0.606289 |
| GO:0044267\_cellular\_protein\_metabolic\_process | 559 | 7 | 1.988465 | -1.269157 | 98 | 27.564178 | 59.15 | 90.735822 | 0.603571 |
| GO:0006611\_protein\_export\_from\_nucleus | 9 | 1 | 17.643678 | -1.257138 | 107 | 32.158580 | 65.81 | 99.461420 | 0.615047 |
| GO:0007128\_meiotic\_prophase\_I | 9 | 1 | 17.643678 | -1.257138 | 107 | 32.158580 | 65.81 | 99.461420 | 0.615047 |
| GO:0032388\_positive\_regulation\_of\_intracellular\_transport | 9 | 1 | 17.643678 | -1.257138 | 107 | 32.158580 | 65.81 | 99.461420 | 0.615047 |
| GO:0032963\_collagen\_metabolic\_process | 9 | 1 | 17.643678 | -1.257138 | 107 | 32.158580 | 65.81 | 99.461420 | 0.615047 |
| GO:0035162\_embryonic\_hemopoiesis | 9 | 1 | 17.643678 | -1.257138 | 107 | 32.158580 | 65.81 | 99.461420 | 0.615047 |
| GO:0045646\_regulation\_of\_erythrocyte\_differentiation | 9 | 1 | 17.643678 | -1.257138 | 107 | 32.158580 | 65.81 | 99.461420 | 0.615047 |
| GO:0050910\_detection\_of\_mechanical\_stimulus\_involved\_in\_sensory\_perception\_of\_sound | 9 | 1 | 17.643678 | -1.257138 | 107 | 32.158580 | 65.81 | 99.461420 | 0.615047 |
| GO:0051324\_prophase | 9 | 1 | 17.643678 | -1.257138 | 107 | 32.158580 | 65.81 | 99.461420 | 0.615047 |
| GO:0051896\_regulation\_of\_protein\_kinase\_B\_signaling\_cascade | 9 | 1 | 17.643678 | -1.257138 | 107 | 32.158580 | 65.81 | 99.461420 | 0.615047 |
| GO:0021537\_telencephalon\_development | 62 | 2 | 5.122358 | -1.241184 | 109 | 32.546467 | 66.93 | 101.313533 | 0.614037 |
| GO:0050954\_sensory\_perception\_of\_mechanical\_stimulus | 62 | 2 | 5.122358 | -1.241184 | 109 | 32.546467 | 66.93 | 101.313533 | 0.614037 |
| GO:0006793\_phosphorus\_metabolic\_process | 340 | 5 | 2.335193 | -1.234186 | 111 | 32.577656 | 67.23 | 101.882344 | 0.605676 |
| GO:0006796\_phosphate\_metabolic\_process | 340 | 5 | 2.335193 | -1.234186 | 111 | 32.577656 | 67.23 | 101.882344 | 0.605676 |
| GO:0006468\_protein\_amino\_acid\_phosphorylation | 237 | 4 | 2.680052 | -1.228311 | 112 | 32.746620 | 67.68 | 102.613380 | 0.604286 |
| GO:0042060\_wound\_healing | 64 | 2 | 4.962284 | -1.216716 | 113 | 33.056318 | 68.31 | 103.563682 | 0.604513 |
| GO:0001659\_temperature\_homeostasis | 10 | 1 | 15.879310 | -1.212694 | 121 | 38.724483 | 76.37 | 114.015517 | 0.631157 |
| GO:0006081\_cellular\_aldehyde\_metabolic\_process | 10 | 1 | 15.879310 | -1.212694 | 121 | 38.724483 | 76.37 | 114.015517 | 0.631157 |
| GO:0009066\_aspartate\_family\_amino\_acid\_metabolic\_process | 10 | 1 | 15.879310 | -1.212694 | 121 | 38.724483 | 76.37 | 114.015517 | 0.631157 |
| GO:0009110\_vitamin\_biosynthetic\_process | 10 | 1 | 15.879310 | -1.212694 | 121 | 38.724483 | 76.37 | 114.015517 | 0.631157 |
| GO:0021871\_forebrain\_regionalization | 10 | 1 | 15.879310 | -1.212694 | 121 | 38.724483 | 76.37 | 114.015517 | 0.631157 |
| GO:0044259\_multicellular\_organismal\_macromolecule\_metabolic\_process | 10 | 1 | 15.879310 | -1.212694 | 121 | 38.724483 | 76.37 | 114.015517 | 0.631157 |
| GO:0045446\_endothelial\_cell\_differentiation | 10 | 1 | 15.879310 | -1.212694 | 121 | 38.724483 | 76.37 | 114.015517 | 0.631157 |
| GO:0048596\_embryonic\_camera-type\_eye\_morphogenesis | 10 | 1 | 15.879310 | -1.212694 | 121 | 38.724483 | 76.37 | 114.015517 | 0.631157 |
| GO:0001837\_epithelial\_to\_mesenchymal\_transition | 11 | 1 | 14.435737 | -1.172613 | 128 | 43.604962 | 83.58 | 123.555038 | 0.652969 |
| GO:0001952\_regulation\_of\_cell-matrix\_adhesion | 11 | 1 | 14.435737 | -1.172613 | 128 | 43.604962 | 83.58 | 123.555038 | 0.652969 |
| GO:0009064\_glutamine\_family\_amino\_acid\_metabolic\_process | 11 | 1 | 14.435737 | -1.172613 | 128 | 43.604962 | 83.58 | 123.555038 | 0.652969 |
| GO:0014902\_myotube\_differentiation | 11 | 1 | 14.435737 | -1.172613 | 128 | 43.604962 | 83.58 | 123.555038 | 0.652969 |
| GO:0042542\_response\_to\_hydrogen\_peroxide | 11 | 1 | 14.435737 | -1.172613 | 128 | 43.604962 | 83.58 | 123.555038 | 0.652969 |
| GO:0043576\_regulation\_of\_respiratory\_gaseous\_exchange | 11 | 1 | 14.435737 | -1.172613 | 128 | 43.604962 | 83.58 | 123.555038 | 0.652969 |
| GO:0046716\_muscle\_maintenance | 11 | 1 | 14.435737 | -1.172613 | 128 | 43.604962 | 83.58 | 123.555038 | 0.652969 |
| GO:0006281\_DNA\_repair | 71 | 2 | 4.473045 | -1.137520 | 130 | 45.086404 | 86.09 | 127.093596 | 0.662231 |
| GO:0006913\_nucleocytoplasmic\_transport | 71 | 2 | 4.473045 | -1.137520 | 130 | 45.086404 | 86.09 | 127.093596 | 0.662231 |
| GO:0000375\_RNA\_splicing\_\_via\_transesterification\_reactions | 12 | 1 | 13.232759 | -1.136136 | 142 | 48.940203 | 91.73 | 134.519797 | 0.645986 |
| GO:0000377\_RNA\_splicing\_\_via\_transesterification\_reactions\_with\_bulged\_adenosine\_as\_nucleophile | 12 | 1 | 13.232759 | -1.136136 | 142 | 48.940203 | 91.73 | 134.519797 | 0.645986 |
| GO:0000398\_nuclear\_mRNA\_splicing\_\_via\_spliceosome | 12 | 1 | 13.232759 | -1.136136 | 142 | 48.940203 | 91.73 | 134.519797 | 0.645986 |
| GO:0002437\_inflammatory\_response\_to\_antigenic\_stimulus | 12 | 1 | 13.232759 | -1.136136 | 142 | 48.940203 | 91.73 | 134.519797 | 0.645986 |
| GO:0002861\_regulation\_of\_inflammatory\_response\_to\_antigenic\_stimulus | 12 | 1 | 13.232759 | -1.136136 | 142 | 48.940203 | 91.73 | 134.519797 | 0.645986 |
| GO:0006413\_translational\_initiation | 12 | 1 | 13.232759 | -1.136136 | 142 | 48.940203 | 91.73 | 134.519797 | 0.645986 |
| GO:0006446\_regulation\_of\_translational\_initiation | 12 | 1 | 13.232759 | -1.136136 | 142 | 48.940203 | 91.73 | 134.519797 | 0.645986 |
| GO:0006879\_cellular\_iron\_ion\_homeostasis | 12 | 1 | 13.232759 | -1.136136 | 142 | 48.940203 | 91.73 | 134.519797 | 0.645986 |
| GO:0007143\_female\_meiosis | 12 | 1 | 13.232759 | -1.136136 | 142 | 48.940203 | 91.73 | 134.519797 | 0.645986 |
| GO:0042743\_hydrogen\_peroxide\_metabolic\_process | 12 | 1 | 13.232759 | -1.136136 | 142 | 48.940203 | 91.73 | 134.519797 | 0.645986 |
| GO:0048854\_brain\_morphogenesis | 12 | 1 | 13.232759 | -1.136136 | 142 | 48.940203 | 91.73 | 134.519797 | 0.645986 |
| GO:0060042\_retina\_morphogenesis\_in\_camera-type\_eye | 12 | 1 | 13.232759 | -1.136136 | 142 | 48.940203 | 91.73 | 134.519797 | 0.645986 |
| GO:0009966\_regulation\_of\_signal\_transduction | 256 | 4 | 2.481142 | -1.129131 | 143 | 48.909737 | 91.85 | 134.790263 | 0.642308 |
| GO:0030879\_mammary\_gland\_development | 72 | 2 | 4.410920 | -1.126945 | 145 | 49.532951 | 92.81 | 136.087049 | 0.640069 |
| GO:0051169\_nuclear\_transport | 72 | 2 | 4.410920 | -1.126945 | 145 | 49.532951 | 92.81 | 136.087049 | 0.640069 |
| GO:0048771\_tissue\_remodeling | 74 | 2 | 4.291705 | -1.106300 | 146 | 49.961801 | 93.71 | 137.458199 | 0.641849 |
| GO:0003016\_respiratory\_system\_process | 13 | 1 | 12.214854 | -1.102683 | 153 | 53.931303 | 99.61 | 145.288697 | 0.651046 |
| GO:0007566\_embryo\_implantation | 13 | 1 | 12.214854 | -1.102683 | 153 | 53.931303 | 99.61 | 145.288697 | 0.651046 |
| GO:0009410\_response\_to\_xenobiotic\_stimulus | 13 | 1 | 12.214854 | -1.102683 | 153 | 53.931303 | 99.61 | 145.288697 | 0.651046 |
| GO:0009994\_oocyte\_differentiation | 13 | 1 | 12.214854 | -1.102683 | 153 | 53.931303 | 99.61 | 145.288697 | 0.651046 |
| GO:0021879\_forebrain\_neuron\_differentiation | 13 | 1 | 12.214854 | -1.102683 | 153 | 53.931303 | 99.61 | 145.288697 | 0.651046 |
| GO:0030539\_male\_genitalia\_development | 13 | 1 | 12.214854 | -1.102683 | 153 | 53.931303 | 99.61 | 145.288697 | 0.651046 |
| GO:0048599\_oocyte\_development | 13 | 1 | 12.214854 | -1.102683 | 153 | 53.931303 | 99.61 | 145.288697 | 0.651046 |
| GO:0006259\_DNA\_metabolic\_process | 165 | 3 | 2.887147 | -1.077611 | 154 | 54.979791 | 100.95 | 146.920209 | 0.655519 |
| GO:0001890\_placenta\_development | 77 | 2 | 4.124496 | -1.076523 | 155 | 55.136403 | 101.17 | 147.203597 | 0.652710 |
| GO:0010332\_response\_to\_gamma\_radiation | 14 | 1 | 11.342365 | -1.071807 | 162 | 58.614715 | 106.01 | 153.405285 | 0.654383 |
| GO:0021782\_glial\_cell\_development | 14 | 1 | 11.342365 | -1.071807 | 162 | 58.614715 | 106.01 | 153.405285 | 0.654383 |
| GO:0031099\_regeneration | 14 | 1 | 11.342365 | -1.071807 | 162 | 58.614715 | 106.01 | 153.405285 | 0.654383 |
| GO:0042573\_retinoic\_acid\_metabolic\_process | 14 | 1 | 11.342365 | -1.071807 | 162 | 58.614715 | 106.01 | 153.405285 | 0.654383 |
| GO:0043491\_protein\_kinase\_B\_signaling\_cascade | 14 | 1 | 11.342365 | -1.071807 | 162 | 58.614715 | 106.01 | 153.405285 | 0.654383 |
| GO:0044236\_multicellular\_organismal\_metabolic\_process | 14 | 1 | 11.342365 | -1.071807 | 162 | 58.614715 | 106.01 | 153.405285 | 0.654383 |
| GO:0048048\_embryonic\_eye\_morphogenesis | 14 | 1 | 11.342365 | -1.071807 | 162 | 58.614715 | 106.01 | 153.405285 | 0.654383 |
| GO:0045944\_positive\_regulation\_of\_transcription\_from\_RNA\_polymerase\_II\_promoter | 269 | 4 | 2.361236 | -1.066916 | 163 | 58.717292 | 106.32 | 153.922708 | 0.652270 |
| GO:0000278\_mitotic\_cell\_cycle | 80 | 2 | 3.969828 | -1.048077 | 164 | 59.775339 | 107.76 | 155.744661 | 0.657073 |
| GO:0021872\_generation\_of\_neurons\_in\_the\_forebrain | 15 | 1 | 10.586207 | -1.043152 | 169 | 63.377582 | 113.31 | 163.242418 | 0.670473 |
| GO:0031076\_embryonic\_camera-type\_eye\_development | 15 | 1 | 10.586207 | -1.043152 | 169 | 63.377582 | 113.31 | 163.242418 | 0.670473 |
| GO:0045666\_positive\_regulation\_of\_neuron\_differentiation | 15 | 1 | 10.586207 | -1.043152 | 169 | 63.377582 | 113.31 | 163.242418 | 0.670473 |
| GO:0048010\_vascular\_endothelial\_growth\_factor\_receptor\_signaling\_pathway | 15 | 1 | 10.586207 | -1.043152 | 169 | 63.377582 | 113.31 | 163.242418 | 0.670473 |
| GO:0060749\_mammary\_gland\_alveolus\_development | 15 | 1 | 10.586207 | -1.043152 | 169 | 63.377582 | 113.31 | 163.242418 | 0.670473 |
| GO:0050877\_neurological\_system\_process | 390 | 5 | 2.035809 | -1.030052 | 170 | 63.608672 | 113.84 | 164.071328 | 0.669647 |
| GO:0042127\_regulation\_of\_cell\_proliferation | 393 | 5 | 2.020268 | -1.019049 | 171 | 64.343130 | 115.13 | 165.916870 | 0.673275 |
| GO:0000302\_response\_to\_reactive\_oxygen\_species | 16 | 1 | 9.924569 | -1.016429 | 176 | 68.093779 | 119.7 | 171.306221 | 0.680114 |
| GO:0042311\_vasodilation | 16 | 1 | 9.924569 | -1.016429 | 176 | 68.093779 | 119.7 | 171.306221 | 0.680114 |
| GO:0046148\_pigment\_biosynthetic\_process | 16 | 1 | 9.924569 | -1.016429 | 176 | 68.093779 | 119.7 | 171.306221 | 0.680114 |
| GO:0046700\_heterocycle\_catabolic\_process | 16 | 1 | 9.924569 | -1.016429 | 176 | 68.093779 | 119.7 | 171.306221 | 0.680114 |
| GO:0050974\_detection\_of\_mechanical\_stimulus\_involved\_in\_sensory\_perception | 16 | 1 | 9.924569 | -1.016429 | 176 | 68.093779 | 119.7 | 171.306221 | 0.680114 |
| GO:0009887\_organ\_morphogenesis | 642 | 7 | 1.731389 | -1.006531 | 177 | 68.504077 | 120.51 | 172.515923 | 0.680847 |
| GO:0070887\_cellular\_response\_to\_chemical\_stimulus | 85 | 2 | 3.736308 | -1.003361 | 178 | 68.656619 | 120.83 | 173.003381 | 0.678820 |
| GO:0048732\_gland\_development | 179 | 3 | 2.661337 | -0.996042 | 179 | 68.887361 | 121.25 | 173.612639 | 0.677374 |
| GO:0006605\_protein\_targeting | 86 | 2 | 3.692863 | -0.994793 | 180 | 69.208284 | 121.71 | 174.211716 | 0.676167 |
| GO:0008380\_RNA\_splicing | 17 | 1 | 9.340771 | -0.991406 | 185 | 73.040020 | 126.43 | 179.819980 | 0.683405 |
| GO:0042440\_pigment\_metabolic\_process | 17 | 1 | 9.340771 | -0.991406 | 185 | 73.040020 | 126.43 | 179.819980 | 0.683405 |
| GO:0045667\_regulation\_of\_osteoblast\_differentiation | 17 | 1 | 9.340771 | -0.991406 | 185 | 73.040020 | 126.43 | 179.819980 | 0.683405 |
| GO:0048873\_homeostasis\_of\_number\_of\_cells\_within\_a\_tissue | 17 | 1 | 9.340771 | -0.991406 | 185 | 73.040020 | 126.43 | 179.819980 | 0.683405 |
| GO:0055072\_iron\_ion\_homeostasis | 17 | 1 | 9.340771 | -0.991406 | 185 | 73.040020 | 126.43 | 179.819980 | 0.683405 |
| GO:0007417\_central\_nervous\_system\_development | 287 | 4 | 2.213144 | -0.987426 | 186 | 73.107320 | 126.64 | 180.172680 | 0.680860 |
| GO:0043583\_ear\_development | 87 | 2 | 3.650416 | -0.986343 | 187 | 73.501502 | 127.36 | 181.218498 | 0.681070 |
| GO:0009888\_tissue\_development | 525 | 6 | 1.814778 | -0.980844 | 188 | 73.780026 | 127.7 | 181.619974 | 0.679255 |
| GO:0001503\_ossification | 88 | 2 | 3.608934 | -0.978009 | 189 | 74.003952 | 127.98 | 181.956048 | 0.677143 |
| GO:0007140\_male\_meiosis | 18 | 1 | 8.821839 | -0.967886 | 196 | 78.696966 | 134.13 | 189.563034 | 0.684337 |
| GO:0009063\_cellular\_amino\_acid\_catabolic\_process | 18 | 1 | 8.821839 | -0.967886 | 196 | 78.696966 | 134.13 | 189.563034 | 0.684337 |
| GO:0030282\_bone\_mineralization | 18 | 1 | 8.821839 | -0.967886 | 196 | 78.696966 | 134.13 | 189.563034 | 0.684337 |
| GO:0033157\_regulation\_of\_intracellular\_protein\_transport | 18 | 1 | 8.821839 | -0.967886 | 196 | 78.696966 | 134.13 | 189.563034 | 0.684337 |
| GO:0050982\_detection\_of\_mechanical\_stimulus | 18 | 1 | 8.821839 | -0.967886 | 196 | 78.696966 | 134.13 | 189.563034 | 0.684337 |
| GO:0051168\_nuclear\_export | 18 | 1 | 8.821839 | -0.967886 | 196 | 78.696966 | 134.13 | 189.563034 | 0.684337 |
| GO:0051222\_positive\_regulation\_of\_protein\_transport | 18 | 1 | 8.821839 | -0.967886 | 196 | 78.696966 | 134.13 | 189.563034 | 0.684337 |
| GO:0042221\_response\_to\_chemical\_stimulus | 409 | 5 | 1.941236 | -0.962473 | 197 | 78.849828 | 134.42 | 189.990172 | 0.682335 |
| GO:0006338\_chromatin\_remodeling | 19 | 1 | 8.357532 | -0.945708 | 204 | 83.130142 | 139.64 | 196.149858 | 0.684510 |
| GO:0006776\_vitamin\_A\_metabolic\_process | 19 | 1 | 8.357532 | -0.945708 | 204 | 83.130142 | 139.64 | 196.149858 | 0.684510 |
| GO:0007595\_lactation | 19 | 1 | 8.357532 | -0.945708 | 204 | 83.130142 | 139.64 | 196.149858 | 0.684510 |
| GO:0032526\_response\_to\_retinoic\_acid | 19 | 1 | 8.357532 | -0.945708 | 204 | 83.130142 | 139.64 | 196.149858 | 0.684510 |
| GO:0033189\_response\_to\_vitamin\_A | 19 | 1 | 8.357532 | -0.945708 | 204 | 83.130142 | 139.64 | 196.149858 | 0.684510 |
| GO:0050728\_negative\_regulation\_of\_inflammatory\_response | 19 | 1 | 8.357532 | -0.945708 | 204 | 83.130142 | 139.64 | 196.149858 | 0.684510 |
| GO:0060444\_branching\_involved\_in\_mammary\_gland\_duct\_morphogenesis | 19 | 1 | 8.357532 | -0.945708 | 204 | 83.130142 | 139.64 | 196.149858 | 0.684510 |
| GO:0034984\_cellular\_response\_to\_DNA\_damage\_stimulus | 94 | 2 | 3.378577 | -0.930285 | 205 | 83.812820 | 140.85 | 197.887180 | 0.687073 |
| GO:0042592\_homeostatic\_process | 419 | 5 | 1.894906 | -0.928826 | 206 | 83.866448 | 140.94 | 198.013552 | 0.684175 |
| GO:0031214\_biomineral\_formation | 20 | 1 | 7.939655 | -0.924733 | 210 | 86.518890 | 144.44 | 202.361110 | 0.687810 |
| GO:0045639\_positive\_regulation\_of\_myeloid\_cell\_differentiation | 20 | 1 | 7.939655 | -0.924733 | 210 | 86.518890 | 144.44 | 202.361110 | 0.687810 |
| GO:0046822\_regulation\_of\_nucleocytoplasmic\_transport | 20 | 1 | 7.939655 | -0.924733 | 210 | 86.518890 | 144.44 | 202.361110 | 0.687810 |
| GO:0048806\_genitalia\_development | 20 | 1 | 7.939655 | -0.924733 | 210 | 86.518890 | 144.44 | 202.361110 | 0.687810 |
| GO:0046907\_intracellular\_transport | 194 | 3 | 2.455563 | -0.917508 | 211 | 86.792939 | 144.88 | 202.967061 | 0.686635 |
| GO:0006954\_inflammatory\_response | 96 | 2 | 3.308190 | -0.915190 | 212 | 86.963557 | 145.24 | 203.516443 | 0.685094 |
| GO:0045893\_positive\_regulation\_of\_transcription\_\_DNA-dependent | 306 | 4 | 2.075727 | -0.910945 | 214 | 87.380355 | 145.77 | 204.159645 | 0.681168 |
| GO:0051254\_positive\_regulation\_of\_RNA\_metabolic\_process | 306 | 4 | 2.075727 | -0.910945 | 214 | 87.380355 | 145.77 | 204.159645 | 0.681168 |
| GO:0001755\_neural\_crest\_cell\_migration | 21 | 1 | 7.561576 | -0.904844 | 217 | 91.203715 | 150.8 | 210.396285 | 0.694931 |
| GO:0019827\_stem\_cell\_maintenance | 21 | 1 | 7.561576 | -0.904844 | 217 | 91.203715 | 150.8 | 210.396285 | 0.694931 |
| GO:0050777\_negative\_regulation\_of\_immune\_response | 21 | 1 | 7.561576 | -0.904844 | 217 | 91.203715 | 150.8 | 210.396285 | 0.694931 |
| GO:0051094\_positive\_regulation\_of\_developmental\_process | 308 | 4 | 2.062248 | -0.903299 | 218 | 91.262480 | 151.04 | 210.817520 | 0.692844 |
| GO:0016310\_phosphorylation | 309 | 4 | 2.055574 | -0.899504 | 219 | 91.854988 | 151.85 | 211.845012 | 0.693379 |
| GO:0060348\_bone\_development | 99 | 2 | 3.207941 | -0.893253 | 220 | 92.075174 | 152.23 | 212.384826 | 0.691955 |
| GO:0001525\_angiogenesis | 100 | 2 | 3.175862 | -0.886121 | 221 | 92.285551 | 152.49 | 212.694449 | 0.690000 |
| GO:0001523\_retinoid\_metabolic\_process | 22 | 1 | 7.217868 | -0.885940 | 232 | 96.717951 | 158.18 | 219.642049 | 0.681810 |
| GO:0001558\_regulation\_of\_cell\_growth | 22 | 1 | 7.217868 | -0.885940 | 232 | 96.717951 | 158.18 | 219.642049 | 0.681810 |
| GO:0001947\_heart\_looping | 22 | 1 | 7.217868 | -0.885940 | 232 | 96.717951 | 158.18 | 219.642049 | 0.681810 |
| GO:0006721\_terpenoid\_metabolic\_process | 22 | 1 | 7.217868 | -0.885940 | 232 | 96.717951 | 158.18 | 219.642049 | 0.681810 |
| GO:0015918\_sterol\_transport | 22 | 1 | 7.217868 | -0.885940 | 232 | 96.717951 | 158.18 | 219.642049 | 0.681810 |
| GO:0016101\_diterpenoid\_metabolic\_process | 22 | 1 | 7.217868 | -0.885940 | 232 | 96.717951 | 158.18 | 219.642049 | 0.681810 |
| GO:0030301\_cholesterol\_transport | 22 | 1 | 7.217868 | -0.885940 | 232 | 96.717951 | 158.18 | 219.642049 | 0.681810 |
| GO:0030335\_positive\_regulation\_of\_cell\_migration | 22 | 1 | 7.217868 | -0.885940 | 232 | 96.717951 | 158.18 | 219.642049 | 0.681810 |
| GO:0033273\_response\_to\_vitamin | 22 | 1 | 7.217868 | -0.885940 | 232 | 96.717951 | 158.18 | 219.642049 | 0.681810 |
| GO:0048477\_oogenesis | 22 | 1 | 7.217868 | -0.885940 | 232 | 96.717951 | 158.18 | 219.642049 | 0.681810 |
| GO:0048864\_stem\_cell\_development | 22 | 1 | 7.217868 | -0.885940 | 232 | 96.717951 | 158.18 | 219.642049 | 0.681810 |
| GO:0065008\_regulation\_of\_biological\_quality | 693 | 7 | 1.603971 | -0.871965 | 233 | 97.160803 | 159.01 | 220.859197 | 0.682446 |
| GO:0006397\_mRNA\_processing | 23 | 1 | 6.904048 | -0.867934 | 235 | 99.637041 | 162.34 | 225.042959 | 0.690809 |
| GO:0007584\_response\_to\_nutrient | 23 | 1 | 6.904048 | -0.867934 | 235 | 99.637041 | 162.34 | 225.042959 | 0.690809 |
| GO:0050896\_response\_to\_stimulus | 1107 | 10 | 1.434445 | -0.865941 | 236 | 99.700466 | 162.39 | 225.079534 | 0.688093 |
| GO:0009968\_negative\_regulation\_of\_signal\_transduction | 103 | 2 | 3.083361 | -0.865240 | 237 | 99.874569 | 162.73 | 225.585431 | 0.686624 |
| GO:0009790\_embryonic\_development | 567 | 6 | 1.680350 | -0.860367 | 238 | 100.108654 | 163.13 | 226.151346 | 0.685420 |
| GO:0048872\_homeostasis\_of\_number\_of\_cells | 105 | 2 | 3.024631 | -0.851732 | 239 | 100.387759 | 163.73 | 227.072241 | 0.685063 |
| GO:0008629\_induction\_of\_apoptosis\_by\_intracellular\_signals | 24 | 1 | 6.616379 | -0.850747 | 244 | 103.510663 | 167.91 | 232.309337 | 0.688156 |
| GO:0009612\_response\_to\_mechanical\_stimulus | 24 | 1 | 6.616379 | -0.850747 | 244 | 103.510663 | 167.91 | 232.309337 | 0.688156 |
| GO:0032386\_regulation\_of\_intracellular\_transport | 24 | 1 | 6.616379 | -0.850747 | 244 | 103.510663 | 167.91 | 232.309337 | 0.688156 |
| GO:0042632\_cholesterol\_homeostasis | 24 | 1 | 6.616379 | -0.850747 | 244 | 103.510663 | 167.91 | 232.309337 | 0.688156 |
| GO:0055092\_sterol\_homeostasis | 24 | 1 | 6.616379 | -0.850747 | 244 | 103.510663 | 167.91 | 232.309337 | 0.688156 |
| GO:0010926\_anatomical\_structure\_formation | 447 | 5 | 1.776209 | -0.841036 | 245 | 103.767366 | 168.35 | 232.932634 | 0.687143 |
| GO:0006302\_double-strand\_break\_repair | 25 | 1 | 6.351724 | -0.834314 | 250 | 106.570544 | 172.19 | 237.809456 | 0.688760 |
| GO:0006775\_fat-soluble\_vitamin\_metabolic\_process | 25 | 1 | 6.351724 | -0.834314 | 250 | 106.570544 | 172.19 | 237.809456 | 0.688760 |
| GO:0021983\_pituitary\_gland\_development | 25 | 1 | 6.351724 | -0.834314 | 250 | 106.570544 | 172.19 | 237.809456 | 0.688760 |
| GO:0031348\_negative\_regulation\_of\_defense\_response | 25 | 1 | 6.351724 | -0.834314 | 250 | 106.570544 | 172.19 | 237.809456 | 0.688760 |
| GO:0060603\_mammary\_gland\_duct\_morphogenesis | 25 | 1 | 6.351724 | -0.834314 | 250 | 106.570544 | 172.19 | 237.809456 | 0.688760 |
| GO:0010648\_negative\_regulation\_of\_cell\_communication | 110 | 2 | 2.887147 | -0.819317 | 251 | 107.238063 | 173.51 | 239.781937 | 0.691275 |
| GO:0001666\_response\_to\_hypoxia | 26 | 1 | 6.107427 | -0.818576 | 259 | 109.820072 | 176.81 | 243.799928 | 0.682664 |
| GO:0006720\_isoprenoid\_metabolic\_process | 26 | 1 | 6.107427 | -0.818576 | 259 | 109.820072 | 176.81 | 243.799928 | 0.682664 |
| GO:0006800\_oxygen\_and\_reactive\_oxygen\_species\_metabolic\_process | 26 | 1 | 6.107427 | -0.818576 | 259 | 109.820072 | 176.81 | 243.799928 | 0.682664 |
| GO:0007405\_neuroblast\_proliferation | 26 | 1 | 6.107427 | -0.818576 | 259 | 109.820072 | 176.81 | 243.799928 | 0.682664 |
| GO:0009310\_amine\_catabolic\_process | 26 | 1 | 6.107427 | -0.818576 | 259 | 109.820072 | 176.81 | 243.799928 | 0.682664 |
| GO:0009636\_response\_to\_toxin | 26 | 1 | 6.107427 | -0.818576 | 259 | 109.820072 | 176.81 | 243.799928 | 0.682664 |
| GO:0010212\_response\_to\_ionizing\_radiation | 26 | 1 | 6.107427 | -0.818576 | 259 | 109.820072 | 176.81 | 243.799928 | 0.682664 |
| GO:0045665\_negative\_regulation\_of\_neuron\_differentiation | 26 | 1 | 6.107427 | -0.818576 | 259 | 109.820072 | 176.81 | 243.799928 | 0.682664 |
| GO:0040007\_growth | 217 | 3 | 2.195296 | -0.811866 | 260 | 110.298288 | 177.61 | 244.921712 | 0.683115 |
| GO:0006479\_protein\_amino\_acid\_methylation | 27 | 1 | 5.881226 | -0.803479 | 264 | 112.879208 | 181.14 | 249.400792 | 0.686136 |
| GO:0008213\_protein\_amino\_acid\_alkylation | 27 | 1 | 5.881226 | -0.803479 | 264 | 112.879208 | 181.14 | 249.400792 | 0.686136 |
| GO:0051272\_positive\_regulation\_of\_cell\_motion | 27 | 1 | 5.881226 | -0.803479 | 264 | 112.879208 | 181.14 | 249.400792 | 0.686136 |
| GO:0070482\_response\_to\_oxygen\_levels | 27 | 1 | 5.881226 | -0.803479 | 264 | 112.879208 | 181.14 | 249.400792 | 0.686136 |
| GO:0006974\_response\_to\_DNA\_damage\_stimulus | 113 | 2 | 2.810497 | -0.800740 | 266 | 113.077016 | 181.54 | 250.002984 | 0.682481 |
| GO:0040008\_regulation\_of\_growth | 113 | 2 | 2.810497 | -0.800740 | 266 | 113.077016 | 181.54 | 250.002984 | 0.682481 |
| GO:0045941\_positive\_regulation\_of\_transcription | 338 | 4 | 1.879208 | -0.796882 | 267 | 113.292809 | 181.85 | 250.407191 | 0.681086 |
| GO:0006470\_protein\_amino\_acid\_dephosphorylation | 28 | 1 | 5.671182 | -0.788977 | 272 | 115.713504 | 185.47 | 255.226496 | 0.681875 |
| GO:0007127\_meiosis\_I | 28 | 1 | 5.671182 | -0.788977 | 272 | 115.713504 | 185.47 | 255.226496 | 0.681875 |
| GO:0007585\_respiratory\_gaseous\_exchange | 28 | 1 | 5.671182 | -0.788977 | 272 | 115.713504 | 185.47 | 255.226496 | 0.681875 |
| GO:0045926\_negative\_regulation\_of\_growth | 28 | 1 | 5.671182 | -0.788977 | 272 | 115.713504 | 185.47 | 255.226496 | 0.681875 |
| GO:0048863\_stem\_cell\_differentiation | 28 | 1 | 5.671182 | -0.788977 | 272 | 115.713504 | 185.47 | 255.226496 | 0.681875 |
| GO:0006807\_nitrogen\_compound\_metabolic\_process | 1147 | 10 | 1.384421 | -0.788795 | 273 | 115.756580 | 185.54 | 255.323420 | 0.679634 |
| GO:0006417\_regulation\_of\_translation | 29 | 1 | 5.475624 | -0.775028 | 278 | 119.145651 | 190.06 | 260.974349 | 0.683669 |
| GO:0006641\_triglyceride\_metabolic\_process | 29 | 1 | 5.475624 | -0.775028 | 278 | 119.145651 | 190.06 | 260.974349 | 0.683669 |
| GO:0044270\_nitrogen\_compound\_catabolic\_process | 29 | 1 | 5.475624 | -0.775028 | 278 | 119.145651 | 190.06 | 260.974349 | 0.683669 |
| GO:0050769\_positive\_regulation\_of\_neurogenesis | 29 | 1 | 5.475624 | -0.775028 | 278 | 119.145651 | 190.06 | 260.974349 | 0.683669 |
| GO:0060041\_retina\_development\_in\_camera-type\_eye | 29 | 1 | 5.475624 | -0.775028 | 278 | 119.145651 | 190.06 | 260.974349 | 0.683669 |
| GO:0006519\_cellular\_amino\_acid\_and\_derivative\_metabolic\_process | 118 | 2 | 2.691409 | -0.771123 | 279 | 119.354673 | 190.43 | 261.505327 | 0.682545 |
| GO:0010628\_positive\_regulation\_of\_gene\_expression | 346 | 4 | 1.835758 | -0.770912 | 280 | 119.467977 | 190.55 | 261.632023 | 0.680536 |
| GO:0007219\_Notch\_signaling\_pathway | 30 | 1 | 5.293103 | -0.761595 | 286 | 122.667476 | 195.16 | 267.652524 | 0.682378 |
| GO:0014032\_neural\_crest\_cell\_development | 30 | 1 | 5.293103 | -0.761595 | 286 | 122.667476 | 195.16 | 267.652524 | 0.682378 |
| GO:0014033\_neural\_crest\_cell\_differentiation | 30 | 1 | 5.293103 | -0.761595 | 286 | 122.667476 | 195.16 | 267.652524 | 0.682378 |
| GO:0032102\_negative\_regulation\_of\_response\_to\_external\_stimulus | 30 | 1 | 5.293103 | -0.761595 | 286 | 122.667476 | 195.16 | 267.652524 | 0.682378 |
| GO:0033500\_carbohydrate\_homeostasis | 30 | 1 | 5.293103 | -0.761595 | 286 | 122.667476 | 195.16 | 267.652524 | 0.682378 |
| GO:0042593\_glucose\_homeostasis | 30 | 1 | 5.293103 | -0.761595 | 286 | 122.667476 | 195.16 | 267.652524 | 0.682378 |
| GO:0045935\_positive\_regulation\_of\_nucleobase\_\_nucleoside\_\_nucleotide\_and\_nucleic\_acid\_metabolic\_process | 352 | 4 | 1.804467 | -0.752042 | 287 | 123.665885 | 196.28 | 268.894115 | 0.683902 |
| GO:0003018\_vascular\_process\_in\_circulatory\_system | 31 | 1 | 5.122358 | -0.748643 | 295 | 128.374676 | 201.89 | 275.405324 | 0.684373 |
| GO:0006639\_acylglycerol\_metabolic\_process | 31 | 1 | 5.122358 | -0.748643 | 295 | 128.374676 | 201.89 | 275.405324 | 0.684373 |
| GO:0016049\_cell\_growth | 31 | 1 | 5.122358 | -0.748643 | 295 | 128.374676 | 201.89 | 275.405324 | 0.684373 |
| GO:0016311\_dephosphorylation | 31 | 1 | 5.122358 | -0.748643 | 295 | 128.374676 | 201.89 | 275.405324 | 0.684373 |
| GO:0035150\_regulation\_of\_tube\_size | 31 | 1 | 5.122358 | -0.748643 | 295 | 128.374676 | 201.89 | 275.405324 | 0.684373 |
| GO:0048562\_embryonic\_organ\_morphogenesis | 31 | 1 | 5.122358 | -0.748643 | 295 | 128.374676 | 201.89 | 275.405324 | 0.684373 |
| GO:0050880\_regulation\_of\_blood\_vessel\_size | 31 | 1 | 5.122358 | -0.748643 | 295 | 128.374676 | 201.89 | 275.405324 | 0.684373 |
| GO:0055088\_lipid\_homeostasis | 31 | 1 | 5.122358 | -0.748643 | 295 | 128.374676 | 201.89 | 275.405324 | 0.684373 |
| GO:0006886\_intracellular\_protein\_transport | 122 | 2 | 2.603166 | -0.748556 | 296 | 129.002846 | 202.77 | 276.537154 | 0.685034 |
| GO:0050890\_cognition | 233 | 3 | 2.044546 | -0.747193 | 297 | 129.225785 | 203.06 | 276.894215 | 0.683704 |
| GO:0009308\_amine\_metabolic\_process | 124 | 2 | 2.561179 | -0.737625 | 298 | 129.695092 | 203.69 | 277.684908 | 0.683523 |
| GO:0001707\_mesoderm\_formation | 32 | 1 | 4.962284 | -0.736142 | 303 | 131.458645 | 206.13 | 280.801355 | 0.680297 |
| GO:0006638\_neutral\_lipid\_metabolic\_process | 32 | 1 | 4.962284 | -0.736142 | 303 | 131.458645 | 206.13 | 280.801355 | 0.680297 |
| GO:0006662\_glycerol\_ether\_metabolic\_process | 32 | 1 | 4.962284 | -0.736142 | 303 | 131.458645 | 206.13 | 280.801355 | 0.680297 |
| GO:0048332\_mesoderm\_morphogenesis | 32 | 1 | 4.962284 | -0.736142 | 303 | 131.458645 | 206.13 | 280.801355 | 0.680297 |
| GO:0050885\_neuromuscular\_process\_controlling\_balance | 32 | 1 | 4.962284 | -0.736142 | 303 | 131.458645 | 206.13 | 280.801355 | 0.680297 |
| GO:0001501\_skeletal\_system\_development | 236 | 3 | 2.018556 | -0.735772 | 304 | 131.517565 | 206.25 | 280.982435 | 0.678454 |
| GO:0051173\_positive\_regulation\_of\_nitrogen\_compound\_metabolic\_process | 361 | 4 | 1.759480 | -0.724670 | 305 | 132.522708 | 207.48 | 282.437292 | 0.680262 |
| GO:0007565\_female\_pregnancy | 33 | 1 | 4.811912 | -0.724065 | 307 | 134.567096 | 210.17 | 285.772904 | 0.684593 |
| GO:0021987\_cerebral\_cortex\_development | 33 | 1 | 4.811912 | -0.724065 | 307 | 134.567096 | 210.17 | 285.772904 | 0.684593 |
| GO:0045597\_positive\_regulation\_of\_cell\_differentiation | 128 | 2 | 2.481142 | -0.716432 | 308 | 135.030832 | 210.72 | 286.409168 | 0.684156 |
| GO:0010720\_positive\_regulation\_of\_cell\_development | 34 | 1 | 4.670385 | -0.712385 | 312 | 138.055254 | 214.67 | 291.284746 | 0.688045 |
| GO:0016054\_organic\_acid\_catabolic\_process | 34 | 1 | 4.670385 | -0.712385 | 312 | 138.055254 | 214.67 | 291.284746 | 0.688045 |
| GO:0046395\_carboxylic\_acid\_catabolic\_process | 34 | 1 | 4.670385 | -0.712385 | 312 | 138.055254 | 214.67 | 291.284746 | 0.688045 |
| GO:0060443\_mammary\_gland\_morphogenesis | 34 | 1 | 4.670385 | -0.712385 | 312 | 138.055254 | 214.67 | 291.284746 | 0.688045 |
| GO:0045165\_cell\_fate\_commitment | 130 | 2 | 2.442971 | -0.706155 | 313 | 138.912714 | 215.66 | 292.407286 | 0.689010 |
| GO:0006869\_lipid\_transport | 35 | 1 | 4.536946 | -0.701080 | 317 | 141.076455 | 218.19 | 295.303545 | 0.688297 |
| GO:0007292\_female\_gamete\_generation | 35 | 1 | 4.536946 | -0.701080 | 317 | 141.076455 | 218.19 | 295.303545 | 0.688297 |
| GO:0010810\_regulation\_of\_cell-substrate\_adhesion | 35 | 1 | 4.536946 | -0.701080 | 317 | 141.076455 | 218.19 | 295.303545 | 0.688297 |
| GO:0018904\_organic\_ether\_metabolic\_process | 35 | 1 | 4.536946 | -0.701080 | 317 | 141.076455 | 218.19 | 295.303545 | 0.688297 |
| GO:0010557\_positive\_regulation\_of\_macromolecule\_biosynthetic\_process | 371 | 4 | 1.712055 | -0.695509 | 318 | 141.485035 | 218.65 | 295.814965 | 0.687579 |
| GO:0044057\_regulation\_of\_system\_process | 133 | 2 | 2.387866 | -0.691121 | 319 | 141.877305 | 219.34 | 296.802695 | 0.687586 |
| GO:0001704\_formation\_of\_primary\_germ\_layer | 36 | 1 | 4.410920 | -0.690129 | 322 | 144.454936 | 222.87 | 301.285064 | 0.692143 |
| GO:0030278\_regulation\_of\_ossification | 36 | 1 | 4.410920 | -0.690129 | 322 | 144.454936 | 222.87 | 301.285064 | 0.692143 |
| GO:0051223\_regulation\_of\_protein\_transport | 36 | 1 | 4.410920 | -0.690129 | 322 | 144.454936 | 222.87 | 301.285064 | 0.692143 |
| GO:0022414\_reproductive\_process | 376 | 4 | 1.689288 | -0.681401 | 323 | 144.966548 | 223.71 | 302.453452 | 0.692601 |
| GO:0050906\_detection\_of\_stimulus\_involved\_in\_sensory\_perception | 37 | 1 | 4.291705 | -0.679511 | 324 | 146.842900 | 226.09 | 305.337100 | 0.697809 |
| GO:0007267\_cell-cell\_signaling | 252 | 3 | 1.890394 | -0.678257 | 325 | 146.988523 | 226.27 | 305.551477 | 0.696215 |
| GO:0000003\_reproduction | 379 | 4 | 1.675917 | -0.673082 | 326 | 147.348869 | 226.78 | 306.211131 | 0.695644 |
| GO:0001649\_osteoblast\_differentiation | 38 | 1 | 4.178766 | -0.669210 | 331 | 150.304235 | 230.65 | 310.995765 | 0.696828 |
| GO:0032259\_methylation | 38 | 1 | 4.178766 | -0.669210 | 331 | 150.304235 | 230.65 | 310.995765 | 0.696828 |
| GO:0042493\_response\_to\_drug | 38 | 1 | 4.178766 | -0.669210 | 331 | 150.304235 | 230.65 | 310.995765 | 0.696828 |
| GO:0043414\_biopolymer\_methylation | 38 | 1 | 4.178766 | -0.669210 | 331 | 150.304235 | 230.65 | 310.995765 | 0.696828 |
| GO:0050727\_regulation\_of\_inflammatory\_response | 38 | 1 | 4.178766 | -0.669210 | 331 | 150.304235 | 230.65 | 310.995765 | 0.696828 |
| GO:0048729\_tissue\_morphogenesis | 255 | 3 | 1.868154 | -0.668071 | 332 | 150.452795 | 230.79 | 311.127205 | 0.695151 |
| GO:0034613\_cellular\_protein\_localization | 139 | 2 | 2.284793 | -0.662351 | 333 | 150.909565 | 231.5 | 312.090435 | 0.695195 |
| GO:0006730\_one-carbon\_metabolic\_process | 39 | 1 | 4.071618 | -0.659208 | 338 | 153.720248 | 234.96 | 316.199752 | 0.695148 |
| GO:0007160\_cell-matrix\_adhesion | 39 | 1 | 4.071618 | -0.659208 | 338 | 153.720248 | 234.96 | 316.199752 | 0.695148 |
| GO:0007286\_spermatid\_development | 39 | 1 | 4.071618 | -0.659208 | 338 | 153.720248 | 234.96 | 316.199752 | 0.695148 |
| GO:0048663\_neuron\_fate\_commitment | 39 | 1 | 4.071618 | -0.659208 | 338 | 153.720248 | 234.96 | 316.199752 | 0.695148 |
| GO:0070201\_regulation\_of\_establishment\_of\_protein\_localization | 39 | 1 | 4.071618 | -0.659208 | 338 | 153.720248 | 234.96 | 316.199752 | 0.695148 |
| GO:0003008\_system\_process | 516 | 5 | 1.538693 | -0.659098 | 339 | 153.784024 | 235.12 | 316.455976 | 0.693569 |
| GO:0048468\_cell\_development | 654 | 6 | 1.456817 | -0.654585 | 340 | 153.886276 | 235.43 | 316.973724 | 0.692441 |
| GO:0070727\_cellular\_macromolecule\_localization | 141 | 2 | 2.252384 | -0.653124 | 341 | 154.244417 | 235.76 | 317.275583 | 0.691378 |
| GO:0031328\_positive\_regulation\_of\_cellular\_biosynthetic\_process | 387 | 4 | 1.641272 | -0.651418 | 342 | 154.616567 | 236.18 | 317.743433 | 0.690585 |
| GO:0014031\_mesenchymal\_cell\_development | 40 | 1 | 3.969828 | -0.649491 | 345 | 157.054000 | 238.79 | 320.526000 | 0.692145 |
| GO:0016071\_mRNA\_metabolic\_process | 40 | 1 | 3.969828 | -0.649491 | 345 | 157.054000 | 238.79 | 320.526000 | 0.692145 |
| GO:0046850\_regulation\_of\_bone\_remodeling | 40 | 1 | 3.969828 | -0.649491 | 345 | 157.054000 | 238.79 | 320.526000 | 0.692145 |
| GO:0009891\_positive\_regulation\_of\_biosynthetic\_process | 388 | 4 | 1.637042 | -0.648762 | 346 | 157.209126 | 238.94 | 320.670874 | 0.690578 |
| GO:0019748\_secondary\_metabolic\_process | 41 | 1 | 3.873003 | -0.640044 | 348 | 160.888782 | 243.76 | 326.631218 | 0.700460 |
| GO:0032844\_regulation\_of\_homeostatic\_process | 41 | 1 | 3.873003 | -0.640044 | 348 | 160.888782 | 243.76 | 326.631218 | 0.700460 |
| GO:0044255\_cellular\_lipid\_metabolic\_process | 264 | 3 | 1.804467 | -0.638564 | 349 | 161.407357 | 244.39 | 327.372643 | 0.700258 |
| GO:0034961\_cellular\_biopolymer\_biosynthetic\_process | 804 | 7 | 1.382527 | -0.634250 | 350 | 161.700181 | 244.83 | 327.959819 | 0.699514 |
| GO:0006006\_glucose\_metabolic\_process | 42 | 1 | 3.780788 | -0.630855 | 355 | 165.229638 | 248.87 | 332.510362 | 0.701042 |
| GO:0008361\_regulation\_of\_cell\_size | 42 | 1 | 3.780788 | -0.630855 | 355 | 165.229638 | 248.87 | 332.510362 | 0.701042 |
| GO:0010740\_positive\_regulation\_of\_protein\_kinase\_cascade | 42 | 1 | 3.780788 | -0.630855 | 355 | 165.229638 | 248.87 | 332.510362 | 0.701042 |
| GO:0045637\_regulation\_of\_myeloid\_cell\_differentiation | 42 | 1 | 3.780788 | -0.630855 | 355 | 165.229638 | 248.87 | 332.510362 | 0.701042 |
| GO:0048515\_spermatid\_differentiation | 42 | 1 | 3.780788 | -0.630855 | 355 | 165.229638 | 248.87 | 332.510362 | 0.701042 |
| GO:0030900\_forebrain\_development | 146 | 2 | 2.175248 | -0.630805 | 356 | 165.712028 | 249.45 | 333.187972 | 0.700702 |
| GO:0043284\_biopolymer\_biosynthetic\_process | 807 | 7 | 1.377388 | -0.628723 | 357 | 165.822875 | 249.57 | 333.317125 | 0.699076 |
| GO:0001894\_tissue\_homeostasis | 43 | 1 | 3.692863 | -0.621910 | 363 | 169.685211 | 254.13 | 338.574789 | 0.700083 |
| GO:0006766\_vitamin\_metabolic\_process | 43 | 1 | 3.692863 | -0.621910 | 363 | 169.685211 | 254.13 | 338.574789 | 0.700083 |
| GO:0009582\_detection\_of\_abiotic\_stimulus | 43 | 1 | 3.692863 | -0.621910 | 363 | 169.685211 | 254.13 | 338.574789 | 0.700083 |
| GO:0010001\_glial\_cell\_differentiation | 43 | 1 | 3.692863 | -0.621910 | 363 | 169.685211 | 254.13 | 338.574789 | 0.700083 |
| GO:0048762\_mesenchymal\_cell\_differentiation | 43 | 1 | 3.692863 | -0.621910 | 363 | 169.685211 | 254.13 | 338.574789 | 0.700083 |
| GO:0051789\_response\_to\_protein\_stimulus | 43 | 1 | 3.692863 | -0.621910 | 363 | 169.685211 | 254.13 | 338.574789 | 0.700083 |
| GO:0006606\_protein\_import\_into\_nucleus | 44 | 1 | 3.608934 | -0.613199 | 366 | 173.823668 | 259.37 | 344.916332 | 0.708661 |
| GO:0051170\_nuclear\_import | 44 | 1 | 3.608934 | -0.613199 | 366 | 173.823668 | 259.37 | 344.916332 | 0.708661 |
| GO:0060485\_mesenchyme\_development | 44 | 1 | 3.608934 | -0.613199 | 366 | 173.823668 | 259.37 | 344.916332 | 0.708661 |
| GO:0034103\_regulation\_of\_tissue\_remodeling | 45 | 1 | 3.528736 | -0.604712 | 368 | 175.357034 | 261.41 | 347.462966 | 0.710353 |
| GO:0046546\_development\_of\_primary\_male\_sexual\_characteristics | 45 | 1 | 3.528736 | -0.604712 | 368 | 175.357034 | 261.41 | 347.462966 | 0.710353 |
| GO:0007612\_learning | 46 | 1 | 3.452024 | -0.596438 | 372 | 177.857290 | 264.56 | 351.262710 | 0.711183 |
| GO:0009581\_detection\_of\_external\_stimulus | 46 | 1 | 3.452024 | -0.596438 | 372 | 177.857290 | 264.56 | 351.262710 | 0.711183 |
| GO:0030218\_erythrocyte\_differentiation | 46 | 1 | 3.452024 | -0.596438 | 372 | 177.857290 | 264.56 | 351.262710 | 0.711183 |
| GO:0042063\_gliogenesis | 46 | 1 | 3.452024 | -0.596438 | 372 | 177.857290 | 264.56 | 351.262710 | 0.711183 |
| GO:0008285\_negative\_regulation\_of\_cell\_proliferation | 155 | 2 | 2.048943 | -0.593125 | 373 | 178.315503 | 265.15 | 351.984497 | 0.710858 |
| GO:0006396\_RNA\_processing | 47 | 1 | 3.378577 | -0.588367 | 377 | 180.977247 | 268.5 | 356.022753 | 0.712202 |
| GO:0031667\_response\_to\_nutrient\_levels | 47 | 1 | 3.378577 | -0.588367 | 377 | 180.977247 | 268.5 | 356.022753 | 0.712202 |
| GO:0034754\_cellular\_hormone\_metabolic\_process | 47 | 1 | 3.378577 | -0.588367 | 377 | 180.977247 | 268.5 | 356.022753 | 0.712202 |
| GO:0048871\_multicellular\_organismal\_homeostasis | 47 | 1 | 3.378577 | -0.588367 | 377 | 180.977247 | 268.5 | 356.022753 | 0.712202 |
| GO:0006950\_response\_to\_stress | 549 | 5 | 1.446203 | -0.586443 | 378 | 180.991217 | 268.62 | 356.248783 | 0.710635 |
| GO:0051704\_multi-organism\_process | 157 | 2 | 2.022842 | -0.585157 | 379 | 181.182213 | 268.86 | 356.537787 | 0.709393 |
| GO:0048514\_blood\_vessel\_morphogenesis | 158 | 2 | 2.010039 | -0.581225 | 380 | 181.776712 | 269.72 | 357.663288 | 0.709789 |
| GO:0007498\_mesoderm\_development | 48 | 1 | 3.308190 | -0.580493 | 384 | 183.555086 | 271.72 | 359.884914 | 0.707604 |
| GO:0019318\_hexose\_metabolic\_process | 48 | 1 | 3.308190 | -0.580493 | 384 | 183.555086 | 271.72 | 359.884914 | 0.707604 |
| GO:0034504\_protein\_localization\_in\_nucleus | 48 | 1 | 3.308190 | -0.580493 | 384 | 183.555086 | 271.72 | 359.884914 | 0.707604 |
| GO:0046849\_bone\_remodeling | 48 | 1 | 3.308190 | -0.580493 | 384 | 183.555086 | 271.72 | 359.884914 | 0.707604 |
| GO:0006629\_lipid\_metabolic\_process | 285 | 3 | 1.671506 | -0.575338 | 385 | 184.009786 | 272.34 | 360.670214 | 0.707377 |
| GO:0003015\_heart\_process | 49 | 1 | 3.240676 | -0.572805 | 391 | 186.230601 | 275.08 | 363.929399 | 0.703529 |
| GO:0021543\_pallium\_development | 49 | 1 | 3.240676 | -0.572805 | 391 | 186.230601 | 275.08 | 363.929399 | 0.703529 |
| GO:0034101\_erythrocyte\_homeostasis | 49 | 1 | 3.240676 | -0.572805 | 391 | 186.230601 | 275.08 | 363.929399 | 0.703529 |
| GO:0046661\_male\_sex\_differentiation | 49 | 1 | 3.240676 | -0.572805 | 391 | 186.230601 | 275.08 | 363.929399 | 0.703529 |
| GO:0048741\_skeletal\_muscle\_fiber\_development | 49 | 1 | 3.240676 | -0.572805 | 391 | 186.230601 | 275.08 | 363.929399 | 0.703529 |
| GO:0060047\_heart\_contraction | 49 | 1 | 3.240676 | -0.572805 | 391 | 186.230601 | 275.08 | 363.929399 | 0.703529 |
| GO:0009628\_response\_to\_abiotic\_stimulus | 162 | 2 | 1.960409 | -0.565835 | 392 | 186.849483 | 275.93 | 365.010517 | 0.703903 |
| GO:0017038\_protein\_import | 50 | 1 | 3.175862 | -0.565298 | 394 | 188.217194 | 277.73 | 367.242806 | 0.704898 |
| GO:0051606\_detection\_of\_stimulus | 50 | 1 | 3.175862 | -0.565298 | 394 | 188.217194 | 277.73 | 367.242806 | 0.704898 |
| GO:0032880\_regulation\_of\_protein\_localization | 51 | 1 | 3.113590 | -0.557963 | 396 | 191.355404 | 281.3 | 371.244596 | 0.710354 |
| GO:0048747\_muscle\_fiber\_development | 51 | 1 | 3.113590 | -0.557963 | 396 | 191.355404 | 281.3 | 371.244596 | 0.710354 |
| GO:0048518\_positive\_regulation\_of\_biological\_process | 995 | 8 | 1.276728 | -0.555053 | 397 | 191.444452 | 281.41 | 371.375548 | 0.708841 |
| GO:0043065\_positive\_regulation\_of\_apoptosis | 166 | 2 | 1.913170 | -0.550967 | 398 | 192.032583 | 282.5 | 372.967417 | 0.709799 |
| GO:0010608\_posttranscriptional\_regulation\_of\_gene\_expression | 52 | 1 | 3.053714 | -0.550794 | 400 | 193.072597 | 283.75 | 374.427403 | 0.709375 |
| GO:0048585\_negative\_regulation\_of\_response\_to\_stimulus | 52 | 1 | 3.053714 | -0.550794 | 400 | 193.072597 | 283.75 | 374.427403 | 0.709375 |
| GO:0010942\_positive\_regulation\_of\_cell\_death | 167 | 2 | 1.901714 | -0.547328 | 402 | 194.204506 | 285.42 | 376.635494 | 0.710000 |
| GO:0043068\_positive\_regulation\_of\_programmed\_cell\_death | 167 | 2 | 1.901714 | -0.547328 | 402 | 194.204506 | 285.42 | 376.635494 | 0.710000 |
| GO:0006139\_nucleobase\_\_nucleoside\_\_nucleotide\_and\_nucleic\_acid\_metabolic\_process | 1002 | 8 | 1.267809 | -0.544339 | 403 | 194.365905 | 285.57 | 376.774095 | 0.708610 |
| GO:0050905\_neuromuscular\_process | 53 | 1 | 2.996096 | -0.543784 | 404 | 196.273148 | 287.77 | 379.266852 | 0.712302 |
| GO:0010604\_positive\_regulation\_of\_macromolecule\_metabolic\_process | 433 | 4 | 1.466911 | -0.540149 | 405 | 196.449793 | 288.04 | 379.630207 | 0.711210 |
| GO:0007275\_multicellular\_organismal\_development | 1760 | 13 | 1.172904 | -0.537157 | 406 | 196.708516 | 288.3 | 379.891484 | 0.710099 |
| GO:0048598\_embryonic\_morphogenesis | 299 | 3 | 1.593242 | -0.537104 | 407 | 196.873558 | 288.47 | 380.066442 | 0.708771 |
| GO:0006412\_translation | 54 | 1 | 2.940613 | -0.536928 | 408 | 198.866983 | 290.7 | 382.533017 | 0.712500 |
| GO:0006357\_regulation\_of\_transcription\_from\_RNA\_polymerase\_II\_promoter | 435 | 4 | 1.460166 | -0.535778 | 409 | 199.273260 | 291.24 | 383.206740 | 0.712078 |
| GO:0048731\_system\_development | 1609 | 12 | 1.184287 | -0.534421 | 410 | 199.403857 | 291.45 | 383.496143 | 0.710854 |
| GO:0006310\_DNA\_recombination | 55 | 1 | 2.887147 | -0.530219 | 413 | 201.308839 | 293.55 | 385.791161 | 0.710775 |
| GO:0007126\_meiosis | 55 | 1 | 2.887147 | -0.530219 | 413 | 201.308839 | 293.55 | 385.791161 | 0.710775 |
| GO:0051327\_M\_phase\_of\_meiotic\_cell\_cycle | 55 | 1 | 2.887147 | -0.530219 | 413 | 201.308839 | 293.55 | 385.791161 | 0.710775 |
| GO:0007600\_sensory\_perception | 172 | 2 | 1.846431 | -0.529586 | 415 | 201.662692 | 294.16 | 386.657308 | 0.708819 |
| GO:0009611\_response\_to\_wounding | 172 | 2 | 1.846431 | -0.529586 | 415 | 201.662692 | 294.16 | 386.657308 | 0.708819 |
| GO:0044248\_cellular\_catabolic\_process | 173 | 2 | 1.835758 | -0.526126 | 416 | 202.469664 | 294.97 | 387.470336 | 0.709062 |
| GO:0002683\_negative\_regulation\_of\_immune\_system\_process | 56 | 1 | 2.835591 | -0.523653 | 420 | 204.495075 | 297.63 | 390.764925 | 0.708643 |
| GO:0006790\_sulfur\_metabolic\_process | 56 | 1 | 2.835591 | -0.523653 | 420 | 204.495075 | 297.63 | 390.764925 | 0.708643 |
| GO:0046486\_glycerolipid\_metabolic\_process | 56 | 1 | 2.835591 | -0.523653 | 420 | 204.495075 | 297.63 | 390.764925 | 0.708643 |
| GO:0051321\_meiotic\_cell\_cycle | 56 | 1 | 2.835591 | -0.523653 | 420 | 204.495075 | 297.63 | 390.764925 | 0.708643 |
| GO:0031325\_positive\_regulation\_of\_cellular\_metabolic\_process | 442 | 4 | 1.437042 | -0.520758 | 421 | 204.669540 | 297.79 | 390.910460 | 0.707340 |
| GO:0015031\_protein\_transport | 175 | 2 | 1.814778 | -0.519289 | 422 | 205.086856 | 298.33 | 391.573144 | 0.706943 |
| GO:0001892\_embryonic\_placenta\_development | 57 | 1 | 2.785844 | -0.517224 | 430 | 207.613473 | 301.77 | 395.926527 | 0.701791 |
| GO:0008344\_adult\_locomotory\_behavior | 57 | 1 | 2.785844 | -0.517224 | 430 | 207.613473 | 301.77 | 395.926527 | 0.701791 |
| GO:0018108\_peptidyl-tyrosine\_phosphorylation | 57 | 1 | 2.785844 | -0.517224 | 430 | 207.613473 | 301.77 | 395.926527 | 0.701791 |
| GO:0018212\_peptidyl-tyrosine\_modification | 57 | 1 | 2.785844 | -0.517224 | 430 | 207.613473 | 301.77 | 395.926527 | 0.701791 |
| GO:0033365\_protein\_localization\_in\_organelle | 57 | 1 | 2.785844 | -0.517224 | 430 | 207.613473 | 301.77 | 395.926527 | 0.701791 |
| GO:0042472\_inner\_ear\_morphogenesis | 57 | 1 | 2.785844 | -0.517224 | 430 | 207.613473 | 301.77 | 395.926527 | 0.701791 |
| GO:0043523\_regulation\_of\_neuron\_apoptosis | 57 | 1 | 2.785844 | -0.517224 | 430 | 207.613473 | 301.77 | 395.926527 | 0.701791 |
| GO:0045444\_fat\_cell\_differentiation | 57 | 1 | 2.785844 | -0.517224 | 430 | 207.613473 | 301.77 | 395.926527 | 0.701791 |
| GO:0006366\_transcription\_from\_RNA\_polymerase\_II\_promoter | 444 | 4 | 1.430568 | -0.516545 | 431 | 207.747241 | 302.02 | 396.292759 | 0.700742 |
| GO:0050804\_regulation\_of\_synaptic\_transmission | 58 | 1 | 2.737812 | -0.510928 | 432 | 209.681229 | 304.2 | 398.718771 | 0.704167 |
| GO:0030334\_regulation\_of\_cell\_migration | 59 | 1 | 2.691409 | -0.504760 | 435 | 211.392827 | 306.45 | 401.507173 | 0.704483 |
| GO:0035270\_endocrine\_system\_development | 59 | 1 | 2.691409 | -0.504760 | 435 | 211.392827 | 306.45 | 401.507173 | 0.704483 |
| GO:0048469\_cell\_maturation | 59 | 1 | 2.691409 | -0.504760 | 435 | 211.392827 | 306.45 | 401.507173 | 0.704483 |
| GO:0045184\_establishment\_of\_protein\_localization | 180 | 2 | 1.764368 | -0.502678 | 436 | 211.583760 | 306.79 | 401.996240 | 0.703647 |
| GO:0009991\_response\_to\_extracellular\_stimulus | 61 | 1 | 2.603166 | -0.492789 | 438 | 214.108260 | 309.8 | 405.491740 | 0.707306 |
| GO:0051969\_regulation\_of\_transmission\_of\_nerve\_impulse | 61 | 1 | 2.603166 | -0.492789 | 438 | 214.108260 | 309.8 | 405.491740 | 0.707306 |
| GO:0009893\_positive\_regulation\_of\_metabolic\_process | 458 | 4 | 1.386839 | -0.487995 | 439 | 215.251254 | 310.95 | 406.648746 | 0.708314 |
| GO:0030155\_regulation\_of\_cell\_adhesion | 62 | 1 | 2.561179 | -0.486980 | 440 | 217.048197 | 313.19 | 409.331803 | 0.711795 |
| GO:0048522\_positive\_regulation\_of\_cellular\_process | 895 | 7 | 1.241957 | -0.484216 | 441 | 217.416930 | 313.64 | 409.863070 | 0.711202 |
| GO:0007369\_gastrulation | 63 | 1 | 2.520525 | -0.481282 | 443 | 219.215004 | 315.96 | 412.704996 | 0.713228 |
| GO:0051216\_cartilage\_development | 63 | 1 | 2.520525 | -0.481282 | 443 | 219.215004 | 315.96 | 412.704996 | 0.713228 |
| GO:0006952\_defense\_response | 187 | 2 | 1.698322 | -0.480516 | 444 | 219.299676 | 316.17 | 413.040324 | 0.712095 |
| GO:0031644\_regulation\_of\_neurological\_system\_process | 64 | 1 | 2.481142 | -0.475692 | 445 | 220.424673 | 317.61 | 414.795327 | 0.713730 |
| GO:0034645\_cellular\_macromolecule\_biosynthetic\_process | 901 | 7 | 1.233687 | -0.475507 | 446 | 220.537292 | 317.75 | 414.962708 | 0.712444 |
| GO:0042471\_ear\_morphogenesis | 65 | 1 | 2.442971 | -0.470207 | 447 | 221.256849 | 318.68 | 416.103151 | 0.712931 |
| GO:0010467\_gene\_expression | 905 | 7 | 1.228234 | -0.469776 | 448 | 221.663617 | 319.04 | 416.416383 | 0.712143 |
| GO:0031589\_cell-substrate\_adhesion | 66 | 1 | 2.405956 | -0.464824 | 450 | 223.018998 | 320.67 | 418.321002 | 0.712600 |
| GO:0051402\_neuron\_apoptosis | 66 | 1 | 2.405956 | -0.464824 | 450 | 223.018998 | 320.67 | 418.321002 | 0.712600 |
| GO:0009059\_macromolecule\_biosynthetic\_process | 910 | 7 | 1.221485 | -0.462695 | 451 | 223.283973 | 321.04 | 418.796027 | 0.711840 |
| GO:0003007\_heart\_morphogenesis | 67 | 1 | 2.370046 | -0.459539 | 454 | 225.710096 | 323.87 | 422.029904 | 0.713370 |
| GO:0031347\_regulation\_of\_defense\_response | 67 | 1 | 2.370046 | -0.459539 | 454 | 225.710096 | 323.87 | 422.029904 | 0.713370 |
| GO:0042445\_hormone\_metabolic\_process | 67 | 1 | 2.370046 | -0.459539 | 454 | 225.710096 | 323.87 | 422.029904 | 0.713370 |
| GO:0048513\_organ\_development | 1365 | 10 | 1.163319 | -0.458859 | 455 | 225.888684 | 324.05 | 422.211316 | 0.712198 |
| GO:0042692\_muscle\_cell\_differentiation | 68 | 1 | 2.335193 | -0.454350 | 456 | 227.716572 | 326.21 | 424.703428 | 0.715373 |
| GO:0005996\_monosaccharide\_metabolic\_process | 69 | 1 | 2.301349 | -0.449253 | 458 | 229.435942 | 328.26 | 427.084058 | 0.716725 |
| GO:0032101\_regulation\_of\_response\_to\_external\_stimulus | 69 | 1 | 2.301349 | -0.449253 | 458 | 229.435942 | 328.26 | 427.084058 | 0.716725 |
| GO:0002009\_morphogenesis\_of\_an\_epithelium | 198 | 2 | 1.603971 | -0.448051 | 460 | 229.989766 | 328.88 | 427.770234 | 0.714957 |
| GO:0060429\_epithelium\_development | 198 | 2 | 1.603971 | -0.448051 | 460 | 229.989766 | 328.88 | 427.770234 | 0.714957 |
| GO:0007611\_learning\_or\_memory | 70 | 1 | 2.268473 | -0.444247 | 462 | 231.728977 | 330.85 | 429.971023 | 0.716126 |
| GO:0009617\_response\_to\_bacterium | 70 | 1 | 2.268473 | -0.444247 | 462 | 231.728977 | 330.85 | 429.971023 | 0.716126 |
| GO:0009605\_response\_to\_external\_stimulus | 339 | 3 | 1.405249 | -0.442352 | 463 | 231.822303 | 330.99 | 430.157697 | 0.714881 |
| GO:0048856\_anatomical\_structure\_development | 1688 | 12 | 1.128861 | -0.440176 | 464 | 232.601393 | 331.8 | 430.998607 | 0.715086 |
| GO:0009100\_glycoprotein\_metabolic\_process | 71 | 1 | 2.236523 | -0.439328 | 466 | 233.921951 | 333.28 | 432.638049 | 0.715193 |
| GO:0016331\_morphogenesis\_of\_embryonic\_epithelium | 71 | 1 | 2.236523 | -0.439328 | 466 | 233.921951 | 333.28 | 432.638049 | 0.715193 |
| GO:0010468\_regulation\_of\_gene\_expression | 778 | 6 | 1.224625 | -0.438876 | 467 | 234.018473 | 333.43 | 432.841527 | 0.713983 |
| GO:0051649\_establishment\_of\_localization\_in\_cell | 342 | 3 | 1.392922 | -0.436006 | 468 | 234.161421 | 333.59 | 433.018579 | 0.712799 |
| GO:0016568\_chromatin\_modification | 72 | 1 | 2.205460 | -0.434494 | 472 | 237.193815 | 336.9 | 436.606185 | 0.713771 |
| GO:0021915\_neural\_tube\_development | 72 | 1 | 2.205460 | -0.434494 | 472 | 237.193815 | 336.9 | 436.606185 | 0.713771 |
| GO:0040012\_regulation\_of\_locomotion | 72 | 1 | 2.205460 | -0.434494 | 472 | 237.193815 | 336.9 | 436.606185 | 0.713771 |
| GO:0048839\_inner\_ear\_development | 72 | 1 | 2.205460 | -0.434494 | 472 | 237.193815 | 336.9 | 436.606185 | 0.713771 |
| GO:0001568\_blood\_vessel\_development | 203 | 2 | 1.564464 | -0.434173 | 473 | 237.415965 | 337.12 | 436.824035 | 0.712727 |
| GO:0051270\_regulation\_of\_cell\_motion | 73 | 1 | 2.175248 | -0.429742 | 474 | 239.117685 | 339.01 | 438.902315 | 0.715211 |
| GO:0001944\_vasculature\_development | 208 | 2 | 1.526857 | -0.420805 | 476 | 240.846185 | 341.28 | 441.713815 | 0.716975 |
| GO:0008284\_positive\_regulation\_of\_cell\_proliferation | 208 | 2 | 1.526857 | -0.420805 | 476 | 240.846185 | 341.28 | 441.713815 | 0.716975 |
| GO:0007281\_germ\_cell\_development | 75 | 1 | 2.117241 | -0.420478 | 479 | 241.670252 | 342.35 | 443.029748 | 0.714718 |
| GO:0048589\_developmental\_growth | 75 | 1 | 2.117241 | -0.420478 | 479 | 241.670252 | 342.35 | 443.029748 | 0.714718 |
| GO:0051050\_positive\_regulation\_of\_transport | 75 | 1 | 2.117241 | -0.420478 | 479 | 241.670252 | 342.35 | 443.029748 | 0.714718 |
| GO:0030182\_neuron\_differentiation | 356 | 3 | 1.338144 | -0.407638 | 480 | 244.124195 | 345.19 | 446.255805 | 0.719146 |
| GO:0007519\_skeletal\_muscle\_tissue\_development | 78 | 1 | 2.035809 | -0.407147 | 482 | 246.604272 | 347.62 | 448.635728 | 0.721203 |
| GO:0060538\_skeletal\_muscle\_organ\_development | 78 | 1 | 2.035809 | -0.407147 | 482 | 246.604272 | 347.62 | 448.635728 | 0.721203 |
| GO:0042981\_regulation\_of\_apoptosis | 360 | 3 | 1.323276 | -0.399893 | 483 | 248.145324 | 349.58 | 451.014676 | 0.723768 |
| GO:0009653\_anatomical\_structure\_morphogenesis | 958 | 7 | 1.160284 | -0.399212 | 484 | 248.236990 | 349.74 | 451.243010 | 0.722603 |
| GO:0006631\_fatty\_acid\_metabolic\_process | 80 | 1 | 1.984914 | -0.398614 | 485 | 249.780312 | 351.53 | 453.279688 | 0.724804 |
| GO:0016070\_RNA\_metabolic\_process | 658 | 5 | 1.206635 | -0.396751 | 486 | 250.500866 | 352.36 | 454.219134 | 0.725021 |
| GO:0021700\_developmental\_maturation | 81 | 1 | 1.960409 | -0.394448 | 487 | 251.149328 | 353.07 | 454.990672 | 0.724990 |
| GO:0010941\_regulation\_of\_cell\_death | 365 | 3 | 1.305149 | -0.390426 | 489 | 252.470689 | 354.73 | 456.989311 | 0.725419 |
| GO:0043067\_regulation\_of\_programmed\_cell\_death | 365 | 3 | 1.305149 | -0.390426 | 489 | 252.470689 | 354.73 | 456.989311 | 0.725419 |
| GO:0010627\_regulation\_of\_protein\_kinase\_cascade | 82 | 1 | 1.936501 | -0.390347 | 491 | 253.611752 | 356.19 | 458.768248 | 0.725438 |
| GO:0045664\_regulation\_of\_neuron\_differentiation | 82 | 1 | 1.936501 | -0.390347 | 491 | 253.611752 | 356.19 | 458.768248 | 0.725438 |
| GO:0006325\_chromatin\_organization | 83 | 1 | 1.913170 | -0.386310 | 494 | 255.740765 | 358.62 | 461.499235 | 0.725951 |
| GO:0006575\_cellular\_amino\_acid\_derivative\_metabolic\_process | 83 | 1 | 1.913170 | -0.386310 | 494 | 255.740765 | 358.62 | 461.499235 | 0.725951 |
| GO:0030534\_adult\_behavior | 83 | 1 | 1.913170 | -0.386310 | 494 | 255.740765 | 358.62 | 461.499235 | 0.725951 |
| GO:0030005\_cellular\_di-\_\_tri-valent\_inorganic\_cation\_homeostasis | 84 | 1 | 1.890394 | -0.382334 | 496 | 256.729183 | 359.61 | 462.490817 | 0.725020 |
| GO:0045137\_development\_of\_primary\_sexual\_characteristics | 84 | 1 | 1.890394 | -0.382334 | 496 | 256.729183 | 359.61 | 462.490817 | 0.725020 |
| GO:0051641\_cellular\_localization | 370 | 3 | 1.287512 | -0.381191 | 497 | 256.902679 | 359.8 | 462.697321 | 0.723944 |
| GO:0032502\_developmental\_process | 2060 | 14 | 1.079176 | -0.377304 | 498 | 258.232139 | 361.17 | 464.107861 | 0.725241 |
| GO:0032504\_multicellular\_organism\_reproduction | 86 | 1 | 1.846431 | -0.374563 | 501 | 260.338769 | 363.41 | 466.481231 | 0.725369 |
| GO:0034641\_cellular\_nitrogen\_compound\_metabolic\_process | 86 | 1 | 1.846431 | -0.374563 | 501 | 260.338769 | 363.41 | 466.481231 | 0.725369 |
| GO:0048609\_reproductive\_process\_in\_a\_multicellular\_organism | 86 | 1 | 1.846431 | -0.374563 | 501 | 260.338769 | 363.41 | 466.481231 | 0.725369 |
| GO:0002682\_regulation\_of\_immune\_system\_process | 228 | 2 | 1.392922 | -0.371950 | 502 | 260.950555 | 364.1 | 467.249445 | 0.725299 |
| GO:0022612\_gland\_morphogenesis | 87 | 1 | 1.825208 | -0.370764 | 503 | 263.135781 | 366.67 | 470.204219 | 0.728966 |
| GO:0048754\_branching\_morphogenesis\_of\_a\_tube | 88 | 1 | 1.804467 | -0.367022 | 504 | 264.622593 | 368.27 | 471.917407 | 0.730694 |
| GO:0007420\_brain\_development | 231 | 2 | 1.374832 | -0.365204 | 505 | 264.994561 | 368.7 | 472.405439 | 0.730099 |
| GO:0030003\_cellular\_cation\_homeostasis | 90 | 1 | 1.764368 | -0.359700 | 506 | 266.816661 | 370.82 | 474.823339 | 0.732846 |
| GO:0016477\_cell\_migration | 234 | 2 | 1.357206 | -0.358597 | 507 | 267.012493 | 371.07 | 475.127507 | 0.731893 |
| GO:0044249\_cellular\_biosynthetic\_process | 1150 | 8 | 1.104648 | -0.354705 | 508 | 268.610532 | 372.81 | 477.009468 | 0.733878 |
| GO:0055066\_di-\_\_tri-valent\_inorganic\_cation\_homeostasis | 93 | 1 | 1.707453 | -0.349108 | 509 | 272.376106 | 376.81 | 481.243894 | 0.740295 |
| GO:0008610\_lipid\_biosynthetic\_process | 94 | 1 | 1.689288 | -0.345676 | 510 | 274.279944 | 379.28 | 484.280056 | 0.743686 |
| GO:0051707\_response\_to\_other\_organism | 95 | 1 | 1.671506 | -0.342293 | 511 | 275.015311 | 380.12 | 485.224689 | 0.743875 |
| GO:0009056\_catabolic\_process | 243 | 2 | 1.306939 | -0.339579 | 512 | 275.417118 | 380.63 | 485.842882 | 0.743418 |
| GO:0048699\_generation\_of\_neurons | 396 | 3 | 1.202978 | -0.336659 | 513 | 277.932265 | 383.26 | 488.587735 | 0.747096 |
| GO:0018193\_peptidyl-amino\_acid\_modification | 97 | 1 | 1.637042 | -0.335666 | 515 | 278.665808 | 384.16 | 489.654192 | 0.745942 |
| GO:0060341\_regulation\_of\_cellular\_localization | 97 | 1 | 1.637042 | -0.335666 | 515 | 278.665808 | 384.16 | 489.654192 | 0.745942 |
| GO:0048519\_negative\_regulation\_of\_biological\_process | 859 | 6 | 1.109149 | -0.334645 | 516 | 278.867679 | 384.4 | 489.932321 | 0.744961 |
| GO:0007548\_sex\_differentiation | 98 | 1 | 1.620338 | -0.332420 | 519 | 280.171329 | 385.92 | 491.668671 | 0.743584 |
| GO:0009314\_response\_to\_radiation | 98 | 1 | 1.620338 | -0.332420 | 519 | 280.171329 | 385.92 | 491.668671 | 0.743584 |
| GO:0060541\_respiratory\_system\_development | 98 | 1 | 1.620338 | -0.332420 | 519 | 280.171329 | 385.92 | 491.668671 | 0.743584 |
| GO:0032879\_regulation\_of\_localization | 248 | 2 | 1.280590 | -0.329507 | 520 | 280.614042 | 386.47 | 492.325958 | 0.743212 |
| GO:0060562\_epithelial\_tube\_morphogenesis | 99 | 1 | 1.603971 | -0.329219 | 521 | 281.928925 | 387.9 | 493.871075 | 0.744530 |
| GO:0009058\_biosynthetic\_process | 1175 | 8 | 1.081145 | -0.328830 | 522 | 282.074884 | 388.06 | 494.045116 | 0.743410 |
| GO:0008104\_protein\_localization | 251 | 2 | 1.265284 | -0.323624 | 523 | 282.947078 | 389.01 | 495.072922 | 0.743805 |
| GO:0016481\_negative\_regulation\_of\_transcription | 253 | 2 | 1.255281 | -0.319768 | 524 | 284.717764 | 390.82 | 496.922236 | 0.745840 |
| GO:0048878\_chemical\_homeostasis | 254 | 2 | 1.250339 | -0.317859 | 525 | 285.025170 | 391.21 | 497.394830 | 0.745162 |
| GO:0003013\_circulatory\_system\_process | 103 | 1 | 1.541681 | -0.316835 | 527 | 285.886459 | 392.32 | 498.753541 | 0.744440 |
| GO:0008015\_blood\_circulation | 103 | 1 | 1.541681 | -0.316835 | 527 | 285.886459 | 392.32 | 498.753541 | 0.744440 |
| GO:0050767\_regulation\_of\_neurogenesis | 104 | 1 | 1.526857 | -0.313841 | 528 | 287.069395 | 393.69 | 500.310605 | 0.745625 |
| GO:0048870\_cell\_motility | 257 | 2 | 1.235744 | -0.312208 | 529 | 287.778249 | 394.51 | 501.241751 | 0.745766 |
| GO:0010817\_regulation\_of\_hormone\_levels | 106 | 1 | 1.498048 | -0.307968 | 530 | 288.452808 | 395.37 | 502.287192 | 0.745981 |
| GO:0009987\_cellular\_process | 3868 | 25 | 1.026326 | -0.305589 | 531 | 288.568347 | 395.52 | 502.471653 | 0.744859 |
| GO:0010629\_negative\_regulation\_of\_gene\_expression | 262 | 2 | 1.212161 | -0.303035 | 533 | 289.608811 | 396.81 | 504.011189 | 0.744484 |
| GO:0048666\_neuron\_development | 262 | 2 | 1.212161 | -0.303035 | 533 | 289.608811 | 396.81 | 504.011189 | 0.744484 |
| GO:0006355\_regulation\_of\_transcription\_\_DNA-dependent | 575 | 4 | 1.104648 | -0.302450 | 534 | 289.834897 | 397.05 | 504.265103 | 0.743539 |
| GO:0030099\_myeloid\_cell\_differentiation | 108 | 1 | 1.470307 | -0.302244 | 535 | 290.463938 | 397.8 | 505.136062 | 0.743551 |
| GO:0055080\_cation\_homeostasis | 110 | 1 | 1.443574 | -0.296665 | 536 | 292.746240 | 400.44 | 508.133760 | 0.747090 |
| GO:0022008\_neurogenesis | 423 | 3 | 1.126192 | -0.295972 | 537 | 292.945992 | 400.66 | 508.374008 | 0.746108 |
| GO:0006915\_apoptosis | 427 | 3 | 1.115642 | -0.290378 | 538 | 294.213589 | 402.44 | 510.666411 | 0.748030 |
| GO:0045934\_negative\_regulation\_of\_nucleobase\_\_nucleoside\_\_nucleotide\_and\_nucleic\_acid\_metabolic\_process | 270 | 2 | 1.176245 | -0.288970 | 539 | 294.946771 | 403.18 | 511.413229 | 0.748015 |
| GO:0030154\_cell\_differentiation | 1060 | 7 | 1.048634 | -0.288293 | 540 | 295.698662 | 403.98 | 512.261338 | 0.748111 |
| GO:0051239\_regulation\_of\_multicellular\_organismal\_process | 587 | 4 | 1.082065 | -0.287785 | 541 | 295.819833 | 404.17 | 512.520167 | 0.747079 |
| GO:0010556\_regulation\_of\_macromolecule\_biosynthetic\_process | 745 | 5 | 1.065726 | -0.287762 | 542 | 295.982664 | 404.36 | 512.737336 | 0.746052 |
| GO:0051172\_negative\_regulation\_of\_nitrogen\_compound\_metabolic\_process | 271 | 2 | 1.171905 | -0.287263 | 543 | 296.257964 | 404.63 | 513.002036 | 0.745175 |
| GO:0009607\_response\_to\_biotic\_stimulus | 114 | 1 | 1.392922 | -0.285918 | 544 | 296.938334 | 405.31 | 513.681666 | 0.745055 |
| GO:0051252\_regulation\_of\_RNA\_metabolic\_process | 590 | 4 | 1.076563 | -0.284223 | 545 | 297.300156 | 405.76 | 514.219844 | 0.744514 |
| GO:0010558\_negative\_regulation\_of\_macromolecule\_biosynthetic\_process | 274 | 2 | 1.159074 | -0.282207 | 547 | 298.766063 | 407.32 | 515.873937 | 0.744644 |
| GO:0033036\_macromolecule\_localization | 274 | 2 | 1.159074 | -0.282207 | 547 | 298.766063 | 407.32 | 515.873937 | 0.744644 |
| GO:0012501\_programmed\_cell\_death | 433 | 3 | 1.100183 | -0.282183 | 548 | 299.065003 | 407.74 | 516.414997 | 0.744051 |
| GO:0046483\_heterocycle\_metabolic\_process | 116 | 1 | 1.368906 | -0.280740 | 550 | 300.217900 | 408.97 | 517.722100 | 0.743582 |
| GO:0080134\_regulation\_of\_response\_to\_stress | 116 | 1 | 1.368906 | -0.280740 | 550 | 300.217900 | 408.97 | 517.722100 | 0.743582 |
| GO:0006351\_transcription\_\_DNA-dependent | 594 | 4 | 1.069314 | -0.279539 | 551 | 300.632700 | 409.38 | 518.127300 | 0.742976 |
| GO:0032774\_RNA\_biosynthetic\_process | 595 | 4 | 1.067517 | -0.278379 | 552 | 301.098577 | 409.89 | 518.681423 | 0.742554 |
| GO:0051960\_regulation\_of\_nervous\_system\_development | 118 | 1 | 1.345704 | -0.275687 | 553 | 303.080373 | 412.04 | 520.999627 | 0.745099 |
| GO:0007610\_behavior | 279 | 2 | 1.138302 | -0.273995 | 554 | 303.889547 | 413.0 | 522.110453 | 0.745487 |
| GO:0032501\_multicellular\_organismal\_process | 2183 | 14 | 1.018371 | -0.271327 | 555 | 304.536976 | 413.68 | 522.823024 | 0.745369 |
| GO:0014706\_striated\_muscle\_tissue\_development | 120 | 1 | 1.323276 | -0.270753 | 556 | 304.979076 | 414.09 | 523.200924 | 0.744766 |
| GO:0031327\_negative\_regulation\_of\_cellular\_biosynthetic\_process | 282 | 2 | 1.126192 | -0.269192 | 557 | 305.343045 | 414.51 | 523.676955 | 0.744183 |
| GO:0006917\_induction\_of\_apoptosis | 121 | 1 | 1.312340 | -0.268330 | 560 | 306.565715 | 415.96 | 525.354285 | 0.742786 |
| GO:0012502\_induction\_of\_programmed\_cell\_death | 121 | 1 | 1.312340 | -0.268330 | 560 | 306.565715 | 415.96 | 525.354285 | 0.742786 |
| GO:0051726\_regulation\_of\_cell\_cycle | 121 | 1 | 1.312340 | -0.268330 | 560 | 306.565715 | 415.96 | 525.354285 | 0.742786 |
| GO:0008219\_cell\_death | 444 | 3 | 1.072926 | -0.267748 | 561 | 306.964885 | 416.46 | 525.955115 | 0.742353 |
| GO:0009890\_negative\_regulation\_of\_biosynthetic\_process | 284 | 2 | 1.118261 | -0.266040 | 562 | 307.484582 | 416.96 | 526.435418 | 0.741922 |
| GO:0060284\_regulation\_of\_cell\_development | 122 | 1 | 1.301583 | -0.265935 | 563 | 309.318905 | 419.1 | 528.881095 | 0.744405 |
| GO:0006996\_organelle\_organization | 449 | 3 | 1.060978 | -0.261430 | 564 | 310.478929 | 420.22 | 529.961071 | 0.745071 |
| GO:0016265\_death | 450 | 3 | 1.058621 | -0.260184 | 565 | 311.532078 | 421.52 | 531.507922 | 0.746053 |
| GO:0001763\_morphogenesis\_of\_a\_branching\_structure | 125 | 1 | 1.270345 | -0.258917 | 566 | 312.147548 | 422.21 | 532.272452 | 0.745954 |
| GO:0048523\_negative\_regulation\_of\_cellular\_process | 774 | 5 | 1.025795 | -0.257911 | 567 | 312.568419 | 422.56 | 532.551581 | 0.745256 |
| GO:0060255\_regulation\_of\_macromolecule\_metabolic\_process | 936 | 6 | 1.017905 | -0.256160 | 568 | 312.618151 | 422.69 | 532.761849 | 0.744173 |
| GO:0060537\_muscle\_tissue\_development | 128 | 1 | 1.240571 | -0.252138 | 569 | 314.249895 | 424.37 | 534.490105 | 0.745817 |
| GO:0051276\_chromosome\_organization | 129 | 1 | 1.230954 | -0.249929 | 570 | 315.291591 | 425.47 | 535.648409 | 0.746439 |
| GO:0007399\_nervous\_system\_development | 621 | 4 | 1.022822 | -0.249743 | 571 | 315.584975 | 425.71 | 535.835025 | 0.745552 |
| GO:0040011\_locomotion | 295 | 2 | 1.076563 | -0.249402 | 573 | 316.354136 | 426.58 | 536.805864 | 0.744468 |
| GO:0045595\_regulation\_of\_cell\_differentiation | 295 | 2 | 1.076563 | -0.249402 | 573 | 316.354136 | 426.58 | 536.805864 | 0.744468 |
| GO:0050776\_regulation\_of\_immune\_response | 130 | 1 | 1.221485 | -0.247746 | 574 | 317.250275 | 427.66 | 538.069725 | 0.745052 |
| GO:0048869\_cellular\_developmental\_process | 1113 | 7 | 0.998699 | -0.241674 | 575 | 318.219927 | 428.53 | 538.840073 | 0.745270 |
| GO:0009952\_anterior\_posterior\_pattern\_formation | 133 | 1 | 1.193933 | -0.241340 | 576 | 318.919149 | 429.26 | 539.600851 | 0.745243 |
| GO:0007283\_spermatogenesis | 134 | 1 | 1.185023 | -0.239252 | 578 | 319.584545 | 429.92 | 540.255455 | 0.743806 |
| GO:0048232\_male\_gamete\_generation | 134 | 1 | 1.185023 | -0.239252 | 578 | 319.584545 | 429.92 | 540.255455 | 0.743806 |
| GO:0007169\_transmembrane\_receptor\_protein\_tyrosine\_kinase\_signaling\_pathway | 139 | 1 | 1.142396 | -0.229150 | 579 | 322.815324 | 433.37 | 543.924676 | 0.748480 |
| GO:0003006\_reproductive\_developmental\_process | 141 | 1 | 1.126192 | -0.225260 | 580 | 324.282118 | 434.92 | 545.557882 | 0.749862 |
| GO:0031326\_regulation\_of\_cellular\_biosynthetic\_process | 812 | 5 | 0.977790 | -0.222954 | 581 | 324.427462 | 435.11 | 545.792538 | 0.748898 |
| GO:0035239\_tube\_morphogenesis | 143 | 1 | 1.110441 | -0.221453 | 582 | 324.852548 | 435.5 | 546.147452 | 0.748282 |
| GO:0009889\_regulation\_of\_biosynthetic\_process | 815 | 5 | 0.974191 | -0.220381 | 583 | 324.996530 | 435.69 | 546.383470 | 0.747324 |
| GO:0045596\_negative\_regulation\_of\_cell\_differentiation | 144 | 1 | 1.102730 | -0.219580 | 584 | 325.793028 | 436.42 | 547.046972 | 0.747295 |
| GO:0005975\_carbohydrate\_metabolic\_process | 146 | 1 | 1.087624 | -0.215892 | 585 | 327.177966 | 437.76 | 548.342034 | 0.748308 |
| GO:0032268\_regulation\_of\_cellular\_protein\_metabolic\_process | 152 | 1 | 1.044691 | -0.205279 | 586 | 329.470767 | 440.21 | 550.949233 | 0.751212 |
| GO:0065007\_biological\_regulation | 2593 | 16 | 0.979826 | -0.204338 | 587 | 329.650132 | 440.34 | 551.029868 | 0.750153 |
| GO:0007517\_muscle\_organ\_development | 153 | 1 | 1.037863 | -0.203573 | 588 | 330.072803 | 440.75 | 551.427197 | 0.749575 |
| GO:0006928\_cell\_motion | 330 | 2 | 0.962382 | -0.203413 | 590 | 331.035586 | 441.63 | 552.224414 | 0.748525 |
| GO:0051674\_localization\_of\_cell | 330 | 2 | 0.962382 | -0.203413 | 590 | 331.035586 | 441.63 | 552.224414 | 0.748525 |
| GO:0010605\_negative\_regulation\_of\_macromolecule\_metabolic\_process | 331 | 2 | 0.959475 | -0.202238 | 592 | 331.573048 | 442.14 | 552.706952 | 0.746858 |
| GO:0051093\_negative\_regulation\_of\_developmental\_process | 331 | 2 | 0.959475 | -0.202238 | 592 | 331.573048 | 442.14 | 552.706952 | 0.746858 |
| GO:0007268\_synaptic\_transmission | 154 | 1 | 1.031124 | -0.201885 | 593 | 332.037601 | 442.69 | 553.342399 | 0.746526 |
| GO:0031324\_negative\_regulation\_of\_cellular\_metabolic\_process | 332 | 2 | 0.956585 | -0.201070 | 594 | 332.405345 | 443.09 | 553.774655 | 0.745943 |
| GO:0045449\_regulation\_of\_transcription | 676 | 4 | 0.939604 | -0.197870 | 595 | 333.810418 | 444.43 | 555.049582 | 0.746941 |
| GO:0006066\_alcohol\_metabolic\_process | 158 | 1 | 1.005020 | -0.195300 | 596 | 335.485135 | 446.09 | 556.694865 | 0.748473 |
| GO:0007626\_locomotory\_behavior | 163 | 1 | 0.974191 | -0.187431 | 597 | 338.853092 | 449.68 | 560.506908 | 0.753233 |
| GO:0009892\_negative\_regulation\_of\_metabolic\_process | 348 | 2 | 0.912604 | -0.183296 | 598 | 341.238116 | 452.02 | 562.801884 | 0.755886 |
| GO:0051049\_regulation\_of\_transport | 167 | 1 | 0.950857 | -0.181410 | 599 | 342.917623 | 453.64 | 564.362377 | 0.757329 |
| GO:0006350\_transcription | 701 | 4 | 0.906095 | -0.177710 | 600 | 343.762744 | 454.38 | 564.997256 | 0.757300 |
| GO:0051246\_regulation\_of\_protein\_metabolic\_process | 170 | 1 | 0.934077 | -0.177046 | 601 | 344.594380 | 455.18 | 565.765620 | 0.757371 |
| GO:0050793\_regulation\_of\_developmental\_process | 703 | 4 | 0.903517 | -0.176181 | 602 | 344.827112 | 455.4 | 565.972888 | 0.756478 |
| GO:0000122\_negative\_regulation\_of\_transcription\_from\_RNA\_polymerase\_II\_promoter | 175 | 1 | 0.907389 | -0.170046 | 603 | 348.580669 | 458.93 | 569.279331 | 0.761078 |
| GO:0006873\_cellular\_ion\_homeostasis | 176 | 1 | 0.902234 | -0.168686 | 605 | 350.026712 | 460.4 | 570.773288 | 0.760992 |
| GO:0043066\_negative\_regulation\_of\_apoptosis | 176 | 1 | 0.902234 | -0.168686 | 605 | 350.026712 | 460.4 | 570.773288 | 0.760992 |
| GO:0043009\_chordate\_embryonic\_development | 365 | 2 | 0.870099 | -0.166168 | 606 | 350.857000 | 461.13 | 571.403000 | 0.760941 |
| GO:0043069\_negative\_regulation\_of\_programmed\_cell\_death | 179 | 1 | 0.887112 | -0.164680 | 608 | 352.346293 | 462.73 | 573.113707 | 0.761069 |
| GO:0060548\_negative\_regulation\_of\_cell\_death | 179 | 1 | 0.887112 | -0.164680 | 608 | 352.346293 | 462.73 | 573.113707 | 0.761069 |
| GO:0009792\_embryonic\_development\_ending\_in\_birth\_or\_egg\_hatching | 368 | 2 | 0.863006 | -0.163319 | 609 | 353.460304 | 463.68 | 573.899696 | 0.761379 |
| GO:0055082\_cellular\_chemical\_homeostasis | 181 | 1 | 0.877310 | -0.162072 | 610 | 354.677314 | 464.88 | 575.082686 | 0.762098 |
| GO:0050789\_regulation\_of\_biological\_process | 2357 | 14 | 0.943192 | -0.160110 | 611 | 356.060979 | 466.24 | 576.419021 | 0.763077 |
| GO:0007155\_cell\_adhesion | 186 | 1 | 0.853726 | -0.155761 | 613 | 359.019467 | 469.39 | 579.760533 | 0.765726 |
| GO:0022610\_biological\_adhesion | 186 | 1 | 0.853726 | -0.155761 | 613 | 359.019467 | 469.39 | 579.760533 | 0.765726 |
| GO:0007276\_gamete\_generation | 188 | 1 | 0.844644 | -0.153316 | 614 | 360.106097 | 470.47 | 580.833903 | 0.766238 |
| GO:0019226\_transmission\_of\_nerve\_impulse | 189 | 1 | 0.840175 | -0.152111 | 615 | 360.817502 | 471.15 | 581.482498 | 0.766098 |
| GO:0007165\_signal\_transduction | 915 | 5 | 0.867722 | -0.148164 | 616 | 361.831869 | 472.1 | 582.368131 | 0.766396 |
| GO:0019222\_regulation\_of\_metabolic\_process | 1088 | 6 | 0.875697 | -0.146181 | 617 | 363.080072 | 473.29 | 583.499928 | 0.767083 |
| GO:0003002\_regionalization | 195 | 1 | 0.814324 | -0.145103 | 620 | 364.285657 | 474.56 | 584.834343 | 0.765419 |
| GO:0007507\_heart\_development | 195 | 1 | 0.814324 | -0.145103 | 620 | 364.285657 | 474.56 | 584.834343 | 0.765419 |
| GO:0019725\_cellular\_homeostasis | 195 | 1 | 0.814324 | -0.145103 | 620 | 364.285657 | 474.56 | 584.834343 | 0.765419 |
| GO:0031175\_neuron\_projection\_development | 197 | 1 | 0.806056 | -0.142850 | 622 | 365.906653 | 476.02 | 586.133347 | 0.765305 |
| GO:0050801\_ion\_homeostasis | 197 | 1 | 0.806056 | -0.142850 | 622 | 365.906653 | 476.02 | 586.133347 | 0.765305 |
| GO:0007154\_cell\_communication | 1096 | 6 | 0.869305 | -0.141731 | 623 | 367.242103 | 477.17 | 587.097897 | 0.765923 |
| GO:0080090\_regulation\_of\_primary\_metabolic\_process | 926 | 5 | 0.857414 | -0.141651 | 624 | 367.328672 | 477.28 | 587.231328 | 0.764872 |
| GO:0000904\_cell\_morphogenesis\_involved\_in\_differentiation | 199 | 1 | 0.797955 | -0.140638 | 625 | 368.047304 | 477.97 | 587.892696 | 0.764752 |
| GO:0019219\_regulation\_of\_nucleobase\_\_nucleoside\_\_nucleotide\_and\_nucleic\_acid\_metabolic\_process | 757 | 4 | 0.839065 | -0.139110 | 626 | 368.698021 | 478.42 | 588.141979 | 0.764249 |
| GO:0006955\_immune\_response | 205 | 1 | 0.774601 | -0.134230 | 628 | 370.960425 | 480.69 | 590.419575 | 0.765430 |
| GO:0007243\_protein\_kinase\_cascade | 205 | 1 | 0.774601 | -0.134230 | 628 | 370.960425 | 480.69 | 590.419575 | 0.765430 |
| GO:0051171\_regulation\_of\_nitrogen\_compound\_metabolic\_process | 771 | 4 | 0.823829 | -0.130722 | 629 | 372.773447 | 482.31 | 591.846553 | 0.766789 |
| GO:0007166\_cell\_surface\_receptor\_linked\_signal\_transduction | 597 | 3 | 0.797955 | -0.127627 | 630 | 373.904564 | 483.31 | 592.715436 | 0.767159 |
| GO:0035295\_tube\_development | 212 | 1 | 0.749024 | -0.127168 | 631 | 374.479743 | 483.93 | 593.380257 | 0.766926 |
| GO:0010033\_response\_to\_organic\_substance | 216 | 1 | 0.735153 | -0.123321 | 632 | 375.228302 | 484.64 | 594.051698 | 0.766835 |
| GO:0048583\_regulation\_of\_response\_to\_stimulus | 217 | 1 | 0.731765 | -0.122380 | 633 | 375.912785 | 485.37 | 594.827215 | 0.766777 |
| GO:0045892\_negative\_regulation\_of\_transcription\_\_DNA-dependent | 218 | 1 | 0.728409 | -0.121446 | 634 | 376.930585 | 486.16 | 595.389415 | 0.766814 |
| GO:0016043\_cellular\_component\_organization | 964 | 5 | 0.823616 | -0.121021 | 635 | 377.196825 | 486.39 | 595.583175 | 0.765969 |
| GO:0051253\_negative\_regulation\_of\_RNA\_metabolic\_process | 220 | 1 | 0.721787 | -0.119604 | 636 | 377.887463 | 486.99 | 596.092537 | 0.765708 |
| GO:0001701\_in\_utero\_embryonic\_development | 221 | 1 | 0.718521 | -0.118694 | 637 | 378.491460 | 487.53 | 596.568540 | 0.765353 |
| GO:0019953\_sexual\_reproduction | 228 | 1 | 0.696461 | -0.112537 | 638 | 381.201753 | 489.81 | 598.418247 | 0.767727 |
| GO:0007167\_enzyme\_linked\_receptor\_protein\_signaling\_pathway | 229 | 1 | 0.693420 | -0.111687 | 639 | 382.025789 | 490.62 | 599.214211 | 0.767793 |
| GO:0050790\_regulation\_of\_catalytic\_activity | 233 | 1 | 0.681515 | -0.108356 | 640 | 383.858316 | 492.32 | 600.781684 | 0.769250 |
| GO:0031323\_regulation\_of\_cellular\_metabolic\_process | 1015 | 5 | 0.782232 | -0.097447 | 641 | 388.937283 | 496.73 | 604.522717 | 0.774930 |
| GO:0007389\_pattern\_specification\_process | 250 | 1 | 0.635172 | -0.095364 | 642 | 391.367706 | 498.75 | 606.132294 | 0.776869 |
| GO:0050794\_regulation\_of\_cellular\_process | 2190 | 12 | 0.870099 | -0.095170 | 643 | 391.519643 | 498.85 | 606.180357 | 0.775816 |
| GO:0030097\_hemopoiesis | 253 | 1 | 0.627641 | -0.093252 | 644 | 393.222774 | 500.32 | 607.417226 | 0.776894 |
| GO:0030030\_cell\_projection\_organization | 263 | 1 | 0.603776 | -0.086570 | 645 | 396.743601 | 503.34 | 609.936399 | 0.780372 |
| GO:0051179\_localization | 1058 | 5 | 0.750440 | -0.080767 | 646 | 399.053621 | 505.45 | 611.846379 | 0.782430 |
| GO:0048534\_hemopoietic\_or\_lymphoid\_organ\_development | 277 | 1 | 0.573260 | -0.078062 | 647 | 401.196311 | 507.31 | 613.423689 | 0.784096 |
| GO:0065009\_regulation\_of\_molecular\_function | 279 | 1 | 0.569151 | -0.076921 | 648 | 401.881598 | 507.91 | 613.938402 | 0.783812 |
| GO:0000902\_cell\_morphogenesis | 283 | 1 | 0.561106 | -0.074692 | 649 | 403.074347 | 508.93 | 614.785653 | 0.784176 |
| GO:0002376\_immune\_system\_process | 505 | 2 | 0.628884 | -0.073961 | 650 | 403.709647 | 509.43 | 615.150353 | 0.783738 |
| GO:0006810\_transport | 718 | 3 | 0.663481 | -0.069215 | 651 | 405.691267 | 511.13 | 616.568733 | 0.785146 |
| GO:0002520\_immune\_system\_development | 295 | 1 | 0.538282 | -0.068405 | 652 | 407.110576 | 512.28 | 617.449424 | 0.785706 |
| GO:0051234\_establishment\_of\_localization | 729 | 3 | 0.653470 | -0.065371 | 653 | 408.411531 | 513.38 | 618.348469 | 0.786187 |
| GO:0032989\_cellular\_component\_morphogenesis | 307 | 1 | 0.517241 | -0.062670 | 654 | 410.231555 | 514.86 | 619.488445 | 0.787248 |
| GO:0007242\_intracellular\_signaling\_cascade | 411 | 1 | 0.386358 | -0.029600 | 655 | 428.175758 | 529.38 | 630.584242 | 0.808214 |
| GO:0030029\_actin\_filament-based\_process | 109 | 0 | 0.000000 | -0.000000 | 656 | 442.845809 | 539.84 | 636.834191 | 0.822927 |
| GO:0000910\_cytokinesis | 8 | 0 | 0.000000 | -0.000000 | 783 | 569.997785 | 665.06 | 760.122215 | 0.849374 |
| GO:0001783\_B\_cell\_apoptosis | 8 | 0 | 0.000000 | -0.000000 | 783 | 569.997785 | 665.06 | 760.122215 | 0.849374 |
| GO:0001833\_inner\_cell\_mass\_cell\_proliferation | 8 | 0 | 0.000000 | -0.000000 | 783 | 569.997785 | 665.06 | 760.122215 | 0.849374 |
| GO:0001840\_neural\_plate\_development | 8 | 0 | 0.000000 | -0.000000 | 783 | 569.997785 | 665.06 | 760.122215 | 0.849374 |
| GO:0001893\_maternal\_placenta\_development | 8 | 0 | 0.000000 | -0.000000 | 783 | 569.997785 | 665.06 | 760.122215 | 0.849374 |
| GO:0001911\_negative\_regulation\_of\_leukocyte\_mediated\_cytotoxicity | 8 | 0 | 0.000000 | -0.000000 | 783 | 569.997785 | 665.06 | 760.122215 | 0.849374 |
| GO:0001916\_positive\_regulation\_of\_T\_cell\_mediated\_cytotoxicity | 8 | 0 | 0.000000 | -0.000000 | 783 | 569.997785 | 665.06 | 760.122215 | 0.849374 |
| GO:0002065\_columnar\_cuboidal\_epithelial\_cell\_differentiation | 8 | 0 | 0.000000 | -0.000000 | 783 | 569.997785 | 665.06 | 760.122215 | 0.849374 |
| GO:0002320\_lymphoid\_progenitor\_cell\_differentiation | 8 | 0 | 0.000000 | -0.000000 | 783 | 569.997785 | 665.06 | 760.122215 | 0.849374 |
| GO:0002438\_acute\_inflammatory\_response\_to\_antigenic\_stimulus | 8 | 0 | 0.000000 | -0.000000 | 783 | 569.997785 | 665.06 | 760.122215 | 0.849374 |
| GO:0002524\_hypersensitivity | 8 | 0 | 0.000000 | -0.000000 | 783 | 569.997785 | 665.06 | 760.122215 | 0.849374 |
| GO:0002566\_somatic\_diversification\_of\_immune\_receptors\_via\_somatic\_mutation | 8 | 0 | 0.000000 | -0.000000 | 783 | 569.997785 | 665.06 | 760.122215 | 0.849374 |
| GO:0002864\_regulation\_of\_acute\_inflammatory\_response\_to\_antigenic\_stimulus | 8 | 0 | 0.000000 | -0.000000 | 783 | 569.997785 | 665.06 | 760.122215 | 0.849374 |
| GO:0002883\_regulation\_of\_hypersensitivity | 8 | 0 | 0.000000 | -0.000000 | 783 | 569.997785 | 665.06 | 760.122215 | 0.849374 |
| GO:0003081\_regulation\_of\_systemic\_arterial\_blood\_pressure\_by\_renin-angiotensin | 8 | 0 | 0.000000 | -0.000000 | 783 | 569.997785 | 665.06 | 760.122215 | 0.849374 |
| GO:0006020\_inositol\_metabolic\_process | 8 | 0 | 0.000000 | -0.000000 | 783 | 569.997785 | 665.06 | 760.122215 | 0.849374 |
| GO:0006195\_purine\_nucleotide\_catabolic\_process | 8 | 0 | 0.000000 | -0.000000 | 783 | 569.997785 | 665.06 | 760.122215 | 0.849374 |
| GO:0006349\_genetic\_imprinting | 8 | 0 | 0.000000 | -0.000000 | 783 | 569.997785 | 665.06 | 760.122215 | 0.849374 |
| GO:0006360\_transcription\_from\_RNA\_polymerase\_I\_promoter | 8 | 0 | 0.000000 | -0.000000 | 783 | 569.997785 | 665.06 | 760.122215 | 0.849374 |
| GO:0006399\_tRNA\_metabolic\_process | 8 | 0 | 0.000000 | -0.000000 | 783 | 569.997785 | 665.06 | 760.122215 | 0.849374 |
| GO:0006458\_'de\_novo'\_protein\_folding | 8 | 0 | 0.000000 | -0.000000 | 783 | 569.997785 | 665.06 | 760.122215 | 0.849374 |
| GO:0006493\_protein\_amino\_acid\_O-linked\_glycosylation | 8 | 0 | 0.000000 | -0.000000 | 783 | 569.997785 | 665.06 | 760.122215 | 0.849374 |
| GO:0006582\_melanin\_metabolic\_process | 8 | 0 | 0.000000 | -0.000000 | 783 | 569.997785 | 665.06 | 760.122215 | 0.849374 |
| GO:0006733\_oxidoreduction\_coenzyme\_metabolic\_process | 8 | 0 | 0.000000 | -0.000000 | 783 | 569.997785 | 665.06 | 760.122215 | 0.849374 |
| GO:0006829\_zinc\_ion\_transport | 8 | 0 | 0.000000 | -0.000000 | 783 | 569.997785 | 665.06 | 760.122215 | 0.849374 |
| GO:0007009\_plasma\_membrane\_organization | 8 | 0 | 0.000000 | -0.000000 | 783 | 569.997785 | 665.06 | 760.122215 | 0.849374 |
| GO:0007098\_centrosome\_cycle | 8 | 0 | 0.000000 | -0.000000 | 783 | 569.997785 | 665.06 | 760.122215 | 0.849374 |
| GO:0007625\_grooming\_behavior | 8 | 0 | 0.000000 | -0.000000 | 783 | 569.997785 | 665.06 | 760.122215 | 0.849374 |
| GO:0008105\_asymmetric\_protein\_localization | 8 | 0 | 0.000000 | -0.000000 | 783 | 569.997785 | 665.06 | 760.122215 | 0.849374 |
| GO:0009072\_aromatic\_amino\_acid\_family\_metabolic\_process | 8 | 0 | 0.000000 | -0.000000 | 783 | 569.997785 | 665.06 | 760.122215 | 0.849374 |
| GO:0009144\_purine\_nucleoside\_triphosphate\_metabolic\_process | 8 | 0 | 0.000000 | -0.000000 | 783 | 569.997785 | 665.06 | 760.122215 | 0.849374 |
| GO:0009746\_response\_to\_hexose\_stimulus | 8 | 0 | 0.000000 | -0.000000 | 783 | 569.997785 | 665.06 | 760.122215 | 0.849374 |
| GO:0009749\_response\_to\_glucose\_stimulus | 8 | 0 | 0.000000 | -0.000000 | 783 | 569.997785 | 665.06 | 760.122215 | 0.849374 |
| GO:0014014\_negative\_regulation\_of\_gliogenesis | 8 | 0 | 0.000000 | -0.000000 | 783 | 569.997785 | 665.06 | 760.122215 | 0.849374 |
| GO:0014046\_dopamine\_secretion | 8 | 0 | 0.000000 | -0.000000 | 783 | 569.997785 | 665.06 | 760.122215 | 0.849374 |
| GO:0014059\_regulation\_of\_dopamine\_secretion | 8 | 0 | 0.000000 | -0.000000 | 783 | 569.997785 | 665.06 | 760.122215 | 0.849374 |
| GO:0014065\_phosphoinositide\_3-kinase\_cascade | 8 | 0 | 0.000000 | -0.000000 | 783 | 569.997785 | 665.06 | 760.122215 | 0.849374 |
| GO:0015800\_acidic\_amino\_acid\_transport | 8 | 0 | 0.000000 | -0.000000 | 783 | 569.997785 | 665.06 | 760.122215 | 0.849374 |
| GO:0015804\_neutral\_amino\_acid\_transport | 8 | 0 | 0.000000 | -0.000000 | 783 | 569.997785 | 665.06 | 760.122215 | 0.849374 |
| GO:0016236\_macroautophagy | 8 | 0 | 0.000000 | -0.000000 | 783 | 569.997785 | 665.06 | 760.122215 | 0.849374 |
| GO:0016446\_somatic\_hypermutation\_of\_immunoglobulin\_genes | 8 | 0 | 0.000000 | -0.000000 | 783 | 569.997785 | 665.06 | 760.122215 | 0.849374 |
| GO:0018107\_peptidyl-threonine\_phosphorylation | 8 | 0 | 0.000000 | -0.000000 | 783 | 569.997785 | 665.06 | 760.122215 | 0.849374 |
| GO:0018210\_peptidyl-threonine\_modification | 8 | 0 | 0.000000 | -0.000000 | 783 | 569.997785 | 665.06 | 760.122215 | 0.849374 |
| GO:0018345\_protein\_palmitoylation | 8 | 0 | 0.000000 | -0.000000 | 783 | 569.997785 | 665.06 | 760.122215 | 0.849374 |
| GO:0019229\_regulation\_of\_vasoconstriction | 8 | 0 | 0.000000 | -0.000000 | 783 | 569.997785 | 665.06 | 760.122215 | 0.849374 |
| GO:0019400\_alditol\_metabolic\_process | 8 | 0 | 0.000000 | -0.000000 | 783 | 569.997785 | 665.06 | 760.122215 | 0.849374 |
| GO:0021692\_cerebellar\_Purkinje\_cell\_layer\_morphogenesis | 8 | 0 | 0.000000 | -0.000000 | 783 | 569.997785 | 665.06 | 760.122215 | 0.849374 |
| GO:0021694\_cerebellar\_Purkinje\_cell\_layer\_formation | 8 | 0 | 0.000000 | -0.000000 | 783 | 569.997785 | 665.06 | 760.122215 | 0.849374 |
| GO:0021702\_cerebellar\_Purkinje\_cell\_differentiation | 8 | 0 | 0.000000 | -0.000000 | 783 | 569.997785 | 665.06 | 760.122215 | 0.849374 |
| GO:0021781\_glial\_cell\_fate\_commitment | 8 | 0 | 0.000000 | -0.000000 | 783 | 569.997785 | 665.06 | 760.122215 | 0.849374 |
| GO:0021799\_cerebral\_cortex\_radially\_oriented\_cell\_migration | 8 | 0 | 0.000000 | -0.000000 | 783 | 569.997785 | 665.06 | 760.122215 | 0.849374 |
| GO:0022898\_regulation\_of\_transmembrane\_transporter\_activity | 8 | 0 | 0.000000 | -0.000000 | 783 | 569.997785 | 665.06 | 760.122215 | 0.849374 |
| GO:0030035\_microspike\_assembly | 8 | 0 | 0.000000 | -0.000000 | 783 | 569.997785 | 665.06 | 760.122215 | 0.849374 |
| GO:0030193\_regulation\_of\_blood\_coagulation | 8 | 0 | 0.000000 | -0.000000 | 783 | 569.997785 | 665.06 | 760.122215 | 0.849374 |
| GO:0030204\_chondroitin\_sulfate\_metabolic\_process | 8 | 0 | 0.000000 | -0.000000 | 783 | 569.997785 | 665.06 | 760.122215 | 0.849374 |
| GO:0030500\_regulation\_of\_bone\_mineralization | 8 | 0 | 0.000000 | -0.000000 | 783 | 569.997785 | 665.06 | 760.122215 | 0.849374 |
| GO:0030511\_positive\_regulation\_of\_transforming\_growth\_factor\_beta\_receptor\_signaling\_pathway | 8 | 0 | 0.000000 | -0.000000 | 783 | 569.997785 | 665.06 | 760.122215 | 0.849374 |
| GO:0031102\_neuron\_projection\_regeneration | 8 | 0 | 0.000000 | -0.000000 | 783 | 569.997785 | 665.06 | 760.122215 | 0.849374 |
| GO:0031103\_axon\_regeneration | 8 | 0 | 0.000000 | -0.000000 | 783 | 569.997785 | 665.06 | 760.122215 | 0.849374 |
| GO:0031111\_negative\_regulation\_of\_microtubule\_polymerization\_or\_depolymerization | 8 | 0 | 0.000000 | -0.000000 | 783 | 569.997785 | 665.06 | 760.122215 | 0.849374 |
| GO:0031123\_RNA\_3'-end\_processing | 8 | 0 | 0.000000 | -0.000000 | 783 | 569.997785 | 665.06 | 760.122215 | 0.849374 |
| GO:0031294\_lymphocyte\_costimulation | 8 | 0 | 0.000000 | -0.000000 | 783 | 569.997785 | 665.06 | 760.122215 | 0.849374 |
| GO:0031295\_T\_cell\_costimulation | 8 | 0 | 0.000000 | -0.000000 | 783 | 569.997785 | 665.06 | 760.122215 | 0.849374 |
| GO:0031334\_positive\_regulation\_of\_protein\_complex\_assembly | 8 | 0 | 0.000000 | -0.000000 | 783 | 569.997785 | 665.06 | 760.122215 | 0.849374 |
| GO:0031342\_negative\_regulation\_of\_cell\_killing | 8 | 0 | 0.000000 | -0.000000 | 783 | 569.997785 | 665.06 | 760.122215 | 0.849374 |
| GO:0031396\_regulation\_of\_protein\_ubiquitination | 8 | 0 | 0.000000 | -0.000000 | 783 | 569.997785 | 665.06 | 760.122215 | 0.849374 |
| GO:0032094\_response\_to\_food | 8 | 0 | 0.000000 | -0.000000 | 783 | 569.997785 | 665.06 | 760.122215 | 0.849374 |
| GO:0032273\_positive\_regulation\_of\_protein\_polymerization | 8 | 0 | 0.000000 | -0.000000 | 783 | 569.997785 | 665.06 | 760.122215 | 0.849374 |
| GO:0032409\_regulation\_of\_transporter\_activity | 8 | 0 | 0.000000 | -0.000000 | 783 | 569.997785 | 665.06 | 760.122215 | 0.849374 |
| GO:0032412\_regulation\_of\_ion\_transmembrane\_transporter\_activity | 8 | 0 | 0.000000 | -0.000000 | 783 | 569.997785 | 665.06 | 760.122215 | 0.849374 |
| GO:0032613\_interleukin-10\_production | 8 | 0 | 0.000000 | -0.000000 | 783 | 569.997785 | 665.06 | 760.122215 | 0.849374 |
| GO:0033198\_response\_to\_ATP | 8 | 0 | 0.000000 | -0.000000 | 783 | 569.997785 | 665.06 | 760.122215 | 0.849374 |
| GO:0034284\_response\_to\_monosaccharide\_stimulus | 8 | 0 | 0.000000 | -0.000000 | 783 | 569.997785 | 665.06 | 760.122215 | 0.849374 |
| GO:0034728\_nucleosome\_organization | 8 | 0 | 0.000000 | -0.000000 | 783 | 569.997785 | 665.06 | 760.122215 | 0.849374 |
| GO:0035023\_regulation\_of\_Rho\_protein\_signal\_transduction | 8 | 0 | 0.000000 | -0.000000 | 783 | 569.997785 | 665.06 | 760.122215 | 0.849374 |
| GO:0035112\_genitalia\_morphogenesis | 8 | 0 | 0.000000 | -0.000000 | 783 | 569.997785 | 665.06 | 760.122215 | 0.849374 |
| GO:0040017\_positive\_regulation\_of\_locomotion | 8 | 0 | 0.000000 | -0.000000 | 783 | 569.997785 | 665.06 | 760.122215 | 0.849374 |
| GO:0040034\_regulation\_of\_development\_\_heterochronic | 8 | 0 | 0.000000 | -0.000000 | 783 | 569.997785 | 665.06 | 760.122215 | 0.849374 |
| GO:0042074\_cell\_migration\_involved\_in\_gastrulation | 8 | 0 | 0.000000 | -0.000000 | 783 | 569.997785 | 665.06 | 760.122215 | 0.849374 |
| GO:0042090\_interleukin-12\_biosynthetic\_process | 8 | 0 | 0.000000 | -0.000000 | 783 | 569.997785 | 665.06 | 760.122215 | 0.849374 |
| GO:0042092\_T-helper\_2\_type\_immune\_response | 8 | 0 | 0.000000 | -0.000000 | 783 | 569.997785 | 665.06 | 760.122215 | 0.849374 |
| GO:0042095\_interferon-gamma\_biosynthetic\_process | 8 | 0 | 0.000000 | -0.000000 | 783 | 569.997785 | 665.06 | 760.122215 | 0.849374 |
| GO:0042104\_positive\_regulation\_of\_activated\_T\_cell\_proliferation | 8 | 0 | 0.000000 | -0.000000 | 783 | 569.997785 | 665.06 | 760.122215 | 0.849374 |
| GO:0042226\_interleukin-6\_biosynthetic\_process | 8 | 0 | 0.000000 | -0.000000 | 783 | 569.997785 | 665.06 | 760.122215 | 0.849374 |
| GO:0042304\_regulation\_of\_fatty\_acid\_biosynthetic\_process | 8 | 0 | 0.000000 | -0.000000 | 783 | 569.997785 | 665.06 | 760.122215 | 0.849374 |
| GO:0042423\_catecholamine\_biosynthetic\_process | 8 | 0 | 0.000000 | -0.000000 | 783 | 569.997785 | 665.06 | 760.122215 | 0.849374 |
| GO:0042771\_DNA\_damage\_response\_\_signal\_transduction\_by\_p53\_class\_mediator\_resulting\_in\_induction\_of\_apoptosis | 8 | 0 | 0.000000 | -0.000000 | 783 | 569.997785 | 665.06 | 760.122215 | 0.849374 |
| GO:0042990\_regulation\_of\_transcription\_factor\_import\_into\_nucleus | 8 | 0 | 0.000000 | -0.000000 | 783 | 569.997785 | 665.06 | 760.122215 | 0.849374 |
| GO:0042991\_transcription\_factor\_import\_into\_nucleus | 8 | 0 | 0.000000 | -0.000000 | 783 | 569.997785 | 665.06 | 760.122215 | 0.849374 |
| GO:0043011\_myeloid\_dendritic\_cell\_differentiation | 8 | 0 | 0.000000 | -0.000000 | 783 | 569.997785 | 665.06 | 760.122215 | 0.849374 |
| GO:0043368\_positive\_T\_cell\_selection | 8 | 0 | 0.000000 | -0.000000 | 783 | 569.997785 | 665.06 | 760.122215 | 0.849374 |
| GO:0043370\_regulation\_of\_CD4-positive\_\_alpha\_beta\_T\_cell\_differentiation | 8 | 0 | 0.000000 | -0.000000 | 783 | 569.997785 | 665.06 | 760.122215 | 0.849374 |
| GO:0043616\_keratinocyte\_proliferation | 8 | 0 | 0.000000 | -0.000000 | 783 | 569.997785 | 665.06 | 760.122215 | 0.849374 |
| GO:0045075\_regulation\_of\_interleukin-12\_biosynthetic\_process | 8 | 0 | 0.000000 | -0.000000 | 783 | 569.997785 | 665.06 | 760.122215 | 0.849374 |
| GO:0045086\_positive\_regulation\_of\_interleukin-2\_biosynthetic\_process | 8 | 0 | 0.000000 | -0.000000 | 783 | 569.997785 | 665.06 | 760.122215 | 0.849374 |
| GO:0045351\_type\_I\_interferon\_biosynthetic\_process | 8 | 0 | 0.000000 | -0.000000 | 783 | 569.997785 | 665.06 | 760.122215 | 0.849374 |
| GO:0045408\_regulation\_of\_interleukin-6\_biosynthetic\_process | 8 | 0 | 0.000000 | -0.000000 | 783 | 569.997785 | 665.06 | 760.122215 | 0.849374 |
| GO:0045429\_positive\_regulation\_of\_nitric\_oxide\_biosynthetic\_process | 8 | 0 | 0.000000 | -0.000000 | 783 | 569.997785 | 665.06 | 760.122215 | 0.849374 |
| GO:0045494\_photoreceptor\_cell\_maintenance | 8 | 0 | 0.000000 | -0.000000 | 783 | 569.997785 | 665.06 | 760.122215 | 0.849374 |
| GO:0045686\_negative\_regulation\_of\_glial\_cell\_differentiation | 8 | 0 | 0.000000 | -0.000000 | 783 | 569.997785 | 665.06 | 760.122215 | 0.849374 |
| GO:0045910\_negative\_regulation\_of\_DNA\_recombination | 8 | 0 | 0.000000 | -0.000000 | 783 | 569.997785 | 665.06 | 760.122215 | 0.849374 |
| GO:0045921\_positive\_regulation\_of\_exocytosis | 8 | 0 | 0.000000 | -0.000000 | 783 | 569.997785 | 665.06 | 760.122215 | 0.849374 |
| GO:0045932\_negative\_regulation\_of\_muscle\_contraction | 8 | 0 | 0.000000 | -0.000000 | 783 | 569.997785 | 665.06 | 760.122215 | 0.849374 |
| GO:0046470\_phosphatidylcholine\_metabolic\_process | 8 | 0 | 0.000000 | -0.000000 | 783 | 569.997785 | 665.06 | 760.122215 | 0.849374 |
| GO:0048266\_behavioral\_response\_to\_pain | 8 | 0 | 0.000000 | -0.000000 | 783 | 569.997785 | 665.06 | 760.122215 | 0.849374 |
| GO:0048505\_regulation\_of\_timing\_of\_cell\_differentiation | 8 | 0 | 0.000000 | -0.000000 | 783 | 569.997785 | 665.06 | 760.122215 | 0.849374 |
| GO:0048520\_positive\_regulation\_of\_behavior | 8 | 0 | 0.000000 | -0.000000 | 783 | 569.997785 | 665.06 | 760.122215 | 0.849374 |
| GO:0048557\_embryonic\_digestive\_tract\_morphogenesis | 8 | 0 | 0.000000 | -0.000000 | 783 | 569.997785 | 665.06 | 760.122215 | 0.849374 |
| GO:0048638\_regulation\_of\_developmental\_growth | 8 | 0 | 0.000000 | -0.000000 | 783 | 569.997785 | 665.06 | 760.122215 | 0.849374 |
| GO:0048742\_regulation\_of\_skeletal\_muscle\_fiber\_development | 8 | 0 | 0.000000 | -0.000000 | 783 | 569.997785 | 665.06 | 760.122215 | 0.849374 |
| GO:0050707\_regulation\_of\_cytokine\_secretion | 8 | 0 | 0.000000 | -0.000000 | 783 | 569.997785 | 665.06 | 760.122215 | 0.849374 |
| GO:0050909\_sensory\_perception\_of\_taste | 8 | 0 | 0.000000 | -0.000000 | 783 | 569.997785 | 665.06 | 760.122215 | 0.849374 |
| GO:0050920\_regulation\_of\_chemotaxis | 8 | 0 | 0.000000 | -0.000000 | 783 | 569.997785 | 665.06 | 760.122215 | 0.849374 |
| GO:0050921\_positive\_regulation\_of\_chemotaxis | 8 | 0 | 0.000000 | -0.000000 | 783 | 569.997785 | 665.06 | 760.122215 | 0.849374 |
| GO:0050926\_regulation\_of\_positive\_chemotaxis | 8 | 0 | 0.000000 | -0.000000 | 783 | 569.997785 | 665.06 | 760.122215 | 0.849374 |
| GO:0050927\_positive\_regulation\_of\_positive\_chemotaxis | 8 | 0 | 0.000000 | -0.000000 | 783 | 569.997785 | 665.06 | 760.122215 | 0.849374 |
| GO:0050930\_induction\_of\_positive\_chemotaxis | 8 | 0 | 0.000000 | -0.000000 | 783 | 569.997785 | 665.06 | 760.122215 | 0.849374 |
| GO:0051084\_'de\_novo'\_posttranslational\_protein\_folding | 8 | 0 | 0.000000 | -0.000000 | 783 | 569.997785 | 665.06 | 760.122215 | 0.849374 |
| GO:0051181\_cofactor\_transport | 8 | 0 | 0.000000 | -0.000000 | 783 | 569.997785 | 665.06 | 760.122215 | 0.849374 |
| GO:0060043\_regulation\_of\_cardiac\_muscle\_cell\_proliferation | 8 | 0 | 0.000000 | -0.000000 | 783 | 569.997785 | 665.06 | 760.122215 | 0.849374 |
| GO:0060347\_heart\_trabecula\_formation | 8 | 0 | 0.000000 | -0.000000 | 783 | 569.997785 | 665.06 | 760.122215 | 0.849374 |
| GO:0060670\_branching\_involved\_in\_embryonic\_placenta\_morphogenesis | 8 | 0 | 0.000000 | -0.000000 | 783 | 569.997785 | 665.06 | 760.122215 | 0.849374 |
| GO:0060712\_spongiotrophoblast\_layer\_development | 8 | 0 | 0.000000 | -0.000000 | 783 | 569.997785 | 665.06 | 760.122215 | 0.849374 |
| GO:0070167\_regulation\_of\_biomineral\_formation | 8 | 0 | 0.000000 | -0.000000 | 783 | 569.997785 | 665.06 | 760.122215 | 0.849374 |
| GO:0070193\_synaptonemal\_complex\_organization | 8 | 0 | 0.000000 | -0.000000 | 783 | 569.997785 | 665.06 | 760.122215 | 0.849374 |
| GO:0070231\_T\_cell\_apoptosis | 8 | 0 | 0.000000 | -0.000000 | 783 | 569.997785 | 665.06 | 760.122215 | 0.849374 |
| GO:0070584\_mitochondrion\_morphogenesis | 8 | 0 | 0.000000 | -0.000000 | 783 | 569.997785 | 665.06 | 760.122215 | 0.849374 |
| GO:0015674\_di-\_\_tri-valent\_inorganic\_cation\_transport | 79 | 0 | 0.000000 | -0.000000 | 785 | 571.528252 | 666.25 | 760.971748 | 0.848726 |
| GO:0051046\_regulation\_of\_secretion | 79 | 0 | 0.000000 | -0.000000 | 785 | 571.528252 | 666.25 | 760.971748 | 0.848726 |
| GO:0048736\_appendage\_development | 96 | 0 | 0.000000 | -0.000000 | 788 | 574.352132 | 668.72 | 763.087868 | 0.848629 |
| GO:0060173\_limb\_development | 96 | 0 | 0.000000 | -0.000000 | 788 | 574.352132 | 668.72 | 763.087868 | 0.848629 |
| GO:0070661\_leukocyte\_proliferation | 96 | 0 | 0.000000 | -0.000000 | 788 | 574.352132 | 668.72 | 763.087868 | 0.848629 |
| GO:0001759\_induction\_of\_an\_organ | 15 | 0 | 0.000000 | -0.000000 | 839 | 626.807968 | 719.35 | 811.892032 | 0.857390 |
| GO:0001782\_B\_cell\_homeostasis | 15 | 0 | 0.000000 | -0.000000 | 839 | 626.807968 | 719.35 | 811.892032 | 0.857390 |
| GO:0001964\_startle\_response | 15 | 0 | 0.000000 | -0.000000 | 839 | 626.807968 | 719.35 | 811.892032 | 0.857390 |
| GO:0002286\_T\_cell\_activation\_during\_immune\_response | 15 | 0 | 0.000000 | -0.000000 | 839 | 626.807968 | 719.35 | 811.892032 | 0.857390 |
| GO:0002495\_antigen\_processing\_and\_presentation\_of\_peptide\_antigen\_via\_MHC\_class\_II | 15 | 0 | 0.000000 | -0.000000 | 839 | 626.807968 | 719.35 | 811.892032 | 0.857390 |
| GO:0002504\_antigen\_processing\_and\_presentation\_of\_peptide\_or\_polysaccharide\_antigen\_via\_MHC\_class\_II | 15 | 0 | 0.000000 | -0.000000 | 839 | 626.807968 | 719.35 | 811.892032 | 0.857390 |
| GO:0002709\_regulation\_of\_T\_cell\_mediated\_immunity | 15 | 0 | 0.000000 | -0.000000 | 839 | 626.807968 | 719.35 | 811.892032 | 0.857390 |
| GO:0006473\_protein\_amino\_acid\_acetylation | 15 | 0 | 0.000000 | -0.000000 | 839 | 626.807968 | 719.35 | 811.892032 | 0.857390 |
| GO:0006487\_protein\_amino\_acid\_N-linked\_glycosylation | 15 | 0 | 0.000000 | -0.000000 | 839 | 626.807968 | 719.35 | 811.892032 | 0.857390 |
| GO:0006749\_glutathione\_metabolic\_process | 15 | 0 | 0.000000 | -0.000000 | 839 | 626.807968 | 719.35 | 811.892032 | 0.857390 |
| GO:0006885\_regulation\_of\_pH | 15 | 0 | 0.000000 | -0.000000 | 839 | 626.807968 | 719.35 | 811.892032 | 0.857390 |
| GO:0007040\_lysosome\_organization | 15 | 0 | 0.000000 | -0.000000 | 839 | 626.807968 | 719.35 | 811.892032 | 0.857390 |
| GO:0007173\_epidermal\_growth\_factor\_receptor\_signaling\_pathway | 15 | 0 | 0.000000 | -0.000000 | 839 | 626.807968 | 719.35 | 811.892032 | 0.857390 |
| GO:0007200\_activation\_of\_phospholipase\_C\_activity\_by\_G-protein\_coupled\_receptor\_protein\_signaling\_pathway\_coupled\_to\_IP3\_second\_messenger | 15 | 0 | 0.000000 | -0.000000 | 839 | 626.807968 | 719.35 | 811.892032 | 0.857390 |
| GO:0007202\_activation\_of\_phospholipase\_C\_activity | 15 | 0 | 0.000000 | -0.000000 | 839 | 626.807968 | 719.35 | 811.892032 | 0.857390 |
| GO:0007218\_neuropeptide\_signaling\_pathway | 15 | 0 | 0.000000 | -0.000000 | 839 | 626.807968 | 719.35 | 811.892032 | 0.857390 |
| GO:0007588\_excretion | 15 | 0 | 0.000000 | -0.000000 | 839 | 626.807968 | 719.35 | 811.892032 | 0.857390 |
| GO:0007618\_mating | 15 | 0 | 0.000000 | -0.000000 | 839 | 626.807968 | 719.35 | 811.892032 | 0.857390 |
| GO:0008543\_fibroblast\_growth\_factor\_receptor\_signaling\_pathway | 15 | 0 | 0.000000 | -0.000000 | 839 | 626.807968 | 719.35 | 811.892032 | 0.857390 |
| GO:0009062\_fatty\_acid\_catabolic\_process | 15 | 0 | 0.000000 | -0.000000 | 839 | 626.807968 | 719.35 | 811.892032 | 0.857390 |
| GO:0009116\_nucleoside\_metabolic\_process | 15 | 0 | 0.000000 | -0.000000 | 839 | 626.807968 | 719.35 | 811.892032 | 0.857390 |
| GO:0010092\_specification\_of\_organ\_identity | 15 | 0 | 0.000000 | -0.000000 | 839 | 626.807968 | 719.35 | 811.892032 | 0.857390 |
| GO:0010171\_body\_morphogenesis | 15 | 0 | 0.000000 | -0.000000 | 839 | 626.807968 | 719.35 | 811.892032 | 0.857390 |
| GO:0010518\_positive\_regulation\_of\_phospholipase\_activity | 15 | 0 | 0.000000 | -0.000000 | 839 | 626.807968 | 719.35 | 811.892032 | 0.857390 |
| GO:0010863\_positive\_regulation\_of\_phospholipase\_C\_activity | 15 | 0 | 0.000000 | -0.000000 | 839 | 626.807968 | 719.35 | 811.892032 | 0.857390 |
| GO:0015931\_nucleobase\_\_nucleoside\_\_nucleotide\_and\_nucleic\_acid\_transport | 15 | 0 | 0.000000 | -0.000000 | 839 | 626.807968 | 719.35 | 811.892032 | 0.857390 |
| GO:0019886\_antigen\_processing\_and\_presentation\_of\_exogenous\_peptide\_antigen\_via\_MHC\_class\_II | 15 | 0 | 0.000000 | -0.000000 | 839 | 626.807968 | 719.35 | 811.892032 | 0.857390 |
| GO:0021795\_cerebral\_cortex\_cell\_migration | 15 | 0 | 0.000000 | -0.000000 | 839 | 626.807968 | 719.35 | 811.892032 | 0.857390 |
| GO:0022600\_digestive\_system\_process | 15 | 0 | 0.000000 | -0.000000 | 839 | 626.807968 | 719.35 | 811.892032 | 0.857390 |
| GO:0030041\_actin\_filament\_polymerization | 15 | 0 | 0.000000 | -0.000000 | 839 | 626.807968 | 719.35 | 811.892032 | 0.857390 |
| GO:0031069\_hair\_follicle\_morphogenesis | 15 | 0 | 0.000000 | -0.000000 | 839 | 626.807968 | 719.35 | 811.892032 | 0.857390 |
| GO:0031329\_regulation\_of\_cellular\_catabolic\_process | 15 | 0 | 0.000000 | -0.000000 | 839 | 626.807968 | 719.35 | 811.892032 | 0.857390 |
| GO:0035116\_embryonic\_hindlimb\_morphogenesis | 15 | 0 | 0.000000 | -0.000000 | 839 | 626.807968 | 719.35 | 811.892032 | 0.857390 |
| GO:0035249\_synaptic\_transmission\_\_glutamatergic | 15 | 0 | 0.000000 | -0.000000 | 839 | 626.807968 | 719.35 | 811.892032 | 0.857390 |
| GO:0042306\_regulation\_of\_protein\_import\_into\_nucleus | 15 | 0 | 0.000000 | -0.000000 | 839 | 626.807968 | 719.35 | 811.892032 | 0.857390 |
| GO:0046164\_alcohol\_catabolic\_process | 15 | 0 | 0.000000 | -0.000000 | 839 | 626.807968 | 719.35 | 811.892032 | 0.857390 |
| GO:0046638\_positive\_regulation\_of\_alpha-beta\_T\_cell\_differentiation | 15 | 0 | 0.000000 | -0.000000 | 839 | 626.807968 | 719.35 | 811.892032 | 0.857390 |
| GO:0048008\_platelet-derived\_growth\_factor\_receptor\_signaling\_pathway | 15 | 0 | 0.000000 | -0.000000 | 839 | 626.807968 | 719.35 | 811.892032 | 0.857390 |
| GO:0048144\_fibroblast\_proliferation | 15 | 0 | 0.000000 | -0.000000 | 839 | 626.807968 | 719.35 | 811.892032 | 0.857390 |
| GO:0048145\_regulation\_of\_fibroblast\_proliferation | 15 | 0 | 0.000000 | -0.000000 | 839 | 626.807968 | 719.35 | 811.892032 | 0.857390 |
| GO:0048610\_reproductive\_cellular\_process | 15 | 0 | 0.000000 | -0.000000 | 839 | 626.807968 | 719.35 | 811.892032 | 0.857390 |
| GO:0048709\_oligodendrocyte\_differentiation | 15 | 0 | 0.000000 | -0.000000 | 839 | 626.807968 | 719.35 | 811.892032 | 0.857390 |
| GO:0050729\_positive\_regulation\_of\_inflammatory\_response | 15 | 0 | 0.000000 | -0.000000 | 839 | 626.807968 | 719.35 | 811.892032 | 0.857390 |
| GO:0050796\_regulation\_of\_insulin\_secretion | 15 | 0 | 0.000000 | -0.000000 | 839 | 626.807968 | 719.35 | 811.892032 | 0.857390 |
| GO:0050798\_activated\_T\_cell\_proliferation | 15 | 0 | 0.000000 | -0.000000 | 839 | 626.807968 | 719.35 | 811.892032 | 0.857390 |
| GO:0055010\_ventricular\_cardiac\_muscle\_morphogenesis | 15 | 0 | 0.000000 | -0.000000 | 839 | 626.807968 | 719.35 | 811.892032 | 0.857390 |
| GO:0060322\_head\_development | 15 | 0 | 0.000000 | -0.000000 | 839 | 626.807968 | 719.35 | 811.892032 | 0.857390 |
| GO:0060425\_lung\_morphogenesis | 15 | 0 | 0.000000 | -0.000000 | 839 | 626.807968 | 719.35 | 811.892032 | 0.857390 |
| GO:0060442\_branching\_involved\_in\_prostate\_gland\_morphogenesis | 15 | 0 | 0.000000 | -0.000000 | 839 | 626.807968 | 719.35 | 811.892032 | 0.857390 |
| GO:0070227\_lymphocyte\_apoptosis | 15 | 0 | 0.000000 | -0.000000 | 839 | 626.807968 | 719.35 | 811.892032 | 0.857390 |
| GO:0070507\_regulation\_of\_microtubule\_cytoskeleton\_organization | 15 | 0 | 0.000000 | -0.000000 | 839 | 626.807968 | 719.35 | 811.892032 | 0.857390 |
| GO:0002757\_immune\_response-activating\_signal\_transduction | 47 | 0 | 0.000000 | -0.000000 | 847 | 636.663365 | 728.29 | 819.916635 | 0.859847 |
| GO:0006140\_regulation\_of\_nucleotide\_metabolic\_process | 47 | 0 | 0.000000 | -0.000000 | 847 | 636.663365 | 728.29 | 819.916635 | 0.859847 |
| GO:0016570\_histone\_modification | 47 | 0 | 0.000000 | -0.000000 | 847 | 636.663365 | 728.29 | 819.916635 | 0.859847 |
| GO:0030183\_B\_cell\_differentiation | 47 | 0 | 0.000000 | -0.000000 | 847 | 636.663365 | 728.29 | 819.916635 | 0.859847 |
| GO:0030799\_regulation\_of\_cyclic\_nucleotide\_metabolic\_process | 47 | 0 | 0.000000 | -0.000000 | 847 | 636.663365 | 728.29 | 819.916635 | 0.859847 |
| GO:0045087\_innate\_immune\_response | 47 | 0 | 0.000000 | -0.000000 | 847 | 636.663365 | 728.29 | 819.916635 | 0.859847 |
| GO:0045619\_regulation\_of\_lymphocyte\_differentiation | 47 | 0 | 0.000000 | -0.000000 | 847 | 636.663365 | 728.29 | 819.916635 | 0.859847 |
| GO:0060627\_regulation\_of\_vesicle-mediated\_transport | 47 | 0 | 0.000000 | -0.000000 | 847 | 636.663365 | 728.29 | 819.916635 | 0.859847 |
| GO:0002703\_regulation\_of\_leukocyte\_mediated\_immunity | 56 | 0 | 0.000000 | -0.000000 | 852 | 644.604828 | 735.31 | 826.015172 | 0.863040 |
| GO:0009187\_cyclic\_nucleotide\_metabolic\_process | 56 | 0 | 0.000000 | -0.000000 | 852 | 644.604828 | 735.31 | 826.015172 | 0.863040 |
| GO:0042089\_cytokine\_biosynthetic\_process | 56 | 0 | 0.000000 | -0.000000 | 852 | 644.604828 | 735.31 | 826.015172 | 0.863040 |
| GO:0042107\_cytokine\_metabolic\_process | 56 | 0 | 0.000000 | -0.000000 | 852 | 644.604828 | 735.31 | 826.015172 | 0.863040 |
| GO:0050678\_regulation\_of\_epithelial\_cell\_proliferation | 56 | 0 | 0.000000 | -0.000000 | 852 | 644.604828 | 735.31 | 826.015172 | 0.863040 |
| GO:0000027\_ribosomal\_large\_subunit\_assembly | 1 | 0 |  |  |  |  |  |  |  |  |
| GO:0000042\_protein\_targeting\_to\_Golgi | 1 | 0 |  |  |  |  |  |  |  |  |
| GO:0000046\_autophagic\_vacuole\_fusion | 1 | 0 |  |  |  |  |  |  |  |  |
| GO:0000050\_urea\_cycle | 1 | 0 |  |  |  |  |  |  |  |  |
| GO:0000054\_ribosome\_export\_from\_nucleus | 1 | 0 |  |  |  |  |  |  |  |  |
| GO:0000055\_ribosomal\_large\_subunit\_export\_from\_nucleus | 1 | 0 |  |  |  |  |  |  |  |  |
| GO:0000056\_ribosomal\_small\_subunit\_export\_from\_nucleus | 1 | 0 |  |  |  |  |  |  |  |  |
| GO:0000072\_M\_phase\_specific\_microtubule\_process | 1 | 0 |  |  |  |  |  |  |  |  |
| GO:0000101\_sulfur\_amino\_acid\_transport | 1 | 0 |  |  |  |  |  |  |  |  |
| GO:0000147\_actin\_cortical\_patch\_assembly | 1 | 0 |  |  |  |  |  |  |  |  |
| GO:0000154\_rRNA\_modification | 1 | 0 |  |  |  |  |  |  |  |  |
| GO:0000183\_chromatin\_silencing\_at\_rDNA | 1 | 0 |  |  |  |  |  |  |  |  |
| GO:0000185\_activation\_of\_MAPKKK\_activity | 1 | 0 |  |  |  |  |  |  |  |  |
| GO:0000238\_zygotene | 1 | 0 |  |  |  |  |  |  |  |  |
| GO:0000255\_allantoin\_metabolic\_process | 1 | 0 |  |  |  |  |  |  |  |  |
| GO:0000266\_mitochondrial\_fission | 1 | 0 |  |  |  |  |  |  |  |  |
| GO:0000273\_lipoic\_acid\_metabolic\_process | 1 | 0 |  |  |  |  |  |  |  |  |
| GO:0000301\_retrograde\_transport\_\_vesicle\_recycling\_within\_Golgi | 1 | 0 |  |  |  |  |  |  |  |  |
| GO:0000394\_RNA\_splicing\_\_via\_endonucleolytic\_cleavage\_and\_ligation | 1 | 0 |  |  |  |  |  |  |  |  |
| GO:0000429\_regulation\_of\_transcription\_from\_RNA\_polymerase\_II\_promoter\_by\_carbon\_catabolites | 1 | 0 |  |  |  |  |  |  |  |  |
| GO:0000430\_regulation\_of\_transcription\_from\_RNA\_polymerase\_II\_promoter\_by\_glucose | 1 | 0 |  |  |  |  |  |  |  |  |
| GO:0000432\_positive\_regulation\_of\_transcription\_from\_RNA\_polymerase\_II\_promoter\_by\_glucose | 1 | 0 |  |  |  |  |  |  |  |  |
| GO:0000436\_positive\_regulation\_of\_transcription\_from\_RNA\_polymerase\_II\_promoter\_by\_carbon\_catabolites | 1 | 0 |  |  |  |  |  |  |  |  |
| GO:0000448\_cleavage\_in\_ITS2\_between\_5.8S\_rRNA\_and\_LSU-rRNA\_of\_tricistronic\_rRNA\_transcript\_(SSU-rRNA\_\_5.8S\_rRNA\_\_LSU-rRNA) | 1 | 0 |  |  |  |  |  |  |  |  |
| GO:0000460\_maturation\_of\_5.8S\_rRNA | 1 | 0 |  |  |  |  |  |  |  |  |
| GO:0000463\_maturation\_of\_LSU-rRNA\_from\_tricistronic\_rRNA\_transcript\_(SSU-rRNA\_\_5.8S\_rRNA\_\_LSU-rRNA) | 1 | 0 |  |  |  |  |  |  |  |  |
| GO:0000466\_maturation\_of\_5.8S\_rRNA\_from\_tricistronic\_rRNA\_transcript\_(SSU-rRNA\_\_5.8S\_rRNA\_\_LSU-rRNA) | 1 | 0 |  |  |  |  |  |  |  |  |
| GO:0000469\_cleavages\_during\_rRNA\_processing | 1 | 0 |  |  |  |  |  |  |  |  |
| GO:0000470\_maturation\_of\_LSU-rRNA | 1 | 0 |  |  |  |  |  |  |  |  |
| GO:0000478\_endonucleolytic\_cleavages\_during\_rRNA\_processing | 1 | 0 |  |  |  |  |  |  |  |  |
| GO:0000479\_endonucleolytic\_cleavage\_of\_tricistronic\_rRNA\_transcript\_(SSU-rRNA\_\_5.8S\_rRNA\_\_LSU-rRNA) | 1 | 0 |  |  |  |  |  |  |  |  |
| GO:0000705\_achiasmate\_meiosis\_I | 1 | 0 |  |  |  |  |  |  |  |  |
| GO:0000966\_RNA\_5'-end\_processing | 1 | 0 |  |  |  |  |  |  |  |  |
| GO:0001300\_chronological\_cell\_aging | 1 | 0 |  |  |  |  |  |  |  |  |
| GO:0001547\_antral\_ovarian\_follicle\_growth | 1 | 0 |  |  |  |  |  |  |  |  |
| GO:0001555\_oocyte\_growth | 1 | 0 |  |  |  |  |  |  |  |  |
| GO:0001560\_regulation\_of\_cell\_growth\_by\_extracellular\_stimulus | 1 | 0 |  |  |  |  |  |  |  |  |
| GO:0001660\_fever | 1 | 0 |  |  |  |  |  |  |  |  |
| GO:0001696\_gastric\_acid\_secretion | 1 | 0 |  |  |  |  |  |  |  |  |
| GO:0001712\_ectodermal\_cell\_fate\_commitment | 1 | 0 |  |  |  |  |  |  |  |  |
| GO:0001714\_endodermal\_cell\_fate\_specification | 1 | 0 |  |  |  |  |  |  |  |  |
| GO:0001762\_beta-alanine\_transport | 1 | 0 |  |  |  |  |  |  |  |  |
| GO:0001766\_membrane\_raft\_polarization | 1 | 0 |  |  |  |  |  |  |  |  |
| GO:0001811\_negative\_regulation\_of\_type\_I\_hypersensitivity | 1 | 0 |  |  |  |  |  |  |  |  |
| GO:0001821\_histamine\_secretion | 1 | 0 |  |  |  |  |  |  |  |  |
| GO:0001826\_inner\_cell\_mass\_cell\_differentiation | 1 | 0 |  |  |  |  |  |  |  |  |
| GO:0001830\_trophectodermal\_cell\_fate\_commitment | 1 | 0 |  |  |  |  |  |  |  |  |
| GO:0001834\_trophectodermal\_cell\_proliferation | 1 | 0 |  |  |  |  |  |  |  |  |
| GO:0001867\_complement\_activation\_\_lectin\_pathway | 1 | 0 |  |  |  |  |  |  |  |  |
| GO:0001880\_Mullerian\_duct\_regression | 1 | 0 |  |  |  |  |  |  |  |  |
| GO:0001887\_selenium\_metabolic\_process | 1 | 0 |  |  |  |  |  |  |  |  |
| GO:0001922\_B-1\_B\_cell\_homeostasis | 1 | 0 |  |  |  |  |  |  |  |  |
| GO:0001923\_B-1\_B\_cell\_differentiation | 1 | 0 |  |  |  |  |  |  |  |  |
| GO:0001941\_postsynaptic\_membrane\_organization | 1 | 0 |  |  |  |  |  |  |  |  |
| GO:0001946\_lymphangiogenesis | 1 | 0 |  |  |  |  |  |  |  |  |
| GO:0001956\_positive\_regulation\_of\_neurotransmitter\_secretion | 1 | 0 |  |  |  |  |  |  |  |  |
| GO:0001961\_positive\_regulation\_of\_cytokine-mediated\_signaling\_pathway | 1 | 0 |  |  |  |  |  |  |  |  |
| GO:0001979\_regulation\_of\_systemic\_arterial\_blood\_pressure\_by\_chemoreceptor\_signaling | 1 | 0 |  |  |  |  |  |  |  |  |
| GO:0001980\_regulation\_of\_systemic\_arterial\_blood\_pressure\_by\_ischemic\_conditions | 1 | 0 |  |  |  |  |  |  |  |  |
| GO:0001984\_vasodilation\_of\_artery\_during\_baroreceptor\_response\_to\_increased\_systemic\_arterial\_blood\_pressure | 1 | 0 |  |  |  |  |  |  |  |  |
| GO:0001985\_negative\_regulation\_of\_heart\_rate\_in\_baroreceptor\_response\_to\_increased\_systemic\_arterial\_blood\_pressure | 1 | 0 |  |  |  |  |  |  |  |  |
| GO:0001987\_vasoconstriction\_of\_artery\_involved\_in\_baroreceptor\_response\_to\_lowering\_of\_systemic\_arterial\_blood\_pressure | 1 | 0 |  |  |  |  |  |  |  |  |
| GO:0001988\_positive\_regulation\_of\_heart\_rate\_in\_baroreceptor\_response\_to\_decreased\_systemic\_arterial\_blood\_pressure | 1 | 0 |  |  |  |  |  |  |  |  |
| GO:0001994\_norepinephrine-epinephrine\_vasoconstriction\_involved\_in\_regulation\_of\_systemic\_arterial\_blood\_pressure | 1 | 0 |  |  |  |  |  |  |  |  |
| GO:0002001\_renin\_secretion\_into\_blood\_stream | 1 | 0 |  |  |  |  |  |  |  |  |
| GO:0002002\_regulation\_of\_angiotensin\_levels\_in\_blood | 1 | 0 |  |  |  |  |  |  |  |  |
| GO:0002003\_angiotensin\_maturation | 1 | 0 |  |  |  |  |  |  |  |  |
| GO:0002007\_detection\_of\_hypoxic\_conditions\_in\_blood\_by\_chemoreceptor\_signaling | 1 | 0 |  |  |  |  |  |  |  |  |
| GO:0002017\_regulation\_of\_blood\_volume\_by\_renal\_aldosterone | 1 | 0 |  |  |  |  |  |  |  |  |
| GO:0002023\_reduction\_of\_food\_intake\_in\_response\_to\_dietary\_excess | 1 | 0 |  |  |  |  |  |  |  |  |
| GO:0002031\_G-protein\_coupled\_receptor\_internalization | 1 | 0 |  |  |  |  |  |  |  |  |
| GO:0002036\_regulation\_of\_L-glutamate\_transport | 1 | 0 |  |  |  |  |  |  |  |  |
| GO:0002040\_sprouting\_angiogenesis | 1 | 0 |  |  |  |  |  |  |  |  |
| GO:0002041\_intussusceptive\_angiogenesis | 1 | 0 |  |  |  |  |  |  |  |  |
| GO:0002068\_glandular\_epithelial\_cell\_development | 1 | 0 |  |  |  |  |  |  |  |  |
| GO:0002069\_columnar\_cuboidal\_epithelial\_cell\_maturation | 1 | 0 |  |  |  |  |  |  |  |  |
| GO:0002071\_glandular\_epithelial\_cell\_maturation | 1 | 0 |  |  |  |  |  |  |  |  |
| GO:0002082\_regulation\_of\_oxidative\_phosphorylation | 1 | 0 |  |  |  |  |  |  |  |  |
| GO:0002084\_protein\_depalmitoylation | 1 | 0 |  |  |  |  |  |  |  |  |
| GO:0002085\_inhibition\_of\_neuroepithelial\_cell\_differentiation | 1 | 0 |  |  |  |  |  |  |  |  |
| GO:0002086\_diaphragm\_contraction | 1 | 0 |  |  |  |  |  |  |  |  |
| GO:0002118\_aggressive\_behavior | 1 | 0 |  |  |  |  |  |  |  |  |
| GO:0002121\_inter-male\_aggressive\_behavior | 1 | 0 |  |  |  |  |  |  |  |  |
| GO:0002124\_territorial\_aggressive\_behavior | 1 | 0 |  |  |  |  |  |  |  |  |
| GO:0002227\_innate\_immune\_response\_in\_mucosa | 1 | 0 |  |  |  |  |  |  |  |  |
| GO:0002232\_leukocyte\_chemotaxis\_during\_inflammatory\_response | 1 | 0 |  |  |  |  |  |  |  |  |
| GO:0002282\_microglial\_cell\_activation\_during\_immune\_response | 1 | 0 |  |  |  |  |  |  |  |  |
| GO:0002287\_alpha-beta\_T\_cell\_activation\_during\_immune\_response | 1 | 0 |  |  |  |  |  |  |  |  |
| GO:0002314\_germinal\_center\_B\_cell\_differentiation | 1 | 0 |  |  |  |  |  |  |  |  |
| GO:0002315\_marginal\_zone\_B\_cell\_differentiation | 1 | 0 |  |  |  |  |  |  |  |  |
| GO:0002316\_follicular\_B\_cell\_differentiation | 1 | 0 |  |  |  |  |  |  |  |  |
| GO:0002317\_plasma\_cell\_differentiation | 1 | 0 |  |  |  |  |  |  |  |  |
| GO:0002349\_histamine\_production\_during\_acute\_inflammatory\_response | 1 | 0 |  |  |  |  |  |  |  |  |
| GO:0002351\_serotonin\_production\_during\_acute\_inflammatory\_response | 1 | 0 |  |  |  |  |  |  |  |  |
| GO:0002355\_detection\_of\_tumor\_cell | 1 | 0 |  |  |  |  |  |  |  |  |
| GO:0002370\_natural\_killer\_cell\_cytokine\_production | 1 | 0 |  |  |  |  |  |  |  |  |
| GO:0002371\_dendritic\_cell\_cytokine\_production | 1 | 0 |  |  |  |  |  |  |  |  |
| GO:0002380\_immunoglobulin\_secretion\_during\_immune\_response | 1 | 0 |  |  |  |  |  |  |  |  |
| GO:0002396\_MHC\_protein\_complex\_assembly | 1 | 0 |  |  |  |  |  |  |  |  |
| GO:0002397\_MHC\_class\_I\_protein\_complex\_assembly | 1 | 0 |  |  |  |  |  |  |  |  |
| GO:0002420\_natural\_killer\_cell\_mediated\_cytotoxicity\_directed\_against\_tumor\_cell\_target | 1 | 0 |  |  |  |  |  |  |  |  |
| GO:0002423\_natural\_killer\_cell\_mediated\_immune\_response\_to\_tumor\_cell | 1 | 0 |  |  |  |  |  |  |  |  |
| GO:0002424\_T\_cell\_mediated\_immune\_response\_to\_tumor\_cell | 1 | 0 |  |  |  |  |  |  |  |  |
| GO:0002426\_immunoglobulin\_production\_in\_mucosal\_tissue | 1 | 0 |  |  |  |  |  |  |  |  |
| GO:0002431\_Fc\_receptor\_mediated\_stimulatory\_signaling\_pathway | 1 | 0 |  |  |  |  |  |  |  |  |
| GO:0002432\_granuloma\_formation | 1 | 0 |  |  |  |  |  |  |  |  |
| GO:0002441\_histamine\_secretion\_during\_acute\_inflammatory\_response | 1 | 0 |  |  |  |  |  |  |  |  |
| GO:0002442\_serotonin\_secretion\_during\_acute\_inflammatory\_response | 1 | 0 |  |  |  |  |  |  |  |  |
| GO:0002457\_T\_cell\_antigen\_processing\_and\_presentation | 1 | 0 |  |  |  |  |  |  |  |  |
| GO:0002458\_peripheral\_T\_cell\_tolerance\_induction | 1 | 0 |  |  |  |  |  |  |  |  |
| GO:0002461\_tolerance\_induction\_dependent\_upon\_immune\_response | 1 | 0 |  |  |  |  |  |  |  |  |
| GO:0002465\_peripheral\_tolerance\_induction | 1 | 0 |  |  |  |  |  |  |  |  |
| GO:0002468\_dendritic\_cell\_antigen\_processing\_and\_presentation | 1 | 0 |  |  |  |  |  |  |  |  |
| GO:0002476\_antigen\_processing\_and\_presentation\_of\_endogenous\_peptide\_antigen\_via\_MHC\_class\_Ib | 1 | 0 |  |  |  |  |  |  |  |  |
| GO:0002479\_antigen\_processing\_and\_presentation\_of\_exogenous\_peptide\_antigen\_via\_MHC\_class\_I\_\_TAP-dependent | 1 | 0 |  |  |  |  |  |  |  |  |
| GO:0002483\_antigen\_processing\_and\_presentation\_of\_endogenous\_peptide\_antigen | 1 | 0 |  |  |  |  |  |  |  |  |
| GO:0002501\_peptide\_antigen\_assembly\_with\_MHC\_protein\_complex | 1 | 0 |  |  |  |  |  |  |  |  |
| GO:0002502\_peptide\_antigen\_assembly\_with\_MHC\_class\_I\_protein\_complex | 1 | 0 |  |  |  |  |  |  |  |  |
| GO:0002508\_central\_tolerance\_induction | 1 | 0 |  |  |  |  |  |  |  |  |
| GO:0002510\_central\_B\_cell\_tolerance\_induction | 1 | 0 |  |  |  |  |  |  |  |  |
| GO:0002545\_chronic\_inflammatory\_response\_to\_non-antigenic\_stimulus | 1 | 0 |  |  |  |  |  |  |  |  |
| GO:0002553\_histamine\_secretion\_by\_mast\_cell | 1 | 0 |  |  |  |  |  |  |  |  |
| GO:0002554\_serotonin\_secretion\_by\_platelet | 1 | 0 |  |  |  |  |  |  |  |  |
| GO:0002572\_pro-T\_cell\_differentiation | 1 | 0 |  |  |  |  |  |  |  |  |
| GO:0002577\_regulation\_of\_antigen\_processing\_and\_presentation | 1 | 0 |  |  |  |  |  |  |  |  |
| GO:0002579\_positive\_regulation\_of\_antigen\_processing\_and\_presentation | 1 | 0 |  |  |  |  |  |  |  |  |
| GO:0002604\_regulation\_of\_dendritic\_cell\_antigen\_processing\_and\_presentation | 1 | 0 |  |  |  |  |  |  |  |  |
| GO:0002606\_positive\_regulation\_of\_dendritic\_cell\_antigen\_processing\_and\_presentation | 1 | 0 |  |  |  |  |  |  |  |  |
| GO:0002635\_negative\_regulation\_of\_germinal\_center\_formation | 1 | 0 |  |  |  |  |  |  |  |  |
| GO:0002646\_regulation\_of\_central\_tolerance\_induction | 1 | 0 |  |  |  |  |  |  |  |  |
| GO:0002648\_positive\_regulation\_of\_central\_tolerance\_induction | 1 | 0 |  |  |  |  |  |  |  |  |
| GO:0002649\_regulation\_of\_tolerance\_induction\_to\_self\_antigen | 1 | 0 |  |  |  |  |  |  |  |  |
| GO:0002651\_positive\_regulation\_of\_tolerance\_induction\_to\_self\_antigen | 1 | 0 |  |  |  |  |  |  |  |  |
| GO:0002652\_regulation\_of\_tolerance\_induction\_dependent\_upon\_immune\_response | 1 | 0 |  |  |  |  |  |  |  |  |
| GO:0002654\_positive\_regulation\_of\_tolerance\_induction\_dependent\_upon\_immune\_response | 1 | 0 |  |  |  |  |  |  |  |  |
| GO:0002658\_regulation\_of\_peripheral\_tolerance\_induction | 1 | 0 |  |  |  |  |  |  |  |  |
| GO:0002660\_positive\_regulation\_of\_peripheral\_tolerance\_induction | 1 | 0 |  |  |  |  |  |  |  |  |
| GO:0002677\_negative\_regulation\_of\_chronic\_inflammatory\_response | 1 | 0 |  |  |  |  |  |  |  |  |
| GO:0002678\_positive\_regulation\_of\_chronic\_inflammatory\_response | 1 | 0 |  |  |  |  |  |  |  |  |
| GO:0002701\_negative\_regulation\_of\_production\_of\_molecular\_mediator\_of\_immune\_response | 1 | 0 |  |  |  |  |  |  |  |  |
| GO:0002719\_negative\_regulation\_of\_cytokine\_production\_during\_immune\_response | 1 | 0 |  |  |  |  |  |  |  |  |
| GO:0002724\_regulation\_of\_T\_cell\_cytokine\_production | 1 | 0 |  |  |  |  |  |  |  |  |
| GO:0002727\_regulation\_of\_natural\_killer\_cell\_cytokine\_production | 1 | 0 |  |  |  |  |  |  |  |  |
| GO:0002729\_positive\_regulation\_of\_natural\_killer\_cell\_cytokine\_production | 1 | 0 |  |  |  |  |  |  |  |  |
| GO:0002730\_regulation\_of\_dendritic\_cell\_cytokine\_production | 1 | 0 |  |  |  |  |  |  |  |  |
| GO:0002756\_MyD88-independent\_toll-like\_receptor\_signaling\_pathway | 1 | 0 |  |  |  |  |  |  |  |  |
| GO:0002767\_immune\_response-inhibiting\_cell\_surface\_receptor\_signaling\_pathway | 1 | 0 |  |  |  |  |  |  |  |  |
| GO:0002769\_natural\_killer\_cell\_inhibitory\_signaling\_pathway | 1 | 0 |  |  |  |  |  |  |  |  |
| GO:0002840\_regulation\_of\_T\_cell\_mediated\_immune\_response\_to\_tumor\_cell | 1 | 0 |  |  |  |  |  |  |  |  |
| GO:0002842\_positive\_regulation\_of\_T\_cell\_mediated\_immune\_response\_to\_tumor\_cell | 1 | 0 |  |  |  |  |  |  |  |  |
| GO:0002849\_regulation\_of\_peripheral\_T\_cell\_tolerance\_induction | 1 | 0 |  |  |  |  |  |  |  |  |
| GO:0002851\_positive\_regulation\_of\_peripheral\_T\_cell\_tolerance\_induction | 1 | 0 |  |  |  |  |  |  |  |  |
| GO:0002855\_regulation\_of\_natural\_killer\_cell\_mediated\_immune\_response\_to\_tumor\_cell | 1 | 0 |  |  |  |  |  |  |  |  |
| GO:0002857\_positive\_regulation\_of\_natural\_killer\_cell\_mediated\_immune\_response\_to\_tumor\_cell | 1 | 0 |  |  |  |  |  |  |  |  |
| GO:0002858\_regulation\_of\_natural\_killer\_cell\_mediated\_cytotoxicity\_directed\_against\_tumor\_cell\_target | 1 | 0 |  |  |  |  |  |  |  |  |
| GO:0002860\_positive\_regulation\_of\_natural\_killer\_cell\_mediated\_cytotoxicity\_directed\_against\_tumor\_cell\_target | 1 | 0 |  |  |  |  |  |  |  |  |
| GO:0002880\_regulation\_of\_chronic\_inflammatory\_response\_to\_non-antigenic\_stimulus | 1 | 0 |  |  |  |  |  |  |  |  |
| GO:0002882\_positive\_regulation\_of\_chronic\_inflammatory\_response\_to\_non-antigenic\_stimulus | 1 | 0 |  |  |  |  |  |  |  |  |
| GO:0002895\_regulation\_of\_central\_B\_cell\_tolerance\_induction | 1 | 0 |  |  |  |  |  |  |  |  |
| GO:0002897\_positive\_regulation\_of\_central\_B\_cell\_tolerance\_induction | 1 | 0 |  |  |  |  |  |  |  |  |
| GO:0002901\_mature\_B\_cell\_apoptosis | 1 | 0 |  |  |  |  |  |  |  |  |
| GO:0002903\_negative\_regulation\_of\_B\_cell\_apoptosis | 1 | 0 |  |  |  |  |  |  |  |  |
| GO:0002905\_regulation\_of\_mature\_B\_cell\_apoptosis | 1 | 0 |  |  |  |  |  |  |  |  |
| GO:0002906\_negative\_regulation\_of\_mature\_B\_cell\_apoptosis | 1 | 0 |  |  |  |  |  |  |  |  |
| GO:0003011\_involuntary\_skeletal\_muscle\_contraction | 1 | 0 |  |  |  |  |  |  |  |  |
| GO:0003027\_regulation\_of\_systemic\_arterial\_blood\_pressure\_by\_carotid\_body\_chemoreceptor\_signaling | 1 | 0 |  |  |  |  |  |  |  |  |
| GO:0003029\_detection\_of\_hypoxic\_conditions\_in\_blood\_by\_carotid\_body\_chemoreceptor\_signaling | 1 | 0 |  |  |  |  |  |  |  |  |
| GO:0003032\_detection\_of\_oxygen | 1 | 0 |  |  |  |  |  |  |  |  |
| GO:0003056\_regulation\_of\_vascular\_smooth\_muscle\_contraction | 1 | 0 |  |  |  |  |  |  |  |  |
| GO:0003062\_regulation\_of\_heart\_rate\_by\_chemical\_signal | 1 | 0 |  |  |  |  |  |  |  |  |
| GO:0003065\_positive\_regulation\_of\_heart\_rate\_by\_epinephrine | 1 | 0 |  |  |  |  |  |  |  |  |
| GO:0003068\_regulation\_of\_systemic\_arterial\_blood\_pressure\_by\_acetylcholine | 1 | 0 |  |  |  |  |  |  |  |  |
| GO:0003069\_vasodilation\_by\_acetylcholine\_involved\_in\_regulation\_of\_systemic\_arterial\_blood\_pressure | 1 | 0 |  |  |  |  |  |  |  |  |
| GO:0003070\_regulation\_of\_systemic\_arterial\_blood\_pressure\_by\_neurotransmitter | 1 | 0 |  |  |  |  |  |  |  |  |
| GO:0003097\_renal\_water\_transport | 1 | 0 |  |  |  |  |  |  |  |  |
| GO:0005979\_regulation\_of\_glycogen\_biosynthetic\_process | 1 | 0 |  |  |  |  |  |  |  |  |
| GO:0005984\_disaccharide\_metabolic\_process | 1 | 0 |  |  |  |  |  |  |  |  |
| GO:0005988\_lactose\_metabolic\_process | 1 | 0 |  |  |  |  |  |  |  |  |
| GO:0005989\_lactose\_biosynthetic\_process | 1 | 0 |  |  |  |  |  |  |  |  |
| GO:0005997\_xylulose\_metabolic\_process | 1 | 0 |  |  |  |  |  |  |  |  |
| GO:0006000\_fructose\_metabolic\_process | 1 | 0 |  |  |  |  |  |  |  |  |
| GO:0006002\_fructose\_6-phosphate\_metabolic\_process | 1 | 0 |  |  |  |  |  |  |  |  |
| GO:0006004\_fucose\_metabolic\_process | 1 | 0 |  |  |  |  |  |  |  |  |
| GO:0006013\_mannose\_metabolic\_process | 1 | 0 |  |  |  |  |  |  |  |  |
| GO:0006060\_sorbitol\_metabolic\_process | 1 | 0 |  |  |  |  |  |  |  |  |
| GO:0006064\_glucuronate\_catabolic\_process | 1 | 0 |  |  |  |  |  |  |  |  |
| GO:0006086\_acetyl-CoA\_biosynthetic\_process\_from\_pyruvate | 1 | 0 |  |  |  |  |  |  |  |  |
| GO:0006098\_pentose-phosphate\_shunt | 1 | 0 |  |  |  |  |  |  |  |  |
| GO:0006101\_citrate\_metabolic\_process | 1 | 0 |  |  |  |  |  |  |  |  |
| GO:0006104\_succinyl-CoA\_metabolic\_process | 1 | 0 |  |  |  |  |  |  |  |  |
| GO:0006116\_NADH\_oxidation | 1 | 0 |  |  |  |  |  |  |  |  |
| GO:0006120\_mitochondrial\_electron\_transport\_\_NADH\_to\_ubiquinone | 1 | 0 |  |  |  |  |  |  |  |  |
| GO:0006154\_adenosine\_catabolic\_process | 1 | 0 |  |  |  |  |  |  |  |  |
| GO:0006157\_deoxyadenosine\_catabolic\_process | 1 | 0 |  |  |  |  |  |  |  |  |
| GO:0006167\_AMP\_biosynthetic\_process | 1 | 0 |  |  |  |  |  |  |  |  |
| GO:0006175\_dATP\_biosynthetic\_process | 1 | 0 |  |  |  |  |  |  |  |  |
| GO:0006178\_guanine\_salvage | 1 | 0 |  |  |  |  |  |  |  |  |
| GO:0006196\_AMP\_catabolic\_process | 1 | 0 |  |  |  |  |  |  |  |  |
| GO:0006203\_dGTP\_catabolic\_process | 1 | 0 |  |  |  |  |  |  |  |  |
| GO:0006208\_pyrimidine\_base\_catabolic\_process | 1 | 0 |  |  |  |  |  |  |  |  |
| GO:0006221\_pyrimidine\_nucleotide\_biosynthetic\_process | 1 | 0 |  |  |  |  |  |  |  |  |
| GO:0006235\_dTTP\_biosynthetic\_process | 1 | 0 |  |  |  |  |  |  |  |  |
| GO:0006244\_pyrimidine\_nucleotide\_catabolic\_process | 1 | 0 |  |  |  |  |  |  |  |  |
| GO:0006269\_DNA\_replication\_\_synthesis\_of\_RNA\_primer | 1 | 0 |  |  |  |  |  |  |  |  |
| GO:0006283\_transcription-coupled\_nucleotide-excision\_repair | 1 | 0 |  |  |  |  |  |  |  |  |
| GO:0006296\_nucleotide-excision\_repair\_\_DNA\_incision\_\_5'-to\_lesion | 1 | 0 |  |  |  |  |  |  |  |  |
| GO:0006307\_DNA\_dealkylation | 1 | 0 |  |  |  |  |  |  |  |  |
| GO:0006337\_nucleosome\_disassembly | 1 | 0 |  |  |  |  |  |  |  |  |
| GO:0006344\_maintenance\_of\_chromatin\_silencing | 1 | 0 |  |  |  |  |  |  |  |  |
| GO:0006356\_regulation\_of\_transcription\_from\_RNA\_polymerase\_I\_promoter | 1 | 0 |  |  |  |  |  |  |  |  |
| GO:0006388\_tRNA\_splicing\_\_via\_endonucleolytic\_cleavage\_and\_ligation | 1 | 0 |  |  |  |  |  |  |  |  |
| GO:0006407\_rRNA\_export\_from\_nucleus | 1 | 0 |  |  |  |  |  |  |  |  |
| GO:0006419\_alanyl-tRNA\_aminoacylation | 1 | 0 |  |  |  |  |  |  |  |  |
| GO:0006434\_seryl-tRNA\_aminoacylation | 1 | 0 |  |  |  |  |  |  |  |  |
| GO:0006447\_regulation\_of\_translational\_initiation\_by\_iron | 1 | 0 |  |  |  |  |  |  |  |  |
| GO:0006463\_steroid\_hormone\_receptor\_complex\_assembly | 1 | 0 |  |  |  |  |  |  |  |  |
| GO:0006467\_protein\_thiol-disulfide\_exchange | 1 | 0 |  |  |  |  |  |  |  |  |
| GO:0006474\_N-terminal\_protein\_amino\_acid\_acetylation | 1 | 0 |  |  |  |  |  |  |  |  |
| GO:0006481\_C-terminal\_protein\_amino\_acid\_methylation | 1 | 0 |  |  |  |  |  |  |  |  |
| GO:0006488\_dolichol-linked\_oligosaccharide\_biosynthetic\_process | 1 | 0 |  |  |  |  |  |  |  |  |
| GO:0006494\_protein\_amino\_acid\_terminal\_glycosylation | 1 | 0 |  |  |  |  |  |  |  |  |
| GO:0006496\_protein\_amino\_acid\_terminal\_N-glycosylation | 1 | 0 |  |  |  |  |  |  |  |  |
| GO:0006500\_N-terminal\_protein\_palmitoylation | 1 | 0 |  |  |  |  |  |  |  |  |
| GO:0006507\_GPI\_anchor\_release | 1 | 0 |  |  |  |  |  |  |  |  |
| GO:0006537\_glutamate\_biosynthetic\_process | 1 | 0 |  |  |  |  |  |  |  |  |
| GO:0006549\_isoleucine\_metabolic\_process | 1 | 0 |  |  |  |  |  |  |  |  |
| GO:0006553\_lysine\_metabolic\_process | 1 | 0 |  |  |  |  |  |  |  |  |
| GO:0006554\_lysine\_catabolic\_process | 1 | 0 |  |  |  |  |  |  |  |  |
| GO:0006556\_S-adenosylmethionine\_biosynthetic\_process | 1 | 0 |  |  |  |  |  |  |  |  |
| GO:0006559\_L-phenylalanine\_catabolic\_process | 1 | 0 |  |  |  |  |  |  |  |  |
| GO:0006569\_tryptophan\_catabolic\_process | 1 | 0 |  |  |  |  |  |  |  |  |
| GO:0006572\_tyrosine\_catabolic\_process | 1 | 0 |  |  |  |  |  |  |  |  |
| GO:0006573\_valine\_metabolic\_process | 1 | 0 |  |  |  |  |  |  |  |  |
| GO:0006581\_acetylcholine\_catabolic\_process | 1 | 0 |  |  |  |  |  |  |  |  |
| GO:0006585\_dopamine\_biosynthetic\_process\_from\_tyrosine | 1 | 0 |  |  |  |  |  |  |  |  |
| GO:0006590\_thyroid\_hormone\_generation | 1 | 0 |  |  |  |  |  |  |  |  |
| GO:0006591\_ornithine\_metabolic\_process | 1 | 0 |  |  |  |  |  |  |  |  |
| GO:0006596\_polyamine\_biosynthetic\_process | 1 | 0 |  |  |  |  |  |  |  |  |
| GO:0006597\_spermine\_biosynthetic\_process | 1 | 0 |  |  |  |  |  |  |  |  |
| GO:0006601\_creatine\_biosynthetic\_process | 1 | 0 |  |  |  |  |  |  |  |  |
| GO:0006613\_cotranslational\_protein\_targeting\_to\_membrane | 1 | 0 |  |  |  |  |  |  |  |  |
| GO:0006622\_protein\_targeting\_to\_lysosome | 1 | 0 |  |  |  |  |  |  |  |  |
| GO:0006627\_mitochondrial\_protein\_processing\_during\_import | 1 | 0 |  |  |  |  |  |  |  |  |
| GO:0006653\_lecithin\_metabolic\_process | 1 | 0 |  |  |  |  |  |  |  |  |
| GO:0006654\_phosphatidic\_acid\_biosynthetic\_process | 1 | 0 |  |  |  |  |  |  |  |  |
| GO:0006658\_phosphatidylserine\_metabolic\_process | 1 | 0 |  |  |  |  |  |  |  |  |
| GO:0006659\_phosphatidylserine\_biosynthetic\_process | 1 | 0 |  |  |  |  |  |  |  |  |
| GO:0006667\_sphinganine\_metabolic\_process | 1 | 0 |  |  |  |  |  |  |  |  |
| GO:0006668\_sphinganine-1-phosphate\_metabolic\_process | 1 | 0 |  |  |  |  |  |  |  |  |
| GO:0006678\_glucosylceramide\_metabolic\_process | 1 | 0 |  |  |  |  |  |  |  |  |
| GO:0006682\_galactosylceramide\_biosynthetic\_process | 1 | 0 |  |  |  |  |  |  |  |  |
| GO:0006685\_sphingomyelin\_catabolic\_process | 1 | 0 |  |  |  |  |  |  |  |  |
| GO:0006700\_C21-steroid\_hormone\_biosynthetic\_process | 1 | 0 |  |  |  |  |  |  |  |  |
| GO:0006705\_mineralocorticoid\_biosynthetic\_process | 1 | 0 |  |  |  |  |  |  |  |  |
| GO:0006709\_progesterone\_catabolic\_process | 1 | 0 |  |  |  |  |  |  |  |  |
| GO:0006729\_tetrahydrobiopterin\_biosynthetic\_process | 1 | 0 |  |  |  |  |  |  |  |  |
| GO:0006734\_NADH\_metabolic\_process | 1 | 0 |  |  |  |  |  |  |  |  |
| GO:0006740\_NADPH\_regeneration | 1 | 0 |  |  |  |  |  |  |  |  |
| GO:0006741\_NADP\_biosynthetic\_process | 1 | 0 |  |  |  |  |  |  |  |  |
| GO:0006743\_ubiquinone\_metabolic\_process | 1 | 0 |  |  |  |  |  |  |  |  |
| GO:0006744\_ubiquinone\_biosynthetic\_process | 1 | 0 |  |  |  |  |  |  |  |  |
| GO:0006772\_thiamin\_metabolic\_process | 1 | 0 |  |  |  |  |  |  |  |  |
| GO:0006784\_heme\_a\_biosynthetic\_process | 1 | 0 |  |  |  |  |  |  |  |  |
| GO:0006797\_polyphosphate\_metabolic\_process | 1 | 0 |  |  |  |  |  |  |  |  |
| GO:0006798\_polyphosphate\_catabolic\_process | 1 | 0 |  |  |  |  |  |  |  |  |
| GO:0006824\_cobalt\_ion\_transport | 1 | 0 |  |  |  |  |  |  |  |  |
| GO:0006842\_tricarboxylic\_acid\_transport | 1 | 0 |  |  |  |  |  |  |  |  |
| GO:0006844\_acyl\_carnitine\_transport | 1 | 0 |  |  |  |  |  |  |  |  |
| GO:0006855\_multidrug\_transport | 1 | 0 |  |  |  |  |  |  |  |  |
| GO:0006863\_purine\_transport | 1 | 0 |  |  |  |  |  |  |  |  |
| GO:0006890\_retrograde\_vesicle-mediated\_transport\_\_Golgi\_to\_ER | 1 | 0 |  |  |  |  |  |  |  |  |
| GO:0006891\_intra-Golgi\_vesicle-mediated\_transport | 1 | 0 |  |  |  |  |  |  |  |  |
| GO:0006893\_Golgi\_to\_plasma\_membrane\_transport | 1 | 0 |  |  |  |  |  |  |  |  |
| GO:0006895\_Golgi\_to\_endosome\_transport | 1 | 0 |  |  |  |  |  |  |  |  |
| GO:0006896\_Golgi\_to\_vacuole\_transport | 1 | 0 |  |  |  |  |  |  |  |  |
| GO:0006900\_membrane\_budding | 1 | 0 |  |  |  |  |  |  |  |  |
| GO:0006930\_substrate-bound\_cell\_migration\_\_cell\_extension | 1 | 0 |  |  |  |  |  |  |  |  |
| GO:0006931\_substrate-bound\_cell\_migration\_\_cell\_attachment\_to\_substrate | 1 | 0 |  |  |  |  |  |  |  |  |
| GO:0006933\_negative\_regulation\_of\_cell\_adhesion\_involved\_in\_substrate-bound\_cell\_migration | 1 | 0 |  |  |  |  |  |  |  |  |
| GO:0006957\_complement\_activation\_\_alternative\_pathway | 1 | 0 |  |  |  |  |  |  |  |  |
| GO:0006958\_complement\_activation\_\_classical\_pathway | 1 | 0 |  |  |  |  |  |  |  |  |
| GO:0006978\_DNA\_damage\_response\_\_signal\_transduction\_by\_p53\_class\_mediator\_resulting\_in\_transcription\_of\_p21\_class\_mediator | 1 | 0 |  |  |  |  |  |  |  |  |
| GO:0007016\_cytoskeletal\_anchoring\_at\_plasma\_membrane | 1 | 0 |  |  |  |  |  |  |  |  |
| GO:0007021\_tubulin\_complex\_assembly | 1 | 0 |  |  |  |  |  |  |  |  |
| GO:0007052\_mitotic\_spindle\_organization | 1 | 0 |  |  |  |  |  |  |  |  |
| GO:0007056\_spindle\_assembly\_involved\_in\_female\_meiosis | 1 | 0 |  |  |  |  |  |  |  |  |
| GO:0007057\_spindle\_assembly\_involved\_in\_female\_meiosis\_I | 1 | 0 |  |  |  |  |  |  |  |  |
| GO:0007063\_regulation\_of\_sister\_chromatid\_cohesion | 1 | 0 |  |  |  |  |  |  |  |  |
| GO:0007065\_male\_meiosis\_sister\_chromatid\_cohesion | 1 | 0 |  |  |  |  |  |  |  |  |
| GO:0007076\_mitotic\_chromosome\_condensation | 1 | 0 |  |  |  |  |  |  |  |  |
| GO:0007095\_mitotic\_cell\_cycle\_G2\_M\_transition\_DNA\_damage\_checkpoint | 1 | 0 |  |  |  |  |  |  |  |  |
| GO:0007096\_regulation\_of\_exit\_from\_mitosis | 1 | 0 |  |  |  |  |  |  |  |  |
| GO:0007158\_neuron\_adhesion | 1 | 0 |  |  |  |  |  |  |  |  |
| GO:0007168\_receptor\_guanylyl\_cyclase\_signaling\_pathway | 1 | 0 |  |  |  |  |  |  |  |  |
| GO:0007197\_inhibition\_of\_adenylate\_cyclase\_activity\_by\_muscarinic\_acetylcholine\_receptor\_signaling\_pathway | 1 | 0 |  |  |  |  |  |  |  |  |
| GO:0007207\_activation\_of\_phospholipase\_C\_activity\_by\_muscarinic\_acetylcholine\_receptor\_signaling\_pathway | 1 | 0 |  |  |  |  |  |  |  |  |
| GO:0007208\_activation\_of\_phospholipase\_C\_activity\_by\_serotonin\_receptor\_signaling\_pathway | 1 | 0 |  |  |  |  |  |  |  |  |
| GO:0007217\_tachykinin\_receptor\_signaling\_pathway | 1 | 0 |  |  |  |  |  |  |  |  |
| GO:0007221\_positive\_regulation\_of\_transcription\_of\_Notch\_receptor\_target | 1 | 0 |  |  |  |  |  |  |  |  |
| GO:0007223\_Wnt\_receptor\_signaling\_pathway\_\_calcium\_modulating\_pathway | 1 | 0 |  |  |  |  |  |  |  |  |
| GO:0007225\_patched\_ligand\_processing | 1 | 0 |  |  |  |  |  |  |  |  |
| GO:0007227\_signal\_transduction\_downstream\_of\_smoothened | 1 | 0 |  |  |  |  |  |  |  |  |
| GO:0007228\_positive\_regulation\_of\_hh\_target\_transcription\_factor\_activity | 1 | 0 |  |  |  |  |  |  |  |  |
| GO:0007231\_osmosensory\_signaling\_pathway | 1 | 0 |  |  |  |  |  |  |  |  |
| GO:0007284\_spermatogonial\_cell\_division | 1 | 0 |  |  |  |  |  |  |  |  |
| GO:0007290\_spermatid\_nucleus\_elongation | 1 | 0 |  |  |  |  |  |  |  |  |
| GO:0007296\_vitellogenesis | 1 | 0 |  |  |  |  |  |  |  |  |
| GO:0007321\_sperm\_displacement | 1 | 0 |  |  |  |  |  |  |  |  |
| GO:0007380\_specification\_of\_segmental\_identity\_\_head | 1 | 0 |  |  |  |  |  |  |  |  |
| GO:0007382\_specification\_of\_segmental\_identity\_\_maxillary\_segment | 1 | 0 |  |  |  |  |  |  |  |  |
| GO:0007400\_neuroblast\_fate\_determination | 1 | 0 |  |  |  |  |  |  |  |  |
| GO:0007402\_ganglion\_mother\_cell\_fate\_determination | 1 | 0 |  |  |  |  |  |  |  |  |
| GO:0007495\_visceral\_mesoderm-endoderm\_interaction\_involved\_in\_midgut\_development | 1 | 0 |  |  |  |  |  |  |  |  |
| GO:0007497\_posterior\_midgut\_development | 1 | 0 |  |  |  |  |  |  |  |  |
| GO:0007499\_ectoderm\_and\_mesoderm\_interaction | 1 | 0 |  |  |  |  |  |  |  |  |
| GO:0007500\_mesodermal\_cell\_fate\_determination | 1 | 0 |  |  |  |  |  |  |  |  |
| GO:0007509\_mesoderm\_migration | 1 | 0 |  |  |  |  |  |  |  |  |
| GO:0007518\_myoblast\_cell\_fate\_determination | 1 | 0 |  |  |  |  |  |  |  |  |
| GO:0007521\_muscle\_cell\_fate\_determination | 1 | 0 |  |  |  |  |  |  |  |  |
| GO:0007522\_visceral\_muscle\_development | 1 | 0 |  |  |  |  |  |  |  |  |
| GO:0007529\_establishment\_of\_synaptic\_specificity\_at\_neuromuscular\_junction | 1 | 0 |  |  |  |  |  |  |  |  |
| GO:0007538\_primary\_sex\_determination | 1 | 0 |  |  |  |  |  |  |  |  |
| GO:0007542\_primary\_sex\_determination\_\_germ-line | 1 | 0 |  |  |  |  |  |  |  |  |
| GO:0007567\_parturition | 1 | 0 |  |  |  |  |  |  |  |  |
| GO:0007614\_short-term\_memory | 1 | 0 |  |  |  |  |  |  |  |  |
| GO:0007621\_negative\_regulation\_of\_female\_receptivity | 1 | 0 |  |  |  |  |  |  |  |  |
| GO:0008049\_male\_courtship\_behavior | 1 | 0 |  |  |  |  |  |  |  |  |
| GO:0008050\_female\_courtship\_behavior | 1 | 0 |  |  |  |  |  |  |  |  |
| GO:0008052\_sensory\_organ\_boundary\_specification | 1 | 0 |  |  |  |  |  |  |  |  |
| GO:0008054\_cyclin\_catabolic\_process | 1 | 0 |  |  |  |  |  |  |  |  |
| GO:0008057\_eye\_pigment\_granule\_organization | 1 | 0 |  |  |  |  |  |  |  |  |
| GO:0008078\_mesodermal\_cell\_migration | 1 | 0 |  |  |  |  |  |  |  |  |
| GO:0008208\_C21-steroid\_hormone\_catabolic\_process | 1 | 0 |  |  |  |  |  |  |  |  |
| GO:0008216\_spermidine\_metabolic\_process | 1 | 0 |  |  |  |  |  |  |  |  |
| GO:0008292\_acetylcholine\_biosynthetic\_process | 1 | 0 |  |  |  |  |  |  |  |  |
| GO:0008295\_spermidine\_biosynthetic\_process | 1 | 0 |  |  |  |  |  |  |  |  |
| GO:0008300\_isoprenoid\_catabolic\_process | 1 | 0 |  |  |  |  |  |  |  |  |
| GO:0008333\_endosome\_to\_lysosome\_transport | 1 | 0 |  |  |  |  |  |  |  |  |
| GO:0008355\_olfactory\_learning | 1 | 0 |  |  |  |  |  |  |  |  |
| GO:0008611\_ether\_lipid\_biosynthetic\_process | 1 | 0 |  |  |  |  |  |  |  |  |
| GO:0008626\_induction\_of\_apoptosis\_by\_granzyme | 1 | 0 |  |  |  |  |  |  |  |  |
| GO:0008633\_activation\_of\_pro-apoptotic\_gene\_products | 1 | 0 |  |  |  |  |  |  |  |  |
| GO:0008653\_lipopolysaccharide\_metabolic\_process | 1 | 0 |  |  |  |  |  |  |  |  |
| GO:0009068\_aspartate\_family\_amino\_acid\_catabolic\_process | 1 | 0 |  |  |  |  |  |  |  |  |
| GO:0009084\_glutamine\_family\_amino\_acid\_biosynthetic\_process | 1 | 0 |  |  |  |  |  |  |  |  |
| GO:0009088\_threonine\_biosynthetic\_process | 1 | 0 |  |  |  |  |  |  |  |  |
| GO:0009105\_lipoic\_acid\_biosynthetic\_process | 1 | 0 |  |  |  |  |  |  |  |  |
| GO:0009109\_coenzyme\_catabolic\_process | 1 | 0 |  |  |  |  |  |  |  |  |
| GO:0009111\_vitamin\_catabolic\_process | 1 | 0 |  |  |  |  |  |  |  |  |
| GO:0009113\_purine\_base\_biosynthetic\_process | 1 | 0 |  |  |  |  |  |  |  |  |
| GO:0009127\_purine\_nucleoside\_monophosphate\_biosynthetic\_process | 1 | 0 |  |  |  |  |  |  |  |  |
| GO:0009128\_purine\_nucleoside\_monophosphate\_catabolic\_process | 1 | 0 |  |  |  |  |  |  |  |  |
| GO:0009129\_pyrimidine\_nucleoside\_monophosphate\_metabolic\_process | 1 | 0 |  |  |  |  |  |  |  |  |
| GO:0009131\_pyrimidine\_nucleoside\_monophosphate\_catabolic\_process | 1 | 0 |  |  |  |  |  |  |  |  |
| GO:0009133\_nucleoside\_diphosphate\_biosynthetic\_process | 1 | 0 |  |  |  |  |  |  |  |  |
| GO:0009145\_purine\_nucleoside\_triphosphate\_biosynthetic\_process | 1 | 0 |  |  |  |  |  |  |  |  |
| GO:0009147\_pyrimidine\_nucleoside\_triphosphate\_metabolic\_process | 1 | 0 |  |  |  |  |  |  |  |  |
| GO:0009148\_pyrimidine\_nucleoside\_triphosphate\_biosynthetic\_process | 1 | 0 |  |  |  |  |  |  |  |  |
| GO:0009152\_purine\_ribonucleotide\_biosynthetic\_process | 1 | 0 |  |  |  |  |  |  |  |  |
| GO:0009153\_purine\_deoxyribonucleotide\_biosynthetic\_process | 1 | 0 |  |  |  |  |  |  |  |  |
| GO:0009156\_ribonucleoside\_monophosphate\_biosynthetic\_process | 1 | 0 |  |  |  |  |  |  |  |  |
| GO:0009158\_ribonucleoside\_monophosphate\_catabolic\_process | 1 | 0 |  |  |  |  |  |  |  |  |
| GO:0009159\_deoxyribonucleoside\_monophosphate\_catabolic\_process | 1 | 0 |  |  |  |  |  |  |  |  |
| GO:0009162\_deoxyribonucleoside\_monophosphate\_metabolic\_process | 1 | 0 |  |  |  |  |  |  |  |  |
| GO:0009168\_purine\_ribonucleoside\_monophosphate\_biosynthetic\_process | 1 | 0 |  |  |  |  |  |  |  |  |
| GO:0009169\_purine\_ribonucleoside\_monophosphate\_catabolic\_process | 1 | 0 |  |  |  |  |  |  |  |  |
| GO:0009176\_pyrimidine\_deoxyribonucleoside\_monophosphate\_metabolic\_process | 1 | 0 |  |  |  |  |  |  |  |  |
| GO:0009178\_pyrimidine\_deoxyribonucleoside\_monophosphate\_catabolic\_process | 1 | 0 |  |  |  |  |  |  |  |  |
| GO:0009211\_pyrimidine\_deoxyribonucleoside\_triphosphate\_metabolic\_process | 1 | 0 |  |  |  |  |  |  |  |  |
| GO:0009212\_pyrimidine\_deoxyribonucleoside\_triphosphate\_biosynthetic\_process | 1 | 0 |  |  |  |  |  |  |  |  |
| GO:0009216\_purine\_deoxyribonucleoside\_triphosphate\_biosynthetic\_process | 1 | 0 |  |  |  |  |  |  |  |  |
| GO:0009221\_pyrimidine\_deoxyribonucleotide\_biosynthetic\_process | 1 | 0 |  |  |  |  |  |  |  |  |
| GO:0009223\_pyrimidine\_deoxyribonucleotide\_catabolic\_process | 1 | 0 |  |  |  |  |  |  |  |  |
| GO:0009260\_ribonucleotide\_biosynthetic\_process | 1 | 0 |  |  |  |  |  |  |  |  |
| GO:0009405\_pathogenesis | 1 | 0 |  |  |  |  |  |  |  |  |
| GO:0009414\_response\_to\_water\_deprivation | 1 | 0 |  |  |  |  |  |  |  |  |
| GO:0009415\_response\_to\_water | 1 | 0 |  |  |  |  |  |  |  |  |
| GO:0009449\_gamma-aminobutyric\_acid\_biosynthetic\_process | 1 | 0 |  |  |  |  |  |  |  |  |
| GO:0009450\_gamma-aminobutyric\_acid\_catabolic\_process | 1 | 0 |  |  |  |  |  |  |  |  |
| GO:0009589\_detection\_of\_UV | 1 | 0 |  |  |  |  |  |  |  |  |
| GO:0009590\_detection\_of\_gravity | 1 | 0 |  |  |  |  |  |  |  |  |
| GO:0009624\_response\_to\_nematode | 1 | 0 |  |  |  |  |  |  |  |  |
| GO:0009629\_response\_to\_gravity | 1 | 0 |  |  |  |  |  |  |  |  |
| GO:0009648\_photoperiodism | 1 | 0 |  |  |  |  |  |  |  |  |
| GO:0009690\_cytokinin\_metabolic\_process | 1 | 0 |  |  |  |  |  |  |  |  |
| GO:0009691\_cytokinin\_biosynthetic\_process | 1 | 0 |  |  |  |  |  |  |  |  |
| GO:0009786\_regulation\_of\_asymmetric\_cell\_division | 1 | 0 |  |  |  |  |  |  |  |  |
| GO:0009794\_regulation\_of\_mitotic\_cell\_cycle\_\_embryonic | 1 | 0 |  |  |  |  |  |  |  |  |
| GO:0009956\_radial\_pattern\_formation | 1 | 0 |  |  |  |  |  |  |  |  |
| GO:0009957\_epidermal\_cell\_fate\_specification | 1 | 0 |  |  |  |  |  |  |  |  |
| GO:0009992\_cellular\_water\_homeostasis | 1 | 0 |  |  |  |  |  |  |  |  |
| GO:0010032\_meiotic\_chromosome\_condensation | 1 | 0 |  |  |  |  |  |  |  |  |
| GO:0010039\_response\_to\_iron\_ion | 1 | 0 |  |  |  |  |  |  |  |  |
| GO:0010042\_response\_to\_manganese\_ion | 1 | 0 |  |  |  |  |  |  |  |  |
| GO:0010045\_response\_to\_nickel\_ion | 1 | 0 |  |  |  |  |  |  |  |  |
| GO:0010046\_response\_to\_mycotoxin | 1 | 0 |  |  |  |  |  |  |  |  |
| GO:0010107\_potassium\_ion\_import | 1 | 0 |  |  |  |  |  |  |  |  |
| GO:0010155\_regulation\_of\_proton\_transport | 1 | 0 |  |  |  |  |  |  |  |  |
| GO:0010160\_formation\_of\_organ\_boundary | 1 | 0 |  |  |  |  |  |  |  |  |
| GO:0010260\_organ\_senescence | 1 | 0 |  |  |  |  |  |  |  |  |
| GO:0010310\_regulation\_of\_hydrogen\_peroxide\_metabolic\_process | 1 | 0 |  |  |  |  |  |  |  |  |
| GO:0010447\_response\_to\_acidity | 1 | 0 |  |  |  |  |  |  |  |  |
| GO:0010452\_histone\_H3-K36\_methylation | 1 | 0 |  |  |  |  |  |  |  |  |
| GO:0010455\_positive\_regulation\_of\_cell\_fate\_commitment | 1 | 0 |  |  |  |  |  |  |  |  |
| GO:0010470\_regulation\_of\_gastrulation | 1 | 0 |  |  |  |  |  |  |  |  |
| GO:0010508\_positive\_regulation\_of\_autophagy | 1 | 0 |  |  |  |  |  |  |  |  |
| GO:0010519\_negative\_regulation\_of\_phospholipase\_activity | 1 | 0 |  |  |  |  |  |  |  |  |
| GO:0010520\_regulation\_of\_reciprocal\_meiotic\_recombination | 1 | 0 |  |  |  |  |  |  |  |  |
| GO:0010523\_negative\_regulation\_of\_calcium\_ion\_transport\_into\_cytosol | 1 | 0 |  |  |  |  |  |  |  |  |
| GO:0010543\_regulation\_of\_platelet\_activation | 1 | 0 |  |  |  |  |  |  |  |  |
| GO:0010561\_negative\_regulation\_of\_glycoprotein\_biosynthetic\_process | 1 | 0 |  |  |  |  |  |  |  |  |
| GO:0010569\_regulation\_of\_double-strand\_break\_repair\_via\_homologous\_recombination | 1 | 0 |  |  |  |  |  |  |  |  |
| GO:0010572\_positive\_regulation\_of\_platelet\_activation | 1 | 0 |  |  |  |  |  |  |  |  |
| GO:0010594\_regulation\_of\_endothelial\_cell\_migration | 1 | 0 |  |  |  |  |  |  |  |  |
| GO:0010596\_negative\_regulation\_of\_endothelial\_cell\_migration | 1 | 0 |  |  |  |  |  |  |  |  |
| GO:0010611\_regulation\_of\_cardiac\_muscle\_hypertrophy | 1 | 0 |  |  |  |  |  |  |  |  |
| GO:0010612\_regulation\_of\_cardiac\_muscle\_adaptation | 1 | 0 |  |  |  |  |  |  |  |  |
| GO:0010614\_negative\_regulation\_of\_cardiac\_muscle\_hypertrophy | 1 | 0 |  |  |  |  |  |  |  |  |
| GO:0010616\_negative\_regulation\_of\_cardiac\_muscle\_adaptation | 1 | 0 |  |  |  |  |  |  |  |  |
| GO:0010656\_negative\_regulation\_of\_muscle\_cell\_apoptosis | 1 | 0 |  |  |  |  |  |  |  |  |
| GO:0010657\_muscle\_cell\_apoptosis | 1 | 0 |  |  |  |  |  |  |  |  |
| GO:0010658\_striated\_muscle\_cell\_apoptosis | 1 | 0 |  |  |  |  |  |  |  |  |
| GO:0010659\_cardiac\_muscle\_cell\_apoptosis | 1 | 0 |  |  |  |  |  |  |  |  |
| GO:0010660\_regulation\_of\_muscle\_cell\_apoptosis | 1 | 0 |  |  |  |  |  |  |  |  |
| GO:0010662\_regulation\_of\_striated\_muscle\_cell\_apoptosis | 1 | 0 |  |  |  |  |  |  |  |  |
| GO:0010664\_negative\_regulation\_of\_striated\_muscle\_cell\_apoptosis | 1 | 0 |  |  |  |  |  |  |  |  |
| GO:0010665\_regulation\_of\_cardiac\_muscle\_cell\_apoptosis | 1 | 0 |  |  |  |  |  |  |  |  |
| GO:0010667\_negative\_regulation\_of\_cardiac\_muscle\_cell\_apoptosis | 1 | 0 |  |  |  |  |  |  |  |  |
| GO:0010668\_ectodermal\_cell\_differentiation | 1 | 0 |  |  |  |  |  |  |  |  |
| GO:0010671\_negative\_regulation\_of\_oxygen\_and\_reactive\_oxygen\_species\_metabolic\_process | 1 | 0 |  |  |  |  |  |  |  |  |
| GO:0010719\_negative\_regulation\_of\_epithelial\_to\_mesenchymal\_transition | 1 | 0 |  |  |  |  |  |  |  |  |
| GO:0010735\_positive\_regulation\_of\_transcription\_via\_serum\_response\_element\_binding | 1 | 0 |  |  |  |  |  |  |  |  |
| GO:0010825\_positive\_regulation\_of\_centrosome\_duplication | 1 | 0 |  |  |  |  |  |  |  |  |
| GO:0010845\_positive\_regulation\_of\_reciprocal\_meiotic\_recombination | 1 | 0 |  |  |  |  |  |  |  |  |
| GO:0010850\_chemoreceptor\_signaling\_pathway\_involved\_in\_regulation\_of\_blood\_pressure | 1 | 0 |  |  |  |  |  |  |  |  |
| GO:0010873\_positive\_regulation\_of\_cholesterol\_esterification | 1 | 0 |  |  |  |  |  |  |  |  |
| GO:0010880\_regulation\_of\_release\_of\_sequestered\_calcium\_ion\_into\_cytosol\_by\_sarcoplasmic\_reticulum | 1 | 0 |  |  |  |  |  |  |  |  |
| GO:0010881\_regulation\_of\_cardiac\_muscle\_contraction\_by\_regulation\_of\_the\_release\_of\_sequestered\_calcium\_ion | 1 | 0 |  |  |  |  |  |  |  |  |
| GO:0010882\_regulation\_of\_cardiac\_muscle\_contraction\_by\_calcium\_ion\_signaling | 1 | 0 |  |  |  |  |  |  |  |  |
| GO:0010890\_positive\_regulation\_of\_sequestering\_of\_triglyceride | 1 | 0 |  |  |  |  |  |  |  |  |
| GO:0010919\_regulation\_of\_inositol\_phosphate\_biosynthetic\_process | 1 | 0 |  |  |  |  |  |  |  |  |
| GO:0010931\_macrophage\_tolerance\_induction | 1 | 0 |  |  |  |  |  |  |  |  |
| GO:0010932\_regulation\_of\_macrophage\_tolerance\_induction | 1 | 0 |  |  |  |  |  |  |  |  |
| GO:0010933\_positive\_regulation\_of\_macrophage\_tolerance\_induction | 1 | 0 |  |  |  |  |  |  |  |  |
| GO:0010934\_macrophage\_cytokine\_production | 1 | 0 |  |  |  |  |  |  |  |  |
| GO:0010935\_regulation\_of\_macrophage\_cytokine\_production | 1 | 0 |  |  |  |  |  |  |  |  |
| GO:0010936\_negative\_regulation\_of\_macrophage\_cytokine\_production | 1 | 0 |  |  |  |  |  |  |  |  |
| GO:0010953\_regulation\_of\_protein\_maturation\_by\_peptide\_bond\_cleavage | 1 | 0 |  |  |  |  |  |  |  |  |
| GO:0010962\_regulation\_of\_glucan\_biosynthetic\_process | 1 | 0 |  |  |  |  |  |  |  |  |
| GO:0010966\_regulation\_of\_phosphate\_transport | 1 | 0 |  |  |  |  |  |  |  |  |
| GO:0014012\_axon\_regeneration\_in\_the\_peripheral\_nervous\_system | 1 | 0 |  |  |  |  |  |  |  |  |
| GO:0014016\_neuroblast\_differentiation | 1 | 0 |  |  |  |  |  |  |  |  |
| GO:0014017\_neuroblast\_fate\_commitment | 1 | 0 |  |  |  |  |  |  |  |  |
| GO:0014041\_regulation\_of\_neuron\_maturation | 1 | 0 |  |  |  |  |  |  |  |  |
| GO:0014042\_positive\_regulation\_of\_neuron\_maturation | 1 | 0 |  |  |  |  |  |  |  |  |
| GO:0014049\_positive\_regulation\_of\_glutamate\_secretion | 1 | 0 |  |  |  |  |  |  |  |  |
| GO:0014061\_regulation\_of\_norepinephrine\_secretion | 1 | 0 |  |  |  |  |  |  |  |  |
| GO:0014071\_response\_to\_cycloalkane | 1 | 0 |  |  |  |  |  |  |  |  |
| GO:0014707\_branchiomeric\_skeletal\_muscle\_development | 1 | 0 |  |  |  |  |  |  |  |  |
| GO:0014738\_regulation\_of\_muscle\_hyperplasia | 1 | 0 |  |  |  |  |  |  |  |  |
| GO:0014740\_negative\_regulation\_of\_muscle\_hyperplasia | 1 | 0 |  |  |  |  |  |  |  |  |
| GO:0014741\_negative\_regulation\_of\_muscle\_hypertrophy | 1 | 0 |  |  |  |  |  |  |  |  |
| GO:0014743\_regulation\_of\_muscle\_hypertrophy | 1 | 0 |  |  |  |  |  |  |  |  |
| GO:0014805\_smooth\_muscle\_adaptation | 1 | 0 |  |  |  |  |  |  |  |  |
| GO:0014806\_smooth\_muscle\_hyperplasia | 1 | 0 |  |  |  |  |  |  |  |  |
| GO:0014807\_regulation\_of\_somitogenesis | 1 | 0 |  |  |  |  |  |  |  |  |
| GO:0014808\_release\_of\_sequestered\_calcium\_ion\_into\_cytosol\_by\_sarcoplasmic\_reticulum | 1 | 0 |  |  |  |  |  |  |  |  |
| GO:0014813\_satellite\_cell\_commitment | 1 | 0 |  |  |  |  |  |  |  |  |
| GO:0014816\_satellite\_cell\_differentiation | 1 | 0 |  |  |  |  |  |  |  |  |
| GO:0014819\_regulation\_of\_skeletal\_muscle\_contraction | 1 | 0 |  |  |  |  |  |  |  |  |
| GO:0014852\_regulation\_of\_skeletal\_muscle\_contraction\_by\_neural\_stimulation\_via\_neuromuscular\_junction | 1 | 0 |  |  |  |  |  |  |  |  |
| GO:0014853\_regulation\_of\_excitatory\_postsynaptic\_membrane\_potential\_involved\_in\_skeletal\_muscle\_contraction | 1 | 0 |  |  |  |  |  |  |  |  |
| GO:0014856\_skeletal\_muscle\_cell\_proliferation | 1 | 0 |  |  |  |  |  |  |  |  |
| GO:0014857\_regulation\_of\_skeletal\_muscle\_cell\_proliferation | 1 | 0 |  |  |  |  |  |  |  |  |
| GO:0014858\_positive\_regulation\_of\_skeletal\_muscle\_cell\_proliferation | 1 | 0 |  |  |  |  |  |  |  |  |
| GO:0014887\_cardiac\_muscle\_adaptation | 1 | 0 |  |  |  |  |  |  |  |  |
| GO:0014889\_muscle\_atrophy | 1 | 0 |  |  |  |  |  |  |  |  |
| GO:0014896\_muscle\_hypertrophy | 1 | 0 |  |  |  |  |  |  |  |  |
| GO:0014897\_striated\_muscle\_hypertrophy | 1 | 0 |  |  |  |  |  |  |  |  |
| GO:0014898\_cardiac\_muscle\_hypertrophy | 1 | 0 |  |  |  |  |  |  |  |  |
| GO:0014900\_muscle\_hyperplasia | 1 | 0 |  |  |  |  |  |  |  |  |
| GO:0014910\_regulation\_of\_smooth\_muscle\_cell\_migration | 1 | 0 |  |  |  |  |  |  |  |  |
| GO:0014911\_positive\_regulation\_of\_smooth\_muscle\_cell\_migration | 1 | 0 |  |  |  |  |  |  |  |  |
| GO:0015014\_heparan\_sulfate\_proteoglycan\_biosynthetic\_process\_\_polysaccharide\_chain\_biosynthetic\_process | 1 | 0 |  |  |  |  |  |  |  |  |
| GO:0015074\_DNA\_integration | 1 | 0 |  |  |  |  |  |  |  |  |
| GO:0015670\_carbon\_dioxide\_transport | 1 | 0 |  |  |  |  |  |  |  |  |
| GO:0015677\_copper\_ion\_import | 1 | 0 |  |  |  |  |  |  |  |  |
| GO:0015680\_intracellular\_copper\_ion\_transport | 1 | 0 |  |  |  |  |  |  |  |  |
| GO:0015684\_ferrous\_iron\_transport | 1 | 0 |  |  |  |  |  |  |  |  |
| GO:0015707\_nitrite\_transport | 1 | 0 |  |  |  |  |  |  |  |  |
| GO:0015724\_formate\_transport | 1 | 0 |  |  |  |  |  |  |  |  |
| GO:0015734\_taurine\_transport | 1 | 0 |  |  |  |  |  |  |  |  |
| GO:0015740\_C4-dicarboxylate\_transport | 1 | 0 |  |  |  |  |  |  |  |  |
| GO:0015744\_succinate\_transport | 1 | 0 |  |  |  |  |  |  |  |  |
| GO:0015746\_citrate\_transport | 1 | 0 |  |  |  |  |  |  |  |  |
| GO:0015747\_urate\_transport | 1 | 0 |  |  |  |  |  |  |  |  |
| GO:0015791\_polyol\_transport | 1 | 0 |  |  |  |  |  |  |  |  |
| GO:0015798\_myo-inositol\_transport | 1 | 0 |  |  |  |  |  |  |  |  |
| GO:0015808\_L-alanine\_transport | 1 | 0 |  |  |  |  |  |  |  |  |
| GO:0015810\_aspartate\_transport | 1 | 0 |  |  |  |  |  |  |  |  |
| GO:0015811\_L-cystine\_transport | 1 | 0 |  |  |  |  |  |  |  |  |
| GO:0015817\_histidine\_transport | 1 | 0 |  |  |  |  |  |  |  |  |
| GO:0015822\_ornithine\_transport | 1 | 0 |  |  |  |  |  |  |  |  |
| GO:0015824\_proline\_transport | 1 | 0 |  |  |  |  |  |  |  |  |
| GO:0015851\_nucleobase\_transport | 1 | 0 |  |  |  |  |  |  |  |  |
| GO:0015864\_pyrimidine\_nucleoside\_transport | 1 | 0 |  |  |  |  |  |  |  |  |
| GO:0015874\_norepinephrine\_transport | 1 | 0 |  |  |  |  |  |  |  |  |
| GO:0015881\_creatine\_transport | 1 | 0 |  |  |  |  |  |  |  |  |
| GO:0015884\_folic\_acid\_transport | 1 | 0 |  |  |  |  |  |  |  |  |
| GO:0015886\_heme\_transport | 1 | 0 |  |  |  |  |  |  |  |  |
| GO:0015888\_thiamin\_transport | 1 | 0 |  |  |  |  |  |  |  |  |
| GO:0015938\_coenzyme\_A\_catabolic\_process | 1 | 0 |  |  |  |  |  |  |  |  |
| GO:0015939\_pantothenate\_metabolic\_process | 1 | 0 |  |  |  |  |  |  |  |  |
| GO:0016073\_snRNA\_metabolic\_process | 1 | 0 |  |  |  |  |  |  |  |  |
| GO:0016074\_snoRNA\_metabolic\_process | 1 | 0 |  |  |  |  |  |  |  |  |
| GO:0016082\_synaptic\_vesicle\_priming | 1 | 0 |  |  |  |  |  |  |  |  |
| GO:0016090\_prenol\_metabolic\_process | 1 | 0 |  |  |  |  |  |  |  |  |
| GO:0016093\_polyprenol\_metabolic\_process | 1 | 0 |  |  |  |  |  |  |  |  |
| GO:0016180\_snRNA\_processing | 1 | 0 |  |  |  |  |  |  |  |  |
| GO:0016239\_positive\_regulation\_of\_macroautophagy | 1 | 0 |  |  |  |  |  |  |  |  |
| GO:0016246\_RNA\_interference | 1 | 0 |  |  |  |  |  |  |  |  |
| GO:0016255\_attachment\_of\_GPI\_anchor\_to\_protein | 1 | 0 |  |  |  |  |  |  |  |  |
| GO:0016333\_morphogenesis\_of\_follicular\_epithelium | 1 | 0 |  |  |  |  |  |  |  |  |
| GO:0016340\_calcium-dependent\_cell-matrix\_adhesion | 1 | 0 |  |  |  |  |  |  |  |  |
| GO:0016344\_meiotic\_chromosome\_movement\_towards\_spindle\_pole | 1 | 0 |  |  |  |  |  |  |  |  |
| GO:0016482\_cytoplasmic\_transport | 1 | 0 |  |  |  |  |  |  |  |  |
| GO:0016553\_base\_conversion\_or\_substitution\_editing | 1 | 0 |  |  |  |  |  |  |  |  |
| GO:0016554\_cytidine\_to\_uridine\_editing | 1 | 0 |  |  |  |  |  |  |  |  |
| GO:0016560\_protein\_import\_into\_peroxisome\_matrix\_\_docking | 1 | 0 |  |  |  |  |  |  |  |  |
| GO:0016578\_histone\_deubiquitination | 1 | 0 |  |  |  |  |  |  |  |  |
| GO:0016598\_protein\_arginylation | 1 | 0 |  |  |  |  |  |  |  |  |
| GO:0017004\_cytochrome\_complex\_assembly | 1 | 0 |  |  |  |  |  |  |  |  |
| GO:0018022\_peptidyl-lysine\_methylation | 1 | 0 |  |  |  |  |  |  |  |  |
| GO:0018023\_peptidyl-lysine\_trimethylation | 1 | 0 |  |  |  |  |  |  |  |  |
| GO:0018120\_peptidyl-arginine\_ADP-ribosylation | 1 | 0 |  |  |  |  |  |  |  |  |
| GO:0018126\_protein\_amino\_acid\_hydroxylation | 1 | 0 |  |  |  |  |  |  |  |  |
| GO:0018146\_keratan\_sulfate\_biosynthetic\_process | 1 | 0 |  |  |  |  |  |  |  |  |
| GO:0018195\_peptidyl-arginine\_modification | 1 | 0 |  |  |  |  |  |  |  |  |
| GO:0018197\_peptidyl-aspartic\_acid\_modification | 1 | 0 |  |  |  |  |  |  |  |  |
| GO:0018282\_metal\_incorporation\_into\_metallo-sulfur\_cluster | 1 | 0 |  |  |  |  |  |  |  |  |
| GO:0018283\_iron\_incorporation\_into\_metallo-sulfur\_cluster | 1 | 0 |  |  |  |  |  |  |  |  |
| GO:0018318\_protein\_amino\_acid\_palmitoylation | 1 | 0 |  |  |  |  |  |  |  |  |
| GO:0018342\_protein\_prenylation | 1 | 0 |  |  |  |  |  |  |  |  |
| GO:0018344\_protein\_geranylgeranylation | 1 | 0 |  |  |  |  |  |  |  |  |
| GO:0018410\_peptide\_or\_protein\_carboxyl-terminal\_blocking | 1 | 0 |  |  |  |  |  |  |  |  |
| GO:0018916\_nitrobenzene\_metabolic\_process | 1 | 0 |  |  |  |  |  |  |  |  |
| GO:0018931\_naphthalene\_metabolic\_process | 1 | 0 |  |  |  |  |  |  |  |  |
| GO:0018992\_germ-line\_sex\_determination | 1 | 0 |  |  |  |  |  |  |  |  |
| GO:0019042\_latent\_virus\_infection | 1 | 0 |  |  |  |  |  |  |  |  |
| GO:0019046\_reactivation\_of\_latent\_virus | 1 | 0 |  |  |  |  |  |  |  |  |
| GO:0019047\_provirus\_integration | 1 | 0 |  |  |  |  |  |  |  |  |
| GO:0019076\_release\_of\_virus\_from\_host | 1 | 0 |  |  |  |  |  |  |  |  |
| GO:0019079\_viral\_genome\_replication | 1 | 0 |  |  |  |  |  |  |  |  |
| GO:0019100\_male\_germ-line\_sex\_determination | 1 | 0 |  |  |  |  |  |  |  |  |
| GO:0019101\_female\_somatic\_sex\_determination | 1 | 0 |  |  |  |  |  |  |  |  |
| GO:0019102\_male\_somatic\_sex\_determination | 1 | 0 |  |  |  |  |  |  |  |  |
| GO:0019255\_glucose\_1-phosphate\_metabolic\_process | 1 | 0 |  |  |  |  |  |  |  |  |
| GO:0019276\_UDP-N-acetylgalactosamine\_metabolic\_process | 1 | 0 |  |  |  |  |  |  |  |  |
| GO:0019344\_cysteine\_biosynthetic\_process | 1 | 0 |  |  |  |  |  |  |  |  |
| GO:0019348\_dolichol\_metabolic\_process | 1 | 0 |  |  |  |  |  |  |  |  |
| GO:0019375\_galactolipid\_biosynthetic\_process | 1 | 0 |  |  |  |  |  |  |  |  |
| GO:0019402\_galactitol\_metabolic\_process | 1 | 0 |  |  |  |  |  |  |  |  |
| GO:0019441\_tryptophan\_catabolic\_process\_to\_kynurenine | 1 | 0 |  |  |  |  |  |  |  |  |
| GO:0019477\_L-lysine\_catabolic\_process | 1 | 0 |  |  |  |  |  |  |  |  |
| GO:0019510\_S-adenosylhomocysteine\_catabolic\_process | 1 | 0 |  |  |  |  |  |  |  |  |
| GO:0019532\_oxalate\_transport | 1 | 0 |  |  |  |  |  |  |  |  |
| GO:0019626\_short-chain\_fatty\_acid\_catabolic\_process | 1 | 0 |  |  |  |  |  |  |  |  |
| GO:0019627\_urea\_metabolic\_process | 1 | 0 |  |  |  |  |  |  |  |  |
| GO:0019676\_ammonia\_assimilation\_cycle | 1 | 0 |  |  |  |  |  |  |  |  |
| GO:0019695\_choline\_metabolic\_process | 1 | 0 |  |  |  |  |  |  |  |  |
| GO:0019731\_antibacterial\_humoral\_response | 1 | 0 |  |  |  |  |  |  |  |  |
| GO:0019794\_nonprotein\_amino\_acid\_metabolic\_process | 1 | 0 |  |  |  |  |  |  |  |  |
| GO:0019858\_cytosine\_metabolic\_process | 1 | 0 |  |  |  |  |  |  |  |  |
| GO:0019883\_antigen\_processing\_and\_presentation\_of\_endogenous\_antigen | 1 | 0 |  |  |  |  |  |  |  |  |
| GO:0019889\_pteridine\_metabolic\_process | 1 | 0 |  |  |  |  |  |  |  |  |
| GO:0019896\_axon\_transport\_of\_mitochondrion | 1 | 0 |  |  |  |  |  |  |  |  |
| GO:0021508\_floor\_plate\_formation | 1 | 0 |  |  |  |  |  |  |  |  |
| GO:0021528\_commissural\_neuron\_differentiation\_in\_the\_spinal\_cord | 1 | 0 |  |  |  |  |  |  |  |  |
| GO:0021572\_rhombomere\_6\_development | 1 | 0 |  |  |  |  |  |  |  |  |
| GO:0021577\_hindbrain\_structural\_organization | 1 | 0 |  |  |  |  |  |  |  |  |
| GO:0021586\_pons\_maturation | 1 | 0 |  |  |  |  |  |  |  |  |
| GO:0021589\_cerebellum\_structural\_organization | 1 | 0 |  |  |  |  |  |  |  |  |
| GO:0021590\_cerebellum\_maturation | 1 | 0 |  |  |  |  |  |  |  |  |
| GO:0021592\_fourth\_ventricle\_development | 1 | 0 |  |  |  |  |  |  |  |  |
| GO:0021594\_rhombomere\_formation | 1 | 0 |  |  |  |  |  |  |  |  |
| GO:0021660\_rhombomere\_3\_formation | 1 | 0 |  |  |  |  |  |  |  |  |
| GO:0021664\_rhombomere\_5\_morphogenesis | 1 | 0 |  |  |  |  |  |  |  |  |
| GO:0021666\_rhombomere\_5\_formation | 1 | 0 |  |  |  |  |  |  |  |  |
| GO:0021670\_lateral\_ventricle\_development | 1 | 0 |  |  |  |  |  |  |  |  |
| GO:0021678\_third\_ventricle\_development | 1 | 0 |  |  |  |  |  |  |  |  |
| GO:0021679\_cerebellar\_molecular\_layer\_development | 1 | 0 |  |  |  |  |  |  |  |  |
| GO:0021703\_locus\_ceruleus\_development | 1 | 0 |  |  |  |  |  |  |  |  |
| GO:0021732\_midbrain-hindbrain\_boundary\_maturation | 1 | 0 |  |  |  |  |  |  |  |  |
| GO:0021747\_cochlear\_nucleus\_development | 1 | 0 |  |  |  |  |  |  |  |  |
| GO:0021750\_vestibular\_nucleus\_development | 1 | 0 |  |  |  |  |  |  |  |  |
| GO:0021759\_globus\_pallidus\_development | 1 | 0 |  |  |  |  |  |  |  |  |
| GO:0021768\_nucleus\_accumbens\_development | 1 | 0 |  |  |  |  |  |  |  |  |
| GO:0021771\_lateral\_geniculate\_nucleus\_development | 1 | 0 |  |  |  |  |  |  |  |  |
| GO:0021812\_neuronal-glial\_interaction\_involved\_in\_cerebral\_cortex\_radial\_glia\_guided\_migration | 1 | 0 |  |  |  |  |  |  |  |  |
| GO:0021813\_cell-cell\_adhesion\_involved\_in\_neuronal-glial\_interactions\_involved\_in\_cerebral\_cortex\_radial\_glia\_guided\_migration | 1 | 0 |  |  |  |  |  |  |  |  |
| GO:0021870\_Cajal-Retzius\_cell\_differentiation | 1 | 0 |  |  |  |  |  |  |  |  |
| GO:0021874\_Wnt\_receptor\_signaling\_pathway\_in\_forebrain\_neuroblast\_division | 1 | 0 |  |  |  |  |  |  |  |  |
| GO:0021896\_forebrain\_astrocyte\_differentiation | 1 | 0 |  |  |  |  |  |  |  |  |
| GO:0021897\_forebrain\_astrocyte\_development | 1 | 0 |  |  |  |  |  |  |  |  |
| GO:0021902\_commitment\_of\_a\_neuronal\_cell\_to\_a\_specific\_type\_of\_neuron\_in\_the\_forebrain | 1 | 0 |  |  |  |  |  |  |  |  |
| GO:0021905\_forebrain-midbrain\_boundary\_formation | 1 | 0 |  |  |  |  |  |  |  |  |
| GO:0021914\_negative\_regulation\_of\_smoothened\_signaling\_pathway\_involved\_in\_ventral\_spinal\_cord\_patterning | 1 | 0 |  |  |  |  |  |  |  |  |
| GO:0021917\_somatic\_motor\_neuron\_fate\_commitment | 1 | 0 |  |  |  |  |  |  |  |  |
| GO:0021918\_regulation\_of\_transcription\_from\_RNA\_polymerase\_II\_promoter\_involved\_in\_somatic\_motor\_neuron\_fate\_commitment | 1 | 0 |  |  |  |  |  |  |  |  |
| GO:0021933\_radial\_glia\_guided\_migration\_of\_granule\_cell | 1 | 0 |  |  |  |  |  |  |  |  |
| GO:0021934\_hindbrain\_tangential\_cell\_migration | 1 | 0 |  |  |  |  |  |  |  |  |
| GO:0021935\_granule\_cell\_precursor\_tangential\_migration | 1 | 0 |  |  |  |  |  |  |  |  |
| GO:0021942\_radial\_glia\_guided\_migration\_of\_Purkinje\_cell | 1 | 0 |  |  |  |  |  |  |  |  |
| GO:0021960\_anterior\_commissure\_morphogenesis | 1 | 0 |  |  |  |  |  |  |  |  |
| GO:0021997\_neural\_plate\_axis\_specification | 1 | 0 |  |  |  |  |  |  |  |  |
| GO:0021999\_neural\_plate\_anterior\_posterior\_pattern\_formation | 1 | 0 |  |  |  |  |  |  |  |  |
| GO:0022004\_midbrain-hindbrain\_boundary\_maturation\_during\_brain\_development | 1 | 0 |  |  |  |  |  |  |  |  |
| GO:0022038\_corpus\_callosum\_development | 1 | 0 |  |  |  |  |  |  |  |  |
| GO:0022605\_oogenesis\_stage | 1 | 0 |  |  |  |  |  |  |  |  |
| GO:0030011\_maintenance\_of\_cell\_polarity | 1 | 0 |  |  |  |  |  |  |  |  |
| GO:0030069\_lysogeny | 1 | 0 |  |  |  |  |  |  |  |  |
| GO:0030070\_insulin\_processing | 1 | 0 |  |  |  |  |  |  |  |  |
| GO:0030092\_regulation\_of\_flagellum\_assembly | 1 | 0 |  |  |  |  |  |  |  |  |
| GO:0030103\_vasopressin\_secretion | 1 | 0 |  |  |  |  |  |  |  |  |
| GO:0030194\_positive\_regulation\_of\_blood\_coagulation | 1 | 0 |  |  |  |  |  |  |  |  |
| GO:0030206\_chondroitin\_sulfate\_biosynthetic\_process | 1 | 0 |  |  |  |  |  |  |  |  |
| GO:0030210\_heparin\_biosynthetic\_process | 1 | 0 |  |  |  |  |  |  |  |  |
| GO:0030220\_platelet\_formation | 1 | 0 |  |  |  |  |  |  |  |  |
| GO:0030222\_eosinophil\_differentiation | 1 | 0 |  |  |  |  |  |  |  |  |
| GO:0030237\_female\_sex\_determination | 1 | 0 |  |  |  |  |  |  |  |  |
| GO:0030264\_nuclear\_fragmentation\_during\_apoptosis | 1 | 0 |  |  |  |  |  |  |  |  |
| GO:0030322\_stabilization\_of\_membrane\_potential | 1 | 0 |  |  |  |  |  |  |  |  |
| GO:0030327\_prenylated\_protein\_catabolic\_process | 1 | 0 |  |  |  |  |  |  |  |  |
| GO:0030328\_prenylcysteine\_catabolic\_process | 1 | 0 |  |  |  |  |  |  |  |  |
| GO:0030329\_prenylcysteine\_metabolic\_process | 1 | 0 |  |  |  |  |  |  |  |  |
| GO:0030382\_sperm\_mitochondrion\_organization | 1 | 0 |  |  |  |  |  |  |  |  |
| GO:0030389\_fructosamine\_metabolic\_process | 1 | 0 |  |  |  |  |  |  |  |  |
| GO:0030422\_RNA\_interference\_\_production\_of\_siRNA | 1 | 0 |  |  |  |  |  |  |  |  |
| GO:0030449\_regulation\_of\_complement\_activation | 1 | 0 |  |  |  |  |  |  |  |  |
| GO:0030497\_fatty\_acid\_elongation | 1 | 0 |  |  |  |  |  |  |  |  |
| GO:0030575\_nuclear\_body\_organization | 1 | 0 |  |  |  |  |  |  |  |  |
| GO:0030578\_PML\_body\_organization | 1 | 0 |  |  |  |  |  |  |  |  |
| GO:0030853\_negative\_regulation\_of\_granulocyte\_differentiation | 1 | 0 |  |  |  |  |  |  |  |  |
| GO:0030854\_positive\_regulation\_of\_granulocyte\_differentiation | 1 | 0 |  |  |  |  |  |  |  |  |
| GO:0030886\_negative\_regulation\_of\_myeloid\_dendritic\_cell\_activation | 1 | 0 |  |  |  |  |  |  |  |  |
| GO:0030913\_paranodal\_junction\_assembly | 1 | 0 |  |  |  |  |  |  |  |  |
| GO:0031033\_myosin\_filament\_assembly\_or\_disassembly | 1 | 0 |  |  |  |  |  |  |  |  |
| GO:0031034\_myosin\_filament\_assembly | 1 | 0 |  |  |  |  |  |  |  |  |
| GO:0031055\_chromatin\_remodeling\_at\_centromere | 1 | 0 |  |  |  |  |  |  |  |  |
| GO:0031062\_positive\_regulation\_of\_histone\_methylation | 1 | 0 |  |  |  |  |  |  |  |  |
| GO:0031115\_negative\_regulation\_of\_microtubule\_polymerization | 1 | 0 |  |  |  |  |  |  |  |  |
| GO:0031129\_inductive\_cell-cell\_signaling | 1 | 0 |  |  |  |  |  |  |  |  |
| GO:0031284\_positive\_regulation\_of\_guanylate\_cyclase\_activity | 1 | 0 |  |  |  |  |  |  |  |  |
| GO:0031498\_chromatin\_disassembly | 1 | 0 |  |  |  |  |  |  |  |  |
| GO:0031507\_heterochromatin\_formation | 1 | 0 |  |  |  |  |  |  |  |  |
| GO:0031508\_centromeric\_heterochromatin\_formation | 1 | 0 |  |  |  |  |  |  |  |  |
| GO:0031529\_ruffle\_organization | 1 | 0 |  |  |  |  |  |  |  |  |
| GO:0031536\_positive\_regulation\_of\_exit\_from\_mitosis | 1 | 0 |  |  |  |  |  |  |  |  |
| GO:0031572\_G2\_M\_transition\_DNA\_damage\_checkpoint | 1 | 0 |  |  |  |  |  |  |  |  |
| GO:0031576\_G2\_M\_transition\_checkpoint | 1 | 0 |  |  |  |  |  |  |  |  |
| GO:0031580\_membrane\_raft\_distribution | 1 | 0 |  |  |  |  |  |  |  |  |
| GO:0031583\_activation\_of\_phospholipase\_D\_activity\_by\_G-protein\_coupled\_receptor\_protein\_signaling\_pathway | 1 | 0 |  |  |  |  |  |  |  |  |
| GO:0031584\_activation\_of\_phospholipase\_D\_activity | 1 | 0 |  |  |  |  |  |  |  |  |
| GO:0031585\_regulation\_of\_inositol-1\_4\_5-triphosphate\_receptor\_activity | 1 | 0 |  |  |  |  |  |  |  |  |
| GO:0031639\_plasminogen\_activation | 1 | 0 |  |  |  |  |  |  |  |  |
| GO:0031648\_protein\_destabilization | 1 | 0 |  |  |  |  |  |  |  |  |
| GO:0031665\_negative\_regulation\_of\_lipopolysaccharide-mediated\_signaling\_pathway | 1 | 0 |  |  |  |  |  |  |  |  |
| GO:0031914\_negative\_regulation\_of\_synaptic\_plasticity | 1 | 0 |  |  |  |  |  |  |  |  |
| GO:0031944\_negative\_regulation\_of\_glucocorticoid\_metabolic\_process | 1 | 0 |  |  |  |  |  |  |  |  |
| GO:0031947\_negative\_regulation\_of\_glucocorticoid\_biosynthetic\_process | 1 | 0 |  |  |  |  |  |  |  |  |
| GO:0032025\_response\_to\_cobalt\_ion | 1 | 0 |  |  |  |  |  |  |  |  |
| GO:0032026\_response\_to\_magnesium\_ion | 1 | 0 |  |  |  |  |  |  |  |  |
| GO:0032048\_cardiolipin\_metabolic\_process | 1 | 0 |  |  |  |  |  |  |  |  |
| GO:0032066\_nucleolus\_to\_nucleoplasm\_transport | 1 | 0 |  |  |  |  |  |  |  |  |
| GO:0032091\_negative\_regulation\_of\_protein\_binding | 1 | 0 |  |  |  |  |  |  |  |  |
| GO:0032092\_positive\_regulation\_of\_protein\_binding | 1 | 0 |  |  |  |  |  |  |  |  |
| GO:0032097\_positive\_regulation\_of\_response\_to\_food | 1 | 0 |  |  |  |  |  |  |  |  |
| GO:0032100\_positive\_regulation\_of\_appetite | 1 | 0 |  |  |  |  |  |  |  |  |
| GO:0032204\_regulation\_of\_telomere\_maintenance | 1 | 0 |  |  |  |  |  |  |  |  |
| GO:0032206\_positive\_regulation\_of\_telomere\_maintenance | 1 | 0 |  |  |  |  |  |  |  |  |
| GO:0032222\_regulation\_of\_synaptic\_transmission\_\_cholinergic | 1 | 0 |  |  |  |  |  |  |  |  |
| GO:0032224\_positive\_regulation\_of\_synaptic\_transmission\_\_cholinergic | 1 | 0 |  |  |  |  |  |  |  |  |
| GO:0032229\_negative\_regulation\_of\_synaptic\_transmission\_\_GABAergic | 1 | 0 |  |  |  |  |  |  |  |  |
| GO:0032237\_activation\_of\_store-operated\_calcium\_channel\_activity | 1 | 0 |  |  |  |  |  |  |  |  |
| GO:0032239\_regulation\_of\_nucleobase\_\_nucleoside\_\_nucleotide\_and\_nucleic\_acid\_transport | 1 | 0 |  |  |  |  |  |  |  |  |
| GO:0032252\_secretory\_granule\_localization | 1 | 0 |  |  |  |  |  |  |  |  |
| GO:0032274\_gonadotropin\_secretion | 1 | 0 |  |  |  |  |  |  |  |  |
| GO:0032275\_luteinizing\_hormone\_secretion | 1 | 0 |  |  |  |  |  |  |  |  |
| GO:0032287\_myelin\_maintenance\_in\_the\_peripheral\_nervous\_system | 1 | 0 |  |  |  |  |  |  |  |  |
| GO:0032289\_myelin\_formation\_in\_the\_central\_nervous\_system | 1 | 0 |  |  |  |  |  |  |  |  |
| GO:0032303\_regulation\_of\_icosanoid\_secretion | 1 | 0 |  |  |  |  |  |  |  |  |
| GO:0032305\_positive\_regulation\_of\_icosanoid\_secretion | 1 | 0 |  |  |  |  |  |  |  |  |
| GO:0032306\_regulation\_of\_prostaglandin\_secretion | 1 | 0 |  |  |  |  |  |  |  |  |
| GO:0032308\_positive\_regulation\_of\_prostaglandin\_secretion | 1 | 0 |  |  |  |  |  |  |  |  |
| GO:0032310\_prostaglandin\_secretion | 1 | 0 |  |  |  |  |  |  |  |  |
| GO:0032313\_regulation\_of\_Rab\_GTPase\_activity | 1 | 0 |  |  |  |  |  |  |  |  |
| GO:0032314\_regulation\_of\_Rac\_GTPase\_activity | 1 | 0 |  |  |  |  |  |  |  |  |
| GO:0032317\_regulation\_of\_Rap\_GTPase\_activity | 1 | 0 |  |  |  |  |  |  |  |  |
| GO:0032324\_molybdopterin\_cofactor\_biosynthetic\_process | 1 | 0 |  |  |  |  |  |  |  |  |
| GO:0032329\_serine\_transport | 1 | 0 |  |  |  |  |  |  |  |  |
| GO:0032342\_aldosterone\_biosynthetic\_process | 1 | 0 |  |  |  |  |  |  |  |  |
| GO:0032344\_regulation\_of\_aldosterone\_metabolic\_process | 1 | 0 |  |  |  |  |  |  |  |  |
| GO:0032370\_positive\_regulation\_of\_lipid\_transport | 1 | 0 |  |  |  |  |  |  |  |  |
| GO:0032410\_negative\_regulation\_of\_transporter\_activity | 1 | 0 |  |  |  |  |  |  |  |  |
| GO:0032413\_negative\_regulation\_of\_ion\_transmembrane\_transporter\_activity | 1 | 0 |  |  |  |  |  |  |  |  |
| GO:0032429\_regulation\_of\_phospholipase\_A2\_activity | 1 | 0 |  |  |  |  |  |  |  |  |
| GO:0032474\_otolith\_morphogenesis | 1 | 0 |  |  |  |  |  |  |  |  |
| GO:0032482\_Rab\_protein\_signal\_transduction | 1 | 0 |  |  |  |  |  |  |  |  |
| GO:0032483\_regulation\_of\_Rab\_protein\_signal\_transduction | 1 | 0 |  |  |  |  |  |  |  |  |
| GO:0032486\_Rap\_protein\_signal\_transduction | 1 | 0 |  |  |  |  |  |  |  |  |
| GO:0032487\_regulation\_of\_Rap\_protein\_signal\_transduction | 1 | 0 |  |  |  |  |  |  |  |  |
| GO:0032594\_protein\_transport\_within\_lipid\_bilayer | 1 | 0 |  |  |  |  |  |  |  |  |
| GO:0032599\_protein\_transport\_out\_of\_membrane\_raft | 1 | 0 |  |  |  |  |  |  |  |  |
| GO:0032600\_chemokine\_receptor\_transport\_out\_of\_membrane\_raft | 1 | 0 |  |  |  |  |  |  |  |  |
| GO:0032607\_interferon-alpha\_production | 1 | 0 |  |  |  |  |  |  |  |  |
| GO:0032621\_interleukin-18\_production | 1 | 0 |  |  |  |  |  |  |  |  |
| GO:0032647\_regulation\_of\_interferon-alpha\_production | 1 | 0 |  |  |  |  |  |  |  |  |
| GO:0032656\_regulation\_of\_interleukin-13\_production | 1 | 0 |  |  |  |  |  |  |  |  |
| GO:0032682\_negative\_regulation\_of\_chemokine\_production | 1 | 0 |  |  |  |  |  |  |  |  |
| GO:0032691\_negative\_regulation\_of\_interleukin-1\_beta\_production | 1 | 0 |  |  |  |  |  |  |  |  |
| GO:0032692\_negative\_regulation\_of\_interleukin-1\_production | 1 | 0 |  |  |  |  |  |  |  |  |
| GO:0032693\_negative\_regulation\_of\_interleukin-10\_production | 1 | 0 |  |  |  |  |  |  |  |  |
| GO:0032696\_negative\_regulation\_of\_interleukin-13\_production | 1 | 0 |  |  |  |  |  |  |  |  |
| GO:0032727\_positive\_regulation\_of\_interferon-alpha\_production | 1 | 0 |  |  |  |  |  |  |  |  |
| GO:0032731\_positive\_regulation\_of\_interleukin-1\_beta\_production | 1 | 0 |  |  |  |  |  |  |  |  |
| GO:0032732\_positive\_regulation\_of\_interleukin-1\_production | 1 | 0 |  |  |  |  |  |  |  |  |
| GO:0032735\_positive\_regulation\_of\_interleukin-12\_production | 1 | 0 |  |  |  |  |  |  |  |  |
| GO:0032764\_negative\_regulation\_of\_mast\_cell\_cytokine\_production | 1 | 0 |  |  |  |  |  |  |  |  |
| GO:0032765\_positive\_regulation\_of\_mast\_cell\_cytokine\_production | 1 | 0 |  |  |  |  |  |  |  |  |
| GO:0032769\_negative\_regulation\_of\_monooxygenase\_activity | 1 | 0 |  |  |  |  |  |  |  |  |
| GO:0032781\_positive\_regulation\_of\_ATPase\_activity | 1 | 0 |  |  |  |  |  |  |  |  |
| GO:0032790\_ribosome\_disassembly | 1 | 0 |  |  |  |  |  |  |  |  |
| GO:0032799\_low-density\_lipoprotein\_receptor\_metabolic\_process | 1 | 0 |  |  |  |  |  |  |  |  |
| GO:0032802\_low-density\_lipoprotein\_receptor\_catabolic\_process | 1 | 0 |  |  |  |  |  |  |  |  |
| GO:0032803\_regulation\_of\_low-density\_lipoprotein\_receptor\_catabolic\_process | 1 | 0 |  |  |  |  |  |  |  |  |
| GO:0032817\_regulation\_of\_natural\_killer\_cell\_proliferation | 1 | 0 |  |  |  |  |  |  |  |  |
| GO:0032819\_positive\_regulation\_of\_natural\_killer\_cell\_proliferation | 1 | 0 |  |  |  |  |  |  |  |  |
| GO:0032836\_glomerular\_basement\_membrane\_development | 1 | 0 |  |  |  |  |  |  |  |  |
| GO:0032855\_positive\_regulation\_of\_Rac\_GTPase\_activity | 1 | 0 |  |  |  |  |  |  |  |  |
| GO:0032863\_activation\_of\_Rac\_GTPase\_activity | 1 | 0 |  |  |  |  |  |  |  |  |
| GO:0032864\_activation\_of\_Cdc42\_GTPase\_activity | 1 | 0 |  |  |  |  |  |  |  |  |
| GO:0032885\_regulation\_of\_polysaccharide\_biosynthetic\_process | 1 | 0 |  |  |  |  |  |  |  |  |
| GO:0032907\_transforming\_growth\_factor-beta3\_production | 1 | 0 |  |  |  |  |  |  |  |  |
| GO:0032910\_regulation\_of\_transforming\_growth\_factor-beta3\_production | 1 | 0 |  |  |  |  |  |  |  |  |
| GO:0032913\_negative\_regulation\_of\_transforming\_growth\_factor-beta3\_production | 1 | 0 |  |  |  |  |  |  |  |  |
| GO:0032924\_activin\_receptor\_signaling\_pathway | 1 | 0 |  |  |  |  |  |  |  |  |
| GO:0032925\_regulation\_of\_activin\_receptor\_signaling\_pathway | 1 | 0 |  |  |  |  |  |  |  |  |
| GO:0032960\_regulation\_of\_inositol\_trisphosphate\_biosynthetic\_process | 1 | 0 |  |  |  |  |  |  |  |  |
| GO:0032962\_positive\_regulation\_of\_inositol\_trisphosphate\_biosynthetic\_process | 1 | 0 |  |  |  |  |  |  |  |  |
| GO:0032964\_collagen\_biosynthetic\_process | 1 | 0 |  |  |  |  |  |  |  |  |
| GO:0032971\_regulation\_of\_muscle\_filament\_sliding | 1 | 0 |  |  |  |  |  |  |  |  |
| GO:0032972\_regulation\_of\_muscle\_filament\_sliding\_speed | 1 | 0 |  |  |  |  |  |  |  |  |
| GO:0032986\_protein-DNA\_complex\_disassembly | 1 | 0 |  |  |  |  |  |  |  |  |
| GO:0032988\_ribonucleoprotein\_complex\_disassembly | 1 | 0 |  |  |  |  |  |  |  |  |
| GO:0033037\_polysaccharide\_localization | 1 | 0 |  |  |  |  |  |  |  |  |
| GO:0033078\_extrathymic\_T\_cell\_differentiation | 1 | 0 |  |  |  |  |  |  |  |  |
| GO:0033085\_negative\_regulation\_of\_T\_cell\_differentiation\_in\_the\_thymus | 1 | 0 |  |  |  |  |  |  |  |  |
| GO:0033087\_negative\_regulation\_of\_immature\_T\_cell\_proliferation | 1 | 0 |  |  |  |  |  |  |  |  |
| GO:0033088\_negative\_regulation\_of\_immature\_T\_cell\_proliferation\_in\_the\_thymus | 1 | 0 |  |  |  |  |  |  |  |  |
| GO:0033108\_mitochondrial\_respiratory\_chain\_complex\_assembly | 1 | 0 |  |  |  |  |  |  |  |  |
| GO:0033127\_regulation\_of\_histone\_phosphorylation | 1 | 0 |  |  |  |  |  |  |  |  |
| GO:0033128\_negative\_regulation\_of\_histone\_phosphorylation | 1 | 0 |  |  |  |  |  |  |  |  |
| GO:0033138\_positive\_regulation\_of\_peptidyl-serine\_phosphorylation | 1 | 0 |  |  |  |  |  |  |  |  |
| GO:0033158\_regulation\_of\_protein\_import\_into\_nucleus\_\_translocation | 1 | 0 |  |  |  |  |  |  |  |  |
| GO:0033160\_positive\_regulation\_of\_protein\_import\_into\_nucleus\_\_translocation | 1 | 0 |  |  |  |  |  |  |  |  |
| GO:0033169\_histone\_H3-K9\_demethylation | 1 | 0 |  |  |  |  |  |  |  |  |
| GO:0033206\_cytokinesis\_after\_meiosis | 1 | 0 |  |  |  |  |  |  |  |  |
| GO:0033240\_positive\_regulation\_of\_cellular\_amine\_metabolic\_process | 1 | 0 |  |  |  |  |  |  |  |  |
| GO:0033313\_meiotic\_cell\_cycle\_checkpoint | 1 | 0 |  |  |  |  |  |  |  |  |
| GO:0033315\_meiotic\_cell\_cycle\_DNA\_replication\_checkpoint | 1 | 0 |  |  |  |  |  |  |  |  |
| GO:0033326\_cerebrospinal\_fluid\_secretion | 1 | 0 |  |  |  |  |  |  |  |  |
| GO:0033366\_protein\_localization\_in\_secretory\_granule | 1 | 0 |  |  |  |  |  |  |  |  |
| GO:0033367\_protein\_localization\_in\_mast\_cell\_secretory\_granule | 1 | 0 |  |  |  |  |  |  |  |  |
| GO:0033368\_protease\_localization\_in\_mast\_cell\_secretory\_granule | 1 | 0 |  |  |  |  |  |  |  |  |
| GO:0033370\_maintenance\_of\_protein\_location\_in\_mast\_cell\_secretory\_granule | 1 | 0 |  |  |  |  |  |  |  |  |
| GO:0033371\_T\_cell\_secretory\_granule\_organization | 1 | 0 |  |  |  |  |  |  |  |  |
| GO:0033373\_maintenance\_of\_protease\_location\_in\_mast\_cell\_secretory\_granule | 1 | 0 |  |  |  |  |  |  |  |  |
| GO:0033374\_protein\_localization\_in\_T\_cell\_secretory\_granule | 1 | 0 |  |  |  |  |  |  |  |  |
| GO:0033375\_protease\_localization\_in\_T\_cell\_secretory\_granule | 1 | 0 |  |  |  |  |  |  |  |  |
| GO:0033377\_maintenance\_of\_protein\_location\_in\_T\_cell\_secretory\_granule | 1 | 0 |  |  |  |  |  |  |  |  |
| GO:0033379\_maintenance\_of\_protease\_location\_in\_T\_cell\_secretory\_granule | 1 | 0 |  |  |  |  |  |  |  |  |
| GO:0033380\_granzyme\_B\_localization\_in\_T\_cell\_secretory\_granule | 1 | 0 |  |  |  |  |  |  |  |  |
| GO:0033382\_maintenance\_of\_granzyme\_B\_location\_in\_T\_cell\_secretory\_granule | 1 | 0 |  |  |  |  |  |  |  |  |
| GO:0033483\_gas\_homeostasis | 1 | 0 |  |  |  |  |  |  |  |  |
| GO:0033484\_nitric\_oxide\_homeostasis | 1 | 0 |  |  |  |  |  |  |  |  |
| GO:0033505\_floor\_plate\_morphogenesis | 1 | 0 |  |  |  |  |  |  |  |  |
| GO:0033522\_histone\_H2A\_ubiquitination | 1 | 0 |  |  |  |  |  |  |  |  |
| GO:0033523\_histone\_H2B\_ubiquitination | 1 | 0 |  |  |  |  |  |  |  |  |
| GO:0033574\_response\_to\_testosterone\_stimulus | 1 | 0 |  |  |  |  |  |  |  |  |
| GO:0033606\_chemokine\_receptor\_transport\_within\_lipid\_bilayer | 1 | 0 |  |  |  |  |  |  |  |  |
| GO:0033628\_regulation\_of\_cell\_adhesion\_mediated\_by\_integrin | 1 | 0 |  |  |  |  |  |  |  |  |
| GO:0033630\_positive\_regulation\_of\_cell\_adhesion\_mediated\_by\_integrin | 1 | 0 |  |  |  |  |  |  |  |  |
| GO:0033632\_regulation\_of\_cell-cell\_adhesion\_mediated\_by\_integrin | 1 | 0 |  |  |  |  |  |  |  |  |
| GO:0033634\_positive\_regulation\_of\_cell-cell\_adhesion\_mediated\_by\_integrin | 1 | 0 |  |  |  |  |  |  |  |  |
| GO:0033683\_nucleotide-excision\_repair\_\_DNA\_incision | 1 | 0 |  |  |  |  |  |  |  |  |
| GO:0033687\_osteoblast\_proliferation | 1 | 0 |  |  |  |  |  |  |  |  |
| GO:0033688\_regulation\_of\_osteoblast\_proliferation | 1 | 0 |  |  |  |  |  |  |  |  |
| GO:0033689\_negative\_regulation\_of\_osteoblast\_proliferation | 1 | 0 |  |  |  |  |  |  |  |  |
| GO:0033750\_ribosome\_localization | 1 | 0 |  |  |  |  |  |  |  |  |
| GO:0033753\_establishment\_of\_ribosome\_localization | 1 | 0 |  |  |  |  |  |  |  |  |
| GO:0033866\_nucleoside\_bisphosphate\_biosynthetic\_process | 1 | 0 |  |  |  |  |  |  |  |  |
| GO:0033875\_ribonucleoside\_bisphosphate\_metabolic\_process | 1 | 0 |  |  |  |  |  |  |  |  |
| GO:0034030\_ribonucleoside\_bisphosphate\_biosynthetic\_process | 1 | 0 |  |  |  |  |  |  |  |  |
| GO:0034032\_purine\_nucleoside\_bisphosphate\_metabolic\_process | 1 | 0 |  |  |  |  |  |  |  |  |
| GO:0034033\_purine\_nucleoside\_bisphosphate\_biosynthetic\_process | 1 | 0 |  |  |  |  |  |  |  |  |
| GO:0034035\_purine\_ribonucleoside\_bisphosphate\_metabolic\_process | 1 | 0 |  |  |  |  |  |  |  |  |
| GO:0034036\_purine\_ribonucleoside\_bisphosphate\_biosynthetic\_process | 1 | 0 |  |  |  |  |  |  |  |  |
| GO:0034067\_protein\_localization\_in\_Golgi\_apparatus | 1 | 0 |  |  |  |  |  |  |  |  |
| GO:0034102\_erythrocyte\_clearance | 1 | 0 |  |  |  |  |  |  |  |  |
| GO:0034106\_regulation\_of\_erythrocyte\_clearance | 1 | 0 |  |  |  |  |  |  |  |  |
| GO:0034107\_negative\_regulation\_of\_erythrocyte\_clearance | 1 | 0 |  |  |  |  |  |  |  |  |
| GO:0034110\_regulation\_of\_homotypic\_cell-cell\_adhesion | 1 | 0 |  |  |  |  |  |  |  |  |
| GO:0034111\_negative\_regulation\_of\_homotypic\_cell-cell\_adhesion | 1 | 0 |  |  |  |  |  |  |  |  |
| GO:0034113\_heterotypic\_cell-cell\_adhesion | 1 | 0 |  |  |  |  |  |  |  |  |
| GO:0034117\_erythrocyte\_aggregation | 1 | 0 |  |  |  |  |  |  |  |  |
| GO:0034118\_regulation\_of\_erythrocyte\_aggregation | 1 | 0 |  |  |  |  |  |  |  |  |
| GO:0034119\_negative\_regulation\_of\_erythrocyte\_aggregation | 1 | 0 |  |  |  |  |  |  |  |  |
| GO:0034121\_regulation\_of\_toll-like\_receptor\_signaling\_pathway | 1 | 0 |  |  |  |  |  |  |  |  |
| GO:0034122\_negative\_regulation\_of\_toll-like\_receptor\_signaling\_pathway | 1 | 0 |  |  |  |  |  |  |  |  |
| GO:0034230\_enkephalin\_processing | 1 | 0 |  |  |  |  |  |  |  |  |
| GO:0034372\_very-low-density\_lipoprotein\_particle\_remodeling | 1 | 0 |  |  |  |  |  |  |  |  |
| GO:0034379\_very-low-density\_lipoprotein\_particle\_assembly | 1 | 0 |  |  |  |  |  |  |  |  |
| GO:0034380\_high-density\_lipoprotein\_particle\_assembly | 1 | 0 |  |  |  |  |  |  |  |  |
| GO:0034394\_protein\_localization\_at\_cell\_surface | 1 | 0 |  |  |  |  |  |  |  |  |
| GO:0034405\_response\_to\_fluid\_shear\_stress | 1 | 0 |  |  |  |  |  |  |  |  |
| GO:0034472\_snRNA\_3'-end\_processing | 1 | 0 |  |  |  |  |  |  |  |  |
| GO:0034474\_U2\_snRNA\_3'-end\_processing | 1 | 0 |  |  |  |  |  |  |  |  |
| GO:0034502\_protein\_localization\_to\_chromosome | 1 | 0 |  |  |  |  |  |  |  |  |
| GO:0034505\_tooth\_mineralization | 1 | 0 |  |  |  |  |  |  |  |  |
| GO:0034508\_centromere\_complex\_assembly | 1 | 0 |  |  |  |  |  |  |  |  |
| GO:0034633\_retinol\_transport | 1 | 0 |  |  |  |  |  |  |  |  |
| GO:0034643\_mitochondrion\_localization\_\_microtubule-mediated | 1 | 0 |  |  |  |  |  |  |  |  |
| GO:0034969\_histone\_arginine\_methylation | 1 | 0 |  |  |  |  |  |  |  |  |
| GO:0034982\_mitochondrial\_protein\_processing | 1 | 0 |  |  |  |  |  |  |  |  |
| GO:0035022\_positive\_regulation\_of\_Rac\_protein\_signal\_transduction | 1 | 0 |  |  |  |  |  |  |  |  |
| GO:0035024\_negative\_regulation\_of\_Rho\_protein\_signal\_transduction | 1 | 0 |  |  |  |  |  |  |  |  |
| GO:0035026\_leading\_edge\_cell\_differentiation | 1 | 0 |  |  |  |  |  |  |  |  |
| GO:0035037\_sperm\_entry | 1 | 0 |  |  |  |  |  |  |  |  |
| GO:0035039\_male\_pronucleus\_formation | 1 | 0 |  |  |  |  |  |  |  |  |
| GO:0035066\_positive\_regulation\_of\_histone\_acetylation | 1 | 0 |  |  |  |  |  |  |  |  |
| GO:0035083\_cilium\_axoneme\_assembly | 1 | 0 |  |  |  |  |  |  |  |  |
| GO:0035090\_maintenance\_of\_apical\_basal\_cell\_polarity | 1 | 0 |  |  |  |  |  |  |  |  |
| GO:0035106\_operant\_conditioning | 1 | 0 |  |  |  |  |  |  |  |  |
| GO:0035172\_hemocyte\_proliferation | 1 | 0 |  |  |  |  |  |  |  |  |
| GO:0035227\_regulation\_of\_glutamate-cysteine\_ligase\_activity | 1 | 0 |  |  |  |  |  |  |  |  |
| GO:0035229\_positive\_regulation\_of\_glutamate-cysteine\_ligase\_activity | 1 | 0 |  |  |  |  |  |  |  |  |
| GO:0035260\_internal\_genitalia\_morphogenesis | 1 | 0 |  |  |  |  |  |  |  |  |
| GO:0035262\_gonad\_morphogenesis | 1 | 0 |  |  |  |  |  |  |  |  |
| GO:0035287\_head\_segmentation | 1 | 0 |  |  |  |  |  |  |  |  |
| GO:0035289\_posterior\_head\_segmentation | 1 | 0 |  |  |  |  |  |  |  |  |
| GO:0035303\_regulation\_of\_dephosphorylation | 1 | 0 |  |  |  |  |  |  |  |  |
| GO:0035304\_regulation\_of\_protein\_amino\_acid\_dephosphorylation | 1 | 0 |  |  |  |  |  |  |  |  |
| GO:0035305\_negative\_regulation\_of\_dephosphorylation | 1 | 0 |  |  |  |  |  |  |  |  |
| GO:0035308\_negative\_regulation\_of\_protein\_amino\_acid\_dephosphorylation | 1 | 0 |  |  |  |  |  |  |  |  |
| GO:0035313\_wound\_healing\_\_spreading\_of\_epidermal\_cells | 1 | 0 |  |  |  |  |  |  |  |  |
| GO:0040013\_negative\_regulation\_of\_locomotion | 1 | 0 |  |  |  |  |  |  |  |  |
| GO:0040019\_positive\_regulation\_of\_embryonic\_development | 1 | 0 |  |  |  |  |  |  |  |  |
| GO:0040032\_post-embryonic\_body\_morphogenesis | 1 | 0 |  |  |  |  |  |  |  |  |
| GO:0040038\_polar\_body\_extrusion\_after\_meiotic\_divisions | 1 | 0 |  |  |  |  |  |  |  |  |
| GO:0042026\_protein\_refolding | 1 | 0 |  |  |  |  |  |  |  |  |
| GO:0042048\_olfactory\_behavior | 1 | 0 |  |  |  |  |  |  |  |  |
| GO:0042059\_negative\_regulation\_of\_epidermal\_growth\_factor\_receptor\_signaling\_pathway | 1 | 0 |  |  |  |  |  |  |  |  |
| GO:0042073\_intraflagellar\_transport | 1 | 0 |  |  |  |  |  |  |  |  |
| GO:0042078\_germ-line\_stem\_cell\_division | 1 | 0 |  |  |  |  |  |  |  |  |
| GO:0042091\_interleukin-10\_biosynthetic\_process | 1 | 0 |  |  |  |  |  |  |  |  |
| GO:0042103\_positive\_regulation\_of\_T\_cell\_homeostatic\_proliferation | 1 | 0 |  |  |  |  |  |  |  |  |
| GO:0042136\_neurotransmitter\_biosynthetic\_process | 1 | 0 |  |  |  |  |  |  |  |  |
| GO:0042137\_sequestering\_of\_neurotransmitter | 1 | 0 |  |  |  |  |  |  |  |  |
| GO:0042138\_meiotic\_DNA\_double-strand\_break\_formation | 1 | 0 |  |  |  |  |  |  |  |  |
| GO:0042178\_xenobiotic\_catabolic\_process | 1 | 0 |  |  |  |  |  |  |  |  |
| GO:0042225\_interleukin-5\_biosynthetic\_process | 1 | 0 |  |  |  |  |  |  |  |  |
| GO:0042231\_interleukin-13\_biosynthetic\_process | 1 | 0 |  |  |  |  |  |  |  |  |
| GO:0042255\_ribosome\_assembly | 1 | 0 |  |  |  |  |  |  |  |  |
| GO:0042257\_ribosomal\_subunit\_assembly | 1 | 0 |  |  |  |  |  |  |  |  |
| GO:0042264\_peptidyl-aspartic\_acid\_hydroxylation | 1 | 0 |  |  |  |  |  |  |  |  |
| GO:0042276\_error-prone\_postreplication\_DNA\_repair | 1 | 0 |  |  |  |  |  |  |  |  |
| GO:0042297\_vocal\_learning | 1 | 0 |  |  |  |  |  |  |  |  |
| GO:0042309\_homoiothermy | 1 | 0 |  |  |  |  |  |  |  |  |
| GO:0042320\_regulation\_of\_circadian\_sleep\_wake\_cycle\_\_REM\_sleep | 1 | 0 |  |  |  |  |  |  |  |  |
| GO:0042339\_keratan\_sulfate\_metabolic\_process | 1 | 0 |  |  |  |  |  |  |  |  |
| GO:0042347\_negative\_regulation\_of\_NF-kappaB\_import\_into\_nucleus | 1 | 0 |  |  |  |  |  |  |  |  |
| GO:0042360\_vitamin\_E\_metabolic\_process | 1 | 0 |  |  |  |  |  |  |  |  |
| GO:0042363\_fat-soluble\_vitamin\_catabolic\_process | 1 | 0 |  |  |  |  |  |  |  |  |
| GO:0042369\_vitamin\_D\_catabolic\_process | 1 | 0 |  |  |  |  |  |  |  |  |
| GO:0042373\_vitamin\_K\_metabolic\_process | 1 | 0 |  |  |  |  |  |  |  |  |
| GO:0042404\_thyroid\_hormone\_catabolic\_process | 1 | 0 |  |  |  |  |  |  |  |  |
| GO:0042414\_epinephrine\_metabolic\_process | 1 | 0 |  |  |  |  |  |  |  |  |
| GO:0042436\_indole\_derivative\_catabolic\_process | 1 | 0 |  |  |  |  |  |  |  |  |
| GO:0042489\_negative\_regulation\_of\_odontogenesis\_of\_dentine-containing\_tooth | 1 | 0 |  |  |  |  |  |  |  |  |
| GO:0042508\_tyrosine\_phosphorylation\_of\_Stat1\_protein | 1 | 0 |  |  |  |  |  |  |  |  |
| GO:0042518\_negative\_regulation\_of\_tyrosine\_phosphorylation\_of\_Stat3\_protein | 1 | 0 |  |  |  |  |  |  |  |  |
| GO:0042524\_negative\_regulation\_of\_tyrosine\_phosphorylation\_of\_Stat5\_protein | 1 | 0 |  |  |  |  |  |  |  |  |
| GO:0042536\_negative\_regulation\_of\_tumor\_necrosis\_factor\_biosynthetic\_process | 1 | 0 |  |  |  |  |  |  |  |  |
| GO:0042538\_hyperosmotic\_salinity\_response | 1 | 0 |  |  |  |  |  |  |  |  |
| GO:0042628\_mating\_plug\_formation | 1 | 0 |  |  |  |  |  |  |  |  |
| GO:0042631\_cellular\_response\_to\_water\_deprivation | 1 | 0 |  |  |  |  |  |  |  |  |
| GO:0042637\_catagen | 1 | 0 |  |  |  |  |  |  |  |  |
| GO:0042660\_positive\_regulation\_of\_cell\_fate\_specification | 1 | 0 |  |  |  |  |  |  |  |  |
| GO:0042663\_regulation\_of\_endodermal\_cell\_fate\_specification | 1 | 0 |  |  |  |  |  |  |  |  |
| GO:0042664\_negative\_regulation\_of\_endodermal\_cell\_fate\_specification | 1 | 0 |  |  |  |  |  |  |  |  |
| GO:0042667\_auditory\_receptor\_cell\_fate\_specification | 1 | 0 |  |  |  |  |  |  |  |  |
| GO:0042694\_muscle\_cell\_fate\_specification | 1 | 0 |  |  |  |  |  |  |  |  |
| GO:0042706\_eye\_photoreceptor\_cell\_fate\_commitment | 1 | 0 |  |  |  |  |  |  |  |  |
| GO:0042713\_sperm\_ejaculation | 1 | 0 |  |  |  |  |  |  |  |  |
| GO:0042723\_thiamin\_and\_derivative\_metabolic\_process | 1 | 0 |  |  |  |  |  |  |  |  |
| GO:0042737\_drug\_catabolic\_process | 1 | 0 |  |  |  |  |  |  |  |  |
| GO:0042738\_exogenous\_drug\_catabolic\_process | 1 | 0 |  |  |  |  |  |  |  |  |
| GO:0042747\_circadian\_sleep\_wake\_cycle\_\_REM\_sleep | 1 | 0 |  |  |  |  |  |  |  |  |
| GO:0042748\_circadian\_sleep\_wake\_cycle\_\_non-REM\_sleep | 1 | 0 |  |  |  |  |  |  |  |  |
| GO:0042772\_DNA\_damage\_response\_\_signal\_transduction\_resulting\_in\_transcription | 1 | 0 |  |  |  |  |  |  |  |  |
| GO:0042790\_transcription\_of\_nuclear\_rRNA\_large\_RNA\_polymerase\_I\_transcript | 1 | 0 |  |  |  |  |  |  |  |  |
| GO:0042839\_D-glucuronate\_metabolic\_process | 1 | 0 |  |  |  |  |  |  |  |  |
| GO:0042840\_D-glucuronate\_catabolic\_process | 1 | 0 |  |  |  |  |  |  |  |  |
| GO:0042891\_antibiotic\_transport | 1 | 0 |  |  |  |  |  |  |  |  |
| GO:0042892\_chloramphenicol\_transport | 1 | 0 |  |  |  |  |  |  |  |  |
| GO:0042940\_D-amino\_acid\_transport | 1 | 0 |  |  |  |  |  |  |  |  |
| GO:0042941\_D-alanine\_transport | 1 | 0 |  |  |  |  |  |  |  |  |
| GO:0042942\_D-serine\_transport | 1 | 0 |  |  |  |  |  |  |  |  |
| GO:0042983\_amyloid\_precursor\_protein\_biosynthetic\_process | 1 | 0 |  |  |  |  |  |  |  |  |
| GO:0042984\_regulation\_of\_amyloid\_precursor\_protein\_biosynthetic\_process | 1 | 0 |  |  |  |  |  |  |  |  |
| GO:0042985\_negative\_regulation\_of\_amyloid\_precursor\_protein\_biosynthetic\_process | 1 | 0 |  |  |  |  |  |  |  |  |
| GO:0042989\_sequestering\_of\_actin\_monomers | 1 | 0 |  |  |  |  |  |  |  |  |
| GO:0043044\_ATP-dependent\_chromatin\_remodeling | 1 | 0 |  |  |  |  |  |  |  |  |
| GO:0043056\_forward\_locomotion | 1 | 0 |  |  |  |  |  |  |  |  |
| GO:0043060\_meiotic\_metaphase\_I\_plate\_congression | 1 | 0 |  |  |  |  |  |  |  |  |
| GO:0043091\_L-arginine\_import | 1 | 0 |  |  |  |  |  |  |  |  |
| GO:0043124\_negative\_regulation\_of\_I-kappaB\_kinase\_NF-kappaB\_cascade | 1 | 0 |  |  |  |  |  |  |  |  |
| GO:0043132\_NAD\_transport | 1 | 0 |  |  |  |  |  |  |  |  |
| GO:0043153\_entrainment\_of\_circadian\_clock\_by\_photoperiod | 1 | 0 |  |  |  |  |  |  |  |  |
| GO:0043171\_peptide\_catabolic\_process | 1 | 0 |  |  |  |  |  |  |  |  |
| GO:0043179\_rhythmic\_excitation | 1 | 0 |  |  |  |  |  |  |  |  |
| GO:0043206\_fibril\_organization | 1 | 0 |  |  |  |  |  |  |  |  |
| GO:0043217\_myelin\_maintenance | 1 | 0 |  |  |  |  |  |  |  |  |
| GO:0043313\_regulation\_of\_neutrophil\_degranulation | 1 | 0 |  |  |  |  |  |  |  |  |
| GO:0043316\_cytotoxic\_T\_cell\_degranulation | 1 | 0 |  |  |  |  |  |  |  |  |
| GO:0043369\_CD4-positive\_or\_CD8-positive\_\_alpha-beta\_T\_cell\_lineage\_commitment | 1 | 0 |  |  |  |  |  |  |  |  |
| GO:0043375\_CD8-positive\_\_alpha-beta\_T\_cell\_lineage\_commitment | 1 | 0 |  |  |  |  |  |  |  |  |
| GO:0043379\_memory\_T\_cell\_differentiation | 1 | 0 |  |  |  |  |  |  |  |  |
| GO:0043380\_regulation\_of\_memory\_T\_cell\_differentiation | 1 | 0 |  |  |  |  |  |  |  |  |
| GO:0043400\_cortisol\_secretion | 1 | 0 |  |  |  |  |  |  |  |  |
| GO:0043415\_positive\_regulation\_of\_skeletal\_muscle\_regeneration | 1 | 0 |  |  |  |  |  |  |  |  |
| GO:0043416\_regulation\_of\_skeletal\_muscle\_regeneration | 1 | 0 |  |  |  |  |  |  |  |  |
| GO:0043437\_butanoic\_acid\_metabolic\_process | 1 | 0 |  |  |  |  |  |  |  |  |
| GO:0043438\_acetoacetic\_acid\_metabolic\_process | 1 | 0 |  |  |  |  |  |  |  |  |
| GO:0043480\_pigment\_accumulation\_in\_tissues | 1 | 0 |  |  |  |  |  |  |  |  |
| GO:0043482\_cellular\_pigment\_accumulation | 1 | 0 |  |  |  |  |  |  |  |  |
| GO:0043486\_histone\_exchange | 1 | 0 |  |  |  |  |  |  |  |  |
| GO:0043496\_regulation\_of\_protein\_homodimerization\_activity | 1 | 0 |  |  |  |  |  |  |  |  |
| GO:0043501\_skeletal\_muscle\_adaptation | 1 | 0 |  |  |  |  |  |  |  |  |
| GO:0043508\_negative\_regulation\_of\_JUN\_kinase\_activity | 1 | 0 |  |  |  |  |  |  |  |  |
| GO:0043517\_positive\_regulation\_of\_DNA\_damage\_response\_\_signal\_transduction\_by\_p53\_class\_mediator | 1 | 0 |  |  |  |  |  |  |  |  |
| GO:0043535\_regulation\_of\_blood\_vessel\_endothelial\_cell\_migration | 1 | 0 |  |  |  |  |  |  |  |  |
| GO:0043537\_negative\_regulation\_of\_blood\_vessel\_endothelial\_cell\_migration | 1 | 0 |  |  |  |  |  |  |  |  |
| GO:0043545\_molybdopterin\_cofactor\_metabolic\_process | 1 | 0 |  |  |  |  |  |  |  |  |
| GO:0043587\_tongue\_morphogenesis | 1 | 0 |  |  |  |  |  |  |  |  |
| GO:0043604\_amide\_biosynthetic\_process | 1 | 0 |  |  |  |  |  |  |  |  |
| GO:0043628\_ncRNA\_3'-end\_processing | 1 | 0 |  |  |  |  |  |  |  |  |
| GO:0044254\_multicellular\_organismal\_protein\_catabolic\_process | 1 | 0 |  |  |  |  |  |  |  |  |
| GO:0044256\_protein\_digestion | 1 | 0 |  |  |  |  |  |  |  |  |
| GO:0044266\_multicellular\_organismal\_macromolecule\_catabolic\_process | 1 | 0 |  |  |  |  |  |  |  |  |
| GO:0045019\_negative\_regulation\_of\_nitric\_oxide\_biosynthetic\_process | 1 | 0 |  |  |  |  |  |  |  |  |
| GO:0045020\_error-prone\_DNA\_repair | 1 | 0 |  |  |  |  |  |  |  |  |
| GO:0045022\_early\_endosome\_to\_late\_endosome\_transport | 1 | 0 |  |  |  |  |  |  |  |  |
| GO:0045062\_extrathymic\_T\_cell\_selection | 1 | 0 |  |  |  |  |  |  |  |  |
| GO:0045069\_regulation\_of\_viral\_genome\_replication | 1 | 0 |  |  |  |  |  |  |  |  |
| GO:0045074\_regulation\_of\_interleukin-10\_biosynthetic\_process | 1 | 0 |  |  |  |  |  |  |  |  |
| GO:0045082\_positive\_regulation\_of\_interleukin-10\_biosynthetic\_process | 1 | 0 |  |  |  |  |  |  |  |  |
| GO:0045083\_negative\_regulation\_of\_interleukin-12\_biosynthetic\_process | 1 | 0 |  |  |  |  |  |  |  |  |
| GO:0045112\_integrin\_biosynthetic\_process | 1 | 0 |  |  |  |  |  |  |  |  |
| GO:0045113\_regulation\_of\_integrin\_biosynthetic\_process | 1 | 0 |  |  |  |  |  |  |  |  |
| GO:0045188\_regulation\_of\_circadian\_sleep\_wake\_cycle\_\_non-REM\_sleep | 1 | 0 |  |  |  |  |  |  |  |  |
| GO:0045210\_FasL\_biosynthetic\_process | 1 | 0 |  |  |  |  |  |  |  |  |
| GO:0045297\_post-mating\_behavior | 1 | 0 |  |  |  |  |  |  |  |  |
| GO:0045299\_otolith\_mineralization | 1 | 0 |  |  |  |  |  |  |  |  |
| GO:0045329\_carnitine\_biosynthetic\_process | 1 | 0 |  |  |  |  |  |  |  |  |
| GO:0045341\_MHC\_class\_I\_biosynthetic\_process | 1 | 0 |  |  |  |  |  |  |  |  |
| GO:0045343\_regulation\_of\_MHC\_class\_I\_biosynthetic\_process | 1 | 0 |  |  |  |  |  |  |  |  |
| GO:0045347\_negative\_regulation\_of\_MHC\_class\_II\_biosynthetic\_process | 1 | 0 |  |  |  |  |  |  |  |  |
| GO:0045405\_regulation\_of\_interleukin-5\_biosynthetic\_process | 1 | 0 |  |  |  |  |  |  |  |  |
| GO:0045407\_positive\_regulation\_of\_interleukin-5\_biosynthetic\_process | 1 | 0 |  |  |  |  |  |  |  |  |
| GO:0045426\_quinone\_cofactor\_biosynthetic\_process | 1 | 0 |  |  |  |  |  |  |  |  |
| GO:0045448\_mitotic\_cell\_cycle\_\_embryonic | 1 | 0 |  |  |  |  |  |  |  |  |
| GO:0045454\_cell\_redox\_homeostasis | 1 | 0 |  |  |  |  |  |  |  |  |
| GO:0045583\_regulation\_of\_cytotoxic\_T\_cell\_differentiation | 1 | 0 |  |  |  |  |  |  |  |  |
| GO:0045585\_positive\_regulation\_of\_cytotoxic\_T\_cell\_differentiation | 1 | 0 |  |  |  |  |  |  |  |  |
| GO:0045601\_regulation\_of\_endothelial\_cell\_differentiation | 1 | 0 |  |  |  |  |  |  |  |  |
| GO:0045602\_negative\_regulation\_of\_endothelial\_cell\_differentiation | 1 | 0 |  |  |  |  |  |  |  |  |
| GO:0045605\_negative\_regulation\_of\_epidermal\_cell\_differentiation | 1 | 0 |  |  |  |  |  |  |  |  |
| GO:0045606\_positive\_regulation\_of\_epidermal\_cell\_differentiation | 1 | 0 |  |  |  |  |  |  |  |  |
| GO:0045609\_positive\_regulation\_of\_auditory\_receptor\_cell\_differentiation | 1 | 0 |  |  |  |  |  |  |  |  |
| GO:0045617\_negative\_regulation\_of\_keratinocyte\_differentiation | 1 | 0 |  |  |  |  |  |  |  |  |
| GO:0045618\_positive\_regulation\_of\_keratinocyte\_differentiation | 1 | 0 |  |  |  |  |  |  |  |  |
| GO:0045626\_negative\_regulation\_of\_T-helper\_1\_cell\_differentiation | 1 | 0 |  |  |  |  |  |  |  |  |
| GO:0045633\_positive\_regulation\_of\_mechanoreceptor\_differentiation | 1 | 0 |  |  |  |  |  |  |  |  |
| GO:0045650\_negative\_regulation\_of\_macrophage\_differentiation | 1 | 0 |  |  |  |  |  |  |  |  |
| GO:0045656\_negative\_regulation\_of\_monocyte\_differentiation | 1 | 0 |  |  |  |  |  |  |  |  |
| GO:0045657\_positive\_regulation\_of\_monocyte\_differentiation | 1 | 0 |  |  |  |  |  |  |  |  |
| GO:0045659\_negative\_regulation\_of\_neutrophil\_differentiation | 1 | 0 |  |  |  |  |  |  |  |  |
| GO:0045660\_positive\_regulation\_of\_neutrophil\_differentiation | 1 | 0 |  |  |  |  |  |  |  |  |
| GO:0045721\_negative\_regulation\_of\_gluconeogenesis | 1 | 0 |  |  |  |  |  |  |  |  |
| GO:0045724\_positive\_regulation\_of\_flagellum\_assembly | 1 | 0 |  |  |  |  |  |  |  |  |
| GO:0045725\_positive\_regulation\_of\_glycogen\_biosynthetic\_process | 1 | 0 |  |  |  |  |  |  |  |  |
| GO:0045740\_positive\_regulation\_of\_DNA\_replication | 1 | 0 |  |  |  |  |  |  |  |  |
| GO:0045759\_negative\_regulation\_of\_action\_potential | 1 | 0 |  |  |  |  |  |  |  |  |
| GO:0045768\_positive\_regulation\_of\_anti-apoptosis | 1 | 0 |  |  |  |  |  |  |  |  |
| GO:0045769\_negative\_regulation\_of\_asymmetric\_cell\_division | 1 | 0 |  |  |  |  |  |  |  |  |
| GO:0045794\_negative\_regulation\_of\_cell\_volume | 1 | 0 |  |  |  |  |  |  |  |  |
| GO:0045815\_positive\_regulation\_of\_gene\_expression\_\_epigenetic | 1 | 0 |  |  |  |  |  |  |  |  |
| GO:0045818\_negative\_regulation\_of\_glycogen\_catabolic\_process | 1 | 0 |  |  |  |  |  |  |  |  |
| GO:0045842\_positive\_regulation\_of\_mitotic\_metaphase\_anaphase\_transition | 1 | 0 |  |  |  |  |  |  |  |  |
| GO:0045875\_negative\_regulation\_of\_sister\_chromatid\_cohesion | 1 | 0 |  |  |  |  |  |  |  |  |
| GO:0045898\_regulation\_of\_transcriptional\_preinitiation\_complex\_assembly | 1 | 0 |  |  |  |  |  |  |  |  |
| GO:0045899\_positive\_regulation\_of\_transcriptional\_preinitiation\_complex\_assembly | 1 | 0 |  |  |  |  |  |  |  |  |
| GO:0045906\_negative\_regulation\_of\_vasoconstriction | 1 | 0 |  |  |  |  |  |  |  |  |
| GO:0045908\_negative\_regulation\_of\_vasodilation | 1 | 0 |  |  |  |  |  |  |  |  |
| GO:0045909\_positive\_regulation\_of\_vasodilation | 1 | 0 |  |  |  |  |  |  |  |  |
| GO:0045915\_positive\_regulation\_of\_catecholamine\_metabolic\_process | 1 | 0 |  |  |  |  |  |  |  |  |
| GO:0045920\_negative\_regulation\_of\_exocytosis | 1 | 0 |  |  |  |  |  |  |  |  |
| GO:0045924\_regulation\_of\_female\_receptivity | 1 | 0 |  |  |  |  |  |  |  |  |
| GO:0045947\_negative\_regulation\_of\_translational\_initiation | 1 | 0 |  |  |  |  |  |  |  |  |
| GO:0045955\_negative\_regulation\_of\_calcium\_ion-dependent\_exocytosis | 1 | 0 |  |  |  |  |  |  |  |  |
| GO:0045956\_positive\_regulation\_of\_calcium\_ion-dependent\_exocytosis | 1 | 0 |  |  |  |  |  |  |  |  |
| GO:0045964\_positive\_regulation\_of\_dopamine\_metabolic\_process | 1 | 0 |  |  |  |  |  |  |  |  |
| GO:0045988\_negative\_regulation\_of\_striated\_muscle\_contraction | 1 | 0 |  |  |  |  |  |  |  |  |
| GO:0045989\_positive\_regulation\_of\_striated\_muscle\_contraction | 1 | 0 |  |  |  |  |  |  |  |  |
| GO:0045990\_regulation\_of\_transcription\_by\_carbon\_catabolites | 1 | 0 |  |  |  |  |  |  |  |  |
| GO:0045991\_positive\_regulation\_of\_transcription\_by\_carbon\_catabolites | 1 | 0 |  |  |  |  |  |  |  |  |
| GO:0045994\_positive\_regulation\_of\_translational\_initiation\_by\_iron | 1 | 0 |  |  |  |  |  |  |  |  |
| GO:0046007\_negative\_regulation\_of\_activated\_T\_cell\_proliferation | 1 | 0 |  |  |  |  |  |  |  |  |
| GO:0046014\_negative\_regulation\_of\_T\_cell\_homeostatic\_proliferation | 1 | 0 |  |  |  |  |  |  |  |  |
| GO:0046015\_regulation\_of\_transcription\_by\_glucose | 1 | 0 |  |  |  |  |  |  |  |  |
| GO:0046016\_positive\_regulation\_of\_transcription\_by\_glucose | 1 | 0 |  |  |  |  |  |  |  |  |
| GO:0046031\_ADP\_metabolic\_process | 1 | 0 |  |  |  |  |  |  |  |  |
| GO:0046032\_ADP\_catabolic\_process | 1 | 0 |  |  |  |  |  |  |  |  |
| GO:0046061\_dATP\_catabolic\_process | 1 | 0 |  |  |  |  |  |  |  |  |
| GO:0046075\_dTTP\_metabolic\_process | 1 | 0 |  |  |  |  |  |  |  |  |
| GO:0046078\_dUMP\_metabolic\_process | 1 | 0 |  |  |  |  |  |  |  |  |
| GO:0046079\_dUMP\_catabolic\_process | 1 | 0 |  |  |  |  |  |  |  |  |
| GO:0046086\_adenosine\_biosynthetic\_process | 1 | 0 |  |  |  |  |  |  |  |  |
| GO:0046090\_deoxyadenosine\_metabolic\_process | 1 | 0 |  |  |  |  |  |  |  |  |
| GO:0046098\_guanine\_metabolic\_process | 1 | 0 |  |  |  |  |  |  |  |  |
| GO:0046101\_hypoxanthine\_biosynthetic\_process | 1 | 0 |  |  |  |  |  |  |  |  |
| GO:0046102\_inosine\_metabolic\_process | 1 | 0 |  |  |  |  |  |  |  |  |
| GO:0046103\_inosine\_biosynthetic\_process | 1 | 0 |  |  |  |  |  |  |  |  |
| GO:0046108\_uridine\_metabolic\_process | 1 | 0 |  |  |  |  |  |  |  |  |
| GO:0046110\_xanthine\_metabolic\_process | 1 | 0 |  |  |  |  |  |  |  |  |
| GO:0046111\_xanthine\_biosynthetic\_process | 1 | 0 |  |  |  |  |  |  |  |  |
| GO:0046112\_nucleobase\_biosynthetic\_process | 1 | 0 |  |  |  |  |  |  |  |  |
| GO:0046113\_nucleobase\_catabolic\_process | 1 | 0 |  |  |  |  |  |  |  |  |
| GO:0046121\_deoxyribonucleoside\_catabolic\_process | 1 | 0 |  |  |  |  |  |  |  |  |
| GO:0046122\_purine\_deoxyribonucleoside\_metabolic\_process | 1 | 0 |  |  |  |  |  |  |  |  |
| GO:0046124\_purine\_deoxyribonucleoside\_catabolic\_process | 1 | 0 |  |  |  |  |  |  |  |  |
| GO:0046125\_pyrimidine\_deoxyribonucleoside\_metabolic\_process | 1 | 0 |  |  |  |  |  |  |  |  |
| GO:0046131\_pyrimidine\_ribonucleoside\_metabolic\_process | 1 | 0 |  |  |  |  |  |  |  |  |
| GO:0046160\_heme\_a\_metabolic\_process | 1 | 0 |  |  |  |  |  |  |  |  |
| GO:0046218\_indolalkylamine\_catabolic\_process | 1 | 0 |  |  |  |  |  |  |  |  |
| GO:0046292\_formaldehyde\_metabolic\_process | 1 | 0 |  |  |  |  |  |  |  |  |
| GO:0046294\_formaldehyde\_catabolic\_process | 1 | 0 |  |  |  |  |  |  |  |  |
| GO:0046314\_phosphocreatine\_biosynthetic\_process | 1 | 0 |  |  |  |  |  |  |  |  |
| GO:0046327\_glycerol\_biosynthetic\_process\_from\_pyruvate | 1 | 0 |  |  |  |  |  |  |  |  |
| GO:0046329\_negative\_regulation\_of\_JNK\_cascade | 1 | 0 |  |  |  |  |  |  |  |  |
| GO:0046340\_diacylglycerol\_catabolic\_process | 1 | 0 |  |  |  |  |  |  |  |  |
| GO:0046351\_disaccharide\_biosynthetic\_process | 1 | 0 |  |  |  |  |  |  |  |  |
| GO:0046356\_acetyl-CoA\_catabolic\_process | 1 | 0 |  |  |  |  |  |  |  |  |
| GO:0046358\_butyrate\_biosynthetic\_process | 1 | 0 |  |  |  |  |  |  |  |  |
| GO:0046359\_butyrate\_catabolic\_process | 1 | 0 |  |  |  |  |  |  |  |  |
| GO:0046381\_CMP-N-acetylneuraminate\_metabolic\_process | 1 | 0 |  |  |  |  |  |  |  |  |
| GO:0046415\_urate\_metabolic\_process | 1 | 0 |  |  |  |  |  |  |  |  |
| GO:0046416\_D-amino\_acid\_metabolic\_process | 1 | 0 |  |  |  |  |  |  |  |  |
| GO:0046434\_organophosphate\_catabolic\_process | 1 | 0 |  |  |  |  |  |  |  |  |
| GO:0046437\_D-amino\_acid\_biosynthetic\_process | 1 | 0 |  |  |  |  |  |  |  |  |
| GO:0046440\_L-lysine\_metabolic\_process | 1 | 0 |  |  |  |  |  |  |  |  |
| GO:0046449\_creatinine\_metabolic\_process | 1 | 0 |  |  |  |  |  |  |  |  |
| GO:0046471\_phosphatidylglycerol\_metabolic\_process | 1 | 0 |  |  |  |  |  |  |  |  |
| GO:0046473\_phosphatidic\_acid\_metabolic\_process | 1 | 0 |  |  |  |  |  |  |  |  |
| GO:0046476\_glycosylceramide\_biosynthetic\_process | 1 | 0 |  |  |  |  |  |  |  |  |
| GO:0046477\_glycosylceramide\_catabolic\_process | 1 | 0 |  |  |  |  |  |  |  |  |
| GO:0046485\_ether\_lipid\_metabolic\_process | 1 | 0 |  |  |  |  |  |  |  |  |
| GO:0046487\_glyoxylate\_metabolic\_process | 1 | 0 |  |  |  |  |  |  |  |  |
| GO:0046498\_S-adenosylhomocysteine\_metabolic\_process | 1 | 0 |  |  |  |  |  |  |  |  |
| GO:0046552\_photoreceptor\_cell\_fate\_commitment | 1 | 0 |  |  |  |  |  |  |  |  |
| GO:0046586\_regulation\_of\_calcium-dependent\_cell-cell\_adhesion | 1 | 0 |  |  |  |  |  |  |  |  |
| GO:0046587\_positive\_regulation\_of\_calcium-dependent\_cell-cell\_adhesion | 1 | 0 |  |  |  |  |  |  |  |  |
| GO:0046602\_regulation\_of\_mitotic\_centrosome\_separation | 1 | 0 |  |  |  |  |  |  |  |  |
| GO:0046604\_positive\_regulation\_of\_mitotic\_centrosome\_separation | 1 | 0 |  |  |  |  |  |  |  |  |
| GO:0046607\_positive\_regulation\_of\_centrosome\_cycle | 1 | 0 |  |  |  |  |  |  |  |  |
| GO:0046655\_folic\_acid\_metabolic\_process | 1 | 0 |  |  |  |  |  |  |  |  |
| GO:0046671\_negative\_regulation\_of\_retinal\_cell\_programmed\_cell\_death | 1 | 0 |  |  |  |  |  |  |  |  |
| GO:0046685\_response\_to\_arsenic | 1 | 0 |  |  |  |  |  |  |  |  |
| GO:0046692\_sperm\_competition | 1 | 0 |  |  |  |  |  |  |  |  |
| GO:0046707\_IDP\_metabolic\_process | 1 | 0 |  |  |  |  |  |  |  |  |
| GO:0046709\_IDP\_catabolic\_process | 1 | 0 |  |  |  |  |  |  |  |  |
| GO:0046724\_oxalic\_acid\_secretion | 1 | 0 |  |  |  |  |  |  |  |  |
| GO:0046753\_non-lytic\_viral\_release | 1 | 0 |  |  |  |  |  |  |  |  |
| GO:0046755\_non-lytic\_virus\_budding | 1 | 0 |  |  |  |  |  |  |  |  |
| GO:0046826\_negative\_regulation\_of\_protein\_export\_from\_nucleus | 1 | 0 |  |  |  |  |  |  |  |  |
| GO:0046831\_regulation\_of\_RNA\_export\_from\_nucleus | 1 | 0 |  |  |  |  |  |  |  |  |
| GO:0046834\_lipid\_phosphorylation | 1 | 0 |  |  |  |  |  |  |  |  |
| GO:0046853\_inositol\_and\_derivative\_phosphorylation | 1 | 0 |  |  |  |  |  |  |  |  |
| GO:0046864\_isoprenoid\_transport | 1 | 0 |  |  |  |  |  |  |  |  |
| GO:0046865\_terpenoid\_transport | 1 | 0 |  |  |  |  |  |  |  |  |
| GO:0046877\_regulation\_of\_saliva\_secretion | 1 | 0 |  |  |  |  |  |  |  |  |
| GO:0046878\_positive\_regulation\_of\_saliva\_secretion | 1 | 0 |  |  |  |  |  |  |  |  |
| GO:0046884\_follicle-stimulating\_hormone\_secretion | 1 | 0 |  |  |  |  |  |  |  |  |
| GO:0046898\_response\_to\_cycloheximide | 1 | 0 |  |  |  |  |  |  |  |  |
| GO:0046929\_negative\_regulation\_of\_neurotransmitter\_secretion | 1 | 0 |  |  |  |  |  |  |  |  |
| GO:0046931\_pore\_complex\_biogenesis | 1 | 0 |  |  |  |  |  |  |  |  |
| GO:0046949\_acyl-CoA\_biosynthetic\_process | 1 | 0 |  |  |  |  |  |  |  |  |
| GO:0046958\_nonassociative\_learning | 1 | 0 |  |  |  |  |  |  |  |  |
| GO:0046960\_sensitization | 1 | 0 |  |  |  |  |  |  |  |  |
| GO:0046986\_negative\_regulation\_of\_hemoglobin\_biosynthetic\_process | 1 | 0 |  |  |  |  |  |  |  |  |
| GO:0047497\_mitochondrion\_transport\_along\_microtubule | 1 | 0 |  |  |  |  |  |  |  |  |
| GO:0048047\_mating\_behavior\_\_sex\_discrimination | 1 | 0 |  |  |  |  |  |  |  |  |
| GO:0048133\_male\_germ-line\_stem\_cell\_division | 1 | 0 |  |  |  |  |  |  |  |  |
| GO:0048137\_spermatocyte\_division | 1 | 0 |  |  |  |  |  |  |  |  |
| GO:0048143\_astrocyte\_activation | 1 | 0 |  |  |  |  |  |  |  |  |
| GO:0048170\_positive\_regulation\_of\_long-term\_neuronal\_synaptic\_plasticity | 1 | 0 |  |  |  |  |  |  |  |  |
| GO:0048199\_vesicle\_targeting\_\_to\_\_from\_or\_within\_Golgi | 1 | 0 |  |  |  |  |  |  |  |  |
| GO:0048241\_epinephrine\_transport | 1 | 0 |  |  |  |  |  |  |  |  |
| GO:0048242\_epinephrine\_secretion | 1 | 0 |  |  |  |  |  |  |  |  |
| GO:0048243\_norepinephrine\_secretion | 1 | 0 |  |  |  |  |  |  |  |  |
| GO:0048247\_lymphocyte\_chemotaxis | 1 | 0 |  |  |  |  |  |  |  |  |
| GO:0048250\_mitochondrial\_iron\_ion\_transport | 1 | 0 |  |  |  |  |  |  |  |  |
| GO:0048259\_regulation\_of\_receptor-mediated\_endocytosis | 1 | 0 |  |  |  |  |  |  |  |  |
| GO:0048260\_positive\_regulation\_of\_receptor-mediated\_endocytosis | 1 | 0 |  |  |  |  |  |  |  |  |
| GO:0048290\_isotype\_switching\_to\_IgA\_isotypes | 1 | 0 |  |  |  |  |  |  |  |  |
| GO:0048296\_regulation\_of\_isotype\_switching\_to\_IgA\_isotypes | 1 | 0 |  |  |  |  |  |  |  |  |
| GO:0048298\_positive\_regulation\_of\_isotype\_switching\_to\_IgA\_isotypes | 1 | 0 |  |  |  |  |  |  |  |  |
| GO:0048319\_axial\_mesoderm\_morphogenesis | 1 | 0 |  |  |  |  |  |  |  |  |
| GO:0048320\_axial\_mesoderm\_formation | 1 | 0 |  |  |  |  |  |  |  |  |
| GO:0048385\_regulation\_of\_retinoic\_acid\_receptor\_signaling\_pathway | 1 | 0 |  |  |  |  |  |  |  |  |
| GO:0048387\_negative\_regulation\_of\_retinoic\_acid\_receptor\_signaling\_pathway | 1 | 0 |  |  |  |  |  |  |  |  |
| GO:0048388\_endosomal\_lumen\_acidification | 1 | 0 |  |  |  |  |  |  |  |  |
| GO:0048389\_intermediate\_mesoderm\_development | 1 | 0 |  |  |  |  |  |  |  |  |
| GO:0048478\_replication\_fork\_protection | 1 | 0 |  |  |  |  |  |  |  |  |
| GO:0048496\_maintenance\_of\_organ\_identity | 1 | 0 |  |  |  |  |  |  |  |  |
| GO:0048525\_negative\_regulation\_of\_viral\_reproduction | 1 | 0 |  |  |  |  |  |  |  |  |
| GO:0048539\_bone\_marrow\_development | 1 | 0 |  |  |  |  |  |  |  |  |
| GO:0048548\_regulation\_of\_pinocytosis | 1 | 0 |  |  |  |  |  |  |  |  |
| GO:0048549\_positive\_regulation\_of\_pinocytosis | 1 | 0 |  |  |  |  |  |  |  |  |
| GO:0048553\_negative\_regulation\_of\_metalloenzyme\_activity | 1 | 0 |  |  |  |  |  |  |  |  |
| GO:0048588\_developmental\_cell\_growth | 1 | 0 |  |  |  |  |  |  |  |  |
| GO:0048601\_oocyte\_morphogenesis | 1 | 0 |  |  |  |  |  |  |  |  |
| GO:0048621\_post-embryonic\_gut\_morphogenesis | 1 | 0 |  |  |  |  |  |  |  |  |
| GO:0048640\_negative\_regulation\_of\_developmental\_growth | 1 | 0 |  |  |  |  |  |  |  |  |
| GO:0048642\_negative\_regulation\_of\_skeletal\_muscle\_tissue\_development | 1 | 0 |  |  |  |  |  |  |  |  |
| GO:0048669\_collateral\_sprouting\_in\_the\_absence\_of\_injury | 1 | 0 |  |  |  |  |  |  |  |  |
| GO:0048680\_positive\_regulation\_of\_axon\_regeneration | 1 | 0 |  |  |  |  |  |  |  |  |
| GO:0048681\_negative\_regulation\_of\_axon\_regeneration | 1 | 0 |  |  |  |  |  |  |  |  |
| GO:0048686\_regulation\_of\_sprouting\_of\_injured\_axon | 1 | 0 |  |  |  |  |  |  |  |  |
| GO:0048687\_positive\_regulation\_of\_sprouting\_of\_injured\_axon | 1 | 0 |  |  |  |  |  |  |  |  |
| GO:0048690\_regulation\_of\_axon\_extension\_involved\_in\_regeneration | 1 | 0 |  |  |  |  |  |  |  |  |
| GO:0048691\_positive\_regulation\_of\_axon\_extension\_involved\_in\_regeneration | 1 | 0 |  |  |  |  |  |  |  |  |
| GO:0048714\_positive\_regulation\_of\_oligodendrocyte\_differentiation | 1 | 0 |  |  |  |  |  |  |  |  |
| GO:0048733\_sebaceous\_gland\_development | 1 | 0 |  |  |  |  |  |  |  |  |
| GO:0048743\_positive\_regulation\_of\_skeletal\_muscle\_fiber\_development | 1 | 0 |  |  |  |  |  |  |  |  |
| GO:0048752\_semicircular\_canal\_morphogenesis | 1 | 0 |  |  |  |  |  |  |  |  |
| GO:0048773\_erythrophore\_differentiation | 1 | 0 |  |  |  |  |  |  |  |  |
| GO:0048790\_maintenance\_of\_presynaptic\_active\_zone\_structure | 1 | 0 |  |  |  |  |  |  |  |  |
| GO:0048791\_calcium\_ion-dependent\_exocytosis\_of\_neurotransmitter | 1 | 0 |  |  |  |  |  |  |  |  |
| GO:0048822\_enucleate\_erythrocyte\_development | 1 | 0 |  |  |  |  |  |  |  |  |
| GO:0048866\_stem\_cell\_fate\_specification | 1 | 0 |  |  |  |  |  |  |  |  |
| GO:0048936\_peripheral\_nervous\_system\_neuron\_axonogenesis | 1 | 0 |  |  |  |  |  |  |  |  |
| GO:0050427\_3'-phosphoadenosine\_5'-phosphosulfate\_metabolic\_process | 1 | 0 |  |  |  |  |  |  |  |  |
| GO:0050428\_3'-phosphoadenosine\_5'-phosphosulfate\_biosynthetic\_process | 1 | 0 |  |  |  |  |  |  |  |  |
| GO:0050482\_arachidonic\_acid\_secretion | 1 | 0 |  |  |  |  |  |  |  |  |
| GO:0050667\_homocysteine\_metabolic\_process | 1 | 0 |  |  |  |  |  |  |  |  |
| GO:0050674\_urothelial\_cell\_proliferation | 1 | 0 |  |  |  |  |  |  |  |  |
| GO:0050675\_regulation\_of\_urothelial\_cell\_proliferation | 1 | 0 |  |  |  |  |  |  |  |  |
| GO:0050677\_positive\_regulation\_of\_urothelial\_cell\_proliferation | 1 | 0 |  |  |  |  |  |  |  |  |
| GO:0050691\_regulation\_of\_defense\_response\_to\_virus\_by\_host | 1 | 0 |  |  |  |  |  |  |  |  |
| GO:0050748\_negative\_regulation\_of\_lipoprotein\_metabolic\_process | 1 | 0 |  |  |  |  |  |  |  |  |
| GO:0050757\_thymidylate\_synthase\_biosynthetic\_process | 1 | 0 |  |  |  |  |  |  |  |  |
| GO:0050758\_regulation\_of\_thymidylate\_synthase\_biosynthetic\_process | 1 | 0 |  |  |  |  |  |  |  |  |
| GO:0050760\_negative\_regulation\_of\_thymidylate\_synthase\_biosynthetic\_process | 1 | 0 |  |  |  |  |  |  |  |  |
| GO:0050812\_regulation\_of\_acyl-CoA\_biosynthetic\_process | 1 | 0 |  |  |  |  |  |  |  |  |
| GO:0050832\_defense\_response\_to\_fungus | 1 | 0 |  |  |  |  |  |  |  |  |
| GO:0050861\_positive\_regulation\_of\_B\_cell\_receptor\_signaling\_pathway | 1 | 0 |  |  |  |  |  |  |  |  |
| GO:0050862\_positive\_regulation\_of\_T\_cell\_receptor\_signaling\_pathway | 1 | 0 |  |  |  |  |  |  |  |  |
| GO:0050916\_sensory\_perception\_of\_sweet\_taste | 1 | 0 |  |  |  |  |  |  |  |  |
| GO:0050975\_sensory\_perception\_of\_touch | 1 | 0 |  |  |  |  |  |  |  |  |
| GO:0050995\_negative\_regulation\_of\_lipid\_catabolic\_process | 1 | 0 |  |  |  |  |  |  |  |  |
| GO:0051001\_negative\_regulation\_of\_nitric-oxide\_synthase\_activity | 1 | 0 |  |  |  |  |  |  |  |  |
| GO:0051005\_negative\_regulation\_of\_lipoprotein\_lipase\_activity | 1 | 0 |  |  |  |  |  |  |  |  |
| GO:0051006\_positive\_regulation\_of\_lipoprotein\_lipase\_activity | 1 | 0 |  |  |  |  |  |  |  |  |
| GO:0051016\_barbed-end\_actin\_filament\_capping | 1 | 0 |  |  |  |  |  |  |  |  |
| GO:0051029\_rRNA\_transport | 1 | 0 |  |  |  |  |  |  |  |  |
| GO:0051043\_regulation\_of\_membrane\_protein\_ectodomain\_proteolysis | 1 | 0 |  |  |  |  |  |  |  |  |
| GO:0051044\_positive\_regulation\_of\_membrane\_protein\_ectodomain\_proteolysis | 1 | 0 |  |  |  |  |  |  |  |  |
| GO:0051088\_PMA-inducible\_membrane\_protein\_ectodomain\_proteolysis | 1 | 0 |  |  |  |  |  |  |  |  |
| GO:0051102\_DNA\_ligation\_during\_DNA\_recombination | 1 | 0 |  |  |  |  |  |  |  |  |
| GO:0051103\_DNA\_ligation\_during\_DNA\_repair | 1 | 0 |  |  |  |  |  |  |  |  |
| GO:0051123\_transcriptional\_preinitiation\_complex\_assembly | 1 | 0 |  |  |  |  |  |  |  |  |
| GO:0051125\_regulation\_of\_actin\_nucleation | 1 | 0 |  |  |  |  |  |  |  |  |
| GO:0051127\_positive\_regulation\_of\_actin\_nucleation | 1 | 0 |  |  |  |  |  |  |  |  |
| GO:0051151\_negative\_regulation\_of\_smooth\_muscle\_cell\_differentiation | 1 | 0 |  |  |  |  |  |  |  |  |
| GO:0051154\_negative\_regulation\_of\_striated\_muscle\_cell\_differentiation | 1 | 0 |  |  |  |  |  |  |  |  |
| GO:0051155\_positive\_regulation\_of\_striated\_muscle\_cell\_differentiation | 1 | 0 |  |  |  |  |  |  |  |  |
| GO:0051156\_glucose\_6-phosphate\_metabolic\_process | 1 | 0 |  |  |  |  |  |  |  |  |
| GO:0051187\_cofactor\_catabolic\_process | 1 | 0 |  |  |  |  |  |  |  |  |
| GO:0051189\_prosthetic\_group\_metabolic\_process | 1 | 0 |  |  |  |  |  |  |  |  |
| GO:0051193\_regulation\_of\_cofactor\_metabolic\_process | 1 | 0 |  |  |  |  |  |  |  |  |
| GO:0051196\_regulation\_of\_coenzyme\_metabolic\_process | 1 | 0 |  |  |  |  |  |  |  |  |
| GO:0051255\_spindle\_midzone\_assembly | 1 | 0 |  |  |  |  |  |  |  |  |
| GO:0051257\_spindle\_midzone\_assembly\_involved\_in\_meiosis | 1 | 0 |  |  |  |  |  |  |  |  |
| GO:0051281\_positive\_regulation\_of\_release\_of\_sequestered\_calcium\_ion\_into\_cytosol | 1 | 0 |  |  |  |  |  |  |  |  |
| GO:0051290\_protein\_heterotetramerization | 1 | 0 |  |  |  |  |  |  |  |  |
| GO:0051305\_chromosome\_movement\_towards\_spindle\_pole | 1 | 0 |  |  |  |  |  |  |  |  |
| GO:0051310\_metaphase\_plate\_congression | 1 | 0 |  |  |  |  |  |  |  |  |
| GO:0051311\_meiotic\_metaphase\_plate\_congression | 1 | 0 |  |  |  |  |  |  |  |  |
| GO:0051340\_regulation\_of\_ligase\_activity | 1 | 0 |  |  |  |  |  |  |  |  |
| GO:0051351\_positive\_regulation\_of\_ligase\_activity | 1 | 0 |  |  |  |  |  |  |  |  |
| GO:0051354\_negative\_regulation\_of\_oxidoreductase\_activity | 1 | 0 |  |  |  |  |  |  |  |  |
| GO:0051355\_proprioception\_during\_equilibrioception | 1 | 0 |  |  |  |  |  |  |  |  |
| GO:0051383\_kinetochore\_organization | 1 | 0 |  |  |  |  |  |  |  |  |
| GO:0051386\_regulation\_of\_nerve\_growth\_factor\_receptor\_signaling\_pathway | 1 | 0 |  |  |  |  |  |  |  |  |
| GO:0051409\_response\_to\_nitrosative\_stress | 1 | 0 |  |  |  |  |  |  |  |  |
| GO:0051457\_maintenance\_of\_protein\_location\_in\_nucleus | 1 | 0 |  |  |  |  |  |  |  |  |
| GO:0051462\_regulation\_of\_cortisol\_secretion | 1 | 0 |  |  |  |  |  |  |  |  |
| GO:0051463\_negative\_regulation\_of\_cortisol\_secretion | 1 | 0 |  |  |  |  |  |  |  |  |
| GO:0051481\_reduction\_of\_cytosolic\_calcium\_ion\_concentration | 1 | 0 |  |  |  |  |  |  |  |  |
| GO:0051482\_elevation\_of\_cytosolic\_calcium\_ion\_concentration\_during\_G-protein\_signaling\_\_coupled\_to\_IP3\_second\_messenger\_(phospholipase\_C\_activating) | 1 | 0 |  |  |  |  |  |  |  |  |
| GO:0051542\_elastin\_biosynthetic\_process | 1 | 0 |  |  |  |  |  |  |  |  |
| GO:0051568\_histone\_H3-K4\_methylation | 1 | 0 |  |  |  |  |  |  |  |  |
| GO:0051569\_regulation\_of\_histone\_H3-K4\_methylation | 1 | 0 |  |  |  |  |  |  |  |  |
| GO:0051570\_regulation\_of\_histone\_H3-K9\_methylation | 1 | 0 |  |  |  |  |  |  |  |  |
| GO:0051573\_negative\_regulation\_of\_histone\_H3-K9\_methylation | 1 | 0 |  |  |  |  |  |  |  |  |
| GO:0051580\_regulation\_of\_neurotransmitter\_uptake | 1 | 0 |  |  |  |  |  |  |  |  |
| GO:0051582\_positive\_regulation\_of\_neurotransmitter\_uptake | 1 | 0 |  |  |  |  |  |  |  |  |
| GO:0051584\_regulation\_of\_dopamine\_uptake | 1 | 0 |  |  |  |  |  |  |  |  |
| GO:0051586\_positive\_regulation\_of\_dopamine\_uptake | 1 | 0 |  |  |  |  |  |  |  |  |
| GO:0051589\_negative\_regulation\_of\_neurotransmitter\_transport | 1 | 0 |  |  |  |  |  |  |  |  |
| GO:0051593\_response\_to\_folic\_acid | 1 | 0 |  |  |  |  |  |  |  |  |
| GO:0051615\_histamine\_uptake | 1 | 0 |  |  |  |  |  |  |  |  |
| GO:0051646\_mitochondrion\_localization | 1 | 0 |  |  |  |  |  |  |  |  |
| GO:0051654\_establishment\_of\_mitochondrion\_localization | 1 | 0 |  |  |  |  |  |  |  |  |
| GO:0051661\_maintenance\_of\_centrosome\_location | 1 | 0 |  |  |  |  |  |  |  |  |
| GO:0051665\_membrane\_raft\_localization | 1 | 0 |  |  |  |  |  |  |  |  |
| GO:0051685\_maintenance\_of\_ER\_location | 1 | 0 |  |  |  |  |  |  |  |  |
| GO:0051693\_actin\_filament\_capping | 1 | 0 |  |  |  |  |  |  |  |  |
| GO:0051701\_interaction\_with\_host | 1 | 0 |  |  |  |  |  |  |  |  |
| GO:0051754\_meiotic\_sister\_chromatid\_cohesion\_\_centromeric | 1 | 0 |  |  |  |  |  |  |  |  |
| GO:0051782\_negative\_regulation\_of\_cell\_division | 1 | 0 |  |  |  |  |  |  |  |  |
| GO:0051790\_short-chain\_fatty\_acid\_biosynthetic\_process | 1 | 0 |  |  |  |  |  |  |  |  |
| GO:0051799\_negative\_regulation\_of\_hair\_follicle\_development | 1 | 0 |  |  |  |  |  |  |  |  |
| GO:0051823\_regulation\_of\_synapse\_structural\_plasticity | 1 | 0 |  |  |  |  |  |  |  |  |
| GO:0051865\_protein\_autoubiquitination | 1 | 0 |  |  |  |  |  |  |  |  |
| GO:0051901\_positive\_regulation\_of\_mitochondrial\_depolarization | 1 | 0 |  |  |  |  |  |  |  |  |
| GO:0051917\_regulation\_of\_fibrinolysis | 1 | 0 |  |  |  |  |  |  |  |  |
| GO:0051918\_negative\_regulation\_of\_fibrinolysis | 1 | 0 |  |  |  |  |  |  |  |  |
| GO:0051929\_positive\_regulation\_of\_calcium\_ion\_transport\_via\_voltage-gated\_calcium\_channel\_activity | 1 | 0 |  |  |  |  |  |  |  |  |
| GO:0051933\_amino\_acid\_uptake\_during\_transmission\_of\_nerve\_impulse | 1 | 0 |  |  |  |  |  |  |  |  |
| GO:0051935\_glutamate\_uptake\_during\_transmission\_of\_nerve\_impulse | 1 | 0 |  |  |  |  |  |  |  |  |
| GO:0051940\_regulation\_of\_catecholamine\_uptake\_during\_transmission\_of\_nerve\_impulse | 1 | 0 |  |  |  |  |  |  |  |  |
| GO:0051944\_positive\_regulation\_of\_catecholamine\_uptake\_during\_transmission\_of\_nerve\_impulse | 1 | 0 |  |  |  |  |  |  |  |  |
| GO:0051961\_negative\_regulation\_of\_nervous\_system\_development | 1 | 0 |  |  |  |  |  |  |  |  |
| GO:0051964\_negative\_regulation\_of\_synaptogenesis | 1 | 0 |  |  |  |  |  |  |  |  |
| GO:0051968\_positive\_regulation\_of\_synaptic\_transmission\_\_glutamatergic | 1 | 0 |  |  |  |  |  |  |  |  |
| GO:0051984\_positive\_regulation\_of\_chromosome\_segregation | 1 | 0 |  |  |  |  |  |  |  |  |
| GO:0051987\_positive\_regulation\_of\_attachment\_of\_spindle\_microtubules\_to\_kinetochore | 1 | 0 |  |  |  |  |  |  |  |  |
| GO:0052173\_response\_to\_defenses\_of\_other\_organism\_during\_symbiotic\_interaction | 1 | 0 |  |  |  |  |  |  |  |  |
| GO:0052200\_response\_to\_host\_defenses | 1 | 0 |  |  |  |  |  |  |  |  |
| GO:0052551\_response\_to\_defense-related\_nitric\_oxide\_production\_by\_other\_organism\_during\_symbiotic\_interaction | 1 | 0 |  |  |  |  |  |  |  |  |
| GO:0052564\_response\_to\_immune\_response\_of\_other\_organism\_during\_symbiotic\_interaction | 1 | 0 |  |  |  |  |  |  |  |  |
| GO:0052565\_response\_to\_defense-related\_host\_nitric\_oxide\_production | 1 | 0 |  |  |  |  |  |  |  |  |
| GO:0052572\_response\_to\_host\_immune\_response | 1 | 0 |  |  |  |  |  |  |  |  |
| GO:0055005\_ventricular\_cardiac\_myofibril\_development | 1 | 0 |  |  |  |  |  |  |  |  |
| GO:0055011\_atrial\_cardiac\_muscle\_cell\_differentiation | 1 | 0 |  |  |  |  |  |  |  |  |
| GO:0055014\_atrial\_cardiac\_muscle\_cell\_development | 1 | 0 |  |  |  |  |  |  |  |  |
| GO:0055078\_sodium\_ion\_homeostasis | 1 | 0 |  |  |  |  |  |  |  |  |
| GO:0055089\_fatty\_acid\_homeostasis | 1 | 0 |  |  |  |  |  |  |  |  |
| GO:0055093\_response\_to\_hyperoxia | 1 | 0 |  |  |  |  |  |  |  |  |
| GO:0060003\_copper\_ion\_export | 1 | 0 |  |  |  |  |  |  |  |  |
| GO:0060005\_vestibular\_reflex | 1 | 0 |  |  |  |  |  |  |  |  |
| GO:0060014\_granulosa\_cell\_differentiation | 1 | 0 |  |  |  |  |  |  |  |  |
| GO:0060018\_astrocyte\_fate\_commitment | 1 | 0 |  |  |  |  |  |  |  |  |
| GO:0060020\_Bergmann\_glial\_cell\_differentiation | 1 | 0 |  |  |  |  |  |  |  |  |
| GO:0060022\_hard\_palate\_development | 1 | 0 |  |  |  |  |  |  |  |  |
| GO:0060034\_notochord\_cell\_differentiation | 1 | 0 |  |  |  |  |  |  |  |  |
| GO:0060035\_notochord\_cell\_development | 1 | 0 |  |  |  |  |  |  |  |  |
| GO:0060046\_regulation\_of\_acrosome\_reaction | 1 | 0 |  |  |  |  |  |  |  |  |
| GO:0060054\_positive\_regulation\_of\_epithelial\_cell\_proliferation\_involved\_in\_wound\_healing | 1 | 0 |  |  |  |  |  |  |  |  |
| GO:0060059\_embryonic\_retina\_morphogenesis\_in\_camera-type\_eye | 1 | 0 |  |  |  |  |  |  |  |  |
| GO:0060061\_Spemann\_organizer\_formation | 1 | 0 |  |  |  |  |  |  |  |  |
| GO:0060064\_Spemann\_organizer\_formation\_at\_the\_anterior\_end\_of\_the\_primitive\_streak | 1 | 0 |  |  |  |  |  |  |  |  |
| GO:0060071\_Wnt\_receptor\_signaling\_pathway\_\_planar\_cell\_polarity\_pathway | 1 | 0 |  |  |  |  |  |  |  |  |
| GO:0060075\_regulation\_of\_resting\_membrane\_potential | 1 | 0 |  |  |  |  |  |  |  |  |
| GO:0060082\_eye\_blink\_reflex | 1 | 0 |  |  |  |  |  |  |  |  |
| GO:0060112\_generation\_of\_ovulation\_cycle\_rhythm | 1 | 0 |  |  |  |  |  |  |  |  |
| GO:0060125\_negative\_regulation\_of\_growth\_hormone\_secretion | 1 | 0 |  |  |  |  |  |  |  |  |
| GO:0060151\_peroxisome\_localization | 1 | 0 |  |  |  |  |  |  |  |  |
| GO:0060152\_microtubule-based\_peroxisome\_localization | 1 | 0 |  |  |  |  |  |  |  |  |
| GO:0060161\_positive\_regulation\_of\_dopamine\_receptor\_signaling\_pathway | 1 | 0 |  |  |  |  |  |  |  |  |
| GO:0060163\_subpallium\_neuron\_fate\_commitment | 1 | 0 |  |  |  |  |  |  |  |  |
| GO:0060165\_regulation\_of\_timing\_of\_subpallium\_neuron\_differentiation | 1 | 0 |  |  |  |  |  |  |  |  |
| GO:0060174\_limb\_bud\_formation | 1 | 0 |  |  |  |  |  |  |  |  |
| GO:0060177\_regulation\_of\_angiotensin\_metabolic\_process | 1 | 0 |  |  |  |  |  |  |  |  |
| GO:0060197\_cloacal\_septation | 1 | 0 |  |  |  |  |  |  |  |  |
| GO:0060215\_primitive\_hemopoiesis | 1 | 0 |  |  |  |  |  |  |  |  |
| GO:0060231\_mesenchymal\_to\_epithelial\_transition | 1 | 0 |  |  |  |  |  |  |  |  |
| GO:0060254\_regulation\_of\_N-terminal\_protein\_palmitoylation | 1 | 0 |  |  |  |  |  |  |  |  |
| GO:0060261\_positive\_regulation\_of\_transcription\_initiation\_from\_RNA\_polymerase\_II\_promoter | 1 | 0 |  |  |  |  |  |  |  |  |
| GO:0060262\_negative\_regulation\_of\_N-terminal\_protein\_palmitoylation | 1 | 0 |  |  |  |  |  |  |  |  |
| GO:0060263\_regulation\_of\_respiratory\_burst | 1 | 0 |  |  |  |  |  |  |  |  |
| GO:0060264\_regulation\_of\_respiratory\_burst\_during\_acute\_inflammatory\_response | 1 | 0 |  |  |  |  |  |  |  |  |
| GO:0060265\_positive\_regulation\_of\_respiratory\_burst\_during\_acute\_inflammatory\_response | 1 | 0 |  |  |  |  |  |  |  |  |
| GO:0060267\_positive\_regulation\_of\_respiratory\_burst | 1 | 0 |  |  |  |  |  |  |  |  |
| GO:0060272\_embryonic\_skeletal\_joint\_morphogenesis | 1 | 0 |  |  |  |  |  |  |  |  |
| GO:0060297\_regulation\_of\_sarcomere\_organization | 1 | 0 |  |  |  |  |  |  |  |  |
| GO:0060298\_positive\_regulation\_of\_sarcomere\_organization | 1 | 0 |  |  |  |  |  |  |  |  |
| GO:0060315\_negative\_regulation\_of\_ryanodine-sensitive\_calcium-release\_channel\_activity | 1 | 0 |  |  |  |  |  |  |  |  |
| GO:0060319\_primitive\_erythrocyte\_differentiation | 1 | 0 |  |  |  |  |  |  |  |  |
| GO:0060371\_regulation\_of\_atrial\_cardiomyocyte\_membrane\_depolarization | 1 | 0 |  |  |  |  |  |  |  |  |
| GO:0060374\_mast\_cell\_differentiation | 1 | 0 |  |  |  |  |  |  |  |  |
| GO:0060375\_regulation\_of\_mast\_cell\_differentiation | 1 | 0 |  |  |  |  |  |  |  |  |
| GO:0060376\_positive\_regulation\_of\_mast\_cell\_differentiation | 1 | 0 |  |  |  |  |  |  |  |  |
| GO:0060390\_regulation\_of\_SMAD\_protein\_nuclear\_translocation | 1 | 0 |  |  |  |  |  |  |  |  |
| GO:0060391\_positive\_regulation\_of\_SMAD\_protein\_nuclear\_translocation | 1 | 0 |  |  |  |  |  |  |  |  |
| GO:0060398\_regulation\_of\_growth\_hormone\_receptor\_signaling\_pathway | 1 | 0 |  |  |  |  |  |  |  |  |
| GO:0060399\_positive\_regulation\_of\_growth\_hormone\_receptor\_signaling\_pathway | 1 | 0 |  |  |  |  |  |  |  |  |
| GO:0060405\_regulation\_of\_penile\_erection | 1 | 0 |  |  |  |  |  |  |  |  |
| GO:0060407\_negative\_regulation\_of\_penile\_erection | 1 | 0 |  |  |  |  |  |  |  |  |
| GO:0060413\_atrial\_septum\_morphogenesis | 1 | 0 |  |  |  |  |  |  |  |  |
| GO:0060414\_aorta\_smooth\_muscle\_tissue\_morphogenesis | 1 | 0 |  |  |  |  |  |  |  |  |
| GO:0060419\_heart\_growth | 1 | 0 |  |  |  |  |  |  |  |  |
| GO:0060420\_regulation\_of\_heart\_growth | 1 | 0 |  |  |  |  |  |  |  |  |
| GO:0060421\_positive\_regulation\_of\_heart\_growth | 1 | 0 |  |  |  |  |  |  |  |  |
| GO:0060431\_primary\_lung\_bud\_formation | 1 | 0 |  |  |  |  |  |  |  |  |
| GO:0060436\_bronchiole\_morphogenesis | 1 | 0 |  |  |  |  |  |  |  |  |
| GO:0060440\_trachea\_formation | 1 | 0 |  |  |  |  |  |  |  |  |
| GO:0060449\_bud\_elongation\_involved\_in\_lung\_branching | 1 | 0 |  |  |  |  |  |  |  |  |
| GO:0060456\_positive\_regulation\_of\_digestive\_system\_process | 1 | 0 |  |  |  |  |  |  |  |  |
| GO:0060461\_right\_lung\_morphogenesis | 1 | 0 |  |  |  |  |  |  |  |  |
| GO:0060481\_lobar\_bronchus\_epithelium\_development | 1 | 0 |  |  |  |  |  |  |  |  |
| GO:0060482\_lobar\_bronchus\_development | 1 | 0 |  |  |  |  |  |  |  |  |
| GO:0060484\_lung-associated\_mesenchyme\_development | 1 | 0 |  |  |  |  |  |  |  |  |
| GO:0060486\_Clara\_cell\_differentiation | 1 | 0 |  |  |  |  |  |  |  |  |
| GO:0060510\_Type\_II\_pneumocyte\_differentiation | 1 | 0 |  |  |  |  |  |  |  |  |
| GO:0060514\_prostate\_induction | 1 | 0 |  |  |  |  |  |  |  |  |
| GO:0060515\_prostate\_field\_specification | 1 | 0 |  |  |  |  |  |  |  |  |
| GO:0060517\_epithelial\_cell\_proliferation\_involved\_in\_prostatic\_bud\_elongation | 1 | 0 |  |  |  |  |  |  |  |  |
| GO:0060520\_activation\_of\_prostate\_induction\_by\_androgen\_receptor\_signaling\_pathway | 1 | 0 |  |  |  |  |  |  |  |  |
| GO:0060535\_trachea\_cartilage\_morphogenesis | 1 | 0 |  |  |  |  |  |  |  |  |
| GO:0060536\_cartilage\_morphogenesis | 1 | 0 |  |  |  |  |  |  |  |  |
| GO:0060563\_neuroepithelial\_cell\_differentiation | 1 | 0 |  |  |  |  |  |  |  |  |
| GO:0060577\_pulmonary\_vein\_morphogenesis | 1 | 0 |  |  |  |  |  |  |  |  |
| GO:0060578\_superior\_vena\_cava\_morphogenesis | 1 | 0 |  |  |  |  |  |  |  |  |
| GO:0060584\_regulation\_of\_prostaglandin-endoperoxide\_synthase\_activity | 1 | 0 |  |  |  |  |  |  |  |  |
| GO:0060585\_positive\_regulation\_of\_prostaglandin-endoperoxidase\_synthase\_activity | 1 | 0 |  |  |  |  |  |  |  |  |
| GO:0060598\_dichotomous\_subdivision\_of\_terminal\_units\_involved\_in\_mammary\_gland\_duct\_morphogenesis | 1 | 0 |  |  |  |  |  |  |  |  |
| GO:0060611\_mammary\_gland\_fat\_development | 1 | 0 |  |  |  |  |  |  |  |  |
| GO:0060618\_nipple\_development | 1 | 0 |  |  |  |  |  |  |  |  |
| GO:0060631\_regulation\_of\_meiosis\_I | 1 | 0 |  |  |  |  |  |  |  |  |
| GO:0060649\_mammary\_gland\_bud\_elongation | 1 | 0 |  |  |  |  |  |  |  |  |
| GO:0060658\_nipple\_morphogenesis | 1 | 0 |  |  |  |  |  |  |  |  |
| GO:0060659\_nipple\_sheath\_formation | 1 | 0 |  |  |  |  |  |  |  |  |
| GO:0060668\_regulation\_of\_branching\_involved\_in\_salivary\_gland\_morphogenesis\_by\_extracellular\_matrix-epithelial\_cell\_signaling | 1 | 0 |  |  |  |  |  |  |  |  |
| GO:0060683\_regulation\_of\_branching\_involved\_in\_salivary\_gland\_morphogenesis\_by\_epithelial-mesenchymal\_signaling | 1 | 0 |  |  |  |  |  |  |  |  |
| GO:0060691\_epithelial\_cell\_maturation\_involved\_in\_salivary\_gland\_development | 1 | 0 |  |  |  |  |  |  |  |  |
| GO:0060709\_glycogen\_cell\_development\_involved\_in\_embryonic\_placenta\_development | 1 | 0 |  |  |  |  |  |  |  |  |
| GO:0060732\_positive\_regulation\_of\_inositol\_phosphate\_biosynthetic\_process | 1 | 0 |  |  |  |  |  |  |  |  |
| GO:0060739\_mesenchymal-epithelial\_cell\_signaling\_involved\_in\_prostate\_gland\_development | 1 | 0 |  |  |  |  |  |  |  |  |
| GO:0060781\_mesenchymal\_cell\_proliferation\_involved\_in\_prostate\_gland\_development | 1 | 0 |  |  |  |  |  |  |  |  |
| GO:0060782\_regulation\_of\_mesenchymal\_cell\_proliferation\_involved\_in\_prostate\_gland\_development | 1 | 0 |  |  |  |  |  |  |  |  |
| GO:0060783\_mesenchymal\_smoothened\_signaling\_pathway\_involved\_in\_prostate\_gland\_development | 1 | 0 |  |  |  |  |  |  |  |  |
| GO:0060872\_semicircular\_canal\_development | 1 | 0 |  |  |  |  |  |  |  |  |
| GO:0060896\_neural\_plate\_pattern\_specification | 1 | 0 |  |  |  |  |  |  |  |  |
| GO:0070091\_glucagon\_secretion | 1 | 0 |  |  |  |  |  |  |  |  |
| GO:0070162\_adiponectin\_secretion | 1 | 0 |  |  |  |  |  |  |  |  |
| GO:0070163\_regulation\_of\_adiponectin\_secretion | 1 | 0 |  |  |  |  |  |  |  |  |
| GO:0070164\_negative\_regulation\_of\_adiponectin\_secretion | 1 | 0 |  |  |  |  |  |  |  |  |
| GO:0070178\_D-serine\_metabolic\_process | 1 | 0 |  |  |  |  |  |  |  |  |
| GO:0070179\_D-serine\_biosynthetic\_process | 1 | 0 |  |  |  |  |  |  |  |  |
| GO:0070296\_sarcoplasmic\_reticulum\_calcium\_ion\_transport | 1 | 0 |  |  |  |  |  |  |  |  |
| GO:0070303\_negative\_regulation\_of\_stress-activated\_protein\_kinase\_signaling\_pathway | 1 | 0 |  |  |  |  |  |  |  |  |
| GO:0070328\_triglyceride\_homeostasis | 1 | 0 |  |  |  |  |  |  |  |  |
| GO:0070365\_hepatocyte\_differentiation | 1 | 0 |  |  |  |  |  |  |  |  |
| GO:0070384\_Harderian\_gland\_development | 1 | 0 |  |  |  |  |  |  |  |  |
| GO:0070391\_response\_to\_lipoteichoic\_acid | 1 | 0 |  |  |  |  |  |  |  |  |
| GO:0070424\_regulation\_of\_nucleotide-binding\_oligomerization\_domain\_containing\_signaling\_pathway | 1 | 0 |  |  |  |  |  |  |  |  |
| GO:0070426\_positive\_regulation\_of\_nucleotide-binding\_oligomerization\_domain\_containing\_signaling\_pathway | 1 | 0 |  |  |  |  |  |  |  |  |
| GO:0070428\_regulation\_of\_nucleotide-binding\_oligomerization\_domain\_containing\_1\_signaling\_pathway | 1 | 0 |  |  |  |  |  |  |  |  |
| GO:0070430\_positive\_regulation\_of\_nucleotide-binding\_oligomerization\_domain\_containing\_1\_signaling\_pathway | 1 | 0 |  |  |  |  |  |  |  |  |
| GO:0070432\_regulation\_of\_nucleotide-binding\_oligomerization\_domain\_containing\_2\_signaling\_pathway | 1 | 0 |  |  |  |  |  |  |  |  |
| GO:0070434\_positive\_regulation\_of\_nucleotide-binding\_oligomerization\_domain\_containing\_2\_signaling\_pathway | 1 | 0 |  |  |  |  |  |  |  |  |
| GO:0070493\_thrombin\_receptor\_signaling\_pathway | 1 | 0 |  |  |  |  |  |  |  |  |
| GO:0070508\_cholesterol\_import | 1 | 0 |  |  |  |  |  |  |  |  |
| GO:0070527\_platelet\_aggregation | 1 | 0 |  |  |  |  |  |  |  |  |
| GO:0070528\_protein\_kinase\_C\_signaling\_cascade | 1 | 0 |  |  |  |  |  |  |  |  |
| GO:0070555\_response\_to\_interleukin-1 | 1 | 0 |  |  |  |  |  |  |  |  |
| GO:0070560\_protein\_secretion\_by\_platelet | 1 | 0 |  |  |  |  |  |  |  |  |
| GO:0070561\_vitamin\_D\_receptor\_signaling\_pathway | 1 | 0 |  |  |  |  |  |  |  |  |
| GO:0070562\_regulation\_of\_vitamin\_D\_receptor\_signaling\_pathway | 1 | 0 |  |  |  |  |  |  |  |  |
| GO:0070571\_negative\_regulation\_of\_neuron\_projection\_regeneration | 1 | 0 |  |  |  |  |  |  |  |  |
| GO:0070572\_positive\_regulation\_of\_neuron\_projection\_regeneration | 1 | 0 |  |  |  |  |  |  |  |  |
| GO:0070613\_regulation\_of\_protein\_processing | 1 | 0 |  |  |  |  |  |  |  |  |
| GO:0070627\_ferrous\_iron\_import | 1 | 0 |  |  |  |  |  |  |  |  |
| GO:0070669\_response\_to\_interleukin-2 | 1 | 0 |  |  |  |  |  |  |  |  |
| GO:0070670\_response\_to\_interleukin-4 | 1 | 0 |  |  |  |  |  |  |  |  |
| GO:0070671\_response\_to\_interleukin-12 | 1 | 0 |  |  |  |  |  |  |  |  |
| GO:0070672\_response\_to\_interleukin-15 | 1 | 0 |  |  |  |  |  |  |  |  |
| GO:0070673\_response\_to\_interleukin-18 | 1 | 0 |  |  |  |  |  |  |  |  |
| GO:0070828\_heterochromatin\_organization | 1 | 0 |  |  |  |  |  |  |  |  |
| GO:0070874\_negative\_regulation\_of\_glycogen\_metabolic\_process | 1 | 0 |  |  |  |  |  |  |  |  |
| GO:0075136\_response\_to\_host | 1 | 0 |  |  |  |  |  |  |  |  |
| GO:0080010\_regulation\_of\_oxygen\_and\_reactive\_oxygen\_species\_metabolic\_process | 1 | 0 |  |  |  |  |  |  |  |  |
| GO:0090032\_negative\_regulation\_of\_steroid\_hormone\_biosynthetic\_process | 1 | 0 |  |  |  |  |  |  |  |  |
| GO:0001843\_neural\_tube\_closure | 33 | 0 | 0.000000 | -0.000000 | 864 | 660.165371 | 749.45 | 838.734629 | 0.867419 |
| GO:0002562\_somatic\_diversification\_of\_immune\_receptors\_via\_germline\_recombination\_within\_a\_single\_locus | 33 | 0 | 0.000000 | -0.000000 | 864 | 660.165371 | 749.45 | 838.734629 | 0.867419 |
| GO:0006643\_membrane\_lipid\_metabolic\_process | 33 | 0 | 0.000000 | -0.000000 | 864 | 660.165371 | 749.45 | 838.734629 | 0.867419 |
| GO:0007188\_G-protein\_signaling\_\_coupled\_to\_cAMP\_nucleotide\_second\_messenger | 33 | 0 | 0.000000 | -0.000000 | 864 | 660.165371 | 749.45 | 838.734629 | 0.867419 |
| GO:0007270\_nerve-nerve\_synaptic\_transmission | 33 | 0 | 0.000000 | -0.000000 | 864 | 660.165371 | 749.45 | 838.734629 | 0.867419 |
| GO:0007431\_salivary\_gland\_development | 33 | 0 | 0.000000 | -0.000000 | 864 | 660.165371 | 749.45 | 838.734629 | 0.867419 |
| GO:0008584\_male\_gonad\_development | 33 | 0 | 0.000000 | -0.000000 | 864 | 660.165371 | 749.45 | 838.734629 | 0.867419 |
| GO:0008643\_carbohydrate\_transport | 33 | 0 | 0.000000 | -0.000000 | 864 | 660.165371 | 749.45 | 838.734629 | 0.867419 |
| GO:0016444\_somatic\_cell\_DNA\_recombination | 33 | 0 | 0.000000 | -0.000000 | 864 | 660.165371 | 749.45 | 838.734629 | 0.867419 |
| GO:0022037\_metencephalon\_development | 33 | 0 | 0.000000 | -0.000000 | 864 | 660.165371 | 749.45 | 838.734629 | 0.867419 |
| GO:0042108\_positive\_regulation\_of\_cytokine\_biosynthetic\_process | 33 | 0 | 0.000000 | -0.000000 | 864 | 660.165371 | 749.45 | 838.734629 | 0.867419 |
| GO:0060606\_tube\_closure | 33 | 0 | 0.000000 | -0.000000 | 864 | 660.165371 | 749.45 | 838.734629 | 0.867419 |
| GO:0001932\_regulation\_of\_protein\_amino\_acid\_phosphorylation | 69 | 0 | 0.000000 | -0.000000 | 867 | 664.344654 | 753.07 | 841.795346 | 0.868593 |
| GO:0006816\_calcium\_ion\_transport | 69 | 0 | 0.000000 | -0.000000 | 867 | 664.344654 | 753.07 | 841.795346 | 0.868593 |
| GO:0055065\_metal\_ion\_homeostasis | 69 | 0 | 0.000000 | -0.000000 | 867 | 664.344654 | 753.07 | 841.795346 | 0.868593 |
| GO:0001658\_branching\_involved\_in\_ureteric\_bud\_morphogenesis | 26 | 0 | 0.000000 | -0.000000 | 883 | 685.782043 | 773.52 | 861.257957 | 0.876014 |
| GO:0007613\_memory | 26 | 0 | 0.000000 | -0.000000 | 883 | 685.782043 | 773.52 | 861.257957 | 0.876014 |
| GO:0007623\_circadian\_rhythm | 26 | 0 | 0.000000 | -0.000000 | 883 | 685.782043 | 773.52 | 861.257957 | 0.876014 |
| GO:0009880\_embryonic\_pattern\_specification | 26 | 0 | 0.000000 | -0.000000 | 883 | 685.782043 | 773.52 | 861.257957 | 0.876014 |
| GO:0010959\_regulation\_of\_metal\_ion\_transport | 26 | 0 | 0.000000 | -0.000000 | 883 | 685.782043 | 773.52 | 861.257957 | 0.876014 |
| GO:0019233\_sensory\_perception\_of\_pain | 26 | 0 | 0.000000 | -0.000000 | 883 | 685.782043 | 773.52 | 861.257957 | 0.876014 |
| GO:0032609\_interferon-gamma\_production | 26 | 0 | 0.000000 | -0.000000 | 883 | 685.782043 | 773.52 | 861.257957 | 0.876014 |
| GO:0040029\_regulation\_of\_gene\_expression\_\_epigenetic | 26 | 0 | 0.000000 | -0.000000 | 883 | 685.782043 | 773.52 | 861.257957 | 0.876014 |
| GO:0045765\_regulation\_of\_angiogenesis | 26 | 0 | 0.000000 | -0.000000 | 883 | 685.782043 | 773.52 | 861.257957 | 0.876014 |
| GO:0046530\_photoreceptor\_cell\_differentiation | 26 | 0 | 0.000000 | -0.000000 | 883 | 685.782043 | 773.52 | 861.257957 | 0.876014 |
| GO:0048645\_organ\_formation | 26 | 0 | 0.000000 | -0.000000 | 883 | 685.782043 | 773.52 | 861.257957 | 0.876014 |
| GO:0050680\_negative\_regulation\_of\_epithelial\_cell\_proliferation | 26 | 0 | 0.000000 | -0.000000 | 883 | 685.782043 | 773.52 | 861.257957 | 0.876014 |
| GO:0050873\_brown\_fat\_cell\_differentiation | 26 | 0 | 0.000000 | -0.000000 | 883 | 685.782043 | 773.52 | 861.257957 | 0.876014 |
| GO:0051235\_maintenance\_of\_location | 26 | 0 | 0.000000 | -0.000000 | 883 | 685.782043 | 773.52 | 861.257957 | 0.876014 |
| GO:0051480\_cytosolic\_calcium\_ion\_homeostasis | 26 | 0 | 0.000000 | -0.000000 | 883 | 685.782043 | 773.52 | 861.257957 | 0.876014 |
| GO:0060675\_ureteric\_bud\_morphogenesis | 26 | 0 | 0.000000 | -0.000000 | 883 | 685.782043 | 773.52 | 861.257957 | 0.876014 |
| GO:0000084\_S\_phase\_of\_mitotic\_cell\_cycle | 3 | 0 |  |  |  |  |  |  |  |  |
| GO:0000089\_mitotic\_metaphase | 3 | 0 |  |  |  |  |  |  |  |  |
| GO:0000098\_sulfur\_amino\_acid\_catabolic\_process | 3 | 0 |  |  |  |  |  |  |  |  |
| GO:0000103\_sulfate\_assimilation | 3 | 0 |  |  |  |  |  |  |  |  |
| GO:0000212\_meiotic\_spindle\_organization | 3 | 0 |  |  |  |  |  |  |  |  |
| GO:0000281\_cytokinesis\_after\_mitosis | 3 | 0 |  |  |  |  |  |  |  |  |
| GO:0000303\_response\_to\_superoxide | 3 | 0 |  |  |  |  |  |  |  |  |
| GO:0000320\_re-entry\_into\_mitotic\_cell\_cycle | 3 | 0 |  |  |  |  |  |  |  |  |
| GO:0000380\_alternative\_nuclear\_mRNA\_splicing\_\_via\_spliceosome | 3 | 0 |  |  |  |  |  |  |  |  |
| GO:0001516\_prostaglandin\_biosynthetic\_process | 3 | 0 |  |  |  |  |  |  |  |  |
| GO:0001553\_luteinization | 3 | 0 |  |  |  |  |  |  |  |  |
| GO:0001574\_ganglioside\_biosynthetic\_process | 3 | 0 |  |  |  |  |  |  |  |  |
| GO:0001705\_ectoderm\_formation | 3 | 0 |  |  |  |  |  |  |  |  |
| GO:0001711\_endodermal\_cell\_fate\_commitment | 3 | 0 |  |  |  |  |  |  |  |  |
| GO:0001757\_somite\_specification | 3 | 0 |  |  |  |  |  |  |  |  |
| GO:0001778\_plasma\_membrane\_repair | 3 | 0 |  |  |  |  |  |  |  |  |
| GO:0001780\_neutrophil\_homeostasis | 3 | 0 |  |  |  |  |  |  |  |  |
| GO:0001802\_type\_III\_hypersensitivity | 3 | 0 |  |  |  |  |  |  |  |  |
| GO:0001803\_regulation\_of\_type\_III\_hypersensitivity | 3 | 0 |  |  |  |  |  |  |  |  |
| GO:0001805\_positive\_regulation\_of\_type\_III\_hypersensitivity | 3 | 0 |  |  |  |  |  |  |  |  |
| GO:0001812\_positive\_regulation\_of\_type\_I\_hypersensitivity | 3 | 0 |  |  |  |  |  |  |  |  |
| GO:0001831\_trophectodermal\_cellular\_morphogenesis | 3 | 0 |  |  |  |  |  |  |  |  |
| GO:0001844\_protein\_insertion\_into\_mitochondrial\_membrane\_during\_induction\_of\_apoptosis | 3 | 0 |  |  |  |  |  |  |  |  |
| GO:0001878\_response\_to\_yeast | 3 | 0 |  |  |  |  |  |  |  |  |
| GO:0001895\_retina\_homeostasis | 3 | 0 |  |  |  |  |  |  |  |  |
| GO:0001915\_negative\_regulation\_of\_T\_cell\_mediated\_cytotoxicity | 3 | 0 |  |  |  |  |  |  |  |  |
| GO:0001937\_negative\_regulation\_of\_endothelial\_cell\_proliferation | 3 | 0 |  |  |  |  |  |  |  |  |
| GO:0001953\_negative\_regulation\_of\_cell-matrix\_adhesion | 3 | 0 |  |  |  |  |  |  |  |  |
| GO:0001955\_blood\_vessel\_maturation | 3 | 0 |  |  |  |  |  |  |  |  |
| GO:0001960\_negative\_regulation\_of\_cytokine-mediated\_signaling\_pathway | 3 | 0 |  |  |  |  |  |  |  |  |
| GO:0001973\_adenosine\_receptor\_signaling\_pathway | 3 | 0 |  |  |  |  |  |  |  |  |
| GO:0001996\_positive\_regulation\_of\_heart\_rate\_by\_epinephrine-norepinephrine | 3 | 0 |  |  |  |  |  |  |  |  |
| GO:0002034\_regulation\_of\_blood\_vessel\_size\_by\_renin-angiotensin | 3 | 0 |  |  |  |  |  |  |  |  |
| GO:0002238\_response\_to\_molecule\_of\_fungal\_origin | 3 | 0 |  |  |  |  |  |  |  |  |
| GO:0002275\_myeloid\_cell\_activation\_during\_immune\_response | 3 | 0 |  |  |  |  |  |  |  |  |
| GO:0002281\_macrophage\_activation\_during\_immune\_response | 3 | 0 |  |  |  |  |  |  |  |  |
| GO:0002309\_T\_cell\_proliferation\_during\_immune\_response | 3 | 0 |  |  |  |  |  |  |  |  |
| GO:0002361\_CD4-positive\_\_CD25-positive\_\_alpha-beta\_regulatory\_T\_cell\_differentiation | 3 | 0 |  |  |  |  |  |  |  |  |
| GO:0002369\_T\_cell\_cytokine\_production | 3 | 0 |  |  |  |  |  |  |  |  |
| GO:0002428\_antigen\_processing\_and\_presentation\_of\_peptide\_antigen\_via\_MHC\_class\_Ib | 3 | 0 |  |  |  |  |  |  |  |  |
| GO:0002446\_neutrophil\_mediated\_immunity | 3 | 0 |  |  |  |  |  |  |  |  |
| GO:0002477\_antigen\_processing\_and\_presentation\_of\_exogenous\_peptide\_antigen\_via\_MHC\_class\_Ib | 3 | 0 |  |  |  |  |  |  |  |  |
| GO:0002481\_antigen\_processing\_and\_presentation\_of\_exogenous\_protein\_antigen\_via\_MHC\_class\_Ib\_\_TAP-dependent | 3 | 0 |  |  |  |  |  |  |  |  |
| GO:0002513\_tolerance\_induction\_to\_self\_antigen | 3 | 0 |  |  |  |  |  |  |  |  |
| GO:0002568\_somatic\_diversification\_of\_T\_cell\_receptor\_genes | 3 | 0 |  |  |  |  |  |  |  |  |
| GO:0002674\_negative\_regulation\_of\_acute\_inflammatory\_response | 3 | 0 |  |  |  |  |  |  |  |  |
| GO:0002681\_somatic\_recombination\_of\_T\_cell\_receptor\_gene\_segments | 3 | 0 |  |  |  |  |  |  |  |  |
| GO:0002713\_negative\_regulation\_of\_B\_cell\_mediated\_immunity | 3 | 0 |  |  |  |  |  |  |  |  |
| GO:0002827\_positive\_regulation\_of\_T-helper\_1\_type\_immune\_response | 3 | 0 |  |  |  |  |  |  |  |  |
| GO:0002865\_negative\_regulation\_of\_acute\_inflammatory\_response\_to\_antigenic\_stimulus | 3 | 0 |  |  |  |  |  |  |  |  |
| GO:0002884\_negative\_regulation\_of\_hypersensitivity | 3 | 0 |  |  |  |  |  |  |  |  |
| GO:0002890\_negative\_regulation\_of\_immunoglobulin\_mediated\_immune\_response | 3 | 0 |  |  |  |  |  |  |  |  |
| GO:0002904\_positive\_regulation\_of\_B\_cell\_apoptosis | 3 | 0 |  |  |  |  |  |  |  |  |
| GO:0003009\_skeletal\_muscle\_contraction | 3 | 0 |  |  |  |  |  |  |  |  |
| GO:0003072\_renal\_control\_of\_peripheral\_vascular\_resistance\_involved\_in\_regulation\_of\_systemic\_arterial\_blood\_pressure | 3 | 0 |  |  |  |  |  |  |  |  |
| GO:0006047\_UDP-N-acetylglucosamine\_metabolic\_process | 3 | 0 |  |  |  |  |  |  |  |  |
| GO:0006067\_ethanol\_metabolic\_process | 3 | 0 |  |  |  |  |  |  |  |  |
| GO:0006072\_glycerol-3-phosphate\_metabolic\_process | 3 | 0 |  |  |  |  |  |  |  |  |
| GO:0006103\_2-oxoglutarate\_metabolic\_process | 3 | 0 |  |  |  |  |  |  |  |  |
| GO:0006107\_oxaloacetate\_metabolic\_process | 3 | 0 |  |  |  |  |  |  |  |  |
| GO:0006166\_purine\_ribonucleoside\_salvage | 3 | 0 |  |  |  |  |  |  |  |  |
| GO:0006220\_pyrimidine\_nucleotide\_metabolic\_process | 3 | 0 |  |  |  |  |  |  |  |  |
| GO:0006266\_DNA\_ligation | 3 | 0 |  |  |  |  |  |  |  |  |
| GO:0006282\_regulation\_of\_DNA\_repair | 3 | 0 |  |  |  |  |  |  |  |  |
| GO:0006301\_postreplication\_repair | 3 | 0 |  |  |  |  |  |  |  |  |
| GO:0006361\_transcription\_initiation\_from\_RNA\_polymerase\_I\_promoter | 3 | 0 |  |  |  |  |  |  |  |  |
| GO:0006367\_transcription\_initiation\_from\_RNA\_polymerase\_II\_promoter | 3 | 0 |  |  |  |  |  |  |  |  |
| GO:0006414\_translational\_elongation | 3 | 0 |  |  |  |  |  |  |  |  |
| GO:0006491\_N-glycan\_processing | 3 | 0 |  |  |  |  |  |  |  |  |
| GO:0006498\_N-terminal\_protein\_lipidation | 3 | 0 |  |  |  |  |  |  |  |  |
| GO:0006531\_aspartate\_metabolic\_process | 3 | 0 |  |  |  |  |  |  |  |  |
| GO:0006598\_polyamine\_catabolic\_process | 3 | 0 |  |  |  |  |  |  |  |  |
| GO:0006620\_posttranslational\_protein\_targeting\_to\_membrane | 3 | 0 |  |  |  |  |  |  |  |  |
| GO:0006625\_protein\_targeting\_to\_peroxisome | 3 | 0 |  |  |  |  |  |  |  |  |
| GO:0006651\_diacylglycerol\_biosynthetic\_process | 3 | 0 |  |  |  |  |  |  |  |  |
| GO:0006670\_sphingosine\_metabolic\_process | 3 | 0 |  |  |  |  |  |  |  |  |
| GO:0006677\_glycosylceramide\_metabolic\_process | 3 | 0 |  |  |  |  |  |  |  |  |
| GO:0006689\_ganglioside\_catabolic\_process | 3 | 0 |  |  |  |  |  |  |  |  |
| GO:0006699\_bile\_acid\_biosynthetic\_process | 3 | 0 |  |  |  |  |  |  |  |  |
| GO:0006791\_sulfur\_utilization | 3 | 0 |  |  |  |  |  |  |  |  |
| GO:0006817\_phosphate\_transport | 3 | 0 |  |  |  |  |  |  |  |  |
| GO:0006825\_copper\_ion\_transport | 3 | 0 |  |  |  |  |  |  |  |  |
| GO:0006828\_manganese\_ion\_transport | 3 | 0 |  |  |  |  |  |  |  |  |
| GO:0006857\_oligopeptide\_transport | 3 | 0 |  |  |  |  |  |  |  |  |
| GO:0006892\_post-Golgi\_vesicle-mediated\_transport | 3 | 0 |  |  |  |  |  |  |  |  |
| GO:0006904\_vesicle\_docking\_during\_exocytosis | 3 | 0 |  |  |  |  |  |  |  |  |
| GO:0006926\_virus-infected\_cell\_apoptosis | 3 | 0 |  |  |  |  |  |  |  |  |
| GO:0006953\_acute-phase\_response | 3 | 0 |  |  |  |  |  |  |  |  |
| GO:0007000\_nucleolus\_organization | 3 | 0 |  |  |  |  |  |  |  |  |
| GO:0007041\_lysosomal\_transport | 3 | 0 |  |  |  |  |  |  |  |  |
| GO:0007043\_cell-cell\_junction\_assembly | 3 | 0 |  |  |  |  |  |  |  |  |
| GO:0007090\_regulation\_of\_S\_phase\_of\_mitotic\_cell\_cycle | 3 | 0 |  |  |  |  |  |  |  |  |
| GO:0007195\_inhibition\_of\_adenylate\_cyclase\_activity\_by\_dopamine\_receptor\_signaling\_pathway | 3 | 0 |  |  |  |  |  |  |  |  |
| GO:0007199\_G-protein\_signaling\_\_coupled\_to\_cGMP\_nucleotide\_second\_messenger | 3 | 0 |  |  |  |  |  |  |  |  |
| GO:0007213\_muscarinic\_acetylcholine\_receptor\_signaling\_pathway | 3 | 0 |  |  |  |  |  |  |  |  |
| GO:0007250\_activation\_of\_NF-kappaB-inducing\_kinase\_activity | 3 | 0 |  |  |  |  |  |  |  |  |
| GO:0007252\_I-kappaB\_phosphorylation | 3 | 0 |  |  |  |  |  |  |  |  |
| GO:0007262\_STAT\_protein\_nuclear\_translocation | 3 | 0 |  |  |  |  |  |  |  |  |
| GO:0007288\_sperm\_axoneme\_assembly | 3 | 0 |  |  |  |  |  |  |  |  |
| GO:0007350\_blastoderm\_segmentation | 3 | 0 |  |  |  |  |  |  |  |  |
| GO:0007403\_glial\_cell\_fate\_determination | 3 | 0 |  |  |  |  |  |  |  |  |
| GO:0007412\_axon\_target\_recognition | 3 | 0 |  |  |  |  |  |  |  |  |
| GO:0007468\_regulation\_of\_rhodopsin\_gene\_expression | 3 | 0 |  |  |  |  |  |  |  |  |
| GO:0007525\_somatic\_muscle\_development | 3 | 0 |  |  |  |  |  |  |  |  |
| GO:0007635\_chemosensory\_behavior | 3 | 0 |  |  |  |  |  |  |  |  |
| GO:0008090\_retrograde\_axon\_cargo\_transport | 3 | 0 |  |  |  |  |  |  |  |  |
| GO:0008347\_glial\_cell\_migration | 3 | 0 |  |  |  |  |  |  |  |  |
| GO:0008635\_activation\_of\_caspase\_activity\_by\_cytochrome\_c | 3 | 0 |  |  |  |  |  |  |  |  |
| GO:0009060\_aerobic\_respiration | 3 | 0 |  |  |  |  |  |  |  |  |
| GO:0009081\_branched\_chain\_family\_amino\_acid\_metabolic\_process | 3 | 0 |  |  |  |  |  |  |  |  |
| GO:0009086\_methionine\_biosynthetic\_process | 3 | 0 |  |  |  |  |  |  |  |  |
| GO:0009135\_purine\_nucleoside\_diphosphate\_metabolic\_process | 3 | 0 |  |  |  |  |  |  |  |  |
| GO:0009137\_purine\_nucleoside\_diphosphate\_catabolic\_process | 3 | 0 |  |  |  |  |  |  |  |  |
| GO:0009155\_purine\_deoxyribonucleotide\_catabolic\_process | 3 | 0 |  |  |  |  |  |  |  |  |
| GO:0009179\_purine\_ribonucleoside\_diphosphate\_metabolic\_process | 3 | 0 |  |  |  |  |  |  |  |  |
| GO:0009181\_purine\_ribonucleoside\_diphosphate\_catabolic\_process | 3 | 0 |  |  |  |  |  |  |  |  |
| GO:0009185\_ribonucleoside\_diphosphate\_metabolic\_process | 3 | 0 |  |  |  |  |  |  |  |  |
| GO:0009191\_ribonucleoside\_diphosphate\_catabolic\_process | 3 | 0 |  |  |  |  |  |  |  |  |
| GO:0009199\_ribonucleoside\_triphosphate\_metabolic\_process | 3 | 0 |  |  |  |  |  |  |  |  |
| GO:0009204\_deoxyribonucleoside\_triphosphate\_catabolic\_process | 3 | 0 |  |  |  |  |  |  |  |  |
| GO:0009205\_purine\_ribonucleoside\_triphosphate\_metabolic\_process | 3 | 0 |  |  |  |  |  |  |  |  |
| GO:0009217\_purine\_deoxyribonucleoside\_triphosphate\_catabolic\_process | 3 | 0 |  |  |  |  |  |  |  |  |
| GO:0010043\_response\_to\_zinc\_ion | 3 | 0 |  |  |  |  |  |  |  |  |
| GO:0010159\_specification\_of\_organ\_position | 3 | 0 |  |  |  |  |  |  |  |  |
| GO:0010172\_embryonic\_body\_morphogenesis | 3 | 0 |  |  |  |  |  |  |  |  |
| GO:0010216\_maintenance\_of\_DNA\_methylation | 3 | 0 |  |  |  |  |  |  |  |  |
| GO:0010273\_detoxification\_of\_copper\_ion | 3 | 0 |  |  |  |  |  |  |  |  |
| GO:0010454\_negative\_regulation\_of\_cell\_fate\_commitment | 3 | 0 |  |  |  |  |  |  |  |  |
| GO:0010507\_negative\_regulation\_of\_autophagy | 3 | 0 |  |  |  |  |  |  |  |  |
| GO:0010524\_positive\_regulation\_of\_calcium\_ion\_transport\_into\_cytosol | 3 | 0 |  |  |  |  |  |  |  |  |
| GO:0010573\_vascular\_endothelial\_growth\_factor\_production | 3 | 0 |  |  |  |  |  |  |  |  |
| GO:0010574\_regulation\_of\_vascular\_endothelial\_growth\_factor\_production | 3 | 0 |  |  |  |  |  |  |  |  |
| GO:0010575\_positive\_regulation\_vascular\_endothelial\_growth\_factor\_production | 3 | 0 |  |  |  |  |  |  |  |  |
| GO:0010717\_regulation\_of\_epithelial\_to\_mesenchymal\_transition | 3 | 0 |  |  |  |  |  |  |  |  |
| GO:0010884\_positive\_regulation\_of\_lipid\_storage | 3 | 0 |  |  |  |  |  |  |  |  |
| GO:0010888\_negative\_regulation\_of\_lipid\_storage | 3 | 0 |  |  |  |  |  |  |  |  |
| GO:0010889\_regulation\_of\_sequestering\_of\_triglyceride | 3 | 0 |  |  |  |  |  |  |  |  |
| GO:0010893\_positive\_regulation\_of\_steroid\_biosynthetic\_process | 3 | 0 |  |  |  |  |  |  |  |  |
| GO:0010894\_negative\_regulation\_of\_steroid\_biosynthetic\_process | 3 | 0 |  |  |  |  |  |  |  |  |
| GO:0010998\_regulation\_of\_translational\_initiation\_by\_eIF2\_alpha\_phosphorylation | 3 | 0 |  |  |  |  |  |  |  |  |
| GO:0010999\_regulation\_of\_eIF2\_alpha\_phosphorylation\_by\_heme | 3 | 0 |  |  |  |  |  |  |  |  |
| GO:0014074\_response\_to\_purine | 3 | 0 |  |  |  |  |  |  |  |  |
| GO:0014909\_smooth\_muscle\_cell\_migration | 3 | 0 |  |  |  |  |  |  |  |  |
| GO:0015669\_gas\_transport | 3 | 0 |  |  |  |  |  |  |  |  |
| GO:0015760\_glucose-6-phosphate\_transport | 3 | 0 |  |  |  |  |  |  |  |  |
| GO:0015816\_glycine\_transport | 3 | 0 |  |  |  |  |  |  |  |  |
| GO:0015838\_betaine\_transport | 3 | 0 |  |  |  |  |  |  |  |  |
| GO:0015871\_choline\_transport | 3 | 0 |  |  |  |  |  |  |  |  |
| GO:0015879\_carnitine\_transport | 3 | 0 |  |  |  |  |  |  |  |  |
| GO:0015893\_drug\_transport | 3 | 0 |  |  |  |  |  |  |  |  |
| GO:0015909\_long-chain\_fatty\_acid\_transport | 3 | 0 |  |  |  |  |  |  |  |  |
| GO:0015936\_coenzyme\_A\_metabolic\_process | 3 | 0 |  |  |  |  |  |  |  |  |
| GO:0015988\_energy\_coupled\_proton\_transport\_\_against\_electrochemical\_gradient | 3 | 0 |  |  |  |  |  |  |  |  |
| GO:0015991\_ATP\_hydrolysis\_coupled\_proton\_transport | 3 | 0 |  |  |  |  |  |  |  |  |
| GO:0016241\_regulation\_of\_macroautophagy | 3 | 0 |  |  |  |  |  |  |  |  |
| GO:0016322\_neuron\_remodeling | 3 | 0 |  |  |  |  |  |  |  |  |
| GO:0016556\_mRNA\_modification | 3 | 0 |  |  |  |  |  |  |  |  |
| GO:0016973\_poly(A)+\_mRNA\_export\_from\_nucleus | 3 | 0 |  |  |  |  |  |  |  |  |
| GO:0018196\_peptidyl-asparagine\_modification | 3 | 0 |  |  |  |  |  |  |  |  |
| GO:0018208\_peptidyl-proline\_modification | 3 | 0 |  |  |  |  |  |  |  |  |
| GO:0018279\_protein\_amino\_acid\_N-linked\_glycosylation\_via\_asparagine | 3 | 0 |  |  |  |  |  |  |  |  |
| GO:0018894\_dibenzo-p-dioxin\_metabolic\_process | 3 | 0 |  |  |  |  |  |  |  |  |
| GO:0019058\_viral\_infectious\_cycle | 3 | 0 |  |  |  |  |  |  |  |  |
| GO:0019230\_proprioception | 3 | 0 |  |  |  |  |  |  |  |  |
| GO:0019236\_response\_to\_pheromone | 3 | 0 |  |  |  |  |  |  |  |  |
| GO:0019359\_nicotinamide\_nucleotide\_biosynthetic\_process | 3 | 0 |  |  |  |  |  |  |  |  |
| GO:0019363\_pyridine\_nucleotide\_biosynthetic\_process | 3 | 0 |  |  |  |  |  |  |  |  |
| GO:0019438\_aromatic\_compound\_biosynthetic\_process | 3 | 0 |  |  |  |  |  |  |  |  |
| GO:0019439\_aromatic\_compound\_catabolic\_process | 3 | 0 |  |  |  |  |  |  |  |  |
| GO:0019614\_catechol\_catabolic\_process | 3 | 0 |  |  |  |  |  |  |  |  |
| GO:0019674\_NAD\_metabolic\_process | 3 | 0 |  |  |  |  |  |  |  |  |
| GO:0019852\_L-ascorbic\_acid\_metabolic\_process | 3 | 0 |  |  |  |  |  |  |  |  |
| GO:0019934\_cGMP-mediated\_signaling | 3 | 0 |  |  |  |  |  |  |  |  |
| GO:0019987\_negative\_regulation\_of\_anti-apoptosis | 3 | 0 |  |  |  |  |  |  |  |  |
| GO:0021527\_spinal\_cord\_association\_neuron\_differentiation | 3 | 0 |  |  |  |  |  |  |  |  |
| GO:0021529\_spinal\_cord\_oligodendrocyte\_cell\_differentiation | 3 | 0 |  |  |  |  |  |  |  |  |
| GO:0021530\_spinal\_cord\_oligodendrocyte\_cell\_fate\_specification | 3 | 0 |  |  |  |  |  |  |  |  |
| GO:0021555\_midbrain-hindbrain\_boundary\_morphogenesis | 3 | 0 |  |  |  |  |  |  |  |  |
| GO:0021563\_glossopharyngeal\_nerve\_development | 3 | 0 |  |  |  |  |  |  |  |  |
| GO:0021570\_rhombomere\_4\_development | 3 | 0 |  |  |  |  |  |  |  |  |
| GO:0021591\_ventricular\_system\_development | 3 | 0 |  |  |  |  |  |  |  |  |
| GO:0021615\_glossopharyngeal\_nerve\_morphogenesis | 3 | 0 |  |  |  |  |  |  |  |  |
| GO:0021794\_thalamus\_development | 3 | 0 |  |  |  |  |  |  |  |  |
| GO:0021798\_forebrain\_dorsal\_ventral\_pattern\_formation | 3 | 0 |  |  |  |  |  |  |  |  |
| GO:0021800\_cerebral\_cortex\_tangential\_migration | 3 | 0 |  |  |  |  |  |  |  |  |
| GO:0021819\_layer\_formation\_in\_the\_cerebral\_cortex | 3 | 0 |  |  |  |  |  |  |  |  |
| GO:0021859\_pyramidal\_neuron\_differentiation | 3 | 0 |  |  |  |  |  |  |  |  |
| GO:0021860\_pyramidal\_neuron\_development | 3 | 0 |  |  |  |  |  |  |  |  |
| GO:0021889\_olfactory\_bulb\_interneuron\_differentiation | 3 | 0 |  |  |  |  |  |  |  |  |
| GO:0021891\_olfactory\_bulb\_interneuron\_development | 3 | 0 |  |  |  |  |  |  |  |  |
| GO:0021912\_regulation\_of\_transcription\_from\_RNA\_polymerase\_II\_promoter\_involved\_in\_spinal\_cord\_motor\_neuron\_fate\_specification | 3 | 0 |  |  |  |  |  |  |  |  |
| GO:0021979\_hypothalamus\_cell\_differentiation | 3 | 0 |  |  |  |  |  |  |  |  |
| GO:0022010\_myelination\_in\_the\_central\_nervous\_system | 3 | 0 |  |  |  |  |  |  |  |  |
| GO:0022027\_interkinetic\_nuclear\_migration | 3 | 0 |  |  |  |  |  |  |  |  |
| GO:0022406\_membrane\_docking | 3 | 0 |  |  |  |  |  |  |  |  |
| GO:0030033\_microvillus\_assembly | 3 | 0 |  |  |  |  |  |  |  |  |
| GO:0030091\_protein\_repair | 3 | 0 |  |  |  |  |  |  |  |  |
| GO:0030195\_negative\_regulation\_of\_blood\_coagulation | 3 | 0 |  |  |  |  |  |  |  |  |
| GO:0030224\_monocyte\_differentiation | 3 | 0 |  |  |  |  |  |  |  |  |
| GO:0030307\_positive\_regulation\_of\_cell\_growth | 3 | 0 |  |  |  |  |  |  |  |  |
| GO:0030319\_cellular\_di-\_\_tri-valent\_inorganic\_anion\_homeostasis | 3 | 0 |  |  |  |  |  |  |  |  |
| GO:0030320\_cellular\_monovalent\_inorganic\_anion\_homeostasis | 3 | 0 |  |  |  |  |  |  |  |  |
| GO:0030321\_transepithelial\_chloride\_transport | 3 | 0 |  |  |  |  |  |  |  |  |
| GO:0030501\_positive\_regulation\_of\_bone\_mineralization | 3 | 0 |  |  |  |  |  |  |  |  |
| GO:0030513\_positive\_regulation\_of\_BMP\_signaling\_pathway | 3 | 0 |  |  |  |  |  |  |  |  |
| GO:0030538\_embryonic\_genitalia\_morphogenesis | 3 | 0 |  |  |  |  |  |  |  |  |
| GO:0030540\_female\_genitalia\_development | 3 | 0 |  |  |  |  |  |  |  |  |
| GO:0030574\_collagen\_catabolic\_process | 3 | 0 |  |  |  |  |  |  |  |  |
| GO:0030643\_cellular\_phosphate\_ion\_homeostasis | 3 | 0 |  |  |  |  |  |  |  |  |
| GO:0030718\_germ-line\_stem\_cell\_maintenance | 3 | 0 |  |  |  |  |  |  |  |  |
| GO:0030730\_sequestering\_of\_triglyceride | 3 | 0 |  |  |  |  |  |  |  |  |
| GO:0030836\_positive\_regulation\_of\_actin\_filament\_depolymerization | 3 | 0 |  |  |  |  |  |  |  |  |
| GO:0030857\_negative\_regulation\_of\_epithelial\_cell\_differentiation | 3 | 0 |  |  |  |  |  |  |  |  |
| GO:0030916\_otic\_vesicle\_formation | 3 | 0 |  |  |  |  |  |  |  |  |
| GO:0031000\_response\_to\_caffeine | 3 | 0 |  |  |  |  |  |  |  |  |
| GO:0031063\_regulation\_of\_histone\_deacetylation | 3 | 0 |  |  |  |  |  |  |  |  |
| GO:0031065\_positive\_regulation\_of\_histone\_deacetylation | 3 | 0 |  |  |  |  |  |  |  |  |
| GO:0031112\_positive\_regulation\_of\_microtubule\_polymerization\_or\_depolymerization | 3 | 0 |  |  |  |  |  |  |  |  |
| GO:0031116\_positive\_regulation\_of\_microtubule\_polymerization | 3 | 0 |  |  |  |  |  |  |  |  |
| GO:0031133\_regulation\_of\_axon\_diameter | 3 | 0 |  |  |  |  |  |  |  |  |
| GO:0031282\_regulation\_of\_guanylate\_cyclase\_activity | 3 | 0 |  |  |  |  |  |  |  |  |
| GO:0031333\_negative\_regulation\_of\_protein\_complex\_assembly | 3 | 0 |  |  |  |  |  |  |  |  |
| GO:0031397\_negative\_regulation\_of\_protein\_ubiquitination | 3 | 0 |  |  |  |  |  |  |  |  |
| GO:0031398\_positive\_regulation\_of\_protein\_ubiquitination | 3 | 0 |  |  |  |  |  |  |  |  |
| GO:0031503\_protein\_complex\_localization | 3 | 0 |  |  |  |  |  |  |  |  |
| GO:0031571\_G1\_DNA\_damage\_checkpoint | 3 | 0 |  |  |  |  |  |  |  |  |
| GO:0031579\_membrane\_raft\_organization | 3 | 0 |  |  |  |  |  |  |  |  |
| GO:0031638\_zymogen\_activation | 3 | 0 |  |  |  |  |  |  |  |  |
| GO:0031641\_regulation\_of\_myelination | 3 | 0 |  |  |  |  |  |  |  |  |
| GO:0031642\_negative\_regulation\_of\_myelination | 3 | 0 |  |  |  |  |  |  |  |  |
| GO:0031649\_heat\_generation | 3 | 0 |  |  |  |  |  |  |  |  |
| GO:0031943\_regulation\_of\_glucocorticoid\_metabolic\_process | 3 | 0 |  |  |  |  |  |  |  |  |
| GO:0032020\_ISG15-protein\_conjugation | 3 | 0 |  |  |  |  |  |  |  |  |
| GO:0032060\_bleb\_formation | 3 | 0 |  |  |  |  |  |  |  |  |
| GO:0032095\_regulation\_of\_response\_to\_food | 3 | 0 |  |  |  |  |  |  |  |  |
| GO:0032272\_negative\_regulation\_of\_protein\_polymerization | 3 | 0 |  |  |  |  |  |  |  |  |
| GO:0032288\_myelin\_assembly | 3 | 0 |  |  |  |  |  |  |  |  |
| GO:0032291\_ensheathment\_of\_axons\_in\_the\_central\_nervous\_system | 3 | 0 |  |  |  |  |  |  |  |  |
| GO:0032355\_response\_to\_estradiol\_stimulus | 3 | 0 |  |  |  |  |  |  |  |  |
| GO:0032402\_melanosome\_transport | 3 | 0 |  |  |  |  |  |  |  |  |
| GO:0032411\_positive\_regulation\_of\_transporter\_activity | 3 | 0 |  |  |  |  |  |  |  |  |
| GO:0032414\_positive\_regulation\_of\_ion\_transmembrane\_transporter\_activity | 3 | 0 |  |  |  |  |  |  |  |  |
| GO:0032436\_positive\_regulation\_of\_proteasomal\_ubiquitin-dependent\_protein\_catabolic\_process | 3 | 0 |  |  |  |  |  |  |  |  |
| GO:0032528\_microvillus\_organization | 3 | 0 |  |  |  |  |  |  |  |  |
| GO:0032536\_regulation\_of\_cell\_projection\_size | 3 | 0 |  |  |  |  |  |  |  |  |
| GO:0032632\_interleukin-3\_production | 3 | 0 |  |  |  |  |  |  |  |  |
| GO:0032634\_interleukin-5\_production | 3 | 0 |  |  |  |  |  |  |  |  |
| GO:0032674\_regulation\_of\_interleukin-5\_production | 3 | 0 |  |  |  |  |  |  |  |  |
| GO:0032703\_negative\_regulation\_of\_interleukin-2\_production | 3 | 0 |  |  |  |  |  |  |  |  |
| GO:0032753\_positive\_regulation\_of\_interleukin-4\_production | 3 | 0 |  |  |  |  |  |  |  |  |
| GO:0032823\_regulation\_of\_natural\_killer\_cell\_differentiation | 3 | 0 |  |  |  |  |  |  |  |  |
| GO:0032825\_positive\_regulation\_of\_natural\_killer\_cell\_differentiation | 3 | 0 |  |  |  |  |  |  |  |  |
| GO:0032856\_activation\_of\_Ras\_GTPase\_activity | 3 | 0 |  |  |  |  |  |  |  |  |
| GO:0032862\_activation\_of\_Rho\_GTPase\_activity | 3 | 0 |  |  |  |  |  |  |  |  |
| GO:0032874\_positive\_regulation\_of\_stress-activated\_MAPK\_cascade | 3 | 0 |  |  |  |  |  |  |  |  |
| GO:0032881\_regulation\_of\_polysaccharide\_metabolic\_process | 3 | 0 |  |  |  |  |  |  |  |  |
| GO:0032890\_regulation\_of\_organic\_acid\_transport | 3 | 0 |  |  |  |  |  |  |  |  |
| GO:0033058\_directional\_locomotion | 3 | 0 |  |  |  |  |  |  |  |  |
| GO:0033080\_immature\_T\_cell\_proliferation\_in\_the\_thymus | 3 | 0 |  |  |  |  |  |  |  |  |
| GO:0033084\_regulation\_of\_immature\_T\_cell\_proliferation\_in\_the\_thymus | 3 | 0 |  |  |  |  |  |  |  |  |
| GO:0033091\_positive\_regulation\_of\_immature\_T\_cell\_proliferation | 3 | 0 |  |  |  |  |  |  |  |  |
| GO:0033137\_negative\_regulation\_of\_peptidyl-serine\_phosphorylation | 3 | 0 |  |  |  |  |  |  |  |  |
| GO:0033153\_T\_cell\_receptor\_V(D)J\_recombination | 3 | 0 |  |  |  |  |  |  |  |  |
| GO:0033209\_tumor\_necrosis\_factor-mediated\_signaling\_pathway | 3 | 0 |  |  |  |  |  |  |  |  |
| GO:0033261\_regulation\_of\_S\_phase | 3 | 0 |  |  |  |  |  |  |  |  |
| GO:0033600\_negative\_regulation\_of\_mammary\_gland\_epithelial\_cell\_proliferation | 3 | 0 |  |  |  |  |  |  |  |  |
| GO:0033631\_cell-cell\_adhesion\_mediated\_by\_integrin | 3 | 0 |  |  |  |  |  |  |  |  |
| GO:0033993\_response\_to\_lipid | 3 | 0 |  |  |  |  |  |  |  |  |
| GO:0034220\_ion\_transmembrane\_transport | 3 | 0 |  |  |  |  |  |  |  |  |
| GO:0034308\_monohydric\_alcohol\_metabolic\_process | 3 | 0 |  |  |  |  |  |  |  |  |
| GO:0034313\_diol\_catabolic\_process | 3 | 0 |  |  |  |  |  |  |  |  |
| GO:0034331\_cell\_junction\_maintenance | 3 | 0 |  |  |  |  |  |  |  |  |
| GO:0034332\_adherens\_junction\_organization | 3 | 0 |  |  |  |  |  |  |  |  |
| GO:0034375\_high-density\_lipoprotein\_particle\_remodeling | 3 | 0 |  |  |  |  |  |  |  |  |
| GO:0034381\_lipoprotein\_particle\_clearance | 3 | 0 |  |  |  |  |  |  |  |  |
| GO:0034612\_response\_to\_tumor\_necrosis\_factor | 3 | 0 |  |  |  |  |  |  |  |  |
| GO:0034655\_nucleobase\_\_nucleoside\_\_nucleotide\_and\_nucleic\_acid\_catabolic\_process | 3 | 0 |  |  |  |  |  |  |  |  |
| GO:0034656\_nucleobase\_\_nucleoside\_and\_nucleotide\_catabolic\_process | 3 | 0 |  |  |  |  |  |  |  |  |
| GO:0035067\_negative\_regulation\_of\_histone\_acetylation | 3 | 0 |  |  |  |  |  |  |  |  |
| GO:0035084\_flagellar\_axoneme\_assembly | 3 | 0 |  |  |  |  |  |  |  |  |
| GO:0035166\_post-embryonic\_hemopoiesis | 3 | 0 |  |  |  |  |  |  |  |  |
| GO:0035283\_central\_nervous\_system\_segmentation | 3 | 0 |  |  |  |  |  |  |  |  |
| GO:0035284\_brain\_segmentation | 3 | 0 |  |  |  |  |  |  |  |  |
| GO:0042097\_interleukin-4\_biosynthetic\_process | 3 | 0 |  |  |  |  |  |  |  |  |
| GO:0042135\_neurotransmitter\_catabolic\_process | 3 | 0 |  |  |  |  |  |  |  |  |
| GO:0042271\_susceptibility\_to\_natural\_killer\_cell\_mediated\_cytotoxicity | 3 | 0 |  |  |  |  |  |  |  |  |
| GO:0042273\_ribosomal\_large\_subunit\_biogenesis | 3 | 0 |  |  |  |  |  |  |  |  |
| GO:0042375\_quinone\_cofactor\_metabolic\_process | 3 | 0 |  |  |  |  |  |  |  |  |
| GO:0042420\_dopamine\_catabolic\_process | 3 | 0 |  |  |  |  |  |  |  |  |
| GO:0042421\_norepinephrine\_biosynthetic\_process | 3 | 0 |  |  |  |  |  |  |  |  |
| GO:0042424\_catecholamine\_catabolic\_process | 3 | 0 |  |  |  |  |  |  |  |  |
| GO:0042447\_hormone\_catabolic\_process | 3 | 0 |  |  |  |  |  |  |  |  |
| GO:0042448\_progesterone\_metabolic\_process | 3 | 0 |  |  |  |  |  |  |  |  |
| GO:0042523\_positive\_regulation\_of\_tyrosine\_phosphorylation\_of\_Stat5\_protein | 3 | 0 |  |  |  |  |  |  |  |  |
| GO:0042659\_regulation\_of\_cell\_fate\_specification | 3 | 0 |  |  |  |  |  |  |  |  |
| GO:0042668\_auditory\_receptor\_cell\_fate\_determination | 3 | 0 |  |  |  |  |  |  |  |  |
| GO:0042670\_retinal\_cone\_cell\_differentiation | 3 | 0 |  |  |  |  |  |  |  |  |
| GO:0042693\_muscle\_cell\_fate\_commitment | 3 | 0 |  |  |  |  |  |  |  |  |
| GO:0042711\_maternal\_behavior | 3 | 0 |  |  |  |  |  |  |  |  |
| GO:0042745\_circadian\_sleep\_wake\_cycle | 3 | 0 |  |  |  |  |  |  |  |  |
| GO:0042759\_long-chain\_fatty\_acid\_biosynthetic\_process | 3 | 0 |  |  |  |  |  |  |  |  |
| GO:0042787\_protein\_ubiquitination\_during\_ubiquitin-dependent\_protein\_catabolic\_process | 3 | 0 |  |  |  |  |  |  |  |  |
| GO:0043045\_DNA\_methylation\_during\_embryonic\_development | 3 | 0 |  |  |  |  |  |  |  |  |
| GO:0043090\_amino\_acid\_import | 3 | 0 |  |  |  |  |  |  |  |  |
| GO:0043092\_L-amino\_acid\_import | 3 | 0 |  |  |  |  |  |  |  |  |
| GO:0043094\_cellular\_metabolic\_compound\_salvage | 3 | 0 |  |  |  |  |  |  |  |  |
| GO:0043101\_purine\_salvage | 3 | 0 |  |  |  |  |  |  |  |  |
| GO:0043149\_stress\_fiber\_formation | 3 | 0 |  |  |  |  |  |  |  |  |
| GO:0043174\_nucleoside\_salvage | 3 | 0 |  |  |  |  |  |  |  |  |
| GO:0043200\_response\_to\_amino\_acid\_stimulus | 3 | 0 |  |  |  |  |  |  |  |  |
| GO:0043243\_positive\_regulation\_of\_protein\_complex\_disassembly | 3 | 0 |  |  |  |  |  |  |  |  |
| GO:0043249\_erythrocyte\_maturation | 3 | 0 |  |  |  |  |  |  |  |  |
| GO:0043267\_negative\_regulation\_of\_potassium\_ion\_transport | 3 | 0 |  |  |  |  |  |  |  |  |
| GO:0043371\_negative\_regulation\_of\_CD4-positive\_\_alpha\_beta\_T\_cell\_differentiation | 3 | 0 |  |  |  |  |  |  |  |  |
| GO:0043462\_regulation\_of\_ATPase\_activity | 3 | 0 |  |  |  |  |  |  |  |  |
| GO:0043569\_negative\_regulation\_of\_insulin-like\_growth\_factor\_receptor\_signaling\_pathway | 3 | 0 |  |  |  |  |  |  |  |  |
| GO:0043574\_peroxisomal\_transport | 3 | 0 |  |  |  |  |  |  |  |  |
| GO:0043900\_regulation\_of\_multi-organism\_process | 3 | 0 |  |  |  |  |  |  |  |  |
| GO:0043954\_cellular\_component\_maintenance | 3 | 0 |  |  |  |  |  |  |  |  |
| GO:0044030\_regulation\_of\_DNA\_methylation | 3 | 0 |  |  |  |  |  |  |  |  |
| GO:0044089\_positive\_regulation\_of\_cellular\_component\_biogenesis | 3 | 0 |  |  |  |  |  |  |  |  |
| GO:0044273\_sulfur\_compound\_catabolic\_process | 3 | 0 |  |  |  |  |  |  |  |  |
| GO:0045047\_protein\_targeting\_to\_ER | 3 | 0 |  |  |  |  |  |  |  |  |
| GO:0045085\_negative\_regulation\_of\_interleukin-2\_biosynthetic\_process | 3 | 0 |  |  |  |  |  |  |  |  |
| GO:0045110\_intermediate\_filament\_bundle\_assembly | 3 | 0 |  |  |  |  |  |  |  |  |
| GO:0045143\_homologous\_chromosome\_segregation | 3 | 0 |  |  |  |  |  |  |  |  |
| GO:0045198\_establishment\_of\_epithelial\_cell\_apical\_basal\_polarity | 3 | 0 |  |  |  |  |  |  |  |  |
| GO:0045217\_cell-cell\_junction\_maintenance | 3 | 0 |  |  |  |  |  |  |  |  |
| GO:0045348\_positive\_regulation\_of\_MHC\_class\_II\_biosynthetic\_process | 3 | 0 |  |  |  |  |  |  |  |  |
| GO:0045402\_regulation\_of\_interleukin-4\_biosynthetic\_process | 3 | 0 |  |  |  |  |  |  |  |  |
| GO:0045404\_positive\_regulation\_of\_interleukin-4\_biosynthetic\_process | 3 | 0 |  |  |  |  |  |  |  |  |
| GO:0045542\_positive\_regulation\_of\_cholesterol\_biosynthetic\_process | 3 | 0 |  |  |  |  |  |  |  |  |
| GO:0045607\_regulation\_of\_auditory\_receptor\_cell\_differentiation | 3 | 0 |  |  |  |  |  |  |  |  |
| GO:0045623\_negative\_regulation\_of\_T-helper\_cell\_differentiation | 3 | 0 |  |  |  |  |  |  |  |  |
| GO:0045625\_regulation\_of\_T-helper\_1\_cell\_differentiation | 3 | 0 |  |  |  |  |  |  |  |  |
| GO:0045631\_regulation\_of\_mechanoreceptor\_differentiation | 3 | 0 |  |  |  |  |  |  |  |  |
| GO:0045717\_negative\_regulation\_of\_fatty\_acid\_biosynthetic\_process | 3 | 0 |  |  |  |  |  |  |  |  |
| GO:0045723\_positive\_regulation\_of\_fatty\_acid\_biosynthetic\_process | 3 | 0 |  |  |  |  |  |  |  |  |
| GO:0045746\_negative\_regulation\_of\_Notch\_signaling\_pathway | 3 | 0 |  |  |  |  |  |  |  |  |
| GO:0045806\_negative\_regulation\_of\_endocytosis | 3 | 0 |  |  |  |  |  |  |  |  |
| GO:0045829\_negative\_regulation\_of\_isotype\_switching | 3 | 0 |  |  |  |  |  |  |  |  |
| GO:0045844\_positive\_regulation\_of\_striated\_muscle\_development | 3 | 0 |  |  |  |  |  |  |  |  |
| GO:0045907\_positive\_regulation\_of\_vasoconstriction | 3 | 0 |  |  |  |  |  |  |  |  |
| GO:0045922\_negative\_regulation\_of\_fatty\_acid\_metabolic\_process | 3 | 0 |  |  |  |  |  |  |  |  |
| GO:0045939\_negative\_regulation\_of\_steroid\_metabolic\_process | 3 | 0 |  |  |  |  |  |  |  |  |
| GO:0046013\_regulation\_of\_T\_cell\_homeostatic\_proliferation | 3 | 0 |  |  |  |  |  |  |  |  |
| GO:0046034\_ATP\_metabolic\_process | 3 | 0 |  |  |  |  |  |  |  |  |
| GO:0046325\_negative\_regulation\_of\_glucose\_import | 3 | 0 |  |  |  |  |  |  |  |  |
| GO:0046426\_negative\_regulation\_of\_JAK-STAT\_cascade | 3 | 0 |  |  |  |  |  |  |  |  |
| GO:0046457\_prostanoid\_biosynthetic\_process | 3 | 0 |  |  |  |  |  |  |  |  |
| GO:0046479\_glycosphingolipid\_catabolic\_process | 3 | 0 |  |  |  |  |  |  |  |  |
| GO:0046488\_phosphatidylinositol\_metabolic\_process | 3 | 0 |  |  |  |  |  |  |  |  |
| GO:0046549\_retinal\_cone\_cell\_development | 3 | 0 |  |  |  |  |  |  |  |  |
| GO:0046605\_regulation\_of\_centrosome\_cycle | 3 | 0 |  |  |  |  |  |  |  |  |
| GO:0046688\_response\_to\_copper\_ion | 3 | 0 |  |  |  |  |  |  |  |  |
| GO:0046717\_acid\_secretion | 3 | 0 |  |  |  |  |  |  |  |  |
| GO:0046885\_regulation\_of\_hormone\_biosynthetic\_process | 3 | 0 |  |  |  |  |  |  |  |  |
| GO:0048003\_antigen\_processing\_and\_presentation\_of\_lipid\_antigen\_via\_MHC\_class\_Ib | 3 | 0 |  |  |  |  |  |  |  |  |
| GO:0048007\_antigen\_processing\_and\_presentation\_\_exogenous\_lipid\_antigen\_via\_MHC\_class\_Ib | 3 | 0 |  |  |  |  |  |  |  |  |
| GO:0048012\_hepatocyte\_growth\_factor\_receptor\_signaling\_pathway | 3 | 0 |  |  |  |  |  |  |  |  |
| GO:0048050\_post-embryonic\_eye\_morphogenesis | 3 | 0 |  |  |  |  |  |  |  |  |
| GO:0048087\_positive\_regulation\_of\_pigmentation\_during\_development | 3 | 0 |  |  |  |  |  |  |  |  |
| GO:0048246\_macrophage\_chemotaxis | 3 | 0 |  |  |  |  |  |  |  |  |
| GO:0048251\_elastic\_fiber\_assembly | 3 | 0 |  |  |  |  |  |  |  |  |
| GO:0048278\_vesicle\_docking | 3 | 0 |  |  |  |  |  |  |  |  |
| GO:0048294\_negative\_regulation\_of\_isotype\_switching\_to\_IgE\_isotypes | 3 | 0 |  |  |  |  |  |  |  |  |
| GO:0048318\_axial\_mesoderm\_development | 3 | 0 |  |  |  |  |  |  |  |  |
| GO:0048597\_post-embryonic\_camera-type\_eye\_morphogenesis | 3 | 0 |  |  |  |  |  |  |  |  |
| GO:0048636\_positive\_regulation\_of\_muscle\_development | 3 | 0 |  |  |  |  |  |  |  |  |
| GO:0048660\_regulation\_of\_smooth\_muscle\_cell\_proliferation | 3 | 0 |  |  |  |  |  |  |  |  |
| GO:0048668\_collateral\_sprouting | 3 | 0 |  |  |  |  |  |  |  |  |
| GO:0048676\_axon\_extension\_involved\_in\_development | 3 | 0 |  |  |  |  |  |  |  |  |
| GO:0048755\_branching\_morphogenesis\_of\_a\_nerve | 3 | 0 |  |  |  |  |  |  |  |  |
| GO:0048845\_venous\_blood\_vessel\_morphogenesis | 3 | 0 |  |  |  |  |  |  |  |  |
| GO:0048859\_formation\_of\_anatomical\_boundary | 3 | 0 |  |  |  |  |  |  |  |  |
| GO:0048865\_stem\_cell\_fate\_commitment | 3 | 0 |  |  |  |  |  |  |  |  |
| GO:0050435\_beta-amyloid\_metabolic\_process | 3 | 0 |  |  |  |  |  |  |  |  |
| GO:0050650\_chondroitin\_sulfate\_proteoglycan\_biosynthetic\_process | 3 | 0 |  |  |  |  |  |  |  |  |
| GO:0050703\_interleukin-1\_alpha\_secretion | 3 | 0 |  |  |  |  |  |  |  |  |
| GO:0050705\_regulation\_of\_interleukin-1\_alpha\_secretion | 3 | 0 |  |  |  |  |  |  |  |  |
| GO:0050709\_negative\_regulation\_of\_protein\_secretion | 3 | 0 |  |  |  |  |  |  |  |  |
| GO:0050710\_negative\_regulation\_of\_cytokine\_secretion | 3 | 0 |  |  |  |  |  |  |  |  |
| GO:0050717\_positive\_regulation\_of\_interleukin-1\_alpha\_secretion | 3 | 0 |  |  |  |  |  |  |  |  |
| GO:0050774\_negative\_regulation\_of\_dendrite\_morphogenesis | 3 | 0 |  |  |  |  |  |  |  |  |
| GO:0050857\_positive\_regulation\_of\_antigen\_receptor-mediated\_signaling\_pathway | 3 | 0 |  |  |  |  |  |  |  |  |
| GO:0050882\_voluntary\_musculoskeletal\_movement | 3 | 0 |  |  |  |  |  |  |  |  |
| GO:0050913\_sensory\_perception\_of\_bitter\_taste | 3 | 0 |  |  |  |  |  |  |  |  |
| GO:0050996\_positive\_regulation\_of\_lipid\_catabolic\_process | 3 | 0 |  |  |  |  |  |  |  |  |
| GO:0051149\_positive\_regulation\_of\_muscle\_cell\_differentiation | 3 | 0 |  |  |  |  |  |  |  |  |
| GO:0051153\_regulation\_of\_striated\_muscle\_cell\_differentiation | 3 | 0 |  |  |  |  |  |  |  |  |
| GO:0051204\_protein\_insertion\_into\_mitochondrial\_membrane | 3 | 0 |  |  |  |  |  |  |  |  |
| GO:0051291\_protein\_heterooligomerization | 3 | 0 |  |  |  |  |  |  |  |  |
| GO:0051302\_regulation\_of\_cell\_division | 3 | 0 |  |  |  |  |  |  |  |  |
| GO:0051320\_S\_phase | 3 | 0 |  |  |  |  |  |  |  |  |
| GO:0051583\_dopamine\_uptake | 3 | 0 |  |  |  |  |  |  |  |  |
| GO:0051798\_positive\_regulation\_of\_hair\_follicle\_development | 3 | 0 |  |  |  |  |  |  |  |  |
| GO:0051882\_mitochondrial\_depolarization | 3 | 0 |  |  |  |  |  |  |  |  |
| GO:0051900\_regulation\_of\_mitochondrial\_depolarization | 3 | 0 |  |  |  |  |  |  |  |  |
| GO:0051925\_regulation\_of\_calcium\_ion\_transport\_via\_voltage-gated\_calcium\_channel\_activity | 3 | 0 |  |  |  |  |  |  |  |  |
| GO:0051926\_negative\_regulation\_of\_calcium\_ion\_transport | 3 | 0 |  |  |  |  |  |  |  |  |
| GO:0051930\_regulation\_of\_sensory\_perception\_of\_pain | 3 | 0 |  |  |  |  |  |  |  |  |
| GO:0051931\_regulation\_of\_sensory\_perception | 3 | 0 |  |  |  |  |  |  |  |  |
| GO:0051934\_catecholamine\_uptake\_during\_transmission\_of\_nerve\_impulse | 3 | 0 |  |  |  |  |  |  |  |  |
| GO:0051955\_regulation\_of\_amino\_acid\_transport | 3 | 0 |  |  |  |  |  |  |  |  |
| GO:0051962\_positive\_regulation\_of\_nervous\_system\_development | 3 | 0 |  |  |  |  |  |  |  |  |
| GO:0051965\_positive\_regulation\_of\_synaptogenesis | 3 | 0 |  |  |  |  |  |  |  |  |
| GO:0051967\_negative\_regulation\_of\_synaptic\_transmission\_\_glutamatergic | 3 | 0 |  |  |  |  |  |  |  |  |
| GO:0051983\_regulation\_of\_chromosome\_segregation | 3 | 0 |  |  |  |  |  |  |  |  |
| GO:0055061\_di-\_\_tri-valent\_inorganic\_anion\_homeostasis | 3 | 0 |  |  |  |  |  |  |  |  |
| GO:0055062\_phosphate\_ion\_homeostasis | 3 | 0 |  |  |  |  |  |  |  |  |
| GO:0055083\_monovalent\_inorganic\_anion\_homeostasis | 3 | 0 |  |  |  |  |  |  |  |  |
| GO:0055117\_regulation\_of\_cardiac\_muscle\_contraction | 3 | 0 |  |  |  |  |  |  |  |  |
| GO:0060009\_Sertoli\_cell\_development | 3 | 0 |  |  |  |  |  |  |  |  |
| GO:0060024\_rhythmic\_synaptic\_transmission | 3 | 0 |  |  |  |  |  |  |  |  |
| GO:0060033\_anatomical\_structure\_regression | 3 | 0 |  |  |  |  |  |  |  |  |
| GO:0060040\_retinal\_bipolar\_neuron\_differentiation | 3 | 0 |  |  |  |  |  |  |  |  |
| GO:0060084\_synaptic\_transmission\_involved\_in\_micturition | 3 | 0 |  |  |  |  |  |  |  |  |
| GO:0060123\_regulation\_of\_growth\_hormone\_secretion | 3 | 0 |  |  |  |  |  |  |  |  |
| GO:0060126\_somatotropin\_secreting\_cell\_differentiation | 3 | 0 |  |  |  |  |  |  |  |  |
| GO:0060192\_negative\_regulation\_of\_lipase\_activity | 3 | 0 |  |  |  |  |  |  |  |  |
| GO:0060219\_camera-type\_eye\_photoreceptor\_cell\_differentiation | 3 | 0 |  |  |  |  |  |  |  |  |
| GO:0060285\_ciliary\_cell\_motility | 3 | 0 |  |  |  |  |  |  |  |  |
| GO:0060294\_cilium\_movement\_involved\_in\_ciliary\_motility | 3 | 0 |  |  |  |  |  |  |  |  |
| GO:0060295\_regulation\_of\_cilium\_movement\_involved\_in\_ciliary\_motility | 3 | 0 |  |  |  |  |  |  |  |  |
| GO:0060296\_regulation\_of\_cilium\_beat\_frequency\_involved\_in\_ciliary\_motility | 3 | 0 |  |  |  |  |  |  |  |  |
| GO:0060314\_regulation\_of\_ryanodine-sensitive\_calcium-release\_channel\_activity | 3 | 0 |  |  |  |  |  |  |  |  |
| GO:0060396\_growth\_hormone\_receptor\_signaling\_pathway | 3 | 0 |  |  |  |  |  |  |  |  |
| GO:0060416\_response\_to\_growth\_hormone\_stimulus | 3 | 0 |  |  |  |  |  |  |  |  |
| GO:0060428\_lung\_epithelium\_development | 3 | 0 |  |  |  |  |  |  |  |  |
| GO:0060433\_bronchus\_development | 3 | 0 |  |  |  |  |  |  |  |  |
| GO:0060435\_bronchiole\_development | 3 | 0 |  |  |  |  |  |  |  |  |
| GO:0060460\_left\_lung\_morphogenesis | 3 | 0 |  |  |  |  |  |  |  |  |
| GO:0060491\_regulation\_of\_cell\_projection\_assembly | 3 | 0 |  |  |  |  |  |  |  |  |
| GO:0060523\_prostate\_epithelial\_cord\_elongation | 3 | 0 |  |  |  |  |  |  |  |  |
| GO:0060586\_multicellular\_organismal\_iron\_ion\_homeostasis | 3 | 0 |  |  |  |  |  |  |  |  |
| GO:0060596\_mammary\_placode\_formation | 3 | 0 |  |  |  |  |  |  |  |  |
| GO:0060632\_regulation\_of\_microtubule-based\_movement | 3 | 0 |  |  |  |  |  |  |  |  |
| GO:0060648\_mammary\_gland\_bud\_morphogenesis | 3 | 0 |  |  |  |  |  |  |  |  |
| GO:0060684\_epithelial-mesenchymal\_cell\_signaling | 3 | 0 |  |  |  |  |  |  |  |  |
| GO:0060686\_negative\_regulation\_of\_prostatic\_bud\_formation | 3 | 0 |  |  |  |  |  |  |  |  |
| GO:0060689\_cell\_differentiation\_involved\_in\_salivary\_gland\_development | 3 | 0 |  |  |  |  |  |  |  |  |
| GO:0060708\_spongiotrophoblast\_differentiation | 3 | 0 |  |  |  |  |  |  |  |  |
| GO:0060746\_parental\_behavior | 3 | 0 |  |  |  |  |  |  |  |  |
| GO:0060748\_tertiary\_branching\_involved\_in\_mammary\_gland\_duct\_morphogenesis | 3 | 0 |  |  |  |  |  |  |  |  |
| GO:0060750\_epithelial\_cell\_proliferation\_involved\_in\_mammary\_gland\_duct\_elongation | 3 | 0 |  |  |  |  |  |  |  |  |
| GO:0060841\_venous\_blood\_vessel\_development | 3 | 0 |  |  |  |  |  |  |  |  |
| GO:0070102\_interleukin-6-mediated\_signaling\_pathway | 3 | 0 |  |  |  |  |  |  |  |  |
| GO:0070169\_positive\_regulation\_of\_biomineral\_formation | 3 | 0 |  |  |  |  |  |  |  |  |
| GO:0070206\_protein\_trimerization | 3 | 0 |  |  |  |  |  |  |  |  |
| GO:0070207\_protein\_homotrimerization | 3 | 0 |  |  |  |  |  |  |  |  |
| GO:0070229\_negative\_regulation\_of\_lymphocyte\_apoptosis | 3 | 0 |  |  |  |  |  |  |  |  |
| GO:0070230\_positive\_regulation\_of\_lymphocyte\_apoptosis | 3 | 0 |  |  |  |  |  |  |  |  |
| GO:0070232\_regulation\_of\_T\_cell\_apoptosis | 3 | 0 |  |  |  |  |  |  |  |  |
| GO:0070233\_negative\_regulation\_of\_T\_cell\_apoptosis | 3 | 0 |  |  |  |  |  |  |  |  |
| GO:0070242\_thymocyte\_apoptosis | 3 | 0 |  |  |  |  |  |  |  |  |
| GO:0070243\_regulation\_of\_thymocyte\_apoptosis | 3 | 0 |  |  |  |  |  |  |  |  |
| GO:0070244\_negative\_regulation\_of\_thymocyte\_apoptosis | 3 | 0 |  |  |  |  |  |  |  |  |
| GO:0070307\_lens\_fiber\_cell\_development | 3 | 0 |  |  |  |  |  |  |  |  |
| GO:0070309\_lens\_fiber\_cell\_morphogenesis | 3 | 0 |  |  |  |  |  |  |  |  |
| GO:0070423\_nucleotide-binding\_oligomerization\_domain\_containing\_signaling\_pathway | 3 | 0 |  |  |  |  |  |  |  |  |
| GO:0070427\_nucleotide-binding\_oligomerization\_domain\_containing\_1\_signaling\_pathway | 3 | 0 |  |  |  |  |  |  |  |  |
| GO:0070431\_nucleotide-binding\_oligomerization\_domain\_containing\_2\_signaling\_pathway | 3 | 0 |  |  |  |  |  |  |  |  |
| GO:0070633\_transepithelial\_transport | 3 | 0 |  |  |  |  |  |  |  |  |
| GO:0070846\_Hsp90\_deacetylation | 3 | 0 |  |  |  |  |  |  |  |  |
| GO:0070873\_regulation\_of\_glycogen\_metabolic\_process | 3 | 0 |  |  |  |  |  |  |  |  |
| GO:0070875\_positive\_regulation\_of\_glycogen\_metabolic\_process | 3 | 0 |  |  |  |  |  |  |  |  |
| GO:0006753\_nucleoside\_phosphate\_metabolic\_process | 94 | 0 | 0.000000 | -0.000000 | 887 | 691.479878 | 778.12 | 864.760122 | 0.877249 |
| GO:0009117\_nucleotide\_metabolic\_process | 94 | 0 | 0.000000 | -0.000000 | 887 | 691.479878 | 778.12 | 864.760122 | 0.877249 |
| GO:0032943\_mononuclear\_cell\_proliferation | 94 | 0 | 0.000000 | -0.000000 | 887 | 691.479878 | 778.12 | 864.760122 | 0.877249 |
| GO:0046651\_lymphocyte\_proliferation | 94 | 0 | 0.000000 | -0.000000 | 887 | 691.479878 | 778.12 | 864.760122 | 0.877249 |
| GO:0001933\_negative\_regulation\_of\_protein\_amino\_acid\_phosphorylation | 16 | 0 | 0.000000 | -0.000000 | 930 | 736.237404 | 821.67 | 907.102596 | 0.883516 |
| GO:0003044\_regulation\_of\_systemic\_arterial\_blood\_pressure\_mediated\_by\_a\_chemical\_signal | 16 | 0 | 0.000000 | -0.000000 | 930 | 736.237404 | 821.67 | 907.102596 | 0.883516 |
| GO:0006664\_glycolipid\_metabolic\_process | 16 | 0 | 0.000000 | -0.000000 | 930 | 736.237404 | 821.67 | 907.102596 | 0.883516 |
| GO:0006821\_chloride\_transport | 16 | 0 | 0.000000 | -0.000000 | 930 | 736.237404 | 821.67 | 907.102596 | 0.883516 |
| GO:0007033\_vacuole\_organization | 16 | 0 | 0.000000 | -0.000000 | 930 | 736.237404 | 821.67 | 907.102596 | 0.883516 |
| GO:0007156\_homophilic\_cell\_adhesion | 16 | 0 | 0.000000 | -0.000000 | 930 | 736.237404 | 821.67 | 907.102596 | 0.883516 |
| GO:0007602\_phototransduction | 16 | 0 | 0.000000 | -0.000000 | 930 | 736.237404 | 821.67 | 907.102596 | 0.883516 |
| GO:0008654\_phospholipid\_biosynthetic\_process | 16 | 0 | 0.000000 | -0.000000 | 930 | 736.237404 | 821.67 | 907.102596 | 0.883516 |
| GO:0009988\_cell-cell\_recognition | 16 | 0 | 0.000000 | -0.000000 | 930 | 736.237404 | 821.67 | 907.102596 | 0.883516 |
| GO:0010038\_response\_to\_metal\_ion | 16 | 0 | 0.000000 | -0.000000 | 930 | 736.237404 | 821.67 | 907.102596 | 0.883516 |
| GO:0010243\_response\_to\_organic\_nitrogen | 16 | 0 | 0.000000 | -0.000000 | 930 | 736.237404 | 821.67 | 907.102596 | 0.883516 |
| GO:0010876\_lipid\_localization | 16 | 0 | 0.000000 | -0.000000 | 930 | 736.237404 | 821.67 | 907.102596 | 0.883516 |
| GO:0014075\_response\_to\_amine\_stimulus | 16 | 0 | 0.000000 | -0.000000 | 930 | 736.237404 | 821.67 | 907.102596 | 0.883516 |
| GO:0016126\_sterol\_biosynthetic\_process | 16 | 0 | 0.000000 | -0.000000 | 930 | 736.237404 | 821.67 | 907.102596 | 0.883516 |
| GO:0019722\_calcium-mediated\_signaling | 16 | 0 | 0.000000 | -0.000000 | 930 | 736.237404 | 821.67 | 907.102596 | 0.883516 |
| GO:0019751\_polyol\_metabolic\_process | 16 | 0 | 0.000000 | -0.000000 | 930 | 736.237404 | 821.67 | 907.102596 | 0.883516 |
| GO:0019915\_lipid\_storage | 16 | 0 | 0.000000 | -0.000000 | 930 | 736.237404 | 821.67 | 907.102596 | 0.883516 |
| GO:0021522\_spinal\_cord\_motor\_neuron\_differentiation | 16 | 0 | 0.000000 | -0.000000 | 930 | 736.237404 | 821.67 | 907.102596 | 0.883516 |
| GO:0021696\_cerebellar\_cortex\_morphogenesis | 16 | 0 | 0.000000 | -0.000000 | 930 | 736.237404 | 821.67 | 907.102596 | 0.883516 |
| GO:0030890\_positive\_regulation\_of\_B\_cell\_proliferation | 16 | 0 | 0.000000 | -0.000000 | 930 | 736.237404 | 821.67 | 907.102596 | 0.883516 |
| GO:0031345\_negative\_regulation\_of\_cell\_projection\_organization | 16 | 0 | 0.000000 | -0.000000 | 930 | 736.237404 | 821.67 | 907.102596 | 0.883516 |
| GO:0031570\_DNA\_integrity\_checkpoint | 16 | 0 | 0.000000 | -0.000000 | 930 | 736.237404 | 821.67 | 907.102596 | 0.883516 |
| GO:0031669\_cellular\_response\_to\_nutrient\_levels | 16 | 0 | 0.000000 | -0.000000 | 930 | 736.237404 | 821.67 | 907.102596 | 0.883516 |
| GO:0032663\_regulation\_of\_interleukin-2\_production | 16 | 0 | 0.000000 | -0.000000 | 930 | 736.237404 | 821.67 | 907.102596 | 0.883516 |
| GO:0032956\_regulation\_of\_actin\_cytoskeleton\_organization | 16 | 0 | 0.000000 | -0.000000 | 930 | 736.237404 | 821.67 | 907.102596 | 0.883516 |
| GO:0034976\_response\_to\_endoplasmic\_reticulum\_stress | 16 | 0 | 0.000000 | -0.000000 | 930 | 736.237404 | 821.67 | 907.102596 | 0.883516 |
| GO:0042594\_response\_to\_starvation | 16 | 0 | 0.000000 | -0.000000 | 930 | 736.237404 | 821.67 | 907.102596 | 0.883516 |
| GO:0042596\_fear\_response | 16 | 0 | 0.000000 | -0.000000 | 930 | 736.237404 | 821.67 | 907.102596 | 0.883516 |
| GO:0043087\_regulation\_of\_GTPase\_activity | 16 | 0 | 0.000000 | -0.000000 | 930 | 736.237404 | 821.67 | 907.102596 | 0.883516 |
| GO:0043122\_regulation\_of\_I-kappaB\_kinase\_NF-kappaB\_cascade | 16 | 0 | 0.000000 | -0.000000 | 930 | 736.237404 | 821.67 | 907.102596 | 0.883516 |
| GO:0043367\_CD4-positive\_\_alpha\_beta\_T\_cell\_differentiation | 16 | 0 | 0.000000 | -0.000000 | 930 | 736.237404 | 821.67 | 907.102596 | 0.883516 |
| GO:0045104\_intermediate\_filament\_cytoskeleton\_organization | 16 | 0 | 0.000000 | -0.000000 | 930 | 736.237404 | 821.67 | 907.102596 | 0.883516 |
| GO:0046364\_monosaccharide\_biosynthetic\_process | 16 | 0 | 0.000000 | -0.000000 | 930 | 736.237404 | 821.67 | 907.102596 | 0.883516 |
| GO:0046467\_membrane\_lipid\_biosynthetic\_process | 16 | 0 | 0.000000 | -0.000000 | 930 | 736.237404 | 821.67 | 907.102596 | 0.883516 |
| GO:0046633\_alpha-beta\_T\_cell\_proliferation | 16 | 0 | 0.000000 | -0.000000 | 930 | 736.237404 | 821.67 | 907.102596 | 0.883516 |
| GO:0048015\_phosphoinositide-mediated\_signaling | 16 | 0 | 0.000000 | -0.000000 | 930 | 736.237404 | 821.67 | 907.102596 | 0.883516 |
| GO:0048286\_lung\_alveolus\_development | 16 | 0 | 0.000000 | -0.000000 | 930 | 736.237404 | 821.67 | 907.102596 | 0.883516 |
| GO:0048483\_autonomic\_nervous\_system\_development | 16 | 0 | 0.000000 | -0.000000 | 930 | 736.237404 | 821.67 | 907.102596 | 0.883516 |
| GO:0051048\_negative\_regulation\_of\_secretion | 16 | 0 | 0.000000 | -0.000000 | 930 | 736.237404 | 821.67 | 907.102596 | 0.883516 |
| GO:0051937\_catecholamine\_transport | 16 | 0 | 0.000000 | -0.000000 | 930 | 736.237404 | 821.67 | 907.102596 | 0.883516 |
| GO:0055007\_cardiac\_muscle\_cell\_differentiation | 16 | 0 | 0.000000 | -0.000000 | 930 | 736.237404 | 821.67 | 907.102596 | 0.883516 |
| GO:0060193\_positive\_regulation\_of\_lipase\_activity | 16 | 0 | 0.000000 | -0.000000 | 930 | 736.237404 | 821.67 | 907.102596 | 0.883516 |
| GO:0060713\_labyrinthine\_layer\_morphogenesis | 16 | 0 | 0.000000 | -0.000000 | 930 | 736.237404 | 821.67 | 907.102596 | 0.883516 |
| GO:0006576\_biogenic\_amine\_metabolic\_process | 53 | 0 | 0.000000 | -0.000000 | 937 | 742.422946 | 827.31 | 912.197054 | 0.882935 |
| GO:0006935\_chemotaxis | 53 | 0 | 0.000000 | -0.000000 | 937 | 742.422946 | 827.31 | 912.197054 | 0.882935 |
| GO:0030031\_cell\_projection\_assembly | 53 | 0 | 0.000000 | -0.000000 | 937 | 742.422946 | 827.31 | 912.197054 | 0.882935 |
| GO:0042330\_taxis | 53 | 0 | 0.000000 | -0.000000 | 937 | 742.422946 | 827.31 | 912.197054 | 0.882935 |
| GO:0046942\_carboxylic\_acid\_transport | 53 | 0 | 0.000000 | -0.000000 | 937 | 742.422946 | 827.31 | 912.197054 | 0.882935 |
| GO:0051248\_negative\_regulation\_of\_protein\_metabolic\_process | 53 | 0 | 0.000000 | -0.000000 | 937 | 742.422946 | 827.31 | 912.197054 | 0.882935 |
| GO:0055085\_transmembrane\_transport | 53 | 0 | 0.000000 | -0.000000 | 937 | 742.422946 | 827.31 | 912.197054 | 0.882935 |
| GO:0006812\_cation\_transport | 146 | 0 | 0.000000 | -0.000000 | 938 | 743.837738 | 828.54 | 913.242262 | 0.883305 |
| GO:0002200\_somatic\_diversification\_of\_immune\_receptors | 34 | 0 | 0.000000 | -0.000000 | 952 | 758.891644 | 842.65 | 926.408356 | 0.885137 |
| GO:0002237\_response\_to\_molecule\_of\_bacterial\_origin | 34 | 0 | 0.000000 | -0.000000 | 952 | 758.891644 | 842.65 | 926.408356 | 0.885137 |
| GO:0002699\_positive\_regulation\_of\_immune\_effector\_process | 34 | 0 | 0.000000 | -0.000000 | 952 | 758.891644 | 842.65 | 926.408356 | 0.885137 |
| GO:0007269\_neurotransmitter\_secretion | 34 | 0 | 0.000000 | -0.000000 | 952 | 758.891644 | 842.65 | 926.408356 | 0.885137 |
| GO:0007338\_single\_fertilization | 34 | 0 | 0.000000 | -0.000000 | 952 | 758.891644 | 842.65 | 926.408356 | 0.885137 |
| GO:0007568\_aging | 34 | 0 | 0.000000 | -0.000000 | 952 | 758.891644 | 842.65 | 926.408356 | 0.885137 |
| GO:0010721\_negative\_regulation\_of\_cell\_development | 34 | 0 | 0.000000 | -0.000000 | 952 | 758.891644 | 842.65 | 926.408356 | 0.885137 |
| GO:0019882\_antigen\_processing\_and\_presentation | 34 | 0 | 0.000000 | -0.000000 | 952 | 758.891644 | 842.65 | 926.408356 | 0.885137 |
| GO:0030509\_BMP\_signaling\_pathway | 34 | 0 | 0.000000 | -0.000000 | 952 | 758.891644 | 842.65 | 926.408356 | 0.885137 |
| GO:0045927\_positive\_regulation\_of\_growth | 34 | 0 | 0.000000 | -0.000000 | 952 | 758.891644 | 842.65 | 926.408356 | 0.885137 |
| GO:0050730\_regulation\_of\_peptidyl-tyrosine\_phosphorylation | 34 | 0 | 0.000000 | -0.000000 | 952 | 758.891644 | 842.65 | 926.408356 | 0.885137 |
| GO:0051047\_positive\_regulation\_of\_secretion | 34 | 0 | 0.000000 | -0.000000 | 952 | 758.891644 | 842.65 | 926.408356 | 0.885137 |
| GO:0051052\_regulation\_of\_DNA\_metabolic\_process | 34 | 0 | 0.000000 | -0.000000 | 952 | 758.891644 | 842.65 | 926.408356 | 0.885137 |
| GO:0060711\_labyrinthine\_layer\_development | 34 | 0 | 0.000000 | -0.000000 | 952 | 758.891644 | 842.65 | 926.408356 | 0.885137 |
| GO:0002764\_immune\_response-regulating\_signal\_transduction | 51 | 0 | 0.000000 | -0.000000 | 958 | 766.385481 | 849.81 | 933.234519 | 0.887067 |
| GO:0006887\_exocytosis | 51 | 0 | 0.000000 | -0.000000 | 958 | 766.385481 | 849.81 | 933.234519 | 0.887067 |
| GO:0007601\_visual\_perception | 51 | 0 | 0.000000 | -0.000000 | 958 | 766.385481 | 849.81 | 933.234519 | 0.887067 |
| GO:0016569\_covalent\_chromatin\_modification | 51 | 0 | 0.000000 | -0.000000 | 958 | 766.385481 | 849.81 | 933.234519 | 0.887067 |
| GO:0032583\_regulation\_of\_gene-specific\_transcription | 51 | 0 | 0.000000 | -0.000000 | 958 | 766.385481 | 849.81 | 933.234519 | 0.887067 |
| GO:0043408\_regulation\_of\_MAPKKK\_cascade | 51 | 0 | 0.000000 | -0.000000 | 958 | 766.385481 | 849.81 | 933.234519 | 0.887067 |
| GO:0044265\_cellular\_macromolecule\_catabolic\_process | 75 | 0 | 0.000000 | -0.000000 | 959 | 769.365896 | 852.47 | 935.574104 | 0.888916 |
| GO:0001819\_positive\_regulation\_of\_cytokine\_production | 36 | 0 | 0.000000 | -0.000000 | 973 | 785.813988 | 867.56 | 949.306012 | 0.891634 |
| GO:0001889\_liver\_development | 36 | 0 | 0.000000 | -0.000000 | 973 | 785.813988 | 867.56 | 949.306012 | 0.891634 |
| GO:0006469\_negative\_regulation\_of\_protein\_kinase\_activity | 36 | 0 | 0.000000 | -0.000000 | 973 | 785.813988 | 867.56 | 949.306012 | 0.891634 |
| GO:0007187\_G-protein\_signaling\_\_coupled\_to\_cyclic\_nucleotide\_second\_messenger | 36 | 0 | 0.000000 | -0.000000 | 973 | 785.813988 | 867.56 | 949.306012 | 0.891634 |
| GO:0007368\_determination\_of\_left\_right\_symmetry | 36 | 0 | 0.000000 | -0.000000 | 973 | 785.813988 | 867.56 | 949.306012 | 0.891634 |
| GO:0007631\_feeding\_behavior | 36 | 0 | 0.000000 | -0.000000 | 973 | 785.813988 | 867.56 | 949.306012 | 0.891634 |
| GO:0014020\_primary\_neural\_tube\_formation | 36 | 0 | 0.000000 | -0.000000 | 973 | 785.813988 | 867.56 | 949.306012 | 0.891634 |
| GO:0019228\_regulation\_of\_action\_potential\_in\_neuron | 36 | 0 | 0.000000 | -0.000000 | 973 | 785.813988 | 867.56 | 949.306012 | 0.891634 |
| GO:0022602\_ovulation\_cycle\_process | 36 | 0 | 0.000000 | -0.000000 | 973 | 785.813988 | 867.56 | 949.306012 | 0.891634 |
| GO:0030072\_peptide\_hormone\_secretion | 36 | 0 | 0.000000 | -0.000000 | 973 | 785.813988 | 867.56 | 949.306012 | 0.891634 |
| GO:0033673\_negative\_regulation\_of\_kinase\_activity | 36 | 0 | 0.000000 | -0.000000 | 973 | 785.813988 | 867.56 | 949.306012 | 0.891634 |
| GO:0042742\_defense\_response\_to\_bacterium | 36 | 0 | 0.000000 | -0.000000 | 973 | 785.813988 | 867.56 | 949.306012 | 0.891634 |
| GO:0050851\_antigen\_receptor-mediated\_signaling\_pathway | 36 | 0 | 0.000000 | -0.000000 | 973 | 785.813988 | 867.56 | 949.306012 | 0.891634 |
| GO:0050900\_leukocyte\_migration | 36 | 0 | 0.000000 | -0.000000 | 973 | 785.813988 | 867.56 | 949.306012 | 0.891634 |
| GO:0001934\_positive\_regulation\_of\_protein\_amino\_acid\_phosphorylation | 29 | 0 | 0.000000 | -0.000000 | 990 | 805.211543 | 885.97 | 966.728457 | 0.894919 |
| GO:0006909\_phagocytosis | 29 | 0 | 0.000000 | -0.000000 | 990 | 805.211543 | 885.97 | 966.728457 | 0.894919 |
| GO:0007190\_activation\_of\_adenylate\_cyclase\_activity | 29 | 0 | 0.000000 | -0.000000 | 990 | 805.211543 | 885.97 | 966.728457 | 0.894919 |
| GO:0010564\_regulation\_of\_cell\_cycle\_process | 29 | 0 | 0.000000 | -0.000000 | 990 | 805.211543 | 885.97 | 966.728457 | 0.894919 |
| GO:0016447\_somatic\_recombination\_of\_immunoglobulin\_gene\_segments | 29 | 0 | 0.000000 | -0.000000 | 990 | 805.211543 | 885.97 | 966.728457 | 0.894919 |
| GO:0021761\_limbic\_system\_development | 29 | 0 | 0.000000 | -0.000000 | 990 | 805.211543 | 885.97 | 966.728457 | 0.894919 |
| GO:0042176\_regulation\_of\_protein\_catabolic\_process | 29 | 0 | 0.000000 | -0.000000 | 990 | 805.211543 | 885.97 | 966.728457 | 0.894919 |
| GO:0042490\_mechanoreceptor\_differentiation | 29 | 0 | 0.000000 | -0.000000 | 990 | 805.211543 | 885.97 | 966.728457 | 0.894919 |
| GO:0042770\_DNA\_damage\_response\_\_signal\_transduction | 29 | 0 | 0.000000 | -0.000000 | 990 | 805.211543 | 885.97 | 966.728457 | 0.894919 |
| GO:0043281\_regulation\_of\_caspase\_activity | 29 | 0 | 0.000000 | -0.000000 | 990 | 805.211543 | 885.97 | 966.728457 | 0.894919 |
| GO:0044087\_regulation\_of\_cellular\_component\_biogenesis | 29 | 0 | 0.000000 | -0.000000 | 990 | 805.211543 | 885.97 | 966.728457 | 0.894919 |
| GO:0045621\_positive\_regulation\_of\_lymphocyte\_differentiation | 29 | 0 | 0.000000 | -0.000000 | 990 | 805.211543 | 885.97 | 966.728457 | 0.894919 |
| GO:0046634\_regulation\_of\_alpha-beta\_T\_cell\_activation | 29 | 0 | 0.000000 | -0.000000 | 990 | 805.211543 | 885.97 | 966.728457 | 0.894919 |
| GO:0048066\_pigmentation\_during\_development | 29 | 0 | 0.000000 | -0.000000 | 990 | 805.211543 | 885.97 | 966.728457 | 0.894919 |
| GO:0051301\_cell\_division | 29 | 0 | 0.000000 | -0.000000 | 990 | 805.211543 | 885.97 | 966.728457 | 0.894919 |
| GO:0052548\_regulation\_of\_endopeptidase\_activity | 29 | 0 | 0.000000 | -0.000000 | 990 | 805.211543 | 885.97 | 966.728457 | 0.894919 |
| GO:0070302\_regulation\_of\_stress-activated\_protein\_kinase\_signaling\_pathway | 29 | 0 | 0.000000 | -0.000000 | 990 | 805.211543 | 885.97 | 966.728457 | 0.894919 |
| GO:0006171\_cAMP\_biosynthetic\_process | 42 | 0 | 0.000000 | -0.000000 | 1004 | 820.652890 | 900.53 | 980.407110 | 0.896942 |
| GO:0010769\_regulation\_of\_cell\_morphogenesis\_involved\_in\_differentiation | 42 | 0 | 0.000000 | -0.000000 | 1004 | 820.652890 | 900.53 | 980.407110 | 0.896942 |
| GO:0015672\_monovalent\_inorganic\_cation\_transport | 42 | 0 | 0.000000 | -0.000000 | 1004 | 820.652890 | 900.53 | 980.407110 | 0.896942 |
| GO:0016125\_sterol\_metabolic\_process | 42 | 0 | 0.000000 | -0.000000 | 1004 | 820.652890 | 900.53 | 980.407110 | 0.896942 |
| GO:0019221\_cytokine-mediated\_signaling\_pathway | 42 | 0 | 0.000000 | -0.000000 | 1004 | 820.652890 | 900.53 | 980.407110 | 0.896942 |
| GO:0019941\_modification-dependent\_protein\_catabolic\_process | 42 | 0 | 0.000000 | -0.000000 | 1004 | 820.652890 | 900.53 | 980.407110 | 0.896942 |
| GO:0032946\_positive\_regulation\_of\_mononuclear\_cell\_proliferation | 42 | 0 | 0.000000 | -0.000000 | 1004 | 820.652890 | 900.53 | 980.407110 | 0.896942 |
| GO:0042476\_odontogenesis | 42 | 0 | 0.000000 | -0.000000 | 1004 | 820.652890 | 900.53 | 980.407110 | 0.896942 |
| GO:0043632\_modification-dependent\_macromolecule\_catabolic\_process | 42 | 0 | 0.000000 | -0.000000 | 1004 | 820.652890 | 900.53 | 980.407110 | 0.896942 |
| GO:0050671\_positive\_regulation\_of\_lymphocyte\_proliferation | 42 | 0 | 0.000000 | -0.000000 | 1004 | 820.652890 | 900.53 | 980.407110 | 0.896942 |
| GO:0050817\_coagulation | 42 | 0 | 0.000000 | -0.000000 | 1004 | 820.652890 | 900.53 | 980.407110 | 0.896942 |
| GO:0051345\_positive\_regulation\_of\_hydrolase\_activity | 42 | 0 | 0.000000 | -0.000000 | 1004 | 820.652890 | 900.53 | 980.407110 | 0.896942 |
| GO:0051603\_proteolysis\_involved\_in\_cellular\_protein\_catabolic\_process | 42 | 0 | 0.000000 | -0.000000 | 1004 | 820.652890 | 900.53 | 980.407110 | 0.896942 |
| GO:0080135\_regulation\_of\_cellular\_response\_to\_stress | 42 | 0 | 0.000000 | -0.000000 | 1004 | 820.652890 | 900.53 | 980.407110 | 0.896942 |
| GO:0001776\_leukocyte\_homeostasis | 41 | 0 | 0.000000 | -0.000000 | 1020 | 837.348895 | 916.03 | 994.711105 | 0.898069 |
| GO:0002429\_immune\_response-activating\_cell\_surface\_receptor\_signaling\_pathway | 41 | 0 | 0.000000 | -0.000000 | 1020 | 837.348895 | 916.03 | 994.711105 | 0.898069 |
| GO:0006836\_neurotransmitter\_transport | 41 | 0 | 0.000000 | -0.000000 | 1020 | 837.348895 | 916.03 | 994.711105 | 0.898069 |
| GO:0006865\_amino\_acid\_transport | 41 | 0 | 0.000000 | -0.000000 | 1020 | 837.348895 | 916.03 | 994.711105 | 0.898069 |
| GO:0007254\_JNK\_cascade | 41 | 0 | 0.000000 | -0.000000 | 1020 | 837.348895 | 916.03 | 994.711105 | 0.898069 |
| GO:0008585\_female\_gonad\_development | 41 | 0 | 0.000000 | -0.000000 | 1020 | 837.348895 | 916.03 | 994.711105 | 0.898069 |
| GO:0009894\_regulation\_of\_catabolic\_process | 41 | 0 | 0.000000 | -0.000000 | 1020 | 837.348895 | 916.03 | 994.711105 | 0.898069 |
| GO:0010551\_regulation\_of\_specific\_transcription\_from\_RNA\_polymerase\_II\_promoter | 41 | 0 | 0.000000 | -0.000000 | 1020 | 837.348895 | 916.03 | 994.711105 | 0.898069 |
| GO:0015833\_peptide\_transport | 41 | 0 | 0.000000 | -0.000000 | 1020 | 837.348895 | 916.03 | 994.711105 | 0.898069 |
| GO:0015980\_energy\_derivation\_by\_oxidation\_of\_organic\_compounds | 41 | 0 | 0.000000 | -0.000000 | 1020 | 837.348895 | 916.03 | 994.711105 | 0.898069 |
| GO:0019216\_regulation\_of\_lipid\_metabolic\_process | 41 | 0 | 0.000000 | -0.000000 | 1020 | 837.348895 | 916.03 | 994.711105 | 0.898069 |
| GO:0030817\_regulation\_of\_cAMP\_biosynthetic\_process | 41 | 0 | 0.000000 | -0.000000 | 1020 | 837.348895 | 916.03 | 994.711105 | 0.898069 |
| GO:0031344\_regulation\_of\_cell\_projection\_organization | 41 | 0 | 0.000000 | -0.000000 | 1020 | 837.348895 | 916.03 | 994.711105 | 0.898069 |
| GO:0032569\_specific\_transcription\_from\_RNA\_polymerase\_II\_promoter | 41 | 0 | 0.000000 | -0.000000 | 1020 | 837.348895 | 916.03 | 994.711105 | 0.898069 |
| GO:0033077\_T\_cell\_differentiation\_in\_the\_thymus | 41 | 0 | 0.000000 | -0.000000 | 1020 | 837.348895 | 916.03 | 994.711105 | 0.898069 |
| GO:0050864\_regulation\_of\_B\_cell\_activation | 41 | 0 | 0.000000 | -0.000000 | 1020 | 837.348895 | 916.03 | 994.711105 | 0.898069 |
| GO:0002062\_chondrocyte\_differentiation | 28 | 0 | 0.000000 | -0.000000 | 1031 | 853.316489 | 931.13 | 1008.943511 | 0.903133 |
| GO:0002705\_positive\_regulation\_of\_leukocyte\_mediated\_immunity | 28 | 0 | 0.000000 | -0.000000 | 1031 | 853.316489 | 931.13 | 1008.943511 | 0.903133 |
| GO:0002708\_positive\_regulation\_of\_lymphocyte\_mediated\_immunity | 28 | 0 | 0.000000 | -0.000000 | 1031 | 853.316489 | 931.13 | 1008.943511 | 0.903133 |
| GO:0006997\_nucleus\_organization | 28 | 0 | 0.000000 | -0.000000 | 1031 | 853.316489 | 931.13 | 1008.943511 | 0.903133 |
| GO:0021549\_cerebellum\_development | 28 | 0 | 0.000000 | -0.000000 | 1031 | 853.316489 | 931.13 | 1008.943511 | 0.903133 |
| GO:0030073\_insulin\_secretion | 28 | 0 | 0.000000 | -0.000000 | 1031 | 853.316489 | 931.13 | 1008.943511 | 0.903133 |
| GO:0042100\_B\_cell\_proliferation | 28 | 0 | 0.000000 | -0.000000 | 1031 | 853.316489 | 931.13 | 1008.943511 | 0.903133 |
| GO:0043193\_positive\_regulation\_of\_gene-specific\_transcription | 28 | 0 | 0.000000 | -0.000000 | 1031 | 853.316489 | 931.13 | 1008.943511 | 0.903133 |
| GO:0046328\_regulation\_of\_JNK\_cascade | 28 | 0 | 0.000000 | -0.000000 | 1031 | 853.316489 | 931.13 | 1008.943511 | 0.903133 |
| GO:0050871\_positive\_regulation\_of\_B\_cell\_activation | 28 | 0 | 0.000000 | -0.000000 | 1031 | 853.316489 | 931.13 | 1008.943511 | 0.903133 |
| GO:0051188\_cofactor\_biosynthetic\_process | 28 | 0 | 0.000000 | -0.000000 | 1031 | 853.316489 | 931.13 | 1008.943511 | 0.903133 |
| GO:0007264\_small\_GTPase\_mediated\_signal\_transduction | 72 | 0 | 0.000000 | -0.000000 | 1036 | 861.882784 | 938.94 | 1015.997216 | 0.906313 |
| GO:0042098\_T\_cell\_proliferation | 72 | 0 | 0.000000 | -0.000000 | 1036 | 861.882784 | 938.94 | 1015.997216 | 0.906313 |
| GO:0044262\_cellular\_carbohydrate\_metabolic\_process | 72 | 0 | 0.000000 | -0.000000 | 1036 | 861.882784 | 938.94 | 1015.997216 | 0.906313 |
| GO:0050673\_epithelial\_cell\_proliferation | 72 | 0 | 0.000000 | -0.000000 | 1036 | 861.882784 | 938.94 | 1015.997216 | 0.906313 |
| GO:0051347\_positive\_regulation\_of\_transferase\_activity | 72 | 0 | 0.000000 | -0.000000 | 1036 | 861.882784 | 938.94 | 1015.997216 | 0.906313 |
| GO:0000082\_G1\_S\_transition\_of\_mitotic\_cell\_cycle | 23 | 0 | 0.000000 | -0.000000 | 1058 | 884.343467 | 960.09 | 1035.836533 | 0.907457 |
| GO:0002204\_somatic\_recombination\_of\_immunoglobulin\_genes\_during\_immune\_response | 23 | 0 | 0.000000 | -0.000000 | 1058 | 884.343467 | 960.09 | 1035.836533 | 0.907457 |
| GO:0002208\_somatic\_diversification\_of\_immunoglobulins\_during\_immune\_response | 23 | 0 | 0.000000 | -0.000000 | 1058 | 884.343467 | 960.09 | 1035.836533 | 0.907457 |
| GO:0002228\_natural\_killer\_cell\_mediated\_immunity | 23 | 0 | 0.000000 | -0.000000 | 1058 | 884.343467 | 960.09 | 1035.836533 | 0.907457 |
| GO:0002821\_positive\_regulation\_of\_adaptive\_immune\_response | 23 | 0 | 0.000000 | -0.000000 | 1058 | 884.343467 | 960.09 | 1035.836533 | 0.907457 |
| GO:0002824\_positive\_regulation\_of\_adaptive\_immune\_response\_based\_on\_somatic\_recombination\_of\_immune\_receptors\_built\_from\_immunoglobulin\_superfamily\_domains | 23 | 0 | 0.000000 | -0.000000 | 1058 | 884.343467 | 960.09 | 1035.836533 | 0.907457 |
| GO:0003073\_regulation\_of\_systemic\_arterial\_blood\_pressure | 23 | 0 | 0.000000 | -0.000000 | 1058 | 884.343467 | 960.09 | 1035.836533 | 0.907457 |
| GO:0007018\_microtubule-based\_movement | 23 | 0 | 0.000000 | -0.000000 | 1058 | 884.343467 | 960.09 | 1035.836533 | 0.907457 |
| GO:0007163\_establishment\_or\_maintenance\_of\_cell\_polarity | 23 | 0 | 0.000000 | -0.000000 | 1058 | 884.343467 | 960.09 | 1035.836533 | 0.907457 |
| GO:0008542\_visual\_learning | 23 | 0 | 0.000000 | -0.000000 | 1058 | 884.343467 | 960.09 | 1035.836533 | 0.907457 |
| GO:0009954\_proximal\_distal\_pattern\_formation | 23 | 0 | 0.000000 | -0.000000 | 1058 | 884.343467 | 960.09 | 1035.836533 | 0.907457 |
| GO:0015698\_inorganic\_anion\_transport | 23 | 0 | 0.000000 | -0.000000 | 1058 | 884.343467 | 960.09 | 1035.836533 | 0.907457 |
| GO:0022613\_ribonucleoprotein\_complex\_biogenesis | 23 | 0 | 0.000000 | -0.000000 | 1058 | 884.343467 | 960.09 | 1035.836533 | 0.907457 |
| GO:0030512\_negative\_regulation\_of\_transforming\_growth\_factor\_beta\_receptor\_signaling\_pathway | 23 | 0 | 0.000000 | -0.000000 | 1058 | 884.343467 | 960.09 | 1035.836533 | 0.907457 |
| GO:0032635\_interleukin-6\_production | 23 | 0 | 0.000000 | -0.000000 | 1058 | 884.343467 | 960.09 | 1035.836533 | 0.907457 |
| GO:0032675\_regulation\_of\_interleukin-6\_production | 23 | 0 | 0.000000 | -0.000000 | 1058 | 884.343467 | 960.09 | 1035.836533 | 0.907457 |
| GO:0042267\_natural\_killer\_cell\_mediated\_cytotoxicity | 23 | 0 | 0.000000 | -0.000000 | 1058 | 884.343467 | 960.09 | 1035.836533 | 0.907457 |
| GO:0043388\_positive\_regulation\_of\_DNA\_binding | 23 | 0 | 0.000000 | -0.000000 | 1058 | 884.343467 | 960.09 | 1035.836533 | 0.907457 |
| GO:0045190\_isotype\_switching | 23 | 0 | 0.000000 | -0.000000 | 1058 | 884.343467 | 960.09 | 1035.836533 | 0.907457 |
| GO:0051705\_behavioral\_interaction\_between\_organisms | 23 | 0 | 0.000000 | -0.000000 | 1058 | 884.343467 | 960.09 | 1035.836533 | 0.907457 |
| GO:0060349\_bone\_morphogenesis | 23 | 0 | 0.000000 | -0.000000 | 1058 | 884.343467 | 960.09 | 1035.836533 | 0.907457 |
| GO:0060445\_branching\_involved\_in\_salivary\_gland\_morphogenesis | 23 | 0 | 0.000000 | -0.000000 | 1058 | 884.343467 | 960.09 | 1035.836533 | 0.907457 |
| GO:0006163\_purine\_nucleotide\_metabolic\_process | 73 | 0 | 0.000000 | -0.000000 | 1062 | 887.560389 | 962.99 | 1038.419611 | 0.906770 |
| GO:0006936\_muscle\_contraction | 73 | 0 | 0.000000 | -0.000000 | 1062 | 887.560389 | 962.99 | 1038.419611 | 0.906770 |
| GO:0048706\_embryonic\_skeletal\_system\_development | 73 | 0 | 0.000000 | -0.000000 | 1062 | 887.560389 | 962.99 | 1038.419611 | 0.906770 |
| GO:0051336\_regulation\_of\_hydrolase\_activity | 73 | 0 | 0.000000 | -0.000000 | 1062 | 887.560389 | 962.99 | 1038.419611 | 0.906770 |
| GO:0001508\_regulation\_of\_action\_potential | 43 | 0 | 0.000000 | -0.000000 | 1074 | 902.350253 | 976.76 | 1051.169747 | 0.909460 |
| GO:0001841\_neural\_tube\_formation | 43 | 0 | 0.000000 | -0.000000 | 1074 | 902.350253 | 976.76 | 1051.169747 | 0.909460 |
| GO:0002819\_regulation\_of\_adaptive\_immune\_response | 43 | 0 | 0.000000 | -0.000000 | 1074 | 902.350253 | 976.76 | 1051.169747 | 0.909460 |
| GO:0002822\_regulation\_of\_adaptive\_immune\_response\_based\_on\_somatic\_recombination\_of\_immune\_receptors\_built\_from\_immunoglobulin\_superfamily\_domains | 43 | 0 | 0.000000 | -0.000000 | 1074 | 902.350253 | 976.76 | 1051.169747 | 0.909460 |
| GO:0007224\_smoothened\_signaling\_pathway | 43 | 0 | 0.000000 | -0.000000 | 1074 | 902.350253 | 976.76 | 1051.169747 | 0.909460 |
| GO:0019637\_organophosphate\_metabolic\_process | 43 | 0 | 0.000000 | -0.000000 | 1074 | 902.350253 | 976.76 | 1051.169747 | 0.909460 |
| GO:0030814\_regulation\_of\_cAMP\_metabolic\_process | 43 | 0 | 0.000000 | -0.000000 | 1074 | 902.350253 | 976.76 | 1051.169747 | 0.909460 |
| GO:0031098\_stress-activated\_protein\_kinase\_signaling\_pathway | 43 | 0 | 0.000000 | -0.000000 | 1074 | 902.350253 | 976.76 | 1051.169747 | 0.909460 |
| GO:0032446\_protein\_modification\_by\_small\_protein\_conjugation | 43 | 0 | 0.000000 | -0.000000 | 1074 | 902.350253 | 976.76 | 1051.169747 | 0.909460 |
| GO:0032868\_response\_to\_insulin\_stimulus | 43 | 0 | 0.000000 | -0.000000 | 1074 | 902.350253 | 976.76 | 1051.169747 | 0.909460 |
| GO:0046879\_hormone\_secretion | 43 | 0 | 0.000000 | -0.000000 | 1074 | 902.350253 | 976.76 | 1051.169747 | 0.909460 |
| GO:0051604\_protein\_maturation | 43 | 0 | 0.000000 | -0.000000 | 1074 | 902.350253 | 976.76 | 1051.169747 | 0.909460 |
| GO:0001817\_regulation\_of\_cytokine\_production | 99 | 0 | 0.000000 | -0.000000 | 1076 | 904.863914 | 978.99 | 1053.116086 | 0.909842 |
| GO:0007398\_ectoderm\_development | 99 | 0 | 0.000000 | -0.000000 | 1076 | 904.863914 | 978.99 | 1053.116086 | 0.909842 |
| GO:0009416\_response\_to\_light\_stimulus | 74 | 0 | 0.000000 | -0.000000 | 1077 | 906.384555 | 980.39 | 1054.395445 | 0.910297 |
| GO:0000060\_protein\_import\_into\_nucleus\_\_translocation | 14 | 0 | 0.000000 | -0.000000 | 1135 | 967.695898 | 1040.46 | 1113.224102 | 0.916705 |
| GO:0000077\_DNA\_damage\_checkpoint | 14 | 0 | 0.000000 | -0.000000 | 1135 | 967.695898 | 1040.46 | 1113.224102 | 0.916705 |
| GO:0001502\_cartilage\_condensation | 14 | 0 | 0.000000 | -0.000000 | 1135 | 967.695898 | 1040.46 | 1113.224102 | 0.916705 |
| GO:0001829\_trophectodermal\_cell\_differentiation | 14 | 0 | 0.000000 | -0.000000 | 1135 | 967.695898 | 1040.46 | 1113.224102 | 0.916705 |
| GO:0002027\_regulation\_of\_heart\_rate | 14 | 0 | 0.000000 | -0.000000 | 1135 | 967.695898 | 1040.46 | 1113.224102 | 0.916705 |
| GO:0002262\_myeloid\_cell\_homeostasis | 14 | 0 | 0.000000 | -0.000000 | 1135 | 967.695898 | 1040.46 | 1113.224102 | 0.916705 |
| GO:0002698\_negative\_regulation\_of\_immune\_effector\_process | 14 | 0 | 0.000000 | -0.000000 | 1135 | 967.695898 | 1040.46 | 1113.224102 | 0.916705 |
| GO:0006304\_DNA\_modification | 14 | 0 | 0.000000 | -0.000000 | 1135 | 967.695898 | 1040.46 | 1113.224102 | 0.916705 |
| GO:0006305\_DNA\_alkylation | 14 | 0 | 0.000000 | -0.000000 | 1135 | 967.695898 | 1040.46 | 1113.224102 | 0.916705 |
| GO:0006306\_DNA\_methylation | 14 | 0 | 0.000000 | -0.000000 | 1135 | 967.695898 | 1040.46 | 1113.224102 | 0.916705 |
| GO:0006695\_cholesterol\_biosynthetic\_process | 14 | 0 | 0.000000 | -0.000000 | 1135 | 967.695898 | 1040.46 | 1113.224102 | 0.916705 |
| GO:0006809\_nitric\_oxide\_biosynthetic\_process | 14 | 0 | 0.000000 | -0.000000 | 1135 | 967.695898 | 1040.46 | 1113.224102 | 0.916705 |
| GO:0006914\_autophagy | 14 | 0 | 0.000000 | -0.000000 | 1135 | 967.695898 | 1040.46 | 1113.224102 | 0.916705 |
| GO:0006970\_response\_to\_osmotic\_stress | 14 | 0 | 0.000000 | -0.000000 | 1135 | 967.695898 | 1040.46 | 1113.224102 | 0.916705 |
| GO:0007157\_heterophilic\_cell\_adhesion | 14 | 0 | 0.000000 | -0.000000 | 1135 | 967.695898 | 1040.46 | 1113.224102 | 0.916705 |
| GO:0007530\_sex\_determination | 14 | 0 | 0.000000 | -0.000000 | 1135 | 967.695898 | 1040.46 | 1113.224102 | 0.916705 |
| GO:0007589\_body\_fluid\_secretion | 14 | 0 | 0.000000 | -0.000000 | 1135 | 967.695898 | 1040.46 | 1113.224102 | 0.916705 |
| GO:0008064\_regulation\_of\_actin\_polymerization\_or\_depolymerization | 14 | 0 | 0.000000 | -0.000000 | 1135 | 967.695898 | 1040.46 | 1113.224102 | 0.916705 |
| GO:0008306\_associative\_learning | 14 | 0 | 0.000000 | -0.000000 | 1135 | 967.695898 | 1040.46 | 1113.224102 | 0.916705 |
| GO:0008630\_DNA\_damage\_response\_\_signal\_transduction\_resulting\_in\_induction\_of\_apoptosis | 14 | 0 | 0.000000 | -0.000000 | 1135 | 967.695898 | 1040.46 | 1113.224102 | 0.916705 |
| GO:0009108\_coenzyme\_biosynthetic\_process | 14 | 0 | 0.000000 | -0.000000 | 1135 | 967.695898 | 1040.46 | 1113.224102 | 0.916705 |
| GO:0009267\_cellular\_response\_to\_starvation | 14 | 0 | 0.000000 | -0.000000 | 1135 | 967.695898 | 1040.46 | 1113.224102 | 0.916705 |
| GO:0009895\_negative\_regulation\_of\_catabolic\_process | 14 | 0 | 0.000000 | -0.000000 | 1135 | 967.695898 | 1040.46 | 1113.224102 | 0.916705 |
| GO:0014855\_striated\_muscle\_cell\_proliferation | 14 | 0 | 0.000000 | -0.000000 | 1135 | 967.695898 | 1040.46 | 1113.224102 | 0.916705 |
| GO:0016573\_histone\_acetylation | 14 | 0 | 0.000000 | -0.000000 | 1135 | 967.695898 | 1040.46 | 1113.224102 | 0.916705 |
| GO:0018130\_heterocycle\_biosynthetic\_process | 14 | 0 | 0.000000 | -0.000000 | 1135 | 967.695898 | 1040.46 | 1113.224102 | 0.916705 |
| GO:0019217\_regulation\_of\_fatty\_acid\_metabolic\_process | 14 | 0 | 0.000000 | -0.000000 | 1135 | 967.695898 | 1040.46 | 1113.224102 | 0.916705 |
| GO:0021904\_dorsal\_ventral\_neural\_tube\_patterning | 14 | 0 | 0.000000 | -0.000000 | 1135 | 967.695898 | 1040.46 | 1113.224102 | 0.916705 |
| GO:0030032\_lamellipodium\_assembly | 14 | 0 | 0.000000 | -0.000000 | 1135 | 967.695898 | 1040.46 | 1113.224102 | 0.916705 |
| GO:0030148\_sphingolipid\_biosynthetic\_process | 14 | 0 | 0.000000 | -0.000000 | 1135 | 967.695898 | 1040.46 | 1113.224102 | 0.916705 |
| GO:0030162\_regulation\_of\_proteolysis | 14 | 0 | 0.000000 | -0.000000 | 1135 | 967.695898 | 1040.46 | 1113.224102 | 0.916705 |
| GO:0030832\_regulation\_of\_actin\_filament\_length | 14 | 0 | 0.000000 | -0.000000 | 1135 | 967.695898 | 1040.46 | 1113.224102 | 0.916705 |
| GO:0031346\_positive\_regulation\_of\_cell\_projection\_organization | 14 | 0 | 0.000000 | -0.000000 | 1135 | 967.695898 | 1040.46 | 1113.224102 | 0.916705 |
| GO:0031663\_lipopolysaccharide-mediated\_signaling\_pathway | 14 | 0 | 0.000000 | -0.000000 | 1135 | 967.695898 | 1040.46 | 1113.224102 | 0.916705 |
| GO:0032271\_regulation\_of\_protein\_polymerization | 14 | 0 | 0.000000 | -0.000000 | 1135 | 967.695898 | 1040.46 | 1113.224102 | 0.916705 |
| GO:0033044\_regulation\_of\_chromosome\_organization | 14 | 0 | 0.000000 | -0.000000 | 1135 | 967.695898 | 1040.46 | 1113.224102 | 0.916705 |
| GO:0034104\_negative\_regulation\_of\_tissue\_remodeling | 14 | 0 | 0.000000 | -0.000000 | 1135 | 967.695898 | 1040.46 | 1113.224102 | 0.916705 |
| GO:0034623\_cellular\_macromolecular\_complex\_disassembly | 14 | 0 | 0.000000 | -0.000000 | 1135 | 967.695898 | 1040.46 | 1113.224102 | 0.916705 |
| GO:0035036\_sperm-egg\_recognition | 14 | 0 | 0.000000 | -0.000000 | 1135 | 967.695898 | 1040.46 | 1113.224102 | 0.916705 |
| GO:0042310\_vasoconstriction | 14 | 0 | 0.000000 | -0.000000 | 1135 | 967.695898 | 1040.46 | 1113.224102 | 0.916705 |
| GO:0043123\_positive\_regulation\_of\_I-kappaB\_kinase\_NF-kappaB\_cascade | 14 | 0 | 0.000000 | -0.000000 | 1135 | 967.695898 | 1040.46 | 1113.224102 | 0.916705 |
| GO:0043254\_regulation\_of\_protein\_complex\_assembly | 14 | 0 | 0.000000 | -0.000000 | 1135 | 967.695898 | 1040.46 | 1113.224102 | 0.916705 |
| GO:0045061\_thymic\_T\_cell\_selection | 14 | 0 | 0.000000 | -0.000000 | 1135 | 967.695898 | 1040.46 | 1113.224102 | 0.916705 |
| GO:0045453\_bone\_resorption | 14 | 0 | 0.000000 | -0.000000 | 1135 | 967.695898 | 1040.46 | 1113.224102 | 0.916705 |
| GO:0045598\_regulation\_of\_fat\_cell\_differentiation | 14 | 0 | 0.000000 | -0.000000 | 1135 | 967.695898 | 1040.46 | 1113.224102 | 0.916705 |
| GO:0045732\_positive\_regulation\_of\_protein\_catabolic\_process | 14 | 0 | 0.000000 | -0.000000 | 1135 | 967.695898 | 1040.46 | 1113.224102 | 0.916705 |
| GO:0046209\_nitric\_oxide\_metabolic\_process | 14 | 0 | 0.000000 | -0.000000 | 1135 | 967.695898 | 1040.46 | 1113.224102 | 0.916705 |
| GO:0048545\_response\_to\_steroid\_hormone\_stimulus | 14 | 0 | 0.000000 | -0.000000 | 1135 | 967.695898 | 1040.46 | 1113.224102 | 0.916705 |
| GO:0048665\_neuron\_fate\_specification | 14 | 0 | 0.000000 | -0.000000 | 1135 | 967.695898 | 1040.46 | 1113.224102 | 0.916705 |
| GO:0048844\_artery\_morphogenesis | 14 | 0 | 0.000000 | -0.000000 | 1135 | 967.695898 | 1040.46 | 1113.224102 | 0.916705 |
| GO:0050810\_regulation\_of\_steroid\_biosynthetic\_process | 14 | 0 | 0.000000 | -0.000000 | 1135 | 967.695898 | 1040.46 | 1113.224102 | 0.916705 |
| GO:0051017\_actin\_filament\_bundle\_formation | 14 | 0 | 0.000000 | -0.000000 | 1135 | 967.695898 | 1040.46 | 1113.224102 | 0.916705 |
| GO:0051053\_negative\_regulation\_of\_DNA\_metabolic\_process | 14 | 0 | 0.000000 | -0.000000 | 1135 | 967.695898 | 1040.46 | 1113.224102 | 0.916705 |
| GO:0051054\_positive\_regulation\_of\_DNA\_metabolic\_process | 14 | 0 | 0.000000 | -0.000000 | 1135 | 967.695898 | 1040.46 | 1113.224102 | 0.916705 |
| GO:0051100\_negative\_regulation\_of\_binding | 14 | 0 | 0.000000 | -0.000000 | 1135 | 967.695898 | 1040.46 | 1113.224102 | 0.916705 |
| GO:0051952\_regulation\_of\_amine\_transport | 14 | 0 | 0.000000 | -0.000000 | 1135 | 967.695898 | 1040.46 | 1113.224102 | 0.916705 |
| GO:0060716\_labyrinthine\_layer\_blood\_vessel\_development | 14 | 0 | 0.000000 | -0.000000 | 1135 | 967.695898 | 1040.46 | 1113.224102 | 0.916705 |
| GO:0060840\_artery\_development | 14 | 0 | 0.000000 | -0.000000 | 1135 | 967.695898 | 1040.46 | 1113.224102 | 0.916705 |
| GO:0000002\_mitochondrial\_genome\_maintenance | 9 | 0 | 0.000000 | -0.000000 | 1246 | 1083.686804 | 1153.95 | 1224.213196 | 0.926124 |
| GO:0000186\_activation\_of\_MAPKK\_activity | 9 | 0 | 0.000000 | -0.000000 | 1246 | 1083.686804 | 1153.95 | 1224.213196 | 0.926124 |
| GO:0001539\_ciliary\_or\_flagellar\_motility | 9 | 0 | 0.000000 | -0.000000 | 1246 | 1083.686804 | 1153.95 | 1224.213196 | 0.926124 |
| GO:0001542\_ovulation\_from\_ovarian\_follicle | 9 | 0 | 0.000000 | -0.000000 | 1246 | 1083.686804 | 1153.95 | 1224.213196 | 0.926124 |
| GO:0001667\_ameboidal\_cell\_migration | 9 | 0 | 0.000000 | -0.000000 | 1246 | 1083.686804 | 1153.95 | 1224.213196 | 0.926124 |
| GO:0001676\_long-chain\_fatty\_acid\_metabolic\_process | 9 | 0 | 0.000000 | -0.000000 | 1246 | 1083.686804 | 1153.95 | 1224.213196 | 0.926124 |
| GO:0001935\_endothelial\_cell\_proliferation | 9 | 0 | 0.000000 | -0.000000 | 1246 | 1083.686804 | 1153.95 | 1224.213196 | 0.926124 |
| GO:0002021\_response\_to\_dietary\_excess | 9 | 0 | 0.000000 | -0.000000 | 1246 | 1083.686804 | 1153.95 | 1224.213196 | 0.926124 |
| GO:0002028\_regulation\_of\_sodium\_ion\_transport | 9 | 0 | 0.000000 | -0.000000 | 1246 | 1083.686804 | 1153.95 | 1224.213196 | 0.926124 |
| GO:0002221\_pattern\_recognition\_receptor\_signaling\_pathway | 9 | 0 | 0.000000 | -0.000000 | 1246 | 1083.686804 | 1153.95 | 1224.213196 | 0.926124 |
| GO:0002292\_T\_cell\_differentiation\_during\_immune\_response | 9 | 0 | 0.000000 | -0.000000 | 1246 | 1083.686804 | 1153.95 | 1224.213196 | 0.926124 |
| GO:0002293\_alpha-beta\_T\_cell\_differentiation\_during\_immune\_response | 9 | 0 | 0.000000 | -0.000000 | 1246 | 1083.686804 | 1153.95 | 1224.213196 | 0.926124 |
| GO:0002294\_CD4-positive\_\_alpha-beta\_T\_cell\_differentiation\_during\_immune\_response | 9 | 0 | 0.000000 | -0.000000 | 1246 | 1083.686804 | 1153.95 | 1224.213196 | 0.926124 |
| GO:0002507\_tolerance\_induction | 9 | 0 | 0.000000 | -0.000000 | 1246 | 1083.686804 | 1153.95 | 1224.213196 | 0.926124 |
| GO:0002886\_regulation\_of\_myeloid\_leukocyte\_mediated\_immunity | 9 | 0 | 0.000000 | -0.000000 | 1246 | 1083.686804 | 1153.95 | 1224.213196 | 0.926124 |
| GO:0006007\_glucose\_catabolic\_process | 9 | 0 | 0.000000 | -0.000000 | 1246 | 1083.686804 | 1153.95 | 1224.213196 | 0.926124 |
| GO:0006182\_cGMP\_biosynthetic\_process | 9 | 0 | 0.000000 | -0.000000 | 1246 | 1083.686804 | 1153.95 | 1224.213196 | 0.926124 |
| GO:0006309\_DNA\_fragmentation\_involved\_in\_apoptosis | 9 | 0 | 0.000000 | -0.000000 | 1246 | 1083.686804 | 1153.95 | 1224.213196 | 0.926124 |
| GO:0006364\_rRNA\_processing | 9 | 0 | 0.000000 | -0.000000 | 1246 | 1083.686804 | 1153.95 | 1224.213196 | 0.926124 |
| GO:0006476\_protein\_amino\_acid\_deacetylation | 9 | 0 | 0.000000 | -0.000000 | 1246 | 1083.686804 | 1153.95 | 1224.213196 | 0.926124 |
| GO:0006595\_polyamine\_metabolic\_process | 9 | 0 | 0.000000 | -0.000000 | 1246 | 1083.686804 | 1153.95 | 1224.213196 | 0.926124 |
| GO:0006910\_phagocytosis\_\_recognition | 9 | 0 | 0.000000 | -0.000000 | 1246 | 1083.686804 | 1153.95 | 1224.213196 | 0.926124 |
| GO:0006911\_phagocytosis\_\_engulfment | 9 | 0 | 0.000000 | -0.000000 | 1246 | 1083.686804 | 1153.95 | 1224.213196 | 0.926124 |
| GO:0007193\_inhibition\_of\_adenylate\_cyclase\_activity\_by\_G-protein\_signaling | 9 | 0 | 0.000000 | -0.000000 | 1246 | 1083.686804 | 1153.95 | 1224.213196 | 0.926124 |
| GO:0007379\_segment\_specification | 9 | 0 | 0.000000 | -0.000000 | 1246 | 1083.686804 | 1153.95 | 1224.213196 | 0.926124 |
| GO:0007617\_mating\_behavior | 9 | 0 | 0.000000 | -0.000000 | 1246 | 1083.686804 | 1153.95 | 1224.213196 | 0.926124 |
| GO:0009451\_RNA\_modification | 9 | 0 | 0.000000 | -0.000000 | 1246 | 1083.686804 | 1153.95 | 1224.213196 | 0.926124 |
| GO:0010165\_response\_to\_X-ray | 9 | 0 | 0.000000 | -0.000000 | 1246 | 1083.686804 | 1153.95 | 1224.213196 | 0.926124 |
| GO:0010675\_regulation\_of\_cellular\_carbohydrate\_metabolic\_process | 9 | 0 | 0.000000 | -0.000000 | 1246 | 1083.686804 | 1153.95 | 1224.213196 | 0.926124 |
| GO:0014037\_Schwann\_cell\_differentiation | 9 | 0 | 0.000000 | -0.000000 | 1246 | 1083.686804 | 1153.95 | 1224.213196 | 0.926124 |
| GO:0014073\_response\_to\_tropane | 9 | 0 | 0.000000 | -0.000000 | 1246 | 1083.686804 | 1153.95 | 1224.213196 | 0.926124 |
| GO:0015695\_organic\_cation\_transport | 9 | 0 | 0.000000 | -0.000000 | 1246 | 1083.686804 | 1153.95 | 1224.213196 | 0.926124 |
| GO:0016072\_rRNA\_metabolic\_process | 9 | 0 | 0.000000 | -0.000000 | 1246 | 1083.686804 | 1153.95 | 1224.213196 | 0.926124 |
| GO:0016601\_Rac\_protein\_signal\_transduction | 9 | 0 | 0.000000 | -0.000000 | 1246 | 1083.686804 | 1153.95 | 1224.213196 | 0.926124 |
| GO:0017145\_stem\_cell\_division | 9 | 0 | 0.000000 | -0.000000 | 1246 | 1083.686804 | 1153.95 | 1224.213196 | 0.926124 |
| GO:0019320\_hexose\_catabolic\_process | 9 | 0 | 0.000000 | -0.000000 | 1246 | 1083.686804 | 1153.95 | 1224.213196 | 0.926124 |
| GO:0021544\_subpallium\_development | 9 | 0 | 0.000000 | -0.000000 | 1246 | 1083.686804 | 1153.95 | 1224.213196 | 0.926124 |
| GO:0021936\_regulation\_of\_granule\_cell\_precursor\_proliferation | 9 | 0 | 0.000000 | -0.000000 | 1246 | 1083.686804 | 1153.95 | 1224.213196 | 0.926124 |
| GO:0021940\_positive\_regulation\_of\_granule\_cell\_precursor\_proliferation | 9 | 0 | 0.000000 | -0.000000 | 1246 | 1083.686804 | 1153.95 | 1224.213196 | 0.926124 |
| GO:0030048\_actin\_filament-based\_movement | 9 | 0 | 0.000000 | -0.000000 | 1246 | 1083.686804 | 1153.95 | 1224.213196 | 0.926124 |
| GO:0030279\_negative\_regulation\_of\_ossification | 9 | 0 | 0.000000 | -0.000000 | 1246 | 1083.686804 | 1153.95 | 1224.213196 | 0.926124 |
| GO:0030325\_adrenal\_gland\_development | 9 | 0 | 0.000000 | -0.000000 | 1246 | 1083.686804 | 1153.95 | 1224.213196 | 0.926124 |
| GO:0030728\_ovulation | 9 | 0 | 0.000000 | -0.000000 | 1246 | 1083.686804 | 1153.95 | 1224.213196 | 0.926124 |
| GO:0031023\_microtubule\_organizing\_center\_organization | 9 | 0 | 0.000000 | -0.000000 | 1246 | 1083.686804 | 1153.95 | 1224.213196 | 0.926124 |
| GO:0032606\_type\_I\_interferon\_production | 9 | 0 | 0.000000 | -0.000000 | 1246 | 1083.686804 | 1153.95 | 1224.213196 | 0.926124 |
| GO:0032814\_regulation\_of\_natural\_killer\_cell\_activation | 9 | 0 | 0.000000 | -0.000000 | 1246 | 1083.686804 | 1153.95 | 1224.213196 | 0.926124 |
| GO:0032816\_positive\_regulation\_of\_natural\_killer\_cell\_activation | 9 | 0 | 0.000000 | -0.000000 | 1246 | 1083.686804 | 1153.95 | 1224.213196 | 0.926124 |
| GO:0033028\_myeloid\_cell\_apoptosis | 9 | 0 | 0.000000 | -0.000000 | 1246 | 1083.686804 | 1153.95 | 1224.213196 | 0.926124 |
| GO:0033143\_regulation\_of\_steroid\_hormone\_receptor\_signaling\_pathway | 9 | 0 | 0.000000 | -0.000000 | 1246 | 1083.686804 | 1153.95 | 1224.213196 | 0.926124 |
| GO:0033151\_V(D)J\_recombination | 9 | 0 | 0.000000 | -0.000000 | 1246 | 1083.686804 | 1153.95 | 1224.213196 | 0.926124 |
| GO:0033344\_cholesterol\_efflux | 9 | 0 | 0.000000 | -0.000000 | 1246 | 1083.686804 | 1153.95 | 1224.213196 | 0.926124 |
| GO:0034605\_cellular\_response\_to\_heat | 9 | 0 | 0.000000 | -0.000000 | 1246 | 1083.686804 | 1153.95 | 1224.213196 | 0.926124 |
| GO:0035088\_establishment\_or\_maintenance\_of\_apical\_basal\_cell\_polarity | 9 | 0 | 0.000000 | -0.000000 | 1246 | 1083.686804 | 1153.95 | 1224.213196 | 0.926124 |
| GO:0040020\_regulation\_of\_meiosis | 9 | 0 | 0.000000 | -0.000000 | 1246 | 1083.686804 | 1153.95 | 1224.213196 | 0.926124 |
| GO:0042058\_regulation\_of\_epidermal\_growth\_factor\_receptor\_signaling\_pathway | 9 | 0 | 0.000000 | -0.000000 | 1246 | 1083.686804 | 1153.95 | 1224.213196 | 0.926124 |
| GO:0042093\_T-helper\_cell\_differentiation | 9 | 0 | 0.000000 | -0.000000 | 1246 | 1083.686804 | 1153.95 | 1224.213196 | 0.926124 |
| GO:0042220\_response\_to\_cocaine | 9 | 0 | 0.000000 | -0.000000 | 1246 | 1083.686804 | 1153.95 | 1224.213196 | 0.926124 |
| GO:0042402\_biogenic\_amine\_catabolic\_process | 9 | 0 | 0.000000 | -0.000000 | 1246 | 1083.686804 | 1153.95 | 1224.213196 | 0.926124 |
| GO:0042509\_regulation\_of\_tyrosine\_phosphorylation\_of\_STAT\_protein | 9 | 0 | 0.000000 | -0.000000 | 1246 | 1083.686804 | 1153.95 | 1224.213196 | 0.926124 |
| GO:0042640\_anagen | 9 | 0 | 0.000000 | -0.000000 | 1246 | 1083.686804 | 1153.95 | 1224.213196 | 0.926124 |
| GO:0043242\_negative\_regulation\_of\_protein\_complex\_disassembly | 9 | 0 | 0.000000 | -0.000000 | 1246 | 1083.686804 | 1153.95 | 1224.213196 | 0.926124 |
| GO:0043299\_leukocyte\_degranulation | 9 | 0 | 0.000000 | -0.000000 | 1246 | 1083.686804 | 1153.95 | 1224.213196 | 0.926124 |
| GO:0043383\_negative\_T\_cell\_selection | 9 | 0 | 0.000000 | -0.000000 | 1246 | 1083.686804 | 1153.95 | 1224.213196 | 0.926124 |
| GO:0043409\_negative\_regulation\_of\_MAPKKK\_cascade | 9 | 0 | 0.000000 | -0.000000 | 1246 | 1083.686804 | 1153.95 | 1224.213196 | 0.926124 |
| GO:0043433\_negative\_regulation\_of\_transcription\_factor\_activity | 9 | 0 | 0.000000 | -0.000000 | 1246 | 1083.686804 | 1153.95 | 1224.213196 | 0.926124 |
| GO:0043603\_cellular\_amide\_metabolic\_process | 9 | 0 | 0.000000 | -0.000000 | 1246 | 1083.686804 | 1153.95 | 1224.213196 | 0.926124 |
| GO:0045060\_negative\_thymic\_T\_cell\_selection | 9 | 0 | 0.000000 | -0.000000 | 1246 | 1083.686804 | 1153.95 | 1224.213196 | 0.926124 |
| GO:0045109\_intermediate\_filament\_organization | 9 | 0 | 0.000000 | -0.000000 | 1246 | 1083.686804 | 1153.95 | 1224.213196 | 0.926124 |
| GO:0045136\_development\_of\_secondary\_sexual\_characteristics | 9 | 0 | 0.000000 | -0.000000 | 1246 | 1083.686804 | 1153.95 | 1224.213196 | 0.926124 |
| GO:0045185\_maintenance\_of\_protein\_location | 9 | 0 | 0.000000 | -0.000000 | 1246 | 1083.686804 | 1153.95 | 1224.213196 | 0.926124 |
| GO:0045214\_sarcomere\_organization | 9 | 0 | 0.000000 | -0.000000 | 1246 | 1083.686804 | 1153.95 | 1224.213196 | 0.926124 |
| GO:0045428\_regulation\_of\_nitric\_oxide\_biosynthetic\_process | 9 | 0 | 0.000000 | -0.000000 | 1246 | 1083.686804 | 1153.95 | 1224.213196 | 0.926124 |
| GO:0045620\_negative\_regulation\_of\_lymphocyte\_differentiation | 9 | 0 | 0.000000 | -0.000000 | 1246 | 1083.686804 | 1153.95 | 1224.213196 | 0.926124 |
| GO:0045671\_negative\_regulation\_of\_osteoclast\_differentiation | 9 | 0 | 0.000000 | -0.000000 | 1246 | 1083.686804 | 1153.95 | 1224.213196 | 0.926124 |
| GO:0045766\_positive\_regulation\_of\_angiogenesis | 9 | 0 | 0.000000 | -0.000000 | 1246 | 1083.686804 | 1153.95 | 1224.213196 | 0.926124 |
| GO:0045830\_positive\_regulation\_of\_isotype\_switching | 9 | 0 | 0.000000 | -0.000000 | 1246 | 1083.686804 | 1153.95 | 1224.213196 | 0.926124 |
| GO:0045884\_regulation\_of\_survival\_gene\_product\_expression | 9 | 0 | 0.000000 | -0.000000 | 1246 | 1083.686804 | 1153.95 | 1224.213196 | 0.926124 |
| GO:0046006\_regulation\_of\_activated\_T\_cell\_proliferation | 9 | 0 | 0.000000 | -0.000000 | 1246 | 1083.686804 | 1153.95 | 1224.213196 | 0.926124 |
| GO:0046324\_regulation\_of\_glucose\_import | 9 | 0 | 0.000000 | -0.000000 | 1246 | 1083.686804 | 1153.95 | 1224.213196 | 0.926124 |
| GO:0046365\_monosaccharide\_catabolic\_process | 9 | 0 | 0.000000 | -0.000000 | 1246 | 1083.686804 | 1153.95 | 1224.213196 | 0.926124 |
| GO:0046636\_negative\_regulation\_of\_alpha-beta\_T\_cell\_activation | 9 | 0 | 0.000000 | -0.000000 | 1246 | 1083.686804 | 1153.95 | 1224.213196 | 0.926124 |
| GO:0046641\_positive\_regulation\_of\_alpha-beta\_T\_cell\_proliferation | 9 | 0 | 0.000000 | -0.000000 | 1246 | 1083.686804 | 1153.95 | 1224.213196 | 0.926124 |
| GO:0046888\_negative\_regulation\_of\_hormone\_secretion | 9 | 0 | 0.000000 | -0.000000 | 1246 | 1083.686804 | 1153.95 | 1224.213196 | 0.926124 |
| GO:0048070\_regulation\_of\_pigmentation\_during\_development | 9 | 0 | 0.000000 | -0.000000 | 1246 | 1083.686804 | 1153.95 | 1224.213196 | 0.926124 |
| GO:0048146\_positive\_regulation\_of\_fibroblast\_proliferation | 9 | 0 | 0.000000 | -0.000000 | 1246 | 1083.686804 | 1153.95 | 1224.213196 | 0.926124 |
| GO:0048284\_organelle\_fusion | 9 | 0 | 0.000000 | -0.000000 | 1246 | 1083.686804 | 1153.95 | 1224.213196 | 0.926124 |
| GO:0048488\_synaptic\_vesicle\_endocytosis | 9 | 0 | 0.000000 | -0.000000 | 1246 | 1083.686804 | 1153.95 | 1224.213196 | 0.926124 |
| GO:0048569\_post-embryonic\_organ\_development | 9 | 0 | 0.000000 | -0.000000 | 1246 | 1083.686804 | 1153.95 | 1224.213196 | 0.926124 |
| GO:0048708\_astrocyte\_differentiation | 9 | 0 | 0.000000 | -0.000000 | 1246 | 1083.686804 | 1153.95 | 1224.213196 | 0.926124 |
| GO:0050433\_regulation\_of\_catecholamine\_secretion | 9 | 0 | 0.000000 | -0.000000 | 1246 | 1083.686804 | 1153.95 | 1224.213196 | 0.926124 |
| GO:0050856\_regulation\_of\_T\_cell\_receptor\_signaling\_pathway | 9 | 0 | 0.000000 | -0.000000 | 1246 | 1083.686804 | 1153.95 | 1224.213196 | 0.926124 |
| GO:0050884\_neuromuscular\_process\_controlling\_posture | 9 | 0 | 0.000000 | -0.000000 | 1246 | 1083.686804 | 1153.95 | 1224.213196 | 0.926124 |
| GO:0050918\_positive\_chemotaxis | 9 | 0 | 0.000000 | -0.000000 | 1246 | 1083.686804 | 1153.95 | 1224.213196 | 0.926124 |
| GO:0051023\_regulation\_of\_immunoglobulin\_secretion | 9 | 0 | 0.000000 | -0.000000 | 1246 | 1083.686804 | 1153.95 | 1224.213196 | 0.926124 |
| GO:0051297\_centrosome\_organization | 9 | 0 | 0.000000 | -0.000000 | 1246 | 1083.686804 | 1153.95 | 1224.213196 | 0.926124 |
| GO:0051607\_defense\_response\_to\_virus | 9 | 0 | 0.000000 | -0.000000 | 1246 | 1083.686804 | 1153.95 | 1224.213196 | 0.926124 |
| GO:0051647\_nucleus\_localization | 9 | 0 | 0.000000 | -0.000000 | 1246 | 1083.686804 | 1153.95 | 1224.213196 | 0.926124 |
| GO:0051932\_synaptic\_transmission\_\_GABAergic | 9 | 0 | 0.000000 | -0.000000 | 1246 | 1083.686804 | 1153.95 | 1224.213196 | 0.926124 |
| GO:0051963\_regulation\_of\_synaptogenesis | 9 | 0 | 0.000000 | -0.000000 | 1246 | 1083.686804 | 1153.95 | 1224.213196 | 0.926124 |
| GO:0055012\_ventricular\_cardiac\_muscle\_cell\_differentiation | 9 | 0 | 0.000000 | -0.000000 | 1246 | 1083.686804 | 1153.95 | 1224.213196 | 0.926124 |
| GO:0055013\_cardiac\_muscle\_cell\_development | 9 | 0 | 0.000000 | -0.000000 | 1246 | 1083.686804 | 1153.95 | 1224.213196 | 0.926124 |
| GO:0060052\_neurofilament\_cytoskeleton\_organization | 9 | 0 | 0.000000 | -0.000000 | 1246 | 1083.686804 | 1153.95 | 1224.213196 | 0.926124 |
| GO:0060081\_membrane\_hyperpolarization | 9 | 0 | 0.000000 | -0.000000 | 1246 | 1083.686804 | 1153.95 | 1224.213196 | 0.926124 |
| GO:0060119\_inner\_ear\_receptor\_cell\_development | 9 | 0 | 0.000000 | -0.000000 | 1246 | 1083.686804 | 1153.95 | 1224.213196 | 0.926124 |
| GO:0060122\_inner\_ear\_receptor\_stereocilium\_organization | 9 | 0 | 0.000000 | -0.000000 | 1246 | 1083.686804 | 1153.95 | 1224.213196 | 0.926124 |
| GO:0060325\_face\_morphogenesis | 9 | 0 | 0.000000 | -0.000000 | 1246 | 1083.686804 | 1153.95 | 1224.213196 | 0.926124 |
| GO:0060513\_prostatic\_bud\_formation | 9 | 0 | 0.000000 | -0.000000 | 1246 | 1083.686804 | 1153.95 | 1224.213196 | 0.926124 |
| GO:0060602\_branch\_elongation\_of\_an\_epithelium | 9 | 0 | 0.000000 | -0.000000 | 1246 | 1083.686804 | 1153.95 | 1224.213196 | 0.926124 |
| GO:0060693\_regulation\_of\_branching\_involved\_in\_salivary\_gland\_morphogenesis | 9 | 0 | 0.000000 | -0.000000 | 1246 | 1083.686804 | 1153.95 | 1224.213196 | 0.926124 |
| GO:0070306\_lens\_fiber\_cell\_differentiation | 9 | 0 | 0.000000 | -0.000000 | 1246 | 1083.686804 | 1153.95 | 1224.213196 | 0.926124 |
| GO:0090048\_negative\_regulation\_of\_transcription\_regulator\_activity | 9 | 0 | 0.000000 | -0.000000 | 1246 | 1083.686804 | 1153.95 | 1224.213196 | 0.926124 |
| GO:0009165\_nucleotide\_biosynthetic\_process | 63 | 0 | 0.000000 | -0.000000 | 1250 | 1088.291305 | 1157.92 | 1227.548695 | 0.926336 |
| GO:0051186\_cofactor\_metabolic\_process | 63 | 0 | 0.000000 | -0.000000 | 1250 | 1088.291305 | 1157.92 | 1227.548695 | 0.926336 |
| GO:0070662\_mast\_cell\_proliferation | 63 | 0 | 0.000000 | -0.000000 | 1250 | 1088.291305 | 1157.92 | 1227.548695 | 0.926336 |
| GO:0070666\_regulation\_of\_mast\_cell\_proliferation | 63 | 0 | 0.000000 | -0.000000 | 1250 | 1088.291305 | 1157.92 | 1227.548695 | 0.926336 |
| GO:0006461\_protein\_complex\_assembly | 78 | 0 | 0.000000 | -0.000000 | 1255 | 1092.390826 | 1162.02 | 1231.649174 | 0.925912 |
| GO:0030326\_embryonic\_limb\_morphogenesis | 78 | 0 | 0.000000 | -0.000000 | 1255 | 1092.390826 | 1162.02 | 1231.649174 | 0.925912 |
| GO:0035113\_embryonic\_appendage\_morphogenesis | 78 | 0 | 0.000000 | -0.000000 | 1255 | 1092.390826 | 1162.02 | 1231.649174 | 0.925912 |
| GO:0051251\_positive\_regulation\_of\_lymphocyte\_activation | 78 | 0 | 0.000000 | -0.000000 | 1255 | 1092.390826 | 1162.02 | 1231.649174 | 0.925912 |
| GO:0070271\_protein\_complex\_biogenesis | 78 | 0 | 0.000000 | -0.000000 | 1255 | 1092.390826 | 1162.02 | 1231.649174 | 0.925912 |
| GO:0050863\_regulation\_of\_T\_cell\_activation | 88 | 0 | 0.000000 | -0.000000 | 1256 | 1095.685290 | 1164.76 | 1233.834710 | 0.927357 |
| GO:0000723\_telomere\_maintenance | 13 | 0 | 0.000000 | -0.000000 | 1324 | 1166.564854 | 1233.71 | 1300.855146 | 0.931805 |
| GO:0001836\_release\_of\_cytochrome\_c\_from\_mitochondria | 13 | 0 | 0.000000 | -0.000000 | 1324 | 1166.564854 | 1233.71 | 1300.855146 | 0.931805 |
| GO:0001958\_endochondral\_ossification | 13 | 0 | 0.000000 | -0.000000 | 1324 | 1166.564854 | 1233.71 | 1300.855146 | 0.931805 |
| GO:0001975\_response\_to\_amphetamine | 13 | 0 | 0.000000 | -0.000000 | 1324 | 1166.564854 | 1233.71 | 1300.855146 | 0.931805 |
| GO:0001976\_neurological\_system\_process\_involved\_in\_regulation\_of\_systemic\_arterial\_blood\_pressure | 13 | 0 | 0.000000 | -0.000000 | 1324 | 1166.564854 | 1233.71 | 1300.855146 | 0.931805 |
| GO:0002704\_negative\_regulation\_of\_leukocyte\_mediated\_immunity | 13 | 0 | 0.000000 | -0.000000 | 1324 | 1166.564854 | 1233.71 | 1300.855146 | 0.931805 |
| GO:0002707\_negative\_regulation\_of\_lymphocyte\_mediated\_immunity | 13 | 0 | 0.000000 | -0.000000 | 1324 | 1166.564854 | 1233.71 | 1300.855146 | 0.931805 |
| GO:0002717\_positive\_regulation\_of\_natural\_killer\_cell\_mediated\_immunity | 13 | 0 | 0.000000 | -0.000000 | 1324 | 1166.564854 | 1233.71 | 1300.855146 | 0.931805 |
| GO:0006090\_pyruvate\_metabolic\_process | 13 | 0 | 0.000000 | -0.000000 | 1324 | 1166.564854 | 1233.71 | 1300.855146 | 0.931805 |
| GO:0006687\_glycosphingolipid\_metabolic\_process | 13 | 0 | 0.000000 | -0.000000 | 1324 | 1166.564854 | 1233.71 | 1300.855146 | 0.931805 |
| GO:0006778\_porphyrin\_metabolic\_process | 13 | 0 | 0.000000 | -0.000000 | 1324 | 1166.564854 | 1233.71 | 1300.855146 | 0.931805 |
| GO:0006833\_water\_transport | 13 | 0 | 0.000000 | -0.000000 | 1324 | 1166.564854 | 1233.71 | 1300.855146 | 0.931805 |
| GO:0006898\_receptor-mediated\_endocytosis | 13 | 0 | 0.000000 | -0.000000 | 1324 | 1166.564854 | 1233.71 | 1300.855146 | 0.931805 |
| GO:0006986\_response\_to\_unfolded\_protein | 13 | 0 | 0.000000 | -0.000000 | 1324 | 1166.564854 | 1233.71 | 1300.855146 | 0.931805 |
| GO:0007129\_synapsis | 13 | 0 | 0.000000 | -0.000000 | 1324 | 1166.564854 | 1233.71 | 1300.855146 | 0.931805 |
| GO:0007212\_dopamine\_receptor\_signaling\_pathway | 13 | 0 | 0.000000 | -0.000000 | 1324 | 1166.564854 | 1233.71 | 1300.855146 | 0.931805 |
| GO:0007274\_neuromuscular\_synaptic\_transmission | 13 | 0 | 0.000000 | -0.000000 | 1324 | 1166.564854 | 1233.71 | 1300.855146 | 0.931805 |
| GO:0007339\_binding\_of\_sperm\_to\_zona\_pellucida | 13 | 0 | 0.000000 | -0.000000 | 1324 | 1166.564854 | 1233.71 | 1300.855146 | 0.931805 |
| GO:0007439\_ectodermal\_gut\_development | 13 | 0 | 0.000000 | -0.000000 | 1324 | 1166.564854 | 1233.71 | 1300.855146 | 0.931805 |
| GO:0007512\_adult\_heart\_development | 13 | 0 | 0.000000 | -0.000000 | 1324 | 1166.564854 | 1233.71 | 1300.855146 | 0.931805 |
| GO:0009119\_ribonucleoside\_metabolic\_process | 13 | 0 | 0.000000 | -0.000000 | 1324 | 1166.564854 | 1233.71 | 1300.855146 | 0.931805 |
| GO:0010623\_developmental\_programmed\_cell\_death | 13 | 0 | 0.000000 | -0.000000 | 1324 | 1166.564854 | 1233.71 | 1300.855146 | 0.931805 |
| GO:0010970\_microtubule-based\_transport | 13 | 0 | 0.000000 | -0.000000 | 1324 | 1166.564854 | 1233.71 | 1300.855146 | 0.931805 |
| GO:0016525\_negative\_regulation\_of\_angiogenesis | 13 | 0 | 0.000000 | -0.000000 | 1324 | 1166.564854 | 1233.71 | 1300.855146 | 0.931805 |
| GO:0018105\_peptidyl-serine\_phosphorylation | 13 | 0 | 0.000000 | -0.000000 | 1324 | 1166.564854 | 1233.71 | 1300.855146 | 0.931805 |
| GO:0019098\_reproductive\_behavior | 13 | 0 | 0.000000 | -0.000000 | 1324 | 1166.564854 | 1233.71 | 1300.855146 | 0.931805 |
| GO:0021511\_spinal\_cord\_patterning | 13 | 0 | 0.000000 | -0.000000 | 1324 | 1166.564854 | 1233.71 | 1300.855146 | 0.931805 |
| GO:0021533\_cell\_differentiation\_in\_hindbrain | 13 | 0 | 0.000000 | -0.000000 | 1324 | 1166.564854 | 1233.71 | 1300.855146 | 0.931805 |
| GO:0021955\_central\_nervous\_system\_neuron\_axonogenesis | 13 | 0 | 0.000000 | -0.000000 | 1324 | 1166.564854 | 1233.71 | 1300.855146 | 0.931805 |
| GO:0030384\_phosphoinositide\_metabolic\_process | 13 | 0 | 0.000000 | -0.000000 | 1324 | 1166.564854 | 1233.71 | 1300.855146 | 0.931805 |
| GO:0030516\_regulation\_of\_axon\_extension | 13 | 0 | 0.000000 | -0.000000 | 1324 | 1166.564854 | 1233.71 | 1300.855146 | 0.931805 |
| GO:0031032\_actomyosin\_structure\_organization | 13 | 0 | 0.000000 | -0.000000 | 1324 | 1166.564854 | 1233.71 | 1300.855146 | 0.931805 |
| GO:0031290\_retinal\_ganglion\_cell\_axon\_guidance | 13 | 0 | 0.000000 | -0.000000 | 1324 | 1166.564854 | 1233.71 | 1300.855146 | 0.931805 |
| GO:0032200\_telomere\_organization | 13 | 0 | 0.000000 | -0.000000 | 1324 | 1166.564854 | 1233.71 | 1300.855146 | 0.931805 |
| GO:0032330\_regulation\_of\_chondrocyte\_differentiation | 13 | 0 | 0.000000 | -0.000000 | 1324 | 1166.564854 | 1233.71 | 1300.855146 | 0.931805 |
| GO:0032615\_interleukin-12\_production | 13 | 0 | 0.000000 | -0.000000 | 1324 | 1166.564854 | 1233.71 | 1300.855146 | 0.931805 |
| GO:0032729\_positive\_regulation\_of\_interferon-gamma\_production | 13 | 0 | 0.000000 | -0.000000 | 1324 | 1166.564854 | 1233.71 | 1300.855146 | 0.931805 |
| GO:0033013\_tetrapyrrole\_metabolic\_process | 13 | 0 | 0.000000 | -0.000000 | 1324 | 1166.564854 | 1233.71 | 1300.855146 | 0.931805 |
| GO:0034329\_cell\_junction\_assembly | 13 | 0 | 0.000000 | -0.000000 | 1324 | 1166.564854 | 1233.71 | 1300.855146 | 0.931805 |
| GO:0042044\_fluid\_transport | 13 | 0 | 0.000000 | -0.000000 | 1324 | 1166.564854 | 1233.71 | 1300.855146 | 0.931805 |
| GO:0042094\_interleukin-2\_biosynthetic\_process | 13 | 0 | 0.000000 | -0.000000 | 1324 | 1166.564854 | 1233.71 | 1300.855146 | 0.931805 |
| GO:0042474\_middle\_ear\_morphogenesis | 13 | 0 | 0.000000 | -0.000000 | 1324 | 1166.564854 | 1233.71 | 1300.855146 | 0.931805 |
| GO:0043241\_protein\_complex\_disassembly | 13 | 0 | 0.000000 | -0.000000 | 1324 | 1166.564854 | 1233.71 | 1300.855146 | 0.931805 |
| GO:0043244\_regulation\_of\_protein\_complex\_disassembly | 13 | 0 | 0.000000 | -0.000000 | 1324 | 1166.564854 | 1233.71 | 1300.855146 | 0.931805 |
| GO:0045191\_regulation\_of\_isotype\_switching | 13 | 0 | 0.000000 | -0.000000 | 1324 | 1166.564854 | 1233.71 | 1300.855146 | 0.931805 |
| GO:0045577\_regulation\_of\_B\_cell\_differentiation | 13 | 0 | 0.000000 | -0.000000 | 1324 | 1166.564854 | 1233.71 | 1300.855146 | 0.931805 |
| GO:0045682\_regulation\_of\_epidermis\_development | 13 | 0 | 0.000000 | -0.000000 | 1324 | 1166.564854 | 1233.71 | 1300.855146 | 0.931805 |
| GO:0045954\_positive\_regulation\_of\_natural\_killer\_cell\_mediated\_cytotoxicity | 13 | 0 | 0.000000 | -0.000000 | 1324 | 1166.564854 | 1233.71 | 1300.855146 | 0.931805 |
| GO:0046474\_glycerophospholipid\_biosynthetic\_process | 13 | 0 | 0.000000 | -0.000000 | 1324 | 1166.564854 | 1233.71 | 1300.855146 | 0.931805 |
| GO:0046640\_regulation\_of\_alpha-beta\_T\_cell\_proliferation | 13 | 0 | 0.000000 | -0.000000 | 1324 | 1166.564854 | 1233.71 | 1300.855146 | 0.931805 |
| GO:0046851\_negative\_regulation\_of\_bone\_remodeling | 13 | 0 | 0.000000 | -0.000000 | 1324 | 1166.564854 | 1233.71 | 1300.855146 | 0.931805 |
| GO:0048305\_immunoglobulin\_secretion | 13 | 0 | 0.000000 | -0.000000 | 1324 | 1166.564854 | 1233.71 | 1300.855146 | 0.931805 |
| GO:0048566\_embryonic\_gut\_development | 13 | 0 | 0.000000 | -0.000000 | 1324 | 1166.564854 | 1233.71 | 1300.855146 | 0.931805 |
| GO:0048567\_ectodermal\_gut\_morphogenesis | 13 | 0 | 0.000000 | -0.000000 | 1324 | 1166.564854 | 1233.71 | 1300.855146 | 0.931805 |
| GO:0050764\_regulation\_of\_phagocytosis | 13 | 0 | 0.000000 | -0.000000 | 1324 | 1166.564854 | 1233.71 | 1300.855146 | 0.931805 |
| GO:0050766\_positive\_regulation\_of\_phagocytosis | 13 | 0 | 0.000000 | -0.000000 | 1324 | 1166.564854 | 1233.71 | 1300.855146 | 0.931805 |
| GO:0050771\_negative\_regulation\_of\_axonogenesis | 13 | 0 | 0.000000 | -0.000000 | 1324 | 1166.564854 | 1233.71 | 1300.855146 | 0.931805 |
| GO:0050818\_regulation\_of\_coagulation | 13 | 0 | 0.000000 | -0.000000 | 1324 | 1166.564854 | 1233.71 | 1300.855146 | 0.931805 |
| GO:0051346\_negative\_regulation\_of\_hydrolase\_activity | 13 | 0 | 0.000000 | -0.000000 | 1324 | 1166.564854 | 1233.71 | 1300.855146 | 0.931805 |
| GO:0051495\_positive\_regulation\_of\_cytoskeleton\_organization | 13 | 0 | 0.000000 | -0.000000 | 1324 | 1166.564854 | 1233.71 | 1300.855146 | 0.931805 |
| GO:0060038\_cardiac\_muscle\_cell\_proliferation | 13 | 0 | 0.000000 | -0.000000 | 1324 | 1166.564854 | 1233.71 | 1300.855146 | 0.931805 |
| GO:0060070\_Wnt\_receptor\_signaling\_pathway\_through\_beta-catenin | 13 | 0 | 0.000000 | -0.000000 | 1324 | 1166.564854 | 1233.71 | 1300.855146 | 0.931805 |
| GO:0060324\_face\_development | 13 | 0 | 0.000000 | -0.000000 | 1324 | 1166.564854 | 1233.71 | 1300.855146 | 0.931805 |
| GO:0060401\_cytosolic\_calcium\_ion\_transport | 13 | 0 | 0.000000 | -0.000000 | 1324 | 1166.564854 | 1233.71 | 1300.855146 | 0.931805 |
| GO:0060402\_calcium\_ion\_transport\_into\_cytosol | 13 | 0 | 0.000000 | -0.000000 | 1324 | 1166.564854 | 1233.71 | 1300.855146 | 0.931805 |
| GO:0060560\_developmental\_growth\_involved\_in\_morphogenesis | 13 | 0 | 0.000000 | -0.000000 | 1324 | 1166.564854 | 1233.71 | 1300.855146 | 0.931805 |
| GO:0060742\_epithelial\_cell\_differentiation\_involved\_in\_prostate\_gland\_development | 13 | 0 | 0.000000 | -0.000000 | 1324 | 1166.564854 | 1233.71 | 1300.855146 | 0.931805 |
| GO:0070192\_chromosome\_organization\_involved\_in\_meiosis | 13 | 0 | 0.000000 | -0.000000 | 1324 | 1166.564854 | 1233.71 | 1300.855146 | 0.931805 |
| GO:0002253\_activation\_of\_immune\_response | 54 | 0 | 0.000000 | -0.000000 | 1332 | 1173.361681 | 1240.09 | 1306.818319 | 0.930998 |
| GO:0006091\_generation\_of\_precursor\_metabolites\_and\_energy | 54 | 0 | 0.000000 | -0.000000 | 1332 | 1173.361681 | 1240.09 | 1306.818319 | 0.930998 |
| GO:0006164\_purine\_nucleotide\_biosynthetic\_process | 54 | 0 | 0.000000 | -0.000000 | 1332 | 1173.361681 | 1240.09 | 1306.818319 | 0.930998 |
| GO:0007265\_Ras\_protein\_signal\_transduction | 54 | 0 | 0.000000 | -0.000000 | 1332 | 1173.361681 | 1240.09 | 1306.818319 | 0.930998 |
| GO:0009566\_fertilization | 54 | 0 | 0.000000 | -0.000000 | 1332 | 1173.361681 | 1240.09 | 1306.818319 | 0.930998 |
| GO:0015849\_organic\_acid\_transport | 54 | 0 | 0.000000 | -0.000000 | 1332 | 1173.361681 | 1240.09 | 1306.818319 | 0.930998 |
| GO:0043405\_regulation\_of\_MAP\_kinase\_activity | 54 | 0 | 0.000000 | -0.000000 | 1332 | 1173.361681 | 1240.09 | 1306.818319 | 0.930998 |
| GO:0044271\_nitrogen\_compound\_biosynthetic\_process | 54 | 0 | 0.000000 | -0.000000 | 1332 | 1173.361681 | 1240.09 | 1306.818319 | 0.930998 |
| GO:0002694\_regulation\_of\_leukocyte\_activation | 121 | 0 | 0.000000 | -0.000000 | 1333 | 1175.600166 | 1241.98 | 1308.359834 | 0.931718 |
| GO:0000038\_very-long-chain\_fatty\_acid\_metabolic\_process | 6 | 0 | 0.000000 | -0.000000 | 1513 | 1358.717479 | 1422.86 | 1487.002521 | 0.940423 |
| GO:0000245\_spliceosome\_assembly | 6 | 0 | 0.000000 | -0.000000 | 1513 | 1358.717479 | 1422.86 | 1487.002521 | 0.940423 |
| GO:0000768\_syncytium\_formation\_by\_plasma\_membrane\_fusion | 6 | 0 | 0.000000 | -0.000000 | 1513 | 1358.717479 | 1422.86 | 1487.002521 | 0.940423 |
| GO:0001710\_mesodermal\_cell\_fate\_commitment | 6 | 0 | 0.000000 | -0.000000 | 1513 | 1358.717479 | 1422.86 | 1487.002521 | 0.940423 |
| GO:0001779\_natural\_killer\_cell\_differentiation | 6 | 0 | 0.000000 | -0.000000 | 1513 | 1358.717479 | 1422.86 | 1487.002521 | 0.940423 |
| GO:0002016\_regulation\_of\_blood\_volume\_by\_renin-angiotensin | 6 | 0 | 0.000000 | -0.000000 | 1513 | 1358.717479 | 1422.86 | 1487.002521 | 0.940423 |
| GO:0002335\_mature\_B\_cell\_differentiation | 6 | 0 | 0.000000 | -0.000000 | 1513 | 1358.717479 | 1422.86 | 1487.002521 | 0.940423 |
| GO:0002360\_T\_cell\_lineage\_commitment | 6 | 0 | 0.000000 | -0.000000 | 1513 | 1358.717479 | 1422.86 | 1487.002521 | 0.940423 |
| GO:0002367\_cytokine\_production\_during\_immune\_response | 6 | 0 | 0.000000 | -0.000000 | 1513 | 1358.717479 | 1422.86 | 1487.002521 | 0.940423 |
| GO:0002474\_antigen\_processing\_and\_presentation\_of\_peptide\_antigen\_via\_MHC\_class\_I | 6 | 0 | 0.000000 | -0.000000 | 1513 | 1358.717479 | 1422.86 | 1487.002521 | 0.940423 |
| GO:0002475\_antigen\_processing\_and\_presentation\_via\_MHC\_class\_Ib | 6 | 0 | 0.000000 | -0.000000 | 1513 | 1358.717479 | 1422.86 | 1487.002521 | 0.940423 |
| GO:0002532\_production\_of\_molecular\_mediator\_of\_acute\_inflammatory\_response | 6 | 0 | 0.000000 | -0.000000 | 1513 | 1358.717479 | 1422.86 | 1487.002521 | 0.940423 |
| GO:0002541\_activation\_of\_plasma\_proteins\_involved\_in\_acute\_inflammatory\_response | 6 | 0 | 0.000000 | -0.000000 | 1513 | 1358.717479 | 1422.86 | 1487.002521 | 0.940423 |
| GO:0002675\_positive\_regulation\_of\_acute\_inflammatory\_response | 6 | 0 | 0.000000 | -0.000000 | 1513 | 1358.717479 | 1422.86 | 1487.002521 | 0.940423 |
| GO:0002685\_regulation\_of\_leukocyte\_migration | 6 | 0 | 0.000000 | -0.000000 | 1513 | 1358.717479 | 1422.86 | 1487.002521 | 0.940423 |
| GO:0002831\_regulation\_of\_response\_to\_biotic\_stimulus | 6 | 0 | 0.000000 | -0.000000 | 1513 | 1358.717479 | 1422.86 | 1487.002521 | 0.940423 |
| GO:0002920\_regulation\_of\_humoral\_immune\_response | 6 | 0 | 0.000000 | -0.000000 | 1513 | 1358.717479 | 1422.86 | 1487.002521 | 0.940423 |
| GO:0006071\_glycerol\_metabolic\_process | 6 | 0 | 0.000000 | -0.000000 | 1513 | 1358.717479 | 1422.86 | 1487.002521 | 0.940423 |
| GO:0006084\_acetyl-CoA\_metabolic\_process | 6 | 0 | 0.000000 | -0.000000 | 1513 | 1358.717479 | 1422.86 | 1487.002521 | 0.940423 |
| GO:0006264\_mitochondrial\_DNA\_replication | 6 | 0 | 0.000000 | -0.000000 | 1513 | 1358.717479 | 1422.86 | 1487.002521 | 0.940423 |
| GO:0006402\_mRNA\_catabolic\_process | 6 | 0 | 0.000000 | -0.000000 | 1513 | 1358.717479 | 1422.86 | 1487.002521 | 0.940423 |
| GO:0006471\_protein\_amino\_acid\_ADP-ribosylation | 6 | 0 | 0.000000 | -0.000000 | 1513 | 1358.717479 | 1422.86 | 1487.002521 | 0.940423 |
| GO:0006536\_glutamate\_metabolic\_process | 6 | 0 | 0.000000 | -0.000000 | 1513 | 1358.717479 | 1422.86 | 1487.002521 | 0.940423 |
| GO:0006656\_phosphatidylcholine\_biosynthetic\_process | 6 | 0 | 0.000000 | -0.000000 | 1513 | 1358.717479 | 1422.86 | 1487.002521 | 0.940423 |
| GO:0006692\_prostanoid\_metabolic\_process | 6 | 0 | 0.000000 | -0.000000 | 1513 | 1358.717479 | 1422.86 | 1487.002521 | 0.940423 |
| GO:0006693\_prostaglandin\_metabolic\_process | 6 | 0 | 0.000000 | -0.000000 | 1513 | 1358.717479 | 1422.86 | 1487.002521 | 0.940423 |
| GO:0006706\_steroid\_catabolic\_process | 6 | 0 | 0.000000 | -0.000000 | 1513 | 1358.717479 | 1422.86 | 1487.002521 | 0.940423 |
| GO:0006752\_group\_transfer\_coenzyme\_metabolic\_process | 6 | 0 | 0.000000 | -0.000000 | 1513 | 1358.717479 | 1422.86 | 1487.002521 | 0.940423 |
| GO:0006882\_cellular\_zinc\_ion\_homeostasis | 6 | 0 | 0.000000 | -0.000000 | 1513 | 1358.717479 | 1422.86 | 1487.002521 | 0.940423 |
| GO:0006942\_regulation\_of\_striated\_muscle\_contraction | 6 | 0 | 0.000000 | -0.000000 | 1513 | 1358.717479 | 1422.86 | 1487.002521 | 0.940423 |
| GO:0006956\_complement\_activation | 6 | 0 | 0.000000 | -0.000000 | 1513 | 1358.717479 | 1422.86 | 1487.002521 | 0.940423 |
| GO:0006998\_nuclear\_envelope\_organization | 6 | 0 | 0.000000 | -0.000000 | 1513 | 1358.717479 | 1422.86 | 1487.002521 | 0.940423 |
| GO:0007032\_endosome\_organization | 6 | 0 | 0.000000 | -0.000000 | 1513 | 1358.717479 | 1422.86 | 1487.002521 | 0.940423 |
| GO:0007176\_regulation\_of\_epidermal\_growth\_factor\_receptor\_activity | 6 | 0 | 0.000000 | -0.000000 | 1513 | 1358.717479 | 1422.86 | 1487.002521 | 0.940423 |
| GO:0007214\_gamma-aminobutyric\_acid\_signaling\_pathway | 6 | 0 | 0.000000 | -0.000000 | 1513 | 1358.717479 | 1422.86 | 1487.002521 | 0.940423 |
| GO:0007257\_activation\_of\_JUN\_kinase\_activity | 6 | 0 | 0.000000 | -0.000000 | 1513 | 1358.717479 | 1422.86 | 1487.002521 | 0.940423 |
| GO:0007341\_penetration\_of\_zona\_pellucida | 6 | 0 | 0.000000 | -0.000000 | 1513 | 1358.717479 | 1422.86 | 1487.002521 | 0.940423 |
| GO:0007406\_negative\_regulation\_of\_neuroblast\_proliferation | 6 | 0 | 0.000000 | -0.000000 | 1513 | 1358.717479 | 1422.86 | 1487.002521 | 0.940423 |
| GO:0007442\_hindgut\_morphogenesis | 6 | 0 | 0.000000 | -0.000000 | 1513 | 1358.717479 | 1422.86 | 1487.002521 | 0.940423 |
| GO:0007520\_myoblast\_fusion | 6 | 0 | 0.000000 | -0.000000 | 1513 | 1358.717479 | 1422.86 | 1487.002521 | 0.940423 |
| GO:0007620\_copulation | 6 | 0 | 0.000000 | -0.000000 | 1513 | 1358.717479 | 1422.86 | 1487.002521 | 0.940423 |
| GO:0008156\_negative\_regulation\_of\_DNA\_replication | 6 | 0 | 0.000000 | -0.000000 | 1513 | 1358.717479 | 1422.86 | 1487.002521 | 0.940423 |
| GO:0008209\_androgen\_metabolic\_process | 6 | 0 | 0.000000 | -0.000000 | 1513 | 1358.717479 | 1422.86 | 1487.002521 | 0.940423 |
| GO:0008625\_induction\_of\_apoptosis\_via\_death\_domain\_receptors | 6 | 0 | 0.000000 | -0.000000 | 1513 | 1358.717479 | 1422.86 | 1487.002521 | 0.940423 |
| GO:0009067\_aspartate\_family\_amino\_acid\_biosynthetic\_process | 6 | 0 | 0.000000 | -0.000000 | 1513 | 1358.717479 | 1422.86 | 1487.002521 | 0.940423 |
| GO:0009112\_nucleobase\_metabolic\_process | 6 | 0 | 0.000000 | -0.000000 | 1513 | 1358.717479 | 1422.86 | 1487.002521 | 0.940423 |
| GO:0009143\_nucleoside\_triphosphate\_catabolic\_process | 6 | 0 | 0.000000 | -0.000000 | 1513 | 1358.717479 | 1422.86 | 1487.002521 | 0.940423 |
| GO:0009247\_glycolipid\_biosynthetic\_process | 6 | 0 | 0.000000 | -0.000000 | 1513 | 1358.717479 | 1422.86 | 1487.002521 | 0.940423 |
| GO:0009650\_UV\_protection | 6 | 0 | 0.000000 | -0.000000 | 1513 | 1358.717479 | 1422.86 | 1487.002521 | 0.940423 |
| GO:0009651\_response\_to\_salt\_stress | 6 | 0 | 0.000000 | -0.000000 | 1513 | 1358.717479 | 1422.86 | 1487.002521 | 0.940423 |
| GO:0010466\_negative\_regulation\_of\_peptidase\_activity | 6 | 0 | 0.000000 | -0.000000 | 1513 | 1358.717479 | 1422.86 | 1487.002521 | 0.940423 |
| GO:0010883\_regulation\_of\_lipid\_storage | 6 | 0 | 0.000000 | -0.000000 | 1513 | 1358.717479 | 1422.86 | 1487.002521 | 0.940423 |
| GO:0010906\_regulation\_of\_glucose\_metabolic\_process | 6 | 0 | 0.000000 | -0.000000 | 1513 | 1358.717479 | 1422.86 | 1487.002521 | 0.940423 |
| GO:0014003\_oligodendrocyte\_development | 6 | 0 | 0.000000 | -0.000000 | 1513 | 1358.717479 | 1422.86 | 1487.002521 | 0.940423 |
| GO:0014051\_gamma-aminobutyric\_acid\_secretion | 6 | 0 | 0.000000 | -0.000000 | 1513 | 1358.717479 | 1422.86 | 1487.002521 | 0.940423 |
| GO:0014072\_response\_to\_isoquinoline\_alkaloid | 6 | 0 | 0.000000 | -0.000000 | 1513 | 1358.717479 | 1422.86 | 1487.002521 | 0.940423 |
| GO:0014812\_muscle\_cell\_migration | 6 | 0 | 0.000000 | -0.000000 | 1513 | 1358.717479 | 1422.86 | 1487.002521 | 0.940423 |
| GO:0015012\_heparan\_sulfate\_proteoglycan\_biosynthetic\_process | 6 | 0 | 0.000000 | -0.000000 | 1513 | 1358.717479 | 1422.86 | 1487.002521 | 0.940423 |
| GO:0015812\_gamma-aminobutyric\_acid\_transport | 6 | 0 | 0.000000 | -0.000000 | 1513 | 1358.717479 | 1422.86 | 1487.002521 | 0.940423 |
| GO:0016032\_viral\_reproduction | 6 | 0 | 0.000000 | -0.000000 | 1513 | 1358.717479 | 1422.86 | 1487.002521 | 0.940423 |
| GO:0016574\_histone\_ubiquitination | 6 | 0 | 0.000000 | -0.000000 | 1513 | 1358.717479 | 1422.86 | 1487.002521 | 0.940423 |
| GO:0016925\_protein\_sumoylation | 6 | 0 | 0.000000 | -0.000000 | 1513 | 1358.717479 | 1422.86 | 1487.002521 | 0.940423 |
| GO:0019433\_triglyceride\_catabolic\_process | 6 | 0 | 0.000000 | -0.000000 | 1513 | 1358.717479 | 1422.86 | 1487.002521 | 0.940423 |
| GO:0019835\_cytolysis | 6 | 0 | 0.000000 | -0.000000 | 1513 | 1358.717479 | 1422.86 | 1487.002521 | 0.940423 |
| GO:0021548\_pons\_development | 6 | 0 | 0.000000 | -0.000000 | 1513 | 1358.717479 | 1422.86 | 1487.002521 | 0.940423 |
| GO:0021783\_preganglionic\_parasympathetic\_nervous\_system\_development | 6 | 0 | 0.000000 | -0.000000 | 1513 | 1358.717479 | 1422.86 | 1487.002521 | 0.940423 |
| GO:0021892\_cerebral\_cortex\_GABAergic\_interneuron\_differentiation | 6 | 0 | 0.000000 | -0.000000 | 1513 | 1358.717479 | 1422.86 | 1487.002521 | 0.940423 |
| GO:0021937\_Purkinje\_cell-granule\_cell\_precursor\_cell\_signaling\_involved\_in\_regulation\_of\_granule\_cell\_precursor\_cell\_proliferation | 6 | 0 | 0.000000 | -0.000000 | 1513 | 1358.717479 | 1422.86 | 1487.002521 | 0.940423 |
| GO:0022409\_positive\_regulation\_of\_cell-cell\_adhesion | 6 | 0 | 0.000000 | -0.000000 | 1513 | 1358.717479 | 1422.86 | 1487.002521 | 0.940423 |
| GO:0030002\_cellular\_anion\_homeostasis | 6 | 0 | 0.000000 | -0.000000 | 1513 | 1358.717479 | 1422.86 | 1487.002521 | 0.940423 |
| GO:0030149\_sphingolipid\_catabolic\_process | 6 | 0 | 0.000000 | -0.000000 | 1513 | 1358.717479 | 1422.86 | 1487.002521 | 0.940423 |
| GO:0030252\_growth\_hormone\_secretion | 6 | 0 | 0.000000 | -0.000000 | 1513 | 1358.717479 | 1422.86 | 1487.002521 | 0.940423 |
| GO:0030865\_cortical\_cytoskeleton\_organization | 6 | 0 | 0.000000 | -0.000000 | 1513 | 1358.717479 | 1422.86 | 1487.002521 | 0.940423 |
| GO:0031077\_post-embryonic\_camera-type\_eye\_development | 6 | 0 | 0.000000 | -0.000000 | 1513 | 1358.717479 | 1422.86 | 1487.002521 | 0.940423 |
| GO:0031330\_negative\_regulation\_of\_cellular\_catabolic\_process | 6 | 0 | 0.000000 | -0.000000 | 1513 | 1358.717479 | 1422.86 | 1487.002521 | 0.940423 |
| GO:0031575\_G1\_S\_transition\_checkpoint | 6 | 0 | 0.000000 | -0.000000 | 1513 | 1358.717479 | 1422.86 | 1487.002521 | 0.940423 |
| GO:0031960\_response\_to\_corticosteroid\_stimulus | 6 | 0 | 0.000000 | -0.000000 | 1513 | 1358.717479 | 1422.86 | 1487.002521 | 0.940423 |
| GO:0032042\_mitochondrial\_DNA\_metabolic\_process | 6 | 0 | 0.000000 | -0.000000 | 1513 | 1358.717479 | 1422.86 | 1487.002521 | 0.940423 |
| GO:0032331\_negative\_regulation\_of\_chondrocyte\_differentiation | 6 | 0 | 0.000000 | -0.000000 | 1513 | 1358.717479 | 1422.86 | 1487.002521 | 0.940423 |
| GO:0032438\_melanosome\_organization | 6 | 0 | 0.000000 | -0.000000 | 1513 | 1358.717479 | 1422.86 | 1487.002521 | 0.940423 |
| GO:0032469\_endoplasmic\_reticulum\_calcium\_ion\_homeostasis | 6 | 0 | 0.000000 | -0.000000 | 1513 | 1358.717479 | 1422.86 | 1487.002521 | 0.940423 |
| GO:0032653\_regulation\_of\_interleukin-10\_production | 6 | 0 | 0.000000 | -0.000000 | 1513 | 1358.717479 | 1422.86 | 1487.002521 | 0.940423 |
| GO:0033238\_regulation\_of\_cellular\_amine\_metabolic\_process | 6 | 0 | 0.000000 | -0.000000 | 1513 | 1358.717479 | 1422.86 | 1487.002521 | 0.940423 |
| GO:0034968\_histone\_lysine\_methylation | 6 | 0 | 0.000000 | -0.000000 | 1513 | 1358.717479 | 1422.86 | 1487.002521 | 0.940423 |
| GO:0035019\_somatic\_stem\_cell\_maintenance | 6 | 0 | 0.000000 | -0.000000 | 1513 | 1358.717479 | 1422.86 | 1487.002521 | 0.940423 |
| GO:0035094\_response\_to\_nicotine | 6 | 0 | 0.000000 | -0.000000 | 1513 | 1358.717479 | 1422.86 | 1487.002521 | 0.940423 |
| GO:0035121\_tail\_morphogenesis | 6 | 0 | 0.000000 | -0.000000 | 1513 | 1358.717479 | 1422.86 | 1487.002521 | 0.940423 |
| GO:0040016\_embryonic\_cleavage | 6 | 0 | 0.000000 | -0.000000 | 1513 | 1358.717479 | 1422.86 | 1487.002521 | 0.940423 |
| GO:0040023\_establishment\_of\_nucleus\_localization | 6 | 0 | 0.000000 | -0.000000 | 1513 | 1358.717479 | 1422.86 | 1487.002521 | 0.940423 |
| GO:0040036\_regulation\_of\_fibroblast\_growth\_factor\_receptor\_signaling\_pathway | 6 | 0 | 0.000000 | -0.000000 | 1513 | 1358.717479 | 1422.86 | 1487.002521 | 0.940423 |
| GO:0042053\_regulation\_of\_dopamine\_metabolic\_process | 6 | 0 | 0.000000 | -0.000000 | 1513 | 1358.717479 | 1422.86 | 1487.002521 | 0.940423 |
| GO:0042069\_regulation\_of\_catecholamine\_metabolic\_process | 6 | 0 | 0.000000 | -0.000000 | 1513 | 1358.717479 | 1422.86 | 1487.002521 | 0.940423 |
| GO:0042307\_positive\_regulation\_of\_protein\_import\_into\_nucleus | 6 | 0 | 0.000000 | -0.000000 | 1513 | 1358.717479 | 1422.86 | 1487.002521 | 0.940423 |
| GO:0042308\_negative\_regulation\_of\_protein\_import\_into\_nucleus | 6 | 0 | 0.000000 | -0.000000 | 1513 | 1358.717479 | 1422.86 | 1487.002521 | 0.940423 |
| GO:0042403\_thyroid\_hormone\_metabolic\_process | 6 | 0 | 0.000000 | -0.000000 | 1513 | 1358.717479 | 1422.86 | 1487.002521 | 0.940423 |
| GO:0042481\_regulation\_of\_odontogenesis | 6 | 0 | 0.000000 | -0.000000 | 1513 | 1358.717479 | 1422.86 | 1487.002521 | 0.940423 |
| GO:0042492\_gamma-delta\_T\_cell\_differentiation | 6 | 0 | 0.000000 | -0.000000 | 1513 | 1358.717479 | 1422.86 | 1487.002521 | 0.940423 |
| GO:0042953\_lipoprotein\_transport | 6 | 0 | 0.000000 | -0.000000 | 1513 | 1358.717479 | 1422.86 | 1487.002521 | 0.940423 |
| GO:0043064\_flagellum\_organization | 6 | 0 | 0.000000 | -0.000000 | 1513 | 1358.717479 | 1422.86 | 1487.002521 | 0.940423 |
| GO:0043154\_negative\_regulation\_of\_caspase\_activity | 6 | 0 | 0.000000 | -0.000000 | 1513 | 1358.717479 | 1422.86 | 1487.002521 | 0.940423 |
| GO:0043255\_regulation\_of\_carbohydrate\_biosynthetic\_process | 6 | 0 | 0.000000 | -0.000000 | 1513 | 1358.717479 | 1422.86 | 1487.002521 | 0.940423 |
| GO:0043271\_negative\_regulation\_of\_ion\_transport | 6 | 0 | 0.000000 | -0.000000 | 1513 | 1358.717479 | 1422.86 | 1487.002521 | 0.940423 |
| GO:0043278\_response\_to\_morphine | 6 | 0 | 0.000000 | -0.000000 | 1513 | 1358.717479 | 1422.86 | 1487.002521 | 0.940423 |
| GO:0043300\_regulation\_of\_leukocyte\_degranulation | 6 | 0 | 0.000000 | -0.000000 | 1513 | 1358.717479 | 1422.86 | 1487.002521 | 0.940423 |
| GO:0043467\_regulation\_of\_generation\_of\_precursor\_metabolites\_and\_energy | 6 | 0 | 0.000000 | -0.000000 | 1513 | 1358.717479 | 1422.86 | 1487.002521 | 0.940423 |
| GO:0043547\_positive\_regulation\_of\_GTPase\_activity | 6 | 0 | 0.000000 | -0.000000 | 1513 | 1358.717479 | 1422.86 | 1487.002521 | 0.940423 |
| GO:0043627\_response\_to\_estrogen\_stimulus | 6 | 0 | 0.000000 | -0.000000 | 1513 | 1358.717479 | 1422.86 | 1487.002521 | 0.940423 |
| GO:0044269\_glycerol\_ether\_catabolic\_process | 6 | 0 | 0.000000 | -0.000000 | 1513 | 1358.717479 | 1422.86 | 1487.002521 | 0.940423 |
| GO:0045072\_regulation\_of\_interferon-gamma\_biosynthetic\_process | 6 | 0 | 0.000000 | -0.000000 | 1513 | 1358.717479 | 1422.86 | 1487.002521 | 0.940423 |
| GO:0045084\_positive\_regulation\_of\_interleukin-12\_biosynthetic\_process | 6 | 0 | 0.000000 | -0.000000 | 1513 | 1358.717479 | 1422.86 | 1487.002521 | 0.940423 |
| GO:0045124\_regulation\_of\_bone\_resorption | 6 | 0 | 0.000000 | -0.000000 | 1513 | 1358.717479 | 1422.86 | 1487.002521 | 0.940423 |
| GO:0045176\_apical\_protein\_localization | 6 | 0 | 0.000000 | -0.000000 | 1513 | 1358.717479 | 1422.86 | 1487.002521 | 0.940423 |
| GO:0045540\_regulation\_of\_cholesterol\_biosynthetic\_process | 6 | 0 | 0.000000 | -0.000000 | 1513 | 1358.717479 | 1422.86 | 1487.002521 | 0.940423 |
| GO:0045579\_positive\_regulation\_of\_B\_cell\_differentiation | 6 | 0 | 0.000000 | -0.000000 | 1513 | 1358.717479 | 1422.86 | 1487.002521 | 0.940423 |
| GO:0045649\_regulation\_of\_macrophage\_differentiation | 6 | 0 | 0.000000 | -0.000000 | 1513 | 1358.717479 | 1422.86 | 1487.002521 | 0.940423 |
| GO:0045727\_positive\_regulation\_of\_translation | 6 | 0 | 0.000000 | -0.000000 | 1513 | 1358.717479 | 1422.86 | 1487.002521 | 0.940423 |
| GO:0045778\_positive\_regulation\_of\_ossification | 6 | 0 | 0.000000 | -0.000000 | 1513 | 1358.717479 | 1422.86 | 1487.002521 | 0.940423 |
| GO:0045822\_negative\_regulation\_of\_heart\_contraction | 6 | 0 | 0.000000 | -0.000000 | 1513 | 1358.717479 | 1422.86 | 1487.002521 | 0.940423 |
| GO:0045824\_negative\_regulation\_of\_innate\_immune\_response | 6 | 0 | 0.000000 | -0.000000 | 1513 | 1358.717479 | 1422.86 | 1487.002521 | 0.940423 |
| GO:0045833\_negative\_regulation\_of\_lipid\_metabolic\_process | 6 | 0 | 0.000000 | -0.000000 | 1513 | 1358.717479 | 1422.86 | 1487.002521 | 0.940423 |
| GO:0045843\_negative\_regulation\_of\_striated\_muscle\_development | 6 | 0 | 0.000000 | -0.000000 | 1513 | 1358.717479 | 1422.86 | 1487.002521 | 0.940423 |
| GO:0045861\_negative\_regulation\_of\_proteolysis | 6 | 0 | 0.000000 | -0.000000 | 1513 | 1358.717479 | 1422.86 | 1487.002521 | 0.940423 |
| GO:0045913\_positive\_regulation\_of\_carbohydrate\_metabolic\_process | 6 | 0 | 0.000000 | -0.000000 | 1513 | 1358.717479 | 1422.86 | 1487.002521 | 0.940423 |
| GO:0045931\_positive\_regulation\_of\_mitotic\_cell\_cycle | 6 | 0 | 0.000000 | -0.000000 | 1513 | 1358.717479 | 1422.86 | 1487.002521 | 0.940423 |
| GO:0045933\_positive\_regulation\_of\_muscle\_contraction | 6 | 0 | 0.000000 | -0.000000 | 1513 | 1358.717479 | 1422.86 | 1487.002521 | 0.940423 |
| GO:0046427\_positive\_regulation\_of\_JAK-STAT\_cascade | 6 | 0 | 0.000000 | -0.000000 | 1513 | 1358.717479 | 1422.86 | 1487.002521 | 0.940423 |
| GO:0046460\_neutral\_lipid\_biosynthetic\_process | 6 | 0 | 0.000000 | -0.000000 | 1513 | 1358.717479 | 1422.86 | 1487.002521 | 0.940423 |
| GO:0046461\_neutral\_lipid\_catabolic\_process | 6 | 0 | 0.000000 | -0.000000 | 1513 | 1358.717479 | 1422.86 | 1487.002521 | 0.940423 |
| GO:0046463\_acylglycerol\_biosynthetic\_process | 6 | 0 | 0.000000 | -0.000000 | 1513 | 1358.717479 | 1422.86 | 1487.002521 | 0.940423 |
| GO:0046464\_acylglycerol\_catabolic\_process | 6 | 0 | 0.000000 | -0.000000 | 1513 | 1358.717479 | 1422.86 | 1487.002521 | 0.940423 |
| GO:0046466\_membrane\_lipid\_catabolic\_process | 6 | 0 | 0.000000 | -0.000000 | 1513 | 1358.717479 | 1422.86 | 1487.002521 | 0.940423 |
| GO:0046503\_glycerolipid\_catabolic\_process | 6 | 0 | 0.000000 | -0.000000 | 1513 | 1358.717479 | 1422.86 | 1487.002521 | 0.940423 |
| GO:0046580\_negative\_regulation\_of\_Ras\_protein\_signal\_transduction | 6 | 0 | 0.000000 | -0.000000 | 1513 | 1358.717479 | 1422.86 | 1487.002521 | 0.940423 |
| GO:0046627\_negative\_regulation\_of\_insulin\_receptor\_signaling\_pathway | 6 | 0 | 0.000000 | -0.000000 | 1513 | 1358.717479 | 1422.86 | 1487.002521 | 0.940423 |
| GO:0046629\_gamma-delta\_T\_cell\_activation | 6 | 0 | 0.000000 | -0.000000 | 1513 | 1358.717479 | 1422.86 | 1487.002521 | 0.940423 |
| GO:0046666\_retinal\_cell\_programmed\_cell\_death | 6 | 0 | 0.000000 | -0.000000 | 1513 | 1358.717479 | 1422.86 | 1487.002521 | 0.940423 |
| GO:0046852\_positive\_regulation\_of\_bone\_remodeling | 6 | 0 | 0.000000 | -0.000000 | 1513 | 1358.717479 | 1422.86 | 1487.002521 | 0.940423 |
| GO:0046889\_positive\_regulation\_of\_lipid\_biosynthetic\_process | 6 | 0 | 0.000000 | -0.000000 | 1513 | 1358.717479 | 1422.86 | 1487.002521 | 0.940423 |
| GO:0048041\_focal\_adhesion\_formation | 6 | 0 | 0.000000 | -0.000000 | 1513 | 1358.717479 | 1422.86 | 1487.002521 | 0.940423 |
| GO:0048103\_somatic\_stem\_cell\_division | 6 | 0 | 0.000000 | -0.000000 | 1513 | 1358.717479 | 1422.86 | 1487.002521 | 0.940423 |
| GO:0048147\_negative\_regulation\_of\_fibroblast\_proliferation | 6 | 0 | 0.000000 | -0.000000 | 1513 | 1358.717479 | 1422.86 | 1487.002521 | 0.940423 |
| GO:0048333\_mesodermal\_cell\_differentiation | 6 | 0 | 0.000000 | -0.000000 | 1513 | 1358.717479 | 1422.86 | 1487.002521 | 0.940423 |
| GO:0048340\_paraxial\_mesoderm\_morphogenesis | 6 | 0 | 0.000000 | -0.000000 | 1513 | 1358.717479 | 1422.86 | 1487.002521 | 0.940423 |
| GO:0048541\_Peyer's\_patch\_development | 6 | 0 | 0.000000 | -0.000000 | 1513 | 1358.717479 | 1422.86 | 1487.002521 | 0.940423 |
| GO:0048563\_post-embryonic\_organ\_morphogenesis | 6 | 0 | 0.000000 | -0.000000 | 1513 | 1358.717479 | 1422.86 | 1487.002521 | 0.940423 |
| GO:0048617\_embryonic\_foregut\_morphogenesis | 6 | 0 | 0.000000 | -0.000000 | 1513 | 1358.717479 | 1422.86 | 1487.002521 | 0.940423 |
| GO:0048635\_negative\_regulation\_of\_muscle\_development | 6 | 0 | 0.000000 | -0.000000 | 1513 | 1358.717479 | 1422.86 | 1487.002521 | 0.940423 |
| GO:0048644\_muscle\_organ\_morphogenesis | 6 | 0 | 0.000000 | -0.000000 | 1513 | 1358.717479 | 1422.86 | 1487.002521 | 0.940423 |
| GO:0048703\_embryonic\_viscerocranium\_morphogenesis | 6 | 0 | 0.000000 | -0.000000 | 1513 | 1358.717479 | 1422.86 | 1487.002521 | 0.940423 |
| GO:0048713\_regulation\_of\_oligodendrocyte\_differentiation | 6 | 0 | 0.000000 | -0.000000 | 1513 | 1358.717479 | 1422.86 | 1487.002521 | 0.940423 |
| GO:0050684\_regulation\_of\_mRNA\_processing | 6 | 0 | 0.000000 | -0.000000 | 1513 | 1358.717479 | 1422.86 | 1487.002521 | 0.940423 |
| GO:0050732\_negative\_regulation\_of\_peptidyl-tyrosine\_phosphorylation | 6 | 0 | 0.000000 | -0.000000 | 1513 | 1358.717479 | 1422.86 | 1487.002521 | 0.940423 |
| GO:0050805\_negative\_regulation\_of\_synaptic\_transmission | 6 | 0 | 0.000000 | -0.000000 | 1513 | 1358.717479 | 1422.86 | 1487.002521 | 0.940423 |
| GO:0050821\_protein\_stabilization | 6 | 0 | 0.000000 | -0.000000 | 1513 | 1358.717479 | 1422.86 | 1487.002521 | 0.940423 |
| GO:0050829\_defense\_response\_to\_Gram-negative\_bacterium | 6 | 0 | 0.000000 | -0.000000 | 1513 | 1358.717479 | 1422.86 | 1487.002521 | 0.940423 |
| GO:0050872\_white\_fat\_cell\_differentiation | 6 | 0 | 0.000000 | -0.000000 | 1513 | 1358.717479 | 1422.86 | 1487.002521 | 0.940423 |
| GO:0050951\_sensory\_perception\_of\_temperature\_stimulus | 6 | 0 | 0.000000 | -0.000000 | 1513 | 1358.717479 | 1422.86 | 1487.002521 | 0.940423 |
| GO:0050966\_detection\_of\_mechanical\_stimulus\_involved\_in\_sensory\_perception\_of\_pain | 6 | 0 | 0.000000 | -0.000000 | 1513 | 1358.717479 | 1422.86 | 1487.002521 | 0.940423 |
| GO:0051058\_negative\_regulation\_of\_small\_GTPase\_mediated\_signal\_transduction | 6 | 0 | 0.000000 | -0.000000 | 1513 | 1358.717479 | 1422.86 | 1487.002521 | 0.940423 |
| GO:0051085\_chaperone\_mediated\_protein\_folding\_requiring\_cofactor | 6 | 0 | 0.000000 | -0.000000 | 1513 | 1358.717479 | 1422.86 | 1487.002521 | 0.940423 |
| GO:0051180\_vitamin\_transport | 6 | 0 | 0.000000 | -0.000000 | 1513 | 1358.717479 | 1422.86 | 1487.002521 | 0.940423 |
| GO:0051384\_response\_to\_glucocorticoid\_stimulus | 6 | 0 | 0.000000 | -0.000000 | 1513 | 1358.717479 | 1422.86 | 1487.002521 | 0.940423 |
| GO:0051592\_response\_to\_calcium\_ion | 6 | 0 | 0.000000 | -0.000000 | 1513 | 1358.717479 | 1422.86 | 1487.002521 | 0.940423 |
| GO:0051875\_pigment\_granule\_localization | 6 | 0 | 0.000000 | -0.000000 | 1513 | 1358.717479 | 1422.86 | 1487.002521 | 0.940423 |
| GO:0051881\_regulation\_of\_mitochondrial\_membrane\_potential | 6 | 0 | 0.000000 | -0.000000 | 1513 | 1358.717479 | 1422.86 | 1487.002521 | 0.940423 |
| GO:0051970\_negative\_regulation\_of\_transmission\_of\_nerve\_impulse | 6 | 0 | 0.000000 | -0.000000 | 1513 | 1358.717479 | 1422.86 | 1487.002521 | 0.940423 |
| GO:0055081\_anion\_homeostasis | 6 | 0 | 0.000000 | -0.000000 | 1513 | 1358.717479 | 1422.86 | 1487.002521 | 0.940423 |
| GO:0060013\_righting\_reflex | 6 | 0 | 0.000000 | -0.000000 | 1513 | 1358.717479 | 1422.86 | 1487.002521 | 0.940423 |
| GO:0060017\_parathyroid\_gland\_development | 6 | 0 | 0.000000 | -0.000000 | 1513 | 1358.717479 | 1422.86 | 1487.002521 | 0.940423 |
| GO:0060056\_mammary\_gland\_involution | 6 | 0 | 0.000000 | -0.000000 | 1513 | 1358.717479 | 1422.86 | 1487.002521 | 0.940423 |
| GO:0060068\_vagina\_development | 6 | 0 | 0.000000 | -0.000000 | 1513 | 1358.717479 | 1422.86 | 1487.002521 | 0.940423 |
| GO:0060134\_prepulse\_inhibition | 6 | 0 | 0.000000 | -0.000000 | 1513 | 1358.717479 | 1422.86 | 1487.002521 | 0.940423 |
| GO:0060136\_embryonic\_process\_involved\_in\_female\_pregnancy | 6 | 0 | 0.000000 | -0.000000 | 1513 | 1358.717479 | 1422.86 | 1487.002521 | 0.940423 |
| GO:0060271\_cilium\_morphogenesis | 6 | 0 | 0.000000 | -0.000000 | 1513 | 1358.717479 | 1422.86 | 1487.002521 | 0.940423 |
| GO:0060389\_pathway-restricted\_SMAD\_protein\_phosphorylation | 6 | 0 | 0.000000 | -0.000000 | 1513 | 1358.717479 | 1422.86 | 1487.002521 | 0.940423 |
| GO:0060411\_heart\_septum\_morphogenesis | 6 | 0 | 0.000000 | -0.000000 | 1513 | 1358.717479 | 1422.86 | 1487.002521 | 0.940423 |
| GO:0060638\_mesenchymal-epithelial\_cell\_signaling | 6 | 0 | 0.000000 | -0.000000 | 1513 | 1358.717479 | 1422.86 | 1487.002521 | 0.940423 |
| GO:0060685\_regulation\_of\_prostatic\_bud\_formation | 6 | 0 | 0.000000 | -0.000000 | 1513 | 1358.717479 | 1422.86 | 1487.002521 | 0.940423 |
| GO:0060710\_chorio-allantoic\_fusion | 6 | 0 | 0.000000 | -0.000000 | 1513 | 1358.717479 | 1422.86 | 1487.002521 | 0.940423 |
| GO:0065004\_protein-DNA\_complex\_assembly | 6 | 0 | 0.000000 | -0.000000 | 1513 | 1358.717479 | 1422.86 | 1487.002521 | 0.940423 |
| GO:0032940\_secretion\_by\_cell | 149 | 0 | 0.000000 | -0.000000 | 1514 | 1360.821518 | 1424.82 | 1488.818482 | 0.941096 |
| GO:0000012\_single\_strand\_break\_repair | 2 | 0 |  |  |  |  |  |  |  |  |
| GO:0000019\_regulation\_of\_mitotic\_recombination | 2 | 0 |  |  |  |  |  |  |  |  |
| GO:0000076\_DNA\_replication\_checkpoint | 2 | 0 |  |  |  |  |  |  |  |  |
| GO:0000080\_G1\_phase\_of\_mitotic\_cell\_cycle | 2 | 0 |  |  |  |  |  |  |  |  |
| GO:0000083\_regulation\_of\_transcription\_of\_G1\_S-phase\_of\_mitotic\_cell\_cycle | 2 | 0 |  |  |  |  |  |  |  |  |
| GO:0000085\_G2\_phase\_of\_mitotic\_cell\_cycle | 2 | 0 |  |  |  |  |  |  |  |  |
| GO:0000289\_nuclear-transcribed\_mRNA\_poly(A)\_tail\_shortening | 2 | 0 |  |  |  |  |  |  |  |  |
| GO:0000381\_regulation\_of\_alternative\_nuclear\_mRNA\_splicing\_\_via\_spliceosome | 2 | 0 |  |  |  |  |  |  |  |  |
| GO:0000712\_resolution\_of\_meiotic\_joint\_molecules\_as\_recombinants | 2 | 0 |  |  |  |  |  |  |  |  |
| GO:0000720\_pyrimidine\_dimer\_repair\_by\_nucleotide-excision\_repair | 2 | 0 |  |  |  |  |  |  |  |  |
| GO:0001302\_replicative\_cell\_aging | 2 | 0 |  |  |  |  |  |  |  |  |
| GO:0001306\_age-dependent\_response\_to\_oxidative\_stress | 2 | 0 |  |  |  |  |  |  |  |  |
| GO:0001514\_selenocysteine\_incorporation | 2 | 0 |  |  |  |  |  |  |  |  |
| GO:0001522\_pseudouridine\_synthesis | 2 | 0 |  |  |  |  |  |  |  |  |
| GO:0001543\_ovarian\_follicle\_rupture | 2 | 0 |  |  |  |  |  |  |  |  |
| GO:0001561\_fatty\_acid\_alpha-oxidation | 2 | 0 |  |  |  |  |  |  |  |  |
| GO:0001675\_acrosome\_assembly | 2 | 0 |  |  |  |  |  |  |  |  |
| GO:0001743\_optic\_placode\_formation | 2 | 0 |  |  |  |  |  |  |  |  |
| GO:0001767\_establishment\_of\_lymphocyte\_polarity | 2 | 0 |  |  |  |  |  |  |  |  |
| GO:0001768\_establishment\_of\_T\_cell\_polarity | 2 | 0 |  |  |  |  |  |  |  |  |
| GO:0001771\_formation\_of\_immunological\_synapse | 2 | 0 |  |  |  |  |  |  |  |  |
| GO:0001774\_microglial\_cell\_activation | 2 | 0 |  |  |  |  |  |  |  |  |
| GO:0001781\_neutrophil\_apoptosis | 2 | 0 |  |  |  |  |  |  |  |  |
| GO:0001787\_natural\_killer\_cell\_proliferation | 2 | 0 |  |  |  |  |  |  |  |  |
| GO:0001788\_antibody-dependent\_cellular\_cytotoxicity | 2 | 0 |  |  |  |  |  |  |  |  |
| GO:0001806\_type\_IV\_hypersensitivity | 2 | 0 |  |  |  |  |  |  |  |  |
| GO:0001807\_regulation\_of\_type\_IV\_hypersensitivity | 2 | 0 |  |  |  |  |  |  |  |  |
| GO:0001808\_negative\_regulation\_of\_type\_IV\_hypersensitivity | 2 | 0 |  |  |  |  |  |  |  |  |
| GO:0001823\_mesonephros\_development | 2 | 0 |  |  |  |  |  |  |  |  |
| GO:0001845\_phagolysosome\_formation | 2 | 0 |  |  |  |  |  |  |  |  |
| GO:0001866\_NK\_T\_cell\_proliferation | 2 | 0 |  |  |  |  |  |  |  |  |
| GO:0001879\_detection\_of\_yeast | 2 | 0 |  |  |  |  |  |  |  |  |
| GO:0001886\_endothelial\_cell\_morphogenesis | 2 | 0 |  |  |  |  |  |  |  |  |
| GO:0001919\_regulation\_of\_receptor\_recycling | 2 | 0 |  |  |  |  |  |  |  |  |
| GO:0001954\_positive\_regulation\_of\_cell-matrix\_adhesion | 2 | 0 |  |  |  |  |  |  |  |  |
| GO:0001977\_renal\_system\_process\_involved\_in\_regulation\_of\_blood\_volume | 2 | 0 |  |  |  |  |  |  |  |  |
| GO:0001982\_baroreceptor\_response\_to\_decreased\_systemic\_arterial\_blood\_pressure | 2 | 0 |  |  |  |  |  |  |  |  |
| GO:0001983\_baroreceptor\_response\_to\_increased\_systemic\_arterial\_blood\_pressure | 2 | 0 |  |  |  |  |  |  |  |  |
| GO:0001992\_regulation\_of\_systemic\_arterial\_blood\_pressure\_by\_vasopressin | 2 | 0 |  |  |  |  |  |  |  |  |
| GO:0001997\_positive\_regulation\_of\_the\_force\_of\_heart\_contraction\_by\_epinephrine-norepinephrine | 2 | 0 |  |  |  |  |  |  |  |  |
| GO:0001998\_angiotensin\_mediated\_vasoconstriction\_involved\_in\_regulation\_of\_systemic\_arterial\_blood\_pressure | 2 | 0 |  |  |  |  |  |  |  |  |
| GO:0001999\_renal\_response\_to\_blood\_flow\_during\_renin-angiotensin\_regulation\_of\_systemic\_arterial\_blood\_pressure | 2 | 0 |  |  |  |  |  |  |  |  |
| GO:0002018\_renin-angiotensin\_regulation\_of\_aldosterone\_production | 2 | 0 |  |  |  |  |  |  |  |  |
| GO:0002019\_regulation\_of\_renal\_output\_by\_angiotensin | 2 | 0 |  |  |  |  |  |  |  |  |
| GO:0002024\_diet\_induced\_thermogenesis | 2 | 0 |  |  |  |  |  |  |  |  |
| GO:0002025\_vasodilation\_by\_norepinephrine-epinephrine\_involved\_in\_regulation\_of\_systemic\_arterial\_blood\_pressure | 2 | 0 |  |  |  |  |  |  |  |  |
| GO:0002029\_desensitization\_of\_G-protein\_coupled\_receptor\_protein\_signaling\_pathway | 2 | 0 |  |  |  |  |  |  |  |  |
| GO:0002033\_vasodilation\_by\_angiotensin\_involved\_in\_regulation\_of\_systemic\_arterial\_blood\_pressure | 2 | 0 |  |  |  |  |  |  |  |  |
| GO:0002066\_columnar\_cuboidal\_epithelial\_cell\_development | 2 | 0 |  |  |  |  |  |  |  |  |
| GO:0002074\_extraocular\_skeletal\_muscle\_development | 2 | 0 |  |  |  |  |  |  |  |  |
| GO:0002138\_retinoic\_acid\_biosynthetic\_process | 2 | 0 |  |  |  |  |  |  |  |  |
| GO:0002223\_stimulatory\_C-type\_lectin\_receptor\_signaling\_pathway | 2 | 0 |  |  |  |  |  |  |  |  |
| GO:0002251\_organ\_or\_tissue\_specific\_immune\_response | 2 | 0 |  |  |  |  |  |  |  |  |
| GO:0002266\_follicular\_dendritic\_cell\_activation | 2 | 0 |  |  |  |  |  |  |  |  |
| GO:0002268\_follicular\_dendritic\_cell\_differentiation | 2 | 0 |  |  |  |  |  |  |  |  |
| GO:0002327\_immature\_B\_cell\_differentiation | 2 | 0 |  |  |  |  |  |  |  |  |
| GO:0002329\_pre-B\_cell\_differentiation | 2 | 0 |  |  |  |  |  |  |  |  |
| GO:0002339\_B\_cell\_selection | 2 | 0 |  |  |  |  |  |  |  |  |
| GO:0002352\_B\_cell\_negative\_selection | 2 | 0 |  |  |  |  |  |  |  |  |
| GO:0002358\_B\_cell\_homeostatic\_proliferation | 2 | 0 |  |  |  |  |  |  |  |  |
| GO:0002385\_mucosal\_immune\_response | 2 | 0 |  |  |  |  |  |  |  |  |
| GO:0002514\_B\_cell\_tolerance\_induction | 2 | 0 |  |  |  |  |  |  |  |  |
| GO:0002523\_leukocyte\_migration\_during\_inflammatory\_response | 2 | 0 |  |  |  |  |  |  |  |  |
| GO:0002536\_respiratory\_burst\_during\_acute\_inflammatory\_response | 2 | 0 |  |  |  |  |  |  |  |  |
| GO:0002537\_production\_of\_nitric\_oxide\_during\_acute\_inflammatory\_response | 2 | 0 |  |  |  |  |  |  |  |  |
| GO:0002576\_platelet\_degranulation | 2 | 0 |  |  |  |  |  |  |  |  |
| GO:0002639\_positive\_regulation\_of\_immunoglobulin\_production | 2 | 0 |  |  |  |  |  |  |  |  |
| GO:0002661\_regulation\_of\_B\_cell\_tolerance\_induction | 2 | 0 |  |  |  |  |  |  |  |  |
| GO:0002663\_positive\_regulation\_of\_B\_cell\_tolerance\_induction | 2 | 0 |  |  |  |  |  |  |  |  |
| GO:0002676\_regulation\_of\_chronic\_inflammatory\_response | 2 | 0 |  |  |  |  |  |  |  |  |
| GO:0002679\_respiratory\_burst\_during\_defense\_response | 2 | 0 |  |  |  |  |  |  |  |  |
| GO:0002686\_negative\_regulation\_of\_leukocyte\_migration | 2 | 0 |  |  |  |  |  |  |  |  |
| GO:0002720\_positive\_regulation\_of\_cytokine\_production\_during\_immune\_response | 2 | 0 |  |  |  |  |  |  |  |  |
| GO:0002752\_cell\_surface\_pattern\_recognition\_receptor\_signaling\_pathway | 2 | 0 |  |  |  |  |  |  |  |  |
| GO:0002755\_MyD88-dependent\_toll-like\_receptor\_signaling\_pathway | 2 | 0 |  |  |  |  |  |  |  |  |
| GO:0002765\_immune\_response-inhibiting\_signal\_transduction | 2 | 0 |  |  |  |  |  |  |  |  |
| GO:0002921\_negative\_regulation\_of\_humoral\_immune\_response | 2 | 0 |  |  |  |  |  |  |  |  |
| GO:0002922\_positive\_regulation\_of\_humoral\_immune\_response | 2 | 0 |  |  |  |  |  |  |  |  |
| GO:0002924\_negative\_regulation\_of\_humoral\_immune\_response\_mediated\_by\_circulating\_immunoglobulin | 2 | 0 |  |  |  |  |  |  |  |  |
| GO:0002925\_positive\_regulation\_of\_humoral\_immune\_response\_mediated\_by\_circulating\_immunoglobulin | 2 | 0 |  |  |  |  |  |  |  |  |
| GO:0003057\_regulation\_of\_the\_force\_of\_heart\_contraction\_by\_chemical\_signal | 2 | 0 |  |  |  |  |  |  |  |  |
| GO:0003099\_positive\_regulation\_of\_the\_force\_of\_heart\_contraction\_by\_chemical\_signal | 2 | 0 |  |  |  |  |  |  |  |  |
| GO:0005981\_regulation\_of\_glycogen\_catabolic\_process | 2 | 0 |  |  |  |  |  |  |  |  |
| GO:0006021\_inositol\_biosynthetic\_process | 2 | 0 |  |  |  |  |  |  |  |  |
| GO:0006042\_glucosamine\_biosynthetic\_process | 2 | 0 |  |  |  |  |  |  |  |  |
| GO:0006045\_N-acetylglucosamine\_biosynthetic\_process | 2 | 0 |  |  |  |  |  |  |  |  |
| GO:0006048\_UDP-N-acetylglucosamine\_biosynthetic\_process | 2 | 0 |  |  |  |  |  |  |  |  |
| GO:0006054\_N-acetylneuraminate\_metabolic\_process | 2 | 0 |  |  |  |  |  |  |  |  |
| GO:0006059\_hexitol\_metabolic\_process | 2 | 0 |  |  |  |  |  |  |  |  |
| GO:0006063\_uronic\_acid\_metabolic\_process | 2 | 0 |  |  |  |  |  |  |  |  |
| GO:0006068\_ethanol\_catabolic\_process | 2 | 0 |  |  |  |  |  |  |  |  |
| GO:0006083\_acetate\_metabolic\_process | 2 | 0 |  |  |  |  |  |  |  |  |
| GO:0006105\_succinate\_metabolic\_process | 2 | 0 |  |  |  |  |  |  |  |  |
| GO:0006106\_fumarate\_metabolic\_process | 2 | 0 |  |  |  |  |  |  |  |  |
| GO:0006110\_regulation\_of\_glycolysis | 2 | 0 |  |  |  |  |  |  |  |  |
| GO:0006113\_fermentation | 2 | 0 |  |  |  |  |  |  |  |  |
| GO:0006114\_glycerol\_biosynthetic\_process | 2 | 0 |  |  |  |  |  |  |  |  |
| GO:0006122\_mitochondrial\_electron\_transport\_\_ubiquinol\_to\_cytochrome\_c | 2 | 0 |  |  |  |  |  |  |  |  |
| GO:0006152\_purine\_nucleoside\_catabolic\_process | 2 | 0 |  |  |  |  |  |  |  |  |
| GO:0006168\_adenine\_salvage | 2 | 0 |  |  |  |  |  |  |  |  |
| GO:0006200\_ATP\_catabolic\_process | 2 | 0 |  |  |  |  |  |  |  |  |
| GO:0006206\_pyrimidine\_base\_metabolic\_process | 2 | 0 |  |  |  |  |  |  |  |  |
| GO:0006213\_pyrimidine\_nucleoside\_metabolic\_process | 2 | 0 |  |  |  |  |  |  |  |  |
| GO:0006265\_DNA\_topological\_change | 2 | 0 |  |  |  |  |  |  |  |  |
| GO:0006278\_RNA-dependent\_DNA\_replication | 2 | 0 |  |  |  |  |  |  |  |  |
| GO:0006312\_mitotic\_recombination | 2 | 0 |  |  |  |  |  |  |  |  |
| GO:0006398\_histone\_mRNA\_3'-end\_processing | 2 | 0 |  |  |  |  |  |  |  |  |
| GO:0006418\_tRNA\_aminoacylation\_for\_protein\_translation | 2 | 0 |  |  |  |  |  |  |  |  |
| GO:0006451\_translational\_readthrough | 2 | 0 |  |  |  |  |  |  |  |  |
| GO:0006477\_protein\_amino\_acid\_sulfation | 2 | 0 |  |  |  |  |  |  |  |  |
| GO:0006482\_protein\_amino\_acid\_demethylation | 2 | 0 |  |  |  |  |  |  |  |  |
| GO:0006499\_N-terminal\_protein\_myristoylation | 2 | 0 |  |  |  |  |  |  |  |  |
| GO:0006525\_arginine\_metabolic\_process | 2 | 0 |  |  |  |  |  |  |  |  |
| GO:0006527\_arginine\_catabolic\_process | 2 | 0 |  |  |  |  |  |  |  |  |
| GO:0006532\_aspartate\_biosynthetic\_process | 2 | 0 |  |  |  |  |  |  |  |  |
| GO:0006538\_glutamate\_catabolic\_process | 2 | 0 |  |  |  |  |  |  |  |  |
| GO:0006558\_L-phenylalanine\_metabolic\_process | 2 | 0 |  |  |  |  |  |  |  |  |
| GO:0006568\_tryptophan\_metabolic\_process | 2 | 0 |  |  |  |  |  |  |  |  |
| GO:0006583\_melanin\_biosynthetic\_process\_from\_tyrosine | 2 | 0 |  |  |  |  |  |  |  |  |
| GO:0006600\_creatine\_metabolic\_process | 2 | 0 |  |  |  |  |  |  |  |  |
| GO:0006603\_phosphocreatine\_metabolic\_process | 2 | 0 |  |  |  |  |  |  |  |  |
| GO:0006610\_ribosomal\_protein\_import\_into\_nucleus | 2 | 0 |  |  |  |  |  |  |  |  |
| GO:0006642\_triglyceride\_mobilization | 2 | 0 |  |  |  |  |  |  |  |  |
| GO:0006649\_phospholipid\_transfer\_to\_membrane | 2 | 0 |  |  |  |  |  |  |  |  |
| GO:0006681\_galactosylceramide\_metabolic\_process | 2 | 0 |  |  |  |  |  |  |  |  |
| GO:0006686\_sphingomyelin\_biosynthetic\_process | 2 | 0 |  |  |  |  |  |  |  |  |
| GO:0006702\_androgen\_biosynthetic\_process | 2 | 0 |  |  |  |  |  |  |  |  |
| GO:0006750\_glutathione\_biosynthetic\_process | 2 | 0 |  |  |  |  |  |  |  |  |
| GO:0006760\_folic\_acid\_and\_derivative\_metabolic\_process | 2 | 0 |  |  |  |  |  |  |  |  |
| GO:0006808\_regulation\_of\_nitrogen\_utilization | 2 | 0 |  |  |  |  |  |  |  |  |
| GO:0006868\_glutamine\_transport | 2 | 0 |  |  |  |  |  |  |  |  |
| GO:0006907\_pinocytosis | 2 | 0 |  |  |  |  |  |  |  |  |
| GO:0006925\_inflammatory\_cell\_apoptosis | 2 | 0 |  |  |  |  |  |  |  |  |
| GO:0006977\_DNA\_damage\_response\_\_signal\_transduction\_by\_p53\_class\_mediator\_resulting\_in\_cell\_cycle\_arrest | 2 | 0 |  |  |  |  |  |  |  |  |
| GO:0006991\_response\_to\_sterol\_depletion | 2 | 0 |  |  |  |  |  |  |  |  |
| GO:0007004\_telomere\_maintenance\_via\_telomerase | 2 | 0 |  |  |  |  |  |  |  |  |
| GO:0007020\_microtubule\_nucleation | 2 | 0 |  |  |  |  |  |  |  |  |
| GO:0007030\_Golgi\_organization | 2 | 0 |  |  |  |  |  |  |  |  |
| GO:0007035\_vacuolar\_acidification | 2 | 0 |  |  |  |  |  |  |  |  |
| GO:0007042\_lysosomal\_lumen\_acidification | 2 | 0 |  |  |  |  |  |  |  |  |
| GO:0007060\_male\_meiosis\_chromosome\_segregation | 2 | 0 |  |  |  |  |  |  |  |  |
| GO:0007089\_traversing\_start\_control\_point\_of\_mitotic\_cell\_cycle | 2 | 0 |  |  |  |  |  |  |  |  |
| GO:0007094\_mitotic\_cell\_cycle\_spindle\_assembly\_checkpoint | 2 | 0 |  |  |  |  |  |  |  |  |
| GO:0007097\_nuclear\_migration | 2 | 0 |  |  |  |  |  |  |  |  |
| GO:0007100\_mitotic\_centrosome\_separation | 2 | 0 |  |  |  |  |  |  |  |  |
| GO:0007132\_meiotic\_metaphase\_I | 2 | 0 |  |  |  |  |  |  |  |  |
| GO:0007171\_activation\_of\_transmembrane\_receptor\_protein\_tyrosine\_kinase\_activity | 2 | 0 |  |  |  |  |  |  |  |  |
| GO:0007182\_common-partner\_SMAD\_protein\_phosphorylation | 2 | 0 |  |  |  |  |  |  |  |  |
| GO:0007185\_transmembrane\_receptor\_protein\_tyrosine\_phosphatase\_signaling\_pathway | 2 | 0 |  |  |  |  |  |  |  |  |
| GO:0007205\_activation\_of\_protein\_kinase\_C\_activity\_by\_G-protein\_coupled\_receptor\_protein\_signaling\_pathway | 2 | 0 |  |  |  |  |  |  |  |  |
| GO:0007210\_serotonin\_receptor\_signaling\_pathway | 2 | 0 |  |  |  |  |  |  |  |  |
| GO:0007220\_Notch\_receptor\_processing | 2 | 0 |  |  |  |  |  |  |  |  |
| GO:0007256\_activation\_of\_JNKK\_activity | 2 | 0 |  |  |  |  |  |  |  |  |
| GO:0007258\_JUN\_phosphorylation | 2 | 0 |  |  |  |  |  |  |  |  |
| GO:0007263\_nitric\_oxide\_mediated\_signal\_transduction | 2 | 0 |  |  |  |  |  |  |  |  |
| GO:0007289\_spermatid\_nucleus\_differentiation | 2 | 0 |  |  |  |  |  |  |  |  |
| GO:0007343\_egg\_activation | 2 | 0 |  |  |  |  |  |  |  |  |
| GO:0007351\_tripartite\_regional\_subdivision | 2 | 0 |  |  |  |  |  |  |  |  |
| GO:0007418\_ventral\_midline\_development | 2 | 0 |  |  |  |  |  |  |  |  |
| GO:0007494\_midgut\_development | 2 | 0 |  |  |  |  |  |  |  |  |
| GO:0007527\_adult\_somatic\_muscle\_development | 2 | 0 |  |  |  |  |  |  |  |  |
| GO:0007549\_dosage\_compensation | 2 | 0 |  |  |  |  |  |  |  |  |
| GO:0007571\_age-dependent\_general\_metabolic\_decline | 2 | 0 |  |  |  |  |  |  |  |  |
| GO:0007603\_phototransduction\_\_visible\_light | 2 | 0 |  |  |  |  |  |  |  |  |
| GO:0007619\_courtship\_behavior | 2 | 0 |  |  |  |  |  |  |  |  |
| GO:0008065\_establishment\_of\_blood-nerve\_barrier | 2 | 0 |  |  |  |  |  |  |  |  |
[truncated: 255,992 more chars]
