# Supplementary material for: RedundancyMiner: De-replication of redundant GO categories in microarray and proteomics analysis
Source: BMC Bioinformatics. 2011 Feb 10;12:52. doi: 10.1186/1471-2105-12-52 (PMC3223614; doi:10.1186/1471-2105-12-52)
Supplement: Additional file 8 — Retinal development HTGM download. compressed package of the results of running HTGM on the retinal development genes list. [file 1471-2105-12-52-S8.ZIP › SCENARIO_2_MODIFIED/total.txt.total.txt.dir/Exp1_BestClusterMap_LEIGS_KM_24.csv.join.14.txt.dir/Exp1_BestClusterMap_LEIGS_KM_24.csv.join.14.txt.change.gce.html]

Gene Category Report for Exp1\_BestClusterMap\_LEIGS\_KM\_24.csv.join.14.txt

# Gene Category Report for Exp1\_BestClusterMap\_LEIGS\_KM\_24.csv.join.14.txt

| HYPERLINKED GO CATEGORY | HYPERLINKED GENE NAME | TOTAL GENES | CHANGED GENES | ENRICHMENT | LOG10(p) | CUMULATIVE NUMBER OF CATEGORIES | CUMULATIVE RANDOMS MEAN | FALSE DISCOVERY RATE |
| --- | --- | --- | --- | --- | --- | --- | --- | --- |
| GO:0008088\_axon\_cargo\_transport | KLC1 | 10 | 2 | 31.758621 | -2.777194 | 1 | 2.12 | 2.120000 |
| GO:0008088\_axon\_cargo\_transport | PAFAH1B1 | 10 | 2 | 31.758621 | -2.777194 | 1 | 2.12 | 2.120000 |
| GO:0010970\_microtubule-based\_transport | KLC1 | 13 | 2 | 24.429708 | -2.543401 | 3 | 3.54 | 1.180000 |
| GO:0010970\_microtubule-based\_transport | PAFAH1B1 | 13 | 2 | 24.429708 | -2.543401 | 3 | 3.54 | 1.180000 |
| GO:0042474\_middle\_ear\_morphogenesis | INSIG1 | 13 | 2 | 24.429708 | -2.543401 | 3 | 3.54 | 1.180000 |
| GO:0042474\_middle\_ear\_morphogenesis | GAS1 | 13 | 2 | 24.429708 | -2.543401 | 3 | 3.54 | 1.180000 |
| GO:0001764\_neuron\_migration | NAV1 | 57 | 3 | 8.357532 | -2.281483 | 4 | 5.53 | 1.382500 |
| GO:0001764\_neuron\_migration | FYN | 57 | 3 | 8.357532 | -2.281483 | 4 | 5.53 | 1.382500 |
| GO:0001764\_neuron\_migration | PAFAH1B1 | 57 | 3 | 8.357532 | -2.281483 | 4 | 5.53 | 1.382500 |
| GO:0021587\_cerebellum\_morphogenesis | AGTPBP1 | 19 | 2 | 16.715064 | -2.212664 | 5 | 6.46 | 1.292000 |
| GO:0021587\_cerebellum\_morphogenesis | GAS1 | 19 | 2 | 16.715064 | -2.212664 | 5 | 6.46 | 1.292000 |
| GO:0021575\_hindbrain\_morphogenesis | AGTPBP1 | 22 | 2 | 14.435737 | -2.087122 | 7 | 8.1 | 1.157143 |
| GO:0021575\_hindbrain\_morphogenesis | GAS1 | 22 | 2 | 14.435737 | -2.087122 | 7 | 8.1 | 1.157143 |
| GO:0030705\_cytoskeleton-dependent\_intracellular\_transport | KLC1 | 22 | 2 | 14.435737 | -2.087122 | 7 | 8.1 | 1.157143 |
| GO:0030705\_cytoskeleton-dependent\_intracellular\_transport | PAFAH1B1 | 22 | 2 | 14.435737 | -2.087122 | 7 | 8.1 | 1.157143 |
| GO:0007018\_microtubule-based\_movement | KLC1 | 23 | 2 | 13.808096 | -2.049304 | 8 | 8.72 | 1.090000 |
| GO:0007018\_microtubule-based\_movement | PAFAH1B1 | 23 | 2 | 13.808096 | -2.049304 | 8 | 8.72 | 1.090000 |
| GO:0001675\_acrosome\_assembly | PAFAH1B1 | 2 | 1 |  |  |  |  |  |  |
| GO:0006991\_response\_to\_sterol\_depletion | INSIG1 | 2 | 1 |  |  |  |  |  |  |
| GO:0030397\_membrane\_disassembly | PAFAH1B1 | 2 | 1 |  |  |  |  |  |  |
| GO:0045065\_cytotoxic\_T\_cell\_differentiation | CD8A | 2 | 1 |  |  |  |  |  |  |
| GO:0048739\_cardiac\_muscle\_fiber\_development | CXADR | 2 | 1 |  |  |  |  |  |  |
| GO:0051081\_nuclear\_envelope\_disassembly | PAFAH1B1 | 2 | 1 |  |  |  |  |  |  |
| GO:0060044\_negative\_regulation\_of\_cardiac\_muscle\_cell\_proliferation | CXADR | 2 | 1 |  |  |  |  |  |  |
| GO:0060363\_cranial\_suture\_morphogenesis | INSIG1 | 2 | 1 |  |  |  |  |  |  |
| GO:0021549\_cerebellum\_development | AGTPBP1 | 28 | 2 | 11.342365 | -1.883375 | 9 | 11.37 | 1.263333 |
| GO:0021549\_cerebellum\_development | GAS1 | 28 | 2 | 11.342365 | -1.883375 | 9 | 11.37 | 1.263333 |
| GO:0006641\_triglyceride\_metabolic\_process | MOGAT2 | 29 | 2 | 10.951249 | -1.854027 | 10 | 12.03 | 1.203000 |
| GO:0006641\_triglyceride\_metabolic\_process | INSIG1 | 29 | 2 | 10.951249 | -1.854027 | 10 | 12.03 | 1.203000 |
| GO:0007017\_microtubule-based\_process | NAV1 | 83 | 3 | 5.739510 | -1.832079 | 11 | 12.3 | 1.118182 |
| GO:0007017\_microtubule-based\_process | KLC1 | 83 | 3 | 5.739510 | -1.832079 | 11 | 12.3 | 1.118182 |
| GO:0007017\_microtubule-based\_process | PAFAH1B1 | 83 | 3 | 5.739510 | -1.832079 | 11 | 12.3 | 1.118182 |
| GO:0060021\_palate\_development | INSIG1 | 30 | 2 | 10.586207 | -1.825750 | 12 | 12.69 | 1.057500 |
| GO:0060021\_palate\_development | GAS1 | 30 | 2 | 10.586207 | -1.825750 | 12 | 12.69 | 1.057500 |
| GO:0006639\_acylglycerol\_metabolic\_process | MOGAT2 | 31 | 2 | 10.244716 | -1.798472 | 14 | 13.54 | 0.967143 |
| GO:0006639\_acylglycerol\_metabolic\_process | INSIG1 | 31 | 2 | 10.244716 | -1.798472 | 14 | 13.54 | 0.967143 |
| GO:0042157\_lipoprotein\_metabolic\_process | ZDHHC17 | 31 | 2 | 10.244716 | -1.798472 | 14 | 13.54 | 0.967143 |
| GO:0042157\_lipoprotein\_metabolic\_process | LDLR | 31 | 2 | 10.244716 | -1.798472 | 14 | 13.54 | 0.967143 |
| GO:0006638\_neutral\_lipid\_metabolic\_process | MOGAT2 | 32 | 2 | 9.924569 | -1.772128 | 16 | 14.35 | 0.896875 |
| GO:0006638\_neutral\_lipid\_metabolic\_process | INSIG1 | 32 | 2 | 9.924569 | -1.772128 | 16 | 14.35 | 0.896875 |
| GO:0006662\_glycerol\_ether\_metabolic\_process | MOGAT2 | 32 | 2 | 9.924569 | -1.772128 | 16 | 14.35 | 0.896875 |
| GO:0006662\_glycerol\_ether\_metabolic\_process | INSIG1 | 32 | 2 | 9.924569 | -1.772128 | 16 | 14.35 | 0.896875 |
| GO:0022037\_metencephalon\_development | AGTPBP1 | 33 | 2 | 9.623824 | -1.746660 | 17 | 15.16 | 0.891765 |
| GO:0022037\_metencephalon\_development | GAS1 | 33 | 2 | 9.623824 | -1.746660 | 17 | 15.16 | 0.891765 |
| GO:0006651\_diacylglycerol\_biosynthetic\_process | MOGAT2 | 3 | 1 |  |  |  |  |  |  |
| GO:0008090\_retrograde\_axon\_cargo\_transport | PAFAH1B1 | 3 | 1 |  |  |  |  |  |  |
| GO:0010894\_negative\_regulation\_of\_steroid\_biosynthetic\_process | INSIG1 | 3 | 1 |  |  |  |  |  |  |
| GO:0016556\_mRNA\_modification | DNAJB11 | 3 | 1 |  |  |  |  |  |  |
| GO:0021819\_layer\_formation\_in\_the\_cerebral\_cortex | PAFAH1B1 | 3 | 1 |  |  |  |  |  |  |
| GO:0045717\_negative\_regulation\_of\_fatty\_acid\_biosynthetic\_process | INSIG1 | 3 | 1 |  |  |  |  |  |  |
| GO:0045922\_negative\_regulation\_of\_fatty\_acid\_metabolic\_process | INSIG1 | 3 | 1 |  |  |  |  |  |  |
| GO:0045939\_negative\_regulation\_of\_steroid\_metabolic\_process | INSIG1 | 3 | 1 |  |  |  |  |  |  |
| GO:0018904\_organic\_ether\_metabolic\_process | MOGAT2 | 35 | 2 | 9.073892 | -1.698144 | 18 | 16.5 | 0.916667 |
| GO:0018904\_organic\_ether\_metabolic\_process | INSIG1 | 35 | 2 | 9.073892 | -1.698144 | 18 | 16.5 | 0.916667 |
| GO:0030154\_cell\_differentiation | NAV1 | 1060 | 12 | 1.797658 | -1.680415 | 19 | 16.79 | 0.883684 |
| GO:0030154\_cell\_differentiation | CD8A | 1060 | 12 | 1.797658 | -1.680415 | 19 | 16.79 | 0.883684 |
| GO:0030154\_cell\_differentiation | FYN | 1060 | 12 | 1.797658 | -1.680415 | 19 | 16.79 | 0.883684 |
| GO:0030154\_cell\_differentiation | AGTPBP1 | 1060 | 12 | 1.797658 | -1.680415 | 19 | 16.79 | 0.883684 |
| GO:0030154\_cell\_differentiation | GNA11 | 1060 | 12 | 1.797658 | -1.680415 | 19 | 16.79 | 0.883684 |
| GO:0030154\_cell\_differentiation | LGALS1 | 1060 | 12 | 1.797658 | -1.680415 | 19 | 16.79 | 0.883684 |
| GO:0030154\_cell\_differentiation | FST | 1060 | 12 | 1.797658 | -1.680415 | 19 | 16.79 | 0.883684 |
| GO:0030154\_cell\_differentiation | INSIG1 | 1060 | 12 | 1.797658 | -1.680415 | 19 | 16.79 | 0.883684 |
| GO:0030154\_cell\_differentiation | PAFAH1B1 | 1060 | 12 | 1.797658 | -1.680415 | 19 | 16.79 | 0.883684 |
| GO:0030154\_cell\_differentiation | FZD2 | 1060 | 12 | 1.797658 | -1.680415 | 19 | 16.79 | 0.883684 |
| GO:0030154\_cell\_differentiation | GAS1 | 1060 | 12 | 1.797658 | -1.680415 | 19 | 16.79 | 0.883684 |
| GO:0030154\_cell\_differentiation | IGFBP5 | 1060 | 12 | 1.797658 | -1.680415 | 19 | 16.79 | 0.883684 |
| GO:0019432\_triglyceride\_biosynthetic\_process | MOGAT2 | 4 | 1 |  |  |  |  |  |  |
| GO:0021801\_cerebral\_cortex\_radial\_glia\_guided\_migration | PAFAH1B1 | 4 | 1 |  |  |  |  |  |  |
| GO:0042473\_outer\_ear\_morphogenesis | GAS1 | 4 | 1 |  |  |  |  |  |  |
| GO:0045634\_regulation\_of\_melanocyte\_differentiation | GNA11 | 4 | 1 |  |  |  |  |  |  |
| GO:0047496\_vesicle\_transport\_along\_microtubule | PAFAH1B1 | 4 | 1 |  |  |  |  |  |  |
| GO:0050932\_regulation\_of\_pigment\_cell\_differentiation | GNA11 | 4 | 1 |  |  |  |  |  |  |
| GO:0051055\_negative\_regulation\_of\_lipid\_biosynthetic\_process | INSIG1 | 4 | 1 |  |  |  |  |  |  |
| GO:0060158\_activation\_of\_phospholipase\_C\_activity\_by\_dopamine\_receptor\_signaling\_pathway | GNA11 | 4 | 1 |  |  |  |  |  |  |
| GO:0008203\_cholesterol\_metabolic\_process | LDLR | 40 | 2 | 7.939655 | -1.588973 | 20 | 20.12 | 1.006000 |
| GO:0008203\_cholesterol\_metabolic\_process | INSIG1 | 40 | 2 | 7.939655 | -1.588973 | 20 | 20.12 | 1.006000 |
| GO:0016125\_sterol\_metabolic\_process | LDLR | 42 | 2 | 7.561576 | -1.549423 | 21 | 22.05 | 1.050000 |
| GO:0016125\_sterol\_metabolic\_process | INSIG1 | 42 | 2 | 7.561576 | -1.549423 | 21 | 22.05 | 1.050000 |
| GO:0001508\_regulation\_of\_action\_potential | FYN | 43 | 2 | 7.385726 | -1.530417 | 22 | 22.74 | 1.033636 |
| GO:0001508\_regulation\_of\_action\_potential | GNA11 | 43 | 2 | 7.385726 | -1.530417 | 22 | 22.74 | 1.033636 |
| GO:0048869\_cellular\_developmental\_process | NAV1 | 1113 | 12 | 1.712055 | -1.521001 | 23 | 22.76 | 0.989565 |
| GO:0048869\_cellular\_developmental\_process | CD8A | 1113 | 12 | 1.712055 | -1.521001 | 23 | 22.76 | 0.989565 |
| GO:0048869\_cellular\_developmental\_process | FYN | 1113 | 12 | 1.712055 | -1.521001 | 23 | 22.76 | 0.989565 |
| GO:0048869\_cellular\_developmental\_process | AGTPBP1 | 1113 | 12 | 1.712055 | -1.521001 | 23 | 22.76 | 0.989565 |
| GO:0048869\_cellular\_developmental\_process | GNA11 | 1113 | 12 | 1.712055 | -1.521001 | 23 | 22.76 | 0.989565 |
| GO:0048869\_cellular\_developmental\_process | LGALS1 | 1113 | 12 | 1.712055 | -1.521001 | 23 | 22.76 | 0.989565 |
| GO:0048869\_cellular\_developmental\_process | FST | 1113 | 12 | 1.712055 | -1.521001 | 23 | 22.76 | 0.989565 |
| GO:0048869\_cellular\_developmental\_process | INSIG1 | 1113 | 12 | 1.712055 | -1.521001 | 23 | 22.76 | 0.989565 |
| GO:0048869\_cellular\_developmental\_process | PAFAH1B1 | 1113 | 12 | 1.712055 | -1.521001 | 23 | 22.76 | 0.989565 |
| GO:0048869\_cellular\_developmental\_process | FZD2 | 1113 | 12 | 1.712055 | -1.521001 | 23 | 22.76 | 0.989565 |
| GO:0048869\_cellular\_developmental\_process | GAS1 | 1113 | 12 | 1.712055 | -1.521001 | 23 | 22.76 | 0.989565 |
| GO:0048869\_cellular\_developmental\_process | IGFBP5 | 1113 | 12 | 1.712055 | -1.521001 | 23 | 22.76 | 0.989565 |
| GO:0042159\_lipoprotein\_catabolic\_process | LDLR | 5 | 1 | 31.758621 | -1.507145 | 27 | 32.86 | 1.217037 |
| GO:0046339\_diacylglycerol\_metabolic\_process | MOGAT2 | 5 | 1 | 31.758621 | -1.507145 | 27 | 32.86 | 1.217037 |
| GO:0060605\_tube\_lumen\_cavitation | TGM2 | 5 | 1 | 31.758621 | -1.507145 | 27 | 32.86 | 1.217037 |
| GO:0060662\_salivary\_gland\_cavitation | TGM2 | 5 | 1 | 31.758621 | -1.507145 | 27 | 32.86 | 1.217037 |
| GO:0006998\_nuclear\_envelope\_organization | PAFAH1B1 | 6 | 1 | 26.465517 | -1.429282 | 32 | 42.18 | 1.318125 |
| GO:0045833\_negative\_regulation\_of\_lipid\_metabolic\_process | INSIG1 | 6 | 1 | 26.465517 | -1.429282 | 32 | 42.18 | 1.318125 |
| GO:0046460\_neutral\_lipid\_biosynthetic\_process | MOGAT2 | 6 | 1 | 26.465517 | -1.429282 | 32 | 42.18 | 1.318125 |
| GO:0046463\_acylglycerol\_biosynthetic\_process | MOGAT2 | 6 | 1 | 26.465517 | -1.429282 | 32 | 42.18 | 1.318125 |
| GO:0050966\_detection\_of\_mechanical\_stimulus\_involved\_in\_sensory\_perception\_of\_pain | FYN | 6 | 1 | 26.465517 | -1.429282 | 32 | 42.18 | 1.318125 |
| GO:0006878\_cellular\_copper\_ion\_homeostasis | PRNP | 7 | 1 | 22.684729 | -1.363652 | 40 | 53.74 | 1.343500 |
| GO:0032319\_regulation\_of\_Rho\_GTPase\_activity | PAFAH1B1 | 7 | 1 | 22.684729 | -1.363652 | 40 | 53.74 | 1.343500 |
| GO:0042133\_neurotransmitter\_metabolic\_process | AGTPBP1 | 7 | 1 | 22.684729 | -1.363652 | 40 | 53.74 | 1.343500 |
| GO:0045599\_negative\_regulation\_of\_fat\_cell\_differentiation | INSIG1 | 7 | 1 | 22.684729 | -1.363652 | 40 | 53.74 | 1.343500 |
| GO:0045879\_negative\_regulation\_of\_smoothened\_signaling\_pathway | GAS1 | 7 | 1 | 22.684729 | -1.363652 | 40 | 53.74 | 1.343500 |
| GO:0045880\_positive\_regulation\_of\_smoothened\_signaling\_pathway | GAS1 | 7 | 1 | 22.684729 | -1.363652 | 40 | 53.74 | 1.343500 |
| GO:0046504\_glycerol\_ether\_biosynthetic\_process | MOGAT2 | 7 | 1 | 22.684729 | -1.363652 | 40 | 53.74 | 1.343500 |
| GO:0055070\_copper\_ion\_homeostasis | PRNP | 7 | 1 | 22.684729 | -1.363652 | 40 | 53.74 | 1.343500 |
| GO:0050905\_neuromuscular\_process | AGTPBP1 | 53 | 2 | 5.992193 | -1.363605 | 41 | 54.02 | 1.317561 |
| GO:0050905\_neuromuscular\_process | PAFAH1B1 | 53 | 2 | 5.992193 | -1.363605 | 41 | 54.02 | 1.317561 |
| GO:0048731\_system\_development | CD8A | 1609 | 15 | 1.480358 | -1.333248 | 42 | 55.36 | 1.318095 |
| GO:0048731\_system\_development | AGTPBP1 | 1609 | 15 | 1.480358 | -1.333248 | 42 | 55.36 | 1.318095 |
| GO:0048731\_system\_development | LGALS1 | 1609 | 15 | 1.480358 | -1.333248 | 42 | 55.36 | 1.318095 |
| GO:0048731\_system\_development | GNA11 | 1609 | 15 | 1.480358 | -1.333248 | 42 | 55.36 | 1.318095 |
| GO:0048731\_system\_development | FST | 1609 | 15 | 1.480358 | -1.333248 | 42 | 55.36 | 1.318095 |
| GO:0048731\_system\_development | GAS1 | 1609 | 15 | 1.480358 | -1.333248 | 42 | 55.36 | 1.318095 |
| GO:0048731\_system\_development | FZD2 | 1609 | 15 | 1.480358 | -1.333248 | 42 | 55.36 | 1.318095 |
| GO:0048731\_system\_development | CXADR | 1609 | 15 | 1.480358 | -1.333248 | 42 | 55.36 | 1.318095 |
| GO:0048731\_system\_development | NAV1 | 1609 | 15 | 1.480358 | -1.333248 | 42 | 55.36 | 1.318095 |
| GO:0048731\_system\_development | FYN | 1609 | 15 | 1.480358 | -1.333248 | 42 | 55.36 | 1.318095 |
| GO:0048731\_system\_development | INSIG1 | 1609 | 15 | 1.480358 | -1.333248 | 42 | 55.36 | 1.318095 |
| GO:0048731\_system\_development | PRKAR1A | 1609 | 15 | 1.480358 | -1.333248 | 42 | 55.36 | 1.318095 |
| GO:0048731\_system\_development | TGM2 | 1609 | 15 | 1.480358 | -1.333248 | 42 | 55.36 | 1.318095 |
| GO:0048731\_system\_development | PAFAH1B1 | 1609 | 15 | 1.480358 | -1.333248 | 42 | 55.36 | 1.318095 |
| GO:0048731\_system\_development | IGFBP5 | 1609 | 15 | 1.480358 | -1.333248 | 42 | 55.36 | 1.318095 |
| GO:0007275\_multicellular\_organismal\_development | CD8A | 1760 | 16 | 1.443574 | -1.328351 | 43 | 55.45 | 1.289535 |
| GO:0007275\_multicellular\_organismal\_development | AGTPBP1 | 1760 | 16 | 1.443574 | -1.328351 | 43 | 55.45 | 1.289535 |
| GO:0007275\_multicellular\_organismal\_development | LGALS1 | 1760 | 16 | 1.443574 | -1.328351 | 43 | 55.45 | 1.289535 |
| GO:0007275\_multicellular\_organismal\_development | GNA11 | 1760 | 16 | 1.443574 | -1.328351 | 43 | 55.45 | 1.289535 |
| GO:0007275\_multicellular\_organismal\_development | PITPNB | 1760 | 16 | 1.443574 | -1.328351 | 43 | 55.45 | 1.289535 |
| GO:0007275\_multicellular\_organismal\_development | FST | 1760 | 16 | 1.443574 | -1.328351 | 43 | 55.45 | 1.289535 |
| GO:0007275\_multicellular\_organismal\_development | FZD2 | 1760 | 16 | 1.443574 | -1.328351 | 43 | 55.45 | 1.289535 |
| GO:0007275\_multicellular\_organismal\_development | GAS1 | 1760 | 16 | 1.443574 | -1.328351 | 43 | 55.45 | 1.289535 |
| GO:0007275\_multicellular\_organismal\_development | CXADR | 1760 | 16 | 1.443574 | -1.328351 | 43 | 55.45 | 1.289535 |
| GO:0007275\_multicellular\_organismal\_development | NAV1 | 1760 | 16 | 1.443574 | -1.328351 | 43 | 55.45 | 1.289535 |
| GO:0007275\_multicellular\_organismal\_development | FYN | 1760 | 16 | 1.443574 | -1.328351 | 43 | 55.45 | 1.289535 |
| GO:0007275\_multicellular\_organismal\_development | PRKAR1A | 1760 | 16 | 1.443574 | -1.328351 | 43 | 55.45 | 1.289535 |
| GO:0007275\_multicellular\_organismal\_development | INSIG1 | 1760 | 16 | 1.443574 | -1.328351 | 43 | 55.45 | 1.289535 |
| GO:0007275\_multicellular\_organismal\_development | TGM2 | 1760 | 16 | 1.443574 | -1.328351 | 43 | 55.45 | 1.289535 |
| GO:0007275\_multicellular\_organismal\_development | PAFAH1B1 | 1760 | 16 | 1.443574 | -1.328351 | 43 | 55.45 | 1.289535 |
| GO:0007275\_multicellular\_organismal\_development | IGFBP5 | 1760 | 16 | 1.443574 | -1.328351 | 43 | 55.45 | 1.289535 |
| GO:0046486\_glycerolipid\_metabolic\_process | MOGAT2 | 56 | 2 | 5.671182 | -1.320346 | 44 | 56.12 | 1.275455 |
| GO:0046486\_glycerolipid\_metabolic\_process | INSIG1 | 56 | 2 | 5.671182 | -1.320346 | 44 | 56.12 | 1.275455 |
| GO:0018345\_protein\_palmitoylation | ZDHHC17 | 8 | 1 | 19.849138 | -1.306976 | 53 | 62.58 | 1.180755 |
| GO:0021692\_cerebellar\_Purkinje\_cell\_layer\_morphogenesis | AGTPBP1 | 8 | 1 | 19.849138 | -1.306976 | 53 | 62.58 | 1.180755 |
| GO:0021694\_cerebellar\_Purkinje\_cell\_layer\_formation | AGTPBP1 | 8 | 1 | 19.849138 | -1.306976 | 53 | 62.58 | 1.180755 |
| GO:0021702\_cerebellar\_Purkinje\_cell\_differentiation | AGTPBP1 | 8 | 1 | 19.849138 | -1.306976 | 53 | 62.58 | 1.180755 |
| GO:0021799\_cerebral\_cortex\_radially\_oriented\_cell\_migration | PAFAH1B1 | 8 | 1 | 19.849138 | -1.306976 | 53 | 62.58 | 1.180755 |
| GO:0035023\_regulation\_of\_Rho\_protein\_signal\_transduction | PAFAH1B1 | 8 | 1 | 19.849138 | -1.306976 | 53 | 62.58 | 1.180755 |
| GO:0042304\_regulation\_of\_fatty\_acid\_biosynthetic\_process | INSIG1 | 8 | 1 | 19.849138 | -1.306976 | 53 | 62.58 | 1.180755 |
| GO:0043616\_keratinocyte\_proliferation | FST | 8 | 1 | 19.849138 | -1.306976 | 53 | 62.58 | 1.180755 |
| GO:0060043\_regulation\_of\_cardiac\_muscle\_cell\_proliferation | CXADR | 8 | 1 | 19.849138 | -1.306976 | 53 | 62.58 | 1.180755 |
| GO:0000226\_microtubule\_cytoskeleton\_organization | NAV1 | 57 | 2 | 5.571688 | -1.306503 | 54 | 63.18 | 1.170000 |
| GO:0000226\_microtubule\_cytoskeleton\_organization | PAFAH1B1 | 57 | 2 | 5.571688 | -1.306503 | 54 | 63.18 | 1.170000 |
| GO:0030902\_hindbrain\_development | AGTPBP1 | 58 | 2 | 5.475624 | -1.292933 | 55 | 63.83 | 1.160545 |
| GO:0030902\_hindbrain\_development | GAS1 | 58 | 2 | 5.475624 | -1.292933 | 55 | 63.83 | 1.160545 |
| GO:0007420\_brain\_development | AGTPBP1 | 231 | 4 | 2.749664 | -1.261889 | 56 | 65.2 | 1.164286 |
| GO:0007420\_brain\_development | FYN | 231 | 4 | 2.749664 | -1.261889 | 56 | 65.2 | 1.164286 |
| GO:0007420\_brain\_development | PAFAH1B1 | 231 | 4 | 2.749664 | -1.261889 | 56 | 65.2 | 1.164286 |
| GO:0007420\_brain\_development | GAS1 | 231 | 4 | 2.749664 | -1.261889 | 56 | 65.2 | 1.164286 |
| GO:0009451\_RNA\_modification | DNAJB11 | 9 | 1 | 17.643678 | -1.257138 | 59 | 72.29 | 1.225254 |
| GO:0048070\_regulation\_of\_pigmentation\_during\_development | GNA11 | 9 | 1 | 17.643678 | -1.257138 | 59 | 72.29 | 1.225254 |
| GO:0048488\_synaptic\_vesicle\_endocytosis | AMPH | 9 | 1 | 17.643678 | -1.257138 | 59 | 72.29 | 1.225254 |
| GO:0016044\_membrane\_organization | SGCG | 140 | 3 | 3.402709 | -1.248003 | 60 | 72.93 | 1.215500 |
| GO:0016044\_membrane\_organization | PAFAH1B1 | 140 | 3 | 3.402709 | -1.248003 | 60 | 72.93 | 1.215500 |
| GO:0016044\_membrane\_organization | AMPH | 140 | 3 | 3.402709 | -1.248003 | 60 | 72.93 | 1.215500 |
| GO:0001501\_skeletal\_system\_development | GNA11 | 236 | 4 | 2.691409 | -1.233828 | 61 | 74.0 | 1.213115 |
| GO:0001501\_skeletal\_system\_development | INSIG1 | 236 | 4 | 2.691409 | -1.233828 | 61 | 74.0 | 1.213115 |
| GO:0001501\_skeletal\_system\_development | GAS1 | 236 | 4 | 2.691409 | -1.233828 | 61 | 74.0 | 1.213115 |
| GO:0001501\_skeletal\_system\_development | IGFBP5 | 236 | 4 | 2.691409 | -1.233828 | 61 | 74.0 | 1.213115 |
| GO:0048513\_organ\_development | CD8A | 1365 | 13 | 1.512315 | -1.227527 | 62 | 74.54 | 1.202258 |
| GO:0048513\_organ\_development | AGTPBP1 | 1365 | 13 | 1.512315 | -1.227527 | 62 | 74.54 | 1.202258 |
| GO:0048513\_organ\_development | LGALS1 | 1365 | 13 | 1.512315 | -1.227527 | 62 | 74.54 | 1.202258 |
| GO:0048513\_organ\_development | GNA11 | 1365 | 13 | 1.512315 | -1.227527 | 62 | 74.54 | 1.202258 |
| GO:0048513\_organ\_development | FST | 1365 | 13 | 1.512315 | -1.227527 | 62 | 74.54 | 1.202258 |
| GO:0048513\_organ\_development | GAS1 | 1365 | 13 | 1.512315 | -1.227527 | 62 | 74.54 | 1.202258 |
| GO:0048513\_organ\_development | FZD2 | 1365 | 13 | 1.512315 | -1.227527 | 62 | 74.54 | 1.202258 |
| GO:0048513\_organ\_development | CXADR | 1365 | 13 | 1.512315 | -1.227527 | 62 | 74.54 | 1.202258 |
| GO:0048513\_organ\_development | FYN | 1365 | 13 | 1.512315 | -1.227527 | 62 | 74.54 | 1.202258 |
| GO:0048513\_organ\_development | INSIG1 | 1365 | 13 | 1.512315 | -1.227527 | 62 | 74.54 | 1.202258 |
| GO:0048513\_organ\_development | PRKAR1A | 1365 | 13 | 1.512315 | -1.227527 | 62 | 74.54 | 1.202258 |
| GO:0048513\_organ\_development | TGM2 | 1365 | 13 | 1.512315 | -1.227527 | 62 | 74.54 | 1.202258 |
| GO:0048513\_organ\_development | PAFAH1B1 | 1365 | 13 | 1.512315 | -1.227527 | 62 | 74.54 | 1.202258 |
| GO:0000209\_protein\_polyubiquitination | CHFR | 10 | 1 | 15.879310 | -1.212694 | 68 | 82.65 | 1.215441 |
| GO:0001578\_microtubule\_bundle\_formation | NAV1 | 10 | 1 | 15.879310 | -1.212694 | 68 | 82.65 | 1.215441 |
| GO:0018149\_peptide\_cross-linking | TGM2 | 10 | 1 | 15.879310 | -1.212694 | 68 | 82.65 | 1.215441 |
| GO:0032318\_regulation\_of\_Ras\_GTPase\_activity | PAFAH1B1 | 10 | 1 | 15.879310 | -1.212694 | 68 | 82.65 | 1.215441 |
| GO:0048738\_cardiac\_muscle\_tissue\_development | CXADR | 10 | 1 | 15.879310 | -1.212694 | 68 | 82.65 | 1.215441 |
| GO:0050892\_intestinal\_absorption | MOGAT2 | 10 | 1 | 15.879310 | -1.212694 | 68 | 82.65 | 1.215441 |
| GO:0043412\_biopolymer\_modification | ZDHHC17 | 458 | 6 | 2.080259 | -1.209334 | 69 | 82.76 | 1.199420 |
| GO:0043412\_biopolymer\_modification | DNAJB11 | 458 | 6 | 2.080259 | -1.209334 | 69 | 82.76 | 1.199420 |
| GO:0043412\_biopolymer\_modification | FYN | 458 | 6 | 2.080259 | -1.209334 | 69 | 82.76 | 1.199420 |
| GO:0043412\_biopolymer\_modification | PRKAR1A | 458 | 6 | 2.080259 | -1.209334 | 69 | 82.76 | 1.199420 |
| GO:0043412\_biopolymer\_modification | TGM2 | 458 | 6 | 2.080259 | -1.209334 | 69 | 82.76 | 1.199420 |
| GO:0043412\_biopolymer\_modification | CHFR | 458 | 6 | 2.080259 | -1.209334 | 69 | 82.76 | 1.199420 |
| GO:0042471\_ear\_morphogenesis | INSIG1 | 65 | 2 | 4.885942 | -1.204808 | 70 | 83.05 | 1.186429 |
| GO:0042471\_ear\_morphogenesis | GAS1 | 65 | 2 | 4.885942 | -1.204808 | 70 | 83.05 | 1.186429 |
| GO:0007215\_glutamate\_signaling\_pathway | GNA11 | 11 | 1 | 14.435737 | -1.172613 | 72 | 90.1 | 1.251389 |
| GO:0045216\_cell-cell\_junction\_organization | CXADR | 11 | 1 | 14.435737 | -1.172613 | 72 | 90.1 | 1.251389 |
| GO:0019932\_second-messenger-mediated\_signaling | CD8A | 68 | 2 | 4.670385 | -1.170308 | 73 | 90.27 | 1.236575 |
| GO:0019932\_second-messenger-mediated\_signaling | GNA11 | 68 | 2 | 4.670385 | -1.170308 | 73 | 90.27 | 1.236575 |
| GO:0048856\_anatomical\_structure\_development | CD8A | 1688 | 15 | 1.411076 | -1.159475 | 74 | 90.65 | 1.225000 |
| GO:0048856\_anatomical\_structure\_development | AGTPBP1 | 1688 | 15 | 1.411076 | -1.159475 | 74 | 90.65 | 1.225000 |
| GO:0048856\_anatomical\_structure\_development | LGALS1 | 1688 | 15 | 1.411076 | -1.159475 | 74 | 90.65 | 1.225000 |
| GO:0048856\_anatomical\_structure\_development | GNA11 | 1688 | 15 | 1.411076 | -1.159475 | 74 | 90.65 | 1.225000 |
| GO:0048856\_anatomical\_structure\_development | FST | 1688 | 15 | 1.411076 | -1.159475 | 74 | 90.65 | 1.225000 |
| GO:0048856\_anatomical\_structure\_development | GAS1 | 1688 | 15 | 1.411076 | -1.159475 | 74 | 90.65 | 1.225000 |
| GO:0048856\_anatomical\_structure\_development | FZD2 | 1688 | 15 | 1.411076 | -1.159475 | 74 | 90.65 | 1.225000 |
| GO:0048856\_anatomical\_structure\_development | CXADR | 1688 | 15 | 1.411076 | -1.159475 | 74 | 90.65 | 1.225000 |
| GO:0048856\_anatomical\_structure\_development | NAV1 | 1688 | 15 | 1.411076 | -1.159475 | 74 | 90.65 | 1.225000 |
| GO:0048856\_anatomical\_structure\_development | FYN | 1688 | 15 | 1.411076 | -1.159475 | 74 | 90.65 | 1.225000 |
| GO:0048856\_anatomical\_structure\_development | INSIG1 | 1688 | 15 | 1.411076 | -1.159475 | 74 | 90.65 | 1.225000 |
| GO:0048856\_anatomical\_structure\_development | PRKAR1A | 1688 | 15 | 1.411076 | -1.159475 | 74 | 90.65 | 1.225000 |
| GO:0048856\_anatomical\_structure\_development | TGM2 | 1688 | 15 | 1.411076 | -1.159475 | 74 | 90.65 | 1.225000 |
| GO:0048856\_anatomical\_structure\_development | PAFAH1B1 | 1688 | 15 | 1.411076 | -1.159475 | 74 | 90.65 | 1.225000 |
| GO:0048856\_anatomical\_structure\_development | IGFBP5 | 1688 | 15 | 1.411076 | -1.159475 | 74 | 90.65 | 1.225000 |
| GO:0007611\_learning\_or\_memory | PAFAH1B1 | 70 | 2 | 4.536946 | -1.148268 | 75 | 91.77 | 1.223600 |
| GO:0007611\_learning\_or\_memory | AMPH | 70 | 2 | 4.536946 | -1.148268 | 75 | 91.77 | 1.223600 |
| GO:0048878\_chemical\_homeostasis | LDLR | 254 | 4 | 2.500679 | -1.139091 | 76 | 92.39 | 1.215658 |
| GO:0048878\_chemical\_homeostasis | FYN | 254 | 4 | 2.500679 | -1.139091 | 76 | 92.39 | 1.215658 |
| GO:0048878\_chemical\_homeostasis | GNA11 | 254 | 4 | 2.500679 | -1.139091 | 76 | 92.39 | 1.215658 |
| GO:0048878\_chemical\_homeostasis | PRNP | 254 | 4 | 2.500679 | -1.139091 | 76 | 92.39 | 1.215658 |
| GO:0021680\_cerebellar\_Purkinje\_cell\_layer\_development | AGTPBP1 | 12 | 1 | 13.232759 | -1.136136 | 82 | 98.94 | 1.206585 |
| GO:0021697\_cerebellar\_cortex\_formation | AGTPBP1 | 12 | 1 | 13.232759 | -1.136136 | 82 | 98.94 | 1.206585 |
| GO:0045445\_myoblast\_differentiation | LGALS1 | 12 | 1 | 13.232759 | -1.136136 | 82 | 98.94 | 1.206585 |
| GO:0045471\_response\_to\_ethanol | FYN | 12 | 1 | 13.232759 | -1.136136 | 82 | 98.94 | 1.206585 |
| GO:0050848\_regulation\_of\_calcium-mediated\_signaling | CD8A | 12 | 1 | 13.232759 | -1.136136 | 82 | 98.94 | 1.206585 |
| GO:0050850\_positive\_regulation\_of\_calcium-mediated\_signaling | CD8A | 12 | 1 | 13.232759 | -1.136136 | 82 | 98.94 | 1.206585 |
| GO:0051336\_regulation\_of\_hydrolase\_activity | GNA11 | 73 | 2 | 4.350496 | -1.116540 | 83 | 100.77 | 1.214096 |
| GO:0051336\_regulation\_of\_hydrolase\_activity | PAFAH1B1 | 73 | 2 | 4.350496 | -1.116540 | 83 | 100.77 | 1.214096 |
| GO:0007212\_dopamine\_receptor\_signaling\_pathway | GNA11 | 13 | 1 | 12.214854 | -1.102683 | 86 | 106.62 | 1.239767 |
| GO:0021533\_cell\_differentiation\_in\_hindbrain | AGTPBP1 | 13 | 1 | 12.214854 | -1.102683 | 86 | 106.62 | 1.239767 |
| GO:0060038\_cardiac\_muscle\_cell\_proliferation | CXADR | 13 | 1 | 12.214854 | -1.102683 | 86 | 106.62 | 1.239767 |
| GO:0044265\_cellular\_macromolecule\_catabolic\_process | LDLR | 75 | 2 | 4.234483 | -1.096220 | 87 | 107.23 | 1.232529 |
| GO:0044265\_cellular\_macromolecule\_catabolic\_process | CHFR | 75 | 2 | 4.234483 | -1.096220 | 87 | 107.23 | 1.232529 |
| GO:0032501\_multicellular\_organismal\_process | MOGAT2 | 2183 | 18 | 1.309334 | -1.093291 | 88 | 107.29 | 1.219205 |
| GO:0032501\_multicellular\_organismal\_process | CD8A | 2183 | 18 | 1.309334 | -1.093291 | 88 | 107.29 | 1.219205 |
| GO:0032501\_multicellular\_organismal\_process | AGTPBP1 | 2183 | 18 | 1.309334 | -1.093291 | 88 | 107.29 | 1.219205 |
| GO:0032501\_multicellular\_organismal\_process | LGALS1 | 2183 | 18 | 1.309334 | -1.093291 | 88 | 107.29 | 1.219205 |
| GO:0032501\_multicellular\_organismal\_process | GNA11 | 2183 | 18 | 1.309334 | -1.093291 | 88 | 107.29 | 1.219205 |
| GO:0032501\_multicellular\_organismal\_process | PITPNB | 2183 | 18 | 1.309334 | -1.093291 | 88 | 107.29 | 1.219205 |
| GO:0032501\_multicellular\_organismal\_process | FST | 2183 | 18 | 1.309334 | -1.093291 | 88 | 107.29 | 1.219205 |
| GO:0032501\_multicellular\_organismal\_process | FZD2 | 2183 | 18 | 1.309334 | -1.093291 | 88 | 107.29 | 1.219205 |
| GO:0032501\_multicellular\_organismal\_process | GAS1 | 2183 | 18 | 1.309334 | -1.093291 | 88 | 107.29 | 1.219205 |
| GO:0032501\_multicellular\_organismal\_process | CXADR | 2183 | 18 | 1.309334 | -1.093291 | 88 | 107.29 | 1.219205 |
| GO:0032501\_multicellular\_organismal\_process | AMPH | 2183 | 18 | 1.309334 | -1.093291 | 88 | 107.29 | 1.219205 |
| GO:0032501\_multicellular\_organismal\_process | NAV1 | 2183 | 18 | 1.309334 | -1.093291 | 88 | 107.29 | 1.219205 |
| GO:0032501\_multicellular\_organismal\_process | FYN | 2183 | 18 | 1.309334 | -1.093291 | 88 | 107.29 | 1.219205 |
| GO:0032501\_multicellular\_organismal\_process | PRKAR1A | 2183 | 18 | 1.309334 | -1.093291 | 88 | 107.29 | 1.219205 |
| GO:0032501\_multicellular\_organismal\_process | INSIG1 | 2183 | 18 | 1.309334 | -1.093291 | 88 | 107.29 | 1.219205 |
| GO:0032501\_multicellular\_organismal\_process | TGM2 | 2183 | 18 | 1.309334 | -1.093291 | 88 | 107.29 | 1.219205 |
| GO:0032501\_multicellular\_organismal\_process | PAFAH1B1 | 2183 | 18 | 1.309334 | -1.093291 | 88 | 107.29 | 1.219205 |
| GO:0032501\_multicellular\_organismal\_process | IGFBP5 | 2183 | 18 | 1.309334 | -1.093291 | 88 | 107.29 | 1.219205 |
| GO:0006695\_cholesterol\_biosynthetic\_process | INSIG1 | 14 | 1 | 11.342365 | -1.071807 | 94 | 113.7 | 1.209574 |
| GO:0014855\_striated\_muscle\_cell\_proliferation | CXADR | 14 | 1 | 11.342365 | -1.071807 | 94 | 113.7 | 1.209574 |
| GO:0019217\_regulation\_of\_fatty\_acid\_metabolic\_process | INSIG1 | 14 | 1 | 11.342365 | -1.071807 | 94 | 113.7 | 1.209574 |
| GO:0021904\_dorsal\_ventral\_neural\_tube\_patterning | GAS1 | 14 | 1 | 11.342365 | -1.071807 | 94 | 113.7 | 1.209574 |
| GO:0045598\_regulation\_of\_fat\_cell\_differentiation | INSIG1 | 14 | 1 | 11.342365 | -1.071807 | 94 | 113.7 | 1.209574 |
| GO:0050810\_regulation\_of\_steroid\_biosynthetic\_process | INSIG1 | 14 | 1 | 11.342365 | -1.071807 | 94 | 113.7 | 1.209574 |
| GO:0007200\_activation\_of\_phospholipase\_C\_activity\_by\_G-protein\_coupled\_receptor\_protein\_signaling\_pathway\_coupled\_to\_IP3\_second\_messenger | GNA11 | 15 | 1 | 10.586207 | -1.043152 | 102 | 120.86 | 1.184902 |
| GO:0007202\_activation\_of\_phospholipase\_C\_activity | GNA11 | 15 | 1 | 10.586207 | -1.043152 | 102 | 120.86 | 1.184902 |
| GO:0010518\_positive\_regulation\_of\_phospholipase\_activity | GNA11 | 15 | 1 | 10.586207 | -1.043152 | 102 | 120.86 | 1.184902 |
| GO:0010863\_positive\_regulation\_of\_phospholipase\_C\_activity | GNA11 | 15 | 1 | 10.586207 | -1.043152 | 102 | 120.86 | 1.184902 |
| GO:0021795\_cerebral\_cortex\_cell\_migration | PAFAH1B1 | 15 | 1 | 10.586207 | -1.043152 | 102 | 120.86 | 1.184902 |
| GO:0022600\_digestive\_system\_process | MOGAT2 | 15 | 1 | 10.586207 | -1.043152 | 102 | 120.86 | 1.184902 |
| GO:0031069\_hair\_follicle\_morphogenesis | FST | 15 | 1 | 10.586207 | -1.043152 | 102 | 120.86 | 1.184902 |
| GO:0050798\_activated\_T\_cell\_proliferation | FYN | 15 | 1 | 10.586207 | -1.043152 | 102 | 120.86 | 1.184902 |
| GO:0048646\_anatomical\_structure\_formation\_involved\_in\_morphogenesis | AGTPBP1 | 277 | 4 | 2.293041 | -1.030683 | 103 | 121.31 | 1.177767 |
| GO:0048646\_anatomical\_structure\_formation\_involved\_in\_morphogenesis | PRKAR1A | 277 | 4 | 2.293041 | -1.030683 | 103 | 121.31 | 1.177767 |
| GO:0048646\_anatomical\_structure\_formation\_involved\_in\_morphogenesis | TGM2 | 277 | 4 | 2.293041 | -1.030683 | 103 | 121.31 | 1.177767 |
| GO:0048646\_anatomical\_structure\_formation\_involved\_in\_morphogenesis | PAFAH1B1 | 277 | 4 | 2.293041 | -1.030683 | 103 | 121.31 | 1.177767 |
| GO:0008202\_steroid\_metabolic\_process | LDLR | 82 | 2 | 3.873003 | -1.029804 | 104 | 122.04 | 1.173462 |
| GO:0008202\_steroid\_metabolic\_process | INSIG1 | 82 | 2 | 3.873003 | -1.029804 | 104 | 122.04 | 1.173462 |
| GO:0032502\_developmental\_process | CD8A | 2060 | 17 | 1.310429 | -1.029059 | 105 | 122.17 | 1.163524 |
| GO:0032502\_developmental\_process | AGTPBP1 | 2060 | 17 | 1.310429 | -1.029059 | 105 | 122.17 | 1.163524 |
| GO:0032502\_developmental\_process | LGALS1 | 2060 | 17 | 1.310429 | -1.029059 | 105 | 122.17 | 1.163524 |
| GO:0032502\_developmental\_process | GNA11 | 2060 | 17 | 1.310429 | -1.029059 | 105 | 122.17 | 1.163524 |
| GO:0032502\_developmental\_process | FST | 2060 | 17 | 1.310429 | -1.029059 | 105 | 122.17 | 1.163524 |
| GO:0032502\_developmental\_process | PITPNB | 2060 | 17 | 1.310429 | -1.029059 | 105 | 122.17 | 1.163524 |
| GO:0032502\_developmental\_process | FZD2 | 2060 | 17 | 1.310429 | -1.029059 | 105 | 122.17 | 1.163524 |
| GO:0032502\_developmental\_process | GAS1 | 2060 | 17 | 1.310429 | -1.029059 | 105 | 122.17 | 1.163524 |
| GO:0032502\_developmental\_process | CXADR | 2060 | 17 | 1.310429 | -1.029059 | 105 | 122.17 | 1.163524 |
| GO:0032502\_developmental\_process | NAV1 | 2060 | 17 | 1.310429 | -1.029059 | 105 | 122.17 | 1.163524 |
| GO:0032502\_developmental\_process | FYN | 2060 | 17 | 1.310429 | -1.029059 | 105 | 122.17 | 1.163524 |
| GO:0032502\_developmental\_process | PRKAR1A | 2060 | 17 | 1.310429 | -1.029059 | 105 | 122.17 | 1.163524 |
| GO:0032502\_developmental\_process | INSIG1 | 2060 | 17 | 1.310429 | -1.029059 | 105 | 122.17 | 1.163524 |
| GO:0032502\_developmental\_process | TGM2 | 2060 | 17 | 1.310429 | -1.029059 | 105 | 122.17 | 1.163524 |
| GO:0032502\_developmental\_process | PAFAH1B1 | 2060 | 17 | 1.310429 | -1.029059 | 105 | 122.17 | 1.163524 |
| GO:0032502\_developmental\_process | PRNP | 2060 | 17 | 1.310429 | -1.029059 | 105 | 122.17 | 1.163524 |
| GO:0032502\_developmental\_process | IGFBP5 | 2060 | 17 | 1.310429 | -1.029059 | 105 | 122.17 | 1.163524 |
| GO:0016126\_sterol\_biosynthetic\_process | INSIG1 | 16 | 1 | 9.924569 | -1.016429 | 112 | 127.78 | 1.140893 |
| GO:0019722\_calcium-mediated\_signaling | CD8A | 16 | 1 | 9.924569 | -1.016429 | 112 | 127.78 | 1.140893 |
| GO:0021696\_cerebellar\_cortex\_morphogenesis | AGTPBP1 | 16 | 1 | 9.924569 | -1.016429 | 112 | 127.78 | 1.140893 |
| GO:0043087\_regulation\_of\_GTPase\_activity | PAFAH1B1 | 16 | 1 | 9.924569 | -1.016429 | 112 | 127.78 | 1.140893 |
| GO:0048015\_phosphoinositide-mediated\_signaling | GNA11 | 16 | 1 | 9.924569 | -1.016429 | 112 | 127.78 | 1.140893 |
| GO:0050974\_detection\_of\_mechanical\_stimulus\_involved\_in\_sensory\_perception | FYN | 16 | 1 | 9.924569 | -1.016429 | 112 | 127.78 | 1.140893 |
| GO:0060193\_positive\_regulation\_of\_lipase\_activity | GNA11 | 16 | 1 | 9.924569 | -1.016429 | 112 | 127.78 | 1.140893 |
| GO:0006873\_cellular\_ion\_homeostasis | FYN | 176 | 3 | 2.706701 | -1.012803 | 113 | 128.21 | 1.134602 |
| GO:0006873\_cellular\_ion\_homeostasis | GNA11 | 176 | 3 | 2.706701 | -1.012803 | 113 | 128.21 | 1.134602 |
| GO:0006873\_cellular\_ion\_homeostasis | PRNP | 176 | 3 | 2.706701 | -1.012803 | 113 | 128.21 | 1.134602 |
| GO:0048699\_generation\_of\_neurons | NAV1 | 396 | 5 | 2.004963 | -1.008174 | 114 | 128.59 | 1.127982 |
| GO:0048699\_generation\_of\_neurons | AGTPBP1 | 396 | 5 | 2.004963 | -1.008174 | 114 | 128.59 | 1.127982 |
| GO:0048699\_generation\_of\_neurons | FYN | 396 | 5 | 2.004963 | -1.008174 | 114 | 128.59 | 1.127982 |
| GO:0048699\_generation\_of\_neurons | PAFAH1B1 | 396 | 5 | 2.004963 | -1.008174 | 114 | 128.59 | 1.127982 |
| GO:0048699\_generation\_of\_neurons | GAS1 | 396 | 5 | 2.004963 | -1.008174 | 114 | 128.59 | 1.127982 |
| GO:0010517\_regulation\_of\_phospholipase\_activity | GNA11 | 17 | 1 | 9.340771 | -0.991406 | 117 | 135.32 | 1.156581 |
| GO:0010565\_regulation\_of\_cellular\_ketone\_metabolic\_process | INSIG1 | 17 | 1 | 9.340771 | -0.991406 | 117 | 135.32 | 1.156581 |
| GO:0022029\_telencephalon\_cell\_migration | PAFAH1B1 | 17 | 1 | 9.340771 | -0.991406 | 117 | 135.32 | 1.156581 |
| GO:0007417\_central\_nervous\_system\_development | AGTPBP1 | 287 | 4 | 2.213144 | -0.987426 | 118 | 135.6 | 1.149153 |
| GO:0007417\_central\_nervous\_system\_development | FYN | 287 | 4 | 2.213144 | -0.987426 | 118 | 135.6 | 1.149153 |
| GO:0007417\_central\_nervous\_system\_development | PAFAH1B1 | 287 | 4 | 2.213144 | -0.987426 | 118 | 135.6 | 1.149153 |
| GO:0007417\_central\_nervous\_system\_development | GAS1 | 287 | 4 | 2.213144 | -0.987426 | 118 | 135.6 | 1.149153 |
| GO:0043583\_ear\_development | INSIG1 | 87 | 2 | 3.650416 | -0.986343 | 119 | 136.23 | 1.144790 |
| GO:0043583\_ear\_development | GAS1 | 87 | 2 | 3.650416 | -0.986343 | 119 | 136.23 | 1.144790 |
| GO:0055082\_cellular\_chemical\_homeostasis | FYN | 181 | 3 | 2.631930 | -0.985073 | 120 | 136.47 | 1.137250 |
| GO:0055082\_cellular\_chemical\_homeostasis | GNA11 | 181 | 3 | 2.631930 | -0.985073 | 120 | 136.47 | 1.137250 |
| GO:0055082\_cellular\_chemical\_homeostasis | PRNP | 181 | 3 | 2.631930 | -0.985073 | 120 | 136.47 | 1.137250 |
| GO:0008589\_regulation\_of\_smoothened\_signaling\_pathway | GAS1 | 18 | 1 | 8.821839 | -0.967886 | 126 | 142.92 | 1.134286 |
| GO:0021885\_forebrain\_cell\_migration | PAFAH1B1 | 18 | 1 | 8.821839 | -0.967886 | 126 | 142.92 | 1.134286 |
| GO:0030318\_melanocyte\_differentiation | GNA11 | 18 | 1 | 8.821839 | -0.967886 | 126 | 142.92 | 1.134286 |
| GO:0046578\_regulation\_of\_Ras\_protein\_signal\_transduction | PAFAH1B1 | 18 | 1 | 8.821839 | -0.967886 | 126 | 142.92 | 1.134286 |
| GO:0048730\_epidermis\_morphogenesis | FST | 18 | 1 | 8.821839 | -0.967886 | 126 | 142.92 | 1.134286 |
| GO:0050982\_detection\_of\_mechanical\_stimulus | FYN | 18 | 1 | 8.821839 | -0.967886 | 126 | 142.92 | 1.134286 |
| GO:0007276\_gamete\_generation | FST | 188 | 3 | 2.533933 | -0.947914 | 127 | 144.64 | 1.138898 |
| GO:0007276\_gamete\_generation | PAFAH1B1 | 188 | 3 | 2.533933 | -0.947914 | 127 | 144.64 | 1.138898 |
| GO:0007276\_gamete\_generation | HERPUD2 | 188 | 3 | 2.533933 | -0.947914 | 127 | 144.64 | 1.138898 |
| GO:0006497\_protein\_amino\_acid\_lipidation | ZDHHC17 | 19 | 1 | 8.357532 | -0.945708 | 134 | 148.76 | 1.110149 |
| GO:0019218\_regulation\_of\_steroid\_metabolic\_process | INSIG1 | 19 | 1 | 8.357532 | -0.945708 | 134 | 148.76 | 1.110149 |
| GO:0033002\_muscle\_cell\_proliferation | CXADR | 19 | 1 | 8.357532 | -0.945708 | 134 | 148.76 | 1.110149 |
| GO:0046890\_regulation\_of\_lipid\_biosynthetic\_process | INSIG1 | 19 | 1 | 8.357532 | -0.945708 | 134 | 148.76 | 1.110149 |
| GO:0048701\_embryonic\_cranial\_skeleton\_morphogenesis | GAS1 | 19 | 1 | 8.357532 | -0.945708 | 134 | 148.76 | 1.110149 |
| GO:0050931\_pigment\_cell\_differentiation | GNA11 | 19 | 1 | 8.357532 | -0.945708 | 134 | 148.76 | 1.110149 |
| GO:0051056\_regulation\_of\_small\_GTPase\_mediated\_signal\_transduction | PAFAH1B1 | 19 | 1 | 8.357532 | -0.945708 | 134 | 148.76 | 1.110149 |
| GO:0019226\_transmission\_of\_nerve\_impulse | FYN | 189 | 3 | 2.520525 | -0.942757 | 135 | 148.89 | 1.102889 |
| GO:0019226\_transmission\_of\_nerve\_impulse | AGTPBP1 | 189 | 3 | 2.520525 | -0.942757 | 135 | 148.89 | 1.102889 |
| GO:0019226\_transmission\_of\_nerve\_impulse | PAFAH1B1 | 189 | 3 | 2.520525 | -0.942757 | 135 | 148.89 | 1.102889 |
| GO:0008610\_lipid\_biosynthetic\_process | MOGAT2 | 94 | 2 | 3.378577 | -0.930285 | 136 | 149.97 | 1.102721 |
| GO:0008610\_lipid\_biosynthetic\_process | INSIG1 | 94 | 2 | 3.378577 | -0.930285 | 136 | 149.97 | 1.102721 |
| GO:0007586\_digestion | MOGAT2 | 20 | 1 | 7.939655 | -0.924733 | 142 | 153.69 | 1.082324 |
| GO:0008360\_regulation\_of\_cell\_shape | FYN | 20 | 1 | 7.939655 | -0.924733 | 142 | 153.69 | 1.082324 |
| GO:0010927\_cellular\_component\_assembly\_involved\_in\_morphogenesis | PAFAH1B1 | 20 | 1 | 7.939655 | -0.924733 | 142 | 153.69 | 1.082324 |
| GO:0021695\_cerebellar\_cortex\_development | AGTPBP1 | 20 | 1 | 7.939655 | -0.924733 | 142 | 153.69 | 1.082324 |
| GO:0045017\_glycerolipid\_biosynthetic\_process | MOGAT2 | 20 | 1 | 7.939655 | -0.924733 | 142 | 153.69 | 1.082324 |
| GO:0060191\_regulation\_of\_lipase\_activity | GNA11 | 20 | 1 | 7.939655 | -0.924733 | 142 | 153.69 | 1.082324 |
| GO:0008283\_cell\_proliferation | FYN | 544 | 6 | 1.751395 | -0.924406 | 143 | 153.77 | 1.075315 |
| GO:0008283\_cell\_proliferation | PRKAR1A | 544 | 6 | 1.751395 | -0.924406 | 143 | 153.77 | 1.075315 |
| GO:0008283\_cell\_proliferation | FST | 544 | 6 | 1.751395 | -0.924406 | 143 | 153.77 | 1.075315 |
| GO:0008283\_cell\_proliferation | PAFAH1B1 | 544 | 6 | 1.751395 | -0.924406 | 143 | 153.77 | 1.075315 |
| GO:0008283\_cell\_proliferation | GAS1 | 544 | 6 | 1.751395 | -0.924406 | 143 | 153.77 | 1.075315 |
| GO:0008283\_cell\_proliferation | CXADR | 544 | 6 | 1.751395 | -0.924406 | 143 | 153.77 | 1.075315 |
| GO:0042391\_regulation\_of\_membrane\_potential | FYN | 95 | 2 | 3.343013 | -0.922689 | 144 | 153.95 | 1.069097 |
| GO:0042391\_regulation\_of\_membrane\_potential | GNA11 | 95 | 2 | 3.343013 | -0.922689 | 144 | 153.95 | 1.069097 |
| GO:0022008\_neurogenesis | NAV1 | 423 | 5 | 1.876987 | -0.915719 | 145 | 154.29 | 1.064069 |
| GO:0022008\_neurogenesis | AGTPBP1 | 423 | 5 | 1.876987 | -0.915719 | 145 | 154.29 | 1.064069 |
| GO:0022008\_neurogenesis | FYN | 423 | 5 | 1.876987 | -0.915719 | 145 | 154.29 | 1.064069 |
| GO:0022008\_neurogenesis | PAFAH1B1 | 423 | 5 | 1.876987 | -0.915719 | 145 | 154.29 | 1.064069 |
| GO:0022008\_neurogenesis | GAS1 | 423 | 5 | 1.876987 | -0.915719 | 145 | 154.29 | 1.064069 |
| GO:0019725\_cellular\_homeostasis | FYN | 195 | 3 | 2.442971 | -0.912564 | 146 | 155.1 | 1.062329 |
| GO:0019725\_cellular\_homeostasis | GNA11 | 195 | 3 | 2.442971 | -0.912564 | 146 | 155.1 | 1.062329 |
| GO:0019725\_cellular\_homeostasis | PRNP | 195 | 3 | 2.442971 | -0.912564 | 146 | 155.1 | 1.062329 |
| GO:0002053\_positive\_regulation\_of\_mesenchymal\_cell\_proliferation | GAS1 | 21 | 1 | 7.561576 | -0.904844 | 151 | 160.43 | 1.062450 |
| GO:0002456\_T\_cell\_mediated\_immunity | CD8A | 21 | 1 | 7.561576 | -0.904844 | 151 | 160.43 | 1.062450 |
| GO:0006633\_fatty\_acid\_biosynthetic\_process | INSIG1 | 21 | 1 | 7.561576 | -0.904844 | 151 | 160.43 | 1.062450 |
| GO:0021532\_neural\_tube\_patterning | GAS1 | 21 | 1 | 7.561576 | -0.904844 | 151 | 160.43 | 1.062450 |
| GO:0034330\_cell\_junction\_organization | CXADR | 21 | 1 | 7.561576 | -0.904844 | 151 | 160.43 | 1.062450 |
| GO:0050801\_ion\_homeostasis | FYN | 197 | 3 | 2.418169 | -0.902777 | 152 | 160.92 | 1.058684 |
| GO:0050801\_ion\_homeostasis | GNA11 | 197 | 3 | 2.418169 | -0.902777 | 152 | 160.92 | 1.058684 |
| GO:0050801\_ion\_homeostasis | PRNP | 197 | 3 | 2.418169 | -0.902777 | 152 | 160.92 | 1.058684 |
| GO:0009967\_positive\_regulation\_of\_signal\_transduction | CD8A | 98 | 2 | 3.240676 | -0.900474 | 153 | 161.38 | 1.054771 |
| GO:0009967\_positive\_regulation\_of\_signal\_transduction | GAS1 | 98 | 2 | 3.240676 | -0.900474 | 153 | 161.38 | 1.054771 |
| GO:0060348\_bone\_development | INSIG1 | 99 | 2 | 3.207941 | -0.893253 | 154 | 162.16 | 1.052987 |
| GO:0060348\_bone\_development | IGFBP5 | 99 | 2 | 3.207941 | -0.893253 | 154 | 162.16 | 1.052987 |
| GO:0007154\_cell\_communication | CD8A | 1096 | 10 | 1.448842 | -0.888203 | 155 | 162.35 | 1.047419 |
| GO:0007154\_cell\_communication | AGTPBP1 | 1096 | 10 | 1.448842 | -0.888203 | 155 | 162.35 | 1.047419 |
| GO:0007154\_cell\_communication | FYN | 1096 | 10 | 1.448842 | -0.888203 | 155 | 162.35 | 1.047419 |
| GO:0007154\_cell\_communication | GNA11 | 1096 | 10 | 1.448842 | -0.888203 | 155 | 162.35 | 1.047419 |
| GO:0007154\_cell\_communication | FST | 1096 | 10 | 1.448842 | -0.888203 | 155 | 162.35 | 1.047419 |
| GO:0007154\_cell\_communication | TGM2 | 1096 | 10 | 1.448842 | -0.888203 | 155 | 162.35 | 1.047419 |
| GO:0007154\_cell\_communication | PAFAH1B1 | 1096 | 10 | 1.448842 | -0.888203 | 155 | 162.35 | 1.047419 |
| GO:0007154\_cell\_communication | FZD2 | 1096 | 10 | 1.448842 | -0.888203 | 155 | 162.35 | 1.047419 |
| GO:0007154\_cell\_communication | GAS1 | 1096 | 10 | 1.448842 | -0.888203 | 155 | 162.35 | 1.047419 |
| GO:0007154\_cell\_communication | AZI2 | 1096 | 10 | 1.448842 | -0.888203 | 155 | 162.35 | 1.047419 |
| GO:0010463\_mesenchymal\_cell\_proliferation | GAS1 | 22 | 1 | 7.217868 | -0.885940 | 162 | 168.13 | 1.037840 |
| GO:0010464\_regulation\_of\_mesenchymal\_cell\_proliferation | GAS1 | 22 | 1 | 7.217868 | -0.885940 | 162 | 168.13 | 1.037840 |
| GO:0015918\_sterol\_transport | LDLR | 22 | 1 | 7.217868 | -0.885940 | 162 | 168.13 | 1.037840 |
| GO:0021766\_hippocampus\_development | PAFAH1B1 | 22 | 1 | 7.217868 | -0.885940 | 162 | 168.13 | 1.037840 |
| GO:0030301\_cholesterol\_transport | LDLR | 22 | 1 | 7.217868 | -0.885940 | 162 | 168.13 | 1.037840 |
| GO:0042733\_embryonic\_digit\_morphogenesis | GAS1 | 22 | 1 | 7.217868 | -0.885940 | 162 | 168.13 | 1.037840 |
| GO:0048489\_synaptic\_vesicle\_transport | AMPH | 22 | 1 | 7.217868 | -0.885940 | 162 | 168.13 | 1.037840 |
| GO:0009653\_anatomical\_structure\_morphogenesis | FYN | 958 | 9 | 1.491793 | -0.884940 | 163 | 168.21 | 1.031963 |
| GO:0009653\_anatomical\_structure\_morphogenesis | AGTPBP1 | 958 | 9 | 1.491793 | -0.884940 | 163 | 168.21 | 1.031963 |
| GO:0009653\_anatomical\_structure\_morphogenesis | PRKAR1A | 958 | 9 | 1.491793 | -0.884940 | 163 | 168.21 | 1.031963 |
| GO:0009653\_anatomical\_structure\_morphogenesis | FST | 958 | 9 | 1.491793 | -0.884940 | 163 | 168.21 | 1.031963 |
| GO:0009653\_anatomical\_structure\_morphogenesis | INSIG1 | 958 | 9 | 1.491793 | -0.884940 | 163 | 168.21 | 1.031963 |
| GO:0009653\_anatomical\_structure\_morphogenesis | TGM2 | 958 | 9 | 1.491793 | -0.884940 | 163 | 168.21 | 1.031963 |
| GO:0009653\_anatomical\_structure\_morphogenesis | PAFAH1B1 | 958 | 9 | 1.491793 | -0.884940 | 163 | 168.21 | 1.031963 |
| GO:0009653\_anatomical\_structure\_morphogenesis | FZD2 | 958 | 9 | 1.491793 | -0.884940 | 163 | 168.21 | 1.031963 |
| GO:0009653\_anatomical\_structure\_morphogenesis | GAS1 | 958 | 9 | 1.491793 | -0.884940 | 163 | 168.21 | 1.031963 |
| GO:0044267\_cellular\_protein\_metabolic\_process | ZDHHC17 | 559 | 6 | 1.704398 | -0.882131 | 164 | 168.48 | 1.027317 |
| GO:0044267\_cellular\_protein\_metabolic\_process | MRPL12 | 559 | 6 | 1.704398 | -0.882131 | 164 | 168.48 | 1.027317 |
| GO:0044267\_cellular\_protein\_metabolic\_process | FYN | 559 | 6 | 1.704398 | -0.882131 | 164 | 168.48 | 1.027317 |
| GO:0044267\_cellular\_protein\_metabolic\_process | PRKAR1A | 559 | 6 | 1.704398 | -0.882131 | 164 | 168.48 | 1.027317 |
| GO:0044267\_cellular\_protein\_metabolic\_process | TGM2 | 559 | 6 | 1.704398 | -0.882131 | 164 | 168.48 | 1.027317 |
| GO:0044267\_cellular\_protein\_metabolic\_process | CHFR | 559 | 6 | 1.704398 | -0.882131 | 164 | 168.48 | 1.027317 |
| GO:0060349\_bone\_morphogenesis | INSIG1 | 23 | 1 | 6.904048 | -0.867934 | 166 | 172.18 | 1.037229 |
| GO:0060445\_branching\_involved\_in\_salivary\_gland\_morphogenesis | TGM2 | 23 | 1 | 6.904048 | -0.867934 | 166 | 172.18 | 1.037229 |
| GO:0006464\_protein\_modification\_process | ZDHHC17 | 439 | 5 | 1.808577 | -0.865199 | 167 | 172.82 | 1.034850 |
| GO:0006464\_protein\_modification\_process | FYN | 439 | 5 | 1.808577 | -0.865199 | 167 | 172.82 | 1.034850 |
| GO:0006464\_protein\_modification\_process | PRKAR1A | 439 | 5 | 1.808577 | -0.865199 | 167 | 172.82 | 1.034850 |
| GO:0006464\_protein\_modification\_process | TGM2 | 439 | 5 | 1.808577 | -0.865199 | 167 | 172.82 | 1.034850 |
| GO:0006464\_protein\_modification\_process | CHFR | 439 | 5 | 1.808577 | -0.865199 | 167 | 172.82 | 1.034850 |
| GO:0007050\_cell\_cycle\_arrest | GAS1 | 24 | 1 | 6.616379 | -0.850747 | 174 | 177.87 | 1.022241 |
| GO:0007266\_Rho\_protein\_signal\_transduction | PAFAH1B1 | 24 | 1 | 6.616379 | -0.850747 | 174 | 177.87 | 1.022241 |
| GO:0009612\_response\_to\_mechanical\_stimulus | FYN | 24 | 1 | 6.616379 | -0.850747 | 174 | 177.87 | 1.022241 |
| GO:0042158\_lipoprotein\_biosynthetic\_process | ZDHHC17 | 24 | 1 | 6.616379 | -0.850747 | 174 | 177.87 | 1.022241 |
| GO:0042632\_cholesterol\_homeostasis | LDLR | 24 | 1 | 6.616379 | -0.850747 | 174 | 177.87 | 1.022241 |
| GO:0050679\_positive\_regulation\_of\_epithelial\_cell\_proliferation | GAS1 | 24 | 1 | 6.616379 | -0.850747 | 174 | 177.87 | 1.022241 |
| GO:0055092\_sterol\_homeostasis | LDLR | 24 | 1 | 6.616379 | -0.850747 | 174 | 177.87 | 1.022241 |
| GO:0050793\_regulation\_of\_developmental\_process | FYN | 703 | 7 | 1.581155 | -0.847634 | 175 | 178.15 | 1.018000 |
| GO:0050793\_regulation\_of\_developmental\_process | GNA11 | 703 | 7 | 1.581155 | -0.847634 | 175 | 178.15 | 1.018000 |
| GO:0050793\_regulation\_of\_developmental\_process | FST | 703 | 7 | 1.581155 | -0.847634 | 175 | 178.15 | 1.018000 |
| GO:0050793\_regulation\_of\_developmental\_process | INSIG1 | 703 | 7 | 1.581155 | -0.847634 | 175 | 178.15 | 1.018000 |
| GO:0050793\_regulation\_of\_developmental\_process | GAS1 | 703 | 7 | 1.581155 | -0.847634 | 175 | 178.15 | 1.018000 |
| GO:0050793\_regulation\_of\_developmental\_process | CXADR | 703 | 7 | 1.581155 | -0.847634 | 175 | 178.15 | 1.018000 |
| GO:0050793\_regulation\_of\_developmental\_process | PRNP | 703 | 7 | 1.581155 | -0.847634 | 175 | 178.15 | 1.018000 |
| GO:0043543\_protein\_amino\_acid\_acylation | ZDHHC17 | 25 | 1 | 6.351724 | -0.834314 | 176 | 182.12 | 1.034773 |
| GO:0006928\_cell\_motion | NAV1 | 330 | 4 | 1.924765 | -0.823819 | 178 | 183.05 | 1.028371 |
| GO:0006928\_cell\_motion | FYN | 330 | 4 | 1.924765 | -0.823819 | 178 | 183.05 | 1.028371 |
| GO:0006928\_cell\_motion | PAFAH1B1 | 330 | 4 | 1.924765 | -0.823819 | 178 | 183.05 | 1.028371 |
| GO:0006928\_cell\_motion | GAS1 | 330 | 4 | 1.924765 | -0.823819 | 178 | 183.05 | 1.028371 |
| GO:0051674\_localization\_of\_cell | NAV1 | 330 | 4 | 1.924765 | -0.823819 | 178 | 183.05 | 1.028371 |
| GO:0051674\_localization\_of\_cell | FYN | 330 | 4 | 1.924765 | -0.823819 | 178 | 183.05 | 1.028371 |
| GO:0051674\_localization\_of\_cell | PAFAH1B1 | 330 | 4 | 1.924765 | -0.823819 | 178 | 183.05 | 1.028371 |
| GO:0051674\_localization\_of\_cell | GAS1 | 330 | 4 | 1.924765 | -0.823819 | 178 | 183.05 | 1.028371 |
| GO:0051093\_negative\_regulation\_of\_developmental\_process | FST | 331 | 4 | 1.918950 | -0.820397 | 179 | 183.28 | 1.023911 |
| GO:0051093\_negative\_regulation\_of\_developmental\_process | INSIG1 | 331 | 4 | 1.918950 | -0.820397 | 179 | 183.28 | 1.023911 |
| GO:0051093\_negative\_regulation\_of\_developmental\_process | CXADR | 331 | 4 | 1.918950 | -0.820397 | 179 | 183.28 | 1.023911 |
| GO:0051093\_negative\_regulation\_of\_developmental\_process | PRNP | 331 | 4 | 1.918950 | -0.820397 | 179 | 183.28 | 1.023911 |
| GO:0010647\_positive\_regulation\_of\_cell\_communication | CD8A | 110 | 2 | 2.887147 | -0.819317 | 180 | 183.9 | 1.021667 |
| GO:0010647\_positive\_regulation\_of\_cell\_communication | GAS1 | 110 | 2 | 2.887147 | -0.819317 | 180 | 183.9 | 1.021667 |
| GO:0007405\_neuroblast\_proliferation | PAFAH1B1 | 26 | 1 | 6.107427 | -0.818576 | 183 | 187.2 | 1.022951 |
| GO:0019233\_sensory\_perception\_of\_pain | FYN | 26 | 1 | 6.107427 | -0.818576 | 183 | 187.2 | 1.022951 |
| GO:0050680\_negative\_regulation\_of\_epithelial\_cell\_proliferation | GAS1 | 26 | 1 | 6.107427 | -0.818576 | 183 | 187.2 | 1.022951 |
| GO:0048705\_skeletal\_system\_morphogenesis | INSIG1 | 111 | 2 | 2.861137 | -0.813055 | 184 | 187.62 | 1.019674 |
| GO:0048705\_skeletal\_system\_morphogenesis | GAS1 | 111 | 2 | 2.861137 | -0.813055 | 184 | 187.62 | 1.019674 |
| GO:0006997\_nucleus\_organization | PAFAH1B1 | 28 | 1 | 5.671182 | -0.788977 | 185 | 196.16 | 1.060324 |
| GO:0007166\_cell\_surface\_receptor\_linked\_signal\_transduction | CD8A | 597 | 6 | 1.595911 | -0.783292 | 186 | 196.85 | 1.058333 |
| GO:0007166\_cell\_surface\_receptor\_linked\_signal\_transduction | FYN | 597 | 6 | 1.595911 | -0.783292 | 186 | 196.85 | 1.058333 |
| GO:0007166\_cell\_surface\_receptor\_linked\_signal\_transduction | GNA11 | 597 | 6 | 1.595911 | -0.783292 | 186 | 196.85 | 1.058333 |
| GO:0007166\_cell\_surface\_receptor\_linked\_signal\_transduction | FST | 597 | 6 | 1.595911 | -0.783292 | 186 | 196.85 | 1.058333 |
| GO:0007166\_cell\_surface\_receptor\_linked\_signal\_transduction | TGM2 | 597 | 6 | 1.595911 | -0.783292 | 186 | 196.85 | 1.058333 |
| GO:0007166\_cell\_surface\_receptor\_linked\_signal\_transduction | GAS1 | 597 | 6 | 1.595911 | -0.783292 | 186 | 196.85 | 1.058333 |
| GO:0021761\_limbic\_system\_development | PAFAH1B1 | 29 | 1 | 5.475624 | -0.775028 | 188 | 200.89 | 1.068564 |
| GO:0048066\_pigmentation\_during\_development | GNA11 | 29 | 1 | 5.475624 | -0.775028 | 188 | 200.89 | 1.068564 |
| GO:0019953\_sexual\_reproduction | FST | 228 | 3 | 2.089383 | -0.766706 | 189 | 201.75 | 1.067460 |
| GO:0019953\_sexual\_reproduction | PAFAH1B1 | 228 | 3 | 2.089383 | -0.766706 | 189 | 201.75 | 1.067460 |
| GO:0019953\_sexual\_reproduction | HERPUD2 | 228 | 3 | 2.089383 | -0.766706 | 189 | 201.75 | 1.067460 |
| GO:0007435\_salivary\_gland\_morphogenesis | TGM2 | 30 | 1 | 5.293103 | -0.761595 | 192 | 205.63 | 1.070990 |
| GO:0022411\_cellular\_component\_disassembly | PAFAH1B1 | 30 | 1 | 5.293103 | -0.761595 | 192 | 205.63 | 1.070990 |
| GO:0042552\_myelination | FYN | 30 | 1 | 5.293103 | -0.761595 | 192 | 205.63 | 1.070990 |
| GO:0014706\_striated\_muscle\_tissue\_development | LGALS1 | 120 | 2 | 2.646552 | -0.759719 | 193 | 205.79 | 1.066269 |
| GO:0014706\_striated\_muscle\_tissue\_development | CXADR | 120 | 2 | 2.646552 | -0.759719 | 193 | 205.79 | 1.066269 |
| GO:0006694\_steroid\_biosynthetic\_process | INSIG1 | 31 | 1 | 5.122358 | -0.748643 | 195 | 212.04 | 1.087385 |
| GO:0055088\_lipid\_homeostasis | LDLR | 31 | 1 | 5.122358 | -0.748643 | 195 | 212.04 | 1.087385 |
| GO:0050890\_cognition | FYN | 233 | 3 | 2.044546 | -0.747193 | 196 | 213.17 | 1.087602 |
| GO:0050890\_cognition | PAFAH1B1 | 233 | 3 | 2.044546 | -0.747193 | 196 | 213.17 | 1.087602 |
| GO:0050890\_cognition | AMPH | 233 | 3 | 2.044546 | -0.747193 | 196 | 213.17 | 1.087602 |
| GO:0016477\_cell\_migration | NAV1 | 234 | 3 | 2.035809 | -0.743362 | 197 | 213.34 | 1.082944 |
| GO:0016477\_cell\_migration | FYN | 234 | 3 | 2.035809 | -0.743362 | 197 | 213.34 | 1.082944 |
| GO:0016477\_cell\_migration | PAFAH1B1 | 234 | 3 | 2.035809 | -0.743362 | 197 | 213.34 | 1.082944 |
| GO:0001707\_mesoderm\_formation | PRKAR1A | 32 | 1 | 4.962284 | -0.736142 | 203 | 216.52 | 1.066601 |
| GO:0007249\_I-kappaB\_kinase\_NF-kappaB\_cascade | AZI2 | 32 | 1 | 4.962284 | -0.736142 | 203 | 216.52 | 1.066601 |
| GO:0007272\_ensheathment\_of\_neurons | FYN | 32 | 1 | 4.962284 | -0.736142 | 203 | 216.52 | 1.066601 |
| GO:0008366\_axon\_ensheathment | FYN | 32 | 1 | 4.962284 | -0.736142 | 203 | 216.52 | 1.066601 |
| GO:0048332\_mesoderm\_morphogenesis | PRKAR1A | 32 | 1 | 4.962284 | -0.736142 | 203 | 216.52 | 1.066601 |
| GO:0050885\_neuromuscular\_process\_controlling\_balance | PAFAH1B1 | 32 | 1 | 4.962284 | -0.736142 | 203 | 216.52 | 1.066601 |
| GO:0007431\_salivary\_gland\_development | TGM2 | 33 | 1 | 4.811912 | -0.724065 | 205 | 220.5 | 1.075610 |
| GO:0021987\_cerebral\_cortex\_development | PAFAH1B1 | 33 | 1 | 4.811912 | -0.724065 | 205 | 220.5 | 1.075610 |
| GO:0060537\_muscle\_tissue\_development | LGALS1 | 128 | 2 | 2.481142 | -0.716432 | 206 | 221.07 | 1.073155 |
| GO:0060537\_muscle\_tissue\_development | CXADR | 128 | 2 | 2.481142 | -0.716432 | 206 | 221.07 | 1.073155 |
| GO:0030509\_BMP\_signaling\_pathway | FST | 34 | 1 | 4.670385 | -0.712385 | 207 | 224.59 | 1.084976 |
| GO:0006869\_lipid\_transport | LDLR | 35 | 1 | 4.536946 | -0.701080 | 209 | 228.18 | 1.091770 |
| GO:0016567\_protein\_ubiquitination | CHFR | 35 | 1 | 4.536946 | -0.701080 | 209 | 228.18 | 1.091770 |
| GO:0007165\_signal\_transduction | CD8A | 915 | 8 | 1.388355 | -0.690694 | 210 | 229.49 | 1.092810 |
| GO:0007165\_signal\_transduction | FYN | 915 | 8 | 1.388355 | -0.690694 | 210 | 229.49 | 1.092810 |
| GO:0007165\_signal\_transduction | GNA11 | 915 | 8 | 1.388355 | -0.690694 | 210 | 229.49 | 1.092810 |
| GO:0007165\_signal\_transduction | FST | 915 | 8 | 1.388355 | -0.690694 | 210 | 229.49 | 1.092810 |
| GO:0007165\_signal\_transduction | TGM2 | 915 | 8 | 1.388355 | -0.690694 | 210 | 229.49 | 1.092810 |
| GO:0007165\_signal\_transduction | PAFAH1B1 | 915 | 8 | 1.388355 | -0.690694 | 210 | 229.49 | 1.092810 |
| GO:0007165\_signal\_transduction | GAS1 | 915 | 8 | 1.388355 | -0.690694 | 210 | 229.49 | 1.092810 |
| GO:0007165\_signal\_transduction | AZI2 | 915 | 8 | 1.388355 | -0.690694 | 210 | 229.49 | 1.092810 |
| GO:0001704\_formation\_of\_primary\_germ\_layer | PRKAR1A | 36 | 1 | 4.410920 | -0.690129 | 212 | 233.08 | 1.099434 |
| GO:0019228\_regulation\_of\_action\_potential\_in\_neuron | FYN | 36 | 1 | 4.410920 | -0.690129 | 212 | 233.08 | 1.099434 |
| GO:0007283\_spermatogenesis | PAFAH1B1 | 134 | 2 | 2.370046 | -0.686209 | 214 | 233.45 | 1.090888 |
| GO:0007283\_spermatogenesis | HERPUD2 | 134 | 2 | 2.370046 | -0.686209 | 214 | 233.45 | 1.090888 |
| GO:0048232\_male\_gamete\_generation | PAFAH1B1 | 134 | 2 | 2.370046 | -0.686209 | 214 | 233.45 | 1.090888 |
| GO:0048232\_male\_gamete\_generation | HERPUD2 | 134 | 2 | 2.370046 | -0.686209 | 214 | 233.45 | 1.090888 |
| GO:0045785\_positive\_regulation\_of\_cell\_adhesion | TGM2 | 37 | 1 | 4.291705 | -0.679511 | 216 | 236.33 | 1.094120 |
| GO:0050906\_detection\_of\_stimulus\_involved\_in\_sensory\_perception | FYN | 37 | 1 | 4.291705 | -0.679511 | 216 | 236.33 | 1.094120 |
| GO:0007267\_cell-cell\_signaling | AGTPBP1 | 252 | 3 | 1.890394 | -0.678257 | 217 | 236.52 | 1.089954 |
| GO:0007267\_cell-cell\_signaling | PAFAH1B1 | 252 | 3 | 1.890394 | -0.678257 | 217 | 236.52 | 1.089954 |
| GO:0007267\_cell-cell\_signaling | FZD2 | 252 | 3 | 1.890394 | -0.678257 | 217 | 236.52 | 1.089954 |
| GO:0009057\_macromolecule\_catabolic\_process | LDLR | 137 | 2 | 2.318147 | -0.671755 | 218 | 237.32 | 1.088624 |
| GO:0009057\_macromolecule\_catabolic\_process | CHFR | 137 | 2 | 2.318147 | -0.671755 | 218 | 237.32 | 1.088624 |
| GO:0001649\_osteoblast\_differentiation | IGFBP5 | 38 | 1 | 4.178766 | -0.669210 | 222 | 241.21 | 1.086532 |
| GO:0016053\_organic\_acid\_biosynthetic\_process | INSIG1 | 38 | 1 | 4.178766 | -0.669210 | 222 | 241.21 | 1.086532 |
| GO:0046394\_carboxylic\_acid\_biosynthetic\_process | INSIG1 | 38 | 1 | 4.178766 | -0.669210 | 222 | 241.21 | 1.086532 |
| GO:0046777\_protein\_amino\_acid\_autophosphorylation | FYN | 38 | 1 | 4.178766 | -0.669210 | 222 | 241.21 | 1.086532 |
| GO:0048729\_tissue\_morphogenesis | FST | 255 | 3 | 1.868154 | -0.668071 | 223 | 241.3 | 1.082063 |
| GO:0048729\_tissue\_morphogenesis | PRKAR1A | 255 | 3 | 1.868154 | -0.668071 | 223 | 241.3 | 1.082063 |
| GO:0048729\_tissue\_morphogenesis | FZD2 | 255 | 3 | 1.868154 | -0.668071 | 223 | 241.3 | 1.082063 |
| GO:0009966\_regulation\_of\_signal\_transduction | CD8A | 256 | 3 | 1.860857 | -0.664715 | 224 | 241.6 | 1.078571 |
| GO:0009966\_regulation\_of\_signal\_transduction | PAFAH1B1 | 256 | 3 | 1.860857 | -0.664715 | 224 | 241.6 | 1.078571 |
| GO:0009966\_regulation\_of\_signal\_transduction | GAS1 | 256 | 3 | 1.860857 | -0.664715 | 224 | 241.6 | 1.078571 |
| GO:0048870\_cell\_motility | NAV1 | 257 | 3 | 1.853616 | -0.661380 | 225 | 242.25 | 1.076667 |
| GO:0048870\_cell\_motility | FYN | 257 | 3 | 1.853616 | -0.661380 | 225 | 242.25 | 1.076667 |
| GO:0048870\_cell\_motility | PAFAH1B1 | 257 | 3 | 1.853616 | -0.661380 | 225 | 242.25 | 1.076667 |
| GO:0043687\_post-translational\_protein\_modification | FYN | 384 | 4 | 1.654095 | -0.659454 | 226 | 242.38 | 1.072478 |
| GO:0043687\_post-translational\_protein\_modification | PRKAR1A | 384 | 4 | 1.654095 | -0.659454 | 226 | 242.38 | 1.072478 |
| GO:0043687\_post-translational\_protein\_modification | TGM2 | 384 | 4 | 1.654095 | -0.659454 | 226 | 242.38 | 1.072478 |
| GO:0043687\_post-translational\_protein\_modification | CHFR | 384 | 4 | 1.654095 | -0.659454 | 226 | 242.38 | 1.072478 |
| GO:0006511\_ubiquitin-dependent\_protein\_catabolic\_process | CHFR | 39 | 1 | 4.071618 | -0.659208 | 230 | 245.02 | 1.065304 |
| GO:0007286\_spermatid\_development | PAFAH1B1 | 39 | 1 | 4.071618 | -0.659208 | 230 | 245.02 | 1.065304 |
| GO:0035148\_tube\_lumen\_formation | TGM2 | 39 | 1 | 4.071618 | -0.659208 | 230 | 245.02 | 1.065304 |
| GO:0042475\_odontogenesis\_of\_dentine-containing\_tooth | FST | 39 | 1 | 4.071618 | -0.659208 | 230 | 245.02 | 1.065304 |
| GO:0003008\_system\_process | MOGAT2 | 516 | 5 | 1.538693 | -0.659098 | 231 | 245.19 | 1.061429 |
| GO:0003008\_system\_process | FYN | 516 | 5 | 1.538693 | -0.659098 | 231 | 245.19 | 1.061429 |
| GO:0003008\_system\_process | AGTPBP1 | 516 | 5 | 1.538693 | -0.659098 | 231 | 245.19 | 1.061429 |
| GO:0003008\_system\_process | PAFAH1B1 | 516 | 5 | 1.538693 | -0.659098 | 231 | 245.19 | 1.061429 |
| GO:0003008\_system\_process | AMPH | 516 | 5 | 1.538693 | -0.659098 | 231 | 245.19 | 1.061429 |
| GO:0048468\_cell\_development | NAV1 | 654 | 6 | 1.456817 | -0.654585 | 232 | 245.62 | 1.058707 |
| GO:0048468\_cell\_development | AGTPBP1 | 654 | 6 | 1.456817 | -0.654585 | 232 | 245.62 | 1.058707 |
| GO:0048468\_cell\_development | FYN | 654 | 6 | 1.456817 | -0.654585 | 232 | 245.62 | 1.058707 |
| GO:0048468\_cell\_development | LGALS1 | 654 | 6 | 1.456817 | -0.654585 | 232 | 245.62 | 1.058707 |
| GO:0048468\_cell\_development | PAFAH1B1 | 654 | 6 | 1.456817 | -0.654585 | 232 | 245.62 | 1.058707 |
| GO:0048468\_cell\_development | GAS1 | 654 | 6 | 1.456817 | -0.654585 | 232 | 245.62 | 1.058707 |
| GO:0019538\_protein\_metabolic\_process | ZDHHC17 | 655 | 6 | 1.454593 | -0.652515 | 233 | 246.26 | 1.056910 |
| GO:0019538\_protein\_metabolic\_process | MRPL12 | 655 | 6 | 1.454593 | -0.652515 | 233 | 246.26 | 1.056910 |
| GO:0019538\_protein\_metabolic\_process | FYN | 655 | 6 | 1.454593 | -0.652515 | 233 | 246.26 | 1.056910 |
| GO:0019538\_protein\_metabolic\_process | PRKAR1A | 655 | 6 | 1.454593 | -0.652515 | 233 | 246.26 | 1.056910 |
| GO:0019538\_protein\_metabolic\_process | TGM2 | 655 | 6 | 1.454593 | -0.652515 | 233 | 246.26 | 1.056910 |
| GO:0019538\_protein\_metabolic\_process | CHFR | 655 | 6 | 1.454593 | -0.652515 | 233 | 246.26 | 1.056910 |
| GO:0016071\_mRNA\_metabolic\_process | DNAJB11 | 40 | 1 | 3.969828 | -0.649491 | 235 | 248.92 | 1.059234 |
| GO:0035272\_exocrine\_system\_development | TGM2 | 40 | 1 | 3.969828 | -0.649491 | 235 | 248.92 | 1.059234 |
| GO:0050877\_neurological\_system\_process | FYN | 390 | 4 | 1.628647 | -0.643484 | 236 | 249.95 | 1.059110 |
| GO:0050877\_neurological\_system\_process | AGTPBP1 | 390 | 4 | 1.628647 | -0.643484 | 236 | 249.95 | 1.059110 |
| GO:0050877\_neurological\_system\_process | PAFAH1B1 | 390 | 4 | 1.628647 | -0.643484 | 236 | 249.95 | 1.059110 |
| GO:0050877\_neurological\_system\_process | AMPH | 390 | 4 | 1.628647 | -0.643484 | 236 | 249.95 | 1.059110 |
| GO:0006979\_response\_to\_oxidative\_stress | PRNP | 41 | 1 | 3.873003 | -0.640044 | 239 | 253.95 | 1.062552 |
| GO:0008585\_female\_gonad\_development | FST | 41 | 1 | 3.873003 | -0.640044 | 239 | 253.95 | 1.062552 |
| GO:0019216\_regulation\_of\_lipid\_metabolic\_process | INSIG1 | 41 | 1 | 3.873003 | -0.640044 | 239 | 253.95 | 1.062552 |
| GO:0007186\_G-protein\_coupled\_receptor\_protein\_signaling\_pathway | GNA11 | 144 | 2 | 2.205460 | -0.639608 | 241 | 254.27 | 1.055062 |
| GO:0007186\_G-protein\_coupled\_receptor\_protein\_signaling\_pathway | TGM2 | 144 | 2 | 2.205460 | -0.639608 | 241 | 254.27 | 1.055062 |
| GO:0045596\_negative\_regulation\_of\_cell\_differentiation | FST | 144 | 2 | 2.205460 | -0.639608 | 241 | 254.27 | 1.055062 |
| GO:0045596\_negative\_regulation\_of\_cell\_differentiation | INSIG1 | 144 | 2 | 2.205460 | -0.639608 | 241 | 254.27 | 1.055062 |
| GO:0044255\_cellular\_lipid\_metabolic\_process | MOGAT2 | 264 | 3 | 1.804467 | -0.638564 | 242 | 254.53 | 1.051777 |
| GO:0044255\_cellular\_lipid\_metabolic\_process | LDLR | 264 | 3 | 1.804467 | -0.638564 | 242 | 254.53 | 1.051777 |
| GO:0044255\_cellular\_lipid\_metabolic\_process | INSIG1 | 264 | 3 | 1.804467 | -0.638564 | 242 | 254.53 | 1.051777 |
| GO:0009888\_tissue\_development | LGALS1 | 525 | 5 | 1.512315 | -0.638458 | 243 | 254.67 | 1.048025 |
| GO:0009888\_tissue\_development | PRKAR1A | 525 | 5 | 1.512315 | -0.638458 | 243 | 254.67 | 1.048025 |
| GO:0009888\_tissue\_development | FST | 525 | 5 | 1.512315 | -0.638458 | 243 | 254.67 | 1.048025 |
| GO:0009888\_tissue\_development | FZD2 | 525 | 5 | 1.512315 | -0.638458 | 243 | 254.67 | 1.048025 |
| GO:0009888\_tissue\_development | CXADR | 525 | 5 | 1.512315 | -0.638458 | 243 | 254.67 | 1.048025 |
| GO:0019941\_modification-dependent\_protein\_catabolic\_process | CHFR | 42 | 1 | 3.780788 | -0.630855 | 249 | 258.66 | 1.038795 |
| GO:0042476\_odontogenesis | FST | 42 | 1 | 3.780788 | -0.630855 | 249 | 258.66 | 1.038795 |
| GO:0043632\_modification-dependent\_macromolecule\_catabolic\_process | CHFR | 42 | 1 | 3.780788 | -0.630855 | 249 | 258.66 | 1.038795 |
| GO:0048515\_spermatid\_differentiation | PAFAH1B1 | 42 | 1 | 3.780788 | -0.630855 | 249 | 258.66 | 1.038795 |
| GO:0051345\_positive\_regulation\_of\_hydrolase\_activity | GNA11 | 42 | 1 | 3.780788 | -0.630855 | 249 | 258.66 | 1.038795 |
| GO:0051603\_proteolysis\_involved\_in\_cellular\_protein\_catabolic\_process | CHFR | 42 | 1 | 3.780788 | -0.630855 | 249 | 258.66 | 1.038795 |
| GO:0030900\_forebrain\_development | FYN | 146 | 2 | 2.175248 | -0.630805 | 250 | 259.19 | 1.036760 |
| GO:0030900\_forebrain\_development | PAFAH1B1 | 146 | 2 | 2.175248 | -0.630805 | 250 | 259.19 | 1.036760 |
| GO:0007224\_smoothened\_signaling\_pathway | GAS1 | 43 | 1 | 3.692863 | -0.621910 | 253 | 264.09 | 1.043834 |
| GO:0009582\_detection\_of\_abiotic\_stimulus | FYN | 43 | 1 | 3.692863 | -0.621910 | 253 | 264.09 | 1.043834 |
| GO:0032446\_protein\_modification\_by\_small\_protein\_conjugation | CHFR | 43 | 1 | 3.692863 | -0.621910 | 253 | 264.09 | 1.043834 |
| GO:0001942\_hair\_follicle\_development | FST | 44 | 1 | 3.608934 | -0.613199 | 260 | 269.22 | 1.035462 |
| GO:0022404\_molting\_cycle\_process | FST | 44 | 1 | 3.608934 | -0.613199 | 260 | 269.22 | 1.035462 |
| GO:0022405\_hair\_cycle\_process | FST | 44 | 1 | 3.608934 | -0.613199 | 260 | 269.22 | 1.035462 |
| GO:0042303\_molting\_cycle | FST | 44 | 1 | 3.608934 | -0.613199 | 260 | 269.22 | 1.035462 |
| GO:0042633\_hair\_cycle | FST | 44 | 1 | 3.608934 | -0.613199 | 260 | 269.22 | 1.035462 |
| GO:0044257\_cellular\_protein\_catabolic\_process | CHFR | 44 | 1 | 3.608934 | -0.613199 | 260 | 269.22 | 1.035462 |
| GO:0046545\_development\_of\_primary\_female\_sexual\_characteristics | FST | 44 | 1 | 3.608934 | -0.613199 | 260 | 269.22 | 1.035462 |
| GO:0007517\_muscle\_organ\_development | LGALS1 | 153 | 2 | 2.075727 | -0.601236 | 261 | 271.45 | 1.040038 |
| GO:0007517\_muscle\_organ\_development | CXADR | 153 | 2 | 2.075727 | -0.601236 | 261 | 271.45 | 1.040038 |
| GO:0007268\_synaptic\_transmission | AGTPBP1 | 154 | 2 | 2.062248 | -0.597163 | 262 | 271.98 | 1.038092 |
| GO:0007268\_synaptic\_transmission | PAFAH1B1 | 154 | 2 | 2.062248 | -0.597163 | 262 | 271.98 | 1.038092 |
| GO:0007612\_learning | AMPH | 46 | 1 | 3.452024 | -0.596438 | 264 | 274.46 | 1.039621 |
| GO:0009581\_detection\_of\_external\_stimulus | FYN | 46 | 1 | 3.452024 | -0.596438 | 264 | 274.46 | 1.039621 |
| GO:0008285\_negative\_regulation\_of\_cell\_proliferation | GAS1 | 155 | 2 | 2.048943 | -0.593125 | 265 | 274.94 | 1.037509 |
| GO:0008285\_negative\_regulation\_of\_cell\_proliferation | CXADR | 155 | 2 | 2.048943 | -0.593125 | 265 | 274.94 | 1.037509 |
| GO:0007242\_intracellular\_signaling\_cascade | CD8A | 411 | 4 | 1.545432 | -0.590692 | 266 | 275.65 | 1.036278 |
| GO:0007242\_intracellular\_signaling\_cascade | GNA11 | 411 | 4 | 1.545432 | -0.590692 | 266 | 275.65 | 1.036278 |
| GO:0007242\_intracellular\_signaling\_cascade | PAFAH1B1 | 411 | 4 | 1.545432 | -0.590692 | 266 | 275.65 | 1.036278 |
| GO:0007242\_intracellular\_signaling\_cascade | AZI2 | 411 | 4 | 1.545432 | -0.590692 | 266 | 275.65 | 1.036278 |
| GO:0006066\_alcohol\_metabolic\_process | LDLR | 158 | 2 | 2.010039 | -0.581225 | 267 | 279.22 | 1.045768 |
| GO:0006066\_alcohol\_metabolic\_process | INSIG1 | 158 | 2 | 2.010039 | -0.581225 | 267 | 279.22 | 1.045768 |
| GO:0001505\_regulation\_of\_neurotransmitter\_levels | AGTPBP1 | 48 | 1 | 3.308190 | -0.580493 | 269 | 281.1 | 1.044981 |
| GO:0007498\_mesoderm\_development | PRKAR1A | 48 | 1 | 3.308190 | -0.580493 | 269 | 281.1 | 1.044981 |
| GO:0006629\_lipid\_metabolic\_process | MOGAT2 | 285 | 3 | 1.671506 | -0.575338 | 270 | 281.59 | 1.042926 |
| GO:0006629\_lipid\_metabolic\_process | LDLR | 285 | 3 | 1.671506 | -0.575338 | 270 | 281.59 | 1.042926 |
| GO:0006629\_lipid\_metabolic\_process | INSIG1 | 285 | 3 | 1.671506 | -0.575338 | 270 | 281.59 | 1.042926 |
| GO:0021543\_pallium\_development | PAFAH1B1 | 49 | 1 | 3.240676 | -0.572805 | 274 | 284.68 | 1.038978 |
| GO:0043473\_pigmentation | GNA11 | 49 | 1 | 3.240676 | -0.572805 | 274 | 284.68 | 1.038978 |
| GO:0046660\_female\_sex\_differentiation | FST | 49 | 1 | 3.240676 | -0.572805 | 274 | 284.68 | 1.038978 |
| GO:0048741\_skeletal\_muscle\_fiber\_development | LGALS1 | 49 | 1 | 3.240676 | -0.572805 | 274 | 284.68 | 1.038978 |
| GO:0042592\_homeostatic\_process | LDLR | 419 | 4 | 1.515925 | -0.571776 | 275 | 284.79 | 1.035600 |
| GO:0042592\_homeostatic\_process | FYN | 419 | 4 | 1.515925 | -0.571776 | 275 | 284.79 | 1.035600 |
| GO:0042592\_homeostatic\_process | GNA11 | 419 | 4 | 1.515925 | -0.571776 | 275 | 284.79 | 1.035600 |
| GO:0042592\_homeostatic\_process | PRNP | 419 | 4 | 1.515925 | -0.571776 | 275 | 284.79 | 1.035600 |
| GO:0051606\_detection\_of\_stimulus | FYN | 50 | 1 | 3.175862 | -0.565298 | 277 | 287.35 | 1.037365 |
| GO:0070647\_protein\_modification\_by\_small\_protein\_conjugation\_or\_removal | CHFR | 50 | 1 | 3.175862 | -0.565298 | 277 | 287.35 | 1.037365 |
| GO:0042110\_T\_cell\_activation | CD8A | 163 | 2 | 1.948382 | -0.562071 | 278 | 288.11 | 1.036367 |
| GO:0042110\_T\_cell\_activation | FYN | 163 | 2 | 1.948382 | -0.562071 | 278 | 288.11 | 1.036367 |
| GO:0048747\_muscle\_fiber\_development | LGALS1 | 51 | 1 | 3.113590 | -0.557963 | 279 | 290.93 | 1.042760 |
| GO:0040011\_locomotion | NAV1 | 295 | 3 | 1.614845 | -0.547732 | 281 | 294.03 | 1.046370 |
| GO:0040011\_locomotion | FYN | 295 | 3 | 1.614845 | -0.547732 | 281 | 294.03 | 1.046370 |
| GO:0040011\_locomotion | PAFAH1B1 | 295 | 3 | 1.614845 | -0.547732 | 281 | 294.03 | 1.046370 |
| GO:0045595\_regulation\_of\_cell\_differentiation | GNA11 | 295 | 3 | 1.614845 | -0.547732 | 281 | 294.03 | 1.046370 |
| GO:0045595\_regulation\_of\_cell\_differentiation | FST | 295 | 3 | 1.614845 | -0.547732 | 281 | 294.03 | 1.046370 |
| GO:0045595\_regulation\_of\_cell\_differentiation | INSIG1 | 295 | 3 | 1.614845 | -0.547732 | 281 | 294.03 | 1.046370 |
| GO:0048598\_embryonic\_morphogenesis | INSIG1 | 299 | 3 | 1.593242 | -0.537104 | 282 | 297.59 | 1.055284 |
| GO:0048598\_embryonic\_morphogenesis | PRKAR1A | 299 | 3 | 1.593242 | -0.537104 | 282 | 297.59 | 1.055284 |
| GO:0048598\_embryonic\_morphogenesis | GAS1 | 299 | 3 | 1.593242 | -0.537104 | 282 | 297.59 | 1.055284 |
| GO:0006412\_translation | MRPL12 | 54 | 1 | 2.940613 | -0.536928 | 284 | 299.73 | 1.055387 |
| GO:0007265\_Ras\_protein\_signal\_transduction | PAFAH1B1 | 54 | 1 | 2.940613 | -0.536928 | 284 | 299.73 | 1.055387 |
| GO:0048704\_embryonic\_skeletal\_system\_morphogenesis | GAS1 | 55 | 1 | 2.887147 | -0.530219 | 285 | 302.48 | 1.061333 |
| GO:0009987\_cellular\_process | CD8A | 3868 | 26 | 1.067379 | -0.529892 | 286 | 302.57 | 1.057937 |
| GO:0009987\_cellular\_process | LDLR | 3868 | 26 | 1.067379 | -0.529892 | 286 | 302.57 | 1.057937 |
| GO:0009987\_cellular\_process | AGTPBP1 | 3868 | 26 | 1.067379 | -0.529892 | 286 | 302.57 | 1.057937 |
| GO:0009987\_cellular\_process | GNA11 | 3868 | 26 | 1.067379 | -0.529892 | 286 | 302.57 | 1.057937 |
| GO:0009987\_cellular\_process | FST | 3868 | 26 | 1.067379 | -0.529892 | 286 | 302.57 | 1.057937 |
| GO:0009987\_cellular\_process | CXADR | 3868 | 26 | 1.067379 | -0.529892 | 286 | 302.57 | 1.057937 |
| GO:0009987\_cellular\_process | AZI2 | 3868 | 26 | 1.067379 | -0.529892 | 286 | 302.57 | 1.057937 |
| GO:0009987\_cellular\_process | AMPH | 3868 | 26 | 1.067379 | -0.529892 | 286 | 302.57 | 1.057937 |
| GO:0009987\_cellular\_process | MRPL12 | 3868 | 26 | 1.067379 | -0.529892 | 286 | 302.57 | 1.057937 |
| GO:0009987\_cellular\_process | DNAJB11 | 3868 | 26 | 1.067379 | -0.529892 | 286 | 302.57 | 1.057937 |
| GO:0009987\_cellular\_process | KLC1 | 3868 | 26 | 1.067379 | -0.529892 | 286 | 302.57 | 1.057937 |
| GO:0009987\_cellular\_process | INSIG1 | 3868 | 26 | 1.067379 | -0.529892 | 286 | 302.57 | 1.057937 |
| GO:0009987\_cellular\_process | TGM2 | 3868 | 26 | 1.067379 | -0.529892 | 286 | 302.57 | 1.057937 |
| GO:0009987\_cellular\_process | PAFAH1B1 | 3868 | 26 | 1.067379 | -0.529892 | 286 | 302.57 | 1.057937 |
| GO:0009987\_cellular\_process | MOGAT2 | 3868 | 26 | 1.067379 | -0.529892 | 286 | 302.57 | 1.057937 |
| GO:0009987\_cellular\_process | LGALS1 | 3868 | 26 | 1.067379 | -0.529892 | 286 | 302.57 | 1.057937 |
| GO:0009987\_cellular\_process | GAS1 | 3868 | 26 | 1.067379 | -0.529892 | 286 | 302.57 | 1.057937 |
| GO:0009987\_cellular\_process | FZD2 | 3868 | 26 | 1.067379 | -0.529892 | 286 | 302.57 | 1.057937 |
| GO:0009987\_cellular\_process | ZDHHC17 | 3868 | 26 | 1.067379 | -0.529892 | 286 | 302.57 | 1.057937 |
| GO:0009987\_cellular\_process | NAV1 | 3868 | 26 | 1.067379 | -0.529892 | 286 | 302.57 | 1.057937 |
| GO:0009987\_cellular\_process | SGCG | 3868 | 26 | 1.067379 | -0.529892 | 286 | 302.57 | 1.057937 |
| GO:0009987\_cellular\_process | FYN | 3868 | 26 | 1.067379 | -0.529892 | 286 | 302.57 | 1.057937 |
| GO:0009987\_cellular\_process | PRKAR1A | 3868 | 26 | 1.067379 | -0.529892 | 286 | 302.57 | 1.057937 |
| GO:0009987\_cellular\_process | CHFR | 3868 | 26 | 1.067379 | -0.529892 | 286 | 302.57 | 1.057937 |
| GO:0009987\_cellular\_process | PRNP | 3868 | 26 | 1.067379 | -0.529892 | 286 | 302.57 | 1.057937 |
| GO:0009987\_cellular\_process | IGFBP5 | 3868 | 26 | 1.067379 | -0.529892 | 286 | 302.57 | 1.057937 |
| GO:0044248\_cellular\_catabolic\_process | LDLR | 173 | 2 | 1.835758 | -0.526126 | 287 | 303.72 | 1.058258 |
| GO:0044248\_cellular\_catabolic\_process | CHFR | 173 | 2 | 1.835758 | -0.526126 | 287 | 303.72 | 1.058258 |
| GO:0050678\_regulation\_of\_epithelial\_cell\_proliferation | GAS1 | 56 | 1 | 2.835591 | -0.523653 | 288 | 306.16 | 1.063056 |
| GO:0008344\_adult\_locomotory\_behavior | PAFAH1B1 | 57 | 1 | 2.785844 | -0.517224 | 294 | 310.44 | 1.055918 |
| GO:0009953\_dorsal\_ventral\_pattern\_formation | GAS1 | 57 | 1 | 2.785844 | -0.517224 | 294 | 310.44 | 1.055918 |
| GO:0018108\_peptidyl-tyrosine\_phosphorylation | FYN | 57 | 1 | 2.785844 | -0.517224 | 294 | 310.44 | 1.055918 |
| GO:0018212\_peptidyl-tyrosine\_modification | FYN | 57 | 1 | 2.785844 | -0.517224 | 294 | 310.44 | 1.055918 |
| GO:0042472\_inner\_ear\_morphogenesis | INSIG1 | 57 | 1 | 2.785844 | -0.517224 | 294 | 310.44 | 1.055918 |
| GO:0045444\_fat\_cell\_differentiation | INSIG1 | 57 | 1 | 2.785844 | -0.517224 | 294 | 310.44 | 1.055918 |
| GO:0032989\_cellular\_component\_morphogenesis | FYN | 307 | 3 | 1.551724 | -0.516522 | 295 | 310.95 | 1.054068 |
| GO:0032989\_cellular\_component\_morphogenesis | PAFAH1B1 | 307 | 3 | 1.551724 | -0.516522 | 295 | 310.95 | 1.054068 |
| GO:0032989\_cellular\_component\_morphogenesis | GAS1 | 307 | 3 | 1.551724 | -0.516522 | 295 | 310.95 | 1.054068 |
| GO:0010926\_anatomical\_structure\_formation | AGTPBP1 | 447 | 4 | 1.420967 | -0.510290 | 296 | 313.32 | 1.058514 |
| GO:0010926\_anatomical\_structure\_formation | PRKAR1A | 447 | 4 | 1.420967 | -0.510290 | 296 | 313.32 | 1.058514 |
| GO:0010926\_anatomical\_structure\_formation | TGM2 | 447 | 4 | 1.420967 | -0.510290 | 296 | 313.32 | 1.058514 |
| GO:0010926\_anatomical\_structure\_formation | PAFAH1B1 | 447 | 4 | 1.420967 | -0.510290 | 296 | 313.32 | 1.058514 |
| GO:0007005\_mitochondrion\_organization | CXADR | 61 | 1 | 2.603166 | -0.492789 | 297 | 319.18 | 1.074680 |
| GO:0021537\_telencephalon\_development | PAFAH1B1 | 62 | 1 | 2.561179 | -0.486980 | 302 | 322.79 | 1.068841 |
| GO:0022604\_regulation\_of\_cell\_morphogenesis | FYN | 62 | 1 | 2.561179 | -0.486980 | 302 | 322.79 | 1.068841 |
| GO:0030155\_regulation\_of\_cell\_adhesion | TGM2 | 62 | 1 | 2.561179 | -0.486980 | 302 | 322.79 | 1.068841 |
| GO:0030855\_epithelial\_cell\_differentiation | FZD2 | 62 | 1 | 2.561179 | -0.486980 | 302 | 322.79 | 1.068841 |
| GO:0050954\_sensory\_perception\_of\_mechanical\_stimulus | FYN | 62 | 1 | 2.561179 | -0.486980 | 302 | 322.79 | 1.068841 |
| GO:0007010\_cytoskeleton\_organization | NAV1 | 185 | 2 | 1.716682 | -0.486723 | 303 | 322.98 | 1.065941 |
| GO:0007010\_cytoskeleton\_organization | PAFAH1B1 | 185 | 2 | 1.716682 | -0.486723 | 303 | 322.98 | 1.065941 |
| GO:0007369\_gastrulation | PRKAR1A | 63 | 1 | 2.520525 | -0.481282 | 304 | 325.05 | 1.069243 |
| GO:0010646\_regulation\_of\_cell\_communication | CD8A | 330 | 3 | 1.443574 | -0.461982 | 305 | 331.62 | 1.087279 |
| GO:0010646\_regulation\_of\_cell\_communication | PAFAH1B1 | 330 | 3 | 1.443574 | -0.461982 | 305 | 331.62 | 1.087279 |
| GO:0010646\_regulation\_of\_cell\_communication | GAS1 | 330 | 3 | 1.443574 | -0.461982 | 305 | 331.62 | 1.087279 |
| GO:0046907\_intracellular\_transport | KLC1 | 194 | 2 | 1.637042 | -0.459539 | 306 | 332.16 | 1.085490 |
| GO:0046907\_intracellular\_transport | PAFAH1B1 | 194 | 2 | 1.637042 | -0.459539 | 306 | 332.16 | 1.085490 |
| GO:0007507\_heart\_development | GNA11 | 195 | 2 | 1.628647 | -0.456634 | 307 | 334.36 | 1.089121 |
| GO:0007507\_heart\_development | CXADR | 195 | 2 | 1.628647 | -0.456634 | 307 | 334.36 | 1.089121 |
| GO:0034962\_cellular\_biopolymer\_catabolic\_process | CHFR | 68 | 1 | 2.335193 | -0.454350 | 309 | 335.56 | 1.085955 |
| GO:0042692\_muscle\_cell\_differentiation | LGALS1 | 68 | 1 | 2.335193 | -0.454350 | 309 | 335.56 | 1.085955 |
| GO:0007399\_nervous\_system\_development | NAV1 | 621 | 5 | 1.278527 | -0.453550 | 310 | 336.0 | 1.083871 |
| GO:0007399\_nervous\_system\_development | AGTPBP1 | 621 | 5 | 1.278527 | -0.453550 | 310 | 336.0 | 1.083871 |
| GO:0007399\_nervous\_system\_development | FYN | 621 | 5 | 1.278527 | -0.453550 | 310 | 336.0 | 1.083871 |
| GO:0007399\_nervous\_system\_development | PAFAH1B1 | 621 | 5 | 1.278527 | -0.453550 | 310 | 336.0 | 1.083871 |
| GO:0007399\_nervous\_system\_development | GAS1 | 621 | 5 | 1.278527 | -0.453550 | 310 | 336.0 | 1.083871 |
| GO:0008406\_gonad\_development | FST | 70 | 1 | 2.268473 | -0.444247 | 312 | 340.84 | 1.092436 |
| GO:0048592\_eye\_morphogenesis | GAS1 | 70 | 1 | 2.268473 | -0.444247 | 312 | 340.84 | 1.092436 |
| GO:0007264\_small\_GTPase\_mediated\_signal\_transduction | PAFAH1B1 | 72 | 1 | 2.205460 | -0.434494 | 317 | 346.87 | 1.094227 |
| GO:0021915\_neural\_tube\_development | GAS1 | 72 | 1 | 2.205460 | -0.434494 | 317 | 346.87 | 1.094227 |
| GO:0042098\_T\_cell\_proliferation | FYN | 72 | 1 | 2.205460 | -0.434494 | 317 | 346.87 | 1.094227 |
| GO:0048839\_inner\_ear\_development | INSIG1 | 72 | 1 | 2.205460 | -0.434494 | 317 | 346.87 | 1.094227 |
| GO:0050673\_epithelial\_cell\_proliferation | GAS1 | 72 | 1 | 2.205460 | -0.434494 | 317 | 346.87 | 1.094227 |
| GO:0048706\_embryonic\_skeletal\_system\_development | GAS1 | 73 | 1 | 2.175248 | -0.429742 | 318 | 348.89 | 1.097138 |
| GO:0007281\_germ\_cell\_development | PAFAH1B1 | 75 | 1 | 2.117241 | -0.420478 | 320 | 352.48 | 1.101500 |
| GO:0048589\_developmental\_growth | GAS1 | 75 | 1 | 2.117241 | -0.420478 | 320 | 352.48 | 1.101500 |
| GO:0009887\_organ\_morphogenesis | PRKAR1A | 642 | 5 | 1.236706 | -0.420457 | 321 | 352.61 | 1.098474 |
| GO:0009887\_organ\_morphogenesis | FST | 642 | 5 | 1.236706 | -0.420457 | 321 | 352.61 | 1.098474 |
| GO:0009887\_organ\_morphogenesis | INSIG1 | 642 | 5 | 1.236706 | -0.420457 | 321 | 352.61 | 1.098474 |
| GO:0009887\_organ\_morphogenesis | FZD2 | 642 | 5 | 1.236706 | -0.420457 | 321 | 352.61 | 1.098474 |
| GO:0009887\_organ\_morphogenesis | GAS1 | 642 | 5 | 1.236706 | -0.420457 | 321 | 352.61 | 1.098474 |
| GO:0006508\_proteolysis | CHFR | 76 | 1 | 2.089383 | -0.415961 | 322 | 354.12 | 1.099752 |
| GO:0051241\_negative\_regulation\_of\_multicellular\_organismal\_process | CXADR | 77 | 1 | 2.062248 | -0.411518 | 323 | 354.91 | 1.098793 |
| GO:0007519\_skeletal\_muscle\_tissue\_development | LGALS1 | 78 | 1 | 2.035809 | -0.407147 | 327 | 357.73 | 1.093976 |
| GO:0030326\_embryonic\_limb\_morphogenesis | GAS1 | 78 | 1 | 2.035809 | -0.407147 | 327 | 357.73 | 1.093976 |
| GO:0035113\_embryonic\_appendage\_morphogenesis | GAS1 | 78 | 1 | 2.035809 | -0.407147 | 327 | 357.73 | 1.093976 |
| GO:0060538\_skeletal\_muscle\_organ\_development | LGALS1 | 78 | 1 | 2.035809 | -0.407147 | 327 | 357.73 | 1.093976 |
| GO:0000278\_mitotic\_cell\_cycle | CHFR | 80 | 1 | 1.984914 | -0.398614 | 331 | 361.7 | 1.092749 |
| GO:0002250\_adaptive\_immune\_response | CD8A | 80 | 1 | 1.984914 | -0.398614 | 331 | 361.7 | 1.092749 |
| GO:0002460\_adaptive\_immune\_response\_based\_on\_somatic\_recombination\_of\_immune\_receptors\_built\_from\_immunoglobulin\_superfamily\_domains | CD8A | 80 | 1 | 1.984914 | -0.398614 | 331 | 361.7 | 1.092749 |
| GO:0006631\_fatty\_acid\_metabolic\_process | INSIG1 | 80 | 1 | 1.984914 | -0.398614 | 331 | 361.7 | 1.092749 |
| GO:0007423\_sensory\_organ\_development | INSIG1 | 219 | 2 | 1.450165 | -0.393068 | 332 | 364.07 | 1.096596 |
| GO:0007423\_sensory\_organ\_development | GAS1 | 219 | 2 | 1.450165 | -0.393068 | 332 | 364.07 | 1.096596 |
| GO:0016043\_cellular\_component\_organization | SGCG | 964 | 7 | 1.153062 | -0.391823 | 333 | 364.37 | 1.094204 |
| GO:0016043\_cellular\_component\_organization | NAV1 | 964 | 7 | 1.153062 | -0.391823 | 333 | 364.37 | 1.094204 |
| GO:0016043\_cellular\_component\_organization | FYN | 964 | 7 | 1.153062 | -0.391823 | 333 | 364.37 | 1.094204 |
| GO:0016043\_cellular\_component\_organization | PAFAH1B1 | 964 | 7 | 1.153062 | -0.391823 | 333 | 364.37 | 1.094204 |
| GO:0016043\_cellular\_component\_organization | GAS1 | 964 | 7 | 1.153062 | -0.391823 | 333 | 364.37 | 1.094204 |
| GO:0016043\_cellular\_component\_organization | CXADR | 964 | 7 | 1.153062 | -0.391823 | 333 | 364.37 | 1.094204 |
| GO:0016043\_cellular\_component\_organization | AMPH | 964 | 7 | 1.153062 | -0.391823 | 333 | 364.37 | 1.094204 |
| GO:0007411\_axon\_guidance | GAS1 | 82 | 1 | 1.936501 | -0.390347 | 334 | 366.85 | 1.098353 |
| GO:0030534\_adult\_behavior | PAFAH1B1 | 83 | 1 | 1.913170 | -0.386310 | 335 | 369.01 | 1.101522 |
| GO:0030005\_cellular\_di-\_\_tri-valent\_inorganic\_cation\_homeostasis | PRNP | 84 | 1 | 1.890394 | -0.382334 | 337 | 370.06 | 1.098101 |
| GO:0045137\_development\_of\_primary\_sexual\_characteristics | FST | 84 | 1 | 1.890394 | -0.382334 | 337 | 370.06 | 1.098101 |
| GO:0002449\_lymphocyte\_mediated\_immunity | CD8A | 85 | 1 | 1.868154 | -0.378419 | 338 | 371.41 | 1.098846 |
| GO:0006897\_endocytosis | AMPH | 86 | 1 | 1.846431 | -0.374563 | 340 | 373.49 | 1.098500 |
| GO:0010324\_membrane\_invagination | AMPH | 86 | 1 | 1.846431 | -0.374563 | 340 | 373.49 | 1.098500 |
| GO:0046649\_lymphocyte\_activation | CD8A | 228 | 2 | 1.392922 | -0.371950 | 341 | 374.27 | 1.097566 |
| GO:0046649\_lymphocyte\_activation | FYN | 228 | 2 | 1.392922 | -0.371950 | 341 | 374.27 | 1.097566 |
| GO:0007178\_transmembrane\_receptor\_protein\_serine\_threonine\_kinase\_signaling\_pathway | FST | 87 | 1 | 1.825208 | -0.370764 | 343 | 376.9 | 1.098834 |
| GO:0022612\_gland\_morphogenesis | TGM2 | 87 | 1 | 1.825208 | -0.370764 | 343 | 376.9 | 1.098834 |
| GO:0022414\_reproductive\_process | FST | 376 | 3 | 1.266966 | -0.370406 | 344 | 377.18 | 1.096453 |
| GO:0022414\_reproductive\_process | PAFAH1B1 | 376 | 3 | 1.266966 | -0.370406 | 344 | 377.18 | 1.096453 |
| GO:0022414\_reproductive\_process | HERPUD2 | 376 | 3 | 1.266966 | -0.370406 | 344 | 377.18 | 1.096453 |
| GO:0001503\_ossification | IGFBP5 | 88 | 1 | 1.804467 | -0.367022 | 345 | 378.5 | 1.097101 |
| GO:0000003\_reproduction | FST | 379 | 3 | 1.256937 | -0.365131 | 346 | 379.24 | 1.096069 |
| GO:0000003\_reproduction | PAFAH1B1 | 379 | 3 | 1.256937 | -0.365131 | 346 | 379.24 | 1.096069 |
| GO:0000003\_reproduction | HERPUD2 | 379 | 3 | 1.256937 | -0.365131 | 346 | 379.24 | 1.096069 |
| GO:0050790\_regulation\_of\_catalytic\_activity | GNA11 | 233 | 2 | 1.363031 | -0.360784 | 347 | 380.04 | 1.095216 |
| GO:0050790\_regulation\_of\_catalytic\_activity | PAFAH1B1 | 233 | 2 | 1.363031 | -0.360784 | 347 | 380.04 | 1.095216 |
| GO:0030003\_cellular\_cation\_homeostasis | PRNP | 90 | 1 | 1.764368 | -0.359700 | 348 | 381.39 | 1.095948 |
| GO:0002443\_leukocyte\_mediated\_immunity | CD8A | 91 | 1 | 1.744979 | -0.356118 | 350 | 383.0 | 1.094286 |
| GO:0008544\_epidermis\_development | FST | 91 | 1 | 1.744979 | -0.356118 | 350 | 383.0 | 1.094286 |
| GO:0030217\_T\_cell\_differentiation | CD8A | 92 | 1 | 1.726012 | -0.352588 | 351 | 384.58 | 1.095670 |
| GO:0006468\_protein\_amino\_acid\_phosphorylation | FYN | 237 | 2 | 1.340026 | -0.352126 | 352 | 385.17 | 1.094233 |
| GO:0006468\_protein\_amino\_acid\_phosphorylation | PRKAR1A | 237 | 2 | 1.340026 | -0.352126 | 352 | 385.17 | 1.094233 |
| GO:0007049\_cell\_cycle | GAS1 | 238 | 2 | 1.334396 | -0.349999 | 353 | 385.71 | 1.092663 |
| GO:0007049\_cell\_cycle | CHFR | 238 | 2 | 1.334396 | -0.349999 | 353 | 385.71 | 1.092663 |
| GO:0035107\_appendage\_morphogenesis | GAS1 | 93 | 1 | 1.707453 | -0.349108 | 356 | 387.3 | 1.087921 |
| GO:0035108\_limb\_morphogenesis | GAS1 | 93 | 1 | 1.707453 | -0.349108 | 356 | 387.3 | 1.087921 |
| GO:0055066\_di-\_\_tri-valent\_inorganic\_cation\_homeostasis | PRNP | 93 | 1 | 1.707453 | -0.349108 | 356 | 387.3 | 1.087921 |
| GO:0065008\_regulation\_of\_biological\_quality | LDLR | 693 | 5 | 1.145693 | -0.349086 | 357 | 387.41 | 1.085182 |
| GO:0065008\_regulation\_of\_biological\_quality | FYN | 693 | 5 | 1.145693 | -0.349086 | 357 | 387.41 | 1.085182 |
| GO:0065008\_regulation\_of\_biological\_quality | AGTPBP1 | 693 | 5 | 1.145693 | -0.349086 | 357 | 387.41 | 1.085182 |
| GO:0065008\_regulation\_of\_biological\_quality | GNA11 | 693 | 5 | 1.145693 | -0.349086 | 357 | 387.41 | 1.085182 |
| GO:0065008\_regulation\_of\_biological\_quality | PRNP | 693 | 5 | 1.145693 | -0.349086 | 357 | 387.41 | 1.085182 |
| GO:0032943\_mononuclear\_cell\_proliferation | FYN | 94 | 1 | 1.689288 | -0.345676 | 359 | 389.66 | 1.085404 |
| GO:0046651\_lymphocyte\_proliferation | FYN | 94 | 1 | 1.689288 | -0.345676 | 359 | 389.66 | 1.085404 |
| GO:0009056\_catabolic\_process | LDLR | 243 | 2 | 1.306939 | -0.339579 | 360 | 390.9 | 1.085833 |
| GO:0009056\_catabolic\_process | CHFR | 243 | 2 | 1.306939 | -0.339579 | 360 | 390.9 | 1.085833 |
| GO:0048736\_appendage\_development | GAS1 | 96 | 1 | 1.654095 | -0.338956 | 363 | 392.55 | 1.081405 |
| GO:0060173\_limb\_development | GAS1 | 96 | 1 | 1.654095 | -0.338956 | 363 | 392.55 | 1.081405 |
| GO:0070661\_leukocyte\_proliferation | FYN | 96 | 1 | 1.654095 | -0.338956 | 363 | 392.55 | 1.081405 |
| GO:0018193\_peptidyl-amino\_acid\_modification | FYN | 97 | 1 | 1.637042 | -0.335666 | 364 | 394.0 | 1.082418 |
| GO:0007548\_sex\_differentiation | FST | 98 | 1 | 1.620338 | -0.332420 | 365 | 395.68 | 1.084055 |
| GO:0045321\_leukocyte\_activation | CD8A | 248 | 2 | 1.280590 | -0.329507 | 366 | 396.18 | 1.082459 |
| GO:0045321\_leukocyte\_activation | FYN | 248 | 2 | 1.280590 | -0.329507 | 366 | 396.18 | 1.082459 |
| GO:0007398\_ectoderm\_development | FST | 99 | 1 | 1.603971 | -0.329219 | 367 | 397.56 | 1.083270 |
| GO:0007389\_pattern\_specification\_process | FST | 250 | 2 | 1.270345 | -0.325572 | 368 | 398.56 | 1.083043 |
| GO:0007389\_pattern\_specification\_process | GAS1 | 250 | 2 | 1.270345 | -0.325572 | 368 | 398.56 | 1.083043 |
| GO:0030163\_protein\_catabolic\_process | CHFR | 101 | 1 | 1.572209 | -0.322944 | 369 | 399.34 | 1.082222 |
| GO:0030036\_actin\_cytoskeleton\_organization | PAFAH1B1 | 102 | 1 | 1.556795 | -0.319869 | 370 | 400.03 | 1.081162 |
| GO:0009968\_negative\_regulation\_of\_signal\_transduction | GAS1 | 103 | 1 | 1.541681 | -0.316835 | 371 | 401.94 | 1.083396 |
| GO:0009790\_embryonic\_development | INSIG1 | 567 | 4 | 1.120234 | -0.312613 | 372 | 404.08 | 1.086237 |
| GO:0009790\_embryonic\_development | PRKAR1A | 567 | 4 | 1.120234 | -0.312613 | 372 | 404.08 | 1.086237 |
| GO:0009790\_embryonic\_development | PITPNB | 567 | 4 | 1.120234 | -0.312613 | 372 | 404.08 | 1.086237 |
| GO:0009790\_embryonic\_development | GAS1 | 567 | 4 | 1.120234 | -0.312613 | 372 | 404.08 | 1.086237 |
| GO:0001775\_cell\_activation | CD8A | 262 | 2 | 1.212161 | -0.303035 | 373 | 406.79 | 1.090590 |
| GO:0001775\_cell\_activation | FYN | 262 | 2 | 1.212161 | -0.303035 | 373 | 406.79 | 1.090590 |
| GO:0030029\_actin\_filament-based\_process | PAFAH1B1 | 109 | 1 | 1.456817 | -0.299437 | 374 | 408.89 | 1.093289 |
| GO:0010648\_negative\_regulation\_of\_cell\_communication | GAS1 | 110 | 1 | 1.443574 | -0.296665 | 377 | 410.59 | 1.089098 |
| GO:0043010\_camera-type\_eye\_development | GAS1 | 110 | 1 | 1.443574 | -0.296665 | 377 | 410.59 | 1.089098 |
| GO:0055080\_cation\_homeostasis | PRNP | 110 | 1 | 1.443574 | -0.296665 | 377 | 410.59 | 1.089098 |
| GO:0051179\_localization | NAV1 | 1058 | 7 | 1.050616 | -0.290189 | 378 | 412.77 | 1.091984 |
| GO:0051179\_localization | LDLR | 1058 | 7 | 1.050616 | -0.290189 | 378 | 412.77 | 1.091984 |
| GO:0051179\_localization | FYN | 1058 | 7 | 1.050616 | -0.290189 | 378 | 412.77 | 1.091984 |
| GO:0051179\_localization | KLC1 | 1058 | 7 | 1.050616 | -0.290189 | 378 | 412.77 | 1.091984 |
| GO:0051179\_localization | PAFAH1B1 | 1058 | 7 | 1.050616 | -0.290189 | 378 | 412.77 | 1.091984 |
| GO:0051179\_localization | GAS1 | 1058 | 7 | 1.050616 | -0.290189 | 378 | 412.77 | 1.091984 |
| GO:0051179\_localization | AMPH | 1058 | 7 | 1.050616 | -0.290189 | 378 | 412.77 | 1.091984 |
| GO:0048608\_reproductive\_structure\_development | FST | 116 | 1 | 1.368906 | -0.280740 | 379 | 419.29 | 1.106306 |
| GO:0007610\_behavior | PAFAH1B1 | 279 | 2 | 1.138302 | -0.273995 | 381 | 423.25 | 1.110892 |
| GO:0007610\_behavior | AMPH | 279 | 2 | 1.138302 | -0.273995 | 381 | 423.25 | 1.110892 |
| GO:0065009\_regulation\_of\_molecular\_function | GNA11 | 279 | 2 | 1.138302 | -0.273995 | 381 | 423.25 | 1.110892 |
| GO:0065009\_regulation\_of\_molecular\_function | PAFAH1B1 | 279 | 2 | 1.138302 | -0.273995 | 381 | 423.25 | 1.110892 |
| GO:0000902\_cell\_morphogenesis | FYN | 283 | 2 | 1.122213 | -0.267611 | 382 | 427.0 | 1.117801 |
| GO:0000902\_cell\_morphogenesis | GAS1 | 283 | 2 | 1.122213 | -0.267611 | 382 | 427.0 | 1.117801 |
| GO:0002252\_immune\_effector\_process | CD8A | 122 | 1 | 1.301583 | -0.265935 | 383 | 429.46 | 1.121305 |
| GO:0006996\_organelle\_organization | NAV1 | 449 | 3 | 1.060978 | -0.261430 | 384 | 430.52 | 1.121146 |
| GO:0006996\_organelle\_organization | PAFAH1B1 | 449 | 3 | 1.060978 | -0.261430 | 384 | 430.52 | 1.121146 |
| GO:0006996\_organelle\_organization | CXADR | 449 | 3 | 1.060978 | -0.261430 | 384 | 430.52 | 1.121146 |
| GO:0030098\_lymphocyte\_differentiation | CD8A | 124 | 1 | 1.280590 | -0.261229 | 385 | 431.46 | 1.120675 |
| GO:0001763\_morphogenesis\_of\_a\_branching\_structure | TGM2 | 125 | 1 | 1.270345 | -0.258917 | 386 | 432.72 | 1.121036 |
| GO:0048523\_negative\_regulation\_of\_cellular\_process | FST | 774 | 5 | 1.025795 | -0.257911 | 387 | 433.04 | 1.118966 |
| GO:0048523\_negative\_regulation\_of\_cellular\_process | INSIG1 | 774 | 5 | 1.025795 | -0.257911 | 387 | 433.04 | 1.118966 |
| GO:0048523\_negative\_regulation\_of\_cellular\_process | GAS1 | 774 | 5 | 1.025795 | -0.257911 | 387 | 433.04 | 1.118966 |
| GO:0048523\_negative\_regulation\_of\_cellular\_process | PRNP | 774 | 5 | 1.025795 | -0.257911 | 387 | 433.04 | 1.118966 |
| GO:0048523\_negative\_regulation\_of\_cellular\_process | CXADR | 774 | 5 | 1.025795 | -0.257911 | 387 | 433.04 | 1.118966 |
| GO:0043285\_biopolymer\_catabolic\_process | CHFR | 129 | 1 | 1.230954 | -0.249929 | 388 | 435.95 | 1.123582 |
| GO:0032787\_monocarboxylic\_acid\_metabolic\_process | INSIG1 | 130 | 1 | 1.221485 | -0.247746 | 390 | 438.08 | 1.123282 |
| GO:0045165\_cell\_fate\_commitment | GAS1 | 130 | 1 | 1.221485 | -0.247746 | 390 | 438.08 | 1.123282 |
| GO:0050896\_response\_to\_stimulus | CD8A | 1107 | 7 | 1.004112 | -0.246615 | 391 | 438.32 | 1.121023 |
| GO:0050896\_response\_to\_stimulus | FYN | 1107 | 7 | 1.004112 | -0.246615 | 391 | 438.32 | 1.121023 |
| GO:0050896\_response\_to\_stimulus | INSIG1 | 1107 | 7 | 1.004112 | -0.246615 | 391 | 438.32 | 1.121023 |
| GO:0050896\_response\_to\_stimulus | PAFAH1B1 | 1107 | 7 | 1.004112 | -0.246615 | 391 | 438.32 | 1.121023 |
| GO:0050896\_response\_to\_stimulus | PDIA5 | 1107 | 7 | 1.004112 | -0.246615 | 391 | 438.32 | 1.121023 |
| GO:0050896\_response\_to\_stimulus | PRNP | 1107 | 7 | 1.004112 | -0.246615 | 391 | 438.32 | 1.121023 |
| GO:0050896\_response\_to\_stimulus | AMPH | 1107 | 7 | 1.004112 | -0.246615 | 391 | 438.32 | 1.121023 |
| GO:0001654\_eye\_development | GAS1 | 136 | 1 | 1.167596 | -0.235145 | 392 | 441.11 | 1.125281 |
| GO:0016310\_phosphorylation | FYN | 309 | 2 | 1.027787 | -0.229815 | 393 | 443.53 | 1.128575 |
| GO:0016310\_phosphorylation | PRKAR1A | 309 | 2 | 1.027787 | -0.229815 | 393 | 443.53 | 1.128575 |
| GO:0003006\_reproductive\_developmental\_process | FST | 141 | 1 | 1.126192 | -0.225260 | 394 | 445.95 | 1.131853 |
| GO:0035239\_tube\_morphogenesis | TGM2 | 143 | 1 | 1.110441 | -0.221453 | 395 | 446.45 | 1.130253 |
| GO:0022603\_regulation\_of\_anatomical\_structure\_morphogenesis | FYN | 147 | 1 | 1.080225 | -0.214077 | 396 | 449.32 | 1.134646 |
| GO:0043085\_positive\_regulation\_of\_catalytic\_activity | GNA11 | 148 | 1 | 1.072926 | -0.212280 | 397 | 450.39 | 1.134484 |
| GO:0022402\_cell\_cycle\_process | GAS1 | 155 | 1 | 1.024472 | -0.200214 | 398 | 454.97 | 1.143141 |
| GO:0044237\_cellular\_metabolic\_process | MOGAT2 | 1974 | 12 | 0.965308 | -0.198108 | 399 | 455.29 | 1.141078 |
| GO:0044237\_cellular\_metabolic\_process | ZDHHC17 | 1974 | 12 | 0.965308 | -0.198108 | 399 | 455.29 | 1.141078 |
| GO:0044237\_cellular\_metabolic\_process | MRPL12 | 1974 | 12 | 0.965308 | -0.198108 | 399 | 455.29 | 1.141078 |
| GO:0044237\_cellular\_metabolic\_process | LDLR | 1974 | 12 | 0.965308 | -0.198108 | 399 | 455.29 | 1.141078 |
| GO:0044237\_cellular\_metabolic\_process | DNAJB11 | 1974 | 12 | 0.965308 | -0.198108 | 399 | 455.29 | 1.141078 |
| GO:0044237\_cellular\_metabolic\_process | AGTPBP1 | 1974 | 12 | 0.965308 | -0.198108 | 399 | 455.29 | 1.141078 |
| GO:0044237\_cellular\_metabolic\_process | FYN | 1974 | 12 | 0.965308 | -0.198108 | 399 | 455.29 | 1.141078 |
| GO:0044237\_cellular\_metabolic\_process | PRKAR1A | 1974 | 12 | 0.965308 | -0.198108 | 399 | 455.29 | 1.141078 |
| GO:0044237\_cellular\_metabolic\_process | INSIG1 | 1974 | 12 | 0.965308 | -0.198108 | 399 | 455.29 | 1.141078 |
| GO:0044237\_cellular\_metabolic\_process | TGM2 | 1974 | 12 | 0.965308 | -0.198108 | 399 | 455.29 | 1.141078 |
| GO:0044237\_cellular\_metabolic\_process | CHFR | 1974 | 12 | 0.965308 | -0.198108 | 399 | 455.29 | 1.141078 |
| GO:0044237\_cellular\_metabolic\_process | PRNP | 1974 | 12 | 0.965308 | -0.198108 | 399 | 455.29 | 1.141078 |
| GO:0007409\_axonogenesis | GAS1 | 158 | 1 | 1.005020 | -0.195300 | 400 | 456.99 | 1.142475 |
| GO:0051128\_regulation\_of\_cellular\_component\_organization | FYN | 160 | 1 | 0.992457 | -0.192106 | 401 | 458.11 | 1.142419 |
| GO:0006793\_phosphorus\_metabolic\_process | FYN | 340 | 2 | 0.934077 | -0.191971 | 403 | 458.67 | 1.138139 |
| GO:0006793\_phosphorus\_metabolic\_process | PRKAR1A | 340 | 2 | 0.934077 | -0.191971 | 403 | 458.67 | 1.138139 |
| GO:0006796\_phosphate\_metabolic\_process | FYN | 340 | 2 | 0.934077 | -0.191971 | 403 | 458.67 | 1.138139 |
| GO:0006796\_phosphate\_metabolic\_process | PRKAR1A | 340 | 2 | 0.934077 | -0.191971 | 403 | 458.67 | 1.138139 |
| GO:0002521\_leukocyte\_differentiation | CD8A | 161 | 1 | 0.986293 | -0.190532 | 404 | 459.01 | 1.136163 |
| GO:0051649\_establishment\_of\_localization\_in\_cell | KLC1 | 342 | 2 | 0.928615 | -0.189763 | 405 | 459.31 | 1.134099 |
| GO:0051649\_establishment\_of\_localization\_in\_cell | PAFAH1B1 | 342 | 2 | 0.928615 | -0.189763 | 405 | 459.31 | 1.134099 |
| GO:0009628\_response\_to\_abiotic\_stimulus | FYN | 162 | 1 | 0.980204 | -0.188974 | 406 | 460.0 | 1.133005 |
| GO:0007626\_locomotory\_behavior | PAFAH1B1 | 163 | 1 | 0.974191 | -0.187431 | 407 | 460.69 | 1.131916 |
| GO:0048519\_negative\_regulation\_of\_biological\_process | FST | 859 | 5 | 0.924290 | -0.185517 | 408 | 461.26 | 1.130539 |
| GO:0048519\_negative\_regulation\_of\_biological\_process | INSIG1 | 859 | 5 | 0.924290 | -0.185517 | 408 | 461.26 | 1.130539 |
| GO:0048519\_negative\_regulation\_of\_biological\_process | GAS1 | 859 | 5 | 0.924290 | -0.185517 | 408 | 461.26 | 1.130539 |
| GO:0048519\_negative\_regulation\_of\_biological\_process | PRNP | 859 | 5 | 0.924290 | -0.185517 | 408 | 461.26 | 1.130539 |
| GO:0048519\_negative\_regulation\_of\_biological\_process | CXADR | 859 | 5 | 0.924290 | -0.185517 | 408 | 461.26 | 1.130539 |
| GO:0048812\_neuron\_projection\_morphogenesis | GAS1 | 170 | 1 | 0.934077 | -0.177046 | 409 | 466.46 | 1.140489 |
| GO:0030182\_neuron\_differentiation | AGTPBP1 | 356 | 2 | 0.892096 | -0.175022 | 410 | 466.84 | 1.138634 |
| GO:0030182\_neuron\_differentiation | GAS1 | 356 | 2 | 0.892096 | -0.175022 | 410 | 466.84 | 1.138634 |
| GO:0007600\_sensory\_perception | FYN | 172 | 1 | 0.923216 | -0.174206 | 411 | 467.59 | 1.137689 |
| GO:0044093\_positive\_regulation\_of\_molecular\_function | GNA11 | 173 | 1 | 0.917879 | -0.172806 | 413 | 468.75 | 1.134988 |
| GO:0048667\_cell\_morphogenesis\_involved\_in\_neuron\_differentiation | GAS1 | 173 | 1 | 0.917879 | -0.172806 | 413 | 468.75 | 1.134988 |
| GO:0042981\_regulation\_of\_apoptosis | GAS1 | 360 | 2 | 0.882184 | -0.171029 | 414 | 469.05 | 1.132971 |
| GO:0042981\_regulation\_of\_apoptosis | PRNP | 360 | 2 | 0.882184 | -0.171029 | 414 | 469.05 | 1.132971 |
| GO:0043066\_negative\_regulation\_of\_apoptosis | PRNP | 176 | 1 | 0.902234 | -0.168686 | 416 | 471.77 | 1.134062 |
| GO:0048858\_cell\_projection\_morphogenesis | GAS1 | 176 | 1 | 0.902234 | -0.168686 | 416 | 471.77 | 1.134062 |
| GO:0010941\_regulation\_of\_cell\_death | GAS1 | 365 | 2 | 0.870099 | -0.166168 | 419 | 472.66 | 1.128067 |
| GO:0010941\_regulation\_of\_cell\_death | PRNP | 365 | 2 | 0.870099 | -0.166168 | 419 | 472.66 | 1.128067 |
| GO:0043009\_chordate\_embryonic\_development | PITPNB | 365 | 2 | 0.870099 | -0.166168 | 419 | 472.66 | 1.128067 |
| GO:0043009\_chordate\_embryonic\_development | GAS1 | 365 | 2 | 0.870099 | -0.166168 | 419 | 472.66 | 1.128067 |
| GO:0043067\_regulation\_of\_programmed\_cell\_death | GAS1 | 365 | 2 | 0.870099 | -0.166168 | 419 | 472.66 | 1.128067 |
| GO:0043067\_regulation\_of\_programmed\_cell\_death | PRNP | 365 | 2 | 0.870099 | -0.166168 | 419 | 472.66 | 1.128067 |
| GO:0006810\_transport | LDLR | 718 | 4 | 0.884641 | -0.165083 | 420 | 473.28 | 1.126857 |
| GO:0006810\_transport | KLC1 | 718 | 4 | 0.884641 | -0.165083 | 420 | 473.28 | 1.126857 |
| GO:0006810\_transport | PAFAH1B1 | 718 | 4 | 0.884641 | -0.165083 | 420 | 473.28 | 1.126857 |
| GO:0006810\_transport | AMPH | 718 | 4 | 0.884641 | -0.165083 | 420 | 473.28 | 1.126857 |
| GO:0043069\_negative\_regulation\_of\_programmed\_cell\_death | PRNP | 179 | 1 | 0.887112 | -0.164680 | 423 | 474.52 | 1.121797 |
| GO:0048732\_gland\_development | TGM2 | 179 | 1 | 0.887112 | -0.164680 | 423 | 474.52 | 1.121797 |
| GO:0060548\_negative\_regulation\_of\_cell\_death | PRNP | 179 | 1 | 0.887112 | -0.164680 | 423 | 474.52 | 1.121797 |
| GO:0009792\_embryonic\_development\_ending\_in\_birth\_or\_egg\_hatching | PITPNB | 368 | 2 | 0.863006 | -0.163319 | 424 | 475.63 | 1.121769 |
| GO:0009792\_embryonic\_development\_ending\_in\_birth\_or\_egg\_hatching | GAS1 | 368 | 2 | 0.863006 | -0.163319 | 424 | 475.63 | 1.121769 |
| GO:0019752\_carboxylic\_acid\_metabolic\_process | INSIG1 | 181 | 1 | 0.877310 | -0.162072 | 426 | 476.89 | 1.119460 |
| GO:0043436\_oxoacid\_metabolic\_process | INSIG1 | 181 | 1 | 0.877310 | -0.162072 | 426 | 476.89 | 1.119460 |
| GO:0006950\_response\_to\_stress | INSIG1 | 549 | 3 | 0.867722 | -0.161509 | 427 | 477.15 | 1.117447 |
| GO:0006950\_response\_to\_stress | PDIA5 | 549 | 3 | 0.867722 | -0.161509 | 427 | 477.15 | 1.117447 |
| GO:0006950\_response\_to\_stress | PRNP | 549 | 3 | 0.867722 | -0.161509 | 427 | 477.15 | 1.117447 |
| GO:0051641\_cellular\_localization | KLC1 | 370 | 2 | 0.858341 | -0.161447 | 428 | 477.5 | 1.115654 |
| GO:0051641\_cellular\_localization | PAFAH1B1 | 370 | 2 | 0.858341 | -0.161447 | 428 | 477.5 | 1.115654 |
| GO:0006082\_organic\_acid\_metabolic\_process | INSIG1 | 182 | 1 | 0.872490 | -0.160787 | 429 | 477.84 | 1.113846 |
| GO:0042180\_cellular\_ketone\_metabolic\_process | INSIG1 | 183 | 1 | 0.867722 | -0.159513 | 430 | 478.79 | 1.113465 |
| GO:0016192\_vesicle-mediated\_transport | AMPH | 184 | 1 | 0.863006 | -0.158250 | 432 | 479.44 | 1.109815 |
| GO:0032990\_cell\_part\_morphogenesis | GAS1 | 184 | 1 | 0.863006 | -0.158250 | 432 | 479.44 | 1.109815 |
| GO:0051234\_establishment\_of\_localization | LDLR | 729 | 4 | 0.871293 | -0.157349 | 433 | 479.63 | 1.107691 |
| GO:0051234\_establishment\_of\_localization | KLC1 | 729 | 4 | 0.871293 | -0.157349 | 433 | 479.63 | 1.107691 |
| GO:0051234\_establishment\_of\_localization | PAFAH1B1 | 729 | 4 | 0.871293 | -0.157349 | 433 | 479.63 | 1.107691 |
| GO:0051234\_establishment\_of\_localization | AMPH | 729 | 4 | 0.871293 | -0.157349 | 433 | 479.63 | 1.107691 |
| GO:0007155\_cell\_adhesion | TGM2 | 186 | 1 | 0.853726 | -0.155761 | 435 | 481.62 | 1.107172 |
| GO:0022610\_biological\_adhesion | TGM2 | 186 | 1 | 0.853726 | -0.155761 | 435 | 481.62 | 1.107172 |
| GO:0044238\_primary\_metabolic\_process | MOGAT2 | 1905 | 11 | 0.916916 | -0.147893 | 436 | 484.35 | 1.110894 |
| GO:0044238\_primary\_metabolic\_process | ZDHHC17 | 1905 | 11 | 0.916916 | -0.147893 | 436 | 484.35 | 1.110894 |
| GO:0044238\_primary\_metabolic\_process | MRPL12 | 1905 | 11 | 0.916916 | -0.147893 | 436 | 484.35 | 1.110894 |
| GO:0044238\_primary\_metabolic\_process | LDLR | 1905 | 11 | 0.916916 | -0.147893 | 436 | 484.35 | 1.110894 |
| GO:0044238\_primary\_metabolic\_process | DNAJB11 | 1905 | 11 | 0.916916 | -0.147893 | 436 | 484.35 | 1.110894 |
| GO:0044238\_primary\_metabolic\_process | FYN | 1905 | 11 | 0.916916 | -0.147893 | 436 | 484.35 | 1.110894 |
| GO:0044238\_primary\_metabolic\_process | PRKAR1A | 1905 | 11 | 0.916916 | -0.147893 | 436 | 484.35 | 1.110894 |
| GO:0044238\_primary\_metabolic\_process | INSIG1 | 1905 | 11 | 0.916916 | -0.147893 | 436 | 484.35 | 1.110894 |
| GO:0044238\_primary\_metabolic\_process | TGM2 | 1905 | 11 | 0.916916 | -0.147893 | 436 | 484.35 | 1.110894 |
| GO:0044238\_primary\_metabolic\_process | CHFR | 1905 | 11 | 0.916916 | -0.147893 | 436 | 484.35 | 1.110894 |
| GO:0044238\_primary\_metabolic\_process | PRNP | 1905 | 11 | 0.916916 | -0.147893 | 436 | 484.35 | 1.110894 |
| GO:0003002\_regionalization | GAS1 | 195 | 1 | 0.814324 | -0.145103 | 437 | 486.71 | 1.113753 |
| GO:0031175\_neuron\_projection\_development | GAS1 | 197 | 1 | 0.806056 | -0.142850 | 438 | 488.1 | 1.114384 |
| GO:0002009\_morphogenesis\_of\_an\_epithelium | FZD2 | 198 | 1 | 0.801985 | -0.141739 | 440 | 489.09 | 1.111568 |
| GO:0060429\_epithelium\_development | FZD2 | 198 | 1 | 0.801985 | -0.141739 | 440 | 489.09 | 1.111568 |
| GO:0042127\_regulation\_of\_cell\_proliferation | GAS1 | 393 | 2 | 0.808107 | -0.141414 | 441 | 489.63 | 1.110272 |
| GO:0042127\_regulation\_of\_cell\_proliferation | CXADR | 393 | 2 | 0.808107 | -0.141414 | 441 | 489.63 | 1.110272 |
| GO:0000904\_cell\_morphogenesis\_involved\_in\_differentiation | GAS1 | 199 | 1 | 0.797955 | -0.140638 | 442 | 489.97 | 1.108529 |
| GO:0022607\_cellular\_component\_assembly | PAFAH1B1 | 204 | 1 | 0.778398 | -0.135274 | 443 | 491.59 | 1.109684 |
| GO:0006955\_immune\_response | CD8A | 205 | 1 | 0.774601 | -0.134230 | 445 | 492.37 | 1.106449 |
| GO:0007243\_protein\_kinase\_cascade | AZI2 | 205 | 1 | 0.774601 | -0.134230 | 445 | 492.37 | 1.106449 |
| GO:0044260\_cellular\_macromolecule\_metabolic\_process | ZDHHC17 | 1447 | 8 | 0.877916 | -0.133110 | 446 | 492.7 | 1.104709 |
| GO:0044260\_cellular\_macromolecule\_metabolic\_process | MRPL12 | 1447 | 8 | 0.877916 | -0.133110 | 446 | 492.7 | 1.104709 |
| GO:0044260\_cellular\_macromolecule\_metabolic\_process | LDLR | 1447 | 8 | 0.877916 | -0.133110 | 446 | 492.7 | 1.104709 |
| GO:0044260\_cellular\_macromolecule\_metabolic\_process | DNAJB11 | 1447 | 8 | 0.877916 | -0.133110 | 446 | 492.7 | 1.104709 |
| GO:0044260\_cellular\_macromolecule\_metabolic\_process | FYN | 1447 | 8 | 0.877916 | -0.133110 | 446 | 492.7 | 1.104709 |
| GO:0044260\_cellular\_macromolecule\_metabolic\_process | PRKAR1A | 1447 | 8 | 0.877916 | -0.133110 | 446 | 492.7 | 1.104709 |
| GO:0044260\_cellular\_macromolecule\_metabolic\_process | TGM2 | 1447 | 8 | 0.877916 | -0.133110 | 446 | 492.7 | 1.104709 |
| GO:0044260\_cellular\_macromolecule\_metabolic\_process | CHFR | 1447 | 8 | 0.877916 | -0.133110 | 446 | 492.7 | 1.104709 |
| GO:0008284\_positive\_regulation\_of\_cell\_proliferation | GAS1 | 208 | 1 | 0.763428 | -0.131150 | 447 | 493.78 | 1.104653 |
| GO:0042221\_response\_to\_chemical\_stimulus | FYN | 409 | 2 | 0.776494 | -0.128965 | 448 | 494.76 | 1.104375 |
| GO:0042221\_response\_to\_chemical\_stimulus | PRNP | 409 | 2 | 0.776494 | -0.128965 | 448 | 494.76 | 1.104375 |
| GO:0035295\_tube\_development | TGM2 | 212 | 1 | 0.749024 | -0.127168 | 449 | 495.72 | 1.104053 |
| GO:0010033\_response\_to\_organic\_substance | FYN | 216 | 1 | 0.735153 | -0.123321 | 450 | 496.34 | 1.102978 |
| GO:0040007\_growth | GAS1 | 217 | 1 | 0.731765 | -0.122380 | 451 | 497.01 | 1.102018 |
| GO:0001701\_in\_utero\_embryonic\_development | PITPNB | 221 | 1 | 0.718521 | -0.118694 | 452 | 498.96 | 1.103894 |
| GO:0008152\_metabolic\_process | MOGAT2 | 2133 | 12 | 0.893351 | -0.117009 | 453 | 499.1 | 1.101766 |
| GO:0008152\_metabolic\_process | ZDHHC17 | 2133 | 12 | 0.893351 | -0.117009 | 453 | 499.1 | 1.101766 |
| GO:0008152\_metabolic\_process | MRPL12 | 2133 | 12 | 0.893351 | -0.117009 | 453 | 499.1 | 1.101766 |
| GO:0008152\_metabolic\_process | DNAJB11 | 2133 | 12 | 0.893351 | -0.117009 | 453 | 499.1 | 1.101766 |
| GO:0008152\_metabolic\_process | LDLR | 2133 | 12 | 0.893351 | -0.117009 | 453 | 499.1 | 1.101766 |
| GO:0008152\_metabolic\_process | AGTPBP1 | 2133 | 12 | 0.893351 | -0.117009 | 453 | 499.1 | 1.101766 |
| GO:0008152\_metabolic\_process | FYN | 2133 | 12 | 0.893351 | -0.117009 | 453 | 499.1 | 1.101766 |
| GO:0008152\_metabolic\_process | PRKAR1A | 2133 | 12 | 0.893351 | -0.117009 | 453 | 499.1 | 1.101766 |
| GO:0008152\_metabolic\_process | INSIG1 | 2133 | 12 | 0.893351 | -0.117009 | 453 | 499.1 | 1.101766 |
| GO:0008152\_metabolic\_process | TGM2 | 2133 | 12 | 0.893351 | -0.117009 | 453 | 499.1 | 1.101766 |
| GO:0008152\_metabolic\_process | CHFR | 2133 | 12 | 0.893351 | -0.117009 | 453 | 499.1 | 1.101766 |
| GO:0008152\_metabolic\_process | PRNP | 2133 | 12 | 0.893351 | -0.117009 | 453 | 499.1 | 1.101766 |
| GO:0006915\_apoptosis | GAS1 | 427 | 2 | 0.743762 | -0.116256 | 454 | 499.51 | 1.100242 |
| GO:0006915\_apoptosis | PRNP | 427 | 2 | 0.743762 | -0.116256 | 454 | 499.51 | 1.100242 |
| GO:0012501\_programmed\_cell\_death | GAS1 | 433 | 2 | 0.733455 | -0.112301 | 455 | 501.72 | 1.102681 |
| GO:0012501\_programmed\_cell\_death | PRNP | 433 | 2 | 0.733455 | -0.112301 | 455 | 501.72 | 1.102681 |
| GO:0007167\_enzyme\_linked\_receptor\_protein\_signaling\_pathway | FST | 229 | 1 | 0.693420 | -0.111687 | 456 | 502.07 | 1.101031 |
| GO:0008219\_cell\_death | GAS1 | 444 | 2 | 0.715284 | -0.105390 | 457 | 505.58 | 1.106302 |
| GO:0008219\_cell\_death | PRNP | 444 | 2 | 0.715284 | -0.105390 | 457 | 505.58 | 1.106302 |
| GO:0044085\_cellular\_component\_biogenesis | PAFAH1B1 | 237 | 1 | 0.670013 | -0.105134 | 458 | 506.26 | 1.105371 |
| GO:0016265\_death | GAS1 | 450 | 2 | 0.705747 | -0.101798 | 459 | 507.77 | 1.106253 |
| GO:0016265\_death | PRNP | 450 | 2 | 0.705747 | -0.101798 | 459 | 507.77 | 1.106253 |
| GO:0030097\_hemopoiesis | CD8A | 253 | 1 | 0.627641 | -0.093252 | 460 | 511.55 | 1.112065 |
| GO:0048666\_neuron\_development | GAS1 | 262 | 1 | 0.606081 | -0.087214 | 461 | 514.1 | 1.115184 |
| GO:0030030\_cell\_projection\_organization | GAS1 | 263 | 1 | 0.603776 | -0.086570 | 462 | 514.39 | 1.113398 |
| GO:0034960\_cellular\_biopolymer\_metabolic\_process | ZDHHC17 | 1395 | 7 | 0.796811 | -0.085216 | 463 | 515.0 | 1.112311 |
| GO:0034960\_cellular\_biopolymer\_metabolic\_process | MRPL12 | 1395 | 7 | 0.796811 | -0.085216 | 463 | 515.0 | 1.112311 |
| GO:0034960\_cellular\_biopolymer\_metabolic\_process | DNAJB11 | 1395 | 7 | 0.796811 | -0.085216 | 463 | 515.0 | 1.112311 |
| GO:0034960\_cellular\_biopolymer\_metabolic\_process | FYN | 1395 | 7 | 0.796811 | -0.085216 | 463 | 515.0 | 1.112311 |
| GO:0034960\_cellular\_biopolymer\_metabolic\_process | PRKAR1A | 1395 | 7 | 0.796811 | -0.085216 | 463 | 515.0 | 1.112311 |
| GO:0034960\_cellular\_biopolymer\_metabolic\_process | TGM2 | 1395 | 7 | 0.796811 | -0.085216 | 463 | 515.0 | 1.112311 |
| GO:0034960\_cellular\_biopolymer\_metabolic\_process | CHFR | 1395 | 7 | 0.796811 | -0.085216 | 463 | 515.0 | 1.112311 |
| GO:0043170\_macromolecule\_metabolic\_process | ZDHHC17 | 1576 | 8 | 0.806056 | -0.081476 | 464 | 516.15 | 1.112392 |
| GO:0043170\_macromolecule\_metabolic\_process | MRPL12 | 1576 | 8 | 0.806056 | -0.081476 | 464 | 516.15 | 1.112392 |
| GO:0043170\_macromolecule\_metabolic\_process | DNAJB11 | 1576 | 8 | 0.806056 | -0.081476 | 464 | 516.15 | 1.112392 |
| GO:0043170\_macromolecule\_metabolic\_process | LDLR | 1576 | 8 | 0.806056 | -0.081476 | 464 | 516.15 | 1.112392 |
| GO:0043170\_macromolecule\_metabolic\_process | FYN | 1576 | 8 | 0.806056 | -0.081476 | 464 | 516.15 | 1.112392 |
| GO:0043170\_macromolecule\_metabolic\_process | PRKAR1A | 1576 | 8 | 0.806056 | -0.081476 | 464 | 516.15 | 1.112392 |
| GO:0043170\_macromolecule\_metabolic\_process | TGM2 | 1576 | 8 | 0.806056 | -0.081476 | 464 | 516.15 | 1.112392 |
| GO:0043170\_macromolecule\_metabolic\_process | CHFR | 1576 | 8 | 0.806056 | -0.081476 | 464 | 516.15 | 1.112392 |
| GO:0048534\_hemopoietic\_or\_lymphoid\_organ\_development | CD8A | 277 | 1 | 0.573260 | -0.078062 | 465 | 518.38 | 1.114796 |
| GO:0031327\_negative\_regulation\_of\_cellular\_biosynthetic\_process | INSIG1 | 282 | 1 | 0.563096 | -0.075243 | 466 | 519.5 | 1.114807 |
| GO:0009890\_negative\_regulation\_of\_biosynthetic\_process | INSIG1 | 284 | 1 | 0.559131 | -0.074146 | 467 | 520.26 | 1.114047 |
| GO:0002376\_immune\_system\_process | CD8A | 505 | 2 | 0.628884 | -0.073961 | 468 | 520.45 | 1.112073 |
| GO:0002376\_immune\_system\_process | FYN | 505 | 2 | 0.628884 | -0.073961 | 468 | 520.45 | 1.112073 |
| GO:0002520\_immune\_system\_development | CD8A | 295 | 1 | 0.538282 | -0.068405 | 469 | 523.01 | 1.115160 |
| GO:0043283\_biopolymer\_metabolic\_process | ZDHHC17 | 1490 | 7 | 0.746008 | -0.057233 | 470 | 526.56 | 1.120340 |
| GO:0043283\_biopolymer\_metabolic\_process | MRPL12 | 1490 | 7 | 0.746008 | -0.057233 | 470 | 526.56 | 1.120340 |
| GO:0043283\_biopolymer\_metabolic\_process | DNAJB11 | 1490 | 7 | 0.746008 | -0.057233 | 470 | 526.56 | 1.120340 |
| GO:0043283\_biopolymer\_metabolic\_process | FYN | 1490 | 7 | 0.746008 | -0.057233 | 470 | 526.56 | 1.120340 |
| GO:0043283\_biopolymer\_metabolic\_process | PRKAR1A | 1490 | 7 | 0.746008 | -0.057233 | 470 | 526.56 | 1.120340 |
| GO:0043283\_biopolymer\_metabolic\_process | TGM2 | 1490 | 7 | 0.746008 | -0.057233 | 470 | 526.56 | 1.120340 |
| GO:0043283\_biopolymer\_metabolic\_process | CHFR | 1490 | 7 | 0.746008 | -0.057233 | 470 | 526.56 | 1.120340 |
| GO:0044249\_cellular\_biosynthetic\_process | MOGAT2 | 1150 | 5 | 0.690405 | -0.053153 | 471 | 527.33 | 1.119597 |
| GO:0044249\_cellular\_biosynthetic\_process | ZDHHC17 | 1150 | 5 | 0.690405 | -0.053153 | 471 | 527.33 | 1.119597 |
| GO:0044249\_cellular\_biosynthetic\_process | MRPL12 | 1150 | 5 | 0.690405 | -0.053153 | 471 | 527.33 | 1.119597 |
| GO:0044249\_cellular\_biosynthetic\_process | LDLR | 1150 | 5 | 0.690405 | -0.053153 | 471 | 527.33 | 1.119597 |
| GO:0044249\_cellular\_biosynthetic\_process | INSIG1 | 1150 | 5 | 0.690405 | -0.053153 | 471 | 527.33 | 1.119597 |
| GO:0031324\_negative\_regulation\_of\_cellular\_metabolic\_process | INSIG1 | 332 | 1 | 0.478292 | -0.052271 | 472 | 529.11 | 1.120996 |
| GO:0050794\_regulation\_of\_cellular\_process | CD8A | 2190 | 11 | 0.797591 | -0.050201 | 473 | 529.63 | 1.119725 |
| GO:0050794\_regulation\_of\_cellular\_process | FYN | 2190 | 11 | 0.797591 | -0.050201 | 473 | 529.63 | 1.119725 |
| GO:0050794\_regulation\_of\_cellular\_process | GNA11 | 2190 | 11 | 0.797591 | -0.050201 | 473 | 529.63 | 1.119725 |
| GO:0050794\_regulation\_of\_cellular\_process | INSIG1 | 2190 | 11 | 0.797591 | -0.050201 | 473 | 529.63 | 1.119725 |
| GO:0050794\_regulation\_of\_cellular\_process | FST | 2190 | 11 | 0.797591 | -0.050201 | 473 | 529.63 | 1.119725 |
| GO:0050794\_regulation\_of\_cellular\_process | TGM2 | 2190 | 11 | 0.797591 | -0.050201 | 473 | 529.63 | 1.119725 |
| GO:0050794\_regulation\_of\_cellular\_process | PAFAH1B1 | 2190 | 11 | 0.797591 | -0.050201 | 473 | 529.63 | 1.119725 |
| GO:0050794\_regulation\_of\_cellular\_process | GAS1 | 2190 | 11 | 0.797591 | -0.050201 | 473 | 529.63 | 1.119725 |
| GO:0050794\_regulation\_of\_cellular\_process | CXADR | 2190 | 11 | 0.797591 | -0.050201 | 473 | 529.63 | 1.119725 |
| GO:0050794\_regulation\_of\_cellular\_process | PRNP | 2190 | 11 | 0.797591 | -0.050201 | 473 | 529.63 | 1.119725 |
| GO:0050794\_regulation\_of\_cellular\_process | AZI2 | 2190 | 11 | 0.797591 | -0.050201 | 473 | 529.63 | 1.119725 |
| GO:0009605\_response\_to\_external\_stimulus | FYN | 339 | 1 | 0.468416 | -0.049692 | 474 | 530.11 | 1.118376 |
| GO:0009058\_biosynthetic\_process | MOGAT2 | 1175 | 5 | 0.675715 | -0.047245 | 475 | 531.27 | 1.118463 |
| GO:0009058\_biosynthetic\_process | ZDHHC17 | 1175 | 5 | 0.675715 | -0.047245 | 475 | 531.27 | 1.118463 |
| GO:0009058\_biosynthetic\_process | MRPL12 | 1175 | 5 | 0.675715 | -0.047245 | 475 | 531.27 | 1.118463 |
| GO:0009058\_biosynthetic\_process | LDLR | 1175 | 5 | 0.675715 | -0.047245 | 475 | 531.27 | 1.118463 |
| GO:0009058\_biosynthetic\_process | INSIG1 | 1175 | 5 | 0.675715 | -0.047245 | 475 | 531.27 | 1.118463 |
| GO:0009892\_negative\_regulation\_of\_metabolic\_process | INSIG1 | 348 | 1 | 0.456302 | -0.046565 | 476 | 531.69 | 1.116996 |
| GO:0065007\_biological\_regulation | CD8A | 2593 | 13 | 0.796109 | -0.034213 | 477 | 537.14 | 1.126080 |
| GO:0065007\_biological\_regulation | LDLR | 2593 | 13 | 0.796109 | -0.034213 | 477 | 537.14 | 1.126080 |
| GO:0065007\_biological\_regulation | AGTPBP1 | 2593 | 13 | 0.796109 | -0.034213 | 477 | 537.14 | 1.126080 |
| GO:0065007\_biological\_regulation | GNA11 | 2593 | 13 | 0.796109 | -0.034213 | 477 | 537.14 | 1.126080 |
| GO:0065007\_biological\_regulation | FST | 2593 | 13 | 0.796109 | -0.034213 | 477 | 537.14 | 1.126080 |
| GO:0065007\_biological\_regulation | GAS1 | 2593 | 13 | 0.796109 | -0.034213 | 477 | 537.14 | 1.126080 |
| GO:0065007\_biological\_regulation | CXADR | 2593 | 13 | 0.796109 | -0.034213 | 477 | 537.14 | 1.126080 |
| GO:0065007\_biological\_regulation | AZI2 | 2593 | 13 | 0.796109 | -0.034213 | 477 | 537.14 | 1.126080 |
| GO:0065007\_biological\_regulation | FYN | 2593 | 13 | 0.796109 | -0.034213 | 477 | 537.14 | 1.126080 |
| GO:0065007\_biological\_regulation | INSIG1 | 2593 | 13 | 0.796109 | -0.034213 | 477 | 537.14 | 1.126080 |
| GO:0065007\_biological\_regulation | TGM2 | 2593 | 13 | 0.796109 | -0.034213 | 477 | 537.14 | 1.126080 |
| GO:0065007\_biological\_regulation | PAFAH1B1 | 2593 | 13 | 0.796109 | -0.034213 | 477 | 537.14 | 1.126080 |
| GO:0065007\_biological\_regulation | PRNP | 2593 | 13 | 0.796109 | -0.034213 | 477 | 537.14 | 1.126080 |
| GO:0048522\_positive\_regulation\_of\_cellular\_process | CD8A | 895 | 3 | 0.532267 | -0.026616 | 478 | 539.52 | 1.128703 |
| GO:0048522\_positive\_regulation\_of\_cellular\_process | TGM2 | 895 | 3 | 0.532267 | -0.026616 | 478 | 539.52 | 1.128703 |
| GO:0048522\_positive\_regulation\_of\_cellular\_process | GAS1 | 895 | 3 | 0.532267 | -0.026616 | 478 | 539.52 | 1.128703 |
| GO:0050789\_regulation\_of\_biological\_process | CD8A | 2357 | 11 | 0.741079 | -0.023296 | 479 | 541.63 | 1.130752 |
| GO:0050789\_regulation\_of\_biological\_process | FYN | 2357 | 11 | 0.741079 | -0.023296 | 479 | 541.63 | 1.130752 |
| GO:0050789\_regulation\_of\_biological\_process | GNA11 | 2357 | 11 | 0.741079 | -0.023296 | 479 | 541.63 | 1.130752 |
| GO:0050789\_regulation\_of\_biological\_process | FST | 2357 | 11 | 0.741079 | -0.023296 | 479 | 541.63 | 1.130752 |
| GO:0050789\_regulation\_of\_biological\_process | INSIG1 | 2357 | 11 | 0.741079 | -0.023296 | 479 | 541.63 | 1.130752 |
| GO:0050789\_regulation\_of\_biological\_process | TGM2 | 2357 | 11 | 0.741079 | -0.023296 | 479 | 541.63 | 1.130752 |
| GO:0050789\_regulation\_of\_biological\_process | PAFAH1B1 | 2357 | 11 | 0.741079 | -0.023296 | 479 | 541.63 | 1.130752 |
| GO:0050789\_regulation\_of\_biological\_process | GAS1 | 2357 | 11 | 0.741079 | -0.023296 | 479 | 541.63 | 1.130752 |
| GO:0050789\_regulation\_of\_biological\_process | PRNP | 2357 | 11 | 0.741079 | -0.023296 | 479 | 541.63 | 1.130752 |
| GO:0050789\_regulation\_of\_biological\_process | CXADR | 2357 | 11 | 0.741079 | -0.023296 | 479 | 541.63 | 1.130752 |
| GO:0050789\_regulation\_of\_biological\_process | AZI2 | 2357 | 11 | 0.741079 | -0.023296 | 479 | 541.63 | 1.130752 |
| GO:0048518\_positive\_regulation\_of\_biological\_process | CD8A | 995 | 3 | 0.478773 | -0.014943 | 480 | 543.97 | 1.133271 |
| GO:0048518\_positive\_regulation\_of\_biological\_process | TGM2 | 995 | 3 | 0.478773 | -0.014943 | 480 | 543.97 | 1.133271 |
| GO:0048518\_positive\_regulation\_of\_biological\_process | GAS1 | 995 | 3 | 0.478773 | -0.014943 | 480 | 543.97 | 1.133271 |
| GO:0051239\_regulation\_of\_multicellular\_organismal\_process | CXADR | 587 | 1 | 0.270516 | -0.008297 | 481 | 545.82 | 1.134761 |
| GO:0034645\_cellular\_macromolecule\_biosynthetic\_process | ZDHHC17 | 901 | 2 | 0.352482 | -0.006285 | 482 | 546.3 | 1.133402 |
| GO:0034645\_cellular\_macromolecule\_biosynthetic\_process | MRPL12 | 901 | 2 | 0.352482 | -0.006285 | 482 | 546.3 | 1.133402 |
| GO:0009059\_macromolecule\_biosynthetic\_process | ZDHHC17 | 910 | 2 | 0.348996 | -0.005916 | 483 | 546.34 | 1.131139 |
| GO:0009059\_macromolecule\_biosynthetic\_process | MRPL12 | 910 | 2 | 0.348996 | -0.005916 | 483 | 546.34 | 1.131139 |
| GO:0016070\_RNA\_metabolic\_process | DNAJB11 | 658 | 1 | 0.241327 | -0.004919 | 484 | 546.87 | 1.129897 |
| GO:0006139\_nucleobase\_\_nucleoside\_\_nucleotide\_and\_nucleic\_acid\_metabolic\_process | DNAJB11 | 1002 | 2 | 0.316952 | -0.003152 | 485 | 547.16 | 1.128165 |
| GO:0006139\_nucleobase\_\_nucleoside\_\_nucleotide\_and\_nucleic\_acid\_metabolic\_process | PRNP | 1002 | 2 | 0.316952 | -0.003152 | 485 | 547.16 | 1.128165 |
| GO:0034961\_cellular\_biopolymer\_biosynthetic\_process | MRPL12 | 804 | 1 | 0.197504 | -0.001636 | 486 | 547.47 | 1.126481 |
| GO:0043284\_biopolymer\_biosynthetic\_process | MRPL12 | 807 | 1 | 0.196770 | -0.001599 | 487 | 547.53 | 1.124292 |
| GO:0031326\_regulation\_of\_cellular\_biosynthetic\_process | INSIG1 | 812 | 1 | 0.195558 | -0.001539 | 488 | 547.54 | 1.122008 |
| GO:0009889\_regulation\_of\_biosynthetic\_process | INSIG1 | 815 | 1 | 0.194838 | -0.001504 | 489 | 547.55 | 1.119734 |
| GO:0006807\_nitrogen\_compound\_metabolic\_process | DNAJB11 | 1147 | 2 | 0.276884 | -0.001115 | 490 | 547.61 | 1.117571 |
| GO:0006807\_nitrogen\_compound\_metabolic\_process | PRNP | 1147 | 2 | 0.276884 | -0.001115 | 490 | 547.61 | 1.117571 |
| GO:0010467\_gene\_expression | MRPL12 | 905 | 1 | 0.175462 | -0.000746 | 491 | 547.65 | 1.115377 |
| GO:0080090\_regulation\_of\_primary\_metabolic\_process | INSIG1 | 926 | 1 | 0.171483 | -0.000632 | 492 | 547.72 | 1.113252 |
| GO:0031323\_regulation\_of\_cellular\_metabolic\_process | INSIG1 | 1015 | 1 | 0.156446 | -0.000310 | 493 | 547.77 | 1.111095 |
| GO:0019222\_regulation\_of\_metabolic\_process | INSIG1 | 1088 | 1 | 0.145950 | -0.000170 | 494 | 547.79 | 1.108887 |
| GO:0008150\_biological\_process | LDLR | 4605 | 29 | 1.000000 | 0.000000 | 2310 | 2318.39 | 1.003632 |
| GO:0008150\_biological\_process | CD8A | 4605 | 29 | 1.000000 | 0.000000 | 2310 | 2318.39 | 1.003632 |
| GO:0008150\_biological\_process | AGTPBP1 | 4605 | 29 | 1.000000 | 0.000000 | 2310 | 2318.39 | 1.003632 |
| GO:0008150\_biological\_process | GNA11 | 4605 | 29 | 1.000000 | 0.000000 | 2310 | 2318.39 | 1.003632 |
| GO:0008150\_biological\_process | PITPNB | 4605 | 29 | 1.000000 | 0.000000 | 2310 | 2318.39 | 1.003632 |
| GO:0008150\_biological\_process | FST | 4605 | 29 | 1.000000 | 0.000000 | 2310 | 2318.39 | 1.003632 |
| GO:0008150\_biological\_process | PDIA5 | 4605 | 29 | 1.000000 | 0.000000 | 2310 | 2318.39 | 1.003632 |
| GO:0008150\_biological\_process | CXADR | 4605 | 29 | 1.000000 | 0.000000 | 2310 | 2318.39 | 1.003632 |
| GO:0008150\_biological\_process | AZI2 | 4605 | 29 | 1.000000 | 0.000000 | 2310 | 2318.39 | 1.003632 |
| GO:0008150\_biological\_process | AMPH | 4605 | 29 | 1.000000 | 0.000000 | 2310 | 2318.39 | 1.003632 |
| GO:0008150\_biological\_process | MRPL12 | 4605 | 29 | 1.000000 | 0.000000 | 2310 | 2318.39 | 1.003632 |
| GO:0008150\_biological\_process | DNAJB11 | 4605 | 29 | 1.000000 | 0.000000 | 2310 | 2318.39 | 1.003632 |
| GO:0008150\_biological\_process | KLC1 | 4605 | 29 | 1.000000 | 0.000000 | 2310 | 2318.39 | 1.003632 |
| GO:0008150\_biological\_process | INSIG1 | 4605 | 29 | 1.000000 | 0.000000 | 2310 | 2318.39 | 1.003632 |
| GO:0008150\_biological\_process | TGM2 | 4605 | 29 | 1.000000 | 0.000000 | 2310 | 2318.39 | 1.003632 |
| GO:0008150\_biological\_process | PAFAH1B1 | 4605 | 29 | 1.000000 | 0.000000 | 2310 | 2318.39 | 1.003632 |
| GO:0008150\_biological\_process | MOGAT2 | 4605 | 29 | 1.000000 | 0.000000 | 2310 | 2318.39 | 1.003632 |
| GO:0008150\_biological\_process | LGALS1 | 4605 | 29 | 1.000000 | 0.000000 | 2310 | 2318.39 | 1.003632 |
| GO:0008150\_biological\_process | GAS1 | 4605 | 29 | 1.000000 | 0.000000 | 2310 | 2318.39 | 1.003632 |
| GO:0008150\_biological\_process | FZD2 | 4605 | 29 | 1.000000 | 0.000000 | 2310 | 2318.39 | 1.003632 |
| GO:0008150\_biological\_process | HERPUD2 | 4605 | 29 | 1.000000 | 0.000000 | 2310 | 2318.39 | 1.003632 |
| GO:0008150\_biological\_process | ZDHHC17 | 4605 | 29 | 1.000000 | 0.000000 | 2310 | 2318.39 | 1.003632 |
| GO:0008150\_biological\_process | NAV1 | 4605 | 29 | 1.000000 | 0.000000 | 2310 | 2318.39 | 1.003632 |
| GO:0008150\_biological\_process | SGCG | 4605 | 29 | 1.000000 | 0.000000 | 2310 | 2318.39 | 1.003632 |
| GO:0008150\_biological\_process | FYN | 4605 | 29 | 1.000000 | 0.000000 | 2310 | 2318.39 | 1.003632 |
| GO:0008150\_biological\_process | PRKAR1A | 4605 | 29 | 1.000000 | 0.000000 | 2310 | 2318.39 | 1.003632 |
| GO:0008150\_biological\_process | CHFR | 4605 | 29 | 1.000000 | 0.000000 | 2310 | 2318.39 | 1.003632 |
| GO:0008150\_biological\_process | PRNP | 4605 | 29 | 1.000000 | 0.000000 | 2310 | 2318.39 | 1.003632 |
| GO:0008150\_biological\_process | IGFBP5 | 4605 | 29 | 1.000000 | 0.000000 | 2310 | 2318.39 | 1.003632 |
