# Supplementary material for: RedundancyMiner: De-replication of redundant GO categories in microarray and proteomics analysis
Source: BMC Bioinformatics. 2011 Feb 10;12:52. doi: 10.1186/1471-2105-12-52 (PMC3223614; doi:10.1186/1471-2105-12-52)
Supplement: Additional file 8 — Retinal development HTGM download. compressed package of the results of running HTGM on the retinal development genes list. [file 1471-2105-12-52-S8.ZIP › SCENARIO_2_MODIFIED/total.txt.total.txt.dir/Exp1_BestClusterMap_LEIGS_KM_24.csv.join.15.txt.dir/Exp1_BestClusterMap_LEIGS_KM_24.csv.join.15.txt.change.gce.html]

Gene Category Report for Exp1\_BestClusterMap\_LEIGS\_KM\_24.csv.join.15.txt

# Gene Category Report for Exp1\_BestClusterMap\_LEIGS\_KM\_24.csv.join.15.txt

| HYPERLINKED GO CATEGORY | HYPERLINKED GENE NAME | TOTAL GENES | CHANGED GENES | ENRICHMENT | LOG10(p) | CUMULATIVE NUMBER OF CATEGORIES | CUMULATIVE RANDOMS MEAN | FALSE DISCOVERY RATE |
| --- | --- | --- | --- | --- | --- | --- | --- | --- |
| GO:0016074\_snoRNA\_metabolic\_process | FBL | 1 | 1 |  |  |  |  |  |  |
| GO:0045947\_negative\_regulation\_of\_translational\_initiation | EIF4EBP1 | 1 | 1 |  |  |  |  |  |  |
| GO:0048199\_vesicle\_targeting\_\_to\_\_from\_or\_within\_Golgi | TMED10 | 1 | 1 |  |  |  |  |  |  |
| GO:0006168\_adenine\_salvage | APRT | 2 | 1 |  |  |  |  |  |  |
| GO:0006610\_ribosomal\_protein\_import\_into\_nucleus | KPNB1 | 2 | 1 |  |  |  |  |  |  |
| GO:0043096\_purine\_base\_salvage | APRT | 2 | 1 |  |  |  |  |  |  |
| GO:0046083\_adenine\_metabolic\_process | APRT | 2 | 1 |  |  |  |  |  |  |
| GO:0006166\_purine\_ribonucleoside\_salvage | APRT | 3 | 1 |  |  |  |  |  |  |
| GO:0007199\_G-protein\_signaling\_\_coupled\_to\_cGMP\_nucleotide\_second\_messenger | GKAP1 | 3 | 1 |  |  |  |  |  |  |
| GO:0010172\_embryonic\_body\_morphogenesis | MAB21L2 | 3 | 1 |  |  |  |  |  |  |
| GO:0019934\_cGMP-mediated\_signaling | GKAP1 | 3 | 1 |  |  |  |  |  |  |
| GO:0043094\_cellular\_metabolic\_compound\_salvage | APRT | 3 | 1 |  |  |  |  |  |  |
| GO:0043101\_purine\_salvage | APRT | 3 | 1 |  |  |  |  |  |  |
| GO:0043174\_nucleoside\_salvage | APRT | 3 | 1 |  |  |  |  |  |  |
| GO:0043010\_camera-type\_eye\_development | YY1 | 110 | 3 | 5.232955 | -1.729701 | 1 | 10.81 | 10.810000 |
| GO:0043010\_camera-type\_eye\_development | SKIL | 110 | 3 | 5.232955 | -1.729701 | 1 | 10.81 | 10.810000 |
| GO:0043010\_camera-type\_eye\_development | MAB21L2 | 110 | 3 | 5.232955 | -1.729701 | 1 | 10.81 | 10.810000 |
| GO:0006144\_purine\_base\_metabolic\_process | APRT | 4 | 1 |  |  |  |  |  |  |
| GO:0006606\_protein\_import\_into\_nucleus | PKIG | 44 | 2 | 8.721591 | -1.666456 | 3 | 13.11 | 4.370000 |
| GO:0006606\_protein\_import\_into\_nucleus | KPNB1 | 44 | 2 | 8.721591 | -1.666456 | 3 | 13.11 | 4.370000 |
| GO:0051170\_nuclear\_import | PKIG | 44 | 2 | 8.721591 | -1.666456 | 3 | 13.11 | 4.370000 |
| GO:0051170\_nuclear\_import | KPNB1 | 44 | 2 | 8.721591 | -1.666456 | 3 | 13.11 | 4.370000 |
| GO:0016570\_histone\_modification | RNF2 | 47 | 2 | 8.164894 | -1.612635 | 4 | 14.07 | 3.517500 |
| GO:0016570\_histone\_modification | EED | 47 | 2 | 8.164894 | -1.612635 | 4 | 14.07 | 3.517500 |
| GO:0006886\_intracellular\_protein\_transport | PKIG | 122 | 3 | 4.718238 | -1.611336 | 5 | 14.24 | 2.848000 |
| GO:0006886\_intracellular\_protein\_transport | TMED10 | 122 | 3 | 4.718238 | -1.611336 | 5 | 14.24 | 2.848000 |
| GO:0006886\_intracellular\_protein\_transport | KPNB1 | 122 | 3 | 4.718238 | -1.611336 | 5 | 14.24 | 2.848000 |
| GO:0034504\_protein\_localization\_in\_nucleus | PKIG | 48 | 2 | 7.994792 | -1.595522 | 6 | 14.65 | 2.441667 |
| GO:0034504\_protein\_localization\_in\_nucleus | KPNB1 | 48 | 2 | 7.994792 | -1.595522 | 6 | 14.65 | 2.441667 |
| GO:0006903\_vesicle\_targeting | TMED10 | 5 | 1 | 38.375000 | -1.588388 | 9 | 21.39 | 2.376667 |
| GO:0006983\_ER\_overload\_response | PPP1R15B | 5 | 1 | 38.375000 | -1.588388 | 9 | 21.39 | 2.376667 |
| GO:0017148\_negative\_regulation\_of\_translation | EIF4EBP1 | 5 | 1 | 38.375000 | -1.588388 | 9 | 21.39 | 2.376667 |
| GO:0017038\_protein\_import | PKIG | 50 | 2 | 7.675000 | -1.562435 | 10 | 21.99 | 2.199000 |
| GO:0017038\_protein\_import | KPNB1 | 50 | 2 | 7.675000 | -1.562435 | 10 | 21.99 | 2.199000 |
| GO:0016569\_covalent\_chromatin\_modification | RNF2 | 51 | 2 | 7.524510 | -1.546431 | 11 | 22.65 | 2.059091 |
| GO:0016569\_covalent\_chromatin\_modification | EED | 51 | 2 | 7.524510 | -1.546431 | 11 | 22.65 | 2.059091 |
| GO:0009112\_nucleobase\_metabolic\_process | APRT | 6 | 1 | 31.979167 | -1.510290 | 14 | 28.74 | 2.052857 |
| GO:0016574\_histone\_ubiquitination | RNF2 | 6 | 1 | 31.979167 | -1.510290 | 14 | 28.74 | 2.052857 |
| GO:0042308\_negative\_regulation\_of\_protein\_import\_into\_nucleus | PKIG | 6 | 1 | 31.979167 | -1.510290 | 14 | 28.74 | 2.052857 |
| GO:0006412\_translation | EIF4EBP1 | 54 | 2 | 7.106481 | -1.500407 | 15 | 29.13 | 1.942000 |
| GO:0006412\_translation | MRPL3 | 54 | 2 | 7.106481 | -1.500407 | 15 | 29.13 | 1.942000 |
| GO:0010467\_gene\_expression | EIF4EBP1 | 905 | 9 | 1.908149 | -1.491403 | 16 | 29.3 | 1.831250 |
| GO:0010467\_gene\_expression | MRPL3 | 905 | 9 | 1.908149 | -1.491403 | 16 | 29.3 | 1.831250 |
| GO:0010467\_gene\_expression | YY1 | 905 | 9 | 1.908149 | -1.491403 | 16 | 29.3 | 1.831250 |
| GO:0010467\_gene\_expression | RNF2 | 905 | 9 | 1.908149 | -1.491403 | 16 | 29.3 | 1.831250 |
| GO:0010467\_gene\_expression | PKIG | 905 | 9 | 1.908149 | -1.491403 | 16 | 29.3 | 1.831250 |
| GO:0010467\_gene\_expression | EED | 905 | 9 | 1.908149 | -1.491403 | 16 | 29.3 | 1.831250 |
| GO:0010467\_gene\_expression | SKIL | 905 | 9 | 1.908149 | -1.491403 | 16 | 29.3 | 1.831250 |
| GO:0010467\_gene\_expression | FBL | 905 | 9 | 1.908149 | -1.491403 | 16 | 29.3 | 1.831250 |
| GO:0010467\_gene\_expression | CBFB | 905 | 9 | 1.908149 | -1.491403 | 16 | 29.3 | 1.831250 |
| GO:0001654\_eye\_development | YY1 | 136 | 3 | 4.232537 | -1.489332 | 17 | 29.36 | 1.727059 |
| GO:0001654\_eye\_development | SKIL | 136 | 3 | 4.232537 | -1.489332 | 17 | 29.36 | 1.727059 |
| GO:0001654\_eye\_development | MAB21L2 | 136 | 3 | 4.232537 | -1.489332 | 17 | 29.36 | 1.727059 |
| GO:0034613\_cellular\_protein\_localization | PKIG | 139 | 3 | 4.141187 | -1.465115 | 18 | 30.11 | 1.672778 |
| GO:0034613\_cellular\_protein\_localization | TMED10 | 139 | 3 | 4.141187 | -1.465115 | 18 | 30.11 | 1.672778 |
| GO:0034613\_cellular\_protein\_localization | KPNB1 | 139 | 3 | 4.141187 | -1.465115 | 18 | 30.11 | 1.672778 |
| GO:0033365\_protein\_localization\_in\_organelle | PKIG | 57 | 2 | 6.732456 | -1.457117 | 19 | 30.52 | 1.606316 |
| GO:0033365\_protein\_localization\_in\_organelle | KPNB1 | 57 | 2 | 6.732456 | -1.457117 | 19 | 30.52 | 1.606316 |
| GO:0070727\_cellular\_macromolecule\_localization | PKIG | 141 | 3 | 4.082447 | -1.449314 | 20 | 30.68 | 1.534000 |
| GO:0070727\_cellular\_macromolecule\_localization | TMED10 | 141 | 3 | 4.082447 | -1.449314 | 20 | 30.68 | 1.534000 |
| GO:0070727\_cellular\_macromolecule\_localization | KPNB1 | 141 | 3 | 4.082447 | -1.449314 | 20 | 30.68 | 1.534000 |
| GO:0006783\_heme\_biosynthetic\_process | UROD | 7 | 1 | 27.410714 | -1.444426 | 24 | 37.07 | 1.544583 |
| GO:0032387\_negative\_regulation\_of\_intracellular\_transport | PKIG | 7 | 1 | 27.410714 | -1.444426 | 24 | 37.07 | 1.544583 |
| GO:0042168\_heme\_metabolic\_process | UROD | 7 | 1 | 27.410714 | -1.444426 | 24 | 37.07 | 1.544583 |
| GO:0046823\_negative\_regulation\_of\_nucleocytoplasmic\_transport | PKIG | 7 | 1 | 27.410714 | -1.444426 | 24 | 37.07 | 1.544583 |
| GO:0002566\_somatic\_diversification\_of\_immune\_receptors\_via\_somatic\_mutation | UNG | 8 | 1 | 23.984375 | -1.387516 | 30 | 43.89 | 1.463000 |
| GO:0006349\_genetic\_imprinting | EED | 8 | 1 | 23.984375 | -1.387516 | 30 | 43.89 | 1.463000 |
| GO:0007625\_grooming\_behavior | APRT | 8 | 1 | 23.984375 | -1.387516 | 30 | 43.89 | 1.463000 |
| GO:0016446\_somatic\_hypermutation\_of\_immunoglobulin\_genes | UNG | 8 | 1 | 23.984375 | -1.387516 | 30 | 43.89 | 1.463000 |
| GO:0018107\_peptidyl-threonine\_phosphorylation | CLK1 | 8 | 1 | 23.984375 | -1.387516 | 30 | 43.89 | 1.463000 |
| GO:0018210\_peptidyl-threonine\_modification | CLK1 | 8 | 1 | 23.984375 | -1.387516 | 30 | 43.89 | 1.463000 |
| GO:0006364\_rRNA\_processing | FBL | 9 | 1 | 21.319444 | -1.337444 | 33 | 50.82 | 1.540000 |
| GO:0016072\_rRNA\_metabolic\_process | FBL | 9 | 1 | 21.319444 | -1.337444 | 33 | 50.82 | 1.540000 |
| GO:0070306\_lens\_fiber\_cell\_differentiation | SKIL | 9 | 1 | 21.319444 | -1.337444 | 33 | 50.82 | 1.540000 |
| GO:0051716\_cellular\_response\_to\_stimulus | EIF4EBP1 | 273 | 4 | 2.811355 | -1.298865 | 34 | 52.0 | 1.529412 |
| GO:0051716\_cellular\_response\_to\_stimulus | UNG | 273 | 4 | 2.811355 | -1.298865 | 34 | 52.0 | 1.529412 |
| GO:0051716\_cellular\_response\_to\_stimulus | PPP1R15B | 273 | 4 | 2.811355 | -1.298865 | 34 | 52.0 | 1.529412 |
| GO:0051716\_cellular\_response\_to\_stimulus | MIF | 273 | 4 | 2.811355 | -1.298865 | 34 | 52.0 | 1.529412 |
| GO:0010558\_negative\_regulation\_of\_macromolecule\_biosynthetic\_process | EIF4EBP1 | 274 | 4 | 2.801095 | -1.293995 | 35 | 52.25 | 1.492857 |
| GO:0010558\_negative\_regulation\_of\_macromolecule\_biosynthetic\_process | RNF2 | 274 | 4 | 2.801095 | -1.293995 | 35 | 52.25 | 1.492857 |
| GO:0010558\_negative\_regulation\_of\_macromolecule\_biosynthetic\_process | PKIG | 274 | 4 | 2.801095 | -1.293995 | 35 | 52.25 | 1.492857 |
| GO:0010558\_negative\_regulation\_of\_macromolecule\_biosynthetic\_process | SKIL | 274 | 4 | 2.801095 | -1.293995 | 35 | 52.25 | 1.492857 |
| GO:0009948\_anterior\_posterior\_axis\_specification | RNF2 | 10 | 1 | 19.187500 | -1.292767 | 39 | 58.29 | 1.494615 |
| GO:0051224\_negative\_regulation\_of\_protein\_transport | PKIG | 10 | 1 | 19.187500 | -1.292767 | 39 | 58.29 | 1.494615 |
| GO:0051650\_establishment\_of\_vesicle\_localization | TMED10 | 10 | 1 | 19.187500 | -1.292767 | 39 | 58.29 | 1.494615 |
| GO:0060216\_definitive\_hemopoiesis | CBFB | 10 | 1 | 19.187500 | -1.292767 | 39 | 58.29 | 1.494615 |
| GO:0006913\_nucleocytoplasmic\_transport | PKIG | 71 | 2 | 5.404930 | -1.283926 | 40 | 58.59 | 1.464750 |
| GO:0006913\_nucleocytoplasmic\_transport | KPNB1 | 71 | 2 | 5.404930 | -1.283926 | 40 | 58.59 | 1.464750 |
| GO:0006807\_nitrogen\_compound\_metabolic\_process | YY1 | 1147 | 10 | 1.672842 | -1.275904 | 41 | 58.83 | 1.434878 |
| GO:0006807\_nitrogen\_compound\_metabolic\_process | RNF2 | 1147 | 10 | 1.672842 | -1.275904 | 41 | 58.83 | 1.434878 |
| GO:0006807\_nitrogen\_compound\_metabolic\_process | UNG | 1147 | 10 | 1.672842 | -1.275904 | 41 | 58.83 | 1.434878 |
| GO:0006807\_nitrogen\_compound\_metabolic\_process | PKIG | 1147 | 10 | 1.672842 | -1.275904 | 41 | 58.83 | 1.434878 |
| GO:0006807\_nitrogen\_compound\_metabolic\_process | EED | 1147 | 10 | 1.672842 | -1.275904 | 41 | 58.83 | 1.434878 |
| GO:0006807\_nitrogen\_compound\_metabolic\_process | SKIL | 1147 | 10 | 1.672842 | -1.275904 | 41 | 58.83 | 1.434878 |
| GO:0006807\_nitrogen\_compound\_metabolic\_process | UROD | 1147 | 10 | 1.672842 | -1.275904 | 41 | 58.83 | 1.434878 |
| GO:0006807\_nitrogen\_compound\_metabolic\_process | FBL | 1147 | 10 | 1.672842 | -1.275904 | 41 | 58.83 | 1.434878 |
| GO:0006807\_nitrogen\_compound\_metabolic\_process | CBFB | 1147 | 10 | 1.672842 | -1.275904 | 41 | 58.83 | 1.434878 |
| GO:0006807\_nitrogen\_compound\_metabolic\_process | APRT | 1147 | 10 | 1.672842 | -1.275904 | 41 | 58.83 | 1.434878 |
| GO:0016568\_chromatin\_modification | RNF2 | 72 | 2 | 5.329861 | -1.273053 | 43 | 59.38 | 1.380930 |
| GO:0016568\_chromatin\_modification | EED | 72 | 2 | 5.329861 | -1.273053 | 43 | 59.38 | 1.380930 |
| GO:0051169\_nuclear\_transport | PKIG | 72 | 2 | 5.329861 | -1.273053 | 43 | 59.38 | 1.380930 |
| GO:0051169\_nuclear\_transport | KPNB1 | 72 | 2 | 5.329861 | -1.273053 | 43 | 59.38 | 1.380930 |
| GO:0031327\_negative\_regulation\_of\_cellular\_biosynthetic\_process | EIF4EBP1 | 282 | 4 | 2.721631 | -1.255856 | 44 | 59.98 | 1.363182 |
| GO:0031327\_negative\_regulation\_of\_cellular\_biosynthetic\_process | RNF2 | 282 | 4 | 2.721631 | -1.255856 | 44 | 59.98 | 1.363182 |
| GO:0031327\_negative\_regulation\_of\_cellular\_biosynthetic\_process | PKIG | 282 | 4 | 2.721631 | -1.255856 | 44 | 59.98 | 1.363182 |
| GO:0031327\_negative\_regulation\_of\_cellular\_biosynthetic\_process | SKIL | 282 | 4 | 2.721631 | -1.255856 | 44 | 59.98 | 1.363182 |
| GO:0006779\_porphyrin\_biosynthetic\_process | UROD | 11 | 1 | 17.443182 | -1.252453 | 50 | 64.66 | 1.293200 |
| GO:0033014\_tetrapyrrole\_biosynthetic\_process | UROD | 11 | 1 | 17.443182 | -1.252453 | 50 | 64.66 | 1.293200 |
| GO:0042542\_response\_to\_hydrogen\_peroxide | PPP1R15B | 11 | 1 | 17.443182 | -1.252453 | 50 | 64.66 | 1.293200 |
| GO:0045055\_regulated\_secretory\_pathway | TMED10 | 11 | 1 | 17.443182 | -1.252453 | 50 | 64.66 | 1.293200 |
| GO:0048193\_Golgi\_vesicle\_transport | TMED10 | 11 | 1 | 17.443182 | -1.252453 | 50 | 64.66 | 1.293200 |
| GO:0051648\_vesicle\_localization | TMED10 | 11 | 1 | 17.443182 | -1.252453 | 50 | 64.66 | 1.293200 |
| GO:0009890\_negative\_regulation\_of\_biosynthetic\_process | EIF4EBP1 | 284 | 4 | 2.702465 | -1.246545 | 51 | 64.86 | 1.271765 |
| GO:0009890\_negative\_regulation\_of\_biosynthetic\_process | RNF2 | 284 | 4 | 2.702465 | -1.246545 | 51 | 64.86 | 1.271765 |
| GO:0009890\_negative\_regulation\_of\_biosynthetic\_process | PKIG | 284 | 4 | 2.702465 | -1.246545 | 51 | 64.86 | 1.271765 |
| GO:0009890\_negative\_regulation\_of\_biosynthetic\_process | SKIL | 284 | 4 | 2.702465 | -1.246545 | 51 | 64.86 | 1.271765 |
| GO:0006139\_nucleobase\_\_nucleoside\_\_nucleotide\_and\_nucleic\_acid\_metabolic\_process | YY1 | 1002 | 9 | 1.723428 | -1.236395 | 52 | 65.4 | 1.257692 |
| GO:0006139\_nucleobase\_\_nucleoside\_\_nucleotide\_and\_nucleic\_acid\_metabolic\_process | RNF2 | 1002 | 9 | 1.723428 | -1.236395 | 52 | 65.4 | 1.257692 |
| GO:0006139\_nucleobase\_\_nucleoside\_\_nucleotide\_and\_nucleic\_acid\_metabolic\_process | UNG | 1002 | 9 | 1.723428 | -1.236395 | 52 | 65.4 | 1.257692 |
| GO:0006139\_nucleobase\_\_nucleoside\_\_nucleotide\_and\_nucleic\_acid\_metabolic\_process | PKIG | 1002 | 9 | 1.723428 | -1.236395 | 52 | 65.4 | 1.257692 |
| GO:0006139\_nucleobase\_\_nucleoside\_\_nucleotide\_and\_nucleic\_acid\_metabolic\_process | EED | 1002 | 9 | 1.723428 | -1.236395 | 52 | 65.4 | 1.257692 |
| GO:0006139\_nucleobase\_\_nucleoside\_\_nucleotide\_and\_nucleic\_acid\_metabolic\_process | SKIL | 1002 | 9 | 1.723428 | -1.236395 | 52 | 65.4 | 1.257692 |
| GO:0006139\_nucleobase\_\_nucleoside\_\_nucleotide\_and\_nucleic\_acid\_metabolic\_process | FBL | 1002 | 9 | 1.723428 | -1.236395 | 52 | 65.4 | 1.257692 |
| GO:0006139\_nucleobase\_\_nucleoside\_\_nucleotide\_and\_nucleic\_acid\_metabolic\_process | CBFB | 1002 | 9 | 1.723428 | -1.236395 | 52 | 65.4 | 1.257692 |
| GO:0006139\_nucleobase\_\_nucleoside\_\_nucleotide\_and\_nucleic\_acid\_metabolic\_process | APRT | 1002 | 9 | 1.723428 | -1.236395 | 52 | 65.4 | 1.257692 |
| GO:0000122\_negative\_regulation\_of\_transcription\_from\_RNA\_polymerase\_II\_promoter | RNF2 | 175 | 3 | 3.289286 | -1.216032 | 54 | 66.4 | 1.229630 |
| GO:0000122\_negative\_regulation\_of\_transcription\_from\_RNA\_polymerase\_II\_promoter | PKIG | 175 | 3 | 3.289286 | -1.216032 | 54 | 66.4 | 1.229630 |
| GO:0000122\_negative\_regulation\_of\_transcription\_from\_RNA\_polymerase\_II\_promoter | SKIL | 175 | 3 | 3.289286 | -1.216032 | 54 | 66.4 | 1.229630 |
| GO:0015031\_protein\_transport | PKIG | 175 | 3 | 3.289286 | -1.216032 | 54 | 66.4 | 1.229630 |
| GO:0015031\_protein\_transport | TMED10 | 175 | 3 | 3.289286 | -1.216032 | 54 | 66.4 | 1.229630 |
| GO:0015031\_protein\_transport | KPNB1 | 175 | 3 | 3.289286 | -1.216032 | 54 | 66.4 | 1.229630 |
| GO:0006413\_translational\_initiation | EIF4EBP1 | 12 | 1 | 15.989583 | -1.215743 | 59 | 71.15 | 1.205932 |
| GO:0006446\_regulation\_of\_translational\_initiation | EIF4EBP1 | 12 | 1 | 15.989583 | -1.215743 | 59 | 71.15 | 1.205932 |
| GO:0030330\_DNA\_damage\_response\_\_signal\_transduction\_by\_p53\_class\_mediator | MIF | 12 | 1 | 15.989583 | -1.215743 | 59 | 71.15 | 1.205932 |
| GO:0042278\_purine\_nucleoside\_metabolic\_process | APRT | 12 | 1 | 15.989583 | -1.215743 | 59 | 71.15 | 1.205932 |
| GO:0046128\_purine\_ribonucleoside\_metabolic\_process | APRT | 12 | 1 | 15.989583 | -1.215743 | 59 | 71.15 | 1.205932 |
| GO:0045184\_establishment\_of\_protein\_localization | PKIG | 180 | 3 | 3.197917 | -1.186455 | 60 | 72.8 | 1.213333 |
| GO:0045184\_establishment\_of\_protein\_localization | TMED10 | 180 | 3 | 3.197917 | -1.186455 | 60 | 72.8 | 1.213333 |
| GO:0045184\_establishment\_of\_protein\_localization | KPNB1 | 180 | 3 | 3.197917 | -1.186455 | 60 | 72.8 | 1.213333 |
| GO:0006778\_porphyrin\_metabolic\_process | UROD | 13 | 1 | 14.759615 | -1.182059 | 64 | 77.53 | 1.211406 |
| GO:0009119\_ribonucleoside\_metabolic\_process | APRT | 13 | 1 | 14.759615 | -1.182059 | 64 | 77.53 | 1.211406 |
| GO:0018105\_peptidyl-serine\_phosphorylation | CLK1 | 13 | 1 | 14.759615 | -1.182059 | 64 | 77.53 | 1.211406 |
| GO:0033013\_tetrapyrrole\_metabolic\_process | UROD | 13 | 1 | 14.759615 | -1.182059 | 64 | 77.53 | 1.211406 |
| GO:0006325\_chromatin\_organization | RNF2 | 83 | 2 | 4.623494 | -1.163705 | 65 | 78.48 | 1.207385 |
| GO:0006325\_chromatin\_organization | EED | 83 | 2 | 4.623494 | -1.163705 | 65 | 78.48 | 1.207385 |
| GO:0018130\_heterocycle\_biosynthetic\_process | UROD | 14 | 1 | 13.705357 | -1.150952 | 66 | 83.69 | 1.268030 |
| GO:0006605\_protein\_targeting | PKIG | 86 | 2 | 4.462209 | -1.136750 | 67 | 84.68 | 1.263881 |
| GO:0006605\_protein\_targeting | KPNB1 | 86 | 2 | 4.462209 | -1.136750 | 67 | 84.68 | 1.263881 |
| GO:0009116\_nucleoside\_metabolic\_process | APRT | 15 | 1 | 12.791667 | -1.122065 | 70 | 89.78 | 1.282571 |
| GO:0010171\_body\_morphogenesis | MAB21L2 | 15 | 1 | 12.791667 | -1.122065 | 70 | 89.78 | 1.282571 |
| GO:0042306\_regulation\_of\_protein\_import\_into\_nucleus | PKIG | 15 | 1 | 12.791667 | -1.122065 | 70 | 89.78 | 1.282571 |
| GO:0046907\_intracellular\_transport | PKIG | 194 | 3 | 2.967139 | -1.108856 | 71 | 90.31 | 1.271972 |
| GO:0046907\_intracellular\_transport | TMED10 | 194 | 3 | 2.967139 | -1.108856 | 71 | 90.31 | 1.271972 |
| GO:0046907\_intracellular\_transport | KPNB1 | 194 | 3 | 2.967139 | -1.108856 | 71 | 90.31 | 1.271972 |
| GO:0034960\_cellular\_biopolymer\_metabolic\_process | EIF4EBP1 | 1395 | 11 | 1.512993 | -1.105678 | 72 | 90.42 | 1.255833 |
| GO:0034960\_cellular\_biopolymer\_metabolic\_process | MRPL3 | 1395 | 11 | 1.512993 | -1.105678 | 72 | 90.42 | 1.255833 |
| GO:0034960\_cellular\_biopolymer\_metabolic\_process | YY1 | 1395 | 11 | 1.512993 | -1.105678 | 72 | 90.42 | 1.255833 |
| GO:0034960\_cellular\_biopolymer\_metabolic\_process | RNF2 | 1395 | 11 | 1.512993 | -1.105678 | 72 | 90.42 | 1.255833 |
| GO:0034960\_cellular\_biopolymer\_metabolic\_process | UNG | 1395 | 11 | 1.512993 | -1.105678 | 72 | 90.42 | 1.255833 |
| GO:0034960\_cellular\_biopolymer\_metabolic\_process | PKIG | 1395 | 11 | 1.512993 | -1.105678 | 72 | 90.42 | 1.255833 |
| GO:0034960\_cellular\_biopolymer\_metabolic\_process | EED | 1395 | 11 | 1.512993 | -1.105678 | 72 | 90.42 | 1.255833 |
| GO:0034960\_cellular\_biopolymer\_metabolic\_process | CLK1 | 1395 | 11 | 1.512993 | -1.105678 | 72 | 90.42 | 1.255833 |
| GO:0034960\_cellular\_biopolymer\_metabolic\_process | SKIL | 1395 | 11 | 1.512993 | -1.105678 | 72 | 90.42 | 1.255833 |
| GO:0034960\_cellular\_biopolymer\_metabolic\_process | CBFB | 1395 | 11 | 1.512993 | -1.105678 | 72 | 90.42 | 1.255833 |
| GO:0034960\_cellular\_biopolymer\_metabolic\_process | FBL | 1395 | 11 | 1.512993 | -1.105678 | 72 | 90.42 | 1.255833 |
| GO:0033554\_cellular\_response\_to\_stress | UNG | 196 | 3 | 2.936862 | -1.098352 | 73 | 91.27 | 1.250274 |
| GO:0033554\_cellular\_response\_to\_stress | PPP1R15B | 196 | 3 | 2.936862 | -1.098352 | 73 | 91.27 | 1.250274 |
| GO:0033554\_cellular\_response\_to\_stress | MIF | 196 | 3 | 2.936862 | -1.098352 | 73 | 91.27 | 1.250274 |
| GO:0000302\_response\_to\_reactive\_oxygen\_species | PPP1R15B | 16 | 1 | 11.992188 | -1.095112 | 76 | 94.63 | 1.245132 |
| GO:0034976\_response\_to\_endoplasmic\_reticulum\_stress | PPP1R15B | 16 | 1 | 11.992188 | -1.095112 | 76 | 94.63 | 1.245132 |
| GO:0046148\_pigment\_biosynthetic\_process | UROD | 16 | 1 | 11.992188 | -1.095112 | 76 | 94.63 | 1.245132 |
| GO:0034984\_cellular\_response\_to\_DNA\_damage\_stimulus | UNG | 94 | 2 | 4.082447 | -1.069892 | 77 | 96.47 | 1.252857 |
| GO:0034984\_cellular\_response\_to\_DNA\_damage\_stimulus | MIF | 94 | 2 | 4.082447 | -1.069892 | 77 | 96.47 | 1.252857 |
| GO:0006984\_ER-nuclear\_signaling\_pathway | PPP1R15B | 17 | 1 | 11.286765 | -1.069858 | 81 | 100.13 | 1.236173 |
| GO:0034470\_ncRNA\_processing | FBL | 17 | 1 | 11.286765 | -1.069858 | 81 | 100.13 | 1.236173 |
| GO:0042254\_ribosome\_biogenesis | FBL | 17 | 1 | 11.286765 | -1.069858 | 81 | 100.13 | 1.236173 |
| GO:0042440\_pigment\_metabolic\_process | UROD | 17 | 1 | 11.286765 | -1.069858 | 81 | 100.13 | 1.236173 |
| GO:0010605\_negative\_regulation\_of\_macromolecule\_metabolic\_process | EIF4EBP1 | 331 | 4 | 2.318731 | -1.050381 | 82 | 101.4 | 1.236585 |
| GO:0010605\_negative\_regulation\_of\_macromolecule\_metabolic\_process | RNF2 | 331 | 4 | 2.318731 | -1.050381 | 82 | 101.4 | 1.236585 |
| GO:0010605\_negative\_regulation\_of\_macromolecule\_metabolic\_process | PKIG | 331 | 4 | 2.318731 | -1.050381 | 82 | 101.4 | 1.236585 |
| GO:0010605\_negative\_regulation\_of\_macromolecule\_metabolic\_process | SKIL | 331 | 4 | 2.318731 | -1.050381 | 82 | 101.4 | 1.236585 |
| GO:0031324\_negative\_regulation\_of\_cellular\_metabolic\_process | EIF4EBP1 | 332 | 4 | 2.311747 | -1.046630 | 83 | 101.46 | 1.222410 |
| GO:0031324\_negative\_regulation\_of\_cellular\_metabolic\_process | RNF2 | 332 | 4 | 2.311747 | -1.046630 | 83 | 101.46 | 1.222410 |
| GO:0031324\_negative\_regulation\_of\_cellular\_metabolic\_process | PKIG | 332 | 4 | 2.311747 | -1.046630 | 83 | 101.46 | 1.222410 |
| GO:0031324\_negative\_regulation\_of\_cellular\_metabolic\_process | SKIL | 332 | 4 | 2.311747 | -1.046630 | 83 | 101.46 | 1.222410 |
| GO:0001825\_blastocyst\_formation | SKIL | 18 | 1 | 10.659722 | -1.046108 | 85 | 106.32 | 1.250824 |
| GO:0033157\_regulation\_of\_intracellular\_protein\_transport | PKIG | 18 | 1 | 10.659722 | -1.046108 | 85 | 106.32 | 1.250824 |
| GO:0007569\_cell\_aging | MIF | 19 | 1 | 10.098684 | -1.023700 | 87 | 111.14 | 1.277471 |
| GO:0009798\_axis\_specification | RNF2 | 19 | 1 | 10.098684 | -1.023700 | 87 | 111.14 | 1.277471 |
| GO:0010468\_regulation\_of\_gene\_expression | EIF4EBP1 | 778 | 7 | 1.726382 | -1.017525 | 88 | 111.43 | 1.266250 |
| GO:0010468\_regulation\_of\_gene\_expression | YY1 | 778 | 7 | 1.726382 | -1.017525 | 88 | 111.43 | 1.266250 |
| GO:0010468\_regulation\_of\_gene\_expression | RNF2 | 778 | 7 | 1.726382 | -1.017525 | 88 | 111.43 | 1.266250 |
| GO:0010468\_regulation\_of\_gene\_expression | PKIG | 778 | 7 | 1.726382 | -1.017525 | 88 | 111.43 | 1.266250 |
| GO:0010468\_regulation\_of\_gene\_expression | EED | 778 | 7 | 1.726382 | -1.017525 | 88 | 111.43 | 1.266250 |
| GO:0010468\_regulation\_of\_gene\_expression | SKIL | 778 | 7 | 1.726382 | -1.017525 | 88 | 111.43 | 1.266250 |
| GO:0010468\_regulation\_of\_gene\_expression | CBFB | 778 | 7 | 1.726382 | -1.017525 | 88 | 111.43 | 1.266250 |
| GO:0044260\_cellular\_macromolecule\_metabolic\_process | EIF4EBP1 | 1447 | 11 | 1.458621 | -1.006989 | 89 | 111.78 | 1.255955 |
| GO:0044260\_cellular\_macromolecule\_metabolic\_process | MRPL3 | 1447 | 11 | 1.458621 | -1.006989 | 89 | 111.78 | 1.255955 |
| GO:0044260\_cellular\_macromolecule\_metabolic\_process | UNG | 1447 | 11 | 1.458621 | -1.006989 | 89 | 111.78 | 1.255955 |
| GO:0044260\_cellular\_macromolecule\_metabolic\_process | YY1 | 1447 | 11 | 1.458621 | -1.006989 | 89 | 111.78 | 1.255955 |
| GO:0044260\_cellular\_macromolecule\_metabolic\_process | RNF2 | 1447 | 11 | 1.458621 | -1.006989 | 89 | 111.78 | 1.255955 |
| GO:0044260\_cellular\_macromolecule\_metabolic\_process | PKIG | 1447 | 11 | 1.458621 | -1.006989 | 89 | 111.78 | 1.255955 |
| GO:0044260\_cellular\_macromolecule\_metabolic\_process | EED | 1447 | 11 | 1.458621 | -1.006989 | 89 | 111.78 | 1.255955 |
| GO:0044260\_cellular\_macromolecule\_metabolic\_process | CLK1 | 1447 | 11 | 1.458621 | -1.006989 | 89 | 111.78 | 1.255955 |
| GO:0044260\_cellular\_macromolecule\_metabolic\_process | SKIL | 1447 | 11 | 1.458621 | -1.006989 | 89 | 111.78 | 1.255955 |
| GO:0044260\_cellular\_macromolecule\_metabolic\_process | FBL | 1447 | 11 | 1.458621 | -1.006989 | 89 | 111.78 | 1.255955 |
| GO:0044260\_cellular\_macromolecule\_metabolic\_process | CBFB | 1447 | 11 | 1.458621 | -1.006989 | 89 | 111.78 | 1.255955 |
| GO:0016571\_histone\_methylation | EED | 20 | 1 | 9.593750 | -1.002496 | 92 | 114.64 | 1.246087 |
| GO:0018209\_peptidyl-serine\_modification | CLK1 | 20 | 1 | 9.593750 | -1.002496 | 92 | 114.64 | 1.246087 |
| GO:0046822\_regulation\_of\_nucleocytoplasmic\_transport | PKIG | 20 | 1 | 9.593750 | -1.002496 | 92 | 114.64 | 1.246087 |
| GO:0045892\_negative\_regulation\_of\_transcription\_\_DNA-dependent | RNF2 | 218 | 3 | 2.640482 | -0.991233 | 93 | 115.51 | 1.242043 |
| GO:0045892\_negative\_regulation\_of\_transcription\_\_DNA-dependent | PKIG | 218 | 3 | 2.640482 | -0.991233 | 93 | 115.51 | 1.242043 |
| GO:0045892\_negative\_regulation\_of\_transcription\_\_DNA-dependent | SKIL | 218 | 3 | 2.640482 | -0.991233 | 93 | 115.51 | 1.242043 |
| GO:0009892\_negative\_regulation\_of\_metabolic\_process | EIF4EBP1 | 348 | 4 | 2.205460 | -0.988707 | 94 | 115.61 | 1.229894 |
| GO:0009892\_negative\_regulation\_of\_metabolic\_process | RNF2 | 348 | 4 | 2.205460 | -0.988707 | 94 | 115.61 | 1.229894 |
| GO:0009892\_negative\_regulation\_of\_metabolic\_process | PKIG | 348 | 4 | 2.205460 | -0.988707 | 94 | 115.61 | 1.229894 |
| GO:0009892\_negative\_regulation\_of\_metabolic\_process | SKIL | 348 | 4 | 2.205460 | -0.988707 | 94 | 115.61 | 1.229894 |
| GO:0007423\_sensory\_organ\_development | YY1 | 219 | 3 | 2.628425 | -0.986702 | 95 | 115.81 | 1.219053 |
| GO:0007423\_sensory\_organ\_development | SKIL | 219 | 3 | 2.628425 | -0.986702 | 95 | 115.81 | 1.219053 |
| GO:0007423\_sensory\_organ\_development | MAB21L2 | 219 | 3 | 2.628425 | -0.986702 | 95 | 115.81 | 1.219053 |
| GO:0001702\_gastrulation\_with\_mouth\_forming\_second | RNF2 | 21 | 1 | 9.136905 | -0.982379 | 97 | 119.52 | 1.232165 |
| GO:0051656\_establishment\_of\_organelle\_localization | TMED10 | 21 | 1 | 9.136905 | -0.982379 | 97 | 119.52 | 1.232165 |
| GO:0051253\_negative\_regulation\_of\_RNA\_metabolic\_process | RNF2 | 220 | 3 | 2.616477 | -0.982198 | 98 | 119.6 | 1.220408 |
| GO:0051253\_negative\_regulation\_of\_RNA\_metabolic\_process | PKIG | 220 | 3 | 2.616477 | -0.982198 | 98 | 119.6 | 1.220408 |
| GO:0051253\_negative\_regulation\_of\_RNA\_metabolic\_process | SKIL | 220 | 3 | 2.616477 | -0.982198 | 98 | 119.6 | 1.220408 |
| GO:0034660\_ncRNA\_metabolic\_process | FBL | 22 | 1 | 8.721591 | -0.963247 | 99 | 124.85 | 1.261111 |
| GO:0034961\_cellular\_biopolymer\_biosynthetic\_process | EIF4EBP1 | 804 | 7 | 1.670553 | -0.957424 | 100 | 125.03 | 1.250300 |
| GO:0034961\_cellular\_biopolymer\_biosynthetic\_process | MRPL3 | 804 | 7 | 1.670553 | -0.957424 | 100 | 125.03 | 1.250300 |
| GO:0034961\_cellular\_biopolymer\_biosynthetic\_process | YY1 | 804 | 7 | 1.670553 | -0.957424 | 100 | 125.03 | 1.250300 |
| GO:0034961\_cellular\_biopolymer\_biosynthetic\_process | RNF2 | 804 | 7 | 1.670553 | -0.957424 | 100 | 125.03 | 1.250300 |
| GO:0034961\_cellular\_biopolymer\_biosynthetic\_process | PKIG | 804 | 7 | 1.670553 | -0.957424 | 100 | 125.03 | 1.250300 |
| GO:0034961\_cellular\_biopolymer\_biosynthetic\_process | SKIL | 804 | 7 | 1.670553 | -0.957424 | 100 | 125.03 | 1.250300 |
| GO:0034961\_cellular\_biopolymer\_biosynthetic\_process | CBFB | 804 | 7 | 1.670553 | -0.957424 | 100 | 125.03 | 1.250300 |
| GO:0043284\_biopolymer\_biosynthetic\_process | EIF4EBP1 | 807 | 7 | 1.664343 | -0.950705 | 101 | 125.41 | 1.241683 |
| GO:0043284\_biopolymer\_biosynthetic\_process | MRPL3 | 807 | 7 | 1.664343 | -0.950705 | 101 | 125.41 | 1.241683 |
| GO:0043284\_biopolymer\_biosynthetic\_process | YY1 | 807 | 7 | 1.664343 | -0.950705 | 101 | 125.41 | 1.241683 |
| GO:0043284\_biopolymer\_biosynthetic\_process | RNF2 | 807 | 7 | 1.664343 | -0.950705 | 101 | 125.41 | 1.241683 |
| GO:0043284\_biopolymer\_biosynthetic\_process | PKIG | 807 | 7 | 1.664343 | -0.950705 | 101 | 125.41 | 1.241683 |
| GO:0043284\_biopolymer\_biosynthetic\_process | SKIL | 807 | 7 | 1.664343 | -0.950705 | 101 | 125.41 | 1.241683 |
| GO:0043284\_biopolymer\_biosynthetic\_process | CBFB | 807 | 7 | 1.664343 | -0.950705 | 101 | 125.41 | 1.241683 |
| GO:0022613\_ribonucleoprotein\_complex\_biogenesis | FBL | 23 | 1 | 8.342391 | -0.945012 | 103 | 128.25 | 1.245146 |
| GO:0030512\_negative\_regulation\_of\_transforming\_growth\_factor\_beta\_receptor\_signaling\_pathway | SKIL | 23 | 1 | 8.342391 | -0.945012 | 103 | 128.25 | 1.245146 |
| GO:0006974\_response\_to\_DNA\_damage\_stimulus | UNG | 113 | 2 | 3.396018 | -0.934841 | 104 | 129.1 | 1.241346 |
| GO:0006974\_response\_to\_DNA\_damage\_stimulus | MIF | 113 | 2 | 3.396018 | -0.934841 | 104 | 129.1 | 1.241346 |
| GO:0016070\_RNA\_metabolic\_process | YY1 | 658 | 6 | 1.749620 | -0.933632 | 105 | 129.35 | 1.231905 |
| GO:0016070\_RNA\_metabolic\_process | RNF2 | 658 | 6 | 1.749620 | -0.933632 | 105 | 129.35 | 1.231905 |
| GO:0016070\_RNA\_metabolic\_process | PKIG | 658 | 6 | 1.749620 | -0.933632 | 105 | 129.35 | 1.231905 |
| GO:0016070\_RNA\_metabolic\_process | SKIL | 658 | 6 | 1.749620 | -0.933632 | 105 | 129.35 | 1.231905 |
| GO:0016070\_RNA\_metabolic\_process | CBFB | 658 | 6 | 1.749620 | -0.933632 | 105 | 129.35 | 1.231905 |
| GO:0016070\_RNA\_metabolic\_process | FBL | 658 | 6 | 1.749620 | -0.933632 | 105 | 129.35 | 1.231905 |
| GO:0043283\_biopolymer\_metabolic\_process | EIF4EBP1 | 1490 | 11 | 1.416527 | -0.930646 | 106 | 129.81 | 1.224623 |
| GO:0043283\_biopolymer\_metabolic\_process | MRPL3 | 1490 | 11 | 1.416527 | -0.930646 | 106 | 129.81 | 1.224623 |
| GO:0043283\_biopolymer\_metabolic\_process | YY1 | 1490 | 11 | 1.416527 | -0.930646 | 106 | 129.81 | 1.224623 |
| GO:0043283\_biopolymer\_metabolic\_process | UNG | 1490 | 11 | 1.416527 | -0.930646 | 106 | 129.81 | 1.224623 |
| GO:0043283\_biopolymer\_metabolic\_process | RNF2 | 1490 | 11 | 1.416527 | -0.930646 | 106 | 129.81 | 1.224623 |
| GO:0043283\_biopolymer\_metabolic\_process | PKIG | 1490 | 11 | 1.416527 | -0.930646 | 106 | 129.81 | 1.224623 |
| GO:0043283\_biopolymer\_metabolic\_process | EED | 1490 | 11 | 1.416527 | -0.930646 | 106 | 129.81 | 1.224623 |
| GO:0043283\_biopolymer\_metabolic\_process | SKIL | 1490 | 11 | 1.416527 | -0.930646 | 106 | 129.81 | 1.224623 |
| GO:0043283\_biopolymer\_metabolic\_process | CLK1 | 1490 | 11 | 1.416527 | -0.930646 | 106 | 129.81 | 1.224623 |
| GO:0043283\_biopolymer\_metabolic\_process | CBFB | 1490 | 11 | 1.416527 | -0.930646 | 106 | 129.81 | 1.224623 |
| GO:0043283\_biopolymer\_metabolic\_process | FBL | 1490 | 11 | 1.416527 | -0.930646 | 106 | 129.81 | 1.224623 |
| GO:0007050\_cell\_cycle\_arrest | SKIL | 24 | 1 | 7.994792 | -0.927598 | 108 | 132.98 | 1.231296 |
| GO:0032386\_regulation\_of\_intracellular\_transport | PKIG | 24 | 1 | 7.994792 | -0.927598 | 108 | 132.98 | 1.231296 |
| GO:0046483\_heterocycle\_metabolic\_process | UROD | 116 | 2 | 3.308190 | -0.916016 | 109 | 134.03 | 1.229633 |
| GO:0046483\_heterocycle\_metabolic\_process | APRT | 116 | 2 | 3.308190 | -0.916016 | 109 | 134.03 | 1.229633 |
| GO:0007049\_cell\_cycle | CCND2 | 238 | 3 | 2.418592 | -0.905519 | 110 | 137.83 | 1.253000 |
| GO:0007049\_cell\_cycle | RNF2 | 238 | 3 | 2.418592 | -0.905519 | 110 | 137.83 | 1.253000 |
| GO:0007049\_cell\_cycle | SKIL | 238 | 3 | 2.418592 | -0.905519 | 110 | 137.83 | 1.253000 |
| GO:0040029\_regulation\_of\_gene\_expression\_\_epigenetic | EED | 26 | 1 | 7.379808 | -0.894972 | 111 | 141.16 | 1.271712 |
| GO:0006479\_protein\_amino\_acid\_methylation | EED | 27 | 1 | 7.106481 | -0.879649 | 114 | 144.56 | 1.268070 |
| GO:0008213\_protein\_amino\_acid\_alkylation | EED | 27 | 1 | 7.106481 | -0.879649 | 114 | 144.56 | 1.268070 |
| GO:0008286\_insulin\_receptor\_signaling\_pathway | EIF4EBP1 | 27 | 1 | 7.106481 | -0.879649 | 114 | 144.56 | 1.268070 |
| GO:0002088\_lens\_development\_in\_camera-type\_eye | SKIL | 28 | 1 | 6.852679 | -0.864921 | 116 | 147.35 | 1.270259 |
| GO:0051188\_cofactor\_biosynthetic\_process | UROD | 28 | 1 | 6.852679 | -0.864921 | 116 | 147.35 | 1.270259 |
| GO:0008104\_protein\_localization | PKIG | 251 | 3 | 2.293327 | -0.854849 | 117 | 148.03 | 1.265214 |
| GO:0008104\_protein\_localization | TMED10 | 251 | 3 | 2.293327 | -0.854849 | 117 | 148.03 | 1.265214 |
| GO:0008104\_protein\_localization | KPNB1 | 251 | 3 | 2.293327 | -0.854849 | 117 | 148.03 | 1.265214 |
| GO:0006417\_regulation\_of\_translation | EIF4EBP1 | 29 | 1 | 6.616379 | -0.850747 | 120 | 151.29 | 1.260750 |
| GO:0016447\_somatic\_recombination\_of\_immunoglobulin\_gene\_segments | UNG | 29 | 1 | 6.616379 | -0.850747 | 120 | 151.29 | 1.260750 |
| GO:0042770\_DNA\_damage\_response\_\_signal\_transduction | MIF | 29 | 1 | 6.616379 | -0.850747 | 120 | 151.29 | 1.260750 |
| GO:0016481\_negative\_regulation\_of\_transcription | RNF2 | 253 | 3 | 2.275198 | -0.847373 | 121 | 151.47 | 1.251818 |
| GO:0016481\_negative\_regulation\_of\_transcription | PKIG | 253 | 3 | 2.275198 | -0.847373 | 121 | 151.47 | 1.251818 |
| GO:0016481\_negative\_regulation\_of\_transcription | SKIL | 253 | 3 | 2.275198 | -0.847373 | 121 | 151.47 | 1.251818 |
| GO:0051276\_chromosome\_organization | RNF2 | 129 | 2 | 2.974806 | -0.840811 | 122 | 152.4 | 1.249180 |
| GO:0051276\_chromosome\_organization | EED | 129 | 2 | 2.974806 | -0.840811 | 122 | 152.4 | 1.249180 |
| GO:0002260\_lymphocyte\_homeostasis | SKIL | 30 | 1 | 6.395833 | -0.837089 | 124 | 155.97 | 1.257823 |
| GO:0016445\_somatic\_diversification\_of\_immunoglobulins | UNG | 30 | 1 | 6.395833 | -0.837089 | 124 | 155.97 | 1.257823 |
| GO:0051640\_organelle\_localization | TMED10 | 31 | 1 | 6.189516 | -0.823913 | 125 | 161.52 | 1.292160 |
| GO:0009952\_anterior\_posterior\_pattern\_formation | YY1 | 133 | 2 | 2.885338 | -0.819537 | 126 | 161.79 | 1.284048 |
| GO:0009952\_anterior\_posterior\_pattern\_formation | RNF2 | 133 | 2 | 2.885338 | -0.819537 | 126 | 161.79 | 1.284048 |
| GO:0010629\_negative\_regulation\_of\_gene\_expression | RNF2 | 262 | 3 | 2.197042 | -0.814716 | 127 | 162.05 | 1.275984 |
| GO:0010629\_negative\_regulation\_of\_gene\_expression | PKIG | 262 | 3 | 2.197042 | -0.814716 | 127 | 162.05 | 1.275984 |
| GO:0010629\_negative\_regulation\_of\_gene\_expression | SKIL | 262 | 3 | 2.197042 | -0.814716 | 127 | 162.05 | 1.275984 |
| GO:0044267\_cellular\_protein\_metabolic\_process | EIF4EBP1 | 559 | 5 | 1.716234 | -0.801793 | 128 | 164.65 | 1.286328 |
| GO:0044267\_cellular\_protein\_metabolic\_process | MRPL3 | 559 | 5 | 1.716234 | -0.801793 | 128 | 164.65 | 1.286328 |
| GO:0044267\_cellular\_protein\_metabolic\_process | RNF2 | 559 | 5 | 1.716234 | -0.801793 | 128 | 164.65 | 1.286328 |
| GO:0044267\_cellular\_protein\_metabolic\_process | EED | 559 | 5 | 1.716234 | -0.801793 | 128 | 164.65 | 1.286328 |
| GO:0044267\_cellular\_protein\_metabolic\_process | CLK1 | 559 | 5 | 1.716234 | -0.801793 | 128 | 164.65 | 1.286328 |
| GO:0002562\_somatic\_diversification\_of\_immune\_receptors\_via\_germline\_recombination\_within\_a\_single\_locus | UNG | 33 | 1 | 5.814394 | -0.798886 | 130 | 167.27 | 1.286692 |
| GO:0016444\_somatic\_cell\_DNA\_recombination | UNG | 33 | 1 | 5.814394 | -0.798886 | 130 | 167.27 | 1.286692 |
| GO:0043170\_macromolecule\_metabolic\_process | EIF4EBP1 | 1576 | 11 | 1.339229 | -0.791241 | 131 | 167.59 | 1.279313 |
| GO:0043170\_macromolecule\_metabolic\_process | MRPL3 | 1576 | 11 | 1.339229 | -0.791241 | 131 | 167.59 | 1.279313 |
| GO:0043170\_macromolecule\_metabolic\_process | UNG | 1576 | 11 | 1.339229 | -0.791241 | 131 | 167.59 | 1.279313 |
| GO:0043170\_macromolecule\_metabolic\_process | YY1 | 1576 | 11 | 1.339229 | -0.791241 | 131 | 167.59 | 1.279313 |
| GO:0043170\_macromolecule\_metabolic\_process | RNF2 | 1576 | 11 | 1.339229 | -0.791241 | 131 | 167.59 | 1.279313 |
| GO:0043170\_macromolecule\_metabolic\_process | PKIG | 1576 | 11 | 1.339229 | -0.791241 | 131 | 167.59 | 1.279313 |
| GO:0043170\_macromolecule\_metabolic\_process | EED | 1576 | 11 | 1.339229 | -0.791241 | 131 | 167.59 | 1.279313 |
| GO:0043170\_macromolecule\_metabolic\_process | CLK1 | 1576 | 11 | 1.339229 | -0.791241 | 131 | 167.59 | 1.279313 |
| GO:0043170\_macromolecule\_metabolic\_process | SKIL | 1576 | 11 | 1.339229 | -0.791241 | 131 | 167.59 | 1.279313 |
| GO:0043170\_macromolecule\_metabolic\_process | FBL | 1576 | 11 | 1.339229 | -0.791241 | 131 | 167.59 | 1.279313 |
| GO:0043170\_macromolecule\_metabolic\_process | CBFB | 1576 | 11 | 1.339229 | -0.791241 | 131 | 167.59 | 1.279313 |
| GO:0002200\_somatic\_diversification\_of\_immune\_receptors | UNG | 34 | 1 | 5.643382 | -0.786983 | 133 | 170.78 | 1.284060 |
| GO:0007568\_aging | MIF | 34 | 1 | 5.643382 | -0.786983 | 133 | 170.78 | 1.284060 |
| GO:0045934\_negative\_regulation\_of\_nucleobase\_\_nucleoside\_\_nucleotide\_and\_nucleic\_acid\_metabolic\_process | RNF2 | 270 | 3 | 2.131944 | -0.786978 | 134 | 170.94 | 1.275672 |
| GO:0045934\_negative\_regulation\_of\_nucleobase\_\_nucleoside\_\_nucleotide\_and\_nucleic\_acid\_metabolic\_process | PKIG | 270 | 3 | 2.131944 | -0.786978 | 134 | 170.94 | 1.275672 |
| GO:0045934\_negative\_regulation\_of\_nucleobase\_\_nucleoside\_\_nucleotide\_and\_nucleic\_acid\_metabolic\_process | SKIL | 270 | 3 | 2.131944 | -0.786978 | 134 | 170.94 | 1.275672 |
| GO:0051172\_negative\_regulation\_of\_nitrogen\_compound\_metabolic\_process | RNF2 | 271 | 3 | 2.124077 | -0.783592 | 135 | 171.28 | 1.268741 |
| GO:0051172\_negative\_regulation\_of\_nitrogen\_compound\_metabolic\_process | PKIG | 271 | 3 | 2.124077 | -0.783592 | 135 | 171.28 | 1.268741 |
| GO:0051172\_negative\_regulation\_of\_nitrogen\_compound\_metabolic\_process | SKIL | 271 | 3 | 2.124077 | -0.783592 | 135 | 171.28 | 1.268741 |
| GO:0016567\_protein\_ubiquitination | RNF2 | 35 | 1 | 5.482143 | -0.775455 | 137 | 173.93 | 1.269562 |
| GO:0051051\_negative\_regulation\_of\_transport | PKIG | 35 | 1 | 5.482143 | -0.775455 | 137 | 173.93 | 1.269562 |
| GO:0033036\_macromolecule\_localization | PKIG | 274 | 3 | 2.100821 | -0.773539 | 138 | 174.18 | 1.262174 |
| GO:0033036\_macromolecule\_localization | TMED10 | 274 | 3 | 2.100821 | -0.773539 | 138 | 174.18 | 1.262174 |
| GO:0033036\_macromolecule\_localization | KPNB1 | 274 | 3 | 2.100821 | -0.773539 | 138 | 174.18 | 1.262174 |
| GO:0006355\_regulation\_of\_transcription\_\_DNA-dependent | YY1 | 575 | 5 | 1.668478 | -0.764900 | 139 | 175.06 | 1.259424 |
| GO:0006355\_regulation\_of\_transcription\_\_DNA-dependent | RNF2 | 575 | 5 | 1.668478 | -0.764900 | 139 | 175.06 | 1.259424 |
| GO:0006355\_regulation\_of\_transcription\_\_DNA-dependent | PKIG | 575 | 5 | 1.668478 | -0.764900 | 139 | 175.06 | 1.259424 |
| GO:0006355\_regulation\_of\_transcription\_\_DNA-dependent | SKIL | 575 | 5 | 1.668478 | -0.764900 | 139 | 175.06 | 1.259424 |
| GO:0006355\_regulation\_of\_transcription\_\_DNA-dependent | CBFB | 575 | 5 | 1.668478 | -0.764900 | 139 | 175.06 | 1.259424 |
| GO:0006469\_negative\_regulation\_of\_protein\_kinase\_activity | PKIG | 36 | 1 | 5.329861 | -0.764281 | 143 | 177.96 | 1.244476 |
| GO:0007187\_G-protein\_signaling\_\_coupled\_to\_cyclic\_nucleotide\_second\_messenger | GKAP1 | 36 | 1 | 5.329861 | -0.764281 | 143 | 177.96 | 1.244476 |
| GO:0033673\_negative\_regulation\_of\_kinase\_activity | PKIG | 36 | 1 | 5.329861 | -0.764281 | 143 | 177.96 | 1.244476 |
| GO:0051223\_regulation\_of\_protein\_transport | PKIG | 36 | 1 | 5.329861 | -0.764281 | 143 | 177.96 | 1.244476 |
| GO:0034645\_cellular\_macromolecule\_biosynthetic\_process | EIF4EBP1 | 901 | 7 | 1.490705 | -0.760677 | 144 | 178.41 | 1.238958 |
| GO:0034645\_cellular\_macromolecule\_biosynthetic\_process | MRPL3 | 901 | 7 | 1.490705 | -0.760677 | 144 | 178.41 | 1.238958 |
| GO:0034645\_cellular\_macromolecule\_biosynthetic\_process | YY1 | 901 | 7 | 1.490705 | -0.760677 | 144 | 178.41 | 1.238958 |
| GO:0034645\_cellular\_macromolecule\_biosynthetic\_process | RNF2 | 901 | 7 | 1.490705 | -0.760677 | 144 | 178.41 | 1.238958 |
| GO:0034645\_cellular\_macromolecule\_biosynthetic\_process | PKIG | 901 | 7 | 1.490705 | -0.760677 | 144 | 178.41 | 1.238958 |
| GO:0034645\_cellular\_macromolecule\_biosynthetic\_process | SKIL | 901 | 7 | 1.490705 | -0.760677 | 144 | 178.41 | 1.238958 |
| GO:0034645\_cellular\_macromolecule\_biosynthetic\_process | CBFB | 901 | 7 | 1.490705 | -0.760677 | 144 | 178.41 | 1.238958 |
| GO:0032869\_cellular\_response\_to\_insulin\_stimulus | EIF4EBP1 | 37 | 1 | 5.185811 | -0.753441 | 145 | 180.96 | 1.248000 |
| GO:0044237\_cellular\_metabolic\_process | MRPL3 | 1974 | 13 | 1.263614 | -0.745067 | 146 | 181.9 | 1.245890 |
| GO:0044237\_cellular\_metabolic\_process | UNG | 1974 | 13 | 1.263614 | -0.745067 | 146 | 181.9 | 1.245890 |
| GO:0044237\_cellular\_metabolic\_process | YY1 | 1974 | 13 | 1.263614 | -0.745067 | 146 | 181.9 | 1.245890 |
| GO:0044237\_cellular\_metabolic\_process | PKIG | 1974 | 13 | 1.263614 | -0.745067 | 146 | 181.9 | 1.245890 |
| GO:0044237\_cellular\_metabolic\_process | CLK1 | 1974 | 13 | 1.263614 | -0.745067 | 146 | 181.9 | 1.245890 |
| GO:0044237\_cellular\_metabolic\_process | CBFB | 1974 | 13 | 1.263614 | -0.745067 | 146 | 181.9 | 1.245890 |
| GO:0044237\_cellular\_metabolic\_process | FBL | 1974 | 13 | 1.263614 | -0.745067 | 146 | 181.9 | 1.245890 |
| GO:0044237\_cellular\_metabolic\_process | APRT | 1974 | 13 | 1.263614 | -0.745067 | 146 | 181.9 | 1.245890 |
| GO:0044237\_cellular\_metabolic\_process | EIF4EBP1 | 1974 | 13 | 1.263614 | -0.745067 | 146 | 181.9 | 1.245890 |
| GO:0044237\_cellular\_metabolic\_process | RNF2 | 1974 | 13 | 1.263614 | -0.745067 | 146 | 181.9 | 1.245890 |
| GO:0044237\_cellular\_metabolic\_process | EED | 1974 | 13 | 1.263614 | -0.745067 | 146 | 181.9 | 1.245890 |
| GO:0044237\_cellular\_metabolic\_process | SKIL | 1974 | 13 | 1.263614 | -0.745067 | 146 | 181.9 | 1.245890 |
| GO:0044237\_cellular\_metabolic\_process | UROD | 1974 | 13 | 1.263614 | -0.745067 | 146 | 181.9 | 1.245890 |
| GO:0009059\_macromolecule\_biosynthetic\_process | EIF4EBP1 | 910 | 7 | 1.475962 | -0.744402 | 147 | 182.01 | 1.238163 |
| GO:0009059\_macromolecule\_biosynthetic\_process | MRPL3 | 910 | 7 | 1.475962 | -0.744402 | 147 | 182.01 | 1.238163 |
| GO:0009059\_macromolecule\_biosynthetic\_process | YY1 | 910 | 7 | 1.475962 | -0.744402 | 147 | 182.01 | 1.238163 |
| GO:0009059\_macromolecule\_biosynthetic\_process | RNF2 | 910 | 7 | 1.475962 | -0.744402 | 147 | 182.01 | 1.238163 |
| GO:0009059\_macromolecule\_biosynthetic\_process | PKIG | 910 | 7 | 1.475962 | -0.744402 | 147 | 182.01 | 1.238163 |
| GO:0009059\_macromolecule\_biosynthetic\_process | SKIL | 910 | 7 | 1.475962 | -0.744402 | 147 | 182.01 | 1.238163 |
| GO:0009059\_macromolecule\_biosynthetic\_process | CBFB | 910 | 7 | 1.475962 | -0.744402 | 147 | 182.01 | 1.238163 |
| GO:0010556\_regulation\_of\_macromolecule\_biosynthetic\_process | EIF4EBP1 | 745 | 6 | 1.545302 | -0.743883 | 148 | 182.24 | 1.231351 |
| GO:0010556\_regulation\_of\_macromolecule\_biosynthetic\_process | YY1 | 745 | 6 | 1.545302 | -0.743883 | 148 | 182.24 | 1.231351 |
| GO:0010556\_regulation\_of\_macromolecule\_biosynthetic\_process | RNF2 | 745 | 6 | 1.545302 | -0.743883 | 148 | 182.24 | 1.231351 |
| GO:0010556\_regulation\_of\_macromolecule\_biosynthetic\_process | PKIG | 745 | 6 | 1.545302 | -0.743883 | 148 | 182.24 | 1.231351 |
| GO:0010556\_regulation\_of\_macromolecule\_biosynthetic\_process | SKIL | 745 | 6 | 1.545302 | -0.743883 | 148 | 182.24 | 1.231351 |
| GO:0010556\_regulation\_of\_macromolecule\_biosynthetic\_process | CBFB | 745 | 6 | 1.545302 | -0.743883 | 148 | 182.24 | 1.231351 |
| GO:0001649\_osteoblast\_differentiation | CBFB | 38 | 1 | 5.049342 | -0.742918 | 153 | 185.49 | 1.212353 |
| GO:0032259\_methylation | EED | 38 | 1 | 5.049342 | -0.742918 | 153 | 185.49 | 1.212353 |
| GO:0043414\_biopolymer\_methylation | EED | 38 | 1 | 5.049342 | -0.742918 | 153 | 185.49 | 1.212353 |
| GO:0046777\_protein\_amino\_acid\_autophosphorylation | CLK1 | 38 | 1 | 5.049342 | -0.742918 | 153 | 185.49 | 1.212353 |
| GO:0051348\_negative\_regulation\_of\_transferase\_activity | PKIG | 38 | 1 | 5.049342 | -0.742918 | 153 | 185.49 | 1.212353 |
| GO:0006730\_one-carbon\_metabolic\_process | EED | 39 | 1 | 4.919872 | -0.732695 | 155 | 189.09 | 1.219935 |
| GO:0070201\_regulation\_of\_establishment\_of\_protein\_localization | PKIG | 39 | 1 | 4.919872 | -0.732695 | 155 | 189.09 | 1.219935 |
| GO:0051252\_regulation\_of\_RNA\_metabolic\_process | YY1 | 590 | 5 | 1.626059 | -0.731849 | 156 | 189.21 | 1.212885 |
| GO:0051252\_regulation\_of\_RNA\_metabolic\_process | RNF2 | 590 | 5 | 1.626059 | -0.731849 | 156 | 189.21 | 1.212885 |
| GO:0051252\_regulation\_of\_RNA\_metabolic\_process | PKIG | 590 | 5 | 1.626059 | -0.731849 | 156 | 189.21 | 1.212885 |
| GO:0051252\_regulation\_of\_RNA\_metabolic\_process | SKIL | 590 | 5 | 1.626059 | -0.731849 | 156 | 189.21 | 1.212885 |
| GO:0051252\_regulation\_of\_RNA\_metabolic\_process | CBFB | 590 | 5 | 1.626059 | -0.731849 | 156 | 189.21 | 1.212885 |
| GO:0006357\_regulation\_of\_transcription\_from\_RNA\_polymerase\_II\_promoter | RNF2 | 435 | 4 | 1.764368 | -0.730820 | 157 | 189.47 | 1.206815 |
| GO:0006357\_regulation\_of\_transcription\_from\_RNA\_polymerase\_II\_promoter | PKIG | 435 | 4 | 1.764368 | -0.730820 | 157 | 189.47 | 1.206815 |
| GO:0006357\_regulation\_of\_transcription\_from\_RNA\_polymerase\_II\_promoter | SKIL | 435 | 4 | 1.764368 | -0.730820 | 157 | 189.47 | 1.206815 |
| GO:0006357\_regulation\_of\_transcription\_from\_RNA\_polymerase\_II\_promoter | CBFB | 435 | 4 | 1.764368 | -0.730820 | 157 | 189.47 | 1.206815 |
| GO:0006351\_transcription\_\_DNA-dependent | YY1 | 594 | 5 | 1.615109 | -0.723276 | 158 | 190.02 | 1.202658 |
| GO:0006351\_transcription\_\_DNA-dependent | RNF2 | 594 | 5 | 1.615109 | -0.723276 | 158 | 190.02 | 1.202658 |
| GO:0006351\_transcription\_\_DNA-dependent | PKIG | 594 | 5 | 1.615109 | -0.723276 | 158 | 190.02 | 1.202658 |
| GO:0006351\_transcription\_\_DNA-dependent | SKIL | 594 | 5 | 1.615109 | -0.723276 | 158 | 190.02 | 1.202658 |
| GO:0006351\_transcription\_\_DNA-dependent | CBFB | 594 | 5 | 1.615109 | -0.723276 | 158 | 190.02 | 1.202658 |
| GO:0001824\_blastocyst\_development | SKIL | 40 | 1 | 4.796875 | -0.722757 | 161 | 192.16 | 1.193540 |
| GO:0017015\_regulation\_of\_transforming\_growth\_factor\_beta\_receptor\_signaling\_pathway | SKIL | 40 | 1 | 4.796875 | -0.722757 | 161 | 192.16 | 1.193540 |
| GO:0019935\_cyclic-nucleotide-mediated\_signaling | GKAP1 | 40 | 1 | 4.796875 | -0.722757 | 161 | 192.16 | 1.193540 |
| GO:0032774\_RNA\_biosynthetic\_process | YY1 | 595 | 5 | 1.612395 | -0.721148 | 162 | 192.33 | 1.187222 |
| GO:0032774\_RNA\_biosynthetic\_process | RNF2 | 595 | 5 | 1.612395 | -0.721148 | 162 | 192.33 | 1.187222 |
| GO:0032774\_RNA\_biosynthetic\_process | PKIG | 595 | 5 | 1.612395 | -0.721148 | 162 | 192.33 | 1.187222 |
| GO:0032774\_RNA\_biosynthetic\_process | SKIL | 595 | 5 | 1.612395 | -0.721148 | 162 | 192.33 | 1.187222 |
| GO:0032774\_RNA\_biosynthetic\_process | CBFB | 595 | 5 | 1.612395 | -0.721148 | 162 | 192.33 | 1.187222 |
| GO:0001776\_leukocyte\_homeostasis | SKIL | 41 | 1 | 4.679878 | -0.713090 | 164 | 197.08 | 1.201707 |
| GO:0006979\_response\_to\_oxidative\_stress | PPP1R15B | 41 | 1 | 4.679878 | -0.713090 | 164 | 197.08 | 1.201707 |
| GO:0006366\_transcription\_from\_RNA\_polymerase\_II\_promoter | RNF2 | 444 | 4 | 1.728604 | -0.708660 | 165 | 197.3 | 1.195758 |
| GO:0006366\_transcription\_from\_RNA\_polymerase\_II\_promoter | PKIG | 444 | 4 | 1.728604 | -0.708660 | 165 | 197.3 | 1.195758 |
| GO:0006366\_transcription\_from\_RNA\_polymerase\_II\_promoter | SKIL | 444 | 4 | 1.728604 | -0.708660 | 165 | 197.3 | 1.195758 |
| GO:0006366\_transcription\_from\_RNA\_polymerase\_II\_promoter | CBFB | 444 | 4 | 1.728604 | -0.708660 | 165 | 197.3 | 1.195758 |
| GO:0060255\_regulation\_of\_macromolecule\_metabolic\_process | EIF4EBP1 | 936 | 7 | 1.434963 | -0.699092 | 166 | 201.87 | 1.216084 |
| GO:0060255\_regulation\_of\_macromolecule\_metabolic\_process | YY1 | 936 | 7 | 1.434963 | -0.699092 | 166 | 201.87 | 1.216084 |
| GO:0060255\_regulation\_of\_macromolecule\_metabolic\_process | RNF2 | 936 | 7 | 1.434963 | -0.699092 | 166 | 201.87 | 1.216084 |
| GO:0060255\_regulation\_of\_macromolecule\_metabolic\_process | PKIG | 936 | 7 | 1.434963 | -0.699092 | 166 | 201.87 | 1.216084 |
| GO:0060255\_regulation\_of\_macromolecule\_metabolic\_process | EED | 936 | 7 | 1.434963 | -0.699092 | 166 | 201.87 | 1.216084 |
| GO:0060255\_regulation\_of\_macromolecule\_metabolic\_process | SKIL | 936 | 7 | 1.434963 | -0.699092 | 166 | 201.87 | 1.216084 |
| GO:0060255\_regulation\_of\_macromolecule\_metabolic\_process | CBFB | 936 | 7 | 1.434963 | -0.699092 | 166 | 201.87 | 1.216084 |
| GO:0032446\_protein\_modification\_by\_small\_protein\_conjugation | RNF2 | 43 | 1 | 4.462209 | -0.694516 | 168 | 205.35 | 1.222321 |
| GO:0032868\_response\_to\_insulin\_stimulus | EIF4EBP1 | 43 | 1 | 4.462209 | -0.694516 | 168 | 205.35 | 1.222321 |
| GO:0002377\_immunoglobulin\_production | UNG | 44 | 1 | 4.360795 | -0.685586 | 170 | 209.96 | 1.235059 |
| GO:0048593\_camera-type\_eye\_morphogenesis | YY1 | 44 | 1 | 4.360795 | -0.685586 | 170 | 209.96 | 1.235059 |
| GO:0032870\_cellular\_response\_to\_hormone\_stimulus | EIF4EBP1 | 45 | 1 | 4.263889 | -0.676880 | 171 | 211.76 | 1.238363 |
| GO:0006396\_RNA\_processing | FBL | 47 | 1 | 4.082447 | -0.660099 | 172 | 217.79 | 1.266221 |
| GO:0032269\_negative\_regulation\_of\_cellular\_protein\_metabolic\_process | EIF4EBP1 | 48 | 1 | 3.997396 | -0.652007 | 173 | 219.7 | 1.269942 |
| GO:0002440\_production\_of\_molecular\_mediator\_of\_immune\_response | UNG | 49 | 1 | 3.915816 | -0.644102 | 175 | 222.44 | 1.271086 |
| GO:0006725\_cellular\_aromatic\_compound\_metabolic\_process | APRT | 49 | 1 | 3.915816 | -0.644102 | 175 | 222.44 | 1.271086 |
| GO:0070647\_protein\_modification\_by\_small\_protein\_conjugation\_or\_removal | RNF2 | 50 | 1 | 3.837500 | -0.636378 | 176 | 224.69 | 1.276648 |
| GO:0044249\_cellular\_biosynthetic\_process | EIF4EBP1 | 1150 | 8 | 1.334783 | -0.633899 | 177 | 225.33 | 1.273051 |
| GO:0044249\_cellular\_biosynthetic\_process | MRPL3 | 1150 | 8 | 1.334783 | -0.633899 | 177 | 225.33 | 1.273051 |
| GO:0044249\_cellular\_biosynthetic\_process | YY1 | 1150 | 8 | 1.334783 | -0.633899 | 177 | 225.33 | 1.273051 |
| GO:0044249\_cellular\_biosynthetic\_process | RNF2 | 1150 | 8 | 1.334783 | -0.633899 | 177 | 225.33 | 1.273051 |
| GO:0044249\_cellular\_biosynthetic\_process | PKIG | 1150 | 8 | 1.334783 | -0.633899 | 177 | 225.33 | 1.273051 |
| GO:0044249\_cellular\_biosynthetic\_process | SKIL | 1150 | 8 | 1.334783 | -0.633899 | 177 | 225.33 | 1.273051 |
| GO:0044249\_cellular\_biosynthetic\_process | UROD | 1150 | 8 | 1.334783 | -0.633899 | 177 | 225.33 | 1.273051 |
| GO:0044249\_cellular\_biosynthetic\_process | CBFB | 1150 | 8 | 1.334783 | -0.633899 | 177 | 225.33 | 1.273051 |
| GO:0006887\_exocytosis | TMED10 | 51 | 1 | 3.762255 | -0.628826 | 179 | 227.87 | 1.273017 |
| GO:0032880\_regulation\_of\_protein\_localization | PKIG | 51 | 1 | 3.762255 | -0.628826 | 179 | 227.87 | 1.273017 |
| GO:0009987\_cellular\_process | ZFAND6 | 3868 | 22 | 1.091326 | -0.626246 | 180 | 228.09 | 1.267167 |
| GO:0009987\_cellular\_process | MRPL3 | 3868 | 22 | 1.091326 | -0.626246 | 180 | 228.09 | 1.267167 |
| GO:0009987\_cellular\_process | YY1 | 3868 | 22 | 1.091326 | -0.626246 | 180 | 228.09 | 1.267167 |
| GO:0009987\_cellular\_process | UNG | 3868 | 22 | 1.091326 | -0.626246 | 180 | 228.09 | 1.267167 |
| GO:0009987\_cellular\_process | PKIG | 3868 | 22 | 1.091326 | -0.626246 | 180 | 228.09 | 1.267167 |
| GO:0009987\_cellular\_process | CLK1 | 3868 | 22 | 1.091326 | -0.626246 | 180 | 228.09 | 1.267167 |
| GO:0009987\_cellular\_process | FBL | 3868 | 22 | 1.091326 | -0.626246 | 180 | 228.09 | 1.267167 |
| GO:0009987\_cellular\_process | CBFB | 3868 | 22 | 1.091326 | -0.626246 | 180 | 228.09 | 1.267167 |
| GO:0009987\_cellular\_process | APRT | 3868 | 22 | 1.091326 | -0.626246 | 180 | 228.09 | 1.267167 |
| GO:0009987\_cellular\_process | MIF | 3868 | 22 | 1.091326 | -0.626246 | 180 | 228.09 | 1.267167 |
| GO:0009987\_cellular\_process | EIF4EBP1 | 3868 | 22 | 1.091326 | -0.626246 | 180 | 228.09 | 1.267167 |
| GO:0009987\_cellular\_process | CCND2 | 3868 | 22 | 1.091326 | -0.626246 | 180 | 228.09 | 1.267167 |
| GO:0009987\_cellular\_process | RNF2 | 3868 | 22 | 1.091326 | -0.626246 | 180 | 228.09 | 1.267167 |
| GO:0009987\_cellular\_process | EED | 3868 | 22 | 1.091326 | -0.626246 | 180 | 228.09 | 1.267167 |
| GO:0009987\_cellular\_process | TMED10 | 3868 | 22 | 1.091326 | -0.626246 | 180 | 228.09 | 1.267167 |
| GO:0009987\_cellular\_process | GKAP1 | 3868 | 22 | 1.091326 | -0.626246 | 180 | 228.09 | 1.267167 |
| GO:0009987\_cellular\_process | SKIL | 3868 | 22 | 1.091326 | -0.626246 | 180 | 228.09 | 1.267167 |
| GO:0009987\_cellular\_process | UROD | 3868 | 22 | 1.091326 | -0.626246 | 180 | 228.09 | 1.267167 |
| GO:0009987\_cellular\_process | TOP2B | 3868 | 22 | 1.091326 | -0.626246 | 180 | 228.09 | 1.267167 |
| GO:0009987\_cellular\_process | PPP1R15B | 3868 | 22 | 1.091326 | -0.626246 | 180 | 228.09 | 1.267167 |
| GO:0009987\_cellular\_process | KPNB1 | 3868 | 22 | 1.091326 | -0.626246 | 180 | 228.09 | 1.267167 |
| GO:0009987\_cellular\_process | MAB21L2 | 3868 | 22 | 1.091326 | -0.626246 | 180 | 228.09 | 1.267167 |
| GO:0031326\_regulation\_of\_cellular\_biosynthetic\_process | EIF4EBP1 | 812 | 6 | 1.417796 | -0.622983 | 181 | 228.2 | 1.260773 |
| GO:0031326\_regulation\_of\_cellular\_biosynthetic\_process | YY1 | 812 | 6 | 1.417796 | -0.622983 | 181 | 228.2 | 1.260773 |
| GO:0031326\_regulation\_of\_cellular\_biosynthetic\_process | RNF2 | 812 | 6 | 1.417796 | -0.622983 | 181 | 228.2 | 1.260773 |
| GO:0031326\_regulation\_of\_cellular\_biosynthetic\_process | PKIG | 812 | 6 | 1.417796 | -0.622983 | 181 | 228.2 | 1.260773 |
| GO:0031326\_regulation\_of\_cellular\_biosynthetic\_process | SKIL | 812 | 6 | 1.417796 | -0.622983 | 181 | 228.2 | 1.260773 |
| GO:0031326\_regulation\_of\_cellular\_biosynthetic\_process | CBFB | 812 | 6 | 1.417796 | -0.622983 | 181 | 228.2 | 1.260773 |
| GO:0010608\_posttranscriptional\_regulation\_of\_gene\_expression | EIF4EBP1 | 52 | 1 | 3.689904 | -0.621441 | 182 | 229.68 | 1.261978 |
| GO:0009889\_regulation\_of\_biosynthetic\_process | EIF4EBP1 | 815 | 6 | 1.412577 | -0.618014 | 183 | 229.98 | 1.256721 |
| GO:0009889\_regulation\_of\_biosynthetic\_process | YY1 | 815 | 6 | 1.412577 | -0.618014 | 183 | 229.98 | 1.256721 |
| GO:0009889\_regulation\_of\_biosynthetic\_process | RNF2 | 815 | 6 | 1.412577 | -0.618014 | 183 | 229.98 | 1.256721 |
| GO:0009889\_regulation\_of\_biosynthetic\_process | PKIG | 815 | 6 | 1.412577 | -0.618014 | 183 | 229.98 | 1.256721 |
| GO:0009889\_regulation\_of\_biosynthetic\_process | SKIL | 815 | 6 | 1.412577 | -0.618014 | 183 | 229.98 | 1.256721 |
| GO:0009889\_regulation\_of\_biosynthetic\_process | CBFB | 815 | 6 | 1.412577 | -0.618014 | 183 | 229.98 | 1.256721 |
| GO:0051248\_negative\_regulation\_of\_protein\_metabolic\_process | EIF4EBP1 | 53 | 1 | 3.620283 | -0.614216 | 184 | 231.9 | 1.260326 |
| GO:0044271\_nitrogen\_compound\_biosynthetic\_process | UROD | 54 | 1 | 3.553241 | -0.607144 | 185 | 234.7 | 1.268649 |
| GO:0019538\_protein\_metabolic\_process | EIF4EBP1 | 655 | 5 | 1.464695 | -0.604037 | 186 | 235.55 | 1.266398 |
| GO:0019538\_protein\_metabolic\_process | MRPL3 | 655 | 5 | 1.464695 | -0.604037 | 186 | 235.55 | 1.266398 |
| GO:0019538\_protein\_metabolic\_process | RNF2 | 655 | 5 | 1.464695 | -0.604037 | 186 | 235.55 | 1.266398 |
| GO:0019538\_protein\_metabolic\_process | EED | 655 | 5 | 1.464695 | -0.604037 | 186 | 235.55 | 1.266398 |
| GO:0019538\_protein\_metabolic\_process | CLK1 | 655 | 5 | 1.464695 | -0.604037 | 186 | 235.55 | 1.266398 |
| GO:0006310\_DNA\_recombination | UNG | 55 | 1 | 3.488636 | -0.600221 | 188 | 237.26 | 1.262021 |
| GO:0043434\_response\_to\_peptide\_hormone\_stimulus | EIF4EBP1 | 55 | 1 | 3.488636 | -0.600221 | 188 | 237.26 | 1.262021 |
| GO:0009058\_biosynthetic\_process | EIF4EBP1 | 1175 | 8 | 1.306383 | -0.598235 | 189 | 237.56 | 1.256931 |
| GO:0009058\_biosynthetic\_process | MRPL3 | 1175 | 8 | 1.306383 | -0.598235 | 189 | 237.56 | 1.256931 |
| GO:0009058\_biosynthetic\_process | YY1 | 1175 | 8 | 1.306383 | -0.598235 | 189 | 237.56 | 1.256931 |
| GO:0009058\_biosynthetic\_process | RNF2 | 1175 | 8 | 1.306383 | -0.598235 | 189 | 237.56 | 1.256931 |
| GO:0009058\_biosynthetic\_process | PKIG | 1175 | 8 | 1.306383 | -0.598235 | 189 | 237.56 | 1.256931 |
| GO:0009058\_biosynthetic\_process | SKIL | 1175 | 8 | 1.306383 | -0.598235 | 189 | 237.56 | 1.256931 |
| GO:0009058\_biosynthetic\_process | UROD | 1175 | 8 | 1.306383 | -0.598235 | 189 | 237.56 | 1.256931 |
| GO:0009058\_biosynthetic\_process | CBFB | 1175 | 8 | 1.306383 | -0.598235 | 189 | 237.56 | 1.256931 |
| GO:0044238\_primary\_metabolic\_process | EIF4EBP1 | 1905 | 12 | 1.208661 | -0.593388 | 190 | 240.76 | 1.267158 |
| GO:0044238\_primary\_metabolic\_process | MRPL3 | 1905 | 12 | 1.208661 | -0.593388 | 190 | 240.76 | 1.267158 |
| GO:0044238\_primary\_metabolic\_process | YY1 | 1905 | 12 | 1.208661 | -0.593388 | 190 | 240.76 | 1.267158 |
| GO:0044238\_primary\_metabolic\_process | RNF2 | 1905 | 12 | 1.208661 | -0.593388 | 190 | 240.76 | 1.267158 |
| GO:0044238\_primary\_metabolic\_process | UNG | 1905 | 12 | 1.208661 | -0.593388 | 190 | 240.76 | 1.267158 |
| GO:0044238\_primary\_metabolic\_process | PKIG | 1905 | 12 | 1.208661 | -0.593388 | 190 | 240.76 | 1.267158 |
| GO:0044238\_primary\_metabolic\_process | EED | 1905 | 12 | 1.208661 | -0.593388 | 190 | 240.76 | 1.267158 |
| GO:0044238\_primary\_metabolic\_process | CLK1 | 1905 | 12 | 1.208661 | -0.593388 | 190 | 240.76 | 1.267158 |
| GO:0044238\_primary\_metabolic\_process | SKIL | 1905 | 12 | 1.208661 | -0.593388 | 190 | 240.76 | 1.267158 |
| GO:0044238\_primary\_metabolic\_process | FBL | 1905 | 12 | 1.208661 | -0.593388 | 190 | 240.76 | 1.267158 |
| GO:0044238\_primary\_metabolic\_process | CBFB | 1905 | 12 | 1.208661 | -0.593388 | 190 | 240.76 | 1.267158 |
| GO:0044238\_primary\_metabolic\_process | APRT | 1905 | 12 | 1.208661 | -0.593388 | 190 | 240.76 | 1.267158 |
| GO:0001764\_neuron\_migration | TOP2B | 57 | 1 | 3.366228 | -0.586798 | 193 | 244.03 | 1.264404 |
| GO:0018108\_peptidyl-tyrosine\_phosphorylation | CLK1 | 57 | 1 | 3.366228 | -0.586798 | 193 | 244.03 | 1.264404 |
| GO:0018212\_peptidyl-tyrosine\_modification | CLK1 | 57 | 1 | 3.366228 | -0.586798 | 193 | 244.03 | 1.264404 |
| GO:0051649\_establishment\_of\_localization\_in\_cell | PKIG | 342 | 3 | 1.683114 | -0.581658 | 194 | 244.86 | 1.262165 |
| GO:0051649\_establishment\_of\_localization\_in\_cell | TMED10 | 342 | 3 | 1.683114 | -0.581658 | 194 | 244.86 | 1.262165 |
| GO:0051649\_establishment\_of\_localization\_in\_cell | KPNB1 | 342 | 3 | 1.683114 | -0.581658 | 194 | 244.86 | 1.262165 |
| GO:0048469\_cell\_maturation | CBFB | 59 | 1 | 3.252119 | -0.573907 | 195 | 247.28 | 1.268103 |
| GO:0003002\_regionalization | YY1 | 195 | 2 | 1.967949 | -0.568185 | 196 | 248.26 | 1.266633 |
| GO:0003002\_regionalization | RNF2 | 195 | 2 | 1.967949 | -0.568185 | 196 | 248.26 | 1.266633 |
| GO:0045449\_regulation\_of\_transcription | YY1 | 676 | 5 | 1.419194 | -0.567531 | 197 | 248.64 | 1.262132 |
| GO:0045449\_regulation\_of\_transcription | RNF2 | 676 | 5 | 1.419194 | -0.567531 | 197 | 248.64 | 1.262132 |
| GO:0045449\_regulation\_of\_transcription | PKIG | 676 | 5 | 1.419194 | -0.567531 | 197 | 248.64 | 1.262132 |
| GO:0045449\_regulation\_of\_transcription | SKIL | 676 | 5 | 1.419194 | -0.567531 | 197 | 248.64 | 1.262132 |
| GO:0045449\_regulation\_of\_transcription | CBFB | 676 | 5 | 1.419194 | -0.567531 | 197 | 248.64 | 1.262132 |
| GO:0007369\_gastrulation | RNF2 | 63 | 1 | 3.045635 | -0.549581 | 199 | 255.28 | 1.282814 |
| GO:0051186\_cofactor\_metabolic\_process | UROD | 63 | 1 | 3.045635 | -0.549581 | 199 | 255.28 | 1.282814 |
| GO:0008152\_metabolic\_process | MRPL3 | 2133 | 13 | 1.169421 | -0.546069 | 200 | 255.79 | 1.278950 |
| GO:0008152\_metabolic\_process | UNG | 2133 | 13 | 1.169421 | -0.546069 | 200 | 255.79 | 1.278950 |
| GO:0008152\_metabolic\_process | YY1 | 2133 | 13 | 1.169421 | -0.546069 | 200 | 255.79 | 1.278950 |
| GO:0008152\_metabolic\_process | PKIG | 2133 | 13 | 1.169421 | -0.546069 | 200 | 255.79 | 1.278950 |
| GO:0008152\_metabolic\_process | CLK1 | 2133 | 13 | 1.169421 | -0.546069 | 200 | 255.79 | 1.278950 |
| GO:0008152\_metabolic\_process | CBFB | 2133 | 13 | 1.169421 | -0.546069 | 200 | 255.79 | 1.278950 |
| GO:0008152\_metabolic\_process | FBL | 2133 | 13 | 1.169421 | -0.546069 | 200 | 255.79 | 1.278950 |
| GO:0008152\_metabolic\_process | APRT | 2133 | 13 | 1.169421 | -0.546069 | 200 | 255.79 | 1.278950 |
| GO:0008152\_metabolic\_process | EIF4EBP1 | 2133 | 13 | 1.169421 | -0.546069 | 200 | 255.79 | 1.278950 |
| GO:0008152\_metabolic\_process | RNF2 | 2133 | 13 | 1.169421 | -0.546069 | 200 | 255.79 | 1.278950 |
| GO:0008152\_metabolic\_process | EED | 2133 | 13 | 1.169421 | -0.546069 | 200 | 255.79 | 1.278950 |
| GO:0008152\_metabolic\_process | SKIL | 2133 | 13 | 1.169421 | -0.546069 | 200 | 255.79 | 1.278950 |
| GO:0008152\_metabolic\_process | UROD | 2133 | 13 | 1.169421 | -0.546069 | 200 | 255.79 | 1.278950 |
| GO:0043086\_negative\_regulation\_of\_catalytic\_activity | PKIG | 65 | 1 | 2.951923 | -0.538085 | 201 | 259.02 | 1.288657 |
| GO:0007179\_transforming\_growth\_factor\_beta\_receptor\_signaling\_pathway | SKIL | 66 | 1 | 2.907197 | -0.532491 | 202 | 260.93 | 1.291733 |
| GO:0006350\_transcription | YY1 | 701 | 5 | 1.368581 | -0.526778 | 203 | 263.19 | 1.296502 |
| GO:0006350\_transcription | RNF2 | 701 | 5 | 1.368581 | -0.526778 | 203 | 263.19 | 1.296502 |
| GO:0006350\_transcription | PKIG | 701 | 5 | 1.368581 | -0.526778 | 203 | 263.19 | 1.296502 |
| GO:0006350\_transcription | SKIL | 701 | 5 | 1.368581 | -0.526778 | 203 | 263.19 | 1.296502 |
| GO:0006350\_transcription | CBFB | 701 | 5 | 1.368581 | -0.526778 | 203 | 263.19 | 1.296502 |
| GO:0019932\_second-messenger-mediated\_signaling | GKAP1 | 68 | 1 | 2.821691 | -0.521598 | 204 | 264.57 | 1.296912 |
| GO:0051641\_cellular\_localization | PKIG | 370 | 3 | 1.555743 | -0.518801 | 205 | 264.81 | 1.291756 |
| GO:0051641\_cellular\_localization | TMED10 | 370 | 3 | 1.555743 | -0.518801 | 205 | 264.81 | 1.291756 |
| GO:0051641\_cellular\_localization | KPNB1 | 370 | 3 | 1.555743 | -0.518801 | 205 | 264.81 | 1.291756 |
| GO:0048592\_eye\_morphogenesis | YY1 | 70 | 1 | 2.741071 | -0.511078 | 206 | 268.16 | 1.301748 |
| GO:0006281\_DNA\_repair | UNG | 71 | 1 | 2.702465 | -0.505951 | 207 | 270.12 | 1.304928 |
| GO:0006950\_response\_to\_stress | UNG | 549 | 4 | 1.397996 | -0.495928 | 208 | 276.45 | 1.329087 |
| GO:0006950\_response\_to\_stress | PPP1R15B | 549 | 4 | 1.397996 | -0.495928 | 208 | 276.45 | 1.329087 |
| GO:0006950\_response\_to\_stress | MDK | 549 | 4 | 1.397996 | -0.495928 | 208 | 276.45 | 1.329087 |
| GO:0006950\_response\_to\_stress | MIF | 549 | 4 | 1.397996 | -0.495928 | 208 | 276.45 | 1.329087 |
| GO:0001701\_in\_utero\_embryonic\_development | BTF3 | 221 | 2 | 1.736425 | -0.493096 | 209 | 276.86 | 1.324689 |
| GO:0001701\_in\_utero\_embryonic\_development | SKIL | 221 | 2 | 1.736425 | -0.493096 | 209 | 276.86 | 1.324689 |
| GO:0043687\_post-translational\_protein\_modification | RNF2 | 384 | 3 | 1.499023 | -0.490180 | 210 | 277.8 | 1.322857 |
| GO:0043687\_post-translational\_protein\_modification | EED | 384 | 3 | 1.499023 | -0.490180 | 210 | 277.8 | 1.322857 |
| GO:0043687\_post-translational\_protein\_modification | CLK1 | 384 | 3 | 1.499023 | -0.490180 | 210 | 277.8 | 1.322857 |
| GO:0009725\_response\_to\_hormone\_stimulus | EIF4EBP1 | 76 | 1 | 2.524671 | -0.481550 | 211 | 281.19 | 1.332654 |
| GO:0019222\_regulation\_of\_metabolic\_process | EIF4EBP1 | 1088 | 7 | 1.234490 | -0.478794 | 212 | 281.49 | 1.327783 |
| GO:0019222\_regulation\_of\_metabolic\_process | YY1 | 1088 | 7 | 1.234490 | -0.478794 | 212 | 281.49 | 1.327783 |
| GO:0019222\_regulation\_of\_metabolic\_process | RNF2 | 1088 | 7 | 1.234490 | -0.478794 | 212 | 281.49 | 1.327783 |
| GO:0019222\_regulation\_of\_metabolic\_process | PKIG | 1088 | 7 | 1.234490 | -0.478794 | 212 | 281.49 | 1.327783 |
| GO:0019222\_regulation\_of\_metabolic\_process | EED | 1088 | 7 | 1.234490 | -0.478794 | 212 | 281.49 | 1.327783 |
| GO:0019222\_regulation\_of\_metabolic\_process | SKIL | 1088 | 7 | 1.234490 | -0.478794 | 212 | 281.49 | 1.327783 |
| GO:0019222\_regulation\_of\_metabolic\_process | CBFB | 1088 | 7 | 1.234490 | -0.478794 | 212 | 281.49 | 1.327783 |
| GO:0007167\_enzyme\_linked\_receptor\_protein\_signaling\_pathway | EIF4EBP1 | 229 | 2 | 1.675764 | -0.472503 | 213 | 283.38 | 1.330423 |
| GO:0007167\_enzyme\_linked\_receptor\_protein\_signaling\_pathway | SKIL | 229 | 2 | 1.675764 | -0.472503 | 213 | 283.38 | 1.330423 |
| GO:0007165\_signal\_transduction | ZFAND6 | 915 | 6 | 1.258197 | -0.471296 | 214 | 285.42 | 1.333738 |
| GO:0007165\_signal\_transduction | EIF4EBP1 | 915 | 6 | 1.258197 | -0.471296 | 214 | 285.42 | 1.333738 |
| GO:0007165\_signal\_transduction | GKAP1 | 915 | 6 | 1.258197 | -0.471296 | 214 | 285.42 | 1.333738 |
| GO:0007165\_signal\_transduction | SKIL | 915 | 6 | 1.258197 | -0.471296 | 214 | 285.42 | 1.333738 |
| GO:0007165\_signal\_transduction | PPP1R15B | 915 | 6 | 1.258197 | -0.471296 | 214 | 285.42 | 1.333738 |
| GO:0007165\_signal\_transduction | MIF | 915 | 6 | 1.258197 | -0.471296 | 214 | 285.42 | 1.333738 |
| GO:0009790\_embryonic\_development | RNF2 | 567 | 4 | 1.353616 | -0.466505 | 215 | 286.55 | 1.332791 |
| GO:0009790\_embryonic\_development | BTF3 | 567 | 4 | 1.353616 | -0.466505 | 215 | 286.55 | 1.332791 |
| GO:0009790\_embryonic\_development | SKIL | 567 | 4 | 1.353616 | -0.466505 | 215 | 286.55 | 1.332791 |
| GO:0009790\_embryonic\_development | MAB21L2 | 567 | 4 | 1.353616 | -0.466505 | 215 | 286.55 | 1.332791 |
| GO:0000278\_mitotic\_cell\_cycle | RNF2 | 80 | 1 | 2.398438 | -0.463383 | 217 | 287.92 | 1.326820 |
| GO:0044092\_negative\_regulation\_of\_molecular\_function | PKIG | 80 | 1 | 2.398438 | -0.463383 | 217 | 287.92 | 1.326820 |
| GO:0021700\_developmental\_maturation | CBFB | 81 | 1 | 2.368827 | -0.459013 | 218 | 289.15 | 1.326376 |
| GO:0080090\_regulation\_of\_primary\_metabolic\_process | EIF4EBP1 | 926 | 6 | 1.243251 | -0.457202 | 219 | 289.28 | 1.320913 |
| GO:0080090\_regulation\_of\_primary\_metabolic\_process | YY1 | 926 | 6 | 1.243251 | -0.457202 | 219 | 289.28 | 1.320913 |
| GO:0080090\_regulation\_of\_primary\_metabolic\_process | RNF2 | 926 | 6 | 1.243251 | -0.457202 | 219 | 289.28 | 1.320913 |
| GO:0080090\_regulation\_of\_primary\_metabolic\_process | PKIG | 926 | 6 | 1.243251 | -0.457202 | 219 | 289.28 | 1.320913 |
| GO:0080090\_regulation\_of\_primary\_metabolic\_process | SKIL | 926 | 6 | 1.243251 | -0.457202 | 219 | 289.28 | 1.320913 |
| GO:0080090\_regulation\_of\_primary\_metabolic\_process | CBFB | 926 | 6 | 1.243251 | -0.457202 | 219 | 289.28 | 1.320913 |
| GO:0019219\_regulation\_of\_nucleobase\_\_nucleoside\_\_nucleotide\_and\_nucleic\_acid\_metabolic\_process | YY1 | 757 | 5 | 1.267338 | -0.445131 | 220 | 294.58 | 1.339000 |
| GO:0019219\_regulation\_of\_nucleobase\_\_nucleoside\_\_nucleotide\_and\_nucleic\_acid\_metabolic\_process | RNF2 | 757 | 5 | 1.267338 | -0.445131 | 220 | 294.58 | 1.339000 |
| GO:0019219\_regulation\_of\_nucleobase\_\_nucleoside\_\_nucleotide\_and\_nucleic\_acid\_metabolic\_process | PKIG | 757 | 5 | 1.267338 | -0.445131 | 220 | 294.58 | 1.339000 |
| GO:0019219\_regulation\_of\_nucleobase\_\_nucleoside\_\_nucleotide\_and\_nucleic\_acid\_metabolic\_process | SKIL | 757 | 5 | 1.267338 | -0.445131 | 220 | 294.58 | 1.339000 |
| GO:0019219\_regulation\_of\_nucleobase\_\_nucleoside\_\_nucleotide\_and\_nucleic\_acid\_metabolic\_process | CBFB | 757 | 5 | 1.267338 | -0.445131 | 220 | 294.58 | 1.339000 |
| GO:0070887\_cellular\_response\_to\_chemical\_stimulus | EIF4EBP1 | 85 | 1 | 2.257353 | -0.442173 | 221 | 295.68 | 1.337919 |
| GO:0007242\_intracellular\_signaling\_cascade | GKAP1 | 411 | 3 | 1.400547 | -0.439631 | 222 | 295.83 | 1.332568 |
| GO:0007242\_intracellular\_signaling\_cascade | PPP1R15B | 411 | 3 | 1.400547 | -0.439631 | 222 | 295.83 | 1.332568 |
| GO:0007242\_intracellular\_signaling\_cascade | MIF | 411 | 3 | 1.400547 | -0.439631 | 222 | 295.83 | 1.332568 |
| GO:0034641\_cellular\_nitrogen\_compound\_metabolic\_process | UROD | 86 | 1 | 2.231105 | -0.438115 | 223 | 297.83 | 1.335561 |
| GO:0007178\_transmembrane\_receptor\_protein\_serine\_threonine\_kinase\_signaling\_pathway | SKIL | 87 | 1 | 2.205460 | -0.434115 | 224 | 300.17 | 1.340045 |
| GO:0001503\_ossification | CBFB | 88 | 1 | 2.180398 | -0.430171 | 225 | 301.25 | 1.338889 |
| GO:0051171\_regulation\_of\_nitrogen\_compound\_metabolic\_process | YY1 | 771 | 5 | 1.244326 | -0.426617 | 226 | 301.88 | 1.335752 |
| GO:0051171\_regulation\_of\_nitrogen\_compound\_metabolic\_process | RNF2 | 771 | 5 | 1.244326 | -0.426617 | 226 | 301.88 | 1.335752 |
| GO:0051171\_regulation\_of\_nitrogen\_compound\_metabolic\_process | PKIG | 771 | 5 | 1.244326 | -0.426617 | 226 | 301.88 | 1.335752 |
| GO:0051171\_regulation\_of\_nitrogen\_compound\_metabolic\_process | SKIL | 771 | 5 | 1.244326 | -0.426617 | 226 | 301.88 | 1.335752 |
| GO:0051171\_regulation\_of\_nitrogen\_compound\_metabolic\_process | CBFB | 771 | 5 | 1.244326 | -0.426617 | 226 | 301.88 | 1.335752 |
| GO:0007389\_pattern\_specification\_process | YY1 | 250 | 2 | 1.535000 | -0.423185 | 227 | 303.0 | 1.334802 |
| GO:0007389\_pattern\_specification\_process | RNF2 | 250 | 2 | 1.535000 | -0.423185 | 227 | 303.0 | 1.334802 |
| GO:0009719\_response\_to\_endogenous\_stimulus | EIF4EBP1 | 92 | 1 | 2.085598 | -0.414938 | 228 | 308.14 | 1.351491 |
| GO:0018193\_peptidyl-amino\_acid\_modification | CLK1 | 97 | 1 | 1.978093 | -0.397026 | 230 | 316.94 | 1.378000 |
| GO:0060341\_regulation\_of\_cellular\_localization | PKIG | 97 | 1 | 1.978093 | -0.397026 | 230 | 316.94 | 1.378000 |
| GO:0006464\_protein\_modification\_process | RNF2 | 439 | 3 | 1.311219 | -0.392940 | 231 | 319.29 | 1.382208 |
| GO:0006464\_protein\_modification\_process | EED | 439 | 3 | 1.311219 | -0.392940 | 231 | 319.29 | 1.382208 |
| GO:0006464\_protein\_modification\_process | CLK1 | 439 | 3 | 1.311219 | -0.392940 | 231 | 319.29 | 1.382208 |
| GO:0060348\_bone\_development | CBFB | 99 | 1 | 1.938131 | -0.390185 | 232 | 320.52 | 1.381552 |
| GO:0009968\_negative\_regulation\_of\_signal\_transduction | SKIL | 103 | 1 | 1.862864 | -0.377020 | 233 | 325.06 | 1.395107 |
| GO:0055086\_nucleobase\_\_nucleoside\_and\_nucleotide\_metabolic\_process | APRT | 104 | 1 | 1.844952 | -0.373831 | 234 | 327.47 | 1.399444 |
| GO:0048872\_homeostasis\_of\_number\_of\_cells | SKIL | 105 | 1 | 1.827381 | -0.370682 | 235 | 328.06 | 1.396000 |
| GO:0045859\_regulation\_of\_protein\_kinase\_activity | PKIG | 107 | 1 | 1.793224 | -0.364498 | 236 | 329.93 | 1.398008 |
| GO:0043412\_biopolymer\_modification | RNF2 | 458 | 3 | 1.256823 | -0.364190 | 237 | 330.39 | 1.394051 |
| GO:0043412\_biopolymer\_modification | EED | 458 | 3 | 1.256823 | -0.364190 | 237 | 330.39 | 1.394051 |
| GO:0043412\_biopolymer\_modification | CLK1 | 458 | 3 | 1.256823 | -0.364190 | 237 | 330.39 | 1.394051 |
| GO:0030099\_myeloid\_cell\_differentiation | CBFB | 108 | 1 | 1.776620 | -0.361462 | 238 | 331.15 | 1.391387 |
| GO:0031323\_regulation\_of\_cellular\_metabolic\_process | EIF4EBP1 | 1015 | 6 | 1.134236 | -0.355920 | 239 | 332.65 | 1.391841 |
| GO:0031323\_regulation\_of\_cellular\_metabolic\_process | YY1 | 1015 | 6 | 1.134236 | -0.355920 | 239 | 332.65 | 1.391841 |
| GO:0031323\_regulation\_of\_cellular\_metabolic\_process | RNF2 | 1015 | 6 | 1.134236 | -0.355920 | 239 | 332.65 | 1.391841 |
| GO:0031323\_regulation\_of\_cellular\_metabolic\_process | PKIG | 1015 | 6 | 1.134236 | -0.355920 | 239 | 332.65 | 1.391841 |
| GO:0031323\_regulation\_of\_cellular\_metabolic\_process | SKIL | 1015 | 6 | 1.134236 | -0.355920 | 239 | 332.65 | 1.391841 |
| GO:0031323\_regulation\_of\_cellular\_metabolic\_process | CBFB | 1015 | 6 | 1.134236 | -0.355920 | 239 | 332.65 | 1.391841 |
| GO:0010648\_negative\_regulation\_of\_cell\_communication | SKIL | 110 | 1 | 1.744318 | -0.355499 | 240 | 334.2 | 1.392500 |
| GO:0043549\_regulation\_of\_kinase\_activity | PKIG | 112 | 1 | 1.713170 | -0.349676 | 241 | 335.74 | 1.393112 |
| GO:0009607\_response\_to\_biotic\_stimulus | PPP1R15B | 114 | 1 | 1.683114 | -0.343988 | 242 | 338.1 | 1.397107 |
| GO:0051338\_regulation\_of\_transferase\_activity | PKIG | 115 | 1 | 1.668478 | -0.341194 | 243 | 338.93 | 1.394774 |
| GO:0002520\_immune\_system\_development | UNG | 295 | 2 | 1.300847 | -0.336368 | 244 | 340.76 | 1.396557 |
| GO:0002520\_immune\_system\_development | CBFB | 295 | 2 | 1.300847 | -0.336368 | 244 | 340.76 | 1.396557 |
| GO:0050789\_regulation\_of\_biological\_process | ZFAND6 | 2357 | 13 | 1.058284 | -0.332043 | 245 | 342.12 | 1.396408 |
| GO:0050789\_regulation\_of\_biological\_process | YY1 | 2357 | 13 | 1.058284 | -0.332043 | 245 | 342.12 | 1.396408 |
| GO:0050789\_regulation\_of\_biological\_process | PKIG | 2357 | 13 | 1.058284 | -0.332043 | 245 | 342.12 | 1.396408 |
| GO:0050789\_regulation\_of\_biological\_process | CBFB | 2357 | 13 | 1.058284 | -0.332043 | 245 | 342.12 | 1.396408 |
| GO:0050789\_regulation\_of\_biological\_process | MIF | 2357 | 13 | 1.058284 | -0.332043 | 245 | 342.12 | 1.396408 |
| GO:0050789\_regulation\_of\_biological\_process | EIF4EBP1 | 2357 | 13 | 1.058284 | -0.332043 | 245 | 342.12 | 1.396408 |
| GO:0050789\_regulation\_of\_biological\_process | CCND2 | 2357 | 13 | 1.058284 | -0.332043 | 245 | 342.12 | 1.396408 |
| GO:0050789\_regulation\_of\_biological\_process | RNF2 | 2357 | 13 | 1.058284 | -0.332043 | 245 | 342.12 | 1.396408 |
| GO:0050789\_regulation\_of\_biological\_process | EED | 2357 | 13 | 1.058284 | -0.332043 | 245 | 342.12 | 1.396408 |
| GO:0050789\_regulation\_of\_biological\_process | GKAP1 | 2357 | 13 | 1.058284 | -0.332043 | 245 | 342.12 | 1.396408 |
| GO:0050789\_regulation\_of\_biological\_process | SKIL | 2357 | 13 | 1.058284 | -0.332043 | 245 | 342.12 | 1.396408 |
| GO:0050789\_regulation\_of\_biological\_process | PPP1R15B | 2357 | 13 | 1.058284 | -0.332043 | 245 | 342.12 | 1.396408 |
| GO:0050789\_regulation\_of\_biological\_process | MAB21L2 | 2357 | 13 | 1.058284 | -0.332043 | 245 | 342.12 | 1.396408 |
| GO:0048598\_embryonic\_morphogenesis | RNF2 | 299 | 2 | 1.283445 | -0.329684 | 246 | 342.71 | 1.393130 |
| GO:0048598\_embryonic\_morphogenesis | MAB21L2 | 299 | 2 | 1.283445 | -0.329684 | 246 | 342.71 | 1.393130 |
| GO:0051726\_regulation\_of\_cell\_cycle | CCND2 | 121 | 1 | 1.585744 | -0.325081 | 247 | 344.73 | 1.395668 |
| GO:0002252\_immune\_effector\_process | UNG | 122 | 1 | 1.572746 | -0.322499 | 248 | 346.74 | 1.398145 |
| GO:0030098\_lymphocyte\_differentiation | CBFB | 124 | 1 | 1.547379 | -0.317421 | 249 | 348.34 | 1.398956 |
| GO:0050794\_regulation\_of\_cellular\_process | ZFAND6 | 2190 | 12 | 1.051370 | -0.314614 | 250 | 349.87 | 1.399480 |
| GO:0050794\_regulation\_of\_cellular\_process | EIF4EBP1 | 2190 | 12 | 1.051370 | -0.314614 | 250 | 349.87 | 1.399480 |
| GO:0050794\_regulation\_of\_cellular\_process | CCND2 | 2190 | 12 | 1.051370 | -0.314614 | 250 | 349.87 | 1.399480 |
| GO:0050794\_regulation\_of\_cellular\_process | YY1 | 2190 | 12 | 1.051370 | -0.314614 | 250 | 349.87 | 1.399480 |
| GO:0050794\_regulation\_of\_cellular\_process | RNF2 | 2190 | 12 | 1.051370 | -0.314614 | 250 | 349.87 | 1.399480 |
| GO:0050794\_regulation\_of\_cellular\_process | PKIG | 2190 | 12 | 1.051370 | -0.314614 | 250 | 349.87 | 1.399480 |
| GO:0050794\_regulation\_of\_cellular\_process | GKAP1 | 2190 | 12 | 1.051370 | -0.314614 | 250 | 349.87 | 1.399480 |
| GO:0050794\_regulation\_of\_cellular\_process | SKIL | 2190 | 12 | 1.051370 | -0.314614 | 250 | 349.87 | 1.399480 |
| GO:0050794\_regulation\_of\_cellular\_process | PPP1R15B | 2190 | 12 | 1.051370 | -0.314614 | 250 | 349.87 | 1.399480 |
| GO:0050794\_regulation\_of\_cellular\_process | MAB21L2 | 2190 | 12 | 1.051370 | -0.314614 | 250 | 349.87 | 1.399480 |
| GO:0050794\_regulation\_of\_cellular\_process | CBFB | 2190 | 12 | 1.051370 | -0.314614 | 250 | 349.87 | 1.399480 |
| GO:0050794\_regulation\_of\_cellular\_process | MIF | 2190 | 12 | 1.051370 | -0.314614 | 250 | 349.87 | 1.399480 |
| GO:0016310\_phosphorylation | PKIG | 309 | 2 | 1.241909 | -0.313611 | 251 | 350.32 | 1.395697 |
| GO:0016310\_phosphorylation | CLK1 | 309 | 2 | 1.241909 | -0.313611 | 251 | 350.32 | 1.395697 |
| GO:0002376\_immune\_system\_process | UNG | 505 | 3 | 1.139851 | -0.301850 | 252 | 353.75 | 1.403770 |
| GO:0002376\_immune\_system\_process | SKIL | 505 | 3 | 1.139851 | -0.301850 | 252 | 353.75 | 1.403770 |
| GO:0002376\_immune\_system\_process | CBFB | 505 | 3 | 1.139851 | -0.301850 | 252 | 353.75 | 1.403770 |
| GO:0007169\_transmembrane\_receptor\_protein\_tyrosine\_kinase\_signaling\_pathway | EIF4EBP1 | 139 | 1 | 1.380396 | -0.282602 | 253 | 357.79 | 1.414190 |
| GO:0007154\_cell\_communication | ZFAND6 | 1096 | 6 | 1.050411 | -0.281010 | 254 | 359.74 | 1.416299 |
| GO:0007154\_cell\_communication | EIF4EBP1 | 1096 | 6 | 1.050411 | -0.281010 | 254 | 359.74 | 1.416299 |
| GO:0007154\_cell\_communication | GKAP1 | 1096 | 6 | 1.050411 | -0.281010 | 254 | 359.74 | 1.416299 |
| GO:0007154\_cell\_communication | SKIL | 1096 | 6 | 1.050411 | -0.281010 | 254 | 359.74 | 1.416299 |
| GO:0007154\_cell\_communication | PPP1R15B | 1096 | 6 | 1.050411 | -0.281010 | 254 | 359.74 | 1.416299 |
| GO:0007154\_cell\_communication | MIF | 1096 | 6 | 1.050411 | -0.281010 | 254 | 359.74 | 1.416299 |
| GO:0007186\_G-protein\_coupled\_receptor\_protein\_signaling\_pathway | GKAP1 | 144 | 1 | 1.332465 | -0.272139 | 256 | 363.15 | 1.418555 |
| GO:0045596\_negative\_regulation\_of\_cell\_differentiation | SKIL | 144 | 1 | 1.332465 | -0.272139 | 256 | 363.15 | 1.418555 |
| GO:0050896\_response\_to\_stimulus | EIF4EBP1 | 1107 | 6 | 1.039973 | -0.271946 | 257 | 363.4 | 1.414008 |
| GO:0050896\_response\_to\_stimulus | UNG | 1107 | 6 | 1.039973 | -0.271946 | 257 | 363.4 | 1.414008 |
| GO:0050896\_response\_to\_stimulus | PPP1R15B | 1107 | 6 | 1.039973 | -0.271946 | 257 | 363.4 | 1.414008 |
| GO:0050896\_response\_to\_stimulus | MDK | 1107 | 6 | 1.039973 | -0.271946 | 257 | 363.4 | 1.414008 |
| GO:0050896\_response\_to\_stimulus | APRT | 1107 | 6 | 1.039973 | -0.271946 | 257 | 363.4 | 1.414008 |
| GO:0050896\_response\_to\_stimulus | MIF | 1107 | 6 | 1.039973 | -0.271946 | 257 | 363.4 | 1.414008 |
| GO:0006793\_phosphorus\_metabolic\_process | PKIG | 340 | 2 | 1.128676 | -0.268997 | 259 | 364.84 | 1.408649 |
| GO:0006793\_phosphorus\_metabolic\_process | CLK1 | 340 | 2 | 1.128676 | -0.268997 | 259 | 364.84 | 1.408649 |
| GO:0006796\_phosphate\_metabolic\_process | PKIG | 340 | 2 | 1.128676 | -0.268997 | 259 | 364.84 | 1.408649 |
| GO:0006796\_phosphate\_metabolic\_process | CLK1 | 340 | 2 | 1.128676 | -0.268997 | 259 | 364.84 | 1.408649 |
| GO:0030900\_forebrain\_development | TOP2B | 146 | 1 | 1.314212 | -0.268097 | 260 | 365.97 | 1.407577 |
| GO:0032940\_secretion\_by\_cell | TMED10 | 149 | 1 | 1.287752 | -0.262180 | 261 | 368.37 | 1.411379 |
| GO:0032268\_regulation\_of\_cellular\_protein\_metabolic\_process | EIF4EBP1 | 152 | 1 | 1.262336 | -0.256431 | 262 | 369.58 | 1.410611 |
| GO:0022402\_cell\_cycle\_process | SKIL | 155 | 1 | 1.237903 | -0.250845 | 263 | 371.91 | 1.414106 |
| GO:0007409\_axonogenesis | TOP2B | 158 | 1 | 1.214399 | -0.245415 | 264 | 373.82 | 1.415985 |
| GO:0002521\_leukocyte\_differentiation | CBFB | 161 | 1 | 1.191770 | -0.240133 | 265 | 375.99 | 1.418830 |
| GO:0043009\_chordate\_embryonic\_development | BTF3 | 365 | 2 | 1.051370 | -0.237984 | 266 | 377.3 | 1.418421 |
| GO:0043009\_chordate\_embryonic\_development | SKIL | 365 | 2 | 1.051370 | -0.237984 | 266 | 377.3 | 1.418421 |
| GO:0009653\_anatomical\_structure\_morphogenesis | RNF2 | 958 | 5 | 1.001435 | -0.237126 | 267 | 377.47 | 1.413745 |
| GO:0009653\_anatomical\_structure\_morphogenesis | YY1 | 958 | 5 | 1.001435 | -0.237126 | 267 | 377.47 | 1.413745 |
| GO:0009653\_anatomical\_structure\_morphogenesis | SKIL | 958 | 5 | 1.001435 | -0.237126 | 267 | 377.47 | 1.413745 |
| GO:0009653\_anatomical\_structure\_morphogenesis | TOP2B | 958 | 5 | 1.001435 | -0.237126 | 267 | 377.47 | 1.413745 |
| GO:0009653\_anatomical\_structure\_morphogenesis | MAB21L2 | 958 | 5 | 1.001435 | -0.237126 | 267 | 377.47 | 1.413745 |
| GO:0042325\_regulation\_of\_phosphorylation | PKIG | 164 | 1 | 1.169970 | -0.234995 | 268 | 378.88 | 1.413731 |
| GO:0009792\_embryonic\_development\_ending\_in\_birth\_or\_egg\_hatching | BTF3 | 368 | 2 | 1.042799 | -0.234525 | 269 | 379.19 | 1.409628 |
| GO:0009792\_embryonic\_development\_ending\_in\_birth\_or\_egg\_hatching | SKIL | 368 | 2 | 1.042799 | -0.234525 | 269 | 379.19 | 1.409628 |
| GO:0006259\_DNA\_metabolic\_process | UNG | 165 | 1 | 1.162879 | -0.233314 | 272 | 380.3 | 1.398162 |
| GO:0019220\_regulation\_of\_phosphate\_metabolic\_process | PKIG | 165 | 1 | 1.162879 | -0.233314 | 272 | 380.3 | 1.398162 |
| GO:0051174\_regulation\_of\_phosphorus\_metabolic\_process | PKIG | 165 | 1 | 1.162879 | -0.233314 | 272 | 380.3 | 1.398162 |
| GO:0051049\_regulation\_of\_transport | PKIG | 167 | 1 | 1.148952 | -0.229996 | 273 | 382.31 | 1.400403 |
| GO:0048523\_negative\_regulation\_of\_cellular\_process | EIF4EBP1 | 774 | 4 | 0.991602 | -0.227546 | 274 | 383.09 | 1.398139 |
| GO:0048523\_negative\_regulation\_of\_cellular\_process | RNF2 | 774 | 4 | 0.991602 | -0.227546 | 274 | 383.09 | 1.398139 |
| GO:0048523\_negative\_regulation\_of\_cellular\_process | PKIG | 774 | 4 | 0.991602 | -0.227546 | 274 | 383.09 | 1.398139 |
| GO:0048523\_negative\_regulation\_of\_cellular\_process | SKIL | 774 | 4 | 0.991602 | -0.227546 | 274 | 383.09 | 1.398139 |
| GO:0048812\_neuron\_projection\_morphogenesis | TOP2B | 170 | 1 | 1.128676 | -0.225129 | 276 | 384.15 | 1.391848 |
| GO:0051246\_regulation\_of\_protein\_metabolic\_process | EIF4EBP1 | 170 | 1 | 1.128676 | -0.225129 | 276 | 384.15 | 1.391848 |
| GO:0009611\_response\_to\_wounding | MDK | 172 | 1 | 1.115552 | -0.221956 | 277 | 385.75 | 1.392599 |
| GO:0048667\_cell\_morphogenesis\_involved\_in\_neuron\_differentiation | TOP2B | 173 | 1 | 1.109104 | -0.220390 | 278 | 387.07 | 1.392338 |
| GO:0046903\_secretion | TMED10 | 175 | 1 | 1.096429 | -0.217300 | 279 | 388.34 | 1.391900 |
| GO:0048858\_cell\_projection\_morphogenesis | TOP2B | 176 | 1 | 1.090199 | -0.215775 | 280 | 390.02 | 1.392929 |
| GO:0007166\_cell\_surface\_receptor\_linked\_signal\_transduction | EIF4EBP1 | 597 | 3 | 0.964196 | -0.208602 | 281 | 393.63 | 1.400819 |
| GO:0007166\_cell\_surface\_receptor\_linked\_signal\_transduction | GKAP1 | 597 | 3 | 0.964196 | -0.208602 | 281 | 393.63 | 1.400819 |
| GO:0007166\_cell\_surface\_receptor\_linked\_signal\_transduction | SKIL | 597 | 3 | 0.964196 | -0.208602 | 281 | 393.63 | 1.400819 |
| GO:0042127\_regulation\_of\_cell\_proliferation | MAB21L2 | 393 | 2 | 0.976463 | -0.207662 | 282 | 394.94 | 1.400496 |
| GO:0042127\_regulation\_of\_cell\_proliferation | MIF | 393 | 2 | 0.976463 | -0.207662 | 282 | 394.94 | 1.400496 |
| GO:0016192\_vesicle-mediated\_transport | TMED10 | 184 | 1 | 1.042799 | -0.204038 | 284 | 397.51 | 1.399683 |
| GO:0032990\_cell\_part\_morphogenesis | TOP2B | 184 | 1 | 1.042799 | -0.204038 | 284 | 397.51 | 1.399683 |
| GO:0042221\_response\_to\_chemical\_stimulus | EIF4EBP1 | 409 | 2 | 0.938264 | -0.192158 | 285 | 401.72 | 1.409544 |
| GO:0042221\_response\_to\_chemical\_stimulus | PPP1R15B | 409 | 2 | 0.938264 | -0.192158 | 285 | 401.72 | 1.409544 |
| GO:0031175\_neuron\_projection\_development | TOP2B | 197 | 1 | 0.973985 | -0.186577 | 286 | 404.9 | 1.415734 |
| GO:0000904\_cell\_morphogenesis\_involved\_in\_differentiation | TOP2B | 199 | 1 | 0.964196 | -0.184053 | 287 | 405.92 | 1.414355 |
| GO:0065007\_biological\_regulation | ZFAND6 | 2593 | 13 | 0.961965 | -0.177606 | 288 | 407.52 | 1.415000 |
| GO:0065007\_biological\_regulation | YY1 | 2593 | 13 | 0.961965 | -0.177606 | 288 | 407.52 | 1.415000 |
| GO:0065007\_biological\_regulation | PKIG | 2593 | 13 | 0.961965 | -0.177606 | 288 | 407.52 | 1.415000 |
| GO:0065007\_biological\_regulation | CBFB | 2593 | 13 | 0.961965 | -0.177606 | 288 | 407.52 | 1.415000 |
| GO:0065007\_biological\_regulation | MIF | 2593 | 13 | 0.961965 | -0.177606 | 288 | 407.52 | 1.415000 |
| GO:0065007\_biological\_regulation | EIF4EBP1 | 2593 | 13 | 0.961965 | -0.177606 | 288 | 407.52 | 1.415000 |
| GO:0065007\_biological\_regulation | CCND2 | 2593 | 13 | 0.961965 | -0.177606 | 288 | 407.52 | 1.415000 |
| GO:0065007\_biological\_regulation | RNF2 | 2593 | 13 | 0.961965 | -0.177606 | 288 | 407.52 | 1.415000 |
| GO:0065007\_biological\_regulation | EED | 2593 | 13 | 0.961965 | -0.177606 | 288 | 407.52 | 1.415000 |
| GO:0065007\_biological\_regulation | GKAP1 | 2593 | 13 | 0.961965 | -0.177606 | 288 | 407.52 | 1.415000 |
| GO:0065007\_biological\_regulation | SKIL | 2593 | 13 | 0.961965 | -0.177606 | 288 | 407.52 | 1.415000 |
| GO:0065007\_biological\_regulation | PPP1R15B | 2593 | 13 | 0.961965 | -0.177606 | 288 | 407.52 | 1.415000 |
| GO:0065007\_biological\_regulation | MAB21L2 | 2593 | 13 | 0.961965 | -0.177606 | 288 | 407.52 | 1.415000 |
| GO:0006955\_immune\_response | UNG | 205 | 1 | 0.935976 | -0.176721 | 289 | 408.44 | 1.413287 |
| GO:0008284\_positive\_regulation\_of\_cell\_proliferation | MAB21L2 | 208 | 1 | 0.922476 | -0.173186 | 290 | 409.74 | 1.412897 |
| GO:0048519\_negative\_regulation\_of\_biological\_process | EIF4EBP1 | 859 | 4 | 0.893481 | -0.167202 | 291 | 411.62 | 1.414502 |
| GO:0048519\_negative\_regulation\_of\_biological\_process | RNF2 | 859 | 4 | 0.893481 | -0.167202 | 291 | 411.62 | 1.414502 |
| GO:0048519\_negative\_regulation\_of\_biological\_process | PKIG | 859 | 4 | 0.893481 | -0.167202 | 291 | 411.62 | 1.414502 |
| GO:0048519\_negative\_regulation\_of\_biological\_process | SKIL | 859 | 4 | 0.893481 | -0.167202 | 291 | 411.62 | 1.414502 |
| GO:0010033\_response\_to\_organic\_substance | EIF4EBP1 | 216 | 1 | 0.888310 | -0.164156 | 292 | 412.67 | 1.413253 |
| GO:0006996\_organelle\_organization | RNF2 | 449 | 2 | 0.854677 | -0.158350 | 293 | 416.41 | 1.421195 |
| GO:0006996\_organelle\_organization | EED | 449 | 2 | 0.854677 | -0.158350 | 293 | 416.41 | 1.421195 |
| GO:0046649\_lymphocyte\_activation | CBFB | 228 | 1 | 0.841557 | -0.151620 | 294 | 418.36 | 1.422993 |
| GO:0007420\_brain\_development | TOP2B | 231 | 1 | 0.830628 | -0.148661 | 295 | 420.1 | 1.424068 |
| GO:0050790\_regulation\_of\_catalytic\_activity | PKIG | 233 | 1 | 0.823498 | -0.146725 | 296 | 420.89 | 1.421926 |
| GO:0016477\_cell\_migration | TOP2B | 234 | 1 | 0.819979 | -0.145768 | 297 | 421.59 | 1.419495 |
| GO:0001501\_skeletal\_system\_development | CBFB | 236 | 1 | 0.813030 | -0.143875 | 298 | 421.97 | 1.416007 |
| GO:0006468\_protein\_amino\_acid\_phosphorylation | CLK1 | 237 | 1 | 0.809599 | -0.142939 | 300 | 422.93 | 1.409767 |
| GO:0044085\_cellular\_component\_biogenesis | FBL | 237 | 1 | 0.809599 | -0.142939 | 300 | 422.93 | 1.409767 |
| GO:0032879\_regulation\_of\_localization | PKIG | 248 | 1 | 0.773690 | -0.133086 | 302 | 426.04 | 1.410728 |
| GO:0045321\_leukocyte\_activation | CBFB | 248 | 1 | 0.773690 | -0.133086 | 302 | 426.04 | 1.410728 |
| GO:0030097\_hemopoiesis | CBFB | 253 | 1 | 0.758399 | -0.128863 | 303 | 428.05 | 1.412706 |
| GO:0006810\_transport | PKIG | 718 | 3 | 0.801706 | -0.126890 | 304 | 429.03 | 1.411283 |
| GO:0006810\_transport | TMED10 | 718 | 3 | 0.801706 | -0.126890 | 304 | 429.03 | 1.411283 |
| GO:0006810\_transport | KPNB1 | 718 | 3 | 0.801706 | -0.126890 | 304 | 429.03 | 1.411283 |
| GO:0009966\_regulation\_of\_signal\_transduction | SKIL | 256 | 1 | 0.749512 | -0.126401 | 305 | 429.48 | 1.408131 |
| GO:0048870\_cell\_motility | TOP2B | 257 | 1 | 0.746595 | -0.125592 | 306 | 429.88 | 1.404837 |
| GO:0001775\_cell\_activation | CBFB | 262 | 1 | 0.732347 | -0.121633 | 308 | 431.48 | 1.400909 |
| GO:0048666\_neuron\_development | TOP2B | 262 | 1 | 0.732347 | -0.121633 | 308 | 431.48 | 1.400909 |
| GO:0007275\_multicellular\_organismal\_development | UNG | 1760 | 8 | 0.872159 | -0.121293 | 309 | 431.64 | 1.396893 |
| GO:0007275\_multicellular\_organismal\_development | YY1 | 1760 | 8 | 0.872159 | -0.121293 | 309 | 431.64 | 1.396893 |
| GO:0007275\_multicellular\_organismal\_development | RNF2 | 1760 | 8 | 0.872159 | -0.121293 | 309 | 431.64 | 1.396893 |
| GO:0007275\_multicellular\_organismal\_development | BTF3 | 1760 | 8 | 0.872159 | -0.121293 | 309 | 431.64 | 1.396893 |
| GO:0007275\_multicellular\_organismal\_development | SKIL | 1760 | 8 | 0.872159 | -0.121293 | 309 | 431.64 | 1.396893 |
| GO:0007275\_multicellular\_organismal\_development | TOP2B | 1760 | 8 | 0.872159 | -0.121293 | 309 | 431.64 | 1.396893 |
| GO:0007275\_multicellular\_organismal\_development | MAB21L2 | 1760 | 8 | 0.872159 | -0.121293 | 309 | 431.64 | 1.396893 |
| GO:0007275\_multicellular\_organismal\_development | CBFB | 1760 | 8 | 0.872159 | -0.121293 | 309 | 431.64 | 1.396893 |
| GO:0051234\_establishment\_of\_localization | PKIG | 729 | 3 | 0.789609 | -0.121174 | 310 | 431.83 | 1.393000 |
| GO:0051234\_establishment\_of\_localization | TMED10 | 729 | 3 | 0.789609 | -0.121174 | 310 | 431.83 | 1.393000 |
| GO:0051234\_establishment\_of\_localization | KPNB1 | 729 | 3 | 0.789609 | -0.121174 | 310 | 431.83 | 1.393000 |
| GO:0030030\_cell\_projection\_organization | TOP2B | 263 | 1 | 0.729563 | -0.120858 | 311 | 432.24 | 1.389839 |
| GO:0045944\_positive\_regulation\_of\_transcription\_from\_RNA\_polymerase\_II\_promoter | CBFB | 269 | 1 | 0.713290 | -0.116321 | 312 | 433.59 | 1.389712 |
| GO:0048534\_hemopoietic\_or\_lymphoid\_organ\_development | CBFB | 277 | 1 | 0.692690 | -0.110562 | 314 | 437.39 | 1.392962 |
| GO:0048646\_anatomical\_structure\_formation\_involved\_in\_morphogenesis | SKIL | 277 | 1 | 0.692690 | -0.110562 | 314 | 437.39 | 1.392962 |
| GO:0007610\_behavior | APRT | 279 | 1 | 0.687724 | -0.109171 | 316 | 438.4 | 1.387342 |
| GO:0065009\_regulation\_of\_molecular\_function | PKIG | 279 | 1 | 0.687724 | -0.109171 | 316 | 438.4 | 1.387342 |
| GO:0000902\_cell\_morphogenesis | TOP2B | 283 | 1 | 0.678004 | -0.106447 | 317 | 439.6 | 1.386751 |
| GO:0007417\_central\_nervous\_system\_development | TOP2B | 287 | 1 | 0.668554 | -0.103797 | 318 | 440.83 | 1.386258 |
| GO:0008283\_cell\_proliferation | MAB21L2 | 544 | 2 | 0.705423 | -0.099918 | 319 | 441.53 | 1.384107 |
| GO:0008283\_cell\_proliferation | MIF | 544 | 2 | 0.705423 | -0.099918 | 319 | 441.53 | 1.384107 |
| GO:0040011\_locomotion | TOP2B | 295 | 1 | 0.650424 | -0.098710 | 321 | 442.86 | 1.379626 |
| GO:0045595\_regulation\_of\_cell\_differentiation | SKIL | 295 | 1 | 0.650424 | -0.098710 | 321 | 442.86 | 1.379626 |
| GO:0045893\_positive\_regulation\_of\_transcription\_\_DNA-dependent | CBFB | 306 | 1 | 0.627042 | -0.092149 | 323 | 444.89 | 1.377368 |
| GO:0051254\_positive\_regulation\_of\_RNA\_metabolic\_process | CBFB | 306 | 1 | 0.627042 | -0.092149 | 323 | 444.89 | 1.377368 |
| GO:0032989\_cellular\_component\_morphogenesis | TOP2B | 307 | 1 | 0.625000 | -0.091577 | 324 | 445.2 | 1.374074 |
| GO:0032502\_developmental\_process | UNG | 2060 | 9 | 0.838289 | -0.085738 | 325 | 446.9 | 1.375077 |
| GO:0032502\_developmental\_process | YY1 | 2060 | 9 | 0.838289 | -0.085738 | 325 | 446.9 | 1.375077 |
| GO:0032502\_developmental\_process | RNF2 | 2060 | 9 | 0.838289 | -0.085738 | 325 | 446.9 | 1.375077 |
| GO:0032502\_developmental\_process | BTF3 | 2060 | 9 | 0.838289 | -0.085738 | 325 | 446.9 | 1.375077 |
| GO:0032502\_developmental\_process | SKIL | 2060 | 9 | 0.838289 | -0.085738 | 325 | 446.9 | 1.375077 |
| GO:0032502\_developmental\_process | TOP2B | 2060 | 9 | 0.838289 | -0.085738 | 325 | 446.9 | 1.375077 |
| GO:0032502\_developmental\_process | MAB21L2 | 2060 | 9 | 0.838289 | -0.085738 | 325 | 446.9 | 1.375077 |
| GO:0032502\_developmental\_process | CBFB | 2060 | 9 | 0.838289 | -0.085738 | 325 | 446.9 | 1.375077 |
| GO:0032502\_developmental\_process | MIF | 2060 | 9 | 0.838289 | -0.085738 | 325 | 446.9 | 1.375077 |
| GO:0006928\_cell\_motion | TOP2B | 330 | 1 | 0.581439 | -0.079400 | 328 | 448.82 | 1.368354 |
| GO:0010646\_regulation\_of\_cell\_communication | SKIL | 330 | 1 | 0.581439 | -0.079400 | 328 | 448.82 | 1.368354 |
| GO:0051674\_localization\_of\_cell | TOP2B | 330 | 1 | 0.581439 | -0.079400 | 328 | 448.82 | 1.368354 |
| GO:0051093\_negative\_regulation\_of\_developmental\_process | SKIL | 331 | 1 | 0.579683 | -0.078911 | 329 | 449.31 | 1.365684 |
| GO:0048856\_anatomical\_structure\_development | UNG | 1688 | 7 | 0.795690 | -0.078237 | 330 | 449.65 | 1.362576 |
| GO:0048856\_anatomical\_structure\_development | RNF2 | 1688 | 7 | 0.795690 | -0.078237 | 330 | 449.65 | 1.362576 |
| GO:0048856\_anatomical\_structure\_development | YY1 | 1688 | 7 | 0.795690 | -0.078237 | 330 | 449.65 | 1.362576 |
| GO:0048856\_anatomical\_structure\_development | SKIL | 1688 | 7 | 0.795690 | -0.078237 | 330 | 449.65 | 1.362576 |
| GO:0048856\_anatomical\_structure\_development | TOP2B | 1688 | 7 | 0.795690 | -0.078237 | 330 | 449.65 | 1.362576 |
| GO:0048856\_anatomical\_structure\_development | MAB21L2 | 1688 | 7 | 0.795690 | -0.078237 | 330 | 449.65 | 1.362576 |
| GO:0048856\_anatomical\_structure\_development | CBFB | 1688 | 7 | 0.795690 | -0.078237 | 330 | 449.65 | 1.362576 |
| GO:0051179\_localization | PKIG | 1058 | 4 | 0.725425 | -0.077625 | 331 | 450.22 | 1.360181 |
| GO:0051179\_localization | TMED10 | 1058 | 4 | 0.725425 | -0.077625 | 331 | 450.22 | 1.360181 |
| GO:0051179\_localization | TOP2B | 1058 | 4 | 0.725425 | -0.077625 | 331 | 450.22 | 1.360181 |
| GO:0051179\_localization | KPNB1 | 1058 | 4 | 0.725425 | -0.077625 | 331 | 450.22 | 1.360181 |
| GO:0045941\_positive\_regulation\_of\_transcription | CBFB | 338 | 1 | 0.567678 | -0.075577 | 332 | 451.03 | 1.358524 |
| GO:0009605\_response\_to\_external\_stimulus | MDK | 339 | 1 | 0.566003 | -0.075113 | 333 | 451.28 | 1.355195 |
| GO:0010628\_positive\_regulation\_of\_gene\_expression | CBFB | 346 | 1 | 0.554552 | -0.071947 | 334 | 452.4 | 1.354491 |
| GO:0045935\_positive\_regulation\_of\_nucleobase\_\_nucleoside\_\_nucleotide\_and\_nucleic\_acid\_metabolic\_process | CBFB | 352 | 1 | 0.545099 | -0.069344 | 335 | 452.91 | 1.351970 |
| GO:0030182\_neuron\_differentiation | TOP2B | 356 | 1 | 0.538975 | -0.067664 | 336 | 453.76 | 1.350476 |
| GO:0042981\_regulation\_of\_apoptosis | SKIL | 360 | 1 | 0.532986 | -0.066026 | 337 | 454.24 | 1.347893 |
| GO:0051173\_positive\_regulation\_of\_nitrogen\_compound\_metabolic\_process | CBFB | 361 | 1 | 0.531510 | -0.065623 | 338 | 454.51 | 1.344704 |
| GO:0010941\_regulation\_of\_cell\_death | SKIL | 365 | 1 | 0.525685 | -0.064037 | 340 | 455.31 | 1.339147 |
| GO:0043067\_regulation\_of\_programmed\_cell\_death | SKIL | 365 | 1 | 0.525685 | -0.064037 | 340 | 455.31 | 1.339147 |
| GO:0010557\_positive\_regulation\_of\_macromolecule\_biosynthetic\_process | CBFB | 371 | 1 | 0.517183 | -0.061731 | 341 | 456.44 | 1.338534 |
| GO:0048468\_cell\_development | TOP2B | 654 | 2 | 0.586774 | -0.058112 | 342 | 457.5 | 1.337719 |
| GO:0048468\_cell\_development | CBFB | 654 | 2 | 0.586774 | -0.058112 | 342 | 457.5 | 1.337719 |
| GO:0031328\_positive\_regulation\_of\_cellular\_biosynthetic\_process | CBFB | 387 | 1 | 0.495801 | -0.055992 | 343 | 458.68 | 1.337259 |
| GO:0009891\_positive\_regulation\_of\_biosynthetic\_process | CBFB | 388 | 1 | 0.494523 | -0.055653 | 344 | 458.94 | 1.334128 |
| GO:0048513\_organ\_development | YY1 | 1365 | 5 | 0.702839 | -0.054359 | 345 | 459.61 | 1.332203 |
| GO:0048513\_organ\_development | SKIL | 1365 | 5 | 0.702839 | -0.054359 | 345 | 459.61 | 1.332203 |
| GO:0048513\_organ\_development | TOP2B | 1365 | 5 | 0.702839 | -0.054359 | 345 | 459.61 | 1.332203 |
| GO:0048513\_organ\_development | MAB21L2 | 1365 | 5 | 0.702839 | -0.054359 | 345 | 459.61 | 1.332203 |
| GO:0048513\_organ\_development | CBFB | 1365 | 5 | 0.702839 | -0.054359 | 345 | 459.61 | 1.332203 |
| GO:0048699\_generation\_of\_neurons | TOP2B | 396 | 1 | 0.484533 | -0.053008 | 346 | 460.7 | 1.331503 |
| GO:0048731\_system\_development | YY1 | 1609 | 6 | 0.715507 | -0.048127 | 347 | 461.77 | 1.330749 |
| GO:0048731\_system\_development | UNG | 1609 | 6 | 0.715507 | -0.048127 | 347 | 461.77 | 1.330749 |
| GO:0048731\_system\_development | SKIL | 1609 | 6 | 0.715507 | -0.048127 | 347 | 461.77 | 1.330749 |
| GO:0048731\_system\_development | TOP2B | 1609 | 6 | 0.715507 | -0.048127 | 347 | 461.77 | 1.330749 |
| GO:0048731\_system\_development | MAB21L2 | 1609 | 6 | 0.715507 | -0.048127 | 347 | 461.77 | 1.330749 |
| GO:0048731\_system\_development | CBFB | 1609 | 6 | 0.715507 | -0.048127 | 347 | 461.77 | 1.330749 |
| GO:0042592\_homeostatic\_process | SKIL | 419 | 1 | 0.457936 | -0.046098 | 348 | 462.27 | 1.328362 |
| GO:0022008\_neurogenesis | TOP2B | 423 | 1 | 0.453605 | -0.044994 | 349 | 462.87 | 1.326275 |
| GO:0006915\_apoptosis | SKIL | 427 | 1 | 0.449356 | -0.043916 | 350 | 463.22 | 1.323486 |
| GO:0016043\_cellular\_component\_organization | RNF2 | 964 | 3 | 0.597121 | -0.043133 | 351 | 463.37 | 1.320142 |
| GO:0016043\_cellular\_component\_organization | EED | 964 | 3 | 0.597121 | -0.043133 | 351 | 463.37 | 1.320142 |
| GO:0016043\_cellular\_component\_organization | TOP2B | 964 | 3 | 0.597121 | -0.043133 | 351 | 463.37 | 1.320142 |
| GO:0010604\_positive\_regulation\_of\_macromolecule\_metabolic\_process | CBFB | 433 | 1 | 0.443129 | -0.042347 | 353 | 463.8 | 1.313881 |
| GO:0012501\_programmed\_cell\_death | SKIL | 433 | 1 | 0.443129 | -0.042347 | 353 | 463.8 | 1.313881 |
| GO:0031325\_positive\_regulation\_of\_cellular\_metabolic\_process | CBFB | 442 | 1 | 0.434106 | -0.040100 | 354 | 464.64 | 1.312542 |
| GO:0008219\_cell\_death | SKIL | 444 | 1 | 0.432151 | -0.039618 | 355 | 465.25 | 1.310563 |
| GO:0010926\_anatomical\_structure\_formation | SKIL | 447 | 1 | 0.429251 | -0.038904 | 356 | 465.48 | 1.307528 |
| GO:0016265\_death | SKIL | 450 | 1 | 0.426389 | -0.038204 | 357 | 466.06 | 1.305490 |
| GO:0009893\_positive\_regulation\_of\_metabolic\_process | CBFB | 458 | 1 | 0.418941 | -0.036398 | 358 | 466.63 | 1.303436 |
| GO:0030154\_cell\_differentiation | SKIL | 1060 | 3 | 0.543042 | -0.027411 | 359 | 467.94 | 1.303454 |
| GO:0030154\_cell\_differentiation | TOP2B | 1060 | 3 | 0.543042 | -0.027411 | 359 | 467.94 | 1.303454 |
| GO:0030154\_cell\_differentiation | CBFB | 1060 | 3 | 0.543042 | -0.027411 | 359 | 467.94 | 1.303454 |
| GO:0032501\_multicellular\_organismal\_process | UNG | 2183 | 8 | 0.703161 | -0.024308 | 360 | 468.94 | 1.302611 |
| GO:0032501\_multicellular\_organismal\_process | YY1 | 2183 | 8 | 0.703161 | -0.024308 | 360 | 468.94 | 1.302611 |
| GO:0032501\_multicellular\_organismal\_process | RNF2 | 2183 | 8 | 0.703161 | -0.024308 | 360 | 468.94 | 1.302611 |
| GO:0032501\_multicellular\_organismal\_process | BTF3 | 2183 | 8 | 0.703161 | -0.024308 | 360 | 468.94 | 1.302611 |
| GO:0032501\_multicellular\_organismal\_process | SKIL | 2183 | 8 | 0.703161 | -0.024308 | 360 | 468.94 | 1.302611 |
| GO:0032501\_multicellular\_organismal\_process | TOP2B | 2183 | 8 | 0.703161 | -0.024308 | 360 | 468.94 | 1.302611 |
| GO:0032501\_multicellular\_organismal\_process | MAB21L2 | 2183 | 8 | 0.703161 | -0.024308 | 360 | 468.94 | 1.302611 |
| GO:0032501\_multicellular\_organismal\_process | CBFB | 2183 | 8 | 0.703161 | -0.024308 | 360 | 468.94 | 1.302611 |
| GO:0048869\_cellular\_developmental\_process | SKIL | 1113 | 3 | 0.517183 | -0.021153 | 361 | 469.74 | 1.301219 |
| GO:0048869\_cellular\_developmental\_process | TOP2B | 1113 | 3 | 0.517183 | -0.021153 | 361 | 469.74 | 1.301219 |
| GO:0048869\_cellular\_developmental\_process | CBFB | 1113 | 3 | 0.517183 | -0.021153 | 361 | 469.74 | 1.301219 |
| GO:0048522\_positive\_regulation\_of\_cellular\_process | MAB21L2 | 895 | 2 | 0.428771 | -0.016649 | 362 | 470.78 | 1.300497 |
| GO:0048522\_positive\_regulation\_of\_cellular\_process | CBFB | 895 | 2 | 0.428771 | -0.016649 | 362 | 470.78 | 1.300497 |
| GO:0007399\_nervous\_system\_development | TOP2B | 621 | 1 | 0.308977 | -0.013508 | 363 | 471.88 | 1.299945 |
| GO:0009887\_organ\_morphogenesis | YY1 | 642 | 1 | 0.298871 | -0.011872 | 364 | 472.27 | 1.297445 |
| GO:0048518\_positive\_regulation\_of\_biological\_process | MAB21L2 | 995 | 2 | 0.385678 | -0.009597 | 365 | 472.93 | 1.295699 |
| GO:0048518\_positive\_regulation\_of\_biological\_process | CBFB | 995 | 2 | 0.385678 | -0.009597 | 365 | 472.93 | 1.295699 |
| GO:0065008\_regulation\_of\_biological\_quality | SKIL | 693 | 1 | 0.276876 | -0.008660 | 366 | 473.15 | 1.292760 |
| GO:0050793\_regulation\_of\_developmental\_process | SKIL | 703 | 1 | 0.272937 | -0.008138 | 367 | 473.42 | 1.289973 |
| GO:0008150\_biological\_process | ZFAND6 | 4605 | 24 | 1.000000 | 0.000000 | 2718 | 2718.08 | 1.000029 |
| GO:0008150\_biological\_process | MRPL3 | 4605 | 24 | 1.000000 | 0.000000 | 2718 | 2718.08 | 1.000029 |
| GO:0008150\_biological\_process | UNG | 4605 | 24 | 1.000000 | 0.000000 | 2718 | 2718.08 | 1.000029 |
| GO:0008150\_biological\_process | YY1 | 4605 | 24 | 1.000000 | 0.000000 | 2718 | 2718.08 | 1.000029 |
| GO:0008150\_biological\_process | PKIG | 4605 | 24 | 1.000000 | 0.000000 | 2718 | 2718.08 | 1.000029 |
| GO:0008150\_biological\_process | CLK1 | 4605 | 24 | 1.000000 | 0.000000 | 2718 | 2718.08 | 1.000029 |
| GO:0008150\_biological\_process | MDK | 4605 | 24 | 1.000000 | 0.000000 | 2718 | 2718.08 | 1.000029 |
| GO:0008150\_biological\_process | CBFB | 4605 | 24 | 1.000000 | 0.000000 | 2718 | 2718.08 | 1.000029 |
| GO:0008150\_biological\_process | FBL | 4605 | 24 | 1.000000 | 0.000000 | 2718 | 2718.08 | 1.000029 |
| GO:0008150\_biological\_process | MIF | 4605 | 24 | 1.000000 | 0.000000 | 2718 | 2718.08 | 1.000029 |
| GO:0008150\_biological\_process | APRT | 4605 | 24 | 1.000000 | 0.000000 | 2718 | 2718.08 | 1.000029 |
| GO:0008150\_biological\_process | EIF4EBP1 | 4605 | 24 | 1.000000 | 0.000000 | 2718 | 2718.08 | 1.000029 |
| GO:0008150\_biological\_process | CCND2 | 4605 | 24 | 1.000000 | 0.000000 | 2718 | 2718.08 | 1.000029 |
| GO:0008150\_biological\_process | RNF2 | 4605 | 24 | 1.000000 | 0.000000 | 2718 | 2718.08 | 1.000029 |
| GO:0008150\_biological\_process | EED | 4605 | 24 | 1.000000 | 0.000000 | 2718 | 2718.08 | 1.000029 |
| GO:0008150\_biological\_process | BTF3 | 4605 | 24 | 1.000000 | 0.000000 | 2718 | 2718.08 | 1.000029 |
| GO:0008150\_biological\_process | TMED10 | 4605 | 24 | 1.000000 | 0.000000 | 2718 | 2718.08 | 1.000029 |
| GO:0008150\_biological\_process | GKAP1 | 4605 | 24 | 1.000000 | 0.000000 | 2718 | 2718.08 | 1.000029 |
| GO:0008150\_biological\_process | SKIL | 4605 | 24 | 1.000000 | 0.000000 | 2718 | 2718.08 | 1.000029 |
| GO:0008150\_biological\_process | PPP1R15B | 4605 | 24 | 1.000000 | 0.000000 | 2718 | 2718.08 | 1.000029 |
| GO:0008150\_biological\_process | TOP2B | 4605 | 24 | 1.000000 | 0.000000 | 2718 | 2718.08 | 1.000029 |
| GO:0008150\_biological\_process | UROD | 4605 | 24 | 1.000000 | 0.000000 | 2718 | 2718.08 | 1.000029 |
| GO:0008150\_biological\_process | KPNB1 | 4605 | 24 | 1.000000 | 0.000000 | 2718 | 2718.08 | 1.000029 |
| GO:0008150\_biological\_process | MAB21L2 | 4605 | 24 | 1.000000 | 0.000000 | 2718 | 2718.08 | 1.000029 |
