# Supplementary material for: RedundancyMiner: De-replication of redundant GO categories in microarray and proteomics analysis
Source: BMC Bioinformatics. 2011 Feb 10;12:52. doi: 10.1186/1471-2105-12-52 (PMC3223614; doi:10.1186/1471-2105-12-52)
Supplement: Additional file 8 — Retinal development HTGM download. compressed package of the results of running HTGM on the retinal development genes list. [file 1471-2105-12-52-S8.ZIP › SCENARIO_2_MODIFIED/total.txt.total.txt.dir/Exp1_BestClusterMap_LEIGS_KM_24.csv.join.15.txt.dir/Exp1_BestClusterMap_LEIGS_KM_24.csv.join.15.txt.change.html]

Category Summary Report for Exp1\_BestClusterMap\_LEIGS\_KM\_24.csv.join.15.txt

# Category Summary Report for Exp1\_BestClusterMap\_LEIGS\_KM\_24.csv.join.15.txt

| HYPERLINKED GO CATEGORY | TOTAL GENES | CHANGED GENES | ENRICHMENT | LOG10(p) | CUMULATIVE NUMBER OF CATEGORIES | CUMULATIVE RANDOMS LOWER BOUND | CUMULATIVE RANDOMS MEAN | CUMULATIVE RANDOMS UPPER BOUND | FALSE DISCOVERY RATE |
| --- | --- | --- | --- | --- | --- | --- | --- | --- | --- |
| GO:0016074\_snoRNA\_metabolic\_process | 1 | 1 |  |  |  |  |  |  |  |  |
| GO:0045947\_negative\_regulation\_of\_translational\_initiation | 1 | 1 |  |  |  |  |  |  |  |  |
| GO:0048199\_vesicle\_targeting\_\_to\_\_from\_or\_within\_Golgi | 1 | 1 |  |  |  |  |  |  |  |  |
| GO:0006168\_adenine\_salvage | 2 | 1 |  |  |  |  |  |  |  |  |
| GO:0006610\_ribosomal\_protein\_import\_into\_nucleus | 2 | 1 |  |  |  |  |  |  |  |  |
| GO:0043096\_purine\_base\_salvage | 2 | 1 |  |  |  |  |  |  |  |  |
| GO:0046083\_adenine\_metabolic\_process | 2 | 1 |  |  |  |  |  |  |  |  |
| GO:0006166\_purine\_ribonucleoside\_salvage | 3 | 1 |  |  |  |  |  |  |  |  |
| GO:0007199\_G-protein\_signaling\_\_coupled\_to\_cGMP\_nucleotide\_second\_messenger | 3 | 1 |  |  |  |  |  |  |  |  |
| GO:0010172\_embryonic\_body\_morphogenesis | 3 | 1 |  |  |  |  |  |  |  |  |
| GO:0019934\_cGMP-mediated\_signaling | 3 | 1 |  |  |  |  |  |  |  |  |
| GO:0043094\_cellular\_metabolic\_compound\_salvage | 3 | 1 |  |  |  |  |  |  |  |  |
| GO:0043101\_purine\_salvage | 3 | 1 |  |  |  |  |  |  |  |  |
| GO:0043174\_nucleoside\_salvage | 3 | 1 |  |  |  |  |  |  |  |  |
| GO:0043010\_camera-type\_eye\_development | 110 | 3 | 5.232955 | -1.729701 | 1 | -1.410776 | 10.81 | 23.030776 | 10.810000 |
| GO:0006144\_purine\_base\_metabolic\_process | 4 | 1 |  |  |  |  |  |  |  |  |
| GO:0006606\_protein\_import\_into\_nucleus | 44 | 2 | 8.721591 | -1.666456 | 3 | -0.959736 | 13.11 | 27.179736 | 4.370000 |
| GO:0051170\_nuclear\_import | 44 | 2 | 8.721591 | -1.666456 | 3 | -0.959736 | 13.11 | 27.179736 | 4.370000 |
| GO:0016570\_histone\_modification | 47 | 2 | 8.164894 | -1.612635 | 4 | -0.972871 | 14.07 | 29.112871 | 3.517500 |
| GO:0006886\_intracellular\_protein\_transport | 122 | 3 | 4.718238 | -1.611336 | 5 | -0.915837 | 14.24 | 29.395837 | 2.848000 |
| GO:0034504\_protein\_localization\_in\_nucleus | 48 | 2 | 7.994792 | -1.595522 | 6 | -0.803008 | 14.65 | 30.103008 | 2.441667 |
| GO:0006903\_vesicle\_targeting | 5 | 1 | 38.375000 | -1.588388 | 9 | 4.014820 | 21.39 | 38.765180 | 2.376667 |
| GO:0006983\_ER\_overload\_response | 5 | 1 | 38.375000 | -1.588388 | 9 | 4.014820 | 21.39 | 38.765180 | 2.376667 |
| GO:0017148\_negative\_regulation\_of\_translation | 5 | 1 | 38.375000 | -1.588388 | 9 | 4.014820 | 21.39 | 38.765180 | 2.376667 |
| GO:0017038\_protein\_import | 50 | 2 | 7.675000 | -1.562435 | 10 | 4.293059 | 21.99 | 39.686941 | 2.199000 |
| GO:0016569\_covalent\_chromatin\_modification | 51 | 2 | 7.524510 | -1.546431 | 11 | 4.523968 | 22.65 | 40.776032 | 2.059091 |
| GO:0009112\_nucleobase\_metabolic\_process | 6 | 1 | 31.979167 | -1.510290 | 14 | 7.830566 | 28.74 | 49.649434 | 2.052857 |
| GO:0016574\_histone\_ubiquitination | 6 | 1 | 31.979167 | -1.510290 | 14 | 7.830566 | 28.74 | 49.649434 | 2.052857 |
| GO:0042308\_negative\_regulation\_of\_protein\_import\_into\_nucleus | 6 | 1 | 31.979167 | -1.510290 | 14 | 7.830566 | 28.74 | 49.649434 | 2.052857 |
| GO:0006412\_translation | 54 | 2 | 7.106481 | -1.500407 | 15 | 7.929345 | 29.13 | 50.330655 | 1.942000 |
| GO:0010467\_gene\_expression | 905 | 9 | 1.908149 | -1.491403 | 16 | 7.939932 | 29.3 | 50.660068 | 1.831250 |
| GO:0001654\_eye\_development | 136 | 3 | 4.232537 | -1.489332 | 17 | 7.903669 | 29.36 | 50.816331 | 1.727059 |
| GO:0034613\_cellular\_protein\_localization | 139 | 3 | 4.141187 | -1.465115 | 18 | 8.117855 | 30.11 | 52.102145 | 1.672778 |
| GO:0033365\_protein\_localization\_in\_organelle | 57 | 2 | 6.732456 | -1.457117 | 19 | 8.374257 | 30.52 | 52.665743 | 1.606316 |
| GO:0070727\_cellular\_macromolecule\_localization | 141 | 3 | 4.082447 | -1.449314 | 20 | 8.324344 | 30.68 | 53.035656 | 1.534000 |
| GO:0006783\_heme\_biosynthetic\_process | 7 | 1 | 27.410714 | -1.444426 | 24 | 12.729298 | 37.07 | 61.410702 | 1.544583 |
| GO:0032387\_negative\_regulation\_of\_intracellular\_transport | 7 | 1 | 27.410714 | -1.444426 | 24 | 12.729298 | 37.07 | 61.410702 | 1.544583 |
| GO:0042168\_heme\_metabolic\_process | 7 | 1 | 27.410714 | -1.444426 | 24 | 12.729298 | 37.07 | 61.410702 | 1.544583 |
| GO:0046823\_negative\_regulation\_of\_nucleocytoplasmic\_transport | 7 | 1 | 27.410714 | -1.444426 | 24 | 12.729298 | 37.07 | 61.410702 | 1.544583 |
| GO:0002566\_somatic\_diversification\_of\_immune\_receptors\_via\_somatic\_mutation | 8 | 1 | 23.984375 | -1.387516 | 30 | 16.756252 | 43.89 | 71.023748 | 1.463000 |
| GO:0006349\_genetic\_imprinting | 8 | 1 | 23.984375 | -1.387516 | 30 | 16.756252 | 43.89 | 71.023748 | 1.463000 |
| GO:0007625\_grooming\_behavior | 8 | 1 | 23.984375 | -1.387516 | 30 | 16.756252 | 43.89 | 71.023748 | 1.463000 |
| GO:0016446\_somatic\_hypermutation\_of\_immunoglobulin\_genes | 8 | 1 | 23.984375 | -1.387516 | 30 | 16.756252 | 43.89 | 71.023748 | 1.463000 |
| GO:0018107\_peptidyl-threonine\_phosphorylation | 8 | 1 | 23.984375 | -1.387516 | 30 | 16.756252 | 43.89 | 71.023748 | 1.463000 |
| GO:0018210\_peptidyl-threonine\_modification | 8 | 1 | 23.984375 | -1.387516 | 30 | 16.756252 | 43.89 | 71.023748 | 1.463000 |
| GO:0006364\_rRNA\_processing | 9 | 1 | 21.319444 | -1.337444 | 33 | 20.863001 | 50.82 | 80.776999 | 1.540000 |
| GO:0016072\_rRNA\_metabolic\_process | 9 | 1 | 21.319444 | -1.337444 | 33 | 20.863001 | 50.82 | 80.776999 | 1.540000 |
| GO:0070306\_lens\_fiber\_cell\_differentiation | 9 | 1 | 21.319444 | -1.337444 | 33 | 20.863001 | 50.82 | 80.776999 | 1.540000 |
| GO:0051716\_cellular\_response\_to\_stimulus | 273 | 4 | 2.811355 | -1.298865 | 34 | 21.291202 | 52.0 | 82.708798 | 1.529412 |
| GO:0010558\_negative\_regulation\_of\_macromolecule\_biosynthetic\_process | 274 | 4 | 2.801095 | -1.293995 | 35 | 21.375417 | 52.25 | 83.124583 | 1.492857 |
| GO:0009948\_anterior\_posterior\_axis\_specification | 10 | 1 | 19.187500 | -1.292767 | 39 | 25.274150 | 58.29 | 91.305850 | 1.494615 |
| GO:0051224\_negative\_regulation\_of\_protein\_transport | 10 | 1 | 19.187500 | -1.292767 | 39 | 25.274150 | 58.29 | 91.305850 | 1.494615 |
| GO:0051650\_establishment\_of\_vesicle\_localization | 10 | 1 | 19.187500 | -1.292767 | 39 | 25.274150 | 58.29 | 91.305850 | 1.494615 |
| GO:0060216\_definitive\_hemopoiesis | 10 | 1 | 19.187500 | -1.292767 | 39 | 25.274150 | 58.29 | 91.305850 | 1.494615 |
| GO:0006913\_nucleocytoplasmic\_transport | 71 | 2 | 5.404930 | -1.283926 | 40 | 25.470353 | 58.59 | 91.709647 | 1.464750 |
| GO:0006807\_nitrogen\_compound\_metabolic\_process | 1147 | 10 | 1.672842 | -1.275904 | 41 | 25.591330 | 58.83 | 92.068670 | 1.434878 |
| GO:0016568\_chromatin\_modification | 72 | 2 | 5.329861 | -1.273053 | 43 | 25.875054 | 59.38 | 92.884946 | 1.380930 |
| GO:0051169\_nuclear\_transport | 72 | 2 | 5.329861 | -1.273053 | 43 | 25.875054 | 59.38 | 92.884946 | 1.380930 |
| GO:0031327\_negative\_regulation\_of\_cellular\_biosynthetic\_process | 282 | 4 | 2.721631 | -1.255856 | 44 | 25.843323 | 59.98 | 94.116677 | 1.363182 |
| GO:0006779\_porphyrin\_biosynthetic\_process | 11 | 1 | 17.443182 | -1.252453 | 50 | 29.065420 | 64.66 | 100.254580 | 1.293200 |
| GO:0033014\_tetrapyrrole\_biosynthetic\_process | 11 | 1 | 17.443182 | -1.252453 | 50 | 29.065420 | 64.66 | 100.254580 | 1.293200 |
| GO:0042542\_response\_to\_hydrogen\_peroxide | 11 | 1 | 17.443182 | -1.252453 | 50 | 29.065420 | 64.66 | 100.254580 | 1.293200 |
| GO:0045055\_regulated\_secretory\_pathway | 11 | 1 | 17.443182 | -1.252453 | 50 | 29.065420 | 64.66 | 100.254580 | 1.293200 |
| GO:0048193\_Golgi\_vesicle\_transport | 11 | 1 | 17.443182 | -1.252453 | 50 | 29.065420 | 64.66 | 100.254580 | 1.293200 |
| GO:0051648\_vesicle\_localization | 11 | 1 | 17.443182 | -1.252453 | 50 | 29.065420 | 64.66 | 100.254580 | 1.293200 |
| GO:0009890\_negative\_regulation\_of\_biosynthetic\_process | 284 | 4 | 2.702465 | -1.246545 | 51 | 29.080366 | 64.86 | 100.639634 | 1.271765 |
| GO:0006139\_nucleobase\_\_nucleoside\_\_nucleotide\_and\_nucleic\_acid\_metabolic\_process | 1002 | 9 | 1.723428 | -1.236395 | 52 | 29.313684 | 65.4 | 101.486316 | 1.257692 |
| GO:0000122\_negative\_regulation\_of\_transcription\_from\_RNA\_polymerase\_II\_promoter | 175 | 3 | 3.289286 | -1.216032 | 54 | 29.683780 | 66.4 | 103.116220 | 1.229630 |
| GO:0015031\_protein\_transport | 175 | 3 | 3.289286 | -1.216032 | 54 | 29.683780 | 66.4 | 103.116220 | 1.229630 |
| GO:0006413\_translational\_initiation | 12 | 1 | 15.989583 | -1.215743 | 59 | 32.867062 | 71.15 | 109.432938 | 1.205932 |
| GO:0006446\_regulation\_of\_translational\_initiation | 12 | 1 | 15.989583 | -1.215743 | 59 | 32.867062 | 71.15 | 109.432938 | 1.205932 |
| GO:0030330\_DNA\_damage\_response\_\_signal\_transduction\_by\_p53\_class\_mediator | 12 | 1 | 15.989583 | -1.215743 | 59 | 32.867062 | 71.15 | 109.432938 | 1.205932 |
| GO:0042278\_purine\_nucleoside\_metabolic\_process | 12 | 1 | 15.989583 | -1.215743 | 59 | 32.867062 | 71.15 | 109.432938 | 1.205932 |
| GO:0046128\_purine\_ribonucleoside\_metabolic\_process | 12 | 1 | 15.989583 | -1.215743 | 59 | 32.867062 | 71.15 | 109.432938 | 1.205932 |
| GO:0045184\_establishment\_of\_protein\_localization | 180 | 3 | 3.197917 | -1.186455 | 60 | 33.440225 | 72.8 | 112.159775 | 1.213333 |
| GO:0006778\_porphyrin\_metabolic\_process | 13 | 1 | 14.759615 | -1.182059 | 64 | 36.983368 | 77.53 | 118.076632 | 1.211406 |
| GO:0009119\_ribonucleoside\_metabolic\_process | 13 | 1 | 14.759615 | -1.182059 | 64 | 36.983368 | 77.53 | 118.076632 | 1.211406 |
| GO:0018105\_peptidyl-serine\_phosphorylation | 13 | 1 | 14.759615 | -1.182059 | 64 | 36.983368 | 77.53 | 118.076632 | 1.211406 |
| GO:0033013\_tetrapyrrole\_metabolic\_process | 13 | 1 | 14.759615 | -1.182059 | 64 | 36.983368 | 77.53 | 118.076632 | 1.211406 |
| GO:0006325\_chromatin\_organization | 83 | 2 | 4.623494 | -1.163705 | 65 | 37.441098 | 78.48 | 119.518902 | 1.207385 |
| GO:0018130\_heterocycle\_biosynthetic\_process | 14 | 1 | 13.705357 | -1.150952 | 66 | 41.014174 | 83.69 | 126.365826 | 1.268030 |
| GO:0006605\_protein\_targeting | 86 | 2 | 4.462209 | -1.136750 | 67 | 41.587691 | 84.68 | 127.772309 | 1.263881 |
| GO:0009116\_nucleoside\_metabolic\_process | 15 | 1 | 12.791667 | -1.122065 | 70 | 44.884705 | 89.78 | 134.675295 | 1.282571 |
| GO:0010171\_body\_morphogenesis | 15 | 1 | 12.791667 | -1.122065 | 70 | 44.884705 | 89.78 | 134.675295 | 1.282571 |
| GO:0042306\_regulation\_of\_protein\_import\_into\_nucleus | 15 | 1 | 12.791667 | -1.122065 | 70 | 44.884705 | 89.78 | 134.675295 | 1.282571 |
| GO:0046907\_intracellular\_transport | 194 | 3 | 2.967139 | -1.108856 | 71 | 45.246477 | 90.31 | 135.373523 | 1.271972 |
| GO:0034960\_cellular\_biopolymer\_metabolic\_process | 1395 | 11 | 1.512993 | -1.105678 | 72 | 45.226548 | 90.42 | 135.613452 | 1.255833 |
| GO:0033554\_cellular\_response\_to\_stress | 196 | 3 | 2.936862 | -1.098352 | 73 | 45.897945 | 91.27 | 136.642055 | 1.250274 |
| GO:0000302\_response\_to\_reactive\_oxygen\_species | 16 | 1 | 11.992188 | -1.095112 | 76 | 48.425218 | 94.63 | 140.834782 | 1.245132 |
| GO:0034976\_response\_to\_endoplasmic\_reticulum\_stress | 16 | 1 | 11.992188 | -1.095112 | 76 | 48.425218 | 94.63 | 140.834782 | 1.245132 |
| GO:0046148\_pigment\_biosynthetic\_process | 16 | 1 | 11.992188 | -1.095112 | 76 | 48.425218 | 94.63 | 140.834782 | 1.245132 |
| GO:0034984\_cellular\_response\_to\_DNA\_damage\_stimulus | 94 | 2 | 4.082447 | -1.069892 | 77 | 49.419102 | 96.47 | 143.520898 | 1.252857 |
| GO:0006984\_ER-nuclear\_signaling\_pathway | 17 | 1 | 11.286765 | -1.069858 | 81 | 52.045971 | 100.13 | 148.214029 | 1.236173 |
| GO:0034470\_ncRNA\_processing | 17 | 1 | 11.286765 | -1.069858 | 81 | 52.045971 | 100.13 | 148.214029 | 1.236173 |
| GO:0042254\_ribosome\_biogenesis | 17 | 1 | 11.286765 | -1.069858 | 81 | 52.045971 | 100.13 | 148.214029 | 1.236173 |
| GO:0042440\_pigment\_metabolic\_process | 17 | 1 | 11.286765 | -1.069858 | 81 | 52.045971 | 100.13 | 148.214029 | 1.236173 |
| GO:0010605\_negative\_regulation\_of\_macromolecule\_metabolic\_process | 331 | 4 | 2.318731 | -1.050381 | 82 | 52.564676 | 101.4 | 150.235324 | 1.236585 |
| GO:0031324\_negative\_regulation\_of\_cellular\_metabolic\_process | 332 | 4 | 2.311747 | -1.046630 | 83 | 52.575800 | 101.46 | 150.344200 | 1.222410 |
| GO:0001825\_blastocyst\_formation | 18 | 1 | 10.659722 | -1.046108 | 85 | 55.572282 | 106.32 | 157.067718 | 1.250824 |
| GO:0033157\_regulation\_of\_intracellular\_protein\_transport | 18 | 1 | 10.659722 | -1.046108 | 85 | 55.572282 | 106.32 | 157.067718 | 1.250824 |
| GO:0007569\_cell\_aging | 19 | 1 | 10.098684 | -1.023700 | 87 | 59.604777 | 111.14 | 162.675223 | 1.277471 |
| GO:0009798\_axis\_specification | 19 | 1 | 10.098684 | -1.023700 | 87 | 59.604777 | 111.14 | 162.675223 | 1.277471 |
| GO:0010468\_regulation\_of\_gene\_expression | 778 | 7 | 1.726382 | -1.017525 | 88 | 59.817760 | 111.43 | 163.042240 | 1.266250 |
| GO:0044260\_cellular\_macromolecule\_metabolic\_process | 1447 | 11 | 1.458621 | -1.006989 | 89 | 60.047280 | 111.78 | 163.512720 | 1.255955 |
| GO:0016571\_histone\_methylation | 20 | 1 | 9.593750 | -1.002496 | 92 | 61.805109 | 114.64 | 167.474891 | 1.246087 |
| GO:0018209\_peptidyl-serine\_modification | 20 | 1 | 9.593750 | -1.002496 | 92 | 61.805109 | 114.64 | 167.474891 | 1.246087 |
| GO:0046822\_regulation\_of\_nucleocytoplasmic\_transport | 20 | 1 | 9.593750 | -1.002496 | 92 | 61.805109 | 114.64 | 167.474891 | 1.246087 |
| GO:0045892\_negative\_regulation\_of\_transcription\_\_DNA-dependent | 218 | 3 | 2.640482 | -0.991233 | 93 | 62.262817 | 115.51 | 168.757183 | 1.242043 |
| GO:0009892\_negative\_regulation\_of\_metabolic\_process | 348 | 4 | 2.205460 | -0.988707 | 94 | 62.283695 | 115.61 | 168.936305 | 1.229894 |
| GO:0007423\_sensory\_organ\_development | 219 | 3 | 2.628425 | -0.986702 | 95 | 62.364343 | 115.81 | 169.255657 | 1.219053 |
| GO:0001702\_gastrulation\_with\_mouth\_forming\_second | 21 | 1 | 9.136905 | -0.982379 | 97 | 64.625155 | 119.52 | 174.414845 | 1.232165 |
| GO:0051656\_establishment\_of\_organelle\_localization | 21 | 1 | 9.136905 | -0.982379 | 97 | 64.625155 | 119.52 | 174.414845 | 1.232165 |
| GO:0051253\_negative\_regulation\_of\_RNA\_metabolic\_process | 220 | 3 | 2.616477 | -0.982198 | 98 | 64.602663 | 119.6 | 174.597337 | 1.220408 |
| GO:0034660\_ncRNA\_metabolic\_process | 22 | 1 | 8.721591 | -0.963247 | 99 | 68.418335 | 124.85 | 181.281665 | 1.261111 |
| GO:0034961\_cellular\_biopolymer\_biosynthetic\_process | 804 | 7 | 1.670553 | -0.957424 | 100 | 68.589550 | 125.03 | 181.470450 | 1.250300 |
| GO:0043284\_biopolymer\_biosynthetic\_process | 807 | 7 | 1.664343 | -0.950705 | 101 | 68.864658 | 125.41 | 181.955342 | 1.241683 |
| GO:0022613\_ribonucleoprotein\_complex\_biogenesis | 23 | 1 | 8.342391 | -0.945012 | 103 | 71.298953 | 128.25 | 185.201047 | 1.245146 |
| GO:0030512\_negative\_regulation\_of\_transforming\_growth\_factor\_beta\_receptor\_signaling\_pathway | 23 | 1 | 8.342391 | -0.945012 | 103 | 71.298953 | 128.25 | 185.201047 | 1.245146 |
| GO:0006974\_response\_to\_DNA\_damage\_stimulus | 113 | 2 | 3.396018 | -0.934841 | 104 | 71.966539 | 129.1 | 186.233461 | 1.241346 |
| GO:0016070\_RNA\_metabolic\_process | 658 | 6 | 1.749620 | -0.933632 | 105 | 72.100171 | 129.35 | 186.599829 | 1.231905 |
| GO:0043283\_biopolymer\_metabolic\_process | 1490 | 11 | 1.416527 | -0.930646 | 106 | 72.466327 | 129.81 | 187.153673 | 1.224623 |
| GO:0007050\_cell\_cycle\_arrest | 24 | 1 | 7.994792 | -0.927598 | 108 | 74.710168 | 132.98 | 191.249832 | 1.231296 |
| GO:0032386\_regulation\_of\_intracellular\_transport | 24 | 1 | 7.994792 | -0.927598 | 108 | 74.710168 | 132.98 | 191.249832 | 1.231296 |
| GO:0046483\_heterocycle\_metabolic\_process | 116 | 2 | 3.308190 | -0.916016 | 109 | 75.329862 | 134.03 | 192.730138 | 1.229633 |
| GO:0007049\_cell\_cycle | 238 | 3 | 2.418592 | -0.905519 | 110 | 78.296111 | 137.83 | 197.363889 | 1.253000 |
| GO:0040029\_regulation\_of\_gene\_expression\_\_epigenetic | 26 | 1 | 7.379808 | -0.894972 | 111 | 80.515195 | 141.16 | 201.804805 | 1.271712 |
| GO:0006479\_protein\_amino\_acid\_methylation | 27 | 1 | 7.106481 | -0.879649 | 114 | 82.863886 | 144.56 | 206.256114 | 1.268070 |
| GO:0008213\_protein\_amino\_acid\_alkylation | 27 | 1 | 7.106481 | -0.879649 | 114 | 82.863886 | 144.56 | 206.256114 | 1.268070 |
| GO:0008286\_insulin\_receptor\_signaling\_pathway | 27 | 1 | 7.106481 | -0.879649 | 114 | 82.863886 | 144.56 | 206.256114 | 1.268070 |
| GO:0002088\_lens\_development\_in\_camera-type\_eye | 28 | 1 | 6.852679 | -0.864921 | 116 | 84.779736 | 147.35 | 209.920264 | 1.270259 |
| GO:0051188\_cofactor\_biosynthetic\_process | 28 | 1 | 6.852679 | -0.864921 | 116 | 84.779736 | 147.35 | 209.920264 | 1.270259 |
| GO:0008104\_protein\_localization | 251 | 3 | 2.293327 | -0.854849 | 117 | 85.291251 | 148.03 | 210.768749 | 1.265214 |
| GO:0006417\_regulation\_of\_translation | 29 | 1 | 6.616379 | -0.850747 | 120 | 87.448610 | 151.29 | 215.131390 | 1.260750 |
| GO:0016447\_somatic\_recombination\_of\_immunoglobulin\_gene\_segments | 29 | 1 | 6.616379 | -0.850747 | 120 | 87.448610 | 151.29 | 215.131390 | 1.260750 |
| GO:0042770\_DNA\_damage\_response\_\_signal\_transduction | 29 | 1 | 6.616379 | -0.850747 | 120 | 87.448610 | 151.29 | 215.131390 | 1.260750 |
| GO:0016481\_negative\_regulation\_of\_transcription | 253 | 3 | 2.275198 | -0.847373 | 121 | 87.495501 | 151.47 | 215.444499 | 1.251818 |
| GO:0051276\_chromosome\_organization | 129 | 2 | 2.974806 | -0.840811 | 122 | 88.209154 | 152.4 | 216.590846 | 1.249180 |
| GO:0002260\_lymphocyte\_homeostasis | 30 | 1 | 6.395833 | -0.837089 | 124 | 90.645873 | 155.97 | 221.294127 | 1.257823 |
| GO:0016445\_somatic\_diversification\_of\_immunoglobulins | 30 | 1 | 6.395833 | -0.837089 | 124 | 90.645873 | 155.97 | 221.294127 | 1.257823 |
| GO:0051640\_organelle\_localization | 31 | 1 | 6.189516 | -0.823913 | 125 | 94.293190 | 161.52 | 228.746810 | 1.292160 |
| GO:0009952\_anterior\_posterior\_pattern\_formation | 133 | 2 | 2.885338 | -0.819537 | 126 | 94.491585 | 161.79 | 229.088415 | 1.284048 |
| GO:0010629\_negative\_regulation\_of\_gene\_expression | 262 | 3 | 2.197042 | -0.814716 | 127 | 94.626811 | 162.05 | 229.473189 | 1.275984 |
| GO:0044267\_cellular\_protein\_metabolic\_process | 559 | 5 | 1.716234 | -0.801793 | 128 | 96.350988 | 164.65 | 232.949012 | 1.286328 |
| GO:0002562\_somatic\_diversification\_of\_immune\_receptors\_via\_germline\_recombination\_within\_a\_single\_locus | 33 | 1 | 5.814394 | -0.798886 | 130 | 98.575999 | 167.27 | 235.964001 | 1.286692 |
| GO:0016444\_somatic\_cell\_DNA\_recombination | 33 | 1 | 5.814394 | -0.798886 | 130 | 98.575999 | 167.27 | 235.964001 | 1.286692 |
| GO:0043170\_macromolecule\_metabolic\_process | 1576 | 11 | 1.339229 | -0.791241 | 131 | 98.779604 | 167.59 | 236.400396 | 1.279313 |
| GO:0002200\_somatic\_diversification\_of\_immune\_receptors | 34 | 1 | 5.643382 | -0.786983 | 133 | 101.332166 | 170.78 | 240.227834 | 1.284060 |
| GO:0007568\_aging | 34 | 1 | 5.643382 | -0.786983 | 133 | 101.332166 | 170.78 | 240.227834 | 1.284060 |
| GO:0045934\_negative\_regulation\_of\_nucleobase\_\_nucleoside\_\_nucleotide\_and\_nucleic\_acid\_metabolic\_process | 270 | 3 | 2.131944 | -0.786978 | 134 | 101.382405 | 170.94 | 240.497595 | 1.275672 |
| GO:0051172\_negative\_regulation\_of\_nitrogen\_compound\_metabolic\_process | 271 | 3 | 2.124077 | -0.783592 | 135 | 101.518056 | 171.28 | 241.041944 | 1.268741 |
| GO:0016567\_protein\_ubiquitination | 35 | 1 | 5.482143 | -0.775455 | 137 | 103.256436 | 173.93 | 244.603564 | 1.269562 |
| GO:0051051\_negative\_regulation\_of\_transport | 35 | 1 | 5.482143 | -0.775455 | 137 | 103.256436 | 173.93 | 244.603564 | 1.269562 |
| GO:0033036\_macromolecule\_localization | 274 | 3 | 2.100821 | -0.773539 | 138 | 103.338253 | 174.18 | 245.021747 | 1.262174 |
| GO:0006355\_regulation\_of\_transcription\_\_DNA-dependent | 575 | 5 | 1.668478 | -0.764900 | 139 | 103.943391 | 175.06 | 246.176609 | 1.259424 |
| GO:0006469\_negative\_regulation\_of\_protein\_kinase\_activity | 36 | 1 | 5.329861 | -0.764281 | 143 | 106.189192 | 177.96 | 249.730808 | 1.244476 |
| GO:0007187\_G-protein\_signaling\_\_coupled\_to\_cyclic\_nucleotide\_second\_messenger | 36 | 1 | 5.329861 | -0.764281 | 143 | 106.189192 | 177.96 | 249.730808 | 1.244476 |
| GO:0033673\_negative\_regulation\_of\_kinase\_activity | 36 | 1 | 5.329861 | -0.764281 | 143 | 106.189192 | 177.96 | 249.730808 | 1.244476 |
| GO:0051223\_regulation\_of\_protein\_transport | 36 | 1 | 5.329861 | -0.764281 | 143 | 106.189192 | 177.96 | 249.730808 | 1.244476 |
| GO:0034645\_cellular\_macromolecule\_biosynthetic\_process | 901 | 7 | 1.490705 | -0.760677 | 144 | 106.495054 | 178.41 | 250.324946 | 1.238958 |
| GO:0032869\_cellular\_response\_to\_insulin\_stimulus | 37 | 1 | 5.185811 | -0.753441 | 145 | 108.372064 | 180.96 | 253.547936 | 1.248000 |
| GO:0044237\_cellular\_metabolic\_process | 1974 | 13 | 1.263614 | -0.745067 | 146 | 109.093698 | 181.9 | 254.706302 | 1.245890 |
| GO:0009059\_macromolecule\_biosynthetic\_process | 910 | 7 | 1.475962 | -0.744402 | 147 | 109.213272 | 182.01 | 254.806728 | 1.238163 |
| GO:0010556\_regulation\_of\_macromolecule\_biosynthetic\_process | 745 | 6 | 1.545302 | -0.743883 | 148 | 109.326031 | 182.24 | 255.153969 | 1.231351 |
| GO:0001649\_osteoblast\_differentiation | 38 | 1 | 5.049342 | -0.742918 | 153 | 111.623413 | 185.49 | 259.356587 | 1.212353 |
| GO:0032259\_methylation | 38 | 1 | 5.049342 | -0.742918 | 153 | 111.623413 | 185.49 | 259.356587 | 1.212353 |
| GO:0043414\_biopolymer\_methylation | 38 | 1 | 5.049342 | -0.742918 | 153 | 111.623413 | 185.49 | 259.356587 | 1.212353 |
| GO:0046777\_protein\_amino\_acid\_autophosphorylation | 38 | 1 | 5.049342 | -0.742918 | 153 | 111.623413 | 185.49 | 259.356587 | 1.212353 |
| GO:0051348\_negative\_regulation\_of\_transferase\_activity | 38 | 1 | 5.049342 | -0.742918 | 153 | 111.623413 | 185.49 | 259.356587 | 1.212353 |
| GO:0006730\_one-carbon\_metabolic\_process | 39 | 1 | 4.919872 | -0.732695 | 155 | 114.314093 | 189.09 | 263.865907 | 1.219935 |
| GO:0070201\_regulation\_of\_establishment\_of\_protein\_localization | 39 | 1 | 4.919872 | -0.732695 | 155 | 114.314093 | 189.09 | 263.865907 | 1.219935 |
| GO:0051252\_regulation\_of\_RNA\_metabolic\_process | 590 | 5 | 1.626059 | -0.731849 | 156 | 114.390987 | 189.21 | 264.029013 | 1.212885 |
| GO:0006357\_regulation\_of\_transcription\_from\_RNA\_polymerase\_II\_promoter | 435 | 4 | 1.764368 | -0.730820 | 157 | 114.584302 | 189.47 | 264.355698 | 1.206815 |
| GO:0006351\_transcription\_\_DNA-dependent | 594 | 5 | 1.615109 | -0.723276 | 158 | 114.917514 | 190.02 | 265.122486 | 1.202658 |
| GO:0001824\_blastocyst\_development | 40 | 1 | 4.796875 | -0.722757 | 161 | 116.668249 | 192.16 | 267.651751 | 1.193540 |
| GO:0017015\_regulation\_of\_transforming\_growth\_factor\_beta\_receptor\_signaling\_pathway | 40 | 1 | 4.796875 | -0.722757 | 161 | 116.668249 | 192.16 | 267.651751 | 1.193540 |
| GO:0019935\_cyclic-nucleotide-mediated\_signaling | 40 | 1 | 4.796875 | -0.722757 | 161 | 116.668249 | 192.16 | 267.651751 | 1.193540 |
| GO:0032774\_RNA\_biosynthetic\_process | 595 | 5 | 1.612395 | -0.721148 | 162 | 116.784702 | 192.33 | 267.875298 | 1.187222 |
| GO:0001776\_leukocyte\_homeostasis | 41 | 1 | 4.679878 | -0.713090 | 164 | 120.659885 | 197.08 | 273.500115 | 1.201707 |
| GO:0006979\_response\_to\_oxidative\_stress | 41 | 1 | 4.679878 | -0.713090 | 164 | 120.659885 | 197.08 | 273.500115 | 1.201707 |
| GO:0006366\_transcription\_from\_RNA\_polymerase\_II\_promoter | 444 | 4 | 1.728604 | -0.708660 | 165 | 120.851364 | 197.3 | 273.748636 | 1.195758 |
| GO:0060255\_regulation\_of\_macromolecule\_metabolic\_process | 936 | 7 | 1.434963 | -0.699092 | 166 | 124.330873 | 201.87 | 279.409127 | 1.216084 |
| GO:0032446\_protein\_modification\_by\_small\_protein\_conjugation | 43 | 1 | 4.462209 | -0.694516 | 168 | 127.391921 | 205.35 | 283.308079 | 1.222321 |
| GO:0032868\_response\_to\_insulin\_stimulus | 43 | 1 | 4.462209 | -0.694516 | 168 | 127.391921 | 205.35 | 283.308079 | 1.222321 |
| GO:0002377\_immunoglobulin\_production | 44 | 1 | 4.360795 | -0.685586 | 170 | 130.326506 | 209.96 | 289.593494 | 1.235059 |
| GO:0048593\_camera-type\_eye\_morphogenesis | 44 | 1 | 4.360795 | -0.685586 | 170 | 130.326506 | 209.96 | 289.593494 | 1.235059 |
| GO:0032870\_cellular\_response\_to\_hormone\_stimulus | 45 | 1 | 4.263889 | -0.676880 | 171 | 131.978592 | 211.76 | 291.541408 | 1.238363 |
| GO:0006396\_RNA\_processing | 47 | 1 | 4.082447 | -0.660099 | 172 | 137.066217 | 217.79 | 298.513783 | 1.266221 |
| GO:0032269\_negative\_regulation\_of\_cellular\_protein\_metabolic\_process | 48 | 1 | 3.997396 | -0.652007 | 173 | 138.557464 | 219.7 | 300.842536 | 1.269942 |
| GO:0002440\_production\_of\_molecular\_mediator\_of\_immune\_response | 49 | 1 | 3.915816 | -0.644102 | 175 | 140.954261 | 222.44 | 303.925739 | 1.271086 |
| GO:0006725\_cellular\_aromatic\_compound\_metabolic\_process | 49 | 1 | 3.915816 | -0.644102 | 175 | 140.954261 | 222.44 | 303.925739 | 1.271086 |
| GO:0070647\_protein\_modification\_by\_small\_protein\_conjugation\_or\_removal | 50 | 1 | 3.837500 | -0.636378 | 176 | 142.656662 | 224.69 | 306.723338 | 1.276648 |
| GO:0044249\_cellular\_biosynthetic\_process | 1150 | 8 | 1.334783 | -0.633899 | 177 | 143.166815 | 225.33 | 307.493185 | 1.273051 |
| GO:0006887\_exocytosis | 51 | 1 | 3.762255 | -0.628826 | 179 | 145.413932 | 227.87 | 310.326068 | 1.273017 |
| GO:0032880\_regulation\_of\_protein\_localization | 51 | 1 | 3.762255 | -0.628826 | 179 | 145.413932 | 227.87 | 310.326068 | 1.273017 |
| GO:0009987\_cellular\_process | 3868 | 22 | 1.091326 | -0.626246 | 180 | 145.556371 | 228.09 | 310.623629 | 1.267167 |
| GO:0031326\_regulation\_of\_cellular\_biosynthetic\_process | 812 | 6 | 1.417796 | -0.622983 | 181 | 145.611694 | 228.2 | 310.788306 | 1.260773 |
| GO:0010608\_posttranscriptional\_regulation\_of\_gene\_expression | 52 | 1 | 3.689904 | -0.621441 | 182 | 146.748011 | 229.68 | 312.611989 | 1.261978 |
| GO:0009889\_regulation\_of\_biosynthetic\_process | 815 | 6 | 1.412577 | -0.618014 | 183 | 146.939547 | 229.98 | 313.020453 | 1.256721 |
| GO:0051248\_negative\_regulation\_of\_protein\_metabolic\_process | 53 | 1 | 3.620283 | -0.614216 | 184 | 148.512442 | 231.9 | 315.287558 | 1.260326 |
| GO:0044271\_nitrogen\_compound\_biosynthetic\_process | 54 | 1 | 3.553241 | -0.607144 | 185 | 150.845569 | 234.7 | 318.554431 | 1.268649 |
| GO:0019538\_protein\_metabolic\_process | 655 | 5 | 1.464695 | -0.604037 | 186 | 151.507277 | 235.55 | 319.592723 | 1.266398 |
| GO:0006310\_DNA\_recombination | 55 | 1 | 3.488636 | -0.600221 | 188 | 153.031760 | 237.26 | 321.488240 | 1.262021 |
| GO:0043434\_response\_to\_peptide\_hormone\_stimulus | 55 | 1 | 3.488636 | -0.600221 | 188 | 153.031760 | 237.26 | 321.488240 | 1.262021 |
| GO:0009058\_biosynthetic\_process | 1175 | 8 | 1.306383 | -0.598235 | 189 | 153.289953 | 237.56 | 321.830047 | 1.256931 |
| GO:0044238\_primary\_metabolic\_process | 1905 | 12 | 1.208661 | -0.593388 | 190 | 155.987563 | 240.76 | 325.532437 | 1.267158 |
| GO:0001764\_neuron\_migration | 57 | 1 | 3.366228 | -0.586798 | 193 | 158.432934 | 244.03 | 329.627066 | 1.264404 |
| GO:0018108\_peptidyl-tyrosine\_phosphorylation | 57 | 1 | 3.366228 | -0.586798 | 193 | 158.432934 | 244.03 | 329.627066 | 1.264404 |
| GO:0018212\_peptidyl-tyrosine\_modification | 57 | 1 | 3.366228 | -0.586798 | 193 | 158.432934 | 244.03 | 329.627066 | 1.264404 |
| GO:0051649\_establishment\_of\_localization\_in\_cell | 342 | 3 | 1.683114 | -0.581658 | 194 | 158.869042 | 244.86 | 330.850958 | 1.262165 |
| GO:0048469\_cell\_maturation | 59 | 1 | 3.252119 | -0.573907 | 195 | 160.960523 | 247.28 | 333.599477 | 1.268103 |
| GO:0003002\_regionalization | 195 | 2 | 1.967949 | -0.568185 | 196 | 161.683518 | 248.26 | 334.836482 | 1.266633 |
| GO:0045449\_regulation\_of\_transcription | 676 | 5 | 1.419194 | -0.567531 | 197 | 161.993671 | 248.64 | 335.286329 | 1.262132 |
| GO:0007369\_gastrulation | 63 | 1 | 3.045635 | -0.549581 | 199 | 167.243612 | 255.28 | 343.316388 | 1.282814 |
| GO:0051186\_cofactor\_metabolic\_process | 63 | 1 | 3.045635 | -0.549581 | 199 | 167.243612 | 255.28 | 343.316388 | 1.282814 |
| GO:0008152\_metabolic\_process | 2133 | 13 | 1.169421 | -0.546069 | 200 | 167.684313 | 255.79 | 343.895687 | 1.278950 |
| GO:0043086\_negative\_regulation\_of\_catalytic\_activity | 65 | 1 | 2.951923 | -0.538085 | 201 | 170.183528 | 259.02 | 347.856472 | 1.288657 |
| GO:0007179\_transforming\_growth\_factor\_beta\_receptor\_signaling\_pathway | 66 | 1 | 2.907197 | -0.532491 | 202 | 171.704346 | 260.93 | 350.155654 | 1.291733 |
| GO:0006350\_transcription | 701 | 5 | 1.368581 | -0.526778 | 203 | 173.592957 | 263.19 | 352.787043 | 1.296502 |
| GO:0019932\_second-messenger-mediated\_signaling | 68 | 1 | 2.821691 | -0.521598 | 204 | 174.559983 | 264.57 | 354.580017 | 1.296912 |
| GO:0051641\_cellular\_localization | 370 | 3 | 1.555743 | -0.518801 | 205 | 174.658198 | 264.81 | 354.961802 | 1.291756 |
| GO:0048592\_eye\_morphogenesis | 70 | 1 | 2.741071 | -0.511078 | 206 | 177.322514 | 268.16 | 358.997486 | 1.301748 |
| GO:0006281\_DNA\_repair | 71 | 1 | 2.702465 | -0.505951 | 207 | 178.869841 | 270.12 | 361.370159 | 1.304928 |
| GO:0006950\_response\_to\_stress | 549 | 4 | 1.397996 | -0.495928 | 208 | 184.032483 | 276.45 | 368.867517 | 1.329087 |
| GO:0001701\_in\_utero\_embryonic\_development | 221 | 2 | 1.736425 | -0.493096 | 209 | 184.358728 | 276.86 | 369.361272 | 1.324689 |
| GO:0043687\_post-translational\_protein\_modification | 384 | 3 | 1.499023 | -0.490180 | 210 | 185.244366 | 277.8 | 370.355634 | 1.322857 |
| GO:0009725\_response\_to\_hormone\_stimulus | 76 | 1 | 2.524671 | -0.481550 | 211 | 188.247670 | 281.19 | 374.132330 | 1.332654 |
| GO:0019222\_regulation\_of\_metabolic\_process | 1088 | 7 | 1.234490 | -0.478794 | 212 | 188.487068 | 281.49 | 374.492932 | 1.327783 |
| GO:0007167\_enzyme\_linked\_receptor\_protein\_signaling\_pathway | 229 | 2 | 1.675764 | -0.472503 | 213 | 190.068988 | 283.38 | 376.691012 | 1.330423 |
| GO:0007165\_signal\_transduction | 915 | 6 | 1.258197 | -0.471296 | 214 | 191.593793 | 285.42 | 379.246207 | 1.333738 |
| GO:0009790\_embryonic\_development | 567 | 4 | 1.353616 | -0.466505 | 215 | 192.366923 | 286.55 | 380.733077 | 1.332791 |
| GO:0000278\_mitotic\_cell\_cycle | 80 | 1 | 2.398438 | -0.463383 | 217 | 193.392928 | 287.92 | 382.447072 | 1.326820 |
| GO:0044092\_negative\_regulation\_of\_molecular\_function | 80 | 1 | 2.398438 | -0.463383 | 217 | 193.392928 | 287.92 | 382.447072 | 1.326820 |
| GO:0021700\_developmental\_maturation | 81 | 1 | 2.368827 | -0.459013 | 218 | 194.484680 | 289.15 | 383.815320 | 1.326376 |
| GO:0080090\_regulation\_of\_primary\_metabolic\_process | 926 | 6 | 1.243251 | -0.457202 | 219 | 194.551992 | 289.28 | 384.008008 | 1.320913 |
| GO:0019219\_regulation\_of\_nucleobase\_\_nucleoside\_\_nucleotide\_and\_nucleic\_acid\_metabolic\_process | 757 | 5 | 1.267338 | -0.445131 | 220 | 199.296247 | 294.58 | 389.863753 | 1.339000 |
| GO:0070887\_cellular\_response\_to\_chemical\_stimulus | 85 | 1 | 2.257353 | -0.442173 | 221 | 200.254437 | 295.68 | 391.105563 | 1.337919 |
| GO:0007242\_intracellular\_signaling\_cascade | 411 | 3 | 1.400547 | -0.439631 | 222 | 200.352460 | 295.83 | 391.307540 | 1.332568 |
| GO:0034641\_cellular\_nitrogen\_compound\_metabolic\_process | 86 | 1 | 2.231105 | -0.438115 | 223 | 202.200237 | 297.83 | 393.459763 | 1.335561 |
| GO:0007178\_transmembrane\_receptor\_protein\_serine\_threonine\_kinase\_signaling\_pathway | 87 | 1 | 2.205460 | -0.434115 | 224 | 204.269794 | 300.17 | 396.070206 | 1.340045 |
| GO:0001503\_ossification | 88 | 1 | 2.180398 | -0.430171 | 225 | 204.969226 | 301.25 | 397.530774 | 1.338889 |
| GO:0051171\_regulation\_of\_nitrogen\_compound\_metabolic\_process | 771 | 5 | 1.244326 | -0.426617 | 226 | 205.382417 | 301.88 | 398.377583 | 1.335752 |
| GO:0007389\_pattern\_specification\_process | 250 | 2 | 1.535000 | -0.423185 | 227 | 206.417173 | 303.0 | 399.582827 | 1.334802 |
| GO:0009719\_response\_to\_endogenous\_stimulus | 92 | 1 | 2.085598 | -0.414938 | 228 | 210.491794 | 308.14 | 405.788206 | 1.351491 |
| GO:0018193\_peptidyl-amino\_acid\_modification | 97 | 1 | 1.978093 | -0.397026 | 230 | 217.805073 | 316.94 | 416.074927 | 1.378000 |
| GO:0060341\_regulation\_of\_cellular\_localization | 97 | 1 | 1.978093 | -0.397026 | 230 | 217.805073 | 316.94 | 416.074927 | 1.378000 |
| GO:0006464\_protein\_modification\_process | 439 | 3 | 1.311219 | -0.392940 | 231 | 220.108777 | 319.29 | 418.471223 | 1.382208 |
| GO:0060348\_bone\_development | 99 | 1 | 1.938131 | -0.390185 | 232 | 221.053800 | 320.52 | 419.986200 | 1.381552 |
| GO:0009968\_negative\_regulation\_of\_signal\_transduction | 103 | 1 | 1.862864 | -0.377020 | 233 | 224.844795 | 325.06 | 425.275205 | 1.395107 |
| GO:0055086\_nucleobase\_\_nucleoside\_and\_nucleotide\_metabolic\_process | 104 | 1 | 1.844952 | -0.373831 | 234 | 226.977229 | 327.47 | 427.962771 | 1.399444 |
| GO:0048872\_homeostasis\_of\_number\_of\_cells | 105 | 1 | 1.827381 | -0.370682 | 235 | 227.415429 | 328.06 | 428.704571 | 1.396000 |
| GO:0045859\_regulation\_of\_protein\_kinase\_activity | 107 | 1 | 1.793224 | -0.364498 | 236 | 229.256184 | 329.93 | 430.603816 | 1.398008 |
| GO:0043412\_biopolymer\_modification | 458 | 3 | 1.256823 | -0.364190 | 237 | 229.687528 | 330.39 | 431.092472 | 1.394051 |
| GO:0030099\_myeloid\_cell\_differentiation | 108 | 1 | 1.776620 | -0.361462 | 238 | 230.299839 | 331.15 | 432.000161 | 1.391387 |
| GO:0031323\_regulation\_of\_cellular\_metabolic\_process | 1015 | 6 | 1.134236 | -0.355920 | 239 | 231.570836 | 332.65 | 433.729164 | 1.391841 |
| GO:0010648\_negative\_regulation\_of\_cell\_communication | 110 | 1 | 1.744318 | -0.355499 | 240 | 232.917118 | 334.2 | 435.482882 | 1.392500 |
| GO:0043549\_regulation\_of\_kinase\_activity | 112 | 1 | 1.713170 | -0.349676 | 241 | 234.104523 | 335.74 | 437.375477 | 1.393112 |
| GO:0009607\_response\_to\_biotic\_stimulus | 114 | 1 | 1.683114 | -0.343988 | 242 | 236.128871 | 338.1 | 440.071129 | 1.397107 |
| GO:0051338\_regulation\_of\_transferase\_activity | 115 | 1 | 1.668478 | -0.341194 | 243 | 236.866220 | 338.93 | 440.993780 | 1.394774 |
| GO:0002520\_immune\_system\_development | 295 | 2 | 1.300847 | -0.336368 | 244 | 238.618375 | 340.76 | 442.901625 | 1.396557 |
| GO:0050789\_regulation\_of\_biological\_process | 2357 | 13 | 1.058284 | -0.332043 | 245 | 239.817983 | 342.12 | 444.422017 | 1.396408 |
| GO:0048598\_embryonic\_morphogenesis | 299 | 2 | 1.283445 | -0.329684 | 246 | 240.417757 | 342.71 | 445.002243 | 1.393130 |
| GO:0051726\_regulation\_of\_cell\_cycle | 121 | 1 | 1.585744 | -0.325081 | 247 | 241.936016 | 344.73 | 447.523984 | 1.395668 |
| GO:0002252\_immune\_effector\_process | 122 | 1 | 1.572746 | -0.322499 | 248 | 243.587116 | 346.74 | 449.892884 | 1.398145 |
| GO:0030098\_lymphocyte\_differentiation | 124 | 1 | 1.547379 | -0.317421 | 249 | 245.077442 | 348.34 | 451.602558 | 1.398956 |
| GO:0050794\_regulation\_of\_cellular\_process | 2190 | 12 | 1.051370 | -0.314614 | 250 | 246.424835 | 349.87 | 453.315165 | 1.399480 |
| GO:0016310\_phosphorylation | 309 | 2 | 1.241909 | -0.313611 | 251 | 246.768044 | 350.32 | 453.871956 | 1.395697 |
| GO:0002376\_immune\_system\_process | 505 | 3 | 1.139851 | -0.301850 | 252 | 249.978848 | 353.75 | 457.521152 | 1.403770 |
| GO:0007169\_transmembrane\_receptor\_protein\_tyrosine\_kinase\_signaling\_pathway | 139 | 1 | 1.380396 | -0.282602 | 253 | 253.660972 | 357.79 | 461.919028 | 1.414190 |
| GO:0007154\_cell\_communication | 1096 | 6 | 1.050411 | -0.281010 | 254 | 255.473479 | 359.74 | 464.006521 | 1.416299 |
| GO:0007186\_G-protein\_coupled\_receptor\_protein\_signaling\_pathway | 144 | 1 | 1.332465 | -0.272139 | 256 | 258.894450 | 363.15 | 467.405550 | 1.418555 |
| GO:0045596\_negative\_regulation\_of\_cell\_differentiation | 144 | 1 | 1.332465 | -0.272139 | 256 | 258.894450 | 363.15 | 467.405550 | 1.418555 |
| GO:0050896\_response\_to\_stimulus | 1107 | 6 | 1.039973 | -0.271946 | 257 | 259.079592 | 363.4 | 467.720408 | 1.414008 |
| GO:0006793\_phosphorus\_metabolic\_process | 340 | 2 | 1.128676 | -0.268997 | 259 | 260.512745 | 364.84 | 469.167255 | 1.408649 |
| GO:0006796\_phosphate\_metabolic\_process | 340 | 2 | 1.128676 | -0.268997 | 259 | 260.512745 | 364.84 | 469.167255 | 1.408649 |
| GO:0030900\_forebrain\_development | 146 | 1 | 1.314212 | -0.268097 | 260 | 261.499768 | 365.97 | 470.440232 | 1.407577 |
| GO:0032940\_secretion\_by\_cell | 149 | 1 | 1.287752 | -0.262180 | 261 | 263.894624 | 368.37 | 472.845376 | 1.411379 |
| GO:0032268\_regulation\_of\_cellular\_protein\_metabolic\_process | 152 | 1 | 1.262336 | -0.256431 | 262 | 265.203430 | 369.58 | 473.956570 | 1.410611 |
| GO:0022402\_cell\_cycle\_process | 155 | 1 | 1.237903 | -0.250845 | 263 | 267.336494 | 371.91 | 476.483506 | 1.414106 |
| GO:0007409\_axonogenesis | 158 | 1 | 1.214399 | -0.245415 | 264 | 269.143260 | 373.82 | 478.496740 | 1.415985 |
| GO:0002521\_leukocyte\_differentiation | 161 | 1 | 1.191770 | -0.240133 | 265 | 271.427275 | 375.99 | 480.552725 | 1.418830 |
| GO:0043009\_chordate\_embryonic\_development | 365 | 2 | 1.051370 | -0.237984 | 266 | 272.737564 | 377.3 | 481.862436 | 1.418421 |
| GO:0009653\_anatomical\_structure\_morphogenesis | 958 | 5 | 1.001435 | -0.237126 | 267 | 272.858023 | 377.47 | 482.081977 | 1.413745 |
| GO:0042325\_regulation\_of\_phosphorylation | 164 | 1 | 1.169970 | -0.234995 | 268 | 274.044556 | 378.88 | 483.715444 | 1.413731 |
| GO:0009792\_embryonic\_development\_ending\_in\_birth\_or\_egg\_hatching | 368 | 2 | 1.042799 | -0.234525 | 269 | 274.298551 | 379.19 | 484.081449 | 1.409628 |
| GO:0006259\_DNA\_metabolic\_process | 165 | 1 | 1.162879 | -0.233314 | 272 | 275.278934 | 380.3 | 485.321066 | 1.398162 |
| GO:0019220\_regulation\_of\_phosphate\_metabolic\_process | 165 | 1 | 1.162879 | -0.233314 | 272 | 275.278934 | 380.3 | 485.321066 | 1.398162 |
| GO:0051174\_regulation\_of\_phosphorus\_metabolic\_process | 165 | 1 | 1.162879 | -0.233314 | 272 | 275.278934 | 380.3 | 485.321066 | 1.398162 |
| GO:0051049\_regulation\_of\_transport | 167 | 1 | 1.148952 | -0.229996 | 273 | 277.209308 | 382.31 | 487.410692 | 1.400403 |
| GO:0048523\_negative\_regulation\_of\_cellular\_process | 774 | 4 | 0.991602 | -0.227546 | 274 | 278.023297 | 383.09 | 488.156703 | 1.398139 |
| GO:0048812\_neuron\_projection\_morphogenesis | 170 | 1 | 1.128676 | -0.225129 | 276 | 279.150974 | 384.15 | 489.149026 | 1.391848 |
| GO:0051246\_regulation\_of\_protein\_metabolic\_process | 170 | 1 | 1.128676 | -0.225129 | 276 | 279.150974 | 384.15 | 489.149026 | 1.391848 |
| GO:0009611\_response\_to\_wounding | 172 | 1 | 1.115552 | -0.221956 | 277 | 280.605138 | 385.75 | 490.894862 | 1.392599 |
| GO:0048667\_cell\_morphogenesis\_involved\_in\_neuron\_differentiation | 173 | 1 | 1.109104 | -0.220390 | 278 | 282.003474 | 387.07 | 492.136526 | 1.392338 |
| GO:0046903\_secretion | 175 | 1 | 1.096429 | -0.217300 | 279 | 283.325502 | 388.34 | 493.354498 | 1.391900 |
| GO:0048858\_cell\_projection\_morphogenesis | 176 | 1 | 1.090199 | -0.215775 | 280 | 284.808143 | 390.02 | 495.231857 | 1.392929 |
| GO:0007166\_cell\_surface\_receptor\_linked\_signal\_transduction | 597 | 3 | 0.964196 | -0.208602 | 281 | 288.214070 | 393.63 | 499.045930 | 1.400819 |
| GO:0042127\_regulation\_of\_cell\_proliferation | 393 | 2 | 0.976463 | -0.207662 | 282 | 289.530666 | 394.94 | 500.349334 | 1.400496 |
| GO:0016192\_vesicle-mediated\_transport | 184 | 1 | 1.042799 | -0.204038 | 284 | 292.082875 | 397.51 | 502.937125 | 1.399683 |
| GO:0032990\_cell\_part\_morphogenesis | 184 | 1 | 1.042799 | -0.204038 | 284 | 292.082875 | 397.51 | 502.937125 | 1.399683 |
| GO:0042221\_response\_to\_chemical\_stimulus | 409 | 2 | 0.938264 | -0.192158 | 285 | 296.459085 | 401.72 | 506.980915 | 1.409544 |
| GO:0031175\_neuron\_projection\_development | 197 | 1 | 0.973985 | -0.186577 | 286 | 299.655264 | 404.9 | 510.144736 | 1.415734 |
| GO:0000904\_cell\_morphogenesis\_involved\_in\_differentiation | 199 | 1 | 0.964196 | -0.184053 | 287 | 300.823634 | 405.92 | 511.016366 | 1.414355 |
| GO:0065007\_biological\_regulation | 2593 | 13 | 0.961965 | -0.177606 | 288 | 302.497590 | 407.52 | 512.542410 | 1.415000 |
| GO:0006955\_immune\_response | 205 | 1 | 0.935976 | -0.176721 | 289 | 303.452909 | 408.44 | 513.427091 | 1.413287 |
| GO:0008284\_positive\_regulation\_of\_cell\_proliferation | 208 | 1 | 0.922476 | -0.173186 | 290 | 304.769141 | 409.74 | 514.710859 | 1.412897 |
| GO:0048519\_negative\_regulation\_of\_biological\_process | 859 | 4 | 0.893481 | -0.167202 | 291 | 306.830285 | 411.62 | 516.409715 | 1.414502 |
| GO:0010033\_response\_to\_organic\_substance | 216 | 1 | 0.888310 | -0.164156 | 292 | 307.880258 | 412.67 | 517.459742 | 1.413253 |
| GO:0006996\_organelle\_organization | 449 | 2 | 0.854677 | -0.158350 | 293 | 311.828671 | 416.41 | 520.991329 | 1.421195 |
| GO:0046649\_lymphocyte\_activation | 228 | 1 | 0.841557 | -0.151620 | 294 | 313.818430 | 418.36 | 522.901570 | 1.422993 |
| GO:0007420\_brain\_development | 231 | 1 | 0.830628 | -0.148661 | 295 | 315.613425 | 420.1 | 524.586575 | 1.424068 |
| GO:0050790\_regulation\_of\_catalytic\_activity | 233 | 1 | 0.823498 | -0.146725 | 296 | 316.421369 | 420.89 | 525.358631 | 1.421926 |
| GO:0016477\_cell\_migration | 234 | 1 | 0.819979 | -0.145768 | 297 | 317.110618 | 421.59 | 526.069382 | 1.419495 |
| GO:0001501\_skeletal\_system\_development | 236 | 1 | 0.813030 | -0.143875 | 298 | 317.456171 | 421.97 | 526.483829 | 1.416007 |
| GO:0006468\_protein\_amino\_acid\_phosphorylation | 237 | 1 | 0.809599 | -0.142939 | 300 | 318.494214 | 422.93 | 527.365786 | 1.409767 |
| GO:0044085\_cellular\_component\_biogenesis | 237 | 1 | 0.809599 | -0.142939 | 300 | 318.494214 | 422.93 | 527.365786 | 1.409767 |
| GO:0032879\_regulation\_of\_localization | 248 | 1 | 0.773690 | -0.133086 | 302 | 321.936233 | 426.04 | 530.143767 | 1.410728 |
| GO:0045321\_leukocyte\_activation | 248 | 1 | 0.773690 | -0.133086 | 302 | 321.936233 | 426.04 | 530.143767 | 1.410728 |
| GO:0030097\_hemopoiesis | 253 | 1 | 0.758399 | -0.128863 | 303 | 324.144606 | 428.05 | 531.955394 | 1.412706 |
| GO:0006810\_transport | 718 | 3 | 0.801706 | -0.126890 | 304 | 325.189266 | 429.03 | 532.870734 | 1.411283 |
| GO:0009966\_regulation\_of\_signal\_transduction | 256 | 1 | 0.749512 | -0.126401 | 305 | 325.747683 | 429.48 | 533.212317 | 1.408131 |
| GO:0048870\_cell\_motility | 257 | 1 | 0.746595 | -0.125592 | 306 | 326.219494 | 429.88 | 533.540506 | 1.404837 |
| GO:0001775\_cell\_activation | 262 | 1 | 0.732347 | -0.121633 | 308 | 328.071964 | 431.48 | 534.888036 | 1.400909 |
| GO:0048666\_neuron\_development | 262 | 1 | 0.732347 | -0.121633 | 308 | 328.071964 | 431.48 | 534.888036 | 1.400909 |
| GO:0007275\_multicellular\_organismal\_development | 1760 | 8 | 0.872159 | -0.121293 | 309 | 328.243488 | 431.64 | 535.036512 | 1.396893 |
| GO:0051234\_establishment\_of\_localization | 729 | 3 | 0.789609 | -0.121174 | 310 | 328.463333 | 431.83 | 535.196667 | 1.393000 |
| GO:0030030\_cell\_projection\_organization | 263 | 1 | 0.729563 | -0.120858 | 311 | 328.900497 | 432.24 | 535.579503 | 1.389839 |
| GO:0045944\_positive\_regulation\_of\_transcription\_from\_RNA\_polymerase\_II\_promoter | 269 | 1 | 0.713290 | -0.116321 | 312 | 330.235643 | 433.59 | 536.944357 | 1.389712 |
| GO:0048534\_hemopoietic\_or\_lymphoid\_organ\_development | 277 | 1 | 0.692690 | -0.110562 | 314 | 334.391727 | 437.39 | 540.388273 | 1.392962 |
| GO:0048646\_anatomical\_structure\_formation\_involved\_in\_morphogenesis | 277 | 1 | 0.692690 | -0.110562 | 314 | 334.391727 | 437.39 | 540.388273 | 1.392962 |
| GO:0007610\_behavior | 279 | 1 | 0.687724 | -0.109171 | 316 | 335.320350 | 438.4 | 541.479650 | 1.387342 |
| GO:0065009\_regulation\_of\_molecular\_function | 279 | 1 | 0.687724 | -0.109171 | 316 | 335.320350 | 438.4 | 541.479650 | 1.387342 |
| GO:0000902\_cell\_morphogenesis | 283 | 1 | 0.678004 | -0.106447 | 317 | 336.744605 | 439.6 | 542.455395 | 1.386751 |
| GO:0007417\_central\_nervous\_system\_development | 287 | 1 | 0.668554 | -0.103797 | 318 | 338.095759 | 440.83 | 543.564241 | 1.386258 |
| GO:0008283\_cell\_proliferation | 544 | 2 | 0.705423 | -0.099918 | 319 | 338.881590 | 441.53 | 544.178410 | 1.384107 |
| GO:0040011\_locomotion | 295 | 1 | 0.650424 | -0.098710 | 321 | 340.443538 | 442.86 | 545.276462 | 1.379626 |
| GO:0045595\_regulation\_of\_cell\_differentiation | 295 | 1 | 0.650424 | -0.098710 | 321 | 340.443538 | 442.86 | 545.276462 | 1.379626 |
| GO:0045893\_positive\_regulation\_of\_transcription\_\_DNA-dependent | 306 | 1 | 0.627042 | -0.092149 | 323 | 342.678505 | 444.89 | 547.101495 | 1.377368 |
| GO:0051254\_positive\_regulation\_of\_RNA\_metabolic\_process | 306 | 1 | 0.627042 | -0.092149 | 323 | 342.678505 | 444.89 | 547.101495 | 1.377368 |
| GO:0032989\_cellular\_component\_morphogenesis | 307 | 1 | 0.625000 | -0.091577 | 324 | 343.054530 | 445.2 | 547.345470 | 1.374074 |
| GO:0032502\_developmental\_process | 2060 | 9 | 0.838289 | -0.085738 | 325 | 345.178405 | 446.9 | 548.621595 | 1.375077 |
| GO:0006928\_cell\_motion | 330 | 1 | 0.581439 | -0.079400 | 328 | 347.435505 | 448.82 | 550.204495 | 1.368354 |
| GO:0010646\_regulation\_of\_cell\_communication | 330 | 1 | 0.581439 | -0.079400 | 328 | 347.435505 | 448.82 | 550.204495 | 1.368354 |
| GO:0051674\_localization\_of\_cell | 330 | 1 | 0.581439 | -0.079400 | 328 | 347.435505 | 448.82 | 550.204495 | 1.368354 |
| GO:0051093\_negative\_regulation\_of\_developmental\_process | 331 | 1 | 0.579683 | -0.078911 | 329 | 347.929260 | 449.31 | 550.690740 | 1.365684 |
| GO:0048856\_anatomical\_structure\_development | 1688 | 7 | 0.795690 | -0.078237 | 330 | 348.269192 | 449.65 | 551.030808 | 1.362576 |
| GO:0051179\_localization | 1058 | 4 | 0.725425 | -0.077625 | 331 | 349.036141 | 450.22 | 551.403859 | 1.360181 |
| GO:0045941\_positive\_regulation\_of\_transcription | 338 | 1 | 0.567678 | -0.075577 | 332 | 350.005005 | 451.03 | 552.054995 | 1.358524 |
| GO:0009605\_response\_to\_external\_stimulus | 339 | 1 | 0.566003 | -0.075113 | 333 | 350.288543 | 451.28 | 552.271457 | 1.355195 |
| GO:0010628\_positive\_regulation\_of\_gene\_expression | 346 | 1 | 0.554552 | -0.071947 | 334 | 351.605279 | 452.4 | 553.194721 | 1.354491 |
| GO:0045935\_positive\_regulation\_of\_nucleobase\_\_nucleoside\_\_nucleotide\_and\_nucleic\_acid\_metabolic\_process | 352 | 1 | 0.545099 | -0.069344 | 335 | 352.074291 | 452.91 | 553.745709 | 1.351970 |
| GO:0030182\_neuron\_differentiation | 356 | 1 | 0.538975 | -0.067664 | 336 | 353.064026 | 453.76 | 554.455974 | 1.350476 |
| GO:0042981\_regulation\_of\_apoptosis | 360 | 1 | 0.532986 | -0.066026 | 337 | 353.607343 | 454.24 | 554.872657 | 1.347893 |
| GO:0051173\_positive\_regulation\_of\_nitrogen\_compound\_metabolic\_process | 361 | 1 | 0.531510 | -0.065623 | 338 | 353.843284 | 454.51 | 555.176716 | 1.344704 |
| GO:0010941\_regulation\_of\_cell\_death | 365 | 1 | 0.525685 | -0.064037 | 340 | 354.664940 | 455.31 | 555.955060 | 1.339147 |
| GO:0043067\_regulation\_of\_programmed\_cell\_death | 365 | 1 | 0.525685 | -0.064037 | 340 | 354.664940 | 455.31 | 555.955060 | 1.339147 |
| GO:0010557\_positive\_regulation\_of\_macromolecule\_biosynthetic\_process | 371 | 1 | 0.517183 | -0.061731 | 341 | 355.935583 | 456.44 | 556.944417 | 1.338534 |
| GO:0048468\_cell\_development | 654 | 2 | 0.586774 | -0.058112 | 342 | 357.173412 | 457.5 | 557.826588 | 1.337719 |
| GO:0031328\_positive\_regulation\_of\_cellular\_biosynthetic\_process | 387 | 1 | 0.495801 | -0.055992 | 343 | 358.548987 | 458.68 | 558.811013 | 1.337259 |
| GO:0009891\_positive\_regulation\_of\_biosynthetic\_process | 388 | 1 | 0.494523 | -0.055653 | 344 | 358.774095 | 458.94 | 559.105905 | 1.334128 |
| GO:0048513\_organ\_development | 1365 | 5 | 0.702839 | -0.054359 | 345 | 359.499567 | 459.61 | 559.720433 | 1.332203 |
| GO:0048699\_generation\_of\_neurons | 396 | 1 | 0.484533 | -0.053008 | 346 | 360.828011 | 460.7 | 560.571989 | 1.331503 |
| GO:0048731\_system\_development | 1609 | 6 | 0.715507 | -0.048127 | 347 | 362.116575 | 461.77 | 561.423425 | 1.330749 |
| GO:0042592\_homeostatic\_process | 419 | 1 | 0.457936 | -0.046098 | 348 | 362.662908 | 462.27 | 561.877092 | 1.328362 |
| GO:0022008\_neurogenesis | 423 | 1 | 0.453605 | -0.044994 | 349 | 363.404290 | 462.87 | 562.335710 | 1.326275 |
| GO:0006915\_apoptosis | 427 | 1 | 0.449356 | -0.043916 | 350 | 363.747393 | 463.22 | 562.692607 | 1.323486 |
| GO:0016043\_cellular\_component\_organization | 964 | 3 | 0.597121 | -0.043133 | 351 | 363.912618 | 463.37 | 562.827382 | 1.320142 |
| GO:0010604\_positive\_regulation\_of\_macromolecule\_metabolic\_process | 433 | 1 | 0.443129 | -0.042347 | 353 | 364.271110 | 463.8 | 563.328890 | 1.313881 |
| GO:0012501\_programmed\_cell\_death | 433 | 1 | 0.443129 | -0.042347 | 353 | 364.271110 | 463.8 | 563.328890 | 1.313881 |
| GO:0031325\_positive\_regulation\_of\_cellular\_metabolic\_process | 442 | 1 | 0.434106 | -0.040100 | 354 | 365.293192 | 464.64 | 563.986808 | 1.312542 |
| GO:0008219\_cell\_death | 444 | 1 | 0.432151 | -0.039618 | 355 | 365.976846 | 465.25 | 564.523154 | 1.310563 |
| GO:0010926\_anatomical\_structure\_formation | 447 | 1 | 0.429251 | -0.038904 | 356 | 366.228918 | 465.48 | 564.731082 | 1.307528 |
| GO:0016265\_death | 450 | 1 | 0.426389 | -0.038204 | 357 | 366.825880 | 466.06 | 565.294120 | 1.305490 |
| GO:0009893\_positive\_regulation\_of\_metabolic\_process | 458 | 1 | 0.418941 | -0.036398 | 358 | 367.521687 | 466.63 | 565.738313 | 1.303436 |
| GO:0030154\_cell\_differentiation | 1060 | 3 | 0.543042 | -0.027411 | 359 | 369.287481 | 467.94 | 566.592519 | 1.303454 |
| GO:0032501\_multicellular\_organismal\_process | 2183 | 8 | 0.703161 | -0.024308 | 360 | 370.693986 | 468.94 | 567.186014 | 1.302611 |
| GO:0048869\_cellular\_developmental\_process | 1113 | 3 | 0.517183 | -0.021153 | 361 | 371.808915 | 469.74 | 567.671085 | 1.301219 |
| GO:0048522\_positive\_regulation\_of\_cellular\_process | 895 | 2 | 0.428771 | -0.016649 | 362 | 373.259261 | 470.78 | 568.300739 | 1.300497 |
| GO:0007399\_nervous\_system\_development | 621 | 1 | 0.308977 | -0.013508 | 363 | 374.880856 | 471.88 | 568.879144 | 1.299945 |
| GO:0009887\_organ\_morphogenesis | 642 | 1 | 0.298871 | -0.011872 | 364 | 375.535347 | 472.27 | 569.004653 | 1.297445 |
| GO:0048518\_positive\_regulation\_of\_biological\_process | 995 | 2 | 0.385678 | -0.009597 | 365 | 376.446866 | 472.93 | 569.413134 | 1.295699 |
| GO:0065008\_regulation\_of\_biological\_quality | 693 | 1 | 0.276876 | -0.008660 | 366 | 376.783237 | 473.15 | 569.516763 | 1.292760 |
| GO:0050793\_regulation\_of\_developmental\_process | 703 | 1 | 0.272937 | -0.008138 | 367 | 377.192032 | 473.42 | 569.647968 | 1.289973 |
| GO:0043065\_positive\_regulation\_of\_apoptosis | 166 | 0 | 0.000000 | -0.000000 | 368 | 380.597776 | 475.81 | 571.022224 | 1.292962 |
| GO:0001843\_neural\_tube\_closure | 33 | 0 | 0.000000 | -0.000000 | 381 | 393.614182 | 488.3 | 582.985818 | 1.281627 |
| GO:0006643\_membrane\_lipid\_metabolic\_process | 33 | 0 | 0.000000 | -0.000000 | 381 | 393.614182 | 488.3 | 582.985818 | 1.281627 |
| GO:0007188\_G-protein\_signaling\_\_coupled\_to\_cAMP\_nucleotide\_second\_messenger | 33 | 0 | 0.000000 | -0.000000 | 381 | 393.614182 | 488.3 | 582.985818 | 1.281627 |
| GO:0007270\_nerve-nerve\_synaptic\_transmission | 33 | 0 | 0.000000 | -0.000000 | 381 | 393.614182 | 488.3 | 582.985818 | 1.281627 |
| GO:0007431\_salivary\_gland\_development | 33 | 0 | 0.000000 | -0.000000 | 381 | 393.614182 | 488.3 | 582.985818 | 1.281627 |
| GO:0007565\_female\_pregnancy | 33 | 0 | 0.000000 | -0.000000 | 381 | 393.614182 | 488.3 | 582.985818 | 1.281627 |
| GO:0008584\_male\_gonad\_development | 33 | 0 | 0.000000 | -0.000000 | 381 | 393.614182 | 488.3 | 582.985818 | 1.281627 |
| GO:0008643\_carbohydrate\_transport | 33 | 0 | 0.000000 | -0.000000 | 381 | 393.614182 | 488.3 | 582.985818 | 1.281627 |
| GO:0021536\_diencephalon\_development | 33 | 0 | 0.000000 | -0.000000 | 381 | 393.614182 | 488.3 | 582.985818 | 1.281627 |
| GO:0021987\_cerebral\_cortex\_development | 33 | 0 | 0.000000 | -0.000000 | 381 | 393.614182 | 488.3 | 582.985818 | 1.281627 |
| GO:0022037\_metencephalon\_development | 33 | 0 | 0.000000 | -0.000000 | 381 | 393.614182 | 488.3 | 582.985818 | 1.281627 |
| GO:0042108\_positive\_regulation\_of\_cytokine\_biosynthetic\_process | 33 | 0 | 0.000000 | -0.000000 | 381 | 393.614182 | 488.3 | 582.985818 | 1.281627 |
| GO:0060606\_tube\_closure | 33 | 0 | 0.000000 | -0.000000 | 381 | 393.614182 | 488.3 | 582.985818 | 1.281627 |
| GO:0000910\_cytokinesis | 8 | 0 | 0.000000 | -0.000000 | 507 | 521.975576 | 615.29 | 708.604424 | 1.213590 |
| GO:0001783\_B\_cell\_apoptosis | 8 | 0 | 0.000000 | -0.000000 | 507 | 521.975576 | 615.29 | 708.604424 | 1.213590 |
| GO:0001833\_inner\_cell\_mass\_cell\_proliferation | 8 | 0 | 0.000000 | -0.000000 | 507 | 521.975576 | 615.29 | 708.604424 | 1.213590 |
| GO:0001840\_neural\_plate\_development | 8 | 0 | 0.000000 | -0.000000 | 507 | 521.975576 | 615.29 | 708.604424 | 1.213590 |
| GO:0001893\_maternal\_placenta\_development | 8 | 0 | 0.000000 | -0.000000 | 507 | 521.975576 | 615.29 | 708.604424 | 1.213590 |
| GO:0001911\_negative\_regulation\_of\_leukocyte\_mediated\_cytotoxicity | 8 | 0 | 0.000000 | -0.000000 | 507 | 521.975576 | 615.29 | 708.604424 | 1.213590 |
| GO:0001916\_positive\_regulation\_of\_T\_cell\_mediated\_cytotoxicity | 8 | 0 | 0.000000 | -0.000000 | 507 | 521.975576 | 615.29 | 708.604424 | 1.213590 |
| GO:0002065\_columnar\_cuboidal\_epithelial\_cell\_differentiation | 8 | 0 | 0.000000 | -0.000000 | 507 | 521.975576 | 615.29 | 708.604424 | 1.213590 |
| GO:0002320\_lymphoid\_progenitor\_cell\_differentiation | 8 | 0 | 0.000000 | -0.000000 | 507 | 521.975576 | 615.29 | 708.604424 | 1.213590 |
| GO:0002438\_acute\_inflammatory\_response\_to\_antigenic\_stimulus | 8 | 0 | 0.000000 | -0.000000 | 507 | 521.975576 | 615.29 | 708.604424 | 1.213590 |
| GO:0002524\_hypersensitivity | 8 | 0 | 0.000000 | -0.000000 | 507 | 521.975576 | 615.29 | 708.604424 | 1.213590 |
| GO:0002864\_regulation\_of\_acute\_inflammatory\_response\_to\_antigenic\_stimulus | 8 | 0 | 0.000000 | -0.000000 | 507 | 521.975576 | 615.29 | 708.604424 | 1.213590 |
| GO:0002883\_regulation\_of\_hypersensitivity | 8 | 0 | 0.000000 | -0.000000 | 507 | 521.975576 | 615.29 | 708.604424 | 1.213590 |
| GO:0003081\_regulation\_of\_systemic\_arterial\_blood\_pressure\_by\_renin-angiotensin | 8 | 0 | 0.000000 | -0.000000 | 507 | 521.975576 | 615.29 | 708.604424 | 1.213590 |
| GO:0006020\_inositol\_metabolic\_process | 8 | 0 | 0.000000 | -0.000000 | 507 | 521.975576 | 615.29 | 708.604424 | 1.213590 |
| GO:0006195\_purine\_nucleotide\_catabolic\_process | 8 | 0 | 0.000000 | -0.000000 | 507 | 521.975576 | 615.29 | 708.604424 | 1.213590 |
| GO:0006284\_base-excision\_repair | 8 | 0 | 0.000000 | -0.000000 | 507 | 521.975576 | 615.29 | 708.604424 | 1.213590 |
| GO:0006360\_transcription\_from\_RNA\_polymerase\_I\_promoter | 8 | 0 | 0.000000 | -0.000000 | 507 | 521.975576 | 615.29 | 708.604424 | 1.213590 |
| GO:0006399\_tRNA\_metabolic\_process | 8 | 0 | 0.000000 | -0.000000 | 507 | 521.975576 | 615.29 | 708.604424 | 1.213590 |
| GO:0006458\_'de\_novo'\_protein\_folding | 8 | 0 | 0.000000 | -0.000000 | 507 | 521.975576 | 615.29 | 708.604424 | 1.213590 |
| GO:0006493\_protein\_amino\_acid\_O-linked\_glycosylation | 8 | 0 | 0.000000 | -0.000000 | 507 | 521.975576 | 615.29 | 708.604424 | 1.213590 |
| GO:0006582\_melanin\_metabolic\_process | 8 | 0 | 0.000000 | -0.000000 | 507 | 521.975576 | 615.29 | 708.604424 | 1.213590 |
| GO:0006733\_oxidoreduction\_coenzyme\_metabolic\_process | 8 | 0 | 0.000000 | -0.000000 | 507 | 521.975576 | 615.29 | 708.604424 | 1.213590 |
| GO:0006829\_zinc\_ion\_transport | 8 | 0 | 0.000000 | -0.000000 | 507 | 521.975576 | 615.29 | 708.604424 | 1.213590 |
| GO:0007009\_plasma\_membrane\_organization | 8 | 0 | 0.000000 | -0.000000 | 507 | 521.975576 | 615.29 | 708.604424 | 1.213590 |
| GO:0007098\_centrosome\_cycle | 8 | 0 | 0.000000 | -0.000000 | 507 | 521.975576 | 615.29 | 708.604424 | 1.213590 |
| GO:0007131\_reciprocal\_meiotic\_recombination | 8 | 0 | 0.000000 | -0.000000 | 507 | 521.975576 | 615.29 | 708.604424 | 1.213590 |
| GO:0007141\_male\_meiosis\_I | 8 | 0 | 0.000000 | -0.000000 | 507 | 521.975576 | 615.29 | 708.604424 | 1.213590 |
| GO:0008105\_asymmetric\_protein\_localization | 8 | 0 | 0.000000 | -0.000000 | 507 | 521.975576 | 615.29 | 708.604424 | 1.213590 |
| GO:0008593\_regulation\_of\_Notch\_signaling\_pathway | 8 | 0 | 0.000000 | -0.000000 | 507 | 521.975576 | 615.29 | 708.604424 | 1.213590 |
| GO:0009072\_aromatic\_amino\_acid\_family\_metabolic\_process | 8 | 0 | 0.000000 | -0.000000 | 507 | 521.975576 | 615.29 | 708.604424 | 1.213590 |
| GO:0009144\_purine\_nucleoside\_triphosphate\_metabolic\_process | 8 | 0 | 0.000000 | -0.000000 | 507 | 521.975576 | 615.29 | 708.604424 | 1.213590 |
| GO:0009746\_response\_to\_hexose\_stimulus | 8 | 0 | 0.000000 | -0.000000 | 507 | 521.975576 | 615.29 | 708.604424 | 1.213590 |
| GO:0009749\_response\_to\_glucose\_stimulus | 8 | 0 | 0.000000 | -0.000000 | 507 | 521.975576 | 615.29 | 708.604424 | 1.213590 |
| GO:0014014\_negative\_regulation\_of\_gliogenesis | 8 | 0 | 0.000000 | -0.000000 | 507 | 521.975576 | 615.29 | 708.604424 | 1.213590 |
| GO:0014046\_dopamine\_secretion | 8 | 0 | 0.000000 | -0.000000 | 507 | 521.975576 | 615.29 | 708.604424 | 1.213590 |
| GO:0014059\_regulation\_of\_dopamine\_secretion | 8 | 0 | 0.000000 | -0.000000 | 507 | 521.975576 | 615.29 | 708.604424 | 1.213590 |
| GO:0014065\_phosphoinositide\_3-kinase\_cascade | 8 | 0 | 0.000000 | -0.000000 | 507 | 521.975576 | 615.29 | 708.604424 | 1.213590 |
| GO:0015800\_acidic\_amino\_acid\_transport | 8 | 0 | 0.000000 | -0.000000 | 507 | 521.975576 | 615.29 | 708.604424 | 1.213590 |
| GO:0015804\_neutral\_amino\_acid\_transport | 8 | 0 | 0.000000 | -0.000000 | 507 | 521.975576 | 615.29 | 708.604424 | 1.213590 |
| GO:0016236\_macroautophagy | 8 | 0 | 0.000000 | -0.000000 | 507 | 521.975576 | 615.29 | 708.604424 | 1.213590 |
| GO:0018345\_protein\_palmitoylation | 8 | 0 | 0.000000 | -0.000000 | 507 | 521.975576 | 615.29 | 708.604424 | 1.213590 |
| GO:0019229\_regulation\_of\_vasoconstriction | 8 | 0 | 0.000000 | -0.000000 | 507 | 521.975576 | 615.29 | 708.604424 | 1.213590 |
| GO:0019400\_alditol\_metabolic\_process | 8 | 0 | 0.000000 | -0.000000 | 507 | 521.975576 | 615.29 | 708.604424 | 1.213590 |
| GO:0021692\_cerebellar\_Purkinje\_cell\_layer\_morphogenesis | 8 | 0 | 0.000000 | -0.000000 | 507 | 521.975576 | 615.29 | 708.604424 | 1.213590 |
| GO:0021694\_cerebellar\_Purkinje\_cell\_layer\_formation | 8 | 0 | 0.000000 | -0.000000 | 507 | 521.975576 | 615.29 | 708.604424 | 1.213590 |
| GO:0021702\_cerebellar\_Purkinje\_cell\_differentiation | 8 | 0 | 0.000000 | -0.000000 | 507 | 521.975576 | 615.29 | 708.604424 | 1.213590 |
| GO:0021781\_glial\_cell\_fate\_commitment | 8 | 0 | 0.000000 | -0.000000 | 507 | 521.975576 | 615.29 | 708.604424 | 1.213590 |
| GO:0021799\_cerebral\_cortex\_radially\_oriented\_cell\_migration | 8 | 0 | 0.000000 | -0.000000 | 507 | 521.975576 | 615.29 | 708.604424 | 1.213590 |
| GO:0022898\_regulation\_of\_transmembrane\_transporter\_activity | 8 | 0 | 0.000000 | -0.000000 | 507 | 521.975576 | 615.29 | 708.604424 | 1.213590 |
| GO:0030035\_microspike\_assembly | 8 | 0 | 0.000000 | -0.000000 | 507 | 521.975576 | 615.29 | 708.604424 | 1.213590 |
| GO:0030193\_regulation\_of\_blood\_coagulation | 8 | 0 | 0.000000 | -0.000000 | 507 | 521.975576 | 615.29 | 708.604424 | 1.213590 |
| GO:0030204\_chondroitin\_sulfate\_metabolic\_process | 8 | 0 | 0.000000 | -0.000000 | 507 | 521.975576 | 615.29 | 708.604424 | 1.213590 |
| GO:0030500\_regulation\_of\_bone\_mineralization | 8 | 0 | 0.000000 | -0.000000 | 507 | 521.975576 | 615.29 | 708.604424 | 1.213590 |
| GO:0030511\_positive\_regulation\_of\_transforming\_growth\_factor\_beta\_receptor\_signaling\_pathway | 8 | 0 | 0.000000 | -0.000000 | 507 | 521.975576 | 615.29 | 708.604424 | 1.213590 |
| GO:0031102\_neuron\_projection\_regeneration | 8 | 0 | 0.000000 | -0.000000 | 507 | 521.975576 | 615.29 | 708.604424 | 1.213590 |
| GO:0031103\_axon\_regeneration | 8 | 0 | 0.000000 | -0.000000 | 507 | 521.975576 | 615.29 | 708.604424 | 1.213590 |
| GO:0031111\_negative\_regulation\_of\_microtubule\_polymerization\_or\_depolymerization | 8 | 0 | 0.000000 | -0.000000 | 507 | 521.975576 | 615.29 | 708.604424 | 1.213590 |
| GO:0031123\_RNA\_3'-end\_processing | 8 | 0 | 0.000000 | -0.000000 | 507 | 521.975576 | 615.29 | 708.604424 | 1.213590 |
| GO:0031294\_lymphocyte\_costimulation | 8 | 0 | 0.000000 | -0.000000 | 507 | 521.975576 | 615.29 | 708.604424 | 1.213590 |
| GO:0031295\_T\_cell\_costimulation | 8 | 0 | 0.000000 | -0.000000 | 507 | 521.975576 | 615.29 | 708.604424 | 1.213590 |
| GO:0031334\_positive\_regulation\_of\_protein\_complex\_assembly | 8 | 0 | 0.000000 | -0.000000 | 507 | 521.975576 | 615.29 | 708.604424 | 1.213590 |
| GO:0031342\_negative\_regulation\_of\_cell\_killing | 8 | 0 | 0.000000 | -0.000000 | 507 | 521.975576 | 615.29 | 708.604424 | 1.213590 |
| GO:0031396\_regulation\_of\_protein\_ubiquitination | 8 | 0 | 0.000000 | -0.000000 | 507 | 521.975576 | 615.29 | 708.604424 | 1.213590 |
| GO:0032094\_response\_to\_food | 8 | 0 | 0.000000 | -0.000000 | 507 | 521.975576 | 615.29 | 708.604424 | 1.213590 |
| GO:0032273\_positive\_regulation\_of\_protein\_polymerization | 8 | 0 | 0.000000 | -0.000000 | 507 | 521.975576 | 615.29 | 708.604424 | 1.213590 |
| GO:0032409\_regulation\_of\_transporter\_activity | 8 | 0 | 0.000000 | -0.000000 | 507 | 521.975576 | 615.29 | 708.604424 | 1.213590 |
| GO:0032412\_regulation\_of\_ion\_transmembrane\_transporter\_activity | 8 | 0 | 0.000000 | -0.000000 | 507 | 521.975576 | 615.29 | 708.604424 | 1.213590 |
| GO:0032613\_interleukin-10\_production | 8 | 0 | 0.000000 | -0.000000 | 507 | 521.975576 | 615.29 | 708.604424 | 1.213590 |
| GO:0033198\_response\_to\_ATP | 8 | 0 | 0.000000 | -0.000000 | 507 | 521.975576 | 615.29 | 708.604424 | 1.213590 |
| GO:0034284\_response\_to\_monosaccharide\_stimulus | 8 | 0 | 0.000000 | -0.000000 | 507 | 521.975576 | 615.29 | 708.604424 | 1.213590 |
| GO:0034728\_nucleosome\_organization | 8 | 0 | 0.000000 | -0.000000 | 507 | 521.975576 | 615.29 | 708.604424 | 1.213590 |
| GO:0035023\_regulation\_of\_Rho\_protein\_signal\_transduction | 8 | 0 | 0.000000 | -0.000000 | 507 | 521.975576 | 615.29 | 708.604424 | 1.213590 |
| GO:0035112\_genitalia\_morphogenesis | 8 | 0 | 0.000000 | -0.000000 | 507 | 521.975576 | 615.29 | 708.604424 | 1.213590 |
| GO:0040017\_positive\_regulation\_of\_locomotion | 8 | 0 | 0.000000 | -0.000000 | 507 | 521.975576 | 615.29 | 708.604424 | 1.213590 |
| GO:0040034\_regulation\_of\_development\_\_heterochronic | 8 | 0 | 0.000000 | -0.000000 | 507 | 521.975576 | 615.29 | 708.604424 | 1.213590 |
| GO:0042074\_cell\_migration\_involved\_in\_gastrulation | 8 | 0 | 0.000000 | -0.000000 | 507 | 521.975576 | 615.29 | 708.604424 | 1.213590 |
| GO:0042090\_interleukin-12\_biosynthetic\_process | 8 | 0 | 0.000000 | -0.000000 | 507 | 521.975576 | 615.29 | 708.604424 | 1.213590 |
| GO:0042092\_T-helper\_2\_type\_immune\_response | 8 | 0 | 0.000000 | -0.000000 | 507 | 521.975576 | 615.29 | 708.604424 | 1.213590 |
| GO:0042095\_interferon-gamma\_biosynthetic\_process | 8 | 0 | 0.000000 | -0.000000 | 507 | 521.975576 | 615.29 | 708.604424 | 1.213590 |
| GO:0042104\_positive\_regulation\_of\_activated\_T\_cell\_proliferation | 8 | 0 | 0.000000 | -0.000000 | 507 | 521.975576 | 615.29 | 708.604424 | 1.213590 |
| GO:0042226\_interleukin-6\_biosynthetic\_process | 8 | 0 | 0.000000 | -0.000000 | 507 | 521.975576 | 615.29 | 708.604424 | 1.213590 |
| GO:0042304\_regulation\_of\_fatty\_acid\_biosynthetic\_process | 8 | 0 | 0.000000 | -0.000000 | 507 | 521.975576 | 615.29 | 708.604424 | 1.213590 |
| GO:0042423\_catecholamine\_biosynthetic\_process | 8 | 0 | 0.000000 | -0.000000 | 507 | 521.975576 | 615.29 | 708.604424 | 1.213590 |
| GO:0042771\_DNA\_damage\_response\_\_signal\_transduction\_by\_p53\_class\_mediator\_resulting\_in\_induction\_of\_apoptosis | 8 | 0 | 0.000000 | -0.000000 | 507 | 521.975576 | 615.29 | 708.604424 | 1.213590 |
| GO:0042990\_regulation\_of\_transcription\_factor\_import\_into\_nucleus | 8 | 0 | 0.000000 | -0.000000 | 507 | 521.975576 | 615.29 | 708.604424 | 1.213590 |
| GO:0042991\_transcription\_factor\_import\_into\_nucleus | 8 | 0 | 0.000000 | -0.000000 | 507 | 521.975576 | 615.29 | 708.604424 | 1.213590 |
| GO:0043011\_myeloid\_dendritic\_cell\_differentiation | 8 | 0 | 0.000000 | -0.000000 | 507 | 521.975576 | 615.29 | 708.604424 | 1.213590 |
| GO:0043368\_positive\_T\_cell\_selection | 8 | 0 | 0.000000 | -0.000000 | 507 | 521.975576 | 615.29 | 708.604424 | 1.213590 |
| GO:0043370\_regulation\_of\_CD4-positive\_\_alpha\_beta\_T\_cell\_differentiation | 8 | 0 | 0.000000 | -0.000000 | 507 | 521.975576 | 615.29 | 708.604424 | 1.213590 |
| GO:0043542\_endothelial\_cell\_migration | 8 | 0 | 0.000000 | -0.000000 | 507 | 521.975576 | 615.29 | 708.604424 | 1.213590 |
| GO:0043616\_keratinocyte\_proliferation | 8 | 0 | 0.000000 | -0.000000 | 507 | 521.975576 | 615.29 | 708.604424 | 1.213590 |
| GO:0045075\_regulation\_of\_interleukin-12\_biosynthetic\_process | 8 | 0 | 0.000000 | -0.000000 | 507 | 521.975576 | 615.29 | 708.604424 | 1.213590 |
| GO:0045086\_positive\_regulation\_of\_interleukin-2\_biosynthetic\_process | 8 | 0 | 0.000000 | -0.000000 | 507 | 521.975576 | 615.29 | 708.604424 | 1.213590 |
| GO:0045351\_type\_I\_interferon\_biosynthetic\_process | 8 | 0 | 0.000000 | -0.000000 | 507 | 521.975576 | 615.29 | 708.604424 | 1.213590 |
| GO:0045408\_regulation\_of\_interleukin-6\_biosynthetic\_process | 8 | 0 | 0.000000 | -0.000000 | 507 | 521.975576 | 615.29 | 708.604424 | 1.213590 |
| GO:0045429\_positive\_regulation\_of\_nitric\_oxide\_biosynthetic\_process | 8 | 0 | 0.000000 | -0.000000 | 507 | 521.975576 | 615.29 | 708.604424 | 1.213590 |
| GO:0045494\_photoreceptor\_cell\_maintenance | 8 | 0 | 0.000000 | -0.000000 | 507 | 521.975576 | 615.29 | 708.604424 | 1.213590 |
| GO:0045686\_negative\_regulation\_of\_glial\_cell\_differentiation | 8 | 0 | 0.000000 | -0.000000 | 507 | 521.975576 | 615.29 | 708.604424 | 1.213590 |
| GO:0045910\_negative\_regulation\_of\_DNA\_recombination | 8 | 0 | 0.000000 | -0.000000 | 507 | 521.975576 | 615.29 | 708.604424 | 1.213590 |
| GO:0045921\_positive\_regulation\_of\_exocytosis | 8 | 0 | 0.000000 | -0.000000 | 507 | 521.975576 | 615.29 | 708.604424 | 1.213590 |
| GO:0045932\_negative\_regulation\_of\_muscle\_contraction | 8 | 0 | 0.000000 | -0.000000 | 507 | 521.975576 | 615.29 | 708.604424 | 1.213590 |
| GO:0046470\_phosphatidylcholine\_metabolic\_process | 8 | 0 | 0.000000 | -0.000000 | 507 | 521.975576 | 615.29 | 708.604424 | 1.213590 |
| GO:0048266\_behavioral\_response\_to\_pain | 8 | 0 | 0.000000 | -0.000000 | 507 | 521.975576 | 615.29 | 708.604424 | 1.213590 |
| GO:0048505\_regulation\_of\_timing\_of\_cell\_differentiation | 8 | 0 | 0.000000 | -0.000000 | 507 | 521.975576 | 615.29 | 708.604424 | 1.213590 |
| GO:0048520\_positive\_regulation\_of\_behavior | 8 | 0 | 0.000000 | -0.000000 | 507 | 521.975576 | 615.29 | 708.604424 | 1.213590 |
| GO:0048557\_embryonic\_digestive\_tract\_morphogenesis | 8 | 0 | 0.000000 | -0.000000 | 507 | 521.975576 | 615.29 | 708.604424 | 1.213590 |
| GO:0048638\_regulation\_of\_developmental\_growth | 8 | 0 | 0.000000 | -0.000000 | 507 | 521.975576 | 615.29 | 708.604424 | 1.213590 |
| GO:0048742\_regulation\_of\_skeletal\_muscle\_fiber\_development | 8 | 0 | 0.000000 | -0.000000 | 507 | 521.975576 | 615.29 | 708.604424 | 1.213590 |
| GO:0050707\_regulation\_of\_cytokine\_secretion | 8 | 0 | 0.000000 | -0.000000 | 507 | 521.975576 | 615.29 | 708.604424 | 1.213590 |
| GO:0050909\_sensory\_perception\_of\_taste | 8 | 0 | 0.000000 | -0.000000 | 507 | 521.975576 | 615.29 | 708.604424 | 1.213590 |
| GO:0050920\_regulation\_of\_chemotaxis | 8 | 0 | 0.000000 | -0.000000 | 507 | 521.975576 | 615.29 | 708.604424 | 1.213590 |
| GO:0050921\_positive\_regulation\_of\_chemotaxis | 8 | 0 | 0.000000 | -0.000000 | 507 | 521.975576 | 615.29 | 708.604424 | 1.213590 |
| GO:0050926\_regulation\_of\_positive\_chemotaxis | 8 | 0 | 0.000000 | -0.000000 | 507 | 521.975576 | 615.29 | 708.604424 | 1.213590 |
| GO:0050927\_positive\_regulation\_of\_positive\_chemotaxis | 8 | 0 | 0.000000 | -0.000000 | 507 | 521.975576 | 615.29 | 708.604424 | 1.213590 |
| GO:0050930\_induction\_of\_positive\_chemotaxis | 8 | 0 | 0.000000 | -0.000000 | 507 | 521.975576 | 615.29 | 708.604424 | 1.213590 |
| GO:0051084\_'de\_novo'\_posttranslational\_protein\_folding | 8 | 0 | 0.000000 | -0.000000 | 507 | 521.975576 | 615.29 | 708.604424 | 1.213590 |
| GO:0051181\_cofactor\_transport | 8 | 0 | 0.000000 | -0.000000 | 507 | 521.975576 | 615.29 | 708.604424 | 1.213590 |
| GO:0060043\_regulation\_of\_cardiac\_muscle\_cell\_proliferation | 8 | 0 | 0.000000 | -0.000000 | 507 | 521.975576 | 615.29 | 708.604424 | 1.213590 |
| GO:0060347\_heart\_trabecula\_formation | 8 | 0 | 0.000000 | -0.000000 | 507 | 521.975576 | 615.29 | 708.604424 | 1.213590 |
| GO:0060670\_branching\_involved\_in\_embryonic\_placenta\_morphogenesis | 8 | 0 | 0.000000 | -0.000000 | 507 | 521.975576 | 615.29 | 708.604424 | 1.213590 |
| GO:0060712\_spongiotrophoblast\_layer\_development | 8 | 0 | 0.000000 | -0.000000 | 507 | 521.975576 | 615.29 | 708.604424 | 1.213590 |
| GO:0070167\_regulation\_of\_biomineral\_formation | 8 | 0 | 0.000000 | -0.000000 | 507 | 521.975576 | 615.29 | 708.604424 | 1.213590 |
| GO:0070193\_synaptonemal\_complex\_organization | 8 | 0 | 0.000000 | -0.000000 | 507 | 521.975576 | 615.29 | 708.604424 | 1.213590 |
| GO:0070231\_T\_cell\_apoptosis | 8 | 0 | 0.000000 | -0.000000 | 507 | 521.975576 | 615.29 | 708.604424 | 1.213590 |
| GO:0070584\_mitochondrion\_morphogenesis | 8 | 0 | 0.000000 | -0.000000 | 507 | 521.975576 | 615.29 | 708.604424 | 1.213590 |
| GO:0048584\_positive\_regulation\_of\_response\_to\_stimulus | 115 | 0 | 0.000000 | -0.000000 | 508 | 523.383227 | 616.51 | 709.636773 | 1.213602 |
| GO:0002757\_immune\_response-activating\_signal\_transduction | 47 | 0 | 0.000000 | -0.000000 | 518 | 533.380309 | 626.07 | 718.759691 | 1.208629 |
| GO:0006140\_regulation\_of\_nucleotide\_metabolic\_process | 47 | 0 | 0.000000 | -0.000000 | 518 | 533.380309 | 626.07 | 718.759691 | 1.208629 |
| GO:0030183\_B\_cell\_differentiation | 47 | 0 | 0.000000 | -0.000000 | 518 | 533.380309 | 626.07 | 718.759691 | 1.208629 |
| GO:0030799\_regulation\_of\_cyclic\_nucleotide\_metabolic\_process | 47 | 0 | 0.000000 | -0.000000 | 518 | 533.380309 | 626.07 | 718.759691 | 1.208629 |
| GO:0031667\_response\_to\_nutrient\_levels | 47 | 0 | 0.000000 | -0.000000 | 518 | 533.380309 | 626.07 | 718.759691 | 1.208629 |
| GO:0034754\_cellular\_hormone\_metabolic\_process | 47 | 0 | 0.000000 | -0.000000 | 518 | 533.380309 | 626.07 | 718.759691 | 1.208629 |
| GO:0045087\_innate\_immune\_response | 47 | 0 | 0.000000 | -0.000000 | 518 | 533.380309 | 626.07 | 718.759691 | 1.208629 |
| GO:0045619\_regulation\_of\_lymphocyte\_differentiation | 47 | 0 | 0.000000 | -0.000000 | 518 | 533.380309 | 626.07 | 718.759691 | 1.208629 |
| GO:0048871\_multicellular\_organismal\_homeostasis | 47 | 0 | 0.000000 | -0.000000 | 518 | 533.380309 | 626.07 | 718.759691 | 1.208629 |
| GO:0060627\_regulation\_of\_vesicle-mediated\_transport | 47 | 0 | 0.000000 | -0.000000 | 518 | 533.380309 | 626.07 | 718.759691 | 1.208629 |
| GO:0003018\_vascular\_process\_in\_circulatory\_system | 31 | 0 | 0.000000 | -0.000000 | 549 | 562.185119 | 653.18 | 744.174881 | 1.189763 |
| GO:0006486\_protein\_amino\_acid\_glycosylation | 31 | 0 | 0.000000 | -0.000000 | 549 | 562.185119 | 653.18 | 744.174881 | 1.189763 |
| GO:0006639\_acylglycerol\_metabolic\_process | 31 | 0 | 0.000000 | -0.000000 | 549 | 562.185119 | 653.18 | 744.174881 | 1.189763 |
| GO:0006665\_sphingolipid\_metabolic\_process | 31 | 0 | 0.000000 | -0.000000 | 549 | 562.185119 | 653.18 | 744.174881 | 1.189763 |
| GO:0006694\_steroid\_biosynthetic\_process | 31 | 0 | 0.000000 | -0.000000 | 549 | 562.185119 | 653.18 | 744.174881 | 1.189763 |
| GO:0006939\_smooth\_muscle\_contraction | 31 | 0 | 0.000000 | -0.000000 | 549 | 562.185119 | 653.18 | 744.174881 | 1.189763 |
| GO:0008645\_hexose\_transport | 31 | 0 | 0.000000 | -0.000000 | 549 | 562.185119 | 653.18 | 744.174881 | 1.189763 |
| GO:0009306\_protein\_secretion | 31 | 0 | 0.000000 | -0.000000 | 549 | 562.185119 | 653.18 | 744.174881 | 1.189763 |
| GO:0010562\_positive\_regulation\_of\_phosphorus\_metabolic\_process | 31 | 0 | 0.000000 | -0.000000 | 549 | 562.185119 | 653.18 | 744.174881 | 1.189763 |
| GO:0015749\_monosaccharide\_transport | 31 | 0 | 0.000000 | -0.000000 | 549 | 562.185119 | 653.18 | 744.174881 | 1.189763 |
| GO:0015758\_glucose\_transport | 31 | 0 | 0.000000 | -0.000000 | 549 | 562.185119 | 653.18 | 744.174881 | 1.189763 |
| GO:0016049\_cell\_growth | 31 | 0 | 0.000000 | -0.000000 | 549 | 562.185119 | 653.18 | 744.174881 | 1.189763 |
| GO:0016311\_dephosphorylation | 31 | 0 | 0.000000 | -0.000000 | 549 | 562.185119 | 653.18 | 744.174881 | 1.189763 |
| GO:0021954\_central\_nervous\_system\_neuron\_development | 31 | 0 | 0.000000 | -0.000000 | 549 | 562.185119 | 653.18 | 744.174881 | 1.189763 |
| GO:0033555\_multicellular\_organismal\_response\_to\_stress | 31 | 0 | 0.000000 | -0.000000 | 549 | 562.185119 | 653.18 | 744.174881 | 1.189763 |
| GO:0035150\_regulation\_of\_tube\_size | 31 | 0 | 0.000000 | -0.000000 | 549 | 562.185119 | 653.18 | 744.174881 | 1.189763 |
| GO:0042157\_lipoprotein\_metabolic\_process | 31 | 0 | 0.000000 | -0.000000 | 549 | 562.185119 | 653.18 | 744.174881 | 1.189763 |
| GO:0042327\_positive\_regulation\_of\_phosphorylation | 31 | 0 | 0.000000 | -0.000000 | 549 | 562.185119 | 653.18 | 744.174881 | 1.189763 |
| GO:0043269\_regulation\_of\_ion\_transport | 31 | 0 | 0.000000 | -0.000000 | 549 | 562.185119 | 653.18 | 744.174881 | 1.189763 |
| GO:0043413\_biopolymer\_glycosylation | 31 | 0 | 0.000000 | -0.000000 | 549 | 562.185119 | 653.18 | 744.174881 | 1.189763 |
| GO:0045088\_regulation\_of\_innate\_immune\_response | 31 | 0 | 0.000000 | -0.000000 | 549 | 562.185119 | 653.18 | 744.174881 | 1.189763 |
| GO:0045937\_positive\_regulation\_of\_phosphate\_metabolic\_process | 31 | 0 | 0.000000 | -0.000000 | 549 | 562.185119 | 653.18 | 744.174881 | 1.189763 |
| GO:0046632\_alpha-beta\_T\_cell\_differentiation | 31 | 0 | 0.000000 | -0.000000 | 549 | 562.185119 | 653.18 | 744.174881 | 1.189763 |
| GO:0048167\_regulation\_of\_synaptic\_plasticity | 31 | 0 | 0.000000 | -0.000000 | 549 | 562.185119 | 653.18 | 744.174881 | 1.189763 |
| GO:0048562\_embryonic\_organ\_morphogenesis | 31 | 0 | 0.000000 | -0.000000 | 549 | 562.185119 | 653.18 | 744.174881 | 1.189763 |
| GO:0050868\_negative\_regulation\_of\_T\_cell\_activation | 31 | 0 | 0.000000 | -0.000000 | 549 | 562.185119 | 653.18 | 744.174881 | 1.189763 |
| GO:0050880\_regulation\_of\_blood\_vessel\_size | 31 | 0 | 0.000000 | -0.000000 | 549 | 562.185119 | 653.18 | 744.174881 | 1.189763 |
| GO:0051899\_membrane\_depolarization | 31 | 0 | 0.000000 | -0.000000 | 549 | 562.185119 | 653.18 | 744.174881 | 1.189763 |
| GO:0055088\_lipid\_homeostasis | 31 | 0 | 0.000000 | -0.000000 | 549 | 562.185119 | 653.18 | 744.174881 | 1.189763 |
| GO:0060512\_prostate\_gland\_morphogenesis | 31 | 0 | 0.000000 | -0.000000 | 549 | 562.185119 | 653.18 | 744.174881 | 1.189763 |
| GO:0070085\_glycosylation | 31 | 0 | 0.000000 | -0.000000 | 549 | 562.185119 | 653.18 | 744.174881 | 1.189763 |
| GO:0002429\_immune\_response-activating\_cell\_surface\_receptor\_signaling\_pathway | 41 | 0 | 0.000000 | -0.000000 | 567 | 578.976817 | 669.09 | 759.203183 | 1.180053 |
| GO:0006260\_DNA\_replication | 41 | 0 | 0.000000 | -0.000000 | 567 | 578.976817 | 669.09 | 759.203183 | 1.180053 |
| GO:0006836\_neurotransmitter\_transport | 41 | 0 | 0.000000 | -0.000000 | 567 | 578.976817 | 669.09 | 759.203183 | 1.180053 |
| GO:0006865\_amino\_acid\_transport | 41 | 0 | 0.000000 | -0.000000 | 567 | 578.976817 | 669.09 | 759.203183 | 1.180053 |
| GO:0007254\_JNK\_cascade | 41 | 0 | 0.000000 | -0.000000 | 567 | 578.976817 | 669.09 | 759.203183 | 1.180053 |
| GO:0008585\_female\_gonad\_development | 41 | 0 | 0.000000 | -0.000000 | 567 | 578.976817 | 669.09 | 759.203183 | 1.180053 |
| GO:0009894\_regulation\_of\_catabolic\_process | 41 | 0 | 0.000000 | -0.000000 | 567 | 578.976817 | 669.09 | 759.203183 | 1.180053 |
| GO:0010551\_regulation\_of\_specific\_transcription\_from\_RNA\_polymerase\_II\_promoter | 41 | 0 | 0.000000 | -0.000000 | 567 | 578.976817 | 669.09 | 759.203183 | 1.180053 |
| GO:0015833\_peptide\_transport | 41 | 0 | 0.000000 | -0.000000 | 567 | 578.976817 | 669.09 | 759.203183 | 1.180053 |
| GO:0015980\_energy\_derivation\_by\_oxidation\_of\_organic\_compounds | 41 | 0 | 0.000000 | -0.000000 | 567 | 578.976817 | 669.09 | 759.203183 | 1.180053 |
| GO:0019216\_regulation\_of\_lipid\_metabolic\_process | 41 | 0 | 0.000000 | -0.000000 | 567 | 578.976817 | 669.09 | 759.203183 | 1.180053 |
| GO:0019748\_secondary\_metabolic\_process | 41 | 0 | 0.000000 | -0.000000 | 567 | 578.976817 | 669.09 | 759.203183 | 1.180053 |
| GO:0030817\_regulation\_of\_cAMP\_biosynthetic\_process | 41 | 0 | 0.000000 | -0.000000 | 567 | 578.976817 | 669.09 | 759.203183 | 1.180053 |
| GO:0031344\_regulation\_of\_cell\_projection\_organization | 41 | 0 | 0.000000 | -0.000000 | 567 | 578.976817 | 669.09 | 759.203183 | 1.180053 |
| GO:0032569\_specific\_transcription\_from\_RNA\_polymerase\_II\_promoter | 41 | 0 | 0.000000 | -0.000000 | 567 | 578.976817 | 669.09 | 759.203183 | 1.180053 |
| GO:0032844\_regulation\_of\_homeostatic\_process | 41 | 0 | 0.000000 | -0.000000 | 567 | 578.976817 | 669.09 | 759.203183 | 1.180053 |
| GO:0033077\_T\_cell\_differentiation\_in\_the\_thymus | 41 | 0 | 0.000000 | -0.000000 | 567 | 578.976817 | 669.09 | 759.203183 | 1.180053 |
| GO:0050864\_regulation\_of\_B\_cell\_activation | 41 | 0 | 0.000000 | -0.000000 | 567 | 578.976817 | 669.09 | 759.203183 | 1.180053 |
| GO:0000280\_nuclear\_division | 24 | 0 | 0.000000 | -0.000000 | 594 | 605.923293 | 694.86 | 783.796707 | 1.169798 |
| GO:0001541\_ovarian\_follicle\_development | 24 | 0 | 0.000000 | -0.000000 | 594 | 605.923293 | 694.86 | 783.796707 | 1.169798 |
| GO:0002381\_immunoglobulin\_production\_during\_immune\_response | 24 | 0 | 0.000000 | -0.000000 | 594 | 605.923293 | 694.86 | 783.796707 | 1.169798 |
| GO:0006650\_glycerophospholipid\_metabolic\_process | 24 | 0 | 0.000000 | -0.000000 | 594 | 605.923293 | 694.86 | 783.796707 | 1.169798 |
| GO:0006941\_striated\_muscle\_contraction | 24 | 0 | 0.000000 | -0.000000 | 594 | 605.923293 | 694.86 | 783.796707 | 1.169798 |
| GO:0006959\_humoral\_immune\_response | 24 | 0 | 0.000000 | -0.000000 | 594 | 605.923293 | 694.86 | 783.796707 | 1.169798 |
| GO:0007067\_mitosis | 24 | 0 | 0.000000 | -0.000000 | 594 | 605.923293 | 694.86 | 783.796707 | 1.169798 |
| GO:0007204\_elevation\_of\_cytosolic\_calcium\_ion\_concentration | 24 | 0 | 0.000000 | -0.000000 | 594 | 605.923293 | 694.86 | 783.796707 | 1.169798 |
| GO:0007259\_JAK-STAT\_cascade | 24 | 0 | 0.000000 | -0.000000 | 594 | 605.923293 | 694.86 | 783.796707 | 1.169798 |
| GO:0007266\_Rho\_protein\_signal\_transduction | 24 | 0 | 0.000000 | -0.000000 | 594 | 605.923293 | 694.86 | 783.796707 | 1.169798 |
| GO:0007632\_visual\_behavior | 24 | 0 | 0.000000 | -0.000000 | 594 | 605.923293 | 694.86 | 783.796707 | 1.169798 |
| GO:0008629\_induction\_of\_apoptosis\_by\_intracellular\_signals | 24 | 0 | 0.000000 | -0.000000 | 594 | 605.923293 | 694.86 | 783.796707 | 1.169798 |
| GO:0009612\_response\_to\_mechanical\_stimulus | 24 | 0 | 0.000000 | -0.000000 | 594 | 605.923293 | 694.86 | 783.796707 | 1.169798 |
| GO:0014070\_response\_to\_organic\_cyclic\_substance | 24 | 0 | 0.000000 | -0.000000 | 594 | 605.923293 | 694.86 | 783.796707 | 1.169798 |
| GO:0021515\_cell\_differentiation\_in\_spinal\_cord | 24 | 0 | 0.000000 | -0.000000 | 594 | 605.923293 | 694.86 | 783.796707 | 1.169798 |
| GO:0042158\_lipoprotein\_biosynthetic\_process | 24 | 0 | 0.000000 | -0.000000 | 594 | 605.923293 | 694.86 | 783.796707 | 1.169798 |
| GO:0042632\_cholesterol\_homeostasis | 24 | 0 | 0.000000 | -0.000000 | 594 | 605.923293 | 694.86 | 783.796707 | 1.169798 |
| GO:0043410\_positive\_regulation\_of\_MAPKKK\_cascade | 24 | 0 | 0.000000 | -0.000000 | 594 | 605.923293 | 694.86 | 783.796707 | 1.169798 |
| GO:0043588\_skin\_development | 24 | 0 | 0.000000 | -0.000000 | 594 | 605.923293 | 694.86 | 783.796707 | 1.169798 |
| GO:0048002\_antigen\_processing\_and\_presentation\_of\_peptide\_antigen | 24 | 0 | 0.000000 | -0.000000 | 594 | 605.923293 | 694.86 | 783.796707 | 1.169798 |
| GO:0048546\_digestive\_tract\_morphogenesis | 24 | 0 | 0.000000 | -0.000000 | 594 | 605.923293 | 694.86 | 783.796707 | 1.169798 |
| GO:0050679\_positive\_regulation\_of\_epithelial\_cell\_proliferation | 24 | 0 | 0.000000 | -0.000000 | 594 | 605.923293 | 694.86 | 783.796707 | 1.169798 |
| GO:0051099\_positive\_regulation\_of\_binding | 24 | 0 | 0.000000 | -0.000000 | 594 | 605.923293 | 694.86 | 783.796707 | 1.169798 |
| GO:0055092\_sterol\_homeostasis | 24 | 0 | 0.000000 | -0.000000 | 594 | 605.923293 | 694.86 | 783.796707 | 1.169798 |
| GO:0060078\_regulation\_of\_postsynaptic\_membrane\_potential | 24 | 0 | 0.000000 | -0.000000 | 594 | 605.923293 | 694.86 | 783.796707 | 1.169798 |
| GO:0060113\_inner\_ear\_receptor\_cell\_differentiation | 24 | 0 | 0.000000 | -0.000000 | 594 | 605.923293 | 694.86 | 783.796707 | 1.169798 |
| GO:0070667\_negative\_regulation\_of\_mast\_cell\_proliferation | 24 | 0 | 0.000000 | -0.000000 | 594 | 605.923293 | 694.86 | 783.796707 | 1.169798 |
| GO:0001708\_cell\_fate\_specification | 56 | 0 | 0.000000 | -0.000000 | 604 | 614.251063 | 702.46 | 790.668937 | 1.163013 |
| GO:0002683\_negative\_regulation\_of\_immune\_system\_process | 56 | 0 | 0.000000 | -0.000000 | 604 | 614.251063 | 702.46 | 790.668937 | 1.163013 |
| GO:0002703\_regulation\_of\_leukocyte\_mediated\_immunity | 56 | 0 | 0.000000 | -0.000000 | 604 | 614.251063 | 702.46 | 790.668937 | 1.163013 |
| GO:0006790\_sulfur\_metabolic\_process | 56 | 0 | 0.000000 | -0.000000 | 604 | 614.251063 | 702.46 | 790.668937 | 1.163013 |
| GO:0009187\_cyclic\_nucleotide\_metabolic\_process | 56 | 0 | 0.000000 | -0.000000 | 604 | 614.251063 | 702.46 | 790.668937 | 1.163013 |
| GO:0042089\_cytokine\_biosynthetic\_process | 56 | 0 | 0.000000 | -0.000000 | 604 | 614.251063 | 702.46 | 790.668937 | 1.163013 |
| GO:0042107\_cytokine\_metabolic\_process | 56 | 0 | 0.000000 | -0.000000 | 604 | 614.251063 | 702.46 | 790.668937 | 1.163013 |
| GO:0046486\_glycerolipid\_metabolic\_process | 56 | 0 | 0.000000 | -0.000000 | 604 | 614.251063 | 702.46 | 790.668937 | 1.163013 |
| GO:0050678\_regulation\_of\_epithelial\_cell\_proliferation | 56 | 0 | 0.000000 | -0.000000 | 604 | 614.251063 | 702.46 | 790.668937 | 1.163013 |
| GO:0051321\_meiotic\_cell\_cycle | 56 | 0 | 0.000000 | -0.000000 | 604 | 614.251063 | 702.46 | 790.668937 | 1.163013 |
| GO:0000027\_ribosomal\_large\_subunit\_assembly | 1 | 0 |  |  |  |  |  |  |  |  |
| GO:0000042\_protein\_targeting\_to\_Golgi | 1 | 0 |  |  |  |  |  |  |  |  |
| GO:0000046\_autophagic\_vacuole\_fusion | 1 | 0 |  |  |  |  |  |  |  |  |
| GO:0000050\_urea\_cycle | 1 | 0 |  |  |  |  |  |  |  |  |
| GO:0000054\_ribosome\_export\_from\_nucleus | 1 | 0 |  |  |  |  |  |  |  |  |
| GO:0000055\_ribosomal\_large\_subunit\_export\_from\_nucleus | 1 | 0 |  |  |  |  |  |  |  |  |
| GO:0000056\_ribosomal\_small\_subunit\_export\_from\_nucleus | 1 | 0 |  |  |  |  |  |  |  |  |
| GO:0000072\_M\_phase\_specific\_microtubule\_process | 1 | 0 |  |  |  |  |  |  |  |  |
| GO:0000101\_sulfur\_amino\_acid\_transport | 1 | 0 |  |  |  |  |  |  |  |  |
| GO:0000147\_actin\_cortical\_patch\_assembly | 1 | 0 |  |  |  |  |  |  |  |  |
| GO:0000154\_rRNA\_modification | 1 | 0 |  |  |  |  |  |  |  |  |
| GO:0000183\_chromatin\_silencing\_at\_rDNA | 1 | 0 |  |  |  |  |  |  |  |  |
| GO:0000185\_activation\_of\_MAPKKK\_activity | 1 | 0 |  |  |  |  |  |  |  |  |
| GO:0000238\_zygotene | 1 | 0 |  |  |  |  |  |  |  |  |
| GO:0000255\_allantoin\_metabolic\_process | 1 | 0 |  |  |  |  |  |  |  |  |
| GO:0000266\_mitochondrial\_fission | 1 | 0 |  |  |  |  |  |  |  |  |
| GO:0000273\_lipoic\_acid\_metabolic\_process | 1 | 0 |  |  |  |  |  |  |  |  |
| GO:0000301\_retrograde\_transport\_\_vesicle\_recycling\_within\_Golgi | 1 | 0 |  |  |  |  |  |  |  |  |
| GO:0000394\_RNA\_splicing\_\_via\_endonucleolytic\_cleavage\_and\_ligation | 1 | 0 |  |  |  |  |  |  |  |  |
| GO:0000429\_regulation\_of\_transcription\_from\_RNA\_polymerase\_II\_promoter\_by\_carbon\_catabolites | 1 | 0 |  |  |  |  |  |  |  |  |
| GO:0000430\_regulation\_of\_transcription\_from\_RNA\_polymerase\_II\_promoter\_by\_glucose | 1 | 0 |  |  |  |  |  |  |  |  |
| GO:0000432\_positive\_regulation\_of\_transcription\_from\_RNA\_polymerase\_II\_promoter\_by\_glucose | 1 | 0 |  |  |  |  |  |  |  |  |
| GO:0000436\_positive\_regulation\_of\_transcription\_from\_RNA\_polymerase\_II\_promoter\_by\_carbon\_catabolites | 1 | 0 |  |  |  |  |  |  |  |  |
| GO:0000448\_cleavage\_in\_ITS2\_between\_5.8S\_rRNA\_and\_LSU-rRNA\_of\_tricistronic\_rRNA\_transcript\_(SSU-rRNA\_\_5.8S\_rRNA\_\_LSU-rRNA) | 1 | 0 |  |  |  |  |  |  |  |  |
| GO:0000460\_maturation\_of\_5.8S\_rRNA | 1 | 0 |  |  |  |  |  |  |  |  |
| GO:0000463\_maturation\_of\_LSU-rRNA\_from\_tricistronic\_rRNA\_transcript\_(SSU-rRNA\_\_5.8S\_rRNA\_\_LSU-rRNA) | 1 | 0 |  |  |  |  |  |  |  |  |
| GO:0000466\_maturation\_of\_5.8S\_rRNA\_from\_tricistronic\_rRNA\_transcript\_(SSU-rRNA\_\_5.8S\_rRNA\_\_LSU-rRNA) | 1 | 0 |  |  |  |  |  |  |  |  |
| GO:0000469\_cleavages\_during\_rRNA\_processing | 1 | 0 |  |  |  |  |  |  |  |  |
| GO:0000470\_maturation\_of\_LSU-rRNA | 1 | 0 |  |  |  |  |  |  |  |  |
| GO:0000478\_endonucleolytic\_cleavages\_during\_rRNA\_processing | 1 | 0 |  |  |  |  |  |  |  |  |
| GO:0000479\_endonucleolytic\_cleavage\_of\_tricistronic\_rRNA\_transcript\_(SSU-rRNA\_\_5.8S\_rRNA\_\_LSU-rRNA) | 1 | 0 |  |  |  |  |  |  |  |  |
| GO:0000705\_achiasmate\_meiosis\_I | 1 | 0 |  |  |  |  |  |  |  |  |
| GO:0000966\_RNA\_5'-end\_processing | 1 | 0 |  |  |  |  |  |  |  |  |
| GO:0001300\_chronological\_cell\_aging | 1 | 0 |  |  |  |  |  |  |  |  |
| GO:0001547\_antral\_ovarian\_follicle\_growth | 1 | 0 |  |  |  |  |  |  |  |  |
| GO:0001555\_oocyte\_growth | 1 | 0 |  |  |  |  |  |  |  |  |
| GO:0001560\_regulation\_of\_cell\_growth\_by\_extracellular\_stimulus | 1 | 0 |  |  |  |  |  |  |  |  |
| GO:0001660\_fever | 1 | 0 |  |  |  |  |  |  |  |  |
| GO:0001696\_gastric\_acid\_secretion | 1 | 0 |  |  |  |  |  |  |  |  |
| GO:0001712\_ectodermal\_cell\_fate\_commitment | 1 | 0 |  |  |  |  |  |  |  |  |
| GO:0001714\_endodermal\_cell\_fate\_specification | 1 | 0 |  |  |  |  |  |  |  |  |
| GO:0001762\_beta-alanine\_transport | 1 | 0 |  |  |  |  |  |  |  |  |
| GO:0001766\_membrane\_raft\_polarization | 1 | 0 |  |  |  |  |  |  |  |  |
| GO:0001811\_negative\_regulation\_of\_type\_I\_hypersensitivity | 1 | 0 |  |  |  |  |  |  |  |  |
| GO:0001821\_histamine\_secretion | 1 | 0 |  |  |  |  |  |  |  |  |
| GO:0001826\_inner\_cell\_mass\_cell\_differentiation | 1 | 0 |  |  |  |  |  |  |  |  |
| GO:0001830\_trophectodermal\_cell\_fate\_commitment | 1 | 0 |  |  |  |  |  |  |  |  |
| GO:0001834\_trophectodermal\_cell\_proliferation | 1 | 0 |  |  |  |  |  |  |  |  |
| GO:0001867\_complement\_activation\_\_lectin\_pathway | 1 | 0 |  |  |  |  |  |  |  |  |
| GO:0001880\_Mullerian\_duct\_regression | 1 | 0 |  |  |  |  |  |  |  |  |
| GO:0001887\_selenium\_metabolic\_process | 1 | 0 |  |  |  |  |  |  |  |  |
| GO:0001922\_B-1\_B\_cell\_homeostasis | 1 | 0 |  |  |  |  |  |  |  |  |
| GO:0001923\_B-1\_B\_cell\_differentiation | 1 | 0 |  |  |  |  |  |  |  |  |
| GO:0001941\_postsynaptic\_membrane\_organization | 1 | 0 |  |  |  |  |  |  |  |  |
| GO:0001946\_lymphangiogenesis | 1 | 0 |  |  |  |  |  |  |  |  |
| GO:0001956\_positive\_regulation\_of\_neurotransmitter\_secretion | 1 | 0 |  |  |  |  |  |  |  |  |
| GO:0001961\_positive\_regulation\_of\_cytokine-mediated\_signaling\_pathway | 1 | 0 |  |  |  |  |  |  |  |  |
| GO:0001979\_regulation\_of\_systemic\_arterial\_blood\_pressure\_by\_chemoreceptor\_signaling | 1 | 0 |  |  |  |  |  |  |  |  |
| GO:0001980\_regulation\_of\_systemic\_arterial\_blood\_pressure\_by\_ischemic\_conditions | 1 | 0 |  |  |  |  |  |  |  |  |
| GO:0001984\_vasodilation\_of\_artery\_during\_baroreceptor\_response\_to\_increased\_systemic\_arterial\_blood\_pressure | 1 | 0 |  |  |  |  |  |  |  |  |
| GO:0001985\_negative\_regulation\_of\_heart\_rate\_in\_baroreceptor\_response\_to\_increased\_systemic\_arterial\_blood\_pressure | 1 | 0 |  |  |  |  |  |  |  |  |
| GO:0001987\_vasoconstriction\_of\_artery\_involved\_in\_baroreceptor\_response\_to\_lowering\_of\_systemic\_arterial\_blood\_pressure | 1 | 0 |  |  |  |  |  |  |  |  |
| GO:0001988\_positive\_regulation\_of\_heart\_rate\_in\_baroreceptor\_response\_to\_decreased\_systemic\_arterial\_blood\_pressure | 1 | 0 |  |  |  |  |  |  |  |  |
| GO:0001994\_norepinephrine-epinephrine\_vasoconstriction\_involved\_in\_regulation\_of\_systemic\_arterial\_blood\_pressure | 1 | 0 |  |  |  |  |  |  |  |  |
| GO:0002001\_renin\_secretion\_into\_blood\_stream | 1 | 0 |  |  |  |  |  |  |  |  |
| GO:0002002\_regulation\_of\_angiotensin\_levels\_in\_blood | 1 | 0 |  |  |  |  |  |  |  |  |
| GO:0002003\_angiotensin\_maturation | 1 | 0 |  |  |  |  |  |  |  |  |
| GO:0002007\_detection\_of\_hypoxic\_conditions\_in\_blood\_by\_chemoreceptor\_signaling | 1 | 0 |  |  |  |  |  |  |  |  |
| GO:0002017\_regulation\_of\_blood\_volume\_by\_renal\_aldosterone | 1 | 0 |  |  |  |  |  |  |  |  |
| GO:0002023\_reduction\_of\_food\_intake\_in\_response\_to\_dietary\_excess | 1 | 0 |  |  |  |  |  |  |  |  |
| GO:0002031\_G-protein\_coupled\_receptor\_internalization | 1 | 0 |  |  |  |  |  |  |  |  |
| GO:0002036\_regulation\_of\_L-glutamate\_transport | 1 | 0 |  |  |  |  |  |  |  |  |
| GO:0002040\_sprouting\_angiogenesis | 1 | 0 |  |  |  |  |  |  |  |  |
| GO:0002041\_intussusceptive\_angiogenesis | 1 | 0 |  |  |  |  |  |  |  |  |
| GO:0002068\_glandular\_epithelial\_cell\_development | 1 | 0 |  |  |  |  |  |  |  |  |
| GO:0002069\_columnar\_cuboidal\_epithelial\_cell\_maturation | 1 | 0 |  |  |  |  |  |  |  |  |
| GO:0002071\_glandular\_epithelial\_cell\_maturation | 1 | 0 |  |  |  |  |  |  |  |  |
| GO:0002082\_regulation\_of\_oxidative\_phosphorylation | 1 | 0 |  |  |  |  |  |  |  |  |
| GO:0002084\_protein\_depalmitoylation | 1 | 0 |  |  |  |  |  |  |  |  |
| GO:0002085\_inhibition\_of\_neuroepithelial\_cell\_differentiation | 1 | 0 |  |  |  |  |  |  |  |  |
| GO:0002086\_diaphragm\_contraction | 1 | 0 |  |  |  |  |  |  |  |  |
| GO:0002118\_aggressive\_behavior | 1 | 0 |  |  |  |  |  |  |  |  |
| GO:0002121\_inter-male\_aggressive\_behavior | 1 | 0 |  |  |  |  |  |  |  |  |
| GO:0002124\_territorial\_aggressive\_behavior | 1 | 0 |  |  |  |  |  |  |  |  |
| GO:0002227\_innate\_immune\_response\_in\_mucosa | 1 | 0 |  |  |  |  |  |  |  |  |
| GO:0002232\_leukocyte\_chemotaxis\_during\_inflammatory\_response | 1 | 0 |  |  |  |  |  |  |  |  |
| GO:0002248\_connective\_tissue\_replacement\_during\_inflammatory\_response | 1 | 0 |  |  |  |  |  |  |  |  |
| GO:0002282\_microglial\_cell\_activation\_during\_immune\_response | 1 | 0 |  |  |  |  |  |  |  |  |
| GO:0002287\_alpha-beta\_T\_cell\_activation\_during\_immune\_response | 1 | 0 |  |  |  |  |  |  |  |  |
| GO:0002314\_germinal\_center\_B\_cell\_differentiation | 1 | 0 |  |  |  |  |  |  |  |  |
| GO:0002315\_marginal\_zone\_B\_cell\_differentiation | 1 | 0 |  |  |  |  |  |  |  |  |
| GO:0002316\_follicular\_B\_cell\_differentiation | 1 | 0 |  |  |  |  |  |  |  |  |
| GO:0002317\_plasma\_cell\_differentiation | 1 | 0 |  |  |  |  |  |  |  |  |
| GO:0002349\_histamine\_production\_during\_acute\_inflammatory\_response | 1 | 0 |  |  |  |  |  |  |  |  |
| GO:0002351\_serotonin\_production\_during\_acute\_inflammatory\_response | 1 | 0 |  |  |  |  |  |  |  |  |
| GO:0002355\_detection\_of\_tumor\_cell | 1 | 0 |  |  |  |  |  |  |  |  |
| GO:0002370\_natural\_killer\_cell\_cytokine\_production | 1 | 0 |  |  |  |  |  |  |  |  |
| GO:0002371\_dendritic\_cell\_cytokine\_production | 1 | 0 |  |  |  |  |  |  |  |  |
| GO:0002380\_immunoglobulin\_secretion\_during\_immune\_response | 1 | 0 |  |  |  |  |  |  |  |  |
| GO:0002396\_MHC\_protein\_complex\_assembly | 1 | 0 |  |  |  |  |  |  |  |  |
| GO:0002397\_MHC\_class\_I\_protein\_complex\_assembly | 1 | 0 |  |  |  |  |  |  |  |  |
| GO:0002420\_natural\_killer\_cell\_mediated\_cytotoxicity\_directed\_against\_tumor\_cell\_target | 1 | 0 |  |  |  |  |  |  |  |  |
| GO:0002423\_natural\_killer\_cell\_mediated\_immune\_response\_to\_tumor\_cell | 1 | 0 |  |  |  |  |  |  |  |  |
| GO:0002424\_T\_cell\_mediated\_immune\_response\_to\_tumor\_cell | 1 | 0 |  |  |  |  |  |  |  |  |
| GO:0002426\_immunoglobulin\_production\_in\_mucosal\_tissue | 1 | 0 |  |  |  |  |  |  |  |  |
| GO:0002431\_Fc\_receptor\_mediated\_stimulatory\_signaling\_pathway | 1 | 0 |  |  |  |  |  |  |  |  |
| GO:0002432\_granuloma\_formation | 1 | 0 |  |  |  |  |  |  |  |  |
| GO:0002441\_histamine\_secretion\_during\_acute\_inflammatory\_response | 1 | 0 |  |  |  |  |  |  |  |  |
| GO:0002442\_serotonin\_secretion\_during\_acute\_inflammatory\_response | 1 | 0 |  |  |  |  |  |  |  |  |
| GO:0002457\_T\_cell\_antigen\_processing\_and\_presentation | 1 | 0 |  |  |  |  |  |  |  |  |
| GO:0002458\_peripheral\_T\_cell\_tolerance\_induction | 1 | 0 |  |  |  |  |  |  |  |  |
| GO:0002461\_tolerance\_induction\_dependent\_upon\_immune\_response | 1 | 0 |  |  |  |  |  |  |  |  |
| GO:0002465\_peripheral\_tolerance\_induction | 1 | 0 |  |  |  |  |  |  |  |  |
| GO:0002468\_dendritic\_cell\_antigen\_processing\_and\_presentation | 1 | 0 |  |  |  |  |  |  |  |  |
| GO:0002476\_antigen\_processing\_and\_presentation\_of\_endogenous\_peptide\_antigen\_via\_MHC\_class\_Ib | 1 | 0 |  |  |  |  |  |  |  |  |
| GO:0002479\_antigen\_processing\_and\_presentation\_of\_exogenous\_peptide\_antigen\_via\_MHC\_class\_I\_\_TAP-dependent | 1 | 0 |  |  |  |  |  |  |  |  |
| GO:0002483\_antigen\_processing\_and\_presentation\_of\_endogenous\_peptide\_antigen | 1 | 0 |  |  |  |  |  |  |  |  |
| GO:0002501\_peptide\_antigen\_assembly\_with\_MHC\_protein\_complex | 1 | 0 |  |  |  |  |  |  |  |  |
| GO:0002502\_peptide\_antigen\_assembly\_with\_MHC\_class\_I\_protein\_complex | 1 | 0 |  |  |  |  |  |  |  |  |
| GO:0002508\_central\_tolerance\_induction | 1 | 0 |  |  |  |  |  |  |  |  |
| GO:0002510\_central\_B\_cell\_tolerance\_induction | 1 | 0 |  |  |  |  |  |  |  |  |
| GO:0002545\_chronic\_inflammatory\_response\_to\_non-antigenic\_stimulus | 1 | 0 |  |  |  |  |  |  |  |  |
| GO:0002553\_histamine\_secretion\_by\_mast\_cell | 1 | 0 |  |  |  |  |  |  |  |  |
| GO:0002554\_serotonin\_secretion\_by\_platelet | 1 | 0 |  |  |  |  |  |  |  |  |
| GO:0002572\_pro-T\_cell\_differentiation | 1 | 0 |  |  |  |  |  |  |  |  |
| GO:0002577\_regulation\_of\_antigen\_processing\_and\_presentation | 1 | 0 |  |  |  |  |  |  |  |  |
| GO:0002579\_positive\_regulation\_of\_antigen\_processing\_and\_presentation | 1 | 0 |  |  |  |  |  |  |  |  |
| GO:0002604\_regulation\_of\_dendritic\_cell\_antigen\_processing\_and\_presentation | 1 | 0 |  |  |  |  |  |  |  |  |
| GO:0002606\_positive\_regulation\_of\_dendritic\_cell\_antigen\_processing\_and\_presentation | 1 | 0 |  |  |  |  |  |  |  |  |
| GO:0002635\_negative\_regulation\_of\_germinal\_center\_formation | 1 | 0 |  |  |  |  |  |  |  |  |
| GO:0002646\_regulation\_of\_central\_tolerance\_induction | 1 | 0 |  |  |  |  |  |  |  |  |
| GO:0002648\_positive\_regulation\_of\_central\_tolerance\_induction | 1 | 0 |  |  |  |  |  |  |  |  |
| GO:0002649\_regulation\_of\_tolerance\_induction\_to\_self\_antigen | 1 | 0 |  |  |  |  |  |  |  |  |
| GO:0002651\_positive\_regulation\_of\_tolerance\_induction\_to\_self\_antigen | 1 | 0 |  |  |  |  |  |  |  |  |
| GO:0002652\_regulation\_of\_tolerance\_induction\_dependent\_upon\_immune\_response | 1 | 0 |  |  |  |  |  |  |  |  |
| GO:0002654\_positive\_regulation\_of\_tolerance\_induction\_dependent\_upon\_immune\_response | 1 | 0 |  |  |  |  |  |  |  |  |
| GO:0002658\_regulation\_of\_peripheral\_tolerance\_induction | 1 | 0 |  |  |  |  |  |  |  |  |
| GO:0002660\_positive\_regulation\_of\_peripheral\_tolerance\_induction | 1 | 0 |  |  |  |  |  |  |  |  |
| GO:0002677\_negative\_regulation\_of\_chronic\_inflammatory\_response | 1 | 0 |  |  |  |  |  |  |  |  |
| GO:0002678\_positive\_regulation\_of\_chronic\_inflammatory\_response | 1 | 0 |  |  |  |  |  |  |  |  |
| GO:0002701\_negative\_regulation\_of\_production\_of\_molecular\_mediator\_of\_immune\_response | 1 | 0 |  |  |  |  |  |  |  |  |
| GO:0002719\_negative\_regulation\_of\_cytokine\_production\_during\_immune\_response | 1 | 0 |  |  |  |  |  |  |  |  |
| GO:0002724\_regulation\_of\_T\_cell\_cytokine\_production | 1 | 0 |  |  |  |  |  |  |  |  |
| GO:0002727\_regulation\_of\_natural\_killer\_cell\_cytokine\_production | 1 | 0 |  |  |  |  |  |  |  |  |
| GO:0002729\_positive\_regulation\_of\_natural\_killer\_cell\_cytokine\_production | 1 | 0 |  |  |  |  |  |  |  |  |
| GO:0002730\_regulation\_of\_dendritic\_cell\_cytokine\_production | 1 | 0 |  |  |  |  |  |  |  |  |
| GO:0002756\_MyD88-independent\_toll-like\_receptor\_signaling\_pathway | 1 | 0 |  |  |  |  |  |  |  |  |
| GO:0002767\_immune\_response-inhibiting\_cell\_surface\_receptor\_signaling\_pathway | 1 | 0 |  |  |  |  |  |  |  |  |
| GO:0002769\_natural\_killer\_cell\_inhibitory\_signaling\_pathway | 1 | 0 |  |  |  |  |  |  |  |  |
| GO:0002840\_regulation\_of\_T\_cell\_mediated\_immune\_response\_to\_tumor\_cell | 1 | 0 |  |  |  |  |  |  |  |  |
| GO:0002842\_positive\_regulation\_of\_T\_cell\_mediated\_immune\_response\_to\_tumor\_cell | 1 | 0 |  |  |  |  |  |  |  |  |
| GO:0002849\_regulation\_of\_peripheral\_T\_cell\_tolerance\_induction | 1 | 0 |  |  |  |  |  |  |  |  |
| GO:0002851\_positive\_regulation\_of\_peripheral\_T\_cell\_tolerance\_induction | 1 | 0 |  |  |  |  |  |  |  |  |
| GO:0002855\_regulation\_of\_natural\_killer\_cell\_mediated\_immune\_response\_to\_tumor\_cell | 1 | 0 |  |  |  |  |  |  |  |  |
| GO:0002857\_positive\_regulation\_of\_natural\_killer\_cell\_mediated\_immune\_response\_to\_tumor\_cell | 1 | 0 |  |  |  |  |  |  |  |  |
| GO:0002858\_regulation\_of\_natural\_killer\_cell\_mediated\_cytotoxicity\_directed\_against\_tumor\_cell\_target | 1 | 0 |  |  |  |  |  |  |  |  |
| GO:0002860\_positive\_regulation\_of\_natural\_killer\_cell\_mediated\_cytotoxicity\_directed\_against\_tumor\_cell\_target | 1 | 0 |  |  |  |  |  |  |  |  |
| GO:0002880\_regulation\_of\_chronic\_inflammatory\_response\_to\_non-antigenic\_stimulus | 1 | 0 |  |  |  |  |  |  |  |  |
| GO:0002882\_positive\_regulation\_of\_chronic\_inflammatory\_response\_to\_non-antigenic\_stimulus | 1 | 0 |  |  |  |  |  |  |  |  |
| GO:0002895\_regulation\_of\_central\_B\_cell\_tolerance\_induction | 1 | 0 |  |  |  |  |  |  |  |  |
| GO:0002897\_positive\_regulation\_of\_central\_B\_cell\_tolerance\_induction | 1 | 0 |  |  |  |  |  |  |  |  |
| GO:0002901\_mature\_B\_cell\_apoptosis | 1 | 0 |  |  |  |  |  |  |  |  |
| GO:0002903\_negative\_regulation\_of\_B\_cell\_apoptosis | 1 | 0 |  |  |  |  |  |  |  |  |
| GO:0002905\_regulation\_of\_mature\_B\_cell\_apoptosis | 1 | 0 |  |  |  |  |  |  |  |  |
| GO:0002906\_negative\_regulation\_of\_mature\_B\_cell\_apoptosis | 1 | 0 |  |  |  |  |  |  |  |  |
| GO:0003011\_involuntary\_skeletal\_muscle\_contraction | 1 | 0 |  |  |  |  |  |  |  |  |
| GO:0003027\_regulation\_of\_systemic\_arterial\_blood\_pressure\_by\_carotid\_body\_chemoreceptor\_signaling | 1 | 0 |  |  |  |  |  |  |  |  |
| GO:0003029\_detection\_of\_hypoxic\_conditions\_in\_blood\_by\_carotid\_body\_chemoreceptor\_signaling | 1 | 0 |  |  |  |  |  |  |  |  |
| GO:0003032\_detection\_of\_oxygen | 1 | 0 |  |  |  |  |  |  |  |  |
| GO:0003056\_regulation\_of\_vascular\_smooth\_muscle\_contraction | 1 | 0 |  |  |  |  |  |  |  |  |
| GO:0003062\_regulation\_of\_heart\_rate\_by\_chemical\_signal | 1 | 0 |  |  |  |  |  |  |  |  |
| GO:0003065\_positive\_regulation\_of\_heart\_rate\_by\_epinephrine | 1 | 0 |  |  |  |  |  |  |  |  |
| GO:0003068\_regulation\_of\_systemic\_arterial\_blood\_pressure\_by\_acetylcholine | 1 | 0 |  |  |  |  |  |  |  |  |
| GO:0003069\_vasodilation\_by\_acetylcholine\_involved\_in\_regulation\_of\_systemic\_arterial\_blood\_pressure | 1 | 0 |  |  |  |  |  |  |  |  |
| GO:0003070\_regulation\_of\_systemic\_arterial\_blood\_pressure\_by\_neurotransmitter | 1 | 0 |  |  |  |  |  |  |  |  |
| GO:0003097\_renal\_water\_transport | 1 | 0 |  |  |  |  |  |  |  |  |
| GO:0005979\_regulation\_of\_glycogen\_biosynthetic\_process | 1 | 0 |  |  |  |  |  |  |  |  |
| GO:0005984\_disaccharide\_metabolic\_process | 1 | 0 |  |  |  |  |  |  |  |  |
| GO:0005988\_lactose\_metabolic\_process | 1 | 0 |  |  |  |  |  |  |  |  |
| GO:0005989\_lactose\_biosynthetic\_process | 1 | 0 |  |  |  |  |  |  |  |  |
| GO:0005997\_xylulose\_metabolic\_process | 1 | 0 |  |  |  |  |  |  |  |  |
| GO:0006000\_fructose\_metabolic\_process | 1 | 0 |  |  |  |  |  |  |  |  |
| GO:0006002\_fructose\_6-phosphate\_metabolic\_process | 1 | 0 |  |  |  |  |  |  |  |  |
| GO:0006004\_fucose\_metabolic\_process | 1 | 0 |  |  |  |  |  |  |  |  |
| GO:0006013\_mannose\_metabolic\_process | 1 | 0 |  |  |  |  |  |  |  |  |
| GO:0006060\_sorbitol\_metabolic\_process | 1 | 0 |  |  |  |  |  |  |  |  |
| GO:0006064\_glucuronate\_catabolic\_process | 1 | 0 |  |  |  |  |  |  |  |  |
| GO:0006086\_acetyl-CoA\_biosynthetic\_process\_from\_pyruvate | 1 | 0 |  |  |  |  |  |  |  |  |
| GO:0006098\_pentose-phosphate\_shunt | 1 | 0 |  |  |  |  |  |  |  |  |
| GO:0006101\_citrate\_metabolic\_process | 1 | 0 |  |  |  |  |  |  |  |  |
| GO:0006104\_succinyl-CoA\_metabolic\_process | 1 | 0 |  |  |  |  |  |  |  |  |
| GO:0006116\_NADH\_oxidation | 1 | 0 |  |  |  |  |  |  |  |  |
| GO:0006120\_mitochondrial\_electron\_transport\_\_NADH\_to\_ubiquinone | 1 | 0 |  |  |  |  |  |  |  |  |
| GO:0006154\_adenosine\_catabolic\_process | 1 | 0 |  |  |  |  |  |  |  |  |
| GO:0006157\_deoxyadenosine\_catabolic\_process | 1 | 0 |  |  |  |  |  |  |  |  |
| GO:0006167\_AMP\_biosynthetic\_process | 1 | 0 |  |  |  |  |  |  |  |  |
| GO:0006175\_dATP\_biosynthetic\_process | 1 | 0 |  |  |  |  |  |  |  |  |
| GO:0006178\_guanine\_salvage | 1 | 0 |  |  |  |  |  |  |  |  |
| GO:0006196\_AMP\_catabolic\_process | 1 | 0 |  |  |  |  |  |  |  |  |
| GO:0006203\_dGTP\_catabolic\_process | 1 | 0 |  |  |  |  |  |  |  |  |
| GO:0006208\_pyrimidine\_base\_catabolic\_process | 1 | 0 |  |  |  |  |  |  |  |  |
| GO:0006221\_pyrimidine\_nucleotide\_biosynthetic\_process | 1 | 0 |  |  |  |  |  |  |  |  |
| GO:0006235\_dTTP\_biosynthetic\_process | 1 | 0 |  |  |  |  |  |  |  |  |
| GO:0006244\_pyrimidine\_nucleotide\_catabolic\_process | 1 | 0 |  |  |  |  |  |  |  |  |
| GO:0006269\_DNA\_replication\_\_synthesis\_of\_RNA\_primer | 1 | 0 |  |  |  |  |  |  |  |  |
| GO:0006283\_transcription-coupled\_nucleotide-excision\_repair | 1 | 0 |  |  |  |  |  |  |  |  |
| GO:0006296\_nucleotide-excision\_repair\_\_DNA\_incision\_\_5'-to\_lesion | 1 | 0 |  |  |  |  |  |  |  |  |
| GO:0006307\_DNA\_dealkylation | 1 | 0 |  |  |  |  |  |  |  |  |
| GO:0006337\_nucleosome\_disassembly | 1 | 0 |  |  |  |  |  |  |  |  |
| GO:0006344\_maintenance\_of\_chromatin\_silencing | 1 | 0 |  |  |  |  |  |  |  |  |
| GO:0006356\_regulation\_of\_transcription\_from\_RNA\_polymerase\_I\_promoter | 1 | 0 |  |  |  |  |  |  |  |  |
| GO:0006388\_tRNA\_splicing\_\_via\_endonucleolytic\_cleavage\_and\_ligation | 1 | 0 |  |  |  |  |  |  |  |  |
| GO:0006407\_rRNA\_export\_from\_nucleus | 1 | 0 |  |  |  |  |  |  |  |  |
| GO:0006419\_alanyl-tRNA\_aminoacylation | 1 | 0 |  |  |  |  |  |  |  |  |
| GO:0006434\_seryl-tRNA\_aminoacylation | 1 | 0 |  |  |  |  |  |  |  |  |
| GO:0006447\_regulation\_of\_translational\_initiation\_by\_iron | 1 | 0 |  |  |  |  |  |  |  |  |
| GO:0006463\_steroid\_hormone\_receptor\_complex\_assembly | 1 | 0 |  |  |  |  |  |  |  |  |
| GO:0006467\_protein\_thiol-disulfide\_exchange | 1 | 0 |  |  |  |  |  |  |  |  |
| GO:0006474\_N-terminal\_protein\_amino\_acid\_acetylation | 1 | 0 |  |  |  |  |  |  |  |  |
| GO:0006481\_C-terminal\_protein\_amino\_acid\_methylation | 1 | 0 |  |  |  |  |  |  |  |  |
| GO:0006488\_dolichol-linked\_oligosaccharide\_biosynthetic\_process | 1 | 0 |  |  |  |  |  |  |  |  |
| GO:0006494\_protein\_amino\_acid\_terminal\_glycosylation | 1 | 0 |  |  |  |  |  |  |  |  |
| GO:0006496\_protein\_amino\_acid\_terminal\_N-glycosylation | 1 | 0 |  |  |  |  |  |  |  |  |
| GO:0006500\_N-terminal\_protein\_palmitoylation | 1 | 0 |  |  |  |  |  |  |  |  |
| GO:0006507\_GPI\_anchor\_release | 1 | 0 |  |  |  |  |  |  |  |  |
| GO:0006537\_glutamate\_biosynthetic\_process | 1 | 0 |  |  |  |  |  |  |  |  |
| GO:0006544\_glycine\_metabolic\_process | 1 | 0 |  |  |  |  |  |  |  |  |
| GO:0006549\_isoleucine\_metabolic\_process | 1 | 0 |  |  |  |  |  |  |  |  |
| GO:0006553\_lysine\_metabolic\_process | 1 | 0 |  |  |  |  |  |  |  |  |
| GO:0006554\_lysine\_catabolic\_process | 1 | 0 |  |  |  |  |  |  |  |  |
| GO:0006556\_S-adenosylmethionine\_biosynthetic\_process | 1 | 0 |  |  |  |  |  |  |  |  |
| GO:0006559\_L-phenylalanine\_catabolic\_process | 1 | 0 |  |  |  |  |  |  |  |  |
| GO:0006569\_tryptophan\_catabolic\_process | 1 | 0 |  |  |  |  |  |  |  |  |
| GO:0006572\_tyrosine\_catabolic\_process | 1 | 0 |  |  |  |  |  |  |  |  |
| GO:0006573\_valine\_metabolic\_process | 1 | 0 |  |  |  |  |  |  |  |  |
| GO:0006581\_acetylcholine\_catabolic\_process | 1 | 0 |  |  |  |  |  |  |  |  |
| GO:0006585\_dopamine\_biosynthetic\_process\_from\_tyrosine | 1 | 0 |  |  |  |  |  |  |  |  |
| GO:0006590\_thyroid\_hormone\_generation | 1 | 0 |  |  |  |  |  |  |  |  |
| GO:0006591\_ornithine\_metabolic\_process | 1 | 0 |  |  |  |  |  |  |  |  |
| GO:0006596\_polyamine\_biosynthetic\_process | 1 | 0 |  |  |  |  |  |  |  |  |
| GO:0006597\_spermine\_biosynthetic\_process | 1 | 0 |  |  |  |  |  |  |  |  |
| GO:0006601\_creatine\_biosynthetic\_process | 1 | 0 |  |  |  |  |  |  |  |  |
| GO:0006613\_cotranslational\_protein\_targeting\_to\_membrane | 1 | 0 |  |  |  |  |  |  |  |  |
| GO:0006622\_protein\_targeting\_to\_lysosome | 1 | 0 |  |  |  |  |  |  |  |  |
| GO:0006627\_mitochondrial\_protein\_processing\_during\_import | 1 | 0 |  |  |  |  |  |  |  |  |
| GO:0006653\_lecithin\_metabolic\_process | 1 | 0 |  |  |  |  |  |  |  |  |
| GO:0006654\_phosphatidic\_acid\_biosynthetic\_process | 1 | 0 |  |  |  |  |  |  |  |  |
| GO:0006658\_phosphatidylserine\_metabolic\_process | 1 | 0 |  |  |  |  |  |  |  |  |
| GO:0006659\_phosphatidylserine\_biosynthetic\_process | 1 | 0 |  |  |  |  |  |  |  |  |
| GO:0006667\_sphinganine\_metabolic\_process | 1 | 0 |  |  |  |  |  |  |  |  |
| GO:0006668\_sphinganine-1-phosphate\_metabolic\_process | 1 | 0 |  |  |  |  |  |  |  |  |
| GO:0006678\_glucosylceramide\_metabolic\_process | 1 | 0 |  |  |  |  |  |  |  |  |
| GO:0006682\_galactosylceramide\_biosynthetic\_process | 1 | 0 |  |  |  |  |  |  |  |  |
| GO:0006685\_sphingomyelin\_catabolic\_process | 1 | 0 |  |  |  |  |  |  |  |  |
| GO:0006700\_C21-steroid\_hormone\_biosynthetic\_process | 1 | 0 |  |  |  |  |  |  |  |  |
| GO:0006705\_mineralocorticoid\_biosynthetic\_process | 1 | 0 |  |  |  |  |  |  |  |  |
| GO:0006709\_progesterone\_catabolic\_process | 1 | 0 |  |  |  |  |  |  |  |  |
| GO:0006729\_tetrahydrobiopterin\_biosynthetic\_process | 1 | 0 |  |  |  |  |  |  |  |  |
| GO:0006734\_NADH\_metabolic\_process | 1 | 0 |  |  |  |  |  |  |  |  |
| GO:0006740\_NADPH\_regeneration | 1 | 0 |  |  |  |  |  |  |  |  |
| GO:0006741\_NADP\_biosynthetic\_process | 1 | 0 |  |  |  |  |  |  |  |  |
| GO:0006743\_ubiquinone\_metabolic\_process | 1 | 0 |  |  |  |  |  |  |  |  |
| GO:0006744\_ubiquinone\_biosynthetic\_process | 1 | 0 |  |  |  |  |  |  |  |  |
| GO:0006772\_thiamin\_metabolic\_process | 1 | 0 |  |  |  |  |  |  |  |  |
| GO:0006784\_heme\_a\_biosynthetic\_process | 1 | 0 |  |  |  |  |  |  |  |  |
| GO:0006797\_polyphosphate\_metabolic\_process | 1 | 0 |  |  |  |  |  |  |  |  |
| GO:0006798\_polyphosphate\_catabolic\_process | 1 | 0 |  |  |  |  |  |  |  |  |
| GO:0006824\_cobalt\_ion\_transport | 1 | 0 |  |  |  |  |  |  |  |  |
| GO:0006842\_tricarboxylic\_acid\_transport | 1 | 0 |  |  |  |  |  |  |  |  |
| GO:0006844\_acyl\_carnitine\_transport | 1 | 0 |  |  |  |  |  |  |  |  |
| GO:0006855\_multidrug\_transport | 1 | 0 |  |  |  |  |  |  |  |  |
| GO:0006863\_purine\_transport | 1 | 0 |  |  |  |  |  |  |  |  |
| GO:0006890\_retrograde\_vesicle-mediated\_transport\_\_Golgi\_to\_ER | 1 | 0 |  |  |  |  |  |  |  |  |
| GO:0006891\_intra-Golgi\_vesicle-mediated\_transport | 1 | 0 |  |  |  |  |  |  |  |  |
| GO:0006893\_Golgi\_to\_plasma\_membrane\_transport | 1 | 0 |  |  |  |  |  |  |  |  |
| GO:0006895\_Golgi\_to\_endosome\_transport | 1 | 0 |  |  |  |  |  |  |  |  |
| GO:0006896\_Golgi\_to\_vacuole\_transport | 1 | 0 |  |  |  |  |  |  |  |  |
| GO:0006900\_membrane\_budding | 1 | 0 |  |  |  |  |  |  |  |  |
| GO:0006930\_substrate-bound\_cell\_migration\_\_cell\_extension | 1 | 0 |  |  |  |  |  |  |  |  |
| GO:0006931\_substrate-bound\_cell\_migration\_\_cell\_attachment\_to\_substrate | 1 | 0 |  |  |  |  |  |  |  |  |
| GO:0006933\_negative\_regulation\_of\_cell\_adhesion\_involved\_in\_substrate-bound\_cell\_migration | 1 | 0 |  |  |  |  |  |  |  |  |
| GO:0006957\_complement\_activation\_\_alternative\_pathway | 1 | 0 |  |  |  |  |  |  |  |  |
| GO:0006958\_complement\_activation\_\_classical\_pathway | 1 | 0 |  |  |  |  |  |  |  |  |
| GO:0006978\_DNA\_damage\_response\_\_signal\_transduction\_by\_p53\_class\_mediator\_resulting\_in\_transcription\_of\_p21\_class\_mediator | 1 | 0 |  |  |  |  |  |  |  |  |
| GO:0007016\_cytoskeletal\_anchoring\_at\_plasma\_membrane | 1 | 0 |  |  |  |  |  |  |  |  |
| GO:0007021\_tubulin\_complex\_assembly | 1 | 0 |  |  |  |  |  |  |  |  |
| GO:0007052\_mitotic\_spindle\_organization | 1 | 0 |  |  |  |  |  |  |  |  |
| GO:0007056\_spindle\_assembly\_involved\_in\_female\_meiosis | 1 | 0 |  |  |  |  |  |  |  |  |
| GO:0007057\_spindle\_assembly\_involved\_in\_female\_meiosis\_I | 1 | 0 |  |  |  |  |  |  |  |  |
| GO:0007063\_regulation\_of\_sister\_chromatid\_cohesion | 1 | 0 |  |  |  |  |  |  |  |  |
| GO:0007065\_male\_meiosis\_sister\_chromatid\_cohesion | 1 | 0 |  |  |  |  |  |  |  |  |
| GO:0007076\_mitotic\_chromosome\_condensation | 1 | 0 |  |  |  |  |  |  |  |  |
| GO:0007095\_mitotic\_cell\_cycle\_G2\_M\_transition\_DNA\_damage\_checkpoint | 1 | 0 |  |  |  |  |  |  |  |  |
| GO:0007096\_regulation\_of\_exit\_from\_mitosis | 1 | 0 |  |  |  |  |  |  |  |  |
| GO:0007158\_neuron\_adhesion | 1 | 0 |  |  |  |  |  |  |  |  |
| GO:0007168\_receptor\_guanylyl\_cyclase\_signaling\_pathway | 1 | 0 |  |  |  |  |  |  |  |  |
| GO:0007197\_inhibition\_of\_adenylate\_cyclase\_activity\_by\_muscarinic\_acetylcholine\_receptor\_signaling\_pathway | 1 | 0 |  |  |  |  |  |  |  |  |
| GO:0007207\_activation\_of\_phospholipase\_C\_activity\_by\_muscarinic\_acetylcholine\_receptor\_signaling\_pathway | 1 | 0 |  |  |  |  |  |  |  |  |
| GO:0007208\_activation\_of\_phospholipase\_C\_activity\_by\_serotonin\_receptor\_signaling\_pathway | 1 | 0 |  |  |  |  |  |  |  |  |
| GO:0007217\_tachykinin\_receptor\_signaling\_pathway | 1 | 0 |  |  |  |  |  |  |  |  |
| GO:0007221\_positive\_regulation\_of\_transcription\_of\_Notch\_receptor\_target | 1 | 0 |  |  |  |  |  |  |  |  |
| GO:0007223\_Wnt\_receptor\_signaling\_pathway\_\_calcium\_modulating\_pathway | 1 | 0 |  |  |  |  |  |  |  |  |
| GO:0007225\_patched\_ligand\_processing | 1 | 0 |  |  |  |  |  |  |  |  |
| GO:0007227\_signal\_transduction\_downstream\_of\_smoothened | 1 | 0 |  |  |  |  |  |  |  |  |
| GO:0007228\_positive\_regulation\_of\_hh\_target\_transcription\_factor\_activity | 1 | 0 |  |  |  |  |  |  |  |  |
| GO:0007231\_osmosensory\_signaling\_pathway | 1 | 0 |  |  |  |  |  |  |  |  |
| GO:0007284\_spermatogonial\_cell\_division | 1 | 0 |  |  |  |  |  |  |  |  |
| GO:0007290\_spermatid\_nucleus\_elongation | 1 | 0 |  |  |  |  |  |  |  |  |
| GO:0007296\_vitellogenesis | 1 | 0 |  |  |  |  |  |  |  |  |
| GO:0007321\_sperm\_displacement | 1 | 0 |  |  |  |  |  |  |  |  |
| GO:0007380\_specification\_of\_segmental\_identity\_\_head | 1 | 0 |  |  |  |  |  |  |  |  |
| GO:0007382\_specification\_of\_segmental\_identity\_\_maxillary\_segment | 1 | 0 |  |  |  |  |  |  |  |  |
| GO:0007400\_neuroblast\_fate\_determination | 1 | 0 |  |  |  |  |  |  |  |  |
| GO:0007402\_ganglion\_mother\_cell\_fate\_determination | 1 | 0 |  |  |  |  |  |  |  |  |
| GO:0007495\_visceral\_mesoderm-endoderm\_interaction\_involved\_in\_midgut\_development | 1 | 0 |  |  |  |  |  |  |  |  |
| GO:0007497\_posterior\_midgut\_development | 1 | 0 |  |  |  |  |  |  |  |  |
| GO:0007499\_ectoderm\_and\_mesoderm\_interaction | 1 | 0 |  |  |  |  |  |  |  |  |
| GO:0007500\_mesodermal\_cell\_fate\_determination | 1 | 0 |  |  |  |  |  |  |  |  |
| GO:0007509\_mesoderm\_migration | 1 | 0 |  |  |  |  |  |  |  |  |
| GO:0007518\_myoblast\_cell\_fate\_determination | 1 | 0 |  |  |  |  |  |  |  |  |
| GO:0007521\_muscle\_cell\_fate\_determination | 1 | 0 |  |  |  |  |  |  |  |  |
| GO:0007522\_visceral\_muscle\_development | 1 | 0 |  |  |  |  |  |  |  |  |
| GO:0007529\_establishment\_of\_synaptic\_specificity\_at\_neuromuscular\_junction | 1 | 0 |  |  |  |  |  |  |  |  |
| GO:0007538\_primary\_sex\_determination | 1 | 0 |  |  |  |  |  |  |  |  |
| GO:0007542\_primary\_sex\_determination\_\_germ-line | 1 | 0 |  |  |  |  |  |  |  |  |
| GO:0007567\_parturition | 1 | 0 |  |  |  |  |  |  |  |  |
| GO:0007614\_short-term\_memory | 1 | 0 |  |  |  |  |  |  |  |  |
| GO:0007621\_negative\_regulation\_of\_female\_receptivity | 1 | 0 |  |  |  |  |  |  |  |  |
| GO:0008049\_male\_courtship\_behavior | 1 | 0 |  |  |  |  |  |  |  |  |
| GO:0008050\_female\_courtship\_behavior | 1 | 0 |  |  |  |  |  |  |  |  |
| GO:0008052\_sensory\_organ\_boundary\_specification | 1 | 0 |  |  |  |  |  |  |  |  |
| GO:0008054\_cyclin\_catabolic\_process | 1 | 0 |  |  |  |  |  |  |  |  |
| GO:0008057\_eye\_pigment\_granule\_organization | 1 | 0 |  |  |  |  |  |  |  |  |
| GO:0008078\_mesodermal\_cell\_migration | 1 | 0 |  |  |  |  |  |  |  |  |
| GO:0008208\_C21-steroid\_hormone\_catabolic\_process | 1 | 0 |  |  |  |  |  |  |  |  |
| GO:0008216\_spermidine\_metabolic\_process | 1 | 0 |  |  |  |  |  |  |  |  |
| GO:0008292\_acetylcholine\_biosynthetic\_process | 1 | 0 |  |  |  |  |  |  |  |  |
| GO:0008295\_spermidine\_biosynthetic\_process | 1 | 0 |  |  |  |  |  |  |  |  |
| GO:0008300\_isoprenoid\_catabolic\_process | 1 | 0 |  |  |  |  |  |  |  |  |
| GO:0008333\_endosome\_to\_lysosome\_transport | 1 | 0 |  |  |  |  |  |  |  |  |
| GO:0008355\_olfactory\_learning | 1 | 0 |  |  |  |  |  |  |  |  |
| GO:0008611\_ether\_lipid\_biosynthetic\_process | 1 | 0 |  |  |  |  |  |  |  |  |
| GO:0008626\_induction\_of\_apoptosis\_by\_granzyme | 1 | 0 |  |  |  |  |  |  |  |  |
| GO:0008633\_activation\_of\_pro-apoptotic\_gene\_products | 1 | 0 |  |  |  |  |  |  |  |  |
| GO:0008653\_lipopolysaccharide\_metabolic\_process | 1 | 0 |  |  |  |  |  |  |  |  |
| GO:0009068\_aspartate\_family\_amino\_acid\_catabolic\_process | 1 | 0 |  |  |  |  |  |  |  |  |
| GO:0009084\_glutamine\_family\_amino\_acid\_biosynthetic\_process | 1 | 0 |  |  |  |  |  |  |  |  |
| GO:0009088\_threonine\_biosynthetic\_process | 1 | 0 |  |  |  |  |  |  |  |  |
| GO:0009105\_lipoic\_acid\_biosynthetic\_process | 1 | 0 |  |  |  |  |  |  |  |  |
| GO:0009109\_coenzyme\_catabolic\_process | 1 | 0 |  |  |  |  |  |  |  |  |
| GO:0009111\_vitamin\_catabolic\_process | 1 | 0 |  |  |  |  |  |  |  |  |
| GO:0009113\_purine\_base\_biosynthetic\_process | 1 | 0 |  |  |  |  |  |  |  |  |
| GO:0009127\_purine\_nucleoside\_monophosphate\_biosynthetic\_process | 1 | 0 |  |  |  |  |  |  |  |  |
| GO:0009128\_purine\_nucleoside\_monophosphate\_catabolic\_process | 1 | 0 |  |  |  |  |  |  |  |  |
| GO:0009129\_pyrimidine\_nucleoside\_monophosphate\_metabolic\_process | 1 | 0 |  |  |  |  |  |  |  |  |
| GO:0009131\_pyrimidine\_nucleoside\_monophosphate\_catabolic\_process | 1 | 0 |  |  |  |  |  |  |  |  |
| GO:0009133\_nucleoside\_diphosphate\_biosynthetic\_process | 1 | 0 |  |  |  |  |  |  |  |  |
| GO:0009145\_purine\_nucleoside\_triphosphate\_biosynthetic\_process | 1 | 0 |  |  |  |  |  |  |  |  |
| GO:0009147\_pyrimidine\_nucleoside\_triphosphate\_metabolic\_process | 1 | 0 |  |  |  |  |  |  |  |  |
| GO:0009148\_pyrimidine\_nucleoside\_triphosphate\_biosynthetic\_process | 1 | 0 |  |  |  |  |  |  |  |  |
| GO:0009152\_purine\_ribonucleotide\_biosynthetic\_process | 1 | 0 |  |  |  |  |  |  |  |  |
| GO:0009153\_purine\_deoxyribonucleotide\_biosynthetic\_process | 1 | 0 |  |  |  |  |  |  |  |  |
| GO:0009156\_ribonucleoside\_monophosphate\_biosynthetic\_process | 1 | 0 |  |  |  |  |  |  |  |  |
| GO:0009158\_ribonucleoside\_monophosphate\_catabolic\_process | 1 | 0 |  |  |  |  |  |  |  |  |
| GO:0009159\_deoxyribonucleoside\_monophosphate\_catabolic\_process | 1 | 0 |  |  |  |  |  |  |  |  |
| GO:0009162\_deoxyribonucleoside\_monophosphate\_metabolic\_process | 1 | 0 |  |  |  |  |  |  |  |  |
| GO:0009168\_purine\_ribonucleoside\_monophosphate\_biosynthetic\_process | 1 | 0 |  |  |  |  |  |  |  |  |
| GO:0009169\_purine\_ribonucleoside\_monophosphate\_catabolic\_process | 1 | 0 |  |  |  |  |  |  |  |  |
| GO:0009176\_pyrimidine\_deoxyribonucleoside\_monophosphate\_metabolic\_process | 1 | 0 |  |  |  |  |  |  |  |  |
| GO:0009178\_pyrimidine\_deoxyribonucleoside\_monophosphate\_catabolic\_process | 1 | 0 |  |  |  |  |  |  |  |  |
| GO:0009211\_pyrimidine\_deoxyribonucleoside\_triphosphate\_metabolic\_process | 1 | 0 |  |  |  |  |  |  |  |  |
| GO:0009212\_pyrimidine\_deoxyribonucleoside\_triphosphate\_biosynthetic\_process | 1 | 0 |  |  |  |  |  |  |  |  |
| GO:0009216\_purine\_deoxyribonucleoside\_triphosphate\_biosynthetic\_process | 1 | 0 |  |  |  |  |  |  |  |  |
| GO:0009221\_pyrimidine\_deoxyribonucleotide\_biosynthetic\_process | 1 | 0 |  |  |  |  |  |  |  |  |
| GO:0009223\_pyrimidine\_deoxyribonucleotide\_catabolic\_process | 1 | 0 |  |  |  |  |  |  |  |  |
| GO:0009260\_ribonucleotide\_biosynthetic\_process | 1 | 0 |  |  |  |  |  |  |  |  |
| GO:0009405\_pathogenesis | 1 | 0 |  |  |  |  |  |  |  |  |
| GO:0009414\_response\_to\_water\_deprivation | 1 | 0 |  |  |  |  |  |  |  |  |
| GO:0009415\_response\_to\_water | 1 | 0 |  |  |  |  |  |  |  |  |
| GO:0009449\_gamma-aminobutyric\_acid\_biosynthetic\_process | 1 | 0 |  |  |  |  |  |  |  |  |
| GO:0009450\_gamma-aminobutyric\_acid\_catabolic\_process | 1 | 0 |  |  |  |  |  |  |  |  |
| GO:0009589\_detection\_of\_UV | 1 | 0 |  |  |  |  |  |  |  |  |
| GO:0009590\_detection\_of\_gravity | 1 | 0 |  |  |  |  |  |  |  |  |
| GO:0009624\_response\_to\_nematode | 1 | 0 |  |  |  |  |  |  |  |  |
| GO:0009629\_response\_to\_gravity | 1 | 0 |  |  |  |  |  |  |  |  |
| GO:0009648\_photoperiodism | 1 | 0 |  |  |  |  |  |  |  |  |
| GO:0009690\_cytokinin\_metabolic\_process | 1 | 0 |  |  |  |  |  |  |  |  |
| GO:0009691\_cytokinin\_biosynthetic\_process | 1 | 0 |  |  |  |  |  |  |  |  |
| GO:0009786\_regulation\_of\_asymmetric\_cell\_division | 1 | 0 |  |  |  |  |  |  |  |  |
| GO:0009794\_regulation\_of\_mitotic\_cell\_cycle\_\_embryonic | 1 | 0 |  |  |  |  |  |  |  |  |
| GO:0009956\_radial\_pattern\_formation | 1 | 0 |  |  |  |  |  |  |  |  |
| GO:0009957\_epidermal\_cell\_fate\_specification | 1 | 0 |  |  |  |  |  |  |  |  |
| GO:0009992\_cellular\_water\_homeostasis | 1 | 0 |  |  |  |  |  |  |  |  |
| GO:0010032\_meiotic\_chromosome\_condensation | 1 | 0 |  |  |  |  |  |  |  |  |
| GO:0010039\_response\_to\_iron\_ion | 1 | 0 |  |  |  |  |  |  |  |  |
| GO:0010042\_response\_to\_manganese\_ion | 1 | 0 |  |  |  |  |  |  |  |  |
| GO:0010045\_response\_to\_nickel\_ion | 1 | 0 |  |  |  |  |  |  |  |  |
| GO:0010046\_response\_to\_mycotoxin | 1 | 0 |  |  |  |  |  |  |  |  |
| GO:0010107\_potassium\_ion\_import | 1 | 0 |  |  |  |  |  |  |  |  |
| GO:0010155\_regulation\_of\_proton\_transport | 1 | 0 |  |  |  |  |  |  |  |  |
| GO:0010160\_formation\_of\_organ\_boundary | 1 | 0 |  |  |  |  |  |  |  |  |
| GO:0010260\_organ\_senescence | 1 | 0 |  |  |  |  |  |  |  |  |
| GO:0010310\_regulation\_of\_hydrogen\_peroxide\_metabolic\_process | 1 | 0 |  |  |  |  |  |  |  |  |
| GO:0010447\_response\_to\_acidity | 1 | 0 |  |  |  |  |  |  |  |  |
| GO:0010452\_histone\_H3-K36\_methylation | 1 | 0 |  |  |  |  |  |  |  |  |
| GO:0010455\_positive\_regulation\_of\_cell\_fate\_commitment | 1 | 0 |  |  |  |  |  |  |  |  |
| GO:0010470\_regulation\_of\_gastrulation | 1 | 0 |  |  |  |  |  |  |  |  |
| GO:0010508\_positive\_regulation\_of\_autophagy | 1 | 0 |  |  |  |  |  |  |  |  |
| GO:0010519\_negative\_regulation\_of\_phospholipase\_activity | 1 | 0 |  |  |  |  |  |  |  |  |
| GO:0010520\_regulation\_of\_reciprocal\_meiotic\_recombination | 1 | 0 |  |  |  |  |  |  |  |  |
| GO:0010523\_negative\_regulation\_of\_calcium\_ion\_transport\_into\_cytosol | 1 | 0 |  |  |  |  |  |  |  |  |
| GO:0010543\_regulation\_of\_platelet\_activation | 1 | 0 |  |  |  |  |  |  |  |  |
| GO:0010561\_negative\_regulation\_of\_glycoprotein\_biosynthetic\_process | 1 | 0 |  |  |  |  |  |  |  |  |
| GO:0010569\_regulation\_of\_double-strand\_break\_repair\_via\_homologous\_recombination | 1 | 0 |  |  |  |  |  |  |  |  |
| GO:0010572\_positive\_regulation\_of\_platelet\_activation | 1 | 0 |  |  |  |  |  |  |  |  |
| GO:0010594\_regulation\_of\_endothelial\_cell\_migration | 1 | 0 |  |  |  |  |  |  |  |  |
| GO:0010596\_negative\_regulation\_of\_endothelial\_cell\_migration | 1 | 0 |  |  |  |  |  |  |  |  |
| GO:0010611\_regulation\_of\_cardiac\_muscle\_hypertrophy | 1 | 0 |  |  |  |  |  |  |  |  |
| GO:0010612\_regulation\_of\_cardiac\_muscle\_adaptation | 1 | 0 |  |  |  |  |  |  |  |  |
| GO:0010614\_negative\_regulation\_of\_cardiac\_muscle\_hypertrophy | 1 | 0 |  |  |  |  |  |  |  |  |
| GO:0010616\_negative\_regulation\_of\_cardiac\_muscle\_adaptation | 1 | 0 |  |  |  |  |  |  |  |  |
| GO:0010634\_positive\_regulation\_of\_epithelial\_cell\_migration | 1 | 0 |  |  |  |  |  |  |  |  |
| GO:0010656\_negative\_regulation\_of\_muscle\_cell\_apoptosis | 1 | 0 |  |  |  |  |  |  |  |  |
| GO:0010657\_muscle\_cell\_apoptosis | 1 | 0 |  |  |  |  |  |  |  |  |
| GO:0010658\_striated\_muscle\_cell\_apoptosis | 1 | 0 |  |  |  |  |  |  |  |  |
| GO:0010659\_cardiac\_muscle\_cell\_apoptosis | 1 | 0 |  |  |  |  |  |  |  |  |
| GO:0010660\_regulation\_of\_muscle\_cell\_apoptosis | 1 | 0 |  |  |  |  |  |  |  |  |
| GO:0010662\_regulation\_of\_striated\_muscle\_cell\_apoptosis | 1 | 0 |  |  |  |  |  |  |  |  |
| GO:0010664\_negative\_regulation\_of\_striated\_muscle\_cell\_apoptosis | 1 | 0 |  |  |  |  |  |  |  |  |
| GO:0010665\_regulation\_of\_cardiac\_muscle\_cell\_apoptosis | 1 | 0 |  |  |  |  |  |  |  |  |
| GO:0010667\_negative\_regulation\_of\_cardiac\_muscle\_cell\_apoptosis | 1 | 0 |  |  |  |  |  |  |  |  |
| GO:0010668\_ectodermal\_cell\_differentiation | 1 | 0 |  |  |  |  |  |  |  |  |
| GO:0010671\_negative\_regulation\_of\_oxygen\_and\_reactive\_oxygen\_species\_metabolic\_process | 1 | 0 |  |  |  |  |  |  |  |  |
| GO:0010719\_negative\_regulation\_of\_epithelial\_to\_mesenchymal\_transition | 1 | 0 |  |  |  |  |  |  |  |  |
| GO:0010735\_positive\_regulation\_of\_transcription\_via\_serum\_response\_element\_binding | 1 | 0 |  |  |  |  |  |  |  |  |
| GO:0010825\_positive\_regulation\_of\_centrosome\_duplication | 1 | 0 |  |  |  |  |  |  |  |  |
| GO:0010845\_positive\_regulation\_of\_reciprocal\_meiotic\_recombination | 1 | 0 |  |  |  |  |  |  |  |  |
| GO:0010850\_chemoreceptor\_signaling\_pathway\_involved\_in\_regulation\_of\_blood\_pressure | 1 | 0 |  |  |  |  |  |  |  |  |
| GO:0010873\_positive\_regulation\_of\_cholesterol\_esterification | 1 | 0 |  |  |  |  |  |  |  |  |
| GO:0010880\_regulation\_of\_release\_of\_sequestered\_calcium\_ion\_into\_cytosol\_by\_sarcoplasmic\_reticulum | 1 | 0 |  |  |  |  |  |  |  |  |
| GO:0010881\_regulation\_of\_cardiac\_muscle\_contraction\_by\_regulation\_of\_the\_release\_of\_sequestered\_calcium\_ion | 1 | 0 |  |  |  |  |  |  |  |  |
| GO:0010882\_regulation\_of\_cardiac\_muscle\_contraction\_by\_calcium\_ion\_signaling | 1 | 0 |  |  |  |  |  |  |  |  |
| GO:0010890\_positive\_regulation\_of\_sequestering\_of\_triglyceride | 1 | 0 |  |  |  |  |  |  |  |  |
| GO:0010919\_regulation\_of\_inositol\_phosphate\_biosynthetic\_process | 1 | 0 |  |  |  |  |  |  |  |  |
| GO:0010931\_macrophage\_tolerance\_induction | 1 | 0 |  |  |  |  |  |  |  |  |
| GO:0010932\_regulation\_of\_macrophage\_tolerance\_induction | 1 | 0 |  |  |  |  |  |  |  |  |
| GO:0010933\_positive\_regulation\_of\_macrophage\_tolerance\_induction | 1 | 0 |  |  |  |  |  |  |  |  |
| GO:0010934\_macrophage\_cytokine\_production | 1 | 0 |  |  |  |  |  |  |  |  |
| GO:0010935\_regulation\_of\_macrophage\_cytokine\_production | 1 | 0 |  |  |  |  |  |  |  |  |
| GO:0010936\_negative\_regulation\_of\_macrophage\_cytokine\_production | 1 | 0 |  |  |  |  |  |  |  |  |
| GO:0010953\_regulation\_of\_protein\_maturation\_by\_peptide\_bond\_cleavage | 1 | 0 |  |  |  |  |  |  |  |  |
| GO:0010962\_regulation\_of\_glucan\_biosynthetic\_process | 1 | 0 |  |  |  |  |  |  |  |  |
| GO:0010966\_regulation\_of\_phosphate\_transport | 1 | 0 |  |  |  |  |  |  |  |  |
| GO:0014012\_axon\_regeneration\_in\_the\_peripheral\_nervous\_system | 1 | 0 |  |  |  |  |  |  |  |  |
| GO:0014016\_neuroblast\_differentiation | 1 | 0 |  |  |  |  |  |  |  |  |
| GO:0014017\_neuroblast\_fate\_commitment | 1 | 0 |  |  |  |  |  |  |  |  |
| GO:0014041\_regulation\_of\_neuron\_maturation | 1 | 0 |  |  |  |  |  |  |  |  |
| GO:0014042\_positive\_regulation\_of\_neuron\_maturation | 1 | 0 |  |  |  |  |  |  |  |  |
| GO:0014049\_positive\_regulation\_of\_glutamate\_secretion | 1 | 0 |  |  |  |  |  |  |  |  |
| GO:0014061\_regulation\_of\_norepinephrine\_secretion | 1 | 0 |  |  |  |  |  |  |  |  |
| GO:0014071\_response\_to\_cycloalkane | 1 | 0 |  |  |  |  |  |  |  |  |
| GO:0014707\_branchiomeric\_skeletal\_muscle\_development | 1 | 0 |  |  |  |  |  |  |  |  |
| GO:0014738\_regulation\_of\_muscle\_hyperplasia | 1 | 0 |  |  |  |  |  |  |  |  |
| GO:0014740\_negative\_regulation\_of\_muscle\_hyperplasia | 1 | 0 |  |  |  |  |  |  |  |  |
| GO:0014741\_negative\_regulation\_of\_muscle\_hypertrophy | 1 | 0 |  |  |  |  |  |  |  |  |
| GO:0014743\_regulation\_of\_muscle\_hypertrophy | 1 | 0 |  |  |  |  |  |  |  |  |
| GO:0014805\_smooth\_muscle\_adaptation | 1 | 0 |  |  |  |  |  |  |  |  |
| GO:0014806\_smooth\_muscle\_hyperplasia | 1 | 0 |  |  |  |  |  |  |  |  |
| GO:0014807\_regulation\_of\_somitogenesis | 1 | 0 |  |  |  |  |  |  |  |  |
| GO:0014808\_release\_of\_sequestered\_calcium\_ion\_into\_cytosol\_by\_sarcoplasmic\_reticulum | 1 | 0 |  |  |  |  |  |  |  |  |
| GO:0014813\_satellite\_cell\_commitment | 1 | 0 |  |  |  |  |  |  |  |  |
| GO:0014816\_satellite\_cell\_differentiation | 1 | 0 |  |  |  |  |  |  |  |  |
| GO:0014819\_regulation\_of\_skeletal\_muscle\_contraction | 1 | 0 |  |  |  |  |  |  |  |  |
| GO:0014852\_regulation\_of\_skeletal\_muscle\_contraction\_by\_neural\_stimulation\_via\_neuromuscular\_junction | 1 | 0 |  |  |  |  |  |  |  |  |
| GO:0014853\_regulation\_of\_excitatory\_postsynaptic\_membrane\_potential\_involved\_in\_skeletal\_muscle\_contraction | 1 | 0 |  |  |  |  |  |  |  |  |
| GO:0014856\_skeletal\_muscle\_cell\_proliferation | 1 | 0 |  |  |  |  |  |  |  |  |
| GO:0014857\_regulation\_of\_skeletal\_muscle\_cell\_proliferation | 1 | 0 |  |  |  |  |  |  |  |  |
| GO:0014858\_positive\_regulation\_of\_skeletal\_muscle\_cell\_proliferation | 1 | 0 |  |  |  |  |  |  |  |  |
| GO:0014887\_cardiac\_muscle\_adaptation | 1 | 0 |  |  |  |  |  |  |  |  |
| GO:0014889\_muscle\_atrophy | 1 | 0 |  |  |  |  |  |  |  |  |
| GO:0014896\_muscle\_hypertrophy | 1 | 0 |  |  |  |  |  |  |  |  |
| GO:0014897\_striated\_muscle\_hypertrophy | 1 | 0 |  |  |  |  |  |  |  |  |
| GO:0014898\_cardiac\_muscle\_hypertrophy | 1 | 0 |  |  |  |  |  |  |  |  |
| GO:0014900\_muscle\_hyperplasia | 1 | 0 |  |  |  |  |  |  |  |  |
| GO:0014910\_regulation\_of\_smooth\_muscle\_cell\_migration | 1 | 0 |  |  |  |  |  |  |  |  |
| GO:0014911\_positive\_regulation\_of\_smooth\_muscle\_cell\_migration | 1 | 0 |  |  |  |  |  |  |  |  |
| GO:0015014\_heparan\_sulfate\_proteoglycan\_biosynthetic\_process\_\_polysaccharide\_chain\_biosynthetic\_process | 1 | 0 |  |  |  |  |  |  |  |  |
| GO:0015074\_DNA\_integration | 1 | 0 |  |  |  |  |  |  |  |  |
| GO:0015670\_carbon\_dioxide\_transport | 1 | 0 |  |  |  |  |  |  |  |  |
| GO:0015677\_copper\_ion\_import | 1 | 0 |  |  |  |  |  |  |  |  |
| GO:0015680\_intracellular\_copper\_ion\_transport | 1 | 0 |  |  |  |  |  |  |  |  |
| GO:0015684\_ferrous\_iron\_transport | 1 | 0 |  |  |  |  |  |  |  |  |
| GO:0015707\_nitrite\_transport | 1 | 0 |  |  |  |  |  |  |  |  |
| GO:0015724\_formate\_transport | 1 | 0 |  |  |  |  |  |  |  |  |
| GO:0015734\_taurine\_transport | 1 | 0 |  |  |  |  |  |  |  |  |
| GO:0015740\_C4-dicarboxylate\_transport | 1 | 0 |  |  |  |  |  |  |  |  |
| GO:0015744\_succinate\_transport | 1 | 0 |  |  |  |  |  |  |  |  |
| GO:0015746\_citrate\_transport | 1 | 0 |  |  |  |  |  |  |  |  |
| GO:0015747\_urate\_transport | 1 | 0 |  |  |  |  |  |  |  |  |
| GO:0015791\_polyol\_transport | 1 | 0 |  |  |  |  |  |  |  |  |
| GO:0015798\_myo-inositol\_transport | 1 | 0 |  |  |  |  |  |  |  |  |
| GO:0015808\_L-alanine\_transport | 1 | 0 |  |  |  |  |  |  |  |  |
| GO:0015810\_aspartate\_transport | 1 | 0 |  |  |  |  |  |  |  |  |
| GO:0015811\_L-cystine\_transport | 1 | 0 |  |  |  |  |  |  |  |  |
| GO:0015817\_histidine\_transport | 1 | 0 |  |  |  |  |  |  |  |  |
| GO:0015822\_ornithine\_transport | 1 | 0 |  |  |  |  |  |  |  |  |
| GO:0015824\_proline\_transport | 1 | 0 |  |  |  |  |  |  |  |  |
| GO:0015851\_nucleobase\_transport | 1 | 0 |  |  |  |  |  |  |  |  |
| GO:0015864\_pyrimidine\_nucleoside\_transport | 1 | 0 |  |  |  |  |  |  |  |  |
| GO:0015874\_norepinephrine\_transport | 1 | 0 |  |  |  |  |  |  |  |  |
| GO:0015881\_creatine\_transport | 1 | 0 |  |  |  |  |  |  |  |  |
| GO:0015884\_folic\_acid\_transport | 1 | 0 |  |  |  |  |  |  |  |  |
| GO:0015886\_heme\_transport | 1 | 0 |  |  |  |  |  |  |  |  |
| GO:0015888\_thiamin\_transport | 1 | 0 |  |  |  |  |  |  |  |  |
| GO:0015938\_coenzyme\_A\_catabolic\_process | 1 | 0 |  |  |  |  |  |  |  |  |
| GO:0015939\_pantothenate\_metabolic\_process | 1 | 0 |  |  |  |  |  |  |  |  |
| GO:0016073\_snRNA\_metabolic\_process | 1 | 0 |  |  |  |  |  |  |  |  |
| GO:0016082\_synaptic\_vesicle\_priming | 1 | 0 |  |  |  |  |  |  |  |  |
| GO:0016090\_prenol\_metabolic\_process | 1 | 0 |  |  |  |  |  |  |  |  |
| GO:0016093\_polyprenol\_metabolic\_process | 1 | 0 |  |  |  |  |  |  |  |  |
| GO:0016180\_snRNA\_processing | 1 | 0 |  |  |  |  |  |  |  |  |
| GO:0016239\_positive\_regulation\_of\_macroautophagy | 1 | 0 |  |  |  |  |  |  |  |  |
| GO:0016246\_RNA\_interference | 1 | 0 |  |  |  |  |  |  |  |  |
| GO:0016255\_attachment\_of\_GPI\_anchor\_to\_protein | 1 | 0 |  |  |  |  |  |  |  |  |
| GO:0016333\_morphogenesis\_of\_follicular\_epithelium | 1 | 0 |  |  |  |  |  |  |  |  |
| GO:0016340\_calcium-dependent\_cell-matrix\_adhesion | 1 | 0 |  |  |  |  |  |  |  |  |
| GO:0016344\_meiotic\_chromosome\_movement\_towards\_spindle\_pole | 1 | 0 |  |  |  |  |  |  |  |  |
| GO:0016482\_cytoplasmic\_transport | 1 | 0 |  |  |  |  |  |  |  |  |
| GO:0016553\_base\_conversion\_or\_substitution\_editing | 1 | 0 |  |  |  |  |  |  |  |  |
| GO:0016554\_cytidine\_to\_uridine\_editing | 1 | 0 |  |  |  |  |  |  |  |  |
| GO:0016560\_protein\_import\_into\_peroxisome\_matrix\_\_docking | 1 | 0 |  |  |  |  |  |  |  |  |
| GO:0016578\_histone\_deubiquitination | 1 | 0 |  |  |  |  |  |  |  |  |
| GO:0016598\_protein\_arginylation | 1 | 0 |  |  |  |  |  |  |  |  |
| GO:0017004\_cytochrome\_complex\_assembly | 1 | 0 |  |  |  |  |  |  |  |  |
| GO:0018022\_peptidyl-lysine\_methylation | 1 | 0 |  |  |  |  |  |  |  |  |
| GO:0018023\_peptidyl-lysine\_trimethylation | 1 | 0 |  |  |  |  |  |  |  |  |
| GO:0018120\_peptidyl-arginine\_ADP-ribosylation | 1 | 0 |  |  |  |  |  |  |  |  |
| GO:0018126\_protein\_amino\_acid\_hydroxylation | 1 | 0 |  |  |  |  |  |  |  |  |
| GO:0018146\_keratan\_sulfate\_biosynthetic\_process | 1 | 0 |  |  |  |  |  |  |  |  |
| GO:0018158\_protein\_amino\_acid\_oxidation | 1 | 0 |  |  |  |  |  |  |  |  |
| GO:0018195\_peptidyl-arginine\_modification | 1 | 0 |  |  |  |  |  |  |  |  |
| GO:0018197\_peptidyl-aspartic\_acid\_modification | 1 | 0 |  |  |  |  |  |  |  |  |
| GO:0018282\_metal\_incorporation\_into\_metallo-sulfur\_cluster | 1 | 0 |  |  |  |  |  |  |  |  |
| GO:0018283\_iron\_incorporation\_into\_metallo-sulfur\_cluster | 1 | 0 |  |  |  |  |  |  |  |  |
| GO:0018318\_protein\_amino\_acid\_palmitoylation | 1 | 0 |  |  |  |  |  |  |  |  |
| GO:0018342\_protein\_prenylation | 1 | 0 |  |  |  |  |  |  |  |  |
| GO:0018344\_protein\_geranylgeranylation | 1 | 0 |  |  |  |  |  |  |  |  |
| GO:0018410\_peptide\_or\_protein\_carboxyl-terminal\_blocking | 1 | 0 |  |  |  |  |  |  |  |  |
| GO:0018916\_nitrobenzene\_metabolic\_process | 1 | 0 |  |  |  |  |  |  |  |  |
| GO:0018931\_naphthalene\_metabolic\_process | 1 | 0 |  |  |  |  |  |  |  |  |
| GO:0018992\_germ-line\_sex\_determination | 1 | 0 |  |  |  |  |  |  |  |  |
| GO:0019042\_latent\_virus\_infection | 1 | 0 |  |  |  |  |  |  |  |  |
| GO:0019046\_reactivation\_of\_latent\_virus | 1 | 0 |  |  |  |  |  |  |  |  |
| GO:0019047\_provirus\_integration | 1 | 0 |  |  |  |  |  |  |  |  |
| GO:0019076\_release\_of\_virus\_from\_host | 1 | 0 |  |  |  |  |  |  |  |  |
| GO:0019079\_viral\_genome\_replication | 1 | 0 |  |  |  |  |  |  |  |  |
| GO:0019100\_male\_germ-line\_sex\_determination | 1 | 0 |  |  |  |  |  |  |  |  |
| GO:0019101\_female\_somatic\_sex\_determination | 1 | 0 |  |  |  |  |  |  |  |  |
| GO:0019102\_male\_somatic\_sex\_determination | 1 | 0 |  |  |  |  |  |  |  |  |
| GO:0019255\_glucose\_1-phosphate\_metabolic\_process | 1 | 0 |  |  |  |  |  |  |  |  |
| GO:0019276\_UDP-N-acetylgalactosamine\_metabolic\_process | 1 | 0 |  |  |  |  |  |  |  |  |
| GO:0019344\_cysteine\_biosynthetic\_process | 1 | 0 |  |  |  |  |  |  |  |  |
| GO:0019348\_dolichol\_metabolic\_process | 1 | 0 |  |  |  |  |  |  |  |  |
| GO:0019375\_galactolipid\_biosynthetic\_process | 1 | 0 |  |  |  |  |  |  |  |  |
| GO:0019402\_galactitol\_metabolic\_process | 1 | 0 |  |  |  |  |  |  |  |  |
| GO:0019441\_tryptophan\_catabolic\_process\_to\_kynurenine | 1 | 0 |  |  |  |  |  |  |  |  |
| GO:0019477\_L-lysine\_catabolic\_process | 1 | 0 |  |  |  |  |  |  |  |  |
| GO:0019510\_S-adenosylhomocysteine\_catabolic\_process | 1 | 0 |  |  |  |  |  |  |  |  |
| GO:0019532\_oxalate\_transport | 1 | 0 |  |  |  |  |  |  |  |  |
| GO:0019626\_short-chain\_fatty\_acid\_catabolic\_process | 1 | 0 |  |  |  |  |  |  |  |  |
| GO:0019627\_urea\_metabolic\_process | 1 | 0 |  |  |  |  |  |  |  |  |
| GO:0019676\_ammonia\_assimilation\_cycle | 1 | 0 |  |  |  |  |  |  |  |  |
| GO:0019682\_glyceraldehyde-3-phosphate\_metabolic\_process | 1 | 0 |  |  |  |  |  |  |  |  |
| GO:0019695\_choline\_metabolic\_process | 1 | 0 |  |  |  |  |  |  |  |  |
| GO:0019731\_antibacterial\_humoral\_response | 1 | 0 |  |  |  |  |  |  |  |  |
| GO:0019794\_nonprotein\_amino\_acid\_metabolic\_process | 1 | 0 |  |  |  |  |  |  |  |  |
| GO:0019858\_cytosine\_metabolic\_process | 1 | 0 |  |  |  |  |  |  |  |  |
| GO:0019883\_antigen\_processing\_and\_presentation\_of\_endogenous\_antigen | 1 | 0 |  |  |  |  |  |  |  |  |
| GO:0019889\_pteridine\_metabolic\_process | 1 | 0 |  |  |  |  |  |  |  |  |
| GO:0019896\_axon\_transport\_of\_mitochondrion | 1 | 0 |  |  |  |  |  |  |  |  |
| GO:0021508\_floor\_plate\_formation | 1 | 0 |  |  |  |  |  |  |  |  |
| GO:0021528\_commissural\_neuron\_differentiation\_in\_the\_spinal\_cord | 1 | 0 |  |  |  |  |  |  |  |  |
| GO:0021572\_rhombomere\_6\_development | 1 | 0 |  |  |  |  |  |  |  |  |
| GO:0021577\_hindbrain\_structural\_organization | 1 | 0 |  |  |  |  |  |  |  |  |
| GO:0021586\_pons\_maturation | 1 | 0 |  |  |  |  |  |  |  |  |
| GO:0021589\_cerebellum\_structural\_organization | 1 | 0 |  |  |  |  |  |  |  |  |
| GO:0021590\_cerebellum\_maturation | 1 | 0 |  |  |  |  |  |  |  |  |
| GO:0021592\_fourth\_ventricle\_development | 1 | 0 |  |  |  |  |  |  |  |  |
| GO:0021594\_rhombomere\_formation | 1 | 0 |  |  |  |  |  |  |  |  |
| GO:0021660\_rhombomere\_3\_formation | 1 | 0 |  |  |  |  |  |  |  |  |
| GO:0021664\_rhombomere\_5\_morphogenesis | 1 | 0 |  |  |  |  |  |  |  |  |
| GO:0021666\_rhombomere\_5\_formation | 1 | 0 |  |  |  |  |  |  |  |  |
| GO:0021670\_lateral\_ventricle\_development | 1 | 0 |  |  |  |  |  |  |  |  |
| GO:0021678\_third\_ventricle\_development | 1 | 0 |  |  |  |  |  |  |  |  |
| GO:0021679\_cerebellar\_molecular\_layer\_development | 1 | 0 |  |  |  |  |  |  |  |  |
| GO:0021703\_locus\_ceruleus\_development | 1 | 0 |  |  |  |  |  |  |  |  |
| GO:0021732\_midbrain-hindbrain\_boundary\_maturation | 1 | 0 |  |  |  |  |  |  |  |  |
| GO:0021747\_cochlear\_nucleus\_development | 1 | 0 |  |  |  |  |  |  |  |  |
| GO:0021750\_vestibular\_nucleus\_development | 1 | 0 |  |  |  |  |  |  |  |  |
| GO:0021759\_globus\_pallidus\_development | 1 | 0 |  |  |  |  |  |  |  |  |
| GO:0021768\_nucleus\_accumbens\_development | 1 | 0 |  |  |  |  |  |  |  |  |
| GO:0021771\_lateral\_geniculate\_nucleus\_development | 1 | 0 |  |  |  |  |  |  |  |  |
| GO:0021812\_neuronal-glial\_interaction\_involved\_in\_cerebral\_cortex\_radial\_glia\_guided\_migration | 1 | 0 |  |  |  |  |  |  |  |  |
| GO:0021813\_cell-cell\_adhesion\_involved\_in\_neuronal-glial\_interactions\_involved\_in\_cerebral\_cortex\_radial\_glia\_guided\_migration | 1 | 0 |  |  |  |  |  |  |  |  |
| GO:0021870\_Cajal-Retzius\_cell\_differentiation | 1 | 0 |  |  |  |  |  |  |  |  |
| GO:0021874\_Wnt\_receptor\_signaling\_pathway\_in\_forebrain\_neuroblast\_division | 1 | 0 |  |  |  |  |  |  |  |  |
| GO:0021896\_forebrain\_astrocyte\_differentiation | 1 | 0 |  |  |  |  |  |  |  |  |
| GO:0021897\_forebrain\_astrocyte\_development | 1 | 0 |  |  |  |  |  |  |  |  |
| GO:0021902\_commitment\_of\_a\_neuronal\_cell\_to\_a\_specific\_type\_of\_neuron\_in\_the\_forebrain | 1 | 0 |  |  |  |  |  |  |  |  |
| GO:0021905\_forebrain-midbrain\_boundary\_formation | 1 | 0 |  |  |  |  |  |  |  |  |
| GO:0021914\_negative\_regulation\_of\_smoothened\_signaling\_pathway\_involved\_in\_ventral\_spinal\_cord\_patterning | 1 | 0 |  |  |  |  |  |  |  |  |
| GO:0021917\_somatic\_motor\_neuron\_fate\_commitment | 1 | 0 |  |  |  |  |  |  |  |  |
| GO:0021918\_regulation\_of\_transcription\_from\_RNA\_polymerase\_II\_promoter\_involved\_in\_somatic\_motor\_neuron\_fate\_commitment | 1 | 0 |  |  |  |  |  |  |  |  |
| GO:0021933\_radial\_glia\_guided\_migration\_of\_granule\_cell | 1 | 0 |  |  |  |  |  |  |  |  |
| GO:0021934\_hindbrain\_tangential\_cell\_migration | 1 | 0 |  |  |  |  |  |  |  |  |
| GO:0021935\_granule\_cell\_precursor\_tangential\_migration | 1 | 0 |  |  |  |  |  |  |  |  |
| GO:0021942\_radial\_glia\_guided\_migration\_of\_Purkinje\_cell | 1 | 0 |  |  |  |  |  |  |  |  |
| GO:0021960\_anterior\_commissure\_morphogenesis | 1 | 0 |  |  |  |  |  |  |  |  |
| GO:0021997\_neural\_plate\_axis\_specification | 1 | 0 |  |  |  |  |  |  |  |  |
| GO:0021999\_neural\_plate\_anterior\_posterior\_pattern\_formation | 1 | 0 |  |  |  |  |  |  |  |  |
| GO:0022004\_midbrain-hindbrain\_boundary\_maturation\_during\_brain\_development | 1 | 0 |  |  |  |  |  |  |  |  |
| GO:0022038\_corpus\_callosum\_development | 1 | 0 |  |  |  |  |  |  |  |  |
| GO:0022605\_oogenesis\_stage | 1 | 0 |  |  |  |  |  |  |  |  |
| GO:0030011\_maintenance\_of\_cell\_polarity | 1 | 0 |  |  |  |  |  |  |  |  |
| GO:0030069\_lysogeny | 1 | 0 |  |  |  |  |  |  |  |  |
| GO:0030070\_insulin\_processing | 1 | 0 |  |  |  |  |  |  |  |  |
| GO:0030092\_regulation\_of\_flagellum\_assembly | 1 | 0 |  |  |  |  |  |  |  |  |
| GO:0030103\_vasopressin\_secretion | 1 | 0 |  |  |  |  |  |  |  |  |
| GO:0030194\_positive\_regulation\_of\_blood\_coagulation | 1 | 0 |  |  |  |  |  |  |  |  |
| GO:0030206\_chondroitin\_sulfate\_biosynthetic\_process | 1 | 0 |  |  |  |  |  |  |  |  |
| GO:0030210\_heparin\_biosynthetic\_process | 1 | 0 |  |  |  |  |  |  |  |  |
| GO:0030220\_platelet\_formation | 1 | 0 |  |  |  |  |  |  |  |  |
| GO:0030222\_eosinophil\_differentiation | 1 | 0 |  |  |  |  |  |  |  |  |
| GO:0030237\_female\_sex\_determination | 1 | 0 |  |  |  |  |  |  |  |  |
| GO:0030264\_nuclear\_fragmentation\_during\_apoptosis | 1 | 0 |  |  |  |  |  |  |  |  |
| GO:0030322\_stabilization\_of\_membrane\_potential | 1 | 0 |  |  |  |  |  |  |  |  |
| GO:0030327\_prenylated\_protein\_catabolic\_process | 1 | 0 |  |  |  |  |  |  |  |  |
| GO:0030328\_prenylcysteine\_catabolic\_process | 1 | 0 |  |  |  |  |  |  |  |  |
| GO:0030329\_prenylcysteine\_metabolic\_process | 1 | 0 |  |  |  |  |  |  |  |  |
| GO:0030382\_sperm\_mitochondrion\_organization | 1 | 0 |  |  |  |  |  |  |  |  |
| GO:0030389\_fructosamine\_metabolic\_process | 1 | 0 |  |  |  |  |  |  |  |  |
| GO:0030422\_RNA\_interference\_\_production\_of\_siRNA | 1 | 0 |  |  |  |  |  |  |  |  |
| GO:0030449\_regulation\_of\_complement\_activation | 1 | 0 |  |  |  |  |  |  |  |  |
| GO:0030497\_fatty\_acid\_elongation | 1 | 0 |  |  |  |  |  |  |  |  |
| GO:0030575\_nuclear\_body\_organization | 1 | 0 |  |  |  |  |  |  |  |  |
| GO:0030578\_PML\_body\_organization | 1 | 0 |  |  |  |  |  |  |  |  |
| GO:0030853\_negative\_regulation\_of\_granulocyte\_differentiation | 1 | 0 |  |  |  |  |  |  |  |  |
| GO:0030854\_positive\_regulation\_of\_granulocyte\_differentiation | 1 | 0 |  |  |  |  |  |  |  |  |
| GO:0030886\_negative\_regulation\_of\_myeloid\_dendritic\_cell\_activation | 1 | 0 |  |  |  |  |  |  |  |  |
| GO:0030913\_paranodal\_junction\_assembly | 1 | 0 |  |  |  |  |  |  |  |  |
| GO:0031033\_myosin\_filament\_assembly\_or\_disassembly | 1 | 0 |  |  |  |  |  |  |  |  |
| GO:0031034\_myosin\_filament\_assembly | 1 | 0 |  |  |  |  |  |  |  |  |
| GO:0031055\_chromatin\_remodeling\_at\_centromere | 1 | 0 |  |  |  |  |  |  |  |  |
| GO:0031062\_positive\_regulation\_of\_histone\_methylation | 1 | 0 |  |  |  |  |  |  |  |  |
| GO:0031115\_negative\_regulation\_of\_microtubule\_polymerization | 1 | 0 |  |  |  |  |  |  |  |  |
| GO:0031129\_inductive\_cell-cell\_signaling | 1 | 0 |  |  |  |  |  |  |  |  |
| GO:0031284\_positive\_regulation\_of\_guanylate\_cyclase\_activity | 1 | 0 |  |  |  |  |  |  |  |  |
| GO:0031498\_chromatin\_disassembly | 1 | 0 |  |  |  |  |  |  |  |  |
| GO:0031507\_heterochromatin\_formation | 1 | 0 |  |  |  |  |  |  |  |  |
| GO:0031508\_centromeric\_heterochromatin\_formation | 1 | 0 |  |  |  |  |  |  |  |  |
| GO:0031529\_ruffle\_organization | 1 | 0 |  |  |  |  |  |  |  |  |
| GO:0031536\_positive\_regulation\_of\_exit\_from\_mitosis | 1 | 0 |  |  |  |  |  |  |  |  |
| GO:0031572\_G2\_M\_transition\_DNA\_damage\_checkpoint | 1 | 0 |  |  |  |  |  |  |  |  |
| GO:0031576\_G2\_M\_transition\_checkpoint | 1 | 0 |  |  |  |  |  |  |  |  |
| GO:0031580\_membrane\_raft\_distribution | 1 | 0 |  |  |  |  |  |  |  |  |
| GO:0031583\_activation\_of\_phospholipase\_D\_activity\_by\_G-protein\_coupled\_receptor\_protein\_signaling\_pathway | 1 | 0 |  |  |  |  |  |  |  |  |
| GO:0031584\_activation\_of\_phospholipase\_D\_activity | 1 | 0 |  |  |  |  |  |  |  |  |
| GO:0031585\_regulation\_of\_inositol-1\_4\_5-triphosphate\_receptor\_activity | 1 | 0 |  |  |  |  |  |  |  |  |
| GO:0031639\_plasminogen\_activation | 1 | 0 |  |  |  |  |  |  |  |  |
| GO:0031648\_protein\_destabilization | 1 | 0 |  |  |  |  |  |  |  |  |
| GO:0031665\_negative\_regulation\_of\_lipopolysaccharide-mediated\_signaling\_pathway | 1 | 0 |  |  |  |  |  |  |  |  |
| GO:0031914\_negative\_regulation\_of\_synaptic\_plasticity | 1 | 0 |  |  |  |  |  |  |  |  |
| GO:0031944\_negative\_regulation\_of\_glucocorticoid\_metabolic\_process | 1 | 0 |  |  |  |  |  |  |  |  |
| GO:0031947\_negative\_regulation\_of\_glucocorticoid\_biosynthetic\_process | 1 | 0 |  |  |  |  |  |  |  |  |
| GO:0032025\_response\_to\_cobalt\_ion | 1 | 0 |  |  |  |  |  |  |  |  |
| GO:0032026\_response\_to\_magnesium\_ion | 1 | 0 |  |  |  |  |  |  |  |  |
| GO:0032048\_cardiolipin\_metabolic\_process | 1 | 0 |  |  |  |  |  |  |  |  |
| GO:0032066\_nucleolus\_to\_nucleoplasm\_transport | 1 | 0 |  |  |  |  |  |  |  |  |
| GO:0032091\_negative\_regulation\_of\_protein\_binding | 1 | 0 |  |  |  |  |  |  |  |  |
| GO:0032092\_positive\_regulation\_of\_protein\_binding | 1 | 0 |  |  |  |  |  |  |  |  |
| GO:0032097\_positive\_regulation\_of\_response\_to\_food | 1 | 0 |  |  |  |  |  |  |  |  |
| GO:0032100\_positive\_regulation\_of\_appetite | 1 | 0 |  |  |  |  |  |  |  |  |
| GO:0032204\_regulation\_of\_telomere\_maintenance | 1 | 0 |  |  |  |  |  |  |  |  |
| GO:0032206\_positive\_regulation\_of\_telomere\_maintenance | 1 | 0 |  |  |  |  |  |  |  |  |
| GO:0032222\_regulation\_of\_synaptic\_transmission\_\_cholinergic | 1 | 0 |  |  |  |  |  |  |  |  |
| GO:0032224\_positive\_regulation\_of\_synaptic\_transmission\_\_cholinergic | 1 | 0 |  |  |  |  |  |  |  |  |
| GO:0032229\_negative\_regulation\_of\_synaptic\_transmission\_\_GABAergic | 1 | 0 |  |  |  |  |  |  |  |  |
| GO:0032237\_activation\_of\_store-operated\_calcium\_channel\_activity | 1 | 0 |  |  |  |  |  |  |  |  |
| GO:0032239\_regulation\_of\_nucleobase\_\_nucleoside\_\_nucleotide\_and\_nucleic\_acid\_transport | 1 | 0 |  |  |  |  |  |  |  |  |
| GO:0032252\_secretory\_granule\_localization | 1 | 0 |  |  |  |  |  |  |  |  |
| GO:0032274\_gonadotropin\_secretion | 1 | 0 |  |  |  |  |  |  |  |  |
| GO:0032275\_luteinizing\_hormone\_secretion | 1 | 0 |  |  |  |  |  |  |  |  |
| GO:0032287\_myelin\_maintenance\_in\_the\_peripheral\_nervous\_system | 1 | 0 |  |  |  |  |  |  |  |  |
| GO:0032289\_myelin\_formation\_in\_the\_central\_nervous\_system | 1 | 0 |  |  |  |  |  |  |  |  |
| GO:0032303\_regulation\_of\_icosanoid\_secretion | 1 | 0 |  |  |  |  |  |  |  |  |
| GO:0032305\_positive\_regulation\_of\_icosanoid\_secretion | 1 | 0 |  |  |  |  |  |  |  |  |
| GO:0032306\_regulation\_of\_prostaglandin\_secretion | 1 | 0 |  |  |  |  |  |  |  |  |
| GO:0032308\_positive\_regulation\_of\_prostaglandin\_secretion | 1 | 0 |  |  |  |  |  |  |  |  |
| GO:0032310\_prostaglandin\_secretion | 1 | 0 |  |  |  |  |  |  |  |  |
| GO:0032313\_regulation\_of\_Rab\_GTPase\_activity | 1 | 0 |  |  |  |  |  |  |  |  |
| GO:0032314\_regulation\_of\_Rac\_GTPase\_activity | 1 | 0 |  |  |  |  |  |  |  |  |
| GO:0032317\_regulation\_of\_Rap\_GTPase\_activity | 1 | 0 |  |  |  |  |  |  |  |  |
| GO:0032324\_molybdopterin\_cofactor\_biosynthetic\_process | 1 | 0 |  |  |  |  |  |  |  |  |
| GO:0032329\_serine\_transport | 1 | 0 |  |  |  |  |  |  |  |  |
| GO:0032342\_aldosterone\_biosynthetic\_process | 1 | 0 |  |  |  |  |  |  |  |  |
| GO:0032344\_regulation\_of\_aldosterone\_metabolic\_process | 1 | 0 |  |  |  |  |  |  |  |  |
| GO:0032365\_intracellular\_lipid\_transport | 1 | 0 |  |  |  |  |  |  |  |  |
| GO:0032366\_intracellular\_sterol\_transport | 1 | 0 |  |  |  |  |  |  |  |  |
| GO:0032367\_intracellular\_cholesterol\_transport | 1 | 0 |  |  |  |  |  |  |  |  |
| GO:0032370\_positive\_regulation\_of\_lipid\_transport | 1 | 0 |  |  |  |  |  |  |  |  |
| GO:0032410\_negative\_regulation\_of\_transporter\_activity | 1 | 0 |  |  |  |  |  |  |  |  |
| GO:0032413\_negative\_regulation\_of\_ion\_transmembrane\_transporter\_activity | 1 | 0 |  |  |  |  |  |  |  |  |
| GO:0032429\_regulation\_of\_phospholipase\_A2\_activity | 1 | 0 |  |  |  |  |  |  |  |  |
| GO:0032474\_otolith\_morphogenesis | 1 | 0 |  |  |  |  |  |  |  |  |
| GO:0032482\_Rab\_protein\_signal\_transduction | 1 | 0 |  |  |  |  |  |  |  |  |
| GO:0032483\_regulation\_of\_Rab\_protein\_signal\_transduction | 1 | 0 |  |  |  |  |  |  |  |  |
| GO:0032486\_Rap\_protein\_signal\_transduction | 1 | 0 |  |  |  |  |  |  |  |  |
| GO:0032487\_regulation\_of\_Rap\_protein\_signal\_transduction | 1 | 0 |  |  |  |  |  |  |  |  |
| GO:0032594\_protein\_transport\_within\_lipid\_bilayer | 1 | 0 |  |  |  |  |  |  |  |  |
| GO:0032599\_protein\_transport\_out\_of\_membrane\_raft | 1 | 0 |  |  |  |  |  |  |  |  |
| GO:0032600\_chemokine\_receptor\_transport\_out\_of\_membrane\_raft | 1 | 0 |  |  |  |  |  |  |  |  |
| GO:0032607\_interferon-alpha\_production | 1 | 0 |  |  |  |  |  |  |  |  |
| GO:0032621\_interleukin-18\_production | 1 | 0 |  |  |  |  |  |  |  |  |
| GO:0032647\_regulation\_of\_interferon-alpha\_production | 1 | 0 |  |  |  |  |  |  |  |  |
| GO:0032656\_regulation\_of\_interleukin-13\_production | 1 | 0 |  |  |  |  |  |  |  |  |
| GO:0032682\_negative\_regulation\_of\_chemokine\_production | 1 | 0 |  |  |  |  |  |  |  |  |
| GO:0032691\_negative\_regulation\_of\_interleukin-1\_beta\_production | 1 | 0 |  |  |  |  |  |  |  |  |
| GO:0032692\_negative\_regulation\_of\_interleukin-1\_production | 1 | 0 |  |  |  |  |  |  |  |  |
| GO:0032693\_negative\_regulation\_of\_interleukin-10\_production | 1 | 0 |  |  |  |  |  |  |  |  |
| GO:0032696\_negative\_regulation\_of\_interleukin-13\_production | 1 | 0 |  |  |  |  |  |  |  |  |
| GO:0032727\_positive\_regulation\_of\_interferon-alpha\_production | 1 | 0 |  |  |  |  |  |  |  |  |
| GO:0032731\_positive\_regulation\_of\_interleukin-1\_beta\_production | 1 | 0 |  |  |  |  |  |  |  |  |
| GO:0032732\_positive\_regulation\_of\_interleukin-1\_production | 1 | 0 |  |  |  |  |  |  |  |  |
| GO:0032735\_positive\_regulation\_of\_interleukin-12\_production | 1 | 0 |  |  |  |  |  |  |  |  |
| GO:0032764\_negative\_regulation\_of\_mast\_cell\_cytokine\_production | 1 | 0 |  |  |  |  |  |  |  |  |
| GO:0032765\_positive\_regulation\_of\_mast\_cell\_cytokine\_production | 1 | 0 |  |  |  |  |  |  |  |  |
| GO:0032769\_negative\_regulation\_of\_monooxygenase\_activity | 1 | 0 |  |  |  |  |  |  |  |  |
| GO:0032781\_positive\_regulation\_of\_ATPase\_activity | 1 | 0 |  |  |  |  |  |  |  |  |
| GO:0032790\_ribosome\_disassembly | 1 | 0 |  |  |  |  |  |  |  |  |
| GO:0032799\_low-density\_lipoprotein\_receptor\_metabolic\_process | 1 | 0 |  |  |  |  |  |  |  |  |
| GO:0032802\_low-density\_lipoprotein\_receptor\_catabolic\_process | 1 | 0 |  |  |  |  |  |  |  |  |
| GO:0032803\_regulation\_of\_low-density\_lipoprotein\_receptor\_catabolic\_process | 1 | 0 |  |  |  |  |  |  |  |  |
| GO:0032817\_regulation\_of\_natural\_killer\_cell\_proliferation | 1 | 0 |  |  |  |  |  |  |  |  |
| GO:0032819\_positive\_regulation\_of\_natural\_killer\_cell\_proliferation | 1 | 0 |  |  |  |  |  |  |  |  |
| GO:0032836\_glomerular\_basement\_membrane\_development | 1 | 0 |  |  |  |  |  |  |  |  |
| GO:0032855\_positive\_regulation\_of\_Rac\_GTPase\_activity | 1 | 0 |  |  |  |  |  |  |  |  |
| GO:0032863\_activation\_of\_Rac\_GTPase\_activity | 1 | 0 |  |  |  |  |  |  |  |  |
| GO:0032864\_activation\_of\_Cdc42\_GTPase\_activity | 1 | 0 |  |  |  |  |  |  |  |  |
| GO:0032885\_regulation\_of\_polysaccharide\_biosynthetic\_process | 1 | 0 |  |  |  |  |  |  |  |  |
| GO:0032907\_transforming\_growth\_factor-beta3\_production | 1 | 0 |  |  |  |  |  |  |  |  |
| GO:0032910\_regulation\_of\_transforming\_growth\_factor-beta3\_production | 1 | 0 |  |  |  |  |  |  |  |  |
| GO:0032913\_negative\_regulation\_of\_transforming\_growth\_factor-beta3\_production | 1 | 0 |  |  |  |  |  |  |  |  |
| GO:0032924\_activin\_receptor\_signaling\_pathway | 1 | 0 |  |  |  |  |  |  |  |  |
| GO:0032925\_regulation\_of\_activin\_receptor\_signaling\_pathway | 1 | 0 |  |  |  |  |  |  |  |  |
| GO:0032960\_regulation\_of\_inositol\_trisphosphate\_biosynthetic\_process | 1 | 0 |  |  |  |  |  |  |  |  |
| GO:0032962\_positive\_regulation\_of\_inositol\_trisphosphate\_biosynthetic\_process | 1 | 0 |  |  |  |  |  |  |  |  |
| GO:0032964\_collagen\_biosynthetic\_process | 1 | 0 |  |  |  |  |  |  |  |  |
| GO:0032971\_regulation\_of\_muscle\_filament\_sliding | 1 | 0 |  |  |  |  |  |  |  |  |
| GO:0032972\_regulation\_of\_muscle\_filament\_sliding\_speed | 1 | 0 |  |  |  |  |  |  |  |  |
| GO:0032986\_protein-DNA\_complex\_disassembly | 1 | 0 |  |  |  |  |  |  |  |  |
| GO:0032988\_ribonucleoprotein\_complex\_disassembly | 1 | 0 |  |  |  |  |  |  |  |  |
| GO:0033037\_polysaccharide\_localization | 1 | 0 |  |  |  |  |  |  |  |  |
| GO:0033078\_extrathymic\_T\_cell\_differentiation | 1 | 0 |  |  |  |  |  |  |  |  |
| GO:0033085\_negative\_regulation\_of\_T\_cell\_differentiation\_in\_the\_thymus | 1 | 0 |  |  |  |  |  |  |  |  |
| GO:0033087\_negative\_regulation\_of\_immature\_T\_cell\_proliferation | 1 | 0 |  |  |  |  |  |  |  |  |
| GO:0033088\_negative\_regulation\_of\_immature\_T\_cell\_proliferation\_in\_the\_thymus | 1 | 0 |  |  |  |  |  |  |  |  |
| GO:0033108\_mitochondrial\_respiratory\_chain\_complex\_assembly | 1 | 0 |  |  |  |  |  |  |  |  |
| GO:0033127\_regulation\_of\_histone\_phosphorylation | 1 | 0 |  |  |  |  |  |  |  |  |
| GO:0033128\_negative\_regulation\_of\_histone\_phosphorylation | 1 | 0 |  |  |  |  |  |  |  |  |
| GO:0033138\_positive\_regulation\_of\_peptidyl-serine\_phosphorylation | 1 | 0 |  |  |  |  |  |  |  |  |
| GO:0033158\_regulation\_of\_protein\_import\_into\_nucleus\_\_translocation | 1 | 0 |  |  |  |  |  |  |  |  |
| GO:0033160\_positive\_regulation\_of\_protein\_import\_into\_nucleus\_\_translocation | 1 | 0 |  |  |  |  |  |  |  |  |
| GO:0033169\_histone\_H3-K9\_demethylation | 1 | 0 |  |  |  |  |  |  |  |  |
| GO:0033206\_cytokinesis\_after\_meiosis | 1 | 0 |  |  |  |  |  |  |  |  |
| GO:0033240\_positive\_regulation\_of\_cellular\_amine\_metabolic\_process | 1 | 0 |  |  |  |  |  |  |  |  |
| GO:0033313\_meiotic\_cell\_cycle\_checkpoint | 1 | 0 |  |  |  |  |  |  |  |  |
| GO:0033315\_meiotic\_cell\_cycle\_DNA\_replication\_checkpoint | 1 | 0 |  |  |  |  |  |  |  |  |
| GO:0033326\_cerebrospinal\_fluid\_secretion | 1 | 0 |  |  |  |  |  |  |  |  |
| GO:0033366\_protein\_localization\_in\_secretory\_granule | 1 | 0 |  |  |  |  |  |  |  |  |
| GO:0033367\_protein\_localization\_in\_mast\_cell\_secretory\_granule | 1 | 0 |  |  |  |  |  |  |  |  |
| GO:0033368\_protease\_localization\_in\_mast\_cell\_secretory\_granule | 1 | 0 |  |  |  |  |  |  |  |  |
| GO:0033370\_maintenance\_of\_protein\_location\_in\_mast\_cell\_secretory\_granule | 1 | 0 |  |  |  |  |  |  |  |  |
| GO:0033371\_T\_cell\_secretory\_granule\_organization | 1 | 0 |  |  |  |  |  |  |  |  |
| GO:0033373\_maintenance\_of\_protease\_location\_in\_mast\_cell\_secretory\_granule | 1 | 0 |  |  |  |  |  |  |  |  |
| GO:0033374\_protein\_localization\_in\_T\_cell\_secretory\_granule | 1 | 0 |  |  |  |  |  |  |  |  |
| GO:0033375\_protease\_localization\_in\_T\_cell\_secretory\_granule | 1 | 0 |  |  |  |  |  |  |  |  |
| GO:0033377\_maintenance\_of\_protein\_location\_in\_T\_cell\_secretory\_granule | 1 | 0 |  |  |  |  |  |  |  |  |
| GO:0033379\_maintenance\_of\_protease\_location\_in\_T\_cell\_secretory\_granule | 1 | 0 |  |  |  |  |  |  |  |  |
| GO:0033380\_granzyme\_B\_localization\_in\_T\_cell\_secretory\_granule | 1 | 0 |  |  |  |  |  |  |  |  |
| GO:0033382\_maintenance\_of\_granzyme\_B\_location\_in\_T\_cell\_secretory\_granule | 1 | 0 |  |  |  |  |  |  |  |  |
| GO:0033483\_gas\_homeostasis | 1 | 0 |  |  |  |  |  |  |  |  |
| GO:0033484\_nitric\_oxide\_homeostasis | 1 | 0 |  |  |  |  |  |  |  |  |
| GO:0033505\_floor\_plate\_morphogenesis | 1 | 0 |  |  |  |  |  |  |  |  |
| GO:0033522\_histone\_H2A\_ubiquitination | 1 | 0 |  |  |  |  |  |  |  |  |
| GO:0033523\_histone\_H2B\_ubiquitination | 1 | 0 |  |  |  |  |  |  |  |  |
| GO:0033574\_response\_to\_testosterone\_stimulus | 1 | 0 |  |  |  |  |  |  |  |  |
| GO:0033606\_chemokine\_receptor\_transport\_within\_lipid\_bilayer | 1 | 0 |  |  |  |  |  |  |  |  |
| GO:0033628\_regulation\_of\_cell\_adhesion\_mediated\_by\_integrin | 1 | 0 |  |  |  |  |  |  |  |  |
| GO:0033630\_positive\_regulation\_of\_cell\_adhesion\_mediated\_by\_integrin | 1 | 0 |  |  |  |  |  |  |  |  |
| GO:0033632\_regulation\_of\_cell-cell\_adhesion\_mediated\_by\_integrin | 1 | 0 |  |  |  |  |  |  |  |  |
| GO:0033634\_positive\_regulation\_of\_cell-cell\_adhesion\_mediated\_by\_integrin | 1 | 0 |  |  |  |  |  |  |  |  |
| GO:0033683\_nucleotide-excision\_repair\_\_DNA\_incision | 1 | 0 |  |  |  |  |  |  |  |  |
| GO:0033687\_osteoblast\_proliferation | 1 | 0 |  |  |  |  |  |  |  |  |
| GO:0033688\_regulation\_of\_osteoblast\_proliferation | 1 | 0 |  |  |  |  |  |  |  |  |
| GO:0033689\_negative\_regulation\_of\_osteoblast\_proliferation | 1 | 0 |  |  |  |  |  |  |  |  |
| GO:0033750\_ribosome\_localization | 1 | 0 |  |  |  |  |  |  |  |  |
| GO:0033753\_establishment\_of\_ribosome\_localization | 1 | 0 |  |  |  |  |  |  |  |  |
| GO:0033866\_nucleoside\_bisphosphate\_biosynthetic\_process | 1 | 0 |  |  |  |  |  |  |  |  |
| GO:0033875\_ribonucleoside\_bisphosphate\_metabolic\_process | 1 | 0 |  |  |  |  |  |  |  |  |
| GO:0034030\_ribonucleoside\_bisphosphate\_biosynthetic\_process | 1 | 0 |  |  |  |  |  |  |  |  |
| GO:0034032\_purine\_nucleoside\_bisphosphate\_metabolic\_process | 1 | 0 |  |  |  |  |  |  |  |  |
| GO:0034033\_purine\_nucleoside\_bisphosphate\_biosynthetic\_process | 1 | 0 |  |  |  |  |  |  |  |  |
| GO:0034035\_purine\_ribonucleoside\_bisphosphate\_metabolic\_process | 1 | 0 |  |  |  |  |  |  |  |  |
| GO:0034036\_purine\_ribonucleoside\_bisphosphate\_biosynthetic\_process | 1 | 0 |  |  |  |  |  |  |  |  |
| GO:0034067\_protein\_localization\_in\_Golgi\_apparatus | 1 | 0 |  |  |  |  |  |  |  |  |
| GO:0034102\_erythrocyte\_clearance | 1 | 0 |  |  |  |  |  |  |  |  |
| GO:0034106\_regulation\_of\_erythrocyte\_clearance | 1 | 0 |  |  |  |  |  |  |  |  |
| GO:0034107\_negative\_regulation\_of\_erythrocyte\_clearance | 1 | 0 |  |  |  |  |  |  |  |  |
| GO:0034110\_regulation\_of\_homotypic\_cell-cell\_adhesion | 1 | 0 |  |  |  |  |  |  |  |  |
| GO:0034111\_negative\_regulation\_of\_homotypic\_cell-cell\_adhesion | 1 | 0 |  |  |  |  |  |  |  |  |
| GO:0034113\_heterotypic\_cell-cell\_adhesion | 1 | 0 |  |  |  |  |  |  |  |  |
| GO:0034117\_erythrocyte\_aggregation | 1 | 0 |  |  |  |  |  |  |  |  |
| GO:0034118\_regulation\_of\_erythrocyte\_aggregation | 1 | 0 |  |  |  |  |  |  |  |  |
| GO:0034119\_negative\_regulation\_of\_erythrocyte\_aggregation | 1 | 0 |  |  |  |  |  |  |  |  |
| GO:0034121\_regulation\_of\_toll-like\_receptor\_signaling\_pathway | 1 | 0 |  |  |  |  |  |  |  |  |
| GO:0034122\_negative\_regulation\_of\_toll-like\_receptor\_signaling\_pathway | 1 | 0 |  |  |  |  |  |  |  |  |
| GO:0034230\_enkephalin\_processing | 1 | 0 |  |  |  |  |  |  |  |  |
| GO:0034372\_very-low-density\_lipoprotein\_particle\_remodeling | 1 | 0 |  |  |  |  |  |  |  |  |
| GO:0034379\_very-low-density\_lipoprotein\_particle\_assembly | 1 | 0 |  |  |  |  |  |  |  |  |
| GO:0034380\_high-density\_lipoprotein\_particle\_assembly | 1 | 0 |  |  |  |  |  |  |  |  |
| GO:0034394\_protein\_localization\_at\_cell\_surface | 1 | 0 |  |  |  |  |  |  |  |  |
| GO:0034405\_response\_to\_fluid\_shear\_stress | 1 | 0 |  |  |  |  |  |  |  |  |
| GO:0034472\_snRNA\_3'-end\_processing | 1 | 0 |  |  |  |  |  |  |  |  |
| GO:0034474\_U2\_snRNA\_3'-end\_processing | 1 | 0 |  |  |  |  |  |  |  |  |
| GO:0034502\_protein\_localization\_to\_chromosome | 1 | 0 |  |  |  |  |  |  |  |  |
| GO:0034505\_tooth\_mineralization | 1 | 0 |  |  |  |  |  |  |  |  |
| GO:0034508\_centromere\_complex\_assembly | 1 | 0 |  |  |  |  |  |  |  |  |
| GO:0034633\_retinol\_transport | 1 | 0 |  |  |  |  |  |  |  |  |
| GO:0034643\_mitochondrion\_localization\_\_microtubule-mediated | 1 | 0 |  |  |  |  |  |  |  |  |
| GO:0034969\_histone\_arginine\_methylation | 1 | 0 |  |  |  |  |  |  |  |  |
| GO:0034982\_mitochondrial\_protein\_processing | 1 | 0 |  |  |  |  |  |  |  |  |
| GO:0035022\_positive\_regulation\_of\_Rac\_protein\_signal\_transduction | 1 | 0 |  |  |  |  |  |  |  |  |
| GO:0035024\_negative\_regulation\_of\_Rho\_protein\_signal\_transduction | 1 | 0 |  |  |  |  |  |  |  |  |
| GO:0035026\_leading\_edge\_cell\_differentiation | 1 | 0 |  |  |  |  |  |  |  |  |
| GO:0035037\_sperm\_entry | 1 | 0 |  |  |  |  |  |  |  |  |
| GO:0035039\_male\_pronucleus\_formation | 1 | 0 |  |  |  |  |  |  |  |  |
| GO:0035066\_positive\_regulation\_of\_histone\_acetylation | 1 | 0 |  |  |  |  |  |  |  |  |
| GO:0035083\_cilium\_axoneme\_assembly | 1 | 0 |  |  |  |  |  |  |  |  |
| GO:0035090\_maintenance\_of\_apical\_basal\_cell\_polarity | 1 | 0 |  |  |  |  |  |  |  |  |
| GO:0035106\_operant\_conditioning | 1 | 0 |  |  |  |  |  |  |  |  |
| GO:0035172\_hemocyte\_proliferation | 1 | 0 |  |  |  |  |  |  |  |  |
| GO:0035227\_regulation\_of\_glutamate-cysteine\_ligase\_activity | 1 | 0 |  |  |  |  |  |  |  |  |
| GO:0035229\_positive\_regulation\_of\_glutamate-cysteine\_ligase\_activity | 1 | 0 |  |  |  |  |  |  |  |  |
| GO:0035260\_internal\_genitalia\_morphogenesis | 1 | 0 |  |  |  |  |  |  |  |  |
| GO:0035262\_gonad\_morphogenesis | 1 | 0 |  |  |  |  |  |  |  |  |
| GO:0035287\_head\_segmentation | 1 | 0 |  |  |  |  |  |  |  |  |
| GO:0035289\_posterior\_head\_segmentation | 1 | 0 |  |  |  |  |  |  |  |  |
| GO:0035303\_regulation\_of\_dephosphorylation | 1 | 0 |  |  |  |  |  |  |  |  |
| GO:0035304\_regulation\_of\_protein\_amino\_acid\_dephosphorylation | 1 | 0 |  |  |  |  |  |  |  |  |
| GO:0035305\_negative\_regulation\_of\_dephosphorylation | 1 | 0 |  |  |  |  |  |  |  |  |
| GO:0035308\_negative\_regulation\_of\_protein\_amino\_acid\_dephosphorylation | 1 | 0 |  |  |  |  |  |  |  |  |
| GO:0035313\_wound\_healing\_\_spreading\_of\_epidermal\_cells | 1 | 0 |  |  |  |  |  |  |  |  |
| GO:0040013\_negative\_regulation\_of\_locomotion | 1 | 0 |  |  |  |  |  |  |  |  |
| GO:0040019\_positive\_regulation\_of\_embryonic\_development | 1 | 0 |  |  |  |  |  |  |  |  |
| GO:0040032\_post-embryonic\_body\_morphogenesis | 1 | 0 |  |  |  |  |  |  |  |  |
| GO:0040038\_polar\_body\_extrusion\_after\_meiotic\_divisions | 1 | 0 |  |  |  |  |  |  |  |  |
| GO:0042026\_protein\_refolding | 1 | 0 |  |  |  |  |  |  |  |  |
| GO:0042048\_olfactory\_behavior | 1 | 0 |  |  |  |  |  |  |  |  |
| GO:0042059\_negative\_regulation\_of\_epidermal\_growth\_factor\_receptor\_signaling\_pathway | 1 | 0 |  |  |  |  |  |  |  |  |
| GO:0042073\_intraflagellar\_transport | 1 | 0 |  |  |  |  |  |  |  |  |
| GO:0042078\_germ-line\_stem\_cell\_division | 1 | 0 |  |  |  |  |  |  |  |  |
| GO:0042091\_interleukin-10\_biosynthetic\_process | 1 | 0 |  |  |  |  |  |  |  |  |
| GO:0042103\_positive\_regulation\_of\_T\_cell\_homeostatic\_proliferation | 1 | 0 |  |  |  |  |  |  |  |  |
| GO:0042136\_neurotransmitter\_biosynthetic\_process | 1 | 0 |  |  |  |  |  |  |  |  |
| GO:0042137\_sequestering\_of\_neurotransmitter | 1 | 0 |  |  |  |  |  |  |  |  |
| GO:0042138\_meiotic\_DNA\_double-strand\_break\_formation | 1 | 0 |  |  |  |  |  |  |  |  |
| GO:0042178\_xenobiotic\_catabolic\_process | 1 | 0 |  |  |  |  |  |  |  |  |
| GO:0042225\_interleukin-5\_biosynthetic\_process | 1 | 0 |  |  |  |  |  |  |  |  |
| GO:0042231\_interleukin-13\_biosynthetic\_process | 1 | 0 |  |  |  |  |  |  |  |  |
| GO:0042255\_ribosome\_assembly | 1 | 0 |  |  |  |  |  |  |  |  |
| GO:0042257\_ribosomal\_subunit\_assembly | 1 | 0 |  |  |  |  |  |  |  |  |
| GO:0042264\_peptidyl-aspartic\_acid\_hydroxylation | 1 | 0 |  |  |  |  |  |  |  |  |
| GO:0042276\_error-prone\_postreplication\_DNA\_repair | 1 | 0 |  |  |  |  |  |  |  |  |
| GO:0042297\_vocal\_learning | 1 | 0 |  |  |  |  |  |  |  |  |
| GO:0042309\_homoiothermy | 1 | 0 |  |  |  |  |  |  |  |  |
| GO:0042320\_regulation\_of\_circadian\_sleep\_wake\_cycle\_\_REM\_sleep | 1 | 0 |  |  |  |  |  |  |  |  |
| GO:0042339\_keratan\_sulfate\_metabolic\_process | 1 | 0 |  |  |  |  |  |  |  |  |
| GO:0042347\_negative\_regulation\_of\_NF-kappaB\_import\_into\_nucleus | 1 | 0 |  |  |  |  |  |  |  |  |
| GO:0042360\_vitamin\_E\_metabolic\_process | 1 | 0 |  |  |  |  |  |  |  |  |
| GO:0042363\_fat-soluble\_vitamin\_catabolic\_process | 1 | 0 |  |  |  |  |  |  |  |  |
| GO:0042369\_vitamin\_D\_catabolic\_process | 1 | 0 |  |  |  |  |  |  |  |  |
| GO:0042373\_vitamin\_K\_metabolic\_process | 1 | 0 |  |  |  |  |  |  |  |  |
| GO:0042404\_thyroid\_hormone\_catabolic\_process | 1 | 0 |  |  |  |  |  |  |  |  |
| GO:0042414\_epinephrine\_metabolic\_process | 1 | 0 |  |  |  |  |  |  |  |  |
| GO:0042436\_indole\_derivative\_catabolic\_process | 1 | 0 |  |  |  |  |  |  |  |  |
| GO:0042489\_negative\_regulation\_of\_odontogenesis\_of\_dentine-containing\_tooth | 1 | 0 |  |  |  |  |  |  |  |  |
| GO:0042508\_tyrosine\_phosphorylation\_of\_Stat1\_protein | 1 | 0 |  |  |  |  |  |  |  |  |
| GO:0042518\_negative\_regulation\_of\_tyrosine\_phosphorylation\_of\_Stat3\_protein | 1 | 0 |  |  |  |  |  |  |  |  |
| GO:0042524\_negative\_regulation\_of\_tyrosine\_phosphorylation\_of\_Stat5\_protein | 1 | 0 |  |  |  |  |  |  |  |  |
| GO:0042536\_negative\_regulation\_of\_tumor\_necrosis\_factor\_biosynthetic\_process | 1 | 0 |  |  |  |  |  |  |  |  |
| GO:0042538\_hyperosmotic\_salinity\_response | 1 | 0 |  |  |  |  |  |  |  |  |
| GO:0042628\_mating\_plug\_formation | 1 | 0 |  |  |  |  |  |  |  |  |
| GO:0042631\_cellular\_response\_to\_water\_deprivation | 1 | 0 |  |  |  |  |  |  |  |  |
| GO:0042637\_catagen | 1 | 0 |  |  |  |  |  |  |  |  |
| GO:0042660\_positive\_regulation\_of\_cell\_fate\_specification | 1 | 0 |  |  |  |  |  |  |  |  |
| GO:0042663\_regulation\_of\_endodermal\_cell\_fate\_specification | 1 | 0 |  |  |  |  |  |  |  |  |
| GO:0042664\_negative\_regulation\_of\_endodermal\_cell\_fate\_specification | 1 | 0 |  |  |  |  |  |  |  |  |
| GO:0042667\_auditory\_receptor\_cell\_fate\_specification | 1 | 0 |  |  |  |  |  |  |  |  |
| GO:0042694\_muscle\_cell\_fate\_specification | 1 | 0 |  |  |  |  |  |  |  |  |
| GO:0042706\_eye\_photoreceptor\_cell\_fate\_commitment | 1 | 0 |  |  |  |  |  |  |  |  |
| GO:0042713\_sperm\_ejaculation | 1 | 0 |  |  |  |  |  |  |  |  |
| GO:0042723\_thiamin\_and\_derivative\_metabolic\_process | 1 | 0 |  |  |  |  |  |  |  |  |
| GO:0042737\_drug\_catabolic\_process | 1 | 0 |  |  |  |  |  |  |  |  |
| GO:0042738\_exogenous\_drug\_catabolic\_process | 1 | 0 |  |  |  |  |  |  |  |  |
| GO:0042747\_circadian\_sleep\_wake\_cycle\_\_REM\_sleep | 1 | 0 |  |  |  |  |  |  |  |  |
| GO:0042748\_circadian\_sleep\_wake\_cycle\_\_non-REM\_sleep | 1 | 0 |  |  |  |  |  |  |  |  |
| GO:0042772\_DNA\_damage\_response\_\_signal\_transduction\_resulting\_in\_transcription | 1 | 0 |  |  |  |  |  |  |  |  |
| GO:0042790\_transcription\_of\_nuclear\_rRNA\_large\_RNA\_polymerase\_I\_transcript | 1 | 0 |  |  |  |  |  |  |  |  |
| GO:0042839\_D-glucuronate\_metabolic\_process | 1 | 0 |  |  |  |  |  |  |  |  |
| GO:0042840\_D-glucuronate\_catabolic\_process | 1 | 0 |  |  |  |  |  |  |  |  |
| GO:0042891\_antibiotic\_transport | 1 | 0 |  |  |  |  |  |  |  |  |
| GO:0042892\_chloramphenicol\_transport | 1 | 0 |  |  |  |  |  |  |  |  |
| GO:0042940\_D-amino\_acid\_transport | 1 | 0 |  |  |  |  |  |  |  |  |
| GO:0042941\_D-alanine\_transport | 1 | 0 |  |  |  |  |  |  |  |  |
| GO:0042942\_D-serine\_transport | 1 | 0 |  |  |  |  |  |  |  |  |
| GO:0042983\_amyloid\_precursor\_protein\_biosynthetic\_process | 1 | 0 |  |  |  |  |  |  |  |  |
| GO:0042984\_regulation\_of\_amyloid\_precursor\_protein\_biosynthetic\_process | 1 | 0 |  |  |  |  |  |  |  |  |
| GO:0042985\_negative\_regulation\_of\_amyloid\_precursor\_protein\_biosynthetic\_process | 1 | 0 |  |  |  |  |  |  |  |  |
| GO:0042989\_sequestering\_of\_actin\_monomers | 1 | 0 |  |  |  |  |  |  |  |  |
| GO:0043044\_ATP-dependent\_chromatin\_remodeling | 1 | 0 |  |  |  |  |  |  |  |  |
| GO:0043056\_forward\_locomotion | 1 | 0 |  |  |  |  |  |  |  |  |
| GO:0043060\_meiotic\_metaphase\_I\_plate\_congression | 1 | 0 |  |  |  |  |  |  |  |  |
| GO:0043091\_L-arginine\_import | 1 | 0 |  |  |  |  |  |  |  |  |
| GO:0043124\_negative\_regulation\_of\_I-kappaB\_kinase\_NF-kappaB\_cascade | 1 | 0 |  |  |  |  |  |  |  |  |
| GO:0043132\_NAD\_transport | 1 | 0 |  |  |  |  |  |  |  |  |
| GO:0043153\_entrainment\_of\_circadian\_clock\_by\_photoperiod | 1 | 0 |  |  |  |  |  |  |  |  |
| GO:0043171\_peptide\_catabolic\_process | 1 | 0 |  |  |  |  |  |  |  |  |
| GO:0043179\_rhythmic\_excitation | 1 | 0 |  |  |  |  |  |  |  |  |
| GO:0043206\_fibril\_organization | 1 | 0 |  |  |  |  |  |  |  |  |
| GO:0043217\_myelin\_maintenance | 1 | 0 |  |  |  |  |  |  |  |  |
| GO:0043313\_regulation\_of\_neutrophil\_degranulation | 1 | 0 |  |  |  |  |  |  |  |  |
| GO:0043316\_cytotoxic\_T\_cell\_degranulation | 1 | 0 |  |  |  |  |  |  |  |  |
| GO:0043369\_CD4-positive\_or\_CD8-positive\_\_alpha-beta\_T\_cell\_lineage\_commitment | 1 | 0 |  |  |  |  |  |  |  |  |
| GO:0043375\_CD8-positive\_\_alpha-beta\_T\_cell\_lineage\_commitment | 1 | 0 |  |  |  |  |  |  |  |  |
| GO:0043379\_memory\_T\_cell\_differentiation | 1 | 0 |  |  |  |  |  |  |  |  |
| GO:0043380\_regulation\_of\_memory\_T\_cell\_differentiation | 1 | 0 |  |  |  |  |  |  |  |  |
| GO:0043400\_cortisol\_secretion | 1 | 0 |  |  |  |  |  |  |  |  |
| GO:0043415\_positive\_regulation\_of\_skeletal\_muscle\_regeneration | 1 | 0 |  |  |  |  |  |  |  |  |
| GO:0043416\_regulation\_of\_skeletal\_muscle\_regeneration | 1 | 0 |  |  |  |  |  |  |  |  |
| GO:0043437\_butanoic\_acid\_metabolic\_process | 1 | 0 |  |  |  |  |  |  |  |  |
| GO:0043438\_acetoacetic\_acid\_metabolic\_process | 1 | 0 |  |  |  |  |  |  |  |  |
| GO:0043480\_pigment\_accumulation\_in\_tissues | 1 | 0 |  |  |  |  |  |  |  |  |
| GO:0043482\_cellular\_pigment\_accumulation | 1 | 0 |  |  |  |  |  |  |  |  |
| GO:0043486\_histone\_exchange | 1 | 0 |  |  |  |  |  |  |  |  |
| GO:0043496\_regulation\_of\_protein\_homodimerization\_activity | 1 | 0 |  |  |  |  |  |  |  |  |
| GO:0043501\_skeletal\_muscle\_adaptation | 1 | 0 |  |  |  |  |  |  |  |  |
| GO:0043508\_negative\_regulation\_of\_JUN\_kinase\_activity | 1 | 0 |  |  |  |  |  |  |  |  |
| GO:0043517\_positive\_regulation\_of\_DNA\_damage\_response\_\_signal\_transduction\_by\_p53\_class\_mediator | 1 | 0 |  |  |  |  |  |  |  |  |
| GO:0043535\_regulation\_of\_blood\_vessel\_endothelial\_cell\_migration | 1 | 0 |  |  |  |  |  |  |  |  |
| GO:0043537\_negative\_regulation\_of\_blood\_vessel\_endothelial\_cell\_migration | 1 | 0 |  |  |  |  |  |  |  |  |
| GO:0043545\_molybdopterin\_cofactor\_metabolic\_process | 1 | 0 |  |  |  |  |  |  |  |  |
| GO:0043587\_tongue\_morphogenesis | 1 | 0 |  |  |  |  |  |  |  |  |
| GO:0043604\_amide\_biosynthetic\_process | 1 | 0 |  |  |  |  |  |  |  |  |
| GO:0043628\_ncRNA\_3'-end\_processing | 1 | 0 |  |  |  |  |  |  |  |  |
| GO:0044254\_multicellular\_organismal\_protein\_catabolic\_process | 1 | 0 |  |  |  |  |  |  |  |  |
| GO:0044256\_protein\_digestion | 1 | 0 |  |  |  |  |  |  |  |  |
| GO:0044266\_multicellular\_organismal\_macromolecule\_catabolic\_process | 1 | 0 |  |  |  |  |  |  |  |  |
| GO:0045004\_DNA\_replication\_proofreading | 1 | 0 |  |  |  |  |  |  |  |  |
| GO:0045019\_negative\_regulation\_of\_nitric\_oxide\_biosynthetic\_process | 1 | 0 |  |  |  |  |  |  |  |  |
| GO:0045020\_error-prone\_DNA\_repair | 1 | 0 |  |  |  |  |  |  |  |  |
| GO:0045022\_early\_endosome\_to\_late\_endosome\_transport | 1 | 0 |  |  |  |  |  |  |  |  |
| GO:0045062\_extrathymic\_T\_cell\_selection | 1 | 0 |  |  |  |  |  |  |  |  |
| GO:0045069\_regulation\_of\_viral\_genome\_replication | 1 | 0 |  |  |  |  |  |  |  |  |
| GO:0045074\_regulation\_of\_interleukin-10\_biosynthetic\_process | 1 | 0 |  |  |  |  |  |  |  |  |
| GO:0045082\_positive\_regulation\_of\_interleukin-10\_biosynthetic\_process | 1 | 0 |  |  |  |  |  |  |  |  |
| GO:0045083\_negative\_regulation\_of\_interleukin-12\_biosynthetic\_process | 1 | 0 |  |  |  |  |  |  |  |  |
| GO:0045112\_integrin\_biosynthetic\_process | 1 | 0 |  |  |  |  |  |  |  |  |
| GO:0045113\_regulation\_of\_integrin\_biosynthetic\_process | 1 | 0 |  |  |  |  |  |  |  |  |
| GO:0045188\_regulation\_of\_circadian\_sleep\_wake\_cycle\_\_non-REM\_sleep | 1 | 0 |  |  |  |  |  |  |  |  |
| GO:0045210\_FasL\_biosynthetic\_process | 1 | 0 |  |  |  |  |  |  |  |  |
| GO:0045297\_post-mating\_behavior | 1 | 0 |  |  |  |  |  |  |  |  |
| GO:0045299\_otolith\_mineralization | 1 | 0 |  |  |  |  |  |  |  |  |
| GO:0045329\_carnitine\_biosynthetic\_process | 1 | 0 |  |  |  |  |  |  |  |  |
| GO:0045341\_MHC\_class\_I\_biosynthetic\_process | 1 | 0 |  |  |  |  |  |  |  |  |
| GO:0045343\_regulation\_of\_MHC\_class\_I\_biosynthetic\_process | 1 | 0 |  |  |  |  |  |  |  |  |
| GO:0045347\_negative\_regulation\_of\_MHC\_class\_II\_biosynthetic\_process | 1 | 0 |  |  |  |  |  |  |  |  |
| GO:0045405\_regulation\_of\_interleukin-5\_biosynthetic\_process | 1 | 0 |  |  |  |  |  |  |  |  |
| GO:0045407\_positive\_regulation\_of\_interleukin-5\_biosynthetic\_process | 1 | 0 |  |  |  |  |  |  |  |  |
| GO:0045426\_quinone\_cofactor\_biosynthetic\_process | 1 | 0 |  |  |  |  |  |  |  |  |
| GO:0045448\_mitotic\_cell\_cycle\_\_embryonic | 1 | 0 |  |  |  |  |  |  |  |  |
| GO:0045454\_cell\_redox\_homeostasis | 1 | 0 |  |  |  |  |  |  |  |  |
| GO:0045583\_regulation\_of\_cytotoxic\_T\_cell\_differentiation | 1 | 0 |  |  |  |  |  |  |  |  |
| GO:0045585\_positive\_regulation\_of\_cytotoxic\_T\_cell\_differentiation | 1 | 0 |  |  |  |  |  |  |  |  |
| GO:0045601\_regulation\_of\_endothelial\_cell\_differentiation | 1 | 0 |  |  |  |  |  |  |  |  |
| GO:0045602\_negative\_regulation\_of\_endothelial\_cell\_differentiation | 1 | 0 |  |  |  |  |  |  |  |  |
| GO:0045605\_negative\_regulation\_of\_epidermal\_cell\_differentiation | 1 | 0 |  |  |  |  |  |  |  |  |
| GO:0045606\_positive\_regulation\_of\_epidermal\_cell\_differentiation | 1 | 0 |  |  |  |  |  |  |  |  |
| GO:0045609\_positive\_regulation\_of\_auditory\_receptor\_cell\_differentiation | 1 | 0 |  |  |  |  |  |  |  |  |
| GO:0045617\_negative\_regulation\_of\_keratinocyte\_differentiation | 1 | 0 |  |  |  |  |  |  |  |  |
| GO:0045618\_positive\_regulation\_of\_keratinocyte\_differentiation | 1 | 0 |  |  |  |  |  |  |  |  |
| GO:0045626\_negative\_regulation\_of\_T-helper\_1\_cell\_differentiation | 1 | 0 |  |  |  |  |  |  |  |  |
| GO:0045633\_positive\_regulation\_of\_mechanoreceptor\_differentiation | 1 | 0 |  |  |  |  |  |  |  |  |
| GO:0045650\_negative\_regulation\_of\_macrophage\_differentiation | 1 | 0 |  |  |  |  |  |  |  |  |
| GO:0045656\_negative\_regulation\_of\_monocyte\_differentiation | 1 | 0 |  |  |  |  |  |  |  |  |
| GO:0045657\_positive\_regulation\_of\_monocyte\_differentiation | 1 | 0 |  |  |  |  |  |  |  |  |
| GO:0045659\_negative\_regulation\_of\_neutrophil\_differentiation | 1 | 0 |  |  |  |  |  |  |  |  |
| GO:0045660\_positive\_regulation\_of\_neutrophil\_differentiation | 1 | 0 |  |  |  |  |  |  |  |  |
| GO:0045721\_negative\_regulation\_of\_gluconeogenesis | 1 | 0 |  |  |  |  |  |  |  |  |
| GO:0045724\_positive\_regulation\_of\_flagellum\_assembly | 1 | 0 |  |  |  |  |  |  |  |  |
| GO:0045725\_positive\_regulation\_of\_glycogen\_biosynthetic\_process | 1 | 0 |  |  |  |  |  |  |  |  |
| GO:0045740\_positive\_regulation\_of\_DNA\_replication | 1 | 0 |  |  |  |  |  |  |  |  |
| GO:0045759\_negative\_regulation\_of\_action\_potential | 1 | 0 |  |  |  |  |  |  |  |  |
| GO:0045768\_positive\_regulation\_of\_anti-apoptosis | 1 | 0 |  |  |  |  |  |  |  |  |
| GO:0045769\_negative\_regulation\_of\_asymmetric\_cell\_division | 1 | 0 |  |  |  |  |  |  |  |  |
| GO:0045794\_negative\_regulation\_of\_cell\_volume | 1 | 0 |  |  |  |  |  |  |  |  |
| GO:0045815\_positive\_regulation\_of\_gene\_expression\_\_epigenetic | 1 | 0 |  |  |  |  |  |  |  |  |
| GO:0045818\_negative\_regulation\_of\_glycogen\_catabolic\_process | 1 | 0 |  |  |  |  |  |  |  |  |
| GO:0045842\_positive\_regulation\_of\_mitotic\_metaphase\_anaphase\_transition | 1 | 0 |  |  |  |  |  |  |  |  |
| GO:0045875\_negative\_regulation\_of\_sister\_chromatid\_cohesion | 1 | 0 |  |  |  |  |  |  |  |  |
| GO:0045898\_regulation\_of\_transcriptional\_preinitiation\_complex\_assembly | 1 | 0 |  |  |  |  |  |  |  |  |
| GO:0045899\_positive\_regulation\_of\_transcriptional\_preinitiation\_complex\_assembly | 1 | 0 |  |  |  |  |  |  |  |  |
| GO:0045906\_negative\_regulation\_of\_vasoconstriction | 1 | 0 |  |  |  |  |  |  |  |  |
| GO:0045908\_negative\_regulation\_of\_vasodilation | 1 | 0 |  |  |  |  |  |  |  |  |
| GO:0045909\_positive\_regulation\_of\_vasodilation | 1 | 0 |  |  |  |  |  |  |  |  |
| GO:0045915\_positive\_regulation\_of\_catecholamine\_metabolic\_process | 1 | 0 |  |  |  |  |  |  |  |  |
| GO:0045920\_negative\_regulation\_of\_exocytosis | 1 | 0 |  |  |  |  |  |  |  |  |
| GO:0045924\_regulation\_of\_female\_receptivity | 1 | 0 |  |  |  |  |  |  |  |  |
| GO:0045955\_negative\_regulation\_of\_calcium\_ion-dependent\_exocytosis | 1 | 0 |  |  |  |  |  |  |  |  |
| GO:0045956\_positive\_regulation\_of\_calcium\_ion-dependent\_exocytosis | 1 | 0 |  |  |  |  |  |  |  |  |
| GO:0045964\_positive\_regulation\_of\_dopamine\_metabolic\_process | 1 | 0 |  |  |  |  |  |  |  |  |
| GO:0045988\_negative\_regulation\_of\_striated\_muscle\_contraction | 1 | 0 |  |  |  |  |  |  |  |  |
| GO:0045989\_positive\_regulation\_of\_striated\_muscle\_contraction | 1 | 0 |  |  |  |  |  |  |  |  |
| GO:0045990\_regulation\_of\_transcription\_by\_carbon\_catabolites | 1 | 0 |  |  |  |  |  |  |  |  |
| GO:0045991\_positive\_regulation\_of\_transcription\_by\_carbon\_catabolites | 1 | 0 |  |  |  |  |  |  |  |  |
| GO:0045994\_positive\_regulation\_of\_translational\_initiation\_by\_iron | 1 | 0 |  |  |  |  |  |  |  |  |
| GO:0046007\_negative\_regulation\_of\_activated\_T\_cell\_proliferation | 1 | 0 |  |  |  |  |  |  |  |  |
| GO:0046014\_negative\_regulation\_of\_T\_cell\_homeostatic\_proliferation | 1 | 0 |  |  |  |  |  |  |  |  |
| GO:0046015\_regulation\_of\_transcription\_by\_glucose | 1 | 0 |  |  |  |  |  |  |  |  |
| GO:0046016\_positive\_regulation\_of\_transcription\_by\_glucose | 1 | 0 |  |  |  |  |  |  |  |  |
| GO:0046031\_ADP\_metabolic\_process | 1 | 0 |  |  |  |  |  |  |  |  |
| GO:0046032\_ADP\_catabolic\_process | 1 | 0 |  |  |  |  |  |  |  |  |
| GO:0046061\_dATP\_catabolic\_process | 1 | 0 |  |  |  |  |  |  |  |  |
| GO:0046075\_dTTP\_metabolic\_process | 1 | 0 |  |  |  |  |  |  |  |  |
| GO:0046078\_dUMP\_metabolic\_process | 1 | 0 |  |  |  |  |  |  |  |  |
| GO:0046079\_dUMP\_catabolic\_process | 1 | 0 |  |  |  |  |  |  |  |  |
| GO:0046086\_adenosine\_biosynthetic\_process | 1 | 0 |  |  |  |  |  |  |  |  |
| GO:0046090\_deoxyadenosine\_metabolic\_process | 1 | 0 |  |  |  |  |  |  |  |  |
| GO:0046098\_guanine\_metabolic\_process | 1 | 0 |  |  |  |  |  |  |  |  |
| GO:0046101\_hypoxanthine\_biosynthetic\_process | 1 | 0 |  |  |  |  |  |  |  |  |
| GO:0046102\_inosine\_metabolic\_process | 1 | 0 |  |  |  |  |  |  |  |  |
| GO:0046103\_inosine\_biosynthetic\_process | 1 | 0 |  |  |  |  |  |  |  |  |
| GO:0046108\_uridine\_metabolic\_process | 1 | 0 |  |  |  |  |  |  |  |  |
| GO:0046110\_xanthine\_metabolic\_process | 1 | 0 |  |  |  |  |  |  |  |  |
| GO:0046111\_xanthine\_biosynthetic\_process | 1 | 0 |  |  |  |  |  |  |  |  |
| GO:0046112\_nucleobase\_biosynthetic\_process | 1 | 0 |  |  |  |  |  |  |  |  |
| GO:0046113\_nucleobase\_catabolic\_process | 1 | 0 |  |  |  |  |  |  |  |  |
| GO:0046121\_deoxyribonucleoside\_catabolic\_process | 1 | 0 |  |  |  |  |  |  |  |  |
| GO:0046122\_purine\_deoxyribonucleoside\_metabolic\_process | 1 | 0 |  |  |  |  |  |  |  |  |
| GO:0046124\_purine\_deoxyribonucleoside\_catabolic\_process | 1 | 0 |  |  |  |  |  |  |  |  |
| GO:0046125\_pyrimidine\_deoxyribonucleoside\_metabolic\_process | 1 | 0 |  |  |  |  |  |  |  |  |
| GO:0046131\_pyrimidine\_ribonucleoside\_metabolic\_process | 1 | 0 |  |  |  |  |  |  |  |  |
| GO:0046160\_heme\_a\_metabolic\_process | 1 | 0 |  |  |  |  |  |  |  |  |
| GO:0046218\_indolalkylamine\_catabolic\_process | 1 | 0 |  |  |  |  |  |  |  |  |
| GO:0046292\_formaldehyde\_metabolic\_process | 1 | 0 |  |  |  |  |  |  |  |  |
| GO:0046294\_formaldehyde\_catabolic\_process | 1 | 0 |  |  |  |  |  |  |  |  |
| GO:0046314\_phosphocreatine\_biosynthetic\_process | 1 | 0 |  |  |  |  |  |  |  |  |
| GO:0046327\_glycerol\_biosynthetic\_process\_from\_pyruvate | 1 | 0 |  |  |  |  |  |  |  |  |
| GO:0046329\_negative\_regulation\_of\_JNK\_cascade | 1 | 0 |  |  |  |  |  |  |  |  |
| GO:0046340\_diacylglycerol\_catabolic\_process | 1 | 0 |  |  |  |  |  |  |  |  |
| GO:0046351\_disaccharide\_biosynthetic\_process | 1 | 0 |  |  |  |  |  |  |  |  |
| GO:0046356\_acetyl-CoA\_catabolic\_process | 1 | 0 |  |  |  |  |  |  |  |  |
| GO:0046358\_butyrate\_biosynthetic\_process | 1 | 0 |  |  |  |  |  |  |  |  |
| GO:0046359\_butyrate\_catabolic\_process | 1 | 0 |  |  |  |  |  |  |  |  |
| GO:0046381\_CMP-N-acetylneuraminate\_metabolic\_process | 1 | 0 |  |  |  |  |  |  |  |  |
| GO:0046415\_urate\_metabolic\_process | 1 | 0 |  |  |  |  |  |  |  |  |
| GO:0046416\_D-amino\_acid\_metabolic\_process | 1 | 0 |  |  |  |  |  |  |  |  |
| GO:0046434\_organophosphate\_catabolic\_process | 1 | 0 |  |  |  |  |  |  |  |  |
| GO:0046437\_D-amino\_acid\_biosynthetic\_process | 1 | 0 |  |  |  |  |  |  |  |  |
| GO:0046440\_L-lysine\_metabolic\_process | 1 | 0 |  |  |  |  |  |  |  |  |
| GO:0046449\_creatinine\_metabolic\_process | 1 | 0 |  |  |  |  |  |  |  |  |
| GO:0046471\_phosphatidylglycerol\_metabolic\_process | 1 | 0 |  |  |  |  |  |  |  |  |
| GO:0046473\_phosphatidic\_acid\_metabolic\_process | 1 | 0 |  |  |  |  |  |  |  |  |
| GO:0046476\_glycosylceramide\_biosynthetic\_process | 1 | 0 |  |  |  |  |  |  |  |  |
| GO:0046477\_glycosylceramide\_catabolic\_process | 1 | 0 |  |  |  |  |  |  |  |  |
| GO:0046485\_ether\_lipid\_metabolic\_process | 1 | 0 |  |  |  |  |  |  |  |  |
| GO:0046487\_glyoxylate\_metabolic\_process | 1 | 0 |  |  |  |  |  |  |  |  |
| GO:0046498\_S-adenosylhomocysteine\_metabolic\_process | 1 | 0 |  |  |  |  |  |  |  |  |
| GO:0046552\_photoreceptor\_cell\_fate\_commitment | 1 | 0 |  |  |  |  |  |  |  |  |
| GO:0046586\_regulation\_of\_calcium-dependent\_cell-cell\_adhesion | 1 | 0 |  |  |  |  |  |  |  |  |
| GO:0046587\_positive\_regulation\_of\_calcium-dependent\_cell-cell\_adhesion | 1 | 0 |  |  |  |  |  |  |  |  |
| GO:0046602\_regulation\_of\_mitotic\_centrosome\_separation | 1 | 0 |  |  |  |  |  |  |  |  |
| GO:0046604\_positive\_regulation\_of\_mitotic\_centrosome\_separation | 1 | 0 |  |  |  |  |  |  |  |  |
| GO:0046607\_positive\_regulation\_of\_centrosome\_cycle | 1 | 0 |  |  |  |  |  |  |  |  |
| GO:0046655\_folic\_acid\_metabolic\_process | 1 | 0 |  |  |  |  |  |  |  |  |
| GO:0046671\_negative\_regulation\_of\_retinal\_cell\_programmed\_cell\_death | 1 | 0 |  |  |  |  |  |  |  |  |
| GO:0046685\_response\_to\_arsenic | 1 | 0 |  |  |  |  |  |  |  |  |
| GO:0046692\_sperm\_competition | 1 | 0 |  |  |  |  |  |  |  |  |
| GO:0046707\_IDP\_metabolic\_process | 1 | 0 |  |  |  |  |  |  |  |  |
| GO:0046709\_IDP\_catabolic\_process | 1 | 0 |  |  |  |  |  |  |  |  |
| GO:0046724\_oxalic\_acid\_secretion | 1 | 0 |  |  |  |  |  |  |  |  |
| GO:0046753\_non-lytic\_viral\_release | 1 | 0 |  |  |  |  |  |  |  |  |
| GO:0046755\_non-lytic\_virus\_budding | 1 | 0 |  |  |  |  |  |  |  |  |
| GO:0046826\_negative\_regulation\_of\_protein\_export\_from\_nucleus | 1 | 0 |  |  |  |  |  |  |  |  |
| GO:0046827\_positive\_regulation\_of\_protein\_export\_from\_nucleus | 1 | 0 |  |  |  |  |  |  |  |  |
| GO:0046831\_regulation\_of\_RNA\_export\_from\_nucleus | 1 | 0 |  |  |  |  |  |  |  |  |
| GO:0046834\_lipid\_phosphorylation | 1 | 0 |  |  |  |  |  |  |  |  |
| GO:0046853\_inositol\_and\_derivative\_phosphorylation | 1 | 0 |  |  |  |  |  |  |  |  |
| GO:0046864\_isoprenoid\_transport | 1 | 0 |  |  |  |  |  |  |  |  |
| GO:0046865\_terpenoid\_transport | 1 | 0 |  |  |  |  |  |  |  |  |
| GO:0046877\_regulation\_of\_saliva\_secretion | 1 | 0 |  |  |  |  |  |  |  |  |
| GO:0046878\_positive\_regulation\_of\_saliva\_secretion | 1 | 0 |  |  |  |  |  |  |  |  |
| GO:0046884\_follicle-stimulating\_hormone\_secretion | 1 | 0 |  |  |  |  |  |  |  |  |
| GO:0046898\_response\_to\_cycloheximide | 1 | 0 |  |  |  |  |  |  |  |  |
| GO:0046929\_negative\_regulation\_of\_neurotransmitter\_secretion | 1 | 0 |  |  |  |  |  |  |  |  |
| GO:0046931\_pore\_complex\_biogenesis | 1 | 0 |  |  |  |  |  |  |  |  |
| GO:0046949\_acyl-CoA\_biosynthetic\_process | 1 | 0 |  |  |  |  |  |  |  |  |
| GO:0046958\_nonassociative\_learning | 1 | 0 |  |  |  |  |  |  |  |  |
| GO:0046960\_sensitization | 1 | 0 |  |  |  |  |  |  |  |  |
| GO:0046986\_negative\_regulation\_of\_hemoglobin\_biosynthetic\_process | 1 | 0 |  |  |  |  |  |  |  |  |
| GO:0047497\_mitochondrion\_transport\_along\_microtubule | 1 | 0 |  |  |  |  |  |  |  |  |
| GO:0048047\_mating\_behavior\_\_sex\_discrimination | 1 | 0 |  |  |  |  |  |  |  |  |
| GO:0048133\_male\_germ-line\_stem\_cell\_division | 1 | 0 |  |  |  |  |  |  |  |  |
| GO:0048137\_spermatocyte\_division | 1 | 0 |  |  |  |  |  |  |  |  |
| GO:0048143\_astrocyte\_activation | 1 | 0 |  |  |  |  |  |  |  |  |
| GO:0048170\_positive\_regulation\_of\_long-term\_neuronal\_synaptic\_plasticity | 1 | 0 |  |  |  |  |  |  |  |  |
| GO:0048241\_epinephrine\_transport | 1 | 0 |  |  |  |  |  |  |  |  |
| GO:0048242\_epinephrine\_secretion | 1 | 0 |  |  |  |  |  |  |  |  |
| GO:0048243\_norepinephrine\_secretion | 1 | 0 |  |  |  |  |  |  |  |  |
| GO:0048247\_lymphocyte\_chemotaxis | 1 | 0 |  |  |  |  |  |  |  |  |
| GO:0048250\_mitochondrial\_iron\_ion\_transport | 1 | 0 |  |  |  |  |  |  |  |  |
| GO:0048259\_regulation\_of\_receptor-mediated\_endocytosis | 1 | 0 |  |  |  |  |  |  |  |  |
| GO:0048260\_positive\_regulation\_of\_receptor-mediated\_endocytosis | 1 | 0 |  |  |  |  |  |  |  |  |
| GO:0048290\_isotype\_switching\_to\_IgA\_isotypes | 1 | 0 |  |  |  |  |  |  |  |  |
| GO:0048296\_regulation\_of\_isotype\_switching\_to\_IgA\_isotypes | 1 | 0 |  |  |  |  |  |  |  |  |
| GO:0048298\_positive\_regulation\_of\_isotype\_switching\_to\_IgA\_isotypes | 1 | 0 |  |  |  |  |  |  |  |  |
| GO:0048319\_axial\_mesoderm\_morphogenesis | 1 | 0 |  |  |  |  |  |  |  |  |
| GO:0048320\_axial\_mesoderm\_formation | 1 | 0 |  |  |  |  |  |  |  |  |
| GO:0048385\_regulation\_of\_retinoic\_acid\_receptor\_signaling\_pathway | 1 | 0 |  |  |  |  |  |  |  |  |
| GO:0048387\_negative\_regulation\_of\_retinoic\_acid\_receptor\_signaling\_pathway | 1 | 0 |  |  |  |  |  |  |  |  |
| GO:0048388\_endosomal\_lumen\_acidification | 1 | 0 |  |  |  |  |  |  |  |  |
| GO:0048389\_intermediate\_mesoderm\_development | 1 | 0 |  |  |  |  |  |  |  |  |
| GO:0048478\_replication\_fork\_protection | 1 | 0 |  |  |  |  |  |  |  |  |
| GO:0048496\_maintenance\_of\_organ\_identity | 1 | 0 |  |  |  |  |  |  |  |  |
| GO:0048525\_negative\_regulation\_of\_viral\_reproduction | 1 | 0 |  |  |  |  |  |  |  |  |
| GO:0048539\_bone\_marrow\_development | 1 | 0 |  |  |  |  |  |  |  |  |
| GO:0048548\_regulation\_of\_pinocytosis | 1 | 0 |  |  |  |  |  |  |  |  |
| GO:0048549\_positive\_regulation\_of\_pinocytosis | 1 | 0 |  |  |  |  |  |  |  |  |
| GO:0048553\_negative\_regulation\_of\_metalloenzyme\_activity | 1 | 0 |  |  |  |  |  |  |  |  |
| GO:0048588\_developmental\_cell\_growth | 1 | 0 |  |  |  |  |  |  |  |  |
| GO:0048601\_oocyte\_morphogenesis | 1 | 0 |  |  |  |  |  |  |  |  |
| GO:0048621\_post-embryonic\_gut\_morphogenesis | 1 | 0 |  |  |  |  |  |  |  |  |
| GO:0048640\_negative\_regulation\_of\_developmental\_growth | 1 | 0 |  |  |  |  |  |  |  |  |
| GO:0048642\_negative\_regulation\_of\_skeletal\_muscle\_tissue\_development | 1 | 0 |  |  |  |  |  |  |  |  |
| GO:0048669\_collateral\_sprouting\_in\_the\_absence\_of\_injury | 1 | 0 |  |  |  |  |  |  |  |  |
| GO:0048680\_positive\_regulation\_of\_axon\_regeneration | 1 | 0 |  |  |  |  |  |  |  |  |
| GO:0048681\_negative\_regulation\_of\_axon\_regeneration | 1 | 0 |  |  |  |  |  |  |  |  |
| GO:0048686\_regulation\_of\_sprouting\_of\_injured\_axon | 1 | 0 |  |  |  |  |  |  |  |  |
| GO:0048687\_positive\_regulation\_of\_sprouting\_of\_injured\_axon | 1 | 0 |  |  |  |  |  |  |  |  |
| GO:0048690\_regulation\_of\_axon\_extension\_involved\_in\_regeneration | 1 | 0 |  |  |  |  |  |  |  |  |
| GO:0048691\_positive\_regulation\_of\_axon\_extension\_involved\_in\_regeneration | 1 | 0 |  |  |  |  |  |  |  |  |
| GO:0048714\_positive\_regulation\_of\_oligodendrocyte\_differentiation | 1 | 0 |  |  |  |  |  |  |  |  |
| GO:0048733\_sebaceous\_gland\_development | 1 | 0 |  |  |  |  |  |  |  |  |
| GO:0048743\_positive\_regulation\_of\_skeletal\_muscle\_fiber\_development | 1 | 0 |  |  |  |  |  |  |  |  |
| GO:0048752\_semicircular\_canal\_morphogenesis | 1 | 0 |  |  |  |  |  |  |  |  |
| GO:0048773\_erythrophore\_differentiation | 1 | 0 |  |  |  |  |  |  |  |  |
| GO:0048790\_maintenance\_of\_presynaptic\_active\_zone\_structure | 1 | 0 |  |  |  |  |  |  |  |  |
| GO:0048791\_calcium\_ion-dependent\_exocytosis\_of\_neurotransmitter | 1 | 0 |  |  |  |  |  |  |  |  |
| GO:0048822\_enucleate\_erythrocyte\_development | 1 | 0 |  |  |  |  |  |  |  |  |
| GO:0048866\_stem\_cell\_fate\_specification | 1 | 0 |  |  |  |  |  |  |  |  |
| GO:0048936\_peripheral\_nervous\_system\_neuron\_axonogenesis | 1 | 0 |  |  |  |  |  |  |  |  |
| GO:0050427\_3'-phosphoadenosine\_5'-phosphosulfate\_metabolic\_process | 1 | 0 |  |  |  |  |  |  |  |  |
| GO:0050428\_3'-phosphoadenosine\_5'-phosphosulfate\_biosynthetic\_process | 1 | 0 |  |  |  |  |  |  |  |  |
| GO:0050482\_arachidonic\_acid\_secretion | 1 | 0 |  |  |  |  |  |  |  |  |
| GO:0050667\_homocysteine\_metabolic\_process | 1 | 0 |  |  |  |  |  |  |  |  |
| GO:0050674\_urothelial\_cell\_proliferation | 1 | 0 |  |  |  |  |  |  |  |  |
| GO:0050675\_regulation\_of\_urothelial\_cell\_proliferation | 1 | 0 |  |  |  |  |  |  |  |  |
| GO:0050677\_positive\_regulation\_of\_urothelial\_cell\_proliferation | 1 | 0 |  |  |  |  |  |  |  |  |
| GO:0050691\_regulation\_of\_defense\_response\_to\_virus\_by\_host | 1 | 0 |  |  |  |  |  |  |  |  |
| GO:0050748\_negative\_regulation\_of\_lipoprotein\_metabolic\_process | 1 | 0 |  |  |  |  |  |  |  |  |
| GO:0050757\_thymidylate\_synthase\_biosynthetic\_process | 1 | 0 |  |  |  |  |  |  |  |  |
| GO:0050758\_regulation\_of\_thymidylate\_synthase\_biosynthetic\_process | 1 | 0 |  |  |  |  |  |  |  |  |
| GO:0050760\_negative\_regulation\_of\_thymidylate\_synthase\_biosynthetic\_process | 1 | 0 |  |  |  |  |  |  |  |  |
| GO:0050812\_regulation\_of\_acyl-CoA\_biosynthetic\_process | 1 | 0 |  |  |  |  |  |  |  |  |
| GO:0050832\_defense\_response\_to\_fungus | 1 | 0 |  |  |  |  |  |  |  |  |
| GO:0050861\_positive\_regulation\_of\_B\_cell\_receptor\_signaling\_pathway | 1 | 0 |  |  |  |  |  |  |  |  |
| GO:0050862\_positive\_regulation\_of\_T\_cell\_receptor\_signaling\_pathway | 1 | 0 |  |  |  |  |  |  |  |  |
| GO:0050916\_sensory\_perception\_of\_sweet\_taste | 1 | 0 |  |  |  |  |  |  |  |  |
| GO:0050975\_sensory\_perception\_of\_touch | 1 | 0 |  |  |  |  |  |  |  |  |
| GO:0050995\_negative\_regulation\_of\_lipid\_catabolic\_process | 1 | 0 |  |  |  |  |  |  |  |  |
| GO:0051001\_negative\_regulation\_of\_nitric-oxide\_synthase\_activity | 1 | 0 |  |  |  |  |  |  |  |  |
| GO:0051005\_negative\_regulation\_of\_lipoprotein\_lipase\_activity | 1 | 0 |  |  |  |  |  |  |  |  |
| GO:0051006\_positive\_regulation\_of\_lipoprotein\_lipase\_activity | 1 | 0 |  |  |  |  |  |  |  |  |
| GO:0051016\_barbed-end\_actin\_filament\_capping | 1 | 0 |  |  |  |  |  |  |  |  |
| GO:0051029\_rRNA\_transport | 1 | 0 |  |  |  |  |  |  |  |  |
| GO:0051043\_regulation\_of\_membrane\_protein\_ectodomain\_proteolysis | 1 | 0 |  |  |  |  |  |  |  |  |
| GO:0051044\_positive\_regulation\_of\_membrane\_protein\_ectodomain\_proteolysis | 1 | 0 |  |  |  |  |  |  |  |  |
| GO:0051088\_PMA-inducible\_membrane\_protein\_ectodomain\_proteolysis | 1 | 0 |  |  |  |  |  |  |  |  |
| GO:0051102\_DNA\_ligation\_during\_DNA\_recombination | 1 | 0 |  |  |  |  |  |  |  |  |
| GO:0051103\_DNA\_ligation\_during\_DNA\_repair | 1 | 0 |  |  |  |  |  |  |  |  |
| GO:0051123\_transcriptional\_preinitiation\_complex\_assembly | 1 | 0 |  |  |  |  |  |  |  |  |
| GO:0051125\_regulation\_of\_actin\_nucleation | 1 | 0 |  |  |  |  |  |  |  |  |
| GO:0051127\_positive\_regulation\_of\_actin\_nucleation | 1 | 0 |  |  |  |  |  |  |  |  |
| GO:0051151\_negative\_regulation\_of\_smooth\_muscle\_cell\_differentiation | 1 | 0 |  |  |  |  |  |  |  |  |
| GO:0051154\_negative\_regulation\_of\_striated\_muscle\_cell\_differentiation | 1 | 0 |  |  |  |  |  |  |  |  |
| GO:0051155\_positive\_regulation\_of\_striated\_muscle\_cell\_differentiation | 1 | 0 |  |  |  |  |  |  |  |  |
| GO:0051156\_glucose\_6-phosphate\_metabolic\_process | 1 | 0 |  |  |  |  |  |  |  |  |
| GO:0051187\_cofactor\_catabolic\_process | 1 | 0 |  |  |  |  |  |  |  |  |
| GO:0051189\_prosthetic\_group\_metabolic\_process | 1 | 0 |  |  |  |  |  |  |  |  |
| GO:0051193\_regulation\_of\_cofactor\_metabolic\_process | 1 | 0 |  |  |  |  |  |  |  |  |
| GO:0051196\_regulation\_of\_coenzyme\_metabolic\_process | 1 | 0 |  |  |  |  |  |  |  |  |
| GO:0051255\_spindle\_midzone\_assembly | 1 | 0 |  |  |  |  |  |  |  |  |
| GO:0051257\_spindle\_midzone\_assembly\_involved\_in\_meiosis | 1 | 0 |  |  |  |  |  |  |  |  |
| GO:0051281\_positive\_regulation\_of\_release\_of\_sequestered\_calcium\_ion\_into\_cytosol | 1 | 0 |  |  |  |  |  |  |  |  |
| GO:0051290\_protein\_heterotetramerization | 1 | 0 |  |  |  |  |  |  |  |  |
| GO:0051305\_chromosome\_movement\_towards\_spindle\_pole | 1 | 0 |  |  |  |  |  |  |  |  |
| GO:0051310\_metaphase\_plate\_congression | 1 | 0 |  |  |  |  |  |  |  |  |
| GO:0051311\_meiotic\_metaphase\_plate\_congression | 1 | 0 |  |  |  |  |  |  |  |  |
| GO:0051340\_regulation\_of\_ligase\_activity | 1 | 0 |  |  |  |  |  |  |  |  |
| GO:0051351\_positive\_regulation\_of\_ligase\_activity | 1 | 0 |  |  |  |  |  |  |  |  |
| GO:0051354\_negative\_regulation\_of\_oxidoreductase\_activity | 1 | 0 |  |  |  |  |  |  |  |  |
| GO:0051355\_proprioception\_during\_equilibrioception | 1 | 0 |  |  |  |  |  |  |  |  |
| GO:0051383\_kinetochore\_organization | 1 | 0 |  |  |  |  |  |  |  |  |
| GO:0051386\_regulation\_of\_nerve\_growth\_factor\_receptor\_signaling\_pathway | 1 | 0 |  |  |  |  |  |  |  |  |
| GO:0051409\_response\_to\_nitrosative\_stress | 1 | 0 |  |  |  |  |  |  |  |  |
| GO:0051457\_maintenance\_of\_protein\_location\_in\_nucleus | 1 | 0 |  |  |  |  |  |  |  |  |
| GO:0051462\_regulation\_of\_cortisol\_secretion | 1 | 0 |  |  |  |  |  |  |  |  |
| GO:0051463\_negative\_regulation\_of\_cortisol\_secretion | 1 | 0 |  |  |  |  |  |  |  |  |
| GO:0051481\_reduction\_of\_cytosolic\_calcium\_ion\_concentration | 1 | 0 |  |  |  |  |  |  |  |  |
| GO:0051482\_elevation\_of\_cytosolic\_calcium\_ion\_concentration\_during\_G-protein\_signaling\_\_coupled\_to\_IP3\_second\_messenger\_(phospholipase\_C\_activating) | 1 | 0 |  |  |  |  |  |  |  |  |
| GO:0051542\_elastin\_biosynthetic\_process | 1 | 0 |  |  |  |  |  |  |  |  |
| GO:0051568\_histone\_H3-K4\_methylation | 1 | 0 |  |  |  |  |  |  |  |  |
| GO:0051569\_regulation\_of\_histone\_H3-K4\_methylation | 1 | 0 |  |  |  |  |  |  |  |  |
| GO:0051570\_regulation\_of\_histone\_H3-K9\_methylation | 1 | 0 |  |  |  |  |  |  |  |  |
| GO:0051573\_negative\_regulation\_of\_histone\_H3-K9\_methylation | 1 | 0 |  |  |  |  |  |  |  |  |
| GO:0051580\_regulation\_of\_neurotransmitter\_uptake | 1 | 0 |  |  |  |  |  |  |  |  |
| GO:0051582\_positive\_regulation\_of\_neurotransmitter\_uptake | 1 | 0 |  |  |  |  |  |  |  |  |
| GO:0051584\_regulation\_of\_dopamine\_uptake | 1 | 0 |  |  |  |  |  |  |  |  |
| GO:0051586\_positive\_regulation\_of\_dopamine\_uptake | 1 | 0 |  |  |  |  |  |  |  |  |
| GO:0051589\_negative\_regulation\_of\_neurotransmitter\_transport | 1 | 0 |  |  |  |  |  |  |  |  |
| GO:0051593\_response\_to\_folic\_acid | 1 | 0 |  |  |  |  |  |  |  |  |
| GO:0051615\_histamine\_uptake | 1 | 0 |  |  |  |  |  |  |  |  |
| GO:0051646\_mitochondrion\_localization | 1 | 0 |  |  |  |  |  |  |  |  |
| GO:0051654\_establishment\_of\_mitochondrion\_localization | 1 | 0 |  |  |  |  |  |  |  |  |
| GO:0051661\_maintenance\_of\_centrosome\_location | 1 | 0 |  |  |  |  |  |  |  |  |
| GO:0051665\_membrane\_raft\_localization | 1 | 0 |  |  |  |  |  |  |  |  |
| GO:0051685\_maintenance\_of\_ER\_location | 1 | 0 |  |  |  |  |  |  |  |  |
| GO:0051693\_actin\_filament\_capping | 1 | 0 |  |  |  |  |  |  |  |  |
| GO:0051701\_interaction\_with\_host | 1 | 0 |  |  |  |  |  |  |  |  |
| GO:0051754\_meiotic\_sister\_chromatid\_cohesion\_\_centromeric | 1 | 0 |  |  |  |  |  |  |  |  |
| GO:0051782\_negative\_regulation\_of\_cell\_division | 1 | 0 |  |  |  |  |  |  |  |  |
| GO:0051790\_short-chain\_fatty\_acid\_biosynthetic\_process | 1 | 0 |  |  |  |  |  |  |  |  |
| GO:0051799\_negative\_regulation\_of\_hair\_follicle\_development | 1 | 0 |  |  |  |  |  |  |  |  |
| GO:0051823\_regulation\_of\_synapse\_structural\_plasticity | 1 | 0 |  |  |  |  |  |  |  |  |
| GO:0051865\_protein\_autoubiquitination | 1 | 0 |  |  |  |  |  |  |  |  |
| GO:0051901\_positive\_regulation\_of\_mitochondrial\_depolarization | 1 | 0 |  |  |  |  |  |  |  |  |
| GO:0051917\_regulation\_of\_fibrinolysis | 1 | 0 |  |  |  |  |  |  |  |  |
| GO:0051918\_negative\_regulation\_of\_fibrinolysis | 1 | 0 |  |  |  |  |  |  |  |  |
| GO:0051929\_positive\_regulation\_of\_calcium\_ion\_transport\_via\_voltage-gated\_calcium\_channel\_activity | 1 | 0 |  |  |  |  |  |  |  |  |
| GO:0051933\_amino\_acid\_uptake\_during\_transmission\_of\_nerve\_impulse | 1 | 0 |  |  |  |  |  |  |  |  |
| GO:0051935\_glutamate\_uptake\_during\_transmission\_of\_nerve\_impulse | 1 | 0 |  |  |  |  |  |  |  |  |
| GO:0051940\_regulation\_of\_catecholamine\_uptake\_during\_transmission\_of\_nerve\_impulse | 1 | 0 |  |  |  |  |  |  |  |  |
| GO:0051944\_positive\_regulation\_of\_catecholamine\_uptake\_during\_transmission\_of\_nerve\_impulse | 1 | 0 |  |  |  |  |  |  |  |  |
| GO:0051961\_negative\_regulation\_of\_nervous\_system\_development | 1 | 0 |  |  |  |  |  |  |  |  |
| GO:0051964\_negative\_regulation\_of\_synaptogenesis | 1 | 0 |  |  |  |  |  |  |  |  |
| GO:0051968\_positive\_regulation\_of\_synaptic\_transmission\_\_glutamatergic | 1 | 0 |  |  |  |  |  |  |  |  |
| GO:0051984\_positive\_regulation\_of\_chromosome\_segregation | 1 | 0 |  |  |  |  |  |  |  |  |
| GO:0051987\_positive\_regulation\_of\_attachment\_of\_spindle\_microtubules\_to\_kinetochore | 1 | 0 |  |  |  |  |  |  |  |  |
| GO:0052173\_response\_to\_defenses\_of\_other\_organism\_during\_symbiotic\_interaction | 1 | 0 |  |  |  |  |  |  |  |  |
| GO:0052200\_response\_to\_host\_defenses | 1 | 0 |  |  |  |  |  |  |  |  |
| GO:0052551\_response\_to\_defense-related\_nitric\_oxide\_production\_by\_other\_organism\_during\_symbiotic\_interaction | 1 | 0 |  |  |  |  |  |  |  |  |
| GO:0052564\_response\_to\_immune\_response\_of\_other\_organism\_during\_symbiotic\_interaction | 1 | 0 |  |  |  |  |  |  |  |  |
| GO:0052565\_response\_to\_defense-related\_host\_nitric\_oxide\_production | 1 | 0 |  |  |  |  |  |  |  |  |
| GO:0052572\_response\_to\_host\_immune\_response | 1 | 0 |  |  |  |  |  |  |  |  |
| GO:0055005\_ventricular\_cardiac\_myofibril\_development | 1 | 0 |  |  |  |  |  |  |  |  |
| GO:0055011\_atrial\_cardiac\_muscle\_cell\_differentiation | 1 | 0 |  |  |  |  |  |  |  |  |
| GO:0055014\_atrial\_cardiac\_muscle\_cell\_development | 1 | 0 |  |  |  |  |  |  |  |  |
| GO:0055078\_sodium\_ion\_homeostasis | 1 | 0 |  |  |  |  |  |  |  |  |
| GO:0055089\_fatty\_acid\_homeostasis | 1 | 0 |  |  |  |  |  |  |  |  |
| GO:0055093\_response\_to\_hyperoxia | 1 | 0 |  |  |  |  |  |  |  |  |
| GO:0060003\_copper\_ion\_export | 1 | 0 |  |  |  |  |  |  |  |  |
| GO:0060005\_vestibular\_reflex | 1 | 0 |  |  |  |  |  |  |  |  |
| GO:0060014\_granulosa\_cell\_differentiation | 1 | 0 |  |  |  |  |  |  |  |  |
| GO:0060018\_astrocyte\_fate\_commitment | 1 | 0 |  |  |  |  |  |  |  |  |
| GO:0060020\_Bergmann\_glial\_cell\_differentiation | 1 | 0 |  |  |  |  |  |  |  |  |
| GO:0060022\_hard\_palate\_development | 1 | 0 |  |  |  |  |  |  |  |  |
| GO:0060034\_notochord\_cell\_differentiation | 1 | 0 |  |  |  |  |  |  |  |  |
| GO:0060035\_notochord\_cell\_development | 1 | 0 |  |  |  |  |  |  |  |  |
| GO:0060046\_regulation\_of\_acrosome\_reaction | 1 | 0 |  |  |  |  |  |  |  |  |
| GO:0060054\_positive\_regulation\_of\_epithelial\_cell\_proliferation\_involved\_in\_wound\_healing | 1 | 0 |  |  |  |  |  |  |  |  |
| GO:0060059\_embryonic\_retina\_morphogenesis\_in\_camera-type\_eye | 1 | 0 |  |  |  |  |  |  |  |  |
| GO:0060061\_Spemann\_organizer\_formation | 1 | 0 |  |  |  |  |  |  |  |  |
| GO:0060064\_Spemann\_organizer\_formation\_at\_the\_anterior\_end\_of\_the\_primitive\_streak | 1 | 0 |  |  |  |  |  |  |  |  |
| GO:0060071\_Wnt\_receptor\_signaling\_pathway\_\_planar\_cell\_polarity\_pathway | 1 | 0 |  |  |  |  |  |  |  |  |
| GO:0060075\_regulation\_of\_resting\_membrane\_potential | 1 | 0 |  |  |  |  |  |  |  |  |
| GO:0060082\_eye\_blink\_reflex | 1 | 0 |  |  |  |  |  |  |  |  |
| GO:0060112\_generation\_of\_ovulation\_cycle\_rhythm | 1 | 0 |  |  |  |  |  |  |  |  |
| GO:0060125\_negative\_regulation\_of\_growth\_hormone\_secretion | 1 | 0 |  |  |  |  |  |  |  |  |
| GO:0060151\_peroxisome\_localization | 1 | 0 |  |  |  |  |  |  |  |  |
| GO:0060152\_microtubule-based\_peroxisome\_localization | 1 | 0 |  |  |  |  |  |  |  |  |
| GO:0060161\_positive\_regulation\_of\_dopamine\_receptor\_signaling\_pathway | 1 | 0 |  |  |  |  |  |  |  |  |
| GO:0060163\_subpallium\_neuron\_fate\_commitment | 1 | 0 |  |  |  |  |  |  |  |  |
| GO:0060165\_regulation\_of\_timing\_of\_subpallium\_neuron\_differentiation | 1 | 0 |  |  |  |  |  |  |  |  |
| GO:0060174\_limb\_bud\_formation | 1 | 0 |  |  |  |  |  |  |  |  |
| GO:0060177\_regulation\_of\_angiotensin\_metabolic\_process | 1 | 0 |  |  |  |  |  |  |  |  |
| GO:0060197\_cloacal\_septation | 1 | 0 |  |  |  |  |  |  |  |  |
| GO:0060215\_primitive\_hemopoiesis | 1 | 0 |  |  |  |  |  |  |  |  |
| GO:0060231\_mesenchymal\_to\_epithelial\_transition | 1 | 0 |  |  |  |  |  |  |  |  |
| GO:0060254\_regulation\_of\_N-terminal\_protein\_palmitoylation | 1 | 0 |  |  |  |  |  |  |  |  |
| GO:0060261\_positive\_regulation\_of\_transcription\_initiation\_from\_RNA\_polymerase\_II\_promoter | 1 | 0 |  |  |  |  |  |  |  |  |
| GO:0060262\_negative\_regulation\_of\_N-terminal\_protein\_palmitoylation | 1 | 0 |  |  |  |  |  |  |  |  |
| GO:0060263\_regulation\_of\_respiratory\_burst | 1 | 0 |  |  |  |  |  |  |  |  |
| GO:0060264\_regulation\_of\_respiratory\_burst\_during\_acute\_inflammatory\_response | 1 | 0 |  |  |  |  |  |  |  |  |
| GO:0060265\_positive\_regulation\_of\_respiratory\_burst\_during\_acute\_inflammatory\_response | 1 | 0 |  |  |  |  |  |  |  |  |
| GO:0060267\_positive\_regulation\_of\_respiratory\_burst | 1 | 0 |  |  |  |  |  |  |  |  |
| GO:0060272\_embryonic\_skeletal\_joint\_morphogenesis | 1 | 0 |  |  |  |  |  |  |  |  |
| GO:0060297\_regulation\_of\_sarcomere\_organization | 1 | 0 |  |  |  |  |  |  |  |  |
| GO:0060298\_positive\_regulation\_of\_sarcomere\_organization | 1 | 0 |  |  |  |  |  |  |  |  |
| GO:0060315\_negative\_regulation\_of\_ryanodine-sensitive\_calcium-release\_channel\_activity | 1 | 0 |  |  |  |  |  |  |  |  |
| GO:0060319\_primitive\_erythrocyte\_differentiation | 1 | 0 |  |  |  |  |  |  |  |  |
| GO:0060371\_regulation\_of\_atrial\_cardiomyocyte\_membrane\_depolarization | 1 | 0 |  |  |  |  |  |  |  |  |
| GO:0060374\_mast\_cell\_differentiation | 1 | 0 |  |  |  |  |  |  |  |  |
| GO:0060375\_regulation\_of\_mast\_cell\_differentiation | 1 | 0 |  |  |  |  |  |  |  |  |
| GO:0060376\_positive\_regulation\_of\_mast\_cell\_differentiation | 1 | 0 |  |  |  |  |  |  |  |  |
| GO:0060390\_regulation\_of\_SMAD\_protein\_nuclear\_translocation | 1 | 0 |  |  |  |  |  |  |  |  |
| GO:0060391\_positive\_regulation\_of\_SMAD\_protein\_nuclear\_translocation | 1 | 0 |  |  |  |  |  |  |  |  |
| GO:0060398\_regulation\_of\_growth\_hormone\_receptor\_signaling\_pathway | 1 | 0 |  |  |  |  |  |  |  |  |
| GO:0060399\_positive\_regulation\_of\_growth\_hormone\_receptor\_signaling\_pathway | 1 | 0 |  |  |  |  |  |  |  |  |
| GO:0060405\_regulation\_of\_penile\_erection | 1 | 0 |  |  |  |  |  |  |  |  |
| GO:0060407\_negative\_regulation\_of\_penile\_erection | 1 | 0 |  |  |  |  |  |  |  |  |
| GO:0060413\_atrial\_septum\_morphogenesis | 1 | 0 |  |  |  |  |  |  |  |  |
| GO:0060414\_aorta\_smooth\_muscle\_tissue\_morphogenesis | 1 | 0 |  |  |  |  |  |  |  |  |
| GO:0060419\_heart\_growth | 1 | 0 |  |  |  |  |  |  |  |  |
| GO:0060420\_regulation\_of\_heart\_growth | 1 | 0 |  |  |  |  |  |  |  |  |
| GO:0060421\_positive\_regulation\_of\_heart\_growth | 1 | 0 |  |  |  |  |  |  |  |  |
| GO:0060431\_primary\_lung\_bud\_formation | 1 | 0 |  |  |  |  |  |  |  |  |
| GO:0060436\_bronchiole\_morphogenesis | 1 | 0 |  |  |  |  |  |  |  |  |
| GO:0060440\_trachea\_formation | 1 | 0 |  |  |  |  |  |  |  |  |
| GO:0060449\_bud\_elongation\_involved\_in\_lung\_branching | 1 | 0 |  |  |  |  |  |  |  |  |
| GO:0060456\_positive\_regulation\_of\_digestive\_system\_process | 1 | 0 |  |  |  |  |  |  |  |  |
| GO:0060461\_right\_lung\_morphogenesis | 1 | 0 |  |  |  |  |  |  |  |  |
| GO:0060481\_lobar\_bronchus\_epithelium\_development | 1 | 0 |  |  |  |  |  |  |  |  |
| GO:0060482\_lobar\_bronchus\_development | 1 | 0 |  |  |  |  |  |  |  |  |
| GO:0060484\_lung-associated\_mesenchyme\_development | 1 | 0 |  |  |  |  |  |  |  |  |
| GO:0060486\_Clara\_cell\_differentiation | 1 | 0 |  |  |  |  |  |  |  |  |
| GO:0060510\_Type\_II\_pneumocyte\_differentiation | 1 | 0 |  |  |  |  |  |  |  |  |
| GO:0060514\_prostate\_induction | 1 | 0 |  |  |  |  |  |  |  |  |
| GO:0060515\_prostate\_field\_specification | 1 | 0 |  |  |  |  |  |  |  |  |
| GO:0060517\_epithelial\_cell\_proliferation\_involved\_in\_prostatic\_bud\_elongation | 1 | 0 |  |  |  |  |  |  |  |  |
| GO:0060520\_activation\_of\_prostate\_induction\_by\_androgen\_receptor\_signaling\_pathway | 1 | 0 |  |  |  |  |  |  |  |  |
| GO:0060535\_trachea\_cartilage\_morphogenesis | 1 | 0 |  |  |  |  |  |  |  |  |
| GO:0060536\_cartilage\_morphogenesis | 1 | 0 |  |  |  |  |  |  |  |  |
| GO:0060563\_neuroepithelial\_cell\_differentiation | 1 | 0 |  |  |  |  |  |  |  |  |
| GO:0060577\_pulmonary\_vein\_morphogenesis | 1 | 0 |  |  |  |  |  |  |  |  |
| GO:0060578\_superior\_vena\_cava\_morphogenesis | 1 | 0 |  |  |  |  |  |  |  |  |
| GO:0060584\_regulation\_of\_prostaglandin-endoperoxide\_synthase\_activity | 1 | 0 |  |  |  |  |  |  |  |  |
| GO:0060585\_positive\_regulation\_of\_prostaglandin-endoperoxidase\_synthase\_activity | 1 | 0 |  |  |  |  |  |  |  |  |
| GO:0060598\_dichotomous\_subdivision\_of\_terminal\_units\_involved\_in\_mammary\_gland\_duct\_morphogenesis | 1 | 0 |  |  |  |  |  |  |  |  |
| GO:0060611\_mammary\_gland\_fat\_development | 1 | 0 |  |  |  |  |  |  |  |  |
| GO:0060618\_nipple\_development | 1 | 0 |  |  |  |  |  |  |  |  |
| GO:0060631\_regulation\_of\_meiosis\_I | 1 | 0 |  |  |  |  |  |  |  |  |
| GO:0060649\_mammary\_gland\_bud\_elongation | 1 | 0 |  |  |  |  |  |  |  |  |
| GO:0060658\_nipple\_morphogenesis | 1 | 0 |  |  |  |  |  |  |  |  |
| GO:0060659\_nipple\_sheath\_formation | 1 | 0 |  |  |  |  |  |  |  |  |
| GO:0060668\_regulation\_of\_branching\_involved\_in\_salivary\_gland\_morphogenesis\_by\_extracellular\_matrix-epithelial\_cell\_signaling | 1 | 0 |  |  |  |  |  |  |  |  |
| GO:0060683\_regulation\_of\_branching\_involved\_in\_salivary\_gland\_morphogenesis\_by\_epithelial-mesenchymal\_signaling | 1 | 0 |  |  |  |  |  |  |  |  |
| GO:0060691\_epithelial\_cell\_maturation\_involved\_in\_salivary\_gland\_development | 1 | 0 |  |  |  |  |  |  |  |  |
| GO:0060709\_glycogen\_cell\_development\_involved\_in\_embryonic\_placenta\_development | 1 | 0 |  |  |  |  |  |  |  |  |
| GO:0060732\_positive\_regulation\_of\_inositol\_phosphate\_biosynthetic\_process | 1 | 0 |  |  |  |  |  |  |  |  |
| GO:0060739\_mesenchymal-epithelial\_cell\_signaling\_involved\_in\_prostate\_gland\_development | 1 | 0 |  |  |  |  |  |  |  |  |
| GO:0060781\_mesenchymal\_cell\_proliferation\_involved\_in\_prostate\_gland\_development | 1 | 0 |  |  |  |  |  |  |  |  |
| GO:0060782\_regulation\_of\_mesenchymal\_cell\_proliferation\_involved\_in\_prostate\_gland\_development | 1 | 0 |  |  |  |  |  |  |  |  |
| GO:0060783\_mesenchymal\_smoothened\_signaling\_pathway\_involved\_in\_prostate\_gland\_development | 1 | 0 |  |  |  |  |  |  |  |  |
| GO:0060872\_semicircular\_canal\_development | 1 | 0 |  |  |  |  |  |  |  |  |
| GO:0060896\_neural\_plate\_pattern\_specification | 1 | 0 |  |  |  |  |  |  |  |  |
| GO:0070091\_glucagon\_secretion | 1 | 0 |  |  |  |  |  |  |  |  |
| GO:0070162\_adiponectin\_secretion | 1 | 0 |  |  |  |  |  |  |  |  |
| GO:0070163\_regulation\_of\_adiponectin\_secretion | 1 | 0 |  |  |  |  |  |  |  |  |
| GO:0070164\_negative\_regulation\_of\_adiponectin\_secretion | 1 | 0 |  |  |  |  |  |  |  |  |
| GO:0070178\_D-serine\_metabolic\_process | 1 | 0 |  |  |  |  |  |  |  |  |
| GO:0070179\_D-serine\_biosynthetic\_process | 1 | 0 |  |  |  |  |  |  |  |  |
| GO:0070296\_sarcoplasmic\_reticulum\_calcium\_ion\_transport | 1 | 0 |  |  |  |  |  |  |  |  |
| GO:0070303\_negative\_regulation\_of\_stress-activated\_protein\_kinase\_signaling\_pathway | 1 | 0 |  |  |  |  |  |  |  |  |
| GO:0070328\_triglyceride\_homeostasis | 1 | 0 |  |  |  |  |  |  |  |  |
| GO:0070365\_hepatocyte\_differentiation | 1 | 0 |  |  |  |  |  |  |  |  |
| GO:0070384\_Harderian\_gland\_development | 1 | 0 |  |  |  |  |  |  |  |  |
| GO:0070391\_response\_to\_lipoteichoic\_acid | 1 | 0 |  |  |  |  |  |  |  |  |
| GO:0070424\_regulation\_of\_nucleotide-binding\_oligomerization\_domain\_containing\_signaling\_pathway | 1 | 0 |  |  |  |  |  |  |  |  |
| GO:0070426\_positive\_regulation\_of\_nucleotide-binding\_oligomerization\_domain\_containing\_signaling\_pathway | 1 | 0 |  |  |  |  |  |  |  |  |
| GO:0070428\_regulation\_of\_nucleotide-binding\_oligomerization\_domain\_containing\_1\_signaling\_pathway | 1 | 0 |  |  |  |  |  |  |  |  |
| GO:0070430\_positive\_regulation\_of\_nucleotide-binding\_oligomerization\_domain\_containing\_1\_signaling\_pathway | 1 | 0 |  |  |  |  |  |  |  |  |
| GO:0070432\_regulation\_of\_nucleotide-binding\_oligomerization\_domain\_containing\_2\_signaling\_pathway | 1 | 0 |  |  |  |  |  |  |  |  |
| GO:0070434\_positive\_regulation\_of\_nucleotide-binding\_oligomerization\_domain\_containing\_2\_signaling\_pathway | 1 | 0 |  |  |  |  |  |  |  |  |
| GO:0070493\_thrombin\_receptor\_signaling\_pathway | 1 | 0 |  |  |  |  |  |  |  |  |
| GO:0070508\_cholesterol\_import | 1 | 0 |  |  |  |  |  |  |  |  |
| GO:0070527\_platelet\_aggregation | 1 | 0 |  |  |  |  |  |  |  |  |
| GO:0070528\_protein\_kinase\_C\_signaling\_cascade | 1 | 0 |  |  |  |  |  |  |  |  |
| GO:0070555\_response\_to\_interleukin-1 | 1 | 0 |  |  |  |  |  |  |  |  |
| GO:0070560\_protein\_secretion\_by\_platelet | 1 | 0 |  |  |  |  |  |  |  |  |
| GO:0070561\_vitamin\_D\_receptor\_signaling\_pathway | 1 | 0 |  |  |  |  |  |  |  |  |
| GO:0070562\_regulation\_of\_vitamin\_D\_receptor\_signaling\_pathway | 1 | 0 |  |  |  |  |  |  |  |  |
| GO:0070571\_negative\_regulation\_of\_neuron\_projection\_regeneration | 1 | 0 |  |  |  |  |  |  |  |  |
| GO:0070572\_positive\_regulation\_of\_neuron\_projection\_regeneration | 1 | 0 |  |  |  |  |  |  |  |  |
| GO:0070613\_regulation\_of\_protein\_processing | 1 | 0 |  |  |  |  |  |  |  |  |
| GO:0070627\_ferrous\_iron\_import | 1 | 0 |  |  |  |  |  |  |  |  |
| GO:0070669\_response\_to\_interleukin-2 | 1 | 0 |  |  |  |  |  |  |  |  |
| GO:0070670\_response\_to\_interleukin-4 | 1 | 0 |  |  |  |  |  |  |  |  |
| GO:0070671\_response\_to\_interleukin-12 | 1 | 0 |  |  |  |  |  |  |  |  |
| GO:0070672\_response\_to\_interleukin-15 | 1 | 0 |  |  |  |  |  |  |  |  |
| GO:0070673\_response\_to\_interleukin-18 | 1 | 0 |  |  |  |  |  |  |  |  |
| GO:0070828\_heterochromatin\_organization | 1 | 0 |  |  |  |  |  |  |  |  |
| GO:0070874\_negative\_regulation\_of\_glycogen\_metabolic\_process | 1 | 0 |  |  |  |  |  |  |  |  |
| GO:0075136\_response\_to\_host | 1 | 0 |  |  |  |  |  |  |  |  |
| GO:0080010\_regulation\_of\_oxygen\_and\_reactive\_oxygen\_species\_metabolic\_process | 1 | 0 |  |  |  |  |  |  |  |  |
| GO:0090032\_negative\_regulation\_of\_steroid\_hormone\_biosynthetic\_process | 1 | 0 |  |  |  |  |  |  |  |  |
| GO:0007283\_spermatogenesis | 134 | 0 | 0.000000 | -0.000000 | 606 | 615.346638 | 703.54 | 791.733362 | 1.160957 |
| GO:0048232\_male\_gamete\_generation | 134 | 0 | 0.000000 | -0.000000 | 606 | 615.346638 | 703.54 | 791.733362 | 1.160957 |
| GO:0009416\_response\_to\_light\_stimulus | 74 | 0 | 0.000000 | -0.000000 | 608 | 616.836883 | 704.89 | 792.943117 | 1.159359 |
| GO:0048771\_tissue\_remodeling | 74 | 0 | 0.000000 | -0.000000 | 608 | 616.836883 | 704.89 | 792.943117 | 1.159359 |
| GO:0051128\_regulation\_of\_cellular\_component\_organization | 160 | 0 | 0.000000 | -0.000000 | 609 | 617.265988 | 705.28 | 793.294012 | 1.158095 |
| GO:0030029\_actin\_filament-based\_process | 109 | 0 | 0.000000 | -0.000000 | 610 | 617.967002 | 705.9 | 793.832998 | 1.157213 |
| GO:0002237\_response\_to\_molecule\_of\_bacterial\_origin | 34 | 0 | 0.000000 | -0.000000 | 626 | 633.835883 | 721.02 | 808.204117 | 1.151789 |
| GO:0002699\_positive\_regulation\_of\_immune\_effector\_process | 34 | 0 | 0.000000 | -0.000000 | 626 | 633.835883 | 721.02 | 808.204117 | 1.151789 |
| GO:0007269\_neurotransmitter\_secretion | 34 | 0 | 0.000000 | -0.000000 | 626 | 633.835883 | 721.02 | 808.204117 | 1.151789 |
| GO:0007338\_single\_fertilization | 34 | 0 | 0.000000 | -0.000000 | 626 | 633.835883 | 721.02 | 808.204117 | 1.151789 |
| GO:0010720\_positive\_regulation\_of\_cell\_development | 34 | 0 | 0.000000 | -0.000000 | 626 | 633.835883 | 721.02 | 808.204117 | 1.151789 |
| GO:0010721\_negative\_regulation\_of\_cell\_development | 34 | 0 | 0.000000 | -0.000000 | 626 | 633.835883 | 721.02 | 808.204117 | 1.151789 |
| GO:0016054\_organic\_acid\_catabolic\_process | 34 | 0 | 0.000000 | -0.000000 | 626 | 633.835883 | 721.02 | 808.204117 | 1.151789 |
| GO:0019882\_antigen\_processing\_and\_presentation | 34 | 0 | 0.000000 | -0.000000 | 626 | 633.835883 | 721.02 | 808.204117 | 1.151789 |
| GO:0030509\_BMP\_signaling\_pathway | 34 | 0 | 0.000000 | -0.000000 | 626 | 633.835883 | 721.02 | 808.204117 | 1.151789 |
| GO:0045927\_positive\_regulation\_of\_growth | 34 | 0 | 0.000000 | -0.000000 | 626 | 633.835883 | 721.02 | 808.204117 | 1.151789 |
| GO:0046395\_carboxylic\_acid\_catabolic\_process | 34 | 0 | 0.000000 | -0.000000 | 626 | 633.835883 | 721.02 | 808.204117 | 1.151789 |
| GO:0050730\_regulation\_of\_peptidyl-tyrosine\_phosphorylation | 34 | 0 | 0.000000 | -0.000000 | 626 | 633.835883 | 721.02 | 808.204117 | 1.151789 |
| GO:0051047\_positive\_regulation\_of\_secretion | 34 | 0 | 0.000000 | -0.000000 | 626 | 633.835883 | 721.02 | 808.204117 | 1.151789 |
| GO:0051052\_regulation\_of\_DNA\_metabolic\_process | 34 | 0 | 0.000000 | -0.000000 | 626 | 633.835883 | 721.02 | 808.204117 | 1.151789 |
| GO:0060443\_mammary\_gland\_morphogenesis | 34 | 0 | 0.000000 | -0.000000 | 626 | 633.835883 | 721.02 | 808.204117 | 1.151789 |
| GO:0060711\_labyrinthine\_layer\_development | 34 | 0 | 0.000000 | -0.000000 | 626 | 633.835883 | 721.02 | 808.204117 | 1.151789 |
| GO:0001932\_regulation\_of\_protein\_amino\_acid\_phosphorylation | 69 | 0 | 0.000000 | -0.000000 | 631 | 637.836689 | 724.66 | 811.483311 | 1.148431 |
| GO:0005996\_monosaccharide\_metabolic\_process | 69 | 0 | 0.000000 | -0.000000 | 631 | 637.836689 | 724.66 | 811.483311 | 1.148431 |
| GO:0006816\_calcium\_ion\_transport | 69 | 0 | 0.000000 | -0.000000 | 631 | 637.836689 | 724.66 | 811.483311 | 1.148431 |
| GO:0032101\_regulation\_of\_response\_to\_external\_stimulus | 69 | 0 | 0.000000 | -0.000000 | 631 | 637.836689 | 724.66 | 811.483311 | 1.148431 |
| GO:0055065\_metal\_ion\_homeostasis | 69 | 0 | 0.000000 | -0.000000 | 631 | 637.836689 | 724.66 | 811.483311 | 1.148431 |
| GO:0000060\_protein\_import\_into\_nucleus\_\_translocation | 14 | 0 | 0.000000 | -0.000000 | 695 | 699.541747 | 784.8 | 870.058253 | 1.129209 |
| GO:0000077\_DNA\_damage\_checkpoint | 14 | 0 | 0.000000 | -0.000000 | 695 | 699.541747 | 784.8 | 870.058253 | 1.129209 |
| GO:0001502\_cartilage\_condensation | 14 | 0 | 0.000000 | -0.000000 | 695 | 699.541747 | 784.8 | 870.058253 | 1.129209 |
| GO:0001829\_trophectodermal\_cell\_differentiation | 14 | 0 | 0.000000 | -0.000000 | 695 | 699.541747 | 784.8 | 870.058253 | 1.129209 |
| GO:0002027\_regulation\_of\_heart\_rate | 14 | 0 | 0.000000 | -0.000000 | 695 | 699.541747 | 784.8 | 870.058253 | 1.129209 |
| GO:0002262\_myeloid\_cell\_homeostasis | 14 | 0 | 0.000000 | -0.000000 | 695 | 699.541747 | 784.8 | 870.058253 | 1.129209 |
| GO:0002698\_negative\_regulation\_of\_immune\_effector\_process | 14 | 0 | 0.000000 | -0.000000 | 695 | 699.541747 | 784.8 | 870.058253 | 1.129209 |
| GO:0006304\_DNA\_modification | 14 | 0 | 0.000000 | -0.000000 | 695 | 699.541747 | 784.8 | 870.058253 | 1.129209 |
| GO:0006305\_DNA\_alkylation | 14 | 0 | 0.000000 | -0.000000 | 695 | 699.541747 | 784.8 | 870.058253 | 1.129209 |
| GO:0006306\_DNA\_methylation | 14 | 0 | 0.000000 | -0.000000 | 695 | 699.541747 | 784.8 | 870.058253 | 1.129209 |
| GO:0006695\_cholesterol\_biosynthetic\_process | 14 | 0 | 0.000000 | -0.000000 | 695 | 699.541747 | 784.8 | 870.058253 | 1.129209 |
| GO:0006809\_nitric\_oxide\_biosynthetic\_process | 14 | 0 | 0.000000 | -0.000000 | 695 | 699.541747 | 784.8 | 870.058253 | 1.129209 |
| GO:0006914\_autophagy | 14 | 0 | 0.000000 | -0.000000 | 695 | 699.541747 | 784.8 | 870.058253 | 1.129209 |
| GO:0006970\_response\_to\_osmotic\_stress | 14 | 0 | 0.000000 | -0.000000 | 695 | 699.541747 | 784.8 | 870.058253 | 1.129209 |
| GO:0007157\_heterophilic\_cell\_adhesion | 14 | 0 | 0.000000 | -0.000000 | 695 | 699.541747 | 784.8 | 870.058253 | 1.129209 |
| GO:0007530\_sex\_determination | 14 | 0 | 0.000000 | -0.000000 | 695 | 699.541747 | 784.8 | 870.058253 | 1.129209 |
| GO:0007589\_body\_fluid\_secretion | 14 | 0 | 0.000000 | -0.000000 | 695 | 699.541747 | 784.8 | 870.058253 | 1.129209 |
| GO:0008064\_regulation\_of\_actin\_polymerization\_or\_depolymerization | 14 | 0 | 0.000000 | -0.000000 | 695 | 699.541747 | 784.8 | 870.058253 | 1.129209 |
| GO:0008306\_associative\_learning | 14 | 0 | 0.000000 | -0.000000 | 695 | 699.541747 | 784.8 | 870.058253 | 1.129209 |
| GO:0008630\_DNA\_damage\_response\_\_signal\_transduction\_resulting\_in\_induction\_of\_apoptosis | 14 | 0 | 0.000000 | -0.000000 | 695 | 699.541747 | 784.8 | 870.058253 | 1.129209 |
| GO:0009108\_coenzyme\_biosynthetic\_process | 14 | 0 | 0.000000 | -0.000000 | 695 | 699.541747 | 784.8 | 870.058253 | 1.129209 |
| GO:0009267\_cellular\_response\_to\_starvation | 14 | 0 | 0.000000 | -0.000000 | 695 | 699.541747 | 784.8 | 870.058253 | 1.129209 |
| GO:0009895\_negative\_regulation\_of\_catabolic\_process | 14 | 0 | 0.000000 | -0.000000 | 695 | 699.541747 | 784.8 | 870.058253 | 1.129209 |
| GO:0010332\_response\_to\_gamma\_radiation | 14 | 0 | 0.000000 | -0.000000 | 695 | 699.541747 | 784.8 | 870.058253 | 1.129209 |
| GO:0014855\_striated\_muscle\_cell\_proliferation | 14 | 0 | 0.000000 | -0.000000 | 695 | 699.541747 | 784.8 | 870.058253 | 1.129209 |
| GO:0016573\_histone\_acetylation | 14 | 0 | 0.000000 | -0.000000 | 695 | 699.541747 | 784.8 | 870.058253 | 1.129209 |
| GO:0019217\_regulation\_of\_fatty\_acid\_metabolic\_process | 14 | 0 | 0.000000 | -0.000000 | 695 | 699.541747 | 784.8 | 870.058253 | 1.129209 |
| GO:0021782\_glial\_cell\_development | 14 | 0 | 0.000000 | -0.000000 | 695 | 699.541747 | 784.8 | 870.058253 | 1.129209 |
| GO:0021904\_dorsal\_ventral\_neural\_tube\_patterning | 14 | 0 | 0.000000 | -0.000000 | 695 | 699.541747 | 784.8 | 870.058253 | 1.129209 |
| GO:0030032\_lamellipodium\_assembly | 14 | 0 | 0.000000 | -0.000000 | 695 | 699.541747 | 784.8 | 870.058253 | 1.129209 |
| GO:0030148\_sphingolipid\_biosynthetic\_process | 14 | 0 | 0.000000 | -0.000000 | 695 | 699.541747 | 784.8 | 870.058253 | 1.129209 |
| GO:0030162\_regulation\_of\_proteolysis | 14 | 0 | 0.000000 | -0.000000 | 695 | 699.541747 | 784.8 | 870.058253 | 1.129209 |
| GO:0030832\_regulation\_of\_actin\_filament\_length | 14 | 0 | 0.000000 | -0.000000 | 695 | 699.541747 | 784.8 | 870.058253 | 1.129209 |
| GO:0031099\_regeneration | 14 | 0 | 0.000000 | -0.000000 | 695 | 699.541747 | 784.8 | 870.058253 | 1.129209 |
| GO:0031346\_positive\_regulation\_of\_cell\_projection\_organization | 14 | 0 | 0.000000 | -0.000000 | 695 | 699.541747 | 784.8 | 870.058253 | 1.129209 |
| GO:0031663\_lipopolysaccharide-mediated\_signaling\_pathway | 14 | 0 | 0.000000 | -0.000000 | 695 | 699.541747 | 784.8 | 870.058253 | 1.129209 |
| GO:0032271\_regulation\_of\_protein\_polymerization | 14 | 0 | 0.000000 | -0.000000 | 695 | 699.541747 | 784.8 | 870.058253 | 1.129209 |
| GO:0033044\_regulation\_of\_chromosome\_organization | 14 | 0 | 0.000000 | -0.000000 | 695 | 699.541747 | 784.8 | 870.058253 | 1.129209 |
| GO:0034104\_negative\_regulation\_of\_tissue\_remodeling | 14 | 0 | 0.000000 | -0.000000 | 695 | 699.541747 | 784.8 | 870.058253 | 1.129209 |
| GO:0034623\_cellular\_macromolecular\_complex\_disassembly | 14 | 0 | 0.000000 | -0.000000 | 695 | 699.541747 | 784.8 | 870.058253 | 1.129209 |
| GO:0035036\_sperm-egg\_recognition | 14 | 0 | 0.000000 | -0.000000 | 695 | 699.541747 | 784.8 | 870.058253 | 1.129209 |
| GO:0042310\_vasoconstriction | 14 | 0 | 0.000000 | -0.000000 | 695 | 699.541747 | 784.8 | 870.058253 | 1.129209 |
| GO:0042573\_retinoic\_acid\_metabolic\_process | 14 | 0 | 0.000000 | -0.000000 | 695 | 699.541747 | 784.8 | 870.058253 | 1.129209 |
| GO:0043123\_positive\_regulation\_of\_I-kappaB\_kinase\_NF-kappaB\_cascade | 14 | 0 | 0.000000 | -0.000000 | 695 | 699.541747 | 784.8 | 870.058253 | 1.129209 |
| GO:0043254\_regulation\_of\_protein\_complex\_assembly | 14 | 0 | 0.000000 | -0.000000 | 695 | 699.541747 | 784.8 | 870.058253 | 1.129209 |
| GO:0043491\_protein\_kinase\_B\_signaling\_cascade | 14 | 0 | 0.000000 | -0.000000 | 695 | 699.541747 | 784.8 | 870.058253 | 1.129209 |
| GO:0044236\_multicellular\_organismal\_metabolic\_process | 14 | 0 | 0.000000 | -0.000000 | 695 | 699.541747 | 784.8 | 870.058253 | 1.129209 |
| GO:0045061\_thymic\_T\_cell\_selection | 14 | 0 | 0.000000 | -0.000000 | 695 | 699.541747 | 784.8 | 870.058253 | 1.129209 |
| GO:0045453\_bone\_resorption | 14 | 0 | 0.000000 | -0.000000 | 695 | 699.541747 | 784.8 | 870.058253 | 1.129209 |
| GO:0045598\_regulation\_of\_fat\_cell\_differentiation | 14 | 0 | 0.000000 | -0.000000 | 695 | 699.541747 | 784.8 | 870.058253 | 1.129209 |
| GO:0045732\_positive\_regulation\_of\_protein\_catabolic\_process | 14 | 0 | 0.000000 | -0.000000 | 695 | 699.541747 | 784.8 | 870.058253 | 1.129209 |
| GO:0046209\_nitric\_oxide\_metabolic\_process | 14 | 0 | 0.000000 | -0.000000 | 695 | 699.541747 | 784.8 | 870.058253 | 1.129209 |
| GO:0048048\_embryonic\_eye\_morphogenesis | 14 | 0 | 0.000000 | -0.000000 | 695 | 699.541747 | 784.8 | 870.058253 | 1.129209 |
| GO:0048545\_response\_to\_steroid\_hormone\_stimulus | 14 | 0 | 0.000000 | -0.000000 | 695 | 699.541747 | 784.8 | 870.058253 | 1.129209 |
| GO:0048665\_neuron\_fate\_specification | 14 | 0 | 0.000000 | -0.000000 | 695 | 699.541747 | 784.8 | 870.058253 | 1.129209 |
| GO:0048844\_artery\_morphogenesis | 14 | 0 | 0.000000 | -0.000000 | 695 | 699.541747 | 784.8 | 870.058253 | 1.129209 |
| GO:0050810\_regulation\_of\_steroid\_biosynthetic\_process | 14 | 0 | 0.000000 | -0.000000 | 695 | 699.541747 | 784.8 | 870.058253 | 1.129209 |
| GO:0051017\_actin\_filament\_bundle\_formation | 14 | 0 | 0.000000 | -0.000000 | 695 | 699.541747 | 784.8 | 870.058253 | 1.129209 |
| GO:0051053\_negative\_regulation\_of\_DNA\_metabolic\_process | 14 | 0 | 0.000000 | -0.000000 | 695 | 699.541747 | 784.8 | 870.058253 | 1.129209 |
| GO:0051054\_positive\_regulation\_of\_DNA\_metabolic\_process | 14 | 0 | 0.000000 | -0.000000 | 695 | 699.541747 | 784.8 | 870.058253 | 1.129209 |
| GO:0051100\_negative\_regulation\_of\_binding | 14 | 0 | 0.000000 | -0.000000 | 695 | 699.541747 | 784.8 | 870.058253 | 1.129209 |
| GO:0051952\_regulation\_of\_amine\_transport | 14 | 0 | 0.000000 | -0.000000 | 695 | 699.541747 | 784.8 | 870.058253 | 1.129209 |
| GO:0060716\_labyrinthine\_layer\_blood\_vessel\_development | 14 | 0 | 0.000000 | -0.000000 | 695 | 699.541747 | 784.8 | 870.058253 | 1.129209 |
| GO:0060840\_artery\_development | 14 | 0 | 0.000000 | -0.000000 | 695 | 699.541747 | 784.8 | 870.058253 | 1.129209 |
| GO:0000723\_telomere\_maintenance | 13 | 0 | 0.000000 | -0.000000 | 766 | 771.087610 | 855.02 | 938.952390 | 1.116214 |
| GO:0001836\_release\_of\_cytochrome\_c\_from\_mitochondria | 13 | 0 | 0.000000 | -0.000000 | 766 | 771.087610 | 855.02 | 938.952390 | 1.116214 |
| GO:0001958\_endochondral\_ossification | 13 | 0 | 0.000000 | -0.000000 | 766 | 771.087610 | 855.02 | 938.952390 | 1.116214 |
| GO:0001975\_response\_to\_amphetamine | 13 | 0 | 0.000000 | -0.000000 | 766 | 771.087610 | 855.02 | 938.952390 | 1.116214 |
| GO:0001976\_neurological\_system\_process\_involved\_in\_regulation\_of\_systemic\_arterial\_blood\_pressure | 13 | 0 | 0.000000 | -0.000000 | 766 | 771.087610 | 855.02 | 938.952390 | 1.116214 |
| GO:0002704\_negative\_regulation\_of\_leukocyte\_mediated\_immunity | 13 | 0 | 0.000000 | -0.000000 | 766 | 771.087610 | 855.02 | 938.952390 | 1.116214 |
| GO:0002707\_negative\_regulation\_of\_lymphocyte\_mediated\_immunity | 13 | 0 | 0.000000 | -0.000000 | 766 | 771.087610 | 855.02 | 938.952390 | 1.116214 |
| GO:0002717\_positive\_regulation\_of\_natural\_killer\_cell\_mediated\_immunity | 13 | 0 | 0.000000 | -0.000000 | 766 | 771.087610 | 855.02 | 938.952390 | 1.116214 |
| GO:0003016\_respiratory\_system\_process | 13 | 0 | 0.000000 | -0.000000 | 766 | 771.087610 | 855.02 | 938.952390 | 1.116214 |
| GO:0006090\_pyruvate\_metabolic\_process | 13 | 0 | 0.000000 | -0.000000 | 766 | 771.087610 | 855.02 | 938.952390 | 1.116214 |
| GO:0006687\_glycosphingolipid\_metabolic\_process | 13 | 0 | 0.000000 | -0.000000 | 766 | 771.087610 | 855.02 | 938.952390 | 1.116214 |
| GO:0006833\_water\_transport | 13 | 0 | 0.000000 | -0.000000 | 766 | 771.087610 | 855.02 | 938.952390 | 1.116214 |
| GO:0006898\_receptor-mediated\_endocytosis | 13 | 0 | 0.000000 | -0.000000 | 766 | 771.087610 | 855.02 | 938.952390 | 1.116214 |
| GO:0006986\_response\_to\_unfolded\_protein | 13 | 0 | 0.000000 | -0.000000 | 766 | 771.087610 | 855.02 | 938.952390 | 1.116214 |
| GO:0007129\_synapsis | 13 | 0 | 0.000000 | -0.000000 | 766 | 771.087610 | 855.02 | 938.952390 | 1.116214 |
| GO:0007212\_dopamine\_receptor\_signaling\_pathway | 13 | 0 | 0.000000 | -0.000000 | 766 | 771.087610 | 855.02 | 938.952390 | 1.116214 |
| GO:0007274\_neuromuscular\_synaptic\_transmission | 13 | 0 | 0.000000 | -0.000000 | 766 | 771.087610 | 855.02 | 938.952390 | 1.116214 |
| GO:0007339\_binding\_of\_sperm\_to\_zona\_pellucida | 13 | 0 | 0.000000 | -0.000000 | 766 | 771.087610 | 855.02 | 938.952390 | 1.116214 |
| GO:0007439\_ectodermal\_gut\_development | 13 | 0 | 0.000000 | -0.000000 | 766 | 771.087610 | 855.02 | 938.952390 | 1.116214 |
| GO:0007512\_adult\_heart\_development | 13 | 0 | 0.000000 | -0.000000 | 766 | 771.087610 | 855.02 | 938.952390 | 1.116214 |
| GO:0007566\_embryo\_implantation | 13 | 0 | 0.000000 | -0.000000 | 766 | 771.087610 | 855.02 | 938.952390 | 1.116214 |
| GO:0009410\_response\_to\_xenobiotic\_stimulus | 13 | 0 | 0.000000 | -0.000000 | 766 | 771.087610 | 855.02 | 938.952390 | 1.116214 |
| GO:0009994\_oocyte\_differentiation | 13 | 0 | 0.000000 | -0.000000 | 766 | 771.087610 | 855.02 | 938.952390 | 1.116214 |
| GO:0010623\_developmental\_programmed\_cell\_death | 13 | 0 | 0.000000 | -0.000000 | 766 | 771.087610 | 855.02 | 938.952390 | 1.116214 |
| GO:0010970\_microtubule-based\_transport | 13 | 0 | 0.000000 | -0.000000 | 766 | 771.087610 | 855.02 | 938.952390 | 1.116214 |
| GO:0016525\_negative\_regulation\_of\_angiogenesis | 13 | 0 | 0.000000 | -0.000000 | 766 | 771.087610 | 855.02 | 938.952390 | 1.116214 |
| GO:0019098\_reproductive\_behavior | 13 | 0 | 0.000000 | -0.000000 | 766 | 771.087610 | 855.02 | 938.952390 | 1.116214 |
| GO:0021511\_spinal\_cord\_patterning | 13 | 0 | 0.000000 | -0.000000 | 766 | 771.087610 | 855.02 | 938.952390 | 1.116214 |
| GO:0021533\_cell\_differentiation\_in\_hindbrain | 13 | 0 | 0.000000 | -0.000000 | 766 | 771.087610 | 855.02 | 938.952390 | 1.116214 |
| GO:0021879\_forebrain\_neuron\_differentiation | 13 | 0 | 0.000000 | -0.000000 | 766 | 771.087610 | 855.02 | 938.952390 | 1.116214 |
| GO:0021955\_central\_nervous\_system\_neuron\_axonogenesis | 13 | 0 | 0.000000 | -0.000000 | 766 | 771.087610 | 855.02 | 938.952390 | 1.116214 |
| GO:0030384\_phosphoinositide\_metabolic\_process | 13 | 0 | 0.000000 | -0.000000 | 766 | 771.087610 | 855.02 | 938.952390 | 1.116214 |
| GO:0030516\_regulation\_of\_axon\_extension | 13 | 0 | 0.000000 | -0.000000 | 766 | 771.087610 | 855.02 | 938.952390 | 1.116214 |
| GO:0030539\_male\_genitalia\_development | 13 | 0 | 0.000000 | -0.000000 | 766 | 771.087610 | 855.02 | 938.952390 | 1.116214 |
| GO:0031032\_actomyosin\_structure\_organization | 13 | 0 | 0.000000 | -0.000000 | 766 | 771.087610 | 855.02 | 938.952390 | 1.116214 |
| GO:0031290\_retinal\_ganglion\_cell\_axon\_guidance | 13 | 0 | 0.000000 | -0.000000 | 766 | 771.087610 | 855.02 | 938.952390 | 1.116214 |
| GO:0032200\_telomere\_organization | 13 | 0 | 0.000000 | -0.000000 | 766 | 771.087610 | 855.02 | 938.952390 | 1.116214 |
| GO:0032330\_regulation\_of\_chondrocyte\_differentiation | 13 | 0 | 0.000000 | -0.000000 | 766 | 771.087610 | 855.02 | 938.952390 | 1.116214 |
| GO:0032615\_interleukin-12\_production | 13 | 0 | 0.000000 | -0.000000 | 766 | 771.087610 | 855.02 | 938.952390 | 1.116214 |
| GO:0032729\_positive\_regulation\_of\_interferon-gamma\_production | 13 | 0 | 0.000000 | -0.000000 | 766 | 771.087610 | 855.02 | 938.952390 | 1.116214 |
| GO:0034329\_cell\_junction\_assembly | 13 | 0 | 0.000000 | -0.000000 | 766 | 771.087610 | 855.02 | 938.952390 | 1.116214 |
| GO:0042044\_fluid\_transport | 13 | 0 | 0.000000 | -0.000000 | 766 | 771.087610 | 855.02 | 938.952390 | 1.116214 |
| GO:0042094\_interleukin-2\_biosynthetic\_process | 13 | 0 | 0.000000 | -0.000000 | 766 | 771.087610 | 855.02 | 938.952390 | 1.116214 |
| GO:0042474\_middle\_ear\_morphogenesis | 13 | 0 | 0.000000 | -0.000000 | 766 | 771.087610 | 855.02 | 938.952390 | 1.116214 |
| GO:0043241\_protein\_complex\_disassembly | 13 | 0 | 0.000000 | -0.000000 | 766 | 771.087610 | 855.02 | 938.952390 | 1.116214 |
| GO:0043244\_regulation\_of\_protein\_complex\_disassembly | 13 | 0 | 0.000000 | -0.000000 | 766 | 771.087610 | 855.02 | 938.952390 | 1.116214 |
| GO:0045191\_regulation\_of\_isotype\_switching | 13 | 0 | 0.000000 | -0.000000 | 766 | 771.087610 | 855.02 | 938.952390 | 1.116214 |
| GO:0045577\_regulation\_of\_B\_cell\_differentiation | 13 | 0 | 0.000000 | -0.000000 | 766 | 771.087610 | 855.02 | 938.952390 | 1.116214 |
| GO:0045682\_regulation\_of\_epidermis\_development | 13 | 0 | 0.000000 | -0.000000 | 766 | 771.087610 | 855.02 | 938.952390 | 1.116214 |
| GO:0045954\_positive\_regulation\_of\_natural\_killer\_cell\_mediated\_cytotoxicity | 13 | 0 | 0.000000 | -0.000000 | 766 | 771.087610 | 855.02 | 938.952390 | 1.116214 |
| GO:0046474\_glycerophospholipid\_biosynthetic\_process | 13 | 0 | 0.000000 | -0.000000 | 766 | 771.087610 | 855.02 | 938.952390 | 1.116214 |
| GO:0046640\_regulation\_of\_alpha-beta\_T\_cell\_proliferation | 13 | 0 | 0.000000 | -0.000000 | 766 | 771.087610 | 855.02 | 938.952390 | 1.116214 |
| GO:0046851\_negative\_regulation\_of\_bone\_remodeling | 13 | 0 | 0.000000 | -0.000000 | 766 | 771.087610 | 855.02 | 938.952390 | 1.116214 |
| GO:0048305\_immunoglobulin\_secretion | 13 | 0 | 0.000000 | -0.000000 | 766 | 771.087610 | 855.02 | 938.952390 | 1.116214 |
| GO:0048566\_embryonic\_gut\_development | 13 | 0 | 0.000000 | -0.000000 | 766 | 771.087610 | 855.02 | 938.952390 | 1.116214 |
| GO:0048567\_ectodermal\_gut\_morphogenesis | 13 | 0 | 0.000000 | -0.000000 | 766 | 771.087610 | 855.02 | 938.952390 | 1.116214 |
| GO:0048599\_oocyte\_development | 13 | 0 | 0.000000 | -0.000000 | 766 | 771.087610 | 855.02 | 938.952390 | 1.116214 |
| GO:0050764\_regulation\_of\_phagocytosis | 13 | 0 | 0.000000 | -0.000000 | 766 | 771.087610 | 855.02 | 938.952390 | 1.116214 |
| GO:0050766\_positive\_regulation\_of\_phagocytosis | 13 | 0 | 0.000000 | -0.000000 | 766 | 771.087610 | 855.02 | 938.952390 | 1.116214 |
| GO:0050771\_negative\_regulation\_of\_axonogenesis | 13 | 0 | 0.000000 | -0.000000 | 766 | 771.087610 | 855.02 | 938.952390 | 1.116214 |
| GO:0050818\_regulation\_of\_coagulation | 13 | 0 | 0.000000 | -0.000000 | 766 | 771.087610 | 855.02 | 938.952390 | 1.116214 |
| GO:0051346\_negative\_regulation\_of\_hydrolase\_activity | 13 | 0 | 0.000000 | -0.000000 | 766 | 771.087610 | 855.02 | 938.952390 | 1.116214 |
| GO:0051495\_positive\_regulation\_of\_cytoskeleton\_organization | 13 | 0 | 0.000000 | -0.000000 | 766 | 771.087610 | 855.02 | 938.952390 | 1.116214 |
| GO:0060038\_cardiac\_muscle\_cell\_proliferation | 13 | 0 | 0.000000 | -0.000000 | 766 | 771.087610 | 855.02 | 938.952390 | 1.116214 |
| GO:0060070\_Wnt\_receptor\_signaling\_pathway\_through\_beta-catenin | 13 | 0 | 0.000000 | -0.000000 | 766 | 771.087610 | 855.02 | 938.952390 | 1.116214 |
| GO:0060324\_face\_development | 13 | 0 | 0.000000 | -0.000000 | 766 | 771.087610 | 855.02 | 938.952390 | 1.116214 |
| GO:0060401\_cytosolic\_calcium\_ion\_transport | 13 | 0 | 0.000000 | -0.000000 | 766 | 771.087610 | 855.02 | 938.952390 | 1.116214 |
| GO:0060402\_calcium\_ion\_transport\_into\_cytosol | 13 | 0 | 0.000000 | -0.000000 | 766 | 771.087610 | 855.02 | 938.952390 | 1.116214 |
| GO:0060560\_developmental\_growth\_involved\_in\_morphogenesis | 13 | 0 | 0.000000 | -0.000000 | 766 | 771.087610 | 855.02 | 938.952390 | 1.116214 |
| GO:0060742\_epithelial\_cell\_differentiation\_involved\_in\_prostate\_gland\_development | 13 | 0 | 0.000000 | -0.000000 | 766 | 771.087610 | 855.02 | 938.952390 | 1.116214 |
| GO:0070192\_chromosome\_organization\_involved\_in\_meiosis | 13 | 0 | 0.000000 | -0.000000 | 766 | 771.087610 | 855.02 | 938.952390 | 1.116214 |
| GO:0007346\_regulation\_of\_mitotic\_cell\_cycle | 40 | 0 | 0.000000 | -0.000000 | 776 | 782.220225 | 865.58 | 948.939775 | 1.115438 |
| GO:0007599\_hemostasis | 40 | 0 | 0.000000 | -0.000000 | 776 | 782.220225 | 865.58 | 948.939775 | 1.115438 |
| GO:0008203\_cholesterol\_metabolic\_process | 40 | 0 | 0.000000 | -0.000000 | 776 | 782.220225 | 865.58 | 948.939775 | 1.115438 |
| GO:0014031\_mesenchymal\_cell\_development | 40 | 0 | 0.000000 | -0.000000 | 776 | 782.220225 | 865.58 | 948.939775 | 1.115438 |
| GO:0016071\_mRNA\_metabolic\_process | 40 | 0 | 0.000000 | -0.000000 | 776 | 782.220225 | 865.58 | 948.939775 | 1.115438 |
| GO:0016358\_dendrite\_development | 40 | 0 | 0.000000 | -0.000000 | 776 | 782.220225 | 865.58 | 948.939775 | 1.115438 |
| GO:0016485\_protein\_processing | 40 | 0 | 0.000000 | -0.000000 | 776 | 782.220225 | 865.58 | 948.939775 | 1.115438 |
| GO:0035272\_exocrine\_system\_development | 40 | 0 | 0.000000 | -0.000000 | 776 | 782.220225 | 865.58 | 948.939775 | 1.115438 |
| GO:0046850\_regulation\_of\_bone\_remodeling | 40 | 0 | 0.000000 | -0.000000 | 776 | 782.220225 | 865.58 | 948.939775 | 1.115438 |
| GO:0051129\_negative\_regulation\_of\_cellular\_component\_organization | 40 | 0 | 0.000000 | -0.000000 | 776 | 782.220225 | 865.58 | 948.939775 | 1.115438 |
| GO:0006753\_nucleoside\_phosphate\_metabolic\_process | 94 | 0 | 0.000000 | -0.000000 | 781 | 786.464349 | 869.42 | 952.375651 | 1.113214 |
| GO:0008610\_lipid\_biosynthetic\_process | 94 | 0 | 0.000000 | -0.000000 | 781 | 786.464349 | 869.42 | 952.375651 | 1.113214 |
| GO:0009117\_nucleotide\_metabolic\_process | 94 | 0 | 0.000000 | -0.000000 | 781 | 786.464349 | 869.42 | 952.375651 | 1.113214 |
| GO:0032943\_mononuclear\_cell\_proliferation | 94 | 0 | 0.000000 | -0.000000 | 781 | 786.464349 | 869.42 | 952.375651 | 1.113214 |
| GO:0046651\_lymphocyte\_proliferation | 94 | 0 | 0.000000 | -0.000000 | 781 | 786.464349 | 869.42 | 952.375651 | 1.113214 |
| GO:0002697\_regulation\_of\_immune\_effector\_process | 68 | 0 | 0.000000 | -0.000000 | 784 | 790.443717 | 872.85 | 955.256283 | 1.113329 |
| GO:0034962\_cellular\_biopolymer\_catabolic\_process | 68 | 0 | 0.000000 | -0.000000 | 784 | 790.443717 | 872.85 | 955.256283 | 1.113329 |
| GO:0042692\_muscle\_cell\_differentiation | 68 | 0 | 0.000000 | -0.000000 | 784 | 790.443717 | 872.85 | 955.256283 | 1.113329 |
| GO:0040008\_regulation\_of\_growth | 113 | 0 | 0.000000 | -0.000000 | 785 | 791.670358 | 873.92 | 956.169642 | 1.113274 |
| GO:0000165\_MAPKKK\_cascade | 114 | 0 | 0.000000 | -0.000000 | 786 | 792.956601 | 874.98 | 957.003399 | 1.113206 |
| GO:0003013\_circulatory\_system\_process | 103 | 0 | 0.000000 | -0.000000 | 788 | 795.005858 | 876.76 | 958.514142 | 1.112640 |
| GO:0008015\_blood\_circulation | 103 | 0 | 0.000000 | -0.000000 | 788 | 795.005858 | 876.76 | 958.514142 | 1.112640 |
| GO:0007281\_germ\_cell\_development | 75 | 0 | 0.000000 | -0.000000 | 792 | 797.968693 | 879.43 | 960.891307 | 1.110391 |
| GO:0044265\_cellular\_macromolecule\_catabolic\_process | 75 | 0 | 0.000000 | -0.000000 | 792 | 797.968693 | 879.43 | 960.891307 | 1.110391 |
| GO:0048589\_developmental\_growth | 75 | 0 | 0.000000 | -0.000000 | 792 | 797.968693 | 879.43 | 960.891307 | 1.110391 |
| GO:0051050\_positive\_regulation\_of\_transport | 75 | 0 | 0.000000 | -0.000000 | 792 | 797.968693 | 879.43 | 960.891307 | 1.110391 |
| GO:0035239\_tube\_morphogenesis | 143 | 0 | 0.000000 | -0.000000 | 793 | 798.409668 | 879.81 | 961.210332 | 1.109470 |
| GO:0044057\_regulation\_of\_system\_process | 133 | 0 | 0.000000 | -0.000000 | 794 | 799.479066 | 880.73 | 961.980934 | 1.109232 |
| GO:0001759\_induction\_of\_an\_organ | 15 | 0 | 0.000000 | -0.000000 | 847 | 852.878491 | 932.29 | 1011.701509 | 1.100697 |
| GO:0001782\_B\_cell\_homeostasis | 15 | 0 | 0.000000 | -0.000000 | 847 | 852.878491 | 932.29 | 1011.701509 | 1.100697 |
| GO:0001964\_startle\_response | 15 | 0 | 0.000000 | -0.000000 | 847 | 852.878491 | 932.29 | 1011.701509 | 1.100697 |
| GO:0002286\_T\_cell\_activation\_during\_immune\_response | 15 | 0 | 0.000000 | -0.000000 | 847 | 852.878491 | 932.29 | 1011.701509 | 1.100697 |
| GO:0002495\_antigen\_processing\_and\_presentation\_of\_peptide\_antigen\_via\_MHC\_class\_II | 15 | 0 | 0.000000 | -0.000000 | 847 | 852.878491 | 932.29 | 1011.701509 | 1.100697 |
| GO:0002504\_antigen\_processing\_and\_presentation\_of\_peptide\_or\_polysaccharide\_antigen\_via\_MHC\_class\_II | 15 | 0 | 0.000000 | -0.000000 | 847 | 852.878491 | 932.29 | 1011.701509 | 1.100697 |
| GO:0002709\_regulation\_of\_T\_cell\_mediated\_immunity | 15 | 0 | 0.000000 | -0.000000 | 847 | 852.878491 | 932.29 | 1011.701509 | 1.100697 |
| GO:0006473\_protein\_amino\_acid\_acetylation | 15 | 0 | 0.000000 | -0.000000 | 847 | 852.878491 | 932.29 | 1011.701509 | 1.100697 |
| GO:0006487\_protein\_amino\_acid\_N-linked\_glycosylation | 15 | 0 | 0.000000 | -0.000000 | 847 | 852.878491 | 932.29 | 1011.701509 | 1.100697 |
| GO:0006749\_glutathione\_metabolic\_process | 15 | 0 | 0.000000 | -0.000000 | 847 | 852.878491 | 932.29 | 1011.701509 | 1.100697 |
| GO:0006885\_regulation\_of\_pH | 15 | 0 | 0.000000 | -0.000000 | 847 | 852.878491 | 932.29 | 1011.701509 | 1.100697 |
| GO:0007040\_lysosome\_organization | 15 | 0 | 0.000000 | -0.000000 | 847 | 852.878491 | 932.29 | 1011.701509 | 1.100697 |
| GO:0007173\_epidermal\_growth\_factor\_receptor\_signaling\_pathway | 15 | 0 | 0.000000 | -0.000000 | 847 | 852.878491 | 932.29 | 1011.701509 | 1.100697 |
| GO:0007200\_activation\_of\_phospholipase\_C\_activity\_by\_G-protein\_coupled\_receptor\_protein\_signaling\_pathway\_coupled\_to\_IP3\_second\_messenger | 15 | 0 | 0.000000 | -0.000000 | 847 | 852.878491 | 932.29 | 1011.701509 | 1.100697 |
| GO:0007202\_activation\_of\_phospholipase\_C\_activity | 15 | 0 | 0.000000 | -0.000000 | 847 | 852.878491 | 932.29 | 1011.701509 | 1.100697 |
| GO:0007218\_neuropeptide\_signaling\_pathway | 15 | 0 | 0.000000 | -0.000000 | 847 | 852.878491 | 932.29 | 1011.701509 | 1.100697 |
| GO:0007588\_excretion | 15 | 0 | 0.000000 | -0.000000 | 847 | 852.878491 | 932.29 | 1011.701509 | 1.100697 |
| GO:0007618\_mating | 15 | 0 | 0.000000 | -0.000000 | 847 | 852.878491 | 932.29 | 1011.701509 | 1.100697 |
| GO:0008543\_fibroblast\_growth\_factor\_receptor\_signaling\_pathway | 15 | 0 | 0.000000 | -0.000000 | 847 | 852.878491 | 932.29 | 1011.701509 | 1.100697 |
| GO:0009062\_fatty\_acid\_catabolic\_process | 15 | 0 | 0.000000 | -0.000000 | 847 | 852.878491 | 932.29 | 1011.701509 | 1.100697 |
| GO:0010092\_specification\_of\_organ\_identity | 15 | 0 | 0.000000 | -0.000000 | 847 | 852.878491 | 932.29 | 1011.701509 | 1.100697 |
| GO:0010518\_positive\_regulation\_of\_phospholipase\_activity | 15 | 0 | 0.000000 | -0.000000 | 847 | 852.878491 | 932.29 | 1011.701509 | 1.100697 |
| GO:0010863\_positive\_regulation\_of\_phospholipase\_C\_activity | 15 | 0 | 0.000000 | -0.000000 | 847 | 852.878491 | 932.29 | 1011.701509 | 1.100697 |
| GO:0015931\_nucleobase\_\_nucleoside\_\_nucleotide\_and\_nucleic\_acid\_transport | 15 | 0 | 0.000000 | -0.000000 | 847 | 852.878491 | 932.29 | 1011.701509 | 1.100697 |
| GO:0019886\_antigen\_processing\_and\_presentation\_of\_exogenous\_peptide\_antigen\_via\_MHC\_class\_II | 15 | 0 | 0.000000 | -0.000000 | 847 | 852.878491 | 932.29 | 1011.701509 | 1.100697 |
| GO:0021795\_cerebral\_cortex\_cell\_migration | 15 | 0 | 0.000000 | -0.000000 | 847 | 852.878491 | 932.29 | 1011.701509 | 1.100697 |
| GO:0021872\_generation\_of\_neurons\_in\_the\_forebrain | 15 | 0 | 0.000000 | -0.000000 | 847 | 852.878491 | 932.29 | 1011.701509 | 1.100697 |
| GO:0022600\_digestive\_system\_process | 15 | 0 | 0.000000 | -0.000000 | 847 | 852.878491 | 932.29 | 1011.701509 | 1.100697 |
| GO:0030041\_actin\_filament\_polymerization | 15 | 0 | 0.000000 | -0.000000 | 847 | 852.878491 | 932.29 | 1011.701509 | 1.100697 |
| GO:0031069\_hair\_follicle\_morphogenesis | 15 | 0 | 0.000000 | -0.000000 | 847 | 852.878491 | 932.29 | 1011.701509 | 1.100697 |
| GO:0031076\_embryonic\_camera-type\_eye\_development | 15 | 0 | 0.000000 | -0.000000 | 847 | 852.878491 | 932.29 | 1011.701509 | 1.100697 |
| GO:0031329\_regulation\_of\_cellular\_catabolic\_process | 15 | 0 | 0.000000 | -0.000000 | 847 | 852.878491 | 932.29 | 1011.701509 | 1.100697 |
| GO:0035116\_embryonic\_hindlimb\_morphogenesis | 15 | 0 | 0.000000 | -0.000000 | 847 | 852.878491 | 932.29 | 1011.701509 | 1.100697 |
| GO:0035249\_synaptic\_transmission\_\_glutamatergic | 15 | 0 | 0.000000 | -0.000000 | 847 | 852.878491 | 932.29 | 1011.701509 | 1.100697 |
| GO:0045666\_positive\_regulation\_of\_neuron\_differentiation | 15 | 0 | 0.000000 | -0.000000 | 847 | 852.878491 | 932.29 | 1011.701509 | 1.100697 |
| GO:0046164\_alcohol\_catabolic\_process | 15 | 0 | 0.000000 | -0.000000 | 847 | 852.878491 | 932.29 | 1011.701509 | 1.100697 |
| GO:0046638\_positive\_regulation\_of\_alpha-beta\_T\_cell\_differentiation | 15 | 0 | 0.000000 | -0.000000 | 847 | 852.878491 | 932.29 | 1011.701509 | 1.100697 |
| GO:0048008\_platelet-derived\_growth\_factor\_receptor\_signaling\_pathway | 15 | 0 | 0.000000 | -0.000000 | 847 | 852.878491 | 932.29 | 1011.701509 | 1.100697 |
| GO:0048010\_vascular\_endothelial\_growth\_factor\_receptor\_signaling\_pathway | 15 | 0 | 0.000000 | -0.000000 | 847 | 852.878491 | 932.29 | 1011.701509 | 1.100697 |
| GO:0048144\_fibroblast\_proliferation | 15 | 0 | 0.000000 | -0.000000 | 847 | 852.878491 | 932.29 | 1011.701509 | 1.100697 |
| GO:0048145\_regulation\_of\_fibroblast\_proliferation | 15 | 0 | 0.000000 | -0.000000 | 847 | 852.878491 | 932.29 | 1011.701509 | 1.100697 |
| GO:0048610\_reproductive\_cellular\_process | 15 | 0 | 0.000000 | -0.000000 | 847 | 852.878491 | 932.29 | 1011.701509 | 1.100697 |
| GO:0048709\_oligodendrocyte\_differentiation | 15 | 0 | 0.000000 | -0.000000 | 847 | 852.878491 | 932.29 | 1011.701509 | 1.100697 |
| GO:0050729\_positive\_regulation\_of\_inflammatory\_response | 15 | 0 | 0.000000 | -0.000000 | 847 | 852.878491 | 932.29 | 1011.701509 | 1.100697 |
| GO:0050796\_regulation\_of\_insulin\_secretion | 15 | 0 | 0.000000 | -0.000000 | 847 | 852.878491 | 932.29 | 1011.701509 | 1.100697 |
| GO:0050798\_activated\_T\_cell\_proliferation | 15 | 0 | 0.000000 | -0.000000 | 847 | 852.878491 | 932.29 | 1011.701509 | 1.100697 |
| GO:0055010\_ventricular\_cardiac\_muscle\_morphogenesis | 15 | 0 | 0.000000 | -0.000000 | 847 | 852.878491 | 932.29 | 1011.701509 | 1.100697 |
| GO:0060322\_head\_development | 15 | 0 | 0.000000 | -0.000000 | 847 | 852.878491 | 932.29 | 1011.701509 | 1.100697 |
| GO:0060425\_lung\_morphogenesis | 15 | 0 | 0.000000 | -0.000000 | 847 | 852.878491 | 932.29 | 1011.701509 | 1.100697 |
| GO:0060442\_branching\_involved\_in\_prostate\_gland\_morphogenesis | 15 | 0 | 0.000000 | -0.000000 | 847 | 852.878491 | 932.29 | 1011.701509 | 1.100697 |
| GO:0060749\_mammary\_gland\_alveolus\_development | 15 | 0 | 0.000000 | -0.000000 | 847 | 852.878491 | 932.29 | 1011.701509 | 1.100697 |
| GO:0070227\_lymphocyte\_apoptosis | 15 | 0 | 0.000000 | -0.000000 | 847 | 852.878491 | 932.29 | 1011.701509 | 1.100697 |
| GO:0070507\_regulation\_of\_microtubule\_cytoskeleton\_organization | 15 | 0 | 0.000000 | -0.000000 | 847 | 852.878491 | 932.29 | 1011.701509 | 1.100697 |
| GO:0000079\_regulation\_of\_cyclin-dependent\_protein\_kinase\_activity | 7 | 0 | 0.000000 | -0.000000 | 1025 | 1030.440375 | 1107.85 | 1185.259625 | 1.080829 |
| GO:0000188\_inactivation\_of\_MAPK\_activity | 7 | 0 | 0.000000 | -0.000000 | 1025 | 1030.440375 | 1107.85 | 1185.259625 | 1.080829 |
| GO:0001504\_neurotransmitter\_uptake | 7 | 0 | 0.000000 | -0.000000 | 1025 | 1030.440375 | 1107.85 | 1185.259625 | 1.080829 |
| GO:0001556\_oocyte\_maturation | 7 | 0 | 0.000000 | -0.000000 | 1025 | 1030.440375 | 1107.85 | 1185.259625 | 1.080829 |
| GO:0001573\_ganglioside\_metabolic\_process | 7 | 0 | 0.000000 | -0.000000 | 1025 | 1030.440375 | 1107.85 | 1185.259625 | 1.080829 |
| GO:0001736\_establishment\_of\_planar\_polarity | 7 | 0 | 0.000000 | -0.000000 | 1025 | 1030.440375 | 1107.85 | 1185.259625 | 1.080829 |
| GO:0001839\_neural\_plate\_morphogenesis | 7 | 0 | 0.000000 | -0.000000 | 1025 | 1030.440375 | 1107.85 | 1185.259625 | 1.080829 |
| GO:0001936\_regulation\_of\_endothelial\_cell\_proliferation | 7 | 0 | 0.000000 | -0.000000 | 1025 | 1030.440375 | 1107.85 | 1185.259625 | 1.080829 |
| GO:0001967\_suckling\_behavior | 7 | 0 | 0.000000 | -0.000000 | 1025 | 1030.440375 | 1107.85 | 1185.259625 | 1.080829 |
| GO:0002011\_morphogenesis\_of\_an\_epithelial\_sheet | 7 | 0 | 0.000000 | -0.000000 | 1025 | 1030.440375 | 1107.85 | 1185.259625 | 1.080829 |
| GO:0002052\_positive\_regulation\_of\_neuroblast\_proliferation | 7 | 0 | 0.000000 | -0.000000 | 1025 | 1030.440375 | 1107.85 | 1185.259625 | 1.080829 |
| GO:0002063\_chondrocyte\_development | 7 | 0 | 0.000000 | -0.000000 | 1025 | 1030.440375 | 1107.85 | 1185.259625 | 1.080829 |
| GO:0002067\_glandular\_epithelial\_cell\_differentiation | 7 | 0 | 0.000000 | -0.000000 | 1025 | 1030.440375 | 1107.85 | 1185.259625 | 1.080829 |
| GO:0002076\_osteoblast\_development | 7 | 0 | 0.000000 | -0.000000 | 1025 | 1030.440375 | 1107.85 | 1185.259625 | 1.080829 |
| GO:0002087\_regulation\_of\_respiratory\_gaseous\_exchange\_by\_neurological\_system\_process | 7 | 0 | 0.000000 | -0.000000 | 1025 | 1030.440375 | 1107.85 | 1185.259625 | 1.080829 |
| GO:0002093\_auditory\_receptor\_cell\_morphogenesis | 7 | 0 | 0.000000 | -0.000000 | 1025 | 1030.440375 | 1107.85 | 1185.259625 | 1.080829 |
| GO:0002224\_toll-like\_receptor\_signaling\_pathway | 7 | 0 | 0.000000 | -0.000000 | 1025 | 1030.440375 | 1107.85 | 1185.259625 | 1.080829 |
| GO:0002455\_humoral\_immune\_response\_mediated\_by\_circulating\_immunoglobulin | 7 | 0 | 0.000000 | -0.000000 | 1025 | 1030.440375 | 1107.85 | 1185.259625 | 1.080829 |
| GO:0002643\_regulation\_of\_tolerance\_induction | 7 | 0 | 0.000000 | -0.000000 | 1025 | 1030.440375 | 1107.85 | 1185.259625 | 1.080829 |
| GO:0002645\_positive\_regulation\_of\_tolerance\_induction | 7 | 0 | 0.000000 | -0.000000 | 1025 | 1030.440375 | 1107.85 | 1185.259625 | 1.080829 |
| GO:0002714\_positive\_regulation\_of\_B\_cell\_mediated\_immunity | 7 | 0 | 0.000000 | -0.000000 | 1025 | 1030.440375 | 1107.85 | 1185.259625 | 1.080829 |
| GO:0002792\_negative\_regulation\_of\_peptide\_secretion | 7 | 0 | 0.000000 | -0.000000 | 1025 | 1030.440375 | 1107.85 | 1185.259625 | 1.080829 |
| GO:0002793\_positive\_regulation\_of\_peptide\_secretion | 7 | 0 | 0.000000 | -0.000000 | 1025 | 1030.440375 | 1107.85 | 1185.259625 | 1.080829 |
| GO:0002828\_regulation\_of\_T-helper\_2\_type\_immune\_response | 7 | 0 | 0.000000 | -0.000000 | 1025 | 1030.440375 | 1107.85 | 1185.259625 | 1.080829 |
| GO:0002863\_positive\_regulation\_of\_inflammatory\_response\_to\_antigenic\_stimulus | 7 | 0 | 0.000000 | -0.000000 | 1025 | 1030.440375 | 1107.85 | 1185.259625 | 1.080829 |
| GO:0002891\_positive\_regulation\_of\_immunoglobulin\_mediated\_immune\_response | 7 | 0 | 0.000000 | -0.000000 | 1025 | 1030.440375 | 1107.85 | 1185.259625 | 1.080829 |
| GO:0003084\_positive\_regulation\_of\_systemic\_arterial\_blood\_pressure | 7 | 0 | 0.000000 | -0.000000 | 1025 | 1030.440375 | 1107.85 | 1185.259625 | 1.080829 |
| GO:0003085\_negative\_regulation\_of\_systemic\_arterial\_blood\_pressure | 7 | 0 | 0.000000 | -0.000000 | 1025 | 1030.440375 | 1107.85 | 1185.259625 | 1.080829 |
| GO:0006014\_D-ribose\_metabolic\_process | 7 | 0 | 0.000000 | -0.000000 | 1025 | 1030.440375 | 1107.85 | 1185.259625 | 1.080829 |
| GO:0006041\_glucosamine\_metabolic\_process | 7 | 0 | 0.000000 | -0.000000 | 1025 | 1030.440375 | 1107.85 | 1185.259625 | 1.080829 |
| GO:0006044\_N-acetylglucosamine\_metabolic\_process | 7 | 0 | 0.000000 | -0.000000 | 1025 | 1030.440375 | 1107.85 | 1185.259625 | 1.080829 |
| GO:0006096\_glycolysis | 7 | 0 | 0.000000 | -0.000000 | 1025 | 1030.440375 | 1107.85 | 1185.259625 | 1.080829 |
| GO:0006119\_oxidative\_phosphorylation | 7 | 0 | 0.000000 | -0.000000 | 1025 | 1030.440375 | 1107.85 | 1185.259625 | 1.080829 |
| GO:0006275\_regulation\_of\_DNA\_replication | 7 | 0 | 0.000000 | -0.000000 | 1025 | 1030.440375 | 1107.85 | 1185.259625 | 1.080829 |
| GO:0006298\_mismatch\_repair | 7 | 0 | 0.000000 | -0.000000 | 1025 | 1030.440375 | 1107.85 | 1185.259625 | 1.080829 |
| GO:0006352\_transcription\_initiation | 7 | 0 | 0.000000 | -0.000000 | 1025 | 1030.440375 | 1107.85 | 1185.259625 | 1.080829 |
| GO:0006401\_RNA\_catabolic\_process | 7 | 0 | 0.000000 | -0.000000 | 1025 | 1030.440375 | 1107.85 | 1185.259625 | 1.080829 |
| GO:0006406\_mRNA\_export\_from\_nucleus | 7 | 0 | 0.000000 | -0.000000 | 1025 | 1030.440375 | 1107.85 | 1185.259625 | 1.080829 |
| GO:0006505\_GPI\_anchor\_metabolic\_process | 7 | 0 | 0.000000 | -0.000000 | 1025 | 1030.440375 | 1107.85 | 1185.259625 | 1.080829 |
| GO:0006516\_glycoprotein\_catabolic\_process | 7 | 0 | 0.000000 | -0.000000 | 1025 | 1030.440375 | 1107.85 | 1185.259625 | 1.080829 |
| GO:0006612\_protein\_targeting\_to\_membrane | 7 | 0 | 0.000000 | -0.000000 | 1025 | 1030.440375 | 1107.85 | 1185.259625 | 1.080829 |
| GO:0006769\_nicotinamide\_metabolic\_process | 7 | 0 | 0.000000 | -0.000000 | 1025 | 1030.440375 | 1107.85 | 1185.259625 | 1.080829 |
| GO:0006818\_hydrogen\_transport | 7 | 0 | 0.000000 | -0.000000 | 1025 | 1030.440375 | 1107.85 | 1185.259625 | 1.080829 |
| GO:0006878\_cellular\_copper\_ion\_homeostasis | 7 | 0 | 0.000000 | -0.000000 | 1025 | 1030.440375 | 1107.85 | 1185.259625 | 1.080829 |
| GO:0006884\_cell\_volume\_homeostasis | 7 | 0 | 0.000000 | -0.000000 | 1025 | 1030.440375 | 1107.85 | 1185.259625 | 1.080829 |
| GO:0006949\_syncytium\_formation | 7 | 0 | 0.000000 | -0.000000 | 1025 | 1030.440375 | 1107.85 | 1185.259625 | 1.080829 |
| GO:0007019\_microtubule\_depolymerization | 7 | 0 | 0.000000 | -0.000000 | 1025 | 1030.440375 | 1107.85 | 1185.259625 | 1.080829 |
| GO:0007026\_negative\_regulation\_of\_microtubule\_depolymerization | 7 | 0 | 0.000000 | -0.000000 | 1025 | 1030.440375 | 1107.85 | 1185.259625 | 1.080829 |
| GO:0007034\_vacuolar\_transport | 7 | 0 | 0.000000 | -0.000000 | 1025 | 1030.440375 | 1107.85 | 1185.259625 | 1.080829 |
| GO:0007062\_sister\_chromatid\_cohesion | 7 | 0 | 0.000000 | -0.000000 | 1025 | 1030.440375 | 1107.85 | 1185.259625 | 1.080829 |
| GO:0007130\_synaptonemal\_complex\_assembly | 7 | 0 | 0.000000 | -0.000000 | 1025 | 1030.440375 | 1107.85 | 1185.259625 | 1.080829 |
| GO:0007164\_establishment\_of\_tissue\_polarity | 7 | 0 | 0.000000 | -0.000000 | 1025 | 1030.440375 | 1107.85 | 1185.259625 | 1.080829 |
| GO:0007191\_activation\_of\_adenylate\_cyclase\_activity\_by\_dopamine\_receptor\_signaling\_pathway | 7 | 0 | 0.000000 | -0.000000 | 1025 | 1030.440375 | 1107.85 | 1185.259625 | 1.080829 |
| GO:0007271\_synaptic\_transmission\_\_cholinergic | 7 | 0 | 0.000000 | -0.000000 | 1025 | 1030.440375 | 1107.85 | 1185.259625 | 1.080829 |
| GO:0007413\_axonal\_fasciculation | 7 | 0 | 0.000000 | -0.000000 | 1025 | 1030.440375 | 1107.85 | 1185.259625 | 1.080829 |
| GO:0007440\_foregut\_morphogenesis | 7 | 0 | 0.000000 | -0.000000 | 1025 | 1030.440375 | 1107.85 | 1185.259625 | 1.080829 |
| GO:0007616\_long-term\_memory | 7 | 0 | 0.000000 | -0.000000 | 1025 | 1030.440375 | 1107.85 | 1185.259625 | 1.080829 |
| GO:0008033\_tRNA\_processing | 7 | 0 | 0.000000 | -0.000000 | 1025 | 1030.440375 | 1107.85 | 1185.259625 | 1.080829 |
| GO:0008299\_isoprenoid\_biosynthetic\_process | 7 | 0 | 0.000000 | -0.000000 | 1025 | 1030.440375 | 1107.85 | 1185.259625 | 1.080829 |
| GO:0008340\_determination\_of\_adult\_lifespan | 7 | 0 | 0.000000 | -0.000000 | 1025 | 1030.440375 | 1107.85 | 1185.259625 | 1.080829 |
| GO:0009150\_purine\_ribonucleotide\_metabolic\_process | 7 | 0 | 0.000000 | -0.000000 | 1025 | 1030.440375 | 1107.85 | 1185.259625 | 1.080829 |
| GO:0009200\_deoxyribonucleoside\_triphosphate\_metabolic\_process | 7 | 0 | 0.000000 | -0.000000 | 1025 | 1030.440375 | 1107.85 | 1185.259625 | 1.080829 |
| GO:0009259\_ribonucleotide\_metabolic\_process | 7 | 0 | 0.000000 | -0.000000 | 1025 | 1030.440375 | 1107.85 | 1185.259625 | 1.080829 |
| GO:0009311\_oligosaccharide\_metabolic\_process | 7 | 0 | 0.000000 | -0.000000 | 1025 | 1030.440375 | 1107.85 | 1185.259625 | 1.080829 |
| GO:0009394\_2'-deoxyribonucleotide\_metabolic\_process | 7 | 0 | 0.000000 | -0.000000 | 1025 | 1030.440375 | 1107.85 | 1185.259625 | 1.080829 |
| GO:0009820\_alkaloid\_metabolic\_process | 7 | 0 | 0.000000 | -0.000000 | 1025 | 1030.440375 | 1107.85 | 1185.259625 | 1.080829 |
| GO:0010469\_regulation\_of\_receptor\_activity | 7 | 0 | 0.000000 | -0.000000 | 1025 | 1030.440375 | 1107.85 | 1185.259625 | 1.080829 |
| GO:0010948\_negative\_regulation\_of\_cell\_cycle\_process | 7 | 0 | 0.000000 | -0.000000 | 1025 | 1030.440375 | 1107.85 | 1185.259625 | 1.080829 |
| GO:0014047\_glutamate\_secretion | 7 | 0 | 0.000000 | -0.000000 | 1025 | 1030.440375 | 1107.85 | 1185.259625 | 1.080829 |
| GO:0014066\_regulation\_of\_phosphoinositide\_3-kinase\_cascade | 7 | 0 | 0.000000 | -0.000000 | 1025 | 1030.440375 | 1107.85 | 1185.259625 | 1.080829 |
| GO:0014821\_phasic\_smooth\_muscle\_contraction | 7 | 0 | 0.000000 | -0.000000 | 1025 | 1030.440375 | 1107.85 | 1185.259625 | 1.080829 |
| GO:0015697\_quaternary\_ammonium\_group\_transport | 7 | 0 | 0.000000 | -0.000000 | 1025 | 1030.440375 | 1107.85 | 1185.259625 | 1.080829 |
| GO:0015813\_L-glutamate\_transport | 7 | 0 | 0.000000 | -0.000000 | 1025 | 1030.440375 | 1107.85 | 1185.259625 | 1.080829 |
| GO:0015908\_fatty\_acid\_transport | 7 | 0 | 0.000000 | -0.000000 | 1025 | 1030.440375 | 1107.85 | 1185.259625 | 1.080829 |
| GO:0015914\_phospholipid\_transport | 7 | 0 | 0.000000 | -0.000000 | 1025 | 1030.440375 | 1107.85 | 1185.259625 | 1.080829 |
| GO:0015992\_proton\_transport | 7 | 0 | 0.000000 | -0.000000 | 1025 | 1030.440375 | 1107.85 | 1185.259625 | 1.080829 |
| GO:0016339\_calcium-dependent\_cell-cell\_adhesion | 7 | 0 | 0.000000 | -0.000000 | 1025 | 1030.440375 | 1107.85 | 1185.259625 | 1.080829 |
| GO:0016575\_histone\_deacetylation | 7 | 0 | 0.000000 | -0.000000 | 1025 | 1030.440375 | 1107.85 | 1185.259625 | 1.080829 |
| GO:0019362\_pyridine\_nucleotide\_metabolic\_process | 7 | 0 | 0.000000 | -0.000000 | 1025 | 1030.440375 | 1107.85 | 1185.259625 | 1.080829 |
| GO:0019692\_deoxyribose\_phosphate\_metabolic\_process | 7 | 0 | 0.000000 | -0.000000 | 1025 | 1030.440375 | 1107.85 | 1185.259625 | 1.080829 |
| GO:0019800\_peptide\_cross-linking\_via\_chondroitin\_4-sulfate\_glycosaminoglycan | 7 | 0 | 0.000000 | -0.000000 | 1025 | 1030.440375 | 1107.85 | 1185.259625 | 1.080829 |
| GO:0020027\_hemoglobin\_metabolic\_process | 7 | 0 | 0.000000 | -0.000000 | 1025 | 1030.440375 | 1107.85 | 1185.259625 | 1.080829 |
| GO:0021514\_ventral\_spinal\_cord\_interneuron\_differentiation | 7 | 0 | 0.000000 | -0.000000 | 1025 | 1030.440375 | 1107.85 | 1185.259625 | 1.080829 |
| GO:0021516\_dorsal\_spinal\_cord\_development | 7 | 0 | 0.000000 | -0.000000 | 1025 | 1030.440375 | 1107.85 | 1185.259625 | 1.080829 |
| GO:0021520\_spinal\_cord\_motor\_neuron\_cell\_fate\_specification | 7 | 0 | 0.000000 | -0.000000 | 1025 | 1030.440375 | 1107.85 | 1185.259625 | 1.080829 |
| GO:0021521\_ventral\_spinal\_cord\_interneuron\_specification | 7 | 0 | 0.000000 | -0.000000 | 1025 | 1030.440375 | 1107.85 | 1185.259625 | 1.080829 |
| GO:0021546\_rhombomere\_development | 7 | 0 | 0.000000 | -0.000000 | 1025 | 1030.440375 | 1107.85 | 1185.259625 | 1.080829 |
| GO:0021756\_striatum\_development | 7 | 0 | 0.000000 | -0.000000 | 1025 | 1030.440375 | 1107.85 | 1185.259625 | 1.080829 |
| GO:0021884\_forebrain\_neuron\_development | 7 | 0 | 0.000000 | -0.000000 | 1025 | 1030.440375 | 1107.85 | 1185.259625 | 1.080829 |
| GO:0021903\_rostrocaudal\_neural\_tube\_patterning | 7 | 0 | 0.000000 | -0.000000 | 1025 | 1030.440375 | 1107.85 | 1185.259625 | 1.080829 |
| GO:0021984\_adenohypophysis\_development | 7 | 0 | 0.000000 | -0.000000 | 1025 | 1030.440375 | 1107.85 | 1185.259625 | 1.080829 |
| GO:0022407\_regulation\_of\_cell-cell\_adhesion | 7 | 0 | 0.000000 | -0.000000 | 1025 | 1030.440375 | 1107.85 | 1185.259625 | 1.080829 |
| GO:0022618\_ribonucleoprotein\_complex\_assembly | 7 | 0 | 0.000000 | -0.000000 | 1025 | 1030.440375 | 1107.85 | 1185.259625 | 1.080829 |
| GO:0030104\_water\_homeostasis | 7 | 0 | 0.000000 | -0.000000 | 1025 | 1030.440375 | 1107.85 | 1185.259625 | 1.080829 |
| GO:0030201\_heparan\_sulfate\_proteoglycan\_metabolic\_process | 7 | 0 | 0.000000 | -0.000000 | 1025 | 1030.440375 | 1107.85 | 1185.259625 | 1.080829 |
| GO:0030432\_peristalsis | 7 | 0 | 0.000000 | -0.000000 | 1025 | 1030.440375 | 1107.85 | 1185.259625 | 1.080829 |
| GO:0030517\_negative\_regulation\_of\_axon\_extension | 7 | 0 | 0.000000 | -0.000000 | 1025 | 1030.440375 | 1107.85 | 1185.259625 | 1.080829 |
| GO:0030520\_estrogen\_receptor\_signaling\_pathway | 7 | 0 | 0.000000 | -0.000000 | 1025 | 1030.440375 | 1107.85 | 1185.259625 | 1.080829 |
| GO:0030521\_androgen\_receptor\_signaling\_pathway | 7 | 0 | 0.000000 | -0.000000 | 1025 | 1030.440375 | 1107.85 | 1185.259625 | 1.080829 |
| GO:0030903\_notochord\_development | 7 | 0 | 0.000000 | -0.000000 | 1025 | 1030.440375 | 1107.85 | 1185.259625 | 1.080829 |
| GO:0031017\_exocrine\_pancreas\_development | 7 | 0 | 0.000000 | -0.000000 | 1025 | 1030.440375 | 1107.85 | 1185.259625 | 1.080829 |
| GO:0031114\_regulation\_of\_microtubule\_depolymerization | 7 | 0 | 0.000000 | -0.000000 | 1025 | 1030.440375 | 1107.85 | 1185.259625 | 1.080829 |
| GO:0031124\_mRNA\_3'-end\_processing | 7 | 0 | 0.000000 | -0.000000 | 1025 | 1030.440375 | 1107.85 | 1185.259625 | 1.080829 |
| GO:0031497\_chromatin\_assembly | 7 | 0 | 0.000000 | -0.000000 | 1025 | 1030.440375 | 1107.85 | 1185.259625 | 1.080829 |
| GO:0032104\_regulation\_of\_response\_to\_extracellular\_stimulus | 7 | 0 | 0.000000 | -0.000000 | 1025 | 1030.440375 | 1107.85 | 1185.259625 | 1.080829 |
| GO:0032107\_regulation\_of\_response\_to\_nutrient\_levels | 7 | 0 | 0.000000 | -0.000000 | 1025 | 1030.440375 | 1107.85 | 1185.259625 | 1.080829 |
| GO:0032228\_regulation\_of\_synaptic\_transmission\_\_GABAergic | 7 | 0 | 0.000000 | -0.000000 | 1025 | 1030.440375 | 1107.85 | 1185.259625 | 1.080829 |
| GO:0032319\_regulation\_of\_Rho\_GTPase\_activity | 7 | 0 | 0.000000 | -0.000000 | 1025 | 1030.440375 | 1107.85 | 1185.259625 | 1.080829 |
| GO:0032507\_maintenance\_of\_protein\_location\_in\_cell | 7 | 0 | 0.000000 | -0.000000 | 1025 | 1030.440375 | 1107.85 | 1185.259625 | 1.080829 |
| GO:0033032\_regulation\_of\_myeloid\_cell\_apoptosis | 7 | 0 | 0.000000 | -0.000000 | 1025 | 1030.440375 | 1107.85 | 1185.259625 | 1.080829 |
| GO:0033057\_reproductive\_behavior\_in\_a\_multicellular\_organism | 7 | 0 | 0.000000 | -0.000000 | 1025 | 1030.440375 | 1107.85 | 1185.259625 | 1.080829 |
| GO:0034599\_cellular\_response\_to\_oxidative\_stress | 7 | 0 | 0.000000 | -0.000000 | 1025 | 1030.440375 | 1107.85 | 1185.259625 | 1.080829 |
| GO:0042033\_chemokine\_biosynthetic\_process | 7 | 0 | 0.000000 | -0.000000 | 1025 | 1030.440375 | 1107.85 | 1185.259625 | 1.080829 |
| GO:0042133\_neurotransmitter\_metabolic\_process | 7 | 0 | 0.000000 | -0.000000 | 1025 | 1030.440375 | 1107.85 | 1185.259625 | 1.080829 |
| GO:0042415\_norepinephrine\_metabolic\_process | 7 | 0 | 0.000000 | -0.000000 | 1025 | 1030.440375 | 1107.85 | 1185.259625 | 1.080829 |
| GO:0042438\_melanin\_biosynthetic\_process | 7 | 0 | 0.000000 | -0.000000 | 1025 | 1030.440375 | 1107.85 | 1185.259625 | 1.080829 |
| GO:0042503\_tyrosine\_phosphorylation\_of\_Stat3\_protein | 7 | 0 | 0.000000 | -0.000000 | 1025 | 1030.440375 | 1107.85 | 1185.259625 | 1.080829 |
| GO:0042572\_retinol\_metabolic\_process | 7 | 0 | 0.000000 | -0.000000 | 1025 | 1030.440375 | 1107.85 | 1185.259625 | 1.080829 |
| GO:0043353\_enucleate\_erythrocyte\_differentiation | 7 | 0 | 0.000000 | -0.000000 | 1025 | 1030.440375 | 1107.85 | 1185.259625 | 1.080829 |
| GO:0043372\_positive\_regulation\_of\_CD4-positive\_\_alpha\_beta\_T\_cell\_differentiation | 7 | 0 | 0.000000 | -0.000000 | 1025 | 1030.440375 | 1107.85 | 1185.259625 | 1.080829 |
| GO:0043449\_cellular\_alkene\_metabolic\_process | 7 | 0 | 0.000000 | -0.000000 | 1025 | 1030.440375 | 1107.85 | 1185.259625 | 1.080829 |
| GO:0043507\_positive\_regulation\_of\_JUN\_kinase\_activity | 7 | 0 | 0.000000 | -0.000000 | 1025 | 1030.440375 | 1107.85 | 1185.259625 | 1.080829 |
| GO:0043567\_regulation\_of\_insulin-like\_growth\_factor\_receptor\_signaling\_pathway | 7 | 0 | 0.000000 | -0.000000 | 1025 | 1030.440375 | 1107.85 | 1185.259625 | 1.080829 |
| GO:0043584\_nose\_development | 7 | 0 | 0.000000 | -0.000000 | 1025 | 1030.440375 | 1107.85 | 1185.259625 | 1.080829 |
| GO:0044065\_regulation\_of\_respiratory\_system\_process | 7 | 0 | 0.000000 | -0.000000 | 1025 | 1030.440375 | 1107.85 | 1185.259625 | 1.080829 |
| GO:0044275\_cellular\_carbohydrate\_catabolic\_process | 7 | 0 | 0.000000 | -0.000000 | 1025 | 1030.440375 | 1107.85 | 1185.259625 | 1.080829 |
| GO:0045059\_positive\_thymic\_T\_cell\_selection | 7 | 0 | 0.000000 | -0.000000 | 1025 | 1030.440375 | 1107.85 | 1185.259625 | 1.080829 |
| GO:0045073\_regulation\_of\_chemokine\_biosynthetic\_process | 7 | 0 | 0.000000 | -0.000000 | 1025 | 1030.440375 | 1107.85 | 1185.259625 | 1.080829 |
| GO:0045581\_negative\_regulation\_of\_T\_cell\_differentiation | 7 | 0 | 0.000000 | -0.000000 | 1025 | 1030.440375 | 1107.85 | 1185.259625 | 1.080829 |
| GO:0045599\_negative\_regulation\_of\_fat\_cell\_differentiation | 7 | 0 | 0.000000 | -0.000000 | 1025 | 1030.440375 | 1107.85 | 1185.259625 | 1.080829 |
| GO:0045604\_regulation\_of\_epidermal\_cell\_differentiation | 7 | 0 | 0.000000 | -0.000000 | 1025 | 1030.440375 | 1107.85 | 1185.259625 | 1.080829 |
| GO:0045668\_negative\_regulation\_of\_osteoblast\_differentiation | 7 | 0 | 0.000000 | -0.000000 | 1025 | 1030.440375 | 1107.85 | 1185.259625 | 1.080829 |
| GO:0045823\_positive\_regulation\_of\_heart\_contraction | 7 | 0 | 0.000000 | -0.000000 | 1025 | 1030.440375 | 1107.85 | 1185.259625 | 1.080829 |
| GO:0045840\_positive\_regulation\_of\_mitosis | 7 | 0 | 0.000000 | -0.000000 | 1025 | 1030.440375 | 1107.85 | 1185.259625 | 1.080829 |
| GO:0045862\_positive\_regulation\_of\_proteolysis | 7 | 0 | 0.000000 | -0.000000 | 1025 | 1030.440375 | 1107.85 | 1185.259625 | 1.080829 |
| GO:0045879\_negative\_regulation\_of\_smoothened\_signaling\_pathway | 7 | 0 | 0.000000 | -0.000000 | 1025 | 1030.440375 | 1107.85 | 1185.259625 | 1.080829 |
| GO:0045880\_positive\_regulation\_of\_smoothened\_signaling\_pathway | 7 | 0 | 0.000000 | -0.000000 | 1025 | 1030.440375 | 1107.85 | 1185.259625 | 1.080829 |
| GO:0045986\_negative\_regulation\_of\_smooth\_muscle\_contraction | 7 | 0 | 0.000000 | -0.000000 | 1025 | 1030.440375 | 1107.85 | 1185.259625 | 1.080829 |
| GO:0046496\_nicotinamide\_nucleotide\_metabolic\_process | 7 | 0 | 0.000000 | -0.000000 | 1025 | 1030.440375 | 1107.85 | 1185.259625 | 1.080829 |
| GO:0046504\_glycerol\_ether\_biosynthetic\_process | 7 | 0 | 0.000000 | -0.000000 | 1025 | 1030.440375 | 1107.85 | 1185.259625 | 1.080829 |
| GO:0046513\_ceramide\_biosynthetic\_process | 7 | 0 | 0.000000 | -0.000000 | 1025 | 1030.440375 | 1107.85 | 1185.259625 | 1.080829 |
| GO:0046520\_sphingoid\_biosynthetic\_process | 7 | 0 | 0.000000 | -0.000000 | 1025 | 1030.440375 | 1107.85 | 1185.259625 | 1.080829 |
| GO:0046543\_development\_of\_secondary\_female\_sexual\_characteristics | 7 | 0 | 0.000000 | -0.000000 | 1025 | 1030.440375 | 1107.85 | 1185.259625 | 1.080829 |
| GO:0046622\_positive\_regulation\_of\_organ\_growth | 7 | 0 | 0.000000 | -0.000000 | 1025 | 1030.440375 | 1107.85 | 1185.259625 | 1.080829 |
| GO:0046626\_regulation\_of\_insulin\_receptor\_signaling\_pathway | 7 | 0 | 0.000000 | -0.000000 | 1025 | 1030.440375 | 1107.85 | 1185.259625 | 1.080829 |
| GO:0046676\_negative\_regulation\_of\_insulin\_secretion | 7 | 0 | 0.000000 | -0.000000 | 1025 | 1030.440375 | 1107.85 | 1185.259625 | 1.080829 |
| GO:0046677\_response\_to\_antibiotic | 7 | 0 | 0.000000 | -0.000000 | 1025 | 1030.440375 | 1107.85 | 1185.259625 | 1.080829 |
| GO:0046824\_positive\_regulation\_of\_nucleocytoplasmic\_transport | 7 | 0 | 0.000000 | -0.000000 | 1025 | 1030.440375 | 1107.85 | 1185.259625 | 1.080829 |
| GO:0046847\_filopodium\_assembly | 7 | 0 | 0.000000 | -0.000000 | 1025 | 1030.440375 | 1107.85 | 1185.259625 | 1.080829 |
| GO:0048148\_behavioral\_response\_to\_cocaine | 7 | 0 | 0.000000 | -0.000000 | 1025 | 1030.440375 | 1107.85 | 1185.259625 | 1.080829 |
| GO:0048304\_positive\_regulation\_of\_isotype\_switching\_to\_IgG\_isotypes | 7 | 0 | 0.000000 | -0.000000 | 1025 | 1030.440375 | 1107.85 | 1185.259625 | 1.080829 |
| GO:0048486\_parasympathetic\_nervous\_system\_development | 7 | 0 | 0.000000 | -0.000000 | 1025 | 1030.440375 | 1107.85 | 1185.259625 | 1.080829 |
| GO:0048537\_mucosal-associated\_lymphoid\_tissue\_development | 7 | 0 | 0.000000 | -0.000000 | 1025 | 1030.440375 | 1107.85 | 1185.259625 | 1.080829 |
| GO:0048753\_pigment\_granule\_organization | 7 | 0 | 0.000000 | -0.000000 | 1025 | 1030.440375 | 1107.85 | 1185.259625 | 1.080829 |
| GO:0048814\_regulation\_of\_dendrite\_morphogenesis | 7 | 0 | 0.000000 | -0.000000 | 1025 | 1030.440375 | 1107.85 | 1185.259625 | 1.080829 |
| GO:0048857\_neural\_nucleus\_development | 7 | 0 | 0.000000 | -0.000000 | 1025 | 1030.440375 | 1107.85 | 1185.259625 | 1.080829 |
| GO:0050755\_chemokine\_metabolic\_process | 7 | 0 | 0.000000 | -0.000000 | 1025 | 1030.440375 | 1107.85 | 1185.259625 | 1.080829 |
| GO:0050773\_regulation\_of\_dendrite\_development | 7 | 0 | 0.000000 | -0.000000 | 1025 | 1030.440375 | 1107.85 | 1185.259625 | 1.080829 |
| GO:0051028\_mRNA\_transport | 7 | 0 | 0.000000 | -0.000000 | 1025 | 1030.440375 | 1107.85 | 1185.259625 | 1.080829 |
| GO:0051785\_positive\_regulation\_of\_nuclear\_division | 7 | 0 | 0.000000 | -0.000000 | 1025 | 1030.440375 | 1107.85 | 1185.259625 | 1.080829 |
| GO:0051928\_positive\_regulation\_of\_calcium\_ion\_transport | 7 | 0 | 0.000000 | -0.000000 | 1025 | 1030.440375 | 1107.85 | 1185.259625 | 1.080829 |
| GO:0055069\_zinc\_ion\_homeostasis | 7 | 0 | 0.000000 | -0.000000 | 1025 | 1030.440375 | 1107.85 | 1185.259625 | 1.080829 |
| GO:0055070\_copper\_ion\_homeostasis | 7 | 0 | 0.000000 | -0.000000 | 1025 | 1030.440375 | 1107.85 | 1185.259625 | 1.080829 |
| GO:0060037\_pharyngeal\_system\_development | 7 | 0 | 0.000000 | -0.000000 | 1025 | 1030.440375 | 1107.85 | 1185.259625 | 1.080829 |
| GO:0060080\_regulation\_of\_inhibitory\_postsynaptic\_membrane\_potential | 7 | 0 | 0.000000 | -0.000000 | 1025 | 1030.440375 | 1107.85 | 1185.259625 | 1.080829 |
| GO:0060088\_auditory\_receptor\_cell\_stereocilium\_organization | 7 | 0 | 0.000000 | -0.000000 | 1025 | 1030.440375 | 1107.85 | 1185.259625 | 1.080829 |
| GO:0060117\_auditory\_receptor\_cell\_development | 7 | 0 | 0.000000 | -0.000000 | 1025 | 1030.440375 | 1107.85 | 1185.259625 | 1.080829 |
| GO:0060441\_branching\_involved\_in\_lung\_morphogenesis | 7 | 0 | 0.000000 | -0.000000 | 1025 | 1030.440375 | 1107.85 | 1185.259625 | 1.080829 |
| GO:0060526\_prostate\_glandular\_acinus\_morphogenesis | 7 | 0 | 0.000000 | -0.000000 | 1025 | 1030.440375 | 1107.85 | 1185.259625 | 1.080829 |
| GO:0060527\_prostate\_epithelial\_cord\_arborization\_involved\_in\_prostate\_glandular\_acinus\_morphogenesis | 7 | 0 | 0.000000 | -0.000000 | 1025 | 1030.440375 | 1107.85 | 1185.259625 | 1.080829 |
| GO:0060579\_ventral\_spinal\_cord\_interneuron\_fate\_commitment | 7 | 0 | 0.000000 | -0.000000 | 1025 | 1030.440375 | 1107.85 | 1185.259625 | 1.080829 |
| GO:0060664\_epithelial\_cell\_proliferation\_involved\_in\_salivary\_gland\_morphogenesis | 7 | 0 | 0.000000 | -0.000000 | 1025 | 1030.440375 | 1107.85 | 1185.259625 | 1.080829 |
| GO:0060687\_regulation\_of\_branching\_involved\_in\_prostate\_gland\_morphogenesis | 7 | 0 | 0.000000 | -0.000000 | 1025 | 1030.440375 | 1107.85 | 1185.259625 | 1.080829 |
| GO:0060770\_negative\_regulation\_of\_epithelial\_cell\_proliferation\_involved\_in\_prostate\_gland\_development | 7 | 0 | 0.000000 | -0.000000 | 1025 | 1030.440375 | 1107.85 | 1185.259625 | 1.080829 |
| GO:0060788\_ectodermal\_placode\_formation | 7 | 0 | 0.000000 | -0.000000 | 1025 | 1030.440375 | 1107.85 | 1185.259625 | 1.080829 |
| GO:0060795\_cell\_fate\_commitment\_involved\_in\_the\_formation\_of\_primary\_germ\_layers | 7 | 0 | 0.000000 | -0.000000 | 1025 | 1030.440375 | 1107.85 | 1185.259625 | 1.080829 |
| GO:0070228\_regulation\_of\_lymphocyte\_apoptosis | 7 | 0 | 0.000000 | -0.000000 | 1025 | 1030.440375 | 1107.85 | 1185.259625 | 1.080829 |
| GO:0070646\_protein\_modification\_by\_small\_protein\_removal | 7 | 0 | 0.000000 | -0.000000 | 1025 | 1030.440375 | 1107.85 | 1185.259625 | 1.080829 |
| GO:0019752\_carboxylic\_acid\_metabolic\_process | 181 | 0 | 0.000000 | -0.000000 | 1028 | 1031.740645 | 1109.04 | 1186.339355 | 1.078833 |
| GO:0043436\_oxoacid\_metabolic\_process | 181 | 0 | 0.000000 | -0.000000 | 1028 | 1031.740645 | 1109.04 | 1186.339355 | 1.078833 |
| GO:0055082\_cellular\_chemical\_homeostasis | 181 | 0 | 0.000000 | -0.000000 | 1028 | 1031.740645 | 1109.04 | 1186.339355 | 1.078833 |
| GO:0000084\_S\_phase\_of\_mitotic\_cell\_cycle | 3 | 0 |  |  |  |  |  |  |  |  |
| GO:0000089\_mitotic\_metaphase | 3 | 0 |  |  |  |  |  |  |  |  |
| GO:0000098\_sulfur\_amino\_acid\_catabolic\_process | 3 | 0 |  |  |  |  |  |  |  |  |
| GO:0000103\_sulfate\_assimilation | 3 | 0 |  |  |  |  |  |  |  |  |
| GO:0000212\_meiotic\_spindle\_organization | 3 | 0 |  |  |  |  |  |  |  |  |
| GO:0000281\_cytokinesis\_after\_mitosis | 3 | 0 |  |  |  |  |  |  |  |  |
| GO:0000303\_response\_to\_superoxide | 3 | 0 |  |  |  |  |  |  |  |  |
| GO:0000320\_re-entry\_into\_mitotic\_cell\_cycle | 3 | 0 |  |  |  |  |  |  |  |  |
| GO:0000380\_alternative\_nuclear\_mRNA\_splicing\_\_via\_spliceosome | 3 | 0 |  |  |  |  |  |  |  |  |
| GO:0001516\_prostaglandin\_biosynthetic\_process | 3 | 0 |  |  |  |  |  |  |  |  |
| GO:0001553\_luteinization | 3 | 0 |  |  |  |  |  |  |  |  |
| GO:0001574\_ganglioside\_biosynthetic\_process | 3 | 0 |  |  |  |  |  |  |  |  |
| GO:0001705\_ectoderm\_formation | 3 | 0 |  |  |  |  |  |  |  |  |
| GO:0001711\_endodermal\_cell\_fate\_commitment | 3 | 0 |  |  |  |  |  |  |  |  |
| GO:0001757\_somite\_specification | 3 | 0 |  |  |  |  |  |  |  |  |
| GO:0001778\_plasma\_membrane\_repair | 3 | 0 |  |  |  |  |  |  |  |  |
| GO:0001780\_neutrophil\_homeostasis | 3 | 0 |  |  |  |  |  |  |  |  |
| GO:0001802\_type\_III\_hypersensitivity | 3 | 0 |  |  |  |  |  |  |  |  |
| GO:0001803\_regulation\_of\_type\_III\_hypersensitivity | 3 | 0 |  |  |  |  |  |  |  |  |
| GO:0001805\_positive\_regulation\_of\_type\_III\_hypersensitivity | 3 | 0 |  |  |  |  |  |  |  |  |
| GO:0001812\_positive\_regulation\_of\_type\_I\_hypersensitivity | 3 | 0 |  |  |  |  |  |  |  |  |
| GO:0001831\_trophectodermal\_cellular\_morphogenesis | 3 | 0 |  |  |  |  |  |  |  |  |
| GO:0001844\_protein\_insertion\_into\_mitochondrial\_membrane\_during\_induction\_of\_apoptosis | 3 | 0 |  |  |  |  |  |  |  |  |
| GO:0001878\_response\_to\_yeast | 3 | 0 |  |  |  |  |  |  |  |  |
| GO:0001895\_retina\_homeostasis | 3 | 0 |  |  |  |  |  |  |  |  |
| GO:0001915\_negative\_regulation\_of\_T\_cell\_mediated\_cytotoxicity | 3 | 0 |  |  |  |  |  |  |  |  |
| GO:0001937\_negative\_regulation\_of\_endothelial\_cell\_proliferation | 3 | 0 |  |  |  |  |  |  |  |  |
| GO:0001953\_negative\_regulation\_of\_cell-matrix\_adhesion | 3 | 0 |  |  |  |  |  |  |  |  |
| GO:0001955\_blood\_vessel\_maturation | 3 | 0 |  |  |  |  |  |  |  |  |
| GO:0001960\_negative\_regulation\_of\_cytokine-mediated\_signaling\_pathway | 3 | 0 |  |  |  |  |  |  |  |  |
| GO:0001973\_adenosine\_receptor\_signaling\_pathway | 3 | 0 |  |  |  |  |  |  |  |  |
| GO:0001996\_positive\_regulation\_of\_heart\_rate\_by\_epinephrine-norepinephrine | 3 | 0 |  |  |  |  |  |  |  |  |
| GO:0002034\_regulation\_of\_blood\_vessel\_size\_by\_renin-angiotensin | 3 | 0 |  |  |  |  |  |  |  |  |
| GO:0002238\_response\_to\_molecule\_of\_fungal\_origin | 3 | 0 |  |  |  |  |  |  |  |  |
| GO:0002275\_myeloid\_cell\_activation\_during\_immune\_response | 3 | 0 |  |  |  |  |  |  |  |  |
| GO:0002281\_macrophage\_activation\_during\_immune\_response | 3 | 0 |  |  |  |  |  |  |  |  |
| GO:0002309\_T\_cell\_proliferation\_during\_immune\_response | 3 | 0 |  |  |  |  |  |  |  |  |
| GO:0002361\_CD4-positive\_\_CD25-positive\_\_alpha-beta\_regulatory\_T\_cell\_differentiation | 3 | 0 |  |  |  |  |  |  |  |  |
| GO:0002369\_T\_cell\_cytokine\_production | 3 | 0 |  |  |  |  |  |  |  |  |
| GO:0002428\_antigen\_processing\_and\_presentation\_of\_peptide\_antigen\_via\_MHC\_class\_Ib | 3 | 0 |  |  |  |  |  |  |  |  |
| GO:0002446\_neutrophil\_mediated\_immunity | 3 | 0 |  |  |  |  |  |  |  |  |
| GO:0002477\_antigen\_processing\_and\_presentation\_of\_exogenous\_peptide\_antigen\_via\_MHC\_class\_Ib | 3 | 0 |  |  |  |  |  |  |  |  |
| GO:0002481\_antigen\_processing\_and\_presentation\_of\_exogenous\_protein\_antigen\_via\_MHC\_class\_Ib\_\_TAP-dependent | 3 | 0 |  |  |  |  |  |  |  |  |
| GO:0002513\_tolerance\_induction\_to\_self\_antigen | 3 | 0 |  |  |  |  |  |  |  |  |
| GO:0002568\_somatic\_diversification\_of\_T\_cell\_receptor\_genes | 3 | 0 |  |  |  |  |  |  |  |  |
| GO:0002674\_negative\_regulation\_of\_acute\_inflammatory\_response | 3 | 0 |  |  |  |  |  |  |  |  |
| GO:0002681\_somatic\_recombination\_of\_T\_cell\_receptor\_gene\_segments | 3 | 0 |  |  |  |  |  |  |  |  |
| GO:0002713\_negative\_regulation\_of\_B\_cell\_mediated\_immunity | 3 | 0 |  |  |  |  |  |  |  |  |
| GO:0002827\_positive\_regulation\_of\_T-helper\_1\_type\_immune\_response | 3 | 0 |  |  |  |  |  |  |  |  |
| GO:0002865\_negative\_regulation\_of\_acute\_inflammatory\_response\_to\_antigenic\_stimulus | 3 | 0 |  |  |  |  |  |  |  |  |
| GO:0002884\_negative\_regulation\_of\_hypersensitivity | 3 | 0 |  |  |  |  |  |  |  |  |
| GO:0002890\_negative\_regulation\_of\_immunoglobulin\_mediated\_immune\_response | 3 | 0 |  |  |  |  |  |  |  |  |
| GO:0002904\_positive\_regulation\_of\_B\_cell\_apoptosis | 3 | 0 |  |  |  |  |  |  |  |  |
| GO:0003009\_skeletal\_muscle\_contraction | 3 | 0 |  |  |  |  |  |  |  |  |
| GO:0003072\_renal\_control\_of\_peripheral\_vascular\_resistance\_involved\_in\_regulation\_of\_systemic\_arterial\_blood\_pressure | 3 | 0 |  |  |  |  |  |  |  |  |
| GO:0006047\_UDP-N-acetylglucosamine\_metabolic\_process | 3 | 0 |  |  |  |  |  |  |  |  |
| GO:0006067\_ethanol\_metabolic\_process | 3 | 0 |  |  |  |  |  |  |  |  |
| GO:0006072\_glycerol-3-phosphate\_metabolic\_process | 3 | 0 |  |  |  |  |  |  |  |  |
| GO:0006103\_2-oxoglutarate\_metabolic\_process | 3 | 0 |  |  |  |  |  |  |  |  |
| GO:0006107\_oxaloacetate\_metabolic\_process | 3 | 0 |  |  |  |  |  |  |  |  |
| GO:0006220\_pyrimidine\_nucleotide\_metabolic\_process | 3 | 0 |  |  |  |  |  |  |  |  |
| GO:0006266\_DNA\_ligation | 3 | 0 |  |  |  |  |  |  |  |  |
| GO:0006282\_regulation\_of\_DNA\_repair | 3 | 0 |  |  |  |  |  |  |  |  |
| GO:0006287\_base-excision\_repair\_\_gap-filling | 3 | 0 |  |  |  |  |  |  |  |  |
| GO:0006301\_postreplication\_repair | 3 | 0 |  |  |  |  |  |  |  |  |
| GO:0006361\_transcription\_initiation\_from\_RNA\_polymerase\_I\_promoter | 3 | 0 |  |  |  |  |  |  |  |  |
| GO:0006367\_transcription\_initiation\_from\_RNA\_polymerase\_II\_promoter | 3 | 0 |  |  |  |  |  |  |  |  |
| GO:0006414\_translational\_elongation | 3 | 0 |  |  |  |  |  |  |  |  |
| GO:0006491\_N-glycan\_processing | 3 | 0 |  |  |  |  |  |  |  |  |
| GO:0006498\_N-terminal\_protein\_lipidation | 3 | 0 |  |  |  |  |  |  |  |  |
| GO:0006531\_aspartate\_metabolic\_process | 3 | 0 |  |  |  |  |  |  |  |  |
| GO:0006598\_polyamine\_catabolic\_process | 3 | 0 |  |  |  |  |  |  |  |  |
| GO:0006620\_posttranslational\_protein\_targeting\_to\_membrane | 3 | 0 |  |  |  |  |  |  |  |  |
| GO:0006625\_protein\_targeting\_to\_peroxisome | 3 | 0 |  |  |  |  |  |  |  |  |
| GO:0006651\_diacylglycerol\_biosynthetic\_process | 3 | 0 |  |  |  |  |  |  |  |  |
| GO:0006670\_sphingosine\_metabolic\_process | 3 | 0 |  |  |  |  |  |  |  |  |
| GO:0006677\_glycosylceramide\_metabolic\_process | 3 | 0 |  |  |  |  |  |  |  |  |
| GO:0006689\_ganglioside\_catabolic\_process | 3 | 0 |  |  |  |  |  |  |  |  |
| GO:0006699\_bile\_acid\_biosynthetic\_process | 3 | 0 |  |  |  |  |  |  |  |  |
| GO:0006791\_sulfur\_utilization | 3 | 0 |  |  |  |  |  |  |  |  |
| GO:0006817\_phosphate\_transport | 3 | 0 |  |  |  |  |  |  |  |  |
| GO:0006825\_copper\_ion\_transport | 3 | 0 |  |  |  |  |  |  |  |  |
| GO:0006828\_manganese\_ion\_transport | 3 | 0 |  |  |  |  |  |  |  |  |
| GO:0006857\_oligopeptide\_transport | 3 | 0 |  |  |  |  |  |  |  |  |
| GO:0006892\_post-Golgi\_vesicle-mediated\_transport | 3 | 0 |  |  |  |  |  |  |  |  |
| GO:0006904\_vesicle\_docking\_during\_exocytosis | 3 | 0 |  |  |  |  |  |  |  |  |
| GO:0006926\_virus-infected\_cell\_apoptosis | 3 | 0 |  |  |  |  |  |  |  |  |
| GO:0006953\_acute-phase\_response | 3 | 0 |  |  |  |  |  |  |  |  |
| GO:0007000\_nucleolus\_organization | 3 | 0 |  |  |  |  |  |  |  |  |
| GO:0007041\_lysosomal\_transport | 3 | 0 |  |  |  |  |  |  |  |  |
| GO:0007043\_cell-cell\_junction\_assembly | 3 | 0 |  |  |  |  |  |  |  |  |
| GO:0007090\_regulation\_of\_S\_phase\_of\_mitotic\_cell\_cycle | 3 | 0 |  |  |  |  |  |  |  |  |
| GO:0007195\_inhibition\_of\_adenylate\_cyclase\_activity\_by\_dopamine\_receptor\_signaling\_pathway | 3 | 0 |  |  |  |  |  |  |  |  |
| GO:0007213\_muscarinic\_acetylcholine\_receptor\_signaling\_pathway | 3 | 0 |  |  |  |  |  |  |  |  |
| GO:0007250\_activation\_of\_NF-kappaB-inducing\_kinase\_activity | 3 | 0 |  |  |  |  |  |  |  |  |
| GO:0007252\_I-kappaB\_phosphorylation | 3 | 0 |  |  |  |  |  |  |  |  |
| GO:0007262\_STAT\_protein\_nuclear\_translocation | 3 | 0 |  |  |  |  |  |  |  |  |
| GO:0007288\_sperm\_axoneme\_assembly | 3 | 0 |  |  |  |  |  |  |  |  |
| GO:0007350\_blastoderm\_segmentation | 3 | 0 |  |  |  |  |  |  |  |  |
| GO:0007403\_glial\_cell\_fate\_determination | 3 | 0 |  |  |  |  |  |  |  |  |
| GO:0007412\_axon\_target\_recognition | 3 | 0 |  |  |  |  |  |  |  |  |
| GO:0007468\_regulation\_of\_rhodopsin\_gene\_expression | 3 | 0 |  |  |  |  |  |  |  |  |
| GO:0007525\_somatic\_muscle\_development | 3 | 0 |  |  |  |  |  |  |  |  |
| GO:0007635\_chemosensory\_behavior | 3 | 0 |  |  |  |  |  |  |  |  |
| GO:0008090\_retrograde\_axon\_cargo\_transport | 3 | 0 |  |  |  |  |  |  |  |  |
| GO:0008347\_glial\_cell\_migration | 3 | 0 |  |  |  |  |  |  |  |  |
| GO:0008635\_activation\_of\_caspase\_activity\_by\_cytochrome\_c | 3 | 0 |  |  |  |  |  |  |  |  |
| GO:0009060\_aerobic\_respiration | 3 | 0 |  |  |  |  |  |  |  |  |
| GO:0009081\_branched\_chain\_family\_amino\_acid\_metabolic\_process | 3 | 0 |  |  |  |  |  |  |  |  |
| GO:0009086\_methionine\_biosynthetic\_process | 3 | 0 |  |  |  |  |  |  |  |  |
| GO:0009135\_purine\_nucleoside\_diphosphate\_metabolic\_process | 3 | 0 |  |  |  |  |  |  |  |  |
| GO:0009137\_purine\_nucleoside\_diphosphate\_catabolic\_process | 3 | 0 |  |  |  |  |  |  |  |  |
| GO:0009155\_purine\_deoxyribonucleotide\_catabolic\_process | 3 | 0 |  |  |  |  |  |  |  |  |
| GO:0009179\_purine\_ribonucleoside\_diphosphate\_metabolic\_process | 3 | 0 |  |  |  |  |  |  |  |  |
| GO:0009181\_purine\_ribonucleoside\_diphosphate\_catabolic\_process | 3 | 0 |  |  |  |  |  |  |  |  |
| GO:0009185\_ribonucleoside\_diphosphate\_metabolic\_process | 3 | 0 |  |  |  |  |  |  |  |  |
| GO:0009191\_ribonucleoside\_diphosphate\_catabolic\_process | 3 | 0 |  |  |  |  |  |  |  |  |
| GO:0009199\_ribonucleoside\_triphosphate\_metabolic\_process | 3 | 0 |  |  |  |  |  |  |  |  |
| GO:0009204\_deoxyribonucleoside\_triphosphate\_catabolic\_process | 3 | 0 |  |  |  |  |  |  |  |  |
| GO:0009205\_purine\_ribonucleoside\_triphosphate\_metabolic\_process | 3 | 0 |  |  |  |  |  |  |  |  |
| GO:0009217\_purine\_deoxyribonucleoside\_triphosphate\_catabolic\_process | 3 | 0 |  |  |  |  |  |  |  |  |
| GO:0009448\_gamma-aminobutyric\_acid\_metabolic\_process | 3 | 0 |  |  |  |  |  |  |  |  |
| GO:0010043\_response\_to\_zinc\_ion | 3 | 0 |  |  |  |  |  |  |  |  |
| GO:0010159\_specification\_of\_organ\_position | 3 | 0 |  |  |  |  |  |  |  |  |
| GO:0010216\_maintenance\_of\_DNA\_methylation | 3 | 0 |  |  |  |  |  |  |  |  |
| GO:0010273\_detoxification\_of\_copper\_ion | 3 | 0 |  |  |  |  |  |  |  |  |
| GO:0010454\_negative\_regulation\_of\_cell\_fate\_commitment | 3 | 0 |  |  |  |  |  |  |  |  |
| GO:0010507\_negative\_regulation\_of\_autophagy | 3 | 0 |  |  |  |  |  |  |  |  |
| GO:0010524\_positive\_regulation\_of\_calcium\_ion\_transport\_into\_cytosol | 3 | 0 |  |  |  |  |  |  |  |  |
| GO:0010573\_vascular\_endothelial\_growth\_factor\_production | 3 | 0 |  |  |  |  |  |  |  |  |
| GO:0010574\_regulation\_of\_vascular\_endothelial\_growth\_factor\_production | 3 | 0 |  |  |  |  |  |  |  |  |
| GO:0010575\_positive\_regulation\_vascular\_endothelial\_growth\_factor\_production | 3 | 0 |  |  |  |  |  |  |  |  |
| GO:0010632\_regulation\_of\_epithelial\_cell\_migration | 3 | 0 |  |  |  |  |  |  |  |  |
| GO:0010717\_regulation\_of\_epithelial\_to\_mesenchymal\_transition | 3 | 0 |  |  |  |  |  |  |  |  |
| GO:0010884\_positive\_regulation\_of\_lipid\_storage | 3 | 0 |  |  |  |  |  |  |  |  |
| GO:0010888\_negative\_regulation\_of\_lipid\_storage | 3 | 0 |  |  |  |  |  |  |  |  |
| GO:0010889\_regulation\_of\_sequestering\_of\_triglyceride | 3 | 0 |  |  |  |  |  |  |  |  |
| GO:0010893\_positive\_regulation\_of\_steroid\_biosynthetic\_process | 3 | 0 |  |  |  |  |  |  |  |  |
| GO:0010894\_negative\_regulation\_of\_steroid\_biosynthetic\_process | 3 | 0 |  |  |  |  |  |  |  |  |
| GO:0010998\_regulation\_of\_translational\_initiation\_by\_eIF2\_alpha\_phosphorylation | 3 | 0 |  |  |  |  |  |  |  |  |
| GO:0010999\_regulation\_of\_eIF2\_alpha\_phosphorylation\_by\_heme | 3 | 0 |  |  |  |  |  |  |  |  |
| GO:0014074\_response\_to\_purine | 3 | 0 |  |  |  |  |  |  |  |  |
| GO:0014909\_smooth\_muscle\_cell\_migration | 3 | 0 |  |  |  |  |  |  |  |  |
| GO:0015669\_gas\_transport | 3 | 0 |  |  |  |  |  |  |  |  |
| GO:0015760\_glucose-6-phosphate\_transport | 3 | 0 |  |  |  |  |  |  |  |  |
| GO:0015816\_glycine\_transport | 3 | 0 |  |  |  |  |  |  |  |  |
| GO:0015838\_betaine\_transport | 3 | 0 |  |  |  |  |  |  |  |  |
| GO:0015871\_choline\_transport | 3 | 0 |  |  |  |  |  |  |  |  |
| GO:0015879\_carnitine\_transport | 3 | 0 |  |  |  |  |  |  |  |  |
| GO:0015893\_drug\_transport | 3 | 0 |  |  |  |  |  |  |  |  |
| GO:0015909\_long-chain\_fatty\_acid\_transport | 3 | 0 |  |  |  |  |  |  |  |  |
| GO:0015936\_coenzyme\_A\_metabolic\_process | 3 | 0 |  |  |  |  |  |  |  |  |
| GO:0015988\_energy\_coupled\_proton\_transport\_\_against\_electrochemical\_gradient | 3 | 0 |  |  |  |  |  |  |  |  |
| GO:0015991\_ATP\_hydrolysis\_coupled\_proton\_transport | 3 | 0 |  |  |  |  |  |  |  |  |
| GO:0016241\_regulation\_of\_macroautophagy | 3 | 0 |  |  |  |  |  |  |  |  |
| GO:0016322\_neuron\_remodeling | 3 | 0 |  |  |  |  |  |  |  |  |
| GO:0016556\_mRNA\_modification | 3 | 0 |  |  |  |  |  |  |  |  |
| GO:0016973\_poly(A)+\_mRNA\_export\_from\_nucleus | 3 | 0 |  |  |  |  |  |  |  |  |
| GO:0018196\_peptidyl-asparagine\_modification | 3 | 0 |  |  |  |  |  |  |  |  |
| GO:0018208\_peptidyl-proline\_modification | 3 | 0 |  |  |  |  |  |  |  |  |
| GO:0018279\_protein\_amino\_acid\_N-linked\_glycosylation\_via\_asparagine | 3 | 0 |  |  |  |  |  |  |  |  |
| GO:0018894\_dibenzo-p-dioxin\_metabolic\_process | 3 | 0 |  |  |  |  |  |  |  |  |
| GO:0019058\_viral\_infectious\_cycle | 3 | 0 |  |  |  |  |  |  |  |  |
| GO:0019230\_proprioception | 3 | 0 |  |  |  |  |  |  |  |  |
| GO:0019236\_response\_to\_pheromone | 3 | 0 |  |  |  |  |  |  |  |  |
| GO:0019359\_nicotinamide\_nucleotide\_biosynthetic\_process | 3 | 0 |  |  |  |  |  |  |  |  |
| GO:0019363\_pyridine\_nucleotide\_biosynthetic\_process | 3 | 0 |  |  |  |  |  |  |  |  |
| GO:0019438\_aromatic\_compound\_biosynthetic\_process | 3 | 0 |  |  |  |  |  |  |  |  |
| GO:0019439\_aromatic\_compound\_catabolic\_process | 3 | 0 |  |  |  |  |  |  |  |  |
| GO:0019605\_butyrate\_metabolic\_process | 3 | 0 |  |  |  |  |  |  |  |  |
| GO:0019614\_catechol\_catabolic\_process | 3 | 0 |  |  |  |  |  |  |  |  |
| GO:0019674\_NAD\_metabolic\_process | 3 | 0 |  |  |  |  |  |  |  |  |
| GO:0019852\_L-ascorbic\_acid\_metabolic\_process | 3 | 0 |  |  |  |  |  |  |  |  |
| GO:0019987\_negative\_regulation\_of\_anti-apoptosis | 3 | 0 |  |  |  |  |  |  |  |  |
| GO:0021527\_spinal\_cord\_association\_neuron\_differentiation | 3 | 0 |  |  |  |  |  |  |  |  |
| GO:0021529\_spinal\_cord\_oligodendrocyte\_cell\_differentiation | 3 | 0 |  |  |  |  |  |  |  |  |
| GO:0021530\_spinal\_cord\_oligodendrocyte\_cell\_fate\_specification | 3 | 0 |  |  |  |  |  |  |  |  |
| GO:0021555\_midbrain-hindbrain\_boundary\_morphogenesis | 3 | 0 |  |  |  |  |  |  |  |  |
| GO:0021563\_glossopharyngeal\_nerve\_development | 3 | 0 |  |  |  |  |  |  |  |  |
| GO:0021570\_rhombomere\_4\_development | 3 | 0 |  |  |  |  |  |  |  |  |
| GO:0021591\_ventricular\_system\_development | 3 | 0 |  |  |  |  |  |  |  |  |
| GO:0021615\_glossopharyngeal\_nerve\_morphogenesis | 3 | 0 |  |  |  |  |  |  |  |  |
| GO:0021794\_thalamus\_development | 3 | 0 |  |  |  |  |  |  |  |  |
| GO:0021797\_forebrain\_anterior\_posterior\_pattern\_formation | 3 | 0 |  |  |  |  |  |  |  |  |
| GO:0021798\_forebrain\_dorsal\_ventral\_pattern\_formation | 3 | 0 |  |  |  |  |  |  |  |  |
| GO:0021800\_cerebral\_cortex\_tangential\_migration | 3 | 0 |  |  |  |  |  |  |  |  |
| GO:0021819\_layer\_formation\_in\_the\_cerebral\_cortex | 3 | 0 |  |  |  |  |  |  |  |  |
| GO:0021859\_pyramidal\_neuron\_differentiation | 3 | 0 |  |  |  |  |  |  |  |  |
| GO:0021860\_pyramidal\_neuron\_development | 3 | 0 |  |  |  |  |  |  |  |  |
| GO:0021889\_olfactory\_bulb\_interneuron\_differentiation | 3 | 0 |  |  |  |  |  |  |  |  |
| GO:0021891\_olfactory\_bulb\_interneuron\_development | 3 | 0 |  |  |  |  |  |  |  |  |
| GO:0021912\_regulation\_of\_transcription\_from\_RNA\_polymerase\_II\_promoter\_involved\_in\_spinal\_cord\_motor\_neuron\_fate\_specification | 3 | 0 |  |  |  |  |  |  |  |  |
| GO:0021979\_hypothalamus\_cell\_differentiation | 3 | 0 |  |  |  |  |  |  |  |  |
| GO:0022010\_myelination\_in\_the\_central\_nervous\_system | 3 | 0 |  |  |  |  |  |  |  |  |
| GO:0022027\_interkinetic\_nuclear\_migration | 3 | 0 |  |  |  |  |  |  |  |  |
| GO:0022406\_membrane\_docking | 3 | 0 |  |  |  |  |  |  |  |  |
| GO:0030033\_microvillus\_assembly | 3 | 0 |  |  |  |  |  |  |  |  |
| GO:0030091\_protein\_repair | 3 | 0 |  |  |  |  |  |  |  |  |
| GO:0030195\_negative\_regulation\_of\_blood\_coagulation | 3 | 0 |  |  |  |  |  |  |  |  |
| GO:0030224\_monocyte\_differentiation | 3 | 0 |  |  |  |  |  |  |  |  |
| GO:0030307\_positive\_regulation\_of\_cell\_growth | 3 | 0 |  |  |  |  |  |  |  |  |
| GO:0030319\_cellular\_di-\_\_tri-valent\_inorganic\_anion\_homeostasis | 3 | 0 |  |  |  |  |  |  |  |  |
| GO:0030320\_cellular\_monovalent\_inorganic\_anion\_homeostasis | 3 | 0 |  |  |  |  |  |  |  |  |
| GO:0030321\_transepithelial\_chloride\_transport | 3 | 0 |  |  |  |  |  |  |  |  |
| GO:0030501\_positive\_regulation\_of\_bone\_mineralization | 3 | 0 |  |  |  |  |  |  |  |  |
| GO:0030513\_positive\_regulation\_of\_BMP\_signaling\_pathway | 3 | 0 |  |  |  |  |  |  |  |  |
| GO:0030538\_embryonic\_genitalia\_morphogenesis | 3 | 0 |  |  |  |  |  |  |  |  |
| GO:0030540\_female\_genitalia\_development | 3 | 0 |  |  |  |  |  |  |  |  |
| GO:0030574\_collagen\_catabolic\_process | 3 | 0 |  |  |  |  |  |  |  |  |
| GO:0030643\_cellular\_phosphate\_ion\_homeostasis | 3 | 0 |  |  |  |  |  |  |  |  |
| GO:0030718\_germ-line\_stem\_cell\_maintenance | 3 | 0 |  |  |  |  |  |  |  |  |
| GO:0030730\_sequestering\_of\_triglyceride | 3 | 0 |  |  |  |  |  |  |  |  |
| GO:0030836\_positive\_regulation\_of\_actin\_filament\_depolymerization | 3 | 0 |  |  |  |  |  |  |  |  |
| GO:0030857\_negative\_regulation\_of\_epithelial\_cell\_differentiation | 3 | 0 |  |  |  |  |  |  |  |  |
| GO:0030916\_otic\_vesicle\_formation | 3 | 0 |  |  |  |  |  |  |  |  |
| GO:0031000\_response\_to\_caffeine | 3 | 0 |  |  |  |  |  |  |  |  |
| GO:0031063\_regulation\_of\_histone\_deacetylation | 3 | 0 |  |  |  |  |  |  |  |  |
| GO:0031065\_positive\_regulation\_of\_histone\_deacetylation | 3 | 0 |  |  |  |  |  |  |  |  |
| GO:0031112\_positive\_regulation\_of\_microtubule\_polymerization\_or\_depolymerization | 3 | 0 |  |  |  |  |  |  |  |  |
| GO:0031116\_positive\_regulation\_of\_microtubule\_polymerization | 3 | 0 |  |  |  |  |  |  |  |  |
| GO:0031133\_regulation\_of\_axon\_diameter | 3 | 0 |  |  |  |  |  |  |  |  |
| GO:0031282\_regulation\_of\_guanylate\_cyclase\_activity | 3 | 0 |  |  |  |  |  |  |  |  |
| GO:0031333\_negative\_regulation\_of\_protein\_complex\_assembly | 3 | 0 |  |  |  |  |  |  |  |  |
| GO:0031397\_negative\_regulation\_of\_protein\_ubiquitination | 3 | 0 |  |  |  |  |  |  |  |  |
| GO:0031398\_positive\_regulation\_of\_protein\_ubiquitination | 3 | 0 |  |  |  |  |  |  |  |  |
| GO:0031503\_protein\_complex\_localization | 3 | 0 |  |  |  |  |  |  |  |  |
| GO:0031571\_G1\_DNA\_damage\_checkpoint | 3 | 0 |  |  |  |  |  |  |  |  |
| GO:0031579\_membrane\_raft\_organization | 3 | 0 |  |  |  |  |  |  |  |  |
| GO:0031638\_zymogen\_activation | 3 | 0 |  |  |  |  |  |  |  |  |
| GO:0031641\_regulation\_of\_myelination | 3 | 0 |  |  |  |  |  |  |  |  |
| GO:0031642\_negative\_regulation\_of\_myelination | 3 | 0 |  |  |  |  |  |  |  |  |
| GO:0031649\_heat\_generation | 3 | 0 |  |  |  |  |  |  |  |  |
| GO:0031943\_regulation\_of\_glucocorticoid\_metabolic\_process | 3 | 0 |  |  |  |  |  |  |  |  |
| GO:0032020\_ISG15-protein\_conjugation | 3 | 0 |  |  |  |  |  |  |  |  |
| GO:0032060\_bleb\_formation | 3 | 0 |  |  |  |  |  |  |  |  |
| GO:0032095\_regulation\_of\_response\_to\_food | 3 | 0 |  |  |  |  |  |  |  |  |
| GO:0032272\_negative\_regulation\_of\_protein\_polymerization | 3 | 0 |  |  |  |  |  |  |  |  |
| GO:0032288\_myelin\_assembly | 3 | 0 |  |  |  |  |  |  |  |  |
| GO:0032291\_ensheathment\_of\_axons\_in\_the\_central\_nervous\_system | 3 | 0 |  |  |  |  |  |  |  |  |
| GO:0032355\_response\_to\_estradiol\_stimulus | 3 | 0 |  |  |  |  |  |  |  |  |
| GO:0032402\_melanosome\_transport | 3 | 0 |  |  |  |  |  |  |  |  |
| GO:0032411\_positive\_regulation\_of\_transporter\_activity | 3 | 0 |  |  |  |  |  |  |  |  |
| GO:0032414\_positive\_regulation\_of\_ion\_transmembrane\_transporter\_activity | 3 | 0 |  |  |  |  |  |  |  |  |
| GO:0032436\_positive\_regulation\_of\_proteasomal\_ubiquitin-dependent\_protein\_catabolic\_process | 3 | 0 |  |  |  |  |  |  |  |  |
| GO:0032528\_microvillus\_organization | 3 | 0 |  |  |  |  |  |  |  |  |
| GO:0032536\_regulation\_of\_cell\_projection\_size | 3 | 0 |  |  |  |  |  |  |  |  |
| GO:0032632\_interleukin-3\_production | 3 | 0 |  |  |  |  |  |  |  |  |
| GO:0032634\_interleukin-5\_production | 3 | 0 |  |  |  |  |  |  |  |  |
| GO:0032674\_regulation\_of\_interleukin-5\_production | 3 | 0 |  |  |  |  |  |  |  |  |
| GO:0032703\_negative\_regulation\_of\_interleukin-2\_production | 3 | 0 |  |  |  |  |  |  |  |  |
| GO:0032753\_positive\_regulation\_of\_interleukin-4\_production | 3 | 0 |  |  |  |  |  |  |  |  |
| GO:0032823\_regulation\_of\_natural\_killer\_cell\_differentiation | 3 | 0 |  |  |  |  |  |  |  |  |
| GO:0032825\_positive\_regulation\_of\_natural\_killer\_cell\_differentiation | 3 | 0 |  |  |  |  |  |  |  |  |
| GO:0032856\_activation\_of\_Ras\_GTPase\_activity | 3 | 0 |  |  |  |  |  |  |  |  |
| GO:0032862\_activation\_of\_Rho\_GTPase\_activity | 3 | 0 |  |  |  |  |  |  |  |  |
| GO:0032874\_positive\_regulation\_of\_stress-activated\_MAPK\_cascade | 3 | 0 |  |  |  |  |  |  |  |  |
| GO:0032881\_regulation\_of\_polysaccharide\_metabolic\_process | 3 | 0 |  |  |  |  |  |  |  |  |
| GO:0032890\_regulation\_of\_organic\_acid\_transport | 3 | 0 |  |  |  |  |  |  |  |  |
| GO:0033058\_directional\_locomotion | 3 | 0 |  |  |  |  |  |  |  |  |
| GO:0033080\_immature\_T\_cell\_proliferation\_in\_the\_thymus | 3 | 0 |  |  |  |  |  |  |  |  |
| GO:0033084\_regulation\_of\_immature\_T\_cell\_proliferation\_in\_the\_thymus | 3 | 0 |  |  |  |  |  |  |  |  |
| GO:0033091\_positive\_regulation\_of\_immature\_T\_cell\_proliferation | 3 | 0 |  |  |  |  |  |  |  |  |
| GO:0033137\_negative\_regulation\_of\_peptidyl-serine\_phosphorylation | 3 | 0 |  |  |  |  |  |  |  |  |
| GO:0033153\_T\_cell\_receptor\_V(D)J\_recombination | 3 | 0 |  |  |  |  |  |  |  |  |
| GO:0033209\_tumor\_necrosis\_factor-mediated\_signaling\_pathway | 3 | 0 |  |  |  |  |  |  |  |  |
| GO:0033261\_regulation\_of\_S\_phase | 3 | 0 |  |  |  |  |  |  |  |  |
| GO:0033600\_negative\_regulation\_of\_mammary\_gland\_epithelial\_cell\_proliferation | 3 | 0 |  |  |  |  |  |  |  |  |
| GO:0033631\_cell-cell\_adhesion\_mediated\_by\_integrin | 3 | 0 |  |  |  |  |  |  |  |  |
| GO:0033993\_response\_to\_lipid | 3 | 0 |  |  |  |  |  |  |  |  |
| GO:0034220\_ion\_transmembrane\_transport | 3 | 0 |  |  |  |  |  |  |  |  |
| GO:0034308\_monohydric\_alcohol\_metabolic\_process | 3 | 0 |  |  |  |  |  |  |  |  |
| GO:0034313\_diol\_catabolic\_process | 3 | 0 |  |  |  |  |  |  |  |  |
| GO:0034331\_cell\_junction\_maintenance | 3 | 0 |  |  |  |  |  |  |  |  |
| GO:0034332\_adherens\_junction\_organization | 3 | 0 |  |  |  |  |  |  |  |  |
| GO:0034375\_high-density\_lipoprotein\_particle\_remodeling | 3 | 0 |  |  |  |  |  |  |  |  |
| GO:0034381\_lipoprotein\_particle\_clearance | 3 | 0 |  |  |  |  |  |  |  |  |
| GO:0034612\_response\_to\_tumor\_necrosis\_factor | 3 | 0 |  |  |  |  |  |  |  |  |
| GO:0034655\_nucleobase\_\_nucleoside\_\_nucleotide\_and\_nucleic\_acid\_catabolic\_process | 3 | 0 |  |  |  |  |  |  |  |  |
| GO:0034656\_nucleobase\_\_nucleoside\_and\_nucleotide\_catabolic\_process | 3 | 0 |  |  |  |  |  |  |  |  |
| GO:0035067\_negative\_regulation\_of\_histone\_acetylation | 3 | 0 |  |  |  |  |  |  |  |  |
| GO:0035084\_flagellar\_axoneme\_assembly | 3 | 0 |  |  |  |  |  |  |  |  |
| GO:0035166\_post-embryonic\_hemopoiesis | 3 | 0 |  |  |  |  |  |  |  |  |
| GO:0035283\_central\_nervous\_system\_segmentation | 3 | 0 |  |  |  |  |  |  |  |  |
| GO:0035284\_brain\_segmentation | 3 | 0 |  |  |  |  |  |  |  |  |
| GO:0042097\_interleukin-4\_biosynthetic\_process | 3 | 0 |  |  |  |  |  |  |  |  |
| GO:0042135\_neurotransmitter\_catabolic\_process | 3 | 0 |  |  |  |  |  |  |  |  |
| GO:0042271\_susceptibility\_to\_natural\_killer\_cell\_mediated\_cytotoxicity | 3 | 0 |  |  |  |  |  |  |  |  |
| GO:0042273\_ribosomal\_large\_subunit\_biogenesis | 3 | 0 |  |  |  |  |  |  |  |  |
| GO:0042375\_quinone\_cofactor\_metabolic\_process | 3 | 0 |  |  |  |  |  |  |  |  |
| GO:0042420\_dopamine\_catabolic\_process | 3 | 0 |  |  |  |  |  |  |  |  |
| GO:0042421\_norepinephrine\_biosynthetic\_process | 3 | 0 |  |  |  |  |  |  |  |  |
| GO:0042424\_catecholamine\_catabolic\_process | 3 | 0 |  |  |  |  |  |  |  |  |
| GO:0042447\_hormone\_catabolic\_process | 3 | 0 |  |  |  |  |  |  |  |  |
| GO:0042448\_progesterone\_metabolic\_process | 3 | 0 |  |  |  |  |  |  |  |  |
| GO:0042523\_positive\_regulation\_of\_tyrosine\_phosphorylation\_of\_Stat5\_protein | 3 | 0 |  |  |  |  |  |  |  |  |
| GO:0042659\_regulation\_of\_cell\_fate\_specification | 3 | 0 |  |  |  |  |  |  |  |  |
| GO:0042668\_auditory\_receptor\_cell\_fate\_determination | 3 | 0 |  |  |  |  |  |  |  |  |
| GO:0042670\_retinal\_cone\_cell\_differentiation | 3 | 0 |  |  |  |  |  |  |  |  |
| GO:0042693\_muscle\_cell\_fate\_commitment | 3 | 0 |  |  |  |  |  |  |  |  |
| GO:0042711\_maternal\_behavior | 3 | 0 |  |  |  |  |  |  |  |  |
| GO:0042745\_circadian\_sleep\_wake\_cycle | 3 | 0 |  |  |  |  |  |  |  |  |
| GO:0042759\_long-chain\_fatty\_acid\_biosynthetic\_process | 3 | 0 |  |  |  |  |  |  |  |  |
| GO:0042787\_protein\_ubiquitination\_during\_ubiquitin-dependent\_protein\_catabolic\_process | 3 | 0 |  |  |  |  |  |  |  |  |
| GO:0043045\_DNA\_methylation\_during\_embryonic\_development | 3 | 0 |  |  |  |  |  |  |  |  |
| GO:0043090\_amino\_acid\_import | 3 | 0 |  |  |  |  |  |  |  |  |
| GO:0043092\_L-amino\_acid\_import | 3 | 0 |  |  |  |  |  |  |  |  |
| GO:0043149\_stress\_fiber\_formation | 3 | 0 |  |  |  |  |  |  |  |  |
| GO:0043200\_response\_to\_amino\_acid\_stimulus | 3 | 0 |  |  |  |  |  |  |  |  |
| GO:0043243\_positive\_regulation\_of\_protein\_complex\_disassembly | 3 | 0 |  |  |  |  |  |  |  |  |
| GO:0043249\_erythrocyte\_maturation | 3 | 0 |  |  |  |  |  |  |  |  |
| GO:0043267\_negative\_regulation\_of\_potassium\_ion\_transport | 3 | 0 |  |  |  |  |  |  |  |  |
| GO:0043371\_negative\_regulation\_of\_CD4-positive\_\_alpha\_beta\_T\_cell\_differentiation | 3 | 0 |  |  |  |  |  |  |  |  |
| GO:0043462\_regulation\_of\_ATPase\_activity | 3 | 0 |  |  |  |  |  |  |  |  |
| GO:0043569\_negative\_regulation\_of\_insulin-like\_growth\_factor\_receptor\_signaling\_pathway | 3 | 0 |  |  |  |  |  |  |  |  |
| GO:0043574\_peroxisomal\_transport | 3 | 0 |  |  |  |  |  |  |  |  |
| GO:0043586\_tongue\_development | 3 | 0 |  |  |  |  |  |  |  |  |
| GO:0043900\_regulation\_of\_multi-organism\_process | 3 | 0 |  |  |  |  |  |  |  |  |
| GO:0043954\_cellular\_component\_maintenance | 3 | 0 |  |  |  |  |  |  |  |  |
| GO:0044030\_regulation\_of\_DNA\_methylation | 3 | 0 |  |  |  |  |  |  |  |  |
| GO:0044089\_positive\_regulation\_of\_cellular\_component\_biogenesis | 3 | 0 |  |  |  |  |  |  |  |  |
| GO:0044273\_sulfur\_compound\_catabolic\_process | 3 | 0 |  |  |  |  |  |  |  |  |
| GO:0045047\_protein\_targeting\_to\_ER | 3 | 0 |  |  |  |  |  |  |  |  |
| GO:0045085\_negative\_regulation\_of\_interleukin-2\_biosynthetic\_process | 3 | 0 |  |  |  |  |  |  |  |  |
| GO:0045110\_intermediate\_filament\_bundle\_assembly | 3 | 0 |  |  |  |  |  |  |  |  |
| GO:0045143\_homologous\_chromosome\_segregation | 3 | 0 |  |  |  |  |  |  |  |  |
| GO:0045198\_establishment\_of\_epithelial\_cell\_apical\_basal\_polarity | 3 | 0 |  |  |  |  |  |  |  |  |
| GO:0045217\_cell-cell\_junction\_maintenance | 3 | 0 |  |  |  |  |  |  |  |  |
| GO:0045348\_positive\_regulation\_of\_MHC\_class\_II\_biosynthetic\_process | 3 | 0 |  |  |  |  |  |  |  |  |
| GO:0045402\_regulation\_of\_interleukin-4\_biosynthetic\_process | 3 | 0 |  |  |  |  |  |  |  |  |
| GO:0045404\_positive\_regulation\_of\_interleukin-4\_biosynthetic\_process | 3 | 0 |  |  |  |  |  |  |  |  |
| GO:0045542\_positive\_regulation\_of\_cholesterol\_biosynthetic\_process | 3 | 0 |  |  |  |  |  |  |  |  |
| GO:0045607\_regulation\_of\_auditory\_receptor\_cell\_differentiation | 3 | 0 |  |  |  |  |  |  |  |  |
| GO:0045623\_negative\_regulation\_of\_T-helper\_cell\_differentiation | 3 | 0 |  |  |  |  |  |  |  |  |
| GO:0045625\_regulation\_of\_T-helper\_1\_cell\_differentiation | 3 | 0 |  |  |  |  |  |  |  |  |
| GO:0045631\_regulation\_of\_mechanoreceptor\_differentiation | 3 | 0 |  |  |  |  |  |  |  |  |
| GO:0045717\_negative\_regulation\_of\_fatty\_acid\_biosynthetic\_process | 3 | 0 |  |  |  |  |  |  |  |  |
| GO:0045723\_positive\_regulation\_of\_fatty\_acid\_biosynthetic\_process | 3 | 0 |  |  |  |  |  |  |  |  |
| GO:0045746\_negative\_regulation\_of\_Notch\_signaling\_pathway | 3 | 0 |  |  |  |  |  |  |  |  |
| GO:0045806\_negative\_regulation\_of\_endocytosis | 3 | 0 |  |  |  |  |  |  |  |  |
| GO:0045829\_negative\_regulation\_of\_isotype\_switching | 3 | 0 |  |  |  |  |  |  |  |  |
| GO:0045844\_positive\_regulation\_of\_striated\_muscle\_development | 3 | 0 |  |  |  |  |  |  |  |  |
| GO:0045907\_positive\_regulation\_of\_vasoconstriction | 3 | 0 |  |  |  |  |  |  |  |  |
| GO:0045922\_negative\_regulation\_of\_fatty\_acid\_metabolic\_process | 3 | 0 |  |  |  |  |  |  |  |  |
| GO:0045939\_negative\_regulation\_of\_steroid\_metabolic\_process | 3 | 0 |  |  |  |  |  |  |  |  |
| GO:0046013\_regulation\_of\_T\_cell\_homeostatic\_proliferation | 3 | 0 |  |  |  |  |  |  |  |  |
| GO:0046034\_ATP\_metabolic\_process | 3 | 0 |  |  |  |  |  |  |  |  |
| GO:0046325\_negative\_regulation\_of\_glucose\_import | 3 | 0 |  |  |  |  |  |  |  |  |
| GO:0046426\_negative\_regulation\_of\_JAK-STAT\_cascade | 3 | 0 |  |  |  |  |  |  |  |  |
| GO:0046457\_prostanoid\_biosynthetic\_process | 3 | 0 |  |  |  |  |  |  |  |  |
| GO:0046479\_glycosphingolipid\_catabolic\_process | 3 | 0 |  |  |  |  |  |  |  |  |
| GO:0046488\_phosphatidylinositol\_metabolic\_process | 3 | 0 |  |  |  |  |  |  |  |  |
| GO:0046549\_retinal\_cone\_cell\_development | 3 | 0 |  |  |  |  |  |  |  |  |
| GO:0046605\_regulation\_of\_centrosome\_cycle | 3 | 0 |  |  |  |  |  |  |  |  |
| GO:0046688\_response\_to\_copper\_ion | 3 | 0 |  |  |  |  |  |  |  |  |
| GO:0046717\_acid\_secretion | 3 | 0 |  |  |  |  |  |  |  |  |
| GO:0046825\_regulation\_of\_protein\_export\_from\_nucleus | 3 | 0 |  |  |  |  |  |  |  |  |
| GO:0046885\_regulation\_of\_hormone\_biosynthetic\_process | 3 | 0 |  |  |  |  |  |  |  |  |
| GO:0048003\_antigen\_processing\_and\_presentation\_of\_lipid\_antigen\_via\_MHC\_class\_Ib | 3 | 0 |  |  |  |  |  |  |  |  |
| GO:0048007\_antigen\_processing\_and\_presentation\_\_exogenous\_lipid\_antigen\_via\_MHC\_class\_Ib | 3 | 0 |  |  |  |  |  |  |  |  |
| GO:0048012\_hepatocyte\_growth\_factor\_receptor\_signaling\_pathway | 3 | 0 |  |  |  |  |  |  |  |  |
| GO:0048050\_post-embryonic\_eye\_morphogenesis | 3 | 0 |  |  |  |  |  |  |  |  |
| GO:0048087\_positive\_regulation\_of\_pigmentation\_during\_development | 3 | 0 |  |  |  |  |  |  |  |  |
| GO:0048246\_macrophage\_chemotaxis | 3 | 0 |  |  |  |  |  |  |  |  |
| GO:0048251\_elastic\_fiber\_assembly | 3 | 0 |  |  |  |  |  |  |  |  |
| GO:0048278\_vesicle\_docking | 3 | 0 |  |  |  |  |  |  |  |  |
| GO:0048294\_negative\_regulation\_of\_isotype\_switching\_to\_IgE\_isotypes | 3 | 0 |  |  |  |  |  |  |  |  |
| GO:0048318\_axial\_mesoderm\_development | 3 | 0 |  |  |  |  |  |  |  |  |
| GO:0048597\_post-embryonic\_camera-type\_eye\_morphogenesis | 3 | 0 |  |  |  |  |  |  |  |  |
| GO:0048636\_positive\_regulation\_of\_muscle\_development | 3 | 0 |  |  |  |  |  |  |  |  |
| GO:0048660\_regulation\_of\_smooth\_muscle\_cell\_proliferation | 3 | 0 |  |  |  |  |  |  |  |  |
| GO:0048668\_collateral\_sprouting | 3 | 0 |  |  |  |  |  |  |  |  |
| GO:0048676\_axon\_extension\_involved\_in\_development | 3 | 0 |  |  |  |  |  |  |  |  |
| GO:0048755\_branching\_morphogenesis\_of\_a\_nerve | 3 | 0 |  |  |  |  |  |  |  |  |
| GO:0048845\_venous\_blood\_vessel\_morphogenesis | 3 | 0 |  |  |  |  |  |  |  |  |
| GO:0048852\_diencephalon\_morphogenesis | 3 | 0 |  |  |  |  |  |  |  |  |
| GO:0048859\_formation\_of\_anatomical\_boundary | 3 | 0 |  |  |  |  |  |  |  |  |
| GO:0048865\_stem\_cell\_fate\_commitment | 3 | 0 |  |  |  |  |  |  |  |  |
| GO:0050435\_beta-amyloid\_metabolic\_process | 3 | 0 |  |  |  |  |  |  |  |  |
| GO:0050650\_chondroitin\_sulfate\_proteoglycan\_biosynthetic\_process | 3 | 0 |  |  |  |  |  |  |  |  |
| GO:0050703\_interleukin-1\_alpha\_secretion | 3 | 0 |  |  |  |  |  |  |  |  |
| GO:0050705\_regulation\_of\_interleukin-1\_alpha\_secretion | 3 | 0 |  |  |  |  |  |  |  |  |
| GO:0050709\_negative\_regulation\_of\_protein\_secretion | 3 | 0 |  |  |  |  |  |  |  |  |
| GO:0050710\_negative\_regulation\_of\_cytokine\_secretion | 3 | 0 |  |  |  |  |  |  |  |  |
| GO:0050717\_positive\_regulation\_of\_interleukin-1\_alpha\_secretion | 3 | 0 |  |  |  |  |  |  |  |  |
| GO:0050774\_negative\_regulation\_of\_dendrite\_morphogenesis | 3 | 0 |  |  |  |  |  |  |  |  |
| GO:0050857\_positive\_regulation\_of\_antigen\_receptor-mediated\_signaling\_pathway | 3 | 0 |  |  |  |  |  |  |  |  |
| GO:0050882\_voluntary\_musculoskeletal\_movement | 3 | 0 |  |  |  |  |  |  |  |  |
| GO:0050913\_sensory\_perception\_of\_bitter\_taste | 3 | 0 |  |  |  |  |  |  |  |  |
| GO:0050957\_equilibrioception | 3 | 0 |  |  |  |  |  |  |  |  |
| GO:0050996\_positive\_regulation\_of\_lipid\_catabolic\_process | 3 | 0 |  |  |  |  |  |  |  |  |
| GO:0051149\_positive\_regulation\_of\_muscle\_cell\_differentiation | 3 | 0 |  |  |  |  |  |  |  |  |
| GO:0051153\_regulation\_of\_striated\_muscle\_cell\_differentiation | 3 | 0 |  |  |  |  |  |  |  |  |
| GO:0051204\_protein\_insertion\_into\_mitochondrial\_membrane | 3 | 0 |  |  |  |  |  |  |  |  |
| GO:0051291\_protein\_heterooligomerization | 3 | 0 |  |  |  |  |  |  |  |  |
| GO:0051302\_regulation\_of\_cell\_division | 3 | 0 |  |  |  |  |  |  |  |  |
| GO:0051320\_S\_phase | 3 | 0 |  |  |  |  |  |  |  |  |
| GO:0051450\_myoblast\_proliferation | 3 | 0 |  |  |  |  |  |  |  |  |
| GO:0051583\_dopamine\_uptake | 3 | 0 |  |  |  |  |  |  |  |  |
| GO:0051798\_positive\_regulation\_of\_hair\_follicle\_development | 3 | 0 |  |  |  |  |  |  |  |  |
| GO:0051882\_mitochondrial\_depolarization | 3 | 0 |  |  |  |  |  |  |  |  |
| GO:0051900\_regulation\_of\_mitochondrial\_depolarization | 3 | 0 |  |  |  |  |  |  |  |  |
| GO:0051925\_regulation\_of\_calcium\_ion\_transport\_via\_voltage-gated\_calcium\_channel\_activity | 3 | 0 |  |  |  |  |  |  |  |  |
| GO:0051926\_negative\_regulation\_of\_calcium\_ion\_transport | 3 | 0 |  |  |  |  |  |  |  |  |
| GO:0051930\_regulation\_of\_sensory\_perception\_of\_pain | 3 | 0 |  |  |  |  |  |  |  |  |
| GO:0051931\_regulation\_of\_sensory\_perception | 3 | 0 |  |  |  |  |  |  |  |  |
| GO:0051934\_catecholamine\_uptake\_during\_transmission\_of\_nerve\_impulse | 3 | 0 |  |  |  |  |  |  |  |  |
| GO:0051955\_regulation\_of\_amino\_acid\_transport | 3 | 0 |  |  |  |  |  |  |  |  |
| GO:0051962\_positive\_regulation\_of\_nervous\_system\_development | 3 | 0 |  |  |  |  |  |  |  |  |
| GO:0051965\_positive\_regulation\_of\_synaptogenesis | 3 | 0 |  |  |  |  |  |  |  |  |
| GO:0051967\_negative\_regulation\_of\_synaptic\_transmission\_\_glutamatergic | 3 | 0 |  |  |  |  |  |  |  |  |
| GO:0051983\_regulation\_of\_chromosome\_segregation | 3 | 0 |  |  |  |  |  |  |  |  |
| GO:0055061\_di-\_\_tri-valent\_inorganic\_anion\_homeostasis | 3 | 0 |  |  |  |  |  |  |  |  |
| GO:0055062\_phosphate\_ion\_homeostasis | 3 | 0 |  |  |  |  |  |  |  |  |
| GO:0055083\_monovalent\_inorganic\_anion\_homeostasis | 3 | 0 |  |  |  |  |  |  |  |  |
| GO:0055117\_regulation\_of\_cardiac\_muscle\_contraction | 3 | 0 |  |  |  |  |  |  |  |  |
| GO:0060009\_Sertoli\_cell\_development | 3 | 0 |  |  |  |  |  |  |  |  |
| GO:0060024\_rhythmic\_synaptic\_transmission | 3 | 0 |  |  |  |  |  |  |  |  |
| GO:0060033\_anatomical\_structure\_regression | 3 | 0 |  |  |  |  |  |  |  |  |
| GO:0060040\_retinal\_bipolar\_neuron\_differentiation | 3 | 0 |  |  |  |  |  |  |  |  |
| GO:0060055\_angiogenesis\_involved\_in\_wound\_healing | 3 | 0 |  |  |  |  |  |  |  |  |
| GO:0060084\_synaptic\_transmission\_involved\_in\_micturition | 3 | 0 |  |  |  |  |  |  |  |  |
| GO:0060123\_regulation\_of\_growth\_hormone\_secretion | 3 | 0 |  |  |  |  |  |  |  |  |
| GO:0060126\_somatotropin\_secreting\_cell\_differentiation | 3 | 0 |  |  |  |  |  |  |  |  |
| GO:0060192\_negative\_regulation\_of\_lipase\_activity | 3 | 0 |  |  |  |  |  |  |  |  |
| GO:0060219\_camera-type\_eye\_photoreceptor\_cell\_differentiation | 3 | 0 |  |  |  |  |  |  |  |  |
| GO:0060285\_ciliary\_cell\_motility | 3 | 0 |  |  |  |  |  |  |  |  |
| GO:0060294\_cilium\_movement\_involved\_in\_ciliary\_motility | 3 | 0 |  |  |  |  |  |  |  |  |
| GO:0060295\_regulation\_of\_cilium\_movement\_involved\_in\_ciliary\_motility | 3 | 0 |  |  |  |  |  |  |  |  |
| GO:0060296\_regulation\_of\_cilium\_beat\_frequency\_involved\_in\_ciliary\_motility | 3 | 0 |  |  |  |  |  |  |  |  |
| GO:0060314\_regulation\_of\_ryanodine-sensitive\_calcium-release\_channel\_activity | 3 | 0 |  |  |  |  |  |  |  |  |
| GO:0060396\_growth\_hormone\_receptor\_signaling\_pathway | 3 | 0 |  |  |  |  |  |  |  |  |
| GO:0060416\_response\_to\_growth\_hormone\_stimulus | 3 | 0 |  |  |  |  |  |  |  |  |
| GO:0060428\_lung\_epithelium\_development | 3 | 0 |  |  |  |  |  |  |  |  |
| GO:0060433\_bronchus\_development | 3 | 0 |  |  |  |  |  |  |  |  |
| GO:0060435\_bronchiole\_development | 3 | 0 |  |  |  |  |  |  |  |  |
| GO:0060460\_left\_lung\_morphogenesis | 3 | 0 |  |  |  |  |  |  |  |  |
| GO:0060491\_regulation\_of\_cell\_projection\_assembly | 3 | 0 |  |  |  |  |  |  |  |  |
| GO:0060523\_prostate\_epithelial\_cord\_elongation | 3 | 0 |  |  |  |  |  |  |  |  |
| GO:0060586\_multicellular\_organismal\_iron\_ion\_homeostasis | 3 | 0 |  |  |  |  |  |  |  |  |
| GO:0060596\_mammary\_placode\_formation | 3 | 0 |  |  |  |  |  |  |  |  |
| GO:0060632\_regulation\_of\_microtubule-based\_movement | 3 | 0 |  |  |  |  |  |  |  |  |
| GO:0060648\_mammary\_gland\_bud\_morphogenesis | 3 | 0 |  |  |  |  |  |  |  |  |
| GO:0060684\_epithelial-mesenchymal\_cell\_signaling | 3 | 0 |  |  |  |  |  |  |  |  |
| GO:0060686\_negative\_regulation\_of\_prostatic\_bud\_formation | 3 | 0 |  |  |  |  |  |  |  |  |
| GO:0060689\_cell\_differentiation\_involved\_in\_salivary\_gland\_development | 3 | 0 |  |  |  |  |  |  |  |  |
| GO:0060708\_spongiotrophoblast\_differentiation | 3 | 0 |  |  |  |  |  |  |  |  |
| GO:0060746\_parental\_behavior | 3 | 0 |  |  |  |  |  |  |  |  |
| GO:0060748\_tertiary\_branching\_involved\_in\_mammary\_gland\_duct\_morphogenesis | 3 | 0 |  |  |  |  |  |  |  |  |
| GO:0060750\_epithelial\_cell\_proliferation\_involved\_in\_mammary\_gland\_duct\_elongation | 3 | 0 |  |  |  |  |  |  |  |  |
| GO:0060841\_venous\_blood\_vessel\_development | 3 | 0 |  |  |  |  |  |  |  |  |
| GO:0070102\_interleukin-6-mediated\_signaling\_pathway | 3 | 0 |  |  |  |  |  |  |  |  |
| GO:0070169\_positive\_regulation\_of\_biomineral\_formation | 3 | 0 |  |  |  |  |  |  |  |  |
| GO:0070206\_protein\_trimerization | 3 | 0 |  |  |  |  |  |  |  |  |
| GO:0070207\_protein\_homotrimerization | 3 | 0 |  |  |  |  |  |  |  |  |
| GO:0070229\_negative\_regulation\_of\_lymphocyte\_apoptosis | 3 | 0 |  |  |  |  |  |  |  |  |
| GO:0070230\_positive\_regulation\_of\_lymphocyte\_apoptosis | 3 | 0 |  |  |  |  |  |  |  |  |
| GO:0070232\_regulation\_of\_T\_cell\_apoptosis | 3 | 0 |  |  |  |  |  |  |  |  |
| GO:0070233\_negative\_regulation\_of\_T\_cell\_apoptosis | 3 | 0 |  |  |  |  |  |  |  |  |
| GO:0070242\_thymocyte\_apoptosis | 3 | 0 |  |  |  |  |  |  |  |  |
| GO:0070243\_regulation\_of\_thymocyte\_apoptosis | 3 | 0 |  |  |  |  |  |  |  |  |
| GO:0070244\_negative\_regulation\_of\_thymocyte\_apoptosis | 3 | 0 |  |  |  |  |  |  |  |  |
| GO:0070307\_lens\_fiber\_cell\_development | 3 | 0 |  |  |  |  |  |  |  |  |
| GO:0070309\_lens\_fiber\_cell\_morphogenesis | 3 | 0 |  |  |  |  |  |  |  |  |
| GO:0070423\_nucleotide-binding\_oligomerization\_domain\_containing\_signaling\_pathway | 3 | 0 |  |  |  |  |  |  |  |  |
| GO:0070427\_nucleotide-binding\_oligomerization\_domain\_containing\_1\_signaling\_pathway | 3 | 0 |  |  |  |  |  |  |  |  |
| GO:0070431\_nucleotide-binding\_oligomerization\_domain\_containing\_2\_signaling\_pathway | 3 | 0 |  |  |  |  |  |  |  |  |
| GO:0070633\_transepithelial\_transport | 3 | 0 |  |  |  |  |  |  |  |  |
| GO:0070846\_Hsp90\_deacetylation | 3 | 0 |  |  |  |  |  |  |  |  |
| GO:0070873\_regulation\_of\_glycogen\_metabolic\_process | 3 | 0 |  |  |  |  |  |  |  |  |
| GO:0070875\_positive\_regulation\_of\_glycogen\_metabolic\_process | 3 | 0 |  |  |  |  |  |  |  |  |
| GO:0001890\_placenta\_development | 77 | 0 | 0.000000 | -0.000000 | 1030 | 1033.266154 | 1110.37 | 1187.473846 | 1.078029 |
| GO:0051241\_negative\_regulation\_of\_multicellular\_organismal\_process | 77 | 0 | 0.000000 | -0.000000 | 1030 | 1033.266154 | 1110.37 | 1187.473846 | 1.078029 |
| GO:0006576\_biogenic\_amine\_metabolic\_process | 53 | 0 | 0.000000 | -0.000000 | 1037 | 1040.677204 | 1117.22 | 1193.762796 | 1.077358 |
| GO:0006935\_chemotaxis | 53 | 0 | 0.000000 | -0.000000 | 1037 | 1040.677204 | 1117.22 | 1193.762796 | 1.077358 |
| GO:0030031\_cell\_projection\_assembly | 53 | 0 | 0.000000 | -0.000000 | 1037 | 1040.677204 | 1117.22 | 1193.762796 | 1.077358 |
| GO:0042330\_taxis | 53 | 0 | 0.000000 | -0.000000 | 1037 | 1040.677204 | 1117.22 | 1193.762796 | 1.077358 |
| GO:0046942\_carboxylic\_acid\_transport | 53 | 0 | 0.000000 | -0.000000 | 1037 | 1040.677204 | 1117.22 | 1193.762796 | 1.077358 |
| GO:0050905\_neuromuscular\_process | 53 | 0 | 0.000000 | -0.000000 | 1037 | 1040.677204 | 1117.22 | 1193.762796 | 1.077358 |
| GO:0055085\_transmembrane\_transport | 53 | 0 | 0.000000 | -0.000000 | 1037 | 1040.677204 | 1117.22 | 1193.762796 | 1.077358 |
| GO:0014706\_striated\_muscle\_tissue\_development | 120 | 0 | 0.000000 | -0.000000 | 1038 | 1041.409971 | 1117.82 | 1194.230029 | 1.076898 |
| GO:0000086\_G2\_M\_transition\_of\_mitotic\_cell\_cycle | 4 | 0 |  |  |  |  |  |  |  |  |
| GO:0000305\_response\_to\_oxygen\_radical | 4 | 0 |  |  |  |  |  |  |  |  |
| GO:0001661\_conditioned\_taste\_aversion | 4 | 0 |  |  |  |  |  |  |  |  |
| GO:0001678\_cellular\_glucose\_homeostasis | 4 | 0 |  |  |  |  |  |  |  |  |
| GO:0001777\_T\_cell\_homeostatic\_proliferation | 4 | 0 |  |  |  |  |  |  |  |  |
| GO:0001794\_type\_IIa\_hypersensitivity | 4 | 0 |  |  |  |  |  |  |  |  |
| GO:0001796\_regulation\_of\_type\_IIa\_hypersensitivity | 4 | 0 |  |  |  |  |  |  |  |  |
| GO:0001798\_positive\_regulation\_of\_type\_IIa\_hypersensitivity | 4 | 0 |  |  |  |  |  |  |  |  |
| GO:0001810\_regulation\_of\_type\_I\_hypersensitivity | 4 | 0 |  |  |  |  |  |  |  |  |
| GO:0001820\_serotonin\_secretion | 4 | 0 |  |  |  |  |  |  |  |  |
| GO:0001835\_blastocyst\_hatching | 4 | 0 |  |  |  |  |  |  |  |  |
| GO:0001842\_neural\_fold\_formation | 4 | 0 |  |  |  |  |  |  |  |  |
| GO:0001881\_receptor\_recycling | 4 | 0 |  |  |  |  |  |  |  |  |
| GO:0001938\_positive\_regulation\_of\_endothelial\_cell\_proliferation | 4 | 0 |  |  |  |  |  |  |  |  |
| GO:0001978\_regulation\_of\_systemic\_arterial\_blood\_pressure\_by\_carotid\_sinus\_baroreceptor\_feedback | 4 | 0 |  |  |  |  |  |  |  |  |
| GO:0002035\_brain\_renin-angiotensin\_system | 4 | 0 |  |  |  |  |  |  |  |  |
| GO:0002051\_osteoblast\_fate\_commitment | 4 | 0 |  |  |  |  |  |  |  |  |
| GO:0002220\_innate\_immune\_response\_activating\_cell\_surface\_receptor\_signaling\_pathway | 4 | 0 |  |  |  |  |  |  |  |  |
| GO:0002249\_lymphocyte\_anergy | 4 | 0 |  |  |  |  |  |  |  |  |
| GO:0002312\_B\_cell\_activation\_during\_immune\_response | 4 | 0 |  |  |  |  |  |  |  |  |
| GO:0002313\_mature\_B\_cell\_differentiation\_during\_immune\_response | 4 | 0 |  |  |  |  |  |  |  |  |
| GO:0002318\_myeloid\_progenitor\_cell\_differentiation | 4 | 0 |  |  |  |  |  |  |  |  |
| GO:0002326\_B\_cell\_lineage\_commitment | 4 | 0 |  |  |  |  |  |  |  |  |
| GO:0002347\_response\_to\_tumor\_cell | 4 | 0 |  |  |  |  |  |  |  |  |
| GO:0002418\_immune\_response\_to\_tumor\_cell | 4 | 0 |  |  |  |  |  |  |  |  |
| GO:0002445\_type\_II\_hypersensitivity | 4 | 0 |  |  |  |  |  |  |  |  |
| GO:0002544\_chronic\_inflammatory\_response | 4 | 0 |  |  |  |  |  |  |  |  |
| GO:0002636\_positive\_regulation\_of\_germinal\_center\_formation | 4 | 0 |  |  |  |  |  |  |  |  |
| GO:0002667\_regulation\_of\_T\_cell\_anergy | 4 | 0 |  |  |  |  |  |  |  |  |
| GO:0002669\_positive\_regulation\_of\_T\_cell\_anergy | 4 | 0 |  |  |  |  |  |  |  |  |
| GO:0002687\_positive\_regulation\_of\_leukocyte\_migration | 4 | 0 |  |  |  |  |  |  |  |  |
| GO:0002702\_positive\_regulation\_of\_production\_of\_molecular\_mediator\_of\_immune\_response | 4 | 0 |  |  |  |  |  |  |  |  |
| GO:0002718\_regulation\_of\_cytokine\_production\_during\_immune\_response | 4 | 0 |  |  |  |  |  |  |  |  |
| GO:0002829\_negative\_regulation\_of\_T-helper\_2\_type\_immune\_response | 4 | 0 |  |  |  |  |  |  |  |  |
| GO:0002833\_positive\_regulation\_of\_response\_to\_biotic\_stimulus | 4 | 0 |  |  |  |  |  |  |  |  |
| GO:0002834\_regulation\_of\_response\_to\_tumor\_cell | 4 | 0 |  |  |  |  |  |  |  |  |
| GO:0002836\_positive\_regulation\_of\_response\_to\_tumor\_cell | 4 | 0 |  |  |  |  |  |  |  |  |
| GO:0002837\_regulation\_of\_immune\_response\_to\_tumor\_cell | 4 | 0 |  |  |  |  |  |  |  |  |
| GO:0002839\_positive\_regulation\_of\_immune\_response\_to\_tumor\_cell | 4 | 0 |  |  |  |  |  |  |  |  |
| GO:0002870\_T\_cell\_anergy | 4 | 0 |  |  |  |  |  |  |  |  |
| GO:0002888\_positive\_regulation\_of\_myeloid\_leukocyte\_mediated\_immunity | 4 | 0 |  |  |  |  |  |  |  |  |
| GO:0002892\_regulation\_of\_type\_II\_hypersensitivity | 4 | 0 |  |  |  |  |  |  |  |  |
| GO:0002894\_positive\_regulation\_of\_type\_II\_hypersensitivity | 4 | 0 |  |  |  |  |  |  |  |  |
| GO:0002911\_regulation\_of\_lymphocyte\_anergy | 4 | 0 |  |  |  |  |  |  |  |  |
| GO:0002913\_positive\_regulation\_of\_lymphocyte\_anergy | 4 | 0 |  |  |  |  |  |  |  |  |
| GO:0002923\_regulation\_of\_humoral\_immune\_response\_mediated\_by\_circulating\_immunoglobulin | 4 | 0 |  |  |  |  |  |  |  |  |
| GO:0003025\_regulation\_of\_systemic\_arterial\_blood\_pressure\_by\_baroreceptor\_feedback | 4 | 0 |  |  |  |  |  |  |  |  |
| GO:0003091\_renal\_water\_homeostasis | 4 | 0 |  |  |  |  |  |  |  |  |
| GO:0005978\_glycogen\_biosynthetic\_process | 4 | 0 |  |  |  |  |  |  |  |  |
| GO:0006012\_galactose\_metabolic\_process | 4 | 0 |  |  |  |  |  |  |  |  |
| GO:0006085\_acetyl-CoA\_biosynthetic\_process | 4 | 0 |  |  |  |  |  |  |  |  |
| GO:0006111\_regulation\_of\_gluconeogenesis | 4 | 0 |  |  |  |  |  |  |  |  |
| GO:0006290\_pyrimidine\_dimer\_repair | 4 | 0 |  |  |  |  |  |  |  |  |
| GO:0006334\_nucleosome\_assembly | 4 | 0 |  |  |  |  |  |  |  |  |
| GO:0006534\_cysteine\_metabolic\_process | 4 | 0 |  |  |  |  |  |  |  |  |
| GO:0006547\_histidine\_metabolic\_process | 4 | 0 |  |  |  |  |  |  |  |  |
| GO:0006548\_histidine\_catabolic\_process | 4 | 0 |  |  |  |  |  |  |  |  |
| GO:0006555\_methionine\_metabolic\_process | 4 | 0 |  |  |  |  |  |  |  |  |
| GO:0006599\_phosphagen\_metabolic\_process | 4 | 0 |  |  |  |  |  |  |  |  |
| GO:0006623\_protein\_targeting\_to\_vacuole | 4 | 0 |  |  |  |  |  |  |  |  |
| GO:0006626\_protein\_targeting\_to\_mitochondrion | 4 | 0 |  |  |  |  |  |  |  |  |
| GO:0006684\_sphingomyelin\_metabolic\_process | 4 | 0 |  |  |  |  |  |  |  |  |
| GO:0006688\_glycosphingolipid\_biosynthetic\_process | 4 | 0 |  |  |  |  |  |  |  |  |
| GO:0006707\_cholesterol\_catabolic\_process | 4 | 0 |  |  |  |  |  |  |  |  |
| GO:0006739\_NADP\_metabolic\_process | 4 | 0 |  |  |  |  |  |  |  |  |
| GO:0006835\_dicarboxylic\_acid\_transport | 4 | 0 |  |  |  |  |  |  |  |  |
| GO:0006837\_serotonin\_transport | 4 | 0 |  |  |  |  |  |  |  |  |
| GO:0006888\_ER\_to\_Golgi\_vesicle-mediated\_transport | 4 | 0 |  |  |  |  |  |  |  |  |
| GO:0006906\_vesicle\_fusion | 4 | 0 |  |  |  |  |  |  |  |  |
| GO:0006927\_transformed\_cell\_apoptosis | 4 | 0 |  |  |  |  |  |  |  |  |
| GO:0006972\_hyperosmotic\_response | 4 | 0 |  |  |  |  |  |  |  |  |
| GO:0007028\_cytoplasm\_organization | 4 | 0 |  |  |  |  |  |  |  |  |
| GO:0007031\_peroxisome\_organization | 4 | 0 |  |  |  |  |  |  |  |  |
| GO:0007066\_female\_meiosis\_sister\_chromatid\_cohesion | 4 | 0 |  |  |  |  |  |  |  |  |
| GO:0007144\_female\_meiosis\_I | 4 | 0 |  |  |  |  |  |  |  |  |
| GO:0007184\_SMAD\_protein\_nuclear\_translocation | 4 | 0 |  |  |  |  |  |  |  |  |
| GO:0007216\_metabotropic\_glutamate\_receptor\_signaling\_pathway | 4 | 0 |  |  |  |  |  |  |  |  |
| GO:0007342\_fusion\_of\_sperm\_to\_egg\_plasma\_membrane | 4 | 0 |  |  |  |  |  |  |  |  |
| GO:0007386\_compartment\_specification | 4 | 0 |  |  |  |  |  |  |  |  |
| GO:0008053\_mitochondrial\_fusion | 4 | 0 |  |  |  |  |  |  |  |  |
| GO:0008207\_C21-steroid\_hormone\_metabolic\_process | 4 | 0 |  |  |  |  |  |  |  |  |
| GO:0008215\_spermine\_metabolic\_process | 4 | 0 |  |  |  |  |  |  |  |  |
| GO:0009065\_glutamine\_family\_amino\_acid\_catabolic\_process | 4 | 0 |  |  |  |  |  |  |  |  |
| GO:0009075\_histidine\_family\_amino\_acid\_metabolic\_process | 4 | 0 |  |  |  |  |  |  |  |  |
| GO:0009077\_histidine\_family\_amino\_acid\_catabolic\_process | 4 | 0 |  |  |  |  |  |  |  |  |
| GO:0009134\_nucleoside\_diphosphate\_catabolic\_process | 4 | 0 |  |  |  |  |  |  |  |  |
| GO:0009163\_nucleoside\_biosynthetic\_process | 4 | 0 |  |  |  |  |  |  |  |  |
| GO:0009225\_nucleotide-sugar\_metabolic\_process | 4 | 0 |  |  |  |  |  |  |  |  |
| GO:0009250\_glucan\_biosynthetic\_process | 4 | 0 |  |  |  |  |  |  |  |  |
| GO:0009404\_toxin\_metabolic\_process | 4 | 0 |  |  |  |  |  |  |  |  |
| GO:0009593\_detection\_of\_chemical\_stimulus | 4 | 0 |  |  |  |  |  |  |  |  |
| GO:0009595\_detection\_of\_biotic\_stimulus | 4 | 0 |  |  |  |  |  |  |  |  |
| GO:0009755\_hormone-mediated\_signaling | 4 | 0 |  |  |  |  |  |  |  |  |
| GO:0009912\_auditory\_receptor\_cell\_fate\_commitment | 4 | 0 |  |  |  |  |  |  |  |  |
| GO:0010224\_response\_to\_UV-B | 4 | 0 |  |  |  |  |  |  |  |  |
| GO:0010453\_regulation\_of\_cell\_fate\_commitment | 4 | 0 |  |  |  |  |  |  |  |  |
| GO:0010506\_regulation\_of\_autophagy | 4 | 0 |  |  |  |  |  |  |  |  |
| GO:0010631\_epithelial\_cell\_migration | 4 | 0 |  |  |  |  |  |  |  |  |
| GO:0010812\_negative\_regulation\_of\_cell-substrate\_adhesion | 4 | 0 |  |  |  |  |  |  |  |  |
| GO:0010829\_negative\_regulation\_of\_glucose\_transport | 4 | 0 |  |  |  |  |  |  |  |  |
| GO:0014002\_astrocyte\_development | 4 | 0 |  |  |  |  |  |  |  |  |
| GO:0014832\_urinary\_bladder\_smooth\_muscle\_contraction | 4 | 0 |  |  |  |  |  |  |  |  |
| GO:0014848\_urinary\_tract\_smooth\_muscle\_contraction | 4 | 0 |  |  |  |  |  |  |  |  |
| GO:0015701\_bicarbonate\_transport | 4 | 0 |  |  |  |  |  |  |  |  |
| GO:0015809\_arginine\_transport | 4 | 0 |  |  |  |  |  |  |  |  |
| GO:0015850\_organic\_alcohol\_transport | 4 | 0 |  |  |  |  |  |  |  |  |
| GO:0015858\_nucleoside\_transport | 4 | 0 |  |  |  |  |  |  |  |  |
| GO:0016068\_type\_I\_hypersensitivity | 4 | 0 |  |  |  |  |  |  |  |  |
| GO:0016127\_sterol\_catabolic\_process | 4 | 0 |  |  |  |  |  |  |  |  |
| GO:0016198\_axon\_choice\_point\_recognition | 4 | 0 |  |  |  |  |  |  |  |  |
| GO:0016338\_calcium-independent\_cell-cell\_adhesion | 4 | 0 |  |  |  |  |  |  |  |  |
| GO:0018198\_peptidyl-cysteine\_modification | 4 | 0 |  |  |  |  |  |  |  |  |
| GO:0018409\_peptide\_or\_protein\_amino-terminal\_blocking | 4 | 0 |  |  |  |  |  |  |  |  |
| GO:0019377\_glycolipid\_catabolic\_process | 4 | 0 |  |  |  |  |  |  |  |  |
| GO:0019432\_triglyceride\_biosynthetic\_process | 4 | 0 |  |  |  |  |  |  |  |  |
| GO:0019530\_taurine\_metabolic\_process | 4 | 0 |  |  |  |  |  |  |  |  |
| GO:0021523\_somatic\_motor\_neuron\_differentiation | 4 | 0 |  |  |  |  |  |  |  |  |
| GO:0021535\_cell\_migration\_in\_hindbrain | 4 | 0 |  |  |  |  |  |  |  |  |
| GO:0021542\_dentate\_gyrus\_development | 4 | 0 |  |  |  |  |  |  |  |  |
| GO:0021561\_facial\_nerve\_development | 4 | 0 |  |  |  |  |  |  |  |  |
| GO:0021569\_rhombomere\_3\_development | 4 | 0 |  |  |  |  |  |  |  |  |
| GO:0021571\_rhombomere\_5\_development | 4 | 0 |  |  |  |  |  |  |  |  |
| GO:0021604\_cranial\_nerve\_structural\_organization | 4 | 0 |  |  |  |  |  |  |  |  |
| GO:0021610\_facial\_nerve\_morphogenesis | 4 | 0 |  |  |  |  |  |  |  |  |
| GO:0021612\_facial\_nerve\_structural\_organization | 4 | 0 |  |  |  |  |  |  |  |  |
| GO:0021631\_optic\_nerve\_morphogenesis | 4 | 0 |  |  |  |  |  |  |  |  |
| GO:0021681\_cerebellar\_granular\_layer\_development | 4 | 0 |  |  |  |  |  |  |  |  |
| GO:0021683\_cerebellar\_granular\_layer\_morphogenesis | 4 | 0 |  |  |  |  |  |  |  |  |
| GO:0021684\_cerebellar\_granular\_layer\_formation | 4 | 0 |  |  |  |  |  |  |  |  |
| GO:0021707\_cerebellar\_granule\_cell\_differentiation | 4 | 0 |  |  |  |  |  |  |  |  |
| GO:0021778\_oligodendrocyte\_cell\_fate\_specification | 4 | 0 |  |  |  |  |  |  |  |  |
| GO:0021779\_oligodendrocyte\_cell\_fate\_commitment | 4 | 0 |  |  |  |  |  |  |  |  |
| GO:0021780\_glial\_cell\_fate\_specification | 4 | 0 |  |  |  |  |  |  |  |  |
| GO:0021801\_cerebral\_cortex\_radial\_glia\_guided\_migration | 4 | 0 |  |  |  |  |  |  |  |  |
| GO:0021830\_interneuron\_migration\_from\_the\_subpallium\_to\_the\_cortex | 4 | 0 |  |  |  |  |  |  |  |  |
| GO:0021853\_cerebral\_cortex\_GABAergic\_interneuron\_migration | 4 | 0 |  |  |  |  |  |  |  |  |
| GO:0021877\_forebrain\_neuron\_fate\_commitment | 4 | 0 |  |  |  |  |  |  |  |  |
| GO:0021894\_cerebral\_cortex\_GABAergic\_interneuron\_development | 4 | 0 |  |  |  |  |  |  |  |  |
| GO:0021910\_smoothened\_signaling\_pathway\_involved\_in\_ventral\_spinal\_cord\_patterning | 4 | 0 |  |  |  |  |  |  |  |  |
| GO:0021913\_regulation\_of\_transcription\_from\_RNA\_polymerase\_II\_promoter\_involved\_in\_ventral\_spinal\_cord\_interneuron\_specification | 4 | 0 |  |  |  |  |  |  |  |  |
| GO:0021938\_smoothened\_signaling\_pathway\_involved\_in\_regulation\_of\_granule\_cell\_precursor\_cell\_proliferation | 4 | 0 |  |  |  |  |  |  |  |  |
| GO:0021978\_telencephalon\_regionalization | 4 | 0 |  |  |  |  |  |  |  |  |
| GO:0022011\_myelination\_in\_the\_peripheral\_nervous\_system | 4 | 0 |  |  |  |  |  |  |  |  |
| GO:0030146\_diuresis | 4 | 0 |  |  |  |  |  |  |  |  |
| GO:0030300\_regulation\_of\_intestinal\_cholesterol\_absorption | 4 | 0 |  |  |  |  |  |  |  |  |
| GO:0030800\_negative\_regulation\_of\_cyclic\_nucleotide\_metabolic\_process | 4 | 0 |  |  |  |  |  |  |  |  |
| GO:0030803\_negative\_regulation\_of\_cyclic\_nucleotide\_biosynthetic\_process | 4 | 0 |  |  |  |  |  |  |  |  |
| GO:0030809\_negative\_regulation\_of\_nucleotide\_biosynthetic\_process | 4 | 0 |  |  |  |  |  |  |  |  |
| GO:0030815\_negative\_regulation\_of\_cAMP\_metabolic\_process | 4 | 0 |  |  |  |  |  |  |  |  |
| GO:0030816\_positive\_regulation\_of\_cAMP\_metabolic\_process | 4 | 0 |  |  |  |  |  |  |  |  |
| GO:0030818\_negative\_regulation\_of\_cAMP\_biosynthetic\_process | 4 | 0 |  |  |  |  |  |  |  |  |
| GO:0030819\_positive\_regulation\_of\_cAMP\_biosynthetic\_process | 4 | 0 |  |  |  |  |  |  |  |  |
| GO:0030826\_regulation\_of\_cGMP\_biosynthetic\_process | 4 | 0 |  |  |  |  |  |  |  |  |
| GO:0030858\_positive\_regulation\_of\_epithelial\_cell\_differentiation | 4 | 0 |  |  |  |  |  |  |  |  |
| GO:0030859\_polarized\_epithelial\_cell\_differentiation | 4 | 0 |  |  |  |  |  |  |  |  |
| GO:0030949\_positive\_regulation\_of\_vascular\_endothelial\_growth\_factor\_receptor\_signaling\_pathway | 4 | 0 |  |  |  |  |  |  |  |  |
| GO:0031113\_regulation\_of\_microtubule\_polymerization | 4 | 0 |  |  |  |  |  |  |  |  |
| GO:0031365\_N-terminal\_protein\_amino\_acid\_modification | 4 | 0 |  |  |  |  |  |  |  |  |
| GO:0031424\_keratinization | 4 | 0 |  |  |  |  |  |  |  |  |
| GO:0031557\_induction\_of\_programmed\_cell\_death\_in\_response\_to\_chemical\_stimulus | 4 | 0 |  |  |  |  |  |  |  |  |
| GO:0031558\_induction\_of\_apoptosis\_in\_response\_to\_chemical\_stimulus | 4 | 0 |  |  |  |  |  |  |  |  |
| GO:0031623\_receptor\_internalization | 4 | 0 |  |  |  |  |  |  |  |  |
| GO:0032088\_negative\_regulation\_of\_NF-kappaB\_transcription\_factor\_activity | 4 | 0 |  |  |  |  |  |  |  |  |
| GO:0032098\_regulation\_of\_appetite | 4 | 0 |  |  |  |  |  |  |  |  |
| GO:0032105\_negative\_regulation\_of\_response\_to\_extracellular\_stimulus | 4 | 0 |  |  |  |  |  |  |  |  |
| GO:0032108\_negative\_regulation\_of\_response\_to\_nutrient\_levels | 4 | 0 |  |  |  |  |  |  |  |  |
| GO:0032225\_regulation\_of\_synaptic\_transmission\_\_dopaminergic | 4 | 0 |  |  |  |  |  |  |  |  |
| GO:0032292\_ensheathment\_of\_axons\_in\_the\_peripheral\_nervous\_system | 4 | 0 |  |  |  |  |  |  |  |  |
| GO:0032321\_positive\_regulation\_of\_Rho\_GTPase\_activity | 4 | 0 |  |  |  |  |  |  |  |  |
| GO:0032371\_regulation\_of\_sterol\_transport | 4 | 0 |  |  |  |  |  |  |  |  |
| GO:0032374\_regulation\_of\_cholesterol\_transport | 4 | 0 |  |  |  |  |  |  |  |  |
| GO:0032401\_establishment\_of\_melanosome\_localization | 4 | 0 |  |  |  |  |  |  |  |  |
| GO:0032608\_interferon-beta\_production | 4 | 0 |  |  |  |  |  |  |  |  |
| GO:0032611\_interleukin-1\_beta\_production | 4 | 0 |  |  |  |  |  |  |  |  |
| GO:0032612\_interleukin-1\_production | 4 | 0 |  |  |  |  |  |  |  |  |
| GO:0032648\_regulation\_of\_interferon-beta\_production | 4 | 0 |  |  |  |  |  |  |  |  |
| GO:0032651\_regulation\_of\_interleukin-1\_beta\_production | 4 | 0 |  |  |  |  |  |  |  |  |
| GO:0032652\_regulation\_of\_interleukin-1\_production | 4 | 0 |  |  |  |  |  |  |  |  |
| GO:0032689\_negative\_regulation\_of\_interferon-gamma\_production | 4 | 0 |  |  |  |  |  |  |  |  |
| GO:0032713\_negative\_regulation\_of\_interleukin-4\_production | 4 | 0 |  |  |  |  |  |  |  |  |
| GO:0032715\_negative\_regulation\_of\_interleukin-6\_production | 4 | 0 |  |  |  |  |  |  |  |  |
| GO:0032733\_positive\_regulation\_of\_interleukin-10\_production | 4 | 0 |  |  |  |  |  |  |  |  |
| GO:0032808\_lacrimal\_gland\_development | 4 | 0 |  |  |  |  |  |  |  |  |
| GO:0032835\_glomerulus\_development | 4 | 0 |  |  |  |  |  |  |  |  |
| GO:0032872\_regulation\_of\_stress-activated\_MAPK\_cascade | 4 | 0 |  |  |  |  |  |  |  |  |
| GO:0032922\_circadian\_regulation\_of\_gene\_expression | 4 | 0 |  |  |  |  |  |  |  |  |
| GO:0033026\_negative\_regulation\_of\_mast\_cell\_apoptosis | 4 | 0 |  |  |  |  |  |  |  |  |
| GO:0033079\_immature\_T\_cell\_proliferation | 4 | 0 |  |  |  |  |  |  |  |  |
| GO:0033083\_regulation\_of\_immature\_T\_cell\_proliferation | 4 | 0 |  |  |  |  |  |  |  |  |
| GO:0033089\_positive\_regulation\_of\_T\_cell\_differentiation\_in\_the\_thymus | 4 | 0 |  |  |  |  |  |  |  |  |
| GO:0033135\_regulation\_of\_peptidyl-serine\_phosphorylation | 4 | 0 |  |  |  |  |  |  |  |  |
| GO:0033299\_secretion\_of\_lysosomal\_enzymes | 4 | 0 |  |  |  |  |  |  |  |  |
| GO:0033327\_Leydig\_cell\_differentiation | 4 | 0 |  |  |  |  |  |  |  |  |
| GO:0033363\_secretory\_granule\_organization | 4 | 0 |  |  |  |  |  |  |  |  |
| GO:0033599\_regulation\_of\_mammary\_gland\_epithelial\_cell\_proliferation | 4 | 0 |  |  |  |  |  |  |  |  |
| GO:0033865\_nucleoside\_bisphosphate\_metabolic\_process | 4 | 0 |  |  |  |  |  |  |  |  |
| GO:0034204\_lipid\_translocation | 4 | 0 |  |  |  |  |  |  |  |  |
| GO:0034404\_nucleobase\_\_nucleoside\_and\_nucleotide\_biosynthetic\_process | 4 | 0 |  |  |  |  |  |  |  |  |
| GO:0034587\_piRNA\_metabolic\_process | 4 | 0 |  |  |  |  |  |  |  |  |
| GO:0034614\_cellular\_response\_to\_reactive\_oxygen\_species | 4 | 0 |  |  |  |  |  |  |  |  |
| GO:0034654\_nucleobase\_\_nucleoside\_\_nucleotide\_and\_nucleic\_acid\_biosynthetic\_process | 4 | 0 |  |  |  |  |  |  |  |  |
| GO:0035020\_regulation\_of\_Rac\_protein\_signal\_transduction | 4 | 0 |  |  |  |  |  |  |  |  |
| GO:0035082\_axoneme\_assembly | 4 | 0 |  |  |  |  |  |  |  |  |
| GO:0035188\_hatching | 4 | 0 |  |  |  |  |  |  |  |  |
| GO:0035235\_ionotropic\_glutamate\_receptor\_signaling\_pathway | 4 | 0 |  |  |  |  |  |  |  |  |
| GO:0042345\_regulation\_of\_NF-kappaB\_import\_into\_nucleus | 4 | 0 |  |  |  |  |  |  |  |  |
| GO:0042348\_NF-kappaB\_import\_into\_nucleus | 4 | 0 |  |  |  |  |  |  |  |  |
| GO:0042359\_vitamin\_D\_metabolic\_process | 4 | 0 |  |  |  |  |  |  |  |  |
| GO:0042428\_serotonin\_metabolic\_process | 4 | 0 |  |  |  |  |  |  |  |  |
| GO:0042451\_purine\_nucleoside\_biosynthetic\_process | 4 | 0 |  |  |  |  |  |  |  |  |
| GO:0042455\_ribonucleoside\_biosynthetic\_process | 4 | 0 |  |  |  |  |  |  |  |  |
| GO:0042473\_outer\_ear\_morphogenesis | 4 | 0 |  |  |  |  |  |  |  |  |
| GO:0042522\_regulation\_of\_tyrosine\_phosphorylation\_of\_Stat5\_protein | 4 | 0 |  |  |  |  |  |  |  |  |
| GO:0042535\_positive\_regulation\_of\_tumor\_necrosis\_factor\_biosynthetic\_process | 4 | 0 |  |  |  |  |  |  |  |  |
| GO:0042541\_hemoglobin\_biosynthetic\_process | 4 | 0 |  |  |  |  |  |  |  |  |
| GO:0042558\_pteridine\_and\_derivative\_metabolic\_process | 4 | 0 |  |  |  |  |  |  |  |  |
| GO:0042634\_regulation\_of\_hair\_cycle | 4 | 0 |  |  |  |  |  |  |  |  |
| GO:0042744\_hydrogen\_peroxide\_catabolic\_process | 4 | 0 |  |  |  |  |  |  |  |  |
| GO:0042773\_ATP\_synthesis\_coupled\_electron\_transport | 4 | 0 |  |  |  |  |  |  |  |  |
| GO:0042775\_mitochondrial\_ATP\_synthesis\_coupled\_electron\_transport | 4 | 0 |  |  |  |  |  |  |  |  |
| GO:0042832\_defense\_response\_to\_protozoan | 4 | 0 |  |  |  |  |  |  |  |  |
| GO:0042982\_amyloid\_precursor\_protein\_metabolic\_process | 4 | 0 |  |  |  |  |  |  |  |  |
| GO:0042992\_negative\_regulation\_of\_transcription\_factor\_import\_into\_nucleus | 4 | 0 |  |  |  |  |  |  |  |  |
| GO:0043043\_peptide\_biosynthetic\_process | 4 | 0 |  |  |  |  |  |  |  |  |
| GO:0043129\_surfactant\_homeostasis | 4 | 0 |  |  |  |  |  |  |  |  |
| GO:0043374\_CD8-positive\_\_alpha-beta\_T\_cell\_differentiation | 4 | 0 |  |  |  |  |  |  |  |  |
| GO:0043470\_regulation\_of\_carbohydrate\_catabolic\_process | 4 | 0 |  |  |  |  |  |  |  |  |
| GO:0043471\_regulation\_of\_cellular\_carbohydrate\_catabolic\_process | 4 | 0 |  |  |  |  |  |  |  |  |
| GO:0043484\_regulation\_of\_RNA\_splicing | 4 | 0 |  |  |  |  |  |  |  |  |
| GO:0043500\_muscle\_adaptation | 4 | 0 |  |  |  |  |  |  |  |  |
| GO:0043534\_blood\_vessel\_endothelial\_cell\_migration | 4 | 0 |  |  |  |  |  |  |  |  |
| GO:0043691\_reverse\_cholesterol\_transport | 4 | 0 |  |  |  |  |  |  |  |  |
| GO:0044243\_multicellular\_organismal\_catabolic\_process | 4 | 0 |  |  |  |  |  |  |  |  |
| GO:0044403\_symbiosis\_\_encompassing\_mutualism\_through\_parasitism | 4 | 0 |  |  |  |  |  |  |  |  |
| GO:0044419\_interspecies\_interaction\_between\_organisms | 4 | 0 |  |  |  |  |  |  |  |  |
| GO:0045066\_regulatory\_T\_cell\_differentiation | 4 | 0 |  |  |  |  |  |  |  |  |
| GO:0045078\_positive\_regulation\_of\_interferon-gamma\_biosynthetic\_process | 4 | 0 |  |  |  |  |  |  |  |  |
| GO:0045332\_phospholipid\_translocation | 4 | 0 |  |  |  |  |  |  |  |  |
| GO:0045346\_regulation\_of\_MHC\_class\_II\_biosynthetic\_process | 4 | 0 |  |  |  |  |  |  |  |  |
| GO:0045350\_interferon-beta\_biosynthetic\_process | 4 | 0 |  |  |  |  |  |  |  |  |
| GO:0045357\_regulation\_of\_interferon-beta\_biosynthetic\_process | 4 | 0 |  |  |  |  |  |  |  |  |
| GO:0045359\_positive\_regulation\_of\_interferon-beta\_biosynthetic\_process | 4 | 0 |  |  |  |  |  |  |  |  |
| GO:0045600\_positive\_regulation\_of\_fat\_cell\_differentiation | 4 | 0 |  |  |  |  |  |  |  |  |
| GO:0045616\_regulation\_of\_keratinocyte\_differentiation | 4 | 0 |  |  |  |  |  |  |  |  |
| GO:0045624\_positive\_regulation\_of\_T-helper\_cell\_differentiation | 4 | 0 |  |  |  |  |  |  |  |  |
| GO:0045628\_regulation\_of\_T-helper\_2\_cell\_differentiation | 4 | 0 |  |  |  |  |  |  |  |  |
| GO:0045634\_regulation\_of\_melanocyte\_differentiation | 4 | 0 |  |  |  |  |  |  |  |  |
| GO:0045647\_negative\_regulation\_of\_erythrocyte\_differentiation | 4 | 0 |  |  |  |  |  |  |  |  |
| GO:0045672\_positive\_regulation\_of\_osteoclast\_differentiation | 4 | 0 |  |  |  |  |  |  |  |  |
| GO:0045684\_positive\_regulation\_of\_epidermis\_development | 4 | 0 |  |  |  |  |  |  |  |  |
| GO:0045736\_negative\_regulation\_of\_cyclin-dependent\_protein\_kinase\_activity | 4 | 0 |  |  |  |  |  |  |  |  |
| GO:0045742\_positive\_regulation\_of\_epidermal\_growth\_factor\_receptor\_signaling\_pathway | 4 | 0 |  |  |  |  |  |  |  |  |
| GO:0045747\_positive\_regulation\_of\_Notch\_signaling\_pathway | 4 | 0 |  |  |  |  |  |  |  |  |
| GO:0045767\_regulation\_of\_anti-apoptosis | 4 | 0 |  |  |  |  |  |  |  |  |
| GO:0045779\_negative\_regulation\_of\_bone\_resorption | 4 | 0 |  |  |  |  |  |  |  |  |
| GO:0045923\_positive\_regulation\_of\_fatty\_acid\_metabolic\_process | 4 | 0 |  |  |  |  |  |  |  |  |
| GO:0045930\_negative\_regulation\_of\_mitotic\_cell\_cycle | 4 | 0 |  |  |  |  |  |  |  |  |
| GO:0045940\_positive\_regulation\_of\_steroid\_metabolic\_process | 4 | 0 |  |  |  |  |  |  |  |  |
| GO:0045980\_negative\_regulation\_of\_nucleotide\_metabolic\_process | 4 | 0 |  |  |  |  |  |  |  |  |
| GO:0046129\_purine\_ribonucleoside\_biosynthetic\_process | 4 | 0 |  |  |  |  |  |  |  |  |
| GO:0046173\_polyol\_biosynthetic\_process | 4 | 0 |  |  |  |  |  |  |  |  |
| GO:0046541\_saliva\_secretion | 4 | 0 |  |  |  |  |  |  |  |  |
| GO:0046548\_retinal\_rod\_cell\_development | 4 | 0 |  |  |  |  |  |  |  |  |
| GO:0046579\_positive\_regulation\_of\_Ras\_protein\_signal\_transduction | 4 | 0 |  |  |  |  |  |  |  |  |
| GO:0046639\_negative\_regulation\_of\_alpha-beta\_T\_cell\_differentiation | 4 | 0 |  |  |  |  |  |  |  |  |
| GO:0046642\_negative\_regulation\_of\_alpha-beta\_T\_cell\_proliferation | 4 | 0 |  |  |  |  |  |  |  |  |
| GO:0046668\_regulation\_of\_retinal\_cell\_programmed\_cell\_death | 4 | 0 |  |  |  |  |  |  |  |  |
| GO:0046686\_response\_to\_cadmium\_ion | 4 | 0 |  |  |  |  |  |  |  |  |
| GO:0046835\_carbohydrate\_phosphorylation | 4 | 0 |  |  |  |  |  |  |  |  |
| GO:0046902\_regulation\_of\_mitochondrial\_membrane\_permeability | 4 | 0 |  |  |  |  |  |  |  |  |
| GO:0047496\_vesicle\_transport\_along\_microtubule | 4 | 0 |  |  |  |  |  |  |  |  |
| GO:0048011\_nerve\_growth\_factor\_receptor\_signaling\_pathway | 4 | 0 |  |  |  |  |  |  |  |  |
| GO:0048024\_regulation\_of\_nuclear\_mRNA\_splicing\_\_via\_spliceosome | 4 | 0 |  |  |  |  |  |  |  |  |
| GO:0048240\_sperm\_capacitation | 4 | 0 |  |  |  |  |  |  |  |  |
| GO:0048341\_paraxial\_mesoderm\_formation | 4 | 0 |  |  |  |  |  |  |  |  |
| GO:0048484\_enteric\_nervous\_system\_development | 4 | 0 |  |  |  |  |  |  |  |  |
| GO:0048512\_circadian\_behavior | 4 | 0 |  |  |  |  |  |  |  |  |
| GO:0048558\_embryonic\_gut\_morphogenesis | 4 | 0 |  |  |  |  |  |  |  |  |
| GO:0048639\_positive\_regulation\_of\_developmental\_growth | 4 | 0 |  |  |  |  |  |  |  |  |
| GO:0048710\_regulation\_of\_astrocyte\_differentiation | 4 | 0 |  |  |  |  |  |  |  |  |
| GO:0048841\_regulation\_of\_axon\_extension\_involved\_in\_axon\_guidance | 4 | 0 |  |  |  |  |  |  |  |  |
| GO:0048843\_negative\_regulation\_of\_axon\_extension\_involved\_in\_axon\_guidance | 4 | 0 |  |  |  |  |  |  |  |  |
| GO:0048846\_axon\_extension\_involved\_in\_axon\_guidance | 4 | 0 |  |  |  |  |  |  |  |  |
| GO:0048875\_chemical\_homeostasis\_within\_a\_tissue | 4 | 0 |  |  |  |  |  |  |  |  |
| GO:0048935\_peripheral\_nervous\_system\_neuron\_development | 4 | 0 |  |  |  |  |  |  |  |  |
| GO:0050702\_interleukin-1\_beta\_secretion | 4 | 0 |  |  |  |  |  |  |  |  |
| GO:0050704\_regulation\_of\_interleukin-1\_secretion | 4 | 0 |  |  |  |  |  |  |  |  |
| GO:0050706\_regulation\_of\_interleukin-1\_beta\_secretion | 4 | 0 |  |  |  |  |  |  |  |  |
| GO:0050716\_positive\_regulation\_of\_interleukin-1\_secretion | 4 | 0 |  |  |  |  |  |  |  |  |
| GO:0050718\_positive\_regulation\_of\_interleukin-1\_beta\_secretion | 4 | 0 |  |  |  |  |  |  |  |  |
| GO:0050820\_positive\_regulation\_of\_coagulation | 4 | 0 |  |  |  |  |  |  |  |  |
| GO:0050891\_multicellular\_organismal\_water\_homeostasis | 4 | 0 |  |  |  |  |  |  |  |  |
| GO:0050919\_negative\_chemotaxis | 4 | 0 |  |  |  |  |  |  |  |  |
| GO:0050932\_regulation\_of\_pigment\_cell\_differentiation | 4 | 0 |  |  |  |  |  |  |  |  |
| GO:0050961\_detection\_of\_temperature\_stimulus\_involved\_in\_sensory\_perception | 4 | 0 |  |  |  |  |  |  |  |  |
| GO:0050965\_detection\_of\_temperature\_stimulus\_involved\_in\_sensory\_perception\_of\_pain | 4 | 0 |  |  |  |  |  |  |  |  |
| GO:0050994\_regulation\_of\_lipid\_catabolic\_process | 4 | 0 |  |  |  |  |  |  |  |  |
| GO:0051024\_positive\_regulation\_of\_immunoglobulin\_secretion | 4 | 0 |  |  |  |  |  |  |  |  |
| GO:0051055\_negative\_regulation\_of\_lipid\_biosynthetic\_process | 4 | 0 |  |  |  |  |  |  |  |  |
| GO:0051124\_synaptic\_growth\_at\_neuromuscular\_junction | 4 | 0 |  |  |  |  |  |  |  |  |
| GO:0051148\_negative\_regulation\_of\_muscle\_cell\_differentiation | 4 | 0 |  |  |  |  |  |  |  |  |
| GO:0051205\_protein\_insertion\_into\_membrane | 4 | 0 |  |  |  |  |  |  |  |  |
| GO:0051225\_spindle\_assembly | 4 | 0 |  |  |  |  |  |  |  |  |
| GO:0051341\_regulation\_of\_oxidoreductase\_activity | 4 | 0 |  |  |  |  |  |  |  |  |
| GO:0051452\_intracellular\_pH\_reduction | 4 | 0 |  |  |  |  |  |  |  |  |
| GO:0051567\_histone\_H3-K9\_methylation | 4 | 0 |  |  |  |  |  |  |  |  |
| GO:0051642\_centrosome\_localization | 4 | 0 |  |  |  |  |  |  |  |  |
| GO:0051797\_regulation\_of\_hair\_follicle\_development | 4 | 0 |  |  |  |  |  |  |  |  |
| GO:0051897\_positive\_regulation\_of\_protein\_kinase\_B\_signaling\_cascade | 4 | 0 |  |  |  |  |  |  |  |  |
| GO:0051904\_pigment\_granule\_transport | 4 | 0 |  |  |  |  |  |  |  |  |
| GO:0055009\_atrial\_cardiac\_muscle\_morphogenesis | 4 | 0 |  |  |  |  |  |  |  |  |
| GO:0060008\_Sertoli\_cell\_differentiation | 4 | 0 |  |  |  |  |  |  |  |  |
| GO:0060011\_Sertoli\_cell\_proliferation | 4 | 0 |  |  |  |  |  |  |  |  |
| GO:0060057\_apoptosis\_involved\_in\_mammary\_gland\_involution | 4 | 0 |  |  |  |  |  |  |  |  |
| GO:0060058\_positive\_regulation\_of\_apoptosis\_involved\_in\_mammary\_gland\_involution | 4 | 0 |  |  |  |  |  |  |  |  |
| GO:0060065\_uterus\_development | 4 | 0 |  |  |  |  |  |  |  |  |
| GO:0060087\_relaxation\_of\_vascular\_smooth\_muscle | 4 | 0 |  |  |  |  |  |  |  |  |
| GO:0060120\_inner\_ear\_receptor\_cell\_fate\_commitment | 4 | 0 |  |  |  |  |  |  |  |  |
| GO:0060157\_urinary\_bladder\_development | 4 | 0 |  |  |  |  |  |  |  |  |
| GO:0060158\_activation\_of\_phospholipase\_C\_activity\_by\_dopamine\_receptor\_signaling\_pathway | 4 | 0 |  |  |  |  |  |  |  |  |
| GO:0060164\_regulation\_of\_timing\_of\_neuron\_differentiation | 4 | 0 |  |  |  |  |  |  |  |  |
| GO:0060235\_lens\_induction\_in\_camera-type\_eye | 4 | 0 |  |  |  |  |  |  |  |  |
| GO:0060291\_long-term\_synaptic\_potentiation | 4 | 0 |  |  |  |  |  |  |  |  |
| GO:0060412\_ventricular\_septum\_morphogenesis | 4 | 0 |  |  |  |  |  |  |  |  |
| GO:0060459\_left\_lung\_development | 4 | 0 |  |  |  |  |  |  |  |  |
| GO:0060528\_secretory\_columnal\_luminar\_epithelial\_cell\_differentiation\_involved\_in\_prostate\_glandular\_acinus\_development | 4 | 0 |  |  |  |  |  |  |  |  |
| GO:0060561\_apoptosis\_involved\_in\_morphogenesis | 4 | 0 |  |  |  |  |  |  |  |  |
| GO:0060592\_mammary\_gland\_formation | 4 | 0 |  |  |  |  |  |  |  |  |
| GO:0060644\_mammary\_gland\_epithelial\_cell\_differentiation | 4 | 0 |  |  |  |  |  |  |  |  |
| GO:0060666\_dichotomous\_subdivision\_of\_terminal\_units\_involved\_in\_salivary\_gland\_branching | 4 | 0 |  |  |  |  |  |  |  |  |
| GO:0060737\_prostate\_gland\_morphogenetic\_growth | 4 | 0 |  |  |  |  |  |  |  |  |
| GO:0060743\_epithelial\_cell\_maturation\_involved\_in\_prostate\_gland\_development | 4 | 0 |  |  |  |  |  |  |  |  |
| GO:0060751\_mammary\_gland\_duct\_branch\_elongation | 4 | 0 |  |  |  |  |  |  |  |  |
| GO:0060900\_embryonic\_camera-type\_eye\_formation | 4 | 0 |  |  |  |  |  |  |  |  |
| GO:0070059\_apoptosis\_in\_response\_to\_endoplasmic\_reticulum\_stress | 4 | 0 |  |  |  |  |  |  |  |  |
| GO:0070254\_mucus\_secretion | 4 | 0 |  |  |  |  |  |  |  |  |
| GO:0070255\_regulation\_of\_mucus\_secretion | 4 | 0 |  |  |  |  |  |  |  |  |
| GO:0070301\_cellular\_response\_to\_hydrogen\_peroxide | 4 | 0 |  |  |  |  |  |  |  |  |
| GO:0070585\_protein\_localization\_in\_mitochondrion | 4 | 0 |  |  |  |  |  |  |  |  |
| GO:0006519\_cellular\_amino\_acid\_and\_derivative\_metabolic\_process | 118 | 0 | 0.000000 | -0.000000 | 1040 | 1043.385675 | 1119.62 | 1195.854325 | 1.076558 |
| GO:0051960\_regulation\_of\_nervous\_system\_development | 118 | 0 | 0.000000 | -0.000000 | 1040 | 1043.385675 | 1119.62 | 1195.854325 | 1.076558 |
| GO:0007626\_locomotory\_behavior | 163 | 0 | 0.000000 | -0.000000 | 1042 | 1044.561282 | 1120.52 | 1196.478718 | 1.075355 |
| GO:0042110\_T\_cell\_activation | 163 | 0 | 0.000000 | -0.000000 | 1042 | 1044.561282 | 1120.52 | 1196.478718 | 1.075355 |
| GO:0002706\_regulation\_of\_lymphocyte\_mediated\_immunity | 52 | 0 | 0.000000 | -0.000000 | 1046 | 1048.817715 | 1124.36 | 1199.902285 | 1.074914 |
| GO:0009124\_nucleoside\_monophosphate\_biosynthetic\_process | 52 | 0 | 0.000000 | -0.000000 | 1046 | 1048.817715 | 1124.36 | 1199.902285 | 1.074914 |
| GO:0048585\_negative\_regulation\_of\_response\_to\_stimulus | 52 | 0 | 0.000000 | -0.000000 | 1046 | 1048.817715 | 1124.36 | 1199.902285 | 1.074914 |
| GO:0050953\_sensory\_perception\_of\_light\_stimulus | 52 | 0 | 0.000000 | -0.000000 | 1046 | 1048.817715 | 1124.36 | 1199.902285 | 1.074914 |
| GO:0000012\_single\_strand\_break\_repair | 2 | 0 |  |  |  |  |  |  |  |  |
| GO:0000019\_regulation\_of\_mitotic\_recombination | 2 | 0 |  |  |  |  |  |  |  |  |
| GO:0000076\_DNA\_replication\_checkpoint | 2 | 0 |  |  |  |  |  |  |  |  |
| GO:0000080\_G1\_phase\_of\_mitotic\_cell\_cycle | 2 | 0 |  |  |  |  |  |  |  |  |
| GO:0000083\_regulation\_of\_transcription\_of\_G1\_S-phase\_of\_mitotic\_cell\_cycle | 2 | 0 |  |  |  |  |  |  |  |  |
| GO:0000085\_G2\_phase\_of\_mitotic\_cell\_cycle | 2 | 0 |  |  |  |  |  |  |  |  |
| GO:0000289\_nuclear-transcribed\_mRNA\_poly(A)\_tail\_shortening | 2 | 0 |  |  |  |  |  |  |  |  |
| GO:0000381\_regulation\_of\_alternative\_nuclear\_mRNA\_splicing\_\_via\_spliceosome | 2 | 0 |  |  |  |  |  |  |  |  |
| GO:0000712\_resolution\_of\_meiotic\_joint\_molecules\_as\_recombinants | 2 | 0 |  |  |  |  |  |  |  |  |
| GO:0000720\_pyrimidine\_dimer\_repair\_by\_nucleotide-excision\_repair | 2 | 0 |  |  |  |  |  |  |  |  |
| GO:0001302\_replicative\_cell\_aging | 2 | 0 |  |  |  |  |  |  |  |  |
| GO:0001306\_age-dependent\_response\_to\_oxidative\_stress | 2 | 0 |  |  |  |  |  |  |  |  |
| GO:0001514\_selenocysteine\_incorporation | 2 | 0 |  |  |  |  |  |  |  |  |
| GO:0001522\_pseudouridine\_synthesis | 2 | 0 |  |  |  |  |  |  |  |  |
| GO:0001543\_ovarian\_follicle\_rupture | 2 | 0 |  |  |  |  |  |  |  |  |
| GO:0001561\_fatty\_acid\_alpha-oxidation | 2 | 0 |  |  |  |  |  |  |  |  |
| GO:0001675\_acrosome\_assembly | 2 | 0 |  |  |  |  |  |  |  |  |
| GO:0001743\_optic\_placode\_formation | 2 | 0 |  |  |  |  |  |  |  |  |
| GO:0001767\_establishment\_of\_lymphocyte\_polarity | 2 | 0 |  |  |  |  |  |  |  |  |
| GO:0001768\_establishment\_of\_T\_cell\_polarity | 2 | 0 |  |  |  |  |  |  |  |  |
| GO:0001771\_formation\_of\_immunological\_synapse | 2 | 0 |  |  |  |  |  |  |  |  |
| GO:0001774\_microglial\_cell\_activation | 2 | 0 |  |  |  |  |  |  |  |  |
| GO:0001781\_neutrophil\_apoptosis | 2 | 0 |  |  |  |  |  |  |  |  |
| GO:0001787\_natural\_killer\_cell\_proliferation | 2 | 0 |  |  |  |  |  |  |  |  |
| GO:0001788\_antibody-dependent\_cellular\_cytotoxicity | 2 | 0 |  |  |  |  |  |  |  |  |
| GO:0001806\_type\_IV\_hypersensitivity | 2 | 0 |  |  |  |  |  |  |  |  |
| GO:0001807\_regulation\_of\_type\_IV\_hypersensitivity | 2 | 0 |  |  |  |  |  |  |  |  |
| GO:0001808\_negative\_regulation\_of\_type\_IV\_hypersensitivity | 2 | 0 |  |  |  |  |  |  |  |  |
| GO:0001823\_mesonephros\_development | 2 | 0 |  |  |  |  |  |  |  |  |
| GO:0001845\_phagolysosome\_formation | 2 | 0 |  |  |  |  |  |  |  |  |
| GO:0001866\_NK\_T\_cell\_proliferation | 2 | 0 |  |  |  |  |  |  |  |  |
| GO:0001879\_detection\_of\_yeast | 2 | 0 |  |  |  |  |  |  |  |  |
| GO:0001886\_endothelial\_cell\_morphogenesis | 2 | 0 |  |  |  |  |  |  |  |  |
| GO:0001919\_regulation\_of\_receptor\_recycling | 2 | 0 |  |  |  |  |  |  |  |  |
| GO:0001954\_positive\_regulation\_of\_cell-matrix\_adhesion | 2 | 0 |  |  |  |  |  |  |  |  |
| GO:0001977\_renal\_system\_process\_involved\_in\_regulation\_of\_blood\_volume | 2 | 0 |  |  |  |  |  |  |  |  |
| GO:0001982\_baroreceptor\_response\_to\_decreased\_systemic\_arterial\_blood\_pressure | 2 | 0 |  |  |  |  |  |  |  |  |
| GO:0001983\_baroreceptor\_response\_to\_increased\_systemic\_arterial\_blood\_pressure | 2 | 0 |  |  |  |  |  |  |  |  |
| GO:0001992\_regulation\_of\_systemic\_arterial\_blood\_pressure\_by\_vasopressin | 2 | 0 |  |  |  |  |  |  |  |  |
| GO:0001997\_positive\_regulation\_of\_the\_force\_of\_heart\_contraction\_by\_epinephrine-norepinephrine | 2 | 0 |  |  |  |  |  |  |  |  |
| GO:0001998\_angiotensin\_mediated\_vasoconstriction\_involved\_in\_regulation\_of\_systemic\_arterial\_blood\_pressure | 2 | 0 |  |  |  |  |  |  |  |  |
| GO:0001999\_renal\_response\_to\_blood\_flow\_during\_renin-angiotensin\_regulation\_of\_systemic\_arterial\_blood\_pressure | 2 | 0 |  |  |  |  |  |  |  |  |
| GO:0002018\_renin-angiotensin\_regulation\_of\_aldosterone\_production | 2 | 0 |  |  |  |  |  |  |  |  |
| GO:0002019\_regulation\_of\_renal\_output\_by\_angiotensin | 2 | 0 |  |  |  |  |  |  |  |  |
| GO:0002024\_diet\_induced\_thermogenesis | 2 | 0 |  |  |  |  |  |  |  |  |
| GO:0002025\_vasodilation\_by\_norepinephrine-epinephrine\_involved\_in\_regulation\_of\_systemic\_arterial\_blood\_pressure | 2 | 0 |  |  |  |  |  |  |  |  |
| GO:0002029\_desensitization\_of\_G-protein\_coupled\_receptor\_protein\_signaling\_pathway | 2 | 0 |  |  |  |  |  |  |  |  |
| GO:0002033\_vasodilation\_by\_angiotensin\_involved\_in\_regulation\_of\_systemic\_arterial\_blood\_pressure | 2 | 0 |  |  |  |  |  |  |  |  |
| GO:0002066\_columnar\_cuboidal\_epithelial\_cell\_development | 2 | 0 |  |  |  |  |  |  |  |  |
| GO:0002072\_optic\_cup\_morphogenesis\_involved\_in\_camera-type\_eye\_development | 2 | 0 |  |  |  |  |  |  |  |  |
| GO:0002074\_extraocular\_skeletal\_muscle\_development | 2 | 0 |  |  |  |  |  |  |  |  |
| GO:0002138\_retinoic\_acid\_biosynthetic\_process | 2 | 0 |  |  |  |  |  |  |  |  |
| GO:0002223\_stimulatory\_C-type\_lectin\_receptor\_signaling\_pathway | 2 | 0 |  |  |  |  |  |  |  |  |
| GO:0002246\_healing\_during\_inflammatory\_response | 2 | 0 |  |  |  |  |  |  |  |  |
| GO:0002251\_organ\_or\_tissue\_specific\_immune\_response | 2 | 0 |  |  |  |  |  |  |  |  |
| GO:0002266\_follicular\_dendritic\_cell\_activation | 2 | 0 |  |  |  |  |  |  |  |  |
| GO:0002268\_follicular\_dendritic\_cell\_differentiation | 2 | 0 |  |  |  |  |  |  |  |  |
| GO:0002327\_immature\_B\_cell\_differentiation | 2 | 0 |  |  |  |  |  |  |  |  |
| GO:0002329\_pre-B\_cell\_differentiation | 2 | 0 |  |  |  |  |  |  |  |  |
| GO:0002339\_B\_cell\_selection | 2 | 0 |  |  |  |  |  |  |  |  |
| GO:0002352\_B\_cell\_negative\_selection | 2 | 0 |  |  |  |  |  |  |  |  |
| GO:0002358\_B\_cell\_homeostatic\_proliferation | 2 | 0 |  |  |  |  |  |  |  |  |
| GO:0002385\_mucosal\_immune\_response | 2 | 0 |  |  |  |  |  |  |  |  |
| GO:0002514\_B\_cell\_tolerance\_induction | 2 | 0 |  |  |  |  |  |  |  |  |
| GO:0002523\_leukocyte\_migration\_during\_inflammatory\_response | 2 | 0 |  |  |  |  |  |  |  |  |
| GO:0002536\_respiratory\_burst\_during\_acute\_inflammatory\_response | 2 | 0 |  |  |  |  |  |  |  |  |
| GO:0002537\_production\_of\_nitric\_oxide\_during\_acute\_inflammatory\_response | 2 | 0 |  |  |  |  |  |  |  |  |
| GO:0002576\_platelet\_degranulation | 2 | 0 |  |  |  |  |  |  |  |  |
| GO:0002639\_positive\_regulation\_of\_immunoglobulin\_production | 2 | 0 |  |  |  |  |  |  |  |  |
| GO:0002661\_regulation\_of\_B\_cell\_tolerance\_induction | 2 | 0 |  |  |  |  |  |  |  |  |
| GO:0002663\_positive\_regulation\_of\_B\_cell\_tolerance\_induction | 2 | 0 |  |  |  |  |  |  |  |  |
| GO:0002676\_regulation\_of\_chronic\_inflammatory\_response | 2 | 0 |  |  |  |  |  |  |  |  |
| GO:0002679\_respiratory\_burst\_during\_defense\_response | 2 | 0 |  |  |  |  |  |  |  |  |
| GO:0002686\_negative\_regulation\_of\_leukocyte\_migration | 2 | 0 |  |  |  |  |  |  |  |  |
| GO:0002720\_positive\_regulation\_of\_cytokine\_production\_during\_immune\_response | 2 | 0 |  |  |  |  |  |  |  |  |
| GO:0002752\_cell\_surface\_pattern\_recognition\_receptor\_signaling\_pathway | 2 | 0 |  |  |  |  |  |  |  |  |
| GO:0002755\_MyD88-dependent\_toll-like\_receptor\_signaling\_pathway | 2 | 0 |  |  |  |  |  |  |  |  |
| GO:0002765\_immune\_response-inhibiting\_signal\_transduction | 2 | 0 |  |  |  |  |  |  |  |  |
| GO:0002921\_negative\_regulation\_of\_humoral\_immune\_response | 2 | 0 |  |  |  |  |  |  |  |  |
| GO:0002922\_positive\_regulation\_of\_humoral\_immune\_response | 2 | 0 |  |  |  |  |  |  |  |  |
| GO:0002924\_negative\_regulation\_of\_humoral\_immune\_response\_mediated\_by\_circulating\_immunoglobulin | 2 | 0 |  |  |  |  |  |  |  |  |
| GO:0002925\_positive\_regulation\_of\_humoral\_immune\_response\_mediated\_by\_circulating\_immunoglobulin | 2 | 0 |  |  |  |  |  |  |  |  |
| GO:0003057\_regulation\_of\_the\_force\_of\_heart\_contraction\_by\_chemical\_signal | 2 | 0 |  |  |  |  |  |  |  |  |
| GO:0003099\_positive\_regulation\_of\_the\_force\_of\_heart\_contraction\_by\_chemical\_signal | 2 | 0 |  |  |  |  |  |  |  |  |
| GO:0005981\_regulation\_of\_glycogen\_catabolic\_process | 2 | 0 |  |  |  |  |  |  |  |  |
| GO:0006021\_inositol\_biosynthetic\_process | 2 | 0 |  |  |  |  |  |  |  |  |
| GO:0006042\_glucosamine\_biosynthetic\_process | 2 | 0 |  |  |  |  |  |  |  |  |
| GO:0006045\_N-acetylglucosamine\_biosynthetic\_process | 2 | 0 |  |  |  |  |  |  |  |  |
| GO:0006048\_UDP-N-acetylglucosamine\_biosynthetic\_process | 2 | 0 |  |  |  |  |  |  |  |  |
| GO:0006054\_N-acetylneuraminate\_metabolic\_process | 2 | 0 |  |  |  |  |  |  |  |  |
| GO:0006059\_hexitol\_metabolic\_process | 2 | 0 |  |  |  |  |  |  |  |  |
| GO:0006063\_uronic\_acid\_metabolic\_process | 2 | 0 |  |  |  |  |  |  |  |  |
| GO:0006068\_ethanol\_catabolic\_process | 2 | 0 |  |  |  |  |  |  |  |  |
| GO:0006083\_acetate\_metabolic\_process | 2 | 0 |  |  |  |  |  |  |  |  |
| GO:0006089\_lactate\_metabolic\_process | 2 | 0 |  |  |  |  |  |  |  |  |
| GO:0006105\_succinate\_metabolic\_process | 2 | 0 |  |  |  |  |  |  |  |  |
| GO:0006106\_fumarate\_metabolic\_process | 2 | 0 |  |  |  |  |  |  |  |  |
| GO:0006110\_regulation\_of\_glycolysis | 2 | 0 |  |  |  |  |  |  |  |  |
| GO:0006113\_fermentation | 2 | 0 |  |  |  |  |  |  |  |  |
| GO:0006114\_glycerol\_biosynthetic\_process | 2 | 0 |  |  |  |  |  |  |  |  |
| GO:0006122\_mitochondrial\_electron\_transport\_\_ubiquinol\_to\_cytochrome\_c | 2 | 0 |  |  |  |  |  |  |  |  |
| GO:0006152\_purine\_nucleoside\_catabolic\_process | 2 | 0 |  |  |  |  |  |  |  |  |
| GO:0006200\_ATP\_catabolic\_process | 2 | 0 |  |  |  |  |  |  |  |  |
| GO:0006206\_pyrimidine\_base\_metabolic\_process | 2 | 0 |  |  |  |  |  |  |  |  |
| GO:0006213\_pyrimidine\_nucleoside\_metabolic\_process | 2 | 0 |  |  |  |  |  |  |  |  |
| GO:0006265\_DNA\_topological\_change | 2 | 0 |  |  |  |  |  |  |  |  |
| GO:0006278\_RNA-dependent\_DNA\_replication | 2 | 0 |  |  |  |  |  |  |  |  |
| GO:0006312\_mitotic\_recombination | 2 | 0 |  |  |  |  |  |  |  |  |
| GO:0006398\_histone\_mRNA\_3'-end\_processing | 2 | 0 |  |  |  |  |  |  |  |  |
| GO:0006418\_tRNA\_aminoacylation\_for\_protein\_translation | 2 | 0 |  |  |  |  |  |  |  |  |
| GO:0006451\_translational\_readthrough | 2 | 0 |  |  |  |  |  |  |  |  |
| GO:0006477\_protein\_amino\_acid\_sulfation | 2 | 0 |  |  |  |  |  |  |  |  |
| GO:0006482\_protein\_amino\_acid\_demethylation | 2 | 0 |  |  |  |  |  |  |  |  |
| GO:0006499\_N-terminal\_protein\_myristoylation | 2 | 0 |  |  |  |  |  |  |  |  |
| GO:0006525\_arginine\_metabolic\_process | 2 | 0 |  |  |  |  |  |  |  |  |
| GO:0006527\_arginine\_catabolic\_process | 2 | 0 |  |  |  |  |  |  |  |  |
| GO:0006532\_aspartate\_biosynthetic\_process | 2 | 0 |  |  |  |  |  |  |  |  |
| GO:0006538\_glutamate\_catabolic\_process | 2 | 0 |  |  |  |  |  |  |  |  |
| GO:0006558\_L-phenylalanine\_metabolic\_process | 2 | 0 |  |  |  |  |  |  |  |  |
| GO:0006563\_L-serine\_metabolic\_process | 2 | 0 |  |  |  |  |  |  |  |  |
| GO:0006566\_threonine\_metabolic\_process | 2 | 0 |  |  |  |  |  |  |  |  |
| GO:0006568\_tryptophan\_metabolic\_process | 2 | 0 |  |  |  |  |  |  |  |  |
| GO:0006583\_melanin\_biosynthetic\_process\_from\_tyrosine | 2 | 0 |  |  |  |  |  |  |  |  |
| GO:0006600\_creatine\_metabolic\_process | 2 | 0 |  |  |  |  |  |  |  |  |
| GO:0006603\_phosphocreatine\_metabolic\_process | 2 | 0 |  |  |  |  |  |  |  |  |
| GO:0006642\_triglyceride\_mobilization | 2 | 0 |  |  |  |  |  |  |  |  |
| GO:0006649\_phospholipid\_transfer\_to\_membrane | 2 | 0 |  |  |  |  |  |  |  |  |
| GO:0006681\_galactosylceramide\_metabolic\_process | 2 | 0 |  |  |  |  |  |  |  |  |
| GO:0006686\_sphingomyelin\_biosynthetic\_process | 2 | 0 |  |  |  |  |  |  |  |  |
| GO:0006702\_androgen\_biosynthetic\_process | 2 | 0 |  |  |  |  |  |  |  |  |
| GO:0006750\_glutathione\_biosynthetic\_process | 2 | 0 |  |  |  |  |  |  |  |  |
| GO:0006760\_folic\_acid\_and\_derivative\_metabolic\_process | 2 | 0 |  |  |  |  |  |  |  |  |
| GO:0006808\_regulation\_of\_nitrogen\_utilization | 2 | 0 |  |  |  |  |  |  |  |  |
| GO:0006868\_glutamine\_transport | 2 | 0 |  |  |  |  |  |  |  |  |
| GO:0006907\_pinocytosis | 2 | 0 |  |  |  |  |  |  |  |  |
| GO:0006925\_inflammatory\_cell\_apoptosis | 2 | 0 |  |  |  |  |  |  |  |  |
| GO:0006977\_DNA\_damage\_response\_\_signal\_transduction\_by\_p53\_class\_mediator\_resulting\_in\_cell\_cycle\_arrest | 2 | 0 |  |  |  |  |  |  |  |  |
| GO:0006991\_response\_to\_sterol\_depletion | 2 | 0 |  |  |  |  |  |  |  |  |
| GO:0007004\_telomere\_maintenance\_via\_telomerase | 2 | 0 |  |  |  |  |  |  |  |  |
| GO:0007020\_microtubule\_nucleation | 2 | 0 |  |  |  |  |  |  |  |  |
| GO:0007030\_Golgi\_organization | 2 | 0 |  |  |  |  |  |  |  |  |
| GO:0007035\_vacuolar\_acidification | 2 | 0 |  |  |  |  |  |  |  |  |
| GO:0007042\_lysosomal\_lumen\_acidification | 2 | 0 |  |  |  |  |  |  |  |  |
| GO:0007060\_male\_meiosis\_chromosome\_segregation | 2 | 0 |  |  |  |  |  |  |  |  |
| GO:0007089\_traversing\_start\_control\_point\_of\_mitotic\_cell\_cycle | 2 | 0 |  |  |  |  |  |  |  |  |
| GO:0007094\_mitotic\_cell\_cycle\_spindle\_assembly\_checkpoint | 2 | 0 |  |  |  |  |  |  |  |  |
| GO:0007097\_nuclear\_migration | 2 | 0 |  |  |  |  |  |  |  |  |
| GO:0007100\_mitotic\_centrosome\_separation | 2 | 0 |  |  |  |  |  |  |  |  |
| GO:0007132\_meiotic\_metaphase\_I | 2 | 0 |  |  |  |  |  |  |  |  |
| GO:0007171\_activation\_of\_transmembrane\_receptor\_protein\_tyrosine\_kinase\_activity | 2 | 0 |  |  |  |  |  |  |  |  |
| GO:0007182\_common-partner\_SMAD\_protein\_phosphorylation | 2 | 0 |  |  |  |  |  |  |  |  |
| GO:0007185\_transmembrane\_receptor\_protein\_tyrosine\_phosphatase\_signaling\_pathway | 2 | 0 |  |  |  |  |  |  |  |  |
| GO:0007205\_activation\_of\_protein\_kinase\_C\_activity\_by\_G-protein\_coupled\_receptor\_protein\_signaling\_pathway | 2 | 0 |  |  |  |  |  |  |  |  |
| GO:0007210\_serotonin\_receptor\_signaling\_pathway | 2 | 0 |  |  |  |  |  |  |  |  |
| GO:0007220\_Notch\_receptor\_processing | 2 | 0 |  |  |  |  |  |  |  |  |
| GO:0007256\_activation\_of\_JNKK\_activity | 2 | 0 |  |  |  |  |  |  |  |  |
| GO:0007258\_JUN\_phosphorylation | 2 | 0 |  |  |  |  |  |  |  |  |
| GO:0007263\_nitric\_oxide\_mediated\_signal\_transduction | 2 | 0 |  |  |  |  |  |  |  |  |
| GO:0007289\_spermatid\_nucleus\_differentiation | 2 | 0 |  |  |  |  |  |  |  |  |
| GO:0007343\_egg\_activation | 2 | 0 |  |  |  |  |  |  |  |  |
| GO:0007351\_tripartite\_regional\_subdivision | 2 | 0 |  |  |  |  |  |  |  |  |
| GO:0007418\_ventral\_midline\_development | 2 | 0 |  |  |  |  |  |  |  |  |
| GO:0007494\_midgut\_development | 2 | 0 |  |  |  |  |  |  |  |  |
| GO:0007527\_adult\_somatic\_muscle\_development | 2 | 0 |  |  |  |  |  |  |  |  |
| GO:0007549\_dosage\_compensation | 2 | 0 |  |  |  |  |  |  |  |  |
| GO:0007571\_age-dependent\_general\_metabolic\_decline | 2 | 0 |  |  |  |  |  |  |  |  |
| GO:0007603\_phototransduction\_\_visible\_light | 2 | 0 |  |  |  |  |  |  |  |  |
| GO:0007619\_courtship\_behavior | 2 | 0 |  |  |  |  |  |  |  |  |
| GO:0008065\_establishment\_of\_blood-nerve\_barrier | 2 | 0 |  |  |  |  |  |  |  |  |
| GO:0008089\_anterograde\_axon\_cargo\_transport | 2 | 0 |  |  |  |  |  |  |  |  |
| GO:0008210\_estrogen\_metabolic\_process | 2 | 0 |  |  |  |  |  |  |  |  |
| GO:0008212\_mineralocorticoid\_metabolic\_process | 2 | 0 |  |  |  |  |  |  |  |  |
| GO:0008214\_protein\_amino\_acid\_dealkylation | 2 | 0 |  |  |  |  |  |  |  |  |
| GO:0008228\_opsonization | 2 | 0 |  |  |  |  |  |  |  |  |
| GO:0008272\_sulfate\_transport | 2 | 0 |  |  |  |  |  |  |  |  |
| GO:0008291\_acetylcholine\_metabolic\_process | 2 | 0 |  |  |  |  |  |  |  |  |
| GO:0008298\_intracellular\_mRNA\_localization | 2 | 0 |  |  |  |  |  |  |  |  |
| GO:0008334\_histone\_mRNA\_metabolic\_process | 2 | 0 |  |  |  |  |  |  |  |  |
| GO:0008356\_asymmetric\_cell\_division | 2 | 0 |  |  |  |  |  |  |  |  |
| GO:0008582\_regulation\_of\_synaptic\_growth\_at\_neuromuscular\_junction | 2 | 0 |  |  |  |  |  |  |  |  |
| GO:0008594\_photoreceptor\_cell\_morphogenesis | 2 | 0 |  |  |  |  |  |  |  |  |
| GO:0008595\_determination\_of\_anterior\_posterior\_axis\_\_embryo | 2 | 0 |  |  |  |  |  |  |  |  |
| GO:0008608\_attachment\_of\_spindle\_microtubules\_to\_kinetochore | 2 | 0 |  |  |  |  |  |  |  |  |
| GO:0008616\_queuosine\_biosynthetic\_process | 2 | 0 |  |  |  |  |  |  |  |  |
| GO:0008617\_guanosine\_metabolic\_process | 2 | 0 |  |  |  |  |  |  |  |  |
| GO:0008618\_7-methylguanosine\_metabolic\_process | 2 | 0 |  |  |  |  |  |  |  |  |
| GO:0008634\_negative\_regulation\_of\_survival\_gene\_product\_expression | 2 | 0 |  |  |  |  |  |  |  |  |
| GO:0009048\_dosage\_compensation\_\_by\_inactivation\_of\_X\_chromosome | 2 | 0 |  |  |  |  |  |  |  |  |
| GO:0009070\_serine\_family\_amino\_acid\_biosynthetic\_process | 2 | 0 |  |  |  |  |  |  |  |  |
| GO:0009071\_serine\_family\_amino\_acid\_catabolic\_process | 2 | 0 |  |  |  |  |  |  |  |  |
| GO:0009074\_aromatic\_amino\_acid\_family\_catabolic\_process | 2 | 0 |  |  |  |  |  |  |  |  |
| GO:0009083\_branched\_chain\_family\_amino\_acid\_catabolic\_process | 2 | 0 |  |  |  |  |  |  |  |  |
| GO:0009093\_cysteine\_catabolic\_process | 2 | 0 |  |  |  |  |  |  |  |  |
| GO:0009120\_deoxyribonucleoside\_metabolic\_process | 2 | 0 |  |  |  |  |  |  |  |  |
| GO:0009125\_nucleoside\_monophosphate\_catabolic\_process | 2 | 0 |  |  |  |  |  |  |  |  |
| GO:0009126\_purine\_nucleoside\_monophosphate\_metabolic\_process | 2 | 0 |  |  |  |  |  |  |  |  |
| GO:0009142\_nucleoside\_triphosphate\_biosynthetic\_process | 2 | 0 |  |  |  |  |  |  |  |  |
| GO:0009161\_ribonucleoside\_monophosphate\_metabolic\_process | 2 | 0 |  |  |  |  |  |  |  |  |
| GO:0009164\_nucleoside\_catabolic\_process | 2 | 0 |  |  |  |  |  |  |  |  |
| GO:0009167\_purine\_ribonucleoside\_monophosphate\_metabolic\_process | 2 | 0 |  |  |  |  |  |  |  |  |
| GO:0009202\_deoxyribonucleoside\_triphosphate\_biosynthetic\_process | 2 | 0 |  |  |  |  |  |  |  |  |
| GO:0009203\_ribonucleoside\_triphosphate\_catabolic\_process | 2 | 0 |  |  |  |  |  |  |  |  |
| GO:0009207\_purine\_ribonucleoside\_triphosphate\_catabolic\_process | 2 | 0 |  |  |  |  |  |  |  |  |
| GO:0009219\_pyrimidine\_deoxyribonucleotide\_metabolic\_process | 2 | 0 |  |  |  |  |  |  |  |  |
| GO:0009265\_2'-deoxyribonucleotide\_biosynthetic\_process | 2 | 0 |  |  |  |  |  |  |  |  |
| GO:0009268\_response\_to\_pH | 2 | 0 |  |  |  |  |  |  |  |  |
| GO:0009313\_oligosaccharide\_catabolic\_process | 2 | 0 |  |  |  |  |  |  |  |  |
| GO:0009395\_phospholipid\_catabolic\_process | 2 | 0 |  |  |  |  |  |  |  |  |
| GO:0009435\_NAD\_biosynthetic\_process | 2 | 0 |  |  |  |  |  |  |  |  |
| GO:0009608\_response\_to\_symbiont | 2 | 0 |  |  |  |  |  |  |  |  |
| GO:0009609\_response\_to\_symbiotic\_bacterium | 2 | 0 |  |  |  |  |  |  |  |  |
| GO:0009649\_entrainment\_of\_circadian\_clock | 2 | 0 |  |  |  |  |  |  |  |  |
| GO:0009996\_negative\_regulation\_of\_cell\_fate\_specification | 2 | 0 |  |  |  |  |  |  |  |  |
| GO:0010002\_cardioblast\_differentiation | 2 | 0 |  |  |  |  |  |  |  |  |
| GO:0010149\_senescence | 2 | 0 |  |  |  |  |  |  |  |  |
| GO:0010225\_response\_to\_UV-C | 2 | 0 |  |  |  |  |  |  |  |  |
| GO:0010389\_regulation\_of\_G2\_M\_transition\_of\_mitotic\_cell\_cycle | 2 | 0 |  |  |  |  |  |  |  |  |
| GO:0010458\_exit\_from\_mitosis | 2 | 0 |  |  |  |  |  |  |  |  |
| GO:0010459\_negative\_regulation\_of\_heart\_rate | 2 | 0 |  |  |  |  |  |  |  |  |
| GO:0010559\_regulation\_of\_glycoprotein\_biosynthetic\_process | 2 | 0 |  |  |  |  |  |  |  |  |
| GO:0010633\_negative\_regulation\_of\_epithelial\_cell\_migration | 2 | 0 |  |  |  |  |  |  |  |  |
| GO:0010677\_negative\_regulation\_of\_cellular\_carbohydrate\_metabolic\_process | 2 | 0 |  |  |  |  |  |  |  |  |
| GO:0010718\_positive\_regulation\_of\_epithelial\_to\_mesenchymal\_transition | 2 | 0 |  |  |  |  |  |  |  |  |
| GO:0010742\_foam\_cell\_differentiation | 2 | 0 |  |  |  |  |  |  |  |  |
| GO:0010743\_regulation\_of\_foam\_cell\_differentiation | 2 | 0 |  |  |  |  |  |  |  |  |
| GO:0010744\_positive\_regulation\_of\_foam\_cell\_differentiation | 2 | 0 |  |  |  |  |  |  |  |  |
| GO:0010765\_positive\_regulation\_of\_sodium\_ion\_transport | 2 | 0 |  |  |  |  |  |  |  |  |
| GO:0010766\_negative\_regulation\_of\_sodium\_ion\_transport | 2 | 0 |  |  |  |  |  |  |  |  |
| GO:0010770\_positive\_regulation\_of\_cell\_morphogenesis\_involved\_in\_differentiation | 2 | 0 |  |  |  |  |  |  |  |  |
| GO:0010771\_negative\_regulation\_of\_cell\_morphogenesis\_involved\_in\_differentiation | 2 | 0 |  |  |  |  |  |  |  |  |
| GO:0010824\_regulation\_of\_centrosome\_duplication | 2 | 0 |  |  |  |  |  |  |  |  |
| GO:0010833\_telomere\_maintenance\_via\_telomere\_lengthening | 2 | 0 |  |  |  |  |  |  |  |  |
| GO:0010862\_positive\_regulation\_of\_pathway-restricted\_SMAD\_protein\_phosphorylation | 2 | 0 |  |  |  |  |  |  |  |  |
| GO:0010872\_regulation\_of\_cholesterol\_esterification | 2 | 0 |  |  |  |  |  |  |  |  |
| GO:0010878\_cholesterol\_storage | 2 | 0 |  |  |  |  |  |  |  |  |
| GO:0010885\_regulation\_of\_cholesterol\_storage | 2 | 0 |  |  |  |  |  |  |  |  |
| GO:0010886\_positive\_regulation\_of\_cholesterol\_storage | 2 | 0 |  |  |  |  |  |  |  |  |
| GO:0010891\_negative\_regulation\_of\_sequestering\_of\_triglyceride | 2 | 0 |  |  |  |  |  |  |  |  |
| GO:0010896\_regulation\_of\_triglyceride\_catabolic\_process | 2 | 0 |  |  |  |  |  |  |  |  |
| GO:0010898\_positive\_regulation\_of\_triglyceride\_catabolic\_process | 2 | 0 |  |  |  |  |  |  |  |  |
| GO:0010907\_positive\_regulation\_of\_glucose\_metabolic\_process | 2 | 0 |  |  |  |  |  |  |  |  |
| GO:0014028\_notochord\_formation | 2 | 0 |  |  |  |  |  |  |  |  |
| GO:0014048\_regulation\_of\_glutamate\_secretion | 2 | 0 |  |  |  |  |  |  |  |  |
| GO:0014052\_regulation\_of\_gamma-aminobutyric\_acid\_secretion | 2 | 0 |  |  |  |  |  |  |  |  |
| GO:0014054\_positive\_regulation\_of\_gamma-aminobutyric\_acid\_secretion | 2 | 0 |  |  |  |  |  |  |  |  |
| GO:0014055\_acetylcholine\_secretion | 2 | 0 |  |  |  |  |  |  |  |  |
| GO:0014056\_regulation\_of\_acetylcholine\_secretion | 2 | 0 |  |  |  |  |  |  |  |  |
| GO:0014067\_negative\_regulation\_of\_phosphoinositide\_3-kinase\_cascade | 2 | 0 |  |  |  |  |  |  |  |  |
| GO:0014745\_negative\_regulation\_of\_muscle\_adaptation | 2 | 0 |  |  |  |  |  |  |  |  |
| GO:0014829\_vascular\_smooth\_muscle\_contraction | 2 | 0 |  |  |  |  |  |  |  |  |
| GO:0014850\_response\_to\_muscle\_activity | 2 | 0 |  |  |  |  |  |  |  |  |
| GO:0014866\_skeletal\_myofibril\_assembly | 2 | 0 |  |  |  |  |  |  |  |  |
| GO:0014888\_striated\_muscle\_adaptation | 2 | 0 |  |  |  |  |  |  |  |  |
| GO:0014916\_regulation\_of\_lung\_blood\_pressure | 2 | 0 |  |  |  |  |  |  |  |  |
| GO:0015671\_oxygen\_transport | 2 | 0 |  |  |  |  |  |  |  |  |
| GO:0015696\_ammonium\_transport | 2 | 0 |  |  |  |  |  |  |  |  |
| GO:0015732\_prostaglandin\_transport | 2 | 0 |  |  |  |  |  |  |  |  |
| GO:0015819\_lysine\_transport | 2 | 0 |  |  |  |  |  |  |  |  |
| GO:0015840\_urea\_transport | 2 | 0 |  |  |  |  |  |  |  |  |
| GO:0015860\_purine\_nucleoside\_transport | 2 | 0 |  |  |  |  |  |  |  |  |
| GO:0015870\_acetylcholine\_transport | 2 | 0 |  |  |  |  |  |  |  |  |
| GO:0015937\_coenzyme\_A\_biosynthetic\_process | 2 | 0 |  |  |  |  |  |  |  |  |
| GO:0016045\_detection\_of\_bacterium | 2 | 0 |  |  |  |  |  |  |  |  |
| GO:0016046\_detection\_of\_fungus | 2 | 0 |  |  |  |  |  |  |  |  |
| GO:0016080\_synaptic\_vesicle\_targeting | 2 | 0 |  |  |  |  |  |  |  |  |
| GO:0016199\_axon\_midline\_choice\_point\_recognition | 2 | 0 |  |  |  |  |  |  |  |  |
| GO:0016226\_iron-sulfur\_cluster\_assembly | 2 | 0 |  |  |  |  |  |  |  |  |
| GO:0016233\_telomere\_capping | 2 | 0 |  |  |  |  |  |  |  |  |
| GO:0016242\_negative\_regulation\_of\_macroautophagy | 2 | 0 |  |  |  |  |  |  |  |  |
| GO:0016441\_posttranscriptional\_gene\_silencing | 2 | 0 |  |  |  |  |  |  |  |  |
| GO:0016540\_protein\_autoprocessing | 2 | 0 |  |  |  |  |  |  |  |  |
| GO:0016558\_protein\_import\_into\_peroxisome\_matrix | 2 | 0 |  |  |  |  |  |  |  |  |
| GO:0016572\_histone\_phosphorylation | 2 | 0 |  |  |  |  |  |  |  |  |
| GO:0016577\_histone\_demethylation | 2 | 0 |  |  |  |  |  |  |  |  |
| GO:0016584\_nucleosome\_positioning | 2 | 0 |  |  |  |  |  |  |  |  |
| GO:0016926\_protein\_desumoylation | 2 | 0 |  |  |  |  |  |  |  |  |
| GO:0017014\_protein\_amino\_acid\_nitrosylation | 2 | 0 |  |  |  |  |  |  |  |  |
| GO:0017144\_drug\_metabolic\_process | 2 | 0 |  |  |  |  |  |  |  |  |
| GO:0018094\_protein\_polyglycylation | 2 | 0 |  |  |  |  |  |  |  |  |
| GO:0018119\_peptidyl-cysteine\_S-nitrosylation | 2 | 0 |  |  |  |  |  |  |  |  |
| GO:0018125\_peptidyl-cysteine\_methylation | 2 | 0 |  |  |  |  |  |  |  |  |
| GO:0018205\_peptidyl-lysine\_modification | 2 | 0 |  |  |  |  |  |  |  |  |
| GO:0018319\_protein\_amino\_acid\_myristoylation | 2 | 0 |  |  |  |  |  |  |  |  |
| GO:0018377\_protein\_myristoylation | 2 | 0 |  |  |  |  |  |  |  |  |
| GO:0018401\_peptidyl-proline\_hydroxylation\_to\_4-hydroxy-L-proline | 2 | 0 |  |  |  |  |  |  |  |  |
| GO:0018993\_somatic\_sex\_determination | 2 | 0 |  |  |  |  |  |  |  |  |
| GO:0019067\_viral\_assembly\_\_maturation\_\_egress\_\_and\_release | 2 | 0 |  |  |  |  |  |  |  |  |
| GO:0019322\_pentose\_biosynthetic\_process | 2 | 0 |  |  |  |  |  |  |  |  |
| GO:0019370\_leukotriene\_biosynthetic\_process | 2 | 0 |  |  |  |  |  |  |  |  |
| GO:0019374\_galactolipid\_metabolic\_process | 2 | 0 |  |  |  |  |  |  |  |  |
| GO:0019401\_alditol\_biosynthetic\_process | 2 | 0 |  |  |  |  |  |  |  |  |
| GO:0019448\_L-cysteine\_catabolic\_process | 2 | 0 |  |  |  |  |  |  |  |  |
| GO:0019452\_L-cysteine\_catabolic\_process\_to\_taurine | 2 | 0 |  |  |  |  |  |  |  |  |
| GO:0019471\_4-hydroxyproline\_metabolic\_process | 2 | 0 |  |  |  |  |  |  |  |  |
| GO:0019511\_peptidyl-proline\_hydroxylation | 2 | 0 |  |  |  |  |  |  |  |  |
| GO:0019550\_glutamate\_catabolic\_process\_to\_aspartate | 2 | 0 |  |  |  |  |  |  |  |  |
| GO:0019551\_glutamate\_catabolic\_process\_to\_2-oxoglutarate | 2 | 0 |  |  |  |  |  |  |  |  |
| GO:0019585\_glucuronate\_metabolic\_process | 2 | 0 |  |  |  |  |  |  |  |  |
| GO:0019730\_antimicrobial\_humoral\_response | 2 | 0 |  |  |  |  |  |  |  |  |
| GO:0019740\_nitrogen\_utilization | 2 | 0 |  |  |  |  |  |  |  |  |
| GO:0019853\_L-ascorbic\_acid\_biosynthetic\_process | 2 | 0 |  |  |  |  |  |  |  |  |
| GO:0021506\_anterior\_neuropore\_closure | 2 | 0 |  |  |  |  |  |  |  |  |
| GO:0021524\_visceral\_motor\_neuron\_differentiation | 2 | 0 |  |  |  |  |  |  |  |  |
| GO:0021526\_medial\_motor\_column\_neuron\_differentiation | 2 | 0 |  |  |  |  |  |  |  |  |
| GO:0021557\_oculomotor\_nerve\_development | 2 | 0 |  |  |  |  |  |  |  |  |
| GO:0021558\_trochlear\_nerve\_development | 2 | 0 |  |  |  |  |  |  |  |  |
| GO:0021562\_vestibulocochlear\_nerve\_development | 2 | 0 |  |  |  |  |  |  |  |  |
| GO:0021568\_rhombomere\_2\_development | 2 | 0 |  |  |  |  |  |  |  |  |
| GO:0021578\_hindbrain\_maturation | 2 | 0 |  |  |  |  |  |  |  |  |
| GO:0021593\_rhombomere\_morphogenesis | 2 | 0 |  |  |  |  |  |  |  |  |
| GO:0021626\_central\_nervous\_system\_maturation | 2 | 0 |  |  |  |  |  |  |  |  |
| GO:0021658\_rhombomere\_3\_morphogenesis | 2 | 0 |  |  |  |  |  |  |  |  |
| GO:0021754\_facial\_nucleus\_development | 2 | 0 |  |  |  |  |  |  |  |  |
| GO:0021775\_smoothened\_signaling\_pathway\_involved\_in\_ventral\_spinal\_cord\_interneuron\_specification | 2 | 0 |  |  |  |  |  |  |  |  |
| GO:0021776\_smoothened\_signaling\_pathway\_involved\_in\_spinal\_cord\_motor\_neuron\_cell\_fate\_specification | 2 | 0 |  |  |  |  |  |  |  |  |
| GO:0021796\_cerebral\_cortex\_regionalization | 2 | 0 |  |  |  |  |  |  |  |  |
| GO:0021831\_embryonic\_olfactory\_bulb\_interneuron\_precursor\_migration | 2 | 0 |  |  |  |  |  |  |  |  |
| GO:0021869\_forebrain\_ventricular\_zone\_progenitor\_cell\_division | 2 | 0 |  |  |  |  |  |  |  |  |
| GO:0021873\_forebrain\_neuroblast\_division | 2 | 0 |  |  |  |  |  |  |  |  |
| GO:0021882\_regulation\_of\_transcription\_from\_RNA\_polymerase\_II\_promoter\_involved\_in\_forebrain\_neuron\_fate\_commitment | 2 | 0 |  |  |  |  |  |  |  |  |
| GO:0021893\_cerebral\_cortex\_GABAergic\_interneuron\_fate\_commitment | 2 | 0 |  |  |  |  |  |  |  |  |
| GO:0021898\_commitment\_of\_multipotent\_stem\_cells\_to\_the\_neuronal\_lineage\_in\_the\_forebrain | 2 | 0 |  |  |  |  |  |  |  |  |
| GO:0021932\_hindbrain\_radial\_glia\_guided\_cell\_migration | 2 | 0 |  |  |  |  |  |  |  |  |
| GO:0021965\_spinal\_cord\_ventral\_commissure\_morphogenesis | 2 | 0 |  |  |  |  |  |  |  |  |
| GO:0021985\_neurohypophysis\_development | 2 | 0 |  |  |  |  |  |  |  |  |
| GO:0021990\_neural\_plate\_formation | 2 | 0 |  |  |  |  |  |  |  |  |
| GO:0021995\_neuropore\_closure | 2 | 0 |  |  |  |  |  |  |  |  |
| GO:0022028\_tangential\_migration\_from\_the\_subventricular\_zone\_to\_the\_olfactory\_bulb | 2 | 0 |  |  |  |  |  |  |  |  |
| GO:0022401\_adaptation\_of\_signaling\_pathway | 2 | 0 |  |  |  |  |  |  |  |  |
| GO:0022408\_negative\_regulation\_of\_cell-cell\_adhesion | 2 | 0 |  |  |  |  |  |  |  |  |
| GO:0022410\_circadian\_sleep\_wake\_cycle\_process | 2 | 0 |  |  |  |  |  |  |  |  |
| GO:0030046\_parallel\_actin\_filament\_bundle\_formation | 2 | 0 |  |  |  |  |  |  |  |  |
| GO:0030049\_muscle\_filament\_sliding | 2 | 0 |  |  |  |  |  |  |  |  |
| GO:0030050\_vesicle\_transport\_along\_actin\_filament | 2 | 0 |  |  |  |  |  |  |  |  |
| GO:0030071\_regulation\_of\_mitotic\_metaphase\_anaphase\_transition | 2 | 0 |  |  |  |  |  |  |  |  |
| GO:0030147\_natriuresis | 2 | 0 |  |  |  |  |  |  |  |  |
| GO:0030174\_regulation\_of\_DNA\_replication\_initiation | 2 | 0 |  |  |  |  |  |  |  |  |
| GO:0030202\_heparin\_metabolic\_process | 2 | 0 |  |  |  |  |  |  |  |  |
| GO:0030219\_megakaryocyte\_differentiation | 2 | 0 |  |  |  |  |  |  |  |  |
| GO:0030223\_neutrophil\_differentiation | 2 | 0 |  |  |  |  |  |  |  |  |
| GO:0030240\_muscle\_thin\_filament\_assembly | 2 | 0 |  |  |  |  |  |  |  |  |
| GO:0030259\_lipid\_glycosylation | 2 | 0 |  |  |  |  |  |  |  |  |
| GO:0030397\_membrane\_disassembly | 2 | 0 |  |  |  |  |  |  |  |  |
| GO:0030502\_negative\_regulation\_of\_bone\_mineralization | 2 | 0 |  |  |  |  |  |  |  |  |
| GO:0030644\_cellular\_chloride\_ion\_homeostasis | 2 | 0 |  |  |  |  |  |  |  |  |
| GO:0030825\_positive\_regulation\_of\_cGMP\_metabolic\_process | 2 | 0 |  |  |  |  |  |  |  |  |
| GO:0030828\_positive\_regulation\_of\_cGMP\_biosynthetic\_process | 2 | 0 |  |  |  |  |  |  |  |  |
| GO:0030835\_negative\_regulation\_of\_actin\_filament\_depolymerization | 2 | 0 |  |  |  |  |  |  |  |  |
| GO:0030837\_negative\_regulation\_of\_actin\_filament\_polymerization | 2 | 0 |  |  |  |  |  |  |  |  |
| GO:0030852\_regulation\_of\_granulocyte\_differentiation | 2 | 0 |  |  |  |  |  |  |  |  |
| GO:0030885\_regulation\_of\_myeloid\_dendritic\_cell\_activation | 2 | 0 |  |  |  |  |  |  |  |  |
| GO:0030910\_olfactory\_placode\_formation | 2 | 0 |  |  |  |  |  |  |  |  |
| GO:0030948\_negative\_regulation\_of\_vascular\_endothelial\_growth\_factor\_receptor\_signaling\_pathway | 2 | 0 |  |  |  |  |  |  |  |  |
| GO:0030953\_spindle\_astral\_microtubule\_organization | 2 | 0 |  |  |  |  |  |  |  |  |
| GO:0031050\_dsRNA\_fragmentation | 2 | 0 |  |  |  |  |  |  |  |  |
| GO:0031061\_negative\_regulation\_of\_histone\_methylation | 2 | 0 |  |  |  |  |  |  |  |  |
| GO:0031119\_tRNA\_pseudouridine\_synthesis | 2 | 0 |  |  |  |  |  |  |  |  |
| GO:0031163\_metallo-sulfur\_cluster\_assembly | 2 | 0 |  |  |  |  |  |  |  |  |
| GO:0031223\_auditory\_behavior | 2 | 0 |  |  |  |  |  |  |  |  |
| GO:0031296\_B\_cell\_costimulation | 2 | 0 |  |  |  |  |  |  |  |  |
| GO:0031338\_regulation\_of\_vesicle\_fusion | 2 | 0 |  |  |  |  |  |  |  |  |
| GO:0031573\_intra-S\_DNA\_damage\_checkpoint | 2 | 0 |  |  |  |  |  |  |  |  |
| GO:0031577\_spindle\_checkpoint | 2 | 0 |  |  |  |  |  |  |  |  |
| GO:0031629\_synaptic\_vesicle\_fusion\_to\_presynaptic\_membrane | 2 | 0 |  |  |  |  |  |  |  |  |
| GO:0031630\_regulation\_of\_synaptic\_vesicle\_fusion\_to\_presynaptic\_membrane | 2 | 0 |  |  |  |  |  |  |  |  |
| GO:0031664\_regulation\_of\_lipopolysaccharide-mediated\_signaling\_pathway | 2 | 0 |  |  |  |  |  |  |  |  |
| GO:0031670\_cellular\_response\_to\_nutrient | 2 | 0 |  |  |  |  |  |  |  |  |
| GO:0031848\_protection\_from\_non-homologous\_end\_joining\_at\_telomere | 2 | 0 |  |  |  |  |  |  |  |  |
| GO:0031946\_regulation\_of\_glucocorticoid\_biosynthetic\_process | 2 | 0 |  |  |  |  |  |  |  |  |
| GO:0031952\_regulation\_of\_protein\_amino\_acid\_autophosphorylation | 2 | 0 |  |  |  |  |  |  |  |  |
| GO:0031953\_negative\_regulation\_of\_protein\_amino\_acid\_autophosphorylation | 2 | 0 |  |  |  |  |  |  |  |  |
| GO:0031958\_corticosteroid\_receptor\_signaling\_pathway | 2 | 0 |  |  |  |  |  |  |  |  |
| GO:0031987\_locomotion\_involved\_in\_locomotory\_behavior | 2 | 0 |  |  |  |  |  |  |  |  |
| GO:0032096\_negative\_regulation\_of\_response\_to\_food | 2 | 0 |  |  |  |  |  |  |  |  |
| GO:0032099\_negative\_regulation\_of\_appetite | 2 | 0 |  |  |  |  |  |  |  |  |
| GO:0032106\_positive\_regulation\_of\_response\_to\_extracellular\_stimulus | 2 | 0 |  |  |  |  |  |  |  |  |
| GO:0032109\_positive\_regulation\_of\_response\_to\_nutrient\_levels | 2 | 0 |  |  |  |  |  |  |  |  |
| GO:0032226\_positive\_regulation\_of\_synaptic\_transmission\_\_dopaminergic | 2 | 0 |  |  |  |  |  |  |  |  |
| GO:0032230\_positive\_regulation\_of\_synaptic\_transmission\_\_GABAergic | 2 | 0 |  |  |  |  |  |  |  |  |
| GO:0032234\_regulation\_of\_calcium\_ion\_transport\_via\_store-operated\_calcium\_channel\_activity | 2 | 0 |  |  |  |  |  |  |  |  |
| GO:0032236\_positive\_regulation\_of\_calcium\_ion\_transport\_via\_store-operated\_calcium\_channel\_activity | 2 | 0 |  |  |  |  |  |  |  |  |
| GO:0032297\_negative\_regulation\_of\_DNA\_replication\_initiation | 2 | 0 |  |  |  |  |  |  |  |  |
| GO:0032309\_icosanoid\_secretion | 2 | 0 |  |  |  |  |  |  |  |  |
| GO:0032328\_alanine\_transport | 2 | 0 |  |  |  |  |  |  |  |  |
| GO:0032341\_aldosterone\_metabolic\_process | 2 | 0 |  |  |  |  |  |  |  |  |
| GO:0032351\_negative\_regulation\_of\_hormone\_metabolic\_process | 2 | 0 |  |  |  |  |  |  |  |  |
| GO:0032353\_negative\_regulation\_of\_hormone\_biosynthetic\_process | 2 | 0 |  |  |  |  |  |  |  |  |
| GO:0032435\_negative\_regulation\_of\_proteasomal\_ubiquitin-dependent\_protein\_catabolic\_process | 2 | 0 |  |  |  |  |  |  |  |  |
| GO:0032471\_reduction\_of\_endoplasmic\_reticulum\_calcium\_ion\_concentration | 2 | 0 |  |  |  |  |  |  |  |  |
| GO:0032481\_positive\_regulation\_of\_type\_I\_interferon\_production | 2 | 0 |  |  |  |  |  |  |  |  |
| GO:0032488\_Cdc42\_protein\_signal\_transduction | 2 | 0 |  |  |  |  |  |  |  |  |
| GO:0032489\_regulation\_of\_Cdc42\_protein\_signal\_transduction | 2 | 0 |  |  |  |  |  |  |  |  |
| GO:0032495\_response\_to\_muramyl\_dipeptide | 2 | 0 |  |  |  |  |  |  |  |  |
| GO:0032604\_granulocyte\_macrophage\_colony-stimulating\_factor\_production | 2 | 0 |  |  |  |  |  |  |  |  |
| GO:0032616\_interleukin-13\_production | 2 | 0 |  |  |  |  |  |  |  |  |
| GO:0032645\_regulation\_of\_granulocyte\_macrophage\_colony-stimulating\_factor\_production | 2 | 0 |  |  |  |  |  |  |  |  |
| GO:0032672\_regulation\_of\_interleukin-3\_production | 2 | 0 |  |  |  |  |  |  |  |  |
| GO:0032695\_negative\_regulation\_of\_interleukin-12\_production | 2 | 0 |  |  |  |  |  |  |  |  |
| GO:0032714\_negative\_regulation\_of\_interleukin-5\_production | 2 | 0 |  |  |  |  |  |  |  |  |
| GO:0032722\_positive\_regulation\_of\_chemokine\_production | 2 | 0 |  |  |  |  |  |  |  |  |
| GO:0032743\_positive\_regulation\_of\_interleukin-2\_production | 2 | 0 |  |  |  |  |  |  |  |  |
| GO:0032762\_mast\_cell\_cytokine\_production | 2 | 0 |  |  |  |  |  |  |  |  |
| GO:0032763\_regulation\_of\_mast\_cell\_cytokine\_production | 2 | 0 |  |  |  |  |  |  |  |  |
| GO:0032768\_regulation\_of\_monooxygenase\_activity | 2 | 0 |  |  |  |  |  |  |  |  |
| GO:0032788\_saturated\_monocarboxylic\_acid\_metabolic\_process | 2 | 0 |  |  |  |  |  |  |  |  |
| GO:0032789\_unsaturated\_monocarboxylic\_acid\_metabolic\_process | 2 | 0 |  |  |  |  |  |  |  |  |
| GO:0032796\_uropod\_organization | 2 | 0 |  |  |  |  |  |  |  |  |
| GO:0032800\_receptor\_biosynthetic\_process | 2 | 0 |  |  |  |  |  |  |  |  |
| GO:0032801\_receptor\_catabolic\_process | 2 | 0 |  |  |  |  |  |  |  |  |
| GO:0032829\_regulation\_of\_CD4-positive\_\_CD25-positive\_\_alpha-beta\_regulatory\_T\_cell\_differentiation | 2 | 0 |  |  |  |  |  |  |  |  |
| GO:0032831\_positive\_regulation\_of\_CD4-positive\_\_CD25-positive\_\_alpha-beta\_regulatory\_T\_cell\_differentiation | 2 | 0 |  |  |  |  |  |  |  |  |
| GO:0032892\_positive\_regulation\_of\_organic\_acid\_transport | 2 | 0 |  |  |  |  |  |  |  |  |
| GO:0032905\_transforming\_growth\_factor-beta1\_production | 2 | 0 |  |  |  |  |  |  |  |  |
| GO:0032908\_regulation\_of\_transforming\_growth\_factor-beta1\_production | 2 | 0 |  |  |  |  |  |  |  |  |
| GO:0032914\_positive\_regulation\_of\_transforming\_growth\_factor-beta1\_production | 2 | 0 |  |  |  |  |  |  |  |  |
| GO:0032933\_SREBP-mediated\_signaling\_pathway | 2 | 0 |  |  |  |  |  |  |  |  |
| GO:0032957\_inositol\_trisphosphate\_metabolic\_process | 2 | 0 |  |  |  |  |  |  |  |  |
| GO:0032958\_inositol\_phosphate\_biosynthetic\_process | 2 | 0 |  |  |  |  |  |  |  |  |
| GO:0032959\_inositol\_trisphosphate\_biosynthetic\_process | 2 | 0 |  |  |  |  |  |  |  |  |
| GO:0033092\_positive\_regulation\_of\_immature\_T\_cell\_proliferation\_in\_the\_thymus | 2 | 0 |  |  |  |  |  |  |  |  |
| GO:0033119\_negative\_regulation\_of\_RNA\_splicing | 2 | 0 |  |  |  |  |  |  |  |  |
| GO:0033136\_serine\_phosphorylation\_of\_STAT3\_protein | 2 | 0 |  |  |  |  |  |  |  |  |
| GO:0033145\_positive\_regulation\_of\_steroid\_hormone\_receptor\_signaling\_pathway | 2 | 0 |  |  |  |  |  |  |  |  |
| GO:0033147\_negative\_regulation\_of\_estrogen\_receptor\_signaling\_pathway | 2 | 0 |  |  |  |  |  |  |  |  |
| GO:0033148\_positive\_regulation\_of\_estrogen\_receptor\_signaling\_pathway | 2 | 0 |  |  |  |  |  |  |  |  |
| GO:0033194\_response\_to\_hydroperoxide | 2 | 0 |  |  |  |  |  |  |  |  |
| GO:0033275\_actin-myosin\_filament\_sliding | 2 | 0 |  |  |  |  |  |  |  |  |
| GO:0033280\_response\_to\_vitamin\_D | 2 | 0 |  |  |  |  |  |  |  |  |
| GO:0033364\_mast\_cell\_secretory\_granule\_organization | 2 | 0 |  |  |  |  |  |  |  |  |
| GO:0033504\_floor\_plate\_development | 2 | 0 |  |  |  |  |  |  |  |  |
| GO:0033603\_positive\_regulation\_of\_dopamine\_secretion | 2 | 0 |  |  |  |  |  |  |  |  |
| GO:0033605\_positive\_regulation\_of\_catecholamine\_secretion | 2 | 0 |  |  |  |  |  |  |  |  |
| GO:0033622\_integrin\_activation | 2 | 0 |  |  |  |  |  |  |  |  |
| GO:0033623\_regulation\_of\_integrin\_activation | 2 | 0 |  |  |  |  |  |  |  |  |
| GO:0033625\_positive\_regulation\_of\_integrin\_activation | 2 | 0 |  |  |  |  |  |  |  |  |
| GO:0033700\_phospholipid\_efflux | 2 | 0 |  |  |  |  |  |  |  |  |
| GO:0034142\_toll-like\_receptor\_4\_signaling\_pathway | 2 | 0 |  |  |  |  |  |  |  |  |
| GO:0034310\_monohydric\_alcohol\_catabolic\_process | 2 | 0 |  |  |  |  |  |  |  |  |
| GO:0034341\_response\_to\_interferon-gamma | 2 | 0 |  |  |  |  |  |  |  |  |
| GO:0034370\_triglyceride-rich\_lipoprotein\_particle\_remodeling | 2 | 0 |  |  |  |  |  |  |  |  |
| GO:0034374\_low-density\_lipoprotein\_particle\_remodeling | 2 | 0 |  |  |  |  |  |  |  |  |
| GO:0034377\_plasma\_lipoprotein\_particle\_assembly | 2 | 0 |  |  |  |  |  |  |  |  |
| GO:0034384\_high-density\_lipoprotein\_particle\_clearance | 2 | 0 |  |  |  |  |  |  |  |  |
| GO:0034433\_steroid\_esterification | 2 | 0 |  |  |  |  |  |  |  |  |
| GO:0034434\_sterol\_esterification | 2 | 0 |  |  |  |  |  |  |  |  |
| GO:0034435\_cholesterol\_esterification | 2 | 0 |  |  |  |  |  |  |  |  |
| GO:0034453\_microtubule\_anchoring | 2 | 0 |  |  |  |  |  |  |  |  |
| GO:0034644\_cellular\_response\_to\_UV | 2 | 0 |  |  |  |  |  |  |  |  |
| GO:0034755\_iron\_ion\_transmembrane\_transport | 2 | 0 |  |  |  |  |  |  |  |  |
| GO:0034764\_positive\_regulation\_of\_transmembrane\_transport | 2 | 0 |  |  |  |  |  |  |  |  |
| GO:0035021\_negative\_regulation\_of\_Rac\_protein\_signal\_transduction | 2 | 0 |  |  |  |  |  |  |  |  |
| GO:0035054\_embryonic\_heart\_tube\_anterior\_posterior\_pattern\_formation | 2 | 0 |  |  |  |  |  |  |  |  |
| GO:0035092\_sperm\_chromatin\_condensation | 2 | 0 |  |  |  |  |  |  |  |  |
| GO:0035110\_leg\_morphogenesis | 2 | 0 |  |  |  |  |  |  |  |  |
| GO:0035117\_embryonic\_arm\_morphogenesis | 2 | 0 |  |  |  |  |  |  |  |  |
| GO:0035120\_post-embryonic\_appendage\_morphogenesis | 2 | 0 |  |  |  |  |  |  |  |  |
| GO:0035127\_post-embryonic\_limb\_morphogenesis | 2 | 0 |  |  |  |  |  |  |  |  |
| GO:0035129\_post-embryonic\_hindlimb\_morphogenesis | 2 | 0 |  |  |  |  |  |  |  |  |
| GO:0035140\_arm\_morphogenesis | 2 | 0 |  |  |  |  |  |  |  |  |
| GO:0035194\_posttranscriptional\_gene\_silencing\_by\_RNA | 2 | 0 |  |  |  |  |  |  |  |  |
| GO:0035195\_gene\_silencing\_by\_miRNA | 2 | 0 |  |  |  |  |  |  |  |  |
| GO:0035196\_gene\_silencing\_by\_miRNA\_\_production\_of\_miRNAs | 2 | 0 |  |  |  |  |  |  |  |  |
| GO:0035315\_hair\_cell\_differentiation | 2 | 0 |  |  |  |  |  |  |  |  |
| GO:0040009\_regulation\_of\_growth\_rate | 2 | 0 |  |  |  |  |  |  |  |  |
| GO:0040037\_negative\_regulation\_of\_fibroblast\_growth\_factor\_receptor\_signaling\_pathway | 2 | 0 |  |  |  |  |  |  |  |  |
| GO:0042119\_neutrophil\_activation | 2 | 0 |  |  |  |  |  |  |  |  |
| GO:0042147\_retrograde\_transport\_\_endosome\_to\_Golgi | 2 | 0 |  |  |  |  |  |  |  |  |
| GO:0042223\_interleukin-3\_biosynthetic\_process | 2 | 0 |  |  |  |  |  |  |  |  |
| GO:0042249\_establishment\_of\_polarity\_of\_embryonic\_epithelium | 2 | 0 |  |  |  |  |  |  |  |  |
| GO:0042253\_granulocyte\_macrophage\_colony-stimulating\_factor\_biosynthetic\_process | 2 | 0 |  |  |  |  |  |  |  |  |
| GO:0042270\_protection\_from\_natural\_killer\_cell\_mediated\_cytotoxicity | 2 | 0 |  |  |  |  |  |  |  |  |
| GO:0042274\_ribosomal\_small\_subunit\_biogenesis | 2 | 0 |  |  |  |  |  |  |  |  |
| GO:0042312\_regulation\_of\_vasodilation | 2 | 0 |  |  |  |  |  |  |  |  |
| GO:0042346\_positive\_regulation\_of\_NF-kappaB\_import\_into\_nucleus | 2 | 0 |  |  |  |  |  |  |  |  |
| GO:0042396\_phosphagen\_biosynthetic\_process | 2 | 0 |  |  |  |  |  |  |  |  |
| GO:0042454\_ribonucleoside\_catabolic\_process | 2 | 0 |  |  |  |  |  |  |  |  |
| GO:0042482\_positive\_regulation\_of\_odontogenesis | 2 | 0 |  |  |  |  |  |  |  |  |
| GO:0042483\_negative\_regulation\_of\_odontogenesis | 2 | 0 |  |  |  |  |  |  |  |  |
| GO:0042488\_positive\_regulation\_of\_odontogenesis\_of\_dentine-containing\_tooth | 2 | 0 |  |  |  |  |  |  |  |  |
| GO:0042501\_serine\_phosphorylation\_of\_STAT\_protein | 2 | 0 |  |  |  |  |  |  |  |  |
| GO:0042517\_positive\_regulation\_of\_tyrosine\_phosphorylation\_of\_Stat3\_protein | 2 | 0 |  |  |  |  |  |  |  |  |
| GO:0042532\_negative\_regulation\_of\_tyrosine\_phosphorylation\_of\_STAT\_protein | 2 | 0 |  |  |  |  |  |  |  |  |
| GO:0042559\_pteridine\_and\_derivative\_biosynthetic\_process | 2 | 0 |  |  |  |  |  |  |  |  |
| GO:0042730\_fibrinolysis | 2 | 0 |  |  |  |  |  |  |  |  |
| GO:0042749\_regulation\_of\_circadian\_sleep\_wake\_cycle | 2 | 0 |  |  |  |  |  |  |  |  |
| GO:0042886\_amide\_transport | 2 | 0 |  |  |  |  |  |  |  |  |
| GO:0042921\_glucocorticoid\_receptor\_signaling\_pathway | 2 | 0 |  |  |  |  |  |  |  |  |
| GO:0042987\_amyloid\_precursor\_protein\_catabolic\_process | 2 | 0 |  |  |  |  |  |  |  |  |
| GO:0042993\_positive\_regulation\_of\_transcription\_factor\_import\_into\_nucleus | 2 | 0 |  |  |  |  |  |  |  |  |
| GO:0042994\_cytoplasmic\_sequestering\_of\_transcription\_factor | 2 | 0 |  |  |  |  |  |  |  |  |
| GO:0043032\_positive\_regulation\_of\_macrophage\_activation | 2 | 0 |  |  |  |  |  |  |  |  |
| GO:0043038\_amino\_acid\_activation | 2 | 0 |  |  |  |  |  |  |  |  |
| GO:0043039\_tRNA\_aminoacylation | 2 | 0 |  |  |  |  |  |  |  |  |
| GO:0043084\_penile\_erection | 2 | 0 |  |  |  |  |  |  |  |  |
| GO:0043088\_regulation\_of\_Cdc42\_GTPase\_activity | 2 | 0 |  |  |  |  |  |  |  |  |
| GO:0043089\_positive\_regulation\_of\_Cdc42\_GTPase\_activity | 2 | 0 |  |  |  |  |  |  |  |  |
| GO:0043247\_telomere\_maintenance\_in\_response\_to\_DNA\_damage | 2 | 0 |  |  |  |  |  |  |  |  |
| GO:0043297\_apical\_junction\_assembly | 2 | 0 |  |  |  |  |  |  |  |  |
| GO:0043312\_neutrophil\_degranulation | 2 | 0 |  |  |  |  |  |  |  |  |
| GO:0043320\_natural\_killer\_cell\_degranulation | 2 | 0 |  |  |  |  |  |  |  |  |
| GO:0043366\_beta\_selection | 2 | 0 |  |  |  |  |  |  |  |  |
| GO:0043450\_alkene\_biosynthetic\_process | 2 | 0 |  |  |  |  |  |  |  |  |
| GO:0043476\_pigment\_accumulation | 2 | 0 |  |  |  |  |  |  |  |  |
| GO:0043490\_malate-aspartate\_shuttle | 2 | 0 |  |  |  |  |  |  |  |  |
| GO:0043502\_regulation\_of\_muscle\_adaptation | 2 | 0 |  |  |  |  |  |  |  |  |
| GO:0043516\_regulation\_of\_DNA\_damage\_response\_\_signal\_transduction\_by\_p53\_class\_mediator | 2 | 0 |  |  |  |  |  |  |  |  |
| GO:0043568\_positive\_regulation\_of\_insulin-like\_growth\_factor\_receptor\_signaling\_pathway | 2 | 0 |  |  |  |  |  |  |  |  |
| GO:0043589\_skin\_morphogenesis | 2 | 0 |  |  |  |  |  |  |  |  |
| GO:0043618\_regulation\_of\_transcription\_from\_RNA\_polymerase\_II\_promoter\_in\_response\_to\_stress | 2 | 0 |  |  |  |  |  |  |  |  |
| GO:0043619\_regulation\_of\_transcription\_from\_RNA\_polymerase\_II\_promoter\_in\_response\_to\_oxidative\_stress | 2 | 0 |  |  |  |  |  |  |  |  |
| GO:0043620\_regulation\_of\_transcription\_in\_response\_to\_stress | 2 | 0 |  |  |  |  |  |  |  |  |
| GO:0043647\_inositol\_phosphate\_metabolic\_process | 2 | 0 |  |  |  |  |  |  |  |  |
| GO:0043654\_recognition\_of\_apoptotic\_cell | 2 | 0 |  |  |  |  |  |  |  |  |
| GO:0043966\_histone\_H3\_acetylation | 2 | 0 |  |  |  |  |  |  |  |  |
| GO:0043967\_histone\_H4\_acetylation | 2 | 0 |  |  |  |  |  |  |  |  |
| GO:0044070\_regulation\_of\_anion\_transport | 2 | 0 |  |  |  |  |  |  |  |  |
| GO:0044246\_regulation\_of\_multicellular\_organismal\_metabolic\_process | 2 | 0 |  |  |  |  |  |  |  |  |
| GO:0044253\_positive\_regulation\_of\_multicellular\_organismal\_metabolic\_process | 2 | 0 |  |  |  |  |  |  |  |  |
| GO:0044268\_multicellular\_organismal\_protein\_metabolic\_process | 2 | 0 |  |  |  |  |  |  |  |  |
| GO:0045005\_maintenance\_of\_fidelity\_during\_DNA-dependent\_DNA\_replication | 2 | 0 |  |  |  |  |  |  |  |  |
| GO:0045010\_actin\_nucleation | 2 | 0 |  |  |  |  |  |  |  |  |
| GO:0045065\_cytotoxic\_T\_cell\_differentiation | 2 | 0 |  |  |  |  |  |  |  |  |
| GO:0045077\_negative\_regulation\_of\_interferon-gamma\_biosynthetic\_process | 2 | 0 |  |  |  |  |  |  |  |  |
| GO:0045079\_negative\_regulation\_of\_chemokine\_biosynthetic\_process | 2 | 0 |  |  |  |  |  |  |  |  |
| GO:0045116\_protein\_neddylation | 2 | 0 |  |  |  |  |  |  |  |  |
| GO:0045187\_regulation\_of\_circadian\_sleep\_wake\_cycle\_\_sleep | 2 | 0 |  |  |  |  |  |  |  |  |
| GO:0045212\_neurotransmitter\_receptor\_biosynthetic\_process | 2 | 0 |  |  |  |  |  |  |  |  |
| GO:0045399\_regulation\_of\_interleukin-3\_biosynthetic\_process | 2 | 0 |  |  |  |  |  |  |  |  |
| GO:0045401\_positive\_regulation\_of\_interleukin-3\_biosynthetic\_process | 2 | 0 |  |  |  |  |  |  |  |  |
| GO:0045409\_negative\_regulation\_of\_interleukin-6\_biosynthetic\_process | 2 | 0 |  |  |  |  |  |  |  |  |
| GO:0045423\_regulation\_of\_granulocyte\_macrophage\_colony-stimulating\_factor\_biosynthetic\_process | 2 | 0 |  |  |  |  |  |  |  |  |
| GO:0045425\_positive\_regulation\_of\_granulocyte\_macrophage\_colony-stimulating\_factor\_biosynthetic\_process | 2 | 0 |  |  |  |  |  |  |  |  |
| GO:0045475\_locomotor\_rhythm | 2 | 0 |  |  |  |  |  |  |  |  |
| GO:0045578\_negative\_regulation\_of\_B\_cell\_differentiation | 2 | 0 |  |  |  |  |  |  |  |  |
| GO:0045589\_regulation\_of\_regulatory\_T\_cell\_differentiation | 2 | 0 |  |  |  |  |  |  |  |  |
| GO:0045591\_positive\_regulation\_of\_regulatory\_T\_cell\_differentiation | 2 | 0 |  |  |  |  |  |  |  |  |
| GO:0045608\_negative\_regulation\_of\_auditory\_receptor\_cell\_differentiation | 2 | 0 |  |  |  |  |  |  |  |  |
| GO:0045627\_positive\_regulation\_of\_T-helper\_1\_cell\_differentiation | 2 | 0 |  |  |  |  |  |  |  |  |
| GO:0045629\_negative\_regulation\_of\_T-helper\_2\_cell\_differentiation | 2 | 0 |  |  |  |  |  |  |  |  |
| GO:0045630\_positive\_regulation\_of\_T-helper\_2\_cell\_differentiation | 2 | 0 |  |  |  |  |  |  |  |  |
| GO:0045632\_negative\_regulation\_of\_mechanoreceptor\_differentiation | 2 | 0 |  |  |  |  |  |  |  |  |
| GO:0045636\_positive\_regulation\_of\_melanocyte\_differentiation | 2 | 0 |  |  |  |  |  |  |  |  |
| GO:0045655\_regulation\_of\_monocyte\_differentiation | 2 | 0 |  |  |  |  |  |  |  |  |
| GO:0045658\_regulation\_of\_neutrophil\_differentiation | 2 | 0 |  |  |  |  |  |  |  |  |
| GO:0045662\_negative\_regulation\_of\_myoblast\_differentiation | 2 | 0 |  |  |  |  |  |  |  |  |
| GO:0045663\_positive\_regulation\_of\_myoblast\_differentiation | 2 | 0 |  |  |  |  |  |  |  |  |
| GO:0045683\_negative\_regulation\_of\_epidermis\_development | 2 | 0 |  |  |  |  |  |  |  |  |
| GO:0045737\_positive\_regulation\_of\_cyclin-dependent\_protein\_kinase\_activity | 2 | 0 |  |  |  |  |  |  |  |  |
| GO:0045739\_positive\_regulation\_of\_DNA\_repair | 2 | 0 |  |  |  |  |  |  |  |  |
| GO:0045741\_positive\_regulation\_of\_epidermal\_growth\_factor\_receptor\_activity | 2 | 0 |  |  |  |  |  |  |  |  |
| GO:0045743\_positive\_regulation\_of\_fibroblast\_growth\_factor\_receptor\_signaling\_pathway | 2 | 0 |  |  |  |  |  |  |  |  |
| GO:0045749\_negative\_regulation\_of\_S\_phase\_of\_mitotic\_cell\_cycle | 2 | 0 |  |  |  |  |  |  |  |  |
| GO:0045819\_positive\_regulation\_of\_glycogen\_catabolic\_process | 2 | 0 |  |  |  |  |  |  |  |  |
| GO:0045821\_positive\_regulation\_of\_glycolysis | 2 | 0 |  |  |  |  |  |  |  |  |
| GO:0045835\_negative\_regulation\_of\_meiosis | 2 | 0 |  |  |  |  |  |  |  |  |
| GO:0045836\_positive\_regulation\_of\_meiosis | 2 | 0 |  |  |  |  |  |  |  |  |
[truncated: 257,470 more chars]
