# Supplementary material for: RedundancyMiner: De-replication of redundant GO categories in microarray and proteomics analysis
Source: BMC Bioinformatics. 2011 Feb 10;12:52. doi: 10.1186/1471-2105-12-52 (PMC3223614; doi:10.1186/1471-2105-12-52)
Supplement: Additional file 8 — Retinal development HTGM download. compressed package of the results of running HTGM on the retinal development genes list. [file 1471-2105-12-52-S8.ZIP › SCENARIO_2_MODIFIED/total.txt.total.txt.dir/Exp1_BestClusterMap_LEIGS_KM_24.csv.join.16.txt.dir/Exp1_BestClusterMap_LEIGS_KM_24.csv.join.16.txt.change.gce.html]

Gene Category Report for Exp1\_BestClusterMap\_LEIGS\_KM\_24.csv.join.16.txt

# Gene Category Report for Exp1\_BestClusterMap\_LEIGS\_KM\_24.csv.join.16.txt

| HYPERLINKED GO CATEGORY | HYPERLINKED GENE NAME | TOTAL GENES | CHANGED GENES | ENRICHMENT | LOG10(p) | CUMULATIVE NUMBER OF CATEGORIES | CUMULATIVE RANDOMS MEAN | FALSE DISCOVERY RATE |
| --- | --- | --- | --- | --- | --- | --- | --- | --- |
| GO:0045785\_positive\_regulation\_of\_cell\_adhesion | ITGA6 | 37 | 3 | 7.778716 | -2.190980 | 1 | 7.59 | 7.590000 |
| GO:0045785\_positive\_regulation\_of\_cell\_adhesion | NID1 | 37 | 3 | 7.778716 | -2.190980 | 1 | 7.59 | 7.590000 |
| GO:0045785\_positive\_regulation\_of\_cell\_adhesion | COL8A1 | 37 | 3 | 7.778716 | -2.190980 | 1 | 7.59 | 7.590000 |
| GO:0000375\_RNA\_splicing\_\_via\_transesterification\_reactions | FUSIP1 | 12 | 2 | 15.989583 | -2.182424 | 4 | 8.13 | 2.032500 |
| GO:0000375\_RNA\_splicing\_\_via\_transesterification\_reactions | CUGBP2 | 12 | 2 | 15.989583 | -2.182424 | 4 | 8.13 | 2.032500 |
| GO:0000377\_RNA\_splicing\_\_via\_transesterification\_reactions\_with\_bulged\_adenosine\_as\_nucleophile | FUSIP1 | 12 | 2 | 15.989583 | -2.182424 | 4 | 8.13 | 2.032500 |
| GO:0000377\_RNA\_splicing\_\_via\_transesterification\_reactions\_with\_bulged\_adenosine\_as\_nucleophile | CUGBP2 | 12 | 2 | 15.989583 | -2.182424 | 4 | 8.13 | 2.032500 |
| GO:0000398\_nuclear\_mRNA\_splicing\_\_via\_spliceosome | FUSIP1 | 12 | 2 | 15.989583 | -2.182424 | 4 | 8.13 | 2.032500 |
| GO:0000398\_nuclear\_mRNA\_splicing\_\_via\_spliceosome | CUGBP2 | 12 | 2 | 15.989583 | -2.182424 | 4 | 8.13 | 2.032500 |
| GO:0002316\_follicular\_B\_cell\_differentiation | PLCG2 | 1 | 1 |  |  |  |  |  |  |
| GO:0006596\_polyamine\_biosynthetic\_process | AMD1 | 1 | 1 |  |  |  |  |  |  |
| GO:0006597\_spermine\_biosynthetic\_process | AMD1 | 1 | 1 |  |  |  |  |  |  |
| GO:0008216\_spermidine\_metabolic\_process | AMD1 | 1 | 1 |  |  |  |  |  |  |
| GO:0008295\_spermidine\_biosynthetic\_process | AMD1 | 1 | 1 |  |  |  |  |  |  |
| GO:0016482\_cytoplasmic\_transport | FUSIP1 | 1 | 1 |  |  |  |  |  |  |
| GO:0019255\_glucose\_1-phosphate\_metabolic\_process | PGM3 | 1 | 1 |  |  |  |  |  |  |
| GO:0032237\_activation\_of\_store-operated\_calcium\_channel\_activity | PLCG2 | 1 | 1 |  |  |  |  |  |  |
| GO:0032836\_glomerular\_basement\_membrane\_development | NID1 | 1 | 1 |  |  |  |  |  |  |
| GO:0006396\_RNA\_processing | FUSIP1 | 47 | 3 | 6.123670 | -1.903030 | 5 | 13.17 | 2.634000 |
| GO:0006396\_RNA\_processing | WDR55 | 47 | 3 | 6.123670 | -1.903030 | 5 | 13.17 | 2.634000 |
| GO:0006396\_RNA\_processing | CUGBP2 | 47 | 3 | 6.123670 | -1.903030 | 5 | 13.17 | 2.634000 |
| GO:0008380\_RNA\_splicing | FUSIP1 | 17 | 2 | 11.286765 | -1.882824 | 6 | 13.9 | 2.316667 |
| GO:0008380\_RNA\_splicing | CUGBP2 | 17 | 2 | 11.286765 | -1.882824 | 6 | 13.9 | 2.316667 |
| GO:0007601\_visual\_perception | GUCA1B | 51 | 3 | 5.643382 | -1.806947 | 7 | 15.7 | 2.242857 |
| GO:0007601\_visual\_perception | ABCA4 | 51 | 3 | 5.643382 | -1.806947 | 7 | 15.7 | 2.242857 |
| GO:0007601\_visual\_perception | MFRP | 51 | 3 | 5.643382 | -1.806947 | 7 | 15.7 | 2.242857 |
| GO:0046165\_alcohol\_biosynthetic\_process | PGM3 | 19 | 2 | 10.098684 | -1.789111 | 8 | 16.32 | 2.040000 |
| GO:0046165\_alcohol\_biosynthetic\_process | PLCG2 | 19 | 2 | 10.098684 | -1.789111 | 8 | 16.32 | 2.040000 |
| GO:0050953\_sensory\_perception\_of\_light\_stimulus | GUCA1B | 52 | 3 | 5.534856 | -1.784283 | 9 | 16.41 | 1.823333 |
| GO:0050953\_sensory\_perception\_of\_light\_stimulus | ABCA4 | 52 | 3 | 5.534856 | -1.784283 | 9 | 16.41 | 1.823333 |
| GO:0050953\_sensory\_perception\_of\_light\_stimulus | MFRP | 52 | 3 | 5.534856 | -1.784283 | 9 | 16.41 | 1.823333 |
| GO:0006021\_inositol\_biosynthetic\_process | PLCG2 | 2 | 1 |  |  |  |  |  |  |
| GO:0006042\_glucosamine\_biosynthetic\_process | PGM3 | 2 | 1 |  |  |  |  |  |  |
| GO:0006045\_N-acetylglucosamine\_biosynthetic\_process | PGM3 | 2 | 1 |  |  |  |  |  |  |
| GO:0006048\_UDP-N-acetylglucosamine\_biosynthetic\_process | PGM3 | 2 | 1 |  |  |  |  |  |  |
| GO:0006649\_phospholipid\_transfer\_to\_membrane | ABCA4 | 2 | 1 |  |  |  |  |  |  |
| GO:0032234\_regulation\_of\_calcium\_ion\_transport\_via\_store-operated\_calcium\_channel\_activity | PLCG2 | 2 | 1 |  |  |  |  |  |  |
| GO:0032236\_positive\_regulation\_of\_calcium\_ion\_transport\_via\_store-operated\_calcium\_channel\_activity | PLCG2 | 2 | 1 |  |  |  |  |  |  |
| GO:0032957\_inositol\_trisphosphate\_metabolic\_process | PLCG2 | 2 | 1 |  |  |  |  |  |  |
| GO:0032958\_inositol\_phosphate\_biosynthetic\_process | PLCG2 | 2 | 1 |  |  |  |  |  |  |
| GO:0032959\_inositol\_trisphosphate\_biosynthetic\_process | PLCG2 | 2 | 1 |  |  |  |  |  |  |
| GO:0033119\_negative\_regulation\_of\_RNA\_splicing | FUSIP1 | 2 | 1 |  |  |  |  |  |  |
| GO:0043647\_inositol\_phosphate\_metabolic\_process | PLCG2 | 2 | 1 |  |  |  |  |  |  |
| GO:0046349\_amino\_sugar\_biosynthetic\_process | PGM3 | 2 | 1 |  |  |  |  |  |  |
| GO:0048025\_negative\_regulation\_of\_nuclear\_mRNA\_splicing\_\_via\_spliceosome | FUSIP1 | 2 | 1 |  |  |  |  |  |  |
| GO:0048670\_regulation\_of\_collateral\_sprouting | ULK2 | 2 | 1 |  |  |  |  |  |  |
| GO:0048671\_negative\_regulation\_of\_collateral\_sprouting | ULK2 | 2 | 1 |  |  |  |  |  |  |
| GO:0050686\_negative\_regulation\_of\_mRNA\_processing | FUSIP1 | 2 | 1 |  |  |  |  |  |  |
| GO:0006397\_mRNA\_processing | FUSIP1 | 23 | 2 | 8.342391 | -1.630450 | 12 | 20.97 | 1.747500 |
| GO:0006397\_mRNA\_processing | CUGBP2 | 23 | 2 | 8.342391 | -1.630450 | 12 | 20.97 | 1.747500 |
| GO:0007163\_establishment\_or\_maintenance\_of\_cell\_polarity | SYNE2 | 23 | 2 | 8.342391 | -1.630450 | 12 | 20.97 | 1.747500 |
| GO:0007163\_establishment\_or\_maintenance\_of\_cell\_polarity | BRSK2 | 23 | 2 | 8.342391 | -1.630450 | 12 | 20.97 | 1.747500 |
| GO:0022613\_ribonucleoprotein\_complex\_biogenesis | WDR55 | 23 | 2 | 8.342391 | -1.630450 | 12 | 20.97 | 1.747500 |
| GO:0022613\_ribonucleoprotein\_complex\_biogenesis | CUGBP2 | 23 | 2 | 8.342391 | -1.630450 | 12 | 20.97 | 1.747500 |
| GO:0000902\_cell\_morphogenesis | SYNE2 | 283 | 7 | 2.373012 | -1.588059 | 13 | 22.36 | 1.720000 |
| GO:0000902\_cell\_morphogenesis | NTF3 | 283 | 7 | 2.373012 | -1.588059 | 13 | 22.36 | 1.720000 |
| GO:0000902\_cell\_morphogenesis | BCL11B | 283 | 7 | 2.373012 | -1.588059 | 13 | 22.36 | 1.720000 |
| GO:0000902\_cell\_morphogenesis | ULK2 | 283 | 7 | 2.373012 | -1.588059 | 13 | 22.36 | 1.720000 |
| GO:0000902\_cell\_morphogenesis | BRSK2 | 283 | 7 | 2.373012 | -1.588059 | 13 | 22.36 | 1.720000 |
| GO:0000902\_cell\_morphogenesis | SOX6 | 283 | 7 | 2.373012 | -1.588059 | 13 | 22.36 | 1.720000 |
| GO:0000902\_cell\_morphogenesis | DST | 283 | 7 | 2.373012 | -1.588059 | 13 | 22.36 | 1.720000 |
| GO:0030155\_regulation\_of\_cell\_adhesion | ITGA6 | 62 | 3 | 4.642137 | -1.582320 | 14 | 22.54 | 1.610000 |
| GO:0030155\_regulation\_of\_cell\_adhesion | NID1 | 62 | 3 | 4.642137 | -1.582320 | 14 | 22.54 | 1.610000 |
| GO:0030155\_regulation\_of\_cell\_adhesion | COL8A1 | 62 | 3 | 4.642137 | -1.582320 | 14 | 22.54 | 1.610000 |
| GO:0010811\_positive\_regulation\_of\_cell-substrate\_adhesion | NID1 | 25 | 2 | 7.675000 | -1.562169 | 15 | 23.51 | 1.567333 |
| GO:0010811\_positive\_regulation\_of\_cell-substrate\_adhesion | COL8A1 | 25 | 2 | 7.675000 | -1.562169 | 15 | 23.51 | 1.567333 |
| GO:0031589\_cell-substrate\_adhesion | ITGA6 | 66 | 3 | 4.360795 | -1.512067 | 16 | 25.11 | 1.569375 |
| GO:0031589\_cell-substrate\_adhesion | NID1 | 66 | 3 | 4.360795 | -1.512067 | 16 | 25.11 | 1.569375 |
| GO:0031589\_cell-substrate\_adhesion | COL8A1 | 66 | 3 | 4.360795 | -1.512067 | 16 | 25.11 | 1.569375 |
| GO:0000320\_re-entry\_into\_mitotic\_cell\_cycle | CCNF | 3 | 1 |  |  |  |  |  |  |
| GO:0002568\_somatic\_diversification\_of\_T\_cell\_receptor\_genes | BCL11B | 3 | 1 |  |  |  |  |  |  |
| GO:0002681\_somatic\_recombination\_of\_T\_cell\_receptor\_gene\_segments | BCL11B | 3 | 1 |  |  |  |  |  |  |
| GO:0006047\_UDP-N-acetylglucosamine\_metabolic\_process | PGM3 | 3 | 1 |  |  |  |  |  |  |
| GO:0007000\_nucleolus\_organization | NOLC1 | 3 | 1 |  |  |  |  |  |  |
| GO:0007403\_glial\_cell\_fate\_determination | NTF3 | 3 | 1 |  |  |  |  |  |  |
| GO:0008090\_retrograde\_axon\_cargo\_transport | DST | 3 | 1 |  |  |  |  |  |  |
| GO:0031282\_regulation\_of\_guanylate\_cyclase\_activity | GUCA1B | 3 | 1 |  |  |  |  |  |  |
| GO:0032411\_positive\_regulation\_of\_transporter\_activity | PLCG2 | 3 | 1 |  |  |  |  |  |  |
| GO:0032414\_positive\_regulation\_of\_ion\_transmembrane\_transporter\_activity | PLCG2 | 3 | 1 |  |  |  |  |  |  |
| GO:0033153\_T\_cell\_receptor\_V(D)J\_recombination | BCL11B | 3 | 1 |  |  |  |  |  |  |
| GO:0046488\_phosphatidylinositol\_metabolic\_process | PIP5K1B | 3 | 1 |  |  |  |  |  |  |
| GO:0048668\_collateral\_sprouting | ULK2 | 3 | 1 |  |  |  |  |  |  |
| GO:0006997\_nucleus\_organization | SYNE2 | 28 | 2 | 6.852679 | -1.470362 | 17 | 27.04 | 1.590588 |
| GO:0006997\_nucleus\_organization | NOLC1 | 28 | 2 | 6.852679 | -1.470362 | 17 | 27.04 | 1.590588 |
| GO:0032989\_cellular\_component\_morphogenesis | SYNE2 | 307 | 7 | 2.187500 | -1.420292 | 18 | 29.31 | 1.628333 |
| GO:0032989\_cellular\_component\_morphogenesis | NTF3 | 307 | 7 | 2.187500 | -1.420292 | 18 | 29.31 | 1.628333 |
| GO:0032989\_cellular\_component\_morphogenesis | BCL11B | 307 | 7 | 2.187500 | -1.420292 | 18 | 29.31 | 1.628333 |
| GO:0032989\_cellular\_component\_morphogenesis | ULK2 | 307 | 7 | 2.187500 | -1.420292 | 18 | 29.31 | 1.628333 |
| GO:0032989\_cellular\_component\_morphogenesis | BRSK2 | 307 | 7 | 2.187500 | -1.420292 | 18 | 29.31 | 1.628333 |
| GO:0032989\_cellular\_component\_morphogenesis | SOX6 | 307 | 7 | 2.187500 | -1.420292 | 18 | 29.31 | 1.628333 |
| GO:0032989\_cellular\_component\_morphogenesis | DST | 307 | 7 | 2.187500 | -1.420292 | 18 | 29.31 | 1.628333 |
| GO:0044262\_cellular\_carbohydrate\_metabolic\_process | PGM3 | 72 | 3 | 3.997396 | -1.415744 | 19 | 29.67 | 1.561579 |
| GO:0044262\_cellular\_carbohydrate\_metabolic\_process | GALNT10 | 72 | 3 | 3.997396 | -1.415744 | 19 | 29.67 | 1.561579 |
| GO:0044262\_cellular\_carbohydrate\_metabolic\_process | HK2 | 72 | 3 | 3.997396 | -1.415744 | 19 | 29.67 | 1.561579 |
| GO:0002312\_B\_cell\_activation\_during\_immune\_response | PLCG2 | 4 | 1 |  |  |  |  |  |  |
| GO:0002313\_mature\_B\_cell\_differentiation\_during\_immune\_response | PLCG2 | 4 | 1 |  |  |  |  |  |  |
| GO:0006835\_dicarboxylic\_acid\_transport | SLC13A3 | 4 | 1 |  |  |  |  |  |  |
| GO:0008215\_spermine\_metabolic\_process | AMD1 | 4 | 1 |  |  |  |  |  |  |
| GO:0009225\_nucleotide-sugar\_metabolic\_process | PGM3 | 4 | 1 |  |  |  |  |  |  |
| GO:0030826\_regulation\_of\_cGMP\_biosynthetic\_process | GUCA1B | 4 | 1 |  |  |  |  |  |  |
| GO:0032835\_glomerulus\_development | NID1 | 4 | 1 |  |  |  |  |  |  |
| GO:0043129\_surfactant\_homeostasis | KDR | 4 | 1 |  |  |  |  |  |  |
| GO:0043484\_regulation\_of\_RNA\_splicing | FUSIP1 | 4 | 1 |  |  |  |  |  |  |
| GO:0046173\_polyol\_biosynthetic\_process | PLCG2 | 4 | 1 |  |  |  |  |  |  |
| GO:0046835\_carbohydrate\_phosphorylation | HK2 | 4 | 1 |  |  |  |  |  |  |
| GO:0048024\_regulation\_of\_nuclear\_mRNA\_splicing\_\_via\_spliceosome | FUSIP1 | 4 | 1 |  |  |  |  |  |  |
| GO:0048484\_enteric\_nervous\_system\_development | NTF3 | 4 | 1 |  |  |  |  |  |  |
| GO:0048875\_chemical\_homeostasis\_within\_a\_tissue | KDR | 4 | 1 |  |  |  |  |  |  |
| GO:0010810\_regulation\_of\_cell-substrate\_adhesion | NID1 | 35 | 2 | 5.482143 | -1.293244 | 20 | 38.23 | 1.911500 |
| GO:0010810\_regulation\_of\_cell-substrate\_adhesion | COL8A1 | 35 | 2 | 5.482143 | -1.293244 | 20 | 38.23 | 1.911500 |
| GO:0006376\_mRNA\_splice\_site\_selection | CUGBP2 | 5 | 1 | 19.187500 | -1.291887 | 25 | 52.17 | 2.086800 |
| GO:0010761\_fibroblast\_migration | SYNE2 | 5 | 1 | 19.187500 | -1.291887 | 25 | 52.17 | 2.086800 |
| GO:0030823\_regulation\_of\_cGMP\_metabolic\_process | GUCA1B | 5 | 1 | 19.187500 | -1.291887 | 25 | 52.17 | 2.086800 |
| GO:0031122\_cytoplasmic\_microtubule\_organization | DST | 5 | 1 | 19.187500 | -1.291887 | 25 | 52.17 | 2.086800 |
| GO:0033627\_cell\_adhesion\_mediated\_by\_integrin | ITGA6 | 5 | 1 | 19.187500 | -1.291887 | 25 | 52.17 | 2.086800 |
| GO:0022602\_ovulation\_cycle\_process | ADAMTS1 | 36 | 2 | 5.329861 | -1.271256 | 26 | 53.87 | 2.071923 |
| GO:0022602\_ovulation\_cycle\_process | KDR | 36 | 2 | 5.329861 | -1.271256 | 26 | 53.87 | 2.071923 |
| GO:0042698\_ovulation\_cycle | ADAMTS1 | 37 | 2 | 5.185811 | -1.249956 | 27 | 55.22 | 2.045185 |
| GO:0042698\_ovulation\_cycle | KDR | 37 | 2 | 5.185811 | -1.249956 | 27 | 55.22 | 2.045185 |
| GO:0000245\_spliceosome\_assembly | CUGBP2 | 6 | 1 | 15.989583 | -1.214914 | 34 | 68.69 | 2.020294 |
| GO:0002335\_mature\_B\_cell\_differentiation | PLCG2 | 6 | 1 | 15.989583 | -1.214914 | 34 | 68.69 | 2.020294 |
| GO:0006998\_nuclear\_envelope\_organization | SYNE2 | 6 | 1 | 15.989583 | -1.214914 | 34 | 68.69 | 2.020294 |
| GO:0022409\_positive\_regulation\_of\_cell-cell\_adhesion | ITGA6 | 6 | 1 | 15.989583 | -1.214914 | 34 | 68.69 | 2.020294 |
| GO:0031077\_post-embryonic\_camera-type\_eye\_development | BCL11B | 6 | 1 | 15.989583 | -1.214914 | 34 | 68.69 | 2.020294 |
| GO:0042403\_thyroid\_hormone\_metabolic\_process | CRYM | 6 | 1 | 15.989583 | -1.214914 | 34 | 68.69 | 2.020294 |
| GO:0050684\_regulation\_of\_mRNA\_processing | FUSIP1 | 6 | 1 | 15.989583 | -1.214914 | 34 | 68.69 | 2.020294 |
| GO:0007160\_cell-matrix\_adhesion | ITGA6 | 39 | 2 | 4.919872 | -1.209272 | 35 | 70.07 | 2.002000 |
| GO:0007160\_cell-matrix\_adhesion | NID1 | 39 | 2 | 4.919872 | -1.209272 | 35 | 70.07 | 2.002000 |
| GO:0016071\_mRNA\_metabolic\_process | FUSIP1 | 40 | 2 | 4.796875 | -1.189823 | 36 | 71.64 | 1.990000 |
| GO:0016071\_mRNA\_metabolic\_process | CUGBP2 | 40 | 2 | 4.796875 | -1.189823 | 36 | 71.64 | 1.990000 |
| GO:0008585\_female\_gonad\_development | ADAMTS1 | 41 | 2 | 4.679878 | -1.170928 | 37 | 73.25 | 1.979730 |
| GO:0008585\_female\_gonad\_development | KDR | 41 | 2 | 4.679878 | -1.170928 | 37 | 73.25 | 1.979730 |
| GO:0006006\_glucose\_metabolic\_process | PGM3 | 42 | 2 | 4.568452 | -1.152562 | 38 | 75.12 | 1.976842 |
| GO:0006006\_glucose\_metabolic\_process | HK2 | 42 | 2 | 4.568452 | -1.152562 | 38 | 75.12 | 1.976842 |
| GO:0006041\_glucosamine\_metabolic\_process | PGM3 | 7 | 1 | 13.705357 | -1.150173 | 46 | 87.01 | 1.891522 |
| GO:0006044\_N-acetylglucosamine\_metabolic\_process | PGM3 | 7 | 1 | 13.705357 | -1.150173 | 46 | 87.01 | 1.891522 |
| GO:0015914\_phospholipid\_transport | ABCA4 | 7 | 1 | 13.705357 | -1.150173 | 46 | 87.01 | 1.891522 |
| GO:0022407\_regulation\_of\_cell-cell\_adhesion | ITGA6 | 7 | 1 | 13.705357 | -1.150173 | 46 | 87.01 | 1.891522 |
| GO:0022618\_ribonucleoprotein\_complex\_assembly | CUGBP2 | 7 | 1 | 13.705357 | -1.150173 | 46 | 87.01 | 1.891522 |
| GO:0042438\_melanin\_biosynthetic\_process | TYR | 7 | 1 | 13.705357 | -1.150173 | 46 | 87.01 | 1.891522 |
| GO:0046847\_filopodium\_assembly | ITGA6 | 7 | 1 | 13.705357 | -1.150173 | 46 | 87.01 | 1.891522 |
| GO:0051928\_positive\_regulation\_of\_calcium\_ion\_transport | PLCG2 | 7 | 1 | 13.705357 | -1.150173 | 46 | 87.01 | 1.891522 |
| GO:0010001\_glial\_cell\_differentiation | NTF3 | 43 | 2 | 4.462209 | -1.134698 | 47 | 88.59 | 1.884894 |
| GO:0010001\_glial\_cell\_differentiation | DTX1 | 43 | 2 | 4.462209 | -1.134698 | 47 | 88.59 | 1.884894 |
| GO:0046545\_development\_of\_primary\_female\_sexual\_characteristics | ADAMTS1 | 44 | 2 | 4.360795 | -1.117313 | 48 | 91.07 | 1.897292 |
| GO:0046545\_development\_of\_primary\_female\_sexual\_characteristics | KDR | 44 | 2 | 4.360795 | -1.117313 | 48 | 91.07 | 1.897292 |
| GO:0006020\_inositol\_metabolic\_process | PLCG2 | 8 | 1 | 11.992188 | -1.094382 | 60 | 101.82 | 1.697000 |
| GO:0006493\_protein\_amino\_acid\_O-linked\_glycosylation | GALNT10 | 8 | 1 | 11.992188 | -1.094382 | 60 | 101.82 | 1.697000 |
| GO:0006582\_melanin\_metabolic\_process | TYR | 8 | 1 | 11.992188 | -1.094382 | 60 | 101.82 | 1.697000 |
| GO:0021781\_glial\_cell\_fate\_commitment | NTF3 | 8 | 1 | 11.992188 | -1.094382 | 60 | 101.82 | 1.697000 |
| GO:0022898\_regulation\_of\_transmembrane\_transporter\_activity | PLCG2 | 8 | 1 | 11.992188 | -1.094382 | 60 | 101.82 | 1.697000 |
| GO:0030035\_microspike\_assembly | ITGA6 | 8 | 1 | 11.992188 | -1.094382 | 60 | 101.82 | 1.697000 |
| GO:0030500\_regulation\_of\_bone\_mineralization | ANK | 8 | 1 | 11.992188 | -1.094382 | 60 | 101.82 | 1.697000 |
| GO:0032409\_regulation\_of\_transporter\_activity | PLCG2 | 8 | 1 | 11.992188 | -1.094382 | 60 | 101.82 | 1.697000 |
| GO:0032412\_regulation\_of\_ion\_transmembrane\_transporter\_activity | PLCG2 | 8 | 1 | 11.992188 | -1.094382 | 60 | 101.82 | 1.697000 |
| GO:0045494\_photoreceptor\_cell\_maintenance | ABCA4 | 8 | 1 | 11.992188 | -1.094382 | 60 | 101.82 | 1.697000 |
| GO:0060347\_heart\_trabecula\_formation | ADAMTS1 | 8 | 1 | 11.992188 | -1.094382 | 60 | 101.82 | 1.697000 |
| GO:0070167\_regulation\_of\_biomineral\_formation | ANK | 8 | 1 | 11.992188 | -1.094382 | 60 | 101.82 | 1.697000 |
| GO:0006066\_alcohol\_metabolic\_process | PGM3 | 158 | 4 | 2.428797 | -1.091704 | 62 | 102.06 | 1.646129 |
| GO:0006066\_alcohol\_metabolic\_process | HMGCR | 158 | 4 | 2.428797 | -1.091704 | 62 | 102.06 | 1.646129 |
| GO:0006066\_alcohol\_metabolic\_process | PLCG2 | 158 | 4 | 2.428797 | -1.091704 | 62 | 102.06 | 1.646129 |
| GO:0006066\_alcohol\_metabolic\_process | HK2 | 158 | 4 | 2.428797 | -1.091704 | 62 | 102.06 | 1.646129 |
| GO:0007409\_axonogenesis | NTF3 | 158 | 4 | 2.428797 | -1.091704 | 62 | 102.06 | 1.646129 |
| GO:0007409\_axonogenesis | BCL11B | 158 | 4 | 2.428797 | -1.091704 | 62 | 102.06 | 1.646129 |
| GO:0007409\_axonogenesis | ULK2 | 158 | 4 | 2.428797 | -1.091704 | 62 | 102.06 | 1.646129 |
| GO:0007409\_axonogenesis | DST | 158 | 4 | 2.428797 | -1.091704 | 62 | 102.06 | 1.646129 |
| GO:0042063\_gliogenesis | NTF3 | 46 | 2 | 4.171196 | -1.083893 | 63 | 103.26 | 1.639048 |
| GO:0042063\_gliogenesis | DTX1 | 46 | 2 | 4.171196 | -1.083893 | 63 | 103.26 | 1.639048 |
| GO:0019318\_hexose\_metabolic\_process | PGM3 | 48 | 2 | 3.997396 | -1.052146 | 64 | 106.6 | 1.665625 |
| GO:0019318\_hexose\_metabolic\_process | HK2 | 48 | 2 | 3.997396 | -1.052146 | 64 | 106.6 | 1.665625 |
| GO:0001542\_ovulation\_from\_ovarian\_follicle | ADAMTS1 | 9 | 1 | 10.659722 | -1.045427 | 73 | 116.95 | 1.602055 |
| GO:0001667\_ameboidal\_cell\_migration | SYNE2 | 9 | 1 | 10.659722 | -1.045427 | 73 | 116.95 | 1.602055 |
| GO:0006182\_cGMP\_biosynthetic\_process | GUCA1B | 9 | 1 | 10.659722 | -1.045427 | 73 | 116.95 | 1.602055 |
| GO:0006364\_rRNA\_processing | WDR55 | 9 | 1 | 10.659722 | -1.045427 | 73 | 116.95 | 1.602055 |
| GO:0006595\_polyamine\_metabolic\_process | AMD1 | 9 | 1 | 10.659722 | -1.045427 | 73 | 116.95 | 1.602055 |
| GO:0006611\_protein\_export\_from\_nucleus | XPO5 | 9 | 1 | 10.659722 | -1.045427 | 73 | 116.95 | 1.602055 |
| GO:0016072\_rRNA\_metabolic\_process | WDR55 | 9 | 1 | 10.659722 | -1.045427 | 73 | 116.95 | 1.602055 |
| GO:0030728\_ovulation | ADAMTS1 | 9 | 1 | 10.659722 | -1.045427 | 73 | 116.95 | 1.602055 |
| GO:0033151\_V(D)J\_recombination | BCL11B | 9 | 1 | 10.659722 | -1.045427 | 73 | 116.95 | 1.602055 |
| GO:0043473\_pigmentation | SLC45A2 | 49 | 2 | 3.915816 | -1.036855 | 75 | 118.4 | 1.578667 |
| GO:0043473\_pigmentation | TYR | 49 | 2 | 3.915816 | -1.036855 | 75 | 118.4 | 1.578667 |
| GO:0046660\_female\_sex\_differentiation | ADAMTS1 | 49 | 2 | 3.915816 | -1.036855 | 75 | 118.4 | 1.578667 |
| GO:0046660\_female\_sex\_differentiation | KDR | 49 | 2 | 3.915816 | -1.036855 | 75 | 118.4 | 1.578667 |
| GO:0048812\_neuron\_projection\_morphogenesis | NTF3 | 170 | 4 | 2.257353 | -1.002051 | 76 | 121.52 | 1.598947 |
| GO:0048812\_neuron\_projection\_morphogenesis | BCL11B | 170 | 4 | 2.257353 | -1.002051 | 76 | 121.52 | 1.598947 |
| GO:0048812\_neuron\_projection\_morphogenesis | ULK2 | 170 | 4 | 2.257353 | -1.002051 | 76 | 121.52 | 1.598947 |
| GO:0048812\_neuron\_projection\_morphogenesis | DST | 170 | 4 | 2.257353 | -1.002051 | 76 | 121.52 | 1.598947 |
| GO:0006040\_amino\_sugar\_metabolic\_process | PGM3 | 10 | 1 | 9.593750 | -1.001864 | 81 | 133.06 | 1.642716 |
| GO:0008088\_axon\_cargo\_transport | DST | 10 | 1 | 9.593750 | -1.001864 | 81 | 133.06 | 1.642716 |
| GO:0034637\_cellular\_carbohydrate\_biosynthetic\_process | PGM3 | 10 | 1 | 9.593750 | -1.001864 | 81 | 133.06 | 1.642716 |
| GO:0045446\_endothelial\_cell\_differentiation | KDR | 10 | 1 | 9.593750 | -1.001864 | 81 | 133.06 | 1.642716 |
| GO:0060343\_trabecula\_formation | ADAMTS1 | 10 | 1 | 9.593750 | -1.001864 | 81 | 133.06 | 1.642716 |
| GO:0048667\_cell\_morphogenesis\_involved\_in\_neuron\_differentiation | NTF3 | 173 | 4 | 2.218208 | -0.981045 | 82 | 134.48 | 1.640000 |
| GO:0048667\_cell\_morphogenesis\_involved\_in\_neuron\_differentiation | BCL11B | 173 | 4 | 2.218208 | -0.981045 | 82 | 134.48 | 1.640000 |
| GO:0048667\_cell\_morphogenesis\_involved\_in\_neuron\_differentiation | ULK2 | 173 | 4 | 2.218208 | -0.981045 | 82 | 134.48 | 1.640000 |
| GO:0048667\_cell\_morphogenesis\_involved\_in\_neuron\_differentiation | DST | 173 | 4 | 2.218208 | -0.981045 | 82 | 134.48 | 1.640000 |
| GO:0006576\_biogenic\_amine\_metabolic\_process | AMD1 | 53 | 2 | 3.620283 | -0.979221 | 83 | 135.25 | 1.629518 |
| GO:0006576\_biogenic\_amine\_metabolic\_process | CRYM | 53 | 2 | 3.620283 | -0.979221 | 83 | 135.25 | 1.629518 |
| GO:0043010\_camera-type\_eye\_development | CRYAB | 110 | 3 | 2.616477 | -0.974695 | 84 | 135.67 | 1.615119 |
| GO:0043010\_camera-type\_eye\_development | BCL11B | 110 | 3 | 2.616477 | -0.974695 | 84 | 135.67 | 1.615119 |
| GO:0043010\_camera-type\_eye\_development | COL8A1 | 110 | 3 | 2.616477 | -0.974695 | 84 | 135.67 | 1.615119 |
| GO:0031110\_regulation\_of\_microtubule\_polymerization\_or\_depolymerization | DST | 11 | 1 | 8.721591 | -0.962663 | 88 | 145.24 | 1.650455 |
| GO:0034762\_regulation\_of\_transmembrane\_transport | PLCG2 | 11 | 1 | 8.721591 | -0.962663 | 88 | 145.24 | 1.650455 |
| GO:0042401\_biogenic\_amine\_biosynthetic\_process | AMD1 | 11 | 1 | 8.721591 | -0.962663 | 88 | 145.24 | 1.650455 |
| GO:0043270\_positive\_regulation\_of\_ion\_transport | PLCG2 | 11 | 1 | 8.721591 | -0.962663 | 88 | 145.24 | 1.650455 |
| GO:0048858\_cell\_projection\_morphogenesis | NTF3 | 176 | 4 | 2.180398 | -0.960562 | 89 | 145.47 | 1.634494 |
| GO:0048858\_cell\_projection\_morphogenesis | BCL11B | 176 | 4 | 2.180398 | -0.960562 | 89 | 145.47 | 1.634494 |
| GO:0048858\_cell\_projection\_morphogenesis | ULK2 | 176 | 4 | 2.180398 | -0.960562 | 89 | 145.47 | 1.634494 |
| GO:0048858\_cell\_projection\_morphogenesis | DST | 176 | 4 | 2.180398 | -0.960562 | 89 | 145.47 | 1.634494 |
| GO:0030010\_establishment\_of\_cell\_polarity | BRSK2 | 12 | 1 | 7.994792 | -0.927062 | 95 | 157.75 | 1.660526 |
| GO:0030514\_negative\_regulation\_of\_BMP\_signaling\_pathway | HTRA1 | 12 | 1 | 7.994792 | -0.927062 | 95 | 157.75 | 1.660526 |
| GO:0031109\_microtubule\_polymerization\_or\_depolymerization | DST | 12 | 1 | 7.994792 | -0.927062 | 95 | 157.75 | 1.660526 |
| GO:0046068\_cGMP\_metabolic\_process | GUCA1B | 12 | 1 | 7.994792 | -0.927062 | 95 | 157.75 | 1.660526 |
| GO:0048821\_erythrocyte\_development | SOX6 | 12 | 1 | 7.994792 | -0.927062 | 95 | 157.75 | 1.660526 |
| GO:0051145\_smooth\_muscle\_cell\_differentiation | NTF3 | 12 | 1 | 7.994792 | -0.927062 | 95 | 157.75 | 1.660526 |
| GO:0030097\_hemopoiesis | PGM3 | 253 | 5 | 1.895998 | -0.916615 | 96 | 159.51 | 1.661562 |
| GO:0030097\_hemopoiesis | BCL11B | 253 | 5 | 1.895998 | -0.916615 | 96 | 159.51 | 1.661562 |
| GO:0030097\_hemopoiesis | PLCG2 | 253 | 5 | 1.895998 | -0.916615 | 96 | 159.51 | 1.661562 |
| GO:0030097\_hemopoiesis | SOX6 | 253 | 5 | 1.895998 | -0.916615 | 96 | 159.51 | 1.661562 |
| GO:0030097\_hemopoiesis | KDR | 253 | 5 | 1.895998 | -0.916615 | 96 | 159.51 | 1.661562 |
| GO:0032990\_cell\_part\_morphogenesis | NTF3 | 184 | 4 | 2.085598 | -0.908363 | 97 | 160.41 | 1.653711 |
| GO:0032990\_cell\_part\_morphogenesis | BCL11B | 184 | 4 | 2.085598 | -0.908363 | 97 | 160.41 | 1.653711 |
| GO:0032990\_cell\_part\_morphogenesis | ULK2 | 184 | 4 | 2.085598 | -0.908363 | 97 | 160.41 | 1.653711 |
| GO:0032990\_cell\_part\_morphogenesis | DST | 184 | 4 | 2.085598 | -0.908363 | 97 | 160.41 | 1.653711 |
| GO:0007155\_cell\_adhesion | ITGA6 | 186 | 4 | 2.063172 | -0.895836 | 99 | 162.15 | 1.637879 |
| GO:0007155\_cell\_adhesion | POSTN | 186 | 4 | 2.063172 | -0.895836 | 99 | 162.15 | 1.637879 |
| GO:0007155\_cell\_adhesion | NID1 | 186 | 4 | 2.063172 | -0.895836 | 99 | 162.15 | 1.637879 |
| GO:0007155\_cell\_adhesion | COL8A1 | 186 | 4 | 2.063172 | -0.895836 | 99 | 162.15 | 1.637879 |
| GO:0022610\_biological\_adhesion | ITGA6 | 186 | 4 | 2.063172 | -0.895836 | 99 | 162.15 | 1.637879 |
| GO:0022610\_biological\_adhesion | POSTN | 186 | 4 | 2.063172 | -0.895836 | 99 | 162.15 | 1.637879 |
| GO:0022610\_biological\_adhesion | NID1 | 186 | 4 | 2.063172 | -0.895836 | 99 | 162.15 | 1.637879 |
| GO:0022610\_biological\_adhesion | COL8A1 | 186 | 4 | 2.063172 | -0.895836 | 99 | 162.15 | 1.637879 |
| GO:0007274\_neuromuscular\_synaptic\_transmission | NTF3 | 13 | 1 | 7.379808 | -0.894483 | 104 | 171.39 | 1.647981 |
| GO:0007566\_embryo\_implantation | LAMB1-1 | 13 | 1 | 7.379808 | -0.894483 | 104 | 171.39 | 1.647981 |
| GO:0010970\_microtubule-based\_transport | DST | 13 | 1 | 7.379808 | -0.894483 | 104 | 171.39 | 1.647981 |
| GO:0030384\_phosphoinositide\_metabolic\_process | PIP5K1B | 13 | 1 | 7.379808 | -0.894483 | 104 | 171.39 | 1.647981 |
| GO:0050771\_negative\_regulation\_of\_axonogenesis | ULK2 | 13 | 1 | 7.379808 | -0.894483 | 104 | 171.39 | 1.647981 |
| GO:0007276\_gamete\_generation | PGM3 | 188 | 4 | 2.041223 | -0.883510 | 105 | 172.06 | 1.638667 |
| GO:0007276\_gamete\_generation | TDRD7 | 188 | 4 | 2.041223 | -0.883510 | 105 | 172.06 | 1.638667 |
| GO:0007276\_gamete\_generation | CRTAP | 188 | 4 | 2.041223 | -0.883510 | 105 | 172.06 | 1.638667 |
| GO:0007276\_gamete\_generation | ADAMTS1 | 188 | 4 | 2.041223 | -0.883510 | 105 | 172.06 | 1.638667 |
| GO:0048468\_cell\_development | IRX3 | 654 | 10 | 1.466934 | -0.873657 | 106 | 173.52 | 1.636981 |
| GO:0048468\_cell\_development | NTF3 | 654 | 10 | 1.466934 | -0.873657 | 106 | 173.52 | 1.636981 |
| GO:0048468\_cell\_development | DTX1 | 654 | 10 | 1.466934 | -0.873657 | 106 | 173.52 | 1.636981 |
| GO:0048468\_cell\_development | TDRD7 | 654 | 10 | 1.466934 | -0.873657 | 106 | 173.52 | 1.636981 |
| GO:0048468\_cell\_development | BCL11B | 654 | 10 | 1.466934 | -0.873657 | 106 | 173.52 | 1.636981 |
| GO:0048468\_cell\_development | ULK2 | 654 | 10 | 1.466934 | -0.873657 | 106 | 173.52 | 1.636981 |
| GO:0048468\_cell\_development | BRSK2 | 654 | 10 | 1.466934 | -0.873657 | 106 | 173.52 | 1.636981 |
| GO:0048468\_cell\_development | SOX6 | 654 | 10 | 1.466934 | -0.873657 | 106 | 173.52 | 1.636981 |
| GO:0048468\_cell\_development | DST | 654 | 10 | 1.466934 | -0.873657 | 106 | 173.52 | 1.636981 |
| GO:0048468\_cell\_development | KDR | 654 | 10 | 1.466934 | -0.873657 | 106 | 173.52 | 1.636981 |
| GO:0030030\_cell\_projection\_organization | NTF3 | 263 | 5 | 1.823907 | -0.864752 | 107 | 174.88 | 1.634393 |
| GO:0030030\_cell\_projection\_organization | ITGA6 | 263 | 5 | 1.823907 | -0.864752 | 107 | 174.88 | 1.634393 |
| GO:0030030\_cell\_projection\_organization | BCL11B | 263 | 5 | 1.823907 | -0.864752 | 107 | 174.88 | 1.634393 |
| GO:0030030\_cell\_projection\_organization | ULK2 | 263 | 5 | 1.823907 | -0.864752 | 107 | 174.88 | 1.634393 |
| GO:0030030\_cell\_projection\_organization | DST | 263 | 5 | 1.823907 | -0.864752 | 107 | 174.88 | 1.634393 |
| GO:0006695\_cholesterol\_biosynthetic\_process | HMGCR | 14 | 1 | 6.852679 | -0.864479 | 108 | 183.49 | 1.698981 |
| GO:0045666\_positive\_regulation\_of\_neuron\_differentiation | IRX3 | 15 | 1 | 6.395833 | -0.836693 | 111 | 193.72 | 1.745225 |
| GO:0048010\_vascular\_endothelial\_growth\_factor\_receptor\_signaling\_pathway | KDR | 15 | 1 | 6.395833 | -0.836693 | 111 | 193.72 | 1.745225 |
| GO:0070507\_regulation\_of\_microtubule\_cytoskeleton\_organization | DST | 15 | 1 | 6.395833 | -0.836693 | 111 | 193.72 | 1.745225 |
| GO:0048511\_rhythmic\_process | ADAMTS1 | 65 | 2 | 2.951923 | -0.833817 | 112 | 194.31 | 1.734911 |
| GO:0048511\_rhythmic\_process | KDR | 65 | 2 | 2.951923 | -0.833817 | 112 | 194.31 | 1.734911 |
| GO:0022008\_neurogenesis | IRX3 | 423 | 7 | 1.587618 | -0.833803 | 113 | 194.39 | 1.720265 |
| GO:0022008\_neurogenesis | NTF3 | 423 | 7 | 1.587618 | -0.833803 | 113 | 194.39 | 1.720265 |
| GO:0022008\_neurogenesis | DTX1 | 423 | 7 | 1.587618 | -0.833803 | 113 | 194.39 | 1.720265 |
| GO:0022008\_neurogenesis | ULK2 | 423 | 7 | 1.587618 | -0.833803 | 113 | 194.39 | 1.720265 |
| GO:0022008\_neurogenesis | BCL11B | 423 | 7 | 1.587618 | -0.833803 | 113 | 194.39 | 1.720265 |
| GO:0022008\_neurogenesis | BRSK2 | 423 | 7 | 1.587618 | -0.833803 | 113 | 194.39 | 1.720265 |
| GO:0022008\_neurogenesis | DST | 423 | 7 | 1.587618 | -0.833803 | 113 | 194.39 | 1.720265 |
| GO:0031175\_neuron\_projection\_development | NTF3 | 197 | 4 | 1.947970 | -0.830404 | 114 | 195.0 | 1.710526 |
| GO:0031175\_neuron\_projection\_development | BCL11B | 197 | 4 | 1.947970 | -0.830404 | 114 | 195.0 | 1.710526 |
| GO:0031175\_neuron\_projection\_development | ULK2 | 197 | 4 | 1.947970 | -0.830404 | 114 | 195.0 | 1.710526 |
| GO:0031175\_neuron\_projection\_development | DST | 197 | 4 | 1.947970 | -0.830404 | 114 | 195.0 | 1.710526 |
| GO:0000904\_cell\_morphogenesis\_involved\_in\_differentiation | NTF3 | 199 | 4 | 1.928392 | -0.819103 | 115 | 196.9 | 1.712174 |
| GO:0000904\_cell\_morphogenesis\_involved\_in\_differentiation | BCL11B | 199 | 4 | 1.928392 | -0.819103 | 115 | 196.9 | 1.712174 |
| GO:0000904\_cell\_morphogenesis\_involved\_in\_differentiation | ULK2 | 199 | 4 | 1.928392 | -0.819103 | 115 | 196.9 | 1.712174 |
| GO:0000904\_cell\_morphogenesis\_involved\_in\_differentiation | DST | 199 | 4 | 1.928392 | -0.819103 | 115 | 196.9 | 1.712174 |
| GO:0045165\_cell\_fate\_commitment | NTF3 | 130 | 3 | 2.213942 | -0.816216 | 116 | 197.3 | 1.700862 |
| GO:0045165\_cell\_fate\_commitment | SOX6 | 130 | 3 | 2.213942 | -0.816216 | 116 | 197.3 | 1.700862 |
| GO:0045165\_cell\_fate\_commitment | KDR | 130 | 3 | 2.213942 | -0.816216 | 116 | 197.3 | 1.700862 |
| GO:0009791\_post-embryonic\_development | BCL11B | 67 | 2 | 2.863806 | -0.812828 | 117 | 198.21 | 1.694103 |
| GO:0009791\_post-embryonic\_development | SOX6 | 67 | 2 | 2.863806 | -0.812828 | 117 | 198.21 | 1.694103 |
| GO:0007602\_phototransduction | GUCA1B | 16 | 1 | 5.996094 | -0.810837 | 126 | 204.89 | 1.626111 |
| GO:0016126\_sterol\_biosynthetic\_process | HMGCR | 16 | 1 | 5.996094 | -0.810837 | 126 | 204.89 | 1.626111 |
| GO:0019751\_polyol\_metabolic\_process | PLCG2 | 16 | 1 | 5.996094 | -0.810837 | 126 | 204.89 | 1.626111 |
| GO:0031345\_negative\_regulation\_of\_cell\_projection\_organization | ULK2 | 16 | 1 | 5.996094 | -0.810837 | 126 | 204.89 | 1.626111 |
| GO:0046148\_pigment\_biosynthetic\_process | TYR | 16 | 1 | 5.996094 | -0.810837 | 126 | 204.89 | 1.626111 |
| GO:0046364\_monosaccharide\_biosynthetic\_process | PGM3 | 16 | 1 | 5.996094 | -0.810837 | 126 | 204.89 | 1.626111 |
| GO:0048015\_phosphoinositide-mediated\_signaling | CAR8 | 16 | 1 | 5.996094 | -0.810837 | 126 | 204.89 | 1.626111 |
| GO:0048286\_lung\_alveolus\_development | KDR | 16 | 1 | 5.996094 | -0.810837 | 126 | 204.89 | 1.626111 |
| GO:0048483\_autonomic\_nervous\_system\_development | NTF3 | 16 | 1 | 5.996094 | -0.810837 | 126 | 204.89 | 1.626111 |
| GO:0042692\_muscle\_cell\_differentiation | NTF3 | 68 | 2 | 2.821691 | -0.802628 | 127 | 205.58 | 1.618740 |
| GO:0042692\_muscle\_cell\_differentiation | SOX6 | 68 | 2 | 2.821691 | -0.802628 | 127 | 205.58 | 1.618740 |
| GO:0048534\_hemopoietic\_or\_lymphoid\_organ\_development | PGM3 | 277 | 5 | 1.731724 | -0.797329 | 128 | 205.94 | 1.608906 |
| GO:0048534\_hemopoietic\_or\_lymphoid\_organ\_development | BCL11B | 277 | 5 | 1.731724 | -0.797329 | 128 | 205.94 | 1.608906 |
| GO:0048534\_hemopoietic\_or\_lymphoid\_organ\_development | PLCG2 | 277 | 5 | 1.731724 | -0.797329 | 128 | 205.94 | 1.608906 |
| GO:0048534\_hemopoietic\_or\_lymphoid\_organ\_development | SOX6 | 277 | 5 | 1.731724 | -0.797329 | 128 | 205.94 | 1.608906 |
| GO:0048534\_hemopoietic\_or\_lymphoid\_organ\_development | KDR | 277 | 5 | 1.731724 | -0.797329 | 128 | 205.94 | 1.608906 |
| GO:0005996\_monosaccharide\_metabolic\_process | PGM3 | 69 | 2 | 2.780797 | -0.792617 | 129 | 206.96 | 1.604341 |
| GO:0005996\_monosaccharide\_metabolic\_process | HK2 | 69 | 2 | 2.780797 | -0.792617 | 129 | 206.96 | 1.604341 |
| GO:0030182\_neuron\_differentiation | IRX3 | 356 | 6 | 1.616924 | -0.788418 | 130 | 207.54 | 1.596462 |
| GO:0030182\_neuron\_differentiation | NTF3 | 356 | 6 | 1.616924 | -0.788418 | 130 | 207.54 | 1.596462 |
| GO:0030182\_neuron\_differentiation | ULK2 | 356 | 6 | 1.616924 | -0.788418 | 130 | 207.54 | 1.596462 |
| GO:0030182\_neuron\_differentiation | BCL11B | 356 | 6 | 1.616924 | -0.788418 | 130 | 207.54 | 1.596462 |
| GO:0030182\_neuron\_differentiation | BRSK2 | 356 | 6 | 1.616924 | -0.788418 | 130 | 207.54 | 1.596462 |
| GO:0030182\_neuron\_differentiation | DST | 356 | 6 | 1.616924 | -0.788418 | 130 | 207.54 | 1.596462 |
| GO:0034470\_ncRNA\_processing | WDR55 | 17 | 1 | 5.643382 | -0.786678 | 135 | 214.72 | 1.590519 |
| GO:0042254\_ribosome\_biogenesis | WDR55 | 17 | 1 | 5.643382 | -0.786678 | 135 | 214.72 | 1.590519 |
| GO:0042398\_cellular\_amino\_acid\_derivative\_biosynthetic\_process | AMD1 | 17 | 1 | 5.643382 | -0.786678 | 135 | 214.72 | 1.590519 |
| GO:0042440\_pigment\_metabolic\_process | TYR | 17 | 1 | 5.643382 | -0.786678 | 135 | 214.72 | 1.590519 |
| GO:0043407\_negative\_regulation\_of\_MAP\_kinase\_activity | HMGCR | 17 | 1 | 5.643382 | -0.786678 | 135 | 214.72 | 1.590519 |
| GO:0008406\_gonad\_development | ADAMTS1 | 70 | 2 | 2.741071 | -0.782788 | 136 | 215.57 | 1.585074 |
| GO:0008406\_gonad\_development | KDR | 70 | 2 | 2.741071 | -0.782788 | 136 | 215.57 | 1.585074 |
| GO:0001654\_eye\_development | CRYAB | 136 | 3 | 2.116268 | -0.775171 | 137 | 215.7 | 1.574453 |
| GO:0001654\_eye\_development | BCL11B | 136 | 3 | 2.116268 | -0.775171 | 137 | 215.7 | 1.574453 |
| GO:0001654\_eye\_development | COL8A1 | 136 | 3 | 2.116268 | -0.775171 | 137 | 215.7 | 1.574453 |
| GO:0002285\_lymphocyte\_activation\_during\_immune\_response | PLCG2 | 18 | 1 | 5.329861 | -0.764021 | 144 | 225.74 | 1.567639 |
| GO:0016458\_gene\_silencing | SOX6 | 18 | 1 | 5.329861 | -0.764021 | 144 | 225.74 | 1.567639 |
| GO:0030282\_bone\_mineralization | ANK | 18 | 1 | 5.329861 | -0.764021 | 144 | 225.74 | 1.567639 |
| GO:0030510\_regulation\_of\_BMP\_signaling\_pathway | HTRA1 | 18 | 1 | 5.329861 | -0.764021 | 144 | 225.74 | 1.567639 |
| GO:0035051\_cardiac\_cell\_differentiation | SOX6 | 18 | 1 | 5.329861 | -0.764021 | 144 | 225.74 | 1.567639 |
| GO:0051168\_nuclear\_export | XPO5 | 18 | 1 | 5.329861 | -0.764021 | 144 | 225.74 | 1.567639 |
| GO:0051924\_regulation\_of\_calcium\_ion\_transport | PLCG2 | 18 | 1 | 5.329861 | -0.764021 | 144 | 225.74 | 1.567639 |
| GO:0050673\_epithelial\_cell\_proliferation | COL8A1 | 72 | 2 | 2.664931 | -0.763658 | 145 | 227.27 | 1.567379 |
| GO:0050673\_epithelial\_cell\_proliferation | KDR | 72 | 2 | 2.664931 | -0.763658 | 145 | 227.27 | 1.567379 |
| GO:0007275\_multicellular\_organismal\_development | IRX3 | 1760 | 22 | 1.199219 | -0.762532 | 146 | 227.34 | 1.557123 |
| GO:0007275\_multicellular\_organismal\_development | NTF3 | 1760 | 22 | 1.199219 | -0.762532 | 146 | 227.34 | 1.557123 |
| GO:0007275\_multicellular\_organismal\_development | CRYAB | 1760 | 22 | 1.199219 | -0.762532 | 146 | 227.34 | 1.557123 |
| GO:0007275\_multicellular\_organismal\_development | HMGCR | 1760 | 22 | 1.199219 | -0.762532 | 146 | 227.34 | 1.557123 |
| GO:0007275\_multicellular\_organismal\_development | DTX1 | 1760 | 22 | 1.199219 | -0.762532 | 146 | 227.34 | 1.557123 |
| GO:0007275\_multicellular\_organismal\_development | CCNF | 1760 | 22 | 1.199219 | -0.762532 | 146 | 227.34 | 1.557123 |
| GO:0007275\_multicellular\_organismal\_development | BRSK2 | 1760 | 22 | 1.199219 | -0.762532 | 146 | 227.34 | 1.557123 |
| GO:0007275\_multicellular\_organismal\_development | POSTN | 1760 | 22 | 1.199219 | -0.762532 | 146 | 227.34 | 1.557123 |
| GO:0007275\_multicellular\_organismal\_development | NID1 | 1760 | 22 | 1.199219 | -0.762532 | 146 | 227.34 | 1.557123 |
| GO:0007275\_multicellular\_organismal\_development | SOX6 | 1760 | 22 | 1.199219 | -0.762532 | 146 | 227.34 | 1.557123 |
| GO:0007275\_multicellular\_organismal\_development | KDR | 1760 | 22 | 1.199219 | -0.762532 | 146 | 227.34 | 1.557123 |
| GO:0007275\_multicellular\_organismal\_development | PGM3 | 1760 | 22 | 1.199219 | -0.762532 | 146 | 227.34 | 1.557123 |
| GO:0007275\_multicellular\_organismal\_development | ANK | 1760 | 22 | 1.199219 | -0.762532 | 146 | 227.34 | 1.557123 |
| GO:0007275\_multicellular\_organismal\_development | ITGA6 | 1760 | 22 | 1.199219 | -0.762532 | 146 | 227.34 | 1.557123 |
| GO:0007275\_multicellular\_organismal\_development | ULK2 | 1760 | 22 | 1.199219 | -0.762532 | 146 | 227.34 | 1.557123 |
| GO:0007275\_multicellular\_organismal\_development | BCL11B | 1760 | 22 | 1.199219 | -0.762532 | 146 | 227.34 | 1.557123 |
| GO:0007275\_multicellular\_organismal\_development | LAMB1-1 | 1760 | 22 | 1.199219 | -0.762532 | 146 | 227.34 | 1.557123 |
| GO:0007275\_multicellular\_organismal\_development | PLCG2 | 1760 | 22 | 1.199219 | -0.762532 | 146 | 227.34 | 1.557123 |
| GO:0007275\_multicellular\_organismal\_development | ADAMTS1 | 1760 | 22 | 1.199219 | -0.762532 | 146 | 227.34 | 1.557123 |
| GO:0007275\_multicellular\_organismal\_development | COL8A1 | 1760 | 22 | 1.199219 | -0.762532 | 146 | 227.34 | 1.557123 |
| GO:0007275\_multicellular\_organismal\_development | AMD1 | 1760 | 22 | 1.199219 | -0.762532 | 146 | 227.34 | 1.557123 |
| GO:0007275\_multicellular\_organismal\_development | DST | 1760 | 22 | 1.199219 | -0.762532 | 146 | 227.34 | 1.557123 |
| GO:0009416\_response\_to\_light\_stimulus | GUCA1B | 74 | 2 | 2.592905 | -0.745199 | 147 | 229.71 | 1.562653 |
| GO:0009416\_response\_to\_light\_stimulus | HMGCR | 74 | 2 | 2.592905 | -0.745199 | 147 | 229.71 | 1.562653 |
| GO:0009584\_detection\_of\_visible\_light | GUCA1B | 19 | 1 | 5.049342 | -0.742703 | 150 | 235.27 | 1.568467 |
| GO:0050908\_detection\_of\_light\_stimulus\_involved\_in\_visual\_perception | GUCA1B | 19 | 1 | 5.049342 | -0.742703 | 150 | 235.27 | 1.568467 |
| GO:0050962\_detection\_of\_light\_stimulus\_involved\_in\_sensory\_perception | GUCA1B | 19 | 1 | 5.049342 | -0.742703 | 150 | 235.27 | 1.568467 |
| GO:0032502\_developmental\_process | IRX3 | 2060 | 25 | 1.164290 | -0.725230 | 151 | 237.17 | 1.570662 |
| GO:0032502\_developmental\_process | SLC45A2 | 2060 | 25 | 1.164290 | -0.725230 | 151 | 237.17 | 1.570662 |
| GO:0032502\_developmental\_process | HMGCR | 2060 | 25 | 1.164290 | -0.725230 | 151 | 237.17 | 1.570662 |
| GO:0032502\_developmental\_process | TDRD7 | 2060 | 25 | 1.164290 | -0.725230 | 151 | 237.17 | 1.570662 |
| GO:0032502\_developmental\_process | BRSK2 | 2060 | 25 | 1.164290 | -0.725230 | 151 | 237.17 | 1.570662 |
| GO:0032502\_developmental\_process | POSTN | 2060 | 25 | 1.164290 | -0.725230 | 151 | 237.17 | 1.570662 |
| GO:0032502\_developmental\_process | SOX6 | 2060 | 25 | 1.164290 | -0.725230 | 151 | 237.17 | 1.570662 |
| GO:0032502\_developmental\_process | ANK | 2060 | 25 | 1.164290 | -0.725230 | 151 | 237.17 | 1.570662 |
| GO:0032502\_developmental\_process | BCL11B | 2060 | 25 | 1.164290 | -0.725230 | 151 | 237.17 | 1.570662 |
| GO:0032502\_developmental\_process | AMD1 | 2060 | 25 | 1.164290 | -0.725230 | 151 | 237.17 | 1.570662 |
| GO:0032502\_developmental\_process | COL8A1 | 2060 | 25 | 1.164290 | -0.725230 | 151 | 237.17 | 1.570662 |
| GO:0032502\_developmental\_process | NTF3 | 2060 | 25 | 1.164290 | -0.725230 | 151 | 237.17 | 1.570662 |
| GO:0032502\_developmental\_process | DTX1 | 2060 | 25 | 1.164290 | -0.725230 | 151 | 237.17 | 1.570662 |
| GO:0032502\_developmental\_process | CRYAB | 2060 | 25 | 1.164290 | -0.725230 | 151 | 237.17 | 1.570662 |
| GO:0032502\_developmental\_process | CCNF | 2060 | 25 | 1.164290 | -0.725230 | 151 | 237.17 | 1.570662 |
| GO:0032502\_developmental\_process | NID1 | 2060 | 25 | 1.164290 | -0.725230 | 151 | 237.17 | 1.570662 |
| GO:0032502\_developmental\_process | KDR | 2060 | 25 | 1.164290 | -0.725230 | 151 | 237.17 | 1.570662 |
| GO:0032502\_developmental\_process | PGM3 | 2060 | 25 | 1.164290 | -0.725230 | 151 | 237.17 | 1.570662 |
| GO:0032502\_developmental\_process | SYNE2 | 2060 | 25 | 1.164290 | -0.725230 | 151 | 237.17 | 1.570662 |
| GO:0032502\_developmental\_process | ITGA6 | 2060 | 25 | 1.164290 | -0.725230 | 151 | 237.17 | 1.570662 |
| GO:0032502\_developmental\_process | ULK2 | 2060 | 25 | 1.164290 | -0.725230 | 151 | 237.17 | 1.570662 |
| GO:0032502\_developmental\_process | PLCG2 | 2060 | 25 | 1.164290 | -0.725230 | 151 | 237.17 | 1.570662 |
| GO:0032502\_developmental\_process | LAMB1-1 | 2060 | 25 | 1.164290 | -0.725230 | 151 | 237.17 | 1.570662 |
| GO:0032502\_developmental\_process | ADAMTS1 | 2060 | 25 | 1.164290 | -0.725230 | 151 | 237.17 | 1.570662 |
| GO:0032502\_developmental\_process | DST | 2060 | 25 | 1.164290 | -0.725230 | 151 | 237.17 | 1.570662 |
| GO:0031214\_biomineral\_formation | ANK | 20 | 1 | 4.796875 | -0.722586 | 152 | 242.57 | 1.595855 |
| GO:0002520\_immune\_system\_development | PGM3 | 295 | 5 | 1.626059 | -0.718629 | 153 | 243.37 | 1.590654 |
| GO:0002520\_immune\_system\_development | BCL11B | 295 | 5 | 1.626059 | -0.718629 | 153 | 243.37 | 1.590654 |
| GO:0002520\_immune\_system\_development | PLCG2 | 295 | 5 | 1.626059 | -0.718629 | 153 | 243.37 | 1.590654 |
| GO:0002520\_immune\_system\_development | SOX6 | 295 | 5 | 1.626059 | -0.718629 | 153 | 243.37 | 1.590654 |
| GO:0002520\_immune\_system\_development | KDR | 295 | 5 | 1.626059 | -0.718629 | 153 | 243.37 | 1.590654 |
| GO:0005975\_carbohydrate\_metabolic\_process | PGM3 | 146 | 3 | 1.971318 | -0.712256 | 154 | 243.93 | 1.583961 |
| GO:0005975\_carbohydrate\_metabolic\_process | GALNT10 | 146 | 3 | 1.971318 | -0.712256 | 154 | 243.93 | 1.583961 |
| GO:0005975\_carbohydrate\_metabolic\_process | HK2 | 146 | 3 | 1.971318 | -0.712256 | 154 | 243.93 | 1.583961 |
| GO:0022414\_reproductive\_process | PGM3 | 376 | 6 | 1.530918 | -0.711877 | 155 | 244.04 | 1.574452 |
| GO:0022414\_reproductive\_process | TDRD7 | 376 | 6 | 1.530918 | -0.711877 | 155 | 244.04 | 1.574452 |
| GO:0022414\_reproductive\_process | LAMB1-1 | 376 | 6 | 1.530918 | -0.711877 | 155 | 244.04 | 1.574452 |
| GO:0022414\_reproductive\_process | CRTAP | 376 | 6 | 1.530918 | -0.711877 | 155 | 244.04 | 1.574452 |
| GO:0022414\_reproductive\_process | ADAMTS1 | 376 | 6 | 1.530918 | -0.711877 | 155 | 244.04 | 1.574452 |
| GO:0022414\_reproductive\_process | KDR | 376 | 6 | 1.530918 | -0.711877 | 155 | 244.04 | 1.574452 |
| GO:0001709\_cell\_fate\_determination | NTF3 | 21 | 1 | 4.568452 | -0.703552 | 162 | 253.02 | 1.561852 |
| GO:0002053\_positive\_regulation\_of\_mesenchymal\_cell\_proliferation | KDR | 21 | 1 | 4.568452 | -0.703552 | 162 | 253.02 | 1.561852 |
| GO:0002263\_cell\_activation\_during\_immune\_response | PLCG2 | 21 | 1 | 4.568452 | -0.703552 | 162 | 253.02 | 1.561852 |
| GO:0002366\_leukocyte\_activation\_during\_immune\_response | PLCG2 | 21 | 1 | 4.568452 | -0.703552 | 162 | 253.02 | 1.561852 |
| GO:0009583\_detection\_of\_light\_stimulus | GUCA1B | 21 | 1 | 4.568452 | -0.703552 | 162 | 253.02 | 1.561852 |
| GO:0048538\_thymus\_development | BCL11B | 21 | 1 | 4.568452 | -0.703552 | 162 | 253.02 | 1.561852 |
| GO:0048675\_axon\_extension | ULK2 | 21 | 1 | 4.568452 | -0.703552 | 162 | 253.02 | 1.561852 |
| GO:0000003\_reproduction | PGM3 | 379 | 6 | 1.518799 | -0.701041 | 163 | 253.52 | 1.555337 |
| GO:0000003\_reproduction | TDRD7 | 379 | 6 | 1.518799 | -0.701041 | 163 | 253.52 | 1.555337 |
| GO:0000003\_reproduction | LAMB1-1 | 379 | 6 | 1.518799 | -0.701041 | 163 | 253.52 | 1.555337 |
| GO:0000003\_reproduction | CRTAP | 379 | 6 | 1.518799 | -0.701041 | 163 | 253.52 | 1.555337 |
| GO:0000003\_reproduction | ADAMTS1 | 379 | 6 | 1.518799 | -0.701041 | 163 | 253.52 | 1.555337 |
| GO:0000003\_reproduction | KDR | 379 | 6 | 1.518799 | -0.701041 | 163 | 253.52 | 1.555337 |
| GO:0009309\_amine\_biosynthetic\_process | AMD1 | 22 | 1 | 4.360795 | -0.685501 | 170 | 263.37 | 1.549235 |
| GO:0010463\_mesenchymal\_cell\_proliferation | KDR | 22 | 1 | 4.360795 | -0.685501 | 170 | 263.37 | 1.549235 |
| GO:0010464\_regulation\_of\_mesenchymal\_cell\_proliferation | KDR | 22 | 1 | 4.360795 | -0.685501 | 170 | 263.37 | 1.549235 |
| GO:0021675\_nerve\_development | NTF3 | 22 | 1 | 4.360795 | -0.685501 | 170 | 263.37 | 1.549235 |
| GO:0030705\_cytoskeleton-dependent\_intracellular\_transport | DST | 22 | 1 | 4.360795 | -0.685501 | 170 | 263.37 | 1.549235 |
| GO:0032886\_regulation\_of\_microtubule-based\_process | DST | 22 | 1 | 4.360795 | -0.685501 | 170 | 263.37 | 1.549235 |
| GO:0034660\_ncRNA\_metabolic\_process | WDR55 | 22 | 1 | 4.360795 | -0.685501 | 170 | 263.37 | 1.549235 |
| GO:0045664\_regulation\_of\_neuron\_differentiation | IRX3 | 82 | 2 | 2.339939 | -0.677381 | 171 | 264.77 | 1.548363 |
| GO:0045664\_regulation\_of\_neuron\_differentiation | ULK2 | 82 | 2 | 2.339939 | -0.677381 | 171 | 264.77 | 1.548363 |
| GO:0019953\_sexual\_reproduction | PGM3 | 228 | 4 | 1.683114 | -0.673134 | 172 | 265.23 | 1.542035 |
| GO:0019953\_sexual\_reproduction | TDRD7 | 228 | 4 | 1.683114 | -0.673134 | 172 | 265.23 | 1.542035 |
| GO:0019953\_sexual\_reproduction | CRTAP | 228 | 4 | 1.683114 | -0.673134 | 172 | 265.23 | 1.542035 |
| GO:0019953\_sexual\_reproduction | ADAMTS1 | 228 | 4 | 1.683114 | -0.673134 | 172 | 265.23 | 1.542035 |
| GO:0032501\_multicellular\_organismal\_process | IRX3 | 2183 | 26 | 1.142636 | -0.672898 | 173 | 265.37 | 1.533931 |
| GO:0032501\_multicellular\_organismal\_process | HMGCR | 2183 | 26 | 1.142636 | -0.672898 | 173 | 265.37 | 1.533931 |
| GO:0032501\_multicellular\_organismal\_process | BRSK2 | 2183 | 26 | 1.142636 | -0.672898 | 173 | 265.37 | 1.533931 |
| GO:0032501\_multicellular\_organismal\_process | POSTN | 2183 | 26 | 1.142636 | -0.672898 | 173 | 265.37 | 1.533931 |
| GO:0032501\_multicellular\_organismal\_process | SOX6 | 2183 | 26 | 1.142636 | -0.672898 | 173 | 265.37 | 1.533931 |
| GO:0032501\_multicellular\_organismal\_process | ABCA4 | 2183 | 26 | 1.142636 | -0.672898 | 173 | 265.37 | 1.533931 |
| GO:0032501\_multicellular\_organismal\_process | TPM2 | 2183 | 26 | 1.142636 | -0.672898 | 173 | 265.37 | 1.533931 |
| GO:0032501\_multicellular\_organismal\_process | MFRP | 2183 | 26 | 1.142636 | -0.672898 | 173 | 265.37 | 1.533931 |
| GO:0032501\_multicellular\_organismal\_process | ANK | 2183 | 26 | 1.142636 | -0.672898 | 173 | 265.37 | 1.533931 |
| GO:0032501\_multicellular\_organismal\_process | BCL11B | 2183 | 26 | 1.142636 | -0.672898 | 173 | 265.37 | 1.533931 |
| GO:0032501\_multicellular\_organismal\_process | AMD1 | 2183 | 26 | 1.142636 | -0.672898 | 173 | 265.37 | 1.533931 |
| GO:0032501\_multicellular\_organismal\_process | COL8A1 | 2183 | 26 | 1.142636 | -0.672898 | 173 | 265.37 | 1.533931 |
| GO:0032501\_multicellular\_organismal\_process | GUCA1B | 2183 | 26 | 1.142636 | -0.672898 | 173 | 265.37 | 1.533931 |
| GO:0032501\_multicellular\_organismal\_process | NTF3 | 2183 | 26 | 1.142636 | -0.672898 | 173 | 265.37 | 1.533931 |
| GO:0032501\_multicellular\_organismal\_process | CRYAB | 2183 | 26 | 1.142636 | -0.672898 | 173 | 265.37 | 1.533931 |
| GO:0032501\_multicellular\_organismal\_process | DTX1 | 2183 | 26 | 1.142636 | -0.672898 | 173 | 265.37 | 1.533931 |
| GO:0032501\_multicellular\_organismal\_process | CCNF | 2183 | 26 | 1.142636 | -0.672898 | 173 | 265.37 | 1.533931 |
| GO:0032501\_multicellular\_organismal\_process | NID1 | 2183 | 26 | 1.142636 | -0.672898 | 173 | 265.37 | 1.533931 |
| GO:0032501\_multicellular\_organismal\_process | KDR | 2183 | 26 | 1.142636 | -0.672898 | 173 | 265.37 | 1.533931 |
| GO:0032501\_multicellular\_organismal\_process | PGM3 | 2183 | 26 | 1.142636 | -0.672898 | 173 | 265.37 | 1.533931 |
| GO:0032501\_multicellular\_organismal\_process | ITGA6 | 2183 | 26 | 1.142636 | -0.672898 | 173 | 265.37 | 1.533931 |
| GO:0032501\_multicellular\_organismal\_process | ULK2 | 2183 | 26 | 1.142636 | -0.672898 | 173 | 265.37 | 1.533931 |
| GO:0032501\_multicellular\_organismal\_process | LAMB1-1 | 2183 | 26 | 1.142636 | -0.672898 | 173 | 265.37 | 1.533931 |
| GO:0032501\_multicellular\_organismal\_process | PLCG2 | 2183 | 26 | 1.142636 | -0.672898 | 173 | 265.37 | 1.533931 |
| GO:0032501\_multicellular\_organismal\_process | ADAMTS1 | 2183 | 26 | 1.142636 | -0.672898 | 173 | 265.37 | 1.533931 |
| GO:0032501\_multicellular\_organismal\_process | DST | 2183 | 26 | 1.142636 | -0.672898 | 173 | 265.37 | 1.533931 |
| GO:0006575\_cellular\_amino\_acid\_derivative\_metabolic\_process | AMD1 | 83 | 2 | 2.311747 | -0.669518 | 175 | 266.53 | 1.523029 |
| GO:0006575\_cellular\_amino\_acid\_derivative\_metabolic\_process | CRYM | 83 | 2 | 2.311747 | -0.669518 | 175 | 266.53 | 1.523029 |
| GO:0030198\_extracellular\_matrix\_organization | POSTN | 83 | 2 | 2.311747 | -0.669518 | 175 | 266.53 | 1.523029 |
| GO:0030198\_extracellular\_matrix\_organization | NID1 | 83 | 2 | 2.311747 | -0.669518 | 175 | 266.53 | 1.523029 |
| GO:0007018\_microtubule-based\_movement | DST | 23 | 1 | 4.171196 | -0.668345 | 178 | 271.06 | 1.522809 |
| GO:0008542\_visual\_learning | HMGCR | 23 | 1 | 4.171196 | -0.668345 | 178 | 271.06 | 1.522809 |
| GO:0030512\_negative\_regulation\_of\_transforming\_growth\_factor\_beta\_receptor\_signaling\_pathway | HTRA1 | 23 | 1 | 4.171196 | -0.668345 | 178 | 271.06 | 1.522809 |
| GO:0045137\_development\_of\_primary\_sexual\_characteristics | ADAMTS1 | 84 | 2 | 2.284226 | -0.661781 | 179 | 272.02 | 1.519665 |
| GO:0045137\_development\_of\_primary\_sexual\_characteristics | KDR | 84 | 2 | 2.284226 | -0.661781 | 179 | 272.02 | 1.519665 |
| GO:0001541\_ovarian\_follicle\_development | KDR | 24 | 1 | 3.997396 | -0.652007 | 183 | 279.16 | 1.525464 |
| GO:0006650\_glycerophospholipid\_metabolic\_process | PIP5K1B | 24 | 1 | 3.997396 | -0.652007 | 183 | 279.16 | 1.525464 |
| GO:0007632\_visual\_behavior | HMGCR | 24 | 1 | 3.997396 | -0.652007 | 183 | 279.16 | 1.525464 |
| GO:0050679\_positive\_regulation\_of\_epithelial\_cell\_proliferation | KDR | 24 | 1 | 3.997396 | -0.652007 | 183 | 279.16 | 1.525464 |
| GO:0050890\_cognition | GUCA1B | 233 | 4 | 1.646996 | -0.650975 | 184 | 279.59 | 1.519511 |
| GO:0050890\_cognition | HMGCR | 233 | 4 | 1.646996 | -0.650975 | 184 | 279.59 | 1.519511 |
| GO:0050890\_cognition | ABCA4 | 233 | 4 | 1.646996 | -0.650975 | 184 | 279.59 | 1.519511 |
| GO:0050890\_cognition | MFRP | 233 | 4 | 1.646996 | -0.650975 | 184 | 279.59 | 1.519511 |
| GO:0032504\_multicellular\_organism\_reproduction | ADAMTS1 | 86 | 2 | 2.231105 | -0.646670 | 186 | 280.86 | 1.510000 |
| GO:0032504\_multicellular\_organism\_reproduction | KDR | 86 | 2 | 2.231105 | -0.646670 | 186 | 280.86 | 1.510000 |
| GO:0048609\_reproductive\_process\_in\_a\_multicellular\_organism | ADAMTS1 | 86 | 2 | 2.231105 | -0.646670 | 186 | 280.86 | 1.510000 |
| GO:0048609\_reproductive\_process\_in\_a\_multicellular\_organism | KDR | 86 | 2 | 2.231105 | -0.646670 | 186 | 280.86 | 1.510000 |
| GO:0048699\_generation\_of\_neurons | IRX3 | 396 | 6 | 1.453598 | -0.642585 | 187 | 281.72 | 1.506524 |
| GO:0048699\_generation\_of\_neurons | NTF3 | 396 | 6 | 1.453598 | -0.642585 | 187 | 281.72 | 1.506524 |
| GO:0048699\_generation\_of\_neurons | ULK2 | 396 | 6 | 1.453598 | -0.642585 | 187 | 281.72 | 1.506524 |
| GO:0048699\_generation\_of\_neurons | BCL11B | 396 | 6 | 1.453598 | -0.642585 | 187 | 281.72 | 1.506524 |
| GO:0048699\_generation\_of\_neurons | BRSK2 | 396 | 6 | 1.453598 | -0.642585 | 187 | 281.72 | 1.506524 |
| GO:0048699\_generation\_of\_neurons | DST | 396 | 6 | 1.453598 | -0.642585 | 187 | 281.72 | 1.506524 |
| GO:0001822\_kidney\_development | ADAMTS1 | 87 | 2 | 2.205460 | -0.639290 | 188 | 282.95 | 1.505053 |
| GO:0001822\_kidney\_development | NID1 | 87 | 2 | 2.205460 | -0.639290 | 188 | 282.95 | 1.505053 |
| GO:0050852\_T\_cell\_receptor\_signaling\_pathway | PLCG2 | 25 | 1 | 3.837500 | -0.636419 | 189 | 288.17 | 1.524709 |
| GO:0010959\_regulation\_of\_metal\_ion\_transport | PLCG2 | 26 | 1 | 3.689904 | -0.621524 | 192 | 295.23 | 1.537656 |
| GO:0045665\_negative\_regulation\_of\_neuron\_differentiation | IRX3 | 26 | 1 | 3.689904 | -0.621524 | 192 | 295.23 | 1.537656 |
| GO:0050873\_brown\_fat\_cell\_differentiation | ITGA6 | 26 | 1 | 3.689904 | -0.621524 | 192 | 295.23 | 1.537656 |
| GO:0007422\_peripheral\_nervous\_system\_development | NTF3 | 27 | 1 | 3.553241 | -0.607268 | 194 | 301.48 | 1.554021 |
| GO:0032496\_response\_to\_lipopolysaccharide | PLCG2 | 27 | 1 | 3.553241 | -0.607268 | 194 | 301.48 | 1.554021 |
| GO:0006470\_protein\_amino\_acid\_dephosphorylation | DUSP6 | 28 | 1 | 3.426339 | -0.593605 | 195 | 306.98 | 1.574256 |
| GO:0042490\_mechanoreceptor\_differentiation | NTF3 | 29 | 1 | 3.308190 | -0.580493 | 197 | 314.47 | 1.596294 |
| GO:0048066\_pigmentation\_during\_development | SLC45A2 | 29 | 1 | 3.308190 | -0.580493 | 197 | 314.47 | 1.596294 |
| GO:0060249\_anatomical\_structure\_homeostasis | ABCA4 | 96 | 2 | 1.998698 | -0.577694 | 198 | 315.43 | 1.593081 |
| GO:0060249\_anatomical\_structure\_homeostasis | KDR | 96 | 2 | 1.998698 | -0.577694 | 198 | 315.43 | 1.593081 |
| GO:0007600\_sensory\_perception | GUCA1B | 172 | 3 | 1.673328 | -0.575106 | 199 | 315.91 | 1.587487 |
| GO:0007600\_sensory\_perception | ABCA4 | 172 | 3 | 1.673328 | -0.575106 | 199 | 315.91 | 1.587487 |
| GO:0007600\_sensory\_perception | MFRP | 172 | 3 | 1.673328 | -0.575106 | 199 | 315.91 | 1.587487 |
| GO:0007219\_Notch\_signaling\_pathway | DTX1 | 30 | 1 | 3.197917 | -0.567894 | 201 | 323.36 | 1.608756 |
| GO:0031668\_cellular\_response\_to\_extracellular\_stimulus | ITGA6 | 30 | 1 | 3.197917 | -0.567894 | 201 | 323.36 | 1.608756 |
| GO:0007548\_sex\_differentiation | ADAMTS1 | 98 | 2 | 1.957908 | -0.565083 | 203 | 324.25 | 1.597291 |
| GO:0007548\_sex\_differentiation | KDR | 98 | 2 | 1.957908 | -0.565083 | 203 | 324.25 | 1.597291 |
| GO:0009314\_response\_to\_radiation | GUCA1B | 98 | 2 | 1.957908 | -0.565083 | 203 | 324.25 | 1.597291 |
| GO:0009314\_response\_to\_radiation | HMGCR | 98 | 2 | 1.957908 | -0.565083 | 203 | 324.25 | 1.597291 |
| GO:0006486\_protein\_amino\_acid\_glycosylation | GALNT10 | 31 | 1 | 3.094758 | -0.555774 | 210 | 334.21 | 1.591476 |
| GO:0006694\_steroid\_biosynthetic\_process | HMGCR | 31 | 1 | 3.094758 | -0.555774 | 210 | 334.21 | 1.591476 |
| GO:0016311\_dephosphorylation | DUSP6 | 31 | 1 | 3.094758 | -0.555774 | 210 | 334.21 | 1.591476 |
| GO:0043269\_regulation\_of\_ion\_transport | PLCG2 | 31 | 1 | 3.094758 | -0.555774 | 210 | 334.21 | 1.591476 |
| GO:0043413\_biopolymer\_glycosylation | GALNT10 | 31 | 1 | 3.094758 | -0.555774 | 210 | 334.21 | 1.591476 |
| GO:0046632\_alpha-beta\_T\_cell\_differentiation | BCL11B | 31 | 1 | 3.094758 | -0.555774 | 210 | 334.21 | 1.591476 |
| GO:0070085\_glycosylation | GALNT10 | 31 | 1 | 3.094758 | -0.555774 | 210 | 334.21 | 1.591476 |
| GO:0006793\_phosphorus\_metabolic\_process | HMGCR | 340 | 5 | 1.410846 | -0.554636 | 212 | 334.66 | 1.578585 |
| GO:0006793\_phosphorus\_metabolic\_process | HK2 | 340 | 5 | 1.410846 | -0.554636 | 212 | 334.66 | 1.578585 |
| GO:0006793\_phosphorus\_metabolic\_process | BRSK2 | 340 | 5 | 1.410846 | -0.554636 | 212 | 334.66 | 1.578585 |
| GO:0006793\_phosphorus\_metabolic\_process | DUSP6 | 340 | 5 | 1.410846 | -0.554636 | 212 | 334.66 | 1.578585 |
| GO:0006793\_phosphorus\_metabolic\_process | VLDLR | 340 | 5 | 1.410846 | -0.554636 | 212 | 334.66 | 1.578585 |
| GO:0006796\_phosphate\_metabolic\_process | HMGCR | 340 | 5 | 1.410846 | -0.554636 | 212 | 334.66 | 1.578585 |
| GO:0006796\_phosphate\_metabolic\_process | HK2 | 340 | 5 | 1.410846 | -0.554636 | 212 | 334.66 | 1.578585 |
| GO:0006796\_phosphate\_metabolic\_process | BRSK2 | 340 | 5 | 1.410846 | -0.554636 | 212 | 334.66 | 1.578585 |
| GO:0006796\_phosphate\_metabolic\_process | DUSP6 | 340 | 5 | 1.410846 | -0.554636 | 212 | 334.66 | 1.578585 |
| GO:0006796\_phosphate\_metabolic\_process | VLDLR | 340 | 5 | 1.410846 | -0.554636 | 212 | 334.66 | 1.578585 |
| GO:0048856\_anatomical\_structure\_development | IRX3 | 1688 | 20 | 1.136700 | -0.552357 | 213 | 335.03 | 1.572911 |
| GO:0048856\_anatomical\_structure\_development | NTF3 | 1688 | 20 | 1.136700 | -0.552357 | 213 | 335.03 | 1.572911 |
| GO:0048856\_anatomical\_structure\_development | DTX1 | 1688 | 20 | 1.136700 | -0.552357 | 213 | 335.03 | 1.572911 |
| GO:0048856\_anatomical\_structure\_development | CRYAB | 1688 | 20 | 1.136700 | -0.552357 | 213 | 335.03 | 1.572911 |
| GO:0048856\_anatomical\_structure\_development | CCNF | 1688 | 20 | 1.136700 | -0.552357 | 213 | 335.03 | 1.572911 |
| GO:0048856\_anatomical\_structure\_development | BRSK2 | 1688 | 20 | 1.136700 | -0.552357 | 213 | 335.03 | 1.572911 |
| GO:0048856\_anatomical\_structure\_development | NID1 | 1688 | 20 | 1.136700 | -0.552357 | 213 | 335.03 | 1.572911 |
| GO:0048856\_anatomical\_structure\_development | POSTN | 1688 | 20 | 1.136700 | -0.552357 | 213 | 335.03 | 1.572911 |
| GO:0048856\_anatomical\_structure\_development | SOX6 | 1688 | 20 | 1.136700 | -0.552357 | 213 | 335.03 | 1.572911 |
| GO:0048856\_anatomical\_structure\_development | KDR | 1688 | 20 | 1.136700 | -0.552357 | 213 | 335.03 | 1.572911 |
| GO:0048856\_anatomical\_structure\_development | PGM3 | 1688 | 20 | 1.136700 | -0.552357 | 213 | 335.03 | 1.572911 |
| GO:0048856\_anatomical\_structure\_development | ANK | 1688 | 20 | 1.136700 | -0.552357 | 213 | 335.03 | 1.572911 |
| GO:0048856\_anatomical\_structure\_development | SYNE2 | 1688 | 20 | 1.136700 | -0.552357 | 213 | 335.03 | 1.572911 |
| GO:0048856\_anatomical\_structure\_development | ITGA6 | 1688 | 20 | 1.136700 | -0.552357 | 213 | 335.03 | 1.572911 |
| GO:0048856\_anatomical\_structure\_development | ULK2 | 1688 | 20 | 1.136700 | -0.552357 | 213 | 335.03 | 1.572911 |
| GO:0048856\_anatomical\_structure\_development | BCL11B | 1688 | 20 | 1.136700 | -0.552357 | 213 | 335.03 | 1.572911 |
| GO:0048856\_anatomical\_structure\_development | PLCG2 | 1688 | 20 | 1.136700 | -0.552357 | 213 | 335.03 | 1.572911 |
| GO:0048856\_anatomical\_structure\_development | ADAMTS1 | 1688 | 20 | 1.136700 | -0.552357 | 213 | 335.03 | 1.572911 |
| GO:0048856\_anatomical\_structure\_development | COL8A1 | 1688 | 20 | 1.136700 | -0.552357 | 213 | 335.03 | 1.572911 |
| GO:0048856\_anatomical\_structure\_development | DST | 1688 | 20 | 1.136700 | -0.552357 | 213 | 335.03 | 1.572911 |
| GO:0050768\_negative\_regulation\_of\_neurogenesis | ULK2 | 32 | 1 | 2.998047 | -0.544102 | 216 | 339.12 | 1.570000 |
| GO:0050770\_regulation\_of\_axonogenesis | ULK2 | 32 | 1 | 2.998047 | -0.544102 | 216 | 339.12 | 1.570000 |
| GO:0051493\_regulation\_of\_cytoskeleton\_organization | DST | 32 | 1 | 2.998047 | -0.544102 | 216 | 339.12 | 1.570000 |
| GO:0048666\_neuron\_development | NTF3 | 262 | 4 | 1.464695 | -0.536835 | 217 | 340.85 | 1.570737 |
| GO:0048666\_neuron\_development | BCL11B | 262 | 4 | 1.464695 | -0.536835 | 217 | 340.85 | 1.570737 |
| GO:0048666\_neuron\_development | ULK2 | 262 | 4 | 1.464695 | -0.536835 | 217 | 340.85 | 1.570737 |
| GO:0048666\_neuron\_development | DST | 262 | 4 | 1.464695 | -0.536835 | 217 | 340.85 | 1.570737 |
| GO:0002562\_somatic\_diversification\_of\_immune\_receptors\_via\_germline\_recombination\_within\_a\_single\_locus | BCL11B | 33 | 1 | 2.907197 | -0.532852 | 220 | 346.27 | 1.573955 |
| GO:0007565\_female\_pregnancy | LAMB1-1 | 33 | 1 | 2.907197 | -0.532852 | 220 | 346.27 | 1.573955 |
| GO:0016444\_somatic\_cell\_DNA\_recombination | BCL11B | 33 | 1 | 2.907197 | -0.532852 | 220 | 346.27 | 1.573955 |
| GO:0016043\_cellular\_component\_organization | SYNE2 | 964 | 12 | 1.194243 | -0.531837 | 221 | 346.36 | 1.567240 |
| GO:0016043\_cellular\_component\_organization | NTF3 | 964 | 12 | 1.194243 | -0.531837 | 221 | 346.36 | 1.567240 |
| GO:0016043\_cellular\_component\_organization | ITGA6 | 964 | 12 | 1.194243 | -0.531837 | 221 | 346.36 | 1.567240 |
| GO:0016043\_cellular\_component\_organization | NOLC1 | 964 | 12 | 1.194243 | -0.531837 | 221 | 346.36 | 1.567240 |
| GO:0016043\_cellular\_component\_organization | BCL11B | 964 | 12 | 1.194243 | -0.531837 | 221 | 346.36 | 1.567240 |
| GO:0016043\_cellular\_component\_organization | ULK2 | 964 | 12 | 1.194243 | -0.531837 | 221 | 346.36 | 1.567240 |
| GO:0016043\_cellular\_component\_organization | CUGBP2 | 964 | 12 | 1.194243 | -0.531837 | 221 | 346.36 | 1.567240 |
| GO:0016043\_cellular\_component\_organization | BRSK2 | 964 | 12 | 1.194243 | -0.531837 | 221 | 346.36 | 1.567240 |
| GO:0016043\_cellular\_component\_organization | POSTN | 964 | 12 | 1.194243 | -0.531837 | 221 | 346.36 | 1.567240 |
| GO:0016043\_cellular\_component\_organization | NID1 | 964 | 12 | 1.194243 | -0.531837 | 221 | 346.36 | 1.567240 |
| GO:0016043\_cellular\_component\_organization | SOX6 | 964 | 12 | 1.194243 | -0.531837 | 221 | 346.36 | 1.567240 |
| GO:0016043\_cellular\_component\_organization | DST | 964 | 12 | 1.194243 | -0.531837 | 221 | 346.36 | 1.567240 |
| GO:0050767\_regulation\_of\_neurogenesis | IRX3 | 104 | 2 | 1.844952 | -0.529327 | 222 | 347.63 | 1.565901 |
| GO:0050767\_regulation\_of\_neurogenesis | ULK2 | 104 | 2 | 1.844952 | -0.529327 | 222 | 347.63 | 1.565901 |
| GO:0048731\_system\_development | IRX3 | 1609 | 19 | 1.132885 | -0.528908 | 223 | 347.72 | 1.559283 |
| GO:0048731\_system\_development | NTF3 | 1609 | 19 | 1.132885 | -0.528908 | 223 | 347.72 | 1.559283 |
| GO:0048731\_system\_development | CRYAB | 1609 | 19 | 1.132885 | -0.528908 | 223 | 347.72 | 1.559283 |
| GO:0048731\_system\_development | DTX1 | 1609 | 19 | 1.132885 | -0.528908 | 223 | 347.72 | 1.559283 |
| GO:0048731\_system\_development | CCNF | 1609 | 19 | 1.132885 | -0.528908 | 223 | 347.72 | 1.559283 |
| GO:0048731\_system\_development | BRSK2 | 1609 | 19 | 1.132885 | -0.528908 | 223 | 347.72 | 1.559283 |
| GO:0048731\_system\_development | POSTN | 1609 | 19 | 1.132885 | -0.528908 | 223 | 347.72 | 1.559283 |
| GO:0048731\_system\_development | NID1 | 1609 | 19 | 1.132885 | -0.528908 | 223 | 347.72 | 1.559283 |
| GO:0048731\_system\_development | SOX6 | 1609 | 19 | 1.132885 | -0.528908 | 223 | 347.72 | 1.559283 |
| GO:0048731\_system\_development | KDR | 1609 | 19 | 1.132885 | -0.528908 | 223 | 347.72 | 1.559283 |
| GO:0048731\_system\_development | ANK | 1609 | 19 | 1.132885 | -0.528908 | 223 | 347.72 | 1.559283 |
| GO:0048731\_system\_development | PGM3 | 1609 | 19 | 1.132885 | -0.528908 | 223 | 347.72 | 1.559283 |
| GO:0048731\_system\_development | ITGA6 | 1609 | 19 | 1.132885 | -0.528908 | 223 | 347.72 | 1.559283 |
| GO:0048731\_system\_development | BCL11B | 1609 | 19 | 1.132885 | -0.528908 | 223 | 347.72 | 1.559283 |
| GO:0048731\_system\_development | ULK2 | 1609 | 19 | 1.132885 | -0.528908 | 223 | 347.72 | 1.559283 |
| GO:0048731\_system\_development | PLCG2 | 1609 | 19 | 1.132885 | -0.528908 | 223 | 347.72 | 1.559283 |
| GO:0048731\_system\_development | ADAMTS1 | 1609 | 19 | 1.132885 | -0.528908 | 223 | 347.72 | 1.559283 |
| GO:0048731\_system\_development | COL8A1 | 1609 | 19 | 1.132885 | -0.528908 | 223 | 347.72 | 1.559283 |
| GO:0048731\_system\_development | DST | 1609 | 19 | 1.132885 | -0.528908 | 223 | 347.72 | 1.559283 |
| GO:0002200\_somatic\_diversification\_of\_immune\_receptors | BCL11B | 34 | 1 | 2.821691 | -0.521996 | 227 | 353.41 | 1.556872 |
| GO:0002237\_response\_to\_molecule\_of\_bacterial\_origin | PLCG2 | 34 | 1 | 2.821691 | -0.521996 | 227 | 353.41 | 1.556872 |
| GO:0010721\_negative\_regulation\_of\_cell\_development | ULK2 | 34 | 1 | 2.821691 | -0.521996 | 227 | 353.41 | 1.556872 |
| GO:0030509\_BMP\_signaling\_pathway | HTRA1 | 34 | 1 | 2.821691 | -0.521996 | 227 | 353.41 | 1.556872 |
| GO:0045859\_regulation\_of\_protein\_kinase\_activity | HMGCR | 107 | 2 | 1.793224 | -0.512534 | 228 | 355.52 | 1.559298 |
| GO:0045859\_regulation\_of\_protein\_kinase\_activity | VLDLR | 107 | 2 | 1.793224 | -0.512534 | 228 | 355.52 | 1.559298 |
| GO:0006869\_lipid\_transport | ABCA4 | 35 | 1 | 2.741071 | -0.511514 | 231 | 359.17 | 1.554848 |
| GO:0007292\_female\_gamete\_generation | ADAMTS1 | 35 | 1 | 2.741071 | -0.511514 | 231 | 359.17 | 1.554848 |
| GO:0016051\_carbohydrate\_biosynthetic\_process | PGM3 | 35 | 1 | 2.741071 | -0.511514 | 231 | 359.17 | 1.554848 |
| GO:0006469\_negative\_regulation\_of\_protein\_kinase\_activity | HMGCR | 36 | 1 | 2.664931 | -0.501382 | 236 | 365.92 | 1.550508 |
| GO:0030278\_regulation\_of\_ossification | ANK | 36 | 1 | 2.664931 | -0.501382 | 236 | 365.92 | 1.550508 |
| GO:0033673\_negative\_regulation\_of\_kinase\_activity | HMGCR | 36 | 1 | 2.664931 | -0.501382 | 236 | 365.92 | 1.550508 |
| GO:0050851\_antigen\_receptor-mediated\_signaling\_pathway | PLCG2 | 36 | 1 | 2.664931 | -0.501382 | 236 | 365.92 | 1.550508 |
| GO:0050900\_leukocyte\_migration | ITGA6 | 36 | 1 | 2.664931 | -0.501382 | 236 | 365.92 | 1.550508 |
| GO:0050906\_detection\_of\_stimulus\_involved\_in\_sensory\_perception | GUCA1B | 37 | 1 | 2.592905 | -0.491582 | 237 | 370.35 | 1.562658 |
| GO:0043549\_regulation\_of\_kinase\_activity | HMGCR | 112 | 2 | 1.713170 | -0.486010 | 238 | 371.98 | 1.562941 |
| GO:0043549\_regulation\_of\_kinase\_activity | VLDLR | 112 | 2 | 1.713170 | -0.486010 | 238 | 371.98 | 1.562941 |
| GO:0046907\_intracellular\_transport | FUSIP1 | 194 | 3 | 1.483570 | -0.482307 | 239 | 372.34 | 1.557908 |
| GO:0046907\_intracellular\_transport | XPO5 | 194 | 3 | 1.483570 | -0.482307 | 239 | 372.34 | 1.557908 |
| GO:0046907\_intracellular\_transport | DST | 194 | 3 | 1.483570 | -0.482307 | 239 | 372.34 | 1.557908 |
| GO:0001570\_vasculogenesis | KDR | 38 | 1 | 2.524671 | -0.482096 | 242 | 377.83 | 1.561281 |
| GO:0010975\_regulation\_of\_neuron\_projection\_development | ULK2 | 38 | 1 | 2.524671 | -0.482096 | 242 | 377.83 | 1.561281 |
| GO:0051348\_negative\_regulation\_of\_transferase\_activity | HMGCR | 38 | 1 | 2.524671 | -0.482096 | 242 | 377.83 | 1.561281 |
| GO:0065009\_regulation\_of\_molecular\_function | GUCA1B | 279 | 4 | 1.375448 | -0.479799 | 243 | 379.09 | 1.560041 |
| GO:0065009\_regulation\_of\_molecular\_function | HMGCR | 279 | 4 | 1.375448 | -0.479799 | 243 | 379.09 | 1.560041 |
| GO:0065009\_regulation\_of\_molecular\_function | PLCG2 | 279 | 4 | 1.375448 | -0.479799 | 243 | 379.09 | 1.560041 |
| GO:0065009\_regulation\_of\_molecular\_function | VLDLR | 279 | 4 | 1.375448 | -0.479799 | 243 | 379.09 | 1.560041 |
| GO:0006644\_phospholipid\_metabolic\_process | PIP5K1B | 39 | 1 | 2.459936 | -0.472907 | 248 | 384.73 | 1.551331 |
| GO:0021953\_central\_nervous\_system\_neuron\_differentiation | BCL11B | 39 | 1 | 2.459936 | -0.472907 | 248 | 384.73 | 1.551331 |
| GO:0031279\_regulation\_of\_cyclase\_activity | GUCA1B | 39 | 1 | 2.459936 | -0.472907 | 248 | 384.73 | 1.551331 |
| GO:0042475\_odontogenesis\_of\_dentine-containing\_tooth | ITGA6 | 39 | 1 | 2.459936 | -0.472907 | 248 | 384.73 | 1.551331 |
| GO:0051339\_regulation\_of\_lyase\_activity | GUCA1B | 39 | 1 | 2.459936 | -0.472907 | 248 | 384.73 | 1.551331 |
| GO:0051338\_regulation\_of\_transferase\_activity | HMGCR | 115 | 2 | 1.668478 | -0.470915 | 249 | 386.19 | 1.550964 |
| GO:0051338\_regulation\_of\_transferase\_activity | VLDLR | 115 | 2 | 1.668478 | -0.470915 | 249 | 386.19 | 1.550964 |
| GO:0048608\_reproductive\_structure\_development | ADAMTS1 | 116 | 2 | 1.654095 | -0.466012 | 250 | 388.12 | 1.552480 |
| GO:0048608\_reproductive\_structure\_development | KDR | 116 | 2 | 1.654095 | -0.466012 | 250 | 388.12 | 1.552480 |
| GO:0008203\_cholesterol\_metabolic\_process | HMGCR | 40 | 1 | 2.398438 | -0.464000 | 254 | 391.9 | 1.542913 |
| GO:0017015\_regulation\_of\_transforming\_growth\_factor\_beta\_receptor\_signaling\_pathway | HTRA1 | 40 | 1 | 2.398438 | -0.464000 | 254 | 391.9 | 1.542913 |
| GO:0046850\_regulation\_of\_bone\_remodeling | ANK | 40 | 1 | 2.398438 | -0.464000 | 254 | 391.9 | 1.542913 |
| GO:0051129\_negative\_regulation\_of\_cellular\_component\_organization | ULK2 | 40 | 1 | 2.398438 | -0.464000 | 254 | 391.9 | 1.542913 |
| GO:0006519\_cellular\_amino\_acid\_and\_derivative\_metabolic\_process | AMD1 | 118 | 2 | 1.626059 | -0.456394 | 256 | 393.68 | 1.537812 |
| GO:0006519\_cellular\_amino\_acid\_and\_derivative\_metabolic\_process | CRYM | 118 | 2 | 1.626059 | -0.456394 | 256 | 393.68 | 1.537812 |
| GO:0051960\_regulation\_of\_nervous\_system\_development | IRX3 | 118 | 2 | 1.626059 | -0.456394 | 256 | 393.68 | 1.537812 |
| GO:0051960\_regulation\_of\_nervous\_system\_development | ULK2 | 118 | 2 | 1.626059 | -0.456394 | 256 | 393.68 | 1.537812 |
| GO:0002429\_immune\_response-activating\_cell\_surface\_receptor\_signaling\_pathway | PLCG2 | 41 | 1 | 2.339939 | -0.455361 | 259 | 399.21 | 1.541351 |
| GO:0019748\_secondary\_metabolic\_process | TYR | 41 | 1 | 2.339939 | -0.455361 | 259 | 399.21 | 1.541351 |
| GO:0031344\_regulation\_of\_cell\_projection\_organization | ULK2 | 41 | 1 | 2.339939 | -0.455361 | 259 | 399.21 | 1.541351 |
| GO:0010769\_regulation\_of\_cell\_morphogenesis\_involved\_in\_differentiation | ULK2 | 42 | 1 | 2.284226 | -0.446977 | 262 | 405.79 | 1.548817 |
| GO:0016125\_sterol\_metabolic\_process | HMGCR | 42 | 1 | 2.284226 | -0.446977 | 262 | 405.79 | 1.548817 |
| GO:0042476\_odontogenesis | ITGA6 | 42 | 1 | 2.284226 | -0.446977 | 262 | 405.79 | 1.548817 |
| GO:0001894\_tissue\_homeostasis | KDR | 43 | 1 | 2.231105 | -0.438836 | 265 | 412.68 | 1.557283 |
| GO:0009582\_detection\_of\_abiotic\_stimulus | GUCA1B | 43 | 1 | 2.231105 | -0.438836 | 265 | 412.68 | 1.557283 |
| GO:0019637\_organophosphate\_metabolic\_process | PIP5K1B | 43 | 1 | 2.231105 | -0.438836 | 265 | 412.68 | 1.557283 |
| GO:0060284\_regulation\_of\_cell\_development | IRX3 | 122 | 2 | 1.572746 | -0.437874 | 266 | 414.06 | 1.556617 |
| GO:0060284\_regulation\_of\_cell\_development | ULK2 | 122 | 2 | 1.572746 | -0.437874 | 266 | 414.06 | 1.556617 |
| GO:0048869\_cellular\_developmental\_process | IRX3 | 1113 | 13 | 1.120564 | -0.431058 | 267 | 415.46 | 1.556030 |
| GO:0048869\_cellular\_developmental\_process | NTF3 | 1113 | 13 | 1.120564 | -0.431058 | 267 | 415.46 | 1.556030 |
| GO:0048869\_cellular\_developmental\_process | DTX1 | 1113 | 13 | 1.120564 | -0.431058 | 267 | 415.46 | 1.556030 |
| GO:0048869\_cellular\_developmental\_process | TDRD7 | 1113 | 13 | 1.120564 | -0.431058 | 267 | 415.46 | 1.556030 |
| GO:0048869\_cellular\_developmental\_process | BRSK2 | 1113 | 13 | 1.120564 | -0.431058 | 267 | 415.46 | 1.556030 |
| GO:0048869\_cellular\_developmental\_process | SOX6 | 1113 | 13 | 1.120564 | -0.431058 | 267 | 415.46 | 1.556030 |
| GO:0048869\_cellular\_developmental\_process | KDR | 1113 | 13 | 1.120564 | -0.431058 | 267 | 415.46 | 1.556030 |
| GO:0048869\_cellular\_developmental\_process | SYNE2 | 1113 | 13 | 1.120564 | -0.431058 | 267 | 415.46 | 1.556030 |
| GO:0048869\_cellular\_developmental\_process | ITGA6 | 1113 | 13 | 1.120564 | -0.431058 | 267 | 415.46 | 1.556030 |
| GO:0048869\_cellular\_developmental\_process | BCL11B | 1113 | 13 | 1.120564 | -0.431058 | 267 | 415.46 | 1.556030 |
| GO:0048869\_cellular\_developmental\_process | ULK2 | 1113 | 13 | 1.120564 | -0.431058 | 267 | 415.46 | 1.556030 |
| GO:0048869\_cellular\_developmental\_process | PLCG2 | 1113 | 13 | 1.120564 | -0.431058 | 267 | 415.46 | 1.556030 |
| GO:0048869\_cellular\_developmental\_process | DST | 1113 | 13 | 1.120564 | -0.431058 | 267 | 415.46 | 1.556030 |
| GO:0002768\_immune\_response-regulating\_cell\_surface\_receptor\_signaling\_pathway | PLCG2 | 44 | 1 | 2.180398 | -0.430926 | 271 | 420.96 | 1.553358 |
| GO:0030802\_regulation\_of\_cyclic\_nucleotide\_biosynthetic\_process | GUCA1B | 44 | 1 | 2.180398 | -0.430926 | 271 | 420.96 | 1.553358 |
| GO:0030808\_regulation\_of\_nucleotide\_biosynthetic\_process | GUCA1B | 44 | 1 | 2.180398 | -0.430926 | 271 | 420.96 | 1.553358 |
| GO:0048593\_camera-type\_eye\_morphogenesis | COL8A1 | 44 | 1 | 2.180398 | -0.430926 | 271 | 420.96 | 1.553358 |
| GO:0009308\_amine\_metabolic\_process | AMD1 | 124 | 2 | 1.547379 | -0.428957 | 273 | 421.96 | 1.545641 |
| GO:0009308\_amine\_metabolic\_process | CRYM | 124 | 2 | 1.547379 | -0.428957 | 273 | 421.96 | 1.545641 |
| GO:0030098\_lymphocyte\_differentiation | BCL11B | 124 | 2 | 1.547379 | -0.428957 | 273 | 421.96 | 1.545641 |
| GO:0030098\_lymphocyte\_differentiation | PLCG2 | 124 | 2 | 1.547379 | -0.428957 | 273 | 421.96 | 1.545641 |
| GO:0043062\_extracellular\_structure\_organization | POSTN | 125 | 2 | 1.535000 | -0.424580 | 274 | 422.9 | 1.543431 |
| GO:0043062\_extracellular\_structure\_organization | NID1 | 125 | 2 | 1.535000 | -0.424580 | 274 | 422.9 | 1.543431 |
| GO:0034103\_regulation\_of\_tissue\_remodeling | ANK | 45 | 1 | 2.131944 | -0.423237 | 275 | 424.46 | 1.543491 |
| GO:0007612\_learning | HMGCR | 46 | 1 | 2.085598 | -0.415759 | 279 | 428.46 | 1.535699 |
| GO:0009581\_detection\_of\_external\_stimulus | GUCA1B | 46 | 1 | 2.085598 | -0.415759 | 279 | 428.46 | 1.535699 |
| GO:0030218\_erythrocyte\_differentiation | SOX6 | 46 | 1 | 2.085598 | -0.415759 | 279 | 428.46 | 1.535699 |
| GO:0046631\_alpha-beta\_T\_cell\_activation | BCL11B | 46 | 1 | 2.085598 | -0.415759 | 279 | 428.46 | 1.535699 |
| GO:0050877\_neurological\_system\_process | GUCA1B | 390 | 5 | 1.229968 | -0.415312 | 280 | 428.58 | 1.530643 |
| GO:0050877\_neurological\_system\_process | NTF3 | 390 | 5 | 1.229968 | -0.415312 | 280 | 428.58 | 1.530643 |
| GO:0050877\_neurological\_system\_process | HMGCR | 390 | 5 | 1.229968 | -0.415312 | 280 | 428.58 | 1.530643 |
| GO:0050877\_neurological\_system\_process | ABCA4 | 390 | 5 | 1.229968 | -0.415312 | 280 | 428.58 | 1.530643 |
| GO:0050877\_neurological\_system\_process | MFRP | 390 | 5 | 1.229968 | -0.415312 | 280 | 428.58 | 1.530643 |
| GO:0001655\_urogenital\_system\_development | ADAMTS1 | 128 | 2 | 1.499023 | -0.411767 | 281 | 429.26 | 1.527616 |
| GO:0001655\_urogenital\_system\_development | NID1 | 128 | 2 | 1.499023 | -0.411767 | 281 | 429.26 | 1.527616 |
| GO:0002757\_immune\_response-activating\_signal\_transduction | PLCG2 | 47 | 1 | 2.041223 | -0.408483 | 287 | 433.03 | 1.508815 |
| GO:0006140\_regulation\_of\_nucleotide\_metabolic\_process | GUCA1B | 47 | 1 | 2.041223 | -0.408483 | 287 | 433.03 | 1.508815 |
| GO:0030183\_B\_cell\_differentiation | PLCG2 | 47 | 1 | 2.041223 | -0.408483 | 287 | 433.03 | 1.508815 |
| GO:0030799\_regulation\_of\_cyclic\_nucleotide\_metabolic\_process | GUCA1B | 47 | 1 | 2.041223 | -0.408483 | 287 | 433.03 | 1.508815 |
| GO:0034754\_cellular\_hormone\_metabolic\_process | CRYM | 47 | 1 | 2.041223 | -0.408483 | 287 | 433.03 | 1.508815 |
| GO:0048871\_multicellular\_organismal\_homeostasis | KDR | 47 | 1 | 2.041223 | -0.408483 | 287 | 433.03 | 1.508815 |
| GO:0009101\_glycoprotein\_biosynthetic\_process | GALNT10 | 48 | 1 | 1.998698 | -0.401400 | 290 | 437.88 | 1.509931 |
| GO:0034504\_protein\_localization\_in\_nucleus | SYNE2 | 48 | 1 | 1.998698 | -0.401400 | 290 | 437.88 | 1.509931 |
| GO:0046849\_bone\_remodeling | ANK | 48 | 1 | 1.998698 | -0.401400 | 290 | 437.88 | 1.509931 |
| GO:0007423\_sensory\_organ\_development | CRYAB | 219 | 3 | 1.314212 | -0.396294 | 291 | 438.65 | 1.507388 |
| GO:0007423\_sensory\_organ\_development | BCL11B | 219 | 3 | 1.314212 | -0.396294 | 291 | 438.65 | 1.507388 |
| GO:0007423\_sensory\_organ\_development | COL8A1 | 219 | 3 | 1.314212 | -0.396294 | 291 | 438.65 | 1.507388 |
| GO:0034101\_erythrocyte\_homeostasis | SOX6 | 49 | 1 | 1.957908 | -0.394502 | 292 | 442.61 | 1.515788 |
| GO:0016310\_phosphorylation | HMGCR | 309 | 4 | 1.241909 | -0.393669 | 293 | 442.83 | 1.511365 |
| GO:0016310\_phosphorylation | HK2 | 309 | 4 | 1.241909 | -0.393669 | 293 | 442.83 | 1.511365 |
| GO:0016310\_phosphorylation | BRSK2 | 309 | 4 | 1.241909 | -0.393669 | 293 | 442.83 | 1.511365 |
| GO:0016310\_phosphorylation | VLDLR | 309 | 4 | 1.241909 | -0.393669 | 293 | 442.83 | 1.511365 |
| GO:0009190\_cyclic\_nucleotide\_biosynthetic\_process | GUCA1B | 50 | 1 | 1.918750 | -0.387781 | 295 | 446.66 | 1.514102 |
| GO:0051606\_detection\_of\_stimulus | GUCA1B | 50 | 1 | 1.918750 | -0.387781 | 295 | 446.66 | 1.514102 |
| GO:0007283\_spermatogenesis | PGM3 | 134 | 2 | 1.431903 | -0.387495 | 297 | 447.14 | 1.505522 |
| GO:0007283\_spermatogenesis | CRTAP | 134 | 2 | 1.431903 | -0.387495 | 297 | 447.14 | 1.505522 |
| GO:0048232\_male\_gamete\_generation | PGM3 | 134 | 2 | 1.431903 | -0.387495 | 297 | 447.14 | 1.505522 |
| GO:0048232\_male\_gamete\_generation | CRTAP | 134 | 2 | 1.431903 | -0.387495 | 297 | 447.14 | 1.505522 |
| GO:0009653\_anatomical\_structure\_morphogenesis | SYNE2 | 958 | 11 | 1.101579 | -0.382778 | 298 | 447.6 | 1.502013 |
| GO:0009653\_anatomical\_structure\_morphogenesis | NTF3 | 958 | 11 | 1.101579 | -0.382778 | 298 | 447.6 | 1.502013 |
| GO:0009653\_anatomical\_structure\_morphogenesis | ITGA6 | 958 | 11 | 1.101579 | -0.382778 | 298 | 447.6 | 1.502013 |
| GO:0009653\_anatomical\_structure\_morphogenesis | BCL11B | 958 | 11 | 1.101579 | -0.382778 | 298 | 447.6 | 1.502013 |
| GO:0009653\_anatomical\_structure\_morphogenesis | ULK2 | 958 | 11 | 1.101579 | -0.382778 | 298 | 447.6 | 1.502013 |
| GO:0009653\_anatomical\_structure\_morphogenesis | BRSK2 | 958 | 11 | 1.101579 | -0.382778 | 298 | 447.6 | 1.502013 |
| GO:0009653\_anatomical\_structure\_morphogenesis | ADAMTS1 | 958 | 11 | 1.101579 | -0.382778 | 298 | 447.6 | 1.502013 |
| GO:0009653\_anatomical\_structure\_morphogenesis | SOX6 | 958 | 11 | 1.101579 | -0.382778 | 298 | 447.6 | 1.502013 |
| GO:0009653\_anatomical\_structure\_morphogenesis | COL8A1 | 958 | 11 | 1.101579 | -0.382778 | 298 | 447.6 | 1.502013 |
| GO:0009653\_anatomical\_structure\_morphogenesis | DST | 958 | 11 | 1.101579 | -0.382778 | 298 | 447.6 | 1.502013 |
| GO:0009653\_anatomical\_structure\_morphogenesis | KDR | 958 | 11 | 1.101579 | -0.382778 | 298 | 447.6 | 1.502013 |
| GO:0002764\_immune\_response-regulating\_signal\_transduction | PLCG2 | 51 | 1 | 1.881127 | -0.381230 | 299 | 450.69 | 1.507324 |
| GO:0009124\_nucleoside\_monophosphate\_biosynthetic\_process | GUCA1B | 52 | 1 | 1.844952 | -0.374843 | 300 | 453.44 | 1.511467 |
| GO:0030154\_cell\_differentiation | IRX3 | 1060 | 12 | 1.086085 | -0.370007 | 301 | 454.26 | 1.509169 |
| GO:0030154\_cell\_differentiation | ITGA6 | 1060 | 12 | 1.086085 | -0.370007 | 301 | 454.26 | 1.509169 |
| GO:0030154\_cell\_differentiation | NTF3 | 1060 | 12 | 1.086085 | -0.370007 | 301 | 454.26 | 1.509169 |
| GO:0030154\_cell\_differentiation | TDRD7 | 1060 | 12 | 1.086085 | -0.370007 | 301 | 454.26 | 1.509169 |
| GO:0030154\_cell\_differentiation | DTX1 | 1060 | 12 | 1.086085 | -0.370007 | 301 | 454.26 | 1.509169 |
| GO:0030154\_cell\_differentiation | ULK2 | 1060 | 12 | 1.086085 | -0.370007 | 301 | 454.26 | 1.509169 |
| GO:0030154\_cell\_differentiation | BCL11B | 1060 | 12 | 1.086085 | -0.370007 | 301 | 454.26 | 1.509169 |
| GO:0030154\_cell\_differentiation | PLCG2 | 1060 | 12 | 1.086085 | -0.370007 | 301 | 454.26 | 1.509169 |
| GO:0030154\_cell\_differentiation | BRSK2 | 1060 | 12 | 1.086085 | -0.370007 | 301 | 454.26 | 1.509169 |
| GO:0030154\_cell\_differentiation | SOX6 | 1060 | 12 | 1.086085 | -0.370007 | 301 | 454.26 | 1.509169 |
| GO:0030154\_cell\_differentiation | DST | 1060 | 12 | 1.086085 | -0.370007 | 301 | 454.26 | 1.509169 |
| GO:0030154\_cell\_differentiation | KDR | 1060 | 12 | 1.086085 | -0.370007 | 301 | 454.26 | 1.509169 |
| GO:0030031\_cell\_projection\_assembly | ITGA6 | 53 | 1 | 1.810142 | -0.368613 | 304 | 457.62 | 1.505329 |
| GO:0046942\_carboxylic\_acid\_transport | SLC13A3 | 53 | 1 | 1.810142 | -0.368613 | 304 | 457.62 | 1.505329 |
| GO:0055085\_transmembrane\_transport | PLCG2 | 53 | 1 | 1.810142 | -0.368613 | 304 | 457.62 | 1.505329 |
| GO:0034613\_cellular\_protein\_localization | SYNE2 | 139 | 2 | 1.380396 | -0.368546 | 305 | 458.23 | 1.502393 |
| GO:0034613\_cellular\_protein\_localization | XPO5 | 139 | 2 | 1.380396 | -0.368546 | 305 | 458.23 | 1.502393 |
| GO:0002376\_immune\_system\_process | PGM3 | 505 | 6 | 1.139851 | -0.363571 | 306 | 459.32 | 1.501046 |
| GO:0002376\_immune\_system\_process | ITGA6 | 505 | 6 | 1.139851 | -0.363571 | 306 | 459.32 | 1.501046 |
| GO:0002376\_immune\_system\_process | BCL11B | 505 | 6 | 1.139851 | -0.363571 | 306 | 459.32 | 1.501046 |
| GO:0002376\_immune\_system\_process | PLCG2 | 505 | 6 | 1.139851 | -0.363571 | 306 | 459.32 | 1.501046 |
| GO:0002376\_immune\_system\_process | SOX6 | 505 | 6 | 1.139851 | -0.363571 | 306 | 459.32 | 1.501046 |
| GO:0002376\_immune\_system\_process | KDR | 505 | 6 | 1.139851 | -0.363571 | 306 | 459.32 | 1.501046 |
| GO:0002253\_activation\_of\_immune\_response | PLCG2 | 54 | 1 | 1.776620 | -0.362534 | 311 | 462.21 | 1.486206 |
| GO:0006164\_purine\_nucleotide\_biosynthetic\_process | GUCA1B | 54 | 1 | 1.776620 | -0.362534 | 311 | 462.21 | 1.486206 |
| GO:0015849\_organic\_acid\_transport | SLC13A3 | 54 | 1 | 1.776620 | -0.362534 | 311 | 462.21 | 1.486206 |
| GO:0043405\_regulation\_of\_MAP\_kinase\_activity | HMGCR | 54 | 1 | 1.776620 | -0.362534 | 311 | 462.21 | 1.486206 |
| GO:0044271\_nitrogen\_compound\_biosynthetic\_process | AMD1 | 54 | 1 | 1.776620 | -0.362534 | 311 | 462.21 | 1.486206 |
| GO:0003006\_reproductive\_developmental\_process | ADAMTS1 | 141 | 2 | 1.360816 | -0.361270 | 313 | 462.82 | 1.478658 |
| GO:0003006\_reproductive\_developmental\_process | KDR | 141 | 2 | 1.360816 | -0.361270 | 313 | 462.82 | 1.478658 |
| GO:0070727\_cellular\_macromolecule\_localization | SYNE2 | 141 | 2 | 1.360816 | -0.361270 | 313 | 462.82 | 1.478658 |
| GO:0070727\_cellular\_macromolecule\_localization | XPO5 | 141 | 2 | 1.360816 | -0.361270 | 313 | 462.82 | 1.478658 |
| GO:0006310\_DNA\_recombination | BCL11B | 55 | 1 | 1.744318 | -0.356600 | 314 | 465.53 | 1.482580 |
| GO:0050790\_regulation\_of\_catalytic\_activity | GUCA1B | 233 | 3 | 1.235247 | -0.355394 | 315 | 466.09 | 1.479651 |
| GO:0050790\_regulation\_of\_catalytic\_activity | HMGCR | 233 | 3 | 1.235247 | -0.355394 | 315 | 466.09 | 1.479651 |
| GO:0050790\_regulation\_of\_catalytic\_activity | VLDLR | 233 | 3 | 1.235247 | -0.355394 | 315 | 466.09 | 1.479651 |
| GO:0016477\_cell\_migration | SYNE2 | 234 | 3 | 1.229968 | -0.352647 | 316 | 466.56 | 1.476456 |
| GO:0016477\_cell\_migration | ITGA6 | 234 | 3 | 1.229968 | -0.352647 | 316 | 466.56 | 1.476456 |
| GO:0016477\_cell\_migration | KDR | 234 | 3 | 1.229968 | -0.352647 | 316 | 466.56 | 1.476456 |
| GO:0009187\_cyclic\_nucleotide\_metabolic\_process | GUCA1B | 56 | 1 | 1.713170 | -0.350807 | 319 | 469.56 | 1.471975 |
| GO:0046486\_glycerolipid\_metabolic\_process | PIP5K1B | 56 | 1 | 1.713170 | -0.350807 | 319 | 469.56 | 1.471975 |
| GO:0050678\_regulation\_of\_epithelial\_cell\_proliferation | KDR | 56 | 1 | 1.713170 | -0.350807 | 319 | 469.56 | 1.471975 |
| GO:0045596\_negative\_regulation\_of\_cell\_differentiation | IRX3 | 144 | 2 | 1.332465 | -0.350666 | 320 | 470.03 | 1.468844 |
| GO:0045596\_negative\_regulation\_of\_cell\_differentiation | ULK2 | 144 | 2 | 1.332465 | -0.350666 | 320 | 470.03 | 1.468844 |
| GO:0000226\_microtubule\_cytoskeleton\_organization | DST | 57 | 1 | 1.683114 | -0.345148 | 324 | 475.14 | 1.466481 |
| GO:0033365\_protein\_localization\_in\_organelle | SYNE2 | 57 | 1 | 1.683114 | -0.345148 | 324 | 475.14 | 1.466481 |
| GO:0043523\_regulation\_of\_neuron\_apoptosis | NTF3 | 57 | 1 | 1.683114 | -0.345148 | 324 | 475.14 | 1.466481 |
| GO:0045444\_fat\_cell\_differentiation | ITGA6 | 57 | 1 | 1.683114 | -0.345148 | 324 | 475.14 | 1.466481 |
| GO:0044085\_cellular\_component\_biogenesis | WDR55 | 237 | 3 | 1.214399 | -0.344538 | 325 | 475.62 | 1.463446 |
| GO:0044085\_cellular\_component\_biogenesis | ITGA6 | 237 | 3 | 1.214399 | -0.344538 | 325 | 475.62 | 1.463446 |
| GO:0044085\_cellular\_component\_biogenesis | CUGBP2 | 237 | 3 | 1.214399 | -0.344538 | 325 | 475.62 | 1.463446 |
| GO:0003008\_system\_process | GUCA1B | 516 | 6 | 1.115552 | -0.342731 | 326 | 476.65 | 1.462117 |
| GO:0003008\_system\_process | NTF3 | 516 | 6 | 1.115552 | -0.342731 | 326 | 476.65 | 1.462117 |
| GO:0003008\_system\_process | HMGCR | 516 | 6 | 1.115552 | -0.342731 | 326 | 476.65 | 1.462117 |
| GO:0003008\_system\_process | ABCA4 | 516 | 6 | 1.115552 | -0.342731 | 326 | 476.65 | 1.462117 |
| GO:0003008\_system\_process | TPM2 | 516 | 6 | 1.115552 | -0.342731 | 326 | 476.65 | 1.462117 |
| GO:0003008\_system\_process | MFRP | 516 | 6 | 1.115552 | -0.342731 | 326 | 476.65 | 1.462117 |
| GO:0006928\_cell\_motion | SYNE2 | 330 | 4 | 1.162879 | -0.342680 | 328 | 477.17 | 1.454787 |
| GO:0006928\_cell\_motion | ITGA6 | 330 | 4 | 1.162879 | -0.342680 | 328 | 477.17 | 1.454787 |
| GO:0006928\_cell\_motion | NTF3 | 330 | 4 | 1.162879 | -0.342680 | 328 | 477.17 | 1.454787 |
| GO:0006928\_cell\_motion | KDR | 330 | 4 | 1.162879 | -0.342680 | 328 | 477.17 | 1.454787 |
| GO:0051674\_localization\_of\_cell | SYNE2 | 330 | 4 | 1.162879 | -0.342680 | 328 | 477.17 | 1.454787 |
| GO:0051674\_localization\_of\_cell | ITGA6 | 330 | 4 | 1.162879 | -0.342680 | 328 | 477.17 | 1.454787 |
| GO:0051674\_localization\_of\_cell | NTF3 | 330 | 4 | 1.162879 | -0.342680 | 328 | 477.17 | 1.454787 |
| GO:0051674\_localization\_of\_cell | KDR | 330 | 4 | 1.162879 | -0.342680 | 328 | 477.17 | 1.454787 |
| GO:0051093\_negative\_regulation\_of\_developmental\_process | IRX3 | 331 | 4 | 1.159366 | -0.340421 | 329 | 478.2 | 1.453495 |
| GO:0051093\_negative\_regulation\_of\_developmental\_process | BCL11B | 331 | 4 | 1.159366 | -0.340421 | 329 | 478.2 | 1.453495 |
| GO:0051093\_negative\_regulation\_of\_developmental\_process | ULK2 | 331 | 4 | 1.159366 | -0.340421 | 329 | 478.2 | 1.453495 |
| GO:0051093\_negative\_regulation\_of\_developmental\_process | PLCG2 | 331 | 4 | 1.159366 | -0.340421 | 329 | 478.2 | 1.453495 |
| GO:0033043\_regulation\_of\_organelle\_organization | DST | 58 | 1 | 1.654095 | -0.339620 | 331 | 479.73 | 1.449335 |
| GO:0034622\_cellular\_macromolecular\_complex\_assembly | CUGBP2 | 58 | 1 | 1.654095 | -0.339620 | 331 | 479.73 | 1.449335 |
| GO:0048469\_cell\_maturation | KDR | 59 | 1 | 1.626059 | -0.334217 | 332 | 482.34 | 1.452831 |
| GO:0009123\_nucleoside\_monophosphate\_metabolic\_process | GUCA1B | 60 | 1 | 1.598958 | -0.328935 | 333 | 483.25 | 1.451201 |
| GO:0009991\_response\_to\_extracellular\_stimulus | ITGA6 | 61 | 1 | 1.572746 | -0.323771 | 334 | 486.13 | 1.455479 |
| GO:0007399\_nervous\_system\_development | IRX3 | 621 | 7 | 1.081421 | -0.323439 | 335 | 486.23 | 1.451433 |
| GO:0007399\_nervous\_system\_development | NTF3 | 621 | 7 | 1.081421 | -0.323439 | 335 | 486.23 | 1.451433 |
| GO:0007399\_nervous\_system\_development | DTX1 | 621 | 7 | 1.081421 | -0.323439 | 335 | 486.23 | 1.451433 |
| GO:0007399\_nervous\_system\_development | ULK2 | 621 | 7 | 1.081421 | -0.323439 | 335 | 486.23 | 1.451433 |
| GO:0007399\_nervous\_system\_development | BCL11B | 621 | 7 | 1.081421 | -0.323439 | 335 | 486.23 | 1.451433 |
| GO:0007399\_nervous\_system\_development | BRSK2 | 621 | 7 | 1.081421 | -0.323439 | 335 | 486.23 | 1.451433 |
| GO:0007399\_nervous\_system\_development | DST | 621 | 7 | 1.081421 | -0.323439 | 335 | 486.23 | 1.451433 |
| GO:0022604\_regulation\_of\_cell\_morphogenesis | ULK2 | 62 | 1 | 1.547379 | -0.318719 | 336 | 492.07 | 1.464494 |
| GO:0009165\_nucleotide\_biosynthetic\_process | GUCA1B | 63 | 1 | 1.522817 | -0.313777 | 338 | 496.1 | 1.467751 |
| GO:0051216\_cartilage\_development | SOX6 | 63 | 1 | 1.522817 | -0.313777 | 338 | 496.1 | 1.467751 |
| GO:0055074\_calcium\_ion\_homeostasis | KDR | 64 | 1 | 1.499023 | -0.308941 | 339 | 498.82 | 1.471445 |
| GO:0051704\_multi-organism\_process | PLCG2 | 157 | 2 | 1.222134 | -0.308640 | 340 | 499.14 | 1.468059 |
| GO:0051704\_multi-organism\_process | LAMB1-1 | 157 | 2 | 1.222134 | -0.308640 | 340 | 499.14 | 1.468059 |
| GO:0043086\_negative\_regulation\_of\_catalytic\_activity | HMGCR | 65 | 1 | 1.475962 | -0.304208 | 341 | 502.16 | 1.472610 |
| GO:0051128\_regulation\_of\_cellular\_component\_organization | ULK2 | 160 | 2 | 1.199219 | -0.299769 | 342 | 503.84 | 1.473216 |
| GO:0051128\_regulation\_of\_cellular\_component\_organization | DST | 160 | 2 | 1.199219 | -0.299769 | 342 | 503.84 | 1.473216 |
| GO:0007179\_transforming\_growth\_factor\_beta\_receptor\_signaling\_pathway | HTRA1 | 66 | 1 | 1.453598 | -0.299574 | 345 | 506.22 | 1.467304 |
| GO:0045860\_positive\_regulation\_of\_protein\_kinase\_activity | VLDLR | 66 | 1 | 1.453598 | -0.299574 | 345 | 506.22 | 1.467304 |
| GO:0051402\_neuron\_apoptosis | NTF3 | 66 | 1 | 1.453598 | -0.299574 | 345 | 506.22 | 1.467304 |
| GO:0002521\_leukocyte\_differentiation | BCL11B | 161 | 2 | 1.191770 | -0.296876 | 346 | 507.04 | 1.465434 |
| GO:0002521\_leukocyte\_differentiation | PLCG2 | 161 | 2 | 1.191770 | -0.296876 | 346 | 507.04 | 1.465434 |
| GO:0048870\_cell\_motility | SYNE2 | 257 | 3 | 1.119893 | -0.295158 | 347 | 507.79 | 1.463372 |
| GO:0048870\_cell\_motility | ITGA6 | 257 | 3 | 1.119893 | -0.295158 | 347 | 507.79 | 1.463372 |
| GO:0048870\_cell\_motility | KDR | 257 | 3 | 1.119893 | -0.295158 | 347 | 507.79 | 1.463372 |
| GO:0003007\_heart\_morphogenesis | ADAMTS1 | 67 | 1 | 1.431903 | -0.295035 | 349 | 509.47 | 1.459799 |
| GO:0042445\_hormone\_metabolic\_process | CRYM | 67 | 1 | 1.431903 | -0.295035 | 349 | 509.47 | 1.459799 |
| GO:0009628\_response\_to\_abiotic\_stimulus | GUCA1B | 162 | 2 | 1.184414 | -0.294014 | 350 | 509.99 | 1.457114 |
| GO:0009628\_response\_to\_abiotic\_stimulus | HMGCR | 162 | 2 | 1.184414 | -0.294014 | 350 | 509.99 | 1.457114 |
| GO:0019932\_second-messenger-mediated\_signaling | CAR8 | 68 | 1 | 1.410846 | -0.290591 | 351 | 512.45 | 1.459972 |
| GO:0042325\_regulation\_of\_phosphorylation | HMGCR | 164 | 2 | 1.169970 | -0.288381 | 352 | 512.93 | 1.457187 |
| GO:0042325\_regulation\_of\_phosphorylation | VLDLR | 164 | 2 | 1.169970 | -0.288381 | 352 | 512.93 | 1.457187 |
| GO:0006816\_calcium\_ion\_transport | PLCG2 | 69 | 1 | 1.390399 | -0.286236 | 354 | 514.76 | 1.454124 |
| GO:0055065\_metal\_ion\_homeostasis | KDR | 69 | 1 | 1.390399 | -0.286236 | 354 | 514.76 | 1.454124 |
| GO:0019220\_regulation\_of\_phosphate\_metabolic\_process | HMGCR | 165 | 2 | 1.162879 | -0.285609 | 356 | 515.68 | 1.448539 |
| GO:0019220\_regulation\_of\_phosphate\_metabolic\_process | VLDLR | 165 | 2 | 1.162879 | -0.285609 | 356 | 515.68 | 1.448539 |
| GO:0051174\_regulation\_of\_phosphorus\_metabolic\_process | HMGCR | 165 | 2 | 1.162879 | -0.285609 | 356 | 515.68 | 1.448539 |
| GO:0051174\_regulation\_of\_phosphorus\_metabolic\_process | VLDLR | 165 | 2 | 1.162879 | -0.285609 | 356 | 515.68 | 1.448539 |
| GO:0007611\_learning\_or\_memory | HMGCR | 70 | 1 | 1.370536 | -0.281969 | 360 | 518.47 | 1.440194 |
| GO:0009617\_response\_to\_bacterium | PLCG2 | 70 | 1 | 1.370536 | -0.281969 | 360 | 518.47 | 1.440194 |
| GO:0048592\_eye\_morphogenesis | COL8A1 | 70 | 1 | 1.370536 | -0.281969 | 360 | 518.47 | 1.440194 |
| GO:0070838\_divalent\_metal\_ion\_transport | PLCG2 | 70 | 1 | 1.370536 | -0.281969 | 360 | 518.47 | 1.440194 |
| GO:0006913\_nucleocytoplasmic\_transport | XPO5 | 71 | 1 | 1.351232 | -0.277787 | 363 | 522.94 | 1.440606 |
| GO:0009100\_glycoprotein\_metabolic\_process | GALNT10 | 71 | 1 | 1.351232 | -0.277787 | 363 | 522.94 | 1.440606 |
| GO:0033674\_positive\_regulation\_of\_kinase\_activity | VLDLR | 71 | 1 | 1.351232 | -0.277787 | 363 | 522.94 | 1.440606 |
| GO:0051169\_nuclear\_transport | XPO5 | 72 | 1 | 1.332465 | -0.273688 | 365 | 526.63 | 1.442822 |
| GO:0051347\_positive\_regulation\_of\_transferase\_activity | VLDLR | 72 | 1 | 1.332465 | -0.273688 | 365 | 526.63 | 1.442822 |
| GO:0006163\_purine\_nucleotide\_metabolic\_process | GUCA1B | 73 | 1 | 1.314212 | -0.269669 | 367 | 530.21 | 1.444714 |
| GO:0006936\_muscle\_contraction | TPM2 | 73 | 1 | 1.314212 | -0.269669 | 367 | 530.21 | 1.444714 |
| GO:0045944\_positive\_regulation\_of\_transcription\_from\_RNA\_polymerase\_II\_promoter | NTF3 | 269 | 3 | 1.069935 | -0.269051 | 368 | 530.45 | 1.441440 |
| GO:0045944\_positive\_regulation\_of\_transcription\_from\_RNA\_polymerase\_II\_promoter | BCL11B | 269 | 3 | 1.069935 | -0.269051 | 368 | 530.45 | 1.441440 |
| GO:0045944\_positive\_regulation\_of\_transcription\_from\_RNA\_polymerase\_II\_promoter | SOX6 | 269 | 3 | 1.069935 | -0.269051 | 368 | 530.45 | 1.441440 |
| GO:0048771\_tissue\_remodeling | ANK | 74 | 1 | 1.296453 | -0.265728 | 369 | 532.32 | 1.442602 |
| GO:0044093\_positive\_regulation\_of\_molecular\_function | PLCG2 | 173 | 2 | 1.109104 | -0.264465 | 370 | 533.52 | 1.441946 |
| GO:0044093\_positive\_regulation\_of\_molecular\_function | VLDLR | 173 | 2 | 1.109104 | -0.264465 | 370 | 533.52 | 1.441946 |
| GO:0051641\_cellular\_localization | FUSIP1 | 370 | 4 | 1.037162 | -0.262671 | 371 | 533.76 | 1.438706 |
| GO:0051641\_cellular\_localization | SYNE2 | 370 | 4 | 1.037162 | -0.262671 | 371 | 533.76 | 1.438706 |
| GO:0051641\_cellular\_localization | XPO5 | 370 | 4 | 1.037162 | -0.262671 | 371 | 533.76 | 1.438706 |
| GO:0051641\_cellular\_localization | DST | 370 | 4 | 1.037162 | -0.262671 | 371 | 533.76 | 1.438706 |
| GO:0007281\_germ\_cell\_development | TDRD7 | 75 | 1 | 1.279167 | -0.261863 | 373 | 535.19 | 1.434826 |
| GO:0051050\_positive\_regulation\_of\_transport | PLCG2 | 75 | 1 | 1.279167 | -0.261863 | 373 | 535.19 | 1.434826 |
| GO:0003012\_muscle\_system\_process | TPM2 | 76 | 1 | 1.262336 | -0.258072 | 375 | 538.49 | 1.435973 |
| GO:0034621\_cellular\_macromolecular\_complex\_subunit\_organization | CUGBP2 | 76 | 1 | 1.262336 | -0.258072 | 375 | 538.49 | 1.435973 |
| GO:0001890\_placenta\_development | CCNF | 77 | 1 | 1.245942 | -0.254352 | 376 | 540.27 | 1.436888 |
| GO:0043069\_negative\_regulation\_of\_programmed\_cell\_death | BCL11B | 179 | 2 | 1.071927 | -0.249732 | 378 | 544.24 | 1.439788 |
| GO:0043069\_negative\_regulation\_of\_programmed\_cell\_death | PLCG2 | 179 | 2 | 1.071927 | -0.249732 | 378 | 544.24 | 1.439788 |
| GO:0060548\_negative\_regulation\_of\_cell\_death | BCL11B | 179 | 2 | 1.071927 | -0.249732 | 378 | 544.24 | 1.439788 |
| GO:0060548\_negative\_regulation\_of\_cell\_death | PLCG2 | 179 | 2 | 1.071927 | -0.249732 | 378 | 544.24 | 1.439788 |
| GO:0015674\_di-\_\_tri-valent\_inorganic\_cation\_transport | PLCG2 | 79 | 1 | 1.214399 | -0.247119 | 379 | 546.46 | 1.441847 |
| GO:0000278\_mitotic\_cell\_cycle | PLK2 | 80 | 1 | 1.199219 | -0.243603 | 381 | 549.23 | 1.441549 |
| GO:0044092\_negative\_regulation\_of\_molecular\_function | HMGCR | 80 | 1 | 1.199219 | -0.243603 | 381 | 549.23 | 1.441549 |
| GO:0021700\_developmental\_maturation | KDR | 81 | 1 | 1.184414 | -0.240151 | 382 | 550.53 | 1.441178 |
| GO:0007411\_axon\_guidance | NTF3 | 82 | 1 | 1.169970 | -0.236762 | 384 | 553.78 | 1.442135 |
| GO:0008202\_steroid\_metabolic\_process | HMGCR | 82 | 1 | 1.169970 | -0.236762 | 384 | 553.78 | 1.442135 |
| GO:0048513\_organ\_development | NTF3 | 1365 | 14 | 0.983974 | -0.234036 | 385 | 554.82 | 1.441091 |
| GO:0048513\_organ\_development | CRYAB | 1365 | 14 | 0.983974 | -0.234036 | 385 | 554.82 | 1.441091 |
| GO:0048513\_organ\_development | CCNF | 1365 | 14 | 0.983974 | -0.234036 | 385 | 554.82 | 1.441091 |
| GO:0048513\_organ\_development | NID1 | 1365 | 14 | 0.983974 | -0.234036 | 385 | 554.82 | 1.441091 |
| GO:0048513\_organ\_development | POSTN | 1365 | 14 | 0.983974 | -0.234036 | 385 | 554.82 | 1.441091 |
| GO:0048513\_organ\_development | SOX6 | 1365 | 14 | 0.983974 | -0.234036 | 385 | 554.82 | 1.441091 |
| GO:0048513\_organ\_development | KDR | 1365 | 14 | 0.983974 | -0.234036 | 385 | 554.82 | 1.441091 |
| GO:0048513\_organ\_development | PGM3 | 1365 | 14 | 0.983974 | -0.234036 | 385 | 554.82 | 1.441091 |
| GO:0048513\_organ\_development | ANK | 1365 | 14 | 0.983974 | -0.234036 | 385 | 554.82 | 1.441091 |
| GO:0048513\_organ\_development | ITGA6 | 1365 | 14 | 0.983974 | -0.234036 | 385 | 554.82 | 1.441091 |
| GO:0048513\_organ\_development | BCL11B | 1365 | 14 | 0.983974 | -0.234036 | 385 | 554.82 | 1.441091 |
| GO:0048513\_organ\_development | PLCG2 | 1365 | 14 | 0.983974 | -0.234036 | 385 | 554.82 | 1.441091 |
| GO:0048513\_organ\_development | ADAMTS1 | 1365 | 14 | 0.983974 | -0.234036 | 385 | 554.82 | 1.441091 |
| GO:0048513\_organ\_development | COL8A1 | 1365 | 14 | 0.983974 | -0.234036 | 385 | 554.82 | 1.441091 |
| GO:0007017\_microtubule-based\_process | DST | 83 | 1 | 1.155873 | -0.233434 | 386 | 557.86 | 1.445233 |
| GO:0006605\_protein\_targeting | XPO5 | 86 | 1 | 1.115552 | -0.223800 | 388 | 563.85 | 1.453222 |
| GO:0034641\_cellular\_nitrogen\_compound\_metabolic\_process | AMD1 | 86 | 1 | 1.115552 | -0.223800 | 388 | 563.85 | 1.453222 |
| GO:0007178\_transmembrane\_receptor\_protein\_serine\_threonine\_kinase\_signaling\_pathway | HTRA1 | 87 | 1 | 1.102730 | -0.220702 | 391 | 566.86 | 1.449770 |
| GO:0016337\_cell-cell\_adhesion | ITGA6 | 87 | 1 | 1.102730 | -0.220702 | 391 | 566.86 | 1.449770 |
| GO:0050778\_positive\_regulation\_of\_immune\_response | PLCG2 | 87 | 1 | 1.102730 | -0.220702 | 391 | 566.86 | 1.449770 |
| GO:0040011\_locomotion | SYNE2 | 295 | 3 | 0.975636 | -0.220142 | 392 | 568.04 | 1.449082 |
| GO:0040011\_locomotion | ITGA6 | 295 | 3 | 0.975636 | -0.220142 | 392 | 568.04 | 1.449082 |
| GO:0040011\_locomotion | KDR | 295 | 3 | 0.975636 | -0.220142 | 392 | 568.04 | 1.449082 |
| GO:0001503\_ossification | ANK | 88 | 1 | 1.090199 | -0.217657 | 394 | 569.46 | 1.445330 |
| GO:0048754\_branching\_morphogenesis\_of\_a\_tube | KDR | 88 | 1 | 1.090199 | -0.217657 | 394 | 569.46 | 1.445330 |
| GO:0007507\_heart\_development | ADAMTS1 | 195 | 2 | 0.983974 | -0.214601 | 395 | 570.97 | 1.445494 |
| GO:0007507\_heart\_development | SOX6 | 195 | 2 | 0.983974 | -0.214601 | 395 | 570.97 | 1.445494 |
| GO:0030324\_lung\_development | KDR | 90 | 1 | 1.065972 | -0.211723 | 397 | 573.31 | 1.444106 |
| GO:0042113\_B\_cell\_activation | PLCG2 | 90 | 1 | 1.065972 | -0.211723 | 397 | 573.31 | 1.444106 |
| GO:0008544\_epidermis\_development | NTF3 | 91 | 1 | 1.054258 | -0.208832 | 398 | 575.63 | 1.446307 |
| GO:0030217\_T\_cell\_differentiation | BCL11B | 92 | 1 | 1.042799 | -0.205990 | 400 | 578.23 | 1.445575 |
| GO:0030323\_respiratory\_tube\_development | KDR | 92 | 1 | 1.042799 | -0.205990 | 400 | 578.23 | 1.445575 |
| GO:0055066\_di-\_\_tri-valent\_inorganic\_cation\_homeostasis | KDR | 93 | 1 | 1.031586 | -0.203195 | 402 | 579.88 | 1.442488 |
| GO:0065003\_macromolecular\_complex\_assembly | CUGBP2 | 93 | 1 | 1.031586 | -0.203195 | 402 | 579.88 | 1.442488 |
| GO:0045893\_positive\_regulation\_of\_transcription\_\_DNA-dependent | NTF3 | 306 | 3 | 0.940564 | -0.202199 | 404 | 580.34 | 1.436485 |
| GO:0045893\_positive\_regulation\_of\_transcription\_\_DNA-dependent | BCL11B | 306 | 3 | 0.940564 | -0.202199 | 404 | 580.34 | 1.436485 |
| GO:0045893\_positive\_regulation\_of\_transcription\_\_DNA-dependent | SOX6 | 306 | 3 | 0.940564 | -0.202199 | 404 | 580.34 | 1.436485 |
| GO:0051254\_positive\_regulation\_of\_RNA\_metabolic\_process | NTF3 | 306 | 3 | 0.940564 | -0.202199 | 404 | 580.34 | 1.436485 |
| GO:0051254\_positive\_regulation\_of\_RNA\_metabolic\_process | BCL11B | 306 | 3 | 0.940564 | -0.202199 | 404 | 580.34 | 1.436485 |
| GO:0051254\_positive\_regulation\_of\_RNA\_metabolic\_process | SOX6 | 306 | 3 | 0.940564 | -0.202199 | 404 | 580.34 | 1.436485 |
| GO:0006753\_nucleoside\_phosphate\_metabolic\_process | GUCA1B | 94 | 1 | 1.020612 | -0.200448 | 407 | 583.65 | 1.434029 |
| GO:0008610\_lipid\_biosynthetic\_process | HMGCR | 94 | 1 | 1.020612 | -0.200448 | 407 | 583.65 | 1.434029 |
| GO:0009117\_nucleotide\_metabolic\_process | GUCA1B | 94 | 1 | 1.020612 | -0.200448 | 407 | 583.65 | 1.434029 |
| GO:0051707\_response\_to\_other\_organism | PLCG2 | 95 | 1 | 1.009868 | -0.197746 | 408 | 585.08 | 1.434020 |
| GO:0022607\_cellular\_component\_assembly | ITGA6 | 204 | 2 | 0.940564 | -0.197186 | 409 | 585.69 | 1.432005 |
| GO:0022607\_cellular\_component\_assembly | CUGBP2 | 204 | 2 | 0.940564 | -0.197186 | 409 | 585.69 | 1.432005 |
| GO:0060541\_respiratory\_system\_development | KDR | 98 | 1 | 0.978954 | -0.189903 | 410 | 592.09 | 1.444122 |
| GO:0007398\_ectoderm\_development | NTF3 | 99 | 1 | 0.969066 | -0.187374 | 412 | 593.83 | 1.441335 |
| GO:0060348\_bone\_development | ANK | 99 | 1 | 0.969066 | -0.187374 | 412 | 593.83 | 1.441335 |
| GO:0009968\_negative\_regulation\_of\_signal\_transduction | HTRA1 | 103 | 1 | 0.931432 | -0.177655 | 413 | 597.35 | 1.446368 |
| GO:0010033\_response\_to\_organic\_substance | PLCG2 | 216 | 2 | 0.888310 | -0.176240 | 414 | 597.58 | 1.443430 |
| GO:0010033\_response\_to\_organic\_substance | ANXA5 | 216 | 2 | 0.888310 | -0.176240 | 414 | 597.58 | 1.443430 |
| GO:0055086\_nucleobase\_\_nucleoside\_and\_nucleotide\_metabolic\_process | GUCA1B | 104 | 1 | 0.922476 | -0.175321 | 415 | 598.31 | 1.441711 |
| GO:0048872\_homeostasis\_of\_number\_of\_cells | SOX6 | 105 | 1 | 0.913690 | -0.173024 | 416 | 599.24 | 1.440481 |
| GO:0010817\_regulation\_of\_hormone\_levels | CRYM | 106 | 1 | 0.905071 | -0.170763 | 417 | 600.8 | 1.440767 |
| GO:0051253\_negative\_regulation\_of\_RNA\_metabolic\_process | FUSIP1 | 220 | 2 | 0.872159 | -0.169783 | 418 | 601.08 | 1.437990 |
| GO:0051253\_negative\_regulation\_of\_RNA\_metabolic\_process | SOX6 | 220 | 2 | 0.872159 | -0.169783 | 418 | 601.08 | 1.437990 |
| GO:0030099\_myeloid\_cell\_differentiation | SOX6 | 108 | 1 | 0.888310 | -0.166344 | 419 | 604.39 | 1.442458 |
| GO:0010648\_negative\_regulation\_of\_cell\_communication | HTRA1 | 110 | 1 | 0.872159 | -0.162061 | 421 | 607.09 | 1.442019 |
| GO:0055080\_cation\_homeostasis | KDR | 110 | 1 | 0.872159 | -0.162061 | 421 | 607.09 | 1.442019 |
| GO:0051179\_localization | FUSIP1 | 1058 | 10 | 0.906782 | -0.159411 | 422 | 607.91 | 1.440545 |
| GO:0051179\_localization | SYNE2 | 1058 | 10 | 0.906782 | -0.159411 | 422 | 607.91 | 1.440545 |
| GO:0051179\_localization | ITGA6 | 1058 | 10 | 0.906782 | -0.159411 | 422 | 607.91 | 1.440545 |
| GO:0051179\_localization | NTF3 | 1058 | 10 | 0.906782 | -0.159411 | 422 | 607.91 | 1.440545 |
| GO:0051179\_localization | XPO5 | 1058 | 10 | 0.906782 | -0.159411 | 422 | 607.91 | 1.440545 |
| GO:0051179\_localization | PLCG2 | 1058 | 10 | 0.906782 | -0.159411 | 422 | 607.91 | 1.440545 |
| GO:0051179\_localization | SLC13A3 | 1058 | 10 | 0.906782 | -0.159411 | 422 | 607.91 | 1.440545 |
| GO:0051179\_localization | ABCA4 | 1058 | 10 | 0.906782 | -0.159411 | 422 | 607.91 | 1.440545 |
| GO:0051179\_localization | DST | 1058 | 10 | 0.906782 | -0.159411 | 422 | 607.91 | 1.440545 |
| GO:0051179\_localization | KDR | 1058 | 10 | 0.906782 | -0.159411 | 422 | 607.91 | 1.440545 |
| GO:0045941\_positive\_regulation\_of\_transcription | NTF3 | 338 | 3 | 0.851516 | -0.157727 | 423 | 610.23 | 1.442624 |
| GO:0045941\_positive\_regulation\_of\_transcription | BCL11B | 338 | 3 | 0.851516 | -0.157727 | 423 | 610.23 | 1.442624 |
| GO:0045941\_positive\_regulation\_of\_transcription | SOX6 | 338 | 3 | 0.851516 | -0.157727 | 423 | 610.23 | 1.442624 |
| GO:0046649\_lymphocyte\_activation | BCL11B | 228 | 2 | 0.841557 | -0.157593 | 424 | 611.09 | 1.441250 |
| GO:0046649\_lymphocyte\_activation | PLCG2 | 228 | 2 | 0.841557 | -0.157593 | 424 | 611.09 | 1.441250 |
| GO:0007167\_enzyme\_linked\_receptor\_protein\_signaling\_pathway | HTRA1 | 229 | 2 | 0.837882 | -0.156134 | 425 | 612.03 | 1.440071 |
| GO:0007167\_enzyme\_linked\_receptor\_protein\_signaling\_pathway | KDR | 229 | 2 | 0.837882 | -0.156134 | 425 | 612.03 | 1.440071 |
| GO:0016070\_RNA\_metabolic\_process | FUSIP1 | 658 | 6 | 0.874810 | -0.154113 | 426 | 613.88 | 1.441033 |
| GO:0016070\_RNA\_metabolic\_process | WDR55 | 658 | 6 | 0.874810 | -0.154113 | 426 | 613.88 | 1.441033 |
| GO:0016070\_RNA\_metabolic\_process | NTF3 | 658 | 6 | 0.874810 | -0.154113 | 426 | 613.88 | 1.441033 |
| GO:0016070\_RNA\_metabolic\_process | BCL11B | 658 | 6 | 0.874810 | -0.154113 | 426 | 613.88 | 1.441033 |
| GO:0016070\_RNA\_metabolic\_process | CUGBP2 | 658 | 6 | 0.874810 | -0.154113 | 426 | 613.88 | 1.441033 |
| GO:0016070\_RNA\_metabolic\_process | SOX6 | 658 | 6 | 0.874810 | -0.154113 | 426 | 613.88 | 1.441033 |
| GO:0009607\_response\_to\_biotic\_stimulus | PLCG2 | 114 | 1 | 0.841557 | -0.153879 | 427 | 614.6 | 1.439344 |
| GO:0051649\_establishment\_of\_localization\_in\_cell | FUSIP1 | 342 | 3 | 0.841557 | -0.152883 | 428 | 615.39 | 1.437827 |
| GO:0051649\_establishment\_of\_localization\_in\_cell | XPO5 | 342 | 3 | 0.841557 | -0.152883 | 428 | 615.39 | 1.437827 |
| GO:0051649\_establishment\_of\_localization\_in\_cell | DST | 342 | 3 | 0.841557 | -0.152883 | 428 | 615.39 | 1.437827 |
| GO:0048584\_positive\_regulation\_of\_response\_to\_stimulus | PLCG2 | 115 | 1 | 0.834239 | -0.151910 | 429 | 616.35 | 1.436713 |
| GO:0046483\_heterocycle\_metabolic\_process | GUCA1B | 116 | 1 | 0.827047 | -0.149970 | 430 | 617.87 | 1.436907 |
| GO:0010628\_positive\_regulation\_of\_gene\_expression | NTF3 | 346 | 3 | 0.831828 | -0.148182 | 431 | 618.66 | 1.435406 |
| GO:0010628\_positive\_regulation\_of\_gene\_expression | BCL11B | 346 | 3 | 0.831828 | -0.148182 | 431 | 618.66 | 1.435406 |
| GO:0010628\_positive\_regulation\_of\_gene\_expression | SOX6 | 346 | 3 | 0.831828 | -0.148182 | 431 | 618.66 | 1.435406 |
| GO:0043933\_macromolecular\_complex\_subunit\_organization | CUGBP2 | 117 | 1 | 0.819979 | -0.148059 | 432 | 618.92 | 1.432685 |
| GO:0001501\_skeletal\_system\_development | ANK | 236 | 2 | 0.813030 | -0.146299 | 433 | 619.3 | 1.430254 |
| GO:0001501\_skeletal\_system\_development | SOX6 | 236 | 2 | 0.813030 | -0.146299 | 433 | 619.3 | 1.430254 |
| GO:0007049\_cell\_cycle | PLK2 | 238 | 2 | 0.806197 | -0.143606 | 434 | 621.97 | 1.433111 |
| GO:0007049\_cell\_cycle | CCNF | 238 | 2 | 0.806197 | -0.143606 | 434 | 621.97 | 1.433111 |
| GO:0048523\_negative\_regulation\_of\_cellular\_process | FUSIP1 | 774 | 7 | 0.867652 | -0.143221 | 435 | 622.21 | 1.430368 |
| GO:0048523\_negative\_regulation\_of\_cellular\_process | IRX3 | 774 | 7 | 0.867652 | -0.143221 | 435 | 622.21 | 1.430368 |
| GO:0048523\_negative\_regulation\_of\_cellular\_process | HTRA1 | 774 | 7 | 0.867652 | -0.143221 | 435 | 622.21 | 1.430368 |
| GO:0048523\_negative\_regulation\_of\_cellular\_process | ULK2 | 774 | 7 | 0.867652 | -0.143221 | 435 | 622.21 | 1.430368 |
| GO:0048523\_negative\_regulation\_of\_cellular\_process | BCL11B | 774 | 7 | 0.867652 | -0.143221 | 435 | 622.21 | 1.430368 |
| GO:0048523\_negative\_regulation\_of\_cellular\_process | PLCG2 | 774 | 7 | 0.867652 | -0.143221 | 435 | 622.21 | 1.430368 |
| GO:0048523\_negative\_regulation\_of\_cellular\_process | SOX6 | 774 | 7 | 0.867652 | -0.143221 | 435 | 622.21 | 1.430368 |
| GO:0045935\_positive\_regulation\_of\_nucleobase\_\_nucleoside\_\_nucleotide\_and\_nucleic\_acid\_metabolic\_process | NTF3 | 352 | 3 | 0.817649 | -0.141389 | 436 | 623.02 | 1.428945 |
| GO:0045935\_positive\_regulation\_of\_nucleobase\_\_nucleoside\_\_nucleotide\_and\_nucleic\_acid\_metabolic\_process | BCL11B | 352 | 3 | 0.817649 | -0.141389 | 436 | 623.02 | 1.428945 |
| GO:0045935\_positive\_regulation\_of\_nucleobase\_\_nucleoside\_\_nucleotide\_and\_nucleic\_acid\_metabolic\_process | SOX6 | 352 | 3 | 0.817649 | -0.141389 | 436 | 623.02 | 1.428945 |
| GO:0006886\_intracellular\_protein\_transport | XPO5 | 122 | 1 | 0.786373 | -0.138917 | 438 | 627.15 | 1.431849 |
| GO:0030001\_metal\_ion\_transport | PLCG2 | 122 | 1 | 0.786373 | -0.138917 | 438 | 627.15 | 1.431849 |
| GO:0001763\_morphogenesis\_of\_a\_branching\_structure | KDR | 125 | 1 | 0.767500 | -0.133744 | 439 | 629.7 | 1.434396 |
| GO:0051173\_positive\_regulation\_of\_nitrogen\_compound\_metabolic\_process | NTF3 | 361 | 3 | 0.797265 | -0.131755 | 440 | 630.36 | 1.432636 |
| GO:0051173\_positive\_regulation\_of\_nitrogen\_compound\_metabolic\_process | BCL11B | 361 | 3 | 0.797265 | -0.131755 | 440 | 630.36 | 1.432636 |
| GO:0051173\_positive\_regulation\_of\_nitrogen\_compound\_metabolic\_process | SOX6 | 361 | 3 | 0.797265 | -0.131755 | 440 | 630.36 | 1.432636 |
| GO:0048518\_positive\_regulation\_of\_biological\_process | IRX3 | 995 | 9 | 0.867776 | -0.131388 | 441 | 630.52 | 1.429751 |
| GO:0048518\_positive\_regulation\_of\_biological\_process | ITGA6 | 995 | 9 | 0.867776 | -0.131388 | 441 | 630.52 | 1.429751 |
| GO:0048518\_positive\_regulation\_of\_biological\_process | NTF3 | 995 | 9 | 0.867776 | -0.131388 | 441 | 630.52 | 1.429751 |
| GO:0048518\_positive\_regulation\_of\_biological\_process | BCL11B | 995 | 9 | 0.867776 | -0.131388 | 441 | 630.52 | 1.429751 |
| GO:0048518\_positive\_regulation\_of\_biological\_process | PLCG2 | 995 | 9 | 0.867776 | -0.131388 | 441 | 630.52 | 1.429751 |
| GO:0048518\_positive\_regulation\_of\_biological\_process | NID1 | 995 | 9 | 0.867776 | -0.131388 | 441 | 630.52 | 1.429751 |
| GO:0048518\_positive\_regulation\_of\_biological\_process | SOX6 | 995 | 9 | 0.867776 | -0.131388 | 441 | 630.52 | 1.429751 |
| GO:0048518\_positive\_regulation\_of\_biological\_process | COL8A1 | 995 | 9 | 0.867776 | -0.131388 | 441 | 630.52 | 1.429751 |
| GO:0048518\_positive\_regulation\_of\_biological\_process | KDR | 995 | 9 | 0.867776 | -0.131388 | 441 | 630.52 | 1.429751 |
| GO:0045321\_leukocyte\_activation | BCL11B | 248 | 2 | 0.773690 | -0.130881 | 442 | 631.12 | 1.427873 |
| GO:0045321\_leukocyte\_activation | PLCG2 | 248 | 2 | 0.773690 | -0.130881 | 442 | 631.12 | 1.427873 |
| GO:0048522\_positive\_regulation\_of\_cellular\_process | IRX3 | 895 | 8 | 0.857542 | -0.129647 | 443 | 631.3 | 1.425056 |
| GO:0048522\_positive\_regulation\_of\_cellular\_process | ITGA6 | 895 | 8 | 0.857542 | -0.129647 | 443 | 631.3 | 1.425056 |
| GO:0048522\_positive\_regulation\_of\_cellular\_process | NTF3 | 895 | 8 | 0.857542 | -0.129647 | 443 | 631.3 | 1.425056 |
| GO:0048522\_positive\_regulation\_of\_cellular\_process | BCL11B | 895 | 8 | 0.857542 | -0.129647 | 443 | 631.3 | 1.425056 |
| GO:0048522\_positive\_regulation\_of\_cellular\_process | NID1 | 895 | 8 | 0.857542 | -0.129647 | 443 | 631.3 | 1.425056 |
| GO:0048522\_positive\_regulation\_of\_cellular\_process | SOX6 | 895 | 8 | 0.857542 | -0.129647 | 443 | 631.3 | 1.425056 |
| GO:0048522\_positive\_regulation\_of\_cellular\_process | COL8A1 | 895 | 8 | 0.857542 | -0.129647 | 443 | 631.3 | 1.425056 |
| GO:0048522\_positive\_regulation\_of\_cellular\_process | KDR | 895 | 8 | 0.857542 | -0.129647 | 443 | 631.3 | 1.425056 |
| GO:0045597\_positive\_regulation\_of\_cell\_differentiation | IRX3 | 128 | 1 | 0.749512 | -0.128791 | 444 | 632.51 | 1.424572 |
| GO:0010941\_regulation\_of\_cell\_death | NTF3 | 365 | 3 | 0.788527 | -0.127677 | 446 | 633.36 | 1.420090 |
| GO:0010941\_regulation\_of\_cell\_death | BCL11B | 365 | 3 | 0.788527 | -0.127677 | 446 | 633.36 | 1.420090 |
| GO:0010941\_regulation\_of\_cell\_death | PLCG2 | 365 | 3 | 0.788527 | -0.127677 | 446 | 633.36 | 1.420090 |
| GO:0043067\_regulation\_of\_programmed\_cell\_death | NTF3 | 365 | 3 | 0.788527 | -0.127677 | 446 | 633.36 | 1.420090 |
| GO:0043067\_regulation\_of\_programmed\_cell\_death | BCL11B | 365 | 3 | 0.788527 | -0.127677 | 446 | 633.36 | 1.420090 |
| GO:0043067\_regulation\_of\_programmed\_cell\_death | PLCG2 | 365 | 3 | 0.788527 | -0.127677 | 446 | 633.36 | 1.420090 |
| GO:0008104\_protein\_localization | SYNE2 | 251 | 2 | 0.764442 | -0.127291 | 447 | 633.69 | 1.417651 |
| GO:0008104\_protein\_localization | XPO5 | 251 | 2 | 0.764442 | -0.127291 | 447 | 633.69 | 1.417651 |
| GO:0050776\_regulation\_of\_immune\_response | PLCG2 | 130 | 1 | 0.737981 | -0.125605 | 448 | 635.9 | 1.419420 |
| GO:0010557\_positive\_regulation\_of\_macromolecule\_biosynthetic\_process | NTF3 | 371 | 3 | 0.775775 | -0.121785 | 449 | 638.75 | 1.422606 |
| GO:0010557\_positive\_regulation\_of\_macromolecule\_biosynthetic\_process | BCL11B | 371 | 3 | 0.775775 | -0.121785 | 449 | 638.75 | 1.422606 |
| GO:0010557\_positive\_regulation\_of\_macromolecule\_biosynthetic\_process | SOX6 | 371 | 3 | 0.775775 | -0.121785 | 449 | 638.75 | 1.422606 |
| GO:0050793\_regulation\_of\_developmental\_process | IRX3 | 703 | 6 | 0.818812 | -0.117592 | 450 | 642.46 | 1.427689 |
| GO:0050793\_regulation\_of\_developmental\_process | ANK | 703 | 6 | 0.818812 | -0.117592 | 450 | 642.46 | 1.427689 |
| GO:0050793\_regulation\_of\_developmental\_process | NTF3 | 703 | 6 | 0.818812 | -0.117592 | 450 | 642.46 | 1.427689 |
| GO:0050793\_regulation\_of\_developmental\_process | ULK2 | 703 | 6 | 0.818812 | -0.117592 | 450 | 642.46 | 1.427689 |
| GO:0050793\_regulation\_of\_developmental\_process | BCL11B | 703 | 6 | 0.818812 | -0.117592 | 450 | 642.46 | 1.427689 |
| GO:0050793\_regulation\_of\_developmental\_process | PLCG2 | 703 | 6 | 0.818812 | -0.117592 | 450 | 642.46 | 1.427689 |
| GO:0007166\_cell\_surface\_receptor\_linked\_signal\_transduction | DTX1 | 597 | 5 | 0.803497 | -0.116843 | 451 | 642.8 | 1.425277 |
| GO:0007166\_cell\_surface\_receptor\_linked\_signal\_transduction | GNAI1 | 597 | 5 | 0.803497 | -0.116843 | 451 | 642.8 | 1.425277 |
| GO:0007166\_cell\_surface\_receptor\_linked\_signal\_transduction | HTRA1 | 597 | 5 | 0.803497 | -0.116843 | 451 | 642.8 | 1.425277 |
| GO:0007166\_cell\_surface\_receptor\_linked\_signal\_transduction | PLCG2 | 597 | 5 | 0.803497 | -0.116843 | 451 | 642.8 | 1.425277 |
| GO:0007166\_cell\_surface\_receptor\_linked\_signal\_transduction | KDR | 597 | 5 | 0.803497 | -0.116843 | 451 | 642.8 | 1.425277 |
| GO:0001775\_cell\_activation | BCL11B | 262 | 2 | 0.732347 | -0.114953 | 452 | 644.56 | 1.426018 |
| GO:0001775\_cell\_activation | PLCG2 | 262 | 2 | 0.732347 | -0.114953 | 452 | 644.56 | 1.426018 |
| GO:0044255\_cellular\_lipid\_metabolic\_process | HMGCR | 264 | 2 | 0.726799 | -0.112843 | 453 | 645.58 | 1.425121 |
| GO:0044255\_cellular\_lipid\_metabolic\_process | PIP5K1B | 264 | 2 | 0.726799 | -0.112843 | 453 | 645.58 | 1.425121 |
| GO:0007169\_transmembrane\_receptor\_protein\_tyrosine\_kinase\_signaling\_pathway | KDR | 139 | 1 | 0.690198 | -0.112326 | 454 | 646.31 | 1.423590 |
| GO:0016044\_membrane\_organization | SYNE2 | 140 | 1 | 0.685268 | -0.110950 | 455 | 646.64 | 1.421187 |
| GO:0031328\_positive\_regulation\_of\_cellular\_biosynthetic\_process | NTF3 | 387 | 3 | 0.743702 | -0.107308 | 456 | 648.11 | 1.421294 |
| GO:0031328\_positive\_regulation\_of\_cellular\_biosynthetic\_process | BCL11B | 387 | 3 | 0.743702 | -0.107308 | 456 | 648.11 | 1.421294 |
| GO:0031328\_positive\_regulation\_of\_cellular\_biosynthetic\_process | SOX6 | 387 | 3 | 0.743702 | -0.107308 | 456 | 648.11 | 1.421294 |
| GO:0006810\_transport | FUSIP1 | 718 | 6 | 0.801706 | -0.107229 | 457 | 648.24 | 1.418468 |
| GO:0006810\_transport | XPO5 | 718 | 6 | 0.801706 | -0.107229 | 457 | 648.24 | 1.418468 |
| GO:0006810\_transport | PLCG2 | 718 | 6 | 0.801706 | -0.107229 | 457 | 648.24 | 1.418468 |
| GO:0006810\_transport | SLC13A3 | 718 | 6 | 0.801706 | -0.107229 | 457 | 648.24 | 1.418468 |
| GO:0006810\_transport | ABCA4 | 718 | 6 | 0.801706 | -0.107229 | 457 | 648.24 | 1.418468 |
| GO:0006810\_transport | DST | 718 | 6 | 0.801706 | -0.107229 | 457 | 648.24 | 1.418468 |
| GO:0035239\_tube\_morphogenesis | KDR | 143 | 1 | 0.670892 | -0.106935 | 458 | 648.56 | 1.416070 |
| GO:0045934\_negative\_regulation\_of\_nucleobase\_\_nucleoside\_\_nucleotide\_and\_nucleic\_acid\_metabolic\_process | FUSIP1 | 270 | 2 | 0.710648 | -0.106740 | 459 | 648.84 | 1.413595 |
| GO:0045934\_negative\_regulation\_of\_nucleobase\_\_nucleoside\_\_nucleotide\_and\_nucleic\_acid\_metabolic\_process | SOX6 | 270 | 2 | 0.710648 | -0.106740 | 459 | 648.84 | 1.413595 |
| GO:0009891\_positive\_regulation\_of\_biosynthetic\_process | NTF3 | 388 | 3 | 0.741785 | -0.106460 | 460 | 648.99 | 1.410848 |
| GO:0009891\_positive\_regulation\_of\_biosynthetic\_process | BCL11B | 388 | 3 | 0.741785 | -0.106460 | 460 | 648.99 | 1.410848 |
| GO:0009891\_positive\_regulation\_of\_biosynthetic\_process | SOX6 | 388 | 3 | 0.741785 | -0.106460 | 460 | 648.99 | 1.410848 |
| GO:0051172\_negative\_regulation\_of\_nitrogen\_compound\_metabolic\_process | FUSIP1 | 271 | 2 | 0.708026 | -0.105755 | 461 | 649.27 | 1.408395 |
| GO:0051172\_negative\_regulation\_of\_nitrogen\_compound\_metabolic\_process | SOX6 | 271 | 2 | 0.708026 | -0.105755 | 461 | 649.27 | 1.408395 |
| GO:0007186\_G-protein\_coupled\_receptor\_protein\_signaling\_pathway | GNAI1 | 144 | 1 | 0.666233 | -0.105632 | 462 | 649.96 | 1.406840 |
| GO:0006812\_cation\_transport | PLCG2 | 146 | 1 | 0.657106 | -0.103080 | 463 | 651.86 | 1.407905 |
| GO:0033036\_macromolecule\_localization | SYNE2 | 274 | 2 | 0.700274 | -0.102855 | 464 | 652.37 | 1.405970 |
| GO:0033036\_macromolecule\_localization | XPO5 | 274 | 2 | 0.700274 | -0.102855 | 464 | 652.37 | 1.405970 |
| GO:0022603\_regulation\_of\_anatomical\_structure\_morphogenesis | ULK2 | 147 | 1 | 0.652636 | -0.101829 | 465 | 652.87 | 1.404022 |
| GO:0006807\_nitrogen\_compound\_metabolic\_process | GUCA1B | 1147 | 10 | 0.836421 | -0.101079 | 466 | 652.95 | 1.401180 |
| GO:0006807\_nitrogen\_compound\_metabolic\_process | FUSIP1 | 1147 | 10 | 0.836421 | -0.101079 | 466 | 652.95 | 1.401180 |
| GO:0006807\_nitrogen\_compound\_metabolic\_process | PGM3 | 1147 | 10 | 0.836421 | -0.101079 | 466 | 652.95 | 1.401180 |
| GO:0006807\_nitrogen\_compound\_metabolic\_process | WDR55 | 1147 | 10 | 0.836421 | -0.101079 | 466 | 652.95 | 1.401180 |
| GO:0006807\_nitrogen\_compound\_metabolic\_process | NTF3 | 1147 | 10 | 0.836421 | -0.101079 | 466 | 652.95 | 1.401180 |
| GO:0006807\_nitrogen\_compound\_metabolic\_process | BCL11B | 1147 | 10 | 0.836421 | -0.101079 | 466 | 652.95 | 1.401180 |
| GO:0006807\_nitrogen\_compound\_metabolic\_process | CUGBP2 | 1147 | 10 | 0.836421 | -0.101079 | 466 | 652.95 | 1.401180 |
| GO:0006807\_nitrogen\_compound\_metabolic\_process | SOX6 | 1147 | 10 | 0.836421 | -0.101079 | 466 | 652.95 | 1.401180 |
| GO:0006807\_nitrogen\_compound\_metabolic\_process | AMD1 | 1147 | 10 | 0.836421 | -0.101079 | 466 | 652.95 | 1.401180 |
| GO:0006807\_nitrogen\_compound\_metabolic\_process | CRYM | 1147 | 10 | 0.836421 | -0.101079 | 466 | 652.95 | 1.401180 |
| GO:0002684\_positive\_regulation\_of\_immune\_system\_process | PLCG2 | 148 | 1 | 0.648226 | -0.100595 | 468 | 653.65 | 1.396688 |
| GO:0043085\_positive\_regulation\_of\_catalytic\_activity | VLDLR | 148 | 1 | 0.648226 | -0.100595 | 468 | 653.65 | 1.396688 |
| GO:0051234\_establishment\_of\_localization | FUSIP1 | 729 | 6 | 0.789609 | -0.100145 | 469 | 653.78 | 1.393987 |
| GO:0051234\_establishment\_of\_localization | XPO5 | 729 | 6 | 0.789609 | -0.100145 | 469 | 653.78 | 1.393987 |
| GO:0051234\_establishment\_of\_localization | PLCG2 | 729 | 6 | 0.789609 | -0.100145 | 469 | 653.78 | 1.393987 |
| GO:0051234\_establishment\_of\_localization | SLC13A3 | 729 | 6 | 0.789609 | -0.100145 | 469 | 653.78 | 1.393987 |
| GO:0051234\_establishment\_of\_localization | ABCA4 | 729 | 6 | 0.789609 | -0.100145 | 469 | 653.78 | 1.393987 |
| GO:0051234\_establishment\_of\_localization | DST | 729 | 6 | 0.789609 | -0.100145 | 469 | 653.78 | 1.393987 |
| GO:0007517\_muscle\_organ\_development | CRYAB | 153 | 1 | 0.627042 | -0.094668 | 470 | 656.73 | 1.397298 |
| GO:0007268\_synaptic\_transmission | NTF3 | 154 | 1 | 0.622971 | -0.093529 | 471 | 657.66 | 1.396306 |
| GO:0006629\_lipid\_metabolic\_process | HMGCR | 285 | 2 | 0.673246 | -0.092882 | 472 | 658.03 | 1.394131 |
| GO:0006629\_lipid\_metabolic\_process | PIP5K1B | 285 | 2 | 0.673246 | -0.092882 | 472 | 658.03 | 1.394131 |
| GO:0008285\_negative\_regulation\_of\_cell\_proliferation | BCL11B | 155 | 1 | 0.618952 | -0.092405 | 474 | 658.78 | 1.389831 |
| GO:0022402\_cell\_cycle\_process | CCNF | 155 | 1 | 0.618952 | -0.092405 | 474 | 658.78 | 1.389831 |
| GO:0007417\_central\_nervous\_system\_development | NTF3 | 287 | 2 | 0.668554 | -0.091174 | 475 | 659.0 | 1.387368 |
| GO:0007417\_central\_nervous\_system\_development | BCL11B | 287 | 2 | 0.668554 | -0.091174 | 475 | 659.0 | 1.387368 |
| GO:0009888\_tissue\_development | ANK | 525 | 4 | 0.730952 | -0.089628 | 476 | 660.01 | 1.386576 |
| GO:0009888\_tissue\_development | NTF3 | 525 | 4 | 0.730952 | -0.089628 | 476 | 660.01 | 1.386576 |
| GO:0009888\_tissue\_development | POSTN | 525 | 4 | 0.730952 | -0.089628 | 476 | 660.01 | 1.386576 |
| GO:0009888\_tissue\_development | SOX6 | 525 | 4 | 0.730952 | -0.089628 | 476 | 660.01 | 1.386576 |
| GO:0048514\_blood\_vessel\_morphogenesis | KDR | 158 | 1 | 0.607199 | -0.089120 | 477 | 661.02 | 1.385786 |
| GO:0048519\_negative\_regulation\_of\_biological\_process | FUSIP1 | 859 | 7 | 0.781796 | -0.087167 | 478 | 661.57 | 1.384038 |
| GO:0048519\_negative\_regulation\_of\_biological\_process | IRX3 | 859 | 7 | 0.781796 | -0.087167 | 478 | 661.57 | 1.384038 |
| GO:0048519\_negative\_regulation\_of\_biological\_process | HTRA1 | 859 | 7 | 0.781796 | -0.087167 | 478 | 661.57 | 1.384038 |
| GO:0048519\_negative\_regulation\_of\_biological\_process | ULK2 | 859 | 7 | 0.781796 | -0.087167 | 478 | 661.57 | 1.384038 |
| GO:0048519\_negative\_regulation\_of\_biological\_process | BCL11B | 859 | 7 | 0.781796 | -0.087167 | 478 | 661.57 | 1.384038 |
| GO:0048519\_negative\_regulation\_of\_biological\_process | PLCG2 | 859 | 7 | 0.781796 | -0.087167 | 478 | 661.57 | 1.384038 |
| GO:0048519\_negative\_regulation\_of\_biological\_process | SOX6 | 859 | 7 | 0.781796 | -0.087167 | 478 | 661.57 | 1.384038 |
| GO:0009058\_biosynthetic\_process | GUCA1B | 1175 | 10 | 0.816489 | -0.086902 | 479 | 662.03 | 1.382109 |
| GO:0009058\_biosynthetic\_process | PGM3 | 1175 | 10 | 0.816489 | -0.086902 | 479 | 662.03 | 1.382109 |
| GO:0009058\_biosynthetic\_process | TYR | 1175 | 10 | 0.816489 | -0.086902 | 479 | 662.03 | 1.382109 |
| GO:0009058\_biosynthetic\_process | GALNT10 | 1175 | 10 | 0.816489 | -0.086902 | 479 | 662.03 | 1.382109 |
| GO:0009058\_biosynthetic\_process | NTF3 | 1175 | 10 | 0.816489 | -0.086902 | 479 | 662.03 | 1.382109 |
| GO:0009058\_biosynthetic\_process | HMGCR | 1175 | 10 | 0.816489 | -0.086902 | 479 | 662.03 | 1.382109 |
| GO:0009058\_biosynthetic\_process | BCL11B | 1175 | 10 | 0.816489 | -0.086902 | 479 | 662.03 | 1.382109 |
| GO:0009058\_biosynthetic\_process | PLCG2 | 1175 | 10 | 0.816489 | -0.086902 | 479 | 662.03 | 1.382109 |
| GO:0009058\_biosynthetic\_process | SOX6 | 1175 | 10 | 0.816489 | -0.086902 | 479 | 662.03 | 1.382109 |
| GO:0009058\_biosynthetic\_process | AMD1 | 1175 | 10 | 0.816489 | -0.086902 | 479 | 662.03 | 1.382109 |
| GO:0045595\_regulation\_of\_cell\_differentiation | IRX3 | 295 | 2 | 0.650424 | -0.084649 | 480 | 663.42 | 1.382125 |
| GO:0045595\_regulation\_of\_cell\_differentiation | ULK2 | 295 | 2 | 0.650424 | -0.084649 | 480 | 663.42 | 1.382125 |
| GO:0042110\_T\_cell\_activation | BCL11B | 163 | 1 | 0.588574 | -0.083924 | 481 | 664.33 | 1.381143 |
| GO:0042592\_homeostatic\_process | SOX6 | 419 | 3 | 0.686903 | -0.083089 | 482 | 664.58 | 1.378797 |
| GO:0042592\_homeostatic\_process | ABCA4 | 419 | 3 | 0.686903 | -0.083089 | 482 | 664.58 | 1.378797 |
| GO:0042592\_homeostatic\_process | KDR | 419 | 3 | 0.686903 | -0.083089 | 482 | 664.58 | 1.378797 |
| GO:0006259\_DNA\_metabolic\_process | BCL11B | 165 | 1 | 0.581439 | -0.081939 | 483 | 665.92 | 1.378716 |
| GO:0051049\_regulation\_of\_transport | PLCG2 | 167 | 1 | 0.574476 | -0.080003 | 484 | 667.65 | 1.379442 |
| GO:0008283\_cell\_proliferation | TYR | 544 | 4 | 0.705423 | -0.078030 | 485 | 668.34 | 1.378021 |
| GO:0008283\_cell\_proliferation | BCL11B | 544 | 4 | 0.705423 | -0.078030 | 485 | 668.34 | 1.378021 |
| GO:0008283\_cell\_proliferation | COL8A1 | 544 | 4 | 0.705423 | -0.078030 | 485 | 668.34 | 1.378021 |
| GO:0008283\_cell\_proliferation | KDR | 544 | 4 | 0.705423 | -0.078030 | 485 | 668.34 | 1.378021 |
| GO:0010604\_positive\_regulation\_of\_macromolecule\_metabolic\_process | NTF3 | 433 | 3 | 0.664694 | -0.074200 | 487 | 672.87 | 1.381663 |
| GO:0010604\_positive\_regulation\_of\_macromolecule\_metabolic\_process | BCL11B | 433 | 3 | 0.664694 | -0.074200 | 487 | 672.87 | 1.381663 |
| GO:0010604\_positive\_regulation\_of\_macromolecule\_metabolic\_process | SOX6 | 433 | 3 | 0.664694 | -0.074200 | 487 | 672.87 | 1.381663 |
| GO:0012501\_programmed\_cell\_death | NTF3 | 433 | 3 | 0.664694 | -0.074200 | 487 | 672.87 | 1.381663 |
| GO:0012501\_programmed\_cell\_death | BCL11B | 433 | 3 | 0.664694 | -0.074200 | 487 | 672.87 | 1.381663 |
| GO:0012501\_programmed\_cell\_death | PLCG2 | 433 | 3 | 0.664694 | -0.074200 | 487 | 672.87 | 1.381663 |
| GO:0006357\_regulation\_of\_transcription\_from\_RNA\_polymerase\_II\_promoter | NTF3 | 435 | 3 | 0.661638 | -0.073005 | 488 | 673.33 | 1.379775 |
| GO:0006357\_regulation\_of\_transcription\_from\_RNA\_polymerase\_II\_promoter | BCL11B | 435 | 3 | 0.661638 | -0.073005 | 488 | 673.33 | 1.379775 |
| GO:0006357\_regulation\_of\_transcription\_from\_RNA\_polymerase\_II\_promoter | SOX6 | 435 | 3 | 0.661638 | -0.073005 | 488 | 673.33 | 1.379775 |
| GO:0000122\_negative\_regulation\_of\_transcription\_from\_RNA\_polymerase\_II\_promoter | SOX6 | 175 | 1 | 0.548214 | -0.072740 | 490 | 674.28 | 1.376082 |
| GO:0015031\_protein\_transport | XPO5 | 175 | 1 | 0.548214 | -0.072740 | 490 | 674.28 | 1.376082 |
| GO:0043066\_negative\_regulation\_of\_apoptosis | BCL11B | 176 | 1 | 0.545099 | -0.071882 | 491 | 675.23 | 1.375214 |
| GO:0006464\_protein\_modification\_process | GALNT10 | 439 | 3 | 0.655609 | -0.070670 | 492 | 675.52 | 1.373008 |
| GO:0006464\_protein\_modification\_process | BRSK2 | 439 | 3 | 0.655609 | -0.070670 | 492 | 675.52 | 1.373008 |
| GO:0006464\_protein\_modification\_process | DUSP6 | 439 | 3 | 0.655609 | -0.070670 | 492 | 675.52 | 1.373008 |
| GO:0006139\_nucleobase\_\_nucleoside\_\_nucleotide\_and\_nucleic\_acid\_metabolic\_process | GUCA1B | 1002 | 8 | 0.765968 | -0.070218 | 493 | 675.62 | 1.370426 |
| GO:0006139\_nucleobase\_\_nucleoside\_\_nucleotide\_and\_nucleic\_acid\_metabolic\_process | FUSIP1 | 1002 | 8 | 0.765968 | -0.070218 | 493 | 675.62 | 1.370426 |
| GO:0006139\_nucleobase\_\_nucleoside\_\_nucleotide\_and\_nucleic\_acid\_metabolic\_process | PGM3 | 1002 | 8 | 0.765968 | -0.070218 | 493 | 675.62 | 1.370426 |
| GO:0006139\_nucleobase\_\_nucleoside\_\_nucleotide\_and\_nucleic\_acid\_metabolic\_process | WDR55 | 1002 | 8 | 0.765968 | -0.070218 | 493 | 675.62 | 1.370426 |
| GO:0006139\_nucleobase\_\_nucleoside\_\_nucleotide\_and\_nucleic\_acid\_metabolic\_process | NTF3 | 1002 | 8 | 0.765968 | -0.070218 | 493 | 675.62 | 1.370426 |
| GO:0006139\_nucleobase\_\_nucleoside\_\_nucleotide\_and\_nucleic\_acid\_metabolic\_process | BCL11B | 1002 | 8 | 0.765968 | -0.070218 | 493 | 675.62 | 1.370426 |
| GO:0006139\_nucleobase\_\_nucleoside\_\_nucleotide\_and\_nucleic\_acid\_metabolic\_process | CUGBP2 | 1002 | 8 | 0.765968 | -0.070218 | 493 | 675.62 | 1.370426 |
| GO:0006139\_nucleobase\_\_nucleoside\_\_nucleotide\_and\_nucleic\_acid\_metabolic\_process | SOX6 | 1002 | 8 | 0.765968 | -0.070218 | 493 | 675.62 | 1.370426 |
| GO:0031325\_positive\_regulation\_of\_cellular\_metabolic\_process | NTF3 | 442 | 3 | 0.651160 | -0.068965 | 494 | 676.8 | 1.370040 |
| GO:0031325\_positive\_regulation\_of\_cellular\_metabolic\_process | BCL11B | 442 | 3 | 0.651160 | -0.068965 | 494 | 676.8 | 1.370040 |
| GO:0031325\_positive\_regulation\_of\_cellular\_metabolic\_process | SOX6 | 442 | 3 | 0.651160 | -0.068965 | 494 | 676.8 | 1.370040 |
| GO:0045184\_establishment\_of\_protein\_localization | XPO5 | 180 | 1 | 0.532986 | -0.068560 | 495 | 677.3 | 1.368283 |
| GO:0006366\_transcription\_from\_RNA\_polymerase\_II\_promoter | NTF3 | 444 | 3 | 0.648226 | -0.067849 | 497 | 677.69 | 1.363561 |
| GO:0006366\_transcription\_from\_RNA\_polymerase\_II\_promoter | BCL11B | 444 | 3 | 0.648226 | -0.067849 | 497 | 677.69 | 1.363561 |
| GO:0006366\_transcription\_from\_RNA\_polymerase\_II\_promoter | SOX6 | 444 | 3 | 0.648226 | -0.067849 | 497 | 677.69 | 1.363561 |
| GO:0008219\_cell\_death | NTF3 | 444 | 3 | 0.648226 | -0.067849 | 497 | 677.69 | 1.363561 |
| GO:0008219\_cell\_death | BCL11B | 444 | 3 | 0.648226 | -0.067849 | 497 | 677.69 | 1.363561 |
| GO:0008219\_cell\_death | PLCG2 | 444 | 3 | 0.648226 | -0.067849 | 497 | 677.69 | 1.363561 |
| GO:0010926\_anatomical\_structure\_formation | ITGA6 | 447 | 3 | 0.643876 | -0.066208 | 498 | 679.47 | 1.364398 |
| GO:0010926\_anatomical\_structure\_formation | CUGBP2 | 447 | 3 | 0.643876 | -0.066208 | 498 | 679.47 | 1.364398 |
| GO:0010926\_anatomical\_structure\_formation | ADAMTS1 | 447 | 3 | 0.643876 | -0.066208 | 498 | 679.47 | 1.364398 |
| GO:0010467\_gene\_expression | FUSIP1 | 905 | 7 | 0.742058 | -0.065630 | 499 | 680.13 | 1.362986 |
| GO:0010467\_gene\_expression | WDR55 | 905 | 7 | 0.742058 | -0.065630 | 499 | 680.13 | 1.362986 |
| GO:0010467\_gene\_expression | NTF3 | 905 | 7 | 0.742058 | -0.065630 | 499 | 680.13 | 1.362986 |
| GO:0010467\_gene\_expression | BCL11B | 905 | 7 | 0.742058 | -0.065630 | 499 | 680.13 | 1.362986 |
| GO:0010467\_gene\_expression | PLCG2 | 905 | 7 | 0.742058 | -0.065630 | 499 | 680.13 | 1.362986 |
| GO:0010467\_gene\_expression | CUGBP2 | 905 | 7 | 0.742058 | -0.065630 | 499 | 680.13 | 1.362986 |
| GO:0010467\_gene\_expression | SOX6 | 905 | 7 | 0.742058 | -0.065630 | 499 | 680.13 | 1.362986 |
| GO:0006996\_organelle\_organization | SYNE2 | 449 | 3 | 0.641008 | -0.065134 | 500 | 680.84 | 1.361680 |
| GO:0006996\_organelle\_organization | NOLC1 | 449 | 3 | 0.641008 | -0.065134 | 500 | 680.84 | 1.361680 |
| GO:0006996\_organelle\_organization | DST | 449 | 3 | 0.641008 | -0.065134 | 500 | 680.84 | 1.361680 |
| GO:0008152\_metabolic\_process | FUSIP1 | 2133 | 19 | 0.854577 | -0.064728 | 501 | 681.05 | 1.359381 |
| GO:0008152\_metabolic\_process | GUCA1B | 2133 | 19 | 0.854577 | -0.064728 | 501 | 681.05 | 1.359381 |
| GO:0008152\_metabolic\_process | NTF3 | 2133 | 19 | 0.854577 | -0.064728 | 501 | 681.05 | 1.359381 |
| GO:0008152\_metabolic\_process | HMGCR | 2133 | 19 | 0.854577 | -0.064728 | 501 | 681.05 | 1.359381 |
| GO:0008152\_metabolic\_process | CUGBP2 | 2133 | 19 | 0.854577 | -0.064728 | 501 | 681.05 | 1.359381 |
| GO:0008152\_metabolic\_process | BRSK2 | 2133 | 19 | 0.854577 | -0.064728 | 501 | 681.05 | 1.359381 |
| GO:0008152\_metabolic\_process | PIP5K1B | 2133 | 19 | 0.854577 | -0.064728 | 501 | 681.05 | 1.359381 |
| GO:0008152\_metabolic\_process | HK2 | 2133 | 19 | 0.854577 | -0.064728 | 501 | 681.05 | 1.359381 |
| GO:0008152\_metabolic\_process | SOX6 | 2133 | 19 | 0.854577 | -0.064728 | 501 | 681.05 | 1.359381 |
| GO:0008152\_metabolic\_process | PGM3 | 2133 | 19 | 0.854577 | -0.064728 | 501 | 681.05 | 1.359381 |
| GO:0008152\_metabolic\_process | TYR | 2133 | 19 | 0.854577 | -0.064728 | 501 | 681.05 | 1.359381 |
| GO:0008152\_metabolic\_process | GALNT10 | 2133 | 19 | 0.854577 | -0.064728 | 501 | 681.05 | 1.359381 |
| GO:0008152\_metabolic\_process | WDR55 | 2133 | 19 | 0.854577 | -0.064728 | 501 | 681.05 | 1.359381 |
| GO:0008152\_metabolic\_process | BCL11B | 2133 | 19 | 0.854577 | -0.064728 | 501 | 681.05 | 1.359381 |
| GO:0008152\_metabolic\_process | PLCG2 | 2133 | 19 | 0.854577 | -0.064728 | 501 | 681.05 | 1.359381 |
| GO:0008152\_metabolic\_process | AMD1 | 2133 | 19 | 0.854577 | -0.064728 | 501 | 681.05 | 1.359381 |
| GO:0008152\_metabolic\_process | CRYM | 2133 | 19 | 0.854577 | -0.064728 | 501 | 681.05 | 1.359381 |
| GO:0008152\_metabolic\_process | DUSP6 | 2133 | 19 | 0.854577 | -0.064728 | 501 | 681.05 | 1.359381 |
| GO:0008152\_metabolic\_process | VLDLR | 2133 | 19 | 0.854577 | -0.064728 | 501 | 681.05 | 1.359381 |
| GO:0007010\_cytoskeleton\_organization | DST | 185 | 1 | 0.518581 | -0.064634 | 502 | 681.31 | 1.357191 |
| GO:0016265\_death | NTF3 | 450 | 3 | 0.639583 | -0.064604 | 503 | 681.4 | 1.354672 |
| GO:0016265\_death | BCL11B | 450 | 3 | 0.639583 | -0.064604 | 503 | 681.4 | 1.354672 |
| GO:0016265\_death | PLCG2 | 450 | 3 | 0.639583 | -0.064604 | 503 | 681.4 | 1.354672 |
| GO:0006811\_ion\_transport | PLCG2 | 186 | 1 | 0.515793 | -0.063878 | 504 | 682.37 | 1.353909 |
| GO:0019226\_transmission\_of\_nerve\_impulse | NTF3 | 189 | 1 | 0.507606 | -0.061665 | 505 | 683.74 | 1.353941 |
| GO:0010605\_negative\_regulation\_of\_macromolecule\_metabolic\_process | FUSIP1 | 331 | 2 | 0.579683 | -0.060523 | 506 | 685.18 | 1.354111 |
| GO:0010605\_negative\_regulation\_of\_macromolecule\_metabolic\_process | SOX6 | 331 | 2 | 0.579683 | -0.060523 | 506 | 685.18 | 1.354111 |
| GO:0009893\_positive\_regulation\_of\_metabolic\_process | NTF3 | 458 | 3 | 0.628412 | -0.060502 | 508 | 685.49 | 1.349390 |
| GO:0009893\_positive\_regulation\_of\_metabolic\_process | BCL11B | 458 | 3 | 0.628412 | -0.060502 | 508 | 685.49 | 1.349390 |
| GO:0009893\_positive\_regulation\_of\_metabolic\_process | SOX6 | 458 | 3 | 0.628412 | -0.060502 | 508 | 685.49 | 1.349390 |
| GO:0043412\_biopolymer\_modification | GALNT10 | 458 | 3 | 0.628412 | -0.060502 | 508 | 685.49 | 1.349390 |
| GO:0043412\_biopolymer\_modification | BRSK2 | 458 | 3 | 0.628412 | -0.060502 | 508 | 685.49 | 1.349390 |
| GO:0043412\_biopolymer\_modification | DUSP6 | 458 | 3 | 0.628412 | -0.060502 | 508 | 685.49 | 1.349390 |
| GO:0031324\_negative\_regulation\_of\_cellular\_metabolic\_process | FUSIP1 | 332 | 2 | 0.577937 | -0.059959 | 509 | 685.67 | 1.347092 |
| GO:0031324\_negative\_regulation\_of\_cellular\_metabolic\_process | SOX6 | 332 | 2 | 0.577937 | -0.059959 | 509 | 685.67 | 1.347092 |
| GO:0009605\_response\_to\_external\_stimulus | GUCA1B | 339 | 2 | 0.566003 | -0.056153 | 510 | 688.37 | 1.349745 |
| GO:0009605\_response\_to\_external\_stimulus | ITGA6 | 339 | 2 | 0.566003 | -0.056153 | 510 | 688.37 | 1.349745 |
| GO:0050801\_ion\_homeostasis | KDR | 197 | 1 | 0.486992 | -0.056149 | 511 | 688.89 | 1.348121 |
| GO:0051252\_regulation\_of\_RNA\_metabolic\_process | FUSIP1 | 590 | 4 | 0.650424 | -0.055386 | 512 | 690.01 | 1.347676 |
| GO:0051252\_regulation\_of\_RNA\_metabolic\_process | NTF3 | 590 | 4 | 0.650424 | -0.055386 | 512 | 690.01 | 1.347676 |
| GO:0051252\_regulation\_of\_RNA\_metabolic\_process | BCL11B | 590 | 4 | 0.650424 | -0.055386 | 512 | 690.01 | 1.347676 |
| GO:0051252\_regulation\_of\_RNA\_metabolic\_process | SOX6 | 590 | 4 | 0.650424 | -0.055386 | 512 | 690.01 | 1.347676 |
| GO:0044237\_cellular\_metabolic\_process | FUSIP1 | 1974 | 17 | 0.826209 | -0.053193 | 513 | 691.18 | 1.347329 |
| GO:0044237\_cellular\_metabolic\_process | GUCA1B | 1974 | 17 | 0.826209 | -0.053193 | 513 | 691.18 | 1.347329 |
| GO:0044237\_cellular\_metabolic\_process | NTF3 | 1974 | 17 | 0.826209 | -0.053193 | 513 | 691.18 | 1.347329 |
| GO:0044237\_cellular\_metabolic\_process | HMGCR | 1974 | 17 | 0.826209 | -0.053193 | 513 | 691.18 | 1.347329 |
| GO:0044237\_cellular\_metabolic\_process | CUGBP2 | 1974 | 17 | 0.826209 | -0.053193 | 513 | 691.18 | 1.347329 |
| GO:0044237\_cellular\_metabolic\_process | PIP5K1B | 1974 | 17 | 0.826209 | -0.053193 | 513 | 691.18 | 1.347329 |
| GO:0044237\_cellular\_metabolic\_process | HK2 | 1974 | 17 | 0.826209 | -0.053193 | 513 | 691.18 | 1.347329 |
| GO:0044237\_cellular\_metabolic\_process | BRSK2 | 1974 | 17 | 0.826209 | -0.053193 | 513 | 691.18 | 1.347329 |
| GO:0044237\_cellular\_metabolic\_process | SOX6 | 1974 | 17 | 0.826209 | -0.053193 | 513 | 691.18 | 1.347329 |
| GO:0044237\_cellular\_metabolic\_process | PGM3 | 1974 | 17 | 0.826209 | -0.053193 | 513 | 691.18 | 1.347329 |
| GO:0044237\_cellular\_metabolic\_process | GALNT10 | 1974 | 17 | 0.826209 | -0.053193 | 513 | 691.18 | 1.347329 |
| GO:0044237\_cellular\_metabolic\_process | WDR55 | 1974 | 17 | 0.826209 | -0.053193 | 513 | 691.18 | 1.347329 |
| GO:0044237\_cellular\_metabolic\_process | BCL11B | 1974 | 17 | 0.826209 | -0.053193 | 513 | 691.18 | 1.347329 |
| GO:0044237\_cellular\_metabolic\_process | AMD1 | 1974 | 17 | 0.826209 | -0.053193 | 513 | 691.18 | 1.347329 |
| GO:0044237\_cellular\_metabolic\_process | CRYM | 1974 | 17 | 0.826209 | -0.053193 | 513 | 691.18 | 1.347329 |
| GO:0044237\_cellular\_metabolic\_process | DUSP6 | 1974 | 17 | 0.826209 | -0.053193 | 513 | 691.18 | 1.347329 |
| GO:0044237\_cellular\_metabolic\_process | VLDLR | 1974 | 17 | 0.826209 | -0.053193 | 513 | 691.18 | 1.347329 |
| GO:0001568\_blood\_vessel\_development | KDR | 203 | 1 | 0.472599 | -0.052352 | 514 | 691.91 | 1.346128 |
| GO:0009892\_negative\_regulation\_of\_metabolic\_process | FUSIP1 | 348 | 2 | 0.551365 | -0.051604 | 515 | 692.46 | 1.344583 |
| GO:0009892\_negative\_regulation\_of\_metabolic\_process | SOX6 | 348 | 2 | 0.551365 | -0.051604 | 515 | 692.46 | 1.344583 |
| GO:0006955\_immune\_response | PLCG2 | 205 | 1 | 0.467988 | -0.051147 | 516 | 693.06 | 1.343140 |
| GO:0001944\_vasculature\_development | KDR | 208 | 1 | 0.461238 | -0.049393 | 518 | 694.1 | 1.339961 |
| GO:0008284\_positive\_regulation\_of\_cell\_proliferation | KDR | 208 | 1 | 0.461238 | -0.049393 | 518 | 694.1 | 1.339961 |
| GO:0050794\_regulation\_of\_cellular\_process | FUSIP1 | 2190 | 19 | 0.832334 | -0.047625 | 519 | 694.58 | 1.338304 |
| GO:0050794\_regulation\_of\_cellular\_process | GUCA1B | 2190 | 19 | 0.832334 | -0.047625 | 519 | 694.58 | 1.338304 |
| GO:0050794\_regulation\_of\_cellular\_process | IRX3 | 2190 | 19 | 0.832334 | -0.047625 | 519 | 694.58 | 1.338304 |
| GO:0050794\_regulation\_of\_cellular\_process | NTF3 | 2190 | 19 | 0.832334 | -0.047625 | 519 | 694.58 | 1.338304 |
| GO:0050794\_regulation\_of\_cellular\_process | HMGCR | 2190 | 19 | 0.832334 | -0.047625 | 519 | 694.58 | 1.338304 |
| GO:0050794\_regulation\_of\_cellular\_process | GNAI1 | 2190 | 19 | 0.832334 | -0.047625 | 519 | 694.58 | 1.338304 |
| GO:0050794\_regulation\_of\_cellular\_process | DTX1 | 2190 | 19 | 0.832334 | -0.047625 | 519 | 694.58 | 1.338304 |
| GO:0050794\_regulation\_of\_cellular\_process | NID1 | 2190 | 19 | 0.832334 | -0.047625 | 519 | 694.58 | 1.338304 |
| GO:0050794\_regulation\_of\_cellular\_process | SOX6 | 2190 | 19 | 0.832334 | -0.047625 | 519 | 694.58 | 1.338304 |
| GO:0050794\_regulation\_of\_cellular\_process | KDR | 2190 | 19 | 0.832334 | -0.047625 | 519 | 694.58 | 1.338304 |
| GO:0050794\_regulation\_of\_cellular\_process | ITGA6 | 2190 | 19 | 0.832334 | -0.047625 | 519 | 694.58 | 1.338304 |
| GO:0050794\_regulation\_of\_cellular\_process | HTRA1 | 2190 | 19 | 0.832334 | -0.047625 | 519 | 694.58 | 1.338304 |
| GO:0050794\_regulation\_of\_cellular\_process | BCL11B | 2190 | 19 | 0.832334 | -0.047625 | 519 | 694.58 | 1.338304 |
| GO:0050794\_regulation\_of\_cellular\_process | ULK2 | 2190 | 19 | 0.832334 | -0.047625 | 519 | 694.58 | 1.338304 |
| GO:0050794\_regulation\_of\_cellular\_process | PLCG2 | 2190 | 19 | 0.832334 | -0.047625 | 519 | 694.58 | 1.338304 |
| GO:0050794\_regulation\_of\_cellular\_process | CAR8 | 2190 | 19 | 0.832334 | -0.047625 | 519 | 694.58 | 1.338304 |
| GO:0050794\_regulation\_of\_cellular\_process | COL8A1 | 2190 | 19 | 0.832334 | -0.047625 | 519 | 694.58 | 1.338304 |
| GO:0050794\_regulation\_of\_cellular\_process | DST | 2190 | 19 | 0.832334 | -0.047625 | 519 | 694.58 | 1.338304 |
| GO:0050794\_regulation\_of\_cellular\_process | VLDLR | 2190 | 19 | 0.832334 | -0.047625 | 519 | 694.58 | 1.338304 |
| GO:0035295\_tube\_development | KDR | 212 | 1 | 0.452535 | -0.047151 | 520 | 695.0 | 1.336538 |
| GO:0042981\_regulation\_of\_apoptosis | NTF3 | 360 | 2 | 0.532986 | -0.046089 | 521 | 695.27 | 1.334491 |
| GO:0042981\_regulation\_of\_apoptosis | BCL11B | 360 | 2 | 0.532986 | -0.046089 | 521 | 695.27 | 1.334491 |
| GO:0044238\_primary\_metabolic\_process | FUSIP1 | 1905 | 16 | 0.805774 | -0.044991 | 522 | 695.72 | 1.332797 |
| GO:0044238\_primary\_metabolic\_process | GUCA1B | 1905 | 16 | 0.805774 | -0.044991 | 522 | 695.72 | 1.332797 |
| GO:0044238\_primary\_metabolic\_process | NTF3 | 1905 | 16 | 0.805774 | -0.044991 | 522 | 695.72 | 1.332797 |
| GO:0044238\_primary\_metabolic\_process | HMGCR | 1905 | 16 | 0.805774 | -0.044991 | 522 | 695.72 | 1.332797 |
| GO:0044238\_primary\_metabolic\_process | CUGBP2 | 1905 | 16 | 0.805774 | -0.044991 | 522 | 695.72 | 1.332797 |
| GO:0044238\_primary\_metabolic\_process | BRSK2 | 1905 | 16 | 0.805774 | -0.044991 | 522 | 695.72 | 1.332797 |
| GO:0044238\_primary\_metabolic\_process | PIP5K1B | 1905 | 16 | 0.805774 | -0.044991 | 522 | 695.72 | 1.332797 |
| GO:0044238\_primary\_metabolic\_process | HK2 | 1905 | 16 | 0.805774 | -0.044991 | 522 | 695.72 | 1.332797 |
| GO:0044238\_primary\_metabolic\_process | SOX6 | 1905 | 16 | 0.805774 | -0.044991 | 522 | 695.72 | 1.332797 |
| GO:0044238\_primary\_metabolic\_process | PGM3 | 1905 | 16 | 0.805774 | -0.044991 | 522 | 695.72 | 1.332797 |
| GO:0044238\_primary\_metabolic\_process | GALNT10 | 1905 | 16 | 0.805774 | -0.044991 | 522 | 695.72 | 1.332797 |
| GO:0044238\_primary\_metabolic\_process | WDR55 | 1905 | 16 | 0.805774 | -0.044991 | 522 | 695.72 | 1.332797 |
| GO:0044238\_primary\_metabolic\_process | BCL11B | 1905 | 16 | 0.805774 | -0.044991 | 522 | 695.72 | 1.332797 |
| GO:0044238\_primary\_metabolic\_process | AMD1 | 1905 | 16 | 0.805774 | -0.044991 | 522 | 695.72 | 1.332797 |
| GO:0044238\_primary\_metabolic\_process | CRYM | 1905 | 16 | 0.805774 | -0.044991 | 522 | 695.72 | 1.332797 |
| GO:0044238\_primary\_metabolic\_process | DUSP6 | 1905 | 16 | 0.805774 | -0.044991 | 522 | 695.72 | 1.332797 |
| GO:0048583\_regulation\_of\_response\_to\_stimulus | PLCG2 | 217 | 1 | 0.442108 | -0.044496 | 523 | 696.36 | 1.331472 |
| GO:0045892\_negative\_regulation\_of\_transcription\_\_DNA-dependent | SOX6 | 218 | 1 | 0.440080 | -0.043984 | 524 | 696.6 | 1.329389 |
| GO:0001701\_in\_utero\_embryonic\_development | AMD1 | 221 | 1 | 0.434106 | -0.042483 | 525 | 698.16 | 1.329829 |
| GO:0019222\_regulation\_of\_metabolic\_process | FUSIP1 | 1088 | 8 | 0.705423 | -0.041073 | 526 | 698.74 | 1.328403 |
| GO:0019222\_regulation\_of\_metabolic\_process | GUCA1B | 1088 | 8 | 0.705423 | -0.041073 | 526 | 698.74 | 1.328403 |
| GO:0019222\_regulation\_of\_metabolic\_process | NTF3 | 1088 | 8 | 0.705423 | -0.041073 | 526 | 698.74 | 1.328403 |
| GO:0019222\_regulation\_of\_metabolic\_process | HMGCR | 1088 | 8 | 0.705423 | -0.041073 | 526 | 698.74 | 1.328403 |
| GO:0019222\_regulation\_of\_metabolic\_process | BCL11B | 1088 | 8 | 0.705423 | -0.041073 | 526 | 698.74 | 1.328403 |
| GO:0019222\_regulation\_of\_metabolic\_process | PLCG2 | 1088 | 8 | 0.705423 | -0.041073 | 526 | 698.74 | 1.328403 |
| GO:0019222\_regulation\_of\_metabolic\_process | SOX6 | 1088 | 8 | 0.705423 | -0.041073 | 526 | 698.74 | 1.328403 |
| GO:0019222\_regulation\_of\_metabolic\_process | VLDLR | 1088 | 8 | 0.705423 | -0.041073 | 526 | 698.74 | 1.328403 |
| GO:0002682\_regulation\_of\_immune\_system\_process | PLCG2 | 228 | 1 | 0.420779 | -0.039183 | 527 | 699.62 | 1.327552 |
| GO:0007154\_cell\_communication | ITGA6 | 1096 | 8 | 0.700274 | -0.038993 | 528 | 699.78 | 1.325341 |
| GO:0007154\_cell\_communication | NTF3 | 1096 | 8 | 0.700274 | -0.038993 | 528 | 699.78 | 1.325341 |
| GO:0007154\_cell\_communication | DTX1 | 1096 | 8 | 0.700274 | -0.038993 | 528 | 699.78 | 1.325341 |
| GO:0007154\_cell\_communication | GNAI1 | 1096 | 8 | 0.700274 | -0.038993 | 528 | 699.78 | 1.325341 |
| GO:0007154\_cell\_communication | HTRA1 | 1096 | 8 | 0.700274 | -0.038993 | 528 | 699.78 | 1.325341 |
| GO:0007154\_cell\_communication | PLCG2 | 1096 | 8 | 0.700274 | -0.038993 | 528 | 699.78 | 1.325341 |
| GO:0007154\_cell\_communication | CAR8 | 1096 | 8 | 0.700274 | -0.038993 | 528 | 699.78 | 1.325341 |
| GO:0007154\_cell\_communication | KDR | 1096 | 8 | 0.700274 | -0.038993 | 528 | 699.78 | 1.325341 |
| GO:0019219\_regulation\_of\_nucleobase\_\_nucleoside\_\_nucleotide\_and\_nucleic\_acid\_metabolic\_process | FUSIP1 | 757 | 5 | 0.633669 | -0.038566 | 529 | 700.12 | 1.323478 |
| GO:0019219\_regulation\_of\_nucleobase\_\_nucleoside\_\_nucleotide\_and\_nucleic\_acid\_metabolic\_process | GUCA1B | 757 | 5 | 0.633669 | -0.038566 | 529 | 700.12 | 1.323478 |
| GO:0019219\_regulation\_of\_nucleobase\_\_nucleoside\_\_nucleotide\_and\_nucleic\_acid\_metabolic\_process | NTF3 | 757 | 5 | 0.633669 | -0.038566 | 529 | 700.12 | 1.323478 |
| GO:0019219\_regulation\_of\_nucleobase\_\_nucleoside\_\_nucleotide\_and\_nucleic\_acid\_metabolic\_process | BCL11B | 757 | 5 | 0.633669 | -0.038566 | 529 | 700.12 | 1.323478 |
| GO:0019219\_regulation\_of\_nucleobase\_\_nucleoside\_\_nucleotide\_and\_nucleic\_acid\_metabolic\_process | SOX6 | 757 | 5 | 0.633669 | -0.038566 | 529 | 700.12 | 1.323478 |
| GO:0007420\_brain\_development | NTF3 | 231 | 1 | 0.415314 | -0.037851 | 530 | 700.44 | 1.321585 |
| GO:0009887\_organ\_morphogenesis | ITGA6 | 642 | 4 | 0.597741 | -0.037103 | 531 | 700.64 | 1.319473 |
| GO:0009887\_organ\_morphogenesis | ADAMTS1 | 642 | 4 | 0.597741 | -0.037103 | 531 | 700.64 | 1.319473 |
| GO:0009887\_organ\_morphogenesis | COL8A1 | 642 | 4 | 0.597741 | -0.037103 | 531 | 700.64 | 1.319473 |
| GO:0009887\_organ\_morphogenesis | KDR | 642 | 4 | 0.597741 | -0.037103 | 531 | 700.64 | 1.319473 |
| GO:0043687\_post-translational\_protein\_modification | BRSK2 | 384 | 2 | 0.499674 | -0.036716 | 532 | 701.21 | 1.318064 |
| GO:0043687\_post-translational\_protein\_modification | DUSP6 | 384 | 2 | 0.499674 | -0.036716 | 532 | 701.21 | 1.318064 |
| GO:0006468\_protein\_amino\_acid\_phosphorylation | BRSK2 | 237 | 1 | 0.404800 | -0.035323 | 533 | 702.65 | 1.318293 |
| GO:0051171\_regulation\_of\_nitrogen\_compound\_metabolic\_process | FUSIP1 | 771 | 5 | 0.622163 | -0.034773 | 534 | 703.08 | 1.316629 |
| GO:0051171\_regulation\_of\_nitrogen\_compound\_metabolic\_process | GUCA1B | 771 | 5 | 0.622163 | -0.034773 | 534 | 703.08 | 1.316629 |
| GO:0051171\_regulation\_of\_nitrogen\_compound\_metabolic\_process | NTF3 | 771 | 5 | 0.622163 | -0.034773 | 534 | 703.08 | 1.316629 |
| GO:0051171\_regulation\_of\_nitrogen\_compound\_metabolic\_process | BCL11B | 771 | 5 | 0.622163 | -0.034773 | 534 | 703.08 | 1.316629 |
| GO:0051171\_regulation\_of\_nitrogen\_compound\_metabolic\_process | SOX6 | 771 | 5 | 0.622163 | -0.034773 | 534 | 703.08 | 1.316629 |
| GO:0042127\_regulation\_of\_cell\_proliferation | BCL11B | 393 | 2 | 0.488232 | -0.033698 | 535 | 703.79 | 1.315495 |
| GO:0042127\_regulation\_of\_cell\_proliferation | KDR | 393 | 2 | 0.488232 | -0.033698 | 535 | 703.79 | 1.315495 |
| GO:0010468\_regulation\_of\_gene\_expression | FUSIP1 | 778 | 5 | 0.616565 | -0.033005 | 536 | 704.0 | 1.313433 |
| GO:0010468\_regulation\_of\_gene\_expression | NTF3 | 778 | 5 | 0.616565 | -0.033005 | 536 | 704.0 | 1.313433 |
| GO:0010468\_regulation\_of\_gene\_expression | BCL11B | 778 | 5 | 0.616565 | -0.033005 | 536 | 704.0 | 1.313433 |
| GO:0010468\_regulation\_of\_gene\_expression | PLCG2 | 778 | 5 | 0.616565 | -0.033005 | 536 | 704.0 | 1.313433 |
| GO:0010468\_regulation\_of\_gene\_expression | SOX6 | 778 | 5 | 0.616565 | -0.033005 | 536 | 704.0 | 1.313433 |
| GO:0009987\_cellular\_process | FUSIP1 | 3868 | 37 | 0.917706 | -0.031993 | 537 | 704.62 | 1.312142 |
| GO:0009987\_cellular\_process | IRX3 | 3868 | 37 | 0.917706 | -0.031993 | 537 | 704.62 | 1.312142 |
| GO:0009987\_cellular\_process | XPO5 | 3868 | 37 | 0.917706 | -0.031993 | 537 | 704.62 | 1.312142 |
| GO:0009987\_cellular\_process | TDRD7 | 3868 | 37 | 0.917706 | -0.031993 | 537 | 704.62 | 1.312142 |
| GO:0009987\_cellular\_process | HMGCR | 3868 | 37 | 0.917706 | -0.031993 | 537 | 704.62 | 1.312142 |
| GO:0009987\_cellular\_process | GNAI1 | 3868 | 37 | 0.917706 | -0.031993 | 537 | 704.62 | 1.312142 |
| GO:0009987\_cellular\_process | PIP5K1B | 3868 | 37 | 0.917706 | -0.031993 | 537 | 704.62 | 1.312142 |
| GO:0009987\_cellular\_process | HK2 | 3868 | 37 | 0.917706 | -0.031993 | 537 | 704.62 | 1.312142 |
| GO:0009987\_cellular\_process | BRSK2 | 3868 | 37 | 0.917706 | -0.031993 | 537 | 704.62 | 1.312142 |
| GO:0009987\_cellular\_process | POSTN | 3868 | 37 | 0.917706 | -0.031993 | 537 | 704.62 | 1.312142 |
| GO:0009987\_cellular\_process | SOX6 | 3868 | 37 | 0.917706 | -0.031993 | 537 | 704.62 | 1.312142 |
| GO:0009987\_cellular\_process | TYR | 3868 | 37 | 0.917706 | -0.031993 | 537 | 704.62 | 1.312142 |
| GO:0009987\_cellular\_process | GALNT10 | 3868 | 37 | 0.917706 | -0.031993 | 537 | 704.62 | 1.312142 |
| GO:0009987\_cellular\_process | WDR55 | 3868 | 37 | 0.917706 | -0.031993 | 537 | 704.62 | 1.312142 |
| GO:0009987\_cellular\_process | HTRA1 | 3868 | 37 | 0.917706 | -0.031993 | 537 | 704.62 | 1.312142 |
| GO:0009987\_cellular\_process | BCL11B | 3868 | 37 | 0.917706 | -0.031993 | 537 | 704.62 | 1.312142 |
| GO:0009987\_cellular\_process | AMD1 | 3868 | 37 | 0.917706 | -0.031993 | 537 | 704.62 | 1.312142 |
| GO:0009987\_cellular\_process | COL8A1 | 3868 | 37 | 0.917706 | -0.031993 | 537 | 704.62 | 1.312142 |
| GO:0009987\_cellular\_process | GUCA1B | 3868 | 37 | 0.917706 | -0.031993 | 537 | 704.62 | 1.312142 |
| GO:0009987\_cellular\_process | NTF3 | 3868 | 37 | 0.917706 | -0.031993 | 537 | 704.62 | 1.312142 |
| GO:0009987\_cellular\_process | DTX1 | 3868 | 37 | 0.917706 | -0.031993 | 537 | 704.62 | 1.312142 |
| GO:0009987\_cellular\_process | CCNF | 3868 | 37 | 0.917706 | -0.031993 | 537 | 704.62 | 1.312142 |
| GO:0009987\_cellular\_process | CUGBP2 | 3868 | 37 | 0.917706 | -0.031993 | 537 | 704.62 | 1.312142 |
| GO:0009987\_cellular\_process | NID1 | 3868 | 37 | 0.917706 | -0.031993 | 537 | 704.62 | 1.312142 |
| GO:0009987\_cellular\_process | KDR | 3868 | 37 | 0.917706 | -0.031993 | 537 | 704.62 | 1.312142 |
| GO:0009987\_cellular\_process | PGM3 | 3868 | 37 | 0.917706 | -0.031993 | 537 | 704.62 | 1.312142 |
| GO:0009987\_cellular\_process | SYNE2 | 3868 | 37 | 0.917706 | -0.031993 | 537 | 704.62 | 1.312142 |
| GO:0009987\_cellular\_process | PLK2 | 3868 | 37 | 0.917706 | -0.031993 | 537 | 704.62 | 1.312142 |
| GO:0009987\_cellular\_process | NOLC1 | 3868 | 37 | 0.917706 | -0.031993 | 537 | 704.62 | 1.312142 |
| GO:0009987\_cellular\_process | ITGA6 | 3868 | 37 | 0.917706 | -0.031993 | 537 | 704.62 | 1.312142 |
| GO:0009987\_cellular\_process | ULK2 | 3868 | 37 | 0.917706 | -0.031993 | 537 | 704.62 | 1.312142 |
| GO:0009987\_cellular\_process | PLCG2 | 3868 | 37 | 0.917706 | -0.031993 | 537 | 704.62 | 1.312142 |
| GO:0009987\_cellular\_process | CAR8 | 3868 | 37 | 0.917706 | -0.031993 | 537 | 704.62 | 1.312142 |
| GO:0009987\_cellular\_process | DST | 3868 | 37 | 0.917706 | -0.031993 | 537 | 704.62 | 1.312142 |
| GO:0009987\_cellular\_process | CRYM | 3868 | 37 | 0.917706 | -0.031993 | 537 | 704.62 | 1.312142 |
| GO:0009987\_cellular\_process | DUSP6 | 3868 | 37 | 0.917706 | -0.031993 | 537 | 704.62 | 1.312142 |
| GO:0009987\_cellular\_process | VLDLR | 3868 | 37 | 0.917706 | -0.031993 | 537 | 704.62 | 1.312142 |
| GO:0031323\_regulation\_of\_cellular\_metabolic\_process | FUSIP1 | 1015 | 7 | 0.661638 | -0.031777 | 538 | 704.66 | 1.309777 |
| GO:0031323\_regulation\_of\_cellular\_metabolic\_process | GUCA1B | 1015 | 7 | 0.661638 | -0.031777 | 538 | 704.66 | 1.309777 |
| GO:0031323\_regulation\_of\_cellular\_metabolic\_process | NTF3 | 1015 | 7 | 0.661638 | -0.031777 | 538 | 704.66 | 1.309777 |
| GO:0031323\_regulation\_of\_cellular\_metabolic\_process | HMGCR | 1015 | 7 | 0.661638 | -0.031777 | 538 | 704.66 | 1.309777 |
| GO:0031323\_regulation\_of\_cellular\_metabolic\_process | BCL11B | 1015 | 7 | 0.661638 | -0.031777 | 538 | 704.66 | 1.309777 |
| GO:0031323\_regulation\_of\_cellular\_metabolic\_process | SOX6 | 1015 | 7 | 0.661638 | -0.031777 | 538 | 704.66 | 1.309777 |
| GO:0031323\_regulation\_of\_cellular\_metabolic\_process | VLDLR | 1015 | 7 | 0.661638 | -0.031777 | 538 | 704.66 | 1.309777 |
| GO:0050789\_regulation\_of\_biological\_process | GUCA1B | 2357 | 20 | 0.814064 | -0.031712 | 539 | 704.7 | 1.307421 |
| GO:0050789\_regulation\_of\_biological\_process | FUSIP1 | 2357 | 20 | 0.814064 | -0.031712 | 539 | 704.7 | 1.307421 |
| GO:0050789\_regulation\_of\_biological\_process | IRX3 | 2357 | 20 | 0.814064 | -0.031712 | 539 | 704.7 | 1.307421 |
| GO:0050789\_regulation\_of\_biological\_process | NTF3 | 2357 | 20 | 0.814064 | -0.031712 | 539 | 704.7 | 1.307421 |
| GO:0050789\_regulation\_of\_biological\_process | DTX1 | 2357 | 20 | 0.814064 | -0.031712 | 539 | 704.7 | 1.307421 |
| GO:0050789\_regulation\_of\_biological\_process | GNAI1 | 2357 | 20 | 0.814064 | -0.031712 | 539 | 704.7 | 1.307421 |
| GO:0050789\_regulation\_of\_biological\_process | HMGCR | 2357 | 20 | 0.814064 | -0.031712 | 539 | 704.7 | 1.307421 |
| GO:0050789\_regulation\_of\_biological\_process | NID1 | 2357 | 20 | 0.814064 | -0.031712 | 539 | 704.7 | 1.307421 |
| GO:0050789\_regulation\_of\_biological\_process | SOX6 | 2357 | 20 | 0.814064 | -0.031712 | 539 | 704.7 | 1.307421 |
| GO:0050789\_regulation\_of\_biological\_process | KDR | 2357 | 20 | 0.814064 | -0.031712 | 539 | 704.7 | 1.307421 |
| GO:0050789\_regulation\_of\_biological\_process | ANK | 2357 | 20 | 0.814064 | -0.031712 | 539 | 704.7 | 1.307421 |
| GO:0050789\_regulation\_of\_biological\_process | ITGA6 | 2357 | 20 | 0.814064 | -0.031712 | 539 | 704.7 | 1.307421 |
| GO:0050789\_regulation\_of\_biological\_process | HTRA1 | 2357 | 20 | 0.814064 | -0.031712 | 539 | 704.7 | 1.307421 |
| GO:0050789\_regulation\_of\_biological\_process | ULK2 | 2357 | 20 | 0.814064 | -0.031712 | 539 | 704.7 | 1.307421 |
| GO:0050789\_regulation\_of\_biological\_process | BCL11B | 2357 | 20 | 0.814064 | -0.031712 | 539 | 704.7 | 1.307421 |
| GO:0050789\_regulation\_of\_biological\_process | PLCG2 | 2357 | 20 | 0.814064 | -0.031712 | 539 | 704.7 | 1.307421 |
| GO:0050789\_regulation\_of\_biological\_process | CAR8 | 2357 | 20 | 0.814064 | -0.031712 | 539 | 704.7 | 1.307421 |
| GO:0050789\_regulation\_of\_biological\_process | COL8A1 | 2357 | 20 | 0.814064 | -0.031712 | 539 | 704.7 | 1.307421 |
| GO:0050789\_regulation\_of\_biological\_process | DST | 2357 | 20 | 0.814064 | -0.031712 | 539 | 704.7 | 1.307421 |
| GO:0050789\_regulation\_of\_biological\_process | VLDLR | 2357 | 20 | 0.814064 | -0.031712 | 539 | 704.7 | 1.307421 |
| GO:0032879\_regulation\_of\_localization | PLCG2 | 248 | 1 | 0.386845 | -0.031127 | 540 | 705.14 | 1.305815 |
| GO:0007267\_cell-cell\_signaling | NTF3 | 252 | 1 | 0.380704 | -0.029730 | 541 | 705.75 | 1.304529 |
| GO:0016481\_negative\_regulation\_of\_transcription | SOX6 | 253 | 1 | 0.379200 | -0.029391 | 542 | 706.18 | 1.302915 |
| GO:0048878\_chemical\_homeostasis | KDR | 254 | 1 | 0.377707 | -0.029056 | 543 | 706.46 | 1.301031 |
| GO:0042221\_response\_to\_chemical\_stimulus | PLCG2 | 409 | 2 | 0.469132 | -0.028912 | 544 | 706.57 | 1.298842 |
| GO:0042221\_response\_to\_chemical\_stimulus | ANXA5 | 409 | 2 | 0.469132 | -0.028912 | 544 | 706.57 | 1.298842 |
| GO:0007165\_signal\_transduction | DTX1 | 915 | 6 | 0.629098 | -0.028636 | 545 | 706.85 | 1.296972 |
| GO:0007165\_signal\_transduction | GNAI1 | 915 | 6 | 0.629098 | -0.028636 | 545 | 706.85 | 1.296972 |
| GO:0007165\_signal\_transduction | HTRA1 | 915 | 6 | 0.629098 | -0.028636 | 545 | 706.85 | 1.296972 |
| GO:0007165\_signal\_transduction | PLCG2 | 915 | 6 | 0.629098 | -0.028636 | 545 | 706.85 | 1.296972 |
| GO:0007165\_signal\_transduction | CAR8 | 915 | 6 | 0.629098 | -0.028636 | 545 | 706.85 | 1.296972 |
| GO:0007165\_signal\_transduction | KDR | 915 | 6 | 0.629098 | -0.028636 | 545 | 706.85 | 1.296972 |
| GO:0009966\_regulation\_of\_signal\_transduction | HTRA1 | 256 | 1 | 0.374756 | -0.028397 | 546 | 707.08 | 1.295018 |
| GO:0044249\_cellular\_biosynthetic\_process | GUCA1B | 1150 | 8 | 0.667391 | -0.027190 | 547 | 707.93 | 1.294205 |
| GO:0044249\_cellular\_biosynthetic\_process | PGM3 | 1150 | 8 | 0.667391 | -0.027190 | 547 | 707.93 | 1.294205 |
| GO:0044249\_cellular\_biosynthetic\_process | GALNT10 | 1150 | 8 | 0.667391 | -0.027190 | 547 | 707.93 | 1.294205 |
| GO:0044249\_cellular\_biosynthetic\_process | NTF3 | 1150 | 8 | 0.667391 | -0.027190 | 547 | 707.93 | 1.294205 |
| GO:0044249\_cellular\_biosynthetic\_process | HMGCR | 1150 | 8 | 0.667391 | -0.027190 | 547 | 707.93 | 1.294205 |
| GO:0044249\_cellular\_biosynthetic\_process | BCL11B | 1150 | 8 | 0.667391 | -0.027190 | 547 | 707.93 | 1.294205 |
| GO:0044249\_cellular\_biosynthetic\_process | SOX6 | 1150 | 8 | 0.667391 | -0.027190 | 547 | 707.93 | 1.294205 |
| GO:0044249\_cellular\_biosynthetic\_process | AMD1 | 1150 | 8 | 0.667391 | -0.027190 | 547 | 707.93 | 1.294205 |
| GO:0010629\_negative\_regulation\_of\_gene\_expression | SOX6 | 262 | 1 | 0.366174 | -0.026509 | 548 | 708.67 | 1.293193 |
| GO:0044267\_cellular\_protein\_metabolic\_process | GALNT10 | 559 | 3 | 0.514870 | -0.025754 | 549 | 709.25 | 1.291894 |
| GO:0044267\_cellular\_protein\_metabolic\_process | BRSK2 | 559 | 3 | 0.514870 | -0.025754 | 549 | 709.25 | 1.291894 |
| GO:0044267\_cellular\_protein\_metabolic\_process | DUSP6 | 559 | 3 | 0.514870 | -0.025754 | 549 | 709.25 | 1.291894 |
| GO:0065008\_regulation\_of\_biological\_quality | SOX6 | 693 | 4 | 0.553752 | -0.024693 | 550 | 709.69 | 1.290345 |
| GO:0065008\_regulation\_of\_biological\_quality | ABCA4 | 693 | 4 | 0.553752 | -0.024693 | 550 | 709.69 | 1.290345 |
| GO:0065008\_regulation\_of\_biological\_quality | CRYM | 693 | 4 | 0.553752 | -0.024693 | 550 | 709.69 | 1.290345 |
| GO:0065008\_regulation\_of\_biological\_quality | KDR | 693 | 4 | 0.553752 | -0.024693 | 550 | 709.69 | 1.290345 |
| GO:0006915\_apoptosis | NTF3 | 427 | 2 | 0.449356 | -0.024308 | 551 | 709.93 | 1.288439 |
| GO:0006915\_apoptosis | BCL11B | 427 | 2 | 0.449356 | -0.024308 | 551 | 709.93 | 1.288439 |
| GO:0009790\_embryonic\_development | PGM3 | 567 | 3 | 0.507606 | -0.024018 | 552 | 710.16 | 1.286522 |
| GO:0009790\_embryonic\_development | HMGCR | 567 | 3 | 0.507606 | -0.024018 | 552 | 710.16 | 1.286522 |
| GO:0009790\_embryonic\_development | AMD1 | 567 | 3 | 0.507606 | -0.024018 | 552 | 710.16 | 1.286522 |
| GO:0065007\_biological\_regulation | GUCA1B | 2593 | 22 | 0.813970 | -0.023877 | 553 | 710.37 | 1.284575 |
| GO:0065007\_biological\_regulation | FUSIP1 | 2593 | 22 | 0.813970 | -0.023877 | 553 | 710.37 | 1.284575 |
| GO:0065007\_biological\_regulation | IRX3 | 2593 | 22 | 0.813970 | -0.023877 | 553 | 710.37 | 1.284575 |
| GO:0065007\_biological\_regulation | NTF3 | 2593 | 22 | 0.813970 | -0.023877 | 553 | 710.37 | 1.284575 |
| GO:0065007\_biological\_regulation | DTX1 | 2593 | 22 | 0.813970 | -0.023877 | 553 | 710.37 | 1.284575 |
| GO:0065007\_biological\_regulation | GNAI1 | 2593 | 22 | 0.813970 | -0.023877 | 553 | 710.37 | 1.284575 |
| GO:0065007\_biological\_regulation | HMGCR | 2593 | 22 | 0.813970 | -0.023877 | 553 | 710.37 | 1.284575 |
| GO:0065007\_biological\_regulation | NID1 | 2593 | 22 | 0.813970 | -0.023877 | 553 | 710.37 | 1.284575 |
| GO:0065007\_biological\_regulation | SOX6 | 2593 | 22 | 0.813970 | -0.023877 | 553 | 710.37 | 1.284575 |
| GO:0065007\_biological\_regulation | ABCA4 | 2593 | 22 | 0.813970 | -0.023877 | 553 | 710.37 | 1.284575 |
| GO:0065007\_biological\_regulation | KDR | 2593 | 22 | 0.813970 | -0.023877 | 553 | 710.37 | 1.284575 |
| GO:0065007\_biological\_regulation | ANK | 2593 | 22 | 0.813970 | -0.023877 | 553 | 710.37 | 1.284575 |
| GO:0065007\_biological\_regulation | ITGA6 | 2593 | 22 | 0.813970 | -0.023877 | 553 | 710.37 | 1.284575 |
| GO:0065007\_biological\_regulation | HTRA1 | 2593 | 22 | 0.813970 | -0.023877 | 553 | 710.37 | 1.284575 |
| GO:0065007\_biological\_regulation | ULK2 | 2593 | 22 | 0.813970 | -0.023877 | 553 | 710.37 | 1.284575 |
| GO:0065007\_biological\_regulation | BCL11B | 2593 | 22 | 0.813970 | -0.023877 | 553 | 710.37 | 1.284575 |
| GO:0065007\_biological\_regulation | PLCG2 | 2593 | 22 | 0.813970 | -0.023877 | 553 | 710.37 | 1.284575 |
| GO:0065007\_biological\_regulation | CAR8 | 2593 | 22 | 0.813970 | -0.023877 | 553 | 710.37 | 1.284575 |
| GO:0065007\_biological\_regulation | COL8A1 | 2593 | 22 | 0.813970 | -0.023877 | 553 | 710.37 | 1.284575 |
| GO:0065007\_biological\_regulation | DST | 2593 | 22 | 0.813970 | -0.023877 | 553 | 710.37 | 1.284575 |
| GO:0065007\_biological\_regulation | CRYM | 2593 | 22 | 0.813970 | -0.023877 | 553 | 710.37 | 1.284575 |
| GO:0065007\_biological\_regulation | VLDLR | 2593 | 22 | 0.813970 | -0.023877 | 553 | 710.37 | 1.284575 |
| GO:0051716\_cellular\_response\_to\_stimulus | ITGA6 | 273 | 1 | 0.351419 | -0.023372 | 554 | 710.61 | 1.282690 |
| GO:0010558\_negative\_regulation\_of\_macromolecule\_biosynthetic\_process | SOX6 | 274 | 1 | 0.350137 | -0.023106 | 555 | 711.03 | 1.281135 |
| GO:0006355\_regulation\_of\_transcription\_\_DNA-dependent | NTF3 | 575 | 3 | 0.500543 | -0.022391 | 556 | 711.58 | 1.279820 |
| GO:0006355\_regulation\_of\_transcription\_\_DNA-dependent | BCL11B | 575 | 3 | 0.500543 | -0.022391 | 556 | 711.58 | 1.279820 |
| GO:0006355\_regulation\_of\_transcription\_\_DNA-dependent | SOX6 | 575 | 3 | 0.500543 | -0.022391 | 556 | 711.58 | 1.279820 |
| GO:0048646\_anatomical\_structure\_formation\_involved\_in\_morphogenesis | ADAMTS1 | 277 | 1 | 0.346345 | -0.022326 | 557 | 711.93 | 1.278151 |
| GO:0007610\_behavior | HMGCR | 279 | 1 | 0.343862 | -0.021821 | 558 | 712.17 | 1.276290 |
| GO:0031327\_negative\_regulation\_of\_cellular\_biosynthetic\_process | SOX6 | 282 | 1 | 0.340204 | -0.021085 | 559 | 712.39 | 1.274401 |
| GO:0009890\_negative\_regulation\_of\_biosynthetic\_process | SOX6 | 284 | 1 | 0.337808 | -0.020608 | 560 | 713.09 | 1.273375 |
| GO:0051239\_regulation\_of\_multicellular\_organismal\_process | IRX3 | 587 | 3 | 0.490311 | -0.020142 | 561 | 713.33 | 1.271533 |
| GO:0051239\_regulation\_of\_multicellular\_organismal\_process | ANK | 587 | 3 | 0.490311 | -0.020142 | 561 | 713.33 | 1.271533 |
| GO:0051239\_regulation\_of\_multicellular\_organismal\_process | ULK2 | 587 | 3 | 0.490311 | -0.020142 | 561 | 713.33 | 1.271533 |
| GO:0006351\_transcription\_\_DNA-dependent | NTF3 | 594 | 3 | 0.484533 | -0.018929 | 562 | 713.98 | 1.270427 |
| GO:0006351\_transcription\_\_DNA-dependent | BCL11B | 594 | 3 | 0.484533 | -0.018929 | 562 | 713.98 | 1.270427 |
| GO:0006351\_transcription\_\_DNA-dependent | SOX6 | 594 | 3 | 0.484533 | -0.018929 | 562 | 713.98 | 1.270427 |
| GO:0032774\_RNA\_biosynthetic\_process | NTF3 | 595 | 3 | 0.483718 | -0.018762 | 563 | 714.09 | 1.268366 |
| GO:0032774\_RNA\_biosynthetic\_process | BCL11B | 595 | 3 | 0.483718 | -0.018762 | 563 | 714.09 | 1.268366 |
| GO:0032774\_RNA\_biosynthetic\_process | SOX6 | 595 | 3 | 0.483718 | -0.018762 | 563 | 714.09 | 1.268366 |
| GO:0051094\_positive\_regulation\_of\_developmental\_process | IRX3 | 308 | 1 | 0.311485 | -0.015661 | 564 | 715.9 | 1.269326 |
| GO:0010646\_regulation\_of\_cell\_communication | HTRA1 | 330 | 1 | 0.290720 | -0.012176 | 565 | 717.11 | 1.269221 |
| GO:0019538\_protein\_metabolic\_process | GALNT10 | 655 | 3 | 0.439408 | -0.010899 | 566 | 718.03 | 1.268604 |
| GO:0019538\_protein\_metabolic\_process | BRSK2 | 655 | 3 | 0.439408 | -0.010899 | 566 | 718.03 | 1.268604 |
| GO:0019538\_protein\_metabolic\_process | DUSP6 | 655 | 3 | 0.439408 | -0.010899 | 566 | 718.03 | 1.268604 |
| GO:0034960\_cellular\_biopolymer\_metabolic\_process | FUSIP1 | 1395 | 9 | 0.618952 | -0.010483 | 567 | 718.45 | 1.267108 |
| GO:0034960\_cellular\_biopolymer\_metabolic\_process | GALNT10 | 1395 | 9 | 0.618952 | -0.010483 | 567 | 718.45 | 1.267108 |
| GO:0034960\_cellular\_biopolymer\_metabolic\_process | WDR55 | 1395 | 9 | 0.618952 | -0.010483 | 567 | 718.45 | 1.267108 |
| GO:0034960\_cellular\_biopolymer\_metabolic\_process | NTF3 | 1395 | 9 | 0.618952 | -0.010483 | 567 | 718.45 | 1.267108 |
| GO:0034960\_cellular\_biopolymer\_metabolic\_process | BCL11B | 1395 | 9 | 0.618952 | -0.010483 | 567 | 718.45 | 1.267108 |
| GO:0034960\_cellular\_biopolymer\_metabolic\_process | CUGBP2 | 1395 | 9 | 0.618952 | -0.010483 | 567 | 718.45 | 1.267108 |
| GO:0034960\_cellular\_biopolymer\_metabolic\_process | BRSK2 | 1395 | 9 | 0.618952 | -0.010483 | 567 | 718.45 | 1.267108 |
| GO:0034960\_cellular\_biopolymer\_metabolic\_process | SOX6 | 1395 | 9 | 0.618952 | -0.010483 | 567 | 718.45 | 1.267108 |
| GO:0034960\_cellular\_biopolymer\_metabolic\_process | DUSP6 | 1395 | 9 | 0.618952 | -0.010483 | 567 | 718.45 | 1.267108 |
| GO:0080090\_regulation\_of\_primary\_metabolic\_process | FUSIP1 | 926 | 5 | 0.518021 | -0.010245 | 568 | 718.47 | 1.264912 |
| GO:0080090\_regulation\_of\_primary\_metabolic\_process | GUCA1B | 926 | 5 | 0.518021 | -0.010245 | 568 | 718.47 | 1.264912 |
| GO:0080090\_regulation\_of\_primary\_metabolic\_process | NTF3 | 926 | 5 | 0.518021 | -0.010245 | 568 | 718.47 | 1.264912 |
| GO:0080090\_regulation\_of\_primary\_metabolic\_process | BCL11B | 926 | 5 | 0.518021 | -0.010245 | 568 | 718.47 | 1.264912 |
| GO:0080090\_regulation\_of\_primary\_metabolic\_process | SOX6 | 926 | 5 | 0.518021 | -0.010245 | 568 | 718.47 | 1.264912 |
| GO:0060255\_regulation\_of\_macromolecule\_metabolic\_process | FUSIP1 | 936 | 5 | 0.512487 | -0.009423 | 569 | 718.97 | 1.263568 |
| GO:0060255\_regulation\_of\_macromolecule\_metabolic\_process | NTF3 | 936 | 5 | 0.512487 | -0.009423 | 569 | 718.97 | 1.263568 |
| GO:0060255\_regulation\_of\_macromolecule\_metabolic\_process | BCL11B | 936 | 5 | 0.512487 | -0.009423 | 569 | 718.97 | 1.263568 |
| GO:0060255\_regulation\_of\_macromolecule\_metabolic\_process | PLCG2 | 936 | 5 | 0.512487 | -0.009423 | 569 | 718.97 | 1.263568 |
| GO:0060255\_regulation\_of\_macromolecule\_metabolic\_process | SOX6 | 936 | 5 | 0.512487 | -0.009423 | 569 | 718.97 | 1.263568 |
| GO:0031326\_regulation\_of\_cellular\_biosynthetic\_process | GUCA1B | 812 | 4 | 0.472599 | -0.009019 | 570 | 719.12 | 1.261614 |
| GO:0031326\_regulation\_of\_cellular\_biosynthetic\_process | NTF3 | 812 | 4 | 0.472599 | -0.009019 | 570 | 719.12 | 1.261614 |
| GO:0031326\_regulation\_of\_cellular\_biosynthetic\_process | BCL11B | 812 | 4 | 0.472599 | -0.009019 | 570 | 719.12 | 1.261614 |
| GO:0031326\_regulation\_of\_cellular\_biosynthetic\_process | SOX6 | 812 | 4 | 0.472599 | -0.009019 | 570 | 719.12 | 1.261614 |
| GO:0045449\_regulation\_of\_transcription | NTF3 | 676 | 3 | 0.425758 | -0.008972 | 571 | 719.12 | 1.259405 |
| GO:0045449\_regulation\_of\_transcription | BCL11B | 676 | 3 | 0.425758 | -0.008972 | 571 | 719.12 | 1.259405 |
| GO:0045449\_regulation\_of\_transcription | SOX6 | 676 | 3 | 0.425758 | -0.008972 | 571 | 719.12 | 1.259405 |
| GO:0009889\_regulation\_of\_biosynthetic\_process | GUCA1B | 815 | 4 | 0.470859 | -0.008784 | 572 | 719.15 | 1.257255 |
| GO:0009889\_regulation\_of\_biosynthetic\_process | NTF3 | 815 | 4 | 0.470859 | -0.008784 | 572 | 719.15 | 1.257255 |
| GO:0009889\_regulation\_of\_biosynthetic\_process | BCL11B | 815 | 4 | 0.470859 | -0.008784 | 572 | 719.15 | 1.257255 |
| GO:0009889\_regulation\_of\_biosynthetic\_process | SOX6 | 815 | 4 | 0.470859 | -0.008784 | 572 | 719.15 | 1.257255 |
| GO:0043009\_chordate\_embryonic\_development | AMD1 | 365 | 1 | 0.262842 | -0.008150 | 573 | 719.57 | 1.255794 |
| GO:0009792\_embryonic\_development\_ending\_in\_birth\_or\_egg\_hatching | AMD1 | 368 | 1 | 0.260700 | -0.007874 | 574 | 719.67 | 1.253780 |
| GO:0006350\_transcription | NTF3 | 701 | 3 | 0.410574 | -0.007094 | 575 | 720.12 | 1.252383 |
| GO:0006350\_transcription | BCL11B | 701 | 3 | 0.410574 | -0.007094 | 575 | 720.12 | 1.252383 |
| GO:0006350\_transcription | SOX6 | 701 | 3 | 0.410574 | -0.007094 | 575 | 720.12 | 1.252383 |
| GO:0044260\_cellular\_macromolecule\_metabolic\_process | FUSIP1 | 1447 | 9 | 0.596709 | -0.007035 | 576 | 720.15 | 1.250260 |
| GO:0044260\_cellular\_macromolecule\_metabolic\_process | WDR55 | 1447 | 9 | 0.596709 | -0.007035 | 576 | 720.15 | 1.250260 |
| GO:0044260\_cellular\_macromolecule\_metabolic\_process | GALNT10 | 1447 | 9 | 0.596709 | -0.007035 | 576 | 720.15 | 1.250260 |
| GO:0044260\_cellular\_macromolecule\_metabolic\_process | NTF3 | 1447 | 9 | 0.596709 | -0.007035 | 576 | 720.15 | 1.250260 |
| GO:0044260\_cellular\_macromolecule\_metabolic\_process | BCL11B | 1447 | 9 | 0.596709 | -0.007035 | 576 | 720.15 | 1.250260 |
| GO:0044260\_cellular\_macromolecule\_metabolic\_process | CUGBP2 | 1447 | 9 | 0.596709 | -0.007035 | 576 | 720.15 | 1.250260 |
| GO:0044260\_cellular\_macromolecule\_metabolic\_process | BRSK2 | 1447 | 9 | 0.596709 | -0.007035 | 576 | 720.15 | 1.250260 |
| GO:0044260\_cellular\_macromolecule\_metabolic\_process | SOX6 | 1447 | 9 | 0.596709 | -0.007035 | 576 | 720.15 | 1.250260 |
| GO:0044260\_cellular\_macromolecule\_metabolic\_process | DUSP6 | 1447 | 9 | 0.596709 | -0.007035 | 576 | 720.15 | 1.250260 |
| GO:0043170\_macromolecule\_metabolic\_process | FUSIP1 | 1576 | 10 | 0.608740 | -0.006141 | 577 | 720.66 | 1.248977 |
| GO:0043170\_macromolecule\_metabolic\_process | WDR55 | 1576 | 10 | 0.608740 | -0.006141 | 577 | 720.66 | 1.248977 |
| GO:0043170\_macromolecule\_metabolic\_process | GALNT10 | 1576 | 10 | 0.608740 | -0.006141 | 577 | 720.66 | 1.248977 |
| GO:0043170\_macromolecule\_metabolic\_process | NTF3 | 1576 | 10 | 0.608740 | -0.006141 | 577 | 720.66 | 1.248977 |
| GO:0043170\_macromolecule\_metabolic\_process | BCL11B | 1576 | 10 | 0.608740 | -0.006141 | 577 | 720.66 | 1.248977 |
| GO:0043170\_macromolecule\_metabolic\_process | PLCG2 | 1576 | 10 | 0.608740 | -0.006141 | 577 | 720.66 | 1.248977 |
| GO:0043170\_macromolecule\_metabolic\_process | CUGBP2 | 1576 | 10 | 0.608740 | -0.006141 | 577 | 720.66 | 1.248977 |
| GO:0043170\_macromolecule\_metabolic\_process | BRSK2 | 1576 | 10 | 0.608740 | -0.006141 | 577 | 720.66 | 1.248977 |
| GO:0043170\_macromolecule\_metabolic\_process | SOX6 | 1576 | 10 | 0.608740 | -0.006141 | 577 | 720.66 | 1.248977 |
| GO:0043170\_macromolecule\_metabolic\_process | DUSP6 | 1576 | 10 | 0.608740 | -0.006141 | 577 | 720.66 | 1.248977 |
| GO:0043283\_biopolymer\_metabolic\_process | FUSIP1 | 1490 | 9 | 0.579488 | -0.004991 | 578 | 721.13 | 1.247630 |
| GO:0043283\_biopolymer\_metabolic\_process | WDR55 | 1490 | 9 | 0.579488 | -0.004991 | 578 | 721.13 | 1.247630 |
| GO:0043283\_biopolymer\_metabolic\_process | GALNT10 | 1490 | 9 | 0.579488 | -0.004991 | 578 | 721.13 | 1.247630 |
| GO:0043283\_biopolymer\_metabolic\_process | NTF3 | 1490 | 9 | 0.579488 | -0.004991 | 578 | 721.13 | 1.247630 |
| GO:0043283\_biopolymer\_metabolic\_process | BCL11B | 1490 | 9 | 0.579488 | -0.004991 | 578 | 721.13 | 1.247630 |
| GO:0043283\_biopolymer\_metabolic\_process | CUGBP2 | 1490 | 9 | 0.579488 | -0.004991 | 578 | 721.13 | 1.247630 |
| GO:0043283\_biopolymer\_metabolic\_process | BRSK2 | 1490 | 9 | 0.579488 | -0.004991 | 578 | 721.13 | 1.247630 |
| GO:0043283\_biopolymer\_metabolic\_process | SOX6 | 1490 | 9 | 0.579488 | -0.004991 | 578 | 721.13 | 1.247630 |
| GO:0043283\_biopolymer\_metabolic\_process | DUSP6 | 1490 | 9 | 0.579488 | -0.004991 | 578 | 721.13 | 1.247630 |
| GO:0007242\_intracellular\_signaling\_cascade | CAR8 | 411 | 1 | 0.233425 | -0.004795 | 579 | 721.28 | 1.245734 |
| GO:0010556\_regulation\_of\_macromolecule\_biosynthetic\_process | NTF3 | 745 | 3 | 0.386326 | -0.004655 | 580 | 721.34 | 1.243690 |
| GO:0010556\_regulation\_of\_macromolecule\_biosynthetic\_process | BCL11B | 745 | 3 | 0.386326 | -0.004655 | 580 | 721.34 | 1.243690 |
| GO:0010556\_regulation\_of\_macromolecule\_biosynthetic\_process | SOX6 | 745 | 3 | 0.386326 | -0.004655 | 580 | 721.34 | 1.243690 |
| GO:0034645\_cellular\_macromolecule\_biosynthetic\_process | GALNT10 | 901 | 4 | 0.425916 | -0.004026 | 581 | 721.52 | 1.241859 |
| GO:0034645\_cellular\_macromolecule\_biosynthetic\_process | NTF3 | 901 | 4 | 0.425916 | -0.004026 | 581 | 721.52 | 1.241859 |
| GO:0034645\_cellular\_macromolecule\_biosynthetic\_process | BCL11B | 901 | 4 | 0.425916 | -0.004026 | 581 | 721.52 | 1.241859 |
| GO:0034645\_cellular\_macromolecule\_biosynthetic\_process | SOX6 | 901 | 4 | 0.425916 | -0.004026 | 581 | 721.52 | 1.241859 |
| GO:0009059\_macromolecule\_biosynthetic\_process | GALNT10 | 910 | 4 | 0.421703 | -0.003701 | 582 | 721.7 | 1.240034 |
| GO:0009059\_macromolecule\_biosynthetic\_process | NTF3 | 910 | 4 | 0.421703 | -0.003701 | 582 | 721.7 | 1.240034 |
| GO:0009059\_macromolecule\_biosynthetic\_process | BCL11B | 910 | 4 | 0.421703 | -0.003701 | 582 | 721.7 | 1.240034 |
| GO:0009059\_macromolecule\_biosynthetic\_process | SOX6 | 910 | 4 | 0.421703 | -0.003701 | 582 | 721.7 | 1.240034 |
| GO:0034961\_cellular\_biopolymer\_biosynthetic\_process | NTF3 | 804 | 3 | 0.357976 | -0.002603 | 583 | 722.33 | 1.238988 |
| GO:0034961\_cellular\_biopolymer\_biosynthetic\_process | BCL11B | 804 | 3 | 0.357976 | -0.002603 | 583 | 722.33 | 1.238988 |
| GO:0034961\_cellular\_biopolymer\_biosynthetic\_process | SOX6 | 804 | 3 | 0.357976 | -0.002603 | 583 | 722.33 | 1.238988 |
| GO:0043284\_biopolymer\_biosynthetic\_process | NTF3 | 807 | 3 | 0.356645 | -0.002526 | 584 | 722.34 | 1.236884 |
| GO:0043284\_biopolymer\_biosynthetic\_process | BCL11B | 807 | 3 | 0.356645 | -0.002526 | 584 | 722.34 | 1.236884 |
| GO:0043284\_biopolymer\_biosynthetic\_process | SOX6 | 807 | 3 | 0.356645 | -0.002526 | 584 | 722.34 | 1.236884 |
| GO:0050896\_response\_to\_stimulus | GUCA1B | 1107 | 5 | 0.433322 | -0.002051 | 585 | 722.46 | 1.234974 |
| GO:0050896\_response\_to\_stimulus | ITGA6 | 1107 | 5 | 0.433322 | -0.002051 | 585 | 722.46 | 1.234974 |
| GO:0050896\_response\_to\_stimulus | HMGCR | 1107 | 5 | 0.433322 | -0.002051 | 585 | 722.46 | 1.234974 |
| GO:0050896\_response\_to\_stimulus | PLCG2 | 1107 | 5 | 0.433322 | -0.002051 | 585 | 722.46 | 1.234974 |
| GO:0050896\_response\_to\_stimulus | ANXA5 | 1107 | 5 | 0.433322 | -0.002051 | 585 | 722.46 | 1.234974 |
| GO:0008150\_biological\_process | FUSIP1 | 4605 | 48 | 1.000000 | 0.000000 | 1600 | 1656.53 | 1.035331 |
| GO:0008150\_biological\_process | SLC45A2 | 4605 | 48 | 1.000000 | 0.000000 | 1600 | 1656.53 | 1.035331 |
| GO:0008150\_biological\_process | IRX3 | 4605 | 48 | 1.000000 | 0.000000 | 1600 | 1656.53 | 1.035331 |
| GO:0008150\_biological\_process | XPO5 | 4605 | 48 | 1.000000 | 0.000000 | 1600 | 1656.53 | 1.035331 |
| GO:0008150\_biological\_process | GNAI1 | 4605 | 48 | 1.000000 | 0.000000 | 1600 | 1656.53 | 1.035331 |
| GO:0008150\_biological\_process | HMGCR | 4605 | 48 | 1.000000 | 0.000000 | 1600 | 1656.53 | 1.035331 |
| GO:0008150\_biological\_process | TDRD7 | 4605 | 48 | 1.000000 | 0.000000 | 1600 | 1656.53 | 1.035331 |
| GO:0008150\_biological\_process | HK2 | 4605 | 48 | 1.000000 | 0.000000 | 1600 | 1656.53 | 1.035331 |
| GO:0008150\_biological\_process | PIP5K1B | 4605 | 48 | 1.000000 | 0.000000 | 1600 | 1656.53 | 1.035331 |
| GO:0008150\_biological\_process | BRSK2 | 4605 | 48 | 1.000000 | 0.000000 | 1600 | 1656.53 | 1.035331 |
| GO:0008150\_biological\_process | POSTN | 4605 | 48 | 1.000000 | 0.000000 | 1600 | 1656.53 | 1.035331 |
| GO:0008150\_biological\_process | SOX6 | 4605 | 48 | 1.000000 | 0.000000 | 1600 | 1656.53 | 1.035331 |
| GO:0008150\_biological\_process | ABCA4 | 4605 | 48 | 1.000000 | 0.000000 | 1600 | 1656.53 | 1.035331 |
| GO:0008150\_biological\_process | TPM2 | 4605 | 48 | 1.000000 | 0.000000 | 1600 | 1656.53 | 1.035331 |
| GO:0008150\_biological\_process | MFRP | 4605 | 48 | 1.000000 | 0.000000 | 1600 | 1656.53 | 1.035331 |
| GO:0008150\_biological\_process | ANK | 4605 | 48 | 1.000000 | 0.000000 | 1600 | 1656.53 | 1.035331 |
| GO:0008150\_biological\_process | TYR | 4605 | 48 | 1.000000 | 0.000000 | 1600 | 1656.53 | 1.035331 |
| GO:0008150\_biological\_process | WDR55 | 4605 | 48 | 1.000000 | 0.000000 | 1600 | 1656.53 | 1.035331 |
| GO:0008150\_biological\_process | GALNT10 | 4605 | 48 | 1.000000 | 0.000000 | 1600 | 1656.53 | 1.035331 |
| GO:0008150\_biological\_process | HTRA1 | 4605 | 48 | 1.000000 | 0.000000 | 1600 | 1656.53 | 1.035331 |
| GO:0008150\_biological\_process | BCL11B | 4605 | 48 | 1.000000 | 0.000000 | 1600 | 1656.53 | 1.035331 |
| GO:0008150\_biological\_process | COL8A1 | 4605 | 48 | 1.000000 | 0.000000 | 1600 | 1656.53 | 1.035331 |
| GO:0008150\_biological\_process | AMD1 | 4605 | 48 | 1.000000 | 0.000000 | 1600 | 1656.53 | 1.035331 |
| GO:0008150\_biological\_process | GUCA1B | 4605 | 48 | 1.000000 | 0.000000 | 1600 | 1656.53 | 1.035331 |
| GO:0008150\_biological\_process | NTF3 | 4605 | 48 | 1.000000 | 0.000000 | 1600 | 1656.53 | 1.035331 |
| GO:0008150\_biological\_process | CRYAB | 4605 | 48 | 1.000000 | 0.000000 | 1600 | 1656.53 | 1.035331 |
| GO:0008150\_biological\_process | DTX1 | 4605 | 48 | 1.000000 | 0.000000 | 1600 | 1656.53 | 1.035331 |
| GO:0008150\_biological\_process | CCNF | 4605 | 48 | 1.000000 | 0.000000 | 1600 | 1656.53 | 1.035331 |
| GO:0008150\_biological\_process | CUGBP2 | 4605 | 48 | 1.000000 | 0.000000 | 1600 | 1656.53 | 1.035331 |
| GO:0008150\_biological\_process | CRTAP | 4605 | 48 | 1.000000 | 0.000000 | 1600 | 1656.53 | 1.035331 |
| GO:0008150\_biological\_process | NID1 | 4605 | 48 | 1.000000 | 0.000000 | 1600 | 1656.53 | 1.035331 |
| GO:0008150\_biological\_process | ANXA5 | 4605 | 48 | 1.000000 | 0.000000 | 1600 | 1656.53 | 1.035331 |
| GO:0008150\_biological\_process | KDR | 4605 | 48 | 1.000000 | 0.000000 | 1600 | 1656.53 | 1.035331 |
| GO:0008150\_biological\_process | PGM3 | 4605 | 48 | 1.000000 | 0.000000 | 1600 | 1656.53 | 1.035331 |
| GO:0008150\_biological\_process | SYNE2 | 4605 | 48 | 1.000000 | 0.000000 | 1600 | 1656.53 | 1.035331 |
| GO:0008150\_biological\_process | PLK2 | 4605 | 48 | 1.000000 | 0.000000 | 1600 | 1656.53 | 1.035331 |
| GO:0008150\_biological\_process | NOLC1 | 4605 | 48 | 1.000000 | 0.000000 | 1600 | 1656.53 | 1.035331 |
| GO:0008150\_biological\_process | ITGA6 | 4605 | 48 | 1.000000 | 0.000000 | 1600 | 1656.53 | 1.035331 |
| GO:0008150\_biological\_process | ULK2 | 4605 | 48 | 1.000000 | 0.000000 | 1600 | 1656.53 | 1.035331 |
| GO:0008150\_biological\_process | PLCG2 | 4605 | 48 | 1.000000 | 0.000000 | 1600 | 1656.53 | 1.035331 |
| GO:0008150\_biological\_process | LAMB1-1 | 4605 | 48 | 1.000000 | 0.000000 | 1600 | 1656.53 | 1.035331 |
| GO:0008150\_biological\_process | CAR8 | 4605 | 48 | 1.000000 | 0.000000 | 1600 | 1656.53 | 1.035331 |
| GO:0008150\_biological\_process | SLC13A3 | 4605 | 48 | 1.000000 | 0.000000 | 1600 | 1656.53 | 1.035331 |
| GO:0008150\_biological\_process | ADAMTS1 | 4605 | 48 | 1.000000 | 0.000000 | 1600 | 1656.53 | 1.035331 |
| GO:0008150\_biological\_process | DST | 4605 | 48 | 1.000000 | 0.000000 | 1600 | 1656.53 | 1.035331 |
| GO:0008150\_biological\_process | CRYM | 4605 | 48 | 1.000000 | 0.000000 | 1600 | 1656.53 | 1.035331 |
| GO:0008150\_biological\_process | VLDLR | 4605 | 48 | 1.000000 | 0.000000 | 1600 | 1656.53 | 1.035331 |
| GO:0008150\_biological\_process | DUSP6 | 4605 | 48 | 1.000000 | 0.000000 | 1600 | 1656.53 | 1.035331 |
