# Supplementary material for: RedundancyMiner: De-replication of redundant GO categories in microarray and proteomics analysis
Source: BMC Bioinformatics. 2011 Feb 10;12:52. doi: 10.1186/1471-2105-12-52 (PMC3223614; doi:10.1186/1471-2105-12-52)
Supplement: Additional file 8 — Retinal development HTGM download. compressed package of the results of running HTGM on the retinal development genes list. [file 1471-2105-12-52-S8.ZIP › SCENARIO_2_MODIFIED/total.txt.total.txt.dir/Exp1_BestClusterMap_LEIGS_KM_24.csv.join.16.txt.dir/Exp1_BestClusterMap_LEIGS_KM_24.csv.join.16.txt.change.html]

Category Summary Report for Exp1\_BestClusterMap\_LEIGS\_KM\_24.csv.join.16.txt

# Category Summary Report for Exp1\_BestClusterMap\_LEIGS\_KM\_24.csv.join.16.txt

| HYPERLINKED GO CATEGORY | TOTAL GENES | CHANGED GENES | ENRICHMENT | LOG10(p) | CUMULATIVE NUMBER OF CATEGORIES | CUMULATIVE RANDOMS LOWER BOUND | CUMULATIVE RANDOMS MEAN | CUMULATIVE RANDOMS UPPER BOUND | FALSE DISCOVERY RATE |
| --- | --- | --- | --- | --- | --- | --- | --- | --- | --- |
| GO:0045785\_positive\_regulation\_of\_cell\_adhesion | 37 | 3 | 7.778716 | -2.190980 | 1 | 0.817962 | 7.59 | 14.362038 | 7.590000 |
| GO:0000375\_RNA\_splicing\_\_via\_transesterification\_reactions | 12 | 2 | 15.989583 | -2.182424 | 4 | 0.881656 | 8.13 | 15.378344 | 2.032500 |
| GO:0000377\_RNA\_splicing\_\_via\_transesterification\_reactions\_with\_bulged\_adenosine\_as\_nucleophile | 12 | 2 | 15.989583 | -2.182424 | 4 | 0.881656 | 8.13 | 15.378344 | 2.032500 |
| GO:0000398\_nuclear\_mRNA\_splicing\_\_via\_spliceosome | 12 | 2 | 15.989583 | -2.182424 | 4 | 0.881656 | 8.13 | 15.378344 | 2.032500 |
| GO:0002316\_follicular\_B\_cell\_differentiation | 1 | 1 |  |  |  |  |  |  |  |  |
| GO:0006596\_polyamine\_biosynthetic\_process | 1 | 1 |  |  |  |  |  |  |  |  |
| GO:0006597\_spermine\_biosynthetic\_process | 1 | 1 |  |  |  |  |  |  |  |  |
| GO:0008216\_spermidine\_metabolic\_process | 1 | 1 |  |  |  |  |  |  |  |  |
| GO:0008295\_spermidine\_biosynthetic\_process | 1 | 1 |  |  |  |  |  |  |  |  |
| GO:0016482\_cytoplasmic\_transport | 1 | 1 |  |  |  |  |  |  |  |  |
| GO:0019255\_glucose\_1-phosphate\_metabolic\_process | 1 | 1 |  |  |  |  |  |  |  |  |
| GO:0032237\_activation\_of\_store-operated\_calcium\_channel\_activity | 1 | 1 |  |  |  |  |  |  |  |  |
| GO:0032836\_glomerular\_basement\_membrane\_development | 1 | 1 |  |  |  |  |  |  |  |  |
| GO:0006396\_RNA\_processing | 47 | 3 | 6.123670 | -1.903030 | 5 | 2.772783 | 13.17 | 23.567217 | 2.634000 |
| GO:0008380\_RNA\_splicing | 17 | 2 | 11.286765 | -1.882824 | 6 | 3.178079 | 13.9 | 24.621921 | 2.316667 |
| GO:0007601\_visual\_perception | 51 | 3 | 5.643382 | -1.806947 | 7 | 3.915316 | 15.7 | 27.484684 | 2.242857 |
| GO:0046165\_alcohol\_biosynthetic\_process | 19 | 2 | 10.098684 | -1.789111 | 8 | 4.216173 | 16.32 | 28.423827 | 2.040000 |
| GO:0050953\_sensory\_perception\_of\_light\_stimulus | 52 | 3 | 5.534856 | -1.784283 | 9 | 4.242736 | 16.41 | 28.577264 | 1.823333 |
| GO:0006021\_inositol\_biosynthetic\_process | 2 | 1 |  |  |  |  |  |  |  |  |
| GO:0006042\_glucosamine\_biosynthetic\_process | 2 | 1 |  |  |  |  |  |  |  |  |
| GO:0006045\_N-acetylglucosamine\_biosynthetic\_process | 2 | 1 |  |  |  |  |  |  |  |  |
| GO:0006048\_UDP-N-acetylglucosamine\_biosynthetic\_process | 2 | 1 |  |  |  |  |  |  |  |  |
| GO:0006649\_phospholipid\_transfer\_to\_membrane | 2 | 1 |  |  |  |  |  |  |  |  |
| GO:0032234\_regulation\_of\_calcium\_ion\_transport\_via\_store-operated\_calcium\_channel\_activity | 2 | 1 |  |  |  |  |  |  |  |  |
| GO:0032236\_positive\_regulation\_of\_calcium\_ion\_transport\_via\_store-operated\_calcium\_channel\_activity | 2 | 1 |  |  |  |  |  |  |  |  |
| GO:0032957\_inositol\_trisphosphate\_metabolic\_process | 2 | 1 |  |  |  |  |  |  |  |  |
| GO:0032958\_inositol\_phosphate\_biosynthetic\_process | 2 | 1 |  |  |  |  |  |  |  |  |
| GO:0032959\_inositol\_trisphosphate\_biosynthetic\_process | 2 | 1 |  |  |  |  |  |  |  |  |
| GO:0033119\_negative\_regulation\_of\_RNA\_splicing | 2 | 1 |  |  |  |  |  |  |  |  |
| GO:0043647\_inositol\_phosphate\_metabolic\_process | 2 | 1 |  |  |  |  |  |  |  |  |
| GO:0046349\_amino\_sugar\_biosynthetic\_process | 2 | 1 |  |  |  |  |  |  |  |  |
| GO:0048025\_negative\_regulation\_of\_nuclear\_mRNA\_splicing\_\_via\_spliceosome | 2 | 1 |  |  |  |  |  |  |  |  |
| GO:0048670\_regulation\_of\_collateral\_sprouting | 2 | 1 |  |  |  |  |  |  |  |  |
| GO:0048671\_negative\_regulation\_of\_collateral\_sprouting | 2 | 1 |  |  |  |  |  |  |  |  |
| GO:0050686\_negative\_regulation\_of\_mRNA\_processing | 2 | 1 |  |  |  |  |  |  |  |  |
| GO:0006397\_mRNA\_processing | 23 | 2 | 8.342391 | -1.630450 | 12 | 6.928607 | 20.97 | 35.011393 | 1.747500 |
| GO:0007163\_establishment\_or\_maintenance\_of\_cell\_polarity | 23 | 2 | 8.342391 | -1.630450 | 12 | 6.928607 | 20.97 | 35.011393 | 1.747500 |
| GO:0022613\_ribonucleoprotein\_complex\_biogenesis | 23 | 2 | 8.342391 | -1.630450 | 12 | 6.928607 | 20.97 | 35.011393 | 1.747500 |
| GO:0000902\_cell\_morphogenesis | 283 | 7 | 2.373012 | -1.588059 | 13 | 7.506833 | 22.36 | 37.213167 | 1.720000 |
| GO:0030155\_regulation\_of\_cell\_adhesion | 62 | 3 | 4.642137 | -1.582320 | 14 | 7.564991 | 22.54 | 37.515009 | 1.610000 |
| GO:0010811\_positive\_regulation\_of\_cell-substrate\_adhesion | 25 | 2 | 7.675000 | -1.562169 | 15 | 7.971521 | 23.51 | 39.048479 | 1.567333 |
| GO:0031589\_cell-substrate\_adhesion | 66 | 3 | 4.360795 | -1.512067 | 16 | 8.911175 | 25.11 | 41.308825 | 1.569375 |
| GO:0000320\_re-entry\_into\_mitotic\_cell\_cycle | 3 | 1 |  |  |  |  |  |  |  |  |
| GO:0002568\_somatic\_diversification\_of\_T\_cell\_receptor\_genes | 3 | 1 |  |  |  |  |  |  |  |  |
| GO:0002681\_somatic\_recombination\_of\_T\_cell\_receptor\_gene\_segments | 3 | 1 |  |  |  |  |  |  |  |  |
| GO:0006047\_UDP-N-acetylglucosamine\_metabolic\_process | 3 | 1 |  |  |  |  |  |  |  |  |
| GO:0007000\_nucleolus\_organization | 3 | 1 |  |  |  |  |  |  |  |  |
| GO:0007403\_glial\_cell\_fate\_determination | 3 | 1 |  |  |  |  |  |  |  |  |
| GO:0008090\_retrograde\_axon\_cargo\_transport | 3 | 1 |  |  |  |  |  |  |  |  |
| GO:0031282\_regulation\_of\_guanylate\_cyclase\_activity | 3 | 1 |  |  |  |  |  |  |  |  |
| GO:0032411\_positive\_regulation\_of\_transporter\_activity | 3 | 1 |  |  |  |  |  |  |  |  |
| GO:0032414\_positive\_regulation\_of\_ion\_transmembrane\_transporter\_activity | 3 | 1 |  |  |  |  |  |  |  |  |
| GO:0033153\_T\_cell\_receptor\_V(D)J\_recombination | 3 | 1 |  |  |  |  |  |  |  |  |
| GO:0046488\_phosphatidylinositol\_metabolic\_process | 3 | 1 |  |  |  |  |  |  |  |  |
| GO:0048668\_collateral\_sprouting | 3 | 1 |  |  |  |  |  |  |  |  |
| GO:0006997\_nucleus\_organization | 28 | 2 | 6.852679 | -1.470362 | 17 | 9.854194 | 27.04 | 44.225806 | 1.590588 |
| GO:0032989\_cellular\_component\_morphogenesis | 307 | 7 | 2.187500 | -1.420292 | 18 | 11.042241 | 29.31 | 47.577759 | 1.628333 |
| GO:0044262\_cellular\_carbohydrate\_metabolic\_process | 72 | 3 | 3.997396 | -1.415744 | 19 | 11.370552 | 29.67 | 47.969448 | 1.561579 |
| GO:0002312\_B\_cell\_activation\_during\_immune\_response | 4 | 1 |  |  |  |  |  |  |  |  |
| GO:0002313\_mature\_B\_cell\_differentiation\_during\_immune\_response | 4 | 1 |  |  |  |  |  |  |  |  |
| GO:0006835\_dicarboxylic\_acid\_transport | 4 | 1 |  |  |  |  |  |  |  |  |
| GO:0008215\_spermine\_metabolic\_process | 4 | 1 |  |  |  |  |  |  |  |  |
| GO:0009225\_nucleotide-sugar\_metabolic\_process | 4 | 1 |  |  |  |  |  |  |  |  |
| GO:0030826\_regulation\_of\_cGMP\_biosynthetic\_process | 4 | 1 |  |  |  |  |  |  |  |  |
| GO:0032835\_glomerulus\_development | 4 | 1 |  |  |  |  |  |  |  |  |
| GO:0043129\_surfactant\_homeostasis | 4 | 1 |  |  |  |  |  |  |  |  |
| GO:0043484\_regulation\_of\_RNA\_splicing | 4 | 1 |  |  |  |  |  |  |  |  |
| GO:0046173\_polyol\_biosynthetic\_process | 4 | 1 |  |  |  |  |  |  |  |  |
| GO:0046835\_carbohydrate\_phosphorylation | 4 | 1 |  |  |  |  |  |  |  |  |
| GO:0048024\_regulation\_of\_nuclear\_mRNA\_splicing\_\_via\_spliceosome | 4 | 1 |  |  |  |  |  |  |  |  |
| GO:0048484\_enteric\_nervous\_system\_development | 4 | 1 |  |  |  |  |  |  |  |  |
| GO:0048875\_chemical\_homeostasis\_within\_a\_tissue | 4 | 1 |  |  |  |  |  |  |  |  |
| GO:0010810\_regulation\_of\_cell-substrate\_adhesion | 35 | 2 | 5.482143 | -1.293244 | 20 | 16.229607 | 38.23 | 60.230393 | 1.911500 |
| GO:0006376\_mRNA\_splice\_site\_selection | 5 | 1 | 19.187500 | -1.291887 | 25 | 26.870559 | 52.17 | 77.469441 | 2.086800 |
| GO:0010761\_fibroblast\_migration | 5 | 1 | 19.187500 | -1.291887 | 25 | 26.870559 | 52.17 | 77.469441 | 2.086800 |
| GO:0030823\_regulation\_of\_cGMP\_metabolic\_process | 5 | 1 | 19.187500 | -1.291887 | 25 | 26.870559 | 52.17 | 77.469441 | 2.086800 |
| GO:0031122\_cytoplasmic\_microtubule\_organization | 5 | 1 | 19.187500 | -1.291887 | 25 | 26.870559 | 52.17 | 77.469441 | 2.086800 |
| GO:0033627\_cell\_adhesion\_mediated\_by\_integrin | 5 | 1 | 19.187500 | -1.291887 | 25 | 26.870559 | 52.17 | 77.469441 | 2.086800 |
| GO:0022602\_ovulation\_cycle\_process | 36 | 2 | 5.329861 | -1.271256 | 26 | 27.767384 | 53.87 | 79.972616 | 2.071923 |
| GO:0042698\_ovulation\_cycle | 37 | 2 | 5.185811 | -1.249956 | 27 | 28.377180 | 55.22 | 82.062820 | 2.045185 |
| GO:0000245\_spliceosome\_assembly | 6 | 1 | 15.989583 | -1.214914 | 34 | 37.574803 | 68.69 | 99.805197 | 2.020294 |
| GO:0002335\_mature\_B\_cell\_differentiation | 6 | 1 | 15.989583 | -1.214914 | 34 | 37.574803 | 68.69 | 99.805197 | 2.020294 |
| GO:0006998\_nuclear\_envelope\_organization | 6 | 1 | 15.989583 | -1.214914 | 34 | 37.574803 | 68.69 | 99.805197 | 2.020294 |
| GO:0022409\_positive\_regulation\_of\_cell-cell\_adhesion | 6 | 1 | 15.989583 | -1.214914 | 34 | 37.574803 | 68.69 | 99.805197 | 2.020294 |
| GO:0031077\_post-embryonic\_camera-type\_eye\_development | 6 | 1 | 15.989583 | -1.214914 | 34 | 37.574803 | 68.69 | 99.805197 | 2.020294 |
| GO:0042403\_thyroid\_hormone\_metabolic\_process | 6 | 1 | 15.989583 | -1.214914 | 34 | 37.574803 | 68.69 | 99.805197 | 2.020294 |
| GO:0050684\_regulation\_of\_mRNA\_processing | 6 | 1 | 15.989583 | -1.214914 | 34 | 37.574803 | 68.69 | 99.805197 | 2.020294 |
| GO:0007160\_cell-matrix\_adhesion | 39 | 2 | 4.919872 | -1.209272 | 35 | 38.545036 | 70.07 | 101.594964 | 2.002000 |
| GO:0016071\_mRNA\_metabolic\_process | 40 | 2 | 4.796875 | -1.189823 | 36 | 39.469834 | 71.64 | 103.810166 | 1.990000 |
| GO:0008585\_female\_gonad\_development | 41 | 2 | 4.679878 | -1.170928 | 37 | 40.349361 | 73.25 | 106.150639 | 1.979730 |
| GO:0006006\_glucose\_metabolic\_process | 42 | 2 | 4.568452 | -1.152562 | 38 | 41.432104 | 75.12 | 108.807896 | 1.976842 |
| GO:0006041\_glucosamine\_metabolic\_process | 7 | 1 | 13.705357 | -1.150173 | 46 | 51.220352 | 87.01 | 122.799648 | 1.891522 |
| GO:0006044\_N-acetylglucosamine\_metabolic\_process | 7 | 1 | 13.705357 | -1.150173 | 46 | 51.220352 | 87.01 | 122.799648 | 1.891522 |
| GO:0015914\_phospholipid\_transport | 7 | 1 | 13.705357 | -1.150173 | 46 | 51.220352 | 87.01 | 122.799648 | 1.891522 |
| GO:0022407\_regulation\_of\_cell-cell\_adhesion | 7 | 1 | 13.705357 | -1.150173 | 46 | 51.220352 | 87.01 | 122.799648 | 1.891522 |
| GO:0022618\_ribonucleoprotein\_complex\_assembly | 7 | 1 | 13.705357 | -1.150173 | 46 | 51.220352 | 87.01 | 122.799648 | 1.891522 |
| GO:0042438\_melanin\_biosynthetic\_process | 7 | 1 | 13.705357 | -1.150173 | 46 | 51.220352 | 87.01 | 122.799648 | 1.891522 |
| GO:0046847\_filopodium\_assembly | 7 | 1 | 13.705357 | -1.150173 | 46 | 51.220352 | 87.01 | 122.799648 | 1.891522 |
| GO:0051928\_positive\_regulation\_of\_calcium\_ion\_transport | 7 | 1 | 13.705357 | -1.150173 | 46 | 51.220352 | 87.01 | 122.799648 | 1.891522 |
| GO:0010001\_glial\_cell\_differentiation | 43 | 2 | 4.462209 | -1.134698 | 47 | 52.358395 | 88.59 | 124.821605 | 1.884894 |
| GO:0046545\_development\_of\_primary\_female\_sexual\_characteristics | 44 | 2 | 4.360795 | -1.117313 | 48 | 53.322767 | 91.07 | 128.817233 | 1.897292 |
| GO:0006020\_inositol\_metabolic\_process | 8 | 1 | 11.992188 | -1.094382 | 60 | 61.829501 | 101.82 | 141.810499 | 1.697000 |
| GO:0006493\_protein\_amino\_acid\_O-linked\_glycosylation | 8 | 1 | 11.992188 | -1.094382 | 60 | 61.829501 | 101.82 | 141.810499 | 1.697000 |
| GO:0006582\_melanin\_metabolic\_process | 8 | 1 | 11.992188 | -1.094382 | 60 | 61.829501 | 101.82 | 141.810499 | 1.697000 |
| GO:0021781\_glial\_cell\_fate\_commitment | 8 | 1 | 11.992188 | -1.094382 | 60 | 61.829501 | 101.82 | 141.810499 | 1.697000 |
| GO:0022898\_regulation\_of\_transmembrane\_transporter\_activity | 8 | 1 | 11.992188 | -1.094382 | 60 | 61.829501 | 101.82 | 141.810499 | 1.697000 |
| GO:0030035\_microspike\_assembly | 8 | 1 | 11.992188 | -1.094382 | 60 | 61.829501 | 101.82 | 141.810499 | 1.697000 |
| GO:0030500\_regulation\_of\_bone\_mineralization | 8 | 1 | 11.992188 | -1.094382 | 60 | 61.829501 | 101.82 | 141.810499 | 1.697000 |
| GO:0032409\_regulation\_of\_transporter\_activity | 8 | 1 | 11.992188 | -1.094382 | 60 | 61.829501 | 101.82 | 141.810499 | 1.697000 |
| GO:0032412\_regulation\_of\_ion\_transmembrane\_transporter\_activity | 8 | 1 | 11.992188 | -1.094382 | 60 | 61.829501 | 101.82 | 141.810499 | 1.697000 |
| GO:0045494\_photoreceptor\_cell\_maintenance | 8 | 1 | 11.992188 | -1.094382 | 60 | 61.829501 | 101.82 | 141.810499 | 1.697000 |
| GO:0060347\_heart\_trabecula\_formation | 8 | 1 | 11.992188 | -1.094382 | 60 | 61.829501 | 101.82 | 141.810499 | 1.697000 |
| GO:0070167\_regulation\_of\_biomineral\_formation | 8 | 1 | 11.992188 | -1.094382 | 60 | 61.829501 | 101.82 | 141.810499 | 1.697000 |
| GO:0006066\_alcohol\_metabolic\_process | 158 | 4 | 2.428797 | -1.091704 | 62 | 61.933730 | 102.06 | 142.186270 | 1.646129 |
| GO:0007409\_axonogenesis | 158 | 4 | 2.428797 | -1.091704 | 62 | 61.933730 | 102.06 | 142.186270 | 1.646129 |
| GO:0042063\_gliogenesis | 46 | 2 | 4.171196 | -1.083893 | 63 | 62.684439 | 103.26 | 143.835561 | 1.639048 |
| GO:0019318\_hexose\_metabolic\_process | 48 | 2 | 3.997396 | -1.052146 | 64 | 64.719458 | 106.6 | 148.480542 | 1.665625 |
| GO:0001542\_ovulation\_from\_ovarian\_follicle | 9 | 1 | 10.659722 | -1.045427 | 73 | 72.562274 | 116.95 | 161.337726 | 1.602055 |
| GO:0001667\_ameboidal\_cell\_migration | 9 | 1 | 10.659722 | -1.045427 | 73 | 72.562274 | 116.95 | 161.337726 | 1.602055 |
| GO:0006182\_cGMP\_biosynthetic\_process | 9 | 1 | 10.659722 | -1.045427 | 73 | 72.562274 | 116.95 | 161.337726 | 1.602055 |
| GO:0006364\_rRNA\_processing | 9 | 1 | 10.659722 | -1.045427 | 73 | 72.562274 | 116.95 | 161.337726 | 1.602055 |
| GO:0006595\_polyamine\_metabolic\_process | 9 | 1 | 10.659722 | -1.045427 | 73 | 72.562274 | 116.95 | 161.337726 | 1.602055 |
| GO:0006611\_protein\_export\_from\_nucleus | 9 | 1 | 10.659722 | -1.045427 | 73 | 72.562274 | 116.95 | 161.337726 | 1.602055 |
| GO:0016072\_rRNA\_metabolic\_process | 9 | 1 | 10.659722 | -1.045427 | 73 | 72.562274 | 116.95 | 161.337726 | 1.602055 |
| GO:0030728\_ovulation | 9 | 1 | 10.659722 | -1.045427 | 73 | 72.562274 | 116.95 | 161.337726 | 1.602055 |
| GO:0033151\_V(D)J\_recombination | 9 | 1 | 10.659722 | -1.045427 | 73 | 72.562274 | 116.95 | 161.337726 | 1.602055 |
| GO:0043473\_pigmentation | 49 | 2 | 3.915816 | -1.036855 | 75 | 73.654028 | 118.4 | 163.145972 | 1.578667 |
| GO:0046660\_female\_sex\_differentiation | 49 | 2 | 3.915816 | -1.036855 | 75 | 73.654028 | 118.4 | 163.145972 | 1.578667 |
| GO:0048812\_neuron\_projection\_morphogenesis | 170 | 4 | 2.257353 | -1.002051 | 76 | 75.686864 | 121.52 | 167.353136 | 1.598947 |
| GO:0006040\_amino\_sugar\_metabolic\_process | 10 | 1 | 9.593750 | -1.001864 | 81 | 84.840003 | 133.06 | 181.279997 | 1.642716 |
| GO:0008088\_axon\_cargo\_transport | 10 | 1 | 9.593750 | -1.001864 | 81 | 84.840003 | 133.06 | 181.279997 | 1.642716 |
| GO:0034637\_cellular\_carbohydrate\_biosynthetic\_process | 10 | 1 | 9.593750 | -1.001864 | 81 | 84.840003 | 133.06 | 181.279997 | 1.642716 |
| GO:0045446\_endothelial\_cell\_differentiation | 10 | 1 | 9.593750 | -1.001864 | 81 | 84.840003 | 133.06 | 181.279997 | 1.642716 |
| GO:0060343\_trabecula\_formation | 10 | 1 | 9.593750 | -1.001864 | 81 | 84.840003 | 133.06 | 181.279997 | 1.642716 |
| GO:0048667\_cell\_morphogenesis\_involved\_in\_neuron\_differentiation | 173 | 4 | 2.218208 | -0.981045 | 82 | 85.842715 | 134.48 | 183.117285 | 1.640000 |
| GO:0006576\_biogenic\_amine\_metabolic\_process | 53 | 2 | 3.620283 | -0.979221 | 83 | 86.518956 | 135.25 | 183.981044 | 1.629518 |
| GO:0043010\_camera-type\_eye\_development | 110 | 3 | 2.616477 | -0.974695 | 84 | 86.765836 | 135.67 | 184.574164 | 1.615119 |
| GO:0031110\_regulation\_of\_microtubule\_polymerization\_or\_depolymerization | 11 | 1 | 8.721591 | -0.962663 | 88 | 94.096486 | 145.24 | 196.383514 | 1.650455 |
| GO:0034762\_regulation\_of\_transmembrane\_transport | 11 | 1 | 8.721591 | -0.962663 | 88 | 94.096486 | 145.24 | 196.383514 | 1.650455 |
| GO:0042401\_biogenic\_amine\_biosynthetic\_process | 11 | 1 | 8.721591 | -0.962663 | 88 | 94.096486 | 145.24 | 196.383514 | 1.650455 |
| GO:0043270\_positive\_regulation\_of\_ion\_transport | 11 | 1 | 8.721591 | -0.962663 | 88 | 94.096486 | 145.24 | 196.383514 | 1.650455 |
| GO:0048858\_cell\_projection\_morphogenesis | 176 | 4 | 2.180398 | -0.960562 | 89 | 94.164920 | 145.47 | 196.775080 | 1.634494 |
| GO:0030010\_establishment\_of\_cell\_polarity | 12 | 1 | 7.994792 | -0.927062 | 95 | 103.054077 | 157.75 | 212.445923 | 1.660526 |
| GO:0030514\_negative\_regulation\_of\_BMP\_signaling\_pathway | 12 | 1 | 7.994792 | -0.927062 | 95 | 103.054077 | 157.75 | 212.445923 | 1.660526 |
| GO:0031109\_microtubule\_polymerization\_or\_depolymerization | 12 | 1 | 7.994792 | -0.927062 | 95 | 103.054077 | 157.75 | 212.445923 | 1.660526 |
| GO:0046068\_cGMP\_metabolic\_process | 12 | 1 | 7.994792 | -0.927062 | 95 | 103.054077 | 157.75 | 212.445923 | 1.660526 |
| GO:0048821\_erythrocyte\_development | 12 | 1 | 7.994792 | -0.927062 | 95 | 103.054077 | 157.75 | 212.445923 | 1.660526 |
| GO:0051145\_smooth\_muscle\_cell\_differentiation | 12 | 1 | 7.994792 | -0.927062 | 95 | 103.054077 | 157.75 | 212.445923 | 1.660526 |
| GO:0030097\_hemopoiesis | 253 | 5 | 1.895998 | -0.916615 | 96 | 104.248913 | 159.51 | 214.771087 | 1.661562 |
| GO:0032990\_cell\_part\_morphogenesis | 184 | 4 | 2.085598 | -0.908363 | 97 | 104.767183 | 160.41 | 216.052817 | 1.653711 |
| GO:0007155\_cell\_adhesion | 186 | 4 | 2.063172 | -0.895836 | 99 | 106.100351 | 162.15 | 218.199649 | 1.637879 |
| GO:0022610\_biological\_adhesion | 186 | 4 | 2.063172 | -0.895836 | 99 | 106.100351 | 162.15 | 218.199649 | 1.637879 |
| GO:0007274\_neuromuscular\_synaptic\_transmission | 13 | 1 | 7.379808 | -0.894483 | 104 | 113.292225 | 171.39 | 229.487775 | 1.647981 |
| GO:0007566\_embryo\_implantation | 13 | 1 | 7.379808 | -0.894483 | 104 | 113.292225 | 171.39 | 229.487775 | 1.647981 |
| GO:0010970\_microtubule-based\_transport | 13 | 1 | 7.379808 | -0.894483 | 104 | 113.292225 | 171.39 | 229.487775 | 1.647981 |
| GO:0030384\_phosphoinositide\_metabolic\_process | 13 | 1 | 7.379808 | -0.894483 | 104 | 113.292225 | 171.39 | 229.487775 | 1.647981 |
| GO:0050771\_negative\_regulation\_of\_axonogenesis | 13 | 1 | 7.379808 | -0.894483 | 104 | 113.292225 | 171.39 | 229.487775 | 1.647981 |
| GO:0007276\_gamete\_generation | 188 | 4 | 2.041223 | -0.883510 | 105 | 113.828692 | 172.06 | 230.291308 | 1.638667 |
| GO:0048468\_cell\_development | 654 | 10 | 1.466934 | -0.873657 | 106 | 114.844126 | 173.52 | 232.195874 | 1.636981 |
| GO:0030030\_cell\_projection\_organization | 263 | 5 | 1.823907 | -0.864752 | 107 | 115.468700 | 174.88 | 234.291300 | 1.634393 |
| GO:0006695\_cholesterol\_biosynthetic\_process | 14 | 1 | 6.852679 | -0.864479 | 108 | 121.896104 | 183.49 | 245.083896 | 1.698981 |
| GO:0045666\_positive\_regulation\_of\_neuron\_differentiation | 15 | 1 | 6.395833 | -0.836693 | 111 | 129.044887 | 193.72 | 258.395113 | 1.745225 |
| GO:0048010\_vascular\_endothelial\_growth\_factor\_receptor\_signaling\_pathway | 15 | 1 | 6.395833 | -0.836693 | 111 | 129.044887 | 193.72 | 258.395113 | 1.745225 |
| GO:0070507\_regulation\_of\_microtubule\_cytoskeleton\_organization | 15 | 1 | 6.395833 | -0.836693 | 111 | 129.044887 | 193.72 | 258.395113 | 1.745225 |
| GO:0048511\_rhythmic\_process | 65 | 2 | 2.951923 | -0.833817 | 112 | 129.585612 | 194.31 | 259.034388 | 1.734911 |
| GO:0022008\_neurogenesis | 423 | 7 | 1.587618 | -0.833803 | 113 | 129.630009 | 194.39 | 259.149991 | 1.720265 |
| GO:0031175\_neuron\_projection\_development | 197 | 4 | 1.947970 | -0.830404 | 114 | 129.876031 | 195.0 | 260.123969 | 1.710526 |
| GO:0000904\_cell\_morphogenesis\_involved\_in\_differentiation | 199 | 4 | 1.928392 | -0.819103 | 115 | 131.075394 | 196.9 | 262.724606 | 1.712174 |
| GO:0045165\_cell\_fate\_commitment | 130 | 3 | 2.213942 | -0.816216 | 116 | 131.317220 | 197.3 | 263.282780 | 1.700862 |
| GO:0009791\_post-embryonic\_development | 67 | 2 | 2.863806 | -0.812828 | 117 | 131.918435 | 198.21 | 264.501565 | 1.694103 |
| GO:0007602\_phototransduction | 16 | 1 | 5.996094 | -0.810837 | 126 | 136.570800 | 204.89 | 273.209200 | 1.626111 |
| GO:0016126\_sterol\_biosynthetic\_process | 16 | 1 | 5.996094 | -0.810837 | 126 | 136.570800 | 204.89 | 273.209200 | 1.626111 |
| GO:0019751\_polyol\_metabolic\_process | 16 | 1 | 5.996094 | -0.810837 | 126 | 136.570800 | 204.89 | 273.209200 | 1.626111 |
| GO:0031345\_negative\_regulation\_of\_cell\_projection\_organization | 16 | 1 | 5.996094 | -0.810837 | 126 | 136.570800 | 204.89 | 273.209200 | 1.626111 |
| GO:0046148\_pigment\_biosynthetic\_process | 16 | 1 | 5.996094 | -0.810837 | 126 | 136.570800 | 204.89 | 273.209200 | 1.626111 |
| GO:0046364\_monosaccharide\_biosynthetic\_process | 16 | 1 | 5.996094 | -0.810837 | 126 | 136.570800 | 204.89 | 273.209200 | 1.626111 |
| GO:0048015\_phosphoinositide-mediated\_signaling | 16 | 1 | 5.996094 | -0.810837 | 126 | 136.570800 | 204.89 | 273.209200 | 1.626111 |
| GO:0048286\_lung\_alveolus\_development | 16 | 1 | 5.996094 | -0.810837 | 126 | 136.570800 | 204.89 | 273.209200 | 1.626111 |
| GO:0048483\_autonomic\_nervous\_system\_development | 16 | 1 | 5.996094 | -0.810837 | 126 | 136.570800 | 204.89 | 273.209200 | 1.626111 |
| GO:0042692\_muscle\_cell\_differentiation | 68 | 2 | 2.821691 | -0.802628 | 127 | 136.970112 | 205.58 | 274.189888 | 1.618740 |
| GO:0048534\_hemopoietic\_or\_lymphoid\_organ\_development | 277 | 5 | 1.731724 | -0.797329 | 128 | 137.087380 | 205.94 | 274.792620 | 1.608906 |
| GO:0005996\_monosaccharide\_metabolic\_process | 69 | 2 | 2.780797 | -0.792617 | 129 | 137.829335 | 206.96 | 276.090665 | 1.604341 |
| GO:0030182\_neuron\_differentiation | 356 | 6 | 1.616924 | -0.788418 | 130 | 138.338140 | 207.54 | 276.741860 | 1.596462 |
| GO:0034470\_ncRNA\_processing | 17 | 1 | 5.643382 | -0.786678 | 135 | 144.398648 | 214.72 | 285.041352 | 1.590519 |
| GO:0042254\_ribosome\_biogenesis | 17 | 1 | 5.643382 | -0.786678 | 135 | 144.398648 | 214.72 | 285.041352 | 1.590519 |
| GO:0042398\_cellular\_amino\_acid\_derivative\_biosynthetic\_process | 17 | 1 | 5.643382 | -0.786678 | 135 | 144.398648 | 214.72 | 285.041352 | 1.590519 |
| GO:0042440\_pigment\_metabolic\_process | 17 | 1 | 5.643382 | -0.786678 | 135 | 144.398648 | 214.72 | 285.041352 | 1.590519 |
| GO:0043407\_negative\_regulation\_of\_MAP\_kinase\_activity | 17 | 1 | 5.643382 | -0.786678 | 135 | 144.398648 | 214.72 | 285.041352 | 1.590519 |
| GO:0008406\_gonad\_development | 70 | 2 | 2.741071 | -0.782788 | 136 | 144.969795 | 215.57 | 286.170205 | 1.585074 |
| GO:0001654\_eye\_development | 136 | 3 | 2.116268 | -0.775171 | 137 | 145.108058 | 215.7 | 286.291942 | 1.574453 |
| GO:0002285\_lymphocyte\_activation\_during\_immune\_response | 18 | 1 | 5.329861 | -0.764021 | 144 | 152.795906 | 225.74 | 298.684094 | 1.567639 |
| GO:0016458\_gene\_silencing | 18 | 1 | 5.329861 | -0.764021 | 144 | 152.795906 | 225.74 | 298.684094 | 1.567639 |
| GO:0030282\_bone\_mineralization | 18 | 1 | 5.329861 | -0.764021 | 144 | 152.795906 | 225.74 | 298.684094 | 1.567639 |
| GO:0030510\_regulation\_of\_BMP\_signaling\_pathway | 18 | 1 | 5.329861 | -0.764021 | 144 | 152.795906 | 225.74 | 298.684094 | 1.567639 |
| GO:0035051\_cardiac\_cell\_differentiation | 18 | 1 | 5.329861 | -0.764021 | 144 | 152.795906 | 225.74 | 298.684094 | 1.567639 |
| GO:0051168\_nuclear\_export | 18 | 1 | 5.329861 | -0.764021 | 144 | 152.795906 | 225.74 | 298.684094 | 1.567639 |
| GO:0051924\_regulation\_of\_calcium\_ion\_transport | 18 | 1 | 5.329861 | -0.764021 | 144 | 152.795906 | 225.74 | 298.684094 | 1.567639 |
| GO:0050673\_epithelial\_cell\_proliferation | 72 | 2 | 2.664931 | -0.763658 | 145 | 153.747399 | 227.27 | 300.792601 | 1.567379 |
| GO:0007275\_multicellular\_organismal\_development | 1760 | 22 | 1.199219 | -0.762532 | 146 | 153.845244 | 227.34 | 300.834756 | 1.557123 |
| GO:0009416\_response\_to\_light\_stimulus | 74 | 2 | 2.592905 | -0.745199 | 147 | 155.129788 | 229.71 | 304.290212 | 1.562653 |
| GO:0009584\_detection\_of\_visible\_light | 19 | 1 | 5.049342 | -0.742703 | 150 | 159.851859 | 235.27 | 310.688141 | 1.568467 |
| GO:0050908\_detection\_of\_light\_stimulus\_involved\_in\_visual\_perception | 19 | 1 | 5.049342 | -0.742703 | 150 | 159.851859 | 235.27 | 310.688141 | 1.568467 |
| GO:0050962\_detection\_of\_light\_stimulus\_involved\_in\_sensory\_perception | 19 | 1 | 5.049342 | -0.742703 | 150 | 159.851859 | 235.27 | 310.688141 | 1.568467 |
| GO:0032502\_developmental\_process | 2060 | 25 | 1.164290 | -0.725230 | 151 | 161.190596 | 237.17 | 313.149404 | 1.570662 |
| GO:0031214\_biomineral\_formation | 20 | 1 | 4.796875 | -0.722586 | 152 | 166.206138 | 242.57 | 318.933862 | 1.595855 |
| GO:0002520\_immune\_system\_development | 295 | 5 | 1.626059 | -0.718629 | 153 | 166.743706 | 243.37 | 319.996294 | 1.590654 |
| GO:0005975\_carbohydrate\_metabolic\_process | 146 | 3 | 1.971318 | -0.712256 | 154 | 167.313118 | 243.93 | 320.546882 | 1.583961 |
| GO:0022414\_reproductive\_process | 376 | 6 | 1.530918 | -0.711877 | 155 | 167.370972 | 244.04 | 320.709028 | 1.574452 |
| GO:0001709\_cell\_fate\_determination | 21 | 1 | 4.568452 | -0.703552 | 162 | 174.388911 | 253.02 | 331.651089 | 1.561852 |
| GO:0002053\_positive\_regulation\_of\_mesenchymal\_cell\_proliferation | 21 | 1 | 4.568452 | -0.703552 | 162 | 174.388911 | 253.02 | 331.651089 | 1.561852 |
| GO:0002263\_cell\_activation\_during\_immune\_response | 21 | 1 | 4.568452 | -0.703552 | 162 | 174.388911 | 253.02 | 331.651089 | 1.561852 |
| GO:0002366\_leukocyte\_activation\_during\_immune\_response | 21 | 1 | 4.568452 | -0.703552 | 162 | 174.388911 | 253.02 | 331.651089 | 1.561852 |
| GO:0009583\_detection\_of\_light\_stimulus | 21 | 1 | 4.568452 | -0.703552 | 162 | 174.388911 | 253.02 | 331.651089 | 1.561852 |
| GO:0048538\_thymus\_development | 21 | 1 | 4.568452 | -0.703552 | 162 | 174.388911 | 253.02 | 331.651089 | 1.561852 |
| GO:0048675\_axon\_extension | 21 | 1 | 4.568452 | -0.703552 | 162 | 174.388911 | 253.02 | 331.651089 | 1.561852 |
| GO:0000003\_reproduction | 379 | 6 | 1.518799 | -0.701041 | 163 | 174.543532 | 253.52 | 332.496468 | 1.555337 |
| GO:0009309\_amine\_biosynthetic\_process | 22 | 1 | 4.360795 | -0.685501 | 170 | 182.263803 | 263.37 | 344.476197 | 1.549235 |
| GO:0010463\_mesenchymal\_cell\_proliferation | 22 | 1 | 4.360795 | -0.685501 | 170 | 182.263803 | 263.37 | 344.476197 | 1.549235 |
| GO:0010464\_regulation\_of\_mesenchymal\_cell\_proliferation | 22 | 1 | 4.360795 | -0.685501 | 170 | 182.263803 | 263.37 | 344.476197 | 1.549235 |
| GO:0021675\_nerve\_development | 22 | 1 | 4.360795 | -0.685501 | 170 | 182.263803 | 263.37 | 344.476197 | 1.549235 |
| GO:0030705\_cytoskeleton-dependent\_intracellular\_transport | 22 | 1 | 4.360795 | -0.685501 | 170 | 182.263803 | 263.37 | 344.476197 | 1.549235 |
| GO:0032886\_regulation\_of\_microtubule-based\_process | 22 | 1 | 4.360795 | -0.685501 | 170 | 182.263803 | 263.37 | 344.476197 | 1.549235 |
| GO:0034660\_ncRNA\_metabolic\_process | 22 | 1 | 4.360795 | -0.685501 | 170 | 182.263803 | 263.37 | 344.476197 | 1.549235 |
| GO:0045664\_regulation\_of\_neuron\_differentiation | 82 | 2 | 2.339939 | -0.677381 | 171 | 183.266470 | 264.77 | 346.273530 | 1.548363 |
| GO:0019953\_sexual\_reproduction | 228 | 4 | 1.683114 | -0.673134 | 172 | 183.589268 | 265.23 | 346.870732 | 1.542035 |
| GO:0032501\_multicellular\_organismal\_process | 2183 | 26 | 1.142636 | -0.672898 | 173 | 183.661890 | 265.37 | 347.078110 | 1.533931 |
| GO:0006575\_cellular\_amino\_acid\_derivative\_metabolic\_process | 83 | 2 | 2.311747 | -0.669518 | 175 | 184.555940 | 266.53 | 348.504060 | 1.523029 |
| GO:0030198\_extracellular\_matrix\_organization | 83 | 2 | 2.311747 | -0.669518 | 175 | 184.555940 | 266.53 | 348.504060 | 1.523029 |
| GO:0007018\_microtubule-based\_movement | 23 | 1 | 4.171196 | -0.668345 | 178 | 188.184431 | 271.06 | 353.935569 | 1.522809 |
| GO:0008542\_visual\_learning | 23 | 1 | 4.171196 | -0.668345 | 178 | 188.184431 | 271.06 | 353.935569 | 1.522809 |
| GO:0030512\_negative\_regulation\_of\_transforming\_growth\_factor\_beta\_receptor\_signaling\_pathway | 23 | 1 | 4.171196 | -0.668345 | 178 | 188.184431 | 271.06 | 353.935569 | 1.522809 |
| GO:0045137\_development\_of\_primary\_sexual\_characteristics | 84 | 2 | 2.284226 | -0.661781 | 179 | 188.799351 | 272.02 | 355.240649 | 1.519665 |
| GO:0001541\_ovarian\_follicle\_development | 24 | 1 | 3.997396 | -0.652007 | 183 | 194.509474 | 279.16 | 363.810526 | 1.525464 |
| GO:0006650\_glycerophospholipid\_metabolic\_process | 24 | 1 | 3.997396 | -0.652007 | 183 | 194.509474 | 279.16 | 363.810526 | 1.525464 |
| GO:0007632\_visual\_behavior | 24 | 1 | 3.997396 | -0.652007 | 183 | 194.509474 | 279.16 | 363.810526 | 1.525464 |
| GO:0050679\_positive\_regulation\_of\_epithelial\_cell\_proliferation | 24 | 1 | 3.997396 | -0.652007 | 183 | 194.509474 | 279.16 | 363.810526 | 1.525464 |
| GO:0050890\_cognition | 233 | 4 | 1.646996 | -0.650975 | 184 | 194.899584 | 279.59 | 364.280416 | 1.519511 |
| GO:0032504\_multicellular\_organism\_reproduction | 86 | 2 | 2.231105 | -0.646670 | 186 | 196.020875 | 280.86 | 365.699125 | 1.510000 |
| GO:0048609\_reproductive\_process\_in\_a\_multicellular\_organism | 86 | 2 | 2.231105 | -0.646670 | 186 | 196.020875 | 280.86 | 365.699125 | 1.510000 |
| GO:0048699\_generation\_of\_neurons | 396 | 6 | 1.453598 | -0.642585 | 187 | 196.705078 | 281.72 | 366.734922 | 1.506524 |
| GO:0001822\_kidney\_development | 87 | 2 | 2.205460 | -0.639290 | 188 | 197.774557 | 282.95 | 368.125443 | 1.505053 |
| GO:0050852\_T\_cell\_receptor\_signaling\_pathway | 25 | 1 | 3.837500 | -0.636419 | 189 | 201.835432 | 288.17 | 374.504568 | 1.524709 |
| GO:0010959\_regulation\_of\_metal\_ion\_transport | 26 | 1 | 3.689904 | -0.621524 | 192 | 207.319134 | 295.23 | 383.140866 | 1.537656 |
| GO:0045665\_negative\_regulation\_of\_neuron\_differentiation | 26 | 1 | 3.689904 | -0.621524 | 192 | 207.319134 | 295.23 | 383.140866 | 1.537656 |
| GO:0050873\_brown\_fat\_cell\_differentiation | 26 | 1 | 3.689904 | -0.621524 | 192 | 207.319134 | 295.23 | 383.140866 | 1.537656 |
| GO:0007422\_peripheral\_nervous\_system\_development | 27 | 1 | 3.553241 | -0.607268 | 194 | 212.004031 | 301.48 | 390.955969 | 1.554021 |
| GO:0032496\_response\_to\_lipopolysaccharide | 27 | 1 | 3.553241 | -0.607268 | 194 | 212.004031 | 301.48 | 390.955969 | 1.554021 |
| GO:0006470\_protein\_amino\_acid\_dephosphorylation | 28 | 1 | 3.426339 | -0.593605 | 195 | 216.556643 | 306.98 | 397.403357 | 1.574256 |
| GO:0042490\_mechanoreceptor\_differentiation | 29 | 1 | 3.308190 | -0.580493 | 197 | 222.340156 | 314.47 | 406.599844 | 1.596294 |
| GO:0048066\_pigmentation\_during\_development | 29 | 1 | 3.308190 | -0.580493 | 197 | 222.340156 | 314.47 | 406.599844 | 1.596294 |
| GO:0060249\_anatomical\_structure\_homeostasis | 96 | 2 | 1.998698 | -0.577694 | 198 | 223.098772 | 315.43 | 407.761228 | 1.593081 |
| GO:0007600\_sensory\_perception | 172 | 3 | 1.673328 | -0.575106 | 199 | 223.582072 | 315.91 | 408.237928 | 1.587487 |
| GO:0007219\_Notch\_signaling\_pathway | 30 | 1 | 3.197917 | -0.567894 | 201 | 229.404012 | 323.36 | 417.315988 | 1.608756 |
| GO:0031668\_cellular\_response\_to\_extracellular\_stimulus | 30 | 1 | 3.197917 | -0.567894 | 201 | 229.404012 | 323.36 | 417.315988 | 1.608756 |
| GO:0007548\_sex\_differentiation | 98 | 2 | 1.957908 | -0.565083 | 203 | 229.979341 | 324.25 | 418.520659 | 1.597291 |
| GO:0009314\_response\_to\_radiation | 98 | 2 | 1.957908 | -0.565083 | 203 | 229.979341 | 324.25 | 418.520659 | 1.597291 |
| GO:0006486\_protein\_amino\_acid\_glycosylation | 31 | 1 | 3.094758 | -0.555774 | 210 | 238.576620 | 334.21 | 429.843380 | 1.591476 |
| GO:0006694\_steroid\_biosynthetic\_process | 31 | 1 | 3.094758 | -0.555774 | 210 | 238.576620 | 334.21 | 429.843380 | 1.591476 |
| GO:0016311\_dephosphorylation | 31 | 1 | 3.094758 | -0.555774 | 210 | 238.576620 | 334.21 | 429.843380 | 1.591476 |
| GO:0043269\_regulation\_of\_ion\_transport | 31 | 1 | 3.094758 | -0.555774 | 210 | 238.576620 | 334.21 | 429.843380 | 1.591476 |
| GO:0043413\_biopolymer\_glycosylation | 31 | 1 | 3.094758 | -0.555774 | 210 | 238.576620 | 334.21 | 429.843380 | 1.591476 |
| GO:0046632\_alpha-beta\_T\_cell\_differentiation | 31 | 1 | 3.094758 | -0.555774 | 210 | 238.576620 | 334.21 | 429.843380 | 1.591476 |
| GO:0070085\_glycosylation | 31 | 1 | 3.094758 | -0.555774 | 210 | 238.576620 | 334.21 | 429.843380 | 1.591476 |
| GO:0006793\_phosphorus\_metabolic\_process | 340 | 5 | 1.410846 | -0.554636 | 212 | 238.902813 | 334.66 | 430.417187 | 1.578585 |
| GO:0006796\_phosphate\_metabolic\_process | 340 | 5 | 1.410846 | -0.554636 | 212 | 238.902813 | 334.66 | 430.417187 | 1.578585 |
| GO:0048856\_anatomical\_structure\_development | 1688 | 20 | 1.136700 | -0.552357 | 213 | 239.245683 | 335.03 | 430.814317 | 1.572911 |
| GO:0050768\_negative\_regulation\_of\_neurogenesis | 32 | 1 | 2.998047 | -0.544102 | 216 | 242.534320 | 339.12 | 435.705680 | 1.570000 |
| GO:0050770\_regulation\_of\_axonogenesis | 32 | 1 | 2.998047 | -0.544102 | 216 | 242.534320 | 339.12 | 435.705680 | 1.570000 |
| GO:0051493\_regulation\_of\_cytoskeleton\_organization | 32 | 1 | 2.998047 | -0.544102 | 216 | 242.534320 | 339.12 | 435.705680 | 1.570000 |
| GO:0048666\_neuron\_development | 262 | 4 | 1.464695 | -0.536835 | 217 | 243.951076 | 340.85 | 437.748924 | 1.570737 |
| GO:0002562\_somatic\_diversification\_of\_immune\_receptors\_via\_germline\_recombination\_within\_a\_single\_locus | 33 | 1 | 2.907197 | -0.532852 | 220 | 248.629259 | 346.27 | 443.910741 | 1.573955 |
| GO:0007565\_female\_pregnancy | 33 | 1 | 2.907197 | -0.532852 | 220 | 248.629259 | 346.27 | 443.910741 | 1.573955 |
| GO:0016444\_somatic\_cell\_DNA\_recombination | 33 | 1 | 2.907197 | -0.532852 | 220 | 248.629259 | 346.27 | 443.910741 | 1.573955 |
| GO:0016043\_cellular\_component\_organization | 964 | 12 | 1.194243 | -0.531837 | 221 | 248.705122 | 346.36 | 444.014878 | 1.567240 |
| GO:0050767\_regulation\_of\_neurogenesis | 104 | 2 | 1.844952 | -0.529327 | 222 | 249.630036 | 347.63 | 445.629964 | 1.565901 |
| GO:0048731\_system\_development | 1609 | 19 | 1.132885 | -0.528908 | 223 | 249.707624 | 347.72 | 445.732376 | 1.559283 |
| GO:0002200\_somatic\_diversification\_of\_immune\_receptors | 34 | 1 | 2.821691 | -0.521996 | 227 | 254.615744 | 353.41 | 452.204256 | 1.556872 |
| GO:0002237\_response\_to\_molecule\_of\_bacterial\_origin | 34 | 1 | 2.821691 | -0.521996 | 227 | 254.615744 | 353.41 | 452.204256 | 1.556872 |
| GO:0010721\_negative\_regulation\_of\_cell\_development | 34 | 1 | 2.821691 | -0.521996 | 227 | 254.615744 | 353.41 | 452.204256 | 1.556872 |
| GO:0030509\_BMP\_signaling\_pathway | 34 | 1 | 2.821691 | -0.521996 | 227 | 254.615744 | 353.41 | 452.204256 | 1.556872 |
| GO:0045859\_regulation\_of\_protein\_kinase\_activity | 107 | 2 | 1.793224 | -0.512534 | 228 | 256.247649 | 355.52 | 454.792351 | 1.559298 |
| GO:0006869\_lipid\_transport | 35 | 1 | 2.741071 | -0.511514 | 231 | 259.801585 | 359.17 | 458.538415 | 1.554848 |
| GO:0007292\_female\_gamete\_generation | 35 | 1 | 2.741071 | -0.511514 | 231 | 259.801585 | 359.17 | 458.538415 | 1.554848 |
| GO:0016051\_carbohydrate\_biosynthetic\_process | 35 | 1 | 2.741071 | -0.511514 | 231 | 259.801585 | 359.17 | 458.538415 | 1.554848 |
| GO:0006469\_negative\_regulation\_of\_protein\_kinase\_activity | 36 | 1 | 2.664931 | -0.501382 | 236 | 265.324231 | 365.92 | 466.515769 | 1.550508 |
| GO:0030278\_regulation\_of\_ossification | 36 | 1 | 2.664931 | -0.501382 | 236 | 265.324231 | 365.92 | 466.515769 | 1.550508 |
| GO:0033673\_negative\_regulation\_of\_kinase\_activity | 36 | 1 | 2.664931 | -0.501382 | 236 | 265.324231 | 365.92 | 466.515769 | 1.550508 |
| GO:0050851\_antigen\_receptor-mediated\_signaling\_pathway | 36 | 1 | 2.664931 | -0.501382 | 236 | 265.324231 | 365.92 | 466.515769 | 1.550508 |
| GO:0050900\_leukocyte\_migration | 36 | 1 | 2.664931 | -0.501382 | 236 | 265.324231 | 365.92 | 466.515769 | 1.550508 |
| GO:0050906\_detection\_of\_stimulus\_involved\_in\_sensory\_perception | 37 | 1 | 2.592905 | -0.491582 | 237 | 269.132625 | 370.35 | 471.567375 | 1.562658 |
| GO:0043549\_regulation\_of\_kinase\_activity | 112 | 2 | 1.713170 | -0.486010 | 238 | 270.426414 | 371.98 | 473.533586 | 1.562941 |
| GO:0046907\_intracellular\_transport | 194 | 3 | 1.483570 | -0.482307 | 239 | 270.631939 | 372.34 | 474.048061 | 1.557908 |
| GO:0001570\_vasculogenesis | 38 | 1 | 2.524671 | -0.482096 | 242 | 275.389176 | 377.83 | 480.270824 | 1.561281 |
| GO:0010975\_regulation\_of\_neuron\_projection\_development | 38 | 1 | 2.524671 | -0.482096 | 242 | 275.389176 | 377.83 | 480.270824 | 1.561281 |
| GO:0051348\_negative\_regulation\_of\_transferase\_activity | 38 | 1 | 2.524671 | -0.482096 | 242 | 275.389176 | 377.83 | 480.270824 | 1.561281 |
| GO:0065009\_regulation\_of\_molecular\_function | 279 | 4 | 1.375448 | -0.479799 | 243 | 276.312502 | 379.09 | 481.867498 | 1.560041 |
| GO:0006644\_phospholipid\_metabolic\_process | 39 | 1 | 2.459936 | -0.472907 | 248 | 281.276809 | 384.73 | 488.183191 | 1.551331 |
| GO:0021953\_central\_nervous\_system\_neuron\_differentiation | 39 | 1 | 2.459936 | -0.472907 | 248 | 281.276809 | 384.73 | 488.183191 | 1.551331 |
| GO:0031279\_regulation\_of\_cyclase\_activity | 39 | 1 | 2.459936 | -0.472907 | 248 | 281.276809 | 384.73 | 488.183191 | 1.551331 |
| GO:0042475\_odontogenesis\_of\_dentine-containing\_tooth | 39 | 1 | 2.459936 | -0.472907 | 248 | 281.276809 | 384.73 | 488.183191 | 1.551331 |
| GO:0051339\_regulation\_of\_lyase\_activity | 39 | 1 | 2.459936 | -0.472907 | 248 | 281.276809 | 384.73 | 488.183191 | 1.551331 |
| GO:0051338\_regulation\_of\_transferase\_activity | 115 | 2 | 1.668478 | -0.470915 | 249 | 282.518053 | 386.19 | 489.861947 | 1.550964 |
| GO:0048608\_reproductive\_structure\_development | 116 | 2 | 1.654095 | -0.466012 | 250 | 284.274906 | 388.12 | 491.965094 | 1.552480 |
| GO:0008203\_cholesterol\_metabolic\_process | 40 | 1 | 2.398438 | -0.464000 | 254 | 287.463707 | 391.9 | 496.336293 | 1.542913 |
| GO:0017015\_regulation\_of\_transforming\_growth\_factor\_beta\_receptor\_signaling\_pathway | 40 | 1 | 2.398438 | -0.464000 | 254 | 287.463707 | 391.9 | 496.336293 | 1.542913 |
| GO:0046850\_regulation\_of\_bone\_remodeling | 40 | 1 | 2.398438 | -0.464000 | 254 | 287.463707 | 391.9 | 496.336293 | 1.542913 |
| GO:0051129\_negative\_regulation\_of\_cellular\_component\_organization | 40 | 1 | 2.398438 | -0.464000 | 254 | 287.463707 | 391.9 | 496.336293 | 1.542913 |
| GO:0006519\_cellular\_amino\_acid\_and\_derivative\_metabolic\_process | 118 | 2 | 1.626059 | -0.456394 | 256 | 289.009581 | 393.68 | 498.350419 | 1.537812 |
| GO:0051960\_regulation\_of\_nervous\_system\_development | 118 | 2 | 1.626059 | -0.456394 | 256 | 289.009581 | 393.68 | 498.350419 | 1.537812 |
| GO:0002429\_immune\_response-activating\_cell\_surface\_receptor\_signaling\_pathway | 41 | 1 | 2.339939 | -0.455361 | 259 | 293.671621 | 399.21 | 504.748379 | 1.541351 |
| GO:0019748\_secondary\_metabolic\_process | 41 | 1 | 2.339939 | -0.455361 | 259 | 293.671621 | 399.21 | 504.748379 | 1.541351 |
| GO:0031344\_regulation\_of\_cell\_projection\_organization | 41 | 1 | 2.339939 | -0.455361 | 259 | 293.671621 | 399.21 | 504.748379 | 1.541351 |
| GO:0010769\_regulation\_of\_cell\_morphogenesis\_involved\_in\_differentiation | 42 | 1 | 2.284226 | -0.446977 | 262 | 299.068679 | 405.79 | 512.511321 | 1.548817 |
| GO:0016125\_sterol\_metabolic\_process | 42 | 1 | 2.284226 | -0.446977 | 262 | 299.068679 | 405.79 | 512.511321 | 1.548817 |
| GO:0042476\_odontogenesis | 42 | 1 | 2.284226 | -0.446977 | 262 | 299.068679 | 405.79 | 512.511321 | 1.548817 |
| GO:0001894\_tissue\_homeostasis | 43 | 1 | 2.231105 | -0.438836 | 265 | 304.621572 | 412.68 | 520.738428 | 1.557283 |
| GO:0009582\_detection\_of\_abiotic\_stimulus | 43 | 1 | 2.231105 | -0.438836 | 265 | 304.621572 | 412.68 | 520.738428 | 1.557283 |
| GO:0019637\_organophosphate\_metabolic\_process | 43 | 1 | 2.231105 | -0.438836 | 265 | 304.621572 | 412.68 | 520.738428 | 1.557283 |
| GO:0060284\_regulation\_of\_cell\_development | 122 | 2 | 1.572746 | -0.437874 | 266 | 305.786327 | 414.06 | 522.333673 | 1.556617 |
| GO:0048869\_cellular\_developmental\_process | 1113 | 13 | 1.120564 | -0.431058 | 267 | 306.929650 | 415.46 | 523.990350 | 1.556030 |
| GO:0002768\_immune\_response-regulating\_cell\_surface\_receptor\_signaling\_pathway | 44 | 1 | 2.180398 | -0.430926 | 271 | 311.630462 | 420.96 | 530.289538 | 1.553358 |
| GO:0030802\_regulation\_of\_cyclic\_nucleotide\_biosynthetic\_process | 44 | 1 | 2.180398 | -0.430926 | 271 | 311.630462 | 420.96 | 530.289538 | 1.553358 |
| GO:0030808\_regulation\_of\_nucleotide\_biosynthetic\_process | 44 | 1 | 2.180398 | -0.430926 | 271 | 311.630462 | 420.96 | 530.289538 | 1.553358 |
| GO:0048593\_camera-type\_eye\_morphogenesis | 44 | 1 | 2.180398 | -0.430926 | 271 | 311.630462 | 420.96 | 530.289538 | 1.553358 |
| GO:0009308\_amine\_metabolic\_process | 124 | 2 | 1.547379 | -0.428957 | 273 | 312.580028 | 421.96 | 531.339972 | 1.545641 |
| GO:0030098\_lymphocyte\_differentiation | 124 | 2 | 1.547379 | -0.428957 | 273 | 312.580028 | 421.96 | 531.339972 | 1.545641 |
| GO:0043062\_extracellular\_structure\_organization | 125 | 2 | 1.535000 | -0.424580 | 274 | 313.437262 | 422.9 | 532.362738 | 1.543431 |
| GO:0034103\_regulation\_of\_tissue\_remodeling | 45 | 1 | 2.131944 | -0.423237 | 275 | 314.965992 | 424.46 | 533.954008 | 1.543491 |
| GO:0007612\_learning | 46 | 1 | 2.085598 | -0.415759 | 279 | 318.677808 | 428.46 | 538.242192 | 1.535699 |
| GO:0009581\_detection\_of\_external\_stimulus | 46 | 1 | 2.085598 | -0.415759 | 279 | 318.677808 | 428.46 | 538.242192 | 1.535699 |
| GO:0030218\_erythrocyte\_differentiation | 46 | 1 | 2.085598 | -0.415759 | 279 | 318.677808 | 428.46 | 538.242192 | 1.535699 |
| GO:0046631\_alpha-beta\_T\_cell\_activation | 46 | 1 | 2.085598 | -0.415759 | 279 | 318.677808 | 428.46 | 538.242192 | 1.535699 |
| GO:0050877\_neurological\_system\_process | 390 | 5 | 1.229968 | -0.415312 | 280 | 318.820651 | 428.58 | 538.339349 | 1.530643 |
| GO:0001655\_urogenital\_system\_development | 128 | 2 | 1.499023 | -0.411767 | 281 | 319.408436 | 429.26 | 539.111564 | 1.527616 |
| GO:0002757\_immune\_response-activating\_signal\_transduction | 47 | 1 | 2.041223 | -0.408483 | 287 | 322.760958 | 433.03 | 543.299042 | 1.508815 |
| GO:0006140\_regulation\_of\_nucleotide\_metabolic\_process | 47 | 1 | 2.041223 | -0.408483 | 287 | 322.760958 | 433.03 | 543.299042 | 1.508815 |
| GO:0030183\_B\_cell\_differentiation | 47 | 1 | 2.041223 | -0.408483 | 287 | 322.760958 | 433.03 | 543.299042 | 1.508815 |
| GO:0030799\_regulation\_of\_cyclic\_nucleotide\_metabolic\_process | 47 | 1 | 2.041223 | -0.408483 | 287 | 322.760958 | 433.03 | 543.299042 | 1.508815 |
| GO:0034754\_cellular\_hormone\_metabolic\_process | 47 | 1 | 2.041223 | -0.408483 | 287 | 322.760958 | 433.03 | 543.299042 | 1.508815 |
| GO:0048871\_multicellular\_organismal\_homeostasis | 47 | 1 | 2.041223 | -0.408483 | 287 | 322.760958 | 433.03 | 543.299042 | 1.508815 |
| GO:0009101\_glycoprotein\_biosynthetic\_process | 48 | 1 | 1.998698 | -0.401400 | 290 | 327.017606 | 437.88 | 548.742394 | 1.509931 |
| GO:0034504\_protein\_localization\_in\_nucleus | 48 | 1 | 1.998698 | -0.401400 | 290 | 327.017606 | 437.88 | 548.742394 | 1.509931 |
| GO:0046849\_bone\_remodeling | 48 | 1 | 1.998698 | -0.401400 | 290 | 327.017606 | 437.88 | 548.742394 | 1.509931 |
| GO:0007423\_sensory\_organ\_development | 219 | 3 | 1.314212 | -0.396294 | 291 | 327.776118 | 438.65 | 549.523882 | 1.507388 |
| GO:0034101\_erythrocyte\_homeostasis | 49 | 1 | 1.957908 | -0.394502 | 292 | 331.455462 | 442.61 | 553.764538 | 1.515788 |
| GO:0016310\_phosphorylation | 309 | 4 | 1.241909 | -0.393669 | 293 | 331.681900 | 442.83 | 553.978100 | 1.511365 |
| GO:0009190\_cyclic\_nucleotide\_biosynthetic\_process | 50 | 1 | 1.918750 | -0.387781 | 295 | 335.120432 | 446.66 | 558.199568 | 1.514102 |
| GO:0051606\_detection\_of\_stimulus | 50 | 1 | 1.918750 | -0.387781 | 295 | 335.120432 | 446.66 | 558.199568 | 1.514102 |
| GO:0007283\_spermatogenesis | 134 | 2 | 1.431903 | -0.387495 | 297 | 335.492646 | 447.14 | 558.787354 | 1.505522 |
| GO:0048232\_male\_gamete\_generation | 134 | 2 | 1.431903 | -0.387495 | 297 | 335.492646 | 447.14 | 558.787354 | 1.505522 |
| GO:0009653\_anatomical\_structure\_morphogenesis | 958 | 11 | 1.101579 | -0.382778 | 298 | 335.918545 | 447.6 | 559.281455 | 1.502013 |
| GO:0002764\_immune\_response-regulating\_signal\_transduction | 51 | 1 | 1.881127 | -0.381230 | 299 | 338.841103 | 450.69 | 562.538897 | 1.507324 |
| GO:0009124\_nucleoside\_monophosphate\_biosynthetic\_process | 52 | 1 | 1.844952 | -0.374843 | 300 | 341.389928 | 453.44 | 565.490072 | 1.511467 |
| GO:0030154\_cell\_differentiation | 1060 | 12 | 1.086085 | -0.370007 | 301 | 342.155105 | 454.26 | 566.364895 | 1.509169 |
| GO:0030031\_cell\_projection\_assembly | 53 | 1 | 1.810142 | -0.368613 | 304 | 345.077054 | 457.62 | 570.162946 | 1.505329 |
| GO:0046942\_carboxylic\_acid\_transport | 53 | 1 | 1.810142 | -0.368613 | 304 | 345.077054 | 457.62 | 570.162946 | 1.505329 |
| GO:0055085\_transmembrane\_transport | 53 | 1 | 1.810142 | -0.368613 | 304 | 345.077054 | 457.62 | 570.162946 | 1.505329 |
| GO:0034613\_cellular\_protein\_localization | 139 | 2 | 1.380396 | -0.368546 | 305 | 345.605671 | 458.23 | 570.854329 | 1.502393 |
| GO:0002376\_immune\_system\_process | 505 | 6 | 1.139851 | -0.363571 | 306 | 346.695310 | 459.32 | 571.944690 | 1.501046 |
| GO:0002253\_activation\_of\_immune\_response | 54 | 1 | 1.776620 | -0.362534 | 311 | 349.567785 | 462.21 | 574.852215 | 1.486206 |
| GO:0006164\_purine\_nucleotide\_biosynthetic\_process | 54 | 1 | 1.776620 | -0.362534 | 311 | 349.567785 | 462.21 | 574.852215 | 1.486206 |
| GO:0015849\_organic\_acid\_transport | 54 | 1 | 1.776620 | -0.362534 | 311 | 349.567785 | 462.21 | 574.852215 | 1.486206 |
| GO:0043405\_regulation\_of\_MAP\_kinase\_activity | 54 | 1 | 1.776620 | -0.362534 | 311 | 349.567785 | 462.21 | 574.852215 | 1.486206 |
| GO:0044271\_nitrogen\_compound\_biosynthetic\_process | 54 | 1 | 1.776620 | -0.362534 | 311 | 349.567785 | 462.21 | 574.852215 | 1.486206 |
| GO:0003006\_reproductive\_developmental\_process | 141 | 2 | 1.360816 | -0.361270 | 313 | 350.208271 | 462.82 | 575.431729 | 1.478658 |
| GO:0070727\_cellular\_macromolecule\_localization | 141 | 2 | 1.360816 | -0.361270 | 313 | 350.208271 | 462.82 | 575.431729 | 1.478658 |
| GO:0006310\_DNA\_recombination | 55 | 1 | 1.744318 | -0.356600 | 314 | 352.459498 | 465.53 | 578.600502 | 1.482580 |
| GO:0050790\_regulation\_of\_catalytic\_activity | 233 | 3 | 1.235247 | -0.355394 | 315 | 352.888821 | 466.09 | 579.291179 | 1.479651 |
| GO:0016477\_cell\_migration | 234 | 3 | 1.229968 | -0.352647 | 316 | 353.291719 | 466.56 | 579.828281 | 1.476456 |
| GO:0009187\_cyclic\_nucleotide\_metabolic\_process | 56 | 1 | 1.713170 | -0.350807 | 319 | 356.002439 | 469.56 | 583.117561 | 1.471975 |
| GO:0046486\_glycerolipid\_metabolic\_process | 56 | 1 | 1.713170 | -0.350807 | 319 | 356.002439 | 469.56 | 583.117561 | 1.471975 |
| GO:0050678\_regulation\_of\_epithelial\_cell\_proliferation | 56 | 1 | 1.713170 | -0.350807 | 319 | 356.002439 | 469.56 | 583.117561 | 1.471975 |
| GO:0045596\_negative\_regulation\_of\_cell\_differentiation | 144 | 2 | 1.332465 | -0.350666 | 320 | 356.397200 | 470.03 | 583.662800 | 1.468844 |
| GO:0000226\_microtubule\_cytoskeleton\_organization | 57 | 1 | 1.683114 | -0.345148 | 324 | 361.047532 | 475.14 | 589.232468 | 1.466481 |
| GO:0033365\_protein\_localization\_in\_organelle | 57 | 1 | 1.683114 | -0.345148 | 324 | 361.047532 | 475.14 | 589.232468 | 1.466481 |
| GO:0043523\_regulation\_of\_neuron\_apoptosis | 57 | 1 | 1.683114 | -0.345148 | 324 | 361.047532 | 475.14 | 589.232468 | 1.466481 |
| GO:0045444\_fat\_cell\_differentiation | 57 | 1 | 1.683114 | -0.345148 | 324 | 361.047532 | 475.14 | 589.232468 | 1.466481 |
| GO:0044085\_cellular\_component\_biogenesis | 237 | 3 | 1.214399 | -0.344538 | 325 | 361.533396 | 475.62 | 589.706604 | 1.463446 |
| GO:0003008\_system\_process | 516 | 6 | 1.115552 | -0.342731 | 326 | 362.243197 | 476.65 | 591.056803 | 1.462117 |
| GO:0006928\_cell\_motion | 330 | 4 | 1.162879 | -0.342680 | 328 | 362.665088 | 477.17 | 591.674912 | 1.454787 |
| GO:0051674\_localization\_of\_cell | 330 | 4 | 1.162879 | -0.342680 | 328 | 362.665088 | 477.17 | 591.674912 | 1.454787 |
| GO:0051093\_negative\_regulation\_of\_developmental\_process | 331 | 4 | 1.159366 | -0.340421 | 329 | 363.622339 | 478.2 | 592.777661 | 1.453495 |
| GO:0033043\_regulation\_of\_organelle\_organization | 58 | 1 | 1.654095 | -0.339620 | 331 | 365.076120 | 479.73 | 594.383880 | 1.449335 |
| GO:0034622\_cellular\_macromolecular\_complex\_assembly | 58 | 1 | 1.654095 | -0.339620 | 331 | 365.076120 | 479.73 | 594.383880 | 1.449335 |
| GO:0048469\_cell\_maturation | 59 | 1 | 1.626059 | -0.334217 | 332 | 367.523042 | 482.34 | 597.156958 | 1.452831 |
| GO:0009123\_nucleoside\_monophosphate\_metabolic\_process | 60 | 1 | 1.598958 | -0.328935 | 333 | 368.356165 | 483.25 | 598.143835 | 1.451201 |
| GO:0009991\_response\_to\_extracellular\_stimulus | 61 | 1 | 1.572746 | -0.323771 | 334 | 370.944453 | 486.13 | 601.315547 | 1.455479 |
| GO:0007399\_nervous\_system\_development | 621 | 7 | 1.081421 | -0.323439 | 335 | 371.031195 | 486.23 | 601.428805 | 1.451433 |
| GO:0022604\_regulation\_of\_cell\_morphogenesis | 62 | 1 | 1.547379 | -0.318719 | 336 | 376.060051 | 492.07 | 608.079949 | 1.464494 |
| GO:0009165\_nucleotide\_biosynthetic\_process | 63 | 1 | 1.522817 | -0.313777 | 338 | 379.867344 | 496.1 | 612.332656 | 1.467751 |
| GO:0051216\_cartilage\_development | 63 | 1 | 1.522817 | -0.313777 | 338 | 379.867344 | 496.1 | 612.332656 | 1.467751 |
| GO:0055074\_calcium\_ion\_homeostasis | 64 | 1 | 1.499023 | -0.308941 | 339 | 382.342892 | 498.82 | 615.297108 | 1.471445 |
| GO:0051704\_multi-organism\_process | 157 | 2 | 1.222134 | -0.308640 | 340 | 382.656679 | 499.14 | 615.623321 | 1.468059 |
| GO:0043086\_negative\_regulation\_of\_catalytic\_activity | 65 | 1 | 1.475962 | -0.304208 | 341 | 385.367538 | 502.16 | 618.952462 | 1.472610 |
| GO:0051128\_regulation\_of\_cellular\_component\_organization | 160 | 2 | 1.199219 | -0.299769 | 342 | 387.048835 | 503.84 | 620.631165 | 1.473216 |
| GO:0007179\_transforming\_growth\_factor\_beta\_receptor\_signaling\_pathway | 66 | 1 | 1.453598 | -0.299574 | 345 | 389.131880 | 506.22 | 623.308120 | 1.467304 |
| GO:0045860\_positive\_regulation\_of\_protein\_kinase\_activity | 66 | 1 | 1.453598 | -0.299574 | 345 | 389.131880 | 506.22 | 623.308120 | 1.467304 |
| GO:0051402\_neuron\_apoptosis | 66 | 1 | 1.453598 | -0.299574 | 345 | 389.131880 | 506.22 | 623.308120 | 1.467304 |
| GO:0002521\_leukocyte\_differentiation | 161 | 2 | 1.191770 | -0.296876 | 346 | 389.887598 | 507.04 | 624.192402 | 1.465434 |
| GO:0048870\_cell\_motility | 257 | 3 | 1.119893 | -0.295158 | 347 | 390.581536 | 507.79 | 624.998464 | 1.463372 |
| GO:0003007\_heart\_morphogenesis | 67 | 1 | 1.431903 | -0.295035 | 349 | 392.381977 | 509.47 | 626.558023 | 1.459799 |
| GO:0042445\_hormone\_metabolic\_process | 67 | 1 | 1.431903 | -0.295035 | 349 | 392.381977 | 509.47 | 626.558023 | 1.459799 |
| GO:0009628\_response\_to\_abiotic\_stimulus | 162 | 2 | 1.184414 | -0.294014 | 350 | 392.908358 | 509.99 | 627.071642 | 1.457114 |
| GO:0019932\_second-messenger-mediated\_signaling | 68 | 1 | 1.410846 | -0.290591 | 351 | 395.009676 | 512.45 | 629.890324 | 1.459972 |
| GO:0042325\_regulation\_of\_phosphorylation | 164 | 2 | 1.169970 | -0.288381 | 352 | 395.649342 | 512.93 | 630.210658 | 1.457187 |
| GO:0006816\_calcium\_ion\_transport | 69 | 1 | 1.390399 | -0.286236 | 354 | 397.387235 | 514.76 | 632.132765 | 1.454124 |
| GO:0055065\_metal\_ion\_homeostasis | 69 | 1 | 1.390399 | -0.286236 | 354 | 397.387235 | 514.76 | 632.132765 | 1.454124 |
| GO:0019220\_regulation\_of\_phosphate\_metabolic\_process | 165 | 2 | 1.162879 | -0.285609 | 356 | 398.547089 | 515.68 | 632.812911 | 1.448539 |
| GO:0051174\_regulation\_of\_phosphorus\_metabolic\_process | 165 | 2 | 1.162879 | -0.285609 | 356 | 398.547089 | 515.68 | 632.812911 | 1.448539 |
| GO:0007611\_learning\_or\_memory | 70 | 1 | 1.370536 | -0.281969 | 360 | 401.307637 | 518.47 | 635.632363 | 1.440194 |
| GO:0009617\_response\_to\_bacterium | 70 | 1 | 1.370536 | -0.281969 | 360 | 401.307637 | 518.47 | 635.632363 | 1.440194 |
| GO:0048592\_eye\_morphogenesis | 70 | 1 | 1.370536 | -0.281969 | 360 | 401.307637 | 518.47 | 635.632363 | 1.440194 |
| GO:0070838\_divalent\_metal\_ion\_transport | 70 | 1 | 1.370536 | -0.281969 | 360 | 401.307637 | 518.47 | 635.632363 | 1.440194 |
| GO:0006913\_nucleocytoplasmic\_transport | 71 | 1 | 1.351232 | -0.277787 | 363 | 405.313402 | 522.94 | 640.566598 | 1.440606 |
| GO:0009100\_glycoprotein\_metabolic\_process | 71 | 1 | 1.351232 | -0.277787 | 363 | 405.313402 | 522.94 | 640.566598 | 1.440606 |
| GO:0033674\_positive\_regulation\_of\_kinase\_activity | 71 | 1 | 1.351232 | -0.277787 | 363 | 405.313402 | 522.94 | 640.566598 | 1.440606 |
| GO:0051169\_nuclear\_transport | 72 | 1 | 1.332465 | -0.273688 | 365 | 408.650833 | 526.63 | 644.609167 | 1.442822 |
| GO:0051347\_positive\_regulation\_of\_transferase\_activity | 72 | 1 | 1.332465 | -0.273688 | 365 | 408.650833 | 526.63 | 644.609167 | 1.442822 |
| GO:0006163\_purine\_nucleotide\_metabolic\_process | 73 | 1 | 1.314212 | -0.269669 | 367 | 411.918006 | 530.21 | 648.501994 | 1.444714 |
| GO:0006936\_muscle\_contraction | 73 | 1 | 1.314212 | -0.269669 | 367 | 411.918006 | 530.21 | 648.501994 | 1.444714 |
| GO:0045944\_positive\_regulation\_of\_transcription\_from\_RNA\_polymerase\_II\_promoter | 269 | 3 | 1.069935 | -0.269051 | 368 | 412.196089 | 530.45 | 648.703911 | 1.441440 |
| GO:0048771\_tissue\_remodeling | 74 | 1 | 1.296453 | -0.265728 | 369 | 413.863608 | 532.32 | 650.776392 | 1.442602 |
| GO:0044093\_positive\_regulation\_of\_molecular\_function | 173 | 2 | 1.109104 | -0.264465 | 370 | 414.925156 | 533.52 | 652.114844 | 1.441946 |
| GO:0051641\_cellular\_localization | 370 | 4 | 1.037162 | -0.262671 | 371 | 415.162972 | 533.76 | 652.357028 | 1.438706 |
| GO:0007281\_germ\_cell\_development | 75 | 1 | 1.279167 | -0.261863 | 373 | 416.445159 | 535.19 | 653.934841 | 1.434826 |
| GO:0051050\_positive\_regulation\_of\_transport | 75 | 1 | 1.279167 | -0.261863 | 373 | 416.445159 | 535.19 | 653.934841 | 1.434826 |
| GO:0003012\_muscle\_system\_process | 76 | 1 | 1.262336 | -0.258072 | 375 | 419.635068 | 538.49 | 657.344932 | 1.435973 |
| GO:0034621\_cellular\_macromolecular\_complex\_subunit\_organization | 76 | 1 | 1.262336 | -0.258072 | 375 | 419.635068 | 538.49 | 657.344932 | 1.435973 |
| GO:0001890\_placenta\_development | 77 | 1 | 1.245942 | -0.254352 | 376 | 421.280071 | 540.27 | 659.259929 | 1.436888 |
| GO:0043069\_negative\_regulation\_of\_programmed\_cell\_death | 179 | 2 | 1.071927 | -0.249732 | 378 | 425.233666 | 544.24 | 663.246334 | 1.439788 |
| GO:0060548\_negative\_regulation\_of\_cell\_death | 179 | 2 | 1.071927 | -0.249732 | 378 | 425.233666 | 544.24 | 663.246334 | 1.439788 |
| GO:0015674\_di-\_\_tri-valent\_inorganic\_cation\_transport | 79 | 1 | 1.214399 | -0.247119 | 379 | 427.656927 | 546.46 | 665.263073 | 1.441847 |
| GO:0000278\_mitotic\_cell\_cycle | 80 | 1 | 1.199219 | -0.243603 | 381 | 430.241430 | 549.23 | 668.218570 | 1.441549 |
| GO:0044092\_negative\_regulation\_of\_molecular\_function | 80 | 1 | 1.199219 | -0.243603 | 381 | 430.241430 | 549.23 | 668.218570 | 1.441549 |
| GO:0021700\_developmental\_maturation | 81 | 1 | 1.184414 | -0.240151 | 382 | 431.471534 | 550.53 | 669.588466 | 1.441178 |
| GO:0007411\_axon\_guidance | 82 | 1 | 1.169970 | -0.236762 | 384 | 434.603569 | 553.78 | 672.956431 | 1.442135 |
| GO:0008202\_steroid\_metabolic\_process | 82 | 1 | 1.169970 | -0.236762 | 384 | 434.603569 | 553.78 | 672.956431 | 1.442135 |
| GO:0048513\_organ\_development | 1365 | 14 | 0.983974 | -0.234036 | 385 | 435.525916 | 554.82 | 674.114084 | 1.441091 |
| GO:0007017\_microtubule-based\_process | 83 | 1 | 1.155873 | -0.233434 | 386 | 438.326646 | 557.86 | 677.393354 | 1.445233 |
| GO:0006605\_protein\_targeting | 86 | 1 | 1.115552 | -0.223800 | 388 | 444.297013 | 563.85 | 683.402987 | 1.453222 |
| GO:0034641\_cellular\_nitrogen\_compound\_metabolic\_process | 86 | 1 | 1.115552 | -0.223800 | 388 | 444.297013 | 563.85 | 683.402987 | 1.453222 |
| GO:0007178\_transmembrane\_receptor\_protein\_serine\_threonine\_kinase\_signaling\_pathway | 87 | 1 | 1.102730 | -0.220702 | 391 | 447.206880 | 566.86 | 686.513120 | 1.449770 |
| GO:0016337\_cell-cell\_adhesion | 87 | 1 | 1.102730 | -0.220702 | 391 | 447.206880 | 566.86 | 686.513120 | 1.449770 |
| GO:0050778\_positive\_regulation\_of\_immune\_response | 87 | 1 | 1.102730 | -0.220702 | 391 | 447.206880 | 566.86 | 686.513120 | 1.449770 |
| GO:0040011\_locomotion | 295 | 3 | 0.975636 | -0.220142 | 392 | 448.441351 | 568.04 | 687.638649 | 1.449082 |
| GO:0001503\_ossification | 88 | 1 | 1.090199 | -0.217657 | 394 | 449.778485 | 569.46 | 689.141515 | 1.445330 |
| GO:0048754\_branching\_morphogenesis\_of\_a\_tube | 88 | 1 | 1.090199 | -0.217657 | 394 | 449.778485 | 569.46 | 689.141515 | 1.445330 |
| GO:0007507\_heart\_development | 195 | 2 | 0.983974 | -0.214601 | 395 | 451.505246 | 570.97 | 690.434754 | 1.445494 |
| GO:0030324\_lung\_development | 90 | 1 | 1.065972 | -0.211723 | 397 | 453.785125 | 573.31 | 692.834875 | 1.444106 |
| GO:0042113\_B\_cell\_activation | 90 | 1 | 1.065972 | -0.211723 | 397 | 453.785125 | 573.31 | 692.834875 | 1.444106 |
| GO:0008544\_epidermis\_development | 91 | 1 | 1.054258 | -0.208832 | 398 | 456.232975 | 575.63 | 695.027025 | 1.446307 |
| GO:0030217\_T\_cell\_differentiation | 92 | 1 | 1.042799 | -0.205990 | 400 | 459.004905 | 578.23 | 697.455095 | 1.445575 |
| GO:0030323\_respiratory\_tube\_development | 92 | 1 | 1.042799 | -0.205990 | 400 | 459.004905 | 578.23 | 697.455095 | 1.445575 |
| GO:0055066\_di-\_\_tri-valent\_inorganic\_cation\_homeostasis | 93 | 1 | 1.031586 | -0.203195 | 402 | 460.687322 | 579.88 | 699.072678 | 1.442488 |
| GO:0065003\_macromolecular\_complex\_assembly | 93 | 1 | 1.031586 | -0.203195 | 402 | 460.687322 | 579.88 | 699.072678 | 1.442488 |
| GO:0045893\_positive\_regulation\_of\_transcription\_\_DNA-dependent | 306 | 3 | 0.940564 | -0.202199 | 404 | 461.137751 | 580.34 | 699.542249 | 1.436485 |
| GO:0051254\_positive\_regulation\_of\_RNA\_metabolic\_process | 306 | 3 | 0.940564 | -0.202199 | 404 | 461.137751 | 580.34 | 699.542249 | 1.436485 |
| GO:0006753\_nucleoside\_phosphate\_metabolic\_process | 94 | 1 | 1.020612 | -0.200448 | 407 | 464.460534 | 583.65 | 702.839466 | 1.434029 |
| GO:0008610\_lipid\_biosynthetic\_process | 94 | 1 | 1.020612 | -0.200448 | 407 | 464.460534 | 583.65 | 702.839466 | 1.434029 |
| GO:0009117\_nucleotide\_metabolic\_process | 94 | 1 | 1.020612 | -0.200448 | 407 | 464.460534 | 583.65 | 702.839466 | 1.434029 |
| GO:0051707\_response\_to\_other\_organism | 95 | 1 | 1.009868 | -0.197746 | 408 | 465.721980 | 585.08 | 704.438020 | 1.434020 |
| GO:0022607\_cellular\_component\_assembly | 204 | 2 | 0.940564 | -0.197186 | 409 | 466.264042 | 585.69 | 705.115958 | 1.432005 |
| GO:0060541\_respiratory\_system\_development | 98 | 1 | 0.978954 | -0.189903 | 410 | 472.953706 | 592.09 | 711.226294 | 1.444122 |
| GO:0007398\_ectoderm\_development | 99 | 1 | 0.969066 | -0.187374 | 412 | 474.595569 | 593.83 | 713.064431 | 1.441335 |
| GO:0060348\_bone\_development | 99 | 1 | 0.969066 | -0.187374 | 412 | 474.595569 | 593.83 | 713.064431 | 1.441335 |
| GO:0009968\_negative\_regulation\_of\_signal\_transduction | 103 | 1 | 0.931432 | -0.177655 | 413 | 477.918064 | 597.35 | 716.781936 | 1.446368 |
| GO:0010033\_response\_to\_organic\_substance | 216 | 2 | 0.888310 | -0.176240 | 414 | 478.121949 | 597.58 | 717.038051 | 1.443430 |
| GO:0055086\_nucleobase\_\_nucleoside\_and\_nucleotide\_metabolic\_process | 104 | 1 | 0.922476 | -0.175321 | 415 | 478.915848 | 598.31 | 717.704152 | 1.441711 |
| GO:0048872\_homeostasis\_of\_number\_of\_cells | 105 | 1 | 0.913690 | -0.173024 | 416 | 479.910042 | 599.24 | 718.569958 | 1.440481 |
| GO:0010817\_regulation\_of\_hormone\_levels | 106 | 1 | 0.905071 | -0.170763 | 417 | 481.492571 | 600.8 | 720.107429 | 1.440767 |
| GO:0051253\_negative\_regulation\_of\_RNA\_metabolic\_process | 220 | 2 | 0.872159 | -0.169783 | 418 | 481.797661 | 601.08 | 720.362339 | 1.437990 |
| GO:0030099\_myeloid\_cell\_differentiation | 108 | 1 | 0.888310 | -0.166344 | 419 | 485.096889 | 604.39 | 723.683111 | 1.442458 |
| GO:0010648\_negative\_regulation\_of\_cell\_communication | 110 | 1 | 0.872159 | -0.162061 | 421 | 487.503584 | 607.09 | 726.676416 | 1.442019 |
| GO:0055080\_cation\_homeostasis | 110 | 1 | 0.872159 | -0.162061 | 421 | 487.503584 | 607.09 | 726.676416 | 1.442019 |
| GO:0051179\_localization | 1058 | 10 | 0.906782 | -0.159411 | 422 | 488.412813 | 607.91 | 727.407187 | 1.440545 |
| GO:0045941\_positive\_regulation\_of\_transcription | 338 | 3 | 0.851516 | -0.157727 | 423 | 490.914879 | 610.23 | 729.545121 | 1.442624 |
| GO:0046649\_lymphocyte\_activation | 228 | 2 | 0.841557 | -0.157593 | 424 | 491.942430 | 611.09 | 730.237570 | 1.441250 |
| GO:0007167\_enzyme\_linked\_receptor\_protein\_signaling\_pathway | 229 | 2 | 0.837882 | -0.156134 | 425 | 492.933108 | 612.03 | 731.126892 | 1.440071 |
| GO:0016070\_RNA\_metabolic\_process | 658 | 6 | 0.874810 | -0.154113 | 426 | 495.023132 | 613.88 | 732.736868 | 1.441033 |
| GO:0009607\_response\_to\_biotic\_stimulus | 114 | 1 | 0.841557 | -0.153879 | 427 | 495.570422 | 614.6 | 733.629578 | 1.439344 |
| GO:0051649\_establishment\_of\_localization\_in\_cell | 342 | 3 | 0.841557 | -0.152883 | 428 | 496.344987 | 615.39 | 734.435013 | 1.437827 |
| GO:0048584\_positive\_regulation\_of\_response\_to\_stimulus | 115 | 1 | 0.834239 | -0.151910 | 429 | 497.309444 | 616.35 | 735.390556 | 1.436713 |
| GO:0046483\_heterocycle\_metabolic\_process | 116 | 1 | 0.827047 | -0.149970 | 430 | 499.031373 | 617.87 | 736.708627 | 1.436907 |
| GO:0010628\_positive\_regulation\_of\_gene\_expression | 346 | 3 | 0.831828 | -0.148182 | 431 | 499.935276 | 618.66 | 737.384724 | 1.435406 |
| GO:0043933\_macromolecular\_complex\_subunit\_organization | 117 | 1 | 0.819979 | -0.148059 | 432 | 500.259404 | 618.92 | 737.580596 | 1.432685 |
| GO:0001501\_skeletal\_system\_development | 236 | 2 | 0.813030 | -0.146299 | 433 | 500.571424 | 619.3 | 738.028576 | 1.430254 |
| GO:0007049\_cell\_cycle | 238 | 2 | 0.806197 | -0.143606 | 434 | 503.218034 | 621.97 | 740.721966 | 1.433111 |
| GO:0048523\_negative\_regulation\_of\_cellular\_process | 774 | 7 | 0.867652 | -0.143221 | 435 | 503.528498 | 622.21 | 740.891502 | 1.430368 |
| GO:0045935\_positive\_regulation\_of\_nucleobase\_\_nucleoside\_\_nucleotide\_and\_nucleic\_acid\_metabolic\_process | 352 | 3 | 0.817649 | -0.141389 | 436 | 504.508868 | 623.02 | 741.531132 | 1.428945 |
| GO:0006886\_intracellular\_protein\_transport | 122 | 1 | 0.786373 | -0.138917 | 438 | 508.885327 | 627.15 | 745.414673 | 1.431849 |
| GO:0030001\_metal\_ion\_transport | 122 | 1 | 0.786373 | -0.138917 | 438 | 508.885327 | 627.15 | 745.414673 | 1.431849 |
| GO:0001763\_morphogenesis\_of\_a\_branching\_structure | 125 | 1 | 0.767500 | -0.133744 | 439 | 511.553925 | 629.7 | 747.846075 | 1.434396 |
| GO:0051173\_positive\_regulation\_of\_nitrogen\_compound\_metabolic\_process | 361 | 3 | 0.797265 | -0.131755 | 440 | 512.329228 | 630.36 | 748.390772 | 1.432636 |
| GO:0048518\_positive\_regulation\_of\_biological\_process | 995 | 9 | 0.867776 | -0.131388 | 441 | 512.509002 | 630.52 | 748.530998 | 1.429751 |
| GO:0045321\_leukocyte\_activation | 248 | 2 | 0.773690 | -0.130881 | 442 | 513.204410 | 631.12 | 749.035590 | 1.427873 |
| GO:0048522\_positive\_regulation\_of\_cellular\_process | 895 | 8 | 0.857542 | -0.129647 | 443 | 513.444799 | 631.3 | 749.155201 | 1.425056 |
| GO:0045597\_positive\_regulation\_of\_cell\_differentiation | 128 | 1 | 0.749512 | -0.128791 | 444 | 514.840587 | 632.51 | 750.179413 | 1.424572 |
| GO:0010941\_regulation\_of\_cell\_death | 365 | 3 | 0.788527 | -0.127677 | 446 | 515.755413 | 633.36 | 750.964587 | 1.420090 |
| GO:0043067\_regulation\_of\_programmed\_cell\_death | 365 | 3 | 0.788527 | -0.127677 | 446 | 515.755413 | 633.36 | 750.964587 | 1.420090 |
| GO:0008104\_protein\_localization | 251 | 2 | 0.764442 | -0.127291 | 447 | 516.125344 | 633.69 | 751.254656 | 1.417651 |
| GO:0050776\_regulation\_of\_immune\_response | 130 | 1 | 0.737981 | -0.125605 | 448 | 518.595983 | 635.9 | 753.204017 | 1.419420 |
| GO:0010557\_positive\_regulation\_of\_macromolecule\_biosynthetic\_process | 371 | 3 | 0.775775 | -0.121785 | 449 | 521.769006 | 638.75 | 755.730994 | 1.422606 |
| GO:0050793\_regulation\_of\_developmental\_process | 703 | 6 | 0.818812 | -0.117592 | 450 | 525.779705 | 642.46 | 759.140295 | 1.427689 |
| GO:0007166\_cell\_surface\_receptor\_linked\_signal\_transduction | 597 | 5 | 0.803497 | -0.116843 | 451 | 526.135498 | 642.8 | 759.464502 | 1.425277 |
| GO:0001775\_cell\_activation | 262 | 2 | 0.732347 | -0.114953 | 452 | 528.073618 | 644.56 | 761.046382 | 1.426018 |
| GO:0044255\_cellular\_lipid\_metabolic\_process | 264 | 2 | 0.726799 | -0.112843 | 453 | 529.204330 | 645.58 | 761.955670 | 1.425121 |
| GO:0007169\_transmembrane\_receptor\_protein\_tyrosine\_kinase\_signaling\_pathway | 139 | 1 | 0.690198 | -0.112326 | 454 | 530.003048 | 646.31 | 762.616952 | 1.423590 |
| GO:0016044\_membrane\_organization | 140 | 1 | 0.685268 | -0.110950 | 455 | 530.292166 | 646.64 | 762.987834 | 1.421187 |
| GO:0031328\_positive\_regulation\_of\_cellular\_biosynthetic\_process | 387 | 3 | 0.743702 | -0.107308 | 456 | 531.827525 | 648.11 | 764.392475 | 1.421294 |
| GO:0006810\_transport | 718 | 6 | 0.801706 | -0.107229 | 457 | 532.013460 | 648.24 | 764.466540 | 1.418468 |
| GO:0035239\_tube\_morphogenesis | 143 | 1 | 0.670892 | -0.106935 | 458 | 532.329272 | 648.56 | 764.790728 | 1.416070 |
| GO:0045934\_negative\_regulation\_of\_nucleobase\_\_nucleoside\_\_nucleotide\_and\_nucleic\_acid\_metabolic\_process | 270 | 2 | 0.710648 | -0.106740 | 459 | 532.639571 | 648.84 | 765.040429 | 1.413595 |
| GO:0009891\_positive\_regulation\_of\_biosynthetic\_process | 388 | 3 | 0.741785 | -0.106460 | 460 | 532.870200 | 648.99 | 765.109800 | 1.410848 |
| GO:0051172\_negative\_regulation\_of\_nitrogen\_compound\_metabolic\_process | 271 | 2 | 0.708026 | -0.105755 | 461 | 533.178878 | 649.27 | 765.361122 | 1.408395 |
| GO:0007186\_G-protein\_coupled\_receptor\_protein\_signaling\_pathway | 144 | 1 | 0.666233 | -0.105632 | 462 | 533.954957 | 649.96 | 765.965043 | 1.406840 |
| GO:0006812\_cation\_transport | 146 | 1 | 0.657106 | -0.103080 | 463 | 536.100582 | 651.86 | 767.619418 | 1.407905 |
| GO:0033036\_macromolecule\_localization | 274 | 2 | 0.700274 | -0.102855 | 464 | 536.728997 | 652.37 | 768.011003 | 1.405970 |
| GO:0022603\_regulation\_of\_anatomical\_structure\_morphogenesis | 147 | 1 | 0.652636 | -0.101829 | 465 | 537.363417 | 652.87 | 768.376583 | 1.404022 |
| GO:0006807\_nitrogen\_compound\_metabolic\_process | 1147 | 10 | 0.836421 | -0.101079 | 466 | 537.482888 | 652.95 | 768.417112 | 1.401180 |
| GO:0002684\_positive\_regulation\_of\_immune\_system\_process | 148 | 1 | 0.648226 | -0.100595 | 468 | 538.220335 | 653.65 | 769.079665 | 1.396688 |
| GO:0043085\_positive\_regulation\_of\_catalytic\_activity | 148 | 1 | 0.648226 | -0.100595 | 468 | 538.220335 | 653.65 | 769.079665 | 1.396688 |
| GO:0051234\_establishment\_of\_localization | 729 | 6 | 0.789609 | -0.100145 | 469 | 538.391978 | 653.78 | 769.168022 | 1.393987 |
| GO:0007517\_muscle\_organ\_development | 153 | 1 | 0.627042 | -0.094668 | 470 | 541.826979 | 656.73 | 771.633021 | 1.397298 |
| GO:0007268\_synaptic\_transmission | 154 | 1 | 0.622971 | -0.093529 | 471 | 542.726271 | 657.66 | 772.593729 | 1.396306 |
| GO:0006629\_lipid\_metabolic\_process | 285 | 2 | 0.673246 | -0.092882 | 472 | 543.075336 | 658.03 | 772.984664 | 1.394131 |
| GO:0008285\_negative\_regulation\_of\_cell\_proliferation | 155 | 1 | 0.618952 | -0.092405 | 474 | 543.800285 | 658.78 | 773.759715 | 1.389831 |
| GO:0022402\_cell\_cycle\_process | 155 | 1 | 0.618952 | -0.092405 | 474 | 543.800285 | 658.78 | 773.759715 | 1.389831 |
| GO:0007417\_central\_nervous\_system\_development | 287 | 2 | 0.668554 | -0.091174 | 475 | 544.007422 | 659.0 | 773.992578 | 1.387368 |
| GO:0009888\_tissue\_development | 525 | 4 | 0.730952 | -0.089628 | 476 | 545.338533 | 660.01 | 774.681467 | 1.386576 |
| GO:0048514\_blood\_vessel\_morphogenesis | 158 | 1 | 0.607199 | -0.089120 | 477 | 546.332724 | 661.02 | 775.707276 | 1.385786 |
| GO:0048519\_negative\_regulation\_of\_biological\_process | 859 | 7 | 0.781796 | -0.087167 | 478 | 547.102656 | 661.57 | 776.037344 | 1.384038 |
| GO:0009058\_biosynthetic\_process | 1175 | 10 | 0.816489 | -0.086902 | 479 | 547.671141 | 662.03 | 776.388859 | 1.382109 |
| GO:0045595\_regulation\_of\_cell\_differentiation | 295 | 2 | 0.650424 | -0.084649 | 480 | 549.270074 | 663.42 | 777.569926 | 1.382125 |
| GO:0042110\_T\_cell\_activation | 163 | 1 | 0.588574 | -0.083924 | 481 | 550.312984 | 664.33 | 778.347016 | 1.381143 |
| GO:0042592\_homeostatic\_process | 419 | 3 | 0.686903 | -0.083089 | 482 | 550.656565 | 664.58 | 778.503435 | 1.378797 |
| GO:0006259\_DNA\_metabolic\_process | 165 | 1 | 0.581439 | -0.081939 | 483 | 552.013457 | 665.92 | 779.826543 | 1.378716 |
| GO:0051049\_regulation\_of\_transport | 167 | 1 | 0.574476 | -0.080003 | 484 | 553.956866 | 667.65 | 781.343134 | 1.379442 |
| GO:0008283\_cell\_proliferation | 544 | 4 | 0.705423 | -0.078030 | 485 | 554.694066 | 668.34 | 781.985934 | 1.378021 |
| GO:0010604\_positive\_regulation\_of\_macromolecule\_metabolic\_process | 433 | 3 | 0.664694 | -0.074200 | 487 | 560.017217 | 672.87 | 785.722783 | 1.381663 |
| GO:0012501\_programmed\_cell\_death | 433 | 3 | 0.664694 | -0.074200 | 487 | 560.017217 | 672.87 | 785.722783 | 1.381663 |
| GO:0006357\_regulation\_of\_transcription\_from\_RNA\_polymerase\_II\_promoter | 435 | 3 | 0.661638 | -0.073005 | 488 | 560.548003 | 673.33 | 786.111997 | 1.379775 |
| GO:0000122\_negative\_regulation\_of\_transcription\_from\_RNA\_polymerase\_II\_promoter | 175 | 1 | 0.548214 | -0.072740 | 490 | 561.531412 | 674.28 | 787.028588 | 1.376082 |
| GO:0015031\_protein\_transport | 175 | 1 | 0.548214 | -0.072740 | 490 | 561.531412 | 674.28 | 787.028588 | 1.376082 |
| GO:0043066\_negative\_regulation\_of\_apoptosis | 176 | 1 | 0.545099 | -0.071882 | 491 | 562.673764 | 675.23 | 787.786236 | 1.375214 |
| GO:0006464\_protein\_modification\_process | 439 | 3 | 0.655609 | -0.070670 | 492 | 563.039296 | 675.52 | 788.000704 | 1.373008 |
| GO:0006139\_nucleobase\_\_nucleoside\_\_nucleotide\_and\_nucleic\_acid\_metabolic\_process | 1002 | 8 | 0.765968 | -0.070218 | 493 | 563.148070 | 675.62 | 788.091930 | 1.370426 |
| GO:0031325\_positive\_regulation\_of\_cellular\_metabolic\_process | 442 | 3 | 0.651160 | -0.068965 | 494 | 564.710209 | 676.8 | 788.889791 | 1.370040 |
| GO:0045184\_establishment\_of\_protein\_localization | 180 | 1 | 0.532986 | -0.068560 | 495 | 565.196828 | 677.3 | 789.403172 | 1.368283 |
| GO:0006366\_transcription\_from\_RNA\_polymerase\_II\_promoter | 444 | 3 | 0.648226 | -0.067849 | 497 | 565.633043 | 677.69 | 789.746957 | 1.363561 |
| GO:0008219\_cell\_death | 444 | 3 | 0.648226 | -0.067849 | 497 | 565.633043 | 677.69 | 789.746957 | 1.363561 |
| GO:0010926\_anatomical\_structure\_formation | 447 | 3 | 0.643876 | -0.066208 | 498 | 567.652557 | 679.47 | 791.287443 | 1.364398 |
| GO:0010467\_gene\_expression | 905 | 7 | 0.742058 | -0.065630 | 499 | 568.424249 | 680.13 | 791.835751 | 1.362986 |
| GO:0006996\_organelle\_organization | 449 | 3 | 0.641008 | -0.065134 | 500 | 569.310168 | 680.84 | 792.369832 | 1.361680 |
| GO:0008152\_metabolic\_process | 2133 | 19 | 0.854577 | -0.064728 | 501 | 569.577362 | 681.05 | 792.522638 | 1.359381 |
| GO:0007010\_cytoskeleton\_organization | 185 | 1 | 0.518581 | -0.064634 | 502 | 569.805170 | 681.31 | 792.814830 | 1.357191 |
| GO:0016265\_death | 450 | 3 | 0.639583 | -0.064604 | 503 | 569.939358 | 681.4 | 792.860642 | 1.354672 |
| GO:0006811\_ion\_transport | 186 | 1 | 0.515793 | -0.063878 | 504 | 571.040690 | 682.37 | 793.699310 | 1.353909 |
| GO:0019226\_transmission\_of\_nerve\_impulse | 189 | 1 | 0.507606 | -0.061665 | 505 | 572.757462 | 683.74 | 794.722538 | 1.353941 |
| GO:0010605\_negative\_regulation\_of\_macromolecule\_metabolic\_process | 331 | 2 | 0.579683 | -0.060523 | 506 | 574.640699 | 685.18 | 795.719301 | 1.354111 |
| GO:0009893\_positive\_regulation\_of\_metabolic\_process | 458 | 3 | 0.628412 | -0.060502 | 508 | 575.031041 | 685.49 | 795.948959 | 1.349390 |
| GO:0043412\_biopolymer\_modification | 458 | 3 | 0.628412 | -0.060502 | 508 | 575.031041 | 685.49 | 795.948959 | 1.349390 |
| GO:0031324\_negative\_regulation\_of\_cellular\_metabolic\_process | 332 | 2 | 0.577937 | -0.059959 | 509 | 575.247574 | 685.67 | 796.092426 | 1.347092 |
| GO:0009605\_response\_to\_external\_stimulus | 339 | 2 | 0.566003 | -0.056153 | 510 | 578.237054 | 688.37 | 798.502946 | 1.349745 |
| GO:0050801\_ion\_homeostasis | 197 | 1 | 0.486992 | -0.056149 | 511 | 578.806020 | 688.89 | 798.973980 | 1.348121 |
| GO:0051252\_regulation\_of\_RNA\_metabolic\_process | 590 | 4 | 0.650424 | -0.055386 | 512 | 580.214184 | 690.01 | 799.805816 | 1.347676 |
| GO:0044237\_cellular\_metabolic\_process | 1974 | 17 | 0.826209 | -0.053193 | 513 | 581.606411 | 691.18 | 800.753589 | 1.347329 |
| GO:0001568\_blood\_vessel\_development | 203 | 1 | 0.472599 | -0.052352 | 514 | 582.373963 | 691.91 | 801.446037 | 1.346128 |
| GO:0009892\_negative\_regulation\_of\_metabolic\_process | 348 | 2 | 0.551365 | -0.051604 | 515 | 583.026064 | 692.46 | 801.893936 | 1.344583 |
| GO:0006955\_immune\_response | 205 | 1 | 0.467988 | -0.051147 | 516 | 583.708207 | 693.06 | 802.411793 | 1.343140 |
| GO:0001944\_vasculature\_development | 208 | 1 | 0.461238 | -0.049393 | 518 | 584.884755 | 694.1 | 803.315245 | 1.339961 |
| GO:0008284\_positive\_regulation\_of\_cell\_proliferation | 208 | 1 | 0.461238 | -0.049393 | 518 | 584.884755 | 694.1 | 803.315245 | 1.339961 |
| GO:0050794\_regulation\_of\_cellular\_process | 2190 | 19 | 0.832334 | -0.047625 | 519 | 585.505549 | 694.58 | 803.654451 | 1.338304 |
| GO:0035295\_tube\_development | 212 | 1 | 0.452535 | -0.047151 | 520 | 586.055106 | 695.0 | 803.944894 | 1.336538 |
| GO:0042981\_regulation\_of\_apoptosis | 360 | 2 | 0.532986 | -0.046089 | 521 | 586.420474 | 695.27 | 804.119526 | 1.334491 |
| GO:0044238\_primary\_metabolic\_process | 1905 | 16 | 0.805774 | -0.044991 | 522 | 586.957718 | 695.72 | 804.482282 | 1.332797 |
| GO:0048583\_regulation\_of\_response\_to\_stimulus | 217 | 1 | 0.442108 | -0.044496 | 523 | 587.774278 | 696.36 | 804.945722 | 1.331472 |
| GO:0045892\_negative\_regulation\_of\_transcription\_\_DNA-dependent | 218 | 1 | 0.440080 | -0.043984 | 524 | 588.027816 | 696.6 | 805.172184 | 1.329389 |
| GO:0001701\_in\_utero\_embryonic\_development | 221 | 1 | 0.434106 | -0.042483 | 525 | 589.976773 | 698.16 | 806.343227 | 1.329829 |
| GO:0019222\_regulation\_of\_metabolic\_process | 1088 | 8 | 0.705423 | -0.041073 | 526 | 590.684681 | 698.74 | 806.795319 | 1.328403 |
| GO:0002682\_regulation\_of\_immune\_system\_process | 228 | 1 | 0.420779 | -0.039183 | 527 | 591.861874 | 699.62 | 807.378126 | 1.327552 |
| GO:0007154\_cell\_communication | 1096 | 8 | 0.700274 | -0.038993 | 528 | 592.135563 | 699.78 | 807.424437 | 1.325341 |
| GO:0019219\_regulation\_of\_nucleobase\_\_nucleoside\_\_nucleotide\_and\_nucleic\_acid\_metabolic\_process | 757 | 5 | 0.633669 | -0.038566 | 529 | 592.558575 | 700.12 | 807.681425 | 1.323478 |
| GO:0007420\_brain\_development | 231 | 1 | 0.415314 | -0.037851 | 530 | 592.994141 | 700.44 | 807.885859 | 1.321585 |
| GO:0009887\_organ\_morphogenesis | 642 | 4 | 0.597741 | -0.037103 | 531 | 593.251860 | 700.64 | 808.028140 | 1.319473 |
| GO:0043687\_post-translational\_protein\_modification | 384 | 2 | 0.499674 | -0.036716 | 532 | 593.865535 | 701.21 | 808.554465 | 1.318064 |
| GO:0006468\_protein\_amino\_acid\_phosphorylation | 237 | 1 | 0.404800 | -0.035323 | 533 | 595.734346 | 702.65 | 809.565654 | 1.318293 |
| GO:0051171\_regulation\_of\_nitrogen\_compound\_metabolic\_process | 771 | 5 | 0.622163 | -0.034773 | 534 | 596.311424 | 703.08 | 809.848576 | 1.316629 |
| GO:0042127\_regulation\_of\_cell\_proliferation | 393 | 2 | 0.488232 | -0.033698 | 535 | 597.193973 | 703.79 | 810.386027 | 1.315495 |
| GO:0010468\_regulation\_of\_gene\_expression | 778 | 5 | 0.616565 | -0.033005 | 536 | 597.500225 | 704.0 | 810.499775 | 1.313433 |
| GO:0009987\_cellular\_process | 3868 | 37 | 0.917706 | -0.031993 | 537 | 598.209153 | 704.62 | 811.030847 | 1.312142 |
| GO:0031323\_regulation\_of\_cellular\_metabolic\_process | 1015 | 7 | 0.661638 | -0.031777 | 538 | 598.262402 | 704.66 | 811.057598 | 1.309777 |
| GO:0050789\_regulation\_of\_biological\_process | 2357 | 20 | 0.814064 | -0.031712 | 539 | 598.332759 | 704.7 | 811.067241 | 1.307421 |
| GO:0032879\_regulation\_of\_localization | 248 | 1 | 0.386845 | -0.031127 | 540 | 598.926662 | 705.14 | 811.353338 | 1.305815 |
| GO:0007267\_cell-cell\_signaling | 252 | 1 | 0.380704 | -0.029730 | 541 | 599.542049 | 705.75 | 811.957951 | 1.304529 |
| GO:0016481\_negative\_regulation\_of\_transcription | 253 | 1 | 0.379200 | -0.029391 | 542 | 600.079763 | 706.18 | 812.280237 | 1.302915 |
| GO:0048878\_chemical\_homeostasis | 254 | 1 | 0.377707 | -0.029056 | 543 | 600.405181 | 706.46 | 812.514819 | 1.301031 |
| GO:0042221\_response\_to\_chemical\_stimulus | 409 | 2 | 0.469132 | -0.028912 | 544 | 600.569595 | 706.57 | 812.570405 | 1.298842 |
| GO:0007165\_signal\_transduction | 915 | 6 | 0.629098 | -0.028636 | 545 | 600.970483 | 706.85 | 812.729517 | 1.296972 |
| GO:0009966\_regulation\_of\_signal\_transduction | 256 | 1 | 0.374756 | -0.028397 | 546 | 601.220871 | 707.08 | 812.939129 | 1.295018 |
| GO:0044249\_cellular\_biosynthetic\_process | 1150 | 8 | 0.667391 | -0.027190 | 547 | 602.402728 | 707.93 | 813.457272 | 1.294205 |
| GO:0010629\_negative\_regulation\_of\_gene\_expression | 262 | 1 | 0.366174 | -0.026509 | 548 | 603.280290 | 708.67 | 814.059710 | 1.293193 |
| GO:0044267\_cellular\_protein\_metabolic\_process | 559 | 3 | 0.514870 | -0.025754 | 549 | 603.933414 | 709.25 | 814.566586 | 1.291894 |
| GO:0065008\_regulation\_of\_biological\_quality | 693 | 4 | 0.553752 | -0.024693 | 550 | 604.508032 | 709.69 | 814.871968 | 1.290345 |
| GO:0006915\_apoptosis | 427 | 2 | 0.449356 | -0.024308 | 551 | 604.829254 | 709.93 | 815.030746 | 1.288439 |
| GO:0009790\_embryonic\_development | 567 | 3 | 0.507606 | -0.024018 | 552 | 605.027018 | 710.16 | 815.292982 | 1.286522 |
| GO:0065007\_biological\_regulation | 2593 | 22 | 0.813970 | -0.023877 | 553 | 605.224631 | 710.37 | 815.515369 | 1.284575 |
| GO:0051716\_cellular\_response\_to\_stimulus | 273 | 1 | 0.351419 | -0.023372 | 554 | 605.512076 | 710.61 | 815.707924 | 1.282690 |
| GO:0010558\_negative\_regulation\_of\_macromolecule\_biosynthetic\_process | 274 | 1 | 0.350137 | -0.023106 | 555 | 606.022597 | 711.03 | 816.037403 | 1.281135 |
| GO:0006355\_regulation\_of\_transcription\_\_DNA-dependent | 575 | 3 | 0.500543 | -0.022391 | 556 | 606.778101 | 711.58 | 816.381899 | 1.279820 |
| GO:0048646\_anatomical\_structure\_formation\_involved\_in\_morphogenesis | 277 | 1 | 0.346345 | -0.022326 | 557 | 607.242658 | 711.93 | 816.617342 | 1.278151 |
| GO:0007610\_behavior | 279 | 1 | 0.343862 | -0.021821 | 558 | 607.616205 | 712.17 | 816.723795 | 1.276290 |
| GO:0031327\_negative\_regulation\_of\_cellular\_biosynthetic\_process | 282 | 1 | 0.340204 | -0.021085 | 559 | 607.792755 | 712.39 | 816.987245 | 1.274401 |
| GO:0009890\_negative\_regulation\_of\_biosynthetic\_process | 284 | 1 | 0.337808 | -0.020608 | 560 | 608.639529 | 713.09 | 817.540471 | 1.273375 |
| GO:0051239\_regulation\_of\_multicellular\_organismal\_process | 587 | 3 | 0.490311 | -0.020142 | 561 | 608.951991 | 713.33 | 817.708009 | 1.271533 |
| GO:0006351\_transcription\_\_DNA-dependent | 594 | 3 | 0.484533 | -0.018929 | 562 | 609.757920 | 713.98 | 818.202080 | 1.270427 |
| GO:0032774\_RNA\_biosynthetic\_process | 595 | 3 | 0.483718 | -0.018762 | 563 | 609.925785 | 714.09 | 818.254215 | 1.268366 |
| GO:0051094\_positive\_regulation\_of\_developmental\_process | 308 | 1 | 0.311485 | -0.015661 | 564 | 612.269294 | 715.9 | 819.530706 | 1.269326 |
| GO:0010646\_regulation\_of\_cell\_communication | 330 | 1 | 0.290720 | -0.012176 | 565 | 613.893850 | 717.11 | 820.326150 | 1.269221 |
| GO:0019538\_protein\_metabolic\_process | 655 | 3 | 0.439408 | -0.010899 | 566 | 614.969808 | 718.03 | 821.090192 | 1.268604 |
| GO:0034960\_cellular\_biopolymer\_metabolic\_process | 1395 | 9 | 0.618952 | -0.010483 | 567 | 615.512010 | 718.45 | 821.387990 | 1.267108 |
| GO:0080090\_regulation\_of\_primary\_metabolic\_process | 926 | 5 | 0.518021 | -0.010245 | 568 | 615.546918 | 718.47 | 821.393082 | 1.264912 |
| GO:0060255\_regulation\_of\_macromolecule\_metabolic\_process | 936 | 5 | 0.512487 | -0.009423 | 569 | 616.143240 | 718.97 | 821.796760 | 1.263568 |
| GO:0031326\_regulation\_of\_cellular\_biosynthetic\_process | 812 | 4 | 0.472599 | -0.009019 | 570 | 616.346514 | 719.12 | 821.893486 | 1.261614 |
| GO:0045449\_regulation\_of\_transcription | 676 | 3 | 0.425758 | -0.008972 | 571 | 616.346514 | 719.12 | 821.893486 | 1.259405 |
| GO:0009889\_regulation\_of\_biosynthetic\_process | 815 | 4 | 0.470859 | -0.008784 | 572 | 616.389675 | 719.15 | 821.910325 | 1.257255 |
| GO:0043009\_chordate\_embryonic\_development | 365 | 1 | 0.262842 | -0.008150 | 573 | 617.087850 | 719.57 | 822.052150 | 1.255794 |
| GO:0009792\_embryonic\_development\_ending\_in\_birth\_or\_egg\_hatching | 368 | 1 | 0.260700 | -0.007874 | 574 | 617.174171 | 719.67 | 822.165829 | 1.253780 |
| GO:0006350\_transcription | 701 | 3 | 0.410574 | -0.007094 | 575 | 617.886727 | 720.12 | 822.353273 | 1.252383 |
| GO:0044260\_cellular\_macromolecule\_metabolic\_process | 1447 | 9 | 0.596709 | -0.007035 | 576 | 617.935394 | 720.15 | 822.364606 | 1.250260 |
| GO:0043170\_macromolecule\_metabolic\_process | 1576 | 10 | 0.608740 | -0.006141 | 577 | 618.658196 | 720.66 | 822.661804 | 1.248977 |
| GO:0043283\_biopolymer\_metabolic\_process | 1490 | 9 | 0.579488 | -0.004991 | 578 | 619.342277 | 721.13 | 822.917723 | 1.247630 |
| GO:0007242\_intracellular\_signaling\_cascade | 411 | 1 | 0.233425 | -0.004795 | 579 | 619.565001 | 721.28 | 822.994999 | 1.245734 |
| GO:0010556\_regulation\_of\_macromolecule\_biosynthetic\_process | 745 | 3 | 0.386326 | -0.004655 | 580 | 619.664420 | 721.34 | 823.015580 | 1.243690 |
| GO:0034645\_cellular\_macromolecule\_biosynthetic\_process | 901 | 4 | 0.425916 | -0.004026 | 581 | 619.919726 | 721.52 | 823.120274 | 1.241859 |
| GO:0009059\_macromolecule\_biosynthetic\_process | 910 | 4 | 0.421703 | -0.003701 | 582 | 620.257226 | 721.7 | 823.142774 | 1.240034 |
| GO:0034961\_cellular\_biopolymer\_biosynthetic\_process | 804 | 3 | 0.357976 | -0.002603 | 583 | 621.351949 | 722.33 | 823.308051 | 1.238988 |
| GO:0043284\_biopolymer\_biosynthetic\_process | 807 | 3 | 0.356645 | -0.002526 | 584 | 621.377939 | 722.34 | 823.302061 | 1.236884 |
| GO:0050896\_response\_to\_stimulus | 1107 | 5 | 0.433322 | -0.002051 | 585 | 621.561970 | 722.46 | 823.358030 | 1.234974 |
| GO:0000079\_regulation\_of\_cyclin-dependent\_protein\_kinase\_activity | 7 | 0 | 0.000000 | -0.000000 | 759 | 795.008199 | 892.92 | 990.831801 | 1.176443 |
| GO:0000188\_inactivation\_of\_MAPK\_activity | 7 | 0 | 0.000000 | -0.000000 | 759 | 795.008199 | 892.92 | 990.831801 | 1.176443 |
| GO:0001504\_neurotransmitter\_uptake | 7 | 0 | 0.000000 | -0.000000 | 759 | 795.008199 | 892.92 | 990.831801 | 1.176443 |
| GO:0001556\_oocyte\_maturation | 7 | 0 | 0.000000 | -0.000000 | 759 | 795.008199 | 892.92 | 990.831801 | 1.176443 |
| GO:0001573\_ganglioside\_metabolic\_process | 7 | 0 | 0.000000 | -0.000000 | 759 | 795.008199 | 892.92 | 990.831801 | 1.176443 |
| GO:0001736\_establishment\_of\_planar\_polarity | 7 | 0 | 0.000000 | -0.000000 | 759 | 795.008199 | 892.92 | 990.831801 | 1.176443 |
| GO:0001839\_neural\_plate\_morphogenesis | 7 | 0 | 0.000000 | -0.000000 | 759 | 795.008199 | 892.92 | 990.831801 | 1.176443 |
| GO:0001936\_regulation\_of\_endothelial\_cell\_proliferation | 7 | 0 | 0.000000 | -0.000000 | 759 | 795.008199 | 892.92 | 990.831801 | 1.176443 |
| GO:0001967\_suckling\_behavior | 7 | 0 | 0.000000 | -0.000000 | 759 | 795.008199 | 892.92 | 990.831801 | 1.176443 |
| GO:0002011\_morphogenesis\_of\_an\_epithelial\_sheet | 7 | 0 | 0.000000 | -0.000000 | 759 | 795.008199 | 892.92 | 990.831801 | 1.176443 |
| GO:0002052\_positive\_regulation\_of\_neuroblast\_proliferation | 7 | 0 | 0.000000 | -0.000000 | 759 | 795.008199 | 892.92 | 990.831801 | 1.176443 |
| GO:0002063\_chondrocyte\_development | 7 | 0 | 0.000000 | -0.000000 | 759 | 795.008199 | 892.92 | 990.831801 | 1.176443 |
| GO:0002067\_glandular\_epithelial\_cell\_differentiation | 7 | 0 | 0.000000 | -0.000000 | 759 | 795.008199 | 892.92 | 990.831801 | 1.176443 |
| GO:0002076\_osteoblast\_development | 7 | 0 | 0.000000 | -0.000000 | 759 | 795.008199 | 892.92 | 990.831801 | 1.176443 |
| GO:0002087\_regulation\_of\_respiratory\_gaseous\_exchange\_by\_neurological\_system\_process | 7 | 0 | 0.000000 | -0.000000 | 759 | 795.008199 | 892.92 | 990.831801 | 1.176443 |
| GO:0002093\_auditory\_receptor\_cell\_morphogenesis | 7 | 0 | 0.000000 | -0.000000 | 759 | 795.008199 | 892.92 | 990.831801 | 1.176443 |
| GO:0002224\_toll-like\_receptor\_signaling\_pathway | 7 | 0 | 0.000000 | -0.000000 | 759 | 795.008199 | 892.92 | 990.831801 | 1.176443 |
| GO:0002455\_humoral\_immune\_response\_mediated\_by\_circulating\_immunoglobulin | 7 | 0 | 0.000000 | -0.000000 | 759 | 795.008199 | 892.92 | 990.831801 | 1.176443 |
| GO:0002643\_regulation\_of\_tolerance\_induction | 7 | 0 | 0.000000 | -0.000000 | 759 | 795.008199 | 892.92 | 990.831801 | 1.176443 |
| GO:0002645\_positive\_regulation\_of\_tolerance\_induction | 7 | 0 | 0.000000 | -0.000000 | 759 | 795.008199 | 892.92 | 990.831801 | 1.176443 |
| GO:0002714\_positive\_regulation\_of\_B\_cell\_mediated\_immunity | 7 | 0 | 0.000000 | -0.000000 | 759 | 795.008199 | 892.92 | 990.831801 | 1.176443 |
| GO:0002792\_negative\_regulation\_of\_peptide\_secretion | 7 | 0 | 0.000000 | -0.000000 | 759 | 795.008199 | 892.92 | 990.831801 | 1.176443 |
| GO:0002793\_positive\_regulation\_of\_peptide\_secretion | 7 | 0 | 0.000000 | -0.000000 | 759 | 795.008199 | 892.92 | 990.831801 | 1.176443 |
| GO:0002828\_regulation\_of\_T-helper\_2\_type\_immune\_response | 7 | 0 | 0.000000 | -0.000000 | 759 | 795.008199 | 892.92 | 990.831801 | 1.176443 |
| GO:0002863\_positive\_regulation\_of\_inflammatory\_response\_to\_antigenic\_stimulus | 7 | 0 | 0.000000 | -0.000000 | 759 | 795.008199 | 892.92 | 990.831801 | 1.176443 |
| GO:0002891\_positive\_regulation\_of\_immunoglobulin\_mediated\_immune\_response | 7 | 0 | 0.000000 | -0.000000 | 759 | 795.008199 | 892.92 | 990.831801 | 1.176443 |
| GO:0003084\_positive\_regulation\_of\_systemic\_arterial\_blood\_pressure | 7 | 0 | 0.000000 | -0.000000 | 759 | 795.008199 | 892.92 | 990.831801 | 1.176443 |
| GO:0003085\_negative\_regulation\_of\_systemic\_arterial\_blood\_pressure | 7 | 0 | 0.000000 | -0.000000 | 759 | 795.008199 | 892.92 | 990.831801 | 1.176443 |
| GO:0006014\_D-ribose\_metabolic\_process | 7 | 0 | 0.000000 | -0.000000 | 759 | 795.008199 | 892.92 | 990.831801 | 1.176443 |
| GO:0006096\_glycolysis | 7 | 0 | 0.000000 | -0.000000 | 759 | 795.008199 | 892.92 | 990.831801 | 1.176443 |
| GO:0006119\_oxidative\_phosphorylation | 7 | 0 | 0.000000 | -0.000000 | 759 | 795.008199 | 892.92 | 990.831801 | 1.176443 |
| GO:0006275\_regulation\_of\_DNA\_replication | 7 | 0 | 0.000000 | -0.000000 | 759 | 795.008199 | 892.92 | 990.831801 | 1.176443 |
| GO:0006298\_mismatch\_repair | 7 | 0 | 0.000000 | -0.000000 | 759 | 795.008199 | 892.92 | 990.831801 | 1.176443 |
| GO:0006352\_transcription\_initiation | 7 | 0 | 0.000000 | -0.000000 | 759 | 795.008199 | 892.92 | 990.831801 | 1.176443 |
| GO:0006401\_RNA\_catabolic\_process | 7 | 0 | 0.000000 | -0.000000 | 759 | 795.008199 | 892.92 | 990.831801 | 1.176443 |
| GO:0006406\_mRNA\_export\_from\_nucleus | 7 | 0 | 0.000000 | -0.000000 | 759 | 795.008199 | 892.92 | 990.831801 | 1.176443 |
| GO:0006505\_GPI\_anchor\_metabolic\_process | 7 | 0 | 0.000000 | -0.000000 | 759 | 795.008199 | 892.92 | 990.831801 | 1.176443 |
| GO:0006516\_glycoprotein\_catabolic\_process | 7 | 0 | 0.000000 | -0.000000 | 759 | 795.008199 | 892.92 | 990.831801 | 1.176443 |
| GO:0006612\_protein\_targeting\_to\_membrane | 7 | 0 | 0.000000 | -0.000000 | 759 | 795.008199 | 892.92 | 990.831801 | 1.176443 |
| GO:0006769\_nicotinamide\_metabolic\_process | 7 | 0 | 0.000000 | -0.000000 | 759 | 795.008199 | 892.92 | 990.831801 | 1.176443 |
| GO:0006783\_heme\_biosynthetic\_process | 7 | 0 | 0.000000 | -0.000000 | 759 | 795.008199 | 892.92 | 990.831801 | 1.176443 |
| GO:0006818\_hydrogen\_transport | 7 | 0 | 0.000000 | -0.000000 | 759 | 795.008199 | 892.92 | 990.831801 | 1.176443 |
| GO:0006878\_cellular\_copper\_ion\_homeostasis | 7 | 0 | 0.000000 | -0.000000 | 759 | 795.008199 | 892.92 | 990.831801 | 1.176443 |
| GO:0006884\_cell\_volume\_homeostasis | 7 | 0 | 0.000000 | -0.000000 | 759 | 795.008199 | 892.92 | 990.831801 | 1.176443 |
| GO:0006949\_syncytium\_formation | 7 | 0 | 0.000000 | -0.000000 | 759 | 795.008199 | 892.92 | 990.831801 | 1.176443 |
| GO:0007019\_microtubule\_depolymerization | 7 | 0 | 0.000000 | -0.000000 | 759 | 795.008199 | 892.92 | 990.831801 | 1.176443 |
| GO:0007026\_negative\_regulation\_of\_microtubule\_depolymerization | 7 | 0 | 0.000000 | -0.000000 | 759 | 795.008199 | 892.92 | 990.831801 | 1.176443 |
| GO:0007034\_vacuolar\_transport | 7 | 0 | 0.000000 | -0.000000 | 759 | 795.008199 | 892.92 | 990.831801 | 1.176443 |
| GO:0007062\_sister\_chromatid\_cohesion | 7 | 0 | 0.000000 | -0.000000 | 759 | 795.008199 | 892.92 | 990.831801 | 1.176443 |
| GO:0007130\_synaptonemal\_complex\_assembly | 7 | 0 | 0.000000 | -0.000000 | 759 | 795.008199 | 892.92 | 990.831801 | 1.176443 |
| GO:0007164\_establishment\_of\_tissue\_polarity | 7 | 0 | 0.000000 | -0.000000 | 759 | 795.008199 | 892.92 | 990.831801 | 1.176443 |
| GO:0007191\_activation\_of\_adenylate\_cyclase\_activity\_by\_dopamine\_receptor\_signaling\_pathway | 7 | 0 | 0.000000 | -0.000000 | 759 | 795.008199 | 892.92 | 990.831801 | 1.176443 |
| GO:0007271\_synaptic\_transmission\_\_cholinergic | 7 | 0 | 0.000000 | -0.000000 | 759 | 795.008199 | 892.92 | 990.831801 | 1.176443 |
| GO:0007413\_axonal\_fasciculation | 7 | 0 | 0.000000 | -0.000000 | 759 | 795.008199 | 892.92 | 990.831801 | 1.176443 |
| GO:0007440\_foregut\_morphogenesis | 7 | 0 | 0.000000 | -0.000000 | 759 | 795.008199 | 892.92 | 990.831801 | 1.176443 |
| GO:0007616\_long-term\_memory | 7 | 0 | 0.000000 | -0.000000 | 759 | 795.008199 | 892.92 | 990.831801 | 1.176443 |
| GO:0008033\_tRNA\_processing | 7 | 0 | 0.000000 | -0.000000 | 759 | 795.008199 | 892.92 | 990.831801 | 1.176443 |
| GO:0008299\_isoprenoid\_biosynthetic\_process | 7 | 0 | 0.000000 | -0.000000 | 759 | 795.008199 | 892.92 | 990.831801 | 1.176443 |
| GO:0008340\_determination\_of\_adult\_lifespan | 7 | 0 | 0.000000 | -0.000000 | 759 | 795.008199 | 892.92 | 990.831801 | 1.176443 |
| GO:0009150\_purine\_ribonucleotide\_metabolic\_process | 7 | 0 | 0.000000 | -0.000000 | 759 | 795.008199 | 892.92 | 990.831801 | 1.176443 |
| GO:0009200\_deoxyribonucleoside\_triphosphate\_metabolic\_process | 7 | 0 | 0.000000 | -0.000000 | 759 | 795.008199 | 892.92 | 990.831801 | 1.176443 |
| GO:0009259\_ribonucleotide\_metabolic\_process | 7 | 0 | 0.000000 | -0.000000 | 759 | 795.008199 | 892.92 | 990.831801 | 1.176443 |
| GO:0009311\_oligosaccharide\_metabolic\_process | 7 | 0 | 0.000000 | -0.000000 | 759 | 795.008199 | 892.92 | 990.831801 | 1.176443 |
| GO:0009394\_2'-deoxyribonucleotide\_metabolic\_process | 7 | 0 | 0.000000 | -0.000000 | 759 | 795.008199 | 892.92 | 990.831801 | 1.176443 |
| GO:0009820\_alkaloid\_metabolic\_process | 7 | 0 | 0.000000 | -0.000000 | 759 | 795.008199 | 892.92 | 990.831801 | 1.176443 |
| GO:0010469\_regulation\_of\_receptor\_activity | 7 | 0 | 0.000000 | -0.000000 | 759 | 795.008199 | 892.92 | 990.831801 | 1.176443 |
| GO:0010948\_negative\_regulation\_of\_cell\_cycle\_process | 7 | 0 | 0.000000 | -0.000000 | 759 | 795.008199 | 892.92 | 990.831801 | 1.176443 |
| GO:0014047\_glutamate\_secretion | 7 | 0 | 0.000000 | -0.000000 | 759 | 795.008199 | 892.92 | 990.831801 | 1.176443 |
| GO:0014066\_regulation\_of\_phosphoinositide\_3-kinase\_cascade | 7 | 0 | 0.000000 | -0.000000 | 759 | 795.008199 | 892.92 | 990.831801 | 1.176443 |
| GO:0014821\_phasic\_smooth\_muscle\_contraction | 7 | 0 | 0.000000 | -0.000000 | 759 | 795.008199 | 892.92 | 990.831801 | 1.176443 |
| GO:0015697\_quaternary\_ammonium\_group\_transport | 7 | 0 | 0.000000 | -0.000000 | 759 | 795.008199 | 892.92 | 990.831801 | 1.176443 |
| GO:0015813\_L-glutamate\_transport | 7 | 0 | 0.000000 | -0.000000 | 759 | 795.008199 | 892.92 | 990.831801 | 1.176443 |
| GO:0015908\_fatty\_acid\_transport | 7 | 0 | 0.000000 | -0.000000 | 759 | 795.008199 | 892.92 | 990.831801 | 1.176443 |
| GO:0015992\_proton\_transport | 7 | 0 | 0.000000 | -0.000000 | 759 | 795.008199 | 892.92 | 990.831801 | 1.176443 |
| GO:0016339\_calcium-dependent\_cell-cell\_adhesion | 7 | 0 | 0.000000 | -0.000000 | 759 | 795.008199 | 892.92 | 990.831801 | 1.176443 |
| GO:0016575\_histone\_deacetylation | 7 | 0 | 0.000000 | -0.000000 | 759 | 795.008199 | 892.92 | 990.831801 | 1.176443 |
| GO:0019362\_pyridine\_nucleotide\_metabolic\_process | 7 | 0 | 0.000000 | -0.000000 | 759 | 795.008199 | 892.92 | 990.831801 | 1.176443 |
| GO:0019692\_deoxyribose\_phosphate\_metabolic\_process | 7 | 0 | 0.000000 | -0.000000 | 759 | 795.008199 | 892.92 | 990.831801 | 1.176443 |
| GO:0019800\_peptide\_cross-linking\_via\_chondroitin\_4-sulfate\_glycosaminoglycan | 7 | 0 | 0.000000 | -0.000000 | 759 | 795.008199 | 892.92 | 990.831801 | 1.176443 |
| GO:0020027\_hemoglobin\_metabolic\_process | 7 | 0 | 0.000000 | -0.000000 | 759 | 795.008199 | 892.92 | 990.831801 | 1.176443 |
| GO:0021514\_ventral\_spinal\_cord\_interneuron\_differentiation | 7 | 0 | 0.000000 | -0.000000 | 759 | 795.008199 | 892.92 | 990.831801 | 1.176443 |
| GO:0021516\_dorsal\_spinal\_cord\_development | 7 | 0 | 0.000000 | -0.000000 | 759 | 795.008199 | 892.92 | 990.831801 | 1.176443 |
| GO:0021520\_spinal\_cord\_motor\_neuron\_cell\_fate\_specification | 7 | 0 | 0.000000 | -0.000000 | 759 | 795.008199 | 892.92 | 990.831801 | 1.176443 |
| GO:0021521\_ventral\_spinal\_cord\_interneuron\_specification | 7 | 0 | 0.000000 | -0.000000 | 759 | 795.008199 | 892.92 | 990.831801 | 1.176443 |
| GO:0021546\_rhombomere\_development | 7 | 0 | 0.000000 | -0.000000 | 759 | 795.008199 | 892.92 | 990.831801 | 1.176443 |
| GO:0021756\_striatum\_development | 7 | 0 | 0.000000 | -0.000000 | 759 | 795.008199 | 892.92 | 990.831801 | 1.176443 |
| GO:0021884\_forebrain\_neuron\_development | 7 | 0 | 0.000000 | -0.000000 | 759 | 795.008199 | 892.92 | 990.831801 | 1.176443 |
| GO:0021903\_rostrocaudal\_neural\_tube\_patterning | 7 | 0 | 0.000000 | -0.000000 | 759 | 795.008199 | 892.92 | 990.831801 | 1.176443 |
| GO:0021984\_adenohypophysis\_development | 7 | 0 | 0.000000 | -0.000000 | 759 | 795.008199 | 892.92 | 990.831801 | 1.176443 |
| GO:0030104\_water\_homeostasis | 7 | 0 | 0.000000 | -0.000000 | 759 | 795.008199 | 892.92 | 990.831801 | 1.176443 |
| GO:0030201\_heparan\_sulfate\_proteoglycan\_metabolic\_process | 7 | 0 | 0.000000 | -0.000000 | 759 | 795.008199 | 892.92 | 990.831801 | 1.176443 |
| GO:0030432\_peristalsis | 7 | 0 | 0.000000 | -0.000000 | 759 | 795.008199 | 892.92 | 990.831801 | 1.176443 |
| GO:0030517\_negative\_regulation\_of\_axon\_extension | 7 | 0 | 0.000000 | -0.000000 | 759 | 795.008199 | 892.92 | 990.831801 | 1.176443 |
| GO:0030520\_estrogen\_receptor\_signaling\_pathway | 7 | 0 | 0.000000 | -0.000000 | 759 | 795.008199 | 892.92 | 990.831801 | 1.176443 |
| GO:0030521\_androgen\_receptor\_signaling\_pathway | 7 | 0 | 0.000000 | -0.000000 | 759 | 795.008199 | 892.92 | 990.831801 | 1.176443 |
| GO:0030903\_notochord\_development | 7 | 0 | 0.000000 | -0.000000 | 759 | 795.008199 | 892.92 | 990.831801 | 1.176443 |
| GO:0031017\_exocrine\_pancreas\_development | 7 | 0 | 0.000000 | -0.000000 | 759 | 795.008199 | 892.92 | 990.831801 | 1.176443 |
| GO:0031114\_regulation\_of\_microtubule\_depolymerization | 7 | 0 | 0.000000 | -0.000000 | 759 | 795.008199 | 892.92 | 990.831801 | 1.176443 |
| GO:0031124\_mRNA\_3'-end\_processing | 7 | 0 | 0.000000 | -0.000000 | 759 | 795.008199 | 892.92 | 990.831801 | 1.176443 |
| GO:0031497\_chromatin\_assembly | 7 | 0 | 0.000000 | -0.000000 | 759 | 795.008199 | 892.92 | 990.831801 | 1.176443 |
| GO:0032104\_regulation\_of\_response\_to\_extracellular\_stimulus | 7 | 0 | 0.000000 | -0.000000 | 759 | 795.008199 | 892.92 | 990.831801 | 1.176443 |
| GO:0032107\_regulation\_of\_response\_to\_nutrient\_levels | 7 | 0 | 0.000000 | -0.000000 | 759 | 795.008199 | 892.92 | 990.831801 | 1.176443 |
| GO:0032228\_regulation\_of\_synaptic\_transmission\_\_GABAergic | 7 | 0 | 0.000000 | -0.000000 | 759 | 795.008199 | 892.92 | 990.831801 | 1.176443 |
| GO:0032319\_regulation\_of\_Rho\_GTPase\_activity | 7 | 0 | 0.000000 | -0.000000 | 759 | 795.008199 | 892.92 | 990.831801 | 1.176443 |
| GO:0032387\_negative\_regulation\_of\_intracellular\_transport | 7 | 0 | 0.000000 | -0.000000 | 759 | 795.008199 | 892.92 | 990.831801 | 1.176443 |
| GO:0032507\_maintenance\_of\_protein\_location\_in\_cell | 7 | 0 | 0.000000 | -0.000000 | 759 | 795.008199 | 892.92 | 990.831801 | 1.176443 |
| GO:0033032\_regulation\_of\_myeloid\_cell\_apoptosis | 7 | 0 | 0.000000 | -0.000000 | 759 | 795.008199 | 892.92 | 990.831801 | 1.176443 |
| GO:0033057\_reproductive\_behavior\_in\_a\_multicellular\_organism | 7 | 0 | 0.000000 | -0.000000 | 759 | 795.008199 | 892.92 | 990.831801 | 1.176443 |
| GO:0034599\_cellular\_response\_to\_oxidative\_stress | 7 | 0 | 0.000000 | -0.000000 | 759 | 795.008199 | 892.92 | 990.831801 | 1.176443 |
| GO:0042033\_chemokine\_biosynthetic\_process | 7 | 0 | 0.000000 | -0.000000 | 759 | 795.008199 | 892.92 | 990.831801 | 1.176443 |
| GO:0042133\_neurotransmitter\_metabolic\_process | 7 | 0 | 0.000000 | -0.000000 | 759 | 795.008199 | 892.92 | 990.831801 | 1.176443 |
| GO:0042168\_heme\_metabolic\_process | 7 | 0 | 0.000000 | -0.000000 | 759 | 795.008199 | 892.92 | 990.831801 | 1.176443 |
| GO:0042415\_norepinephrine\_metabolic\_process | 7 | 0 | 0.000000 | -0.000000 | 759 | 795.008199 | 892.92 | 990.831801 | 1.176443 |
| GO:0042503\_tyrosine\_phosphorylation\_of\_Stat3\_protein | 7 | 0 | 0.000000 | -0.000000 | 759 | 795.008199 | 892.92 | 990.831801 | 1.176443 |
| GO:0042572\_retinol\_metabolic\_process | 7 | 0 | 0.000000 | -0.000000 | 759 | 795.008199 | 892.92 | 990.831801 | 1.176443 |
| GO:0043353\_enucleate\_erythrocyte\_differentiation | 7 | 0 | 0.000000 | -0.000000 | 759 | 795.008199 | 892.92 | 990.831801 | 1.176443 |
| GO:0043372\_positive\_regulation\_of\_CD4-positive\_\_alpha\_beta\_T\_cell\_differentiation | 7 | 0 | 0.000000 | -0.000000 | 759 | 795.008199 | 892.92 | 990.831801 | 1.176443 |
| GO:0043449\_cellular\_alkene\_metabolic\_process | 7 | 0 | 0.000000 | -0.000000 | 759 | 795.008199 | 892.92 | 990.831801 | 1.176443 |
| GO:0043507\_positive\_regulation\_of\_JUN\_kinase\_activity | 7 | 0 | 0.000000 | -0.000000 | 759 | 795.008199 | 892.92 | 990.831801 | 1.176443 |
| GO:0043567\_regulation\_of\_insulin-like\_growth\_factor\_receptor\_signaling\_pathway | 7 | 0 | 0.000000 | -0.000000 | 759 | 795.008199 | 892.92 | 990.831801 | 1.176443 |
| GO:0043584\_nose\_development | 7 | 0 | 0.000000 | -0.000000 | 759 | 795.008199 | 892.92 | 990.831801 | 1.176443 |
| GO:0044065\_regulation\_of\_respiratory\_system\_process | 7 | 0 | 0.000000 | -0.000000 | 759 | 795.008199 | 892.92 | 990.831801 | 1.176443 |
| GO:0044275\_cellular\_carbohydrate\_catabolic\_process | 7 | 0 | 0.000000 | -0.000000 | 759 | 795.008199 | 892.92 | 990.831801 | 1.176443 |
| GO:0045059\_positive\_thymic\_T\_cell\_selection | 7 | 0 | 0.000000 | -0.000000 | 759 | 795.008199 | 892.92 | 990.831801 | 1.176443 |
| GO:0045073\_regulation\_of\_chemokine\_biosynthetic\_process | 7 | 0 | 0.000000 | -0.000000 | 759 | 795.008199 | 892.92 | 990.831801 | 1.176443 |
| GO:0045581\_negative\_regulation\_of\_T\_cell\_differentiation | 7 | 0 | 0.000000 | -0.000000 | 759 | 795.008199 | 892.92 | 990.831801 | 1.176443 |
| GO:0045599\_negative\_regulation\_of\_fat\_cell\_differentiation | 7 | 0 | 0.000000 | -0.000000 | 759 | 795.008199 | 892.92 | 990.831801 | 1.176443 |
| GO:0045604\_regulation\_of\_epidermal\_cell\_differentiation | 7 | 0 | 0.000000 | -0.000000 | 759 | 795.008199 | 892.92 | 990.831801 | 1.176443 |
| GO:0045668\_negative\_regulation\_of\_osteoblast\_differentiation | 7 | 0 | 0.000000 | -0.000000 | 759 | 795.008199 | 892.92 | 990.831801 | 1.176443 |
| GO:0045823\_positive\_regulation\_of\_heart\_contraction | 7 | 0 | 0.000000 | -0.000000 | 759 | 795.008199 | 892.92 | 990.831801 | 1.176443 |
| GO:0045840\_positive\_regulation\_of\_mitosis | 7 | 0 | 0.000000 | -0.000000 | 759 | 795.008199 | 892.92 | 990.831801 | 1.176443 |
| GO:0045862\_positive\_regulation\_of\_proteolysis | 7 | 0 | 0.000000 | -0.000000 | 759 | 795.008199 | 892.92 | 990.831801 | 1.176443 |
| GO:0045879\_negative\_regulation\_of\_smoothened\_signaling\_pathway | 7 | 0 | 0.000000 | -0.000000 | 759 | 795.008199 | 892.92 | 990.831801 | 1.176443 |
| GO:0045880\_positive\_regulation\_of\_smoothened\_signaling\_pathway | 7 | 0 | 0.000000 | -0.000000 | 759 | 795.008199 | 892.92 | 990.831801 | 1.176443 |
| GO:0045986\_negative\_regulation\_of\_smooth\_muscle\_contraction | 7 | 0 | 0.000000 | -0.000000 | 759 | 795.008199 | 892.92 | 990.831801 | 1.176443 |
| GO:0046496\_nicotinamide\_nucleotide\_metabolic\_process | 7 | 0 | 0.000000 | -0.000000 | 759 | 795.008199 | 892.92 | 990.831801 | 1.176443 |
| GO:0046504\_glycerol\_ether\_biosynthetic\_process | 7 | 0 | 0.000000 | -0.000000 | 759 | 795.008199 | 892.92 | 990.831801 | 1.176443 |
| GO:0046513\_ceramide\_biosynthetic\_process | 7 | 0 | 0.000000 | -0.000000 | 759 | 795.008199 | 892.92 | 990.831801 | 1.176443 |
| GO:0046520\_sphingoid\_biosynthetic\_process | 7 | 0 | 0.000000 | -0.000000 | 759 | 795.008199 | 892.92 | 990.831801 | 1.176443 |
| GO:0046543\_development\_of\_secondary\_female\_sexual\_characteristics | 7 | 0 | 0.000000 | -0.000000 | 759 | 795.008199 | 892.92 | 990.831801 | 1.176443 |
| GO:0046622\_positive\_regulation\_of\_organ\_growth | 7 | 0 | 0.000000 | -0.000000 | 759 | 795.008199 | 892.92 | 990.831801 | 1.176443 |
| GO:0046626\_regulation\_of\_insulin\_receptor\_signaling\_pathway | 7 | 0 | 0.000000 | -0.000000 | 759 | 795.008199 | 892.92 | 990.831801 | 1.176443 |
| GO:0046676\_negative\_regulation\_of\_insulin\_secretion | 7 | 0 | 0.000000 | -0.000000 | 759 | 795.008199 | 892.92 | 990.831801 | 1.176443 |
| GO:0046677\_response\_to\_antibiotic | 7 | 0 | 0.000000 | -0.000000 | 759 | 795.008199 | 892.92 | 990.831801 | 1.176443 |
| GO:0046823\_negative\_regulation\_of\_nucleocytoplasmic\_transport | 7 | 0 | 0.000000 | -0.000000 | 759 | 795.008199 | 892.92 | 990.831801 | 1.176443 |
| GO:0046824\_positive\_regulation\_of\_nucleocytoplasmic\_transport | 7 | 0 | 0.000000 | -0.000000 | 759 | 795.008199 | 892.92 | 990.831801 | 1.176443 |
| GO:0048148\_behavioral\_response\_to\_cocaine | 7 | 0 | 0.000000 | -0.000000 | 759 | 795.008199 | 892.92 | 990.831801 | 1.176443 |
| GO:0048304\_positive\_regulation\_of\_isotype\_switching\_to\_IgG\_isotypes | 7 | 0 | 0.000000 | -0.000000 | 759 | 795.008199 | 892.92 | 990.831801 | 1.176443 |
| GO:0048486\_parasympathetic\_nervous\_system\_development | 7 | 0 | 0.000000 | -0.000000 | 759 | 795.008199 | 892.92 | 990.831801 | 1.176443 |
| GO:0048537\_mucosal-associated\_lymphoid\_tissue\_development | 7 | 0 | 0.000000 | -0.000000 | 759 | 795.008199 | 892.92 | 990.831801 | 1.176443 |
| GO:0048753\_pigment\_granule\_organization | 7 | 0 | 0.000000 | -0.000000 | 759 | 795.008199 | 892.92 | 990.831801 | 1.176443 |
| GO:0048814\_regulation\_of\_dendrite\_morphogenesis | 7 | 0 | 0.000000 | -0.000000 | 759 | 795.008199 | 892.92 | 990.831801 | 1.176443 |
| GO:0048857\_neural\_nucleus\_development | 7 | 0 | 0.000000 | -0.000000 | 759 | 795.008199 | 892.92 | 990.831801 | 1.176443 |
| GO:0050755\_chemokine\_metabolic\_process | 7 | 0 | 0.000000 | -0.000000 | 759 | 795.008199 | 892.92 | 990.831801 | 1.176443 |
| GO:0050773\_regulation\_of\_dendrite\_development | 7 | 0 | 0.000000 | -0.000000 | 759 | 795.008199 | 892.92 | 990.831801 | 1.176443 |
| GO:0051028\_mRNA\_transport | 7 | 0 | 0.000000 | -0.000000 | 759 | 795.008199 | 892.92 | 990.831801 | 1.176443 |
| GO:0051785\_positive\_regulation\_of\_nuclear\_division | 7 | 0 | 0.000000 | -0.000000 | 759 | 795.008199 | 892.92 | 990.831801 | 1.176443 |
| GO:0055069\_zinc\_ion\_homeostasis | 7 | 0 | 0.000000 | -0.000000 | 759 | 795.008199 | 892.92 | 990.831801 | 1.176443 |
| GO:0055070\_copper\_ion\_homeostasis | 7 | 0 | 0.000000 | -0.000000 | 759 | 795.008199 | 892.92 | 990.831801 | 1.176443 |
| GO:0060037\_pharyngeal\_system\_development | 7 | 0 | 0.000000 | -0.000000 | 759 | 795.008199 | 892.92 | 990.831801 | 1.176443 |
| GO:0060080\_regulation\_of\_inhibitory\_postsynaptic\_membrane\_potential | 7 | 0 | 0.000000 | -0.000000 | 759 | 795.008199 | 892.92 | 990.831801 | 1.176443 |
| GO:0060088\_auditory\_receptor\_cell\_stereocilium\_organization | 7 | 0 | 0.000000 | -0.000000 | 759 | 795.008199 | 892.92 | 990.831801 | 1.176443 |
| GO:0060117\_auditory\_receptor\_cell\_development | 7 | 0 | 0.000000 | -0.000000 | 759 | 795.008199 | 892.92 | 990.831801 | 1.176443 |
| GO:0060441\_branching\_involved\_in\_lung\_morphogenesis | 7 | 0 | 0.000000 | -0.000000 | 759 | 795.008199 | 892.92 | 990.831801 | 1.176443 |
| GO:0060526\_prostate\_glandular\_acinus\_morphogenesis | 7 | 0 | 0.000000 | -0.000000 | 759 | 795.008199 | 892.92 | 990.831801 | 1.176443 |
| GO:0060527\_prostate\_epithelial\_cord\_arborization\_involved\_in\_prostate\_glandular\_acinus\_morphogenesis | 7 | 0 | 0.000000 | -0.000000 | 759 | 795.008199 | 892.92 | 990.831801 | 1.176443 |
| GO:0060579\_ventral\_spinal\_cord\_interneuron\_fate\_commitment | 7 | 0 | 0.000000 | -0.000000 | 759 | 795.008199 | 892.92 | 990.831801 | 1.176443 |
| GO:0060664\_epithelial\_cell\_proliferation\_involved\_in\_salivary\_gland\_morphogenesis | 7 | 0 | 0.000000 | -0.000000 | 759 | 795.008199 | 892.92 | 990.831801 | 1.176443 |
| GO:0060687\_regulation\_of\_branching\_involved\_in\_prostate\_gland\_morphogenesis | 7 | 0 | 0.000000 | -0.000000 | 759 | 795.008199 | 892.92 | 990.831801 | 1.176443 |
| GO:0060770\_negative\_regulation\_of\_epithelial\_cell\_proliferation\_involved\_in\_prostate\_gland\_development | 7 | 0 | 0.000000 | -0.000000 | 759 | 795.008199 | 892.92 | 990.831801 | 1.176443 |
| GO:0060788\_ectodermal\_placode\_formation | 7 | 0 | 0.000000 | -0.000000 | 759 | 795.008199 | 892.92 | 990.831801 | 1.176443 |
| GO:0060795\_cell\_fate\_commitment\_involved\_in\_the\_formation\_of\_primary\_germ\_layers | 7 | 0 | 0.000000 | -0.000000 | 759 | 795.008199 | 892.92 | 990.831801 | 1.176443 |
| GO:0070228\_regulation\_of\_lymphocyte\_apoptosis | 7 | 0 | 0.000000 | -0.000000 | 759 | 795.008199 | 892.92 | 990.831801 | 1.176443 |
| GO:0070646\_protein\_modification\_by\_small\_protein\_removal | 7 | 0 | 0.000000 | -0.000000 | 759 | 795.008199 | 892.92 | 990.831801 | 1.176443 |
| GO:0000002\_mitochondrial\_genome\_maintenance | 9 | 0 | 0.000000 | -0.000000 | 870 | 907.273520 | 1002.18 | 1097.086480 | 1.151931 |
| GO:0000186\_activation\_of\_MAPKK\_activity | 9 | 0 | 0.000000 | -0.000000 | 870 | 907.273520 | 1002.18 | 1097.086480 | 1.151931 |
| GO:0001539\_ciliary\_or\_flagellar\_motility | 9 | 0 | 0.000000 | -0.000000 | 870 | 907.273520 | 1002.18 | 1097.086480 | 1.151931 |
| GO:0001676\_long-chain\_fatty\_acid\_metabolic\_process | 9 | 0 | 0.000000 | -0.000000 | 870 | 907.273520 | 1002.18 | 1097.086480 | 1.151931 |
| GO:0001935\_endothelial\_cell\_proliferation | 9 | 0 | 0.000000 | -0.000000 | 870 | 907.273520 | 1002.18 | 1097.086480 | 1.151931 |
| GO:0002021\_response\_to\_dietary\_excess | 9 | 0 | 0.000000 | -0.000000 | 870 | 907.273520 | 1002.18 | 1097.086480 | 1.151931 |
| GO:0002028\_regulation\_of\_sodium\_ion\_transport | 9 | 0 | 0.000000 | -0.000000 | 870 | 907.273520 | 1002.18 | 1097.086480 | 1.151931 |
| GO:0002221\_pattern\_recognition\_receptor\_signaling\_pathway | 9 | 0 | 0.000000 | -0.000000 | 870 | 907.273520 | 1002.18 | 1097.086480 | 1.151931 |
| GO:0002292\_T\_cell\_differentiation\_during\_immune\_response | 9 | 0 | 0.000000 | -0.000000 | 870 | 907.273520 | 1002.18 | 1097.086480 | 1.151931 |
| GO:0002293\_alpha-beta\_T\_cell\_differentiation\_during\_immune\_response | 9 | 0 | 0.000000 | -0.000000 | 870 | 907.273520 | 1002.18 | 1097.086480 | 1.151931 |
| GO:0002294\_CD4-positive\_\_alpha-beta\_T\_cell\_differentiation\_during\_immune\_response | 9 | 0 | 0.000000 | -0.000000 | 870 | 907.273520 | 1002.18 | 1097.086480 | 1.151931 |
| GO:0002507\_tolerance\_induction | 9 | 0 | 0.000000 | -0.000000 | 870 | 907.273520 | 1002.18 | 1097.086480 | 1.151931 |
| GO:0002886\_regulation\_of\_myeloid\_leukocyte\_mediated\_immunity | 9 | 0 | 0.000000 | -0.000000 | 870 | 907.273520 | 1002.18 | 1097.086480 | 1.151931 |
| GO:0006007\_glucose\_catabolic\_process | 9 | 0 | 0.000000 | -0.000000 | 870 | 907.273520 | 1002.18 | 1097.086480 | 1.151931 |
| GO:0006309\_DNA\_fragmentation\_involved\_in\_apoptosis | 9 | 0 | 0.000000 | -0.000000 | 870 | 907.273520 | 1002.18 | 1097.086480 | 1.151931 |
| GO:0006476\_protein\_amino\_acid\_deacetylation | 9 | 0 | 0.000000 | -0.000000 | 870 | 907.273520 | 1002.18 | 1097.086480 | 1.151931 |
| GO:0006910\_phagocytosis\_\_recognition | 9 | 0 | 0.000000 | -0.000000 | 870 | 907.273520 | 1002.18 | 1097.086480 | 1.151931 |
| GO:0006911\_phagocytosis\_\_engulfment | 9 | 0 | 0.000000 | -0.000000 | 870 | 907.273520 | 1002.18 | 1097.086480 | 1.151931 |
| GO:0007128\_meiotic\_prophase\_I | 9 | 0 | 0.000000 | -0.000000 | 870 | 907.273520 | 1002.18 | 1097.086480 | 1.151931 |
| GO:0007193\_inhibition\_of\_adenylate\_cyclase\_activity\_by\_G-protein\_signaling | 9 | 0 | 0.000000 | -0.000000 | 870 | 907.273520 | 1002.18 | 1097.086480 | 1.151931 |
| GO:0007379\_segment\_specification | 9 | 0 | 0.000000 | -0.000000 | 870 | 907.273520 | 1002.18 | 1097.086480 | 1.151931 |
| GO:0007617\_mating\_behavior | 9 | 0 | 0.000000 | -0.000000 | 870 | 907.273520 | 1002.18 | 1097.086480 | 1.151931 |
| GO:0009451\_RNA\_modification | 9 | 0 | 0.000000 | -0.000000 | 870 | 907.273520 | 1002.18 | 1097.086480 | 1.151931 |
| GO:0010165\_response\_to\_X-ray | 9 | 0 | 0.000000 | -0.000000 | 870 | 907.273520 | 1002.18 | 1097.086480 | 1.151931 |
| GO:0010675\_regulation\_of\_cellular\_carbohydrate\_metabolic\_process | 9 | 0 | 0.000000 | -0.000000 | 870 | 907.273520 | 1002.18 | 1097.086480 | 1.151931 |
| GO:0014037\_Schwann\_cell\_differentiation | 9 | 0 | 0.000000 | -0.000000 | 870 | 907.273520 | 1002.18 | 1097.086480 | 1.151931 |
| GO:0014073\_response\_to\_tropane | 9 | 0 | 0.000000 | -0.000000 | 870 | 907.273520 | 1002.18 | 1097.086480 | 1.151931 |
| GO:0015695\_organic\_cation\_transport | 9 | 0 | 0.000000 | -0.000000 | 870 | 907.273520 | 1002.18 | 1097.086480 | 1.151931 |
| GO:0016601\_Rac\_protein\_signal\_transduction | 9 | 0 | 0.000000 | -0.000000 | 870 | 907.273520 | 1002.18 | 1097.086480 | 1.151931 |
| GO:0017145\_stem\_cell\_division | 9 | 0 | 0.000000 | -0.000000 | 870 | 907.273520 | 1002.18 | 1097.086480 | 1.151931 |
| GO:0019320\_hexose\_catabolic\_process | 9 | 0 | 0.000000 | -0.000000 | 870 | 907.273520 | 1002.18 | 1097.086480 | 1.151931 |
| GO:0021544\_subpallium\_development | 9 | 0 | 0.000000 | -0.000000 | 870 | 907.273520 | 1002.18 | 1097.086480 | 1.151931 |
| GO:0021936\_regulation\_of\_granule\_cell\_precursor\_proliferation | 9 | 0 | 0.000000 | -0.000000 | 870 | 907.273520 | 1002.18 | 1097.086480 | 1.151931 |
| GO:0021940\_positive\_regulation\_of\_granule\_cell\_precursor\_proliferation | 9 | 0 | 0.000000 | -0.000000 | 870 | 907.273520 | 1002.18 | 1097.086480 | 1.151931 |
| GO:0030048\_actin\_filament-based\_movement | 9 | 0 | 0.000000 | -0.000000 | 870 | 907.273520 | 1002.18 | 1097.086480 | 1.151931 |
| GO:0030279\_negative\_regulation\_of\_ossification | 9 | 0 | 0.000000 | -0.000000 | 870 | 907.273520 | 1002.18 | 1097.086480 | 1.151931 |
| GO:0030325\_adrenal\_gland\_development | 9 | 0 | 0.000000 | -0.000000 | 870 | 907.273520 | 1002.18 | 1097.086480 | 1.151931 |
| GO:0031023\_microtubule\_organizing\_center\_organization | 9 | 0 | 0.000000 | -0.000000 | 870 | 907.273520 | 1002.18 | 1097.086480 | 1.151931 |
| GO:0032388\_positive\_regulation\_of\_intracellular\_transport | 9 | 0 | 0.000000 | -0.000000 | 870 | 907.273520 | 1002.18 | 1097.086480 | 1.151931 |
| GO:0032606\_type\_I\_interferon\_production | 9 | 0 | 0.000000 | -0.000000 | 870 | 907.273520 | 1002.18 | 1097.086480 | 1.151931 |
| GO:0032814\_regulation\_of\_natural\_killer\_cell\_activation | 9 | 0 | 0.000000 | -0.000000 | 870 | 907.273520 | 1002.18 | 1097.086480 | 1.151931 |
| GO:0032816\_positive\_regulation\_of\_natural\_killer\_cell\_activation | 9 | 0 | 0.000000 | -0.000000 | 870 | 907.273520 | 1002.18 | 1097.086480 | 1.151931 |
| GO:0032963\_collagen\_metabolic\_process | 9 | 0 | 0.000000 | -0.000000 | 870 | 907.273520 | 1002.18 | 1097.086480 | 1.151931 |
| GO:0033028\_myeloid\_cell\_apoptosis | 9 | 0 | 0.000000 | -0.000000 | 870 | 907.273520 | 1002.18 | 1097.086480 | 1.151931 |
| GO:0033143\_regulation\_of\_steroid\_hormone\_receptor\_signaling\_pathway | 9 | 0 | 0.000000 | -0.000000 | 870 | 907.273520 | 1002.18 | 1097.086480 | 1.151931 |
| GO:0033344\_cholesterol\_efflux | 9 | 0 | 0.000000 | -0.000000 | 870 | 907.273520 | 1002.18 | 1097.086480 | 1.151931 |
| GO:0034605\_cellular\_response\_to\_heat | 9 | 0 | 0.000000 | -0.000000 | 870 | 907.273520 | 1002.18 | 1097.086480 | 1.151931 |
| GO:0035088\_establishment\_or\_maintenance\_of\_apical\_basal\_cell\_polarity | 9 | 0 | 0.000000 | -0.000000 | 870 | 907.273520 | 1002.18 | 1097.086480 | 1.151931 |
| GO:0035162\_embryonic\_hemopoiesis | 9 | 0 | 0.000000 | -0.000000 | 870 | 907.273520 | 1002.18 | 1097.086480 | 1.151931 |
| GO:0040020\_regulation\_of\_meiosis | 9 | 0 | 0.000000 | -0.000000 | 870 | 907.273520 | 1002.18 | 1097.086480 | 1.151931 |
| GO:0042058\_regulation\_of\_epidermal\_growth\_factor\_receptor\_signaling\_pathway | 9 | 0 | 0.000000 | -0.000000 | 870 | 907.273520 | 1002.18 | 1097.086480 | 1.151931 |
| GO:0042093\_T-helper\_cell\_differentiation | 9 | 0 | 0.000000 | -0.000000 | 870 | 907.273520 | 1002.18 | 1097.086480 | 1.151931 |
| GO:0042220\_response\_to\_cocaine | 9 | 0 | 0.000000 | -0.000000 | 870 | 907.273520 | 1002.18 | 1097.086480 | 1.151931 |
| GO:0042402\_biogenic\_amine\_catabolic\_process | 9 | 0 | 0.000000 | -0.000000 | 870 | 907.273520 | 1002.18 | 1097.086480 | 1.151931 |
| GO:0042509\_regulation\_of\_tyrosine\_phosphorylation\_of\_STAT\_protein | 9 | 0 | 0.000000 | -0.000000 | 870 | 907.273520 | 1002.18 | 1097.086480 | 1.151931 |
| GO:0042640\_anagen | 9 | 0 | 0.000000 | -0.000000 | 870 | 907.273520 | 1002.18 | 1097.086480 | 1.151931 |
| GO:0043242\_negative\_regulation\_of\_protein\_complex\_disassembly | 9 | 0 | 0.000000 | -0.000000 | 870 | 907.273520 | 1002.18 | 1097.086480 | 1.151931 |
| GO:0043299\_leukocyte\_degranulation | 9 | 0 | 0.000000 | -0.000000 | 870 | 907.273520 | 1002.18 | 1097.086480 | 1.151931 |
| GO:0043383\_negative\_T\_cell\_selection | 9 | 0 | 0.000000 | -0.000000 | 870 | 907.273520 | 1002.18 | 1097.086480 | 1.151931 |
| GO:0043409\_negative\_regulation\_of\_MAPKKK\_cascade | 9 | 0 | 0.000000 | -0.000000 | 870 | 907.273520 | 1002.18 | 1097.086480 | 1.151931 |
| GO:0043433\_negative\_regulation\_of\_transcription\_factor\_activity | 9 | 0 | 0.000000 | -0.000000 | 870 | 907.273520 | 1002.18 | 1097.086480 | 1.151931 |
| GO:0043603\_cellular\_amide\_metabolic\_process | 9 | 0 | 0.000000 | -0.000000 | 870 | 907.273520 | 1002.18 | 1097.086480 | 1.151931 |
| GO:0045060\_negative\_thymic\_T\_cell\_selection | 9 | 0 | 0.000000 | -0.000000 | 870 | 907.273520 | 1002.18 | 1097.086480 | 1.151931 |
| GO:0045109\_intermediate\_filament\_organization | 9 | 0 | 0.000000 | -0.000000 | 870 | 907.273520 | 1002.18 | 1097.086480 | 1.151931 |
| GO:0045136\_development\_of\_secondary\_sexual\_characteristics | 9 | 0 | 0.000000 | -0.000000 | 870 | 907.273520 | 1002.18 | 1097.086480 | 1.151931 |
| GO:0045185\_maintenance\_of\_protein\_location | 9 | 0 | 0.000000 | -0.000000 | 870 | 907.273520 | 1002.18 | 1097.086480 | 1.151931 |
| GO:0045214\_sarcomere\_organization | 9 | 0 | 0.000000 | -0.000000 | 870 | 907.273520 | 1002.18 | 1097.086480 | 1.151931 |
| GO:0045428\_regulation\_of\_nitric\_oxide\_biosynthetic\_process | 9 | 0 | 0.000000 | -0.000000 | 870 | 907.273520 | 1002.18 | 1097.086480 | 1.151931 |
| GO:0045620\_negative\_regulation\_of\_lymphocyte\_differentiation | 9 | 0 | 0.000000 | -0.000000 | 870 | 907.273520 | 1002.18 | 1097.086480 | 1.151931 |
| GO:0045646\_regulation\_of\_erythrocyte\_differentiation | 9 | 0 | 0.000000 | -0.000000 | 870 | 907.273520 | 1002.18 | 1097.086480 | 1.151931 |
| GO:0045671\_negative\_regulation\_of\_osteoclast\_differentiation | 9 | 0 | 0.000000 | -0.000000 | 870 | 907.273520 | 1002.18 | 1097.086480 | 1.151931 |
| GO:0045766\_positive\_regulation\_of\_angiogenesis | 9 | 0 | 0.000000 | -0.000000 | 870 | 907.273520 | 1002.18 | 1097.086480 | 1.151931 |
| GO:0045830\_positive\_regulation\_of\_isotype\_switching | 9 | 0 | 0.000000 | -0.000000 | 870 | 907.273520 | 1002.18 | 1097.086480 | 1.151931 |
| GO:0045884\_regulation\_of\_survival\_gene\_product\_expression | 9 | 0 | 0.000000 | -0.000000 | 870 | 907.273520 | 1002.18 | 1097.086480 | 1.151931 |
| GO:0046006\_regulation\_of\_activated\_T\_cell\_proliferation | 9 | 0 | 0.000000 | -0.000000 | 870 | 907.273520 | 1002.18 | 1097.086480 | 1.151931 |
| GO:0046324\_regulation\_of\_glucose\_import | 9 | 0 | 0.000000 | -0.000000 | 870 | 907.273520 | 1002.18 | 1097.086480 | 1.151931 |
| GO:0046365\_monosaccharide\_catabolic\_process | 9 | 0 | 0.000000 | -0.000000 | 870 | 907.273520 | 1002.18 | 1097.086480 | 1.151931 |
| GO:0046636\_negative\_regulation\_of\_alpha-beta\_T\_cell\_activation | 9 | 0 | 0.000000 | -0.000000 | 870 | 907.273520 | 1002.18 | 1097.086480 | 1.151931 |
| GO:0046641\_positive\_regulation\_of\_alpha-beta\_T\_cell\_proliferation | 9 | 0 | 0.000000 | -0.000000 | 870 | 907.273520 | 1002.18 | 1097.086480 | 1.151931 |
| GO:0046888\_negative\_regulation\_of\_hormone\_secretion | 9 | 0 | 0.000000 | -0.000000 | 870 | 907.273520 | 1002.18 | 1097.086480 | 1.151931 |
| GO:0048070\_regulation\_of\_pigmentation\_during\_development | 9 | 0 | 0.000000 | -0.000000 | 870 | 907.273520 | 1002.18 | 1097.086480 | 1.151931 |
| GO:0048146\_positive\_regulation\_of\_fibroblast\_proliferation | 9 | 0 | 0.000000 | -0.000000 | 870 | 907.273520 | 1002.18 | 1097.086480 | 1.151931 |
| GO:0048284\_organelle\_fusion | 9 | 0 | 0.000000 | -0.000000 | 870 | 907.273520 | 1002.18 | 1097.086480 | 1.151931 |
| GO:0048488\_synaptic\_vesicle\_endocytosis | 9 | 0 | 0.000000 | -0.000000 | 870 | 907.273520 | 1002.18 | 1097.086480 | 1.151931 |
| GO:0048569\_post-embryonic\_organ\_development | 9 | 0 | 0.000000 | -0.000000 | 870 | 907.273520 | 1002.18 | 1097.086480 | 1.151931 |
| GO:0048708\_astrocyte\_differentiation | 9 | 0 | 0.000000 | -0.000000 | 870 | 907.273520 | 1002.18 | 1097.086480 | 1.151931 |
| GO:0050433\_regulation\_of\_catecholamine\_secretion | 9 | 0 | 0.000000 | -0.000000 | 870 | 907.273520 | 1002.18 | 1097.086480 | 1.151931 |
| GO:0050856\_regulation\_of\_T\_cell\_receptor\_signaling\_pathway | 9 | 0 | 0.000000 | -0.000000 | 870 | 907.273520 | 1002.18 | 1097.086480 | 1.151931 |
| GO:0050884\_neuromuscular\_process\_controlling\_posture | 9 | 0 | 0.000000 | -0.000000 | 870 | 907.273520 | 1002.18 | 1097.086480 | 1.151931 |
| GO:0050910\_detection\_of\_mechanical\_stimulus\_involved\_in\_sensory\_perception\_of\_sound | 9 | 0 | 0.000000 | -0.000000 | 870 | 907.273520 | 1002.18 | 1097.086480 | 1.151931 |
| GO:0050918\_positive\_chemotaxis | 9 | 0 | 0.000000 | -0.000000 | 870 | 907.273520 | 1002.18 | 1097.086480 | 1.151931 |
| GO:0051023\_regulation\_of\_immunoglobulin\_secretion | 9 | 0 | 0.000000 | -0.000000 | 870 | 907.273520 | 1002.18 | 1097.086480 | 1.151931 |
| GO:0051297\_centrosome\_organization | 9 | 0 | 0.000000 | -0.000000 | 870 | 907.273520 | 1002.18 | 1097.086480 | 1.151931 |
| GO:0051324\_prophase | 9 | 0 | 0.000000 | -0.000000 | 870 | 907.273520 | 1002.18 | 1097.086480 | 1.151931 |
| GO:0051607\_defense\_response\_to\_virus | 9 | 0 | 0.000000 | -0.000000 | 870 | 907.273520 | 1002.18 | 1097.086480 | 1.151931 |
| GO:0051647\_nucleus\_localization | 9 | 0 | 0.000000 | -0.000000 | 870 | 907.273520 | 1002.18 | 1097.086480 | 1.151931 |
| GO:0051896\_regulation\_of\_protein\_kinase\_B\_signaling\_cascade | 9 | 0 | 0.000000 | -0.000000 | 870 | 907.273520 | 1002.18 | 1097.086480 | 1.151931 |
| GO:0051932\_synaptic\_transmission\_\_GABAergic | 9 | 0 | 0.000000 | -0.000000 | 870 | 907.273520 | 1002.18 | 1097.086480 | 1.151931 |
| GO:0051963\_regulation\_of\_synaptogenesis | 9 | 0 | 0.000000 | -0.000000 | 870 | 907.273520 | 1002.18 | 1097.086480 | 1.151931 |
| GO:0055012\_ventricular\_cardiac\_muscle\_cell\_differentiation | 9 | 0 | 0.000000 | -0.000000 | 870 | 907.273520 | 1002.18 | 1097.086480 | 1.151931 |
| GO:0055013\_cardiac\_muscle\_cell\_development | 9 | 0 | 0.000000 | -0.000000 | 870 | 907.273520 | 1002.18 | 1097.086480 | 1.151931 |
| GO:0060052\_neurofilament\_cytoskeleton\_organization | 9 | 0 | 0.000000 | -0.000000 | 870 | 907.273520 | 1002.18 | 1097.086480 | 1.151931 |
| GO:0060081\_membrane\_hyperpolarization | 9 | 0 | 0.000000 | -0.000000 | 870 | 907.273520 | 1002.18 | 1097.086480 | 1.151931 |
| GO:0060119\_inner\_ear\_receptor\_cell\_development | 9 | 0 | 0.000000 | -0.000000 | 870 | 907.273520 | 1002.18 | 1097.086480 | 1.151931 |
| GO:0060122\_inner\_ear\_receptor\_stereocilium\_organization | 9 | 0 | 0.000000 | -0.000000 | 870 | 907.273520 | 1002.18 | 1097.086480 | 1.151931 |
| GO:0060325\_face\_morphogenesis | 9 | 0 | 0.000000 | -0.000000 | 870 | 907.273520 | 1002.18 | 1097.086480 | 1.151931 |
| GO:0060513\_prostatic\_bud\_formation | 9 | 0 | 0.000000 | -0.000000 | 870 | 907.273520 | 1002.18 | 1097.086480 | 1.151931 |
| GO:0060602\_branch\_elongation\_of\_an\_epithelium | 9 | 0 | 0.000000 | -0.000000 | 870 | 907.273520 | 1002.18 | 1097.086480 | 1.151931 |
| GO:0060693\_regulation\_of\_branching\_involved\_in\_salivary\_gland\_morphogenesis | 9 | 0 | 0.000000 | -0.000000 | 870 | 907.273520 | 1002.18 | 1097.086480 | 1.151931 |
| GO:0070306\_lens\_fiber\_cell\_differentiation | 9 | 0 | 0.000000 | -0.000000 | 870 | 907.273520 | 1002.18 | 1097.086480 | 1.151931 |
| GO:0090048\_negative\_regulation\_of\_transcription\_regulator\_activity | 9 | 0 | 0.000000 | -0.000000 | 870 | 907.273520 | 1002.18 | 1097.086480 | 1.151931 |
| GO:0000082\_G1\_S\_transition\_of\_mitotic\_cell\_cycle | 23 | 0 | 0.000000 | -0.000000 | 888 | 928.048292 | 1021.88 | 1115.711708 | 1.150766 |
| GO:0002204\_somatic\_recombination\_of\_immunoglobulin\_genes\_during\_immune\_response | 23 | 0 | 0.000000 | -0.000000 | 888 | 928.048292 | 1021.88 | 1115.711708 | 1.150766 |
| GO:0002208\_somatic\_diversification\_of\_immunoglobulins\_during\_immune\_response | 23 | 0 | 0.000000 | -0.000000 | 888 | 928.048292 | 1021.88 | 1115.711708 | 1.150766 |
| GO:0002228\_natural\_killer\_cell\_mediated\_immunity | 23 | 0 | 0.000000 | -0.000000 | 888 | 928.048292 | 1021.88 | 1115.711708 | 1.150766 |
| GO:0002821\_positive\_regulation\_of\_adaptive\_immune\_response | 23 | 0 | 0.000000 | -0.000000 | 888 | 928.048292 | 1021.88 | 1115.711708 | 1.150766 |
| GO:0002824\_positive\_regulation\_of\_adaptive\_immune\_response\_based\_on\_somatic\_recombination\_of\_immune\_receptors\_built\_from\_immunoglobulin\_superfamily\_domains | 23 | 0 | 0.000000 | -0.000000 | 888 | 928.048292 | 1021.88 | 1115.711708 | 1.150766 |
| GO:0003073\_regulation\_of\_systemic\_arterial\_blood\_pressure | 23 | 0 | 0.000000 | -0.000000 | 888 | 928.048292 | 1021.88 | 1115.711708 | 1.150766 |
| GO:0007584\_response\_to\_nutrient | 23 | 0 | 0.000000 | -0.000000 | 888 | 928.048292 | 1021.88 | 1115.711708 | 1.150766 |
| GO:0009954\_proximal\_distal\_pattern\_formation | 23 | 0 | 0.000000 | -0.000000 | 888 | 928.048292 | 1021.88 | 1115.711708 | 1.150766 |
| GO:0015698\_inorganic\_anion\_transport | 23 | 0 | 0.000000 | -0.000000 | 888 | 928.048292 | 1021.88 | 1115.711708 | 1.150766 |
| GO:0032635\_interleukin-6\_production | 23 | 0 | 0.000000 | -0.000000 | 888 | 928.048292 | 1021.88 | 1115.711708 | 1.150766 |
| GO:0032675\_regulation\_of\_interleukin-6\_production | 23 | 0 | 0.000000 | -0.000000 | 888 | 928.048292 | 1021.88 | 1115.711708 | 1.150766 |
| GO:0042267\_natural\_killer\_cell\_mediated\_cytotoxicity | 23 | 0 | 0.000000 | -0.000000 | 888 | 928.048292 | 1021.88 | 1115.711708 | 1.150766 |
| GO:0043388\_positive\_regulation\_of\_DNA\_binding | 23 | 0 | 0.000000 | -0.000000 | 888 | 928.048292 | 1021.88 | 1115.711708 | 1.150766 |
| GO:0045190\_isotype\_switching | 23 | 0 | 0.000000 | -0.000000 | 888 | 928.048292 | 1021.88 | 1115.711708 | 1.150766 |
| GO:0051705\_behavioral\_interaction\_between\_organisms | 23 | 0 | 0.000000 | -0.000000 | 888 | 928.048292 | 1021.88 | 1115.711708 | 1.150766 |
| GO:0060349\_bone\_morphogenesis | 23 | 0 | 0.000000 | -0.000000 | 888 | 928.048292 | 1021.88 | 1115.711708 | 1.150766 |
| GO:0060445\_branching\_involved\_in\_salivary\_gland\_morphogenesis | 23 | 0 | 0.000000 | -0.000000 | 888 | 928.048292 | 1021.88 | 1115.711708 | 1.150766 |
| GO:0001708\_cell\_fate\_specification | 56 | 0 | 0.000000 | -0.000000 | 895 | 934.723951 | 1027.86 | 1120.996049 | 1.148447 |
| GO:0002683\_negative\_regulation\_of\_immune\_system\_process | 56 | 0 | 0.000000 | -0.000000 | 895 | 934.723951 | 1027.86 | 1120.996049 | 1.148447 |
| GO:0002703\_regulation\_of\_leukocyte\_mediated\_immunity | 56 | 0 | 0.000000 | -0.000000 | 895 | 934.723951 | 1027.86 | 1120.996049 | 1.148447 |
| GO:0006790\_sulfur\_metabolic\_process | 56 | 0 | 0.000000 | -0.000000 | 895 | 934.723951 | 1027.86 | 1120.996049 | 1.148447 |
| GO:0042089\_cytokine\_biosynthetic\_process | 56 | 0 | 0.000000 | -0.000000 | 895 | 934.723951 | 1027.86 | 1120.996049 | 1.148447 |
| GO:0042107\_cytokine\_metabolic\_process | 56 | 0 | 0.000000 | -0.000000 | 895 | 934.723951 | 1027.86 | 1120.996049 | 1.148447 |
| GO:0051321\_meiotic\_cell\_cycle | 56 | 0 | 0.000000 | -0.000000 | 895 | 934.723951 | 1027.86 | 1120.996049 | 1.148447 |
| GO:0001776\_leukocyte\_homeostasis | 41 | 0 | 0.000000 | -0.000000 | 911 | 949.444543 | 1041.12 | 1132.795457 | 1.142832 |
| GO:0006260\_DNA\_replication | 41 | 0 | 0.000000 | -0.000000 | 911 | 949.444543 | 1041.12 | 1132.795457 | 1.142832 |
| GO:0006836\_neurotransmitter\_transport | 41 | 0 | 0.000000 | -0.000000 | 911 | 949.444543 | 1041.12 | 1132.795457 | 1.142832 |
| GO:0006865\_amino\_acid\_transport | 41 | 0 | 0.000000 | -0.000000 | 911 | 949.444543 | 1041.12 | 1132.795457 | 1.142832 |
| GO:0006979\_response\_to\_oxidative\_stress | 41 | 0 | 0.000000 | -0.000000 | 911 | 949.444543 | 1041.12 | 1132.795457 | 1.142832 |
| GO:0007254\_JNK\_cascade | 41 | 0 | 0.000000 | -0.000000 | 911 | 949.444543 | 1041.12 | 1132.795457 | 1.142832 |
| GO:0009894\_regulation\_of\_catabolic\_process | 41 | 0 | 0.000000 | -0.000000 | 911 | 949.444543 | 1041.12 | 1132.795457 | 1.142832 |
| GO:0010551\_regulation\_of\_specific\_transcription\_from\_RNA\_polymerase\_II\_promoter | 41 | 0 | 0.000000 | -0.000000 | 911 | 949.444543 | 1041.12 | 1132.795457 | 1.142832 |
| GO:0015833\_peptide\_transport | 41 | 0 | 0.000000 | -0.000000 | 911 | 949.444543 | 1041.12 | 1132.795457 | 1.142832 |
| GO:0015980\_energy\_derivation\_by\_oxidation\_of\_organic\_compounds | 41 | 0 | 0.000000 | -0.000000 | 911 | 949.444543 | 1041.12 | 1132.795457 | 1.142832 |
| GO:0019216\_regulation\_of\_lipid\_metabolic\_process | 41 | 0 | 0.000000 | -0.000000 | 911 | 949.444543 | 1041.12 | 1132.795457 | 1.142832 |
| GO:0030817\_regulation\_of\_cAMP\_biosynthetic\_process | 41 | 0 | 0.000000 | -0.000000 | 911 | 949.444543 | 1041.12 | 1132.795457 | 1.142832 |
| GO:0032569\_specific\_transcription\_from\_RNA\_polymerase\_II\_promoter | 41 | 0 | 0.000000 | -0.000000 | 911 | 949.444543 | 1041.12 | 1132.795457 | 1.142832 |
| GO:0032844\_regulation\_of\_homeostatic\_process | 41 | 0 | 0.000000 | -0.000000 | 911 | 949.444543 | 1041.12 | 1132.795457 | 1.142832 |
| GO:0033077\_T\_cell\_differentiation\_in\_the\_thymus | 41 | 0 | 0.000000 | -0.000000 | 911 | 949.444543 | 1041.12 | 1132.795457 | 1.142832 |
| GO:0050864\_regulation\_of\_B\_cell\_activation | 41 | 0 | 0.000000 | -0.000000 | 911 | 949.444543 | 1041.12 | 1132.795457 | 1.142832 |
| GO:0006520\_cellular\_amino\_acid\_metabolic\_process | 51 | 0 | 0.000000 | -0.000000 | 919 | 956.018035 | 1047.29 | 1138.561965 | 1.139597 |
| GO:0006887\_exocytosis | 51 | 0 | 0.000000 | -0.000000 | 919 | 956.018035 | 1047.29 | 1138.561965 | 1.139597 |
| GO:0016569\_covalent\_chromatin\_modification | 51 | 0 | 0.000000 | -0.000000 | 919 | 956.018035 | 1047.29 | 1138.561965 | 1.139597 |
| GO:0032583\_regulation\_of\_gene-specific\_transcription | 51 | 0 | 0.000000 | -0.000000 | 919 | 956.018035 | 1047.29 | 1138.561965 | 1.139597 |
| GO:0032880\_regulation\_of\_protein\_localization | 51 | 0 | 0.000000 | -0.000000 | 919 | 956.018035 | 1047.29 | 1138.561965 | 1.139597 |
| GO:0043408\_regulation\_of\_MAPKKK\_cascade | 51 | 0 | 0.000000 | -0.000000 | 919 | 956.018035 | 1047.29 | 1138.561965 | 1.139597 |
| GO:0044106\_cellular\_amine\_metabolic\_process | 51 | 0 | 0.000000 | -0.000000 | 919 | 956.018035 | 1047.29 | 1138.561965 | 1.139597 |
| GO:0048747\_muscle\_fiber\_development | 51 | 0 | 0.000000 | -0.000000 | 919 | 956.018035 | 1047.29 | 1138.561965 | 1.139597 |
| GO:0002699\_positive\_regulation\_of\_immune\_effector\_process | 34 | 0 | 0.000000 | -0.000000 | 933 | 969.669242 | 1059.82 | 1149.970758 | 1.135927 |
| GO:0007269\_neurotransmitter\_secretion | 34 | 0 | 0.000000 | -0.000000 | 933 | 969.669242 | 1059.82 | 1149.970758 | 1.135927 |
| GO:0007338\_single\_fertilization | 34 | 0 | 0.000000 | -0.000000 | 933 | 969.669242 | 1059.82 | 1149.970758 | 1.135927 |
| GO:0007568\_aging | 34 | 0 | 0.000000 | -0.000000 | 933 | 969.669242 | 1059.82 | 1149.970758 | 1.135927 |
| GO:0010720\_positive\_regulation\_of\_cell\_development | 34 | 0 | 0.000000 | -0.000000 | 933 | 969.669242 | 1059.82 | 1149.970758 | 1.135927 |
| GO:0016054\_organic\_acid\_catabolic\_process | 34 | 0 | 0.000000 | -0.000000 | 933 | 969.669242 | 1059.82 | 1149.970758 | 1.135927 |
| GO:0019882\_antigen\_processing\_and\_presentation | 34 | 0 | 0.000000 | -0.000000 | 933 | 969.669242 | 1059.82 | 1149.970758 | 1.135927 |
| GO:0045927\_positive\_regulation\_of\_growth | 34 | 0 | 0.000000 | -0.000000 | 933 | 969.669242 | 1059.82 | 1149.970758 | 1.135927 |
| GO:0046395\_carboxylic\_acid\_catabolic\_process | 34 | 0 | 0.000000 | -0.000000 | 933 | 969.669242 | 1059.82 | 1149.970758 | 1.135927 |
| GO:0050730\_regulation\_of\_peptidyl-tyrosine\_phosphorylation | 34 | 0 | 0.000000 | -0.000000 | 933 | 969.669242 | 1059.82 | 1149.970758 | 1.135927 |
| GO:0051047\_positive\_regulation\_of\_secretion | 34 | 0 | 0.000000 | -0.000000 | 933 | 969.669242 | 1059.82 | 1149.970758 | 1.135927 |
| GO:0051052\_regulation\_of\_DNA\_metabolic\_process | 34 | 0 | 0.000000 | -0.000000 | 933 | 969.669242 | 1059.82 | 1149.970758 | 1.135927 |
| GO:0060443\_mammary\_gland\_morphogenesis | 34 | 0 | 0.000000 | -0.000000 | 933 | 969.669242 | 1059.82 | 1149.970758 | 1.135927 |
| GO:0060711\_labyrinthine\_layer\_development | 34 | 0 | 0.000000 | -0.000000 | 933 | 969.669242 | 1059.82 | 1149.970758 | 1.135927 |
| GO:0000060\_protein\_import\_into\_nucleus\_\_translocation | 14 | 0 | 0.000000 | -0.000000 | 997 | 1027.572945 | 1115.61 | 1203.647055 | 1.118967 |
| GO:0000077\_DNA\_damage\_checkpoint | 14 | 0 | 0.000000 | -0.000000 | 997 | 1027.572945 | 1115.61 | 1203.647055 | 1.118967 |
| GO:0001502\_cartilage\_condensation | 14 | 0 | 0.000000 | -0.000000 | 997 | 1027.572945 | 1115.61 | 1203.647055 | 1.118967 |
| GO:0001829\_trophectodermal\_cell\_differentiation | 14 | 0 | 0.000000 | -0.000000 | 997 | 1027.572945 | 1115.61 | 1203.647055 | 1.118967 |
| GO:0002027\_regulation\_of\_heart\_rate | 14 | 0 | 0.000000 | -0.000000 | 997 | 1027.572945 | 1115.61 | 1203.647055 | 1.118967 |
| GO:0002262\_myeloid\_cell\_homeostasis | 14 | 0 | 0.000000 | -0.000000 | 997 | 1027.572945 | 1115.61 | 1203.647055 | 1.118967 |
| GO:0002698\_negative\_regulation\_of\_immune\_effector\_process | 14 | 0 | 0.000000 | -0.000000 | 997 | 1027.572945 | 1115.61 | 1203.647055 | 1.118967 |
| GO:0006304\_DNA\_modification | 14 | 0 | 0.000000 | -0.000000 | 997 | 1027.572945 | 1115.61 | 1203.647055 | 1.118967 |
| GO:0006305\_DNA\_alkylation | 14 | 0 | 0.000000 | -0.000000 | 997 | 1027.572945 | 1115.61 | 1203.647055 | 1.118967 |
| GO:0006306\_DNA\_methylation | 14 | 0 | 0.000000 | -0.000000 | 997 | 1027.572945 | 1115.61 | 1203.647055 | 1.118967 |
| GO:0006809\_nitric\_oxide\_biosynthetic\_process | 14 | 0 | 0.000000 | -0.000000 | 997 | 1027.572945 | 1115.61 | 1203.647055 | 1.118967 |
| GO:0006914\_autophagy | 14 | 0 | 0.000000 | -0.000000 | 997 | 1027.572945 | 1115.61 | 1203.647055 | 1.118967 |
| GO:0006970\_response\_to\_osmotic\_stress | 14 | 0 | 0.000000 | -0.000000 | 997 | 1027.572945 | 1115.61 | 1203.647055 | 1.118967 |
| GO:0007157\_heterophilic\_cell\_adhesion | 14 | 0 | 0.000000 | -0.000000 | 997 | 1027.572945 | 1115.61 | 1203.647055 | 1.118967 |
| GO:0007530\_sex\_determination | 14 | 0 | 0.000000 | -0.000000 | 997 | 1027.572945 | 1115.61 | 1203.647055 | 1.118967 |
| GO:0007589\_body\_fluid\_secretion | 14 | 0 | 0.000000 | -0.000000 | 997 | 1027.572945 | 1115.61 | 1203.647055 | 1.118967 |
| GO:0008064\_regulation\_of\_actin\_polymerization\_or\_depolymerization | 14 | 0 | 0.000000 | -0.000000 | 997 | 1027.572945 | 1115.61 | 1203.647055 | 1.118967 |
| GO:0008306\_associative\_learning | 14 | 0 | 0.000000 | -0.000000 | 997 | 1027.572945 | 1115.61 | 1203.647055 | 1.118967 |
| GO:0008630\_DNA\_damage\_response\_\_signal\_transduction\_resulting\_in\_induction\_of\_apoptosis | 14 | 0 | 0.000000 | -0.000000 | 997 | 1027.572945 | 1115.61 | 1203.647055 | 1.118967 |
| GO:0009108\_coenzyme\_biosynthetic\_process | 14 | 0 | 0.000000 | -0.000000 | 997 | 1027.572945 | 1115.61 | 1203.647055 | 1.118967 |
| GO:0009267\_cellular\_response\_to\_starvation | 14 | 0 | 0.000000 | -0.000000 | 997 | 1027.572945 | 1115.61 | 1203.647055 | 1.118967 |
| GO:0009895\_negative\_regulation\_of\_catabolic\_process | 14 | 0 | 0.000000 | -0.000000 | 997 | 1027.572945 | 1115.61 | 1203.647055 | 1.118967 |
| GO:0010332\_response\_to\_gamma\_radiation | 14 | 0 | 0.000000 | -0.000000 | 997 | 1027.572945 | 1115.61 | 1203.647055 | 1.118967 |
| GO:0014855\_striated\_muscle\_cell\_proliferation | 14 | 0 | 0.000000 | -0.000000 | 997 | 1027.572945 | 1115.61 | 1203.647055 | 1.118967 |
| GO:0016573\_histone\_acetylation | 14 | 0 | 0.000000 | -0.000000 | 997 | 1027.572945 | 1115.61 | 1203.647055 | 1.118967 |
| GO:0018130\_heterocycle\_biosynthetic\_process | 14 | 0 | 0.000000 | -0.000000 | 997 | 1027.572945 | 1115.61 | 1203.647055 | 1.118967 |
| GO:0019217\_regulation\_of\_fatty\_acid\_metabolic\_process | 14 | 0 | 0.000000 | -0.000000 | 997 | 1027.572945 | 1115.61 | 1203.647055 | 1.118967 |
| GO:0021782\_glial\_cell\_development | 14 | 0 | 0.000000 | -0.000000 | 997 | 1027.572945 | 1115.61 | 1203.647055 | 1.118967 |
| GO:0021904\_dorsal\_ventral\_neural\_tube\_patterning | 14 | 0 | 0.000000 | -0.000000 | 997 | 1027.572945 | 1115.61 | 1203.647055 | 1.118967 |
| GO:0030032\_lamellipodium\_assembly | 14 | 0 | 0.000000 | -0.000000 | 997 | 1027.572945 | 1115.61 | 1203.647055 | 1.118967 |
| GO:0030148\_sphingolipid\_biosynthetic\_process | 14 | 0 | 0.000000 | -0.000000 | 997 | 1027.572945 | 1115.61 | 1203.647055 | 1.118967 |
| GO:0030162\_regulation\_of\_proteolysis | 14 | 0 | 0.000000 | -0.000000 | 997 | 1027.572945 | 1115.61 | 1203.647055 | 1.118967 |
| GO:0030832\_regulation\_of\_actin\_filament\_length | 14 | 0 | 0.000000 | -0.000000 | 997 | 1027.572945 | 1115.61 | 1203.647055 | 1.118967 |
| GO:0031099\_regeneration | 14 | 0 | 0.000000 | -0.000000 | 997 | 1027.572945 | 1115.61 | 1203.647055 | 1.118967 |
| GO:0031346\_positive\_regulation\_of\_cell\_projection\_organization | 14 | 0 | 0.000000 | -0.000000 | 997 | 1027.572945 | 1115.61 | 1203.647055 | 1.118967 |
| GO:0031663\_lipopolysaccharide-mediated\_signaling\_pathway | 14 | 0 | 0.000000 | -0.000000 | 997 | 1027.572945 | 1115.61 | 1203.647055 | 1.118967 |
| GO:0032271\_regulation\_of\_protein\_polymerization | 14 | 0 | 0.000000 | -0.000000 | 997 | 1027.572945 | 1115.61 | 1203.647055 | 1.118967 |
| GO:0033044\_regulation\_of\_chromosome\_organization | 14 | 0 | 0.000000 | -0.000000 | 997 | 1027.572945 | 1115.61 | 1203.647055 | 1.118967 |
| GO:0034104\_negative\_regulation\_of\_tissue\_remodeling | 14 | 0 | 0.000000 | -0.000000 | 997 | 1027.572945 | 1115.61 | 1203.647055 | 1.118967 |
| GO:0034623\_cellular\_macromolecular\_complex\_disassembly | 14 | 0 | 0.000000 | -0.000000 | 997 | 1027.572945 | 1115.61 | 1203.647055 | 1.118967 |
| GO:0035036\_sperm-egg\_recognition | 14 | 0 | 0.000000 | -0.000000 | 997 | 1027.572945 | 1115.61 | 1203.647055 | 1.118967 |
| GO:0042310\_vasoconstriction | 14 | 0 | 0.000000 | -0.000000 | 997 | 1027.572945 | 1115.61 | 1203.647055 | 1.118967 |
| GO:0042573\_retinoic\_acid\_metabolic\_process | 14 | 0 | 0.000000 | -0.000000 | 997 | 1027.572945 | 1115.61 | 1203.647055 | 1.118967 |
| GO:0043123\_positive\_regulation\_of\_I-kappaB\_kinase\_NF-kappaB\_cascade | 14 | 0 | 0.000000 | -0.000000 | 997 | 1027.572945 | 1115.61 | 1203.647055 | 1.118967 |
| GO:0043254\_regulation\_of\_protein\_complex\_assembly | 14 | 0 | 0.000000 | -0.000000 | 997 | 1027.572945 | 1115.61 | 1203.647055 | 1.118967 |
| GO:0043491\_protein\_kinase\_B\_signaling\_cascade | 14 | 0 | 0.000000 | -0.000000 | 997 | 1027.572945 | 1115.61 | 1203.647055 | 1.118967 |
| GO:0044236\_multicellular\_organismal\_metabolic\_process | 14 | 0 | 0.000000 | -0.000000 | 997 | 1027.572945 | 1115.61 | 1203.647055 | 1.118967 |
| GO:0045061\_thymic\_T\_cell\_selection | 14 | 0 | 0.000000 | -0.000000 | 997 | 1027.572945 | 1115.61 | 1203.647055 | 1.118967 |
| GO:0045453\_bone\_resorption | 14 | 0 | 0.000000 | -0.000000 | 997 | 1027.572945 | 1115.61 | 1203.647055 | 1.118967 |
| GO:0045598\_regulation\_of\_fat\_cell\_differentiation | 14 | 0 | 0.000000 | -0.000000 | 997 | 1027.572945 | 1115.61 | 1203.647055 | 1.118967 |
| GO:0045732\_positive\_regulation\_of\_protein\_catabolic\_process | 14 | 0 | 0.000000 | -0.000000 | 997 | 1027.572945 | 1115.61 | 1203.647055 | 1.118967 |
| GO:0046209\_nitric\_oxide\_metabolic\_process | 14 | 0 | 0.000000 | -0.000000 | 997 | 1027.572945 | 1115.61 | 1203.647055 | 1.118967 |
| GO:0048048\_embryonic\_eye\_morphogenesis | 14 | 0 | 0.000000 | -0.000000 | 997 | 1027.572945 | 1115.61 | 1203.647055 | 1.118967 |
| GO:0048545\_response\_to\_steroid\_hormone\_stimulus | 14 | 0 | 0.000000 | -0.000000 | 997 | 1027.572945 | 1115.61 | 1203.647055 | 1.118967 |
| GO:0048665\_neuron\_fate\_specification | 14 | 0 | 0.000000 | -0.000000 | 997 | 1027.572945 | 1115.61 | 1203.647055 | 1.118967 |
| GO:0048844\_artery\_morphogenesis | 14 | 0 | 0.000000 | -0.000000 | 997 | 1027.572945 | 1115.61 | 1203.647055 | 1.118967 |
| GO:0050810\_regulation\_of\_steroid\_biosynthetic\_process | 14 | 0 | 0.000000 | -0.000000 | 997 | 1027.572945 | 1115.61 | 1203.647055 | 1.118967 |
| GO:0051017\_actin\_filament\_bundle\_formation | 14 | 0 | 0.000000 | -0.000000 | 997 | 1027.572945 | 1115.61 | 1203.647055 | 1.118967 |
| GO:0051053\_negative\_regulation\_of\_DNA\_metabolic\_process | 14 | 0 | 0.000000 | -0.000000 | 997 | 1027.572945 | 1115.61 | 1203.647055 | 1.118967 |
| GO:0051054\_positive\_regulation\_of\_DNA\_metabolic\_process | 14 | 0 | 0.000000 | -0.000000 | 997 | 1027.572945 | 1115.61 | 1203.647055 | 1.118967 |
| GO:0051100\_negative\_regulation\_of\_binding | 14 | 0 | 0.000000 | -0.000000 | 997 | 1027.572945 | 1115.61 | 1203.647055 | 1.118967 |
| GO:0051952\_regulation\_of\_amine\_transport | 14 | 0 | 0.000000 | -0.000000 | 997 | 1027.572945 | 1115.61 | 1203.647055 | 1.118967 |
| GO:0060716\_labyrinthine\_layer\_blood\_vessel\_development | 14 | 0 | 0.000000 | -0.000000 | 997 | 1027.572945 | 1115.61 | 1203.647055 | 1.118967 |
| GO:0060840\_artery\_development | 14 | 0 | 0.000000 | -0.000000 | 997 | 1027.572945 | 1115.61 | 1203.647055 | 1.118967 |
| GO:0001932\_regulation\_of\_protein\_amino\_acid\_phosphorylation | 69 | 0 | 0.000000 | -0.000000 | 999 | 1030.620873 | 1118.17 | 1205.719127 | 1.119289 |
| GO:0032101\_regulation\_of\_response\_to\_external\_stimulus | 69 | 0 | 0.000000 | -0.000000 | 999 | 1030.620873 | 1118.17 | 1205.719127 | 1.119289 |
| GO:0006935\_chemotaxis | 53 | 0 | 0.000000 | -0.000000 | 1003 | 1035.561589 | 1122.65 | 1209.738411 | 1.119292 |
| GO:0042330\_taxis | 53 | 0 | 0.000000 | -0.000000 | 1003 | 1035.561589 | 1122.65 | 1209.738411 | 1.119292 |
| GO:0050905\_neuromuscular\_process | 53 | 0 | 0.000000 | -0.000000 | 1003 | 1035.561589 | 1122.65 | 1209.738411 | 1.119292 |
| GO:0051248\_negative\_regulation\_of\_protein\_metabolic\_process | 53 | 0 | 0.000000 | -0.000000 | 1003 | 1035.561589 | 1122.65 | 1209.738411 | 1.119292 |
| GO:0001843\_neural\_tube\_closure | 33 | 0 | 0.000000 | -0.000000 | 1015 | 1047.057898 | 1133.17 | 1219.282102 | 1.116424 |
| GO:0006643\_membrane\_lipid\_metabolic\_process | 33 | 0 | 0.000000 | -0.000000 | 1015 | 1047.057898 | 1133.17 | 1219.282102 | 1.116424 |
| GO:0007188\_G-protein\_signaling\_\_coupled\_to\_cAMP\_nucleotide\_second\_messenger | 33 | 0 | 0.000000 | -0.000000 | 1015 | 1047.057898 | 1133.17 | 1219.282102 | 1.116424 |
| GO:0007270\_nerve-nerve\_synaptic\_transmission | 33 | 0 | 0.000000 | -0.000000 | 1015 | 1047.057898 | 1133.17 | 1219.282102 | 1.116424 |
| GO:0007431\_salivary\_gland\_development | 33 | 0 | 0.000000 | -0.000000 | 1015 | 1047.057898 | 1133.17 | 1219.282102 | 1.116424 |
| GO:0008584\_male\_gonad\_development | 33 | 0 | 0.000000 | -0.000000 | 1015 | 1047.057898 | 1133.17 | 1219.282102 | 1.116424 |
| GO:0008643\_carbohydrate\_transport | 33 | 0 | 0.000000 | -0.000000 | 1015 | 1047.057898 | 1133.17 | 1219.282102 | 1.116424 |
| GO:0021536\_diencephalon\_development | 33 | 0 | 0.000000 | -0.000000 | 1015 | 1047.057898 | 1133.17 | 1219.282102 | 1.116424 |
| GO:0021987\_cerebral\_cortex\_development | 33 | 0 | 0.000000 | -0.000000 | 1015 | 1047.057898 | 1133.17 | 1219.282102 | 1.116424 |
| GO:0022037\_metencephalon\_development | 33 | 0 | 0.000000 | -0.000000 | 1015 | 1047.057898 | 1133.17 | 1219.282102 | 1.116424 |
| GO:0042108\_positive\_regulation\_of\_cytokine\_biosynthetic\_process | 33 | 0 | 0.000000 | -0.000000 | 1015 | 1047.057898 | 1133.17 | 1219.282102 | 1.116424 |
| GO:0060606\_tube\_closure | 33 | 0 | 0.000000 | -0.000000 | 1015 | 1047.057898 | 1133.17 | 1219.282102 | 1.116424 |
| GO:0000280\_nuclear\_division | 24 | 0 | 0.000000 | -0.000000 | 1040 | 1071.384671 | 1156.13 | 1240.875329 | 1.111663 |
| GO:0002381\_immunoglobulin\_production\_during\_immune\_response | 24 | 0 | 0.000000 | -0.000000 | 1040 | 1071.384671 | 1156.13 | 1240.875329 | 1.111663 |
| GO:0006941\_striated\_muscle\_contraction | 24 | 0 | 0.000000 | -0.000000 | 1040 | 1071.384671 | 1156.13 | 1240.875329 | 1.111663 |
| GO:0006959\_humoral\_immune\_response | 24 | 0 | 0.000000 | -0.000000 | 1040 | 1071.384671 | 1156.13 | 1240.875329 | 1.111663 |
| GO:0007050\_cell\_cycle\_arrest | 24 | 0 | 0.000000 | -0.000000 | 1040 | 1071.384671 | 1156.13 | 1240.875329 | 1.111663 |
| GO:0007067\_mitosis | 24 | 0 | 0.000000 | -0.000000 | 1040 | 1071.384671 | 1156.13 | 1240.875329 | 1.111663 |
| GO:0007204\_elevation\_of\_cytosolic\_calcium\_ion\_concentration | 24 | 0 | 0.000000 | -0.000000 | 1040 | 1071.384671 | 1156.13 | 1240.875329 | 1.111663 |
| GO:0007259\_JAK-STAT\_cascade | 24 | 0 | 0.000000 | -0.000000 | 1040 | 1071.384671 | 1156.13 | 1240.875329 | 1.111663 |
| GO:0007266\_Rho\_protein\_signal\_transduction | 24 | 0 | 0.000000 | -0.000000 | 1040 | 1071.384671 | 1156.13 | 1240.875329 | 1.111663 |
| GO:0008629\_induction\_of\_apoptosis\_by\_intracellular\_signals | 24 | 0 | 0.000000 | -0.000000 | 1040 | 1071.384671 | 1156.13 | 1240.875329 | 1.111663 |
| GO:0009612\_response\_to\_mechanical\_stimulus | 24 | 0 | 0.000000 | -0.000000 | 1040 | 1071.384671 | 1156.13 | 1240.875329 | 1.111663 |
| GO:0014070\_response\_to\_organic\_cyclic\_substance | 24 | 0 | 0.000000 | -0.000000 | 1040 | 1071.384671 | 1156.13 | 1240.875329 | 1.111663 |
| GO:0021515\_cell\_differentiation\_in\_spinal\_cord | 24 | 0 | 0.000000 | -0.000000 | 1040 | 1071.384671 | 1156.13 | 1240.875329 | 1.111663 |
| GO:0032386\_regulation\_of\_intracellular\_transport | 24 | 0 | 0.000000 | -0.000000 | 1040 | 1071.384671 | 1156.13 | 1240.875329 | 1.111663 |
| GO:0042158\_lipoprotein\_biosynthetic\_process | 24 | 0 | 0.000000 | -0.000000 | 1040 | 1071.384671 | 1156.13 | 1240.875329 | 1.111663 |
| GO:0042632\_cholesterol\_homeostasis | 24 | 0 | 0.000000 | -0.000000 | 1040 | 1071.384671 | 1156.13 | 1240.875329 | 1.111663 |
| GO:0043410\_positive\_regulation\_of\_MAPKKK\_cascade | 24 | 0 | 0.000000 | -0.000000 | 1040 | 1071.384671 | 1156.13 | 1240.875329 | 1.111663 |
| GO:0043588\_skin\_development | 24 | 0 | 0.000000 | -0.000000 | 1040 | 1071.384671 | 1156.13 | 1240.875329 | 1.111663 |
| GO:0048002\_antigen\_processing\_and\_presentation\_of\_peptide\_antigen | 24 | 0 | 0.000000 | -0.000000 | 1040 | 1071.384671 | 1156.13 | 1240.875329 | 1.111663 |
| GO:0048546\_digestive\_tract\_morphogenesis | 24 | 0 | 0.000000 | -0.000000 | 1040 | 1071.384671 | 1156.13 | 1240.875329 | 1.111663 |
| GO:0051099\_positive\_regulation\_of\_binding | 24 | 0 | 0.000000 | -0.000000 | 1040 | 1071.384671 | 1156.13 | 1240.875329 | 1.111663 |
| GO:0055092\_sterol\_homeostasis | 24 | 0 | 0.000000 | -0.000000 | 1040 | 1071.384671 | 1156.13 | 1240.875329 | 1.111663 |
| GO:0060078\_regulation\_of\_postsynaptic\_membrane\_potential | 24 | 0 | 0.000000 | -0.000000 | 1040 | 1071.384671 | 1156.13 | 1240.875329 | 1.111663 |
| GO:0060113\_inner\_ear\_receptor\_cell\_differentiation | 24 | 0 | 0.000000 | -0.000000 | 1040 | 1071.384671 | 1156.13 | 1240.875329 | 1.111663 |
| GO:0070667\_negative\_regulation\_of\_mast\_cell\_proliferation | 24 | 0 | 0.000000 | -0.000000 | 1040 | 1071.384671 | 1156.13 | 1240.875329 | 1.111663 |
| GO:0001656\_metanephros\_development | 50 | 0 | 0.000000 | -0.000000 | 1046 | 1077.779355 | 1161.59 | 1245.400645 | 1.110507 |
| GO:0002573\_myeloid\_leukocyte\_differentiation | 50 | 0 | 0.000000 | -0.000000 | 1046 | 1077.779355 | 1161.59 | 1245.400645 | 1.110507 |
| GO:0007015\_actin\_filament\_organization | 50 | 0 | 0.000000 | -0.000000 | 1046 | 1077.779355 | 1161.59 | 1245.400645 | 1.110507 |
| GO:0017038\_protein\_import | 50 | 0 | 0.000000 | -0.000000 | 1046 | 1077.779355 | 1161.59 | 1245.400645 | 1.110507 |
| GO:0042129\_regulation\_of\_T\_cell\_proliferation | 50 | 0 | 0.000000 | -0.000000 | 1046 | 1077.779355 | 1161.59 | 1245.400645 | 1.110507 |
| GO:0070647\_protein\_modification\_by\_small\_protein\_conjugation\_or\_removal | 50 | 0 | 0.000000 | -0.000000 | 1046 | 1077.779355 | 1161.59 | 1245.400645 | 1.110507 |
| GO:0044265\_cellular\_macromolecule\_catabolic\_process | 75 | 0 | 0.000000 | -0.000000 | 1048 | 1080.008496 | 1163.52 | 1247.031504 | 1.110229 |
| GO:0048589\_developmental\_growth | 75 | 0 | 0.000000 | -0.000000 | 1048 | 1080.008496 | 1163.52 | 1247.031504 | 1.110229 |
| GO:0016570\_histone\_modification | 47 | 0 | 0.000000 | -0.000000 | 1053 | 1088.347390 | 1170.92 | 1253.492610 | 1.111985 |
| GO:0031667\_response\_to\_nutrient\_levels | 47 | 0 | 0.000000 | -0.000000 | 1053 | 1088.347390 | 1170.92 | 1253.492610 | 1.111985 |
| GO:0045087\_innate\_immune\_response | 47 | 0 | 0.000000 | -0.000000 | 1053 | 1088.347390 | 1170.92 | 1253.492610 | 1.111985 |
| GO:0045619\_regulation\_of\_lymphocyte\_differentiation | 47 | 0 | 0.000000 | -0.000000 | 1053 | 1088.347390 | 1170.92 | 1253.492610 | 1.111985 |
| GO:0060627\_regulation\_of\_vesicle-mediated\_transport | 47 | 0 | 0.000000 | -0.000000 | 1053 | 1088.347390 | 1170.92 | 1253.492610 | 1.111985 |
| GO:0051046\_regulation\_of\_secretion | 79 | 0 | 0.000000 | -0.000000 | 1054 | 1089.514784 | 1171.93 | 1254.345216 | 1.111888 |
| GO:0002697\_regulation\_of\_immune\_effector\_process | 68 | 0 | 0.000000 | -0.000000 | 1056 | 1092.018404 | 1173.94 | 1255.861596 | 1.111686 |
| GO:0034962\_cellular\_biopolymer\_catabolic\_process | 68 | 0 | 0.000000 | -0.000000 | 1056 | 1092.018404 | 1173.94 | 1255.861596 | 1.111686 |
| GO:0001759\_induction\_of\_an\_organ | 15 | 0 | 0.000000 | -0.000000 | 1109 | 1142.465066 | 1222.42 | 1302.374934 | 1.102272 |
| GO:0001782\_B\_cell\_homeostasis | 15 | 0 | 0.000000 | -0.000000 | 1109 | 1142.465066 | 1222.42 | 1302.374934 | 1.102272 |
| GO:0001964\_startle\_response | 15 | 0 | 0.000000 | -0.000000 | 1109 | 1142.465066 | 1222.42 | 1302.374934 | 1.102272 |
| GO:0002286\_T\_cell\_activation\_during\_immune\_response | 15 | 0 | 0.000000 | -0.000000 | 1109 | 1142.465066 | 1222.42 | 1302.374934 | 1.102272 |
| GO:0002495\_antigen\_processing\_and\_presentation\_of\_peptide\_antigen\_via\_MHC\_class\_II | 15 | 0 | 0.000000 | -0.000000 | 1109 | 1142.465066 | 1222.42 | 1302.374934 | 1.102272 |
| GO:0002504\_antigen\_processing\_and\_presentation\_of\_peptide\_or\_polysaccharide\_antigen\_via\_MHC\_class\_II | 15 | 0 | 0.000000 | -0.000000 | 1109 | 1142.465066 | 1222.42 | 1302.374934 | 1.102272 |
| GO:0002709\_regulation\_of\_T\_cell\_mediated\_immunity | 15 | 0 | 0.000000 | -0.000000 | 1109 | 1142.465066 | 1222.42 | 1302.374934 | 1.102272 |
| GO:0006473\_protein\_amino\_acid\_acetylation | 15 | 0 | 0.000000 | -0.000000 | 1109 | 1142.465066 | 1222.42 | 1302.374934 | 1.102272 |
| GO:0006487\_protein\_amino\_acid\_N-linked\_glycosylation | 15 | 0 | 0.000000 | -0.000000 | 1109 | 1142.465066 | 1222.42 | 1302.374934 | 1.102272 |
| GO:0006749\_glutathione\_metabolic\_process | 15 | 0 | 0.000000 | -0.000000 | 1109 | 1142.465066 | 1222.42 | 1302.374934 | 1.102272 |
| GO:0006885\_regulation\_of\_pH | 15 | 0 | 0.000000 | -0.000000 | 1109 | 1142.465066 | 1222.42 | 1302.374934 | 1.102272 |
| GO:0007040\_lysosome\_organization | 15 | 0 | 0.000000 | -0.000000 | 1109 | 1142.465066 | 1222.42 | 1302.374934 | 1.102272 |
| GO:0007173\_epidermal\_growth\_factor\_receptor\_signaling\_pathway | 15 | 0 | 0.000000 | -0.000000 | 1109 | 1142.465066 | 1222.42 | 1302.374934 | 1.102272 |
| GO:0007200\_activation\_of\_phospholipase\_C\_activity\_by\_G-protein\_coupled\_receptor\_protein\_signaling\_pathway\_coupled\_to\_IP3\_second\_messenger | 15 | 0 | 0.000000 | -0.000000 | 1109 | 1142.465066 | 1222.42 | 1302.374934 | 1.102272 |
| GO:0007202\_activation\_of\_phospholipase\_C\_activity | 15 | 0 | 0.000000 | -0.000000 | 1109 | 1142.465066 | 1222.42 | 1302.374934 | 1.102272 |
| GO:0007218\_neuropeptide\_signaling\_pathway | 15 | 0 | 0.000000 | -0.000000 | 1109 | 1142.465066 | 1222.42 | 1302.374934 | 1.102272 |
| GO:0007588\_excretion | 15 | 0 | 0.000000 | -0.000000 | 1109 | 1142.465066 | 1222.42 | 1302.374934 | 1.102272 |
| GO:0007618\_mating | 15 | 0 | 0.000000 | -0.000000 | 1109 | 1142.465066 | 1222.42 | 1302.374934 | 1.102272 |
| GO:0008543\_fibroblast\_growth\_factor\_receptor\_signaling\_pathway | 15 | 0 | 0.000000 | -0.000000 | 1109 | 1142.465066 | 1222.42 | 1302.374934 | 1.102272 |
| GO:0009062\_fatty\_acid\_catabolic\_process | 15 | 0 | 0.000000 | -0.000000 | 1109 | 1142.465066 | 1222.42 | 1302.374934 | 1.102272 |
| GO:0009116\_nucleoside\_metabolic\_process | 15 | 0 | 0.000000 | -0.000000 | 1109 | 1142.465066 | 1222.42 | 1302.374934 | 1.102272 |
| GO:0010092\_specification\_of\_organ\_identity | 15 | 0 | 0.000000 | -0.000000 | 1109 | 1142.465066 | 1222.42 | 1302.374934 | 1.102272 |
| GO:0010171\_body\_morphogenesis | 15 | 0 | 0.000000 | -0.000000 | 1109 | 1142.465066 | 1222.42 | 1302.374934 | 1.102272 |
| GO:0010518\_positive\_regulation\_of\_phospholipase\_activity | 15 | 0 | 0.000000 | -0.000000 | 1109 | 1142.465066 | 1222.42 | 1302.374934 | 1.102272 |
| GO:0010863\_positive\_regulation\_of\_phospholipase\_C\_activity | 15 | 0 | 0.000000 | -0.000000 | 1109 | 1142.465066 | 1222.42 | 1302.374934 | 1.102272 |
| GO:0015931\_nucleobase\_\_nucleoside\_\_nucleotide\_and\_nucleic\_acid\_transport | 15 | 0 | 0.000000 | -0.000000 | 1109 | 1142.465066 | 1222.42 | 1302.374934 | 1.102272 |
| GO:0019886\_antigen\_processing\_and\_presentation\_of\_exogenous\_peptide\_antigen\_via\_MHC\_class\_II | 15 | 0 | 0.000000 | -0.000000 | 1109 | 1142.465066 | 1222.42 | 1302.374934 | 1.102272 |
| GO:0021795\_cerebral\_cortex\_cell\_migration | 15 | 0 | 0.000000 | -0.000000 | 1109 | 1142.465066 | 1222.42 | 1302.374934 | 1.102272 |
| GO:0021872\_generation\_of\_neurons\_in\_the\_forebrain | 15 | 0 | 0.000000 | -0.000000 | 1109 | 1142.465066 | 1222.42 | 1302.374934 | 1.102272 |
| GO:0022600\_digestive\_system\_process | 15 | 0 | 0.000000 | -0.000000 | 1109 | 1142.465066 | 1222.42 | 1302.374934 | 1.102272 |
| GO:0030041\_actin\_filament\_polymerization | 15 | 0 | 0.000000 | -0.000000 | 1109 | 1142.465066 | 1222.42 | 1302.374934 | 1.102272 |
| GO:0031069\_hair\_follicle\_morphogenesis | 15 | 0 | 0.000000 | -0.000000 | 1109 | 1142.465066 | 1222.42 | 1302.374934 | 1.102272 |
| GO:0031076\_embryonic\_camera-type\_eye\_development | 15 | 0 | 0.000000 | -0.000000 | 1109 | 1142.465066 | 1222.42 | 1302.374934 | 1.102272 |
| GO:0031329\_regulation\_of\_cellular\_catabolic\_process | 15 | 0 | 0.000000 | -0.000000 | 1109 | 1142.465066 | 1222.42 | 1302.374934 | 1.102272 |
| GO:0035116\_embryonic\_hindlimb\_morphogenesis | 15 | 0 | 0.000000 | -0.000000 | 1109 | 1142.465066 | 1222.42 | 1302.374934 | 1.102272 |
| GO:0035249\_synaptic\_transmission\_\_glutamatergic | 15 | 0 | 0.000000 | -0.000000 | 1109 | 1142.465066 | 1222.42 | 1302.374934 | 1.102272 |
| GO:0042306\_regulation\_of\_protein\_import\_into\_nucleus | 15 | 0 | 0.000000 | -0.000000 | 1109 | 1142.465066 | 1222.42 | 1302.374934 | 1.102272 |
| GO:0046164\_alcohol\_catabolic\_process | 15 | 0 | 0.000000 | -0.000000 | 1109 | 1142.465066 | 1222.42 | 1302.374934 | 1.102272 |
| GO:0046638\_positive\_regulation\_of\_alpha-beta\_T\_cell\_differentiation | 15 | 0 | 0.000000 | -0.000000 | 1109 | 1142.465066 | 1222.42 | 1302.374934 | 1.102272 |
| GO:0048008\_platelet-derived\_growth\_factor\_receptor\_signaling\_pathway | 15 | 0 | 0.000000 | -0.000000 | 1109 | 1142.465066 | 1222.42 | 1302.374934 | 1.102272 |
| GO:0048144\_fibroblast\_proliferation | 15 | 0 | 0.000000 | -0.000000 | 1109 | 1142.465066 | 1222.42 | 1302.374934 | 1.102272 |
| GO:0048145\_regulation\_of\_fibroblast\_proliferation | 15 | 0 | 0.000000 | -0.000000 | 1109 | 1142.465066 | 1222.42 | 1302.374934 | 1.102272 |
| GO:0048610\_reproductive\_cellular\_process | 15 | 0 | 0.000000 | -0.000000 | 1109 | 1142.465066 | 1222.42 | 1302.374934 | 1.102272 |
| GO:0048709\_oligodendrocyte\_differentiation | 15 | 0 | 0.000000 | -0.000000 | 1109 | 1142.465066 | 1222.42 | 1302.374934 | 1.102272 |
| GO:0050729\_positive\_regulation\_of\_inflammatory\_response | 15 | 0 | 0.000000 | -0.000000 | 1109 | 1142.465066 | 1222.42 | 1302.374934 | 1.102272 |
| GO:0050796\_regulation\_of\_insulin\_secretion | 15 | 0 | 0.000000 | -0.000000 | 1109 | 1142.465066 | 1222.42 | 1302.374934 | 1.102272 |
| GO:0050798\_activated\_T\_cell\_proliferation | 15 | 0 | 0.000000 | -0.000000 | 1109 | 1142.465066 | 1222.42 | 1302.374934 | 1.102272 |
| GO:0055010\_ventricular\_cardiac\_muscle\_morphogenesis | 15 | 0 | 0.000000 | -0.000000 | 1109 | 1142.465066 | 1222.42 | 1302.374934 | 1.102272 |
| GO:0060322\_head\_development | 15 | 0 | 0.000000 | -0.000000 | 1109 | 1142.465066 | 1222.42 | 1302.374934 | 1.102272 |
| GO:0060425\_lung\_morphogenesis | 15 | 0 | 0.000000 | -0.000000 | 1109 | 1142.465066 | 1222.42 | 1302.374934 | 1.102272 |
| GO:0060442\_branching\_involved\_in\_prostate\_gland\_morphogenesis | 15 | 0 | 0.000000 | -0.000000 | 1109 | 1142.465066 | 1222.42 | 1302.374934 | 1.102272 |
| GO:0060749\_mammary\_gland\_alveolus\_development | 15 | 0 | 0.000000 | -0.000000 | 1109 | 1142.465066 | 1222.42 | 1302.374934 | 1.102272 |
| GO:0070227\_lymphocyte\_apoptosis | 15 | 0 | 0.000000 | -0.000000 | 1109 | 1142.465066 | 1222.42 | 1302.374934 | 1.102272 |
| GO:0000302\_response\_to\_reactive\_oxygen\_species | 16 | 0 | 0.000000 | -0.000000 | 1148 | 1185.502867 | 1263.3 | 1341.097133 | 1.100436 |
| GO:0001933\_negative\_regulation\_of\_protein\_amino\_acid\_phosphorylation | 16 | 0 | 0.000000 | -0.000000 | 1148 | 1185.502867 | 1263.3 | 1341.097133 | 1.100436 |
| GO:0003044\_regulation\_of\_systemic\_arterial\_blood\_pressure\_mediated\_by\_a\_chemical\_signal | 16 | 0 | 0.000000 | -0.000000 | 1148 | 1185.502867 | 1263.3 | 1341.097133 | 1.100436 |
| GO:0006664\_glycolipid\_metabolic\_process | 16 | 0 | 0.000000 | -0.000000 | 1148 | 1185.502867 | 1263.3 | 1341.097133 | 1.100436 |
| GO:0006821\_chloride\_transport | 16 | 0 | 0.000000 | -0.000000 | 1148 | 1185.502867 | 1263.3 | 1341.097133 | 1.100436 |
| GO:0007033\_vacuole\_organization | 16 | 0 | 0.000000 | -0.000000 | 1148 | 1185.502867 | 1263.3 | 1341.097133 | 1.100436 |
| GO:0007156\_homophilic\_cell\_adhesion | 16 | 0 | 0.000000 | -0.000000 | 1148 | 1185.502867 | 1263.3 | 1341.097133 | 1.100436 |
| GO:0008654\_phospholipid\_biosynthetic\_process | 16 | 0 | 0.000000 | -0.000000 | 1148 | 1185.502867 | 1263.3 | 1341.097133 | 1.100436 |
| GO:0009988\_cell-cell\_recognition | 16 | 0 | 0.000000 | -0.000000 | 1148 | 1185.502867 | 1263.3 | 1341.097133 | 1.100436 |
| GO:0010038\_response\_to\_metal\_ion | 16 | 0 | 0.000000 | -0.000000 | 1148 | 1185.502867 | 1263.3 | 1341.097133 | 1.100436 |
| GO:0010243\_response\_to\_organic\_nitrogen | 16 | 0 | 0.000000 | -0.000000 | 1148 | 1185.502867 | 1263.3 | 1341.097133 | 1.100436 |
| GO:0010876\_lipid\_localization | 16 | 0 | 0.000000 | -0.000000 | 1148 | 1185.502867 | 1263.3 | 1341.097133 | 1.100436 |
| GO:0014075\_response\_to\_amine\_stimulus | 16 | 0 | 0.000000 | -0.000000 | 1148 | 1185.502867 | 1263.3 | 1341.097133 | 1.100436 |
| GO:0019722\_calcium-mediated\_signaling | 16 | 0 | 0.000000 | -0.000000 | 1148 | 1185.502867 | 1263.3 | 1341.097133 | 1.100436 |
| GO:0019915\_lipid\_storage | 16 | 0 | 0.000000 | -0.000000 | 1148 | 1185.502867 | 1263.3 | 1341.097133 | 1.100436 |
| GO:0021522\_spinal\_cord\_motor\_neuron\_differentiation | 16 | 0 | 0.000000 | -0.000000 | 1148 | 1185.502867 | 1263.3 | 1341.097133 | 1.100436 |
| GO:0021696\_cerebellar\_cortex\_morphogenesis | 16 | 0 | 0.000000 | -0.000000 | 1148 | 1185.502867 | 1263.3 | 1341.097133 | 1.100436 |
| GO:0030890\_positive\_regulation\_of\_B\_cell\_proliferation | 16 | 0 | 0.000000 | -0.000000 | 1148 | 1185.502867 | 1263.3 | 1341.097133 | 1.100436 |
| GO:0031570\_DNA\_integrity\_checkpoint | 16 | 0 | 0.000000 | -0.000000 | 1148 | 1185.502867 | 1263.3 | 1341.097133 | 1.100436 |
| GO:0031669\_cellular\_response\_to\_nutrient\_levels | 16 | 0 | 0.000000 | -0.000000 | 1148 | 1185.502867 | 1263.3 | 1341.097133 | 1.100436 |
| GO:0032663\_regulation\_of\_interleukin-2\_production | 16 | 0 | 0.000000 | -0.000000 | 1148 | 1185.502867 | 1263.3 | 1341.097133 | 1.100436 |
| GO:0032956\_regulation\_of\_actin\_cytoskeleton\_organization | 16 | 0 | 0.000000 | -0.000000 | 1148 | 1185.502867 | 1263.3 | 1341.097133 | 1.100436 |
| GO:0034976\_response\_to\_endoplasmic\_reticulum\_stress | 16 | 0 | 0.000000 | -0.000000 | 1148 | 1185.502867 | 1263.3 | 1341.097133 | 1.100436 |
| GO:0042311\_vasodilation | 16 | 0 | 0.000000 | -0.000000 | 1148 | 1185.502867 | 1263.3 | 1341.097133 | 1.100436 |
| GO:0042594\_response\_to\_starvation | 16 | 0 | 0.000000 | -0.000000 | 1148 | 1185.502867 | 1263.3 | 1341.097133 | 1.100436 |
| GO:0042596\_fear\_response | 16 | 0 | 0.000000 | -0.000000 | 1148 | 1185.502867 | 1263.3 | 1341.097133 | 1.100436 |
| GO:0043087\_regulation\_of\_GTPase\_activity | 16 | 0 | 0.000000 | -0.000000 | 1148 | 1185.502867 | 1263.3 | 1341.097133 | 1.100436 |
| GO:0043122\_regulation\_of\_I-kappaB\_kinase\_NF-kappaB\_cascade | 16 | 0 | 0.000000 | -0.000000 | 1148 | 1185.502867 | 1263.3 | 1341.097133 | 1.100436 |
| GO:0043367\_CD4-positive\_\_alpha\_beta\_T\_cell\_differentiation | 16 | 0 | 0.000000 | -0.000000 | 1148 | 1185.502867 | 1263.3 | 1341.097133 | 1.100436 |
| GO:0045104\_intermediate\_filament\_cytoskeleton\_organization | 16 | 0 | 0.000000 | -0.000000 | 1148 | 1185.502867 | 1263.3 | 1341.097133 | 1.100436 |
| GO:0046467\_membrane\_lipid\_biosynthetic\_process | 16 | 0 | 0.000000 | -0.000000 | 1148 | 1185.502867 | 1263.3 | 1341.097133 | 1.100436 |
| GO:0046633\_alpha-beta\_T\_cell\_proliferation | 16 | 0 | 0.000000 | -0.000000 | 1148 | 1185.502867 | 1263.3 | 1341.097133 | 1.100436 |
| GO:0046700\_heterocycle\_catabolic\_process | 16 | 0 | 0.000000 | -0.000000 | 1148 | 1185.502867 | 1263.3 | 1341.097133 | 1.100436 |
| GO:0050974\_detection\_of\_mechanical\_stimulus\_involved\_in\_sensory\_perception | 16 | 0 | 0.000000 | -0.000000 | 1148 | 1185.502867 | 1263.3 | 1341.097133 | 1.100436 |
| GO:0051048\_negative\_regulation\_of\_secretion | 16 | 0 | 0.000000 | -0.000000 | 1148 | 1185.502867 | 1263.3 | 1341.097133 | 1.100436 |
| GO:0051937\_catecholamine\_transport | 16 | 0 | 0.000000 | -0.000000 | 1148 | 1185.502867 | 1263.3 | 1341.097133 | 1.100436 |
| GO:0055007\_cardiac\_muscle\_cell\_differentiation | 16 | 0 | 0.000000 | -0.000000 | 1148 | 1185.502867 | 1263.3 | 1341.097133 | 1.100436 |
| GO:0060193\_positive\_regulation\_of\_lipase\_activity | 16 | 0 | 0.000000 | -0.000000 | 1148 | 1185.502867 | 1263.3 | 1341.097133 | 1.100436 |
| GO:0060713\_labyrinthine\_layer\_morphogenesis | 16 | 0 | 0.000000 | -0.000000 | 1148 | 1185.502867 | 1263.3 | 1341.097133 | 1.100436 |
| GO:0001508\_regulation\_of\_action\_potential | 43 | 0 | 0.000000 | -0.000000 | 1162 | 1198.595448 | 1275.27 | 1351.944552 | 1.097478 |
| GO:0001841\_neural\_tube\_formation | 43 | 0 | 0.000000 | -0.000000 | 1162 | 1198.595448 | 1275.27 | 1351.944552 | 1.097478 |
| GO:0002819\_regulation\_of\_adaptive\_immune\_response | 43 | 0 | 0.000000 | -0.000000 | 1162 | 1198.595448 | 1275.27 | 1351.944552 | 1.097478 |
| GO:0002822\_regulation\_of\_adaptive\_immune\_response\_based\_on\_somatic\_recombination\_of\_immune\_receptors\_built\_from\_immunoglobulin\_superfamily\_domains | 43 | 0 | 0.000000 | -0.000000 | 1162 | 1198.595448 | 1275.27 | 1351.944552 | 1.097478 |
| GO:0006766\_vitamin\_metabolic\_process | 43 | 0 | 0.000000 | -0.000000 | 1162 | 1198.595448 | 1275.27 | 1351.944552 | 1.097478 |
| GO:0007224\_smoothened\_signaling\_pathway | 43 | 0 | 0.000000 | -0.000000 | 1162 | 1198.595448 | 1275.27 | 1351.944552 | 1.097478 |
| GO:0030814\_regulation\_of\_cAMP\_metabolic\_process | 43 | 0 | 0.000000 | -0.000000 | 1162 | 1198.595448 | 1275.27 | 1351.944552 | 1.097478 |
| GO:0031098\_stress-activated\_protein\_kinase\_signaling\_pathway | 43 | 0 | 0.000000 | -0.000000 | 1162 | 1198.595448 | 1275.27 | 1351.944552 | 1.097478 |
| GO:0032446\_protein\_modification\_by\_small\_protein\_conjugation | 43 | 0 | 0.000000 | -0.000000 | 1162 | 1198.595448 | 1275.27 | 1351.944552 | 1.097478 |
| GO:0032868\_response\_to\_insulin\_stimulus | 43 | 0 | 0.000000 | -0.000000 | 1162 | 1198.595448 | 1275.27 | 1351.944552 | 1.097478 |
| GO:0046879\_hormone\_secretion | 43 | 0 | 0.000000 | -0.000000 | 1162 | 1198.595448 | 1275.27 | 1351.944552 | 1.097478 |
| GO:0048762\_mesenchymal\_cell\_differentiation | 43 | 0 | 0.000000 | -0.000000 | 1162 | 1198.595448 | 1275.27 | 1351.944552 | 1.097478 |
| GO:0051604\_protein\_maturation | 43 | 0 | 0.000000 | -0.000000 | 1162 | 1198.595448 | 1275.27 | 1351.944552 | 1.097478 |
| GO:0051789\_response\_to\_protein\_stimulus | 43 | 0 | 0.000000 | -0.000000 | 1162 | 1198.595448 | 1275.27 | 1351.944552 | 1.097478 |
| GO:0002062\_chondrocyte\_differentiation | 28 | 0 | 0.000000 | -0.000000 | 1178 | 1214.031999 | 1289.95 | 1365.868001 | 1.095034 |
| GO:0002088\_lens\_development\_in\_camera-type\_eye | 28 | 0 | 0.000000 | -0.000000 | 1178 | 1214.031999 | 1289.95 | 1365.868001 | 1.095034 |
| GO:0002705\_positive\_regulation\_of\_leukocyte\_mediated\_immunity | 28 | 0 | 0.000000 | -0.000000 | 1178 | 1214.031999 | 1289.95 | 1365.868001 | 1.095034 |
| GO:0002708\_positive\_regulation\_of\_lymphocyte\_mediated\_immunity | 28 | 0 | 0.000000 | -0.000000 | 1178 | 1214.031999 | 1289.95 | 1365.868001 | 1.095034 |
| GO:0007127\_meiosis\_I | 28 | 0 | 0.000000 | -0.000000 | 1178 | 1214.031999 | 1289.95 | 1365.868001 | 1.095034 |
| GO:0007585\_respiratory\_gaseous\_exchange | 28 | 0 | 0.000000 | -0.000000 | 1178 | 1214.031999 | 1289.95 | 1365.868001 | 1.095034 |
| GO:0021549\_cerebellum\_development | 28 | 0 | 0.000000 | -0.000000 | 1178 | 1214.031999 | 1289.95 | 1365.868001 | 1.095034 |
| GO:0030073\_insulin\_secretion | 28 | 0 | 0.000000 | -0.000000 | 1178 | 1214.031999 | 1289.95 | 1365.868001 | 1.095034 |
| GO:0030111\_regulation\_of\_Wnt\_receptor\_signaling\_pathway | 28 | 0 | 0.000000 | -0.000000 | 1178 | 1214.031999 | 1289.95 | 1365.868001 | 1.095034 |
| GO:0042100\_B\_cell\_proliferation | 28 | 0 | 0.000000 | -0.000000 | 1178 | 1214.031999 | 1289.95 | 1365.868001 | 1.095034 |
| GO:0043193\_positive\_regulation\_of\_gene-specific\_transcription | 28 | 0 | 0.000000 | -0.000000 | 1178 | 1214.031999 | 1289.95 | 1365.868001 | 1.095034 |
| GO:0045926\_negative\_regulation\_of\_growth | 28 | 0 | 0.000000 | -0.000000 | 1178 | 1214.031999 | 1289.95 | 1365.868001 | 1.095034 |
| GO:0046328\_regulation\_of\_JNK\_cascade | 28 | 0 | 0.000000 | -0.000000 | 1178 | 1214.031999 | 1289.95 | 1365.868001 | 1.095034 |
| GO:0048863\_stem\_cell\_differentiation | 28 | 0 | 0.000000 | -0.000000 | 1178 | 1214.031999 | 1289.95 | 1365.868001 | 1.095034 |
| GO:0050871\_positive\_regulation\_of\_B\_cell\_activation | 28 | 0 | 0.000000 | -0.000000 | 1178 | 1214.031999 | 1289.95 | 1365.868001 | 1.095034 |
| GO:0051188\_cofactor\_biosynthetic\_process | 28 | 0 | 0.000000 | -0.000000 | 1178 | 1214.031999 | 1289.95 | 1365.868001 | 1.095034 |
| GO:0001824\_blastocyst\_development | 40 | 0 | 0.000000 | -0.000000 | 1186 | 1223.404706 | 1298.61 | 1373.815294 | 1.094949 |
| GO:0007346\_regulation\_of\_mitotic\_cell\_cycle | 40 | 0 | 0.000000 | -0.000000 | 1186 | 1223.404706 | 1298.61 | 1373.815294 | 1.094949 |
| GO:0007599\_hemostasis | 40 | 0 | 0.000000 | -0.000000 | 1186 | 1223.404706 | 1298.61 | 1373.815294 | 1.094949 |
| GO:0014031\_mesenchymal\_cell\_development | 40 | 0 | 0.000000 | -0.000000 | 1186 | 1223.404706 | 1298.61 | 1373.815294 | 1.094949 |
| GO:0016358\_dendrite\_development | 40 | 0 | 0.000000 | -0.000000 | 1186 | 1223.404706 | 1298.61 | 1373.815294 | 1.094949 |
| GO:0016485\_protein\_processing | 40 | 0 | 0.000000 | -0.000000 | 1186 | 1223.404706 | 1298.61 | 1373.815294 | 1.094949 |
| GO:0019935\_cyclic-nucleotide-mediated\_signaling | 40 | 0 | 0.000000 | -0.000000 | 1186 | 1223.404706 | 1298.61 | 1373.815294 | 1.094949 |
| GO:0035272\_exocrine\_system\_development | 40 | 0 | 0.000000 | -0.000000 | 1186 | 1223.404706 | 1298.61 | 1373.815294 | 1.094949 |
| GO:0002250\_adaptive\_immune\_response | 80 | 0 | 0.000000 | -0.000000 | 1189 | 1225.952531 | 1300.77 | 1375.587469 | 1.094003 |
| GO:0002460\_adaptive\_immune\_response\_based\_on\_somatic\_recombination\_of\_immune\_receptors\_built\_from\_immunoglobulin\_superfamily\_domains | 80 | 0 | 0.000000 | -0.000000 | 1189 | 1225.952531 | 1300.77 | 1375.587469 | 1.094003 |
| GO:0006631\_fatty\_acid\_metabolic\_process | 80 | 0 | 0.000000 | -0.000000 | 1189 | 1225.952531 | 1300.77 | 1375.587469 | 1.094003 |
| GO:0051241\_negative\_regulation\_of\_multicellular\_organismal\_process | 77 | 0 | 0.000000 | -0.000000 | 1190 | 1226.944304 | 1301.7 | 1376.455696 | 1.093866 |
| GO:0001934\_positive\_regulation\_of\_protein\_amino\_acid\_phosphorylation | 29 | 0 | 0.000000 | -0.000000 | 1210 | 1244.676222 | 1318.31 | 1391.943778 | 1.089512 |
| GO:0006417\_regulation\_of\_translation | 29 | 0 | 0.000000 | -0.000000 | 1210 | 1244.676222 | 1318.31 | 1391.943778 | 1.089512 |
| GO:0006641\_triglyceride\_metabolic\_process | 29 | 0 | 0.000000 | -0.000000 | 1210 | 1244.676222 | 1318.31 | 1391.943778 | 1.089512 |
| GO:0006909\_phagocytosis | 29 | 0 | 0.000000 | -0.000000 | 1210 | 1244.676222 | 1318.31 | 1391.943778 | 1.089512 |
| GO:0007190\_activation\_of\_adenylate\_cyclase\_activity | 29 | 0 | 0.000000 | -0.000000 | 1210 | 1244.676222 | 1318.31 | 1391.943778 | 1.089512 |
| GO:0010564\_regulation\_of\_cell\_cycle\_process | 29 | 0 | 0.000000 | -0.000000 | 1210 | 1244.676222 | 1318.31 | 1391.943778 | 1.089512 |
| GO:0016447\_somatic\_recombination\_of\_immunoglobulin\_gene\_segments | 29 | 0 | 0.000000 | -0.000000 | 1210 | 1244.676222 | 1318.31 | 1391.943778 | 1.089512 |
| GO:0021761\_limbic\_system\_development | 29 | 0 | 0.000000 | -0.000000 | 1210 | 1244.676222 | 1318.31 | 1391.943778 | 1.089512 |
| GO:0042176\_regulation\_of\_protein\_catabolic\_process | 29 | 0 | 0.000000 | -0.000000 | 1210 | 1244.676222 | 1318.31 | 1391.943778 | 1.089512 |
| GO:0042770\_DNA\_damage\_response\_\_signal\_transduction | 29 | 0 | 0.000000 | -0.000000 | 1210 | 1244.676222 | 1318.31 | 1391.943778 | 1.089512 |
| GO:0043281\_regulation\_of\_caspase\_activity | 29 | 0 | 0.000000 | -0.000000 | 1210 | 1244.676222 | 1318.31 | 1391.943778 | 1.089512 |
| GO:0044087\_regulation\_of\_cellular\_component\_biogenesis | 29 | 0 | 0.000000 | -0.000000 | 1210 | 1244.676222 | 1318.31 | 1391.943778 | 1.089512 |
| GO:0044270\_nitrogen\_compound\_catabolic\_process | 29 | 0 | 0.000000 | -0.000000 | 1210 | 1244.676222 | 1318.31 | 1391.943778 | 1.089512 |
| GO:0045621\_positive\_regulation\_of\_lymphocyte\_differentiation | 29 | 0 | 0.000000 | -0.000000 | 1210 | 1244.676222 | 1318.31 | 1391.943778 | 1.089512 |
| GO:0046634\_regulation\_of\_alpha-beta\_T\_cell\_activation | 29 | 0 | 0.000000 | -0.000000 | 1210 | 1244.676222 | 1318.31 | 1391.943778 | 1.089512 |
| GO:0050769\_positive\_regulation\_of\_neurogenesis | 29 | 0 | 0.000000 | -0.000000 | 1210 | 1244.676222 | 1318.31 | 1391.943778 | 1.089512 |
| GO:0051301\_cell\_division | 29 | 0 | 0.000000 | -0.000000 | 1210 | 1244.676222 | 1318.31 | 1391.943778 | 1.089512 |
| GO:0052548\_regulation\_of\_endopeptidase\_activity | 29 | 0 | 0.000000 | -0.000000 | 1210 | 1244.676222 | 1318.31 | 1391.943778 | 1.089512 |
| GO:0060041\_retina\_development\_in\_camera-type\_eye | 29 | 0 | 0.000000 | -0.000000 | 1210 | 1244.676222 | 1318.31 | 1391.943778 | 1.089512 |
| GO:0070302\_regulation\_of\_stress-activated\_protein\_kinase\_signaling\_pathway | 29 | 0 | 0.000000 | -0.000000 | 1210 | 1244.676222 | 1318.31 | 1391.943778 | 1.089512 |
| GO:0000086\_G2\_M\_transition\_of\_mitotic\_cell\_cycle | 4 | 0 |  |  |  |  |  |  |  |  |
| GO:0000305\_response\_to\_oxygen\_radical | 4 | 0 |  |  |  |  |  |  |  |  |
| GO:0001661\_conditioned\_taste\_aversion | 4 | 0 |  |  |  |  |  |  |  |  |
| GO:0001678\_cellular\_glucose\_homeostasis | 4 | 0 |  |  |  |  |  |  |  |  |
| GO:0001777\_T\_cell\_homeostatic\_proliferation | 4 | 0 |  |  |  |  |  |  |  |  |
| GO:0001794\_type\_IIa\_hypersensitivity | 4 | 0 |  |  |  |  |  |  |  |  |
| GO:0001796\_regulation\_of\_type\_IIa\_hypersensitivity | 4 | 0 |  |  |  |  |  |  |  |  |
| GO:0001798\_positive\_regulation\_of\_type\_IIa\_hypersensitivity | 4 | 0 |  |  |  |  |  |  |  |  |
| GO:0001810\_regulation\_of\_type\_I\_hypersensitivity | 4 | 0 |  |  |  |  |  |  |  |  |
| GO:0001820\_serotonin\_secretion | 4 | 0 |  |  |  |  |  |  |  |  |
| GO:0001835\_blastocyst\_hatching | 4 | 0 |  |  |  |  |  |  |  |  |
| GO:0001842\_neural\_fold\_formation | 4 | 0 |  |  |  |  |  |  |  |  |
| GO:0001881\_receptor\_recycling | 4 | 0 |  |  |  |  |  |  |  |  |
| GO:0001938\_positive\_regulation\_of\_endothelial\_cell\_proliferation | 4 | 0 |  |  |  |  |  |  |  |  |
| GO:0001978\_regulation\_of\_systemic\_arterial\_blood\_pressure\_by\_carotid\_sinus\_baroreceptor\_feedback | 4 | 0 |  |  |  |  |  |  |  |  |
| GO:0002035\_brain\_renin-angiotensin\_system | 4 | 0 |  |  |  |  |  |  |  |  |
| GO:0002051\_osteoblast\_fate\_commitment | 4 | 0 |  |  |  |  |  |  |  |  |
| GO:0002220\_innate\_immune\_response\_activating\_cell\_surface\_receptor\_signaling\_pathway | 4 | 0 |  |  |  |  |  |  |  |  |
| GO:0002249\_lymphocyte\_anergy | 4 | 0 |  |  |  |  |  |  |  |  |
| GO:0002318\_myeloid\_progenitor\_cell\_differentiation | 4 | 0 |  |  |  |  |  |  |  |  |
| GO:0002326\_B\_cell\_lineage\_commitment | 4 | 0 |  |  |  |  |  |  |  |  |
| GO:0002347\_response\_to\_tumor\_cell | 4 | 0 |  |  |  |  |  |  |  |  |
| GO:0002418\_immune\_response\_to\_tumor\_cell | 4 | 0 |  |  |  |  |  |  |  |  |
| GO:0002445\_type\_II\_hypersensitivity | 4 | 0 |  |  |  |  |  |  |  |  |
| GO:0002544\_chronic\_inflammatory\_response | 4 | 0 |  |  |  |  |  |  |  |  |
| GO:0002636\_positive\_regulation\_of\_germinal\_center\_formation | 4 | 0 |  |  |  |  |  |  |  |  |
| GO:0002667\_regulation\_of\_T\_cell\_anergy | 4 | 0 |  |  |  |  |  |  |  |  |
| GO:0002669\_positive\_regulation\_of\_T\_cell\_anergy | 4 | 0 |  |  |  |  |  |  |  |  |
| GO:0002687\_positive\_regulation\_of\_leukocyte\_migration | 4 | 0 |  |  |  |  |  |  |  |  |
| GO:0002702\_positive\_regulation\_of\_production\_of\_molecular\_mediator\_of\_immune\_response | 4 | 0 |  |  |  |  |  |  |  |  |
| GO:0002718\_regulation\_of\_cytokine\_production\_during\_immune\_response | 4 | 0 |  |  |  |  |  |  |  |  |
| GO:0002829\_negative\_regulation\_of\_T-helper\_2\_type\_immune\_response | 4 | 0 |  |  |  |  |  |  |  |  |
| GO:0002833\_positive\_regulation\_of\_response\_to\_biotic\_stimulus | 4 | 0 |  |  |  |  |  |  |  |  |
| GO:0002834\_regulation\_of\_response\_to\_tumor\_cell | 4 | 0 |  |  |  |  |  |  |  |  |
| GO:0002836\_positive\_regulation\_of\_response\_to\_tumor\_cell | 4 | 0 |  |  |  |  |  |  |  |  |
| GO:0002837\_regulation\_of\_immune\_response\_to\_tumor\_cell | 4 | 0 |  |  |  |  |  |  |  |  |
| GO:0002839\_positive\_regulation\_of\_immune\_response\_to\_tumor\_cell | 4 | 0 |  |  |  |  |  |  |  |  |
| GO:0002870\_T\_cell\_anergy | 4 | 0 |  |  |  |  |  |  |  |  |
| GO:0002888\_positive\_regulation\_of\_myeloid\_leukocyte\_mediated\_immunity | 4 | 0 |  |  |  |  |  |  |  |  |
| GO:0002892\_regulation\_of\_type\_II\_hypersensitivity | 4 | 0 |  |  |  |  |  |  |  |  |
| GO:0002894\_positive\_regulation\_of\_type\_II\_hypersensitivity | 4 | 0 |  |  |  |  |  |  |  |  |
| GO:0002911\_regulation\_of\_lymphocyte\_anergy | 4 | 0 |  |  |  |  |  |  |  |  |
| GO:0002913\_positive\_regulation\_of\_lymphocyte\_anergy | 4 | 0 |  |  |  |  |  |  |  |  |
| GO:0002923\_regulation\_of\_humoral\_immune\_response\_mediated\_by\_circulating\_immunoglobulin | 4 | 0 |  |  |  |  |  |  |  |  |
| GO:0003025\_regulation\_of\_systemic\_arterial\_blood\_pressure\_by\_baroreceptor\_feedback | 4 | 0 |  |  |  |  |  |  |  |  |
| GO:0003091\_renal\_water\_homeostasis | 4 | 0 |  |  |  |  |  |  |  |  |
| GO:0005978\_glycogen\_biosynthetic\_process | 4 | 0 |  |  |  |  |  |  |  |  |
| GO:0006012\_galactose\_metabolic\_process | 4 | 0 |  |  |  |  |  |  |  |  |
| GO:0006085\_acetyl-CoA\_biosynthetic\_process | 4 | 0 |  |  |  |  |  |  |  |  |
| GO:0006111\_regulation\_of\_gluconeogenesis | 4 | 0 |  |  |  |  |  |  |  |  |
| GO:0006144\_purine\_base\_metabolic\_process | 4 | 0 |  |  |  |  |  |  |  |  |
| GO:0006290\_pyrimidine\_dimer\_repair | 4 | 0 |  |  |  |  |  |  |  |  |
| GO:0006334\_nucleosome\_assembly | 4 | 0 |  |  |  |  |  |  |  |  |
| GO:0006534\_cysteine\_metabolic\_process | 4 | 0 |  |  |  |  |  |  |  |  |
| GO:0006547\_histidine\_metabolic\_process | 4 | 0 |  |  |  |  |  |  |  |  |
| GO:0006548\_histidine\_catabolic\_process | 4 | 0 |  |  |  |  |  |  |  |  |
| GO:0006555\_methionine\_metabolic\_process | 4 | 0 |  |  |  |  |  |  |  |  |
| GO:0006599\_phosphagen\_metabolic\_process | 4 | 0 |  |  |  |  |  |  |  |  |
| GO:0006623\_protein\_targeting\_to\_vacuole | 4 | 0 |  |  |  |  |  |  |  |  |
| GO:0006626\_protein\_targeting\_to\_mitochondrion | 4 | 0 |  |  |  |  |  |  |  |  |
| GO:0006684\_sphingomyelin\_metabolic\_process | 4 | 0 |  |  |  |  |  |  |  |  |
| GO:0006688\_glycosphingolipid\_biosynthetic\_process | 4 | 0 |  |  |  |  |  |  |  |  |
| GO:0006707\_cholesterol\_catabolic\_process | 4 | 0 |  |  |  |  |  |  |  |  |
| GO:0006739\_NADP\_metabolic\_process | 4 | 0 |  |  |  |  |  |  |  |  |
| GO:0006837\_serotonin\_transport | 4 | 0 |  |  |  |  |  |  |  |  |
| GO:0006888\_ER\_to\_Golgi\_vesicle-mediated\_transport | 4 | 0 |  |  |  |  |  |  |  |  |
| GO:0006906\_vesicle\_fusion | 4 | 0 |  |  |  |  |  |  |  |  |
| GO:0006927\_transformed\_cell\_apoptosis | 4 | 0 |  |  |  |  |  |  |  |  |
| GO:0006972\_hyperosmotic\_response | 4 | 0 |  |  |  |  |  |  |  |  |
| GO:0007028\_cytoplasm\_organization | 4 | 0 |  |  |  |  |  |  |  |  |
| GO:0007031\_peroxisome\_organization | 4 | 0 |  |  |  |  |  |  |  |  |
| GO:0007066\_female\_meiosis\_sister\_chromatid\_cohesion | 4 | 0 |  |  |  |  |  |  |  |  |
| GO:0007144\_female\_meiosis\_I | 4 | 0 |  |  |  |  |  |  |  |  |
| GO:0007184\_SMAD\_protein\_nuclear\_translocation | 4 | 0 |  |  |  |  |  |  |  |  |
| GO:0007216\_metabotropic\_glutamate\_receptor\_signaling\_pathway | 4 | 0 |  |  |  |  |  |  |  |  |
| GO:0007342\_fusion\_of\_sperm\_to\_egg\_plasma\_membrane | 4 | 0 |  |  |  |  |  |  |  |  |
| GO:0007386\_compartment\_specification | 4 | 0 |  |  |  |  |  |  |  |  |
| GO:0008053\_mitochondrial\_fusion | 4 | 0 |  |  |  |  |  |  |  |  |
| GO:0008207\_C21-steroid\_hormone\_metabolic\_process | 4 | 0 |  |  |  |  |  |  |  |  |
| GO:0009065\_glutamine\_family\_amino\_acid\_catabolic\_process | 4 | 0 |  |  |  |  |  |  |  |  |
| GO:0009075\_histidine\_family\_amino\_acid\_metabolic\_process | 4 | 0 |  |  |  |  |  |  |  |  |
| GO:0009077\_histidine\_family\_amino\_acid\_catabolic\_process | 4 | 0 |  |  |  |  |  |  |  |  |
| GO:0009134\_nucleoside\_diphosphate\_catabolic\_process | 4 | 0 |  |  |  |  |  |  |  |  |
| GO:0009163\_nucleoside\_biosynthetic\_process | 4 | 0 |  |  |  |  |  |  |  |  |
| GO:0009250\_glucan\_biosynthetic\_process | 4 | 0 |  |  |  |  |  |  |  |  |
| GO:0009404\_toxin\_metabolic\_process | 4 | 0 |  |  |  |  |  |  |  |  |
| GO:0009593\_detection\_of\_chemical\_stimulus | 4 | 0 |  |  |  |  |  |  |  |  |
| GO:0009595\_detection\_of\_biotic\_stimulus | 4 | 0 |  |  |  |  |  |  |  |  |
| GO:0009755\_hormone-mediated\_signaling | 4 | 0 |  |  |  |  |  |  |  |  |
| GO:0009912\_auditory\_receptor\_cell\_fate\_commitment | 4 | 0 |  |  |  |  |  |  |  |  |
| GO:0010224\_response\_to\_UV-B | 4 | 0 |  |  |  |  |  |  |  |  |
| GO:0010453\_regulation\_of\_cell\_fate\_commitment | 4 | 0 |  |  |  |  |  |  |  |  |
| GO:0010506\_regulation\_of\_autophagy | 4 | 0 |  |  |  |  |  |  |  |  |
| GO:0010631\_epithelial\_cell\_migration | 4 | 0 |  |  |  |  |  |  |  |  |
| GO:0010812\_negative\_regulation\_of\_cell-substrate\_adhesion | 4 | 0 |  |  |  |  |  |  |  |  |
| GO:0010829\_negative\_regulation\_of\_glucose\_transport | 4 | 0 |  |  |  |  |  |  |  |  |
| GO:0014002\_astrocyte\_development | 4 | 0 |  |  |  |  |  |  |  |  |
| GO:0014832\_urinary\_bladder\_smooth\_muscle\_contraction | 4 | 0 |  |  |  |  |  |  |  |  |
| GO:0014848\_urinary\_tract\_smooth\_muscle\_contraction | 4 | 0 |  |  |  |  |  |  |  |  |
| GO:0015701\_bicarbonate\_transport | 4 | 0 |  |  |  |  |  |  |  |  |
| GO:0015809\_arginine\_transport | 4 | 0 |  |  |  |  |  |  |  |  |
| GO:0015850\_organic\_alcohol\_transport | 4 | 0 |  |  |  |  |  |  |  |  |
| GO:0015858\_nucleoside\_transport | 4 | 0 |  |  |  |  |  |  |  |  |
| GO:0016068\_type\_I\_hypersensitivity | 4 | 0 |  |  |  |  |  |  |  |  |
| GO:0016127\_sterol\_catabolic\_process | 4 | 0 |  |  |  |  |  |  |  |  |
| GO:0016198\_axon\_choice\_point\_recognition | 4 | 0 |  |  |  |  |  |  |  |  |
| GO:0016338\_calcium-independent\_cell-cell\_adhesion | 4 | 0 |  |  |  |  |  |  |  |  |
| GO:0018198\_peptidyl-cysteine\_modification | 4 | 0 |  |  |  |  |  |  |  |  |
| GO:0018409\_peptide\_or\_protein\_amino-terminal\_blocking | 4 | 0 |  |  |  |  |  |  |  |  |
| GO:0019377\_glycolipid\_catabolic\_process | 4 | 0 |  |  |  |  |  |  |  |  |
| GO:0019432\_triglyceride\_biosynthetic\_process | 4 | 0 |  |  |  |  |  |  |  |  |
| GO:0019530\_taurine\_metabolic\_process | 4 | 0 |  |  |  |  |  |  |  |  |
| GO:0021523\_somatic\_motor\_neuron\_differentiation | 4 | 0 |  |  |  |  |  |  |  |  |
| GO:0021535\_cell\_migration\_in\_hindbrain | 4 | 0 |  |  |  |  |  |  |  |  |
| GO:0021542\_dentate\_gyrus\_development | 4 | 0 |  |  |  |  |  |  |  |  |
| GO:0021561\_facial\_nerve\_development | 4 | 0 |  |  |  |  |  |  |  |  |
| GO:0021569\_rhombomere\_3\_development | 4 | 0 |  |  |  |  |  |  |  |  |
| GO:0021571\_rhombomere\_5\_development | 4 | 0 |  |  |  |  |  |  |  |  |
| GO:0021604\_cranial\_nerve\_structural\_organization | 4 | 0 |  |  |  |  |  |  |  |  |
| GO:0021610\_facial\_nerve\_morphogenesis | 4 | 0 |  |  |  |  |  |  |  |  |
| GO:0021612\_facial\_nerve\_structural\_organization | 4 | 0 |  |  |  |  |  |  |  |  |
| GO:0021631\_optic\_nerve\_morphogenesis | 4 | 0 |  |  |  |  |  |  |  |  |
| GO:0021681\_cerebellar\_granular\_layer\_development | 4 | 0 |  |  |  |  |  |  |  |  |
| GO:0021683\_cerebellar\_granular\_layer\_morphogenesis | 4 | 0 |  |  |  |  |  |  |  |  |
| GO:0021684\_cerebellar\_granular\_layer\_formation | 4 | 0 |  |  |  |  |  |  |  |  |
| GO:0021707\_cerebellar\_granule\_cell\_differentiation | 4 | 0 |  |  |  |  |  |  |  |  |
| GO:0021778\_oligodendrocyte\_cell\_fate\_specification | 4 | 0 |  |  |  |  |  |  |  |  |
| GO:0021779\_oligodendrocyte\_cell\_fate\_commitment | 4 | 0 |  |  |  |  |  |  |  |  |
| GO:0021780\_glial\_cell\_fate\_specification | 4 | 0 |  |  |  |  |  |  |  |  |
| GO:0021801\_cerebral\_cortex\_radial\_glia\_guided\_migration | 4 | 0 |  |  |  |  |  |  |  |  |
| GO:0021830\_interneuron\_migration\_from\_the\_subpallium\_to\_the\_cortex | 4 | 0 |  |  |  |  |  |  |  |  |
| GO:0021853\_cerebral\_cortex\_GABAergic\_interneuron\_migration | 4 | 0 |  |  |  |  |  |  |  |  |
| GO:0021877\_forebrain\_neuron\_fate\_commitment | 4 | 0 |  |  |  |  |  |  |  |  |
| GO:0021894\_cerebral\_cortex\_GABAergic\_interneuron\_development | 4 | 0 |  |  |  |  |  |  |  |  |
| GO:0021910\_smoothened\_signaling\_pathway\_involved\_in\_ventral\_spinal\_cord\_patterning | 4 | 0 |  |  |  |  |  |  |  |  |
| GO:0021913\_regulation\_of\_transcription\_from\_RNA\_polymerase\_II\_promoter\_involved\_in\_ventral\_spinal\_cord\_interneuron\_specification | 4 | 0 |  |  |  |  |  |  |  |  |
| GO:0021938\_smoothened\_signaling\_pathway\_involved\_in\_regulation\_of\_granule\_cell\_precursor\_cell\_proliferation | 4 | 0 |  |  |  |  |  |  |  |  |
| GO:0021978\_telencephalon\_regionalization | 4 | 0 |  |  |  |  |  |  |  |  |
| GO:0022011\_myelination\_in\_the\_peripheral\_nervous\_system | 4 | 0 |  |  |  |  |  |  |  |  |
| GO:0030146\_diuresis | 4 | 0 |  |  |  |  |  |  |  |  |
| GO:0030300\_regulation\_of\_intestinal\_cholesterol\_absorption | 4 | 0 |  |  |  |  |  |  |  |  |
| GO:0030800\_negative\_regulation\_of\_cyclic\_nucleotide\_metabolic\_process | 4 | 0 |  |  |  |  |  |  |  |  |
| GO:0030803\_negative\_regulation\_of\_cyclic\_nucleotide\_biosynthetic\_process | 4 | 0 |  |  |  |  |  |  |  |  |
| GO:0030809\_negative\_regulation\_of\_nucleotide\_biosynthetic\_process | 4 | 0 |  |  |  |  |  |  |  |  |
| GO:0030815\_negative\_regulation\_of\_cAMP\_metabolic\_process | 4 | 0 |  |  |  |  |  |  |  |  |
| GO:0030816\_positive\_regulation\_of\_cAMP\_metabolic\_process | 4 | 0 |  |  |  |  |  |  |  |  |
| GO:0030818\_negative\_regulation\_of\_cAMP\_biosynthetic\_process | 4 | 0 |  |  |  |  |  |  |  |  |
| GO:0030819\_positive\_regulation\_of\_cAMP\_biosynthetic\_process | 4 | 0 |  |  |  |  |  |  |  |  |
| GO:0030858\_positive\_regulation\_of\_epithelial\_cell\_differentiation | 4 | 0 |  |  |  |  |  |  |  |  |
| GO:0030859\_polarized\_epithelial\_cell\_differentiation | 4 | 0 |  |  |  |  |  |  |  |  |
| GO:0030949\_positive\_regulation\_of\_vascular\_endothelial\_growth\_factor\_receptor\_signaling\_pathway | 4 | 0 |  |  |  |  |  |  |  |  |
| GO:0031113\_regulation\_of\_microtubule\_polymerization | 4 | 0 |  |  |  |  |  |  |  |  |
| GO:0031365\_N-terminal\_protein\_amino\_acid\_modification | 4 | 0 |  |  |  |  |  |  |  |  |
| GO:0031424\_keratinization | 4 | 0 |  |  |  |  |  |  |  |  |
| GO:0031557\_induction\_of\_programmed\_cell\_death\_in\_response\_to\_chemical\_stimulus | 4 | 0 |  |  |  |  |  |  |  |  |
| GO:0031558\_induction\_of\_apoptosis\_in\_response\_to\_chemical\_stimulus | 4 | 0 |  |  |  |  |  |  |  |  |
| GO:0031623\_receptor\_internalization | 4 | 0 |  |  |  |  |  |  |  |  |
| GO:0032088\_negative\_regulation\_of\_NF-kappaB\_transcription\_factor\_activity | 4 | 0 |  |  |  |  |  |  |  |  |
| GO:0032098\_regulation\_of\_appetite | 4 | 0 |  |  |  |  |  |  |  |  |
| GO:0032105\_negative\_regulation\_of\_response\_to\_extracellular\_stimulus | 4 | 0 |  |  |  |  |  |  |  |  |
| GO:0032108\_negative\_regulation\_of\_response\_to\_nutrient\_levels | 4 | 0 |  |  |  |  |  |  |  |  |
| GO:0032225\_regulation\_of\_synaptic\_transmission\_\_dopaminergic | 4 | 0 |  |  |  |  |  |  |  |  |
| GO:0032292\_ensheathment\_of\_axons\_in\_the\_peripheral\_nervous\_system | 4 | 0 |  |  |  |  |  |  |  |  |
| GO:0032321\_positive\_regulation\_of\_Rho\_GTPase\_activity | 4 | 0 |  |  |  |  |  |  |  |  |
| GO:0032371\_regulation\_of\_sterol\_transport | 4 | 0 |  |  |  |  |  |  |  |  |
| GO:0032374\_regulation\_of\_cholesterol\_transport | 4 | 0 |  |  |  |  |  |  |  |  |
| GO:0032401\_establishment\_of\_melanosome\_localization | 4 | 0 |  |  |  |  |  |  |  |  |
| GO:0032608\_interferon-beta\_production | 4 | 0 |  |  |  |  |  |  |  |  |
| GO:0032611\_interleukin-1\_beta\_production | 4 | 0 |  |  |  |  |  |  |  |  |
| GO:0032612\_interleukin-1\_production | 4 | 0 |  |  |  |  |  |  |  |  |
| GO:0032648\_regulation\_of\_interferon-beta\_production | 4 | 0 |  |  |  |  |  |  |  |  |
| GO:0032651\_regulation\_of\_interleukin-1\_beta\_production | 4 | 0 |  |  |  |  |  |  |  |  |
| GO:0032652\_regulation\_of\_interleukin-1\_production | 4 | 0 |  |  |  |  |  |  |  |  |
| GO:0032689\_negative\_regulation\_of\_interferon-gamma\_production | 4 | 0 |  |  |  |  |  |  |  |  |
| GO:0032713\_negative\_regulation\_of\_interleukin-4\_production | 4 | 0 |  |  |  |  |  |  |  |  |
| GO:0032715\_negative\_regulation\_of\_interleukin-6\_production | 4 | 0 |  |  |  |  |  |  |  |  |
| GO:0032733\_positive\_regulation\_of\_interleukin-10\_production | 4 | 0 |  |  |  |  |  |  |  |  |
| GO:0032808\_lacrimal\_gland\_development | 4 | 0 |  |  |  |  |  |  |  |  |
| GO:0032872\_regulation\_of\_stress-activated\_MAPK\_cascade | 4 | 0 |  |  |  |  |  |  |  |  |
| GO:0032922\_circadian\_regulation\_of\_gene\_expression | 4 | 0 |  |  |  |  |  |  |  |  |
| GO:0033026\_negative\_regulation\_of\_mast\_cell\_apoptosis | 4 | 0 |  |  |  |  |  |  |  |  |
| GO:0033079\_immature\_T\_cell\_proliferation | 4 | 0 |  |  |  |  |  |  |  |  |
| GO:0033083\_regulation\_of\_immature\_T\_cell\_proliferation | 4 | 0 |  |  |  |  |  |  |  |  |
| GO:0033089\_positive\_regulation\_of\_T\_cell\_differentiation\_in\_the\_thymus | 4 | 0 |  |  |  |  |  |  |  |  |
| GO:0033135\_regulation\_of\_peptidyl-serine\_phosphorylation | 4 | 0 |  |  |  |  |  |  |  |  |
| GO:0033299\_secretion\_of\_lysosomal\_enzymes | 4 | 0 |  |  |  |  |  |  |  |  |
| GO:0033327\_Leydig\_cell\_differentiation | 4 | 0 |  |  |  |  |  |  |  |  |
| GO:0033363\_secretory\_granule\_organization | 4 | 0 |  |  |  |  |  |  |  |  |
| GO:0033599\_regulation\_of\_mammary\_gland\_epithelial\_cell\_proliferation | 4 | 0 |  |  |  |  |  |  |  |  |
| GO:0033865\_nucleoside\_bisphosphate\_metabolic\_process | 4 | 0 |  |  |  |  |  |  |  |  |
| GO:0034204\_lipid\_translocation | 4 | 0 |  |  |  |  |  |  |  |  |
| GO:0034404\_nucleobase\_\_nucleoside\_and\_nucleotide\_biosynthetic\_process | 4 | 0 |  |  |  |  |  |  |  |  |
| GO:0034587\_piRNA\_metabolic\_process | 4 | 0 |  |  |  |  |  |  |  |  |
| GO:0034614\_cellular\_response\_to\_reactive\_oxygen\_species | 4 | 0 |  |  |  |  |  |  |  |  |
| GO:0034654\_nucleobase\_\_nucleoside\_\_nucleotide\_and\_nucleic\_acid\_biosynthetic\_process | 4 | 0 |  |  |  |  |  |  |  |  |
| GO:0035020\_regulation\_of\_Rac\_protein\_signal\_transduction | 4 | 0 |  |  |  |  |  |  |  |  |
| GO:0035082\_axoneme\_assembly | 4 | 0 |  |  |  |  |  |  |  |  |
| GO:0035188\_hatching | 4 | 0 |  |  |  |  |  |  |  |  |
| GO:0035235\_ionotropic\_glutamate\_receptor\_signaling\_pathway | 4 | 0 |  |  |  |  |  |  |  |  |
| GO:0042345\_regulation\_of\_NF-kappaB\_import\_into\_nucleus | 4 | 0 |  |  |  |  |  |  |  |  |
| GO:0042348\_NF-kappaB\_import\_into\_nucleus | 4 | 0 |  |  |  |  |  |  |  |  |
| GO:0042359\_vitamin\_D\_metabolic\_process | 4 | 0 |  |  |  |  |  |  |  |  |
| GO:0042428\_serotonin\_metabolic\_process | 4 | 0 |  |  |  |  |  |  |  |  |
| GO:0042451\_purine\_nucleoside\_biosynthetic\_process | 4 | 0 |  |  |  |  |  |  |  |  |
| GO:0042455\_ribonucleoside\_biosynthetic\_process | 4 | 0 |  |  |  |  |  |  |  |  |
| GO:0042473\_outer\_ear\_morphogenesis | 4 | 0 |  |  |  |  |  |  |  |  |
| GO:0042522\_regulation\_of\_tyrosine\_phosphorylation\_of\_Stat5\_protein | 4 | 0 |  |  |  |  |  |  |  |  |
| GO:0042535\_positive\_regulation\_of\_tumor\_necrosis\_factor\_biosynthetic\_process | 4 | 0 |  |  |  |  |  |  |  |  |
| GO:0042541\_hemoglobin\_biosynthetic\_process | 4 | 0 |  |  |  |  |  |  |  |  |
| GO:0042558\_pteridine\_and\_derivative\_metabolic\_process | 4 | 0 |  |  |  |  |  |  |  |  |
| GO:0042634\_regulation\_of\_hair\_cycle | 4 | 0 |  |  |  |  |  |  |  |  |
| GO:0042744\_hydrogen\_peroxide\_catabolic\_process | 4 | 0 |  |  |  |  |  |  |  |  |
| GO:0042773\_ATP\_synthesis\_coupled\_electron\_transport | 4 | 0 |  |  |  |  |  |  |  |  |
| GO:0042775\_mitochondrial\_ATP\_synthesis\_coupled\_electron\_transport | 4 | 0 |  |  |  |  |  |  |  |  |
| GO:0042832\_defense\_response\_to\_protozoan | 4 | 0 |  |  |  |  |  |  |  |  |
| GO:0042982\_amyloid\_precursor\_protein\_metabolic\_process | 4 | 0 |  |  |  |  |  |  |  |  |
| GO:0042992\_negative\_regulation\_of\_transcription\_factor\_import\_into\_nucleus | 4 | 0 |  |  |  |  |  |  |  |  |
| GO:0043043\_peptide\_biosynthetic\_process | 4 | 0 |  |  |  |  |  |  |  |  |
| GO:0043374\_CD8-positive\_\_alpha-beta\_T\_cell\_differentiation | 4 | 0 |  |  |  |  |  |  |  |  |
| GO:0043470\_regulation\_of\_carbohydrate\_catabolic\_process | 4 | 0 |  |  |  |  |  |  |  |  |
| GO:0043471\_regulation\_of\_cellular\_carbohydrate\_catabolic\_process | 4 | 0 |  |  |  |  |  |  |  |  |
| GO:0043500\_muscle\_adaptation | 4 | 0 |  |  |  |  |  |  |  |  |
| GO:0043534\_blood\_vessel\_endothelial\_cell\_migration | 4 | 0 |  |  |  |  |  |  |  |  |
| GO:0043691\_reverse\_cholesterol\_transport | 4 | 0 |  |  |  |  |  |  |  |  |
| GO:0044243\_multicellular\_organismal\_catabolic\_process | 4 | 0 |  |  |  |  |  |  |  |  |
| GO:0044403\_symbiosis\_\_encompassing\_mutualism\_through\_parasitism | 4 | 0 |  |  |  |  |  |  |  |  |
| GO:0044419\_interspecies\_interaction\_between\_organisms | 4 | 0 |  |  |  |  |  |  |  |  |
| GO:0045066\_regulatory\_T\_cell\_differentiation | 4 | 0 |  |  |  |  |  |  |  |  |
| GO:0045078\_positive\_regulation\_of\_interferon-gamma\_biosynthetic\_process | 4 | 0 |  |  |  |  |  |  |  |  |
| GO:0045332\_phospholipid\_translocation | 4 | 0 |  |  |  |  |  |  |  |  |
| GO:0045346\_regulation\_of\_MHC\_class\_II\_biosynthetic\_process | 4 | 0 |  |  |  |  |  |  |  |  |
| GO:0045350\_interferon-beta\_biosynthetic\_process | 4 | 0 |  |  |  |  |  |  |  |  |
| GO:0045357\_regulation\_of\_interferon-beta\_biosynthetic\_process | 4 | 0 |  |  |  |  |  |  |  |  |
| GO:0045359\_positive\_regulation\_of\_interferon-beta\_biosynthetic\_process | 4 | 0 |  |  |  |  |  |  |  |  |
| GO:0045600\_positive\_regulation\_of\_fat\_cell\_differentiation | 4 | 0 |  |  |  |  |  |  |  |  |
| GO:0045616\_regulation\_of\_keratinocyte\_differentiation | 4 | 0 |  |  |  |  |  |  |  |  |
| GO:0045624\_positive\_regulation\_of\_T-helper\_cell\_differentiation | 4 | 0 |  |  |  |  |  |  |  |  |
| GO:0045628\_regulation\_of\_T-helper\_2\_cell\_differentiation | 4 | 0 |  |  |  |  |  |  |  |  |
| GO:0045634\_regulation\_of\_melanocyte\_differentiation | 4 | 0 |  |  |  |  |  |  |  |  |
| GO:0045647\_negative\_regulation\_of\_erythrocyte\_differentiation | 4 | 0 |  |  |  |  |  |  |  |  |
| GO:0045672\_positive\_regulation\_of\_osteoclast\_differentiation | 4 | 0 |  |  |  |  |  |  |  |  |
| GO:0045684\_positive\_regulation\_of\_epidermis\_development | 4 | 0 |  |  |  |  |  |  |  |  |
| GO:0045736\_negative\_regulation\_of\_cyclin-dependent\_protein\_kinase\_activity | 4 | 0 |  |  |  |  |  |  |  |  |
| GO:0045742\_positive\_regulation\_of\_epidermal\_growth\_factor\_receptor\_signaling\_pathway | 4 | 0 |  |  |  |  |  |  |  |  |
| GO:0045747\_positive\_regulation\_of\_Notch\_signaling\_pathway | 4 | 0 |  |  |  |  |  |  |  |  |
| GO:0045767\_regulation\_of\_anti-apoptosis | 4 | 0 |  |  |  |  |  |  |  |  |
| GO:0045779\_negative\_regulation\_of\_bone\_resorption | 4 | 0 |  |  |  |  |  |  |  |  |
| GO:0045923\_positive\_regulation\_of\_fatty\_acid\_metabolic\_process | 4 | 0 |  |  |  |  |  |  |  |  |
| GO:0045930\_negative\_regulation\_of\_mitotic\_cell\_cycle | 4 | 0 |  |  |  |  |  |  |  |  |
| GO:0045940\_positive\_regulation\_of\_steroid\_metabolic\_process | 4 | 0 |  |  |  |  |  |  |  |  |
| GO:0045980\_negative\_regulation\_of\_nucleotide\_metabolic\_process | 4 | 0 |  |  |  |  |  |  |  |  |
| GO:0046129\_purine\_ribonucleoside\_biosynthetic\_process | 4 | 0 |  |  |  |  |  |  |  |  |
| GO:0046541\_saliva\_secretion | 4 | 0 |  |  |  |  |  |  |  |  |
| GO:0046548\_retinal\_rod\_cell\_development | 4 | 0 |  |  |  |  |  |  |  |  |
| GO:0046579\_positive\_regulation\_of\_Ras\_protein\_signal\_transduction | 4 | 0 |  |  |  |  |  |  |  |  |
| GO:0046639\_negative\_regulation\_of\_alpha-beta\_T\_cell\_differentiation | 4 | 0 |  |  |  |  |  |  |  |  |
| GO:0046642\_negative\_regulation\_of\_alpha-beta\_T\_cell\_proliferation | 4 | 0 |  |  |  |  |  |  |  |  |
| GO:0046668\_regulation\_of\_retinal\_cell\_programmed\_cell\_death | 4 | 0 |  |  |  |  |  |  |  |  |
| GO:0046686\_response\_to\_cadmium\_ion | 4 | 0 |  |  |  |  |  |  |  |  |
| GO:0046902\_regulation\_of\_mitochondrial\_membrane\_permeability | 4 | 0 |  |  |  |  |  |  |  |  |
| GO:0047496\_vesicle\_transport\_along\_microtubule | 4 | 0 |  |  |  |  |  |  |  |  |
| GO:0048011\_nerve\_growth\_factor\_receptor\_signaling\_pathway | 4 | 0 |  |  |  |  |  |  |  |  |
| GO:0048240\_sperm\_capacitation | 4 | 0 |  |  |  |  |  |  |  |  |
| GO:0048341\_paraxial\_mesoderm\_formation | 4 | 0 |  |  |  |  |  |  |  |  |
| GO:0048512\_circadian\_behavior | 4 | 0 |  |  |  |  |  |  |  |  |
| GO:0048558\_embryonic\_gut\_morphogenesis | 4 | 0 |  |  |  |  |  |  |  |  |
| GO:0048639\_positive\_regulation\_of\_developmental\_growth | 4 | 0 |  |  |  |  |  |  |  |  |
| GO:0048710\_regulation\_of\_astrocyte\_differentiation | 4 | 0 |  |  |  |  |  |  |  |  |
| GO:0048841\_regulation\_of\_axon\_extension\_involved\_in\_axon\_guidance | 4 | 0 |  |  |  |  |  |  |  |  |
| GO:0048843\_negative\_regulation\_of\_axon\_extension\_involved\_in\_axon\_guidance | 4 | 0 |  |  |  |  |  |  |  |  |
| GO:0048846\_axon\_extension\_involved\_in\_axon\_guidance | 4 | 0 |  |  |  |  |  |  |  |  |
| GO:0048935\_peripheral\_nervous\_system\_neuron\_development | 4 | 0 |  |  |  |  |  |  |  |  |
| GO:0050702\_interleukin-1\_beta\_secretion | 4 | 0 |  |  |  |  |  |  |  |  |
| GO:0050704\_regulation\_of\_interleukin-1\_secretion | 4 | 0 |  |  |  |  |  |  |  |  |
| GO:0050706\_regulation\_of\_interleukin-1\_beta\_secretion | 4 | 0 |  |  |  |  |  |  |  |  |
| GO:0050716\_positive\_regulation\_of\_interleukin-1\_secretion | 4 | 0 |  |  |  |  |  |  |  |  |
| GO:0050718\_positive\_regulation\_of\_interleukin-1\_beta\_secretion | 4 | 0 |  |  |  |  |  |  |  |  |
| GO:0050820\_positive\_regulation\_of\_coagulation | 4 | 0 |  |  |  |  |  |  |  |  |
| GO:0050891\_multicellular\_organismal\_water\_homeostasis | 4 | 0 |  |  |  |  |  |  |  |  |
| GO:0050919\_negative\_chemotaxis | 4 | 0 |  |  |  |  |  |  |  |  |
| GO:0050932\_regulation\_of\_pigment\_cell\_differentiation | 4 | 0 |  |  |  |  |  |  |  |  |
| GO:0050961\_detection\_of\_temperature\_stimulus\_involved\_in\_sensory\_perception | 4 | 0 |  |  |  |  |  |  |  |  |
| GO:0050965\_detection\_of\_temperature\_stimulus\_involved\_in\_sensory\_perception\_of\_pain | 4 | 0 |  |  |  |  |  |  |  |  |
| GO:0050994\_regulation\_of\_lipid\_catabolic\_process | 4 | 0 |  |  |  |  |  |  |  |  |
| GO:0051024\_positive\_regulation\_of\_immunoglobulin\_secretion | 4 | 0 |  |  |  |  |  |  |  |  |
| GO:0051055\_negative\_regulation\_of\_lipid\_biosynthetic\_process | 4 | 0 |  |  |  |  |  |  |  |  |
| GO:0051124\_synaptic\_growth\_at\_neuromuscular\_junction | 4 | 0 |  |  |  |  |  |  |  |  |
| GO:0051148\_negative\_regulation\_of\_muscle\_cell\_differentiation | 4 | 0 |  |  |  |  |  |  |  |  |
| GO:0051205\_protein\_insertion\_into\_membrane | 4 | 0 |  |  |  |  |  |  |  |  |
| GO:0051225\_spindle\_assembly | 4 | 0 |  |  |  |  |  |  |  |  |
| GO:0051341\_regulation\_of\_oxidoreductase\_activity | 4 | 0 |  |  |  |  |  |  |  |  |
| GO:0051452\_intracellular\_pH\_reduction | 4 | 0 |  |  |  |  |  |  |  |  |
| GO:0051567\_histone\_H3-K9\_methylation | 4 | 0 |  |  |  |  |  |  |  |  |
| GO:0051642\_centrosome\_localization | 4 | 0 |  |  |  |  |  |  |  |  |
| GO:0051797\_regulation\_of\_hair\_follicle\_development | 4 | 0 |  |  |  |  |  |  |  |  |
| GO:0051897\_positive\_regulation\_of\_protein\_kinase\_B\_signaling\_cascade | 4 | 0 |  |  |  |  |  |  |  |  |
| GO:0051904\_pigment\_granule\_transport | 4 | 0 |  |  |  |  |  |  |  |  |
| GO:0055009\_atrial\_cardiac\_muscle\_morphogenesis | 4 | 0 |  |  |  |  |  |  |  |  |
| GO:0060008\_Sertoli\_cell\_differentiation | 4 | 0 |  |  |  |  |  |  |  |  |
| GO:0060011\_Sertoli\_cell\_proliferation | 4 | 0 |  |  |  |  |  |  |  |  |
| GO:0060057\_apoptosis\_involved\_in\_mammary\_gland\_involution | 4 | 0 |  |  |  |  |  |  |  |  |
| GO:0060058\_positive\_regulation\_of\_apoptosis\_involved\_in\_mammary\_gland\_involution | 4 | 0 |  |  |  |  |  |  |  |  |
| GO:0060065\_uterus\_development | 4 | 0 |  |  |  |  |  |  |  |  |
| GO:0060087\_relaxation\_of\_vascular\_smooth\_muscle | 4 | 0 |  |  |  |  |  |  |  |  |
| GO:0060120\_inner\_ear\_receptor\_cell\_fate\_commitment | 4 | 0 |  |  |  |  |  |  |  |  |
| GO:0060157\_urinary\_bladder\_development | 4 | 0 |  |  |  |  |  |  |  |  |
| GO:0060158\_activation\_of\_phospholipase\_C\_activity\_by\_dopamine\_receptor\_signaling\_pathway | 4 | 0 |  |  |  |  |  |  |  |  |
| GO:0060164\_regulation\_of\_timing\_of\_neuron\_differentiation | 4 | 0 |  |  |  |  |  |  |  |  |
| GO:0060235\_lens\_induction\_in\_camera-type\_eye | 4 | 0 |  |  |  |  |  |  |  |  |
| GO:0060291\_long-term\_synaptic\_potentiation | 4 | 0 |  |  |  |  |  |  |  |  |
| GO:0060412\_ventricular\_septum\_morphogenesis | 4 | 0 |  |  |  |  |  |  |  |  |
| GO:0060459\_left\_lung\_development | 4 | 0 |  |  |  |  |  |  |  |  |
| GO:0060528\_secretory\_columnal\_luminar\_epithelial\_cell\_differentiation\_involved\_in\_prostate\_glandular\_acinus\_development | 4 | 0 |  |  |  |  |  |  |  |  |
| GO:0060561\_apoptosis\_involved\_in\_morphogenesis | 4 | 0 |  |  |  |  |  |  |  |  |
| GO:0060592\_mammary\_gland\_formation | 4 | 0 |  |  |  |  |  |  |  |  |
| GO:0060644\_mammary\_gland\_epithelial\_cell\_differentiation | 4 | 0 |  |  |  |  |  |  |  |  |
| GO:0060666\_dichotomous\_subdivision\_of\_terminal\_units\_involved\_in\_salivary\_gland\_branching | 4 | 0 |  |  |  |  |  |  |  |  |
| GO:0060737\_prostate\_gland\_morphogenetic\_growth | 4 | 0 |  |  |  |  |  |  |  |  |
| GO:0060743\_epithelial\_cell\_maturation\_involved\_in\_prostate\_gland\_development | 4 | 0 |  |  |  |  |  |  |  |  |
| GO:0060751\_mammary\_gland\_duct\_branch\_elongation | 4 | 0 |  |  |  |  |  |  |  |  |
| GO:0060900\_embryonic\_camera-type\_eye\_formation | 4 | 0 |  |  |  |  |  |  |  |  |
| GO:0070059\_apoptosis\_in\_response\_to\_endoplasmic\_reticulum\_stress | 4 | 0 |  |  |  |  |  |  |  |  |
| GO:0070254\_mucus\_secretion | 4 | 0 |  |  |  |  |  |  |  |  |
| GO:0070255\_regulation\_of\_mucus\_secretion | 4 | 0 |  |  |  |  |  |  |  |  |
| GO:0070301\_cellular\_response\_to\_hydrogen\_peroxide | 4 | 0 |  |  |  |  |  |  |  |  |
| GO:0070585\_protein\_localization\_in\_mitochondrion | 4 | 0 |  |  |  |  |  |  |  |  |
| GO:0007369\_gastrulation | 63 | 0 | 0.000000 | -0.000000 | 1214 | 1248.468610 | 1321.41 | 1394.351390 | 1.088476 |
| GO:0051186\_cofactor\_metabolic\_process | 63 | 0 | 0.000000 | -0.000000 | 1214 | 1248.468610 | 1321.41 | 1394.351390 | 1.088476 |
| GO:0070662\_mast\_cell\_proliferation | 63 | 0 | 0.000000 | -0.000000 | 1214 | 1248.468610 | 1321.41 | 1394.351390 | 1.088476 |
| GO:0070666\_regulation\_of\_mast\_cell\_proliferation | 63 | 0 | 0.000000 | -0.000000 | 1214 | 1248.468610 | 1321.41 | 1394.351390 | 1.088476 |
| GO:0006091\_generation\_of\_precursor\_metabolites\_and\_energy | 54 | 0 | 0.000000 | -0.000000 | 1218 | 1253.980896 | 1326.58 | 1399.179104 | 1.089146 |
| GO:0006412\_translation | 54 | 0 | 0.000000 | -0.000000 | 1218 | 1253.980896 | 1326.58 | 1399.179104 | 1.089146 |
| GO:0007265\_Ras\_protein\_signal\_transduction | 54 | 0 | 0.000000 | -0.000000 | 1218 | 1253.980896 | 1326.58 | 1399.179104 | 1.089146 |
| GO:0009566\_fertilization | 54 | 0 | 0.000000 | -0.000000 | 1218 | 1253.980896 | 1326.58 | 1399.179104 | 1.089146 |
| GO:0030003\_cellular\_cation\_homeostasis | 90 | 0 | 0.000000 | -0.000000 | 1220 | 1258.693030 | 1330.65 | 1402.606970 | 1.090697 |
| GO:0035264\_multicellular\_organism\_growth | 90 | 0 | 0.000000 | -0.000000 | 1220 | 1258.693030 | 1330.65 | 1402.606970 | 1.090697 |
| GO:0050863\_regulation\_of\_T\_cell\_activation | 88 | 0 | 0.000000 | -0.000000 | 1221 | 1260.339311 | 1331.87 | 1403.400689 | 1.090803 |
| GO:0000209\_protein\_polyubiquitination | 10 | 0 | 0.000000 | -0.000000 | 1340 | 1374.912679 | 1443.83 | 1512.747321 | 1.077485 |
| GO:0000724\_double-strand\_break\_repair\_via\_homologous\_recombination | 10 | 0 | 0.000000 | -0.000000 | 1340 | 1374.912679 | 1443.83 | 1512.747321 | 1.077485 |
| GO:0000725\_recombinational\_repair | 10 | 0 | 0.000000 | -0.000000 | 1340 | 1374.912679 | 1443.83 | 1512.747321 | 1.077485 |
| GO:0001578\_microtubule\_bundle\_formation | 10 | 0 | 0.000000 | -0.000000 | 1340 | 1374.912679 | 1443.83 | 1512.747321 | 1.077485 |
| GO:0001659\_temperature\_homeostasis | 10 | 0 | 0.000000 | -0.000000 | 1340 | 1374.912679 | 1443.83 | 1512.747321 | 1.077485 |
| GO:0001773\_myeloid\_dendritic\_cell\_activation | 10 | 0 | 0.000000 | -0.000000 | 1340 | 1374.912679 | 1443.83 | 1512.747321 | 1.077485 |
| GO:0001832\_blastocyst\_growth | 10 | 0 | 0.000000 | -0.000000 | 1340 | 1374.912679 | 1443.83 | 1512.747321 | 1.077485 |
| GO:0001914\_regulation\_of\_T\_cell\_mediated\_cytotoxicity | 10 | 0 | 0.000000 | -0.000000 | 1340 | 1374.912679 | 1443.83 | 1512.747321 | 1.077485 |
| GO:0001990\_regulation\_of\_systemic\_arterial\_blood\_pressure\_by\_hormone | 10 | 0 | 0.000000 | -0.000000 | 1340 | 1374.912679 | 1443.83 | 1512.747321 | 1.077485 |
| GO:0002070\_epithelial\_cell\_maturation | 10 | 0 | 0.000000 | -0.000000 | 1340 | 1374.912679 | 1443.83 | 1512.747321 | 1.077485 |
| GO:0002673\_regulation\_of\_acute\_inflammatory\_response | 10 | 0 | 0.000000 | -0.000000 | 1340 | 1374.912679 | 1443.83 | 1512.747321 | 1.077485 |
| GO:0002711\_positive\_regulation\_of\_T\_cell\_mediated\_immunity | 10 | 0 | 0.000000 | -0.000000 | 1340 | 1374.912679 | 1443.83 | 1512.747321 | 1.077485 |
| GO:0002762\_negative\_regulation\_of\_myeloid\_leukocyte\_differentiation | 10 | 0 | 0.000000 | -0.000000 | 1340 | 1374.912679 | 1443.83 | 1512.747321 | 1.077485 |
| GO:0006081\_cellular\_aldehyde\_metabolic\_process | 10 | 0 | 0.000000 | -0.000000 | 1340 | 1374.912679 | 1443.83 | 1512.747321 | 1.077485 |
| GO:0006109\_regulation\_of\_carbohydrate\_metabolic\_process | 10 | 0 | 0.000000 | -0.000000 | 1340 | 1374.912679 | 1443.83 | 1512.747321 | 1.077485 |
| GO:0006289\_nucleotide-excision\_repair | 10 | 0 | 0.000000 | -0.000000 | 1340 | 1374.912679 | 1443.83 | 1512.747321 | 1.077485 |
| GO:0006342\_chromatin\_silencing | 10 | 0 | 0.000000 | -0.000000 | 1340 | 1374.912679 | 1443.83 | 1512.747321 | 1.077485 |
| GO:0006405\_RNA\_export\_from\_nucleus | 10 | 0 | 0.000000 | -0.000000 | 1340 | 1374.912679 | 1443.83 | 1512.747321 | 1.077485 |
| GO:0006801\_superoxide\_metabolic\_process | 10 | 0 | 0.000000 | -0.000000 | 1340 | 1374.912679 | 1443.83 | 1512.747321 | 1.077485 |
| GO:0006805\_xenobiotic\_metabolic\_process | 10 | 0 | 0.000000 | -0.000000 | 1340 | 1374.912679 | 1443.83 | 1512.747321 | 1.077485 |
| GO:0006826\_iron\_ion\_transport | 10 | 0 | 0.000000 | -0.000000 | 1340 | 1374.912679 | 1443.83 | 1512.747321 | 1.077485 |
| GO:0006921\_cell\_structure\_disassembly\_during\_apoptosis | 10 | 0 | 0.000000 | -0.000000 | 1340 | 1374.912679 | 1443.83 | 1512.747321 | 1.077485 |
| GO:0006968\_cellular\_defense\_response | 10 | 0 | 0.000000 | -0.000000 | 1340 | 1374.912679 | 1443.83 | 1512.747321 | 1.077485 |
| GO:0007006\_mitochondrial\_membrane\_organization | 10 | 0 | 0.000000 | -0.000000 | 1340 | 1374.912679 | 1443.83 | 1512.747321 | 1.077485 |
| GO:0007044\_cell-substrate\_junction\_assembly | 10 | 0 | 0.000000 | -0.000000 | 1340 | 1374.912679 | 1443.83 | 1512.747321 | 1.077485 |
| GO:0007093\_mitotic\_cell\_cycle\_checkpoint | 10 | 0 | 0.000000 | -0.000000 | 1340 | 1374.912679 | 1443.83 | 1512.747321 | 1.077485 |
| GO:0007172\_signal\_complex\_assembly | 10 | 0 | 0.000000 | -0.000000 | 1340 | 1374.912679 | 1443.83 | 1512.747321 | 1.077485 |
| GO:0007194\_negative\_regulation\_of\_adenylate\_cyclase\_activity | 10 | 0 | 0.000000 | -0.000000 | 1340 | 1374.912679 | 1443.83 | 1512.747321 | 1.077485 |
| GO:0008206\_bile\_acid\_metabolic\_process | 10 | 0 | 0.000000 | -0.000000 | 1340 | 1374.912679 | 1443.83 | 1512.747321 | 1.077485 |
| GO:0008211\_glucocorticoid\_metabolic\_process | 10 | 0 | 0.000000 | -0.000000 | 1340 | 1374.912679 | 1443.83 | 1512.747321 | 1.077485 |
| GO:0009066\_aspartate\_family\_amino\_acid\_metabolic\_process | 10 | 0 | 0.000000 | -0.000000 | 1340 | 1374.912679 | 1443.83 | 1512.747321 | 1.077485 |
| GO:0009110\_vitamin\_biosynthetic\_process | 10 | 0 | 0.000000 | -0.000000 | 1340 | 1374.912679 | 1443.83 | 1512.747321 | 1.077485 |
| GO:0009620\_response\_to\_fungus | 10 | 0 | 0.000000 | -0.000000 | 1340 | 1374.912679 | 1443.83 | 1512.747321 | 1.077485 |
| GO:0009743\_response\_to\_carbohydrate\_stimulus | 10 | 0 | 0.000000 | -0.000000 | 1340 | 1374.912679 | 1443.83 | 1512.747321 | 1.077485 |
| GO:0009948\_anterior\_posterior\_axis\_specification | 10 | 0 | 0.000000 | -0.000000 | 1340 | 1374.912679 | 1443.83 | 1512.747321 | 1.077485 |
| GO:0010827\_regulation\_of\_glucose\_transport | 10 | 0 | 0.000000 | -0.000000 | 1340 | 1374.912679 | 1443.83 | 1512.747321 | 1.077485 |
| GO:0015718\_monocarboxylic\_acid\_transport | 10 | 0 | 0.000000 | -0.000000 | 1340 | 1374.912679 | 1443.83 | 1512.747321 | 1.077485 |
| GO:0016197\_endosome\_transport | 10 | 0 | 0.000000 | -0.000000 | 1340 | 1374.912679 | 1443.83 | 1512.747321 | 1.077485 |
| GO:0016486\_peptide\_hormone\_processing | 10 | 0 | 0.000000 | -0.000000 | 1340 | 1374.912679 | 1443.83 | 1512.747321 | 1.077485 |
| GO:0017156\_calcium\_ion-dependent\_exocytosis | 10 | 0 | 0.000000 | -0.000000 | 1340 | 1374.912679 | 1443.83 | 1512.747321 | 1.077485 |
| GO:0018149\_peptide\_cross-linking | 10 | 0 | 0.000000 | -0.000000 | 1340 | 1374.912679 | 1443.83 | 1512.747321 | 1.077485 |
| GO:0019321\_pentose\_metabolic\_process | 10 | 0 | 0.000000 | -0.000000 | 1340 | 1374.912679 | 1443.83 | 1512.747321 | 1.077485 |
| GO:0021534\_cell\_proliferation\_in\_hindbrain | 10 | 0 | 0.000000 | -0.000000 | 1340 | 1374.912679 | 1443.83 | 1512.747321 | 1.077485 |
| GO:0021871\_forebrain\_regionalization | 10 | 0 | 0.000000 | -0.000000 | 1340 | 1374.912679 | 1443.83 | 1512.747321 | 1.077485 |
| GO:0021895\_cerebral\_cortex\_neuron\_differentiation | 10 | 0 | 0.000000 | -0.000000 | 1340 | 1374.912679 | 1443.83 | 1512.747321 | 1.077485 |
| GO:0021924\_cell\_proliferation\_in\_the\_external\_granule\_layer | 10 | 0 | 0.000000 | -0.000000 | 1340 | 1374.912679 | 1443.83 | 1512.747321 | 1.077485 |
| GO:0021930\_granule\_cell\_precursor\_proliferation | 10 | 0 | 0.000000 | -0.000000 | 1340 | 1374.912679 | 1443.83 | 1512.747321 | 1.077485 |
| GO:0021952\_central\_nervous\_system\_projection\_neuron\_axonogenesis | 10 | 0 | 0.000000 | -0.000000 | 1340 | 1374.912679 | 1443.83 | 1512.747321 | 1.077485 |
| GO:0022900\_electron\_transport\_chain | 10 | 0 | 0.000000 | -0.000000 | 1340 | 1374.912679 | 1443.83 | 1512.747321 | 1.077485 |
| GO:0022904\_respiratory\_electron\_transport\_chain | 10 | 0 | 0.000000 | -0.000000 | 1340 | 1374.912679 | 1443.83 | 1512.747321 | 1.077485 |
| GO:0030168\_platelet\_activation | 10 | 0 | 0.000000 | -0.000000 | 1340 | 1374.912679 | 1443.83 | 1512.747321 | 1.077485 |
| GO:0030833\_regulation\_of\_actin\_filament\_polymerization | 10 | 0 | 0.000000 | -0.000000 | 1340 | 1374.912679 | 1443.83 | 1512.747321 | 1.077485 |
| GO:0031018\_endocrine\_pancreas\_development | 10 | 0 | 0.000000 | -0.000000 | 1340 | 1374.912679 | 1443.83 | 1512.747321 | 1.077485 |
| GO:0031280\_negative\_regulation\_of\_cyclase\_activity | 10 | 0 | 0.000000 | -0.000000 | 1340 | 1374.912679 | 1443.83 | 1512.747321 | 1.077485 |
| GO:0031331\_positive\_regulation\_of\_cellular\_catabolic\_process | 10 | 0 | 0.000000 | -0.000000 | 1340 | 1374.912679 | 1443.83 | 1512.747321 | 1.077485 |
| GO:0031645\_negative\_regulation\_of\_neurological\_system\_process | 10 | 0 | 0.000000 | -0.000000 | 1340 | 1374.912679 | 1443.83 | 1512.747321 | 1.077485 |
| GO:0032318\_regulation\_of\_Ras\_GTPase\_activity | 10 | 0 | 0.000000 | -0.000000 | 1340 | 1374.912679 | 1443.83 | 1512.747321 | 1.077485 |
| GO:0032602\_chemokine\_production | 10 | 0 | 0.000000 | -0.000000 | 1340 | 1374.912679 | 1443.83 | 1512.747321 | 1.077485 |
| GO:0032633\_interleukin-4\_production | 10 | 0 | 0.000000 | -0.000000 | 1340 | 1374.912679 | 1443.83 | 1512.747321 | 1.077485 |
| GO:0032642\_regulation\_of\_chemokine\_production | 10 | 0 | 0.000000 | -0.000000 | 1340 | 1374.912679 | 1443.83 | 1512.747321 | 1.077485 |
| GO:0032673\_regulation\_of\_interleukin-4\_production | 10 | 0 | 0.000000 | -0.000000 | 1340 | 1374.912679 | 1443.83 | 1512.747321 | 1.077485 |
| GO:0032760\_positive\_regulation\_of\_tumor\_necrosis\_factor\_production | 10 | 0 | 0.000000 | -0.000000 | 1340 | 1374.912679 | 1443.83 | 1512.747321 | 1.077485 |
| GO:0033081\_regulation\_of\_T\_cell\_differentiation\_in\_the\_thymus | 10 | 0 | 0.000000 | -0.000000 | 1340 | 1374.912679 | 1443.83 | 1512.747321 | 1.077485 |
| GO:0034105\_positive\_regulation\_of\_tissue\_remodeling | 10 | 0 | 0.000000 | -0.000000 | 1340 | 1374.912679 | 1443.83 | 1512.747321 | 1.077485 |
| GO:0040015\_negative\_regulation\_of\_multicellular\_organism\_growth | 10 | 0 | 0.000000 | -0.000000 | 1340 | 1374.912679 | 1443.83 | 1512.747321 | 1.077485 |
| GO:0042088\_T-helper\_1\_type\_immune\_response | 10 | 0 | 0.000000 | -0.000000 | 1340 | 1374.912679 | 1443.83 | 1512.747321 | 1.077485 |
| GO:0042116\_macrophage\_activation | 10 | 0 | 0.000000 | -0.000000 | 1340 | 1374.912679 | 1443.83 | 1512.747321 | 1.077485 |
| GO:0042177\_negative\_regulation\_of\_protein\_catabolic\_process | 10 | 0 | 0.000000 | -0.000000 | 1340 | 1374.912679 | 1443.83 | 1512.747321 | 1.077485 |
| GO:0042755\_eating\_behavior | 10 | 0 | 0.000000 | -0.000000 | 1340 | 1374.912679 | 1443.83 | 1512.747321 | 1.077485 |
| GO:0043113\_receptor\_clustering | 10 | 0 | 0.000000 | -0.000000 | 1340 | 1374.912679 | 1443.83 | 1512.747321 | 1.077485 |
| GO:0043330\_response\_to\_exogenous\_dsRNA | 10 | 0 | 0.000000 | -0.000000 | 1340 | 1374.912679 | 1443.83 | 1512.747321 | 1.077485 |
| GO:0043488\_regulation\_of\_mRNA\_stability | 10 | 0 | 0.000000 | -0.000000 | 1340 | 1374.912679 | 1443.83 | 1512.747321 | 1.077485 |
| GO:0043506\_regulation\_of\_JUN\_kinase\_activity | 10 | 0 | 0.000000 | -0.000000 | 1340 | 1374.912679 | 1443.83 | 1512.747321 | 1.077485 |
| GO:0043525\_positive\_regulation\_of\_neuron\_apoptosis | 10 | 0 | 0.000000 | -0.000000 | 1340 | 1374.912679 | 1443.83 | 1512.747321 | 1.077485 |
| GO:0044259\_multicellular\_organismal\_macromolecule\_metabolic\_process | 10 | 0 | 0.000000 | -0.000000 | 1340 | 1374.912679 | 1443.83 | 1512.747321 | 1.077485 |
| GO:0045132\_meiotic\_chromosome\_segregation | 10 | 0 | 0.000000 | -0.000000 | 1340 | 1374.912679 | 1443.83 | 1512.747321 | 1.077485 |
| GO:0045576\_mast\_cell\_activation | 10 | 0 | 0.000000 | -0.000000 | 1340 | 1374.912679 | 1443.83 | 1512.747321 | 1.077485 |
| GO:0045669\_positive\_regulation\_of\_osteoblast\_differentiation | 10 | 0 | 0.000000 | -0.000000 | 1340 | 1374.912679 | 1443.83 | 1512.747321 | 1.077485 |
| GO:0045776\_negative\_regulation\_of\_blood\_pressure | 10 | 0 | 0.000000 | -0.000000 | 1340 | 1374.912679 | 1443.83 | 1512.747321 | 1.077485 |
| GO:0045777\_positive\_regulation\_of\_blood\_pressure | 10 | 0 | 0.000000 | -0.000000 | 1340 | 1374.912679 | 1443.83 | 1512.747321 | 1.077485 |
| GO:0045814\_negative\_regulation\_of\_gene\_expression\_\_epigenetic | 10 | 0 | 0.000000 | -0.000000 | 1340 | 1374.912679 | 1443.83 | 1512.747321 | 1.077485 |
| GO:0045911\_positive\_regulation\_of\_DNA\_recombination | 10 | 0 | 0.000000 | -0.000000 | 1340 | 1374.912679 | 1443.83 | 1512.747321 | 1.077485 |
| GO:0046887\_positive\_regulation\_of\_hormone\_secretion | 10 | 0 | 0.000000 | -0.000000 | 1340 | 1374.912679 | 1443.83 | 1512.747321 | 1.077485 |
| GO:0048291\_isotype\_switching\_to\_IgG\_isotypes | 10 | 0 | 0.000000 | -0.000000 | 1340 | 1374.912679 | 1443.83 | 1512.747321 | 1.077485 |
| GO:0048302\_regulation\_of\_isotype\_switching\_to\_IgG\_isotypes | 10 | 0 | 0.000000 | -0.000000 | 1340 | 1374.912679 | 1443.83 | 1512.747321 | 1.077485 |
| GO:0048339\_paraxial\_mesoderm\_development | 10 | 0 | 0.000000 | -0.000000 | 1340 | 1374.912679 | 1443.83 | 1512.747321 | 1.077485 |
| GO:0048384\_retinoic\_acid\_receptor\_signaling\_pathway | 10 | 0 | 0.000000 | -0.000000 | 1340 | 1374.912679 | 1443.83 | 1512.747321 | 1.077485 |
| GO:0048596\_embryonic\_camera-type\_eye\_morphogenesis | 10 | 0 | 0.000000 | -0.000000 | 1340 | 1374.912679 | 1443.83 | 1512.747321 | 1.077485 |
| GO:0048641\_regulation\_of\_skeletal\_muscle\_tissue\_development | 10 | 0 | 0.000000 | -0.000000 | 1340 | 1374.912679 | 1443.83 | 1512.747321 | 1.077485 |
| GO:0048738\_cardiac\_muscle\_tissue\_development | 10 | 0 | 0.000000 | -0.000000 | 1340 | 1374.912679 | 1443.83 | 1512.747321 | 1.077485 |
| GO:0050654\_chondroitin\_sulfate\_proteoglycan\_metabolic\_process | 10 | 0 | 0.000000 | -0.000000 | 1340 | 1374.912679 | 1443.83 | 1512.747321 | 1.077485 |
| GO:0050657\_nucleic\_acid\_transport | 10 | 0 | 0.000000 | -0.000000 | 1340 | 1374.912679 | 1443.83 | 1512.747321 | 1.077485 |
| GO:0050658\_RNA\_transport | 10 | 0 | 0.000000 | -0.000000 | 1340 | 1374.912679 | 1443.83 | 1512.747321 | 1.077485 |
| GO:0050663\_cytokine\_secretion | 10 | 0 | 0.000000 | -0.000000 | 1340 | 1374.912679 | 1443.83 | 1512.747321 | 1.077485 |
| GO:0050714\_positive\_regulation\_of\_protein\_secretion | 10 | 0 | 0.000000 | -0.000000 | 1340 | 1374.912679 | 1443.83 | 1512.747321 | 1.077485 |
| GO:0050879\_multicellular\_organismal\_movement | 10 | 0 | 0.000000 | -0.000000 | 1340 | 1374.912679 | 1443.83 | 1512.747321 | 1.077485 |
| GO:0050881\_musculoskeletal\_movement | 10 | 0 | 0.000000 | -0.000000 | 1340 | 1374.912679 | 1443.83 | 1512.747321 | 1.077485 |
| GO:0050886\_endocrine\_process | 10 | 0 | 0.000000 | -0.000000 | 1340 | 1374.912679 | 1443.83 | 1512.747321 | 1.077485 |
| GO:0050892\_intestinal\_absorption | 10 | 0 | 0.000000 | -0.000000 | 1340 | 1374.912679 | 1443.83 | 1512.747321 | 1.077485 |
| GO:0051147\_regulation\_of\_muscle\_cell\_differentiation | 10 | 0 | 0.000000 | -0.000000 | 1340 | 1374.912679 | 1443.83 | 1512.747321 | 1.077485 |
| GO:0051208\_sequestering\_of\_calcium\_ion | 10 | 0 | 0.000000 | -0.000000 | 1340 | 1374.912679 | 1443.83 | 1512.747321 | 1.077485 |
| GO:0051209\_release\_of\_sequestered\_calcium\_ion\_into\_cytosol | 10 | 0 | 0.000000 | -0.000000 | 1340 | 1374.912679 | 1443.83 | 1512.747321 | 1.077485 |
| GO:0051224\_negative\_regulation\_of\_protein\_transport | 10 | 0 | 0.000000 | -0.000000 | 1340 | 1374.912679 | 1443.83 | 1512.747321 | 1.077485 |
| GO:0051236\_establishment\_of\_RNA\_localization | 10 | 0 | 0.000000 | -0.000000 | 1340 | 1374.912679 | 1443.83 | 1512.747321 | 1.077485 |
| GO:0051238\_sequestering\_of\_metal\_ion | 10 | 0 | 0.000000 | -0.000000 | 1340 | 1374.912679 | 1443.83 | 1512.747321 | 1.077485 |
| GO:0051262\_protein\_tetramerization | 10 | 0 | 0.000000 | -0.000000 | 1340 | 1374.912679 | 1443.83 | 1512.747321 | 1.077485 |
| GO:0051282\_regulation\_of\_sequestering\_of\_calcium\_ion | 10 | 0 | 0.000000 | -0.000000 | 1340 | 1374.912679 | 1443.83 | 1512.747321 | 1.077485 |
| GO:0051283\_negative\_regulation\_of\_sequestering\_of\_calcium\_ion | 10 | 0 | 0.000000 | -0.000000 | 1340 | 1374.912679 | 1443.83 | 1512.747321 | 1.077485 |
| GO:0051350\_negative\_regulation\_of\_lyase\_activity | 10 | 0 | 0.000000 | -0.000000 | 1340 | 1374.912679 | 1443.83 | 1512.747321 | 1.077485 |
| GO:0051445\_regulation\_of\_meiotic\_cell\_cycle | 10 | 0 | 0.000000 | -0.000000 | 1340 | 1374.912679 | 1443.83 | 1512.747321 | 1.077485 |
| GO:0051650\_establishment\_of\_vesicle\_localization | 10 | 0 | 0.000000 | -0.000000 | 1340 | 1374.912679 | 1443.83 | 1512.747321 | 1.077485 |
| GO:0051651\_maintenance\_of\_location\_in\_cell | 10 | 0 | 0.000000 | -0.000000 | 1340 | 1374.912679 | 1443.83 | 1512.747321 | 1.077485 |
| GO:0060135\_maternal\_process\_involved\_in\_female\_pregnancy | 10 | 0 | 0.000000 | -0.000000 | 1340 | 1374.912679 | 1443.83 | 1512.747321 | 1.077485 |
| GO:0060216\_definitive\_hemopoiesis | 10 | 0 | 0.000000 | -0.000000 | 1340 | 1374.912679 | 1443.83 | 1512.747321 | 1.077485 |
| GO:0060323\_head\_morphogenesis | 10 | 0 | 0.000000 | -0.000000 | 1340 | 1374.912679 | 1443.83 | 1512.747321 | 1.077485 |
| GO:0060601\_lateral\_sprouting\_from\_an\_epithelium | 10 | 0 | 0.000000 | -0.000000 | 1340 | 1374.912679 | 1443.83 | 1512.747321 | 1.077485 |
| GO:0060669\_embryonic\_placenta\_morphogenesis | 10 | 0 | 0.000000 | -0.000000 | 1340 | 1374.912679 | 1443.83 | 1512.747321 | 1.077485 |
| GO:0060706\_cell\_differentiation\_involved\_in\_embryonic\_placenta\_development | 10 | 0 | 0.000000 | -0.000000 | 1340 | 1374.912679 | 1443.83 | 1512.747321 | 1.077485 |
| GO:0060768\_regulation\_of\_epithelial\_cell\_proliferation\_involved\_in\_prostate\_gland\_development | 10 | 0 | 0.000000 | -0.000000 | 1340 | 1374.912679 | 1443.83 | 1512.747321 | 1.077485 |
| GO:0000723\_telomere\_maintenance | 13 | 0 | 0.000000 | -0.000000 | 1410 | 1442.704262 | 1509.23 | 1575.755738 | 1.070376 |
| GO:0001836\_release\_of\_cytochrome\_c\_from\_mitochondria | 13 | 0 | 0.000000 | -0.000000 | 1410 | 1442.704262 | 1509.23 | 1575.755738 | 1.070376 |
| GO:0001958\_endochondral\_ossification | 13 | 0 | 0.000000 | -0.000000 | 1410 | 1442.704262 | 1509.23 | 1575.755738 | 1.070376 |
| GO:0001975\_response\_to\_amphetamine | 13 | 0 | 0.000000 | -0.000000 | 1410 | 1442.704262 | 1509.23 | 1575.755738 | 1.070376 |
| GO:0001976\_neurological\_system\_process\_involved\_in\_regulation\_of\_systemic\_arterial\_blood\_pressure | 13 | 0 | 0.000000 | -0.000000 | 1410 | 1442.704262 | 1509.23 | 1575.755738 | 1.070376 |
| GO:0002704\_negative\_regulation\_of\_leukocyte\_mediated\_immunity | 13 | 0 | 0.000000 | -0.000000 | 1410 | 1442.704262 | 1509.23 | 1575.755738 | 1.070376 |
| GO:0002707\_negative\_regulation\_of\_lymphocyte\_mediated\_immunity | 13 | 0 | 0.000000 | -0.000000 | 1410 | 1442.704262 | 1509.23 | 1575.755738 | 1.070376 |
| GO:0002717\_positive\_regulation\_of\_natural\_killer\_cell\_mediated\_immunity | 13 | 0 | 0.000000 | -0.000000 | 1410 | 1442.704262 | 1509.23 | 1575.755738 | 1.070376 |
| GO:0003016\_respiratory\_system\_process | 13 | 0 | 0.000000 | -0.000000 | 1410 | 1442.704262 | 1509.23 | 1575.755738 | 1.070376 |
| GO:0006090\_pyruvate\_metabolic\_process | 13 | 0 | 0.000000 | -0.000000 | 1410 | 1442.704262 | 1509.23 | 1575.755738 | 1.070376 |
| GO:0006687\_glycosphingolipid\_metabolic\_process | 13 | 0 | 0.000000 | -0.000000 | 1410 | 1442.704262 | 1509.23 | 1575.755738 | 1.070376 |
| GO:0006778\_porphyrin\_metabolic\_process | 13 | 0 | 0.000000 | -0.000000 | 1410 | 1442.704262 | 1509.23 | 1575.755738 | 1.070376 |
| GO:0006833\_water\_transport | 13 | 0 | 0.000000 | -0.000000 | 1410 | 1442.704262 | 1509.23 | 1575.755738 | 1.070376 |
| GO:0006898\_receptor-mediated\_endocytosis | 13 | 0 | 0.000000 | -0.000000 | 1410 | 1442.704262 | 1509.23 | 1575.755738 | 1.070376 |
| GO:0006986\_response\_to\_unfolded\_protein | 13 | 0 | 0.000000 | -0.000000 | 1410 | 1442.704262 | 1509.23 | 1575.755738 | 1.070376 |
| GO:0007129\_synapsis | 13 | 0 | 0.000000 | -0.000000 | 1410 | 1442.704262 | 1509.23 | 1575.755738 | 1.070376 |
| GO:0007212\_dopamine\_receptor\_signaling\_pathway | 13 | 0 | 0.000000 | -0.000000 | 1410 | 1442.704262 | 1509.23 | 1575.755738 | 1.070376 |
| GO:0007339\_binding\_of\_sperm\_to\_zona\_pellucida | 13 | 0 | 0.000000 | -0.000000 | 1410 | 1442.704262 | 1509.23 | 1575.755738 | 1.070376 |
| GO:0007439\_ectodermal\_gut\_development | 13 | 0 | 0.000000 | -0.000000 | 1410 | 1442.704262 | 1509.23 | 1575.755738 | 1.070376 |
| GO:0007512\_adult\_heart\_development | 13 | 0 | 0.000000 | -0.000000 | 1410 | 1442.704262 | 1509.23 | 1575.755738 | 1.070376 |
| GO:0009119\_ribonucleoside\_metabolic\_process | 13 | 0 | 0.000000 | -0.000000 | 1410 | 1442.704262 | 1509.23 | 1575.755738 | 1.070376 |
| GO:0009410\_response\_to\_xenobiotic\_stimulus | 13 | 0 | 0.000000 | -0.000000 | 1410 | 1442.704262 | 1509.23 | 1575.755738 | 1.070376 |
| GO:0009994\_oocyte\_differentiation | 13 | 0 | 0.000000 | -0.000000 | 1410 | 1442.704262 | 1509.23 | 1575.755738 | 1.070376 |
| GO:0010623\_developmental\_programmed\_cell\_death | 13 | 0 | 0.000000 | -0.000000 | 1410 | 1442.704262 | 1509.23 | 1575.755738 | 1.070376 |
| GO:0016525\_negative\_regulation\_of\_angiogenesis | 13 | 0 | 0.000000 | -0.000000 | 1410 | 1442.704262 | 1509.23 | 1575.755738 | 1.070376 |
| GO:0018105\_peptidyl-serine\_phosphorylation | 13 | 0 | 0.000000 | -0.000000 | 1410 | 1442.704262 | 1509.23 | 1575.755738 | 1.070376 |
| GO:0019098\_reproductive\_behavior | 13 | 0 | 0.000000 | -0.000000 | 1410 | 1442.704262 | 1509.23 | 1575.755738 | 1.070376 |
| GO:0021511\_spinal\_cord\_patterning | 13 | 0 | 0.000000 | -0.000000 | 1410 | 1442.704262 | 1509.23 | 1575.755738 | 1.070376 |
| GO:0021533\_cell\_differentiation\_in\_hindbrain | 13 | 0 | 0.000000 | -0.000000 | 1410 | 1442.704262 | 1509.23 | 1575.755738 | 1.070376 |
| GO:0021879\_forebrain\_neuron\_differentiation | 13 | 0 | 0.000000 | -0.000000 | 1410 | 1442.704262 | 1509.23 | 1575.755738 | 1.070376 |
| GO:0021955\_central\_nervous\_system\_neuron\_axonogenesis | 13 | 0 | 0.000000 | -0.000000 | 1410 | 1442.704262 | 1509.23 | 1575.755738 | 1.070376 |
| GO:0030516\_regulation\_of\_axon\_extension | 13 | 0 | 0.000000 | -0.000000 | 1410 | 1442.704262 | 1509.23 | 1575.755738 | 1.070376 |
| GO:0030539\_male\_genitalia\_development | 13 | 0 | 0.000000 | -0.000000 | 1410 | 1442.704262 | 1509.23 | 1575.755738 | 1.070376 |
| GO:0031032\_actomyosin\_structure\_organization | 13 | 0 | 0.000000 | -0.000000 | 1410 | 1442.704262 | 1509.23 | 1575.755738 | 1.070376 |
| GO:0031290\_retinal\_ganglion\_cell\_axon\_guidance | 13 | 0 | 0.000000 | -0.000000 | 1410 | 1442.704262 | 1509.23 | 1575.755738 | 1.070376 |
| GO:0032200\_telomere\_organization | 13 | 0 | 0.000000 | -0.000000 | 1410 | 1442.704262 | 1509.23 | 1575.755738 | 1.070376 |
| GO:0032330\_regulation\_of\_chondrocyte\_differentiation | 13 | 0 | 0.000000 | -0.000000 | 1410 | 1442.704262 | 1509.23 | 1575.755738 | 1.070376 |
| GO:0032615\_interleukin-12\_production | 13 | 0 | 0.000000 | -0.000000 | 1410 | 1442.704262 | 1509.23 | 1575.755738 | 1.070376 |
| GO:0032729\_positive\_regulation\_of\_interferon-gamma\_production | 13 | 0 | 0.000000 | -0.000000 | 1410 | 1442.704262 | 1509.23 | 1575.755738 | 1.070376 |
| GO:0033013\_tetrapyrrole\_metabolic\_process | 13 | 0 | 0.000000 | -0.000000 | 1410 | 1442.704262 | 1509.23 | 1575.755738 | 1.070376 |
| GO:0034329\_cell\_junction\_assembly | 13 | 0 | 0.000000 | -0.000000 | 1410 | 1442.704262 | 1509.23 | 1575.755738 | 1.070376 |
| GO:0042044\_fluid\_transport | 13 | 0 | 0.000000 | -0.000000 | 1410 | 1442.704262 | 1509.23 | 1575.755738 | 1.070376 |
| GO:0042094\_interleukin-2\_biosynthetic\_process | 13 | 0 | 0.000000 | -0.000000 | 1410 | 1442.704262 | 1509.23 | 1575.755738 | 1.070376 |
| GO:0042474\_middle\_ear\_morphogenesis | 13 | 0 | 0.000000 | -0.000000 | 1410 | 1442.704262 | 1509.23 | 1575.755738 | 1.070376 |
| GO:0043241\_protein\_complex\_disassembly | 13 | 0 | 0.000000 | -0.000000 | 1410 | 1442.704262 | 1509.23 | 1575.755738 | 1.070376 |
| GO:0043244\_regulation\_of\_protein\_complex\_disassembly | 13 | 0 | 0.000000 | -0.000000 | 1410 | 1442.704262 | 1509.23 | 1575.755738 | 1.070376 |
| GO:0045191\_regulation\_of\_isotype\_switching | 13 | 0 | 0.000000 | -0.000000 | 1410 | 1442.704262 | 1509.23 | 1575.755738 | 1.070376 |
| GO:0045577\_regulation\_of\_B\_cell\_differentiation | 13 | 0 | 0.000000 | -0.000000 | 1410 | 1442.704262 | 1509.23 | 1575.755738 | 1.070376 |
| GO:0045682\_regulation\_of\_epidermis\_development | 13 | 0 | 0.000000 | -0.000000 | 1410 | 1442.704262 | 1509.23 | 1575.755738 | 1.070376 |
| GO:0045954\_positive\_regulation\_of\_natural\_killer\_cell\_mediated\_cytotoxicity | 13 | 0 | 0.000000 | -0.000000 | 1410 | 1442.704262 | 1509.23 | 1575.755738 | 1.070376 |
| GO:0046474\_glycerophospholipid\_biosynthetic\_process | 13 | 0 | 0.000000 | -0.000000 | 1410 | 1442.704262 | 1509.23 | 1575.755738 | 1.070376 |
| GO:0046640\_regulation\_of\_alpha-beta\_T\_cell\_proliferation | 13 | 0 | 0.000000 | -0.000000 | 1410 | 1442.704262 | 1509.23 | 1575.755738 | 1.070376 |
| GO:0046851\_negative\_regulation\_of\_bone\_remodeling | 13 | 0 | 0.000000 | -0.000000 | 1410 | 1442.704262 | 1509.23 | 1575.755738 | 1.070376 |
| GO:0048305\_immunoglobulin\_secretion | 13 | 0 | 0.000000 | -0.000000 | 1410 | 1442.704262 | 1509.23 | 1575.755738 | 1.070376 |
| GO:0048566\_embryonic\_gut\_development | 13 | 0 | 0.000000 | -0.000000 | 1410 | 1442.704262 | 1509.23 | 1575.755738 | 1.070376 |
| GO:0048567\_ectodermal\_gut\_morphogenesis | 13 | 0 | 0.000000 | -0.000000 | 1410 | 1442.704262 | 1509.23 | 1575.755738 | 1.070376 |
| GO:0048599\_oocyte\_development | 13 | 0 | 0.000000 | -0.000000 | 1410 | 1442.704262 | 1509.23 | 1575.755738 | 1.070376 |
| GO:0050764\_regulation\_of\_phagocytosis | 13 | 0 | 0.000000 | -0.000000 | 1410 | 1442.704262 | 1509.23 | 1575.755738 | 1.070376 |
| GO:0050766\_positive\_regulation\_of\_phagocytosis | 13 | 0 | 0.000000 | -0.000000 | 1410 | 1442.704262 | 1509.23 | 1575.755738 | 1.070376 |
| GO:0050818\_regulation\_of\_coagulation | 13 | 0 | 0.000000 | -0.000000 | 1410 | 1442.704262 | 1509.23 | 1575.755738 | 1.070376 |
| GO:0051346\_negative\_regulation\_of\_hydrolase\_activity | 13 | 0 | 0.000000 | -0.000000 | 1410 | 1442.704262 | 1509.23 | 1575.755738 | 1.070376 |
| GO:0051495\_positive\_regulation\_of\_cytoskeleton\_organization | 13 | 0 | 0.000000 | -0.000000 | 1410 | 1442.704262 | 1509.23 | 1575.755738 | 1.070376 |
| GO:0060038\_cardiac\_muscle\_cell\_proliferation | 13 | 0 | 0.000000 | -0.000000 | 1410 | 1442.704262 | 1509.23 | 1575.755738 | 1.070376 |
| GO:0060070\_Wnt\_receptor\_signaling\_pathway\_through\_beta-catenin | 13 | 0 | 0.000000 | -0.000000 | 1410 | 1442.704262 | 1509.23 | 1575.755738 | 1.070376 |
| GO:0060324\_face\_development | 13 | 0 | 0.000000 | -0.000000 | 1410 | 1442.704262 | 1509.23 | 1575.755738 | 1.070376 |
| GO:0060401\_cytosolic\_calcium\_ion\_transport | 13 | 0 | 0.000000 | -0.000000 | 1410 | 1442.704262 | 1509.23 | 1575.755738 | 1.070376 |
| GO:0060402\_calcium\_ion\_transport\_into\_cytosol | 13 | 0 | 0.000000 | -0.000000 | 1410 | 1442.704262 | 1509.23 | 1575.755738 | 1.070376 |
| GO:0060560\_developmental\_growth\_involved\_in\_morphogenesis | 13 | 0 | 0.000000 | -0.000000 | 1410 | 1442.704262 | 1509.23 | 1575.755738 | 1.070376 |
| GO:0060742\_epithelial\_cell\_differentiation\_involved\_in\_prostate\_gland\_development | 13 | 0 | 0.000000 | -0.000000 | 1410 | 1442.704262 | 1509.23 | 1575.755738 | 1.070376 |
| GO:0070192\_chromosome\_organization\_involved\_in\_meiosis | 13 | 0 | 0.000000 | -0.000000 | 1410 | 1442.704262 | 1509.23 | 1575.755738 | 1.070376 |
| GO:0002440\_production\_of\_molecular\_mediator\_of\_immune\_response | 49 | 0 | 0.000000 | -0.000000 | 1419 | 1450.563633 | 1516.37 | 1582.176367 | 1.068619 |
| GO:0003015\_heart\_process | 49 | 0 | 0.000000 | -0.000000 | 1419 | 1450.563633 | 1516.37 | 1582.176367 | 1.068619 |
| GO:0006725\_cellular\_aromatic\_compound\_metabolic\_process | 49 | 0 | 0.000000 | -0.000000 | 1419 | 1450.563633 | 1516.37 | 1582.176367 | 1.068619 |
| GO:0007606\_sensory\_perception\_of\_chemical\_stimulus | 49 | 0 | 0.000000 | -0.000000 | 1419 | 1450.563633 | 1516.37 | 1582.176367 | 1.068619 |
| GO:0021543\_pallium\_development | 49 | 0 | 0.000000 | -0.000000 | 1419 | 1450.563633 | 1516.37 | 1582.176367 | 1.068619 |
| GO:0042035\_regulation\_of\_cytokine\_biosynthetic\_process | 49 | 0 | 0.000000 | -0.000000 | 1419 | 1450.563633 | 1516.37 | 1582.176367 | 1.068619 |
| GO:0046661\_male\_sex\_differentiation | 49 | 0 | 0.000000 | -0.000000 | 1419 | 1450.563633 | 1516.37 | 1582.176367 | 1.068619 |
| GO:0048741\_skeletal\_muscle\_fiber\_development | 49 | 0 | 0.000000 | -0.000000 | 1419 | 1450.563633 | 1516.37 | 1582.176367 | 1.068619 |
| GO:0060047\_heart\_contraction | 49 | 0 | 0.000000 | -0.000000 | 1419 | 1450.563633 | 1516.37 | 1582.176367 | 1.068619 |
| GO:0001704\_formation\_of\_primary\_germ\_layer | 36 | 0 | 0.000000 | -0.000000 | 1431 | 1464.514260 | 1528.99 | 1593.465740 | 1.068477 |
| GO:0001819\_positive\_regulation\_of\_cytokine\_production | 36 | 0 | 0.000000 | -0.000000 | 1431 | 1464.514260 | 1528.99 | 1593.465740 | 1.068477 |
| GO:0001889\_liver\_development | 36 | 0 | 0.000000 | -0.000000 | 1431 | 1464.514260 | 1528.99 | 1593.465740 | 1.068477 |
| GO:0007187\_G-protein\_signaling\_\_coupled\_to\_cyclic\_nucleotide\_second\_messenger | 36 | 0 | 0.000000 | -0.000000 | 1431 | 1464.514260 | 1528.99 | 1593.465740 | 1.068477 |
| GO:0007368\_determination\_of\_left\_right\_symmetry | 36 | 0 | 0.000000 | -0.000000 | 1431 | 1464.514260 | 1528.99 | 1593.465740 | 1.068477 |
| GO:0007631\_feeding\_behavior | 36 | 0 | 0.000000 | -0.000000 | 1431 | 1464.514260 | 1528.99 | 1593.465740 | 1.068477 |
| GO:0014020\_primary\_neural\_tube\_formation | 36 | 0 | 0.000000 | -0.000000 | 1431 | 1464.514260 | 1528.99 | 1593.465740 | 1.068477 |
| GO:0019228\_regulation\_of\_action\_potential\_in\_neuron | 36 | 0 | 0.000000 | -0.000000 | 1431 | 1464.514260 | 1528.99 | 1593.465740 | 1.068477 |
| GO:0021510\_spinal\_cord\_development | 36 | 0 | 0.000000 | -0.000000 | 1431 | 1464.514260 | 1528.99 | 1593.465740 | 1.068477 |
| GO:0030072\_peptide\_hormone\_secretion | 36 | 0 | 0.000000 | -0.000000 | 1431 | 1464.514260 | 1528.99 | 1593.465740 | 1.068477 |
| GO:0042742\_defense\_response\_to\_bacterium | 36 | 0 | 0.000000 | -0.000000 | 1431 | 1464.514260 | 1528.99 | 1593.465740 | 1.068477 |
| GO:0051223\_regulation\_of\_protein\_transport | 36 | 0 | 0.000000 | -0.000000 | 1431 | 1464.514260 | 1528.99 | 1593.465740 | 1.068477 |
| GO:0000027\_ribosomal\_large\_subunit\_assembly | 1 | 0 |  |  |  |  |  |  |  |  |
| GO:0000042\_protein\_targeting\_to\_Golgi | 1 | 0 |  |  |  |  |  |  |  |  |
| GO:0000046\_autophagic\_vacuole\_fusion | 1 | 0 |  |  |  |  |  |  |  |  |
| GO:0000050\_urea\_cycle | 1 | 0 |  |  |  |  |  |  |  |  |
| GO:0000054\_ribosome\_export\_from\_nucleus | 1 | 0 |  |  |  |  |  |  |  |  |
| GO:0000055\_ribosomal\_large\_subunit\_export\_from\_nucleus | 1 | 0 |  |  |  |  |  |  |  |  |
| GO:0000056\_ribosomal\_small\_subunit\_export\_from\_nucleus | 1 | 0 |  |  |  |  |  |  |  |  |
| GO:0000072\_M\_phase\_specific\_microtubule\_process | 1 | 0 |  |  |  |  |  |  |  |  |
| GO:0000101\_sulfur\_amino\_acid\_transport | 1 | 0 |  |  |  |  |  |  |  |  |
| GO:0000147\_actin\_cortical\_patch\_assembly | 1 | 0 |  |  |  |  |  |  |  |  |
| GO:0000154\_rRNA\_modification | 1 | 0 |  |  |  |  |  |  |  |  |
| GO:0000183\_chromatin\_silencing\_at\_rDNA | 1 | 0 |  |  |  |  |  |  |  |  |
| GO:0000185\_activation\_of\_MAPKKK\_activity | 1 | 0 |  |  |  |  |  |  |  |  |
| GO:0000238\_zygotene | 1 | 0 |  |  |  |  |  |  |  |  |
| GO:0000255\_allantoin\_metabolic\_process | 1 | 0 |  |  |  |  |  |  |  |  |
| GO:0000266\_mitochondrial\_fission | 1 | 0 |  |  |  |  |  |  |  |  |
| GO:0000273\_lipoic\_acid\_metabolic\_process | 1 | 0 |  |  |  |  |  |  |  |  |
| GO:0000301\_retrograde\_transport\_\_vesicle\_recycling\_within\_Golgi | 1 | 0 |  |  |  |  |  |  |  |  |
| GO:0000394\_RNA\_splicing\_\_via\_endonucleolytic\_cleavage\_and\_ligation | 1 | 0 |  |  |  |  |  |  |  |  |
| GO:0000429\_regulation\_of\_transcription\_from\_RNA\_polymerase\_II\_promoter\_by\_carbon\_catabolites | 1 | 0 |  |  |  |  |  |  |  |  |
| GO:0000430\_regulation\_of\_transcription\_from\_RNA\_polymerase\_II\_promoter\_by\_glucose | 1 | 0 |  |  |  |  |  |  |  |  |
| GO:0000432\_positive\_regulation\_of\_transcription\_from\_RNA\_polymerase\_II\_promoter\_by\_glucose | 1 | 0 |  |  |  |  |  |  |  |  |
| GO:0000436\_positive\_regulation\_of\_transcription\_from\_RNA\_polymerase\_II\_promoter\_by\_carbon\_catabolites | 1 | 0 |  |  |  |  |  |  |  |  |
| GO:0000448\_cleavage\_in\_ITS2\_between\_5.8S\_rRNA\_and\_LSU-rRNA\_of\_tricistronic\_rRNA\_transcript\_(SSU-rRNA\_\_5.8S\_rRNA\_\_LSU-rRNA) | 1 | 0 |  |  |  |  |  |  |  |  |
| GO:0000460\_maturation\_of\_5.8S\_rRNA | 1 | 0 |  |  |  |  |  |  |  |  |
| GO:0000463\_maturation\_of\_LSU-rRNA\_from\_tricistronic\_rRNA\_transcript\_(SSU-rRNA\_\_5.8S\_rRNA\_\_LSU-rRNA) | 1 | 0 |  |  |  |  |  |  |  |  |
| GO:0000466\_maturation\_of\_5.8S\_rRNA\_from\_tricistronic\_rRNA\_transcript\_(SSU-rRNA\_\_5.8S\_rRNA\_\_LSU-rRNA) | 1 | 0 |  |  |  |  |  |  |  |  |
| GO:0000469\_cleavages\_during\_rRNA\_processing | 1 | 0 |  |  |  |  |  |  |  |  |
| GO:0000470\_maturation\_of\_LSU-rRNA | 1 | 0 |  |  |  |  |  |  |  |  |
| GO:0000478\_endonucleolytic\_cleavages\_during\_rRNA\_processing | 1 | 0 |  |  |  |  |  |  |  |  |
| GO:0000479\_endonucleolytic\_cleavage\_of\_tricistronic\_rRNA\_transcript\_(SSU-rRNA\_\_5.8S\_rRNA\_\_LSU-rRNA) | 1 | 0 |  |  |  |  |  |  |  |  |
| GO:0000705\_achiasmate\_meiosis\_I | 1 | 0 |  |  |  |  |  |  |  |  |
| GO:0000966\_RNA\_5'-end\_processing | 1 | 0 |  |  |  |  |  |  |  |  |
| GO:0001300\_chronological\_cell\_aging | 1 | 0 |  |  |  |  |  |  |  |  |
| GO:0001547\_antral\_ovarian\_follicle\_growth | 1 | 0 |  |  |  |  |  |  |  |  |
| GO:0001555\_oocyte\_growth | 1 | 0 |  |  |  |  |  |  |  |  |
| GO:0001560\_regulation\_of\_cell\_growth\_by\_extracellular\_stimulus | 1 | 0 |  |  |  |  |  |  |  |  |
| GO:0001660\_fever | 1 | 0 |  |  |  |  |  |  |  |  |
| GO:0001696\_gastric\_acid\_secretion | 1 | 0 |  |  |  |  |  |  |  |  |
| GO:0001712\_ectodermal\_cell\_fate\_commitment | 1 | 0 |  |  |  |  |  |  |  |  |
| GO:0001714\_endodermal\_cell\_fate\_specification | 1 | 0 |  |  |  |  |  |  |  |  |
| GO:0001762\_beta-alanine\_transport | 1 | 0 |  |  |  |  |  |  |  |  |
| GO:0001766\_membrane\_raft\_polarization | 1 | 0 |  |  |  |  |  |  |  |  |
| GO:0001811\_negative\_regulation\_of\_type\_I\_hypersensitivity | 1 | 0 |  |  |  |  |  |  |  |  |
| GO:0001821\_histamine\_secretion | 1 | 0 |  |  |  |  |  |  |  |  |
| GO:0001826\_inner\_cell\_mass\_cell\_differentiation | 1 | 0 |  |  |  |  |  |  |  |  |
| GO:0001830\_trophectodermal\_cell\_fate\_commitment | 1 | 0 |  |  |  |  |  |  |  |  |
| GO:0001834\_trophectodermal\_cell\_proliferation | 1 | 0 |  |  |  |  |  |  |  |  |
| GO:0001867\_complement\_activation\_\_lectin\_pathway | 1 | 0 |  |  |  |  |  |  |  |  |
| GO:0001880\_Mullerian\_duct\_regression | 1 | 0 |  |  |  |  |  |  |  |  |
| GO:0001887\_selenium\_metabolic\_process | 1 | 0 |  |  |  |  |  |  |  |  |
| GO:0001922\_B-1\_B\_cell\_homeostasis | 1 | 0 |  |  |  |  |  |  |  |  |
| GO:0001923\_B-1\_B\_cell\_differentiation | 1 | 0 |  |  |  |  |  |  |  |  |
| GO:0001941\_postsynaptic\_membrane\_organization | 1 | 0 |  |  |  |  |  |  |  |  |
| GO:0001946\_lymphangiogenesis | 1 | 0 |  |  |  |  |  |  |  |  |
| GO:0001956\_positive\_regulation\_of\_neurotransmitter\_secretion | 1 | 0 |  |  |  |  |  |  |  |  |
| GO:0001961\_positive\_regulation\_of\_cytokine-mediated\_signaling\_pathway | 1 | 0 |  |  |  |  |  |  |  |  |
| GO:0001979\_regulation\_of\_systemic\_arterial\_blood\_pressure\_by\_chemoreceptor\_signaling | 1 | 0 |  |  |  |  |  |  |  |  |
| GO:0001980\_regulation\_of\_systemic\_arterial\_blood\_pressure\_by\_ischemic\_conditions | 1 | 0 |  |  |  |  |  |  |  |  |
| GO:0001984\_vasodilation\_of\_artery\_during\_baroreceptor\_response\_to\_increased\_systemic\_arterial\_blood\_pressure | 1 | 0 |  |  |  |  |  |  |  |  |
| GO:0001985\_negative\_regulation\_of\_heart\_rate\_in\_baroreceptor\_response\_to\_increased\_systemic\_arterial\_blood\_pressure | 1 | 0 |  |  |  |  |  |  |  |  |
| GO:0001987\_vasoconstriction\_of\_artery\_involved\_in\_baroreceptor\_response\_to\_lowering\_of\_systemic\_arterial\_blood\_pressure | 1 | 0 |  |  |  |  |  |  |  |  |
| GO:0001988\_positive\_regulation\_of\_heart\_rate\_in\_baroreceptor\_response\_to\_decreased\_systemic\_arterial\_blood\_pressure | 1 | 0 |  |  |  |  |  |  |  |  |
| GO:0001994\_norepinephrine-epinephrine\_vasoconstriction\_involved\_in\_regulation\_of\_systemic\_arterial\_blood\_pressure | 1 | 0 |  |  |  |  |  |  |  |  |
| GO:0002001\_renin\_secretion\_into\_blood\_stream | 1 | 0 |  |  |  |  |  |  |  |  |
| GO:0002002\_regulation\_of\_angiotensin\_levels\_in\_blood | 1 | 0 |  |  |  |  |  |  |  |  |
| GO:0002003\_angiotensin\_maturation | 1 | 0 |  |  |  |  |  |  |  |  |
| GO:0002007\_detection\_of\_hypoxic\_conditions\_in\_blood\_by\_chemoreceptor\_signaling | 1 | 0 |  |  |  |  |  |  |  |  |
| GO:0002017\_regulation\_of\_blood\_volume\_by\_renal\_aldosterone | 1 | 0 |  |  |  |  |  |  |  |  |
| GO:0002023\_reduction\_of\_food\_intake\_in\_response\_to\_dietary\_excess | 1 | 0 |  |  |  |  |  |  |  |  |
| GO:0002031\_G-protein\_coupled\_receptor\_internalization | 1 | 0 |  |  |  |  |  |  |  |  |
| GO:0002036\_regulation\_of\_L-glutamate\_transport | 1 | 0 |  |  |  |  |  |  |  |  |
| GO:0002040\_sprouting\_angiogenesis | 1 | 0 |  |  |  |  |  |  |  |  |
| GO:0002041\_intussusceptive\_angiogenesis | 1 | 0 |  |  |  |  |  |  |  |  |
| GO:0002068\_glandular\_epithelial\_cell\_development | 1 | 0 |  |  |  |  |  |  |  |  |
| GO:0002069\_columnar\_cuboidal\_epithelial\_cell\_maturation | 1 | 0 |  |  |  |  |  |  |  |  |
| GO:0002071\_glandular\_epithelial\_cell\_maturation | 1 | 0 |  |  |  |  |  |  |  |  |
| GO:0002082\_regulation\_of\_oxidative\_phosphorylation | 1 | 0 |  |  |  |  |  |  |  |  |
| GO:0002084\_protein\_depalmitoylation | 1 | 0 |  |  |  |  |  |  |  |  |
| GO:0002085\_inhibition\_of\_neuroepithelial\_cell\_differentiation | 1 | 0 |  |  |  |  |  |  |  |  |
| GO:0002086\_diaphragm\_contraction | 1 | 0 |  |  |  |  |  |  |  |  |
| GO:0002118\_aggressive\_behavior | 1 | 0 |  |  |  |  |  |  |  |  |
| GO:0002121\_inter-male\_aggressive\_behavior | 1 | 0 |  |  |  |  |  |  |  |  |
| GO:0002124\_territorial\_aggressive\_behavior | 1 | 0 |  |  |  |  |  |  |  |  |
| GO:0002227\_innate\_immune\_response\_in\_mucosa | 1 | 0 |  |  |  |  |  |  |  |  |
| GO:0002232\_leukocyte\_chemotaxis\_during\_inflammatory\_response | 1 | 0 |  |  |  |  |  |  |  |  |
| GO:0002248\_connective\_tissue\_replacement\_during\_inflammatory\_response | 1 | 0 |  |  |  |  |  |  |  |  |
| GO:0002282\_microglial\_cell\_activation\_during\_immune\_response | 1 | 0 |  |  |  |  |  |  |  |  |
| GO:0002287\_alpha-beta\_T\_cell\_activation\_during\_immune\_response | 1 | 0 |  |  |  |  |  |  |  |  |
| GO:0002314\_germinal\_center\_B\_cell\_differentiation | 1 | 0 |  |  |  |  |  |  |  |  |
| GO:0002315\_marginal\_zone\_B\_cell\_differentiation | 1 | 0 |  |  |  |  |  |  |  |  |
| GO:0002317\_plasma\_cell\_differentiation | 1 | 0 |  |  |  |  |  |  |  |  |
| GO:0002349\_histamine\_production\_during\_acute\_inflammatory\_response | 1 | 0 |  |  |  |  |  |  |  |  |
| GO:0002351\_serotonin\_production\_during\_acute\_inflammatory\_response | 1 | 0 |  |  |  |  |  |  |  |  |
| GO:0002355\_detection\_of\_tumor\_cell | 1 | 0 |  |  |  |  |  |  |  |  |
| GO:0002370\_natural\_killer\_cell\_cytokine\_production | 1 | 0 |  |  |  |  |  |  |  |  |
| GO:0002371\_dendritic\_cell\_cytokine\_production | 1 | 0 |  |  |  |  |  |  |  |  |
| GO:0002380\_immunoglobulin\_secretion\_during\_immune\_response | 1 | 0 |  |  |  |  |  |  |  |  |
| GO:0002396\_MHC\_protein\_complex\_assembly | 1 | 0 |  |  |  |  |  |  |  |  |
| GO:0002397\_MHC\_class\_I\_protein\_complex\_assembly | 1 | 0 |  |  |  |  |  |  |  |  |
| GO:0002420\_natural\_killer\_cell\_mediated\_cytotoxicity\_directed\_against\_tumor\_cell\_target | 1 | 0 |  |  |  |  |  |  |  |  |
| GO:0002423\_natural\_killer\_cell\_mediated\_immune\_response\_to\_tumor\_cell | 1 | 0 |  |  |  |  |  |  |  |  |
| GO:0002424\_T\_cell\_mediated\_immune\_response\_to\_tumor\_cell | 1 | 0 |  |  |  |  |  |  |  |  |
| GO:0002426\_immunoglobulin\_production\_in\_mucosal\_tissue | 1 | 0 |  |  |  |  |  |  |  |  |
| GO:0002431\_Fc\_receptor\_mediated\_stimulatory\_signaling\_pathway | 1 | 0 |  |  |  |  |  |  |  |  |
| GO:0002432\_granuloma\_formation | 1 | 0 |  |  |  |  |  |  |  |  |
| GO:0002441\_histamine\_secretion\_during\_acute\_inflammatory\_response | 1 | 0 |  |  |  |  |  |  |  |  |
| GO:0002442\_serotonin\_secretion\_during\_acute\_inflammatory\_response | 1 | 0 |  |  |  |  |  |  |  |  |
| GO:0002457\_T\_cell\_antigen\_processing\_and\_presentation | 1 | 0 |  |  |  |  |  |  |  |  |
| GO:0002458\_peripheral\_T\_cell\_tolerance\_induction | 1 | 0 |  |  |  |  |  |  |  |  |
| GO:0002461\_tolerance\_induction\_dependent\_upon\_immune\_response | 1 | 0 |  |  |  |  |  |  |  |  |
| GO:0002465\_peripheral\_tolerance\_induction | 1 | 0 |  |  |  |  |  |  |  |  |
| GO:0002468\_dendritic\_cell\_antigen\_processing\_and\_presentation | 1 | 0 |  |  |  |  |  |  |  |  |
| GO:0002476\_antigen\_processing\_and\_presentation\_of\_endogenous\_peptide\_antigen\_via\_MHC\_class\_Ib | 1 | 0 |  |  |  |  |  |  |  |  |
| GO:0002479\_antigen\_processing\_and\_presentation\_of\_exogenous\_peptide\_antigen\_via\_MHC\_class\_I\_\_TAP-dependent | 1 | 0 |  |  |  |  |  |  |  |  |
| GO:0002483\_antigen\_processing\_and\_presentation\_of\_endogenous\_peptide\_antigen | 1 | 0 |  |  |  |  |  |  |  |  |
| GO:0002501\_peptide\_antigen\_assembly\_with\_MHC\_protein\_complex | 1 | 0 |  |  |  |  |  |  |  |  |
| GO:0002502\_peptide\_antigen\_assembly\_with\_MHC\_class\_I\_protein\_complex | 1 | 0 |  |  |  |  |  |  |  |  |
| GO:0002508\_central\_tolerance\_induction | 1 | 0 |  |  |  |  |  |  |  |  |
| GO:0002510\_central\_B\_cell\_tolerance\_induction | 1 | 0 |  |  |  |  |  |  |  |  |
| GO:0002545\_chronic\_inflammatory\_response\_to\_non-antigenic\_stimulus | 1 | 0 |  |  |  |  |  |  |  |  |
| GO:0002553\_histamine\_secretion\_by\_mast\_cell | 1 | 0 |  |  |  |  |  |  |  |  |
| GO:0002554\_serotonin\_secretion\_by\_platelet | 1 | 0 |  |  |  |  |  |  |  |  |
| GO:0002572\_pro-T\_cell\_differentiation | 1 | 0 |  |  |  |  |  |  |  |  |
| GO:0002577\_regulation\_of\_antigen\_processing\_and\_presentation | 1 | 0 |  |  |  |  |  |  |  |  |
| GO:0002579\_positive\_regulation\_of\_antigen\_processing\_and\_presentation | 1 | 0 |  |  |  |  |  |  |  |  |
| GO:0002604\_regulation\_of\_dendritic\_cell\_antigen\_processing\_and\_presentation | 1 | 0 |  |  |  |  |  |  |  |  |
| GO:0002606\_positive\_regulation\_of\_dendritic\_cell\_antigen\_processing\_and\_presentation | 1 | 0 |  |  |  |  |  |  |  |  |
| GO:0002635\_negative\_regulation\_of\_germinal\_center\_formation | 1 | 0 |  |  |  |  |  |  |  |  |
| GO:0002646\_regulation\_of\_central\_tolerance\_induction | 1 | 0 |  |  |  |  |  |  |  |  |
| GO:0002648\_positive\_regulation\_of\_central\_tolerance\_induction | 1 | 0 |  |  |  |  |  |  |  |  |
| GO:0002649\_regulation\_of\_tolerance\_induction\_to\_self\_antigen | 1 | 0 |  |  |  |  |  |  |  |  |
| GO:0002651\_positive\_regulation\_of\_tolerance\_induction\_to\_self\_antigen | 1 | 0 |  |  |  |  |  |  |  |  |
| GO:0002652\_regulation\_of\_tolerance\_induction\_dependent\_upon\_immune\_response | 1 | 0 |  |  |  |  |  |  |  |  |
| GO:0002654\_positive\_regulation\_of\_tolerance\_induction\_dependent\_upon\_immune\_response | 1 | 0 |  |  |  |  |  |  |  |  |
| GO:0002658\_regulation\_of\_peripheral\_tolerance\_induction | 1 | 0 |  |  |  |  |  |  |  |  |
| GO:0002660\_positive\_regulation\_of\_peripheral\_tolerance\_induction | 1 | 0 |  |  |  |  |  |  |  |  |
| GO:0002677\_negative\_regulation\_of\_chronic\_inflammatory\_response | 1 | 0 |  |  |  |  |  |  |  |  |
| GO:0002678\_positive\_regulation\_of\_chronic\_inflammatory\_response | 1 | 0 |  |  |  |  |  |  |  |  |
| GO:0002701\_negative\_regulation\_of\_production\_of\_molecular\_mediator\_of\_immune\_response | 1 | 0 |  |  |  |  |  |  |  |  |
| GO:0002719\_negative\_regulation\_of\_cytokine\_production\_during\_immune\_response | 1 | 0 |  |  |  |  |  |  |  |  |
| GO:0002724\_regulation\_of\_T\_cell\_cytokine\_production | 1 | 0 |  |  |  |  |  |  |  |  |
| GO:0002727\_regulation\_of\_natural\_killer\_cell\_cytokine\_production | 1 | 0 |  |  |  |  |  |  |  |  |
| GO:0002729\_positive\_regulation\_of\_natural\_killer\_cell\_cytokine\_production | 1 | 0 |  |  |  |  |  |  |  |  |
| GO:0002730\_regulation\_of\_dendritic\_cell\_cytokine\_production | 1 | 0 |  |  |  |  |  |  |  |  |
| GO:0002756\_MyD88-independent\_toll-like\_receptor\_signaling\_pathway | 1 | 0 |  |  |  |  |  |  |  |  |
| GO:0002767\_immune\_response-inhibiting\_cell\_surface\_receptor\_signaling\_pathway | 1 | 0 |  |  |  |  |  |  |  |  |
| GO:0002769\_natural\_killer\_cell\_inhibitory\_signaling\_pathway | 1 | 0 |  |  |  |  |  |  |  |  |
| GO:0002840\_regulation\_of\_T\_cell\_mediated\_immune\_response\_to\_tumor\_cell | 1 | 0 |  |  |  |  |  |  |  |  |
| GO:0002842\_positive\_regulation\_of\_T\_cell\_mediated\_immune\_response\_to\_tumor\_cell | 1 | 0 |  |  |  |  |  |  |  |  |
| GO:0002849\_regulation\_of\_peripheral\_T\_cell\_tolerance\_induction | 1 | 0 |  |  |  |  |  |  |  |  |
| GO:0002851\_positive\_regulation\_of\_peripheral\_T\_cell\_tolerance\_induction | 1 | 0 |  |  |  |  |  |  |  |  |
| GO:0002855\_regulation\_of\_natural\_killer\_cell\_mediated\_immune\_response\_to\_tumor\_cell | 1 | 0 |  |  |  |  |  |  |  |  |
| GO:0002857\_positive\_regulation\_of\_natural\_killer\_cell\_mediated\_immune\_response\_to\_tumor\_cell | 1 | 0 |  |  |  |  |  |  |  |  |
| GO:0002858\_regulation\_of\_natural\_killer\_cell\_mediated\_cytotoxicity\_directed\_against\_tumor\_cell\_target | 1 | 0 |  |  |  |  |  |  |  |  |
| GO:0002860\_positive\_regulation\_of\_natural\_killer\_cell\_mediated\_cytotoxicity\_directed\_against\_tumor\_cell\_target | 1 | 0 |  |  |  |  |  |  |  |  |
| GO:0002880\_regulation\_of\_chronic\_inflammatory\_response\_to\_non-antigenic\_stimulus | 1 | 0 |  |  |  |  |  |  |  |  |
| GO:0002882\_positive\_regulation\_of\_chronic\_inflammatory\_response\_to\_non-antigenic\_stimulus | 1 | 0 |  |  |  |  |  |  |  |  |
| GO:0002895\_regulation\_of\_central\_B\_cell\_tolerance\_induction | 1 | 0 |  |  |  |  |  |  |  |  |
| GO:0002897\_positive\_regulation\_of\_central\_B\_cell\_tolerance\_induction | 1 | 0 |  |  |  |  |  |  |  |  |
| GO:0002901\_mature\_B\_cell\_apoptosis | 1 | 0 |  |  |  |  |  |  |  |  |
| GO:0002903\_negative\_regulation\_of\_B\_cell\_apoptosis | 1 | 0 |  |  |  |  |  |  |  |  |
| GO:0002905\_regulation\_of\_mature\_B\_cell\_apoptosis | 1 | 0 |  |  |  |  |  |  |  |  |
| GO:0002906\_negative\_regulation\_of\_mature\_B\_cell\_apoptosis | 1 | 0 |  |  |  |  |  |  |  |  |
| GO:0003011\_involuntary\_skeletal\_muscle\_contraction | 1 | 0 |  |  |  |  |  |  |  |  |
| GO:0003027\_regulation\_of\_systemic\_arterial\_blood\_pressure\_by\_carotid\_body\_chemoreceptor\_signaling | 1 | 0 |  |  |  |  |  |  |  |  |
| GO:0003029\_detection\_of\_hypoxic\_conditions\_in\_blood\_by\_carotid\_body\_chemoreceptor\_signaling | 1 | 0 |  |  |  |  |  |  |  |  |
| GO:0003032\_detection\_of\_oxygen | 1 | 0 |  |  |  |  |  |  |  |  |
| GO:0003056\_regulation\_of\_vascular\_smooth\_muscle\_contraction | 1 | 0 |  |  |  |  |  |  |  |  |
| GO:0003062\_regulation\_of\_heart\_rate\_by\_chemical\_signal | 1 | 0 |  |  |  |  |  |  |  |  |
| GO:0003065\_positive\_regulation\_of\_heart\_rate\_by\_epinephrine | 1 | 0 |  |  |  |  |  |  |  |  |
| GO:0003068\_regulation\_of\_systemic\_arterial\_blood\_pressure\_by\_acetylcholine | 1 | 0 |  |  |  |  |  |  |  |  |
| GO:0003069\_vasodilation\_by\_acetylcholine\_involved\_in\_regulation\_of\_systemic\_arterial\_blood\_pressure | 1 | 0 |  |  |  |  |  |  |  |  |
| GO:0003070\_regulation\_of\_systemic\_arterial\_blood\_pressure\_by\_neurotransmitter | 1 | 0 |  |  |  |  |  |  |  |  |
| GO:0003097\_renal\_water\_transport | 1 | 0 |  |  |  |  |  |  |  |  |
| GO:0005979\_regulation\_of\_glycogen\_biosynthetic\_process | 1 | 0 |  |  |  |  |  |  |  |  |
| GO:0005984\_disaccharide\_metabolic\_process | 1 | 0 |  |  |  |  |  |  |  |  |
| GO:0005988\_lactose\_metabolic\_process | 1 | 0 |  |  |  |  |  |  |  |  |
| GO:0005989\_lactose\_biosynthetic\_process | 1 | 0 |  |  |  |  |  |  |  |  |
| GO:0005997\_xylulose\_metabolic\_process | 1 | 0 |  |  |  |  |  |  |  |  |
| GO:0006000\_fructose\_metabolic\_process | 1 | 0 |  |  |  |  |  |  |  |  |
| GO:0006002\_fructose\_6-phosphate\_metabolic\_process | 1 | 0 |  |  |  |  |  |  |  |  |
| GO:0006004\_fucose\_metabolic\_process | 1 | 0 |  |  |  |  |  |  |  |  |
| GO:0006013\_mannose\_metabolic\_process | 1 | 0 |  |  |  |  |  |  |  |  |
| GO:0006060\_sorbitol\_metabolic\_process | 1 | 0 |  |  |  |  |  |  |  |  |
| GO:0006064\_glucuronate\_catabolic\_process | 1 | 0 |  |  |  |  |  |  |  |  |
| GO:0006086\_acetyl-CoA\_biosynthetic\_process\_from\_pyruvate | 1 | 0 |  |  |  |  |  |  |  |  |
| GO:0006098\_pentose-phosphate\_shunt | 1 | 0 |  |  |  |  |  |  |  |  |
| GO:0006101\_citrate\_metabolic\_process | 1 | 0 |  |  |  |  |  |  |  |  |
| GO:0006104\_succinyl-CoA\_metabolic\_process | 1 | 0 |  |  |  |  |  |  |  |  |
| GO:0006116\_NADH\_oxidation | 1 | 0 |  |  |  |  |  |  |  |  |
| GO:0006120\_mitochondrial\_electron\_transport\_\_NADH\_to\_ubiquinone | 1 | 0 |  |  |  |  |  |  |  |  |
| GO:0006154\_adenosine\_catabolic\_process | 1 | 0 |  |  |  |  |  |  |  |  |
| GO:0006157\_deoxyadenosine\_catabolic\_process | 1 | 0 |  |  |  |  |  |  |  |  |
| GO:0006167\_AMP\_biosynthetic\_process | 1 | 0 |  |  |  |  |  |  |  |  |
| GO:0006175\_dATP\_biosynthetic\_process | 1 | 0 |  |  |  |  |  |  |  |  |
| GO:0006178\_guanine\_salvage | 1 | 0 |  |  |  |  |  |  |  |  |
| GO:0006196\_AMP\_catabolic\_process | 1 | 0 |  |  |  |  |  |  |  |  |
| GO:0006203\_dGTP\_catabolic\_process | 1 | 0 |  |  |  |  |  |  |  |  |
| GO:0006208\_pyrimidine\_base\_catabolic\_process | 1 | 0 |  |  |  |  |  |  |  |  |
| GO:0006221\_pyrimidine\_nucleotide\_biosynthetic\_process | 1 | 0 |  |  |  |  |  |  |  |  |
| GO:0006235\_dTTP\_biosynthetic\_process | 1 | 0 |  |  |  |  |  |  |  |  |
| GO:0006244\_pyrimidine\_nucleotide\_catabolic\_process | 1 | 0 |  |  |  |  |  |  |  |  |
| GO:0006269\_DNA\_replication\_\_synthesis\_of\_RNA\_primer | 1 | 0 |  |  |  |  |  |  |  |  |
| GO:0006283\_transcription-coupled\_nucleotide-excision\_repair | 1 | 0 |  |  |  |  |  |  |  |  |
| GO:0006296\_nucleotide-excision\_repair\_\_DNA\_incision\_\_5'-to\_lesion | 1 | 0 |  |  |  |  |  |  |  |  |
| GO:0006307\_DNA\_dealkylation | 1 | 0 |  |  |  |  |  |  |  |  |
| GO:0006337\_nucleosome\_disassembly | 1 | 0 |  |  |  |  |  |  |  |  |
| GO:0006344\_maintenance\_of\_chromatin\_silencing | 1 | 0 |  |  |  |  |  |  |  |  |
| GO:0006356\_regulation\_of\_transcription\_from\_RNA\_polymerase\_I\_promoter | 1 | 0 |  |  |  |  |  |  |  |  |
| GO:0006388\_tRNA\_splicing\_\_via\_endonucleolytic\_cleavage\_and\_ligation | 1 | 0 |  |  |  |  |  |  |  |  |
| GO:0006407\_rRNA\_export\_from\_nucleus | 1 | 0 |  |  |  |  |  |  |  |  |
| GO:0006419\_alanyl-tRNA\_aminoacylation | 1 | 0 |  |  |  |  |  |  |  |  |
| GO:0006434\_seryl-tRNA\_aminoacylation | 1 | 0 |  |  |  |  |  |  |  |  |
| GO:0006447\_regulation\_of\_translational\_initiation\_by\_iron | 1 | 0 |  |  |  |  |  |  |  |  |
| GO:0006463\_steroid\_hormone\_receptor\_complex\_assembly | 1 | 0 |  |  |  |  |  |  |  |  |
| GO:0006467\_protein\_thiol-disulfide\_exchange | 1 | 0 |  |  |  |  |  |  |  |  |
| GO:0006474\_N-terminal\_protein\_amino\_acid\_acetylation | 1 | 0 |  |  |  |  |  |  |  |  |
| GO:0006481\_C-terminal\_protein\_amino\_acid\_methylation | 1 | 0 |  |  |  |  |  |  |  |  |
| GO:0006488\_dolichol-linked\_oligosaccharide\_biosynthetic\_process | 1 | 0 |  |  |  |  |  |  |  |  |
| GO:0006494\_protein\_amino\_acid\_terminal\_glycosylation | 1 | 0 |  |  |  |  |  |  |  |  |
| GO:0006496\_protein\_amino\_acid\_terminal\_N-glycosylation | 1 | 0 |  |  |  |  |  |  |  |  |
| GO:0006500\_N-terminal\_protein\_palmitoylation | 1 | 0 |  |  |  |  |  |  |  |  |
| GO:0006507\_GPI\_anchor\_release | 1 | 0 |  |  |  |  |  |  |  |  |
| GO:0006537\_glutamate\_biosynthetic\_process | 1 | 0 |  |  |  |  |  |  |  |  |
| GO:0006544\_glycine\_metabolic\_process | 1 | 0 |  |  |  |  |  |  |  |  |
| GO:0006549\_isoleucine\_metabolic\_process | 1 | 0 |  |  |  |  |  |  |  |  |
| GO:0006553\_lysine\_metabolic\_process | 1 | 0 |  |  |  |  |  |  |  |  |
| GO:0006554\_lysine\_catabolic\_process | 1 | 0 |  |  |  |  |  |  |  |  |
| GO:0006556\_S-adenosylmethionine\_biosynthetic\_process | 1 | 0 |  |  |  |  |  |  |  |  |
| GO:0006559\_L-phenylalanine\_catabolic\_process | 1 | 0 |  |  |  |  |  |  |  |  |
| GO:0006569\_tryptophan\_catabolic\_process | 1 | 0 |  |  |  |  |  |  |  |  |
| GO:0006572\_tyrosine\_catabolic\_process | 1 | 0 |  |  |  |  |  |  |  |  |
| GO:0006573\_valine\_metabolic\_process | 1 | 0 |  |  |  |  |  |  |  |  |
| GO:0006581\_acetylcholine\_catabolic\_process | 1 | 0 |  |  |  |  |  |  |  |  |
| GO:0006585\_dopamine\_biosynthetic\_process\_from\_tyrosine | 1 | 0 |  |  |  |  |  |  |  |  |
| GO:0006590\_thyroid\_hormone\_generation | 1 | 0 |  |  |  |  |  |  |  |  |
| GO:0006591\_ornithine\_metabolic\_process | 1 | 0 |  |  |  |  |  |  |  |  |
| GO:0006601\_creatine\_biosynthetic\_process | 1 | 0 |  |  |  |  |  |  |  |  |
| GO:0006613\_cotranslational\_protein\_targeting\_to\_membrane | 1 | 0 |  |  |  |  |  |  |  |  |
| GO:0006622\_protein\_targeting\_to\_lysosome | 1 | 0 |  |  |  |  |  |  |  |  |
| GO:0006627\_mitochondrial\_protein\_processing\_during\_import | 1 | 0 |  |  |  |  |  |  |  |  |
| GO:0006653\_lecithin\_metabolic\_process | 1 | 0 |  |  |  |  |  |  |  |  |
| GO:0006654\_phosphatidic\_acid\_biosynthetic\_process | 1 | 0 |  |  |  |  |  |  |  |  |
| GO:0006658\_phosphatidylserine\_metabolic\_process | 1 | 0 |  |  |  |  |  |  |  |  |
| GO:0006659\_phosphatidylserine\_biosynthetic\_process | 1 | 0 |  |  |  |  |  |  |  |  |
| GO:0006667\_sphinganine\_metabolic\_process | 1 | 0 |  |  |  |  |  |  |  |  |
| GO:0006668\_sphinganine-1-phosphate\_metabolic\_process | 1 | 0 |  |  |  |  |  |  |  |  |
| GO:0006678\_glucosylceramide\_metabolic\_process | 1 | 0 |  |  |  |  |  |  |  |  |
| GO:0006682\_galactosylceramide\_biosynthetic\_process | 1 | 0 |  |  |  |  |  |  |  |  |
| GO:0006685\_sphingomyelin\_catabolic\_process | 1 | 0 |  |  |  |  |  |  |  |  |
| GO:0006700\_C21-steroid\_hormone\_biosynthetic\_process | 1 | 0 |  |  |  |  |  |  |  |  |
| GO:0006705\_mineralocorticoid\_biosynthetic\_process | 1 | 0 |  |  |  |  |  |  |  |  |
| GO:0006709\_progesterone\_catabolic\_process | 1 | 0 |  |  |  |  |  |  |  |  |
| GO:0006729\_tetrahydrobiopterin\_biosynthetic\_process | 1 | 0 |  |  |  |  |  |  |  |  |
| GO:0006734\_NADH\_metabolic\_process | 1 | 0 |  |  |  |  |  |  |  |  |
| GO:0006740\_NADPH\_regeneration | 1 | 0 |  |  |  |  |  |  |  |  |
| GO:0006741\_NADP\_biosynthetic\_process | 1 | 0 |  |  |  |  |  |  |  |  |
| GO:0006743\_ubiquinone\_metabolic\_process | 1 | 0 |  |  |  |  |  |  |  |  |
| GO:0006744\_ubiquinone\_biosynthetic\_process | 1 | 0 |  |  |  |  |  |  |  |  |
| GO:0006772\_thiamin\_metabolic\_process | 1 | 0 |  |  |  |  |  |  |  |  |
| GO:0006784\_heme\_a\_biosynthetic\_process | 1 | 0 |  |  |  |  |  |  |  |  |
| GO:0006797\_polyphosphate\_metabolic\_process | 1 | 0 |  |  |  |  |  |  |  |  |
| GO:0006798\_polyphosphate\_catabolic\_process | 1 | 0 |  |  |  |  |  |  |  |  |
| GO:0006824\_cobalt\_ion\_transport | 1 | 0 |  |  |  |  |  |  |  |  |
| GO:0006842\_tricarboxylic\_acid\_transport | 1 | 0 |  |  |  |  |  |  |  |  |
| GO:0006844\_acyl\_carnitine\_transport | 1 | 0 |  |  |  |  |  |  |  |  |
| GO:0006855\_multidrug\_transport | 1 | 0 |  |  |  |  |  |  |  |  |
| GO:0006863\_purine\_transport | 1 | 0 |  |  |  |  |  |  |  |  |
| GO:0006890\_retrograde\_vesicle-mediated\_transport\_\_Golgi\_to\_ER | 1 | 0 |  |  |  |  |  |  |  |  |
| GO:0006891\_intra-Golgi\_vesicle-mediated\_transport | 1 | 0 |  |  |  |  |  |  |  |  |
| GO:0006893\_Golgi\_to\_plasma\_membrane\_transport | 1 | 0 |  |  |  |  |  |  |  |  |
| GO:0006895\_Golgi\_to\_endosome\_transport | 1 | 0 |  |  |  |  |  |  |  |  |
| GO:0006896\_Golgi\_to\_vacuole\_transport | 1 | 0 |  |  |  |  |  |  |  |  |
| GO:0006900\_membrane\_budding | 1 | 0 |  |  |  |  |  |  |  |  |
| GO:0006930\_substrate-bound\_cell\_migration\_\_cell\_extension | 1 | 0 |  |  |  |  |  |  |  |  |
| GO:0006931\_substrate-bound\_cell\_migration\_\_cell\_attachment\_to\_substrate | 1 | 0 |  |  |  |  |  |  |  |  |
| GO:0006933\_negative\_regulation\_of\_cell\_adhesion\_involved\_in\_substrate-bound\_cell\_migration | 1 | 0 |  |  |  |  |  |  |  |  |
| GO:0006957\_complement\_activation\_\_alternative\_pathway | 1 | 0 |  |  |  |  |  |  |  |  |
| GO:0006958\_complement\_activation\_\_classical\_pathway | 1 | 0 |  |  |  |  |  |  |  |  |
| GO:0006978\_DNA\_damage\_response\_\_signal\_transduction\_by\_p53\_class\_mediator\_resulting\_in\_transcription\_of\_p21\_class\_mediator | 1 | 0 |  |  |  |  |  |  |  |  |
| GO:0007016\_cytoskeletal\_anchoring\_at\_plasma\_membrane | 1 | 0 |  |  |  |  |  |  |  |  |
| GO:0007021\_tubulin\_complex\_assembly | 1 | 0 |  |  |  |  |  |  |  |  |
| GO:0007052\_mitotic\_spindle\_organization | 1 | 0 |  |  |  |  |  |  |  |  |
| GO:0007056\_spindle\_assembly\_involved\_in\_female\_meiosis | 1 | 0 |  |  |  |  |  |  |  |  |
| GO:0007057\_spindle\_assembly\_involved\_in\_female\_meiosis\_I | 1 | 0 |  |  |  |  |  |  |  |  |
| GO:0007063\_regulation\_of\_sister\_chromatid\_cohesion | 1 | 0 |  |  |  |  |  |  |  |  |
| GO:0007065\_male\_meiosis\_sister\_chromatid\_cohesion | 1 | 0 |  |  |  |  |  |  |  |  |
| GO:0007076\_mitotic\_chromosome\_condensation | 1 | 0 |  |  |  |  |  |  |  |  |
| GO:0007095\_mitotic\_cell\_cycle\_G2\_M\_transition\_DNA\_damage\_checkpoint | 1 | 0 |  |  |  |  |  |  |  |  |
| GO:0007096\_regulation\_of\_exit\_from\_mitosis | 1 | 0 |  |  |  |  |  |  |  |  |
| GO:0007158\_neuron\_adhesion | 1 | 0 |  |  |  |  |  |  |  |  |
| GO:0007168\_receptor\_guanylyl\_cyclase\_signaling\_pathway | 1 | 0 |  |  |  |  |  |  |  |  |
| GO:0007197\_inhibition\_of\_adenylate\_cyclase\_activity\_by\_muscarinic\_acetylcholine\_receptor\_signaling\_pathway | 1 | 0 |  |  |  |  |  |  |  |  |
| GO:0007207\_activation\_of\_phospholipase\_C\_activity\_by\_muscarinic\_acetylcholine\_receptor\_signaling\_pathway | 1 | 0 |  |  |  |  |  |  |  |  |
| GO:0007208\_activation\_of\_phospholipase\_C\_activity\_by\_serotonin\_receptor\_signaling\_pathway | 1 | 0 |  |  |  |  |  |  |  |  |
| GO:0007217\_tachykinin\_receptor\_signaling\_pathway | 1 | 0 |  |  |  |  |  |  |  |  |
| GO:0007221\_positive\_regulation\_of\_transcription\_of\_Notch\_receptor\_target | 1 | 0 |  |  |  |  |  |  |  |  |
| GO:0007223\_Wnt\_receptor\_signaling\_pathway\_\_calcium\_modulating\_pathway | 1 | 0 |  |  |  |  |  |  |  |  |
| GO:0007225\_patched\_ligand\_processing | 1 | 0 |  |  |  |  |  |  |  |  |
| GO:0007227\_signal\_transduction\_downstream\_of\_smoothened | 1 | 0 |  |  |  |  |  |  |  |  |
| GO:0007228\_positive\_regulation\_of\_hh\_target\_transcription\_factor\_activity | 1 | 0 |  |  |  |  |  |  |  |  |
| GO:0007231\_osmosensory\_signaling\_pathway | 1 | 0 |  |  |  |  |  |  |  |  |
| GO:0007284\_spermatogonial\_cell\_division | 1 | 0 |  |  |  |  |  |  |  |  |
| GO:0007290\_spermatid\_nucleus\_elongation | 1 | 0 |  |  |  |  |  |  |  |  |
| GO:0007296\_vitellogenesis | 1 | 0 |  |  |  |  |  |  |  |  |
| GO:0007321\_sperm\_displacement | 1 | 0 |  |  |  |  |  |  |  |  |
| GO:0007380\_specification\_of\_segmental\_identity\_\_head | 1 | 0 |  |  |  |  |  |  |  |  |
| GO:0007382\_specification\_of\_segmental\_identity\_\_maxillary\_segment | 1 | 0 |  |  |  |  |  |  |  |  |
| GO:0007400\_neuroblast\_fate\_determination | 1 | 0 |  |  |  |  |  |  |  |  |
| GO:0007402\_ganglion\_mother\_cell\_fate\_determination | 1 | 0 |  |  |  |  |  |  |  |  |
| GO:0007495\_visceral\_mesoderm-endoderm\_interaction\_involved\_in\_midgut\_development | 1 | 0 |  |  |  |  |  |  |  |  |
| GO:0007497\_posterior\_midgut\_development | 1 | 0 |  |  |  |  |  |  |  |  |
| GO:0007499\_ectoderm\_and\_mesoderm\_interaction | 1 | 0 |  |  |  |  |  |  |  |  |
| GO:0007500\_mesodermal\_cell\_fate\_determination | 1 | 0 |  |  |  |  |  |  |  |  |
| GO:0007509\_mesoderm\_migration | 1 | 0 |  |  |  |  |  |  |  |  |
| GO:0007518\_myoblast\_cell\_fate\_determination | 1 | 0 |  |  |  |  |  |  |  |  |
| GO:0007521\_muscle\_cell\_fate\_determination | 1 | 0 |  |  |  |  |  |  |  |  |
| GO:0007522\_visceral\_muscle\_development | 1 | 0 |  |  |  |  |  |  |  |  |
| GO:0007529\_establishment\_of\_synaptic\_specificity\_at\_neuromuscular\_junction | 1 | 0 |  |  |  |  |  |  |  |  |
| GO:0007538\_primary\_sex\_determination | 1 | 0 |  |  |  |  |  |  |  |  |
| GO:0007542\_primary\_sex\_determination\_\_germ-line | 1 | 0 |  |  |  |  |  |  |  |  |
| GO:0007567\_parturition | 1 | 0 |  |  |  |  |  |  |  |  |
| GO:0007614\_short-term\_memory | 1 | 0 |  |  |  |  |  |  |  |  |
| GO:0007621\_negative\_regulation\_of\_female\_receptivity | 1 | 0 |  |  |  |  |  |  |  |  |
| GO:0008049\_male\_courtship\_behavior | 1 | 0 |  |  |  |  |  |  |  |  |
| GO:0008050\_female\_courtship\_behavior | 1 | 0 |  |  |  |  |  |  |  |  |
| GO:0008052\_sensory\_organ\_boundary\_specification | 1 | 0 |  |  |  |  |  |  |  |  |
| GO:0008054\_cyclin\_catabolic\_process | 1 | 0 |  |  |  |  |  |  |  |  |
| GO:0008057\_eye\_pigment\_granule\_organization | 1 | 0 |  |  |  |  |  |  |  |  |
| GO:0008078\_mesodermal\_cell\_migration | 1 | 0 |  |  |  |  |  |  |  |  |
| GO:0008208\_C21-steroid\_hormone\_catabolic\_process | 1 | 0 |  |  |  |  |  |  |  |  |
| GO:0008292\_acetylcholine\_biosynthetic\_process | 1 | 0 |  |  |  |  |  |  |  |  |
| GO:0008300\_isoprenoid\_catabolic\_process | 1 | 0 |  |  |  |  |  |  |  |  |
| GO:0008333\_endosome\_to\_lysosome\_transport | 1 | 0 |  |  |  |  |  |  |  |  |
| GO:0008355\_olfactory\_learning | 1 | 0 |  |  |  |  |  |  |  |  |
| GO:0008611\_ether\_lipid\_biosynthetic\_process | 1 | 0 |  |  |  |  |  |  |  |  |
| GO:0008626\_induction\_of\_apoptosis\_by\_granzyme | 1 | 0 |  |  |  |  |  |  |  |  |
| GO:0008633\_activation\_of\_pro-apoptotic\_gene\_products | 1 | 0 |  |  |  |  |  |  |  |  |
| GO:0008653\_lipopolysaccharide\_metabolic\_process | 1 | 0 |  |  |  |  |  |  |  |  |
| GO:0009068\_aspartate\_family\_amino\_acid\_catabolic\_process | 1 | 0 |  |  |  |  |  |  |  |  |
| GO:0009084\_glutamine\_family\_amino\_acid\_biosynthetic\_process | 1 | 0 |  |  |  |  |  |  |  |  |
| GO:0009088\_threonine\_biosynthetic\_process | 1 | 0 |  |  |  |  |  |  |  |  |
| GO:0009105\_lipoic\_acid\_biosynthetic\_process | 1 | 0 |  |  |  |  |  |  |  |  |
| GO:0009109\_coenzyme\_catabolic\_process | 1 | 0 |  |  |  |  |  |  |  |  |
| GO:0009111\_vitamin\_catabolic\_process | 1 | 0 |  |  |  |  |  |  |  |  |
| GO:0009113\_purine\_base\_biosynthetic\_process | 1 | 0 |  |  |  |  |  |  |  |  |
| GO:0009127\_purine\_nucleoside\_monophosphate\_biosynthetic\_process | 1 | 0 |  |  |  |  |  |  |  |  |
| GO:0009128\_purine\_nucleoside\_monophosphate\_catabolic\_process | 1 | 0 |  |  |  |  |  |  |  |  |
| GO:0009129\_pyrimidine\_nucleoside\_monophosphate\_metabolic\_process | 1 | 0 |  |  |  |  |  |  |  |  |
| GO:0009131\_pyrimidine\_nucleoside\_monophosphate\_catabolic\_process | 1 | 0 |  |  |  |  |  |  |  |  |
| GO:0009133\_nucleoside\_diphosphate\_biosynthetic\_process | 1 | 0 |  |  |  |  |  |  |  |  |
| GO:0009145\_purine\_nucleoside\_triphosphate\_biosynthetic\_process | 1 | 0 |  |  |  |  |  |  |  |  |
| GO:0009147\_pyrimidine\_nucleoside\_triphosphate\_metabolic\_process | 1 | 0 |  |  |  |  |  |  |  |  |
| GO:0009148\_pyrimidine\_nucleoside\_triphosphate\_biosynthetic\_process | 1 | 0 |  |  |  |  |  |  |  |  |
| GO:0009152\_purine\_ribonucleotide\_biosynthetic\_process | 1 | 0 |  |  |  |  |  |  |  |  |
| GO:0009153\_purine\_deoxyribonucleotide\_biosynthetic\_process | 1 | 0 |  |  |  |  |  |  |  |  |
| GO:0009156\_ribonucleoside\_monophosphate\_biosynthetic\_process | 1 | 0 |  |  |  |  |  |  |  |  |
| GO:0009158\_ribonucleoside\_monophosphate\_catabolic\_process | 1 | 0 |  |  |  |  |  |  |  |  |
| GO:0009159\_deoxyribonucleoside\_monophosphate\_catabolic\_process | 1 | 0 |  |  |  |  |  |  |  |  |
| GO:0009162\_deoxyribonucleoside\_monophosphate\_metabolic\_process | 1 | 0 |  |  |  |  |  |  |  |  |
| GO:0009168\_purine\_ribonucleoside\_monophosphate\_biosynthetic\_process | 1 | 0 |  |  |  |  |  |  |  |  |
| GO:0009169\_purine\_ribonucleoside\_monophosphate\_catabolic\_process | 1 | 0 |  |  |  |  |  |  |  |  |
| GO:0009176\_pyrimidine\_deoxyribonucleoside\_monophosphate\_metabolic\_process | 1 | 0 |  |  |  |  |  |  |  |  |
| GO:0009178\_pyrimidine\_deoxyribonucleoside\_monophosphate\_catabolic\_process | 1 | 0 |  |  |  |  |  |  |  |  |
| GO:0009211\_pyrimidine\_deoxyribonucleoside\_triphosphate\_metabolic\_process | 1 | 0 |  |  |  |  |  |  |  |  |
| GO:0009212\_pyrimidine\_deoxyribonucleoside\_triphosphate\_biosynthetic\_process | 1 | 0 |  |  |  |  |  |  |  |  |
| GO:0009216\_purine\_deoxyribonucleoside\_triphosphate\_biosynthetic\_process | 1 | 0 |  |  |  |  |  |  |  |  |
| GO:0009221\_pyrimidine\_deoxyribonucleotide\_biosynthetic\_process | 1 | 0 |  |  |  |  |  |  |  |  |
| GO:0009223\_pyrimidine\_deoxyribonucleotide\_catabolic\_process | 1 | 0 |  |  |  |  |  |  |  |  |
| GO:0009260\_ribonucleotide\_biosynthetic\_process | 1 | 0 |  |  |  |  |  |  |  |  |
| GO:0009405\_pathogenesis | 1 | 0 |  |  |  |  |  |  |  |  |
| GO:0009414\_response\_to\_water\_deprivation | 1 | 0 |  |  |  |  |  |  |  |  |
| GO:0009415\_response\_to\_water | 1 | 0 |  |  |  |  |  |  |  |  |
| GO:0009449\_gamma-aminobutyric\_acid\_biosynthetic\_process | 1 | 0 |  |  |  |  |  |  |  |  |
| GO:0009450\_gamma-aminobutyric\_acid\_catabolic\_process | 1 | 0 |  |  |  |  |  |  |  |  |
| GO:0009589\_detection\_of\_UV | 1 | 0 |  |  |  |  |  |  |  |  |
| GO:0009590\_detection\_of\_gravity | 1 | 0 |  |  |  |  |  |  |  |  |
| GO:0009624\_response\_to\_nematode | 1 | 0 |  |  |  |  |  |  |  |  |
| GO:0009629\_response\_to\_gravity | 1 | 0 |  |  |  |  |  |  |  |  |
| GO:0009648\_photoperiodism | 1 | 0 |  |  |  |  |  |  |  |  |
| GO:0009690\_cytokinin\_metabolic\_process | 1 | 0 |  |  |  |  |  |  |  |  |
| GO:0009691\_cytokinin\_biosynthetic\_process | 1 | 0 |  |  |  |  |  |  |  |  |
| GO:0009786\_regulation\_of\_asymmetric\_cell\_division | 1 | 0 |  |  |  |  |  |  |  |  |
| GO:0009794\_regulation\_of\_mitotic\_cell\_cycle\_\_embryonic | 1 | 0 |  |  |  |  |  |  |  |  |
| GO:0009956\_radial\_pattern\_formation | 1 | 0 |  |  |  |  |  |  |  |  |
| GO:0009957\_epidermal\_cell\_fate\_specification | 1 | 0 |  |  |  |  |  |  |  |  |
| GO:0009992\_cellular\_water\_homeostasis | 1 | 0 |  |  |  |  |  |  |  |  |
| GO:0010032\_meiotic\_chromosome\_condensation | 1 | 0 |  |  |  |  |  |  |  |  |
| GO:0010039\_response\_to\_iron\_ion | 1 | 0 |  |  |  |  |  |  |  |  |
| GO:0010042\_response\_to\_manganese\_ion | 1 | 0 |  |  |  |  |  |  |  |  |
| GO:0010045\_response\_to\_nickel\_ion | 1 | 0 |  |  |  |  |  |  |  |  |
| GO:0010046\_response\_to\_mycotoxin | 1 | 0 |  |  |  |  |  |  |  |  |
| GO:0010107\_potassium\_ion\_import | 1 | 0 |  |  |  |  |  |  |  |  |
| GO:0010155\_regulation\_of\_proton\_transport | 1 | 0 |  |  |  |  |  |  |  |  |
| GO:0010160\_formation\_of\_organ\_boundary | 1 | 0 |  |  |  |  |  |  |  |  |
| GO:0010260\_organ\_senescence | 1 | 0 |  |  |  |  |  |  |  |  |
| GO:0010310\_regulation\_of\_hydrogen\_peroxide\_metabolic\_process | 1 | 0 |  |  |  |  |  |  |  |  |
| GO:0010447\_response\_to\_acidity | 1 | 0 |  |  |  |  |  |  |  |  |
| GO:0010452\_histone\_H3-K36\_methylation | 1 | 0 |  |  |  |  |  |  |  |  |
| GO:0010455\_positive\_regulation\_of\_cell\_fate\_commitment | 1 | 0 |  |  |  |  |  |  |  |  |
| GO:0010470\_regulation\_of\_gastrulation | 1 | 0 |  |  |  |  |  |  |  |  |
| GO:0010508\_positive\_regulation\_of\_autophagy | 1 | 0 |  |  |  |  |  |  |  |  |
| GO:0010519\_negative\_regulation\_of\_phospholipase\_activity | 1 | 0 |  |  |  |  |  |  |  |  |
| GO:0010520\_regulation\_of\_reciprocal\_meiotic\_recombination | 1 | 0 |  |  |  |  |  |  |  |  |
| GO:0010523\_negative\_regulation\_of\_calcium\_ion\_transport\_into\_cytosol | 1 | 0 |  |  |  |  |  |  |  |  |
| GO:0010543\_regulation\_of\_platelet\_activation | 1 | 0 |  |  |  |  |  |  |  |  |
| GO:0010561\_negative\_regulation\_of\_glycoprotein\_biosynthetic\_process | 1 | 0 |  |  |  |  |  |  |  |  |
| GO:0010569\_regulation\_of\_double-strand\_break\_repair\_via\_homologous\_recombination | 1 | 0 |  |  |  |  |  |  |  |  |
| GO:0010572\_positive\_regulation\_of\_platelet\_activation | 1 | 0 |  |  |  |  |  |  |  |  |
| GO:0010594\_regulation\_of\_endothelial\_cell\_migration | 1 | 0 |  |  |  |  |  |  |  |  |
| GO:0010596\_negative\_regulation\_of\_endothelial\_cell\_migration | 1 | 0 |  |  |  |  |  |  |  |  |
| GO:0010611\_regulation\_of\_cardiac\_muscle\_hypertrophy | 1 | 0 |  |  |  |  |  |  |  |  |
| GO:0010612\_regulation\_of\_cardiac\_muscle\_adaptation | 1 | 0 |  |  |  |  |  |  |  |  |
| GO:0010614\_negative\_regulation\_of\_cardiac\_muscle\_hypertrophy | 1 | 0 |  |  |  |  |  |  |  |  |
| GO:0010616\_negative\_regulation\_of\_cardiac\_muscle\_adaptation | 1 | 0 |  |  |  |  |  |  |  |  |
| GO:0010634\_positive\_regulation\_of\_epithelial\_cell\_migration | 1 | 0 |  |  |  |  |  |  |  |  |
| GO:0010656\_negative\_regulation\_of\_muscle\_cell\_apoptosis | 1 | 0 |  |  |  |  |  |  |  |  |
| GO:0010657\_muscle\_cell\_apoptosis | 1 | 0 |  |  |  |  |  |  |  |  |
| GO:0010658\_striated\_muscle\_cell\_apoptosis | 1 | 0 |  |  |  |  |  |  |  |  |
| GO:0010659\_cardiac\_muscle\_cell\_apoptosis | 1 | 0 |  |  |  |  |  |  |  |  |
| GO:0010660\_regulation\_of\_muscle\_cell\_apoptosis | 1 | 0 |  |  |  |  |  |  |  |  |
| GO:0010662\_regulation\_of\_striated\_muscle\_cell\_apoptosis | 1 | 0 |  |  |  |  |  |  |  |  |
| GO:0010664\_negative\_regulation\_of\_striated\_muscle\_cell\_apoptosis | 1 | 0 |  |  |  |  |  |  |  |  |
| GO:0010665\_regulation\_of\_cardiac\_muscle\_cell\_apoptosis | 1 | 0 |  |  |  |  |  |  |  |  |
| GO:0010667\_negative\_regulation\_of\_cardiac\_muscle\_cell\_apoptosis | 1 | 0 |  |  |  |  |  |  |  |  |
| GO:0010668\_ectodermal\_cell\_differentiation | 1 | 0 |  |  |  |  |  |  |  |  |
| GO:0010671\_negative\_regulation\_of\_oxygen\_and\_reactive\_oxygen\_species\_metabolic\_process | 1 | 0 |  |  |  |  |  |  |  |  |
| GO:0010719\_negative\_regulation\_of\_epithelial\_to\_mesenchymal\_transition | 1 | 0 |  |  |  |  |  |  |  |  |
| GO:0010735\_positive\_regulation\_of\_transcription\_via\_serum\_response\_element\_binding | 1 | 0 |  |  |  |  |  |  |  |  |
| GO:0010825\_positive\_regulation\_of\_centrosome\_duplication | 1 | 0 |  |  |  |  |  |  |  |  |
| GO:0010845\_positive\_regulation\_of\_reciprocal\_meiotic\_recombination | 1 | 0 |  |  |  |  |  |  |  |  |
| GO:0010850\_chemoreceptor\_signaling\_pathway\_involved\_in\_regulation\_of\_blood\_pressure | 1 | 0 |  |  |  |  |  |  |  |  |
| GO:0010873\_positive\_regulation\_of\_cholesterol\_esterification | 1 | 0 |  |  |  |  |  |  |  |  |
| GO:0010880\_regulation\_of\_release\_of\_sequestered\_calcium\_ion\_into\_cytosol\_by\_sarcoplasmic\_reticulum | 1 | 0 |  |  |  |  |  |  |  |  |
| GO:0010881\_regulation\_of\_cardiac\_muscle\_contraction\_by\_regulation\_of\_the\_release\_of\_sequestered\_calcium\_ion | 1 | 0 |  |  |  |  |  |  |  |  |
| GO:0010882\_regulation\_of\_cardiac\_muscle\_contraction\_by\_calcium\_ion\_signaling | 1 | 0 |  |  |  |  |  |  |  |  |
| GO:0010890\_positive\_regulation\_of\_sequestering\_of\_triglyceride | 1 | 0 |  |  |  |  |  |  |  |  |
| GO:0010919\_regulation\_of\_inositol\_phosphate\_biosynthetic\_process | 1 | 0 |  |  |  |  |  |  |  |  |
| GO:0010931\_macrophage\_tolerance\_induction | 1 | 0 |  |  |  |  |  |  |  |  |
| GO:0010932\_regulation\_of\_macrophage\_tolerance\_induction | 1 | 0 |  |  |  |  |  |  |  |  |
| GO:0010933\_positive\_regulation\_of\_macrophage\_tolerance\_induction | 1 | 0 |  |  |  |  |  |  |  |  |
| GO:0010934\_macrophage\_cytokine\_production | 1 | 0 |  |  |  |  |  |  |  |  |
| GO:0010935\_regulation\_of\_macrophage\_cytokine\_production | 1 | 0 |  |  |  |  |  |  |  |  |
| GO:0010936\_negative\_regulation\_of\_macrophage\_cytokine\_production | 1 | 0 |  |  |  |  |  |  |  |  |
| GO:0010953\_regulation\_of\_protein\_maturation\_by\_peptide\_bond\_cleavage | 1 | 0 |  |  |  |  |  |  |  |  |
| GO:0010962\_regulation\_of\_glucan\_biosynthetic\_process | 1 | 0 |  |  |  |  |  |  |  |  |
| GO:0010966\_regulation\_of\_phosphate\_transport | 1 | 0 |  |  |  |  |  |  |  |  |
| GO:0014012\_axon\_regeneration\_in\_the\_peripheral\_nervous\_system | 1 | 0 |  |  |  |  |  |  |  |  |
| GO:0014016\_neuroblast\_differentiation | 1 | 0 |  |  |  |  |  |  |  |  |
| GO:0014017\_neuroblast\_fate\_commitment | 1 | 0 |  |  |  |  |  |  |  |  |
| GO:0014041\_regulation\_of\_neuron\_maturation | 1 | 0 |  |  |  |  |  |  |  |  |
| GO:0014042\_positive\_regulation\_of\_neuron\_maturation | 1 | 0 |  |  |  |  |  |  |  |  |
| GO:0014049\_positive\_regulation\_of\_glutamate\_secretion | 1 | 0 |  |  |  |  |  |  |  |  |
| GO:0014061\_regulation\_of\_norepinephrine\_secretion | 1 | 0 |  |  |  |  |  |  |  |  |
| GO:0014071\_response\_to\_cycloalkane | 1 | 0 |  |  |  |  |  |  |  |  |
| GO:0014707\_branchiomeric\_skeletal\_muscle\_development | 1 | 0 |  |  |  |  |  |  |  |  |
| GO:0014738\_regulation\_of\_muscle\_hyperplasia | 1 | 0 |  |  |  |  |  |  |  |  |
| GO:0014740\_negative\_regulation\_of\_muscle\_hyperplasia | 1 | 0 |  |  |  |  |  |  |  |  |
| GO:0014741\_negative\_regulation\_of\_muscle\_hypertrophy | 1 | 0 |  |  |  |  |  |  |  |  |
| GO:0014743\_regulation\_of\_muscle\_hypertrophy | 1 | 0 |  |  |  |  |  |  |  |  |
| GO:0014805\_smooth\_muscle\_adaptation | 1 | 0 |  |  |  |  |  |  |  |  |
| GO:0014806\_smooth\_muscle\_hyperplasia | 1 | 0 |  |  |  |  |  |  |  |  |
| GO:0014807\_regulation\_of\_somitogenesis | 1 | 0 |  |  |  |  |  |  |  |  |
| GO:0014808\_release\_of\_sequestered\_calcium\_ion\_into\_cytosol\_by\_sarcoplasmic\_reticulum | 1 | 0 |  |  |  |  |  |  |  |  |
| GO:0014813\_satellite\_cell\_commitment | 1 | 0 |  |  |  |  |  |  |  |  |
| GO:0014816\_satellite\_cell\_differentiation | 1 | 0 |  |  |  |  |  |  |  |  |
| GO:0014819\_regulation\_of\_skeletal\_muscle\_contraction | 1 | 0 |  |  |  |  |  |  |  |  |
| GO:0014852\_regulation\_of\_skeletal\_muscle\_contraction\_by\_neural\_stimulation\_via\_neuromuscular\_junction | 1 | 0 |  |  |  |  |  |  |  |  |
| GO:0014853\_regulation\_of\_excitatory\_postsynaptic\_membrane\_potential\_involved\_in\_skeletal\_muscle\_contraction | 1 | 0 |  |  |  |  |  |  |  |  |
| GO:0014856\_skeletal\_muscle\_cell\_proliferation | 1 | 0 |  |  |  |  |  |  |  |  |
| GO:0014857\_regulation\_of\_skeletal\_muscle\_cell\_proliferation | 1 | 0 |  |  |  |  |  |  |  |  |
| GO:0014858\_positive\_regulation\_of\_skeletal\_muscle\_cell\_proliferation | 1 | 0 |  |  |  |  |  |  |  |  |
| GO:0014887\_cardiac\_muscle\_adaptation | 1 | 0 |  |  |  |  |  |  |  |  |
| GO:0014889\_muscle\_atrophy | 1 | 0 |  |  |  |  |  |  |  |  |
| GO:0014896\_muscle\_hypertrophy | 1 | 0 |  |  |  |  |  |  |  |  |
| GO:0014897\_striated\_muscle\_hypertrophy | 1 | 0 |  |  |  |  |  |  |  |  |
| GO:0014898\_cardiac\_muscle\_hypertrophy | 1 | 0 |  |  |  |  |  |  |  |  |
| GO:0014900\_muscle\_hyperplasia | 1 | 0 |  |  |  |  |  |  |  |  |
| GO:0014910\_regulation\_of\_smooth\_muscle\_cell\_migration | 1 | 0 |  |  |  |  |  |  |  |  |
| GO:0014911\_positive\_regulation\_of\_smooth\_muscle\_cell\_migration | 1 | 0 |  |  |  |  |  |  |  |  |
| GO:0015014\_heparan\_sulfate\_proteoglycan\_biosynthetic\_process\_\_polysaccharide\_chain\_biosynthetic\_process | 1 | 0 |  |  |  |  |  |  |  |  |
| GO:0015074\_DNA\_integration | 1 | 0 |  |  |  |  |  |  |  |  |
| GO:0015670\_carbon\_dioxide\_transport | 1 | 0 |  |  |  |  |  |  |  |  |
| GO:0015677\_copper\_ion\_import | 1 | 0 |  |  |  |  |  |  |  |  |
| GO:0015680\_intracellular\_copper\_ion\_transport | 1 | 0 |  |  |  |  |  |  |  |  |
| GO:0015684\_ferrous\_iron\_transport | 1 | 0 |  |  |  |  |  |  |  |  |
| GO:0015707\_nitrite\_transport | 1 | 0 |  |  |  |  |  |  |  |  |
| GO:0015724\_formate\_transport | 1 | 0 |  |  |  |  |  |  |  |  |
| GO:0015734\_taurine\_transport | 1 | 0 |  |  |  |  |  |  |  |  |
| GO:0015740\_C4-dicarboxylate\_transport | 1 | 0 |  |  |  |  |  |  |  |  |
| GO:0015744\_succinate\_transport | 1 | 0 |  |  |  |  |  |  |  |  |
| GO:0015746\_citrate\_transport | 1 | 0 |  |  |  |  |  |  |  |  |
| GO:0015747\_urate\_transport | 1 | 0 |  |  |  |  |  |  |  |  |
| GO:0015791\_polyol\_transport | 1 | 0 |  |  |  |  |  |  |  |  |
| GO:0015798\_myo-inositol\_transport | 1 | 0 |  |  |  |  |  |  |  |  |
| GO:0015808\_L-alanine\_transport | 1 | 0 |  |  |  |  |  |  |  |  |
| GO:0015810\_aspartate\_transport | 1 | 0 |  |  |  |  |  |  |  |  |
| GO:0015811\_L-cystine\_transport | 1 | 0 |  |  |  |  |  |  |  |  |
| GO:0015817\_histidine\_transport | 1 | 0 |  |  |  |  |  |  |  |  |
| GO:0015822\_ornithine\_transport | 1 | 0 |  |  |  |  |  |  |  |  |
| GO:0015824\_proline\_transport | 1 | 0 |  |  |  |  |  |  |  |  |
| GO:0015851\_nucleobase\_transport | 1 | 0 |  |  |  |  |  |  |  |  |
| GO:0015864\_pyrimidine\_nucleoside\_transport | 1 | 0 |  |  |  |  |  |  |  |  |
| GO:0015874\_norepinephrine\_transport | 1 | 0 |  |  |  |  |  |  |  |  |
| GO:0015881\_creatine\_transport | 1 | 0 |  |  |  |  |  |  |  |  |
| GO:0015884\_folic\_acid\_transport | 1 | 0 |  |  |  |  |  |  |  |  |
| GO:0015886\_heme\_transport | 1 | 0 |  |  |  |  |  |  |  |  |
| GO:0015888\_thiamin\_transport | 1 | 0 |  |  |  |  |  |  |  |  |
| GO:0015938\_coenzyme\_A\_catabolic\_process | 1 | 0 |  |  |  |  |  |  |  |  |
| GO:0015939\_pantothenate\_metabolic\_process | 1 | 0 |  |  |  |  |  |  |  |  |
| GO:0016073\_snRNA\_metabolic\_process | 1 | 0 |  |  |  |  |  |  |  |  |
| GO:0016074\_snoRNA\_metabolic\_process | 1 | 0 |  |  |  |  |  |  |  |  |
| GO:0016082\_synaptic\_vesicle\_priming | 1 | 0 |  |  |  |  |  |  |  |  |
| GO:0016090\_prenol\_metabolic\_process | 1 | 0 |  |  |  |  |  |  |  |  |
| GO:0016093\_polyprenol\_metabolic\_process | 1 | 0 |  |  |  |  |  |  |  |  |
| GO:0016180\_snRNA\_processing | 1 | 0 |  |  |  |  |  |  |  |  |
| GO:0016239\_positive\_regulation\_of\_macroautophagy | 1 | 0 |  |  |  |  |  |  |  |  |
| GO:0016246\_RNA\_interference | 1 | 0 |  |  |  |  |  |  |  |  |
| GO:0016255\_attachment\_of\_GPI\_anchor\_to\_protein | 1 | 0 |  |  |  |  |  |  |  |  |
| GO:0016333\_morphogenesis\_of\_follicular\_epithelium | 1 | 0 |  |  |  |  |  |  |  |  |
| GO:0016340\_calcium-dependent\_cell-matrix\_adhesion | 1 | 0 |  |  |  |  |  |  |  |  |
| GO:0016344\_meiotic\_chromosome\_movement\_towards\_spindle\_pole | 1 | 0 |  |  |  |  |  |  |  |  |
| GO:0016553\_base\_conversion\_or\_substitution\_editing | 1 | 0 |  |  |  |  |  |  |  |  |
| GO:0016554\_cytidine\_to\_uridine\_editing | 1 | 0 |  |  |  |  |  |  |  |  |
| GO:0016560\_protein\_import\_into\_peroxisome\_matrix\_\_docking | 1 | 0 |  |  |  |  |  |  |  |  |
| GO:0016578\_histone\_deubiquitination | 1 | 0 |  |  |  |  |  |  |  |  |
| GO:0016598\_protein\_arginylation | 1 | 0 |  |  |  |  |  |  |  |  |
| GO:0017004\_cytochrome\_complex\_assembly | 1 | 0 |  |  |  |  |  |  |  |  |
| GO:0018022\_peptidyl-lysine\_methylation | 1 | 0 |  |  |  |  |  |  |  |  |
| GO:0018023\_peptidyl-lysine\_trimethylation | 1 | 0 |  |  |  |  |  |  |  |  |
| GO:0018120\_peptidyl-arginine\_ADP-ribosylation | 1 | 0 |  |  |  |  |  |  |  |  |
| GO:0018126\_protein\_amino\_acid\_hydroxylation | 1 | 0 |  |  |  |  |  |  |  |  |
| GO:0018146\_keratan\_sulfate\_biosynthetic\_process | 1 | 0 |  |  |  |  |  |  |  |  |
| GO:0018158\_protein\_amino\_acid\_oxidation | 1 | 0 |  |  |  |  |  |  |  |  |
| GO:0018195\_peptidyl-arginine\_modification | 1 | 0 |  |  |  |  |  |  |  |  |
| GO:0018197\_peptidyl-aspartic\_acid\_modification | 1 | 0 |  |  |  |  |  |  |  |  |
| GO:0018282\_metal\_incorporation\_into\_metallo-sulfur\_cluster | 1 | 0 |  |  |  |  |  |  |  |  |
| GO:0018283\_iron\_incorporation\_into\_metallo-sulfur\_cluster | 1 | 0 |  |  |  |  |  |  |  |  |
| GO:0018318\_protein\_amino\_acid\_palmitoylation | 1 | 0 |  |  |  |  |  |  |  |  |
| GO:0018342\_protein\_prenylation | 1 | 0 |  |  |  |  |  |  |  |  |
| GO:0018344\_protein\_geranylgeranylation | 1 | 0 |  |  |  |  |  |  |  |  |
| GO:0018410\_peptide\_or\_protein\_carboxyl-terminal\_blocking | 1 | 0 |  |  |  |  |  |  |  |  |
| GO:0018916\_nitrobenzene\_metabolic\_process | 1 | 0 |  |  |  |  |  |  |  |  |
| GO:0018931\_naphthalene\_metabolic\_process | 1 | 0 |  |  |  |  |  |  |  |  |
| GO:0018992\_germ-line\_sex\_determination | 1 | 0 |  |  |  |  |  |  |  |  |
| GO:0019042\_latent\_virus\_infection | 1 | 0 |  |  |  |  |  |  |  |  |
| GO:0019046\_reactivation\_of\_latent\_virus | 1 | 0 |  |  |  |  |  |  |  |  |
| GO:0019047\_provirus\_integration | 1 | 0 |  |  |  |  |  |  |  |  |
| GO:0019076\_release\_of\_virus\_from\_host | 1 | 0 |  |  |  |  |  |  |  |  |
| GO:0019079\_viral\_genome\_replication | 1 | 0 |  |  |  |  |  |  |  |  |
| GO:0019100\_male\_germ-line\_sex\_determination | 1 | 0 |  |  |  |  |  |  |  |  |
| GO:0019101\_female\_somatic\_sex\_determination | 1 | 0 |  |  |  |  |  |  |  |  |
| GO:0019102\_male\_somatic\_sex\_determination | 1 | 0 |  |  |  |  |  |  |  |  |
| GO:0019276\_UDP-N-acetylgalactosamine\_metabolic\_process | 1 | 0 |  |  |  |  |  |  |  |  |
| GO:0019344\_cysteine\_biosynthetic\_process | 1 | 0 |  |  |  |  |  |  |  |  |
| GO:0019348\_dolichol\_metabolic\_process | 1 | 0 |  |  |  |  |  |  |  |  |
| GO:0019375\_galactolipid\_biosynthetic\_process | 1 | 0 |  |  |  |  |  |  |  |  |
| GO:0019402\_galactitol\_metabolic\_process | 1 | 0 |  |  |  |  |  |  |  |  |
| GO:0019441\_tryptophan\_catabolic\_process\_to\_kynurenine | 1 | 0 |  |  |  |  |  |  |  |  |
| GO:0019477\_L-lysine\_catabolic\_process | 1 | 0 |  |  |  |  |  |  |  |  |
| GO:0019510\_S-adenosylhomocysteine\_catabolic\_process | 1 | 0 |  |  |  |  |  |  |  |  |
| GO:0019532\_oxalate\_transport | 1 | 0 |  |  |  |  |  |  |  |  |
| GO:0019626\_short-chain\_fatty\_acid\_catabolic\_process | 1 | 0 |  |  |  |  |  |  |  |  |
| GO:0019627\_urea\_metabolic\_process | 1 | 0 |  |  |  |  |  |  |  |  |
| GO:0019676\_ammonia\_assimilation\_cycle | 1 | 0 |  |  |  |  |  |  |  |  |
| GO:0019682\_glyceraldehyde-3-phosphate\_metabolic\_process | 1 | 0 |  |  |  |  |  |  |  |  |
| GO:0019695\_choline\_metabolic\_process | 1 | 0 |  |  |  |  |  |  |  |  |
| GO:0019731\_antibacterial\_humoral\_response | 1 | 0 |  |  |  |  |  |  |  |  |
| GO:0019794\_nonprotein\_amino\_acid\_metabolic\_process | 1 | 0 |  |  |  |  |  |  |  |  |
| GO:0019858\_cytosine\_metabolic\_process | 1 | 0 |  |  |  |  |  |  |  |  |
| GO:0019883\_antigen\_processing\_and\_presentation\_of\_endogenous\_antigen | 1 | 0 |  |  |  |  |  |  |  |  |
| GO:0019889\_pteridine\_metabolic\_process | 1 | 0 |  |  |  |  |  |  |  |  |
| GO:0019896\_axon\_transport\_of\_mitochondrion | 1 | 0 |  |  |  |  |  |  |  |  |
| GO:0021508\_floor\_plate\_formation | 1 | 0 |  |  |  |  |  |  |  |  |
| GO:0021528\_commissural\_neuron\_differentiation\_in\_the\_spinal\_cord | 1 | 0 |  |  |  |  |  |  |  |  |
| GO:0021572\_rhombomere\_6\_development | 1 | 0 |  |  |  |  |  |  |  |  |
| GO:0021577\_hindbrain\_structural\_organization | 1 | 0 |  |  |  |  |  |  |  |  |
| GO:0021586\_pons\_maturation | 1 | 0 |  |  |  |  |  |  |  |  |
| GO:0021589\_cerebellum\_structural\_organization | 1 | 0 |  |  |  |  |  |  |  |  |
| GO:0021590\_cerebellum\_maturation | 1 | 0 |  |  |  |  |  |  |  |  |
| GO:0021592\_fourth\_ventricle\_development | 1 | 0 |  |  |  |  |  |  |  |  |
| GO:0021594\_rhombomere\_formation | 1 | 0 |  |  |  |  |  |  |  |  |
| GO:0021660\_rhombomere\_3\_formation | 1 | 0 |  |  |  |  |  |  |  |  |
| GO:0021664\_rhombomere\_5\_morphogenesis | 1 | 0 |  |  |  |  |  |  |  |  |
| GO:0021666\_rhombomere\_5\_formation | 1 | 0 |  |  |  |  |  |  |  |  |
| GO:0021670\_lateral\_ventricle\_development | 1 | 0 |  |  |  |  |  |  |  |  |
| GO:0021678\_third\_ventricle\_development | 1 | 0 |  |  |  |  |  |  |  |  |
| GO:0021679\_cerebellar\_molecular\_layer\_development | 1 | 0 |  |  |  |  |  |  |  |  |
| GO:0021703\_locus\_ceruleus\_development | 1 | 0 |  |  |  |  |  |  |  |  |
| GO:0021732\_midbrain-hindbrain\_boundary\_maturation | 1 | 0 |  |  |  |  |  |  |  |  |
| GO:0021747\_cochlear\_nucleus\_development | 1 | 0 |  |  |  |  |  |  |  |  |
| GO:0021750\_vestibular\_nucleus\_development | 1 | 0 |  |  |  |  |  |  |  |  |
| GO:0021759\_globus\_pallidus\_development | 1 | 0 |  |  |  |  |  |  |  |  |
| GO:0021768\_nucleus\_accumbens\_development | 1 | 0 |  |  |  |  |  |  |  |  |
| GO:0021771\_lateral\_geniculate\_nucleus\_development | 1 | 0 |  |  |  |  |  |  |  |  |
| GO:0021812\_neuronal-glial\_interaction\_involved\_in\_cerebral\_cortex\_radial\_glia\_guided\_migration | 1 | 0 |  |  |  |  |  |  |  |  |
| GO:0021813\_cell-cell\_adhesion\_involved\_in\_neuronal-glial\_interactions\_involved\_in\_cerebral\_cortex\_radial\_glia\_guided\_migration | 1 | 0 |  |  |  |  |  |  |  |  |
| GO:0021870\_Cajal-Retzius\_cell\_differentiation | 1 | 0 |  |  |  |  |  |  |  |  |
| GO:0021874\_Wnt\_receptor\_signaling\_pathway\_in\_forebrain\_neuroblast\_division | 1 | 0 |  |  |  |  |  |  |  |  |
| GO:0021896\_forebrain\_astrocyte\_differentiation | 1 | 0 |  |  |  |  |  |  |  |  |
| GO:0021897\_forebrain\_astrocyte\_development | 1 | 0 |  |  |  |  |  |  |  |  |
| GO:0021902\_commitment\_of\_a\_neuronal\_cell\_to\_a\_specific\_type\_of\_neuron\_in\_the\_forebrain | 1 | 0 |  |  |  |  |  |  |  |  |
| GO:0021905\_forebrain-midbrain\_boundary\_formation | 1 | 0 |  |  |  |  |  |  |  |  |
| GO:0021914\_negative\_regulation\_of\_smoothened\_signaling\_pathway\_involved\_in\_ventral\_spinal\_cord\_patterning | 1 | 0 |  |  |  |  |  |  |  |  |
| GO:0021917\_somatic\_motor\_neuron\_fate\_commitment | 1 | 0 |  |  |  |  |  |  |  |  |
| GO:0021918\_regulation\_of\_transcription\_from\_RNA\_polymerase\_II\_promoter\_involved\_in\_somatic\_motor\_neuron\_fate\_commitment | 1 | 0 |  |  |  |  |  |  |  |  |
| GO:0021933\_radial\_glia\_guided\_migration\_of\_granule\_cell | 1 | 0 |  |  |  |  |  |  |  |  |
| GO:0021934\_hindbrain\_tangential\_cell\_migration | 1 | 0 |  |  |  |  |  |  |  |  |
| GO:0021935\_granule\_cell\_precursor\_tangential\_migration | 1 | 0 |  |  |  |  |  |  |  |  |
| GO:0021942\_radial\_glia\_guided\_migration\_of\_Purkinje\_cell | 1 | 0 |  |  |  |  |  |  |  |  |
| GO:0021960\_anterior\_commissure\_morphogenesis | 1 | 0 |  |  |  |  |  |  |  |  |
| GO:0021997\_neural\_plate\_axis\_specification | 1 | 0 |  |  |  |  |  |  |  |  |
| GO:0021999\_neural\_plate\_anterior\_posterior\_pattern\_formation | 1 | 0 |  |  |  |  |  |  |  |  |
| GO:0022004\_midbrain-hindbrain\_boundary\_maturation\_during\_brain\_development | 1 | 0 |  |  |  |  |  |  |  |  |
| GO:0022038\_corpus\_callosum\_development | 1 | 0 |  |  |  |  |  |  |  |  |
| GO:0022605\_oogenesis\_stage | 1 | 0 |  |  |  |  |  |  |  |  |
| GO:0030011\_maintenance\_of\_cell\_polarity | 1 | 0 |  |  |  |  |  |  |  |  |
| GO:0030069\_lysogeny | 1 | 0 |  |  |  |  |  |  |  |  |
| GO:0030070\_insulin\_processing | 1 | 0 |  |  |  |  |  |  |  |  |
| GO:0030092\_regulation\_of\_flagellum\_assembly | 1 | 0 |  |  |  |  |  |  |  |  |
| GO:0030103\_vasopressin\_secretion | 1 | 0 |  |  |  |  |  |  |  |  |
| GO:0030194\_positive\_regulation\_of\_blood\_coagulation | 1 | 0 |  |  |  |  |  |  |  |  |
| GO:0030206\_chondroitin\_sulfate\_biosynthetic\_process | 1 | 0 |  |  |  |  |  |  |  |  |
| GO:0030210\_heparin\_biosynthetic\_process | 1 | 0 |  |  |  |  |  |  |  |  |
| GO:0030220\_platelet\_formation | 1 | 0 |  |  |  |  |  |  |  |  |
| GO:0030222\_eosinophil\_differentiation | 1 | 0 |  |  |  |  |  |  |  |  |
| GO:0030237\_female\_sex\_determination | 1 | 0 |  |  |  |  |  |  |  |  |
| GO:0030264\_nuclear\_fragmentation\_during\_apoptosis | 1 | 0 |  |  |  |  |  |  |  |  |
| GO:0030322\_stabilization\_of\_membrane\_potential | 1 | 0 |  |  |  |  |  |  |  |  |
| GO:0030327\_prenylated\_protein\_catabolic\_process | 1 | 0 |  |  |  |  |  |  |  |  |
| GO:0030328\_prenylcysteine\_catabolic\_process | 1 | 0 |  |  |  |  |  |  |  |  |
| GO:0030329\_prenylcysteine\_metabolic\_process | 1 | 0 |  |  |  |  |  |  |  |  |
| GO:0030382\_sperm\_mitochondrion\_organization | 1 | 0 |  |  |  |  |  |  |  |  |
| GO:0030389\_fructosamine\_metabolic\_process | 1 | 0 |  |  |  |  |  |  |  |  |
| GO:0030422\_RNA\_interference\_\_production\_of\_siRNA | 1 | 0 |  |  |  |  |  |  |  |  |
| GO:0030449\_regulation\_of\_complement\_activation | 1 | 0 |  |  |  |  |  |  |  |  |
| GO:0030497\_fatty\_acid\_elongation | 1 | 0 |  |  |  |  |  |  |  |  |
| GO:0030575\_nuclear\_body\_organization | 1 | 0 |  |  |  |  |  |  |  |  |
| GO:0030578\_PML\_body\_organization | 1 | 0 |  |  |  |  |  |  |  |  |
| GO:0030853\_negative\_regulation\_of\_granulocyte\_differentiation | 1 | 0 |  |  |  |  |  |  |  |  |
| GO:0030854\_positive\_regulation\_of\_granulocyte\_differentiation | 1 | 0 |  |  |  |  |  |  |  |  |
| GO:0030886\_negative\_regulation\_of\_myeloid\_dendritic\_cell\_activation | 1 | 0 |  |  |  |  |  |  |  |  |
| GO:0030913\_paranodal\_junction\_assembly | 1 | 0 |  |  |  |  |  |  |  |  |
| GO:0031033\_myosin\_filament\_assembly\_or\_disassembly | 1 | 0 |  |  |  |  |  |  |  |  |
| GO:0031034\_myosin\_filament\_assembly | 1 | 0 |  |  |  |  |  |  |  |  |
| GO:0031055\_chromatin\_remodeling\_at\_centromere | 1 | 0 |  |  |  |  |  |  |  |  |
| GO:0031062\_positive\_regulation\_of\_histone\_methylation | 1 | 0 |  |  |  |  |  |  |  |  |
| GO:0031115\_negative\_regulation\_of\_microtubule\_polymerization | 1 | 0 |  |  |  |  |  |  |  |  |
| GO:0031129\_inductive\_cell-cell\_signaling | 1 | 0 |  |  |  |  |  |  |  |  |
| GO:0031284\_positive\_regulation\_of\_guanylate\_cyclase\_activity | 1 | 0 |  |  |  |  |  |  |  |  |
| GO:0031498\_chromatin\_disassembly | 1 | 0 |  |  |  |  |  |  |  |  |
| GO:0031507\_heterochromatin\_formation | 1 | 0 |  |  |  |  |  |  |  |  |
| GO:0031508\_centromeric\_heterochromatin\_formation | 1 | 0 |  |  |  |  |  |  |  |  |
| GO:0031529\_ruffle\_organization | 1 | 0 |  |  |  |  |  |  |  |  |
| GO:0031536\_positive\_regulation\_of\_exit\_from\_mitosis | 1 | 0 |  |  |  |  |  |  |  |  |
| GO:0031572\_G2\_M\_transition\_DNA\_damage\_checkpoint | 1 | 0 |  |  |  |  |  |  |  |  |
| GO:0031576\_G2\_M\_transition\_checkpoint | 1 | 0 |  |  |  |  |  |  |  |  |
| GO:0031580\_membrane\_raft\_distribution | 1 | 0 |  |  |  |  |  |  |  |  |
| GO:0031583\_activation\_of\_phospholipase\_D\_activity\_by\_G-protein\_coupled\_receptor\_protein\_signaling\_pathway | 1 | 0 |  |  |  |  |  |  |  |  |
| GO:0031584\_activation\_of\_phospholipase\_D\_activity | 1 | 0 |  |  |  |  |  |  |  |  |
| GO:0031585\_regulation\_of\_inositol-1\_4\_5-triphosphate\_receptor\_activity | 1 | 0 |  |  |  |  |  |  |  |  |
| GO:0031639\_plasminogen\_activation | 1 | 0 |  |  |  |  |  |  |  |  |
| GO:0031648\_protein\_destabilization | 1 | 0 |  |  |  |  |  |  |  |  |
| GO:0031665\_negative\_regulation\_of\_lipopolysaccharide-mediated\_signaling\_pathway | 1 | 0 |  |  |  |  |  |  |  |  |
| GO:0031914\_negative\_regulation\_of\_synaptic\_plasticity | 1 | 0 |  |  |  |  |  |  |  |  |
| GO:0031944\_negative\_regulation\_of\_glucocorticoid\_metabolic\_process | 1 | 0 |  |  |  |  |  |  |  |  |
| GO:0031947\_negative\_regulation\_of\_glucocorticoid\_biosynthetic\_process | 1 | 0 |  |  |  |  |  |  |  |  |
| GO:0032025\_response\_to\_cobalt\_ion | 1 | 0 |  |  |  |  |  |  |  |  |
| GO:0032026\_response\_to\_magnesium\_ion | 1 | 0 |  |  |  |  |  |  |  |  |
| GO:0032048\_cardiolipin\_metabolic\_process | 1 | 0 |  |  |  |  |  |  |  |  |
| GO:0032066\_nucleolus\_to\_nucleoplasm\_transport | 1 | 0 |  |  |  |  |  |  |  |  |
| GO:0032091\_negative\_regulation\_of\_protein\_binding | 1 | 0 |  |  |  |  |  |  |  |  |
| GO:0032092\_positive\_regulation\_of\_protein\_binding | 1 | 0 |  |  |  |  |  |  |  |  |
| GO:0032097\_positive\_regulation\_of\_response\_to\_food | 1 | 0 |  |  |  |  |  |  |  |  |
| GO:0032100\_positive\_regulation\_of\_appetite | 1 | 0 |  |  |  |  |  |  |  |  |
| GO:0032204\_regulation\_of\_telomere\_maintenance | 1 | 0 |  |  |  |  |  |  |  |  |
| GO:0032206\_positive\_regulation\_of\_telomere\_maintenance | 1 | 0 |  |  |  |  |  |  |  |  |
| GO:0032222\_regulation\_of\_synaptic\_transmission\_\_cholinergic | 1 | 0 |  |  |  |  |  |  |  |  |
| GO:0032224\_positive\_regulation\_of\_synaptic\_transmission\_\_cholinergic | 1 | 0 |  |  |  |  |  |  |  |  |
| GO:0032229\_negative\_regulation\_of\_synaptic\_transmission\_\_GABAergic | 1 | 0 |  |  |  |  |  |  |  |  |
| GO:0032239\_regulation\_of\_nucleobase\_\_nucleoside\_\_nucleotide\_and\_nucleic\_acid\_transport | 1 | 0 |  |  |  |  |  |  |  |  |
| GO:0032252\_secretory\_granule\_localization | 1 | 0 |  |  |  |  |  |  |  |  |
| GO:0032274\_gonadotropin\_secretion | 1 | 0 |  |  |  |  |  |  |  |  |
| GO:0032275\_luteinizing\_hormone\_secretion | 1 | 0 |  |  |  |  |  |  |  |  |
| GO:0032287\_myelin\_maintenance\_in\_the\_peripheral\_nervous\_system | 1 | 0 |  |  |  |  |  |  |  |  |
| GO:0032289\_myelin\_formation\_in\_the\_central\_nervous\_system | 1 | 0 |  |  |  |  |  |  |  |  |
| GO:0032303\_regulation\_of\_icosanoid\_secretion | 1 | 0 |  |  |  |  |  |  |  |  |
| GO:0032305\_positive\_regulation\_of\_icosanoid\_secretion | 1 | 0 |  |  |  |  |  |  |  |  |
| GO:0032306\_regulation\_of\_prostaglandin\_secretion | 1 | 0 |  |  |  |  |  |  |  |  |
| GO:0032308\_positive\_regulation\_of\_prostaglandin\_secretion | 1 | 0 |  |  |  |  |  |  |  |  |
| GO:0032310\_prostaglandin\_secretion | 1 | 0 |  |  |  |  |  |  |  |  |
| GO:0032313\_regulation\_of\_Rab\_GTPase\_activity | 1 | 0 |  |  |  |  |  |  |  |  |
| GO:0032314\_regulation\_of\_Rac\_GTPase\_activity | 1 | 0 |  |  |  |  |  |  |  |  |
| GO:0032317\_regulation\_of\_Rap\_GTPase\_activity | 1 | 0 |  |  |  |  |  |  |  |  |
| GO:0032324\_molybdopterin\_cofactor\_biosynthetic\_process | 1 | 0 |  |  |  |  |  |  |  |  |
| GO:0032329\_serine\_transport | 1 | 0 |  |  |  |  |  |  |  |  |
| GO:0032342\_aldosterone\_biosynthetic\_process | 1 | 0 |  |  |  |  |  |  |  |  |
| GO:0032344\_regulation\_of\_aldosterone\_metabolic\_process | 1 | 0 |  |  |  |  |  |  |  |  |
| GO:0032365\_intracellular\_lipid\_transport | 1 | 0 |  |  |  |  |  |  |  |  |
| GO:0032366\_intracellular\_sterol\_transport | 1 | 0 |  |  |  |  |  |  |  |  |
| GO:0032367\_intracellular\_cholesterol\_transport | 1 | 0 |  |  |  |  |  |  |  |  |
| GO:0032370\_positive\_regulation\_of\_lipid\_transport | 1 | 0 |  |  |  |  |  |  |  |  |
| GO:0032410\_negative\_regulation\_of\_transporter\_activity | 1 | 0 |  |  |  |  |  |  |  |  |
| GO:0032413\_negative\_regulation\_of\_ion\_transmembrane\_transporter\_activity | 1 | 0 |  |  |  |  |  |  |  |  |
| GO:0032429\_regulation\_of\_phospholipase\_A2\_activity | 1 | 0 |  |  |  |  |  |  |  |  |
| GO:0032474\_otolith\_morphogenesis | 1 | 0 |  |  |  |  |  |  |  |  |
| GO:0032482\_Rab\_protein\_signal\_transduction | 1 | 0 |  |  |  |  |  |  |  |  |
| GO:0032483\_regulation\_of\_Rab\_protein\_signal\_transduction | 1 | 0 |  |  |  |  |  |  |  |  |
| GO:0032486\_Rap\_protein\_signal\_transduction | 1 | 0 |  |  |  |  |  |  |  |  |
| GO:0032487\_regulation\_of\_Rap\_protein\_signal\_transduction | 1 | 0 |  |  |  |  |  |  |  |  |
| GO:0032594\_protein\_transport\_within\_lipid\_bilayer | 1 | 0 |  |  |  |  |  |  |  |  |
| GO:0032599\_protein\_transport\_out\_of\_membrane\_raft | 1 | 0 |  |  |  |  |  |  |  |  |
| GO:0032600\_chemokine\_receptor\_transport\_out\_of\_membrane\_raft | 1 | 0 |  |  |  |  |  |  |  |  |
| GO:0032607\_interferon-alpha\_production | 1 | 0 |  |  |  |  |  |  |  |  |
| GO:0032621\_interleukin-18\_production | 1 | 0 |  |  |  |  |  |  |  |  |
| GO:0032647\_regulation\_of\_interferon-alpha\_production | 1 | 0 |  |  |  |  |  |  |  |  |
| GO:0032656\_regulation\_of\_interleukin-13\_production | 1 | 0 |  |  |  |  |  |  |  |  |
| GO:0032682\_negative\_regulation\_of\_chemokine\_production | 1 | 0 |  |  |  |  |  |  |  |  |
| GO:0032691\_negative\_regulation\_of\_interleukin-1\_beta\_production | 1 | 0 |  |  |  |  |  |  |  |  |
| GO:0032692\_negative\_regulation\_of\_interleukin-1\_production | 1 | 0 |  |  |  |  |  |  |  |  |
| GO:0032693\_negative\_regulation\_of\_interleukin-10\_production | 1 | 0 |  |  |  |  |  |  |  |  |
| GO:0032696\_negative\_regulation\_of\_interleukin-13\_production | 1 | 0 |  |  |  |  |  |  |  |  |
| GO:0032727\_positive\_regulation\_of\_interferon-alpha\_production | 1 | 0 |  |  |  |  |  |  |  |  |
| GO:0032731\_positive\_regulation\_of\_interleukin-1\_beta\_production | 1 | 0 |  |  |  |  |  |  |  |  |
| GO:0032732\_positive\_regulation\_of\_interleukin-1\_production | 1 | 0 |  |  |  |  |  |  |  |  |
| GO:0032735\_positive\_regulation\_of\_interleukin-12\_production | 1 | 0 |  |  |  |  |  |  |  |  |
| GO:0032764\_negative\_regulation\_of\_mast\_cell\_cytokine\_production | 1 | 0 |  |  |  |  |  |  |  |  |
| GO:0032765\_positive\_regulation\_of\_mast\_cell\_cytokine\_production | 1 | 0 |  |  |  |  |  |  |  |  |
| GO:0032769\_negative\_regulation\_of\_monooxygenase\_activity | 1 | 0 |  |  |  |  |  |  |  |  |
| GO:0032781\_positive\_regulation\_of\_ATPase\_activity | 1 | 0 |  |  |  |  |  |  |  |  |
| GO:0032790\_ribosome\_disassembly | 1 | 0 |  |  |  |  |  |  |  |  |
| GO:0032799\_low-density\_lipoprotein\_receptor\_metabolic\_process | 1 | 0 |  |  |  |  |  |  |  |  |
| GO:0032802\_low-density\_lipoprotein\_receptor\_catabolic\_process | 1 | 0 |  |  |  |  |  |  |  |  |
| GO:0032803\_regulation\_of\_low-density\_lipoprotein\_receptor\_catabolic\_process | 1 | 0 |  |  |  |  |  |  |  |  |
| GO:0032817\_regulation\_of\_natural\_killer\_cell\_proliferation | 1 | 0 |  |  |  |  |  |  |  |  |
| GO:0032819\_positive\_regulation\_of\_natural\_killer\_cell\_proliferation | 1 | 0 |  |  |  |  |  |  |  |  |
| GO:0032855\_positive\_regulation\_of\_Rac\_GTPase\_activity | 1 | 0 |  |  |  |  |  |  |  |  |
| GO:0032863\_activation\_of\_Rac\_GTPase\_activity | 1 | 0 |  |  |  |  |  |  |  |  |
| GO:0032864\_activation\_of\_Cdc42\_GTPase\_activity | 1 | 0 |  |  |  |  |  |  |  |  |
| GO:0032885\_regulation\_of\_polysaccharide\_biosynthetic\_process | 1 | 0 |  |  |  |  |  |  |  |  |
| GO:0032907\_transforming\_growth\_factor-beta3\_production | 1 | 0 |  |  |  |  |  |  |  |  |
| GO:0032910\_regulation\_of\_transforming\_growth\_factor-beta3\_production | 1 | 0 |  |  |  |  |  |  |  |  |
| GO:0032913\_negative\_regulation\_of\_transforming\_growth\_factor-beta3\_production | 1 | 0 |  |  |  |  |  |  |  |  |
| GO:0032924\_activin\_receptor\_signaling\_pathway | 1 | 0 |  |  |  |  |  |  |  |  |
| GO:0032925\_regulation\_of\_activin\_receptor\_signaling\_pathway | 1 | 0 |  |  |  |  |  |  |  |  |
| GO:0032960\_regulation\_of\_inositol\_trisphosphate\_biosynthetic\_process | 1 | 0 |  |  |  |  |  |  |  |  |
| GO:0032962\_positive\_regulation\_of\_inositol\_trisphosphate\_biosynthetic\_process | 1 | 0 |  |  |  |  |  |  |  |  |
| GO:0032964\_collagen\_biosynthetic\_process | 1 | 0 |  |  |  |  |  |  |  |  |
| GO:0032971\_regulation\_of\_muscle\_filament\_sliding | 1 | 0 |  |  |  |  |  |  |  |  |
| GO:0032972\_regulation\_of\_muscle\_filament\_sliding\_speed | 1 | 0 |  |  |  |  |  |  |  |  |
| GO:0032986\_protein-DNA\_complex\_disassembly | 1 | 0 |  |  |  |  |  |  |  |  |
| GO:0032988\_ribonucleoprotein\_complex\_disassembly | 1 | 0 |  |  |  |  |  |  |  |  |
| GO:0033037\_polysaccharide\_localization | 1 | 0 |  |  |  |  |  |  |  |  |
| GO:0033078\_extrathymic\_T\_cell\_differentiation | 1 | 0 |  |  |  |  |  |  |  |  |
| GO:0033085\_negative\_regulation\_of\_T\_cell\_differentiation\_in\_the\_thymus | 1 | 0 |  |  |  |  |  |  |  |  |
| GO:0033087\_negative\_regulation\_of\_immature\_T\_cell\_proliferation | 1 | 0 |  |  |  |  |  |  |  |  |
| GO:0033088\_negative\_regulation\_of\_immature\_T\_cell\_proliferation\_in\_the\_thymus | 1 | 0 |  |  |  |  |  |  |  |  |
| GO:0033108\_mitochondrial\_respiratory\_chain\_complex\_assembly | 1 | 0 |  |  |  |  |  |  |  |  |
| GO:0033127\_regulation\_of\_histone\_phosphorylation | 1 | 0 |  |  |  |  |  |  |  |  |
| GO:0033128\_negative\_regulation\_of\_histone\_phosphorylation | 1 | 0 |  |  |  |  |  |  |  |  |
| GO:0033138\_positive\_regulation\_of\_peptidyl-serine\_phosphorylation | 1 | 0 |  |  |  |  |  |  |  |  |
| GO:0033158\_regulation\_of\_protein\_import\_into\_nucleus\_\_translocation | 1 | 0 |  |  |  |  |  |  |  |  |
| GO:0033160\_positive\_regulation\_of\_protein\_import\_into\_nucleus\_\_translocation | 1 | 0 |  |  |  |  |  |  |  |  |
| GO:0033169\_histone\_H3-K9\_demethylation | 1 | 0 |  |  |  |  |  |  |  |  |
| GO:0033206\_cytokinesis\_after\_meiosis | 1 | 0 |  |  |  |  |  |  |  |  |
| GO:0033240\_positive\_regulation\_of\_cellular\_amine\_metabolic\_process | 1 | 0 |  |  |  |  |  |  |  |  |
| GO:0033313\_meiotic\_cell\_cycle\_checkpoint | 1 | 0 |  |  |  |  |  |  |  |  |
| GO:0033315\_meiotic\_cell\_cycle\_DNA\_replication\_checkpoint | 1 | 0 |  |  |  |  |  |  |  |  |
| GO:0033326\_cerebrospinal\_fluid\_secretion | 1 | 0 |  |  |  |  |  |  |  |  |
| GO:0033366\_protein\_localization\_in\_secretory\_granule | 1 | 0 |  |  |  |  |  |  |  |  |
| GO:0033367\_protein\_localization\_in\_mast\_cell\_secretory\_granule | 1 | 0 |  |  |  |  |  |  |  |  |
| GO:0033368\_protease\_localization\_in\_mast\_cell\_secretory\_granule | 1 | 0 |  |  |  |  |  |  |  |  |
| GO:0033370\_maintenance\_of\_protein\_location\_in\_mast\_cell\_secretory\_granule | 1 | 0 |  |  |  |  |  |  |  |  |
| GO:0033371\_T\_cell\_secretory\_granule\_organization | 1 | 0 |  |  |  |  |  |  |  |  |
| GO:0033373\_maintenance\_of\_protease\_location\_in\_mast\_cell\_secretory\_granule | 1 | 0 |  |  |  |  |  |  |  |  |
| GO:0033374\_protein\_localization\_in\_T\_cell\_secretory\_granule | 1 | 0 |  |  |  |  |  |  |  |  |
| GO:0033375\_protease\_localization\_in\_T\_cell\_secretory\_granule | 1 | 0 |  |  |  |  |  |  |  |  |
| GO:0033377\_maintenance\_of\_protein\_location\_in\_T\_cell\_secretory\_granule | 1 | 0 |  |  |  |  |  |  |  |  |
| GO:0033379\_maintenance\_of\_protease\_location\_in\_T\_cell\_secretory\_granule | 1 | 0 |  |  |  |  |  |  |  |  |
| GO:0033380\_granzyme\_B\_localization\_in\_T\_cell\_secretory\_granule | 1 | 0 |  |  |  |  |  |  |  |  |
| GO:0033382\_maintenance\_of\_granzyme\_B\_location\_in\_T\_cell\_secretory\_granule | 1 | 0 |  |  |  |  |  |  |  |  |
| GO:0033483\_gas\_homeostasis | 1 | 0 |  |  |  |  |  |  |  |  |
| GO:0033484\_nitric\_oxide\_homeostasis | 1 | 0 |  |  |  |  |  |  |  |  |
| GO:0033505\_floor\_plate\_morphogenesis | 1 | 0 |  |  |  |  |  |  |  |  |
| GO:0033522\_histone\_H2A\_ubiquitination | 1 | 0 |  |  |  |  |  |  |  |  |
| GO:0033523\_histone\_H2B\_ubiquitination | 1 | 0 |  |  |  |  |  |  |  |  |
| GO:0033574\_response\_to\_testosterone\_stimulus | 1 | 0 |  |  |  |  |  |  |  |  |
| GO:0033606\_chemokine\_receptor\_transport\_within\_lipid\_bilayer | 1 | 0 |  |  |  |  |  |  |  |  |
| GO:0033628\_regulation\_of\_cell\_adhesion\_mediated\_by\_integrin | 1 | 0 |  |  |  |  |  |  |  |  |
| GO:0033630\_positive\_regulation\_of\_cell\_adhesion\_mediated\_by\_integrin | 1 | 0 |  |  |  |  |  |  |  |  |
| GO:0033632\_regulation\_of\_cell-cell\_adhesion\_mediated\_by\_integrin | 1 | 0 |  |  |  |  |  |  |  |  |
| GO:0033634\_positive\_regulation\_of\_cell-cell\_adhesion\_mediated\_by\_integrin | 1 | 0 |  |  |  |  |  |  |  |  |
| GO:0033683\_nucleotide-excision\_repair\_\_DNA\_incision | 1 | 0 |  |  |  |  |  |  |  |  |
| GO:0033687\_osteoblast\_proliferation | 1 | 0 |  |  |  |  |  |  |  |  |
| GO:0033688\_regulation\_of\_osteoblast\_proliferation | 1 | 0 |  |  |  |  |  |  |  |  |
| GO:0033689\_negative\_regulation\_of\_osteoblast\_proliferation | 1 | 0 |  |  |  |  |  |  |  |  |
| GO:0033750\_ribosome\_localization | 1 | 0 |  |  |  |  |  |  |  |  |
| GO:0033753\_establishment\_of\_ribosome\_localization | 1 | 0 |  |  |  |  |  |  |  |  |
| GO:0033866\_nucleoside\_bisphosphate\_biosynthetic\_process | 1 | 0 |  |  |  |  |  |  |  |  |
| GO:0033875\_ribonucleoside\_bisphosphate\_metabolic\_process | 1 | 0 |  |  |  |  |  |  |  |  |
| GO:0034030\_ribonucleoside\_bisphosphate\_biosynthetic\_process | 1 | 0 |  |  |  |  |  |  |  |  |
| GO:0034032\_purine\_nucleoside\_bisphosphate\_metabolic\_process | 1 | 0 |  |  |  |  |  |  |  |  |
| GO:0034033\_purine\_nucleoside\_bisphosphate\_biosynthetic\_process | 1 | 0 |  |  |  |  |  |  |  |  |
| GO:0034035\_purine\_ribonucleoside\_bisphosphate\_metabolic\_process | 1 | 0 |  |  |  |  |  |  |  |  |
| GO:0034036\_purine\_ribonucleoside\_bisphosphate\_biosynthetic\_process | 1 | 0 |  |  |  |  |  |  |  |  |
| GO:0034067\_protein\_localization\_in\_Golgi\_apparatus | 1 | 0 |  |  |  |  |  |  |  |  |
| GO:0034102\_erythrocyte\_clearance | 1 | 0 |  |  |  |  |  |  |  |  |
| GO:0034106\_regulation\_of\_erythrocyte\_clearance | 1 | 0 |  |  |  |  |  |  |  |  |
| GO:0034107\_negative\_regulation\_of\_erythrocyte\_clearance | 1 | 0 |  |  |  |  |  |  |  |  |
| GO:0034110\_regulation\_of\_homotypic\_cell-cell\_adhesion | 1 | 0 |  |  |  |  |  |  |  |  |
| GO:0034111\_negative\_regulation\_of\_homotypic\_cell-cell\_adhesion | 1 | 0 |  |  |  |  |  |  |  |  |
| GO:0034113\_heterotypic\_cell-cell\_adhesion | 1 | 0 |  |  |  |  |  |  |  |  |
| GO:0034117\_erythrocyte\_aggregation | 1 | 0 |  |  |  |  |  |  |  |  |
| GO:0034118\_regulation\_of\_erythrocyte\_aggregation | 1 | 0 |  |  |  |  |  |  |  |  |
| GO:0034119\_negative\_regulation\_of\_erythrocyte\_aggregation | 1 | 0 |  |  |  |  |  |  |  |  |
| GO:0034121\_regulation\_of\_toll-like\_receptor\_signaling\_pathway | 1 | 0 |  |  |  |  |  |  |  |  |
| GO:0034122\_negative\_regulation\_of\_toll-like\_receptor\_signaling\_pathway | 1 | 0 |  |  |  |  |  |  |  |  |
| GO:0034230\_enkephalin\_processing | 1 | 0 |  |  |  |  |  |  |  |  |
| GO:0034372\_very-low-density\_lipoprotein\_particle\_remodeling | 1 | 0 |  |  |  |  |  |  |  |  |
| GO:0034379\_very-low-density\_lipoprotein\_particle\_assembly | 1 | 0 |  |  |  |  |  |  |  |  |
| GO:0034380\_high-density\_lipoprotein\_particle\_assembly | 1 | 0 |  |  |  |  |  |  |  |  |
| GO:0034394\_protein\_localization\_at\_cell\_surface | 1 | 0 |  |  |  |  |  |  |  |  |
| GO:0034405\_response\_to\_fluid\_shear\_stress | 1 | 0 |  |  |  |  |  |  |  |  |
| GO:0034472\_snRNA\_3'-end\_processing | 1 | 0 |  |  |  |  |  |  |  |  |
| GO:0034474\_U2\_snRNA\_3'-end\_processing | 1 | 0 |  |  |  |  |  |  |  |  |
| GO:0034502\_protein\_localization\_to\_chromosome | 1 | 0 |  |  |  |  |  |  |  |  |
| GO:0034505\_tooth\_mineralization | 1 | 0 |  |  |  |  |  |  |  |  |
| GO:0034508\_centromere\_complex\_assembly | 1 | 0 |  |  |  |  |  |  |  |  |
| GO:0034633\_retinol\_transport | 1 | 0 |  |  |  |  |  |  |  |  |
| GO:0034643\_mitochondrion\_localization\_\_microtubule-mediated | 1 | 0 |  |  |  |  |  |  |  |  |
| GO:0034969\_histone\_arginine\_methylation | 1 | 0 |  |  |  |  |  |  |  |  |
| GO:0034982\_mitochondrial\_protein\_processing | 1 | 0 |  |  |  |  |  |  |  |  |
| GO:0035022\_positive\_regulation\_of\_Rac\_protein\_signal\_transduction | 1 | 0 |  |  |  |  |  |  |  |  |
| GO:0035024\_negative\_regulation\_of\_Rho\_protein\_signal\_transduction | 1 | 0 |  |  |  |  |  |  |  |  |
| GO:0035026\_leading\_edge\_cell\_differentiation | 1 | 0 |  |  |  |  |  |  |  |  |
| GO:0035037\_sperm\_entry | 1 | 0 |  |  |  |  |  |  |  |  |
| GO:0035039\_male\_pronucleus\_formation | 1 | 0 |  |  |  |  |  |  |  |  |
| GO:0035066\_positive\_regulation\_of\_histone\_acetylation | 1 | 0 |  |  |  |  |  |  |  |  |
| GO:0035083\_cilium\_axoneme\_assembly | 1 | 0 |  |  |  |  |  |  |  |  |
| GO:0035090\_maintenance\_of\_apical\_basal\_cell\_polarity | 1 | 0 |  |  |  |  |  |  |  |  |
| GO:0035106\_operant\_conditioning | 1 | 0 |  |  |  |  |  |  |  |  |
| GO:0035172\_hemocyte\_proliferation | 1 | 0 |  |  |  |  |  |  |  |  |
| GO:0035227\_regulation\_of\_glutamate-cysteine\_ligase\_activity | 1 | 0 |  |  |  |  |  |  |  |  |
| GO:0035229\_positive\_regulation\_of\_glutamate-cysteine\_ligase\_activity | 1 | 0 |  |  |  |  |  |  |  |  |
| GO:0035260\_internal\_genitalia\_morphogenesis | 1 | 0 |  |  |  |  |  |  |  |  |
| GO:0035262\_gonad\_morphogenesis | 1 | 0 |  |  |  |  |  |  |  |  |
| GO:0035287\_head\_segmentation | 1 | 0 |  |  |  |  |  |  |  |  |
| GO:0035289\_posterior\_head\_segmentation | 1 | 0 |  |  |  |  |  |  |  |  |
| GO:0035303\_regulation\_of\_dephosphorylation | 1 | 0 |  |  |  |  |  |  |  |  |
| GO:0035304\_regulation\_of\_protein\_amino\_acid\_dephosphorylation | 1 | 0 |  |  |  |  |  |  |  |  |
| GO:0035305\_negative\_regulation\_of\_dephosphorylation | 1 | 0 |  |  |  |  |  |  |  |  |
| GO:0035308\_negative\_regulation\_of\_protein\_amino\_acid\_dephosphorylation | 1 | 0 |  |  |  |  |  |  |  |  |
| GO:0035313\_wound\_healing\_\_spreading\_of\_epidermal\_cells | 1 | 0 |  |  |  |  |  |  |  |  |
| GO:0040013\_negative\_regulation\_of\_locomotion | 1 | 0 |  |  |  |  |  |  |  |  |
| GO:0040019\_positive\_regulation\_of\_embryonic\_development | 1 | 0 |  |  |  |  |  |  |  |  |
| GO:0040032\_post-embryonic\_body\_morphogenesis | 1 | 0 |  |  |  |  |  |  |  |  |
| GO:0040038\_polar\_body\_extrusion\_after\_meiotic\_divisions | 1 | 0 |  |  |  |  |  |  |  |  |
| GO:0042026\_protein\_refolding | 1 | 0 |  |  |  |  |  |  |  |  |
| GO:0042048\_olfactory\_behavior | 1 | 0 |  |  |  |  |  |  |  |  |
| GO:0042059\_negative\_regulation\_of\_epidermal\_growth\_factor\_receptor\_signaling\_pathway | 1 | 0 |  |  |  |  |  |  |  |  |
| GO:0042073\_intraflagellar\_transport | 1 | 0 |  |  |  |  |  |  |  |  |
| GO:0042078\_germ-line\_stem\_cell\_division | 1 | 0 |  |  |  |  |  |  |  |  |
| GO:0042091\_interleukin-10\_biosynthetic\_process | 1 | 0 |  |  |  |  |  |  |  |  |
| GO:0042103\_positive\_regulation\_of\_T\_cell\_homeostatic\_proliferation | 1 | 0 |  |  |  |  |  |  |  |  |
| GO:0042136\_neurotransmitter\_biosynthetic\_process | 1 | 0 |  |  |  |  |  |  |  |  |
| GO:0042137\_sequestering\_of\_neurotransmitter | 1 | 0 |  |  |  |  |  |  |  |  |
| GO:0042138\_meiotic\_DNA\_double-strand\_break\_formation | 1 | 0 |  |  |  |  |  |  |  |  |
| GO:0042178\_xenobiotic\_catabolic\_process | 1 | 0 |  |  |  |  |  |  |  |  |
| GO:0042225\_interleukin-5\_biosynthetic\_process | 1 | 0 |  |  |  |  |  |  |  |  |
| GO:0042231\_interleukin-13\_biosynthetic\_process | 1 | 0 |  |  |  |  |  |  |  |  |
| GO:0042255\_ribosome\_assembly | 1 | 0 |  |  |  |  |  |  |  |  |
| GO:0042257\_ribosomal\_subunit\_assembly | 1 | 0 |  |  |  |  |  |  |  |  |
| GO:0042264\_peptidyl-aspartic\_acid\_hydroxylation | 1 | 0 |  |  |  |  |  |  |  |  |
| GO:0042276\_error-prone\_postreplication\_DNA\_repair | 1 | 0 |  |  |  |  |  |  |  |  |
| GO:0042297\_vocal\_learning | 1 | 0 |  |  |  |  |  |  |  |  |
| GO:0042309\_homoiothermy | 1 | 0 |  |  |  |  |  |  |  |  |
| GO:0042320\_regulation\_of\_circadian\_sleep\_wake\_cycle\_\_REM\_sleep | 1 | 0 |  |  |  |  |  |  |  |  |
| GO:0042339\_keratan\_sulfate\_metabolic\_process | 1 | 0 |  |  |  |  |  |  |  |  |
| GO:0042347\_negative\_regulation\_of\_NF-kappaB\_import\_into\_nucleus | 1 | 0 |  |  |  |  |  |  |  |  |
| GO:0042360\_vitamin\_E\_metabolic\_process | 1 | 0 |  |  |  |  |  |  |  |  |
| GO:0042363\_fat-soluble\_vitamin\_catabolic\_process | 1 | 0 |  |  |  |  |  |  |  |  |
| GO:0042369\_vitamin\_D\_catabolic\_process | 1 | 0 |  |  |  |  |  |  |  |  |
| GO:0042373\_vitamin\_K\_metabolic\_process | 1 | 0 |  |  |  |  |  |  |  |  |
| GO:0042404\_thyroid\_hormone\_catabolic\_process | 1 | 0 |  |  |  |  |  |  |  |  |
| GO:0042414\_epinephrine\_metabolic\_process | 1 | 0 |  |  |  |  |  |  |  |  |
| GO:0042436\_indole\_derivative\_catabolic\_process | 1 | 0 |  |  |  |  |  |  |  |  |
| GO:0042489\_negative\_regulation\_of\_odontogenesis\_of\_dentine-containing\_tooth | 1 | 0 |  |  |  |  |  |  |  |  |
| GO:0042508\_tyrosine\_phosphorylation\_of\_Stat1\_protein | 1 | 0 |  |  |  |  |  |  |  |  |
| GO:0042518\_negative\_regulation\_of\_tyrosine\_phosphorylation\_of\_Stat3\_protein | 1 | 0 |  |  |  |  |  |  |  |  |
| GO:0042524\_negative\_regulation\_of\_tyrosine\_phosphorylation\_of\_Stat5\_protein | 1 | 0 |  |  |  |  |  |  |  |  |
| GO:0042536\_negative\_regulation\_of\_tumor\_necrosis\_factor\_biosynthetic\_process | 1 | 0 |  |  |  |  |  |  |  |  |
| GO:0042538\_hyperosmotic\_salinity\_response | 1 | 0 |  |  |  |  |  |  |  |  |
| GO:0042628\_mating\_plug\_formation | 1 | 0 |  |  |  |  |  |  |  |  |
| GO:0042631\_cellular\_response\_to\_water\_deprivation | 1 | 0 |  |  |  |  |  |  |  |  |
| GO:0042637\_catagen | 1 | 0 |  |  |  |  |  |  |  |  |
| GO:0042660\_positive\_regulation\_of\_cell\_fate\_specification | 1 | 0 |  |  |  |  |  |  |  |  |
| GO:0042663\_regulation\_of\_endodermal\_cell\_fate\_specification | 1 | 0 |  |  |  |  |  |  |  |  |
| GO:0042664\_negative\_regulation\_of\_endodermal\_cell\_fate\_specification | 1 | 0 |  |  |  |  |  |  |  |  |
| GO:0042667\_auditory\_receptor\_cell\_fate\_specification | 1 | 0 |  |  |  |  |  |  |  |  |
| GO:0042694\_muscle\_cell\_fate\_specification | 1 | 0 |  |  |  |  |  |  |  |  |
| GO:0042706\_eye\_photoreceptor\_cell\_fate\_commitment | 1 | 0 |  |  |  |  |  |  |  |  |
| GO:0042713\_sperm\_ejaculation | 1 | 0 |  |  |  |  |  |  |  |  |
| GO:0042723\_thiamin\_and\_derivative\_metabolic\_process | 1 | 0 |  |  |  |  |  |  |  |  |
| GO:0042737\_drug\_catabolic\_process | 1 | 0 |  |  |  |  |  |  |  |  |
| GO:0042738\_exogenous\_drug\_catabolic\_process | 1 | 0 |  |  |  |  |  |  |  |  |
| GO:0042747\_circadian\_sleep\_wake\_cycle\_\_REM\_sleep | 1 | 0 |  |  |  |  |  |  |  |  |
| GO:0042748\_circadian\_sleep\_wake\_cycle\_\_non-REM\_sleep | 1 | 0 |  |  |  |  |  |  |  |  |
| GO:0042772\_DNA\_damage\_response\_\_signal\_transduction\_resulting\_in\_transcription | 1 | 0 |  |  |  |  |  |  |  |  |
| GO:0042790\_transcription\_of\_nuclear\_rRNA\_large\_RNA\_polymerase\_I\_transcript | 1 | 0 |  |  |  |  |  |  |  |  |
| GO:0042839\_D-glucuronate\_metabolic\_process | 1 | 0 |  |  |  |  |  |  |  |  |
| GO:0042840\_D-glucuronate\_catabolic\_process | 1 | 0 |  |  |  |  |  |  |  |  |
| GO:0042891\_antibiotic\_transport | 1 | 0 |  |  |  |  |  |  |  |  |
| GO:0042892\_chloramphenicol\_transport | 1 | 0 |  |  |  |  |  |  |  |  |
| GO:0042940\_D-amino\_acid\_transport | 1 | 0 |  |  |  |  |  |  |  |  |
| GO:0042941\_D-alanine\_transport | 1 | 0 |  |  |  |  |  |  |  |  |
| GO:0042942\_D-serine\_transport | 1 | 0 |  |  |  |  |  |  |  |  |
| GO:0042983\_amyloid\_precursor\_protein\_biosynthetic\_process | 1 | 0 |  |  |  |  |  |  |  |  |
| GO:0042984\_regulation\_of\_amyloid\_precursor\_protein\_biosynthetic\_process | 1 | 0 |  |  |  |  |  |  |  |  |
| GO:0042985\_negative\_regulation\_of\_amyloid\_precursor\_protein\_biosynthetic\_process | 1 | 0 |  |  |  |  |  |  |  |  |
| GO:0042989\_sequestering\_of\_actin\_monomers | 1 | 0 |  |  |  |  |  |  |  |  |
| GO:0043044\_ATP-dependent\_chromatin\_remodeling | 1 | 0 |  |  |  |  |  |  |  |  |
| GO:0043056\_forward\_locomotion | 1 | 0 |  |  |  |  |  |  |  |  |
| GO:0043060\_meiotic\_metaphase\_I\_plate\_congression | 1 | 0 |  |  |  |  |  |  |  |  |
| GO:0043091\_L-arginine\_import | 1 | 0 |  |  |  |  |  |  |  |  |
| GO:0043124\_negative\_regulation\_of\_I-kappaB\_kinase\_NF-kappaB\_cascade | 1 | 0 |  |  |  |  |  |  |  |  |
| GO:0043132\_NAD\_transport | 1 | 0 |  |  |  |  |  |  |  |  |
| GO:0043153\_entrainment\_of\_circadian\_clock\_by\_photoperiod | 1 | 0 |  |  |  |  |  |  |  |  |
| GO:0043171\_peptide\_catabolic\_process | 1 | 0 |  |  |  |  |  |  |  |  |
| GO:0043179\_rhythmic\_excitation | 1 | 0 |  |  |  |  |  |  |  |  |
| GO:0043206\_fibril\_organization | 1 | 0 |  |  |  |  |  |  |  |  |
| GO:0043217\_myelin\_maintenance | 1 | 0 |  |  |  |  |  |  |  |  |
| GO:0043313\_regulation\_of\_neutrophil\_degranulation | 1 | 0 |  |  |  |  |  |  |  |  |
| GO:0043316\_cytotoxic\_T\_cell\_degranulation | 1 | 0 |  |  |  |  |  |  |  |  |
| GO:0043369\_CD4-positive\_or\_CD8-positive\_\_alpha-beta\_T\_cell\_lineage\_commitment | 1 | 0 |  |  |  |  |  |  |  |  |
| GO:0043375\_CD8-positive\_\_alpha-beta\_T\_cell\_lineage\_commitment | 1 | 0 |  |  |  |  |  |  |  |  |
| GO:0043379\_memory\_T\_cell\_differentiation | 1 | 0 |  |  |  |  |  |  |  |  |
| GO:0043380\_regulation\_of\_memory\_T\_cell\_differentiation | 1 | 0 |  |  |  |  |  |  |  |  |
| GO:0043400\_cortisol\_secretion | 1 | 0 |  |  |  |  |  |  |  |  |
| GO:0043415\_positive\_regulation\_of\_skeletal\_muscle\_regeneration | 1 | 0 |  |  |  |  |  |  |  |  |
| GO:0043416\_regulation\_of\_skeletal\_muscle\_regeneration | 1 | 0 |  |  |  |  |  |  |  |  |
| GO:0043437\_butanoic\_acid\_metabolic\_process | 1 | 0 |  |  |  |  |  |  |  |  |
| GO:0043438\_acetoacetic\_acid\_metabolic\_process | 1 | 0 |  |  |  |  |  |  |  |  |
| GO:0043480\_pigment\_accumulation\_in\_tissues | 1 | 0 |  |  |  |  |  |  |  |  |
| GO:0043482\_cellular\_pigment\_accumulation | 1 | 0 |  |  |  |  |  |  |  |  |
| GO:0043486\_histone\_exchange | 1 | 0 |  |  |  |  |  |  |  |  |
| GO:0043496\_regulation\_of\_protein\_homodimerization\_activity | 1 | 0 |  |  |  |  |  |  |  |  |
| GO:0043501\_skeletal\_muscle\_adaptation | 1 | 0 |  |  |  |  |  |  |  |  |
| GO:0043508\_negative\_regulation\_of\_JUN\_kinase\_activity | 1 | 0 |  |  |  |  |  |  |  |  |
| GO:0043517\_positive\_regulation\_of\_DNA\_damage\_response\_\_signal\_transduction\_by\_p53\_class\_mediator | 1 | 0 |  |  |  |  |  |  |  |  |
| GO:0043535\_regulation\_of\_blood\_vessel\_endothelial\_cell\_migration | 1 | 0 |  |  |  |  |  |  |  |  |
| GO:0043537\_negative\_regulation\_of\_blood\_vessel\_endothelial\_cell\_migration | 1 | 0 |  |  |  |  |  |  |  |  |
| GO:0043545\_molybdopterin\_cofactor\_metabolic\_process | 1 | 0 |  |  |  |  |  |  |  |  |
| GO:0043587\_tongue\_morphogenesis | 1 | 0 |  |  |  |  |  |  |  |  |
| GO:0043604\_amide\_biosynthetic\_process | 1 | 0 |  |  |  |  |  |  |  |  |
| GO:0043628\_ncRNA\_3'-end\_processing | 1 | 0 |  |  |  |  |  |  |  |  |
| GO:0044254\_multicellular\_organismal\_protein\_catabolic\_process | 1 | 0 |  |  |  |  |  |  |  |  |
| GO:0044256\_protein\_digestion | 1 | 0 |  |  |  |  |  |  |  |  |
| GO:0044266\_multicellular\_organismal\_macromolecule\_catabolic\_process | 1 | 0 |  |  |  |  |  |  |  |  |
| GO:0045004\_DNA\_replication\_proofreading | 1 | 0 |  |  |  |  |  |  |  |  |
| GO:0045019\_negative\_regulation\_of\_nitric\_oxide\_biosynthetic\_process | 1 | 0 |  |  |  |  |  |  |  |  |
| GO:0045020\_error-prone\_DNA\_repair | 1 | 0 |  |  |  |  |  |  |  |  |
| GO:0045022\_early\_endosome\_to\_late\_endosome\_transport | 1 | 0 |  |  |  |  |  |  |  |  |
| GO:0045062\_extrathymic\_T\_cell\_selection | 1 | 0 |  |  |  |  |  |  |  |  |
| GO:0045069\_regulation\_of\_viral\_genome\_replication | 1 | 0 |  |  |  |  |  |  |  |  |
| GO:0045074\_regulation\_of\_interleukin-10\_biosynthetic\_process | 1 | 0 |  |  |  |  |  |  |  |  |
| GO:0045082\_positive\_regulation\_of\_interleukin-10\_biosynthetic\_process | 1 | 0 |  |  |  |  |  |  |  |  |
| GO:0045083\_negative\_regulation\_of\_interleukin-12\_biosynthetic\_process | 1 | 0 |  |  |  |  |  |  |  |  |
| GO:0045112\_integrin\_biosynthetic\_process | 1 | 0 |  |  |  |  |  |  |  |  |
| GO:0045113\_regulation\_of\_integrin\_biosynthetic\_process | 1 | 0 |  |  |  |  |  |  |  |  |
| GO:0045188\_regulation\_of\_circadian\_sleep\_wake\_cycle\_\_non-REM\_sleep | 1 | 0 |  |  |  |  |  |  |  |  |
| GO:0045210\_FasL\_biosynthetic\_process | 1 | 0 |  |  |  |  |  |  |  |  |
| GO:0045297\_post-mating\_behavior | 1 | 0 |  |  |  |  |  |  |  |  |
| GO:0045299\_otolith\_mineralization | 1 | 0 |  |  |  |  |  |  |  |  |
| GO:0045329\_carnitine\_biosynthetic\_process | 1 | 0 |  |  |  |  |  |  |  |  |
| GO:0045341\_MHC\_class\_I\_biosynthetic\_process | 1 | 0 |  |  |  |  |  |  |  |  |
| GO:0045343\_regulation\_of\_MHC\_class\_I\_biosynthetic\_process | 1 | 0 |  |  |  |  |  |  |  |  |
| GO:0045347\_negative\_regulation\_of\_MHC\_class\_II\_biosynthetic\_process | 1 | 0 |  |  |  |  |  |  |  |  |
| GO:0045405\_regulation\_of\_interleukin-5\_biosynthetic\_process | 1 | 0 |  |  |  |  |  |  |  |  |
| GO:0045407\_positive\_regulation\_of\_interleukin-5\_biosynthetic\_process | 1 | 0 |  |  |  |  |  |  |  |  |
| GO:0045426\_quinone\_cofactor\_biosynthetic\_process | 1 | 0 |  |  |  |  |  |  |  |  |
| GO:0045448\_mitotic\_cell\_cycle\_\_embryonic | 1 | 0 |  |  |  |  |  |  |  |  |
| GO:0045454\_cell\_redox\_homeostasis | 1 | 0 |  |  |  |  |  |  |  |  |
| GO:0045583\_regulation\_of\_cytotoxic\_T\_cell\_differentiation | 1 | 0 |  |  |  |  |  |  |  |  |
| GO:0045585\_positive\_regulation\_of\_cytotoxic\_T\_cell\_differentiation | 1 | 0 |  |  |  |  |  |  |  |  |
| GO:0045601\_regulation\_of\_endothelial\_cell\_differentiation | 1 | 0 |  |  |  |  |  |  |  |  |
| GO:0045602\_negative\_regulation\_of\_endothelial\_cell\_differentiation | 1 | 0 |  |  |  |  |  |  |  |  |
| GO:0045605\_negative\_regulation\_of\_epidermal\_cell\_differentiation | 1 | 0 |  |  |  |  |  |  |  |  |
| GO:0045606\_positive\_regulation\_of\_epidermal\_cell\_differentiation | 1 | 0 |  |  |  |  |  |  |  |  |
| GO:0045609\_positive\_regulation\_of\_auditory\_receptor\_cell\_differentiation | 1 | 0 |  |  |  |  |  |  |  |  |
| GO:0045617\_negative\_regulation\_of\_keratinocyte\_differentiation | 1 | 0 |  |  |  |  |  |  |  |  |
| GO:0045618\_positive\_regulation\_of\_keratinocyte\_differentiation | 1 | 0 |  |  |  |  |  |  |  |  |
| GO:0045626\_negative\_regulation\_of\_T-helper\_1\_cell\_differentiation | 1 | 0 |  |  |  |  |  |  |  |  |
| GO:0045633\_positive\_regulation\_of\_mechanoreceptor\_differentiation | 1 | 0 |  |  |  |  |  |  |  |  |
| GO:0045650\_negative\_regulation\_of\_macrophage\_differentiation | 1 | 0 |  |  |  |  |  |  |  |  |
| GO:0045656\_negative\_regulation\_of\_monocyte\_differentiation | 1 | 0 |  |  |  |  |  |  |  |  |
| GO:0045657\_positive\_regulation\_of\_monocyte\_differentiation | 1 | 0 |  |  |  |  |  |  |  |  |
| GO:0045659\_negative\_regulation\_of\_neutrophil\_differentiation | 1 | 0 |  |  |  |  |  |  |  |  |
| GO:0045660\_positive\_regulation\_of\_neutrophil\_differentiation | 1 | 0 |  |  |  |  |  |  |  |  |
| GO:0045721\_negative\_regulation\_of\_gluconeogenesis | 1 | 0 |  |  |  |  |  |  |  |  |
| GO:0045724\_positive\_regulation\_of\_flagellum\_assembly | 1 | 0 |  |  |  |  |  |  |  |  |
| GO:0045725\_positive\_regulation\_of\_glycogen\_biosynthetic\_process | 1 | 0 |  |  |  |  |  |  |  |  |
| GO:0045740\_positive\_regulation\_of\_DNA\_replication | 1 | 0 |  |  |  |  |  |  |  |  |
| GO:0045759\_negative\_regulation\_of\_action\_potential | 1 | 0 |  |  |  |  |  |  |  |  |
| GO:0045768\_positive\_regulation\_of\_anti-apoptosis | 1 | 0 |  |  |  |  |  |  |  |  |
| GO:0045769\_negative\_regulation\_of\_asymmetric\_cell\_division | 1 | 0 |  |  |  |  |  |  |  |  |
| GO:0045794\_negative\_regulation\_of\_cell\_volume | 1 | 0 |  |  |  |  |  |  |  |  |
| GO:0045815\_positive\_regulation\_of\_gene\_expression\_\_epigenetic | 1 | 0 |  |  |  |  |  |  |  |  |
| GO:0045818\_negative\_regulation\_of\_glycogen\_catabolic\_process | 1 | 0 |  |  |  |  |  |  |  |  |
| GO:0045842\_positive\_regulation\_of\_mitotic\_metaphase\_anaphase\_transition | 1 | 0 |  |  |  |  |  |  |  |  |
| GO:0045875\_negative\_regulation\_of\_sister\_chromatid\_cohesion | 1 | 0 |  |  |  |  |  |  |  |  |
| GO:0045898\_regulation\_of\_transcriptional\_preinitiation\_complex\_assembly | 1 | 0 |  |  |  |  |  |  |  |  |
| GO:0045899\_positive\_regulation\_of\_transcriptional\_preinitiation\_complex\_assembly | 1 | 0 |  |  |  |  |  |  |  |  |
| GO:0045906\_negative\_regulation\_of\_vasoconstriction | 1 | 0 |  |  |  |  |  |  |  |  |
| GO:0045908\_negative\_regulation\_of\_vasodilation | 1 | 0 |  |  |  |  |  |  |  |  |
| GO:0045909\_positive\_regulation\_of\_vasodilation | 1 | 0 |  |  |  |  |  |  |  |  |
| GO:0045915\_positive\_regulation\_of\_catecholamine\_metabolic\_process | 1 | 0 |  |  |  |  |  |  |  |  |
| GO:0045920\_negative\_regulation\_of\_exocytosis | 1 | 0 |  |  |  |  |  |  |  |  |
| GO:0045924\_regulation\_of\_female\_receptivity | 1 | 0 |  |  |  |  |  |  |  |  |
| GO:0045947\_negative\_regulation\_of\_translational\_initiation | 1 | 0 |  |  |  |  |  |  |  |  |
| GO:0045955\_negative\_regulation\_of\_calcium\_ion-dependent\_exocytosis | 1 | 0 |  |  |  |  |  |  |  |  |
| GO:0045956\_positive\_regulation\_of\_calcium\_ion-dependent\_exocytosis | 1 | 0 |  |  |  |  |  |  |  |  |
| GO:0045964\_positive\_regulation\_of\_dopamine\_metabolic\_process | 1 | 0 |  |  |  |  |  |  |  |  |
| GO:0045988\_negative\_regulation\_of\_striated\_muscle\_contraction | 1 | 0 |  |  |  |  |  |  |  |  |
| GO:0045989\_positive\_regulation\_of\_striated\_muscle\_contraction | 1 | 0 |  |  |  |  |  |  |  |  |
| GO:0045990\_regulation\_of\_transcription\_by\_carbon\_catabolites | 1 | 0 |  |  |  |  |  |  |  |  |
| GO:0045991\_positive\_regulation\_of\_transcription\_by\_carbon\_catabolites | 1 | 0 |  |  |  |  |  |  |  |  |
| GO:0045994\_positive\_regulation\_of\_translational\_initiation\_by\_iron | 1 | 0 |  |  |  |  |  |  |  |  |
| GO:0046007\_negative\_regulation\_of\_activated\_T\_cell\_proliferation | 1 | 0 |  |  |  |  |  |  |  |  |
| GO:0046014\_negative\_regulation\_of\_T\_cell\_homeostatic\_proliferation | 1 | 0 |  |  |  |  |  |  |  |  |
| GO:0046015\_regulation\_of\_transcription\_by\_glucose | 1 | 0 |  |  |  |  |  |  |  |  |
| GO:0046016\_positive\_regulation\_of\_transcription\_by\_glucose | 1 | 0 |  |  |  |  |  |  |  |  |
| GO:0046031\_ADP\_metabolic\_process | 1 | 0 |  |  |  |  |  |  |  |  |
| GO:0046032\_ADP\_catabolic\_process | 1 | 0 |  |  |  |  |  |  |  |  |
| GO:0046061\_dATP\_catabolic\_process | 1 | 0 |  |  |  |  |  |  |  |  |
| GO:0046075\_dTTP\_metabolic\_process | 1 | 0 |  |  |  |  |  |  |  |  |
| GO:0046078\_dUMP\_metabolic\_process | 1 | 0 |  |  |  |  |  |  |  |  |
| GO:0046079\_dUMP\_catabolic\_process | 1 | 0 |  |  |  |  |  |  |  |  |
| GO:0046086\_adenosine\_biosynthetic\_process | 1 | 0 |  |  |  |  |  |  |  |  |
| GO:0046090\_deoxyadenosine\_metabolic\_process | 1 | 0 |  |  |  |  |  |  |  |  |
| GO:0046098\_guanine\_metabolic\_process | 1 | 0 |  |  |  |  |  |  |  |  |
| GO:0046101\_hypoxanthine\_biosynthetic\_process | 1 | 0 |  |  |  |  |  |  |  |  |
| GO:0046102\_inosine\_metabolic\_process | 1 | 0 |  |  |  |  |  |  |  |  |
| GO:0046103\_inosine\_biosynthetic\_process | 1 | 0 |  |  |  |  |  |  |  |  |
| GO:0046108\_uridine\_metabolic\_process | 1 | 0 |  |  |  |  |  |  |  |  |
| GO:0046110\_xanthine\_metabolic\_process | 1 | 0 |  |  |  |  |  |  |  |  |
| GO:0046111\_xanthine\_biosynthetic\_process | 1 | 0 |  |  |  |  |  |  |  |  |
| GO:0046112\_nucleobase\_biosynthetic\_process | 1 | 0 |  |  |  |  |  |  |  |  |
| GO:0046113\_nucleobase\_catabolic\_process | 1 | 0 |  |  |  |  |  |  |  |  |
| GO:0046121\_deoxyribonucleoside\_catabolic\_process | 1 | 0 |  |  |  |  |  |  |  |  |
| GO:0046122\_purine\_deoxyribonucleoside\_metabolic\_process | 1 | 0 |  |  |  |  |  |  |  |  |
| GO:0046124\_purine\_deoxyribonucleoside\_catabolic\_process | 1 | 0 |  |  |  |  |  |  |  |  |
| GO:0046125\_pyrimidine\_deoxyribonucleoside\_metabolic\_process | 1 | 0 |  |  |  |  |  |  |  |  |
| GO:0046131\_pyrimidine\_ribonucleoside\_metabolic\_process | 1 | 0 |  |  |  |  |  |  |  |  |
| GO:0046160\_heme\_a\_metabolic\_process | 1 | 0 |  |  |  |  |  |  |  |  |
| GO:0046218\_indolalkylamine\_catabolic\_process | 1 | 0 |  |  |  |  |  |  |  |  |
| GO:0046292\_formaldehyde\_metabolic\_process | 1 | 0 |  |  |  |  |  |  |  |  |
| GO:0046294\_formaldehyde\_catabolic\_process | 1 | 0 |  |  |  |  |  |  |  |  |
| GO:0046314\_phosphocreatine\_biosynthetic\_process | 1 | 0 |  |  |  |  |  |  |  |  |
| GO:0046327\_glycerol\_biosynthetic\_process\_from\_pyruvate | 1 | 0 |  |  |  |  |  |  |  |  |
| GO:0046329\_negative\_regulation\_of\_JNK\_cascade | 1 | 0 |  |  |  |  |  |  |  |  |
| GO:0046340\_diacylglycerol\_catabolic\_process | 1 | 0 |  |  |  |  |  |  |  |  |
| GO:0046351\_disaccharide\_biosynthetic\_process | 1 | 0 |  |  |  |  |  |  |  |  |
| GO:0046356\_acetyl-CoA\_catabolic\_process | 1 | 0 |  |  |  |  |  |  |  |  |
| GO:0046358\_butyrate\_biosynthetic\_process | 1 | 0 |  |  |  |  |  |  |  |  |
| GO:0046359\_butyrate\_catabolic\_process | 1 | 0 |  |  |  |  |  |  |  |  |
| GO:0046381\_CMP-N-acetylneuraminate\_metabolic\_process | 1 | 0 |  |  |  |  |  |  |  |  |
| GO:0046415\_urate\_metabolic\_process | 1 | 0 |  |  |  |  |  |  |  |  |
| GO:0046416\_D-amino\_acid\_metabolic\_process | 1 | 0 |  |  |  |  |  |  |  |  |
| GO:0046434\_organophosphate\_catabolic\_process | 1 | 0 |  |  |  |  |  |  |  |  |
| GO:0046437\_D-amino\_acid\_biosynthetic\_process | 1 | 0 |  |  |  |  |  |  |  |  |
| GO:0046440\_L-lysine\_metabolic\_process | 1 | 0 |  |  |  |  |  |  |  |  |
| GO:0046449\_creatinine\_metabolic\_process | 1 | 0 |  |  |  |  |  |  |  |  |
| GO:0046471\_phosphatidylglycerol\_metabolic\_process | 1 | 0 |  |  |  |  |  |  |  |  |
| GO:0046473\_phosphatidic\_acid\_metabolic\_process | 1 | 0 |  |  |  |  |  |  |  |  |
| GO:0046476\_glycosylceramide\_biosynthetic\_process | 1 | 0 |  |  |  |  |  |  |  |  |
| GO:0046477\_glycosylceramide\_catabolic\_process | 1 | 0 |  |  |  |  |  |  |  |  |
| GO:0046485\_ether\_lipid\_metabolic\_process | 1 | 0 |  |  |  |  |  |  |  |  |
| GO:0046487\_glyoxylate\_metabolic\_process | 1 | 0 |  |  |  |  |  |  |  |  |
| GO:0046498\_S-adenosylhomocysteine\_metabolic\_process | 1 | 0 |  |  |  |  |  |  |  |  |
| GO:0046552\_photoreceptor\_cell\_fate\_commitment | 1 | 0 |  |  |  |  |  |  |  |  |
| GO:0046586\_regulation\_of\_calcium-dependent\_cell-cell\_adhesion | 1 | 0 |  |  |  |  |  |  |  |  |
| GO:0046587\_positive\_regulation\_of\_calcium-dependent\_cell-cell\_adhesion | 1 | 0 |  |  |  |  |  |  |  |  |
| GO:0046602\_regulation\_of\_mitotic\_centrosome\_separation | 1 | 0 |  |  |  |  |  |  |  |  |
| GO:0046604\_positive\_regulation\_of\_mitotic\_centrosome\_separation | 1 | 0 |  |  |  |  |  |  |  |  |
| GO:0046607\_positive\_regulation\_of\_centrosome\_cycle | 1 | 0 |  |  |  |  |  |  |  |  |
| GO:0046655\_folic\_acid\_metabolic\_process | 1 | 0 |  |  |  |  |  |  |  |  |
| GO:0046671\_negative\_regulation\_of\_retinal\_cell\_programmed\_cell\_death | 1 | 0 |  |  |  |  |  |  |  |  |
| GO:0046685\_response\_to\_arsenic | 1 | 0 |  |  |  |  |  |  |  |  |
| GO:0046692\_sperm\_competition | 1 | 0 |  |  |  |  |  |  |  |  |
| GO:0046707\_IDP\_metabolic\_process | 1 | 0 |  |  |  |  |  |  |  |  |
| GO:0046709\_IDP\_catabolic\_process | 1 | 0 |  |  |  |  |  |  |  |  |
| GO:0046724\_oxalic\_acid\_secretion | 1 | 0 |  |  |  |  |  |  |  |  |
| GO:0046753\_non-lytic\_viral\_release | 1 | 0 |  |  |  |  |  |  |  |  |
| GO:0046755\_non-lytic\_virus\_budding | 1 | 0 |  |  |  |  |  |  |  |  |
| GO:0046826\_negative\_regulation\_of\_protein\_export\_from\_nucleus | 1 | 0 |  |  |  |  |  |  |  |  |
| GO:0046827\_positive\_regulation\_of\_protein\_export\_from\_nucleus | 1 | 0 |  |  |  |  |  |  |  |  |
| GO:0046831\_regulation\_of\_RNA\_export\_from\_nucleus | 1 | 0 |  |  |  |  |  |  |  |  |
| GO:0046834\_lipid\_phosphorylation | 1 | 0 |  |  |  |  |  |  |  |  |
| GO:0046853\_inositol\_and\_derivative\_phosphorylation | 1 | 0 |  |  |  |  |  |  |  |  |
| GO:0046864\_isoprenoid\_transport | 1 | 0 |  |  |  |  |  |  |  |  |
| GO:0046865\_terpenoid\_transport | 1 | 0 |  |  |  |  |  |  |  |  |
| GO:0046877\_regulation\_of\_saliva\_secretion | 1 | 0 |  |  |  |  |  |  |  |  |
| GO:0046878\_positive\_regulation\_of\_saliva\_secretion | 1 | 0 |  |  |  |  |  |  |  |  |
| GO:0046884\_follicle-stimulating\_hormone\_secretion | 1 | 0 |  |  |  |  |  |  |  |  |
| GO:0046898\_response\_to\_cycloheximide | 1 | 0 |  |  |  |  |  |  |  |  |
| GO:0046929\_negative\_regulation\_of\_neurotransmitter\_secretion | 1 | 0 |  |  |  |  |  |  |  |  |
| GO:0046931\_pore\_complex\_biogenesis | 1 | 0 |  |  |  |  |  |  |  |  |
| GO:0046949\_acyl-CoA\_biosynthetic\_process | 1 | 0 |  |  |  |  |  |  |  |  |
| GO:0046958\_nonassociative\_learning | 1 | 0 |  |  |  |  |  |  |  |  |
| GO:0046960\_sensitization | 1 | 0 |  |  |  |  |  |  |  |  |
| GO:0046986\_negative\_regulation\_of\_hemoglobin\_biosynthetic\_process | 1 | 0 |  |  |  |  |  |  |  |  |
| GO:0047497\_mitochondrion\_transport\_along\_microtubule | 1 | 0 |  |  |  |  |  |  |  |  |
| GO:0048047\_mating\_behavior\_\_sex\_discrimination | 1 | 0 |  |  |  |  |  |  |  |  |
| GO:0048133\_male\_germ-line\_stem\_cell\_division | 1 | 0 |  |  |  |  |  |  |  |  |
| GO:0048137\_spermatocyte\_division | 1 | 0 |  |  |  |  |  |  |  |  |
| GO:0048143\_astrocyte\_activation | 1 | 0 |  |  |  |  |  |  |  |  |
| GO:0048170\_positive\_regulation\_of\_long-term\_neuronal\_synaptic\_plasticity | 1 | 0 |  |  |  |  |  |  |  |  |
| GO:0048199\_vesicle\_targeting\_\_to\_\_from\_or\_within\_Golgi | 1 | 0 |  |  |  |  |  |  |  |  |
| GO:0048241\_epinephrine\_transport | 1 | 0 |  |  |  |  |  |  |  |  |
| GO:0048242\_epinephrine\_secretion | 1 | 0 |  |  |  |  |  |  |  |  |
| GO:0048243\_norepinephrine\_secretion | 1 | 0 |  |  |  |  |  |  |  |  |
| GO:0048247\_lymphocyte\_chemotaxis | 1 | 0 |  |  |  |  |  |  |  |  |
| GO:0048250\_mitochondrial\_iron\_ion\_transport | 1 | 0 |  |  |  |  |  |  |  |  |
| GO:0048259\_regulation\_of\_receptor-mediated\_endocytosis | 1 | 0 |  |  |  |  |  |  |  |  |
| GO:0048260\_positive\_regulation\_of\_receptor-mediated\_endocytosis | 1 | 0 |  |  |  |  |  |  |  |  |
| GO:0048290\_isotype\_switching\_to\_IgA\_isotypes | 1 | 0 |  |  |  |  |  |  |  |  |
| GO:0048296\_regulation\_of\_isotype\_switching\_to\_IgA\_isotypes | 1 | 0 |  |  |  |  |  |  |  |  |
| GO:0048298\_positive\_regulation\_of\_isotype\_switching\_to\_IgA\_isotypes | 1 | 0 |  |  |  |  |  |  |  |  |
| GO:0048319\_axial\_mesoderm\_morphogenesis | 1 | 0 |  |  |  |  |  |  |  |  |
| GO:0048320\_axial\_mesoderm\_formation | 1 | 0 |  |  |  |  |  |  |  |  |
| GO:0048385\_regulation\_of\_retinoic\_acid\_receptor\_signaling\_pathway | 1 | 0 |  |  |  |  |  |  |  |  |
| GO:0048387\_negative\_regulation\_of\_retinoic\_acid\_receptor\_signaling\_pathway | 1 | 0 |  |  |  |  |  |  |  |  |
| GO:0048388\_endosomal\_lumen\_acidification | 1 | 0 |  |  |  |  |  |  |  |  |
| GO:0048389\_intermediate\_mesoderm\_development | 1 | 0 |  |  |  |  |  |  |  |  |
| GO:0048478\_replication\_fork\_protection | 1 | 0 |  |  |  |  |  |  |  |  |
| GO:0048496\_maintenance\_of\_organ\_identity | 1 | 0 |  |  |  |  |  |  |  |  |
| GO:0048525\_negative\_regulation\_of\_viral\_reproduction | 1 | 0 |  |  |  |  |  |  |  |  |
| GO:0048539\_bone\_marrow\_development | 1 | 0 |  |  |  |  |  |  |  |  |
| GO:0048548\_regulation\_of\_pinocytosis | 1 | 0 |  |  |  |  |  |  |  |  |
| GO:0048549\_positive\_regulation\_of\_pinocytosis | 1 | 0 |  |  |  |  |  |  |  |  |
| GO:0048553\_negative\_regulation\_of\_metalloenzyme\_activity | 1 | 0 |  |  |  |  |  |  |  |  |
| GO:0048588\_developmental\_cell\_growth | 1 | 0 |  |  |  |  |  |  |  |  |
| GO:0048601\_oocyte\_morphogenesis | 1 | 0 |  |  |  |  |  |  |  |  |
| GO:0048621\_post-embryonic\_gut\_morphogenesis | 1 | 0 |  |  |  |  |  |  |  |  |
| GO:0048640\_negative\_regulation\_of\_developmental\_growth | 1 | 0 |  |  |  |  |  |  |  |  |
| GO:0048642\_negative\_regulation\_of\_skeletal\_muscle\_tissue\_development | 1 | 0 |  |  |  |  |  |  |  |  |
| GO:0048669\_collateral\_sprouting\_in\_the\_absence\_of\_injury | 1 | 0 |  |  |  |  |  |  |  |  |
| GO:0048680\_positive\_regulation\_of\_axon\_regeneration | 1 | 0 |  |  |  |  |  |  |  |  |
| GO:0048681\_negative\_regulation\_of\_axon\_regeneration | 1 | 0 |  |  |  |  |  |  |  |  |
| GO:0048686\_regulation\_of\_sprouting\_of\_injured\_axon | 1 | 0 |  |  |  |  |  |  |  |  |
| GO:0048687\_positive\_regulation\_of\_sprouting\_of\_injured\_axon | 1 | 0 |  |  |  |  |  |  |  |  |
| GO:0048690\_regulation\_of\_axon\_extension\_involved\_in\_regeneration | 1 | 0 |  |  |  |  |  |  |  |  |
| GO:0048691\_positive\_regulation\_of\_axon\_extension\_involved\_in\_regeneration | 1 | 0 |  |  |  |  |  |  |  |  |
| GO:0048714\_positive\_regulation\_of\_oligodendrocyte\_differentiation | 1 | 0 |  |  |  |  |  |  |  |  |
| GO:0048733\_sebaceous\_gland\_development | 1 | 0 |  |  |  |  |  |  |  |  |
| GO:0048743\_positive\_regulation\_of\_skeletal\_muscle\_fiber\_development | 1 | 0 |  |  |  |  |  |  |  |  |
| GO:0048752\_semicircular\_canal\_morphogenesis | 1 | 0 |  |  |  |  |  |  |  |  |
| GO:0048773\_erythrophore\_differentiation | 1 | 0 |  |  |  |  |  |  |  |  |
| GO:0048790\_maintenance\_of\_presynaptic\_active\_zone\_structure | 1 | 0 |  |  |  |  |  |  |  |  |
| GO:0048791\_calcium\_ion-dependent\_exocytosis\_of\_neurotransmitter | 1 | 0 |  |  |  |  |  |  |  |  |
| GO:0048822\_enucleate\_erythrocyte\_development | 1 | 0 |  |  |  |  |  |  |  |  |
| GO:0048866\_stem\_cell\_fate\_specification | 1 | 0 |  |  |  |  |  |  |  |  |
| GO:0048936\_peripheral\_nervous\_system\_neuron\_axonogenesis | 1 | 0 |  |  |  |  |  |  |  |  |
| GO:0050427\_3'-phosphoadenosine\_5'-phosphosulfate\_metabolic\_process | 1 | 0 |  |  |  |  |  |  |  |  |
| GO:0050428\_3'-phosphoadenosine\_5'-phosphosulfate\_biosynthetic\_process | 1 | 0 |  |  |  |  |  |  |  |  |
| GO:0050482\_arachidonic\_acid\_secretion | 1 | 0 |  |  |  |  |  |  |  |  |
| GO:0050667\_homocysteine\_metabolic\_process | 1 | 0 |  |  |  |  |  |  |  |  |
| GO:0050674\_urothelial\_cell\_proliferation | 1 | 0 |  |  |  |  |  |  |  |  |
| GO:0050675\_regulation\_of\_urothelial\_cell\_proliferation | 1 | 0 |  |  |  |  |  |  |  |  |
| GO:0050677\_positive\_regulation\_of\_urothelial\_cell\_proliferation | 1 | 0 |  |  |  |  |  |  |  |  |
| GO:0050691\_regulation\_of\_defense\_response\_to\_virus\_by\_host | 1 | 0 |  |  |  |  |  |  |  |  |
| GO:0050748\_negative\_regulation\_of\_lipoprotein\_metabolic\_process | 1 | 0 |  |  |  |  |  |  |  |  |
| GO:0050757\_thymidylate\_synthase\_biosynthetic\_process | 1 | 0 |  |  |  |  |  |  |  |  |
| GO:0050758\_regulation\_of\_thymidylate\_synthase\_biosynthetic\_process | 1 | 0 |  |  |  |  |  |  |  |  |
| GO:0050760\_negative\_regulation\_of\_thymidylate\_synthase\_biosynthetic\_process | 1 | 0 |  |  |  |  |  |  |  |  |
| GO:0050812\_regulation\_of\_acyl-CoA\_biosynthetic\_process | 1 | 0 |  |  |  |  |  |  |  |  |
| GO:0050832\_defense\_response\_to\_fungus | 1 | 0 |  |  |  |  |  |  |  |  |
| GO:0050861\_positive\_regulation\_of\_B\_cell\_receptor\_signaling\_pathway | 1 | 0 |  |  |  |  |  |  |  |  |
| GO:0050862\_positive\_regulation\_of\_T\_cell\_receptor\_signaling\_pathway | 1 | 0 |  |  |  |  |  |  |  |  |
| GO:0050916\_sensory\_perception\_of\_sweet\_taste | 1 | 0 |  |  |  |  |  |  |  |  |
| GO:0050975\_sensory\_perception\_of\_touch | 1 | 0 |  |  |  |  |  |  |  |  |
| GO:0050995\_negative\_regulation\_of\_lipid\_catabolic\_process | 1 | 0 |  |  |  |  |  |  |  |  |
| GO:0051001\_negative\_regulation\_of\_nitric-oxide\_synthase\_activity | 1 | 0 |  |  |  |  |  |  |  |  |
| GO:0051005\_negative\_regulation\_of\_lipoprotein\_lipase\_activity | 1 | 0 |  |  |  |  |  |  |  |  |
| GO:0051006\_positive\_regulation\_of\_lipoprotein\_lipase\_activity | 1 | 0 |  |  |  |  |  |  |  |  |
| GO:0051016\_barbed-end\_actin\_filament\_capping | 1 | 0 |  |  |  |  |  |  |  |  |
| GO:0051029\_rRNA\_transport | 1 | 0 |  |  |  |  |  |  |  |  |
| GO:0051043\_regulation\_of\_membrane\_protein\_ectodomain\_proteolysis | 1 | 0 |  |  |  |  |  |  |  |  |
| GO:0051044\_positive\_regulation\_of\_membrane\_protein\_ectodomain\_proteolysis | 1 | 0 |  |  |  |  |  |  |  |  |
| GO:0051088\_PMA-inducible\_membrane\_protein\_ectodomain\_proteolysis | 1 | 0 |  |  |  |  |  |  |  |  |
| GO:0051102\_DNA\_ligation\_during\_DNA\_recombination | 1 | 0 |  |  |  |  |  |  |  |  |
| GO:0051103\_DNA\_ligation\_during\_DNA\_repair | 1 | 0 |  |  |  |  |  |  |  |  |
| GO:0051123\_transcriptional\_preinitiation\_complex\_assembly | 1 | 0 |  |  |  |  |  |  |  |  |
| GO:0051125\_regulation\_of\_actin\_nucleation | 1 | 0 |  |  |  |  |  |  |  |  |
| GO:0051127\_positive\_regulation\_of\_actin\_nucleation | 1 | 0 |  |  |  |  |  |  |  |  |
| GO:0051151\_negative\_regulation\_of\_smooth\_muscle\_cell\_differentiation | 1 | 0 |  |  |  |  |  |  |  |  |
| GO:0051154\_negative\_regulation\_of\_striated\_muscle\_cell\_differentiation | 1 | 0 |  |  |  |  |  |  |  |  |
| GO:0051155\_positive\_regulation\_of\_striated\_muscle\_cell\_differentiation | 1 | 0 |  |  |  |  |  |  |  |  |
| GO:0051156\_glucose\_6-phosphate\_metabolic\_process | 1 | 0 |  |  |  |  |  |  |  |  |
| GO:0051187\_cofactor\_catabolic\_process | 1 | 0 |  |  |  |  |  |  |  |  |
| GO:0051189\_prosthetic\_group\_metabolic\_process | 1 | 0 |  |  |  |  |  |  |  |  |
| GO:0051193\_regulation\_of\_cofactor\_metabolic\_process | 1 | 0 |  |  |  |  |  |  |  |  |
| GO:0051196\_regulation\_of\_coenzyme\_metabolic\_process | 1 | 0 |  |  |  |  |  |  |  |  |
| GO:0051255\_spindle\_midzone\_assembly | 1 | 0 |  |  |  |  |  |  |  |  |
| GO:0051257\_spindle\_midzone\_assembly\_involved\_in\_meiosis | 1 | 0 |  |  |  |  |  |  |  |  |
| GO:0051281\_positive\_regulation\_of\_release\_of\_sequestered\_calcium\_ion\_into\_cytosol | 1 | 0 |  |  |  |  |  |  |  |  |
| GO:0051290\_protein\_heterotetramerization | 1 | 0 |  |  |  |  |  |  |  |  |
| GO:0051305\_chromosome\_movement\_towards\_spindle\_pole | 1 | 0 |  |  |  |  |  |  |  |  |
| GO:0051310\_metaphase\_plate\_congression | 1 | 0 |  |  |  |  |  |  |  |  |
| GO:0051311\_meiotic\_metaphase\_plate\_congression | 1 | 0 |  |  |  |  |  |  |  |  |
| GO:0051340\_regulation\_of\_ligase\_activity | 1 | 0 |  |  |  |  |  |  |  |  |
| GO:0051351\_positive\_regulation\_of\_ligase\_activity | 1 | 0 |  |  |  |  |  |  |  |  |
| GO:0051354\_negative\_regulation\_of\_oxidoreductase\_activity | 1 | 0 |  |  |  |  |  |  |  |  |
| GO:0051355\_proprioception\_during\_equilibrioception | 1 | 0 |  |  |  |  |  |  |  |  |
| GO:0051383\_kinetochore\_organization | 1 | 0 |  |  |  |  |  |  |  |  |
| GO:0051386\_regulation\_of\_nerve\_growth\_factor\_receptor\_signaling\_pathway | 1 | 0 |  |  |  |  |  |  |  |  |
| GO:0051409\_response\_to\_nitrosative\_stress | 1 | 0 |  |  |  |  |  |  |  |  |
| GO:0051457\_maintenance\_of\_protein\_location\_in\_nucleus | 1 | 0 |  |  |  |  |  |  |  |  |
| GO:0051462\_regulation\_of\_cortisol\_secretion | 1 | 0 |  |  |  |  |  |  |  |  |
| GO:0051463\_negative\_regulation\_of\_cortisol\_secretion | 1 | 0 |  |  |  |  |  |  |  |  |
| GO:0051481\_reduction\_of\_cytosolic\_calcium\_ion\_concentration | 1 | 0 |  |  |  |  |  |  |  |  |
| GO:0051482\_elevation\_of\_cytosolic\_calcium\_ion\_concentration\_during\_G-protein\_signaling\_\_coupled\_to\_IP3\_second\_messenger\_(phospholipase\_C\_activating) | 1 | 0 |  |  |  |  |  |  |  |  |
| GO:0051542\_elastin\_biosynthetic\_process | 1 | 0 |  |  |  |  |  |  |  |  |
| GO:0051568\_histone\_H3-K4\_methylation | 1 | 0 |  |  |  |  |  |  |  |  |
| GO:0051569\_regulation\_of\_histone\_H3-K4\_methylation | 1 | 0 |  |  |  |  |  |  |  |  |
| GO:0051570\_regulation\_of\_histone\_H3-K9\_methylation | 1 | 0 |  |  |  |  |  |  |  |  |
| GO:0051573\_negative\_regulation\_of\_histone\_H3-K9\_methylation | 1 | 0 |  |  |  |  |  |  |  |  |
| GO:0051580\_regulation\_of\_neurotransmitter\_uptake | 1 | 0 |  |  |  |  |  |  |  |  |
| GO:0051582\_positive\_regulation\_of\_neurotransmitter\_uptake | 1 | 0 |  |  |  |  |  |  |  |  |
| GO:0051584\_regulation\_of\_dopamine\_uptake | 1 | 0 |  |  |  |  |  |  |  |  |
| GO:0051586\_positive\_regulation\_of\_dopamine\_uptake | 1 | 0 |  |  |  |  |  |  |  |  |
| GO:0051589\_negative\_regulation\_of\_neurotransmitter\_transport | 1 | 0 |  |  |  |  |  |  |  |  |
| GO:0051593\_response\_to\_folic\_acid | 1 | 0 |  |  |  |  |  |  |  |  |
| GO:0051615\_histamine\_uptake | 1 | 0 |  |  |  |  |  |  |  |  |
| GO:0051646\_mitochondrion\_localization | 1 | 0 |  |  |  |  |  |  |  |  |
| GO:0051654\_establishment\_of\_mitochondrion\_localization | 1 | 0 |  |  |  |  |  |  |  |  |
| GO:0051661\_maintenance\_of\_centrosome\_location | 1 | 0 |  |  |  |  |  |  |  |  |
| GO:0051665\_membrane\_raft\_localization | 1 | 0 |  |  |  |  |  |  |  |  |
| GO:0051685\_maintenance\_of\_ER\_location | 1 | 0 |  |  |  |  |  |  |  |  |
| GO:0051693\_actin\_filament\_capping | 1 | 0 |  |  |  |  |  |  |  |  |
| GO:0051701\_interaction\_with\_host | 1 | 0 |  |  |  |  |  |  |  |  |
| GO:0051754\_meiotic\_sister\_chromatid\_cohesion\_\_centromeric | 1 | 0 |  |  |  |  |  |  |  |  |
| GO:0051782\_negative\_regulation\_of\_cell\_division | 1 | 0 |  |  |  |  |  |  |  |  |
| GO:0051790\_short-chain\_fatty\_acid\_biosynthetic\_process | 1 | 0 |  |  |  |  |  |  |  |  |
| GO:0051799\_negative\_regulation\_of\_hair\_follicle\_development | 1 | 0 |  |  |  |  |  |  |  |  |
| GO:0051823\_regulation\_of\_synapse\_structural\_plasticity | 1 | 0 |  |  |  |  |  |  |  |  |
| GO:0051865\_protein\_autoubiquitination | 1 | 0 |  |  |  |  |  |  |  |  |
| GO:0051901\_positive\_regulation\_of\_mitochondrial\_depolarization | 1 | 0 |  |  |  |  |  |  |  |  |
| GO:0051917\_regulation\_of\_fibrinolysis | 1 | 0 |  |  |  |  |  |  |  |  |
| GO:0051918\_negative\_regulation\_of\_fibrinolysis | 1 | 0 |  |  |  |  |  |  |  |  |
| GO:0051929\_positive\_regulation\_of\_calcium\_ion\_transport\_via\_voltage-gated\_calcium\_channel\_activity | 1 | 0 |  |  |  |  |  |  |  |  |
| GO:0051933\_amino\_acid\_uptake\_during\_transmission\_of\_nerve\_impulse | 1 | 0 |  |  |  |  |  |  |  |  |
| GO:0051935\_glutamate\_uptake\_during\_transmission\_of\_nerve\_impulse | 1 | 0 |  |  |  |  |  |  |  |  |
| GO:0051940\_regulation\_of\_catecholamine\_uptake\_during\_transmission\_of\_nerve\_impulse | 1 | 0 |  |  |  |  |  |  |  |  |
| GO:0051944\_positive\_regulation\_of\_catecholamine\_uptake\_during\_transmission\_of\_nerve\_impulse | 1 | 0 |  |  |  |  |  |  |  |  |
| GO:0051961\_negative\_regulation\_of\_nervous\_system\_development | 1 | 0 |  |  |  |  |  |  |  |  |
| GO:0051964\_negative\_regulation\_of\_synaptogenesis | 1 | 0 |  |  |  |  |  |  |  |  |
| GO:0051968\_positive\_regulation\_of\_synaptic\_transmission\_\_glutamatergic | 1 | 0 |  |  |  |  |  |  |  |  |
| GO:0051984\_positive\_regulation\_of\_chromosome\_segregation | 1 | 0 |  |  |  |  |  |  |  |  |
| GO:0051987\_positive\_regulation\_of\_attachment\_of\_spindle\_microtubules\_to\_kinetochore | 1 | 0 |  |  |  |  |  |  |  |  |
| GO:0052173\_response\_to\_defenses\_of\_other\_organism\_during\_symbiotic\_interaction | 1 | 0 |  |  |  |  |  |  |  |  |
| GO:0052200\_response\_to\_host\_defenses | 1 | 0 |  |  |  |  |  |  |  |  |
| GO:0052551\_response\_to\_defense-related\_nitric\_oxide\_production\_by\_other\_organism\_during\_symbiotic\_interaction | 1 | 0 |  |  |  |  |  |  |  |  |
| GO:0052564\_response\_to\_immune\_response\_of\_other\_organism\_during\_symbiotic\_interaction | 1 | 0 |  |  |  |  |  |  |  |  |
| GO:0052565\_response\_to\_defense-related\_host\_nitric\_oxide\_production | 1 | 0 |  |  |  |  |  |  |  |  |
| GO:0052572\_response\_to\_host\_immune\_response | 1 | 0 |  |  |  |  |  |  |  |  |
| GO:0055005\_ventricular\_cardiac\_myofibril\_development | 1 | 0 |  |  |  |  |  |  |  |  |
| GO:0055011\_atrial\_cardiac\_muscle\_cell\_differentiation | 1 | 0 |  |  |  |  |  |  |  |  |
| GO:0055014\_atrial\_cardiac\_muscle\_cell\_development | 1 | 0 |  |  |  |  |  |  |  |  |
| GO:0055078\_sodium\_ion\_homeostasis | 1 | 0 |  |  |  |  |  |  |  |  |
| GO:0055089\_fatty\_acid\_homeostasis | 1 | 0 |  |  |  |  |  |  |  |  |
| GO:0055093\_response\_to\_hyperoxia | 1 | 0 |  |  |  |  |  |  |  |  |
| GO:0060003\_copper\_ion\_export | 1 | 0 |  |  |  |  |  |  |  |  |
| GO:0060005\_vestibular\_reflex | 1 | 0 |  |  |  |  |  |  |  |  |
| GO:0060014\_granulosa\_cell\_differentiation | 1 | 0 |  |  |  |  |  |  |  |  |
| GO:0060018\_astrocyte\_fate\_commitment | 1 | 0 |  |  |  |  |  |  |  |  |
| GO:0060020\_Bergmann\_glial\_cell\_differentiation | 1 | 0 |  |  |  |  |  |  |  |  |
| GO:0060022\_hard\_palate\_development | 1 | 0 |  |  |  |  |  |  |  |  |
| GO:0060034\_notochord\_cell\_differentiation | 1 | 0 |  |  |  |  |  |  |  |  |
| GO:0060035\_notochord\_cell\_development | 1 | 0 |  |  |  |  |  |  |  |  |
| GO:0060046\_regulation\_of\_acrosome\_reaction | 1 | 0 |  |  |  |  |  |  |  |  |
| GO:0060054\_positive\_regulation\_of\_epithelial\_cell\_proliferation\_involved\_in\_wound\_healing | 1 | 0 |  |  |  |  |  |  |  |  |
| GO:0060059\_embryonic\_retina\_morphogenesis\_in\_camera-type\_eye | 1 | 0 |  |  |  |  |  |  |  |  |
| GO:0060061\_Spemann\_organizer\_formation | 1 | 0 |  |  |  |  |  |  |  |  |
| GO:0060064\_Spemann\_organizer\_formation\_at\_the\_anterior\_end\_of\_the\_primitive\_streak | 1 | 0 |  |  |  |  |  |  |  |  |
| GO:0060071\_Wnt\_receptor\_signaling\_pathway\_\_planar\_cell\_polarity\_pathway | 1 | 0 |  |  |  |  |  |  |  |  |
| GO:0060075\_regulation\_of\_resting\_membrane\_potential | 1 | 0 |  |  |  |  |  |  |  |  |
| GO:0060082\_eye\_blink\_reflex | 1 | 0 |  |  |  |  |  |  |  |  |
| GO:0060112\_generation\_of\_ovulation\_cycle\_rhythm | 1 | 0 |  |  |  |  |  |  |  |  |
| GO:0060125\_negative\_regulation\_of\_growth\_hormone\_secretion | 1 | 0 |  |  |  |  |  |  |  |  |
| GO:0060151\_peroxisome\_localization | 1 | 0 |  |  |  |  |  |  |  |  |
| GO:0060152\_microtubule-based\_peroxisome\_localization | 1 | 0 |  |  |  |  |  |  |  |  |
| GO:0060161\_positive\_regulation\_of\_dopamine\_receptor\_signaling\_pathway | 1 | 0 |  |  |  |  |  |  |  |  |
| GO:0060163\_subpallium\_neuron\_fate\_commitment | 1 | 0 |  |  |  |  |  |  |  |  |
| GO:0060165\_regulation\_of\_timing\_of\_subpallium\_neuron\_differentiation | 1 | 0 |  |  |  |  |  |  |  |  |
| GO:0060174\_limb\_bud\_formation | 1 | 0 |  |  |  |  |  |  |  |  |
| GO:0060177\_regulation\_of\_angiotensin\_metabolic\_process | 1 | 0 |  |  |  |  |  |  |  |  |
| GO:0060197\_cloacal\_septation | 1 | 0 |  |  |  |  |  |  |  |  |
| GO:0060215\_primitive\_hemopoiesis | 1 | 0 |  |  |  |  |  |  |  |  |
| GO:0060231\_mesenchymal\_to\_epithelial\_transition | 1 | 0 |  |  |  |  |  |  |  |  |
| GO:0060254\_regulation\_of\_N-terminal\_protein\_palmitoylation | 1 | 0 |  |  |  |  |  |  |  |  |
| GO:0060261\_positive\_regulation\_of\_transcription\_initiation\_from\_RNA\_polymerase\_II\_promoter | 1 | 0 |  |  |  |  |  |  |  |  |
| GO:0060262\_negative\_regulation\_of\_N-terminal\_protein\_palmitoylation | 1 | 0 |  |  |  |  |  |  |  |  |
| GO:0060263\_regulation\_of\_respiratory\_burst | 1 | 0 |  |  |  |  |  |  |  |  |
| GO:0060264\_regulation\_of\_respiratory\_burst\_during\_acute\_inflammatory\_response | 1 | 0 |  |  |  |  |  |  |  |  |
| GO:0060265\_positive\_regulation\_of\_respiratory\_burst\_during\_acute\_inflammatory\_response | 1 | 0 |  |  |  |  |  |  |  |  |
| GO:0060267\_positive\_regulation\_of\_respiratory\_burst | 1 | 0 |  |  |  |  |  |  |  |  |
| GO:0060272\_embryonic\_skeletal\_joint\_morphogenesis | 1 | 0 |  |  |  |  |  |  |  |  |
| GO:0060297\_regulation\_of\_sarcomere\_organization | 1 | 0 |  |  |  |  |  |  |  |  |
| GO:0060298\_positive\_regulation\_of\_sarcomere\_organization | 1 | 0 |  |  |  |  |  |  |  |  |
| GO:0060315\_negative\_regulation\_of\_ryanodine-sensitive\_calcium-release\_channel\_activity | 1 | 0 |  |  |  |  |  |  |  |  |
| GO:0060319\_primitive\_erythrocyte\_differentiation | 1 | 0 |  |  |  |  |  |  |  |  |
| GO:0060371\_regulation\_of\_atrial\_cardiomyocyte\_membrane\_depolarization | 1 | 0 |  |  |  |  |  |  |  |  |
| GO:0060374\_mast\_cell\_differentiation | 1 | 0 |  |  |  |  |  |  |  |  |
| GO:0060375\_regulation\_of\_mast\_cell\_differentiation | 1 | 0 |  |  |  |  |  |  |  |  |
| GO:0060376\_positive\_regulation\_of\_mast\_cell\_differentiation | 1 | 0 |  |  |  |  |  |  |  |  |
| GO:0060390\_regulation\_of\_SMAD\_protein\_nuclear\_translocation | 1 | 0 |  |  |  |  |  |  |  |  |
| GO:0060391\_positive\_regulation\_of\_SMAD\_protein\_nuclear\_translocation | 1 | 0 |  |  |  |  |  |  |  |  |
| GO:0060398\_regulation\_of\_growth\_hormone\_receptor\_signaling\_pathway | 1 | 0 |  |  |  |  |  |  |  |  |
| GO:0060399\_positive\_regulation\_of\_growth\_hormone\_receptor\_signaling\_pathway | 1 | 0 |  |  |  |  |  |  |  |  |
| GO:0060405\_regulation\_of\_penile\_erection | 1 | 0 |  |  |  |  |  |  |  |  |
| GO:0060407\_negative\_regulation\_of\_penile\_erection | 1 | 0 |  |  |  |  |  |  |  |  |
| GO:0060413\_atrial\_septum\_morphogenesis | 1 | 0 |  |  |  |  |  |  |  |  |
| GO:0060414\_aorta\_smooth\_muscle\_tissue\_morphogenesis | 1 | 0 |  |  |  |  |  |  |  |  |
| GO:0060419\_heart\_growth | 1 | 0 |  |  |  |  |  |  |  |  |
| GO:0060420\_regulation\_of\_heart\_growth | 1 | 0 |  |  |  |  |  |  |  |  |
| GO:0060421\_positive\_regulation\_of\_heart\_growth | 1 | 0 |  |  |  |  |  |  |  |  |
| GO:0060431\_primary\_lung\_bud\_formation | 1 | 0 |  |  |  |  |  |  |  |  |
| GO:0060436\_bronchiole\_morphogenesis | 1 | 0 |  |  |  |  |  |  |  |  |
| GO:0060440\_trachea\_formation | 1 | 0 |  |  |  |  |  |  |  |  |
| GO:0060449\_bud\_elongation\_involved\_in\_lung\_branching | 1 | 0 |  |  |  |  |  |  |  |  |
| GO:0060456\_positive\_regulation\_of\_digestive\_system\_process | 1 | 0 |  |  |  |  |  |  |  |  |
| GO:0060461\_right\_lung\_morphogenesis | 1 | 0 |  |  |  |  |  |  |  |  |
| GO:0060481\_lobar\_bronchus\_epithelium\_development | 1 | 0 |  |  |  |  |  |  |  |  |
| GO:0060482\_lobar\_bronchus\_development | 1 | 0 |  |  |  |  |  |  |  |  |
| GO:0060484\_lung-associated\_mesenchyme\_development | 1 | 0 |  |  |  |  |  |  |  |  |
| GO:0060486\_Clara\_cell\_differentiation | 1 | 0 |  |  |  |  |  |  |  |  |
| GO:0060510\_Type\_II\_pneumocyte\_differentiation | 1 | 0 |  |  |  |  |  |  |  |  |
| GO:0060514\_prostate\_induction | 1 | 0 |  |  |  |  |  |  |  |  |
| GO:0060515\_prostate\_field\_specification | 1 | 0 |  |  |  |  |  |  |  |  |
| GO:0060517\_epithelial\_cell\_proliferation\_involved\_in\_prostatic\_bud\_elongation | 1 | 0 |  |  |  |  |  |  |  |  |
| GO:0060520\_activation\_of\_prostate\_induction\_by\_androgen\_receptor\_signaling\_pathway | 1 | 0 |  |  |  |  |  |  |  |  |
| GO:0060535\_trachea\_cartilage\_morphogenesis | 1 | 0 |  |  |  |  |  |  |  |  |
| GO:0060536\_cartilage\_morphogenesis | 1 | 0 |  |  |  |  |  |  |  |  |
| GO:0060563\_neuroepithelial\_cell\_differentiation | 1 | 0 |  |  |  |  |  |  |  |  |
| GO:0060577\_pulmonary\_vein\_morphogenesis | 1 | 0 |  |  |  |  |  |  |  |  |
| GO:0060578\_superior\_vena\_cava\_morphogenesis | 1 | 0 |  |  |  |  |  |  |  |  |
| GO:0060584\_regulation\_of\_prostaglandin-endoperoxide\_synthase\_activity | 1 | 0 |  |  |  |  |  |  |  |  |
| GO:0060585\_positive\_regulation\_of\_prostaglandin-endoperoxidase\_synthase\_activity | 1 | 0 |  |  |  |  |  |  |  |  |
| GO:0060598\_dichotomous\_subdivision\_of\_terminal\_units\_involved\_in\_mammary\_gland\_duct\_morphogenesis | 1 | 0 |  |  |  |  |  |  |  |  |
| GO:0060611\_mammary\_gland\_fat\_development | 1 | 0 |  |  |  |  |  |  |  |  |
| GO:0060618\_nipple\_development | 1 | 0 |  |  |  |  |  |  |  |  |
| GO:0060631\_regulation\_of\_meiosis\_I | 1 | 0 |  |  |  |  |  |  |  |  |
| GO:0060649\_mammary\_gland\_bud\_elongation | 1 | 0 |  |  |  |  |  |  |  |  |
| GO:0060658\_nipple\_morphogenesis | 1 | 0 |  |  |  |  |  |  |  |  |
| GO:0060659\_nipple\_sheath\_formation | 1 | 0 |  |  |  |  |  |  |  |  |
| GO:0060668\_regulation\_of\_branching\_involved\_in\_salivary\_gland\_morphogenesis\_by\_extracellular\_matrix-epithelial\_cell\_signaling | 1 | 0 |  |  |  |  |  |  |  |  |
| GO:0060683\_regulation\_of\_branching\_involved\_in\_salivary\_gland\_morphogenesis\_by\_epithelial-mesenchymal\_signaling | 1 | 0 |  |  |  |  |  |  |  |  |
| GO:0060691\_epithelial\_cell\_maturation\_involved\_in\_salivary\_gland\_development | 1 | 0 |  |  |  |  |  |  |  |  |
| GO:0060709\_glycogen\_cell\_development\_involved\_in\_embryonic\_placenta\_development | 1 | 0 |  |  |  |  |  |  |  |  |
| GO:0060732\_positive\_regulation\_of\_inositol\_phosphate\_biosynthetic\_process | 1 | 0 |  |  |  |  |  |  |  |  |
| GO:0060739\_mesenchymal-epithelial\_cell\_signaling\_involved\_in\_prostate\_gland\_development | 1 | 0 |  |  |  |  |  |  |  |  |
| GO:0060781\_mesenchymal\_cell\_proliferation\_involved\_in\_prostate\_gland\_development | 1 | 0 |  |  |  |  |  |  |  |  |
| GO:0060782\_regulation\_of\_mesenchymal\_cell\_proliferation\_involved\_in\_prostate\_gland\_development | 1 | 0 |  |  |  |  |  |  |  |  |
| GO:0060783\_mesenchymal\_smoothened\_signaling\_pathway\_involved\_in\_prostate\_gland\_development | 1 | 0 |  |  |  |  |  |  |  |  |
| GO:0060872\_semicircular\_canal\_development | 1 | 0 |  |  |  |  |  |  |  |  |
| GO:0060896\_neural\_plate\_pattern\_specification | 1 | 0 |  |  |  |  |  |  |  |  |
| GO:0070091\_glucagon\_secretion | 1 | 0 |  |  |  |  |  |  |  |  |
| GO:0070162\_adiponectin\_secretion | 1 | 0 |  |  |  |  |  |  |  |  |
| GO:0070163\_regulation\_of\_adiponectin\_secretion | 1 | 0 |  |  |  |  |  |  |  |  |
| GO:0070164\_negative\_regulation\_of\_adiponectin\_secretion | 1 | 0 |  |  |  |  |  |  |  |  |
| GO:0070178\_D-serine\_metabolic\_process | 1 | 0 |  |  |  |  |  |  |  |  |
| GO:0070179\_D-serine\_biosynthetic\_process | 1 | 0 |  |  |  |  |  |  |  |  |
| GO:0070296\_sarcoplasmic\_reticulum\_calcium\_ion\_transport | 1 | 0 |  |  |  |  |  |  |  |  |
| GO:0070303\_negative\_regulation\_of\_stress-activated\_protein\_kinase\_signaling\_pathway | 1 | 0 |  |  |  |  |  |  |  |  |
| GO:0070328\_triglyceride\_homeostasis | 1 | 0 |  |  |  |  |  |  |  |  |
| GO:0070365\_hepatocyte\_differentiation | 1 | 0 |  |  |  |  |  |  |  |  |
| GO:0070384\_Harderian\_gland\_development | 1 | 0 |  |  |  |  |  |  |  |  |
| GO:0070391\_response\_to\_lipoteichoic\_acid | 1 | 0 |  |  |  |  |  |  |  |  |
| GO:0070424\_regulation\_of\_nucleotide-binding\_oligomerization\_domain\_containing\_signaling\_pathway | 1 | 0 |  |  |  |  |  |  |  |  |
| GO:0070426\_positive\_regulation\_of\_nucleotide-binding\_oligomerization\_domain\_containing\_signaling\_pathway | 1 | 0 |  |  |  |  |  |  |  |  |
| GO:0070428\_regulation\_of\_nucleotide-binding\_oligomerization\_domain\_containing\_1\_signaling\_pathway | 1 | 0 |  |  |  |  |  |  |  |  |
| GO:0070430\_positive\_regulation\_of\_nucleotide-binding\_oligomerization\_domain\_containing\_1\_signaling\_pathway | 1 | 0 |  |  |  |  |  |  |  |  |
| GO:0070432\_regulation\_of\_nucleotide-binding\_oligomerization\_domain\_containing\_2\_signaling\_pathway | 1 | 0 |  |  |  |  |  |  |  |  |
| GO:0070434\_positive\_regulation\_of\_nucleotide-binding\_oligomerization\_domain\_containing\_2\_signaling\_pathway | 1 | 0 |  |  |  |  |  |  |  |  |
| GO:0070493\_thrombin\_receptor\_signaling\_pathway | 1 | 0 |  |  |  |  |  |  |  |  |
| GO:0070508\_cholesterol\_import | 1 | 0 |  |  |  |  |  |  |  |  |
| GO:0070527\_platelet\_aggregation | 1 | 0 |  |  |  |  |  |  |  |  |
| GO:0070528\_protein\_kinase\_C\_signaling\_cascade | 1 | 0 |  |  |  |  |  |  |  |  |
| GO:0070555\_response\_to\_interleukin-1 | 1 | 0 |  |  |  |  |  |  |  |  |
| GO:0070560\_protein\_secretion\_by\_platelet | 1 | 0 |  |  |  |  |  |  |  |  |
| GO:0070561\_vitamin\_D\_receptor\_signaling\_pathway | 1 | 0 |  |  |  |  |  |  |  |  |
| GO:0070562\_regulation\_of\_vitamin\_D\_receptor\_signaling\_pathway | 1 | 0 |  |  |  |  |  |  |  |  |
| GO:0070571\_negative\_regulation\_of\_neuron\_projection\_regeneration | 1 | 0 |  |  |  |  |  |  |  |  |
| GO:0070572\_positive\_regulation\_of\_neuron\_projection\_regeneration | 1 | 0 |  |  |  |  |  |  |  |  |
| GO:0070613\_regulation\_of\_protein\_processing | 1 | 0 |  |  |  |  |  |  |  |  |
| GO:0070627\_ferrous\_iron\_import | 1 | 0 |  |  |  |  |  |  |  |  |
| GO:0070669\_response\_to\_interleukin-2 | 1 | 0 |  |  |  |  |  |  |  |  |
| GO:0070670\_response\_to\_interleukin-4 | 1 | 0 |  |  |  |  |  |  |  |  |
| GO:0070671\_response\_to\_interleukin-12 | 1 | 0 |  |  |  |  |  |  |  |  |
| GO:0070672\_response\_to\_interleukin-15 | 1 | 0 |  |  |  |  |  |  |  |  |
| GO:0070673\_response\_to\_interleukin-18 | 1 | 0 |  |  |  |  |  |  |  |  |
| GO:0070828\_heterochromatin\_organization | 1 | 0 |  |  |  |  |  |  |  |  |
| GO:0070874\_negative\_regulation\_of\_glycogen\_metabolic\_process | 1 | 0 |  |  |  |  |  |  |  |  |
| GO:0075136\_response\_to\_host | 1 | 0 |  |  |  |  |  |  |  |  |
| GO:0080010\_regulation\_of\_oxygen\_and\_reactive\_oxygen\_species\_metabolic\_process | 1 | 0 |  |  |  |  |  |  |  |  |
| GO:0090032\_negative\_regulation\_of\_steroid\_hormone\_biosynthetic\_process | 1 | 0 |  |  |  |  |  |  |  |  |
| GO:0001649\_osteoblast\_differentiation | 38 | 0 | 0.000000 | -0.000000 | 1448 | 1479.206998 | 1542.28 | 1605.353002 | 1.065110 |
| GO:0001657\_ureteric\_bud\_development | 38 | 0 | 0.000000 | -0.000000 | 1448 | 1479.206998 | 1542.28 | 1605.353002 | 1.065110 |
| GO:0002695\_negative\_regulation\_of\_leukocyte\_activation | 38 | 0 | 0.000000 | -0.000000 | 1448 | 1479.206998 | 1542.28 | 1605.353002 | 1.065110 |
| GO:0006820\_anion\_transport | 38 | 0 | 0.000000 | -0.000000 | 1448 | 1479.206998 | 1542.28 | 1605.353002 | 1.065110 |
| GO:0007596\_blood\_coagulation | 38 | 0 | 0.000000 | -0.000000 | 1448 | 1479.206998 | 1542.28 | 1605.353002 | 1.065110 |
| GO:0008016\_regulation\_of\_heart\_contraction | 38 | 0 | 0.000000 | -0.000000 | 1448 | 1479.206998 | 1542.28 | 1605.353002 | 1.065110 |
| GO:0016042\_lipid\_catabolic\_process | 38 | 0 | 0.000000 | -0.000000 | 1448 | 1479.206998 | 1542.28 | 1605.353002 | 1.065110 |
| GO:0016053\_organic\_acid\_biosynthetic\_process | 38 | 0 | 0.000000 | -0.000000 | 1448 | 1479.206998 | 1542.28 | 1605.353002 | 1.065110 |
| GO:0031401\_positive\_regulation\_of\_protein\_modification\_process | 38 | 0 | 0.000000 | -0.000000 | 1448 | 1479.206998 | 1542.28 | 1605.353002 | 1.065110 |
| GO:0032259\_methylation | 38 | 0 | 0.000000 | -0.000000 | 1448 | 1479.206998 | 1542.28 | 1605.353002 | 1.065110 |
| GO:0042493\_response\_to\_drug | 38 | 0 | 0.000000 | -0.000000 | 1448 | 1479.206998 | 1542.28 | 1605.353002 | 1.065110 |
| GO:0043414\_biopolymer\_methylation | 38 | 0 | 0.000000 | -0.000000 | 1448 | 1479.206998 | 1542.28 | 1605.353002 | 1.065110 |
| GO:0045580\_regulation\_of\_T\_cell\_differentiation | 38 | 0 | 0.000000 | -0.000000 | 1448 | 1479.206998 | 1542.28 | 1605.353002 | 1.065110 |
| GO:0046394\_carboxylic\_acid\_biosynthetic\_process | 38 | 0 | 0.000000 | -0.000000 | 1448 | 1479.206998 | 1542.28 | 1605.353002 | 1.065110 |
| GO:0046777\_protein\_amino\_acid\_autophosphorylation | 38 | 0 | 0.000000 | -0.000000 | 1448 | 1479.206998 | 1542.28 | 1605.353002 | 1.065110 |
| GO:0050727\_regulation\_of\_inflammatory\_response | 38 | 0 | 0.000000 | -0.000000 | 1448 | 1479.206998 | 1542.28 | 1605.353002 | 1.065110 |
| GO:0050866\_negative\_regulation\_of\_cell\_activation | 38 | 0 | 0.000000 | -0.000000 | 1448 | 1479.206998 | 1542.28 | 1605.353002 | 1.065110 |
| GO:0048706\_embryonic\_skeletal\_system\_development | 73 | 0 | 0.000000 | -0.000000 | 1451 | 1482.413899 | 1544.86 | 1607.306101 | 1.064686 |
| GO:0051270\_regulation\_of\_cell\_motion | 73 | 0 | 0.000000 | -0.000000 | 1451 | 1482.413899 | 1544.86 | 1607.306101 | 1.064686 |
| GO:0051336\_regulation\_of\_hydrolase\_activity | 73 | 0 | 0.000000 | -0.000000 | 1451 | 1482.413899 | 1544.86 | 1607.306101 | 1.064686 |
| GO:0006897\_endocytosis | 86 | 0 | 0.000000 | -0.000000 | 1453 | 1485.005175 | 1547.21 | 1609.414825 | 1.064838 |
| GO:0010324\_membrane\_invagination | 86 | 0 | 0.000000 | -0.000000 | 1453 | 1485.005175 | 1547.21 | 1609.414825 | 1.064838 |
| GO:0006461\_protein\_complex\_assembly | 78 | 0 | 0.000000 | -0.000000 | 1460 | 1488.806480 | 1550.57 | 1612.333520 | 1.062034 |
| GO:0007519\_skeletal\_muscle\_tissue\_development | 78 | 0 | 0.000000 | -0.000000 | 1460 | 1488.806480 | 1550.57 | 1612.333520 | 1.062034 |
| GO:0030326\_embryonic\_limb\_morphogenesis | 78 | 0 | 0.000000 | -0.000000 | 1460 | 1488.806480 | 1550.57 | 1612.333520 | 1.062034 |
| GO:0035113\_embryonic\_appendage\_morphogenesis | 78 | 0 | 0.000000 | -0.000000 | 1460 | 1488.806480 | 1550.57 | 1612.333520 | 1.062034 |
| GO:0051251\_positive\_regulation\_of\_lymphocyte\_activation | 78 | 0 | 0.000000 | -0.000000 | 1460 | 1488.806480 | 1550.57 | 1612.333520 | 1.062034 |
| GO:0060538\_skeletal\_muscle\_organ\_development | 78 | 0 | 0.000000 | -0.000000 | 1460 | 1488.806480 | 1550.57 | 1612.333520 | 1.062034 |
| GO:0070271\_protein\_complex\_biogenesis | 78 | 0 | 0.000000 | -0.000000 | 1460 | 1488.806480 | 1550.57 | 1612.333520 | 1.062034 |
| GO:0003001\_generation\_of\_a\_signal\_involved\_in\_cell-cell\_signaling | 87 | 0 | 0.000000 | -0.000000 | 1463 | 1492.286643 | 1553.47 | 1614.653357 | 1.061839 |
| GO:0022612\_gland\_morphogenesis | 87 | 0 | 0.000000 | -0.000000 | 1463 | 1492.286643 | 1553.47 | 1614.653357 | 1.061839 |
| GO:0043583\_ear\_development | 87 | 0 | 0.000000 | -0.000000 | 1463 | 1492.286643 | 1553.47 | 1614.653357 | 1.061839 |
| GO:0001818\_negative\_regulation\_of\_cytokine\_production | 18 | 0 | 0.000000 | -0.000000 | 1509 | 1538.365039 | 1597.18 | 1655.994961 | 1.058436 |
| GO:0001825\_blastocyst\_formation | 18 | 0 | 0.000000 | -0.000000 | 1509 | 1538.365039 | 1597.18 | 1655.994961 | 1.058436 |
| GO:0001974\_blood\_vessel\_remodeling | 18 | 0 | 0.000000 | -0.000000 | 1509 | 1538.365039 | 1597.18 | 1655.994961 | 1.058436 |
| GO:0002064\_epithelial\_cell\_development | 18 | 0 | 0.000000 | -0.000000 | 1509 | 1538.365039 | 1597.18 | 1655.994961 | 1.058436 |
| GO:0002715\_regulation\_of\_natural\_killer\_cell\_mediated\_immunity | 18 | 0 | 0.000000 | -0.000000 | 1509 | 1538.365039 | 1597.18 | 1655.994961 | 1.058436 |
| GO:0003014\_renal\_system\_process | 18 | 0 | 0.000000 | -0.000000 | 1509 | 1538.365039 | 1597.18 | 1655.994961 | 1.058436 |
| GO:0006022\_aminoglycan\_metabolic\_process | 18 | 0 | 0.000000 | -0.000000 | 1509 | 1538.365039 | 1597.18 | 1655.994961 | 1.058436 |
| GO:0006457\_protein\_folding | 18 | 0 | 0.000000 | -0.000000 | 1509 | 1538.365039 | 1597.18 | 1655.994961 | 1.058436 |
| GO:0006940\_regulation\_of\_smooth\_muscle\_contraction | 18 | 0 | 0.000000 | -0.000000 | 1509 | 1538.365039 | 1597.18 | 1655.994961 | 1.058436 |
| GO:0007140\_male\_meiosis | 18 | 0 | 0.000000 | -0.000000 | 1509 | 1538.365039 | 1597.18 | 1655.994961 | 1.058436 |
| GO:0007608\_sensory\_perception\_of\_smell | 18 | 0 | 0.000000 | -0.000000 | 1509 | 1538.365039 | 1597.18 | 1655.994961 | 1.058436 |
| GO:0008589\_regulation\_of\_smoothened\_signaling\_pathway | 18 | 0 | 0.000000 | -0.000000 | 1509 | 1538.365039 | 1597.18 | 1655.994961 | 1.058436 |
| GO:0009063\_cellular\_amino\_acid\_catabolic\_process | 18 | 0 | 0.000000 | -0.000000 | 1509 | 1538.365039 | 1597.18 | 1655.994961 | 1.058436 |
| GO:0010498\_proteasomal\_protein\_catabolic\_process | 18 | 0 | 0.000000 | -0.000000 | 1509 | 1538.365039 | 1597.18 | 1655.994961 | 1.058436 |
| GO:0010553\_negative\_regulation\_of\_specific\_transcription\_from\_RNA\_polymerase\_II\_promoter | 18 | 0 | 0.000000 | -0.000000 | 1509 | 1538.365039 | 1597.18 | 1655.994961 | 1.058436 |
| GO:0015711\_organic\_anion\_transport | 18 | 0 | 0.000000 | -0.000000 | 1509 | 1538.365039 | 1597.18 | 1655.994961 | 1.058436 |
| GO:0021517\_ventral\_spinal\_cord\_development | 18 | 0 | 0.000000 | -0.000000 | 1509 | 1538.365039 | 1597.18 | 1655.994961 | 1.058436 |
| GO:0021885\_forebrain\_cell\_migration | 18 | 0 | 0.000000 | -0.000000 | 1509 | 1538.365039 | 1597.18 | 1655.994961 | 1.058436 |
| GO:0030178\_negative\_regulation\_of\_Wnt\_receptor\_signaling\_pathway | 18 | 0 | 0.000000 | -0.000000 | 1509 | 1538.365039 | 1597.18 | 1655.994961 | 1.058436 |
| GO:0030203\_glycosaminoglycan\_metabolic\_process | 18 | 0 | 0.000000 | -0.000000 | 1509 | 1538.365039 | 1597.18 | 1655.994961 | 1.058436 |
| GO:0030318\_melanocyte\_differentiation | 18 | 0 | 0.000000 | -0.000000 | 1509 | 1538.365039 | 1597.18 | 1655.994961 | 1.058436 |
| GO:0030336\_negative\_regulation\_of\_cell\_migration | 18 | 0 | 0.000000 | -0.000000 | 1509 | 1538.365039 | 1597.18 | 1655.994961 | 1.058436 |
| GO:0030901\_midbrain\_development | 18 | 0 | 0.000000 | -0.000000 | 1509 | 1538.365039 | 1597.18 | 1655.994961 | 1.058436 |
| GO:0032623\_interleukin-2\_production | 18 | 0 | 0.000000 | -0.000000 | 1509 | 1538.365039 | 1597.18 | 1655.994961 | 1.058436 |
| GO:0032984\_macromolecular\_complex\_disassembly | 18 | 0 | 0.000000 | -0.000000 | 1509 | 1538.365039 | 1597.18 | 1655.994961 | 1.058436 |
| GO:0033157\_regulation\_of\_intracellular\_protein\_transport | 18 | 0 | 0.000000 | -0.000000 | 1509 | 1538.365039 | 1597.18 | 1655.994961 | 1.058436 |
| GO:0042269\_regulation\_of\_natural\_killer\_cell\_mediated\_cytotoxicity | 18 | 0 | 0.000000 | -0.000000 | 1509 | 1538.365039 | 1597.18 | 1655.994961 | 1.058436 |
| GO:0043029\_T\_cell\_homeostasis | 18 | 0 | 0.000000 | -0.000000 | 1509 | 1538.365039 | 1597.18 | 1655.994961 | 1.058436 |
| GO:0043161\_proteasomal\_ubiquitin-dependent\_protein\_catabolic\_process | 18 | 0 | 0.000000 | -0.000000 | 1509 | 1538.365039 | 1597.18 | 1655.994961 | 1.058436 |
| GO:0044272\_sulfur\_compound\_biosynthetic\_process | 18 | 0 | 0.000000 | -0.000000 | 1509 | 1538.365039 | 1597.18 | 1655.994961 | 1.058436 |
| GO:0045058\_T\_cell\_selection | 18 | 0 | 0.000000 | -0.000000 | 1509 | 1538.365039 | 1597.18 | 1655.994961 | 1.058436 |
| GO:0045103\_intermediate\_filament-based\_process | 18 | 0 | 0.000000 | -0.000000 | 1509 | 1538.365039 | 1597.18 | 1655.994961 | 1.058436 |
| GO:0045638\_negative\_regulation\_of\_myeloid\_cell\_differentiation | 18 | 0 | 0.000000 | -0.000000 | 1509 | 1538.365039 | 1597.18 | 1655.994961 | 1.058436 |
| GO:0045807\_positive\_regulation\_of\_endocytosis | 18 | 0 | 0.000000 | -0.000000 | 1509 | 1538.365039 | 1597.18 | 1655.994961 | 1.058436 |
| GO:0046578\_regulation\_of\_Ras\_protein\_signal\_transduction | 18 | 0 | 0.000000 | -0.000000 | 1509 | 1538.365039 | 1597.18 | 1655.994961 | 1.058436 |
| GO:0046620\_regulation\_of\_organ\_growth | 18 | 0 | 0.000000 | -0.000000 | 1509 | 1538.365039 | 1597.18 | 1655.994961 | 1.058436 |
| GO:0048535\_lymph\_node\_development | 18 | 0 | 0.000000 | -0.000000 | 1509 | 1538.365039 | 1597.18 | 1655.994961 | 1.058436 |
| GO:0048730\_epidermis\_morphogenesis | 18 | 0 | 0.000000 | -0.000000 | 1509 | 1538.365039 | 1597.18 | 1655.994961 | 1.058436 |
| GO:0048813\_dendrite\_morphogenesis | 18 | 0 | 0.000000 | -0.000000 | 1509 | 1538.365039 | 1597.18 | 1655.994961 | 1.058436 |
| GO:0050731\_positive\_regulation\_of\_peptidyl-tyrosine\_phosphorylation | 18 | 0 | 0.000000 | -0.000000 | 1509 | 1538.365039 | 1597.18 | 1655.994961 | 1.058436 |
| GO:0050982\_detection\_of\_mechanical\_stimulus | 18 | 0 | 0.000000 | -0.000000 | 1509 | 1538.365039 | 1597.18 | 1655.994961 | 1.058436 |
| GO:0051222\_positive\_regulation\_of\_protein\_transport | 18 | 0 | 0.000000 | -0.000000 | 1509 | 1538.365039 | 1597.18 | 1655.994961 | 1.058436 |
| GO:0055008\_cardiac\_muscle\_tissue\_morphogenesis | 18 | 0 | 0.000000 | -0.000000 | 1509 | 1538.365039 | 1597.18 | 1655.994961 | 1.058436 |
| GO:0060415\_muscle\_tissue\_morphogenesis | 18 | 0 | 0.000000 | -0.000000 | 1509 | 1538.365039 | 1597.18 | 1655.994961 | 1.058436 |
| GO:0060571\_morphogenesis\_of\_an\_epithelial\_fold | 18 | 0 | 0.000000 | -0.000000 | 1509 | 1538.365039 | 1597.18 | 1655.994961 | 1.058436 |
| GO:0060674\_placenta\_blood\_vessel\_development | 18 | 0 | 0.000000 | -0.000000 | 1509 | 1538.365039 | 1597.18 | 1655.994961 | 1.058436 |
| GO:0006171\_cAMP\_biosynthetic\_process | 42 | 0 | 0.000000 | -0.000000 | 1524 | 1552.212218 | 1609.47 | 1666.727782 | 1.056083 |
| GO:0008361\_regulation\_of\_cell\_size | 42 | 0 | 0.000000 | -0.000000 | 1524 | 1552.212218 | 1609.47 | 1666.727782 | 1.056083 |
| GO:0010740\_positive\_regulation\_of\_protein\_kinase\_cascade | 42 | 0 | 0.000000 | -0.000000 | 1524 | 1552.212218 | 1609.47 | 1666.727782 | 1.056083 |
| GO:0015672\_monovalent\_inorganic\_cation\_transport | 42 | 0 | 0.000000 | -0.000000 | 1524 | 1552.212218 | 1609.47 | 1666.727782 | 1.056083 |
| GO:0019221\_cytokine-mediated\_signaling\_pathway | 42 | 0 | 0.000000 | -0.000000 | 1524 | 1552.212218 | 1609.47 | 1666.727782 | 1.056083 |
| GO:0019941\_modification-dependent\_protein\_catabolic\_process | 42 | 0 | 0.000000 | -0.000000 | 1524 | 1552.212218 | 1609.47 | 1666.727782 | 1.056083 |
| GO:0032946\_positive\_regulation\_of\_mononuclear\_cell\_proliferation | 42 | 0 | 0.000000 | -0.000000 | 1524 | 1552.212218 | 1609.47 | 1666.727782 | 1.056083 |
| GO:0043632\_modification-dependent\_macromolecule\_catabolic\_process | 42 | 0 | 0.000000 | -0.000000 | 1524 | 1552.212218 | 1609.47 | 1666.727782 | 1.056083 |
| GO:0045637\_regulation\_of\_myeloid\_cell\_differentiation | 42 | 0 | 0.000000 | -0.000000 | 1524 | 1552.212218 | 1609.47 | 1666.727782 | 1.056083 |
| GO:0048515\_spermatid\_differentiation | 42 | 0 | 0.000000 | -0.000000 | 1524 | 1552.212218 | 1609.47 | 1666.727782 | 1.056083 |
| GO:0050671\_positive\_regulation\_of\_lymphocyte\_proliferation | 42 | 0 | 0.000000 | -0.000000 | 1524 | 1552.212218 | 1609.47 | 1666.727782 | 1.056083 |
| GO:0050817\_coagulation | 42 | 0 | 0.000000 | -0.000000 | 1524 | 1552.212218 | 1609.47 | 1666.727782 | 1.056083 |
| GO:0051345\_positive\_regulation\_of\_hydrolase\_activity | 42 | 0 | 0.000000 | -0.000000 | 1524 | 1552.212218 | 1609.47 | 1666.727782 | 1.056083 |
| GO:0051603\_proteolysis\_involved\_in\_cellular\_protein\_catabolic\_process | 42 | 0 | 0.000000 | -0.000000 | 1524 | 1552.212218 | 1609.47 | 1666.727782 | 1.056083 |
| GO:0080135\_regulation\_of\_cellular\_response\_to\_stress | 42 | 0 | 0.000000 | -0.000000 | 1524 | 1552.212218 | 1609.47 | 1666.727782 | 1.056083 |
| GO:0000165\_MAPKKK\_cascade | 114 | 0 | 0.000000 | 0.000000 | 1600 | 1612.997717 | 1656.53 | 1700.062283 | 1.035331 |
| GO:0001525\_angiogenesis | 100 | 0 | 0.000000 | 0.000000 | 1600 | 1612.997717 | 1656.53 | 1700.062283 | 1.035331 |
| GO:0001816\_cytokine\_production | 122 | 0 | 0.000000 | 0.000000 | 1600 | 1612.997717 | 1656.53 | 1700.062283 | 1.035331 |
| GO:0001817\_regulation\_of\_cytokine\_production | 99 | 0 | 0.000000 | 0.000000 | 1600 | 1612.997717 | 1656.53 | 1700.062283 | 1.035331 |
| GO:0002009\_morphogenesis\_of\_an\_epithelium | 198 | 0 | 0.000000 | 0.000000 | 1600 | 1612.997717 | 1656.53 | 1700.062283 | 1.035331 |
| GO:0002252\_immune\_effector\_process | 122 | 0 | 0.000000 | 0.000000 | 1600 | 1612.997717 | 1656.53 | 1700.062283 | 1.035331 |
| GO:0002694\_regulation\_of\_leukocyte\_activation | 121 | 0 | 0.000000 | 0.000000 | 1600 | 1612.997717 | 1656.53 | 1700.062283 | 1.035331 |
| GO:0003002\_regionalization | 195 | 0 | 0.000000 | 0.000000 | 1600 | 1612.997717 | 1656.53 | 1700.062283 | 1.035331 |
| GO:0003013\_circulatory\_system\_process | 103 | 0 | 0.000000 | 0.000000 | 1600 | 1612.997717 | 1656.53 | 1700.062283 | 1.035331 |
| GO:0006082\_organic\_acid\_metabolic\_process | 182 | 0 | 0.000000 | 0.000000 | 1600 | 1612.997717 | 1656.53 | 1700.062283 | 1.035331 |
| GO:0006873\_cellular\_ion\_homeostasis | 176 | 0 | 0.000000 | 0.000000 | 1600 | 1612.997717 | 1656.53 | 1700.062283 | 1.035331 |
| GO:0006917\_induction\_of\_apoptosis | 121 | 0 | 0.000000 | 0.000000 | 1600 | 1612.997717 | 1656.53 | 1700.062283 | 1.035331 |
| GO:0006950\_response\_to\_stress | 549 | 0 | 0.000000 | 0.000000 | 1600 | 1612.997717 | 1656.53 | 1700.062283 | 1.035331 |
| GO:0006952\_defense\_response | 187 | 0 | 0.000000 | 0.000000 | 1600 | 1612.997717 | 1656.53 | 1700.062283 | 1.035331 |
| GO:0006954\_inflammatory\_response | 96 | 0 | 0.000000 | 0.000000 | 1600 | 1612.997717 | 1656.53 | 1700.062283 | 1.035331 |
| GO:0006974\_response\_to\_DNA\_damage\_stimulus | 113 | 0 | 0.000000 | 0.000000 | 1600 | 1612.997717 | 1656.53 | 1700.062283 | 1.035331 |
| GO:0007243\_protein\_kinase\_cascade | 205 | 0 | 0.000000 | 0.000000 | 1600 | 1612.997717 | 1656.53 | 1700.062283 | 1.035331 |
| GO:0007389\_pattern\_specification\_process | 250 | 0 | 0.000000 | 0.000000 | 1600 | 1612.997717 | 1656.53 | 1700.062283 | 1.035331 |
| GO:0007626\_locomotory\_behavior | 163 | 0 | 0.000000 | 0.000000 | 1600 | 1612.997717 | 1656.53 | 1700.062283 | 1.035331 |
| GO:0008015\_blood\_circulation | 103 | 0 | 0.000000 | 0.000000 | 1600 | 1612.997717 | 1656.53 | 1700.062283 | 1.035331 |
| GO:0008150\_biological\_process | 4605 | 48 | 1.000000 | 0.000000 | 1600 | 1612.997717 | 1656.53 | 1700.062283 | 1.035331 |
| GO:0009056\_catabolic\_process | 243 | 0 | 0.000000 | 0.000000 | 1600 | 1612.997717 | 1656.53 | 1700.062283 | 1.035331 |
| GO:0009057\_macromolecule\_catabolic\_process | 137 | 0 | 0.000000 | 0.000000 | 1600 | 1612.997717 | 1656.53 | 1700.062283 | 1.035331 |
| GO:0009611\_response\_to\_wounding | 172 | 0 | 0.000000 | 0.000000 | 1600 | 1612.997717 | 1656.53 | 1700.062283 | 1.035331 |
| GO:0009952\_anterior\_posterior\_pattern\_formation | 133 | 0 | 0.000000 | 0.000000 | 1600 | 1612.997717 | 1656.53 | 1700.062283 | 1.035331 |
| GO:0009967\_positive\_regulation\_of\_signal\_transduction | 98 | 0 | 0.000000 | 0.000000 | 1600 | 1612.997717 | 1656.53 | 1700.062283 | 1.035331 |
| GO:0010647\_positive\_regulation\_of\_cell\_communication | 110 | 0 | 0.000000 | 0.000000 | 1600 | 1612.997717 | 1656.53 | 1700.062283 | 1.035331 |
| GO:0010942\_positive\_regulation\_of\_cell\_death | 167 | 0 | 0.000000 | 0.000000 | 1600 | 1612.997717 | 1656.53 | 1700.062283 | 1.035331 |
| GO:0012502\_induction\_of\_programmed\_cell\_death | 121 | 0 | 0.000000 | 0.000000 | 1600 | 1612.997717 | 1656.53 | 1700.062283 | 1.035331 |
| GO:0014706\_striated\_muscle\_tissue\_development | 120 | 0 | 0.000000 | 0.000000 | 1600 | 1612.997717 | 1656.53 | 1700.062283 | 1.035331 |
| GO:0016192\_vesicle-mediated\_transport | 184 | 0 | 0.000000 | 0.000000 | 1600 | 1612.997717 | 1656.53 | 1700.062283 | 1.035331 |
| GO:0018193\_peptidyl-amino\_acid\_modification | 97 | 0 | 0.000000 | 0.000000 | 1600 | 1612.997717 | 1656.53 | 1700.062283 | 1.035331 |
| GO:0019725\_cellular\_homeostasis | 195 | 0 | 0.000000 | 0.000000 | 1600 | 1612.997717 | 1656.53 | 1700.062283 | 1.035331 |
| GO:0019752\_carboxylic\_acid\_metabolic\_process | 181 | 0 | 0.000000 | 0.000000 | 1600 | 1612.997717 | 1656.53 | 1700.062283 | 1.035331 |
| GO:0022403\_cell\_cycle\_phase | 119 | 0 | 0.000000 | 0.000000 | 1600 | 1612.997717 | 1656.53 | 1700.062283 | 1.035331 |
| GO:0030029\_actin\_filament-based\_process | 109 | 0 | 0.000000 | 0.000000 | 1600 | 1612.997717 | 1656.53 | 1700.062283 | 1.035331 |
| GO:0030036\_actin\_cytoskeleton\_organization | 102 | 0 | 0.000000 | 0.000000 | 1600 | 1612.997717 | 1656.53 | 1700.062283 | 1.035331 |
| GO:0030163\_protein\_catabolic\_process | 101 | 0 | 0.000000 | 0.000000 | 1600 | 1612.997717 | 1656.53 | 1700.062283 | 1.035331 |
| GO:0030900\_forebrain\_development | 146 | 0 | 0.000000 | 0.000000 | 1600 | 1612.997717 | 1656.53 | 1700.062283 | 1.035331 |
| GO:0032268\_regulation\_of\_cellular\_protein\_metabolic\_process | 152 | 0 | 0.000000 | 0.000000 | 1600 | 1612.997717 | 1656.53 | 1700.062283 | 1.035331 |
| GO:0032787\_monocarboxylic\_acid\_metabolic\_process | 130 | 0 | 0.000000 | 0.000000 | 1600 | 1612.997717 | 1656.53 | 1700.062283 | 1.035331 |
| GO:0032940\_secretion\_by\_cell | 149 | 0 | 0.000000 | 0.000000 | 1600 | 1612.997717 | 1656.53 | 1700.062283 | 1.035331 |
| GO:0032943\_mononuclear\_cell\_proliferation | 94 | 0 | 0.000000 | 0.000000 | 1600 | 1612.997717 | 1656.53 | 1700.062283 | 1.035331 |
| GO:0033554\_cellular\_response\_to\_stress | 196 | 0 | 0.000000 | 0.000000 | 1600 | 1612.997717 | 1656.53 | 1700.062283 | 1.035331 |
| GO:0034984\_cellular\_response\_to\_DNA\_damage\_stimulus | 94 | 0 | 0.000000 | 0.000000 | 1600 | 1612.997717 | 1656.53 | 1700.062283 | 1.035331 |
| GO:0040007\_growth | 217 | 0 | 0.000000 | 0.000000 | 1600 | 1612.997717 | 1656.53 | 1700.062283 | 1.035331 |
| GO:0040008\_regulation\_of\_growth | 113 | 0 | 0.000000 | 0.000000 | 1600 | 1612.997717 | 1656.53 | 1700.062283 | 1.035331 |
| GO:0042180\_cellular\_ketone\_metabolic\_process | 183 | 0 | 0.000000 | 0.000000 | 1600 | 1612.997717 | 1656.53 | 1700.062283 | 1.035331 |
| GO:0042391\_regulation\_of\_membrane\_potential | 95 | 0 | 0.000000 | 0.000000 | 1600 | 1612.997717 | 1656.53 | 1700.062283 | 1.035331 |
| GO:0043065\_positive\_regulation\_of\_apoptosis | 166 | 0 | 0.000000 | 0.000000 | 1600 | 1612.997717 | 1656.53 | 1700.062283 | 1.035331 |
| GO:0043068\_positive\_regulation\_of\_programmed\_cell\_death | 167 | 0 | 0.000000 | 0.000000 | 1600 | 1612.997717 | 1656.53 | 1700.062283 | 1.035331 |
| GO:0043285\_biopolymer\_catabolic\_process | 129 | 0 | 0.000000 | 0.000000 | 1600 | 1612.997717 | 1656.53 | 1700.062283 | 1.035331 |
| GO:0043436\_oxoacid\_metabolic\_process | 181 | 0 | 0.000000 | 0.000000 | 1600 | 1612.997717 | 1656.53 | 1700.062283 | 1.035331 |
| GO:0044057\_regulation\_of\_system\_process | 133 | 0 | 0.000000 | 0.000000 | 1600 | 1612.997717 | 1656.53 | 1700.062283 | 1.035331 |
| GO:0044248\_cellular\_catabolic\_process | 173 | 0 | 0.000000 | 0.000000 | 1600 | 1612.997717 | 1656.53 | 1700.062283 | 1.035331 |
| GO:0046651\_lymphocyte\_proliferation | 94 | 0 | 0.000000 | 0.000000 | 1600 | 1612.997717 | 1656.53 | 1700.062283 | 1.035331 |
| GO:0046903\_secretion | 175 | 0 | 0.000000 | 0.000000 | 1600 | 1612.997717 | 1656.53 | 1700.062283 | 1.035331 |
| GO:0048598\_embryonic\_morphogenesis | 299 | 0 | 0.000000 | 0.000000 | 1600 | 1612.997717 | 1656.53 | 1700.062283 | 1.035331 |
| GO:0048705\_skeletal\_system\_morphogenesis | 111 | 0 | 0.000000 | 0.000000 | 1600 | 1612.997717 | 1656.53 | 1700.062283 | 1.035331 |
| GO:0048729\_tissue\_morphogenesis | 255 | 0 | 0.000000 | 0.000000 | 1600 | 1612.997717 | 1656.53 | 1700.062283 | 1.035331 |
| GO:0048732\_gland\_development | 179 | 0 | 0.000000 | 0.000000 | 1600 | 1612.997717 | 1656.53 | 1700.062283 | 1.035331 |
| GO:0048736\_appendage\_development | 96 | 0 | 0.000000 | 0.000000 | 1600 | 1612.997717 | 1656.53 | 1700.062283 | 1.035331 |
| GO:0050865\_regulation\_of\_cell\_activation | 122 | 0 | 0.000000 | 0.000000 | 1600 | 1612.997717 | 1656.53 | 1700.062283 | 1.035331 |
| GO:0051240\_positive\_regulation\_of\_multicellular\_organismal\_process | 108 | 0 | 0.000000 | 0.000000 | 1600 | 1612.997717 | 1656.53 | 1700.062283 | 1.035331 |
| GO:0051246\_regulation\_of\_protein\_metabolic\_process | 170 | 0 | 0.000000 | 0.000000 | 1600 | 1612.997717 | 1656.53 | 1700.062283 | 1.035331 |
| GO:0051249\_regulation\_of\_lymphocyte\_activation | 112 | 0 | 0.000000 | 0.000000 | 1600 | 1612.997717 | 1656.53 | 1700.062283 | 1.035331 |
| GO:0051276\_chromosome\_organization | 129 | 0 | 0.000000 | 0.000000 | 1600 | 1612.997717 | 1656.53 | 1700.062283 | 1.035331 |
| GO:0051726\_regulation\_of\_cell\_cycle | 121 | 0 | 0.000000 | 0.000000 | 1600 | 1612.997717 | 1656.53 | 1700.062283 | 1.035331 |
| GO:0055082\_cellular\_chemical\_homeostasis | 181 | 0 | 0.000000 | 0.000000 | 1600 | 1612.997717 | 1656.53 | 1700.062283 | 1.035331 |
| GO:0060173\_limb\_development | 96 | 0 | 0.000000 | 0.000000 | 1600 | 1612.997717 | 1656.53 | 1700.062283 | 1.035331 |
| GO:0060341\_regulation\_of\_cellular\_localization | 97 | 0 | 0.000000 | 0.000000 | 1600 | 1612.997717 | 1656.53 | 1700.062283 | 1.035331 |
| GO:0060429\_epithelium\_development | 198 | 0 | 0.000000 | 0.000000 | 1600 | 1612.997717 | 1656.53 | 1700.062283 | 1.035331 |
| GO:0060537\_muscle\_tissue\_development | 128 | 0 | 0.000000 | 0.000000 | 1600 | 1612.997717 | 1656.53 | 1700.062283 | 1.035331 |
| GO:0060562\_epithelial\_tube\_morphogenesis | 99 | 0 | 0.000000 | 0.000000 | 1600 | 1612.997717 | 1656.53 | 1700.062283 | 1.035331 |
| GO:0070661\_leukocyte\_proliferation | 96 | 0 | 0.000000 | 0.000000 | 1600 | 1612.997717 | 1656.53 | 1700.062283 | 1.035331 |
| GO:0080134\_regulation\_of\_response\_to\_stress | 116 | 0 | 0.000000 | 0.000000 | 1600 | 1612.997717 | 1656.53 | 1700.062283 | 1.035331 |
| GO:0002706\_regulation\_of\_lymphocyte\_mediated\_immunity | 52 | 0 | 0.000000 | 0.000000 | 1603 | 1616.304026 | 1659.57 | 1702.835974 | 1.035290 |
| GO:0010608\_posttranscriptional\_regulation\_of\_gene\_expression | 52 | 0 | 0.000000 | 0.000000 | 1603 | 1616.304026 | 1659.57 | 1702.835974 | 1.035290 |
| GO:0048585\_negative\_regulation\_of\_response\_to\_stimulus | 52 | 0 | 0.000000 | 0.000000 | 1603 | 1616.304026 | 1659.57 | 1702.835974 | 1.035290 |
| GO:0002443\_leukocyte\_mediated\_immunity | 91 | 0 | 0.000000 | 0.000000 | 1605 | 1617.697608 | 1660.78 | 1703.862392 | 1.034754 |
| GO:0031399\_regulation\_of\_protein\_modification\_process | 91 | 0 | 0.000000 | 0.000000 | 1605 | 1617.697608 | 1660.78 | 1703.862392 | 1.034754 |
| GO:0000096\_sulfur\_amino\_acid\_metabolic\_process | 11 | 0 | 0.000000 | 0.000000 | 1685 | 1695.227359 | 1736.18 | 1777.132641 | 1.030374 |
| GO:0000271\_polysaccharide\_biosynthetic\_process | 11 | 0 | 0.000000 | 0.000000 | 1685 | 1695.227359 | 1736.18 | 1777.132641 | 1.030374 |
| GO:0000737\_DNA\_catabolic\_process\_\_endonucleolytic | 11 | 0 | 0.000000 | 0.000000 | 1685 | 1695.227359 | 1736.18 | 1777.132641 | 1.030374 |
| GO:0001101\_response\_to\_acid | 11 | 0 | 0.000000 | 0.000000 | 1685 | 1695.227359 | 1736.18 | 1777.132641 | 1.030374 |
| GO:0001837\_epithelial\_to\_mesenchymal\_transition | 11 | 0 | 0.000000 | 0.000000 | 1685 | 1695.227359 | 1736.18 | 1777.132641 | 1.030374 |
| GO:0001913\_T\_cell\_mediated\_cytotoxicity | 11 | 0 | 0.000000 | 0.000000 | 1685 | 1695.227359 | 1736.18 | 1777.132641 | 1.030374 |
| GO:0001952\_regulation\_of\_cell-matrix\_adhesion | 11 | 0 | 0.000000 | 0.000000 | 1685 | 1695.227359 | 1736.18 | 1777.132641 | 1.030374 |
| GO:0001963\_synaptic\_transmission\_\_dopaminergic | 11 | 0 | 0.000000 | 0.000000 | 1685 | 1695.227359 | 1736.18 | 1777.132641 | 1.030374 |
| GO:0002444\_myeloid\_leukocyte\_mediated\_immunity | 11 | 0 | 0.000000 | 0.000000 | 1685 | 1695.227359 | 1736.18 | 1777.132641 | 1.030374 |
| GO:0002467\_germinal\_center\_formation | 11 | 0 | 0.000000 | 0.000000 | 1685 | 1695.227359 | 1736.18 | 1777.132641 | 1.030374 |
| GO:0002758\_innate\_immune\_response-activating\_signal\_transduction | 11 | 0 | 0.000000 | 0.000000 | 1685 | 1695.227359 | 1736.18 | 1777.132641 | 1.030374 |
| GO:0006333\_chromatin\_assembly\_or\_disassembly | 11 | 0 | 0.000000 | 0.000000 | 1685 | 1695.227359 | 1736.18 | 1777.132641 | 1.030374 |
| GO:0006635\_fatty\_acid\_beta-oxidation | 11 | 0 | 0.000000 | 0.000000 | 1685 | 1695.227359 | 1736.18 | 1777.132641 | 1.030374 |
| GO:0006637\_acyl-CoA\_metabolic\_process | 11 | 0 | 0.000000 | 0.000000 | 1685 | 1695.227359 | 1736.18 | 1777.132641 | 1.030374 |
| GO:0006690\_icosanoid\_metabolic\_process | 11 | 0 | 0.000000 | 0.000000 | 1685 | 1695.227359 | 1736.18 | 1777.132641 | 1.030374 |
| GO:0006779\_porphyrin\_biosynthetic\_process | 11 | 0 | 0.000000 | 0.000000 | 1685 | 1695.227359 | 1736.18 | 1777.132641 | 1.030374 |
| GO:0007051\_spindle\_organization | 11 | 0 | 0.000000 | 0.000000 | 1685 | 1695.227359 | 1736.18 | 1777.132641 | 1.030374 |
| GO:0007088\_regulation\_of\_mitosis | 11 | 0 | 0.000000 | 0.000000 | 1685 | 1695.227359 | 1736.18 | 1777.132641 | 1.030374 |
| GO:0007159\_leukocyte\_adhesion | 11 | 0 | 0.000000 | 0.000000 | 1685 | 1695.227359 | 1736.18 | 1777.132641 | 1.030374 |
| GO:0007162\_negative\_regulation\_of\_cell\_adhesion | 11 | 0 | 0.000000 | 0.000000 | 1685 | 1695.227359 | 1736.18 | 1777.132641 | 1.030374 |
| GO:0007215\_glutamate\_signaling\_pathway | 11 | 0 | 0.000000 | 0.000000 | 1685 | 1695.227359 | 1736.18 | 1777.132641 | 1.030374 |
| GO:0007229\_integrin-mediated\_signaling\_pathway | 11 | 0 | 0.000000 | 0.000000 | 1685 | 1695.227359 | 1736.18 | 1777.132641 | 1.030374 |
| GO:0007260\_tyrosine\_phosphorylation\_of\_STAT\_protein | 11 | 0 | 0.000000 | 0.000000 | 1685 | 1695.227359 | 1736.18 | 1777.132641 | 1.030374 |
| GO:0008354\_germ\_cell\_migration | 11 | 0 | 0.000000 | 0.000000 | 1685 | 1695.227359 | 1736.18 | 1777.132641 | 1.030374 |
| GO:0008652\_cellular\_amino\_acid\_biosynthetic\_process | 11 | 0 | 0.000000 | 0.000000 | 1685 | 1695.227359 | 1736.18 | 1777.132641 | 1.030374 |
| GO:0009064\_glutamine\_family\_amino\_acid\_metabolic\_process | 11 | 0 | 0.000000 | 0.000000 | 1685 | 1695.227359 | 1736.18 | 1777.132641 | 1.030374 |
| GO:0009141\_nucleoside\_triphosphate\_metabolic\_process | 11 | 0 | 0.000000 | 0.000000 | 1685 | 1695.227359 | 1736.18 | 1777.132641 | 1.030374 |
| GO:0009166\_nucleotide\_catabolic\_process | 11 | 0 | 0.000000 | 0.000000 | 1685 | 1695.227359 | 1736.18 | 1777.132641 | 1.030374 |
| GO:0009409\_response\_to\_cold | 11 | 0 | 0.000000 | 0.000000 | 1685 | 1695.227359 | 1736.18 | 1777.132641 | 1.030374 |
| GO:0010259\_multicellular\_organismal\_aging | 11 | 0 | 0.000000 | 0.000000 | 1685 | 1695.227359 | 1736.18 | 1777.132641 | 1.030374 |
| GO:0014013\_regulation\_of\_gliogenesis | 11 | 0 | 0.000000 | 0.000000 | 1685 | 1695.227359 | 1736.18 | 1777.132641 | 1.030374 |
| GO:0014902\_myotube\_differentiation | 11 | 0 | 0.000000 | 0.000000 | 1685 | 1695.227359 | 1736.18 | 1777.132641 | 1.030374 |
| GO:0016079\_synaptic\_vesicle\_exocytosis | 11 | 0 | 0.000000 | 0.000000 | 1685 | 1695.227359 | 1736.18 | 1777.132641 | 1.030374 |
| GO:0021602\_cranial\_nerve\_morphogenesis | 11 | 0 | 0.000000 | 0.000000 | 1685 | 1695.227359 | 1736.18 | 1777.132641 | 1.030374 |
| GO:0021846\_cell\_proliferation\_in\_forebrain | 11 | 0 | 0.000000 | 0.000000 | 1685 | 1695.227359 | 1736.18 | 1777.132641 | 1.030374 |
| GO:0030238\_male\_sex\_determination | 11 | 0 | 0.000000 | 0.000000 | 1685 | 1695.227359 | 1736.18 | 1777.132641 | 1.030374 |
| GO:0030308\_negative\_regulation\_of\_cell\_growth | 11 | 0 | 0.000000 | 0.000000 | 1685 | 1695.227359 | 1736.18 | 1777.132641 | 1.030374 |
| GO:0030593\_neutrophil\_chemotaxis | 11 | 0 | 0.000000 | 0.000000 | 1685 | 1695.227359 | 1736.18 | 1777.132641 | 1.030374 |
| GO:0030856\_regulation\_of\_epithelial\_cell\_differentiation | 11 | 0 | 0.000000 | 0.000000 | 1685 | 1695.227359 | 1736.18 | 1777.132641 | 1.030374 |
| GO:0030878\_thyroid\_gland\_development | 11 | 0 | 0.000000 | 0.000000 | 1685 | 1695.227359 | 1736.18 | 1777.132641 | 1.030374 |
| GO:0030968\_endoplasmic\_reticulum\_unfolded\_protein\_response | 11 | 0 | 0.000000 | 0.000000 | 1685 | 1695.227359 | 1736.18 | 1777.132641 | 1.030374 |
| GO:0031646\_positive\_regulation\_of\_neurological\_system\_process | 11 | 0 | 0.000000 | 0.000000 | 1685 | 1695.227359 | 1736.18 | 1777.132641 | 1.030374 |
| GO:0031647\_regulation\_of\_protein\_stability | 11 | 0 | 0.000000 | 0.000000 | 1685 | 1695.227359 | 1736.18 | 1777.132641 | 1.030374 |
| GO:0032655\_regulation\_of\_interleukin-12\_production | 11 | 0 | 0.000000 | 0.000000 | 1685 | 1695.227359 | 1736.18 | 1777.132641 | 1.030374 |
| GO:0033014\_tetrapyrrole\_biosynthetic\_process | 11 | 0 | 0.000000 | 0.000000 | 1685 | 1695.227359 | 1736.18 | 1777.132641 | 1.030374 |
| GO:0033059\_cellular\_pigmentation | 11 | 0 | 0.000000 | 0.000000 | 1685 | 1695.227359 | 1736.18 | 1777.132641 | 1.030374 |
| GO:0033559\_unsaturated\_fatty\_acid\_metabolic\_process | 11 | 0 | 0.000000 | 0.000000 | 1685 | 1695.227359 | 1736.18 | 1777.132641 | 1.030374 |
| GO:0034620\_cellular\_response\_to\_unfolded\_protein | 11 | 0 | 0.000000 | 0.000000 | 1685 | 1695.227359 | 1736.18 | 1777.132641 | 1.030374 |
| GO:0035176\_social\_behavior | 11 | 0 | 0.000000 | 0.000000 | 1685 | 1695.227359 | 1736.18 | 1777.132641 | 1.030374 |
| GO:0042036\_negative\_regulation\_of\_cytokine\_biosynthetic\_process | 11 | 0 | 0.000000 | 0.000000 | 1685 | 1695.227359 | 1736.18 | 1777.132641 | 1.030374 |
| GO:0042219\_cellular\_amino\_acid\_derivative\_catabolic\_process | 11 | 0 | 0.000000 | 0.000000 | 1685 | 1695.227359 | 1736.18 | 1777.132641 | 1.030374 |
| GO:0042439\_ethanolamine\_and\_derivative\_metabolic\_process | 11 | 0 | 0.000000 | 0.000000 | 1685 | 1695.227359 | 1736.18 | 1777.132641 | 1.030374 |
| GO:0042542\_response\_to\_hydrogen\_peroxide | 11 | 0 | 0.000000 | 0.000000 | 1685 | 1695.227359 | 1736.18 | 1777.132641 | 1.030374 |
| GO:0042551\_neuron\_maturation | 11 | 0 | 0.000000 | 0.000000 | 1685 | 1695.227359 | 1736.18 | 1777.132641 | 1.030374 |
| GO:0043576\_regulation\_of\_respiratory\_gaseous\_exchange | 11 | 0 | 0.000000 | 0.000000 | 1685 | 1695.227359 | 1736.18 | 1777.132641 | 1.030374 |
| GO:0045026\_plasma\_membrane\_fusion | 11 | 0 | 0.000000 | 0.000000 | 1685 | 1695.227359 | 1736.18 | 1777.132641 | 1.030374 |
| GO:0045055\_regulated\_secretory\_pathway | 11 | 0 | 0.000000 | 0.000000 | 1685 | 1695.227359 | 1736.18 | 1777.132641 | 1.030374 |
| GO:0045216\_cell-cell\_junction\_organization | 11 | 0 | 0.000000 | 0.000000 | 1685 | 1695.227359 | 1736.18 | 1777.132641 | 1.030374 |
| GO:0045685\_regulation\_of\_glial\_cell\_differentiation | 11 | 0 | 0.000000 | 0.000000 | 1685 | 1695.227359 | 1736.18 | 1777.132641 | 1.030374 |
| GO:0045834\_positive\_regulation\_of\_lipid\_metabolic\_process | 11 | 0 | 0.000000 | 0.000000 | 1685 | 1695.227359 | 1736.18 | 1777.132641 | 1.030374 |
| GO:0046323\_glucose\_import | 11 | 0 | 0.000000 | 0.000000 | 1685 | 1695.227359 | 1736.18 | 1777.132641 | 1.030374 |
| GO:0046425\_regulation\_of\_JAK-STAT\_cascade | 11 | 0 | 0.000000 | 0.000000 | 1685 | 1695.227359 | 1736.18 | 1777.132641 | 1.030374 |
| GO:0046716\_muscle\_maintenance | 11 | 0 | 0.000000 | 0.000000 | 1685 | 1695.227359 | 1736.18 | 1777.132641 | 1.030374 |
| GO:0046928\_regulation\_of\_neurotransmitter\_secretion | 11 | 0 | 0.000000 | 0.000000 | 1685 | 1695.227359 | 1736.18 | 1777.132641 | 1.030374 |
| GO:0048009\_insulin-like\_growth\_factor\_receptor\_signaling\_pathway | 11 | 0 | 0.000000 | 0.000000 | 1685 | 1695.227359 | 1736.18 | 1777.132641 | 1.030374 |
| GO:0048193\_Golgi\_vesicle\_transport | 11 | 0 | 0.000000 | 0.000000 | 1685 | 1695.227359 | 1736.18 | 1777.132641 | 1.030374 |
| GO:0048678\_response\_to\_axon\_injury | 11 | 0 | 0.000000 | 0.000000 | 1685 | 1695.227359 | 1736.18 | 1777.132641 | 1.030374 |
| GO:0048745\_smooth\_muscle\_tissue\_development | 11 | 0 | 0.000000 | 0.000000 | 1685 | 1695.227359 | 1736.18 | 1777.132641 | 1.030374 |
| GO:0050772\_positive\_regulation\_of\_axonogenesis | 11 | 0 | 0.000000 | 0.000000 | 1685 | 1695.227359 | 1736.18 | 1777.132641 | 1.030374 |
| GO:0050806\_positive\_regulation\_of\_synaptic\_transmission | 11 | 0 | 0.000000 | 0.000000 | 1685 | 1695.227359 | 1736.18 | 1777.132641 | 1.030374 |
| GO:0050807\_regulation\_of\_synapse\_organization | 11 | 0 | 0.000000 | 0.000000 | 1685 | 1695.227359 | 1736.18 | 1777.132641 | 1.030374 |
| GO:0050830\_defense\_response\_to\_Gram-positive\_bacterium | 11 | 0 | 0.000000 | 0.000000 | 1685 | 1695.227359 | 1736.18 | 1777.132641 | 1.030374 |
| GO:0050854\_regulation\_of\_antigen\_receptor-mediated\_signaling\_pathway | 11 | 0 | 0.000000 | 0.000000 | 1685 | 1695.227359 | 1736.18 | 1777.132641 | 1.030374 |
| GO:0051092\_positive\_regulation\_of\_NF-kappaB\_transcription\_factor\_activity | 11 | 0 | 0.000000 | 0.000000 | 1685 | 1695.227359 | 1736.18 | 1777.132641 | 1.030374 |
| GO:0051494\_negative\_regulation\_of\_cytoskeleton\_organization | 11 | 0 | 0.000000 | 0.000000 | 1685 | 1695.227359 | 1736.18 | 1777.132641 | 1.030374 |
| GO:0051648\_vesicle\_localization | 11 | 0 | 0.000000 | 0.000000 | 1685 | 1695.227359 | 1736.18 | 1777.132641 | 1.030374 |
| GO:0051783\_regulation\_of\_nuclear\_division | 11 | 0 | 0.000000 | 0.000000 | 1685 | 1695.227359 | 1736.18 | 1777.132641 | 1.030374 |
| GO:0051971\_positive\_regulation\_of\_transmission\_of\_nerve\_impulse | 11 | 0 | 0.000000 | 0.000000 | 1685 | 1695.227359 | 1736.18 | 1777.132641 | 1.030374 |
| GO:0060004\_reflex | 11 | 0 | 0.000000 | 0.000000 | 1685 | 1695.227359 | 1736.18 | 1777.132641 | 1.030374 |
| GO:0060767\_epithelial\_cell\_proliferation\_involved\_in\_prostate\_gland\_development | 11 | 0 | 0.000000 | 0.000000 | 1685 | 1695.227359 | 1736.18 | 1777.132641 | 1.030374 |
| GO:0000910\_cytokinesis | 8 | 0 | 0.000000 | 0.000000 | 1805 | 1819.391202 | 1858.41 | 1897.428798 | 1.029590 |
| GO:0001783\_B\_cell\_apoptosis | 8 | 0 | 0.000000 | 0.000000 | 1805 | 1819.391202 | 1858.41 | 1897.428798 | 1.029590 |
| GO:0001833\_inner\_cell\_mass\_cell\_proliferation | 8 | 0 | 0.000000 | 0.000000 | 1805 | 1819.391202 | 1858.41 | 1897.428798 | 1.029590 |
| GO:0001840\_neural\_plate\_development | 8 | 0 | 0.000000 | 0.000000 | 1805 | 1819.391202 | 1858.41 | 1897.428798 | 1.029590 |
| GO:0001893\_maternal\_placenta\_development | 8 | 0 | 0.000000 | 0.000000 | 1805 | 1819.391202 | 1858.41 | 1897.428798 | 1.029590 |
| GO:0001911\_negative\_regulation\_of\_leukocyte\_mediated\_cytotoxicity | 8 | 0 | 0.000000 | 0.000000 | 1805 | 1819.391202 | 1858.41 | 1897.428798 | 1.029590 |
| GO:0001916\_positive\_regulation\_of\_T\_cell\_mediated\_cytotoxicity | 8 | 0 | 0.000000 | 0.000000 | 1805 | 1819.391202 | 1858.41 | 1897.428798 | 1.029590 |
| GO:0002065\_columnar\_cuboidal\_epithelial\_cell\_differentiation | 8 | 0 | 0.000000 | 0.000000 | 1805 | 1819.391202 | 1858.41 | 1897.428798 | 1.029590 |
| GO:0002320\_lymphoid\_progenitor\_cell\_differentiation | 8 | 0 | 0.000000 | 0.000000 | 1805 | 1819.391202 | 1858.41 | 1897.428798 | 1.029590 |
| GO:0002438\_acute\_inflammatory\_response\_to\_antigenic\_stimulus | 8 | 0 | 0.000000 | 0.000000 | 1805 | 1819.391202 | 1858.41 | 1897.428798 | 1.029590 |
| GO:0002524\_hypersensitivity | 8 | 0 | 0.000000 | 0.000000 | 1805 | 1819.391202 | 1858.41 | 1897.428798 | 1.029590 |
| GO:0002566\_somatic\_diversification\_of\_immune\_receptors\_via\_somatic\_mutation | 8 | 0 | 0.000000 | 0.000000 | 1805 | 1819.391202 | 1858.41 | 1897.428798 | 1.029590 |
| GO:0002864\_regulation\_of\_acute\_inflammatory\_response\_to\_antigenic\_stimulus | 8 | 0 | 0.000000 | 0.000000 | 1805 | 1819.391202 | 1858.41 | 1897.428798 | 1.029590 |
[truncated: 256,348 more chars]
