# Supplementary material for: RedundancyMiner: De-replication of redundant GO categories in microarray and proteomics analysis
Source: BMC Bioinformatics. 2011 Feb 10;12:52. doi: 10.1186/1471-2105-12-52 (PMC3223614; doi:10.1186/1471-2105-12-52)
Supplement: Additional file 8 — Retinal development HTGM download. compressed package of the results of running HTGM on the retinal development genes list. [file 1471-2105-12-52-S8.ZIP › SCENARIO_2_MODIFIED/total.txt.total.txt.dir/Exp1_BestClusterMap_LEIGS_KM_24.csv.join.17.txt.dir/Exp1_BestClusterMap_LEIGS_KM_24.csv.join.17.txt.change.gce.html]

Gene Category Report for Exp1\_BestClusterMap\_LEIGS\_KM\_24.csv.join.17.txt

# Gene Category Report for Exp1\_BestClusterMap\_LEIGS\_KM\_24.csv.join.17.txt

| HYPERLINKED GO CATEGORY | HYPERLINKED GENE NAME | TOTAL GENES | CHANGED GENES | ENRICHMENT | LOG10(p) | CUMULATIVE NUMBER OF CATEGORIES | CUMULATIVE RANDOMS MEAN | FALSE DISCOVERY RATE |
| --- | --- | --- | --- | --- | --- | --- | --- | --- |
| GO:0042542\_response\_to\_hydrogen\_peroxide | SOD1 | 11 | 2 | 44.066986 | -3.061606 | 1 | 0.77 | 0.770000 |
| GO:0042542\_response\_to\_hydrogen\_peroxide | PARK7 | 11 | 2 | 44.066986 | -3.061606 | 1 | 0.77 | 0.770000 |
| GO:0042743\_hydrogen\_peroxide\_metabolic\_process | SOD1 | 12 | 2 | 40.394737 | -2.983494 | 2 | 0.88 | 0.440000 |
| GO:0042743\_hydrogen\_peroxide\_metabolic\_process | PARK7 | 12 | 2 | 40.394737 | -2.983494 | 2 | 0.88 | 0.440000 |
| GO:0007566\_embryo\_implantation | GRN | 13 | 2 | 37.287449 | -2.912012 | 3 | 1.01 | 0.336667 |
| GO:0007566\_embryo\_implantation | SOD1 | 13 | 2 | 37.287449 | -2.912012 | 3 | 1.01 | 0.336667 |
| GO:0000302\_response\_to\_reactive\_oxygen\_species | SOD1 | 16 | 2 | 30.296053 | -2.728132 | 4 | 1.43 | 0.357500 |
| GO:0000302\_response\_to\_reactive\_oxygen\_species | PARK7 | 16 | 2 | 30.296053 | -2.728132 | 4 | 1.43 | 0.357500 |
| GO:0045103\_intermediate\_filament-based\_process | VIM | 18 | 2 | 26.929825 | -2.624758 | 5 | 1.81 | 0.362000 |
| GO:0045103\_intermediate\_filament-based\_process | SOD1 | 18 | 2 | 26.929825 | -2.624758 | 5 | 1.81 | 0.362000 |
| GO:0034962\_cellular\_biopolymer\_catabolic\_process | ZFP36L1 | 68 | 3 | 10.692724 | -2.598649 | 6 | 1.86 | 0.310000 |
| GO:0034962\_cellular\_biopolymer\_catabolic\_process | ARIH2 | 68 | 3 | 10.692724 | -2.598649 | 6 | 1.86 | 0.310000 |
| GO:0034962\_cellular\_biopolymer\_catabolic\_process | SOD1 | 68 | 3 | 10.692724 | -2.598649 | 6 | 1.86 | 0.310000 |
| GO:0030518\_steroid\_hormone\_receptor\_signaling\_pathway | PTGES3 | 19 | 2 | 25.512465 | -2.577522 | 7 | 1.91 | 0.272857 |
| GO:0030518\_steroid\_hormone\_receptor\_signaling\_pathway | PHB2 | 19 | 2 | 25.512465 | -2.577522 | 7 | 1.91 | 0.272857 |
| GO:0044265\_cellular\_macromolecule\_catabolic\_process | ZFP36L1 | 75 | 3 | 9.694737 | -2.477094 | 8 | 2.27 | 0.283750 |
| GO:0044265\_cellular\_macromolecule\_catabolic\_process | ARIH2 | 75 | 3 | 9.694737 | -2.477094 | 8 | 2.27 | 0.283750 |
| GO:0044265\_cellular\_macromolecule\_catabolic\_process | SOD1 | 75 | 3 | 9.694737 | -2.477094 | 8 | 2.27 | 0.283750 |
| GO:0032239\_regulation\_of\_nucleobase\_\_nucleoside\_\_nucleotide\_and\_nucleic\_acid\_transport | KHDRBS1 | 1 | 1 |  |  |  |  |  |  |
| GO:0032287\_myelin\_maintenance\_in\_the\_peripheral\_nervous\_system | SOD1 | 1 | 1 |  |  |  |  |  |  |
| GO:0043217\_myelin\_maintenance | SOD1 | 1 | 1 |  |  |  |  |  |  |
| GO:0046416\_D-amino\_acid\_metabolic\_process | SRR | 1 | 1 |  |  |  |  |  |  |
| GO:0046437\_D-amino\_acid\_biosynthetic\_process | SRR | 1 | 1 |  |  |  |  |  |  |
| GO:0046831\_regulation\_of\_RNA\_export\_from\_nucleus | KHDRBS1 | 1 | 1 |  |  |  |  |  |  |
| GO:0070178\_D-serine\_metabolic\_process | SRR | 1 | 1 |  |  |  |  |  |  |
| GO:0070179\_D-serine\_biosynthetic\_process | SRR | 1 | 1 |  |  |  |  |  |  |
| GO:0006800\_oxygen\_and\_reactive\_oxygen\_species\_metabolic\_process | SOD1 | 26 | 2 | 18.643725 | -2.306107 | 9 | 3.4 | 0.377778 |
| GO:0006800\_oxygen\_and\_reactive\_oxygen\_species\_metabolic\_process | PARK7 | 26 | 2 | 18.643725 | -2.306107 | 9 | 3.4 | 0.377778 |
| GO:0030522\_intracellular\_receptor-mediated\_signaling\_pathway | PTGES3 | 30 | 2 | 16.157895 | -2.183767 | 10 | 4.46 | 0.446000 |
| GO:0030522\_intracellular\_receptor-mediated\_signaling\_pathway | PHB2 | 30 | 2 | 16.157895 | -2.183767 | 10 | 4.46 | 0.446000 |
| GO:0006937\_regulation\_of\_muscle\_contraction | ATP2A2 | 32 | 2 | 15.148026 | -2.128906 | 11 | 5.17 | 0.470000 |
| GO:0006937\_regulation\_of\_muscle\_contraction | SOD1 | 32 | 2 | 15.148026 | -2.128906 | 11 | 5.17 | 0.470000 |
| GO:0007565\_female\_pregnancy | GRN | 33 | 2 | 14.688995 | -2.102820 | 12 | 5.36 | 0.446667 |
| GO:0007565\_female\_pregnancy | SOD1 | 33 | 2 | 14.688995 | -2.102820 | 12 | 5.36 | 0.446667 |
| GO:0006563\_L-serine\_metabolic\_process | SRR | 2 | 1 |  |  |  |  |  |  |
| GO:0009070\_serine\_family\_amino\_acid\_biosynthetic\_process | SRR | 2 | 1 |  |  |  |  |  |  |
| GO:0019322\_pentose\_biosynthetic\_process | PGD | 2 | 1 |  |  |  |  |  |  |
| GO:0031958\_corticosteroid\_receptor\_signaling\_pathway | PTGES3 | 2 | 1 |  |  |  |  |  |  |
| GO:0033147\_negative\_regulation\_of\_estrogen\_receptor\_signaling\_pathway | PHB2 | 2 | 1 |  |  |  |  |  |  |
| GO:0042921\_glucocorticoid\_receptor\_signaling\_pathway | PTGES3 | 2 | 1 |  |  |  |  |  |  |
| GO:0060430\_lung\_saccule\_development | PTGES3 | 2 | 1 |  |  |  |  |  |  |
| GO:0016051\_carbohydrate\_biosynthetic\_process | PTGES3 | 35 | 2 | 13.849624 | -2.053068 | 13 | 5.85 | 0.450000 |
| GO:0016051\_carbohydrate\_biosynthetic\_process | PGD | 35 | 2 | 13.849624 | -2.053068 | 13 | 5.85 | 0.450000 |
| GO:0016053\_organic\_acid\_biosynthetic\_process | PTGES3 | 38 | 2 | 12.756233 | -1.983825 | 15 | 6.58 | 0.438667 |
| GO:0016053\_organic\_acid\_biosynthetic\_process | SRR | 38 | 2 | 12.756233 | -1.983825 | 15 | 6.58 | 0.438667 |
| GO:0046394\_carboxylic\_acid\_biosynthetic\_process | PTGES3 | 38 | 2 | 12.756233 | -1.983825 | 15 | 6.58 | 0.438667 |
| GO:0046394\_carboxylic\_acid\_biosynthetic\_process | SRR | 38 | 2 | 12.756233 | -1.983825 | 15 | 6.58 | 0.438667 |
| GO:0016071\_mRNA\_metabolic\_process | ZFP36L1 | 40 | 2 | 12.118421 | -1.940815 | 16 | 7.12 | 0.445000 |
| GO:0016071\_mRNA\_metabolic\_process | PABPN1 | 40 | 2 | 12.118421 | -1.940815 | 16 | 7.12 | 0.445000 |
| GO:0006979\_response\_to\_oxidative\_stress | SOD1 | 41 | 2 | 11.822850 | -1.920160 | 17 | 7.45 | 0.438235 |
| GO:0006979\_response\_to\_oxidative\_stress | PARK7 | 41 | 2 | 11.822850 | -1.920160 | 17 | 7.45 | 0.438235 |
| GO:0000303\_response\_to\_superoxide | SOD1 | 3 | 1 |  |  |  |  |  |  |
| GO:0001516\_prostaglandin\_biosynthetic\_process | PTGES3 | 3 | 1 |  |  |  |  |  |  |
| GO:0001895\_retina\_homeostasis | SOD1 | 3 | 1 |  |  |  |  |  |  |
| GO:0033600\_negative\_regulation\_of\_mammary\_gland\_epithelial\_cell\_proliferation | PHB2 | 3 | 1 |  |  |  |  |  |  |
| GO:0046457\_prostanoid\_biosynthetic\_process | PTGES3 | 3 | 1 |  |  |  |  |  |  |
| GO:0051583\_dopamine\_uptake | PARK7 | 3 | 1 |  |  |  |  |  |  |
| GO:0051934\_catecholamine\_uptake\_during\_transmission\_of\_nerve\_impulse | PARK7 | 3 | 1 |  |  |  |  |  |  |
| GO:0043285\_biopolymer\_catabolic\_process | ZFP36L1 | 129 | 3 | 5.636475 | -1.824276 | 18 | 9.04 | 0.502222 |
| GO:0043285\_biopolymer\_catabolic\_process | ARIH2 | 129 | 3 | 5.636475 | -1.824276 | 18 | 9.04 | 0.502222 |
| GO:0043285\_biopolymer\_catabolic\_process | SOD1 | 129 | 3 | 5.636475 | -1.824276 | 18 | 9.04 | 0.502222 |
| GO:0000305\_response\_to\_oxygen\_radical | SOD1 | 4 | 1 |  |  |  |  |  |  |
| GO:0001835\_blastocyst\_hatching | GRN | 4 | 1 |  |  |  |  |  |  |
| GO:0005978\_glycogen\_biosynthetic\_process | PTGES3 | 4 | 1 |  |  |  |  |  |  |
| GO:0009250\_glucan\_biosynthetic\_process | PTGES3 | 4 | 1 |  |  |  |  |  |  |
| GO:0022011\_myelination\_in\_the\_peripheral\_nervous\_system | SOD1 | 4 | 1 |  |  |  |  |  |  |
| GO:0032292\_ensheathment\_of\_axons\_in\_the\_peripheral\_nervous\_system | SOD1 | 4 | 1 |  |  |  |  |  |  |
| GO:0033599\_regulation\_of\_mammary\_gland\_epithelial\_cell\_proliferation | PHB2 | 4 | 1 |  |  |  |  |  |  |
| GO:0035188\_hatching | GRN | 4 | 1 |  |  |  |  |  |  |
| GO:0060087\_relaxation\_of\_vascular\_smooth\_muscle | SOD1 | 4 | 1 |  |  |  |  |  |  |
| GO:0003015\_heart\_process | ATP2A2 | 49 | 2 | 9.892589 | -1.772076 | 21 | 9.89 | 0.470952 |
| GO:0003015\_heart\_process | SOD1 | 49 | 2 | 9.892589 | -1.772076 | 21 | 9.89 | 0.470952 |
| GO:0046660\_female\_sex\_differentiation | PHB2 | 49 | 2 | 9.892589 | -1.772076 | 21 | 9.89 | 0.470952 |
| GO:0046660\_female\_sex\_differentiation | SOD1 | 49 | 2 | 9.892589 | -1.772076 | 21 | 9.89 | 0.470952 |
| GO:0060047\_heart\_contraction | ATP2A2 | 49 | 2 | 9.892589 | -1.772076 | 21 | 9.89 | 0.470952 |
| GO:0060047\_heart\_contraction | SOD1 | 49 | 2 | 9.892589 | -1.772076 | 21 | 9.89 | 0.470952 |
| GO:0009057\_macromolecule\_catabolic\_process | ZFP36L1 | 137 | 3 | 5.307338 | -1.754341 | 22 | 10.16 | 0.461818 |
| GO:0009057\_macromolecule\_catabolic\_process | ARIH2 | 137 | 3 | 5.307338 | -1.754341 | 22 | 10.16 | 0.461818 |
| GO:0009057\_macromolecule\_catabolic\_process | SOD1 | 137 | 3 | 5.307338 | -1.754341 | 22 | 10.16 | 0.461818 |
| GO:0006412\_translation | MRPL11 | 54 | 2 | 8.976608 | -1.692156 | 23 | 11.37 | 0.494348 |
| GO:0006412\_translation | ZFP36L1 | 54 | 2 | 8.976608 | -1.692156 | 23 | 11.37 | 0.494348 |
| GO:0000288\_nuclear-transcribed\_mRNA\_catabolic\_process\_\_deadenylation-dependent\_decay | ZFP36L1 | 5 | 1 | 48.473684 | -1.688903 | 42 | 17.47 | 0.415952 |
| GO:0000956\_nuclear-transcribed\_mRNA\_catabolic\_process | ZFP36L1 | 5 | 1 | 48.473684 | -1.688903 | 42 | 17.47 | 0.415952 |
| GO:0006268\_DNA\_unwinding\_during\_replication | MCM4 | 5 | 1 | 48.473684 | -1.688903 | 42 | 17.47 | 0.415952 |
| GO:0006378\_mRNA\_polyadenylation | PABPN1 | 5 | 1 | 48.473684 | -1.688903 | 42 | 17.47 | 0.415952 |
| GO:0006636\_unsaturated\_fatty\_acid\_biosynthetic\_process | PTGES3 | 5 | 1 | 48.473684 | -1.688903 | 42 | 17.47 | 0.415952 |
| GO:0014044\_Schwann\_cell\_development | SOD1 | 5 | 1 | 48.473684 | -1.688903 | 42 | 17.47 | 0.415952 |
| GO:0019430\_removal\_of\_superoxide\_radicals | SOD1 | 5 | 1 | 48.473684 | -1.688903 | 42 | 17.47 | 0.415952 |
| GO:0032508\_DNA\_duplex\_unwinding | MCM4 | 5 | 1 | 48.473684 | -1.688903 | 42 | 17.47 | 0.415952 |
| GO:0033144\_negative\_regulation\_of\_steroid\_hormone\_receptor\_signaling\_pathway | PHB2 | 5 | 1 | 48.473684 | -1.688903 | 42 | 17.47 | 0.415952 |
| GO:0033146\_regulation\_of\_estrogen\_receptor\_signaling\_pathway | PHB2 | 5 | 1 | 48.473684 | -1.688903 | 42 | 17.47 | 0.415952 |
| GO:0033692\_cellular\_polysaccharide\_biosynthetic\_process | PTGES3 | 5 | 1 | 48.473684 | -1.688903 | 42 | 17.47 | 0.415952 |
| GO:0042554\_superoxide\_anion\_generation | SOD1 | 5 | 1 | 48.473684 | -1.688903 | 42 | 17.47 | 0.415952 |
| GO:0042695\_thelarche | PHB2 | 5 | 1 | 48.473684 | -1.688903 | 42 | 17.47 | 0.415952 |
| GO:0043631\_RNA\_polyadenylation | PABPN1 | 5 | 1 | 48.473684 | -1.688903 | 42 | 17.47 | 0.415952 |
| GO:0046456\_icosanoid\_biosynthetic\_process | PTGES3 | 5 | 1 | 48.473684 | -1.688903 | 42 | 17.47 | 0.415952 |
| GO:0050665\_hydrogen\_peroxide\_biosynthetic\_process | SOD1 | 5 | 1 | 48.473684 | -1.688903 | 42 | 17.47 | 0.415952 |
| GO:0051289\_protein\_homotetramerization | SRR | 5 | 1 | 48.473684 | -1.688903 | 42 | 17.47 | 0.415952 |
| GO:0060744\_mammary\_gland\_branching\_involved\_in\_thelarche | PHB2 | 5 | 1 | 48.473684 | -1.688903 | 42 | 17.47 | 0.415952 |
| GO:0060762\_regulation\_of\_branching\_involved\_in\_mammary\_gland\_duct\_morphogenesis | PHB2 | 5 | 1 | 48.473684 | -1.688903 | 42 | 17.47 | 0.415952 |
| GO:0050678\_regulation\_of\_epithelial\_cell\_proliferation | GRN | 56 | 2 | 8.656015 | -1.662399 | 43 | 18.17 | 0.422558 |
| GO:0050678\_regulation\_of\_epithelial\_cell\_proliferation | PHB2 | 56 | 2 | 8.656015 | -1.662399 | 43 | 18.17 | 0.422558 |
| GO:0006402\_mRNA\_catabolic\_process | ZFP36L1 | 6 | 1 | 40.394737 | -1.610569 | 49 | 23.9 | 0.487755 |
| GO:0006692\_prostanoid\_metabolic\_process | PTGES3 | 6 | 1 | 40.394737 | -1.610569 | 49 | 23.9 | 0.487755 |
| GO:0006693\_prostaglandin\_metabolic\_process | PTGES3 | 6 | 1 | 40.394737 | -1.610569 | 49 | 23.9 | 0.487755 |
| GO:0009069\_serine\_family\_amino\_acid\_metabolic\_process | SRR | 6 | 1 | 40.394737 | -1.610569 | 49 | 23.9 | 0.487755 |
| GO:0032392\_DNA\_geometric\_change | MCM4 | 6 | 1 | 40.394737 | -1.610569 | 49 | 23.9 | 0.487755 |
| GO:0045822\_negative\_regulation\_of\_heart\_contraction | ATP2A2 | 6 | 1 | 40.394737 | -1.610569 | 49 | 23.9 | 0.487755 |
| GO:0001504\_neurotransmitter\_uptake | PARK7 | 7 | 1 | 34.624060 | -1.544470 | 58 | 30.53 | 0.526379 |
| GO:0002093\_auditory\_receptor\_cell\_morphogenesis | SOD1 | 7 | 1 | 34.624060 | -1.544470 | 58 | 30.53 | 0.526379 |
| GO:0006401\_RNA\_catabolic\_process | ZFP36L1 | 7 | 1 | 34.624060 | -1.544470 | 58 | 30.53 | 0.526379 |
| GO:0030520\_estrogen\_receptor\_signaling\_pathway | PHB2 | 7 | 1 | 34.624060 | -1.544470 | 58 | 30.53 | 0.526379 |
| GO:0031124\_mRNA\_3'-end\_processing | PABPN1 | 7 | 1 | 34.624060 | -1.544470 | 58 | 30.53 | 0.526379 |
| GO:0045986\_negative\_regulation\_of\_smooth\_muscle\_contraction | SOD1 | 7 | 1 | 34.624060 | -1.544470 | 58 | 30.53 | 0.526379 |
| GO:0046543\_development\_of\_secondary\_female\_sexual\_characteristics | PHB2 | 7 | 1 | 34.624060 | -1.544470 | 58 | 30.53 | 0.526379 |
| GO:0060088\_auditory\_receptor\_cell\_stereocilium\_organization | SOD1 | 7 | 1 | 34.624060 | -1.544470 | 58 | 30.53 | 0.526379 |
| GO:0060117\_auditory\_receptor\_cell\_development | SOD1 | 7 | 1 | 34.624060 | -1.544470 | 58 | 30.53 | 0.526379 |
| GO:0009058\_biosynthetic\_process | MRPL11 | 1175 | 9 | 1.856439 | -1.490495 | 59 | 31.89 | 0.540508 |
| GO:0009058\_biosynthetic\_process | KHDRBS1 | 1175 | 9 | 1.856439 | -1.490495 | 59 | 31.89 | 0.540508 |
| GO:0009058\_biosynthetic\_process | ZFP36L1 | 1175 | 9 | 1.856439 | -1.490495 | 59 | 31.89 | 0.540508 |
| GO:0009058\_biosynthetic\_process | PTGES3 | 1175 | 9 | 1.856439 | -1.490495 | 59 | 31.89 | 0.540508 |
| GO:0009058\_biosynthetic\_process | CNBP | 1175 | 9 | 1.856439 | -1.490495 | 59 | 31.89 | 0.540508 |
| GO:0009058\_biosynthetic\_process | PGD | 1175 | 9 | 1.856439 | -1.490495 | 59 | 31.89 | 0.540508 |
| GO:0009058\_biosynthetic\_process | SRR | 1175 | 9 | 1.856439 | -1.490495 | 59 | 31.89 | 0.540508 |
| GO:0009058\_biosynthetic\_process | SOD1 | 1175 | 9 | 1.856439 | -1.490495 | 59 | 31.89 | 0.540508 |
| GO:0009058\_biosynthetic\_process | MCM4 | 1175 | 9 | 1.856439 | -1.490495 | 59 | 31.89 | 0.540508 |
| GO:0044248\_cellular\_catabolic\_process | ZFP36L1 | 173 | 3 | 4.202921 | -1.489087 | 60 | 31.99 | 0.533167 |
| GO:0044248\_cellular\_catabolic\_process | ARIH2 | 173 | 3 | 4.202921 | -1.489087 | 60 | 31.99 | 0.533167 |
| GO:0044248\_cellular\_catabolic\_process | SOD1 | 173 | 3 | 4.202921 | -1.489087 | 60 | 31.99 | 0.533167 |
| GO:0007009\_plasma\_membrane\_organization | SOD1 | 8 | 1 | 30.296053 | -1.487326 | 63 | 36.34 | 0.576825 |
| GO:0031123\_RNA\_3'-end\_processing | PABPN1 | 8 | 1 | 30.296053 | -1.487326 | 63 | 36.34 | 0.576825 |
| GO:0045932\_negative\_regulation\_of\_muscle\_contraction | SOD1 | 8 | 1 | 30.296053 | -1.487326 | 63 | 36.34 | 0.576825 |
| GO:0006873\_cellular\_ion\_homeostasis | ATP2A2 | 176 | 3 | 4.131280 | -1.469949 | 64 | 36.98 | 0.577812 |
| GO:0006873\_cellular\_ion\_homeostasis | SOD1 | 176 | 3 | 4.131280 | -1.469949 | 64 | 36.98 | 0.577812 |
| GO:0006873\_cellular\_ion\_homeostasis | PARK7 | 176 | 3 | 4.131280 | -1.469949 | 64 | 36.98 | 0.577812 |
| GO:0050673\_epithelial\_cell\_proliferation | GRN | 72 | 2 | 6.732456 | -1.459324 | 65 | 37.59 | 0.578308 |
| GO:0050673\_epithelial\_cell\_proliferation | PHB2 | 72 | 2 | 6.732456 | -1.459324 | 65 | 37.59 | 0.578308 |
| GO:0006936\_muscle\_contraction | ATP2A2 | 73 | 2 | 6.640231 | -1.448319 | 66 | 37.91 | 0.574394 |
| GO:0006936\_muscle\_contraction | SOD1 | 73 | 2 | 6.640231 | -1.448319 | 66 | 37.91 | 0.574394 |
| GO:0055082\_cellular\_chemical\_homeostasis | ATP2A2 | 181 | 3 | 4.017156 | -1.438896 | 67 | 38.16 | 0.569552 |
| GO:0055082\_cellular\_chemical\_homeostasis | SOD1 | 181 | 3 | 4.017156 | -1.438896 | 67 | 38.16 | 0.569552 |
| GO:0055082\_cellular\_chemical\_homeostasis | PARK7 | 181 | 3 | 4.017156 | -1.438896 | 67 | 38.16 | 0.569552 |
| GO:0006309\_DNA\_fragmentation\_involved\_in\_apoptosis | SOD1 | 9 | 1 | 26.929825 | -1.437020 | 75 | 42.85 | 0.571333 |
| GO:0014037\_Schwann\_cell\_differentiation | SOD1 | 9 | 1 | 26.929825 | -1.437020 | 75 | 42.85 | 0.571333 |
| GO:0033143\_regulation\_of\_steroid\_hormone\_receptor\_signaling\_pathway | PHB2 | 9 | 1 | 26.929825 | -1.437020 | 75 | 42.85 | 0.571333 |
| GO:0045136\_development\_of\_secondary\_sexual\_characteristics | PHB2 | 9 | 1 | 26.929825 | -1.437020 | 75 | 42.85 | 0.571333 |
| GO:0060052\_neurofilament\_cytoskeleton\_organization | SOD1 | 9 | 1 | 26.929825 | -1.437020 | 75 | 42.85 | 0.571333 |
| GO:0060081\_membrane\_hyperpolarization | PARK7 | 9 | 1 | 26.929825 | -1.437020 | 75 | 42.85 | 0.571333 |
| GO:0060119\_inner\_ear\_receptor\_cell\_development | SOD1 | 9 | 1 | 26.929825 | -1.437020 | 75 | 42.85 | 0.571333 |
| GO:0060122\_inner\_ear\_receptor\_stereocilium\_organization | SOD1 | 9 | 1 | 26.929825 | -1.437020 | 75 | 42.85 | 0.571333 |
| GO:0003012\_muscle\_system\_process | ATP2A2 | 76 | 2 | 6.378116 | -1.416274 | 76 | 43.3 | 0.569737 |
| GO:0003012\_muscle\_system\_process | SOD1 | 76 | 2 | 6.378116 | -1.416274 | 76 | 43.3 | 0.569737 |
| GO:0051241\_negative\_regulation\_of\_multicellular\_organismal\_process | ATP2A2 | 77 | 2 | 6.295284 | -1.405903 | 77 | 43.5 | 0.564935 |
| GO:0051241\_negative\_regulation\_of\_multicellular\_organismal\_process | SOD1 | 77 | 2 | 6.295284 | -1.405903 | 77 | 43.5 | 0.564935 |
| GO:0006405\_RNA\_export\_from\_nucleus | KHDRBS1 | 10 | 1 | 24.236842 | -1.392109 | 86 | 49.21 | 0.572209 |
| GO:0006801\_superoxide\_metabolic\_process | SOD1 | 10 | 1 | 24.236842 | -1.392109 | 86 | 49.21 | 0.572209 |
| GO:0006921\_cell\_structure\_disassembly\_during\_apoptosis | SOD1 | 10 | 1 | 24.236842 | -1.392109 | 86 | 49.21 | 0.572209 |
| GO:0019321\_pentose\_metabolic\_process | PGD | 10 | 1 | 24.236842 | -1.392109 | 86 | 49.21 | 0.572209 |
| GO:0034637\_cellular\_carbohydrate\_biosynthetic\_process | PTGES3 | 10 | 1 | 24.236842 | -1.392109 | 86 | 49.21 | 0.572209 |
| GO:0050657\_nucleic\_acid\_transport | KHDRBS1 | 10 | 1 | 24.236842 | -1.392109 | 86 | 49.21 | 0.572209 |
| GO:0050658\_RNA\_transport | KHDRBS1 | 10 | 1 | 24.236842 | -1.392109 | 86 | 49.21 | 0.572209 |
| GO:0051236\_establishment\_of\_RNA\_localization | KHDRBS1 | 10 | 1 | 24.236842 | -1.392109 | 86 | 49.21 | 0.572209 |
| GO:0051262\_protein\_tetramerization | SRR | 10 | 1 | 24.236842 | -1.392109 | 86 | 49.21 | 0.572209 |
| GO:0019725\_cellular\_homeostasis | ATP2A2 | 195 | 3 | 3.728745 | -1.357125 | 87 | 50.21 | 0.577126 |
| GO:0019725\_cellular\_homeostasis | SOD1 | 195 | 3 | 3.728745 | -1.357125 | 87 | 50.21 | 0.577126 |
| GO:0019725\_cellular\_homeostasis | PARK7 | 195 | 3 | 3.728745 | -1.357125 | 87 | 50.21 | 0.577126 |
| GO:0000271\_polysaccharide\_biosynthetic\_process | PTGES3 | 11 | 1 | 22.033493 | -1.351562 | 95 | 54.4 | 0.572632 |
| GO:0000737\_DNA\_catabolic\_process\_\_endonucleolytic | SOD1 | 11 | 1 | 22.033493 | -1.351562 | 95 | 54.4 | 0.572632 |
| GO:0001963\_synaptic\_transmission\_\_dopaminergic | PARK7 | 11 | 1 | 22.033493 | -1.351562 | 95 | 54.4 | 0.572632 |
| GO:0006690\_icosanoid\_metabolic\_process | PTGES3 | 11 | 1 | 22.033493 | -1.351562 | 95 | 54.4 | 0.572632 |
| GO:0008652\_cellular\_amino\_acid\_biosynthetic\_process | SRR | 11 | 1 | 22.033493 | -1.351562 | 95 | 54.4 | 0.572632 |
| GO:0033559\_unsaturated\_fatty\_acid\_metabolic\_process | PTGES3 | 11 | 1 | 22.033493 | -1.351562 | 95 | 54.4 | 0.572632 |
| GO:0046716\_muscle\_maintenance | SOD1 | 11 | 1 | 22.033493 | -1.351562 | 95 | 54.4 | 0.572632 |
| GO:0048678\_response\_to\_axon\_injury | SOD1 | 11 | 1 | 22.033493 | -1.351562 | 95 | 54.4 | 0.572632 |
| GO:0050801\_ion\_homeostasis | ATP2A2 | 197 | 3 | 3.690890 | -1.346021 | 96 | 54.86 | 0.571458 |
| GO:0050801\_ion\_homeostasis | SOD1 | 197 | 3 | 3.690890 | -1.346021 | 96 | 54.86 | 0.571458 |
| GO:0050801\_ion\_homeostasis | PARK7 | 197 | 3 | 3.690890 | -1.346021 | 96 | 54.86 | 0.571458 |
| GO:0030005\_cellular\_di-\_\_tri-valent\_inorganic\_cation\_homeostasis | ATP2A2 | 84 | 2 | 5.770677 | -1.337251 | 97 | 55.06 | 0.567629 |
| GO:0030005\_cellular\_di-\_\_tri-valent\_inorganic\_cation\_homeostasis | SOD1 | 84 | 2 | 5.770677 | -1.337251 | 97 | 55.06 | 0.567629 |
| GO:0002026\_regulation\_of\_the\_force\_of\_heart\_contraction | ATP2A2 | 12 | 1 | 20.197368 | -1.314619 | 105 | 59.34 | 0.565143 |
| GO:0006308\_DNA\_catabolic\_process | SOD1 | 12 | 1 | 20.197368 | -1.314619 | 105 | 59.34 | 0.565143 |
| GO:0006403\_RNA\_localization | KHDRBS1 | 12 | 1 | 20.197368 | -1.314619 | 105 | 59.34 | 0.565143 |
| GO:0006879\_cellular\_iron\_ion\_homeostasis | SOD1 | 12 | 1 | 20.197368 | -1.314619 | 105 | 59.34 | 0.565143 |
| GO:0015872\_dopamine\_transport | PARK7 | 12 | 1 | 20.197368 | -1.314619 | 105 | 59.34 | 0.565143 |
| GO:0030262\_apoptotic\_nuclear\_changes | SOD1 | 12 | 1 | 20.197368 | -1.314619 | 105 | 59.34 | 0.565143 |
| GO:0033598\_mammary\_gland\_epithelial\_cell\_proliferation | PHB2 | 12 | 1 | 20.197368 | -1.314619 | 105 | 59.34 | 0.565143 |
| GO:0045471\_response\_to\_ethanol | SOD1 | 12 | 1 | 20.197368 | -1.314619 | 105 | 59.34 | 0.565143 |
| GO:0030003\_cellular\_cation\_homeostasis | ATP2A2 | 90 | 2 | 5.385965 | -1.283313 | 106 | 60.41 | 0.569906 |
| GO:0030003\_cellular\_cation\_homeostasis | SOD1 | 90 | 2 | 5.385965 | -1.283313 | 106 | 60.41 | 0.569906 |
| GO:0006898\_receptor-mediated\_endocytosis | SNX17 | 13 | 1 | 18.643725 | -1.280702 | 107 | 64.31 | 0.601028 |
| GO:0055066\_di-\_\_tri-valent\_inorganic\_cation\_homeostasis | ATP2A2 | 93 | 2 | 5.212224 | -1.257841 | 108 | 65.0 | 0.601852 |
| GO:0055066\_di-\_\_tri-valent\_inorganic\_cation\_homeostasis | SOD1 | 93 | 2 | 5.212224 | -1.257841 | 108 | 65.0 | 0.601852 |
| GO:0002262\_myeloid\_cell\_homeostasis | SOD1 | 14 | 1 | 17.312030 | -1.249362 | 110 | 68.67 | 0.624273 |
| GO:0021782\_glial\_cell\_development | SOD1 | 14 | 1 | 17.312030 | -1.249362 | 110 | 68.67 | 0.624273 |
| GO:0042391\_regulation\_of\_membrane\_potential | SOD1 | 95 | 2 | 5.102493 | -1.241369 | 111 | 69.01 | 0.621712 |
| GO:0042391\_regulation\_of\_membrane\_potential | PARK7 | 95 | 2 | 5.102493 | -1.241369 | 111 | 69.01 | 0.621712 |
| GO:0006749\_glutathione\_metabolic\_process | SOD1 | 15 | 1 | 16.157895 | -1.220242 | 114 | 73.13 | 0.641491 |
| GO:0015931\_nucleobase\_\_nucleoside\_\_nucleotide\_and\_nucleic\_acid\_transport | KHDRBS1 | 15 | 1 | 16.157895 | -1.220242 | 114 | 73.13 | 0.641491 |
| GO:0060749\_mammary\_gland\_alveolus\_development | PHB2 | 15 | 1 | 16.157895 | -1.220242 | 114 | 73.13 | 0.641491 |
| GO:0007548\_sex\_differentiation | PHB2 | 98 | 2 | 4.946294 | -1.217386 | 115 | 73.52 | 0.639304 |
| GO:0007548\_sex\_differentiation | SOD1 | 98 | 2 | 4.946294 | -1.217386 | 115 | 73.52 | 0.639304 |
| GO:0042311\_vasodilation | SOD1 | 16 | 1 | 15.148026 | -1.193057 | 119 | 77.15 | 0.648319 |
| GO:0045104\_intermediate\_filament\_cytoskeleton\_organization | SOD1 | 16 | 1 | 15.148026 | -1.193057 | 119 | 77.15 | 0.648319 |
| GO:0046364\_monosaccharide\_biosynthetic\_process | PGD | 16 | 1 | 15.148026 | -1.193057 | 119 | 77.15 | 0.648319 |
| GO:0051937\_catecholamine\_transport | PARK7 | 16 | 1 | 15.148026 | -1.193057 | 119 | 77.15 | 0.648319 |
| GO:0008283\_cell\_proliferation | PTGES3 | 544 | 5 | 2.227651 | -1.190791 | 120 | 77.19 | 0.643250 |
| GO:0008283\_cell\_proliferation | CNBP | 544 | 5 | 2.227651 | -1.190791 | 120 | 77.19 | 0.643250 |
| GO:0008283\_cell\_proliferation | PHB2 | 544 | 5 | 2.227651 | -1.190791 | 120 | 77.19 | 0.643250 |
| GO:0008283\_cell\_proliferation | GRN | 544 | 5 | 2.227651 | -1.190791 | 120 | 77.19 | 0.643250 |
| GO:0008283\_cell\_proliferation | PARK7 | 544 | 5 | 2.227651 | -1.190791 | 120 | 77.19 | 0.643250 |
| GO:0003013\_circulatory\_system\_process | ATP2A2 | 103 | 2 | 4.706183 | -1.179214 | 122 | 77.73 | 0.637131 |
| GO:0003013\_circulatory\_system\_process | SOD1 | 103 | 2 | 4.706183 | -1.179214 | 122 | 77.73 | 0.637131 |
| GO:0008015\_blood\_circulation | ATP2A2 | 103 | 2 | 4.706183 | -1.179214 | 122 | 77.73 | 0.637131 |
| GO:0008015\_blood\_circulation | SOD1 | 103 | 2 | 4.706183 | -1.179214 | 122 | 77.73 | 0.637131 |
| GO:0006261\_DNA-dependent\_DNA\_replication | MCM4 | 17 | 1 | 14.256966 | -1.167572 | 126 | 81.48 | 0.646667 |
| GO:0006984\_ER-nuclear\_signaling\_pathway | ATP2A2 | 17 | 1 | 14.256966 | -1.167572 | 126 | 81.48 | 0.646667 |
| GO:0009408\_response\_to\_heat | SOD1 | 17 | 1 | 14.256966 | -1.167572 | 126 | 81.48 | 0.646667 |
| GO:0055072\_iron\_ion\_homeostasis | SOD1 | 17 | 1 | 14.256966 | -1.167572 | 126 | 81.48 | 0.646667 |
| GO:0006940\_regulation\_of\_smooth\_muscle\_contraction | SOD1 | 18 | 1 | 13.464912 | -1.143591 | 128 | 85.89 | 0.671016 |
| GO:0051168\_nuclear\_export | KHDRBS1 | 18 | 1 | 13.464912 | -1.143591 | 128 | 85.89 | 0.671016 |
| GO:0055080\_cation\_homeostasis | ATP2A2 | 110 | 2 | 4.406699 | -1.129199 | 129 | 86.55 | 0.670930 |
| GO:0055080\_cation\_homeostasis | SOD1 | 110 | 2 | 4.406699 | -1.129199 | 129 | 86.55 | 0.670930 |
| GO:0009056\_catabolic\_process | ZFP36L1 | 243 | 3 | 2.992203 | -1.123239 | 130 | 86.65 | 0.666538 |
| GO:0009056\_catabolic\_process | ARIH2 | 243 | 3 | 2.992203 | -1.123239 | 130 | 86.65 | 0.666538 |
| GO:0009056\_catabolic\_process | SOD1 | 243 | 3 | 2.992203 | -1.123239 | 130 | 86.65 | 0.666538 |
| GO:0042491\_auditory\_receptor\_cell\_differentiation | SOD1 | 19 | 1 | 12.756233 | -1.120952 | 133 | 89.11 | 0.670000 |
| GO:0046165\_alcohol\_biosynthetic\_process | PGD | 19 | 1 | 12.756233 | -1.120952 | 133 | 89.11 | 0.670000 |
| GO:0060444\_branching\_involved\_in\_mammary\_gland\_duct\_morphogenesis | PHB2 | 19 | 1 | 12.756233 | -1.120952 | 133 | 89.11 | 0.670000 |
| GO:0044249\_cellular\_biosynthetic\_process | MRPL11 | 1150 | 8 | 1.686041 | -1.115704 | 134 | 89.23 | 0.665896 |
| GO:0044249\_cellular\_biosynthetic\_process | KHDRBS1 | 1150 | 8 | 1.686041 | -1.115704 | 134 | 89.23 | 0.665896 |
| GO:0044249\_cellular\_biosynthetic\_process | ZFP36L1 | 1150 | 8 | 1.686041 | -1.115704 | 134 | 89.23 | 0.665896 |
| GO:0044249\_cellular\_biosynthetic\_process | PTGES3 | 1150 | 8 | 1.686041 | -1.115704 | 134 | 89.23 | 0.665896 |
| GO:0044249\_cellular\_biosynthetic\_process | CNBP | 1150 | 8 | 1.686041 | -1.115704 | 134 | 89.23 | 0.665896 |
| GO:0044249\_cellular\_biosynthetic\_process | SRR | 1150 | 8 | 1.686041 | -1.115704 | 134 | 89.23 | 0.665896 |
| GO:0044249\_cellular\_biosynthetic\_process | SOD1 | 1150 | 8 | 1.686041 | -1.115704 | 134 | 89.23 | 0.665896 |
| GO:0044249\_cellular\_biosynthetic\_process | MCM4 | 1150 | 8 | 1.686041 | -1.115704 | 134 | 89.23 | 0.665896 |
| GO:0005977\_glycogen\_metabolic\_process | PTGES3 | 20 | 1 | 12.118421 | -1.099518 | 139 | 92.45 | 0.665108 |
| GO:0006073\_cellular\_glucan\_metabolic\_process | PTGES3 | 20 | 1 | 12.118421 | -1.099518 | 139 | 92.45 | 0.665108 |
| GO:0006518\_peptide\_metabolic\_process | SOD1 | 20 | 1 | 12.118421 | -1.099518 | 139 | 92.45 | 0.665108 |
| GO:0044042\_glucan\_metabolic\_process | PTGES3 | 20 | 1 | 12.118421 | -1.099518 | 139 | 92.45 | 0.665108 |
| GO:0046822\_regulation\_of\_nucleocytoplasmic\_transport | KHDRBS1 | 20 | 1 | 12.118421 | -1.099518 | 139 | 92.45 | 0.665108 |
| GO:0007242\_intracellular\_signaling\_cascade | PTGES3 | 411 | 4 | 2.358817 | -1.081881 | 140 | 93.22 | 0.665857 |
| GO:0007242\_intracellular\_signaling\_cascade | ATP2A2 | 411 | 4 | 2.358817 | -1.081881 | 140 | 93.22 | 0.665857 |
| GO:0007242\_intracellular\_signaling\_cascade | PHB2 | 411 | 4 | 2.358817 | -1.081881 | 140 | 93.22 | 0.665857 |
| GO:0007242\_intracellular\_signaling\_cascade | SOD1 | 411 | 4 | 2.358817 | -1.081881 | 140 | 93.22 | 0.665857 |
| GO:0006633\_fatty\_acid\_biosynthetic\_process | PTGES3 | 21 | 1 | 11.541353 | -1.079170 | 142 | 96.86 | 0.682113 |
| GO:0015844\_monoamine\_transport | PARK7 | 21 | 1 | 11.541353 | -1.079170 | 142 | 96.86 | 0.682113 |
| GO:0048878\_chemical\_homeostasis | ATP2A2 | 254 | 3 | 2.862619 | -1.077716 | 143 | 97.01 | 0.678392 |
| GO:0048878\_chemical\_homeostasis | SOD1 | 254 | 3 | 2.862619 | -1.077716 | 143 | 97.01 | 0.678392 |
| GO:0048878\_chemical\_homeostasis | PARK7 | 254 | 3 | 2.862619 | -1.077716 | 143 | 97.01 | 0.678392 |
| GO:0006519\_cellular\_amino\_acid\_and\_derivative\_metabolic\_process | SRR | 118 | 2 | 4.107939 | -1.076358 | 144 | 97.16 | 0.674722 |
| GO:0006519\_cellular\_amino\_acid\_and\_derivative\_metabolic\_process | SOD1 | 118 | 2 | 4.107939 | -1.076358 | 144 | 97.16 | 0.674722 |
| GO:0006112\_energy\_reserve\_metabolic\_process | PTGES3 | 22 | 1 | 11.016746 | -1.059808 | 148 | 101.49 | 0.685743 |
| GO:0009309\_amine\_biosynthetic\_process | SRR | 22 | 1 | 11.016746 | -1.059808 | 148 | 101.49 | 0.685743 |
| GO:0044264\_cellular\_polysaccharide\_metabolic\_process | PTGES3 | 22 | 1 | 11.016746 | -1.059808 | 148 | 101.49 | 0.685743 |
| GO:0051260\_protein\_homooligomerization | SRR | 22 | 1 | 11.016746 | -1.059808 | 148 | 101.49 | 0.685743 |
| GO:0034960\_cellular\_biopolymer\_metabolic\_process | PTGES3 | 1395 | 9 | 1.563667 | -1.055161 | 149 | 101.94 | 0.684161 |
| GO:0034960\_cellular\_biopolymer\_metabolic\_process | MRPL11 | 1395 | 9 | 1.563667 | -1.055161 | 149 | 101.94 | 0.684161 |
| GO:0034960\_cellular\_biopolymer\_metabolic\_process | KHDRBS1 | 1395 | 9 | 1.563667 | -1.055161 | 149 | 101.94 | 0.684161 |
| GO:0034960\_cellular\_biopolymer\_metabolic\_process | ZFP36L1 | 1395 | 9 | 1.563667 | -1.055161 | 149 | 101.94 | 0.684161 |
| GO:0034960\_cellular\_biopolymer\_metabolic\_process | PABPN1 | 1395 | 9 | 1.563667 | -1.055161 | 149 | 101.94 | 0.684161 |
| GO:0034960\_cellular\_biopolymer\_metabolic\_process | ARIH2 | 1395 | 9 | 1.563667 | -1.055161 | 149 | 101.94 | 0.684161 |
| GO:0034960\_cellular\_biopolymer\_metabolic\_process | CNBP | 1395 | 9 | 1.563667 | -1.055161 | 149 | 101.94 | 0.684161 |
| GO:0034960\_cellular\_biopolymer\_metabolic\_process | SOD1 | 1395 | 9 | 1.563667 | -1.055161 | 149 | 101.94 | 0.684161 |
| GO:0034960\_cellular\_biopolymer\_metabolic\_process | MCM4 | 1395 | 9 | 1.563667 | -1.055161 | 149 | 101.94 | 0.684161 |
| GO:0006397\_mRNA\_processing | PABPN1 | 23 | 1 | 10.537757 | -1.041343 | 150 | 105.15 | 0.701000 |
| GO:0001541\_ovarian\_follicle\_development | SOD1 | 24 | 1 | 10.098684 | -1.023700 | 155 | 108.55 | 0.700323 |
| GO:0032386\_regulation\_of\_intracellular\_transport | KHDRBS1 | 24 | 1 | 10.098684 | -1.023700 | 155 | 108.55 | 0.700323 |
| GO:0043588\_skin\_development | PTGES3 | 24 | 1 | 10.098684 | -1.023700 | 155 | 108.55 | 0.700323 |
| GO:0050679\_positive\_regulation\_of\_epithelial\_cell\_proliferation | GRN | 24 | 1 | 10.098684 | -1.023700 | 155 | 108.55 | 0.700323 |
| GO:0060113\_inner\_ear\_receptor\_cell\_differentiation | SOD1 | 24 | 1 | 10.098684 | -1.023700 | 155 | 108.55 | 0.700323 |
| GO:0034961\_cellular\_biopolymer\_biosynthetic\_process | PTGES3 | 804 | 6 | 1.808720 | -1.007081 | 156 | 109.84 | 0.704103 |
| GO:0034961\_cellular\_biopolymer\_biosynthetic\_process | MRPL11 | 804 | 6 | 1.808720 | -1.007081 | 156 | 109.84 | 0.704103 |
| GO:0034961\_cellular\_biopolymer\_biosynthetic\_process | ZFP36L1 | 804 | 6 | 1.808720 | -1.007081 | 156 | 109.84 | 0.704103 |
| GO:0034961\_cellular\_biopolymer\_biosynthetic\_process | KHDRBS1 | 804 | 6 | 1.808720 | -1.007081 | 156 | 109.84 | 0.704103 |
| GO:0034961\_cellular\_biopolymer\_biosynthetic\_process | CNBP | 804 | 6 | 1.808720 | -1.007081 | 156 | 109.84 | 0.704103 |
| GO:0034961\_cellular\_biopolymer\_biosynthetic\_process | MCM4 | 804 | 6 | 1.808720 | -1.007081 | 156 | 109.84 | 0.704103 |
| GO:0006302\_double-strand\_break\_repair | SOD1 | 25 | 1 | 9.694737 | -1.006811 | 159 | 112.24 | 0.705912 |
| GO:0060603\_mammary\_gland\_duct\_morphogenesis | PHB2 | 25 | 1 | 9.694737 | -1.006811 | 159 | 112.24 | 0.705912 |
| GO:0060688\_regulation\_of\_morphogenesis\_of\_a\_branching\_structure | PHB2 | 25 | 1 | 9.694737 | -1.006811 | 159 | 112.24 | 0.705912 |
| GO:0043284\_biopolymer\_biosynthetic\_process | MRPL11 | 807 | 6 | 1.801996 | -1.000835 | 160 | 112.91 | 0.705688 |
| GO:0043284\_biopolymer\_biosynthetic\_process | ZFP36L1 | 807 | 6 | 1.801996 | -1.000835 | 160 | 112.91 | 0.705688 |
| GO:0043284\_biopolymer\_biosynthetic\_process | KHDRBS1 | 807 | 6 | 1.801996 | -1.000835 | 160 | 112.91 | 0.705688 |
| GO:0043284\_biopolymer\_biosynthetic\_process | PTGES3 | 807 | 6 | 1.801996 | -1.000835 | 160 | 112.91 | 0.705688 |
| GO:0043284\_biopolymer\_biosynthetic\_process | CNBP | 807 | 6 | 1.801996 | -1.000835 | 160 | 112.91 | 0.705688 |
| GO:0043284\_biopolymer\_biosynthetic\_process | MCM4 | 807 | 6 | 1.801996 | -1.000835 | 160 | 112.91 | 0.705688 |
| GO:0050680\_negative\_regulation\_of\_epithelial\_cell\_proliferation | PHB2 | 26 | 1 | 9.321862 | -0.990617 | 161 | 115.57 | 0.717826 |
| GO:0044057\_regulation\_of\_system\_process | ATP2A2 | 133 | 2 | 3.644638 | -0.987730 | 162 | 115.96 | 0.715802 |
| GO:0044057\_regulation\_of\_system\_process | SOD1 | 133 | 2 | 3.644638 | -0.987730 | 162 | 115.96 | 0.715802 |
| GO:0007422\_peripheral\_nervous\_system\_development | SOD1 | 27 | 1 | 8.976608 | -0.975066 | 164 | 118.46 | 0.722317 |
| GO:0031016\_pancreas\_development | INVS | 27 | 1 | 8.976608 | -0.975066 | 164 | 118.46 | 0.722317 |
| GO:0008152\_metabolic\_process | MRPL11 | 2133 | 12 | 1.363535 | -0.971715 | 165 | 118.64 | 0.719030 |
| GO:0008152\_metabolic\_process | PTGES3 | 2133 | 12 | 1.363535 | -0.971715 | 165 | 118.64 | 0.719030 |
| GO:0008152\_metabolic\_process | ZFP36L1 | 2133 | 12 | 1.363535 | -0.971715 | 165 | 118.64 | 0.719030 |
| GO:0008152\_metabolic\_process | PABPN1 | 2133 | 12 | 1.363535 | -0.971715 | 165 | 118.64 | 0.719030 |
| GO:0008152\_metabolic\_process | KHDRBS1 | 2133 | 12 | 1.363535 | -0.971715 | 165 | 118.64 | 0.719030 |
| GO:0008152\_metabolic\_process | ARIH2 | 2133 | 12 | 1.363535 | -0.971715 | 165 | 118.64 | 0.719030 |
| GO:0008152\_metabolic\_process | CNBP | 2133 | 12 | 1.363535 | -0.971715 | 165 | 118.64 | 0.719030 |
| GO:0008152\_metabolic\_process | PGD | 2133 | 12 | 1.363535 | -0.971715 | 165 | 118.64 | 0.719030 |
| GO:0008152\_metabolic\_process | SRR | 2133 | 12 | 1.363535 | -0.971715 | 165 | 118.64 | 0.719030 |
| GO:0008152\_metabolic\_process | SOD1 | 2133 | 12 | 1.363535 | -0.971715 | 165 | 118.64 | 0.719030 |
| GO:0008152\_metabolic\_process | MCM4 | 2133 | 12 | 1.363535 | -0.971715 | 165 | 118.64 | 0.719030 |
| GO:0008152\_metabolic\_process | PARK7 | 2133 | 12 | 1.363535 | -0.971715 | 165 | 118.64 | 0.719030 |
| GO:0044260\_cellular\_macromolecule\_metabolic\_process | PABPN1 | 1447 | 9 | 1.507475 | -0.969295 | 166 | 118.84 | 0.715904 |
| GO:0044260\_cellular\_macromolecule\_metabolic\_process | PTGES3 | 1447 | 9 | 1.507475 | -0.969295 | 166 | 118.84 | 0.715904 |
| GO:0044260\_cellular\_macromolecule\_metabolic\_process | MRPL11 | 1447 | 9 | 1.507475 | -0.969295 | 166 | 118.84 | 0.715904 |
| GO:0044260\_cellular\_macromolecule\_metabolic\_process | ZFP36L1 | 1447 | 9 | 1.507475 | -0.969295 | 166 | 118.84 | 0.715904 |
| GO:0044260\_cellular\_macromolecule\_metabolic\_process | KHDRBS1 | 1447 | 9 | 1.507475 | -0.969295 | 166 | 118.84 | 0.715904 |
| GO:0044260\_cellular\_macromolecule\_metabolic\_process | ARIH2 | 1447 | 9 | 1.507475 | -0.969295 | 166 | 118.84 | 0.715904 |
| GO:0044260\_cellular\_macromolecule\_metabolic\_process | CNBP | 1447 | 9 | 1.507475 | -0.969295 | 166 | 118.84 | 0.715904 |
| GO:0044260\_cellular\_macromolecule\_metabolic\_process | SOD1 | 1447 | 9 | 1.507475 | -0.969295 | 166 | 118.84 | 0.715904 |
| GO:0044260\_cellular\_macromolecule\_metabolic\_process | MCM4 | 1447 | 9 | 1.507475 | -0.969295 | 166 | 118.84 | 0.715904 |
| GO:0006997\_nucleus\_organization | SOD1 | 28 | 1 | 8.656015 | -0.960110 | 167 | 121.32 | 0.726467 |
| GO:0044238\_primary\_metabolic\_process | MRPL11 | 1905 | 11 | 1.399503 | -0.959947 | 168 | 121.4 | 0.722619 |
| GO:0044238\_primary\_metabolic\_process | ZFP36L1 | 1905 | 11 | 1.399503 | -0.959947 | 168 | 121.4 | 0.722619 |
| GO:0044238\_primary\_metabolic\_process | PABPN1 | 1905 | 11 | 1.399503 | -0.959947 | 168 | 121.4 | 0.722619 |
| GO:0044238\_primary\_metabolic\_process | KHDRBS1 | 1905 | 11 | 1.399503 | -0.959947 | 168 | 121.4 | 0.722619 |
| GO:0044238\_primary\_metabolic\_process | PTGES3 | 1905 | 11 | 1.399503 | -0.959947 | 168 | 121.4 | 0.722619 |
| GO:0044238\_primary\_metabolic\_process | ARIH2 | 1905 | 11 | 1.399503 | -0.959947 | 168 | 121.4 | 0.722619 |
| GO:0044238\_primary\_metabolic\_process | CNBP | 1905 | 11 | 1.399503 | -0.959947 | 168 | 121.4 | 0.722619 |
| GO:0044238\_primary\_metabolic\_process | PGD | 1905 | 11 | 1.399503 | -0.959947 | 168 | 121.4 | 0.722619 |
| GO:0044238\_primary\_metabolic\_process | SRR | 1905 | 11 | 1.399503 | -0.959947 | 168 | 121.4 | 0.722619 |
| GO:0044238\_primary\_metabolic\_process | SOD1 | 1905 | 11 | 1.399503 | -0.959947 | 168 | 121.4 | 0.722619 |
| GO:0044238\_primary\_metabolic\_process | MCM4 | 1905 | 11 | 1.399503 | -0.959947 | 168 | 121.4 | 0.722619 |
| GO:0016044\_membrane\_organization | SNX17 | 140 | 2 | 3.462406 | -0.950338 | 169 | 121.8 | 0.720710 |
| GO:0016044\_membrane\_organization | SOD1 | 140 | 2 | 3.462406 | -0.950338 | 169 | 121.8 | 0.720710 |
| GO:0006417\_regulation\_of\_translation | ZFP36L1 | 29 | 1 | 8.357532 | -0.945708 | 171 | 124.23 | 0.726491 |
| GO:0042490\_mechanoreceptor\_differentiation | SOD1 | 29 | 1 | 8.357532 | -0.945708 | 171 | 124.23 | 0.726491 |
| GO:0003006\_reproductive\_developmental\_process | PHB2 | 141 | 2 | 3.437850 | -0.945179 | 172 | 124.35 | 0.722965 |
| GO:0003006\_reproductive\_developmental\_process | SOD1 | 141 | 2 | 3.437850 | -0.945179 | 172 | 124.35 | 0.722965 |
| GO:0000187\_activation\_of\_MAPK\_activity | SOD1 | 30 | 1 | 8.078947 | -0.931822 | 176 | 127.19 | 0.722670 |
| GO:0009266\_response\_to\_temperature\_stimulus | SOD1 | 30 | 1 | 8.078947 | -0.931822 | 176 | 127.19 | 0.722670 |
| GO:0022411\_cellular\_component\_disassembly | SOD1 | 30 | 1 | 8.078947 | -0.931822 | 176 | 127.19 | 0.722670 |
| GO:0042552\_myelination | SOD1 | 30 | 1 | 8.078947 | -0.931822 | 176 | 127.19 | 0.722670 |
| GO:0005975\_carbohydrate\_metabolic\_process | PTGES3 | 146 | 2 | 3.320115 | -0.920030 | 177 | 128.07 | 0.723559 |
| GO:0005975\_carbohydrate\_metabolic\_process | PGD | 146 | 2 | 3.320115 | -0.920030 | 177 | 128.07 | 0.723559 |
| GO:0003018\_vascular\_process\_in\_circulatory\_system | SOD1 | 31 | 1 | 7.818336 | -0.918419 | 182 | 131.72 | 0.723736 |
| GO:0006939\_smooth\_muscle\_contraction | SOD1 | 31 | 1 | 7.818336 | -0.918419 | 182 | 131.72 | 0.723736 |
| GO:0035150\_regulation\_of\_tube\_size | SOD1 | 31 | 1 | 7.818336 | -0.918419 | 182 | 131.72 | 0.723736 |
| GO:0050880\_regulation\_of\_blood\_vessel\_size | SOD1 | 31 | 1 | 7.818336 | -0.918419 | 182 | 131.72 | 0.723736 |
| GO:0051899\_membrane\_depolarization | PARK7 | 31 | 1 | 7.818336 | -0.918419 | 182 | 131.72 | 0.723736 |
| GO:0007272\_ensheathment\_of\_neurons | SOD1 | 32 | 1 | 7.574013 | -0.905467 | 185 | 134.18 | 0.725297 |
| GO:0008366\_axon\_ensheathment | SOD1 | 32 | 1 | 7.574013 | -0.905467 | 185 | 134.18 | 0.725297 |
| GO:0051259\_protein\_oligomerization | SRR | 32 | 1 | 7.574013 | -0.905467 | 185 | 134.18 | 0.725297 |
| GO:0043283\_biopolymer\_metabolic\_process | KHDRBS1 | 1490 | 9 | 1.463970 | -0.902554 | 186 | 134.34 | 0.722258 |
| GO:0043283\_biopolymer\_metabolic\_process | PABPN1 | 1490 | 9 | 1.463970 | -0.902554 | 186 | 134.34 | 0.722258 |
| GO:0043283\_biopolymer\_metabolic\_process | MRPL11 | 1490 | 9 | 1.463970 | -0.902554 | 186 | 134.34 | 0.722258 |
| GO:0043283\_biopolymer\_metabolic\_process | ZFP36L1 | 1490 | 9 | 1.463970 | -0.902554 | 186 | 134.34 | 0.722258 |
| GO:0043283\_biopolymer\_metabolic\_process | PTGES3 | 1490 | 9 | 1.463970 | -0.902554 | 186 | 134.34 | 0.722258 |
| GO:0043283\_biopolymer\_metabolic\_process | CNBP | 1490 | 9 | 1.463970 | -0.902554 | 186 | 134.34 | 0.722258 |
| GO:0043283\_biopolymer\_metabolic\_process | ARIH2 | 1490 | 9 | 1.463970 | -0.902554 | 186 | 134.34 | 0.722258 |
| GO:0043283\_biopolymer\_metabolic\_process | SOD1 | 1490 | 9 | 1.463970 | -0.902554 | 186 | 134.34 | 0.722258 |
| GO:0043283\_biopolymer\_metabolic\_process | MCM4 | 1490 | 9 | 1.463970 | -0.902554 | 186 | 134.34 | 0.722258 |
| GO:0007270\_nerve-nerve\_synaptic\_transmission | PARK7 | 33 | 1 | 7.344498 | -0.892940 | 187 | 136.33 | 0.729037 |
| GO:0007568\_aging | SOD1 | 34 | 1 | 7.128483 | -0.880810 | 189 | 139.47 | 0.737937 |
| GO:0060443\_mammary\_gland\_morphogenesis | PHB2 | 34 | 1 | 7.128483 | -0.880810 | 189 | 139.47 | 0.737937 |
| GO:0043406\_positive\_regulation\_of\_MAP\_kinase\_activity | SOD1 | 35 | 1 | 6.924812 | -0.869057 | 190 | 141.58 | 0.745158 |
| GO:0051704\_multi-organism\_process | GRN | 157 | 2 | 3.087496 | -0.868203 | 191 | 141.68 | 0.741780 |
| GO:0051704\_multi-organism\_process | SOD1 | 157 | 2 | 3.087496 | -0.868203 | 191 | 141.68 | 0.741780 |
| GO:0044237\_cellular\_metabolic\_process | MRPL11 | 1974 | 11 | 1.350584 | -0.862592 | 192 | 142.12 | 0.740208 |
| GO:0044237\_cellular\_metabolic\_process | ZFP36L1 | 1974 | 11 | 1.350584 | -0.862592 | 192 | 142.12 | 0.740208 |
| GO:0044237\_cellular\_metabolic\_process | PABPN1 | 1974 | 11 | 1.350584 | -0.862592 | 192 | 142.12 | 0.740208 |
| GO:0044237\_cellular\_metabolic\_process | KHDRBS1 | 1974 | 11 | 1.350584 | -0.862592 | 192 | 142.12 | 0.740208 |
| GO:0044237\_cellular\_metabolic\_process | PTGES3 | 1974 | 11 | 1.350584 | -0.862592 | 192 | 142.12 | 0.740208 |
| GO:0044237\_cellular\_metabolic\_process | ARIH2 | 1974 | 11 | 1.350584 | -0.862592 | 192 | 142.12 | 0.740208 |
| GO:0044237\_cellular\_metabolic\_process | CNBP | 1974 | 11 | 1.350584 | -0.862592 | 192 | 142.12 | 0.740208 |
| GO:0044237\_cellular\_metabolic\_process | SRR | 1974 | 11 | 1.350584 | -0.862592 | 192 | 142.12 | 0.740208 |
| GO:0044237\_cellular\_metabolic\_process | SOD1 | 1974 | 11 | 1.350584 | -0.862592 | 192 | 142.12 | 0.740208 |
| GO:0044237\_cellular\_metabolic\_process | MCM4 | 1974 | 11 | 1.350584 | -0.862592 | 192 | 142.12 | 0.740208 |
| GO:0044237\_cellular\_metabolic\_process | PARK7 | 1974 | 11 | 1.350584 | -0.862592 | 192 | 142.12 | 0.740208 |
| GO:0019228\_regulation\_of\_action\_potential\_in\_neuron | SOD1 | 36 | 1 | 6.732456 | -0.857657 | 194 | 144.72 | 0.745979 |
| GO:0022602\_ovulation\_cycle\_process | SOD1 | 36 | 1 | 6.732456 | -0.857657 | 194 | 144.72 | 0.745979 |
| GO:0042698\_ovulation\_cycle | SOD1 | 37 | 1 | 6.550498 | -0.846592 | 195 | 146.81 | 0.752872 |
| GO:0007626\_locomotory\_behavior | SOD1 | 163 | 2 | 2.973846 | -0.841773 | 196 | 147.29 | 0.751480 |
| GO:0007626\_locomotory\_behavior | PARK7 | 163 | 2 | 2.973846 | -0.841773 | 196 | 147.29 | 0.751480 |
| GO:0001570\_vasculogenesis | ZFP36L1 | 38 | 1 | 6.378116 | -0.835845 | 199 | 150.35 | 0.755528 |
| GO:0008016\_regulation\_of\_heart\_contraction | ATP2A2 | 38 | 1 | 6.378116 | -0.835845 | 199 | 150.35 | 0.755528 |
| GO:0042493\_response\_to\_drug | SOD1 | 38 | 1 | 6.378116 | -0.835845 | 199 | 150.35 | 0.755528 |
| GO:0006259\_DNA\_metabolic\_process | SOD1 | 165 | 2 | 2.937799 | -0.833229 | 200 | 150.78 | 0.753900 |
| GO:0006259\_DNA\_metabolic\_process | MCM4 | 165 | 2 | 2.937799 | -0.833229 | 200 | 150.78 | 0.753900 |
| GO:0005976\_polysaccharide\_metabolic\_process | PTGES3 | 39 | 1 | 6.214575 | -0.825397 | 203 | 153.44 | 0.755862 |
| GO:0006511\_ubiquitin-dependent\_protein\_catabolic\_process | ARIH2 | 39 | 1 | 6.214575 | -0.825397 | 203 | 153.44 | 0.755862 |
| GO:0043524\_negative\_regulation\_of\_neuron\_apoptosis | SOD1 | 39 | 1 | 6.214575 | -0.825397 | 203 | 153.44 | 0.755862 |
| GO:0034645\_cellular\_macromolecule\_biosynthetic\_process | PTGES3 | 901 | 6 | 1.613996 | -0.822694 | 204 | 153.89 | 0.754363 |
| GO:0034645\_cellular\_macromolecule\_biosynthetic\_process | MRPL11 | 901 | 6 | 1.613996 | -0.822694 | 204 | 153.89 | 0.754363 |
| GO:0034645\_cellular\_macromolecule\_biosynthetic\_process | ZFP36L1 | 901 | 6 | 1.613996 | -0.822694 | 204 | 153.89 | 0.754363 |
| GO:0034645\_cellular\_macromolecule\_biosynthetic\_process | KHDRBS1 | 901 | 6 | 1.613996 | -0.822694 | 204 | 153.89 | 0.754363 |
| GO:0034645\_cellular\_macromolecule\_biosynthetic\_process | CNBP | 901 | 6 | 1.613996 | -0.822694 | 204 | 153.89 | 0.754363 |
| GO:0034645\_cellular\_macromolecule\_biosynthetic\_process | MCM4 | 901 | 6 | 1.613996 | -0.822694 | 204 | 153.89 | 0.754363 |
| GO:0001824\_blastocyst\_development | GRN | 40 | 1 | 6.059211 | -0.815235 | 205 | 156.93 | 0.765512 |
| GO:0009059\_macromolecule\_biosynthetic\_process | PTGES3 | 910 | 6 | 1.598034 | -0.807285 | 206 | 157.48 | 0.764466 |
| GO:0009059\_macromolecule\_biosynthetic\_process | MRPL11 | 910 | 6 | 1.598034 | -0.807285 | 206 | 157.48 | 0.764466 |
| GO:0009059\_macromolecule\_biosynthetic\_process | KHDRBS1 | 910 | 6 | 1.598034 | -0.807285 | 206 | 157.48 | 0.764466 |
| GO:0009059\_macromolecule\_biosynthetic\_process | ZFP36L1 | 910 | 6 | 1.598034 | -0.807285 | 206 | 157.48 | 0.764466 |
| GO:0009059\_macromolecule\_biosynthetic\_process | CNBP | 910 | 6 | 1.598034 | -0.807285 | 206 | 157.48 | 0.764466 |
| GO:0009059\_macromolecule\_biosynthetic\_process | MCM4 | 910 | 6 | 1.598034 | -0.807285 | 206 | 157.48 | 0.764466 |
| GO:0001776\_leukocyte\_homeostasis | SOD1 | 41 | 1 | 5.911425 | -0.805344 | 211 | 160.78 | 0.761991 |
| GO:0006260\_DNA\_replication | MCM4 | 41 | 1 | 5.911425 | -0.805344 | 211 | 160.78 | 0.761991 |
| GO:0006836\_neurotransmitter\_transport | PARK7 | 41 | 1 | 5.911425 | -0.805344 | 211 | 160.78 | 0.761991 |
| GO:0008585\_female\_gonad\_development | SOD1 | 41 | 1 | 5.911425 | -0.805344 | 211 | 160.78 | 0.761991 |
| GO:0015980\_energy\_derivation\_by\_oxidation\_of\_organic\_compounds | PTGES3 | 41 | 1 | 5.911425 | -0.805344 | 211 | 160.78 | 0.761991 |
| GO:0019941\_modification-dependent\_protein\_catabolic\_process | ARIH2 | 42 | 1 | 5.770677 | -0.795711 | 214 | 164.51 | 0.768738 |
| GO:0043632\_modification-dependent\_macromolecule\_catabolic\_process | ARIH2 | 42 | 1 | 5.770677 | -0.795711 | 214 | 164.51 | 0.768738 |
| GO:0051603\_proteolysis\_involved\_in\_cellular\_protein\_catabolic\_process | ARIH2 | 42 | 1 | 5.770677 | -0.795711 | 214 | 164.51 | 0.768738 |
| GO:0001508\_regulation\_of\_action\_potential | SOD1 | 43 | 1 | 5.636475 | -0.786323 | 217 | 168.8 | 0.777880 |
| GO:0001894\_tissue\_homeostasis | SOD1 | 43 | 1 | 5.636475 | -0.786323 | 217 | 168.8 | 0.777880 |
| GO:0010001\_glial\_cell\_differentiation | SOD1 | 43 | 1 | 5.636475 | -0.786323 | 217 | 168.8 | 0.777880 |
| GO:0043170\_macromolecule\_metabolic\_process | PABPN1 | 1576 | 9 | 1.384084 | -0.779827 | 218 | 168.99 | 0.775183 |
| GO:0043170\_macromolecule\_metabolic\_process | PTGES3 | 1576 | 9 | 1.384084 | -0.779827 | 218 | 168.99 | 0.775183 |
| GO:0043170\_macromolecule\_metabolic\_process | MRPL11 | 1576 | 9 | 1.384084 | -0.779827 | 218 | 168.99 | 0.775183 |
| GO:0043170\_macromolecule\_metabolic\_process | KHDRBS1 | 1576 | 9 | 1.384084 | -0.779827 | 218 | 168.99 | 0.775183 |
| GO:0043170\_macromolecule\_metabolic\_process | ZFP36L1 | 1576 | 9 | 1.384084 | -0.779827 | 218 | 168.99 | 0.775183 |
| GO:0043170\_macromolecule\_metabolic\_process | CNBP | 1576 | 9 | 1.384084 | -0.779827 | 218 | 168.99 | 0.775183 |
| GO:0043170\_macromolecule\_metabolic\_process | ARIH2 | 1576 | 9 | 1.384084 | -0.779827 | 218 | 168.99 | 0.775183 |
| GO:0043170\_macromolecule\_metabolic\_process | SOD1 | 1576 | 9 | 1.384084 | -0.779827 | 218 | 168.99 | 0.775183 |
| GO:0043170\_macromolecule\_metabolic\_process | MCM4 | 1576 | 9 | 1.384084 | -0.779827 | 218 | 168.99 | 0.775183 |
| GO:0044257\_cellular\_protein\_catabolic\_process | ARIH2 | 44 | 1 | 5.508373 | -0.777170 | 220 | 172.51 | 0.784136 |
| GO:0046545\_development\_of\_primary\_female\_sexual\_characteristics | SOD1 | 44 | 1 | 5.508373 | -0.777170 | 220 | 172.51 | 0.784136 |
| GO:0019752\_carboxylic\_acid\_metabolic\_process | PTGES3 | 181 | 2 | 2.678104 | -0.769224 | 222 | 173.99 | 0.783739 |
| GO:0019752\_carboxylic\_acid\_metabolic\_process | SRR | 181 | 2 | 2.678104 | -0.769224 | 222 | 173.99 | 0.783739 |
| GO:0043436\_oxoacid\_metabolic\_process | PTGES3 | 181 | 2 | 2.678104 | -0.769224 | 222 | 173.99 | 0.783739 |
| GO:0043436\_oxoacid\_metabolic\_process | SRR | 181 | 2 | 2.678104 | -0.769224 | 222 | 173.99 | 0.783739 |
| GO:0006082\_organic\_acid\_metabolic\_process | PTGES3 | 182 | 2 | 2.663389 | -0.765462 | 223 | 175.37 | 0.786413 |
| GO:0006082\_organic\_acid\_metabolic\_process | SRR | 182 | 2 | 2.663389 | -0.765462 | 223 | 175.37 | 0.786413 |
| GO:0006807\_nitrogen\_compound\_metabolic\_process | ZFP36L1 | 1147 | 7 | 1.479145 | -0.764672 | 224 | 175.49 | 0.783438 |
| GO:0006807\_nitrogen\_compound\_metabolic\_process | PABPN1 | 1147 | 7 | 1.479145 | -0.764672 | 224 | 175.49 | 0.783438 |
| GO:0006807\_nitrogen\_compound\_metabolic\_process | KHDRBS1 | 1147 | 7 | 1.479145 | -0.764672 | 224 | 175.49 | 0.783438 |
| GO:0006807\_nitrogen\_compound\_metabolic\_process | CNBP | 1147 | 7 | 1.479145 | -0.764672 | 224 | 175.49 | 0.783438 |
| GO:0006807\_nitrogen\_compound\_metabolic\_process | SRR | 1147 | 7 | 1.479145 | -0.764672 | 224 | 175.49 | 0.783438 |
| GO:0006807\_nitrogen\_compound\_metabolic\_process | SOD1 | 1147 | 7 | 1.479145 | -0.764672 | 224 | 175.49 | 0.783438 |
| GO:0006807\_nitrogen\_compound\_metabolic\_process | MCM4 | 1147 | 7 | 1.479145 | -0.764672 | 224 | 175.49 | 0.783438 |
| GO:0042180\_cellular\_ketone\_metabolic\_process | PTGES3 | 183 | 2 | 2.648835 | -0.761726 | 225 | 175.82 | 0.781422 |
| GO:0042180\_cellular\_ketone\_metabolic\_process | SRR | 183 | 2 | 2.648835 | -0.761726 | 225 | 175.82 | 0.781422 |
| GO:0006732\_coenzyme\_metabolic\_process | SOD1 | 46 | 1 | 5.268879 | -0.759527 | 228 | 177.58 | 0.778860 |
| GO:0008217\_regulation\_of\_blood\_pressure | SOD1 | 46 | 1 | 5.268879 | -0.759527 | 228 | 177.58 | 0.778860 |
| GO:0042063\_gliogenesis | SOD1 | 46 | 1 | 5.268879 | -0.759527 | 228 | 177.58 | 0.778860 |
| GO:0006396\_RNA\_processing | PABPN1 | 47 | 1 | 5.156775 | -0.751017 | 230 | 180.02 | 0.782696 |
| GO:0048871\_multicellular\_organismal\_homeostasis | SOD1 | 47 | 1 | 5.156775 | -0.751017 | 230 | 180.02 | 0.782696 |
| GO:0001505\_regulation\_of\_neurotransmitter\_levels | PARK7 | 48 | 1 | 5.049342 | -0.742703 | 231 | 182.23 | 0.788874 |
| GO:0019226\_transmission\_of\_nerve\_impulse | SOD1 | 189 | 2 | 2.564745 | -0.739843 | 232 | 182.65 | 0.787284 |
| GO:0019226\_transmission\_of\_nerve\_impulse | PARK7 | 189 | 2 | 2.564745 | -0.739843 | 232 | 182.65 | 0.787284 |
| GO:0006520\_cellular\_amino\_acid\_metabolic\_process | SRR | 51 | 1 | 4.752322 | -0.718860 | 234 | 189.51 | 0.809872 |
| GO:0044106\_cellular\_amine\_metabolic\_process | SRR | 51 | 1 | 4.752322 | -0.718860 | 234 | 189.51 | 0.809872 |
| GO:0044267\_cellular\_protein\_metabolic\_process | MRPL11 | 559 | 4 | 1.734300 | -0.717106 | 235 | 189.97 | 0.808383 |
| GO:0044267\_cellular\_protein\_metabolic\_process | ZFP36L1 | 559 | 4 | 1.734300 | -0.717106 | 235 | 189.97 | 0.808383 |
| GO:0044267\_cellular\_protein\_metabolic\_process | ARIH2 | 559 | 4 | 1.734300 | -0.717106 | 235 | 189.97 | 0.808383 |
| GO:0044267\_cellular\_protein\_metabolic\_process | SOD1 | 559 | 4 | 1.734300 | -0.717106 | 235 | 189.97 | 0.808383 |
| GO:0010608\_posttranscriptional\_regulation\_of\_gene\_expression | ZFP36L1 | 52 | 1 | 4.660931 | -0.711255 | 236 | 191.74 | 0.812458 |
| GO:0022414\_reproductive\_process | GRN | 376 | 3 | 1.933791 | -0.701827 | 237 | 193.61 | 0.816920 |
| GO:0022414\_reproductive\_process | PHB2 | 376 | 3 | 1.933791 | -0.701827 | 237 | 193.61 | 0.816920 |
| GO:0022414\_reproductive\_process | SOD1 | 376 | 3 | 1.933791 | -0.701827 | 237 | 193.61 | 0.816920 |
| GO:0006091\_generation\_of\_precursor\_metabolites\_and\_energy | PTGES3 | 54 | 1 | 4.488304 | -0.696519 | 240 | 195.64 | 0.815167 |
| GO:0043405\_regulation\_of\_MAP\_kinase\_activity | SOD1 | 54 | 1 | 4.488304 | -0.696519 | 240 | 195.64 | 0.815167 |
| GO:0044271\_nitrogen\_compound\_biosynthetic\_process | SRR | 54 | 1 | 4.488304 | -0.696519 | 240 | 195.64 | 0.815167 |
| GO:0000003\_reproduction | GRN | 379 | 3 | 1.918484 | -0.694794 | 241 | 195.84 | 0.812614 |
| GO:0000003\_reproduction | PHB2 | 379 | 3 | 1.918484 | -0.694794 | 241 | 195.84 | 0.812614 |
| GO:0000003\_reproduction | SOD1 | 379 | 3 | 1.918484 | -0.694794 | 241 | 195.84 | 0.812614 |
| GO:0007605\_sensory\_perception\_of\_sound | SOD1 | 55 | 1 | 4.406699 | -0.689376 | 242 | 197.45 | 0.815909 |
| GO:0006790\_sulfur\_metabolic\_process | SOD1 | 56 | 1 | 4.328008 | -0.682377 | 243 | 200.18 | 0.823786 |
| GO:0008284\_positive\_regulation\_of\_cell\_proliferation | CNBP | 208 | 2 | 2.330466 | -0.676054 | 244 | 200.61 | 0.822172 |
| GO:0008284\_positive\_regulation\_of\_cell\_proliferation | GRN | 208 | 2 | 2.330466 | -0.676054 | 244 | 200.61 | 0.822172 |
| GO:0008344\_adult\_locomotory\_behavior | PARK7 | 57 | 1 | 4.252078 | -0.675515 | 247 | 202.85 | 0.821255 |
| GO:0042472\_inner\_ear\_morphogenesis | SOD1 | 57 | 1 | 4.252078 | -0.675515 | 247 | 202.85 | 0.821255 |
| GO:0043523\_regulation\_of\_neuron\_apoptosis | SOD1 | 57 | 1 | 4.252078 | -0.675515 | 247 | 202.85 | 0.821255 |
| GO:0006139\_nucleobase\_\_nucleoside\_\_nucleotide\_and\_nucleic\_acid\_metabolic\_process | ZFP36L1 | 1002 | 6 | 1.451308 | -0.664086 | 248 | 204.16 | 0.823226 |
| GO:0006139\_nucleobase\_\_nucleoside\_\_nucleotide\_and\_nucleic\_acid\_metabolic\_process | PABPN1 | 1002 | 6 | 1.451308 | -0.664086 | 248 | 204.16 | 0.823226 |
| GO:0006139\_nucleobase\_\_nucleoside\_\_nucleotide\_and\_nucleic\_acid\_metabolic\_process | KHDRBS1 | 1002 | 6 | 1.451308 | -0.664086 | 248 | 204.16 | 0.823226 |
| GO:0006139\_nucleobase\_\_nucleoside\_\_nucleotide\_and\_nucleic\_acid\_metabolic\_process | CNBP | 1002 | 6 | 1.451308 | -0.664086 | 248 | 204.16 | 0.823226 |
| GO:0006139\_nucleobase\_\_nucleoside\_\_nucleotide\_and\_nucleic\_acid\_metabolic\_process | SOD1 | 1002 | 6 | 1.451308 | -0.664086 | 248 | 204.16 | 0.823226 |
| GO:0006139\_nucleobase\_\_nucleoside\_\_nucleotide\_and\_nucleic\_acid\_metabolic\_process | MCM4 | 1002 | 6 | 1.451308 | -0.664086 | 248 | 204.16 | 0.823226 |
| GO:0035295\_tube\_development | PTGES3 | 212 | 2 | 2.286495 | -0.663591 | 249 | 204.33 | 0.820602 |
| GO:0035295\_tube\_development | PHB2 | 212 | 2 | 2.286495 | -0.663591 | 249 | 204.33 | 0.820602 |
| GO:0042127\_regulation\_of\_cell\_proliferation | CNBP | 393 | 3 | 1.850141 | -0.663026 | 250 | 204.5 | 0.818000 |
| GO:0042127\_regulation\_of\_cell\_proliferation | PHB2 | 393 | 3 | 1.850141 | -0.663026 | 250 | 204.5 | 0.818000 |
| GO:0042127\_regulation\_of\_cell\_proliferation | GRN | 393 | 3 | 1.850141 | -0.663026 | 250 | 204.5 | 0.818000 |
| GO:0006874\_cellular\_calcium\_ion\_homeostasis | ATP2A2 | 61 | 1 | 3.973253 | -0.649358 | 251 | 207.82 | 0.827968 |
| GO:0040014\_regulation\_of\_multicellular\_organism\_growth | SOD1 | 62 | 1 | 3.909168 | -0.643120 | 253 | 210.48 | 0.831937 |
| GO:0050954\_sensory\_perception\_of\_mechanical\_stimulus | SOD1 | 62 | 1 | 3.909168 | -0.643120 | 253 | 210.48 | 0.831937 |
| GO:0051186\_cofactor\_metabolic\_process | SOD1 | 63 | 1 | 3.847118 | -0.636994 | 254 | 212.21 | 0.835472 |
| GO:0006875\_cellular\_metal\_ion\_homeostasis | ATP2A2 | 64 | 1 | 3.787007 | -0.630977 | 256 | 213.48 | 0.833906 |
| GO:0055074\_calcium\_ion\_homeostasis | ATP2A2 | 64 | 1 | 3.787007 | -0.630977 | 256 | 213.48 | 0.833906 |
| GO:0042471\_ear\_morphogenesis | SOD1 | 65 | 1 | 3.728745 | -0.625065 | 258 | 214.57 | 0.831667 |
| GO:0048511\_rhythmic\_process | SOD1 | 65 | 1 | 3.728745 | -0.625065 | 258 | 214.57 | 0.831667 |
| GO:0015837\_amine\_transport | PARK7 | 66 | 1 | 3.672249 | -0.619256 | 261 | 216.02 | 0.827663 |
| GO:0045860\_positive\_regulation\_of\_protein\_kinase\_activity | SOD1 | 66 | 1 | 3.672249 | -0.619256 | 261 | 216.02 | 0.827663 |
| GO:0051402\_neuron\_apoptosis | SOD1 | 66 | 1 | 3.672249 | -0.619256 | 261 | 216.02 | 0.827663 |
| GO:0009791\_post-embryonic\_development | INVS | 67 | 1 | 3.617439 | -0.613546 | 262 | 217.88 | 0.831603 |
| GO:0042592\_homeostatic\_process | ATP2A2 | 419 | 3 | 1.735335 | -0.608299 | 263 | 218.33 | 0.830152 |
| GO:0042592\_homeostatic\_process | SOD1 | 419 | 3 | 1.735335 | -0.608299 | 263 | 218.33 | 0.830152 |
| GO:0042592\_homeostatic\_process | PARK7 | 419 | 3 | 1.735335 | -0.608299 | 263 | 218.33 | 0.830152 |
| GO:0005996\_monosaccharide\_metabolic\_process | PGD | 69 | 1 | 3.512586 | -0.602413 | 265 | 221.13 | 0.834453 |
| GO:0055065\_metal\_ion\_homeostasis | ATP2A2 | 69 | 1 | 3.512586 | -0.602413 | 265 | 221.13 | 0.834453 |
| GO:0008406\_gonad\_development | SOD1 | 70 | 1 | 3.462406 | -0.596984 | 266 | 222.72 | 0.837293 |
| GO:0006281\_DNA\_repair | SOD1 | 71 | 1 | 3.413640 | -0.591643 | 269 | 224.85 | 0.835874 |
| GO:0006913\_nucleocytoplasmic\_transport | KHDRBS1 | 71 | 1 | 3.413640 | -0.591643 | 269 | 224.85 | 0.835874 |
| GO:0033674\_positive\_regulation\_of\_kinase\_activity | SOD1 | 71 | 1 | 3.413640 | -0.591643 | 269 | 224.85 | 0.835874 |
| GO:0030879\_mammary\_gland\_development | PHB2 | 72 | 1 | 3.366228 | -0.586387 | 274 | 227.75 | 0.831204 |
| GO:0044262\_cellular\_carbohydrate\_metabolic\_process | PTGES3 | 72 | 1 | 3.366228 | -0.586387 | 274 | 227.75 | 0.831204 |
| GO:0048839\_inner\_ear\_development | SOD1 | 72 | 1 | 3.366228 | -0.586387 | 274 | 227.75 | 0.831204 |
| GO:0051169\_nuclear\_transport | KHDRBS1 | 72 | 1 | 3.366228 | -0.586387 | 274 | 227.75 | 0.831204 |
| GO:0051347\_positive\_regulation\_of\_transferase\_activity | SOD1 | 72 | 1 | 3.366228 | -0.586387 | 274 | 227.75 | 0.831204 |
| GO:0006508\_proteolysis | ARIH2 | 76 | 1 | 3.189058 | -0.566177 | 275 | 232.99 | 0.847236 |
| GO:0006461\_protein\_complex\_assembly | SRR | 78 | 1 | 3.107287 | -0.556528 | 277 | 236.67 | 0.854404 |
| GO:0070271\_protein\_complex\_biogenesis | SRR | 78 | 1 | 3.107287 | -0.556528 | 277 | 236.67 | 0.854404 |
| GO:0019538\_protein\_metabolic\_process | MRPL11 | 655 | 4 | 1.480112 | -0.552023 | 278 | 237.42 | 0.854029 |
| GO:0019538\_protein\_metabolic\_process | ZFP36L1 | 655 | 4 | 1.480112 | -0.552023 | 278 | 237.42 | 0.854029 |
| GO:0019538\_protein\_metabolic\_process | ARIH2 | 655 | 4 | 1.480112 | -0.552023 | 278 | 237.42 | 0.854029 |
| GO:0019538\_protein\_metabolic\_process | SOD1 | 655 | 4 | 1.480112 | -0.552023 | 278 | 237.42 | 0.854029 |
| GO:0006631\_fatty\_acid\_metabolic\_process | PTGES3 | 80 | 1 | 3.029605 | -0.547164 | 279 | 240.6 | 0.862366 |
| GO:0007154\_cell\_communication | PTGES3 | 1096 | 6 | 1.326834 | -0.541399 | 280 | 241.59 | 0.862821 |
| GO:0007154\_cell\_communication | KHDRBS1 | 1096 | 6 | 1.326834 | -0.541399 | 280 | 241.59 | 0.862821 |
| GO:0007154\_cell\_communication | ATP2A2 | 1096 | 6 | 1.326834 | -0.541399 | 280 | 241.59 | 0.862821 |
| GO:0007154\_cell\_communication | PHB2 | 1096 | 6 | 1.326834 | -0.541399 | 280 | 241.59 | 0.862821 |
| GO:0007154\_cell\_communication | SOD1 | 1096 | 6 | 1.326834 | -0.541399 | 280 | 241.59 | 0.862821 |
| GO:0007154\_cell\_communication | PARK7 | 1096 | 6 | 1.326834 | -0.541399 | 280 | 241.59 | 0.862821 |
| GO:0006575\_cellular\_amino\_acid\_derivative\_metabolic\_process | SOD1 | 83 | 1 | 2.920101 | -0.533619 | 282 | 244.37 | 0.866560 |
| GO:0030534\_adult\_behavior | PARK7 | 83 | 1 | 2.920101 | -0.533619 | 282 | 244.37 | 0.866560 |
| GO:0045137\_development\_of\_primary\_sexual\_characteristics | SOD1 | 84 | 1 | 2.885338 | -0.529231 | 283 | 245.65 | 0.868021 |
| GO:0006897\_endocytosis | SNX17 | 86 | 1 | 2.818237 | -0.520637 | 288 | 249.02 | 0.864653 |
| GO:0010324\_membrane\_invagination | SNX17 | 86 | 1 | 2.818237 | -0.520637 | 288 | 249.02 | 0.864653 |
| GO:0032504\_multicellular\_organism\_reproduction | SOD1 | 86 | 1 | 2.818237 | -0.520637 | 288 | 249.02 | 0.864653 |
| GO:0034641\_cellular\_nitrogen\_compound\_metabolic\_process | SRR | 86 | 1 | 2.818237 | -0.520637 | 288 | 249.02 | 0.864653 |
| GO:0048609\_reproductive\_process\_in\_a\_multicellular\_organism | SOD1 | 86 | 1 | 2.818237 | -0.520637 | 288 | 249.02 | 0.864653 |
| GO:0001822\_kidney\_development | INVS | 87 | 1 | 2.785844 | -0.516428 | 291 | 250.93 | 0.862302 |
| GO:0022612\_gland\_morphogenesis | PHB2 | 87 | 1 | 2.785844 | -0.516428 | 291 | 250.93 | 0.862302 |
| GO:0043583\_ear\_development | SOD1 | 87 | 1 | 2.785844 | -0.516428 | 291 | 250.93 | 0.862302 |
| GO:0048754\_branching\_morphogenesis\_of\_a\_tube | PHB2 | 88 | 1 | 2.754187 | -0.512276 | 292 | 252.1 | 0.863356 |
| GO:0010467\_gene\_expression | MRPL11 | 905 | 5 | 1.339052 | -0.505348 | 293 | 253.02 | 0.863549 |
| GO:0010467\_gene\_expression | ZFP36L1 | 905 | 5 | 1.339052 | -0.505348 | 293 | 253.02 | 0.863549 |
| GO:0010467\_gene\_expression | PABPN1 | 905 | 5 | 1.339052 | -0.505348 | 293 | 253.02 | 0.863549 |
| GO:0010467\_gene\_expression | KHDRBS1 | 905 | 5 | 1.339052 | -0.505348 | 293 | 253.02 | 0.863549 |
| GO:0010467\_gene\_expression | CNBP | 905 | 5 | 1.339052 | -0.505348 | 293 | 253.02 | 0.863549 |
| GO:0030324\_lung\_development | PTGES3 | 90 | 1 | 2.692982 | -0.504138 | 295 | 254.15 | 0.861525 |
| GO:0035264\_multicellular\_organism\_growth | SOD1 | 90 | 1 | 2.692982 | -0.504138 | 295 | 254.15 | 0.861525 |
| GO:0008544\_epidermis\_development | PTGES3 | 91 | 1 | 2.663389 | -0.500149 | 296 | 255.36 | 0.862703 |
| GO:0030323\_respiratory\_tube\_development | PTGES3 | 92 | 1 | 2.634439 | -0.496213 | 297 | 256.88 | 0.864916 |
| GO:0007165\_signal\_transduction | PTGES3 | 915 | 5 | 1.324418 | -0.493222 | 298 | 257.19 | 0.863054 |
| GO:0007165\_signal\_transduction | KHDRBS1 | 915 | 5 | 1.324418 | -0.493222 | 298 | 257.19 | 0.863054 |
| GO:0007165\_signal\_transduction | ATP2A2 | 915 | 5 | 1.324418 | -0.493222 | 298 | 257.19 | 0.863054 |
| GO:0007165\_signal\_transduction | PHB2 | 915 | 5 | 1.324418 | -0.493222 | 298 | 257.19 | 0.863054 |
| GO:0007165\_signal\_transduction | SOD1 | 915 | 5 | 1.324418 | -0.493222 | 298 | 257.19 | 0.863054 |
| GO:0007610\_behavior | SOD1 | 279 | 2 | 1.737408 | -0.493010 | 299 | 257.7 | 0.861873 |
| GO:0007610\_behavior | PARK7 | 279 | 2 | 1.737408 | -0.493010 | 299 | 257.7 | 0.861873 |
| GO:0065003\_macromolecular\_complex\_assembly | SRR | 93 | 1 | 2.606112 | -0.492327 | 300 | 259.06 | 0.863533 |
| GO:0008610\_lipid\_biosynthetic\_process | PTGES3 | 94 | 1 | 2.578387 | -0.488491 | 302 | 260.77 | 0.863477 |
| GO:0034984\_cellular\_response\_to\_DNA\_damage\_stimulus | SOD1 | 94 | 1 | 2.578387 | -0.488491 | 302 | 260.77 | 0.863477 |
| GO:0060249\_anatomical\_structure\_homeostasis | SOD1 | 96 | 1 | 2.524671 | -0.480963 | 303 | 263.82 | 0.870693 |
| GO:0060341\_regulation\_of\_cellular\_localization | KHDRBS1 | 97 | 1 | 2.498644 | -0.477270 | 304 | 265.06 | 0.871908 |
| GO:0060541\_respiratory\_system\_development | PTGES3 | 98 | 1 | 2.473147 | -0.473623 | 305 | 266.4 | 0.873443 |
| GO:0007398\_ectoderm\_development | PTGES3 | 99 | 1 | 2.448166 | -0.470020 | 307 | 267.63 | 0.871759 |
| GO:0060562\_epithelial\_tube\_morphogenesis | PHB2 | 99 | 1 | 2.448166 | -0.470020 | 307 | 267.63 | 0.871759 |
| GO:0030163\_protein\_catabolic\_process | ARIH2 | 101 | 1 | 2.399687 | -0.462946 | 308 | 268.54 | 0.871883 |
| GO:0009968\_negative\_regulation\_of\_signal\_transduction | PHB2 | 103 | 1 | 2.353091 | -0.456040 | 309 | 270.76 | 0.876246 |
| GO:0048872\_homeostasis\_of\_number\_of\_cells | SOD1 | 105 | 1 | 2.308271 | -0.449295 | 310 | 272.33 | 0.878484 |
| GO:0003008\_system\_process | ATP2A2 | 516 | 3 | 1.409119 | -0.443661 | 311 | 273.08 | 0.878071 |
| GO:0003008\_system\_process | SOD1 | 516 | 3 | 1.409119 | -0.443661 | 311 | 273.08 | 0.878071 |
| GO:0003008\_system\_process | PARK7 | 516 | 3 | 1.409119 | -0.443661 | 311 | 273.08 | 0.878071 |
| GO:0045859\_regulation\_of\_protein\_kinase\_activity | SOD1 | 107 | 1 | 2.265125 | -0.442707 | 312 | 273.35 | 0.876122 |
| GO:0010648\_negative\_regulation\_of\_cell\_communication | PHB2 | 110 | 1 | 2.203349 | -0.433104 | 313 | 276.93 | 0.884760 |
| GO:0043549\_regulation\_of\_kinase\_activity | SOD1 | 112 | 1 | 2.164004 | -0.426880 | 314 | 278.25 | 0.886146 |
| GO:0006974\_response\_to\_DNA\_damage\_stimulus | SOD1 | 113 | 1 | 2.144853 | -0.423820 | 316 | 278.95 | 0.882753 |
| GO:0040008\_regulation\_of\_growth | SOD1 | 113 | 1 | 2.144853 | -0.423820 | 316 | 278.95 | 0.882753 |
| GO:0000165\_MAPKKK\_cascade | SOD1 | 114 | 1 | 2.126039 | -0.420793 | 317 | 279.72 | 0.882397 |
| GO:0051338\_regulation\_of\_transferase\_activity | SOD1 | 115 | 1 | 2.107551 | -0.417799 | 318 | 280.32 | 0.881509 |
| GO:0048608\_reproductive\_structure\_development | SOD1 | 116 | 1 | 2.089383 | -0.414837 | 319 | 281.4 | 0.882132 |
| GO:0043933\_macromolecular\_complex\_subunit\_organization | SRR | 117 | 1 | 2.071525 | -0.411908 | 320 | 281.76 | 0.880500 |
| GO:0009308\_amine\_metabolic\_process | SRR | 124 | 1 | 1.954584 | -0.392246 | 321 | 289.89 | 0.903084 |
| GO:0001763\_morphogenesis\_of\_a\_branching\_structure | PHB2 | 125 | 1 | 1.938947 | -0.389552 | 322 | 290.49 | 0.902143 |
| GO:0001655\_urogenital\_system\_development | INVS | 128 | 1 | 1.893503 | -0.381633 | 323 | 293.15 | 0.907585 |
| GO:0032787\_monocarboxylic\_acid\_metabolic\_process | PTGES3 | 130 | 1 | 1.864372 | -0.376484 | 324 | 295.28 | 0.911358 |
| GO:0007283\_spermatogenesis | SOD1 | 134 | 1 | 1.808720 | -0.366487 | 326 | 297.24 | 0.911779 |
| GO:0048232\_male\_gamete\_generation | SOD1 | 134 | 1 | 1.808720 | -0.366487 | 326 | 297.24 | 0.911779 |
| GO:0051239\_regulation\_of\_multicellular\_organismal\_process | ATP2A2 | 587 | 3 | 1.238680 | -0.353042 | 327 | 300.12 | 0.917798 |
| GO:0051239\_regulation\_of\_multicellular\_organismal\_process | PHB2 | 587 | 3 | 1.238680 | -0.353042 | 327 | 300.12 | 0.917798 |
| GO:0051239\_regulation\_of\_multicellular\_organismal\_process | SOD1 | 587 | 3 | 1.238680 | -0.353042 | 327 | 300.12 | 0.917798 |
| GO:0035239\_tube\_morphogenesis | PHB2 | 143 | 1 | 1.694884 | -0.345347 | 328 | 303.65 | 0.925762 |
| GO:0022603\_regulation\_of\_anatomical\_structure\_morphogenesis | PHB2 | 147 | 1 | 1.648765 | -0.336505 | 329 | 306.99 | 0.933100 |
| GO:0043085\_positive\_regulation\_of\_catalytic\_activity | SOD1 | 148 | 1 | 1.637624 | -0.334344 | 330 | 307.62 | 0.932182 |
| GO:0032268\_regulation\_of\_cellular\_protein\_metabolic\_process | ZFP36L1 | 152 | 1 | 1.594529 | -0.325893 | 331 | 309.0 | 0.933535 |
| GO:0007268\_synaptic\_transmission | PARK7 | 154 | 1 | 1.573821 | -0.321778 | 332 | 309.68 | 0.932771 |
| GO:0008285\_negative\_regulation\_of\_cell\_proliferation | PHB2 | 155 | 1 | 1.563667 | -0.319747 | 333 | 310.72 | 0.933093 |
| GO:0048519\_negative\_regulation\_of\_biological\_process | KHDRBS1 | 859 | 4 | 1.128607 | -0.314249 | 334 | 311.91 | 0.933862 |
| GO:0048519\_negative\_regulation\_of\_biological\_process | ATP2A2 | 859 | 4 | 1.128607 | -0.314249 | 334 | 311.91 | 0.933862 |
| GO:0048519\_negative\_regulation\_of\_biological\_process | PHB2 | 859 | 4 | 1.128607 | -0.314249 | 334 | 311.91 | 0.933862 |
| GO:0048519\_negative\_regulation\_of\_biological\_process | SOD1 | 859 | 4 | 1.128607 | -0.314249 | 334 | 311.91 | 0.933862 |
| GO:0006066\_alcohol\_metabolic\_process | PGD | 158 | 1 | 1.533977 | -0.313760 | 336 | 313.16 | 0.932024 |
| GO:0048514\_blood\_vessel\_morphogenesis | ZFP36L1 | 158 | 1 | 1.533977 | -0.313760 | 336 | 313.16 | 0.932024 |
| GO:0050877\_neurological\_system\_process | SOD1 | 390 | 2 | 1.242915 | -0.312427 | 337 | 313.42 | 0.930030 |
| GO:0050877\_neurological\_system\_process | PARK7 | 390 | 2 | 1.242915 | -0.312427 | 337 | 313.42 | 0.930030 |
| GO:0009628\_response\_to\_abiotic\_stimulus | SOD1 | 162 | 1 | 1.496101 | -0.306014 | 338 | 314.68 | 0.931006 |
| GO:0042325\_regulation\_of\_phosphorylation | SOD1 | 164 | 1 | 1.477856 | -0.302238 | 339 | 316.25 | 0.932891 |
| GO:0019220\_regulation\_of\_phosphate\_metabolic\_process | SOD1 | 165 | 1 | 1.468900 | -0.300373 | 341 | 317.22 | 0.930264 |
| GO:0051174\_regulation\_of\_phosphorus\_metabolic\_process | SOD1 | 165 | 1 | 1.468900 | -0.300373 | 341 | 317.22 | 0.930264 |
| GO:0051049\_regulation\_of\_transport | KHDRBS1 | 167 | 1 | 1.451308 | -0.296691 | 342 | 318.82 | 0.932222 |
| GO:0009887\_organ\_morphogenesis | ZFP36L1 | 642 | 3 | 1.132563 | -0.295729 | 343 | 319.05 | 0.930175 |
| GO:0009887\_organ\_morphogenesis | PHB2 | 642 | 3 | 1.132563 | -0.295729 | 343 | 319.05 | 0.930175 |
| GO:0009887\_organ\_morphogenesis | SOD1 | 642 | 3 | 1.132563 | -0.295729 | 343 | 319.05 | 0.930175 |
| GO:0051246\_regulation\_of\_protein\_metabolic\_process | ZFP36L1 | 170 | 1 | 1.425697 | -0.291280 | 344 | 319.65 | 0.929215 |
| GO:0042221\_response\_to\_chemical\_stimulus | SOD1 | 409 | 2 | 1.185176 | -0.289663 | 345 | 320.07 | 0.927739 |
| GO:0042221\_response\_to\_chemical\_stimulus | PARK7 | 409 | 2 | 1.185176 | -0.289663 | 345 | 320.07 | 0.927739 |
| GO:0007600\_sensory\_perception | SOD1 | 172 | 1 | 1.409119 | -0.287746 | 347 | 320.75 | 0.924352 |
| GO:0009611\_response\_to\_wounding | SOD1 | 172 | 1 | 1.409119 | -0.287746 | 347 | 320.75 | 0.924352 |
| GO:0044093\_positive\_regulation\_of\_molecular\_function | SOD1 | 173 | 1 | 1.400974 | -0.286000 | 348 | 322.24 | 0.925977 |
| GO:0043066\_negative\_regulation\_of\_apoptosis | SOD1 | 176 | 1 | 1.377093 | -0.280846 | 349 | 324.98 | 0.931175 |
| GO:0016070\_RNA\_metabolic\_process | ZFP36L1 | 658 | 3 | 1.105023 | -0.280831 | 350 | 325.1 | 0.928857 |
| GO:0016070\_RNA\_metabolic\_process | PABPN1 | 658 | 3 | 1.105023 | -0.280831 | 350 | 325.1 | 0.928857 |
| GO:0016070\_RNA\_metabolic\_process | CNBP | 658 | 3 | 1.105023 | -0.280831 | 350 | 325.1 | 0.928857 |
| GO:0043069\_negative\_regulation\_of\_programmed\_cell\_death | SOD1 | 179 | 1 | 1.354014 | -0.275814 | 353 | 326.46 | 0.924816 |
| GO:0048732\_gland\_development | PHB2 | 179 | 1 | 1.354014 | -0.275814 | 353 | 326.46 | 0.924816 |
| GO:0060548\_negative\_regulation\_of\_cell\_death | SOD1 | 179 | 1 | 1.354014 | -0.275814 | 353 | 326.46 | 0.924816 |
| GO:0016192\_vesicle-mediated\_transport | SNX17 | 184 | 1 | 1.317220 | -0.267688 | 354 | 331.35 | 0.936017 |
| GO:0007010\_cytoskeleton\_organization | SOD1 | 185 | 1 | 1.310100 | -0.266100 | 355 | 332.13 | 0.935577 |
| GO:0007276\_gamete\_generation | SOD1 | 188 | 1 | 1.289194 | -0.261410 | 356 | 334.53 | 0.939691 |
| GO:0046907\_intracellular\_transport | KHDRBS1 | 194 | 1 | 1.249322 | -0.252345 | 357 | 336.45 | 0.942437 |
| GO:0065008\_regulation\_of\_biological\_quality | ATP2A2 | 693 | 3 | 1.049214 | -0.250710 | 358 | 338.64 | 0.945922 |
| GO:0065008\_regulation\_of\_biological\_quality | SOD1 | 693 | 3 | 1.049214 | -0.250710 | 358 | 338.64 | 0.945922 |
| GO:0065008\_regulation\_of\_biological\_quality | PARK7 | 693 | 3 | 1.049214 | -0.250710 | 358 | 338.64 | 0.945922 |
| GO:0033554\_cellular\_response\_to\_stress | SOD1 | 196 | 1 | 1.236574 | -0.249413 | 359 | 339.02 | 0.944345 |
| GO:0002009\_morphogenesis\_of\_an\_epithelium | PHB2 | 198 | 1 | 1.224083 | -0.246524 | 361 | 341.09 | 0.944848 |
| GO:0060429\_epithelium\_development | PHB2 | 198 | 1 | 1.224083 | -0.246524 | 361 | 341.09 | 0.944848 |
| GO:0001568\_blood\_vessel\_development | ZFP36L1 | 203 | 1 | 1.193933 | -0.239486 | 362 | 342.62 | 0.946464 |
| GO:0022607\_cellular\_component\_assembly | SRR | 204 | 1 | 1.188080 | -0.238109 | 363 | 343.44 | 0.946116 |
| GO:0007243\_protein\_kinase\_cascade | SOD1 | 205 | 1 | 1.182285 | -0.236742 | 364 | 344.5 | 0.946429 |
| GO:0001944\_vasculature\_development | ZFP36L1 | 208 | 1 | 1.165233 | -0.232700 | 365 | 345.4 | 0.946301 |
| GO:0006810\_transport | KHDRBS1 | 718 | 3 | 1.012681 | -0.231103 | 366 | 345.84 | 0.944918 |
| GO:0006810\_transport | SNX17 | 718 | 3 | 1.012681 | -0.231103 | 366 | 345.84 | 0.944918 |
| GO:0006810\_transport | PARK7 | 718 | 3 | 1.012681 | -0.231103 | 366 | 345.84 | 0.944918 |
| GO:0032501\_multicellular\_organismal\_process | ZFP36L1 | 2183 | 9 | 0.999228 | -0.228866 | 367 | 346.01 | 0.942807 |
| GO:0032501\_multicellular\_organismal\_process | PTGES3 | 2183 | 9 | 0.999228 | -0.228866 | 367 | 346.01 | 0.942807 |
| GO:0032501\_multicellular\_organismal\_process | INVS | 2183 | 9 | 0.999228 | -0.228866 | 367 | 346.01 | 0.942807 |
| GO:0032501\_multicellular\_organismal\_process | ATP2A2 | 2183 | 9 | 0.999228 | -0.228866 | 367 | 346.01 | 0.942807 |
| GO:0032501\_multicellular\_organismal\_process | PHB2 | 2183 | 9 | 0.999228 | -0.228866 | 367 | 346.01 | 0.942807 |
| GO:0032501\_multicellular\_organismal\_process | GRN | 2183 | 9 | 0.999228 | -0.228866 | 367 | 346.01 | 0.942807 |
| GO:0032501\_multicellular\_organismal\_process | SOD1 | 2183 | 9 | 0.999228 | -0.228866 | 367 | 346.01 | 0.942807 |
| GO:0032501\_multicellular\_organismal\_process | GAPDH | 2183 | 9 | 0.999228 | -0.228866 | 367 | 346.01 | 0.942807 |
| GO:0032501\_multicellular\_organismal\_process | PARK7 | 2183 | 9 | 0.999228 | -0.228866 | 367 | 346.01 | 0.942807 |
| GO:0051234\_establishment\_of\_localization | KHDRBS1 | 729 | 3 | 0.997401 | -0.222942 | 368 | 346.92 | 0.942717 |
| GO:0051234\_establishment\_of\_localization | SNX17 | 729 | 3 | 0.997401 | -0.222942 | 368 | 346.92 | 0.942717 |
| GO:0051234\_establishment\_of\_localization | PARK7 | 729 | 3 | 0.997401 | -0.222942 | 368 | 346.92 | 0.942717 |
| GO:0010033\_response\_to\_organic\_substance | SOD1 | 216 | 1 | 1.122076 | -0.222334 | 369 | 347.32 | 0.941247 |
| GO:0040007\_growth | SOD1 | 217 | 1 | 1.116905 | -0.221079 | 370 | 348.04 | 0.940649 |
| GO:0007423\_sensory\_organ\_development | SOD1 | 219 | 1 | 1.106705 | -0.218595 | 371 | 348.8 | 0.940162 |
| GO:0001701\_in\_utero\_embryonic\_development | GRN | 221 | 1 | 1.096690 | -0.216145 | 372 | 349.6 | 0.939785 |
| GO:0010556\_regulation\_of\_macromolecule\_biosynthetic\_process | KHDRBS1 | 745 | 3 | 0.975980 | -0.211552 | 373 | 350.27 | 0.939062 |
| GO:0010556\_regulation\_of\_macromolecule\_biosynthetic\_process | ZFP36L1 | 745 | 3 | 0.975980 | -0.211552 | 373 | 350.27 | 0.939062 |
| GO:0010556\_regulation\_of\_macromolecule\_biosynthetic\_process | CNBP | 745 | 3 | 0.975980 | -0.211552 | 373 | 350.27 | 0.939062 |
| GO:0019953\_sexual\_reproduction | SOD1 | 228 | 1 | 1.063019 | -0.207832 | 374 | 351.4 | 0.939572 |
| GO:0050790\_regulation\_of\_catalytic\_activity | SOD1 | 233 | 1 | 1.040208 | -0.202132 | 376 | 352.93 | 0.938644 |
| GO:0050890\_cognition | SOD1 | 233 | 1 | 1.040208 | -0.202132 | 376 | 352.93 | 0.938644 |
| GO:0031323\_regulation\_of\_cellular\_metabolic\_process | ZFP36L1 | 1015 | 4 | 0.955146 | -0.200293 | 377 | 353.48 | 0.937613 |
| GO:0031323\_regulation\_of\_cellular\_metabolic\_process | KHDRBS1 | 1015 | 4 | 0.955146 | -0.200293 | 377 | 353.48 | 0.937613 |
| GO:0031323\_regulation\_of\_cellular\_metabolic\_process | CNBP | 1015 | 4 | 0.955146 | -0.200293 | 377 | 353.48 | 0.937613 |
| GO:0031323\_regulation\_of\_cellular\_metabolic\_process | SOD1 | 1015 | 4 | 0.955146 | -0.200293 | 377 | 353.48 | 0.937613 |
| GO:0007275\_multicellular\_organismal\_development | ZFP36L1 | 1760 | 7 | 0.963965 | -0.197971 | 378 | 354.39 | 0.937540 |
| GO:0007275\_multicellular\_organismal\_development | PTGES3 | 1760 | 7 | 0.963965 | -0.197971 | 378 | 354.39 | 0.937540 |
| GO:0007275\_multicellular\_organismal\_development | INVS | 1760 | 7 | 0.963965 | -0.197971 | 378 | 354.39 | 0.937540 |
| GO:0007275\_multicellular\_organismal\_development | PHB2 | 1760 | 7 | 0.963965 | -0.197971 | 378 | 354.39 | 0.937540 |
| GO:0007275\_multicellular\_organismal\_development | GRN | 1760 | 7 | 0.963965 | -0.197971 | 378 | 354.39 | 0.937540 |
| GO:0007275\_multicellular\_organismal\_development | SOD1 | 1760 | 7 | 0.963965 | -0.197971 | 378 | 354.39 | 0.937540 |
| GO:0007275\_multicellular\_organismal\_development | GAPDH | 1760 | 7 | 0.963965 | -0.197971 | 378 | 354.39 | 0.937540 |
| GO:0006468\_protein\_amino\_acid\_phosphorylation | SOD1 | 237 | 1 | 1.022652 | -0.197708 | 380 | 355.04 | 0.934316 |
| GO:0044085\_cellular\_component\_biogenesis | SRR | 237 | 1 | 1.022652 | -0.197708 | 380 | 355.04 | 0.934316 |
| GO:0009987\_cellular\_process | KHDRBS1 | 3868 | 16 | 1.002558 | -0.195266 | 381 | 355.75 | 0.933727 |
| GO:0009987\_cellular\_process | PTGES3 | 3868 | 16 | 1.002558 | -0.195266 | 381 | 355.75 | 0.933727 |
| GO:0009987\_cellular\_process | PABPN1 | 3868 | 16 | 1.002558 | -0.195266 | 381 | 355.75 | 0.933727 |
| GO:0009987\_cellular\_process | CNBP | 3868 | 16 | 1.002558 | -0.195266 | 381 | 355.75 | 0.933727 |
| GO:0009987\_cellular\_process | SNX17 | 3868 | 16 | 1.002558 | -0.195266 | 381 | 355.75 | 0.933727 |
| GO:0009987\_cellular\_process | VIM | 3868 | 16 | 1.002558 | -0.195266 | 381 | 355.75 | 0.933727 |
| GO:0009987\_cellular\_process | SOD1 | 3868 | 16 | 1.002558 | -0.195266 | 381 | 355.75 | 0.933727 |
| GO:0009987\_cellular\_process | MCM4 | 3868 | 16 | 1.002558 | -0.195266 | 381 | 355.75 | 0.933727 |
| GO:0009987\_cellular\_process | PARK7 | 3868 | 16 | 1.002558 | -0.195266 | 381 | 355.75 | 0.933727 |
| GO:0009987\_cellular\_process | MRPL11 | 3868 | 16 | 1.002558 | -0.195266 | 381 | 355.75 | 0.933727 |
| GO:0009987\_cellular\_process | ZFP36L1 | 3868 | 16 | 1.002558 | -0.195266 | 381 | 355.75 | 0.933727 |
| GO:0009987\_cellular\_process | ARIH2 | 3868 | 16 | 1.002558 | -0.195266 | 381 | 355.75 | 0.933727 |
| GO:0009987\_cellular\_process | ATP2A2 | 3868 | 16 | 1.002558 | -0.195266 | 381 | 355.75 | 0.933727 |
| GO:0009987\_cellular\_process | PHB2 | 3868 | 16 | 1.002558 | -0.195266 | 381 | 355.75 | 0.933727 |
| GO:0009987\_cellular\_process | GRN | 3868 | 16 | 1.002558 | -0.195266 | 381 | 355.75 | 0.933727 |
| GO:0009987\_cellular\_process | SRR | 3868 | 16 | 1.002558 | -0.195266 | 381 | 355.75 | 0.933727 |
| GO:0048523\_negative\_regulation\_of\_cellular\_process | KHDRBS1 | 774 | 3 | 0.939412 | -0.192280 | 382 | 356.09 | 0.932173 |
| GO:0048523\_negative\_regulation\_of\_cellular\_process | PHB2 | 774 | 3 | 0.939412 | -0.192280 | 382 | 356.09 | 0.932173 |
| GO:0048523\_negative\_regulation\_of\_cellular\_process | SOD1 | 774 | 3 | 0.939412 | -0.192280 | 382 | 356.09 | 0.932173 |
| GO:0010468\_regulation\_of\_gene\_expression | KHDRBS1 | 778 | 3 | 0.934583 | -0.189753 | 383 | 356.92 | 0.931906 |
| GO:0010468\_regulation\_of\_gene\_expression | ZFP36L1 | 778 | 3 | 0.934583 | -0.189753 | 383 | 356.92 | 0.931906 |
| GO:0010468\_regulation\_of\_gene\_expression | CNBP | 778 | 3 | 0.934583 | -0.189753 | 383 | 356.92 | 0.931906 |
| GO:0032879\_regulation\_of\_localization | KHDRBS1 | 248 | 1 | 0.977292 | -0.186127 | 384 | 357.56 | 0.931146 |
| GO:0009888\_tissue\_development | PTGES3 | 525 | 2 | 0.923308 | -0.183661 | 385 | 358.28 | 0.930597 |
| GO:0009888\_tissue\_development | PHB2 | 525 | 2 | 0.923308 | -0.183661 | 385 | 358.28 | 0.930597 |
| GO:0007267\_cell-cell\_signaling | PARK7 | 252 | 1 | 0.961779 | -0.182117 | 386 | 359.11 | 0.930337 |
| GO:0016481\_negative\_regulation\_of\_transcription | KHDRBS1 | 253 | 1 | 0.957978 | -0.181131 | 387 | 359.75 | 0.929587 |
| GO:0048729\_tissue\_morphogenesis | PHB2 | 255 | 1 | 0.950464 | -0.179177 | 388 | 360.49 | 0.929098 |
| GO:0009966\_regulation\_of\_signal\_transduction | PHB2 | 256 | 1 | 0.946752 | -0.178209 | 389 | 360.87 | 0.927686 |
| GO:0010629\_negative\_regulation\_of\_gene\_expression | KHDRBS1 | 262 | 1 | 0.925070 | -0.172530 | 391 | 362.82 | 0.927928 |
| GO:0048666\_neuron\_development | SOD1 | 262 | 1 | 0.925070 | -0.172530 | 391 | 362.82 | 0.927928 |
| GO:0030030\_cell\_projection\_organization | SOD1 | 263 | 1 | 0.921553 | -0.171605 | 392 | 363.33 | 0.926862 |
| GO:0044255\_cellular\_lipid\_metabolic\_process | PTGES3 | 264 | 1 | 0.918062 | -0.170685 | 393 | 363.76 | 0.925598 |
| GO:0031326\_regulation\_of\_cellular\_biosynthetic\_process | KHDRBS1 | 812 | 3 | 0.895450 | -0.169482 | 394 | 364.15 | 0.924239 |
| GO:0031326\_regulation\_of\_cellular\_biosynthetic\_process | ZFP36L1 | 812 | 3 | 0.895450 | -0.169482 | 394 | 364.15 | 0.924239 |
| GO:0031326\_regulation\_of\_cellular\_biosynthetic\_process | CNBP | 812 | 3 | 0.895450 | -0.169482 | 394 | 364.15 | 0.924239 |
| GO:0009889\_regulation\_of\_biosynthetic\_process | ZFP36L1 | 815 | 3 | 0.892154 | -0.167793 | 395 | 364.44 | 0.922633 |
| GO:0009889\_regulation\_of\_biosynthetic\_process | KHDRBS1 | 815 | 3 | 0.892154 | -0.167793 | 395 | 364.44 | 0.922633 |
| GO:0009889\_regulation\_of\_biosynthetic\_process | CNBP | 815 | 3 | 0.892154 | -0.167793 | 395 | 364.44 | 0.922633 |
| GO:0006950\_response\_to\_stress | SOD1 | 549 | 2 | 0.882945 | -0.167224 | 396 | 364.69 | 0.920934 |
| GO:0006950\_response\_to\_stress | PARK7 | 549 | 2 | 0.882945 | -0.167224 | 396 | 364.69 | 0.920934 |
| GO:0045944\_positive\_regulation\_of\_transcription\_from\_RNA\_polymerase\_II\_promoter | CNBP | 269 | 1 | 0.900998 | -0.166170 | 397 | 365.13 | 0.919723 |
| GO:0045934\_negative\_regulation\_of\_nucleobase\_\_nucleoside\_\_nucleotide\_and\_nucleic\_acid\_metabolic\_process | KHDRBS1 | 270 | 1 | 0.897661 | -0.165284 | 398 | 365.47 | 0.918266 |
| GO:0051172\_negative\_regulation\_of\_nitrogen\_compound\_metabolic\_process | KHDRBS1 | 271 | 1 | 0.894348 | -0.164403 | 399 | 365.81 | 0.916817 |
| GO:0051716\_cellular\_response\_to\_stimulus | SOD1 | 273 | 1 | 0.887796 | -0.162658 | 400 | 366.19 | 0.915475 |
| GO:0010558\_negative\_regulation\_of\_macromolecule\_biosynthetic\_process | KHDRBS1 | 274 | 1 | 0.884556 | -0.161794 | 402 | 366.94 | 0.912786 |
| GO:0033036\_macromolecule\_localization | KHDRBS1 | 274 | 1 | 0.884556 | -0.161794 | 402 | 366.94 | 0.912786 |
| GO:0019222\_regulation\_of\_metabolic\_process | ZFP36L1 | 1088 | 4 | 0.891060 | -0.160829 | 403 | 367.09 | 0.910893 |
| GO:0019222\_regulation\_of\_metabolic\_process | KHDRBS1 | 1088 | 4 | 0.891060 | -0.160829 | 403 | 367.09 | 0.910893 |
| GO:0019222\_regulation\_of\_metabolic\_process | CNBP | 1088 | 4 | 0.891060 | -0.160829 | 403 | 367.09 | 0.910893 |
| GO:0019222\_regulation\_of\_metabolic\_process | SOD1 | 1088 | 4 | 0.891060 | -0.160829 | 403 | 367.09 | 0.910893 |
| GO:0065009\_regulation\_of\_molecular\_function | SOD1 | 279 | 1 | 0.868704 | -0.157550 | 404 | 368.7 | 0.912624 |
| GO:0009790\_embryonic\_development | GRN | 567 | 2 | 0.854915 | -0.155863 | 405 | 369.1 | 0.911358 |
| GO:0009790\_embryonic\_development | SOD1 | 567 | 2 | 0.854915 | -0.155863 | 405 | 369.1 | 0.911358 |
| GO:0031327\_negative\_regulation\_of\_cellular\_biosynthetic\_process | KHDRBS1 | 282 | 1 | 0.859462 | -0.155065 | 406 | 369.43 | 0.909926 |
| GO:0000902\_cell\_morphogenesis | SOD1 | 283 | 1 | 0.856426 | -0.154247 | 407 | 369.68 | 0.908305 |
| GO:0009890\_negative\_regulation\_of\_biosynthetic\_process | KHDRBS1 | 284 | 1 | 0.853410 | -0.153433 | 408 | 370.21 | 0.907377 |
| GO:0006629\_lipid\_metabolic\_process | PTGES3 | 285 | 1 | 0.850416 | -0.152625 | 409 | 370.45 | 0.905746 |
| GO:0048513\_organ\_development | PTGES3 | 1365 | 5 | 0.887796 | -0.150782 | 410 | 371.69 | 0.906561 |
| GO:0048513\_organ\_development | ZFP36L1 | 1365 | 5 | 0.887796 | -0.150782 | 410 | 371.69 | 0.906561 |
| GO:0048513\_organ\_development | INVS | 1365 | 5 | 0.887796 | -0.150782 | 410 | 371.69 | 0.906561 |
| GO:0048513\_organ\_development | PHB2 | 1365 | 5 | 0.887796 | -0.150782 | 410 | 371.69 | 0.906561 |
| GO:0048513\_organ\_development | SOD1 | 1365 | 5 | 0.887796 | -0.150782 | 410 | 371.69 | 0.906561 |
| GO:0048598\_embryonic\_morphogenesis | SOD1 | 299 | 1 | 0.810597 | -0.141800 | 411 | 374.58 | 0.911387 |
| GO:0045893\_positive\_regulation\_of\_transcription\_\_DNA-dependent | CNBP | 306 | 1 | 0.792054 | -0.136715 | 413 | 376.57 | 0.911792 |
| GO:0051254\_positive\_regulation\_of\_RNA\_metabolic\_process | CNBP | 306 | 1 | 0.792054 | -0.136715 | 413 | 376.57 | 0.911792 |
| GO:0032989\_cellular\_component\_morphogenesis | SOD1 | 307 | 1 | 0.789474 | -0.136006 | 414 | 376.89 | 0.910362 |
| GO:0016310\_phosphorylation | SOD1 | 309 | 1 | 0.784364 | -0.134599 | 415 | 377.56 | 0.909783 |
| GO:0048856\_anatomical\_structure\_development | PTGES3 | 1688 | 6 | 0.861499 | -0.122908 | 416 | 379.14 | 0.911394 |
| GO:0048856\_anatomical\_structure\_development | ZFP36L1 | 1688 | 6 | 0.861499 | -0.122908 | 416 | 379.14 | 0.911394 |
| GO:0048856\_anatomical\_structure\_development | INVS | 1688 | 6 | 0.861499 | -0.122908 | 416 | 379.14 | 0.911394 |
| GO:0048856\_anatomical\_structure\_development | PHB2 | 1688 | 6 | 0.861499 | -0.122908 | 416 | 379.14 | 0.911394 |
| GO:0048856\_anatomical\_structure\_development | GRN | 1688 | 6 | 0.861499 | -0.122908 | 416 | 379.14 | 0.911394 |
| GO:0048856\_anatomical\_structure\_development | SOD1 | 1688 | 6 | 0.861499 | -0.122908 | 416 | 379.14 | 0.911394 |
| GO:0010646\_regulation\_of\_cell\_communication | PHB2 | 330 | 1 | 0.734450 | -0.120766 | 417 | 380.6 | 0.912710 |
| GO:0010605\_negative\_regulation\_of\_macromolecule\_metabolic\_process | KHDRBS1 | 331 | 1 | 0.732231 | -0.120147 | 419 | 381.26 | 0.909928 |
| GO:0051093\_negative\_regulation\_of\_developmental\_process | SOD1 | 331 | 1 | 0.732231 | -0.120147 | 419 | 381.26 | 0.909928 |
| GO:0050794\_regulation\_of\_cellular\_process | ZFP36L1 | 2190 | 8 | 0.885364 | -0.119727 | 420 | 381.45 | 0.908214 |
| GO:0050794\_regulation\_of\_cellular\_process | PTGES3 | 2190 | 8 | 0.885364 | -0.119727 | 420 | 381.45 | 0.908214 |
| GO:0050794\_regulation\_of\_cellular\_process | KHDRBS1 | 2190 | 8 | 0.885364 | -0.119727 | 420 | 381.45 | 0.908214 |
| GO:0050794\_regulation\_of\_cellular\_process | CNBP | 2190 | 8 | 0.885364 | -0.119727 | 420 | 381.45 | 0.908214 |
| GO:0050794\_regulation\_of\_cellular\_process | ATP2A2 | 2190 | 8 | 0.885364 | -0.119727 | 420 | 381.45 | 0.908214 |
| GO:0050794\_regulation\_of\_cellular\_process | GRN | 2190 | 8 | 0.885364 | -0.119727 | 420 | 381.45 | 0.908214 |
| GO:0050794\_regulation\_of\_cellular\_process | PHB2 | 2190 | 8 | 0.885364 | -0.119727 | 420 | 381.45 | 0.908214 |
| GO:0050794\_regulation\_of\_cellular\_process | SOD1 | 2190 | 8 | 0.885364 | -0.119727 | 420 | 381.45 | 0.908214 |
| GO:0031324\_negative\_regulation\_of\_cellular\_metabolic\_process | KHDRBS1 | 332 | 1 | 0.730025 | -0.119533 | 421 | 381.94 | 0.907221 |
| GO:0045941\_positive\_regulation\_of\_transcription | CNBP | 338 | 1 | 0.717066 | -0.115915 | 422 | 382.58 | 0.906588 |
| GO:0009605\_response\_to\_external\_stimulus | SOD1 | 339 | 1 | 0.714951 | -0.115324 | 423 | 382.95 | 0.905319 |
| GO:0080090\_regulation\_of\_primary\_metabolic\_process | ZFP36L1 | 926 | 3 | 0.785211 | -0.115083 | 424 | 383.12 | 0.903585 |
| GO:0080090\_regulation\_of\_primary\_metabolic\_process | KHDRBS1 | 926 | 3 | 0.785211 | -0.115083 | 424 | 383.12 | 0.903585 |
| GO:0080090\_regulation\_of\_primary\_metabolic\_process | CNBP | 926 | 3 | 0.785211 | -0.115083 | 424 | 383.12 | 0.903585 |
| GO:0006793\_phosphorus\_metabolic\_process | SOD1 | 340 | 1 | 0.712848 | -0.114736 | 426 | 383.84 | 0.901033 |
| GO:0006796\_phosphate\_metabolic\_process | SOD1 | 340 | 1 | 0.712848 | -0.114736 | 426 | 383.84 | 0.901033 |
| GO:0051649\_establishment\_of\_localization\_in\_cell | KHDRBS1 | 342 | 1 | 0.708680 | -0.113570 | 427 | 384.18 | 0.899719 |
| GO:0010628\_positive\_regulation\_of\_gene\_expression | CNBP | 346 | 1 | 0.700487 | -0.111277 | 428 | 384.54 | 0.898458 |
| GO:0060255\_regulation\_of\_macromolecule\_metabolic\_process | KHDRBS1 | 936 | 3 | 0.776822 | -0.111164 | 429 | 384.73 | 0.896807 |
| GO:0060255\_regulation\_of\_macromolecule\_metabolic\_process | ZFP36L1 | 936 | 3 | 0.776822 | -0.111164 | 429 | 384.73 | 0.896807 |
| GO:0060255\_regulation\_of\_macromolecule\_metabolic\_process | CNBP | 936 | 3 | 0.776822 | -0.111164 | 429 | 384.73 | 0.896807 |
| GO:0009892\_negative\_regulation\_of\_metabolic\_process | KHDRBS1 | 348 | 1 | 0.696461 | -0.110149 | 430 | 385.73 | 0.897047 |
| GO:0045935\_positive\_regulation\_of\_nucleobase\_\_nucleoside\_\_nucleotide\_and\_nucleic\_acid\_metabolic\_process | CNBP | 352 | 1 | 0.688547 | -0.107930 | 431 | 386.41 | 0.896543 |
| GO:0030182\_neuron\_differentiation | SOD1 | 356 | 1 | 0.680810 | -0.105760 | 432 | 386.77 | 0.895301 |
| GO:0042981\_regulation\_of\_apoptosis | SOD1 | 360 | 1 | 0.673246 | -0.103637 | 433 | 387.18 | 0.894180 |
| GO:0051173\_positive\_regulation\_of\_nitrogen\_compound\_metabolic\_process | CNBP | 361 | 1 | 0.671381 | -0.103113 | 434 | 387.53 | 0.892926 |
| GO:0009653\_anatomical\_structure\_morphogenesis | ZFP36L1 | 958 | 3 | 0.758983 | -0.102961 | 435 | 387.77 | 0.891425 |
| GO:0009653\_anatomical\_structure\_morphogenesis | PHB2 | 958 | 3 | 0.758983 | -0.102961 | 435 | 387.77 | 0.891425 |
| GO:0009653\_anatomical\_structure\_morphogenesis | SOD1 | 958 | 3 | 0.758983 | -0.102961 | 435 | 387.77 | 0.891425 |
| GO:0045449\_regulation\_of\_transcription | KHDRBS1 | 676 | 2 | 0.717066 | -0.101539 | 436 | 388.02 | 0.889954 |
| GO:0045449\_regulation\_of\_transcription | CNBP | 676 | 2 | 0.717066 | -0.101539 | 436 | 388.02 | 0.889954 |
| GO:0010941\_regulation\_of\_cell\_death | SOD1 | 365 | 1 | 0.664023 | -0.101047 | 439 | 388.89 | 0.885854 |
| GO:0043009\_chordate\_embryonic\_development | GRN | 365 | 1 | 0.664023 | -0.101047 | 439 | 388.89 | 0.885854 |
| GO:0043067\_regulation\_of\_programmed\_cell\_death | SOD1 | 365 | 1 | 0.664023 | -0.101047 | 439 | 388.89 | 0.885854 |
| GO:0016043\_cellular\_component\_organization | SNX17 | 964 | 3 | 0.754259 | -0.100820 | 440 | 389.02 | 0.884136 |
| GO:0016043\_cellular\_component\_organization | SRR | 964 | 3 | 0.754259 | -0.100820 | 440 | 389.02 | 0.884136 |
| GO:0016043\_cellular\_component\_organization | SOD1 | 964 | 3 | 0.754259 | -0.100820 | 440 | 389.02 | 0.884136 |
| GO:0009792\_embryonic\_development\_ending\_in\_birth\_or\_egg\_hatching | GRN | 368 | 1 | 0.658610 | -0.099527 | 441 | 389.36 | 0.882902 |
| GO:0051641\_cellular\_localization | KHDRBS1 | 370 | 1 | 0.655050 | -0.098527 | 442 | 389.88 | 0.882081 |
| GO:0010557\_positive\_regulation\_of\_macromolecule\_biosynthetic\_process | CNBP | 371 | 1 | 0.653284 | -0.098031 | 443 | 390.25 | 0.880926 |
| GO:0006350\_transcription | KHDRBS1 | 701 | 2 | 0.691493 | -0.091942 | 444 | 391.34 | 0.881396 |
| GO:0006350\_transcription | CNBP | 701 | 2 | 0.691493 | -0.091942 | 444 | 391.34 | 0.881396 |
| GO:0043687\_post-translational\_protein\_modification | SOD1 | 384 | 1 | 0.631168 | -0.091821 | 445 | 391.73 | 0.880292 |
| GO:0050793\_regulation\_of\_developmental\_process | PHB2 | 703 | 2 | 0.689526 | -0.091213 | 446 | 391.88 | 0.878655 |
| GO:0050793\_regulation\_of\_developmental\_process | SOD1 | 703 | 2 | 0.689526 | -0.091213 | 446 | 391.88 | 0.878655 |
| GO:0031328\_positive\_regulation\_of\_cellular\_biosynthetic\_process | CNBP | 387 | 1 | 0.626275 | -0.090449 | 447 | 392.24 | 0.877494 |
| GO:0009891\_positive\_regulation\_of\_biosynthetic\_process | CNBP | 388 | 1 | 0.624661 | -0.089996 | 448 | 392.89 | 0.876987 |
| GO:0048699\_generation\_of\_neurons | SOD1 | 396 | 1 | 0.612041 | -0.086459 | 449 | 394.08 | 0.877684 |
| GO:0032502\_developmental\_process | ZFP36L1 | 2060 | 7 | 0.823582 | -0.085225 | 450 | 394.41 | 0.876467 |
| GO:0032502\_developmental\_process | PTGES3 | 2060 | 7 | 0.823582 | -0.085225 | 450 | 394.41 | 0.876467 |
| GO:0032502\_developmental\_process | INVS | 2060 | 7 | 0.823582 | -0.085225 | 450 | 394.41 | 0.876467 |
| GO:0032502\_developmental\_process | GRN | 2060 | 7 | 0.823582 | -0.085225 | 450 | 394.41 | 0.876467 |
| GO:0032502\_developmental\_process | PHB2 | 2060 | 7 | 0.823582 | -0.085225 | 450 | 394.41 | 0.876467 |
| GO:0032502\_developmental\_process | SOD1 | 2060 | 7 | 0.823582 | -0.085225 | 450 | 394.41 | 0.876467 |
| GO:0032502\_developmental\_process | GAPDH | 2060 | 7 | 0.823582 | -0.085225 | 450 | 394.41 | 0.876467 |
| GO:0022008\_neurogenesis | SOD1 | 423 | 1 | 0.572975 | -0.075564 | 451 | 396.56 | 0.879290 |
| GO:0006915\_apoptosis | SOD1 | 427 | 1 | 0.567608 | -0.074077 | 452 | 396.79 | 0.877854 |
| GO:0019219\_regulation\_of\_nucleobase\_\_nucleoside\_\_nucleotide\_and\_nucleic\_acid\_metabolic\_process | KHDRBS1 | 757 | 2 | 0.640339 | -0.073469 | 453 | 397.02 | 0.876424 |
| GO:0019219\_regulation\_of\_nucleobase\_\_nucleoside\_\_nucleotide\_and\_nucleic\_acid\_metabolic\_process | CNBP | 757 | 2 | 0.640339 | -0.073469 | 453 | 397.02 | 0.876424 |
| GO:0065007\_biological\_regulation | PTGES3 | 2593 | 9 | 0.841232 | -0.072712 | 454 | 397.17 | 0.874824 |
| GO:0065007\_biological\_regulation | ZFP36L1 | 2593 | 9 | 0.841232 | -0.072712 | 454 | 397.17 | 0.874824 |
| GO:0065007\_biological\_regulation | KHDRBS1 | 2593 | 9 | 0.841232 | -0.072712 | 454 | 397.17 | 0.874824 |
| GO:0065007\_biological\_regulation | CNBP | 2593 | 9 | 0.841232 | -0.072712 | 454 | 397.17 | 0.874824 |
| GO:0065007\_biological\_regulation | ATP2A2 | 2593 | 9 | 0.841232 | -0.072712 | 454 | 397.17 | 0.874824 |
| GO:0065007\_biological\_regulation | PHB2 | 2593 | 9 | 0.841232 | -0.072712 | 454 | 397.17 | 0.874824 |
| GO:0065007\_biological\_regulation | GRN | 2593 | 9 | 0.841232 | -0.072712 | 454 | 397.17 | 0.874824 |
| GO:0065007\_biological\_regulation | SOD1 | 2593 | 9 | 0.841232 | -0.072712 | 454 | 397.17 | 0.874824 |
| GO:0065007\_biological\_regulation | PARK7 | 2593 | 9 | 0.841232 | -0.072712 | 454 | 397.17 | 0.874824 |
| GO:0050789\_regulation\_of\_biological\_process | PTGES3 | 2357 | 8 | 0.822634 | -0.072186 | 455 | 397.31 | 0.873209 |
| GO:0050789\_regulation\_of\_biological\_process | ZFP36L1 | 2357 | 8 | 0.822634 | -0.072186 | 455 | 397.31 | 0.873209 |
| GO:0050789\_regulation\_of\_biological\_process | KHDRBS1 | 2357 | 8 | 0.822634 | -0.072186 | 455 | 397.31 | 0.873209 |
| GO:0050789\_regulation\_of\_biological\_process | CNBP | 2357 | 8 | 0.822634 | -0.072186 | 455 | 397.31 | 0.873209 |
| GO:0050789\_regulation\_of\_biological\_process | ATP2A2 | 2357 | 8 | 0.822634 | -0.072186 | 455 | 397.31 | 0.873209 |
| GO:0050789\_regulation\_of\_biological\_process | PHB2 | 2357 | 8 | 0.822634 | -0.072186 | 455 | 397.31 | 0.873209 |
| GO:0050789\_regulation\_of\_biological\_process | GRN | 2357 | 8 | 0.822634 | -0.072186 | 455 | 397.31 | 0.873209 |
| GO:0050789\_regulation\_of\_biological\_process | SOD1 | 2357 | 8 | 0.822634 | -0.072186 | 455 | 397.31 | 0.873209 |
| GO:0051179\_localization | KHDRBS1 | 1058 | 3 | 0.687245 | -0.072086 | 456 | 397.47 | 0.871645 |
| GO:0051179\_localization | SNX17 | 1058 | 3 | 0.687245 | -0.072086 | 456 | 397.47 | 0.871645 |
| GO:0051179\_localization | PARK7 | 1058 | 3 | 0.687245 | -0.072086 | 456 | 397.47 | 0.871645 |
| GO:0010604\_positive\_regulation\_of\_macromolecule\_metabolic\_process | CNBP | 433 | 1 | 0.559742 | -0.071902 | 458 | 398.04 | 0.869083 |
| GO:0012501\_programmed\_cell\_death | SOD1 | 433 | 1 | 0.559742 | -0.071902 | 458 | 398.04 | 0.869083 |
| GO:0006357\_regulation\_of\_transcription\_from\_RNA\_polymerase\_II\_promoter | CNBP | 435 | 1 | 0.557169 | -0.071192 | 459 | 398.64 | 0.868497 |
| GO:0048731\_system\_development | PTGES3 | 1609 | 5 | 0.753165 | -0.070953 | 460 | 398.83 | 0.867022 |
| GO:0048731\_system\_development | ZFP36L1 | 1609 | 5 | 0.753165 | -0.070953 | 460 | 398.83 | 0.867022 |
| GO:0048731\_system\_development | INVS | 1609 | 5 | 0.753165 | -0.070953 | 460 | 398.83 | 0.867022 |
| GO:0048731\_system\_development | PHB2 | 1609 | 5 | 0.753165 | -0.070953 | 460 | 398.83 | 0.867022 |
| GO:0048731\_system\_development | SOD1 | 1609 | 5 | 0.753165 | -0.070953 | 460 | 398.83 | 0.867022 |
| GO:0006464\_protein\_modification\_process | SOD1 | 439 | 1 | 0.552092 | -0.069793 | 461 | 399.18 | 0.865900 |
| GO:0051171\_regulation\_of\_nitrogen\_compound\_metabolic\_process | KHDRBS1 | 771 | 2 | 0.628712 | -0.069431 | 462 | 399.4 | 0.864502 |
| GO:0051171\_regulation\_of\_nitrogen\_compound\_metabolic\_process | CNBP | 771 | 2 | 0.628712 | -0.069431 | 462 | 399.4 | 0.864502 |
| GO:0031325\_positive\_regulation\_of\_cellular\_metabolic\_process | CNBP | 442 | 1 | 0.548345 | -0.068763 | 463 | 399.75 | 0.863391 |
| GO:0006366\_transcription\_from\_RNA\_polymerase\_II\_promoter | CNBP | 444 | 1 | 0.545875 | -0.068085 | 465 | 400.59 | 0.861484 |
| GO:0008219\_cell\_death | SOD1 | 444 | 1 | 0.545875 | -0.068085 | 465 | 400.59 | 0.861484 |
| GO:0010926\_anatomical\_structure\_formation | SRR | 447 | 1 | 0.542211 | -0.067081 | 466 | 401.25 | 0.861052 |
| GO:0006996\_organelle\_organization | SOD1 | 449 | 1 | 0.539796 | -0.066420 | 467 | 401.55 | 0.859850 |
| GO:0016265\_death | SOD1 | 450 | 1 | 0.538596 | -0.066092 | 468 | 401.77 | 0.858483 |
| GO:0009893\_positive\_regulation\_of\_metabolic\_process | CNBP | 458 | 1 | 0.529189 | -0.063527 | 470 | 402.79 | 0.857000 |
| GO:0043412\_biopolymer\_modification | SOD1 | 458 | 1 | 0.529189 | -0.063527 | 470 | 402.79 | 0.857000 |
| GO:0002376\_immune\_system\_process | SOD1 | 505 | 1 | 0.479937 | -0.050381 | 471 | 405.69 | 0.861338 |
| GO:0048522\_positive\_regulation\_of\_cellular\_process | CNBP | 895 | 2 | 0.541605 | -0.041681 | 472 | 407.46 | 0.863263 |
| GO:0048522\_positive\_regulation\_of\_cellular\_process | GRN | 895 | 2 | 0.541605 | -0.041681 | 472 | 407.46 | 0.863263 |
| GO:0006355\_regulation\_of\_transcription\_\_DNA-dependent | CNBP | 575 | 1 | 0.421510 | -0.035697 | 473 | 409.69 | 0.866152 |
| GO:0051252\_regulation\_of\_RNA\_metabolic\_process | CNBP | 590 | 1 | 0.410794 | -0.033154 | 474 | 410.21 | 0.865422 |
| GO:0006351\_transcription\_\_DNA-dependent | CNBP | 594 | 1 | 0.408028 | -0.032507 | 475 | 410.41 | 0.864021 |
| GO:0032774\_RNA\_biosynthetic\_process | CNBP | 595 | 1 | 0.407342 | -0.032347 | 476 | 410.61 | 0.862626 |
| GO:0007166\_cell\_surface\_receptor\_linked\_signal\_transduction | KHDRBS1 | 597 | 1 | 0.405977 | -0.032030 | 477 | 410.88 | 0.861384 |
| GO:0007399\_nervous\_system\_development | SOD1 | 621 | 1 | 0.390287 | -0.028452 | 478 | 411.89 | 0.861695 |
| GO:0048518\_positive\_regulation\_of\_biological\_process | CNBP | 995 | 2 | 0.487173 | -0.027222 | 479 | 412.07 | 0.860271 |
| GO:0048518\_positive\_regulation\_of\_biological\_process | GRN | 995 | 2 | 0.487173 | -0.027222 | 479 | 412.07 | 0.860271 |
| GO:0048468\_cell\_development | SOD1 | 654 | 1 | 0.370594 | -0.024166 | 480 | 412.88 | 0.860167 |
| GO:0030154\_cell\_differentiation | ZFP36L1 | 1060 | 2 | 0.457299 | -0.020475 | 481 | 414.02 | 0.860748 |
| GO:0030154\_cell\_differentiation | SOD1 | 1060 | 2 | 0.457299 | -0.020475 | 481 | 414.02 | 0.860748 |
| GO:0050896\_response\_to\_stimulus | SOD1 | 1107 | 2 | 0.437883 | -0.016595 | 482 | 415.25 | 0.861515 |
| GO:0050896\_response\_to\_stimulus | PARK7 | 1107 | 2 | 0.437883 | -0.016595 | 482 | 415.25 | 0.861515 |
| GO:0048869\_cellular\_developmental\_process | ZFP36L1 | 1113 | 2 | 0.435523 | -0.016151 | 483 | 415.33 | 0.859896 |
| GO:0048869\_cellular\_developmental\_process | SOD1 | 1113 | 2 | 0.435523 | -0.016151 | 483 | 415.33 | 0.859896 |
| GO:0008150\_biological\_process | KHDRBS1 | 4605 | 19 | 1.000000 | 0.000000 | 1980 | 1947.27 | 0.983470 |
| GO:0008150\_biological\_process | PABPN1 | 4605 | 19 | 1.000000 | 0.000000 | 1980 | 1947.27 | 0.983470 |
| GO:0008150\_biological\_process | PTGES3 | 4605 | 19 | 1.000000 | 0.000000 | 1980 | 1947.27 | 0.983470 |
| GO:0008150\_biological\_process | CNBP | 4605 | 19 | 1.000000 | 0.000000 | 1980 | 1947.27 | 0.983470 |
| GO:0008150\_biological\_process | SNX17 | 4605 | 19 | 1.000000 | 0.000000 | 1980 | 1947.27 | 0.983470 |
| GO:0008150\_biological\_process | VIM | 4605 | 19 | 1.000000 | 0.000000 | 1980 | 1947.27 | 0.983470 |
| GO:0008150\_biological\_process | PGD | 4605 | 19 | 1.000000 | 0.000000 | 1980 | 1947.27 | 0.983470 |
| GO:0008150\_biological\_process | SOD1 | 4605 | 19 | 1.000000 | 0.000000 | 1980 | 1947.27 | 0.983470 |
| GO:0008150\_biological\_process | MCM4 | 4605 | 19 | 1.000000 | 0.000000 | 1980 | 1947.27 | 0.983470 |
| GO:0008150\_biological\_process | PARK7 | 4605 | 19 | 1.000000 | 0.000000 | 1980 | 1947.27 | 0.983470 |
| GO:0008150\_biological\_process | ZFP36L1 | 4605 | 19 | 1.000000 | 0.000000 | 1980 | 1947.27 | 0.983470 |
| GO:0008150\_biological\_process | MRPL11 | 4605 | 19 | 1.000000 | 0.000000 | 1980 | 1947.27 | 0.983470 |
| GO:0008150\_biological\_process | ARIH2 | 4605 | 19 | 1.000000 | 0.000000 | 1980 | 1947.27 | 0.983470 |
| GO:0008150\_biological\_process | INVS | 4605 | 19 | 1.000000 | 0.000000 | 1980 | 1947.27 | 0.983470 |
| GO:0008150\_biological\_process | ATP2A2 | 4605 | 19 | 1.000000 | 0.000000 | 1980 | 1947.27 | 0.983470 |
| GO:0008150\_biological\_process | PHB2 | 4605 | 19 | 1.000000 | 0.000000 | 1980 | 1947.27 | 0.983470 |
| GO:0008150\_biological\_process | GRN | 4605 | 19 | 1.000000 | 0.000000 | 1980 | 1947.27 | 0.983470 |
| GO:0008150\_biological\_process | SRR | 4605 | 19 | 1.000000 | 0.000000 | 1980 | 1947.27 | 0.983470 |
| GO:0008150\_biological\_process | GAPDH | 4605 | 19 | 1.000000 | 0.000000 | 1980 | 1947.27 | 0.983470 |
