# Supplementary material for: RedundancyMiner: De-replication of redundant GO categories in microarray and proteomics analysis
Source: BMC Bioinformatics. 2011 Feb 10;12:52. doi: 10.1186/1471-2105-12-52 (PMC3223614; doi:10.1186/1471-2105-12-52)
Supplement: Additional file 8 — Retinal development HTGM download. compressed package of the results of running HTGM on the retinal development genes list. [file 1471-2105-12-52-S8.ZIP › SCENARIO_2_MODIFIED/total.txt.total.txt.dir/Exp1_BestClusterMap_LEIGS_KM_24.csv.join.18.txt.dir/Exp1_BestClusterMap_LEIGS_KM_24.csv.join.18.txt.change.gce.html]

Gene Category Report for Exp1\_BestClusterMap\_LEIGS\_KM\_24.csv.join.18.txt

# Gene Category Report for Exp1\_BestClusterMap\_LEIGS\_KM\_24.csv.join.18.txt

| HYPERLINKED GO CATEGORY | HYPERLINKED GENE NAME | TOTAL GENES | CHANGED GENES | ENRICHMENT | LOG10(p) | CUMULATIVE NUMBER OF CATEGORIES | CUMULATIVE RANDOMS MEAN | FALSE DISCOVERY RATE |
| --- | --- | --- | --- | --- | --- | --- | --- | --- |
| GO:0000122\_negative\_regulation\_of\_transcription\_from\_RNA\_polymerase\_II\_promoter | NCOA2 | 175 | 6 | 4.267181 | -2.618333 | 1 | 2.59 | 2.590000 |
| GO:0000122\_negative\_regulation\_of\_transcription\_from\_RNA\_polymerase\_II\_promoter | TLE4 | 175 | 6 | 4.267181 | -2.618333 | 1 | 2.59 | 2.590000 |
| GO:0000122\_negative\_regulation\_of\_transcription\_from\_RNA\_polymerase\_II\_promoter | VAX2 | 175 | 6 | 4.267181 | -2.618333 | 1 | 2.59 | 2.590000 |
| GO:0000122\_negative\_regulation\_of\_transcription\_from\_RNA\_polymerase\_II\_promoter | NCOR1 | 175 | 6 | 4.267181 | -2.618333 | 1 | 2.59 | 2.590000 |
| GO:0000122\_negative\_regulation\_of\_transcription\_from\_RNA\_polymerase\_II\_promoter | NRIP1 | 175 | 6 | 4.267181 | -2.618333 | 1 | 2.59 | 2.590000 |
| GO:0000122\_negative\_regulation\_of\_transcription\_from\_RNA\_polymerase\_II\_promoter | FOXP2 | 175 | 6 | 4.267181 | -2.618333 | 1 | 2.59 | 2.590000 |
| GO:0010558\_negative\_regulation\_of\_macromolecule\_biosynthetic\_process | NCOA2 | 274 | 7 | 3.179621 | -2.269169 | 2 | 4.92 | 2.460000 |
| GO:0010558\_negative\_regulation\_of\_macromolecule\_biosynthetic\_process | TIA1 | 274 | 7 | 3.179621 | -2.269169 | 2 | 4.92 | 2.460000 |
| GO:0010558\_negative\_regulation\_of\_macromolecule\_biosynthetic\_process | TLE4 | 274 | 7 | 3.179621 | -2.269169 | 2 | 4.92 | 2.460000 |
| GO:0010558\_negative\_regulation\_of\_macromolecule\_biosynthetic\_process | VAX2 | 274 | 7 | 3.179621 | -2.269169 | 2 | 4.92 | 2.460000 |
| GO:0010558\_negative\_regulation\_of\_macromolecule\_biosynthetic\_process | NCOR1 | 274 | 7 | 3.179621 | -2.269169 | 2 | 4.92 | 2.460000 |
| GO:0010558\_negative\_regulation\_of\_macromolecule\_biosynthetic\_process | NRIP1 | 274 | 7 | 3.179621 | -2.269169 | 2 | 4.92 | 2.460000 |
| GO:0010558\_negative\_regulation\_of\_macromolecule\_biosynthetic\_process | FOXP2 | 274 | 7 | 3.179621 | -2.269169 | 2 | 4.92 | 2.460000 |
| GO:0010468\_regulation\_of\_gene\_expression | KLF7 | 778 | 13 | 2.079657 | -2.255588 | 3 | 5.0 | 1.666667 |
| GO:0010468\_regulation\_of\_gene\_expression | RXRB | 778 | 13 | 2.079657 | -2.255588 | 3 | 5.0 | 1.666667 |
| GO:0010468\_regulation\_of\_gene\_expression | E2F7 | 778 | 13 | 2.079657 | -2.255588 | 3 | 5.0 | 1.666667 |
| GO:0010468\_regulation\_of\_gene\_expression | ELAVL1 | 778 | 13 | 2.079657 | -2.255588 | 3 | 5.0 | 1.666667 |
| GO:0010468\_regulation\_of\_gene\_expression | TLE4 | 778 | 13 | 2.079657 | -2.255588 | 3 | 5.0 | 1.666667 |
| GO:0010468\_regulation\_of\_gene\_expression | VAX2 | 778 | 13 | 2.079657 | -2.255588 | 3 | 5.0 | 1.666667 |
| GO:0010468\_regulation\_of\_gene\_expression | HES6 | 778 | 13 | 2.079657 | -2.255588 | 3 | 5.0 | 1.666667 |
| GO:0010468\_regulation\_of\_gene\_expression | DACH1 | 778 | 13 | 2.079657 | -2.255588 | 3 | 5.0 | 1.666667 |
| GO:0010468\_regulation\_of\_gene\_expression | FOXP2 | 778 | 13 | 2.079657 | -2.255588 | 3 | 5.0 | 1.666667 |
| GO:0010468\_regulation\_of\_gene\_expression | NRIP1 | 778 | 13 | 2.079657 | -2.255588 | 3 | 5.0 | 1.666667 |
| GO:0010468\_regulation\_of\_gene\_expression | NCOA2 | 778 | 13 | 2.079657 | -2.255588 | 3 | 5.0 | 1.666667 |
| GO:0010468\_regulation\_of\_gene\_expression | TIA1 | 778 | 13 | 2.079657 | -2.255588 | 3 | 5.0 | 1.666667 |
| GO:0010468\_regulation\_of\_gene\_expression | NCOR1 | 778 | 13 | 2.079657 | -2.255588 | 3 | 5.0 | 1.666667 |
| GO:0031327\_negative\_regulation\_of\_cellular\_biosynthetic\_process | NCOA2 | 282 | 7 | 3.089419 | -2.201059 | 4 | 5.45 | 1.362500 |
| GO:0031327\_negative\_regulation\_of\_cellular\_biosynthetic\_process | TIA1 | 282 | 7 | 3.089419 | -2.201059 | 4 | 5.45 | 1.362500 |
| GO:0031327\_negative\_regulation\_of\_cellular\_biosynthetic\_process | TLE4 | 282 | 7 | 3.089419 | -2.201059 | 4 | 5.45 | 1.362500 |
| GO:0031327\_negative\_regulation\_of\_cellular\_biosynthetic\_process | VAX2 | 282 | 7 | 3.089419 | -2.201059 | 4 | 5.45 | 1.362500 |
| GO:0031327\_negative\_regulation\_of\_cellular\_biosynthetic\_process | NCOR1 | 282 | 7 | 3.089419 | -2.201059 | 4 | 5.45 | 1.362500 |
| GO:0031327\_negative\_regulation\_of\_cellular\_biosynthetic\_process | NRIP1 | 282 | 7 | 3.089419 | -2.201059 | 4 | 5.45 | 1.362500 |
| GO:0031327\_negative\_regulation\_of\_cellular\_biosynthetic\_process | FOXP2 | 282 | 7 | 3.089419 | -2.201059 | 4 | 5.45 | 1.362500 |
| GO:0009890\_negative\_regulation\_of\_biosynthetic\_process | NCOA2 | 284 | 7 | 3.067663 | -2.184430 | 5 | 5.48 | 1.096000 |
| GO:0009890\_negative\_regulation\_of\_biosynthetic\_process | TIA1 | 284 | 7 | 3.067663 | -2.184430 | 5 | 5.48 | 1.096000 |
| GO:0009890\_negative\_regulation\_of\_biosynthetic\_process | TLE4 | 284 | 7 | 3.067663 | -2.184430 | 5 | 5.48 | 1.096000 |
| GO:0009890\_negative\_regulation\_of\_biosynthetic\_process | VAX2 | 284 | 7 | 3.067663 | -2.184430 | 5 | 5.48 | 1.096000 |
| GO:0009890\_negative\_regulation\_of\_biosynthetic\_process | NCOR1 | 284 | 7 | 3.067663 | -2.184430 | 5 | 5.48 | 1.096000 |
| GO:0009890\_negative\_regulation\_of\_biosynthetic\_process | NRIP1 | 284 | 7 | 3.067663 | -2.184430 | 5 | 5.48 | 1.096000 |
| GO:0009890\_negative\_regulation\_of\_biosynthetic\_process | FOXP2 | 284 | 7 | 3.067663 | -2.184430 | 5 | 5.48 | 1.096000 |
| GO:0045892\_negative\_regulation\_of\_transcription\_\_DNA-dependent | NCOA2 | 218 | 6 | 3.425490 | -2.147557 | 6 | 6.05 | 1.008333 |
| GO:0045892\_negative\_regulation\_of\_transcription\_\_DNA-dependent | TLE4 | 218 | 6 | 3.425490 | -2.147557 | 6 | 6.05 | 1.008333 |
| GO:0045892\_negative\_regulation\_of\_transcription\_\_DNA-dependent | VAX2 | 218 | 6 | 3.425490 | -2.147557 | 6 | 6.05 | 1.008333 |
| GO:0045892\_negative\_regulation\_of\_transcription\_\_DNA-dependent | NCOR1 | 218 | 6 | 3.425490 | -2.147557 | 6 | 6.05 | 1.008333 |
| GO:0045892\_negative\_regulation\_of\_transcription\_\_DNA-dependent | NRIP1 | 218 | 6 | 3.425490 | -2.147557 | 6 | 6.05 | 1.008333 |
| GO:0045892\_negative\_regulation\_of\_transcription\_\_DNA-dependent | FOXP2 | 218 | 6 | 3.425490 | -2.147557 | 6 | 6.05 | 1.008333 |
| GO:0051253\_negative\_regulation\_of\_RNA\_metabolic\_process | NCOA2 | 220 | 6 | 3.394349 | -2.128566 | 7 | 6.21 | 0.887143 |
| GO:0051253\_negative\_regulation\_of\_RNA\_metabolic\_process | TLE4 | 220 | 6 | 3.394349 | -2.128566 | 7 | 6.21 | 0.887143 |
| GO:0051253\_negative\_regulation\_of\_RNA\_metabolic\_process | VAX2 | 220 | 6 | 3.394349 | -2.128566 | 7 | 6.21 | 0.887143 |
| GO:0051253\_negative\_regulation\_of\_RNA\_metabolic\_process | NCOR1 | 220 | 6 | 3.394349 | -2.128566 | 7 | 6.21 | 0.887143 |
| GO:0051253\_negative\_regulation\_of\_RNA\_metabolic\_process | NRIP1 | 220 | 6 | 3.394349 | -2.128566 | 7 | 6.21 | 0.887143 |
| GO:0051253\_negative\_regulation\_of\_RNA\_metabolic\_process | FOXP2 | 220 | 6 | 3.394349 | -2.128566 | 7 | 6.21 | 0.887143 |
| GO:0001887\_selenium\_metabolic\_process | SEPP1 | 1 | 1 |  |  |  |  |  |  |
| GO:0006463\_steroid\_hormone\_receptor\_complex\_assembly | FKBP4 | 1 | 1 |  |  |  |  |  |  |
| GO:0006654\_phosphatidic\_acid\_biosynthetic\_process | SH3GLB1 | 1 | 1 |  |  |  |  |  |  |
| GO:0007076\_mitotic\_chromosome\_condensation | AKAP8 | 1 | 1 |  |  |  |  |  |  |
| GO:0019510\_S-adenosylhomocysteine\_catabolic\_process | AHCY | 1 | 1 |  |  |  |  |  |  |
| GO:0042297\_vocal\_learning | FOXP2 | 1 | 1 |  |  |  |  |  |  |
| GO:0046473\_phosphatidic\_acid\_metabolic\_process | SH3GLB1 | 1 | 1 |  |  |  |  |  |  |
| GO:0046498\_S-adenosylhomocysteine\_metabolic\_process | AHCY | 1 | 1 |  |  |  |  |  |  |
| GO:0048813\_dendrite\_morphogenesis | KLF7 | 18 | 2 | 13.828829 | -2.052356 | 8 | 7.46 | 0.932500 |
| GO:0048813\_dendrite\_morphogenesis | YWHAH | 18 | 2 | 13.828829 | -2.052356 | 8 | 7.46 | 0.932500 |
| GO:0010556\_regulation\_of\_macromolecule\_biosynthetic\_process | KLF7 | 745 | 12 | 2.004716 | -1.964703 | 9 | 8.28 | 0.920000 |
| GO:0010556\_regulation\_of\_macromolecule\_biosynthetic\_process | NCOA2 | 745 | 12 | 2.004716 | -1.964703 | 9 | 8.28 | 0.920000 |
| GO:0010556\_regulation\_of\_macromolecule\_biosynthetic\_process | RXRB | 745 | 12 | 2.004716 | -1.964703 | 9 | 8.28 | 0.920000 |
| GO:0010556\_regulation\_of\_macromolecule\_biosynthetic\_process | E2F7 | 745 | 12 | 2.004716 | -1.964703 | 9 | 8.28 | 0.920000 |
| GO:0010556\_regulation\_of\_macromolecule\_biosynthetic\_process | TIA1 | 745 | 12 | 2.004716 | -1.964703 | 9 | 8.28 | 0.920000 |
| GO:0010556\_regulation\_of\_macromolecule\_biosynthetic\_process | TLE4 | 745 | 12 | 2.004716 | -1.964703 | 9 | 8.28 | 0.920000 |
| GO:0010556\_regulation\_of\_macromolecule\_biosynthetic\_process | VAX2 | 745 | 12 | 2.004716 | -1.964703 | 9 | 8.28 | 0.920000 |
| GO:0010556\_regulation\_of\_macromolecule\_biosynthetic\_process | DACH1 | 745 | 12 | 2.004716 | -1.964703 | 9 | 8.28 | 0.920000 |
| GO:0010556\_regulation\_of\_macromolecule\_biosynthetic\_process | HES6 | 745 | 12 | 2.004716 | -1.964703 | 9 | 8.28 | 0.920000 |
| GO:0010556\_regulation\_of\_macromolecule\_biosynthetic\_process | NCOR1 | 745 | 12 | 2.004716 | -1.964703 | 9 | 8.28 | 0.920000 |
| GO:0010556\_regulation\_of\_macromolecule\_biosynthetic\_process | NRIP1 | 745 | 12 | 2.004716 | -1.964703 | 9 | 8.28 | 0.920000 |
| GO:0010556\_regulation\_of\_macromolecule\_biosynthetic\_process | FOXP2 | 745 | 12 | 2.004716 | -1.964703 | 9 | 8.28 | 0.920000 |
| GO:0019219\_regulation\_of\_nucleobase\_\_nucleoside\_\_nucleotide\_and\_nucleic\_acid\_metabolic\_process | KLF7 | 757 | 12 | 1.972937 | -1.910479 | 10 | 9.38 | 0.938000 |
| GO:0019219\_regulation\_of\_nucleobase\_\_nucleoside\_\_nucleotide\_and\_nucleic\_acid\_metabolic\_process | NCOA2 | 757 | 12 | 1.972937 | -1.910479 | 10 | 9.38 | 0.938000 |
| GO:0019219\_regulation\_of\_nucleobase\_\_nucleoside\_\_nucleotide\_and\_nucleic\_acid\_metabolic\_process | RXRB | 757 | 12 | 1.972937 | -1.910479 | 10 | 9.38 | 0.938000 |
| GO:0019219\_regulation\_of\_nucleobase\_\_nucleoside\_\_nucleotide\_and\_nucleic\_acid\_metabolic\_process | E2F7 | 757 | 12 | 1.972937 | -1.910479 | 10 | 9.38 | 0.938000 |
| GO:0019219\_regulation\_of\_nucleobase\_\_nucleoside\_\_nucleotide\_and\_nucleic\_acid\_metabolic\_process | ELAVL1 | 757 | 12 | 1.972937 | -1.910479 | 10 | 9.38 | 0.938000 |
| GO:0019219\_regulation\_of\_nucleobase\_\_nucleoside\_\_nucleotide\_and\_nucleic\_acid\_metabolic\_process | TLE4 | 757 | 12 | 1.972937 | -1.910479 | 10 | 9.38 | 0.938000 |
| GO:0019219\_regulation\_of\_nucleobase\_\_nucleoside\_\_nucleotide\_and\_nucleic\_acid\_metabolic\_process | VAX2 | 757 | 12 | 1.972937 | -1.910479 | 10 | 9.38 | 0.938000 |
| GO:0019219\_regulation\_of\_nucleobase\_\_nucleoside\_\_nucleotide\_and\_nucleic\_acid\_metabolic\_process | DACH1 | 757 | 12 | 1.972937 | -1.910479 | 10 | 9.38 | 0.938000 |
| GO:0019219\_regulation\_of\_nucleobase\_\_nucleoside\_\_nucleotide\_and\_nucleic\_acid\_metabolic\_process | HES6 | 757 | 12 | 1.972937 | -1.910479 | 10 | 9.38 | 0.938000 |
| GO:0019219\_regulation\_of\_nucleobase\_\_nucleoside\_\_nucleotide\_and\_nucleic\_acid\_metabolic\_process | NCOR1 | 757 | 12 | 1.972937 | -1.910479 | 10 | 9.38 | 0.938000 |
| GO:0019219\_regulation\_of\_nucleobase\_\_nucleoside\_\_nucleotide\_and\_nucleic\_acid\_metabolic\_process | NRIP1 | 757 | 12 | 1.972937 | -1.910479 | 10 | 9.38 | 0.938000 |
| GO:0019219\_regulation\_of\_nucleobase\_\_nucleoside\_\_nucleotide\_and\_nucleic\_acid\_metabolic\_process | FOXP2 | 757 | 12 | 1.972937 | -1.910479 | 10 | 9.38 | 0.938000 |
| GO:0051171\_regulation\_of\_nitrogen\_compound\_metabolic\_process | KLF7 | 771 | 12 | 1.937112 | -1.848961 | 11 | 10.51 | 0.955455 |
| GO:0051171\_regulation\_of\_nitrogen\_compound\_metabolic\_process | NCOA2 | 771 | 12 | 1.937112 | -1.848961 | 11 | 10.51 | 0.955455 |
| GO:0051171\_regulation\_of\_nitrogen\_compound\_metabolic\_process | RXRB | 771 | 12 | 1.937112 | -1.848961 | 11 | 10.51 | 0.955455 |
| GO:0051171\_regulation\_of\_nitrogen\_compound\_metabolic\_process | E2F7 | 771 | 12 | 1.937112 | -1.848961 | 11 | 10.51 | 0.955455 |
| GO:0051171\_regulation\_of\_nitrogen\_compound\_metabolic\_process | ELAVL1 | 771 | 12 | 1.937112 | -1.848961 | 11 | 10.51 | 0.955455 |
| GO:0051171\_regulation\_of\_nitrogen\_compound\_metabolic\_process | TLE4 | 771 | 12 | 1.937112 | -1.848961 | 11 | 10.51 | 0.955455 |
| GO:0051171\_regulation\_of\_nitrogen\_compound\_metabolic\_process | VAX2 | 771 | 12 | 1.937112 | -1.848961 | 11 | 10.51 | 0.955455 |
| GO:0051171\_regulation\_of\_nitrogen\_compound\_metabolic\_process | DACH1 | 771 | 12 | 1.937112 | -1.848961 | 11 | 10.51 | 0.955455 |
| GO:0051171\_regulation\_of\_nitrogen\_compound\_metabolic\_process | HES6 | 771 | 12 | 1.937112 | -1.848961 | 11 | 10.51 | 0.955455 |
| GO:0051171\_regulation\_of\_nitrogen\_compound\_metabolic\_process | NCOR1 | 771 | 12 | 1.937112 | -1.848961 | 11 | 10.51 | 0.955455 |
| GO:0051171\_regulation\_of\_nitrogen\_compound\_metabolic\_process | NRIP1 | 771 | 12 | 1.937112 | -1.848961 | 11 | 10.51 | 0.955455 |
| GO:0051171\_regulation\_of\_nitrogen\_compound\_metabolic\_process | FOXP2 | 771 | 12 | 1.937112 | -1.848961 | 11 | 10.51 | 0.955455 |
| GO:0045449\_regulation\_of\_transcription | KLF7 | 676 | 11 | 2.025228 | -1.848757 | 12 | 10.53 | 0.877500 |
| GO:0045449\_regulation\_of\_transcription | NCOA2 | 676 | 11 | 2.025228 | -1.848757 | 12 | 10.53 | 0.877500 |
| GO:0045449\_regulation\_of\_transcription | RXRB | 676 | 11 | 2.025228 | -1.848757 | 12 | 10.53 | 0.877500 |
| GO:0045449\_regulation\_of\_transcription | E2F7 | 676 | 11 | 2.025228 | -1.848757 | 12 | 10.53 | 0.877500 |
| GO:0045449\_regulation\_of\_transcription | TLE4 | 676 | 11 | 2.025228 | -1.848757 | 12 | 10.53 | 0.877500 |
| GO:0045449\_regulation\_of\_transcription | VAX2 | 676 | 11 | 2.025228 | -1.848757 | 12 | 10.53 | 0.877500 |
| GO:0045449\_regulation\_of\_transcription | DACH1 | 676 | 11 | 2.025228 | -1.848757 | 12 | 10.53 | 0.877500 |
| GO:0045449\_regulation\_of\_transcription | HES6 | 676 | 11 | 2.025228 | -1.848757 | 12 | 10.53 | 0.877500 |
| GO:0045449\_regulation\_of\_transcription | NCOR1 | 676 | 11 | 2.025228 | -1.848757 | 12 | 10.53 | 0.877500 |
| GO:0045449\_regulation\_of\_transcription | NRIP1 | 676 | 11 | 2.025228 | -1.848757 | 12 | 10.53 | 0.877500 |
| GO:0045449\_regulation\_of\_transcription | FOXP2 | 676 | 11 | 2.025228 | -1.848757 | 12 | 10.53 | 0.877500 |
| GO:0016481\_negative\_regulation\_of\_transcription | NCOA2 | 253 | 6 | 2.951608 | -1.844342 | 13 | 10.92 | 0.840000 |
| GO:0016481\_negative\_regulation\_of\_transcription | TLE4 | 253 | 6 | 2.951608 | -1.844342 | 13 | 10.92 | 0.840000 |
| GO:0016481\_negative\_regulation\_of\_transcription | VAX2 | 253 | 6 | 2.951608 | -1.844342 | 13 | 10.92 | 0.840000 |
| GO:0016481\_negative\_regulation\_of\_transcription | NCOR1 | 253 | 6 | 2.951608 | -1.844342 | 13 | 10.92 | 0.840000 |
| GO:0016481\_negative\_regulation\_of\_transcription | NRIP1 | 253 | 6 | 2.951608 | -1.844342 | 13 | 10.92 | 0.840000 |
| GO:0016481\_negative\_regulation\_of\_transcription | FOXP2 | 253 | 6 | 2.951608 | -1.844342 | 13 | 10.92 | 0.840000 |
| GO:0010605\_negative\_regulation\_of\_macromolecule\_metabolic\_process | NCOA2 | 331 | 7 | 2.632073 | -1.833918 | 14 | 11.0 | 0.785714 |
| GO:0010605\_negative\_regulation\_of\_macromolecule\_metabolic\_process | TIA1 | 331 | 7 | 2.632073 | -1.833918 | 14 | 11.0 | 0.785714 |
| GO:0010605\_negative\_regulation\_of\_macromolecule\_metabolic\_process | TLE4 | 331 | 7 | 2.632073 | -1.833918 | 14 | 11.0 | 0.785714 |
| GO:0010605\_negative\_regulation\_of\_macromolecule\_metabolic\_process | VAX2 | 331 | 7 | 2.632073 | -1.833918 | 14 | 11.0 | 0.785714 |
| GO:0010605\_negative\_regulation\_of\_macromolecule\_metabolic\_process | NCOR1 | 331 | 7 | 2.632073 | -1.833918 | 14 | 11.0 | 0.785714 |
| GO:0010605\_negative\_regulation\_of\_macromolecule\_metabolic\_process | NRIP1 | 331 | 7 | 2.632073 | -1.833918 | 14 | 11.0 | 0.785714 |
| GO:0010605\_negative\_regulation\_of\_macromolecule\_metabolic\_process | FOXP2 | 331 | 7 | 2.632073 | -1.833918 | 14 | 11.0 | 0.785714 |
| GO:0031324\_negative\_regulation\_of\_cellular\_metabolic\_process | NCOA2 | 332 | 7 | 2.624145 | -1.827213 | 15 | 11.09 | 0.739333 |
| GO:0031324\_negative\_regulation\_of\_cellular\_metabolic\_process | TIA1 | 332 | 7 | 2.624145 | -1.827213 | 15 | 11.09 | 0.739333 |
| GO:0031324\_negative\_regulation\_of\_cellular\_metabolic\_process | TLE4 | 332 | 7 | 2.624145 | -1.827213 | 15 | 11.09 | 0.739333 |
| GO:0031324\_negative\_regulation\_of\_cellular\_metabolic\_process | VAX2 | 332 | 7 | 2.624145 | -1.827213 | 15 | 11.09 | 0.739333 |
| GO:0031324\_negative\_regulation\_of\_cellular\_metabolic\_process | NCOR1 | 332 | 7 | 2.624145 | -1.827213 | 15 | 11.09 | 0.739333 |
| GO:0031324\_negative\_regulation\_of\_cellular\_metabolic\_process | NRIP1 | 332 | 7 | 2.624145 | -1.827213 | 15 | 11.09 | 0.739333 |
| GO:0031324\_negative\_regulation\_of\_cellular\_metabolic\_process | FOXP2 | 332 | 7 | 2.624145 | -1.827213 | 15 | 11.09 | 0.739333 |
| GO:0051252\_regulation\_of\_RNA\_metabolic\_process | KLF7 | 590 | 10 | 2.109482 | -1.818813 | 16 | 11.11 | 0.694375 |
| GO:0051252\_regulation\_of\_RNA\_metabolic\_process | NCOA2 | 590 | 10 | 2.109482 | -1.818813 | 16 | 11.11 | 0.694375 |
| GO:0051252\_regulation\_of\_RNA\_metabolic\_process | RXRB | 590 | 10 | 2.109482 | -1.818813 | 16 | 11.11 | 0.694375 |
| GO:0051252\_regulation\_of\_RNA\_metabolic\_process | ELAVL1 | 590 | 10 | 2.109482 | -1.818813 | 16 | 11.11 | 0.694375 |
| GO:0051252\_regulation\_of\_RNA\_metabolic\_process | TLE4 | 590 | 10 | 2.109482 | -1.818813 | 16 | 11.11 | 0.694375 |
| GO:0051252\_regulation\_of\_RNA\_metabolic\_process | VAX2 | 590 | 10 | 2.109482 | -1.818813 | 16 | 11.11 | 0.694375 |
| GO:0051252\_regulation\_of\_RNA\_metabolic\_process | HES6 | 590 | 10 | 2.109482 | -1.818813 | 16 | 11.11 | 0.694375 |
| GO:0051252\_regulation\_of\_RNA\_metabolic\_process | NCOR1 | 590 | 10 | 2.109482 | -1.818813 | 16 | 11.11 | 0.694375 |
| GO:0051252\_regulation\_of\_RNA\_metabolic\_process | NRIP1 | 590 | 10 | 2.109482 | -1.818813 | 16 | 11.11 | 0.694375 |
| GO:0051252\_regulation\_of\_RNA\_metabolic\_process | FOXP2 | 590 | 10 | 2.109482 | -1.818813 | 16 | 11.11 | 0.694375 |
| GO:0009612\_response\_to\_mechanical\_stimulus | SLC12A2 | 24 | 2 | 10.371622 | -1.809262 | 17 | 11.61 | 0.682941 |
| GO:0009612\_response\_to\_mechanical\_stimulus | FOXP2 | 24 | 2 | 10.371622 | -1.809262 | 17 | 11.61 | 0.682941 |
| GO:0001543\_ovarian\_follicle\_rupture | NRIP1 | 2 | 1 |  |  |  |  |  |  |
| GO:0001767\_establishment\_of\_lymphocyte\_polarity | MYH9 | 2 | 1 |  |  |  |  |  |  |
| GO:0001768\_establishment\_of\_T\_cell\_polarity | MYH9 | 2 | 1 |  |  |  |  |  |  |
| GO:0007132\_meiotic\_metaphase\_I | MYH9 | 2 | 1 |  |  |  |  |  |  |
| GO:0014067\_negative\_regulation\_of\_phosphoinositide\_3-kinase\_cascade | NCOR1 | 2 | 1 |  |  |  |  |  |  |
| GO:0031223\_auditory\_behavior | FOXP2 | 2 | 1 |  |  |  |  |  |  |
| GO:0032796\_uropod\_organization | MYH9 | 2 | 1 |  |  |  |  |  |  |
| GO:0045116\_protein\_neddylation | UBE2F | 2 | 1 |  |  |  |  |  |  |
| GO:0051293\_establishment\_of\_spindle\_localization | MYH9 | 2 | 1 |  |  |  |  |  |  |
| GO:0051295\_establishment\_of\_meiotic\_spindle\_localization | MYH9 | 2 | 1 |  |  |  |  |  |  |
| GO:0051653\_spindle\_localization | MYH9 | 2 | 1 |  |  |  |  |  |  |
| GO:0060318\_definitive\_erythrocyte\_differentiation | NCOR1 | 2 | 1 |  |  |  |  |  |  |
| GO:0010629\_negative\_regulation\_of\_gene\_expression | NCOA2 | 262 | 6 | 2.850217 | -1.775244 | 18 | 11.99 | 0.666111 |
| GO:0010629\_negative\_regulation\_of\_gene\_expression | TLE4 | 262 | 6 | 2.850217 | -1.775244 | 18 | 11.99 | 0.666111 |
| GO:0010629\_negative\_regulation\_of\_gene\_expression | VAX2 | 262 | 6 | 2.850217 | -1.775244 | 18 | 11.99 | 0.666111 |
| GO:0010629\_negative\_regulation\_of\_gene\_expression | NCOR1 | 262 | 6 | 2.850217 | -1.775244 | 18 | 11.99 | 0.666111 |
| GO:0010629\_negative\_regulation\_of\_gene\_expression | NRIP1 | 262 | 6 | 2.850217 | -1.775244 | 18 | 11.99 | 0.666111 |
| GO:0010629\_negative\_regulation\_of\_gene\_expression | FOXP2 | 262 | 6 | 2.850217 | -1.775244 | 18 | 11.99 | 0.666111 |
| GO:0006350\_transcription | KLF7 | 701 | 11 | 1.953002 | -1.736646 | 19 | 13.19 | 0.694211 |
| GO:0006350\_transcription | NCOA2 | 701 | 11 | 1.953002 | -1.736646 | 19 | 13.19 | 0.694211 |
| GO:0006350\_transcription | RXRB | 701 | 11 | 1.953002 | -1.736646 | 19 | 13.19 | 0.694211 |
| GO:0006350\_transcription | E2F7 | 701 | 11 | 1.953002 | -1.736646 | 19 | 13.19 | 0.694211 |
| GO:0006350\_transcription | TLE4 | 701 | 11 | 1.953002 | -1.736646 | 19 | 13.19 | 0.694211 |
| GO:0006350\_transcription | VAX2 | 701 | 11 | 1.953002 | -1.736646 | 19 | 13.19 | 0.694211 |
| GO:0006350\_transcription | DACH1 | 701 | 11 | 1.953002 | -1.736646 | 19 | 13.19 | 0.694211 |
| GO:0006350\_transcription | HES6 | 701 | 11 | 1.953002 | -1.736646 | 19 | 13.19 | 0.694211 |
| GO:0006350\_transcription | NCOR1 | 701 | 11 | 1.953002 | -1.736646 | 19 | 13.19 | 0.694211 |
| GO:0006350\_transcription | NRIP1 | 701 | 11 | 1.953002 | -1.736646 | 19 | 13.19 | 0.694211 |
| GO:0006350\_transcription | FOXP2 | 701 | 11 | 1.953002 | -1.736646 | 19 | 13.19 | 0.694211 |
| GO:0009892\_negative\_regulation\_of\_metabolic\_process | NCOA2 | 348 | 7 | 2.503495 | -1.723651 | 20 | 13.46 | 0.673000 |
| GO:0009892\_negative\_regulation\_of\_metabolic\_process | TIA1 | 348 | 7 | 2.503495 | -1.723651 | 20 | 13.46 | 0.673000 |
| GO:0009892\_negative\_regulation\_of\_metabolic\_process | TLE4 | 348 | 7 | 2.503495 | -1.723651 | 20 | 13.46 | 0.673000 |
| GO:0009892\_negative\_regulation\_of\_metabolic\_process | VAX2 | 348 | 7 | 2.503495 | -1.723651 | 20 | 13.46 | 0.673000 |
| GO:0009892\_negative\_regulation\_of\_metabolic\_process | NCOR1 | 348 | 7 | 2.503495 | -1.723651 | 20 | 13.46 | 0.673000 |
| GO:0009892\_negative\_regulation\_of\_metabolic\_process | NRIP1 | 348 | 7 | 2.503495 | -1.723651 | 20 | 13.46 | 0.673000 |
| GO:0009892\_negative\_regulation\_of\_metabolic\_process | FOXP2 | 348 | 7 | 2.503495 | -1.723651 | 20 | 13.46 | 0.673000 |
| GO:0045934\_negative\_regulation\_of\_nucleobase\_\_nucleoside\_\_nucleotide\_and\_nucleic\_acid\_metabolic\_process | NCOA2 | 270 | 6 | 2.765766 | -1.716464 | 21 | 13.56 | 0.645714 |
| GO:0045934\_negative\_regulation\_of\_nucleobase\_\_nucleoside\_\_nucleotide\_and\_nucleic\_acid\_metabolic\_process | TLE4 | 270 | 6 | 2.765766 | -1.716464 | 21 | 13.56 | 0.645714 |
| GO:0045934\_negative\_regulation\_of\_nucleobase\_\_nucleoside\_\_nucleotide\_and\_nucleic\_acid\_metabolic\_process | VAX2 | 270 | 6 | 2.765766 | -1.716464 | 21 | 13.56 | 0.645714 |
| GO:0045934\_negative\_regulation\_of\_nucleobase\_\_nucleoside\_\_nucleotide\_and\_nucleic\_acid\_metabolic\_process | NCOR1 | 270 | 6 | 2.765766 | -1.716464 | 21 | 13.56 | 0.645714 |
| GO:0045934\_negative\_regulation\_of\_nucleobase\_\_nucleoside\_\_nucleotide\_and\_nucleic\_acid\_metabolic\_process | NRIP1 | 270 | 6 | 2.765766 | -1.716464 | 21 | 13.56 | 0.645714 |
| GO:0045934\_negative\_regulation\_of\_nucleobase\_\_nucleoside\_\_nucleotide\_and\_nucleic\_acid\_metabolic\_process | FOXP2 | 270 | 6 | 2.765766 | -1.716464 | 21 | 13.56 | 0.645714 |
| GO:0034961\_cellular\_biopolymer\_biosynthetic\_process | KLF7 | 804 | 12 | 1.857604 | -1.711024 | 22 | 13.93 | 0.633182 |
| GO:0034961\_cellular\_biopolymer\_biosynthetic\_process | NCOA2 | 804 | 12 | 1.857604 | -1.711024 | 22 | 13.93 | 0.633182 |
| GO:0034961\_cellular\_biopolymer\_biosynthetic\_process | RXRB | 804 | 12 | 1.857604 | -1.711024 | 22 | 13.93 | 0.633182 |
| GO:0034961\_cellular\_biopolymer\_biosynthetic\_process | TIA1 | 804 | 12 | 1.857604 | -1.711024 | 22 | 13.93 | 0.633182 |
| GO:0034961\_cellular\_biopolymer\_biosynthetic\_process | E2F7 | 804 | 12 | 1.857604 | -1.711024 | 22 | 13.93 | 0.633182 |
| GO:0034961\_cellular\_biopolymer\_biosynthetic\_process | TLE4 | 804 | 12 | 1.857604 | -1.711024 | 22 | 13.93 | 0.633182 |
| GO:0034961\_cellular\_biopolymer\_biosynthetic\_process | VAX2 | 804 | 12 | 1.857604 | -1.711024 | 22 | 13.93 | 0.633182 |
| GO:0034961\_cellular\_biopolymer\_biosynthetic\_process | DACH1 | 804 | 12 | 1.857604 | -1.711024 | 22 | 13.93 | 0.633182 |
| GO:0034961\_cellular\_biopolymer\_biosynthetic\_process | HES6 | 804 | 12 | 1.857604 | -1.711024 | 22 | 13.93 | 0.633182 |
| GO:0034961\_cellular\_biopolymer\_biosynthetic\_process | NCOR1 | 804 | 12 | 1.857604 | -1.711024 | 22 | 13.93 | 0.633182 |
| GO:0034961\_cellular\_biopolymer\_biosynthetic\_process | NRIP1 | 804 | 12 | 1.857604 | -1.711024 | 22 | 13.93 | 0.633182 |
| GO:0034961\_cellular\_biopolymer\_biosynthetic\_process | FOXP2 | 804 | 12 | 1.857604 | -1.711024 | 22 | 13.93 | 0.633182 |
| GO:0051172\_negative\_regulation\_of\_nitrogen\_compound\_metabolic\_process | NCOA2 | 271 | 6 | 2.755560 | -1.709283 | 23 | 14.21 | 0.617826 |
| GO:0051172\_negative\_regulation\_of\_nitrogen\_compound\_metabolic\_process | TLE4 | 271 | 6 | 2.755560 | -1.709283 | 23 | 14.21 | 0.617826 |
| GO:0051172\_negative\_regulation\_of\_nitrogen\_compound\_metabolic\_process | VAX2 | 271 | 6 | 2.755560 | -1.709283 | 23 | 14.21 | 0.617826 |
| GO:0051172\_negative\_regulation\_of\_nitrogen\_compound\_metabolic\_process | NCOR1 | 271 | 6 | 2.755560 | -1.709283 | 23 | 14.21 | 0.617826 |
| GO:0051172\_negative\_regulation\_of\_nitrogen\_compound\_metabolic\_process | NRIP1 | 271 | 6 | 2.755560 | -1.709283 | 23 | 14.21 | 0.617826 |
| GO:0051172\_negative\_regulation\_of\_nitrogen\_compound\_metabolic\_process | FOXP2 | 271 | 6 | 2.755560 | -1.709283 | 23 | 14.21 | 0.617826 |
| GO:0010467\_gene\_expression | KLF7 | 905 | 13 | 1.787815 | -1.706080 | 24 | 14.22 | 0.592500 |
| GO:0010467\_gene\_expression | RXRB | 905 | 13 | 1.787815 | -1.706080 | 24 | 14.22 | 0.592500 |
| GO:0010467\_gene\_expression | E2F7 | 905 | 13 | 1.787815 | -1.706080 | 24 | 14.22 | 0.592500 |
| GO:0010467\_gene\_expression | TLE4 | 905 | 13 | 1.787815 | -1.706080 | 24 | 14.22 | 0.592500 |
| GO:0010467\_gene\_expression | ELAVL1 | 905 | 13 | 1.787815 | -1.706080 | 24 | 14.22 | 0.592500 |
| GO:0010467\_gene\_expression | VAX2 | 905 | 13 | 1.787815 | -1.706080 | 24 | 14.22 | 0.592500 |
| GO:0010467\_gene\_expression | HES6 | 905 | 13 | 1.787815 | -1.706080 | 24 | 14.22 | 0.592500 |
| GO:0010467\_gene\_expression | DACH1 | 905 | 13 | 1.787815 | -1.706080 | 24 | 14.22 | 0.592500 |
| GO:0010467\_gene\_expression | FOXP2 | 905 | 13 | 1.787815 | -1.706080 | 24 | 14.22 | 0.592500 |
| GO:0010467\_gene\_expression | NRIP1 | 905 | 13 | 1.787815 | -1.706080 | 24 | 14.22 | 0.592500 |
| GO:0010467\_gene\_expression | NCOA2 | 905 | 13 | 1.787815 | -1.706080 | 24 | 14.22 | 0.592500 |
| GO:0010467\_gene\_expression | TIA1 | 905 | 13 | 1.787815 | -1.706080 | 24 | 14.22 | 0.592500 |
| GO:0010467\_gene\_expression | NCOR1 | 905 | 13 | 1.787815 | -1.706080 | 24 | 14.22 | 0.592500 |
| GO:0006357\_regulation\_of\_transcription\_from\_RNA\_polymerase\_II\_promoter | NCOA2 | 435 | 8 | 2.288910 | -1.704576 | 25 | 14.23 | 0.569200 |
| GO:0006357\_regulation\_of\_transcription\_from\_RNA\_polymerase\_II\_promoter | RXRB | 435 | 8 | 2.288910 | -1.704576 | 25 | 14.23 | 0.569200 |
| GO:0006357\_regulation\_of\_transcription\_from\_RNA\_polymerase\_II\_promoter | TLE4 | 435 | 8 | 2.288910 | -1.704576 | 25 | 14.23 | 0.569200 |
| GO:0006357\_regulation\_of\_transcription\_from\_RNA\_polymerase\_II\_promoter | VAX2 | 435 | 8 | 2.288910 | -1.704576 | 25 | 14.23 | 0.569200 |
| GO:0006357\_regulation\_of\_transcription\_from\_RNA\_polymerase\_II\_promoter | HES6 | 435 | 8 | 2.288910 | -1.704576 | 25 | 14.23 | 0.569200 |
| GO:0006357\_regulation\_of\_transcription\_from\_RNA\_polymerase\_II\_promoter | NCOR1 | 435 | 8 | 2.288910 | -1.704576 | 25 | 14.23 | 0.569200 |
| GO:0006357\_regulation\_of\_transcription\_from\_RNA\_polymerase\_II\_promoter | NRIP1 | 435 | 8 | 2.288910 | -1.704576 | 25 | 14.23 | 0.569200 |
| GO:0006357\_regulation\_of\_transcription\_from\_RNA\_polymerase\_II\_promoter | FOXP2 | 435 | 8 | 2.288910 | -1.704576 | 25 | 14.23 | 0.569200 |
| GO:0043284\_biopolymer\_biosynthetic\_process | KLF7 | 807 | 12 | 1.850698 | -1.698956 | 26 | 14.28 | 0.549231 |
| GO:0043284\_biopolymer\_biosynthetic\_process | NCOA2 | 807 | 12 | 1.850698 | -1.698956 | 26 | 14.28 | 0.549231 |
| GO:0043284\_biopolymer\_biosynthetic\_process | RXRB | 807 | 12 | 1.850698 | -1.698956 | 26 | 14.28 | 0.549231 |
| GO:0043284\_biopolymer\_biosynthetic\_process | TIA1 | 807 | 12 | 1.850698 | -1.698956 | 26 | 14.28 | 0.549231 |
| GO:0043284\_biopolymer\_biosynthetic\_process | E2F7 | 807 | 12 | 1.850698 | -1.698956 | 26 | 14.28 | 0.549231 |
| GO:0043284\_biopolymer\_biosynthetic\_process | TLE4 | 807 | 12 | 1.850698 | -1.698956 | 26 | 14.28 | 0.549231 |
| GO:0043284\_biopolymer\_biosynthetic\_process | VAX2 | 807 | 12 | 1.850698 | -1.698956 | 26 | 14.28 | 0.549231 |
| GO:0043284\_biopolymer\_biosynthetic\_process | DACH1 | 807 | 12 | 1.850698 | -1.698956 | 26 | 14.28 | 0.549231 |
| GO:0043284\_biopolymer\_biosynthetic\_process | HES6 | 807 | 12 | 1.850698 | -1.698956 | 26 | 14.28 | 0.549231 |
| GO:0043284\_biopolymer\_biosynthetic\_process | NCOR1 | 807 | 12 | 1.850698 | -1.698956 | 26 | 14.28 | 0.549231 |
| GO:0043284\_biopolymer\_biosynthetic\_process | NRIP1 | 807 | 12 | 1.850698 | -1.698956 | 26 | 14.28 | 0.549231 |
| GO:0043284\_biopolymer\_biosynthetic\_process | FOXP2 | 807 | 12 | 1.850698 | -1.698956 | 26 | 14.28 | 0.549231 |
| GO:0031326\_regulation\_of\_cellular\_biosynthetic\_process | KLF7 | 812 | 12 | 1.839302 | -1.679013 | 27 | 14.83 | 0.549259 |
| GO:0031326\_regulation\_of\_cellular\_biosynthetic\_process | NCOA2 | 812 | 12 | 1.839302 | -1.679013 | 27 | 14.83 | 0.549259 |
| GO:0031326\_regulation\_of\_cellular\_biosynthetic\_process | RXRB | 812 | 12 | 1.839302 | -1.679013 | 27 | 14.83 | 0.549259 |
| GO:0031326\_regulation\_of\_cellular\_biosynthetic\_process | E2F7 | 812 | 12 | 1.839302 | -1.679013 | 27 | 14.83 | 0.549259 |
| GO:0031326\_regulation\_of\_cellular\_biosynthetic\_process | TIA1 | 812 | 12 | 1.839302 | -1.679013 | 27 | 14.83 | 0.549259 |
| GO:0031326\_regulation\_of\_cellular\_biosynthetic\_process | TLE4 | 812 | 12 | 1.839302 | -1.679013 | 27 | 14.83 | 0.549259 |
| GO:0031326\_regulation\_of\_cellular\_biosynthetic\_process | VAX2 | 812 | 12 | 1.839302 | -1.679013 | 27 | 14.83 | 0.549259 |
| GO:0031326\_regulation\_of\_cellular\_biosynthetic\_process | DACH1 | 812 | 12 | 1.839302 | -1.679013 | 27 | 14.83 | 0.549259 |
| GO:0031326\_regulation\_of\_cellular\_biosynthetic\_process | HES6 | 812 | 12 | 1.839302 | -1.679013 | 27 | 14.83 | 0.549259 |
| GO:0031326\_regulation\_of\_cellular\_biosynthetic\_process | NCOR1 | 812 | 12 | 1.839302 | -1.679013 | 27 | 14.83 | 0.549259 |
| GO:0031326\_regulation\_of\_cellular\_biosynthetic\_process | NRIP1 | 812 | 12 | 1.839302 | -1.679013 | 27 | 14.83 | 0.549259 |
| GO:0031326\_regulation\_of\_cellular\_biosynthetic\_process | FOXP2 | 812 | 12 | 1.839302 | -1.679013 | 27 | 14.83 | 0.549259 |
| GO:0009889\_regulation\_of\_biosynthetic\_process | KLF7 | 815 | 12 | 1.832532 | -1.667147 | 28 | 14.92 | 0.532857 |
| GO:0009889\_regulation\_of\_biosynthetic\_process | NCOA2 | 815 | 12 | 1.832532 | -1.667147 | 28 | 14.92 | 0.532857 |
| GO:0009889\_regulation\_of\_biosynthetic\_process | RXRB | 815 | 12 | 1.832532 | -1.667147 | 28 | 14.92 | 0.532857 |
| GO:0009889\_regulation\_of\_biosynthetic\_process | E2F7 | 815 | 12 | 1.832532 | -1.667147 | 28 | 14.92 | 0.532857 |
| GO:0009889\_regulation\_of\_biosynthetic\_process | TIA1 | 815 | 12 | 1.832532 | -1.667147 | 28 | 14.92 | 0.532857 |
| GO:0009889\_regulation\_of\_biosynthetic\_process | TLE4 | 815 | 12 | 1.832532 | -1.667147 | 28 | 14.92 | 0.532857 |
| GO:0009889\_regulation\_of\_biosynthetic\_process | VAX2 | 815 | 12 | 1.832532 | -1.667147 | 28 | 14.92 | 0.532857 |
| GO:0009889\_regulation\_of\_biosynthetic\_process | DACH1 | 815 | 12 | 1.832532 | -1.667147 | 28 | 14.92 | 0.532857 |
| GO:0009889\_regulation\_of\_biosynthetic\_process | HES6 | 815 | 12 | 1.832532 | -1.667147 | 28 | 14.92 | 0.532857 |
| GO:0009889\_regulation\_of\_biosynthetic\_process | NCOR1 | 815 | 12 | 1.832532 | -1.667147 | 28 | 14.92 | 0.532857 |
| GO:0009889\_regulation\_of\_biosynthetic\_process | NRIP1 | 815 | 12 | 1.832532 | -1.667147 | 28 | 14.92 | 0.532857 |
| GO:0009889\_regulation\_of\_biosynthetic\_process | FOXP2 | 815 | 12 | 1.832532 | -1.667147 | 28 | 14.92 | 0.532857 |
| GO:0006366\_transcription\_from\_RNA\_polymerase\_II\_promoter | NCOA2 | 444 | 8 | 2.242513 | -1.655814 | 29 | 15.04 | 0.518621 |
| GO:0006366\_transcription\_from\_RNA\_polymerase\_II\_promoter | RXRB | 444 | 8 | 2.242513 | -1.655814 | 29 | 15.04 | 0.518621 |
| GO:0006366\_transcription\_from\_RNA\_polymerase\_II\_promoter | TLE4 | 444 | 8 | 2.242513 | -1.655814 | 29 | 15.04 | 0.518621 |
| GO:0006366\_transcription\_from\_RNA\_polymerase\_II\_promoter | VAX2 | 444 | 8 | 2.242513 | -1.655814 | 29 | 15.04 | 0.518621 |
| GO:0006366\_transcription\_from\_RNA\_polymerase\_II\_promoter | HES6 | 444 | 8 | 2.242513 | -1.655814 | 29 | 15.04 | 0.518621 |
| GO:0006366\_transcription\_from\_RNA\_polymerase\_II\_promoter | NCOR1 | 444 | 8 | 2.242513 | -1.655814 | 29 | 15.04 | 0.518621 |
| GO:0006366\_transcription\_from\_RNA\_polymerase\_II\_promoter | NRIP1 | 444 | 8 | 2.242513 | -1.655814 | 29 | 15.04 | 0.518621 |
| GO:0006366\_transcription\_from\_RNA\_polymerase\_II\_promoter | FOXP2 | 444 | 8 | 2.242513 | -1.655814 | 29 | 15.04 | 0.518621 |
| GO:0080090\_regulation\_of\_primary\_metabolic\_process | KLF7 | 926 | 13 | 1.747271 | -1.627644 | 30 | 16.0 | 0.533333 |
| GO:0080090\_regulation\_of\_primary\_metabolic\_process | RXRB | 926 | 13 | 1.747271 | -1.627644 | 30 | 16.0 | 0.533333 |
| GO:0080090\_regulation\_of\_primary\_metabolic\_process | E2F7 | 926 | 13 | 1.747271 | -1.627644 | 30 | 16.0 | 0.533333 |
| GO:0080090\_regulation\_of\_primary\_metabolic\_process | TLE4 | 926 | 13 | 1.747271 | -1.627644 | 30 | 16.0 | 0.533333 |
| GO:0080090\_regulation\_of\_primary\_metabolic\_process | ELAVL1 | 926 | 13 | 1.747271 | -1.627644 | 30 | 16.0 | 0.533333 |
| GO:0080090\_regulation\_of\_primary\_metabolic\_process | VAX2 | 926 | 13 | 1.747271 | -1.627644 | 30 | 16.0 | 0.533333 |
| GO:0080090\_regulation\_of\_primary\_metabolic\_process | HES6 | 926 | 13 | 1.747271 | -1.627644 | 30 | 16.0 | 0.533333 |
| GO:0080090\_regulation\_of\_primary\_metabolic\_process | DACH1 | 926 | 13 | 1.747271 | -1.627644 | 30 | 16.0 | 0.533333 |
| GO:0080090\_regulation\_of\_primary\_metabolic\_process | FOXP2 | 926 | 13 | 1.747271 | -1.627644 | 30 | 16.0 | 0.533333 |
| GO:0080090\_regulation\_of\_primary\_metabolic\_process | NRIP1 | 926 | 13 | 1.747271 | -1.627644 | 30 | 16.0 | 0.533333 |
| GO:0080090\_regulation\_of\_primary\_metabolic\_process | NCOA2 | 926 | 13 | 1.747271 | -1.627644 | 30 | 16.0 | 0.533333 |
| GO:0080090\_regulation\_of\_primary\_metabolic\_process | TIA1 | 926 | 13 | 1.747271 | -1.627644 | 30 | 16.0 | 0.533333 |
| GO:0080090\_regulation\_of\_primary\_metabolic\_process | NCOR1 | 926 | 13 | 1.747271 | -1.627644 | 30 | 16.0 | 0.533333 |
| GO:0030522\_intracellular\_receptor-mediated\_signaling\_pathway | RXRB | 30 | 2 | 8.297297 | -1.624766 | 31 | 16.43 | 0.530000 |
| GO:0030522\_intracellular\_receptor-mediated\_signaling\_pathway | FKBP4 | 30 | 2 | 8.297297 | -1.624766 | 31 | 16.43 | 0.530000 |
| GO:0000098\_sulfur\_amino\_acid\_catabolic\_process | AHCY | 3 | 1 |  |  |  |  |  |  |
| GO:0000212\_meiotic\_spindle\_organization | MYH9 | 3 | 1 |  |  |  |  |  |  |
| GO:0001844\_protein\_insertion\_into\_mitochondrial\_membrane\_during\_induction\_of\_apoptosis | SH3GLB1 | 3 | 1 |  |  |  |  |  |  |
| GO:0002361\_CD4-positive\_\_CD25-positive\_\_alpha-beta\_regulatory\_T\_cell\_differentiation | NCOR1 | 3 | 1 |  |  |  |  |  |  |
| GO:0021794\_thalamus\_development | NCOR1 | 3 | 1 |  |  |  |  |  |  |
| GO:0031063\_regulation\_of\_histone\_deacetylation | NCOR1 | 3 | 1 |  |  |  |  |  |  |
| GO:0031065\_positive\_regulation\_of\_histone\_deacetylation | NCOR1 | 3 | 1 |  |  |  |  |  |  |
| GO:0031503\_protein\_complex\_localization | FKBP4 | 3 | 1 |  |  |  |  |  |  |
| GO:0044273\_sulfur\_compound\_catabolic\_process | AHCY | 3 | 1 |  |  |  |  |  |  |
| GO:0050774\_negative\_regulation\_of\_dendrite\_morphogenesis | YWHAH | 3 | 1 |  |  |  |  |  |  |
| GO:0051204\_protein\_insertion\_into\_mitochondrial\_membrane | SH3GLB1 | 3 | 1 |  |  |  |  |  |  |
| GO:0070846\_Hsp90\_deacetylation | NCOR1 | 3 | 1 |  |  |  |  |  |  |
| GO:0060255\_regulation\_of\_macromolecule\_metabolic\_process | KLF7 | 936 | 13 | 1.728604 | -1.591396 | 32 | 17.83 | 0.557188 |
| GO:0060255\_regulation\_of\_macromolecule\_metabolic\_process | RXRB | 936 | 13 | 1.728604 | -1.591396 | 32 | 17.83 | 0.557188 |
| GO:0060255\_regulation\_of\_macromolecule\_metabolic\_process | E2F7 | 936 | 13 | 1.728604 | -1.591396 | 32 | 17.83 | 0.557188 |
| GO:0060255\_regulation\_of\_macromolecule\_metabolic\_process | ELAVL1 | 936 | 13 | 1.728604 | -1.591396 | 32 | 17.83 | 0.557188 |
| GO:0060255\_regulation\_of\_macromolecule\_metabolic\_process | TLE4 | 936 | 13 | 1.728604 | -1.591396 | 32 | 17.83 | 0.557188 |
| GO:0060255\_regulation\_of\_macromolecule\_metabolic\_process | VAX2 | 936 | 13 | 1.728604 | -1.591396 | 32 | 17.83 | 0.557188 |
| GO:0060255\_regulation\_of\_macromolecule\_metabolic\_process | HES6 | 936 | 13 | 1.728604 | -1.591396 | 32 | 17.83 | 0.557188 |
| GO:0060255\_regulation\_of\_macromolecule\_metabolic\_process | DACH1 | 936 | 13 | 1.728604 | -1.591396 | 32 | 17.83 | 0.557188 |
| GO:0060255\_regulation\_of\_macromolecule\_metabolic\_process | FOXP2 | 936 | 13 | 1.728604 | -1.591396 | 32 | 17.83 | 0.557188 |
| GO:0060255\_regulation\_of\_macromolecule\_metabolic\_process | NRIP1 | 936 | 13 | 1.728604 | -1.591396 | 32 | 17.83 | 0.557188 |
| GO:0060255\_regulation\_of\_macromolecule\_metabolic\_process | NCOA2 | 936 | 13 | 1.728604 | -1.591396 | 32 | 17.83 | 0.557188 |
| GO:0060255\_regulation\_of\_macromolecule\_metabolic\_process | TIA1 | 936 | 13 | 1.728604 | -1.591396 | 32 | 17.83 | 0.557188 |
| GO:0060255\_regulation\_of\_macromolecule\_metabolic\_process | NCOR1 | 936 | 13 | 1.728604 | -1.591396 | 32 | 17.83 | 0.557188 |
| GO:0016070\_RNA\_metabolic\_process | KLF7 | 658 | 10 | 1.891481 | -1.513799 | 33 | 20.54 | 0.622424 |
| GO:0016070\_RNA\_metabolic\_process | NCOA2 | 658 | 10 | 1.891481 | -1.513799 | 33 | 20.54 | 0.622424 |
| GO:0016070\_RNA\_metabolic\_process | RXRB | 658 | 10 | 1.891481 | -1.513799 | 33 | 20.54 | 0.622424 |
| GO:0016070\_RNA\_metabolic\_process | TLE4 | 658 | 10 | 1.891481 | -1.513799 | 33 | 20.54 | 0.622424 |
| GO:0016070\_RNA\_metabolic\_process | ELAVL1 | 658 | 10 | 1.891481 | -1.513799 | 33 | 20.54 | 0.622424 |
| GO:0016070\_RNA\_metabolic\_process | VAX2 | 658 | 10 | 1.891481 | -1.513799 | 33 | 20.54 | 0.622424 |
| GO:0016070\_RNA\_metabolic\_process | HES6 | 658 | 10 | 1.891481 | -1.513799 | 33 | 20.54 | 0.622424 |
| GO:0016070\_RNA\_metabolic\_process | NCOR1 | 658 | 10 | 1.891481 | -1.513799 | 33 | 20.54 | 0.622424 |
| GO:0016070\_RNA\_metabolic\_process | NRIP1 | 658 | 10 | 1.891481 | -1.513799 | 33 | 20.54 | 0.622424 |
| GO:0016070\_RNA\_metabolic\_process | FOXP2 | 658 | 10 | 1.891481 | -1.513799 | 33 | 20.54 | 0.622424 |
| GO:0032922\_circadian\_regulation\_of\_gene\_expression | NCOR1 | 4 | 1 |  |  |  |  |  |  |
| GO:0045066\_regulatory\_T\_cell\_differentiation | NCOR1 | 4 | 1 |  |  |  |  |  |  |
| GO:0051205\_protein\_insertion\_into\_membrane | SH3GLB1 | 4 | 1 |  |  |  |  |  |  |
| GO:0006355\_regulation\_of\_transcription\_\_DNA-dependent | KLF7 | 575 | 9 | 1.948061 | -1.462998 | 34 | 22.49 | 0.661471 |
| GO:0006355\_regulation\_of\_transcription\_\_DNA-dependent | NCOA2 | 575 | 9 | 1.948061 | -1.462998 | 34 | 22.49 | 0.661471 |
| GO:0006355\_regulation\_of\_transcription\_\_DNA-dependent | RXRB | 575 | 9 | 1.948061 | -1.462998 | 34 | 22.49 | 0.661471 |
| GO:0006355\_regulation\_of\_transcription\_\_DNA-dependent | TLE4 | 575 | 9 | 1.948061 | -1.462998 | 34 | 22.49 | 0.661471 |
| GO:0006355\_regulation\_of\_transcription\_\_DNA-dependent | VAX2 | 575 | 9 | 1.948061 | -1.462998 | 34 | 22.49 | 0.661471 |
| GO:0006355\_regulation\_of\_transcription\_\_DNA-dependent | HES6 | 575 | 9 | 1.948061 | -1.462998 | 34 | 22.49 | 0.661471 |
| GO:0006355\_regulation\_of\_transcription\_\_DNA-dependent | NCOR1 | 575 | 9 | 1.948061 | -1.462998 | 34 | 22.49 | 0.661471 |
| GO:0006355\_regulation\_of\_transcription\_\_DNA-dependent | NRIP1 | 575 | 9 | 1.948061 | -1.462998 | 34 | 22.49 | 0.661471 |
| GO:0006355\_regulation\_of\_transcription\_\_DNA-dependent | FOXP2 | 575 | 9 | 1.948061 | -1.462998 | 34 | 22.49 | 0.661471 |
| GO:0000070\_mitotic\_sister\_chromatid\_segregation | AKAP8 | 5 | 1 | 24.891892 | -1.402851 | 46 | 37.71 | 0.819783 |
| GO:0000819\_sister\_chromatid\_segregation | AKAP8 | 5 | 1 | 24.891892 | -1.402851 | 46 | 37.71 | 0.819783 |
| GO:0007638\_mechanosensory\_behavior | FOXP2 | 5 | 1 | 24.891892 | -1.402851 | 46 | 37.71 | 0.819783 |
| GO:0009950\_dorsal\_ventral\_axis\_specification | VAX2 | 5 | 1 | 24.891892 | -1.402851 | 46 | 37.71 | 0.819783 |
| GO:0017148\_negative\_regulation\_of\_translation | TIA1 | 5 | 1 | 24.891892 | -1.402851 | 46 | 37.71 | 0.819783 |
| GO:0030261\_chromosome\_condensation | AKAP8 | 5 | 1 | 24.891892 | -1.402851 | 46 | 37.71 | 0.819783 |
| GO:0031058\_positive\_regulation\_of\_histone\_modification | NCOR1 | 5 | 1 | 24.891892 | -1.402851 | 46 | 37.71 | 0.819783 |
| GO:0034109\_homotypic\_cell-cell\_adhesion | NCAM1 | 5 | 1 | 24.891892 | -1.402851 | 46 | 37.71 | 0.819783 |
| GO:0043489\_RNA\_stabilization | ELAVL1 | 5 | 1 | 24.891892 | -1.402851 | 46 | 37.71 | 0.819783 |
| GO:0048255\_mRNA\_stabilization | ELAVL1 | 5 | 1 | 24.891892 | -1.402851 | 46 | 37.71 | 0.819783 |
| GO:0051323\_metaphase | MYH9 | 5 | 1 | 24.891892 | -1.402851 | 46 | 37.71 | 0.819783 |
| GO:0051668\_localization\_within\_membrane | SH3GLB1 | 5 | 1 | 24.891892 | -1.402851 | 46 | 37.71 | 0.819783 |
| GO:0016358\_dendrite\_development | KLF7 | 40 | 2 | 6.222973 | -1.392878 | 47 | 38.45 | 0.818085 |
| GO:0016358\_dendrite\_development | YWHAH | 40 | 2 | 6.222973 | -1.392878 | 47 | 38.45 | 0.818085 |
| GO:0006351\_transcription\_\_DNA-dependent | KLF7 | 594 | 9 | 1.885749 | -1.383616 | 48 | 38.66 | 0.805417 |
| GO:0006351\_transcription\_\_DNA-dependent | NCOA2 | 594 | 9 | 1.885749 | -1.383616 | 48 | 38.66 | 0.805417 |
| GO:0006351\_transcription\_\_DNA-dependent | RXRB | 594 | 9 | 1.885749 | -1.383616 | 48 | 38.66 | 0.805417 |
| GO:0006351\_transcription\_\_DNA-dependent | TLE4 | 594 | 9 | 1.885749 | -1.383616 | 48 | 38.66 | 0.805417 |
| GO:0006351\_transcription\_\_DNA-dependent | VAX2 | 594 | 9 | 1.885749 | -1.383616 | 48 | 38.66 | 0.805417 |
| GO:0006351\_transcription\_\_DNA-dependent | HES6 | 594 | 9 | 1.885749 | -1.383616 | 48 | 38.66 | 0.805417 |
| GO:0006351\_transcription\_\_DNA-dependent | NCOR1 | 594 | 9 | 1.885749 | -1.383616 | 48 | 38.66 | 0.805417 |
| GO:0006351\_transcription\_\_DNA-dependent | NRIP1 | 594 | 9 | 1.885749 | -1.383616 | 48 | 38.66 | 0.805417 |
| GO:0006351\_transcription\_\_DNA-dependent | FOXP2 | 594 | 9 | 1.885749 | -1.383616 | 48 | 38.66 | 0.805417 |
| GO:0032774\_RNA\_biosynthetic\_process | KLF7 | 595 | 9 | 1.882580 | -1.379555 | 49 | 38.68 | 0.789388 |
| GO:0032774\_RNA\_biosynthetic\_process | NCOA2 | 595 | 9 | 1.882580 | -1.379555 | 49 | 38.68 | 0.789388 |
| GO:0032774\_RNA\_biosynthetic\_process | RXRB | 595 | 9 | 1.882580 | -1.379555 | 49 | 38.68 | 0.789388 |
| GO:0032774\_RNA\_biosynthetic\_process | TLE4 | 595 | 9 | 1.882580 | -1.379555 | 49 | 38.68 | 0.789388 |
| GO:0032774\_RNA\_biosynthetic\_process | VAX2 | 595 | 9 | 1.882580 | -1.379555 | 49 | 38.68 | 0.789388 |
| GO:0032774\_RNA\_biosynthetic\_process | HES6 | 595 | 9 | 1.882580 | -1.379555 | 49 | 38.68 | 0.789388 |
| GO:0032774\_RNA\_biosynthetic\_process | NCOR1 | 595 | 9 | 1.882580 | -1.379555 | 49 | 38.68 | 0.789388 |
| GO:0032774\_RNA\_biosynthetic\_process | NRIP1 | 595 | 9 | 1.882580 | -1.379555 | 49 | 38.68 | 0.789388 |
| GO:0032774\_RNA\_biosynthetic\_process | FOXP2 | 595 | 9 | 1.882580 | -1.379555 | 49 | 38.68 | 0.789388 |
| GO:0034645\_cellular\_macromolecule\_biosynthetic\_process | KLF7 | 901 | 12 | 1.657618 | -1.356855 | 50 | 40.15 | 0.803000 |
| GO:0034645\_cellular\_macromolecule\_biosynthetic\_process | NCOA2 | 901 | 12 | 1.657618 | -1.356855 | 50 | 40.15 | 0.803000 |
| GO:0034645\_cellular\_macromolecule\_biosynthetic\_process | RXRB | 901 | 12 | 1.657618 | -1.356855 | 50 | 40.15 | 0.803000 |
| GO:0034645\_cellular\_macromolecule\_biosynthetic\_process | TIA1 | 901 | 12 | 1.657618 | -1.356855 | 50 | 40.15 | 0.803000 |
| GO:0034645\_cellular\_macromolecule\_biosynthetic\_process | E2F7 | 901 | 12 | 1.657618 | -1.356855 | 50 | 40.15 | 0.803000 |
| GO:0034645\_cellular\_macromolecule\_biosynthetic\_process | TLE4 | 901 | 12 | 1.657618 | -1.356855 | 50 | 40.15 | 0.803000 |
| GO:0034645\_cellular\_macromolecule\_biosynthetic\_process | VAX2 | 901 | 12 | 1.657618 | -1.356855 | 50 | 40.15 | 0.803000 |
| GO:0034645\_cellular\_macromolecule\_biosynthetic\_process | DACH1 | 901 | 12 | 1.657618 | -1.356855 | 50 | 40.15 | 0.803000 |
| GO:0034645\_cellular\_macromolecule\_biosynthetic\_process | HES6 | 901 | 12 | 1.657618 | -1.356855 | 50 | 40.15 | 0.803000 |
| GO:0034645\_cellular\_macromolecule\_biosynthetic\_process | NCOR1 | 901 | 12 | 1.657618 | -1.356855 | 50 | 40.15 | 0.803000 |
| GO:0034645\_cellular\_macromolecule\_biosynthetic\_process | NRIP1 | 901 | 12 | 1.657618 | -1.356855 | 50 | 40.15 | 0.803000 |
| GO:0034645\_cellular\_macromolecule\_biosynthetic\_process | FOXP2 | 901 | 12 | 1.657618 | -1.356855 | 50 | 40.15 | 0.803000 |
| GO:0031323\_regulation\_of\_cellular\_metabolic\_process | KLF7 | 1015 | 13 | 1.594062 | -1.328259 | 51 | 42.36 | 0.830588 |
| GO:0031323\_regulation\_of\_cellular\_metabolic\_process | RXRB | 1015 | 13 | 1.594062 | -1.328259 | 51 | 42.36 | 0.830588 |
| GO:0031323\_regulation\_of\_cellular\_metabolic\_process | E2F7 | 1015 | 13 | 1.594062 | -1.328259 | 51 | 42.36 | 0.830588 |
| GO:0031323\_regulation\_of\_cellular\_metabolic\_process | TLE4 | 1015 | 13 | 1.594062 | -1.328259 | 51 | 42.36 | 0.830588 |
| GO:0031323\_regulation\_of\_cellular\_metabolic\_process | ELAVL1 | 1015 | 13 | 1.594062 | -1.328259 | 51 | 42.36 | 0.830588 |
| GO:0031323\_regulation\_of\_cellular\_metabolic\_process | VAX2 | 1015 | 13 | 1.594062 | -1.328259 | 51 | 42.36 | 0.830588 |
| GO:0031323\_regulation\_of\_cellular\_metabolic\_process | HES6 | 1015 | 13 | 1.594062 | -1.328259 | 51 | 42.36 | 0.830588 |
| GO:0031323\_regulation\_of\_cellular\_metabolic\_process | DACH1 | 1015 | 13 | 1.594062 | -1.328259 | 51 | 42.36 | 0.830588 |
| GO:0031323\_regulation\_of\_cellular\_metabolic\_process | FOXP2 | 1015 | 13 | 1.594062 | -1.328259 | 51 | 42.36 | 0.830588 |
| GO:0031323\_regulation\_of\_cellular\_metabolic\_process | NRIP1 | 1015 | 13 | 1.594062 | -1.328259 | 51 | 42.36 | 0.830588 |
| GO:0031323\_regulation\_of\_cellular\_metabolic\_process | NCOA2 | 1015 | 13 | 1.594062 | -1.328259 | 51 | 42.36 | 0.830588 |
| GO:0031323\_regulation\_of\_cellular\_metabolic\_process | TIA1 | 1015 | 13 | 1.594062 | -1.328259 | 51 | 42.36 | 0.830588 |
| GO:0031323\_regulation\_of\_cellular\_metabolic\_process | NCOR1 | 1015 | 13 | 1.594062 | -1.328259 | 51 | 42.36 | 0.830588 |
| GO:0009059\_macromolecule\_biosynthetic\_process | KLF7 | 910 | 12 | 1.641224 | -1.327479 | 52 | 42.38 | 0.815000 |
| GO:0009059\_macromolecule\_biosynthetic\_process | NCOA2 | 910 | 12 | 1.641224 | -1.327479 | 52 | 42.38 | 0.815000 |
| GO:0009059\_macromolecule\_biosynthetic\_process | RXRB | 910 | 12 | 1.641224 | -1.327479 | 52 | 42.38 | 0.815000 |
| GO:0009059\_macromolecule\_biosynthetic\_process | E2F7 | 910 | 12 | 1.641224 | -1.327479 | 52 | 42.38 | 0.815000 |
| GO:0009059\_macromolecule\_biosynthetic\_process | TIA1 | 910 | 12 | 1.641224 | -1.327479 | 52 | 42.38 | 0.815000 |
| GO:0009059\_macromolecule\_biosynthetic\_process | TLE4 | 910 | 12 | 1.641224 | -1.327479 | 52 | 42.38 | 0.815000 |
| GO:0009059\_macromolecule\_biosynthetic\_process | VAX2 | 910 | 12 | 1.641224 | -1.327479 | 52 | 42.38 | 0.815000 |
| GO:0009059\_macromolecule\_biosynthetic\_process | DACH1 | 910 | 12 | 1.641224 | -1.327479 | 52 | 42.38 | 0.815000 |
| GO:0009059\_macromolecule\_biosynthetic\_process | HES6 | 910 | 12 | 1.641224 | -1.327479 | 52 | 42.38 | 0.815000 |
| GO:0009059\_macromolecule\_biosynthetic\_process | NCOR1 | 910 | 12 | 1.641224 | -1.327479 | 52 | 42.38 | 0.815000 |
| GO:0009059\_macromolecule\_biosynthetic\_process | NRIP1 | 910 | 12 | 1.641224 | -1.327479 | 52 | 42.38 | 0.815000 |
| GO:0009059\_macromolecule\_biosynthetic\_process | FOXP2 | 910 | 12 | 1.641224 | -1.327479 | 52 | 42.38 | 0.815000 |
| GO:0000768\_syncytium\_formation\_by\_plasma\_membrane\_fusion | MYH9 | 6 | 1 | 20.743243 | -1.325363 | 56 | 52.11 | 0.930536 |
| GO:0006882\_cellular\_zinc\_ion\_homeostasis | SLC30A1 | 6 | 1 | 20.743243 | -1.325363 | 56 | 52.11 | 0.930536 |
| GO:0007520\_myoblast\_fusion | MYH9 | 6 | 1 | 20.743243 | -1.325363 | 56 | 52.11 | 0.930536 |
| GO:0060013\_righting\_reflex | FOXP2 | 6 | 1 | 20.743243 | -1.325363 | 56 | 52.11 | 0.930536 |
| GO:0030218\_erythrocyte\_differentiation | HEPH | 46 | 2 | 5.411281 | -1.283008 | 57 | 55.22 | 0.968772 |
| GO:0030218\_erythrocyte\_differentiation | NCOR1 | 46 | 2 | 5.411281 | -1.283008 | 57 | 55.22 | 0.968772 |
| GO:0006949\_syncytium\_formation | MYH9 | 7 | 1 | 17.779923 | -1.260108 | 65 | 66.45 | 1.022308 |
| GO:0014066\_regulation\_of\_phosphoinositide\_3-kinase\_cascade | NCOR1 | 7 | 1 | 17.779923 | -1.260108 | 65 | 66.45 | 1.022308 |
| GO:0016575\_histone\_deacetylation | NCOR1 | 7 | 1 | 17.779923 | -1.260108 | 65 | 66.45 | 1.022308 |
| GO:0030521\_androgen\_receptor\_signaling\_pathway | FKBP4 | 7 | 1 | 17.779923 | -1.260108 | 65 | 66.45 | 1.022308 |
| GO:0046677\_response\_to\_antibiotic | CYB5R4 | 7 | 1 | 17.779923 | -1.260108 | 65 | 66.45 | 1.022308 |
| GO:0048814\_regulation\_of\_dendrite\_morphogenesis | YWHAH | 7 | 1 | 17.779923 | -1.260108 | 65 | 66.45 | 1.022308 |
| GO:0050773\_regulation\_of\_dendrite\_development | YWHAH | 7 | 1 | 17.779923 | -1.260108 | 65 | 66.45 | 1.022308 |
| GO:0055069\_zinc\_ion\_homeostasis | SLC30A1 | 7 | 1 | 17.779923 | -1.260108 | 65 | 66.45 | 1.022308 |
| GO:0043010\_camera-type\_eye\_development | VAX2 | 110 | 3 | 3.394349 | -1.241876 | 66 | 67.64 | 1.024848 |
| GO:0043010\_camera-type\_eye\_development | MAB21L1 | 110 | 3 | 3.394349 | -1.241876 | 66 | 67.64 | 1.024848 |
| GO:0043010\_camera-type\_eye\_development | FOXP2 | 110 | 3 | 3.394349 | -1.241876 | 66 | 67.64 | 1.024848 |
| GO:0034101\_erythrocyte\_homeostasis | HEPH | 49 | 2 | 5.079978 | -1.234014 | 67 | 68.41 | 1.021045 |
| GO:0034101\_erythrocyte\_homeostasis | NCOR1 | 49 | 2 | 5.079978 | -1.234014 | 67 | 68.41 | 1.021045 |
| GO:0006458\_'de\_novo'\_protein\_folding | SH3GLB1 | 8 | 1 | 15.557432 | -1.203805 | 70 | 78.32 | 1.118857 |
| GO:0014065\_phosphoinositide\_3-kinase\_cascade | NCOR1 | 8 | 1 | 15.557432 | -1.203805 | 70 | 78.32 | 1.118857 |
| GO:0051084\_'de\_novo'\_posttranslational\_protein\_folding | SH3GLB1 | 8 | 1 | 15.557432 | -1.203805 | 70 | 78.32 | 1.118857 |
| GO:0034960\_cellular\_biopolymer\_metabolic\_process | KLF7 | 1395 | 16 | 1.427492 | -1.189567 | 71 | 79.44 | 1.118873 |
| GO:0034960\_cellular\_biopolymer\_metabolic\_process | RXRB | 1395 | 16 | 1.427492 | -1.189567 | 71 | 79.44 | 1.118873 |
| GO:0034960\_cellular\_biopolymer\_metabolic\_process | E2F7 | 1395 | 16 | 1.427492 | -1.189567 | 71 | 79.44 | 1.118873 |
| GO:0034960\_cellular\_biopolymer\_metabolic\_process | UBE2F | 1395 | 16 | 1.427492 | -1.189567 | 71 | 79.44 | 1.118873 |
| GO:0034960\_cellular\_biopolymer\_metabolic\_process | ELAVL1 | 1395 | 16 | 1.427492 | -1.189567 | 71 | 79.44 | 1.118873 |
| GO:0034960\_cellular\_biopolymer\_metabolic\_process | TLE4 | 1395 | 16 | 1.427492 | -1.189567 | 71 | 79.44 | 1.118873 |
| GO:0034960\_cellular\_biopolymer\_metabolic\_process | VAX2 | 1395 | 16 | 1.427492 | -1.189567 | 71 | 79.44 | 1.118873 |
| GO:0034960\_cellular\_biopolymer\_metabolic\_process | HES6 | 1395 | 16 | 1.427492 | -1.189567 | 71 | 79.44 | 1.118873 |
| GO:0034960\_cellular\_biopolymer\_metabolic\_process | DACH1 | 1395 | 16 | 1.427492 | -1.189567 | 71 | 79.44 | 1.118873 |
| GO:0034960\_cellular\_biopolymer\_metabolic\_process | FOXP2 | 1395 | 16 | 1.427492 | -1.189567 | 71 | 79.44 | 1.118873 |
| GO:0034960\_cellular\_biopolymer\_metabolic\_process | NRIP1 | 1395 | 16 | 1.427492 | -1.189567 | 71 | 79.44 | 1.118873 |
| GO:0034960\_cellular\_biopolymer\_metabolic\_process | NCOA2 | 1395 | 16 | 1.427492 | -1.189567 | 71 | 79.44 | 1.118873 |
| GO:0034960\_cellular\_biopolymer\_metabolic\_process | SH3GLB1 | 1395 | 16 | 1.427492 | -1.189567 | 71 | 79.44 | 1.118873 |
| GO:0034960\_cellular\_biopolymer\_metabolic\_process | TIA1 | 1395 | 16 | 1.427492 | -1.189567 | 71 | 79.44 | 1.118873 |
| GO:0034960\_cellular\_biopolymer\_metabolic\_process | PPM1M | 1395 | 16 | 1.427492 | -1.189567 | 71 | 79.44 | 1.118873 |
| GO:0034960\_cellular\_biopolymer\_metabolic\_process | NCOR1 | 1395 | 16 | 1.427492 | -1.189567 | 71 | 79.44 | 1.118873 |
| GO:0010608\_posttranscriptional\_regulation\_of\_gene\_expression | TIA1 | 52 | 2 | 4.786902 | -1.188340 | 72 | 79.87 | 1.109306 |
| GO:0010608\_posttranscriptional\_regulation\_of\_gene\_expression | ELAVL1 | 52 | 2 | 4.786902 | -1.188340 | 72 | 79.87 | 1.109306 |
| GO:0001542\_ovulation\_from\_ovarian\_follicle | NRIP1 | 9 | 1 | 13.828829 | -1.154340 | 78 | 91.01 | 1.166795 |
| GO:0006476\_protein\_amino\_acid\_deacetylation | NCOR1 | 9 | 1 | 13.828829 | -1.154340 | 78 | 91.01 | 1.166795 |
| GO:0017145\_stem\_cell\_division | TIAL1 | 9 | 1 | 13.828829 | -1.154340 | 78 | 91.01 | 1.166795 |
| GO:0030728\_ovulation | NRIP1 | 9 | 1 | 13.828829 | -1.154340 | 78 | 91.01 | 1.166795 |
| GO:0050910\_detection\_of\_mechanical\_stimulus\_involved\_in\_sensory\_perception\_of\_sound | SLC12A2 | 9 | 1 | 13.828829 | -1.154340 | 78 | 91.01 | 1.166795 |
| GO:0055012\_ventricular\_cardiac\_muscle\_cell\_differentiation | RXRB | 9 | 1 | 13.828829 | -1.154340 | 78 | 91.01 | 1.166795 |
| GO:0014706\_striated\_muscle\_tissue\_development | RXRB | 120 | 3 | 3.111486 | -1.150995 | 79 | 91.35 | 1.156329 |
| GO:0014706\_striated\_muscle\_tissue\_development | MYH9 | 120 | 3 | 3.111486 | -1.150995 | 79 | 91.35 | 1.156329 |
| GO:0014706\_striated\_muscle\_tissue\_development | FOXP2 | 120 | 3 | 3.111486 | -1.150995 | 79 | 91.35 | 1.156329 |
| GO:0051641\_cellular\_localization | CYB5R4 | 370 | 6 | 2.018262 | -1.143017 | 80 | 91.97 | 1.149625 |
| GO:0051641\_cellular\_localization | YWHAH | 370 | 6 | 2.018262 | -1.143017 | 80 | 91.97 | 1.149625 |
| GO:0051641\_cellular\_localization | SH3GLB1 | 370 | 6 | 2.018262 | -1.143017 | 80 | 91.97 | 1.149625 |
| GO:0051641\_cellular\_localization | RAB1 | 370 | 6 | 2.018262 | -1.143017 | 80 | 91.97 | 1.149625 |
| GO:0051641\_cellular\_localization | LRBA | 370 | 6 | 2.018262 | -1.143017 | 80 | 91.97 | 1.149625 |
| GO:0051641\_cellular\_localization | MYH9 | 370 | 6 | 2.018262 | -1.143017 | 80 | 91.97 | 1.149625 |
| GO:0000904\_cell\_morphogenesis\_involved\_in\_differentiation | KLF7 | 199 | 4 | 2.501698 | -1.133262 | 81 | 92.81 | 1.145802 |
| GO:0000904\_cell\_morphogenesis\_involved\_in\_differentiation | YWHAH | 199 | 4 | 2.501698 | -1.133262 | 81 | 92.81 | 1.145802 |
| GO:0000904\_cell\_morphogenesis\_involved\_in\_differentiation | VAX2 | 199 | 4 | 2.501698 | -1.133262 | 81 | 92.81 | 1.145802 |
| GO:0000904\_cell\_morphogenesis\_involved\_in\_differentiation | MYH9 | 199 | 4 | 2.501698 | -1.133262 | 81 | 92.81 | 1.145802 |
| GO:0006790\_sulfur\_metabolic\_process | AHCY | 56 | 2 | 4.444981 | -1.131962 | 82 | 93.67 | 1.142317 |
| GO:0006790\_sulfur\_metabolic\_process | SEPP1 | 56 | 2 | 4.444981 | -1.131962 | 82 | 93.67 | 1.142317 |
| GO:0019222\_regulation\_of\_metabolic\_process | KLF7 | 1088 | 13 | 1.487108 | -1.118138 | 83 | 94.8 | 1.142169 |
| GO:0019222\_regulation\_of\_metabolic\_process | RXRB | 1088 | 13 | 1.487108 | -1.118138 | 83 | 94.8 | 1.142169 |
| GO:0019222\_regulation\_of\_metabolic\_process | E2F7 | 1088 | 13 | 1.487108 | -1.118138 | 83 | 94.8 | 1.142169 |
| GO:0019222\_regulation\_of\_metabolic\_process | TLE4 | 1088 | 13 | 1.487108 | -1.118138 | 83 | 94.8 | 1.142169 |
| GO:0019222\_regulation\_of\_metabolic\_process | ELAVL1 | 1088 | 13 | 1.487108 | -1.118138 | 83 | 94.8 | 1.142169 |
| GO:0019222\_regulation\_of\_metabolic\_process | VAX2 | 1088 | 13 | 1.487108 | -1.118138 | 83 | 94.8 | 1.142169 |
| GO:0019222\_regulation\_of\_metabolic\_process | HES6 | 1088 | 13 | 1.487108 | -1.118138 | 83 | 94.8 | 1.142169 |
| GO:0019222\_regulation\_of\_metabolic\_process | DACH1 | 1088 | 13 | 1.487108 | -1.118138 | 83 | 94.8 | 1.142169 |
| GO:0019222\_regulation\_of\_metabolic\_process | FOXP2 | 1088 | 13 | 1.487108 | -1.118138 | 83 | 94.8 | 1.142169 |
| GO:0019222\_regulation\_of\_metabolic\_process | NRIP1 | 1088 | 13 | 1.487108 | -1.118138 | 83 | 94.8 | 1.142169 |
| GO:0019222\_regulation\_of\_metabolic\_process | NCOA2 | 1088 | 13 | 1.487108 | -1.118138 | 83 | 94.8 | 1.142169 |
| GO:0019222\_regulation\_of\_metabolic\_process | TIA1 | 1088 | 13 | 1.487108 | -1.118138 | 83 | 94.8 | 1.142169 |
| GO:0019222\_regulation\_of\_metabolic\_process | NCOR1 | 1088 | 13 | 1.487108 | -1.118138 | 83 | 94.8 | 1.142169 |
| GO:0006826\_iron\_ion\_transport | HEPH | 10 | 1 | 12.445946 | -1.110268 | 89 | 104.94 | 1.179101 |
| GO:0007006\_mitochondrial\_membrane\_organization | SH3GLB1 | 10 | 1 | 12.445946 | -1.110268 | 89 | 104.94 | 1.179101 |
| GO:0016197\_endosome\_transport | LRBA | 10 | 1 | 12.445946 | -1.110268 | 89 | 104.94 | 1.179101 |
| GO:0043488\_regulation\_of\_mRNA\_stability | ELAVL1 | 10 | 1 | 12.445946 | -1.110268 | 89 | 104.94 | 1.179101 |
| GO:0048384\_retinoic\_acid\_receptor\_signaling\_pathway | RXRB | 10 | 1 | 12.445946 | -1.110268 | 89 | 104.94 | 1.179101 |
| GO:0060216\_definitive\_hemopoiesis | NCOR1 | 10 | 1 | 12.445946 | -1.110268 | 89 | 104.94 | 1.179101 |
| GO:0060537\_muscle\_tissue\_development | RXRB | 128 | 3 | 2.917019 | -1.084959 | 90 | 106.44 | 1.182667 |
| GO:0060537\_muscle\_tissue\_development | MYH9 | 128 | 3 | 2.917019 | -1.084959 | 90 | 106.44 | 1.182667 |
| GO:0060537\_muscle\_tissue\_development | FOXP2 | 128 | 3 | 2.917019 | -1.084959 | 90 | 106.44 | 1.182667 |
| GO:0000096\_sulfur\_amino\_acid\_metabolic\_process | AHCY | 11 | 1 | 11.314496 | -1.070558 | 97 | 114.58 | 1.181237 |
| GO:0007051\_spindle\_organization | MYH9 | 11 | 1 | 11.314496 | -1.070558 | 97 | 114.58 | 1.181237 |
| GO:0014902\_myotube\_differentiation | MYH9 | 11 | 1 | 11.314496 | -1.070558 | 97 | 114.58 | 1.181237 |
| GO:0042036\_negative\_regulation\_of\_cytokine\_biosynthetic\_process | TIA1 | 11 | 1 | 11.314496 | -1.070558 | 97 | 114.58 | 1.181237 |
| GO:0045026\_plasma\_membrane\_fusion | MYH9 | 11 | 1 | 11.314496 | -1.070558 | 97 | 114.58 | 1.181237 |
| GO:0048745\_smooth\_muscle\_tissue\_development | FOXP2 | 11 | 1 | 11.314496 | -1.070558 | 97 | 114.58 | 1.181237 |
| GO:0060004\_reflex | FOXP2 | 11 | 1 | 11.314496 | -1.070558 | 97 | 114.58 | 1.181237 |
| GO:0044260\_cellular\_macromolecule\_metabolic\_process | KLF7 | 1447 | 16 | 1.376193 | -1.062849 | 98 | 115.31 | 1.176633 |
| GO:0044260\_cellular\_macromolecule\_metabolic\_process | RXRB | 1447 | 16 | 1.376193 | -1.062849 | 98 | 115.31 | 1.176633 |
| GO:0044260\_cellular\_macromolecule\_metabolic\_process | E2F7 | 1447 | 16 | 1.376193 | -1.062849 | 98 | 115.31 | 1.176633 |
| GO:0044260\_cellular\_macromolecule\_metabolic\_process | UBE2F | 1447 | 16 | 1.376193 | -1.062849 | 98 | 115.31 | 1.176633 |
| GO:0044260\_cellular\_macromolecule\_metabolic\_process | TLE4 | 1447 | 16 | 1.376193 | -1.062849 | 98 | 115.31 | 1.176633 |
| GO:0044260\_cellular\_macromolecule\_metabolic\_process | ELAVL1 | 1447 | 16 | 1.376193 | -1.062849 | 98 | 115.31 | 1.176633 |
| GO:0044260\_cellular\_macromolecule\_metabolic\_process | VAX2 | 1447 | 16 | 1.376193 | -1.062849 | 98 | 115.31 | 1.176633 |
| GO:0044260\_cellular\_macromolecule\_metabolic\_process | HES6 | 1447 | 16 | 1.376193 | -1.062849 | 98 | 115.31 | 1.176633 |
| GO:0044260\_cellular\_macromolecule\_metabolic\_process | DACH1 | 1447 | 16 | 1.376193 | -1.062849 | 98 | 115.31 | 1.176633 |
| GO:0044260\_cellular\_macromolecule\_metabolic\_process | FOXP2 | 1447 | 16 | 1.376193 | -1.062849 | 98 | 115.31 | 1.176633 |
| GO:0044260\_cellular\_macromolecule\_metabolic\_process | NRIP1 | 1447 | 16 | 1.376193 | -1.062849 | 98 | 115.31 | 1.176633 |
| GO:0044260\_cellular\_macromolecule\_metabolic\_process | NCOA2 | 1447 | 16 | 1.376193 | -1.062849 | 98 | 115.31 | 1.176633 |
| GO:0044260\_cellular\_macromolecule\_metabolic\_process | SH3GLB1 | 1447 | 16 | 1.376193 | -1.062849 | 98 | 115.31 | 1.176633 |
| GO:0044260\_cellular\_macromolecule\_metabolic\_process | TIA1 | 1447 | 16 | 1.376193 | -1.062849 | 98 | 115.31 | 1.176633 |
| GO:0044260\_cellular\_macromolecule\_metabolic\_process | PPM1M | 1447 | 16 | 1.376193 | -1.062849 | 98 | 115.31 | 1.176633 |
| GO:0044260\_cellular\_macromolecule\_metabolic\_process | NCOR1 | 1447 | 16 | 1.376193 | -1.062849 | 98 | 115.31 | 1.176633 |
| GO:0006139\_nucleobase\_\_nucleoside\_\_nucleotide\_and\_nucleic\_acid\_metabolic\_process | KLF7 | 1002 | 12 | 1.490532 | -1.056615 | 99 | 115.35 | 1.165152 |
| GO:0006139\_nucleobase\_\_nucleoside\_\_nucleotide\_and\_nucleic\_acid\_metabolic\_process | NCOA2 | 1002 | 12 | 1.490532 | -1.056615 | 99 | 115.35 | 1.165152 |
| GO:0006139\_nucleobase\_\_nucleoside\_\_nucleotide\_and\_nucleic\_acid\_metabolic\_process | RXRB | 1002 | 12 | 1.490532 | -1.056615 | 99 | 115.35 | 1.165152 |
| GO:0006139\_nucleobase\_\_nucleoside\_\_nucleotide\_and\_nucleic\_acid\_metabolic\_process | E2F7 | 1002 | 12 | 1.490532 | -1.056615 | 99 | 115.35 | 1.165152 |
| GO:0006139\_nucleobase\_\_nucleoside\_\_nucleotide\_and\_nucleic\_acid\_metabolic\_process | ELAVL1 | 1002 | 12 | 1.490532 | -1.056615 | 99 | 115.35 | 1.165152 |
| GO:0006139\_nucleobase\_\_nucleoside\_\_nucleotide\_and\_nucleic\_acid\_metabolic\_process | TLE4 | 1002 | 12 | 1.490532 | -1.056615 | 99 | 115.35 | 1.165152 |
| GO:0006139\_nucleobase\_\_nucleoside\_\_nucleotide\_and\_nucleic\_acid\_metabolic\_process | VAX2 | 1002 | 12 | 1.490532 | -1.056615 | 99 | 115.35 | 1.165152 |
| GO:0006139\_nucleobase\_\_nucleoside\_\_nucleotide\_and\_nucleic\_acid\_metabolic\_process | DACH1 | 1002 | 12 | 1.490532 | -1.056615 | 99 | 115.35 | 1.165152 |
| GO:0006139\_nucleobase\_\_nucleoside\_\_nucleotide\_and\_nucleic\_acid\_metabolic\_process | HES6 | 1002 | 12 | 1.490532 | -1.056615 | 99 | 115.35 | 1.165152 |
| GO:0006139\_nucleobase\_\_nucleoside\_\_nucleotide\_and\_nucleic\_acid\_metabolic\_process | NCOR1 | 1002 | 12 | 1.490532 | -1.056615 | 99 | 115.35 | 1.165152 |
| GO:0006139\_nucleobase\_\_nucleoside\_\_nucleotide\_and\_nucleic\_acid\_metabolic\_process | NRIP1 | 1002 | 12 | 1.490532 | -1.056615 | 99 | 115.35 | 1.165152 |
| GO:0006139\_nucleobase\_\_nucleoside\_\_nucleotide\_and\_nucleic\_acid\_metabolic\_process | FOXP2 | 1002 | 12 | 1.490532 | -1.056615 | 99 | 115.35 | 1.165152 |
| GO:0022604\_regulation\_of\_cell\_morphogenesis | YWHAH | 62 | 2 | 4.014821 | -1.055656 | 100 | 116.17 | 1.161700 |
| GO:0022604\_regulation\_of\_cell\_morphogenesis | MYH9 | 62 | 2 | 4.014821 | -1.055656 | 100 | 116.17 | 1.161700 |
| GO:0030010\_establishment\_of\_cell\_polarity | MYH9 | 12 | 1 | 10.371622 | -1.034451 | 105 | 125.13 | 1.191714 |
| GO:0031056\_regulation\_of\_histone\_modification | NCOR1 | 12 | 1 | 10.371622 | -1.034451 | 105 | 125.13 | 1.191714 |
| GO:0043487\_regulation\_of\_RNA\_stability | ELAVL1 | 12 | 1 | 10.371622 | -1.034451 | 105 | 125.13 | 1.191714 |
| GO:0050848\_regulation\_of\_calcium-mediated\_signaling | NCAM1 | 12 | 1 | 10.371622 | -1.034451 | 105 | 125.13 | 1.191714 |
| GO:0050850\_positive\_regulation\_of\_calcium-mediated\_signaling | NCAM1 | 12 | 1 | 10.371622 | -1.034451 | 105 | 125.13 | 1.191714 |
| GO:0001654\_eye\_development | VAX2 | 136 | 3 | 2.745429 | -1.024059 | 106 | 125.99 | 1.188585 |
| GO:0001654\_eye\_development | MAB21L1 | 136 | 3 | 2.745429 | -1.024059 | 106 | 125.99 | 1.188585 |
| GO:0001654\_eye\_development | FOXP2 | 136 | 3 | 2.745429 | -1.024059 | 106 | 125.99 | 1.188585 |
| GO:0048511\_rhythmic\_process | NCOR1 | 65 | 2 | 3.829522 | -1.020700 | 107 | 126.43 | 1.181589 |
| GO:0048511\_rhythmic\_process | NRIP1 | 65 | 2 | 3.829522 | -1.020700 | 107 | 126.43 | 1.181589 |
| GO:0034613\_cellular\_protein\_localization | YWHAH | 139 | 3 | 2.686175 | -1.002420 | 108 | 128.41 | 1.188981 |
| GO:0034613\_cellular\_protein\_localization | SH3GLB1 | 139 | 3 | 2.686175 | -1.002420 | 108 | 128.41 | 1.188981 |
| GO:0034613\_cellular\_protein\_localization | RAB1 | 139 | 3 | 2.686175 | -1.002420 | 108 | 128.41 | 1.188981 |
| GO:0007566\_embryo\_implantation | FKBP4 | 13 | 1 | 9.573805 | -1.001368 | 111 | 135.9 | 1.224324 |
| GO:0046474\_glycerophospholipid\_biosynthetic\_process | SH3GLB1 | 13 | 1 | 9.573805 | -1.001368 | 111 | 135.9 | 1.224324 |
| GO:0060038\_cardiac\_muscle\_cell\_proliferation | RXRB | 13 | 1 | 9.573805 | -1.001368 | 111 | 135.9 | 1.224324 |
| GO:0009791\_post-embryonic\_development | SEPP1 | 67 | 2 | 3.715208 | -0.998446 | 112 | 136.47 | 1.218482 |
| GO:0009791\_post-embryonic\_development | FOXP2 | 67 | 2 | 3.715208 | -0.998446 | 112 | 136.47 | 1.218482 |
| GO:0070727\_cellular\_macromolecule\_localization | YWHAH | 141 | 3 | 2.648074 | -0.988334 | 113 | 136.77 | 1.210354 |
| GO:0070727\_cellular\_macromolecule\_localization | SH3GLB1 | 141 | 3 | 2.648074 | -0.988334 | 113 | 136.77 | 1.210354 |
| GO:0070727\_cellular\_macromolecule\_localization | RAB1 | 141 | 3 | 2.648074 | -0.988334 | 113 | 136.77 | 1.210354 |
| GO:0042692\_muscle\_cell\_differentiation | RXRB | 68 | 2 | 3.660572 | -0.987615 | 114 | 137.08 | 1.202456 |
| GO:0042692\_muscle\_cell\_differentiation | MYH9 | 68 | 2 | 3.660572 | -0.987615 | 114 | 137.08 | 1.202456 |
| GO:0014855\_striated\_muscle\_cell\_proliferation | RXRB | 14 | 1 | 8.889961 | -0.970861 | 117 | 144.61 | 1.235983 |
| GO:0033044\_regulation\_of\_chromosome\_organization | NCOR1 | 14 | 1 | 8.889961 | -0.970861 | 117 | 144.61 | 1.235983 |
| GO:0048048\_embryonic\_eye\_morphogenesis | VAX2 | 14 | 1 | 8.889961 | -0.970861 | 117 | 144.61 | 1.235983 |
| GO:0006807\_nitrogen\_compound\_metabolic\_process | KLF7 | 1147 | 13 | 1.410613 | -0.968663 | 118 | 144.72 | 1.226441 |
| GO:0006807\_nitrogen\_compound\_metabolic\_process | AHCY | 1147 | 13 | 1.410613 | -0.968663 | 118 | 144.72 | 1.226441 |
| GO:0006807\_nitrogen\_compound\_metabolic\_process | RXRB | 1147 | 13 | 1.410613 | -0.968663 | 118 | 144.72 | 1.226441 |
| GO:0006807\_nitrogen\_compound\_metabolic\_process | E2F7 | 1147 | 13 | 1.410613 | -0.968663 | 118 | 144.72 | 1.226441 |
| GO:0006807\_nitrogen\_compound\_metabolic\_process | TLE4 | 1147 | 13 | 1.410613 | -0.968663 | 118 | 144.72 | 1.226441 |
| GO:0006807\_nitrogen\_compound\_metabolic\_process | ELAVL1 | 1147 | 13 | 1.410613 | -0.968663 | 118 | 144.72 | 1.226441 |
| GO:0006807\_nitrogen\_compound\_metabolic\_process | VAX2 | 1147 | 13 | 1.410613 | -0.968663 | 118 | 144.72 | 1.226441 |
| GO:0006807\_nitrogen\_compound\_metabolic\_process | HES6 | 1147 | 13 | 1.410613 | -0.968663 | 118 | 144.72 | 1.226441 |
| GO:0006807\_nitrogen\_compound\_metabolic\_process | DACH1 | 1147 | 13 | 1.410613 | -0.968663 | 118 | 144.72 | 1.226441 |
| GO:0006807\_nitrogen\_compound\_metabolic\_process | FOXP2 | 1147 | 13 | 1.410613 | -0.968663 | 118 | 144.72 | 1.226441 |
| GO:0006807\_nitrogen\_compound\_metabolic\_process | NRIP1 | 1147 | 13 | 1.410613 | -0.968663 | 118 | 144.72 | 1.226441 |
| GO:0006807\_nitrogen\_compound\_metabolic\_process | NCOA2 | 1147 | 13 | 1.410613 | -0.968663 | 118 | 144.72 | 1.226441 |
| GO:0006807\_nitrogen\_compound\_metabolic\_process | NCOR1 | 1147 | 13 | 1.410613 | -0.968663 | 118 | 144.72 | 1.226441 |
| GO:0019953\_sexual\_reproduction | NHP2L1 | 228 | 4 | 2.183499 | -0.966007 | 119 | 145.7 | 1.224370 |
| GO:0019953\_sexual\_reproduction | TIAL1 | 228 | 4 | 2.183499 | -0.966007 | 119 | 145.7 | 1.224370 |
| GO:0019953\_sexual\_reproduction | SEPP1 | 228 | 4 | 2.183499 | -0.966007 | 119 | 145.7 | 1.224370 |
| GO:0019953\_sexual\_reproduction | NRIP1 | 228 | 4 | 2.183499 | -0.966007 | 119 | 145.7 | 1.224370 |
| GO:0043283\_biopolymer\_metabolic\_process | KLF7 | 1490 | 16 | 1.336477 | -0.965871 | 120 | 145.72 | 1.214333 |
| GO:0043283\_biopolymer\_metabolic\_process | RXRB | 1490 | 16 | 1.336477 | -0.965871 | 120 | 145.72 | 1.214333 |
| GO:0043283\_biopolymer\_metabolic\_process | E2F7 | 1490 | 16 | 1.336477 | -0.965871 | 120 | 145.72 | 1.214333 |
| GO:0043283\_biopolymer\_metabolic\_process | UBE2F | 1490 | 16 | 1.336477 | -0.965871 | 120 | 145.72 | 1.214333 |
| GO:0043283\_biopolymer\_metabolic\_process | ELAVL1 | 1490 | 16 | 1.336477 | -0.965871 | 120 | 145.72 | 1.214333 |
| GO:0043283\_biopolymer\_metabolic\_process | TLE4 | 1490 | 16 | 1.336477 | -0.965871 | 120 | 145.72 | 1.214333 |
| GO:0043283\_biopolymer\_metabolic\_process | VAX2 | 1490 | 16 | 1.336477 | -0.965871 | 120 | 145.72 | 1.214333 |
| GO:0043283\_biopolymer\_metabolic\_process | DACH1 | 1490 | 16 | 1.336477 | -0.965871 | 120 | 145.72 | 1.214333 |
| GO:0043283\_biopolymer\_metabolic\_process | HES6 | 1490 | 16 | 1.336477 | -0.965871 | 120 | 145.72 | 1.214333 |
| GO:0043283\_biopolymer\_metabolic\_process | FOXP2 | 1490 | 16 | 1.336477 | -0.965871 | 120 | 145.72 | 1.214333 |
| GO:0043283\_biopolymer\_metabolic\_process | NRIP1 | 1490 | 16 | 1.336477 | -0.965871 | 120 | 145.72 | 1.214333 |
| GO:0043283\_biopolymer\_metabolic\_process | NCOA2 | 1490 | 16 | 1.336477 | -0.965871 | 120 | 145.72 | 1.214333 |
| GO:0043283\_biopolymer\_metabolic\_process | SH3GLB1 | 1490 | 16 | 1.336477 | -0.965871 | 120 | 145.72 | 1.214333 |
| GO:0043283\_biopolymer\_metabolic\_process | TIA1 | 1490 | 16 | 1.336477 | -0.965871 | 120 | 145.72 | 1.214333 |
| GO:0043283\_biopolymer\_metabolic\_process | PPM1M | 1490 | 16 | 1.336477 | -0.965871 | 120 | 145.72 | 1.214333 |
| GO:0043283\_biopolymer\_metabolic\_process | NCOR1 | 1490 | 16 | 1.336477 | -0.965871 | 120 | 145.72 | 1.214333 |
| GO:0044249\_cellular\_biosynthetic\_process | KLF7 | 1150 | 13 | 1.406933 | -0.961511 | 121 | 145.75 | 1.204545 |
| GO:0044249\_cellular\_biosynthetic\_process | RXRB | 1150 | 13 | 1.406933 | -0.961511 | 121 | 145.75 | 1.204545 |
| GO:0044249\_cellular\_biosynthetic\_process | E2F7 | 1150 | 13 | 1.406933 | -0.961511 | 121 | 145.75 | 1.204545 |
| GO:0044249\_cellular\_biosynthetic\_process | TLE4 | 1150 | 13 | 1.406933 | -0.961511 | 121 | 145.75 | 1.204545 |
| GO:0044249\_cellular\_biosynthetic\_process | VAX2 | 1150 | 13 | 1.406933 | -0.961511 | 121 | 145.75 | 1.204545 |
| GO:0044249\_cellular\_biosynthetic\_process | HES6 | 1150 | 13 | 1.406933 | -0.961511 | 121 | 145.75 | 1.204545 |
| GO:0044249\_cellular\_biosynthetic\_process | DACH1 | 1150 | 13 | 1.406933 | -0.961511 | 121 | 145.75 | 1.204545 |
| GO:0044249\_cellular\_biosynthetic\_process | FOXP2 | 1150 | 13 | 1.406933 | -0.961511 | 121 | 145.75 | 1.204545 |
| GO:0044249\_cellular\_biosynthetic\_process | NRIP1 | 1150 | 13 | 1.406933 | -0.961511 | 121 | 145.75 | 1.204545 |
| GO:0044249\_cellular\_biosynthetic\_process | NCOA2 | 1150 | 13 | 1.406933 | -0.961511 | 121 | 145.75 | 1.204545 |
| GO:0044249\_cellular\_biosynthetic\_process | SH3GLB1 | 1150 | 13 | 1.406933 | -0.961511 | 121 | 145.75 | 1.204545 |
| GO:0044249\_cellular\_biosynthetic\_process | TIA1 | 1150 | 13 | 1.406933 | -0.961511 | 121 | 145.75 | 1.204545 |
| GO:0044249\_cellular\_biosynthetic\_process | NCOR1 | 1150 | 13 | 1.406933 | -0.961511 | 121 | 145.75 | 1.204545 |
| GO:0008654\_phospholipid\_biosynthetic\_process | SH3GLB1 | 16 | 1 | 7.778716 | -0.916218 | 130 | 161.89 | 1.245308 |
| GO:0010876\_lipid\_localization | NRIP1 | 16 | 1 | 7.778716 | -0.916218 | 130 | 161.89 | 1.245308 |
| GO:0019722\_calcium-mediated\_signaling | NCAM1 | 16 | 1 | 7.778716 | -0.916218 | 130 | 161.89 | 1.245308 |
| GO:0019915\_lipid\_storage | NRIP1 | 16 | 1 | 7.778716 | -0.916218 | 130 | 161.89 | 1.245308 |
| GO:0031345\_negative\_regulation\_of\_cell\_projection\_organization | YWHAH | 16 | 1 | 7.778716 | -0.916218 | 130 | 161.89 | 1.245308 |
| GO:0043367\_CD4-positive\_\_alpha\_beta\_T\_cell\_differentiation | NCOR1 | 16 | 1 | 7.778716 | -0.916218 | 130 | 161.89 | 1.245308 |
| GO:0048286\_lung\_alveolus\_development | FOXP2 | 16 | 1 | 7.778716 | -0.916218 | 130 | 161.89 | 1.245308 |
| GO:0050974\_detection\_of\_mechanical\_stimulus\_involved\_in\_sensory\_perception | SLC12A2 | 16 | 1 | 7.778716 | -0.916218 | 130 | 161.89 | 1.245308 |
| GO:0055007\_cardiac\_muscle\_cell\_differentiation | RXRB | 16 | 1 | 7.778716 | -0.916218 | 130 | 161.89 | 1.245308 |
| GO:0007517\_muscle\_organ\_development | RXRB | 153 | 3 | 2.440382 | -0.909092 | 131 | 162.88 | 1.243359 |
| GO:0007517\_muscle\_organ\_development | MYH9 | 153 | 3 | 2.440382 | -0.909092 | 131 | 162.88 | 1.243359 |
| GO:0007517\_muscle\_organ\_development | FOXP2 | 153 | 3 | 2.440382 | -0.909092 | 131 | 162.88 | 1.243359 |
| GO:0009058\_biosynthetic\_process | KLF7 | 1175 | 13 | 1.376998 | -0.903518 | 132 | 163.44 | 1.238182 |
| GO:0009058\_biosynthetic\_process | RXRB | 1175 | 13 | 1.376998 | -0.903518 | 132 | 163.44 | 1.238182 |
| GO:0009058\_biosynthetic\_process | E2F7 | 1175 | 13 | 1.376998 | -0.903518 | 132 | 163.44 | 1.238182 |
| GO:0009058\_biosynthetic\_process | TLE4 | 1175 | 13 | 1.376998 | -0.903518 | 132 | 163.44 | 1.238182 |
| GO:0009058\_biosynthetic\_process | VAX2 | 1175 | 13 | 1.376998 | -0.903518 | 132 | 163.44 | 1.238182 |
| GO:0009058\_biosynthetic\_process | HES6 | 1175 | 13 | 1.376998 | -0.903518 | 132 | 163.44 | 1.238182 |
| GO:0009058\_biosynthetic\_process | DACH1 | 1175 | 13 | 1.376998 | -0.903518 | 132 | 163.44 | 1.238182 |
| GO:0009058\_biosynthetic\_process | FOXP2 | 1175 | 13 | 1.376998 | -0.903518 | 132 | 163.44 | 1.238182 |
| GO:0009058\_biosynthetic\_process | NRIP1 | 1175 | 13 | 1.376998 | -0.903518 | 132 | 163.44 | 1.238182 |
| GO:0009058\_biosynthetic\_process | NCOA2 | 1175 | 13 | 1.376998 | -0.903518 | 132 | 163.44 | 1.238182 |
| GO:0009058\_biosynthetic\_process | SH3GLB1 | 1175 | 13 | 1.376998 | -0.903518 | 132 | 163.44 | 1.238182 |
| GO:0009058\_biosynthetic\_process | TIA1 | 1175 | 13 | 1.376998 | -0.903518 | 132 | 163.44 | 1.238182 |
| GO:0009058\_biosynthetic\_process | NCOR1 | 1175 | 13 | 1.376998 | -0.903518 | 132 | 163.44 | 1.238182 |
| GO:0006323\_DNA\_packaging | AKAP8 | 17 | 1 | 7.321145 | -0.891560 | 134 | 169.67 | 1.266194 |
| GO:0010741\_negative\_regulation\_of\_protein\_kinase\_cascade | NCOR1 | 17 | 1 | 7.321145 | -0.891560 | 134 | 169.67 | 1.266194 |
| GO:0007519\_skeletal\_muscle\_tissue\_development | MYH9 | 78 | 2 | 3.191268 | -0.888887 | 136 | 170.7 | 1.255147 |
| GO:0007519\_skeletal\_muscle\_tissue\_development | FOXP2 | 78 | 2 | 3.191268 | -0.888887 | 136 | 170.7 | 1.255147 |
| GO:0060538\_skeletal\_muscle\_organ\_development | MYH9 | 78 | 2 | 3.191268 | -0.888887 | 136 | 170.7 | 1.255147 |
| GO:0060538\_skeletal\_muscle\_organ\_development | FOXP2 | 78 | 2 | 3.191268 | -0.888887 | 136 | 170.7 | 1.255147 |
| GO:0006457\_protein\_folding | SH3GLB1 | 18 | 1 | 6.914414 | -0.868406 | 140 | 179.44 | 1.281714 |
| GO:0009063\_cellular\_amino\_acid\_catabolic\_process | AHCY | 18 | 1 | 6.914414 | -0.868406 | 140 | 179.44 | 1.281714 |
| GO:0035051\_cardiac\_cell\_differentiation | RXRB | 18 | 1 | 6.914414 | -0.868406 | 140 | 179.44 | 1.281714 |
| GO:0050982\_detection\_of\_mechanical\_stimulus | SLC12A2 | 18 | 1 | 6.914414 | -0.868406 | 140 | 179.44 | 1.281714 |
| GO:0051128\_regulation\_of\_cellular\_component\_organization | YWHAH | 160 | 3 | 2.333615 | -0.866659 | 141 | 179.78 | 1.275035 |
| GO:0051128\_regulation\_of\_cellular\_component\_organization | MYH9 | 160 | 3 | 2.333615 | -0.866659 | 141 | 179.78 | 1.275035 |
| GO:0051128\_regulation\_of\_cellular\_component\_organization | NCOR1 | 160 | 3 | 2.333615 | -0.866659 | 141 | 179.78 | 1.275035 |
| GO:0051649\_establishment\_of\_localization\_in\_cell | CYB5R4 | 342 | 5 | 1.819583 | -0.866646 | 142 | 179.88 | 1.266761 |
| GO:0051649\_establishment\_of\_localization\_in\_cell | YWHAH | 342 | 5 | 1.819583 | -0.866646 | 142 | 179.88 | 1.266761 |
| GO:0051649\_establishment\_of\_localization\_in\_cell | RAB1 | 342 | 5 | 1.819583 | -0.866646 | 142 | 179.88 | 1.266761 |
| GO:0051649\_establishment\_of\_localization\_in\_cell | LRBA | 342 | 5 | 1.819583 | -0.866646 | 142 | 179.88 | 1.266761 |
| GO:0051649\_establishment\_of\_localization\_in\_cell | MYH9 | 342 | 5 | 1.819583 | -0.866646 | 142 | 179.88 | 1.266761 |
| GO:0009798\_axis\_specification | VAX2 | 19 | 1 | 6.550498 | -0.846592 | 145 | 186.26 | 1.284552 |
| GO:0030518\_steroid\_hormone\_receptor\_signaling\_pathway | FKBP4 | 19 | 1 | 6.550498 | -0.846592 | 145 | 186.26 | 1.284552 |
| GO:0033002\_muscle\_cell\_proliferation | RXRB | 19 | 1 | 6.550498 | -0.846592 | 145 | 186.26 | 1.284552 |
| GO:0000279\_M\_phase | AKAP8 | 85 | 2 | 2.928458 | -0.828592 | 146 | 189.48 | 1.297808 |
| GO:0000279\_M\_phase | MYH9 | 85 | 2 | 2.928458 | -0.828592 | 146 | 189.48 | 1.297808 |
| GO:0008360\_regulation\_of\_cell\_shape | MYH9 | 20 | 1 | 6.222973 | -0.825981 | 148 | 194.63 | 1.315068 |
| GO:0045017\_glycerolipid\_biosynthetic\_process | SH3GLB1 | 20 | 1 | 6.222973 | -0.825981 | 148 | 194.63 | 1.315068 |
| GO:0016337\_cell-cell\_adhesion | NCAM1 | 87 | 2 | 2.861137 | -0.812495 | 149 | 196.48 | 1.318658 |
| GO:0016337\_cell-cell\_adhesion | MYH9 | 87 | 2 | 2.861137 | -0.812495 | 149 | 196.48 | 1.318658 |
| GO:0048812\_neuron\_projection\_morphogenesis | KLF7 | 170 | 3 | 2.196343 | -0.810307 | 150 | 196.81 | 1.312067 |
| GO:0048812\_neuron\_projection\_morphogenesis | YWHAH | 170 | 3 | 2.196343 | -0.810307 | 150 | 196.81 | 1.312067 |
| GO:0048812\_neuron\_projection\_morphogenesis | VAX2 | 170 | 3 | 2.196343 | -0.810307 | 150 | 196.81 | 1.312067 |
| GO:0002053\_positive\_regulation\_of\_mesenchymal\_cell\_proliferation | FOXP2 | 21 | 1 | 5.926641 | -0.806455 | 154 | 203.85 | 1.323701 |
| GO:0006944\_membrane\_fusion | MYH9 | 21 | 1 | 5.926641 | -0.806455 | 154 | 203.85 | 1.323701 |
| GO:0008637\_apoptotic\_mitochondrial\_changes | SH3GLB1 | 21 | 1 | 5.926641 | -0.806455 | 154 | 203.85 | 1.323701 |
| GO:0051656\_establishment\_of\_organelle\_localization | MYH9 | 21 | 1 | 5.926641 | -0.806455 | 154 | 203.85 | 1.323701 |
| GO:0048523\_negative\_regulation\_of\_cellular\_process | YWHAH | 774 | 9 | 1.447203 | -0.805998 | 155 | 203.91 | 1.315548 |
| GO:0048523\_negative\_regulation\_of\_cellular\_process | NCOA2 | 774 | 9 | 1.447203 | -0.805998 | 155 | 203.91 | 1.315548 |
| GO:0048523\_negative\_regulation\_of\_cellular\_process | TIA1 | 774 | 9 | 1.447203 | -0.805998 | 155 | 203.91 | 1.315548 |
| GO:0048523\_negative\_regulation\_of\_cellular\_process | E2F7 | 774 | 9 | 1.447203 | -0.805998 | 155 | 203.91 | 1.315548 |
| GO:0048523\_negative\_regulation\_of\_cellular\_process | TLE4 | 774 | 9 | 1.447203 | -0.805998 | 155 | 203.91 | 1.315548 |
| GO:0048523\_negative\_regulation\_of\_cellular\_process | VAX2 | 774 | 9 | 1.447203 | -0.805998 | 155 | 203.91 | 1.315548 |
| GO:0048523\_negative\_regulation\_of\_cellular\_process | NCOR1 | 774 | 9 | 1.447203 | -0.805998 | 155 | 203.91 | 1.315548 |
| GO:0048523\_negative\_regulation\_of\_cellular\_process | NRIP1 | 774 | 9 | 1.447203 | -0.805998 | 155 | 203.91 | 1.315548 |
| GO:0048523\_negative\_regulation\_of\_cellular\_process | FOXP2 | 774 | 9 | 1.447203 | -0.805998 | 155 | 203.91 | 1.315548 |
| GO:0030030\_cell\_projection\_organization | KLF7 | 263 | 4 | 1.892920 | -0.801268 | 156 | 204.81 | 1.312885 |
| GO:0030030\_cell\_projection\_organization | YWHAH | 263 | 4 | 1.892920 | -0.801268 | 156 | 204.81 | 1.312885 |
| GO:0030030\_cell\_projection\_organization | VAX2 | 263 | 4 | 1.892920 | -0.801268 | 156 | 204.81 | 1.312885 |
| GO:0030030\_cell\_projection\_organization | MYH9 | 263 | 4 | 1.892920 | -0.801268 | 156 | 204.81 | 1.312885 |
| GO:0048667\_cell\_morphogenesis\_involved\_in\_neuron\_differentiation | KLF7 | 173 | 3 | 2.158257 | -0.794302 | 157 | 205.78 | 1.310701 |
| GO:0048667\_cell\_morphogenesis\_involved\_in\_neuron\_differentiation | YWHAH | 173 | 3 | 2.158257 | -0.794302 | 157 | 205.78 | 1.310701 |
| GO:0048667\_cell\_morphogenesis\_involved\_in\_neuron\_differentiation | VAX2 | 173 | 3 | 2.158257 | -0.794302 | 157 | 205.78 | 1.310701 |
| GO:0043170\_macromolecule\_metabolic\_process | KLF7 | 1576 | 16 | 1.263548 | -0.791588 | 158 | 205.91 | 1.303228 |
| GO:0043170\_macromolecule\_metabolic\_process | RXRB | 1576 | 16 | 1.263548 | -0.791588 | 158 | 205.91 | 1.303228 |
| GO:0043170\_macromolecule\_metabolic\_process | E2F7 | 1576 | 16 | 1.263548 | -0.791588 | 158 | 205.91 | 1.303228 |
| GO:0043170\_macromolecule\_metabolic\_process | UBE2F | 1576 | 16 | 1.263548 | -0.791588 | 158 | 205.91 | 1.303228 |
| GO:0043170\_macromolecule\_metabolic\_process | TLE4 | 1576 | 16 | 1.263548 | -0.791588 | 158 | 205.91 | 1.303228 |
| GO:0043170\_macromolecule\_metabolic\_process | ELAVL1 | 1576 | 16 | 1.263548 | -0.791588 | 158 | 205.91 | 1.303228 |
| GO:0043170\_macromolecule\_metabolic\_process | VAX2 | 1576 | 16 | 1.263548 | -0.791588 | 158 | 205.91 | 1.303228 |
| GO:0043170\_macromolecule\_metabolic\_process | HES6 | 1576 | 16 | 1.263548 | -0.791588 | 158 | 205.91 | 1.303228 |
| GO:0043170\_macromolecule\_metabolic\_process | DACH1 | 1576 | 16 | 1.263548 | -0.791588 | 158 | 205.91 | 1.303228 |
| GO:0043170\_macromolecule\_metabolic\_process | FOXP2 | 1576 | 16 | 1.263548 | -0.791588 | 158 | 205.91 | 1.303228 |
| GO:0043170\_macromolecule\_metabolic\_process | NRIP1 | 1576 | 16 | 1.263548 | -0.791588 | 158 | 205.91 | 1.303228 |
| GO:0043170\_macromolecule\_metabolic\_process | NCOA2 | 1576 | 16 | 1.263548 | -0.791588 | 158 | 205.91 | 1.303228 |
| GO:0043170\_macromolecule\_metabolic\_process | SH3GLB1 | 1576 | 16 | 1.263548 | -0.791588 | 158 | 205.91 | 1.303228 |
| GO:0043170\_macromolecule\_metabolic\_process | TIA1 | 1576 | 16 | 1.263548 | -0.791588 | 158 | 205.91 | 1.303228 |
| GO:0043170\_macromolecule\_metabolic\_process | PPM1M | 1576 | 16 | 1.263548 | -0.791588 | 158 | 205.91 | 1.303228 |
| GO:0043170\_macromolecule\_metabolic\_process | NCOR1 | 1576 | 16 | 1.263548 | -0.791588 | 158 | 205.91 | 1.303228 |
| GO:0000041\_transition\_metal\_ion\_transport | HEPH | 22 | 1 | 5.657248 | -0.787913 | 163 | 213.82 | 1.311779 |
| GO:0007059\_chromosome\_segregation | AKAP8 | 22 | 1 | 5.657248 | -0.787913 | 163 | 213.82 | 1.311779 |
| GO:0010463\_mesenchymal\_cell\_proliferation | FOXP2 | 22 | 1 | 5.657248 | -0.787913 | 163 | 213.82 | 1.311779 |
| GO:0010464\_regulation\_of\_mesenchymal\_cell\_proliferation | FOXP2 | 22 | 1 | 5.657248 | -0.787913 | 163 | 213.82 | 1.311779 |
| GO:0048864\_stem\_cell\_development | MSI2 | 22 | 1 | 5.657248 | -0.787913 | 163 | 213.82 | 1.311779 |
| GO:0048858\_cell\_projection\_morphogenesis | KLF7 | 176 | 3 | 2.121468 | -0.778685 | 164 | 215.75 | 1.315549 |
| GO:0048858\_cell\_projection\_morphogenesis | YWHAH | 176 | 3 | 2.121468 | -0.778685 | 164 | 215.75 | 1.315549 |
| GO:0048858\_cell\_projection\_morphogenesis | VAX2 | 176 | 3 | 2.121468 | -0.778685 | 164 | 215.75 | 1.315549 |
| GO:0050794\_regulation\_of\_cellular\_process | KLF7 | 2190 | 21 | 1.193447 | -0.773196 | 165 | 216.32 | 1.311030 |
| GO:0050794\_regulation\_of\_cellular\_process | RXRB | 2190 | 21 | 1.193447 | -0.773196 | 165 | 216.32 | 1.311030 |
| GO:0050794\_regulation\_of\_cellular\_process | E2F7 | 2190 | 21 | 1.193447 | -0.773196 | 165 | 216.32 | 1.311030 |
| GO:0050794\_regulation\_of\_cellular\_process | FKBP4 | 2190 | 21 | 1.193447 | -0.773196 | 165 | 216.32 | 1.311030 |
| GO:0050794\_regulation\_of\_cellular\_process | LRBA | 2190 | 21 | 1.193447 | -0.773196 | 165 | 216.32 | 1.311030 |
| GO:0050794\_regulation\_of\_cellular\_process | ELAVL1 | 2190 | 21 | 1.193447 | -0.773196 | 165 | 216.32 | 1.311030 |
| GO:0050794\_regulation\_of\_cellular\_process | TLE4 | 2190 | 21 | 1.193447 | -0.773196 | 165 | 216.32 | 1.311030 |
| GO:0050794\_regulation\_of\_cellular\_process | VAX2 | 2190 | 21 | 1.193447 | -0.773196 | 165 | 216.32 | 1.311030 |
| GO:0050794\_regulation\_of\_cellular\_process | DACH1 | 2190 | 21 | 1.193447 | -0.773196 | 165 | 216.32 | 1.311030 |
| GO:0050794\_regulation\_of\_cellular\_process | HES6 | 2190 | 21 | 1.193447 | -0.773196 | 165 | 216.32 | 1.311030 |
| GO:0050794\_regulation\_of\_cellular\_process | MYH9 | 2190 | 21 | 1.193447 | -0.773196 | 165 | 216.32 | 1.311030 |
| GO:0050794\_regulation\_of\_cellular\_process | NRIP1 | 2190 | 21 | 1.193447 | -0.773196 | 165 | 216.32 | 1.311030 |
| GO:0050794\_regulation\_of\_cellular\_process | FOXP2 | 2190 | 21 | 1.193447 | -0.773196 | 165 | 216.32 | 1.311030 |
| GO:0050794\_regulation\_of\_cellular\_process | NCAM1 | 2190 | 21 | 1.193447 | -0.773196 | 165 | 216.32 | 1.311030 |
| GO:0050794\_regulation\_of\_cellular\_process | YWHAH | 2190 | 21 | 1.193447 | -0.773196 | 165 | 216.32 | 1.311030 |
| GO:0050794\_regulation\_of\_cellular\_process | NCOA2 | 2190 | 21 | 1.193447 | -0.773196 | 165 | 216.32 | 1.311030 |
| GO:0050794\_regulation\_of\_cellular\_process | SH3GLB1 | 2190 | 21 | 1.193447 | -0.773196 | 165 | 216.32 | 1.311030 |
| GO:0050794\_regulation\_of\_cellular\_process | TIAL1 | 2190 | 21 | 1.193447 | -0.773196 | 165 | 216.32 | 1.311030 |
| GO:0050794\_regulation\_of\_cellular\_process | TIA1 | 2190 | 21 | 1.193447 | -0.773196 | 165 | 216.32 | 1.311030 |
| GO:0050794\_regulation\_of\_cellular\_process | MAB21L1 | 2190 | 21 | 1.193447 | -0.773196 | 165 | 216.32 | 1.311030 |
| GO:0050794\_regulation\_of\_cellular\_process | NCOR1 | 2190 | 21 | 1.193447 | -0.773196 | 165 | 216.32 | 1.311030 |
| GO:0007163\_establishment\_or\_maintenance\_of\_cell\_polarity | MYH9 | 23 | 1 | 5.411281 | -0.770268 | 166 | 220.24 | 1.326747 |
| GO:0033036\_macromolecule\_localization | YWHAH | 274 | 4 | 1.816926 | -0.756238 | 167 | 223.26 | 1.336886 |
| GO:0033036\_macromolecule\_localization | SH3GLB1 | 274 | 4 | 1.816926 | -0.756238 | 167 | 223.26 | 1.336886 |
| GO:0033036\_macromolecule\_localization | RAB1 | 274 | 4 | 1.816926 | -0.756238 | 167 | 223.26 | 1.336886 |
| GO:0033036\_macromolecule\_localization | NRIP1 | 274 | 4 | 1.816926 | -0.756238 | 167 | 223.26 | 1.336886 |
| GO:0000280\_nuclear\_division | AKAP8 | 24 | 1 | 5.185811 | -0.753441 | 173 | 228.49 | 1.320751 |
| GO:0006650\_glycerophospholipid\_metabolic\_process | SH3GLB1 | 24 | 1 | 5.185811 | -0.753441 | 173 | 228.49 | 1.320751 |
| GO:0007067\_mitosis | AKAP8 | 24 | 1 | 5.185811 | -0.753441 | 173 | 228.49 | 1.320751 |
| GO:0042632\_cholesterol\_homeostasis | NCOR1 | 24 | 1 | 5.185811 | -0.753441 | 173 | 228.49 | 1.320751 |
| GO:0050679\_positive\_regulation\_of\_epithelial\_cell\_proliferation | FOXP2 | 24 | 1 | 5.185811 | -0.753441 | 173 | 228.49 | 1.320751 |
| GO:0055092\_sterol\_homeostasis | NCOR1 | 24 | 1 | 5.185811 | -0.753441 | 173 | 228.49 | 1.320751 |
| GO:0032990\_cell\_part\_morphogenesis | KLF7 | 184 | 3 | 2.029230 | -0.738844 | 174 | 230.51 | 1.324770 |
| GO:0032990\_cell\_part\_morphogenesis | YWHAH | 184 | 3 | 2.029230 | -0.738844 | 174 | 230.51 | 1.324770 |
| GO:0032990\_cell\_part\_morphogenesis | VAX2 | 184 | 3 | 2.029230 | -0.738844 | 174 | 230.51 | 1.324770 |
| GO:0000087\_M\_phase\_of\_mitotic\_cell\_cycle | AKAP8 | 25 | 1 | 4.978378 | -0.737368 | 176 | 235.46 | 1.337841 |
| GO:0048285\_organelle\_fission | AKAP8 | 25 | 1 | 4.978378 | -0.737368 | 176 | 235.46 | 1.337841 |
| GO:0000003\_reproduction | NHP2L1 | 379 | 5 | 1.641945 | -0.734225 | 177 | 235.89 | 1.332712 |
| GO:0000003\_reproduction | FKBP4 | 379 | 5 | 1.641945 | -0.734225 | 177 | 235.89 | 1.332712 |
| GO:0000003\_reproduction | TIAL1 | 379 | 5 | 1.641945 | -0.734225 | 177 | 235.89 | 1.332712 |
| GO:0000003\_reproduction | SEPP1 | 379 | 5 | 1.641945 | -0.734225 | 177 | 235.89 | 1.332712 |
| GO:0000003\_reproduction | NRIP1 | 379 | 5 | 1.641945 | -0.734225 | 177 | 235.89 | 1.332712 |
| GO:0007548\_sex\_differentiation | FKBP4 | 98 | 2 | 2.539989 | -0.731649 | 178 | 236.8 | 1.330337 |
| GO:0007548\_sex\_differentiation | NRIP1 | 98 | 2 | 2.539989 | -0.731649 | 178 | 236.8 | 1.330337 |
| GO:0007155\_cell\_adhesion | NCAM1 | 186 | 3 | 2.007411 | -0.729274 | 180 | 237.04 | 1.316889 |
| GO:0007155\_cell\_adhesion | MPDZ | 186 | 3 | 2.007411 | -0.729274 | 180 | 237.04 | 1.316889 |
| GO:0007155\_cell\_adhesion | MYH9 | 186 | 3 | 2.007411 | -0.729274 | 180 | 237.04 | 1.316889 |
| GO:0022610\_biological\_adhesion | NCAM1 | 186 | 3 | 2.007411 | -0.729274 | 180 | 237.04 | 1.316889 |
| GO:0022610\_biological\_adhesion | MPDZ | 186 | 3 | 2.007411 | -0.729274 | 180 | 237.04 | 1.316889 |
| GO:0022610\_biological\_adhesion | MYH9 | 186 | 3 | 2.007411 | -0.729274 | 180 | 237.04 | 1.316889 |
| GO:0065007\_biological\_regulation | CYB5R4 | 2593 | 24 | 1.151958 | -0.725776 | 181 | 237.34 | 1.311271 |
| GO:0065007\_biological\_regulation | KLF7 | 2593 | 24 | 1.151958 | -0.725776 | 181 | 237.34 | 1.311271 |
| GO:0065007\_biological\_regulation | RXRB | 2593 | 24 | 1.151958 | -0.725776 | 181 | 237.34 | 1.311271 |
| GO:0065007\_biological\_regulation | FKBP4 | 2593 | 24 | 1.151958 | -0.725776 | 181 | 237.34 | 1.311271 |
| GO:0065007\_biological\_regulation | E2F7 | 2593 | 24 | 1.151958 | -0.725776 | 181 | 237.34 | 1.311271 |
| GO:0065007\_biological\_regulation | LRBA | 2593 | 24 | 1.151958 | -0.725776 | 181 | 237.34 | 1.311271 |
| GO:0065007\_biological\_regulation | TLE4 | 2593 | 24 | 1.151958 | -0.725776 | 181 | 237.34 | 1.311271 |
| GO:0065007\_biological\_regulation | ELAVL1 | 2593 | 24 | 1.151958 | -0.725776 | 181 | 237.34 | 1.311271 |
| GO:0065007\_biological\_regulation | VAX2 | 2593 | 24 | 1.151958 | -0.725776 | 181 | 237.34 | 1.311271 |
| GO:0065007\_biological\_regulation | HES6 | 2593 | 24 | 1.151958 | -0.725776 | 181 | 237.34 | 1.311271 |
| GO:0065007\_biological\_regulation | DACH1 | 2593 | 24 | 1.151958 | -0.725776 | 181 | 237.34 | 1.311271 |
| GO:0065007\_biological\_regulation | MYH9 | 2593 | 24 | 1.151958 | -0.725776 | 181 | 237.34 | 1.311271 |
| GO:0065007\_biological\_regulation | FOXP2 | 2593 | 24 | 1.151958 | -0.725776 | 181 | 237.34 | 1.311271 |
| GO:0065007\_biological\_regulation | NRIP1 | 2593 | 24 | 1.151958 | -0.725776 | 181 | 237.34 | 1.311271 |
| GO:0065007\_biological\_regulation | NCAM1 | 2593 | 24 | 1.151958 | -0.725776 | 181 | 237.34 | 1.311271 |
| GO:0065007\_biological\_regulation | YWHAH | 2593 | 24 | 1.151958 | -0.725776 | 181 | 237.34 | 1.311271 |
| GO:0065007\_biological\_regulation | NCOA2 | 2593 | 24 | 1.151958 | -0.725776 | 181 | 237.34 | 1.311271 |
| GO:0065007\_biological\_regulation | SLC30A1 | 2593 | 24 | 1.151958 | -0.725776 | 181 | 237.34 | 1.311271 |
| GO:0065007\_biological\_regulation | SH3GLB1 | 2593 | 24 | 1.151958 | -0.725776 | 181 | 237.34 | 1.311271 |
| GO:0065007\_biological\_regulation | TIAL1 | 2593 | 24 | 1.151958 | -0.725776 | 181 | 237.34 | 1.311271 |
| GO:0065007\_biological\_regulation | TIA1 | 2593 | 24 | 1.151958 | -0.725776 | 181 | 237.34 | 1.311271 |
| GO:0065007\_biological\_regulation | HEPH | 2593 | 24 | 1.151958 | -0.725776 | 181 | 237.34 | 1.311271 |
| GO:0065007\_biological\_regulation | MAB21L1 | 2593 | 24 | 1.151958 | -0.725776 | 181 | 237.34 | 1.311271 |
| GO:0065007\_biological\_regulation | NCOR1 | 2593 | 24 | 1.151958 | -0.725776 | 181 | 237.34 | 1.311271 |
| GO:0007623\_circadian\_rhythm | NCOR1 | 26 | 1 | 4.786902 | -0.721988 | 183 | 242.23 | 1.323661 |
| GO:0009310\_amine\_catabolic\_process | AHCY | 26 | 1 | 4.786902 | -0.721988 | 183 | 242.23 | 1.323661 |
| GO:0000902\_cell\_morphogenesis | KLF7 | 283 | 4 | 1.759144 | -0.721464 | 184 | 242.31 | 1.316902 |
| GO:0000902\_cell\_morphogenesis | YWHAH | 283 | 4 | 1.759144 | -0.721464 | 184 | 242.31 | 1.316902 |
| GO:0000902\_cell\_morphogenesis | VAX2 | 283 | 4 | 1.759144 | -0.721464 | 184 | 242.31 | 1.316902 |
| GO:0000902\_cell\_morphogenesis | MYH9 | 283 | 4 | 1.759144 | -0.721464 | 184 | 242.31 | 1.316902 |
| GO:0010638\_positive\_regulation\_of\_organelle\_organization | NCOR1 | 27 | 1 | 4.609610 | -0.707249 | 185 | 247.66 | 1.338703 |
| GO:0006470\_protein\_amino\_acid\_dephosphorylation | PPM1M | 28 | 1 | 4.444981 | -0.693104 | 190 | 252.38 | 1.328316 |
| GO:0007127\_meiosis\_I | MYH9 | 28 | 1 | 4.444981 | -0.693104 | 190 | 252.38 | 1.328316 |
| GO:0021549\_cerebellum\_development | FOXP2 | 28 | 1 | 4.444981 | -0.693104 | 190 | 252.38 | 1.328316 |
| GO:0030073\_insulin\_secretion | CYB5R4 | 28 | 1 | 4.444981 | -0.693104 | 190 | 252.38 | 1.328316 |
| GO:0048863\_stem\_cell\_differentiation | MSI2 | 28 | 1 | 4.444981 | -0.693104 | 190 | 252.38 | 1.328316 |
| GO:0046907\_intracellular\_transport | YWHAH | 194 | 3 | 1.924631 | -0.692450 | 191 | 252.54 | 1.322199 |
| GO:0046907\_intracellular\_transport | RAB1 | 194 | 3 | 1.924631 | -0.692450 | 191 | 252.54 | 1.322199 |
| GO:0046907\_intracellular\_transport | LRBA | 194 | 3 | 1.924631 | -0.692450 | 191 | 252.54 | 1.322199 |
| GO:0048872\_homeostasis\_of\_number\_of\_cells | HEPH | 105 | 2 | 2.370656 | -0.686067 | 192 | 253.55 | 1.320573 |
| GO:0048872\_homeostasis\_of\_number\_of\_cells | NCOR1 | 105 | 2 | 2.370656 | -0.686067 | 192 | 253.55 | 1.320573 |
| GO:0006417\_regulation\_of\_translation | TIA1 | 29 | 1 | 4.291705 | -0.679511 | 196 | 257.94 | 1.316020 |
| GO:0044270\_nitrogen\_compound\_catabolic\_process | AHCY | 29 | 1 | 4.291705 | -0.679511 | 196 | 257.94 | 1.316020 |
| GO:0051301\_cell\_division | TIAL1 | 29 | 1 | 4.291705 | -0.679511 | 196 | 257.94 | 1.316020 |
| GO:0060041\_retina\_development\_in\_camera-type\_eye | VAX2 | 29 | 1 | 4.291705 | -0.679511 | 196 | 257.94 | 1.316020 |
| GO:0031175\_neuron\_projection\_development | KLF7 | 197 | 3 | 1.895322 | -0.679214 | 197 | 258.25 | 1.310914 |
| GO:0031175\_neuron\_projection\_development | YWHAH | 197 | 3 | 1.895322 | -0.679214 | 197 | 258.25 | 1.310914 |
| GO:0031175\_neuron\_projection\_development | VAX2 | 197 | 3 | 1.895322 | -0.679214 | 197 | 258.25 | 1.310914 |
| GO:0051179\_localization | SLC8A3 | 1058 | 11 | 1.294002 | -0.672779 | 198 | 259.35 | 1.309848 |
| GO:0051179\_localization | CYB5R4 | 1058 | 11 | 1.294002 | -0.672779 | 198 | 259.35 | 1.309848 |
| GO:0051179\_localization | KLF7 | 1058 | 11 | 1.294002 | -0.672779 | 198 | 259.35 | 1.309848 |
| GO:0051179\_localization | YWHAH | 1058 | 11 | 1.294002 | -0.672779 | 198 | 259.35 | 1.309848 |
| GO:0051179\_localization | SH3GLB1 | 1058 | 11 | 1.294002 | -0.672779 | 198 | 259.35 | 1.309848 |
| GO:0051179\_localization | RAB1 | 1058 | 11 | 1.294002 | -0.672779 | 198 | 259.35 | 1.309848 |
| GO:0051179\_localization | FKBP4 | 1058 | 11 | 1.294002 | -0.672779 | 198 | 259.35 | 1.309848 |
| GO:0051179\_localization | LRBA | 1058 | 11 | 1.294002 | -0.672779 | 198 | 259.35 | 1.309848 |
| GO:0051179\_localization | HEPH | 1058 | 11 | 1.294002 | -0.672779 | 198 | 259.35 | 1.309848 |
| GO:0051179\_localization | MYH9 | 1058 | 11 | 1.294002 | -0.672779 | 198 | 259.35 | 1.309848 |
| GO:0051179\_localization | NRIP1 | 1058 | 11 | 1.294002 | -0.672779 | 198 | 259.35 | 1.309848 |
| GO:0030154\_cell\_differentiation | CYB5R4 | 1060 | 11 | 1.291560 | -0.668919 | 199 | 259.8 | 1.305528 |
| GO:0030154\_cell\_differentiation | KLF7 | 1060 | 11 | 1.291560 | -0.668919 | 199 | 259.8 | 1.305528 |
| GO:0030154\_cell\_differentiation | YWHAH | 1060 | 11 | 1.291560 | -0.668919 | 199 | 259.8 | 1.305528 |
| GO:0030154\_cell\_differentiation | RXRB | 1060 | 11 | 1.291560 | -0.668919 | 199 | 259.8 | 1.305528 |
| GO:0030154\_cell\_differentiation | TIAL1 | 1060 | 11 | 1.291560 | -0.668919 | 199 | 259.8 | 1.305528 |
| GO:0030154\_cell\_differentiation | MSI2 | 1060 | 11 | 1.291560 | -0.668919 | 199 | 259.8 | 1.305528 |
| GO:0030154\_cell\_differentiation | VAX2 | 1060 | 11 | 1.291560 | -0.668919 | 199 | 259.8 | 1.305528 |
| GO:0030154\_cell\_differentiation | HEPH | 1060 | 11 | 1.291560 | -0.668919 | 199 | 259.8 | 1.305528 |
| GO:0030154\_cell\_differentiation | HES6 | 1060 | 11 | 1.291560 | -0.668919 | 199 | 259.8 | 1.305528 |
| GO:0030154\_cell\_differentiation | MYH9 | 1060 | 11 | 1.291560 | -0.668919 | 199 | 259.8 | 1.305528 |
| GO:0030154\_cell\_differentiation | NCOR1 | 1060 | 11 | 1.291560 | -0.668919 | 199 | 259.8 | 1.305528 |
| GO:0030099\_myeloid\_cell\_differentiation | HEPH | 108 | 2 | 2.304805 | -0.667738 | 200 | 260.1 | 1.300500 |
| GO:0030099\_myeloid\_cell\_differentiation | NCOR1 | 108 | 2 | 2.304805 | -0.667738 | 200 | 260.1 | 1.300500 |
| GO:0033500\_carbohydrate\_homeostasis | CYB5R4 | 30 | 1 | 4.148649 | -0.666433 | 203 | 264.74 | 1.304138 |
| GO:0042593\_glucose\_homeostasis | CYB5R4 | 30 | 1 | 4.148649 | -0.666433 | 203 | 264.74 | 1.304138 |
| GO:0051146\_striated\_muscle\_cell\_differentiation | RXRB | 30 | 1 | 4.148649 | -0.666433 | 203 | 264.74 | 1.304138 |
| GO:0016311\_dephosphorylation | PPM1M | 31 | 1 | 4.014821 | -0.653836 | 208 | 272.77 | 1.311394 |
| GO:0046632\_alpha-beta\_T\_cell\_differentiation | NCOR1 | 31 | 1 | 4.014821 | -0.653836 | 208 | 272.77 | 1.311394 |
| GO:0048562\_embryonic\_organ\_morphogenesis | VAX2 | 31 | 1 | 4.014821 | -0.653836 | 208 | 272.77 | 1.311394 |
| GO:0051640\_organelle\_localization | MYH9 | 31 | 1 | 4.014821 | -0.653836 | 208 | 272.77 | 1.311394 |
| GO:0055088\_lipid\_homeostasis | NCOR1 | 31 | 1 | 4.014821 | -0.653836 | 208 | 272.77 | 1.311394 |
| GO:0007399\_nervous\_system\_development | KLF7 | 621 | 7 | 1.402925 | -0.649236 | 209 | 273.24 | 1.307368 |
| GO:0007399\_nervous\_system\_development | YWHAH | 621 | 7 | 1.402925 | -0.649236 | 209 | 273.24 | 1.307368 |
| GO:0007399\_nervous\_system\_development | VAX2 | 621 | 7 | 1.402925 | -0.649236 | 209 | 273.24 | 1.307368 |
| GO:0007399\_nervous\_system\_development | HES6 | 621 | 7 | 1.402925 | -0.649236 | 209 | 273.24 | 1.307368 |
| GO:0007399\_nervous\_system\_development | SEPP1 | 621 | 7 | 1.402925 | -0.649236 | 209 | 273.24 | 1.307368 |
| GO:0007399\_nervous\_system\_development | NCOR1 | 621 | 7 | 1.402925 | -0.649236 | 209 | 273.24 | 1.307368 |
| GO:0007399\_nervous\_system\_development | FOXP2 | 621 | 7 | 1.402925 | -0.649236 | 209 | 273.24 | 1.307368 |
| GO:0050768\_negative\_regulation\_of\_neurogenesis | YWHAH | 32 | 1 | 3.889358 | -0.641689 | 210 | 277.25 | 1.320238 |
| GO:0032989\_cellular\_component\_morphogenesis | KLF7 | 307 | 4 | 1.621622 | -0.636912 | 211 | 278.09 | 1.317962 |
| GO:0032989\_cellular\_component\_morphogenesis | YWHAH | 307 | 4 | 1.621622 | -0.636912 | 211 | 278.09 | 1.317962 |
| GO:0032989\_cellular\_component\_morphogenesis | VAX2 | 307 | 4 | 1.621622 | -0.636912 | 211 | 278.09 | 1.317962 |
| GO:0032989\_cellular\_component\_morphogenesis | MYH9 | 307 | 4 | 1.621622 | -0.636912 | 211 | 278.09 | 1.317962 |
| GO:0008284\_positive\_regulation\_of\_cell\_proliferation | TIAL1 | 208 | 3 | 1.795088 | -0.633163 | 212 | 278.65 | 1.314387 |
| GO:0008284\_positive\_regulation\_of\_cell\_proliferation | MAB21L1 | 208 | 3 | 1.795088 | -0.633163 | 212 | 278.65 | 1.314387 |
| GO:0008284\_positive\_regulation\_of\_cell\_proliferation | FOXP2 | 208 | 3 | 1.795088 | -0.633163 | 212 | 278.65 | 1.314387 |
| GO:0007565\_female\_pregnancy | FKBP4 | 33 | 1 | 3.771499 | -0.629964 | 215 | 282.39 | 1.313442 |
| GO:0021536\_diencephalon\_development | NCOR1 | 33 | 1 | 3.771499 | -0.629964 | 215 | 282.39 | 1.313442 |
| GO:0022037\_metencephalon\_development | FOXP2 | 33 | 1 | 3.771499 | -0.629964 | 215 | 282.39 | 1.313442 |
| GO:0048608\_reproductive\_structure\_development | FKBP4 | 116 | 2 | 2.145853 | -0.622015 | 216 | 283.35 | 1.311806 |
| GO:0048608\_reproductive\_structure\_development | NRIP1 | 116 | 2 | 2.145853 | -0.622015 | 216 | 283.35 | 1.311806 |
| GO:0007338\_single\_fertilization | NHP2L1 | 34 | 1 | 3.660572 | -0.618636 | 220 | 287.12 | 1.305091 |
| GO:0010721\_negative\_regulation\_of\_cell\_development | YWHAH | 34 | 1 | 3.660572 | -0.618636 | 220 | 287.12 | 1.305091 |
| GO:0016054\_organic\_acid\_catabolic\_process | AHCY | 34 | 1 | 3.660572 | -0.618636 | 220 | 287.12 | 1.305091 |
| GO:0046395\_carboxylic\_acid\_catabolic\_process | AHCY | 34 | 1 | 3.660572 | -0.618636 | 220 | 287.12 | 1.305091 |
| GO:0048519\_negative\_regulation\_of\_biological\_process | YWHAH | 859 | 9 | 1.303999 | -0.616675 | 221 | 287.57 | 1.301222 |
| GO:0048519\_negative\_regulation\_of\_biological\_process | NCOA2 | 859 | 9 | 1.303999 | -0.616675 | 221 | 287.57 | 1.301222 |
| GO:0048519\_negative\_regulation\_of\_biological\_process | E2F7 | 859 | 9 | 1.303999 | -0.616675 | 221 | 287.57 | 1.301222 |
| GO:0048519\_negative\_regulation\_of\_biological\_process | TIA1 | 859 | 9 | 1.303999 | -0.616675 | 221 | 287.57 | 1.301222 |
| GO:0048519\_negative\_regulation\_of\_biological\_process | TLE4 | 859 | 9 | 1.303999 | -0.616675 | 221 | 287.57 | 1.301222 |
| GO:0048519\_negative\_regulation\_of\_biological\_process | VAX2 | 859 | 9 | 1.303999 | -0.616675 | 221 | 287.57 | 1.301222 |
| GO:0048519\_negative\_regulation\_of\_biological\_process | NCOR1 | 859 | 9 | 1.303999 | -0.616675 | 221 | 287.57 | 1.301222 |
| GO:0048519\_negative\_regulation\_of\_biological\_process | NRIP1 | 859 | 9 | 1.303999 | -0.616675 | 221 | 287.57 | 1.301222 |
| GO:0048519\_negative\_regulation\_of\_biological\_process | FOXP2 | 859 | 9 | 1.303999 | -0.616675 | 221 | 287.57 | 1.301222 |
| GO:0007292\_female\_gamete\_generation | NRIP1 | 35 | 1 | 3.555985 | -0.607682 | 222 | 291.46 | 1.312883 |
| GO:0022403\_cell\_cycle\_phase | AKAP8 | 119 | 2 | 2.091756 | -0.605955 | 223 | 291.64 | 1.307803 |
| GO:0022403\_cell\_cycle\_phase | MYH9 | 119 | 2 | 2.091756 | -0.605955 | 223 | 291.64 | 1.307803 |
| GO:0040007\_growth | SEPP1 | 217 | 3 | 1.720638 | -0.598164 | 224 | 292.85 | 1.307366 |
| GO:0040007\_growth | NCOR1 | 217 | 3 | 1.720638 | -0.598164 | 224 | 292.85 | 1.307366 |
| GO:0040007\_growth | FOXP2 | 217 | 3 | 1.720638 | -0.598164 | 224 | 292.85 | 1.307366 |
| GO:0022602\_ovulation\_cycle\_process | NRIP1 | 36 | 1 | 3.457207 | -0.597081 | 226 | 297.07 | 1.314469 |
| GO:0030072\_peptide\_hormone\_secretion | CYB5R4 | 36 | 1 | 3.457207 | -0.597081 | 226 | 297.07 | 1.314469 |
| GO:0007423\_sensory\_organ\_development | VAX2 | 219 | 3 | 1.704924 | -0.590691 | 227 | 298.46 | 1.314802 |
| GO:0007423\_sensory\_organ\_development | MAB21L1 | 219 | 3 | 1.704924 | -0.590691 | 227 | 298.46 | 1.314802 |
| GO:0007423\_sensory\_organ\_development | FOXP2 | 219 | 3 | 1.704924 | -0.590691 | 227 | 298.46 | 1.314802 |
| GO:0006886\_intracellular\_protein\_transport | YWHAH | 122 | 2 | 2.040319 | -0.590440 | 229 | 299.64 | 1.308472 |
| GO:0006886\_intracellular\_protein\_transport | RAB1 | 122 | 2 | 2.040319 | -0.590440 | 229 | 299.64 | 1.308472 |
| GO:0030001\_metal\_ion\_transport | SLC8A3 | 122 | 2 | 2.040319 | -0.590440 | 229 | 299.64 | 1.308472 |
| GO:0030001\_metal\_ion\_transport | HEPH | 122 | 2 | 2.040319 | -0.590440 | 229 | 299.64 | 1.308472 |
| GO:0002790\_peptide\_secretion | CYB5R4 | 37 | 1 | 3.363769 | -0.586813 | 232 | 302.78 | 1.305086 |
| GO:0042698\_ovulation\_cycle | NRIP1 | 37 | 1 | 3.363769 | -0.586813 | 232 | 302.78 | 1.305086 |
| GO:0050906\_detection\_of\_stimulus\_involved\_in\_sensory\_perception | SLC12A2 | 37 | 1 | 3.363769 | -0.586813 | 232 | 302.78 | 1.305086 |
| GO:0010975\_regulation\_of\_neuron\_projection\_development | YWHAH | 38 | 1 | 3.275249 | -0.576860 | 236 | 308.66 | 1.307881 |
| GO:0016053\_organic\_acid\_biosynthetic\_process | SH3GLB1 | 38 | 1 | 3.275249 | -0.576860 | 236 | 308.66 | 1.307881 |
| GO:0031401\_positive\_regulation\_of\_protein\_modification\_process | NCOR1 | 38 | 1 | 3.275249 | -0.576860 | 236 | 308.66 | 1.307881 |
| GO:0046394\_carboxylic\_acid\_biosynthetic\_process | SH3GLB1 | 38 | 1 | 3.275249 | -0.576860 | 236 | 308.66 | 1.307881 |
| GO:0048468\_cell\_development | CYB5R4 | 654 | 7 | 1.332135 | -0.575167 | 237 | 309.7 | 1.306751 |
| GO:0048468\_cell\_development | KLF7 | 654 | 7 | 1.332135 | -0.575167 | 237 | 309.7 | 1.306751 |
| GO:0048468\_cell\_development | YWHAH | 654 | 7 | 1.332135 | -0.575167 | 237 | 309.7 | 1.306751 |
| GO:0048468\_cell\_development | TIAL1 | 654 | 7 | 1.332135 | -0.575167 | 237 | 309.7 | 1.306751 |
| GO:0048468\_cell\_development | MSI2 | 654 | 7 | 1.332135 | -0.575167 | 237 | 309.7 | 1.306751 |
| GO:0048468\_cell\_development | VAX2 | 654 | 7 | 1.332135 | -0.575167 | 237 | 309.7 | 1.306751 |
| GO:0048468\_cell\_development | MYH9 | 654 | 7 | 1.332135 | -0.575167 | 237 | 309.7 | 1.306751 |
| GO:0048869\_cellular\_developmental\_process | CYB5R4 | 1113 | 11 | 1.230058 | -0.572917 | 238 | 309.94 | 1.302269 |
| GO:0048869\_cellular\_developmental\_process | KLF7 | 1113 | 11 | 1.230058 | -0.572917 | 238 | 309.94 | 1.302269 |
| GO:0048869\_cellular\_developmental\_process | YWHAH | 1113 | 11 | 1.230058 | -0.572917 | 238 | 309.94 | 1.302269 |
| GO:0048869\_cellular\_developmental\_process | RXRB | 1113 | 11 | 1.230058 | -0.572917 | 238 | 309.94 | 1.302269 |
| GO:0048869\_cellular\_developmental\_process | TIAL1 | 1113 | 11 | 1.230058 | -0.572917 | 238 | 309.94 | 1.302269 |
| GO:0048869\_cellular\_developmental\_process | MSI2 | 1113 | 11 | 1.230058 | -0.572917 | 238 | 309.94 | 1.302269 |
| GO:0048869\_cellular\_developmental\_process | VAX2 | 1113 | 11 | 1.230058 | -0.572917 | 238 | 309.94 | 1.302269 |
| GO:0048869\_cellular\_developmental\_process | HEPH | 1113 | 11 | 1.230058 | -0.572917 | 238 | 309.94 | 1.302269 |
| GO:0048869\_cellular\_developmental\_process | HES6 | 1113 | 11 | 1.230058 | -0.572917 | 238 | 309.94 | 1.302269 |
| GO:0048869\_cellular\_developmental\_process | MYH9 | 1113 | 11 | 1.230058 | -0.572917 | 238 | 309.94 | 1.302269 |
| GO:0048869\_cellular\_developmental\_process | NCOR1 | 1113 | 11 | 1.230058 | -0.572917 | 238 | 309.94 | 1.302269 |
| GO:0006644\_phospholipid\_metabolic\_process | SH3GLB1 | 39 | 1 | 3.191268 | -0.567206 | 239 | 313.67 | 1.312427 |
| GO:0009987\_cellular\_process | CYB5R4 | 3868 | 33 | 1.061831 | -0.566038 | 240 | 314.03 | 1.308458 |
| GO:0009987\_cellular\_process | AHCY | 3868 | 33 | 1.061831 | -0.566038 | 240 | 314.03 | 1.308458 |
| GO:0009987\_cellular\_process | FKBP4 | 3868 | 33 | 1.061831 | -0.566038 | 240 | 314.03 | 1.308458 |
| GO:0009987\_cellular\_process | E2F7 | 3868 | 33 | 1.061831 | -0.566038 | 240 | 314.03 | 1.308458 |
| GO:0009987\_cellular\_process | LRBA | 3868 | 33 | 1.061831 | -0.566038 | 240 | 314.03 | 1.308458 |
| GO:0009987\_cellular\_process | SH3GLB1 | 3868 | 33 | 1.061831 | -0.566038 | 240 | 314.03 | 1.308458 |
| GO:0009987\_cellular\_process | SLC30A1 | 3868 | 33 | 1.061831 | -0.566038 | 240 | 314.03 | 1.308458 |
| GO:0009987\_cellular\_process | CRISPLD2 | 3868 | 33 | 1.061831 | -0.566038 | 240 | 314.03 | 1.308458 |
| GO:0009987\_cellular\_process | TIA1 | 3868 | 33 | 1.061831 | -0.566038 | 240 | 314.03 | 1.308458 |
| GO:0009987\_cellular\_process | MSI2 | 3868 | 33 | 1.061831 | -0.566038 | 240 | 314.03 | 1.308458 |
| GO:0009987\_cellular\_process | SEPP1 | 3868 | 33 | 1.061831 | -0.566038 | 240 | 314.03 | 1.308458 |
| GO:0009987\_cellular\_process | KLF7 | 3868 | 33 | 1.061831 | -0.566038 | 240 | 314.03 | 1.308458 |
| GO:0009987\_cellular\_process | RXRB | 3868 | 33 | 1.061831 | -0.566038 | 240 | 314.03 | 1.308458 |
| GO:0009987\_cellular\_process | MPDZ | 3868 | 33 | 1.061831 | -0.566038 | 240 | 314.03 | 1.308458 |
| GO:0009987\_cellular\_process | UBE2F | 3868 | 33 | 1.061831 | -0.566038 | 240 | 314.03 | 1.308458 |
| GO:0009987\_cellular\_process | ELAVL1 | 3868 | 33 | 1.061831 | -0.566038 | 240 | 314.03 | 1.308458 |
| GO:0009987\_cellular\_process | TLE4 | 3868 | 33 | 1.061831 | -0.566038 | 240 | 314.03 | 1.308458 |
| GO:0009987\_cellular\_process | VAX2 | 3868 | 33 | 1.061831 | -0.566038 | 240 | 314.03 | 1.308458 |
| GO:0009987\_cellular\_process | DACH1 | 3868 | 33 | 1.061831 | -0.566038 | 240 | 314.03 | 1.308458 |
| GO:0009987\_cellular\_process | HES6 | 3868 | 33 | 1.061831 | -0.566038 | 240 | 314.03 | 1.308458 |
| GO:0009987\_cellular\_process | MYH9 | 3868 | 33 | 1.061831 | -0.566038 | 240 | 314.03 | 1.308458 |
| GO:0009987\_cellular\_process | FOXP2 | 3868 | 33 | 1.061831 | -0.566038 | 240 | 314.03 | 1.308458 |
| GO:0009987\_cellular\_process | NRIP1 | 3868 | 33 | 1.061831 | -0.566038 | 240 | 314.03 | 1.308458 |
| GO:0009987\_cellular\_process | NCAM1 | 3868 | 33 | 1.061831 | -0.566038 | 240 | 314.03 | 1.308458 |
| GO:0009987\_cellular\_process | YWHAH | 3868 | 33 | 1.061831 | -0.566038 | 240 | 314.03 | 1.308458 |
| GO:0009987\_cellular\_process | NCOA2 | 3868 | 33 | 1.061831 | -0.566038 | 240 | 314.03 | 1.308458 |
| GO:0009987\_cellular\_process | RAB1 | 3868 | 33 | 1.061831 | -0.566038 | 240 | 314.03 | 1.308458 |
| GO:0009987\_cellular\_process | TIAL1 | 3868 | 33 | 1.061831 | -0.566038 | 240 | 314.03 | 1.308458 |
| GO:0009987\_cellular\_process | HEPH | 3868 | 33 | 1.061831 | -0.566038 | 240 | 314.03 | 1.308458 |
| GO:0009987\_cellular\_process | PPM1M | 3868 | 33 | 1.061831 | -0.566038 | 240 | 314.03 | 1.308458 |
| GO:0009987\_cellular\_process | AKAP8 | 3868 | 33 | 1.061831 | -0.566038 | 240 | 314.03 | 1.308458 |
| GO:0009987\_cellular\_process | MAB21L1 | 3868 | 33 | 1.061831 | -0.566038 | 240 | 314.03 | 1.308458 |
| GO:0009987\_cellular\_process | NCOR1 | 3868 | 33 | 1.061831 | -0.566038 | 240 | 314.03 | 1.308458 |
| GO:0016071\_mRNA\_metabolic\_process | ELAVL1 | 40 | 1 | 3.111486 | -0.557836 | 242 | 320.09 | 1.322686 |
| GO:0051129\_negative\_regulation\_of\_cellular\_component\_organization | YWHAH | 40 | 1 | 3.111486 | -0.557836 | 242 | 320.09 | 1.322686 |
| GO:0051276\_chromosome\_organization | AKAP8 | 129 | 2 | 1.929604 | -0.556208 | 243 | 320.47 | 1.318807 |
| GO:0051276\_chromosome\_organization | NCOR1 | 129 | 2 | 1.929604 | -0.556208 | 243 | 320.47 | 1.318807 |
| GO:0008585\_female\_gonad\_development | NRIP1 | 41 | 1 | 3.035597 | -0.548735 | 246 | 327.32 | 1.330569 |
| GO:0015833\_peptide\_transport | CYB5R4 | 41 | 1 | 3.035597 | -0.548735 | 246 | 327.32 | 1.330569 |
| GO:0031344\_regulation\_of\_cell\_projection\_organization | YWHAH | 41 | 1 | 3.035597 | -0.548735 | 246 | 327.32 | 1.330569 |
| GO:0048522\_positive\_regulation\_of\_cellular\_process | NCAM1 | 895 | 9 | 1.251548 | -0.548707 | 247 | 327.46 | 1.325749 |
| GO:0048522\_positive\_regulation\_of\_cellular\_process | KLF7 | 895 | 9 | 1.251548 | -0.548707 | 247 | 327.46 | 1.325749 |
| GO:0048522\_positive\_regulation\_of\_cellular\_process | NCOA2 | 895 | 9 | 1.251548 | -0.548707 | 247 | 327.46 | 1.325749 |
| GO:0048522\_positive\_regulation\_of\_cellular\_process | RXRB | 895 | 9 | 1.251548 | -0.548707 | 247 | 327.46 | 1.325749 |
| GO:0048522\_positive\_regulation\_of\_cellular\_process | SH3GLB1 | 895 | 9 | 1.251548 | -0.548707 | 247 | 327.46 | 1.325749 |
| GO:0048522\_positive\_regulation\_of\_cellular\_process | TIAL1 | 895 | 9 | 1.251548 | -0.548707 | 247 | 327.46 | 1.325749 |
| GO:0048522\_positive\_regulation\_of\_cellular\_process | MAB21L1 | 895 | 9 | 1.251548 | -0.548707 | 247 | 327.46 | 1.325749 |
| GO:0048522\_positive\_regulation\_of\_cellular\_process | NCOR1 | 895 | 9 | 1.251548 | -0.548707 | 247 | 327.46 | 1.325749 |
| GO:0048522\_positive\_regulation\_of\_cellular\_process | FOXP2 | 895 | 9 | 1.251548 | -0.548707 | 247 | 327.46 | 1.325749 |
| GO:0007420\_brain\_development | SEPP1 | 231 | 3 | 1.616357 | -0.548012 | 248 | 327.61 | 1.321008 |
| GO:0007420\_brain\_development | NCOR1 | 231 | 3 | 1.616357 | -0.548012 | 248 | 327.61 | 1.321008 |
| GO:0007420\_brain\_development | FOXP2 | 231 | 3 | 1.616357 | -0.548012 | 248 | 327.61 | 1.321008 |
| GO:0010769\_regulation\_of\_cell\_morphogenesis\_involved\_in\_differentiation | YWHAH | 42 | 1 | 2.963320 | -0.539891 | 249 | 333.32 | 1.338635 |
| GO:0044237\_cellular\_metabolic\_process | KLF7 | 1974 | 18 | 1.134889 | -0.536623 | 250 | 334.94 | 1.339760 |
| GO:0044237\_cellular\_metabolic\_process | AHCY | 1974 | 18 | 1.134889 | -0.536623 | 250 | 334.94 | 1.339760 |
| GO:0044237\_cellular\_metabolic\_process | RXRB | 1974 | 18 | 1.134889 | -0.536623 | 250 | 334.94 | 1.339760 |
| GO:0044237\_cellular\_metabolic\_process | E2F7 | 1974 | 18 | 1.134889 | -0.536623 | 250 | 334.94 | 1.339760 |
| GO:0044237\_cellular\_metabolic\_process | UBE2F | 1974 | 18 | 1.134889 | -0.536623 | 250 | 334.94 | 1.339760 |
| GO:0044237\_cellular\_metabolic\_process | ELAVL1 | 1974 | 18 | 1.134889 | -0.536623 | 250 | 334.94 | 1.339760 |
| GO:0044237\_cellular\_metabolic\_process | TLE4 | 1974 | 18 | 1.134889 | -0.536623 | 250 | 334.94 | 1.339760 |
| GO:0044237\_cellular\_metabolic\_process | VAX2 | 1974 | 18 | 1.134889 | -0.536623 | 250 | 334.94 | 1.339760 |
| GO:0044237\_cellular\_metabolic\_process | HES6 | 1974 | 18 | 1.134889 | -0.536623 | 250 | 334.94 | 1.339760 |
| GO:0044237\_cellular\_metabolic\_process | DACH1 | 1974 | 18 | 1.134889 | -0.536623 | 250 | 334.94 | 1.339760 |
| GO:0044237\_cellular\_metabolic\_process | FOXP2 | 1974 | 18 | 1.134889 | -0.536623 | 250 | 334.94 | 1.339760 |
| GO:0044237\_cellular\_metabolic\_process | NRIP1 | 1974 | 18 | 1.134889 | -0.536623 | 250 | 334.94 | 1.339760 |
| GO:0044237\_cellular\_metabolic\_process | NCOA2 | 1974 | 18 | 1.134889 | -0.536623 | 250 | 334.94 | 1.339760 |
| GO:0044237\_cellular\_metabolic\_process | SH3GLB1 | 1974 | 18 | 1.134889 | -0.536623 | 250 | 334.94 | 1.339760 |
| GO:0044237\_cellular\_metabolic\_process | TIA1 | 1974 | 18 | 1.134889 | -0.536623 | 250 | 334.94 | 1.339760 |
| GO:0044237\_cellular\_metabolic\_process | PPM1M | 1974 | 18 | 1.134889 | -0.536623 | 250 | 334.94 | 1.339760 |
| GO:0044237\_cellular\_metabolic\_process | SEPP1 | 1974 | 18 | 1.134889 | -0.536623 | 250 | 334.94 | 1.339760 |
| GO:0044237\_cellular\_metabolic\_process | NCOR1 | 1974 | 18 | 1.134889 | -0.536623 | 250 | 334.94 | 1.339760 |
| GO:0009582\_detection\_of\_abiotic\_stimulus | SLC12A2 | 43 | 1 | 2.894406 | -0.531290 | 254 | 340.57 | 1.340827 |
| GO:0019637\_organophosphate\_metabolic\_process | SH3GLB1 | 43 | 1 | 2.894406 | -0.531290 | 254 | 340.57 | 1.340827 |
| GO:0032446\_protein\_modification\_by\_small\_protein\_conjugation | UBE2F | 43 | 1 | 2.894406 | -0.531290 | 254 | 340.57 | 1.340827 |
| GO:0046879\_hormone\_secretion | CYB5R4 | 43 | 1 | 2.894406 | -0.531290 | 254 | 340.57 | 1.340827 |
| GO:0009914\_hormone\_transport | CYB5R4 | 44 | 1 | 2.828624 | -0.522923 | 256 | 347.41 | 1.357070 |
| GO:0046545\_development\_of\_primary\_female\_sexual\_characteristics | NRIP1 | 44 | 1 | 2.828624 | -0.522923 | 256 | 347.41 | 1.357070 |
| GO:0050789\_regulation\_of\_biological\_process | KLF7 | 2357 | 21 | 1.108888 | -0.517659 | 257 | 348.07 | 1.354358 |
| GO:0050789\_regulation\_of\_biological\_process | RXRB | 2357 | 21 | 1.108888 | -0.517659 | 257 | 348.07 | 1.354358 |
| GO:0050789\_regulation\_of\_biological\_process | FKBP4 | 2357 | 21 | 1.108888 | -0.517659 | 257 | 348.07 | 1.354358 |
| GO:0050789\_regulation\_of\_biological\_process | E2F7 | 2357 | 21 | 1.108888 | -0.517659 | 257 | 348.07 | 1.354358 |
| GO:0050789\_regulation\_of\_biological\_process | LRBA | 2357 | 21 | 1.108888 | -0.517659 | 257 | 348.07 | 1.354358 |
| GO:0050789\_regulation\_of\_biological\_process | TLE4 | 2357 | 21 | 1.108888 | -0.517659 | 257 | 348.07 | 1.354358 |
| GO:0050789\_regulation\_of\_biological\_process | ELAVL1 | 2357 | 21 | 1.108888 | -0.517659 | 257 | 348.07 | 1.354358 |
| GO:0050789\_regulation\_of\_biological\_process | VAX2 | 2357 | 21 | 1.108888 | -0.517659 | 257 | 348.07 | 1.354358 |
| GO:0050789\_regulation\_of\_biological\_process | HES6 | 2357 | 21 | 1.108888 | -0.517659 | 257 | 348.07 | 1.354358 |
| GO:0050789\_regulation\_of\_biological\_process | DACH1 | 2357 | 21 | 1.108888 | -0.517659 | 257 | 348.07 | 1.354358 |
| GO:0050789\_regulation\_of\_biological\_process | MYH9 | 2357 | 21 | 1.108888 | -0.517659 | 257 | 348.07 | 1.354358 |
| GO:0050789\_regulation\_of\_biological\_process | FOXP2 | 2357 | 21 | 1.108888 | -0.517659 | 257 | 348.07 | 1.354358 |
| GO:0050789\_regulation\_of\_biological\_process | NRIP1 | 2357 | 21 | 1.108888 | -0.517659 | 257 | 348.07 | 1.354358 |
| GO:0050789\_regulation\_of\_biological\_process | NCAM1 | 2357 | 21 | 1.108888 | -0.517659 | 257 | 348.07 | 1.354358 |
| GO:0050789\_regulation\_of\_biological\_process | YWHAH | 2357 | 21 | 1.108888 | -0.517659 | 257 | 348.07 | 1.354358 |
| GO:0050789\_regulation\_of\_biological\_process | NCOA2 | 2357 | 21 | 1.108888 | -0.517659 | 257 | 348.07 | 1.354358 |
| GO:0050789\_regulation\_of\_biological\_process | SH3GLB1 | 2357 | 21 | 1.108888 | -0.517659 | 257 | 348.07 | 1.354358 |
| GO:0050789\_regulation\_of\_biological\_process | TIAL1 | 2357 | 21 | 1.108888 | -0.517659 | 257 | 348.07 | 1.354358 |
| GO:0050789\_regulation\_of\_biological\_process | TIA1 | 2357 | 21 | 1.108888 | -0.517659 | 257 | 348.07 | 1.354358 |
| GO:0050789\_regulation\_of\_biological\_process | MAB21L1 | 2357 | 21 | 1.108888 | -0.517659 | 257 | 348.07 | 1.354358 |
| GO:0050789\_regulation\_of\_biological\_process | NCOR1 | 2357 | 21 | 1.108888 | -0.517659 | 257 | 348.07 | 1.354358 |
| GO:0043623\_cellular\_protein\_complex\_assembly | FKBP4 | 45 | 1 | 2.765766 | -0.514778 | 258 | 350.39 | 1.358101 |
| GO:0016044\_membrane\_organization | SH3GLB1 | 140 | 2 | 1.777992 | -0.507397 | 259 | 351.48 | 1.357066 |
| GO:0016044\_membrane\_organization | MYH9 | 140 | 2 | 1.777992 | -0.507397 | 259 | 351.48 | 1.357066 |
| GO:0007612\_learning | FOXP2 | 46 | 1 | 2.705640 | -0.506846 | 263 | 354.13 | 1.346502 |
| GO:0009581\_detection\_of\_external\_stimulus | SLC12A2 | 46 | 1 | 2.705640 | -0.506846 | 263 | 354.13 | 1.346502 |
| GO:0030850\_prostate\_gland\_development | FKBP4 | 46 | 1 | 2.705640 | -0.506846 | 263 | 354.13 | 1.346502 |
| GO:0046631\_alpha-beta\_T\_cell\_activation | NCOR1 | 46 | 1 | 2.705640 | -0.506846 | 263 | 354.13 | 1.346502 |
| GO:0003006\_reproductive\_developmental\_process | FKBP4 | 141 | 2 | 1.765382 | -0.503233 | 264 | 355.0 | 1.344697 |
| GO:0003006\_reproductive\_developmental\_process | NRIP1 | 141 | 2 | 1.765382 | -0.503233 | 264 | 355.0 | 1.344697 |
| GO:0016570\_histone\_modification | NCOR1 | 47 | 1 | 2.648074 | -0.499117 | 265 | 358.66 | 1.353434 |
| GO:0032269\_negative\_regulation\_of\_cellular\_protein\_metabolic\_process | TIA1 | 48 | 1 | 2.592905 | -0.491582 | 266 | 361.77 | 1.360038 |
| GO:0008104\_protein\_localization | YWHAH | 251 | 3 | 1.487563 | -0.484306 | 267 | 362.97 | 1.359438 |
| GO:0008104\_protein\_localization | SH3GLB1 | 251 | 3 | 1.487563 | -0.484306 | 267 | 362.97 | 1.359438 |
| GO:0008104\_protein\_localization | RAB1 | 251 | 3 | 1.487563 | -0.484306 | 267 | 362.97 | 1.359438 |
| GO:0042035\_regulation\_of\_cytokine\_biosynthetic\_process | TIA1 | 49 | 1 | 2.539989 | -0.484234 | 272 | 366.16 | 1.346176 |
| GO:0043473\_pigmentation | DOCK7 | 49 | 1 | 2.539989 | -0.484234 | 272 | 366.16 | 1.346176 |
| GO:0046660\_female\_sex\_differentiation | NRIP1 | 49 | 1 | 2.539989 | -0.484234 | 272 | 366.16 | 1.346176 |
| GO:0046661\_male\_sex\_differentiation | FKBP4 | 49 | 1 | 2.539989 | -0.484234 | 272 | 366.16 | 1.346176 |
| GO:0048741\_skeletal\_muscle\_fiber\_development | MYH9 | 49 | 1 | 2.539989 | -0.484234 | 272 | 366.16 | 1.346176 |
| GO:0006812\_cation\_transport | SLC8A3 | 146 | 2 | 1.704924 | -0.483040 | 273 | 366.8 | 1.343590 |
| GO:0006812\_cation\_transport | HEPH | 146 | 2 | 1.704924 | -0.483040 | 273 | 366.8 | 1.343590 |
| GO:0022603\_regulation\_of\_anatomical\_structure\_morphogenesis | YWHAH | 147 | 2 | 1.693326 | -0.479122 | 274 | 367.77 | 1.342226 |
| GO:0022603\_regulation\_of\_anatomical\_structure\_morphogenesis | MYH9 | 147 | 2 | 1.693326 | -0.479122 | 274 | 367.77 | 1.342226 |
| GO:0051606\_detection\_of\_stimulus | SLC12A2 | 50 | 1 | 2.489189 | -0.477065 | 276 | 370.69 | 1.343080 |
| GO:0070647\_protein\_modification\_by\_small\_protein\_conjugation\_or\_removal | UBE2F | 50 | 1 | 2.489189 | -0.477065 | 276 | 370.69 | 1.343080 |
| GO:0048878\_chemical\_homeostasis | CYB5R4 | 254 | 3 | 1.469994 | -0.475472 | 277 | 370.89 | 1.338953 |
| GO:0048878\_chemical\_homeostasis | SLC30A1 | 254 | 3 | 1.469994 | -0.475472 | 277 | 370.89 | 1.338953 |
| GO:0048878\_chemical\_homeostasis | NCOR1 | 254 | 3 | 1.469994 | -0.475472 | 277 | 370.89 | 1.338953 |
| GO:0006520\_cellular\_amino\_acid\_metabolic\_process | AHCY | 51 | 1 | 2.440382 | -0.470067 | 281 | 375.61 | 1.336690 |
| GO:0016569\_covalent\_chromatin\_modification | NCOR1 | 51 | 1 | 2.440382 | -0.470067 | 281 | 375.61 | 1.336690 |
| GO:0044106\_cellular\_amine\_metabolic\_process | AHCY | 51 | 1 | 2.440382 | -0.470067 | 281 | 375.61 | 1.336690 |
| GO:0048747\_muscle\_fiber\_development | MYH9 | 51 | 1 | 2.440382 | -0.470067 | 281 | 375.61 | 1.336690 |
| GO:0044238\_primary\_metabolic\_process | KLF7 | 1905 | 17 | 1.110662 | -0.466158 | 282 | 376.38 | 1.334681 |
| GO:0044238\_primary\_metabolic\_process | AHCY | 1905 | 17 | 1.110662 | -0.466158 | 282 | 376.38 | 1.334681 |
| GO:0044238\_primary\_metabolic\_process | RXRB | 1905 | 17 | 1.110662 | -0.466158 | 282 | 376.38 | 1.334681 |
| GO:0044238\_primary\_metabolic\_process | E2F7 | 1905 | 17 | 1.110662 | -0.466158 | 282 | 376.38 | 1.334681 |
| GO:0044238\_primary\_metabolic\_process | UBE2F | 1905 | 17 | 1.110662 | -0.466158 | 282 | 376.38 | 1.334681 |
| GO:0044238\_primary\_metabolic\_process | TLE4 | 1905 | 17 | 1.110662 | -0.466158 | 282 | 376.38 | 1.334681 |
| GO:0044238\_primary\_metabolic\_process | ELAVL1 | 1905 | 17 | 1.110662 | -0.466158 | 282 | 376.38 | 1.334681 |
| GO:0044238\_primary\_metabolic\_process | VAX2 | 1905 | 17 | 1.110662 | -0.466158 | 282 | 376.38 | 1.334681 |
| GO:0044238\_primary\_metabolic\_process | HES6 | 1905 | 17 | 1.110662 | -0.466158 | 282 | 376.38 | 1.334681 |
| GO:0044238\_primary\_metabolic\_process | DACH1 | 1905 | 17 | 1.110662 | -0.466158 | 282 | 376.38 | 1.334681 |
| GO:0044238\_primary\_metabolic\_process | FOXP2 | 1905 | 17 | 1.110662 | -0.466158 | 282 | 376.38 | 1.334681 |
| GO:0044238\_primary\_metabolic\_process | NRIP1 | 1905 | 17 | 1.110662 | -0.466158 | 282 | 376.38 | 1.334681 |
| GO:0044238\_primary\_metabolic\_process | NCOA2 | 1905 | 17 | 1.110662 | -0.466158 | 282 | 376.38 | 1.334681 |
| GO:0044238\_primary\_metabolic\_process | SH3GLB1 | 1905 | 17 | 1.110662 | -0.466158 | 282 | 376.38 | 1.334681 |
| GO:0044238\_primary\_metabolic\_process | TIA1 | 1905 | 17 | 1.110662 | -0.466158 | 282 | 376.38 | 1.334681 |
| GO:0044238\_primary\_metabolic\_process | PPM1M | 1905 | 17 | 1.110662 | -0.466158 | 282 | 376.38 | 1.334681 |
| GO:0044238\_primary\_metabolic\_process | NCOR1 | 1905 | 17 | 1.110662 | -0.466158 | 282 | 376.38 | 1.334681 |
| GO:0032268\_regulation\_of\_cellular\_protein\_metabolic\_process | TIA1 | 152 | 2 | 1.637624 | -0.460111 | 283 | 378.74 | 1.338304 |
| GO:0032268\_regulation\_of\_cellular\_protein\_metabolic\_process | NCOR1 | 152 | 2 | 1.637624 | -0.460111 | 283 | 378.74 | 1.338304 |
| GO:0051248\_negative\_regulation\_of\_protein\_metabolic\_process | TIA1 | 53 | 1 | 2.348292 | -0.456561 | 284 | 381.33 | 1.342711 |
| GO:0048666\_neuron\_development | KLF7 | 262 | 3 | 1.425108 | -0.452756 | 285 | 382.43 | 1.341860 |
| GO:0048666\_neuron\_development | YWHAH | 262 | 3 | 1.425108 | -0.452756 | 285 | 382.43 | 1.341860 |
| GO:0048666\_neuron\_development | VAX2 | 262 | 3 | 1.425108 | -0.452756 | 285 | 382.43 | 1.341860 |
| GO:0006412\_translation | TIA1 | 54 | 1 | 2.304805 | -0.450040 | 287 | 385.32 | 1.342578 |
| GO:0009566\_fertilization | NHP2L1 | 54 | 1 | 2.304805 | -0.450040 | 287 | 385.32 | 1.342578 |
| GO:0022402\_cell\_cycle\_process | AKAP8 | 155 | 2 | 1.605929 | -0.449146 | 288 | 385.92 | 1.340000 |
| GO:0022402\_cell\_cycle\_process | MYH9 | 155 | 2 | 1.605929 | -0.449146 | 288 | 385.92 | 1.340000 |
| GO:0022414\_reproductive\_process | NHP2L1 | 376 | 4 | 1.324037 | -0.446724 | 289 | 386.29 | 1.336644 |
| GO:0022414\_reproductive\_process | TIAL1 | 376 | 4 | 1.324037 | -0.446724 | 289 | 386.29 | 1.336644 |
| GO:0022414\_reproductive\_process | FKBP4 | 376 | 4 | 1.324037 | -0.446724 | 289 | 386.29 | 1.336644 |
| GO:0022414\_reproductive\_process | NRIP1 | 376 | 4 | 1.324037 | -0.446724 | 289 | 386.29 | 1.336644 |
| GO:0007126\_meiosis | MYH9 | 55 | 1 | 2.262899 | -0.443665 | 293 | 388.56 | 1.326143 |
| GO:0007605\_sensory\_perception\_of\_sound | SLC12A2 | 55 | 1 | 2.262899 | -0.443665 | 293 | 388.56 | 1.326143 |
| GO:0048568\_embryonic\_organ\_development | VAX2 | 55 | 1 | 2.262899 | -0.443665 | 293 | 388.56 | 1.326143 |
| GO:0051327\_M\_phase\_of\_meiotic\_cell\_cycle | MYH9 | 55 | 1 | 2.262899 | -0.443665 | 293 | 388.56 | 1.326143 |
| GO:0007409\_axonogenesis | KLF7 | 158 | 2 | 1.575436 | -0.438497 | 294 | 389.82 | 1.325918 |
| GO:0007409\_axonogenesis | VAX2 | 158 | 2 | 1.575436 | -0.438497 | 294 | 389.82 | 1.325918 |
| GO:0042089\_cytokine\_biosynthetic\_process | TIA1 | 56 | 1 | 2.222490 | -0.437433 | 299 | 393.16 | 1.314916 |
| GO:0042107\_cytokine\_metabolic\_process | TIA1 | 56 | 1 | 2.222490 | -0.437433 | 299 | 393.16 | 1.314916 |
| GO:0046486\_glycerolipid\_metabolic\_process | SH3GLB1 | 56 | 1 | 2.222490 | -0.437433 | 299 | 393.16 | 1.314916 |
| GO:0050678\_regulation\_of\_epithelial\_cell\_proliferation | FOXP2 | 56 | 1 | 2.222490 | -0.437433 | 299 | 393.16 | 1.314916 |
| GO:0051321\_meiotic\_cell\_cycle | MYH9 | 56 | 1 | 2.222490 | -0.437433 | 299 | 393.16 | 1.314916 |
| GO:0016043\_cellular\_component\_organization | KLF7 | 964 | 9 | 1.161966 | -0.435794 | 300 | 393.29 | 1.310967 |
| GO:0016043\_cellular\_component\_organization | YWHAH | 964 | 9 | 1.161966 | -0.435794 | 300 | 393.29 | 1.310967 |
| GO:0016043\_cellular\_component\_organization | CRISPLD2 | 964 | 9 | 1.161966 | -0.435794 | 300 | 393.29 | 1.310967 |
| GO:0016043\_cellular\_component\_organization | SH3GLB1 | 964 | 9 | 1.161966 | -0.435794 | 300 | 393.29 | 1.310967 |
| GO:0016043\_cellular\_component\_organization | FKBP4 | 964 | 9 | 1.161966 | -0.435794 | 300 | 393.29 | 1.310967 |
| GO:0016043\_cellular\_component\_organization | VAX2 | 964 | 9 | 1.161966 | -0.435794 | 300 | 393.29 | 1.310967 |
| GO:0016043\_cellular\_component\_organization | AKAP8 | 964 | 9 | 1.161966 | -0.435794 | 300 | 393.29 | 1.310967 |
| GO:0016043\_cellular\_component\_organization | MYH9 | 964 | 9 | 1.161966 | -0.435794 | 300 | 393.29 | 1.310967 |
| GO:0016043\_cellular\_component\_organization | NCOR1 | 964 | 9 | 1.161966 | -0.435794 | 300 | 393.29 | 1.310967 |
| GO:0051234\_establishment\_of\_localization | SLC8A3 | 729 | 7 | 1.195084 | -0.434017 | 301 | 393.41 | 1.307010 |
| GO:0051234\_establishment\_of\_localization | CYB5R4 | 729 | 7 | 1.195084 | -0.434017 | 301 | 393.41 | 1.307010 |
| GO:0051234\_establishment\_of\_localization | YWHAH | 729 | 7 | 1.195084 | -0.434017 | 301 | 393.41 | 1.307010 |
| GO:0051234\_establishment\_of\_localization | RAB1 | 729 | 7 | 1.195084 | -0.434017 | 301 | 393.41 | 1.307010 |
| GO:0051234\_establishment\_of\_localization | LRBA | 729 | 7 | 1.195084 | -0.434017 | 301 | 393.41 | 1.307010 |
| GO:0051234\_establishment\_of\_localization | HEPH | 729 | 7 | 1.195084 | -0.434017 | 301 | 393.41 | 1.307010 |
| GO:0051234\_establishment\_of\_localization | MYH9 | 729 | 7 | 1.195084 | -0.434017 | 301 | 393.41 | 1.307010 |
| GO:0000226\_microtubule\_cytoskeleton\_organization | MYH9 | 57 | 1 | 2.183499 | -0.431336 | 303 | 397.5 | 1.311881 |
| GO:0009953\_dorsal\_ventral\_pattern\_formation | VAX2 | 57 | 1 | 2.183499 | -0.431336 | 303 | 397.5 | 1.311881 |
| GO:0032502\_developmental\_process | CYB5R4 | 2060 | 18 | 1.087510 | -0.426314 | 304 | 398.67 | 1.311414 |
| GO:0032502\_developmental\_process | KLF7 | 2060 | 18 | 1.087510 | -0.426314 | 304 | 398.67 | 1.311414 |
| GO:0032502\_developmental\_process | RXRB | 2060 | 18 | 1.087510 | -0.426314 | 304 | 398.67 | 1.311414 |
| GO:0032502\_developmental\_process | FKBP4 | 2060 | 18 | 1.087510 | -0.426314 | 304 | 398.67 | 1.311414 |
| GO:0032502\_developmental\_process | VAX2 | 2060 | 18 | 1.087510 | -0.426314 | 304 | 398.67 | 1.311414 |
| GO:0032502\_developmental\_process | HES6 | 2060 | 18 | 1.087510 | -0.426314 | 304 | 398.67 | 1.311414 |
| GO:0032502\_developmental\_process | MYH9 | 2060 | 18 | 1.087510 | -0.426314 | 304 | 398.67 | 1.311414 |
| GO:0032502\_developmental\_process | NRIP1 | 2060 | 18 | 1.087510 | -0.426314 | 304 | 398.67 | 1.311414 |
| GO:0032502\_developmental\_process | FOXP2 | 2060 | 18 | 1.087510 | -0.426314 | 304 | 398.67 | 1.311414 |
| GO:0032502\_developmental\_process | YWHAH | 2060 | 18 | 1.087510 | -0.426314 | 304 | 398.67 | 1.311414 |
| GO:0032502\_developmental\_process | SH3GLB1 | 2060 | 18 | 1.087510 | -0.426314 | 304 | 398.67 | 1.311414 |
| GO:0032502\_developmental\_process | SLC30A1 | 2060 | 18 | 1.087510 | -0.426314 | 304 | 398.67 | 1.311414 |
| GO:0032502\_developmental\_process | TIAL1 | 2060 | 18 | 1.087510 | -0.426314 | 304 | 398.67 | 1.311414 |
| GO:0032502\_developmental\_process | MSI2 | 2060 | 18 | 1.087510 | -0.426314 | 304 | 398.67 | 1.311414 |
| GO:0032502\_developmental\_process | HEPH | 2060 | 18 | 1.087510 | -0.426314 | 304 | 398.67 | 1.311414 |
| GO:0032502\_developmental\_process | SEPP1 | 2060 | 18 | 1.087510 | -0.426314 | 304 | 398.67 | 1.311414 |
| GO:0032502\_developmental\_process | MAB21L1 | 2060 | 18 | 1.087510 | -0.426314 | 304 | 398.67 | 1.311414 |
| GO:0032502\_developmental\_process | NCOR1 | 2060 | 18 | 1.087510 | -0.426314 | 304 | 398.67 | 1.311414 |
| GO:0030902\_hindbrain\_development | FOXP2 | 58 | 1 | 2.145853 | -0.425372 | 307 | 400.13 | 1.303355 |
| GO:0033043\_regulation\_of\_organelle\_organization | NCOR1 | 58 | 1 | 2.145853 | -0.425372 | 307 | 400.13 | 1.303355 |
| GO:0034622\_cellular\_macromolecular\_complex\_assembly | FKBP4 | 58 | 1 | 2.145853 | -0.425372 | 307 | 400.13 | 1.303355 |
| GO:0009628\_response\_to\_abiotic\_stimulus | SLC12A2 | 162 | 2 | 1.536537 | -0.424766 | 308 | 400.3 | 1.299675 |
| GO:0009628\_response\_to\_abiotic\_stimulus | FOXP2 | 162 | 2 | 1.536537 | -0.424766 | 308 | 400.3 | 1.299675 |
| GO:0042110\_T\_cell\_activation | MYH9 | 163 | 2 | 1.527110 | -0.421414 | 309 | 401.14 | 1.298188 |
| GO:0042110\_T\_cell\_activation | NCOR1 | 163 | 2 | 1.527110 | -0.421414 | 309 | 401.14 | 1.298188 |
| GO:0016055\_Wnt\_receptor\_signaling\_pathway | TLE4 | 59 | 1 | 2.109482 | -0.419534 | 310 | 403.42 | 1.301355 |
| GO:0042127\_regulation\_of\_cell\_proliferation | E2F7 | 393 | 4 | 1.266763 | -0.409412 | 311 | 406.63 | 1.307492 |
| GO:0042127\_regulation\_of\_cell\_proliferation | TIAL1 | 393 | 4 | 1.266763 | -0.409412 | 311 | 406.63 | 1.307492 |
| GO:0042127\_regulation\_of\_cell\_proliferation | MAB21L1 | 393 | 4 | 1.266763 | -0.409412 | 311 | 406.63 | 1.307492 |
| GO:0042127\_regulation\_of\_cell\_proliferation | FOXP2 | 393 | 4 | 1.266763 | -0.409412 | 311 | 406.63 | 1.307492 |
| GO:0007005\_mitochondrion\_organization | SH3GLB1 | 61 | 1 | 2.040319 | -0.408223 | 313 | 409.7 | 1.308946 |
| GO:0032270\_positive\_regulation\_of\_cellular\_protein\_metabolic\_process | NCOR1 | 61 | 1 | 2.040319 | -0.408223 | 313 | 409.7 | 1.308946 |
| GO:0040014\_regulation\_of\_multicellular\_organism\_growth | NCOR1 | 62 | 1 | 2.007411 | -0.402742 | 315 | 413.12 | 1.311492 |
| GO:0050954\_sensory\_perception\_of\_mechanical\_stimulus | SLC12A2 | 62 | 1 | 2.007411 | -0.402742 | 315 | 413.12 | 1.311492 |
| GO:0051246\_regulation\_of\_protein\_metabolic\_process | TIA1 | 170 | 2 | 1.464229 | -0.398810 | 316 | 413.9 | 1.309810 |
| GO:0051246\_regulation\_of\_protein\_metabolic\_process | NCOR1 | 170 | 2 | 1.464229 | -0.398810 | 316 | 413.9 | 1.309810 |
| GO:0048518\_positive\_regulation\_of\_biological\_process | NCAM1 | 995 | 9 | 1.125764 | -0.391726 | 317 | 419.3 | 1.322713 |
| GO:0048518\_positive\_regulation\_of\_biological\_process | KLF7 | 995 | 9 | 1.125764 | -0.391726 | 317 | 419.3 | 1.322713 |
| GO:0048518\_positive\_regulation\_of\_biological\_process | NCOA2 | 995 | 9 | 1.125764 | -0.391726 | 317 | 419.3 | 1.322713 |
| GO:0048518\_positive\_regulation\_of\_biological\_process | SH3GLB1 | 995 | 9 | 1.125764 | -0.391726 | 317 | 419.3 | 1.322713 |
| GO:0048518\_positive\_regulation\_of\_biological\_process | RXRB | 995 | 9 | 1.125764 | -0.391726 | 317 | 419.3 | 1.322713 |
| GO:0048518\_positive\_regulation\_of\_biological\_process | TIAL1 | 995 | 9 | 1.125764 | -0.391726 | 317 | 419.3 | 1.322713 |
| GO:0048518\_positive\_regulation\_of\_biological\_process | MAB21L1 | 995 | 9 | 1.125764 | -0.391726 | 317 | 419.3 | 1.322713 |
| GO:0048518\_positive\_regulation\_of\_biological\_process | NCOR1 | 995 | 9 | 1.125764 | -0.391726 | 317 | 419.3 | 1.322713 |
| GO:0048518\_positive\_regulation\_of\_biological\_process | FOXP2 | 995 | 9 | 1.125764 | -0.391726 | 317 | 419.3 | 1.322713 |
| GO:0007417\_central\_nervous\_system\_development | SEPP1 | 287 | 3 | 1.300970 | -0.388934 | 318 | 420.56 | 1.322516 |
| GO:0007417\_central\_nervous\_system\_development | NCOR1 | 287 | 3 | 1.300970 | -0.388934 | 318 | 420.56 | 1.322516 |
| GO:0007417\_central\_nervous\_system\_development | FOXP2 | 287 | 3 | 1.300970 | -0.388934 | 318 | 420.56 | 1.322516 |
| GO:0015031\_protein\_transport | YWHAH | 175 | 2 | 1.422394 | -0.383536 | 319 | 422.36 | 1.324013 |
| GO:0015031\_protein\_transport | RAB1 | 175 | 2 | 1.422394 | -0.383536 | 319 | 422.36 | 1.324013 |
| GO:0051130\_positive\_regulation\_of\_cellular\_component\_organization | NCOR1 | 66 | 1 | 1.885749 | -0.381890 | 320 | 424.63 | 1.326969 |
| GO:0051247\_positive\_regulation\_of\_protein\_metabolic\_process | NCOR1 | 67 | 1 | 1.857604 | -0.376929 | 321 | 428.01 | 1.333364 |
| GO:0007242\_intracellular\_signaling\_cascade | NCAM1 | 411 | 4 | 1.211284 | -0.373240 | 322 | 428.27 | 1.330031 |
| GO:0007242\_intracellular\_signaling\_cascade | RXRB | 411 | 4 | 1.211284 | -0.373240 | 322 | 428.27 | 1.330031 |
| GO:0007242\_intracellular\_signaling\_cascade | FKBP4 | 411 | 4 | 1.211284 | -0.373240 | 322 | 428.27 | 1.330031 |
| GO:0007242\_intracellular\_signaling\_cascade | NCOR1 | 411 | 4 | 1.211284 | -0.373240 | 322 | 428.27 | 1.330031 |
| GO:0019932\_second-messenger-mediated\_signaling | NCAM1 | 68 | 1 | 1.830286 | -0.372063 | 323 | 429.74 | 1.330464 |
| GO:0045184\_establishment\_of\_protein\_localization | YWHAH | 180 | 2 | 1.382883 | -0.368940 | 324 | 431.61 | 1.332130 |
| GO:0045184\_establishment\_of\_protein\_localization | RAB1 | 180 | 2 | 1.382883 | -0.368940 | 324 | 431.61 | 1.332130 |
| GO:0019752\_carboxylic\_acid\_metabolic\_process | AHCY | 181 | 2 | 1.375243 | -0.366099 | 326 | 434.05 | 1.331442 |
| GO:0019752\_carboxylic\_acid\_metabolic\_process | SH3GLB1 | 181 | 2 | 1.375243 | -0.366099 | 326 | 434.05 | 1.331442 |
| GO:0043436\_oxoacid\_metabolic\_process | AHCY | 181 | 2 | 1.375243 | -0.366099 | 326 | 434.05 | 1.331442 |
| GO:0043436\_oxoacid\_metabolic\_process | SH3GLB1 | 181 | 2 | 1.375243 | -0.366099 | 326 | 434.05 | 1.331442 |
| GO:0006082\_organic\_acid\_metabolic\_process | AHCY | 182 | 2 | 1.367686 | -0.363283 | 327 | 434.94 | 1.330092 |
| GO:0006082\_organic\_acid\_metabolic\_process | SH3GLB1 | 182 | 2 | 1.367686 | -0.363283 | 327 | 434.94 | 1.330092 |
| GO:0007611\_learning\_or\_memory | FOXP2 | 70 | 1 | 1.777992 | -0.362604 | 330 | 436.71 | 1.323364 |
| GO:0008406\_gonad\_development | NRIP1 | 70 | 1 | 1.777992 | -0.362604 | 330 | 436.71 | 1.323364 |
| GO:0048592\_eye\_morphogenesis | VAX2 | 70 | 1 | 1.777992 | -0.362604 | 330 | 436.71 | 1.323364 |
| GO:0042180\_cellular\_ketone\_metabolic\_process | AHCY | 183 | 2 | 1.360213 | -0.360492 | 331 | 437.22 | 1.320906 |
| GO:0042180\_cellular\_ketone\_metabolic\_process | SH3GLB1 | 183 | 2 | 1.360213 | -0.360492 | 331 | 437.22 | 1.320906 |
| GO:0042592\_homeostatic\_process | CYB5R4 | 419 | 4 | 1.188157 | -0.358180 | 332 | 437.81 | 1.318705 |
| GO:0042592\_homeostatic\_process | SLC30A1 | 419 | 4 | 1.188157 | -0.358180 | 332 | 437.81 | 1.318705 |
| GO:0042592\_homeostatic\_process | HEPH | 419 | 4 | 1.188157 | -0.358180 | 332 | 437.81 | 1.318705 |
| GO:0042592\_homeostatic\_process | NCOR1 | 419 | 4 | 1.188157 | -0.358180 | 332 | 437.81 | 1.318705 |
| GO:0016568\_chromatin\_modification | NCOR1 | 72 | 1 | 1.728604 | -0.353492 | 334 | 443.72 | 1.328503 |
| GO:0050673\_epithelial\_cell\_proliferation | FOXP2 | 72 | 1 | 1.728604 | -0.353492 | 334 | 443.72 | 1.328503 |
| GO:0006811\_ion\_transport | SLC8A3 | 186 | 2 | 1.338274 | -0.352265 | 335 | 444.63 | 1.327254 |
| GO:0006811\_ion\_transport | HEPH | 186 | 2 | 1.338274 | -0.352265 | 335 | 444.63 | 1.327254 |
| GO:0008283\_cell\_proliferation | RXRB | 544 | 5 | 1.143929 | -0.348892 | 336 | 447.62 | 1.332202 |
| GO:0008283\_cell\_proliferation | E2F7 | 544 | 5 | 1.143929 | -0.348892 | 336 | 447.62 | 1.332202 |
| GO:0008283\_cell\_proliferation | TIAL1 | 544 | 5 | 1.143929 | -0.348892 | 336 | 447.62 | 1.332202 |
| GO:0008283\_cell\_proliferation | MAB21L1 | 544 | 5 | 1.143929 | -0.348892 | 336 | 447.62 | 1.332202 |
| GO:0008283\_cell\_proliferation | FOXP2 | 544 | 5 | 1.143929 | -0.348892 | 336 | 447.62 | 1.332202 |
| GO:0007276\_gamete\_generation | TIAL1 | 188 | 2 | 1.324037 | -0.346899 | 337 | 448.16 | 1.329852 |
| GO:0007276\_gamete\_generation | NRIP1 | 188 | 2 | 1.324037 | -0.346899 | 337 | 448.16 | 1.329852 |
| GO:0045893\_positive\_regulation\_of\_transcription\_\_DNA-dependent | KLF7 | 306 | 3 | 1.220191 | -0.346776 | 339 | 448.62 | 1.323363 |
| GO:0045893\_positive\_regulation\_of\_transcription\_\_DNA-dependent | NCOA2 | 306 | 3 | 1.220191 | -0.346776 | 339 | 448.62 | 1.323363 |
| GO:0045893\_positive\_regulation\_of\_transcription\_\_DNA-dependent | RXRB | 306 | 3 | 1.220191 | -0.346776 | 339 | 448.62 | 1.323363 |
| GO:0051254\_positive\_regulation\_of\_RNA\_metabolic\_process | KLF7 | 306 | 3 | 1.220191 | -0.346776 | 339 | 448.62 | 1.323363 |
| GO:0051254\_positive\_regulation\_of\_RNA\_metabolic\_process | NCOA2 | 306 | 3 | 1.220191 | -0.346776 | 339 | 448.62 | 1.323363 |
| GO:0051254\_positive\_regulation\_of\_RNA\_metabolic\_process | RXRB | 306 | 3 | 1.220191 | -0.346776 | 339 | 448.62 | 1.323363 |
| GO:0008152\_metabolic\_process | KLF7 | 2133 | 18 | 1.050291 | -0.345966 | 340 | 448.79 | 1.319971 |
| GO:0008152\_metabolic\_process | AHCY | 2133 | 18 | 1.050291 | -0.345966 | 340 | 448.79 | 1.319971 |
| GO:0008152\_metabolic\_process | RXRB | 2133 | 18 | 1.050291 | -0.345966 | 340 | 448.79 | 1.319971 |
| GO:0008152\_metabolic\_process | E2F7 | 2133 | 18 | 1.050291 | -0.345966 | 340 | 448.79 | 1.319971 |
| GO:0008152\_metabolic\_process | UBE2F | 2133 | 18 | 1.050291 | -0.345966 | 340 | 448.79 | 1.319971 |
| GO:0008152\_metabolic\_process | TLE4 | 2133 | 18 | 1.050291 | -0.345966 | 340 | 448.79 | 1.319971 |
| GO:0008152\_metabolic\_process | ELAVL1 | 2133 | 18 | 1.050291 | -0.345966 | 340 | 448.79 | 1.319971 |
| GO:0008152\_metabolic\_process | VAX2 | 2133 | 18 | 1.050291 | -0.345966 | 340 | 448.79 | 1.319971 |
| GO:0008152\_metabolic\_process | HES6 | 2133 | 18 | 1.050291 | -0.345966 | 340 | 448.79 | 1.319971 |
| GO:0008152\_metabolic\_process | DACH1 | 2133 | 18 | 1.050291 | -0.345966 | 340 | 448.79 | 1.319971 |
| GO:0008152\_metabolic\_process | FOXP2 | 2133 | 18 | 1.050291 | -0.345966 | 340 | 448.79 | 1.319971 |
| GO:0008152\_metabolic\_process | NRIP1 | 2133 | 18 | 1.050291 | -0.345966 | 340 | 448.79 | 1.319971 |
| GO:0008152\_metabolic\_process | NCOA2 | 2133 | 18 | 1.050291 | -0.345966 | 340 | 448.79 | 1.319971 |
| GO:0008152\_metabolic\_process | SH3GLB1 | 2133 | 18 | 1.050291 | -0.345966 | 340 | 448.79 | 1.319971 |
| GO:0008152\_metabolic\_process | TIA1 | 2133 | 18 | 1.050291 | -0.345966 | 340 | 448.79 | 1.319971 |
| GO:0008152\_metabolic\_process | SEPP1 | 2133 | 18 | 1.050291 | -0.345966 | 340 | 448.79 | 1.319971 |
| GO:0008152\_metabolic\_process | PPM1M | 2133 | 18 | 1.050291 | -0.345966 | 340 | 448.79 | 1.319971 |
| GO:0008152\_metabolic\_process | NCOR1 | 2133 | 18 | 1.050291 | -0.345966 | 340 | 448.79 | 1.319971 |
| GO:0007281\_germ\_cell\_development | TIAL1 | 75 | 1 | 1.659459 | -0.340431 | 341 | 452.39 | 1.326657 |
| GO:0034621\_cellular\_macromolecular\_complex\_subunit\_organization | FKBP4 | 76 | 1 | 1.637624 | -0.336230 | 342 | 453.94 | 1.327310 |
| GO:0010604\_positive\_regulation\_of\_macromolecule\_metabolic\_process | KLF7 | 433 | 4 | 1.149741 | -0.333225 | 343 | 454.31 | 1.324519 |
| GO:0010604\_positive\_regulation\_of\_macromolecule\_metabolic\_process | NCOA2 | 433 | 4 | 1.149741 | -0.333225 | 343 | 454.31 | 1.324519 |
| GO:0010604\_positive\_regulation\_of\_macromolecule\_metabolic\_process | RXRB | 433 | 4 | 1.149741 | -0.333225 | 343 | 454.31 | 1.324519 |
| GO:0010604\_positive\_regulation\_of\_macromolecule\_metabolic\_process | NCOR1 | 433 | 4 | 1.149741 | -0.333225 | 343 | 454.31 | 1.324519 |
| GO:0006461\_protein\_complex\_assembly | FKBP4 | 78 | 1 | 1.595634 | -0.328046 | 345 | 458.92 | 1.330203 |
| GO:0070271\_protein\_complex\_biogenesis | FKBP4 | 78 | 1 | 1.595634 | -0.328046 | 345 | 458.92 | 1.330203 |
| GO:0044267\_cellular\_protein\_metabolic\_process | SH3GLB1 | 559 | 5 | 1.113233 | -0.325544 | 346 | 459.47 | 1.327948 |
| GO:0044267\_cellular\_protein\_metabolic\_process | TIA1 | 559 | 5 | 1.113233 | -0.325544 | 346 | 459.47 | 1.327948 |
| GO:0044267\_cellular\_protein\_metabolic\_process | UBE2F | 559 | 5 | 1.113233 | -0.325544 | 346 | 459.47 | 1.327948 |
| GO:0044267\_cellular\_protein\_metabolic\_process | PPM1M | 559 | 5 | 1.113233 | -0.325544 | 346 | 459.47 | 1.327948 |
| GO:0044267\_cellular\_protein\_metabolic\_process | NCOR1 | 559 | 5 | 1.113233 | -0.325544 | 346 | 459.47 | 1.327948 |
| GO:0015674\_di-\_\_tri-valent\_inorganic\_cation\_transport | HEPH | 79 | 1 | 1.575436 | -0.324060 | 347 | 460.25 | 1.326369 |
| GO:0000278\_mitotic\_cell\_cycle | AKAP8 | 80 | 1 | 1.555743 | -0.320140 | 348 | 463.8 | 1.332759 |
| GO:0031325\_positive\_regulation\_of\_cellular\_metabolic\_process | KLF7 | 442 | 4 | 1.126330 | -0.318073 | 349 | 464.56 | 1.331117 |
| GO:0031325\_positive\_regulation\_of\_cellular\_metabolic\_process | NCOA2 | 442 | 4 | 1.126330 | -0.318073 | 349 | 464.56 | 1.331117 |
| GO:0031325\_positive\_regulation\_of\_cellular\_metabolic\_process | RXRB | 442 | 4 | 1.126330 | -0.318073 | 349 | 464.56 | 1.331117 |
| GO:0031325\_positive\_regulation\_of\_cellular\_metabolic\_process | NCOR1 | 442 | 4 | 1.126330 | -0.318073 | 349 | 464.56 | 1.331117 |
| GO:0007411\_axon\_guidance | KLF7 | 82 | 1 | 1.517798 | -0.312497 | 352 | 467.56 | 1.328295 |
| GO:0010627\_regulation\_of\_protein\_kinase\_cascade | NCOR1 | 82 | 1 | 1.517798 | -0.312497 | 352 | 467.56 | 1.328295 |
| GO:0045664\_regulation\_of\_neuron\_differentiation | YWHAH | 82 | 1 | 1.517798 | -0.312497 | 352 | 467.56 | 1.328295 |
| GO:0006325\_chromatin\_organization | NCOR1 | 83 | 1 | 1.499512 | -0.308771 | 356 | 470.74 | 1.322303 |
| GO:0006575\_cellular\_amino\_acid\_derivative\_metabolic\_process | AHCY | 83 | 1 | 1.499512 | -0.308771 | 356 | 470.74 | 1.322303 |
| GO:0007017\_microtubule-based\_process | MYH9 | 83 | 1 | 1.499512 | -0.308771 | 356 | 470.74 | 1.322303 |
| GO:0030198\_extracellular\_matrix\_organization | CRISPLD2 | 83 | 1 | 1.499512 | -0.308771 | 356 | 470.74 | 1.322303 |
| GO:0006996\_organelle\_organization | SH3GLB1 | 449 | 4 | 1.108770 | -0.306745 | 357 | 471.22 | 1.319944 |
| GO:0006996\_organelle\_organization | AKAP8 | 449 | 4 | 1.108770 | -0.306745 | 357 | 471.22 | 1.319944 |
| GO:0006996\_organelle\_organization | MYH9 | 449 | 4 | 1.108770 | -0.306745 | 357 | 471.22 | 1.319944 |
| GO:0006996\_organelle\_organization | NCOR1 | 449 | 4 | 1.108770 | -0.306745 | 357 | 471.22 | 1.319944 |
| GO:0030005\_cellular\_di-\_\_tri-valent\_inorganic\_cation\_homeostasis | SLC30A1 | 84 | 1 | 1.481660 | -0.305105 | 359 | 472.15 | 1.315181 |
| GO:0045137\_development\_of\_primary\_sexual\_characteristics | NRIP1 | 84 | 1 | 1.481660 | -0.305105 | 359 | 472.15 | 1.315181 |
| GO:0032504\_multicellular\_organism\_reproduction | NRIP1 | 86 | 1 | 1.447203 | -0.297950 | 362 | 477.97 | 1.320359 |
| GO:0034641\_cellular\_nitrogen\_compound\_metabolic\_process | AHCY | 86 | 1 | 1.447203 | -0.297950 | 362 | 477.97 | 1.320359 |
| GO:0048609\_reproductive\_process\_in\_a\_multicellular\_organism | NRIP1 | 86 | 1 | 1.447203 | -0.297950 | 362 | 477.97 | 1.320359 |
| GO:0003001\_generation\_of\_a\_signal\_involved\_in\_cell-cell\_signaling | CYB5R4 | 87 | 1 | 1.430568 | -0.294459 | 363 | 481.22 | 1.325675 |
| GO:0009893\_positive\_regulation\_of\_metabolic\_process | KLF7 | 458 | 4 | 1.086982 | -0.292743 | 364 | 481.83 | 1.323709 |
| GO:0009893\_positive\_regulation\_of\_metabolic\_process | NCOA2 | 458 | 4 | 1.086982 | -0.292743 | 364 | 481.83 | 1.323709 |
| GO:0009893\_positive\_regulation\_of\_metabolic\_process | RXRB | 458 | 4 | 1.086982 | -0.292743 | 364 | 481.83 | 1.323709 |
| GO:0009893\_positive\_regulation\_of\_metabolic\_process | NCOR1 | 458 | 4 | 1.086982 | -0.292743 | 364 | 481.83 | 1.323709 |
| GO:0045941\_positive\_regulation\_of\_transcription | KLF7 | 338 | 3 | 1.104670 | -0.286052 | 365 | 483.97 | 1.325945 |
| GO:0045941\_positive\_regulation\_of\_transcription | NCOA2 | 338 | 3 | 1.104670 | -0.286052 | 365 | 483.97 | 1.325945 |
| GO:0045941\_positive\_regulation\_of\_transcription | RXRB | 338 | 3 | 1.104670 | -0.286052 | 365 | 483.97 | 1.325945 |
| GO:0030003\_cellular\_cation\_homeostasis | SLC30A1 | 90 | 1 | 1.382883 | -0.284311 | 368 | 485.73 | 1.319918 |
| GO:0030324\_lung\_development | FOXP2 | 90 | 1 | 1.382883 | -0.284311 | 368 | 485.73 | 1.319918 |
| GO:0035264\_multicellular\_organism\_growth | NCOR1 | 90 | 1 | 1.382883 | -0.284311 | 368 | 485.73 | 1.319918 |
| GO:0031399\_regulation\_of\_protein\_modification\_process | NCOR1 | 91 | 1 | 1.367686 | -0.281033 | 369 | 487.48 | 1.321084 |
| GO:0030217\_T\_cell\_differentiation | NCOR1 | 92 | 1 | 1.352820 | -0.277806 | 371 | 489.96 | 1.320647 |
| GO:0030323\_respiratory\_tube\_development | FOXP2 | 92 | 1 | 1.352820 | -0.277806 | 371 | 489.96 | 1.320647 |
| GO:0006810\_transport | SLC8A3 | 718 | 6 | 1.040051 | -0.277467 | 372 | 490.18 | 1.317688 |
| GO:0006810\_transport | CYB5R4 | 718 | 6 | 1.040051 | -0.277467 | 372 | 490.18 | 1.317688 |
| GO:0006810\_transport | YWHAH | 718 | 6 | 1.040051 | -0.277467 | 372 | 490.18 | 1.317688 |
| GO:0006810\_transport | RAB1 | 718 | 6 | 1.040051 | -0.277467 | 372 | 490.18 | 1.317688 |
| GO:0006810\_transport | LRBA | 718 | 6 | 1.040051 | -0.277467 | 372 | 490.18 | 1.317688 |
| GO:0006810\_transport | HEPH | 718 | 6 | 1.040051 | -0.277467 | 372 | 490.18 | 1.317688 |
| GO:0055066\_di-\_\_tri-valent\_inorganic\_cation\_homeostasis | SLC30A1 | 93 | 1 | 1.338274 | -0.274628 | 374 | 492.5 | 1.316845 |
| GO:0065003\_macromolecular\_complex\_assembly | FKBP4 | 93 | 1 | 1.338274 | -0.274628 | 374 | 492.5 | 1.316845 |
| GO:0010628\_positive\_regulation\_of\_gene\_expression | KLF7 | 346 | 3 | 1.079128 | -0.272625 | 375 | 492.96 | 1.314560 |
| GO:0010628\_positive\_regulation\_of\_gene\_expression | NCOA2 | 346 | 3 | 1.079128 | -0.272625 | 375 | 492.96 | 1.314560 |
| GO:0010628\_positive\_regulation\_of\_gene\_expression | RXRB | 346 | 3 | 1.079128 | -0.272625 | 375 | 492.96 | 1.314560 |
| GO:0008610\_lipid\_biosynthetic\_process | SH3GLB1 | 94 | 1 | 1.324037 | -0.271498 | 376 | 495.9 | 1.318883 |
| GO:0001701\_in\_utero\_embryonic\_development | SLC30A1 | 221 | 2 | 1.126330 | -0.270403 | 377 | 496.17 | 1.316101 |
| GO:0001701\_in\_utero\_embryonic\_development | MYH9 | 221 | 2 | 1.126330 | -0.270403 | 377 | 496.17 | 1.316101 |
| GO:0045935\_positive\_regulation\_of\_nucleobase\_\_nucleoside\_\_nucleotide\_and\_nucleic\_acid\_metabolic\_process | KLF7 | 352 | 3 | 1.060734 | -0.262969 | 378 | 499.58 | 1.321640 |
| GO:0045935\_positive\_regulation\_of\_nucleobase\_\_nucleoside\_\_nucleotide\_and\_nucleic\_acid\_metabolic\_process | NCOA2 | 352 | 3 | 1.060734 | -0.262969 | 378 | 499.58 | 1.321640 |
| GO:0045935\_positive\_regulation\_of\_nucleobase\_\_nucleoside\_\_nucleotide\_and\_nucleic\_acid\_metabolic\_process | RXRB | 352 | 3 | 1.060734 | -0.262969 | 378 | 499.58 | 1.321640 |
| GO:0009967\_positive\_regulation\_of\_signal\_transduction | NCAM1 | 98 | 1 | 1.269994 | -0.259439 | 380 | 502.22 | 1.321632 |
| GO:0060541\_respiratory\_system\_development | FOXP2 | 98 | 1 | 1.269994 | -0.259439 | 380 | 502.22 | 1.321632 |
| GO:0048731\_system\_development | KLF7 | 1609 | 13 | 1.005577 | -0.258033 | 381 | 502.51 | 1.318924 |
| GO:0048731\_system\_development | RXRB | 1609 | 13 | 1.005577 | -0.258033 | 381 | 502.51 | 1.318924 |
| GO:0048731\_system\_development | FKBP4 | 1609 | 13 | 1.005577 | -0.258033 | 381 | 502.51 | 1.318924 |
| GO:0048731\_system\_development | VAX2 | 1609 | 13 | 1.005577 | -0.258033 | 381 | 502.51 | 1.318924 |
| GO:0048731\_system\_development | HES6 | 1609 | 13 | 1.005577 | -0.258033 | 381 | 502.51 | 1.318924 |
| GO:0048731\_system\_development | MYH9 | 1609 | 13 | 1.005577 | -0.258033 | 381 | 502.51 | 1.318924 |
| GO:0048731\_system\_development | FOXP2 | 1609 | 13 | 1.005577 | -0.258033 | 381 | 502.51 | 1.318924 |
| GO:0048731\_system\_development | NRIP1 | 1609 | 13 | 1.005577 | -0.258033 | 381 | 502.51 | 1.318924 |
| GO:0048731\_system\_development | YWHAH | 1609 | 13 | 1.005577 | -0.258033 | 381 | 502.51 | 1.318924 |
| GO:0048731\_system\_development | HEPH | 1609 | 13 | 1.005577 | -0.258033 | 381 | 502.51 | 1.318924 |
| GO:0048731\_system\_development | SEPP1 | 1609 | 13 | 1.005577 | -0.258033 | 381 | 502.51 | 1.318924 |
| GO:0048731\_system\_development | MAB21L1 | 1609 | 13 | 1.005577 | -0.258033 | 381 | 502.51 | 1.318924 |
| GO:0048731\_system\_development | NCOR1 | 1609 | 13 | 1.005577 | -0.258033 | 381 | 502.51 | 1.318924 |
| GO:0030182\_neuron\_differentiation | KLF7 | 356 | 3 | 1.048816 | -0.256721 | 382 | 502.77 | 1.316152 |
| GO:0030182\_neuron\_differentiation | YWHAH | 356 | 3 | 1.048816 | -0.256721 | 382 | 502.77 | 1.316152 |
| GO:0030182\_neuron\_differentiation | VAX2 | 356 | 3 | 1.048816 | -0.256721 | 382 | 502.77 | 1.316152 |
| GO:0046649\_lymphocyte\_activation | MYH9 | 228 | 2 | 1.091750 | -0.256692 | 383 | 503.65 | 1.315013 |
| GO:0046649\_lymphocyte\_activation | NCOR1 | 228 | 2 | 1.091750 | -0.256692 | 383 | 503.65 | 1.315013 |
| GO:0001817\_regulation\_of\_cytokine\_production | TIA1 | 99 | 1 | 1.257166 | -0.256535 | 384 | 505.28 | 1.315833 |
| GO:0051173\_positive\_regulation\_of\_nitrogen\_compound\_metabolic\_process | KLF7 | 361 | 3 | 1.034289 | -0.249117 | 385 | 507.68 | 1.318649 |
| GO:0051173\_positive\_regulation\_of\_nitrogen\_compound\_metabolic\_process | NCOA2 | 361 | 3 | 1.034289 | -0.249117 | 385 | 507.68 | 1.318649 |
| GO:0051173\_positive\_regulation\_of\_nitrogen\_compound\_metabolic\_process | RXRB | 361 | 3 | 1.034289 | -0.249117 | 385 | 507.68 | 1.318649 |
| GO:0050890\_cognition | SLC12A2 | 233 | 2 | 1.068322 | -0.247359 | 386 | 508.69 | 1.317850 |
| GO:0050890\_cognition | FOXP2 | 233 | 2 | 1.068322 | -0.247359 | 386 | 508.69 | 1.317850 |
| GO:0009968\_negative\_regulation\_of\_signal\_transduction | NCOR1 | 103 | 1 | 1.208344 | -0.245331 | 387 | 510.07 | 1.318010 |
| GO:0050767\_regulation\_of\_neurogenesis | YWHAH | 104 | 1 | 1.196726 | -0.242630 | 388 | 511.68 | 1.318763 |
| GO:0007049\_cell\_cycle | AKAP8 | 238 | 2 | 1.045878 | -0.238390 | 389 | 513.57 | 1.320231 |
| GO:0007049\_cell\_cycle | MYH9 | 238 | 2 | 1.045878 | -0.238390 | 389 | 513.57 | 1.320231 |
| GO:0010817\_regulation\_of\_hormone\_levels | CYB5R4 | 106 | 1 | 1.174146 | -0.237340 | 390 | 514.04 | 1.318051 |
| GO:0007275\_multicellular\_organismal\_development | KLF7 | 1760 | 14 | 0.990018 | -0.235781 | 391 | 514.49 | 1.315831 |
| GO:0007275\_multicellular\_organismal\_development | RXRB | 1760 | 14 | 0.990018 | -0.235781 | 391 | 514.49 | 1.315831 |
| GO:0007275\_multicellular\_organismal\_development | FKBP4 | 1760 | 14 | 0.990018 | -0.235781 | 391 | 514.49 | 1.315831 |
| GO:0007275\_multicellular\_organismal\_development | VAX2 | 1760 | 14 | 0.990018 | -0.235781 | 391 | 514.49 | 1.315831 |
| GO:0007275\_multicellular\_organismal\_development | HES6 | 1760 | 14 | 0.990018 | -0.235781 | 391 | 514.49 | 1.315831 |
| GO:0007275\_multicellular\_organismal\_development | MYH9 | 1760 | 14 | 0.990018 | -0.235781 | 391 | 514.49 | 1.315831 |
| GO:0007275\_multicellular\_organismal\_development | FOXP2 | 1760 | 14 | 0.990018 | -0.235781 | 391 | 514.49 | 1.315831 |
| GO:0007275\_multicellular\_organismal\_development | NRIP1 | 1760 | 14 | 0.990018 | -0.235781 | 391 | 514.49 | 1.315831 |
| GO:0007275\_multicellular\_organismal\_development | YWHAH | 1760 | 14 | 0.990018 | -0.235781 | 391 | 514.49 | 1.315831 |
| GO:0007275\_multicellular\_organismal\_development | SLC30A1 | 1760 | 14 | 0.990018 | -0.235781 | 391 | 514.49 | 1.315831 |
| GO:0007275\_multicellular\_organismal\_development | HEPH | 1760 | 14 | 0.990018 | -0.235781 | 391 | 514.49 | 1.315831 |
| GO:0007275\_multicellular\_organismal\_development | SEPP1 | 1760 | 14 | 0.990018 | -0.235781 | 391 | 514.49 | 1.315831 |
| GO:0007275\_multicellular\_organismal\_development | MAB21L1 | 1760 | 14 | 0.990018 | -0.235781 | 391 | 514.49 | 1.315831 |
| GO:0007275\_multicellular\_organismal\_development | NCOR1 | 1760 | 14 | 0.990018 | -0.235781 | 391 | 514.49 | 1.315831 |
| GO:0010557\_positive\_regulation\_of\_macromolecule\_biosynthetic\_process | KLF7 | 371 | 3 | 1.006411 | -0.234570 | 392 | 515.12 | 1.314082 |
| GO:0010557\_positive\_regulation\_of\_macromolecule\_biosynthetic\_process | NCOA2 | 371 | 3 | 1.006411 | -0.234570 | 392 | 515.12 | 1.314082 |
| GO:0010557\_positive\_regulation\_of\_macromolecule\_biosynthetic\_process | RXRB | 371 | 3 | 1.006411 | -0.234570 | 392 | 515.12 | 1.314082 |
| GO:0010647\_positive\_regulation\_of\_cell\_communication | NCAM1 | 110 | 1 | 1.131450 | -0.227194 | 395 | 518.68 | 1.313114 |
| GO:0010648\_negative\_regulation\_of\_cell\_communication | NCOR1 | 110 | 1 | 1.131450 | -0.227194 | 395 | 518.68 | 1.313114 |
| GO:0055080\_cation\_homeostasis | SLC30A1 | 110 | 1 | 1.131450 | -0.227194 | 395 | 518.68 | 1.313114 |
| GO:0045321\_leukocyte\_activation | MYH9 | 248 | 2 | 1.003705 | -0.221477 | 396 | 521.02 | 1.315707 |
| GO:0045321\_leukocyte\_activation | NCOR1 | 248 | 2 | 1.003705 | -0.221477 | 396 | 521.02 | 1.315707 |
| GO:0040008\_regulation\_of\_growth | NCOR1 | 113 | 1 | 1.101411 | -0.219943 | 397 | 521.92 | 1.314660 |
| GO:0043687\_post-translational\_protein\_modification | UBE2F | 384 | 3 | 0.972340 | -0.216899 | 398 | 523.8 | 1.316080 |
| GO:0043687\_post-translational\_protein\_modification | PPM1M | 384 | 3 | 0.972340 | -0.216899 | 398 | 523.8 | 1.316080 |
| GO:0043687\_post-translational\_protein\_modification | NCOR1 | 384 | 3 | 0.972340 | -0.216899 | 398 | 523.8 | 1.316080 |
| GO:0030097\_hemopoiesis | HEPH | 253 | 2 | 0.983869 | -0.213501 | 399 | 526.12 | 1.318596 |
| GO:0030097\_hemopoiesis | NCOR1 | 253 | 2 | 0.983869 | -0.213501 | 399 | 526.12 | 1.318596 |
| GO:0031328\_positive\_regulation\_of\_cellular\_biosynthetic\_process | KLF7 | 387 | 3 | 0.964802 | -0.213009 | 400 | 526.26 | 1.315650 |
| GO:0031328\_positive\_regulation\_of\_cellular\_biosynthetic\_process | NCOA2 | 387 | 3 | 0.964802 | -0.213009 | 400 | 526.26 | 1.315650 |
| GO:0031328\_positive\_regulation\_of\_cellular\_biosynthetic\_process | RXRB | 387 | 3 | 0.964802 | -0.213009 | 400 | 526.26 | 1.315650 |
| GO:0009891\_positive\_regulation\_of\_biosynthetic\_process | KLF7 | 388 | 3 | 0.962315 | -0.211728 | 401 | 527.92 | 1.316509 |
| GO:0009891\_positive\_regulation\_of\_biosynthetic\_process | NCOA2 | 388 | 3 | 0.962315 | -0.211728 | 401 | 527.92 | 1.316509 |
| GO:0009891\_positive\_regulation\_of\_biosynthetic\_process | RXRB | 388 | 3 | 0.962315 | -0.211728 | 401 | 527.92 | 1.316509 |
| GO:0043933\_macromolecular\_complex\_subunit\_organization | FKBP4 | 117 | 1 | 1.063756 | -0.210720 | 402 | 528.31 | 1.314204 |
| GO:0009966\_regulation\_of\_signal\_transduction | NCAM1 | 256 | 2 | 0.972340 | -0.208862 | 403 | 529.36 | 1.313548 |
| GO:0009966\_regulation\_of\_signal\_transduction | NCOR1 | 256 | 2 | 0.972340 | -0.208862 | 403 | 529.36 | 1.313548 |
| GO:0006519\_cellular\_amino\_acid\_and\_derivative\_metabolic\_process | AHCY | 118 | 1 | 1.054741 | -0.208489 | 405 | 530.21 | 1.309160 |
| GO:0051960\_regulation\_of\_nervous\_system\_development | YWHAH | 118 | 1 | 1.054741 | -0.208489 | 405 | 530.21 | 1.309160 |
| GO:0019538\_protein\_metabolic\_process | SH3GLB1 | 655 | 5 | 0.950072 | -0.206682 | 406 | 531.06 | 1.308030 |
| GO:0019538\_protein\_metabolic\_process | TIA1 | 655 | 5 | 0.950072 | -0.206682 | 406 | 531.06 | 1.308030 |
| GO:0019538\_protein\_metabolic\_process | UBE2F | 655 | 5 | 0.950072 | -0.206682 | 406 | 531.06 | 1.308030 |
| GO:0019538\_protein\_metabolic\_process | PPM1M | 655 | 5 | 0.950072 | -0.206682 | 406 | 531.06 | 1.308030 |
| GO:0019538\_protein\_metabolic\_process | NCOR1 | 655 | 5 | 0.950072 | -0.206682 | 406 | 531.06 | 1.308030 |
| GO:0006917\_induction\_of\_apoptosis | SH3GLB1 | 121 | 1 | 1.028591 | -0.201972 | 408 | 534.44 | 1.309902 |
| GO:0012502\_induction\_of\_programmed\_cell\_death | SH3GLB1 | 121 | 1 | 1.028591 | -0.201972 | 408 | 534.44 | 1.309902 |
| GO:0048699\_generation\_of\_neurons | KLF7 | 396 | 3 | 0.942875 | -0.201743 | 409 | 534.7 | 1.307335 |
| GO:0048699\_generation\_of\_neurons | YWHAH | 396 | 3 | 0.942875 | -0.201743 | 409 | 534.7 | 1.307335 |
| GO:0048699\_generation\_of\_neurons | VAX2 | 396 | 3 | 0.942875 | -0.201743 | 409 | 534.7 | 1.307335 |
| GO:0001775\_cell\_activation | MYH9 | 262 | 2 | 0.950072 | -0.199898 | 410 | 535.7 | 1.306585 |
| GO:0001775\_cell\_activation | NCOR1 | 262 | 2 | 0.950072 | -0.199898 | 410 | 535.7 | 1.306585 |
| GO:0001816\_cytokine\_production | TIA1 | 122 | 1 | 1.020160 | -0.199855 | 412 | 537.96 | 1.305728 |
| GO:0060284\_regulation\_of\_cell\_development | YWHAH | 122 | 1 | 1.020160 | -0.199855 | 412 | 537.96 | 1.305728 |
| GO:0048856\_anatomical\_structure\_development | KLF7 | 1688 | 13 | 0.958515 | -0.195976 | 413 | 539.09 | 1.305303 |
| GO:0048856\_anatomical\_structure\_development | RXRB | 1688 | 13 | 0.958515 | -0.195976 | 413 | 539.09 | 1.305303 |
| GO:0048856\_anatomical\_structure\_development | FKBP4 | 1688 | 13 | 0.958515 | -0.195976 | 413 | 539.09 | 1.305303 |
| GO:0048856\_anatomical\_structure\_development | VAX2 | 1688 | 13 | 0.958515 | -0.195976 | 413 | 539.09 | 1.305303 |
| GO:0048856\_anatomical\_structure\_development | HES6 | 1688 | 13 | 0.958515 | -0.195976 | 413 | 539.09 | 1.305303 |
| GO:0048856\_anatomical\_structure\_development | MYH9 | 1688 | 13 | 0.958515 | -0.195976 | 413 | 539.09 | 1.305303 |
| GO:0048856\_anatomical\_structure\_development | FOXP2 | 1688 | 13 | 0.958515 | -0.195976 | 413 | 539.09 | 1.305303 |
| GO:0048856\_anatomical\_structure\_development | NRIP1 | 1688 | 13 | 0.958515 | -0.195976 | 413 | 539.09 | 1.305303 |
| GO:0048856\_anatomical\_structure\_development | YWHAH | 1688 | 13 | 0.958515 | -0.195976 | 413 | 539.09 | 1.305303 |
| GO:0048856\_anatomical\_structure\_development | HEPH | 1688 | 13 | 0.958515 | -0.195976 | 413 | 539.09 | 1.305303 |
| GO:0048856\_anatomical\_structure\_development | SEPP1 | 1688 | 13 | 0.958515 | -0.195976 | 413 | 539.09 | 1.305303 |
| GO:0048856\_anatomical\_structure\_development | MAB21L1 | 1688 | 13 | 0.958515 | -0.195976 | 413 | 539.09 | 1.305303 |
| GO:0048856\_anatomical\_structure\_development | NCOR1 | 1688 | 13 | 0.958515 | -0.195976 | 413 | 539.09 | 1.305303 |
| GO:0009308\_amine\_metabolic\_process | AHCY | 124 | 1 | 1.003705 | -0.195702 | 415 | 540.02 | 1.301253 |
| GO:0030098\_lymphocyte\_differentiation | NCOR1 | 124 | 1 | 1.003705 | -0.195702 | 415 | 540.02 | 1.301253 |
| GO:0043062\_extracellular\_structure\_organization | CRISPLD2 | 125 | 1 | 0.995676 | -0.193666 | 416 | 540.75 | 1.299880 |
| GO:0045944\_positive\_regulation\_of\_transcription\_from\_RNA\_polymerase\_II\_promoter | NCOA2 | 269 | 2 | 0.925349 | -0.189945 | 417 | 541.53 | 1.298633 |
| GO:0045944\_positive\_regulation\_of\_transcription\_from\_RNA\_polymerase\_II\_promoter | RXRB | 269 | 2 | 0.925349 | -0.189945 | 417 | 541.53 | 1.298633 |
| GO:0001655\_urogenital\_system\_development | FKBP4 | 128 | 1 | 0.972340 | -0.187708 | 418 | 543.5 | 1.300239 |
| GO:0048534\_hemopoietic\_or\_lymphoid\_organ\_development | HEPH | 277 | 2 | 0.898624 | -0.179197 | 419 | 548.97 | 1.310191 |
| GO:0048534\_hemopoietic\_or\_lymphoid\_organ\_development | NCOR1 | 277 | 2 | 0.898624 | -0.179197 | 419 | 548.97 | 1.310191 |
| GO:0007610\_behavior | SEPP1 | 279 | 2 | 0.892183 | -0.176610 | 420 | 550.42 | 1.310524 |
| GO:0007610\_behavior | FOXP2 | 279 | 2 | 0.892183 | -0.176610 | 420 | 550.42 | 1.310524 |
| GO:0065008\_regulation\_of\_biological\_quality | CYB5R4 | 693 | 5 | 0.897976 | -0.171597 | 421 | 552.3 | 1.311876 |
| GO:0065008\_regulation\_of\_biological\_quality | SLC30A1 | 693 | 5 | 0.897976 | -0.171597 | 421 | 552.3 | 1.311876 |
| GO:0065008\_regulation\_of\_biological\_quality | HEPH | 693 | 5 | 0.897976 | -0.171597 | 421 | 552.3 | 1.311876 |
| GO:0065008\_regulation\_of\_biological\_quality | MYH9 | 693 | 5 | 0.897976 | -0.171597 | 421 | 552.3 | 1.311876 |
| GO:0065008\_regulation\_of\_biological\_quality | NCOR1 | 693 | 5 | 0.897976 | -0.171597 | 421 | 552.3 | 1.311876 |
| GO:0022008\_neurogenesis | KLF7 | 423 | 3 | 0.882691 | -0.171293 | 422 | 553.07 | 1.310592 |
| GO:0022008\_neurogenesis | YWHAH | 423 | 3 | 0.882691 | -0.171293 | 422 | 553.07 | 1.310592 |
| GO:0022008\_neurogenesis | VAX2 | 423 | 3 | 0.882691 | -0.171293 | 422 | 553.07 | 1.310592 |
| GO:0009790\_embryonic\_development | SLC30A1 | 567 | 4 | 0.878021 | -0.164321 | 423 | 557.09 | 1.316998 |
| GO:0009790\_embryonic\_development | VAX2 | 567 | 4 | 0.878021 | -0.164321 | 423 | 557.09 | 1.316998 |
| GO:0009790\_embryonic\_development | MYH9 | 567 | 4 | 0.878021 | -0.164321 | 423 | 557.09 | 1.316998 |
| GO:0009790\_embryonic\_development | FOXP2 | 567 | 4 | 0.878021 | -0.164321 | 423 | 557.09 | 1.316998 |
| GO:0045596\_negative\_regulation\_of\_cell\_differentiation | YWHAH | 144 | 1 | 0.864302 | -0.159408 | 424 | 559.76 | 1.320189 |
| GO:0048513\_organ\_development | RXRB | 1365 | 10 | 0.911791 | -0.157675 | 425 | 560.06 | 1.317788 |
| GO:0048513\_organ\_development | FKBP4 | 1365 | 10 | 0.911791 | -0.157675 | 425 | 560.06 | 1.317788 |
| GO:0048513\_organ\_development | HEPH | 1365 | 10 | 0.911791 | -0.157675 | 425 | 560.06 | 1.317788 |
| GO:0048513\_organ\_development | VAX2 | 1365 | 10 | 0.911791 | -0.157675 | 425 | 560.06 | 1.317788 |
| GO:0048513\_organ\_development | SEPP1 | 1365 | 10 | 0.911791 | -0.157675 | 425 | 560.06 | 1.317788 |
| GO:0048513\_organ\_development | MAB21L1 | 1365 | 10 | 0.911791 | -0.157675 | 425 | 560.06 | 1.317788 |
| GO:0048513\_organ\_development | MYH9 | 1365 | 10 | 0.911791 | -0.157675 | 425 | 560.06 | 1.317788 |
| GO:0048513\_organ\_development | NCOR1 | 1365 | 10 | 0.911791 | -0.157675 | 425 | 560.06 | 1.317788 |
| GO:0048513\_organ\_development | NRIP1 | 1365 | 10 | 0.911791 | -0.157675 | 425 | 560.06 | 1.317788 |
| GO:0048513\_organ\_development | FOXP2 | 1365 | 10 | 0.911791 | -0.157675 | 425 | 560.06 | 1.317788 |
| GO:0002520\_immune\_system\_development | HEPH | 295 | 2 | 0.843793 | -0.157243 | 426 | 561.09 | 1.317113 |
| GO:0002520\_immune\_system\_development | NCOR1 | 295 | 2 | 0.843793 | -0.157243 | 426 | 561.09 | 1.317113 |
| GO:0030900\_forebrain\_development | NCOR1 | 146 | 1 | 0.852462 | -0.156238 | 427 | 562.32 | 1.316909 |
| GO:0006464\_protein\_modification\_process | UBE2F | 439 | 3 | 0.850520 | -0.155384 | 428 | 562.56 | 1.314393 |
| GO:0006464\_protein\_modification\_process | PPM1M | 439 | 3 | 0.850520 | -0.155384 | 428 | 562.56 | 1.314393 |
| GO:0006464\_protein\_modification\_process | NCOR1 | 439 | 3 | 0.850520 | -0.155384 | 428 | 562.56 | 1.314393 |
| GO:0032940\_secretion\_by\_cell | CYB5R4 | 149 | 1 | 0.835298 | -0.151619 | 429 | 564.78 | 1.316503 |
| GO:0008285\_negative\_regulation\_of\_cell\_proliferation | E2F7 | 155 | 1 | 0.802964 | -0.142849 | 430 | 570.18 | 1.326000 |
| GO:0051704\_multi-organism\_process | FKBP4 | 157 | 1 | 0.792735 | -0.140057 | 431 | 570.92 | 1.324640 |
| GO:0043412\_biopolymer\_modification | UBE2F | 458 | 3 | 0.815237 | -0.138316 | 432 | 573.1 | 1.326620 |
| GO:0043412\_biopolymer\_modification | PPM1M | 458 | 3 | 0.815237 | -0.138316 | 432 | 573.1 | 1.326620 |
| GO:0043412\_biopolymer\_modification | NCOR1 | 458 | 3 | 0.815237 | -0.138316 | 432 | 573.1 | 1.326620 |
| GO:0002521\_leukocyte\_differentiation | NCOR1 | 161 | 1 | 0.773040 | -0.134657 | 433 | 573.82 | 1.325219 |
| GO:0007626\_locomotory\_behavior | SEPP1 | 163 | 1 | 0.763555 | -0.132047 | 434 | 575.23 | 1.325415 |
| GO:0043065\_positive\_regulation\_of\_apoptosis | SH3GLB1 | 166 | 1 | 0.749756 | -0.128239 | 435 | 577.16 | 1.326805 |
| GO:0010942\_positive\_regulation\_of\_cell\_death | SH3GLB1 | 167 | 1 | 0.745266 | -0.126997 | 437 | 578.11 | 1.322906 |
| GO:0043068\_positive\_regulation\_of\_programmed\_cell\_death | SH3GLB1 | 167 | 1 | 0.745266 | -0.126997 | 437 | 578.11 | 1.322906 |
| GO:0032501\_multicellular\_organismal\_process | KLF7 | 2183 | 16 | 0.912209 | -0.125421 | 438 | 578.22 | 1.320137 |
| GO:0032501\_multicellular\_organismal\_process | SLC12A2 | 2183 | 16 | 0.912209 | -0.125421 | 438 | 578.22 | 1.320137 |
| GO:0032501\_multicellular\_organismal\_process | RXRB | 2183 | 16 | 0.912209 | -0.125421 | 438 | 578.22 | 1.320137 |
| GO:0032501\_multicellular\_organismal\_process | FKBP4 | 2183 | 16 | 0.912209 | -0.125421 | 438 | 578.22 | 1.320137 |
| GO:0032501\_multicellular\_organismal\_process | VAX2 | 2183 | 16 | 0.912209 | -0.125421 | 438 | 578.22 | 1.320137 |
| GO:0032501\_multicellular\_organismal\_process | HES6 | 2183 | 16 | 0.912209 | -0.125421 | 438 | 578.22 | 1.320137 |
| GO:0032501\_multicellular\_organismal\_process | MYH9 | 2183 | 16 | 0.912209 | -0.125421 | 438 | 578.22 | 1.320137 |
| GO:0032501\_multicellular\_organismal\_process | FOXP2 | 2183 | 16 | 0.912209 | -0.125421 | 438 | 578.22 | 1.320137 |
| GO:0032501\_multicellular\_organismal\_process | NRIP1 | 2183 | 16 | 0.912209 | -0.125421 | 438 | 578.22 | 1.320137 |
| GO:0032501\_multicellular\_organismal\_process | YWHAH | 2183 | 16 | 0.912209 | -0.125421 | 438 | 578.22 | 1.320137 |
| GO:0032501\_multicellular\_organismal\_process | SLC30A1 | 2183 | 16 | 0.912209 | -0.125421 | 438 | 578.22 | 1.320137 |
| GO:0032501\_multicellular\_organismal\_process | TIA1 | 2183 | 16 | 0.912209 | -0.125421 | 438 | 578.22 | 1.320137 |
| GO:0032501\_multicellular\_organismal\_process | HEPH | 2183 | 16 | 0.912209 | -0.125421 | 438 | 578.22 | 1.320137 |
| GO:0032501\_multicellular\_organismal\_process | SEPP1 | 2183 | 16 | 0.912209 | -0.125421 | 438 | 578.22 | 1.320137 |
| GO:0032501\_multicellular\_organismal\_process | MAB21L1 | 2183 | 16 | 0.912209 | -0.125421 | 438 | 578.22 | 1.320137 |
| GO:0032501\_multicellular\_organismal\_process | NCOR1 | 2183 | 16 | 0.912209 | -0.125421 | 438 | 578.22 | 1.320137 |
| GO:0006928\_cell\_motion | KLF7 | 330 | 2 | 0.754300 | -0.122045 | 441 | 580.36 | 1.316009 |
| GO:0006928\_cell\_motion | MYH9 | 330 | 2 | 0.754300 | -0.122045 | 441 | 580.36 | 1.316009 |
| GO:0010646\_regulation\_of\_cell\_communication | NCAM1 | 330 | 2 | 0.754300 | -0.122045 | 441 | 580.36 | 1.316009 |
| GO:0010646\_regulation\_of\_cell\_communication | NCOR1 | 330 | 2 | 0.754300 | -0.122045 | 441 | 580.36 | 1.316009 |
| GO:0051674\_localization\_of\_cell | KLF7 | 330 | 2 | 0.754300 | -0.122045 | 441 | 580.36 | 1.316009 |
| GO:0051674\_localization\_of\_cell | MYH9 | 330 | 2 | 0.754300 | -0.122045 | 441 | 580.36 | 1.316009 |
| GO:0007600\_sensory\_perception | SLC12A2 | 172 | 1 | 0.723602 | -0.120989 | 442 | 581.62 | 1.315882 |
| GO:0044248\_cellular\_catabolic\_process | AHCY | 173 | 1 | 0.719419 | -0.119826 | 443 | 583.13 | 1.316321 |
| GO:0046903\_secretion | CYB5R4 | 175 | 1 | 0.711197 | -0.117537 | 444 | 584.33 | 1.316059 |
| GO:0006873\_cellular\_ion\_homeostasis | SLC30A1 | 176 | 1 | 0.707156 | -0.116411 | 445 | 585.39 | 1.315483 |
| GO:0009605\_response\_to\_external\_stimulus | SLC12A2 | 339 | 2 | 0.734274 | -0.114349 | 446 | 586.14 | 1.314215 |
| GO:0009605\_response\_to\_external\_stimulus | FOXP2 | 339 | 2 | 0.734274 | -0.114349 | 446 | 586.14 | 1.314215 |
| GO:0048732\_gland\_development | FKBP4 | 179 | 1 | 0.695304 | -0.113105 | 447 | 587.77 | 1.314922 |
| GO:0007165\_signal\_transduction | NCAM1 | 915 | 6 | 0.816128 | -0.111951 | 448 | 588.56 | 1.313750 |
| GO:0007165\_signal\_transduction | RXRB | 915 | 6 | 0.816128 | -0.111951 | 448 | 588.56 | 1.313750 |
| GO:0007165\_signal\_transduction | FKBP4 | 915 | 6 | 0.816128 | -0.111951 | 448 | 588.56 | 1.313750 |
| GO:0007165\_signal\_transduction | LRBA | 915 | 6 | 0.816128 | -0.111951 | 448 | 588.56 | 1.313750 |
| GO:0007165\_signal\_transduction | TLE4 | 915 | 6 | 0.816128 | -0.111951 | 448 | 588.56 | 1.313750 |
| GO:0007165\_signal\_transduction | NCOR1 | 915 | 6 | 0.816128 | -0.111951 | 448 | 588.56 | 1.313750 |
| GO:0055082\_cellular\_chemical\_homeostasis | SLC30A1 | 181 | 1 | 0.687621 | -0.110959 | 449 | 589.94 | 1.313898 |
| GO:0016192\_vesicle-mediated\_transport | LRBA | 184 | 1 | 0.676410 | -0.107824 | 450 | 591.76 | 1.315022 |
| GO:0007010\_cytoskeleton\_organization | MYH9 | 185 | 1 | 0.672754 | -0.106801 | 451 | 592.61 | 1.313991 |
| GO:0002376\_immune\_system\_process | HEPH | 505 | 3 | 0.739363 | -0.103380 | 452 | 595.01 | 1.316394 |
| GO:0002376\_immune\_system\_process | MYH9 | 505 | 3 | 0.739363 | -0.103380 | 452 | 595.01 | 1.316394 |
| GO:0002376\_immune\_system\_process | NCOR1 | 505 | 3 | 0.739363 | -0.103380 | 452 | 595.01 | 1.316394 |
| GO:0003002\_regionalization | VAX2 | 195 | 1 | 0.638254 | -0.097138 | 455 | 598.26 | 1.314857 |
| GO:0007507\_heart\_development | RXRB | 195 | 1 | 0.638254 | -0.097138 | 455 | 598.26 | 1.314857 |
| GO:0019725\_cellular\_homeostasis | SLC30A1 | 195 | 1 | 0.638254 | -0.097138 | 455 | 598.26 | 1.314857 |
| GO:0050801\_ion\_homeostasis | SLC30A1 | 197 | 1 | 0.631774 | -0.095322 | 456 | 599.66 | 1.315044 |
| GO:0043009\_chordate\_embryonic\_development | SLC30A1 | 365 | 2 | 0.681970 | -0.094714 | 457 | 600.38 | 1.313742 |
| GO:0043009\_chordate\_embryonic\_development | MYH9 | 365 | 2 | 0.681970 | -0.094714 | 457 | 600.38 | 1.313742 |
| GO:0009792\_embryonic\_development\_ending\_in\_birth\_or\_egg\_hatching | SLC30A1 | 368 | 2 | 0.676410 | -0.092675 | 458 | 602.08 | 1.314585 |
| GO:0009792\_embryonic\_development\_ending\_in\_birth\_or\_egg\_hatching | MYH9 | 368 | 2 | 0.676410 | -0.092675 | 458 | 602.08 | 1.314585 |
| GO:0009888\_tissue\_development | RXRB | 525 | 3 | 0.711197 | -0.091186 | 459 | 602.63 | 1.312919 |
| GO:0009888\_tissue\_development | MYH9 | 525 | 3 | 0.711197 | -0.091186 | 459 | 602.63 | 1.312919 |
| GO:0009888\_tissue\_development | FOXP2 | 525 | 3 | 0.711197 | -0.091186 | 459 | 602.63 | 1.312919 |
| GO:0007154\_cell\_communication | NCAM1 | 1096 | 7 | 0.794905 | -0.090180 | 460 | 603.25 | 1.311413 |
| GO:0007154\_cell\_communication | CYB5R4 | 1096 | 7 | 0.794905 | -0.090180 | 460 | 603.25 | 1.311413 |
| GO:0007154\_cell\_communication | RXRB | 1096 | 7 | 0.794905 | -0.090180 | 460 | 603.25 | 1.311413 |
| GO:0007154\_cell\_communication | FKBP4 | 1096 | 7 | 0.794905 | -0.090180 | 460 | 603.25 | 1.311413 |
| GO:0007154\_cell\_communication | LRBA | 1096 | 7 | 0.794905 | -0.090180 | 460 | 603.25 | 1.311413 |
| GO:0007154\_cell\_communication | TLE4 | 1096 | 7 | 0.794905 | -0.090180 | 460 | 603.25 | 1.311413 |
| GO:0007154\_cell\_communication | NCOR1 | 1096 | 7 | 0.794905 | -0.090180 | 460 | 603.25 | 1.311413 |
| GO:0022607\_cellular\_component\_assembly | FKBP4 | 204 | 1 | 0.610095 | -0.089253 | 461 | 604.13 | 1.310477 |
| GO:0007243\_protein\_kinase\_cascade | NCOR1 | 205 | 1 | 0.607119 | -0.088421 | 462 | 604.85 | 1.309199 |
| GO:0035295\_tube\_development | FOXP2 | 212 | 1 | 0.587073 | -0.082825 | 463 | 606.76 | 1.310497 |
| GO:0050877\_neurological\_system\_process | SLC12A2 | 390 | 2 | 0.638254 | -0.078976 | 464 | 608.79 | 1.312047 |
| GO:0050877\_neurological\_system\_process | FOXP2 | 390 | 2 | 0.638254 | -0.078976 | 464 | 608.79 | 1.312047 |
| GO:0044085\_cellular\_component\_biogenesis | FKBP4 | 237 | 1 | 0.525145 | -0.065731 | 465 | 616.61 | 1.326043 |
| GO:0009056\_catabolic\_process | AHCY | 243 | 1 | 0.512179 | -0.062212 | 466 | 617.67 | 1.325472 |
| GO:0051239\_regulation\_of\_multicellular\_organismal\_process | YWHAH | 587 | 3 | 0.636079 | -0.061357 | 467 | 618.04 | 1.323426 |
| GO:0051239\_regulation\_of\_multicellular\_organismal\_process | TIA1 | 587 | 3 | 0.636079 | -0.061357 | 467 | 618.04 | 1.323426 |
| GO:0051239\_regulation\_of\_multicellular\_organismal\_process | NCOR1 | 587 | 3 | 0.636079 | -0.061357 | 467 | 618.04 | 1.323426 |
| GO:0007389\_pattern\_specification\_process | VAX2 | 250 | 1 | 0.497838 | -0.058355 | 468 | 619.84 | 1.324444 |
| GO:0007267\_cell-cell\_signaling | CYB5R4 | 252 | 1 | 0.493887 | -0.057299 | 469 | 621.29 | 1.324712 |
| GO:0010926\_anatomical\_structure\_formation | FKBP4 | 447 | 2 | 0.556866 | -0.052001 | 470 | 625.74 | 1.331362 |
| GO:0010926\_anatomical\_structure\_formation | MYH9 | 447 | 2 | 0.556866 | -0.052001 | 470 | 625.74 | 1.331362 |
| GO:0044255\_cellular\_lipid\_metabolic\_process | SH3GLB1 | 264 | 1 | 0.471437 | -0.051369 | 471 | 626.17 | 1.329448 |
| GO:0048646\_anatomical\_structure\_formation\_involved\_in\_morphogenesis | MYH9 | 277 | 1 | 0.449312 | -0.045656 | 472 | 629.81 | 1.334343 |
| GO:0006629\_lipid\_metabolic\_process | SH3GLB1 | 285 | 1 | 0.436700 | -0.042470 | 473 | 631.5 | 1.335095 |
| GO:0045595\_regulation\_of\_cell\_differentiation | YWHAH | 295 | 1 | 0.421896 | -0.038804 | 474 | 633.77 | 1.337068 |
| GO:0048598\_embryonic\_morphogenesis | VAX2 | 299 | 1 | 0.416252 | -0.037430 | 475 | 634.08 | 1.334905 |
| GO:0051094\_positive\_regulation\_of\_developmental\_process | SH3GLB1 | 308 | 1 | 0.404089 | -0.034516 | 476 | 634.8 | 1.333613 |
| GO:0003008\_system\_process | SLC12A2 | 516 | 2 | 0.482401 | -0.031065 | 477 | 635.86 | 1.333040 |
| GO:0003008\_system\_process | FOXP2 | 516 | 2 | 0.482401 | -0.031065 | 477 | 635.86 | 1.333040 |
| GO:0050793\_regulation\_of\_developmental\_process | YWHAH | 703 | 3 | 0.531121 | -0.028285 | 478 | 637.08 | 1.332803 |
| GO:0050793\_regulation\_of\_developmental\_process | SH3GLB1 | 703 | 3 | 0.531121 | -0.028285 | 478 | 637.08 | 1.332803 |
| GO:0050793\_regulation\_of\_developmental\_process | MYH9 | 703 | 3 | 0.531121 | -0.028285 | 478 | 637.08 | 1.332803 |
| GO:0051093\_negative\_regulation\_of\_developmental\_process | YWHAH | 331 | 1 | 0.376010 | -0.028069 | 479 | 637.45 | 1.330793 |
| GO:0006793\_phosphorus\_metabolic\_process | PPM1M | 340 | 1 | 0.366057 | -0.025890 | 481 | 638.62 | 1.327692 |
| GO:0006796\_phosphate\_metabolic\_process | PPM1M | 340 | 1 | 0.366057 | -0.025890 | 481 | 638.62 | 1.327692 |
| GO:0042981\_regulation\_of\_apoptosis | SH3GLB1 | 360 | 1 | 0.345721 | -0.021636 | 482 | 640.45 | 1.328734 |
| GO:0010941\_regulation\_of\_cell\_death | SH3GLB1 | 365 | 1 | 0.340985 | -0.020687 | 484 | 641.34 | 1.325083 |
| GO:0043067\_regulation\_of\_programmed\_cell\_death | SH3GLB1 | 365 | 1 | 0.340985 | -0.020687 | 484 | 641.34 | 1.325083 |
| GO:0007166\_cell\_surface\_receptor\_linked\_signal\_transduction | NCAM1 | 597 | 2 | 0.416950 | -0.016692 | 485 | 643.87 | 1.327567 |
| GO:0007166\_cell\_surface\_receptor\_linked\_signal\_transduction | TLE4 | 597 | 2 | 0.416950 | -0.016692 | 485 | 643.87 | 1.327567 |
| GO:0009653\_anatomical\_structure\_morphogenesis | KLF7 | 958 | 4 | 0.519664 | -0.015369 | 486 | 644.45 | 1.326029 |
| GO:0009653\_anatomical\_structure\_morphogenesis | YWHAH | 958 | 4 | 0.519664 | -0.015369 | 486 | 644.45 | 1.326029 |
| GO:0009653\_anatomical\_structure\_morphogenesis | VAX2 | 958 | 4 | 0.519664 | -0.015369 | 486 | 644.45 | 1.326029 |
| GO:0009653\_anatomical\_structure\_morphogenesis | MYH9 | 958 | 4 | 0.519664 | -0.015369 | 486 | 644.45 | 1.326029 |
| GO:0042221\_response\_to\_chemical\_stimulus | CYB5R4 | 409 | 1 | 0.304302 | -0.013931 | 487 | 644.73 | 1.323881 |
| GO:0006915\_apoptosis | SH3GLB1 | 427 | 1 | 0.291474 | -0.011846 | 488 | 645.67 | 1.323094 |
| GO:0012501\_programmed\_cell\_death | SH3GLB1 | 433 | 1 | 0.287435 | -0.011222 | 489 | 646.02 | 1.321104 |
| GO:0008219\_cell\_death | SH3GLB1 | 444 | 1 | 0.280314 | -0.010161 | 490 | 646.82 | 1.320041 |
| GO:0016265\_death | SH3GLB1 | 450 | 1 | 0.276577 | -0.009625 | 491 | 647.1 | 1.317923 |
| GO:0050896\_response\_to\_stimulus | CYB5R4 | 1107 | 4 | 0.449718 | -0.005321 | 492 | 648.32 | 1.317724 |
| GO:0050896\_response\_to\_stimulus | SLC12A2 | 1107 | 4 | 0.449718 | -0.005321 | 492 | 648.32 | 1.317724 |
| GO:0050896\_response\_to\_stimulus | SEPP1 | 1107 | 4 | 0.449718 | -0.005321 | 492 | 648.32 | 1.317724 |
| GO:0050896\_response\_to\_stimulus | FOXP2 | 1107 | 4 | 0.449718 | -0.005321 | 492 | 648.32 | 1.317724 |
| GO:0009887\_organ\_morphogenesis | VAX2 | 642 | 1 | 0.193862 | -0.001643 | 493 | 649.77 | 1.317992 |
| GO:0008150\_biological\_process | SLC8A3 | 4605 | 37 | 1.000000 | 0.000000 | 1329 | 1417.41 | 1.066524 |
| GO:0008150\_biological\_process | CYB5R4 | 4605 | 37 | 1.000000 | 0.000000 | 1329 | 1417.41 | 1.066524 |
| GO:0008150\_biological\_process | AHCY | 4605 | 37 | 1.000000 | 0.000000 | 1329 | 1417.41 | 1.066524 |
| GO:0008150\_biological\_process | NHP2L1 | 4605 | 37 | 1.000000 | 0.000000 | 1329 | 1417.41 | 1.066524 |
| GO:0008150\_biological\_process | FKBP4 | 4605 | 37 | 1.000000 | 0.000000 | 1329 | 1417.41 | 1.066524 |
| GO:0008150\_biological\_process | E2F7 | 4605 | 37 | 1.000000 | 0.000000 | 1329 | 1417.41 | 1.066524 |
| GO:0008150\_biological\_process | LRBA | 4605 | 37 | 1.000000 | 0.000000 | 1329 | 1417.41 | 1.066524 |
| GO:0008150\_biological\_process | CRISPLD2 | 4605 | 37 | 1.000000 | 0.000000 | 1329 | 1417.41 | 1.066524 |
| GO:0008150\_biological\_process | SLC30A1 | 4605 | 37 | 1.000000 | 0.000000 | 1329 | 1417.41 | 1.066524 |
| GO:0008150\_biological\_process | SH3GLB1 | 4605 | 37 | 1.000000 | 0.000000 | 1329 | 1417.41 | 1.066524 |
| GO:0008150\_biological\_process | TIA1 | 4605 | 37 | 1.000000 | 0.000000 | 1329 | 1417.41 | 1.066524 |
| GO:0008150\_biological\_process | MSI2 | 4605 | 37 | 1.000000 | 0.000000 | 1329 | 1417.41 | 1.066524 |
| GO:0008150\_biological\_process | SEPP1 | 4605 | 37 | 1.000000 | 0.000000 | 1329 | 1417.41 | 1.066524 |
| GO:0008150\_biological\_process | KLF7 | 4605 | 37 | 1.000000 | 0.000000 | 1329 | 1417.41 | 1.066524 |
| GO:0008150\_biological\_process | SLC12A2 | 4605 | 37 | 1.000000 | 0.000000 | 1329 | 1417.41 | 1.066524 |
| GO:0008150\_biological\_process | MPDZ | 4605 | 37 | 1.000000 | 0.000000 | 1329 | 1417.41 | 1.066524 |
| GO:0008150\_biological\_process | RXRB | 4605 | 37 | 1.000000 | 0.000000 | 1329 | 1417.41 | 1.066524 |
| GO:0008150\_biological\_process | TLE4 | 4605 | 37 | 1.000000 | 0.000000 | 1329 | 1417.41 | 1.066524 |
| GO:0008150\_biological\_process | ELAVL1 | 4605 | 37 | 1.000000 | 0.000000 | 1329 | 1417.41 | 1.066524 |
| GO:0008150\_biological\_process | UBE2F | 4605 | 37 | 1.000000 | 0.000000 | 1329 | 1417.41 | 1.066524 |
| GO:0008150\_biological\_process | DOCK7 | 4605 | 37 | 1.000000 | 0.000000 | 1329 | 1417.41 | 1.066524 |
| GO:0008150\_biological\_process | VAX2 | 4605 | 37 | 1.000000 | 0.000000 | 1329 | 1417.41 | 1.066524 |
| GO:0008150\_biological\_process | HES6 | 4605 | 37 | 1.000000 | 0.000000 | 1329 | 1417.41 | 1.066524 |
| GO:0008150\_biological\_process | DACH1 | 4605 | 37 | 1.000000 | 0.000000 | 1329 | 1417.41 | 1.066524 |
| GO:0008150\_biological\_process | MYH9 | 4605 | 37 | 1.000000 | 0.000000 | 1329 | 1417.41 | 1.066524 |
| GO:0008150\_biological\_process | FOXP2 | 4605 | 37 | 1.000000 | 0.000000 | 1329 | 1417.41 | 1.066524 |
| GO:0008150\_biological\_process | NRIP1 | 4605 | 37 | 1.000000 | 0.000000 | 1329 | 1417.41 | 1.066524 |
| GO:0008150\_biological\_process | NCAM1 | 4605 | 37 | 1.000000 | 0.000000 | 1329 | 1417.41 | 1.066524 |
| GO:0008150\_biological\_process | YWHAH | 4605 | 37 | 1.000000 | 0.000000 | 1329 | 1417.41 | 1.066524 |
| GO:0008150\_biological\_process | NCOA2 | 4605 | 37 | 1.000000 | 0.000000 | 1329 | 1417.41 | 1.066524 |
| GO:0008150\_biological\_process | RAB1 | 4605 | 37 | 1.000000 | 0.000000 | 1329 | 1417.41 | 1.066524 |
| GO:0008150\_biological\_process | TIAL1 | 4605 | 37 | 1.000000 | 0.000000 | 1329 | 1417.41 | 1.066524 |
| GO:0008150\_biological\_process | HEPH | 4605 | 37 | 1.000000 | 0.000000 | 1329 | 1417.41 | 1.066524 |
| GO:0008150\_biological\_process | PPM1M | 4605 | 37 | 1.000000 | 0.000000 | 1329 | 1417.41 | 1.066524 |
| GO:0008150\_biological\_process | AKAP8 | 4605 | 37 | 1.000000 | 0.000000 | 1329 | 1417.41 | 1.066524 |
| GO:0008150\_biological\_process | MAB21L1 | 4605 | 37 | 1.000000 | 0.000000 | 1329 | 1417.41 | 1.066524 |
| GO:0008150\_biological\_process | NCOR1 | 4605 | 37 | 1.000000 | 0.000000 | 1329 | 1417.41 | 1.066524 |
