# Supplementary material for: RedundancyMiner: De-replication of redundant GO categories in microarray and proteomics analysis
Source: BMC Bioinformatics. 2011 Feb 10;12:52. doi: 10.1186/1471-2105-12-52 (PMC3223614; doi:10.1186/1471-2105-12-52)
Supplement: Additional file 8 — Retinal development HTGM download. compressed package of the results of running HTGM on the retinal development genes list. [file 1471-2105-12-52-S8.ZIP › SCENARIO_2_MODIFIED/total.txt.total.txt.dir/Exp1_BestClusterMap_LEIGS_KM_24.csv.join.18.txt.dir/Exp1_BestClusterMap_LEIGS_KM_24.csv.join.18.txt.change.html]

Category Summary Report for Exp1\_BestClusterMap\_LEIGS\_KM\_24.csv.join.18.txt

# Category Summary Report for Exp1\_BestClusterMap\_LEIGS\_KM\_24.csv.join.18.txt

| HYPERLINKED GO CATEGORY | TOTAL GENES | CHANGED GENES | ENRICHMENT | LOG10(p) | CUMULATIVE NUMBER OF CATEGORIES | CUMULATIVE RANDOMS LOWER BOUND | CUMULATIVE RANDOMS MEAN | CUMULATIVE RANDOMS UPPER BOUND | FALSE DISCOVERY RATE |
| --- | --- | --- | --- | --- | --- | --- | --- | --- | --- |
| GO:0000122\_negative\_regulation\_of\_transcription\_from\_RNA\_polymerase\_II\_promoter | 175 | 6 | 4.267181 | -2.618333 | 1 | -2.384876 | 2.59 | 7.564876 | 2.590000 |
| GO:0010558\_negative\_regulation\_of\_macromolecule\_biosynthetic\_process | 274 | 7 | 3.179621 | -2.269169 | 2 | -3.165003 | 4.92 | 13.005003 | 2.460000 |
| GO:0010468\_regulation\_of\_gene\_expression | 778 | 13 | 2.079657 | -2.255588 | 3 | -3.364185 | 5.0 | 13.364185 | 1.666667 |
| GO:0031327\_negative\_regulation\_of\_cellular\_biosynthetic\_process | 282 | 7 | 3.089419 | -2.201059 | 4 | -3.369601 | 5.45 | 14.269601 | 1.362500 |
| GO:0009890\_negative\_regulation\_of\_biosynthetic\_process | 284 | 7 | 3.067663 | -2.184430 | 5 | -3.358026 | 5.48 | 14.318026 | 1.096000 |
| GO:0045892\_negative\_regulation\_of\_transcription\_\_DNA-dependent | 218 | 6 | 3.425490 | -2.147557 | 6 | -3.194573 | 6.05 | 15.294573 | 1.008333 |
| GO:0051253\_negative\_regulation\_of\_RNA\_metabolic\_process | 220 | 6 | 3.394349 | -2.128566 | 7 | -3.205542 | 6.21 | 15.625542 | 0.887143 |
| GO:0001887\_selenium\_metabolic\_process | 1 | 1 |  |  |  |  |  |  |  |  |
| GO:0006463\_steroid\_hormone\_receptor\_complex\_assembly | 1 | 1 |  |  |  |  |  |  |  |  |
| GO:0006654\_phosphatidic\_acid\_biosynthetic\_process | 1 | 1 |  |  |  |  |  |  |  |  |
| GO:0007076\_mitotic\_chromosome\_condensation | 1 | 1 |  |  |  |  |  |  |  |  |
| GO:0019510\_S-adenosylhomocysteine\_catabolic\_process | 1 | 1 |  |  |  |  |  |  |  |  |
| GO:0042297\_vocal\_learning | 1 | 1 |  |  |  |  |  |  |  |  |
| GO:0046473\_phosphatidic\_acid\_metabolic\_process | 1 | 1 |  |  |  |  |  |  |  |  |
| GO:0046498\_S-adenosylhomocysteine\_metabolic\_process | 1 | 1 |  |  |  |  |  |  |  |  |
| GO:0048813\_dendrite\_morphogenesis | 18 | 2 | 13.828829 | -2.052356 | 8 | -2.735483 | 7.46 | 17.655483 | 0.932500 |
| GO:0010556\_regulation\_of\_macromolecule\_biosynthetic\_process | 745 | 12 | 2.004716 | -1.964703 | 9 | -2.566104 | 8.28 | 19.126104 | 0.920000 |
| GO:0019219\_regulation\_of\_nucleobase\_\_nucleoside\_\_nucleotide\_and\_nucleic\_acid\_metabolic\_process | 757 | 12 | 1.972937 | -1.910479 | 10 | -2.087812 | 9.38 | 20.847812 | 0.938000 |
| GO:0051171\_regulation\_of\_nitrogen\_compound\_metabolic\_process | 771 | 12 | 1.937112 | -1.848961 | 11 | -1.757634 | 10.51 | 22.777634 | 0.955455 |
| GO:0045449\_regulation\_of\_transcription | 676 | 11 | 2.025228 | -1.848757 | 12 | -1.731012 | 10.53 | 22.791012 | 0.877500 |
| GO:0016481\_negative\_regulation\_of\_transcription | 253 | 6 | 2.951608 | -1.844342 | 13 | -1.640705 | 10.92 | 23.480705 | 0.840000 |
| GO:0010605\_negative\_regulation\_of\_macromolecule\_metabolic\_process | 331 | 7 | 2.632073 | -1.833918 | 14 | -1.682606 | 11.0 | 23.682606 | 0.785714 |
| GO:0031324\_negative\_regulation\_of\_cellular\_metabolic\_process | 332 | 7 | 2.624145 | -1.827213 | 15 | -1.622910 | 11.09 | 23.802910 | 0.739333 |
| GO:0051252\_regulation\_of\_RNA\_metabolic\_process | 590 | 10 | 2.109482 | -1.818813 | 16 | -1.599572 | 11.11 | 23.819572 | 0.694375 |
| GO:0009612\_response\_to\_mechanical\_stimulus | 24 | 2 | 10.371622 | -1.809262 | 17 | -1.363863 | 11.61 | 24.583863 | 0.682941 |
| GO:0001543\_ovarian\_follicle\_rupture | 2 | 1 |  |  |  |  |  |  |  |  |
| GO:0001767\_establishment\_of\_lymphocyte\_polarity | 2 | 1 |  |  |  |  |  |  |  |  |
| GO:0001768\_establishment\_of\_T\_cell\_polarity | 2 | 1 |  |  |  |  |  |  |  |  |
| GO:0007132\_meiotic\_metaphase\_I | 2 | 1 |  |  |  |  |  |  |  |  |
| GO:0014067\_negative\_regulation\_of\_phosphoinositide\_3-kinase\_cascade | 2 | 1 |  |  |  |  |  |  |  |  |
| GO:0031223\_auditory\_behavior | 2 | 1 |  |  |  |  |  |  |  |  |
| GO:0032796\_uropod\_organization | 2 | 1 |  |  |  |  |  |  |  |  |
| GO:0045116\_protein\_neddylation | 2 | 1 |  |  |  |  |  |  |  |  |
| GO:0051293\_establishment\_of\_spindle\_localization | 2 | 1 |  |  |  |  |  |  |  |  |
| GO:0051295\_establishment\_of\_meiotic\_spindle\_localization | 2 | 1 |  |  |  |  |  |  |  |  |
| GO:0051653\_spindle\_localization | 2 | 1 |  |  |  |  |  |  |  |  |
| GO:0060318\_definitive\_erythrocyte\_differentiation | 2 | 1 |  |  |  |  |  |  |  |  |
| GO:0010629\_negative\_regulation\_of\_gene\_expression | 262 | 6 | 2.850217 | -1.775244 | 18 | -1.425275 | 11.99 | 25.405275 | 0.666111 |
| GO:0006350\_transcription | 701 | 11 | 1.953002 | -1.736646 | 19 | -0.957631 | 13.19 | 27.337631 | 0.694211 |
| GO:0009892\_negative\_regulation\_of\_metabolic\_process | 348 | 7 | 2.503495 | -1.723651 | 20 | -0.744438 | 13.46 | 27.664438 | 0.673000 |
| GO:0045934\_negative\_regulation\_of\_nucleobase\_\_nucleoside\_\_nucleotide\_and\_nucleic\_acid\_metabolic\_process | 270 | 6 | 2.765766 | -1.716464 | 21 | -0.635119 | 13.56 | 27.755119 | 0.645714 |
| GO:0034961\_cellular\_biopolymer\_biosynthetic\_process | 804 | 12 | 1.857604 | -1.711024 | 22 | -0.569220 | 13.93 | 28.429220 | 0.633182 |
| GO:0051172\_negative\_regulation\_of\_nitrogen\_compound\_metabolic\_process | 271 | 6 | 2.755560 | -1.709283 | 23 | -0.447914 | 14.21 | 28.867914 | 0.617826 |
| GO:0010467\_gene\_expression | 905 | 13 | 1.787815 | -1.706080 | 24 | -0.434665 | 14.22 | 28.874665 | 0.592500 |
| GO:0006357\_regulation\_of\_transcription\_from\_RNA\_polymerase\_II\_promoter | 435 | 8 | 2.288910 | -1.704576 | 25 | -0.422097 | 14.23 | 28.882097 | 0.569200 |
| GO:0043284\_biopolymer\_biosynthetic\_process | 807 | 12 | 1.850698 | -1.698956 | 26 | -0.415619 | 14.28 | 28.975619 | 0.549231 |
| GO:0031326\_regulation\_of\_cellular\_biosynthetic\_process | 812 | 12 | 1.839302 | -1.679013 | 27 | -0.258329 | 14.83 | 29.918329 | 0.549259 |
| GO:0009889\_regulation\_of\_biosynthetic\_process | 815 | 12 | 1.832532 | -1.667147 | 28 | -0.250199 | 14.92 | 30.090199 | 0.532857 |
| GO:0006366\_transcription\_from\_RNA\_polymerase\_II\_promoter | 444 | 8 | 2.242513 | -1.655814 | 29 | -0.209388 | 15.04 | 30.289388 | 0.518621 |
| GO:0080090\_regulation\_of\_primary\_metabolic\_process | 926 | 13 | 1.747271 | -1.627644 | 30 | 0.106414 | 16.0 | 31.893586 | 0.533333 |
| GO:0030522\_intracellular\_receptor-mediated\_signaling\_pathway | 30 | 2 | 8.297297 | -1.624766 | 31 | 0.315978 | 16.43 | 32.544022 | 0.530000 |
| GO:0000098\_sulfur\_amino\_acid\_catabolic\_process | 3 | 1 |  |  |  |  |  |  |  |  |
| GO:0000212\_meiotic\_spindle\_organization | 3 | 1 |  |  |  |  |  |  |  |  |
| GO:0001844\_protein\_insertion\_into\_mitochondrial\_membrane\_during\_induction\_of\_apoptosis | 3 | 1 |  |  |  |  |  |  |  |  |
| GO:0002361\_CD4-positive\_\_CD25-positive\_\_alpha-beta\_regulatory\_T\_cell\_differentiation | 3 | 1 |  |  |  |  |  |  |  |  |
| GO:0021794\_thalamus\_development | 3 | 1 |  |  |  |  |  |  |  |  |
| GO:0031063\_regulation\_of\_histone\_deacetylation | 3 | 1 |  |  |  |  |  |  |  |  |
| GO:0031065\_positive\_regulation\_of\_histone\_deacetylation | 3 | 1 |  |  |  |  |  |  |  |  |
| GO:0031503\_protein\_complex\_localization | 3 | 1 |  |  |  |  |  |  |  |  |
| GO:0044273\_sulfur\_compound\_catabolic\_process | 3 | 1 |  |  |  |  |  |  |  |  |
| GO:0050774\_negative\_regulation\_of\_dendrite\_morphogenesis | 3 | 1 |  |  |  |  |  |  |  |  |
| GO:0051204\_protein\_insertion\_into\_mitochondrial\_membrane | 3 | 1 |  |  |  |  |  |  |  |  |
| GO:0070846\_Hsp90\_deacetylation | 3 | 1 |  |  |  |  |  |  |  |  |
| GO:0060255\_regulation\_of\_macromolecule\_metabolic\_process | 936 | 13 | 1.728604 | -1.591396 | 32 | 0.766213 | 17.83 | 34.893787 | 0.557188 |
| GO:0016070\_RNA\_metabolic\_process | 658 | 10 | 1.891481 | -1.513799 | 33 | 2.030150 | 20.54 | 39.049850 | 0.622424 |
| GO:0032922\_circadian\_regulation\_of\_gene\_expression | 4 | 1 |  |  |  |  |  |  |  |  |
| GO:0045066\_regulatory\_T\_cell\_differentiation | 4 | 1 |  |  |  |  |  |  |  |  |
| GO:0051205\_protein\_insertion\_into\_membrane | 4 | 1 |  |  |  |  |  |  |  |  |
| GO:0006355\_regulation\_of\_transcription\_\_DNA-dependent | 575 | 9 | 1.948061 | -1.462998 | 34 | 3.012225 | 22.49 | 41.967775 | 0.661471 |
| GO:0000070\_mitotic\_sister\_chromatid\_segregation | 5 | 1 | 24.891892 | -1.402851 | 46 | 14.073316 | 37.71 | 61.346684 | 0.819783 |
| GO:0000819\_sister\_chromatid\_segregation | 5 | 1 | 24.891892 | -1.402851 | 46 | 14.073316 | 37.71 | 61.346684 | 0.819783 |
| GO:0007638\_mechanosensory\_behavior | 5 | 1 | 24.891892 | -1.402851 | 46 | 14.073316 | 37.71 | 61.346684 | 0.819783 |
| GO:0009950\_dorsal\_ventral\_axis\_specification | 5 | 1 | 24.891892 | -1.402851 | 46 | 14.073316 | 37.71 | 61.346684 | 0.819783 |
| GO:0017148\_negative\_regulation\_of\_translation | 5 | 1 | 24.891892 | -1.402851 | 46 | 14.073316 | 37.71 | 61.346684 | 0.819783 |
| GO:0030261\_chromosome\_condensation | 5 | 1 | 24.891892 | -1.402851 | 46 | 14.073316 | 37.71 | 61.346684 | 0.819783 |
| GO:0031058\_positive\_regulation\_of\_histone\_modification | 5 | 1 | 24.891892 | -1.402851 | 46 | 14.073316 | 37.71 | 61.346684 | 0.819783 |
| GO:0034109\_homotypic\_cell-cell\_adhesion | 5 | 1 | 24.891892 | -1.402851 | 46 | 14.073316 | 37.71 | 61.346684 | 0.819783 |
| GO:0043489\_RNA\_stabilization | 5 | 1 | 24.891892 | -1.402851 | 46 | 14.073316 | 37.71 | 61.346684 | 0.819783 |
| GO:0048255\_mRNA\_stabilization | 5 | 1 | 24.891892 | -1.402851 | 46 | 14.073316 | 37.71 | 61.346684 | 0.819783 |
| GO:0051323\_metaphase | 5 | 1 | 24.891892 | -1.402851 | 46 | 14.073316 | 37.71 | 61.346684 | 0.819783 |
| GO:0051668\_localization\_within\_membrane | 5 | 1 | 24.891892 | -1.402851 | 46 | 14.073316 | 37.71 | 61.346684 | 0.819783 |
| GO:0016358\_dendrite\_development | 40 | 2 | 6.222973 | -1.392878 | 47 | 14.525719 | 38.45 | 62.374281 | 0.818085 |
| GO:0006351\_transcription\_\_DNA-dependent | 594 | 9 | 1.885749 | -1.383616 | 48 | 14.674643 | 38.66 | 62.645357 | 0.805417 |
| GO:0032774\_RNA\_biosynthetic\_process | 595 | 9 | 1.882580 | -1.379555 | 49 | 14.691417 | 38.68 | 62.668583 | 0.789388 |
| GO:0034645\_cellular\_macromolecule\_biosynthetic\_process | 901 | 12 | 1.657618 | -1.356855 | 50 | 15.439811 | 40.15 | 64.860189 | 0.803000 |
| GO:0031323\_regulation\_of\_cellular\_metabolic\_process | 1015 | 13 | 1.594062 | -1.328259 | 51 | 16.483245 | 42.36 | 68.236755 | 0.830588 |
| GO:0009059\_macromolecule\_biosynthetic\_process | 910 | 12 | 1.641224 | -1.327479 | 52 | 16.505485 | 42.38 | 68.254515 | 0.815000 |
| GO:0000768\_syncytium\_formation\_by\_plasma\_membrane\_fusion | 6 | 1 | 20.743243 | -1.325363 | 56 | 24.236224 | 52.11 | 79.983776 | 0.930536 |
| GO:0006882\_cellular\_zinc\_ion\_homeostasis | 6 | 1 | 20.743243 | -1.325363 | 56 | 24.236224 | 52.11 | 79.983776 | 0.930536 |
| GO:0007520\_myoblast\_fusion | 6 | 1 | 20.743243 | -1.325363 | 56 | 24.236224 | 52.11 | 79.983776 | 0.930536 |
| GO:0060013\_righting\_reflex | 6 | 1 | 20.743243 | -1.325363 | 56 | 24.236224 | 52.11 | 79.983776 | 0.930536 |
| GO:0030218\_erythrocyte\_differentiation | 46 | 2 | 5.411281 | -1.283008 | 57 | 25.941615 | 55.22 | 84.498385 | 0.968772 |
| GO:0006949\_syncytium\_formation | 7 | 1 | 17.779923 | -1.260108 | 65 | 34.408873 | 66.45 | 98.491127 | 1.022308 |
| GO:0014066\_regulation\_of\_phosphoinositide\_3-kinase\_cascade | 7 | 1 | 17.779923 | -1.260108 | 65 | 34.408873 | 66.45 | 98.491127 | 1.022308 |
| GO:0016575\_histone\_deacetylation | 7 | 1 | 17.779923 | -1.260108 | 65 | 34.408873 | 66.45 | 98.491127 | 1.022308 |
| GO:0030521\_androgen\_receptor\_signaling\_pathway | 7 | 1 | 17.779923 | -1.260108 | 65 | 34.408873 | 66.45 | 98.491127 | 1.022308 |
| GO:0046677\_response\_to\_antibiotic | 7 | 1 | 17.779923 | -1.260108 | 65 | 34.408873 | 66.45 | 98.491127 | 1.022308 |
| GO:0048814\_regulation\_of\_dendrite\_morphogenesis | 7 | 1 | 17.779923 | -1.260108 | 65 | 34.408873 | 66.45 | 98.491127 | 1.022308 |
| GO:0050773\_regulation\_of\_dendrite\_development | 7 | 1 | 17.779923 | -1.260108 | 65 | 34.408873 | 66.45 | 98.491127 | 1.022308 |
| GO:0055069\_zinc\_ion\_homeostasis | 7 | 1 | 17.779923 | -1.260108 | 65 | 34.408873 | 66.45 | 98.491127 | 1.022308 |
| GO:0043010\_camera-type\_eye\_development | 110 | 3 | 3.394349 | -1.241876 | 66 | 35.040651 | 67.64 | 100.239349 | 1.024848 |
| GO:0034101\_erythrocyte\_homeostasis | 49 | 2 | 5.079978 | -1.234014 | 67 | 35.518966 | 68.41 | 101.301034 | 1.021045 |
| GO:0006458\_'de\_novo'\_protein\_folding | 8 | 1 | 15.557432 | -1.203805 | 70 | 42.615296 | 78.32 | 114.024704 | 1.118857 |
| GO:0014065\_phosphoinositide\_3-kinase\_cascade | 8 | 1 | 15.557432 | -1.203805 | 70 | 42.615296 | 78.32 | 114.024704 | 1.118857 |
| GO:0051084\_'de\_novo'\_posttranslational\_protein\_folding | 8 | 1 | 15.557432 | -1.203805 | 70 | 42.615296 | 78.32 | 114.024704 | 1.118857 |
| GO:0034960\_cellular\_biopolymer\_metabolic\_process | 1395 | 16 | 1.427492 | -1.189567 | 71 | 43.233990 | 79.44 | 115.646010 | 1.118873 |
| GO:0010608\_posttranscriptional\_regulation\_of\_gene\_expression | 52 | 2 | 4.786902 | -1.188340 | 72 | 43.414505 | 79.87 | 116.325495 | 1.109306 |
| GO:0001542\_ovulation\_from\_ovarian\_follicle | 9 | 1 | 13.828829 | -1.154340 | 78 | 51.434603 | 91.01 | 130.585397 | 1.166795 |
| GO:0006476\_protein\_amino\_acid\_deacetylation | 9 | 1 | 13.828829 | -1.154340 | 78 | 51.434603 | 91.01 | 130.585397 | 1.166795 |
| GO:0017145\_stem\_cell\_division | 9 | 1 | 13.828829 | -1.154340 | 78 | 51.434603 | 91.01 | 130.585397 | 1.166795 |
| GO:0030728\_ovulation | 9 | 1 | 13.828829 | -1.154340 | 78 | 51.434603 | 91.01 | 130.585397 | 1.166795 |
| GO:0050910\_detection\_of\_mechanical\_stimulus\_involved\_in\_sensory\_perception\_of\_sound | 9 | 1 | 13.828829 | -1.154340 | 78 | 51.434603 | 91.01 | 130.585397 | 1.166795 |
| GO:0055012\_ventricular\_cardiac\_muscle\_cell\_differentiation | 9 | 1 | 13.828829 | -1.154340 | 78 | 51.434603 | 91.01 | 130.585397 | 1.166795 |
| GO:0014706\_striated\_muscle\_tissue\_development | 120 | 3 | 3.111486 | -1.150995 | 79 | 51.673435 | 91.35 | 131.026565 | 1.156329 |
| GO:0051641\_cellular\_localization | 370 | 6 | 2.018262 | -1.143017 | 80 | 52.148313 | 91.97 | 131.791687 | 1.149625 |
| GO:0000904\_cell\_morphogenesis\_involved\_in\_differentiation | 199 | 4 | 2.501698 | -1.133262 | 81 | 52.679230 | 92.81 | 132.940770 | 1.145802 |
| GO:0006790\_sulfur\_metabolic\_process | 56 | 2 | 4.444981 | -1.131962 | 82 | 53.115994 | 93.67 | 134.224006 | 1.142317 |
| GO:0019222\_regulation\_of\_metabolic\_process | 1088 | 13 | 1.487108 | -1.118138 | 83 | 53.911386 | 94.8 | 135.688614 | 1.142169 |
| GO:0006826\_iron\_ion\_transport | 10 | 1 | 12.445946 | -1.110268 | 89 | 61.863882 | 104.94 | 148.016118 | 1.179101 |
| GO:0007006\_mitochondrial\_membrane\_organization | 10 | 1 | 12.445946 | -1.110268 | 89 | 61.863882 | 104.94 | 148.016118 | 1.179101 |
| GO:0016197\_endosome\_transport | 10 | 1 | 12.445946 | -1.110268 | 89 | 61.863882 | 104.94 | 148.016118 | 1.179101 |
| GO:0043488\_regulation\_of\_mRNA\_stability | 10 | 1 | 12.445946 | -1.110268 | 89 | 61.863882 | 104.94 | 148.016118 | 1.179101 |
| GO:0048384\_retinoic\_acid\_receptor\_signaling\_pathway | 10 | 1 | 12.445946 | -1.110268 | 89 | 61.863882 | 104.94 | 148.016118 | 1.179101 |
| GO:0060216\_definitive\_hemopoiesis | 10 | 1 | 12.445946 | -1.110268 | 89 | 61.863882 | 104.94 | 148.016118 | 1.179101 |
| GO:0060537\_muscle\_tissue\_development | 128 | 3 | 2.917019 | -1.084959 | 90 | 63.033460 | 106.44 | 149.846540 | 1.182667 |
| GO:0000096\_sulfur\_amino\_acid\_metabolic\_process | 11 | 1 | 11.314496 | -1.070558 | 97 | 69.192292 | 114.58 | 159.967708 | 1.181237 |
| GO:0007051\_spindle\_organization | 11 | 1 | 11.314496 | -1.070558 | 97 | 69.192292 | 114.58 | 159.967708 | 1.181237 |
| GO:0014902\_myotube\_differentiation | 11 | 1 | 11.314496 | -1.070558 | 97 | 69.192292 | 114.58 | 159.967708 | 1.181237 |
| GO:0042036\_negative\_regulation\_of\_cytokine\_biosynthetic\_process | 11 | 1 | 11.314496 | -1.070558 | 97 | 69.192292 | 114.58 | 159.967708 | 1.181237 |
| GO:0045026\_plasma\_membrane\_fusion | 11 | 1 | 11.314496 | -1.070558 | 97 | 69.192292 | 114.58 | 159.967708 | 1.181237 |
| GO:0048745\_smooth\_muscle\_tissue\_development | 11 | 1 | 11.314496 | -1.070558 | 97 | 69.192292 | 114.58 | 159.967708 | 1.181237 |
| GO:0060004\_reflex | 11 | 1 | 11.314496 | -1.070558 | 97 | 69.192292 | 114.58 | 159.967708 | 1.181237 |
| GO:0044260\_cellular\_macromolecule\_metabolic\_process | 1447 | 16 | 1.376193 | -1.062849 | 98 | 69.591119 | 115.31 | 161.028881 | 1.176633 |
| GO:0006139\_nucleobase\_\_nucleoside\_\_nucleotide\_and\_nucleic\_acid\_metabolic\_process | 1002 | 12 | 1.490532 | -1.056615 | 99 | 69.618819 | 115.35 | 161.081181 | 1.165152 |
| GO:0022604\_regulation\_of\_cell\_morphogenesis | 62 | 2 | 4.014821 | -1.055656 | 100 | 70.178992 | 116.17 | 162.161008 | 1.161700 |
| GO:0030010\_establishment\_of\_cell\_polarity | 12 | 1 | 10.371622 | -1.034451 | 105 | 76.782843 | 125.13 | 173.477157 | 1.191714 |
| GO:0031056\_regulation\_of\_histone\_modification | 12 | 1 | 10.371622 | -1.034451 | 105 | 76.782843 | 125.13 | 173.477157 | 1.191714 |
| GO:0043487\_regulation\_of\_RNA\_stability | 12 | 1 | 10.371622 | -1.034451 | 105 | 76.782843 | 125.13 | 173.477157 | 1.191714 |
| GO:0050848\_regulation\_of\_calcium-mediated\_signaling | 12 | 1 | 10.371622 | -1.034451 | 105 | 76.782843 | 125.13 | 173.477157 | 1.191714 |
| GO:0050850\_positive\_regulation\_of\_calcium-mediated\_signaling | 12 | 1 | 10.371622 | -1.034451 | 105 | 76.782843 | 125.13 | 173.477157 | 1.191714 |
| GO:0001654\_eye\_development | 136 | 3 | 2.745429 | -1.024059 | 106 | 77.235721 | 125.99 | 174.744279 | 1.188585 |
| GO:0048511\_rhythmic\_process | 65 | 2 | 3.829522 | -1.020700 | 107 | 77.488217 | 126.43 | 175.371783 | 1.181589 |
| GO:0034613\_cellular\_protein\_localization | 139 | 3 | 2.686175 | -1.002420 | 108 | 78.447571 | 128.41 | 178.372429 | 1.188981 |
| GO:0007566\_embryo\_implantation | 13 | 1 | 9.573805 | -1.001368 | 111 | 84.060407 | 135.9 | 187.739593 | 1.224324 |
| GO:0046474\_glycerophospholipid\_biosynthetic\_process | 13 | 1 | 9.573805 | -1.001368 | 111 | 84.060407 | 135.9 | 187.739593 | 1.224324 |
| GO:0060038\_cardiac\_muscle\_cell\_proliferation | 13 | 1 | 9.573805 | -1.001368 | 111 | 84.060407 | 135.9 | 187.739593 | 1.224324 |
| GO:0009791\_post-embryonic\_development | 67 | 2 | 3.715208 | -0.998446 | 112 | 84.249130 | 136.47 | 188.690870 | 1.218482 |
| GO:0070727\_cellular\_macromolecule\_localization | 141 | 3 | 2.648074 | -0.988334 | 113 | 84.409776 | 136.77 | 189.130224 | 1.210354 |
| GO:0042692\_muscle\_cell\_differentiation | 68 | 2 | 3.660572 | -0.987615 | 114 | 84.571092 | 137.08 | 189.588908 | 1.202456 |
| GO:0014855\_striated\_muscle\_cell\_proliferation | 14 | 1 | 8.889961 | -0.970861 | 117 | 90.217321 | 144.61 | 199.002679 | 1.235983 |
| GO:0033044\_regulation\_of\_chromosome\_organization | 14 | 1 | 8.889961 | -0.970861 | 117 | 90.217321 | 144.61 | 199.002679 | 1.235983 |
| GO:0048048\_embryonic\_eye\_morphogenesis | 14 | 1 | 8.889961 | -0.970861 | 117 | 90.217321 | 144.61 | 199.002679 | 1.235983 |
| GO:0006807\_nitrogen\_compound\_metabolic\_process | 1147 | 13 | 1.410613 | -0.968663 | 118 | 90.248604 | 144.72 | 199.191396 | 1.226441 |
| GO:0019953\_sexual\_reproduction | 228 | 4 | 2.183499 | -0.966007 | 119 | 90.969531 | 145.7 | 200.430469 | 1.224370 |
| GO:0043283\_biopolymer\_metabolic\_process | 1490 | 16 | 1.336477 | -0.965871 | 120 | 90.974477 | 145.72 | 200.465523 | 1.214333 |
| GO:0044249\_cellular\_biosynthetic\_process | 1150 | 13 | 1.406933 | -0.961511 | 121 | 90.999994 | 145.75 | 200.500006 | 1.204545 |
| GO:0008654\_phospholipid\_biosynthetic\_process | 16 | 1 | 7.778716 | -0.916218 | 130 | 103.033747 | 161.89 | 220.746253 | 1.245308 |
| GO:0010876\_lipid\_localization | 16 | 1 | 7.778716 | -0.916218 | 130 | 103.033747 | 161.89 | 220.746253 | 1.245308 |
| GO:0019722\_calcium-mediated\_signaling | 16 | 1 | 7.778716 | -0.916218 | 130 | 103.033747 | 161.89 | 220.746253 | 1.245308 |
| GO:0019915\_lipid\_storage | 16 | 1 | 7.778716 | -0.916218 | 130 | 103.033747 | 161.89 | 220.746253 | 1.245308 |
| GO:0031345\_negative\_regulation\_of\_cell\_projection\_organization | 16 | 1 | 7.778716 | -0.916218 | 130 | 103.033747 | 161.89 | 220.746253 | 1.245308 |
| GO:0043367\_CD4-positive\_\_alpha\_beta\_T\_cell\_differentiation | 16 | 1 | 7.778716 | -0.916218 | 130 | 103.033747 | 161.89 | 220.746253 | 1.245308 |
| GO:0048286\_lung\_alveolus\_development | 16 | 1 | 7.778716 | -0.916218 | 130 | 103.033747 | 161.89 | 220.746253 | 1.245308 |
| GO:0050974\_detection\_of\_mechanical\_stimulus\_involved\_in\_sensory\_perception | 16 | 1 | 7.778716 | -0.916218 | 130 | 103.033747 | 161.89 | 220.746253 | 1.245308 |
| GO:0055007\_cardiac\_muscle\_cell\_differentiation | 16 | 1 | 7.778716 | -0.916218 | 130 | 103.033747 | 161.89 | 220.746253 | 1.245308 |
| GO:0007517\_muscle\_organ\_development | 153 | 3 | 2.440382 | -0.909092 | 131 | 103.669320 | 162.88 | 222.090680 | 1.243359 |
| GO:0009058\_biosynthetic\_process | 1175 | 13 | 1.376998 | -0.903518 | 132 | 104.168101 | 163.44 | 222.711899 | 1.238182 |
| GO:0006323\_DNA\_packaging | 17 | 1 | 7.321145 | -0.891560 | 134 | 109.201784 | 169.67 | 230.138216 | 1.266194 |
| GO:0010741\_negative\_regulation\_of\_protein\_kinase\_cascade | 17 | 1 | 7.321145 | -0.891560 | 134 | 109.201784 | 169.67 | 230.138216 | 1.266194 |
| GO:0007519\_skeletal\_muscle\_tissue\_development | 78 | 2 | 3.191268 | -0.888887 | 136 | 110.250255 | 170.7 | 231.149745 | 1.255147 |
| GO:0060538\_skeletal\_muscle\_organ\_development | 78 | 2 | 3.191268 | -0.888887 | 136 | 110.250255 | 170.7 | 231.149745 | 1.255147 |
| GO:0006457\_protein\_folding | 18 | 1 | 6.914414 | -0.868406 | 140 | 116.987542 | 179.44 | 241.892458 | 1.281714 |
| GO:0009063\_cellular\_amino\_acid\_catabolic\_process | 18 | 1 | 6.914414 | -0.868406 | 140 | 116.987542 | 179.44 | 241.892458 | 1.281714 |
| GO:0035051\_cardiac\_cell\_differentiation | 18 | 1 | 6.914414 | -0.868406 | 140 | 116.987542 | 179.44 | 241.892458 | 1.281714 |
| GO:0050982\_detection\_of\_mechanical\_stimulus | 18 | 1 | 6.914414 | -0.868406 | 140 | 116.987542 | 179.44 | 241.892458 | 1.281714 |
| GO:0051128\_regulation\_of\_cellular\_component\_organization | 160 | 3 | 2.333615 | -0.866659 | 141 | 117.202116 | 179.78 | 242.357884 | 1.275035 |
| GO:0051649\_establishment\_of\_localization\_in\_cell | 342 | 5 | 1.819583 | -0.866646 | 142 | 117.283928 | 179.88 | 242.476072 | 1.266761 |
| GO:0009798\_axis\_specification | 19 | 1 | 6.550498 | -0.846592 | 145 | 122.239072 | 186.26 | 250.280928 | 1.284552 |
| GO:0030518\_steroid\_hormone\_receptor\_signaling\_pathway | 19 | 1 | 6.550498 | -0.846592 | 145 | 122.239072 | 186.26 | 250.280928 | 1.284552 |
| GO:0033002\_muscle\_cell\_proliferation | 19 | 1 | 6.550498 | -0.846592 | 145 | 122.239072 | 186.26 | 250.280928 | 1.284552 |
| GO:0000279\_M\_phase | 85 | 2 | 2.928458 | -0.828592 | 146 | 124.270550 | 189.48 | 254.689450 | 1.297808 |
| GO:0008360\_regulation\_of\_cell\_shape | 20 | 1 | 6.222973 | -0.825981 | 148 | 128.014769 | 194.63 | 261.245231 | 1.315068 |
| GO:0045017\_glycerolipid\_biosynthetic\_process | 20 | 1 | 6.222973 | -0.825981 | 148 | 128.014769 | 194.63 | 261.245231 | 1.315068 |
| GO:0016337\_cell-cell\_adhesion | 87 | 2 | 2.861137 | -0.812495 | 149 | 129.372747 | 196.48 | 263.587253 | 1.318658 |
| GO:0048812\_neuron\_projection\_morphogenesis | 170 | 3 | 2.196343 | -0.810307 | 150 | 129.474762 | 196.81 | 264.145238 | 1.312067 |
| GO:0002053\_positive\_regulation\_of\_mesenchymal\_cell\_proliferation | 21 | 1 | 5.926641 | -0.806455 | 154 | 134.636325 | 203.85 | 273.063675 | 1.323701 |
| GO:0006944\_membrane\_fusion | 21 | 1 | 5.926641 | -0.806455 | 154 | 134.636325 | 203.85 | 273.063675 | 1.323701 |
| GO:0008637\_apoptotic\_mitochondrial\_changes | 21 | 1 | 5.926641 | -0.806455 | 154 | 134.636325 | 203.85 | 273.063675 | 1.323701 |
| GO:0051656\_establishment\_of\_organelle\_localization | 21 | 1 | 5.926641 | -0.806455 | 154 | 134.636325 | 203.85 | 273.063675 | 1.323701 |
| GO:0048523\_negative\_regulation\_of\_cellular\_process | 774 | 9 | 1.447203 | -0.805998 | 155 | 134.704831 | 203.91 | 273.115169 | 1.315548 |
| GO:0030030\_cell\_projection\_organization | 263 | 4 | 1.892920 | -0.801268 | 156 | 135.169408 | 204.81 | 274.450592 | 1.312885 |
| GO:0048667\_cell\_morphogenesis\_involved\_in\_neuron\_differentiation | 173 | 3 | 2.158257 | -0.794302 | 157 | 135.728853 | 205.78 | 275.831147 | 1.310701 |
| GO:0043170\_macromolecule\_metabolic\_process | 1576 | 16 | 1.263548 | -0.791588 | 158 | 135.767275 | 205.91 | 276.052725 | 1.303228 |
| GO:0000041\_transition\_metal\_ion\_transport | 22 | 1 | 5.657248 | -0.787913 | 163 | 142.110234 | 213.82 | 285.529766 | 1.311779 |
| GO:0007059\_chromosome\_segregation | 22 | 1 | 5.657248 | -0.787913 | 163 | 142.110234 | 213.82 | 285.529766 | 1.311779 |
| GO:0010463\_mesenchymal\_cell\_proliferation | 22 | 1 | 5.657248 | -0.787913 | 163 | 142.110234 | 213.82 | 285.529766 | 1.311779 |
| GO:0010464\_regulation\_of\_mesenchymal\_cell\_proliferation | 22 | 1 | 5.657248 | -0.787913 | 163 | 142.110234 | 213.82 | 285.529766 | 1.311779 |
| GO:0048864\_stem\_cell\_development | 22 | 1 | 5.657248 | -0.787913 | 163 | 142.110234 | 213.82 | 285.529766 | 1.311779 |
| GO:0048858\_cell\_projection\_morphogenesis | 176 | 3 | 2.121468 | -0.778685 | 164 | 143.216291 | 215.75 | 288.283709 | 1.315549 |
| GO:0050794\_regulation\_of\_cellular\_process | 2190 | 21 | 1.193447 | -0.773196 | 165 | 143.602909 | 216.32 | 289.037091 | 1.311030 |
| GO:0007163\_establishment\_or\_maintenance\_of\_cell\_polarity | 23 | 1 | 5.411281 | -0.770268 | 166 | 146.629214 | 220.24 | 293.850786 | 1.326747 |
| GO:0033036\_macromolecule\_localization | 274 | 4 | 1.816926 | -0.756238 | 167 | 148.787631 | 223.26 | 297.732369 | 1.336886 |
| GO:0000280\_nuclear\_division | 24 | 1 | 5.185811 | -0.753441 | 173 | 152.890912 | 228.49 | 304.089088 | 1.320751 |
| GO:0006650\_glycerophospholipid\_metabolic\_process | 24 | 1 | 5.185811 | -0.753441 | 173 | 152.890912 | 228.49 | 304.089088 | 1.320751 |
| GO:0007067\_mitosis | 24 | 1 | 5.185811 | -0.753441 | 173 | 152.890912 | 228.49 | 304.089088 | 1.320751 |
| GO:0042632\_cholesterol\_homeostasis | 24 | 1 | 5.185811 | -0.753441 | 173 | 152.890912 | 228.49 | 304.089088 | 1.320751 |
| GO:0050679\_positive\_regulation\_of\_epithelial\_cell\_proliferation | 24 | 1 | 5.185811 | -0.753441 | 173 | 152.890912 | 228.49 | 304.089088 | 1.320751 |
| GO:0055092\_sterol\_homeostasis | 24 | 1 | 5.185811 | -0.753441 | 173 | 152.890912 | 228.49 | 304.089088 | 1.320751 |
| GO:0032990\_cell\_part\_morphogenesis | 184 | 3 | 2.029230 | -0.738844 | 174 | 154.408859 | 230.51 | 306.611141 | 1.324770 |
| GO:0000087\_M\_phase\_of\_mitotic\_cell\_cycle | 25 | 1 | 4.978378 | -0.737368 | 176 | 158.158619 | 235.46 | 312.761381 | 1.337841 |
| GO:0048285\_organelle\_fission | 25 | 1 | 4.978378 | -0.737368 | 176 | 158.158619 | 235.46 | 312.761381 | 1.337841 |
| GO:0000003\_reproduction | 379 | 5 | 1.641945 | -0.734225 | 177 | 158.489181 | 235.89 | 313.290819 | 1.332712 |
| GO:0007548\_sex\_differentiation | 98 | 2 | 2.539989 | -0.731649 | 178 | 159.061251 | 236.8 | 314.538749 | 1.330337 |
| GO:0007155\_cell\_adhesion | 186 | 3 | 2.007411 | -0.729274 | 180 | 159.269563 | 237.04 | 314.810437 | 1.316889 |
| GO:0022610\_biological\_adhesion | 186 | 3 | 2.007411 | -0.729274 | 180 | 159.269563 | 237.04 | 314.810437 | 1.316889 |
| GO:0065007\_biological\_regulation | 2593 | 24 | 1.151958 | -0.725776 | 181 | 159.475028 | 237.34 | 315.204972 | 1.311271 |
| GO:0007623\_circadian\_rhythm | 26 | 1 | 4.786902 | -0.721988 | 183 | 163.543580 | 242.23 | 320.916420 | 1.323661 |
| GO:0009310\_amine\_catabolic\_process | 26 | 1 | 4.786902 | -0.721988 | 183 | 163.543580 | 242.23 | 320.916420 | 1.323661 |
| GO:0000902\_cell\_morphogenesis | 283 | 4 | 1.759144 | -0.721464 | 184 | 163.605631 | 242.31 | 321.014369 | 1.316902 |
| GO:0010638\_positive\_regulation\_of\_organelle\_organization | 27 | 1 | 4.609610 | -0.707249 | 185 | 167.681061 | 247.66 | 327.638939 | 1.338703 |
| GO:0006470\_protein\_amino\_acid\_dephosphorylation | 28 | 1 | 4.444981 | -0.693104 | 190 | 171.517630 | 252.38 | 333.242370 | 1.328316 |
| GO:0007127\_meiosis\_I | 28 | 1 | 4.444981 | -0.693104 | 190 | 171.517630 | 252.38 | 333.242370 | 1.328316 |
| GO:0021549\_cerebellum\_development | 28 | 1 | 4.444981 | -0.693104 | 190 | 171.517630 | 252.38 | 333.242370 | 1.328316 |
| GO:0030073\_insulin\_secretion | 28 | 1 | 4.444981 | -0.693104 | 190 | 171.517630 | 252.38 | 333.242370 | 1.328316 |
| GO:0048863\_stem\_cell\_differentiation | 28 | 1 | 4.444981 | -0.693104 | 190 | 171.517630 | 252.38 | 333.242370 | 1.328316 |
| GO:0046907\_intracellular\_transport | 194 | 3 | 1.924631 | -0.692450 | 191 | 171.643456 | 252.54 | 333.436544 | 1.322199 |
| GO:0048872\_homeostasis\_of\_number\_of\_cells | 105 | 2 | 2.370656 | -0.686067 | 192 | 172.390303 | 253.55 | 334.709697 | 1.320573 |
| GO:0006417\_regulation\_of\_translation | 29 | 1 | 4.291705 | -0.679511 | 196 | 176.133648 | 257.94 | 339.746352 | 1.316020 |
| GO:0044270\_nitrogen\_compound\_catabolic\_process | 29 | 1 | 4.291705 | -0.679511 | 196 | 176.133648 | 257.94 | 339.746352 | 1.316020 |
| GO:0051301\_cell\_division | 29 | 1 | 4.291705 | -0.679511 | 196 | 176.133648 | 257.94 | 339.746352 | 1.316020 |
| GO:0060041\_retina\_development\_in\_camera-type\_eye | 29 | 1 | 4.291705 | -0.679511 | 196 | 176.133648 | 257.94 | 339.746352 | 1.316020 |
| GO:0031175\_neuron\_projection\_development | 197 | 3 | 1.895322 | -0.679214 | 197 | 176.373229 | 258.25 | 340.126771 | 1.310914 |
| GO:0051179\_localization | 1058 | 11 | 1.294002 | -0.672779 | 198 | 176.934770 | 259.35 | 341.765230 | 1.309848 |
| GO:0030154\_cell\_differentiation | 1060 | 11 | 1.291560 | -0.668919 | 199 | 177.177211 | 259.8 | 342.422789 | 1.305528 |
| GO:0030099\_myeloid\_cell\_differentiation | 108 | 2 | 2.304805 | -0.667738 | 200 | 177.394914 | 260.1 | 342.805086 | 1.300500 |
| GO:0033500\_carbohydrate\_homeostasis | 30 | 1 | 4.148649 | -0.666433 | 203 | 181.202720 | 264.74 | 348.277280 | 1.304138 |
| GO:0042593\_glucose\_homeostasis | 30 | 1 | 4.148649 | -0.666433 | 203 | 181.202720 | 264.74 | 348.277280 | 1.304138 |
| GO:0051146\_striated\_muscle\_cell\_differentiation | 30 | 1 | 4.148649 | -0.666433 | 203 | 181.202720 | 264.74 | 348.277280 | 1.304138 |
| GO:0016311\_dephosphorylation | 31 | 1 | 4.014821 | -0.653836 | 208 | 187.646696 | 272.77 | 357.893304 | 1.311394 |
| GO:0046632\_alpha-beta\_T\_cell\_differentiation | 31 | 1 | 4.014821 | -0.653836 | 208 | 187.646696 | 272.77 | 357.893304 | 1.311394 |
| GO:0048562\_embryonic\_organ\_morphogenesis | 31 | 1 | 4.014821 | -0.653836 | 208 | 187.646696 | 272.77 | 357.893304 | 1.311394 |
| GO:0051640\_organelle\_localization | 31 | 1 | 4.014821 | -0.653836 | 208 | 187.646696 | 272.77 | 357.893304 | 1.311394 |
| GO:0055088\_lipid\_homeostasis | 31 | 1 | 4.014821 | -0.653836 | 208 | 187.646696 | 272.77 | 357.893304 | 1.311394 |
| GO:0007399\_nervous\_system\_development | 621 | 7 | 1.402925 | -0.649236 | 209 | 188.041465 | 273.24 | 358.438535 | 1.307368 |
| GO:0050768\_negative\_regulation\_of\_neurogenesis | 32 | 1 | 3.889358 | -0.641689 | 210 | 191.014315 | 277.25 | 363.485685 | 1.320238 |
| GO:0032989\_cellular\_component\_morphogenesis | 307 | 4 | 1.621622 | -0.636912 | 211 | 191.824367 | 278.09 | 364.355633 | 1.317962 |
| GO:0008284\_positive\_regulation\_of\_cell\_proliferation | 208 | 3 | 1.795088 | -0.633163 | 212 | 192.145212 | 278.65 | 365.154788 | 1.314387 |
| GO:0007565\_female\_pregnancy | 33 | 1 | 3.771499 | -0.629964 | 215 | 195.071947 | 282.39 | 369.708053 | 1.313442 |
| GO:0021536\_diencephalon\_development | 33 | 1 | 3.771499 | -0.629964 | 215 | 195.071947 | 282.39 | 369.708053 | 1.313442 |
| GO:0022037\_metencephalon\_development | 33 | 1 | 3.771499 | -0.629964 | 215 | 195.071947 | 282.39 | 369.708053 | 1.313442 |
| GO:0048608\_reproductive\_structure\_development | 116 | 2 | 2.145853 | -0.622015 | 216 | 195.763578 | 283.35 | 370.936422 | 1.311806 |
| GO:0007338\_single\_fertilization | 34 | 1 | 3.660572 | -0.618636 | 220 | 198.935932 | 287.12 | 375.304068 | 1.305091 |
| GO:0010721\_negative\_regulation\_of\_cell\_development | 34 | 1 | 3.660572 | -0.618636 | 220 | 198.935932 | 287.12 | 375.304068 | 1.305091 |
| GO:0016054\_organic\_acid\_catabolic\_process | 34 | 1 | 3.660572 | -0.618636 | 220 | 198.935932 | 287.12 | 375.304068 | 1.305091 |
| GO:0046395\_carboxylic\_acid\_catabolic\_process | 34 | 1 | 3.660572 | -0.618636 | 220 | 198.935932 | 287.12 | 375.304068 | 1.305091 |
| GO:0048519\_negative\_regulation\_of\_biological\_process | 859 | 9 | 1.303999 | -0.616675 | 221 | 199.139778 | 287.57 | 376.000222 | 1.301222 |
| GO:0007292\_female\_gamete\_generation | 35 | 1 | 3.555985 | -0.607682 | 222 | 202.355769 | 291.46 | 380.564231 | 1.312883 |
| GO:0022403\_cell\_cycle\_phase | 119 | 2 | 2.091756 | -0.605955 | 223 | 202.511955 | 291.64 | 380.768045 | 1.307803 |
| GO:0040007\_growth | 217 | 3 | 1.720638 | -0.598164 | 224 | 203.460672 | 292.85 | 382.239328 | 1.307366 |
| GO:0022602\_ovulation\_cycle\_process | 36 | 1 | 3.457207 | -0.597081 | 226 | 206.921721 | 297.07 | 387.218279 | 1.314469 |
| GO:0030072\_peptide\_hormone\_secretion | 36 | 1 | 3.457207 | -0.597081 | 226 | 206.921721 | 297.07 | 387.218279 | 1.314469 |
| GO:0007423\_sensory\_organ\_development | 219 | 3 | 1.704924 | -0.590691 | 227 | 208.102973 | 298.46 | 388.817027 | 1.314802 |
| GO:0006886\_intracellular\_protein\_transport | 122 | 2 | 2.040319 | -0.590440 | 229 | 208.974389 | 299.64 | 390.305611 | 1.308472 |
| GO:0030001\_metal\_ion\_transport | 122 | 2 | 2.040319 | -0.590440 | 229 | 208.974389 | 299.64 | 390.305611 | 1.308472 |
| GO:0002790\_peptide\_secretion | 37 | 1 | 3.363769 | -0.586813 | 232 | 211.772776 | 302.78 | 393.787224 | 1.305086 |
| GO:0042698\_ovulation\_cycle | 37 | 1 | 3.363769 | -0.586813 | 232 | 211.772776 | 302.78 | 393.787224 | 1.305086 |
| GO:0050906\_detection\_of\_stimulus\_involved\_in\_sensory\_perception | 37 | 1 | 3.363769 | -0.586813 | 232 | 211.772776 | 302.78 | 393.787224 | 1.305086 |
| GO:0010975\_regulation\_of\_neuron\_projection\_development | 38 | 1 | 3.275249 | -0.576860 | 236 | 216.227199 | 308.66 | 401.092801 | 1.307881 |
| GO:0016053\_organic\_acid\_biosynthetic\_process | 38 | 1 | 3.275249 | -0.576860 | 236 | 216.227199 | 308.66 | 401.092801 | 1.307881 |
| GO:0031401\_positive\_regulation\_of\_protein\_modification\_process | 38 | 1 | 3.275249 | -0.576860 | 236 | 216.227199 | 308.66 | 401.092801 | 1.307881 |
| GO:0046394\_carboxylic\_acid\_biosynthetic\_process | 38 | 1 | 3.275249 | -0.576860 | 236 | 216.227199 | 308.66 | 401.092801 | 1.307881 |
| GO:0048468\_cell\_development | 654 | 7 | 1.332135 | -0.575167 | 237 | 216.996552 | 309.7 | 402.403448 | 1.306751 |
| GO:0048869\_cellular\_developmental\_process | 1113 | 11 | 1.230058 | -0.572917 | 238 | 217.156792 | 309.94 | 402.723208 | 1.302269 |
| GO:0006644\_phospholipid\_metabolic\_process | 39 | 1 | 3.191268 | -0.567206 | 239 | 220.613152 | 313.67 | 406.726848 | 1.312427 |
| GO:0009987\_cellular\_process | 3868 | 33 | 1.061831 | -0.566038 | 240 | 220.946953 | 314.03 | 407.113047 | 1.308458 |
| GO:0016071\_mRNA\_metabolic\_process | 40 | 1 | 3.111486 | -0.557836 | 242 | 225.917571 | 320.09 | 414.262429 | 1.322686 |
| GO:0051129\_negative\_regulation\_of\_cellular\_component\_organization | 40 | 1 | 3.111486 | -0.557836 | 242 | 225.917571 | 320.09 | 414.262429 | 1.322686 |
| GO:0051276\_chromosome\_organization | 129 | 2 | 1.929604 | -0.556208 | 243 | 226.290025 | 320.47 | 414.649975 | 1.318807 |
| GO:0008585\_female\_gonad\_development | 41 | 1 | 3.035597 | -0.548735 | 246 | 232.269362 | 327.32 | 422.370638 | 1.330569 |
| GO:0015833\_peptide\_transport | 41 | 1 | 3.035597 | -0.548735 | 246 | 232.269362 | 327.32 | 422.370638 | 1.330569 |
| GO:0031344\_regulation\_of\_cell\_projection\_organization | 41 | 1 | 3.035597 | -0.548735 | 246 | 232.269362 | 327.32 | 422.370638 | 1.330569 |
| GO:0048522\_positive\_regulation\_of\_cellular\_process | 895 | 9 | 1.251548 | -0.548707 | 247 | 232.348856 | 327.46 | 422.571144 | 1.325749 |
| GO:0007420\_brain\_development | 231 | 3 | 1.616357 | -0.548012 | 248 | 232.475338 | 327.61 | 422.744662 | 1.321008 |
| GO:0010769\_regulation\_of\_cell\_morphogenesis\_involved\_in\_differentiation | 42 | 1 | 2.963320 | -0.539891 | 249 | 237.139416 | 333.32 | 429.500584 | 1.338635 |
| GO:0044237\_cellular\_metabolic\_process | 1974 | 18 | 1.134889 | -0.536623 | 250 | 238.198355 | 334.94 | 431.681645 | 1.339760 |
| GO:0009582\_detection\_of\_abiotic\_stimulus | 43 | 1 | 2.894406 | -0.531290 | 254 | 243.067008 | 340.57 | 438.072992 | 1.340827 |
| GO:0019637\_organophosphate\_metabolic\_process | 43 | 1 | 2.894406 | -0.531290 | 254 | 243.067008 | 340.57 | 438.072992 | 1.340827 |
| GO:0032446\_protein\_modification\_by\_small\_protein\_conjugation | 43 | 1 | 2.894406 | -0.531290 | 254 | 243.067008 | 340.57 | 438.072992 | 1.340827 |
| GO:0046879\_hormone\_secretion | 43 | 1 | 2.894406 | -0.531290 | 254 | 243.067008 | 340.57 | 438.072992 | 1.340827 |
| GO:0009914\_hormone\_transport | 44 | 1 | 2.828624 | -0.522923 | 256 | 248.579046 | 347.41 | 446.240954 | 1.357070 |
| GO:0046545\_development\_of\_primary\_female\_sexual\_characteristics | 44 | 1 | 2.828624 | -0.522923 | 256 | 248.579046 | 347.41 | 446.240954 | 1.357070 |
| GO:0050789\_regulation\_of\_biological\_process | 2357 | 21 | 1.108888 | -0.517659 | 257 | 249.061659 | 348.07 | 447.078341 | 1.354358 |
| GO:0043623\_cellular\_protein\_complex\_assembly | 45 | 1 | 2.765766 | -0.514778 | 258 | 251.007168 | 350.39 | 449.772832 | 1.358101 |
| GO:0016044\_membrane\_organization | 140 | 2 | 1.777992 | -0.507397 | 259 | 252.066622 | 351.48 | 450.893378 | 1.357066 |
| GO:0007612\_learning | 46 | 1 | 2.705640 | -0.506846 | 263 | 254.228770 | 354.13 | 454.031230 | 1.346502 |
| GO:0009581\_detection\_of\_external\_stimulus | 46 | 1 | 2.705640 | -0.506846 | 263 | 254.228770 | 354.13 | 454.031230 | 1.346502 |
| GO:0030850\_prostate\_gland\_development | 46 | 1 | 2.705640 | -0.506846 | 263 | 254.228770 | 354.13 | 454.031230 | 1.346502 |
| GO:0046631\_alpha-beta\_T\_cell\_activation | 46 | 1 | 2.705640 | -0.506846 | 263 | 254.228770 | 354.13 | 454.031230 | 1.346502 |
| GO:0003006\_reproductive\_developmental\_process | 141 | 2 | 1.765382 | -0.503233 | 264 | 255.120072 | 355.0 | 454.879928 | 1.344697 |
| GO:0016570\_histone\_modification | 47 | 1 | 2.648074 | -0.499117 | 265 | 258.289050 | 358.66 | 459.030950 | 1.353434 |
| GO:0032269\_negative\_regulation\_of\_cellular\_protein\_metabolic\_process | 48 | 1 | 2.592905 | -0.491582 | 266 | 261.029123 | 361.77 | 462.510877 | 1.360038 |
| GO:0008104\_protein\_localization | 251 | 3 | 1.487563 | -0.484306 | 267 | 262.037534 | 362.97 | 463.902466 | 1.359438 |
| GO:0042035\_regulation\_of\_cytokine\_biosynthetic\_process | 49 | 1 | 2.539989 | -0.484234 | 272 | 264.529483 | 366.16 | 467.790517 | 1.346176 |
| GO:0043473\_pigmentation | 49 | 1 | 2.539989 | -0.484234 | 272 | 264.529483 | 366.16 | 467.790517 | 1.346176 |
| GO:0046660\_female\_sex\_differentiation | 49 | 1 | 2.539989 | -0.484234 | 272 | 264.529483 | 366.16 | 467.790517 | 1.346176 |
| GO:0046661\_male\_sex\_differentiation | 49 | 1 | 2.539989 | -0.484234 | 272 | 264.529483 | 366.16 | 467.790517 | 1.346176 |
| GO:0048741\_skeletal\_muscle\_fiber\_development | 49 | 1 | 2.539989 | -0.484234 | 272 | 264.529483 | 366.16 | 467.790517 | 1.346176 |
| GO:0006812\_cation\_transport | 146 | 2 | 1.704924 | -0.483040 | 273 | 265.068724 | 366.8 | 468.531276 | 1.343590 |
| GO:0022603\_regulation\_of\_anatomical\_structure\_morphogenesis | 147 | 2 | 1.693326 | -0.479122 | 274 | 265.815479 | 367.77 | 469.724521 | 1.342226 |
| GO:0051606\_detection\_of\_stimulus | 50 | 1 | 2.489189 | -0.477065 | 276 | 268.253255 | 370.69 | 473.126745 | 1.343080 |
| GO:0070647\_protein\_modification\_by\_small\_protein\_conjugation\_or\_removal | 50 | 1 | 2.489189 | -0.477065 | 276 | 268.253255 | 370.69 | 473.126745 | 1.343080 |
| GO:0048878\_chemical\_homeostasis | 254 | 3 | 1.469994 | -0.475472 | 277 | 268.446136 | 370.89 | 473.333864 | 1.338953 |
| GO:0006520\_cellular\_amino\_acid\_metabolic\_process | 51 | 1 | 2.440382 | -0.470067 | 281 | 272.325662 | 375.61 | 478.894338 | 1.336690 |
| GO:0016569\_covalent\_chromatin\_modification | 51 | 1 | 2.440382 | -0.470067 | 281 | 272.325662 | 375.61 | 478.894338 | 1.336690 |
| GO:0044106\_cellular\_amine\_metabolic\_process | 51 | 1 | 2.440382 | -0.470067 | 281 | 272.325662 | 375.61 | 478.894338 | 1.336690 |
| GO:0048747\_muscle\_fiber\_development | 51 | 1 | 2.440382 | -0.470067 | 281 | 272.325662 | 375.61 | 478.894338 | 1.336690 |
| GO:0044238\_primary\_metabolic\_process | 1905 | 17 | 1.110662 | -0.466158 | 282 | 272.981801 | 376.38 | 479.778199 | 1.334681 |
| GO:0032268\_regulation\_of\_cellular\_protein\_metabolic\_process | 152 | 2 | 1.637624 | -0.460111 | 283 | 274.933690 | 378.74 | 482.546310 | 1.338304 |
| GO:0051248\_negative\_regulation\_of\_protein\_metabolic\_process | 53 | 1 | 2.348292 | -0.456561 | 284 | 277.224861 | 381.33 | 485.435139 | 1.342711 |
| GO:0048666\_neuron\_development | 262 | 3 | 1.425108 | -0.452756 | 285 | 278.223983 | 382.43 | 486.636017 | 1.341860 |
| GO:0006412\_translation | 54 | 1 | 2.304805 | -0.450040 | 287 | 280.662127 | 385.32 | 489.977873 | 1.342578 |
| GO:0009566\_fertilization | 54 | 1 | 2.304805 | -0.450040 | 287 | 280.662127 | 385.32 | 489.977873 | 1.342578 |
| GO:0022402\_cell\_cycle\_process | 155 | 2 | 1.605929 | -0.449146 | 288 | 281.140320 | 385.92 | 490.699680 | 1.340000 |
| GO:0022414\_reproductive\_process | 376 | 4 | 1.324037 | -0.446724 | 289 | 281.503609 | 386.29 | 491.076391 | 1.336644 |
| GO:0007126\_meiosis | 55 | 1 | 2.262899 | -0.443665 | 293 | 283.407363 | 388.56 | 493.712637 | 1.326143 |
| GO:0007605\_sensory\_perception\_of\_sound | 55 | 1 | 2.262899 | -0.443665 | 293 | 283.407363 | 388.56 | 493.712637 | 1.326143 |
| GO:0048568\_embryonic\_organ\_development | 55 | 1 | 2.262899 | -0.443665 | 293 | 283.407363 | 388.56 | 493.712637 | 1.326143 |
| GO:0051327\_M\_phase\_of\_meiotic\_cell\_cycle | 55 | 1 | 2.262899 | -0.443665 | 293 | 283.407363 | 388.56 | 493.712637 | 1.326143 |
| GO:0007409\_axonogenesis | 158 | 2 | 1.575436 | -0.438497 | 294 | 284.564814 | 389.82 | 495.075186 | 1.325918 |
| GO:0042089\_cytokine\_biosynthetic\_process | 56 | 1 | 2.222490 | -0.437433 | 299 | 287.577563 | 393.16 | 498.742437 | 1.314916 |
| GO:0042107\_cytokine\_metabolic\_process | 56 | 1 | 2.222490 | -0.437433 | 299 | 287.577563 | 393.16 | 498.742437 | 1.314916 |
| GO:0046486\_glycerolipid\_metabolic\_process | 56 | 1 | 2.222490 | -0.437433 | 299 | 287.577563 | 393.16 | 498.742437 | 1.314916 |
| GO:0050678\_regulation\_of\_epithelial\_cell\_proliferation | 56 | 1 | 2.222490 | -0.437433 | 299 | 287.577563 | 393.16 | 498.742437 | 1.314916 |
| GO:0051321\_meiotic\_cell\_cycle | 56 | 1 | 2.222490 | -0.437433 | 299 | 287.577563 | 393.16 | 498.742437 | 1.314916 |
| GO:0016043\_cellular\_component\_organization | 964 | 9 | 1.161966 | -0.435794 | 300 | 287.731524 | 393.29 | 498.848476 | 1.310967 |
| GO:0051234\_establishment\_of\_localization | 729 | 7 | 1.195084 | -0.434017 | 301 | 287.883892 | 393.41 | 498.936108 | 1.307010 |
| GO:0000226\_microtubule\_cytoskeleton\_organization | 57 | 1 | 2.183499 | -0.431336 | 303 | 291.275585 | 397.5 | 503.724415 | 1.311881 |
| GO:0009953\_dorsal\_ventral\_pattern\_formation | 57 | 1 | 2.183499 | -0.431336 | 303 | 291.275585 | 397.5 | 503.724415 | 1.311881 |
| GO:0032502\_developmental\_process | 2060 | 18 | 1.087510 | -0.426314 | 304 | 292.094655 | 398.67 | 505.245345 | 1.311414 |
| GO:0030902\_hindbrain\_development | 58 | 1 | 2.145853 | -0.425372 | 307 | 293.265065 | 400.13 | 506.994935 | 1.303355 |
| GO:0033043\_regulation\_of\_organelle\_organization | 58 | 1 | 2.145853 | -0.425372 | 307 | 293.265065 | 400.13 | 506.994935 | 1.303355 |
| GO:0034622\_cellular\_macromolecular\_complex\_assembly | 58 | 1 | 2.145853 | -0.425372 | 307 | 293.265065 | 400.13 | 506.994935 | 1.303355 |
| GO:0009628\_response\_to\_abiotic\_stimulus | 162 | 2 | 1.536537 | -0.424766 | 308 | 293.402003 | 400.3 | 507.197997 | 1.299675 |
| GO:0042110\_T\_cell\_activation | 163 | 2 | 1.527110 | -0.421414 | 309 | 294.109369 | 401.14 | 508.170631 | 1.298188 |
| GO:0016055\_Wnt\_receptor\_signaling\_pathway | 59 | 1 | 2.109482 | -0.419534 | 310 | 296.018160 | 403.42 | 510.821840 | 1.301355 |
| GO:0042127\_regulation\_of\_cell\_proliferation | 393 | 4 | 1.266763 | -0.409412 | 311 | 298.993532 | 406.63 | 514.266468 | 1.307492 |
| GO:0007005\_mitochondrion\_organization | 61 | 1 | 2.040319 | -0.408223 | 313 | 301.541727 | 409.7 | 517.858273 | 1.308946 |
| GO:0032270\_positive\_regulation\_of\_cellular\_protein\_metabolic\_process | 61 | 1 | 2.040319 | -0.408223 | 313 | 301.541727 | 409.7 | 517.858273 | 1.308946 |
| GO:0040014\_regulation\_of\_multicellular\_organism\_growth | 62 | 1 | 2.007411 | -0.402742 | 315 | 304.470319 | 413.12 | 521.769681 | 1.311492 |
| GO:0050954\_sensory\_perception\_of\_mechanical\_stimulus | 62 | 1 | 2.007411 | -0.402742 | 315 | 304.470319 | 413.12 | 521.769681 | 1.311492 |
| GO:0051246\_regulation\_of\_protein\_metabolic\_process | 170 | 2 | 1.464229 | -0.398810 | 316 | 305.170189 | 413.9 | 522.629811 | 1.309810 |
| GO:0048518\_positive\_regulation\_of\_biological\_process | 995 | 9 | 1.125764 | -0.391726 | 317 | 309.764848 | 419.3 | 528.835152 | 1.322713 |
| GO:0007417\_central\_nervous\_system\_development | 287 | 3 | 1.300970 | -0.388934 | 318 | 310.782326 | 420.56 | 530.337674 | 1.322516 |
| GO:0015031\_protein\_transport | 175 | 2 | 1.422394 | -0.383536 | 319 | 312.324237 | 422.36 | 532.395763 | 1.324013 |
| GO:0051130\_positive\_regulation\_of\_cellular\_component\_organization | 66 | 1 | 1.885749 | -0.381890 | 320 | 314.269374 | 424.63 | 534.990626 | 1.326969 |
| GO:0051247\_positive\_regulation\_of\_protein\_metabolic\_process | 67 | 1 | 1.857604 | -0.376929 | 321 | 317.333705 | 428.01 | 538.686295 | 1.333364 |
| GO:0007242\_intracellular\_signaling\_cascade | 411 | 4 | 1.211284 | -0.373240 | 322 | 317.522963 | 428.27 | 539.017037 | 1.330031 |
| GO:0019932\_second-messenger-mediated\_signaling | 68 | 1 | 1.830286 | -0.372063 | 323 | 318.779945 | 429.74 | 540.700055 | 1.330464 |
| GO:0045184\_establishment\_of\_protein\_localization | 180 | 2 | 1.382883 | -0.368940 | 324 | 320.179912 | 431.61 | 543.040088 | 1.332130 |
| GO:0019752\_carboxylic\_acid\_metabolic\_process | 181 | 2 | 1.375243 | -0.366099 | 326 | 322.317602 | 434.05 | 545.782398 | 1.331442 |
| GO:0043436\_oxoacid\_metabolic\_process | 181 | 2 | 1.375243 | -0.366099 | 326 | 322.317602 | 434.05 | 545.782398 | 1.331442 |
| GO:0006082\_organic\_acid\_metabolic\_process | 182 | 2 | 1.367686 | -0.363283 | 327 | 323.190296 | 434.94 | 546.689704 | 1.330092 |
| GO:0007611\_learning\_or\_memory | 70 | 1 | 1.777992 | -0.362604 | 330 | 324.670749 | 436.71 | 548.749251 | 1.323364 |
| GO:0008406\_gonad\_development | 70 | 1 | 1.777992 | -0.362604 | 330 | 324.670749 | 436.71 | 548.749251 | 1.323364 |
| GO:0048592\_eye\_morphogenesis | 70 | 1 | 1.777992 | -0.362604 | 330 | 324.670749 | 436.71 | 548.749251 | 1.323364 |
| GO:0042180\_cellular\_ketone\_metabolic\_process | 183 | 2 | 1.360213 | -0.360492 | 331 | 325.145117 | 437.22 | 549.294883 | 1.320906 |
| GO:0042592\_homeostatic\_process | 419 | 4 | 1.188157 | -0.358180 | 332 | 325.602788 | 437.81 | 550.017212 | 1.318705 |
| GO:0016568\_chromatin\_modification | 72 | 1 | 1.728604 | -0.353492 | 334 | 330.900033 | 443.72 | 556.539967 | 1.328503 |
| GO:0050673\_epithelial\_cell\_proliferation | 72 | 1 | 1.728604 | -0.353492 | 334 | 330.900033 | 443.72 | 556.539967 | 1.328503 |
| GO:0006811\_ion\_transport | 186 | 2 | 1.338274 | -0.352265 | 335 | 331.791091 | 444.63 | 557.468909 | 1.327254 |
| GO:0008283\_cell\_proliferation | 544 | 5 | 1.143929 | -0.348892 | 336 | 334.476149 | 447.62 | 560.763851 | 1.332202 |
| GO:0007276\_gamete\_generation | 188 | 2 | 1.324037 | -0.346899 | 337 | 335.010441 | 448.16 | 561.309559 | 1.329852 |
| GO:0045893\_positive\_regulation\_of\_transcription\_\_DNA-dependent | 306 | 3 | 1.220191 | -0.346776 | 339 | 335.362469 | 448.62 | 561.877531 | 1.323363 |
| GO:0051254\_positive\_regulation\_of\_RNA\_metabolic\_process | 306 | 3 | 1.220191 | -0.346776 | 339 | 335.362469 | 448.62 | 561.877531 | 1.323363 |
| GO:0008152\_metabolic\_process | 2133 | 18 | 1.050291 | -0.345966 | 340 | 335.474021 | 448.79 | 562.105979 | 1.319971 |
| GO:0007281\_germ\_cell\_development | 75 | 1 | 1.659459 | -0.340431 | 341 | 338.720277 | 452.39 | 566.059723 | 1.326657 |
| GO:0034621\_cellular\_macromolecular\_complex\_subunit\_organization | 76 | 1 | 1.637624 | -0.336230 | 342 | 340.294546 | 453.94 | 567.585454 | 1.327310 |
| GO:0010604\_positive\_regulation\_of\_macromolecule\_metabolic\_process | 433 | 4 | 1.149741 | -0.333225 | 343 | 340.592319 | 454.31 | 568.027681 | 1.324519 |
| GO:0006461\_protein\_complex\_assembly | 78 | 1 | 1.595634 | -0.328046 | 345 | 344.764189 | 458.92 | 573.075811 | 1.330203 |
| GO:0070271\_protein\_complex\_biogenesis | 78 | 1 | 1.595634 | -0.328046 | 345 | 344.764189 | 458.92 | 573.075811 | 1.330203 |
| GO:0044267\_cellular\_protein\_metabolic\_process | 559 | 5 | 1.113233 | -0.325544 | 346 | 345.305804 | 459.47 | 573.634196 | 1.327948 |
| GO:0015674\_di-\_\_tri-valent\_inorganic\_cation\_transport | 79 | 1 | 1.575436 | -0.324060 | 347 | 345.858648 | 460.25 | 574.641352 | 1.326369 |
| GO:0000278\_mitotic\_cell\_cycle | 80 | 1 | 1.555743 | -0.320140 | 348 | 349.095637 | 463.8 | 578.504363 | 1.332759 |
| GO:0031325\_positive\_regulation\_of\_cellular\_metabolic\_process | 442 | 4 | 1.126330 | -0.318073 | 349 | 349.737315 | 464.56 | 579.382685 | 1.331117 |
| GO:0007411\_axon\_guidance | 82 | 1 | 1.517798 | -0.312497 | 352 | 352.554570 | 467.56 | 582.565430 | 1.328295 |
| GO:0010627\_regulation\_of\_protein\_kinase\_cascade | 82 | 1 | 1.517798 | -0.312497 | 352 | 352.554570 | 467.56 | 582.565430 | 1.328295 |
| GO:0045664\_regulation\_of\_neuron\_differentiation | 82 | 1 | 1.517798 | -0.312497 | 352 | 352.554570 | 467.56 | 582.565430 | 1.328295 |
| GO:0006325\_chromatin\_organization | 83 | 1 | 1.499512 | -0.308771 | 356 | 355.665266 | 470.74 | 585.814734 | 1.322303 |
| GO:0006575\_cellular\_amino\_acid\_derivative\_metabolic\_process | 83 | 1 | 1.499512 | -0.308771 | 356 | 355.665266 | 470.74 | 585.814734 | 1.322303 |
| GO:0007017\_microtubule-based\_process | 83 | 1 | 1.499512 | -0.308771 | 356 | 355.665266 | 470.74 | 585.814734 | 1.322303 |
| GO:0030198\_extracellular\_matrix\_organization | 83 | 1 | 1.499512 | -0.308771 | 356 | 355.665266 | 470.74 | 585.814734 | 1.322303 |
| GO:0006996\_organelle\_organization | 449 | 4 | 1.108770 | -0.306745 | 357 | 356.076736 | 471.22 | 586.363264 | 1.319944 |
| GO:0030005\_cellular\_di-\_\_tri-valent\_inorganic\_cation\_homeostasis | 84 | 1 | 1.481660 | -0.305105 | 359 | 356.860520 | 472.15 | 587.439480 | 1.315181 |
| GO:0045137\_development\_of\_primary\_sexual\_characteristics | 84 | 1 | 1.481660 | -0.305105 | 359 | 356.860520 | 472.15 | 587.439480 | 1.315181 |
| GO:0032504\_multicellular\_organism\_reproduction | 86 | 1 | 1.447203 | -0.297950 | 362 | 362.256888 | 477.97 | 593.683112 | 1.320359 |
| GO:0034641\_cellular\_nitrogen\_compound\_metabolic\_process | 86 | 1 | 1.447203 | -0.297950 | 362 | 362.256888 | 477.97 | 593.683112 | 1.320359 |
| GO:0048609\_reproductive\_process\_in\_a\_multicellular\_organism | 86 | 1 | 1.447203 | -0.297950 | 362 | 362.256888 | 477.97 | 593.683112 | 1.320359 |
| GO:0003001\_generation\_of\_a\_signal\_involved\_in\_cell-cell\_signaling | 87 | 1 | 1.430568 | -0.294459 | 363 | 365.035665 | 481.22 | 597.404335 | 1.325675 |
| GO:0009893\_positive\_regulation\_of\_metabolic\_process | 458 | 4 | 1.086982 | -0.292743 | 364 | 365.562453 | 481.83 | 598.097547 | 1.323709 |
| GO:0045941\_positive\_regulation\_of\_transcription | 338 | 3 | 1.104670 | -0.286052 | 365 | 367.301389 | 483.97 | 600.638611 | 1.325945 |
| GO:0030003\_cellular\_cation\_homeostasis | 90 | 1 | 1.382883 | -0.284311 | 368 | 368.887115 | 485.73 | 602.572885 | 1.319918 |
| GO:0030324\_lung\_development | 90 | 1 | 1.382883 | -0.284311 | 368 | 368.887115 | 485.73 | 602.572885 | 1.319918 |
| GO:0035264\_multicellular\_organism\_growth | 90 | 1 | 1.382883 | -0.284311 | 368 | 368.887115 | 485.73 | 602.572885 | 1.319918 |
| GO:0031399\_regulation\_of\_protein\_modification\_process | 91 | 1 | 1.367686 | -0.281033 | 369 | 370.649770 | 487.48 | 604.310230 | 1.321084 |
| GO:0030217\_T\_cell\_differentiation | 92 | 1 | 1.352820 | -0.277806 | 371 | 372.995279 | 489.96 | 606.924721 | 1.320647 |
| GO:0030323\_respiratory\_tube\_development | 92 | 1 | 1.352820 | -0.277806 | 371 | 372.995279 | 489.96 | 606.924721 | 1.320647 |
| GO:0006810\_transport | 718 | 6 | 1.040051 | -0.277467 | 372 | 373.256354 | 490.18 | 607.103646 | 1.317688 |
| GO:0055066\_di-\_\_tri-valent\_inorganic\_cation\_homeostasis | 93 | 1 | 1.338274 | -0.274628 | 374 | 375.404295 | 492.5 | 609.595705 | 1.316845 |
| GO:0065003\_macromolecular\_complex\_assembly | 93 | 1 | 1.338274 | -0.274628 | 374 | 375.404295 | 492.5 | 609.595705 | 1.316845 |
| GO:0010628\_positive\_regulation\_of\_gene\_expression | 346 | 3 | 1.079128 | -0.272625 | 375 | 375.825447 | 492.96 | 610.094553 | 1.314560 |
| GO:0008610\_lipid\_biosynthetic\_process | 94 | 1 | 1.324037 | -0.271498 | 376 | 378.809730 | 495.9 | 612.990270 | 1.318883 |
| GO:0001701\_in\_utero\_embryonic\_development | 221 | 2 | 1.126330 | -0.270403 | 377 | 379.035522 | 496.17 | 613.304478 | 1.316101 |
| GO:0045935\_positive\_regulation\_of\_nucleobase\_\_nucleoside\_\_nucleotide\_and\_nucleic\_acid\_metabolic\_process | 352 | 3 | 1.060734 | -0.262969 | 378 | 382.204476 | 499.58 | 616.955524 | 1.321640 |
| GO:0009967\_positive\_regulation\_of\_signal\_transduction | 98 | 1 | 1.269994 | -0.259439 | 380 | 384.589215 | 502.22 | 619.850785 | 1.321632 |
| GO:0060541\_respiratory\_system\_development | 98 | 1 | 1.269994 | -0.259439 | 380 | 384.589215 | 502.22 | 619.850785 | 1.321632 |
| GO:0048731\_system\_development | 1609 | 13 | 1.005577 | -0.258033 | 381 | 384.833205 | 502.51 | 620.186795 | 1.318924 |
| GO:0030182\_neuron\_differentiation | 356 | 3 | 1.048816 | -0.256721 | 382 | 385.191240 | 502.77 | 620.348760 | 1.316152 |
| GO:0046649\_lymphocyte\_activation | 228 | 2 | 1.091750 | -0.256692 | 383 | 386.064753 | 503.65 | 621.235247 | 1.315013 |
| GO:0001817\_regulation\_of\_cytokine\_production | 99 | 1 | 1.257166 | -0.256535 | 384 | 387.441120 | 505.28 | 623.118880 | 1.315833 |
| GO:0051173\_positive\_regulation\_of\_nitrogen\_compound\_metabolic\_process | 361 | 3 | 1.034289 | -0.249117 | 385 | 389.638757 | 507.68 | 625.721243 | 1.318649 |
| GO:0050890\_cognition | 233 | 2 | 1.068322 | -0.247359 | 386 | 390.601290 | 508.69 | 626.778710 | 1.317850 |
| GO:0009968\_negative\_regulation\_of\_signal\_transduction | 103 | 1 | 1.208344 | -0.245331 | 387 | 391.995870 | 510.07 | 628.144130 | 1.318010 |
| GO:0050767\_regulation\_of\_neurogenesis | 104 | 1 | 1.196726 | -0.242630 | 388 | 393.449966 | 511.68 | 629.910034 | 1.318763 |
| GO:0007049\_cell\_cycle | 238 | 2 | 1.045878 | -0.238390 | 389 | 395.103173 | 513.57 | 632.036827 | 1.320231 |
| GO:0010817\_regulation\_of\_hormone\_levels | 106 | 1 | 1.174146 | -0.237340 | 390 | 395.548478 | 514.04 | 632.531522 | 1.318051 |
| GO:0007275\_multicellular\_organismal\_development | 1760 | 14 | 0.990018 | -0.235781 | 391 | 395.942435 | 514.49 | 633.037565 | 1.315831 |
| GO:0010557\_positive\_regulation\_of\_macromolecule\_biosynthetic\_process | 371 | 3 | 1.006411 | -0.234570 | 392 | 396.577822 | 515.12 | 633.662178 | 1.314082 |
| GO:0010647\_positive\_regulation\_of\_cell\_communication | 110 | 1 | 1.131450 | -0.227194 | 395 | 399.941353 | 518.68 | 637.418647 | 1.313114 |
| GO:0010648\_negative\_regulation\_of\_cell\_communication | 110 | 1 | 1.131450 | -0.227194 | 395 | 399.941353 | 518.68 | 637.418647 | 1.313114 |
| GO:0055080\_cation\_homeostasis | 110 | 1 | 1.131450 | -0.227194 | 395 | 399.941353 | 518.68 | 637.418647 | 1.313114 |
| GO:0045321\_leukocyte\_activation | 248 | 2 | 1.003705 | -0.221477 | 396 | 402.251234 | 521.02 | 639.788766 | 1.315707 |
| GO:0040008\_regulation\_of\_growth | 113 | 1 | 1.101411 | -0.219943 | 397 | 403.006257 | 521.92 | 640.833743 | 1.314660 |
| GO:0043687\_post-translational\_protein\_modification | 384 | 3 | 0.972340 | -0.216899 | 398 | 404.680249 | 523.8 | 642.919751 | 1.316080 |
| GO:0030097\_hemopoiesis | 253 | 2 | 0.983869 | -0.213501 | 399 | 406.917322 | 526.12 | 645.322678 | 1.318596 |
| GO:0031328\_positive\_regulation\_of\_cellular\_biosynthetic\_process | 387 | 3 | 0.964802 | -0.213009 | 400 | 407.040771 | 526.26 | 645.479229 | 1.315650 |
| GO:0009891\_positive\_regulation\_of\_biosynthetic\_process | 388 | 3 | 0.962315 | -0.211728 | 401 | 408.509015 | 527.92 | 647.330985 | 1.316509 |
| GO:0043933\_macromolecular\_complex\_subunit\_organization | 117 | 1 | 1.063756 | -0.210720 | 402 | 408.893431 | 528.31 | 647.726569 | 1.314204 |
| GO:0009966\_regulation\_of\_signal\_transduction | 256 | 2 | 0.972340 | -0.208862 | 403 | 409.846380 | 529.36 | 648.873620 | 1.313548 |
| GO:0006519\_cellular\_amino\_acid\_and\_derivative\_metabolic\_process | 118 | 1 | 1.054741 | -0.208489 | 405 | 410.700456 | 530.21 | 649.719544 | 1.309160 |
| GO:0051960\_regulation\_of\_nervous\_system\_development | 118 | 1 | 1.054741 | -0.208489 | 405 | 410.700456 | 530.21 | 649.719544 | 1.309160 |
| GO:0019538\_protein\_metabolic\_process | 655 | 5 | 0.950072 | -0.206682 | 406 | 411.581434 | 531.06 | 650.538566 | 1.308030 |
| GO:0006917\_induction\_of\_apoptosis | 121 | 1 | 1.028591 | -0.201972 | 408 | 414.520471 | 534.44 | 654.359529 | 1.309902 |
| GO:0012502\_induction\_of\_programmed\_cell\_death | 121 | 1 | 1.028591 | -0.201972 | 408 | 414.520471 | 534.44 | 654.359529 | 1.309902 |
| GO:0048699\_generation\_of\_neurons | 396 | 3 | 0.942875 | -0.201743 | 409 | 414.832270 | 534.7 | 654.567730 | 1.307335 |
| GO:0001775\_cell\_activation | 262 | 2 | 0.950072 | -0.199898 | 410 | 415.835725 | 535.7 | 655.564275 | 1.306585 |
| GO:0001816\_cytokine\_production | 122 | 1 | 1.020160 | -0.199855 | 412 | 418.057184 | 537.96 | 657.862816 | 1.305728 |
| GO:0060284\_regulation\_of\_cell\_development | 122 | 1 | 1.020160 | -0.199855 | 412 | 418.057184 | 537.96 | 657.862816 | 1.305728 |
| GO:0048856\_anatomical\_structure\_development | 1688 | 13 | 0.958515 | -0.195976 | 413 | 419.279283 | 539.09 | 658.900717 | 1.305303 |
| GO:0009308\_amine\_metabolic\_process | 124 | 1 | 1.003705 | -0.195702 | 415 | 420.243020 | 540.02 | 659.796980 | 1.301253 |
| GO:0030098\_lymphocyte\_differentiation | 124 | 1 | 1.003705 | -0.195702 | 415 | 420.243020 | 540.02 | 659.796980 | 1.301253 |
| GO:0043062\_extracellular\_structure\_organization | 125 | 1 | 0.995676 | -0.193666 | 416 | 420.931756 | 540.75 | 660.568244 | 1.299880 |
| GO:0045944\_positive\_regulation\_of\_transcription\_from\_RNA\_polymerase\_II\_promoter | 269 | 2 | 0.925349 | -0.189945 | 417 | 421.820887 | 541.53 | 661.239113 | 1.298633 |
| GO:0001655\_urogenital\_system\_development | 128 | 1 | 0.972340 | -0.187708 | 418 | 423.571781 | 543.5 | 663.428219 | 1.300239 |
| GO:0048534\_hemopoietic\_or\_lymphoid\_organ\_development | 277 | 2 | 0.898624 | -0.179197 | 419 | 428.614931 | 548.97 | 669.325069 | 1.310191 |
| GO:0007610\_behavior | 279 | 2 | 0.892183 | -0.176610 | 420 | 429.962943 | 550.42 | 670.877057 | 1.310524 |
| GO:0065008\_regulation\_of\_biological\_quality | 693 | 5 | 0.897976 | -0.171597 | 421 | 431.984716 | 552.3 | 672.615284 | 1.311876 |
| GO:0022008\_neurogenesis | 423 | 3 | 0.882691 | -0.171293 | 422 | 432.797812 | 553.07 | 673.342188 | 1.310592 |
| GO:0009790\_embryonic\_development | 567 | 4 | 0.878021 | -0.164321 | 423 | 437.016109 | 557.09 | 677.163891 | 1.316998 |
| GO:0045596\_negative\_regulation\_of\_cell\_differentiation | 144 | 1 | 0.864302 | -0.159408 | 424 | 439.565787 | 559.76 | 679.954213 | 1.320189 |
| GO:0048513\_organ\_development | 1365 | 10 | 0.911791 | -0.157675 | 425 | 439.827748 | 560.06 | 680.292252 | 1.317788 |
| GO:0002520\_immune\_system\_development | 295 | 2 | 0.843793 | -0.157243 | 426 | 440.877554 | 561.09 | 681.302446 | 1.317113 |
| GO:0030900\_forebrain\_development | 146 | 1 | 0.852462 | -0.156238 | 427 | 442.053975 | 562.32 | 682.586025 | 1.316909 |
| GO:0006464\_protein\_modification\_process | 439 | 3 | 0.850520 | -0.155384 | 428 | 442.281676 | 562.56 | 682.838324 | 1.314393 |
| GO:0032940\_secretion\_by\_cell | 149 | 1 | 0.835298 | -0.151619 | 429 | 444.718519 | 564.78 | 684.841481 | 1.316503 |
| GO:0008285\_negative\_regulation\_of\_cell\_proliferation | 155 | 1 | 0.802964 | -0.142849 | 430 | 450.203876 | 570.18 | 690.156124 | 1.326000 |
| GO:0051704\_multi-organism\_process | 157 | 1 | 0.792735 | -0.140057 | 431 | 451.055963 | 570.92 | 690.784037 | 1.324640 |
| GO:0043412\_biopolymer\_modification | 458 | 3 | 0.815237 | -0.138316 | 432 | 453.438904 | 573.1 | 692.761096 | 1.326620 |
| GO:0002521\_leukocyte\_differentiation | 161 | 1 | 0.773040 | -0.134657 | 433 | 454.276814 | 573.82 | 693.363186 | 1.325219 |
| GO:0007626\_locomotory\_behavior | 163 | 1 | 0.763555 | -0.132047 | 434 | 455.695139 | 575.23 | 694.764861 | 1.325415 |
| GO:0043065\_positive\_regulation\_of\_apoptosis | 166 | 1 | 0.749756 | -0.128239 | 435 | 457.866650 | 577.16 | 696.453350 | 1.326805 |
| GO:0010942\_positive\_regulation\_of\_cell\_death | 167 | 1 | 0.745266 | -0.126997 | 437 | 458.808930 | 578.11 | 697.411070 | 1.322906 |
| GO:0043068\_positive\_regulation\_of\_programmed\_cell\_death | 167 | 1 | 0.745266 | -0.126997 | 437 | 458.808930 | 578.11 | 697.411070 | 1.322906 |
| GO:0032501\_multicellular\_organismal\_process | 2183 | 16 | 0.912209 | -0.125421 | 438 | 458.925391 | 578.22 | 697.514609 | 1.320137 |
| GO:0006928\_cell\_motion | 330 | 2 | 0.754300 | -0.122045 | 441 | 461.464280 | 580.36 | 699.255720 | 1.316009 |
| GO:0010646\_regulation\_of\_cell\_communication | 330 | 2 | 0.754300 | -0.122045 | 441 | 461.464280 | 580.36 | 699.255720 | 1.316009 |
| GO:0051674\_localization\_of\_cell | 330 | 2 | 0.754300 | -0.122045 | 441 | 461.464280 | 580.36 | 699.255720 | 1.316009 |
| GO:0007600\_sensory\_perception | 172 | 1 | 0.723602 | -0.120989 | 442 | 462.842576 | 581.62 | 700.397424 | 1.315882 |
| GO:0044248\_cellular\_catabolic\_process | 173 | 1 | 0.719419 | -0.119826 | 443 | 464.482602 | 583.13 | 701.777398 | 1.316321 |
| GO:0046903\_secretion | 175 | 1 | 0.711197 | -0.117537 | 444 | 465.780257 | 584.33 | 702.879743 | 1.316059 |
| GO:0006873\_cellular\_ion\_homeostasis | 176 | 1 | 0.707156 | -0.116411 | 445 | 466.949724 | 585.39 | 703.830276 | 1.315483 |
| GO:0009605\_response\_to\_external\_stimulus | 339 | 2 | 0.734274 | -0.114349 | 446 | 467.770606 | 586.14 | 704.509394 | 1.314215 |
| GO:0048732\_gland\_development | 179 | 1 | 0.695304 | -0.113105 | 447 | 469.492988 | 587.77 | 706.047012 | 1.314922 |
| GO:0007165\_signal\_transduction | 915 | 6 | 0.816128 | -0.111951 | 448 | 470.401288 | 588.56 | 706.718712 | 1.313750 |
| GO:0055082\_cellular\_chemical\_homeostasis | 181 | 1 | 0.687621 | -0.110959 | 449 | 471.809972 | 589.94 | 708.070028 | 1.313898 |
| GO:0016192\_vesicle-mediated\_transport | 184 | 1 | 0.676410 | -0.107824 | 450 | 473.750574 | 591.76 | 709.769426 | 1.315022 |
| GO:0007010\_cytoskeleton\_organization | 185 | 1 | 0.672754 | -0.106801 | 451 | 474.559771 | 592.61 | 710.660229 | 1.313991 |
| GO:0002376\_immune\_system\_process | 505 | 3 | 0.739363 | -0.103380 | 452 | 476.880855 | 595.01 | 713.139145 | 1.316394 |
| GO:0003002\_regionalization | 195 | 1 | 0.638254 | -0.097138 | 455 | 480.270990 | 598.26 | 716.249010 | 1.314857 |
| GO:0007507\_heart\_development | 195 | 1 | 0.638254 | -0.097138 | 455 | 480.270990 | 598.26 | 716.249010 | 1.314857 |
| GO:0019725\_cellular\_homeostasis | 195 | 1 | 0.638254 | -0.097138 | 455 | 480.270990 | 598.26 | 716.249010 | 1.314857 |
| GO:0050801\_ion\_homeostasis | 197 | 1 | 0.631774 | -0.095322 | 456 | 481.797795 | 599.66 | 717.522205 | 1.315044 |
| GO:0043009\_chordate\_embryonic\_development | 365 | 2 | 0.681970 | -0.094714 | 457 | 482.753835 | 600.38 | 718.006165 | 1.313742 |
| GO:0009792\_embryonic\_development\_ending\_in\_birth\_or\_egg\_hatching | 368 | 2 | 0.676410 | -0.092675 | 458 | 484.882706 | 602.08 | 719.277294 | 1.314585 |
| GO:0009888\_tissue\_development | 525 | 3 | 0.711197 | -0.091186 | 459 | 485.551018 | 602.63 | 719.708982 | 1.312919 |
| GO:0007154\_cell\_communication | 1096 | 7 | 0.794905 | -0.090180 | 460 | 486.206421 | 603.25 | 720.293579 | 1.311413 |
| GO:0022607\_cellular\_component\_assembly | 204 | 1 | 0.610095 | -0.089253 | 461 | 487.200024 | 604.13 | 721.059976 | 1.310477 |
| GO:0007243\_protein\_kinase\_cascade | 205 | 1 | 0.607119 | -0.088421 | 462 | 488.107914 | 604.85 | 721.592086 | 1.309199 |
| GO:0035295\_tube\_development | 212 | 1 | 0.587073 | -0.082825 | 463 | 490.034117 | 606.76 | 723.485883 | 1.310497 |
| GO:0050877\_neurological\_system\_process | 390 | 2 | 0.638254 | -0.078976 | 464 | 492.211306 | 608.79 | 725.368694 | 1.312047 |
| GO:0044085\_cellular\_component\_biogenesis | 237 | 1 | 0.525145 | -0.065731 | 465 | 501.665754 | 616.61 | 731.554246 | 1.326043 |
| GO:0009056\_catabolic\_process | 243 | 1 | 0.512179 | -0.062212 | 466 | 503.032049 | 617.67 | 732.307951 | 1.325472 |
| GO:0051239\_regulation\_of\_multicellular\_organismal\_process | 587 | 3 | 0.636079 | -0.061357 | 467 | 503.514989 | 618.04 | 732.565011 | 1.323426 |
| GO:0007389\_pattern\_specification\_process | 250 | 1 | 0.497838 | -0.058355 | 468 | 505.859697 | 619.84 | 733.820303 | 1.324444 |
| GO:0007267\_cell-cell\_signaling | 252 | 1 | 0.493887 | -0.057299 | 469 | 507.555834 | 621.29 | 735.024166 | 1.324712 |
| GO:0010926\_anatomical\_structure\_formation | 447 | 2 | 0.556866 | -0.052001 | 470 | 513.140000 | 625.74 | 738.340000 | 1.331362 |
| GO:0044255\_cellular\_lipid\_metabolic\_process | 264 | 1 | 0.471437 | -0.051369 | 471 | 513.642468 | 626.17 | 738.697532 | 1.329448 |
| GO:0048646\_anatomical\_structure\_formation\_involved\_in\_morphogenesis | 277 | 1 | 0.449312 | -0.045656 | 472 | 518.381743 | 629.81 | 741.238257 | 1.334343 |
| GO:0006629\_lipid\_metabolic\_process | 285 | 1 | 0.436700 | -0.042470 | 473 | 520.572278 | 631.5 | 742.427722 | 1.335095 |
| GO:0045595\_regulation\_of\_cell\_differentiation | 295 | 1 | 0.421896 | -0.038804 | 474 | 523.756516 | 633.77 | 743.783484 | 1.337068 |
| GO:0048598\_embryonic\_morphogenesis | 299 | 1 | 0.416252 | -0.037430 | 475 | 524.134313 | 634.08 | 744.025687 | 1.334905 |
| GO:0051094\_positive\_regulation\_of\_developmental\_process | 308 | 1 | 0.404089 | -0.034516 | 476 | 525.102252 | 634.8 | 744.497748 | 1.333613 |
| GO:0003008\_system\_process | 516 | 2 | 0.482401 | -0.031065 | 477 | 526.454165 | 635.86 | 745.265835 | 1.333040 |
| GO:0050793\_regulation\_of\_developmental\_process | 703 | 3 | 0.531121 | -0.028285 | 478 | 528.164067 | 637.08 | 745.995933 | 1.332803 |
| GO:0051093\_negative\_regulation\_of\_developmental\_process | 331 | 1 | 0.376010 | -0.028069 | 479 | 528.612119 | 637.45 | 746.287881 | 1.330793 |
| GO:0006793\_phosphorus\_metabolic\_process | 340 | 1 | 0.366057 | -0.025890 | 481 | 530.115587 | 638.62 | 747.124413 | 1.327692 |
| GO:0006796\_phosphate\_metabolic\_process | 340 | 1 | 0.366057 | -0.025890 | 481 | 530.115587 | 638.62 | 747.124413 | 1.327692 |
| GO:0042981\_regulation\_of\_apoptosis | 360 | 1 | 0.345721 | -0.021636 | 482 | 532.646459 | 640.45 | 748.253541 | 1.328734 |
| GO:0010941\_regulation\_of\_cell\_death | 365 | 1 | 0.340985 | -0.020687 | 484 | 533.993189 | 641.34 | 748.686811 | 1.325083 |
| GO:0043067\_regulation\_of\_programmed\_cell\_death | 365 | 1 | 0.340985 | -0.020687 | 484 | 533.993189 | 641.34 | 748.686811 | 1.325083 |
| GO:0007166\_cell\_surface\_receptor\_linked\_signal\_transduction | 597 | 2 | 0.416950 | -0.016692 | 485 | 537.358972 | 643.87 | 750.381028 | 1.327567 |
| GO:0009653\_anatomical\_structure\_morphogenesis | 958 | 4 | 0.519664 | -0.015369 | 486 | 538.135204 | 644.45 | 750.764796 | 1.326029 |
| GO:0042221\_response\_to\_chemical\_stimulus | 409 | 1 | 0.304302 | -0.013931 | 487 | 538.430646 | 644.73 | 751.029354 | 1.323881 |
| GO:0006915\_apoptosis | 427 | 1 | 0.291474 | -0.011846 | 488 | 539.779542 | 645.67 | 751.560458 | 1.323094 |
| GO:0012501\_programmed\_cell\_death | 433 | 1 | 0.287435 | -0.011222 | 489 | 540.369444 | 646.02 | 751.670556 | 1.321104 |
| GO:0008219\_cell\_death | 444 | 1 | 0.280314 | -0.010161 | 490 | 541.496892 | 646.82 | 752.143108 | 1.320041 |
| GO:0016265\_death | 450 | 1 | 0.276577 | -0.009625 | 491 | 541.944560 | 647.1 | 752.255440 | 1.317923 |
| GO:0050896\_response\_to\_stimulus | 1107 | 4 | 0.449718 | -0.005321 | 492 | 543.934653 | 648.32 | 752.705347 | 1.317724 |
| GO:0009887\_organ\_morphogenesis | 642 | 1 | 0.193862 | -0.001643 | 493 | 546.442155 | 649.77 | 753.097845 | 1.317992 |
| GO:0001708\_cell\_fate\_specification | 56 | 0 | 0.000000 | -0.000000 | 497 | 553.726071 | 655.96 | 758.193929 | 1.319839 |
| GO:0002683\_negative\_regulation\_of\_immune\_system\_process | 56 | 0 | 0.000000 | -0.000000 | 497 | 553.726071 | 655.96 | 758.193929 | 1.319839 |
| GO:0002703\_regulation\_of\_leukocyte\_mediated\_immunity | 56 | 0 | 0.000000 | -0.000000 | 497 | 553.726071 | 655.96 | 758.193929 | 1.319839 |
| GO:0009187\_cyclic\_nucleotide\_metabolic\_process | 56 | 0 | 0.000000 | -0.000000 | 497 | 553.726071 | 655.96 | 758.193929 | 1.319839 |
| GO:0002200\_somatic\_diversification\_of\_immune\_receptors | 34 | 0 | 0.000000 | -0.000000 | 511 | 568.281907 | 669.57 | 770.858093 | 1.310313 |
| GO:0002237\_response\_to\_molecule\_of\_bacterial\_origin | 34 | 0 | 0.000000 | -0.000000 | 511 | 568.281907 | 669.57 | 770.858093 | 1.310313 |
| GO:0002699\_positive\_regulation\_of\_immune\_effector\_process | 34 | 0 | 0.000000 | -0.000000 | 511 | 568.281907 | 669.57 | 770.858093 | 1.310313 |
| GO:0007269\_neurotransmitter\_secretion | 34 | 0 | 0.000000 | -0.000000 | 511 | 568.281907 | 669.57 | 770.858093 | 1.310313 |
| GO:0007568\_aging | 34 | 0 | 0.000000 | -0.000000 | 511 | 568.281907 | 669.57 | 770.858093 | 1.310313 |
| GO:0010720\_positive\_regulation\_of\_cell\_development | 34 | 0 | 0.000000 | -0.000000 | 511 | 568.281907 | 669.57 | 770.858093 | 1.310313 |
| GO:0019882\_antigen\_processing\_and\_presentation | 34 | 0 | 0.000000 | -0.000000 | 511 | 568.281907 | 669.57 | 770.858093 | 1.310313 |
| GO:0030509\_BMP\_signaling\_pathway | 34 | 0 | 0.000000 | -0.000000 | 511 | 568.281907 | 669.57 | 770.858093 | 1.310313 |
| GO:0045927\_positive\_regulation\_of\_growth | 34 | 0 | 0.000000 | -0.000000 | 511 | 568.281907 | 669.57 | 770.858093 | 1.310313 |
| GO:0050730\_regulation\_of\_peptidyl-tyrosine\_phosphorylation | 34 | 0 | 0.000000 | -0.000000 | 511 | 568.281907 | 669.57 | 770.858093 | 1.310313 |
| GO:0051047\_positive\_regulation\_of\_secretion | 34 | 0 | 0.000000 | -0.000000 | 511 | 568.281907 | 669.57 | 770.858093 | 1.310313 |
| GO:0051052\_regulation\_of\_DNA\_metabolic\_process | 34 | 0 | 0.000000 | -0.000000 | 511 | 568.281907 | 669.57 | 770.858093 | 1.310313 |
| GO:0060443\_mammary\_gland\_morphogenesis | 34 | 0 | 0.000000 | -0.000000 | 511 | 568.281907 | 669.57 | 770.858093 | 1.310313 |
| GO:0060711\_labyrinthine\_layer\_development | 34 | 0 | 0.000000 | -0.000000 | 511 | 568.281907 | 669.57 | 770.858093 | 1.310313 |
| GO:0000910\_cytokinesis | 8 | 0 | 0.000000 | -0.000000 | 640 | 694.317306 | 793.24 | 892.162694 | 1.239438 |
| GO:0001783\_B\_cell\_apoptosis | 8 | 0 | 0.000000 | -0.000000 | 640 | 694.317306 | 793.24 | 892.162694 | 1.239438 |
| GO:0001833\_inner\_cell\_mass\_cell\_proliferation | 8 | 0 | 0.000000 | -0.000000 | 640 | 694.317306 | 793.24 | 892.162694 | 1.239438 |
| GO:0001840\_neural\_plate\_development | 8 | 0 | 0.000000 | -0.000000 | 640 | 694.317306 | 793.24 | 892.162694 | 1.239438 |
| GO:0001893\_maternal\_placenta\_development | 8 | 0 | 0.000000 | -0.000000 | 640 | 694.317306 | 793.24 | 892.162694 | 1.239438 |
| GO:0001911\_negative\_regulation\_of\_leukocyte\_mediated\_cytotoxicity | 8 | 0 | 0.000000 | -0.000000 | 640 | 694.317306 | 793.24 | 892.162694 | 1.239438 |
| GO:0001916\_positive\_regulation\_of\_T\_cell\_mediated\_cytotoxicity | 8 | 0 | 0.000000 | -0.000000 | 640 | 694.317306 | 793.24 | 892.162694 | 1.239438 |
| GO:0002065\_columnar\_cuboidal\_epithelial\_cell\_differentiation | 8 | 0 | 0.000000 | -0.000000 | 640 | 694.317306 | 793.24 | 892.162694 | 1.239438 |
| GO:0002320\_lymphoid\_progenitor\_cell\_differentiation | 8 | 0 | 0.000000 | -0.000000 | 640 | 694.317306 | 793.24 | 892.162694 | 1.239438 |
| GO:0002438\_acute\_inflammatory\_response\_to\_antigenic\_stimulus | 8 | 0 | 0.000000 | -0.000000 | 640 | 694.317306 | 793.24 | 892.162694 | 1.239438 |
| GO:0002524\_hypersensitivity | 8 | 0 | 0.000000 | -0.000000 | 640 | 694.317306 | 793.24 | 892.162694 | 1.239438 |
| GO:0002566\_somatic\_diversification\_of\_immune\_receptors\_via\_somatic\_mutation | 8 | 0 | 0.000000 | -0.000000 | 640 | 694.317306 | 793.24 | 892.162694 | 1.239438 |
| GO:0002864\_regulation\_of\_acute\_inflammatory\_response\_to\_antigenic\_stimulus | 8 | 0 | 0.000000 | -0.000000 | 640 | 694.317306 | 793.24 | 892.162694 | 1.239438 |
| GO:0002883\_regulation\_of\_hypersensitivity | 8 | 0 | 0.000000 | -0.000000 | 640 | 694.317306 | 793.24 | 892.162694 | 1.239438 |
| GO:0003081\_regulation\_of\_systemic\_arterial\_blood\_pressure\_by\_renin-angiotensin | 8 | 0 | 0.000000 | -0.000000 | 640 | 694.317306 | 793.24 | 892.162694 | 1.239438 |
| GO:0006020\_inositol\_metabolic\_process | 8 | 0 | 0.000000 | -0.000000 | 640 | 694.317306 | 793.24 | 892.162694 | 1.239438 |
| GO:0006195\_purine\_nucleotide\_catabolic\_process | 8 | 0 | 0.000000 | -0.000000 | 640 | 694.317306 | 793.24 | 892.162694 | 1.239438 |
| GO:0006284\_base-excision\_repair | 8 | 0 | 0.000000 | -0.000000 | 640 | 694.317306 | 793.24 | 892.162694 | 1.239438 |
| GO:0006349\_genetic\_imprinting | 8 | 0 | 0.000000 | -0.000000 | 640 | 694.317306 | 793.24 | 892.162694 | 1.239438 |
| GO:0006360\_transcription\_from\_RNA\_polymerase\_I\_promoter | 8 | 0 | 0.000000 | -0.000000 | 640 | 694.317306 | 793.24 | 892.162694 | 1.239438 |
| GO:0006399\_tRNA\_metabolic\_process | 8 | 0 | 0.000000 | -0.000000 | 640 | 694.317306 | 793.24 | 892.162694 | 1.239438 |
| GO:0006493\_protein\_amino\_acid\_O-linked\_glycosylation | 8 | 0 | 0.000000 | -0.000000 | 640 | 694.317306 | 793.24 | 892.162694 | 1.239438 |
| GO:0006582\_melanin\_metabolic\_process | 8 | 0 | 0.000000 | -0.000000 | 640 | 694.317306 | 793.24 | 892.162694 | 1.239438 |
| GO:0006733\_oxidoreduction\_coenzyme\_metabolic\_process | 8 | 0 | 0.000000 | -0.000000 | 640 | 694.317306 | 793.24 | 892.162694 | 1.239438 |
| GO:0006829\_zinc\_ion\_transport | 8 | 0 | 0.000000 | -0.000000 | 640 | 694.317306 | 793.24 | 892.162694 | 1.239438 |
| GO:0007009\_plasma\_membrane\_organization | 8 | 0 | 0.000000 | -0.000000 | 640 | 694.317306 | 793.24 | 892.162694 | 1.239438 |
| GO:0007098\_centrosome\_cycle | 8 | 0 | 0.000000 | -0.000000 | 640 | 694.317306 | 793.24 | 892.162694 | 1.239438 |
| GO:0007131\_reciprocal\_meiotic\_recombination | 8 | 0 | 0.000000 | -0.000000 | 640 | 694.317306 | 793.24 | 892.162694 | 1.239438 |
| GO:0007141\_male\_meiosis\_I | 8 | 0 | 0.000000 | -0.000000 | 640 | 694.317306 | 793.24 | 892.162694 | 1.239438 |
| GO:0007625\_grooming\_behavior | 8 | 0 | 0.000000 | -0.000000 | 640 | 694.317306 | 793.24 | 892.162694 | 1.239438 |
| GO:0008105\_asymmetric\_protein\_localization | 8 | 0 | 0.000000 | -0.000000 | 640 | 694.317306 | 793.24 | 892.162694 | 1.239438 |
| GO:0008593\_regulation\_of\_Notch\_signaling\_pathway | 8 | 0 | 0.000000 | -0.000000 | 640 | 694.317306 | 793.24 | 892.162694 | 1.239438 |
| GO:0009072\_aromatic\_amino\_acid\_family\_metabolic\_process | 8 | 0 | 0.000000 | -0.000000 | 640 | 694.317306 | 793.24 | 892.162694 | 1.239438 |
| GO:0009144\_purine\_nucleoside\_triphosphate\_metabolic\_process | 8 | 0 | 0.000000 | -0.000000 | 640 | 694.317306 | 793.24 | 892.162694 | 1.239438 |
| GO:0009746\_response\_to\_hexose\_stimulus | 8 | 0 | 0.000000 | -0.000000 | 640 | 694.317306 | 793.24 | 892.162694 | 1.239438 |
| GO:0009749\_response\_to\_glucose\_stimulus | 8 | 0 | 0.000000 | -0.000000 | 640 | 694.317306 | 793.24 | 892.162694 | 1.239438 |
| GO:0014014\_negative\_regulation\_of\_gliogenesis | 8 | 0 | 0.000000 | -0.000000 | 640 | 694.317306 | 793.24 | 892.162694 | 1.239438 |
| GO:0014046\_dopamine\_secretion | 8 | 0 | 0.000000 | -0.000000 | 640 | 694.317306 | 793.24 | 892.162694 | 1.239438 |
| GO:0014059\_regulation\_of\_dopamine\_secretion | 8 | 0 | 0.000000 | -0.000000 | 640 | 694.317306 | 793.24 | 892.162694 | 1.239438 |
| GO:0015800\_acidic\_amino\_acid\_transport | 8 | 0 | 0.000000 | -0.000000 | 640 | 694.317306 | 793.24 | 892.162694 | 1.239438 |
| GO:0015804\_neutral\_amino\_acid\_transport | 8 | 0 | 0.000000 | -0.000000 | 640 | 694.317306 | 793.24 | 892.162694 | 1.239438 |
| GO:0016236\_macroautophagy | 8 | 0 | 0.000000 | -0.000000 | 640 | 694.317306 | 793.24 | 892.162694 | 1.239438 |
| GO:0016446\_somatic\_hypermutation\_of\_immunoglobulin\_genes | 8 | 0 | 0.000000 | -0.000000 | 640 | 694.317306 | 793.24 | 892.162694 | 1.239438 |
| GO:0018107\_peptidyl-threonine\_phosphorylation | 8 | 0 | 0.000000 | -0.000000 | 640 | 694.317306 | 793.24 | 892.162694 | 1.239438 |
| GO:0018210\_peptidyl-threonine\_modification | 8 | 0 | 0.000000 | -0.000000 | 640 | 694.317306 | 793.24 | 892.162694 | 1.239438 |
| GO:0018345\_protein\_palmitoylation | 8 | 0 | 0.000000 | -0.000000 | 640 | 694.317306 | 793.24 | 892.162694 | 1.239438 |
| GO:0019229\_regulation\_of\_vasoconstriction | 8 | 0 | 0.000000 | -0.000000 | 640 | 694.317306 | 793.24 | 892.162694 | 1.239438 |
| GO:0019400\_alditol\_metabolic\_process | 8 | 0 | 0.000000 | -0.000000 | 640 | 694.317306 | 793.24 | 892.162694 | 1.239438 |
| GO:0021692\_cerebellar\_Purkinje\_cell\_layer\_morphogenesis | 8 | 0 | 0.000000 | -0.000000 | 640 | 694.317306 | 793.24 | 892.162694 | 1.239438 |
| GO:0021694\_cerebellar\_Purkinje\_cell\_layer\_formation | 8 | 0 | 0.000000 | -0.000000 | 640 | 694.317306 | 793.24 | 892.162694 | 1.239438 |
| GO:0021702\_cerebellar\_Purkinje\_cell\_differentiation | 8 | 0 | 0.000000 | -0.000000 | 640 | 694.317306 | 793.24 | 892.162694 | 1.239438 |
| GO:0021781\_glial\_cell\_fate\_commitment | 8 | 0 | 0.000000 | -0.000000 | 640 | 694.317306 | 793.24 | 892.162694 | 1.239438 |
| GO:0021799\_cerebral\_cortex\_radially\_oriented\_cell\_migration | 8 | 0 | 0.000000 | -0.000000 | 640 | 694.317306 | 793.24 | 892.162694 | 1.239438 |
| GO:0022898\_regulation\_of\_transmembrane\_transporter\_activity | 8 | 0 | 0.000000 | -0.000000 | 640 | 694.317306 | 793.24 | 892.162694 | 1.239438 |
| GO:0030035\_microspike\_assembly | 8 | 0 | 0.000000 | -0.000000 | 640 | 694.317306 | 793.24 | 892.162694 | 1.239438 |
| GO:0030193\_regulation\_of\_blood\_coagulation | 8 | 0 | 0.000000 | -0.000000 | 640 | 694.317306 | 793.24 | 892.162694 | 1.239438 |
| GO:0030204\_chondroitin\_sulfate\_metabolic\_process | 8 | 0 | 0.000000 | -0.000000 | 640 | 694.317306 | 793.24 | 892.162694 | 1.239438 |
| GO:0030500\_regulation\_of\_bone\_mineralization | 8 | 0 | 0.000000 | -0.000000 | 640 | 694.317306 | 793.24 | 892.162694 | 1.239438 |
| GO:0030511\_positive\_regulation\_of\_transforming\_growth\_factor\_beta\_receptor\_signaling\_pathway | 8 | 0 | 0.000000 | -0.000000 | 640 | 694.317306 | 793.24 | 892.162694 | 1.239438 |
| GO:0031102\_neuron\_projection\_regeneration | 8 | 0 | 0.000000 | -0.000000 | 640 | 694.317306 | 793.24 | 892.162694 | 1.239438 |
| GO:0031103\_axon\_regeneration | 8 | 0 | 0.000000 | -0.000000 | 640 | 694.317306 | 793.24 | 892.162694 | 1.239438 |
| GO:0031111\_negative\_regulation\_of\_microtubule\_polymerization\_or\_depolymerization | 8 | 0 | 0.000000 | -0.000000 | 640 | 694.317306 | 793.24 | 892.162694 | 1.239438 |
| GO:0031123\_RNA\_3'-end\_processing | 8 | 0 | 0.000000 | -0.000000 | 640 | 694.317306 | 793.24 | 892.162694 | 1.239438 |
| GO:0031294\_lymphocyte\_costimulation | 8 | 0 | 0.000000 | -0.000000 | 640 | 694.317306 | 793.24 | 892.162694 | 1.239438 |
| GO:0031295\_T\_cell\_costimulation | 8 | 0 | 0.000000 | -0.000000 | 640 | 694.317306 | 793.24 | 892.162694 | 1.239438 |
| GO:0031334\_positive\_regulation\_of\_protein\_complex\_assembly | 8 | 0 | 0.000000 | -0.000000 | 640 | 694.317306 | 793.24 | 892.162694 | 1.239438 |
| GO:0031342\_negative\_regulation\_of\_cell\_killing | 8 | 0 | 0.000000 | -0.000000 | 640 | 694.317306 | 793.24 | 892.162694 | 1.239438 |
| GO:0031396\_regulation\_of\_protein\_ubiquitination | 8 | 0 | 0.000000 | -0.000000 | 640 | 694.317306 | 793.24 | 892.162694 | 1.239438 |
| GO:0032094\_response\_to\_food | 8 | 0 | 0.000000 | -0.000000 | 640 | 694.317306 | 793.24 | 892.162694 | 1.239438 |
| GO:0032273\_positive\_regulation\_of\_protein\_polymerization | 8 | 0 | 0.000000 | -0.000000 | 640 | 694.317306 | 793.24 | 892.162694 | 1.239438 |
| GO:0032409\_regulation\_of\_transporter\_activity | 8 | 0 | 0.000000 | -0.000000 | 640 | 694.317306 | 793.24 | 892.162694 | 1.239438 |
| GO:0032412\_regulation\_of\_ion\_transmembrane\_transporter\_activity | 8 | 0 | 0.000000 | -0.000000 | 640 | 694.317306 | 793.24 | 892.162694 | 1.239438 |
| GO:0032613\_interleukin-10\_production | 8 | 0 | 0.000000 | -0.000000 | 640 | 694.317306 | 793.24 | 892.162694 | 1.239438 |
| GO:0033198\_response\_to\_ATP | 8 | 0 | 0.000000 | -0.000000 | 640 | 694.317306 | 793.24 | 892.162694 | 1.239438 |
| GO:0034284\_response\_to\_monosaccharide\_stimulus | 8 | 0 | 0.000000 | -0.000000 | 640 | 694.317306 | 793.24 | 892.162694 | 1.239438 |
| GO:0034728\_nucleosome\_organization | 8 | 0 | 0.000000 | -0.000000 | 640 | 694.317306 | 793.24 | 892.162694 | 1.239438 |
| GO:0035023\_regulation\_of\_Rho\_protein\_signal\_transduction | 8 | 0 | 0.000000 | -0.000000 | 640 | 694.317306 | 793.24 | 892.162694 | 1.239438 |
| GO:0035112\_genitalia\_morphogenesis | 8 | 0 | 0.000000 | -0.000000 | 640 | 694.317306 | 793.24 | 892.162694 | 1.239438 |
| GO:0040017\_positive\_regulation\_of\_locomotion | 8 | 0 | 0.000000 | -0.000000 | 640 | 694.317306 | 793.24 | 892.162694 | 1.239438 |
| GO:0040034\_regulation\_of\_development\_\_heterochronic | 8 | 0 | 0.000000 | -0.000000 | 640 | 694.317306 | 793.24 | 892.162694 | 1.239438 |
| GO:0042074\_cell\_migration\_involved\_in\_gastrulation | 8 | 0 | 0.000000 | -0.000000 | 640 | 694.317306 | 793.24 | 892.162694 | 1.239438 |
| GO:0042090\_interleukin-12\_biosynthetic\_process | 8 | 0 | 0.000000 | -0.000000 | 640 | 694.317306 | 793.24 | 892.162694 | 1.239438 |
| GO:0042092\_T-helper\_2\_type\_immune\_response | 8 | 0 | 0.000000 | -0.000000 | 640 | 694.317306 | 793.24 | 892.162694 | 1.239438 |
| GO:0042095\_interferon-gamma\_biosynthetic\_process | 8 | 0 | 0.000000 | -0.000000 | 640 | 694.317306 | 793.24 | 892.162694 | 1.239438 |
| GO:0042104\_positive\_regulation\_of\_activated\_T\_cell\_proliferation | 8 | 0 | 0.000000 | -0.000000 | 640 | 694.317306 | 793.24 | 892.162694 | 1.239438 |
| GO:0042226\_interleukin-6\_biosynthetic\_process | 8 | 0 | 0.000000 | -0.000000 | 640 | 694.317306 | 793.24 | 892.162694 | 1.239438 |
| GO:0042304\_regulation\_of\_fatty\_acid\_biosynthetic\_process | 8 | 0 | 0.000000 | -0.000000 | 640 | 694.317306 | 793.24 | 892.162694 | 1.239438 |
| GO:0042423\_catecholamine\_biosynthetic\_process | 8 | 0 | 0.000000 | -0.000000 | 640 | 694.317306 | 793.24 | 892.162694 | 1.239438 |
| GO:0042771\_DNA\_damage\_response\_\_signal\_transduction\_by\_p53\_class\_mediator\_resulting\_in\_induction\_of\_apoptosis | 8 | 0 | 0.000000 | -0.000000 | 640 | 694.317306 | 793.24 | 892.162694 | 1.239438 |
| GO:0042990\_regulation\_of\_transcription\_factor\_import\_into\_nucleus | 8 | 0 | 0.000000 | -0.000000 | 640 | 694.317306 | 793.24 | 892.162694 | 1.239438 |
| GO:0042991\_transcription\_factor\_import\_into\_nucleus | 8 | 0 | 0.000000 | -0.000000 | 640 | 694.317306 | 793.24 | 892.162694 | 1.239438 |
| GO:0043011\_myeloid\_dendritic\_cell\_differentiation | 8 | 0 | 0.000000 | -0.000000 | 640 | 694.317306 | 793.24 | 892.162694 | 1.239438 |
| GO:0043368\_positive\_T\_cell\_selection | 8 | 0 | 0.000000 | -0.000000 | 640 | 694.317306 | 793.24 | 892.162694 | 1.239438 |
| GO:0043370\_regulation\_of\_CD4-positive\_\_alpha\_beta\_T\_cell\_differentiation | 8 | 0 | 0.000000 | -0.000000 | 640 | 694.317306 | 793.24 | 892.162694 | 1.239438 |
| GO:0043542\_endothelial\_cell\_migration | 8 | 0 | 0.000000 | -0.000000 | 640 | 694.317306 | 793.24 | 892.162694 | 1.239438 |
| GO:0043616\_keratinocyte\_proliferation | 8 | 0 | 0.000000 | -0.000000 | 640 | 694.317306 | 793.24 | 892.162694 | 1.239438 |
| GO:0045075\_regulation\_of\_interleukin-12\_biosynthetic\_process | 8 | 0 | 0.000000 | -0.000000 | 640 | 694.317306 | 793.24 | 892.162694 | 1.239438 |
| GO:0045086\_positive\_regulation\_of\_interleukin-2\_biosynthetic\_process | 8 | 0 | 0.000000 | -0.000000 | 640 | 694.317306 | 793.24 | 892.162694 | 1.239438 |
| GO:0045351\_type\_I\_interferon\_biosynthetic\_process | 8 | 0 | 0.000000 | -0.000000 | 640 | 694.317306 | 793.24 | 892.162694 | 1.239438 |
| GO:0045408\_regulation\_of\_interleukin-6\_biosynthetic\_process | 8 | 0 | 0.000000 | -0.000000 | 640 | 694.317306 | 793.24 | 892.162694 | 1.239438 |
| GO:0045429\_positive\_regulation\_of\_nitric\_oxide\_biosynthetic\_process | 8 | 0 | 0.000000 | -0.000000 | 640 | 694.317306 | 793.24 | 892.162694 | 1.239438 |
| GO:0045494\_photoreceptor\_cell\_maintenance | 8 | 0 | 0.000000 | -0.000000 | 640 | 694.317306 | 793.24 | 892.162694 | 1.239438 |
| GO:0045686\_negative\_regulation\_of\_glial\_cell\_differentiation | 8 | 0 | 0.000000 | -0.000000 | 640 | 694.317306 | 793.24 | 892.162694 | 1.239438 |
| GO:0045910\_negative\_regulation\_of\_DNA\_recombination | 8 | 0 | 0.000000 | -0.000000 | 640 | 694.317306 | 793.24 | 892.162694 | 1.239438 |
| GO:0045921\_positive\_regulation\_of\_exocytosis | 8 | 0 | 0.000000 | -0.000000 | 640 | 694.317306 | 793.24 | 892.162694 | 1.239438 |
| GO:0045932\_negative\_regulation\_of\_muscle\_contraction | 8 | 0 | 0.000000 | -0.000000 | 640 | 694.317306 | 793.24 | 892.162694 | 1.239438 |
| GO:0046470\_phosphatidylcholine\_metabolic\_process | 8 | 0 | 0.000000 | -0.000000 | 640 | 694.317306 | 793.24 | 892.162694 | 1.239438 |
| GO:0048266\_behavioral\_response\_to\_pain | 8 | 0 | 0.000000 | -0.000000 | 640 | 694.317306 | 793.24 | 892.162694 | 1.239438 |
| GO:0048505\_regulation\_of\_timing\_of\_cell\_differentiation | 8 | 0 | 0.000000 | -0.000000 | 640 | 694.317306 | 793.24 | 892.162694 | 1.239438 |
| GO:0048520\_positive\_regulation\_of\_behavior | 8 | 0 | 0.000000 | -0.000000 | 640 | 694.317306 | 793.24 | 892.162694 | 1.239438 |
| GO:0048557\_embryonic\_digestive\_tract\_morphogenesis | 8 | 0 | 0.000000 | -0.000000 | 640 | 694.317306 | 793.24 | 892.162694 | 1.239438 |
| GO:0048638\_regulation\_of\_developmental\_growth | 8 | 0 | 0.000000 | -0.000000 | 640 | 694.317306 | 793.24 | 892.162694 | 1.239438 |
| GO:0048742\_regulation\_of\_skeletal\_muscle\_fiber\_development | 8 | 0 | 0.000000 | -0.000000 | 640 | 694.317306 | 793.24 | 892.162694 | 1.239438 |
| GO:0050707\_regulation\_of\_cytokine\_secretion | 8 | 0 | 0.000000 | -0.000000 | 640 | 694.317306 | 793.24 | 892.162694 | 1.239438 |
| GO:0050909\_sensory\_perception\_of\_taste | 8 | 0 | 0.000000 | -0.000000 | 640 | 694.317306 | 793.24 | 892.162694 | 1.239438 |
| GO:0050920\_regulation\_of\_chemotaxis | 8 | 0 | 0.000000 | -0.000000 | 640 | 694.317306 | 793.24 | 892.162694 | 1.239438 |
| GO:0050921\_positive\_regulation\_of\_chemotaxis | 8 | 0 | 0.000000 | -0.000000 | 640 | 694.317306 | 793.24 | 892.162694 | 1.239438 |
| GO:0050926\_regulation\_of\_positive\_chemotaxis | 8 | 0 | 0.000000 | -0.000000 | 640 | 694.317306 | 793.24 | 892.162694 | 1.239438 |
| GO:0050927\_positive\_regulation\_of\_positive\_chemotaxis | 8 | 0 | 0.000000 | -0.000000 | 640 | 694.317306 | 793.24 | 892.162694 | 1.239438 |
| GO:0050930\_induction\_of\_positive\_chemotaxis | 8 | 0 | 0.000000 | -0.000000 | 640 | 694.317306 | 793.24 | 892.162694 | 1.239438 |
| GO:0051181\_cofactor\_transport | 8 | 0 | 0.000000 | -0.000000 | 640 | 694.317306 | 793.24 | 892.162694 | 1.239438 |
| GO:0060043\_regulation\_of\_cardiac\_muscle\_cell\_proliferation | 8 | 0 | 0.000000 | -0.000000 | 640 | 694.317306 | 793.24 | 892.162694 | 1.239438 |
| GO:0060347\_heart\_trabecula\_formation | 8 | 0 | 0.000000 | -0.000000 | 640 | 694.317306 | 793.24 | 892.162694 | 1.239438 |
| GO:0060670\_branching\_involved\_in\_embryonic\_placenta\_morphogenesis | 8 | 0 | 0.000000 | -0.000000 | 640 | 694.317306 | 793.24 | 892.162694 | 1.239438 |
| GO:0060712\_spongiotrophoblast\_layer\_development | 8 | 0 | 0.000000 | -0.000000 | 640 | 694.317306 | 793.24 | 892.162694 | 1.239438 |
| GO:0070167\_regulation\_of\_biomineral\_formation | 8 | 0 | 0.000000 | -0.000000 | 640 | 694.317306 | 793.24 | 892.162694 | 1.239438 |
| GO:0070193\_synaptonemal\_complex\_organization | 8 | 0 | 0.000000 | -0.000000 | 640 | 694.317306 | 793.24 | 892.162694 | 1.239438 |
| GO:0070231\_T\_cell\_apoptosis | 8 | 0 | 0.000000 | -0.000000 | 640 | 694.317306 | 793.24 | 892.162694 | 1.239438 |
| GO:0070584\_mitochondrion\_morphogenesis | 8 | 0 | 0.000000 | -0.000000 | 640 | 694.317306 | 793.24 | 892.162694 | 1.239438 |
| GO:0048584\_positive\_regulation\_of\_response\_to\_stimulus | 115 | 0 | 0.000000 | -0.000000 | 642 | 695.296790 | 793.93 | 892.563210 | 1.236651 |
| GO:0051338\_regulation\_of\_transferase\_activity | 115 | 0 | 0.000000 | -0.000000 | 642 | 695.296790 | 793.93 | 892.563210 | 1.236651 |
| GO:0001818\_negative\_regulation\_of\_cytokine\_production | 18 | 0 | 0.000000 | -0.000000 | 690 | 742.812914 | 839.66 | 936.507086 | 1.216899 |
| GO:0001825\_blastocyst\_formation | 18 | 0 | 0.000000 | -0.000000 | 690 | 742.812914 | 839.66 | 936.507086 | 1.216899 |
| GO:0001974\_blood\_vessel\_remodeling | 18 | 0 | 0.000000 | -0.000000 | 690 | 742.812914 | 839.66 | 936.507086 | 1.216899 |
| GO:0002064\_epithelial\_cell\_development | 18 | 0 | 0.000000 | -0.000000 | 690 | 742.812914 | 839.66 | 936.507086 | 1.216899 |
| GO:0002285\_lymphocyte\_activation\_during\_immune\_response | 18 | 0 | 0.000000 | -0.000000 | 690 | 742.812914 | 839.66 | 936.507086 | 1.216899 |
| GO:0002715\_regulation\_of\_natural\_killer\_cell\_mediated\_immunity | 18 | 0 | 0.000000 | -0.000000 | 690 | 742.812914 | 839.66 | 936.507086 | 1.216899 |
| GO:0003014\_renal\_system\_process | 18 | 0 | 0.000000 | -0.000000 | 690 | 742.812914 | 839.66 | 936.507086 | 1.216899 |
| GO:0006022\_aminoglycan\_metabolic\_process | 18 | 0 | 0.000000 | -0.000000 | 690 | 742.812914 | 839.66 | 936.507086 | 1.216899 |
| GO:0006940\_regulation\_of\_smooth\_muscle\_contraction | 18 | 0 | 0.000000 | -0.000000 | 690 | 742.812914 | 839.66 | 936.507086 | 1.216899 |
| GO:0007140\_male\_meiosis | 18 | 0 | 0.000000 | -0.000000 | 690 | 742.812914 | 839.66 | 936.507086 | 1.216899 |
| GO:0007608\_sensory\_perception\_of\_smell | 18 | 0 | 0.000000 | -0.000000 | 690 | 742.812914 | 839.66 | 936.507086 | 1.216899 |
| GO:0008589\_regulation\_of\_smoothened\_signaling\_pathway | 18 | 0 | 0.000000 | -0.000000 | 690 | 742.812914 | 839.66 | 936.507086 | 1.216899 |
| GO:0010498\_proteasomal\_protein\_catabolic\_process | 18 | 0 | 0.000000 | -0.000000 | 690 | 742.812914 | 839.66 | 936.507086 | 1.216899 |
| GO:0010553\_negative\_regulation\_of\_specific\_transcription\_from\_RNA\_polymerase\_II\_promoter | 18 | 0 | 0.000000 | -0.000000 | 690 | 742.812914 | 839.66 | 936.507086 | 1.216899 |
| GO:0015711\_organic\_anion\_transport | 18 | 0 | 0.000000 | -0.000000 | 690 | 742.812914 | 839.66 | 936.507086 | 1.216899 |
| GO:0016458\_gene\_silencing | 18 | 0 | 0.000000 | -0.000000 | 690 | 742.812914 | 839.66 | 936.507086 | 1.216899 |
| GO:0021517\_ventral\_spinal\_cord\_development | 18 | 0 | 0.000000 | -0.000000 | 690 | 742.812914 | 839.66 | 936.507086 | 1.216899 |
| GO:0021885\_forebrain\_cell\_migration | 18 | 0 | 0.000000 | -0.000000 | 690 | 742.812914 | 839.66 | 936.507086 | 1.216899 |
| GO:0030178\_negative\_regulation\_of\_Wnt\_receptor\_signaling\_pathway | 18 | 0 | 0.000000 | -0.000000 | 690 | 742.812914 | 839.66 | 936.507086 | 1.216899 |
| GO:0030203\_glycosaminoglycan\_metabolic\_process | 18 | 0 | 0.000000 | -0.000000 | 690 | 742.812914 | 839.66 | 936.507086 | 1.216899 |
| GO:0030282\_bone\_mineralization | 18 | 0 | 0.000000 | -0.000000 | 690 | 742.812914 | 839.66 | 936.507086 | 1.216899 |
| GO:0030318\_melanocyte\_differentiation | 18 | 0 | 0.000000 | -0.000000 | 690 | 742.812914 | 839.66 | 936.507086 | 1.216899 |
| GO:0030336\_negative\_regulation\_of\_cell\_migration | 18 | 0 | 0.000000 | -0.000000 | 690 | 742.812914 | 839.66 | 936.507086 | 1.216899 |
| GO:0030510\_regulation\_of\_BMP\_signaling\_pathway | 18 | 0 | 0.000000 | -0.000000 | 690 | 742.812914 | 839.66 | 936.507086 | 1.216899 |
| GO:0030901\_midbrain\_development | 18 | 0 | 0.000000 | -0.000000 | 690 | 742.812914 | 839.66 | 936.507086 | 1.216899 |
| GO:0032623\_interleukin-2\_production | 18 | 0 | 0.000000 | -0.000000 | 690 | 742.812914 | 839.66 | 936.507086 | 1.216899 |
| GO:0032984\_macromolecular\_complex\_disassembly | 18 | 0 | 0.000000 | -0.000000 | 690 | 742.812914 | 839.66 | 936.507086 | 1.216899 |
| GO:0033157\_regulation\_of\_intracellular\_protein\_transport | 18 | 0 | 0.000000 | -0.000000 | 690 | 742.812914 | 839.66 | 936.507086 | 1.216899 |
| GO:0042269\_regulation\_of\_natural\_killer\_cell\_mediated\_cytotoxicity | 18 | 0 | 0.000000 | -0.000000 | 690 | 742.812914 | 839.66 | 936.507086 | 1.216899 |
| GO:0043029\_T\_cell\_homeostasis | 18 | 0 | 0.000000 | -0.000000 | 690 | 742.812914 | 839.66 | 936.507086 | 1.216899 |
| GO:0043161\_proteasomal\_ubiquitin-dependent\_protein\_catabolic\_process | 18 | 0 | 0.000000 | -0.000000 | 690 | 742.812914 | 839.66 | 936.507086 | 1.216899 |
| GO:0044272\_sulfur\_compound\_biosynthetic\_process | 18 | 0 | 0.000000 | -0.000000 | 690 | 742.812914 | 839.66 | 936.507086 | 1.216899 |
| GO:0045058\_T\_cell\_selection | 18 | 0 | 0.000000 | -0.000000 | 690 | 742.812914 | 839.66 | 936.507086 | 1.216899 |
| GO:0045103\_intermediate\_filament-based\_process | 18 | 0 | 0.000000 | -0.000000 | 690 | 742.812914 | 839.66 | 936.507086 | 1.216899 |
| GO:0045638\_negative\_regulation\_of\_myeloid\_cell\_differentiation | 18 | 0 | 0.000000 | -0.000000 | 690 | 742.812914 | 839.66 | 936.507086 | 1.216899 |
| GO:0045807\_positive\_regulation\_of\_endocytosis | 18 | 0 | 0.000000 | -0.000000 | 690 | 742.812914 | 839.66 | 936.507086 | 1.216899 |
| GO:0046578\_regulation\_of\_Ras\_protein\_signal\_transduction | 18 | 0 | 0.000000 | -0.000000 | 690 | 742.812914 | 839.66 | 936.507086 | 1.216899 |
| GO:0046620\_regulation\_of\_organ\_growth | 18 | 0 | 0.000000 | -0.000000 | 690 | 742.812914 | 839.66 | 936.507086 | 1.216899 |
| GO:0048535\_lymph\_node\_development | 18 | 0 | 0.000000 | -0.000000 | 690 | 742.812914 | 839.66 | 936.507086 | 1.216899 |
| GO:0048730\_epidermis\_morphogenesis | 18 | 0 | 0.000000 | -0.000000 | 690 | 742.812914 | 839.66 | 936.507086 | 1.216899 |
| GO:0050731\_positive\_regulation\_of\_peptidyl-tyrosine\_phosphorylation | 18 | 0 | 0.000000 | -0.000000 | 690 | 742.812914 | 839.66 | 936.507086 | 1.216899 |
| GO:0051168\_nuclear\_export | 18 | 0 | 0.000000 | -0.000000 | 690 | 742.812914 | 839.66 | 936.507086 | 1.216899 |
| GO:0051222\_positive\_regulation\_of\_protein\_transport | 18 | 0 | 0.000000 | -0.000000 | 690 | 742.812914 | 839.66 | 936.507086 | 1.216899 |
| GO:0051924\_regulation\_of\_calcium\_ion\_transport | 18 | 0 | 0.000000 | -0.000000 | 690 | 742.812914 | 839.66 | 936.507086 | 1.216899 |
| GO:0055008\_cardiac\_muscle\_tissue\_morphogenesis | 18 | 0 | 0.000000 | -0.000000 | 690 | 742.812914 | 839.66 | 936.507086 | 1.216899 |
| GO:0060415\_muscle\_tissue\_morphogenesis | 18 | 0 | 0.000000 | -0.000000 | 690 | 742.812914 | 839.66 | 936.507086 | 1.216899 |
| GO:0060571\_morphogenesis\_of\_an\_epithelial\_fold | 18 | 0 | 0.000000 | -0.000000 | 690 | 742.812914 | 839.66 | 936.507086 | 1.216899 |
| GO:0060674\_placenta\_blood\_vessel\_development | 18 | 0 | 0.000000 | -0.000000 | 690 | 742.812914 | 839.66 | 936.507086 | 1.216899 |
| GO:0000079\_regulation\_of\_cyclin-dependent\_protein\_kinase\_activity | 7 | 0 | 0.000000 | -0.000000 | 864 | 917.538854 | 1011.5 | 1105.461146 | 1.170718 |
| GO:0000188\_inactivation\_of\_MAPK\_activity | 7 | 0 | 0.000000 | -0.000000 | 864 | 917.538854 | 1011.5 | 1105.461146 | 1.170718 |
| GO:0001504\_neurotransmitter\_uptake | 7 | 0 | 0.000000 | -0.000000 | 864 | 917.538854 | 1011.5 | 1105.461146 | 1.170718 |
| GO:0001556\_oocyte\_maturation | 7 | 0 | 0.000000 | -0.000000 | 864 | 917.538854 | 1011.5 | 1105.461146 | 1.170718 |
| GO:0001573\_ganglioside\_metabolic\_process | 7 | 0 | 0.000000 | -0.000000 | 864 | 917.538854 | 1011.5 | 1105.461146 | 1.170718 |
| GO:0001736\_establishment\_of\_planar\_polarity | 7 | 0 | 0.000000 | -0.000000 | 864 | 917.538854 | 1011.5 | 1105.461146 | 1.170718 |
| GO:0001839\_neural\_plate\_morphogenesis | 7 | 0 | 0.000000 | -0.000000 | 864 | 917.538854 | 1011.5 | 1105.461146 | 1.170718 |
| GO:0001936\_regulation\_of\_endothelial\_cell\_proliferation | 7 | 0 | 0.000000 | -0.000000 | 864 | 917.538854 | 1011.5 | 1105.461146 | 1.170718 |
| GO:0001967\_suckling\_behavior | 7 | 0 | 0.000000 | -0.000000 | 864 | 917.538854 | 1011.5 | 1105.461146 | 1.170718 |
| GO:0002011\_morphogenesis\_of\_an\_epithelial\_sheet | 7 | 0 | 0.000000 | -0.000000 | 864 | 917.538854 | 1011.5 | 1105.461146 | 1.170718 |
| GO:0002052\_positive\_regulation\_of\_neuroblast\_proliferation | 7 | 0 | 0.000000 | -0.000000 | 864 | 917.538854 | 1011.5 | 1105.461146 | 1.170718 |
| GO:0002063\_chondrocyte\_development | 7 | 0 | 0.000000 | -0.000000 | 864 | 917.538854 | 1011.5 | 1105.461146 | 1.170718 |
| GO:0002067\_glandular\_epithelial\_cell\_differentiation | 7 | 0 | 0.000000 | -0.000000 | 864 | 917.538854 | 1011.5 | 1105.461146 | 1.170718 |
| GO:0002076\_osteoblast\_development | 7 | 0 | 0.000000 | -0.000000 | 864 | 917.538854 | 1011.5 | 1105.461146 | 1.170718 |
| GO:0002087\_regulation\_of\_respiratory\_gaseous\_exchange\_by\_neurological\_system\_process | 7 | 0 | 0.000000 | -0.000000 | 864 | 917.538854 | 1011.5 | 1105.461146 | 1.170718 |
| GO:0002093\_auditory\_receptor\_cell\_morphogenesis | 7 | 0 | 0.000000 | -0.000000 | 864 | 917.538854 | 1011.5 | 1105.461146 | 1.170718 |
| GO:0002224\_toll-like\_receptor\_signaling\_pathway | 7 | 0 | 0.000000 | -0.000000 | 864 | 917.538854 | 1011.5 | 1105.461146 | 1.170718 |
| GO:0002455\_humoral\_immune\_response\_mediated\_by\_circulating\_immunoglobulin | 7 | 0 | 0.000000 | -0.000000 | 864 | 917.538854 | 1011.5 | 1105.461146 | 1.170718 |
| GO:0002643\_regulation\_of\_tolerance\_induction | 7 | 0 | 0.000000 | -0.000000 | 864 | 917.538854 | 1011.5 | 1105.461146 | 1.170718 |
| GO:0002645\_positive\_regulation\_of\_tolerance\_induction | 7 | 0 | 0.000000 | -0.000000 | 864 | 917.538854 | 1011.5 | 1105.461146 | 1.170718 |
| GO:0002714\_positive\_regulation\_of\_B\_cell\_mediated\_immunity | 7 | 0 | 0.000000 | -0.000000 | 864 | 917.538854 | 1011.5 | 1105.461146 | 1.170718 |
| GO:0002792\_negative\_regulation\_of\_peptide\_secretion | 7 | 0 | 0.000000 | -0.000000 | 864 | 917.538854 | 1011.5 | 1105.461146 | 1.170718 |
| GO:0002793\_positive\_regulation\_of\_peptide\_secretion | 7 | 0 | 0.000000 | -0.000000 | 864 | 917.538854 | 1011.5 | 1105.461146 | 1.170718 |
| GO:0002828\_regulation\_of\_T-helper\_2\_type\_immune\_response | 7 | 0 | 0.000000 | -0.000000 | 864 | 917.538854 | 1011.5 | 1105.461146 | 1.170718 |
| GO:0002863\_positive\_regulation\_of\_inflammatory\_response\_to\_antigenic\_stimulus | 7 | 0 | 0.000000 | -0.000000 | 864 | 917.538854 | 1011.5 | 1105.461146 | 1.170718 |
| GO:0002891\_positive\_regulation\_of\_immunoglobulin\_mediated\_immune\_response | 7 | 0 | 0.000000 | -0.000000 | 864 | 917.538854 | 1011.5 | 1105.461146 | 1.170718 |
| GO:0003084\_positive\_regulation\_of\_systemic\_arterial\_blood\_pressure | 7 | 0 | 0.000000 | -0.000000 | 864 | 917.538854 | 1011.5 | 1105.461146 | 1.170718 |
| GO:0003085\_negative\_regulation\_of\_systemic\_arterial\_blood\_pressure | 7 | 0 | 0.000000 | -0.000000 | 864 | 917.538854 | 1011.5 | 1105.461146 | 1.170718 |
| GO:0006014\_D-ribose\_metabolic\_process | 7 | 0 | 0.000000 | -0.000000 | 864 | 917.538854 | 1011.5 | 1105.461146 | 1.170718 |
| GO:0006041\_glucosamine\_metabolic\_process | 7 | 0 | 0.000000 | -0.000000 | 864 | 917.538854 | 1011.5 | 1105.461146 | 1.170718 |
| GO:0006044\_N-acetylglucosamine\_metabolic\_process | 7 | 0 | 0.000000 | -0.000000 | 864 | 917.538854 | 1011.5 | 1105.461146 | 1.170718 |
| GO:0006096\_glycolysis | 7 | 0 | 0.000000 | -0.000000 | 864 | 917.538854 | 1011.5 | 1105.461146 | 1.170718 |
| GO:0006119\_oxidative\_phosphorylation | 7 | 0 | 0.000000 | -0.000000 | 864 | 917.538854 | 1011.5 | 1105.461146 | 1.170718 |
| GO:0006275\_regulation\_of\_DNA\_replication | 7 | 0 | 0.000000 | -0.000000 | 864 | 917.538854 | 1011.5 | 1105.461146 | 1.170718 |
| GO:0006298\_mismatch\_repair | 7 | 0 | 0.000000 | -0.000000 | 864 | 917.538854 | 1011.5 | 1105.461146 | 1.170718 |
| GO:0006352\_transcription\_initiation | 7 | 0 | 0.000000 | -0.000000 | 864 | 917.538854 | 1011.5 | 1105.461146 | 1.170718 |
| GO:0006401\_RNA\_catabolic\_process | 7 | 0 | 0.000000 | -0.000000 | 864 | 917.538854 | 1011.5 | 1105.461146 | 1.170718 |
| GO:0006406\_mRNA\_export\_from\_nucleus | 7 | 0 | 0.000000 | -0.000000 | 864 | 917.538854 | 1011.5 | 1105.461146 | 1.170718 |
| GO:0006505\_GPI\_anchor\_metabolic\_process | 7 | 0 | 0.000000 | -0.000000 | 864 | 917.538854 | 1011.5 | 1105.461146 | 1.170718 |
| GO:0006516\_glycoprotein\_catabolic\_process | 7 | 0 | 0.000000 | -0.000000 | 864 | 917.538854 | 1011.5 | 1105.461146 | 1.170718 |
| GO:0006612\_protein\_targeting\_to\_membrane | 7 | 0 | 0.000000 | -0.000000 | 864 | 917.538854 | 1011.5 | 1105.461146 | 1.170718 |
| GO:0006769\_nicotinamide\_metabolic\_process | 7 | 0 | 0.000000 | -0.000000 | 864 | 917.538854 | 1011.5 | 1105.461146 | 1.170718 |
| GO:0006783\_heme\_biosynthetic\_process | 7 | 0 | 0.000000 | -0.000000 | 864 | 917.538854 | 1011.5 | 1105.461146 | 1.170718 |
| GO:0006818\_hydrogen\_transport | 7 | 0 | 0.000000 | -0.000000 | 864 | 917.538854 | 1011.5 | 1105.461146 | 1.170718 |
| GO:0006878\_cellular\_copper\_ion\_homeostasis | 7 | 0 | 0.000000 | -0.000000 | 864 | 917.538854 | 1011.5 | 1105.461146 | 1.170718 |
| GO:0006884\_cell\_volume\_homeostasis | 7 | 0 | 0.000000 | -0.000000 | 864 | 917.538854 | 1011.5 | 1105.461146 | 1.170718 |
| GO:0007019\_microtubule\_depolymerization | 7 | 0 | 0.000000 | -0.000000 | 864 | 917.538854 | 1011.5 | 1105.461146 | 1.170718 |
| GO:0007026\_negative\_regulation\_of\_microtubule\_depolymerization | 7 | 0 | 0.000000 | -0.000000 | 864 | 917.538854 | 1011.5 | 1105.461146 | 1.170718 |
| GO:0007034\_vacuolar\_transport | 7 | 0 | 0.000000 | -0.000000 | 864 | 917.538854 | 1011.5 | 1105.461146 | 1.170718 |
| GO:0007062\_sister\_chromatid\_cohesion | 7 | 0 | 0.000000 | -0.000000 | 864 | 917.538854 | 1011.5 | 1105.461146 | 1.170718 |
| GO:0007130\_synaptonemal\_complex\_assembly | 7 | 0 | 0.000000 | -0.000000 | 864 | 917.538854 | 1011.5 | 1105.461146 | 1.170718 |
| GO:0007164\_establishment\_of\_tissue\_polarity | 7 | 0 | 0.000000 | -0.000000 | 864 | 917.538854 | 1011.5 | 1105.461146 | 1.170718 |
| GO:0007191\_activation\_of\_adenylate\_cyclase\_activity\_by\_dopamine\_receptor\_signaling\_pathway | 7 | 0 | 0.000000 | -0.000000 | 864 | 917.538854 | 1011.5 | 1105.461146 | 1.170718 |
| GO:0007271\_synaptic\_transmission\_\_cholinergic | 7 | 0 | 0.000000 | -0.000000 | 864 | 917.538854 | 1011.5 | 1105.461146 | 1.170718 |
| GO:0007413\_axonal\_fasciculation | 7 | 0 | 0.000000 | -0.000000 | 864 | 917.538854 | 1011.5 | 1105.461146 | 1.170718 |
| GO:0007440\_foregut\_morphogenesis | 7 | 0 | 0.000000 | -0.000000 | 864 | 917.538854 | 1011.5 | 1105.461146 | 1.170718 |
| GO:0007616\_long-term\_memory | 7 | 0 | 0.000000 | -0.000000 | 864 | 917.538854 | 1011.5 | 1105.461146 | 1.170718 |
| GO:0008033\_tRNA\_processing | 7 | 0 | 0.000000 | -0.000000 | 864 | 917.538854 | 1011.5 | 1105.461146 | 1.170718 |
| GO:0008299\_isoprenoid\_biosynthetic\_process | 7 | 0 | 0.000000 | -0.000000 | 864 | 917.538854 | 1011.5 | 1105.461146 | 1.170718 |
| GO:0008340\_determination\_of\_adult\_lifespan | 7 | 0 | 0.000000 | -0.000000 | 864 | 917.538854 | 1011.5 | 1105.461146 | 1.170718 |
| GO:0009150\_purine\_ribonucleotide\_metabolic\_process | 7 | 0 | 0.000000 | -0.000000 | 864 | 917.538854 | 1011.5 | 1105.461146 | 1.170718 |
| GO:0009200\_deoxyribonucleoside\_triphosphate\_metabolic\_process | 7 | 0 | 0.000000 | -0.000000 | 864 | 917.538854 | 1011.5 | 1105.461146 | 1.170718 |
| GO:0009259\_ribonucleotide\_metabolic\_process | 7 | 0 | 0.000000 | -0.000000 | 864 | 917.538854 | 1011.5 | 1105.461146 | 1.170718 |
| GO:0009311\_oligosaccharide\_metabolic\_process | 7 | 0 | 0.000000 | -0.000000 | 864 | 917.538854 | 1011.5 | 1105.461146 | 1.170718 |
| GO:0009394\_2'-deoxyribonucleotide\_metabolic\_process | 7 | 0 | 0.000000 | -0.000000 | 864 | 917.538854 | 1011.5 | 1105.461146 | 1.170718 |
| GO:0009820\_alkaloid\_metabolic\_process | 7 | 0 | 0.000000 | -0.000000 | 864 | 917.538854 | 1011.5 | 1105.461146 | 1.170718 |
| GO:0010469\_regulation\_of\_receptor\_activity | 7 | 0 | 0.000000 | -0.000000 | 864 | 917.538854 | 1011.5 | 1105.461146 | 1.170718 |
| GO:0010948\_negative\_regulation\_of\_cell\_cycle\_process | 7 | 0 | 0.000000 | -0.000000 | 864 | 917.538854 | 1011.5 | 1105.461146 | 1.170718 |
| GO:0014047\_glutamate\_secretion | 7 | 0 | 0.000000 | -0.000000 | 864 | 917.538854 | 1011.5 | 1105.461146 | 1.170718 |
| GO:0014821\_phasic\_smooth\_muscle\_contraction | 7 | 0 | 0.000000 | -0.000000 | 864 | 917.538854 | 1011.5 | 1105.461146 | 1.170718 |
| GO:0015697\_quaternary\_ammonium\_group\_transport | 7 | 0 | 0.000000 | -0.000000 | 864 | 917.538854 | 1011.5 | 1105.461146 | 1.170718 |
| GO:0015813\_L-glutamate\_transport | 7 | 0 | 0.000000 | -0.000000 | 864 | 917.538854 | 1011.5 | 1105.461146 | 1.170718 |
| GO:0015908\_fatty\_acid\_transport | 7 | 0 | 0.000000 | -0.000000 | 864 | 917.538854 | 1011.5 | 1105.461146 | 1.170718 |
| GO:0015914\_phospholipid\_transport | 7 | 0 | 0.000000 | -0.000000 | 864 | 917.538854 | 1011.5 | 1105.461146 | 1.170718 |
| GO:0015992\_proton\_transport | 7 | 0 | 0.000000 | -0.000000 | 864 | 917.538854 | 1011.5 | 1105.461146 | 1.170718 |
| GO:0016339\_calcium-dependent\_cell-cell\_adhesion | 7 | 0 | 0.000000 | -0.000000 | 864 | 917.538854 | 1011.5 | 1105.461146 | 1.170718 |
| GO:0019362\_pyridine\_nucleotide\_metabolic\_process | 7 | 0 | 0.000000 | -0.000000 | 864 | 917.538854 | 1011.5 | 1105.461146 | 1.170718 |
| GO:0019692\_deoxyribose\_phosphate\_metabolic\_process | 7 | 0 | 0.000000 | -0.000000 | 864 | 917.538854 | 1011.5 | 1105.461146 | 1.170718 |
| GO:0019800\_peptide\_cross-linking\_via\_chondroitin\_4-sulfate\_glycosaminoglycan | 7 | 0 | 0.000000 | -0.000000 | 864 | 917.538854 | 1011.5 | 1105.461146 | 1.170718 |
| GO:0020027\_hemoglobin\_metabolic\_process | 7 | 0 | 0.000000 | -0.000000 | 864 | 917.538854 | 1011.5 | 1105.461146 | 1.170718 |
| GO:0021514\_ventral\_spinal\_cord\_interneuron\_differentiation | 7 | 0 | 0.000000 | -0.000000 | 864 | 917.538854 | 1011.5 | 1105.461146 | 1.170718 |
| GO:0021516\_dorsal\_spinal\_cord\_development | 7 | 0 | 0.000000 | -0.000000 | 864 | 917.538854 | 1011.5 | 1105.461146 | 1.170718 |
| GO:0021520\_spinal\_cord\_motor\_neuron\_cell\_fate\_specification | 7 | 0 | 0.000000 | -0.000000 | 864 | 917.538854 | 1011.5 | 1105.461146 | 1.170718 |
| GO:0021521\_ventral\_spinal\_cord\_interneuron\_specification | 7 | 0 | 0.000000 | -0.000000 | 864 | 917.538854 | 1011.5 | 1105.461146 | 1.170718 |
| GO:0021546\_rhombomere\_development | 7 | 0 | 0.000000 | -0.000000 | 864 | 917.538854 | 1011.5 | 1105.461146 | 1.170718 |
| GO:0021756\_striatum\_development | 7 | 0 | 0.000000 | -0.000000 | 864 | 917.538854 | 1011.5 | 1105.461146 | 1.170718 |
| GO:0021884\_forebrain\_neuron\_development | 7 | 0 | 0.000000 | -0.000000 | 864 | 917.538854 | 1011.5 | 1105.461146 | 1.170718 |
| GO:0021903\_rostrocaudal\_neural\_tube\_patterning | 7 | 0 | 0.000000 | -0.000000 | 864 | 917.538854 | 1011.5 | 1105.461146 | 1.170718 |
| GO:0021984\_adenohypophysis\_development | 7 | 0 | 0.000000 | -0.000000 | 864 | 917.538854 | 1011.5 | 1105.461146 | 1.170718 |
| GO:0022407\_regulation\_of\_cell-cell\_adhesion | 7 | 0 | 0.000000 | -0.000000 | 864 | 917.538854 | 1011.5 | 1105.461146 | 1.170718 |
| GO:0022618\_ribonucleoprotein\_complex\_assembly | 7 | 0 | 0.000000 | -0.000000 | 864 | 917.538854 | 1011.5 | 1105.461146 | 1.170718 |
| GO:0030104\_water\_homeostasis | 7 | 0 | 0.000000 | -0.000000 | 864 | 917.538854 | 1011.5 | 1105.461146 | 1.170718 |
| GO:0030201\_heparan\_sulfate\_proteoglycan\_metabolic\_process | 7 | 0 | 0.000000 | -0.000000 | 864 | 917.538854 | 1011.5 | 1105.461146 | 1.170718 |
| GO:0030432\_peristalsis | 7 | 0 | 0.000000 | -0.000000 | 864 | 917.538854 | 1011.5 | 1105.461146 | 1.170718 |
| GO:0030517\_negative\_regulation\_of\_axon\_extension | 7 | 0 | 0.000000 | -0.000000 | 864 | 917.538854 | 1011.5 | 1105.461146 | 1.170718 |
| GO:0030520\_estrogen\_receptor\_signaling\_pathway | 7 | 0 | 0.000000 | -0.000000 | 864 | 917.538854 | 1011.5 | 1105.461146 | 1.170718 |
| GO:0030903\_notochord\_development | 7 | 0 | 0.000000 | -0.000000 | 864 | 917.538854 | 1011.5 | 1105.461146 | 1.170718 |
| GO:0031017\_exocrine\_pancreas\_development | 7 | 0 | 0.000000 | -0.000000 | 864 | 917.538854 | 1011.5 | 1105.461146 | 1.170718 |
| GO:0031114\_regulation\_of\_microtubule\_depolymerization | 7 | 0 | 0.000000 | -0.000000 | 864 | 917.538854 | 1011.5 | 1105.461146 | 1.170718 |
| GO:0031124\_mRNA\_3'-end\_processing | 7 | 0 | 0.000000 | -0.000000 | 864 | 917.538854 | 1011.5 | 1105.461146 | 1.170718 |
| GO:0031497\_chromatin\_assembly | 7 | 0 | 0.000000 | -0.000000 | 864 | 917.538854 | 1011.5 | 1105.461146 | 1.170718 |
| GO:0032104\_regulation\_of\_response\_to\_extracellular\_stimulus | 7 | 0 | 0.000000 | -0.000000 | 864 | 917.538854 | 1011.5 | 1105.461146 | 1.170718 |
| GO:0032107\_regulation\_of\_response\_to\_nutrient\_levels | 7 | 0 | 0.000000 | -0.000000 | 864 | 917.538854 | 1011.5 | 1105.461146 | 1.170718 |
| GO:0032228\_regulation\_of\_synaptic\_transmission\_\_GABAergic | 7 | 0 | 0.000000 | -0.000000 | 864 | 917.538854 | 1011.5 | 1105.461146 | 1.170718 |
| GO:0032319\_regulation\_of\_Rho\_GTPase\_activity | 7 | 0 | 0.000000 | -0.000000 | 864 | 917.538854 | 1011.5 | 1105.461146 | 1.170718 |
| GO:0032387\_negative\_regulation\_of\_intracellular\_transport | 7 | 0 | 0.000000 | -0.000000 | 864 | 917.538854 | 1011.5 | 1105.461146 | 1.170718 |
| GO:0032507\_maintenance\_of\_protein\_location\_in\_cell | 7 | 0 | 0.000000 | -0.000000 | 864 | 917.538854 | 1011.5 | 1105.461146 | 1.170718 |
| GO:0033032\_regulation\_of\_myeloid\_cell\_apoptosis | 7 | 0 | 0.000000 | -0.000000 | 864 | 917.538854 | 1011.5 | 1105.461146 | 1.170718 |
| GO:0033057\_reproductive\_behavior\_in\_a\_multicellular\_organism | 7 | 0 | 0.000000 | -0.000000 | 864 | 917.538854 | 1011.5 | 1105.461146 | 1.170718 |
| GO:0034599\_cellular\_response\_to\_oxidative\_stress | 7 | 0 | 0.000000 | -0.000000 | 864 | 917.538854 | 1011.5 | 1105.461146 | 1.170718 |
| GO:0042033\_chemokine\_biosynthetic\_process | 7 | 0 | 0.000000 | -0.000000 | 864 | 917.538854 | 1011.5 | 1105.461146 | 1.170718 |
| GO:0042133\_neurotransmitter\_metabolic\_process | 7 | 0 | 0.000000 | -0.000000 | 864 | 917.538854 | 1011.5 | 1105.461146 | 1.170718 |
| GO:0042168\_heme\_metabolic\_process | 7 | 0 | 0.000000 | -0.000000 | 864 | 917.538854 | 1011.5 | 1105.461146 | 1.170718 |
| GO:0042415\_norepinephrine\_metabolic\_process | 7 | 0 | 0.000000 | -0.000000 | 864 | 917.538854 | 1011.5 | 1105.461146 | 1.170718 |
| GO:0042438\_melanin\_biosynthetic\_process | 7 | 0 | 0.000000 | -0.000000 | 864 | 917.538854 | 1011.5 | 1105.461146 | 1.170718 |
| GO:0042503\_tyrosine\_phosphorylation\_of\_Stat3\_protein | 7 | 0 | 0.000000 | -0.000000 | 864 | 917.538854 | 1011.5 | 1105.461146 | 1.170718 |
| GO:0042572\_retinol\_metabolic\_process | 7 | 0 | 0.000000 | -0.000000 | 864 | 917.538854 | 1011.5 | 1105.461146 | 1.170718 |
| GO:0043353\_enucleate\_erythrocyte\_differentiation | 7 | 0 | 0.000000 | -0.000000 | 864 | 917.538854 | 1011.5 | 1105.461146 | 1.170718 |
| GO:0043372\_positive\_regulation\_of\_CD4-positive\_\_alpha\_beta\_T\_cell\_differentiation | 7 | 0 | 0.000000 | -0.000000 | 864 | 917.538854 | 1011.5 | 1105.461146 | 1.170718 |
| GO:0043449\_cellular\_alkene\_metabolic\_process | 7 | 0 | 0.000000 | -0.000000 | 864 | 917.538854 | 1011.5 | 1105.461146 | 1.170718 |
| GO:0043507\_positive\_regulation\_of\_JUN\_kinase\_activity | 7 | 0 | 0.000000 | -0.000000 | 864 | 917.538854 | 1011.5 | 1105.461146 | 1.170718 |
| GO:0043567\_regulation\_of\_insulin-like\_growth\_factor\_receptor\_signaling\_pathway | 7 | 0 | 0.000000 | -0.000000 | 864 | 917.538854 | 1011.5 | 1105.461146 | 1.170718 |
| GO:0043584\_nose\_development | 7 | 0 | 0.000000 | -0.000000 | 864 | 917.538854 | 1011.5 | 1105.461146 | 1.170718 |
| GO:0044065\_regulation\_of\_respiratory\_system\_process | 7 | 0 | 0.000000 | -0.000000 | 864 | 917.538854 | 1011.5 | 1105.461146 | 1.170718 |
| GO:0044275\_cellular\_carbohydrate\_catabolic\_process | 7 | 0 | 0.000000 | -0.000000 | 864 | 917.538854 | 1011.5 | 1105.461146 | 1.170718 |
| GO:0045059\_positive\_thymic\_T\_cell\_selection | 7 | 0 | 0.000000 | -0.000000 | 864 | 917.538854 | 1011.5 | 1105.461146 | 1.170718 |
| GO:0045073\_regulation\_of\_chemokine\_biosynthetic\_process | 7 | 0 | 0.000000 | -0.000000 | 864 | 917.538854 | 1011.5 | 1105.461146 | 1.170718 |
| GO:0045581\_negative\_regulation\_of\_T\_cell\_differentiation | 7 | 0 | 0.000000 | -0.000000 | 864 | 917.538854 | 1011.5 | 1105.461146 | 1.170718 |
| GO:0045599\_negative\_regulation\_of\_fat\_cell\_differentiation | 7 | 0 | 0.000000 | -0.000000 | 864 | 917.538854 | 1011.5 | 1105.461146 | 1.170718 |
| GO:0045604\_regulation\_of\_epidermal\_cell\_differentiation | 7 | 0 | 0.000000 | -0.000000 | 864 | 917.538854 | 1011.5 | 1105.461146 | 1.170718 |
| GO:0045668\_negative\_regulation\_of\_osteoblast\_differentiation | 7 | 0 | 0.000000 | -0.000000 | 864 | 917.538854 | 1011.5 | 1105.461146 | 1.170718 |
| GO:0045823\_positive\_regulation\_of\_heart\_contraction | 7 | 0 | 0.000000 | -0.000000 | 864 | 917.538854 | 1011.5 | 1105.461146 | 1.170718 |
| GO:0045840\_positive\_regulation\_of\_mitosis | 7 | 0 | 0.000000 | -0.000000 | 864 | 917.538854 | 1011.5 | 1105.461146 | 1.170718 |
| GO:0045862\_positive\_regulation\_of\_proteolysis | 7 | 0 | 0.000000 | -0.000000 | 864 | 917.538854 | 1011.5 | 1105.461146 | 1.170718 |
| GO:0045879\_negative\_regulation\_of\_smoothened\_signaling\_pathway | 7 | 0 | 0.000000 | -0.000000 | 864 | 917.538854 | 1011.5 | 1105.461146 | 1.170718 |
| GO:0045880\_positive\_regulation\_of\_smoothened\_signaling\_pathway | 7 | 0 | 0.000000 | -0.000000 | 864 | 917.538854 | 1011.5 | 1105.461146 | 1.170718 |
| GO:0045986\_negative\_regulation\_of\_smooth\_muscle\_contraction | 7 | 0 | 0.000000 | -0.000000 | 864 | 917.538854 | 1011.5 | 1105.461146 | 1.170718 |
| GO:0046496\_nicotinamide\_nucleotide\_metabolic\_process | 7 | 0 | 0.000000 | -0.000000 | 864 | 917.538854 | 1011.5 | 1105.461146 | 1.170718 |
| GO:0046504\_glycerol\_ether\_biosynthetic\_process | 7 | 0 | 0.000000 | -0.000000 | 864 | 917.538854 | 1011.5 | 1105.461146 | 1.170718 |
| GO:0046513\_ceramide\_biosynthetic\_process | 7 | 0 | 0.000000 | -0.000000 | 864 | 917.538854 | 1011.5 | 1105.461146 | 1.170718 |
| GO:0046520\_sphingoid\_biosynthetic\_process | 7 | 0 | 0.000000 | -0.000000 | 864 | 917.538854 | 1011.5 | 1105.461146 | 1.170718 |
| GO:0046543\_development\_of\_secondary\_female\_sexual\_characteristics | 7 | 0 | 0.000000 | -0.000000 | 864 | 917.538854 | 1011.5 | 1105.461146 | 1.170718 |
| GO:0046622\_positive\_regulation\_of\_organ\_growth | 7 | 0 | 0.000000 | -0.000000 | 864 | 917.538854 | 1011.5 | 1105.461146 | 1.170718 |
| GO:0046626\_regulation\_of\_insulin\_receptor\_signaling\_pathway | 7 | 0 | 0.000000 | -0.000000 | 864 | 917.538854 | 1011.5 | 1105.461146 | 1.170718 |
| GO:0046676\_negative\_regulation\_of\_insulin\_secretion | 7 | 0 | 0.000000 | -0.000000 | 864 | 917.538854 | 1011.5 | 1105.461146 | 1.170718 |
| GO:0046823\_negative\_regulation\_of\_nucleocytoplasmic\_transport | 7 | 0 | 0.000000 | -0.000000 | 864 | 917.538854 | 1011.5 | 1105.461146 | 1.170718 |
| GO:0046824\_positive\_regulation\_of\_nucleocytoplasmic\_transport | 7 | 0 | 0.000000 | -0.000000 | 864 | 917.538854 | 1011.5 | 1105.461146 | 1.170718 |
| GO:0046847\_filopodium\_assembly | 7 | 0 | 0.000000 | -0.000000 | 864 | 917.538854 | 1011.5 | 1105.461146 | 1.170718 |
| GO:0048148\_behavioral\_response\_to\_cocaine | 7 | 0 | 0.000000 | -0.000000 | 864 | 917.538854 | 1011.5 | 1105.461146 | 1.170718 |
| GO:0048304\_positive\_regulation\_of\_isotype\_switching\_to\_IgG\_isotypes | 7 | 0 | 0.000000 | -0.000000 | 864 | 917.538854 | 1011.5 | 1105.461146 | 1.170718 |
| GO:0048486\_parasympathetic\_nervous\_system\_development | 7 | 0 | 0.000000 | -0.000000 | 864 | 917.538854 | 1011.5 | 1105.461146 | 1.170718 |
| GO:0048537\_mucosal-associated\_lymphoid\_tissue\_development | 7 | 0 | 0.000000 | -0.000000 | 864 | 917.538854 | 1011.5 | 1105.461146 | 1.170718 |
| GO:0048753\_pigment\_granule\_organization | 7 | 0 | 0.000000 | -0.000000 | 864 | 917.538854 | 1011.5 | 1105.461146 | 1.170718 |
| GO:0048857\_neural\_nucleus\_development | 7 | 0 | 0.000000 | -0.000000 | 864 | 917.538854 | 1011.5 | 1105.461146 | 1.170718 |
| GO:0050755\_chemokine\_metabolic\_process | 7 | 0 | 0.000000 | -0.000000 | 864 | 917.538854 | 1011.5 | 1105.461146 | 1.170718 |
| GO:0051028\_mRNA\_transport | 7 | 0 | 0.000000 | -0.000000 | 864 | 917.538854 | 1011.5 | 1105.461146 | 1.170718 |
| GO:0051785\_positive\_regulation\_of\_nuclear\_division | 7 | 0 | 0.000000 | -0.000000 | 864 | 917.538854 | 1011.5 | 1105.461146 | 1.170718 |
| GO:0051928\_positive\_regulation\_of\_calcium\_ion\_transport | 7 | 0 | 0.000000 | -0.000000 | 864 | 917.538854 | 1011.5 | 1105.461146 | 1.170718 |
| GO:0055070\_copper\_ion\_homeostasis | 7 | 0 | 0.000000 | -0.000000 | 864 | 917.538854 | 1011.5 | 1105.461146 | 1.170718 |
| GO:0060037\_pharyngeal\_system\_development | 7 | 0 | 0.000000 | -0.000000 | 864 | 917.538854 | 1011.5 | 1105.461146 | 1.170718 |
| GO:0060080\_regulation\_of\_inhibitory\_postsynaptic\_membrane\_potential | 7 | 0 | 0.000000 | -0.000000 | 864 | 917.538854 | 1011.5 | 1105.461146 | 1.170718 |
| GO:0060088\_auditory\_receptor\_cell\_stereocilium\_organization | 7 | 0 | 0.000000 | -0.000000 | 864 | 917.538854 | 1011.5 | 1105.461146 | 1.170718 |
| GO:0060117\_auditory\_receptor\_cell\_development | 7 | 0 | 0.000000 | -0.000000 | 864 | 917.538854 | 1011.5 | 1105.461146 | 1.170718 |
| GO:0060441\_branching\_involved\_in\_lung\_morphogenesis | 7 | 0 | 0.000000 | -0.000000 | 864 | 917.538854 | 1011.5 | 1105.461146 | 1.170718 |
| GO:0060526\_prostate\_glandular\_acinus\_morphogenesis | 7 | 0 | 0.000000 | -0.000000 | 864 | 917.538854 | 1011.5 | 1105.461146 | 1.170718 |
| GO:0060527\_prostate\_epithelial\_cord\_arborization\_involved\_in\_prostate\_glandular\_acinus\_morphogenesis | 7 | 0 | 0.000000 | -0.000000 | 864 | 917.538854 | 1011.5 | 1105.461146 | 1.170718 |
| GO:0060579\_ventral\_spinal\_cord\_interneuron\_fate\_commitment | 7 | 0 | 0.000000 | -0.000000 | 864 | 917.538854 | 1011.5 | 1105.461146 | 1.170718 |
| GO:0060664\_epithelial\_cell\_proliferation\_involved\_in\_salivary\_gland\_morphogenesis | 7 | 0 | 0.000000 | -0.000000 | 864 | 917.538854 | 1011.5 | 1105.461146 | 1.170718 |
| GO:0060687\_regulation\_of\_branching\_involved\_in\_prostate\_gland\_morphogenesis | 7 | 0 | 0.000000 | -0.000000 | 864 | 917.538854 | 1011.5 | 1105.461146 | 1.170718 |
| GO:0060770\_negative\_regulation\_of\_epithelial\_cell\_proliferation\_involved\_in\_prostate\_gland\_development | 7 | 0 | 0.000000 | -0.000000 | 864 | 917.538854 | 1011.5 | 1105.461146 | 1.170718 |
| GO:0060788\_ectodermal\_placode\_formation | 7 | 0 | 0.000000 | -0.000000 | 864 | 917.538854 | 1011.5 | 1105.461146 | 1.170718 |
| GO:0060795\_cell\_fate\_commitment\_involved\_in\_the\_formation\_of\_primary\_germ\_layers | 7 | 0 | 0.000000 | -0.000000 | 864 | 917.538854 | 1011.5 | 1105.461146 | 1.170718 |
| GO:0070228\_regulation\_of\_lymphocyte\_apoptosis | 7 | 0 | 0.000000 | -0.000000 | 864 | 917.538854 | 1011.5 | 1105.461146 | 1.170718 |
| GO:0070646\_protein\_modification\_by\_small\_protein\_removal | 7 | 0 | 0.000000 | -0.000000 | 864 | 917.538854 | 1011.5 | 1105.461146 | 1.170718 |
| GO:0006954\_inflammatory\_response | 96 | 0 | 0.000000 | -0.000000 | 869 | 920.155833 | 1013.69 | 1107.224167 | 1.166502 |
| GO:0048736\_appendage\_development | 96 | 0 | 0.000000 | -0.000000 | 869 | 920.155833 | 1013.69 | 1107.224167 | 1.166502 |
| GO:0060173\_limb\_development | 96 | 0 | 0.000000 | -0.000000 | 869 | 920.155833 | 1013.69 | 1107.224167 | 1.166502 |
| GO:0060249\_anatomical\_structure\_homeostasis | 96 | 0 | 0.000000 | -0.000000 | 869 | 920.155833 | 1013.69 | 1107.224167 | 1.166502 |
| GO:0070661\_leukocyte\_proliferation | 96 | 0 | 0.000000 | -0.000000 | 869 | 920.155833 | 1013.69 | 1107.224167 | 1.166502 |
| GO:0030036\_actin\_cytoskeleton\_organization | 102 | 0 | 0.000000 | -0.000000 | 870 | 920.617290 | 1014.11 | 1107.602710 | 1.165644 |
| GO:0030029\_actin\_filament-based\_process | 109 | 0 | 0.000000 | -0.000000 | 871 | 921.076132 | 1014.53 | 1107.983868 | 1.164788 |
| GO:0044265\_cellular\_macromolecule\_catabolic\_process | 75 | 0 | 0.000000 | -0.000000 | 874 | 923.685839 | 1016.72 | 1109.754161 | 1.163295 |
| GO:0048589\_developmental\_growth | 75 | 0 | 0.000000 | -0.000000 | 874 | 923.685839 | 1016.72 | 1109.754161 | 1.163295 |
| GO:0051050\_positive\_regulation\_of\_transport | 75 | 0 | 0.000000 | -0.000000 | 874 | 923.685839 | 1016.72 | 1109.754161 | 1.163295 |
| GO:0021700\_developmental\_maturation | 81 | 0 | 0.000000 | -0.000000 | 875 | 924.320078 | 1017.22 | 1110.119922 | 1.162537 |
| GO:0009416\_response\_to\_light\_stimulus | 74 | 0 | 0.000000 | -0.000000 | 877 | 925.728141 | 1018.39 | 1111.051859 | 1.161220 |
| GO:0048771\_tissue\_remodeling | 74 | 0 | 0.000000 | -0.000000 | 877 | 925.728141 | 1018.39 | 1111.051859 | 1.161220 |
| GO:0051046\_regulation\_of\_secretion | 79 | 0 | 0.000000 | -0.000000 | 878 | 927.119370 | 1019.49 | 1111.860630 | 1.161150 |
| GO:0001843\_neural\_tube\_closure | 33 | 0 | 0.000000 | -0.000000 | 890 | 939.660918 | 1030.89 | 1122.119082 | 1.158303 |
| GO:0002562\_somatic\_diversification\_of\_immune\_receptors\_via\_germline\_recombination\_within\_a\_single\_locus | 33 | 0 | 0.000000 | -0.000000 | 890 | 939.660918 | 1030.89 | 1122.119082 | 1.158303 |
| GO:0006643\_membrane\_lipid\_metabolic\_process | 33 | 0 | 0.000000 | -0.000000 | 890 | 939.660918 | 1030.89 | 1122.119082 | 1.158303 |
| GO:0007188\_G-protein\_signaling\_\_coupled\_to\_cAMP\_nucleotide\_second\_messenger | 33 | 0 | 0.000000 | -0.000000 | 890 | 939.660918 | 1030.89 | 1122.119082 | 1.158303 |
| GO:0007270\_nerve-nerve\_synaptic\_transmission | 33 | 0 | 0.000000 | -0.000000 | 890 | 939.660918 | 1030.89 | 1122.119082 | 1.158303 |
| GO:0007431\_salivary\_gland\_development | 33 | 0 | 0.000000 | -0.000000 | 890 | 939.660918 | 1030.89 | 1122.119082 | 1.158303 |
| GO:0008584\_male\_gonad\_development | 33 | 0 | 0.000000 | -0.000000 | 890 | 939.660918 | 1030.89 | 1122.119082 | 1.158303 |
| GO:0008643\_carbohydrate\_transport | 33 | 0 | 0.000000 | -0.000000 | 890 | 939.660918 | 1030.89 | 1122.119082 | 1.158303 |
| GO:0016444\_somatic\_cell\_DNA\_recombination | 33 | 0 | 0.000000 | -0.000000 | 890 | 939.660918 | 1030.89 | 1122.119082 | 1.158303 |
| GO:0021987\_cerebral\_cortex\_development | 33 | 0 | 0.000000 | -0.000000 | 890 | 939.660918 | 1030.89 | 1122.119082 | 1.158303 |
| GO:0042108\_positive\_regulation\_of\_cytokine\_biosynthetic\_process | 33 | 0 | 0.000000 | -0.000000 | 890 | 939.660918 | 1030.89 | 1122.119082 | 1.158303 |
| GO:0060606\_tube\_closure | 33 | 0 | 0.000000 | -0.000000 | 890 | 939.660918 | 1030.89 | 1122.119082 | 1.158303 |
| GO:0000002\_mitochondrial\_genome\_maintenance | 9 | 0 | 0.000000 | -0.000000 | 1004 | 1053.399141 | 1141.97 | 1230.540859 | 1.137420 |
| GO:0000186\_activation\_of\_MAPKK\_activity | 9 | 0 | 0.000000 | -0.000000 | 1004 | 1053.399141 | 1141.97 | 1230.540859 | 1.137420 |
| GO:0001539\_ciliary\_or\_flagellar\_motility | 9 | 0 | 0.000000 | -0.000000 | 1004 | 1053.399141 | 1141.97 | 1230.540859 | 1.137420 |
| GO:0001667\_ameboidal\_cell\_migration | 9 | 0 | 0.000000 | -0.000000 | 1004 | 1053.399141 | 1141.97 | 1230.540859 | 1.137420 |
| GO:0001676\_long-chain\_fatty\_acid\_metabolic\_process | 9 | 0 | 0.000000 | -0.000000 | 1004 | 1053.399141 | 1141.97 | 1230.540859 | 1.137420 |
| GO:0001935\_endothelial\_cell\_proliferation | 9 | 0 | 0.000000 | -0.000000 | 1004 | 1053.399141 | 1141.97 | 1230.540859 | 1.137420 |
| GO:0002021\_response\_to\_dietary\_excess | 9 | 0 | 0.000000 | -0.000000 | 1004 | 1053.399141 | 1141.97 | 1230.540859 | 1.137420 |
| GO:0002028\_regulation\_of\_sodium\_ion\_transport | 9 | 0 | 0.000000 | -0.000000 | 1004 | 1053.399141 | 1141.97 | 1230.540859 | 1.137420 |
| GO:0002221\_pattern\_recognition\_receptor\_signaling\_pathway | 9 | 0 | 0.000000 | -0.000000 | 1004 | 1053.399141 | 1141.97 | 1230.540859 | 1.137420 |
| GO:0002292\_T\_cell\_differentiation\_during\_immune\_response | 9 | 0 | 0.000000 | -0.000000 | 1004 | 1053.399141 | 1141.97 | 1230.540859 | 1.137420 |
| GO:0002293\_alpha-beta\_T\_cell\_differentiation\_during\_immune\_response | 9 | 0 | 0.000000 | -0.000000 | 1004 | 1053.399141 | 1141.97 | 1230.540859 | 1.137420 |
| GO:0002294\_CD4-positive\_\_alpha-beta\_T\_cell\_differentiation\_during\_immune\_response | 9 | 0 | 0.000000 | -0.000000 | 1004 | 1053.399141 | 1141.97 | 1230.540859 | 1.137420 |
| GO:0002507\_tolerance\_induction | 9 | 0 | 0.000000 | -0.000000 | 1004 | 1053.399141 | 1141.97 | 1230.540859 | 1.137420 |
| GO:0002886\_regulation\_of\_myeloid\_leukocyte\_mediated\_immunity | 9 | 0 | 0.000000 | -0.000000 | 1004 | 1053.399141 | 1141.97 | 1230.540859 | 1.137420 |
| GO:0006007\_glucose\_catabolic\_process | 9 | 0 | 0.000000 | -0.000000 | 1004 | 1053.399141 | 1141.97 | 1230.540859 | 1.137420 |
| GO:0006182\_cGMP\_biosynthetic\_process | 9 | 0 | 0.000000 | -0.000000 | 1004 | 1053.399141 | 1141.97 | 1230.540859 | 1.137420 |
| GO:0006309\_DNA\_fragmentation\_involved\_in\_apoptosis | 9 | 0 | 0.000000 | -0.000000 | 1004 | 1053.399141 | 1141.97 | 1230.540859 | 1.137420 |
| GO:0006364\_rRNA\_processing | 9 | 0 | 0.000000 | -0.000000 | 1004 | 1053.399141 | 1141.97 | 1230.540859 | 1.137420 |
| GO:0006595\_polyamine\_metabolic\_process | 9 | 0 | 0.000000 | -0.000000 | 1004 | 1053.399141 | 1141.97 | 1230.540859 | 1.137420 |
| GO:0006611\_protein\_export\_from\_nucleus | 9 | 0 | 0.000000 | -0.000000 | 1004 | 1053.399141 | 1141.97 | 1230.540859 | 1.137420 |
| GO:0006910\_phagocytosis\_\_recognition | 9 | 0 | 0.000000 | -0.000000 | 1004 | 1053.399141 | 1141.97 | 1230.540859 | 1.137420 |
| GO:0006911\_phagocytosis\_\_engulfment | 9 | 0 | 0.000000 | -0.000000 | 1004 | 1053.399141 | 1141.97 | 1230.540859 | 1.137420 |
| GO:0007128\_meiotic\_prophase\_I | 9 | 0 | 0.000000 | -0.000000 | 1004 | 1053.399141 | 1141.97 | 1230.540859 | 1.137420 |
| GO:0007193\_inhibition\_of\_adenylate\_cyclase\_activity\_by\_G-protein\_signaling | 9 | 0 | 0.000000 | -0.000000 | 1004 | 1053.399141 | 1141.97 | 1230.540859 | 1.137420 |
| GO:0007379\_segment\_specification | 9 | 0 | 0.000000 | -0.000000 | 1004 | 1053.399141 | 1141.97 | 1230.540859 | 1.137420 |
| GO:0007617\_mating\_behavior | 9 | 0 | 0.000000 | -0.000000 | 1004 | 1053.399141 | 1141.97 | 1230.540859 | 1.137420 |
| GO:0009451\_RNA\_modification | 9 | 0 | 0.000000 | -0.000000 | 1004 | 1053.399141 | 1141.97 | 1230.540859 | 1.137420 |
| GO:0010165\_response\_to\_X-ray | 9 | 0 | 0.000000 | -0.000000 | 1004 | 1053.399141 | 1141.97 | 1230.540859 | 1.137420 |
| GO:0010675\_regulation\_of\_cellular\_carbohydrate\_metabolic\_process | 9 | 0 | 0.000000 | -0.000000 | 1004 | 1053.399141 | 1141.97 | 1230.540859 | 1.137420 |
| GO:0014037\_Schwann\_cell\_differentiation | 9 | 0 | 0.000000 | -0.000000 | 1004 | 1053.399141 | 1141.97 | 1230.540859 | 1.137420 |
| GO:0014073\_response\_to\_tropane | 9 | 0 | 0.000000 | -0.000000 | 1004 | 1053.399141 | 1141.97 | 1230.540859 | 1.137420 |
| GO:0015695\_organic\_cation\_transport | 9 | 0 | 0.000000 | -0.000000 | 1004 | 1053.399141 | 1141.97 | 1230.540859 | 1.137420 |
| GO:0016072\_rRNA\_metabolic\_process | 9 | 0 | 0.000000 | -0.000000 | 1004 | 1053.399141 | 1141.97 | 1230.540859 | 1.137420 |
| GO:0016601\_Rac\_protein\_signal\_transduction | 9 | 0 | 0.000000 | -0.000000 | 1004 | 1053.399141 | 1141.97 | 1230.540859 | 1.137420 |
| GO:0019320\_hexose\_catabolic\_process | 9 | 0 | 0.000000 | -0.000000 | 1004 | 1053.399141 | 1141.97 | 1230.540859 | 1.137420 |
| GO:0021544\_subpallium\_development | 9 | 0 | 0.000000 | -0.000000 | 1004 | 1053.399141 | 1141.97 | 1230.540859 | 1.137420 |
| GO:0021936\_regulation\_of\_granule\_cell\_precursor\_proliferation | 9 | 0 | 0.000000 | -0.000000 | 1004 | 1053.399141 | 1141.97 | 1230.540859 | 1.137420 |
| GO:0021940\_positive\_regulation\_of\_granule\_cell\_precursor\_proliferation | 9 | 0 | 0.000000 | -0.000000 | 1004 | 1053.399141 | 1141.97 | 1230.540859 | 1.137420 |
| GO:0030048\_actin\_filament-based\_movement | 9 | 0 | 0.000000 | -0.000000 | 1004 | 1053.399141 | 1141.97 | 1230.540859 | 1.137420 |
| GO:0030279\_negative\_regulation\_of\_ossification | 9 | 0 | 0.000000 | -0.000000 | 1004 | 1053.399141 | 1141.97 | 1230.540859 | 1.137420 |
| GO:0030325\_adrenal\_gland\_development | 9 | 0 | 0.000000 | -0.000000 | 1004 | 1053.399141 | 1141.97 | 1230.540859 | 1.137420 |
| GO:0031023\_microtubule\_organizing\_center\_organization | 9 | 0 | 0.000000 | -0.000000 | 1004 | 1053.399141 | 1141.97 | 1230.540859 | 1.137420 |
| GO:0032388\_positive\_regulation\_of\_intracellular\_transport | 9 | 0 | 0.000000 | -0.000000 | 1004 | 1053.399141 | 1141.97 | 1230.540859 | 1.137420 |
| GO:0032606\_type\_I\_interferon\_production | 9 | 0 | 0.000000 | -0.000000 | 1004 | 1053.399141 | 1141.97 | 1230.540859 | 1.137420 |
| GO:0032814\_regulation\_of\_natural\_killer\_cell\_activation | 9 | 0 | 0.000000 | -0.000000 | 1004 | 1053.399141 | 1141.97 | 1230.540859 | 1.137420 |
| GO:0032816\_positive\_regulation\_of\_natural\_killer\_cell\_activation | 9 | 0 | 0.000000 | -0.000000 | 1004 | 1053.399141 | 1141.97 | 1230.540859 | 1.137420 |
| GO:0032963\_collagen\_metabolic\_process | 9 | 0 | 0.000000 | -0.000000 | 1004 | 1053.399141 | 1141.97 | 1230.540859 | 1.137420 |
| GO:0033028\_myeloid\_cell\_apoptosis | 9 | 0 | 0.000000 | -0.000000 | 1004 | 1053.399141 | 1141.97 | 1230.540859 | 1.137420 |
| GO:0033143\_regulation\_of\_steroid\_hormone\_receptor\_signaling\_pathway | 9 | 0 | 0.000000 | -0.000000 | 1004 | 1053.399141 | 1141.97 | 1230.540859 | 1.137420 |
| GO:0033151\_V(D)J\_recombination | 9 | 0 | 0.000000 | -0.000000 | 1004 | 1053.399141 | 1141.97 | 1230.540859 | 1.137420 |
| GO:0033344\_cholesterol\_efflux | 9 | 0 | 0.000000 | -0.000000 | 1004 | 1053.399141 | 1141.97 | 1230.540859 | 1.137420 |
| GO:0034605\_cellular\_response\_to\_heat | 9 | 0 | 0.000000 | -0.000000 | 1004 | 1053.399141 | 1141.97 | 1230.540859 | 1.137420 |
| GO:0035088\_establishment\_or\_maintenance\_of\_apical\_basal\_cell\_polarity | 9 | 0 | 0.000000 | -0.000000 | 1004 | 1053.399141 | 1141.97 | 1230.540859 | 1.137420 |
| GO:0035162\_embryonic\_hemopoiesis | 9 | 0 | 0.000000 | -0.000000 | 1004 | 1053.399141 | 1141.97 | 1230.540859 | 1.137420 |
| GO:0040020\_regulation\_of\_meiosis | 9 | 0 | 0.000000 | -0.000000 | 1004 | 1053.399141 | 1141.97 | 1230.540859 | 1.137420 |
| GO:0042058\_regulation\_of\_epidermal\_growth\_factor\_receptor\_signaling\_pathway | 9 | 0 | 0.000000 | -0.000000 | 1004 | 1053.399141 | 1141.97 | 1230.540859 | 1.137420 |
| GO:0042093\_T-helper\_cell\_differentiation | 9 | 0 | 0.000000 | -0.000000 | 1004 | 1053.399141 | 1141.97 | 1230.540859 | 1.137420 |
| GO:0042220\_response\_to\_cocaine | 9 | 0 | 0.000000 | -0.000000 | 1004 | 1053.399141 | 1141.97 | 1230.540859 | 1.137420 |
| GO:0042402\_biogenic\_amine\_catabolic\_process | 9 | 0 | 0.000000 | -0.000000 | 1004 | 1053.399141 | 1141.97 | 1230.540859 | 1.137420 |
| GO:0042509\_regulation\_of\_tyrosine\_phosphorylation\_of\_STAT\_protein | 9 | 0 | 0.000000 | -0.000000 | 1004 | 1053.399141 | 1141.97 | 1230.540859 | 1.137420 |
| GO:0042640\_anagen | 9 | 0 | 0.000000 | -0.000000 | 1004 | 1053.399141 | 1141.97 | 1230.540859 | 1.137420 |
| GO:0043242\_negative\_regulation\_of\_protein\_complex\_disassembly | 9 | 0 | 0.000000 | -0.000000 | 1004 | 1053.399141 | 1141.97 | 1230.540859 | 1.137420 |
| GO:0043299\_leukocyte\_degranulation | 9 | 0 | 0.000000 | -0.000000 | 1004 | 1053.399141 | 1141.97 | 1230.540859 | 1.137420 |
| GO:0043383\_negative\_T\_cell\_selection | 9 | 0 | 0.000000 | -0.000000 | 1004 | 1053.399141 | 1141.97 | 1230.540859 | 1.137420 |
| GO:0043409\_negative\_regulation\_of\_MAPKKK\_cascade | 9 | 0 | 0.000000 | -0.000000 | 1004 | 1053.399141 | 1141.97 | 1230.540859 | 1.137420 |
| GO:0043433\_negative\_regulation\_of\_transcription\_factor\_activity | 9 | 0 | 0.000000 | -0.000000 | 1004 | 1053.399141 | 1141.97 | 1230.540859 | 1.137420 |
| GO:0043603\_cellular\_amide\_metabolic\_process | 9 | 0 | 0.000000 | -0.000000 | 1004 | 1053.399141 | 1141.97 | 1230.540859 | 1.137420 |
| GO:0045060\_negative\_thymic\_T\_cell\_selection | 9 | 0 | 0.000000 | -0.000000 | 1004 | 1053.399141 | 1141.97 | 1230.540859 | 1.137420 |
| GO:0045109\_intermediate\_filament\_organization | 9 | 0 | 0.000000 | -0.000000 | 1004 | 1053.399141 | 1141.97 | 1230.540859 | 1.137420 |
| GO:0045136\_development\_of\_secondary\_sexual\_characteristics | 9 | 0 | 0.000000 | -0.000000 | 1004 | 1053.399141 | 1141.97 | 1230.540859 | 1.137420 |
| GO:0045185\_maintenance\_of\_protein\_location | 9 | 0 | 0.000000 | -0.000000 | 1004 | 1053.399141 | 1141.97 | 1230.540859 | 1.137420 |
| GO:0045214\_sarcomere\_organization | 9 | 0 | 0.000000 | -0.000000 | 1004 | 1053.399141 | 1141.97 | 1230.540859 | 1.137420 |
| GO:0045428\_regulation\_of\_nitric\_oxide\_biosynthetic\_process | 9 | 0 | 0.000000 | -0.000000 | 1004 | 1053.399141 | 1141.97 | 1230.540859 | 1.137420 |
| GO:0045620\_negative\_regulation\_of\_lymphocyte\_differentiation | 9 | 0 | 0.000000 | -0.000000 | 1004 | 1053.399141 | 1141.97 | 1230.540859 | 1.137420 |
| GO:0045646\_regulation\_of\_erythrocyte\_differentiation | 9 | 0 | 0.000000 | -0.000000 | 1004 | 1053.399141 | 1141.97 | 1230.540859 | 1.137420 |
| GO:0045671\_negative\_regulation\_of\_osteoclast\_differentiation | 9 | 0 | 0.000000 | -0.000000 | 1004 | 1053.399141 | 1141.97 | 1230.540859 | 1.137420 |
| GO:0045766\_positive\_regulation\_of\_angiogenesis | 9 | 0 | 0.000000 | -0.000000 | 1004 | 1053.399141 | 1141.97 | 1230.540859 | 1.137420 |
| GO:0045830\_positive\_regulation\_of\_isotype\_switching | 9 | 0 | 0.000000 | -0.000000 | 1004 | 1053.399141 | 1141.97 | 1230.540859 | 1.137420 |
| GO:0045884\_regulation\_of\_survival\_gene\_product\_expression | 9 | 0 | 0.000000 | -0.000000 | 1004 | 1053.399141 | 1141.97 | 1230.540859 | 1.137420 |
| GO:0046006\_regulation\_of\_activated\_T\_cell\_proliferation | 9 | 0 | 0.000000 | -0.000000 | 1004 | 1053.399141 | 1141.97 | 1230.540859 | 1.137420 |
| GO:0046324\_regulation\_of\_glucose\_import | 9 | 0 | 0.000000 | -0.000000 | 1004 | 1053.399141 | 1141.97 | 1230.540859 | 1.137420 |
| GO:0046365\_monosaccharide\_catabolic\_process | 9 | 0 | 0.000000 | -0.000000 | 1004 | 1053.399141 | 1141.97 | 1230.540859 | 1.137420 |
| GO:0046636\_negative\_regulation\_of\_alpha-beta\_T\_cell\_activation | 9 | 0 | 0.000000 | -0.000000 | 1004 | 1053.399141 | 1141.97 | 1230.540859 | 1.137420 |
| GO:0046641\_positive\_regulation\_of\_alpha-beta\_T\_cell\_proliferation | 9 | 0 | 0.000000 | -0.000000 | 1004 | 1053.399141 | 1141.97 | 1230.540859 | 1.137420 |
| GO:0046888\_negative\_regulation\_of\_hormone\_secretion | 9 | 0 | 0.000000 | -0.000000 | 1004 | 1053.399141 | 1141.97 | 1230.540859 | 1.137420 |
| GO:0048070\_regulation\_of\_pigmentation\_during\_development | 9 | 0 | 0.000000 | -0.000000 | 1004 | 1053.399141 | 1141.97 | 1230.540859 | 1.137420 |
| GO:0048146\_positive\_regulation\_of\_fibroblast\_proliferation | 9 | 0 | 0.000000 | -0.000000 | 1004 | 1053.399141 | 1141.97 | 1230.540859 | 1.137420 |
| GO:0048284\_organelle\_fusion | 9 | 0 | 0.000000 | -0.000000 | 1004 | 1053.399141 | 1141.97 | 1230.540859 | 1.137420 |
| GO:0048488\_synaptic\_vesicle\_endocytosis | 9 | 0 | 0.000000 | -0.000000 | 1004 | 1053.399141 | 1141.97 | 1230.540859 | 1.137420 |
| GO:0048569\_post-embryonic\_organ\_development | 9 | 0 | 0.000000 | -0.000000 | 1004 | 1053.399141 | 1141.97 | 1230.540859 | 1.137420 |
| GO:0048708\_astrocyte\_differentiation | 9 | 0 | 0.000000 | -0.000000 | 1004 | 1053.399141 | 1141.97 | 1230.540859 | 1.137420 |
| GO:0050433\_regulation\_of\_catecholamine\_secretion | 9 | 0 | 0.000000 | -0.000000 | 1004 | 1053.399141 | 1141.97 | 1230.540859 | 1.137420 |
| GO:0050856\_regulation\_of\_T\_cell\_receptor\_signaling\_pathway | 9 | 0 | 0.000000 | -0.000000 | 1004 | 1053.399141 | 1141.97 | 1230.540859 | 1.137420 |
| GO:0050884\_neuromuscular\_process\_controlling\_posture | 9 | 0 | 0.000000 | -0.000000 | 1004 | 1053.399141 | 1141.97 | 1230.540859 | 1.137420 |
| GO:0050918\_positive\_chemotaxis | 9 | 0 | 0.000000 | -0.000000 | 1004 | 1053.399141 | 1141.97 | 1230.540859 | 1.137420 |
| GO:0051023\_regulation\_of\_immunoglobulin\_secretion | 9 | 0 | 0.000000 | -0.000000 | 1004 | 1053.399141 | 1141.97 | 1230.540859 | 1.137420 |
| GO:0051297\_centrosome\_organization | 9 | 0 | 0.000000 | -0.000000 | 1004 | 1053.399141 | 1141.97 | 1230.540859 | 1.137420 |
| GO:0051324\_prophase | 9 | 0 | 0.000000 | -0.000000 | 1004 | 1053.399141 | 1141.97 | 1230.540859 | 1.137420 |
| GO:0051607\_defense\_response\_to\_virus | 9 | 0 | 0.000000 | -0.000000 | 1004 | 1053.399141 | 1141.97 | 1230.540859 | 1.137420 |
| GO:0051647\_nucleus\_localization | 9 | 0 | 0.000000 | -0.000000 | 1004 | 1053.399141 | 1141.97 | 1230.540859 | 1.137420 |
| GO:0051896\_regulation\_of\_protein\_kinase\_B\_signaling\_cascade | 9 | 0 | 0.000000 | -0.000000 | 1004 | 1053.399141 | 1141.97 | 1230.540859 | 1.137420 |
| GO:0051932\_synaptic\_transmission\_\_GABAergic | 9 | 0 | 0.000000 | -0.000000 | 1004 | 1053.399141 | 1141.97 | 1230.540859 | 1.137420 |
| GO:0051963\_regulation\_of\_synaptogenesis | 9 | 0 | 0.000000 | -0.000000 | 1004 | 1053.399141 | 1141.97 | 1230.540859 | 1.137420 |
| GO:0055013\_cardiac\_muscle\_cell\_development | 9 | 0 | 0.000000 | -0.000000 | 1004 | 1053.399141 | 1141.97 | 1230.540859 | 1.137420 |
| GO:0060052\_neurofilament\_cytoskeleton\_organization | 9 | 0 | 0.000000 | -0.000000 | 1004 | 1053.399141 | 1141.97 | 1230.540859 | 1.137420 |
| GO:0060081\_membrane\_hyperpolarization | 9 | 0 | 0.000000 | -0.000000 | 1004 | 1053.399141 | 1141.97 | 1230.540859 | 1.137420 |
| GO:0060119\_inner\_ear\_receptor\_cell\_development | 9 | 0 | 0.000000 | -0.000000 | 1004 | 1053.399141 | 1141.97 | 1230.540859 | 1.137420 |
| GO:0060122\_inner\_ear\_receptor\_stereocilium\_organization | 9 | 0 | 0.000000 | -0.000000 | 1004 | 1053.399141 | 1141.97 | 1230.540859 | 1.137420 |
| GO:0060325\_face\_morphogenesis | 9 | 0 | 0.000000 | -0.000000 | 1004 | 1053.399141 | 1141.97 | 1230.540859 | 1.137420 |
| GO:0060513\_prostatic\_bud\_formation | 9 | 0 | 0.000000 | -0.000000 | 1004 | 1053.399141 | 1141.97 | 1230.540859 | 1.137420 |
| GO:0060602\_branch\_elongation\_of\_an\_epithelium | 9 | 0 | 0.000000 | -0.000000 | 1004 | 1053.399141 | 1141.97 | 1230.540859 | 1.137420 |
| GO:0060693\_regulation\_of\_branching\_involved\_in\_salivary\_gland\_morphogenesis | 9 | 0 | 0.000000 | -0.000000 | 1004 | 1053.399141 | 1141.97 | 1230.540859 | 1.137420 |
| GO:0070306\_lens\_fiber\_cell\_differentiation | 9 | 0 | 0.000000 | -0.000000 | 1004 | 1053.399141 | 1141.97 | 1230.540859 | 1.137420 |
| GO:0090048\_negative\_regulation\_of\_transcription\_regulator\_activity | 9 | 0 | 0.000000 | -0.000000 | 1004 | 1053.399141 | 1141.97 | 1230.540859 | 1.137420 |
| GO:0002764\_immune\_response-regulating\_signal\_transduction | 51 | 0 | 0.000000 | -0.000000 | 1010 | 1060.508875 | 1148.48 | 1236.451125 | 1.137109 |
| GO:0006887\_exocytosis | 51 | 0 | 0.000000 | -0.000000 | 1010 | 1060.508875 | 1148.48 | 1236.451125 | 1.137109 |
| GO:0007601\_visual\_perception | 51 | 0 | 0.000000 | -0.000000 | 1010 | 1060.508875 | 1148.48 | 1236.451125 | 1.137109 |
| GO:0032583\_regulation\_of\_gene-specific\_transcription | 51 | 0 | 0.000000 | -0.000000 | 1010 | 1060.508875 | 1148.48 | 1236.451125 | 1.137109 |
| GO:0032880\_regulation\_of\_protein\_localization | 51 | 0 | 0.000000 | -0.000000 | 1010 | 1060.508875 | 1148.48 | 1236.451125 | 1.137109 |
| GO:0043408\_regulation\_of\_MAPKKK\_cascade | 51 | 0 | 0.000000 | -0.000000 | 1010 | 1060.508875 | 1148.48 | 1236.451125 | 1.137109 |
| GO:0001541\_ovarian\_follicle\_development | 24 | 0 | 0.000000 | -0.000000 | 1032 | 1085.810942 | 1172.27 | 1258.729058 | 1.135921 |
| GO:0002381\_immunoglobulin\_production\_during\_immune\_response | 24 | 0 | 0.000000 | -0.000000 | 1032 | 1085.810942 | 1172.27 | 1258.729058 | 1.135921 |
| GO:0006941\_striated\_muscle\_contraction | 24 | 0 | 0.000000 | -0.000000 | 1032 | 1085.810942 | 1172.27 | 1258.729058 | 1.135921 |
| GO:0006959\_humoral\_immune\_response | 24 | 0 | 0.000000 | -0.000000 | 1032 | 1085.810942 | 1172.27 | 1258.729058 | 1.135921 |
| GO:0007050\_cell\_cycle\_arrest | 24 | 0 | 0.000000 | -0.000000 | 1032 | 1085.810942 | 1172.27 | 1258.729058 | 1.135921 |
| GO:0007204\_elevation\_of\_cytosolic\_calcium\_ion\_concentration | 24 | 0 | 0.000000 | -0.000000 | 1032 | 1085.810942 | 1172.27 | 1258.729058 | 1.135921 |
| GO:0007259\_JAK-STAT\_cascade | 24 | 0 | 0.000000 | -0.000000 | 1032 | 1085.810942 | 1172.27 | 1258.729058 | 1.135921 |
| GO:0007266\_Rho\_protein\_signal\_transduction | 24 | 0 | 0.000000 | -0.000000 | 1032 | 1085.810942 | 1172.27 | 1258.729058 | 1.135921 |
| GO:0007632\_visual\_behavior | 24 | 0 | 0.000000 | -0.000000 | 1032 | 1085.810942 | 1172.27 | 1258.729058 | 1.135921 |
| GO:0008629\_induction\_of\_apoptosis\_by\_intracellular\_signals | 24 | 0 | 0.000000 | -0.000000 | 1032 | 1085.810942 | 1172.27 | 1258.729058 | 1.135921 |
| GO:0014070\_response\_to\_organic\_cyclic\_substance | 24 | 0 | 0.000000 | -0.000000 | 1032 | 1085.810942 | 1172.27 | 1258.729058 | 1.135921 |
| GO:0021515\_cell\_differentiation\_in\_spinal\_cord | 24 | 0 | 0.000000 | -0.000000 | 1032 | 1085.810942 | 1172.27 | 1258.729058 | 1.135921 |
| GO:0032386\_regulation\_of\_intracellular\_transport | 24 | 0 | 0.000000 | -0.000000 | 1032 | 1085.810942 | 1172.27 | 1258.729058 | 1.135921 |
| GO:0042158\_lipoprotein\_biosynthetic\_process | 24 | 0 | 0.000000 | -0.000000 | 1032 | 1085.810942 | 1172.27 | 1258.729058 | 1.135921 |
| GO:0043410\_positive\_regulation\_of\_MAPKKK\_cascade | 24 | 0 | 0.000000 | -0.000000 | 1032 | 1085.810942 | 1172.27 | 1258.729058 | 1.135921 |
| GO:0043588\_skin\_development | 24 | 0 | 0.000000 | -0.000000 | 1032 | 1085.810942 | 1172.27 | 1258.729058 | 1.135921 |
| GO:0048002\_antigen\_processing\_and\_presentation\_of\_peptide\_antigen | 24 | 0 | 0.000000 | -0.000000 | 1032 | 1085.810942 | 1172.27 | 1258.729058 | 1.135921 |
| GO:0048546\_digestive\_tract\_morphogenesis | 24 | 0 | 0.000000 | -0.000000 | 1032 | 1085.810942 | 1172.27 | 1258.729058 | 1.135921 |
| GO:0051099\_positive\_regulation\_of\_binding | 24 | 0 | 0.000000 | -0.000000 | 1032 | 1085.810942 | 1172.27 | 1258.729058 | 1.135921 |
| GO:0060078\_regulation\_of\_postsynaptic\_membrane\_potential | 24 | 0 | 0.000000 | -0.000000 | 1032 | 1085.810942 | 1172.27 | 1258.729058 | 1.135921 |
| GO:0060113\_inner\_ear\_receptor\_cell\_differentiation | 24 | 0 | 0.000000 | -0.000000 | 1032 | 1085.810942 | 1172.27 | 1258.729058 | 1.135921 |
| GO:0070667\_negative\_regulation\_of\_mast\_cell\_proliferation | 24 | 0 | 0.000000 | -0.000000 | 1032 | 1085.810942 | 1172.27 | 1258.729058 | 1.135921 |
| GO:0001759\_induction\_of\_an\_organ | 15 | 0 | 0.000000 | -0.000000 | 1088 | 1139.112920 | 1223.52 | 1307.927080 | 1.124559 |
| GO:0001782\_B\_cell\_homeostasis | 15 | 0 | 0.000000 | -0.000000 | 1088 | 1139.112920 | 1223.52 | 1307.927080 | 1.124559 |
| GO:0001964\_startle\_response | 15 | 0 | 0.000000 | -0.000000 | 1088 | 1139.112920 | 1223.52 | 1307.927080 | 1.124559 |
| GO:0002286\_T\_cell\_activation\_during\_immune\_response | 15 | 0 | 0.000000 | -0.000000 | 1088 | 1139.112920 | 1223.52 | 1307.927080 | 1.124559 |
| GO:0002495\_antigen\_processing\_and\_presentation\_of\_peptide\_antigen\_via\_MHC\_class\_II | 15 | 0 | 0.000000 | -0.000000 | 1088 | 1139.112920 | 1223.52 | 1307.927080 | 1.124559 |
| GO:0002504\_antigen\_processing\_and\_presentation\_of\_peptide\_or\_polysaccharide\_antigen\_via\_MHC\_class\_II | 15 | 0 | 0.000000 | -0.000000 | 1088 | 1139.112920 | 1223.52 | 1307.927080 | 1.124559 |
| GO:0002709\_regulation\_of\_T\_cell\_mediated\_immunity | 15 | 0 | 0.000000 | -0.000000 | 1088 | 1139.112920 | 1223.52 | 1307.927080 | 1.124559 |
| GO:0006473\_protein\_amino\_acid\_acetylation | 15 | 0 | 0.000000 | -0.000000 | 1088 | 1139.112920 | 1223.52 | 1307.927080 | 1.124559 |
| GO:0006487\_protein\_amino\_acid\_N-linked\_glycosylation | 15 | 0 | 0.000000 | -0.000000 | 1088 | 1139.112920 | 1223.52 | 1307.927080 | 1.124559 |
| GO:0006749\_glutathione\_metabolic\_process | 15 | 0 | 0.000000 | -0.000000 | 1088 | 1139.112920 | 1223.52 | 1307.927080 | 1.124559 |
| GO:0006885\_regulation\_of\_pH | 15 | 0 | 0.000000 | -0.000000 | 1088 | 1139.112920 | 1223.52 | 1307.927080 | 1.124559 |
| GO:0007040\_lysosome\_organization | 15 | 0 | 0.000000 | -0.000000 | 1088 | 1139.112920 | 1223.52 | 1307.927080 | 1.124559 |
| GO:0007173\_epidermal\_growth\_factor\_receptor\_signaling\_pathway | 15 | 0 | 0.000000 | -0.000000 | 1088 | 1139.112920 | 1223.52 | 1307.927080 | 1.124559 |
| GO:0007200\_activation\_of\_phospholipase\_C\_activity\_by\_G-protein\_coupled\_receptor\_protein\_signaling\_pathway\_coupled\_to\_IP3\_second\_messenger | 15 | 0 | 0.000000 | -0.000000 | 1088 | 1139.112920 | 1223.52 | 1307.927080 | 1.124559 |
| GO:0007202\_activation\_of\_phospholipase\_C\_activity | 15 | 0 | 0.000000 | -0.000000 | 1088 | 1139.112920 | 1223.52 | 1307.927080 | 1.124559 |
| GO:0007218\_neuropeptide\_signaling\_pathway | 15 | 0 | 0.000000 | -0.000000 | 1088 | 1139.112920 | 1223.52 | 1307.927080 | 1.124559 |
| GO:0007588\_excretion | 15 | 0 | 0.000000 | -0.000000 | 1088 | 1139.112920 | 1223.52 | 1307.927080 | 1.124559 |
| GO:0007618\_mating | 15 | 0 | 0.000000 | -0.000000 | 1088 | 1139.112920 | 1223.52 | 1307.927080 | 1.124559 |
| GO:0008543\_fibroblast\_growth\_factor\_receptor\_signaling\_pathway | 15 | 0 | 0.000000 | -0.000000 | 1088 | 1139.112920 | 1223.52 | 1307.927080 | 1.124559 |
| GO:0009062\_fatty\_acid\_catabolic\_process | 15 | 0 | 0.000000 | -0.000000 | 1088 | 1139.112920 | 1223.52 | 1307.927080 | 1.124559 |
| GO:0009116\_nucleoside\_metabolic\_process | 15 | 0 | 0.000000 | -0.000000 | 1088 | 1139.112920 | 1223.52 | 1307.927080 | 1.124559 |
| GO:0010092\_specification\_of\_organ\_identity | 15 | 0 | 0.000000 | -0.000000 | 1088 | 1139.112920 | 1223.52 | 1307.927080 | 1.124559 |
| GO:0010171\_body\_morphogenesis | 15 | 0 | 0.000000 | -0.000000 | 1088 | 1139.112920 | 1223.52 | 1307.927080 | 1.124559 |
| GO:0010518\_positive\_regulation\_of\_phospholipase\_activity | 15 | 0 | 0.000000 | -0.000000 | 1088 | 1139.112920 | 1223.52 | 1307.927080 | 1.124559 |
| GO:0010863\_positive\_regulation\_of\_phospholipase\_C\_activity | 15 | 0 | 0.000000 | -0.000000 | 1088 | 1139.112920 | 1223.52 | 1307.927080 | 1.124559 |
| GO:0015931\_nucleobase\_\_nucleoside\_\_nucleotide\_and\_nucleic\_acid\_transport | 15 | 0 | 0.000000 | -0.000000 | 1088 | 1139.112920 | 1223.52 | 1307.927080 | 1.124559 |
| GO:0019886\_antigen\_processing\_and\_presentation\_of\_exogenous\_peptide\_antigen\_via\_MHC\_class\_II | 15 | 0 | 0.000000 | -0.000000 | 1088 | 1139.112920 | 1223.52 | 1307.927080 | 1.124559 |
| GO:0021795\_cerebral\_cortex\_cell\_migration | 15 | 0 | 0.000000 | -0.000000 | 1088 | 1139.112920 | 1223.52 | 1307.927080 | 1.124559 |
| GO:0021872\_generation\_of\_neurons\_in\_the\_forebrain | 15 | 0 | 0.000000 | -0.000000 | 1088 | 1139.112920 | 1223.52 | 1307.927080 | 1.124559 |
| GO:0022600\_digestive\_system\_process | 15 | 0 | 0.000000 | -0.000000 | 1088 | 1139.112920 | 1223.52 | 1307.927080 | 1.124559 |
| GO:0030041\_actin\_filament\_polymerization | 15 | 0 | 0.000000 | -0.000000 | 1088 | 1139.112920 | 1223.52 | 1307.927080 | 1.124559 |
| GO:0031069\_hair\_follicle\_morphogenesis | 15 | 0 | 0.000000 | -0.000000 | 1088 | 1139.112920 | 1223.52 | 1307.927080 | 1.124559 |
| GO:0031076\_embryonic\_camera-type\_eye\_development | 15 | 0 | 0.000000 | -0.000000 | 1088 | 1139.112920 | 1223.52 | 1307.927080 | 1.124559 |
| GO:0031329\_regulation\_of\_cellular\_catabolic\_process | 15 | 0 | 0.000000 | -0.000000 | 1088 | 1139.112920 | 1223.52 | 1307.927080 | 1.124559 |
| GO:0035116\_embryonic\_hindlimb\_morphogenesis | 15 | 0 | 0.000000 | -0.000000 | 1088 | 1139.112920 | 1223.52 | 1307.927080 | 1.124559 |
| GO:0035249\_synaptic\_transmission\_\_glutamatergic | 15 | 0 | 0.000000 | -0.000000 | 1088 | 1139.112920 | 1223.52 | 1307.927080 | 1.124559 |
| GO:0042306\_regulation\_of\_protein\_import\_into\_nucleus | 15 | 0 | 0.000000 | -0.000000 | 1088 | 1139.112920 | 1223.52 | 1307.927080 | 1.124559 |
| GO:0045666\_positive\_regulation\_of\_neuron\_differentiation | 15 | 0 | 0.000000 | -0.000000 | 1088 | 1139.112920 | 1223.52 | 1307.927080 | 1.124559 |
| GO:0046164\_alcohol\_catabolic\_process | 15 | 0 | 0.000000 | -0.000000 | 1088 | 1139.112920 | 1223.52 | 1307.927080 | 1.124559 |
| GO:0046638\_positive\_regulation\_of\_alpha-beta\_T\_cell\_differentiation | 15 | 0 | 0.000000 | -0.000000 | 1088 | 1139.112920 | 1223.52 | 1307.927080 | 1.124559 |
| GO:0048008\_platelet-derived\_growth\_factor\_receptor\_signaling\_pathway | 15 | 0 | 0.000000 | -0.000000 | 1088 | 1139.112920 | 1223.52 | 1307.927080 | 1.124559 |
| GO:0048010\_vascular\_endothelial\_growth\_factor\_receptor\_signaling\_pathway | 15 | 0 | 0.000000 | -0.000000 | 1088 | 1139.112920 | 1223.52 | 1307.927080 | 1.124559 |
| GO:0048144\_fibroblast\_proliferation | 15 | 0 | 0.000000 | -0.000000 | 1088 | 1139.112920 | 1223.52 | 1307.927080 | 1.124559 |
| GO:0048145\_regulation\_of\_fibroblast\_proliferation | 15 | 0 | 0.000000 | -0.000000 | 1088 | 1139.112920 | 1223.52 | 1307.927080 | 1.124559 |
| GO:0048610\_reproductive\_cellular\_process | 15 | 0 | 0.000000 | -0.000000 | 1088 | 1139.112920 | 1223.52 | 1307.927080 | 1.124559 |
| GO:0048709\_oligodendrocyte\_differentiation | 15 | 0 | 0.000000 | -0.000000 | 1088 | 1139.112920 | 1223.52 | 1307.927080 | 1.124559 |
| GO:0050729\_positive\_regulation\_of\_inflammatory\_response | 15 | 0 | 0.000000 | -0.000000 | 1088 | 1139.112920 | 1223.52 | 1307.927080 | 1.124559 |
| GO:0050796\_regulation\_of\_insulin\_secretion | 15 | 0 | 0.000000 | -0.000000 | 1088 | 1139.112920 | 1223.52 | 1307.927080 | 1.124559 |
| GO:0050798\_activated\_T\_cell\_proliferation | 15 | 0 | 0.000000 | -0.000000 | 1088 | 1139.112920 | 1223.52 | 1307.927080 | 1.124559 |
| GO:0055010\_ventricular\_cardiac\_muscle\_morphogenesis | 15 | 0 | 0.000000 | -0.000000 | 1088 | 1139.112920 | 1223.52 | 1307.927080 | 1.124559 |
| GO:0060322\_head\_development | 15 | 0 | 0.000000 | -0.000000 | 1088 | 1139.112920 | 1223.52 | 1307.927080 | 1.124559 |
| GO:0060425\_lung\_morphogenesis | 15 | 0 | 0.000000 | -0.000000 | 1088 | 1139.112920 | 1223.52 | 1307.927080 | 1.124559 |
| GO:0060442\_branching\_involved\_in\_prostate\_gland\_morphogenesis | 15 | 0 | 0.000000 | -0.000000 | 1088 | 1139.112920 | 1223.52 | 1307.927080 | 1.124559 |
| GO:0060749\_mammary\_gland\_alveolus\_development | 15 | 0 | 0.000000 | -0.000000 | 1088 | 1139.112920 | 1223.52 | 1307.927080 | 1.124559 |
| GO:0070227\_lymphocyte\_apoptosis | 15 | 0 | 0.000000 | -0.000000 | 1088 | 1139.112920 | 1223.52 | 1307.927080 | 1.124559 |
| GO:0070507\_regulation\_of\_microtubule\_cytoskeleton\_organization | 15 | 0 | 0.000000 | -0.000000 | 1088 | 1139.112920 | 1223.52 | 1307.927080 | 1.124559 |
| GO:0006576\_biogenic\_amine\_metabolic\_process | 53 | 0 | 0.000000 | -0.000000 | 1095 | 1144.556809 | 1228.43 | 1312.303191 | 1.121854 |
| GO:0006935\_chemotaxis | 53 | 0 | 0.000000 | -0.000000 | 1095 | 1144.556809 | 1228.43 | 1312.303191 | 1.121854 |
| GO:0030031\_cell\_projection\_assembly | 53 | 0 | 0.000000 | -0.000000 | 1095 | 1144.556809 | 1228.43 | 1312.303191 | 1.121854 |
| GO:0042330\_taxis | 53 | 0 | 0.000000 | -0.000000 | 1095 | 1144.556809 | 1228.43 | 1312.303191 | 1.121854 |
| GO:0046942\_carboxylic\_acid\_transport | 53 | 0 | 0.000000 | -0.000000 | 1095 | 1144.556809 | 1228.43 | 1312.303191 | 1.121854 |
| GO:0050905\_neuromuscular\_process | 53 | 0 | 0.000000 | -0.000000 | 1095 | 1144.556809 | 1228.43 | 1312.303191 | 1.121854 |
| GO:0055085\_transmembrane\_transport | 53 | 0 | 0.000000 | -0.000000 | 1095 | 1144.556809 | 1228.43 | 1312.303191 | 1.121854 |
| GO:0000027\_ribosomal\_large\_subunit\_assembly | 1 | 0 |  |  |  |  |  |  |  |  |
| GO:0000042\_protein\_targeting\_to\_Golgi | 1 | 0 |  |  |  |  |  |  |  |  |
| GO:0000046\_autophagic\_vacuole\_fusion | 1 | 0 |  |  |  |  |  |  |  |  |
| GO:0000050\_urea\_cycle | 1 | 0 |  |  |  |  |  |  |  |  |
| GO:0000054\_ribosome\_export\_from\_nucleus | 1 | 0 |  |  |  |  |  |  |  |  |
| GO:0000055\_ribosomal\_large\_subunit\_export\_from\_nucleus | 1 | 0 |  |  |  |  |  |  |  |  |
| GO:0000056\_ribosomal\_small\_subunit\_export\_from\_nucleus | 1 | 0 |  |  |  |  |  |  |  |  |
| GO:0000072\_M\_phase\_specific\_microtubule\_process | 1 | 0 |  |  |  |  |  |  |  |  |
| GO:0000101\_sulfur\_amino\_acid\_transport | 1 | 0 |  |  |  |  |  |  |  |  |
| GO:0000147\_actin\_cortical\_patch\_assembly | 1 | 0 |  |  |  |  |  |  |  |  |
| GO:0000154\_rRNA\_modification | 1 | 0 |  |  |  |  |  |  |  |  |
| GO:0000183\_chromatin\_silencing\_at\_rDNA | 1 | 0 |  |  |  |  |  |  |  |  |
| GO:0000185\_activation\_of\_MAPKKK\_activity | 1 | 0 |  |  |  |  |  |  |  |  |
| GO:0000238\_zygotene | 1 | 0 |  |  |  |  |  |  |  |  |
| GO:0000255\_allantoin\_metabolic\_process | 1 | 0 |  |  |  |  |  |  |  |  |
| GO:0000266\_mitochondrial\_fission | 1 | 0 |  |  |  |  |  |  |  |  |
| GO:0000273\_lipoic\_acid\_metabolic\_process | 1 | 0 |  |  |  |  |  |  |  |  |
| GO:0000301\_retrograde\_transport\_\_vesicle\_recycling\_within\_Golgi | 1 | 0 |  |  |  |  |  |  |  |  |
| GO:0000394\_RNA\_splicing\_\_via\_endonucleolytic\_cleavage\_and\_ligation | 1 | 0 |  |  |  |  |  |  |  |  |
| GO:0000429\_regulation\_of\_transcription\_from\_RNA\_polymerase\_II\_promoter\_by\_carbon\_catabolites | 1 | 0 |  |  |  |  |  |  |  |  |
| GO:0000430\_regulation\_of\_transcription\_from\_RNA\_polymerase\_II\_promoter\_by\_glucose | 1 | 0 |  |  |  |  |  |  |  |  |
| GO:0000432\_positive\_regulation\_of\_transcription\_from\_RNA\_polymerase\_II\_promoter\_by\_glucose | 1 | 0 |  |  |  |  |  |  |  |  |
| GO:0000436\_positive\_regulation\_of\_transcription\_from\_RNA\_polymerase\_II\_promoter\_by\_carbon\_catabolites | 1 | 0 |  |  |  |  |  |  |  |  |
| GO:0000448\_cleavage\_in\_ITS2\_between\_5.8S\_rRNA\_and\_LSU-rRNA\_of\_tricistronic\_rRNA\_transcript\_(SSU-rRNA\_\_5.8S\_rRNA\_\_LSU-rRNA) | 1 | 0 |  |  |  |  |  |  |  |  |
| GO:0000460\_maturation\_of\_5.8S\_rRNA | 1 | 0 |  |  |  |  |  |  |  |  |
| GO:0000463\_maturation\_of\_LSU-rRNA\_from\_tricistronic\_rRNA\_transcript\_(SSU-rRNA\_\_5.8S\_rRNA\_\_LSU-rRNA) | 1 | 0 |  |  |  |  |  |  |  |  |
| GO:0000466\_maturation\_of\_5.8S\_rRNA\_from\_tricistronic\_rRNA\_transcript\_(SSU-rRNA\_\_5.8S\_rRNA\_\_LSU-rRNA) | 1 | 0 |  |  |  |  |  |  |  |  |
| GO:0000469\_cleavages\_during\_rRNA\_processing | 1 | 0 |  |  |  |  |  |  |  |  |
| GO:0000470\_maturation\_of\_LSU-rRNA | 1 | 0 |  |  |  |  |  |  |  |  |
| GO:0000478\_endonucleolytic\_cleavages\_during\_rRNA\_processing | 1 | 0 |  |  |  |  |  |  |  |  |
| GO:0000479\_endonucleolytic\_cleavage\_of\_tricistronic\_rRNA\_transcript\_(SSU-rRNA\_\_5.8S\_rRNA\_\_LSU-rRNA) | 1 | 0 |  |  |  |  |  |  |  |  |
| GO:0000705\_achiasmate\_meiosis\_I | 1 | 0 |  |  |  |  |  |  |  |  |
| GO:0000966\_RNA\_5'-end\_processing | 1 | 0 |  |  |  |  |  |  |  |  |
| GO:0001300\_chronological\_cell\_aging | 1 | 0 |  |  |  |  |  |  |  |  |
| GO:0001547\_antral\_ovarian\_follicle\_growth | 1 | 0 |  |  |  |  |  |  |  |  |
| GO:0001555\_oocyte\_growth | 1 | 0 |  |  |  |  |  |  |  |  |
| GO:0001560\_regulation\_of\_cell\_growth\_by\_extracellular\_stimulus | 1 | 0 |  |  |  |  |  |  |  |  |
| GO:0001660\_fever | 1 | 0 |  |  |  |  |  |  |  |  |
| GO:0001696\_gastric\_acid\_secretion | 1 | 0 |  |  |  |  |  |  |  |  |
| GO:0001712\_ectodermal\_cell\_fate\_commitment | 1 | 0 |  |  |  |  |  |  |  |  |
| GO:0001714\_endodermal\_cell\_fate\_specification | 1 | 0 |  |  |  |  |  |  |  |  |
| GO:0001762\_beta-alanine\_transport | 1 | 0 |  |  |  |  |  |  |  |  |
| GO:0001766\_membrane\_raft\_polarization | 1 | 0 |  |  |  |  |  |  |  |  |
| GO:0001811\_negative\_regulation\_of\_type\_I\_hypersensitivity | 1 | 0 |  |  |  |  |  |  |  |  |
| GO:0001821\_histamine\_secretion | 1 | 0 |  |  |  |  |  |  |  |  |
| GO:0001826\_inner\_cell\_mass\_cell\_differentiation | 1 | 0 |  |  |  |  |  |  |  |  |
| GO:0001830\_trophectodermal\_cell\_fate\_commitment | 1 | 0 |  |  |  |  |  |  |  |  |
| GO:0001834\_trophectodermal\_cell\_proliferation | 1 | 0 |  |  |  |  |  |  |  |  |
| GO:0001867\_complement\_activation\_\_lectin\_pathway | 1 | 0 |  |  |  |  |  |  |  |  |
| GO:0001880\_Mullerian\_duct\_regression | 1 | 0 |  |  |  |  |  |  |  |  |
| GO:0001922\_B-1\_B\_cell\_homeostasis | 1 | 0 |  |  |  |  |  |  |  |  |
| GO:0001923\_B-1\_B\_cell\_differentiation | 1 | 0 |  |  |  |  |  |  |  |  |
| GO:0001941\_postsynaptic\_membrane\_organization | 1 | 0 |  |  |  |  |  |  |  |  |
| GO:0001946\_lymphangiogenesis | 1 | 0 |  |  |  |  |  |  |  |  |
| GO:0001956\_positive\_regulation\_of\_neurotransmitter\_secretion | 1 | 0 |  |  |  |  |  |  |  |  |
| GO:0001961\_positive\_regulation\_of\_cytokine-mediated\_signaling\_pathway | 1 | 0 |  |  |  |  |  |  |  |  |
| GO:0001979\_regulation\_of\_systemic\_arterial\_blood\_pressure\_by\_chemoreceptor\_signaling | 1 | 0 |  |  |  |  |  |  |  |  |
| GO:0001980\_regulation\_of\_systemic\_arterial\_blood\_pressure\_by\_ischemic\_conditions | 1 | 0 |  |  |  |  |  |  |  |  |
| GO:0001984\_vasodilation\_of\_artery\_during\_baroreceptor\_response\_to\_increased\_systemic\_arterial\_blood\_pressure | 1 | 0 |  |  |  |  |  |  |  |  |
| GO:0001985\_negative\_regulation\_of\_heart\_rate\_in\_baroreceptor\_response\_to\_increased\_systemic\_arterial\_blood\_pressure | 1 | 0 |  |  |  |  |  |  |  |  |
| GO:0001987\_vasoconstriction\_of\_artery\_involved\_in\_baroreceptor\_response\_to\_lowering\_of\_systemic\_arterial\_blood\_pressure | 1 | 0 |  |  |  |  |  |  |  |  |
| GO:0001988\_positive\_regulation\_of\_heart\_rate\_in\_baroreceptor\_response\_to\_decreased\_systemic\_arterial\_blood\_pressure | 1 | 0 |  |  |  |  |  |  |  |  |
| GO:0001994\_norepinephrine-epinephrine\_vasoconstriction\_involved\_in\_regulation\_of\_systemic\_arterial\_blood\_pressure | 1 | 0 |  |  |  |  |  |  |  |  |
| GO:0002001\_renin\_secretion\_into\_blood\_stream | 1 | 0 |  |  |  |  |  |  |  |  |
| GO:0002002\_regulation\_of\_angiotensin\_levels\_in\_blood | 1 | 0 |  |  |  |  |  |  |  |  |
| GO:0002003\_angiotensin\_maturation | 1 | 0 |  |  |  |  |  |  |  |  |
| GO:0002007\_detection\_of\_hypoxic\_conditions\_in\_blood\_by\_chemoreceptor\_signaling | 1 | 0 |  |  |  |  |  |  |  |  |
| GO:0002017\_regulation\_of\_blood\_volume\_by\_renal\_aldosterone | 1 | 0 |  |  |  |  |  |  |  |  |
| GO:0002023\_reduction\_of\_food\_intake\_in\_response\_to\_dietary\_excess | 1 | 0 |  |  |  |  |  |  |  |  |
| GO:0002031\_G-protein\_coupled\_receptor\_internalization | 1 | 0 |  |  |  |  |  |  |  |  |
| GO:0002036\_regulation\_of\_L-glutamate\_transport | 1 | 0 |  |  |  |  |  |  |  |  |
| GO:0002040\_sprouting\_angiogenesis | 1 | 0 |  |  |  |  |  |  |  |  |
| GO:0002041\_intussusceptive\_angiogenesis | 1 | 0 |  |  |  |  |  |  |  |  |
| GO:0002068\_glandular\_epithelial\_cell\_development | 1 | 0 |  |  |  |  |  |  |  |  |
| GO:0002069\_columnar\_cuboidal\_epithelial\_cell\_maturation | 1 | 0 |  |  |  |  |  |  |  |  |
| GO:0002071\_glandular\_epithelial\_cell\_maturation | 1 | 0 |  |  |  |  |  |  |  |  |
| GO:0002082\_regulation\_of\_oxidative\_phosphorylation | 1 | 0 |  |  |  |  |  |  |  |  |
| GO:0002084\_protein\_depalmitoylation | 1 | 0 |  |  |  |  |  |  |  |  |
| GO:0002085\_inhibition\_of\_neuroepithelial\_cell\_differentiation | 1 | 0 |  |  |  |  |  |  |  |  |
| GO:0002086\_diaphragm\_contraction | 1 | 0 |  |  |  |  |  |  |  |  |
| GO:0002118\_aggressive\_behavior | 1 | 0 |  |  |  |  |  |  |  |  |
| GO:0002121\_inter-male\_aggressive\_behavior | 1 | 0 |  |  |  |  |  |  |  |  |
| GO:0002124\_territorial\_aggressive\_behavior | 1 | 0 |  |  |  |  |  |  |  |  |
| GO:0002227\_innate\_immune\_response\_in\_mucosa | 1 | 0 |  |  |  |  |  |  |  |  |
| GO:0002232\_leukocyte\_chemotaxis\_during\_inflammatory\_response | 1 | 0 |  |  |  |  |  |  |  |  |
| GO:0002248\_connective\_tissue\_replacement\_during\_inflammatory\_response | 1 | 0 |  |  |  |  |  |  |  |  |
| GO:0002282\_microglial\_cell\_activation\_during\_immune\_response | 1 | 0 |  |  |  |  |  |  |  |  |
| GO:0002287\_alpha-beta\_T\_cell\_activation\_during\_immune\_response | 1 | 0 |  |  |  |  |  |  |  |  |
| GO:0002314\_germinal\_center\_B\_cell\_differentiation | 1 | 0 |  |  |  |  |  |  |  |  |
| GO:0002315\_marginal\_zone\_B\_cell\_differentiation | 1 | 0 |  |  |  |  |  |  |  |  |
| GO:0002316\_follicular\_B\_cell\_differentiation | 1 | 0 |  |  |  |  |  |  |  |  |
| GO:0002317\_plasma\_cell\_differentiation | 1 | 0 |  |  |  |  |  |  |  |  |
| GO:0002349\_histamine\_production\_during\_acute\_inflammatory\_response | 1 | 0 |  |  |  |  |  |  |  |  |
| GO:0002351\_serotonin\_production\_during\_acute\_inflammatory\_response | 1 | 0 |  |  |  |  |  |  |  |  |
| GO:0002355\_detection\_of\_tumor\_cell | 1 | 0 |  |  |  |  |  |  |  |  |
| GO:0002370\_natural\_killer\_cell\_cytokine\_production | 1 | 0 |  |  |  |  |  |  |  |  |
| GO:0002371\_dendritic\_cell\_cytokine\_production | 1 | 0 |  |  |  |  |  |  |  |  |
| GO:0002380\_immunoglobulin\_secretion\_during\_immune\_response | 1 | 0 |  |  |  |  |  |  |  |  |
| GO:0002396\_MHC\_protein\_complex\_assembly | 1 | 0 |  |  |  |  |  |  |  |  |
| GO:0002397\_MHC\_class\_I\_protein\_complex\_assembly | 1 | 0 |  |  |  |  |  |  |  |  |
| GO:0002420\_natural\_killer\_cell\_mediated\_cytotoxicity\_directed\_against\_tumor\_cell\_target | 1 | 0 |  |  |  |  |  |  |  |  |
| GO:0002423\_natural\_killer\_cell\_mediated\_immune\_response\_to\_tumor\_cell | 1 | 0 |  |  |  |  |  |  |  |  |
| GO:0002424\_T\_cell\_mediated\_immune\_response\_to\_tumor\_cell | 1 | 0 |  |  |  |  |  |  |  |  |
| GO:0002426\_immunoglobulin\_production\_in\_mucosal\_tissue | 1 | 0 |  |  |  |  |  |  |  |  |
| GO:0002431\_Fc\_receptor\_mediated\_stimulatory\_signaling\_pathway | 1 | 0 |  |  |  |  |  |  |  |  |
| GO:0002432\_granuloma\_formation | 1 | 0 |  |  |  |  |  |  |  |  |
| GO:0002441\_histamine\_secretion\_during\_acute\_inflammatory\_response | 1 | 0 |  |  |  |  |  |  |  |  |
| GO:0002442\_serotonin\_secretion\_during\_acute\_inflammatory\_response | 1 | 0 |  |  |  |  |  |  |  |  |
| GO:0002457\_T\_cell\_antigen\_processing\_and\_presentation | 1 | 0 |  |  |  |  |  |  |  |  |
| GO:0002458\_peripheral\_T\_cell\_tolerance\_induction | 1 | 0 |  |  |  |  |  |  |  |  |
| GO:0002461\_tolerance\_induction\_dependent\_upon\_immune\_response | 1 | 0 |  |  |  |  |  |  |  |  |
| GO:0002465\_peripheral\_tolerance\_induction | 1 | 0 |  |  |  |  |  |  |  |  |
| GO:0002468\_dendritic\_cell\_antigen\_processing\_and\_presentation | 1 | 0 |  |  |  |  |  |  |  |  |
| GO:0002476\_antigen\_processing\_and\_presentation\_of\_endogenous\_peptide\_antigen\_via\_MHC\_class\_Ib | 1 | 0 |  |  |  |  |  |  |  |  |
| GO:0002479\_antigen\_processing\_and\_presentation\_of\_exogenous\_peptide\_antigen\_via\_MHC\_class\_I\_\_TAP-dependent | 1 | 0 |  |  |  |  |  |  |  |  |
| GO:0002483\_antigen\_processing\_and\_presentation\_of\_endogenous\_peptide\_antigen | 1 | 0 |  |  |  |  |  |  |  |  |
| GO:0002501\_peptide\_antigen\_assembly\_with\_MHC\_protein\_complex | 1 | 0 |  |  |  |  |  |  |  |  |
| GO:0002502\_peptide\_antigen\_assembly\_with\_MHC\_class\_I\_protein\_complex | 1 | 0 |  |  |  |  |  |  |  |  |
| GO:0002508\_central\_tolerance\_induction | 1 | 0 |  |  |  |  |  |  |  |  |
| GO:0002510\_central\_B\_cell\_tolerance\_induction | 1 | 0 |  |  |  |  |  |  |  |  |
| GO:0002545\_chronic\_inflammatory\_response\_to\_non-antigenic\_stimulus | 1 | 0 |  |  |  |  |  |  |  |  |
| GO:0002553\_histamine\_secretion\_by\_mast\_cell | 1 | 0 |  |  |  |  |  |  |  |  |
| GO:0002554\_serotonin\_secretion\_by\_platelet | 1 | 0 |  |  |  |  |  |  |  |  |
| GO:0002572\_pro-T\_cell\_differentiation | 1 | 0 |  |  |  |  |  |  |  |  |
| GO:0002577\_regulation\_of\_antigen\_processing\_and\_presentation | 1 | 0 |  |  |  |  |  |  |  |  |
| GO:0002579\_positive\_regulation\_of\_antigen\_processing\_and\_presentation | 1 | 0 |  |  |  |  |  |  |  |  |
| GO:0002604\_regulation\_of\_dendritic\_cell\_antigen\_processing\_and\_presentation | 1 | 0 |  |  |  |  |  |  |  |  |
| GO:0002606\_positive\_regulation\_of\_dendritic\_cell\_antigen\_processing\_and\_presentation | 1 | 0 |  |  |  |  |  |  |  |  |
| GO:0002635\_negative\_regulation\_of\_germinal\_center\_formation | 1 | 0 |  |  |  |  |  |  |  |  |
| GO:0002646\_regulation\_of\_central\_tolerance\_induction | 1 | 0 |  |  |  |  |  |  |  |  |
| GO:0002648\_positive\_regulation\_of\_central\_tolerance\_induction | 1 | 0 |  |  |  |  |  |  |  |  |
| GO:0002649\_regulation\_of\_tolerance\_induction\_to\_self\_antigen | 1 | 0 |  |  |  |  |  |  |  |  |
| GO:0002651\_positive\_regulation\_of\_tolerance\_induction\_to\_self\_antigen | 1 | 0 |  |  |  |  |  |  |  |  |
| GO:0002652\_regulation\_of\_tolerance\_induction\_dependent\_upon\_immune\_response | 1 | 0 |  |  |  |  |  |  |  |  |
| GO:0002654\_positive\_regulation\_of\_tolerance\_induction\_dependent\_upon\_immune\_response | 1 | 0 |  |  |  |  |  |  |  |  |
| GO:0002658\_regulation\_of\_peripheral\_tolerance\_induction | 1 | 0 |  |  |  |  |  |  |  |  |
| GO:0002660\_positive\_regulation\_of\_peripheral\_tolerance\_induction | 1 | 0 |  |  |  |  |  |  |  |  |
| GO:0002677\_negative\_regulation\_of\_chronic\_inflammatory\_response | 1 | 0 |  |  |  |  |  |  |  |  |
| GO:0002678\_positive\_regulation\_of\_chronic\_inflammatory\_response | 1 | 0 |  |  |  |  |  |  |  |  |
| GO:0002701\_negative\_regulation\_of\_production\_of\_molecular\_mediator\_of\_immune\_response | 1 | 0 |  |  |  |  |  |  |  |  |
| GO:0002719\_negative\_regulation\_of\_cytokine\_production\_during\_immune\_response | 1 | 0 |  |  |  |  |  |  |  |  |
| GO:0002724\_regulation\_of\_T\_cell\_cytokine\_production | 1 | 0 |  |  |  |  |  |  |  |  |
| GO:0002727\_regulation\_of\_natural\_killer\_cell\_cytokine\_production | 1 | 0 |  |  |  |  |  |  |  |  |
| GO:0002729\_positive\_regulation\_of\_natural\_killer\_cell\_cytokine\_production | 1 | 0 |  |  |  |  |  |  |  |  |
| GO:0002730\_regulation\_of\_dendritic\_cell\_cytokine\_production | 1 | 0 |  |  |  |  |  |  |  |  |
| GO:0002756\_MyD88-independent\_toll-like\_receptor\_signaling\_pathway | 1 | 0 |  |  |  |  |  |  |  |  |
| GO:0002767\_immune\_response-inhibiting\_cell\_surface\_receptor\_signaling\_pathway | 1 | 0 |  |  |  |  |  |  |  |  |
| GO:0002769\_natural\_killer\_cell\_inhibitory\_signaling\_pathway | 1 | 0 |  |  |  |  |  |  |  |  |
| GO:0002840\_regulation\_of\_T\_cell\_mediated\_immune\_response\_to\_tumor\_cell | 1 | 0 |  |  |  |  |  |  |  |  |
| GO:0002842\_positive\_regulation\_of\_T\_cell\_mediated\_immune\_response\_to\_tumor\_cell | 1 | 0 |  |  |  |  |  |  |  |  |
| GO:0002849\_regulation\_of\_peripheral\_T\_cell\_tolerance\_induction | 1 | 0 |  |  |  |  |  |  |  |  |
| GO:0002851\_positive\_regulation\_of\_peripheral\_T\_cell\_tolerance\_induction | 1 | 0 |  |  |  |  |  |  |  |  |
| GO:0002855\_regulation\_of\_natural\_killer\_cell\_mediated\_immune\_response\_to\_tumor\_cell | 1 | 0 |  |  |  |  |  |  |  |  |
| GO:0002857\_positive\_regulation\_of\_natural\_killer\_cell\_mediated\_immune\_response\_to\_tumor\_cell | 1 | 0 |  |  |  |  |  |  |  |  |
| GO:0002858\_regulation\_of\_natural\_killer\_cell\_mediated\_cytotoxicity\_directed\_against\_tumor\_cell\_target | 1 | 0 |  |  |  |  |  |  |  |  |
| GO:0002860\_positive\_regulation\_of\_natural\_killer\_cell\_mediated\_cytotoxicity\_directed\_against\_tumor\_cell\_target | 1 | 0 |  |  |  |  |  |  |  |  |
| GO:0002880\_regulation\_of\_chronic\_inflammatory\_response\_to\_non-antigenic\_stimulus | 1 | 0 |  |  |  |  |  |  |  |  |
| GO:0002882\_positive\_regulation\_of\_chronic\_inflammatory\_response\_to\_non-antigenic\_stimulus | 1 | 0 |  |  |  |  |  |  |  |  |
| GO:0002895\_regulation\_of\_central\_B\_cell\_tolerance\_induction | 1 | 0 |  |  |  |  |  |  |  |  |
| GO:0002897\_positive\_regulation\_of\_central\_B\_cell\_tolerance\_induction | 1 | 0 |  |  |  |  |  |  |  |  |
| GO:0002901\_mature\_B\_cell\_apoptosis | 1 | 0 |  |  |  |  |  |  |  |  |
| GO:0002903\_negative\_regulation\_of\_B\_cell\_apoptosis | 1 | 0 |  |  |  |  |  |  |  |  |
| GO:0002905\_regulation\_of\_mature\_B\_cell\_apoptosis | 1 | 0 |  |  |  |  |  |  |  |  |
| GO:0002906\_negative\_regulation\_of\_mature\_B\_cell\_apoptosis | 1 | 0 |  |  |  |  |  |  |  |  |
| GO:0003011\_involuntary\_skeletal\_muscle\_contraction | 1 | 0 |  |  |  |  |  |  |  |  |
| GO:0003027\_regulation\_of\_systemic\_arterial\_blood\_pressure\_by\_carotid\_body\_chemoreceptor\_signaling | 1 | 0 |  |  |  |  |  |  |  |  |
| GO:0003029\_detection\_of\_hypoxic\_conditions\_in\_blood\_by\_carotid\_body\_chemoreceptor\_signaling | 1 | 0 |  |  |  |  |  |  |  |  |
| GO:0003032\_detection\_of\_oxygen | 1 | 0 |  |  |  |  |  |  |  |  |
| GO:0003056\_regulation\_of\_vascular\_smooth\_muscle\_contraction | 1 | 0 |  |  |  |  |  |  |  |  |
| GO:0003062\_regulation\_of\_heart\_rate\_by\_chemical\_signal | 1 | 0 |  |  |  |  |  |  |  |  |
| GO:0003065\_positive\_regulation\_of\_heart\_rate\_by\_epinephrine | 1 | 0 |  |  |  |  |  |  |  |  |
| GO:0003068\_regulation\_of\_systemic\_arterial\_blood\_pressure\_by\_acetylcholine | 1 | 0 |  |  |  |  |  |  |  |  |
| GO:0003069\_vasodilation\_by\_acetylcholine\_involved\_in\_regulation\_of\_systemic\_arterial\_blood\_pressure | 1 | 0 |  |  |  |  |  |  |  |  |
| GO:0003070\_regulation\_of\_systemic\_arterial\_blood\_pressure\_by\_neurotransmitter | 1 | 0 |  |  |  |  |  |  |  |  |
| GO:0003097\_renal\_water\_transport | 1 | 0 |  |  |  |  |  |  |  |  |
| GO:0005979\_regulation\_of\_glycogen\_biosynthetic\_process | 1 | 0 |  |  |  |  |  |  |  |  |
| GO:0005984\_disaccharide\_metabolic\_process | 1 | 0 |  |  |  |  |  |  |  |  |
| GO:0005988\_lactose\_metabolic\_process | 1 | 0 |  |  |  |  |  |  |  |  |
| GO:0005989\_lactose\_biosynthetic\_process | 1 | 0 |  |  |  |  |  |  |  |  |
| GO:0005997\_xylulose\_metabolic\_process | 1 | 0 |  |  |  |  |  |  |  |  |
| GO:0006000\_fructose\_metabolic\_process | 1 | 0 |  |  |  |  |  |  |  |  |
| GO:0006002\_fructose\_6-phosphate\_metabolic\_process | 1 | 0 |  |  |  |  |  |  |  |  |
| GO:0006004\_fucose\_metabolic\_process | 1 | 0 |  |  |  |  |  |  |  |  |
| GO:0006013\_mannose\_metabolic\_process | 1 | 0 |  |  |  |  |  |  |  |  |
| GO:0006060\_sorbitol\_metabolic\_process | 1 | 0 |  |  |  |  |  |  |  |  |
| GO:0006064\_glucuronate\_catabolic\_process | 1 | 0 |  |  |  |  |  |  |  |  |
| GO:0006086\_acetyl-CoA\_biosynthetic\_process\_from\_pyruvate | 1 | 0 |  |  |  |  |  |  |  |  |
| GO:0006098\_pentose-phosphate\_shunt | 1 | 0 |  |  |  |  |  |  |  |  |
| GO:0006101\_citrate\_metabolic\_process | 1 | 0 |  |  |  |  |  |  |  |  |
| GO:0006104\_succinyl-CoA\_metabolic\_process | 1 | 0 |  |  |  |  |  |  |  |  |
| GO:0006116\_NADH\_oxidation | 1 | 0 |  |  |  |  |  |  |  |  |
| GO:0006120\_mitochondrial\_electron\_transport\_\_NADH\_to\_ubiquinone | 1 | 0 |  |  |  |  |  |  |  |  |
| GO:0006154\_adenosine\_catabolic\_process | 1 | 0 |  |  |  |  |  |  |  |  |
| GO:0006157\_deoxyadenosine\_catabolic\_process | 1 | 0 |  |  |  |  |  |  |  |  |
| GO:0006167\_AMP\_biosynthetic\_process | 1 | 0 |  |  |  |  |  |  |  |  |
| GO:0006175\_dATP\_biosynthetic\_process | 1 | 0 |  |  |  |  |  |  |  |  |
| GO:0006178\_guanine\_salvage | 1 | 0 |  |  |  |  |  |  |  |  |
| GO:0006196\_AMP\_catabolic\_process | 1 | 0 |  |  |  |  |  |  |  |  |
| GO:0006203\_dGTP\_catabolic\_process | 1 | 0 |  |  |  |  |  |  |  |  |
| GO:0006208\_pyrimidine\_base\_catabolic\_process | 1 | 0 |  |  |  |  |  |  |  |  |
| GO:0006221\_pyrimidine\_nucleotide\_biosynthetic\_process | 1 | 0 |  |  |  |  |  |  |  |  |
| GO:0006235\_dTTP\_biosynthetic\_process | 1 | 0 |  |  |  |  |  |  |  |  |
| GO:0006244\_pyrimidine\_nucleotide\_catabolic\_process | 1 | 0 |  |  |  |  |  |  |  |  |
| GO:0006269\_DNA\_replication\_\_synthesis\_of\_RNA\_primer | 1 | 0 |  |  |  |  |  |  |  |  |
| GO:0006283\_transcription-coupled\_nucleotide-excision\_repair | 1 | 0 |  |  |  |  |  |  |  |  |
| GO:0006296\_nucleotide-excision\_repair\_\_DNA\_incision\_\_5'-to\_lesion | 1 | 0 |  |  |  |  |  |  |  |  |
| GO:0006307\_DNA\_dealkylation | 1 | 0 |  |  |  |  |  |  |  |  |
| GO:0006337\_nucleosome\_disassembly | 1 | 0 |  |  |  |  |  |  |  |  |
| GO:0006344\_maintenance\_of\_chromatin\_silencing | 1 | 0 |  |  |  |  |  |  |  |  |
| GO:0006356\_regulation\_of\_transcription\_from\_RNA\_polymerase\_I\_promoter | 1 | 0 |  |  |  |  |  |  |  |  |
| GO:0006388\_tRNA\_splicing\_\_via\_endonucleolytic\_cleavage\_and\_ligation | 1 | 0 |  |  |  |  |  |  |  |  |
| GO:0006407\_rRNA\_export\_from\_nucleus | 1 | 0 |  |  |  |  |  |  |  |  |
| GO:0006419\_alanyl-tRNA\_aminoacylation | 1 | 0 |  |  |  |  |  |  |  |  |
| GO:0006434\_seryl-tRNA\_aminoacylation | 1 | 0 |  |  |  |  |  |  |  |  |
| GO:0006447\_regulation\_of\_translational\_initiation\_by\_iron | 1 | 0 |  |  |  |  |  |  |  |  |
| GO:0006467\_protein\_thiol-disulfide\_exchange | 1 | 0 |  |  |  |  |  |  |  |  |
| GO:0006474\_N-terminal\_protein\_amino\_acid\_acetylation | 1 | 0 |  |  |  |  |  |  |  |  |
| GO:0006481\_C-terminal\_protein\_amino\_acid\_methylation | 1 | 0 |  |  |  |  |  |  |  |  |
| GO:0006488\_dolichol-linked\_oligosaccharide\_biosynthetic\_process | 1 | 0 |  |  |  |  |  |  |  |  |
| GO:0006494\_protein\_amino\_acid\_terminal\_glycosylation | 1 | 0 |  |  |  |  |  |  |  |  |
| GO:0006496\_protein\_amino\_acid\_terminal\_N-glycosylation | 1 | 0 |  |  |  |  |  |  |  |  |
| GO:0006500\_N-terminal\_protein\_palmitoylation | 1 | 0 |  |  |  |  |  |  |  |  |
| GO:0006507\_GPI\_anchor\_release | 1 | 0 |  |  |  |  |  |  |  |  |
| GO:0006537\_glutamate\_biosynthetic\_process | 1 | 0 |  |  |  |  |  |  |  |  |
| GO:0006544\_glycine\_metabolic\_process | 1 | 0 |  |  |  |  |  |  |  |  |
| GO:0006549\_isoleucine\_metabolic\_process | 1 | 0 |  |  |  |  |  |  |  |  |
| GO:0006553\_lysine\_metabolic\_process | 1 | 0 |  |  |  |  |  |  |  |  |
| GO:0006554\_lysine\_catabolic\_process | 1 | 0 |  |  |  |  |  |  |  |  |
| GO:0006556\_S-adenosylmethionine\_biosynthetic\_process | 1 | 0 |  |  |  |  |  |  |  |  |
| GO:0006559\_L-phenylalanine\_catabolic\_process | 1 | 0 |  |  |  |  |  |  |  |  |
| GO:0006569\_tryptophan\_catabolic\_process | 1 | 0 |  |  |  |  |  |  |  |  |
| GO:0006572\_tyrosine\_catabolic\_process | 1 | 0 |  |  |  |  |  |  |  |  |
| GO:0006573\_valine\_metabolic\_process | 1 | 0 |  |  |  |  |  |  |  |  |
| GO:0006581\_acetylcholine\_catabolic\_process | 1 | 0 |  |  |  |  |  |  |  |  |
| GO:0006585\_dopamine\_biosynthetic\_process\_from\_tyrosine | 1 | 0 |  |  |  |  |  |  |  |  |
| GO:0006590\_thyroid\_hormone\_generation | 1 | 0 |  |  |  |  |  |  |  |  |
| GO:0006591\_ornithine\_metabolic\_process | 1 | 0 |  |  |  |  |  |  |  |  |
| GO:0006596\_polyamine\_biosynthetic\_process | 1 | 0 |  |  |  |  |  |  |  |  |
| GO:0006597\_spermine\_biosynthetic\_process | 1 | 0 |  |  |  |  |  |  |  |  |
| GO:0006601\_creatine\_biosynthetic\_process | 1 | 0 |  |  |  |  |  |  |  |  |
| GO:0006613\_cotranslational\_protein\_targeting\_to\_membrane | 1 | 0 |  |  |  |  |  |  |  |  |
| GO:0006622\_protein\_targeting\_to\_lysosome | 1 | 0 |  |  |  |  |  |  |  |  |
| GO:0006627\_mitochondrial\_protein\_processing\_during\_import | 1 | 0 |  |  |  |  |  |  |  |  |
| GO:0006653\_lecithin\_metabolic\_process | 1 | 0 |  |  |  |  |  |  |  |  |
| GO:0006658\_phosphatidylserine\_metabolic\_process | 1 | 0 |  |  |  |  |  |  |  |  |
| GO:0006659\_phosphatidylserine\_biosynthetic\_process | 1 | 0 |  |  |  |  |  |  |  |  |
| GO:0006667\_sphinganine\_metabolic\_process | 1 | 0 |  |  |  |  |  |  |  |  |
| GO:0006668\_sphinganine-1-phosphate\_metabolic\_process | 1 | 0 |  |  |  |  |  |  |  |  |
| GO:0006678\_glucosylceramide\_metabolic\_process | 1 | 0 |  |  |  |  |  |  |  |  |
| GO:0006682\_galactosylceramide\_biosynthetic\_process | 1 | 0 |  |  |  |  |  |  |  |  |
| GO:0006685\_sphingomyelin\_catabolic\_process | 1 | 0 |  |  |  |  |  |  |  |  |
| GO:0006700\_C21-steroid\_hormone\_biosynthetic\_process | 1 | 0 |  |  |  |  |  |  |  |  |
| GO:0006705\_mineralocorticoid\_biosynthetic\_process | 1 | 0 |  |  |  |  |  |  |  |  |
| GO:0006709\_progesterone\_catabolic\_process | 1 | 0 |  |  |  |  |  |  |  |  |
| GO:0006729\_tetrahydrobiopterin\_biosynthetic\_process | 1 | 0 |  |  |  |  |  |  |  |  |
| GO:0006734\_NADH\_metabolic\_process | 1 | 0 |  |  |  |  |  |  |  |  |
| GO:0006740\_NADPH\_regeneration | 1 | 0 |  |  |  |  |  |  |  |  |
| GO:0006741\_NADP\_biosynthetic\_process | 1 | 0 |  |  |  |  |  |  |  |  |
| GO:0006743\_ubiquinone\_metabolic\_process | 1 | 0 |  |  |  |  |  |  |  |  |
| GO:0006744\_ubiquinone\_biosynthetic\_process | 1 | 0 |  |  |  |  |  |  |  |  |
| GO:0006772\_thiamin\_metabolic\_process | 1 | 0 |  |  |  |  |  |  |  |  |
| GO:0006784\_heme\_a\_biosynthetic\_process | 1 | 0 |  |  |  |  |  |  |  |  |
| GO:0006797\_polyphosphate\_metabolic\_process | 1 | 0 |  |  |  |  |  |  |  |  |
| GO:0006798\_polyphosphate\_catabolic\_process | 1 | 0 |  |  |  |  |  |  |  |  |
| GO:0006824\_cobalt\_ion\_transport | 1 | 0 |  |  |  |  |  |  |  |  |
| GO:0006842\_tricarboxylic\_acid\_transport | 1 | 0 |  |  |  |  |  |  |  |  |
| GO:0006844\_acyl\_carnitine\_transport | 1 | 0 |  |  |  |  |  |  |  |  |
| GO:0006855\_multidrug\_transport | 1 | 0 |  |  |  |  |  |  |  |  |
| GO:0006863\_purine\_transport | 1 | 0 |  |  |  |  |  |  |  |  |
| GO:0006890\_retrograde\_vesicle-mediated\_transport\_\_Golgi\_to\_ER | 1 | 0 |  |  |  |  |  |  |  |  |
| GO:0006891\_intra-Golgi\_vesicle-mediated\_transport | 1 | 0 |  |  |  |  |  |  |  |  |
| GO:0006893\_Golgi\_to\_plasma\_membrane\_transport | 1 | 0 |  |  |  |  |  |  |  |  |
| GO:0006895\_Golgi\_to\_endosome\_transport | 1 | 0 |  |  |  |  |  |  |  |  |
| GO:0006896\_Golgi\_to\_vacuole\_transport | 1 | 0 |  |  |  |  |  |  |  |  |
| GO:0006900\_membrane\_budding | 1 | 0 |  |  |  |  |  |  |  |  |
| GO:0006930\_substrate-bound\_cell\_migration\_\_cell\_extension | 1 | 0 |  |  |  |  |  |  |  |  |
| GO:0006931\_substrate-bound\_cell\_migration\_\_cell\_attachment\_to\_substrate | 1 | 0 |  |  |  |  |  |  |  |  |
| GO:0006933\_negative\_regulation\_of\_cell\_adhesion\_involved\_in\_substrate-bound\_cell\_migration | 1 | 0 |  |  |  |  |  |  |  |  |
| GO:0006957\_complement\_activation\_\_alternative\_pathway | 1 | 0 |  |  |  |  |  |  |  |  |
| GO:0006958\_complement\_activation\_\_classical\_pathway | 1 | 0 |  |  |  |  |  |  |  |  |
| GO:0006978\_DNA\_damage\_response\_\_signal\_transduction\_by\_p53\_class\_mediator\_resulting\_in\_transcription\_of\_p21\_class\_mediator | 1 | 0 |  |  |  |  |  |  |  |  |
| GO:0007016\_cytoskeletal\_anchoring\_at\_plasma\_membrane | 1 | 0 |  |  |  |  |  |  |  |  |
| GO:0007021\_tubulin\_complex\_assembly | 1 | 0 |  |  |  |  |  |  |  |  |
| GO:0007052\_mitotic\_spindle\_organization | 1 | 0 |  |  |  |  |  |  |  |  |
| GO:0007056\_spindle\_assembly\_involved\_in\_female\_meiosis | 1 | 0 |  |  |  |  |  |  |  |  |
| GO:0007057\_spindle\_assembly\_involved\_in\_female\_meiosis\_I | 1 | 0 |  |  |  |  |  |  |  |  |
| GO:0007063\_regulation\_of\_sister\_chromatid\_cohesion | 1 | 0 |  |  |  |  |  |  |  |  |
| GO:0007065\_male\_meiosis\_sister\_chromatid\_cohesion | 1 | 0 |  |  |  |  |  |  |  |  |
| GO:0007095\_mitotic\_cell\_cycle\_G2\_M\_transition\_DNA\_damage\_checkpoint | 1 | 0 |  |  |  |  |  |  |  |  |
| GO:0007096\_regulation\_of\_exit\_from\_mitosis | 1 | 0 |  |  |  |  |  |  |  |  |
| GO:0007158\_neuron\_adhesion | 1 | 0 |  |  |  |  |  |  |  |  |
| GO:0007168\_receptor\_guanylyl\_cyclase\_signaling\_pathway | 1 | 0 |  |  |  |  |  |  |  |  |
| GO:0007197\_inhibition\_of\_adenylate\_cyclase\_activity\_by\_muscarinic\_acetylcholine\_receptor\_signaling\_pathway | 1 | 0 |  |  |  |  |  |  |  |  |
| GO:0007207\_activation\_of\_phospholipase\_C\_activity\_by\_muscarinic\_acetylcholine\_receptor\_signaling\_pathway | 1 | 0 |  |  |  |  |  |  |  |  |
| GO:0007208\_activation\_of\_phospholipase\_C\_activity\_by\_serotonin\_receptor\_signaling\_pathway | 1 | 0 |  |  |  |  |  |  |  |  |
| GO:0007217\_tachykinin\_receptor\_signaling\_pathway | 1 | 0 |  |  |  |  |  |  |  |  |
| GO:0007221\_positive\_regulation\_of\_transcription\_of\_Notch\_receptor\_target | 1 | 0 |  |  |  |  |  |  |  |  |
| GO:0007223\_Wnt\_receptor\_signaling\_pathway\_\_calcium\_modulating\_pathway | 1 | 0 |  |  |  |  |  |  |  |  |
| GO:0007225\_patched\_ligand\_processing | 1 | 0 |  |  |  |  |  |  |  |  |
| GO:0007227\_signal\_transduction\_downstream\_of\_smoothened | 1 | 0 |  |  |  |  |  |  |  |  |
| GO:0007228\_positive\_regulation\_of\_hh\_target\_transcription\_factor\_activity | 1 | 0 |  |  |  |  |  |  |  |  |
| GO:0007231\_osmosensory\_signaling\_pathway | 1 | 0 |  |  |  |  |  |  |  |  |
| GO:0007284\_spermatogonial\_cell\_division | 1 | 0 |  |  |  |  |  |  |  |  |
| GO:0007290\_spermatid\_nucleus\_elongation | 1 | 0 |  |  |  |  |  |  |  |  |
| GO:0007296\_vitellogenesis | 1 | 0 |  |  |  |  |  |  |  |  |
| GO:0007321\_sperm\_displacement | 1 | 0 |  |  |  |  |  |  |  |  |
| GO:0007380\_specification\_of\_segmental\_identity\_\_head | 1 | 0 |  |  |  |  |  |  |  |  |
| GO:0007382\_specification\_of\_segmental\_identity\_\_maxillary\_segment | 1 | 0 |  |  |  |  |  |  |  |  |
| GO:0007400\_neuroblast\_fate\_determination | 1 | 0 |  |  |  |  |  |  |  |  |
| GO:0007402\_ganglion\_mother\_cell\_fate\_determination | 1 | 0 |  |  |  |  |  |  |  |  |
| GO:0007495\_visceral\_mesoderm-endoderm\_interaction\_involved\_in\_midgut\_development | 1 | 0 |  |  |  |  |  |  |  |  |
| GO:0007497\_posterior\_midgut\_development | 1 | 0 |  |  |  |  |  |  |  |  |
| GO:0007499\_ectoderm\_and\_mesoderm\_interaction | 1 | 0 |  |  |  |  |  |  |  |  |
| GO:0007500\_mesodermal\_cell\_fate\_determination | 1 | 0 |  |  |  |  |  |  |  |  |
| GO:0007509\_mesoderm\_migration | 1 | 0 |  |  |  |  |  |  |  |  |
| GO:0007518\_myoblast\_cell\_fate\_determination | 1 | 0 |  |  |  |  |  |  |  |  |
| GO:0007521\_muscle\_cell\_fate\_determination | 1 | 0 |  |  |  |  |  |  |  |  |
| GO:0007522\_visceral\_muscle\_development | 1 | 0 |  |  |  |  |  |  |  |  |
| GO:0007529\_establishment\_of\_synaptic\_specificity\_at\_neuromuscular\_junction | 1 | 0 |  |  |  |  |  |  |  |  |
| GO:0007538\_primary\_sex\_determination | 1 | 0 |  |  |  |  |  |  |  |  |
| GO:0007542\_primary\_sex\_determination\_\_germ-line | 1 | 0 |  |  |  |  |  |  |  |  |
| GO:0007567\_parturition | 1 | 0 |  |  |  |  |  |  |  |  |
| GO:0007614\_short-term\_memory | 1 | 0 |  |  |  |  |  |  |  |  |
| GO:0007621\_negative\_regulation\_of\_female\_receptivity | 1 | 0 |  |  |  |  |  |  |  |  |
| GO:0008049\_male\_courtship\_behavior | 1 | 0 |  |  |  |  |  |  |  |  |
| GO:0008050\_female\_courtship\_behavior | 1 | 0 |  |  |  |  |  |  |  |  |
| GO:0008052\_sensory\_organ\_boundary\_specification | 1 | 0 |  |  |  |  |  |  |  |  |
| GO:0008054\_cyclin\_catabolic\_process | 1 | 0 |  |  |  |  |  |  |  |  |
| GO:0008057\_eye\_pigment\_granule\_organization | 1 | 0 |  |  |  |  |  |  |  |  |
| GO:0008078\_mesodermal\_cell\_migration | 1 | 0 |  |  |  |  |  |  |  |  |
| GO:0008208\_C21-steroid\_hormone\_catabolic\_process | 1 | 0 |  |  |  |  |  |  |  |  |
| GO:0008216\_spermidine\_metabolic\_process | 1 | 0 |  |  |  |  |  |  |  |  |
| GO:0008292\_acetylcholine\_biosynthetic\_process | 1 | 0 |  |  |  |  |  |  |  |  |
| GO:0008295\_spermidine\_biosynthetic\_process | 1 | 0 |  |  |  |  |  |  |  |  |
| GO:0008300\_isoprenoid\_catabolic\_process | 1 | 0 |  |  |  |  |  |  |  |  |
| GO:0008333\_endosome\_to\_lysosome\_transport | 1 | 0 |  |  |  |  |  |  |  |  |
| GO:0008355\_olfactory\_learning | 1 | 0 |  |  |  |  |  |  |  |  |
| GO:0008611\_ether\_lipid\_biosynthetic\_process | 1 | 0 |  |  |  |  |  |  |  |  |
| GO:0008626\_induction\_of\_apoptosis\_by\_granzyme | 1 | 0 |  |  |  |  |  |  |  |  |
| GO:0008633\_activation\_of\_pro-apoptotic\_gene\_products | 1 | 0 |  |  |  |  |  |  |  |  |
| GO:0008653\_lipopolysaccharide\_metabolic\_process | 1 | 0 |  |  |  |  |  |  |  |  |
| GO:0009068\_aspartate\_family\_amino\_acid\_catabolic\_process | 1 | 0 |  |  |  |  |  |  |  |  |
| GO:0009084\_glutamine\_family\_amino\_acid\_biosynthetic\_process | 1 | 0 |  |  |  |  |  |  |  |  |
| GO:0009088\_threonine\_biosynthetic\_process | 1 | 0 |  |  |  |  |  |  |  |  |
| GO:0009105\_lipoic\_acid\_biosynthetic\_process | 1 | 0 |  |  |  |  |  |  |  |  |
| GO:0009109\_coenzyme\_catabolic\_process | 1 | 0 |  |  |  |  |  |  |  |  |
| GO:0009111\_vitamin\_catabolic\_process | 1 | 0 |  |  |  |  |  |  |  |  |
| GO:0009113\_purine\_base\_biosynthetic\_process | 1 | 0 |  |  |  |  |  |  |  |  |
| GO:0009127\_purine\_nucleoside\_monophosphate\_biosynthetic\_process | 1 | 0 |  |  |  |  |  |  |  |  |
| GO:0009128\_purine\_nucleoside\_monophosphate\_catabolic\_process | 1 | 0 |  |  |  |  |  |  |  |  |
| GO:0009129\_pyrimidine\_nucleoside\_monophosphate\_metabolic\_process | 1 | 0 |  |  |  |  |  |  |  |  |
| GO:0009131\_pyrimidine\_nucleoside\_monophosphate\_catabolic\_process | 1 | 0 |  |  |  |  |  |  |  |  |
| GO:0009133\_nucleoside\_diphosphate\_biosynthetic\_process | 1 | 0 |  |  |  |  |  |  |  |  |
| GO:0009145\_purine\_nucleoside\_triphosphate\_biosynthetic\_process | 1 | 0 |  |  |  |  |  |  |  |  |
| GO:0009147\_pyrimidine\_nucleoside\_triphosphate\_metabolic\_process | 1 | 0 |  |  |  |  |  |  |  |  |
| GO:0009148\_pyrimidine\_nucleoside\_triphosphate\_biosynthetic\_process | 1 | 0 |  |  |  |  |  |  |  |  |
| GO:0009152\_purine\_ribonucleotide\_biosynthetic\_process | 1 | 0 |  |  |  |  |  |  |  |  |
| GO:0009153\_purine\_deoxyribonucleotide\_biosynthetic\_process | 1 | 0 |  |  |  |  |  |  |  |  |
| GO:0009156\_ribonucleoside\_monophosphate\_biosynthetic\_process | 1 | 0 |  |  |  |  |  |  |  |  |
| GO:0009158\_ribonucleoside\_monophosphate\_catabolic\_process | 1 | 0 |  |  |  |  |  |  |  |  |
| GO:0009159\_deoxyribonucleoside\_monophosphate\_catabolic\_process | 1 | 0 |  |  |  |  |  |  |  |  |
| GO:0009162\_deoxyribonucleoside\_monophosphate\_metabolic\_process | 1 | 0 |  |  |  |  |  |  |  |  |
| GO:0009168\_purine\_ribonucleoside\_monophosphate\_biosynthetic\_process | 1 | 0 |  |  |  |  |  |  |  |  |
| GO:0009169\_purine\_ribonucleoside\_monophosphate\_catabolic\_process | 1 | 0 |  |  |  |  |  |  |  |  |
| GO:0009176\_pyrimidine\_deoxyribonucleoside\_monophosphate\_metabolic\_process | 1 | 0 |  |  |  |  |  |  |  |  |
| GO:0009178\_pyrimidine\_deoxyribonucleoside\_monophosphate\_catabolic\_process | 1 | 0 |  |  |  |  |  |  |  |  |
| GO:0009211\_pyrimidine\_deoxyribonucleoside\_triphosphate\_metabolic\_process | 1 | 0 |  |  |  |  |  |  |  |  |
| GO:0009212\_pyrimidine\_deoxyribonucleoside\_triphosphate\_biosynthetic\_process | 1 | 0 |  |  |  |  |  |  |  |  |
| GO:0009216\_purine\_deoxyribonucleoside\_triphosphate\_biosynthetic\_process | 1 | 0 |  |  |  |  |  |  |  |  |
| GO:0009221\_pyrimidine\_deoxyribonucleotide\_biosynthetic\_process | 1 | 0 |  |  |  |  |  |  |  |  |
| GO:0009223\_pyrimidine\_deoxyribonucleotide\_catabolic\_process | 1 | 0 |  |  |  |  |  |  |  |  |
| GO:0009260\_ribonucleotide\_biosynthetic\_process | 1 | 0 |  |  |  |  |  |  |  |  |
| GO:0009405\_pathogenesis | 1 | 0 |  |  |  |  |  |  |  |  |
| GO:0009414\_response\_to\_water\_deprivation | 1 | 0 |  |  |  |  |  |  |  |  |
| GO:0009415\_response\_to\_water | 1 | 0 |  |  |  |  |  |  |  |  |
| GO:0009449\_gamma-aminobutyric\_acid\_biosynthetic\_process | 1 | 0 |  |  |  |  |  |  |  |  |
| GO:0009450\_gamma-aminobutyric\_acid\_catabolic\_process | 1 | 0 |  |  |  |  |  |  |  |  |
| GO:0009589\_detection\_of\_UV | 1 | 0 |  |  |  |  |  |  |  |  |
| GO:0009590\_detection\_of\_gravity | 1 | 0 |  |  |  |  |  |  |  |  |
| GO:0009624\_response\_to\_nematode | 1 | 0 |  |  |  |  |  |  |  |  |
| GO:0009629\_response\_to\_gravity | 1 | 0 |  |  |  |  |  |  |  |  |
| GO:0009648\_photoperiodism | 1 | 0 |  |  |  |  |  |  |  |  |
| GO:0009690\_cytokinin\_metabolic\_process | 1 | 0 |  |  |  |  |  |  |  |  |
| GO:0009691\_cytokinin\_biosynthetic\_process | 1 | 0 |  |  |  |  |  |  |  |  |
| GO:0009786\_regulation\_of\_asymmetric\_cell\_division | 1 | 0 |  |  |  |  |  |  |  |  |
| GO:0009794\_regulation\_of\_mitotic\_cell\_cycle\_\_embryonic | 1 | 0 |  |  |  |  |  |  |  |  |
| GO:0009956\_radial\_pattern\_formation | 1 | 0 |  |  |  |  |  |  |  |  |
| GO:0009957\_epidermal\_cell\_fate\_specification | 1 | 0 |  |  |  |  |  |  |  |  |
| GO:0009992\_cellular\_water\_homeostasis | 1 | 0 |  |  |  |  |  |  |  |  |
| GO:0010032\_meiotic\_chromosome\_condensation | 1 | 0 |  |  |  |  |  |  |  |  |
| GO:0010039\_response\_to\_iron\_ion | 1 | 0 |  |  |  |  |  |  |  |  |
| GO:0010042\_response\_to\_manganese\_ion | 1 | 0 |  |  |  |  |  |  |  |  |
| GO:0010045\_response\_to\_nickel\_ion | 1 | 0 |  |  |  |  |  |  |  |  |
| GO:0010046\_response\_to\_mycotoxin | 1 | 0 |  |  |  |  |  |  |  |  |
| GO:0010107\_potassium\_ion\_import | 1 | 0 |  |  |  |  |  |  |  |  |
| GO:0010155\_regulation\_of\_proton\_transport | 1 | 0 |  |  |  |  |  |  |  |  |
| GO:0010160\_formation\_of\_organ\_boundary | 1 | 0 |  |  |  |  |  |  |  |  |
| GO:0010260\_organ\_senescence | 1 | 0 |  |  |  |  |  |  |  |  |
| GO:0010310\_regulation\_of\_hydrogen\_peroxide\_metabolic\_process | 1 | 0 |  |  |  |  |  |  |  |  |
| GO:0010447\_response\_to\_acidity | 1 | 0 |  |  |  |  |  |  |  |  |
| GO:0010452\_histone\_H3-K36\_methylation | 1 | 0 |  |  |  |  |  |  |  |  |
| GO:0010455\_positive\_regulation\_of\_cell\_fate\_commitment | 1 | 0 |  |  |  |  |  |  |  |  |
| GO:0010470\_regulation\_of\_gastrulation | 1 | 0 |  |  |  |  |  |  |  |  |
| GO:0010508\_positive\_regulation\_of\_autophagy | 1 | 0 |  |  |  |  |  |  |  |  |
| GO:0010519\_negative\_regulation\_of\_phospholipase\_activity | 1 | 0 |  |  |  |  |  |  |  |  |
| GO:0010520\_regulation\_of\_reciprocal\_meiotic\_recombination | 1 | 0 |  |  |  |  |  |  |  |  |
| GO:0010523\_negative\_regulation\_of\_calcium\_ion\_transport\_into\_cytosol | 1 | 0 |  |  |  |  |  |  |  |  |
| GO:0010543\_regulation\_of\_platelet\_activation | 1 | 0 |  |  |  |  |  |  |  |  |
| GO:0010561\_negative\_regulation\_of\_glycoprotein\_biosynthetic\_process | 1 | 0 |  |  |  |  |  |  |  |  |
| GO:0010569\_regulation\_of\_double-strand\_break\_repair\_via\_homologous\_recombination | 1 | 0 |  |  |  |  |  |  |  |  |
| GO:0010572\_positive\_regulation\_of\_platelet\_activation | 1 | 0 |  |  |  |  |  |  |  |  |
| GO:0010594\_regulation\_of\_endothelial\_cell\_migration | 1 | 0 |  |  |  |  |  |  |  |  |
| GO:0010596\_negative\_regulation\_of\_endothelial\_cell\_migration | 1 | 0 |  |  |  |  |  |  |  |  |
| GO:0010611\_regulation\_of\_cardiac\_muscle\_hypertrophy | 1 | 0 |  |  |  |  |  |  |  |  |
| GO:0010612\_regulation\_of\_cardiac\_muscle\_adaptation | 1 | 0 |  |  |  |  |  |  |  |  |
| GO:0010614\_negative\_regulation\_of\_cardiac\_muscle\_hypertrophy | 1 | 0 |  |  |  |  |  |  |  |  |
| GO:0010616\_negative\_regulation\_of\_cardiac\_muscle\_adaptation | 1 | 0 |  |  |  |  |  |  |  |  |
| GO:0010634\_positive\_regulation\_of\_epithelial\_cell\_migration | 1 | 0 |  |  |  |  |  |  |  |  |
| GO:0010656\_negative\_regulation\_of\_muscle\_cell\_apoptosis | 1 | 0 |  |  |  |  |  |  |  |  |
| GO:0010657\_muscle\_cell\_apoptosis | 1 | 0 |  |  |  |  |  |  |  |  |
| GO:0010658\_striated\_muscle\_cell\_apoptosis | 1 | 0 |  |  |  |  |  |  |  |  |
| GO:0010659\_cardiac\_muscle\_cell\_apoptosis | 1 | 0 |  |  |  |  |  |  |  |  |
| GO:0010660\_regulation\_of\_muscle\_cell\_apoptosis | 1 | 0 |  |  |  |  |  |  |  |  |
| GO:0010662\_regulation\_of\_striated\_muscle\_cell\_apoptosis | 1 | 0 |  |  |  |  |  |  |  |  |
| GO:0010664\_negative\_regulation\_of\_striated\_muscle\_cell\_apoptosis | 1 | 0 |  |  |  |  |  |  |  |  |
| GO:0010665\_regulation\_of\_cardiac\_muscle\_cell\_apoptosis | 1 | 0 |  |  |  |  |  |  |  |  |
| GO:0010667\_negative\_regulation\_of\_cardiac\_muscle\_cell\_apoptosis | 1 | 0 |  |  |  |  |  |  |  |  |
| GO:0010668\_ectodermal\_cell\_differentiation | 1 | 0 |  |  |  |  |  |  |  |  |
| GO:0010671\_negative\_regulation\_of\_oxygen\_and\_reactive\_oxygen\_species\_metabolic\_process | 1 | 0 |  |  |  |  |  |  |  |  |
| GO:0010719\_negative\_regulation\_of\_epithelial\_to\_mesenchymal\_transition | 1 | 0 |  |  |  |  |  |  |  |  |
| GO:0010735\_positive\_regulation\_of\_transcription\_via\_serum\_response\_element\_binding | 1 | 0 |  |  |  |  |  |  |  |  |
| GO:0010825\_positive\_regulation\_of\_centrosome\_duplication | 1 | 0 |  |  |  |  |  |  |  |  |
| GO:0010845\_positive\_regulation\_of\_reciprocal\_meiotic\_recombination | 1 | 0 |  |  |  |  |  |  |  |  |
| GO:0010850\_chemoreceptor\_signaling\_pathway\_involved\_in\_regulation\_of\_blood\_pressure | 1 | 0 |  |  |  |  |  |  |  |  |
| GO:0010873\_positive\_regulation\_of\_cholesterol\_esterification | 1 | 0 |  |  |  |  |  |  |  |  |
| GO:0010880\_regulation\_of\_release\_of\_sequestered\_calcium\_ion\_into\_cytosol\_by\_sarcoplasmic\_reticulum | 1 | 0 |  |  |  |  |  |  |  |  |
| GO:0010881\_regulation\_of\_cardiac\_muscle\_contraction\_by\_regulation\_of\_the\_release\_of\_sequestered\_calcium\_ion | 1 | 0 |  |  |  |  |  |  |  |  |
| GO:0010882\_regulation\_of\_cardiac\_muscle\_contraction\_by\_calcium\_ion\_signaling | 1 | 0 |  |  |  |  |  |  |  |  |
| GO:0010890\_positive\_regulation\_of\_sequestering\_of\_triglyceride | 1 | 0 |  |  |  |  |  |  |  |  |
| GO:0010919\_regulation\_of\_inositol\_phosphate\_biosynthetic\_process | 1 | 0 |  |  |  |  |  |  |  |  |
| GO:0010931\_macrophage\_tolerance\_induction | 1 | 0 |  |  |  |  |  |  |  |  |
| GO:0010932\_regulation\_of\_macrophage\_tolerance\_induction | 1 | 0 |  |  |  |  |  |  |  |  |
| GO:0010933\_positive\_regulation\_of\_macrophage\_tolerance\_induction | 1 | 0 |  |  |  |  |  |  |  |  |
| GO:0010934\_macrophage\_cytokine\_production | 1 | 0 |  |  |  |  |  |  |  |  |
| GO:0010935\_regulation\_of\_macrophage\_cytokine\_production | 1 | 0 |  |  |  |  |  |  |  |  |
| GO:0010936\_negative\_regulation\_of\_macrophage\_cytokine\_production | 1 | 0 |  |  |  |  |  |  |  |  |
| GO:0010953\_regulation\_of\_protein\_maturation\_by\_peptide\_bond\_cleavage | 1 | 0 |  |  |  |  |  |  |  |  |
| GO:0010962\_regulation\_of\_glucan\_biosynthetic\_process | 1 | 0 |  |  |  |  |  |  |  |  |
| GO:0010966\_regulation\_of\_phosphate\_transport | 1 | 0 |  |  |  |  |  |  |  |  |
| GO:0014012\_axon\_regeneration\_in\_the\_peripheral\_nervous\_system | 1 | 0 |  |  |  |  |  |  |  |  |
| GO:0014016\_neuroblast\_differentiation | 1 | 0 |  |  |  |  |  |  |  |  |
| GO:0014017\_neuroblast\_fate\_commitment | 1 | 0 |  |  |  |  |  |  |  |  |
| GO:0014041\_regulation\_of\_neuron\_maturation | 1 | 0 |  |  |  |  |  |  |  |  |
| GO:0014042\_positive\_regulation\_of\_neuron\_maturation | 1 | 0 |  |  |  |  |  |  |  |  |
| GO:0014049\_positive\_regulation\_of\_glutamate\_secretion | 1 | 0 |  |  |  |  |  |  |  |  |
| GO:0014061\_regulation\_of\_norepinephrine\_secretion | 1 | 0 |  |  |  |  |  |  |  |  |
| GO:0014071\_response\_to\_cycloalkane | 1 | 0 |  |  |  |  |  |  |  |  |
| GO:0014707\_branchiomeric\_skeletal\_muscle\_development | 1 | 0 |  |  |  |  |  |  |  |  |
| GO:0014738\_regulation\_of\_muscle\_hyperplasia | 1 | 0 |  |  |  |  |  |  |  |  |
| GO:0014740\_negative\_regulation\_of\_muscle\_hyperplasia | 1 | 0 |  |  |  |  |  |  |  |  |
| GO:0014741\_negative\_regulation\_of\_muscle\_hypertrophy | 1 | 0 |  |  |  |  |  |  |  |  |
| GO:0014743\_regulation\_of\_muscle\_hypertrophy | 1 | 0 |  |  |  |  |  |  |  |  |
| GO:0014805\_smooth\_muscle\_adaptation | 1 | 0 |  |  |  |  |  |  |  |  |
| GO:0014806\_smooth\_muscle\_hyperplasia | 1 | 0 |  |  |  |  |  |  |  |  |
| GO:0014807\_regulation\_of\_somitogenesis | 1 | 0 |  |  |  |  |  |  |  |  |
| GO:0014808\_release\_of\_sequestered\_calcium\_ion\_into\_cytosol\_by\_sarcoplasmic\_reticulum | 1 | 0 |  |  |  |  |  |  |  |  |
| GO:0014813\_satellite\_cell\_commitment | 1 | 0 |  |  |  |  |  |  |  |  |
| GO:0014816\_satellite\_cell\_differentiation | 1 | 0 |  |  |  |  |  |  |  |  |
| GO:0014819\_regulation\_of\_skeletal\_muscle\_contraction | 1 | 0 |  |  |  |  |  |  |  |  |
| GO:0014852\_regulation\_of\_skeletal\_muscle\_contraction\_by\_neural\_stimulation\_via\_neuromuscular\_junction | 1 | 0 |  |  |  |  |  |  |  |  |
| GO:0014853\_regulation\_of\_excitatory\_postsynaptic\_membrane\_potential\_involved\_in\_skeletal\_muscle\_contraction | 1 | 0 |  |  |  |  |  |  |  |  |
| GO:0014856\_skeletal\_muscle\_cell\_proliferation | 1 | 0 |  |  |  |  |  |  |  |  |
| GO:0014857\_regulation\_of\_skeletal\_muscle\_cell\_proliferation | 1 | 0 |  |  |  |  |  |  |  |  |
| GO:0014858\_positive\_regulation\_of\_skeletal\_muscle\_cell\_proliferation | 1 | 0 |  |  |  |  |  |  |  |  |
| GO:0014887\_cardiac\_muscle\_adaptation | 1 | 0 |  |  |  |  |  |  |  |  |
| GO:0014889\_muscle\_atrophy | 1 | 0 |  |  |  |  |  |  |  |  |
| GO:0014896\_muscle\_hypertrophy | 1 | 0 |  |  |  |  |  |  |  |  |
| GO:0014897\_striated\_muscle\_hypertrophy | 1 | 0 |  |  |  |  |  |  |  |  |
| GO:0014898\_cardiac\_muscle\_hypertrophy | 1 | 0 |  |  |  |  |  |  |  |  |
| GO:0014900\_muscle\_hyperplasia | 1 | 0 |  |  |  |  |  |  |  |  |
| GO:0014910\_regulation\_of\_smooth\_muscle\_cell\_migration | 1 | 0 |  |  |  |  |  |  |  |  |
| GO:0014911\_positive\_regulation\_of\_smooth\_muscle\_cell\_migration | 1 | 0 |  |  |  |  |  |  |  |  |
| GO:0015014\_heparan\_sulfate\_proteoglycan\_biosynthetic\_process\_\_polysaccharide\_chain\_biosynthetic\_process | 1 | 0 |  |  |  |  |  |  |  |  |
| GO:0015074\_DNA\_integration | 1 | 0 |  |  |  |  |  |  |  |  |
| GO:0015670\_carbon\_dioxide\_transport | 1 | 0 |  |  |  |  |  |  |  |  |
| GO:0015677\_copper\_ion\_import | 1 | 0 |  |  |  |  |  |  |  |  |
| GO:0015680\_intracellular\_copper\_ion\_transport | 1 | 0 |  |  |  |  |  |  |  |  |
| GO:0015684\_ferrous\_iron\_transport | 1 | 0 |  |  |  |  |  |  |  |  |
| GO:0015707\_nitrite\_transport | 1 | 0 |  |  |  |  |  |  |  |  |
| GO:0015724\_formate\_transport | 1 | 0 |  |  |  |  |  |  |  |  |
| GO:0015734\_taurine\_transport | 1 | 0 |  |  |  |  |  |  |  |  |
| GO:0015740\_C4-dicarboxylate\_transport | 1 | 0 |  |  |  |  |  |  |  |  |
| GO:0015744\_succinate\_transport | 1 | 0 |  |  |  |  |  |  |  |  |
| GO:0015746\_citrate\_transport | 1 | 0 |  |  |  |  |  |  |  |  |
| GO:0015747\_urate\_transport | 1 | 0 |  |  |  |  |  |  |  |  |
| GO:0015791\_polyol\_transport | 1 | 0 |  |  |  |  |  |  |  |  |
| GO:0015798\_myo-inositol\_transport | 1 | 0 |  |  |  |  |  |  |  |  |
| GO:0015808\_L-alanine\_transport | 1 | 0 |  |  |  |  |  |  |  |  |
| GO:0015810\_aspartate\_transport | 1 | 0 |  |  |  |  |  |  |  |  |
| GO:0015811\_L-cystine\_transport | 1 | 0 |  |  |  |  |  |  |  |  |
| GO:0015817\_histidine\_transport | 1 | 0 |  |  |  |  |  |  |  |  |
| GO:0015822\_ornithine\_transport | 1 | 0 |  |  |  |  |  |  |  |  |
| GO:0015824\_proline\_transport | 1 | 0 |  |  |  |  |  |  |  |  |
| GO:0015851\_nucleobase\_transport | 1 | 0 |  |  |  |  |  |  |  |  |
| GO:0015864\_pyrimidine\_nucleoside\_transport | 1 | 0 |  |  |  |  |  |  |  |  |
| GO:0015874\_norepinephrine\_transport | 1 | 0 |  |  |  |  |  |  |  |  |
| GO:0015881\_creatine\_transport | 1 | 0 |  |  |  |  |  |  |  |  |
| GO:0015884\_folic\_acid\_transport | 1 | 0 |  |  |  |  |  |  |  |  |
| GO:0015886\_heme\_transport | 1 | 0 |  |  |  |  |  |  |  |  |
| GO:0015888\_thiamin\_transport | 1 | 0 |  |  |  |  |  |  |  |  |
| GO:0015938\_coenzyme\_A\_catabolic\_process | 1 | 0 |  |  |  |  |  |  |  |  |
| GO:0015939\_pantothenate\_metabolic\_process | 1 | 0 |  |  |  |  |  |  |  |  |
| GO:0016073\_snRNA\_metabolic\_process | 1 | 0 |  |  |  |  |  |  |  |  |
| GO:0016074\_snoRNA\_metabolic\_process | 1 | 0 |  |  |  |  |  |  |  |  |
| GO:0016082\_synaptic\_vesicle\_priming | 1 | 0 |  |  |  |  |  |  |  |  |
| GO:0016090\_prenol\_metabolic\_process | 1 | 0 |  |  |  |  |  |  |  |  |
| GO:0016093\_polyprenol\_metabolic\_process | 1 | 0 |  |  |  |  |  |  |  |  |
| GO:0016180\_snRNA\_processing | 1 | 0 |  |  |  |  |  |  |  |  |
| GO:0016239\_positive\_regulation\_of\_macroautophagy | 1 | 0 |  |  |  |  |  |  |  |  |
| GO:0016246\_RNA\_interference | 1 | 0 |  |  |  |  |  |  |  |  |
| GO:0016255\_attachment\_of\_GPI\_anchor\_to\_protein | 1 | 0 |  |  |  |  |  |  |  |  |
| GO:0016333\_morphogenesis\_of\_follicular\_epithelium | 1 | 0 |  |  |  |  |  |  |  |  |
| GO:0016340\_calcium-dependent\_cell-matrix\_adhesion | 1 | 0 |  |  |  |  |  |  |  |  |
| GO:0016344\_meiotic\_chromosome\_movement\_towards\_spindle\_pole | 1 | 0 |  |  |  |  |  |  |  |  |
| GO:0016482\_cytoplasmic\_transport | 1 | 0 |  |  |  |  |  |  |  |  |
| GO:0016553\_base\_conversion\_or\_substitution\_editing | 1 | 0 |  |  |  |  |  |  |  |  |
| GO:0016554\_cytidine\_to\_uridine\_editing | 1 | 0 |  |  |  |  |  |  |  |  |
| GO:0016560\_protein\_import\_into\_peroxisome\_matrix\_\_docking | 1 | 0 |  |  |  |  |  |  |  |  |
| GO:0016578\_histone\_deubiquitination | 1 | 0 |  |  |  |  |  |  |  |  |
| GO:0016598\_protein\_arginylation | 1 | 0 |  |  |  |  |  |  |  |  |
| GO:0017004\_cytochrome\_complex\_assembly | 1 | 0 |  |  |  |  |  |  |  |  |
| GO:0018022\_peptidyl-lysine\_methylation | 1 | 0 |  |  |  |  |  |  |  |  |
| GO:0018023\_peptidyl-lysine\_trimethylation | 1 | 0 |  |  |  |  |  |  |  |  |
| GO:0018120\_peptidyl-arginine\_ADP-ribosylation | 1 | 0 |  |  |  |  |  |  |  |  |
| GO:0018126\_protein\_amino\_acid\_hydroxylation | 1 | 0 |  |  |  |  |  |  |  |  |
| GO:0018146\_keratan\_sulfate\_biosynthetic\_process | 1 | 0 |  |  |  |  |  |  |  |  |
| GO:0018158\_protein\_amino\_acid\_oxidation | 1 | 0 |  |  |  |  |  |  |  |  |
| GO:0018195\_peptidyl-arginine\_modification | 1 | 0 |  |  |  |  |  |  |  |  |
| GO:0018197\_peptidyl-aspartic\_acid\_modification | 1 | 0 |  |  |  |  |  |  |  |  |
| GO:0018282\_metal\_incorporation\_into\_metallo-sulfur\_cluster | 1 | 0 |  |  |  |  |  |  |  |  |
| GO:0018283\_iron\_incorporation\_into\_metallo-sulfur\_cluster | 1 | 0 |  |  |  |  |  |  |  |  |
| GO:0018318\_protein\_amino\_acid\_palmitoylation | 1 | 0 |  |  |  |  |  |  |  |  |
| GO:0018342\_protein\_prenylation | 1 | 0 |  |  |  |  |  |  |  |  |
| GO:0018344\_protein\_geranylgeranylation | 1 | 0 |  |  |  |  |  |  |  |  |
| GO:0018410\_peptide\_or\_protein\_carboxyl-terminal\_blocking | 1 | 0 |  |  |  |  |  |  |  |  |
| GO:0018916\_nitrobenzene\_metabolic\_process | 1 | 0 |  |  |  |  |  |  |  |  |
| GO:0018931\_naphthalene\_metabolic\_process | 1 | 0 |  |  |  |  |  |  |  |  |
| GO:0018992\_germ-line\_sex\_determination | 1 | 0 |  |  |  |  |  |  |  |  |
| GO:0019042\_latent\_virus\_infection | 1 | 0 |  |  |  |  |  |  |  |  |
| GO:0019046\_reactivation\_of\_latent\_virus | 1 | 0 |  |  |  |  |  |  |  |  |
| GO:0019047\_provirus\_integration | 1 | 0 |  |  |  |  |  |  |  |  |
| GO:0019076\_release\_of\_virus\_from\_host | 1 | 0 |  |  |  |  |  |  |  |  |
| GO:0019079\_viral\_genome\_replication | 1 | 0 |  |  |  |  |  |  |  |  |
| GO:0019100\_male\_germ-line\_sex\_determination | 1 | 0 |  |  |  |  |  |  |  |  |
| GO:0019101\_female\_somatic\_sex\_determination | 1 | 0 |  |  |  |  |  |  |  |  |
| GO:0019102\_male\_somatic\_sex\_determination | 1 | 0 |  |  |  |  |  |  |  |  |
| GO:0019255\_glucose\_1-phosphate\_metabolic\_process | 1 | 0 |  |  |  |  |  |  |  |  |
| GO:0019276\_UDP-N-acetylgalactosamine\_metabolic\_process | 1 | 0 |  |  |  |  |  |  |  |  |
| GO:0019344\_cysteine\_biosynthetic\_process | 1 | 0 |  |  |  |  |  |  |  |  |
| GO:0019348\_dolichol\_metabolic\_process | 1 | 0 |  |  |  |  |  |  |  |  |
| GO:0019375\_galactolipid\_biosynthetic\_process | 1 | 0 |  |  |  |  |  |  |  |  |
| GO:0019402\_galactitol\_metabolic\_process | 1 | 0 |  |  |  |  |  |  |  |  |
| GO:0019441\_tryptophan\_catabolic\_process\_to\_kynurenine | 1 | 0 |  |  |  |  |  |  |  |  |
| GO:0019477\_L-lysine\_catabolic\_process | 1 | 0 |  |  |  |  |  |  |  |  |
| GO:0019532\_oxalate\_transport | 1 | 0 |  |  |  |  |  |  |  |  |
| GO:0019626\_short-chain\_fatty\_acid\_catabolic\_process | 1 | 0 |  |  |  |  |  |  |  |  |
| GO:0019627\_urea\_metabolic\_process | 1 | 0 |  |  |  |  |  |  |  |  |
| GO:0019676\_ammonia\_assimilation\_cycle | 1 | 0 |  |  |  |  |  |  |  |  |
| GO:0019682\_glyceraldehyde-3-phosphate\_metabolic\_process | 1 | 0 |  |  |  |  |  |  |  |  |
| GO:0019695\_choline\_metabolic\_process | 1 | 0 |  |  |  |  |  |  |  |  |
| GO:0019731\_antibacterial\_humoral\_response | 1 | 0 |  |  |  |  |  |  |  |  |
| GO:0019794\_nonprotein\_amino\_acid\_metabolic\_process | 1 | 0 |  |  |  |  |  |  |  |  |
| GO:0019858\_cytosine\_metabolic\_process | 1 | 0 |  |  |  |  |  |  |  |  |
| GO:0019883\_antigen\_processing\_and\_presentation\_of\_endogenous\_antigen | 1 | 0 |  |  |  |  |  |  |  |  |
| GO:0019889\_pteridine\_metabolic\_process | 1 | 0 |  |  |  |  |  |  |  |  |
| GO:0019896\_axon\_transport\_of\_mitochondrion | 1 | 0 |  |  |  |  |  |  |  |  |
| GO:0021508\_floor\_plate\_formation | 1 | 0 |  |  |  |  |  |  |  |  |
| GO:0021528\_commissural\_neuron\_differentiation\_in\_the\_spinal\_cord | 1 | 0 |  |  |  |  |  |  |  |  |
| GO:0021572\_rhombomere\_6\_development | 1 | 0 |  |  |  |  |  |  |  |  |
| GO:0021577\_hindbrain\_structural\_organization | 1 | 0 |  |  |  |  |  |  |  |  |
| GO:0021586\_pons\_maturation | 1 | 0 |  |  |  |  |  |  |  |  |
| GO:0021589\_cerebellum\_structural\_organization | 1 | 0 |  |  |  |  |  |  |  |  |
| GO:0021590\_cerebellum\_maturation | 1 | 0 |  |  |  |  |  |  |  |  |
| GO:0021592\_fourth\_ventricle\_development | 1 | 0 |  |  |  |  |  |  |  |  |
| GO:0021594\_rhombomere\_formation | 1 | 0 |  |  |  |  |  |  |  |  |
| GO:0021660\_rhombomere\_3\_formation | 1 | 0 |  |  |  |  |  |  |  |  |
| GO:0021664\_rhombomere\_5\_morphogenesis | 1 | 0 |  |  |  |  |  |  |  |  |
| GO:0021666\_rhombomere\_5\_formation | 1 | 0 |  |  |  |  |  |  |  |  |
| GO:0021670\_lateral\_ventricle\_development | 1 | 0 |  |  |  |  |  |  |  |  |
| GO:0021678\_third\_ventricle\_development | 1 | 0 |  |  |  |  |  |  |  |  |
| GO:0021679\_cerebellar\_molecular\_layer\_development | 1 | 0 |  |  |  |  |  |  |  |  |
| GO:0021703\_locus\_ceruleus\_development | 1 | 0 |  |  |  |  |  |  |  |  |
| GO:0021732\_midbrain-hindbrain\_boundary\_maturation | 1 | 0 |  |  |  |  |  |  |  |  |
| GO:0021747\_cochlear\_nucleus\_development | 1 | 0 |  |  |  |  |  |  |  |  |
| GO:0021750\_vestibular\_nucleus\_development | 1 | 0 |  |  |  |  |  |  |  |  |
| GO:0021759\_globus\_pallidus\_development | 1 | 0 |  |  |  |  |  |  |  |  |
| GO:0021768\_nucleus\_accumbens\_development | 1 | 0 |  |  |  |  |  |  |  |  |
| GO:0021771\_lateral\_geniculate\_nucleus\_development | 1 | 0 |  |  |  |  |  |  |  |  |
| GO:0021812\_neuronal-glial\_interaction\_involved\_in\_cerebral\_cortex\_radial\_glia\_guided\_migration | 1 | 0 |  |  |  |  |  |  |  |  |
| GO:0021813\_cell-cell\_adhesion\_involved\_in\_neuronal-glial\_interactions\_involved\_in\_cerebral\_cortex\_radial\_glia\_guided\_migration | 1 | 0 |  |  |  |  |  |  |  |  |
| GO:0021870\_Cajal-Retzius\_cell\_differentiation | 1 | 0 |  |  |  |  |  |  |  |  |
| GO:0021874\_Wnt\_receptor\_signaling\_pathway\_in\_forebrain\_neuroblast\_division | 1 | 0 |  |  |  |  |  |  |  |  |
| GO:0021896\_forebrain\_astrocyte\_differentiation | 1 | 0 |  |  |  |  |  |  |  |  |
| GO:0021897\_forebrain\_astrocyte\_development | 1 | 0 |  |  |  |  |  |  |  |  |
| GO:0021902\_commitment\_of\_a\_neuronal\_cell\_to\_a\_specific\_type\_of\_neuron\_in\_the\_forebrain | 1 | 0 |  |  |  |  |  |  |  |  |
| GO:0021905\_forebrain-midbrain\_boundary\_formation | 1 | 0 |  |  |  |  |  |  |  |  |
| GO:0021914\_negative\_regulation\_of\_smoothened\_signaling\_pathway\_involved\_in\_ventral\_spinal\_cord\_patterning | 1 | 0 |  |  |  |  |  |  |  |  |
| GO:0021917\_somatic\_motor\_neuron\_fate\_commitment | 1 | 0 |  |  |  |  |  |  |  |  |
| GO:0021918\_regulation\_of\_transcription\_from\_RNA\_polymerase\_II\_promoter\_involved\_in\_somatic\_motor\_neuron\_fate\_commitment | 1 | 0 |  |  |  |  |  |  |  |  |
| GO:0021933\_radial\_glia\_guided\_migration\_of\_granule\_cell | 1 | 0 |  |  |  |  |  |  |  |  |
| GO:0021934\_hindbrain\_tangential\_cell\_migration | 1 | 0 |  |  |  |  |  |  |  |  |
| GO:0021935\_granule\_cell\_precursor\_tangential\_migration | 1 | 0 |  |  |  |  |  |  |  |  |
| GO:0021942\_radial\_glia\_guided\_migration\_of\_Purkinje\_cell | 1 | 0 |  |  |  |  |  |  |  |  |
| GO:0021960\_anterior\_commissure\_morphogenesis | 1 | 0 |  |  |  |  |  |  |  |  |
| GO:0021997\_neural\_plate\_axis\_specification | 1 | 0 |  |  |  |  |  |  |  |  |
| GO:0021999\_neural\_plate\_anterior\_posterior\_pattern\_formation | 1 | 0 |  |  |  |  |  |  |  |  |
| GO:0022004\_midbrain-hindbrain\_boundary\_maturation\_during\_brain\_development | 1 | 0 |  |  |  |  |  |  |  |  |
| GO:0022038\_corpus\_callosum\_development | 1 | 0 |  |  |  |  |  |  |  |  |
| GO:0022605\_oogenesis\_stage | 1 | 0 |  |  |  |  |  |  |  |  |
| GO:0030011\_maintenance\_of\_cell\_polarity | 1 | 0 |  |  |  |  |  |  |  |  |
| GO:0030069\_lysogeny | 1 | 0 |  |  |  |  |  |  |  |  |
| GO:0030070\_insulin\_processing | 1 | 0 |  |  |  |  |  |  |  |  |
| GO:0030092\_regulation\_of\_flagellum\_assembly | 1 | 0 |  |  |  |  |  |  |  |  |
| GO:0030103\_vasopressin\_secretion | 1 | 0 |  |  |  |  |  |  |  |  |
| GO:0030194\_positive\_regulation\_of\_blood\_coagulation | 1 | 0 |  |  |  |  |  |  |  |  |
| GO:0030206\_chondroitin\_sulfate\_biosynthetic\_process | 1 | 0 |  |  |  |  |  |  |  |  |
| GO:0030210\_heparin\_biosynthetic\_process | 1 | 0 |  |  |  |  |  |  |  |  |
| GO:0030220\_platelet\_formation | 1 | 0 |  |  |  |  |  |  |  |  |
| GO:0030222\_eosinophil\_differentiation | 1 | 0 |  |  |  |  |  |  |  |  |
| GO:0030237\_female\_sex\_determination | 1 | 0 |  |  |  |  |  |  |  |  |
| GO:0030264\_nuclear\_fragmentation\_during\_apoptosis | 1 | 0 |  |  |  |  |  |  |  |  |
| GO:0030322\_stabilization\_of\_membrane\_potential | 1 | 0 |  |  |  |  |  |  |  |  |
| GO:0030327\_prenylated\_protein\_catabolic\_process | 1 | 0 |  |  |  |  |  |  |  |  |
| GO:0030328\_prenylcysteine\_catabolic\_process | 1 | 0 |  |  |  |  |  |  |  |  |
| GO:0030329\_prenylcysteine\_metabolic\_process | 1 | 0 |  |  |  |  |  |  |  |  |
| GO:0030382\_sperm\_mitochondrion\_organization | 1 | 0 |  |  |  |  |  |  |  |  |
| GO:0030389\_fructosamine\_metabolic\_process | 1 | 0 |  |  |  |  |  |  |  |  |
| GO:0030422\_RNA\_interference\_\_production\_of\_siRNA | 1 | 0 |  |  |  |  |  |  |  |  |
| GO:0030449\_regulation\_of\_complement\_activation | 1 | 0 |  |  |  |  |  |  |  |  |
| GO:0030497\_fatty\_acid\_elongation | 1 | 0 |  |  |  |  |  |  |  |  |
| GO:0030575\_nuclear\_body\_organization | 1 | 0 |  |  |  |  |  |  |  |  |
| GO:0030578\_PML\_body\_organization | 1 | 0 |  |  |  |  |  |  |  |  |
| GO:0030853\_negative\_regulation\_of\_granulocyte\_differentiation | 1 | 0 |  |  |  |  |  |  |  |  |
| GO:0030854\_positive\_regulation\_of\_granulocyte\_differentiation | 1 | 0 |  |  |  |  |  |  |  |  |
| GO:0030886\_negative\_regulation\_of\_myeloid\_dendritic\_cell\_activation | 1 | 0 |  |  |  |  |  |  |  |  |
| GO:0030913\_paranodal\_junction\_assembly | 1 | 0 |  |  |  |  |  |  |  |  |
| GO:0031033\_myosin\_filament\_assembly\_or\_disassembly | 1 | 0 |  |  |  |  |  |  |  |  |
| GO:0031034\_myosin\_filament\_assembly | 1 | 0 |  |  |  |  |  |  |  |  |
| GO:0031055\_chromatin\_remodeling\_at\_centromere | 1 | 0 |  |  |  |  |  |  |  |  |
| GO:0031062\_positive\_regulation\_of\_histone\_methylation | 1 | 0 |  |  |  |  |  |  |  |  |
| GO:0031115\_negative\_regulation\_of\_microtubule\_polymerization | 1 | 0 |  |  |  |  |  |  |  |  |
| GO:0031129\_inductive\_cell-cell\_signaling | 1 | 0 |  |  |  |  |  |  |  |  |
| GO:0031284\_positive\_regulation\_of\_guanylate\_cyclase\_activity | 1 | 0 |  |  |  |  |  |  |  |  |
| GO:0031498\_chromatin\_disassembly | 1 | 0 |  |  |  |  |  |  |  |  |
| GO:0031507\_heterochromatin\_formation | 1 | 0 |  |  |  |  |  |  |  |  |
| GO:0031508\_centromeric\_heterochromatin\_formation | 1 | 0 |  |  |  |  |  |  |  |  |
| GO:0031529\_ruffle\_organization | 1 | 0 |  |  |  |  |  |  |  |  |
| GO:0031536\_positive\_regulation\_of\_exit\_from\_mitosis | 1 | 0 |  |  |  |  |  |  |  |  |
| GO:0031572\_G2\_M\_transition\_DNA\_damage\_checkpoint | 1 | 0 |  |  |  |  |  |  |  |  |
| GO:0031576\_G2\_M\_transition\_checkpoint | 1 | 0 |  |  |  |  |  |  |  |  |
| GO:0031580\_membrane\_raft\_distribution | 1 | 0 |  |  |  |  |  |  |  |  |
| GO:0031583\_activation\_of\_phospholipase\_D\_activity\_by\_G-protein\_coupled\_receptor\_protein\_signaling\_pathway | 1 | 0 |  |  |  |  |  |  |  |  |
| GO:0031584\_activation\_of\_phospholipase\_D\_activity | 1 | 0 |  |  |  |  |  |  |  |  |
| GO:0031585\_regulation\_of\_inositol-1\_4\_5-triphosphate\_receptor\_activity | 1 | 0 |  |  |  |  |  |  |  |  |
| GO:0031639\_plasminogen\_activation | 1 | 0 |  |  |  |  |  |  |  |  |
| GO:0031648\_protein\_destabilization | 1 | 0 |  |  |  |  |  |  |  |  |
| GO:0031665\_negative\_regulation\_of\_lipopolysaccharide-mediated\_signaling\_pathway | 1 | 0 |  |  |  |  |  |  |  |  |
| GO:0031914\_negative\_regulation\_of\_synaptic\_plasticity | 1 | 0 |  |  |  |  |  |  |  |  |
| GO:0031944\_negative\_regulation\_of\_glucocorticoid\_metabolic\_process | 1 | 0 |  |  |  |  |  |  |  |  |
| GO:0031947\_negative\_regulation\_of\_glucocorticoid\_biosynthetic\_process | 1 | 0 |  |  |  |  |  |  |  |  |
| GO:0032025\_response\_to\_cobalt\_ion | 1 | 0 |  |  |  |  |  |  |  |  |
| GO:0032026\_response\_to\_magnesium\_ion | 1 | 0 |  |  |  |  |  |  |  |  |
| GO:0032048\_cardiolipin\_metabolic\_process | 1 | 0 |  |  |  |  |  |  |  |  |
| GO:0032066\_nucleolus\_to\_nucleoplasm\_transport | 1 | 0 |  |  |  |  |  |  |  |  |
| GO:0032091\_negative\_regulation\_of\_protein\_binding | 1 | 0 |  |  |  |  |  |  |  |  |
| GO:0032092\_positive\_regulation\_of\_protein\_binding | 1 | 0 |  |  |  |  |  |  |  |  |
| GO:0032097\_positive\_regulation\_of\_response\_to\_food | 1 | 0 |  |  |  |  |  |  |  |  |
| GO:0032100\_positive\_regulation\_of\_appetite | 1 | 0 |  |  |  |  |  |  |  |  |
| GO:0032204\_regulation\_of\_telomere\_maintenance | 1 | 0 |  |  |  |  |  |  |  |  |
| GO:0032206\_positive\_regulation\_of\_telomere\_maintenance | 1 | 0 |  |  |  |  |  |  |  |  |
| GO:0032222\_regulation\_of\_synaptic\_transmission\_\_cholinergic | 1 | 0 |  |  |  |  |  |  |  |  |
| GO:0032224\_positive\_regulation\_of\_synaptic\_transmission\_\_cholinergic | 1 | 0 |  |  |  |  |  |  |  |  |
| GO:0032229\_negative\_regulation\_of\_synaptic\_transmission\_\_GABAergic | 1 | 0 |  |  |  |  |  |  |  |  |
| GO:0032237\_activation\_of\_store-operated\_calcium\_channel\_activity | 1 | 0 |  |  |  |  |  |  |  |  |
| GO:0032239\_regulation\_of\_nucleobase\_\_nucleoside\_\_nucleotide\_and\_nucleic\_acid\_transport | 1 | 0 |  |  |  |  |  |  |  |  |
| GO:0032252\_secretory\_granule\_localization | 1 | 0 |  |  |  |  |  |  |  |  |
| GO:0032274\_gonadotropin\_secretion | 1 | 0 |  |  |  |  |  |  |  |  |
| GO:0032275\_luteinizing\_hormone\_secretion | 1 | 0 |  |  |  |  |  |  |  |  |
| GO:0032287\_myelin\_maintenance\_in\_the\_peripheral\_nervous\_system | 1 | 0 |  |  |  |  |  |  |  |  |
| GO:0032289\_myelin\_formation\_in\_the\_central\_nervous\_system | 1 | 0 |  |  |  |  |  |  |  |  |
| GO:0032303\_regulation\_of\_icosanoid\_secretion | 1 | 0 |  |  |  |  |  |  |  |  |
| GO:0032305\_positive\_regulation\_of\_icosanoid\_secretion | 1 | 0 |  |  |  |  |  |  |  |  |
| GO:0032306\_regulation\_of\_prostaglandin\_secretion | 1 | 0 |  |  |  |  |  |  |  |  |
| GO:0032308\_positive\_regulation\_of\_prostaglandin\_secretion | 1 | 0 |  |  |  |  |  |  |  |  |
| GO:0032310\_prostaglandin\_secretion | 1 | 0 |  |  |  |  |  |  |  |  |
| GO:0032313\_regulation\_of\_Rab\_GTPase\_activity | 1 | 0 |  |  |  |  |  |  |  |  |
| GO:0032314\_regulation\_of\_Rac\_GTPase\_activity | 1 | 0 |  |  |  |  |  |  |  |  |
| GO:0032317\_regulation\_of\_Rap\_GTPase\_activity | 1 | 0 |  |  |  |  |  |  |  |  |
| GO:0032324\_molybdopterin\_cofactor\_biosynthetic\_process | 1 | 0 |  |  |  |  |  |  |  |  |
| GO:0032329\_serine\_transport | 1 | 0 |  |  |  |  |  |  |  |  |
| GO:0032342\_aldosterone\_biosynthetic\_process | 1 | 0 |  |  |  |  |  |  |  |  |
| GO:0032344\_regulation\_of\_aldosterone\_metabolic\_process | 1 | 0 |  |  |  |  |  |  |  |  |
| GO:0032365\_intracellular\_lipid\_transport | 1 | 0 |  |  |  |  |  |  |  |  |
| GO:0032366\_intracellular\_sterol\_transport | 1 | 0 |  |  |  |  |  |  |  |  |
| GO:0032367\_intracellular\_cholesterol\_transport | 1 | 0 |  |  |  |  |  |  |  |  |
| GO:0032370\_positive\_regulation\_of\_lipid\_transport | 1 | 0 |  |  |  |  |  |  |  |  |
| GO:0032410\_negative\_regulation\_of\_transporter\_activity | 1 | 0 |  |  |  |  |  |  |  |  |
| GO:0032413\_negative\_regulation\_of\_ion\_transmembrane\_transporter\_activity | 1 | 0 |  |  |  |  |  |  |  |  |
| GO:0032429\_regulation\_of\_phospholipase\_A2\_activity | 1 | 0 |  |  |  |  |  |  |  |  |
| GO:0032474\_otolith\_morphogenesis | 1 | 0 |  |  |  |  |  |  |  |  |
| GO:0032482\_Rab\_protein\_signal\_transduction | 1 | 0 |  |  |  |  |  |  |  |  |
| GO:0032483\_regulation\_of\_Rab\_protein\_signal\_transduction | 1 | 0 |  |  |  |  |  |  |  |  |
| GO:0032486\_Rap\_protein\_signal\_transduction | 1 | 0 |  |  |  |  |  |  |  |  |
| GO:0032487\_regulation\_of\_Rap\_protein\_signal\_transduction | 1 | 0 |  |  |  |  |  |  |  |  |
| GO:0032594\_protein\_transport\_within\_lipid\_bilayer | 1 | 0 |  |  |  |  |  |  |  |  |
| GO:0032599\_protein\_transport\_out\_of\_membrane\_raft | 1 | 0 |  |  |  |  |  |  |  |  |
| GO:0032600\_chemokine\_receptor\_transport\_out\_of\_membrane\_raft | 1 | 0 |  |  |  |  |  |  |  |  |
| GO:0032607\_interferon-alpha\_production | 1 | 0 |  |  |  |  |  |  |  |  |
| GO:0032621\_interleukin-18\_production | 1 | 0 |  |  |  |  |  |  |  |  |
| GO:0032647\_regulation\_of\_interferon-alpha\_production | 1 | 0 |  |  |  |  |  |  |  |  |
| GO:0032656\_regulation\_of\_interleukin-13\_production | 1 | 0 |  |  |  |  |  |  |  |  |
| GO:0032682\_negative\_regulation\_of\_chemokine\_production | 1 | 0 |  |  |  |  |  |  |  |  |
| GO:0032691\_negative\_regulation\_of\_interleukin-1\_beta\_production | 1 | 0 |  |  |  |  |  |  |  |  |
| GO:0032692\_negative\_regulation\_of\_interleukin-1\_production | 1 | 0 |  |  |  |  |  |  |  |  |
| GO:0032693\_negative\_regulation\_of\_interleukin-10\_production | 1 | 0 |  |  |  |  |  |  |  |  |
| GO:0032696\_negative\_regulation\_of\_interleukin-13\_production | 1 | 0 |  |  |  |  |  |  |  |  |
| GO:0032727\_positive\_regulation\_of\_interferon-alpha\_production | 1 | 0 |  |  |  |  |  |  |  |  |
| GO:0032731\_positive\_regulation\_of\_interleukin-1\_beta\_production | 1 | 0 |  |  |  |  |  |  |  |  |
| GO:0032732\_positive\_regulation\_of\_interleukin-1\_production | 1 | 0 |  |  |  |  |  |  |  |  |
| GO:0032735\_positive\_regulation\_of\_interleukin-12\_production | 1 | 0 |  |  |  |  |  |  |  |  |
| GO:0032764\_negative\_regulation\_of\_mast\_cell\_cytokine\_production | 1 | 0 |  |  |  |  |  |  |  |  |
| GO:0032765\_positive\_regulation\_of\_mast\_cell\_cytokine\_production | 1 | 0 |  |  |  |  |  |  |  |  |
| GO:0032769\_negative\_regulation\_of\_monooxygenase\_activity | 1 | 0 |  |  |  |  |  |  |  |  |
| GO:0032781\_positive\_regulation\_of\_ATPase\_activity | 1 | 0 |  |  |  |  |  |  |  |  |
| GO:0032790\_ribosome\_disassembly | 1 | 0 |  |  |  |  |  |  |  |  |
| GO:0032799\_low-density\_lipoprotein\_receptor\_metabolic\_process | 1 | 0 |  |  |  |  |  |  |  |  |
| GO:0032802\_low-density\_lipoprotein\_receptor\_catabolic\_process | 1 | 0 |  |  |  |  |  |  |  |  |
| GO:0032803\_regulation\_of\_low-density\_lipoprotein\_receptor\_catabolic\_process | 1 | 0 |  |  |  |  |  |  |  |  |
| GO:0032817\_regulation\_of\_natural\_killer\_cell\_proliferation | 1 | 0 |  |  |  |  |  |  |  |  |
| GO:0032819\_positive\_regulation\_of\_natural\_killer\_cell\_proliferation | 1 | 0 |  |  |  |  |  |  |  |  |
| GO:0032836\_glomerular\_basement\_membrane\_development | 1 | 0 |  |  |  |  |  |  |  |  |
| GO:0032855\_positive\_regulation\_of\_Rac\_GTPase\_activity | 1 | 0 |  |  |  |  |  |  |  |  |
| GO:0032863\_activation\_of\_Rac\_GTPase\_activity | 1 | 0 |  |  |  |  |  |  |  |  |
| GO:0032864\_activation\_of\_Cdc42\_GTPase\_activity | 1 | 0 |  |  |  |  |  |  |  |  |
| GO:0032885\_regulation\_of\_polysaccharide\_biosynthetic\_process | 1 | 0 |  |  |  |  |  |  |  |  |
| GO:0032907\_transforming\_growth\_factor-beta3\_production | 1 | 0 |  |  |  |  |  |  |  |  |
| GO:0032910\_regulation\_of\_transforming\_growth\_factor-beta3\_production | 1 | 0 |  |  |  |  |  |  |  |  |
| GO:0032913\_negative\_regulation\_of\_transforming\_growth\_factor-beta3\_production | 1 | 0 |  |  |  |  |  |  |  |  |
| GO:0032924\_activin\_receptor\_signaling\_pathway | 1 | 0 |  |  |  |  |  |  |  |  |
| GO:0032925\_regulation\_of\_activin\_receptor\_signaling\_pathway | 1 | 0 |  |  |  |  |  |  |  |  |
| GO:0032960\_regulation\_of\_inositol\_trisphosphate\_biosynthetic\_process | 1 | 0 |  |  |  |  |  |  |  |  |
| GO:0032962\_positive\_regulation\_of\_inositol\_trisphosphate\_biosynthetic\_process | 1 | 0 |  |  |  |  |  |  |  |  |
| GO:0032964\_collagen\_biosynthetic\_process | 1 | 0 |  |  |  |  |  |  |  |  |
| GO:0032971\_regulation\_of\_muscle\_filament\_sliding | 1 | 0 |  |  |  |  |  |  |  |  |
| GO:0032972\_regulation\_of\_muscle\_filament\_sliding\_speed | 1 | 0 |  |  |  |  |  |  |  |  |
| GO:0032986\_protein-DNA\_complex\_disassembly | 1 | 0 |  |  |  |  |  |  |  |  |
| GO:0032988\_ribonucleoprotein\_complex\_disassembly | 1 | 0 |  |  |  |  |  |  |  |  |
| GO:0033037\_polysaccharide\_localization | 1 | 0 |  |  |  |  |  |  |  |  |
| GO:0033078\_extrathymic\_T\_cell\_differentiation | 1 | 0 |  |  |  |  |  |  |  |  |
| GO:0033085\_negative\_regulation\_of\_T\_cell\_differentiation\_in\_the\_thymus | 1 | 0 |  |  |  |  |  |  |  |  |
| GO:0033087\_negative\_regulation\_of\_immature\_T\_cell\_proliferation | 1 | 0 |  |  |  |  |  |  |  |  |
| GO:0033088\_negative\_regulation\_of\_immature\_T\_cell\_proliferation\_in\_the\_thymus | 1 | 0 |  |  |  |  |  |  |  |  |
| GO:0033108\_mitochondrial\_respiratory\_chain\_complex\_assembly | 1 | 0 |  |  |  |  |  |  |  |  |
| GO:0033127\_regulation\_of\_histone\_phosphorylation | 1 | 0 |  |  |  |  |  |  |  |  |
| GO:0033128\_negative\_regulation\_of\_histone\_phosphorylation | 1 | 0 |  |  |  |  |  |  |  |  |
| GO:0033138\_positive\_regulation\_of\_peptidyl-serine\_phosphorylation | 1 | 0 |  |  |  |  |  |  |  |  |
| GO:0033158\_regulation\_of\_protein\_import\_into\_nucleus\_\_translocation | 1 | 0 |  |  |  |  |  |  |  |  |
| GO:0033160\_positive\_regulation\_of\_protein\_import\_into\_nucleus\_\_translocation | 1 | 0 |  |  |  |  |  |  |  |  |
| GO:0033169\_histone\_H3-K9\_demethylation | 1 | 0 |  |  |  |  |  |  |  |  |
| GO:0033206\_cytokinesis\_after\_meiosis | 1 | 0 |  |  |  |  |  |  |  |  |
| GO:0033240\_positive\_regulation\_of\_cellular\_amine\_metabolic\_process | 1 | 0 |  |  |  |  |  |  |  |  |
| GO:0033313\_meiotic\_cell\_cycle\_checkpoint | 1 | 0 |  |  |  |  |  |  |  |  |
| GO:0033315\_meiotic\_cell\_cycle\_DNA\_replication\_checkpoint | 1 | 0 |  |  |  |  |  |  |  |  |
| GO:0033326\_cerebrospinal\_fluid\_secretion | 1 | 0 |  |  |  |  |  |  |  |  |
| GO:0033366\_protein\_localization\_in\_secretory\_granule | 1 | 0 |  |  |  |  |  |  |  |  |
| GO:0033367\_protein\_localization\_in\_mast\_cell\_secretory\_granule | 1 | 0 |  |  |  |  |  |  |  |  |
| GO:0033368\_protease\_localization\_in\_mast\_cell\_secretory\_granule | 1 | 0 |  |  |  |  |  |  |  |  |
| GO:0033370\_maintenance\_of\_protein\_location\_in\_mast\_cell\_secretory\_granule | 1 | 0 |  |  |  |  |  |  |  |  |
| GO:0033371\_T\_cell\_secretory\_granule\_organization | 1 | 0 |  |  |  |  |  |  |  |  |
| GO:0033373\_maintenance\_of\_protease\_location\_in\_mast\_cell\_secretory\_granule | 1 | 0 |  |  |  |  |  |  |  |  |
| GO:0033374\_protein\_localization\_in\_T\_cell\_secretory\_granule | 1 | 0 |  |  |  |  |  |  |  |  |
| GO:0033375\_protease\_localization\_in\_T\_cell\_secretory\_granule | 1 | 0 |  |  |  |  |  |  |  |  |
| GO:0033377\_maintenance\_of\_protein\_location\_in\_T\_cell\_secretory\_granule | 1 | 0 |  |  |  |  |  |  |  |  |
| GO:0033379\_maintenance\_of\_protease\_location\_in\_T\_cell\_secretory\_granule | 1 | 0 |  |  |  |  |  |  |  |  |
| GO:0033380\_granzyme\_B\_localization\_in\_T\_cell\_secretory\_granule | 1 | 0 |  |  |  |  |  |  |  |  |
| GO:0033382\_maintenance\_of\_granzyme\_B\_location\_in\_T\_cell\_secretory\_granule | 1 | 0 |  |  |  |  |  |  |  |  |
| GO:0033483\_gas\_homeostasis | 1 | 0 |  |  |  |  |  |  |  |  |
| GO:0033484\_nitric\_oxide\_homeostasis | 1 | 0 |  |  |  |  |  |  |  |  |
| GO:0033505\_floor\_plate\_morphogenesis | 1 | 0 |  |  |  |  |  |  |  |  |
| GO:0033522\_histone\_H2A\_ubiquitination | 1 | 0 |  |  |  |  |  |  |  |  |
| GO:0033523\_histone\_H2B\_ubiquitination | 1 | 0 |  |  |  |  |  |  |  |  |
| GO:0033574\_response\_to\_testosterone\_stimulus | 1 | 0 |  |  |  |  |  |  |  |  |
| GO:0033606\_chemokine\_receptor\_transport\_within\_lipid\_bilayer | 1 | 0 |  |  |  |  |  |  |  |  |
| GO:0033628\_regulation\_of\_cell\_adhesion\_mediated\_by\_integrin | 1 | 0 |  |  |  |  |  |  |  |  |
| GO:0033630\_positive\_regulation\_of\_cell\_adhesion\_mediated\_by\_integrin | 1 | 0 |  |  |  |  |  |  |  |  |
| GO:0033632\_regulation\_of\_cell-cell\_adhesion\_mediated\_by\_integrin | 1 | 0 |  |  |  |  |  |  |  |  |
| GO:0033634\_positive\_regulation\_of\_cell-cell\_adhesion\_mediated\_by\_integrin | 1 | 0 |  |  |  |  |  |  |  |  |
| GO:0033683\_nucleotide-excision\_repair\_\_DNA\_incision | 1 | 0 |  |  |  |  |  |  |  |  |
| GO:0033687\_osteoblast\_proliferation | 1 | 0 |  |  |  |  |  |  |  |  |
| GO:0033688\_regulation\_of\_osteoblast\_proliferation | 1 | 0 |  |  |  |  |  |  |  |  |
| GO:0033689\_negative\_regulation\_of\_osteoblast\_proliferation | 1 | 0 |  |  |  |  |  |  |  |  |
| GO:0033750\_ribosome\_localization | 1 | 0 |  |  |  |  |  |  |  |  |
| GO:0033753\_establishment\_of\_ribosome\_localization | 1 | 0 |  |  |  |  |  |  |  |  |
| GO:0033866\_nucleoside\_bisphosphate\_biosynthetic\_process | 1 | 0 |  |  |  |  |  |  |  |  |
| GO:0033875\_ribonucleoside\_bisphosphate\_metabolic\_process | 1 | 0 |  |  |  |  |  |  |  |  |
| GO:0034030\_ribonucleoside\_bisphosphate\_biosynthetic\_process | 1 | 0 |  |  |  |  |  |  |  |  |
| GO:0034032\_purine\_nucleoside\_bisphosphate\_metabolic\_process | 1 | 0 |  |  |  |  |  |  |  |  |
| GO:0034033\_purine\_nucleoside\_bisphosphate\_biosynthetic\_process | 1 | 0 |  |  |  |  |  |  |  |  |
| GO:0034035\_purine\_ribonucleoside\_bisphosphate\_metabolic\_process | 1 | 0 |  |  |  |  |  |  |  |  |
| GO:0034036\_purine\_ribonucleoside\_bisphosphate\_biosynthetic\_process | 1 | 0 |  |  |  |  |  |  |  |  |
| GO:0034067\_protein\_localization\_in\_Golgi\_apparatus | 1 | 0 |  |  |  |  |  |  |  |  |
| GO:0034102\_erythrocyte\_clearance | 1 | 0 |  |  |  |  |  |  |  |  |
| GO:0034106\_regulation\_of\_erythrocyte\_clearance | 1 | 0 |  |  |  |  |  |  |  |  |
| GO:0034107\_negative\_regulation\_of\_erythrocyte\_clearance | 1 | 0 |  |  |  |  |  |  |  |  |
| GO:0034110\_regulation\_of\_homotypic\_cell-cell\_adhesion | 1 | 0 |  |  |  |  |  |  |  |  |
| GO:0034111\_negative\_regulation\_of\_homotypic\_cell-cell\_adhesion | 1 | 0 |  |  |  |  |  |  |  |  |
| GO:0034113\_heterotypic\_cell-cell\_adhesion | 1 | 0 |  |  |  |  |  |  |  |  |
| GO:0034117\_erythrocyte\_aggregation | 1 | 0 |  |  |  |  |  |  |  |  |
| GO:0034118\_regulation\_of\_erythrocyte\_aggregation | 1 | 0 |  |  |  |  |  |  |  |  |
| GO:0034119\_negative\_regulation\_of\_erythrocyte\_aggregation | 1 | 0 |  |  |  |  |  |  |  |  |
| GO:0034121\_regulation\_of\_toll-like\_receptor\_signaling\_pathway | 1 | 0 |  |  |  |  |  |  |  |  |
| GO:0034122\_negative\_regulation\_of\_toll-like\_receptor\_signaling\_pathway | 1 | 0 |  |  |  |  |  |  |  |  |
| GO:0034230\_enkephalin\_processing | 1 | 0 |  |  |  |  |  |  |  |  |
| GO:0034372\_very-low-density\_lipoprotein\_particle\_remodeling | 1 | 0 |  |  |  |  |  |  |  |  |
| GO:0034379\_very-low-density\_lipoprotein\_particle\_assembly | 1 | 0 |  |  |  |  |  |  |  |  |
| GO:0034380\_high-density\_lipoprotein\_particle\_assembly | 1 | 0 |  |  |  |  |  |  |  |  |
| GO:0034394\_protein\_localization\_at\_cell\_surface | 1 | 0 |  |  |  |  |  |  |  |  |
| GO:0034405\_response\_to\_fluid\_shear\_stress | 1 | 0 |  |  |  |  |  |  |  |  |
| GO:0034472\_snRNA\_3'-end\_processing | 1 | 0 |  |  |  |  |  |  |  |  |
| GO:0034474\_U2\_snRNA\_3'-end\_processing | 1 | 0 |  |  |  |  |  |  |  |  |
| GO:0034502\_protein\_localization\_to\_chromosome | 1 | 0 |  |  |  |  |  |  |  |  |
| GO:0034505\_tooth\_mineralization | 1 | 0 |  |  |  |  |  |  |  |  |
| GO:0034508\_centromere\_complex\_assembly | 1 | 0 |  |  |  |  |  |  |  |  |
| GO:0034633\_retinol\_transport | 1 | 0 |  |  |  |  |  |  |  |  |
| GO:0034643\_mitochondrion\_localization\_\_microtubule-mediated | 1 | 0 |  |  |  |  |  |  |  |  |
| GO:0034969\_histone\_arginine\_methylation | 1 | 0 |  |  |  |  |  |  |  |  |
| GO:0034982\_mitochondrial\_protein\_processing | 1 | 0 |  |  |  |  |  |  |  |  |
| GO:0035022\_positive\_regulation\_of\_Rac\_protein\_signal\_transduction | 1 | 0 |  |  |  |  |  |  |  |  |
| GO:0035024\_negative\_regulation\_of\_Rho\_protein\_signal\_transduction | 1 | 0 |  |  |  |  |  |  |  |  |
| GO:0035026\_leading\_edge\_cell\_differentiation | 1 | 0 |  |  |  |  |  |  |  |  |
| GO:0035037\_sperm\_entry | 1 | 0 |  |  |  |  |  |  |  |  |
| GO:0035039\_male\_pronucleus\_formation | 1 | 0 |  |  |  |  |  |  |  |  |
| GO:0035066\_positive\_regulation\_of\_histone\_acetylation | 1 | 0 |  |  |  |  |  |  |  |  |
| GO:0035083\_cilium\_axoneme\_assembly | 1 | 0 |  |  |  |  |  |  |  |  |
| GO:0035090\_maintenance\_of\_apical\_basal\_cell\_polarity | 1 | 0 |  |  |  |  |  |  |  |  |
| GO:0035106\_operant\_conditioning | 1 | 0 |  |  |  |  |  |  |  |  |
| GO:0035172\_hemocyte\_proliferation | 1 | 0 |  |  |  |  |  |  |  |  |
| GO:0035227\_regulation\_of\_glutamate-cysteine\_ligase\_activity | 1 | 0 |  |  |  |  |  |  |  |  |
| GO:0035229\_positive\_regulation\_of\_glutamate-cysteine\_ligase\_activity | 1 | 0 |  |  |  |  |  |  |  |  |
| GO:0035260\_internal\_genitalia\_morphogenesis | 1 | 0 |  |  |  |  |  |  |  |  |
| GO:0035262\_gonad\_morphogenesis | 1 | 0 |  |  |  |  |  |  |  |  |
| GO:0035287\_head\_segmentation | 1 | 0 |  |  |  |  |  |  |  |  |
| GO:0035289\_posterior\_head\_segmentation | 1 | 0 |  |  |  |  |  |  |  |  |
| GO:0035303\_regulation\_of\_dephosphorylation | 1 | 0 |  |  |  |  |  |  |  |  |
| GO:0035304\_regulation\_of\_protein\_amino\_acid\_dephosphorylation | 1 | 0 |  |  |  |  |  |  |  |  |
| GO:0035305\_negative\_regulation\_of\_dephosphorylation | 1 | 0 |  |  |  |  |  |  |  |  |
| GO:0035308\_negative\_regulation\_of\_protein\_amino\_acid\_dephosphorylation | 1 | 0 |  |  |  |  |  |  |  |  |
| GO:0035313\_wound\_healing\_\_spreading\_of\_epidermal\_cells | 1 | 0 |  |  |  |  |  |  |  |  |
| GO:0040013\_negative\_regulation\_of\_locomotion | 1 | 0 |  |  |  |  |  |  |  |  |
| GO:0040019\_positive\_regulation\_of\_embryonic\_development | 1 | 0 |  |  |  |  |  |  |  |  |
| GO:0040032\_post-embryonic\_body\_morphogenesis | 1 | 0 |  |  |  |  |  |  |  |  |
| GO:0040038\_polar\_body\_extrusion\_after\_meiotic\_divisions | 1 | 0 |  |  |  |  |  |  |  |  |
| GO:0042026\_protein\_refolding | 1 | 0 |  |  |  |  |  |  |  |  |
| GO:0042048\_olfactory\_behavior | 1 | 0 |  |  |  |  |  |  |  |  |
| GO:0042059\_negative\_regulation\_of\_epidermal\_growth\_factor\_receptor\_signaling\_pathway | 1 | 0 |  |  |  |  |  |  |  |  |
| GO:0042073\_intraflagellar\_transport | 1 | 0 |  |  |  |  |  |  |  |  |
| GO:0042078\_germ-line\_stem\_cell\_division | 1 | 0 |  |  |  |  |  |  |  |  |
| GO:0042091\_interleukin-10\_biosynthetic\_process | 1 | 0 |  |  |  |  |  |  |  |  |
| GO:0042103\_positive\_regulation\_of\_T\_cell\_homeostatic\_proliferation | 1 | 0 |  |  |  |  |  |  |  |  |
| GO:0042136\_neurotransmitter\_biosynthetic\_process | 1 | 0 |  |  |  |  |  |  |  |  |
| GO:0042137\_sequestering\_of\_neurotransmitter | 1 | 0 |  |  |  |  |  |  |  |  |
| GO:0042138\_meiotic\_DNA\_double-strand\_break\_formation | 1 | 0 |  |  |  |  |  |  |  |  |
| GO:0042178\_xenobiotic\_catabolic\_process | 1 | 0 |  |  |  |  |  |  |  |  |
| GO:0042225\_interleukin-5\_biosynthetic\_process | 1 | 0 |  |  |  |  |  |  |  |  |
| GO:0042231\_interleukin-13\_biosynthetic\_process | 1 | 0 |  |  |  |  |  |  |  |  |
| GO:0042255\_ribosome\_assembly | 1 | 0 |  |  |  |  |  |  |  |  |
| GO:0042257\_ribosomal\_subunit\_assembly | 1 | 0 |  |  |  |  |  |  |  |  |
| GO:0042264\_peptidyl-aspartic\_acid\_hydroxylation | 1 | 0 |  |  |  |  |  |  |  |  |
| GO:0042276\_error-prone\_postreplication\_DNA\_repair | 1 | 0 |  |  |  |  |  |  |  |  |
| GO:0042309\_homoiothermy | 1 | 0 |  |  |  |  |  |  |  |  |
| GO:0042320\_regulation\_of\_circadian\_sleep\_wake\_cycle\_\_REM\_sleep | 1 | 0 |  |  |  |  |  |  |  |  |
| GO:0042339\_keratan\_sulfate\_metabolic\_process | 1 | 0 |  |  |  |  |  |  |  |  |
| GO:0042347\_negative\_regulation\_of\_NF-kappaB\_import\_into\_nucleus | 1 | 0 |  |  |  |  |  |  |  |  |
| GO:0042360\_vitamin\_E\_metabolic\_process | 1 | 0 |  |  |  |  |  |  |  |  |
| GO:0042363\_fat-soluble\_vitamin\_catabolic\_process | 1 | 0 |  |  |  |  |  |  |  |  |
| GO:0042369\_vitamin\_D\_catabolic\_process | 1 | 0 |  |  |  |  |  |  |  |  |
| GO:0042373\_vitamin\_K\_metabolic\_process | 1 | 0 |  |  |  |  |  |  |  |  |
| GO:0042404\_thyroid\_hormone\_catabolic\_process | 1 | 0 |  |  |  |  |  |  |  |  |
| GO:0042414\_epinephrine\_metabolic\_process | 1 | 0 |  |  |  |  |  |  |  |  |
| GO:0042436\_indole\_derivative\_catabolic\_process | 1 | 0 |  |  |  |  |  |  |  |  |
| GO:0042489\_negative\_regulation\_of\_odontogenesis\_of\_dentine-containing\_tooth | 1 | 0 |  |  |  |  |  |  |  |  |
| GO:0042508\_tyrosine\_phosphorylation\_of\_Stat1\_protein | 1 | 0 |  |  |  |  |  |  |  |  |
| GO:0042518\_negative\_regulation\_of\_tyrosine\_phosphorylation\_of\_Stat3\_protein | 1 | 0 |  |  |  |  |  |  |  |  |
| GO:0042524\_negative\_regulation\_of\_tyrosine\_phosphorylation\_of\_Stat5\_protein | 1 | 0 |  |  |  |  |  |  |  |  |
| GO:0042536\_negative\_regulation\_of\_tumor\_necrosis\_factor\_biosynthetic\_process | 1 | 0 |  |  |  |  |  |  |  |  |
| GO:0042538\_hyperosmotic\_salinity\_response | 1 | 0 |  |  |  |  |  |  |  |  |
| GO:0042628\_mating\_plug\_formation | 1 | 0 |  |  |  |  |  |  |  |  |
| GO:0042631\_cellular\_response\_to\_water\_deprivation | 1 | 0 |  |  |  |  |  |  |  |  |
| GO:0042637\_catagen | 1 | 0 |  |  |  |  |  |  |  |  |
| GO:0042660\_positive\_regulation\_of\_cell\_fate\_specification | 1 | 0 |  |  |  |  |  |  |  |  |
| GO:0042663\_regulation\_of\_endodermal\_cell\_fate\_specification | 1 | 0 |  |  |  |  |  |  |  |  |
| GO:0042664\_negative\_regulation\_of\_endodermal\_cell\_fate\_specification | 1 | 0 |  |  |  |  |  |  |  |  |
| GO:0042667\_auditory\_receptor\_cell\_fate\_specification | 1 | 0 |  |  |  |  |  |  |  |  |
| GO:0042694\_muscle\_cell\_fate\_specification | 1 | 0 |  |  |  |  |  |  |  |  |
| GO:0042706\_eye\_photoreceptor\_cell\_fate\_commitment | 1 | 0 |  |  |  |  |  |  |  |  |
| GO:0042713\_sperm\_ejaculation | 1 | 0 |  |  |  |  |  |  |  |  |
| GO:0042723\_thiamin\_and\_derivative\_metabolic\_process | 1 | 0 |  |  |  |  |  |  |  |  |
| GO:0042737\_drug\_catabolic\_process | 1 | 0 |  |  |  |  |  |  |  |  |
| GO:0042738\_exogenous\_drug\_catabolic\_process | 1 | 0 |  |  |  |  |  |  |  |  |
| GO:0042747\_circadian\_sleep\_wake\_cycle\_\_REM\_sleep | 1 | 0 |  |  |  |  |  |  |  |  |
| GO:0042748\_circadian\_sleep\_wake\_cycle\_\_non-REM\_sleep | 1 | 0 |  |  |  |  |  |  |  |  |
| GO:0042772\_DNA\_damage\_response\_\_signal\_transduction\_resulting\_in\_transcription | 1 | 0 |  |  |  |  |  |  |  |  |
| GO:0042790\_transcription\_of\_nuclear\_rRNA\_large\_RNA\_polymerase\_I\_transcript | 1 | 0 |  |  |  |  |  |  |  |  |
| GO:0042839\_D-glucuronate\_metabolic\_process | 1 | 0 |  |  |  |  |  |  |  |  |
| GO:0042840\_D-glucuronate\_catabolic\_process | 1 | 0 |  |  |  |  |  |  |  |  |
| GO:0042891\_antibiotic\_transport | 1 | 0 |  |  |  |  |  |  |  |  |
| GO:0042892\_chloramphenicol\_transport | 1 | 0 |  |  |  |  |  |  |  |  |
| GO:0042940\_D-amino\_acid\_transport | 1 | 0 |  |  |  |  |  |  |  |  |
| GO:0042941\_D-alanine\_transport | 1 | 0 |  |  |  |  |  |  |  |  |
| GO:0042942\_D-serine\_transport | 1 | 0 |  |  |  |  |  |  |  |  |
| GO:0042983\_amyloid\_precursor\_protein\_biosynthetic\_process | 1 | 0 |  |  |  |  |  |  |  |  |
| GO:0042984\_regulation\_of\_amyloid\_precursor\_protein\_biosynthetic\_process | 1 | 0 |  |  |  |  |  |  |  |  |
| GO:0042985\_negative\_regulation\_of\_amyloid\_precursor\_protein\_biosynthetic\_process | 1 | 0 |  |  |  |  |  |  |  |  |
| GO:0042989\_sequestering\_of\_actin\_monomers | 1 | 0 |  |  |  |  |  |  |  |  |
| GO:0043044\_ATP-dependent\_chromatin\_remodeling | 1 | 0 |  |  |  |  |  |  |  |  |
| GO:0043056\_forward\_locomotion | 1 | 0 |  |  |  |  |  |  |  |  |
| GO:0043060\_meiotic\_metaphase\_I\_plate\_congression | 1 | 0 |  |  |  |  |  |  |  |  |
| GO:0043091\_L-arginine\_import | 1 | 0 |  |  |  |  |  |  |  |  |
| GO:0043124\_negative\_regulation\_of\_I-kappaB\_kinase\_NF-kappaB\_cascade | 1 | 0 |  |  |  |  |  |  |  |  |
| GO:0043132\_NAD\_transport | 1 | 0 |  |  |  |  |  |  |  |  |
| GO:0043153\_entrainment\_of\_circadian\_clock\_by\_photoperiod | 1 | 0 |  |  |  |  |  |  |  |  |
| GO:0043171\_peptide\_catabolic\_process | 1 | 0 |  |  |  |  |  |  |  |  |
| GO:0043179\_rhythmic\_excitation | 1 | 0 |  |  |  |  |  |  |  |  |
| GO:0043206\_fibril\_organization | 1 | 0 |  |  |  |  |  |  |  |  |
| GO:0043217\_myelin\_maintenance | 1 | 0 |  |  |  |  |  |  |  |  |
| GO:0043313\_regulation\_of\_neutrophil\_degranulation | 1 | 0 |  |  |  |  |  |  |  |  |
| GO:0043316\_cytotoxic\_T\_cell\_degranulation | 1 | 0 |  |  |  |  |  |  |  |  |
| GO:0043369\_CD4-positive\_or\_CD8-positive\_\_alpha-beta\_T\_cell\_lineage\_commitment | 1 | 0 |  |  |  |  |  |  |  |  |
| GO:0043375\_CD8-positive\_\_alpha-beta\_T\_cell\_lineage\_commitment | 1 | 0 |  |  |  |  |  |  |  |  |
| GO:0043379\_memory\_T\_cell\_differentiation | 1 | 0 |  |  |  |  |  |  |  |  |
| GO:0043380\_regulation\_of\_memory\_T\_cell\_differentiation | 1 | 0 |  |  |  |  |  |  |  |  |
| GO:0043400\_cortisol\_secretion | 1 | 0 |  |  |  |  |  |  |  |  |
| GO:0043415\_positive\_regulation\_of\_skeletal\_muscle\_regeneration | 1 | 0 |  |  |  |  |  |  |  |  |
| GO:0043416\_regulation\_of\_skeletal\_muscle\_regeneration | 1 | 0 |  |  |  |  |  |  |  |  |
| GO:0043437\_butanoic\_acid\_metabolic\_process | 1 | 0 |  |  |  |  |  |  |  |  |
| GO:0043438\_acetoacetic\_acid\_metabolic\_process | 1 | 0 |  |  |  |  |  |  |  |  |
| GO:0043480\_pigment\_accumulation\_in\_tissues | 1 | 0 |  |  |  |  |  |  |  |  |
| GO:0043482\_cellular\_pigment\_accumulation | 1 | 0 |  |  |  |  |  |  |  |  |
| GO:0043486\_histone\_exchange | 1 | 0 |  |  |  |  |  |  |  |  |
| GO:0043496\_regulation\_of\_protein\_homodimerization\_activity | 1 | 0 |  |  |  |  |  |  |  |  |
| GO:0043501\_skeletal\_muscle\_adaptation | 1 | 0 |  |  |  |  |  |  |  |  |
| GO:0043508\_negative\_regulation\_of\_JUN\_kinase\_activity | 1 | 0 |  |  |  |  |  |  |  |  |
| GO:0043517\_positive\_regulation\_of\_DNA\_damage\_response\_\_signal\_transduction\_by\_p53\_class\_mediator | 1 | 0 |  |  |  |  |  |  |  |  |
| GO:0043535\_regulation\_of\_blood\_vessel\_endothelial\_cell\_migration | 1 | 0 |  |  |  |  |  |  |  |  |
| GO:0043537\_negative\_regulation\_of\_blood\_vessel\_endothelial\_cell\_migration | 1 | 0 |  |  |  |  |  |  |  |  |
| GO:0043545\_molybdopterin\_cofactor\_metabolic\_process | 1 | 0 |  |  |  |  |  |  |  |  |
| GO:0043587\_tongue\_morphogenesis | 1 | 0 |  |  |  |  |  |  |  |  |
| GO:0043604\_amide\_biosynthetic\_process | 1 | 0 |  |  |  |  |  |  |  |  |
| GO:0043628\_ncRNA\_3'-end\_processing | 1 | 0 |  |  |  |  |  |  |  |  |
| GO:0044254\_multicellular\_organismal\_protein\_catabolic\_process | 1 | 0 |  |  |  |  |  |  |  |  |
| GO:0044256\_protein\_digestion | 1 | 0 |  |  |  |  |  |  |  |  |
| GO:0044266\_multicellular\_organismal\_macromolecule\_catabolic\_process | 1 | 0 |  |  |  |  |  |  |  |  |
| GO:0045004\_DNA\_replication\_proofreading | 1 | 0 |  |  |  |  |  |  |  |  |
| GO:0045019\_negative\_regulation\_of\_nitric\_oxide\_biosynthetic\_process | 1 | 0 |  |  |  |  |  |  |  |  |
| GO:0045020\_error-prone\_DNA\_repair | 1 | 0 |  |  |  |  |  |  |  |  |
| GO:0045022\_early\_endosome\_to\_late\_endosome\_transport | 1 | 0 |  |  |  |  |  |  |  |  |
| GO:0045062\_extrathymic\_T\_cell\_selection | 1 | 0 |  |  |  |  |  |  |  |  |
| GO:0045069\_regulation\_of\_viral\_genome\_replication | 1 | 0 |  |  |  |  |  |  |  |  |
| GO:0045074\_regulation\_of\_interleukin-10\_biosynthetic\_process | 1 | 0 |  |  |  |  |  |  |  |  |
| GO:0045082\_positive\_regulation\_of\_interleukin-10\_biosynthetic\_process | 1 | 0 |  |  |  |  |  |  |  |  |
| GO:0045083\_negative\_regulation\_of\_interleukin-12\_biosynthetic\_process | 1 | 0 |  |  |  |  |  |  |  |  |
| GO:0045112\_integrin\_biosynthetic\_process | 1 | 0 |  |  |  |  |  |  |  |  |
| GO:0045113\_regulation\_of\_integrin\_biosynthetic\_process | 1 | 0 |  |  |  |  |  |  |  |  |
| GO:0045188\_regulation\_of\_circadian\_sleep\_wake\_cycle\_\_non-REM\_sleep | 1 | 0 |  |  |  |  |  |  |  |  |
| GO:0045210\_FasL\_biosynthetic\_process | 1 | 0 |  |  |  |  |  |  |  |  |
| GO:0045297\_post-mating\_behavior | 1 | 0 |  |  |  |  |  |  |  |  |
| GO:0045299\_otolith\_mineralization | 1 | 0 |  |  |  |  |  |  |  |  |
| GO:0045329\_carnitine\_biosynthetic\_process | 1 | 0 |  |  |  |  |  |  |  |  |
| GO:0045341\_MHC\_class\_I\_biosynthetic\_process | 1 | 0 |  |  |  |  |  |  |  |  |
| GO:0045343\_regulation\_of\_MHC\_class\_I\_biosynthetic\_process | 1 | 0 |  |  |  |  |  |  |  |  |
| GO:0045347\_negative\_regulation\_of\_MHC\_class\_II\_biosynthetic\_process | 1 | 0 |  |  |  |  |  |  |  |  |
| GO:0045405\_regulation\_of\_interleukin-5\_biosynthetic\_process | 1 | 0 |  |  |  |  |  |  |  |  |
| GO:0045407\_positive\_regulation\_of\_interleukin-5\_biosynthetic\_process | 1 | 0 |  |  |  |  |  |  |  |  |
| GO:0045426\_quinone\_cofactor\_biosynthetic\_process | 1 | 0 |  |  |  |  |  |  |  |  |
| GO:0045448\_mitotic\_cell\_cycle\_\_embryonic | 1 | 0 |  |  |  |  |  |  |  |  |
| GO:0045454\_cell\_redox\_homeostasis | 1 | 0 |  |  |  |  |  |  |  |  |
| GO:0045583\_regulation\_of\_cytotoxic\_T\_cell\_differentiation | 1 | 0 |  |  |  |  |  |  |  |  |
| GO:0045585\_positive\_regulation\_of\_cytotoxic\_T\_cell\_differentiation | 1 | 0 |  |  |  |  |  |  |  |  |
| GO:0045601\_regulation\_of\_endothelial\_cell\_differentiation | 1 | 0 |  |  |  |  |  |  |  |  |
| GO:0045602\_negative\_regulation\_of\_endothelial\_cell\_differentiation | 1 | 0 |  |  |  |  |  |  |  |  |
| GO:0045605\_negative\_regulation\_of\_epidermal\_cell\_differentiation | 1 | 0 |  |  |  |  |  |  |  |  |
| GO:0045606\_positive\_regulation\_of\_epidermal\_cell\_differentiation | 1 | 0 |  |  |  |  |  |  |  |  |
| GO:0045609\_positive\_regulation\_of\_auditory\_receptor\_cell\_differentiation | 1 | 0 |  |  |  |  |  |  |  |  |
| GO:0045617\_negative\_regulation\_of\_keratinocyte\_differentiation | 1 | 0 |  |  |  |  |  |  |  |  |
| GO:0045618\_positive\_regulation\_of\_keratinocyte\_differentiation | 1 | 0 |  |  |  |  |  |  |  |  |
| GO:0045626\_negative\_regulation\_of\_T-helper\_1\_cell\_differentiation | 1 | 0 |  |  |  |  |  |  |  |  |
| GO:0045633\_positive\_regulation\_of\_mechanoreceptor\_differentiation | 1 | 0 |  |  |  |  |  |  |  |  |
| GO:0045650\_negative\_regulation\_of\_macrophage\_differentiation | 1 | 0 |  |  |  |  |  |  |  |  |
| GO:0045656\_negative\_regulation\_of\_monocyte\_differentiation | 1 | 0 |  |  |  |  |  |  |  |  |
| GO:0045657\_positive\_regulation\_of\_monocyte\_differentiation | 1 | 0 |  |  |  |  |  |  |  |  |
| GO:0045659\_negative\_regulation\_of\_neutrophil\_differentiation | 1 | 0 |  |  |  |  |  |  |  |  |
| GO:0045660\_positive\_regulation\_of\_neutrophil\_differentiation | 1 | 0 |  |  |  |  |  |  |  |  |
| GO:0045721\_negative\_regulation\_of\_gluconeogenesis | 1 | 0 |  |  |  |  |  |  |  |  |
| GO:0045724\_positive\_regulation\_of\_flagellum\_assembly | 1 | 0 |  |  |  |  |  |  |  |  |
| GO:0045725\_positive\_regulation\_of\_glycogen\_biosynthetic\_process | 1 | 0 |  |  |  |  |  |  |  |  |
| GO:0045740\_positive\_regulation\_of\_DNA\_replication | 1 | 0 |  |  |  |  |  |  |  |  |
| GO:0045759\_negative\_regulation\_of\_action\_potential | 1 | 0 |  |  |  |  |  |  |  |  |
| GO:0045768\_positive\_regulation\_of\_anti-apoptosis | 1 | 0 |  |  |  |  |  |  |  |  |
| GO:0045769\_negative\_regulation\_of\_asymmetric\_cell\_division | 1 | 0 |  |  |  |  |  |  |  |  |
| GO:0045794\_negative\_regulation\_of\_cell\_volume | 1 | 0 |  |  |  |  |  |  |  |  |
| GO:0045815\_positive\_regulation\_of\_gene\_expression\_\_epigenetic | 1 | 0 |  |  |  |  |  |  |  |  |
| GO:0045818\_negative\_regulation\_of\_glycogen\_catabolic\_process | 1 | 0 |  |  |  |  |  |  |  |  |
| GO:0045842\_positive\_regulation\_of\_mitotic\_metaphase\_anaphase\_transition | 1 | 0 |  |  |  |  |  |  |  |  |
| GO:0045875\_negative\_regulation\_of\_sister\_chromatid\_cohesion | 1 | 0 |  |  |  |  |  |  |  |  |
| GO:0045898\_regulation\_of\_transcriptional\_preinitiation\_complex\_assembly | 1 | 0 |  |  |  |  |  |  |  |  |
| GO:0045899\_positive\_regulation\_of\_transcriptional\_preinitiation\_complex\_assembly | 1 | 0 |  |  |  |  |  |  |  |  |
| GO:0045906\_negative\_regulation\_of\_vasoconstriction | 1 | 0 |  |  |  |  |  |  |  |  |
| GO:0045908\_negative\_regulation\_of\_vasodilation | 1 | 0 |  |  |  |  |  |  |  |  |
| GO:0045909\_positive\_regulation\_of\_vasodilation | 1 | 0 |  |  |  |  |  |  |  |  |
| GO:0045915\_positive\_regulation\_of\_catecholamine\_metabolic\_process | 1 | 0 |  |  |  |  |  |  |  |  |
| GO:0045920\_negative\_regulation\_of\_exocytosis | 1 | 0 |  |  |  |  |  |  |  |  |
| GO:0045924\_regulation\_of\_female\_receptivity | 1 | 0 |  |  |  |  |  |  |  |  |
| GO:0045947\_negative\_regulation\_of\_translational\_initiation | 1 | 0 |  |  |  |  |  |  |  |  |
| GO:0045955\_negative\_regulation\_of\_calcium\_ion-dependent\_exocytosis | 1 | 0 |  |  |  |  |  |  |  |  |
| GO:0045956\_positive\_regulation\_of\_calcium\_ion-dependent\_exocytosis | 1 | 0 |  |  |  |  |  |  |  |  |
| GO:0045964\_positive\_regulation\_of\_dopamine\_metabolic\_process | 1 | 0 |  |  |  |  |  |  |  |  |
| GO:0045988\_negative\_regulation\_of\_striated\_muscle\_contraction | 1 | 0 |  |  |  |  |  |  |  |  |
| GO:0045989\_positive\_regulation\_of\_striated\_muscle\_contraction | 1 | 0 |  |  |  |  |  |  |  |  |
| GO:0045990\_regulation\_of\_transcription\_by\_carbon\_catabolites | 1 | 0 |  |  |  |  |  |  |  |  |
| GO:0045991\_positive\_regulation\_of\_transcription\_by\_carbon\_catabolites | 1 | 0 |  |  |  |  |  |  |  |  |
| GO:0045994\_positive\_regulation\_of\_translational\_initiation\_by\_iron | 1 | 0 |  |  |  |  |  |  |  |  |
| GO:0046007\_negative\_regulation\_of\_activated\_T\_cell\_proliferation | 1 | 0 |  |  |  |  |  |  |  |  |
| GO:0046014\_negative\_regulation\_of\_T\_cell\_homeostatic\_proliferation | 1 | 0 |  |  |  |  |  |  |  |  |
| GO:0046015\_regulation\_of\_transcription\_by\_glucose | 1 | 0 |  |  |  |  |  |  |  |  |
| GO:0046016\_positive\_regulation\_of\_transcription\_by\_glucose | 1 | 0 |  |  |  |  |  |  |  |  |
| GO:0046031\_ADP\_metabolic\_process | 1 | 0 |  |  |  |  |  |  |  |  |
| GO:0046032\_ADP\_catabolic\_process | 1 | 0 |  |  |  |  |  |  |  |  |
| GO:0046061\_dATP\_catabolic\_process | 1 | 0 |  |  |  |  |  |  |  |  |
| GO:0046075\_dTTP\_metabolic\_process | 1 | 0 |  |  |  |  |  |  |  |  |
| GO:0046078\_dUMP\_metabolic\_process | 1 | 0 |  |  |  |  |  |  |  |  |
| GO:0046079\_dUMP\_catabolic\_process | 1 | 0 |  |  |  |  |  |  |  |  |
| GO:0046086\_adenosine\_biosynthetic\_process | 1 | 0 |  |  |  |  |  |  |  |  |
| GO:0046090\_deoxyadenosine\_metabolic\_process | 1 | 0 |  |  |  |  |  |  |  |  |
| GO:0046098\_guanine\_metabolic\_process | 1 | 0 |  |  |  |  |  |  |  |  |
| GO:0046101\_hypoxanthine\_biosynthetic\_process | 1 | 0 |  |  |  |  |  |  |  |  |
| GO:0046102\_inosine\_metabolic\_process | 1 | 0 |  |  |  |  |  |  |  |  |
| GO:0046103\_inosine\_biosynthetic\_process | 1 | 0 |  |  |  |  |  |  |  |  |
| GO:0046108\_uridine\_metabolic\_process | 1 | 0 |  |  |  |  |  |  |  |  |
| GO:0046110\_xanthine\_metabolic\_process | 1 | 0 |  |  |  |  |  |  |  |  |
| GO:0046111\_xanthine\_biosynthetic\_process | 1 | 0 |  |  |  |  |  |  |  |  |
| GO:0046112\_nucleobase\_biosynthetic\_process | 1 | 0 |  |  |  |  |  |  |  |  |
| GO:0046113\_nucleobase\_catabolic\_process | 1 | 0 |  |  |  |  |  |  |  |  |
| GO:0046121\_deoxyribonucleoside\_catabolic\_process | 1 | 0 |  |  |  |  |  |  |  |  |
| GO:0046122\_purine\_deoxyribonucleoside\_metabolic\_process | 1 | 0 |  |  |  |  |  |  |  |  |
| GO:0046124\_purine\_deoxyribonucleoside\_catabolic\_process | 1 | 0 |  |  |  |  |  |  |  |  |
| GO:0046125\_pyrimidine\_deoxyribonucleoside\_metabolic\_process | 1 | 0 |  |  |  |  |  |  |  |  |
| GO:0046131\_pyrimidine\_ribonucleoside\_metabolic\_process | 1 | 0 |  |  |  |  |  |  |  |  |
| GO:0046160\_heme\_a\_metabolic\_process | 1 | 0 |  |  |  |  |  |  |  |  |
| GO:0046218\_indolalkylamine\_catabolic\_process | 1 | 0 |  |  |  |  |  |  |  |  |
| GO:0046292\_formaldehyde\_metabolic\_process | 1 | 0 |  |  |  |  |  |  |  |  |
| GO:0046294\_formaldehyde\_catabolic\_process | 1 | 0 |  |  |  |  |  |  |  |  |
| GO:0046314\_phosphocreatine\_biosynthetic\_process | 1 | 0 |  |  |  |  |  |  |  |  |
| GO:0046327\_glycerol\_biosynthetic\_process\_from\_pyruvate | 1 | 0 |  |  |  |  |  |  |  |  |
| GO:0046329\_negative\_regulation\_of\_JNK\_cascade | 1 | 0 |  |  |  |  |  |  |  |  |
| GO:0046340\_diacylglycerol\_catabolic\_process | 1 | 0 |  |  |  |  |  |  |  |  |
| GO:0046351\_disaccharide\_biosynthetic\_process | 1 | 0 |  |  |  |  |  |  |  |  |
| GO:0046356\_acetyl-CoA\_catabolic\_process | 1 | 0 |  |  |  |  |  |  |  |  |
| GO:0046358\_butyrate\_biosynthetic\_process | 1 | 0 |  |  |  |  |  |  |  |  |
| GO:0046359\_butyrate\_catabolic\_process | 1 | 0 |  |  |  |  |  |  |  |  |
| GO:0046381\_CMP-N-acetylneuraminate\_metabolic\_process | 1 | 0 |  |  |  |  |  |  |  |  |
| GO:0046415\_urate\_metabolic\_process | 1 | 0 |  |  |  |  |  |  |  |  |
| GO:0046416\_D-amino\_acid\_metabolic\_process | 1 | 0 |  |  |  |  |  |  |  |  |
| GO:0046434\_organophosphate\_catabolic\_process | 1 | 0 |  |  |  |  |  |  |  |  |
| GO:0046437\_D-amino\_acid\_biosynthetic\_process | 1 | 0 |  |  |  |  |  |  |  |  |
| GO:0046440\_L-lysine\_metabolic\_process | 1 | 0 |  |  |  |  |  |  |  |  |
| GO:0046449\_creatinine\_metabolic\_process | 1 | 0 |  |  |  |  |  |  |  |  |
| GO:0046471\_phosphatidylglycerol\_metabolic\_process | 1 | 0 |  |  |  |  |  |  |  |  |
| GO:0046476\_glycosylceramide\_biosynthetic\_process | 1 | 0 |  |  |  |  |  |  |  |  |
| GO:0046477\_glycosylceramide\_catabolic\_process | 1 | 0 |  |  |  |  |  |  |  |  |
| GO:0046485\_ether\_lipid\_metabolic\_process | 1 | 0 |  |  |  |  |  |  |  |  |
| GO:0046487\_glyoxylate\_metabolic\_process | 1 | 0 |  |  |  |  |  |  |  |  |
| GO:0046552\_photoreceptor\_cell\_fate\_commitment | 1 | 0 |  |  |  |  |  |  |  |  |
| GO:0046586\_regulation\_of\_calcium-dependent\_cell-cell\_adhesion | 1 | 0 |  |  |  |  |  |  |  |  |
| GO:0046587\_positive\_regulation\_of\_calcium-dependent\_cell-cell\_adhesion | 1 | 0 |  |  |  |  |  |  |  |  |
| GO:0046602\_regulation\_of\_mitotic\_centrosome\_separation | 1 | 0 |  |  |  |  |  |  |  |  |
| GO:0046604\_positive\_regulation\_of\_mitotic\_centrosome\_separation | 1 | 0 |  |  |  |  |  |  |  |  |
| GO:0046607\_positive\_regulation\_of\_centrosome\_cycle | 1 | 0 |  |  |  |  |  |  |  |  |
| GO:0046655\_folic\_acid\_metabolic\_process | 1 | 0 |  |  |  |  |  |  |  |  |
| GO:0046671\_negative\_regulation\_of\_retinal\_cell\_programmed\_cell\_death | 1 | 0 |  |  |  |  |  |  |  |  |
| GO:0046685\_response\_to\_arsenic | 1 | 0 |  |  |  |  |  |  |  |  |
| GO:0046692\_sperm\_competition | 1 | 0 |  |  |  |  |  |  |  |  |
| GO:0046707\_IDP\_metabolic\_process | 1 | 0 |  |  |  |  |  |  |  |  |
| GO:0046709\_IDP\_catabolic\_process | 1 | 0 |  |  |  |  |  |  |  |  |
| GO:0046724\_oxalic\_acid\_secretion | 1 | 0 |  |  |  |  |  |  |  |  |
| GO:0046753\_non-lytic\_viral\_release | 1 | 0 |  |  |  |  |  |  |  |  |
| GO:0046755\_non-lytic\_virus\_budding | 1 | 0 |  |  |  |  |  |  |  |  |
| GO:0046826\_negative\_regulation\_of\_protein\_export\_from\_nucleus | 1 | 0 |  |  |  |  |  |  |  |  |
| GO:0046827\_positive\_regulation\_of\_protein\_export\_from\_nucleus | 1 | 0 |  |  |  |  |  |  |  |  |
| GO:0046831\_regulation\_of\_RNA\_export\_from\_nucleus | 1 | 0 |  |  |  |  |  |  |  |  |
| GO:0046834\_lipid\_phosphorylation | 1 | 0 |  |  |  |  |  |  |  |  |
| GO:0046853\_inositol\_and\_derivative\_phosphorylation | 1 | 0 |  |  |  |  |  |  |  |  |
| GO:0046864\_isoprenoid\_transport | 1 | 0 |  |  |  |  |  |  |  |  |
| GO:0046865\_terpenoid\_transport | 1 | 0 |  |  |  |  |  |  |  |  |
| GO:0046877\_regulation\_of\_saliva\_secretion | 1 | 0 |  |  |  |  |  |  |  |  |
| GO:0046878\_positive\_regulation\_of\_saliva\_secretion | 1 | 0 |  |  |  |  |  |  |  |  |
| GO:0046884\_follicle-stimulating\_hormone\_secretion | 1 | 0 |  |  |  |  |  |  |  |  |
| GO:0046898\_response\_to\_cycloheximide | 1 | 0 |  |  |  |  |  |  |  |  |
| GO:0046929\_negative\_regulation\_of\_neurotransmitter\_secretion | 1 | 0 |  |  |  |  |  |  |  |  |
| GO:0046931\_pore\_complex\_biogenesis | 1 | 0 |  |  |  |  |  |  |  |  |
| GO:0046949\_acyl-CoA\_biosynthetic\_process | 1 | 0 |  |  |  |  |  |  |  |  |
| GO:0046958\_nonassociative\_learning | 1 | 0 |  |  |  |  |  |  |  |  |
| GO:0046960\_sensitization | 1 | 0 |  |  |  |  |  |  |  |  |
| GO:0046986\_negative\_regulation\_of\_hemoglobin\_biosynthetic\_process | 1 | 0 |  |  |  |  |  |  |  |  |
| GO:0047497\_mitochondrion\_transport\_along\_microtubule | 1 | 0 |  |  |  |  |  |  |  |  |
| GO:0048047\_mating\_behavior\_\_sex\_discrimination | 1 | 0 |  |  |  |  |  |  |  |  |
| GO:0048133\_male\_germ-line\_stem\_cell\_division | 1 | 0 |  |  |  |  |  |  |  |  |
| GO:0048137\_spermatocyte\_division | 1 | 0 |  |  |  |  |  |  |  |  |
| GO:0048143\_astrocyte\_activation | 1 | 0 |  |  |  |  |  |  |  |  |
| GO:0048170\_positive\_regulation\_of\_long-term\_neuronal\_synaptic\_plasticity | 1 | 0 |  |  |  |  |  |  |  |  |
| GO:0048199\_vesicle\_targeting\_\_to\_\_from\_or\_within\_Golgi | 1 | 0 |  |  |  |  |  |  |  |  |
| GO:0048241\_epinephrine\_transport | 1 | 0 |  |  |  |  |  |  |  |  |
| GO:0048242\_epinephrine\_secretion | 1 | 0 |  |  |  |  |  |  |  |  |
| GO:0048243\_norepinephrine\_secretion | 1 | 0 |  |  |  |  |  |  |  |  |
| GO:0048247\_lymphocyte\_chemotaxis | 1 | 0 |  |  |  |  |  |  |  |  |
| GO:0048250\_mitochondrial\_iron\_ion\_transport | 1 | 0 |  |  |  |  |  |  |  |  |
| GO:0048259\_regulation\_of\_receptor-mediated\_endocytosis | 1 | 0 |  |  |  |  |  |  |  |  |
| GO:0048260\_positive\_regulation\_of\_receptor-mediated\_endocytosis | 1 | 0 |  |  |  |  |  |  |  |  |
| GO:0048290\_isotype\_switching\_to\_IgA\_isotypes | 1 | 0 |  |  |  |  |  |  |  |  |
| GO:0048296\_regulation\_of\_isotype\_switching\_to\_IgA\_isotypes | 1 | 0 |  |  |  |  |  |  |  |  |
| GO:0048298\_positive\_regulation\_of\_isotype\_switching\_to\_IgA\_isotypes | 1 | 0 |  |  |  |  |  |  |  |  |
| GO:0048319\_axial\_mesoderm\_morphogenesis | 1 | 0 |  |  |  |  |  |  |  |  |
| GO:0048320\_axial\_mesoderm\_formation | 1 | 0 |  |  |  |  |  |  |  |  |
| GO:0048385\_regulation\_of\_retinoic\_acid\_receptor\_signaling\_pathway | 1 | 0 |  |  |  |  |  |  |  |  |
| GO:0048387\_negative\_regulation\_of\_retinoic\_acid\_receptor\_signaling\_pathway | 1 | 0 |  |  |  |  |  |  |  |  |
| GO:0048388\_endosomal\_lumen\_acidification | 1 | 0 |  |  |  |  |  |  |  |  |
| GO:0048389\_intermediate\_mesoderm\_development | 1 | 0 |  |  |  |  |  |  |  |  |
| GO:0048478\_replication\_fork\_protection | 1 | 0 |  |  |  |  |  |  |  |  |
| GO:0048496\_maintenance\_of\_organ\_identity | 1 | 0 |  |  |  |  |  |  |  |  |
| GO:0048525\_negative\_regulation\_of\_viral\_reproduction | 1 | 0 |  |  |  |  |  |  |  |  |
| GO:0048539\_bone\_marrow\_development | 1 | 0 |  |  |  |  |  |  |  |  |
| GO:0048548\_regulation\_of\_pinocytosis | 1 | 0 |  |  |  |  |  |  |  |  |
| GO:0048549\_positive\_regulation\_of\_pinocytosis | 1 | 0 |  |  |  |  |  |  |  |  |
| GO:0048553\_negative\_regulation\_of\_metalloenzyme\_activity | 1 | 0 |  |  |  |  |  |  |  |  |
| GO:0048588\_developmental\_cell\_growth | 1 | 0 |  |  |  |  |  |  |  |  |
| GO:0048601\_oocyte\_morphogenesis | 1 | 0 |  |  |  |  |  |  |  |  |
| GO:0048621\_post-embryonic\_gut\_morphogenesis | 1 | 0 |  |  |  |  |  |  |  |  |
| GO:0048640\_negative\_regulation\_of\_developmental\_growth | 1 | 0 |  |  |  |  |  |  |  |  |
| GO:0048642\_negative\_regulation\_of\_skeletal\_muscle\_tissue\_development | 1 | 0 |  |  |  |  |  |  |  |  |
| GO:0048669\_collateral\_sprouting\_in\_the\_absence\_of\_injury | 1 | 0 |  |  |  |  |  |  |  |  |
| GO:0048680\_positive\_regulation\_of\_axon\_regeneration | 1 | 0 |  |  |  |  |  |  |  |  |
| GO:0048681\_negative\_regulation\_of\_axon\_regeneration | 1 | 0 |  |  |  |  |  |  |  |  |
| GO:0048686\_regulation\_of\_sprouting\_of\_injured\_axon | 1 | 0 |  |  |  |  |  |  |  |  |
| GO:0048687\_positive\_regulation\_of\_sprouting\_of\_injured\_axon | 1 | 0 |  |  |  |  |  |  |  |  |
| GO:0048690\_regulation\_of\_axon\_extension\_involved\_in\_regeneration | 1 | 0 |  |  |  |  |  |  |  |  |
| GO:0048691\_positive\_regulation\_of\_axon\_extension\_involved\_in\_regeneration | 1 | 0 |  |  |  |  |  |  |  |  |
| GO:0048714\_positive\_regulation\_of\_oligodendrocyte\_differentiation | 1 | 0 |  |  |  |  |  |  |  |  |
| GO:0048733\_sebaceous\_gland\_development | 1 | 0 |  |  |  |  |  |  |  |  |
| GO:0048743\_positive\_regulation\_of\_skeletal\_muscle\_fiber\_development | 1 | 0 |  |  |  |  |  |  |  |  |
| GO:0048752\_semicircular\_canal\_morphogenesis | 1 | 0 |  |  |  |  |  |  |  |  |
| GO:0048773\_erythrophore\_differentiation | 1 | 0 |  |  |  |  |  |  |  |  |
| GO:0048790\_maintenance\_of\_presynaptic\_active\_zone\_structure | 1 | 0 |  |  |  |  |  |  |  |  |
| GO:0048791\_calcium\_ion-dependent\_exocytosis\_of\_neurotransmitter | 1 | 0 |  |  |  |  |  |  |  |  |
| GO:0048822\_enucleate\_erythrocyte\_development | 1 | 0 |  |  |  |  |  |  |  |  |
| GO:0048866\_stem\_cell\_fate\_specification | 1 | 0 |  |  |  |  |  |  |  |  |
| GO:0048936\_peripheral\_nervous\_system\_neuron\_axonogenesis | 1 | 0 |  |  |  |  |  |  |  |  |
| GO:0050427\_3'-phosphoadenosine\_5'-phosphosulfate\_metabolic\_process | 1 | 0 |  |  |  |  |  |  |  |  |
| GO:0050428\_3'-phosphoadenosine\_5'-phosphosulfate\_biosynthetic\_process | 1 | 0 |  |  |  |  |  |  |  |  |
| GO:0050482\_arachidonic\_acid\_secretion | 1 | 0 |  |  |  |  |  |  |  |  |
| GO:0050667\_homocysteine\_metabolic\_process | 1 | 0 |  |  |  |  |  |  |  |  |
| GO:0050674\_urothelial\_cell\_proliferation | 1 | 0 |  |  |  |  |  |  |  |  |
| GO:0050675\_regulation\_of\_urothelial\_cell\_proliferation | 1 | 0 |  |  |  |  |  |  |  |  |
| GO:0050677\_positive\_regulation\_of\_urothelial\_cell\_proliferation | 1 | 0 |  |  |  |  |  |  |  |  |
| GO:0050691\_regulation\_of\_defense\_response\_to\_virus\_by\_host | 1 | 0 |  |  |  |  |  |  |  |  |
| GO:0050748\_negative\_regulation\_of\_lipoprotein\_metabolic\_process | 1 | 0 |  |  |  |  |  |  |  |  |
| GO:0050757\_thymidylate\_synthase\_biosynthetic\_process | 1 | 0 |  |  |  |  |  |  |  |  |
| GO:0050758\_regulation\_of\_thymidylate\_synthase\_biosynthetic\_process | 1 | 0 |  |  |  |  |  |  |  |  |
| GO:0050760\_negative\_regulation\_of\_thymidylate\_synthase\_biosynthetic\_process | 1 | 0 |  |  |  |  |  |  |  |  |
| GO:0050812\_regulation\_of\_acyl-CoA\_biosynthetic\_process | 1 | 0 |  |  |  |  |  |  |  |  |
| GO:0050832\_defense\_response\_to\_fungus | 1 | 0 |  |  |  |  |  |  |  |  |
| GO:0050861\_positive\_regulation\_of\_B\_cell\_receptor\_signaling\_pathway | 1 | 0 |  |  |  |  |  |  |  |  |
| GO:0050862\_positive\_regulation\_of\_T\_cell\_receptor\_signaling\_pathway | 1 | 0 |  |  |  |  |  |  |  |  |
| GO:0050916\_sensory\_perception\_of\_sweet\_taste | 1 | 0 |  |  |  |  |  |  |  |  |
| GO:0050975\_sensory\_perception\_of\_touch | 1 | 0 |  |  |  |  |  |  |  |  |
| GO:0050995\_negative\_regulation\_of\_lipid\_catabolic\_process | 1 | 0 |  |  |  |  |  |  |  |  |
| GO:0051001\_negative\_regulation\_of\_nitric-oxide\_synthase\_activity | 1 | 0 |  |  |  |  |  |  |  |  |
| GO:0051005\_negative\_regulation\_of\_lipoprotein\_lipase\_activity | 1 | 0 |  |  |  |  |  |  |  |  |
| GO:0051006\_positive\_regulation\_of\_lipoprotein\_lipase\_activity | 1 | 0 |  |  |  |  |  |  |  |  |
| GO:0051016\_barbed-end\_actin\_filament\_capping | 1 | 0 |  |  |  |  |  |  |  |  |
| GO:0051029\_rRNA\_transport | 1 | 0 |  |  |  |  |  |  |  |  |
| GO:0051043\_regulation\_of\_membrane\_protein\_ectodomain\_proteolysis | 1 | 0 |  |  |  |  |  |  |  |  |
| GO:0051044\_positive\_regulation\_of\_membrane\_protein\_ectodomain\_proteolysis | 1 | 0 |  |  |  |  |  |  |  |  |
| GO:0051088\_PMA-inducible\_membrane\_protein\_ectodomain\_proteolysis | 1 | 0 |  |  |  |  |  |  |  |  |
| GO:0051102\_DNA\_ligation\_during\_DNA\_recombination | 1 | 0 |  |  |  |  |  |  |  |  |
| GO:0051103\_DNA\_ligation\_during\_DNA\_repair | 1 | 0 |  |  |  |  |  |  |  |  |
| GO:0051123\_transcriptional\_preinitiation\_complex\_assembly | 1 | 0 |  |  |  |  |  |  |  |  |
| GO:0051125\_regulation\_of\_actin\_nucleation | 1 | 0 |  |  |  |  |  |  |  |  |
| GO:0051127\_positive\_regulation\_of\_actin\_nucleation | 1 | 0 |  |  |  |  |  |  |  |  |
| GO:0051151\_negative\_regulation\_of\_smooth\_muscle\_cell\_differentiation | 1 | 0 |  |  |  |  |  |  |  |  |
| GO:0051154\_negative\_regulation\_of\_striated\_muscle\_cell\_differentiation | 1 | 0 |  |  |  |  |  |  |  |  |
| GO:0051155\_positive\_regulation\_of\_striated\_muscle\_cell\_differentiation | 1 | 0 |  |  |  |  |  |  |  |  |
| GO:0051156\_glucose\_6-phosphate\_metabolic\_process | 1 | 0 |  |  |  |  |  |  |  |  |
| GO:0051187\_cofactor\_catabolic\_process | 1 | 0 |  |  |  |  |  |  |  |  |
| GO:0051189\_prosthetic\_group\_metabolic\_process | 1 | 0 |  |  |  |  |  |  |  |  |
| GO:0051193\_regulation\_of\_cofactor\_metabolic\_process | 1 | 0 |  |  |  |  |  |  |  |  |
| GO:0051196\_regulation\_of\_coenzyme\_metabolic\_process | 1 | 0 |  |  |  |  |  |  |  |  |
| GO:0051255\_spindle\_midzone\_assembly | 1 | 0 |  |  |  |  |  |  |  |  |
| GO:0051257\_spindle\_midzone\_assembly\_involved\_in\_meiosis | 1 | 0 |  |  |  |  |  |  |  |  |
| GO:0051281\_positive\_regulation\_of\_release\_of\_sequestered\_calcium\_ion\_into\_cytosol | 1 | 0 |  |  |  |  |  |  |  |  |
| GO:0051290\_protein\_heterotetramerization | 1 | 0 |  |  |  |  |  |  |  |  |
| GO:0051305\_chromosome\_movement\_towards\_spindle\_pole | 1 | 0 |  |  |  |  |  |  |  |  |
| GO:0051310\_metaphase\_plate\_congression | 1 | 0 |  |  |  |  |  |  |  |  |
| GO:0051311\_meiotic\_metaphase\_plate\_congression | 1 | 0 |  |  |  |  |  |  |  |  |
| GO:0051340\_regulation\_of\_ligase\_activity | 1 | 0 |  |  |  |  |  |  |  |  |
| GO:0051351\_positive\_regulation\_of\_ligase\_activity | 1 | 0 |  |  |  |  |  |  |  |  |
| GO:0051354\_negative\_regulation\_of\_oxidoreductase\_activity | 1 | 0 |  |  |  |  |  |  |  |  |
| GO:0051355\_proprioception\_during\_equilibrioception | 1 | 0 |  |  |  |  |  |  |  |  |
| GO:0051383\_kinetochore\_organization | 1 | 0 |  |  |  |  |  |  |  |  |
| GO:0051386\_regulation\_of\_nerve\_growth\_factor\_receptor\_signaling\_pathway | 1 | 0 |  |  |  |  |  |  |  |  |
| GO:0051409\_response\_to\_nitrosative\_stress | 1 | 0 |  |  |  |  |  |  |  |  |
| GO:0051457\_maintenance\_of\_protein\_location\_in\_nucleus | 1 | 0 |  |  |  |  |  |  |  |  |
| GO:0051462\_regulation\_of\_cortisol\_secretion | 1 | 0 |  |  |  |  |  |  |  |  |
| GO:0051463\_negative\_regulation\_of\_cortisol\_secretion | 1 | 0 |  |  |  |  |  |  |  |  |
| GO:0051481\_reduction\_of\_cytosolic\_calcium\_ion\_concentration | 1 | 0 |  |  |  |  |  |  |  |  |
| GO:0051482\_elevation\_of\_cytosolic\_calcium\_ion\_concentration\_during\_G-protein\_signaling\_\_coupled\_to\_IP3\_second\_messenger\_(phospholipase\_C\_activating) | 1 | 0 |  |  |  |  |  |  |  |  |
| GO:0051542\_elastin\_biosynthetic\_process | 1 | 0 |  |  |  |  |  |  |  |  |
| GO:0051568\_histone\_H3-K4\_methylation | 1 | 0 |  |  |  |  |  |  |  |  |
| GO:0051569\_regulation\_of\_histone\_H3-K4\_methylation | 1 | 0 |  |  |  |  |  |  |  |  |
| GO:0051570\_regulation\_of\_histone\_H3-K9\_methylation | 1 | 0 |  |  |  |  |  |  |  |  |
| GO:0051573\_negative\_regulation\_of\_histone\_H3-K9\_methylation | 1 | 0 |  |  |  |  |  |  |  |  |
| GO:0051580\_regulation\_of\_neurotransmitter\_uptake | 1 | 0 |  |  |  |  |  |  |  |  |
| GO:0051582\_positive\_regulation\_of\_neurotransmitter\_uptake | 1 | 0 |  |  |  |  |  |  |  |  |
| GO:0051584\_regulation\_of\_dopamine\_uptake | 1 | 0 |  |  |  |  |  |  |  |  |
| GO:0051586\_positive\_regulation\_of\_dopamine\_uptake | 1 | 0 |  |  |  |  |  |  |  |  |
| GO:0051589\_negative\_regulation\_of\_neurotransmitter\_transport | 1 | 0 |  |  |  |  |  |  |  |  |
| GO:0051593\_response\_to\_folic\_acid | 1 | 0 |  |  |  |  |  |  |  |  |
| GO:0051615\_histamine\_uptake | 1 | 0 |  |  |  |  |  |  |  |  |
| GO:0051646\_mitochondrion\_localization | 1 | 0 |  |  |  |  |  |  |  |  |
| GO:0051654\_establishment\_of\_mitochondrion\_localization | 1 | 0 |  |  |  |  |  |  |  |  |
| GO:0051661\_maintenance\_of\_centrosome\_location | 1 | 0 |  |  |  |  |  |  |  |  |
| GO:0051665\_membrane\_raft\_localization | 1 | 0 |  |  |  |  |  |  |  |  |
| GO:0051685\_maintenance\_of\_ER\_location | 1 | 0 |  |  |  |  |  |  |  |  |
| GO:0051693\_actin\_filament\_capping | 1 | 0 |  |  |  |  |  |  |  |  |
| GO:0051701\_interaction\_with\_host | 1 | 0 |  |  |  |  |  |  |  |  |
| GO:0051754\_meiotic\_sister\_chromatid\_cohesion\_\_centromeric | 1 | 0 |  |  |  |  |  |  |  |  |
| GO:0051782\_negative\_regulation\_of\_cell\_division | 1 | 0 |  |  |  |  |  |  |  |  |
| GO:0051790\_short-chain\_fatty\_acid\_biosynthetic\_process | 1 | 0 |  |  |  |  |  |  |  |  |
| GO:0051799\_negative\_regulation\_of\_hair\_follicle\_development | 1 | 0 |  |  |  |  |  |  |  |  |
| GO:0051823\_regulation\_of\_synapse\_structural\_plasticity | 1 | 0 |  |  |  |  |  |  |  |  |
| GO:0051865\_protein\_autoubiquitination | 1 | 0 |  |  |  |  |  |  |  |  |
| GO:0051901\_positive\_regulation\_of\_mitochondrial\_depolarization | 1 | 0 |  |  |  |  |  |  |  |  |
| GO:0051917\_regulation\_of\_fibrinolysis | 1 | 0 |  |  |  |  |  |  |  |  |
| GO:0051918\_negative\_regulation\_of\_fibrinolysis | 1 | 0 |  |  |  |  |  |  |  |  |
| GO:0051929\_positive\_regulation\_of\_calcium\_ion\_transport\_via\_voltage-gated\_calcium\_channel\_activity | 1 | 0 |  |  |  |  |  |  |  |  |
| GO:0051933\_amino\_acid\_uptake\_during\_transmission\_of\_nerve\_impulse | 1 | 0 |  |  |  |  |  |  |  |  |
| GO:0051935\_glutamate\_uptake\_during\_transmission\_of\_nerve\_impulse | 1 | 0 |  |  |  |  |  |  |  |  |
| GO:0051940\_regulation\_of\_catecholamine\_uptake\_during\_transmission\_of\_nerve\_impulse | 1 | 0 |  |  |  |  |  |  |  |  |
| GO:0051944\_positive\_regulation\_of\_catecholamine\_uptake\_during\_transmission\_of\_nerve\_impulse | 1 | 0 |  |  |  |  |  |  |  |  |
| GO:0051961\_negative\_regulation\_of\_nervous\_system\_development | 1 | 0 |  |  |  |  |  |  |  |  |
| GO:0051964\_negative\_regulation\_of\_synaptogenesis | 1 | 0 |  |  |  |  |  |  |  |  |
| GO:0051968\_positive\_regulation\_of\_synaptic\_transmission\_\_glutamatergic | 1 | 0 |  |  |  |  |  |  |  |  |
| GO:0051984\_positive\_regulation\_of\_chromosome\_segregation | 1 | 0 |  |  |  |  |  |  |  |  |
| GO:0051987\_positive\_regulation\_of\_attachment\_of\_spindle\_microtubules\_to\_kinetochore | 1 | 0 |  |  |  |  |  |  |  |  |
| GO:0052173\_response\_to\_defenses\_of\_other\_organism\_during\_symbiotic\_interaction | 1 | 0 |  |  |  |  |  |  |  |  |
| GO:0052200\_response\_to\_host\_defenses | 1 | 0 |  |  |  |  |  |  |  |  |
| GO:0052551\_response\_to\_defense-related\_nitric\_oxide\_production\_by\_other\_organism\_during\_symbiotic\_interaction | 1 | 0 |  |  |  |  |  |  |  |  |
| GO:0052564\_response\_to\_immune\_response\_of\_other\_organism\_during\_symbiotic\_interaction | 1 | 0 |  |  |  |  |  |  |  |  |
| GO:0052565\_response\_to\_defense-related\_host\_nitric\_oxide\_production | 1 | 0 |  |  |  |  |  |  |  |  |
| GO:0052572\_response\_to\_host\_immune\_response | 1 | 0 |  |  |  |  |  |  |  |  |
| GO:0055005\_ventricular\_cardiac\_myofibril\_development | 1 | 0 |  |  |  |  |  |  |  |  |
| GO:0055011\_atrial\_cardiac\_muscle\_cell\_differentiation | 1 | 0 |  |  |  |  |  |  |  |  |
| GO:0055014\_atrial\_cardiac\_muscle\_cell\_development | 1 | 0 |  |  |  |  |  |  |  |  |
| GO:0055078\_sodium\_ion\_homeostasis | 1 | 0 |  |  |  |  |  |  |  |  |
| GO:0055089\_fatty\_acid\_homeostasis | 1 | 0 |  |  |  |  |  |  |  |  |
| GO:0055093\_response\_to\_hyperoxia | 1 | 0 |  |  |  |  |  |  |  |  |
| GO:0060003\_copper\_ion\_export | 1 | 0 |  |  |  |  |  |  |  |  |
| GO:0060005\_vestibular\_reflex | 1 | 0 |  |  |  |  |  |  |  |  |
| GO:0060014\_granulosa\_cell\_differentiation | 1 | 0 |  |  |  |  |  |  |  |  |
| GO:0060018\_astrocyte\_fate\_commitment | 1 | 0 |  |  |  |  |  |  |  |  |
| GO:0060020\_Bergmann\_glial\_cell\_differentiation | 1 | 0 |  |  |  |  |  |  |  |  |
| GO:0060022\_hard\_palate\_development | 1 | 0 |  |  |  |  |  |  |  |  |
| GO:0060034\_notochord\_cell\_differentiation | 1 | 0 |  |  |  |  |  |  |  |  |
| GO:0060035\_notochord\_cell\_development | 1 | 0 |  |  |  |  |  |  |  |  |
| GO:0060046\_regulation\_of\_acrosome\_reaction | 1 | 0 |  |  |  |  |  |  |  |  |
| GO:0060054\_positive\_regulation\_of\_epithelial\_cell\_proliferation\_involved\_in\_wound\_healing | 1 | 0 |  |  |  |  |  |  |  |  |
| GO:0060059\_embryonic\_retina\_morphogenesis\_in\_camera-type\_eye | 1 | 0 |  |  |  |  |  |  |  |  |
| GO:0060061\_Spemann\_organizer\_formation | 1 | 0 |  |  |  |  |  |  |  |  |
| GO:0060064\_Spemann\_organizer\_formation\_at\_the\_anterior\_end\_of\_the\_primitive\_streak | 1 | 0 |  |  |  |  |  |  |  |  |
| GO:0060071\_Wnt\_receptor\_signaling\_pathway\_\_planar\_cell\_polarity\_pathway | 1 | 0 |  |  |  |  |  |  |  |  |
| GO:0060075\_regulation\_of\_resting\_membrane\_potential | 1 | 0 |  |  |  |  |  |  |  |  |
| GO:0060082\_eye\_blink\_reflex | 1 | 0 |  |  |  |  |  |  |  |  |
| GO:0060112\_generation\_of\_ovulation\_cycle\_rhythm | 1 | 0 |  |  |  |  |  |  |  |  |
| GO:0060125\_negative\_regulation\_of\_growth\_hormone\_secretion | 1 | 0 |  |  |  |  |  |  |  |  |
| GO:0060151\_peroxisome\_localization | 1 | 0 |  |  |  |  |  |  |  |  |
| GO:0060152\_microtubule-based\_peroxisome\_localization | 1 | 0 |  |  |  |  |  |  |  |  |
| GO:0060161\_positive\_regulation\_of\_dopamine\_receptor\_signaling\_pathway | 1 | 0 |  |  |  |  |  |  |  |  |
| GO:0060163\_subpallium\_neuron\_fate\_commitment | 1 | 0 |  |  |  |  |  |  |  |  |
| GO:0060165\_regulation\_of\_timing\_of\_subpallium\_neuron\_differentiation | 1 | 0 |  |  |  |  |  |  |  |  |
| GO:0060174\_limb\_bud\_formation | 1 | 0 |  |  |  |  |  |  |  |  |
| GO:0060177\_regulation\_of\_angiotensin\_metabolic\_process | 1 | 0 |  |  |  |  |  |  |  |  |
| GO:0060197\_cloacal\_septation | 1 | 0 |  |  |  |  |  |  |  |  |
| GO:0060215\_primitive\_hemopoiesis | 1 | 0 |  |  |  |  |  |  |  |  |
| GO:0060231\_mesenchymal\_to\_epithelial\_transition | 1 | 0 |  |  |  |  |  |  |  |  |
| GO:0060254\_regulation\_of\_N-terminal\_protein\_palmitoylation | 1 | 0 |  |  |  |  |  |  |  |  |
| GO:0060261\_positive\_regulation\_of\_transcription\_initiation\_from\_RNA\_polymerase\_II\_promoter | 1 | 0 |  |  |  |  |  |  |  |  |
| GO:0060262\_negative\_regulation\_of\_N-terminal\_protein\_palmitoylation | 1 | 0 |  |  |  |  |  |  |  |  |
| GO:0060263\_regulation\_of\_respiratory\_burst | 1 | 0 |  |  |  |  |  |  |  |  |
| GO:0060264\_regulation\_of\_respiratory\_burst\_during\_acute\_inflammatory\_response | 1 | 0 |  |  |  |  |  |  |  |  |
| GO:0060265\_positive\_regulation\_of\_respiratory\_burst\_during\_acute\_inflammatory\_response | 1 | 0 |  |  |  |  |  |  |  |  |
| GO:0060267\_positive\_regulation\_of\_respiratory\_burst | 1 | 0 |  |  |  |  |  |  |  |  |
| GO:0060272\_embryonic\_skeletal\_joint\_morphogenesis | 1 | 0 |  |  |  |  |  |  |  |  |
| GO:0060297\_regulation\_of\_sarcomere\_organization | 1 | 0 |  |  |  |  |  |  |  |  |
| GO:0060298\_positive\_regulation\_of\_sarcomere\_organization | 1 | 0 |  |  |  |  |  |  |  |  |
| GO:0060315\_negative\_regulation\_of\_ryanodine-sensitive\_calcium-release\_channel\_activity | 1 | 0 |  |  |  |  |  |  |  |  |
| GO:0060319\_primitive\_erythrocyte\_differentiation | 1 | 0 |  |  |  |  |  |  |  |  |
| GO:0060371\_regulation\_of\_atrial\_cardiomyocyte\_membrane\_depolarization | 1 | 0 |  |  |  |  |  |  |  |  |
| GO:0060374\_mast\_cell\_differentiation | 1 | 0 |  |  |  |  |  |  |  |  |
| GO:0060375\_regulation\_of\_mast\_cell\_differentiation | 1 | 0 |  |  |  |  |  |  |  |  |
| GO:0060376\_positive\_regulation\_of\_mast\_cell\_differentiation | 1 | 0 |  |  |  |  |  |  |  |  |
| GO:0060390\_regulation\_of\_SMAD\_protein\_nuclear\_translocation | 1 | 0 |  |  |  |  |  |  |  |  |
| GO:0060391\_positive\_regulation\_of\_SMAD\_protein\_nuclear\_translocation | 1 | 0 |  |  |  |  |  |  |  |  |
| GO:0060398\_regulation\_of\_growth\_hormone\_receptor\_signaling\_pathway | 1 | 0 |  |  |  |  |  |  |  |  |
| GO:0060399\_positive\_regulation\_of\_growth\_hormone\_receptor\_signaling\_pathway | 1 | 0 |  |  |  |  |  |  |  |  |
| GO:0060405\_regulation\_of\_penile\_erection | 1 | 0 |  |  |  |  |  |  |  |  |
| GO:0060407\_negative\_regulation\_of\_penile\_erection | 1 | 0 |  |  |  |  |  |  |  |  |
| GO:0060413\_atrial\_septum\_morphogenesis | 1 | 0 |  |  |  |  |  |  |  |  |
| GO:0060414\_aorta\_smooth\_muscle\_tissue\_morphogenesis | 1 | 0 |  |  |  |  |  |  |  |  |
| GO:0060419\_heart\_growth | 1 | 0 |  |  |  |  |  |  |  |  |
| GO:0060420\_regulation\_of\_heart\_growth | 1 | 0 |  |  |  |  |  |  |  |  |
| GO:0060421\_positive\_regulation\_of\_heart\_growth | 1 | 0 |  |  |  |  |  |  |  |  |
| GO:0060431\_primary\_lung\_bud\_formation | 1 | 0 |  |  |  |  |  |  |  |  |
| GO:0060436\_bronchiole\_morphogenesis | 1 | 0 |  |  |  |  |  |  |  |  |
| GO:0060440\_trachea\_formation | 1 | 0 |  |  |  |  |  |  |  |  |
| GO:0060449\_bud\_elongation\_involved\_in\_lung\_branching | 1 | 0 |  |  |  |  |  |  |  |  |
| GO:0060456\_positive\_regulation\_of\_digestive\_system\_process | 1 | 0 |  |  |  |  |  |  |  |  |
| GO:0060461\_right\_lung\_morphogenesis | 1 | 0 |  |  |  |  |  |  |  |  |
| GO:0060481\_lobar\_bronchus\_epithelium\_development | 1 | 0 |  |  |  |  |  |  |  |  |
| GO:0060482\_lobar\_bronchus\_development | 1 | 0 |  |  |  |  |  |  |  |  |
| GO:0060484\_lung-associated\_mesenchyme\_development | 1 | 0 |  |  |  |  |  |  |  |  |
| GO:0060486\_Clara\_cell\_differentiation | 1 | 0 |  |  |  |  |  |  |  |  |
| GO:0060510\_Type\_II\_pneumocyte\_differentiation | 1 | 0 |  |  |  |  |  |  |  |  |
| GO:0060514\_prostate\_induction | 1 | 0 |  |  |  |  |  |  |  |  |
| GO:0060515\_prostate\_field\_specification | 1 | 0 |  |  |  |  |  |  |  |  |
| GO:0060517\_epithelial\_cell\_proliferation\_involved\_in\_prostatic\_bud\_elongation | 1 | 0 |  |  |  |  |  |  |  |  |
| GO:0060520\_activation\_of\_prostate\_induction\_by\_androgen\_receptor\_signaling\_pathway | 1 | 0 |  |  |  |  |  |  |  |  |
| GO:0060535\_trachea\_cartilage\_morphogenesis | 1 | 0 |  |  |  |  |  |  |  |  |
| GO:0060536\_cartilage\_morphogenesis | 1 | 0 |  |  |  |  |  |  |  |  |
| GO:0060563\_neuroepithelial\_cell\_differentiation | 1 | 0 |  |  |  |  |  |  |  |  |
| GO:0060577\_pulmonary\_vein\_morphogenesis | 1 | 0 |  |  |  |  |  |  |  |  |
| GO:0060578\_superior\_vena\_cava\_morphogenesis | 1 | 0 |  |  |  |  |  |  |  |  |
| GO:0060584\_regulation\_of\_prostaglandin-endoperoxide\_synthase\_activity | 1 | 0 |  |  |  |  |  |  |  |  |
| GO:0060585\_positive\_regulation\_of\_prostaglandin-endoperoxidase\_synthase\_activity | 1 | 0 |  |  |  |  |  |  |  |  |
| GO:0060598\_dichotomous\_subdivision\_of\_terminal\_units\_involved\_in\_mammary\_gland\_duct\_morphogenesis | 1 | 0 |  |  |  |  |  |  |  |  |
| GO:0060611\_mammary\_gland\_fat\_development | 1 | 0 |  |  |  |  |  |  |  |  |
| GO:0060618\_nipple\_development | 1 | 0 |  |  |  |  |  |  |  |  |
| GO:0060631\_regulation\_of\_meiosis\_I | 1 | 0 |  |  |  |  |  |  |  |  |
| GO:0060649\_mammary\_gland\_bud\_elongation | 1 | 0 |  |  |  |  |  |  |  |  |
| GO:0060658\_nipple\_morphogenesis | 1 | 0 |  |  |  |  |  |  |  |  |
| GO:0060659\_nipple\_sheath\_formation | 1 | 0 |  |  |  |  |  |  |  |  |
| GO:0060668\_regulation\_of\_branching\_involved\_in\_salivary\_gland\_morphogenesis\_by\_extracellular\_matrix-epithelial\_cell\_signaling | 1 | 0 |  |  |  |  |  |  |  |  |
| GO:0060683\_regulation\_of\_branching\_involved\_in\_salivary\_gland\_morphogenesis\_by\_epithelial-mesenchymal\_signaling | 1 | 0 |  |  |  |  |  |  |  |  |
| GO:0060691\_epithelial\_cell\_maturation\_involved\_in\_salivary\_gland\_development | 1 | 0 |  |  |  |  |  |  |  |  |
| GO:0060709\_glycogen\_cell\_development\_involved\_in\_embryonic\_placenta\_development | 1 | 0 |  |  |  |  |  |  |  |  |
| GO:0060732\_positive\_regulation\_of\_inositol\_phosphate\_biosynthetic\_process | 1 | 0 |  |  |  |  |  |  |  |  |
| GO:0060739\_mesenchymal-epithelial\_cell\_signaling\_involved\_in\_prostate\_gland\_development | 1 | 0 |  |  |  |  |  |  |  |  |
| GO:0060781\_mesenchymal\_cell\_proliferation\_involved\_in\_prostate\_gland\_development | 1 | 0 |  |  |  |  |  |  |  |  |
| GO:0060782\_regulation\_of\_mesenchymal\_cell\_proliferation\_involved\_in\_prostate\_gland\_development | 1 | 0 |  |  |  |  |  |  |  |  |
| GO:0060783\_mesenchymal\_smoothened\_signaling\_pathway\_involved\_in\_prostate\_gland\_development | 1 | 0 |  |  |  |  |  |  |  |  |
| GO:0060872\_semicircular\_canal\_development | 1 | 0 |  |  |  |  |  |  |  |  |
| GO:0060896\_neural\_plate\_pattern\_specification | 1 | 0 |  |  |  |  |  |  |  |  |
| GO:0070091\_glucagon\_secretion | 1 | 0 |  |  |  |  |  |  |  |  |
| GO:0070162\_adiponectin\_secretion | 1 | 0 |  |  |  |  |  |  |  |  |
| GO:0070163\_regulation\_of\_adiponectin\_secretion | 1 | 0 |  |  |  |  |  |  |  |  |
| GO:0070164\_negative\_regulation\_of\_adiponectin\_secretion | 1 | 0 |  |  |  |  |  |  |  |  |
| GO:0070178\_D-serine\_metabolic\_process | 1 | 0 |  |  |  |  |  |  |  |  |
| GO:0070179\_D-serine\_biosynthetic\_process | 1 | 0 |  |  |  |  |  |  |  |  |
| GO:0070296\_sarcoplasmic\_reticulum\_calcium\_ion\_transport | 1 | 0 |  |  |  |  |  |  |  |  |
| GO:0070303\_negative\_regulation\_of\_stress-activated\_protein\_kinase\_signaling\_pathway | 1 | 0 |  |  |  |  |  |  |  |  |
| GO:0070328\_triglyceride\_homeostasis | 1 | 0 |  |  |  |  |  |  |  |  |
| GO:0070365\_hepatocyte\_differentiation | 1 | 0 |  |  |  |  |  |  |  |  |
| GO:0070384\_Harderian\_gland\_development | 1 | 0 |  |  |  |  |  |  |  |  |
| GO:0070391\_response\_to\_lipoteichoic\_acid | 1 | 0 |  |  |  |  |  |  |  |  |
| GO:0070424\_regulation\_of\_nucleotide-binding\_oligomerization\_domain\_containing\_signaling\_pathway | 1 | 0 |  |  |  |  |  |  |  |  |
| GO:0070426\_positive\_regulation\_of\_nucleotide-binding\_oligomerization\_domain\_containing\_signaling\_pathway | 1 | 0 |  |  |  |  |  |  |  |  |
| GO:0070428\_regulation\_of\_nucleotide-binding\_oligomerization\_domain\_containing\_1\_signaling\_pathway | 1 | 0 |  |  |  |  |  |  |  |  |
| GO:0070430\_positive\_regulation\_of\_nucleotide-binding\_oligomerization\_domain\_containing\_1\_signaling\_pathway | 1 | 0 |  |  |  |  |  |  |  |  |
| GO:0070432\_regulation\_of\_nucleotide-binding\_oligomerization\_domain\_containing\_2\_signaling\_pathway | 1 | 0 |  |  |  |  |  |  |  |  |
| GO:0070434\_positive\_regulation\_of\_nucleotide-binding\_oligomerization\_domain\_containing\_2\_signaling\_pathway | 1 | 0 |  |  |  |  |  |  |  |  |
| GO:0070493\_thrombin\_receptor\_signaling\_pathway | 1 | 0 |  |  |  |  |  |  |  |  |
| GO:0070508\_cholesterol\_import | 1 | 0 |  |  |  |  |  |  |  |  |
| GO:0070527\_platelet\_aggregation | 1 | 0 |  |  |  |  |  |  |  |  |
| GO:0070528\_protein\_kinase\_C\_signaling\_cascade | 1 | 0 |  |  |  |  |  |  |  |  |
| GO:0070555\_response\_to\_interleukin-1 | 1 | 0 |  |  |  |  |  |  |  |  |
| GO:0070560\_protein\_secretion\_by\_platelet | 1 | 0 |  |  |  |  |  |  |  |  |
| GO:0070561\_vitamin\_D\_receptor\_signaling\_pathway | 1 | 0 |  |  |  |  |  |  |  |  |
| GO:0070562\_regulation\_of\_vitamin\_D\_receptor\_signaling\_pathway | 1 | 0 |  |  |  |  |  |  |  |  |
| GO:0070571\_negative\_regulation\_of\_neuron\_projection\_regeneration | 1 | 0 |  |  |  |  |  |  |  |  |
| GO:0070572\_positive\_regulation\_of\_neuron\_projection\_regeneration | 1 | 0 |  |  |  |  |  |  |  |  |
| GO:0070613\_regulation\_of\_protein\_processing | 1 | 0 |  |  |  |  |  |  |  |  |
| GO:0070627\_ferrous\_iron\_import | 1 | 0 |  |  |  |  |  |  |  |  |
| GO:0070669\_response\_to\_interleukin-2 | 1 | 0 |  |  |  |  |  |  |  |  |
| GO:0070670\_response\_to\_interleukin-4 | 1 | 0 |  |  |  |  |  |  |  |  |
| GO:0070671\_response\_to\_interleukin-12 | 1 | 0 |  |  |  |  |  |  |  |  |
| GO:0070672\_response\_to\_interleukin-15 | 1 | 0 |  |  |  |  |  |  |  |  |
| GO:0070673\_response\_to\_interleukin-18 | 1 | 0 |  |  |  |  |  |  |  |  |
| GO:0070828\_heterochromatin\_organization | 1 | 0 |  |  |  |  |  |  |  |  |
| GO:0070874\_negative\_regulation\_of\_glycogen\_metabolic\_process | 1 | 0 |  |  |  |  |  |  |  |  |
| GO:0075136\_response\_to\_host | 1 | 0 |  |  |  |  |  |  |  |  |
| GO:0080010\_regulation\_of\_oxygen\_and\_reactive\_oxygen\_species\_metabolic\_process | 1 | 0 |  |  |  |  |  |  |  |  |
| GO:0090032\_negative\_regulation\_of\_steroid\_hormone\_biosynthetic\_process | 1 | 0 |  |  |  |  |  |  |  |  |
| GO:0002440\_production\_of\_molecular\_mediator\_of\_immune\_response | 49 | 0 | 0.000000 | -0.000000 | 1101 | 1153.617707 | 1236.55 | 1319.482293 | 1.123115 |
| GO:0003015\_heart\_process | 49 | 0 | 0.000000 | -0.000000 | 1101 | 1153.617707 | 1236.55 | 1319.482293 | 1.123115 |
| GO:0006725\_cellular\_aromatic\_compound\_metabolic\_process | 49 | 0 | 0.000000 | -0.000000 | 1101 | 1153.617707 | 1236.55 | 1319.482293 | 1.123115 |
| GO:0007606\_sensory\_perception\_of\_chemical\_stimulus | 49 | 0 | 0.000000 | -0.000000 | 1101 | 1153.617707 | 1236.55 | 1319.482293 | 1.123115 |
| GO:0021543\_pallium\_development | 49 | 0 | 0.000000 | -0.000000 | 1101 | 1153.617707 | 1236.55 | 1319.482293 | 1.123115 |
| GO:0060047\_heart\_contraction | 49 | 0 | 0.000000 | -0.000000 | 1101 | 1153.617707 | 1236.55 | 1319.482293 | 1.123115 |
| GO:0002757\_immune\_response-activating\_signal\_transduction | 47 | 0 | 0.000000 | -0.000000 | 1112 | 1162.354369 | 1244.43 | 1326.505631 | 1.119092 |
| GO:0006140\_regulation\_of\_nucleotide\_metabolic\_process | 47 | 0 | 0.000000 | -0.000000 | 1112 | 1162.354369 | 1244.43 | 1326.505631 | 1.119092 |
| GO:0006396\_RNA\_processing | 47 | 0 | 0.000000 | -0.000000 | 1112 | 1162.354369 | 1244.43 | 1326.505631 | 1.119092 |
| GO:0030183\_B\_cell\_differentiation | 47 | 0 | 0.000000 | -0.000000 | 1112 | 1162.354369 | 1244.43 | 1326.505631 | 1.119092 |
| GO:0030799\_regulation\_of\_cyclic\_nucleotide\_metabolic\_process | 47 | 0 | 0.000000 | -0.000000 | 1112 | 1162.354369 | 1244.43 | 1326.505631 | 1.119092 |
| GO:0031667\_response\_to\_nutrient\_levels | 47 | 0 | 0.000000 | -0.000000 | 1112 | 1162.354369 | 1244.43 | 1326.505631 | 1.119092 |
| GO:0034754\_cellular\_hormone\_metabolic\_process | 47 | 0 | 0.000000 | -0.000000 | 1112 | 1162.354369 | 1244.43 | 1326.505631 | 1.119092 |
| GO:0045087\_innate\_immune\_response | 47 | 0 | 0.000000 | -0.000000 | 1112 | 1162.354369 | 1244.43 | 1326.505631 | 1.119092 |
| GO:0045619\_regulation\_of\_lymphocyte\_differentiation | 47 | 0 | 0.000000 | -0.000000 | 1112 | 1162.354369 | 1244.43 | 1326.505631 | 1.119092 |
| GO:0048871\_multicellular\_organismal\_homeostasis | 47 | 0 | 0.000000 | -0.000000 | 1112 | 1162.354369 | 1244.43 | 1326.505631 | 1.119092 |
| GO:0060627\_regulation\_of\_vesicle-mediated\_transport | 47 | 0 | 0.000000 | -0.000000 | 1112 | 1162.354369 | 1244.43 | 1326.505631 | 1.119092 |
| GO:0006753\_nucleoside\_phosphate\_metabolic\_process | 94 | 0 | 0.000000 | -0.000000 | 1117 | 1165.313563 | 1246.99 | 1328.666437 | 1.116374 |
| GO:0009117\_nucleotide\_metabolic\_process | 94 | 0 | 0.000000 | -0.000000 | 1117 | 1165.313563 | 1246.99 | 1328.666437 | 1.116374 |
| GO:0032943\_mononuclear\_cell\_proliferation | 94 | 0 | 0.000000 | -0.000000 | 1117 | 1165.313563 | 1246.99 | 1328.666437 | 1.116374 |
| GO:0034984\_cellular\_response\_to\_DNA\_damage\_stimulus | 94 | 0 | 0.000000 | -0.000000 | 1117 | 1165.313563 | 1246.99 | 1328.666437 | 1.116374 |
| GO:0046651\_lymphocyte\_proliferation | 94 | 0 | 0.000000 | -0.000000 | 1117 | 1165.313563 | 1246.99 | 1328.666437 | 1.116374 |
| GO:0001934\_positive\_regulation\_of\_protein\_amino\_acid\_phosphorylation | 29 | 0 | 0.000000 | -0.000000 | 1135 | 1183.658936 | 1264.37 | 1345.081064 | 1.113982 |
| GO:0006641\_triglyceride\_metabolic\_process | 29 | 0 | 0.000000 | -0.000000 | 1135 | 1183.658936 | 1264.37 | 1345.081064 | 1.113982 |
| GO:0006909\_phagocytosis | 29 | 0 | 0.000000 | -0.000000 | 1135 | 1183.658936 | 1264.37 | 1345.081064 | 1.113982 |
| GO:0007190\_activation\_of\_adenylate\_cyclase\_activity | 29 | 0 | 0.000000 | -0.000000 | 1135 | 1183.658936 | 1264.37 | 1345.081064 | 1.113982 |
| GO:0010564\_regulation\_of\_cell\_cycle\_process | 29 | 0 | 0.000000 | -0.000000 | 1135 | 1183.658936 | 1264.37 | 1345.081064 | 1.113982 |
| GO:0016447\_somatic\_recombination\_of\_immunoglobulin\_gene\_segments | 29 | 0 | 0.000000 | -0.000000 | 1135 | 1183.658936 | 1264.37 | 1345.081064 | 1.113982 |
| GO:0021761\_limbic\_system\_development | 29 | 0 | 0.000000 | -0.000000 | 1135 | 1183.658936 | 1264.37 | 1345.081064 | 1.113982 |
| GO:0042176\_regulation\_of\_protein\_catabolic\_process | 29 | 0 | 0.000000 | -0.000000 | 1135 | 1183.658936 | 1264.37 | 1345.081064 | 1.113982 |
| GO:0042490\_mechanoreceptor\_differentiation | 29 | 0 | 0.000000 | -0.000000 | 1135 | 1183.658936 | 1264.37 | 1345.081064 | 1.113982 |
| GO:0042770\_DNA\_damage\_response\_\_signal\_transduction | 29 | 0 | 0.000000 | -0.000000 | 1135 | 1183.658936 | 1264.37 | 1345.081064 | 1.113982 |
| GO:0043281\_regulation\_of\_caspase\_activity | 29 | 0 | 0.000000 | -0.000000 | 1135 | 1183.658936 | 1264.37 | 1345.081064 | 1.113982 |
| GO:0044087\_regulation\_of\_cellular\_component\_biogenesis | 29 | 0 | 0.000000 | -0.000000 | 1135 | 1183.658936 | 1264.37 | 1345.081064 | 1.113982 |
| GO:0045621\_positive\_regulation\_of\_lymphocyte\_differentiation | 29 | 0 | 0.000000 | -0.000000 | 1135 | 1183.658936 | 1264.37 | 1345.081064 | 1.113982 |
| GO:0046634\_regulation\_of\_alpha-beta\_T\_cell\_activation | 29 | 0 | 0.000000 | -0.000000 | 1135 | 1183.658936 | 1264.37 | 1345.081064 | 1.113982 |
| GO:0048066\_pigmentation\_during\_development | 29 | 0 | 0.000000 | -0.000000 | 1135 | 1183.658936 | 1264.37 | 1345.081064 | 1.113982 |
| GO:0050769\_positive\_regulation\_of\_neurogenesis | 29 | 0 | 0.000000 | -0.000000 | 1135 | 1183.658936 | 1264.37 | 1345.081064 | 1.113982 |
| GO:0052548\_regulation\_of\_endopeptidase\_activity | 29 | 0 | 0.000000 | -0.000000 | 1135 | 1183.658936 | 1264.37 | 1345.081064 | 1.113982 |
| GO:0070302\_regulation\_of\_stress-activated\_protein\_kinase\_signaling\_pathway | 29 | 0 | 0.000000 | -0.000000 | 1135 | 1183.658936 | 1264.37 | 1345.081064 | 1.113982 |
| GO:0000165\_MAPKKK\_cascade | 114 | 0 | 0.000000 | -0.000000 | 1137 | 1184.628419 | 1265.12 | 1345.611581 | 1.112682 |
| GO:0009607\_response\_to\_biotic\_stimulus | 114 | 0 | 0.000000 | -0.000000 | 1137 | 1184.628419 | 1265.12 | 1345.611581 | 1.112682 |
| GO:0001932\_regulation\_of\_protein\_amino\_acid\_phosphorylation | 69 | 0 | 0.000000 | -0.000000 | 1142 | 1187.928829 | 1267.96 | 1347.991171 | 1.110298 |
| GO:0005996\_monosaccharide\_metabolic\_process | 69 | 0 | 0.000000 | -0.000000 | 1142 | 1187.928829 | 1267.96 | 1347.991171 | 1.110298 |
| GO:0006816\_calcium\_ion\_transport | 69 | 0 | 0.000000 | -0.000000 | 1142 | 1187.928829 | 1267.96 | 1347.991171 | 1.110298 |
| GO:0032101\_regulation\_of\_response\_to\_external\_stimulus | 69 | 0 | 0.000000 | -0.000000 | 1142 | 1187.928829 | 1267.96 | 1347.991171 | 1.110298 |
| GO:0055065\_metal\_ion\_homeostasis | 69 | 0 | 0.000000 | -0.000000 | 1142 | 1187.928829 | 1267.96 | 1347.991171 | 1.110298 |
| GO:0002250\_adaptive\_immune\_response | 80 | 0 | 0.000000 | -0.000000 | 1146 | 1190.880688 | 1270.34 | 1349.799312 | 1.108499 |
| GO:0002460\_adaptive\_immune\_response\_based\_on\_somatic\_recombination\_of\_immune\_receptors\_built\_from\_immunoglobulin\_superfamily\_domains | 80 | 0 | 0.000000 | -0.000000 | 1146 | 1190.880688 | 1270.34 | 1349.799312 | 1.108499 |
| GO:0006631\_fatty\_acid\_metabolic\_process | 80 | 0 | 0.000000 | -0.000000 | 1146 | 1190.880688 | 1270.34 | 1349.799312 | 1.108499 |
| GO:0044092\_negative\_regulation\_of\_molecular\_function | 80 | 0 | 0.000000 | -0.000000 | 1146 | 1190.880688 | 1270.34 | 1349.799312 | 1.108499 |
| GO:0018193\_peptidyl-amino\_acid\_modification | 97 | 0 | 0.000000 | -0.000000 | 1148 | 1191.832074 | 1271.1 | 1350.367926 | 1.107230 |
| GO:0060341\_regulation\_of\_cellular\_localization | 97 | 0 | 0.000000 | -0.000000 | 1148 | 1191.832074 | 1271.1 | 1350.367926 | 1.107230 |
| GO:0005976\_polysaccharide\_metabolic\_process | 39 | 0 | 0.000000 | -0.000000 | 1162 | 1203.349584 | 1282.08 | 1360.810416 | 1.103339 |
| GO:0006511\_ubiquitin-dependent\_protein\_catabolic\_process | 39 | 0 | 0.000000 | -0.000000 | 1162 | 1203.349584 | 1282.08 | 1360.810416 | 1.103339 |
| GO:0006730\_one-carbon\_metabolic\_process | 39 | 0 | 0.000000 | -0.000000 | 1162 | 1203.349584 | 1282.08 | 1360.810416 | 1.103339 |
| GO:0007160\_cell-matrix\_adhesion | 39 | 0 | 0.000000 | -0.000000 | 1162 | 1203.349584 | 1282.08 | 1360.810416 | 1.103339 |
| GO:0007286\_spermatid\_development | 39 | 0 | 0.000000 | -0.000000 | 1162 | 1203.349584 | 1282.08 | 1360.810416 | 1.103339 |
| GO:0008037\_cell\_recognition | 39 | 0 | 0.000000 | -0.000000 | 1162 | 1203.349584 | 1282.08 | 1360.810416 | 1.103339 |
| GO:0021953\_central\_nervous\_system\_neuron\_differentiation | 39 | 0 | 0.000000 | -0.000000 | 1162 | 1203.349584 | 1282.08 | 1360.810416 | 1.103339 |
| GO:0031279\_regulation\_of\_cyclase\_activity | 39 | 0 | 0.000000 | -0.000000 | 1162 | 1203.349584 | 1282.08 | 1360.810416 | 1.103339 |
| GO:0035148\_tube\_lumen\_formation | 39 | 0 | 0.000000 | -0.000000 | 1162 | 1203.349584 | 1282.08 | 1360.810416 | 1.103339 |
| GO:0042475\_odontogenesis\_of\_dentine-containing\_tooth | 39 | 0 | 0.000000 | -0.000000 | 1162 | 1203.349584 | 1282.08 | 1360.810416 | 1.103339 |
| GO:0043524\_negative\_regulation\_of\_neuron\_apoptosis | 39 | 0 | 0.000000 | -0.000000 | 1162 | 1203.349584 | 1282.08 | 1360.810416 | 1.103339 |
| GO:0048663\_neuron\_fate\_commitment | 39 | 0 | 0.000000 | -0.000000 | 1162 | 1203.349584 | 1282.08 | 1360.810416 | 1.103339 |
| GO:0051339\_regulation\_of\_lyase\_activity | 39 | 0 | 0.000000 | -0.000000 | 1162 | 1203.349584 | 1282.08 | 1360.810416 | 1.103339 |
| GO:0070201\_regulation\_of\_establishment\_of\_protein\_localization | 39 | 0 | 0.000000 | -0.000000 | 1162 | 1203.349584 | 1282.08 | 1360.810416 | 1.103339 |
| GO:0001824\_blastocyst\_development | 40 | 0 | 0.000000 | -0.000000 | 1172 | 1213.777171 | 1291.89 | 1370.002829 | 1.102295 |
| GO:0007346\_regulation\_of\_mitotic\_cell\_cycle | 40 | 0 | 0.000000 | -0.000000 | 1172 | 1213.777171 | 1291.89 | 1370.002829 | 1.102295 |
| GO:0007599\_hemostasis | 40 | 0 | 0.000000 | -0.000000 | 1172 | 1213.777171 | 1291.89 | 1370.002829 | 1.102295 |
| GO:0008203\_cholesterol\_metabolic\_process | 40 | 0 | 0.000000 | -0.000000 | 1172 | 1213.777171 | 1291.89 | 1370.002829 | 1.102295 |
| GO:0014031\_mesenchymal\_cell\_development | 40 | 0 | 0.000000 | -0.000000 | 1172 | 1213.777171 | 1291.89 | 1370.002829 | 1.102295 |
| GO:0016485\_protein\_processing | 40 | 0 | 0.000000 | -0.000000 | 1172 | 1213.777171 | 1291.89 | 1370.002829 | 1.102295 |
| GO:0017015\_regulation\_of\_transforming\_growth\_factor\_beta\_receptor\_signaling\_pathway | 40 | 0 | 0.000000 | -0.000000 | 1172 | 1213.777171 | 1291.89 | 1370.002829 | 1.102295 |
| GO:0019935\_cyclic-nucleotide-mediated\_signaling | 40 | 0 | 0.000000 | -0.000000 | 1172 | 1213.777171 | 1291.89 | 1370.002829 | 1.102295 |
| GO:0035272\_exocrine\_system\_development | 40 | 0 | 0.000000 | -0.000000 | 1172 | 1213.777171 | 1291.89 | 1370.002829 | 1.102295 |
| GO:0046850\_regulation\_of\_bone\_remodeling | 40 | 0 | 0.000000 | -0.000000 | 1172 | 1213.777171 | 1291.89 | 1370.002829 | 1.102295 |
| GO:0000082\_G1\_S\_transition\_of\_mitotic\_cell\_cycle | 23 | 0 | 0.000000 | -0.000000 | 1195 | 1234.671702 | 1311.81 | 1388.948298 | 1.097749 |
| GO:0002204\_somatic\_recombination\_of\_immunoglobulin\_genes\_during\_immune\_response | 23 | 0 | 0.000000 | -0.000000 | 1195 | 1234.671702 | 1311.81 | 1388.948298 | 1.097749 |
| GO:0002208\_somatic\_diversification\_of\_immunoglobulins\_during\_immune\_response | 23 | 0 | 0.000000 | -0.000000 | 1195 | 1234.671702 | 1311.81 | 1388.948298 | 1.097749 |
| GO:0002228\_natural\_killer\_cell\_mediated\_immunity | 23 | 0 | 0.000000 | -0.000000 | 1195 | 1234.671702 | 1311.81 | 1388.948298 | 1.097749 |
| GO:0002821\_positive\_regulation\_of\_adaptive\_immune\_response | 23 | 0 | 0.000000 | -0.000000 | 1195 | 1234.671702 | 1311.81 | 1388.948298 | 1.097749 |
| GO:0002824\_positive\_regulation\_of\_adaptive\_immune\_response\_based\_on\_somatic\_recombination\_of\_immune\_receptors\_built\_from\_immunoglobulin\_superfamily\_domains | 23 | 0 | 0.000000 | -0.000000 | 1195 | 1234.671702 | 1311.81 | 1388.948298 | 1.097749 |
| GO:0003073\_regulation\_of\_systemic\_arterial\_blood\_pressure | 23 | 0 | 0.000000 | -0.000000 | 1195 | 1234.671702 | 1311.81 | 1388.948298 | 1.097749 |
| GO:0006397\_mRNA\_processing | 23 | 0 | 0.000000 | -0.000000 | 1195 | 1234.671702 | 1311.81 | 1388.948298 | 1.097749 |
| GO:0007018\_microtubule-based\_movement | 23 | 0 | 0.000000 | -0.000000 | 1195 | 1234.671702 | 1311.81 | 1388.948298 | 1.097749 |
| GO:0007584\_response\_to\_nutrient | 23 | 0 | 0.000000 | -0.000000 | 1195 | 1234.671702 | 1311.81 | 1388.948298 | 1.097749 |
| GO:0008542\_visual\_learning | 23 | 0 | 0.000000 | -0.000000 | 1195 | 1234.671702 | 1311.81 | 1388.948298 | 1.097749 |
| GO:0009954\_proximal\_distal\_pattern\_formation | 23 | 0 | 0.000000 | -0.000000 | 1195 | 1234.671702 | 1311.81 | 1388.948298 | 1.097749 |
| GO:0015698\_inorganic\_anion\_transport | 23 | 0 | 0.000000 | -0.000000 | 1195 | 1234.671702 | 1311.81 | 1388.948298 | 1.097749 |
| GO:0022613\_ribonucleoprotein\_complex\_biogenesis | 23 | 0 | 0.000000 | -0.000000 | 1195 | 1234.671702 | 1311.81 | 1388.948298 | 1.097749 |
| GO:0030512\_negative\_regulation\_of\_transforming\_growth\_factor\_beta\_receptor\_signaling\_pathway | 23 | 0 | 0.000000 | -0.000000 | 1195 | 1234.671702 | 1311.81 | 1388.948298 | 1.097749 |
| GO:0032635\_interleukin-6\_production | 23 | 0 | 0.000000 | -0.000000 | 1195 | 1234.671702 | 1311.81 | 1388.948298 | 1.097749 |
| GO:0032675\_regulation\_of\_interleukin-6\_production | 23 | 0 | 0.000000 | -0.000000 | 1195 | 1234.671702 | 1311.81 | 1388.948298 | 1.097749 |
| GO:0042267\_natural\_killer\_cell\_mediated\_cytotoxicity | 23 | 0 | 0.000000 | -0.000000 | 1195 | 1234.671702 | 1311.81 | 1388.948298 | 1.097749 |
| GO:0043388\_positive\_regulation\_of\_DNA\_binding | 23 | 0 | 0.000000 | -0.000000 | 1195 | 1234.671702 | 1311.81 | 1388.948298 | 1.097749 |
| GO:0045190\_isotype\_switching | 23 | 0 | 0.000000 | -0.000000 | 1195 | 1234.671702 | 1311.81 | 1388.948298 | 1.097749 |
| GO:0051705\_behavioral\_interaction\_between\_organisms | 23 | 0 | 0.000000 | -0.000000 | 1195 | 1234.671702 | 1311.81 | 1388.948298 | 1.097749 |
| GO:0060349\_bone\_morphogenesis | 23 | 0 | 0.000000 | -0.000000 | 1195 | 1234.671702 | 1311.81 | 1388.948298 | 1.097749 |
| GO:0060445\_branching\_involved\_in\_salivary\_gland\_morphogenesis | 23 | 0 | 0.000000 | -0.000000 | 1195 | 1234.671702 | 1311.81 | 1388.948298 | 1.097749 |
| GO:0002062\_chondrocyte\_differentiation | 28 | 0 | 0.000000 | -0.000000 | 1208 | 1249.555683 | 1326.01 | 1402.464317 | 1.097690 |
| GO:0002088\_lens\_development\_in\_camera-type\_eye | 28 | 0 | 0.000000 | -0.000000 | 1208 | 1249.555683 | 1326.01 | 1402.464317 | 1.097690 |
| GO:0002705\_positive\_regulation\_of\_leukocyte\_mediated\_immunity | 28 | 0 | 0.000000 | -0.000000 | 1208 | 1249.555683 | 1326.01 | 1402.464317 | 1.097690 |
| GO:0002708\_positive\_regulation\_of\_lymphocyte\_mediated\_immunity | 28 | 0 | 0.000000 | -0.000000 | 1208 | 1249.555683 | 1326.01 | 1402.464317 | 1.097690 |
| GO:0006997\_nucleus\_organization | 28 | 0 | 0.000000 | -0.000000 | 1208 | 1249.555683 | 1326.01 | 1402.464317 | 1.097690 |
| GO:0007585\_respiratory\_gaseous\_exchange | 28 | 0 | 0.000000 | -0.000000 | 1208 | 1249.555683 | 1326.01 | 1402.464317 | 1.097690 |
| GO:0030111\_regulation\_of\_Wnt\_receptor\_signaling\_pathway | 28 | 0 | 0.000000 | -0.000000 | 1208 | 1249.555683 | 1326.01 | 1402.464317 | 1.097690 |
| GO:0042100\_B\_cell\_proliferation | 28 | 0 | 0.000000 | -0.000000 | 1208 | 1249.555683 | 1326.01 | 1402.464317 | 1.097690 |
| GO:0043193\_positive\_regulation\_of\_gene-specific\_transcription | 28 | 0 | 0.000000 | -0.000000 | 1208 | 1249.555683 | 1326.01 | 1402.464317 | 1.097690 |
| GO:0045926\_negative\_regulation\_of\_growth | 28 | 0 | 0.000000 | -0.000000 | 1208 | 1249.555683 | 1326.01 | 1402.464317 | 1.097690 |
| GO:0046328\_regulation\_of\_JNK\_cascade | 28 | 0 | 0.000000 | -0.000000 | 1208 | 1249.555683 | 1326.01 | 1402.464317 | 1.097690 |
| GO:0050871\_positive\_regulation\_of\_B\_cell\_activation | 28 | 0 | 0.000000 | -0.000000 | 1208 | 1249.555683 | 1326.01 | 1402.464317 | 1.097690 |
| GO:0051188\_cofactor\_biosynthetic\_process | 28 | 0 | 0.000000 | -0.000000 | 1208 | 1249.555683 | 1326.01 | 1402.464317 | 1.097690 |
| GO:0030163\_protein\_catabolic\_process | 101 | 0 | 0.000000 | -0.000000 | 1209 | 1250.032139 | 1326.41 | 1402.787861 | 1.097113 |
| GO:0001656\_metanephros\_development | 50 | 0 | 0.000000 | -0.000000 | 1215 | 1255.793095 | 1331.6 | 1407.406905 | 1.095967 |
| GO:0002573\_myeloid\_leukocyte\_differentiation | 50 | 0 | 0.000000 | -0.000000 | 1215 | 1255.793095 | 1331.6 | 1407.406905 | 1.095967 |
| GO:0007015\_actin\_filament\_organization | 50 | 0 | 0.000000 | -0.000000 | 1215 | 1255.793095 | 1331.6 | 1407.406905 | 1.095967 |
| GO:0009190\_cyclic\_nucleotide\_biosynthetic\_process | 50 | 0 | 0.000000 | -0.000000 | 1215 | 1255.793095 | 1331.6 | 1407.406905 | 1.095967 |
| GO:0017038\_protein\_import | 50 | 0 | 0.000000 | -0.000000 | 1215 | 1255.793095 | 1331.6 | 1407.406905 | 1.095967 |
| GO:0042129\_regulation\_of\_T\_cell\_proliferation | 50 | 0 | 0.000000 | -0.000000 | 1215 | 1255.793095 | 1331.6 | 1407.406905 | 1.095967 |
| GO:0003013\_circulatory\_system\_process | 103 | 0 | 0.000000 | -0.000000 | 1217 | 1257.281397 | 1332.96 | 1408.638603 | 1.095283 |
| GO:0008015\_blood\_circulation | 103 | 0 | 0.000000 | -0.000000 | 1217 | 1257.281397 | 1332.96 | 1408.638603 | 1.095283 |
| GO:0002253\_activation\_of\_immune\_response | 54 | 0 | 0.000000 | -0.000000 | 1224 | 1263.438342 | 1338.5 | 1413.561658 | 1.093546 |
| GO:0006091\_generation\_of\_precursor\_metabolites\_and\_energy | 54 | 0 | 0.000000 | -0.000000 | 1224 | 1263.438342 | 1338.5 | 1413.561658 | 1.093546 |
| GO:0006164\_purine\_nucleotide\_biosynthetic\_process | 54 | 0 | 0.000000 | -0.000000 | 1224 | 1263.438342 | 1338.5 | 1413.561658 | 1.093546 |
| GO:0007265\_Ras\_protein\_signal\_transduction | 54 | 0 | 0.000000 | -0.000000 | 1224 | 1263.438342 | 1338.5 | 1413.561658 | 1.093546 |
| GO:0015849\_organic\_acid\_transport | 54 | 0 | 0.000000 | -0.000000 | 1224 | 1263.438342 | 1338.5 | 1413.561658 | 1.093546 |
| GO:0043405\_regulation\_of\_MAP\_kinase\_activity | 54 | 0 | 0.000000 | -0.000000 | 1224 | 1263.438342 | 1338.5 | 1413.561658 | 1.093546 |
| GO:0044271\_nitrogen\_compound\_biosynthetic\_process | 54 | 0 | 0.000000 | -0.000000 | 1224 | 1263.438342 | 1338.5 | 1413.561658 | 1.093546 |
| GO:0001776\_leukocyte\_homeostasis | 41 | 0 | 0.000000 | -0.000000 | 1241 | 1278.765357 | 1352.4 | 1426.034643 | 1.089766 |
| GO:0002429\_immune\_response-activating\_cell\_surface\_receptor\_signaling\_pathway | 41 | 0 | 0.000000 | -0.000000 | 1241 | 1278.765357 | 1352.4 | 1426.034643 | 1.089766 |
| GO:0006260\_DNA\_replication | 41 | 0 | 0.000000 | -0.000000 | 1241 | 1278.765357 | 1352.4 | 1426.034643 | 1.089766 |
| GO:0006836\_neurotransmitter\_transport | 41 | 0 | 0.000000 | -0.000000 | 1241 | 1278.765357 | 1352.4 | 1426.034643 | 1.089766 |
| GO:0006865\_amino\_acid\_transport | 41 | 0 | 0.000000 | -0.000000 | 1241 | 1278.765357 | 1352.4 | 1426.034643 | 1.089766 |
| GO:0006979\_response\_to\_oxidative\_stress | 41 | 0 | 0.000000 | -0.000000 | 1241 | 1278.765357 | 1352.4 | 1426.034643 | 1.089766 |
| GO:0007254\_JNK\_cascade | 41 | 0 | 0.000000 | -0.000000 | 1241 | 1278.765357 | 1352.4 | 1426.034643 | 1.089766 |
| GO:0009894\_regulation\_of\_catabolic\_process | 41 | 0 | 0.000000 | -0.000000 | 1241 | 1278.765357 | 1352.4 | 1426.034643 | 1.089766 |
| GO:0010551\_regulation\_of\_specific\_transcription\_from\_RNA\_polymerase\_II\_promoter | 41 | 0 | 0.000000 | -0.000000 | 1241 | 1278.765357 | 1352.4 | 1426.034643 | 1.089766 |
| GO:0015980\_energy\_derivation\_by\_oxidation\_of\_organic\_compounds | 41 | 0 | 0.000000 | -0.000000 | 1241 | 1278.765357 | 1352.4 | 1426.034643 | 1.089766 |
| GO:0019216\_regulation\_of\_lipid\_metabolic\_process | 41 | 0 | 0.000000 | -0.000000 | 1241 | 1278.765357 | 1352.4 | 1426.034643 | 1.089766 |
| GO:0019748\_secondary\_metabolic\_process | 41 | 0 | 0.000000 | -0.000000 | 1241 | 1278.765357 | 1352.4 | 1426.034643 | 1.089766 |
| GO:0030817\_regulation\_of\_cAMP\_biosynthetic\_process | 41 | 0 | 0.000000 | -0.000000 | 1241 | 1278.765357 | 1352.4 | 1426.034643 | 1.089766 |
| GO:0032569\_specific\_transcription\_from\_RNA\_polymerase\_II\_promoter | 41 | 0 | 0.000000 | -0.000000 | 1241 | 1278.765357 | 1352.4 | 1426.034643 | 1.089766 |
| GO:0032844\_regulation\_of\_homeostatic\_process | 41 | 0 | 0.000000 | -0.000000 | 1241 | 1278.765357 | 1352.4 | 1426.034643 | 1.089766 |
| GO:0033077\_T\_cell\_differentiation\_in\_the\_thymus | 41 | 0 | 0.000000 | -0.000000 | 1241 | 1278.765357 | 1352.4 | 1426.034643 | 1.089766 |
| GO:0050864\_regulation\_of\_B\_cell\_activation | 41 | 0 | 0.000000 | -0.000000 | 1241 | 1278.765357 | 1352.4 | 1426.034643 | 1.089766 |
| GO:0001912\_positive\_regulation\_of\_leukocyte\_mediated\_cytotoxicity | 20 | 0 | 0.000000 | -0.000000 | 1270 | 1306.398049 | 1378.48 | 1450.561951 | 1.085417 |
| GO:0005977\_glycogen\_metabolic\_process | 20 | 0 | 0.000000 | -0.000000 | 1270 | 1306.398049 | 1378.48 | 1450.561951 | 1.085417 |
| GO:0006073\_cellular\_glucan\_metabolic\_process | 20 | 0 | 0.000000 | -0.000000 | 1270 | 1306.398049 | 1378.48 | 1450.561951 | 1.085417 |
| GO:0006518\_peptide\_metabolic\_process | 20 | 0 | 0.000000 | -0.000000 | 1270 | 1306.398049 | 1378.48 | 1450.561951 | 1.085417 |
| GO:0006584\_catecholamine\_metabolic\_process | 20 | 0 | 0.000000 | -0.000000 | 1270 | 1306.398049 | 1378.48 | 1450.561951 | 1.085417 |
| GO:0007416\_synaptogenesis | 20 | 0 | 0.000000 | -0.000000 | 1270 | 1306.398049 | 1378.48 | 1450.561951 | 1.085417 |
| GO:0007528\_neuromuscular\_junction\_development | 20 | 0 | 0.000000 | -0.000000 | 1270 | 1306.398049 | 1378.48 | 1450.561951 | 1.085417 |
| GO:0007586\_digestion | 20 | 0 | 0.000000 | -0.000000 | 1270 | 1306.398049 | 1378.48 | 1450.561951 | 1.085417 |
| GO:0009615\_response\_to\_virus | 20 | 0 | 0.000000 | -0.000000 | 1270 | 1306.398049 | 1378.48 | 1450.561951 | 1.085417 |
| GO:0009712\_catechol\_metabolic\_process | 20 | 0 | 0.000000 | -0.000000 | 1270 | 1306.398049 | 1378.48 | 1450.561951 | 1.085417 |
| GO:0010927\_cellular\_component\_assembly\_involved\_in\_morphogenesis | 20 | 0 | 0.000000 | -0.000000 | 1270 | 1306.398049 | 1378.48 | 1450.561951 | 1.085417 |
| GO:0016571\_histone\_methylation | 20 | 0 | 0.000000 | -0.000000 | 1270 | 1306.398049 | 1378.48 | 1450.561951 | 1.085417 |
| GO:0018209\_peptidyl-serine\_modification | 20 | 0 | 0.000000 | -0.000000 | 1270 | 1306.398049 | 1378.48 | 1450.561951 | 1.085417 |
| GO:0018958\_phenol\_metabolic\_process | 20 | 0 | 0.000000 | -0.000000 | 1270 | 1306.398049 | 1378.48 | 1450.561951 | 1.085417 |
| GO:0021695\_cerebellar\_cortex\_development | 20 | 0 | 0.000000 | -0.000000 | 1270 | 1306.398049 | 1378.48 | 1450.561951 | 1.085417 |
| GO:0031128\_developmental\_induction | 20 | 0 | 0.000000 | -0.000000 | 1270 | 1306.398049 | 1378.48 | 1450.561951 | 1.085417 |
| GO:0031214\_biomineral\_formation | 20 | 0 | 0.000000 | -0.000000 | 1270 | 1306.398049 | 1378.48 | 1450.561951 | 1.085417 |
| GO:0031343\_positive\_regulation\_of\_cell\_killing | 20 | 0 | 0.000000 | -0.000000 | 1270 | 1306.398049 | 1378.48 | 1450.561951 | 1.085417 |
| GO:0032582\_negative\_regulation\_of\_gene-specific\_transcription | 20 | 0 | 0.000000 | -0.000000 | 1270 | 1306.398049 | 1378.48 | 1450.561951 | 1.085417 |
| GO:0032640\_tumor\_necrosis\_factor\_production | 20 | 0 | 0.000000 | -0.000000 | 1270 | 1306.398049 | 1378.48 | 1450.561951 | 1.085417 |
| GO:0032680\_regulation\_of\_tumor\_necrosis\_factor\_production | 20 | 0 | 0.000000 | -0.000000 | 1270 | 1306.398049 | 1378.48 | 1450.561951 | 1.085417 |
| GO:0034311\_diol\_metabolic\_process | 20 | 0 | 0.000000 | -0.000000 | 1270 | 1306.398049 | 1378.48 | 1450.561951 | 1.085417 |
| GO:0042326\_negative\_regulation\_of\_phosphorylation | 20 | 0 | 0.000000 | -0.000000 | 1270 | 1306.398049 | 1378.48 | 1450.561951 | 1.085417 |
| GO:0044042\_glucan\_metabolic\_process | 20 | 0 | 0.000000 | -0.000000 | 1270 | 1306.398049 | 1378.48 | 1450.561951 | 1.085417 |
| GO:0045168\_cell-cell\_signaling\_involved\_in\_cell\_fate\_specification | 20 | 0 | 0.000000 | -0.000000 | 1270 | 1306.398049 | 1378.48 | 1450.561951 | 1.085417 |
| GO:0045639\_positive\_regulation\_of\_myeloid\_cell\_differentiation | 20 | 0 | 0.000000 | -0.000000 | 1270 | 1306.398049 | 1378.48 | 1450.561951 | 1.085417 |
| GO:0046822\_regulation\_of\_nucleocytoplasmic\_transport | 20 | 0 | 0.000000 | -0.000000 | 1270 | 1306.398049 | 1378.48 | 1450.561951 | 1.085417 |
| GO:0048806\_genitalia\_development | 20 | 0 | 0.000000 | -0.000000 | 1270 | 1306.398049 | 1378.48 | 1450.561951 | 1.085417 |
| GO:0060191\_regulation\_of\_lipase\_activity | 20 | 0 | 0.000000 | -0.000000 | 1270 | 1306.398049 | 1378.48 | 1450.561951 | 1.085417 |
| GO:0001501\_skeletal\_system\_development | 236 | 0 | 0.000000 | 0.000000 | 1329 | 1357.625692 | 1417.41 | 1477.194308 | 1.066524 |
| GO:0001568\_blood\_vessel\_development | 203 | 0 | 0.000000 | 0.000000 | 1329 | 1357.625692 | 1417.41 | 1477.194308 | 1.066524 |
| GO:0001763\_morphogenesis\_of\_a\_branching\_structure | 125 | 0 | 0.000000 | 0.000000 | 1329 | 1357.625692 | 1417.41 | 1477.194308 | 1.066524 |
| GO:0001944\_vasculature\_development | 208 | 0 | 0.000000 | 0.000000 | 1329 | 1357.625692 | 1417.41 | 1477.194308 | 1.066524 |
| GO:0002009\_morphogenesis\_of\_an\_epithelium | 198 | 0 | 0.000000 | 0.000000 | 1329 | 1357.625692 | 1417.41 | 1477.194308 | 1.066524 |
| GO:0002252\_immune\_effector\_process | 122 | 0 | 0.000000 | 0.000000 | 1329 | 1357.625692 | 1417.41 | 1477.194308 | 1.066524 |
| GO:0002682\_regulation\_of\_immune\_system\_process | 228 | 0 | 0.000000 | 0.000000 | 1329 | 1357.625692 | 1417.41 | 1477.194308 | 1.066524 |
| GO:0002684\_positive\_regulation\_of\_immune\_system\_process | 148 | 0 | 0.000000 | 0.000000 | 1329 | 1357.625692 | 1417.41 | 1477.194308 | 1.066524 |
| GO:0002694\_regulation\_of\_leukocyte\_activation | 121 | 0 | 0.000000 | 0.000000 | 1329 | 1357.625692 | 1417.41 | 1477.194308 | 1.066524 |
| GO:0005975\_carbohydrate\_metabolic\_process | 146 | 0 | 0.000000 | 0.000000 | 1329 | 1357.625692 | 1417.41 | 1477.194308 | 1.066524 |
| GO:0006066\_alcohol\_metabolic\_process | 158 | 0 | 0.000000 | 0.000000 | 1329 | 1357.625692 | 1417.41 | 1477.194308 | 1.066524 |
| GO:0006259\_DNA\_metabolic\_process | 165 | 0 | 0.000000 | 0.000000 | 1329 | 1357.625692 | 1417.41 | 1477.194308 | 1.066524 |
| GO:0006468\_protein\_amino\_acid\_phosphorylation | 237 | 0 | 0.000000 | 0.000000 | 1329 | 1357.625692 | 1417.41 | 1477.194308 | 1.066524 |
| GO:0006950\_response\_to\_stress | 549 | 0 | 0.000000 | 0.000000 | 1329 | 1357.625692 | 1417.41 | 1477.194308 | 1.066524 |
| GO:0006952\_defense\_response | 187 | 0 | 0.000000 | 0.000000 | 1329 | 1357.625692 | 1417.41 | 1477.194308 | 1.066524 |
| GO:0006955\_immune\_response | 205 | 0 | 0.000000 | 0.000000 | 1329 | 1357.625692 | 1417.41 | 1477.194308 | 1.066524 |
| GO:0007167\_enzyme\_linked\_receptor\_protein\_signaling\_pathway | 229 | 0 | 0.000000 | 0.000000 | 1329 | 1357.625692 | 1417.41 | 1477.194308 | 1.066524 |
| GO:0007169\_transmembrane\_receptor\_protein\_tyrosine\_kinase\_signaling\_pathway | 139 | 0 | 0.000000 | 0.000000 | 1329 | 1357.625692 | 1417.41 | 1477.194308 | 1.066524 |
| GO:0007186\_G-protein\_coupled\_receptor\_protein\_signaling\_pathway | 144 | 0 | 0.000000 | 0.000000 | 1329 | 1357.625692 | 1417.41 | 1477.194308 | 1.066524 |
| GO:0007268\_synaptic\_transmission | 154 | 0 | 0.000000 | 0.000000 | 1329 | 1357.625692 | 1417.41 | 1477.194308 | 1.066524 |
| GO:0007283\_spermatogenesis | 134 | 0 | 0.000000 | 0.000000 | 1329 | 1357.625692 | 1417.41 | 1477.194308 | 1.066524 |
| GO:0008150\_biological\_process | 4605 | 37 | 1.000000 | 0.000000 | 1329 | 1357.625692 | 1417.41 | 1477.194308 | 1.066524 |
| GO:0009057\_macromolecule\_catabolic\_process | 137 | 0 | 0.000000 | 0.000000 | 1329 | 1357.625692 | 1417.41 | 1477.194308 | 1.066524 |
| GO:0009611\_response\_to\_wounding | 172 | 0 | 0.000000 | 0.000000 | 1329 | 1357.625692 | 1417.41 | 1477.194308 | 1.066524 |
| GO:0009952\_anterior\_posterior\_pattern\_formation | 133 | 0 | 0.000000 | 0.000000 | 1329 | 1357.625692 | 1417.41 | 1477.194308 | 1.066524 |
| GO:0010033\_response\_to\_organic\_substance | 216 | 0 | 0.000000 | 0.000000 | 1329 | 1357.625692 | 1417.41 | 1477.194308 | 1.066524 |
| GO:0016310\_phosphorylation | 309 | 0 | 0.000000 | 0.000000 | 1329 | 1357.625692 | 1417.41 | 1477.194308 | 1.066524 |
| GO:0016477\_cell\_migration | 234 | 0 | 0.000000 | 0.000000 | 1329 | 1357.625692 | 1417.41 | 1477.194308 | 1.066524 |
| GO:0019220\_regulation\_of\_phosphate\_metabolic\_process | 165 | 0 | 0.000000 | 0.000000 | 1329 | 1357.625692 | 1417.41 | 1477.194308 | 1.066524 |
| GO:0019226\_transmission\_of\_nerve\_impulse | 189 | 0 | 0.000000 | 0.000000 | 1329 | 1357.625692 | 1417.41 | 1477.194308 | 1.066524 |
| GO:0032787\_monocarboxylic\_acid\_metabolic\_process | 130 | 0 | 0.000000 | 0.000000 | 1329 | 1357.625692 | 1417.41 | 1477.194308 | 1.066524 |
| GO:0032879\_regulation\_of\_localization | 248 | 0 | 0.000000 | 0.000000 | 1329 | 1357.625692 | 1417.41 | 1477.194308 | 1.066524 |
| GO:0033554\_cellular\_response\_to\_stress | 196 | 0 | 0.000000 | 0.000000 | 1329 | 1357.625692 | 1417.41 | 1477.194308 | 1.066524 |
| GO:0035239\_tube\_morphogenesis | 143 | 0 | 0.000000 | 0.000000 | 1329 | 1357.625692 | 1417.41 | 1477.194308 | 1.066524 |
| GO:0040011\_locomotion | 295 | 0 | 0.000000 | 0.000000 | 1329 | 1357.625692 | 1417.41 | 1477.194308 | 1.066524 |
| GO:0042325\_regulation\_of\_phosphorylation | 164 | 0 | 0.000000 | 0.000000 | 1329 | 1357.625692 | 1417.41 | 1477.194308 | 1.066524 |
| GO:0043066\_negative\_regulation\_of\_apoptosis | 176 | 0 | 0.000000 | 0.000000 | 1329 | 1357.625692 | 1417.41 | 1477.194308 | 1.066524 |
| GO:0043069\_negative\_regulation\_of\_programmed\_cell\_death | 179 | 0 | 0.000000 | 0.000000 | 1329 | 1357.625692 | 1417.41 | 1477.194308 | 1.066524 |
| GO:0043085\_positive\_regulation\_of\_catalytic\_activity | 148 | 0 | 0.000000 | 0.000000 | 1329 | 1357.625692 | 1417.41 | 1477.194308 | 1.066524 |
| GO:0043285\_biopolymer\_catabolic\_process | 129 | 0 | 0.000000 | 0.000000 | 1329 | 1357.625692 | 1417.41 | 1477.194308 | 1.066524 |
| GO:0044057\_regulation\_of\_system\_process | 133 | 0 | 0.000000 | 0.000000 | 1329 | 1357.625692 | 1417.41 | 1477.194308 | 1.066524 |
| GO:0044093\_positive\_regulation\_of\_molecular\_function | 173 | 0 | 0.000000 | 0.000000 | 1329 | 1357.625692 | 1417.41 | 1477.194308 | 1.066524 |
| GO:0045165\_cell\_fate\_commitment | 130 | 0 | 0.000000 | 0.000000 | 1329 | 1357.625692 | 1417.41 | 1477.194308 | 1.066524 |
| GO:0045597\_positive\_regulation\_of\_cell\_differentiation | 128 | 0 | 0.000000 | 0.000000 | 1329 | 1357.625692 | 1417.41 | 1477.194308 | 1.066524 |
| GO:0048232\_male\_gamete\_generation | 134 | 0 | 0.000000 | 0.000000 | 1329 | 1357.625692 | 1417.41 | 1477.194308 | 1.066524 |
| GO:0048514\_blood\_vessel\_morphogenesis | 158 | 0 | 0.000000 | 0.000000 | 1329 | 1357.625692 | 1417.41 | 1477.194308 | 1.066524 |
| GO:0048583\_regulation\_of\_response\_to\_stimulus | 217 | 0 | 0.000000 | 0.000000 | 1329 | 1357.625692 | 1417.41 | 1477.194308 | 1.066524 |
| GO:0048729\_tissue\_morphogenesis | 255 | 0 | 0.000000 | 0.000000 | 1329 | 1357.625692 | 1417.41 | 1477.194308 | 1.066524 |
| GO:0048870\_cell\_motility | 257 | 0 | 0.000000 | 0.000000 | 1329 | 1357.625692 | 1417.41 | 1477.194308 | 1.066524 |
| GO:0050776\_regulation\_of\_immune\_response | 130 | 0 | 0.000000 | 0.000000 | 1329 | 1357.625692 | 1417.41 | 1477.194308 | 1.066524 |
| GO:0050790\_regulation\_of\_catalytic\_activity | 233 | 0 | 0.000000 | 0.000000 | 1329 | 1357.625692 | 1417.41 | 1477.194308 | 1.066524 |
| GO:0050865\_regulation\_of\_cell\_activation | 122 | 0 | 0.000000 | 0.000000 | 1329 | 1357.625692 | 1417.41 | 1477.194308 | 1.066524 |
| GO:0051049\_regulation\_of\_transport | 167 | 0 | 0.000000 | 0.000000 | 1329 | 1357.625692 | 1417.41 | 1477.194308 | 1.066524 |
| GO:0051174\_regulation\_of\_phosphorus\_metabolic\_process | 165 | 0 | 0.000000 | 0.000000 | 1329 | 1357.625692 | 1417.41 | 1477.194308 | 1.066524 |
| GO:0051716\_cellular\_response\_to\_stimulus | 273 | 0 | 0.000000 | 0.000000 | 1329 | 1357.625692 | 1417.41 | 1477.194308 | 1.066524 |
| GO:0051726\_regulation\_of\_cell\_cycle | 121 | 0 | 0.000000 | 0.000000 | 1329 | 1357.625692 | 1417.41 | 1477.194308 | 1.066524 |
| GO:0060429\_epithelium\_development | 198 | 0 | 0.000000 | 0.000000 | 1329 | 1357.625692 | 1417.41 | 1477.194308 | 1.066524 |
| GO:0060548\_negative\_regulation\_of\_cell\_death | 179 | 0 | 0.000000 | 0.000000 | 1329 | 1357.625692 | 1417.41 | 1477.194308 | 1.066524 |
| GO:0065009\_regulation\_of\_molecular\_function | 279 | 0 | 0.000000 | 0.000000 | 1329 | 1357.625692 | 1417.41 | 1477.194308 | 1.066524 |
| GO:0007369\_gastrulation | 63 | 0 | 0.000000 | 0.000000 | 1335 | 1361.579209 | 1420.71 | 1479.840791 | 1.064202 |
| GO:0009165\_nucleotide\_biosynthetic\_process | 63 | 0 | 0.000000 | 0.000000 | 1335 | 1361.579209 | 1420.71 | 1479.840791 | 1.064202 |
| GO:0051186\_cofactor\_metabolic\_process | 63 | 0 | 0.000000 | 0.000000 | 1335 | 1361.579209 | 1420.71 | 1479.840791 | 1.064202 |
| GO:0051216\_cartilage\_development | 63 | 0 | 0.000000 | 0.000000 | 1335 | 1361.579209 | 1420.71 | 1479.840791 | 1.064202 |
| GO:0070662\_mast\_cell\_proliferation | 63 | 0 | 0.000000 | 0.000000 | 1335 | 1361.579209 | 1420.71 | 1479.840791 | 1.064202 |
| GO:0070666\_regulation\_of\_mast\_cell\_proliferation | 63 | 0 | 0.000000 | 0.000000 | 1335 | 1361.579209 | 1420.71 | 1479.840791 | 1.064202 |
| GO:0001890\_placenta\_development | 77 | 0 | 0.000000 | 0.000000 | 1337 | 1362.757944 | 1421.81 | 1480.862056 | 1.063433 |
| GO:0051241\_negative\_regulation\_of\_multicellular\_organismal\_process | 77 | 0 | 0.000000 | 0.000000 | 1337 | 1362.757944 | 1421.81 | 1480.862056 | 1.063433 |
| GO:0006974\_response\_to\_DNA\_damage\_stimulus | 113 | 0 | 0.000000 | 0.000000 | 1338 | 1363.689967 | 1422.69 | 1481.690033 | 1.063296 |
| GO:0002697\_regulation\_of\_immune\_effector\_process | 68 | 0 | 0.000000 | 0.000000 | 1340 | 1366.273619 | 1424.86 | 1483.446381 | 1.063328 |
| GO:0034962\_cellular\_biopolymer\_catabolic\_process | 68 | 0 | 0.000000 | 0.000000 | 1340 | 1366.273619 | 1424.86 | 1483.446381 | 1.063328 |
| GO:0001503\_ossification | 88 | 0 | 0.000000 | 0.000000 | 1343 | 1368.212675 | 1426.29 | 1484.367325 | 1.062018 |
| GO:0048754\_branching\_morphogenesis\_of\_a\_tube | 88 | 0 | 0.000000 | 0.000000 | 1343 | 1368.212675 | 1426.29 | 1484.367325 | 1.062018 |
| GO:0050863\_regulation\_of\_T\_cell\_activation | 88 | 0 | 0.000000 | 0.000000 | 1343 | 1368.212675 | 1426.29 | 1484.367325 | 1.062018 |
| GO:0001508\_regulation\_of\_action\_potential | 43 | 0 | 0.000000 | 0.000000 | 1357 | 1381.949119 | 1438.78 | 1495.610881 | 1.060265 |
| GO:0001841\_neural\_tube\_formation | 43 | 0 | 0.000000 | 0.000000 | 1357 | 1381.949119 | 1438.78 | 1495.610881 | 1.060265 |
| GO:0001894\_tissue\_homeostasis | 43 | 0 | 0.000000 | 0.000000 | 1357 | 1381.949119 | 1438.78 | 1495.610881 | 1.060265 |
| GO:0002819\_regulation\_of\_adaptive\_immune\_response | 43 | 0 | 0.000000 | 0.000000 | 1357 | 1381.949119 | 1438.78 | 1495.610881 | 1.060265 |
| GO:0002822\_regulation\_of\_adaptive\_immune\_response\_based\_on\_somatic\_recombination\_of\_immune\_receptors\_built\_from\_immunoglobulin\_superfamily\_domains | 43 | 0 | 0.000000 | 0.000000 | 1357 | 1381.949119 | 1438.78 | 1495.610881 | 1.060265 |
| GO:0006766\_vitamin\_metabolic\_process | 43 | 0 | 0.000000 | 0.000000 | 1357 | 1381.949119 | 1438.78 | 1495.610881 | 1.060265 |
| GO:0007224\_smoothened\_signaling\_pathway | 43 | 0 | 0.000000 | 0.000000 | 1357 | 1381.949119 | 1438.78 | 1495.610881 | 1.060265 |
| GO:0010001\_glial\_cell\_differentiation | 43 | 0 | 0.000000 | 0.000000 | 1357 | 1381.949119 | 1438.78 | 1495.610881 | 1.060265 |
| GO:0030814\_regulation\_of\_cAMP\_metabolic\_process | 43 | 0 | 0.000000 | 0.000000 | 1357 | 1381.949119 | 1438.78 | 1495.610881 | 1.060265 |
| GO:0031098\_stress-activated\_protein\_kinase\_signaling\_pathway | 43 | 0 | 0.000000 | 0.000000 | 1357 | 1381.949119 | 1438.78 | 1495.610881 | 1.060265 |
| GO:0032868\_response\_to\_insulin\_stimulus | 43 | 0 | 0.000000 | 0.000000 | 1357 | 1381.949119 | 1438.78 | 1495.610881 | 1.060265 |
| GO:0048762\_mesenchymal\_cell\_differentiation | 43 | 0 | 0.000000 | 0.000000 | 1357 | 1381.949119 | 1438.78 | 1495.610881 | 1.060265 |
| GO:0051604\_protein\_maturation | 43 | 0 | 0.000000 | 0.000000 | 1357 | 1381.949119 | 1438.78 | 1495.610881 | 1.060265 |
| GO:0051789\_response\_to\_protein\_stimulus | 43 | 0 | 0.000000 | 0.000000 | 1357 | 1381.949119 | 1438.78 | 1495.610881 | 1.060265 |
| GO:0009617\_response\_to\_bacterium | 70 | 0 | 0.000000 | 0.000000 | 1359 | 1385.095956 | 1441.56 | 1498.024044 | 1.060751 |
| GO:0070838\_divalent\_metal\_ion\_transport | 70 | 0 | 0.000000 | 0.000000 | 1359 | 1385.095956 | 1441.56 | 1498.024044 | 1.060751 |
| GO:0001525\_angiogenesis | 100 | 0 | 0.000000 | 0.000000 | 1360 | 1386.221203 | 1442.5 | 1498.778797 | 1.060662 |
| GO:0001822\_kidney\_development | 87 | 0 | 0.000000 | 0.000000 | 1365 | 1390.250303 | 1445.91 | 1501.569697 | 1.059275 |
| GO:0007178\_transmembrane\_receptor\_protein\_serine\_threonine\_kinase\_signaling\_pathway | 87 | 0 | 0.000000 | 0.000000 | 1365 | 1390.250303 | 1445.91 | 1501.569697 | 1.059275 |
| GO:0022612\_gland\_morphogenesis | 87 | 0 | 0.000000 | 0.000000 | 1365 | 1390.250303 | 1445.91 | 1501.569697 | 1.059275 |
| GO:0043583\_ear\_development | 87 | 0 | 0.000000 | 0.000000 | 1365 | 1390.250303 | 1445.91 | 1501.569697 | 1.059275 |
| GO:0050778\_positive\_regulation\_of\_immune\_response | 87 | 0 | 0.000000 | 0.000000 | 1365 | 1390.250303 | 1445.91 | 1501.569697 | 1.059275 |
| GO:0000012\_single\_strand\_break\_repair | 2 | 0 |  |  |  |  |  |  |  |  |
| GO:0000019\_regulation\_of\_mitotic\_recombination | 2 | 0 |  |  |  |  |  |  |  |  |
| GO:0000076\_DNA\_replication\_checkpoint | 2 | 0 |  |  |  |  |  |  |  |  |
| GO:0000080\_G1\_phase\_of\_mitotic\_cell\_cycle | 2 | 0 |  |  |  |  |  |  |  |  |
| GO:0000083\_regulation\_of\_transcription\_of\_G1\_S-phase\_of\_mitotic\_cell\_cycle | 2 | 0 |  |  |  |  |  |  |  |  |
| GO:0000085\_G2\_phase\_of\_mitotic\_cell\_cycle | 2 | 0 |  |  |  |  |  |  |  |  |
| GO:0000289\_nuclear-transcribed\_mRNA\_poly(A)\_tail\_shortening | 2 | 0 |  |  |  |  |  |  |  |  |
| GO:0000381\_regulation\_of\_alternative\_nuclear\_mRNA\_splicing\_\_via\_spliceosome | 2 | 0 |  |  |  |  |  |  |  |  |
| GO:0000712\_resolution\_of\_meiotic\_joint\_molecules\_as\_recombinants | 2 | 0 |  |  |  |  |  |  |  |  |
| GO:0000720\_pyrimidine\_dimer\_repair\_by\_nucleotide-excision\_repair | 2 | 0 |  |  |  |  |  |  |  |  |
| GO:0001302\_replicative\_cell\_aging | 2 | 0 |  |  |  |  |  |  |  |  |
| GO:0001306\_age-dependent\_response\_to\_oxidative\_stress | 2 | 0 |  |  |  |  |  |  |  |  |
| GO:0001514\_selenocysteine\_incorporation | 2 | 0 |  |  |  |  |  |  |  |  |
| GO:0001522\_pseudouridine\_synthesis | 2 | 0 |  |  |  |  |  |  |  |  |
| GO:0001561\_fatty\_acid\_alpha-oxidation | 2 | 0 |  |  |  |  |  |  |  |  |
| GO:0001675\_acrosome\_assembly | 2 | 0 |  |  |  |  |  |  |  |  |
| GO:0001743\_optic\_placode\_formation | 2 | 0 |  |  |  |  |  |  |  |  |
| GO:0001771\_formation\_of\_immunological\_synapse | 2 | 0 |  |  |  |  |  |  |  |  |
| GO:0001774\_microglial\_cell\_activation | 2 | 0 |  |  |  |  |  |  |  |  |
| GO:0001781\_neutrophil\_apoptosis | 2 | 0 |  |  |  |  |  |  |  |  |
| GO:0001787\_natural\_killer\_cell\_proliferation | 2 | 0 |  |  |  |  |  |  |  |  |
| GO:0001788\_antibody-dependent\_cellular\_cytotoxicity | 2 | 0 |  |  |  |  |  |  |  |  |
| GO:0001806\_type\_IV\_hypersensitivity | 2 | 0 |  |  |  |  |  |  |  |  |
| GO:0001807\_regulation\_of\_type\_IV\_hypersensitivity | 2 | 0 |  |  |  |  |  |  |  |  |
| GO:0001808\_negative\_regulation\_of\_type\_IV\_hypersensitivity | 2 | 0 |  |  |  |  |  |  |  |  |
| GO:0001823\_mesonephros\_development | 2 | 0 |  |  |  |  |  |  |  |  |
| GO:0001845\_phagolysosome\_formation | 2 | 0 |  |  |  |  |  |  |  |  |
| GO:0001866\_NK\_T\_cell\_proliferation | 2 | 0 |  |  |  |  |  |  |  |  |
| GO:0001879\_detection\_of\_yeast | 2 | 0 |  |  |  |  |  |  |  |  |
| GO:0001886\_endothelial\_cell\_morphogenesis | 2 | 0 |  |  |  |  |  |  |  |  |
| GO:0001919\_regulation\_of\_receptor\_recycling | 2 | 0 |  |  |  |  |  |  |  |  |
| GO:0001954\_positive\_regulation\_of\_cell-matrix\_adhesion | 2 | 0 |  |  |  |  |  |  |  |  |
| GO:0001977\_renal\_system\_process\_involved\_in\_regulation\_of\_blood\_volume | 2 | 0 |  |  |  |  |  |  |  |  |
| GO:0001982\_baroreceptor\_response\_to\_decreased\_systemic\_arterial\_blood\_pressure | 2 | 0 |  |  |  |  |  |  |  |  |
| GO:0001983\_baroreceptor\_response\_to\_increased\_systemic\_arterial\_blood\_pressure | 2 | 0 |  |  |  |  |  |  |  |  |
| GO:0001992\_regulation\_of\_systemic\_arterial\_blood\_pressure\_by\_vasopressin | 2 | 0 |  |  |  |  |  |  |  |  |
| GO:0001997\_positive\_regulation\_of\_the\_force\_of\_heart\_contraction\_by\_epinephrine-norepinephrine | 2 | 0 |  |  |  |  |  |  |  |  |
| GO:0001998\_angiotensin\_mediated\_vasoconstriction\_involved\_in\_regulation\_of\_systemic\_arterial\_blood\_pressure | 2 | 0 |  |  |  |  |  |  |  |  |
| GO:0001999\_renal\_response\_to\_blood\_flow\_during\_renin-angiotensin\_regulation\_of\_systemic\_arterial\_blood\_pressure | 2 | 0 |  |  |  |  |  |  |  |  |
| GO:0002018\_renin-angiotensin\_regulation\_of\_aldosterone\_production | 2 | 0 |  |  |  |  |  |  |  |  |
| GO:0002019\_regulation\_of\_renal\_output\_by\_angiotensin | 2 | 0 |  |  |  |  |  |  |  |  |
| GO:0002024\_diet\_induced\_thermogenesis | 2 | 0 |  |  |  |  |  |  |  |  |
| GO:0002025\_vasodilation\_by\_norepinephrine-epinephrine\_involved\_in\_regulation\_of\_systemic\_arterial\_blood\_pressure | 2 | 0 |  |  |  |  |  |  |  |  |
| GO:0002029\_desensitization\_of\_G-protein\_coupled\_receptor\_protein\_signaling\_pathway | 2 | 0 |  |  |  |  |  |  |  |  |
| GO:0002033\_vasodilation\_by\_angiotensin\_involved\_in\_regulation\_of\_systemic\_arterial\_blood\_pressure | 2 | 0 |  |  |  |  |  |  |  |  |
| GO:0002066\_columnar\_cuboidal\_epithelial\_cell\_development | 2 | 0 |  |  |  |  |  |  |  |  |
| GO:0002072\_optic\_cup\_morphogenesis\_involved\_in\_camera-type\_eye\_development | 2 | 0 |  |  |  |  |  |  |  |  |
| GO:0002074\_extraocular\_skeletal\_muscle\_development | 2 | 0 |  |  |  |  |  |  |  |  |
| GO:0002138\_retinoic\_acid\_biosynthetic\_process | 2 | 0 |  |  |  |  |  |  |  |  |
| GO:0002223\_stimulatory\_C-type\_lectin\_receptor\_signaling\_pathway | 2 | 0 |  |  |  |  |  |  |  |  |
| GO:0002246\_healing\_during\_inflammatory\_response | 2 | 0 |  |  |  |  |  |  |  |  |
| GO:0002251\_organ\_or\_tissue\_specific\_immune\_response | 2 | 0 |  |  |  |  |  |  |  |  |
| GO:0002266\_follicular\_dendritic\_cell\_activation | 2 | 0 |  |  |  |  |  |  |  |  |
| GO:0002268\_follicular\_dendritic\_cell\_differentiation | 2 | 0 |  |  |  |  |  |  |  |  |
| GO:0002327\_immature\_B\_cell\_differentiation | 2 | 0 |  |  |  |  |  |  |  |  |
| GO:0002329\_pre-B\_cell\_differentiation | 2 | 0 |  |  |  |  |  |  |  |  |
| GO:0002339\_B\_cell\_selection | 2 | 0 |  |  |  |  |  |  |  |  |
| GO:0002352\_B\_cell\_negative\_selection | 2 | 0 |  |  |  |  |  |  |  |  |
| GO:0002358\_B\_cell\_homeostatic\_proliferation | 2 | 0 |  |  |  |  |  |  |  |  |
| GO:0002385\_mucosal\_immune\_response | 2 | 0 |  |  |  |  |  |  |  |  |
| GO:0002514\_B\_cell\_tolerance\_induction | 2 | 0 |  |  |  |  |  |  |  |  |
| GO:0002523\_leukocyte\_migration\_during\_inflammatory\_response | 2 | 0 |  |  |  |  |  |  |  |  |
| GO:0002536\_respiratory\_burst\_during\_acute\_inflammatory\_response | 2 | 0 |  |  |  |  |  |  |  |  |
| GO:0002537\_production\_of\_nitric\_oxide\_during\_acute\_inflammatory\_response | 2 | 0 |  |  |  |  |  |  |  |  |
| GO:0002576\_platelet\_degranulation | 2 | 0 |  |  |  |  |  |  |  |  |
| GO:0002639\_positive\_regulation\_of\_immunoglobulin\_production | 2 | 0 |  |  |  |  |  |  |  |  |
| GO:0002661\_regulation\_of\_B\_cell\_tolerance\_induction | 2 | 0 |  |  |  |  |  |  |  |  |
| GO:0002663\_positive\_regulation\_of\_B\_cell\_tolerance\_induction | 2 | 0 |  |  |  |  |  |  |  |  |
| GO:0002676\_regulation\_of\_chronic\_inflammatory\_response | 2 | 0 |  |  |  |  |  |  |  |  |
| GO:0002679\_respiratory\_burst\_during\_defense\_response | 2 | 0 |  |  |  |  |  |  |  |  |
| GO:0002686\_negative\_regulation\_of\_leukocyte\_migration | 2 | 0 |  |  |  |  |  |  |  |  |
| GO:0002720\_positive\_regulation\_of\_cytokine\_production\_during\_immune\_response | 2 | 0 |  |  |  |  |  |  |  |  |
| GO:0002752\_cell\_surface\_pattern\_recognition\_receptor\_signaling\_pathway | 2 | 0 |  |  |  |  |  |  |  |  |
| GO:0002755\_MyD88-dependent\_toll-like\_receptor\_signaling\_pathway | 2 | 0 |  |  |  |  |  |  |  |  |
| GO:0002765\_immune\_response-inhibiting\_signal\_transduction | 2 | 0 |  |  |  |  |  |  |  |  |
| GO:0002921\_negative\_regulation\_of\_humoral\_immune\_response | 2 | 0 |  |  |  |  |  |  |  |  |
| GO:0002922\_positive\_regulation\_of\_humoral\_immune\_response | 2 | 0 |  |  |  |  |  |  |  |  |
| GO:0002924\_negative\_regulation\_of\_humoral\_immune\_response\_mediated\_by\_circulating\_immunoglobulin | 2 | 0 |  |  |  |  |  |  |  |  |
| GO:0002925\_positive\_regulation\_of\_humoral\_immune\_response\_mediated\_by\_circulating\_immunoglobulin | 2 | 0 |  |  |  |  |  |  |  |  |
| GO:0003057\_regulation\_of\_the\_force\_of\_heart\_contraction\_by\_chemical\_signal | 2 | 0 |  |  |  |  |  |  |  |  |
| GO:0003099\_positive\_regulation\_of\_the\_force\_of\_heart\_contraction\_by\_chemical\_signal | 2 | 0 |  |  |  |  |  |  |  |  |
| GO:0005981\_regulation\_of\_glycogen\_catabolic\_process | 2 | 0 |  |  |  |  |  |  |  |  |
| GO:0006021\_inositol\_biosynthetic\_process | 2 | 0 |  |  |  |  |  |  |  |  |
| GO:0006042\_glucosamine\_biosynthetic\_process | 2 | 0 |  |  |  |  |  |  |  |  |
| GO:0006045\_N-acetylglucosamine\_biosynthetic\_process | 2 | 0 |  |  |  |  |  |  |  |  |
| GO:0006048\_UDP-N-acetylglucosamine\_biosynthetic\_process | 2 | 0 |  |  |  |  |  |  |  |  |
| GO:0006054\_N-acetylneuraminate\_metabolic\_process | 2 | 0 |  |  |  |  |  |  |  |  |
| GO:0006059\_hexitol\_metabolic\_process | 2 | 0 |  |  |  |  |  |  |  |  |
| GO:0006063\_uronic\_acid\_metabolic\_process | 2 | 0 |  |  |  |  |  |  |  |  |
| GO:0006068\_ethanol\_catabolic\_process | 2 | 0 |  |  |  |  |  |  |  |  |
| GO:0006083\_acetate\_metabolic\_process | 2 | 0 |  |  |  |  |  |  |  |  |
| GO:0006089\_lactate\_metabolic\_process | 2 | 0 |  |  |  |  |  |  |  |  |
| GO:0006105\_succinate\_metabolic\_process | 2 | 0 |  |  |  |  |  |  |  |  |
| GO:0006106\_fumarate\_metabolic\_process | 2 | 0 |  |  |  |  |  |  |  |  |
| GO:0006110\_regulation\_of\_glycolysis | 2 | 0 |  |  |  |  |  |  |  |  |
| GO:0006113\_fermentation | 2 | 0 |  |  |  |  |  |  |  |  |
| GO:0006114\_glycerol\_biosynthetic\_process | 2 | 0 |  |  |  |  |  |  |  |  |
| GO:0006122\_mitochondrial\_electron\_transport\_\_ubiquinol\_to\_cytochrome\_c | 2 | 0 |  |  |  |  |  |  |  |  |
| GO:0006152\_purine\_nucleoside\_catabolic\_process | 2 | 0 |  |  |  |  |  |  |  |  |
| GO:0006168\_adenine\_salvage | 2 | 0 |  |  |  |  |  |  |  |  |
| GO:0006200\_ATP\_catabolic\_process | 2 | 0 |  |  |  |  |  |  |  |  |
| GO:0006206\_pyrimidine\_base\_metabolic\_process | 2 | 0 |  |  |  |  |  |  |  |  |
| GO:0006213\_pyrimidine\_nucleoside\_metabolic\_process | 2 | 0 |  |  |  |  |  |  |  |  |
| GO:0006265\_DNA\_topological\_change | 2 | 0 |  |  |  |  |  |  |  |  |
| GO:0006278\_RNA-dependent\_DNA\_replication | 2 | 0 |  |  |  |  |  |  |  |  |
| GO:0006312\_mitotic\_recombination | 2 | 0 |  |  |  |  |  |  |  |  |
| GO:0006398\_histone\_mRNA\_3'-end\_processing | 2 | 0 |  |  |  |  |  |  |  |  |
| GO:0006418\_tRNA\_aminoacylation\_for\_protein\_translation | 2 | 0 |  |  |  |  |  |  |  |  |
| GO:0006451\_translational\_readthrough | 2 | 0 |  |  |  |  |  |  |  |  |
| GO:0006477\_protein\_amino\_acid\_sulfation | 2 | 0 |  |  |  |  |  |  |  |  |
| GO:0006482\_protein\_amino\_acid\_demethylation | 2 | 0 |  |  |  |  |  |  |  |  |
| GO:0006499\_N-terminal\_protein\_myristoylation | 2 | 0 |  |  |  |  |  |  |  |  |
| GO:0006525\_arginine\_metabolic\_process | 2 | 0 |  |  |  |  |  |  |  |  |
| GO:0006527\_arginine\_catabolic\_process | 2 | 0 |  |  |  |  |  |  |  |  |
| GO:0006532\_aspartate\_biosynthetic\_process | 2 | 0 |  |  |  |  |  |  |  |  |
| GO:0006538\_glutamate\_catabolic\_process | 2 | 0 |  |  |  |  |  |  |  |  |
| GO:0006558\_L-phenylalanine\_metabolic\_process | 2 | 0 |  |  |  |  |  |  |  |  |
| GO:0006563\_L-serine\_metabolic\_process | 2 | 0 |  |  |  |  |  |  |  |  |
| GO:0006566\_threonine\_metabolic\_process | 2 | 0 |  |  |  |  |  |  |  |  |
| GO:0006568\_tryptophan\_metabolic\_process | 2 | 0 |  |  |  |  |  |  |  |  |
| GO:0006583\_melanin\_biosynthetic\_process\_from\_tyrosine | 2 | 0 |  |  |  |  |  |  |  |  |
| GO:0006600\_creatine\_metabolic\_process | 2 | 0 |  |  |  |  |  |  |  |  |
| GO:0006603\_phosphocreatine\_metabolic\_process | 2 | 0 |  |  |  |  |  |  |  |  |
| GO:0006610\_ribosomal\_protein\_import\_into\_nucleus | 2 | 0 |  |  |  |  |  |  |  |  |
| GO:0006642\_triglyceride\_mobilization | 2 | 0 |  |  |  |  |  |  |  |  |
| GO:0006649\_phospholipid\_transfer\_to\_membrane | 2 | 0 |  |  |  |  |  |  |  |  |
| GO:0006681\_galactosylceramide\_metabolic\_process | 2 | 0 |  |  |  |  |  |  |  |  |
| GO:0006686\_sphingomyelin\_biosynthetic\_process | 2 | 0 |  |  |  |  |  |  |  |  |
| GO:0006702\_androgen\_biosynthetic\_process | 2 | 0 |  |  |  |  |  |  |  |  |
| GO:0006750\_glutathione\_biosynthetic\_process | 2 | 0 |  |  |  |  |  |  |  |  |
| GO:0006760\_folic\_acid\_and\_derivative\_metabolic\_process | 2 | 0 |  |  |  |  |  |  |  |  |
| GO:0006808\_regulation\_of\_nitrogen\_utilization | 2 | 0 |  |  |  |  |  |  |  |  |
| GO:0006868\_glutamine\_transport | 2 | 0 |  |  |  |  |  |  |  |  |
| GO:0006907\_pinocytosis | 2 | 0 |  |  |  |  |  |  |  |  |
| GO:0006925\_inflammatory\_cell\_apoptosis | 2 | 0 |  |  |  |  |  |  |  |  |
| GO:0006977\_DNA\_damage\_response\_\_signal\_transduction\_by\_p53\_class\_mediator\_resulting\_in\_cell\_cycle\_arrest | 2 | 0 |  |  |  |  |  |  |  |  |
| GO:0006991\_response\_to\_sterol\_depletion | 2 | 0 |  |  |  |  |  |  |  |  |
| GO:0007004\_telomere\_maintenance\_via\_telomerase | 2 | 0 |  |  |  |  |  |  |  |  |
| GO:0007020\_microtubule\_nucleation | 2 | 0 |  |  |  |  |  |  |  |  |
| GO:0007030\_Golgi\_organization | 2 | 0 |  |  |  |  |  |  |  |  |
| GO:0007035\_vacuolar\_acidification | 2 | 0 |  |  |  |  |  |  |  |  |
| GO:0007042\_lysosomal\_lumen\_acidification | 2 | 0 |  |  |  |  |  |  |  |  |
| GO:0007060\_male\_meiosis\_chromosome\_segregation | 2 | 0 |  |  |  |  |  |  |  |  |
| GO:0007089\_traversing\_start\_control\_point\_of\_mitotic\_cell\_cycle | 2 | 0 |  |  |  |  |  |  |  |  |
| GO:0007094\_mitotic\_cell\_cycle\_spindle\_assembly\_checkpoint | 2 | 0 |  |  |  |  |  |  |  |  |
| GO:0007097\_nuclear\_migration | 2 | 0 |  |  |  |  |  |  |  |  |
| GO:0007100\_mitotic\_centrosome\_separation | 2 | 0 |  |  |  |  |  |  |  |  |
| GO:0007171\_activation\_of\_transmembrane\_receptor\_protein\_tyrosine\_kinase\_activity | 2 | 0 |  |  |  |  |  |  |  |  |
| GO:0007182\_common-partner\_SMAD\_protein\_phosphorylation | 2 | 0 |  |  |  |  |  |  |  |  |
| GO:0007185\_transmembrane\_receptor\_protein\_tyrosine\_phosphatase\_signaling\_pathway | 2 | 0 |  |  |  |  |  |  |  |  |
| GO:0007205\_activation\_of\_protein\_kinase\_C\_activity\_by\_G-protein\_coupled\_receptor\_protein\_signaling\_pathway | 2 | 0 |  |  |  |  |  |  |  |  |
| GO:0007210\_serotonin\_receptor\_signaling\_pathway | 2 | 0 |  |  |  |  |  |  |  |  |
| GO:0007220\_Notch\_receptor\_processing | 2 | 0 |  |  |  |  |  |  |  |  |
| GO:0007256\_activation\_of\_JNKK\_activity | 2 | 0 |  |  |  |  |  |  |  |  |
| GO:0007258\_JUN\_phosphorylation | 2 | 0 |  |  |  |  |  |  |  |  |
| GO:0007263\_nitric\_oxide\_mediated\_signal\_transduction | 2 | 0 |  |  |  |  |  |  |  |  |
| GO:0007289\_spermatid\_nucleus\_differentiation | 2 | 0 |  |  |  |  |  |  |  |  |
| GO:0007343\_egg\_activation | 2 | 0 |  |  |  |  |  |  |  |  |
| GO:0007351\_tripartite\_regional\_subdivision | 2 | 0 |  |  |  |  |  |  |  |  |
| GO:0007418\_ventral\_midline\_development | 2 | 0 |  |  |  |  |  |  |  |  |
| GO:0007494\_midgut\_development | 2 | 0 |  |  |  |  |  |  |  |  |
| GO:0007527\_adult\_somatic\_muscle\_development | 2 | 0 |  |  |  |  |  |  |  |  |
| GO:0007549\_dosage\_compensation | 2 | 0 |  |  |  |  |  |  |  |  |
| GO:0007571\_age-dependent\_general\_metabolic\_decline | 2 | 0 |  |  |  |  |  |  |  |  |
| GO:0007603\_phototransduction\_\_visible\_light | 2 | 0 |  |  |  |  |  |  |  |  |
| GO:0007619\_courtship\_behavior | 2 | 0 |  |  |  |  |  |  |  |  |
| GO:0008065\_establishment\_of\_blood-nerve\_barrier | 2 | 0 |  |  |  |  |  |  |  |  |
| GO:0008089\_anterograde\_axon\_cargo\_transport | 2 | 0 |  |  |  |  |  |  |  |  |
| GO:0008210\_estrogen\_metabolic\_process | 2 | 0 |  |  |  |  |  |  |  |  |
| GO:0008212\_mineralocorticoid\_metabolic\_process | 2 | 0 |  |  |  |  |  |  |  |  |
| GO:0008214\_protein\_amino\_acid\_dealkylation | 2 | 0 |  |  |  |  |  |  |  |  |
| GO:0008228\_opsonization | 2 | 0 |  |  |  |  |  |  |  |  |
| GO:0008272\_sulfate\_transport | 2 | 0 |  |  |  |  |  |  |  |  |
| GO:0008291\_acetylcholine\_metabolic\_process | 2 | 0 |  |  |  |  |  |  |  |  |
| GO:0008298\_intracellular\_mRNA\_localization | 2 | 0 |  |  |  |  |  |  |  |  |
| GO:0008334\_histone\_mRNA\_metabolic\_process | 2 | 0 |  |  |  |  |  |  |  |  |
| GO:0008356\_asymmetric\_cell\_division | 2 | 0 |  |  |  |  |  |  |  |  |
| GO:0008582\_regulation\_of\_synaptic\_growth\_at\_neuromuscular\_junction | 2 | 0 |  |  |  |  |  |  |  |  |
| GO:0008594\_photoreceptor\_cell\_morphogenesis | 2 | 0 |  |  |  |  |  |  |  |  |
| GO:0008595\_determination\_of\_anterior\_posterior\_axis\_\_embryo | 2 | 0 |  |  |  |  |  |  |  |  |
| GO:0008608\_attachment\_of\_spindle\_microtubules\_to\_kinetochore | 2 | 0 |  |  |  |  |  |  |  |  |
| GO:0008616\_queuosine\_biosynthetic\_process | 2 | 0 |  |  |  |  |  |  |  |  |
| GO:0008617\_guanosine\_metabolic\_process | 2 | 0 |  |  |  |  |  |  |  |  |
| GO:0008618\_7-methylguanosine\_metabolic\_process | 2 | 0 |  |  |  |  |  |  |  |  |
| GO:0008634\_negative\_regulation\_of\_survival\_gene\_product\_expression | 2 | 0 |  |  |  |  |  |  |  |  |
| GO:0009048\_dosage\_compensation\_\_by\_inactivation\_of\_X\_chromosome | 2 | 0 |  |  |  |  |  |  |  |  |
| GO:0009070\_serine\_family\_amino\_acid\_biosynthetic\_process | 2 | 0 |  |  |  |  |  |  |  |  |
| GO:0009071\_serine\_family\_amino\_acid\_catabolic\_process | 2 | 0 |  |  |  |  |  |  |  |  |
| GO:0009074\_aromatic\_amino\_acid\_family\_catabolic\_process | 2 | 0 |  |  |  |  |  |  |  |  |
| GO:0009083\_branched\_chain\_family\_amino\_acid\_catabolic\_process | 2 | 0 |  |  |  |  |  |  |  |  |
| GO:0009093\_cysteine\_catabolic\_process | 2 | 0 |  |  |  |  |  |  |  |  |
| GO:0009120\_deoxyribonucleoside\_metabolic\_process | 2 | 0 |  |  |  |  |  |  |  |  |
| GO:0009125\_nucleoside\_monophosphate\_catabolic\_process | 2 | 0 |  |  |  |  |  |  |  |  |
| GO:0009126\_purine\_nucleoside\_monophosphate\_metabolic\_process | 2 | 0 |  |  |  |  |  |  |  |  |
| GO:0009142\_nucleoside\_triphosphate\_biosynthetic\_process | 2 | 0 |  |  |  |  |  |  |  |  |
| GO:0009161\_ribonucleoside\_monophosphate\_metabolic\_process | 2 | 0 |  |  |  |  |  |  |  |  |
| GO:0009164\_nucleoside\_catabolic\_process | 2 | 0 |  |  |  |  |  |  |  |  |
| GO:0009167\_purine\_ribonucleoside\_monophosphate\_metabolic\_process | 2 | 0 |  |  |  |  |  |  |  |  |
| GO:0009202\_deoxyribonucleoside\_triphosphate\_biosynthetic\_process | 2 | 0 |  |  |  |  |  |  |  |  |
| GO:0009203\_ribonucleoside\_triphosphate\_catabolic\_process | 2 | 0 |  |  |  |  |  |  |  |  |
| GO:0009207\_purine\_ribonucleoside\_triphosphate\_catabolic\_process | 2 | 0 |  |  |  |  |  |  |  |  |
| GO:0009219\_pyrimidine\_deoxyribonucleotide\_metabolic\_process | 2 | 0 |  |  |  |  |  |  |  |  |
| GO:0009265\_2'-deoxyribonucleotide\_biosynthetic\_process | 2 | 0 |  |  |  |  |  |  |  |  |
| GO:0009268\_response\_to\_pH | 2 | 0 |  |  |  |  |  |  |  |  |
| GO:0009313\_oligosaccharide\_catabolic\_process | 2 | 0 |  |  |  |  |  |  |  |  |
| GO:0009395\_phospholipid\_catabolic\_process | 2 | 0 |  |  |  |  |  |  |  |  |
| GO:0009435\_NAD\_biosynthetic\_process | 2 | 0 |  |  |  |  |  |  |  |  |
| GO:0009608\_response\_to\_symbiont | 2 | 0 |  |  |  |  |  |  |  |  |
| GO:0009609\_response\_to\_symbiotic\_bacterium | 2 | 0 |  |  |  |  |  |  |  |  |
| GO:0009649\_entrainment\_of\_circadian\_clock | 2 | 0 |  |  |  |  |  |  |  |  |
| GO:0009996\_negative\_regulation\_of\_cell\_fate\_specification | 2 | 0 |  |  |  |  |  |  |  |  |
| GO:0010002\_cardioblast\_differentiation | 2 | 0 |  |  |  |  |  |  |  |  |
| GO:0010149\_senescence | 2 | 0 |  |  |  |  |  |  |  |  |
| GO:0010225\_response\_to\_UV-C | 2 | 0 |  |  |  |  |  |  |  |  |
| GO:0010389\_regulation\_of\_G2\_M\_transition\_of\_mitotic\_cell\_cycle | 2 | 0 |  |  |  |  |  |  |  |  |
| GO:0010458\_exit\_from\_mitosis | 2 | 0 |  |  |  |  |  |  |  |  |
| GO:0010459\_negative\_regulation\_of\_heart\_rate | 2 | 0 |  |  |  |  |  |  |  |  |
| GO:0010559\_regulation\_of\_glycoprotein\_biosynthetic\_process | 2 | 0 |  |  |  |  |  |  |  |  |
| GO:0010633\_negative\_regulation\_of\_epithelial\_cell\_migration | 2 | 0 |  |  |  |  |  |  |  |  |
| GO:0010677\_negative\_regulation\_of\_cellular\_carbohydrate\_metabolic\_process | 2 | 0 |  |  |  |  |  |  |  |  |
| GO:0010718\_positive\_regulation\_of\_epithelial\_to\_mesenchymal\_transition | 2 | 0 |  |  |  |  |  |  |  |  |
| GO:0010742\_foam\_cell\_differentiation | 2 | 0 |  |  |  |  |  |  |  |  |
| GO:0010743\_regulation\_of\_foam\_cell\_differentiation | 2 | 0 |  |  |  |  |  |  |  |  |
| GO:0010744\_positive\_regulation\_of\_foam\_cell\_differentiation | 2 | 0 |  |  |  |  |  |  |  |  |
| GO:0010765\_positive\_regulation\_of\_sodium\_ion\_transport | 2 | 0 |  |  |  |  |  |  |  |  |
| GO:0010766\_negative\_regulation\_of\_sodium\_ion\_transport | 2 | 0 |  |  |  |  |  |  |  |  |
| GO:0010770\_positive\_regulation\_of\_cell\_morphogenesis\_involved\_in\_differentiation | 2 | 0 |  |  |  |  |  |  |  |  |
| GO:0010771\_negative\_regulation\_of\_cell\_morphogenesis\_involved\_in\_differentiation | 2 | 0 |  |  |  |  |  |  |  |  |
| GO:0010824\_regulation\_of\_centrosome\_duplication | 2 | 0 |  |  |  |  |  |  |  |  |
| GO:0010833\_telomere\_maintenance\_via\_telomere\_lengthening | 2 | 0 |  |  |  |  |  |  |  |  |
| GO:0010862\_positive\_regulation\_of\_pathway-restricted\_SMAD\_protein\_phosphorylation | 2 | 0 |  |  |  |  |  |  |  |  |
| GO:0010872\_regulation\_of\_cholesterol\_esterification | 2 | 0 |  |  |  |  |  |  |  |  |
| GO:0010878\_cholesterol\_storage | 2 | 0 |  |  |  |  |  |  |  |  |
| GO:0010885\_regulation\_of\_cholesterol\_storage | 2 | 0 |  |  |  |  |  |  |  |  |
| GO:0010886\_positive\_regulation\_of\_cholesterol\_storage | 2 | 0 |  |  |  |  |  |  |  |  |
| GO:0010891\_negative\_regulation\_of\_sequestering\_of\_triglyceride | 2 | 0 |  |  |  |  |  |  |  |  |
| GO:0010896\_regulation\_of\_triglyceride\_catabolic\_process | 2 | 0 |  |  |  |  |  |  |  |  |
| GO:0010898\_positive\_regulation\_of\_triglyceride\_catabolic\_process | 2 | 0 |  |  |  |  |  |  |  |  |
| GO:0010907\_positive\_regulation\_of\_glucose\_metabolic\_process | 2 | 0 |  |  |  |  |  |  |  |  |
| GO:0014028\_notochord\_formation | 2 | 0 |  |  |  |  |  |  |  |  |
| GO:0014048\_regulation\_of\_glutamate\_secretion | 2 | 0 |  |  |  |  |  |  |  |  |
| GO:0014052\_regulation\_of\_gamma-aminobutyric\_acid\_secretion | 2 | 0 |  |  |  |  |  |  |  |  |
| GO:0014054\_positive\_regulation\_of\_gamma-aminobutyric\_acid\_secretion | 2 | 0 |  |  |  |  |  |  |  |  |
| GO:0014055\_acetylcholine\_secretion | 2 | 0 |  |  |  |  |  |  |  |  |
| GO:0014056\_regulation\_of\_acetylcholine\_secretion | 2 | 0 |  |  |  |  |  |  |  |  |
| GO:0014745\_negative\_regulation\_of\_muscle\_adaptation | 2 | 0 |  |  |  |  |  |  |  |  |
| GO:0014829\_vascular\_smooth\_muscle\_contraction | 2 | 0 |  |  |  |  |  |  |  |  |
| GO:0014850\_response\_to\_muscle\_activity | 2 | 0 |  |  |  |  |  |  |  |  |
| GO:0014866\_skeletal\_myofibril\_assembly | 2 | 0 |  |  |  |  |  |  |  |  |
| GO:0014888\_striated\_muscle\_adaptation | 2 | 0 |  |  |  |  |  |  |  |  |
| GO:0014916\_regulation\_of\_lung\_blood\_pressure | 2 | 0 |  |  |  |  |  |  |  |  |
| GO:0015671\_oxygen\_transport | 2 | 0 |  |  |  |  |  |  |  |  |
| GO:0015696\_ammonium\_transport | 2 | 0 |  |  |  |  |  |  |  |  |
| GO:0015732\_prostaglandin\_transport | 2 | 0 |  |  |  |  |  |  |  |  |
| GO:0015819\_lysine\_transport | 2 | 0 |  |  |  |  |  |  |  |  |
| GO:0015840\_urea\_transport | 2 | 0 |  |  |  |  |  |  |  |  |
| GO:0015860\_purine\_nucleoside\_transport | 2 | 0 |  |  |  |  |  |  |  |  |
| GO:0015870\_acetylcholine\_transport | 2 | 0 |  |  |  |  |  |  |  |  |
| GO:0015937\_coenzyme\_A\_biosynthetic\_process | 2 | 0 |  |  |  |  |  |  |  |  |
| GO:0016045\_detection\_of\_bacterium | 2 | 0 |  |  |  |  |  |  |  |  |
| GO:0016046\_detection\_of\_fungus | 2 | 0 |  |  |  |  |  |  |  |  |
| GO:0016080\_synaptic\_vesicle\_targeting | 2 | 0 |  |  |  |  |  |  |  |  |
| GO:0016199\_axon\_midline\_choice\_point\_recognition | 2 | 0 |  |  |  |  |  |  |  |  |
| GO:0016226\_iron-sulfur\_cluster\_assembly | 2 | 0 |  |  |  |  |  |  |  |  |
| GO:0016233\_telomere\_capping | 2 | 0 |  |  |  |  |  |  |  |  |
| GO:0016242\_negative\_regulation\_of\_macroautophagy | 2 | 0 |  |  |  |  |  |  |  |  |
| GO:0016441\_posttranscriptional\_gene\_silencing | 2 | 0 |  |  |  |  |  |  |  |  |
| GO:0016540\_protein\_autoprocessing | 2 | 0 |  |  |  |  |  |  |  |  |
| GO:0016558\_protein\_import\_into\_peroxisome\_matrix | 2 | 0 |  |  |  |  |  |  |  |  |
| GO:0016572\_histone\_phosphorylation | 2 | 0 |  |  |  |  |  |  |  |  |
| GO:0016577\_histone\_demethylation | 2 | 0 |  |  |  |  |  |  |  |  |
| GO:0016584\_nucleosome\_positioning | 2 | 0 |  |  |  |  |  |  |  |  |
| GO:0016926\_protein\_desumoylation | 2 | 0 |  |  |  |  |  |  |  |  |
| GO:0017014\_protein\_amino\_acid\_nitrosylation | 2 | 0 |  |  |  |  |  |  |  |  |
| GO:0017144\_drug\_metabolic\_process | 2 | 0 |  |  |  |  |  |  |  |  |
| GO:0018094\_protein\_polyglycylation | 2 | 0 |  |  |  |  |  |  |  |  |
| GO:0018119\_peptidyl-cysteine\_S-nitrosylation | 2 | 0 |  |  |  |  |  |  |  |  |
| GO:0018125\_peptidyl-cysteine\_methylation | 2 | 0 |  |  |  |  |  |  |  |  |
| GO:0018205\_peptidyl-lysine\_modification | 2 | 0 |  |  |  |  |  |  |  |  |
| GO:0018319\_protein\_amino\_acid\_myristoylation | 2 | 0 |  |  |  |  |  |  |  |  |
| GO:0018377\_protein\_myristoylation | 2 | 0 |  |  |  |  |  |  |  |  |
| GO:0018401\_peptidyl-proline\_hydroxylation\_to\_4-hydroxy-L-proline | 2 | 0 |  |  |  |  |  |  |  |  |
| GO:0018993\_somatic\_sex\_determination | 2 | 0 |  |  |  |  |  |  |  |  |
| GO:0019067\_viral\_assembly\_\_maturation\_\_egress\_\_and\_release | 2 | 0 |  |  |  |  |  |  |  |  |
| GO:0019322\_pentose\_biosynthetic\_process | 2 | 0 |  |  |  |  |  |  |  |  |
| GO:0019370\_leukotriene\_biosynthetic\_process | 2 | 0 |  |  |  |  |  |  |  |  |
| GO:0019374\_galactolipid\_metabolic\_process | 2 | 0 |  |  |  |  |  |  |  |  |
| GO:0019401\_alditol\_biosynthetic\_process | 2 | 0 |  |  |  |  |  |  |  |  |
| GO:0019448\_L-cysteine\_catabolic\_process | 2 | 0 |  |  |  |  |  |  |  |  |
| GO:0019452\_L-cysteine\_catabolic\_process\_to\_taurine | 2 | 0 |  |  |  |  |  |  |  |  |
| GO:0019471\_4-hydroxyproline\_metabolic\_process | 2 | 0 |  |  |  |  |  |  |  |  |
| GO:0019511\_peptidyl-proline\_hydroxylation | 2 | 0 |  |  |  |  |  |  |  |  |
| GO:0019550\_glutamate\_catabolic\_process\_to\_aspartate | 2 | 0 |  |  |  |  |  |  |  |  |
| GO:0019551\_glutamate\_catabolic\_process\_to\_2-oxoglutarate | 2 | 0 |  |  |  |  |  |  |  |  |
| GO:0019585\_glucuronate\_metabolic\_process | 2 | 0 |  |  |  |  |  |  |  |  |
| GO:0019730\_antimicrobial\_humoral\_response | 2 | 0 |  |  |  |  |  |  |  |  |
| GO:0019740\_nitrogen\_utilization | 2 | 0 |  |  |  |  |  |  |  |  |
| GO:0019853\_L-ascorbic\_acid\_biosynthetic\_process | 2 | 0 |  |  |  |  |  |  |  |  |
| GO:0021506\_anterior\_neuropore\_closure | 2 | 0 |  |  |  |  |  |  |  |  |
| GO:0021524\_visceral\_motor\_neuron\_differentiation | 2 | 0 |  |  |  |  |  |  |  |  |
| GO:0021526\_medial\_motor\_column\_neuron\_differentiation | 2 | 0 |  |  |  |  |  |  |  |  |
| GO:0021557\_oculomotor\_nerve\_development | 2 | 0 |  |  |  |  |  |  |  |  |
| GO:0021558\_trochlear\_nerve\_development | 2 | 0 |  |  |  |  |  |  |  |  |
| GO:0021562\_vestibulocochlear\_nerve\_development | 2 | 0 |  |  |  |  |  |  |  |  |
| GO:0021568\_rhombomere\_2\_development | 2 | 0 |  |  |  |  |  |  |  |  |
| GO:0021578\_hindbrain\_maturation | 2 | 0 |  |  |  |  |  |  |  |  |
| GO:0021593\_rhombomere\_morphogenesis | 2 | 0 |  |  |  |  |  |  |  |  |
| GO:0021626\_central\_nervous\_system\_maturation | 2 | 0 |  |  |  |  |  |  |  |  |
| GO:0021658\_rhombomere\_3\_morphogenesis | 2 | 0 |  |  |  |  |  |  |  |  |
| GO:0021754\_facial\_nucleus\_development | 2 | 0 |  |  |  |  |  |  |  |  |
| GO:0021775\_smoothened\_signaling\_pathway\_involved\_in\_ventral\_spinal\_cord\_interneuron\_specification | 2 | 0 |  |  |  |  |  |  |  |  |
| GO:0021776\_smoothened\_signaling\_pathway\_involved\_in\_spinal\_cord\_motor\_neuron\_cell\_fate\_specification | 2 | 0 |  |  |  |  |  |  |  |  |
| GO:0021796\_cerebral\_cortex\_regionalization | 2 | 0 |  |  |  |  |  |  |  |  |
| GO:0021831\_embryonic\_olfactory\_bulb\_interneuron\_precursor\_migration | 2 | 0 |  |  |  |  |  |  |  |  |
| GO:0021869\_forebrain\_ventricular\_zone\_progenitor\_cell\_division | 2 | 0 |  |  |  |  |  |  |  |  |
| GO:0021873\_forebrain\_neuroblast\_division | 2 | 0 |  |  |  |  |  |  |  |  |
| GO:0021882\_regulation\_of\_transcription\_from\_RNA\_polymerase\_II\_promoter\_involved\_in\_forebrain\_neuron\_fate\_commitment | 2 | 0 |  |  |  |  |  |  |  |  |
| GO:0021893\_cerebral\_cortex\_GABAergic\_interneuron\_fate\_commitment | 2 | 0 |  |  |  |  |  |  |  |  |
| GO:0021898\_commitment\_of\_multipotent\_stem\_cells\_to\_the\_neuronal\_lineage\_in\_the\_forebrain | 2 | 0 |  |  |  |  |  |  |  |  |
| GO:0021932\_hindbrain\_radial\_glia\_guided\_cell\_migration | 2 | 0 |  |  |  |  |  |  |  |  |
| GO:0021965\_spinal\_cord\_ventral\_commissure\_morphogenesis | 2 | 0 |  |  |  |  |  |  |  |  |
| GO:0021985\_neurohypophysis\_development | 2 | 0 |  |  |  |  |  |  |  |  |
| GO:0021990\_neural\_plate\_formation | 2 | 0 |  |  |  |  |  |  |  |  |
| GO:0021995\_neuropore\_closure | 2 | 0 |  |  |  |  |  |  |  |  |
| GO:0022028\_tangential\_migration\_from\_the\_subventricular\_zone\_to\_the\_olfactory\_bulb | 2 | 0 |  |  |  |  |  |  |  |  |
| GO:0022401\_adaptation\_of\_signaling\_pathway | 2 | 0 |  |  |  |  |  |  |  |  |
| GO:0022408\_negative\_regulation\_of\_cell-cell\_adhesion | 2 | 0 |  |  |  |  |  |  |  |  |
| GO:0022410\_circadian\_sleep\_wake\_cycle\_process | 2 | 0 |  |  |  |  |  |  |  |  |
| GO:0030046\_parallel\_actin\_filament\_bundle\_formation | 2 | 0 |  |  |  |  |  |  |  |  |
| GO:0030049\_muscle\_filament\_sliding | 2 | 0 |  |  |  |  |  |  |  |  |
| GO:0030050\_vesicle\_transport\_along\_actin\_filament | 2 | 0 |  |  |  |  |  |  |  |  |
| GO:0030071\_regulation\_of\_mitotic\_metaphase\_anaphase\_transition | 2 | 0 |  |  |  |  |  |  |  |  |
| GO:0030147\_natriuresis | 2 | 0 |  |  |  |  |  |  |  |  |
| GO:0030174\_regulation\_of\_DNA\_replication\_initiation | 2 | 0 |  |  |  |  |  |  |  |  |
| GO:0030202\_heparin\_metabolic\_process | 2 | 0 |  |  |  |  |  |  |  |  |
| GO:0030219\_megakaryocyte\_differentiation | 2 | 0 |  |  |  |  |  |  |  |  |
| GO:0030223\_neutrophil\_differentiation | 2 | 0 |  |  |  |  |  |  |  |  |
| GO:0030240\_muscle\_thin\_filament\_assembly | 2 | 0 |  |  |  |  |  |  |  |  |
| GO:0030259\_lipid\_glycosylation | 2 | 0 |  |  |  |  |  |  |  |  |
| GO:0030397\_membrane\_disassembly | 2 | 0 |  |  |  |  |  |  |  |  |
| GO:0030502\_negative\_regulation\_of\_bone\_mineralization | 2 | 0 |  |  |  |  |  |  |  |  |
| GO:0030644\_cellular\_chloride\_ion\_homeostasis | 2 | 0 |  |  |  |  |  |  |  |  |
| GO:0030825\_positive\_regulation\_of\_cGMP\_metabolic\_process | 2 | 0 |  |  |  |  |  |  |  |  |
| GO:0030828\_positive\_regulation\_of\_cGMP\_biosynthetic\_process | 2 | 0 |  |  |  |  |  |  |  |  |
| GO:0030835\_negative\_regulation\_of\_actin\_filament\_depolymerization | 2 | 0 |  |  |  |  |  |  |  |  |
| GO:0030837\_negative\_regulation\_of\_actin\_filament\_polymerization | 2 | 0 |  |  |  |  |  |  |  |  |
| GO:0030852\_regulation\_of\_granulocyte\_differentiation | 2 | 0 |  |  |  |  |  |  |  |  |
| GO:0030885\_regulation\_of\_myeloid\_dendritic\_cell\_activation | 2 | 0 |  |  |  |  |  |  |  |  |
| GO:0030910\_olfactory\_placode\_formation | 2 | 0 |  |  |  |  |  |  |  |  |
| GO:0030948\_negative\_regulation\_of\_vascular\_endothelial\_growth\_factor\_receptor\_signaling\_pathway | 2 | 0 |  |  |  |  |  |  |  |  |
| GO:0030953\_spindle\_astral\_microtubule\_organization | 2 | 0 |  |  |  |  |  |  |  |  |
| GO:0031050\_dsRNA\_fragmentation | 2 | 0 |  |  |  |  |  |  |  |  |
| GO:0031061\_negative\_regulation\_of\_histone\_methylation | 2 | 0 |  |  |  |  |  |  |  |  |
| GO:0031119\_tRNA\_pseudouridine\_synthesis | 2 | 0 |  |  |  |  |  |  |  |  |
| GO:0031163\_metallo-sulfur\_cluster\_assembly | 2 | 0 |  |  |  |  |  |  |  |  |
| GO:0031296\_B\_cell\_costimulation | 2 | 0 |  |  |  |  |  |  |  |  |
| GO:0031338\_regulation\_of\_vesicle\_fusion | 2 | 0 |  |  |  |  |  |  |  |  |
| GO:0031573\_intra-S\_DNA\_damage\_checkpoint | 2 | 0 |  |  |  |  |  |  |  |  |
| GO:0031577\_spindle\_checkpoint | 2 | 0 |  |  |  |  |  |  |  |  |
| GO:0031629\_synaptic\_vesicle\_fusion\_to\_presynaptic\_membrane | 2 | 0 |  |  |  |  |  |  |  |  |
| GO:0031630\_regulation\_of\_synaptic\_vesicle\_fusion\_to\_presynaptic\_membrane | 2 | 0 |  |  |  |  |  |  |  |  |
| GO:0031664\_regulation\_of\_lipopolysaccharide-mediated\_signaling\_pathway | 2 | 0 |  |  |  |  |  |  |  |  |
| GO:0031670\_cellular\_response\_to\_nutrient | 2 | 0 |  |  |  |  |  |  |  |  |
| GO:0031848\_protection\_from\_non-homologous\_end\_joining\_at\_telomere | 2 | 0 |  |  |  |  |  |  |  |  |
| GO:0031946\_regulation\_of\_glucocorticoid\_biosynthetic\_process | 2 | 0 |  |  |  |  |  |  |  |  |
| GO:0031952\_regulation\_of\_protein\_amino\_acid\_autophosphorylation | 2 | 0 |  |  |  |  |  |  |  |  |
| GO:0031953\_negative\_regulation\_of\_protein\_amino\_acid\_autophosphorylation | 2 | 0 |  |  |  |  |  |  |  |  |
| GO:0031958\_corticosteroid\_receptor\_signaling\_pathway | 2 | 0 |  |  |  |  |  |  |  |  |
| GO:0031987\_locomotion\_involved\_in\_locomotory\_behavior | 2 | 0 |  |  |  |  |  |  |  |  |
| GO:0032096\_negative\_regulation\_of\_response\_to\_food | 2 | 0 |  |  |  |  |  |  |  |  |
| GO:0032099\_negative\_regulation\_of\_appetite | 2 | 0 |  |  |  |  |  |  |  |  |
| GO:0032106\_positive\_regulation\_of\_response\_to\_extracellular\_stimulus | 2 | 0 |  |  |  |  |  |  |  |  |
| GO:0032109\_positive\_regulation\_of\_response\_to\_nutrient\_levels | 2 | 0 |  |  |  |  |  |  |  |  |
| GO:0032226\_positive\_regulation\_of\_synaptic\_transmission\_\_dopaminergic | 2 | 0 |  |  |  |  |  |  |  |  |
| GO:0032230\_positive\_regulation\_of\_synaptic\_transmission\_\_GABAergic | 2 | 0 |  |  |  |  |  |  |  |  |
| GO:0032234\_regulation\_of\_calcium\_ion\_transport\_via\_store-operated\_calcium\_channel\_activity | 2 | 0 |  |  |  |  |  |  |  |  |
| GO:0032236\_positive\_regulation\_of\_calcium\_ion\_transport\_via\_store-operated\_calcium\_channel\_activity | 2 | 0 |  |  |  |  |  |  |  |  |
| GO:0032297\_negative\_regulation\_of\_DNA\_replication\_initiation | 2 | 0 |  |  |  |  |  |  |  |  |
| GO:0032309\_icosanoid\_secretion | 2 | 0 |  |  |  |  |  |  |  |  |
| GO:0032328\_alanine\_transport | 2 | 0 |  |  |  |  |  |  |  |  |
| GO:0032341\_aldosterone\_metabolic\_process | 2 | 0 |  |  |  |  |  |  |  |  |
| GO:0032351\_negative\_regulation\_of\_hormone\_metabolic\_process | 2 | 0 |  |  |  |  |  |  |  |  |
| GO:0032353\_negative\_regulation\_of\_hormone\_biosynthetic\_process | 2 | 0 |  |  |  |  |  |  |  |  |
| GO:0032435\_negative\_regulation\_of\_proteasomal\_ubiquitin-dependent\_protein\_catabolic\_process | 2 | 0 |  |  |  |  |  |  |  |  |
| GO:0032471\_reduction\_of\_endoplasmic\_reticulum\_calcium\_ion\_concentration | 2 | 0 |  |  |  |  |  |  |  |  |
| GO:0032481\_positive\_regulation\_of\_type\_I\_interferon\_production | 2 | 0 |  |  |  |  |  |  |  |  |
| GO:0032488\_Cdc42\_protein\_signal\_transduction | 2 | 0 |  |  |  |  |  |  |  |  |
| GO:0032489\_regulation\_of\_Cdc42\_protein\_signal\_transduction | 2 | 0 |  |  |  |  |  |  |  |  |
| GO:0032495\_response\_to\_muramyl\_dipeptide | 2 | 0 |  |  |  |  |  |  |  |  |
| GO:0032604\_granulocyte\_macrophage\_colony-stimulating\_factor\_production | 2 | 0 |  |  |  |  |  |  |  |  |
| GO:0032616\_interleukin-13\_production | 2 | 0 |  |  |  |  |  |  |  |  |
| GO:0032645\_regulation\_of\_granulocyte\_macrophage\_colony-stimulating\_factor\_production | 2 | 0 |  |  |  |  |  |  |  |  |
| GO:0032672\_regulation\_of\_interleukin-3\_production | 2 | 0 |  |  |  |  |  |  |  |  |
| GO:0032695\_negative\_regulation\_of\_interleukin-12\_production | 2 | 0 |  |  |  |  |  |  |  |  |
| GO:0032714\_negative\_regulation\_of\_interleukin-5\_production | 2 | 0 |  |  |  |  |  |  |  |  |
| GO:0032722\_positive\_regulation\_of\_chemokine\_production | 2 | 0 |  |  |  |  |  |  |  |  |
| GO:0032743\_positive\_regulation\_of\_interleukin-2\_production | 2 | 0 |  |  |  |  |  |  |  |  |
| GO:0032762\_mast\_cell\_cytokine\_production | 2 | 0 |  |  |  |  |  |  |  |  |
| GO:0032763\_regulation\_of\_mast\_cell\_cytokine\_production | 2 | 0 |  |  |  |  |  |  |  |  |
| GO:0032768\_regulation\_of\_monooxygenase\_activity | 2 | 0 |  |  |  |  |  |  |  |  |
| GO:0032788\_saturated\_monocarboxylic\_acid\_metabolic\_process | 2 | 0 |  |  |  |  |  |  |  |  |
| GO:0032789\_unsaturated\_monocarboxylic\_acid\_metabolic\_process | 2 | 0 |  |  |  |  |  |  |  |  |
| GO:0032800\_receptor\_biosynthetic\_process | 2 | 0 |  |  |  |  |  |  |  |  |
| GO:0032801\_receptor\_catabolic\_process | 2 | 0 |  |  |  |  |  |  |  |  |
| GO:0032829\_regulation\_of\_CD4-positive\_\_CD25-positive\_\_alpha-beta\_regulatory\_T\_cell\_differentiation | 2 | 0 |  |  |  |  |  |  |  |  |
| GO:0032831\_positive\_regulation\_of\_CD4-positive\_\_CD25-positive\_\_alpha-beta\_regulatory\_T\_cell\_differentiation | 2 | 0 |  |  |  |  |  |  |  |  |
| GO:0032892\_positive\_regulation\_of\_organic\_acid\_transport | 2 | 0 |  |  |  |  |  |  |  |  |
| GO:0032905\_transforming\_growth\_factor-beta1\_production | 2 | 0 |  |  |  |  |  |  |  |  |
| GO:0032908\_regulation\_of\_transforming\_growth\_factor-beta1\_production | 2 | 0 |  |  |  |  |  |  |  |  |
| GO:0032914\_positive\_regulation\_of\_transforming\_growth\_factor-beta1\_production | 2 | 0 |  |  |  |  |  |  |  |  |
| GO:0032933\_SREBP-mediated\_signaling\_pathway | 2 | 0 |  |  |  |  |  |  |  |  |
| GO:0032957\_inositol\_trisphosphate\_metabolic\_process | 2 | 0 |  |  |  |  |  |  |  |  |
| GO:0032958\_inositol\_phosphate\_biosynthetic\_process | 2 | 0 |  |  |  |  |  |  |  |  |
| GO:0032959\_inositol\_trisphosphate\_biosynthetic\_process | 2 | 0 |  |  |  |  |  |  |  |  |
| GO:0033092\_positive\_regulation\_of\_immature\_T\_cell\_proliferation\_in\_the\_thymus | 2 | 0 |  |  |  |  |  |  |  |  |
| GO:0033119\_negative\_regulation\_of\_RNA\_splicing | 2 | 0 |  |  |  |  |  |  |  |  |
| GO:0033136\_serine\_phosphorylation\_of\_STAT3\_protein | 2 | 0 |  |  |  |  |  |  |  |  |
| GO:0033145\_positive\_regulation\_of\_steroid\_hormone\_receptor\_signaling\_pathway | 2 | 0 |  |  |  |  |  |  |  |  |
| GO:0033147\_negative\_regulation\_of\_estrogen\_receptor\_signaling\_pathway | 2 | 0 |  |  |  |  |  |  |  |  |
| GO:0033148\_positive\_regulation\_of\_estrogen\_receptor\_signaling\_pathway | 2 | 0 |  |  |  |  |  |  |  |  |
| GO:0033194\_response\_to\_hydroperoxide | 2 | 0 |  |  |  |  |  |  |  |  |
| GO:0033275\_actin-myosin\_filament\_sliding | 2 | 0 |  |  |  |  |  |  |  |  |
| GO:0033280\_response\_to\_vitamin\_D | 2 | 0 |  |  |  |  |  |  |  |  |
| GO:0033364\_mast\_cell\_secretory\_granule\_organization | 2 | 0 |  |  |  |  |  |  |  |  |
| GO:0033504\_floor\_plate\_development | 2 | 0 |  |  |  |  |  |  |  |  |
| GO:0033603\_positive\_regulation\_of\_dopamine\_secretion | 2 | 0 |  |  |  |  |  |  |  |  |
| GO:0033605\_positive\_regulation\_of\_catecholamine\_secretion | 2 | 0 |  |  |  |  |  |  |  |  |
| GO:0033622\_integrin\_activation | 2 | 0 |  |  |  |  |  |  |  |  |
| GO:0033623\_regulation\_of\_integrin\_activation | 2 | 0 |  |  |  |  |  |  |  |  |
| GO:0033625\_positive\_regulation\_of\_integrin\_activation | 2 | 0 |  |  |  |  |  |  |  |  |
| GO:0033700\_phospholipid\_efflux | 2 | 0 |  |  |  |  |  |  |  |  |
| GO:0034142\_toll-like\_receptor\_4\_signaling\_pathway | 2 | 0 |  |  |  |  |  |  |  |  |
| GO:0034310\_monohydric\_alcohol\_catabolic\_process | 2 | 0 |  |  |  |  |  |  |  |  |
| GO:0034341\_response\_to\_interferon-gamma | 2 | 0 |  |  |  |  |  |  |  |  |
| GO:0034370\_triglyceride-rich\_lipoprotein\_particle\_remodeling | 2 | 0 |  |  |  |  |  |  |  |  |
| GO:0034374\_low-density\_lipoprotein\_particle\_remodeling | 2 | 0 |  |  |  |  |  |  |  |  |
| GO:0034377\_plasma\_lipoprotein\_particle\_assembly | 2 | 0 |  |  |  |  |  |  |  |  |
| GO:0034384\_high-density\_lipoprotein\_particle\_clearance | 2 | 0 |  |  |  |  |  |  |  |  |
| GO:0034433\_steroid\_esterification | 2 | 0 |  |  |  |  |  |  |  |  |
| GO:0034434\_sterol\_esterification | 2 | 0 |  |  |  |  |  |  |  |  |
| GO:0034435\_cholesterol\_esterification | 2 | 0 |  |  |  |  |  |  |  |  |
| GO:0034453\_microtubule\_anchoring | 2 | 0 |  |  |  |  |  |  |  |  |
| GO:0034644\_cellular\_response\_to\_UV | 2 | 0 |  |  |  |  |  |  |  |  |
| GO:0034755\_iron\_ion\_transmembrane\_transport | 2 | 0 |  |  |  |  |  |  |  |  |
| GO:0034764\_positive\_regulation\_of\_transmembrane\_transport | 2 | 0 |  |  |  |  |  |  |  |  |
| GO:0035021\_negative\_regulation\_of\_Rac\_protein\_signal\_transduction | 2 | 0 |  |  |  |  |  |  |  |  |
| GO:0035054\_embryonic\_heart\_tube\_anterior\_posterior\_pattern\_formation | 2 | 0 |  |  |  |  |  |  |  |  |
| GO:0035092\_sperm\_chromatin\_condensation | 2 | 0 |  |  |  |  |  |  |  |  |
| GO:0035110\_leg\_morphogenesis | 2 | 0 |  |  |  |  |  |  |  |  |
| GO:0035117\_embryonic\_arm\_morphogenesis | 2 | 0 |  |  |  |  |  |  |  |  |
| GO:0035120\_post-embryonic\_appendage\_morphogenesis | 2 | 0 |  |  |  |  |  |  |  |  |
| GO:0035127\_post-embryonic\_limb\_morphogenesis | 2 | 0 |  |  |  |  |  |  |  |  |
| GO:0035129\_post-embryonic\_hindlimb\_morphogenesis | 2 | 0 |  |  |  |  |  |  |  |  |
| GO:0035140\_arm\_morphogenesis | 2 | 0 |  |  |  |  |  |  |  |  |
| GO:0035194\_posttranscriptional\_gene\_silencing\_by\_RNA | 2 | 0 |  |  |  |  |  |  |  |  |
| GO:0035195\_gene\_silencing\_by\_miRNA | 2 | 0 |  |  |  |  |  |  |  |  |
| GO:0035196\_gene\_silencing\_by\_miRNA\_\_production\_of\_miRNAs | 2 | 0 |  |  |  |  |  |  |  |  |
| GO:0035315\_hair\_cell\_differentiation | 2 | 0 |  |  |  |  |  |  |  |  |
| GO:0040009\_regulation\_of\_growth\_rate | 2 | 0 |  |  |  |  |  |  |  |  |
| GO:0040037\_negative\_regulation\_of\_fibroblast\_growth\_factor\_receptor\_signaling\_pathway | 2 | 0 |  |  |  |  |  |  |  |  |
| GO:0042119\_neutrophil\_activation | 2 | 0 |  |  |  |  |  |  |  |  |
| GO:0042147\_retrograde\_transport\_\_endosome\_to\_Golgi | 2 | 0 |  |  |  |  |  |  |  |  |
| GO:0042223\_interleukin-3\_biosynthetic\_process | 2 | 0 |  |  |  |  |  |  |  |  |
| GO:0042249\_establishment\_of\_polarity\_of\_embryonic\_epithelium | 2 | 0 |  |  |  |  |  |  |  |  |
| GO:0042253\_granulocyte\_macrophage\_colony-stimulating\_factor\_biosynthetic\_process | 2 | 0 |  |  |  |  |  |  |  |  |
| GO:0042270\_protection\_from\_natural\_killer\_cell\_mediated\_cytotoxicity | 2 | 0 |  |  |  |  |  |  |  |  |
| GO:0042274\_ribosomal\_small\_subunit\_biogenesis | 2 | 0 |  |  |  |  |  |  |  |  |
| GO:0042312\_regulation\_of\_vasodilation | 2 | 0 |  |  |  |  |  |  |  |  |
| GO:0042346\_positive\_regulation\_of\_NF-kappaB\_import\_into\_nucleus | 2 | 0 |  |  |  |  |  |  |  |  |
| GO:0042396\_phosphagen\_biosynthetic\_process | 2 | 0 |  |  |  |  |  |  |  |  |
| GO:0042454\_ribonucleoside\_catabolic\_process | 2 | 0 |  |  |  |  |  |  |  |  |
| GO:0042482\_positive\_regulation\_of\_odontogenesis | 2 | 0 |  |  |  |  |  |  |  |  |
| GO:0042483\_negative\_regulation\_of\_odontogenesis | 2 | 0 |  |  |  |  |  |  |  |  |
| GO:0042488\_positive\_regulation\_of\_odontogenesis\_of\_dentine-containing\_tooth | 2 | 0 |  |  |  |  |  |  |  |  |
| GO:0042501\_serine\_phosphorylation\_of\_STAT\_protein | 2 | 0 |  |  |  |  |  |  |  |  |
| GO:0042517\_positive\_regulation\_of\_tyrosine\_phosphorylation\_of\_Stat3\_protein | 2 | 0 |  |  |  |  |  |  |  |  |
| GO:0042532\_negative\_regulation\_of\_tyrosine\_phosphorylation\_of\_STAT\_protein | 2 | 0 |  |  |  |  |  |  |  |  |
| GO:0042559\_pteridine\_and\_derivative\_biosynthetic\_process | 2 | 0 |  |  |  |  |  |  |  |  |
| GO:0042730\_fibrinolysis | 2 | 0 |  |  |  |  |  |  |  |  |
| GO:0042749\_regulation\_of\_circadian\_sleep\_wake\_cycle | 2 | 0 |  |  |  |  |  |  |  |  |
| GO:0042886\_amide\_transport | 2 | 0 |  |  |  |  |  |  |  |  |
| GO:0042921\_glucocorticoid\_receptor\_signaling\_pathway | 2 | 0 |  |  |  |  |  |  |  |  |
| GO:0042987\_amyloid\_precursor\_protein\_catabolic\_process | 2 | 0 |  |  |  |  |  |  |  |  |
| GO:0042993\_positive\_regulation\_of\_transcription\_factor\_import\_into\_nucleus | 2 | 0 |  |  |  |  |  |  |  |  |
| GO:0042994\_cytoplasmic\_sequestering\_of\_transcription\_factor | 2 | 0 |  |  |  |  |  |  |  |  |
| GO:0043032\_positive\_regulation\_of\_macrophage\_activation | 2 | 0 |  |  |  |  |  |  |  |  |
| GO:0043038\_amino\_acid\_activation | 2 | 0 |  |  |  |  |  |  |  |  |
| GO:0043039\_tRNA\_aminoacylation | 2 | 0 |  |  |  |  |  |  |  |  |
| GO:0043084\_penile\_erection | 2 | 0 |  |  |  |  |  |  |  |  |
| GO:0043088\_regulation\_of\_Cdc42\_GTPase\_activity | 2 | 0 |  |  |  |  |  |  |  |  |
| GO:0043089\_positive\_regulation\_of\_Cdc42\_GTPase\_activity | 2 | 0 |  |  |  |  |  |  |  |  |
| GO:0043096\_purine\_base\_salvage | 2 | 0 |  |  |  |  |  |  |  |  |
| GO:0043247\_telomere\_maintenance\_in\_response\_to\_DNA\_damage | 2 | 0 |  |  |  |  |  |  |  |  |
| GO:0043297\_apical\_junction\_assembly | 2 | 0 |  |  |  |  |  |  |  |  |
| GO:0043312\_neutrophil\_degranulation | 2 | 0 |  |  |  |  |  |  |  |  |
| GO:0043320\_natural\_killer\_cell\_degranulation | 2 | 0 |  |  |  |  |  |  |  |  |
| GO:0043366\_beta\_selection | 2 | 0 |  |  |  |  |  |  |  |  |
| GO:0043450\_alkene\_biosynthetic\_process | 2 | 0 |  |  |  |  |  |  |  |  |
| GO:0043476\_pigment\_accumulation | 2 | 0 |  |  |  |  |  |  |  |  |
| GO:0043490\_malate-aspartate\_shuttle | 2 | 0 |  |  |  |  |  |  |  |  |
| GO:0043502\_regulation\_of\_muscle\_adaptation | 2 | 0 |  |  |  |  |  |  |  |  |
| GO:0043516\_regulation\_of\_DNA\_damage\_response\_\_signal\_transduction\_by\_p53\_class\_mediator | 2 | 0 |  |  |  |  |  |  |  |  |
| GO:0043568\_positive\_regulation\_of\_insulin-like\_growth\_factor\_receptor\_signaling\_pathway | 2 | 0 |  |  |  |  |  |  |  |  |
| GO:0043589\_skin\_morphogenesis | 2 | 0 |  |  |  |  |  |  |  |  |
| GO:0043618\_regulation\_of\_transcription\_from\_RNA\_polymerase\_II\_promoter\_in\_response\_to\_stress | 2 | 0 |  |  |  |  |  |  |  |  |
| GO:0043619\_regulation\_of\_transcription\_from\_RNA\_polymerase\_II\_promoter\_in\_response\_to\_oxidative\_stress | 2 | 0 |  |  |  |  |  |  |  |  |
| GO:0043620\_regulation\_of\_transcription\_in\_response\_to\_stress | 2 | 0 |  |  |  |  |  |  |  |  |
| GO:0043647\_inositol\_phosphate\_metabolic\_process | 2 | 0 |  |  |  |  |  |  |  |  |
| GO:0043654\_recognition\_of\_apoptotic\_cell | 2 | 0 |  |  |  |  |  |  |  |  |
| GO:0043966\_histone\_H3\_acetylation | 2 | 0 |  |  |  |  |  |  |  |  |
| GO:0043967\_histone\_H4\_acetylation | 2 | 0 |  |  |  |  |  |  |  |  |
| GO:0044070\_regulation\_of\_anion\_transport | 2 | 0 |  |  |  |  |  |  |  |  |
| GO:0044246\_regulation\_of\_multicellular\_organismal\_metabolic\_process | 2 | 0 |  |  |  |  |  |  |  |  |
| GO:0044253\_positive\_regulation\_of\_multicellular\_organismal\_metabolic\_process | 2 | 0 |  |  |  |  |  |  |  |  |
| GO:0044268\_multicellular\_organismal\_protein\_metabolic\_process | 2 | 0 |  |  |  |  |  |  |  |  |
| GO:0045005\_maintenance\_of\_fidelity\_during\_DNA-dependent\_DNA\_replication | 2 | 0 |  |  |  |  |  |  |  |  |
| GO:0045010\_actin\_nucleation | 2 | 0 |  |  |  |  |  |  |  |  |
| GO:0045065\_cytotoxic\_T\_cell\_differentiation | 2 | 0 |  |  |  |  |  |  |  |  |
| GO:0045077\_negative\_regulation\_of\_interferon-gamma\_biosynthetic\_process | 2 | 0 |  |  |  |  |  |  |  |  |
| GO:0045079\_negative\_regulation\_of\_chemokine\_biosynthetic\_process | 2 | 0 |  |  |  |  |  |  |  |  |
| GO:0045187\_regulation\_of\_circadian\_sleep\_wake\_cycle\_\_sleep | 2 | 0 |  |  |  |  |  |  |  |  |
| GO:0045212\_neurotransmitter\_receptor\_biosynthetic\_process | 2 | 0 |  |  |  |  |  |  |  |  |
| GO:0045399\_regulation\_of\_interleukin-3\_biosynthetic\_process | 2 | 0 |  |  |  |  |  |  |  |  |
| GO:0045401\_positive\_regulation\_of\_interleukin-3\_biosynthetic\_process | 2 | 0 |  |  |  |  |  |  |  |  |
| GO:0045409\_negative\_regulation\_of\_interleukin-6\_biosynthetic\_process | 2 | 0 |  |  |  |  |  |  |  |  |
| GO:0045423\_regulation\_of\_granulocyte\_macrophage\_colony-stimulating\_factor\_biosynthetic\_process | 2 | 0 |  |  |  |  |  |  |  |  |
| GO:0045425\_positive\_regulation\_of\_granulocyte\_macrophage\_colony-stimulating\_factor\_biosynthetic\_process | 2 | 0 |  |  |  |  |  |  |  |  |
| GO:0045475\_locomotor\_rhythm | 2 | 0 |  |  |  |  |  |  |  |  |
| GO:0045578\_negative\_regulation\_of\_B\_cell\_differentiation | 2 | 0 |  |  |  |  |  |  |  |  |
| GO:0045589\_regulation\_of\_regulatory\_T\_cell\_differentiation | 2 | 0 |  |  |  |  |  |  |  |  |
| GO:0045591\_positive\_regulation\_of\_regulatory\_T\_cell\_differentiation | 2 | 0 |  |  |  |  |  |  |  |  |
| GO:0045608\_negative\_regulation\_of\_auditory\_receptor\_cell\_differentiation | 2 | 0 |  |  |  |  |  |  |  |  |
| GO:0045627\_positive\_regulation\_of\_T-helper\_1\_cell\_differentiation | 2 | 0 |  |  |  |  |  |  |  |  |
| GO:0045629\_negative\_regulation\_of\_T-helper\_2\_cell\_differentiation | 2 | 0 |  |  |  |  |  |  |  |  |
| GO:0045630\_positive\_regulation\_of\_T-helper\_2\_cell\_differentiation | 2 | 0 |  |  |  |  |  |  |  |  |
| GO:0045632\_negative\_regulation\_of\_mechanoreceptor\_differentiation | 2 | 0 |  |  |  |  |  |  |  |  |
| GO:0045636\_positive\_regulation\_of\_melanocyte\_differentiation | 2 | 0 |  |  |  |  |  |  |  |  |
| GO:0045655\_regulation\_of\_monocyte\_differentiation | 2 | 0 |  |  |  |  |  |  |  |  |
| GO:0045658\_regulation\_of\_neutrophil\_differentiation | 2 | 0 |  |  |  |  |  |  |  |  |
| GO:0045662\_negative\_regulation\_of\_myoblast\_differentiation | 2 | 0 |  |  |  |  |  |  |  |  |
| GO:0045663\_positive\_regulation\_of\_myoblast\_differentiation | 2 | 0 |  |  |  |  |  |  |  |  |
| GO:0045683\_negative\_regulation\_of\_epidermis\_development | 2 | 0 |  |  |  |  |  |  |  |  |
| GO:0045737\_positive\_regulation\_of\_cyclin-dependent\_protein\_kinase\_activity | 2 | 0 |  |  |  |  |  |  |  |  |
| GO:0045739\_positive\_regulation\_of\_DNA\_repair | 2 | 0 |  |  |  |  |  |  |  |  |
| GO:0045741\_positive\_regulation\_of\_epidermal\_growth\_factor\_receptor\_activity | 2 | 0 |  |  |  |  |  |  |  |  |
| GO:0045743\_positive\_regulation\_of\_fibroblast\_growth\_factor\_receptor\_signaling\_pathway | 2 | 0 |  |  |  |  |  |  |  |  |
| GO:0045749\_negative\_regulation\_of\_S\_phase\_of\_mitotic\_cell\_cycle | 2 | 0 |  |  |  |  |  |  |  |  |
| GO:0045819\_positive\_regulation\_of\_glycogen\_catabolic\_process | 2 | 0 |  |  |  |  |  |  |  |  |
| GO:0045821\_positive\_regulation\_of\_glycolysis | 2 | 0 |  |  |  |  |  |  |  |  |
| GO:0045835\_negative\_regulation\_of\_meiosis | 2 | 0 |  |  |  |  |  |  |  |  |
| GO:0045836\_positive\_regulation\_of\_meiosis | 2 | 0 |  |  |  |  |  |  |  |  |
| GO:0045839\_negative\_regulation\_of\_mitosis | 2 | 0 |  |  |  |  |  |  |  |  |
| GO:0045841\_negative\_regulation\_of\_mitotic\_metaphase\_anaphase\_transition | 2 | 0 |  |  |  |  |  |  |  |  |
| GO:0045872\_positive\_regulation\_of\_rhodopsin\_gene\_expression | 2 | 0 |  |  |  |  |  |  |  |  |
| GO:0045912\_negative\_regulation\_of\_carbohydrate\_metabolic\_process | 2 | 0 |  |  |  |  |  |  |  |  |
| GO:0045948\_positive\_regulation\_of\_translational\_initiation | 2 | 0 |  |  |  |  |  |  |  |  |
| GO:0045950\_negative\_regulation\_of\_mitotic\_recombination | 2 | 0 |  |  |  |  |  |  |  |  |
| GO:0046033\_AMP\_metabolic\_process | 2 | 0 |  |  |  |  |  |  |  |  |
| GO:0046060\_dATP\_metabolic\_process | 2 | 0 |  |  |  |  |  |  |  |  |
| GO:0046070\_dGTP\_metabolic\_process | 2 | 0 |  |  |  |  |  |  |  |  |
| GO:0046083\_adenine\_metabolic\_process | 2 | 0 |  |  |  |  |  |  |  |  |
| GO:0046085\_adenosine\_metabolic\_process | 2 | 0 |  |  |  |  |  |  |  |  |
| GO:0046100\_hypoxanthine\_metabolic\_process | 2 | 0 |  |  |  |  |  |  |  |  |
| GO:0046114\_guanosine\_biosynthetic\_process | 2 | 0 |  |  |  |  |  |  |  |  |
| GO:0046116\_queuosine\_metabolic\_process | 2 | 0 |  |  |  |  |  |  |  |  |
| GO:0046118\_7-methylguanosine\_biosynthetic\_process | 2 | 0 |  |  |  |  |  |  |  |  |
| GO:0046130\_purine\_ribonucleoside\_catabolic\_process | 2 | 0 |  |  |  |  |  |  |  |  |
| GO:0046146\_tetrahydrobiopterin\_metabolic\_process | 2 | 0 |  |  |  |  |  |  |  |  |
| GO:0046185\_aldehyde\_catabolic\_process | 2 | 0 |  |  |  |  |  |  |  |  |
| GO:0046208\_spermine\_catabolic\_process | 2 | 0 |  |  |  |  |  |  |  |  |
| GO:0046349\_amino\_sugar\_biosynthetic\_process | 2 | 0 |  |  |  |  |  |  |  |  |
| GO:0046439\_L-cysteine\_metabolic\_process | 2 | 0 |  |  |  |  |  |  |  |  |
| GO:0046500\_S-adenosylmethionine\_metabolic\_process | 2 | 0 |  |  |  |  |  |  |  |  |
| GO:0046501\_protoporphyrinogen\_IX\_metabolic\_process | 2 | 0 |  |  |  |  |  |  |  |  |
| GO:0046514\_ceramide\_catabolic\_process | 2 | 0 |  |  |  |  |  |  |  |  |
| GO:0046521\_sphingoid\_catabolic\_process | 2 | 0 |  |  |  |  |  |  |  |  |
| GO:0046532\_regulation\_of\_photoreceptor\_cell\_differentiation | 2 | 0 |  |  |  |  |  |  |  |  |
| GO:0046533\_negative\_regulation\_of\_photoreceptor\_cell\_differentiation | 2 | 0 |  |  |  |  |  |  |  |  |
| GO:0046544\_development\_of\_secondary\_male\_sexual\_characteristics | 2 | 0 |  |  |  |  |  |  |  |  |
| GO:0046619\_optic\_placode\_formation\_involved\_in\_camera-type\_eye | 2 | 0 |  |  |  |  |  |  |  |  |
| GO:0046950\_cellular\_ketone\_body\_metabolic\_process | 2 | 0 |  |  |  |  |  |  |  |  |
| GO:0046984\_regulation\_of\_hemoglobin\_biosynthetic\_process | 2 | 0 |  |  |  |  |  |  |  |  |
| GO:0047484\_regulation\_of\_response\_to\_osmotic\_stress | 2 | 0 |  |  |  |  |  |  |  |  |
| GO:0048025\_negative\_regulation\_of\_nuclear\_mRNA\_splicing\_\_via\_spliceosome | 2 | 0 |  |  |  |  |  |  |  |  |
| GO:0048134\_germ-line\_cyst\_formation | 2 | 0 |  |  |  |  |  |  |  |  |
| GO:0048136\_male\_germ-line\_cyst\_formation | 2 | 0 |  |  |  |  |  |  |  |  |
| GO:0048172\_regulation\_of\_short-term\_neuronal\_synaptic\_plasticity | 2 | 0 |  |  |  |  |  |  |  |  |
| GO:0048295\_positive\_regulation\_of\_isotype\_switching\_to\_IgE\_isotypes | 2 | 0 |  |  |  |  |  |  |  |  |
| GO:0048342\_paraxial\_mesodermal\_cell\_differentiation | 2 | 0 |  |  |  |  |  |  |  |  |
| GO:0048343\_paraxial\_mesodermal\_cell\_fate\_commitment | 2 | 0 |  |  |  |  |  |  |  |  |
| GO:0048382\_mesendoderm\_development | 2 | 0 |  |  |  |  |  |  |  |  |
| GO:0048552\_regulation\_of\_metalloenzyme\_activity | 2 | 0 |  |  |  |  |  |  |  |  |
| GO:0048554\_positive\_regulation\_of\_metalloenzyme\_activity | 2 | 0 |  |  |  |  |  |  |  |  |
| GO:0048619\_embryonic\_hindgut\_morphogenesis | 2 | 0 |  |  |  |  |  |  |  |  |
| GO:0048625\_myoblast\_cell\_fate\_commitment | 2 | 0 |  |  |  |  |  |  |  |  |
| GO:0048627\_myoblast\_development | 2 | 0 |  |  |  |  |  |  |  |  |
| GO:0048643\_positive\_regulation\_of\_skeletal\_muscle\_tissue\_development | 2 | 0 |  |  |  |  |  |  |  |  |
| GO:0048661\_positive\_regulation\_of\_smooth\_muscle\_cell\_proliferation | 2 | 0 |  |  |  |  |  |  |  |  |
| GO:0048670\_regulation\_of\_collateral\_sprouting | 2 | 0 |  |  |  |  |  |  |  |  |
| GO:0048671\_negative\_regulation\_of\_collateral\_sprouting | 2 | 0 |  |  |  |  |  |  |  |  |
| GO:0048677\_axon\_extension\_involved\_in\_regeneration | 2 | 0 |  |  |  |  |  |  |  |  |
| GO:0048679\_regulation\_of\_axon\_regeneration | 2 | 0 |  |  |  |  |  |  |  |  |
| GO:0048682\_sprouting\_of\_injured\_axon | 2 | 0 |  |  |  |  |  |  |  |  |
| GO:0048702\_embryonic\_neurocranium\_morphogenesis | 2 | 0 |  |  |  |  |  |  |  |  |
| GO:0048711\_positive\_regulation\_of\_astrocyte\_differentiation | 2 | 0 |  |  |  |  |  |  |  |  |
| GO:0048712\_negative\_regulation\_of\_astrocyte\_differentiation | 2 | 0 |  |  |  |  |  |  |  |  |
| GO:0048739\_cardiac\_muscle\_fiber\_development | 2 | 0 |  |  |  |  |  |  |  |  |
| GO:0048807\_female\_genitalia\_morphogenesis | 2 | 0 |  |  |  |  |  |  |  |  |
| GO:0048808\_male\_genitalia\_morphogenesis | 2 | 0 |  |  |  |  |  |  |  |  |
| GO:0048840\_otolith\_development | 2 | 0 |  |  |  |  |  |  |  |  |
| GO:0048850\_hypophysis\_morphogenesis | 2 | 0 |  |  |  |  |  |  |  |  |
| GO:0048867\_stem\_cell\_fate\_determination | 2 | 0 |  |  |  |  |  |  |  |  |
| GO:0050000\_chromosome\_localization | 2 | 0 |  |  |  |  |  |  |  |  |
| GO:0050686\_negative\_regulation\_of\_mRNA\_processing | 2 | 0 |  |  |  |  |  |  |  |  |
| GO:0050688\_regulation\_of\_defense\_response\_to\_virus | 2 | 0 |  |  |  |  |  |  |  |  |
| GO:0050746\_regulation\_of\_lipoprotein\_metabolic\_process | 2 | 0 |  |  |  |  |  |  |  |  |
| GO:0050779\_RNA\_destabilization | 2 | 0 |  |  |  |  |  |  |  |  |
| GO:0050792\_regulation\_of\_viral\_reproduction | 2 | 0 |  |  |  |  |  |  |  |  |
| GO:0050802\_circadian\_sleep\_wake\_cycle\_\_sleep | 2 | 0 |  |  |  |  |  |  |  |  |
| GO:0050847\_progesterone\_receptor\_signaling\_pathway | 2 | 0 |  |  |  |  |  |  |  |  |
| GO:0050855\_regulation\_of\_B\_cell\_receptor\_signaling\_pathway | 2 | 0 |  |  |  |  |  |  |  |  |
| GO:0050883\_musculoskeletal\_movement\_\_spinal\_reflex\_action | 2 | 0 |  |  |  |  |  |  |  |  |
| GO:0050901\_leukocyte\_tethering\_or\_rolling | 2 | 0 |  |  |  |  |  |  |  |  |
| GO:0050907\_detection\_of\_chemical\_stimulus\_involved\_in\_sensory\_perception | 2 | 0 |  |  |  |  |  |  |  |  |
| GO:0050917\_sensory\_perception\_of\_umami\_taste | 2 | 0 |  |  |  |  |  |  |  |  |
| GO:0050942\_positive\_regulation\_of\_pigment\_cell\_differentiation | 2 | 0 |  |  |  |  |  |  |  |  |
| GO:0050955\_thermoception | 2 | 0 |  |  |  |  |  |  |  |  |
| GO:0050968\_detection\_of\_chemical\_stimulus\_involved\_in\_sensory\_perception\_of\_pain | 2 | 0 |  |  |  |  |  |  |  |  |
| GO:0050973\_detection\_of\_mechanical\_stimulus\_involved\_in\_equilibrioception | 2 | 0 |  |  |  |  |  |  |  |  |
| GO:0050999\_regulation\_of\_nitric-oxide\_synthase\_activity | 2 | 0 |  |  |  |  |  |  |  |  |
| GO:0051004\_regulation\_of\_lipoprotein\_lipase\_activity | 2 | 0 |  |  |  |  |  |  |  |  |
| GO:0051014\_actin\_filament\_severing | 2 | 0 |  |  |  |  |  |  |  |  |
| GO:0051026\_chiasma\_formation | 2 | 0 |  |  |  |  |  |  |  |  |
| GO:0051081\_nuclear\_envelope\_disassembly | 2 | 0 |  |  |  |  |  |  |  |  |
| GO:0051132\_NK\_T\_cell\_activation | 2 | 0 |  |  |  |  |  |  |  |  |
| GO:0051133\_regulation\_of\_NK\_T\_cell\_activation | 2 | 0 |  |  |  |  |  |  |  |  |
| GO:0051135\_positive\_regulation\_of\_NK\_T\_cell\_activation | 2 | 0 |  |  |  |  |  |  |  |  |
| GO:0051150\_regulation\_of\_smooth\_muscle\_cell\_differentiation | 2 | 0 |  |  |  |  |  |  |  |  |
| GO:0051220\_cytoplasmic\_sequestering\_of\_protein | 2 | 0 |  |  |  |  |  |  |  |  |
| GO:0051279\_regulation\_of\_release\_of\_sequestered\_calcium\_ion\_into\_cytosol | 2 | 0 |  |  |  |  |  |  |  |  |
| GO:0051299\_centrosome\_separation | 2 | 0 |  |  |  |  |  |  |  |  |
| GO:0051303\_establishment\_of\_chromosome\_localization | 2 | 0 |  |  |  |  |  |  |  |  |
| GO:0051304\_chromosome\_separation | 2 | 0 |  |  |  |  |  |  |  |  |
| GO:0051307\_meiotic\_chromosome\_separation | 2 | 0 |  |  |  |  |  |  |  |  |
| GO:0051313\_attachment\_of\_spindle\_microtubules\_to\_chromosome | 2 | 0 |  |  |  |  |  |  |  |  |
| GO:0051318\_G1\_phase | 2 | 0 |  |  |  |  |  |  |  |  |
| GO:0051319\_G2\_phase | 2 | 0 |  |  |  |  |  |  |  |  |
| GO:0051353\_positive\_regulation\_of\_oxidoreductase\_activity | 2 | 0 |  |  |  |  |  |  |  |  |
| GO:0051451\_myoblast\_migration | 2 | 0 |  |  |  |  |  |  |  |  |
| GO:0051489\_regulation\_of\_filopodium\_assembly | 2 | 0 |  |  |  |  |  |  |  |  |
| GO:0051491\_positive\_regulation\_of\_filopodium\_assembly | 2 | 0 |  |  |  |  |  |  |  |  |
| GO:0051541\_elastin\_metabolic\_process | 2 | 0 |  |  |  |  |  |  |  |  |
| GO:0051546\_keratinocyte\_migration | 2 | 0 |  |  |  |  |  |  |  |  |
| GO:0051563\_smooth\_endoplasmic\_reticulum\_calcium\_ion\_homeostasis | 2 | 0 |  |  |  |  |  |  |  |  |
| GO:0051590\_positive\_regulation\_of\_neurotransmitter\_transport | 2 | 0 |  |  |  |  |  |  |  |  |
| GO:0051602\_response\_to\_electrical\_stimulus | 2 | 0 |  |  |  |  |  |  |  |  |
| GO:0051608\_histamine\_transport | 2 | 0 |  |  |  |  |  |  |  |  |
| GO:0051643\_ER\_localization | 2 | 0 |  |  |  |  |  |  |  |  |
| GO:0051657\_maintenance\_of\_organelle\_location | 2 | 0 |  |  |  |  |  |  |  |  |
| GO:0051702\_interaction\_with\_symbiont | 2 | 0 |  |  |  |  |  |  |  |  |
| GO:0051781\_positive\_regulation\_of\_cell\_division | 2 | 0 |  |  |  |  |  |  |  |  |
| GO:0051784\_negative\_regulation\_of\_nuclear\_division | 2 | 0 |  |  |  |  |  |  |  |  |
| GO:0051890\_regulation\_of\_cardioblast\_differentiation | 2 | 0 |  |  |  |  |  |  |  |  |
| GO:0051891\_positive\_regulation\_of\_cardioblast\_differentiation | 2 | 0 |  |  |  |  |  |  |  |  |
| GO:0051923\_sulfation | 2 | 0 |  |  |  |  |  |  |  |  |
| GO:0051938\_L-glutamate\_import | 2 | 0 |  |  |  |  |  |  |  |  |
| GO:0051957\_positive\_regulation\_of\_amino\_acid\_transport | 2 | 0 |  |  |  |  |  |  |  |  |
| GO:0051988\_regulation\_of\_attachment\_of\_spindle\_microtubules\_to\_kinetochore | 2 | 0 |  |  |  |  |  |  |  |  |
| GO:0055057\_neuroblast\_division | 2 | 0 |  |  |  |  |  |  |  |  |
| GO:0055064\_chloride\_ion\_homeostasis | 2 | 0 |  |  |  |  |  |  |  |  |
| GO:0055075\_potassium\_ion\_homeostasis | 2 | 0 |  |  |  |  |  |  |  |  |
| GO:0055090\_acylglycerol\_homeostasis | 2 | 0 |  |  |  |  |  |  |  |  |
| GO:0055091\_phospholipid\_homeostasis | 2 | 0 |  |  |  |  |  |  |  |  |
| GO:0060012\_synaptic\_transmission\_\_glycinergic | 2 | 0 |  |  |  |  |  |  |  |  |
| GO:0060023\_soft\_palate\_development | 2 | 0 |  |  |  |  |  |  |  |  |
| GO:0060032\_notochord\_regression | 2 | 0 |  |  |  |  |  |  |  |  |
| GO:0060039\_pericardium\_development | 2 | 0 |  |  |  |  |  |  |  |  |
| GO:0060044\_negative\_regulation\_of\_cardiac\_muscle\_cell\_proliferation | 2 | 0 |  |  |  |  |  |  |  |  |
| GO:0060060\_post-embryonic\_retina\_morphogenesis\_in\_camera-type\_eye | 2 | 0 |  |  |  |  |  |  |  |  |
| GO:0060083\_smooth\_muscle\_contraction\_involved\_in\_micturition | 2 | 0 |  |  |  |  |  |  |  |  |
| GO:0060124\_positive\_regulation\_of\_growth\_hormone\_secretion | 2 | 0 |  |  |  |  |  |  |  |  |
| GO:0060133\_somatotropin\_secreting\_cell\_development | 2 | 0 |  |  |  |  |  |  |  |  |
| GO:0060155\_platelet\_dense\_granule\_organization | 2 | 0 |  |  |  |  |  |  |  |  |
| GO:0060159\_regulation\_of\_dopamine\_receptor\_signaling\_pathway | 2 | 0 |  |  |  |  |  |  |  |  |
| GO:0060160\_negative\_regulation\_of\_dopamine\_receptor\_signaling\_pathway | 2 | 0 |  |  |  |  |  |  |  |  |
| GO:0060166\_olfactory\_pit\_development | 2 | 0 |  |  |  |  |  |  |  |  |
| GO:0060179\_male\_mating\_behavior | 2 | 0 |  |  |  |  |  |  |  |  |
| GO:0060180\_female\_mating\_behavior | 2 | 0 |  |  |  |  |  |  |  |  |
| GO:0060214\_endocardium\_formation | 2 | 0 |  |  |  |  |  |  |  |  |
| GO:0060218\_hemopoietic\_stem\_cell\_differentiation | 2 | 0 |  |  |  |  |  |  |  |  |
| GO:0060259\_regulation\_of\_feeding\_behavior | 2 | 0 |  |  |  |  |  |  |  |  |
| GO:0060260\_regulation\_of\_transcription\_initiation\_from\_RNA\_polymerase\_II\_promoter | 2 | 0 |  |  |  |  |  |  |  |  |
| GO:0060292\_long\_term\_synaptic\_depression | 2 | 0 |  |  |  |  |  |  |  |  |
| GO:0060346\_bone\_trabecula\_formation | 2 | 0 |  |  |  |  |  |  |  |  |
| GO:0060363\_cranial\_suture\_morphogenesis | 2 | 0 |  |  |  |  |  |  |  |  |
| GO:0060393\_regulation\_of\_pathway-restricted\_SMAD\_protein\_phosphorylation | 2 | 0 |  |  |  |  |  |  |  |  |
| GO:0060397\_JAK-STAT\_cascade\_involved\_in\_growth\_hormone\_signaling\_pathway | 2 | 0 |  |  |  |  |  |  |  |  |
| GO:0060426\_lung\_vasculature\_development | 2 | 0 |  |  |  |  |  |  |  |  |
| GO:0060430\_lung\_saccule\_development | 2 | 0 |  |  |  |  |  |  |  |  |
| GO:0060434\_bronchus\_morphogenesis | 2 | 0 |  |  |  |  |  |  |  |  |
| GO:0060439\_trachea\_morphogenesis | 2 | 0 |  |  |  |  |  |  |  |  |
| GO:0060458\_right\_lung\_development | 2 | 0 |  |  |  |  |  |  |  |  |
| GO:0060462\_lung\_lobe\_development | 2 | 0 |  |  |  |  |  |  |  |  |
| GO:0060463\_lung\_lobe\_morphogenesis | 2 | 0 |  |  |  |  |  |  |  |  |
| GO:0060479\_lung\_cell\_differentiation | 2 | 0 |  |  |  |  |  |  |  |  |
| GO:0060487\_lung\_epithelial\_cell\_differentiation | 2 | 0 |  |  |  |  |  |  |  |  |
| GO:0060516\_primary\_prostatic\_bud\_elongation | 2 | 0 |  |  |  |  |  |  |  |  |
| GO:0060529\_squamous\_basal\_epithelial\_stem\_cell\_differentiation\_involved\_in\_prostate\_gland\_acinus\_development | 2 | 0 |  |  |  |  |  |  |  |  |
| GO:0060534\_trachea\_cartilage\_development | 2 | 0 |  |  |  |  |  |  |  |  |
| GO:0060599\_lateral\_sprouting\_involved\_in\_mammary\_gland\_duct\_morphogenesis | 2 | 0 |  |  |  |  |  |  |  |  |
| GO:0060612\_adipose\_tissue\_development | 2 | 0 |  |  |  |  |  |  |  |  |
| GO:0060615\_mammary\_gland\_bud\_formation | 2 | 0 |  |  |  |  |  |  |  |  |
| GO:0060667\_branch\_elongation\_involved\_in\_salivary\_gland\_morphogenesis | 2 | 0 |  |  |  |  |  |  |  |  |
| GO:0060690\_epithelial\_cell\_differentiation\_involved\_in\_salivary\_gland\_development | 2 | 0 |  |  |  |  |  |  |  |  |
| GO:0060738\_epithelial-mesenchymal\_signaling\_involved\_in\_prostate\_gland\_development | 2 | 0 |  |  |  |  |  |  |  |  |
| GO:0060741\_prostate\_gland\_stromal\_morphogenesis | 2 | 0 |  |  |  |  |  |  |  |  |
| GO:0060763\_mammary\_duct\_terminal\_end\_bud\_growth | 2 | 0 |  |  |  |  |  |  |  |  |
| GO:0060765\_regulation\_of\_androgen\_receptor\_signaling\_pathway | 2 | 0 |  |  |  |  |  |  |  |  |
| GO:0060766\_negative\_regulation\_of\_androgen\_receptor\_signaling\_pathway | 2 | 0 |  |  |  |  |  |  |  |  |
| GO:0060769\_positive\_regulation\_of\_epithelial\_cell\_proliferation\_involved\_in\_prostate\_gland\_development | 2 | 0 |  |  |  |  |  |  |  |  |
| GO:0065005\_protein-lipid\_complex\_assembly | 2 | 0 |  |  |  |  |  |  |  |  |
| GO:0070076\_histone\_lysine\_demethylation | 2 | 0 |  |  |  |  |  |  |  |  |
| GO:0070168\_negative\_regulation\_of\_biomineral\_formation | 2 | 0 |  |  |  |  |  |  |  |  |
| GO:0070252\_actin-mediated\_cell\_contraction | 2 | 0 |  |  |  |  |  |  |  |  |
| GO:0070256\_negative\_regulation\_of\_mucus\_secretion | 2 | 0 |  |  |  |  |  |  |  |  |
| GO:0070257\_positive\_regulation\_of\_mucus\_secretion | 2 | 0 |  |  |  |  |  |  |  |  |
| GO:0070570\_regulation\_of\_neuron\_projection\_regeneration | 2 | 0 |  |  |  |  |  |  |  |  |
| GO:0070723\_response\_to\_cholesterol | 2 | 0 |  |  |  |  |  |  |  |  |
| GO:0090030\_regulation\_of\_steroid\_hormone\_biosynthetic\_process | 2 | 0 |  |  |  |  |  |  |  |  |
| GO:0007398\_ectoderm\_development | 99 | 0 | 0.000000 | 0.000000 | 1368 | 1392.492575 | 1447.6 | 1502.707425 | 1.058187 |
| GO:0060348\_bone\_development | 99 | 0 | 0.000000 | 0.000000 | 1368 | 1392.492575 | 1447.6 | 1502.707425 | 1.058187 |
| GO:0060562\_epithelial\_tube\_morphogenesis | 99 | 0 | 0.000000 | 0.000000 | 1368 | 1392.492575 | 1447.6 | 1502.707425 | 1.058187 |
| GO:0050804\_regulation\_of\_synaptic\_transmission | 58 | 0 | 0.000000 | 0.000000 | 1369 | 1395.218261 | 1449.98 | 1504.741739 | 1.059153 |
| GO:0006163\_purine\_nucleotide\_metabolic\_process | 73 | 0 | 0.000000 | 0.000000 | 1374 | 1398.183572 | 1452.66 | 1507.136428 | 1.057249 |
| GO:0006936\_muscle\_contraction | 73 | 0 | 0.000000 | 0.000000 | 1374 | 1398.183572 | 1452.66 | 1507.136428 | 1.057249 |
| GO:0048706\_embryonic\_skeletal\_system\_development | 73 | 0 | 0.000000 | 0.000000 | 1374 | 1398.183572 | 1452.66 | 1507.136428 | 1.057249 |
| GO:0051270\_regulation\_of\_cell\_motion | 73 | 0 | 0.000000 | 0.000000 | 1374 | 1398.183572 | 1452.66 | 1507.136428 | 1.057249 |
| GO:0051336\_regulation\_of\_hydrolase\_activity | 73 | 0 | 0.000000 | 0.000000 | 1374 | 1398.183572 | 1452.66 | 1507.136428 | 1.057249 |
| GO:0000060\_protein\_import\_into\_nucleus\_\_translocation | 14 | 0 | 0.000000 | 0.000000 | 1436 | 1458.034802 | 1510.51 | 1562.985198 | 1.051887 |
| GO:0000077\_DNA\_damage\_checkpoint | 14 | 0 | 0.000000 | 0.000000 | 1436 | 1458.034802 | 1510.51 | 1562.985198 | 1.051887 |
| GO:0001502\_cartilage\_condensation | 14 | 0 | 0.000000 | 0.000000 | 1436 | 1458.034802 | 1510.51 | 1562.985198 | 1.051887 |
| GO:0001829\_trophectodermal\_cell\_differentiation | 14 | 0 | 0.000000 | 0.000000 | 1436 | 1458.034802 | 1510.51 | 1562.985198 | 1.051887 |
| GO:0002027\_regulation\_of\_heart\_rate | 14 | 0 | 0.000000 | 0.000000 | 1436 | 1458.034802 | 1510.51 | 1562.985198 | 1.051887 |
| GO:0002262\_myeloid\_cell\_homeostasis | 14 | 0 | 0.000000 | 0.000000 | 1436 | 1458.034802 | 1510.51 | 1562.985198 | 1.051887 |
| GO:0002698\_negative\_regulation\_of\_immune\_effector\_process | 14 | 0 | 0.000000 | 0.000000 | 1436 | 1458.034802 | 1510.51 | 1562.985198 | 1.051887 |
| GO:0006304\_DNA\_modification | 14 | 0 | 0.000000 | 0.000000 | 1436 | 1458.034802 | 1510.51 | 1562.985198 | 1.051887 |
| GO:0006305\_DNA\_alkylation | 14 | 0 | 0.000000 | 0.000000 | 1436 | 1458.034802 | 1510.51 | 1562.985198 | 1.051887 |
| GO:0006306\_DNA\_methylation | 14 | 0 | 0.000000 | 0.000000 | 1436 | 1458.034802 | 1510.51 | 1562.985198 | 1.051887 |
| GO:0006695\_cholesterol\_biosynthetic\_process | 14 | 0 | 0.000000 | 0.000000 | 1436 | 1458.034802 | 1510.51 | 1562.985198 | 1.051887 |
| GO:0006809\_nitric\_oxide\_biosynthetic\_process | 14 | 0 | 0.000000 | 0.000000 | 1436 | 1458.034802 | 1510.51 | 1562.985198 | 1.051887 |
| GO:0006914\_autophagy | 14 | 0 | 0.000000 | 0.000000 | 1436 | 1458.034802 | 1510.51 | 1562.985198 | 1.051887 |
| GO:0006970\_response\_to\_osmotic\_stress | 14 | 0 | 0.000000 | 0.000000 | 1436 | 1458.034802 | 1510.51 | 1562.985198 | 1.051887 |
| GO:0007157\_heterophilic\_cell\_adhesion | 14 | 0 | 0.000000 | 0.000000 | 1436 | 1458.034802 | 1510.51 | 1562.985198 | 1.051887 |
| GO:0007530\_sex\_determination | 14 | 0 | 0.000000 | 0.000000 | 1436 | 1458.034802 | 1510.51 | 1562.985198 | 1.051887 |
| GO:0007589\_body\_fluid\_secretion | 14 | 0 | 0.000000 | 0.000000 | 1436 | 1458.034802 | 1510.51 | 1562.985198 | 1.051887 |
| GO:0008064\_regulation\_of\_actin\_polymerization\_or\_depolymerization | 14 | 0 | 0.000000 | 0.000000 | 1436 | 1458.034802 | 1510.51 | 1562.985198 | 1.051887 |
| GO:0008306\_associative\_learning | 14 | 0 | 0.000000 | 0.000000 | 1436 | 1458.034802 | 1510.51 | 1562.985198 | 1.051887 |
| GO:0008630\_DNA\_damage\_response\_\_signal\_transduction\_resulting\_in\_induction\_of\_apoptosis | 14 | 0 | 0.000000 | 0.000000 | 1436 | 1458.034802 | 1510.51 | 1562.985198 | 1.051887 |
| GO:0009108\_coenzyme\_biosynthetic\_process | 14 | 0 | 0.000000 | 0.000000 | 1436 | 1458.034802 | 1510.51 | 1562.985198 | 1.051887 |
| GO:0009267\_cellular\_response\_to\_starvation | 14 | 0 | 0.000000 | 0.000000 | 1436 | 1458.034802 | 1510.51 | 1562.985198 | 1.051887 |
| GO:0009895\_negative\_regulation\_of\_catabolic\_process | 14 | 0 | 0.000000 | 0.000000 | 1436 | 1458.034802 | 1510.51 | 1562.985198 | 1.051887 |
| GO:0010332\_response\_to\_gamma\_radiation | 14 | 0 | 0.000000 | 0.000000 | 1436 | 1458.034802 | 1510.51 | 1562.985198 | 1.051887 |
| GO:0016573\_histone\_acetylation | 14 | 0 | 0.000000 | 0.000000 | 1436 | 1458.034802 | 1510.51 | 1562.985198 | 1.051887 |
| GO:0018130\_heterocycle\_biosynthetic\_process | 14 | 0 | 0.000000 | 0.000000 | 1436 | 1458.034802 | 1510.51 | 1562.985198 | 1.051887 |
| GO:0019217\_regulation\_of\_fatty\_acid\_metabolic\_process | 14 | 0 | 0.000000 | 0.000000 | 1436 | 1458.034802 | 1510.51 | 1562.985198 | 1.051887 |
| GO:0021782\_glial\_cell\_development | 14 | 0 | 0.000000 | 0.000000 | 1436 | 1458.034802 | 1510.51 | 1562.985198 | 1.051887 |
| GO:0021904\_dorsal\_ventral\_neural\_tube\_patterning | 14 | 0 | 0.000000 | 0.000000 | 1436 | 1458.034802 | 1510.51 | 1562.985198 | 1.051887 |
| GO:0030032\_lamellipodium\_assembly | 14 | 0 | 0.000000 | 0.000000 | 1436 | 1458.034802 | 1510.51 | 1562.985198 | 1.051887 |
| GO:0030148\_sphingolipid\_biosynthetic\_process | 14 | 0 | 0.000000 | 0.000000 | 1436 | 1458.034802 | 1510.51 | 1562.985198 | 1.051887 |
| GO:0030162\_regulation\_of\_proteolysis | 14 | 0 | 0.000000 | 0.000000 | 1436 | 1458.034802 | 1510.51 | 1562.985198 | 1.051887 |
| GO:0030832\_regulation\_of\_actin\_filament\_length | 14 | 0 | 0.000000 | 0.000000 | 1436 | 1458.034802 | 1510.51 | 1562.985198 | 1.051887 |
| GO:0031099\_regeneration | 14 | 0 | 0.000000 | 0.000000 | 1436 | 1458.034802 | 1510.51 | 1562.985198 | 1.051887 |
| GO:0031346\_positive\_regulation\_of\_cell\_projection\_organization | 14 | 0 | 0.000000 | 0.000000 | 1436 | 1458.034802 | 1510.51 | 1562.985198 | 1.051887 |
| GO:0031663\_lipopolysaccharide-mediated\_signaling\_pathway | 14 | 0 | 0.000000 | 0.000000 | 1436 | 1458.034802 | 1510.51 | 1562.985198 | 1.051887 |
| GO:0032271\_regulation\_of\_protein\_polymerization | 14 | 0 | 0.000000 | 0.000000 | 1436 | 1458.034802 | 1510.51 | 1562.985198 | 1.051887 |
| GO:0034104\_negative\_regulation\_of\_tissue\_remodeling | 14 | 0 | 0.000000 | 0.000000 | 1436 | 1458.034802 | 1510.51 | 1562.985198 | 1.051887 |
| GO:0034623\_cellular\_macromolecular\_complex\_disassembly | 14 | 0 | 0.000000 | 0.000000 | 1436 | 1458.034802 | 1510.51 | 1562.985198 | 1.051887 |
| GO:0035036\_sperm-egg\_recognition | 14 | 0 | 0.000000 | 0.000000 | 1436 | 1458.034802 | 1510.51 | 1562.985198 | 1.051887 |
| GO:0042310\_vasoconstriction | 14 | 0 | 0.000000 | 0.000000 | 1436 | 1458.034802 | 1510.51 | 1562.985198 | 1.051887 |
| GO:0042573\_retinoic\_acid\_metabolic\_process | 14 | 0 | 0.000000 | 0.000000 | 1436 | 1458.034802 | 1510.51 | 1562.985198 | 1.051887 |
| GO:0043123\_positive\_regulation\_of\_I-kappaB\_kinase\_NF-kappaB\_cascade | 14 | 0 | 0.000000 | 0.000000 | 1436 | 1458.034802 | 1510.51 | 1562.985198 | 1.051887 |
| GO:0043254\_regulation\_of\_protein\_complex\_assembly | 14 | 0 | 0.000000 | 0.000000 | 1436 | 1458.034802 | 1510.51 | 1562.985198 | 1.051887 |
| GO:0043491\_protein\_kinase\_B\_signaling\_cascade | 14 | 0 | 0.000000 | 0.000000 | 1436 | 1458.034802 | 1510.51 | 1562.985198 | 1.051887 |
| GO:0044236\_multicellular\_organismal\_metabolic\_process | 14 | 0 | 0.000000 | 0.000000 | 1436 | 1458.034802 | 1510.51 | 1562.985198 | 1.051887 |
| GO:0045061\_thymic\_T\_cell\_selection | 14 | 0 | 0.000000 | 0.000000 | 1436 | 1458.034802 | 1510.51 | 1562.985198 | 1.051887 |
| GO:0045453\_bone\_resorption | 14 | 0 | 0.000000 | 0.000000 | 1436 | 1458.034802 | 1510.51 | 1562.985198 | 1.051887 |
| GO:0045598\_regulation\_of\_fat\_cell\_differentiation | 14 | 0 | 0.000000 | 0.000000 | 1436 | 1458.034802 | 1510.51 | 1562.985198 | 1.051887 |
| GO:0045732\_positive\_regulation\_of\_protein\_catabolic\_process | 14 | 0 | 0.000000 | 0.000000 | 1436 | 1458.034802 | 1510.51 | 1562.985198 | 1.051887 |
| GO:0046209\_nitric\_oxide\_metabolic\_process | 14 | 0 | 0.000000 | 0.000000 | 1436 | 1458.034802 | 1510.51 | 1562.985198 | 1.051887 |
| GO:0048545\_response\_to\_steroid\_hormone\_stimulus | 14 | 0 | 0.000000 | 0.000000 | 1436 | 1458.034802 | 1510.51 | 1562.985198 | 1.051887 |
| GO:0048665\_neuron\_fate\_specification | 14 | 0 | 0.000000 | 0.000000 | 1436 | 1458.034802 | 1510.51 | 1562.985198 | 1.051887 |
| GO:0048844\_artery\_morphogenesis | 14 | 0 | 0.000000 | 0.000000 | 1436 | 1458.034802 | 1510.51 | 1562.985198 | 1.051887 |
| GO:0050810\_regulation\_of\_steroid\_biosynthetic\_process | 14 | 0 | 0.000000 | 0.000000 | 1436 | 1458.034802 | 1510.51 | 1562.985198 | 1.051887 |
| GO:0051017\_actin\_filament\_bundle\_formation | 14 | 0 | 0.000000 | 0.000000 | 1436 | 1458.034802 | 1510.51 | 1562.985198 | 1.051887 |
| GO:0051053\_negative\_regulation\_of\_DNA\_metabolic\_process | 14 | 0 | 0.000000 | 0.000000 | 1436 | 1458.034802 | 1510.51 | 1562.985198 | 1.051887 |
| GO:0051054\_positive\_regulation\_of\_DNA\_metabolic\_process | 14 | 0 | 0.000000 | 0.000000 | 1436 | 1458.034802 | 1510.51 | 1562.985198 | 1.051887 |
| GO:0051100\_negative\_regulation\_of\_binding | 14 | 0 | 0.000000 | 0.000000 | 1436 | 1458.034802 | 1510.51 | 1562.985198 | 1.051887 |
| GO:0051952\_regulation\_of\_amine\_transport | 14 | 0 | 0.000000 | 0.000000 | 1436 | 1458.034802 | 1510.51 | 1562.985198 | 1.051887 |
| GO:0060716\_labyrinthine\_layer\_blood\_vessel\_development | 14 | 0 | 0.000000 | 0.000000 | 1436 | 1458.034802 | 1510.51 | 1562.985198 | 1.051887 |
| GO:0060840\_artery\_development | 14 | 0 | 0.000000 | 0.000000 | 1436 | 1458.034802 | 1510.51 | 1562.985198 | 1.051887 |
| GO:0006874\_cellular\_calcium\_ion\_homeostasis | 61 | 0 | 0.000000 | 0.000000 | 1439 | 1461.336400 | 1513.36 | 1565.383600 | 1.051675 |
| GO:0009991\_response\_to\_extracellular\_stimulus | 61 | 0 | 0.000000 | 0.000000 | 1439 | 1461.336400 | 1513.36 | 1565.383600 | 1.051675 |
| GO:0051969\_regulation\_of\_transmission\_of\_nerve\_impulse | 61 | 0 | 0.000000 | 0.000000 | 1439 | 1461.336400 | 1513.36 | 1565.383600 | 1.051675 |
| GO:0009719\_response\_to\_endogenous\_stimulus | 92 | 0 | 0.000000 | 0.000000 | 1440 | 1463.099513 | 1514.71 | 1566.320487 | 1.051882 |
| GO:0055086\_nucleobase\_\_nucleoside\_and\_nucleotide\_metabolic\_process | 104 | 0 | 0.000000 | 0.000000 | 1441 | 1464.103034 | 1515.61 | 1567.116966 | 1.051777 |
| GO:0043549\_regulation\_of\_kinase\_activity | 112 | 0 | 0.000000 | 0.000000 | 1443 | 1465.113870 | 1516.33 | 1567.546130 | 1.050818 |
| GO:0051249\_regulation\_of\_lymphocyte\_activation | 112 | 0 | 0.000000 | 0.000000 | 1443 | 1465.113870 | 1516.33 | 1567.546130 | 1.050818 |
| GO:0042471\_ear\_morphogenesis | 65 | 0 | 0.000000 | 0.000000 | 1445 | 1467.087091 | 1518.02 | 1568.952909 | 1.050533 |
| GO:0043086\_negative\_regulation\_of\_catalytic\_activity | 65 | 0 | 0.000000 | 0.000000 | 1445 | 1467.087091 | 1518.02 | 1568.952909 | 1.050533 |
| GO:0030326\_embryonic\_limb\_morphogenesis | 78 | 0 | 0.000000 | 0.000000 | 1448 | 1471.013393 | 1521.48 | 1571.946607 | 1.050746 |
| GO:0035113\_embryonic\_appendage\_morphogenesis | 78 | 0 | 0.000000 | 0.000000 | 1448 | 1471.013393 | 1521.48 | 1571.946607 | 1.050746 |
| GO:0051251\_positive\_regulation\_of\_lymphocyte\_activation | 78 | 0 | 0.000000 | 0.000000 | 1448 | 1471.013393 | 1521.48 | 1571.946607 | 1.050746 |
| GO:0006605\_protein\_targeting | 86 | 0 | 0.000000 | 0.000000 | 1451 | 1474.014043 | 1524.13 | 1574.245957 | 1.050400 |
| GO:0006897\_endocytosis | 86 | 0 | 0.000000 | 0.000000 | 1451 | 1474.014043 | 1524.13 | 1574.245957 | 1.050400 |
| GO:0010324\_membrane\_invagination | 86 | 0 | 0.000000 | 0.000000 | 1451 | 1474.014043 | 1524.13 | 1574.245957 | 1.050400 |
| GO:0003007\_heart\_morphogenesis | 67 | 0 | 0.000000 | 0.000000 | 1454 | 1477.130734 | 1526.9 | 1576.669266 | 1.050138 |
[truncated: 256,165 more chars]
