# Supplementary material for: RedundancyMiner: De-replication of redundant GO categories in microarray and proteomics analysis
Source: BMC Bioinformatics. 2011 Feb 10;12:52. doi: 10.1186/1471-2105-12-52 (PMC3223614; doi:10.1186/1471-2105-12-52)
Supplement: Additional file 8 — Retinal development HTGM download. compressed package of the results of running HTGM on the retinal development genes list. [file 1471-2105-12-52-S8.ZIP › SCENARIO_2_MODIFIED/total.txt.total.txt.dir/Exp1_BestClusterMap_LEIGS_KM_24.csv.join.19.txt.dir/Exp1_BestClusterMap_LEIGS_KM_24.csv.join.19.txt.change.gce.html]

Gene Category Report for Exp1\_BestClusterMap\_LEIGS\_KM\_24.csv.join.19.txt

# Gene Category Report for Exp1\_BestClusterMap\_LEIGS\_KM\_24.csv.join.19.txt

| HYPERLINKED GO CATEGORY | HYPERLINKED GENE NAME | TOTAL GENES | CHANGED GENES | ENRICHMENT | LOG10(p) | CUMULATIVE NUMBER OF CATEGORIES | CUMULATIVE RANDOMS MEAN | FALSE DISCOVERY RATE |
| --- | --- | --- | --- | --- | --- | --- | --- | --- |
| GO:0032507\_maintenance\_of\_protein\_location\_in\_cell | TACC3 | 7 | 2 | 54.821429 | -3.269133 | 1 | 0.44 | 0.440000 |
| GO:0032507\_maintenance\_of\_protein\_location\_in\_cell | APC | 7 | 2 | 54.821429 | -3.269133 | 1 | 0.44 | 0.440000 |
| GO:0045185\_maintenance\_of\_protein\_location | TACC3 | 9 | 2 | 42.638889 | -3.037819 | 2 | 0.69 | 0.345000 |
| GO:0045185\_maintenance\_of\_protein\_location | APC | 9 | 2 | 42.638889 | -3.037819 | 2 | 0.69 | 0.345000 |
| GO:0051651\_maintenance\_of\_location\_in\_cell | TACC3 | 10 | 2 | 38.375000 | -2.942292 | 3 | 0.91 | 0.303333 |
| GO:0051651\_maintenance\_of\_location\_in\_cell | APC | 10 | 2 | 38.375000 | -2.942292 | 3 | 0.91 | 0.303333 |
| GO:0034754\_cellular\_hormone\_metabolic\_process | RDH10 | 47 | 3 | 12.247340 | -2.760554 | 4 | 1.29 | 0.322500 |
| GO:0034754\_cellular\_hormone\_metabolic\_process | RELN | 47 | 3 | 12.247340 | -2.760554 | 4 | 1.29 | 0.322500 |
| GO:0034754\_cellular\_hormone\_metabolic\_process | ATP1A1 | 47 | 3 | 12.247340 | -2.760554 | 4 | 1.29 | 0.322500 |
| GO:0016055\_Wnt\_receptor\_signaling\_pathway | FGF9 | 59 | 3 | 9.756356 | -2.476278 | 5 | 2.14 | 0.428000 |
| GO:0016055\_Wnt\_receptor\_signaling\_pathway | FZD1 | 59 | 3 | 9.756356 | -2.476278 | 5 | 2.14 | 0.428000 |
| GO:0016055\_Wnt\_receptor\_signaling\_pathway | APC | 59 | 3 | 9.756356 | -2.476278 | 5 | 2.14 | 0.428000 |
| GO:0030178\_negative\_regulation\_of\_Wnt\_receptor\_signaling\_pathway | FGF9 | 18 | 2 | 21.319444 | -2.421871 | 6 | 2.52 | 0.420000 |
| GO:0030178\_negative\_regulation\_of\_Wnt\_receptor\_signaling\_pathway | APC | 18 | 2 | 21.319444 | -2.421871 | 6 | 2.52 | 0.420000 |
| GO:0042445\_hormone\_metabolic\_process | RDH10 | 67 | 3 | 8.591418 | -2.319766 | 7 | 3.04 | 0.434286 |
| GO:0042445\_hormone\_metabolic\_process | RELN | 67 | 3 | 8.591418 | -2.319766 | 7 | 3.04 | 0.434286 |
| GO:0042445\_hormone\_metabolic\_process | ATP1A1 | 67 | 3 | 8.591418 | -2.319766 | 7 | 3.04 | 0.434286 |
| GO:0031944\_negative\_regulation\_of\_glucocorticoid\_metabolic\_process | ATP1A1 | 1 | 1 |  |  |  |  |  |  |
| GO:0031947\_negative\_regulation\_of\_glucocorticoid\_biosynthetic\_process | ATP1A1 | 1 | 1 |  |  |  |  |  |  |
| GO:0043437\_butanoic\_acid\_metabolic\_process | TYRP1 | 1 | 1 |  |  |  |  |  |  |
| GO:0043438\_acetoacetic\_acid\_metabolic\_process | TYRP1 | 1 | 1 |  |  |  |  |  |  |
| GO:0045989\_positive\_regulation\_of\_striated\_muscle\_contraction | ATP1A1 | 1 | 1 |  |  |  |  |  |  |
| GO:0060431\_primary\_lung\_bud\_formation | RDH10 | 1 | 1 |  |  |  |  |  |  |
| GO:0060449\_bud\_elongation\_involved\_in\_lung\_branching | RDH10 | 1 | 1 |  |  |  |  |  |  |
| GO:0090032\_negative\_regulation\_of\_steroid\_hormone\_biosynthetic\_process | ATP1A1 | 1 | 1 |  |  |  |  |  |  |
| GO:0007059\_chromosome\_segregation | EBNA1BP2 | 22 | 2 | 17.443182 | -2.248471 | 9 | 3.5 | 0.388889 |
| GO:0007059\_chromosome\_segregation | APC | 22 | 2 | 17.443182 | -2.248471 | 9 | 3.5 | 0.388889 |
| GO:0032886\_regulation\_of\_microtubule-based\_process | TACC3 | 22 | 2 | 17.443182 | -2.248471 | 9 | 3.5 | 0.388889 |
| GO:0032886\_regulation\_of\_microtubule-based\_process | APC | 22 | 2 | 17.443182 | -2.248471 | 9 | 3.5 | 0.388889 |
| GO:0051235\_maintenance\_of\_location | TACC3 | 26 | 2 | 14.759615 | -2.105715 | 10 | 4.58 | 0.458000 |
| GO:0051235\_maintenance\_of\_location | APC | 26 | 2 | 14.759615 | -2.105715 | 10 | 4.58 | 0.458000 |
| GO:0007017\_microtubule-based\_process | TACC3 | 83 | 3 | 6.935241 | -2.060630 | 11 | 4.84 | 0.440000 |
| GO:0007017\_microtubule-based\_process | BICD2 | 83 | 3 | 6.935241 | -2.060630 | 11 | 4.84 | 0.440000 |
| GO:0007017\_microtubule-based\_process | APC | 83 | 3 | 6.935241 | -2.060630 | 11 | 4.84 | 0.440000 |
| GO:0030111\_regulation\_of\_Wnt\_receptor\_signaling\_pathway | FGF9 | 28 | 2 | 13.705357 | -2.042862 | 12 | 4.99 | 0.415833 |
| GO:0030111\_regulation\_of\_Wnt\_receptor\_signaling\_pathway | APC | 28 | 2 | 13.705357 | -2.042862 | 12 | 4.99 | 0.415833 |
| GO:0002138\_retinoic\_acid\_biosynthetic\_process | RDH10 | 2 | 1 |  |  |  |  |  |  |
| GO:0006583\_melanin\_biosynthetic\_process\_from\_tyrosine | TYRP1 | 2 | 1 |  |  |  |  |  |  |
| GO:0008608\_attachment\_of\_spindle\_microtubules\_to\_kinetochore | APC | 2 | 1 |  |  |  |  |  |  |
| GO:0030953\_spindle\_astral\_microtubule\_organization | TACC3 | 2 | 1 |  |  |  |  |  |  |
| GO:0031946\_regulation\_of\_glucocorticoid\_biosynthetic\_process | ATP1A1 | 2 | 1 |  |  |  |  |  |  |
| GO:0032351\_negative\_regulation\_of\_hormone\_metabolic\_process | ATP1A1 | 2 | 1 |  |  |  |  |  |  |
| GO:0032353\_negative\_regulation\_of\_hormone\_biosynthetic\_process | ATP1A1 | 2 | 1 |  |  |  |  |  |  |
| GO:0034453\_microtubule\_anchoring | APC | 2 | 1 |  |  |  |  |  |  |
| GO:0042483\_negative\_regulation\_of\_odontogenesis | APC | 2 | 1 |  |  |  |  |  |  |
| GO:0042994\_cytoplasmic\_sequestering\_of\_transcription\_factor | TACC3 | 2 | 1 |  |  |  |  |  |  |
| GO:0045743\_positive\_regulation\_of\_fibroblast\_growth\_factor\_receptor\_signaling\_pathway | FGF9 | 2 | 1 |  |  |  |  |  |  |
| GO:0051220\_cytoplasmic\_sequestering\_of\_protein | TACC3 | 2 | 1 |  |  |  |  |  |  |
| GO:0051313\_attachment\_of\_spindle\_microtubules\_to\_chromosome | APC | 2 | 1 |  |  |  |  |  |  |
| GO:0051781\_positive\_regulation\_of\_cell\_division | APC | 2 | 1 |  |  |  |  |  |  |
| GO:0051988\_regulation\_of\_attachment\_of\_spindle\_microtubules\_to\_kinetochore | APC | 2 | 1 |  |  |  |  |  |  |
| GO:0090030\_regulation\_of\_steroid\_hormone\_biosynthetic\_process | ATP1A1 | 2 | 1 |  |  |  |  |  |  |
| GO:0021987\_cerebral\_cortex\_development | RELN | 33 | 2 | 11.628788 | -1.904602 | 13 | 6.79 | 0.522308 |
| GO:0021987\_cerebral\_cortex\_development | TACC3 | 33 | 2 | 11.628788 | -1.904602 | 13 | 6.79 | 0.522308 |
| GO:0007269\_neurotransmitter\_secretion | STX4A | 34 | 2 | 11.286765 | -1.879649 | 14 | 7.18 | 0.512857 |
| GO:0007269\_neurotransmitter\_secretion | LIN7C | 34 | 2 | 11.286765 | -1.879649 | 14 | 7.18 | 0.512857 |
| GO:0000281\_cytokinesis\_after\_mitosis | APC | 3 | 1 |  |  |  |  |  |  |
| GO:0010894\_negative\_regulation\_of\_steroid\_biosynthetic\_process | ATP1A1 | 3 | 1 |  |  |  |  |  |  |
| GO:0021800\_cerebral\_cortex\_tangential\_migration | RELN | 3 | 1 |  |  |  |  |  |  |
| GO:0022027\_interkinetic\_nuclear\_migration | TACC3 | 3 | 1 |  |  |  |  |  |  |
| GO:0031112\_positive\_regulation\_of\_microtubule\_polymerization\_or\_depolymerization | APC | 3 | 1 |  |  |  |  |  |  |
| GO:0031116\_positive\_regulation\_of\_microtubule\_polymerization | APC | 3 | 1 |  |  |  |  |  |  |
| GO:0031943\_regulation\_of\_glucocorticoid\_metabolic\_process | ATP1A1 | 3 | 1 |  |  |  |  |  |  |
| GO:0045939\_negative\_regulation\_of\_steroid\_metabolic\_process | ATP1A1 | 3 | 1 |  |  |  |  |  |  |
| GO:0046885\_regulation\_of\_hormone\_biosynthetic\_process | ATP1A1 | 3 | 1 |  |  |  |  |  |  |
| GO:0051302\_regulation\_of\_cell\_division | APC | 3 | 1 |  |  |  |  |  |  |
| GO:0051983\_regulation\_of\_chromosome\_segregation | APC | 3 | 1 |  |  |  |  |  |  |
| GO:0001649\_osteoblast\_differentiation | FGF9 | 38 | 2 | 10.098684 | -1.787155 | 15 | 8.71 | 0.580667 |
| GO:0001649\_osteoblast\_differentiation | APC | 38 | 2 | 10.098684 | -1.787155 | 15 | 8.71 | 0.580667 |
| GO:0010817\_regulation\_of\_hormone\_levels | RDH10 | 106 | 3 | 5.430425 | -1.772505 | 16 | 8.78 | 0.548750 |
| GO:0010817\_regulation\_of\_hormone\_levels | RELN | 106 | 3 | 5.430425 | -1.772505 | 16 | 8.78 | 0.548750 |
| GO:0010817\_regulation\_of\_hormone\_levels | ATP1A1 | 106 | 3 | 5.430425 | -1.772505 | 16 | 8.78 | 0.548750 |
| GO:0006836\_neurotransmitter\_transport | STX4A | 41 | 2 | 9.359756 | -1.724416 | 18 | 9.88 | 0.548889 |
| GO:0006836\_neurotransmitter\_transport | LIN7C | 41 | 2 | 9.359756 | -1.724416 | 18 | 9.88 | 0.548889 |
| GO:0019748\_secondary\_metabolic\_process | RDH10 | 41 | 2 | 9.359756 | -1.724416 | 18 | 9.88 | 0.548889 |
| GO:0019748\_secondary\_metabolic\_process | TYRP1 | 41 | 2 | 9.359756 | -1.724416 | 18 | 9.88 | 0.548889 |
| GO:0030858\_positive\_regulation\_of\_epithelial\_cell\_differentiation | APC | 4 | 1 |  |  |  |  |  |  |
| GO:0030949\_positive\_regulation\_of\_vascular\_endothelial\_growth\_factor\_receptor\_signaling\_pathway | FGF9 | 4 | 1 |  |  |  |  |  |  |
| GO:0031113\_regulation\_of\_microtubule\_polymerization | APC | 4 | 1 |  |  |  |  |  |  |
| GO:0042992\_negative\_regulation\_of\_transcription\_factor\_import\_into\_nucleus | TACC3 | 4 | 1 |  |  |  |  |  |  |
| GO:0051055\_negative\_regulation\_of\_lipid\_biosynthetic\_process | ATP1A1 | 4 | 1 |  |  |  |  |  |  |
| GO:0048608\_reproductive\_structure\_development | RDH10 | 116 | 3 | 4.962284 | -1.668745 | 19 | 10.72 | 0.564211 |
| GO:0048608\_reproductive\_structure\_development | FGF9 | 116 | 3 | 4.962284 | -1.668745 | 19 | 10.72 | 0.564211 |
| GO:0048608\_reproductive\_structure\_development | APC | 116 | 3 | 4.962284 | -1.668745 | 19 | 10.72 | 0.564211 |
| GO:0006606\_protein\_import\_into\_nucleus | FGF9 | 44 | 2 | 8.721591 | -1.666456 | 21 | 11.02 | 0.524762 |
| GO:0006606\_protein\_import\_into\_nucleus | TACC3 | 44 | 2 | 8.721591 | -1.666456 | 21 | 11.02 | 0.524762 |
| GO:0051170\_nuclear\_import | FGF9 | 44 | 2 | 8.721591 | -1.666456 | 21 | 11.02 | 0.524762 |
| GO:0051170\_nuclear\_import | TACC3 | 44 | 2 | 8.721591 | -1.666456 | 21 | 11.02 | 0.524762 |
| GO:0001505\_regulation\_of\_neurotransmitter\_levels | STX4A | 48 | 2 | 7.994792 | -1.595522 | 23 | 12.25 | 0.532609 |
| GO:0001505\_regulation\_of\_neurotransmitter\_levels | LIN7C | 48 | 2 | 7.994792 | -1.595522 | 23 | 12.25 | 0.532609 |
| GO:0034504\_protein\_localization\_in\_nucleus | FGF9 | 48 | 2 | 7.994792 | -1.595522 | 23 | 12.25 | 0.532609 |
| GO:0034504\_protein\_localization\_in\_nucleus | TACC3 | 48 | 2 | 7.994792 | -1.595522 | 23 | 12.25 | 0.532609 |
| GO:0006570\_tyrosine\_metabolic\_process | TYRP1 | 5 | 1 | 38.375000 | -1.588388 | 34 | 18.95 | 0.557353 |
| GO:0006704\_glucocorticoid\_biosynthetic\_process | ATP1A1 | 5 | 1 | 38.375000 | -1.588388 | 34 | 18.95 | 0.557353 |
| GO:0007091\_mitotic\_metaphase\_anaphase\_transition | APC | 5 | 1 | 38.375000 | -1.588388 | 34 | 18.95 | 0.557353 |
| GO:0009263\_deoxyribonucleotide\_biosynthetic\_process | RRM2 | 5 | 1 | 38.375000 | -1.588388 | 34 | 18.95 | 0.557353 |
| GO:0031122\_cytoplasmic\_microtubule\_organization | TACC3 | 5 | 1 | 38.375000 | -1.588388 | 34 | 18.95 | 0.557353 |
| GO:0032350\_regulation\_of\_hormone\_metabolic\_process | ATP1A1 | 5 | 1 | 38.375000 | -1.588388 | 34 | 18.95 | 0.557353 |
| GO:0033205\_cytokinesis\_during\_cell\_cycle | APC | 5 | 1 | 38.375000 | -1.588388 | 34 | 18.95 | 0.557353 |
| GO:0046459\_short-chain\_fatty\_acid\_metabolic\_process | TYRP1 | 5 | 1 | 38.375000 | -1.588388 | 34 | 18.95 | 0.557353 |
| GO:0046785\_microtubule\_polymerization | APC | 5 | 1 | 38.375000 | -1.588388 | 34 | 18.95 | 0.557353 |
| GO:0051057\_positive\_regulation\_of\_small\_GTPase\_mediated\_signal\_transduction | RELN | 5 | 1 | 38.375000 | -1.588388 | 34 | 18.95 | 0.557353 |
| GO:0060045\_positive\_regulation\_of\_cardiac\_muscle\_cell\_proliferation | FGF9 | 5 | 1 | 38.375000 | -1.588388 | 34 | 18.95 | 0.557353 |
| GO:0021543\_pallium\_development | RELN | 49 | 2 | 7.831633 | -1.578794 | 35 | 19.34 | 0.552571 |
| GO:0021543\_pallium\_development | TACC3 | 49 | 2 | 7.831633 | -1.578794 | 35 | 19.34 | 0.552571 |
| GO:0017038\_protein\_import | FGF9 | 50 | 2 | 7.675000 | -1.562435 | 36 | 19.58 | 0.543889 |
| GO:0017038\_protein\_import | TACC3 | 50 | 2 | 7.675000 | -1.562435 | 36 | 19.58 | 0.543889 |
| GO:0032880\_regulation\_of\_protein\_localization | TACC3 | 51 | 2 | 7.524510 | -1.546431 | 38 | 20.19 | 0.531316 |
| GO:0032880\_regulation\_of\_protein\_localization | APC | 51 | 2 | 7.524510 | -1.546431 | 38 | 20.19 | 0.531316 |
| GO:0043408\_regulation\_of\_MAPKKK\_cascade | TIMP2 | 51 | 2 | 7.524510 | -1.546431 | 38 | 20.19 | 0.531316 |
| GO:0043408\_regulation\_of\_MAPKKK\_cascade | APC | 51 | 2 | 7.524510 | -1.546431 | 38 | 20.19 | 0.531316 |
| GO:0006942\_regulation\_of\_striated\_muscle\_contraction | ATP1A1 | 6 | 1 | 31.979167 | -1.510290 | 50 | 26.52 | 0.530400 |
| GO:0030947\_regulation\_of\_vascular\_endothelial\_growth\_factor\_receptor\_signaling\_pathway | FGF9 | 6 | 1 | 31.979167 | -1.510290 | 50 | 26.52 | 0.530400 |
| GO:0032438\_melanosome\_organization | TYRP1 | 6 | 1 | 31.979167 | -1.510290 | 50 | 26.52 | 0.530400 |
| GO:0035019\_somatic\_stem\_cell\_maintenance | APC | 6 | 1 | 31.979167 | -1.510290 | 50 | 26.52 | 0.530400 |
| GO:0040036\_regulation\_of\_fibroblast\_growth\_factor\_receptor\_signaling\_pathway | FGF9 | 6 | 1 | 31.979167 | -1.510290 | 50 | 26.52 | 0.530400 |
| GO:0042308\_negative\_regulation\_of\_protein\_import\_into\_nucleus | TACC3 | 6 | 1 | 31.979167 | -1.510290 | 50 | 26.52 | 0.530400 |
| GO:0042403\_thyroid\_hormone\_metabolic\_process | RELN | 6 | 1 | 31.979167 | -1.510290 | 50 | 26.52 | 0.530400 |
| GO:0042481\_regulation\_of\_odontogenesis | APC | 6 | 1 | 31.979167 | -1.510290 | 50 | 26.52 | 0.530400 |
| GO:0045822\_negative\_regulation\_of\_heart\_contraction | ATP1A1 | 6 | 1 | 31.979167 | -1.510290 | 50 | 26.52 | 0.530400 |
| GO:0045833\_negative\_regulation\_of\_lipid\_metabolic\_process | ATP1A1 | 6 | 1 | 31.979167 | -1.510290 | 50 | 26.52 | 0.530400 |
| GO:0045933\_positive\_regulation\_of\_muscle\_contraction | ATP1A1 | 6 | 1 | 31.979167 | -1.510290 | 50 | 26.52 | 0.530400 |
| GO:0048703\_embryonic\_viscerocranium\_morphogenesis | RDH10 | 6 | 1 | 31.979167 | -1.510290 | 50 | 26.52 | 0.530400 |
| GO:0048568\_embryonic\_organ\_development | RDH10 | 55 | 2 | 6.977273 | -1.485688 | 51 | 27.15 | 0.532353 |
| GO:0048568\_embryonic\_organ\_development | FGF9 | 55 | 2 | 6.977273 | -1.485688 | 51 | 27.15 | 0.532353 |
| GO:0050678\_regulation\_of\_epithelial\_cell\_proliferation | FGF9 | 56 | 2 | 6.852679 | -1.471262 | 52 | 27.54 | 0.529615 |
| GO:0050678\_regulation\_of\_epithelial\_cell\_proliferation | APC | 56 | 2 | 6.852679 | -1.471262 | 52 | 27.54 | 0.529615 |
| GO:0000226\_microtubule\_cytoskeleton\_organization | TACC3 | 57 | 2 | 6.732456 | -1.457117 | 54 | 27.93 | 0.517222 |
| GO:0000226\_microtubule\_cytoskeleton\_organization | APC | 57 | 2 | 6.732456 | -1.457117 | 54 | 27.93 | 0.517222 |
| GO:0033365\_protein\_localization\_in\_organelle | FGF9 | 57 | 2 | 6.732456 | -1.457117 | 54 | 27.93 | 0.517222 |
| GO:0033365\_protein\_localization\_in\_organelle | TACC3 | 57 | 2 | 6.732456 | -1.457117 | 54 | 27.93 | 0.517222 |
| GO:0003006\_reproductive\_developmental\_process | RDH10 | 141 | 3 | 4.082447 | -1.449314 | 55 | 28.06 | 0.510182 |
| GO:0003006\_reproductive\_developmental\_process | FGF9 | 141 | 3 | 4.082447 | -1.449314 | 55 | 28.06 | 0.510182 |
| GO:0003006\_reproductive\_developmental\_process | APC | 141 | 3 | 4.082447 | -1.449314 | 55 | 28.06 | 0.510182 |
| GO:0002455\_humoral\_immune\_response\_mediated\_by\_circulating\_immunoglobulin | EXO1 | 7 | 1 | 27.410714 | -1.444426 | 68 | 34.62 | 0.509118 |
| GO:0007019\_microtubule\_depolymerization | APC | 7 | 1 | 27.410714 | -1.444426 | 68 | 34.62 | 0.509118 |
| GO:0007026\_negative\_regulation\_of\_microtubule\_depolymerization | APC | 7 | 1 | 27.410714 | -1.444426 | 68 | 34.62 | 0.509118 |
| GO:0031114\_regulation\_of\_microtubule\_depolymerization | APC | 7 | 1 | 27.410714 | -1.444426 | 68 | 34.62 | 0.509118 |
| GO:0032387\_negative\_regulation\_of\_intracellular\_transport | TACC3 | 7 | 1 | 27.410714 | -1.444426 | 68 | 34.62 | 0.509118 |
| GO:0042438\_melanin\_biosynthetic\_process | TYRP1 | 7 | 1 | 27.410714 | -1.444426 | 68 | 34.62 | 0.509118 |
| GO:0043584\_nose\_development | RDH10 | 7 | 1 | 27.410714 | -1.444426 | 68 | 34.62 | 0.509118 |
| GO:0045823\_positive\_regulation\_of\_heart\_contraction | ATP1A1 | 7 | 1 | 27.410714 | -1.444426 | 68 | 34.62 | 0.509118 |
| GO:0045880\_positive\_regulation\_of\_smoothened\_signaling\_pathway | FGF9 | 7 | 1 | 27.410714 | -1.444426 | 68 | 34.62 | 0.509118 |
| GO:0046823\_negative\_regulation\_of\_nucleocytoplasmic\_transport | TACC3 | 7 | 1 | 27.410714 | -1.444426 | 68 | 34.62 | 0.509118 |
| GO:0048753\_pigment\_granule\_organization | TYRP1 | 7 | 1 | 27.410714 | -1.444426 | 68 | 34.62 | 0.509118 |
| GO:0060441\_branching\_involved\_in\_lung\_morphogenesis | RDH10 | 7 | 1 | 27.410714 | -1.444426 | 68 | 34.62 | 0.509118 |
| GO:0060770\_negative\_regulation\_of\_epithelial\_cell\_proliferation\_involved\_in\_prostate\_gland\_development | APC | 7 | 1 | 27.410714 | -1.444426 | 68 | 34.62 | 0.509118 |
| GO:0051641\_cellular\_localization | FGF9 | 370 | 5 | 2.592905 | -1.411451 | 69 | 35.3 | 0.511594 |
| GO:0051641\_cellular\_localization | STX4A | 370 | 5 | 2.592905 | -1.411451 | 69 | 35.3 | 0.511594 |
| GO:0051641\_cellular\_localization | LIN7C | 370 | 5 | 2.592905 | -1.411451 | 69 | 35.3 | 0.511594 |
| GO:0051641\_cellular\_localization | TACC3 | 370 | 5 | 2.592905 | -1.411451 | 69 | 35.3 | 0.511594 |
| GO:0051641\_cellular\_localization | APC | 370 | 5 | 2.592905 | -1.411451 | 69 | 35.3 | 0.511594 |
| GO:0007267\_cell-cell\_signaling | FGF9 | 252 | 4 | 3.045635 | -1.406870 | 70 | 35.56 | 0.508000 |
| GO:0007267\_cell-cell\_signaling | STX4A | 252 | 4 | 3.045635 | -1.406870 | 70 | 35.56 | 0.508000 |
| GO:0007267\_cell-cell\_signaling | FZD1 | 252 | 4 | 3.045635 | -1.406870 | 70 | 35.56 | 0.508000 |
| GO:0007267\_cell-cell\_signaling | LIN7C | 252 | 4 | 3.045635 | -1.406870 | 70 | 35.56 | 0.508000 |
| GO:0021537\_telencephalon\_development | RELN | 62 | 2 | 6.189516 | -1.390289 | 72 | 36.43 | 0.505972 |
| GO:0021537\_telencephalon\_development | TACC3 | 62 | 2 | 6.189516 | -1.390289 | 72 | 36.43 | 0.505972 |
| GO:0030855\_epithelial\_cell\_differentiation | FZD1 | 62 | 2 | 6.189516 | -1.390289 | 72 | 36.43 | 0.505972 |
| GO:0030855\_epithelial\_cell\_differentiation | APC | 62 | 2 | 6.189516 | -1.390289 | 72 | 36.43 | 0.505972 |
| GO:0000910\_cytokinesis | APC | 8 | 1 | 23.984375 | -1.387516 | 85 | 42.05 | 0.494706 |
| GO:0002566\_somatic\_diversification\_of\_immune\_receptors\_via\_somatic\_mutation | EXO1 | 8 | 1 | 23.984375 | -1.387516 | 85 | 42.05 | 0.494706 |
| GO:0006582\_melanin\_metabolic\_process | TYRP1 | 8 | 1 | 23.984375 | -1.387516 | 85 | 42.05 | 0.494706 |
| GO:0009072\_aromatic\_amino\_acid\_family\_metabolic\_process | TYRP1 | 8 | 1 | 23.984375 | -1.387516 | 85 | 42.05 | 0.494706 |
| GO:0016446\_somatic\_hypermutation\_of\_immunoglobulin\_genes | EXO1 | 8 | 1 | 23.984375 | -1.387516 | 85 | 42.05 | 0.494706 |
| GO:0031111\_negative\_regulation\_of\_microtubule\_polymerization\_or\_depolymerization | APC | 8 | 1 | 23.984375 | -1.387516 | 85 | 42.05 | 0.494706 |
| GO:0031334\_positive\_regulation\_of\_protein\_complex\_assembly | APC | 8 | 1 | 23.984375 | -1.387516 | 85 | 42.05 | 0.494706 |
| GO:0032273\_positive\_regulation\_of\_protein\_polymerization | APC | 8 | 1 | 23.984375 | -1.387516 | 85 | 42.05 | 0.494706 |
| GO:0040034\_regulation\_of\_development\_\_heterochronic | FGF9 | 8 | 1 | 23.984375 | -1.387516 | 85 | 42.05 | 0.494706 |
| GO:0042990\_regulation\_of\_transcription\_factor\_import\_into\_nucleus | TACC3 | 8 | 1 | 23.984375 | -1.387516 | 85 | 42.05 | 0.494706 |
| GO:0042991\_transcription\_factor\_import\_into\_nucleus | TACC3 | 8 | 1 | 23.984375 | -1.387516 | 85 | 42.05 | 0.494706 |
| GO:0048505\_regulation\_of\_timing\_of\_cell\_differentiation | FGF9 | 8 | 1 | 23.984375 | -1.387516 | 85 | 42.05 | 0.494706 |
| GO:0060043\_regulation\_of\_cardiac\_muscle\_cell\_proliferation | FGF9 | 8 | 1 | 23.984375 | -1.387516 | 85 | 42.05 | 0.494706 |
| GO:0009966\_regulation\_of\_signal\_transduction | FGF9 | 256 | 4 | 2.998047 | -1.385416 | 86 | 42.12 | 0.489767 |
| GO:0009966\_regulation\_of\_signal\_transduction | RELN | 256 | 4 | 2.998047 | -1.385416 | 86 | 42.12 | 0.489767 |
| GO:0009966\_regulation\_of\_signal\_transduction | TIMP2 | 256 | 4 | 2.998047 | -1.385416 | 86 | 42.12 | 0.489767 |
| GO:0009966\_regulation\_of\_signal\_transduction | APC | 256 | 4 | 2.998047 | -1.385416 | 86 | 42.12 | 0.489767 |
| GO:0044255\_cellular\_lipid\_metabolic\_process | RDH10 | 264 | 4 | 2.907197 | -1.343782 | 87 | 43.64 | 0.501609 |
| GO:0044255\_cellular\_lipid\_metabolic\_process | TYRP1 | 264 | 4 | 2.907197 | -1.343782 | 87 | 43.64 | 0.501609 |
| GO:0044255\_cellular\_lipid\_metabolic\_process | B3GNT5 | 264 | 4 | 2.907197 | -1.343782 | 87 | 43.64 | 0.501609 |
| GO:0044255\_cellular\_lipid\_metabolic\_process | ATP1A1 | 264 | 4 | 2.907197 | -1.343782 | 87 | 43.64 | 0.501609 |
| GO:0006364\_rRNA\_processing | EBNA1BP2 | 9 | 1 | 21.319444 | -1.337444 | 93 | 48.71 | 0.523763 |
| GO:0016072\_rRNA\_metabolic\_process | EBNA1BP2 | 9 | 1 | 21.319444 | -1.337444 | 93 | 48.71 | 0.523763 |
| GO:0043242\_negative\_regulation\_of\_protein\_complex\_disassembly | APC | 9 | 1 | 21.319444 | -1.337444 | 93 | 48.71 | 0.523763 |
| GO:0043409\_negative\_regulation\_of\_MAPKKK\_cascade | APC | 9 | 1 | 21.319444 | -1.337444 | 93 | 48.71 | 0.523763 |
| GO:0051647\_nucleus\_localization | TACC3 | 9 | 1 | 21.319444 | -1.337444 | 93 | 48.71 | 0.523763 |
| GO:0060602\_branch\_elongation\_of\_an\_epithelium | RDH10 | 9 | 1 | 21.319444 | -1.337444 | 93 | 48.71 | 0.523763 |
| GO:0008406\_gonad\_development | RDH10 | 70 | 2 | 5.482143 | -1.294973 | 94 | 50.22 | 0.534255 |
| GO:0008406\_gonad\_development | FGF9 | 70 | 2 | 5.482143 | -1.294973 | 94 | 50.22 | 0.534255 |
| GO:0008211\_glucocorticoid\_metabolic\_process | ATP1A1 | 10 | 1 | 19.187500 | -1.292767 | 97 | 56.54 | 0.582887 |
| GO:0051224\_negative\_regulation\_of\_protein\_transport | TACC3 | 10 | 1 | 19.187500 | -1.292767 | 97 | 56.54 | 0.582887 |
| GO:0060768\_regulation\_of\_epithelial\_cell\_proliferation\_involved\_in\_prostate\_gland\_development | APC | 10 | 1 | 19.187500 | -1.292767 | 97 | 56.54 | 0.582887 |
| GO:0006913\_nucleocytoplasmic\_transport | FGF9 | 71 | 2 | 5.404930 | -1.283926 | 98 | 56.82 | 0.579796 |
| GO:0006913\_nucleocytoplasmic\_transport | TACC3 | 71 | 2 | 5.404930 | -1.283926 | 98 | 56.82 | 0.579796 |
| GO:0050673\_epithelial\_cell\_proliferation | FGF9 | 72 | 2 | 5.329861 | -1.273053 | 100 | 57.53 | 0.575300 |
| GO:0050673\_epithelial\_cell\_proliferation | APC | 72 | 2 | 5.329861 | -1.273053 | 100 | 57.53 | 0.575300 |
| GO:0051169\_nuclear\_transport | FGF9 | 72 | 2 | 5.329861 | -1.273053 | 100 | 57.53 | 0.575300 |
| GO:0051169\_nuclear\_transport | TACC3 | 72 | 2 | 5.329861 | -1.273053 | 100 | 57.53 | 0.575300 |
| GO:0048706\_embryonic\_skeletal\_system\_development | RDH10 | 73 | 2 | 5.256849 | -1.262350 | 101 | 57.99 | 0.574158 |
| GO:0048706\_embryonic\_skeletal\_system\_development | FGF9 | 73 | 2 | 5.256849 | -1.262350 | 101 | 57.99 | 0.574158 |
| GO:0007051\_spindle\_organization | TACC3 | 11 | 1 | 17.443182 | -1.252453 | 111 | 62.52 | 0.563243 |
| GO:0016079\_synaptic\_vesicle\_exocytosis | STX4A | 11 | 1 | 17.443182 | -1.252453 | 111 | 62.52 | 0.563243 |
| GO:0021846\_cell\_proliferation\_in\_forebrain | TACC3 | 11 | 1 | 17.443182 | -1.252453 | 111 | 62.52 | 0.563243 |
| GO:0030238\_male\_sex\_determination | FGF9 | 11 | 1 | 17.443182 | -1.252453 | 111 | 62.52 | 0.563243 |
| GO:0030856\_regulation\_of\_epithelial\_cell\_differentiation | APC | 11 | 1 | 17.443182 | -1.252453 | 111 | 62.52 | 0.563243 |
| GO:0031110\_regulation\_of\_microtubule\_polymerization\_or\_depolymerization | APC | 11 | 1 | 17.443182 | -1.252453 | 111 | 62.52 | 0.563243 |
| GO:0033059\_cellular\_pigmentation | TYRP1 | 11 | 1 | 17.443182 | -1.252453 | 111 | 62.52 | 0.563243 |
| GO:0046716\_muscle\_maintenance | APC | 11 | 1 | 17.443182 | -1.252453 | 111 | 62.52 | 0.563243 |
| GO:0051494\_negative\_regulation\_of\_cytoskeleton\_organization | APC | 11 | 1 | 17.443182 | -1.252453 | 111 | 62.52 | 0.563243 |
| GO:0060767\_epithelial\_cell\_proliferation\_involved\_in\_prostate\_gland\_development | APC | 11 | 1 | 17.443182 | -1.252453 | 111 | 62.52 | 0.563243 |
| GO:0006629\_lipid\_metabolic\_process | RDH10 | 285 | 4 | 2.692982 | -1.241922 | 112 | 62.78 | 0.560536 |
| GO:0006629\_lipid\_metabolic\_process | TYRP1 | 285 | 4 | 2.692982 | -1.241922 | 112 | 62.78 | 0.560536 |
| GO:0006629\_lipid\_metabolic\_process | B3GNT5 | 285 | 4 | 2.692982 | -1.241922 | 112 | 62.78 | 0.560536 |
| GO:0006629\_lipid\_metabolic\_process | ATP1A1 | 285 | 4 | 2.692982 | -1.241922 | 112 | 62.78 | 0.560536 |
| GO:0065008\_regulation\_of\_biological\_quality | RDH10 | 693 | 7 | 1.938131 | -1.239872 | 113 | 62.95 | 0.557080 |
| GO:0065008\_regulation\_of\_biological\_quality | STX4A | 693 | 7 | 1.938131 | -1.239872 | 113 | 62.95 | 0.557080 |
| GO:0065008\_regulation\_of\_biological\_quality | LIN7C | 693 | 7 | 1.938131 | -1.239872 | 113 | 62.95 | 0.557080 |
| GO:0065008\_regulation\_of\_biological\_quality | RELN | 693 | 7 | 1.938131 | -1.239872 | 113 | 62.95 | 0.557080 |
| GO:0065008\_regulation\_of\_biological\_quality | ATP1A1 | 693 | 7 | 1.938131 | -1.239872 | 113 | 62.95 | 0.557080 |
| GO:0065008\_regulation\_of\_biological\_quality | TACC3 | 693 | 7 | 1.938131 | -1.239872 | 113 | 62.95 | 0.557080 |
| GO:0065008\_regulation\_of\_biological\_quality | APC | 693 | 7 | 1.938131 | -1.239872 | 113 | 62.95 | 0.557080 |
| GO:0002026\_regulation\_of\_the\_force\_of\_heart\_contraction | ATP1A1 | 12 | 1 | 15.989583 | -1.215743 | 120 | 68.39 | 0.569917 |
| GO:0009262\_deoxyribonucleotide\_metabolic\_process | RRM2 | 12 | 1 | 15.989583 | -1.215743 | 120 | 68.39 | 0.569917 |
| GO:0031109\_microtubule\_polymerization\_or\_depolymerization | APC | 12 | 1 | 15.989583 | -1.215743 | 120 | 68.39 | 0.569917 |
| GO:0042446\_hormone\_biosynthetic\_process | ATP1A1 | 12 | 1 | 15.989583 | -1.215743 | 120 | 68.39 | 0.569917 |
| GO:0043624\_cellular\_protein\_complex\_disassembly | APC | 12 | 1 | 15.989583 | -1.215743 | 120 | 68.39 | 0.569917 |
| GO:0051261\_protein\_depolymerization | APC | 12 | 1 | 15.989583 | -1.215743 | 120 | 68.39 | 0.569917 |
| GO:0060572\_morphogenesis\_of\_an\_epithelial\_bud | RDH10 | 12 | 1 | 15.989583 | -1.215743 | 120 | 68.39 | 0.569917 |
| GO:0006461\_protein\_complex\_assembly | RRM2 | 78 | 2 | 4.919872 | -1.211220 | 124 | 68.91 | 0.555726 |
| GO:0006461\_protein\_complex\_assembly | APC | 78 | 2 | 4.919872 | -1.211220 | 124 | 68.91 | 0.555726 |
| GO:0030326\_embryonic\_limb\_morphogenesis | RDH10 | 78 | 2 | 4.919872 | -1.211220 | 124 | 68.91 | 0.555726 |
| GO:0030326\_embryonic\_limb\_morphogenesis | FGF9 | 78 | 2 | 4.919872 | -1.211220 | 124 | 68.91 | 0.555726 |
| GO:0035113\_embryonic\_appendage\_morphogenesis | RDH10 | 78 | 2 | 4.919872 | -1.211220 | 124 | 68.91 | 0.555726 |
| GO:0035113\_embryonic\_appendage\_morphogenesis | FGF9 | 78 | 2 | 4.919872 | -1.211220 | 124 | 68.91 | 0.555726 |
| GO:0070271\_protein\_complex\_biogenesis | RRM2 | 78 | 2 | 4.919872 | -1.211220 | 124 | 68.91 | 0.555726 |
| GO:0070271\_protein\_complex\_biogenesis | APC | 78 | 2 | 4.919872 | -1.211220 | 124 | 68.91 | 0.555726 |
| GO:0021511\_spinal\_cord\_patterning | RELN | 13 | 1 | 14.759615 | -1.182059 | 132 | 74.44 | 0.563939 |
| GO:0043241\_protein\_complex\_disassembly | APC | 13 | 1 | 14.759615 | -1.182059 | 132 | 74.44 | 0.563939 |
| GO:0043244\_regulation\_of\_protein\_complex\_disassembly | APC | 13 | 1 | 14.759615 | -1.182059 | 132 | 74.44 | 0.563939 |
| GO:0048566\_embryonic\_gut\_development | FGF9 | 13 | 1 | 14.759615 | -1.182059 | 132 | 74.44 | 0.563939 |
| GO:0051495\_positive\_regulation\_of\_cytoskeleton\_organization | APC | 13 | 1 | 14.759615 | -1.182059 | 132 | 74.44 | 0.563939 |
| GO:0060038\_cardiac\_muscle\_cell\_proliferation | FGF9 | 13 | 1 | 14.759615 | -1.182059 | 132 | 74.44 | 0.563939 |
| GO:0060070\_Wnt\_receptor\_signaling\_pathway\_through\_beta-catenin | APC | 13 | 1 | 14.759615 | -1.182059 | 132 | 74.44 | 0.563939 |
| GO:0060560\_developmental\_growth\_involved\_in\_morphogenesis | RDH10 | 13 | 1 | 14.759615 | -1.182059 | 132 | 74.44 | 0.563939 |
| GO:0010627\_regulation\_of\_protein\_kinase\_cascade | TIMP2 | 82 | 2 | 4.679878 | -1.172941 | 133 | 74.89 | 0.563083 |
| GO:0010627\_regulation\_of\_protein\_kinase\_cascade | APC | 82 | 2 | 4.679878 | -1.172941 | 133 | 74.89 | 0.563083 |
| GO:0044238\_primary\_metabolic\_process | EXO1 | 1905 | 14 | 1.410105 | -1.155323 | 134 | 75.69 | 0.564851 |
| GO:0044238\_primary\_metabolic\_process | TYRP1 | 1905 | 14 | 1.410105 | -1.155323 | 134 | 75.69 | 0.564851 |
| GO:0044238\_primary\_metabolic\_process | FZD1 | 1905 | 14 | 1.410105 | -1.155323 | 134 | 75.69 | 0.564851 |
| GO:0044238\_primary\_metabolic\_process | TLE1 | 1905 | 14 | 1.410105 | -1.155323 | 134 | 75.69 | 0.564851 |
| GO:0044238\_primary\_metabolic\_process | ATP1A1 | 1905 | 14 | 1.410105 | -1.155323 | 134 | 75.69 | 0.564851 |
| GO:0044238\_primary\_metabolic\_process | TIMP2 | 1905 | 14 | 1.410105 | -1.155323 | 134 | 75.69 | 0.564851 |
| GO:0044238\_primary\_metabolic\_process | EBNA1BP2 | 1905 | 14 | 1.410105 | -1.155323 | 134 | 75.69 | 0.564851 |
| GO:0044238\_primary\_metabolic\_process | RDH10 | 1905 | 14 | 1.410105 | -1.155323 | 134 | 75.69 | 0.564851 |
| GO:0044238\_primary\_metabolic\_process | CSNK2A1 | 1905 | 14 | 1.410105 | -1.155323 | 134 | 75.69 | 0.564851 |
| GO:0044238\_primary\_metabolic\_process | B3GNT5 | 1905 | 14 | 1.410105 | -1.155323 | 134 | 75.69 | 0.564851 |
| GO:0044238\_primary\_metabolic\_process | RRM2 | 1905 | 14 | 1.410105 | -1.155323 | 134 | 75.69 | 0.564851 |
| GO:0044238\_primary\_metabolic\_process | MRPL19 | 1905 | 14 | 1.410105 | -1.155323 | 134 | 75.69 | 0.564851 |
| GO:0044238\_primary\_metabolic\_process | RELN | 1905 | 14 | 1.410105 | -1.155323 | 134 | 75.69 | 0.564851 |
| GO:0044238\_primary\_metabolic\_process | APC | 1905 | 14 | 1.410105 | -1.155323 | 134 | 75.69 | 0.564851 |
| GO:0045137\_development\_of\_primary\_sexual\_characteristics | RDH10 | 84 | 2 | 4.568452 | -1.154597 | 135 | 75.78 | 0.561333 |
| GO:0045137\_development\_of\_primary\_sexual\_characteristics | FGF9 | 84 | 2 | 4.568452 | -1.154597 | 135 | 75.78 | 0.561333 |
| GO:0007530\_sex\_determination | FGF9 | 14 | 1 | 13.705357 | -1.150952 | 144 | 80.28 | 0.557500 |
| GO:0014855\_striated\_muscle\_cell\_proliferation | FGF9 | 14 | 1 | 13.705357 | -1.150952 | 144 | 80.28 | 0.557500 |
| GO:0030148\_sphingolipid\_biosynthetic\_process | B3GNT5 | 14 | 1 | 13.705357 | -1.150952 | 144 | 80.28 | 0.557500 |
| GO:0032271\_regulation\_of\_protein\_polymerization | APC | 14 | 1 | 13.705357 | -1.150952 | 144 | 80.28 | 0.557500 |
| GO:0034623\_cellular\_macromolecular\_complex\_disassembly | APC | 14 | 1 | 13.705357 | -1.150952 | 144 | 80.28 | 0.557500 |
| GO:0042573\_retinoic\_acid\_metabolic\_process | RDH10 | 14 | 1 | 13.705357 | -1.150952 | 144 | 80.28 | 0.557500 |
| GO:0043254\_regulation\_of\_protein\_complex\_assembly | APC | 14 | 1 | 13.705357 | -1.150952 | 144 | 80.28 | 0.557500 |
| GO:0045732\_positive\_regulation\_of\_protein\_catabolic\_process | APC | 14 | 1 | 13.705357 | -1.150952 | 144 | 80.28 | 0.557500 |
| GO:0050810\_regulation\_of\_steroid\_biosynthetic\_process | ATP1A1 | 14 | 1 | 13.705357 | -1.150952 | 144 | 80.28 | 0.557500 |
| GO:0000279\_M\_phase | TACC3 | 85 | 2 | 4.514706 | -1.145612 | 145 | 80.54 | 0.555448 |
| GO:0000279\_M\_phase | APC | 85 | 2 | 4.514706 | -1.145612 | 145 | 80.54 | 0.555448 |
| GO:0006605\_protein\_targeting | FGF9 | 86 | 2 | 4.462209 | -1.136750 | 146 | 81.23 | 0.556370 |
| GO:0006605\_protein\_targeting | TACC3 | 86 | 2 | 4.462209 | -1.136750 | 146 | 81.23 | 0.556370 |
| GO:0001822\_kidney\_development | RDH10 | 87 | 2 | 4.410920 | -1.128005 | 149 | 81.78 | 0.548859 |
| GO:0001822\_kidney\_development | APC | 87 | 2 | 4.410920 | -1.128005 | 149 | 81.78 | 0.548859 |
| GO:0003001\_generation\_of\_a\_signal\_involved\_in\_cell-cell\_signaling | STX4A | 87 | 2 | 4.410920 | -1.128005 | 149 | 81.78 | 0.548859 |
| GO:0003001\_generation\_of\_a\_signal\_involved\_in\_cell-cell\_signaling | LIN7C | 87 | 2 | 4.410920 | -1.128005 | 149 | 81.78 | 0.548859 |
| GO:0043583\_ear\_development | RDH10 | 87 | 2 | 4.410920 | -1.128005 | 149 | 81.78 | 0.548859 |
| GO:0043583\_ear\_development | FGF9 | 87 | 2 | 4.410920 | -1.128005 | 149 | 81.78 | 0.548859 |
| GO:0008543\_fibroblast\_growth\_factor\_receptor\_signaling\_pathway | FGF9 | 15 | 1 | 12.791667 | -1.122065 | 156 | 85.94 | 0.550897 |
| GO:0021795\_cerebral\_cortex\_cell\_migration | RELN | 15 | 1 | 12.791667 | -1.122065 | 156 | 85.94 | 0.550897 |
| GO:0031076\_embryonic\_camera-type\_eye\_development | RDH10 | 15 | 1 | 12.791667 | -1.122065 | 156 | 85.94 | 0.550897 |
| GO:0042306\_regulation\_of\_protein\_import\_into\_nucleus | TACC3 | 15 | 1 | 12.791667 | -1.122065 | 156 | 85.94 | 0.550897 |
| GO:0048010\_vascular\_endothelial\_growth\_factor\_receptor\_signaling\_pathway | FGF9 | 15 | 1 | 12.791667 | -1.122065 | 156 | 85.94 | 0.550897 |
| GO:0060425\_lung\_morphogenesis | RDH10 | 15 | 1 | 12.791667 | -1.122065 | 156 | 85.94 | 0.550897 |
| GO:0070507\_regulation\_of\_microtubule\_cytoskeleton\_organization | APC | 15 | 1 | 12.791667 | -1.122065 | 156 | 85.94 | 0.550897 |
| GO:0001503\_ossification | FGF9 | 88 | 2 | 4.360795 | -1.119376 | 157 | 86.1 | 0.548408 |
| GO:0001503\_ossification | APC | 88 | 2 | 4.360795 | -1.119376 | 157 | 86.1 | 0.548408 |
| GO:0030324\_lung\_development | RDH10 | 90 | 2 | 4.263889 | -1.102456 | 158 | 86.99 | 0.550570 |
| GO:0030324\_lung\_development | FGF9 | 90 | 2 | 4.263889 | -1.102456 | 158 | 86.99 | 0.550570 |
| GO:0046148\_pigment\_biosynthetic\_process | TYRP1 | 16 | 1 | 11.992188 | -1.095112 | 160 | 91.19 | 0.569938 |
| GO:0046467\_membrane\_lipid\_biosynthetic\_process | B3GNT5 | 16 | 1 | 11.992188 | -1.095112 | 160 | 91.19 | 0.569938 |
| GO:0002009\_morphogenesis\_of\_an\_epithelium | RDH10 | 198 | 3 | 2.907197 | -1.087984 | 162 | 91.8 | 0.566667 |
| GO:0002009\_morphogenesis\_of\_an\_epithelium | FZD1 | 198 | 3 | 2.907197 | -1.087984 | 162 | 91.8 | 0.566667 |
| GO:0002009\_morphogenesis\_of\_an\_epithelium | APC | 198 | 3 | 2.907197 | -1.087984 | 162 | 91.8 | 0.566667 |
| GO:0060429\_epithelium\_development | RDH10 | 198 | 3 | 2.907197 | -1.087984 | 162 | 91.8 | 0.566667 |
| GO:0060429\_epithelium\_development | FZD1 | 198 | 3 | 2.907197 | -1.087984 | 162 | 91.8 | 0.566667 |
| GO:0060429\_epithelium\_development | APC | 198 | 3 | 2.907197 | -1.087984 | 162 | 91.8 | 0.566667 |
| GO:0030323\_respiratory\_tube\_development | RDH10 | 92 | 2 | 4.171196 | -1.085967 | 163 | 91.93 | 0.563988 |
| GO:0030323\_respiratory\_tube\_development | FGF9 | 92 | 2 | 4.171196 | -1.085967 | 163 | 91.93 | 0.563988 |
| GO:0035107\_appendage\_morphogenesis | RDH10 | 93 | 2 | 4.126344 | -1.077879 | 166 | 92.63 | 0.558012 |
| GO:0035107\_appendage\_morphogenesis | FGF9 | 93 | 2 | 4.126344 | -1.077879 | 166 | 92.63 | 0.558012 |
| GO:0035108\_limb\_morphogenesis | RDH10 | 93 | 2 | 4.126344 | -1.077879 | 166 | 92.63 | 0.558012 |
| GO:0035108\_limb\_morphogenesis | FGF9 | 93 | 2 | 4.126344 | -1.077879 | 166 | 92.63 | 0.558012 |
| GO:0065003\_macromolecular\_complex\_assembly | RRM2 | 93 | 2 | 4.126344 | -1.077879 | 166 | 92.63 | 0.558012 |
| GO:0065003\_macromolecular\_complex\_assembly | APC | 93 | 2 | 4.126344 | -1.077879 | 166 | 92.63 | 0.558012 |
| GO:0006753\_nucleoside\_phosphate\_metabolic\_process | RRM2 | 94 | 2 | 4.082447 | -1.069892 | 169 | 93.11 | 0.550947 |
| GO:0006753\_nucleoside\_phosphate\_metabolic\_process | TIMP2 | 94 | 2 | 4.082447 | -1.069892 | 169 | 93.11 | 0.550947 |
| GO:0008610\_lipid\_biosynthetic\_process | B3GNT5 | 94 | 2 | 4.082447 | -1.069892 | 169 | 93.11 | 0.550947 |
| GO:0008610\_lipid\_biosynthetic\_process | ATP1A1 | 94 | 2 | 4.082447 | -1.069892 | 169 | 93.11 | 0.550947 |
| GO:0009117\_nucleotide\_metabolic\_process | RRM2 | 94 | 2 | 4.082447 | -1.069892 | 169 | 93.11 | 0.550947 |
| GO:0009117\_nucleotide\_metabolic\_process | TIMP2 | 94 | 2 | 4.082447 | -1.069892 | 169 | 93.11 | 0.550947 |
| GO:0010741\_negative\_regulation\_of\_protein\_kinase\_cascade | APC | 17 | 1 | 11.286765 | -1.069858 | 178 | 96.89 | 0.544326 |
| GO:0022029\_telencephalon\_cell\_migration | RELN | 17 | 1 | 11.286765 | -1.069858 | 178 | 96.89 | 0.544326 |
| GO:0034470\_ncRNA\_processing | EBNA1BP2 | 17 | 1 | 11.286765 | -1.069858 | 178 | 96.89 | 0.544326 |
| GO:0035115\_embryonic\_forelimb\_morphogenesis | RDH10 | 17 | 1 | 11.286765 | -1.069858 | 178 | 96.89 | 0.544326 |
| GO:0042254\_ribosome\_biogenesis | EBNA1BP2 | 17 | 1 | 11.286765 | -1.069858 | 178 | 96.89 | 0.544326 |
| GO:0042440\_pigment\_metabolic\_process | TYRP1 | 17 | 1 | 11.286765 | -1.069858 | 178 | 96.89 | 0.544326 |
| GO:0045667\_regulation\_of\_osteoblast\_differentiation | APC | 17 | 1 | 11.286765 | -1.069858 | 178 | 96.89 | 0.544326 |
| GO:0045670\_regulation\_of\_osteoclast\_differentiation | APC | 17 | 1 | 11.286765 | -1.069858 | 178 | 96.89 | 0.544326 |
| GO:0048265\_response\_to\_pain | RELN | 17 | 1 | 11.286765 | -1.069858 | 178 | 96.89 | 0.544326 |
| GO:0048736\_appendage\_development | RDH10 | 96 | 2 | 3.997396 | -1.054214 | 180 | 97.63 | 0.542389 |
| GO:0048736\_appendage\_development | FGF9 | 96 | 2 | 3.997396 | -1.054214 | 180 | 97.63 | 0.542389 |
| GO:0060173\_limb\_development | RDH10 | 96 | 2 | 3.997396 | -1.054214 | 180 | 97.63 | 0.542389 |
| GO:0060173\_limb\_development | FGF9 | 96 | 2 | 3.997396 | -1.054214 | 180 | 97.63 | 0.542389 |
| GO:0010646\_regulation\_of\_cell\_communication | FGF9 | 330 | 4 | 2.325758 | -1.054148 | 181 | 97.8 | 0.540331 |
| GO:0010646\_regulation\_of\_cell\_communication | RELN | 330 | 4 | 2.325758 | -1.054148 | 181 | 97.8 | 0.540331 |
| GO:0010646\_regulation\_of\_cell\_communication | TIMP2 | 330 | 4 | 2.325758 | -1.054148 | 181 | 97.8 | 0.540331 |
| GO:0010646\_regulation\_of\_cell\_communication | APC | 330 | 4 | 2.325758 | -1.054148 | 181 | 97.8 | 0.540331 |
| GO:0031324\_negative\_regulation\_of\_cellular\_metabolic\_process | FZD1 | 332 | 4 | 2.311747 | -1.046630 | 182 | 98.06 | 0.538791 |
| GO:0031324\_negative\_regulation\_of\_cellular\_metabolic\_process | ATP1A1 | 332 | 4 | 2.311747 | -1.046630 | 182 | 98.06 | 0.538791 |
| GO:0031324\_negative\_regulation\_of\_cellular\_metabolic\_process | TLE1 | 332 | 4 | 2.311747 | -1.046630 | 182 | 98.06 | 0.538791 |
| GO:0031324\_negative\_regulation\_of\_cellular\_metabolic\_process | APC | 332 | 4 | 2.311747 | -1.046630 | 182 | 98.06 | 0.538791 |
| GO:0060341\_regulation\_of\_cellular\_localization | TACC3 | 97 | 2 | 3.956186 | -1.046517 | 183 | 98.19 | 0.536557 |
| GO:0060341\_regulation\_of\_cellular\_localization | APC | 97 | 2 | 3.956186 | -1.046517 | 183 | 98.19 | 0.536557 |
| GO:0008589\_regulation\_of\_smoothened\_signaling\_pathway | FGF9 | 18 | 1 | 10.659722 | -1.046108 | 189 | 102.31 | 0.541323 |
| GO:0021885\_forebrain\_cell\_migration | RELN | 18 | 1 | 10.659722 | -1.046108 | 189 | 102.31 | 0.541323 |
| GO:0030318\_melanocyte\_differentiation | TYRP1 | 18 | 1 | 10.659722 | -1.046108 | 189 | 102.31 | 0.541323 |
| GO:0032984\_macromolecular\_complex\_disassembly | APC | 18 | 1 | 10.659722 | -1.046108 | 189 | 102.31 | 0.541323 |
| GO:0033157\_regulation\_of\_intracellular\_protein\_transport | TACC3 | 18 | 1 | 10.659722 | -1.046108 | 189 | 102.31 | 0.541323 |
| GO:0060571\_morphogenesis\_of\_an\_epithelial\_fold | RDH10 | 18 | 1 | 10.659722 | -1.046108 | 189 | 102.31 | 0.541323 |
| GO:0007548\_sex\_differentiation | RDH10 | 98 | 2 | 3.915816 | -1.038914 | 192 | 102.7 | 0.534896 |
| GO:0007548\_sex\_differentiation | FGF9 | 98 | 2 | 3.915816 | -1.038914 | 192 | 102.7 | 0.534896 |
| GO:0009967\_positive\_regulation\_of\_signal\_transduction | FGF9 | 98 | 2 | 3.915816 | -1.038914 | 192 | 102.7 | 0.534896 |
| GO:0009967\_positive\_regulation\_of\_signal\_transduction | RELN | 98 | 2 | 3.915816 | -1.038914 | 192 | 102.7 | 0.534896 |
| GO:0060541\_respiratory\_system\_development | RDH10 | 98 | 2 | 3.915816 | -1.038914 | 192 | 102.7 | 0.534896 |
| GO:0060541\_respiratory\_system\_development | FGF9 | 98 | 2 | 3.915816 | -1.038914 | 192 | 102.7 | 0.534896 |
| GO:0044237\_cellular\_metabolic\_process | EXO1 | 1974 | 14 | 1.360816 | -1.032989 | 193 | 102.93 | 0.533316 |
| GO:0044237\_cellular\_metabolic\_process | TYRP1 | 1974 | 14 | 1.360816 | -1.032989 | 193 | 102.93 | 0.533316 |
| GO:0044237\_cellular\_metabolic\_process | FZD1 | 1974 | 14 | 1.360816 | -1.032989 | 193 | 102.93 | 0.533316 |
| GO:0044237\_cellular\_metabolic\_process | TLE1 | 1974 | 14 | 1.360816 | -1.032989 | 193 | 102.93 | 0.533316 |
| GO:0044237\_cellular\_metabolic\_process | ATP1A1 | 1974 | 14 | 1.360816 | -1.032989 | 193 | 102.93 | 0.533316 |
| GO:0044237\_cellular\_metabolic\_process | TIMP2 | 1974 | 14 | 1.360816 | -1.032989 | 193 | 102.93 | 0.533316 |
| GO:0044237\_cellular\_metabolic\_process | EBNA1BP2 | 1974 | 14 | 1.360816 | -1.032989 | 193 | 102.93 | 0.533316 |
| GO:0044237\_cellular\_metabolic\_process | RDH10 | 1974 | 14 | 1.360816 | -1.032989 | 193 | 102.93 | 0.533316 |
| GO:0044237\_cellular\_metabolic\_process | CSNK2A1 | 1974 | 14 | 1.360816 | -1.032989 | 193 | 102.93 | 0.533316 |
| GO:0044237\_cellular\_metabolic\_process | B3GNT5 | 1974 | 14 | 1.360816 | -1.032989 | 193 | 102.93 | 0.533316 |
| GO:0044237\_cellular\_metabolic\_process | RRM2 | 1974 | 14 | 1.360816 | -1.032989 | 193 | 102.93 | 0.533316 |
| GO:0044237\_cellular\_metabolic\_process | MRPL19 | 1974 | 14 | 1.360816 | -1.032989 | 193 | 102.93 | 0.533316 |
| GO:0044237\_cellular\_metabolic\_process | RELN | 1974 | 14 | 1.360816 | -1.032989 | 193 | 102.93 | 0.533316 |
| GO:0044237\_cellular\_metabolic\_process | APC | 1974 | 14 | 1.360816 | -1.032989 | 193 | 102.93 | 0.533316 |
| GO:0060348\_bone\_development | FGF9 | 99 | 2 | 3.876263 | -1.031402 | 194 | 103.27 | 0.532320 |
| GO:0060348\_bone\_development | APC | 99 | 2 | 3.876263 | -1.031402 | 194 | 103.27 | 0.532320 |
| GO:0006776\_vitamin\_A\_metabolic\_process | RDH10 | 19 | 1 | 10.098684 | -1.023700 | 203 | 106.61 | 0.525172 |
| GO:0009798\_axis\_specification | APC | 19 | 1 | 10.098684 | -1.023700 | 203 | 106.61 | 0.525172 |
| GO:0010639\_negative\_regulation\_of\_organelle\_organization | APC | 19 | 1 | 10.098684 | -1.023700 | 203 | 106.61 | 0.525172 |
| GO:0019218\_regulation\_of\_steroid\_metabolic\_process | ATP1A1 | 19 | 1 | 10.098684 | -1.023700 | 203 | 106.61 | 0.525172 |
| GO:0033002\_muscle\_cell\_proliferation | FGF9 | 19 | 1 | 10.098684 | -1.023700 | 203 | 106.61 | 0.525172 |
| GO:0046890\_regulation\_of\_lipid\_biosynthetic\_process | ATP1A1 | 19 | 1 | 10.098684 | -1.023700 | 203 | 106.61 | 0.525172 |
| GO:0048701\_embryonic\_cranial\_skeleton\_morphogenesis | RDH10 | 19 | 1 | 10.098684 | -1.023700 | 203 | 106.61 | 0.525172 |
| GO:0050931\_pigment\_cell\_differentiation | TYRP1 | 19 | 1 | 10.098684 | -1.023700 | 203 | 106.61 | 0.525172 |
| GO:0051056\_regulation\_of\_small\_GTPase\_mediated\_signal\_transduction | RELN | 19 | 1 | 10.098684 | -1.023700 | 203 | 106.61 | 0.525172 |
| GO:0051649\_establishment\_of\_localization\_in\_cell | FGF9 | 342 | 4 | 2.244152 | -1.009977 | 204 | 107.08 | 0.524902 |
| GO:0051649\_establishment\_of\_localization\_in\_cell | STX4A | 342 | 4 | 2.244152 | -1.009977 | 204 | 107.08 | 0.524902 |
| GO:0051649\_establishment\_of\_localization\_in\_cell | LIN7C | 342 | 4 | 2.244152 | -1.009977 | 204 | 107.08 | 0.524902 |
| GO:0051649\_establishment\_of\_localization\_in\_cell | TACC3 | 342 | 4 | 2.244152 | -1.009977 | 204 | 107.08 | 0.524902 |
| GO:0009615\_response\_to\_virus | XPR1 | 20 | 1 | 9.593750 | -1.002496 | 206 | 110.19 | 0.534903 |
| GO:0046822\_regulation\_of\_nucleocytoplasmic\_transport | TACC3 | 20 | 1 | 9.593750 | -1.002496 | 206 | 110.19 | 0.534903 |
| GO:0009968\_negative\_regulation\_of\_signal\_transduction | FGF9 | 103 | 2 | 3.725728 | -1.002227 | 207 | 110.45 | 0.533575 |
| GO:0009968\_negative\_regulation\_of\_signal\_transduction | APC | 103 | 2 | 3.725728 | -1.002227 | 207 | 110.45 | 0.533575 |
| GO:0055086\_nucleobase\_\_nucleoside\_and\_nucleotide\_metabolic\_process | RRM2 | 104 | 2 | 3.689904 | -0.995143 | 208 | 110.86 | 0.532981 |
| GO:0055086\_nucleobase\_\_nucleoside\_and\_nucleotide\_metabolic\_process | TIMP2 | 104 | 2 | 3.689904 | -0.995143 | 208 | 110.86 | 0.532981 |
| GO:0009892\_negative\_regulation\_of\_metabolic\_process | FZD1 | 348 | 4 | 2.205460 | -0.988707 | 209 | 111.02 | 0.531196 |
| GO:0009892\_negative\_regulation\_of\_metabolic\_process | ATP1A1 | 348 | 4 | 2.205460 | -0.988707 | 209 | 111.02 | 0.531196 |
| GO:0009892\_negative\_regulation\_of\_metabolic\_process | TLE1 | 348 | 4 | 2.205460 | -0.988707 | 209 | 111.02 | 0.531196 |
| GO:0009892\_negative\_regulation\_of\_metabolic\_process | APC | 348 | 4 | 2.205460 | -0.988707 | 209 | 111.02 | 0.531196 |
| GO:0007423\_sensory\_organ\_development | RDH10 | 219 | 3 | 2.628425 | -0.986702 | 210 | 111.26 | 0.529810 |
| GO:0007423\_sensory\_organ\_development | FGF9 | 219 | 3 | 2.628425 | -0.986702 | 210 | 111.26 | 0.529810 |
| GO:0007423\_sensory\_organ\_development | APC | 219 | 3 | 2.628425 | -0.986702 | 210 | 111.26 | 0.529810 |
| GO:0002053\_positive\_regulation\_of\_mesenchymal\_cell\_proliferation | FGF9 | 21 | 1 | 9.136905 | -0.982379 | 215 | 115.49 | 0.537163 |
| GO:0019827\_stem\_cell\_maintenance | APC | 21 | 1 | 9.136905 | -0.982379 | 215 | 115.49 | 0.537163 |
| GO:0035136\_forelimb\_morphogenesis | RDH10 | 21 | 1 | 9.136905 | -0.982379 | 215 | 115.49 | 0.537163 |
| GO:0048538\_thymus\_development | APC | 21 | 1 | 9.136905 | -0.982379 | 215 | 115.49 | 0.537163 |
| GO:0051258\_protein\_polymerization | APC | 21 | 1 | 9.136905 | -0.982379 | 215 | 115.49 | 0.537163 |
| GO:0051240\_positive\_regulation\_of\_multicellular\_organismal\_process | FGF9 | 108 | 2 | 3.553241 | -0.967598 | 216 | 116.23 | 0.538102 |
| GO:0051240\_positive\_regulation\_of\_multicellular\_organismal\_process | ATP1A1 | 108 | 2 | 3.553241 | -0.967598 | 216 | 116.23 | 0.538102 |
| GO:0001523\_retinoid\_metabolic\_process | RDH10 | 22 | 1 | 8.721591 | -0.963247 | 227 | 120.65 | 0.531498 |
| GO:0006721\_terpenoid\_metabolic\_process | RDH10 | 22 | 1 | 8.721591 | -0.963247 | 227 | 120.65 | 0.531498 |
| GO:0009896\_positive\_regulation\_of\_catabolic\_process | APC | 22 | 1 | 8.721591 | -0.963247 | 227 | 120.65 | 0.531498 |
| GO:0010463\_mesenchymal\_cell\_proliferation | FGF9 | 22 | 1 | 8.721591 | -0.963247 | 227 | 120.65 | 0.531498 |
| GO:0010464\_regulation\_of\_mesenchymal\_cell\_proliferation | FGF9 | 22 | 1 | 8.721591 | -0.963247 | 227 | 120.65 | 0.531498 |
| GO:0016101\_diterpenoid\_metabolic\_process | RDH10 | 22 | 1 | 8.721591 | -0.963247 | 227 | 120.65 | 0.531498 |
| GO:0030316\_osteoclast\_differentiation | APC | 22 | 1 | 8.721591 | -0.963247 | 227 | 120.65 | 0.531498 |
| GO:0030335\_positive\_regulation\_of\_cell\_migration | APC | 22 | 1 | 8.721591 | -0.963247 | 227 | 120.65 | 0.531498 |
| GO:0034660\_ncRNA\_metabolic\_process | EBNA1BP2 | 22 | 1 | 8.721591 | -0.963247 | 227 | 120.65 | 0.531498 |
| GO:0048489\_synaptic\_vesicle\_transport | STX4A | 22 | 1 | 8.721591 | -0.963247 | 227 | 120.65 | 0.531498 |
| GO:0048864\_stem\_cell\_development | APC | 22 | 1 | 8.721591 | -0.963247 | 227 | 120.65 | 0.531498 |
| GO:0010647\_positive\_regulation\_of\_cell\_communication | FGF9 | 110 | 2 | 3.488636 | -0.954281 | 230 | 121.44 | 0.528000 |
| GO:0010647\_positive\_regulation\_of\_cell\_communication | RELN | 110 | 2 | 3.488636 | -0.954281 | 230 | 121.44 | 0.528000 |
| GO:0010648\_negative\_regulation\_of\_cell\_communication | FGF9 | 110 | 2 | 3.488636 | -0.954281 | 230 | 121.44 | 0.528000 |
| GO:0010648\_negative\_regulation\_of\_cell\_communication | APC | 110 | 2 | 3.488636 | -0.954281 | 230 | 121.44 | 0.528000 |
| GO:0043010\_camera-type\_eye\_development | RDH10 | 110 | 2 | 3.488636 | -0.954281 | 230 | 121.44 | 0.528000 |
| GO:0043010\_camera-type\_eye\_development | APC | 110 | 2 | 3.488636 | -0.954281 | 230 | 121.44 | 0.528000 |
| GO:0002204\_somatic\_recombination\_of\_immunoglobulin\_genes\_during\_immune\_response | EXO1 | 23 | 1 | 8.342391 | -0.945012 | 236 | 124.31 | 0.526737 |
| GO:0002208\_somatic\_diversification\_of\_immunoglobulins\_during\_immune\_response | EXO1 | 23 | 1 | 8.342391 | -0.945012 | 236 | 124.31 | 0.526737 |
| GO:0007018\_microtubule-based\_movement | BICD2 | 23 | 1 | 8.342391 | -0.945012 | 236 | 124.31 | 0.526737 |
| GO:0009954\_proximal\_distal\_pattern\_formation | APC | 23 | 1 | 8.342391 | -0.945012 | 236 | 124.31 | 0.526737 |
| GO:0022613\_ribonucleoprotein\_complex\_biogenesis | EBNA1BP2 | 23 | 1 | 8.342391 | -0.945012 | 236 | 124.31 | 0.526737 |
| GO:0045190\_isotype\_switching | EXO1 | 23 | 1 | 8.342391 | -0.945012 | 236 | 124.31 | 0.526737 |
| GO:0000165\_MAPKKK\_cascade | TIMP2 | 114 | 2 | 3.366228 | -0.928500 | 237 | 125.54 | 0.529705 |
| GO:0000165\_MAPKKK\_cascade | APC | 114 | 2 | 3.366228 | -0.928500 | 237 | 125.54 | 0.529705 |
| GO:0000280\_nuclear\_division | APC | 24 | 1 | 7.994792 | -0.927598 | 245 | 128.51 | 0.524531 |
| GO:0002381\_immunoglobulin\_production\_during\_immune\_response | EXO1 | 24 | 1 | 7.994792 | -0.927598 | 245 | 128.51 | 0.524531 |
| GO:0006941\_striated\_muscle\_contraction | ATP1A1 | 24 | 1 | 7.994792 | -0.927598 | 245 | 128.51 | 0.524531 |
| GO:0006959\_humoral\_immune\_response | EXO1 | 24 | 1 | 7.994792 | -0.927598 | 245 | 128.51 | 0.524531 |
| GO:0007067\_mitosis | APC | 24 | 1 | 7.994792 | -0.927598 | 245 | 128.51 | 0.524531 |
| GO:0032386\_regulation\_of\_intracellular\_transport | TACC3 | 24 | 1 | 7.994792 | -0.927598 | 245 | 128.51 | 0.524531 |
| GO:0043588\_skin\_development | APC | 24 | 1 | 7.994792 | -0.927598 | 245 | 128.51 | 0.524531 |
| GO:0050679\_positive\_regulation\_of\_epithelial\_cell\_proliferation | FGF9 | 24 | 1 | 7.994792 | -0.927598 | 245 | 128.51 | 0.524531 |
| GO:0006807\_nitrogen\_compound\_metabolic\_process | EXO1 | 1147 | 9 | 1.505558 | -0.926174 | 246 | 128.6 | 0.522764 |
| GO:0006807\_nitrogen\_compound\_metabolic\_process | EBNA1BP2 | 1147 | 9 | 1.505558 | -0.926174 | 246 | 128.6 | 0.522764 |
| GO:0006807\_nitrogen\_compound\_metabolic\_process | TYRP1 | 1147 | 9 | 1.505558 | -0.926174 | 246 | 128.6 | 0.522764 |
| GO:0006807\_nitrogen\_compound\_metabolic\_process | RRM2 | 1147 | 9 | 1.505558 | -0.926174 | 246 | 128.6 | 0.522764 |
| GO:0006807\_nitrogen\_compound\_metabolic\_process | FZD1 | 1147 | 9 | 1.505558 | -0.926174 | 246 | 128.6 | 0.522764 |
| GO:0006807\_nitrogen\_compound\_metabolic\_process | RELN | 1147 | 9 | 1.505558 | -0.926174 | 246 | 128.6 | 0.522764 |
| GO:0006807\_nitrogen\_compound\_metabolic\_process | TLE1 | 1147 | 9 | 1.505558 | -0.926174 | 246 | 128.6 | 0.522764 |
| GO:0006807\_nitrogen\_compound\_metabolic\_process | TIMP2 | 1147 | 9 | 1.505558 | -0.926174 | 246 | 128.6 | 0.522764 |
| GO:0006807\_nitrogen\_compound\_metabolic\_process | APC | 1147 | 9 | 1.505558 | -0.926174 | 246 | 128.6 | 0.522764 |
| GO:0001501\_skeletal\_system\_development | RDH10 | 236 | 3 | 2.439089 | -0.913649 | 247 | 129.55 | 0.524494 |
| GO:0001501\_skeletal\_system\_development | FGF9 | 236 | 3 | 2.439089 | -0.913649 | 247 | 129.55 | 0.524494 |
| GO:0001501\_skeletal\_system\_development | APC | 236 | 3 | 2.439089 | -0.913649 | 247 | 129.55 | 0.524494 |
| GO:0000087\_M\_phase\_of\_mitotic\_cell\_cycle | APC | 25 | 1 | 7.675000 | -0.910938 | 250 | 132.18 | 0.528720 |
| GO:0006775\_fat-soluble\_vitamin\_metabolic\_process | RDH10 | 25 | 1 | 7.675000 | -0.910938 | 250 | 132.18 | 0.528720 |
| GO:0048285\_organelle\_fission | APC | 25 | 1 | 7.675000 | -0.910938 | 250 | 132.18 | 0.528720 |
| GO:0043933\_macromolecular\_complex\_subunit\_organization | RRM2 | 117 | 2 | 3.279915 | -0.909872 | 251 | 132.4 | 0.527490 |
| GO:0043933\_macromolecular\_complex\_subunit\_organization | APC | 117 | 2 | 3.279915 | -0.909872 | 251 | 132.4 | 0.527490 |
| GO:0044085\_cellular\_component\_biogenesis | EBNA1BP2 | 237 | 3 | 2.428797 | -0.909572 | 252 | 132.54 | 0.525952 |
| GO:0044085\_cellular\_component\_biogenesis | RRM2 | 237 | 3 | 2.428797 | -0.909572 | 252 | 132.54 | 0.525952 |
| GO:0044085\_cellular\_component\_biogenesis | APC | 237 | 3 | 2.428797 | -0.909572 | 252 | 132.54 | 0.525952 |
| GO:0006519\_cellular\_amino\_acid\_and\_derivative\_metabolic\_process | TYRP1 | 118 | 2 | 3.252119 | -0.903792 | 253 | 132.76 | 0.524743 |
| GO:0006519\_cellular\_amino\_acid\_and\_derivative\_metabolic\_process | RELN | 118 | 2 | 3.252119 | -0.903792 | 253 | 132.76 | 0.524743 |
| GO:0022403\_cell\_cycle\_phase | TACC3 | 119 | 2 | 3.224790 | -0.897774 | 254 | 132.95 | 0.523425 |
| GO:0022403\_cell\_cycle\_phase | APC | 119 | 2 | 3.224790 | -0.897774 | 254 | 132.95 | 0.523425 |
| GO:0022414\_reproductive\_process | RAD23B | 376 | 4 | 2.041223 | -0.896027 | 255 | 133.09 | 0.521922 |
| GO:0022414\_reproductive\_process | RDH10 | 376 | 4 | 2.041223 | -0.896027 | 255 | 133.09 | 0.521922 |
| GO:0022414\_reproductive\_process | FGF9 | 376 | 4 | 2.041223 | -0.896027 | 255 | 133.09 | 0.521922 |
| GO:0022414\_reproductive\_process | APC | 376 | 4 | 2.041223 | -0.896027 | 255 | 133.09 | 0.521922 |
| GO:0006720\_isoprenoid\_metabolic\_process | RDH10 | 26 | 1 | 7.379808 | -0.894972 | 258 | 135.95 | 0.526938 |
| GO:0048645\_organ\_formation | RDH10 | 26 | 1 | 7.379808 | -0.894972 | 258 | 135.95 | 0.526938 |
| GO:0050680\_negative\_regulation\_of\_epithelial\_cell\_proliferation | APC | 26 | 1 | 7.379808 | -0.894972 | 258 | 135.95 | 0.526938 |
| GO:0000003\_reproduction | RAD23B | 379 | 4 | 2.025066 | -0.886696 | 259 | 136.32 | 0.526332 |
| GO:0000003\_reproduction | RDH10 | 379 | 4 | 2.025066 | -0.886696 | 259 | 136.32 | 0.526332 |
| GO:0000003\_reproduction | FGF9 | 379 | 4 | 2.025066 | -0.886696 | 259 | 136.32 | 0.526332 |
| GO:0000003\_reproduction | APC | 379 | 4 | 2.025066 | -0.886696 | 259 | 136.32 | 0.526332 |
| GO:0051726\_regulation\_of\_cell\_cycle | TACC3 | 121 | 2 | 3.171488 | -0.885921 | 260 | 136.77 | 0.526038 |
| GO:0051726\_regulation\_of\_cell\_cycle | APC | 121 | 2 | 3.171488 | -0.885921 | 260 | 136.77 | 0.526038 |
| GO:0006886\_intracellular\_protein\_transport | FGF9 | 122 | 2 | 3.145492 | -0.880084 | 261 | 137.45 | 0.526628 |
| GO:0006886\_intracellular\_protein\_transport | TACC3 | 122 | 2 | 3.145492 | -0.880084 | 261 | 137.45 | 0.526628 |
| GO:0002761\_regulation\_of\_myeloid\_leukocyte\_differentiation | APC | 27 | 1 | 7.106481 | -0.879649 | 265 | 139.6 | 0.526792 |
| GO:0010638\_positive\_regulation\_of\_organelle\_organization | APC | 27 | 1 | 7.106481 | -0.879649 | 265 | 139.6 | 0.526792 |
| GO:0016050\_vesicle\_organization | TYRP1 | 27 | 1 | 7.106481 | -0.879649 | 265 | 139.6 | 0.526792 |
| GO:0051272\_positive\_regulation\_of\_cell\_motion | APC | 27 | 1 | 7.106481 | -0.879649 | 265 | 139.6 | 0.526792 |
| GO:0009308\_amine\_metabolic\_process | TYRP1 | 124 | 2 | 3.094758 | -0.868585 | 266 | 140.01 | 0.526353 |
| GO:0009308\_amine\_metabolic\_process | RELN | 124 | 2 | 3.094758 | -0.868585 | 266 | 140.01 | 0.526353 |
| GO:0002062\_chondrocyte\_differentiation | FGF9 | 28 | 1 | 6.852679 | -0.864921 | 268 | 142.35 | 0.531157 |
| GO:0048863\_stem\_cell\_differentiation | APC | 28 | 1 | 6.852679 | -0.864921 | 268 | 142.35 | 0.531157 |
| GO:0008104\_protein\_localization | FGF9 | 251 | 3 | 2.293327 | -0.854849 | 269 | 142.89 | 0.531190 |
| GO:0008104\_protein\_localization | TACC3 | 251 | 3 | 2.293327 | -0.854849 | 269 | 142.89 | 0.531190 |
| GO:0008104\_protein\_localization | APC | 251 | 3 | 2.293327 | -0.854849 | 269 | 142.89 | 0.531190 |
| GO:0016447\_somatic\_recombination\_of\_immunoglobulin\_gene\_segments | EXO1 | 29 | 1 | 6.616379 | -0.850747 | 275 | 145.92 | 0.530618 |
| GO:0042176\_regulation\_of\_protein\_catabolic\_process | APC | 29 | 1 | 6.616379 | -0.850747 | 275 | 145.92 | 0.530618 |
| GO:0044087\_regulation\_of\_cellular\_component\_biogenesis | APC | 29 | 1 | 6.616379 | -0.850747 | 275 | 145.92 | 0.530618 |
| GO:0048066\_pigmentation\_during\_development | TYRP1 | 29 | 1 | 6.616379 | -0.850747 | 275 | 145.92 | 0.530618 |
| GO:0051301\_cell\_division | APC | 29 | 1 | 6.616379 | -0.850747 | 275 | 145.92 | 0.530618 |
| GO:0060041\_retina\_development\_in\_camera-type\_eye | APC | 29 | 1 | 6.616379 | -0.850747 | 275 | 145.92 | 0.530618 |
| GO:0001655\_urogenital\_system\_development | RDH10 | 128 | 2 | 2.998047 | -0.846258 | 276 | 146.46 | 0.530652 |
| GO:0001655\_urogenital\_system\_development | APC | 128 | 2 | 2.998047 | -0.846258 | 276 | 146.46 | 0.530652 |
| GO:0048519\_negative\_regulation\_of\_biological\_process | FGF9 | 859 | 7 | 1.563591 | -0.840883 | 277 | 146.88 | 0.530253 |
| GO:0048519\_negative\_regulation\_of\_biological\_process | FZD1 | 859 | 7 | 1.563591 | -0.840883 | 277 | 146.88 | 0.530253 |
| GO:0048519\_negative\_regulation\_of\_biological\_process | ATP1A1 | 859 | 7 | 1.563591 | -0.840883 | 277 | 146.88 | 0.530253 |
| GO:0048519\_negative\_regulation\_of\_biological\_process | TLE1 | 859 | 7 | 1.563591 | -0.840883 | 277 | 146.88 | 0.530253 |
| GO:0048519\_negative\_regulation\_of\_biological\_process | TACC3 | 859 | 7 | 1.563591 | -0.840883 | 277 | 146.88 | 0.530253 |
| GO:0048519\_negative\_regulation\_of\_biological\_process | TIMP2 | 859 | 7 | 1.563591 | -0.840883 | 277 | 146.88 | 0.530253 |
| GO:0048519\_negative\_regulation\_of\_biological\_process | APC | 859 | 7 | 1.563591 | -0.840883 | 277 | 146.88 | 0.530253 |
| GO:0048729\_tissue\_morphogenesis | RDH10 | 255 | 3 | 2.257353 | -0.839978 | 278 | 147.13 | 0.529245 |
| GO:0048729\_tissue\_morphogenesis | FZD1 | 255 | 3 | 2.257353 | -0.839978 | 278 | 147.13 | 0.529245 |
| GO:0048729\_tissue\_morphogenesis | APC | 255 | 3 | 2.257353 | -0.839978 | 278 | 147.13 | 0.529245 |
| GO:0008283\_cell\_proliferation | FGF9 | 544 | 5 | 1.763557 | -0.838005 | 279 | 147.2 | 0.527599 |
| GO:0008283\_cell\_proliferation | TACC3 | 544 | 5 | 1.763557 | -0.838005 | 279 | 147.2 | 0.527599 |
| GO:0008283\_cell\_proliferation | TIMP2 | 544 | 5 | 1.763557 | -0.838005 | 279 | 147.2 | 0.527599 |
| GO:0008283\_cell\_proliferation | HDGFRP3 | 544 | 5 | 1.763557 | -0.838005 | 279 | 147.2 | 0.527599 |
| GO:0008283\_cell\_proliferation | APC | 544 | 5 | 1.763557 | -0.838005 | 279 | 147.2 | 0.527599 |
| GO:0014032\_neural\_crest\_cell\_development | RDH10 | 30 | 1 | 6.395833 | -0.837089 | 284 | 150.28 | 0.529155 |
| GO:0014033\_neural\_crest\_cell\_differentiation | RDH10 | 30 | 1 | 6.395833 | -0.837089 | 284 | 150.28 | 0.529155 |
| GO:0016445\_somatic\_diversification\_of\_immunoglobulins | EXO1 | 30 | 1 | 6.395833 | -0.837089 | 284 | 150.28 | 0.529155 |
| GO:0022411\_cellular\_component\_disassembly | APC | 30 | 1 | 6.395833 | -0.837089 | 284 | 150.28 | 0.529155 |
| GO:0048565\_gut\_development | FGF9 | 30 | 1 | 6.395833 | -0.837089 | 284 | 150.28 | 0.529155 |
| GO:0032787\_monocarboxylic\_acid\_metabolic\_process | RDH10 | 130 | 2 | 2.951923 | -0.835416 | 285 | 150.83 | 0.529228 |
| GO:0032787\_monocarboxylic\_acid\_metabolic\_process | TYRP1 | 130 | 2 | 2.951923 | -0.835416 | 285 | 150.83 | 0.529228 |
| GO:0006665\_sphingolipid\_metabolic\_process | B3GNT5 | 31 | 1 | 6.189516 | -0.823913 | 292 | 155.51 | 0.532568 |
| GO:0006694\_steroid\_biosynthetic\_process | ATP1A1 | 31 | 1 | 6.189516 | -0.823913 | 292 | 155.51 | 0.532568 |
| GO:0008645\_hexose\_transport | SLC2A3 | 31 | 1 | 6.189516 | -0.823913 | 292 | 155.51 | 0.532568 |
| GO:0015749\_monosaccharide\_transport | SLC2A3 | 31 | 1 | 6.189516 | -0.823913 | 292 | 155.51 | 0.532568 |
| GO:0015758\_glucose\_transport | SLC2A3 | 31 | 1 | 6.189516 | -0.823913 | 292 | 155.51 | 0.532568 |
| GO:0033555\_multicellular\_organismal\_response\_to\_stress | RELN | 31 | 1 | 6.189516 | -0.823913 | 292 | 155.51 | 0.532568 |
| GO:0051640\_organelle\_localization | TACC3 | 31 | 1 | 6.189516 | -0.823913 | 292 | 155.51 | 0.532568 |
| GO:0006937\_regulation\_of\_muscle\_contraction | ATP1A1 | 32 | 1 | 5.996094 | -0.811188 | 295 | 158.78 | 0.538237 |
| GO:0051259\_protein\_oligomerization | RRM2 | 32 | 1 | 5.996094 | -0.811188 | 295 | 158.78 | 0.538237 |
| GO:0051493\_regulation\_of\_cytoskeleton\_organization | APC | 32 | 1 | 5.996094 | -0.811188 | 295 | 158.78 | 0.538237 |
| GO:0001654\_eye\_development | RDH10 | 136 | 2 | 2.821691 | -0.804099 | 296 | 159.13 | 0.537601 |
| GO:0001654\_eye\_development | APC | 136 | 2 | 2.821691 | -0.804099 | 296 | 159.13 | 0.537601 |
| GO:0002562\_somatic\_diversification\_of\_immune\_receptors\_via\_germline\_recombination\_within\_a\_single\_locus | EXO1 | 33 | 1 | 5.814394 | -0.798886 | 301 | 161.81 | 0.537575 |
| GO:0006643\_membrane\_lipid\_metabolic\_process | B3GNT5 | 33 | 1 | 5.814394 | -0.798886 | 301 | 161.81 | 0.537575 |
| GO:0008584\_male\_gonad\_development | FGF9 | 33 | 1 | 5.814394 | -0.798886 | 301 | 161.81 | 0.537575 |
| GO:0008643\_carbohydrate\_transport | SLC2A3 | 33 | 1 | 5.814394 | -0.798886 | 301 | 161.81 | 0.537575 |
| GO:0016444\_somatic\_cell\_DNA\_recombination | EXO1 | 33 | 1 | 5.814394 | -0.798886 | 301 | 161.81 | 0.537575 |
| GO:0034613\_cellular\_protein\_localization | FGF9 | 139 | 2 | 2.760791 | -0.789083 | 302 | 162.45 | 0.537914 |
| GO:0034613\_cellular\_protein\_localization | TACC3 | 139 | 2 | 2.760791 | -0.789083 | 302 | 162.45 | 0.537914 |
| GO:0002200\_somatic\_diversification\_of\_immune\_receptors | EXO1 | 34 | 1 | 5.643382 | -0.786983 | 303 | 165.13 | 0.544983 |
| GO:0008152\_metabolic\_process | EXO1 | 2133 | 14 | 1.259376 | -0.785176 | 304 | 165.36 | 0.543947 |
| GO:0008152\_metabolic\_process | TYRP1 | 2133 | 14 | 1.259376 | -0.785176 | 304 | 165.36 | 0.543947 |
| GO:0008152\_metabolic\_process | FZD1 | 2133 | 14 | 1.259376 | -0.785176 | 304 | 165.36 | 0.543947 |
| GO:0008152\_metabolic\_process | TLE1 | 2133 | 14 | 1.259376 | -0.785176 | 304 | 165.36 | 0.543947 |
| GO:0008152\_metabolic\_process | ATP1A1 | 2133 | 14 | 1.259376 | -0.785176 | 304 | 165.36 | 0.543947 |
| GO:0008152\_metabolic\_process | TIMP2 | 2133 | 14 | 1.259376 | -0.785176 | 304 | 165.36 | 0.543947 |
| GO:0008152\_metabolic\_process | EBNA1BP2 | 2133 | 14 | 1.259376 | -0.785176 | 304 | 165.36 | 0.543947 |
| GO:0008152\_metabolic\_process | RDH10 | 2133 | 14 | 1.259376 | -0.785176 | 304 | 165.36 | 0.543947 |
| GO:0008152\_metabolic\_process | CSNK2A1 | 2133 | 14 | 1.259376 | -0.785176 | 304 | 165.36 | 0.543947 |
| GO:0008152\_metabolic\_process | B3GNT5 | 2133 | 14 | 1.259376 | -0.785176 | 304 | 165.36 | 0.543947 |
| GO:0008152\_metabolic\_process | RRM2 | 2133 | 14 | 1.259376 | -0.785176 | 304 | 165.36 | 0.543947 |
| GO:0008152\_metabolic\_process | MRPL19 | 2133 | 14 | 1.259376 | -0.785176 | 304 | 165.36 | 0.543947 |
| GO:0008152\_metabolic\_process | RELN | 2133 | 14 | 1.259376 | -0.785176 | 304 | 165.36 | 0.543947 |
| GO:0008152\_metabolic\_process | APC | 2133 | 14 | 1.259376 | -0.785176 | 304 | 165.36 | 0.543947 |
| GO:0070727\_cellular\_macromolecule\_localization | FGF9 | 141 | 2 | 2.721631 | -0.779298 | 305 | 166.14 | 0.544721 |
| GO:0070727\_cellular\_macromolecule\_localization | TACC3 | 141 | 2 | 2.721631 | -0.779298 | 305 | 166.14 | 0.544721 |
| GO:0030154\_cell\_differentiation | TYRP1 | 1060 | 8 | 1.448113 | -0.777227 | 306 | 166.24 | 0.543268 |
| GO:0030154\_cell\_differentiation | RDH10 | 1060 | 8 | 1.448113 | -0.777227 | 306 | 166.24 | 0.543268 |
| GO:0030154\_cell\_differentiation | FGF9 | 1060 | 8 | 1.448113 | -0.777227 | 306 | 166.24 | 0.543268 |
| GO:0030154\_cell\_differentiation | FZD1 | 1060 | 8 | 1.448113 | -0.777227 | 306 | 166.24 | 0.543268 |
| GO:0030154\_cell\_differentiation | RELN | 1060 | 8 | 1.448113 | -0.777227 | 306 | 166.24 | 0.543268 |
| GO:0030154\_cell\_differentiation | TACC3 | 1060 | 8 | 1.448113 | -0.777227 | 306 | 166.24 | 0.543268 |
| GO:0030154\_cell\_differentiation | TIMP2 | 1060 | 8 | 1.448113 | -0.777227 | 306 | 166.24 | 0.543268 |
| GO:0030154\_cell\_differentiation | APC | 1060 | 8 | 1.448113 | -0.777227 | 306 | 166.24 | 0.543268 |
| GO:0051051\_negative\_regulation\_of\_transport | TACC3 | 35 | 1 | 5.482143 | -0.775455 | 307 | 168.57 | 0.549088 |
| GO:0033036\_macromolecule\_localization | FGF9 | 274 | 3 | 2.100821 | -0.773539 | 308 | 168.87 | 0.548279 |
| GO:0033036\_macromolecule\_localization | TACC3 | 274 | 3 | 2.100821 | -0.773539 | 308 | 168.87 | 0.548279 |
| GO:0033036\_macromolecule\_localization | APC | 274 | 3 | 2.100821 | -0.773539 | 308 | 168.87 | 0.548279 |
| GO:0021510\_spinal\_cord\_development | RELN | 36 | 1 | 5.329861 | -0.764281 | 311 | 172.44 | 0.554469 |
| GO:0030278\_regulation\_of\_ossification | APC | 36 | 1 | 5.329861 | -0.764281 | 311 | 172.44 | 0.554469 |
| GO:0051223\_regulation\_of\_protein\_transport | TACC3 | 36 | 1 | 5.329861 | -0.764281 | 311 | 172.44 | 0.554469 |
| GO:0022008\_neurogenesis | RELN | 423 | 4 | 1.814421 | -0.761523 | 312 | 172.71 | 0.553558 |
| GO:0022008\_neurogenesis | TACC3 | 423 | 4 | 1.814421 | -0.761523 | 312 | 172.71 | 0.553558 |
| GO:0022008\_neurogenesis | TIMP2 | 423 | 4 | 1.814421 | -0.761523 | 312 | 172.71 | 0.553558 |
| GO:0022008\_neurogenesis | APC | 423 | 4 | 1.814421 | -0.761523 | 312 | 172.71 | 0.553558 |
| GO:0030900\_forebrain\_development | RELN | 146 | 2 | 2.628425 | -0.755588 | 313 | 173.42 | 0.554058 |
| GO:0030900\_forebrain\_development | TACC3 | 146 | 2 | 2.628425 | -0.755588 | 313 | 173.42 | 0.554058 |
| GO:0045785\_positive\_regulation\_of\_cell\_adhesion | APC | 37 | 1 | 5.185811 | -0.753441 | 314 | 175.36 | 0.558471 |
| GO:0031327\_negative\_regulation\_of\_cellular\_biosynthetic\_process | FZD1 | 282 | 3 | 2.041223 | -0.747484 | 315 | 175.84 | 0.558222 |
| GO:0031327\_negative\_regulation\_of\_cellular\_biosynthetic\_process | ATP1A1 | 282 | 3 | 2.041223 | -0.747484 | 315 | 175.84 | 0.558222 |
| GO:0031327\_negative\_regulation\_of\_cellular\_biosynthetic\_process | TLE1 | 282 | 3 | 2.041223 | -0.747484 | 315 | 175.84 | 0.558222 |
| GO:0008016\_regulation\_of\_heart\_contraction | ATP1A1 | 38 | 1 | 5.049342 | -0.742918 | 318 | 179.93 | 0.565818 |
| GO:0042493\_response\_to\_drug | ATP1A1 | 38 | 1 | 5.049342 | -0.742918 | 318 | 179.93 | 0.565818 |
| GO:0046777\_protein\_amino\_acid\_autophosphorylation | CSNK2A1 | 38 | 1 | 5.049342 | -0.742918 | 318 | 179.93 | 0.565818 |
| GO:0032940\_secretion\_by\_cell | STX4A | 149 | 2 | 2.575503 | -0.741853 | 319 | 180.19 | 0.564859 |
| GO:0032940\_secretion\_by\_cell | LIN7C | 149 | 2 | 2.575503 | -0.741853 | 319 | 180.19 | 0.564859 |
| GO:0009890\_negative\_regulation\_of\_biosynthetic\_process | FZD1 | 284 | 3 | 2.026849 | -0.741135 | 320 | 180.35 | 0.563594 |
| GO:0009890\_negative\_regulation\_of\_biosynthetic\_process | ATP1A1 | 284 | 3 | 2.026849 | -0.741135 | 320 | 180.35 | 0.563594 |
| GO:0009890\_negative\_regulation\_of\_biosynthetic\_process | TLE1 | 284 | 3 | 2.026849 | -0.741135 | 320 | 180.35 | 0.563594 |
| GO:0070201\_regulation\_of\_establishment\_of\_protein\_localization | TACC3 | 39 | 1 | 4.919872 | -0.732695 | 321 | 183.72 | 0.572336 |
| GO:0014031\_mesenchymal\_cell\_development | RDH10 | 40 | 1 | 4.796875 | -0.722757 | 324 | 186.93 | 0.576944 |
| GO:0046850\_regulation\_of\_bone\_remodeling | APC | 40 | 1 | 4.796875 | -0.722757 | 324 | 186.93 | 0.576944 |
| GO:0051129\_negative\_regulation\_of\_cellular\_component\_organization | APC | 40 | 1 | 4.796875 | -0.722757 | 324 | 186.93 | 0.576944 |
| GO:0007268\_synaptic\_transmission | STX4A | 154 | 2 | 2.491883 | -0.719735 | 325 | 187.49 | 0.576892 |
| GO:0007268\_synaptic\_transmission | LIN7C | 154 | 2 | 2.491883 | -0.719735 | 325 | 187.49 | 0.576892 |
| GO:0008285\_negative\_regulation\_of\_cell\_proliferation | TIMP2 | 155 | 2 | 2.475806 | -0.715422 | 327 | 188.3 | 0.575841 |
| GO:0008285\_negative\_regulation\_of\_cell\_proliferation | APC | 155 | 2 | 2.475806 | -0.715422 | 327 | 188.3 | 0.575841 |
| GO:0022402\_cell\_cycle\_process | TACC3 | 155 | 2 | 2.475806 | -0.715422 | 327 | 188.3 | 0.575841 |
| GO:0022402\_cell\_cycle\_process | APC | 155 | 2 | 2.475806 | -0.715422 | 327 | 188.3 | 0.575841 |
| GO:0009894\_regulation\_of\_catabolic\_process | APC | 41 | 1 | 4.679878 | -0.713090 | 330 | 191.51 | 0.580333 |
| GO:0019216\_regulation\_of\_lipid\_metabolic\_process | ATP1A1 | 41 | 1 | 4.679878 | -0.713090 | 330 | 191.51 | 0.580333 |
| GO:0033077\_T\_cell\_differentiation\_in\_the\_thymus | APC | 41 | 1 | 4.679878 | -0.713090 | 330 | 191.51 | 0.580333 |
| GO:0002520\_immune\_system\_development | EXO1 | 295 | 3 | 1.951271 | -0.707338 | 332 | 192.02 | 0.578373 |
| GO:0002520\_immune\_system\_development | TACC3 | 295 | 3 | 1.951271 | -0.707338 | 332 | 192.02 | 0.578373 |
| GO:0002520\_immune\_system\_development | APC | 295 | 3 | 1.951271 | -0.707338 | 332 | 192.02 | 0.578373 |
| GO:0045595\_regulation\_of\_cell\_differentiation | FGF9 | 295 | 3 | 1.951271 | -0.707338 | 332 | 192.02 | 0.578373 |
| GO:0045595\_regulation\_of\_cell\_differentiation | TIMP2 | 295 | 3 | 1.951271 | -0.707338 | 332 | 192.02 | 0.578373 |
| GO:0045595\_regulation\_of\_cell\_differentiation | APC | 295 | 3 | 1.951271 | -0.707338 | 332 | 192.02 | 0.578373 |
| GO:0042476\_odontogenesis | APC | 42 | 1 | 4.568452 | -0.703680 | 334 | 195.64 | 0.585749 |
| GO:0045637\_regulation\_of\_myeloid\_cell\_differentiation | APC | 42 | 1 | 4.568452 | -0.703680 | 334 | 195.64 | 0.585749 |
| GO:0007409\_axonogenesis | RELN | 158 | 2 | 2.428797 | -0.702699 | 335 | 196.08 | 0.585313 |
| GO:0007409\_axonogenesis | APC | 158 | 2 | 2.428797 | -0.702699 | 335 | 196.08 | 0.585313 |
| GO:0010926\_anatomical\_structure\_formation | RDH10 | 447 | 4 | 1.717002 | -0.701432 | 336 | 196.26 | 0.584107 |
| GO:0010926\_anatomical\_structure\_formation | FGF9 | 447 | 4 | 1.717002 | -0.701432 | 336 | 196.26 | 0.584107 |
| GO:0010926\_anatomical\_structure\_formation | RRM2 | 447 | 4 | 1.717002 | -0.701432 | 336 | 196.26 | 0.584107 |
| GO:0010926\_anatomical\_structure\_formation | APC | 447 | 4 | 1.717002 | -0.701432 | 336 | 196.26 | 0.584107 |
| GO:0006766\_vitamin\_metabolic\_process | RDH10 | 43 | 1 | 4.462209 | -0.694516 | 341 | 200.04 | 0.586628 |
| GO:0007224\_smoothened\_signaling\_pathway | FGF9 | 43 | 1 | 4.462209 | -0.694516 | 341 | 200.04 | 0.586628 |
| GO:0010001\_glial\_cell\_differentiation | RELN | 43 | 1 | 4.462209 | -0.694516 | 341 | 200.04 | 0.586628 |
| GO:0030814\_regulation\_of\_cAMP\_metabolic\_process | TIMP2 | 43 | 1 | 4.462209 | -0.694516 | 341 | 200.04 | 0.586628 |
| GO:0048762\_mesenchymal\_cell\_differentiation | RDH10 | 43 | 1 | 4.462209 | -0.694516 | 341 | 200.04 | 0.586628 |
| GO:0048869\_cellular\_developmental\_process | TYRP1 | 1113 | 8 | 1.379155 | -0.689887 | 342 | 200.56 | 0.586433 |
| GO:0048869\_cellular\_developmental\_process | RDH10 | 1113 | 8 | 1.379155 | -0.689887 | 342 | 200.56 | 0.586433 |
| GO:0048869\_cellular\_developmental\_process | FGF9 | 1113 | 8 | 1.379155 | -0.689887 | 342 | 200.56 | 0.586433 |
| GO:0048869\_cellular\_developmental\_process | FZD1 | 1113 | 8 | 1.379155 | -0.689887 | 342 | 200.56 | 0.586433 |
| GO:0048869\_cellular\_developmental\_process | RELN | 1113 | 8 | 1.379155 | -0.689887 | 342 | 200.56 | 0.586433 |
| GO:0048869\_cellular\_developmental\_process | TACC3 | 1113 | 8 | 1.379155 | -0.689887 | 342 | 200.56 | 0.586433 |
| GO:0048869\_cellular\_developmental\_process | TIMP2 | 1113 | 8 | 1.379155 | -0.689887 | 342 | 200.56 | 0.586433 |
| GO:0048869\_cellular\_developmental\_process | APC | 1113 | 8 | 1.379155 | -0.689887 | 342 | 200.56 | 0.586433 |
| GO:0048523\_negative\_regulation\_of\_cellular\_process | FGF9 | 774 | 6 | 1.487403 | -0.689137 | 343 | 200.67 | 0.585044 |
| GO:0048523\_negative\_regulation\_of\_cellular\_process | FZD1 | 774 | 6 | 1.487403 | -0.689137 | 343 | 200.67 | 0.585044 |
| GO:0048523\_negative\_regulation\_of\_cellular\_process | ATP1A1 | 774 | 6 | 1.487403 | -0.689137 | 343 | 200.67 | 0.585044 |
| GO:0048523\_negative\_regulation\_of\_cellular\_process | TLE1 | 774 | 6 | 1.487403 | -0.689137 | 343 | 200.67 | 0.585044 |
| GO:0048523\_negative\_regulation\_of\_cellular\_process | TIMP2 | 774 | 6 | 1.487403 | -0.689137 | 343 | 200.67 | 0.585044 |
| GO:0048523\_negative\_regulation\_of\_cellular\_process | APC | 774 | 6 | 1.487403 | -0.689137 | 343 | 200.67 | 0.585044 |
| GO:0001942\_hair\_follicle\_development | APC | 44 | 1 | 4.360795 | -0.685586 | 351 | 204.72 | 0.583248 |
| GO:0002377\_immunoglobulin\_production | EXO1 | 44 | 1 | 4.360795 | -0.685586 | 351 | 204.72 | 0.583248 |
| GO:0016064\_immunoglobulin\_mediated\_immune\_response | EXO1 | 44 | 1 | 4.360795 | -0.685586 | 351 | 204.72 | 0.583248 |
| GO:0022404\_molting\_cycle\_process | APC | 44 | 1 | 4.360795 | -0.685586 | 351 | 204.72 | 0.583248 |
| GO:0022405\_hair\_cycle\_process | APC | 44 | 1 | 4.360795 | -0.685586 | 351 | 204.72 | 0.583248 |
| GO:0042303\_molting\_cycle | APC | 44 | 1 | 4.360795 | -0.685586 | 351 | 204.72 | 0.583248 |
| GO:0042633\_hair\_cycle | APC | 44 | 1 | 4.360795 | -0.685586 | 351 | 204.72 | 0.583248 |
| GO:0060485\_mesenchyme\_development | RDH10 | 44 | 1 | 4.360795 | -0.685586 | 351 | 204.72 | 0.583248 |
| GO:0034103\_regulation\_of\_tissue\_remodeling | APC | 45 | 1 | 4.263889 | -0.676880 | 355 | 206.67 | 0.582169 |
| GO:0043623\_cellular\_protein\_complex\_assembly | APC | 45 | 1 | 4.263889 | -0.676880 | 355 | 206.67 | 0.582169 |
| GO:0046058\_cAMP\_metabolic\_process | TIMP2 | 45 | 1 | 4.263889 | -0.676880 | 355 | 206.67 | 0.582169 |
| GO:0046546\_development\_of\_primary\_male\_sexual\_characteristics | FGF9 | 45 | 1 | 4.263889 | -0.676880 | 355 | 206.67 | 0.582169 |
| GO:0008217\_regulation\_of\_blood\_pressure | ATP1A1 | 46 | 1 | 4.171196 | -0.668387 | 359 | 209.62 | 0.583900 |
| GO:0019724\_B\_cell\_mediated\_immunity | EXO1 | 46 | 1 | 4.171196 | -0.668387 | 359 | 209.62 | 0.583900 |
| GO:0030850\_prostate\_gland\_development | APC | 46 | 1 | 4.171196 | -0.668387 | 359 | 209.62 | 0.583900 |
| GO:0042063\_gliogenesis | RELN | 46 | 1 | 4.171196 | -0.668387 | 359 | 209.62 | 0.583900 |
| GO:0006140\_regulation\_of\_nucleotide\_metabolic\_process | TIMP2 | 47 | 1 | 4.082447 | -0.660099 | 362 | 212.62 | 0.587348 |
| GO:0006396\_RNA\_processing | EBNA1BP2 | 47 | 1 | 4.082447 | -0.660099 | 362 | 212.62 | 0.587348 |
| GO:0030799\_regulation\_of\_cyclic\_nucleotide\_metabolic\_process | TIMP2 | 47 | 1 | 4.082447 | -0.660099 | 362 | 212.62 | 0.587348 |
| GO:0048812\_neuron\_projection\_morphogenesis | RELN | 170 | 2 | 2.257353 | -0.654803 | 363 | 213.0 | 0.586777 |
| GO:0048812\_neuron\_projection\_morphogenesis | APC | 170 | 2 | 2.257353 | -0.654803 | 363 | 213.0 | 0.586777 |
| GO:0032269\_negative\_regulation\_of\_cellular\_protein\_metabolic\_process | APC | 48 | 1 | 3.997396 | -0.652007 | 365 | 214.5 | 0.587671 |
| GO:0046849\_bone\_remodeling | APC | 48 | 1 | 3.997396 | -0.652007 | 365 | 214.5 | 0.587671 |
| GO:0002440\_production\_of\_molecular\_mediator\_of\_immune\_response | EXO1 | 49 | 1 | 3.915816 | -0.644102 | 371 | 217.17 | 0.585364 |
| GO:0003015\_heart\_process | ATP1A1 | 49 | 1 | 3.915816 | -0.644102 | 371 | 217.17 | 0.585364 |
| GO:0006725\_cellular\_aromatic\_compound\_metabolic\_process | TYRP1 | 49 | 1 | 3.915816 | -0.644102 | 371 | 217.17 | 0.585364 |
| GO:0043473\_pigmentation | TYRP1 | 49 | 1 | 3.915816 | -0.644102 | 371 | 217.17 | 0.585364 |
| GO:0046661\_male\_sex\_differentiation | FGF9 | 49 | 1 | 3.915816 | -0.644102 | 371 | 217.17 | 0.585364 |
| GO:0060047\_heart\_contraction | ATP1A1 | 49 | 1 | 3.915816 | -0.644102 | 371 | 217.17 | 0.585364 |
| GO:0048667\_cell\_morphogenesis\_involved\_in\_neuron\_differentiation | RELN | 173 | 2 | 2.218208 | -0.643525 | 372 | 217.59 | 0.584919 |
| GO:0048667\_cell\_morphogenesis\_involved\_in\_neuron\_differentiation | APC | 173 | 2 | 2.218208 | -0.643525 | 372 | 217.59 | 0.584919 |
| GO:0001656\_metanephros\_development | RDH10 | 50 | 1 | 3.837500 | -0.636378 | 374 | 219.65 | 0.587299 |
| GO:0002573\_myeloid\_leukocyte\_differentiation | APC | 50 | 1 | 3.837500 | -0.636378 | 374 | 219.65 | 0.587299 |
| GO:0015031\_protein\_transport | FGF9 | 175 | 2 | 2.192857 | -0.636151 | 376 | 220.2 | 0.585638 |
| GO:0015031\_protein\_transport | TACC3 | 175 | 2 | 2.192857 | -0.636151 | 376 | 220.2 | 0.585638 |
| GO:0046903\_secretion | STX4A | 175 | 2 | 2.192857 | -0.636151 | 376 | 220.2 | 0.585638 |
| GO:0046903\_secretion | LIN7C | 175 | 2 | 2.192857 | -0.636151 | 376 | 220.2 | 0.585638 |
| GO:0048858\_cell\_projection\_morphogenesis | RELN | 176 | 2 | 2.180398 | -0.632506 | 377 | 220.93 | 0.586021 |
| GO:0048858\_cell\_projection\_morphogenesis | APC | 176 | 2 | 2.180398 | -0.632506 | 377 | 220.93 | 0.586021 |
| GO:0006520\_cellular\_amino\_acid\_metabolic\_process | TYRP1 | 51 | 1 | 3.762255 | -0.628826 | 380 | 223.45 | 0.588026 |
| GO:0006887\_exocytosis | STX4A | 51 | 1 | 3.762255 | -0.628826 | 380 | 223.45 | 0.588026 |
| GO:0044106\_cellular\_amine\_metabolic\_process | TYRP1 | 51 | 1 | 3.762255 | -0.628826 | 380 | 223.45 | 0.588026 |
| GO:0045184\_establishment\_of\_protein\_localization | FGF9 | 180 | 2 | 2.131944 | -0.618201 | 381 | 225.52 | 0.591916 |
| GO:0045184\_establishment\_of\_protein\_localization | TACC3 | 180 | 2 | 2.131944 | -0.618201 | 381 | 225.52 | 0.591916 |
| GO:0019752\_carboxylic\_acid\_metabolic\_process | RDH10 | 181 | 2 | 2.120166 | -0.614691 | 383 | 226.14 | 0.590444 |
| GO:0019752\_carboxylic\_acid\_metabolic\_process | TYRP1 | 181 | 2 | 2.120166 | -0.614691 | 383 | 226.14 | 0.590444 |
| GO:0043436\_oxoacid\_metabolic\_process | RDH10 | 181 | 2 | 2.120166 | -0.614691 | 383 | 226.14 | 0.590444 |
| GO:0043436\_oxoacid\_metabolic\_process | TYRP1 | 181 | 2 | 2.120166 | -0.614691 | 383 | 226.14 | 0.590444 |
| GO:0006576\_biogenic\_amine\_metabolic\_process | RELN | 53 | 1 | 3.620283 | -0.614216 | 385 | 227.83 | 0.591766 |
| GO:0051248\_negative\_regulation\_of\_protein\_metabolic\_process | APC | 53 | 1 | 3.620283 | -0.614216 | 385 | 227.83 | 0.591766 |
| GO:0006082\_organic\_acid\_metabolic\_process | RDH10 | 182 | 2 | 2.108516 | -0.611208 | 386 | 228.01 | 0.590699 |
| GO:0006082\_organic\_acid\_metabolic\_process | TYRP1 | 182 | 2 | 2.108516 | -0.611208 | 386 | 228.01 | 0.590699 |
| GO:0010605\_negative\_regulation\_of\_macromolecule\_metabolic\_process | FZD1 | 331 | 3 | 1.739048 | -0.608623 | 387 | 228.9 | 0.591473 |
| GO:0010605\_negative\_regulation\_of\_macromolecule\_metabolic\_process | TLE1 | 331 | 3 | 1.739048 | -0.608623 | 387 | 228.9 | 0.591473 |
| GO:0010605\_negative\_regulation\_of\_macromolecule\_metabolic\_process | APC | 331 | 3 | 1.739048 | -0.608623 | 387 | 228.9 | 0.591473 |
| GO:0042180\_cellular\_ketone\_metabolic\_process | RDH10 | 183 | 2 | 2.096995 | -0.607751 | 388 | 229.09 | 0.590438 |
| GO:0042180\_cellular\_ketone\_metabolic\_process | TYRP1 | 183 | 2 | 2.096995 | -0.607751 | 388 | 229.09 | 0.590438 |
| GO:0006412\_translation | MRPL19 | 54 | 1 | 3.553241 | -0.607144 | 389 | 231.07 | 0.594010 |
| GO:0048468\_cell\_development | RDH10 | 654 | 5 | 1.466934 | -0.605829 | 390 | 231.47 | 0.593513 |
| GO:0048468\_cell\_development | RELN | 654 | 5 | 1.466934 | -0.605829 | 390 | 231.47 | 0.593513 |
| GO:0048468\_cell\_development | TACC3 | 654 | 5 | 1.466934 | -0.605829 | 390 | 231.47 | 0.593513 |
| GO:0048468\_cell\_development | TIMP2 | 654 | 5 | 1.466934 | -0.605829 | 390 | 231.47 | 0.593513 |
| GO:0048468\_cell\_development | APC | 654 | 5 | 1.466934 | -0.605829 | 390 | 231.47 | 0.593513 |
| GO:0032990\_cell\_part\_morphogenesis | RELN | 184 | 2 | 2.085598 | -0.604320 | 391 | 231.85 | 0.592967 |
| GO:0032990\_cell\_part\_morphogenesis | APC | 184 | 2 | 2.085598 | -0.604320 | 391 | 231.85 | 0.592967 |
| GO:0007010\_cytoskeleton\_organization | TACC3 | 185 | 2 | 2.074324 | -0.600913 | 392 | 232.19 | 0.592321 |
| GO:0007010\_cytoskeleton\_organization | APC | 185 | 2 | 2.074324 | -0.600913 | 392 | 232.19 | 0.592321 |
| GO:0006310\_DNA\_recombination | EXO1 | 55 | 1 | 3.488636 | -0.600221 | 394 | 233.8 | 0.593401 |
| GO:0048704\_embryonic\_skeletal\_system\_morphogenesis | RDH10 | 55 | 1 | 3.488636 | -0.600221 | 394 | 233.8 | 0.593401 |
| GO:0009187\_cyclic\_nucleotide\_metabolic\_process | TIMP2 | 56 | 1 | 3.426339 | -0.593441 | 395 | 236.89 | 0.599722 |
| GO:0019226\_transmission\_of\_nerve\_impulse | STX4A | 189 | 2 | 2.030423 | -0.587536 | 396 | 237.67 | 0.600177 |
| GO:0019226\_transmission\_of\_nerve\_impulse | LIN7C | 189 | 2 | 2.030423 | -0.587536 | 396 | 237.67 | 0.600177 |
| GO:0001764\_neuron\_migration | RELN | 57 | 1 | 3.366228 | -0.586798 | 401 | 240.49 | 0.599726 |
| GO:0009953\_dorsal\_ventral\_pattern\_formation | APC | 57 | 1 | 3.366228 | -0.586798 | 401 | 240.49 | 0.599726 |
| GO:0018108\_peptidyl-tyrosine\_phosphorylation | RELN | 57 | 1 | 3.366228 | -0.586798 | 401 | 240.49 | 0.599726 |
| GO:0018212\_peptidyl-tyrosine\_modification | RELN | 57 | 1 | 3.366228 | -0.586798 | 401 | 240.49 | 0.599726 |
| GO:0042472\_inner\_ear\_morphogenesis | FGF9 | 57 | 1 | 3.366228 | -0.586798 | 401 | 240.49 | 0.599726 |
| GO:0033043\_regulation\_of\_organelle\_organization | APC | 58 | 1 | 3.308190 | -0.580288 | 403 | 242.01 | 0.600521 |
| GO:0034622\_cellular\_macromolecular\_complex\_assembly | APC | 58 | 1 | 3.308190 | -0.580288 | 403 | 242.01 | 0.600521 |
| GO:0030334\_regulation\_of\_cell\_migration | APC | 59 | 1 | 3.252119 | -0.573907 | 404 | 243.51 | 0.602748 |
| GO:0046907\_intracellular\_transport | FGF9 | 194 | 2 | 1.978093 | -0.571353 | 405 | 243.85 | 0.602099 |
| GO:0046907\_intracellular\_transport | TACC3 | 194 | 2 | 1.978093 | -0.571353 | 405 | 243.85 | 0.602099 |
| GO:0003002\_regionalization | RELN | 195 | 2 | 1.967949 | -0.568185 | 406 | 244.48 | 0.602167 |
| GO:0003002\_regionalization | APC | 195 | 2 | 1.967949 | -0.568185 | 406 | 244.48 | 0.602167 |
| GO:0009123\_nucleoside\_monophosphate\_metabolic\_process | TIMP2 | 60 | 1 | 3.197917 | -0.567649 | 407 | 244.7 | 0.601229 |
| GO:0031175\_neuron\_projection\_development | RELN | 197 | 2 | 1.947970 | -0.561917 | 408 | 245.64 | 0.602059 |
| GO:0031175\_neuron\_projection\_development | APC | 197 | 2 | 1.947970 | -0.561917 | 408 | 245.64 | 0.602059 |
| GO:0032270\_positive\_regulation\_of\_cellular\_protein\_metabolic\_process | APC | 61 | 1 | 3.145492 | -0.561512 | 409 | 246.84 | 0.603521 |
| GO:0000904\_cell\_morphogenesis\_involved\_in\_differentiation | RELN | 199 | 2 | 1.928392 | -0.555738 | 410 | 247.48 | 0.603610 |
| GO:0000904\_cell\_morphogenesis\_involved\_in\_differentiation | APC | 199 | 2 | 1.928392 | -0.555738 | 410 | 247.48 | 0.603610 |
| GO:0030155\_regulation\_of\_cell\_adhesion | APC | 62 | 1 | 3.094758 | -0.555490 | 411 | 249.43 | 0.606886 |
| GO:0009165\_nucleotide\_biosynthetic\_process | RRM2 | 63 | 1 | 3.045635 | -0.549581 | 413 | 251.04 | 0.607845 |
| GO:0051216\_cartilage\_development | FGF9 | 63 | 1 | 3.045635 | -0.549581 | 413 | 251.04 | 0.607845 |
| GO:0030182\_neuron\_differentiation | RELN | 356 | 3 | 1.616924 | -0.549243 | 414 | 251.18 | 0.606715 |
| GO:0030182\_neuron\_differentiation | TIMP2 | 356 | 3 | 1.616924 | -0.549243 | 414 | 251.18 | 0.606715 |
| GO:0030182\_neuron\_differentiation | APC | 356 | 3 | 1.616924 | -0.549243 | 414 | 251.18 | 0.606715 |
| GO:0022607\_cellular\_component\_assembly | RRM2 | 204 | 2 | 1.881127 | -0.540662 | 415 | 253.5 | 0.610843 |
| GO:0022607\_cellular\_component\_assembly | APC | 204 | 2 | 1.881127 | -0.540662 | 415 | 253.5 | 0.610843 |
| GO:0042471\_ear\_morphogenesis | FGF9 | 65 | 1 | 2.951923 | -0.538085 | 416 | 254.71 | 0.612284 |
| GO:0009888\_tissue\_development | RDH10 | 525 | 4 | 1.461905 | -0.538018 | 417 | 254.87 | 0.611199 |
| GO:0009888\_tissue\_development | FGF9 | 525 | 4 | 1.461905 | -0.538018 | 417 | 254.87 | 0.611199 |
| GO:0009888\_tissue\_development | FZD1 | 525 | 4 | 1.461905 | -0.538018 | 417 | 254.87 | 0.611199 |
| GO:0009888\_tissue\_development | APC | 525 | 4 | 1.461905 | -0.538018 | 417 | 254.87 | 0.611199 |
| GO:0007243\_protein\_kinase\_cascade | TIMP2 | 205 | 2 | 1.871951 | -0.537710 | 418 | 255.12 | 0.610335 |
| GO:0007243\_protein\_kinase\_cascade | APC | 205 | 2 | 1.871951 | -0.537710 | 418 | 255.12 | 0.610335 |
| GO:0045860\_positive\_regulation\_of\_protein\_kinase\_activity | RELN | 66 | 1 | 2.907197 | -0.532491 | 420 | 256.37 | 0.610405 |
| GO:0051130\_positive\_regulation\_of\_cellular\_component\_organization | APC | 66 | 1 | 2.907197 | -0.532491 | 420 | 256.37 | 0.610405 |
| GO:0051247\_positive\_regulation\_of\_protein\_metabolic\_process | APC | 67 | 1 | 2.863806 | -0.526997 | 421 | 258.61 | 0.614276 |
| GO:0035295\_tube\_development | RDH10 | 212 | 2 | 1.810142 | -0.517598 | 422 | 260.77 | 0.617938 |
| GO:0035295\_tube\_development | FGF9 | 212 | 2 | 1.810142 | -0.517598 | 422 | 260.77 | 0.617938 |
| GO:0051179\_localization | SLC2A3 | 1058 | 7 | 1.269494 | -0.516831 | 423 | 260.89 | 0.616761 |
| GO:0051179\_localization | FGF9 | 1058 | 7 | 1.269494 | -0.516831 | 423 | 260.89 | 0.616761 |
| GO:0051179\_localization | STX4A | 1058 | 7 | 1.269494 | -0.516831 | 423 | 260.89 | 0.616761 |
| GO:0051179\_localization | LIN7C | 1058 | 7 | 1.269494 | -0.516831 | 423 | 260.89 | 0.616761 |
| GO:0051179\_localization | RELN | 1058 | 7 | 1.269494 | -0.516831 | 423 | 260.89 | 0.616761 |
| GO:0051179\_localization | TACC3 | 1058 | 7 | 1.269494 | -0.516831 | 423 | 260.89 | 0.616761 |
| GO:0051179\_localization | APC | 1058 | 7 | 1.269494 | -0.516831 | 423 | 260.89 | 0.616761 |
| GO:0016331\_morphogenesis\_of\_embryonic\_epithelium | RDH10 | 71 | 1 | 2.702465 | -0.505951 | 425 | 265.82 | 0.625459 |
| GO:0033674\_positive\_regulation\_of\_kinase\_activity | RELN | 71 | 1 | 2.702465 | -0.505951 | 425 | 265.82 | 0.625459 |
| GO:0007264\_small\_GTPase\_mediated\_signal\_transduction | RELN | 72 | 1 | 2.664931 | -0.500909 | 429 | 269.6 | 0.628438 |
| GO:0040012\_regulation\_of\_locomotion | APC | 72 | 1 | 2.664931 | -0.500909 | 429 | 269.6 | 0.628438 |
| GO:0048839\_inner\_ear\_development | FGF9 | 72 | 1 | 2.664931 | -0.500909 | 429 | 269.6 | 0.628438 |
| GO:0051347\_positive\_regulation\_of\_transferase\_activity | RELN | 72 | 1 | 2.664931 | -0.500909 | 429 | 269.6 | 0.628438 |
| GO:0006810\_transport | FGF9 | 718 | 5 | 1.336177 | -0.500642 | 430 | 269.76 | 0.627349 |
| GO:0006810\_transport | SLC2A3 | 718 | 5 | 1.336177 | -0.500642 | 430 | 269.76 | 0.627349 |
| GO:0006810\_transport | STX4A | 718 | 5 | 1.336177 | -0.500642 | 430 | 269.76 | 0.627349 |
| GO:0006810\_transport | LIN7C | 718 | 5 | 1.336177 | -0.500642 | 430 | 269.76 | 0.627349 |
| GO:0006810\_transport | TACC3 | 718 | 5 | 1.336177 | -0.500642 | 430 | 269.76 | 0.627349 |
| GO:0006163\_purine\_nucleotide\_metabolic\_process | TIMP2 | 73 | 1 | 2.628425 | -0.495950 | 433 | 271.68 | 0.627436 |
| GO:0006936\_muscle\_contraction | ATP1A1 | 73 | 1 | 2.628425 | -0.495950 | 433 | 271.68 | 0.627436 |
| GO:0051270\_regulation\_of\_cell\_motion | APC | 73 | 1 | 2.628425 | -0.495950 | 433 | 271.68 | 0.627436 |
| GO:0048771\_tissue\_remodeling | APC | 74 | 1 | 2.592905 | -0.491072 | 434 | 272.87 | 0.628733 |
| GO:0048589\_developmental\_growth | RDH10 | 75 | 1 | 2.558333 | -0.486273 | 435 | 274.27 | 0.630506 |
| GO:0051234\_establishment\_of\_localization | FGF9 | 729 | 5 | 1.316015 | -0.484375 | 436 | 274.7 | 0.630046 |
| GO:0051234\_establishment\_of\_localization | SLC2A3 | 729 | 5 | 1.316015 | -0.484375 | 436 | 274.7 | 0.630046 |
| GO:0051234\_establishment\_of\_localization | STX4A | 729 | 5 | 1.316015 | -0.484375 | 436 | 274.7 | 0.630046 |
| GO:0051234\_establishment\_of\_localization | LIN7C | 729 | 5 | 1.316015 | -0.484375 | 436 | 274.7 | 0.630046 |
| GO:0051234\_establishment\_of\_localization | TACC3 | 729 | 5 | 1.316015 | -0.484375 | 436 | 274.7 | 0.630046 |
| GO:0003012\_muscle\_system\_process | ATP1A1 | 76 | 1 | 2.524671 | -0.481550 | 438 | 276.23 | 0.630662 |
| GO:0034621\_cellular\_macromolecular\_complex\_subunit\_organization | APC | 76 | 1 | 2.524671 | -0.481550 | 438 | 276.23 | 0.630662 |
| GO:0044267\_cellular\_protein\_metabolic\_process | CSNK2A1 | 559 | 4 | 1.372987 | -0.479365 | 439 | 276.4 | 0.629613 |
| GO:0044267\_cellular\_protein\_metabolic\_process | MRPL19 | 559 | 4 | 1.372987 | -0.479365 | 439 | 276.4 | 0.629613 |
| GO:0044267\_cellular\_protein\_metabolic\_process | RELN | 559 | 4 | 1.372987 | -0.479365 | 439 | 276.4 | 0.629613 |
| GO:0044267\_cellular\_protein\_metabolic\_process | APC | 559 | 4 | 1.372987 | -0.479365 | 439 | 276.4 | 0.629613 |
| GO:0051241\_negative\_regulation\_of\_multicellular\_organismal\_process | ATP1A1 | 77 | 1 | 2.491883 | -0.476901 | 440 | 277.48 | 0.630636 |
| GO:0046649\_lymphocyte\_activation | EXO1 | 228 | 2 | 1.683114 | -0.475019 | 441 | 278.23 | 0.630907 |
| GO:0046649\_lymphocyte\_activation | APC | 228 | 2 | 1.683114 | -0.475019 | 441 | 278.23 | 0.630907 |
| GO:0042127\_regulation\_of\_cell\_proliferation | FGF9 | 393 | 3 | 1.464695 | -0.472678 | 442 | 278.42 | 0.629910 |
| GO:0042127\_regulation\_of\_cell\_proliferation | TIMP2 | 393 | 3 | 1.464695 | -0.472678 | 442 | 278.42 | 0.629910 |
| GO:0042127\_regulation\_of\_cell\_proliferation | APC | 393 | 3 | 1.464695 | -0.472678 | 442 | 278.42 | 0.629910 |
| GO:0007154\_cell\_communication | FGF9 | 1096 | 7 | 1.225479 | -0.469057 | 443 | 281.12 | 0.634582 |
| GO:0007154\_cell\_communication | STX4A | 1096 | 7 | 1.225479 | -0.469057 | 443 | 281.12 | 0.634582 |
| GO:0007154\_cell\_communication | FZD1 | 1096 | 7 | 1.225479 | -0.469057 | 443 | 281.12 | 0.634582 |
| GO:0007154\_cell\_communication | LIN7C | 1096 | 7 | 1.225479 | -0.469057 | 443 | 281.12 | 0.634582 |
| GO:0007154\_cell\_communication | RELN | 1096 | 7 | 1.225479 | -0.469057 | 443 | 281.12 | 0.634582 |
| GO:0007154\_cell\_communication | TIMP2 | 1096 | 7 | 1.225479 | -0.469057 | 443 | 281.12 | 0.634582 |
| GO:0007154\_cell\_communication | APC | 1096 | 7 | 1.225479 | -0.469057 | 443 | 281.12 | 0.634582 |
| GO:0007420\_brain\_development | RELN | 231 | 2 | 1.661255 | -0.467519 | 444 | 282.03 | 0.635203 |
| GO:0007420\_brain\_development | TACC3 | 231 | 2 | 1.661255 | -0.467519 | 444 | 282.03 | 0.635203 |
| GO:0048699\_generation\_of\_neurons | RELN | 396 | 3 | 1.453598 | -0.466992 | 445 | 282.18 | 0.634112 |
| GO:0048699\_generation\_of\_neurons | TIMP2 | 396 | 3 | 1.453598 | -0.466992 | 445 | 282.18 | 0.634112 |
| GO:0048699\_generation\_of\_neurons | APC | 396 | 3 | 1.453598 | -0.466992 | 445 | 282.18 | 0.634112 |
| GO:0000278\_mitotic\_cell\_cycle | APC | 80 | 1 | 2.398438 | -0.463383 | 449 | 283.71 | 0.631871 |
| GO:0002250\_adaptive\_immune\_response | EXO1 | 80 | 1 | 2.398438 | -0.463383 | 449 | 283.71 | 0.631871 |
| GO:0002460\_adaptive\_immune\_response\_based\_on\_somatic\_recombination\_of\_immune\_receptors\_built\_from\_immunoglobulin\_superfamily\_domains | EXO1 | 80 | 1 | 2.398438 | -0.463383 | 449 | 283.71 | 0.631871 |
| GO:0006631\_fatty\_acid\_metabolic\_process | TYRP1 | 80 | 1 | 2.398438 | -0.463383 | 449 | 283.71 | 0.631871 |
| GO:0016477\_cell\_migration | RELN | 234 | 2 | 1.639957 | -0.460162 | 450 | 284.69 | 0.632644 |
| GO:0016477\_cell\_migration | APC | 234 | 2 | 1.639957 | -0.460162 | 450 | 284.69 | 0.632644 |
| GO:0007411\_axon\_guidance | RELN | 82 | 1 | 2.339939 | -0.454709 | 453 | 286.81 | 0.633135 |
| GO:0008202\_steroid\_metabolic\_process | ATP1A1 | 82 | 1 | 2.339939 | -0.454709 | 453 | 286.81 | 0.633135 |
| GO:0045664\_regulation\_of\_neuron\_differentiation | TIMP2 | 82 | 1 | 2.339939 | -0.454709 | 453 | 286.81 | 0.633135 |
| GO:0006468\_protein\_amino\_acid\_phosphorylation | CSNK2A1 | 237 | 2 | 1.619198 | -0.452944 | 454 | 287.42 | 0.633084 |
| GO:0006468\_protein\_amino\_acid\_phosphorylation | RELN | 237 | 2 | 1.619198 | -0.452944 | 454 | 287.42 | 0.633084 |
| GO:0007049\_cell\_cycle | TACC3 | 238 | 2 | 1.612395 | -0.450568 | 455 | 287.68 | 0.632264 |
| GO:0007049\_cell\_cycle | APC | 238 | 2 | 1.612395 | -0.450568 | 455 | 287.68 | 0.632264 |
| GO:0006575\_cellular\_amino\_acid\_derivative\_metabolic\_process | RELN | 83 | 1 | 2.311747 | -0.450468 | 456 | 289.55 | 0.634978 |
| GO:0002449\_lymphocyte\_mediated\_immunity | EXO1 | 85 | 1 | 2.257353 | -0.442173 | 457 | 291.81 | 0.638534 |
| GO:0007242\_intracellular\_signaling\_cascade | RELN | 411 | 3 | 1.400547 | -0.439631 | 458 | 291.99 | 0.637533 |
| GO:0007242\_intracellular\_signaling\_cascade | TIMP2 | 411 | 3 | 1.400547 | -0.439631 | 458 | 291.99 | 0.637533 |
| GO:0007242\_intracellular\_signaling\_cascade | APC | 411 | 3 | 1.400547 | -0.439631 | 458 | 291.99 | 0.637533 |
| GO:0051239\_regulation\_of\_multicellular\_organismal\_process | FGF9 | 587 | 4 | 1.307496 | -0.435807 | 459 | 294.23 | 0.641024 |
| GO:0051239\_regulation\_of\_multicellular\_organismal\_process | ATP1A1 | 587 | 4 | 1.307496 | -0.435807 | 459 | 294.23 | 0.641024 |
| GO:0051239\_regulation\_of\_multicellular\_organismal\_process | TIMP2 | 587 | 4 | 1.307496 | -0.435807 | 459 | 294.23 | 0.641024 |
| GO:0051239\_regulation\_of\_multicellular\_organismal\_process | APC | 587 | 4 | 1.307496 | -0.435807 | 459 | 294.23 | 0.641024 |
| GO:0048754\_branching\_morphogenesis\_of\_a\_tube | RDH10 | 88 | 1 | 2.180398 | -0.430171 | 460 | 297.35 | 0.646413 |
| GO:0032879\_regulation\_of\_localization | TACC3 | 248 | 2 | 1.547379 | -0.427609 | 462 | 297.83 | 0.644654 |
| GO:0032879\_regulation\_of\_localization | APC | 248 | 2 | 1.547379 | -0.427609 | 462 | 297.83 | 0.644654 |
| GO:0045321\_leukocyte\_activation | EXO1 | 248 | 2 | 1.547379 | -0.427609 | 462 | 297.83 | 0.644654 |
| GO:0045321\_leukocyte\_activation | APC | 248 | 2 | 1.547379 | -0.427609 | 462 | 297.83 | 0.644654 |
| GO:0007389\_pattern\_specification\_process | RELN | 250 | 2 | 1.535000 | -0.423185 | 463 | 299.02 | 0.645832 |
| GO:0007389\_pattern\_specification\_process | APC | 250 | 2 | 1.535000 | -0.423185 | 463 | 299.02 | 0.645832 |
| GO:0042113\_B\_cell\_activation | EXO1 | 90 | 1 | 2.131944 | -0.422449 | 464 | 300.44 | 0.647500 |
| GO:0002443\_leukocyte\_mediated\_immunity | EXO1 | 91 | 1 | 2.108516 | -0.418667 | 466 | 302.13 | 0.648348 |
| GO:0008544\_epidermis\_development | APC | 91 | 1 | 2.108516 | -0.418667 | 466 | 302.13 | 0.648348 |
| GO:0016481\_negative\_regulation\_of\_transcription | FZD1 | 253 | 2 | 1.516798 | -0.416650 | 468 | 303.03 | 0.647500 |
| GO:0016481\_negative\_regulation\_of\_transcription | TLE1 | 253 | 2 | 1.516798 | -0.416650 | 468 | 303.03 | 0.647500 |
| GO:0030097\_hemopoiesis | TACC3 | 253 | 2 | 1.516798 | -0.416650 | 468 | 303.03 | 0.647500 |
| GO:0030097\_hemopoiesis | APC | 253 | 2 | 1.516798 | -0.416650 | 468 | 303.03 | 0.647500 |
| GO:0030217\_T\_cell\_differentiation | APC | 92 | 1 | 2.085598 | -0.414938 | 469 | 303.89 | 0.647953 |
| GO:0048870\_cell\_motility | RELN | 257 | 2 | 1.493191 | -0.408119 | 470 | 306.57 | 0.652277 |
| GO:0048870\_cell\_motility | APC | 257 | 2 | 1.493191 | -0.408119 | 470 | 306.57 | 0.652277 |
| GO:0044249\_cellular\_biosynthetic\_process | TYRP1 | 1150 | 7 | 1.167935 | -0.407538 | 471 | 308.66 | 0.655329 |
| GO:0044249\_cellular\_biosynthetic\_process | B3GNT5 | 1150 | 7 | 1.167935 | -0.407538 | 471 | 308.66 | 0.655329 |
| GO:0044249\_cellular\_biosynthetic\_process | RRM2 | 1150 | 7 | 1.167935 | -0.407538 | 471 | 308.66 | 0.655329 |
| GO:0044249\_cellular\_biosynthetic\_process | MRPL19 | 1150 | 7 | 1.167935 | -0.407538 | 471 | 308.66 | 0.655329 |
| GO:0044249\_cellular\_biosynthetic\_process | FZD1 | 1150 | 7 | 1.167935 | -0.407538 | 471 | 308.66 | 0.655329 |
| GO:0044249\_cellular\_biosynthetic\_process | TLE1 | 1150 | 7 | 1.167935 | -0.407538 | 471 | 308.66 | 0.655329 |
| GO:0044249\_cellular\_biosynthetic\_process | ATP1A1 | 1150 | 7 | 1.167935 | -0.407538 | 471 | 308.66 | 0.655329 |
| GO:0051707\_response\_to\_other\_organism | XPR1 | 95 | 1 | 2.019737 | -0.404047 | 472 | 309.3 | 0.655297 |
| GO:0060249\_anatomical\_structure\_homeostasis | APC | 96 | 1 | 1.998698 | -0.400513 | 473 | 311.28 | 0.658097 |
| GO:0001775\_cell\_activation | EXO1 | 262 | 2 | 1.464695 | -0.397741 | 476 | 312.33 | 0.656155 |
| GO:0001775\_cell\_activation | APC | 262 | 2 | 1.464695 | -0.397741 | 476 | 312.33 | 0.656155 |
| GO:0010629\_negative\_regulation\_of\_gene\_expression | FZD1 | 262 | 2 | 1.464695 | -0.397741 | 476 | 312.33 | 0.656155 |
| GO:0010629\_negative\_regulation\_of\_gene\_expression | TLE1 | 262 | 2 | 1.464695 | -0.397741 | 476 | 312.33 | 0.656155 |
| GO:0048666\_neuron\_development | RELN | 262 | 2 | 1.464695 | -0.397741 | 476 | 312.33 | 0.656155 |
| GO:0048666\_neuron\_development | APC | 262 | 2 | 1.464695 | -0.397741 | 476 | 312.33 | 0.656155 |
| GO:0018193\_peptidyl-amino\_acid\_modification | RELN | 97 | 1 | 1.978093 | -0.397026 | 477 | 313.02 | 0.656226 |
| GO:0030030\_cell\_projection\_organization | RELN | 263 | 2 | 1.459125 | -0.395703 | 478 | 313.39 | 0.655628 |
| GO:0030030\_cell\_projection\_organization | APC | 263 | 2 | 1.459125 | -0.395703 | 478 | 313.39 | 0.655628 |
| GO:0007398\_ectoderm\_development | APC | 99 | 1 | 1.938131 | -0.390185 | 480 | 316.16 | 0.658667 |
| GO:0060562\_epithelial\_tube\_morphogenesis | RDH10 | 99 | 1 | 1.938131 | -0.390185 | 480 | 316.16 | 0.658667 |
| GO:0007399\_nervous\_system\_development | RELN | 621 | 4 | 1.235910 | -0.388026 | 481 | 316.61 | 0.658233 |
| GO:0007399\_nervous\_system\_development | TIMP2 | 621 | 4 | 1.235910 | -0.388026 | 481 | 316.61 | 0.658233 |
| GO:0007399\_nervous\_system\_development | TACC3 | 621 | 4 | 1.235910 | -0.388026 | 481 | 316.61 | 0.658233 |
| GO:0007399\_nervous\_system\_development | APC | 621 | 4 | 1.235910 | -0.388026 | 481 | 316.61 | 0.658233 |
| GO:0001525\_angiogenesis | FGF9 | 100 | 1 | 1.918750 | -0.386831 | 482 | 316.92 | 0.657510 |
| GO:0030163\_protein\_catabolic\_process | APC | 101 | 1 | 1.899752 | -0.383519 | 483 | 318.11 | 0.658613 |
| GO:0045934\_negative\_regulation\_of\_nucleobase\_\_nucleoside\_\_nucleotide\_and\_nucleic\_acid\_metabolic\_process | FZD1 | 270 | 2 | 1.421296 | -0.381765 | 484 | 318.51 | 0.658079 |
| GO:0045934\_negative\_regulation\_of\_nucleobase\_\_nucleoside\_\_nucleotide\_and\_nucleic\_acid\_metabolic\_process | TLE1 | 270 | 2 | 1.421296 | -0.381765 | 484 | 318.51 | 0.658079 |
| GO:0009058\_biosynthetic\_process | TYRP1 | 1175 | 7 | 1.143085 | -0.381408 | 485 | 318.61 | 0.656928 |
| GO:0009058\_biosynthetic\_process | B3GNT5 | 1175 | 7 | 1.143085 | -0.381408 | 485 | 318.61 | 0.656928 |
| GO:0009058\_biosynthetic\_process | RRM2 | 1175 | 7 | 1.143085 | -0.381408 | 485 | 318.61 | 0.656928 |
| GO:0009058\_biosynthetic\_process | MRPL19 | 1175 | 7 | 1.143085 | -0.381408 | 485 | 318.61 | 0.656928 |
| GO:0009058\_biosynthetic\_process | FZD1 | 1175 | 7 | 1.143085 | -0.381408 | 485 | 318.61 | 0.656928 |
| GO:0009058\_biosynthetic\_process | TLE1 | 1175 | 7 | 1.143085 | -0.381408 | 485 | 318.61 | 0.656928 |
| GO:0009058\_biosynthetic\_process | ATP1A1 | 1175 | 7 | 1.143085 | -0.381408 | 485 | 318.61 | 0.656928 |
| GO:0051172\_negative\_regulation\_of\_nitrogen\_compound\_metabolic\_process | FZD1 | 271 | 2 | 1.416052 | -0.379821 | 486 | 319.35 | 0.657099 |
| GO:0051172\_negative\_regulation\_of\_nitrogen\_compound\_metabolic\_process | TLE1 | 271 | 2 | 1.416052 | -0.379821 | 486 | 319.35 | 0.657099 |
| GO:0006996\_organelle\_organization | TYRP1 | 449 | 3 | 1.282016 | -0.377531 | 487 | 319.62 | 0.656304 |
| GO:0006996\_organelle\_organization | TACC3 | 449 | 3 | 1.282016 | -0.377531 | 487 | 319.62 | 0.656304 |
| GO:0006996\_organelle\_organization | APC | 449 | 3 | 1.282016 | -0.377531 | 487 | 319.62 | 0.656304 |
| GO:0003013\_circulatory\_system\_process | ATP1A1 | 103 | 1 | 1.862864 | -0.377020 | 489 | 320.52 | 0.655460 |
| GO:0008015\_blood\_circulation | ATP1A1 | 103 | 1 | 1.862864 | -0.377020 | 489 | 320.52 | 0.655460 |
| GO:0010558\_negative\_regulation\_of\_macromolecule\_biosynthetic\_process | FZD1 | 274 | 2 | 1.400547 | -0.374055 | 490 | 321.93 | 0.657000 |
| GO:0010558\_negative\_regulation\_of\_macromolecule\_biosynthetic\_process | TLE1 | 274 | 2 | 1.400547 | -0.374055 | 490 | 321.93 | 0.657000 |
| GO:0050767\_regulation\_of\_neurogenesis | TIMP2 | 104 | 1 | 1.844952 | -0.373831 | 491 | 322.57 | 0.656965 |
| GO:0006139\_nucleobase\_\_nucleoside\_\_nucleotide\_and\_nucleic\_acid\_metabolic\_process | EXO1 | 1002 | 6 | 1.148952 | -0.369389 | 492 | 323.26 | 0.657033 |
| GO:0006139\_nucleobase\_\_nucleoside\_\_nucleotide\_and\_nucleic\_acid\_metabolic\_process | EBNA1BP2 | 1002 | 6 | 1.148952 | -0.369389 | 492 | 323.26 | 0.657033 |
| GO:0006139\_nucleobase\_\_nucleoside\_\_nucleotide\_and\_nucleic\_acid\_metabolic\_process | RRM2 | 1002 | 6 | 1.148952 | -0.369389 | 492 | 323.26 | 0.657033 |
| GO:0006139\_nucleobase\_\_nucleoside\_\_nucleotide\_and\_nucleic\_acid\_metabolic\_process | FZD1 | 1002 | 6 | 1.148952 | -0.369389 | 492 | 323.26 | 0.657033 |
| GO:0006139\_nucleobase\_\_nucleoside\_\_nucleotide\_and\_nucleic\_acid\_metabolic\_process | TLE1 | 1002 | 6 | 1.148952 | -0.369389 | 492 | 323.26 | 0.657033 |
| GO:0006139\_nucleobase\_\_nucleoside\_\_nucleotide\_and\_nucleic\_acid\_metabolic\_process | TIMP2 | 1002 | 6 | 1.148952 | -0.369389 | 492 | 323.26 | 0.657033 |
| GO:0048534\_hemopoietic\_or\_lymphoid\_organ\_development | TACC3 | 277 | 2 | 1.385379 | -0.368389 | 494 | 323.8 | 0.655466 |
| GO:0048534\_hemopoietic\_or\_lymphoid\_organ\_development | APC | 277 | 2 | 1.385379 | -0.368389 | 494 | 323.8 | 0.655466 |
| GO:0048646\_anatomical\_structure\_formation\_involved\_in\_morphogenesis | RDH10 | 277 | 2 | 1.385379 | -0.368389 | 494 | 323.8 | 0.655466 |
| GO:0048646\_anatomical\_structure\_formation\_involved\_in\_morphogenesis | FGF9 | 277 | 2 | 1.385379 | -0.368389 | 494 | 323.8 | 0.655466 |
| GO:0045859\_regulation\_of\_protein\_kinase\_activity | RELN | 107 | 1 | 1.793224 | -0.364498 | 495 | 324.91 | 0.656384 |
| GO:0030099\_myeloid\_cell\_differentiation | APC | 108 | 1 | 1.776620 | -0.361462 | 496 | 325.86 | 0.656976 |
| GO:0009887\_organ\_morphogenesis | RDH10 | 642 | 4 | 1.195483 | -0.361050 | 497 | 326.08 | 0.656097 |
| GO:0009887\_organ\_morphogenesis | FGF9 | 642 | 4 | 1.195483 | -0.361050 | 497 | 326.08 | 0.656097 |
| GO:0009887\_organ\_morphogenesis | FZD1 | 642 | 4 | 1.195483 | -0.361050 | 497 | 326.08 | 0.656097 |
| GO:0009887\_organ\_morphogenesis | APC | 642 | 4 | 1.195483 | -0.361050 | 497 | 326.08 | 0.656097 |
| GO:0000902\_cell\_morphogenesis | RELN | 283 | 2 | 1.356007 | -0.357348 | 498 | 327.26 | 0.657149 |
| GO:0000902\_cell\_morphogenesis | APC | 283 | 2 | 1.356007 | -0.357348 | 498 | 327.26 | 0.657149 |
| GO:0031323\_regulation\_of\_cellular\_metabolic\_process | FZD1 | 1015 | 6 | 1.134236 | -0.355920 | 499 | 327.36 | 0.656032 |
| GO:0031323\_regulation\_of\_cellular\_metabolic\_process | RELN | 1015 | 6 | 1.134236 | -0.355920 | 499 | 327.36 | 0.656032 |
| GO:0031323\_regulation\_of\_cellular\_metabolic\_process | ATP1A1 | 1015 | 6 | 1.134236 | -0.355920 | 499 | 327.36 | 0.656032 |
| GO:0031323\_regulation\_of\_cellular\_metabolic\_process | TLE1 | 1015 | 6 | 1.134236 | -0.355920 | 499 | 327.36 | 0.656032 |
| GO:0031323\_regulation\_of\_cellular\_metabolic\_process | TIMP2 | 1015 | 6 | 1.134236 | -0.355920 | 499 | 327.36 | 0.656032 |
| GO:0031323\_regulation\_of\_cellular\_metabolic\_process | APC | 1015 | 6 | 1.134236 | -0.355920 | 499 | 327.36 | 0.656032 |
| GO:0048705\_skeletal\_system\_morphogenesis | RDH10 | 111 | 1 | 1.728604 | -0.352570 | 500 | 329.42 | 0.658840 |
| GO:0007417\_central\_nervous\_system\_development | RELN | 287 | 2 | 1.337108 | -0.350195 | 501 | 329.76 | 0.658204 |
| GO:0007417\_central\_nervous\_system\_development | TACC3 | 287 | 2 | 1.337108 | -0.350195 | 501 | 329.76 | 0.658204 |
| GO:0043549\_regulation\_of\_kinase\_activity | RELN | 112 | 1 | 1.713170 | -0.349676 | 502 | 330.42 | 0.658207 |
| GO:0034960\_cellular\_biopolymer\_metabolic\_process | EXO1 | 1395 | 8 | 1.100358 | -0.348864 | 503 | 330.55 | 0.657157 |
| GO:0034960\_cellular\_biopolymer\_metabolic\_process | EBNA1BP2 | 1395 | 8 | 1.100358 | -0.348864 | 503 | 330.55 | 0.657157 |
| GO:0034960\_cellular\_biopolymer\_metabolic\_process | CSNK2A1 | 1395 | 8 | 1.100358 | -0.348864 | 503 | 330.55 | 0.657157 |
| GO:0034960\_cellular\_biopolymer\_metabolic\_process | MRPL19 | 1395 | 8 | 1.100358 | -0.348864 | 503 | 330.55 | 0.657157 |
| GO:0034960\_cellular\_biopolymer\_metabolic\_process | FZD1 | 1395 | 8 | 1.100358 | -0.348864 | 503 | 330.55 | 0.657157 |
| GO:0034960\_cellular\_biopolymer\_metabolic\_process | RELN | 1395 | 8 | 1.100358 | -0.348864 | 503 | 330.55 | 0.657157 |
| GO:0034960\_cellular\_biopolymer\_metabolic\_process | TLE1 | 1395 | 8 | 1.100358 | -0.348864 | 503 | 330.55 | 0.657157 |
| GO:0034960\_cellular\_biopolymer\_metabolic\_process | APC | 1395 | 8 | 1.100358 | -0.348864 | 503 | 330.55 | 0.657157 |
| GO:0009987\_cellular\_process | EXO1 | 3868 | 21 | 1.041721 | -0.347016 | 504 | 330.74 | 0.656230 |
| GO:0009987\_cellular\_process | TYRP1 | 3868 | 21 | 1.041721 | -0.347016 | 504 | 330.74 | 0.656230 |
| GO:0009987\_cellular\_process | FGF9 | 3868 | 21 | 1.041721 | -0.347016 | 504 | 330.74 | 0.656230 |
| GO:0009987\_cellular\_process | STX4A | 3868 | 21 | 1.041721 | -0.347016 | 504 | 330.74 | 0.656230 |
| GO:0009987\_cellular\_process | FZD1 | 3868 | 21 | 1.041721 | -0.347016 | 504 | 330.74 | 0.656230 |
| GO:0009987\_cellular\_process | LIN7C | 3868 | 21 | 1.041721 | -0.347016 | 504 | 330.74 | 0.656230 |
| GO:0009987\_cellular\_process | ATP1A1 | 3868 | 21 | 1.041721 | -0.347016 | 504 | 330.74 | 0.656230 |
| GO:0009987\_cellular\_process | TLE1 | 3868 | 21 | 1.041721 | -0.347016 | 504 | 330.74 | 0.656230 |
| GO:0009987\_cellular\_process | TACC3 | 3868 | 21 | 1.041721 | -0.347016 | 504 | 330.74 | 0.656230 |
| GO:0009987\_cellular\_process | TIMP2 | 3868 | 21 | 1.041721 | -0.347016 | 504 | 330.74 | 0.656230 |
| GO:0009987\_cellular\_process | BICD2 | 3868 | 21 | 1.041721 | -0.347016 | 504 | 330.74 | 0.656230 |
| GO:0009987\_cellular\_process | HDGFRP3 | 3868 | 21 | 1.041721 | -0.347016 | 504 | 330.74 | 0.656230 |
| GO:0009987\_cellular\_process | EBNA1BP2 | 3868 | 21 | 1.041721 | -0.347016 | 504 | 330.74 | 0.656230 |
| GO:0009987\_cellular\_process | RDH10 | 3868 | 21 | 1.041721 | -0.347016 | 504 | 330.74 | 0.656230 |
| GO:0009987\_cellular\_process | CSNK2A1 | 3868 | 21 | 1.041721 | -0.347016 | 504 | 330.74 | 0.656230 |
| GO:0009987\_cellular\_process | B3GNT5 | 3868 | 21 | 1.041721 | -0.347016 | 504 | 330.74 | 0.656230 |
| GO:0009987\_cellular\_process | RRM2 | 3868 | 21 | 1.041721 | -0.347016 | 504 | 330.74 | 0.656230 |
| GO:0009987\_cellular\_process | MRPL19 | 3868 | 21 | 1.041721 | -0.347016 | 504 | 330.74 | 0.656230 |
| GO:0009987\_cellular\_process | RELN | 3868 | 21 | 1.041721 | -0.347016 | 504 | 330.74 | 0.656230 |
| GO:0009987\_cellular\_process | BMF | 3868 | 21 | 1.041721 | -0.347016 | 504 | 330.74 | 0.656230 |
| GO:0009987\_cellular\_process | APC | 3868 | 21 | 1.041721 | -0.347016 | 504 | 330.74 | 0.656230 |
| GO:0006974\_response\_to\_DNA\_damage\_stimulus | RAD23B | 113 | 1 | 1.698009 | -0.346816 | 505 | 331.41 | 0.656257 |
| GO:0019538\_protein\_metabolic\_process | CSNK2A1 | 655 | 4 | 1.171756 | -0.345246 | 506 | 331.74 | 0.655613 |
| GO:0019538\_protein\_metabolic\_process | MRPL19 | 655 | 4 | 1.171756 | -0.345246 | 506 | 331.74 | 0.655613 |
| GO:0019538\_protein\_metabolic\_process | RELN | 655 | 4 | 1.171756 | -0.345246 | 506 | 331.74 | 0.655613 |
| GO:0019538\_protein\_metabolic\_process | APC | 655 | 4 | 1.171756 | -0.345246 | 506 | 331.74 | 0.655613 |
| GO:0009607\_response\_to\_biotic\_stimulus | XPR1 | 114 | 1 | 1.683114 | -0.343988 | 507 | 332.3 | 0.655424 |
| GO:0051338\_regulation\_of\_transferase\_activity | RELN | 115 | 1 | 1.668478 | -0.341194 | 508 | 333.11 | 0.655728 |
| GO:0046483\_heterocycle\_metabolic\_process | TIMP2 | 116 | 1 | 1.654095 | -0.338432 | 509 | 334.19 | 0.656562 |
| GO:0040011\_locomotion | RELN | 295 | 2 | 1.300847 | -0.336368 | 510 | 335.02 | 0.656902 |
| GO:0040011\_locomotion | APC | 295 | 2 | 1.300847 | -0.336368 | 510 | 335.02 | 0.656902 |
| GO:0051960\_regulation\_of\_nervous\_system\_development | TIMP2 | 118 | 1 | 1.626059 | -0.333001 | 511 | 336.04 | 0.657613 |
| GO:0048598\_embryonic\_morphogenesis | RDH10 | 299 | 2 | 1.283445 | -0.329684 | 512 | 336.81 | 0.657832 |
| GO:0048598\_embryonic\_morphogenesis | FGF9 | 299 | 2 | 1.283445 | -0.329684 | 512 | 336.81 | 0.657832 |
| GO:0002252\_immune\_effector\_process | EXO1 | 122 | 1 | 1.572746 | -0.322499 | 514 | 341.15 | 0.663716 |
| GO:0060284\_regulation\_of\_cell\_development | TIMP2 | 122 | 1 | 1.572746 | -0.322499 | 514 | 341.15 | 0.663716 |
| GO:0030098\_lymphocyte\_differentiation | APC | 124 | 1 | 1.547379 | -0.317421 | 515 | 342.65 | 0.665340 |
| GO:0032989\_cellular\_component\_morphogenesis | RELN | 307 | 2 | 1.250000 | -0.316755 | 516 | 342.87 | 0.664477 |
| GO:0032989\_cellular\_component\_morphogenesis | APC | 307 | 2 | 1.250000 | -0.316755 | 516 | 342.87 | 0.664477 |
| GO:0051094\_positive\_regulation\_of\_developmental\_process | FGF9 | 308 | 2 | 1.245942 | -0.315179 | 517 | 343.12 | 0.663675 |
| GO:0051094\_positive\_regulation\_of\_developmental\_process | APC | 308 | 2 | 1.245942 | -0.315179 | 517 | 343.12 | 0.663675 |
| GO:0001763\_morphogenesis\_of\_a\_branching\_structure | RDH10 | 125 | 1 | 1.535000 | -0.314923 | 518 | 343.92 | 0.663938 |
| GO:0016310\_phosphorylation | CSNK2A1 | 309 | 2 | 1.241909 | -0.313611 | 519 | 344.47 | 0.663719 |
| GO:0016310\_phosphorylation | RELN | 309 | 2 | 1.241909 | -0.313611 | 519 | 344.47 | 0.663719 |
| GO:0045597\_positive\_regulation\_of\_cell\_differentiation | APC | 128 | 1 | 1.499023 | -0.307590 | 520 | 345.78 | 0.664962 |
| GO:0043285\_biopolymer\_catabolic\_process | APC | 129 | 1 | 1.487403 | -0.305197 | 522 | 346.62 | 0.664023 |
| GO:0051276\_chromosome\_organization | APC | 129 | 1 | 1.487403 | -0.305197 | 522 | 346.62 | 0.664023 |
| GO:0044260\_cellular\_macromolecule\_metabolic\_process | EXO1 | 1447 | 8 | 1.060815 | -0.304309 | 523 | 346.76 | 0.663021 |
| GO:0044260\_cellular\_macromolecule\_metabolic\_process | EBNA1BP2 | 1447 | 8 | 1.060815 | -0.304309 | 523 | 346.76 | 0.663021 |
| GO:0044260\_cellular\_macromolecule\_metabolic\_process | CSNK2A1 | 1447 | 8 | 1.060815 | -0.304309 | 523 | 346.76 | 0.663021 |
| GO:0044260\_cellular\_macromolecule\_metabolic\_process | MRPL19 | 1447 | 8 | 1.060815 | -0.304309 | 523 | 346.76 | 0.663021 |
| GO:0044260\_cellular\_macromolecule\_metabolic\_process | FZD1 | 1447 | 8 | 1.060815 | -0.304309 | 523 | 346.76 | 0.663021 |
| GO:0044260\_cellular\_macromolecule\_metabolic\_process | RELN | 1447 | 8 | 1.060815 | -0.304309 | 523 | 346.76 | 0.663021 |
| GO:0044260\_cellular\_macromolecule\_metabolic\_process | TLE1 | 1447 | 8 | 1.060815 | -0.304309 | 523 | 346.76 | 0.663021 |
| GO:0044260\_cellular\_macromolecule\_metabolic\_process | APC | 1447 | 8 | 1.060815 | -0.304309 | 523 | 346.76 | 0.663021 |
| GO:0002376\_immune\_system\_process | EXO1 | 505 | 3 | 1.139851 | -0.301850 | 524 | 348.28 | 0.664656 |
| GO:0002376\_immune\_system\_process | TACC3 | 505 | 3 | 1.139851 | -0.301850 | 524 | 348.28 | 0.664656 |
| GO:0002376\_immune\_system\_process | APC | 505 | 3 | 1.139851 | -0.301850 | 524 | 348.28 | 0.664656 |
| GO:0009952\_anterior\_posterior\_pattern\_formation | APC | 133 | 1 | 1.442669 | -0.295877 | 526 | 349.13 | 0.663745 |
| GO:0044057\_regulation\_of\_system\_process | ATP1A1 | 133 | 1 | 1.442669 | -0.295877 | 526 | 349.13 | 0.663745 |
| GO:0007283\_spermatogenesis | RAD23B | 134 | 1 | 1.431903 | -0.293607 | 528 | 350.03 | 0.662936 |
| GO:0048232\_male\_gamete\_generation | RAD23B | 134 | 1 | 1.431903 | -0.293607 | 528 | 350.03 | 0.662936 |
| GO:0050793\_regulation\_of\_developmental\_process | FGF9 | 703 | 4 | 1.091750 | -0.292305 | 529 | 350.16 | 0.661928 |
| GO:0050793\_regulation\_of\_developmental\_process | TIMP2 | 703 | 4 | 1.091750 | -0.292305 | 529 | 350.16 | 0.661928 |
| GO:0050793\_regulation\_of\_developmental\_process | BMF | 703 | 4 | 1.091750 | -0.292305 | 529 | 350.16 | 0.661928 |
| GO:0050793\_regulation\_of\_developmental\_process | APC | 703 | 4 | 1.091750 | -0.292305 | 529 | 350.16 | 0.661928 |
| GO:0003008\_system\_process | STX4A | 516 | 3 | 1.115552 | -0.288864 | 530 | 350.97 | 0.662208 |
| GO:0003008\_system\_process | LIN7C | 516 | 3 | 1.115552 | -0.288864 | 530 | 350.97 | 0.662208 |
| GO:0003008\_system\_process | ATP1A1 | 516 | 3 | 1.115552 | -0.288864 | 530 | 350.97 | 0.662208 |
| GO:0019222\_regulation\_of\_metabolic\_process | FZD1 | 1088 | 6 | 1.058134 | -0.287759 | 531 | 351.08 | 0.661168 |
| GO:0019222\_regulation\_of\_metabolic\_process | RELN | 1088 | 6 | 1.058134 | -0.287759 | 531 | 351.08 | 0.661168 |
| GO:0019222\_regulation\_of\_metabolic\_process | ATP1A1 | 1088 | 6 | 1.058134 | -0.287759 | 531 | 351.08 | 0.661168 |
| GO:0019222\_regulation\_of\_metabolic\_process | TLE1 | 1088 | 6 | 1.058134 | -0.287759 | 531 | 351.08 | 0.661168 |
| GO:0019222\_regulation\_of\_metabolic\_process | TIMP2 | 1088 | 6 | 1.058134 | -0.287759 | 531 | 351.08 | 0.661168 |
| GO:0019222\_regulation\_of\_metabolic\_process | APC | 1088 | 6 | 1.058134 | -0.287759 | 531 | 351.08 | 0.661168 |
| GO:0009057\_macromolecule\_catabolic\_process | APC | 137 | 1 | 1.400547 | -0.286937 | 532 | 351.52 | 0.660752 |
| GO:0007169\_transmembrane\_receptor\_protein\_tyrosine\_kinase\_signaling\_pathway | FGF9 | 139 | 1 | 1.380396 | -0.282602 | 533 | 352.38 | 0.661126 |
| GO:0006928\_cell\_motion | RELN | 330 | 2 | 1.162879 | -0.282586 | 535 | 353.06 | 0.659925 |
| GO:0006928\_cell\_motion | APC | 330 | 2 | 1.162879 | -0.282586 | 535 | 353.06 | 0.659925 |
| GO:0051674\_localization\_of\_cell | RELN | 330 | 2 | 1.162879 | -0.282586 | 535 | 353.06 | 0.659925 |
| GO:0051674\_localization\_of\_cell | APC | 330 | 2 | 1.162879 | -0.282586 | 535 | 353.06 | 0.659925 |
| GO:0035239\_tube\_morphogenesis | RDH10 | 143 | 1 | 1.341783 | -0.274190 | 536 | 356.36 | 0.664851 |
| GO:0007165\_signal\_transduction | FGF9 | 915 | 5 | 1.048497 | -0.272436 | 537 | 356.49 | 0.663855 |
| GO:0007165\_signal\_transduction | FZD1 | 915 | 5 | 1.048497 | -0.272436 | 537 | 356.49 | 0.663855 |
| GO:0007165\_signal\_transduction | RELN | 915 | 5 | 1.048497 | -0.272436 | 537 | 356.49 | 0.663855 |
| GO:0007165\_signal\_transduction | TIMP2 | 915 | 5 | 1.048497 | -0.272436 | 537 | 356.49 | 0.663855 |
| GO:0007165\_signal\_transduction | APC | 915 | 5 | 1.048497 | -0.272436 | 537 | 356.49 | 0.663855 |
| GO:0045596\_negative\_regulation\_of\_cell\_differentiation | APC | 144 | 1 | 1.332465 | -0.272139 | 538 | 357.26 | 0.664052 |
| GO:0050896\_response\_to\_stimulus | EXO1 | 1107 | 6 | 1.039973 | -0.271946 | 539 | 357.44 | 0.663154 |
| GO:0050896\_response\_to\_stimulus | RAD23B | 1107 | 6 | 1.039973 | -0.271946 | 539 | 357.44 | 0.663154 |
| GO:0050896\_response\_to\_stimulus | XPR1 | 1107 | 6 | 1.039973 | -0.271946 | 539 | 357.44 | 0.663154 |
| GO:0050896\_response\_to\_stimulus | ATP1A1 | 1107 | 6 | 1.039973 | -0.271946 | 539 | 357.44 | 0.663154 |
| GO:0050896\_response\_to\_stimulus | RELN | 1107 | 6 | 1.039973 | -0.271946 | 539 | 357.44 | 0.663154 |
| GO:0050896\_response\_to\_stimulus | TACC3 | 1107 | 6 | 1.039973 | -0.271946 | 539 | 357.44 | 0.663154 |
| GO:0043283\_biopolymer\_metabolic\_process | EXO1 | 1490 | 8 | 1.030201 | -0.270961 | 540 | 358.1 | 0.663148 |
| GO:0043283\_biopolymer\_metabolic\_process | EBNA1BP2 | 1490 | 8 | 1.030201 | -0.270961 | 540 | 358.1 | 0.663148 |
| GO:0043283\_biopolymer\_metabolic\_process | CSNK2A1 | 1490 | 8 | 1.030201 | -0.270961 | 540 | 358.1 | 0.663148 |
| GO:0043283\_biopolymer\_metabolic\_process | FZD1 | 1490 | 8 | 1.030201 | -0.270961 | 540 | 358.1 | 0.663148 |
| GO:0043283\_biopolymer\_metabolic\_process | MRPL19 | 1490 | 8 | 1.030201 | -0.270961 | 540 | 358.1 | 0.663148 |
| GO:0043283\_biopolymer\_metabolic\_process | TLE1 | 1490 | 8 | 1.030201 | -0.270961 | 540 | 358.1 | 0.663148 |
| GO:0043283\_biopolymer\_metabolic\_process | RELN | 1490 | 8 | 1.030201 | -0.270961 | 540 | 358.1 | 0.663148 |
| GO:0043283\_biopolymer\_metabolic\_process | APC | 1490 | 8 | 1.030201 | -0.270961 | 540 | 358.1 | 0.663148 |
| GO:0006793\_phosphorus\_metabolic\_process | CSNK2A1 | 340 | 2 | 1.128676 | -0.268997 | 542 | 358.98 | 0.662325 |
| GO:0006793\_phosphorus\_metabolic\_process | RELN | 340 | 2 | 1.128676 | -0.268997 | 542 | 358.98 | 0.662325 |
| GO:0006796\_phosphate\_metabolic\_process | CSNK2A1 | 340 | 2 | 1.128676 | -0.268997 | 542 | 358.98 | 0.662325 |
| GO:0006796\_phosphate\_metabolic\_process | RELN | 340 | 2 | 1.128676 | -0.268997 | 542 | 358.98 | 0.662325 |
| GO:0022603\_regulation\_of\_anatomical\_structure\_morphogenesis | APC | 147 | 1 | 1.305272 | -0.266105 | 543 | 360.94 | 0.664715 |
| GO:0043085\_positive\_regulation\_of\_catalytic\_activity | RELN | 148 | 1 | 1.296453 | -0.264133 | 544 | 361.87 | 0.665202 |
| GO:0080090\_regulation\_of\_primary\_metabolic\_process | FZD1 | 926 | 5 | 1.036042 | -0.262995 | 545 | 362.05 | 0.664312 |
| GO:0080090\_regulation\_of\_primary\_metabolic\_process | TLE1 | 926 | 5 | 1.036042 | -0.262995 | 545 | 362.05 | 0.664312 |
| GO:0080090\_regulation\_of\_primary\_metabolic\_process | ATP1A1 | 926 | 5 | 1.036042 | -0.262995 | 545 | 362.05 | 0.664312 |
| GO:0080090\_regulation\_of\_primary\_metabolic\_process | TIMP2 | 926 | 5 | 1.036042 | -0.262995 | 545 | 362.05 | 0.664312 |
| GO:0080090\_regulation\_of\_primary\_metabolic\_process | APC | 926 | 5 | 1.036042 | -0.262995 | 545 | 362.05 | 0.664312 |
| GO:0032268\_regulation\_of\_cellular\_protein\_metabolic\_process | APC | 152 | 1 | 1.262336 | -0.256431 | 546 | 363.74 | 0.666190 |
| GO:0006950\_response\_to\_stress | RAD23B | 549 | 3 | 1.048497 | -0.253098 | 547 | 364.91 | 0.667112 |
| GO:0006950\_response\_to\_stress | RELN | 549 | 3 | 1.048497 | -0.253098 | 547 | 364.91 | 0.667112 |
| GO:0006950\_response\_to\_stress | TACC3 | 549 | 3 | 1.048497 | -0.253098 | 547 | 364.91 | 0.667112 |
| GO:0051704\_multi-organism\_process | XPR1 | 157 | 1 | 1.222134 | -0.247208 | 548 | 367.16 | 0.670000 |
| GO:0048514\_blood\_vessel\_morphogenesis | FGF9 | 158 | 1 | 1.214399 | -0.245415 | 549 | 368.21 | 0.670692 |
| GO:0042981\_regulation\_of\_apoptosis | BMF | 360 | 2 | 1.065972 | -0.243869 | 550 | 368.5 | 0.670000 |
| GO:0042981\_regulation\_of\_apoptosis | APC | 360 | 2 | 1.065972 | -0.243869 | 550 | 368.5 | 0.670000 |
| GO:0051128\_regulation\_of\_cellular\_component\_organization | APC | 160 | 1 | 1.199219 | -0.241877 | 551 | 369.26 | 0.670163 |
| GO:0002521\_leukocyte\_differentiation | APC | 161 | 1 | 1.191770 | -0.240133 | 552 | 370.04 | 0.670362 |
| GO:0010941\_regulation\_of\_cell\_death | BMF | 365 | 2 | 1.051370 | -0.237984 | 555 | 371.41 | 0.669207 |
| GO:0010941\_regulation\_of\_cell\_death | APC | 365 | 2 | 1.051370 | -0.237984 | 555 | 371.41 | 0.669207 |
| GO:0043009\_chordate\_embryonic\_development | RDH10 | 365 | 2 | 1.051370 | -0.237984 | 555 | 371.41 | 0.669207 |
| GO:0043009\_chordate\_embryonic\_development | FGF9 | 365 | 2 | 1.051370 | -0.237984 | 555 | 371.41 | 0.669207 |
| GO:0043067\_regulation\_of\_programmed\_cell\_death | BMF | 365 | 2 | 1.051370 | -0.237984 | 555 | 371.41 | 0.669207 |
| GO:0043067\_regulation\_of\_programmed\_cell\_death | APC | 365 | 2 | 1.051370 | -0.237984 | 555 | 371.41 | 0.669207 |
| GO:0009653\_anatomical\_structure\_morphogenesis | RDH10 | 958 | 5 | 1.001435 | -0.237126 | 556 | 371.6 | 0.668345 |
| GO:0009653\_anatomical\_structure\_morphogenesis | FGF9 | 958 | 5 | 1.001435 | -0.237126 | 556 | 371.6 | 0.668345 |
| GO:0009653\_anatomical\_structure\_morphogenesis | FZD1 | 958 | 5 | 1.001435 | -0.237126 | 556 | 371.6 | 0.668345 |
| GO:0009653\_anatomical\_structure\_morphogenesis | RELN | 958 | 5 | 1.001435 | -0.237126 | 556 | 371.6 | 0.668345 |
| GO:0009653\_anatomical\_structure\_morphogenesis | APC | 958 | 5 | 1.001435 | -0.237126 | 556 | 371.6 | 0.668345 |
| GO:0007626\_locomotory\_behavior | RELN | 163 | 1 | 1.177147 | -0.236692 | 558 | 372.33 | 0.667258 |
| GO:0042110\_T\_cell\_activation | APC | 163 | 1 | 1.177147 | -0.236692 | 558 | 372.33 | 0.667258 |
| GO:0042325\_regulation\_of\_phosphorylation | RELN | 164 | 1 | 1.169970 | -0.234995 | 559 | 372.88 | 0.667048 |
| GO:0009792\_embryonic\_development\_ending\_in\_birth\_or\_egg\_hatching | RDH10 | 368 | 2 | 1.042799 | -0.234525 | 560 | 373.22 | 0.666464 |
| GO:0009792\_embryonic\_development\_ending\_in\_birth\_or\_egg\_hatching | FGF9 | 368 | 2 | 1.042799 | -0.234525 | 560 | 373.22 | 0.666464 |
| GO:0006259\_DNA\_metabolic\_process | EXO1 | 165 | 1 | 1.162879 | -0.233314 | 563 | 374.42 | 0.665044 |
| GO:0019220\_regulation\_of\_phosphate\_metabolic\_process | RELN | 165 | 1 | 1.162879 | -0.233314 | 563 | 374.42 | 0.665044 |
| GO:0051174\_regulation\_of\_phosphorus\_metabolic\_process | RELN | 165 | 1 | 1.162879 | -0.233314 | 563 | 374.42 | 0.665044 |
| GO:0016043\_cellular\_component\_organization | TYRP1 | 964 | 5 | 0.995202 | -0.232529 | 564 | 374.58 | 0.664149 |
| GO:0016043\_cellular\_component\_organization | RRM2 | 964 | 5 | 0.995202 | -0.232529 | 564 | 374.58 | 0.664149 |
| GO:0016043\_cellular\_component\_organization | RELN | 964 | 5 | 0.995202 | -0.232529 | 564 | 374.58 | 0.664149 |
| GO:0016043\_cellular\_component\_organization | TACC3 | 964 | 5 | 0.995202 | -0.232529 | 564 | 374.58 | 0.664149 |
| GO:0016043\_cellular\_component\_organization | APC | 964 | 5 | 0.995202 | -0.232529 | 564 | 374.58 | 0.664149 |
| GO:0043065\_positive\_regulation\_of\_apoptosis | APC | 166 | 1 | 1.155873 | -0.231647 | 565 | 375.19 | 0.664053 |
| GO:0010942\_positive\_regulation\_of\_cell\_death | APC | 167 | 1 | 1.148952 | -0.229996 | 568 | 376.61 | 0.663046 |
| GO:0043068\_positive\_regulation\_of\_programmed\_cell\_death | APC | 167 | 1 | 1.148952 | -0.229996 | 568 | 376.61 | 0.663046 |
| GO:0051049\_regulation\_of\_transport | TACC3 | 167 | 1 | 1.148952 | -0.229996 | 568 | 376.61 | 0.663046 |
| GO:0051171\_regulation\_of\_nitrogen\_compound\_metabolic\_process | FZD1 | 771 | 4 | 0.995460 | -0.229994 | 569 | 376.85 | 0.662302 |
| GO:0051171\_regulation\_of\_nitrogen\_compound\_metabolic\_process | TLE1 | 771 | 4 | 0.995460 | -0.229994 | 569 | 376.85 | 0.662302 |
| GO:0051171\_regulation\_of\_nitrogen\_compound\_metabolic\_process | TIMP2 | 771 | 4 | 0.995460 | -0.229994 | 569 | 376.85 | 0.662302 |
| GO:0051171\_regulation\_of\_nitrogen\_compound\_metabolic\_process | APC | 771 | 4 | 0.995460 | -0.229994 | 569 | 376.85 | 0.662302 |
| GO:0051246\_regulation\_of\_protein\_metabolic\_process | APC | 170 | 1 | 1.128676 | -0.225129 | 570 | 378.49 | 0.664018 |
| GO:0044093\_positive\_regulation\_of\_molecular\_function | RELN | 173 | 1 | 1.109104 | -0.220390 | 571 | 381.54 | 0.668196 |
| GO:0000122\_negative\_regulation\_of\_transcription\_from\_RNA\_polymerase\_II\_promoter | TLE1 | 175 | 1 | 1.096429 | -0.217300 | 572 | 382.89 | 0.669388 |
| GO:0043687\_post-translational\_protein\_modification | CSNK2A1 | 384 | 2 | 0.999349 | -0.216944 | 573 | 383.26 | 0.668866 |
| GO:0043687\_post-translational\_protein\_modification | RELN | 384 | 2 | 0.999349 | -0.216944 | 573 | 383.26 | 0.668866 |
| GO:0043066\_negative\_regulation\_of\_apoptosis | APC | 176 | 1 | 1.090199 | -0.215775 | 574 | 384.25 | 0.669425 |
| GO:0043170\_macromolecule\_metabolic\_process | EXO1 | 1576 | 8 | 0.973985 | -0.212887 | 575 | 384.92 | 0.669426 |
| GO:0043170\_macromolecule\_metabolic\_process | EBNA1BP2 | 1576 | 8 | 0.973985 | -0.212887 | 575 | 384.92 | 0.669426 |
| GO:0043170\_macromolecule\_metabolic\_process | CSNK2A1 | 1576 | 8 | 0.973985 | -0.212887 | 575 | 384.92 | 0.669426 |
| GO:0043170\_macromolecule\_metabolic\_process | MRPL19 | 1576 | 8 | 0.973985 | -0.212887 | 575 | 384.92 | 0.669426 |
| GO:0043170\_macromolecule\_metabolic\_process | FZD1 | 1576 | 8 | 0.973985 | -0.212887 | 575 | 384.92 | 0.669426 |
| GO:0043170\_macromolecule\_metabolic\_process | RELN | 1576 | 8 | 0.973985 | -0.212887 | 575 | 384.92 | 0.669426 |
| GO:0043170\_macromolecule\_metabolic\_process | TLE1 | 1576 | 8 | 0.973985 | -0.212887 | 575 | 384.92 | 0.669426 |
| GO:0043170\_macromolecule\_metabolic\_process | APC | 1576 | 8 | 0.973985 | -0.212887 | 575 | 384.92 | 0.669426 |
| GO:0043069\_negative\_regulation\_of\_programmed\_cell\_death | APC | 179 | 1 | 1.071927 | -0.211279 | 578 | 386.07 | 0.667941 |
| GO:0048732\_gland\_development | APC | 179 | 1 | 1.071927 | -0.211279 | 578 | 386.07 | 0.667941 |
| GO:0060548\_negative\_regulation\_of\_cell\_death | APC | 179 | 1 | 1.071927 | -0.211279 | 578 | 386.07 | 0.667941 |
| GO:0050877\_neurological\_system\_process | STX4A | 390 | 2 | 0.983974 | -0.210709 | 579 | 386.63 | 0.667755 |
| GO:0050877\_neurological\_system\_process | LIN7C | 390 | 2 | 0.983974 | -0.210709 | 579 | 386.63 | 0.667755 |
| GO:0007166\_cell\_surface\_receptor\_linked\_signal\_transduction | FGF9 | 597 | 3 | 0.964196 | -0.208602 | 580 | 387.7 | 0.668448 |
| GO:0007166\_cell\_surface\_receptor\_linked\_signal\_transduction | FZD1 | 597 | 3 | 0.964196 | -0.208602 | 580 | 387.7 | 0.668448 |
| GO:0007166\_cell\_surface\_receptor\_linked\_signal\_transduction | APC | 597 | 3 | 0.964196 | -0.208602 | 580 | 387.7 | 0.668448 |
| GO:0016192\_vesicle-mediated\_transport | STX4A | 184 | 1 | 1.042799 | -0.204038 | 581 | 391.71 | 0.674200 |
| GO:0007155\_cell\_adhesion | APC | 186 | 1 | 1.031586 | -0.201226 | 583 | 393.68 | 0.675266 |
| GO:0022610\_biological\_adhesion | APC | 186 | 1 | 1.031586 | -0.201226 | 583 | 393.68 | 0.675266 |
| GO:0007276\_gamete\_generation | RAD23B | 188 | 1 | 1.020612 | -0.198462 | 584 | 394.73 | 0.675908 |
| GO:0032501\_multicellular\_organismal\_process | EXO1 | 2183 | 11 | 0.966846 | -0.194634 | 585 | 395.57 | 0.676188 |
| GO:0032501\_multicellular\_organismal\_process | RDH10 | 2183 | 11 | 0.966846 | -0.194634 | 585 | 395.57 | 0.676188 |
| GO:0032501\_multicellular\_organismal\_process | FGF9 | 2183 | 11 | 0.966846 | -0.194634 | 585 | 395.57 | 0.676188 |
| GO:0032501\_multicellular\_organismal\_process | STX4A | 2183 | 11 | 0.966846 | -0.194634 | 585 | 395.57 | 0.676188 |
| GO:0032501\_multicellular\_organismal\_process | FZD1 | 2183 | 11 | 0.966846 | -0.194634 | 585 | 395.57 | 0.676188 |
| GO:0032501\_multicellular\_organismal\_process | LIN7C | 2183 | 11 | 0.966846 | -0.194634 | 585 | 395.57 | 0.676188 |
| GO:0032501\_multicellular\_organismal\_process | RELN | 2183 | 11 | 0.966846 | -0.194634 | 585 | 395.57 | 0.676188 |
| GO:0032501\_multicellular\_organismal\_process | ATP1A1 | 2183 | 11 | 0.966846 | -0.194634 | 585 | 395.57 | 0.676188 |
| GO:0032501\_multicellular\_organismal\_process | TIMP2 | 2183 | 11 | 0.966846 | -0.194634 | 585 | 395.57 | 0.676188 |
| GO:0032501\_multicellular\_organismal\_process | TACC3 | 2183 | 11 | 0.966846 | -0.194634 | 585 | 395.57 | 0.676188 |
| GO:0032501\_multicellular\_organismal\_process | APC | 2183 | 11 | 0.966846 | -0.194634 | 585 | 395.57 | 0.676188 |
| GO:0048731\_system\_development | EXO1 | 1609 | 8 | 0.954009 | -0.193399 | 586 | 395.65 | 0.675171 |
| GO:0048731\_system\_development | RDH10 | 1609 | 8 | 0.954009 | -0.193399 | 586 | 395.65 | 0.675171 |
| GO:0048731\_system\_development | FGF9 | 1609 | 8 | 0.954009 | -0.193399 | 586 | 395.65 | 0.675171 |
| GO:0048731\_system\_development | FZD1 | 1609 | 8 | 0.954009 | -0.193399 | 586 | 395.65 | 0.675171 |
| GO:0048731\_system\_development | RELN | 1609 | 8 | 0.954009 | -0.193399 | 586 | 395.65 | 0.675171 |
| GO:0048731\_system\_development | TIMP2 | 1609 | 8 | 0.954009 | -0.193399 | 586 | 395.65 | 0.675171 |
| GO:0048731\_system\_development | TACC3 | 1609 | 8 | 0.954009 | -0.193399 | 586 | 395.65 | 0.675171 |
| GO:0048731\_system\_development | APC | 1609 | 8 | 0.954009 | -0.193399 | 586 | 395.65 | 0.675171 |
| GO:0007507\_heart\_development | FGF9 | 195 | 1 | 0.983974 | -0.189142 | 588 | 397.96 | 0.676803 |
| GO:0019725\_cellular\_homeostasis | APC | 195 | 1 | 0.983974 | -0.189142 | 588 | 397.96 | 0.676803 |
| GO:0001568\_blood\_vessel\_development | FGF9 | 203 | 1 | 0.945197 | -0.179126 | 589 | 401.09 | 0.680968 |
| GO:0006955\_immune\_response | EXO1 | 205 | 1 | 0.935976 | -0.176721 | 590 | 402.38 | 0.682000 |
| GO:0006915\_apoptosis | BMF | 427 | 2 | 0.898712 | -0.176124 | 591 | 402.71 | 0.681404 |
| GO:0006915\_apoptosis | APC | 427 | 2 | 0.898712 | -0.176124 | 591 | 402.71 | 0.681404 |
| GO:0001944\_vasculature\_development | FGF9 | 208 | 1 | 0.922476 | -0.173186 | 593 | 403.84 | 0.681012 |
| GO:0008284\_positive\_regulation\_of\_cell\_proliferation | FGF9 | 208 | 1 | 0.922476 | -0.173186 | 593 | 403.84 | 0.681012 |
| GO:0012501\_programmed\_cell\_death | BMF | 433 | 2 | 0.886259 | -0.171086 | 594 | 404.42 | 0.680842 |
| GO:0012501\_programmed\_cell\_death | APC | 433 | 2 | 0.886259 | -0.171086 | 594 | 404.42 | 0.680842 |
| GO:0006464\_protein\_modification\_process | CSNK2A1 | 439 | 2 | 0.874146 | -0.166194 | 595 | 405.89 | 0.682168 |
| GO:0006464\_protein\_modification\_process | RELN | 439 | 2 | 0.874146 | -0.166194 | 595 | 405.89 | 0.682168 |
| GO:0040007\_growth | RDH10 | 217 | 1 | 0.884217 | -0.163067 | 596 | 407.67 | 0.684010 |
| GO:0008219\_cell\_death | BMF | 444 | 2 | 0.864302 | -0.162224 | 597 | 408.53 | 0.684305 |
| GO:0008219\_cell\_death | APC | 444 | 2 | 0.864302 | -0.162224 | 597 | 408.53 | 0.684305 |
| GO:0045892\_negative\_regulation\_of\_transcription\_\_DNA-dependent | TLE1 | 218 | 1 | 0.880161 | -0.161986 | 598 | 408.94 | 0.683846 |
| GO:0051253\_negative\_regulation\_of\_RNA\_metabolic\_process | TLE1 | 220 | 1 | 0.872159 | -0.159848 | 599 | 410.01 | 0.684491 |
| GO:0032502\_developmental\_process | EXO1 | 2060 | 10 | 0.931432 | -0.159802 | 600 | 410.15 | 0.683583 |
| GO:0032502\_developmental\_process | TYRP1 | 2060 | 10 | 0.931432 | -0.159802 | 600 | 410.15 | 0.683583 |
| GO:0032502\_developmental\_process | RDH10 | 2060 | 10 | 0.931432 | -0.159802 | 600 | 410.15 | 0.683583 |
| GO:0032502\_developmental\_process | FGF9 | 2060 | 10 | 0.931432 | -0.159802 | 600 | 410.15 | 0.683583 |
| GO:0032502\_developmental\_process | FZD1 | 2060 | 10 | 0.931432 | -0.159802 | 600 | 410.15 | 0.683583 |
| GO:0032502\_developmental\_process | RELN | 2060 | 10 | 0.931432 | -0.159802 | 600 | 410.15 | 0.683583 |
| GO:0032502\_developmental\_process | TIMP2 | 2060 | 10 | 0.931432 | -0.159802 | 600 | 410.15 | 0.683583 |
| GO:0032502\_developmental\_process | TACC3 | 2060 | 10 | 0.931432 | -0.159802 | 600 | 410.15 | 0.683583 |
| GO:0032502\_developmental\_process | BMF | 2060 | 10 | 0.931432 | -0.159802 | 600 | 410.15 | 0.683583 |
| GO:0032502\_developmental\_process | APC | 2060 | 10 | 0.931432 | -0.159802 | 600 | 410.15 | 0.683583 |
| GO:0001701\_in\_utero\_embryonic\_development | RDH10 | 221 | 1 | 0.868213 | -0.158792 | 601 | 410.52 | 0.683062 |
| GO:0016265\_death | BMF | 450 | 2 | 0.852778 | -0.157587 | 602 | 411.12 | 0.682924 |
| GO:0016265\_death | APC | 450 | 2 | 0.852778 | -0.157587 | 602 | 411.12 | 0.682924 |
| GO:0048856\_anatomical\_structure\_development | EXO1 | 1688 | 8 | 0.909360 | -0.152398 | 603 | 411.6 | 0.682587 |
| GO:0048856\_anatomical\_structure\_development | RDH10 | 1688 | 8 | 0.909360 | -0.152398 | 603 | 411.6 | 0.682587 |
| GO:0048856\_anatomical\_structure\_development | FGF9 | 1688 | 8 | 0.909360 | -0.152398 | 603 | 411.6 | 0.682587 |
| GO:0048856\_anatomical\_structure\_development | FZD1 | 1688 | 8 | 0.909360 | -0.152398 | 603 | 411.6 | 0.682587 |
| GO:0048856\_anatomical\_structure\_development | RELN | 1688 | 8 | 0.909360 | -0.152398 | 603 | 411.6 | 0.682587 |
| GO:0048856\_anatomical\_structure\_development | TIMP2 | 1688 | 8 | 0.909360 | -0.152398 | 603 | 411.6 | 0.682587 |
| GO:0048856\_anatomical\_structure\_development | TACC3 | 1688 | 8 | 0.909360 | -0.152398 | 603 | 411.6 | 0.682587 |
| GO:0048856\_anatomical\_structure\_development | APC | 1688 | 8 | 0.909360 | -0.152398 | 603 | 411.6 | 0.682587 |
| GO:0002682\_regulation\_of\_immune\_system\_process | APC | 228 | 1 | 0.841557 | -0.151620 | 605 | 412.68 | 0.682116 |
| GO:0019953\_sexual\_reproduction | RAD23B | 228 | 1 | 0.841557 | -0.151620 | 605 | 412.68 | 0.682116 |
| GO:0043412\_biopolymer\_modification | CSNK2A1 | 458 | 2 | 0.837882 | -0.151609 | 606 | 413.21 | 0.681865 |
| GO:0043412\_biopolymer\_modification | RELN | 458 | 2 | 0.837882 | -0.151609 | 606 | 413.21 | 0.681865 |
| GO:0007167\_enzyme\_linked\_receptor\_protein\_signaling\_pathway | FGF9 | 229 | 1 | 0.837882 | -0.150626 | 607 | 413.89 | 0.681862 |
| GO:0050790\_regulation\_of\_catalytic\_activity | RELN | 233 | 1 | 0.823498 | -0.146725 | 608 | 415.17 | 0.682845 |
| GO:0010467\_gene\_expression | EBNA1BP2 | 905 | 4 | 0.848066 | -0.140891 | 609 | 417.92 | 0.686240 |
| GO:0010467\_gene\_expression | MRPL19 | 905 | 4 | 0.848066 | -0.140891 | 609 | 417.92 | 0.686240 |
| GO:0010467\_gene\_expression | FZD1 | 905 | 4 | 0.848066 | -0.140891 | 609 | 417.92 | 0.686240 |
| GO:0010467\_gene\_expression | TLE1 | 905 | 4 | 0.848066 | -0.140891 | 609 | 417.92 | 0.686240 |
| GO:0009056\_catabolic\_process | APC | 243 | 1 | 0.789609 | -0.137466 | 610 | 418.8 | 0.686557 |
| GO:0007275\_multicellular\_organismal\_development | EXO1 | 1760 | 8 | 0.872159 | -0.121293 | 611 | 425.36 | 0.696170 |
| GO:0007275\_multicellular\_organismal\_development | RDH10 | 1760 | 8 | 0.872159 | -0.121293 | 611 | 425.36 | 0.696170 |
| GO:0007275\_multicellular\_organismal\_development | FGF9 | 1760 | 8 | 0.872159 | -0.121293 | 611 | 425.36 | 0.696170 |
| GO:0007275\_multicellular\_organismal\_development | FZD1 | 1760 | 8 | 0.872159 | -0.121293 | 611 | 425.36 | 0.696170 |
| GO:0007275\_multicellular\_organismal\_development | RELN | 1760 | 8 | 0.872159 | -0.121293 | 611 | 425.36 | 0.696170 |
| GO:0007275\_multicellular\_organismal\_development | TIMP2 | 1760 | 8 | 0.872159 | -0.121293 | 611 | 425.36 | 0.696170 |
| GO:0007275\_multicellular\_organismal\_development | TACC3 | 1760 | 8 | 0.872159 | -0.121293 | 611 | 425.36 | 0.696170 |
| GO:0007275\_multicellular\_organismal\_development | APC | 1760 | 8 | 0.872159 | -0.121293 | 611 | 425.36 | 0.696170 |
| GO:0048513\_organ\_development | RDH10 | 1365 | 6 | 0.843407 | -0.119067 | 612 | 426.7 | 0.697222 |
| GO:0048513\_organ\_development | FGF9 | 1365 | 6 | 0.843407 | -0.119067 | 612 | 426.7 | 0.697222 |
| GO:0048513\_organ\_development | FZD1 | 1365 | 6 | 0.843407 | -0.119067 | 612 | 426.7 | 0.697222 |
| GO:0048513\_organ\_development | RELN | 1365 | 6 | 0.843407 | -0.119067 | 612 | 426.7 | 0.697222 |
| GO:0048513\_organ\_development | TACC3 | 1365 | 6 | 0.843407 | -0.119067 | 612 | 426.7 | 0.697222 |
| GO:0048513\_organ\_development | APC | 1365 | 6 | 0.843407 | -0.119067 | 612 | 426.7 | 0.697222 |
| GO:0007610\_behavior | RELN | 279 | 1 | 0.687724 | -0.109171 | 614 | 431.96 | 0.703518 |
| GO:0065009\_regulation\_of\_molecular\_function | RELN | 279 | 1 | 0.687724 | -0.109171 | 614 | 431.96 | 0.703518 |
| GO:0019219\_regulation\_of\_nucleobase\_\_nucleoside\_\_nucleotide\_and\_nucleic\_acid\_metabolic\_process | FZD1 | 757 | 3 | 0.760403 | -0.107675 | 615 | 432.27 | 0.702878 |
| GO:0019219\_regulation\_of\_nucleobase\_\_nucleoside\_\_nucleotide\_and\_nucleic\_acid\_metabolic\_process | TLE1 | 757 | 3 | 0.760403 | -0.107675 | 615 | 432.27 | 0.702878 |
| GO:0019219\_regulation\_of\_nucleobase\_\_nucleoside\_\_nucleotide\_and\_nucleic\_acid\_metabolic\_process | TIMP2 | 757 | 3 | 0.760403 | -0.107675 | 615 | 432.27 | 0.702878 |
| GO:0048518\_positive\_regulation\_of\_biological\_process | FGF9 | 995 | 4 | 0.771357 | -0.099747 | 616 | 435.22 | 0.706526 |
| GO:0048518\_positive\_regulation\_of\_biological\_process | ATP1A1 | 995 | 4 | 0.771357 | -0.099747 | 616 | 435.22 | 0.706526 |
| GO:0048518\_positive\_regulation\_of\_biological\_process | RELN | 995 | 4 | 0.771357 | -0.099747 | 616 | 435.22 | 0.706526 |
| GO:0048518\_positive\_regulation\_of\_biological\_process | APC | 995 | 4 | 0.771357 | -0.099747 | 616 | 435.22 | 0.706526 |
| GO:0065007\_biological\_regulation | RDH10 | 2593 | 12 | 0.887968 | -0.098259 | 617 | 436.5 | 0.707455 |
| GO:0065007\_biological\_regulation | FGF9 | 2593 | 12 | 0.887968 | -0.098259 | 617 | 436.5 | 0.707455 |
| GO:0065007\_biological\_regulation | STX4A | 2593 | 12 | 0.887968 | -0.098259 | 617 | 436.5 | 0.707455 |
| GO:0065007\_biological\_regulation | FZD1 | 2593 | 12 | 0.887968 | -0.098259 | 617 | 436.5 | 0.707455 |
| GO:0065007\_biological\_regulation | LIN7C | 2593 | 12 | 0.887968 | -0.098259 | 617 | 436.5 | 0.707455 |
| GO:0065007\_biological\_regulation | RELN | 2593 | 12 | 0.887968 | -0.098259 | 617 | 436.5 | 0.707455 |
| GO:0065007\_biological\_regulation | ATP1A1 | 2593 | 12 | 0.887968 | -0.098259 | 617 | 436.5 | 0.707455 |
| GO:0065007\_biological\_regulation | TLE1 | 2593 | 12 | 0.887968 | -0.098259 | 617 | 436.5 | 0.707455 |
| GO:0065007\_biological\_regulation | TACC3 | 2593 | 12 | 0.887968 | -0.098259 | 617 | 436.5 | 0.707455 |
| GO:0065007\_biological\_regulation | TIMP2 | 2593 | 12 | 0.887968 | -0.098259 | 617 | 436.5 | 0.707455 |
| GO:0065007\_biological\_regulation | BMF | 2593 | 12 | 0.887968 | -0.098259 | 617 | 436.5 | 0.707455 |
| GO:0065007\_biological\_regulation | APC | 2593 | 12 | 0.887968 | -0.098259 | 617 | 436.5 | 0.707455 |
| GO:0009790\_embryonic\_development | RDH10 | 567 | 2 | 0.676808 | -0.089306 | 618 | 439.55 | 0.711246 |
| GO:0009790\_embryonic\_development | FGF9 | 567 | 2 | 0.676808 | -0.089306 | 618 | 439.55 | 0.711246 |
| GO:0034961\_cellular\_biopolymer\_biosynthetic\_process | MRPL19 | 804 | 3 | 0.715951 | -0.088069 | 619 | 440.06 | 0.710921 |
| GO:0034961\_cellular\_biopolymer\_biosynthetic\_process | FZD1 | 804 | 3 | 0.715951 | -0.088069 | 619 | 440.06 | 0.710921 |
| GO:0034961\_cellular\_biopolymer\_biosynthetic\_process | TLE1 | 804 | 3 | 0.715951 | -0.088069 | 619 | 440.06 | 0.710921 |
| GO:0043284\_biopolymer\_biosynthetic\_process | MRPL19 | 807 | 3 | 0.713290 | -0.086936 | 620 | 440.37 | 0.710274 |
| GO:0043284\_biopolymer\_biosynthetic\_process | FZD1 | 807 | 3 | 0.713290 | -0.086936 | 620 | 440.37 | 0.710274 |
| GO:0043284\_biopolymer\_biosynthetic\_process | TLE1 | 807 | 3 | 0.713290 | -0.086936 | 620 | 440.37 | 0.710274 |
| GO:0031326\_regulation\_of\_cellular\_biosynthetic\_process | FZD1 | 812 | 3 | 0.708898 | -0.085076 | 621 | 440.91 | 0.710000 |
| GO:0031326\_regulation\_of\_cellular\_biosynthetic\_process | TLE1 | 812 | 3 | 0.708898 | -0.085076 | 621 | 440.91 | 0.710000 |
| GO:0031326\_regulation\_of\_cellular\_biosynthetic\_process | ATP1A1 | 812 | 3 | 0.708898 | -0.085076 | 621 | 440.91 | 0.710000 |
| GO:0009889\_regulation\_of\_biosynthetic\_process | FZD1 | 815 | 3 | 0.706288 | -0.083978 | 622 | 441.14 | 0.709228 |
| GO:0009889\_regulation\_of\_biosynthetic\_process | ATP1A1 | 815 | 3 | 0.706288 | -0.083978 | 622 | 441.14 | 0.709228 |
| GO:0009889\_regulation\_of\_biosynthetic\_process | TLE1 | 815 | 3 | 0.706288 | -0.083978 | 622 | 441.14 | 0.709228 |
| GO:0051093\_negative\_regulation\_of\_developmental\_process | APC | 331 | 1 | 0.579683 | -0.078911 | 623 | 443.21 | 0.711413 |
| GO:0048522\_positive\_regulation\_of\_cellular\_process | FGF9 | 895 | 3 | 0.643156 | -0.059035 | 624 | 452.2 | 0.724679 |
| GO:0048522\_positive\_regulation\_of\_cellular\_process | RELN | 895 | 3 | 0.643156 | -0.059035 | 624 | 452.2 | 0.724679 |
| GO:0048522\_positive\_regulation\_of\_cellular\_process | APC | 895 | 3 | 0.643156 | -0.059035 | 624 | 452.2 | 0.724679 |
| GO:0034645\_cellular\_macromolecule\_biosynthetic\_process | MRPL19 | 901 | 3 | 0.638873 | -0.057467 | 625 | 453.18 | 0.725088 |
| GO:0034645\_cellular\_macromolecule\_biosynthetic\_process | FZD1 | 901 | 3 | 0.638873 | -0.057467 | 625 | 453.18 | 0.725088 |
| GO:0034645\_cellular\_macromolecule\_biosynthetic\_process | TLE1 | 901 | 3 | 0.638873 | -0.057467 | 625 | 453.18 | 0.725088 |
| GO:0016070\_RNA\_metabolic\_process | EBNA1BP2 | 658 | 2 | 0.583207 | -0.056962 | 626 | 453.8 | 0.724920 |
| GO:0016070\_RNA\_metabolic\_process | TLE1 | 658 | 2 | 0.583207 | -0.056962 | 626 | 453.8 | 0.724920 |
| GO:0009059\_macromolecule\_biosynthetic\_process | MRPL19 | 910 | 3 | 0.632555 | -0.055186 | 627 | 454.88 | 0.725486 |
| GO:0009059\_macromolecule\_biosynthetic\_process | FZD1 | 910 | 3 | 0.632555 | -0.055186 | 627 | 454.88 | 0.725486 |
| GO:0009059\_macromolecule\_biosynthetic\_process | TLE1 | 910 | 3 | 0.632555 | -0.055186 | 627 | 454.88 | 0.725486 |
| GO:0050794\_regulation\_of\_cellular\_process | FGF9 | 2190 | 9 | 0.788527 | -0.053371 | 628 | 456.05 | 0.726194 |
| GO:0050794\_regulation\_of\_cellular\_process | FZD1 | 2190 | 9 | 0.788527 | -0.053371 | 628 | 456.05 | 0.726194 |
| GO:0050794\_regulation\_of\_cellular\_process | ATP1A1 | 2190 | 9 | 0.788527 | -0.053371 | 628 | 456.05 | 0.726194 |
| GO:0050794\_regulation\_of\_cellular\_process | TLE1 | 2190 | 9 | 0.788527 | -0.053371 | 628 | 456.05 | 0.726194 |
| GO:0050794\_regulation\_of\_cellular\_process | RELN | 2190 | 9 | 0.788527 | -0.053371 | 628 | 456.05 | 0.726194 |
| GO:0050794\_regulation\_of\_cellular\_process | TIMP2 | 2190 | 9 | 0.788527 | -0.053371 | 628 | 456.05 | 0.726194 |
| GO:0050794\_regulation\_of\_cellular\_process | TACC3 | 2190 | 9 | 0.788527 | -0.053371 | 628 | 456.05 | 0.726194 |
| GO:0050794\_regulation\_of\_cellular\_process | BMF | 2190 | 9 | 0.788527 | -0.053371 | 628 | 456.05 | 0.726194 |
| GO:0050794\_regulation\_of\_cellular\_process | APC | 2190 | 9 | 0.788527 | -0.053371 | 628 | 456.05 | 0.726194 |
| GO:0045449\_regulation\_of\_transcription | FZD1 | 676 | 2 | 0.567678 | -0.052050 | 629 | 456.65 | 0.725994 |
| GO:0045449\_regulation\_of\_transcription | TLE1 | 676 | 2 | 0.567678 | -0.052050 | 629 | 456.65 | 0.725994 |
| GO:0060255\_regulation\_of\_macromolecule\_metabolic\_process | FZD1 | 936 | 3 | 0.614984 | -0.049048 | 630 | 457.27 | 0.725825 |
| GO:0060255\_regulation\_of\_macromolecule\_metabolic\_process | TLE1 | 936 | 3 | 0.614984 | -0.049048 | 630 | 457.27 | 0.725825 |
| GO:0060255\_regulation\_of\_macromolecule\_metabolic\_process | APC | 936 | 3 | 0.614984 | -0.049048 | 630 | 457.27 | 0.725825 |
| GO:0042221\_response\_to\_chemical\_stimulus | ATP1A1 | 409 | 1 | 0.469132 | -0.048982 | 631 | 457.55 | 0.725119 |
| GO:0042592\_homeostatic\_process | APC | 419 | 1 | 0.457936 | -0.046098 | 632 | 458.65 | 0.725712 |
| GO:0006350\_transcription | FZD1 | 701 | 2 | 0.547432 | -0.045887 | 633 | 458.84 | 0.724866 |
| GO:0006350\_transcription | TLE1 | 701 | 2 | 0.547432 | -0.045887 | 633 | 458.84 | 0.724866 |
| GO:0010604\_positive\_regulation\_of\_macromolecule\_metabolic\_process | APC | 433 | 1 | 0.443129 | -0.042347 | 634 | 460.43 | 0.726230 |
| GO:0006357\_regulation\_of\_transcription\_from\_RNA\_polymerase\_II\_promoter | TLE1 | 435 | 1 | 0.441092 | -0.041837 | 635 | 460.87 | 0.725780 |
| GO:0031325\_positive\_regulation\_of\_cellular\_metabolic\_process | APC | 442 | 1 | 0.434106 | -0.040100 | 636 | 461.45 | 0.725550 |
| GO:0006366\_transcription\_from\_RNA\_polymerase\_II\_promoter | TLE1 | 444 | 1 | 0.432151 | -0.039618 | 637 | 462.12 | 0.725463 |
| GO:0010556\_regulation\_of\_macromolecule\_biosynthetic\_process | FZD1 | 745 | 2 | 0.515101 | -0.036673 | 638 | 463.24 | 0.726082 |
| GO:0010556\_regulation\_of\_macromolecule\_biosynthetic\_process | TLE1 | 745 | 2 | 0.515101 | -0.036673 | 638 | 463.24 | 0.726082 |
| GO:0009893\_positive\_regulation\_of\_metabolic\_process | APC | 458 | 1 | 0.418941 | -0.036398 | 639 | 463.75 | 0.725743 |
| GO:0010468\_regulation\_of\_gene\_expression | FZD1 | 778 | 2 | 0.493252 | -0.030933 | 640 | 465.23 | 0.726922 |
| GO:0010468\_regulation\_of\_gene\_expression | TLE1 | 778 | 2 | 0.493252 | -0.030933 | 640 | 465.23 | 0.726922 |
| GO:0050789\_regulation\_of\_biological\_process | FGF9 | 2357 | 9 | 0.732658 | -0.026892 | 641 | 466.0 | 0.726989 |
| GO:0050789\_regulation\_of\_biological\_process | FZD1 | 2357 | 9 | 0.732658 | -0.026892 | 641 | 466.0 | 0.726989 |
| GO:0050789\_regulation\_of\_biological\_process | RELN | 2357 | 9 | 0.732658 | -0.026892 | 641 | 466.0 | 0.726989 |
| GO:0050789\_regulation\_of\_biological\_process | ATP1A1 | 2357 | 9 | 0.732658 | -0.026892 | 641 | 466.0 | 0.726989 |
| GO:0050789\_regulation\_of\_biological\_process | TLE1 | 2357 | 9 | 0.732658 | -0.026892 | 641 | 466.0 | 0.726989 |
| GO:0050789\_regulation\_of\_biological\_process | TACC3 | 2357 | 9 | 0.732658 | -0.026892 | 641 | 466.0 | 0.726989 |
| GO:0050789\_regulation\_of\_biological\_process | TIMP2 | 2357 | 9 | 0.732658 | -0.026892 | 641 | 466.0 | 0.726989 |
| GO:0050789\_regulation\_of\_biological\_process | BMF | 2357 | 9 | 0.732658 | -0.026892 | 641 | 466.0 | 0.726989 |
| GO:0050789\_regulation\_of\_biological\_process | APC | 2357 | 9 | 0.732658 | -0.026892 | 641 | 466.0 | 0.726989 |
| GO:0006355\_regulation\_of\_transcription\_\_DNA-dependent | TLE1 | 575 | 1 | 0.333696 | -0.017897 | 642 | 468.88 | 0.730343 |
| GO:0051252\_regulation\_of\_RNA\_metabolic\_process | TLE1 | 590 | 1 | 0.325212 | -0.016332 | 643 | 469.72 | 0.730513 |
| GO:0006351\_transcription\_\_DNA-dependent | TLE1 | 594 | 1 | 0.323022 | -0.015937 | 644 | 470.03 | 0.729860 |
| GO:0032774\_RNA\_biosynthetic\_process | TLE1 | 595 | 1 | 0.322479 | -0.015840 | 645 | 470.24 | 0.729054 |
| GO:0008150\_biological\_process | EXO1 | 4605 | 24 | 1.000000 | 0.000000 | 2725 | 2718.63 | 0.997662 |
| GO:0008150\_biological\_process | RAD23B | 4605 | 24 | 1.000000 | 0.000000 | 2725 | 2718.63 | 0.997662 |
| GO:0008150\_biological\_process | TYRP1 | 4605 | 24 | 1.000000 | 0.000000 | 2725 | 2718.63 | 0.997662 |
| GO:0008150\_biological\_process | FGF9 | 4605 | 24 | 1.000000 | 0.000000 | 2725 | 2718.63 | 0.997662 |
| GO:0008150\_biological\_process | STX4A | 4605 | 24 | 1.000000 | 0.000000 | 2725 | 2718.63 | 0.997662 |
| GO:0008150\_biological\_process | FZD1 | 4605 | 24 | 1.000000 | 0.000000 | 2725 | 2718.63 | 0.997662 |
| GO:0008150\_biological\_process | LIN7C | 4605 | 24 | 1.000000 | 0.000000 | 2725 | 2718.63 | 0.997662 |
| GO:0008150\_biological\_process | TLE1 | 4605 | 24 | 1.000000 | 0.000000 | 2725 | 2718.63 | 0.997662 |
| GO:0008150\_biological\_process | ATP1A1 | 4605 | 24 | 1.000000 | 0.000000 | 2725 | 2718.63 | 0.997662 |
| GO:0008150\_biological\_process | TACC3 | 4605 | 24 | 1.000000 | 0.000000 | 2725 | 2718.63 | 0.997662 |
| GO:0008150\_biological\_process | TIMP2 | 4605 | 24 | 1.000000 | 0.000000 | 2725 | 2718.63 | 0.997662 |
| GO:0008150\_biological\_process | BICD2 | 4605 | 24 | 1.000000 | 0.000000 | 2725 | 2718.63 | 0.997662 |
| GO:0008150\_biological\_process | HDGFRP3 | 4605 | 24 | 1.000000 | 0.000000 | 2725 | 2718.63 | 0.997662 |
| GO:0008150\_biological\_process | EBNA1BP2 | 4605 | 24 | 1.000000 | 0.000000 | 2725 | 2718.63 | 0.997662 |
| GO:0008150\_biological\_process | RDH10 | 4605 | 24 | 1.000000 | 0.000000 | 2725 | 2718.63 | 0.997662 |
| GO:0008150\_biological\_process | CSNK2A1 | 4605 | 24 | 1.000000 | 0.000000 | 2725 | 2718.63 | 0.997662 |
| GO:0008150\_biological\_process | B3GNT5 | 4605 | 24 | 1.000000 | 0.000000 | 2725 | 2718.63 | 0.997662 |
| GO:0008150\_biological\_process | SLC2A3 | 4605 | 24 | 1.000000 | 0.000000 | 2725 | 2718.63 | 0.997662 |
| GO:0008150\_biological\_process | XPR1 | 4605 | 24 | 1.000000 | 0.000000 | 2725 | 2718.63 | 0.997662 |
| GO:0008150\_biological\_process | RRM2 | 4605 | 24 | 1.000000 | 0.000000 | 2725 | 2718.63 | 0.997662 |
| GO:0008150\_biological\_process | MRPL19 | 4605 | 24 | 1.000000 | 0.000000 | 2725 | 2718.63 | 0.997662 |
| GO:0008150\_biological\_process | RELN | 4605 | 24 | 1.000000 | 0.000000 | 2725 | 2718.63 | 0.997662 |
| GO:0008150\_biological\_process | BMF | 4605 | 24 | 1.000000 | 0.000000 | 2725 | 2718.63 | 0.997662 |
| GO:0008150\_biological\_process | APC | 4605 | 24 | 1.000000 | 0.000000 | 2725 | 2718.63 | 0.997662 |
