# Supplementary material for: RedundancyMiner: De-replication of redundant GO categories in microarray and proteomics analysis
Source: BMC Bioinformatics. 2011 Feb 10;12:52. doi: 10.1186/1471-2105-12-52 (PMC3223614; doi:10.1186/1471-2105-12-52)
Supplement: Additional file 8 — Retinal development HTGM download. compressed package of the results of running HTGM on the retinal development genes list. [file 1471-2105-12-52-S8.ZIP › SCENARIO_2_MODIFIED/total.txt.total.txt.dir/Exp1_BestClusterMap_LEIGS_KM_24.csv.join.2.txt.dir/Exp1_BestClusterMap_LEIGS_KM_24.csv.join.2.txt.change.gce.html]

Gene Category Report for Exp1\_BestClusterMap\_LEIGS\_KM\_24.csv.join.2.txt

# Gene Category Report for Exp1\_BestClusterMap\_LEIGS\_KM\_24.csv.join.2.txt

| HYPERLINKED GO CATEGORY | HYPERLINKED GENE NAME | TOTAL GENES | CHANGED GENES | ENRICHMENT | LOG10(p) | CUMULATIVE NUMBER OF CATEGORIES | CUMULATIVE RANDOMS MEAN | FALSE DISCOVERY RATE |
| --- | --- | --- | --- | --- | --- | --- | --- | --- |
| GO:0006695\_cholesterol\_biosynthetic\_process | CYP51 | 14 | 2 | 36.547619 | -2.893684 | 1 | 1.38 | 1.380000 |
| GO:0006695\_cholesterol\_biosynthetic\_process | HSD17B7 | 14 | 2 | 36.547619 | -2.893684 | 1 | 1.38 | 1.380000 |
| GO:0016126\_sterol\_biosynthetic\_process | CYP51 | 16 | 2 | 31.979167 | -2.775556 | 2 | 1.71 | 0.855000 |
| GO:0016126\_sterol\_biosynthetic\_process | HSD17B7 | 16 | 2 | 31.979167 | -2.775556 | 2 | 1.71 | 0.855000 |
| GO:0008202\_steroid\_metabolic\_process | CYP51 | 82 | 3 | 9.359756 | -2.436321 | 3 | 3.02 | 1.006667 |
| GO:0008202\_steroid\_metabolic\_process | STUB1 | 82 | 3 | 9.359756 | -2.436321 | 3 | 3.02 | 1.006667 |
| GO:0008202\_steroid\_metabolic\_process | HSD17B7 | 82 | 3 | 9.359756 | -2.436321 | 3 | 3.02 | 1.006667 |
| GO:0033108\_mitochondrial\_respiratory\_chain\_complex\_assembly | TFAM | 1 | 1 |  |  |  |  |  |  |
| GO:0009266\_response\_to\_temperature\_stimulus | LXN | 30 | 2 | 17.055556 | -2.230317 | 4 | 4.4 | 1.100000 |
| GO:0009266\_response\_to\_temperature\_stimulus | ST8SIA1 | 30 | 2 | 17.055556 | -2.230317 | 4 | 4.4 | 1.100000 |
| GO:0006694\_steroid\_biosynthetic\_process | CYP51 | 31 | 2 | 16.505376 | -2.202357 | 5 | 4.83 | 0.966000 |
| GO:0006694\_steroid\_biosynthetic\_process | HSD17B7 | 31 | 2 | 16.505376 | -2.202357 | 5 | 4.83 | 0.966000 |
| GO:0019067\_viral\_assembly\_\_maturation\_\_egress\_\_and\_release | PCSK5 | 2 | 1 |  |  |  |  |  |  |
| GO:0033092\_positive\_regulation\_of\_immature\_T\_cell\_proliferation\_in\_the\_thymus | BMI1 | 2 | 1 |  |  |  |  |  |  |
| GO:0016567\_protein\_ubiquitination | BMI1 | 35 | 2 | 14.619048 | -2.099307 | 6 | 5.64 | 0.940000 |
| GO:0016567\_protein\_ubiquitination | STUB1 | 35 | 2 | 14.619048 | -2.099307 | 6 | 5.64 | 0.940000 |
| GO:0008203\_cholesterol\_metabolic\_process | CYP51 | 40 | 2 | 12.791667 | -1.986744 | 7 | 6.7 | 0.957143 |
| GO:0008203\_cholesterol\_metabolic\_process | HSD17B7 | 40 | 2 | 12.791667 | -1.986744 | 7 | 6.7 | 0.957143 |
| GO:0016125\_sterol\_metabolic\_process | CYP51 | 42 | 2 | 12.182540 | -1.945840 | 8 | 7.53 | 0.941250 |
| GO:0016125\_sterol\_metabolic\_process | HSD17B7 | 42 | 2 | 12.182540 | -1.945840 | 8 | 7.53 | 0.941250 |
| GO:0019058\_viral\_infectious\_cycle | PCSK5 | 3 | 1 |  |  |  |  |  |  |
| GO:0031398\_positive\_regulation\_of\_protein\_ubiquitination | STUB1 | 3 | 1 |  |  |  |  |  |  |
| GO:0031943\_regulation\_of\_glucocorticoid\_metabolic\_process | STUB1 | 3 | 1 |  |  |  |  |  |  |
| GO:0032436\_positive\_regulation\_of\_proteasomal\_ubiquitin-dependent\_protein\_catabolic\_process | STUB1 | 3 | 1 |  |  |  |  |  |  |
| GO:0033080\_immature\_T\_cell\_proliferation\_in\_the\_thymus | BMI1 | 3 | 1 |  |  |  |  |  |  |
| GO:0033084\_regulation\_of\_immature\_T\_cell\_proliferation\_in\_the\_thymus | BMI1 | 3 | 1 |  |  |  |  |  |  |
| GO:0033091\_positive\_regulation\_of\_immature\_T\_cell\_proliferation | BMI1 | 3 | 1 |  |  |  |  |  |  |
| GO:0042271\_susceptibility\_to\_natural\_killer\_cell\_mediated\_cytotoxicity | CADM1 | 3 | 1 |  |  |  |  |  |  |
| GO:0032446\_protein\_modification\_by\_small\_protein\_conjugation | BMI1 | 43 | 2 | 11.899225 | -1.926157 | 10 | 7.79 | 0.779000 |
| GO:0032446\_protein\_modification\_by\_small\_protein\_conjugation | STUB1 | 43 | 2 | 11.899225 | -1.926157 | 10 | 7.79 | 0.779000 |
| GO:0051604\_protein\_maturation | STUB1 | 43 | 2 | 11.899225 | -1.926157 | 10 | 7.79 | 0.779000 |
| GO:0051604\_protein\_maturation | PCSK5 | 43 | 2 | 11.899225 | -1.926157 | 10 | 7.79 | 0.779000 |
| GO:0016338\_calcium-independent\_cell-cell\_adhesion | CADM1 | 4 | 1 |  |  |  |  |  |  |
| GO:0033079\_immature\_T\_cell\_proliferation | BMI1 | 4 | 1 |  |  |  |  |  |  |
| GO:0033083\_regulation\_of\_immature\_T\_cell\_proliferation | BMI1 | 4 | 1 |  |  |  |  |  |  |
| GO:0043043\_peptide\_biosynthetic\_process | PCSK5 | 4 | 1 |  |  |  |  |  |  |
| GO:0048011\_nerve\_growth\_factor\_receptor\_signaling\_pathway | RAF1 | 4 | 1 |  |  |  |  |  |  |
| GO:0050961\_detection\_of\_temperature\_stimulus\_involved\_in\_sensory\_perception | LXN | 4 | 1 |  |  |  |  |  |  |
| GO:0050965\_detection\_of\_temperature\_stimulus\_involved\_in\_sensory\_perception\_of\_pain | LXN | 4 | 1 |  |  |  |  |  |  |
| GO:0051606\_detection\_of\_stimulus | CADM1 | 50 | 2 | 10.233333 | -1.800720 | 12 | 9.41 | 0.784167 |
| GO:0051606\_detection\_of\_stimulus | LXN | 50 | 2 | 10.233333 | -1.800720 | 12 | 9.41 | 0.784167 |
| GO:0070647\_protein\_modification\_by\_small\_protein\_conjugation\_or\_removal | BMI1 | 50 | 2 | 10.233333 | -1.800720 | 12 | 9.41 | 0.784167 |
| GO:0070647\_protein\_modification\_by\_small\_protein\_conjugation\_or\_removal | STUB1 | 50 | 2 | 10.233333 | -1.800720 | 12 | 9.41 | 0.784167 |
| GO:0016048\_detection\_of\_temperature\_stimulus | LXN | 5 | 1 | 51.166667 | -1.712195 | 16 | 15.62 | 0.976250 |
| GO:0022415\_viral\_reproductive\_process | PCSK5 | 5 | 1 | 51.166667 | -1.712195 | 16 | 15.62 | 0.976250 |
| GO:0032350\_regulation\_of\_hormone\_metabolic\_process | STUB1 | 5 | 1 | 51.166667 | -1.712195 | 16 | 15.62 | 0.976250 |
| GO:0032434\_regulation\_of\_proteasomal\_ubiquitin-dependent\_protein\_catabolic\_process | STUB1 | 5 | 1 | 51.166667 | -1.712195 | 16 | 15.62 | 0.976250 |
| GO:0016032\_viral\_reproduction | PCSK5 | 6 | 1 | 42.638889 | -1.633815 | 20 | 21.11 | 1.055500 |
| GO:0016574\_histone\_ubiquitination | BMI1 | 6 | 1 | 42.638889 | -1.633815 | 20 | 21.11 | 1.055500 |
| GO:0048103\_somatic\_stem\_cell\_division | BMI1 | 6 | 1 | 42.638889 | -1.633815 | 20 | 21.11 | 1.055500 |
| GO:0050951\_sensory\_perception\_of\_temperature\_stimulus | LXN | 6 | 1 | 42.638889 | -1.633815 | 20 | 21.11 | 1.055500 |
| GO:0021903\_rostrocaudal\_neural\_tube\_patterning | BMI1 | 7 | 1 | 36.547619 | -1.567669 | 22 | 27.18 | 1.235455 |
| GO:0045862\_positive\_regulation\_of\_proteolysis | STUB1 | 7 | 1 | 36.547619 | -1.567669 | 22 | 27.18 | 1.235455 |
| GO:0042445\_hormone\_metabolic\_process | STUB1 | 67 | 2 | 7.636816 | -1.561266 | 23 | 27.33 | 1.188261 |
| GO:0042445\_hormone\_metabolic\_process | PCSK5 | 67 | 2 | 7.636816 | -1.561266 | 23 | 27.33 | 1.188261 |
| GO:0001833\_inner\_cell\_mass\_cell\_proliferation | NCAPG2 | 8 | 1 | 31.979167 | -1.510477 | 25 | 32.48 | 1.299200 |
| GO:0031396\_regulation\_of\_protein\_ubiquitination | STUB1 | 8 | 1 | 31.979167 | -1.510477 | 25 | 32.48 | 1.299200 |
| GO:0048706\_embryonic\_skeletal\_system\_development | BMI1 | 73 | 2 | 7.009132 | -1.492218 | 26 | 33.07 | 1.271923 |
| GO:0048706\_embryonic\_skeletal\_system\_development | PCSK5 | 73 | 2 | 7.009132 | -1.492218 | 26 | 33.07 | 1.271923 |
| GO:0017145\_stem\_cell\_division | BMI1 | 9 | 1 | 28.425926 | -1.460124 | 28 | 37.46 | 1.337857 |
| GO:0034605\_cellular\_response\_to\_heat | ST8SIA1 | 9 | 1 | 28.425926 | -1.460124 | 28 | 37.46 | 1.337857 |
| GO:0000209\_protein\_polyubiquitination | STUB1 | 10 | 1 | 25.583333 | -1.415166 | 34 | 42.9 | 1.261765 |
| GO:0001832\_blastocyst\_growth | NCAPG2 | 10 | 1 | 25.583333 | -1.415166 | 34 | 42.9 | 1.261765 |
| GO:0008211\_glucocorticoid\_metabolic\_process | STUB1 | 10 | 1 | 25.583333 | -1.415166 | 34 | 42.9 | 1.261765 |
| GO:0016486\_peptide\_hormone\_processing | PCSK5 | 10 | 1 | 25.583333 | -1.415166 | 34 | 42.9 | 1.261765 |
| GO:0031331\_positive\_regulation\_of\_cellular\_catabolic\_process | STUB1 | 10 | 1 | 25.583333 | -1.415166 | 34 | 42.9 | 1.261765 |
| GO:0033081\_regulation\_of\_T\_cell\_differentiation\_in\_the\_thymus | BMI1 | 10 | 1 | 25.583333 | -1.415166 | 34 | 42.9 | 1.261765 |
| GO:0002717\_positive\_regulation\_of\_natural\_killer\_cell\_mediated\_immunity | CADM1 | 13 | 1 | 19.679487 | -1.303619 | 38 | 55.9 | 1.471053 |
| GO:0007566\_embryo\_implantation | PCSK5 | 13 | 1 | 19.679487 | -1.303619 | 38 | 55.9 | 1.471053 |
| GO:0045954\_positive\_regulation\_of\_natural\_killer\_cell\_mediated\_cytotoxicity | CADM1 | 13 | 1 | 19.679487 | -1.303619 | 38 | 55.9 | 1.471053 |
| GO:0048566\_embryonic\_gut\_development | PCSK5 | 13 | 1 | 19.679487 | -1.303619 | 38 | 55.9 | 1.471053 |
| GO:0008610\_lipid\_biosynthetic\_process | CYP51 | 94 | 2 | 5.443262 | -1.292182 | 39 | 56.65 | 1.452564 |
| GO:0008610\_lipid\_biosynthetic\_process | HSD17B7 | 94 | 2 | 5.443262 | -1.292182 | 39 | 56.65 | 1.452564 |
| GO:0007157\_heterophilic\_cell\_adhesion | CADM1 | 14 | 1 | 18.273810 | -1.272233 | 43 | 60.71 | 1.411860 |
| GO:0016573\_histone\_acetylation | BMI1 | 14 | 1 | 18.273810 | -1.272233 | 43 | 60.71 | 1.411860 |
| GO:0030162\_regulation\_of\_proteolysis | STUB1 | 14 | 1 | 18.273810 | -1.272233 | 43 | 60.71 | 1.411860 |
| GO:0045732\_positive\_regulation\_of\_protein\_catabolic\_process | STUB1 | 14 | 1 | 18.273810 | -1.272233 | 43 | 60.71 | 1.411860 |
| GO:0006473\_protein\_amino\_acid\_acetylation | BMI1 | 15 | 1 | 17.055556 | -1.243067 | 45 | 65.05 | 1.445556 |
| GO:0031329\_regulation\_of\_cellular\_catabolic\_process | STUB1 | 15 | 1 | 17.055556 | -1.243067 | 45 | 65.05 | 1.445556 |
| GO:0007156\_homophilic\_cell\_adhesion | CADM1 | 16 | 1 | 15.989583 | -1.215835 | 47 | 68.47 | 1.456809 |
| GO:0030890\_positive\_regulation\_of\_B\_cell\_proliferation | BMI1 | 16 | 1 | 15.989583 | -1.215835 | 47 | 68.47 | 1.456809 |
| GO:0010817\_regulation\_of\_hormone\_levels | STUB1 | 106 | 2 | 4.827044 | -1.199220 | 48 | 69.09 | 1.439375 |
| GO:0010817\_regulation\_of\_hormone\_levels | PCSK5 | 106 | 2 | 4.827044 | -1.199220 | 48 | 69.09 | 1.439375 |
| GO:0009408\_response\_to\_heat | ST8SIA1 | 17 | 1 | 15.049020 | -1.190303 | 50 | 72.15 | 1.443000 |
| GO:0051605\_protein\_maturation\_by\_peptide\_bond\_cleavage | PCSK5 | 17 | 1 | 15.049020 | -1.190303 | 50 | 72.15 | 1.443000 |
| GO:0002715\_regulation\_of\_natural\_killer\_cell\_mediated\_immunity | CADM1 | 18 | 1 | 14.212963 | -1.166276 | 55 | 75.94 | 1.380727 |
| GO:0006457\_protein\_folding | STUB1 | 18 | 1 | 14.212963 | -1.166276 | 55 | 75.94 | 1.380727 |
| GO:0010498\_proteasomal\_protein\_catabolic\_process | STUB1 | 18 | 1 | 14.212963 | -1.166276 | 55 | 75.94 | 1.380727 |
| GO:0042269\_regulation\_of\_natural\_killer\_cell\_mediated\_cytotoxicity | CADM1 | 18 | 1 | 14.212963 | -1.166276 | 55 | 75.94 | 1.380727 |
| GO:0043161\_proteasomal\_ubiquitin-dependent\_protein\_catabolic\_process | STUB1 | 18 | 1 | 14.212963 | -1.166276 | 55 | 75.94 | 1.380727 |
| GO:0019218\_regulation\_of\_steroid\_metabolic\_process | STUB1 | 19 | 1 | 13.464912 | -1.143591 | 56 | 78.87 | 1.408393 |
| GO:0001912\_positive\_regulation\_of\_leukocyte\_mediated\_cytotoxicity | CADM1 | 20 | 1 | 12.791667 | -1.122110 | 60 | 82.19 | 1.369833 |
| GO:0006518\_peptide\_metabolic\_process | PCSK5 | 20 | 1 | 12.791667 | -1.122110 | 60 | 82.19 | 1.369833 |
| GO:0007416\_synaptogenesis | CADM1 | 20 | 1 | 12.791667 | -1.122110 | 60 | 82.19 | 1.369833 |
| GO:0031343\_positive\_regulation\_of\_cell\_killing | CADM1 | 20 | 1 | 12.791667 | -1.122110 | 60 | 82.19 | 1.369833 |
| GO:0006915\_apoptosis | BMI1 | 427 | 4 | 2.396565 | -1.104977 | 61 | 82.62 | 1.354426 |
| GO:0006915\_apoptosis | CADM1 | 427 | 4 | 2.396565 | -1.104977 | 61 | 82.62 | 1.354426 |
| GO:0006915\_apoptosis | RAF1 | 427 | 4 | 2.396565 | -1.104977 | 61 | 82.62 | 1.354426 |
| GO:0006915\_apoptosis | BIRC5 | 427 | 4 | 2.396565 | -1.104977 | 61 | 82.62 | 1.354426 |
| GO:0021532\_neural\_tube\_patterning | BMI1 | 21 | 1 | 12.182540 | -1.101716 | 63 | 86.08 | 1.366349 |
| GO:0030888\_regulation\_of\_B\_cell\_proliferation | BMI1 | 21 | 1 | 12.182540 | -1.101716 | 63 | 86.08 | 1.366349 |
| GO:0044255\_cellular\_lipid\_metabolic\_process | CYP51 | 264 | 3 | 2.907197 | -1.095095 | 64 | 86.57 | 1.352656 |
| GO:0044255\_cellular\_lipid\_metabolic\_process | STUB1 | 264 | 3 | 2.907197 | -1.095095 | 64 | 86.57 | 1.352656 |
| GO:0044255\_cellular\_lipid\_metabolic\_process | HSD17B7 | 264 | 3 | 2.907197 | -1.095095 | 64 | 86.57 | 1.352656 |
| GO:0012501\_programmed\_cell\_death | BMI1 | 433 | 4 | 2.363356 | -1.087157 | 65 | 87.15 | 1.340769 |
| GO:0012501\_programmed\_cell\_death | CADM1 | 433 | 4 | 2.363356 | -1.087157 | 65 | 87.15 | 1.340769 |
| GO:0012501\_programmed\_cell\_death | RAF1 | 433 | 4 | 2.363356 | -1.087157 | 65 | 87.15 | 1.340769 |
| GO:0012501\_programmed\_cell\_death | BIRC5 | 433 | 4 | 2.363356 | -1.087157 | 65 | 87.15 | 1.340769 |
| GO:0009896\_positive\_regulation\_of\_catabolic\_process | STUB1 | 22 | 1 | 11.628788 | -1.082308 | 66 | 90.87 | 1.376818 |
| GO:0002228\_natural\_killer\_cell\_mediated\_immunity | CADM1 | 23 | 1 | 11.123188 | -1.063797 | 68 | 93.6 | 1.376471 |
| GO:0042267\_natural\_killer\_cell\_mediated\_cytotoxicity | CADM1 | 23 | 1 | 11.123188 | -1.063797 | 68 | 93.6 | 1.376471 |
| GO:0008219\_cell\_death | BMI1 | 444 | 4 | 2.304805 | -1.055356 | 69 | 94.15 | 1.364493 |
| GO:0008219\_cell\_death | CADM1 | 444 | 4 | 2.304805 | -1.055356 | 69 | 94.15 | 1.364493 |
| GO:0008219\_cell\_death | RAF1 | 444 | 4 | 2.304805 | -1.055356 | 69 | 94.15 | 1.364493 |
| GO:0008219\_cell\_death | BIRC5 | 444 | 4 | 2.304805 | -1.055356 | 69 | 94.15 | 1.364493 |
| GO:0006959\_humoral\_immune\_response | BMI1 | 24 | 1 | 10.659722 | -1.046108 | 70 | 96.47 | 1.378143 |
| GO:0006996\_organelle\_organization | BMI1 | 449 | 4 | 2.279139 | -1.041261 | 71 | 96.93 | 1.365211 |
| GO:0006996\_organelle\_organization | TFAM | 449 | 4 | 2.279139 | -1.041261 | 71 | 96.93 | 1.365211 |
| GO:0006996\_organelle\_organization | RAF1 | 449 | 4 | 2.279139 | -1.041261 | 71 | 96.93 | 1.365211 |
| GO:0006996\_organelle\_organization | BIRC5 | 449 | 4 | 2.279139 | -1.041261 | 71 | 96.93 | 1.365211 |
| GO:0016265\_death | BMI1 | 450 | 4 | 2.274074 | -1.038468 | 72 | 97.14 | 1.349167 |
| GO:0016265\_death | CADM1 | 450 | 4 | 2.274074 | -1.038468 | 72 | 97.14 | 1.349167 |
| GO:0016265\_death | RAF1 | 450 | 4 | 2.274074 | -1.038468 | 72 | 97.14 | 1.349167 |
| GO:0016265\_death | BIRC5 | 450 | 4 | 2.274074 | -1.038468 | 72 | 97.14 | 1.349167 |
| GO:0043543\_protein\_amino\_acid\_acylation | BMI1 | 25 | 1 | 10.233333 | -1.029173 | 74 | 99.2 | 1.340541 |
| GO:0045089\_positive\_regulation\_of\_innate\_immune\_response | CADM1 | 25 | 1 | 10.233333 | -1.029173 | 74 | 99.2 | 1.340541 |
| GO:0009952\_anterior\_posterior\_pattern\_formation | BMI1 | 133 | 2 | 3.847118 | -1.028030 | 75 | 99.33 | 1.324400 |
| GO:0009952\_anterior\_posterior\_pattern\_formation | PCSK5 | 133 | 2 | 3.847118 | -1.028030 | 75 | 99.33 | 1.324400 |
| GO:0006629\_lipid\_metabolic\_process | CYP51 | 285 | 3 | 2.692982 | -1.017238 | 76 | 99.93 | 1.314868 |
| GO:0006629\_lipid\_metabolic\_process | STUB1 | 285 | 3 | 2.692982 | -1.017238 | 76 | 99.93 | 1.314868 |
| GO:0006629\_lipid\_metabolic\_process | HSD17B7 | 285 | 3 | 2.692982 | -1.017238 | 76 | 99.93 | 1.314868 |
| GO:0019233\_sensory\_perception\_of\_pain | LXN | 26 | 1 | 9.839744 | -1.012933 | 77 | 102.2 | 1.327273 |
| GO:0001910\_regulation\_of\_leukocyte\_mediated\_cytotoxicity | CADM1 | 27 | 1 | 9.475309 | -0.997336 | 79 | 104.17 | 1.318608 |
| GO:0031341\_regulation\_of\_cell\_killing | CADM1 | 27 | 1 | 9.475309 | -0.997336 | 79 | 104.17 | 1.318608 |
| GO:0002705\_positive\_regulation\_of\_leukocyte\_mediated\_immunity | CADM1 | 28 | 1 | 9.136905 | -0.982334 | 83 | 106.85 | 1.287349 |
| GO:0002708\_positive\_regulation\_of\_lymphocyte\_mediated\_immunity | CADM1 | 28 | 1 | 9.136905 | -0.982334 | 83 | 106.85 | 1.287349 |
| GO:0042100\_B\_cell\_proliferation | BMI1 | 28 | 1 | 9.136905 | -0.982334 | 83 | 106.85 | 1.287349 |
| GO:0050871\_positive\_regulation\_of\_B\_cell\_activation | BMI1 | 28 | 1 | 9.136905 | -0.982334 | 83 | 106.85 | 1.287349 |
| GO:0042176\_regulation\_of\_protein\_catabolic\_process | STUB1 | 29 | 1 | 8.821839 | -0.967886 | 85 | 109.56 | 1.288941 |
| GO:0051301\_cell\_division | BMI1 | 29 | 1 | 8.821839 | -0.967886 | 85 | 109.56 | 1.288941 |
| GO:0048565\_gut\_development | PCSK5 | 30 | 1 | 8.527778 | -0.953955 | 86 | 112.35 | 1.306395 |
| GO:0002684\_positive\_regulation\_of\_immune\_system\_process | BMI1 | 148 | 2 | 3.457207 | -0.949679 | 87 | 112.62 | 1.294483 |
| GO:0002684\_positive\_regulation\_of\_immune\_system\_process | CADM1 | 148 | 2 | 3.457207 | -0.949679 | 87 | 112.62 | 1.294483 |
| GO:0045088\_regulation\_of\_innate\_immune\_response | CADM1 | 31 | 1 | 8.252688 | -0.940506 | 88 | 116.15 | 1.319886 |
| GO:0042102\_positive\_regulation\_of\_T\_cell\_proliferation | BMI1 | 32 | 1 | 7.994792 | -0.927508 | 89 | 117.82 | 1.323820 |
| GO:0007565\_female\_pregnancy | PCSK5 | 33 | 1 | 7.752525 | -0.914935 | 90 | 120.13 | 1.334778 |
| GO:0002699\_positive\_regulation\_of\_immune\_effector\_process | CADM1 | 34 | 1 | 7.524510 | -0.902760 | 91 | 122.67 | 1.348022 |
| GO:0006066\_alcohol\_metabolic\_process | CYP51 | 158 | 2 | 3.238397 | -0.902547 | 92 | 122.87 | 1.335543 |
| GO:0006066\_alcohol\_metabolic\_process | HSD17B7 | 158 | 2 | 3.238397 | -0.902547 | 92 | 122.87 | 1.335543 |
| GO:0001906\_cell\_killing | CADM1 | 35 | 1 | 7.309524 | -0.890961 | 94 | 125.05 | 1.330319 |
| GO:0001909\_leukocyte\_mediated\_cytotoxicity | CADM1 | 35 | 1 | 7.309524 | -0.890961 | 94 | 125.05 | 1.330319 |
| GO:0009628\_response\_to\_abiotic\_stimulus | LXN | 162 | 2 | 3.158436 | -0.884697 | 95 | 125.41 | 1.320105 |
| GO:0009628\_response\_to\_abiotic\_stimulus | ST8SIA1 | 162 | 2 | 3.158436 | -0.884697 | 95 | 125.41 | 1.320105 |
| GO:0031349\_positive\_regulation\_of\_defense\_response | CADM1 | 37 | 1 | 6.914414 | -0.868406 | 97 | 129.88 | 1.338969 |
| GO:0050906\_detection\_of\_stimulus\_involved\_in\_sensory\_perception | LXN | 37 | 1 | 6.914414 | -0.868406 | 97 | 129.88 | 1.338969 |
| GO:0044249\_cellular\_biosynthetic\_process | BMI1 | 1150 | 7 | 1.557246 | -0.860358 | 98 | 130.75 | 1.334184 |
| GO:0044249\_cellular\_biosynthetic\_process | CYP51 | 1150 | 7 | 1.557246 | -0.860358 | 98 | 130.75 | 1.334184 |
| GO:0044249\_cellular\_biosynthetic\_process | AHCTF1 | 1150 | 7 | 1.557246 | -0.860358 | 98 | 130.75 | 1.334184 |
| GO:0044249\_cellular\_biosynthetic\_process | PCSK5 | 1150 | 7 | 1.557246 | -0.860358 | 98 | 130.75 | 1.334184 |
| GO:0044249\_cellular\_biosynthetic\_process | CBFA2T2 | 1150 | 7 | 1.557246 | -0.860358 | 98 | 130.75 | 1.334184 |
| GO:0044249\_cellular\_biosynthetic\_process | STUB1 | 1150 | 7 | 1.557246 | -0.860358 | 98 | 130.75 | 1.334184 |
| GO:0044249\_cellular\_biosynthetic\_process | HSD17B7 | 1150 | 7 | 1.557246 | -0.860358 | 98 | 130.75 | 1.334184 |
| GO:0031401\_positive\_regulation\_of\_protein\_modification\_process | STUB1 | 38 | 1 | 6.732456 | -0.857613 | 100 | 133.1 | 1.331000 |
| GO:0045580\_regulation\_of\_T\_cell\_differentiation | BMI1 | 38 | 1 | 6.732456 | -0.857613 | 100 | 133.1 | 1.331000 |
| GO:0006511\_ubiquitin-dependent\_protein\_catabolic\_process | STUB1 | 39 | 1 | 6.559829 | -0.847120 | 103 | 135.84 | 1.318835 |
| GO:0008037\_cell\_recognition | CADM1 | 39 | 1 | 6.559829 | -0.847120 | 103 | 135.84 | 1.318835 |
| GO:0043524\_negative\_regulation\_of\_neuron\_apoptosis | BIRC5 | 39 | 1 | 6.559829 | -0.847120 | 103 | 135.84 | 1.318835 |
| GO:0001824\_blastocyst\_development | NCAPG2 | 40 | 1 | 6.395833 | -0.836913 | 105 | 138.16 | 1.315810 |
| GO:0016485\_protein\_processing | PCSK5 | 40 | 1 | 6.395833 | -0.836913 | 105 | 138.16 | 1.315810 |
| GO:0009894\_regulation\_of\_catabolic\_process | STUB1 | 41 | 1 | 6.239837 | -0.826977 | 109 | 141.3 | 1.296330 |
| GO:0019216\_regulation\_of\_lipid\_metabolic\_process | STUB1 | 41 | 1 | 6.239837 | -0.826977 | 109 | 141.3 | 1.296330 |
| GO:0033077\_T\_cell\_differentiation\_in\_the\_thymus | BMI1 | 41 | 1 | 6.239837 | -0.826977 | 109 | 141.3 | 1.296330 |
| GO:0050864\_regulation\_of\_B\_cell\_activation | BMI1 | 41 | 1 | 6.239837 | -0.826977 | 109 | 141.3 | 1.296330 |
| GO:0043066\_negative\_regulation\_of\_apoptosis | BMI1 | 176 | 2 | 2.907197 | -0.826232 | 110 | 141.58 | 1.287091 |
| GO:0043066\_negative\_regulation\_of\_apoptosis | BIRC5 | 176 | 2 | 2.907197 | -0.826232 | 110 | 141.58 | 1.287091 |
| GO:0009058\_biosynthetic\_process | BMI1 | 1175 | 7 | 1.524113 | -0.821945 | 111 | 141.81 | 1.277568 |
| GO:0009058\_biosynthetic\_process | CYP51 | 1175 | 7 | 1.524113 | -0.821945 | 111 | 141.81 | 1.277568 |
| GO:0009058\_biosynthetic\_process | AHCTF1 | 1175 | 7 | 1.524113 | -0.821945 | 111 | 141.81 | 1.277568 |
| GO:0009058\_biosynthetic\_process | STUB1 | 1175 | 7 | 1.524113 | -0.821945 | 111 | 141.81 | 1.277568 |
| GO:0009058\_biosynthetic\_process | PCSK5 | 1175 | 7 | 1.524113 | -0.821945 | 111 | 141.81 | 1.277568 |
| GO:0009058\_biosynthetic\_process | CBFA2T2 | 1175 | 7 | 1.524113 | -0.821945 | 111 | 141.81 | 1.277568 |
| GO:0009058\_biosynthetic\_process | HSD17B7 | 1175 | 7 | 1.524113 | -0.821945 | 111 | 141.81 | 1.277568 |
| GO:0019941\_modification-dependent\_protein\_catabolic\_process | STUB1 | 42 | 1 | 6.091270 | -0.817298 | 116 | 143.97 | 1.241121 |
| GO:0032946\_positive\_regulation\_of\_mononuclear\_cell\_proliferation | BMI1 | 42 | 1 | 6.091270 | -0.817298 | 116 | 143.97 | 1.241121 |
| GO:0043632\_modification-dependent\_macromolecule\_catabolic\_process | STUB1 | 42 | 1 | 6.091270 | -0.817298 | 116 | 143.97 | 1.241121 |
| GO:0050671\_positive\_regulation\_of\_lymphocyte\_proliferation | BMI1 | 42 | 1 | 6.091270 | -0.817298 | 116 | 143.97 | 1.241121 |
| GO:0051603\_proteolysis\_involved\_in\_cellular\_protein\_catabolic\_process | STUB1 | 42 | 1 | 6.091270 | -0.817298 | 116 | 143.97 | 1.241121 |
| GO:0043069\_negative\_regulation\_of\_programmed\_cell\_death | BMI1 | 179 | 2 | 2.858473 | -0.814451 | 118 | 144.5 | 1.224576 |
| GO:0043069\_negative\_regulation\_of\_programmed\_cell\_death | BIRC5 | 179 | 2 | 2.858473 | -0.814451 | 118 | 144.5 | 1.224576 |
| GO:0060548\_negative\_regulation\_of\_cell\_death | BMI1 | 179 | 2 | 2.858473 | -0.814451 | 118 | 144.5 | 1.224576 |
| GO:0060548\_negative\_regulation\_of\_cell\_death | BIRC5 | 179 | 2 | 2.858473 | -0.814451 | 118 | 144.5 | 1.224576 |
| GO:0009582\_detection\_of\_abiotic\_stimulus | LXN | 43 | 1 | 5.949612 | -0.807866 | 120 | 147.4 | 1.228333 |
| GO:0051789\_response\_to\_protein\_stimulus | EIF4A2 | 43 | 1 | 5.949612 | -0.807866 | 120 | 147.4 | 1.228333 |
| GO:0044257\_cellular\_protein\_catabolic\_process | STUB1 | 44 | 1 | 5.814394 | -0.798668 | 124 | 151.06 | 1.218226 |
| GO:0050808\_synapse\_organization | CADM1 | 44 | 1 | 5.814394 | -0.798668 | 124 | 151.06 | 1.218226 |
| GO:0070665\_positive\_regulation\_of\_leukocyte\_proliferation | BMI1 | 44 | 1 | 5.814394 | -0.798668 | 124 | 151.06 | 1.218226 |
| GO:0070668\_positive\_regulation\_of\_mast\_cell\_proliferation | BMI1 | 44 | 1 | 5.814394 | -0.798668 | 124 | 151.06 | 1.218226 |
| GO:0007010\_cytoskeleton\_organization | RAF1 | 185 | 2 | 2.765766 | -0.791610 | 125 | 151.49 | 1.211920 |
| GO:0007010\_cytoskeleton\_organization | BIRC5 | 185 | 2 | 2.765766 | -0.791610 | 125 | 151.49 | 1.211920 |
| GO:0042981\_regulation\_of\_apoptosis | BMI1 | 360 | 3 | 2.131944 | -0.791112 | 126 | 151.59 | 1.203095 |
| GO:0042981\_regulation\_of\_apoptosis | CADM1 | 360 | 3 | 2.131944 | -0.791112 | 126 | 151.59 | 1.203095 |
| GO:0042981\_regulation\_of\_apoptosis | BIRC5 | 360 | 3 | 2.131944 | -0.791112 | 126 | 151.59 | 1.203095 |
| GO:0043623\_cellular\_protein\_complex\_assembly | TFAM | 45 | 1 | 5.685185 | -0.789694 | 127 | 152.61 | 1.201654 |
| GO:0009581\_detection\_of\_external\_stimulus | LXN | 46 | 1 | 5.561594 | -0.780935 | 128 | 154.71 | 1.208672 |
| GO:0010941\_regulation\_of\_cell\_death | BMI1 | 365 | 3 | 2.102740 | -0.778360 | 131 | 155.22 | 1.184885 |
| GO:0010941\_regulation\_of\_cell\_death | CADM1 | 365 | 3 | 2.102740 | -0.778360 | 131 | 155.22 | 1.184885 |
| GO:0010941\_regulation\_of\_cell\_death | BIRC5 | 365 | 3 | 2.102740 | -0.778360 | 131 | 155.22 | 1.184885 |
| GO:0043009\_chordate\_embryonic\_development | BMI1 | 365 | 3 | 2.102740 | -0.778360 | 131 | 155.22 | 1.184885 |
| GO:0043009\_chordate\_embryonic\_development | NCAPG2 | 365 | 3 | 2.102740 | -0.778360 | 131 | 155.22 | 1.184885 |
| GO:0043009\_chordate\_embryonic\_development | PCSK5 | 365 | 3 | 2.102740 | -0.778360 | 131 | 155.22 | 1.184885 |
| GO:0043067\_regulation\_of\_programmed\_cell\_death | BMI1 | 365 | 3 | 2.102740 | -0.778360 | 131 | 155.22 | 1.184885 |
| GO:0043067\_regulation\_of\_programmed\_cell\_death | CADM1 | 365 | 3 | 2.102740 | -0.778360 | 131 | 155.22 | 1.184885 |
| GO:0043067\_regulation\_of\_programmed\_cell\_death | BIRC5 | 365 | 3 | 2.102740 | -0.778360 | 131 | 155.22 | 1.184885 |
| GO:0016570\_histone\_modification | BMI1 | 47 | 1 | 5.443262 | -0.772380 | 135 | 157.06 | 1.163407 |
| GO:0034754\_cellular\_hormone\_metabolic\_process | STUB1 | 47 | 1 | 5.443262 | -0.772380 | 135 | 157.06 | 1.163407 |
| GO:0045087\_innate\_immune\_response | CADM1 | 47 | 1 | 5.443262 | -0.772380 | 135 | 157.06 | 1.163407 |
| GO:0045619\_regulation\_of\_lymphocyte\_differentiation | BMI1 | 47 | 1 | 5.443262 | -0.772380 | 135 | 157.06 | 1.163407 |
| GO:0009792\_embryonic\_development\_ending\_in\_birth\_or\_egg\_hatching | BMI1 | 368 | 3 | 2.085598 | -0.770825 | 136 | 157.14 | 1.155441 |
| GO:0009792\_embryonic\_development\_ending\_in\_birth\_or\_egg\_hatching | NCAPG2 | 368 | 3 | 2.085598 | -0.770825 | 136 | 157.14 | 1.155441 |
| GO:0009792\_embryonic\_development\_ending\_in\_birth\_or\_egg\_hatching | PCSK5 | 368 | 3 | 2.085598 | -0.770825 | 136 | 157.14 | 1.155441 |
| GO:0009790\_embryonic\_development | BMI1 | 567 | 4 | 1.804821 | -0.762848 | 137 | 158.94 | 1.160146 |
| GO:0009790\_embryonic\_development | NCAPG2 | 567 | 4 | 1.804821 | -0.762848 | 137 | 158.94 | 1.160146 |
| GO:0009790\_embryonic\_development | BIRC5 | 567 | 4 | 1.804821 | -0.762848 | 137 | 158.94 | 1.160146 |
| GO:0009790\_embryonic\_development | PCSK5 | 567 | 4 | 1.804821 | -0.762848 | 137 | 158.94 | 1.160146 |
| GO:0003002\_regionalization | BMI1 | 195 | 2 | 2.623932 | -0.755543 | 138 | 161.81 | 1.172536 |
| GO:0003002\_regionalization | PCSK5 | 195 | 2 | 2.623932 | -0.755543 | 138 | 161.81 | 1.172536 |
| GO:0042129\_regulation\_of\_T\_cell\_proliferation | BMI1 | 50 | 1 | 5.116667 | -0.747861 | 139 | 163.53 | 1.176475 |
| GO:0016569\_covalent\_chromatin\_modification | BMI1 | 51 | 1 | 5.016340 | -0.740044 | 140 | 165.69 | 1.183500 |
| GO:0002706\_regulation\_of\_lymphocyte\_mediated\_immunity | CADM1 | 52 | 1 | 4.919872 | -0.732394 | 141 | 166.46 | 1.180567 |
| GO:0006955\_immune\_response | BMI1 | 205 | 2 | 2.495935 | -0.721755 | 142 | 168.96 | 1.189859 |
| GO:0006955\_immune\_response | CADM1 | 205 | 2 | 2.495935 | -0.721755 | 142 | 168.96 | 1.189859 |
| GO:0008284\_positive\_regulation\_of\_cell\_proliferation | BMI1 | 208 | 2 | 2.459936 | -0.712029 | 143 | 171.32 | 1.198042 |
| GO:0008284\_positive\_regulation\_of\_cell\_proliferation | ST8SIA1 | 208 | 2 | 2.459936 | -0.712029 | 143 | 171.32 | 1.198042 |
| GO:0048568\_embryonic\_organ\_development | PCSK5 | 55 | 1 | 4.651515 | -0.710382 | 145 | 172.7 | 1.191034 |
| GO:0048704\_embryonic\_skeletal\_system\_morphogenesis | BMI1 | 55 | 1 | 4.651515 | -0.710382 | 145 | 172.7 | 1.191034 |
| GO:0002703\_regulation\_of\_leukocyte\_mediated\_immunity | CADM1 | 56 | 1 | 4.568452 | -0.703339 | 148 | 175.07 | 1.182905 |
| GO:0042089\_cytokine\_biosynthetic\_process | PCSK5 | 56 | 1 | 4.568452 | -0.703339 | 148 | 175.07 | 1.182905 |
| GO:0042107\_cytokine\_metabolic\_process | PCSK5 | 56 | 1 | 4.568452 | -0.703339 | 148 | 175.07 | 1.182905 |
| GO:0000226\_microtubule\_cytoskeleton\_organization | BIRC5 | 57 | 1 | 4.488304 | -0.696433 | 150 | 177.55 | 1.183667 |
| GO:0043523\_regulation\_of\_neuron\_apoptosis | BIRC5 | 57 | 1 | 4.488304 | -0.696433 | 150 | 177.55 | 1.183667 |
| GO:0034622\_cellular\_macromolecular\_complex\_assembly | TFAM | 58 | 1 | 4.410920 | -0.689661 | 151 | 178.36 | 1.181192 |
| GO:0050870\_positive\_regulation\_of\_T\_cell\_activation | BMI1 | 59 | 1 | 4.336158 | -0.683018 | 152 | 179.72 | 1.182368 |
| GO:0045892\_negative\_regulation\_of\_transcription\_\_DNA-dependent | BMI1 | 218 | 2 | 2.347095 | -0.680882 | 153 | 179.87 | 1.175621 |
| GO:0045892\_negative\_regulation\_of\_transcription\_\_DNA-dependent | CBFA2T2 | 218 | 2 | 2.347095 | -0.680882 | 153 | 179.87 | 1.175621 |
| GO:0051253\_negative\_regulation\_of\_RNA\_metabolic\_process | BMI1 | 220 | 2 | 2.325758 | -0.674876 | 154 | 180.49 | 1.172013 |
| GO:0051253\_negative\_regulation\_of\_RNA\_metabolic\_process | CBFA2T2 | 220 | 2 | 2.325758 | -0.674876 | 154 | 180.49 | 1.172013 |
| GO:0001701\_in\_utero\_embryonic\_development | BMI1 | 221 | 2 | 2.315234 | -0.671900 | 155 | 180.68 | 1.165677 |
| GO:0001701\_in\_utero\_embryonic\_development | NCAPG2 | 221 | 2 | 2.315234 | -0.671900 | 155 | 180.68 | 1.165677 |
| GO:0007005\_mitochondrion\_organization | TFAM | 61 | 1 | 4.193989 | -0.670100 | 157 | 181.83 | 1.158153 |
| GO:0032270\_positive\_regulation\_of\_cellular\_protein\_metabolic\_process | STUB1 | 61 | 1 | 4.193989 | -0.670100 | 157 | 181.83 | 1.158153 |
| GO:0006916\_anti-apoptosis | BIRC5 | 62 | 1 | 4.126344 | -0.663817 | 161 | 183.32 | 1.138634 |
| GO:0030855\_epithelial\_cell\_differentiation | CBFA2T2 | 62 | 1 | 4.126344 | -0.663817 | 161 | 183.32 | 1.138634 |
| GO:0032944\_regulation\_of\_mononuclear\_cell\_proliferation | BMI1 | 62 | 1 | 4.126344 | -0.663817 | 161 | 183.32 | 1.138634 |
| GO:0050670\_regulation\_of\_lymphocyte\_proliferation | BMI1 | 62 | 1 | 4.126344 | -0.663817 | 161 | 183.32 | 1.138634 |
| GO:0070662\_mast\_cell\_proliferation | BMI1 | 63 | 1 | 4.060847 | -0.657647 | 163 | 184.56 | 1.132270 |
| GO:0070666\_regulation\_of\_mast\_cell\_proliferation | BMI1 | 63 | 1 | 4.060847 | -0.657647 | 163 | 184.56 | 1.132270 |
| GO:0070663\_regulation\_of\_leukocyte\_proliferation | BMI1 | 64 | 1 | 3.997396 | -0.651586 | 164 | 185.55 | 1.131402 |
| GO:0002682\_regulation\_of\_immune\_system\_process | BMI1 | 228 | 2 | 2.244152 | -0.651552 | 165 | 185.9 | 1.126667 |
| GO:0002682\_regulation\_of\_immune\_system\_process | CADM1 | 228 | 2 | 2.244152 | -0.651552 | 165 | 185.9 | 1.126667 |
| GO:0051402\_neuron\_apoptosis | BIRC5 | 66 | 1 | 3.876263 | -0.639778 | 166 | 188.1 | 1.133133 |
| GO:0031347\_regulation\_of\_defense\_response | CADM1 | 67 | 1 | 3.818408 | -0.634025 | 168 | 189.63 | 1.128750 |
| GO:0051247\_positive\_regulation\_of\_protein\_metabolic\_process | STUB1 | 67 | 1 | 3.818408 | -0.634025 | 168 | 189.63 | 1.128750 |
| GO:0001501\_skeletal\_system\_development | BMI1 | 236 | 2 | 2.168079 | -0.629291 | 169 | 189.98 | 1.124142 |
| GO:0001501\_skeletal\_system\_development | PCSK5 | 236 | 2 | 2.168079 | -0.629291 | 169 | 189.98 | 1.124142 |
| GO:0002697\_regulation\_of\_immune\_effector\_process | CADM1 | 68 | 1 | 3.762255 | -0.628367 | 171 | 190.62 | 1.114737 |
| GO:0034962\_cellular\_biopolymer\_catabolic\_process | STUB1 | 68 | 1 | 3.762255 | -0.628367 | 171 | 190.62 | 1.114737 |
| GO:0044085\_cellular\_component\_biogenesis | TFAM | 237 | 2 | 2.158931 | -0.626579 | 172 | 191.34 | 1.112442 |
| GO:0044085\_cellular\_component\_biogenesis | CADM1 | 237 | 2 | 2.158931 | -0.626579 | 172 | 191.34 | 1.112442 |
| GO:0016568\_chromatin\_modification | BMI1 | 72 | 1 | 3.553241 | -0.606648 | 175 | 198.18 | 1.132457 |
| GO:0021915\_neural\_tube\_development | BMI1 | 72 | 1 | 3.553241 | -0.606648 | 175 | 198.18 | 1.132457 |
| GO:0042098\_T\_cell\_proliferation | BMI1 | 72 | 1 | 3.553241 | -0.606648 | 175 | 198.18 | 1.132457 |
| GO:0007389\_pattern\_specification\_process | BMI1 | 250 | 2 | 2.046667 | -0.592675 | 176 | 201.43 | 1.144489 |
| GO:0007389\_pattern\_specification\_process | PCSK5 | 250 | 2 | 2.046667 | -0.592675 | 176 | 201.43 | 1.144489 |
| GO:0044265\_cellular\_macromolecule\_catabolic\_process | STUB1 | 75 | 1 | 3.411111 | -0.591242 | 178 | 202.15 | 1.135674 |
| GO:0048589\_developmental\_growth | NCAPG2 | 75 | 1 | 3.411111 | -0.591242 | 178 | 202.15 | 1.135674 |
| GO:0006508\_proteolysis | STUB1 | 76 | 1 | 3.366228 | -0.586264 | 180 | 203.31 | 1.129500 |
| GO:0034621\_cellular\_macromolecular\_complex\_subunit\_organization | TFAM | 76 | 1 | 3.366228 | -0.586264 | 180 | 203.31 | 1.129500 |
| GO:0016481\_negative\_regulation\_of\_transcription | BMI1 | 253 | 2 | 2.022398 | -0.585191 | 181 | 203.69 | 1.125359 |
| GO:0016481\_negative\_regulation\_of\_transcription | CBFA2T2 | 253 | 2 | 2.022398 | -0.585191 | 181 | 203.69 | 1.125359 |
| GO:0006461\_protein\_complex\_assembly | TFAM | 78 | 1 | 3.279915 | -0.576529 | 184 | 207.0 | 1.125000 |
| GO:0051251\_positive\_regulation\_of\_lymphocyte\_activation | BMI1 | 78 | 1 | 3.279915 | -0.576529 | 184 | 207.0 | 1.125000 |
| GO:0070271\_protein\_complex\_biogenesis | TFAM | 78 | 1 | 3.279915 | -0.576529 | 184 | 207.0 | 1.125000 |
| GO:0010467\_gene\_expression | BMI1 | 905 | 5 | 1.413444 | -0.568034 | 185 | 208.21 | 1.125459 |
| GO:0010467\_gene\_expression | AHCTF1 | 905 | 5 | 1.413444 | -0.568034 | 185 | 208.21 | 1.125459 |
| GO:0010467\_gene\_expression | STUB1 | 905 | 5 | 1.413444 | -0.568034 | 185 | 208.21 | 1.125459 |
| GO:0010467\_gene\_expression | PCSK5 | 905 | 5 | 1.413444 | -0.568034 | 185 | 208.21 | 1.125459 |
| GO:0010467\_gene\_expression | CBFA2T2 | 905 | 5 | 1.413444 | -0.568034 | 185 | 208.21 | 1.125459 |
| GO:0010629\_negative\_regulation\_of\_gene\_expression | BMI1 | 262 | 2 | 1.952926 | -0.563450 | 186 | 209.93 | 1.128656 |
| GO:0010629\_negative\_regulation\_of\_gene\_expression | CBFA2T2 | 262 | 2 | 1.952926 | -0.563450 | 186 | 209.93 | 1.128656 |
| GO:0002696\_positive\_regulation\_of\_leukocyte\_activation | BMI1 | 82 | 1 | 3.119919 | -0.557898 | 187 | 211.82 | 1.132727 |
| GO:0006325\_chromatin\_organization | BMI1 | 83 | 1 | 3.082329 | -0.553405 | 190 | 213.59 | 1.124158 |
| GO:0007017\_microtubule-based\_process | BIRC5 | 83 | 1 | 3.082329 | -0.553405 | 190 | 213.59 | 1.124158 |
| GO:0050867\_positive\_regulation\_of\_cell\_activation | BMI1 | 83 | 1 | 3.082329 | -0.553405 | 190 | 213.59 | 1.124158 |
| GO:0045934\_negative\_regulation\_of\_nucleobase\_\_nucleoside\_\_nucleotide\_and\_nucleic\_acid\_metabolic\_process | BMI1 | 270 | 2 | 1.895062 | -0.544976 | 191 | 214.77 | 1.124450 |
| GO:0045934\_negative\_regulation\_of\_nucleobase\_\_nucleoside\_\_nucleotide\_and\_nucleic\_acid\_metabolic\_process | CBFA2T2 | 270 | 2 | 1.895062 | -0.544976 | 191 | 214.77 | 1.124450 |
| GO:0002449\_lymphocyte\_mediated\_immunity | CADM1 | 85 | 1 | 3.009804 | -0.544604 | 192 | 215.42 | 1.121979 |
| GO:0051172\_negative\_regulation\_of\_nitrogen\_compound\_metabolic\_process | BMI1 | 271 | 2 | 1.888069 | -0.542721 | 193 | 215.58 | 1.116995 |
| GO:0051172\_negative\_regulation\_of\_nitrogen\_compound\_metabolic\_process | CBFA2T2 | 271 | 2 | 1.888069 | -0.542721 | 193 | 215.58 | 1.116995 |
| GO:0001822\_kidney\_development | PCSK5 | 87 | 1 | 2.940613 | -0.536043 | 196 | 219.67 | 1.120765 |
| GO:0016337\_cell-cell\_adhesion | CADM1 | 87 | 1 | 2.940613 | -0.536043 | 196 | 219.67 | 1.120765 |
| GO:0050778\_positive\_regulation\_of\_immune\_response | CADM1 | 87 | 1 | 2.940613 | -0.536043 | 196 | 219.67 | 1.120765 |
| GO:0010558\_negative\_regulation\_of\_macromolecule\_biosynthetic\_process | BMI1 | 274 | 2 | 1.867397 | -0.536025 | 197 | 219.97 | 1.116599 |
| GO:0010558\_negative\_regulation\_of\_macromolecule\_biosynthetic\_process | CBFA2T2 | 274 | 2 | 1.867397 | -0.536025 | 197 | 219.97 | 1.116599 |
| GO:0050863\_regulation\_of\_T\_cell\_activation | BMI1 | 88 | 1 | 2.907197 | -0.531849 | 198 | 220.64 | 1.114343 |
| GO:0042113\_B\_cell\_activation | BMI1 | 90 | 1 | 2.842593 | -0.523626 | 199 | 222.58 | 1.118492 |
| GO:0002443\_leukocyte\_mediated\_immunity | CADM1 | 91 | 1 | 2.811355 | -0.519595 | 201 | 223.39 | 1.111393 |
| GO:0031399\_regulation\_of\_protein\_modification\_process | STUB1 | 91 | 1 | 2.811355 | -0.519595 | 201 | 223.39 | 1.111393 |
| GO:0031327\_negative\_regulation\_of\_cellular\_biosynthetic\_process | BMI1 | 282 | 2 | 1.814421 | -0.518663 | 202 | 223.53 | 1.106584 |
| GO:0031327\_negative\_regulation\_of\_cellular\_biosynthetic\_process | CBFA2T2 | 282 | 2 | 1.814421 | -0.518663 | 202 | 223.53 | 1.106584 |
| GO:0030217\_T\_cell\_differentiation | BMI1 | 92 | 1 | 2.780797 | -0.515616 | 204 | 224.76 | 1.101765 |
| GO:0030323\_respiratory\_tube\_development | PCSK5 | 92 | 1 | 2.780797 | -0.515616 | 204 | 224.76 | 1.101765 |
| GO:0009890\_negative\_regulation\_of\_biosynthetic\_process | BMI1 | 284 | 2 | 1.801643 | -0.514431 | 205 | 224.9 | 1.097073 |
| GO:0009890\_negative\_regulation\_of\_biosynthetic\_process | CBFA2T2 | 284 | 2 | 1.801643 | -0.514431 | 205 | 224.9 | 1.097073 |
| GO:0035107\_appendage\_morphogenesis | PCSK5 | 93 | 1 | 2.750896 | -0.511687 | 208 | 226.33 | 1.088125 |
| GO:0035108\_limb\_morphogenesis | PCSK5 | 93 | 1 | 2.750896 | -0.511687 | 208 | 226.33 | 1.088125 |
| GO:0065003\_macromolecular\_complex\_assembly | TFAM | 93 | 1 | 2.750896 | -0.511687 | 208 | 226.33 | 1.088125 |
| GO:0032943\_mononuclear\_cell\_proliferation | BMI1 | 94 | 1 | 2.721631 | -0.507809 | 210 | 227.82 | 1.084857 |
| GO:0046651\_lymphocyte\_proliferation | BMI1 | 94 | 1 | 2.721631 | -0.507809 | 210 | 227.82 | 1.084857 |
| GO:0048736\_appendage\_development | PCSK5 | 96 | 1 | 2.664931 | -0.500198 | 213 | 230.1 | 1.080282 |
| GO:0060173\_limb\_development | PCSK5 | 96 | 1 | 2.664931 | -0.500198 | 213 | 230.1 | 1.080282 |
| GO:0070661\_leukocyte\_proliferation | BMI1 | 96 | 1 | 2.664931 | -0.500198 | 213 | 230.1 | 1.080282 |
| GO:0016043\_cellular\_component\_organization | BMI1 | 964 | 5 | 1.326936 | -0.495927 | 214 | 230.68 | 1.077944 |
| GO:0016043\_cellular\_component\_organization | TFAM | 964 | 5 | 1.326936 | -0.495927 | 214 | 230.68 | 1.077944 |
| GO:0016043\_cellular\_component\_organization | CADM1 | 964 | 5 | 1.326936 | -0.495927 | 214 | 230.68 | 1.077944 |
| GO:0016043\_cellular\_component\_organization | RAF1 | 964 | 5 | 1.326936 | -0.495927 | 214 | 230.68 | 1.077944 |
| GO:0016043\_cellular\_component\_organization | BIRC5 | 964 | 5 | 1.326936 | -0.495927 | 214 | 230.68 | 1.077944 |
| GO:0030163\_protein\_catabolic\_process | STUB1 | 101 | 1 | 2.533003 | -0.481970 | 215 | 234.67 | 1.091488 |
| GO:0048705\_skeletal\_system\_morphogenesis | BMI1 | 111 | 1 | 2.304805 | -0.448584 | 216 | 241.79 | 1.119398 |
| GO:0051249\_regulation\_of\_lymphocyte\_activation | BMI1 | 112 | 1 | 2.284226 | -0.445448 | 217 | 242.65 | 1.118203 |
| GO:0008283\_cell\_proliferation | BMI1 | 544 | 3 | 1.410846 | -0.444460 | 218 | 242.83 | 1.113899 |
| GO:0008283\_cell\_proliferation | NCAPG2 | 544 | 3 | 1.410846 | -0.444460 | 218 | 242.83 | 1.113899 |
| GO:0008283\_cell\_proliferation | ST8SIA1 | 544 | 3 | 1.410846 | -0.444460 | 218 | 242.83 | 1.113899 |
| GO:0048584\_positive\_regulation\_of\_response\_to\_stimulus | CADM1 | 115 | 1 | 2.224638 | -0.436244 | 219 | 245.02 | 1.118813 |
| GO:0080134\_regulation\_of\_response\_to\_stress | CADM1 | 116 | 1 | 2.205460 | -0.433241 | 220 | 245.89 | 1.117682 |
| GO:0043933\_macromolecular\_complex\_subunit\_organization | TFAM | 117 | 1 | 2.186610 | -0.430270 | 221 | 246.17 | 1.113891 |
| GO:0010605\_negative\_regulation\_of\_macromolecule\_metabolic\_process | BMI1 | 331 | 2 | 1.545821 | -0.426006 | 223 | 247.72 | 1.110852 |
| GO:0010605\_negative\_regulation\_of\_macromolecule\_metabolic\_process | CBFA2T2 | 331 | 2 | 1.545821 | -0.426006 | 223 | 247.72 | 1.110852 |
| GO:0051093\_negative\_regulation\_of\_developmental\_process | BMI1 | 331 | 2 | 1.545821 | -0.426006 | 223 | 247.72 | 1.110852 |
| GO:0051093\_negative\_regulation\_of\_developmental\_process | BIRC5 | 331 | 2 | 1.545821 | -0.426006 | 223 | 247.72 | 1.110852 |
| GO:0031324\_negative\_regulation\_of\_cellular\_metabolic\_process | BMI1 | 332 | 2 | 1.541165 | -0.424329 | 224 | 248.53 | 1.109509 |
| GO:0031324\_negative\_regulation\_of\_cellular\_metabolic\_process | CBFA2T2 | 332 | 2 | 1.541165 | -0.424329 | 224 | 248.53 | 1.109509 |
| GO:0002694\_regulation\_of\_leukocyte\_activation | BMI1 | 121 | 1 | 2.114325 | -0.418696 | 227 | 249.94 | 1.101057 |
| GO:0006917\_induction\_of\_apoptosis | CADM1 | 121 | 1 | 2.114325 | -0.418696 | 227 | 249.94 | 1.101057 |
| GO:0012502\_induction\_of\_programmed\_cell\_death | CADM1 | 121 | 1 | 2.114325 | -0.418696 | 227 | 249.94 | 1.101057 |
| GO:0001816\_cytokine\_production | PCSK5 | 122 | 1 | 2.096995 | -0.415877 | 231 | 251.47 | 1.088615 |
| GO:0002252\_immune\_effector\_process | CADM1 | 122 | 1 | 2.096995 | -0.415877 | 231 | 251.47 | 1.088615 |
| GO:0006886\_intracellular\_protein\_transport | AP3S1 | 122 | 1 | 2.096995 | -0.415877 | 231 | 251.47 | 1.088615 |
| GO:0050865\_regulation\_of\_cell\_activation | BMI1 | 122 | 1 | 2.096995 | -0.415877 | 231 | 251.47 | 1.088615 |
| GO:0030098\_lymphocyte\_differentiation | BMI1 | 124 | 1 | 2.063172 | -0.410324 | 232 | 253.3 | 1.091810 |
| GO:0043062\_extracellular\_structure\_organization | CADM1 | 125 | 1 | 2.046667 | -0.407590 | 233 | 254.36 | 1.091674 |
| GO:0031326\_regulation\_of\_cellular\_biosynthetic\_process | BMI1 | 812 | 4 | 1.260263 | -0.403446 | 234 | 254.83 | 1.089017 |
| GO:0031326\_regulation\_of\_cellular\_biosynthetic\_process | AHCTF1 | 812 | 4 | 1.260263 | -0.403446 | 234 | 254.83 | 1.089017 |
| GO:0031326\_regulation\_of\_cellular\_biosynthetic\_process | CBFA2T2 | 812 | 4 | 1.260263 | -0.403446 | 234 | 254.83 | 1.089017 |
| GO:0031326\_regulation\_of\_cellular\_biosynthetic\_process | STUB1 | 812 | 4 | 1.260263 | -0.403446 | 234 | 254.83 | 1.089017 |
| GO:0009889\_regulation\_of\_biosynthetic\_process | BMI1 | 815 | 4 | 1.255624 | -0.400272 | 235 | 255.27 | 1.086255 |
| GO:0009889\_regulation\_of\_biosynthetic\_process | AHCTF1 | 815 | 4 | 1.255624 | -0.400272 | 235 | 255.27 | 1.086255 |
| GO:0009889\_regulation\_of\_biosynthetic\_process | STUB1 | 815 | 4 | 1.255624 | -0.400272 | 235 | 255.27 | 1.086255 |
| GO:0009889\_regulation\_of\_biosynthetic\_process | CBFA2T2 | 815 | 4 | 1.255624 | -0.400272 | 235 | 255.27 | 1.086255 |
| GO:0001655\_urogenital\_system\_development | PCSK5 | 128 | 1 | 1.998698 | -0.399549 | 236 | 256.17 | 1.085466 |
| GO:0009892\_negative\_regulation\_of\_metabolic\_process | BMI1 | 348 | 2 | 1.470307 | -0.398521 | 237 | 256.33 | 1.081561 |
| GO:0009892\_negative\_regulation\_of\_metabolic\_process | CBFA2T2 | 348 | 2 | 1.470307 | -0.398521 | 237 | 256.33 | 1.081561 |
| GO:0043285\_biopolymer\_catabolic\_process | STUB1 | 129 | 1 | 1.983204 | -0.396922 | 239 | 257.03 | 1.075439 |
| GO:0051276\_chromosome\_organization | BMI1 | 129 | 1 | 1.983204 | -0.396922 | 239 | 257.03 | 1.075439 |
| GO:0050776\_regulation\_of\_immune\_response | CADM1 | 130 | 1 | 1.967949 | -0.394320 | 240 | 258.14 | 1.075583 |
| GO:0007283\_spermatogenesis | IFT81 | 134 | 1 | 1.909204 | -0.384162 | 242 | 260.15 | 1.075000 |
| GO:0048232\_male\_gamete\_generation | IFT81 | 134 | 1 | 1.909204 | -0.384162 | 242 | 260.15 | 1.075000 |
| GO:0009057\_macromolecule\_catabolic\_process | STUB1 | 137 | 1 | 1.867397 | -0.376795 | 243 | 262.23 | 1.079136 |
| GO:0007169\_transmembrane\_receptor\_protein\_tyrosine\_kinase\_signaling\_pathway | RAF1 | 139 | 1 | 1.840528 | -0.371998 | 245 | 263.88 | 1.077061 |
| GO:0034613\_cellular\_protein\_localization | AP3S1 | 139 | 1 | 1.840528 | -0.371998 | 245 | 263.88 | 1.077061 |
| GO:0070727\_cellular\_macromolecule\_localization | AP3S1 | 141 | 1 | 1.814421 | -0.367288 | 246 | 265.23 | 1.078171 |
| GO:0022414\_reproductive\_process | IFT81 | 376 | 2 | 1.360816 | -0.357548 | 247 | 267.37 | 1.082470 |
| GO:0022414\_reproductive\_process | PCSK5 | 376 | 2 | 1.360816 | -0.357548 | 247 | 267.37 | 1.082470 |
| GO:0050896\_response\_to\_stimulus | BMI1 | 1107 | 5 | 1.155525 | -0.353623 | 248 | 269.01 | 1.084718 |
| GO:0050896\_response\_to\_stimulus | CADM1 | 1107 | 5 | 1.155525 | -0.353623 | 248 | 269.01 | 1.084718 |
| GO:0050896\_response\_to\_stimulus | LXN | 1107 | 5 | 1.155525 | -0.353623 | 248 | 269.01 | 1.084718 |
| GO:0050896\_response\_to\_stimulus | EIF4A2 | 1107 | 5 | 1.155525 | -0.353623 | 248 | 269.01 | 1.084718 |
| GO:0050896\_response\_to\_stimulus | ST8SIA1 | 1107 | 5 | 1.155525 | -0.353623 | 248 | 269.01 | 1.084718 |
| GO:0000003\_reproduction | IFT81 | 379 | 2 | 1.350044 | -0.353446 | 249 | 269.23 | 1.081245 |
| GO:0000003\_reproduction | PCSK5 | 379 | 2 | 1.350044 | -0.353446 | 249 | 269.23 | 1.081245 |
| GO:0043687\_post-translational\_protein\_modification | BMI1 | 384 | 2 | 1.332465 | -0.346726 | 250 | 271.32 | 1.085280 |
| GO:0043687\_post-translational\_protein\_modification | STUB1 | 384 | 2 | 1.332465 | -0.346726 | 250 | 271.32 | 1.085280 |
| GO:0032268\_regulation\_of\_cellular\_protein\_metabolic\_process | STUB1 | 152 | 1 | 1.683114 | -0.342858 | 251 | 271.82 | 1.082948 |
| GO:0042127\_regulation\_of\_cell\_proliferation | BMI1 | 393 | 2 | 1.301951 | -0.334984 | 252 | 274.08 | 1.087619 |
| GO:0042127\_regulation\_of\_cell\_proliferation | ST8SIA1 | 393 | 2 | 1.301951 | -0.334984 | 252 | 274.08 | 1.087619 |
| GO:0051704\_multi-organism\_process | PCSK5 | 157 | 1 | 1.629512 | -0.332510 | 253 | 274.35 | 1.084387 |
| GO:0002521\_leukocyte\_differentiation | BMI1 | 161 | 1 | 1.589027 | -0.324543 | 254 | 276.53 | 1.088701 |
| GO:0048522\_positive\_regulation\_of\_cellular\_process | BMI1 | 895 | 4 | 1.143389 | -0.323632 | 255 | 276.7 | 1.085098 |
| GO:0048522\_positive\_regulation\_of\_cellular\_process | CADM1 | 895 | 4 | 1.143389 | -0.323632 | 255 | 276.7 | 1.085098 |
| GO:0048522\_positive\_regulation\_of\_cellular\_process | ST8SIA1 | 895 | 4 | 1.143389 | -0.323632 | 255 | 276.7 | 1.085098 |
| GO:0048522\_positive\_regulation\_of\_cellular\_process | STUB1 | 895 | 4 | 1.143389 | -0.323632 | 255 | 276.7 | 1.085098 |
| GO:0042110\_T\_cell\_activation | BMI1 | 163 | 1 | 1.569530 | -0.320659 | 256 | 278.12 | 1.086406 |
| GO:0034645\_cellular\_macromolecule\_biosynthetic\_process | BMI1 | 901 | 4 | 1.135775 | -0.318461 | 257 | 278.73 | 1.084553 |
| GO:0034645\_cellular\_macromolecule\_biosynthetic\_process | AHCTF1 | 901 | 4 | 1.135775 | -0.318461 | 257 | 278.73 | 1.084553 |
| GO:0034645\_cellular\_macromolecule\_biosynthetic\_process | PCSK5 | 901 | 4 | 1.135775 | -0.318461 | 257 | 278.73 | 1.084553 |
| GO:0034645\_cellular\_macromolecule\_biosynthetic\_process | CBFA2T2 | 901 | 4 | 1.135775 | -0.318461 | 257 | 278.73 | 1.084553 |
| GO:0019538\_protein\_metabolic\_process | BMI1 | 655 | 3 | 1.171756 | -0.316394 | 258 | 280.27 | 1.086318 |
| GO:0019538\_protein\_metabolic\_process | PCSK5 | 655 | 3 | 1.171756 | -0.316394 | 258 | 280.27 | 1.086318 |
| GO:0019538\_protein\_metabolic\_process | STUB1 | 655 | 3 | 1.171756 | -0.316394 | 258 | 280.27 | 1.086318 |
| GO:0043065\_positive\_regulation\_of\_apoptosis | CADM1 | 166 | 1 | 1.541165 | -0.314951 | 259 | 281.34 | 1.086255 |
| GO:0010942\_positive\_regulation\_of\_cell\_death | CADM1 | 167 | 1 | 1.531936 | -0.313079 | 261 | 282.63 | 1.082874 |
| GO:0043068\_positive\_regulation\_of\_programmed\_cell\_death | CADM1 | 167 | 1 | 1.531936 | -0.313079 | 261 | 282.63 | 1.082874 |
| GO:0009059\_macromolecule\_biosynthetic\_process | BMI1 | 910 | 4 | 1.124542 | -0.310845 | 262 | 283.07 | 1.080420 |
| GO:0009059\_macromolecule\_biosynthetic\_process | AHCTF1 | 910 | 4 | 1.124542 | -0.310845 | 262 | 283.07 | 1.080420 |
| GO:0009059\_macromolecule\_biosynthetic\_process | PCSK5 | 910 | 4 | 1.124542 | -0.310845 | 262 | 283.07 | 1.080420 |
| GO:0009059\_macromolecule\_biosynthetic\_process | CBFA2T2 | 910 | 4 | 1.124542 | -0.310845 | 262 | 283.07 | 1.080420 |
| GO:0051246\_regulation\_of\_protein\_metabolic\_process | STUB1 | 170 | 1 | 1.504902 | -0.307554 | 263 | 283.76 | 1.078935 |
| GO:0007600\_sensory\_perception | LXN | 172 | 1 | 1.487403 | -0.303944 | 264 | 284.67 | 1.078295 |
| GO:0044248\_cellular\_catabolic\_process | STUB1 | 173 | 1 | 1.478805 | -0.302160 | 265 | 286.01 | 1.079283 |
| GO:0000122\_negative\_regulation\_of\_transcription\_from\_RNA\_polymerase\_II\_promoter | BMI1 | 175 | 1 | 1.461905 | -0.298635 | 267 | 287.57 | 1.077041 |
| GO:0015031\_protein\_transport | AP3S1 | 175 | 1 | 1.461905 | -0.298635 | 267 | 287.57 | 1.077041 |
| GO:0080090\_regulation\_of\_primary\_metabolic\_process | BMI1 | 926 | 4 | 1.105112 | -0.297708 | 268 | 287.8 | 1.073881 |
| GO:0080090\_regulation\_of\_primary\_metabolic\_process | AHCTF1 | 926 | 4 | 1.105112 | -0.297708 | 268 | 287.8 | 1.073881 |
| GO:0080090\_regulation\_of\_primary\_metabolic\_process | CBFA2T2 | 926 | 4 | 1.105112 | -0.297708 | 268 | 287.8 | 1.073881 |
| GO:0080090\_regulation\_of\_primary\_metabolic\_process | STUB1 | 926 | 4 | 1.105112 | -0.297708 | 268 | 287.8 | 1.073881 |
| GO:0045449\_regulation\_of\_transcription | BMI1 | 676 | 3 | 1.135355 | -0.296635 | 269 | 289.25 | 1.075279 |
| GO:0045449\_regulation\_of\_transcription | AHCTF1 | 676 | 3 | 1.135355 | -0.296635 | 269 | 289.25 | 1.075279 |
| GO:0045449\_regulation\_of\_transcription | CBFA2T2 | 676 | 3 | 1.135355 | -0.296635 | 269 | 289.25 | 1.075279 |
| GO:0045184\_establishment\_of\_protein\_localization | AP3S1 | 180 | 1 | 1.421296 | -0.290061 | 270 | 291.45 | 1.079444 |
| GO:0060255\_regulation\_of\_macromolecule\_metabolic\_process | BMI1 | 936 | 4 | 1.093305 | -0.289752 | 271 | 291.65 | 1.076199 |
| GO:0060255\_regulation\_of\_macromolecule\_metabolic\_process | AHCTF1 | 936 | 4 | 1.093305 | -0.289752 | 271 | 291.65 | 1.076199 |
| GO:0060255\_regulation\_of\_macromolecule\_metabolic\_process | STUB1 | 936 | 4 | 1.093305 | -0.289752 | 271 | 291.65 | 1.076199 |
| GO:0060255\_regulation\_of\_macromolecule\_metabolic\_process | CBFA2T2 | 936 | 4 | 1.093305 | -0.289752 | 271 | 291.65 | 1.076199 |
| GO:0016192\_vesicle-mediated\_transport | AP3S1 | 184 | 1 | 1.390399 | -0.283437 | 272 | 295.42 | 1.086103 |
| GO:0006464\_protein\_modification\_process | BMI1 | 439 | 2 | 1.165528 | -0.281362 | 273 | 296.36 | 1.085568 |
| GO:0006464\_protein\_modification\_process | STUB1 | 439 | 2 | 1.165528 | -0.281362 | 273 | 296.36 | 1.085568 |
| GO:0007155\_cell\_adhesion | CADM1 | 186 | 1 | 1.375448 | -0.280199 | 275 | 297.29 | 1.081055 |
| GO:0022610\_biological\_adhesion | CADM1 | 186 | 1 | 1.375448 | -0.280199 | 275 | 297.29 | 1.081055 |
| GO:0006952\_defense\_response | CADM1 | 187 | 1 | 1.368093 | -0.278599 | 276 | 297.67 | 1.078514 |
| GO:0007276\_gamete\_generation | IFT81 | 188 | 1 | 1.360816 | -0.277011 | 277 | 298.31 | 1.076931 |
| GO:0006350\_transcription | BMI1 | 701 | 3 | 1.094864 | -0.274660 | 278 | 299.64 | 1.077842 |
| GO:0006350\_transcription | AHCTF1 | 701 | 3 | 1.094864 | -0.274660 | 278 | 299.64 | 1.077842 |
| GO:0006350\_transcription | CBFA2T2 | 701 | 3 | 1.094864 | -0.274660 | 278 | 299.64 | 1.077842 |
| GO:0050793\_regulation\_of\_developmental\_process | BMI1 | 703 | 3 | 1.091750 | -0.272971 | 279 | 300.18 | 1.075914 |
| GO:0050793\_regulation\_of\_developmental\_process | CADM1 | 703 | 3 | 1.091750 | -0.272971 | 279 | 300.18 | 1.075914 |
| GO:0050793\_regulation\_of\_developmental\_process | BIRC5 | 703 | 3 | 1.091750 | -0.272971 | 279 | 300.18 | 1.075914 |
| GO:0046907\_intracellular\_transport | AP3S1 | 194 | 1 | 1.318729 | -0.267725 | 280 | 301.75 | 1.077679 |
| GO:0007507\_heart\_development | PCSK5 | 195 | 1 | 1.311966 | -0.266217 | 281 | 302.95 | 1.078114 |
| GO:0033554\_cellular\_response\_to\_stress | ST8SIA1 | 196 | 1 | 1.305272 | -0.264720 | 282 | 303.28 | 1.075461 |
| GO:0043412\_biopolymer\_modification | BMI1 | 458 | 2 | 1.117176 | -0.261966 | 283 | 304.57 | 1.076219 |
| GO:0043412\_biopolymer\_modification | STUB1 | 458 | 2 | 1.117176 | -0.261966 | 283 | 304.57 | 1.076219 |
| GO:0002009\_morphogenesis\_of\_an\_epithelium | CBFA2T2 | 198 | 1 | 1.292088 | -0.261759 | 285 | 305.39 | 1.071544 |
| GO:0060429\_epithelium\_development | CBFA2T2 | 198 | 1 | 1.292088 | -0.261759 | 285 | 305.39 | 1.071544 |
| GO:0022607\_cellular\_component\_assembly | TFAM | 204 | 1 | 1.254085 | -0.253127 | 286 | 307.14 | 1.073916 |
| GO:0007243\_protein\_kinase\_cascade | RAF1 | 205 | 1 | 1.247967 | -0.251724 | 287 | 308.09 | 1.073484 |
| GO:0048518\_positive\_regulation\_of\_biological\_process | BMI1 | 995 | 4 | 1.028476 | -0.246543 | 288 | 309.32 | 1.074028 |
| GO:0048518\_positive\_regulation\_of\_biological\_process | CADM1 | 995 | 4 | 1.028476 | -0.246543 | 288 | 309.32 | 1.074028 |
| GO:0048518\_positive\_regulation\_of\_biological\_process | ST8SIA1 | 995 | 4 | 1.028476 | -0.246543 | 288 | 309.32 | 1.074028 |
| GO:0048518\_positive\_regulation\_of\_biological\_process | STUB1 | 995 | 4 | 1.028476 | -0.246543 | 288 | 309.32 | 1.074028 |
| GO:0035295\_tube\_development | PCSK5 | 212 | 1 | 1.206761 | -0.242175 | 289 | 309.72 | 1.071696 |
| GO:0010556\_regulation\_of\_macromolecule\_biosynthetic\_process | BMI1 | 745 | 3 | 1.030201 | -0.239698 | 290 | 310.21 | 1.069690 |
| GO:0010556\_regulation\_of\_macromolecule\_biosynthetic\_process | AHCTF1 | 745 | 3 | 1.030201 | -0.239698 | 290 | 310.21 | 1.069690 |
| GO:0010556\_regulation\_of\_macromolecule\_biosynthetic\_process | CBFA2T2 | 745 | 3 | 1.030201 | -0.239698 | 290 | 310.21 | 1.069690 |
| GO:0010033\_response\_to\_organic\_substance | EIF4A2 | 216 | 1 | 1.184414 | -0.236924 | 291 | 310.58 | 1.067285 |
| GO:0040007\_growth | NCAPG2 | 217 | 1 | 1.178955 | -0.235634 | 293 | 311.25 | 1.062287 |
| GO:0048583\_regulation\_of\_response\_to\_stimulus | CADM1 | 217 | 1 | 1.178955 | -0.235634 | 293 | 311.25 | 1.062287 |
| GO:0031323\_regulation\_of\_cellular\_metabolic\_process | BMI1 | 1015 | 4 | 1.008210 | -0.233247 | 294 | 311.78 | 1.060476 |
| GO:0031323\_regulation\_of\_cellular\_metabolic\_process | AHCTF1 | 1015 | 4 | 1.008210 | -0.233247 | 294 | 311.78 | 1.060476 |
| GO:0031323\_regulation\_of\_cellular\_metabolic\_process | STUB1 | 1015 | 4 | 1.008210 | -0.233247 | 294 | 311.78 | 1.060476 |
| GO:0031323\_regulation\_of\_cellular\_metabolic\_process | CBFA2T2 | 1015 | 4 | 1.008210 | -0.233247 | 294 | 311.78 | 1.060476 |
| GO:0019219\_regulation\_of\_nucleobase\_\_nucleoside\_\_nucleotide\_and\_nucleic\_acid\_metabolic\_process | BMI1 | 757 | 3 | 1.013871 | -0.230918 | 295 | 312.77 | 1.060237 |
| GO:0019219\_regulation\_of\_nucleobase\_\_nucleoside\_\_nucleotide\_and\_nucleic\_acid\_metabolic\_process | AHCTF1 | 757 | 3 | 1.013871 | -0.230918 | 295 | 312.77 | 1.060237 |
| GO:0019219\_regulation\_of\_nucleobase\_\_nucleoside\_\_nucleotide\_and\_nucleic\_acid\_metabolic\_process | CBFA2T2 | 757 | 3 | 1.013871 | -0.230918 | 295 | 312.77 | 1.060237 |
| GO:0019953\_sexual\_reproduction | IFT81 | 228 | 1 | 1.122076 | -0.222003 | 297 | 314.27 | 1.058148 |
| GO:0046649\_lymphocyte\_activation | BMI1 | 228 | 1 | 1.122076 | -0.222003 | 297 | 314.27 | 1.058148 |
| GO:0051171\_regulation\_of\_nitrogen\_compound\_metabolic\_process | BMI1 | 771 | 3 | 0.995460 | -0.221055 | 298 | 314.75 | 1.056208 |
| GO:0051171\_regulation\_of\_nitrogen\_compound\_metabolic\_process | AHCTF1 | 771 | 3 | 0.995460 | -0.221055 | 298 | 314.75 | 1.056208 |
| GO:0051171\_regulation\_of\_nitrogen\_compound\_metabolic\_process | CBFA2T2 | 771 | 3 | 0.995460 | -0.221055 | 298 | 314.75 | 1.056208 |
| GO:0007167\_enzyme\_linked\_receptor\_protein\_signaling\_pathway | RAF1 | 229 | 1 | 1.117176 | -0.220813 | 299 | 315.16 | 1.054047 |
| GO:0002376\_immune\_system\_process | BMI1 | 505 | 2 | 1.013201 | -0.219754 | 300 | 315.38 | 1.051267 |
| GO:0002376\_immune\_system\_process | CADM1 | 505 | 2 | 1.013201 | -0.219754 | 300 | 315.38 | 1.051267 |
| GO:0048523\_negative\_regulation\_of\_cellular\_process | BMI1 | 774 | 3 | 0.991602 | -0.218994 | 301 | 315.52 | 1.048239 |
| GO:0048523\_negative\_regulation\_of\_cellular\_process | BIRC5 | 774 | 3 | 0.991602 | -0.218994 | 301 | 315.52 | 1.048239 |
| GO:0048523\_negative\_regulation\_of\_cellular\_process | CBFA2T2 | 774 | 3 | 0.991602 | -0.218994 | 301 | 315.52 | 1.048239 |
| GO:0007420\_brain\_development | BMI1 | 231 | 1 | 1.107504 | -0.218456 | 302 | 315.91 | 1.046060 |
| GO:0010468\_regulation\_of\_gene\_expression | BMI1 | 778 | 3 | 0.986504 | -0.216273 | 303 | 316.21 | 1.043597 |
| GO:0010468\_regulation\_of\_gene\_expression | AHCTF1 | 778 | 3 | 0.986504 | -0.216273 | 303 | 316.21 | 1.043597 |
| GO:0010468\_regulation\_of\_gene\_expression | CBFA2T2 | 778 | 3 | 0.986504 | -0.216273 | 303 | 316.21 | 1.043597 |
| GO:0050890\_cognition | LXN | 233 | 1 | 1.097997 | -0.216130 | 304 | 317.04 | 1.042895 |
| GO:0016477\_cell\_migration | DOCK1 | 234 | 1 | 1.093305 | -0.214979 | 305 | 317.41 | 1.040689 |
| GO:0009056\_catabolic\_process | STUB1 | 243 | 1 | 1.052812 | -0.204945 | 306 | 320.55 | 1.047549 |
| GO:0045321\_leukocyte\_activation | BMI1 | 248 | 1 | 1.031586 | -0.199616 | 307 | 321.57 | 1.047459 |
| GO:0034961\_cellular\_biopolymer\_biosynthetic\_process | BMI1 | 804 | 3 | 0.954602 | -0.199338 | 308 | 321.76 | 1.044675 |
| GO:0034961\_cellular\_biopolymer\_biosynthetic\_process | AHCTF1 | 804 | 3 | 0.954602 | -0.199338 | 308 | 321.76 | 1.044675 |
| GO:0034961\_cellular\_biopolymer\_biosynthetic\_process | CBFA2T2 | 804 | 3 | 0.954602 | -0.199338 | 308 | 321.76 | 1.044675 |
| GO:0043284\_biopolymer\_biosynthetic\_process | BMI1 | 807 | 3 | 0.951053 | -0.197466 | 309 | 322.57 | 1.043916 |
| GO:0043284\_biopolymer\_biosynthetic\_process | AHCTF1 | 807 | 3 | 0.951053 | -0.197466 | 309 | 322.57 | 1.043916 |
| GO:0043284\_biopolymer\_biosynthetic\_process | CBFA2T2 | 807 | 3 | 0.951053 | -0.197466 | 309 | 322.57 | 1.043916 |
| GO:0008104\_protein\_localization | AP3S1 | 251 | 1 | 1.019256 | -0.196499 | 310 | 322.94 | 1.041742 |
| GO:0030097\_hemopoiesis | BMI1 | 253 | 1 | 1.011199 | -0.194453 | 311 | 323.93 | 1.041576 |
| GO:0048729\_tissue\_morphogenesis | CBFA2T2 | 255 | 1 | 1.003268 | -0.192433 | 312 | 325.21 | 1.042340 |
| GO:0048870\_cell\_motility | DOCK1 | 257 | 1 | 0.995460 | -0.190437 | 313 | 326.2 | 1.042173 |
| GO:0019222\_regulation\_of\_metabolic\_process | BMI1 | 1088 | 4 | 0.940564 | -0.189887 | 314 | 326.68 | 1.040382 |
| GO:0019222\_regulation\_of\_metabolic\_process | AHCTF1 | 1088 | 4 | 0.940564 | -0.189887 | 314 | 326.68 | 1.040382 |
| GO:0019222\_regulation\_of\_metabolic\_process | STUB1 | 1088 | 4 | 0.940564 | -0.189887 | 314 | 326.68 | 1.040382 |
| GO:0019222\_regulation\_of\_metabolic\_process | CBFA2T2 | 1088 | 4 | 0.940564 | -0.189887 | 314 | 326.68 | 1.040382 |
| GO:0006950\_response\_to\_stress | CADM1 | 549 | 2 | 0.931998 | -0.186549 | 315 | 326.98 | 1.038032 |
| GO:0006950\_response\_to\_stress | ST8SIA1 | 549 | 2 | 0.931998 | -0.186549 | 315 | 326.98 | 1.038032 |
| GO:0001775\_cell\_activation | BMI1 | 262 | 1 | 0.976463 | -0.185556 | 316 | 328.65 | 1.040032 |
| GO:0044267\_cellular\_protein\_metabolic\_process | BMI1 | 559 | 2 | 0.915325 | -0.179736 | 317 | 330.22 | 1.041703 |
| GO:0044267\_cellular\_protein\_metabolic\_process | STUB1 | 559 | 2 | 0.915325 | -0.179736 | 317 | 330.22 | 1.041703 |
| GO:0051716\_cellular\_response\_to\_stimulus | ST8SIA1 | 273 | 1 | 0.937118 | -0.175327 | 318 | 331.86 | 1.043585 |
| GO:0033036\_macromolecule\_localization | AP3S1 | 274 | 1 | 0.933698 | -0.174431 | 319 | 332.85 | 1.043417 |
| GO:0044238\_primary\_metabolic\_process | CYP51 | 1905 | 7 | 0.940070 | -0.173760 | 320 | 333.0 | 1.040625 |
| GO:0044238\_primary\_metabolic\_process | BMI1 | 1905 | 7 | 0.940070 | -0.173760 | 320 | 333.0 | 1.040625 |
| GO:0044238\_primary\_metabolic\_process | AHCTF1 | 1905 | 7 | 0.940070 | -0.173760 | 320 | 333.0 | 1.040625 |
| GO:0044238\_primary\_metabolic\_process | PCSK5 | 1905 | 7 | 0.940070 | -0.173760 | 320 | 333.0 | 1.040625 |
| GO:0044238\_primary\_metabolic\_process | STUB1 | 1905 | 7 | 0.940070 | -0.173760 | 320 | 333.0 | 1.040625 |
| GO:0044238\_primary\_metabolic\_process | CBFA2T2 | 1905 | 7 | 0.940070 | -0.173760 | 320 | 333.0 | 1.040625 |
| GO:0044238\_primary\_metabolic\_process | HSD17B7 | 1905 | 7 | 0.940070 | -0.173760 | 320 | 333.0 | 1.040625 |
| GO:0048534\_hemopoietic\_or\_lymphoid\_organ\_development | BMI1 | 277 | 1 | 0.923586 | -0.171773 | 321 | 333.72 | 1.039626 |
| GO:0009987\_cellular\_process | CYP51 | 3868 | 15 | 0.992115 | -0.169364 | 322 | 334.87 | 1.039969 |
| GO:0009987\_cellular\_process | BMI1 | 3868 | 15 | 0.992115 | -0.169364 | 322 | 334.87 | 1.039969 |
| GO:0009987\_cellular\_process | CADM1 | 3868 | 15 | 0.992115 | -0.169364 | 322 | 334.87 | 1.039969 |
| GO:0009987\_cellular\_process | ST8SIA1 | 3868 | 15 | 0.992115 | -0.169364 | 322 | 334.87 | 1.039969 |
| GO:0009987\_cellular\_process | AP3S1 | 3868 | 15 | 0.992115 | -0.169364 | 322 | 334.87 | 1.039969 |
| GO:0009987\_cellular\_process | RAF1 | 3868 | 15 | 0.992115 | -0.169364 | 322 | 334.87 | 1.039969 |
| GO:0009987\_cellular\_process | AHCTF1 | 3868 | 15 | 0.992115 | -0.169364 | 322 | 334.87 | 1.039969 |
| GO:0009987\_cellular\_process | BIRC5 | 3868 | 15 | 0.992115 | -0.169364 | 322 | 334.87 | 1.039969 |
| GO:0009987\_cellular\_process | CBFA2T2 | 3868 | 15 | 0.992115 | -0.169364 | 322 | 334.87 | 1.039969 |
| GO:0009987\_cellular\_process | STUB1 | 3868 | 15 | 0.992115 | -0.169364 | 322 | 334.87 | 1.039969 |
| GO:0009987\_cellular\_process | TFAM | 3868 | 15 | 0.992115 | -0.169364 | 322 | 334.87 | 1.039969 |
| GO:0009987\_cellular\_process | DOCK1 | 3868 | 15 | 0.992115 | -0.169364 | 322 | 334.87 | 1.039969 |
| GO:0009987\_cellular\_process | NCAPG2 | 3868 | 15 | 0.992115 | -0.169364 | 322 | 334.87 | 1.039969 |
| GO:0009987\_cellular\_process | PCSK5 | 3868 | 15 | 0.992115 | -0.169364 | 322 | 334.87 | 1.039969 |
| GO:0009987\_cellular\_process | HSD17B7 | 3868 | 15 | 0.992115 | -0.169364 | 322 | 334.87 | 1.039969 |
| GO:0006355\_regulation\_of\_transcription\_\_DNA-dependent | BMI1 | 575 | 2 | 0.889855 | -0.169348 | 323 | 335.19 | 1.037740 |
| GO:0006355\_regulation\_of\_transcription\_\_DNA-dependent | CBFA2T2 | 575 | 2 | 0.889855 | -0.169348 | 323 | 335.19 | 1.037740 |
| GO:0048519\_negative\_regulation\_of\_biological\_process | BMI1 | 859 | 3 | 0.893481 | -0.167469 | 324 | 335.52 | 1.035556 |
| GO:0048519\_negative\_regulation\_of\_biological\_process | BIRC5 | 859 | 3 | 0.893481 | -0.167469 | 324 | 335.52 | 1.035556 |
| GO:0048519\_negative\_regulation\_of\_biological\_process | CBFA2T2 | 859 | 3 | 0.893481 | -0.167469 | 324 | 335.52 | 1.035556 |
| GO:0007417\_central\_nervous\_system\_development | BMI1 | 287 | 1 | 0.891405 | -0.163248 | 325 | 337.52 | 1.038523 |
| GO:0050794\_regulation\_of\_cellular\_process | BMI1 | 2190 | 8 | 0.934551 | -0.161004 | 326 | 338.06 | 1.036994 |
| GO:0050794\_regulation\_of\_cellular\_process | CADM1 | 2190 | 8 | 0.934551 | -0.161004 | 326 | 338.06 | 1.036994 |
| GO:0050794\_regulation\_of\_cellular\_process | ST8SIA1 | 2190 | 8 | 0.934551 | -0.161004 | 326 | 338.06 | 1.036994 |
| GO:0050794\_regulation\_of\_cellular\_process | AHCTF1 | 2190 | 8 | 0.934551 | -0.161004 | 326 | 338.06 | 1.036994 |
| GO:0050794\_regulation\_of\_cellular\_process | RAF1 | 2190 | 8 | 0.934551 | -0.161004 | 326 | 338.06 | 1.036994 |
| GO:0050794\_regulation\_of\_cellular\_process | BIRC5 | 2190 | 8 | 0.934551 | -0.161004 | 326 | 338.06 | 1.036994 |
| GO:0050794\_regulation\_of\_cellular\_process | STUB1 | 2190 | 8 | 0.934551 | -0.161004 | 326 | 338.06 | 1.036994 |
| GO:0050794\_regulation\_of\_cellular\_process | CBFA2T2 | 2190 | 8 | 0.934551 | -0.161004 | 326 | 338.06 | 1.036994 |
| GO:0051252\_regulation\_of\_RNA\_metabolic\_process | BMI1 | 590 | 2 | 0.867232 | -0.160150 | 327 | 338.38 | 1.034801 |
| GO:0051252\_regulation\_of\_RNA\_metabolic\_process | CBFA2T2 | 590 | 2 | 0.867232 | -0.160150 | 327 | 338.38 | 1.034801 |
| GO:0006351\_transcription\_\_DNA-dependent | BMI1 | 594 | 2 | 0.861392 | -0.157782 | 328 | 339.07 | 1.033750 |
| GO:0006351\_transcription\_\_DNA-dependent | CBFA2T2 | 594 | 2 | 0.861392 | -0.157782 | 328 | 339.07 | 1.033750 |
| GO:0032774\_RNA\_biosynthetic\_process | BMI1 | 595 | 2 | 0.859944 | -0.157195 | 329 | 339.36 | 1.031489 |
| GO:0032774\_RNA\_biosynthetic\_process | CBFA2T2 | 595 | 2 | 0.859944 | -0.157195 | 329 | 339.36 | 1.031489 |
| GO:0002520\_immune\_system\_development | BMI1 | 295 | 1 | 0.867232 | -0.156778 | 332 | 340.41 | 1.025331 |
| GO:0040011\_locomotion | DOCK1 | 295 | 1 | 0.867232 | -0.156778 | 332 | 340.41 | 1.025331 |
| GO:0045595\_regulation\_of\_cell\_differentiation | BMI1 | 295 | 1 | 0.867232 | -0.156778 | 332 | 340.41 | 1.025331 |
| GO:0048598\_embryonic\_morphogenesis | BMI1 | 299 | 1 | 0.855630 | -0.153654 | 333 | 341.28 | 1.024865 |
| GO:0051094\_positive\_regulation\_of\_developmental\_process | CADM1 | 308 | 1 | 0.830628 | -0.146880 | 334 | 343.11 | 1.027275 |
| GO:0044260\_cellular\_macromolecule\_metabolic\_process | BMI1 | 1447 | 5 | 0.884013 | -0.146439 | 335 | 343.26 | 1.024657 |
| GO:0044260\_cellular\_macromolecule\_metabolic\_process | AHCTF1 | 1447 | 5 | 0.884013 | -0.146439 | 335 | 343.26 | 1.024657 |
| GO:0044260\_cellular\_macromolecule\_metabolic\_process | STUB1 | 1447 | 5 | 0.884013 | -0.146439 | 335 | 343.26 | 1.024657 |
| GO:0044260\_cellular\_macromolecule\_metabolic\_process | PCSK5 | 1447 | 5 | 0.884013 | -0.146439 | 335 | 343.26 | 1.024657 |
| GO:0044260\_cellular\_macromolecule\_metabolic\_process | CBFA2T2 | 1447 | 5 | 0.884013 | -0.146439 | 335 | 343.26 | 1.024657 |
| GO:0044237\_cellular\_metabolic\_process | CYP51 | 1974 | 7 | 0.907210 | -0.145196 | 336 | 344.03 | 1.023899 |
| GO:0044237\_cellular\_metabolic\_process | BMI1 | 1974 | 7 | 0.907210 | -0.145196 | 336 | 344.03 | 1.023899 |
| GO:0044237\_cellular\_metabolic\_process | AHCTF1 | 1974 | 7 | 0.907210 | -0.145196 | 336 | 344.03 | 1.023899 |
| GO:0044237\_cellular\_metabolic\_process | STUB1 | 1974 | 7 | 0.907210 | -0.145196 | 336 | 344.03 | 1.023899 |
| GO:0044237\_cellular\_metabolic\_process | PCSK5 | 1974 | 7 | 0.907210 | -0.145196 | 336 | 344.03 | 1.023899 |
| GO:0044237\_cellular\_metabolic\_process | CBFA2T2 | 1974 | 7 | 0.907210 | -0.145196 | 336 | 344.03 | 1.023899 |
| GO:0044237\_cellular\_metabolic\_process | HSD17B7 | 1974 | 7 | 0.907210 | -0.145196 | 336 | 344.03 | 1.023899 |
| GO:0007399\_nervous\_system\_development | BMI1 | 621 | 2 | 0.823940 | -0.142671 | 337 | 344.49 | 1.022226 |
| GO:0007399\_nervous\_system\_development | CADM1 | 621 | 2 | 0.823940 | -0.142671 | 337 | 344.49 | 1.022226 |
| GO:0009887\_organ\_morphogenesis | BMI1 | 642 | 2 | 0.796989 | -0.131903 | 338 | 345.41 | 1.021923 |
| GO:0009887\_organ\_morphogenesis | CBFA2T2 | 642 | 2 | 0.796989 | -0.131903 | 338 | 345.41 | 1.021923 |
| GO:0006928\_cell\_motion | DOCK1 | 330 | 1 | 0.775253 | -0.131695 | 340 | 346.63 | 1.019500 |
| GO:0051674\_localization\_of\_cell | DOCK1 | 330 | 1 | 0.775253 | -0.131695 | 340 | 346.63 | 1.019500 |
| GO:0043283\_biopolymer\_metabolic\_process | BMI1 | 1490 | 5 | 0.858501 | -0.129667 | 341 | 348.11 | 1.020850 |
| GO:0043283\_biopolymer\_metabolic\_process | AHCTF1 | 1490 | 5 | 0.858501 | -0.129667 | 341 | 348.11 | 1.020850 |
| GO:0043283\_biopolymer\_metabolic\_process | CBFA2T2 | 1490 | 5 | 0.858501 | -0.129667 | 341 | 348.11 | 1.020850 |
| GO:0043283\_biopolymer\_metabolic\_process | PCSK5 | 1490 | 5 | 0.858501 | -0.129667 | 341 | 348.11 | 1.020850 |
| GO:0043283\_biopolymer\_metabolic\_process | STUB1 | 1490 | 5 | 0.858501 | -0.129667 | 341 | 348.11 | 1.020850 |
| GO:0007275\_multicellular\_organismal\_development | BMI1 | 1760 | 6 | 0.872159 | -0.128096 | 342 | 348.29 | 1.018392 |
| GO:0007275\_multicellular\_organismal\_development | CADM1 | 1760 | 6 | 0.872159 | -0.128096 | 342 | 348.29 | 1.018392 |
| GO:0007275\_multicellular\_organismal\_development | NCAPG2 | 1760 | 6 | 0.872159 | -0.128096 | 342 | 348.29 | 1.018392 |
| GO:0007275\_multicellular\_organismal\_development | BIRC5 | 1760 | 6 | 0.872159 | -0.128096 | 342 | 348.29 | 1.018392 |
| GO:0007275\_multicellular\_organismal\_development | CBFA2T2 | 1760 | 6 | 0.872159 | -0.128096 | 342 | 348.29 | 1.018392 |
| GO:0007275\_multicellular\_organismal\_development | PCSK5 | 1760 | 6 | 0.872159 | -0.128096 | 342 | 348.29 | 1.018392 |
| GO:0009605\_response\_to\_external\_stimulus | LXN | 339 | 1 | 0.754671 | -0.125995 | 343 | 349.33 | 1.018455 |
| GO:0016070\_RNA\_metabolic\_process | BMI1 | 658 | 2 | 0.777609 | -0.124232 | 344 | 350.65 | 1.019331 |
| GO:0016070\_RNA\_metabolic\_process | CBFA2T2 | 658 | 2 | 0.777609 | -0.124232 | 344 | 350.65 | 1.019331 |
| GO:0051649\_establishment\_of\_localization\_in\_cell | AP3S1 | 342 | 1 | 0.748051 | -0.124156 | 345 | 351.05 | 1.017536 |
| GO:0009653\_anatomical\_structure\_morphogenesis | BMI1 | 958 | 3 | 0.801148 | -0.121533 | 346 | 351.73 | 1.016561 |
| GO:0009653\_anatomical\_structure\_morphogenesis | PCSK5 | 958 | 3 | 0.801148 | -0.121533 | 346 | 351.73 | 1.016561 |
| GO:0009653\_anatomical\_structure\_morphogenesis | CBFA2T2 | 958 | 3 | 0.801148 | -0.121533 | 346 | 351.73 | 1.016561 |
| GO:0065008\_regulation\_of\_biological\_quality | STUB1 | 693 | 2 | 0.738336 | -0.108919 | 347 | 356.21 | 1.026542 |
| GO:0065008\_regulation\_of\_biological\_quality | PCSK5 | 693 | 2 | 0.738336 | -0.108919 | 347 | 356.21 | 1.026542 |
| GO:0051641\_cellular\_localization | AP3S1 | 370 | 1 | 0.691441 | -0.108341 | 348 | 356.63 | 1.024799 |
| GO:0065007\_biological\_regulation | BMI1 | 2593 | 9 | 0.887968 | -0.106427 | 349 | 357.35 | 1.023926 |
| GO:0065007\_biological\_regulation | CADM1 | 2593 | 9 | 0.887968 | -0.106427 | 349 | 357.35 | 1.023926 |
| GO:0065007\_biological\_regulation | ST8SIA1 | 2593 | 9 | 0.887968 | -0.106427 | 349 | 357.35 | 1.023926 |
| GO:0065007\_biological\_regulation | RAF1 | 2593 | 9 | 0.887968 | -0.106427 | 349 | 357.35 | 1.023926 |
| GO:0065007\_biological\_regulation | AHCTF1 | 2593 | 9 | 0.887968 | -0.106427 | 349 | 357.35 | 1.023926 |
| GO:0065007\_biological\_regulation | BIRC5 | 2593 | 9 | 0.887968 | -0.106427 | 349 | 357.35 | 1.023926 |
| GO:0065007\_biological\_regulation | PCSK5 | 2593 | 9 | 0.887968 | -0.106427 | 349 | 357.35 | 1.023926 |
| GO:0065007\_biological\_regulation | STUB1 | 2593 | 9 | 0.887968 | -0.106427 | 349 | 357.35 | 1.023926 |
| GO:0065007\_biological\_regulation | CBFA2T2 | 2593 | 9 | 0.887968 | -0.106427 | 349 | 357.35 | 1.023926 |
| GO:0006139\_nucleobase\_\_nucleoside\_\_nucleotide\_and\_nucleic\_acid\_metabolic\_process | BMI1 | 1002 | 3 | 0.765968 | -0.105038 | 350 | 358.18 | 1.023371 |
| GO:0006139\_nucleobase\_\_nucleoside\_\_nucleotide\_and\_nucleic\_acid\_metabolic\_process | AHCTF1 | 1002 | 3 | 0.765968 | -0.105038 | 350 | 358.18 | 1.023371 |
| GO:0006139\_nucleobase\_\_nucleoside\_\_nucleotide\_and\_nucleic\_acid\_metabolic\_process | CBFA2T2 | 1002 | 3 | 0.765968 | -0.105038 | 350 | 358.18 | 1.023371 |
| GO:0050789\_regulation\_of\_biological\_process | BMI1 | 2357 | 8 | 0.868335 | -0.102053 | 351 | 358.9 | 1.022507 |
| GO:0050789\_regulation\_of\_biological\_process | CADM1 | 2357 | 8 | 0.868335 | -0.102053 | 351 | 358.9 | 1.022507 |
| GO:0050789\_regulation\_of\_biological\_process | ST8SIA1 | 2357 | 8 | 0.868335 | -0.102053 | 351 | 358.9 | 1.022507 |
| GO:0050789\_regulation\_of\_biological\_process | RAF1 | 2357 | 8 | 0.868335 | -0.102053 | 351 | 358.9 | 1.022507 |
| GO:0050789\_regulation\_of\_biological\_process | AHCTF1 | 2357 | 8 | 0.868335 | -0.102053 | 351 | 358.9 | 1.022507 |
| GO:0050789\_regulation\_of\_biological\_process | BIRC5 | 2357 | 8 | 0.868335 | -0.102053 | 351 | 358.9 | 1.022507 |
| GO:0050789\_regulation\_of\_biological\_process | STUB1 | 2357 | 8 | 0.868335 | -0.102053 | 351 | 358.9 | 1.022507 |
| GO:0050789\_regulation\_of\_biological\_process | CBFA2T2 | 2357 | 8 | 0.868335 | -0.102053 | 351 | 358.9 | 1.022507 |
| GO:0043170\_macromolecule\_metabolic\_process | BMI1 | 1576 | 5 | 0.811654 | -0.100828 | 352 | 359.34 | 1.020852 |
| GO:0043170\_macromolecule\_metabolic\_process | AHCTF1 | 1576 | 5 | 0.811654 | -0.100828 | 352 | 359.34 | 1.020852 |
| GO:0043170\_macromolecule\_metabolic\_process | PCSK5 | 1576 | 5 | 0.811654 | -0.100828 | 352 | 359.34 | 1.020852 |
| GO:0043170\_macromolecule\_metabolic\_process | CBFA2T2 | 1576 | 5 | 0.811654 | -0.100828 | 352 | 359.34 | 1.020852 |
| GO:0043170\_macromolecule\_metabolic\_process | STUB1 | 1576 | 5 | 0.811654 | -0.100828 | 352 | 359.34 | 1.020852 |
| GO:0050877\_neurological\_system\_process | LXN | 390 | 1 | 0.655983 | -0.098386 | 353 | 360.73 | 1.021898 |
| GO:0008152\_metabolic\_process | BMI1 | 2133 | 7 | 0.839584 | -0.093045 | 354 | 361.75 | 1.021893 |
| GO:0008152\_metabolic\_process | CYP51 | 2133 | 7 | 0.839584 | -0.093045 | 354 | 361.75 | 1.021893 |
| GO:0008152\_metabolic\_process | AHCTF1 | 2133 | 7 | 0.839584 | -0.093045 | 354 | 361.75 | 1.021893 |
| GO:0008152\_metabolic\_process | PCSK5 | 2133 | 7 | 0.839584 | -0.093045 | 354 | 361.75 | 1.021893 |
| GO:0008152\_metabolic\_process | STUB1 | 2133 | 7 | 0.839584 | -0.093045 | 354 | 361.75 | 1.021893 |
| GO:0008152\_metabolic\_process | CBFA2T2 | 2133 | 7 | 0.839584 | -0.093045 | 354 | 361.75 | 1.021893 |
| GO:0008152\_metabolic\_process | HSD17B7 | 2133 | 7 | 0.839584 | -0.093045 | 354 | 361.75 | 1.021893 |
| GO:0042221\_response\_to\_chemical\_stimulus | EIF4A2 | 409 | 1 | 0.625509 | -0.089830 | 355 | 362.14 | 1.020113 |
| GO:0007242\_intracellular\_signaling\_cascade | RAF1 | 411 | 1 | 0.622466 | -0.088976 | 356 | 362.64 | 1.018652 |
| GO:0010604\_positive\_regulation\_of\_macromolecule\_metabolic\_process | STUB1 | 433 | 1 | 0.590839 | -0.080130 | 357 | 365.53 | 1.023894 |
| GO:0032501\_multicellular\_organismal\_process | BMI1 | 2183 | 7 | 0.820354 | -0.080092 | 358 | 365.65 | 1.021369 |
| GO:0032501\_multicellular\_organismal\_process | CADM1 | 2183 | 7 | 0.820354 | -0.080092 | 358 | 365.65 | 1.021369 |
| GO:0032501\_multicellular\_organismal\_process | NCAPG2 | 2183 | 7 | 0.820354 | -0.080092 | 358 | 365.65 | 1.021369 |
| GO:0032501\_multicellular\_organismal\_process | LXN | 2183 | 7 | 0.820354 | -0.080092 | 358 | 365.65 | 1.021369 |
| GO:0032501\_multicellular\_organismal\_process | BIRC5 | 2183 | 7 | 0.820354 | -0.080092 | 358 | 365.65 | 1.021369 |
| GO:0032501\_multicellular\_organismal\_process | CBFA2T2 | 2183 | 7 | 0.820354 | -0.080092 | 358 | 365.65 | 1.021369 |
| GO:0032501\_multicellular\_organismal\_process | PCSK5 | 2183 | 7 | 0.820354 | -0.080092 | 358 | 365.65 | 1.021369 |
| GO:0006357\_regulation\_of\_transcription\_from\_RNA\_polymerase\_II\_promoter | BMI1 | 435 | 1 | 0.588123 | -0.079373 | 359 | 366.12 | 1.019833 |
| GO:0031325\_positive\_regulation\_of\_cellular\_metabolic\_process | STUB1 | 442 | 1 | 0.578808 | -0.076781 | 360 | 367.19 | 1.019972 |
| GO:0006366\_transcription\_from\_RNA\_polymerase\_II\_promoter | BMI1 | 444 | 1 | 0.576201 | -0.076057 | 361 | 367.87 | 1.019030 |
| GO:0010926\_anatomical\_structure\_formation | TFAM | 447 | 1 | 0.572334 | -0.074984 | 362 | 368.18 | 1.017072 |
| GO:0034960\_cellular\_biopolymer\_metabolic\_process | BMI1 | 1395 | 4 | 0.733572 | -0.074465 | 363 | 368.31 | 1.014628 |
| GO:0034960\_cellular\_biopolymer\_metabolic\_process | AHCTF1 | 1395 | 4 | 0.733572 | -0.074465 | 363 | 368.31 | 1.014628 |
| GO:0034960\_cellular\_biopolymer\_metabolic\_process | STUB1 | 1395 | 4 | 0.733572 | -0.074465 | 363 | 368.31 | 1.014628 |
| GO:0034960\_cellular\_biopolymer\_metabolic\_process | CBFA2T2 | 1395 | 4 | 0.733572 | -0.074465 | 363 | 368.31 | 1.014628 |
| GO:0048856\_anatomical\_structure\_development | BMI1 | 1688 | 5 | 0.757800 | -0.071366 | 364 | 369.44 | 1.014945 |
| GO:0048856\_anatomical\_structure\_development | CADM1 | 1688 | 5 | 0.757800 | -0.071366 | 364 | 369.44 | 1.014945 |
| GO:0048856\_anatomical\_structure\_development | NCAPG2 | 1688 | 5 | 0.757800 | -0.071366 | 364 | 369.44 | 1.014945 |
| GO:0048856\_anatomical\_structure\_development | PCSK5 | 1688 | 5 | 0.757800 | -0.071366 | 364 | 369.44 | 1.014945 |
| GO:0048856\_anatomical\_structure\_development | CBFA2T2 | 1688 | 5 | 0.757800 | -0.071366 | 364 | 369.44 | 1.014945 |
| GO:0009893\_positive\_regulation\_of\_metabolic\_process | STUB1 | 458 | 1 | 0.558588 | -0.071182 | 365 | 370.2 | 1.014247 |
| GO:0006807\_nitrogen\_compound\_metabolic\_process | BMI1 | 1147 | 3 | 0.669137 | -0.063838 | 366 | 371.19 | 1.014180 |
| GO:0006807\_nitrogen\_compound\_metabolic\_process | AHCTF1 | 1147 | 3 | 0.669137 | -0.063838 | 366 | 371.19 | 1.014180 |
| GO:0006807\_nitrogen\_compound\_metabolic\_process | CBFA2T2 | 1147 | 3 | 0.669137 | -0.063838 | 366 | 371.19 | 1.014180 |
| GO:0003008\_system\_process | LXN | 516 | 1 | 0.495801 | -0.054167 | 367 | 372.6 | 1.015259 |
| GO:0009888\_tissue\_development | CBFA2T2 | 525 | 1 | 0.487302 | -0.051925 | 368 | 372.91 | 1.013342 |
| GO:0032502\_developmental\_process | BMI1 | 2060 | 6 | 0.745146 | -0.051336 | 369 | 373.03 | 1.010921 |
| GO:0032502\_developmental\_process | CADM1 | 2060 | 6 | 0.745146 | -0.051336 | 369 | 373.03 | 1.010921 |
| GO:0032502\_developmental\_process | NCAPG2 | 2060 | 6 | 0.745146 | -0.051336 | 369 | 373.03 | 1.010921 |
| GO:0032502\_developmental\_process | BIRC5 | 2060 | 6 | 0.745146 | -0.051336 | 369 | 373.03 | 1.010921 |
| GO:0032502\_developmental\_process | CBFA2T2 | 2060 | 6 | 0.745146 | -0.051336 | 369 | 373.03 | 1.010921 |
| GO:0032502\_developmental\_process | PCSK5 | 2060 | 6 | 0.745146 | -0.051336 | 369 | 373.03 | 1.010921 |
| GO:0051239\_regulation\_of\_multicellular\_organismal\_process | BMI1 | 587 | 1 | 0.435832 | -0.038813 | 370 | 376.23 | 1.016838 |
| GO:0007166\_cell\_surface\_receptor\_linked\_signal\_transduction | RAF1 | 597 | 1 | 0.428532 | -0.037032 | 371 | 377.09 | 1.016415 |
| GO:0048731\_system\_development | BMI1 | 1609 | 4 | 0.636006 | -0.035560 | 372 | 377.22 | 1.014032 |
| GO:0048731\_system\_development | CADM1 | 1609 | 4 | 0.636006 | -0.035560 | 372 | 377.22 | 1.014032 |
| GO:0048731\_system\_development | CBFA2T2 | 1609 | 4 | 0.636006 | -0.035560 | 372 | 377.22 | 1.014032 |
| GO:0048731\_system\_development | PCSK5 | 1609 | 4 | 0.636006 | -0.035560 | 372 | 377.22 | 1.014032 |
| GO:0048513\_organ\_development | BMI1 | 1365 | 3 | 0.562271 | -0.028463 | 373 | 378.38 | 1.014424 |
| GO:0048513\_organ\_development | CBFA2T2 | 1365 | 3 | 0.562271 | -0.028463 | 373 | 378.38 | 1.014424 |
| GO:0048513\_organ\_development | PCSK5 | 1365 | 3 | 0.562271 | -0.028463 | 373 | 378.38 | 1.014424 |
| GO:0051179\_localization | DOCK1 | 1058 | 2 | 0.483617 | -0.025793 | 374 | 379.27 | 1.014091 |
| GO:0051179\_localization | AP3S1 | 1058 | 2 | 0.483617 | -0.025793 | 374 | 379.27 | 1.014091 |
| GO:0030154\_cell\_differentiation | BMI1 | 1060 | 2 | 0.482704 | -0.025579 | 375 | 379.36 | 1.011627 |
| GO:0030154\_cell\_differentiation | CBFA2T2 | 1060 | 2 | 0.482704 | -0.025579 | 375 | 379.36 | 1.011627 |
| GO:0006810\_transport | AP3S1 | 718 | 1 | 0.356314 | -0.020915 | 376 | 380.64 | 1.012340 |
| GO:0048869\_cellular\_developmental\_process | BMI1 | 1113 | 2 | 0.459718 | -0.020457 | 377 | 380.83 | 1.010159 |
| GO:0048869\_cellular\_developmental\_process | CBFA2T2 | 1113 | 2 | 0.459718 | -0.020457 | 377 | 380.83 | 1.010159 |
| GO:0051234\_establishment\_of\_localization | AP3S1 | 729 | 1 | 0.350937 | -0.019848 | 378 | 381.09 | 1.008175 |
| GO:0007165\_signal\_transduction | RAF1 | 915 | 1 | 0.279599 | -0.008064 | 379 | 383.71 | 1.012427 |
| GO:0007154\_cell\_communication | RAF1 | 1096 | 1 | 0.233425 | -0.003236 | 380 | 384.71 | 1.012395 |
| GO:0008150\_biological\_process | CYP51 | 4605 | 18 | 1.000000 | 0.000000 | 1894 | 1893.87 | 0.999931 |
| GO:0008150\_biological\_process | BMI1 | 4605 | 18 | 1.000000 | 0.000000 | 1894 | 1893.87 | 0.999931 |
| GO:0008150\_biological\_process | CADM1 | 4605 | 18 | 1.000000 | 0.000000 | 1894 | 1893.87 | 0.999931 |
| GO:0008150\_biological\_process | LXN | 4605 | 18 | 1.000000 | 0.000000 | 1894 | 1893.87 | 0.999931 |
| GO:0008150\_biological\_process | ST8SIA1 | 4605 | 18 | 1.000000 | 0.000000 | 1894 | 1893.87 | 0.999931 |
| GO:0008150\_biological\_process | RAF1 | 4605 | 18 | 1.000000 | 0.000000 | 1894 | 1893.87 | 0.999931 |
| GO:0008150\_biological\_process | AHCTF1 | 4605 | 18 | 1.000000 | 0.000000 | 1894 | 1893.87 | 0.999931 |
| GO:0008150\_biological\_process | AP3S1 | 4605 | 18 | 1.000000 | 0.000000 | 1894 | 1893.87 | 0.999931 |
| GO:0008150\_biological\_process | BIRC5 | 4605 | 18 | 1.000000 | 0.000000 | 1894 | 1893.87 | 0.999931 |
| GO:0008150\_biological\_process | CBFA2T2 | 4605 | 18 | 1.000000 | 0.000000 | 1894 | 1893.87 | 0.999931 |
| GO:0008150\_biological\_process | STUB1 | 4605 | 18 | 1.000000 | 0.000000 | 1894 | 1893.87 | 0.999931 |
| GO:0008150\_biological\_process | TFAM | 4605 | 18 | 1.000000 | 0.000000 | 1894 | 1893.87 | 0.999931 |
| GO:0008150\_biological\_process | DOCK1 | 4605 | 18 | 1.000000 | 0.000000 | 1894 | 1893.87 | 0.999931 |
| GO:0008150\_biological\_process | NCAPG2 | 4605 | 18 | 1.000000 | 0.000000 | 1894 | 1893.87 | 0.999931 |
| GO:0008150\_biological\_process | EIF4A2 | 4605 | 18 | 1.000000 | 0.000000 | 1894 | 1893.87 | 0.999931 |
| GO:0008150\_biological\_process | IFT81 | 4605 | 18 | 1.000000 | 0.000000 | 1894 | 1893.87 | 0.999931 |
| GO:0008150\_biological\_process | PCSK5 | 4605 | 18 | 1.000000 | 0.000000 | 1894 | 1893.87 | 0.999931 |
| GO:0008150\_biological\_process | HSD17B7 | 4605 | 18 | 1.000000 | 0.000000 | 1894 | 1893.87 | 0.999931 |
