# Supplementary material for: RedundancyMiner: De-replication of redundant GO categories in microarray and proteomics analysis
Source: BMC Bioinformatics. 2011 Feb 10;12:52. doi: 10.1186/1471-2105-12-52 (PMC3223614; doi:10.1186/1471-2105-12-52)
Supplement: Additional file 8 — Retinal development HTGM download. compressed package of the results of running HTGM on the retinal development genes list. [file 1471-2105-12-52-S8.ZIP › SCENARIO_2_MODIFIED/total.txt.total.txt.dir/Exp1_BestClusterMap_LEIGS_KM_24.csv.join.20.txt.dir/Exp1_BestClusterMap_LEIGS_KM_24.csv.join.20.txt.change.gce.html]

Gene Category Report for Exp1\_BestClusterMap\_LEIGS\_KM\_24.csv.join.20.txt

# Gene Category Report for Exp1\_BestClusterMap\_LEIGS\_KM\_24.csv.join.20.txt

| HYPERLINKED GO CATEGORY | HYPERLINKED GENE NAME | TOTAL GENES | CHANGED GENES | ENRICHMENT | LOG10(p) | CUMULATIVE NUMBER OF CATEGORIES | CUMULATIVE RANDOMS MEAN | FALSE DISCOVERY RATE |
| --- | --- | --- | --- | --- | --- | --- | --- | --- |
| GO:0006508\_proteolysis | UBE2N | 76 | 4 | 9.694737 | -3.176313 | 1 | 0.66 | 0.660000 |
| GO:0006508\_proteolysis | CTSS | 76 | 4 | 9.694737 | -3.176313 | 1 | 0.66 | 0.660000 |
| GO:0006508\_proteolysis | PCOLCE | 76 | 4 | 9.694737 | -3.176313 | 1 | 0.66 | 0.660000 |
| GO:0006508\_proteolysis | HDAC6 | 76 | 4 | 9.694737 | -3.176313 | 1 | 0.66 | 0.660000 |
| GO:0006476\_protein\_amino\_acid\_deacetylation | SIRT1 | 9 | 2 | 40.933333 | -3.002047 | 2 | 1.06 | 0.530000 |
| GO:0006476\_protein\_amino\_acid\_deacetylation | HDAC6 | 9 | 2 | 40.933333 | -3.002047 | 2 | 1.06 | 0.530000 |
| GO:0030163\_protein\_catabolic\_process | UBE2N | 101 | 4 | 7.295050 | -2.713343 | 3 | 1.98 | 0.660000 |
| GO:0030163\_protein\_catabolic\_process | CTSS | 101 | 4 | 7.295050 | -2.713343 | 3 | 1.98 | 0.660000 |
| GO:0030163\_protein\_catabolic\_process | PCOLCE | 101 | 4 | 7.295050 | -2.713343 | 3 | 1.98 | 0.660000 |
| GO:0030163\_protein\_catabolic\_process | HDAC6 | 101 | 4 | 7.295050 | -2.713343 | 3 | 1.98 | 0.660000 |
| GO:0010639\_negative\_regulation\_of\_organelle\_organization | RECQL4 | 19 | 2 | 19.389474 | -2.339802 | 4 | 3.79 | 0.947500 |
| GO:0010639\_negative\_regulation\_of\_organelle\_organization | HDAC6 | 19 | 2 | 19.389474 | -2.339802 | 4 | 3.79 | 0.947500 |
| GO:0043285\_biopolymer\_catabolic\_process | UBE2N | 129 | 4 | 5.711628 | -2.327118 | 5 | 3.82 | 0.764000 |
| GO:0043285\_biopolymer\_catabolic\_process | CTSS | 129 | 4 | 5.711628 | -2.327118 | 5 | 3.82 | 0.764000 |
| GO:0043285\_biopolymer\_catabolic\_process | PCOLCE | 129 | 4 | 5.711628 | -2.327118 | 5 | 3.82 | 0.764000 |
| GO:0043285\_biopolymer\_catabolic\_process | HDAC6 | 129 | 4 | 5.711628 | -2.327118 | 5 | 3.82 | 0.764000 |
| GO:0003032\_detection\_of\_oxygen | SOD2 | 1 | 1 |  |  |  |  |  |  |
| GO:0003068\_regulation\_of\_systemic\_arterial\_blood\_pressure\_by\_acetylcholine | SOD2 | 1 | 1 |  |  |  |  |  |  |
| GO:0003069\_vasodilation\_by\_acetylcholine\_involved\_in\_regulation\_of\_systemic\_arterial\_blood\_pressure | SOD2 | 1 | 1 |  |  |  |  |  |  |
| GO:0003070\_regulation\_of\_systemic\_arterial\_blood\_pressure\_by\_neurotransmitter | SOD2 | 1 | 1 |  |  |  |  |  |  |
| GO:0007063\_regulation\_of\_sister\_chromatid\_cohesion | RECQL4 | 1 | 1 |  |  |  |  |  |  |
| GO:0010260\_organ\_senescence | SOD2 | 1 | 1 |  |  |  |  |  |  |
| GO:0030210\_heparin\_biosynthetic\_process | GLCE | 1 | 1 |  |  |  |  |  |  |
| GO:0045875\_negative\_regulation\_of\_sister\_chromatid\_cohesion | RECQL4 | 1 | 1 |  |  |  |  |  |  |
| GO:0048773\_erythrophore\_differentiation | SOD2 | 1 | 1 |  |  |  |  |  |  |
| GO:0055093\_response\_to\_hyperoxia | SOD2 | 1 | 1 |  |  |  |  |  |  |
| GO:0009057\_macromolecule\_catabolic\_process | UBE2N | 137 | 4 | 5.378102 | -2.234127 | 6 | 4.36 | 0.726667 |
| GO:0009057\_macromolecule\_catabolic\_process | CTSS | 137 | 4 | 5.378102 | -2.234127 | 6 | 4.36 | 0.726667 |
| GO:0009057\_macromolecule\_catabolic\_process | PCOLCE | 137 | 4 | 5.378102 | -2.234127 | 6 | 4.36 | 0.726667 |
| GO:0009057\_macromolecule\_catabolic\_process | HDAC6 | 137 | 4 | 5.378102 | -2.234127 | 6 | 4.36 | 0.726667 |
| GO:0001306\_age-dependent\_response\_to\_oxidative\_stress | SOD2 | 2 | 1 |  |  |  |  |  |  |
| GO:0007571\_age-dependent\_general\_metabolic\_decline | SOD2 | 2 | 1 |  |  |  |  |  |  |
| GO:0010149\_senescence | SOD2 | 2 | 1 |  |  |  |  |  |  |
| GO:0016584\_nucleosome\_positioning | HIST1H1C | 2 | 1 |  |  |  |  |  |  |
| GO:0030202\_heparin\_metabolic\_process | GLCE | 2 | 1 |  |  |  |  |  |  |
| GO:0000303\_response\_to\_superoxide | SOD2 | 3 | 1 |  |  |  |  |  |  |
| GO:0018196\_peptidyl-asparagine\_modification | STT3A | 3 | 1 |  |  |  |  |  |  |
| GO:0018279\_protein\_amino\_acid\_N-linked\_glycosylation\_via\_asparagine | STT3A | 3 | 1 |  |  |  |  |  |  |
| GO:0051983\_regulation\_of\_chromosome\_segregation | RECQL4 | 3 | 1 |  |  |  |  |  |  |
| GO:0006511\_ubiquitin-dependent\_protein\_catabolic\_process | UBE2N | 39 | 2 | 9.446154 | -1.731760 | 7 | 10.68 | 1.525714 |
| GO:0006511\_ubiquitin-dependent\_protein\_catabolic\_process | HDAC6 | 39 | 2 | 9.446154 | -1.731760 | 7 | 10.68 | 1.525714 |
| GO:0051129\_negative\_regulation\_of\_cellular\_component\_organization | RECQL4 | 40 | 2 | 9.210000 | -1.710919 | 8 | 11.08 | 1.385000 |
| GO:0051129\_negative\_regulation\_of\_cellular\_component\_organization | HDAC6 | 40 | 2 | 9.210000 | -1.710919 | 8 | 11.08 | 1.385000 |
| GO:0019941\_modification-dependent\_protein\_catabolic\_process | UBE2N | 42 | 2 | 8.771429 | -1.670879 | 11 | 12.14 | 1.103636 |
| GO:0019941\_modification-dependent\_protein\_catabolic\_process | HDAC6 | 42 | 2 | 8.771429 | -1.670879 | 11 | 12.14 | 1.103636 |
| GO:0043632\_modification-dependent\_macromolecule\_catabolic\_process | UBE2N | 42 | 2 | 8.771429 | -1.670879 | 11 | 12.14 | 1.103636 |
| GO:0043632\_modification-dependent\_macromolecule\_catabolic\_process | HDAC6 | 42 | 2 | 8.771429 | -1.670879 | 11 | 12.14 | 1.103636 |
| GO:0051603\_proteolysis\_involved\_in\_cellular\_protein\_catabolic\_process | UBE2N | 42 | 2 | 8.771429 | -1.670879 | 11 | 12.14 | 1.103636 |
| GO:0051603\_proteolysis\_involved\_in\_cellular\_protein\_catabolic\_process | HDAC6 | 42 | 2 | 8.771429 | -1.670879 | 11 | 12.14 | 1.103636 |
| GO:0000305\_response\_to\_oxygen\_radical | SOD2 | 4 | 1 |  |  |  |  |  |  |
| GO:0006972\_hyperosmotic\_response | PDPK1 | 4 | 1 |  |  |  |  |  |  |
| GO:0009593\_detection\_of\_chemical\_stimulus | SOD2 | 4 | 1 |  |  |  |  |  |  |
| GO:0051897\_positive\_regulation\_of\_protein\_kinase\_B\_signaling\_cascade | IGF1R | 4 | 1 |  |  |  |  |  |  |
| GO:0044257\_cellular\_protein\_catabolic\_process | UBE2N | 44 | 2 | 8.372727 | -1.632858 | 12 | 13.24 | 1.103333 |
| GO:0044257\_cellular\_protein\_catabolic\_process | HDAC6 | 44 | 2 | 8.372727 | -1.632858 | 12 | 13.24 | 1.103333 |
| GO:0006023\_aminoglycan\_biosynthetic\_process | GLCE | 5 | 1 | 36.840000 | -1.570848 | 17 | 21.68 | 1.275294 |
| GO:0006024\_glycosaminoglycan\_biosynthetic\_process | GLCE | 5 | 1 | 36.840000 | -1.570848 | 17 | 21.68 | 1.275294 |
| GO:0019430\_removal\_of\_superoxide\_radicals | SOD2 | 5 | 1 | 36.840000 | -1.570848 | 17 | 21.68 | 1.275294 |
| GO:0042554\_superoxide\_anion\_generation | SOD2 | 5 | 1 | 36.840000 | -1.570848 | 17 | 21.68 | 1.275294 |
| GO:0051898\_negative\_regulation\_of\_protein\_kinase\_B\_signaling\_cascade | IGF1R | 5 | 1 | 36.840000 | -1.570848 | 17 | 21.68 | 1.275294 |
| GO:0009101\_glycoprotein\_biosynthetic\_process | STT3A | 48 | 2 | 7.675000 | -1.562169 | 18 | 22.05 | 1.225000 |
| GO:0009101\_glycoprotein\_biosynthetic\_process | GLCE | 48 | 2 | 7.675000 | -1.562169 | 18 | 22.05 | 1.225000 |
| GO:0043473\_pigmentation | RECQL4 | 49 | 2 | 7.518367 | -1.545502 | 19 | 22.46 | 1.182105 |
| GO:0043473\_pigmentation | SOD2 | 49 | 2 | 7.518367 | -1.545502 | 19 | 22.46 | 1.182105 |
| GO:0006996\_organelle\_organization | RECQL4 | 449 | 6 | 2.461470 | -1.529323 | 20 | 22.71 | 1.135500 |
| GO:0006996\_organelle\_organization | HIST1H1C | 449 | 6 | 2.461470 | -1.529323 | 20 | 22.71 | 1.135500 |
| GO:0006996\_organelle\_organization | RHOU | 449 | 6 | 2.461470 | -1.529323 | 20 | 22.71 | 1.135500 |
| GO:0006996\_organelle\_organization | SIRT1 | 449 | 6 | 2.461470 | -1.529323 | 20 | 22.71 | 1.135500 |
| GO:0006996\_organelle\_organization | SOD2 | 449 | 6 | 2.461470 | -1.529323 | 20 | 22.71 | 1.135500 |
| GO:0006996\_organelle\_organization | HDAC6 | 449 | 6 | 2.461470 | -1.529323 | 20 | 22.71 | 1.135500 |
| GO:0051276\_chromosome\_organization | RECQL4 | 129 | 3 | 4.283721 | -1.501631 | 21 | 23.6 | 1.123810 |
| GO:0051276\_chromosome\_organization | HIST1H1C | 129 | 3 | 4.283721 | -1.501631 | 21 | 23.6 | 1.123810 |
| GO:0051276\_chromosome\_organization | SIRT1 | 129 | 3 | 4.283721 | -1.501631 | 21 | 23.6 | 1.123810 |
| GO:0014823\_response\_to\_activity | SOD2 | 6 | 1 | 30.700000 | -1.492797 | 25 | 29.64 | 1.185600 |
| GO:0015012\_heparan\_sulfate\_proteoglycan\_biosynthetic\_process | GLCE | 6 | 1 | 30.700000 | -1.492797 | 25 | 29.64 | 1.185600 |
| GO:0048147\_negative\_regulation\_of\_fibroblast\_proliferation | SOD2 | 6 | 1 | 30.700000 | -1.492797 | 25 | 29.64 | 1.185600 |
| GO:0051881\_regulation\_of\_mitochondrial\_membrane\_potential | SOD2 | 6 | 1 | 30.700000 | -1.492797 | 25 | 29.64 | 1.185600 |
| GO:0007265\_Ras\_protein\_signal\_transduction | NRAS | 54 | 2 | 6.822222 | -1.467420 | 26 | 30.87 | 1.187308 |
| GO:0007265\_Ras\_protein\_signal\_transduction | RHOU | 54 | 2 | 6.822222 | -1.467420 | 26 | 30.87 | 1.187308 |
| GO:0006790\_sulfur\_metabolic\_process | GLCE | 56 | 2 | 6.578571 | -1.438396 | 27 | 31.57 | 1.169259 |
| GO:0006790\_sulfur\_metabolic\_process | SOD2 | 56 | 2 | 6.578571 | -1.438396 | 27 | 31.57 | 1.169259 |
| GO:0000188\_inactivation\_of\_MAPK\_activity | DUSP16 | 7 | 1 | 26.314286 | -1.426980 | 39 | 38.09 | 0.976667 |
| GO:0003085\_negative\_regulation\_of\_systemic\_arterial\_blood\_pressure | SOD2 | 7 | 1 | 26.314286 | -1.426980 | 39 | 38.09 | 0.976667 |
| GO:0007019\_microtubule\_depolymerization | HDAC6 | 7 | 1 | 26.314286 | -1.426980 | 39 | 38.09 | 0.976667 |
| GO:0007026\_negative\_regulation\_of\_microtubule\_depolymerization | HDAC6 | 7 | 1 | 26.314286 | -1.426980 | 39 | 38.09 | 0.976667 |
| GO:0007062\_sister\_chromatid\_cohesion | RECQL4 | 7 | 1 | 26.314286 | -1.426980 | 39 | 38.09 | 0.976667 |
| GO:0010948\_negative\_regulation\_of\_cell\_cycle\_process | RECQL4 | 7 | 1 | 26.314286 | -1.426980 | 39 | 38.09 | 0.976667 |
| GO:0016575\_histone\_deacetylation | SIRT1 | 7 | 1 | 26.314286 | -1.426980 | 39 | 38.09 | 0.976667 |
| GO:0030201\_heparan\_sulfate\_proteoglycan\_metabolic\_process | GLCE | 7 | 1 | 26.314286 | -1.426980 | 39 | 38.09 | 0.976667 |
| GO:0031017\_exocrine\_pancreas\_development | IGF1R | 7 | 1 | 26.314286 | -1.426980 | 39 | 38.09 | 0.976667 |
| GO:0031114\_regulation\_of\_microtubule\_depolymerization | HDAC6 | 7 | 1 | 26.314286 | -1.426980 | 39 | 38.09 | 0.976667 |
| GO:0031497\_chromatin\_assembly | HIST1H1C | 7 | 1 | 26.314286 | -1.426980 | 39 | 38.09 | 0.976667 |
| GO:0045599\_negative\_regulation\_of\_fat\_cell\_differentiation | SOD2 | 7 | 1 | 26.314286 | -1.426980 | 39 | 38.09 | 0.976667 |
| GO:0033043\_regulation\_of\_organelle\_organization | RECQL4 | 58 | 2 | 6.351724 | -1.410499 | 40 | 38.96 | 0.974000 |
| GO:0033043\_regulation\_of\_organelle\_organization | HDAC6 | 58 | 2 | 6.351724 | -1.410499 | 40 | 38.96 | 0.974000 |
| GO:0009056\_catabolic\_process | UBE2N | 243 | 4 | 3.032099 | -1.398814 | 41 | 39.14 | 0.954634 |
| GO:0009056\_catabolic\_process | CTSS | 243 | 4 | 3.032099 | -1.398814 | 41 | 39.14 | 0.954634 |
| GO:0009056\_catabolic\_process | PCOLCE | 243 | 4 | 3.032099 | -1.398814 | 41 | 39.14 | 0.954634 |
| GO:0009056\_catabolic\_process | HDAC6 | 243 | 4 | 3.032099 | -1.398814 | 41 | 39.14 | 0.954634 |
| GO:0031111\_negative\_regulation\_of\_microtubule\_polymerization\_or\_depolymerization | HDAC6 | 8 | 1 | 23.025000 | -1.370116 | 44 | 45.04 | 1.023636 |
| GO:0034728\_nucleosome\_organization | HIST1H1C | 8 | 1 | 23.025000 | -1.370116 | 44 | 45.04 | 1.023636 |
| GO:0045429\_positive\_regulation\_of\_nitric\_oxide\_biosynthetic\_process | SOD2 | 8 | 1 | 23.025000 | -1.370116 | 44 | 45.04 | 1.023636 |
| GO:0001542\_ovulation\_from\_ovarian\_follicle | SIRT1 | 9 | 1 | 20.466667 | -1.320091 | 51 | 52.48 | 1.029020 |
| GO:0016601\_Rac\_protein\_signal\_transduction | RHOU | 9 | 1 | 20.466667 | -1.320091 | 51 | 52.48 | 1.029020 |
| GO:0030728\_ovulation | SIRT1 | 9 | 1 | 20.466667 | -1.320091 | 51 | 52.48 | 1.029020 |
| GO:0043242\_negative\_regulation\_of\_protein\_complex\_disassembly | HDAC6 | 9 | 1 | 20.466667 | -1.320091 | 51 | 52.48 | 1.029020 |
| GO:0043409\_negative\_regulation\_of\_MAPKKK\_cascade | IGF1R | 9 | 1 | 20.466667 | -1.320091 | 51 | 52.48 | 1.029020 |
| GO:0045428\_regulation\_of\_nitric\_oxide\_biosynthetic\_process | SOD2 | 9 | 1 | 20.466667 | -1.320091 | 51 | 52.48 | 1.029020 |
| GO:0051896\_regulation\_of\_protein\_kinase\_B\_signaling\_cascade | IGF1R | 9 | 1 | 20.466667 | -1.320091 | 51 | 52.48 | 1.029020 |
| GO:0034962\_cellular\_biopolymer\_catabolic\_process | UBE2N | 68 | 2 | 5.417647 | -1.285471 | 52 | 53.61 | 1.030962 |
| GO:0034962\_cellular\_biopolymer\_catabolic\_process | HDAC6 | 68 | 2 | 5.417647 | -1.285471 | 52 | 53.61 | 1.030962 |
| GO:0000209\_protein\_polyubiquitination | HDAC6 | 10 | 1 | 18.420000 | -1.275461 | 57 | 60.08 | 1.054035 |
| GO:0006801\_superoxide\_metabolic\_process | SOD2 | 10 | 1 | 18.420000 | -1.275461 | 57 | 60.08 | 1.054035 |
| GO:0022900\_electron\_transport\_chain | SOD2 | 10 | 1 | 18.420000 | -1.275461 | 57 | 60.08 | 1.054035 |
| GO:0022904\_respiratory\_electron\_transport\_chain | SOD2 | 10 | 1 | 18.420000 | -1.275461 | 57 | 60.08 | 1.054035 |
| GO:0045776\_negative\_regulation\_of\_blood\_pressure | SOD2 | 10 | 1 | 18.420000 | -1.275461 | 57 | 60.08 | 1.054035 |
| GO:0051128\_regulation\_of\_cellular\_component\_organization | RECQL4 | 160 | 3 | 3.453750 | -1.266794 | 58 | 60.43 | 1.041897 |
| GO:0051128\_regulation\_of\_cellular\_component\_organization | RHOU | 160 | 3 | 3.453750 | -1.266794 | 58 | 60.43 | 1.041897 |
| GO:0051128\_regulation\_of\_cellular\_component\_organization | HDAC6 | 160 | 3 | 3.453750 | -1.266794 | 58 | 60.43 | 1.041897 |
| GO:0019538\_protein\_metabolic\_process | UBE2N | 655 | 7 | 1.968550 | -1.265801 | 59 | 60.48 | 1.025085 |
| GO:0019538\_protein\_metabolic\_process | IGF1R | 655 | 7 | 1.968550 | -1.265801 | 59 | 60.48 | 1.025085 |
| GO:0019538\_protein\_metabolic\_process | STT3A | 655 | 7 | 1.968550 | -1.265801 | 59 | 60.48 | 1.025085 |
| GO:0019538\_protein\_metabolic\_process | CTSS | 655 | 7 | 1.968550 | -1.265801 | 59 | 60.48 | 1.025085 |
| GO:0019538\_protein\_metabolic\_process | SIRT1 | 655 | 7 | 1.968550 | -1.265801 | 59 | 60.48 | 1.025085 |
| GO:0019538\_protein\_metabolic\_process | PCOLCE | 655 | 7 | 1.968550 | -1.265801 | 59 | 60.48 | 1.025085 |
| GO:0019538\_protein\_metabolic\_process | HDAC6 | 655 | 7 | 1.968550 | -1.265801 | 59 | 60.48 | 1.025085 |
| GO:0006281\_DNA\_repair | TRP53BP1 | 71 | 2 | 5.188732 | -1.251966 | 61 | 61.18 | 1.002951 |
| GO:0006281\_DNA\_repair | SOD2 | 71 | 2 | 5.188732 | -1.251966 | 61 | 61.18 | 1.002951 |
| GO:0009100\_glycoprotein\_metabolic\_process | STT3A | 71 | 2 | 5.188732 | -1.251966 | 61 | 61.18 | 1.002951 |
| GO:0009100\_glycoprotein\_metabolic\_process | GLCE | 71 | 2 | 5.188732 | -1.251966 | 61 | 61.18 | 1.002951 |
| GO:0007264\_small\_GTPase\_mediated\_signal\_transduction | NRAS | 72 | 2 | 5.116667 | -1.241153 | 62 | 62.21 | 1.003387 |
| GO:0007264\_small\_GTPase\_mediated\_signal\_transduction | RHOU | 72 | 2 | 5.116667 | -1.241153 | 62 | 62.21 | 1.003387 |
| GO:0000271\_polysaccharide\_biosynthetic\_process | GLCE | 11 | 1 | 16.745455 | -1.235194 | 70 | 67.04 | 0.957714 |
| GO:0006333\_chromatin\_assembly\_or\_disassembly | HIST1H1C | 11 | 1 | 16.745455 | -1.235194 | 70 | 67.04 | 0.957714 |
| GO:0030238\_male\_sex\_determination | IGF1R | 11 | 1 | 16.745455 | -1.235194 | 70 | 67.04 | 0.957714 |
| GO:0031110\_regulation\_of\_microtubule\_polymerization\_or\_depolymerization | HDAC6 | 11 | 1 | 16.745455 | -1.235194 | 70 | 67.04 | 0.957714 |
| GO:0042542\_response\_to\_hydrogen\_peroxide | SOD2 | 11 | 1 | 16.745455 | -1.235194 | 70 | 67.04 | 0.957714 |
| GO:0048678\_response\_to\_axon\_injury | SOD2 | 11 | 1 | 16.745455 | -1.235194 | 70 | 67.04 | 0.957714 |
| GO:0051092\_positive\_regulation\_of\_NF-kappaB\_transcription\_factor\_activity | UBE2N | 11 | 1 | 16.745455 | -1.235194 | 70 | 67.04 | 0.957714 |
| GO:0051494\_negative\_regulation\_of\_cytoskeleton\_organization | HDAC6 | 11 | 1 | 16.745455 | -1.235194 | 70 | 67.04 | 0.957714 |
| GO:0044265\_cellular\_macromolecule\_catabolic\_process | UBE2N | 75 | 2 | 4.912000 | -1.209714 | 71 | 68.25 | 0.961268 |
| GO:0044265\_cellular\_macromolecule\_catabolic\_process | HDAC6 | 75 | 2 | 4.912000 | -1.209714 | 71 | 68.25 | 0.961268 |
| GO:0034621\_cellular\_macromolecular\_complex\_subunit\_organization | HIST1H1C | 76 | 2 | 4.847368 | -1.199552 | 72 | 68.6 | 0.952778 |
| GO:0034621\_cellular\_macromolecular\_complex\_subunit\_organization | HDAC6 | 76 | 2 | 4.847368 | -1.199552 | 72 | 68.6 | 0.952778 |
| GO:0030166\_proteoglycan\_biosynthetic\_process | GLCE | 12 | 1 | 15.350000 | -1.198531 | 77 | 73.2 | 0.950649 |
| GO:0031109\_microtubule\_polymerization\_or\_depolymerization | HDAC6 | 12 | 1 | 15.350000 | -1.198531 | 77 | 73.2 | 0.950649 |
| GO:0043624\_cellular\_protein\_complex\_disassembly | HDAC6 | 12 | 1 | 15.350000 | -1.198531 | 77 | 73.2 | 0.950649 |
| GO:0051261\_protein\_depolymerization | HDAC6 | 12 | 1 | 15.350000 | -1.198531 | 77 | 73.2 | 0.950649 |
| GO:0055114\_oxidation\_reduction | SOD2 | 12 | 1 | 15.350000 | -1.198531 | 77 | 73.2 | 0.950649 |
| GO:0001836\_release\_of\_cytochrome\_c\_from\_mitochondria | SOD2 | 13 | 1 | 14.169231 | -1.164893 | 82 | 79.31 | 0.967195 |
| GO:0001976\_neurological\_system\_process\_involved\_in\_regulation\_of\_systemic\_arterial\_blood\_pressure | SOD2 | 13 | 1 | 14.169231 | -1.164893 | 82 | 79.31 | 0.967195 |
| GO:0031290\_retinal\_ganglion\_cell\_axon\_guidance | BMPR1B | 13 | 1 | 14.169231 | -1.164893 | 82 | 79.31 | 0.967195 |
| GO:0043241\_protein\_complex\_disassembly | HDAC6 | 13 | 1 | 14.169231 | -1.164893 | 82 | 79.31 | 0.967195 |
| GO:0043244\_regulation\_of\_protein\_complex\_disassembly | HDAC6 | 13 | 1 | 14.169231 | -1.164893 | 82 | 79.31 | 0.967195 |
| GO:0001502\_cartilage\_condensation | BMPR1B | 14 | 1 | 13.157143 | -1.133832 | 92 | 84.73 | 0.920978 |
| GO:0006809\_nitric\_oxide\_biosynthetic\_process | SOD2 | 14 | 1 | 13.157143 | -1.133832 | 92 | 84.73 | 0.920978 |
| GO:0006970\_response\_to\_osmotic\_stress | PDPK1 | 14 | 1 | 13.157143 | -1.133832 | 92 | 84.73 | 0.920978 |
| GO:0007530\_sex\_determination | IGF1R | 14 | 1 | 13.157143 | -1.133832 | 92 | 84.73 | 0.920978 |
| GO:0010332\_response\_to\_gamma\_radiation | SOD2 | 14 | 1 | 13.157143 | -1.133832 | 92 | 84.73 | 0.920978 |
| GO:0033044\_regulation\_of\_chromosome\_organization | RECQL4 | 14 | 1 | 13.157143 | -1.133832 | 92 | 84.73 | 0.920978 |
| GO:0034623\_cellular\_macromolecular\_complex\_disassembly | HDAC6 | 14 | 1 | 13.157143 | -1.133832 | 92 | 84.73 | 0.920978 |
| GO:0043491\_protein\_kinase\_B\_signaling\_cascade | IGF1R | 14 | 1 | 13.157143 | -1.133832 | 92 | 84.73 | 0.920978 |
| GO:0045598\_regulation\_of\_fat\_cell\_differentiation | SOD2 | 14 | 1 | 13.157143 | -1.133832 | 92 | 84.73 | 0.920978 |
| GO:0046209\_nitric\_oxide\_metabolic\_process | SOD2 | 14 | 1 | 13.157143 | -1.133832 | 92 | 84.73 | 0.920978 |
| GO:0006325\_chromatin\_organization | HIST1H1C | 83 | 2 | 4.438554 | -1.132463 | 93 | 85.32 | 0.917419 |
| GO:0006325\_chromatin\_organization | SIRT1 | 83 | 2 | 4.438554 | -1.132463 | 93 | 85.32 | 0.917419 |
| GO:0043283\_biopolymer\_metabolic\_process | UBE2N | 1490 | 12 | 1.483490 | -1.126677 | 94 | 85.46 | 0.909149 |
| GO:0043283\_biopolymer\_metabolic\_process | IGF1R | 1490 | 12 | 1.483490 | -1.126677 | 94 | 85.46 | 0.909149 |
| GO:0043283\_biopolymer\_metabolic\_process | TRP53BP1 | 1490 | 12 | 1.483490 | -1.126677 | 94 | 85.46 | 0.909149 |
| GO:0043283\_biopolymer\_metabolic\_process | SALL4 | 1490 | 12 | 1.483490 | -1.126677 | 94 | 85.46 | 0.909149 |
| GO:0043283\_biopolymer\_metabolic\_process | STT3A | 1490 | 12 | 1.483490 | -1.126677 | 94 | 85.46 | 0.909149 |
| GO:0043283\_biopolymer\_metabolic\_process | FOXK1 | 1490 | 12 | 1.483490 | -1.126677 | 94 | 85.46 | 0.909149 |
| GO:0043283\_biopolymer\_metabolic\_process | CTSS | 1490 | 12 | 1.483490 | -1.126677 | 94 | 85.46 | 0.909149 |
| GO:0043283\_biopolymer\_metabolic\_process | SIRT1 | 1490 | 12 | 1.483490 | -1.126677 | 94 | 85.46 | 0.909149 |
| GO:0043283\_biopolymer\_metabolic\_process | PCOLCE | 1490 | 12 | 1.483490 | -1.126677 | 94 | 85.46 | 0.909149 |
| GO:0043283\_biopolymer\_metabolic\_process | GLCE | 1490 | 12 | 1.483490 | -1.126677 | 94 | 85.46 | 0.909149 |
| GO:0043283\_biopolymer\_metabolic\_process | SOD2 | 1490 | 12 | 1.483490 | -1.126677 | 94 | 85.46 | 0.909149 |
| GO:0043283\_biopolymer\_metabolic\_process | HDAC6 | 1490 | 12 | 1.483490 | -1.126677 | 94 | 85.46 | 0.909149 |
| GO:0006487\_protein\_amino\_acid\_N-linked\_glycosylation | STT3A | 15 | 1 | 12.280000 | -1.104991 | 99 | 90.91 | 0.918283 |
| GO:0006749\_glutathione\_metabolic\_process | SOD2 | 15 | 1 | 12.280000 | -1.104991 | 99 | 90.91 | 0.918283 |
| GO:0048144\_fibroblast\_proliferation | SOD2 | 15 | 1 | 12.280000 | -1.104991 | 99 | 90.91 | 0.918283 |
| GO:0048145\_regulation\_of\_fibroblast\_proliferation | SOD2 | 15 | 1 | 12.280000 | -1.104991 | 99 | 90.91 | 0.918283 |
| GO:0070507\_regulation\_of\_microtubule\_cytoskeleton\_organization | HDAC6 | 15 | 1 | 12.280000 | -1.104991 | 99 | 90.91 | 0.918283 |
| GO:0000302\_response\_to\_reactive\_oxygen\_species | SOD2 | 16 | 1 | 11.512500 | -1.078084 | 103 | 95.95 | 0.931553 |
| GO:0003044\_regulation\_of\_systemic\_arterial\_blood\_pressure\_mediated\_by\_a\_chemical\_signal | SOD2 | 16 | 1 | 11.512500 | -1.078084 | 103 | 95.95 | 0.931553 |
| GO:0006821\_chloride\_transport | SLC4A1 | 16 | 1 | 11.512500 | -1.078084 | 103 | 95.95 | 0.931553 |
| GO:0042311\_vasodilation | SOD2 | 16 | 1 | 11.512500 | -1.078084 | 103 | 95.95 | 0.931553 |
| GO:0006323\_DNA\_packaging | HIST1H1C | 17 | 1 | 10.835294 | -1.052876 | 110 | 100.79 | 0.916273 |
| GO:0010741\_negative\_regulation\_of\_protein\_kinase\_cascade | IGF1R | 17 | 1 | 10.835294 | -1.052876 | 110 | 100.79 | 0.916273 |
| GO:0043407\_negative\_regulation\_of\_MAP\_kinase\_activity | DUSP16 | 17 | 1 | 10.835294 | -1.052876 | 110 | 100.79 | 0.916273 |
| GO:0045333\_cellular\_respiration | SOD2 | 17 | 1 | 10.835294 | -1.052876 | 110 | 100.79 | 0.916273 |
| GO:0051091\_positive\_regulation\_of\_transcription\_factor\_activity | UBE2N | 17 | 1 | 10.835294 | -1.052876 | 110 | 100.79 | 0.916273 |
| GO:0055072\_iron\_ion\_homeostasis | SOD2 | 17 | 1 | 10.835294 | -1.052876 | 110 | 100.79 | 0.916273 |
| GO:0090047\_positive\_regulation\_of\_transcription\_regulator\_activity | UBE2N | 17 | 1 | 10.835294 | -1.052876 | 110 | 100.79 | 0.916273 |
| GO:0034984\_cellular\_response\_to\_DNA\_damage\_stimulus | TRP53BP1 | 94 | 2 | 3.919149 | -1.039301 | 111 | 101.92 | 0.918198 |
| GO:0034984\_cellular\_response\_to\_DNA\_damage\_stimulus | SOD2 | 94 | 2 | 3.919149 | -1.039301 | 111 | 101.92 | 0.918198 |
| GO:0006022\_aminoglycan\_metabolic\_process | GLCE | 18 | 1 | 10.233333 | -1.029173 | 115 | 106.52 | 0.926261 |
| GO:0030203\_glycosaminoglycan\_metabolic\_process | GLCE | 18 | 1 | 10.233333 | -1.029173 | 115 | 106.52 | 0.926261 |
| GO:0032984\_macromolecular\_complex\_disassembly | HDAC6 | 18 | 1 | 10.233333 | -1.029173 | 115 | 106.52 | 0.926261 |
| GO:0044272\_sulfur\_compound\_biosynthetic\_process | GLCE | 18 | 1 | 10.233333 | -1.029173 | 115 | 106.52 | 0.926261 |
| GO:0050931\_pigment\_cell\_differentiation | SOD2 | 19 | 1 | 9.694737 | -1.006811 | 116 | 110.95 | 0.956466 |
| GO:0010605\_negative\_regulation\_of\_macromolecule\_metabolic\_process | SALL4 | 331 | 4 | 2.225982 | -0.998687 | 117 | 111.91 | 0.956496 |
| GO:0010605\_negative\_regulation\_of\_macromolecule\_metabolic\_process | FOXK1 | 331 | 4 | 2.225982 | -0.998687 | 117 | 111.91 | 0.956496 |
| GO:0010605\_negative\_regulation\_of\_macromolecule\_metabolic\_process | SIRT1 | 331 | 4 | 2.225982 | -0.998687 | 117 | 111.91 | 0.956496 |
| GO:0010605\_negative\_regulation\_of\_macromolecule\_metabolic\_process | HDAC6 | 331 | 4 | 2.225982 | -0.998687 | 117 | 111.91 | 0.956496 |
| GO:0031324\_negative\_regulation\_of\_cellular\_metabolic\_process | SALL4 | 332 | 4 | 2.219277 | -0.995007 | 118 | 112.15 | 0.950424 |
| GO:0031324\_negative\_regulation\_of\_cellular\_metabolic\_process | FOXK1 | 332 | 4 | 2.219277 | -0.995007 | 118 | 112.15 | 0.950424 |
| GO:0031324\_negative\_regulation\_of\_cellular\_metabolic\_process | SIRT1 | 332 | 4 | 2.219277 | -0.995007 | 118 | 112.15 | 0.950424 |
| GO:0031324\_negative\_regulation\_of\_cellular\_metabolic\_process | HDAC6 | 332 | 4 | 2.219277 | -0.995007 | 118 | 112.15 | 0.950424 |
| GO:0006518\_peptide\_metabolic\_process | SOD2 | 20 | 1 | 9.210000 | -0.985653 | 120 | 115.32 | 0.961000 |
| GO:0008360\_regulation\_of\_cell\_shape | RHOU | 20 | 1 | 9.210000 | -0.985653 | 120 | 115.32 | 0.961000 |
| GO:0008637\_apoptotic\_mitochondrial\_changes | SOD2 | 21 | 1 | 8.771429 | -0.965582 | 121 | 120.0 | 0.991736 |
| GO:0043170\_macromolecule\_metabolic\_process | UBE2N | 1576 | 12 | 1.402538 | -0.965487 | 122 | 120.09 | 0.984344 |
| GO:0043170\_macromolecule\_metabolic\_process | IGF1R | 1576 | 12 | 1.402538 | -0.965487 | 122 | 120.09 | 0.984344 |
| GO:0043170\_macromolecule\_metabolic\_process | TRP53BP1 | 1576 | 12 | 1.402538 | -0.965487 | 122 | 120.09 | 0.984344 |
| GO:0043170\_macromolecule\_metabolic\_process | STT3A | 1576 | 12 | 1.402538 | -0.965487 | 122 | 120.09 | 0.984344 |
| GO:0043170\_macromolecule\_metabolic\_process | SALL4 | 1576 | 12 | 1.402538 | -0.965487 | 122 | 120.09 | 0.984344 |
| GO:0043170\_macromolecule\_metabolic\_process | FOXK1 | 1576 | 12 | 1.402538 | -0.965487 | 122 | 120.09 | 0.984344 |
| GO:0043170\_macromolecule\_metabolic\_process | CTSS | 1576 | 12 | 1.402538 | -0.965487 | 122 | 120.09 | 0.984344 |
| GO:0043170\_macromolecule\_metabolic\_process | SIRT1 | 1576 | 12 | 1.402538 | -0.965487 | 122 | 120.09 | 0.984344 |
| GO:0043170\_macromolecule\_metabolic\_process | GLCE | 1576 | 12 | 1.402538 | -0.965487 | 122 | 120.09 | 0.984344 |
| GO:0043170\_macromolecule\_metabolic\_process | PCOLCE | 1576 | 12 | 1.402538 | -0.965487 | 122 | 120.09 | 0.984344 |
| GO:0043170\_macromolecule\_metabolic\_process | SOD2 | 1576 | 12 | 1.402538 | -0.965487 | 122 | 120.09 | 0.984344 |
| GO:0043170\_macromolecule\_metabolic\_process | HDAC6 | 1576 | 12 | 1.402538 | -0.965487 | 122 | 120.09 | 0.984344 |
| GO:0048523\_negative\_regulation\_of\_cellular\_process | RECQL4 | 774 | 7 | 1.665891 | -0.948841 | 123 | 121.19 | 0.985285 |
| GO:0048523\_negative\_regulation\_of\_cellular\_process | IGF1R | 774 | 7 | 1.665891 | -0.948841 | 123 | 121.19 | 0.985285 |
| GO:0048523\_negative\_regulation\_of\_cellular\_process | SALL4 | 774 | 7 | 1.665891 | -0.948841 | 123 | 121.19 | 0.985285 |
| GO:0048523\_negative\_regulation\_of\_cellular\_process | FOXK1 | 774 | 7 | 1.665891 | -0.948841 | 123 | 121.19 | 0.985285 |
| GO:0048523\_negative\_regulation\_of\_cellular\_process | SIRT1 | 774 | 7 | 1.665891 | -0.948841 | 123 | 121.19 | 0.985285 |
| GO:0048523\_negative\_regulation\_of\_cellular\_process | HDAC6 | 774 | 7 | 1.665891 | -0.948841 | 123 | 121.19 | 0.985285 |
| GO:0048523\_negative\_regulation\_of\_cellular\_process | SOD2 | 774 | 7 | 1.665891 | -0.948841 | 123 | 121.19 | 0.985285 |
| GO:0006029\_proteoglycan\_metabolic\_process | GLCE | 22 | 1 | 8.372727 | -0.946495 | 126 | 126.24 | 1.001905 |
| GO:0007059\_chromosome\_segregation | RECQL4 | 22 | 1 | 8.372727 | -0.946495 | 126 | 126.24 | 1.001905 |
| GO:0032886\_regulation\_of\_microtubule-based\_process | HDAC6 | 22 | 1 | 8.372727 | -0.946495 | 126 | 126.24 | 1.001905 |
| GO:0009892\_negative\_regulation\_of\_metabolic\_process | SALL4 | 348 | 4 | 2.117241 | -0.938203 | 127 | 126.88 | 0.999055 |
| GO:0009892\_negative\_regulation\_of\_metabolic\_process | FOXK1 | 348 | 4 | 2.117241 | -0.938203 | 127 | 126.88 | 0.999055 |
| GO:0009892\_negative\_regulation\_of\_metabolic\_process | SIRT1 | 348 | 4 | 2.117241 | -0.938203 | 127 | 126.88 | 0.999055 |
| GO:0009892\_negative\_regulation\_of\_metabolic\_process | HDAC6 | 348 | 4 | 2.117241 | -0.938203 | 127 | 126.88 | 0.999055 |
| GO:0000082\_G1\_S\_transition\_of\_mitotic\_cell\_cycle | RHOU | 23 | 1 | 8.008696 | -0.928306 | 131 | 130.0 | 0.992366 |
| GO:0003073\_regulation\_of\_systemic\_arterial\_blood\_pressure | SOD2 | 23 | 1 | 8.008696 | -0.928306 | 131 | 130.0 | 0.992366 |
| GO:0015698\_inorganic\_anion\_transport | SLC4A1 | 23 | 1 | 8.008696 | -0.928306 | 131 | 130.0 | 0.992366 |
| GO:0043388\_positive\_regulation\_of\_DNA\_binding | UBE2N | 23 | 1 | 8.008696 | -0.928306 | 131 | 130.0 | 0.992366 |
| GO:0048705\_skeletal\_system\_morphogenesis | RECQL4 | 111 | 2 | 3.318919 | -0.918135 | 132 | 130.51 | 0.988712 |
| GO:0048705\_skeletal\_system\_morphogenesis | BMPR1B | 111 | 2 | 3.318919 | -0.918135 | 132 | 130.51 | 0.988712 |
| GO:0043410\_positive\_regulation\_of\_MAPKKK\_cascade | IGF1R | 24 | 1 | 7.675000 | -0.910938 | 134 | 134.5 | 1.003731 |
| GO:0051099\_positive\_regulation\_of\_binding | UBE2N | 24 | 1 | 7.675000 | -0.910938 | 134 | 134.5 | 1.003731 |
| GO:0006974\_response\_to\_DNA\_damage\_stimulus | TRP53BP1 | 113 | 2 | 3.260177 | -0.905361 | 135 | 135.06 | 1.000444 |
| GO:0006974\_response\_to\_DNA\_damage\_stimulus | SOD2 | 113 | 2 | 3.260177 | -0.905361 | 135 | 135.06 | 1.000444 |
| GO:0000165\_MAPKKK\_cascade | IGF1R | 114 | 2 | 3.231579 | -0.899078 | 136 | 135.33 | 0.995074 |
| GO:0000165\_MAPKKK\_cascade | DUSP16 | 114 | 2 | 3.231579 | -0.899078 | 136 | 135.33 | 0.995074 |
| GO:0006302\_double-strand\_break\_repair | SOD2 | 25 | 1 | 7.368000 | -0.894323 | 140 | 139.02 | 0.993000 |
| GO:0050852\_T\_cell\_receptor\_signaling\_pathway | UBE2N | 25 | 1 | 7.368000 | -0.894323 | 140 | 139.02 | 0.993000 |
| GO:0051090\_regulation\_of\_transcription\_factor\_activity | UBE2N | 25 | 1 | 7.368000 | -0.894323 | 140 | 139.02 | 0.993000 |
| GO:0090046\_regulation\_of\_transcription\_regulator\_activity | UBE2N | 25 | 1 | 7.368000 | -0.894323 | 140 | 139.02 | 0.993000 |
| GO:0048608\_reproductive\_structure\_development | IGF1R | 116 | 2 | 3.175862 | -0.886710 | 141 | 139.73 | 0.990993 |
| GO:0048608\_reproductive\_structure\_development | SIRT1 | 116 | 2 | 3.175862 | -0.886710 | 141 | 139.73 | 0.990993 |
| GO:0043933\_macromolecular\_complex\_subunit\_organization | HIST1H1C | 117 | 2 | 3.148718 | -0.880624 | 142 | 140.46 | 0.989155 |
| GO:0043933\_macromolecular\_complex\_subunit\_organization | HDAC6 | 117 | 2 | 3.148718 | -0.880624 | 142 | 140.46 | 0.989155 |
| GO:0006800\_oxygen\_and\_reactive\_oxygen\_species\_metabolic\_process | SOD2 | 26 | 1 | 7.084615 | -0.878403 | 144 | 143.13 | 0.993958 |
| GO:0010212\_response\_to\_ionizing\_radiation | SOD2 | 26 | 1 | 7.084615 | -0.878403 | 144 | 143.13 | 0.993958 |
| GO:0016043\_cellular\_component\_organization | RECQL4 | 964 | 8 | 1.528631 | -0.875193 | 145 | 143.26 | 0.988000 |
| GO:0016043\_cellular\_component\_organization | HIST1H1C | 964 | 8 | 1.528631 | -0.875193 | 145 | 143.26 | 0.988000 |
| GO:0016043\_cellular\_component\_organization | PTPRZ1 | 964 | 8 | 1.528631 | -0.875193 | 145 | 143.26 | 0.988000 |
| GO:0016043\_cellular\_component\_organization | BMPR1B | 964 | 8 | 1.528631 | -0.875193 | 145 | 143.26 | 0.988000 |
| GO:0016043\_cellular\_component\_organization | RHOU | 964 | 8 | 1.528631 | -0.875193 | 145 | 143.26 | 0.988000 |
| GO:0016043\_cellular\_component\_organization | SIRT1 | 964 | 8 | 1.528631 | -0.875193 | 145 | 143.26 | 0.988000 |
| GO:0016043\_cellular\_component\_organization | SOD2 | 964 | 8 | 1.528631 | -0.875193 | 145 | 143.26 | 0.988000 |
| GO:0016043\_cellular\_component\_organization | HDAC6 | 964 | 8 | 1.528631 | -0.875193 | 145 | 143.26 | 0.988000 |
| GO:0007049\_cell\_cycle | RECQL4 | 238 | 3 | 2.321849 | -0.865969 | 146 | 144.49 | 0.989658 |
| GO:0007049\_cell\_cycle | NRAS | 238 | 3 | 2.321849 | -0.865969 | 146 | 144.49 | 0.989658 |
| GO:0007049\_cell\_cycle | RHOU | 238 | 3 | 2.321849 | -0.865969 | 146 | 144.49 | 0.989658 |
| GO:0031016\_pancreas\_development | IGF1R | 27 | 1 | 6.822222 | -0.863126 | 148 | 146.66 | 0.990946 |
| GO:0070482\_response\_to\_oxygen\_levels | SOD2 | 27 | 1 | 6.822222 | -0.863126 | 148 | 146.66 | 0.990946 |
| GO:0051726\_regulation\_of\_cell\_cycle | RECQL4 | 121 | 2 | 3.044628 | -0.856903 | 149 | 147.36 | 0.988993 |
| GO:0051726\_regulation\_of\_cell\_cycle | NRAS | 121 | 2 | 3.044628 | -0.856903 | 149 | 147.36 | 0.988993 |
| GO:0010564\_regulation\_of\_cell\_cycle\_process | RECQL4 | 29 | 1 | 6.351724 | -0.834314 | 152 | 153.8 | 1.011842 |
| GO:0048066\_pigmentation\_during\_development | SOD2 | 29 | 1 | 6.351724 | -0.834314 | 152 | 153.8 | 1.011842 |
| GO:0060041\_retina\_development\_in\_camera-type\_eye | BMPR1B | 29 | 1 | 6.351724 | -0.834314 | 152 | 153.8 | 1.011842 |
| GO:0050794\_regulation\_of\_cellular\_process | RECQL4 | 2190 | 15 | 1.261644 | -0.831931 | 153 | 154.11 | 1.007255 |
| GO:0050794\_regulation\_of\_cellular\_process | FOXK1 | 2190 | 15 | 1.261644 | -0.831931 | 153 | 154.11 | 1.007255 |
| GO:0050794\_regulation\_of\_cellular\_process | RHOU | 2190 | 15 | 1.261644 | -0.831931 | 153 | 154.11 | 1.007255 |
| GO:0050794\_regulation\_of\_cellular\_process | SIRT1 | 2190 | 15 | 1.261644 | -0.831931 | 153 | 154.11 | 1.007255 |
| GO:0050794\_regulation\_of\_cellular\_process | PIN1 | 2190 | 15 | 1.261644 | -0.831931 | 153 | 154.11 | 1.007255 |
| GO:0050794\_regulation\_of\_cellular\_process | SOD2 | 2190 | 15 | 1.261644 | -0.831931 | 153 | 154.11 | 1.007255 |
| GO:0050794\_regulation\_of\_cellular\_process | UBE2N | 2190 | 15 | 1.261644 | -0.831931 | 153 | 154.11 | 1.007255 |
| GO:0050794\_regulation\_of\_cellular\_process | NRAS | 2190 | 15 | 1.261644 | -0.831931 | 153 | 154.11 | 1.007255 |
| GO:0050794\_regulation\_of\_cellular\_process | IGF1R | 2190 | 15 | 1.261644 | -0.831931 | 153 | 154.11 | 1.007255 |
| GO:0050794\_regulation\_of\_cellular\_process | PDPK1 | 2190 | 15 | 1.261644 | -0.831931 | 153 | 154.11 | 1.007255 |
| GO:0050794\_regulation\_of\_cellular\_process | TRP53BP1 | 2190 | 15 | 1.261644 | -0.831931 | 153 | 154.11 | 1.007255 |
| GO:0050794\_regulation\_of\_cellular\_process | SALL4 | 2190 | 15 | 1.261644 | -0.831931 | 153 | 154.11 | 1.007255 |
| GO:0050794\_regulation\_of\_cellular\_process | DUSP16 | 2190 | 15 | 1.261644 | -0.831931 | 153 | 154.11 | 1.007255 |
| GO:0050794\_regulation\_of\_cellular\_process | BMPR1B | 2190 | 15 | 1.261644 | -0.831931 | 153 | 154.11 | 1.007255 |
| GO:0050794\_regulation\_of\_cellular\_process | HDAC6 | 2190 | 15 | 1.261644 | -0.831931 | 153 | 154.11 | 1.007255 |
| GO:0022411\_cellular\_component\_disassembly | HDAC6 | 30 | 1 | 6.140000 | -0.820701 | 155 | 158.2 | 1.020645 |
| GO:0060740\_prostate\_gland\_epithelium\_morphogenesis | IGF1R | 30 | 1 | 6.140000 | -0.820701 | 155 | 158.2 | 1.020645 |
| GO:0016481\_negative\_regulation\_of\_transcription | SALL4 | 253 | 3 | 2.184190 | -0.808781 | 156 | 159.63 | 1.023269 |
| GO:0016481\_negative\_regulation\_of\_transcription | FOXK1 | 253 | 3 | 2.184190 | -0.808781 | 156 | 159.63 | 1.023269 |
| GO:0016481\_negative\_regulation\_of\_transcription | SIRT1 | 253 | 3 | 2.184190 | -0.808781 | 156 | 159.63 | 1.023269 |
| GO:0003018\_vascular\_process\_in\_circulatory\_system | SOD2 | 31 | 1 | 5.941935 | -0.807570 | 163 | 164.26 | 1.007730 |
| GO:0006486\_protein\_amino\_acid\_glycosylation | STT3A | 31 | 1 | 5.941935 | -0.807570 | 163 | 164.26 | 1.007730 |
| GO:0035150\_regulation\_of\_tube\_size | SOD2 | 31 | 1 | 5.941935 | -0.807570 | 163 | 164.26 | 1.007730 |
| GO:0043413\_biopolymer\_glycosylation | STT3A | 31 | 1 | 5.941935 | -0.807570 | 163 | 164.26 | 1.007730 |
| GO:0050880\_regulation\_of\_blood\_vessel\_size | SOD2 | 31 | 1 | 5.941935 | -0.807570 | 163 | 164.26 | 1.007730 |
| GO:0060512\_prostate\_gland\_morphogenesis | IGF1R | 31 | 1 | 5.941935 | -0.807570 | 163 | 164.26 | 1.007730 |
| GO:0070085\_glycosylation | STT3A | 31 | 1 | 5.941935 | -0.807570 | 163 | 164.26 | 1.007730 |
| GO:0051493\_regulation\_of\_cytoskeleton\_organization | HDAC6 | 32 | 1 | 5.756250 | -0.794890 | 164 | 167.36 | 1.020488 |
| GO:0031323\_regulation\_of\_cellular\_metabolic\_process | UBE2N | 1015 | 8 | 1.451823 | -0.778672 | 165 | 170.78 | 1.035030 |
| GO:0031323\_regulation\_of\_cellular\_metabolic\_process | TRP53BP1 | 1015 | 8 | 1.451823 | -0.778672 | 165 | 170.78 | 1.035030 |
| GO:0031323\_regulation\_of\_cellular\_metabolic\_process | SALL4 | 1015 | 8 | 1.451823 | -0.778672 | 165 | 170.78 | 1.035030 |
| GO:0031323\_regulation\_of\_cellular\_metabolic\_process | FOXK1 | 1015 | 8 | 1.451823 | -0.778672 | 165 | 170.78 | 1.035030 |
| GO:0031323\_regulation\_of\_cellular\_metabolic\_process | DUSP16 | 1015 | 8 | 1.451823 | -0.778672 | 165 | 170.78 | 1.035030 |
| GO:0031323\_regulation\_of\_cellular\_metabolic\_process | SIRT1 | 1015 | 8 | 1.451823 | -0.778672 | 165 | 170.78 | 1.035030 |
| GO:0031323\_regulation\_of\_cellular\_metabolic\_process | SOD2 | 1015 | 8 | 1.451823 | -0.778672 | 165 | 170.78 | 1.035030 |
| GO:0031323\_regulation\_of\_cellular\_metabolic\_process | HDAC6 | 1015 | 8 | 1.451823 | -0.778672 | 165 | 170.78 | 1.035030 |
| GO:0010629\_negative\_regulation\_of\_gene\_expression | SALL4 | 262 | 3 | 2.109160 | -0.776696 | 167 | 171.23 | 1.025329 |
| GO:0010629\_negative\_regulation\_of\_gene\_expression | FOXK1 | 262 | 3 | 2.109160 | -0.776696 | 167 | 171.23 | 1.025329 |
| GO:0010629\_negative\_regulation\_of\_gene\_expression | SIRT1 | 262 | 3 | 2.109160 | -0.776696 | 167 | 171.23 | 1.025329 |
| GO:0048666\_neuron\_development | PTPRZ1 | 262 | 3 | 2.109160 | -0.776696 | 167 | 171.23 | 1.025329 |
| GO:0048666\_neuron\_development | BMPR1B | 262 | 3 | 2.109160 | -0.776696 | 167 | 171.23 | 1.025329 |
| GO:0048666\_neuron\_development | SOD2 | 262 | 3 | 2.109160 | -0.776696 | 167 | 171.23 | 1.025329 |
| GO:0007568\_aging | SOD2 | 34 | 1 | 5.417647 | -0.770776 | 169 | 174.19 | 1.030710 |
| GO:0030509\_BMP\_signaling\_pathway | BMPR1B | 34 | 1 | 5.417647 | -0.770776 | 169 | 174.19 | 1.030710 |
| GO:0048519\_negative\_regulation\_of\_biological\_process | RECQL4 | 859 | 7 | 1.501048 | -0.769608 | 170 | 174.46 | 1.026235 |
| GO:0048519\_negative\_regulation\_of\_biological\_process | IGF1R | 859 | 7 | 1.501048 | -0.769608 | 170 | 174.46 | 1.026235 |
| GO:0048519\_negative\_regulation\_of\_biological\_process | SALL4 | 859 | 7 | 1.501048 | -0.769608 | 170 | 174.46 | 1.026235 |
| GO:0048519\_negative\_regulation\_of\_biological\_process | FOXK1 | 859 | 7 | 1.501048 | -0.769608 | 170 | 174.46 | 1.026235 |
| GO:0048519\_negative\_regulation\_of\_biological\_process | SIRT1 | 859 | 7 | 1.501048 | -0.769608 | 170 | 174.46 | 1.026235 |
| GO:0048519\_negative\_regulation\_of\_biological\_process | SOD2 | 859 | 7 | 1.501048 | -0.769608 | 170 | 174.46 | 1.026235 |
| GO:0048519\_negative\_regulation\_of\_biological\_process | HDAC6 | 859 | 7 | 1.501048 | -0.769608 | 170 | 174.46 | 1.026235 |
| GO:0001756\_somitogenesis | ABI1 | 35 | 1 | 5.262857 | -0.759292 | 176 | 177.08 | 1.006136 |
| GO:0007292\_female\_gamete\_generation | SIRT1 | 35 | 1 | 5.262857 | -0.759292 | 176 | 177.08 | 1.006136 |
| GO:0016051\_carbohydrate\_biosynthetic\_process | GLCE | 35 | 1 | 5.262857 | -0.759292 | 176 | 177.08 | 1.006136 |
| GO:0016567\_protein\_ubiquitination | HDAC6 | 35 | 1 | 5.262857 | -0.759292 | 176 | 177.08 | 1.006136 |
| GO:0051325\_interphase | RHOU | 35 | 1 | 5.262857 | -0.759292 | 176 | 177.08 | 1.006136 |
| GO:0051329\_interphase\_of\_mitotic\_cell\_cycle | RHOU | 35 | 1 | 5.262857 | -0.759292 | 176 | 177.08 | 1.006136 |
| GO:0003006\_reproductive\_developmental\_process | IGF1R | 141 | 2 | 2.612766 | -0.751422 | 177 | 177.85 | 1.004802 |
| GO:0003006\_reproductive\_developmental\_process | SIRT1 | 141 | 2 | 2.612766 | -0.751422 | 177 | 177.85 | 1.004802 |
| GO:0045934\_negative\_regulation\_of\_nucleobase\_\_nucleoside\_\_nucleotide\_and\_nucleic\_acid\_metabolic\_process | SALL4 | 270 | 3 | 2.046667 | -0.749462 | 178 | 178.03 | 1.000169 |
| GO:0045934\_negative\_regulation\_of\_nucleobase\_\_nucleoside\_\_nucleotide\_and\_nucleic\_acid\_metabolic\_process | FOXK1 | 270 | 3 | 2.046667 | -0.749462 | 178 | 178.03 | 1.000169 |
| GO:0045934\_negative\_regulation\_of\_nucleobase\_\_nucleoside\_\_nucleotide\_and\_nucleic\_acid\_metabolic\_process | SIRT1 | 270 | 3 | 2.046667 | -0.749462 | 178 | 178.03 | 1.000169 |
| GO:0001889\_liver\_development | SOD2 | 36 | 1 | 5.116667 | -0.748163 | 183 | 181.14 | 0.989836 |
| GO:0006469\_negative\_regulation\_of\_protein\_kinase\_activity | DUSP16 | 36 | 1 | 5.116667 | -0.748163 | 183 | 181.14 | 0.989836 |
| GO:0022602\_ovulation\_cycle\_process | SIRT1 | 36 | 1 | 5.116667 | -0.748163 | 183 | 181.14 | 0.989836 |
| GO:0033673\_negative\_regulation\_of\_kinase\_activity | DUSP16 | 36 | 1 | 5.116667 | -0.748163 | 183 | 181.14 | 0.989836 |
| GO:0050851\_antigen\_receptor-mediated\_signaling\_pathway | UBE2N | 36 | 1 | 5.116667 | -0.748163 | 183 | 181.14 | 0.989836 |
| GO:0007242\_intracellular\_signaling\_cascade | NRAS | 411 | 4 | 1.792701 | -0.747451 | 184 | 181.23 | 0.984946 |
| GO:0007242\_intracellular\_signaling\_cascade | IGF1R | 411 | 4 | 1.792701 | -0.747451 | 184 | 181.23 | 0.984946 |
| GO:0007242\_intracellular\_signaling\_cascade | DUSP16 | 411 | 4 | 1.792701 | -0.747451 | 184 | 181.23 | 0.984946 |
| GO:0007242\_intracellular\_signaling\_cascade | RHOU | 411 | 4 | 1.792701 | -0.747451 | 184 | 181.23 | 0.984946 |
| GO:0044267\_cellular\_protein\_metabolic\_process | UBE2N | 559 | 5 | 1.647585 | -0.747414 | 185 | 181.29 | 0.979946 |
| GO:0044267\_cellular\_protein\_metabolic\_process | IGF1R | 559 | 5 | 1.647585 | -0.747414 | 185 | 181.29 | 0.979946 |
| GO:0044267\_cellular\_protein\_metabolic\_process | STT3A | 559 | 5 | 1.647585 | -0.747414 | 185 | 181.29 | 0.979946 |
| GO:0044267\_cellular\_protein\_metabolic\_process | SIRT1 | 559 | 5 | 1.647585 | -0.747414 | 185 | 181.29 | 0.979946 |
| GO:0044267\_cellular\_protein\_metabolic\_process | HDAC6 | 559 | 5 | 1.647585 | -0.747414 | 185 | 181.29 | 0.979946 |
| GO:0051172\_negative\_regulation\_of\_nitrogen\_compound\_metabolic\_process | SALL4 | 271 | 3 | 2.039114 | -0.746139 | 186 | 181.47 | 0.975645 |
| GO:0051172\_negative\_regulation\_of\_nitrogen\_compound\_metabolic\_process | FOXK1 | 271 | 3 | 2.039114 | -0.746139 | 186 | 181.47 | 0.975645 |
| GO:0051172\_negative\_regulation\_of\_nitrogen\_compound\_metabolic\_process | SIRT1 | 271 | 3 | 2.039114 | -0.746139 | 186 | 181.47 | 0.975645 |
| GO:0042698\_ovulation\_cycle | SIRT1 | 37 | 1 | 4.978378 | -0.737368 | 188 | 183.71 | 0.977181 |
| GO:0051101\_regulation\_of\_DNA\_binding | UBE2N | 37 | 1 | 4.978378 | -0.737368 | 188 | 183.71 | 0.977181 |
| GO:0010558\_negative\_regulation\_of\_macromolecule\_biosynthetic\_process | SALL4 | 274 | 3 | 2.016788 | -0.736275 | 189 | 184.38 | 0.975556 |
| GO:0010558\_negative\_regulation\_of\_macromolecule\_biosynthetic\_process | FOXK1 | 274 | 3 | 2.016788 | -0.736275 | 189 | 184.38 | 0.975556 |
| GO:0010558\_negative\_regulation\_of\_macromolecule\_biosynthetic\_process | SIRT1 | 274 | 3 | 2.016788 | -0.736275 | 189 | 184.38 | 0.975556 |
| GO:0005975\_carbohydrate\_metabolic\_process | STT3A | 146 | 2 | 2.523288 | -0.727993 | 190 | 184.88 | 0.973053 |
| GO:0005975\_carbohydrate\_metabolic\_process | GLCE | 146 | 2 | 2.523288 | -0.727993 | 190 | 184.88 | 0.973053 |
| GO:0006820\_anion\_transport | SLC4A1 | 38 | 1 | 4.847368 | -0.726890 | 192 | 188.34 | 0.980938 |
| GO:0051348\_negative\_regulation\_of\_transferase\_activity | DUSP16 | 38 | 1 | 4.847368 | -0.726890 | 192 | 188.34 | 0.980938 |
| GO:0065009\_regulation\_of\_molecular\_function | UBE2N | 279 | 3 | 1.980645 | -0.720179 | 193 | 189.16 | 0.980104 |
| GO:0065009\_regulation\_of\_molecular\_function | DUSP16 | 279 | 3 | 1.980645 | -0.720179 | 193 | 189.16 | 0.980104 |
| GO:0065009\_regulation\_of\_molecular\_function | SOD2 | 279 | 3 | 1.980645 | -0.720179 | 193 | 189.16 | 0.980104 |
| GO:0005976\_polysaccharide\_metabolic\_process | GLCE | 39 | 1 | 4.723077 | -0.716711 | 194 | 191.98 | 0.989588 |
| GO:0031327\_negative\_regulation\_of\_cellular\_biosynthetic\_process | SALL4 | 282 | 3 | 1.959574 | -0.710721 | 195 | 192.71 | 0.988256 |
| GO:0031327\_negative\_regulation\_of\_cellular\_biosynthetic\_process | FOXK1 | 282 | 3 | 1.959574 | -0.710721 | 195 | 192.71 | 0.988256 |
| GO:0031327\_negative\_regulation\_of\_cellular\_biosynthetic\_process | SIRT1 | 282 | 3 | 1.959574 | -0.710721 | 195 | 192.71 | 0.988256 |
| GO:0000902\_cell\_morphogenesis | PTPRZ1 | 283 | 3 | 1.952650 | -0.707600 | 196 | 192.8 | 0.983673 |
| GO:0000902\_cell\_morphogenesis | BMPR1B | 283 | 3 | 1.952650 | -0.707600 | 196 | 192.8 | 0.983673 |
| GO:0000902\_cell\_morphogenesis | RHOU | 283 | 3 | 1.952650 | -0.707600 | 196 | 192.8 | 0.983673 |
| GO:0035272\_exocrine\_system\_development | IGF1R | 40 | 1 | 4.605000 | -0.706817 | 197 | 195.01 | 0.989898 |
| GO:0009890\_negative\_regulation\_of\_biosynthetic\_process | SALL4 | 284 | 3 | 1.945775 | -0.704496 | 198 | 195.24 | 0.986061 |
| GO:0009890\_negative\_regulation\_of\_biosynthetic\_process | FOXK1 | 284 | 3 | 1.945775 | -0.704496 | 198 | 195.24 | 0.986061 |
| GO:0009890\_negative\_regulation\_of\_biosynthetic\_process | SIRT1 | 284 | 3 | 1.945775 | -0.704496 | 198 | 195.24 | 0.986061 |
| GO:0034960\_cellular\_biopolymer\_metabolic\_process | UBE2N | 1395 | 10 | 1.320430 | -0.703527 | 199 | 195.48 | 0.982312 |
| GO:0034960\_cellular\_biopolymer\_metabolic\_process | IGF1R | 1395 | 10 | 1.320430 | -0.703527 | 199 | 195.48 | 0.982312 |
| GO:0034960\_cellular\_biopolymer\_metabolic\_process | TRP53BP1 | 1395 | 10 | 1.320430 | -0.703527 | 199 | 195.48 | 0.982312 |
| GO:0034960\_cellular\_biopolymer\_metabolic\_process | STT3A | 1395 | 10 | 1.320430 | -0.703527 | 199 | 195.48 | 0.982312 |
| GO:0034960\_cellular\_biopolymer\_metabolic\_process | SALL4 | 1395 | 10 | 1.320430 | -0.703527 | 199 | 195.48 | 0.982312 |
| GO:0034960\_cellular\_biopolymer\_metabolic\_process | FOXK1 | 1395 | 10 | 1.320430 | -0.703527 | 199 | 195.48 | 0.982312 |
| GO:0034960\_cellular\_biopolymer\_metabolic\_process | SIRT1 | 1395 | 10 | 1.320430 | -0.703527 | 199 | 195.48 | 0.982312 |
| GO:0034960\_cellular\_biopolymer\_metabolic\_process | GLCE | 1395 | 10 | 1.320430 | -0.703527 | 199 | 195.48 | 0.982312 |
| GO:0034960\_cellular\_biopolymer\_metabolic\_process | HDAC6 | 1395 | 10 | 1.320430 | -0.703527 | 199 | 195.48 | 0.982312 |
| GO:0034960\_cellular\_biopolymer\_metabolic\_process | SOD2 | 1395 | 10 | 1.320430 | -0.703527 | 199 | 195.48 | 0.982312 |
| GO:0002429\_immune\_response-activating\_cell\_surface\_receptor\_signaling\_pathway | UBE2N | 41 | 1 | 4.492683 | -0.697194 | 203 | 199.59 | 0.983202 |
| GO:0006979\_response\_to\_oxidative\_stress | SOD2 | 41 | 1 | 4.492683 | -0.697194 | 203 | 199.59 | 0.983202 |
| GO:0008585\_female\_gonad\_development | SIRT1 | 41 | 1 | 4.492683 | -0.697194 | 203 | 199.59 | 0.983202 |
| GO:0015980\_energy\_derivation\_by\_oxidation\_of\_organic\_compounds | SOD2 | 41 | 1 | 4.492683 | -0.697194 | 203 | 199.59 | 0.983202 |
| GO:0034645\_cellular\_macromolecule\_biosynthetic\_process | UBE2N | 901 | 7 | 1.431077 | -0.692796 | 204 | 200.04 | 0.980588 |
| GO:0034645\_cellular\_macromolecule\_biosynthetic\_process | TRP53BP1 | 901 | 7 | 1.431077 | -0.692796 | 204 | 200.04 | 0.980588 |
| GO:0034645\_cellular\_macromolecule\_biosynthetic\_process | SALL4 | 901 | 7 | 1.431077 | -0.692796 | 204 | 200.04 | 0.980588 |
| GO:0034645\_cellular\_macromolecule\_biosynthetic\_process | STT3A | 901 | 7 | 1.431077 | -0.692796 | 204 | 200.04 | 0.980588 |
| GO:0034645\_cellular\_macromolecule\_biosynthetic\_process | FOXK1 | 901 | 7 | 1.431077 | -0.692796 | 204 | 200.04 | 0.980588 |
| GO:0034645\_cellular\_macromolecule\_biosynthetic\_process | SIRT1 | 901 | 7 | 1.431077 | -0.692796 | 204 | 200.04 | 0.980588 |
| GO:0034645\_cellular\_macromolecule\_biosynthetic\_process | GLCE | 901 | 7 | 1.431077 | -0.692796 | 204 | 200.04 | 0.980588 |
| GO:0022402\_cell\_cycle\_process | RECQL4 | 155 | 2 | 2.376774 | -0.688332 | 205 | 200.81 | 0.979561 |
| GO:0022402\_cell\_cycle\_process | RHOU | 155 | 2 | 2.376774 | -0.688332 | 205 | 200.81 | 0.979561 |
| GO:0010740\_positive\_regulation\_of\_protein\_kinase\_cascade | IGF1R | 42 | 1 | 4.385714 | -0.687829 | 206 | 204.44 | 0.992427 |
| GO:0032446\_protein\_modification\_by\_small\_protein\_conjugation | HDAC6 | 43 | 1 | 4.283721 | -0.678709 | 207 | 208.75 | 1.008454 |
| GO:0009059\_macromolecule\_biosynthetic\_process | UBE2N | 910 | 7 | 1.416923 | -0.677244 | 208 | 208.94 | 1.004519 |
| GO:0009059\_macromolecule\_biosynthetic\_process | TRP53BP1 | 910 | 7 | 1.416923 | -0.677244 | 208 | 208.94 | 1.004519 |
| GO:0009059\_macromolecule\_biosynthetic\_process | STT3A | 910 | 7 | 1.416923 | -0.677244 | 208 | 208.94 | 1.004519 |
| GO:0009059\_macromolecule\_biosynthetic\_process | SALL4 | 910 | 7 | 1.416923 | -0.677244 | 208 | 208.94 | 1.004519 |
| GO:0009059\_macromolecule\_biosynthetic\_process | FOXK1 | 910 | 7 | 1.416923 | -0.677244 | 208 | 208.94 | 1.004519 |
| GO:0009059\_macromolecule\_biosynthetic\_process | SIRT1 | 910 | 7 | 1.416923 | -0.677244 | 208 | 208.94 | 1.004519 |
| GO:0009059\_macromolecule\_biosynthetic\_process | GLCE | 910 | 7 | 1.416923 | -0.677244 | 208 | 208.94 | 1.004519 |
| GO:0006464\_protein\_modification\_process | IGF1R | 439 | 4 | 1.678360 | -0.676599 | 209 | 209.07 | 1.000335 |
| GO:0006464\_protein\_modification\_process | STT3A | 439 | 4 | 1.678360 | -0.676599 | 209 | 209.07 | 1.000335 |
| GO:0006464\_protein\_modification\_process | SIRT1 | 439 | 4 | 1.678360 | -0.676599 | 209 | 209.07 | 1.000335 |
| GO:0006464\_protein\_modification\_process | HDAC6 | 439 | 4 | 1.678360 | -0.676599 | 209 | 209.07 | 1.000335 |
| GO:0007409\_axonogenesis | PTPRZ1 | 158 | 2 | 2.331646 | -0.675776 | 210 | 209.53 | 0.997762 |
| GO:0007409\_axonogenesis | BMPR1B | 158 | 2 | 2.331646 | -0.675776 | 210 | 209.53 | 0.997762 |
| GO:0002768\_immune\_response-regulating\_cell\_surface\_receptor\_signaling\_pathway | UBE2N | 44 | 1 | 4.186364 | -0.669823 | 213 | 214.77 | 1.008310 |
| GO:0035282\_segmentation | ABI1 | 44 | 1 | 4.186364 | -0.669823 | 213 | 214.77 | 1.008310 |
| GO:0046545\_development\_of\_primary\_female\_sexual\_characteristics | SIRT1 | 44 | 1 | 4.186364 | -0.669823 | 213 | 214.77 | 1.008310 |
| GO:0007165\_signal\_transduction | UBE2N | 915 | 7 | 1.409180 | -0.668736 | 214 | 215.15 | 1.005374 |
| GO:0007165\_signal\_transduction | NRAS | 915 | 7 | 1.409180 | -0.668736 | 214 | 215.15 | 1.005374 |
| GO:0007165\_signal\_transduction | IGF1R | 915 | 7 | 1.409180 | -0.668736 | 214 | 215.15 | 1.005374 |
| GO:0007165\_signal\_transduction | PDPK1 | 915 | 7 | 1.409180 | -0.668736 | 214 | 215.15 | 1.005374 |
| GO:0007165\_signal\_transduction | DUSP16 | 915 | 7 | 1.409180 | -0.668736 | 214 | 215.15 | 1.005374 |
| GO:0007165\_signal\_transduction | BMPR1B | 915 | 7 | 1.409180 | -0.668736 | 214 | 215.15 | 1.005374 |
| GO:0007165\_signal\_transduction | RHOU | 915 | 7 | 1.409180 | -0.668736 | 214 | 215.15 | 1.005374 |
| GO:0009628\_response\_to\_abiotic\_stimulus | PDPK1 | 162 | 2 | 2.274074 | -0.659516 | 215 | 217.41 | 1.011209 |
| GO:0009628\_response\_to\_abiotic\_stimulus | SOD2 | 162 | 2 | 2.274074 | -0.659516 | 215 | 217.41 | 1.011209 |
| GO:0019222\_regulation\_of\_metabolic\_process | UBE2N | 1088 | 8 | 1.354412 | -0.656346 | 216 | 217.77 | 1.008194 |
| GO:0019222\_regulation\_of\_metabolic\_process | TRP53BP1 | 1088 | 8 | 1.354412 | -0.656346 | 216 | 217.77 | 1.008194 |
| GO:0019222\_regulation\_of\_metabolic\_process | SALL4 | 1088 | 8 | 1.354412 | -0.656346 | 216 | 217.77 | 1.008194 |
| GO:0019222\_regulation\_of\_metabolic\_process | FOXK1 | 1088 | 8 | 1.354412 | -0.656346 | 216 | 217.77 | 1.008194 |
| GO:0019222\_regulation\_of\_metabolic\_process | DUSP16 | 1088 | 8 | 1.354412 | -0.656346 | 216 | 217.77 | 1.008194 |
| GO:0019222\_regulation\_of\_metabolic\_process | SIRT1 | 1088 | 8 | 1.354412 | -0.656346 | 216 | 217.77 | 1.008194 |
| GO:0019222\_regulation\_of\_metabolic\_process | HDAC6 | 1088 | 8 | 1.354412 | -0.656346 | 216 | 217.77 | 1.008194 |
| GO:0019222\_regulation\_of\_metabolic\_process | SOD2 | 1088 | 8 | 1.354412 | -0.656346 | 216 | 217.77 | 1.008194 |
| GO:0006732\_coenzyme\_metabolic\_process | SOD2 | 46 | 1 | 4.004348 | -0.652712 | 220 | 220.04 | 1.000182 |
| GO:0008217\_regulation\_of\_blood\_pressure | SOD2 | 46 | 1 | 4.004348 | -0.652712 | 220 | 220.04 | 1.000182 |
| GO:0030850\_prostate\_gland\_development | IGF1R | 46 | 1 | 4.004348 | -0.652712 | 220 | 220.04 | 1.000182 |
| GO:0051098\_regulation\_of\_binding | UBE2N | 46 | 1 | 4.004348 | -0.652712 | 220 | 220.04 | 1.000182 |
| GO:0006259\_DNA\_metabolic\_process | TRP53BP1 | 165 | 2 | 2.232727 | -0.647666 | 221 | 221.01 | 1.000045 |
| GO:0006259\_DNA\_metabolic\_process | SOD2 | 165 | 2 | 2.232727 | -0.647666 | 221 | 221.01 | 1.000045 |
| GO:0002757\_immune\_response-activating\_signal\_transduction | UBE2N | 47 | 1 | 3.919149 | -0.644468 | 223 | 223.45 | 1.002018 |
| GO:0016570\_histone\_modification | SIRT1 | 47 | 1 | 3.919149 | -0.644468 | 223 | 223.45 | 1.002018 |
| GO:0032989\_cellular\_component\_morphogenesis | PTPRZ1 | 307 | 3 | 1.800000 | -0.637299 | 224 | 224.56 | 1.002500 |
| GO:0032989\_cellular\_component\_morphogenesis | BMPR1B | 307 | 3 | 1.800000 | -0.637299 | 224 | 224.56 | 1.002500 |
| GO:0032989\_cellular\_component\_morphogenesis | RHOU | 307 | 3 | 1.800000 | -0.637299 | 224 | 224.56 | 1.002500 |
| GO:0051171\_regulation\_of\_nitrogen\_compound\_metabolic\_process | UBE2N | 771 | 6 | 1.433463 | -0.636681 | 225 | 224.67 | 0.998533 |
| GO:0051171\_regulation\_of\_nitrogen\_compound\_metabolic\_process | TRP53BP1 | 771 | 6 | 1.433463 | -0.636681 | 225 | 224.67 | 0.998533 |
| GO:0051171\_regulation\_of\_nitrogen\_compound\_metabolic\_process | SALL4 | 771 | 6 | 1.433463 | -0.636681 | 225 | 224.67 | 0.998533 |
| GO:0051171\_regulation\_of\_nitrogen\_compound\_metabolic\_process | FOXK1 | 771 | 6 | 1.433463 | -0.636681 | 225 | 224.67 | 0.998533 |
| GO:0051171\_regulation\_of\_nitrogen\_compound\_metabolic\_process | SIRT1 | 771 | 6 | 1.433463 | -0.636681 | 225 | 224.67 | 0.998533 |
| GO:0051171\_regulation\_of\_nitrogen\_compound\_metabolic\_process | SOD2 | 771 | 6 | 1.433463 | -0.636681 | 225 | 224.67 | 0.998533 |
| GO:0032269\_negative\_regulation\_of\_cellular\_protein\_metabolic\_process | HDAC6 | 48 | 1 | 3.837500 | -0.636419 | 226 | 225.94 | 0.999735 |
| GO:0043412\_biopolymer\_modification | IGF1R | 458 | 4 | 1.608734 | -0.632571 | 227 | 226.46 | 0.997621 |
| GO:0043412\_biopolymer\_modification | STT3A | 458 | 4 | 1.608734 | -0.632571 | 227 | 226.46 | 0.997621 |
| GO:0043412\_biopolymer\_modification | SIRT1 | 458 | 4 | 1.608734 | -0.632571 | 227 | 226.46 | 0.997621 |
| GO:0043412\_biopolymer\_modification | HDAC6 | 458 | 4 | 1.608734 | -0.632571 | 227 | 226.46 | 0.997621 |
| GO:0044260\_cellular\_macromolecule\_metabolic\_process | UBE2N | 1447 | 10 | 1.272979 | -0.629126 | 228 | 226.87 | 0.995044 |
| GO:0044260\_cellular\_macromolecule\_metabolic\_process | IGF1R | 1447 | 10 | 1.272979 | -0.629126 | 228 | 226.87 | 0.995044 |
| GO:0044260\_cellular\_macromolecule\_metabolic\_process | TRP53BP1 | 1447 | 10 | 1.272979 | -0.629126 | 228 | 226.87 | 0.995044 |
| GO:0044260\_cellular\_macromolecule\_metabolic\_process | SALL4 | 1447 | 10 | 1.272979 | -0.629126 | 228 | 226.87 | 0.995044 |
| GO:0044260\_cellular\_macromolecule\_metabolic\_process | STT3A | 1447 | 10 | 1.272979 | -0.629126 | 228 | 226.87 | 0.995044 |
| GO:0044260\_cellular\_macromolecule\_metabolic\_process | FOXK1 | 1447 | 10 | 1.272979 | -0.629126 | 228 | 226.87 | 0.995044 |
| GO:0044260\_cellular\_macromolecule\_metabolic\_process | SIRT1 | 1447 | 10 | 1.272979 | -0.629126 | 228 | 226.87 | 0.995044 |
| GO:0044260\_cellular\_macromolecule\_metabolic\_process | GLCE | 1447 | 10 | 1.272979 | -0.629126 | 228 | 226.87 | 0.995044 |
| GO:0044260\_cellular\_macromolecule\_metabolic\_process | HDAC6 | 1447 | 10 | 1.272979 | -0.629126 | 228 | 226.87 | 0.995044 |
| GO:0044260\_cellular\_macromolecule\_metabolic\_process | SOD2 | 1447 | 10 | 1.272979 | -0.629126 | 228 | 226.87 | 0.995044 |
| GO:0046660\_female\_sex\_differentiation | SIRT1 | 49 | 1 | 3.759184 | -0.628559 | 229 | 229.37 | 1.001616 |
| GO:0048812\_neuron\_projection\_morphogenesis | PTPRZ1 | 170 | 2 | 2.167059 | -0.628544 | 230 | 229.75 | 0.998913 |
| GO:0048812\_neuron\_projection\_morphogenesis | BMPR1B | 170 | 2 | 2.167059 | -0.628544 | 230 | 229.75 | 0.998913 |
| GO:0051606\_detection\_of\_stimulus | SOD2 | 50 | 1 | 3.684000 | -0.620878 | 232 | 232.19 | 1.000819 |
| GO:0070647\_protein\_modification\_by\_small\_protein\_conjugation\_or\_removal | HDAC6 | 50 | 1 | 3.684000 | -0.620878 | 232 | 232.19 | 1.000819 |
| GO:0007399\_nervous\_system\_development | ATRX | 621 | 5 | 1.483092 | -0.617976 | 233 | 232.49 | 0.997811 |
| GO:0007399\_nervous\_system\_development | IGF1R | 621 | 5 | 1.483092 | -0.617976 | 233 | 232.49 | 0.997811 |
| GO:0007399\_nervous\_system\_development | PTPRZ1 | 621 | 5 | 1.483092 | -0.617976 | 233 | 232.49 | 0.997811 |
| GO:0007399\_nervous\_system\_development | BMPR1B | 621 | 5 | 1.483092 | -0.617976 | 233 | 232.49 | 0.997811 |
| GO:0007399\_nervous\_system\_development | SOD2 | 621 | 5 | 1.483092 | -0.617976 | 233 | 232.49 | 0.997811 |
| GO:0044248\_cellular\_catabolic\_process | UBE2N | 173 | 2 | 2.129480 | -0.617431 | 235 | 233.02 | 0.991574 |
| GO:0044248\_cellular\_catabolic\_process | HDAC6 | 173 | 2 | 2.129480 | -0.617431 | 235 | 233.02 | 0.991574 |
| GO:0048667\_cell\_morphogenesis\_involved\_in\_neuron\_differentiation | PTPRZ1 | 173 | 2 | 2.129480 | -0.617431 | 235 | 233.02 | 0.991574 |
| GO:0048667\_cell\_morphogenesis\_involved\_in\_neuron\_differentiation | BMPR1B | 173 | 2 | 2.129480 | -0.617431 | 235 | 233.02 | 0.991574 |
| GO:0002764\_immune\_response-regulating\_signal\_transduction | UBE2N | 51 | 1 | 3.611765 | -0.613370 | 238 | 235.08 | 0.987731 |
| GO:0016569\_covalent\_chromatin\_modification | SIRT1 | 51 | 1 | 3.611765 | -0.613370 | 238 | 235.08 | 0.987731 |
| GO:0043408\_regulation\_of\_MAPKKK\_cascade | IGF1R | 51 | 1 | 3.611765 | -0.613370 | 238 | 235.08 | 0.987731 |
| GO:0048858\_cell\_projection\_morphogenesis | PTPRZ1 | 176 | 2 | 2.093182 | -0.606576 | 239 | 236.22 | 0.988368 |
| GO:0048858\_cell\_projection\_morphogenesis | BMPR1B | 176 | 2 | 2.093182 | -0.606576 | 239 | 236.22 | 0.988368 |
| GO:0050789\_regulation\_of\_biological\_process | RECQL4 | 2357 | 15 | 1.172253 | -0.605940 | 240 | 237.31 | 0.988792 |
| GO:0050789\_regulation\_of\_biological\_process | FOXK1 | 2357 | 15 | 1.172253 | -0.605940 | 240 | 237.31 | 0.988792 |
| GO:0050789\_regulation\_of\_biological\_process | SIRT1 | 2357 | 15 | 1.172253 | -0.605940 | 240 | 237.31 | 0.988792 |
| GO:0050789\_regulation\_of\_biological\_process | RHOU | 2357 | 15 | 1.172253 | -0.605940 | 240 | 237.31 | 0.988792 |
| GO:0050789\_regulation\_of\_biological\_process | PIN1 | 2357 | 15 | 1.172253 | -0.605940 | 240 | 237.31 | 0.988792 |
| GO:0050789\_regulation\_of\_biological\_process | SOD2 | 2357 | 15 | 1.172253 | -0.605940 | 240 | 237.31 | 0.988792 |
| GO:0050789\_regulation\_of\_biological\_process | UBE2N | 2357 | 15 | 1.172253 | -0.605940 | 240 | 237.31 | 0.988792 |
| GO:0050789\_regulation\_of\_biological\_process | IGF1R | 2357 | 15 | 1.172253 | -0.605940 | 240 | 237.31 | 0.988792 |
| GO:0050789\_regulation\_of\_biological\_process | NRAS | 2357 | 15 | 1.172253 | -0.605940 | 240 | 237.31 | 0.988792 |
| GO:0050789\_regulation\_of\_biological\_process | PDPK1 | 2357 | 15 | 1.172253 | -0.605940 | 240 | 237.31 | 0.988792 |
| GO:0050789\_regulation\_of\_biological\_process | TRP53BP1 | 2357 | 15 | 1.172253 | -0.605940 | 240 | 237.31 | 0.988792 |
| GO:0050789\_regulation\_of\_biological\_process | SALL4 | 2357 | 15 | 1.172253 | -0.605940 | 240 | 237.31 | 0.988792 |
| GO:0050789\_regulation\_of\_biological\_process | DUSP16 | 2357 | 15 | 1.172253 | -0.605940 | 240 | 237.31 | 0.988792 |
| GO:0050789\_regulation\_of\_biological\_process | BMPR1B | 2357 | 15 | 1.172253 | -0.605940 | 240 | 237.31 | 0.988792 |
| GO:0050789\_regulation\_of\_biological\_process | HDAC6 | 2357 | 15 | 1.172253 | -0.605940 | 240 | 237.31 | 0.988792 |
| GO:0051248\_negative\_regulation\_of\_protein\_metabolic\_process | HDAC6 | 53 | 1 | 3.475472 | -0.598847 | 241 | 239.0 | 0.991701 |
| GO:0002253\_activation\_of\_immune\_response | UBE2N | 54 | 1 | 3.411111 | -0.591819 | 245 | 241.69 | 0.986490 |
| GO:0006091\_generation\_of\_precursor\_metabolites\_and\_energy | SOD2 | 54 | 1 | 3.411111 | -0.591819 | 245 | 241.69 | 0.986490 |
| GO:0043405\_regulation\_of\_MAP\_kinase\_activity | DUSP16 | 54 | 1 | 3.411111 | -0.591819 | 245 | 241.69 | 0.986490 |
| GO:0044271\_nitrogen\_compound\_biosynthetic\_process | SOD2 | 54 | 1 | 3.411111 | -0.591819 | 245 | 241.69 | 0.986490 |
| GO:0007605\_sensory\_perception\_of\_sound | SOD2 | 55 | 1 | 3.349091 | -0.584939 | 246 | 244.12 | 0.992358 |
| GO:0034961\_cellular\_biopolymer\_biosynthetic\_process | UBE2N | 804 | 6 | 1.374627 | -0.580935 | 247 | 244.36 | 0.989312 |
| GO:0034961\_cellular\_biopolymer\_biosynthetic\_process | TRP53BP1 | 804 | 6 | 1.374627 | -0.580935 | 247 | 244.36 | 0.989312 |
| GO:0034961\_cellular\_biopolymer\_biosynthetic\_process | SALL4 | 804 | 6 | 1.374627 | -0.580935 | 247 | 244.36 | 0.989312 |
| GO:0034961\_cellular\_biopolymer\_biosynthetic\_process | FOXK1 | 804 | 6 | 1.374627 | -0.580935 | 247 | 244.36 | 0.989312 |
| GO:0034961\_cellular\_biopolymer\_biosynthetic\_process | SIRT1 | 804 | 6 | 1.374627 | -0.580935 | 247 | 244.36 | 0.989312 |
| GO:0034961\_cellular\_biopolymer\_biosynthetic\_process | GLCE | 804 | 6 | 1.374627 | -0.580935 | 247 | 244.36 | 0.989312 |
| GO:0032990\_cell\_part\_morphogenesis | PTPRZ1 | 184 | 2 | 2.002174 | -0.578825 | 248 | 244.95 | 0.987702 |
| GO:0032990\_cell\_part\_morphogenesis | BMPR1B | 184 | 2 | 2.002174 | -0.578825 | 248 | 244.95 | 0.987702 |
| GO:0043284\_biopolymer\_biosynthetic\_process | UBE2N | 807 | 6 | 1.369517 | -0.576093 | 249 | 247.98 | 0.995904 |
| GO:0043284\_biopolymer\_biosynthetic\_process | TRP53BP1 | 807 | 6 | 1.369517 | -0.576093 | 249 | 247.98 | 0.995904 |
| GO:0043284\_biopolymer\_biosynthetic\_process | SALL4 | 807 | 6 | 1.369517 | -0.576093 | 249 | 247.98 | 0.995904 |
| GO:0043284\_biopolymer\_biosynthetic\_process | FOXK1 | 807 | 6 | 1.369517 | -0.576093 | 249 | 247.98 | 0.995904 |
| GO:0043284\_biopolymer\_biosynthetic\_process | SIRT1 | 807 | 6 | 1.369517 | -0.576093 | 249 | 247.98 | 0.995904 |
| GO:0043284\_biopolymer\_biosynthetic\_process | GLCE | 807 | 6 | 1.369517 | -0.576093 | 249 | 247.98 | 0.995904 |
| GO:0007010\_cytoskeleton\_organization | RHOU | 185 | 2 | 1.991351 | -0.575473 | 250 | 248.13 | 0.992520 |
| GO:0007010\_cytoskeleton\_organization | HDAC6 | 185 | 2 | 1.991351 | -0.575473 | 250 | 248.13 | 0.992520 |
| GO:0000226\_microtubule\_cytoskeleton\_organization | HDAC6 | 57 | 1 | 3.231579 | -0.571602 | 253 | 251.91 | 0.995692 |
| GO:0009953\_dorsal\_ventral\_pattern\_formation | BMPR1B | 57 | 1 | 3.231579 | -0.571602 | 253 | 251.91 | 0.995692 |
| GO:0045444\_fat\_cell\_differentiation | SOD2 | 57 | 1 | 3.231579 | -0.571602 | 253 | 251.91 | 0.995692 |
| GO:0031326\_regulation\_of\_cellular\_biosynthetic\_process | UBE2N | 812 | 6 | 1.361084 | -0.568102 | 254 | 252.31 | 0.993346 |
| GO:0031326\_regulation\_of\_cellular\_biosynthetic\_process | TRP53BP1 | 812 | 6 | 1.361084 | -0.568102 | 254 | 252.31 | 0.993346 |
| GO:0031326\_regulation\_of\_cellular\_biosynthetic\_process | SALL4 | 812 | 6 | 1.361084 | -0.568102 | 254 | 252.31 | 0.993346 |
| GO:0031326\_regulation\_of\_cellular\_biosynthetic\_process | FOXK1 | 812 | 6 | 1.361084 | -0.568102 | 254 | 252.31 | 0.993346 |
| GO:0031326\_regulation\_of\_cellular\_biosynthetic\_process | SIRT1 | 812 | 6 | 1.361084 | -0.568102 | 254 | 252.31 | 0.993346 |
| GO:0031326\_regulation\_of\_cellular\_biosynthetic\_process | SOD2 | 812 | 6 | 1.361084 | -0.568102 | 254 | 252.31 | 0.993346 |
| GO:0007276\_gamete\_generation | SIRT1 | 188 | 2 | 1.959574 | -0.565565 | 255 | 252.44 | 0.989961 |
| GO:0007276\_gamete\_generation | ZFP37 | 188 | 2 | 1.959574 | -0.565565 | 255 | 252.44 | 0.989961 |
| GO:0044249\_cellular\_biosynthetic\_process | UBE2N | 1150 | 8 | 1.281391 | -0.565460 | 256 | 252.57 | 0.986602 |
| GO:0044249\_cellular\_biosynthetic\_process | TRP53BP1 | 1150 | 8 | 1.281391 | -0.565460 | 256 | 252.57 | 0.986602 |
| GO:0044249\_cellular\_biosynthetic\_process | STT3A | 1150 | 8 | 1.281391 | -0.565460 | 256 | 252.57 | 0.986602 |
| GO:0044249\_cellular\_biosynthetic\_process | SALL4 | 1150 | 8 | 1.281391 | -0.565460 | 256 | 252.57 | 0.986602 |
| GO:0044249\_cellular\_biosynthetic\_process | FOXK1 | 1150 | 8 | 1.281391 | -0.565460 | 256 | 252.57 | 0.986602 |
| GO:0044249\_cellular\_biosynthetic\_process | SIRT1 | 1150 | 8 | 1.281391 | -0.565460 | 256 | 252.57 | 0.986602 |
| GO:0044249\_cellular\_biosynthetic\_process | GLCE | 1150 | 8 | 1.281391 | -0.565460 | 256 | 252.57 | 0.986602 |
| GO:0044249\_cellular\_biosynthetic\_process | SOD2 | 1150 | 8 | 1.281391 | -0.565460 | 256 | 252.57 | 0.986602 |
| GO:0009889\_regulation\_of\_biosynthetic\_process | UBE2N | 815 | 6 | 1.356074 | -0.563355 | 257 | 253.38 | 0.985914 |
| GO:0009889\_regulation\_of\_biosynthetic\_process | TRP53BP1 | 815 | 6 | 1.356074 | -0.563355 | 257 | 253.38 | 0.985914 |
| GO:0009889\_regulation\_of\_biosynthetic\_process | SALL4 | 815 | 6 | 1.356074 | -0.563355 | 257 | 253.38 | 0.985914 |
| GO:0009889\_regulation\_of\_biosynthetic\_process | FOXK1 | 815 | 6 | 1.356074 | -0.563355 | 257 | 253.38 | 0.985914 |
| GO:0009889\_regulation\_of\_biosynthetic\_process | SIRT1 | 815 | 6 | 1.356074 | -0.563355 | 257 | 253.38 | 0.985914 |
| GO:0009889\_regulation\_of\_biosynthetic\_process | SOD2 | 815 | 6 | 1.356074 | -0.563355 | 257 | 253.38 | 0.985914 |
| GO:0007005\_mitochondrion\_organization | SOD2 | 61 | 1 | 3.019672 | -0.546488 | 258 | 257.41 | 0.997713 |
| GO:0003002\_regionalization | ABI1 | 195 | 2 | 1.889231 | -0.543283 | 260 | 258.65 | 0.994808 |
| GO:0003002\_regionalization | BMPR1B | 195 | 2 | 1.889231 | -0.543283 | 260 | 258.65 | 0.994808 |
| GO:0007507\_heart\_development | SALL4 | 195 | 2 | 1.889231 | -0.543283 | 260 | 258.65 | 0.994808 |
| GO:0007507\_heart\_development | SOD2 | 195 | 2 | 1.889231 | -0.543283 | 260 | 258.65 | 0.994808 |
| GO:0022604\_regulation\_of\_cell\_morphogenesis | RHOU | 62 | 1 | 2.970968 | -0.540509 | 262 | 260.84 | 0.995573 |
| GO:0050954\_sensory\_perception\_of\_mechanical\_stimulus | SOD2 | 62 | 1 | 2.970968 | -0.540509 | 262 | 260.84 | 0.995573 |
| GO:0033554\_cellular\_response\_to\_stress | TRP53BP1 | 196 | 2 | 1.879592 | -0.540191 | 263 | 261.02 | 0.992471 |
| GO:0033554\_cellular\_response\_to\_stress | SOD2 | 196 | 2 | 1.879592 | -0.540191 | 263 | 261.02 | 0.992471 |
| GO:0031175\_neuron\_projection\_development | PTPRZ1 | 197 | 2 | 1.870051 | -0.537122 | 264 | 261.54 | 0.990682 |
| GO:0031175\_neuron\_projection\_development | BMPR1B | 197 | 2 | 1.870051 | -0.537122 | 264 | 261.54 | 0.990682 |
| GO:0051186\_cofactor\_metabolic\_process | SOD2 | 63 | 1 | 2.923810 | -0.534642 | 266 | 263.55 | 0.990789 |
| GO:0051216\_cartilage\_development | BMPR1B | 63 | 1 | 2.923810 | -0.534642 | 266 | 263.55 | 0.990789 |
| GO:0009058\_biosynthetic\_process | UBE2N | 1175 | 8 | 1.254128 | -0.531875 | 267 | 264.02 | 0.988839 |
| GO:0009058\_biosynthetic\_process | TRP53BP1 | 1175 | 8 | 1.254128 | -0.531875 | 267 | 264.02 | 0.988839 |
| GO:0009058\_biosynthetic\_process | STT3A | 1175 | 8 | 1.254128 | -0.531875 | 267 | 264.02 | 0.988839 |
| GO:0009058\_biosynthetic\_process | SALL4 | 1175 | 8 | 1.254128 | -0.531875 | 267 | 264.02 | 0.988839 |
| GO:0009058\_biosynthetic\_process | FOXK1 | 1175 | 8 | 1.254128 | -0.531875 | 267 | 264.02 | 0.988839 |
| GO:0009058\_biosynthetic\_process | SIRT1 | 1175 | 8 | 1.254128 | -0.531875 | 267 | 264.02 | 0.988839 |
| GO:0009058\_biosynthetic\_process | GLCE | 1175 | 8 | 1.254128 | -0.531875 | 267 | 264.02 | 0.988839 |
| GO:0009058\_biosynthetic\_process | SOD2 | 1175 | 8 | 1.254128 | -0.531875 | 267 | 264.02 | 0.988839 |
| GO:0000904\_cell\_morphogenesis\_involved\_in\_differentiation | PTPRZ1 | 199 | 2 | 1.851256 | -0.531049 | 268 | 264.26 | 0.986045 |
| GO:0000904\_cell\_morphogenesis\_involved\_in\_differentiation | BMPR1B | 199 | 2 | 1.851256 | -0.531049 | 268 | 264.26 | 0.986045 |
| GO:0043086\_negative\_regulation\_of\_catalytic\_activity | DUSP16 | 65 | 1 | 2.833846 | -0.523231 | 270 | 266.44 | 0.986815 |
| GO:0048511\_rhythmic\_process | SIRT1 | 65 | 1 | 2.833846 | -0.523231 | 270 | 266.44 | 0.986815 |
| GO:0045449\_regulation\_of\_transcription | UBE2N | 676 | 5 | 1.362426 | -0.521407 | 271 | 266.54 | 0.983542 |
| GO:0045449\_regulation\_of\_transcription | TRP53BP1 | 676 | 5 | 1.362426 | -0.521407 | 271 | 266.54 | 0.983542 |
| GO:0045449\_regulation\_of\_transcription | SALL4 | 676 | 5 | 1.362426 | -0.521407 | 271 | 266.54 | 0.983542 |
| GO:0045449\_regulation\_of\_transcription | FOXK1 | 676 | 5 | 1.362426 | -0.521407 | 271 | 266.54 | 0.983542 |
| GO:0045449\_regulation\_of\_transcription | SIRT1 | 676 | 5 | 1.362426 | -0.521407 | 271 | 266.54 | 0.983542 |
| GO:0007179\_transforming\_growth\_factor\_beta\_receptor\_signaling\_pathway | BMPR1B | 66 | 1 | 2.790909 | -0.517680 | 272 | 268.21 | 0.986066 |
| GO:0030182\_neuron\_differentiation | PTPRZ1 | 356 | 3 | 1.552247 | -0.516984 | 273 | 268.49 | 0.983480 |
| GO:0030182\_neuron\_differentiation | BMPR1B | 356 | 3 | 1.552247 | -0.516984 | 273 | 268.49 | 0.983480 |
| GO:0030182\_neuron\_differentiation | SOD2 | 356 | 3 | 1.552247 | -0.516984 | 273 | 268.49 | 0.983480 |
| GO:0007243\_protein\_kinase\_cascade | IGF1R | 205 | 2 | 1.797073 | -0.513340 | 274 | 269.42 | 0.983285 |
| GO:0007243\_protein\_kinase\_cascade | DUSP16 | 205 | 2 | 1.797073 | -0.513340 | 274 | 269.42 | 0.983285 |
| GO:0009791\_post-embryonic\_development | SOD2 | 67 | 1 | 2.749254 | -0.512228 | 275 | 270.72 | 0.984436 |
| GO:0051173\_positive\_regulation\_of\_nitrogen\_compound\_metabolic\_process | SALL4 | 361 | 3 | 1.530748 | -0.506189 | 276 | 272.22 | 0.986304 |
| GO:0051173\_positive\_regulation\_of\_nitrogen\_compound\_metabolic\_process | FOXK1 | 361 | 3 | 1.530748 | -0.506189 | 276 | 272.22 | 0.986304 |
| GO:0051173\_positive\_regulation\_of\_nitrogen\_compound\_metabolic\_process | SOD2 | 361 | 3 | 1.530748 | -0.506189 | 276 | 272.22 | 0.986304 |
| GO:0044238\_primary\_metabolic\_process | UBE2N | 1905 | 12 | 1.160315 | -0.500570 | 277 | 274.18 | 0.989819 |
| GO:0044238\_primary\_metabolic\_process | IGF1R | 1905 | 12 | 1.160315 | -0.500570 | 277 | 274.18 | 0.989819 |
| GO:0044238\_primary\_metabolic\_process | TRP53BP1 | 1905 | 12 | 1.160315 | -0.500570 | 277 | 274.18 | 0.989819 |
| GO:0044238\_primary\_metabolic\_process | SALL4 | 1905 | 12 | 1.160315 | -0.500570 | 277 | 274.18 | 0.989819 |
| GO:0044238\_primary\_metabolic\_process | STT3A | 1905 | 12 | 1.160315 | -0.500570 | 277 | 274.18 | 0.989819 |
| GO:0044238\_primary\_metabolic\_process | FOXK1 | 1905 | 12 | 1.160315 | -0.500570 | 277 | 274.18 | 0.989819 |
| GO:0044238\_primary\_metabolic\_process | CTSS | 1905 | 12 | 1.160315 | -0.500570 | 277 | 274.18 | 0.989819 |
| GO:0044238\_primary\_metabolic\_process | SIRT1 | 1905 | 12 | 1.160315 | -0.500570 | 277 | 274.18 | 0.989819 |
| GO:0044238\_primary\_metabolic\_process | PCOLCE | 1905 | 12 | 1.160315 | -0.500570 | 277 | 274.18 | 0.989819 |
| GO:0044238\_primary\_metabolic\_process | GLCE | 1905 | 12 | 1.160315 | -0.500570 | 277 | 274.18 | 0.989819 |
| GO:0044238\_primary\_metabolic\_process | HDAC6 | 1905 | 12 | 1.160315 | -0.500570 | 277 | 274.18 | 0.989819 |
| GO:0044238\_primary\_metabolic\_process | SOD2 | 1905 | 12 | 1.160315 | -0.500570 | 277 | 274.18 | 0.989819 |
| GO:0008406\_gonad\_development | SIRT1 | 70 | 1 | 2.631429 | -0.496436 | 278 | 276.3 | 0.993885 |
| GO:0016568\_chromatin\_modification | SIRT1 | 72 | 1 | 2.558333 | -0.486351 | 281 | 281.18 | 1.000641 |
| GO:0030879\_mammary\_gland\_development | IGF1R | 72 | 1 | 2.558333 | -0.486351 | 281 | 281.18 | 1.000641 |
| GO:0044262\_cellular\_carbohydrate\_metabolic\_process | STT3A | 72 | 1 | 2.558333 | -0.486351 | 281 | 281.18 | 1.000641 |
| GO:0006350\_transcription | UBE2N | 701 | 5 | 1.313837 | -0.482361 | 282 | 281.68 | 0.998865 |
| GO:0006350\_transcription | TRP53BP1 | 701 | 5 | 1.313837 | -0.482361 | 282 | 281.68 | 0.998865 |
| GO:0006350\_transcription | SALL4 | 701 | 5 | 1.313837 | -0.482361 | 282 | 281.68 | 0.998865 |
| GO:0006350\_transcription | FOXK1 | 701 | 5 | 1.313837 | -0.482361 | 282 | 281.68 | 0.998865 |
| GO:0006350\_transcription | SIRT1 | 701 | 5 | 1.313837 | -0.482361 | 282 | 281.68 | 0.998865 |
| GO:0045892\_negative\_regulation\_of\_transcription\_\_DNA-dependent | SALL4 | 218 | 2 | 1.689908 | -0.477415 | 283 | 283.89 | 1.003145 |
| GO:0045892\_negative\_regulation\_of\_transcription\_\_DNA-dependent | FOXK1 | 218 | 2 | 1.689908 | -0.477415 | 283 | 283.89 | 1.003145 |
| GO:0022414\_reproductive\_process | IGF1R | 376 | 3 | 1.469681 | -0.475236 | 284 | 284.66 | 1.002324 |
| GO:0022414\_reproductive\_process | SIRT1 | 376 | 3 | 1.469681 | -0.475236 | 284 | 284.66 | 1.002324 |
| GO:0022414\_reproductive\_process | ZFP37 | 376 | 3 | 1.469681 | -0.475236 | 284 | 284.66 | 1.002324 |
| GO:0051253\_negative\_regulation\_of\_RNA\_metabolic\_process | SALL4 | 220 | 2 | 1.674545 | -0.472165 | 285 | 285.08 | 1.000281 |
| GO:0051253\_negative\_regulation\_of\_RNA\_metabolic\_process | FOXK1 | 220 | 2 | 1.674545 | -0.472165 | 285 | 285.08 | 1.000281 |
| GO:0007281\_germ\_cell\_development | ZFP37 | 75 | 1 | 2.456000 | -0.471840 | 286 | 286.22 | 1.000769 |
| GO:0000003\_reproduction | IGF1R | 379 | 3 | 1.458047 | -0.469292 | 287 | 286.69 | 0.998920 |
| GO:0000003\_reproduction | SIRT1 | 379 | 3 | 1.458047 | -0.469292 | 287 | 286.69 | 0.998920 |
| GO:0000003\_reproduction | ZFP37 | 379 | 3 | 1.458047 | -0.469292 | 287 | 286.69 | 0.998920 |
| GO:0043687\_post-translational\_protein\_modification | IGF1R | 384 | 3 | 1.439063 | -0.459560 | 288 | 289.04 | 1.003611 |
| GO:0043687\_post-translational\_protein\_modification | SIRT1 | 384 | 3 | 1.439063 | -0.459560 | 288 | 289.04 | 1.003611 |
| GO:0043687\_post-translational\_protein\_modification | HDAC6 | 384 | 3 | 1.439063 | -0.459560 | 288 | 289.04 | 1.003611 |
| GO:0030326\_embryonic\_limb\_morphogenesis | SALL4 | 78 | 1 | 2.361538 | -0.458018 | 290 | 291.28 | 1.004414 |
| GO:0035113\_embryonic\_appendage\_morphogenesis | SALL4 | 78 | 1 | 2.361538 | -0.458018 | 290 | 291.28 | 1.004414 |
| GO:0031328\_positive\_regulation\_of\_cellular\_biosynthetic\_process | SALL4 | 387 | 3 | 1.427907 | -0.453824 | 291 | 291.57 | 1.001959 |
| GO:0031328\_positive\_regulation\_of\_cellular\_biosynthetic\_process | FOXK1 | 387 | 3 | 1.427907 | -0.453824 | 291 | 291.57 | 1.001959 |
| GO:0031328\_positive\_regulation\_of\_cellular\_biosynthetic\_process | SOD2 | 387 | 3 | 1.427907 | -0.453824 | 291 | 291.57 | 1.001959 |
| GO:0009891\_positive\_regulation\_of\_biosynthetic\_process | SALL4 | 388 | 3 | 1.424227 | -0.451929 | 292 | 292.35 | 1.001199 |
| GO:0009891\_positive\_regulation\_of\_biosynthetic\_process | FOXK1 | 388 | 3 | 1.424227 | -0.451929 | 292 | 292.35 | 1.001199 |
| GO:0009891\_positive\_regulation\_of\_biosynthetic\_process | SOD2 | 388 | 3 | 1.424227 | -0.451929 | 292 | 292.35 | 1.001199 |
| GO:0019953\_sexual\_reproduction | SIRT1 | 228 | 2 | 1.615789 | -0.451852 | 293 | 293.01 | 1.000034 |
| GO:0019953\_sexual\_reproduction | ZFP37 | 228 | 2 | 1.615789 | -0.451852 | 293 | 293.01 | 1.000034 |
| GO:0008152\_metabolic\_process | FOXK1 | 2133 | 13 | 1.122644 | -0.450411 | 294 | 293.15 | 0.997109 |
| GO:0008152\_metabolic\_process | CTSS | 2133 | 13 | 1.122644 | -0.450411 | 294 | 293.15 | 0.997109 |
| GO:0008152\_metabolic\_process | SIRT1 | 2133 | 13 | 1.122644 | -0.450411 | 294 | 293.15 | 0.997109 |
| GO:0008152\_metabolic\_process | GLCE | 2133 | 13 | 1.122644 | -0.450411 | 294 | 293.15 | 0.997109 |
| GO:0008152\_metabolic\_process | PCOLCE | 2133 | 13 | 1.122644 | -0.450411 | 294 | 293.15 | 0.997109 |
| GO:0008152\_metabolic\_process | SOD2 | 2133 | 13 | 1.122644 | -0.450411 | 294 | 293.15 | 0.997109 |
| GO:0008152\_metabolic\_process | UBE2N | 2133 | 13 | 1.122644 | -0.450411 | 294 | 293.15 | 0.997109 |
| GO:0008152\_metabolic\_process | IGF1R | 2133 | 13 | 1.122644 | -0.450411 | 294 | 293.15 | 0.997109 |
| GO:0008152\_metabolic\_process | TRP53BP1 | 2133 | 13 | 1.122644 | -0.450411 | 294 | 293.15 | 0.997109 |
| GO:0008152\_metabolic\_process | SALL4 | 2133 | 13 | 1.122644 | -0.450411 | 294 | 293.15 | 0.997109 |
| GO:0008152\_metabolic\_process | STT3A | 2133 | 13 | 1.122644 | -0.450411 | 294 | 293.15 | 0.997109 |
| GO:0008152\_metabolic\_process | DUSP16 | 2133 | 13 | 1.122644 | -0.450411 | 294 | 293.15 | 0.997109 |
| GO:0008152\_metabolic\_process | HDAC6 | 2133 | 13 | 1.122644 | -0.450411 | 294 | 293.15 | 0.997109 |
| GO:0000278\_mitotic\_cell\_cycle | RHOU | 80 | 1 | 2.302500 | -0.449158 | 296 | 294.95 | 0.996453 |
| GO:0044092\_negative\_regulation\_of\_molecular\_function | DUSP16 | 80 | 1 | 2.302500 | -0.449158 | 296 | 294.95 | 0.996453 |
| GO:0007420\_brain\_development | ATRX | 231 | 2 | 1.594805 | -0.444507 | 297 | 295.95 | 0.996465 |
| GO:0007420\_brain\_development | IGF1R | 231 | 2 | 1.594805 | -0.444507 | 297 | 295.95 | 0.996465 |
| GO:0042127\_regulation\_of\_cell\_proliferation | RECQL4 | 393 | 3 | 1.406107 | -0.442577 | 298 | 296.3 | 0.994295 |
| GO:0042127\_regulation\_of\_cell\_proliferation | PIN1 | 393 | 3 | 1.406107 | -0.442577 | 298 | 296.3 | 0.994295 |
| GO:0042127\_regulation\_of\_cell\_proliferation | SOD2 | 393 | 3 | 1.406107 | -0.442577 | 298 | 296.3 | 0.994295 |
| GO:0007411\_axon\_guidance | BMPR1B | 82 | 1 | 2.246341 | -0.440567 | 300 | 297.97 | 0.993233 |
| GO:0010627\_regulation\_of\_protein\_kinase\_cascade | IGF1R | 82 | 1 | 2.246341 | -0.440567 | 300 | 297.97 | 0.993233 |
| GO:0050790\_regulation\_of\_catalytic\_activity | DUSP16 | 233 | 2 | 1.581116 | -0.439690 | 301 | 298.4 | 0.991362 |
| GO:0050790\_regulation\_of\_catalytic\_activity | SOD2 | 233 | 2 | 1.581116 | -0.439690 | 301 | 298.4 | 0.991362 |
| GO:0048699\_generation\_of\_neurons | PTPRZ1 | 396 | 3 | 1.395455 | -0.437064 | 302 | 298.86 | 0.989603 |
| GO:0048699\_generation\_of\_neurons | BMPR1B | 396 | 3 | 1.395455 | -0.437064 | 302 | 298.86 | 0.989603 |
| GO:0048699\_generation\_of\_neurons | SOD2 | 396 | 3 | 1.395455 | -0.437064 | 302 | 298.86 | 0.989603 |
| GO:0006575\_cellular\_amino\_acid\_derivative\_metabolic\_process | SOD2 | 83 | 1 | 2.219277 | -0.436368 | 304 | 300.55 | 0.988651 |
| GO:0007017\_microtubule-based\_process | HDAC6 | 83 | 1 | 2.219277 | -0.436368 | 304 | 300.55 | 0.988651 |
| GO:0001501\_skeletal\_system\_development | RECQL4 | 236 | 2 | 1.561017 | -0.432579 | 305 | 301.08 | 0.987148 |
| GO:0001501\_skeletal\_system\_development | BMPR1B | 236 | 2 | 1.561017 | -0.432579 | 305 | 301.08 | 0.987148 |
| GO:0045137\_development\_of\_primary\_sexual\_characteristics | SIRT1 | 84 | 1 | 2.192857 | -0.432231 | 306 | 301.72 | 0.986013 |
| GO:0032504\_multicellular\_organism\_reproduction | SIRT1 | 86 | 1 | 2.141860 | -0.424137 | 309 | 305.79 | 0.989612 |
| GO:0034641\_cellular\_nitrogen\_compound\_metabolic\_process | SOD2 | 86 | 1 | 2.141860 | -0.424137 | 309 | 305.79 | 0.989612 |
| GO:0048609\_reproductive\_process\_in\_a\_multicellular\_organism | SIRT1 | 86 | 1 | 2.141860 | -0.424137 | 309 | 305.79 | 0.989612 |
| GO:0007178\_transmembrane\_receptor\_protein\_serine\_threonine\_kinase\_signaling\_pathway | BMPR1B | 87 | 1 | 2.117241 | -0.420178 | 313 | 307.92 | 0.983770 |
| GO:0016337\_cell-cell\_adhesion | BMPR1B | 87 | 1 | 2.117241 | -0.420178 | 313 | 307.92 | 0.983770 |
| GO:0022612\_gland\_morphogenesis | IGF1R | 87 | 1 | 2.117241 | -0.420178 | 313 | 307.92 | 0.983770 |
| GO:0050778\_positive\_regulation\_of\_immune\_response | UBE2N | 87 | 1 | 2.117241 | -0.420178 | 313 | 307.92 | 0.983770 |
| GO:0010556\_regulation\_of\_macromolecule\_biosynthetic\_process | UBE2N | 745 | 5 | 1.236242 | -0.420112 | 314 | 308.07 | 0.981115 |
| GO:0010556\_regulation\_of\_macromolecule\_biosynthetic\_process | TRP53BP1 | 745 | 5 | 1.236242 | -0.420112 | 314 | 308.07 | 0.981115 |
| GO:0010556\_regulation\_of\_macromolecule\_biosynthetic\_process | SALL4 | 745 | 5 | 1.236242 | -0.420112 | 314 | 308.07 | 0.981115 |
| GO:0010556\_regulation\_of\_macromolecule\_biosynthetic\_process | FOXK1 | 745 | 5 | 1.236242 | -0.420112 | 314 | 308.07 | 0.981115 |
| GO:0010556\_regulation\_of\_macromolecule\_biosynthetic\_process | SIRT1 | 745 | 5 | 1.236242 | -0.420112 | 314 | 308.07 | 0.981115 |
| GO:0007154\_cell\_communication | UBE2N | 1096 | 7 | 1.176460 | -0.416389 | 315 | 308.85 | 0.980476 |
| GO:0007154\_cell\_communication | NRAS | 1096 | 7 | 1.176460 | -0.416389 | 315 | 308.85 | 0.980476 |
| GO:0007154\_cell\_communication | IGF1R | 1096 | 7 | 1.176460 | -0.416389 | 315 | 308.85 | 0.980476 |
| GO:0007154\_cell\_communication | PDPK1 | 1096 | 7 | 1.176460 | -0.416389 | 315 | 308.85 | 0.980476 |
| GO:0007154\_cell\_communication | DUSP16 | 1096 | 7 | 1.176460 | -0.416389 | 315 | 308.85 | 0.980476 |
| GO:0007154\_cell\_communication | BMPR1B | 1096 | 7 | 1.176460 | -0.416389 | 315 | 308.85 | 0.980476 |
| GO:0007154\_cell\_communication | RHOU | 1096 | 7 | 1.176460 | -0.416389 | 315 | 308.85 | 0.980476 |
| GO:0080090\_regulation\_of\_primary\_metabolic\_process | UBE2N | 926 | 6 | 1.193521 | -0.410563 | 316 | 310.24 | 0.981772 |
| GO:0080090\_regulation\_of\_primary\_metabolic\_process | TRP53BP1 | 926 | 6 | 1.193521 | -0.410563 | 316 | 310.24 | 0.981772 |
| GO:0080090\_regulation\_of\_primary\_metabolic\_process | SALL4 | 926 | 6 | 1.193521 | -0.410563 | 316 | 310.24 | 0.981772 |
| GO:0080090\_regulation\_of\_primary\_metabolic\_process | FOXK1 | 926 | 6 | 1.193521 | -0.410563 | 316 | 310.24 | 0.981772 |
| GO:0080090\_regulation\_of\_primary\_metabolic\_process | SIRT1 | 926 | 6 | 1.193521 | -0.410563 | 316 | 310.24 | 0.981772 |
| GO:0080090\_regulation\_of\_primary\_metabolic\_process | HDAC6 | 926 | 6 | 1.193521 | -0.410563 | 316 | 310.24 | 0.981772 |
| GO:0019219\_regulation\_of\_nucleobase\_\_nucleoside\_\_nucleotide\_and\_nucleic\_acid\_metabolic\_process | UBE2N | 757 | 5 | 1.216645 | -0.404455 | 317 | 313.36 | 0.988517 |
| GO:0019219\_regulation\_of\_nucleobase\_\_nucleoside\_\_nucleotide\_and\_nucleic\_acid\_metabolic\_process | TRP53BP1 | 757 | 5 | 1.216645 | -0.404455 | 317 | 313.36 | 0.988517 |
| GO:0019219\_regulation\_of\_nucleobase\_\_nucleoside\_\_nucleotide\_and\_nucleic\_acid\_metabolic\_process | SALL4 | 757 | 5 | 1.216645 | -0.404455 | 317 | 313.36 | 0.988517 |
| GO:0019219\_regulation\_of\_nucleobase\_\_nucleoside\_\_nucleotide\_and\_nucleic\_acid\_metabolic\_process | FOXK1 | 757 | 5 | 1.216645 | -0.404455 | 317 | 313.36 | 0.988517 |
| GO:0019219\_regulation\_of\_nucleobase\_\_nucleoside\_\_nucleotide\_and\_nucleic\_acid\_metabolic\_process | SIRT1 | 757 | 5 | 1.216645 | -0.404455 | 317 | 313.36 | 0.988517 |
| GO:0007389\_pattern\_specification\_process | ABI1 | 250 | 2 | 1.473600 | -0.401140 | 318 | 314.95 | 0.990409 |
| GO:0007389\_pattern\_specification\_process | BMPR1B | 250 | 2 | 1.473600 | -0.401140 | 318 | 314.95 | 0.990409 |
| GO:0060255\_regulation\_of\_macromolecule\_metabolic\_process | UBE2N | 936 | 6 | 1.180769 | -0.398771 | 319 | 315.44 | 0.988840 |
| GO:0060255\_regulation\_of\_macromolecule\_metabolic\_process | TRP53BP1 | 936 | 6 | 1.180769 | -0.398771 | 319 | 315.44 | 0.988840 |
| GO:0060255\_regulation\_of\_macromolecule\_metabolic\_process | SALL4 | 936 | 6 | 1.180769 | -0.398771 | 319 | 315.44 | 0.988840 |
| GO:0060255\_regulation\_of\_macromolecule\_metabolic\_process | FOXK1 | 936 | 6 | 1.180769 | -0.398771 | 319 | 315.44 | 0.988840 |
| GO:0060255\_regulation\_of\_macromolecule\_metabolic\_process | SIRT1 | 936 | 6 | 1.180769 | -0.398771 | 319 | 315.44 | 0.988840 |
| GO:0060255\_regulation\_of\_macromolecule\_metabolic\_process | HDAC6 | 936 | 6 | 1.180769 | -0.398771 | 319 | 315.44 | 0.988840 |
| GO:0035107\_appendage\_morphogenesis | SALL4 | 93 | 1 | 1.980645 | -0.397565 | 322 | 316.73 | 0.983634 |
| GO:0035108\_limb\_morphogenesis | SALL4 | 93 | 1 | 1.980645 | -0.397565 | 322 | 316.73 | 0.983634 |
| GO:0055066\_di-\_\_tri-valent\_inorganic\_cation\_homeostasis | SOD2 | 93 | 1 | 1.980645 | -0.397565 | 322 | 316.73 | 0.983634 |
| GO:0022008\_neurogenesis | PTPRZ1 | 423 | 3 | 1.306383 | -0.390553 | 323 | 320.83 | 0.993282 |
| GO:0022008\_neurogenesis | BMPR1B | 423 | 3 | 1.306383 | -0.390553 | 323 | 320.83 | 0.993282 |
| GO:0022008\_neurogenesis | SOD2 | 423 | 3 | 1.306383 | -0.390553 | 323 | 320.83 | 0.993282 |
| GO:0042391\_regulation\_of\_membrane\_potential | SOD2 | 95 | 1 | 1.938947 | -0.390435 | 324 | 321.39 | 0.991944 |
| GO:0048736\_appendage\_development | SALL4 | 96 | 1 | 1.918750 | -0.386941 | 326 | 323.71 | 0.992975 |
| GO:0060173\_limb\_development | SALL4 | 96 | 1 | 1.918750 | -0.386941 | 326 | 323.71 | 0.992975 |
| GO:0018193\_peptidyl-amino\_acid\_modification | STT3A | 97 | 1 | 1.898969 | -0.383493 | 327 | 324.82 | 0.993333 |
| GO:0007548\_sex\_differentiation | SIRT1 | 98 | 1 | 1.879592 | -0.380091 | 330 | 326.57 | 0.989606 |
| GO:0009314\_response\_to\_radiation | SOD2 | 98 | 1 | 1.879592 | -0.380091 | 330 | 326.57 | 0.989606 |
| GO:0009967\_positive\_regulation\_of\_signal\_transduction | IGF1R | 98 | 1 | 1.879592 | -0.380091 | 330 | 326.57 | 0.989606 |
| GO:0010468\_regulation\_of\_gene\_expression | UBE2N | 778 | 5 | 1.183805 | -0.378319 | 331 | 326.69 | 0.986979 |
| GO:0010468\_regulation\_of\_gene\_expression | TRP53BP1 | 778 | 5 | 1.183805 | -0.378319 | 331 | 326.69 | 0.986979 |
| GO:0010468\_regulation\_of\_gene\_expression | SALL4 | 778 | 5 | 1.183805 | -0.378319 | 331 | 326.69 | 0.986979 |
| GO:0010468\_regulation\_of\_gene\_expression | FOXK1 | 778 | 5 | 1.183805 | -0.378319 | 331 | 326.69 | 0.986979 |
| GO:0010468\_regulation\_of\_gene\_expression | SIRT1 | 778 | 5 | 1.183805 | -0.378319 | 331 | 326.69 | 0.986979 |
| GO:0030030\_cell\_projection\_organization | PTPRZ1 | 263 | 2 | 1.400760 | -0.374307 | 332 | 329.57 | 0.992681 |
| GO:0030030\_cell\_projection\_organization | BMPR1B | 263 | 2 | 1.400760 | -0.374307 | 332 | 329.57 | 0.992681 |
| GO:0009653\_anatomical\_structure\_morphogenesis | RECQL4 | 958 | 6 | 1.153653 | -0.373851 | 333 | 329.69 | 0.990060 |
| GO:0009653\_anatomical\_structure\_morphogenesis | IGF1R | 958 | 6 | 1.153653 | -0.373851 | 333 | 329.69 | 0.990060 |
| GO:0009653\_anatomical\_structure\_morphogenesis | SALL4 | 958 | 6 | 1.153653 | -0.373851 | 333 | 329.69 | 0.990060 |
| GO:0009653\_anatomical\_structure\_morphogenesis | PTPRZ1 | 958 | 6 | 1.153653 | -0.373851 | 333 | 329.69 | 0.990060 |
| GO:0009653\_anatomical\_structure\_morphogenesis | BMPR1B | 958 | 6 | 1.153653 | -0.373851 | 333 | 329.69 | 0.990060 |
| GO:0009653\_anatomical\_structure\_morphogenesis | RHOU | 958 | 6 | 1.153653 | -0.373851 | 333 | 329.69 | 0.990060 |
| GO:0030036\_actin\_cytoskeleton\_organization | RHOU | 102 | 1 | 1.805882 | -0.366916 | 334 | 331.3 | 0.991916 |
| GO:0003013\_circulatory\_system\_process | SOD2 | 103 | 1 | 1.788350 | -0.363727 | 337 | 332.46 | 0.986528 |
| GO:0008015\_blood\_circulation | SOD2 | 103 | 1 | 1.788350 | -0.363727 | 337 | 332.46 | 0.986528 |
| GO:0009968\_negative\_regulation\_of\_signal\_transduction | IGF1R | 103 | 1 | 1.788350 | -0.363727 | 337 | 332.46 | 0.986528 |
| GO:0006807\_nitrogen\_compound\_metabolic\_process | UBE2N | 1147 | 7 | 1.124150 | -0.361894 | 338 | 332.84 | 0.984734 |
| GO:0006807\_nitrogen\_compound\_metabolic\_process | TRP53BP1 | 1147 | 7 | 1.124150 | -0.361894 | 338 | 332.84 | 0.984734 |
| GO:0006807\_nitrogen\_compound\_metabolic\_process | SALL4 | 1147 | 7 | 1.124150 | -0.361894 | 338 | 332.84 | 0.984734 |
| GO:0006807\_nitrogen\_compound\_metabolic\_process | FOXK1 | 1147 | 7 | 1.124150 | -0.361894 | 338 | 332.84 | 0.984734 |
| GO:0006807\_nitrogen\_compound\_metabolic\_process | SIRT1 | 1147 | 7 | 1.124150 | -0.361894 | 338 | 332.84 | 0.984734 |
| GO:0006807\_nitrogen\_compound\_metabolic\_process | GLCE | 1147 | 7 | 1.124150 | -0.361894 | 338 | 332.84 | 0.984734 |
| GO:0006807\_nitrogen\_compound\_metabolic\_process | SOD2 | 1147 | 7 | 1.124150 | -0.361894 | 338 | 332.84 | 0.984734 |
| GO:0031325\_positive\_regulation\_of\_cellular\_metabolic\_process | SALL4 | 442 | 3 | 1.250226 | -0.360904 | 339 | 333.04 | 0.982419 |
| GO:0031325\_positive\_regulation\_of\_cellular\_metabolic\_process | FOXK1 | 442 | 3 | 1.250226 | -0.360904 | 339 | 333.04 | 0.982419 |
| GO:0031325\_positive\_regulation\_of\_cellular\_metabolic\_process | SOD2 | 442 | 3 | 1.250226 | -0.360904 | 339 | 333.04 | 0.982419 |
| GO:0065007\_biological\_regulation | RECQL4 | 2593 | 15 | 1.065561 | -0.360891 | 340 | 333.17 | 0.979912 |
| GO:0065007\_biological\_regulation | FOXK1 | 2593 | 15 | 1.065561 | -0.360891 | 340 | 333.17 | 0.979912 |
| GO:0065007\_biological\_regulation | SIRT1 | 2593 | 15 | 1.065561 | -0.360891 | 340 | 333.17 | 0.979912 |
| GO:0065007\_biological\_regulation | RHOU | 2593 | 15 | 1.065561 | -0.360891 | 340 | 333.17 | 0.979912 |
| GO:0065007\_biological\_regulation | PIN1 | 2593 | 15 | 1.065561 | -0.360891 | 340 | 333.17 | 0.979912 |
| GO:0065007\_biological\_regulation | SOD2 | 2593 | 15 | 1.065561 | -0.360891 | 340 | 333.17 | 0.979912 |
| GO:0065007\_biological\_regulation | UBE2N | 2593 | 15 | 1.065561 | -0.360891 | 340 | 333.17 | 0.979912 |
| GO:0065007\_biological\_regulation | IGF1R | 2593 | 15 | 1.065561 | -0.360891 | 340 | 333.17 | 0.979912 |
| GO:0065007\_biological\_regulation | NRAS | 2593 | 15 | 1.065561 | -0.360891 | 340 | 333.17 | 0.979912 |
| GO:0065007\_biological\_regulation | PDPK1 | 2593 | 15 | 1.065561 | -0.360891 | 340 | 333.17 | 0.979912 |
| GO:0065007\_biological\_regulation | TRP53BP1 | 2593 | 15 | 1.065561 | -0.360891 | 340 | 333.17 | 0.979912 |
| GO:0065007\_biological\_regulation | SALL4 | 2593 | 15 | 1.065561 | -0.360891 | 340 | 333.17 | 0.979912 |
| GO:0065007\_biological\_regulation | DUSP16 | 2593 | 15 | 1.065561 | -0.360891 | 340 | 333.17 | 0.979912 |
| GO:0065007\_biological\_regulation | BMPR1B | 2593 | 15 | 1.065561 | -0.360891 | 340 | 333.17 | 0.979912 |
| GO:0065007\_biological\_regulation | HDAC6 | 2593 | 15 | 1.065561 | -0.360891 | 340 | 333.17 | 0.979912 |
| GO:0051716\_cellular\_response\_to\_stimulus | TRP53BP1 | 273 | 2 | 1.349451 | -0.355062 | 341 | 335.85 | 0.984897 |
| GO:0051716\_cellular\_response\_to\_stimulus | SOD2 | 273 | 2 | 1.349451 | -0.355062 | 341 | 335.85 | 0.984897 |
| GO:0045859\_regulation\_of\_protein\_kinase\_activity | DUSP16 | 107 | 1 | 1.721495 | -0.351362 | 342 | 337.38 | 0.986491 |
| GO:0030029\_actin\_filament-based\_process | RHOU | 109 | 1 | 1.689908 | -0.345405 | 343 | 339.58 | 0.990029 |
| GO:0010647\_positive\_regulation\_of\_cell\_communication | IGF1R | 110 | 1 | 1.674545 | -0.342480 | 347 | 341.65 | 0.984582 |
| GO:0010648\_negative\_regulation\_of\_cell\_communication | IGF1R | 110 | 1 | 1.674545 | -0.342480 | 347 | 341.65 | 0.984582 |
| GO:0043010\_camera-type\_eye\_development | BMPR1B | 110 | 1 | 1.674545 | -0.342480 | 347 | 341.65 | 0.984582 |
| GO:0055080\_cation\_homeostasis | SOD2 | 110 | 1 | 1.674545 | -0.342480 | 347 | 341.65 | 0.984582 |
| GO:0009893\_positive\_regulation\_of\_metabolic\_process | SALL4 | 458 | 3 | 1.206550 | -0.337715 | 348 | 342.9 | 0.985345 |
| GO:0009893\_positive\_regulation\_of\_metabolic\_process | FOXK1 | 458 | 3 | 1.206550 | -0.337715 | 348 | 342.9 | 0.985345 |
| GO:0009893\_positive\_regulation\_of\_metabolic\_process | SOD2 | 458 | 3 | 1.206550 | -0.337715 | 348 | 342.9 | 0.985345 |
| GO:0043549\_regulation\_of\_kinase\_activity | DUSP16 | 112 | 1 | 1.644643 | -0.336736 | 349 | 343.9 | 0.985387 |
| GO:0048518\_positive\_regulation\_of\_biological\_process | RECQL4 | 995 | 6 | 1.110754 | -0.334946 | 350 | 344.58 | 0.984514 |
| GO:0048518\_positive\_regulation\_of\_biological\_process | UBE2N | 995 | 6 | 1.110754 | -0.334946 | 350 | 344.58 | 0.984514 |
| GO:0048518\_positive\_regulation\_of\_biological\_process | IGF1R | 995 | 6 | 1.110754 | -0.334946 | 350 | 344.58 | 0.984514 |
| GO:0048518\_positive\_regulation\_of\_biological\_process | SALL4 | 995 | 6 | 1.110754 | -0.334946 | 350 | 344.58 | 0.984514 |
| GO:0048518\_positive\_regulation\_of\_biological\_process | FOXK1 | 995 | 6 | 1.110754 | -0.334946 | 350 | 344.58 | 0.984514 |
| GO:0048518\_positive\_regulation\_of\_biological\_process | SOD2 | 995 | 6 | 1.110754 | -0.334946 | 350 | 344.58 | 0.984514 |
| GO:0007417\_central\_nervous\_system\_development | ATRX | 287 | 2 | 1.283624 | -0.329970 | 351 | 346.67 | 0.987664 |
| GO:0007417\_central\_nervous\_system\_development | IGF1R | 287 | 2 | 1.283624 | -0.329970 | 351 | 346.67 | 0.987664 |
| GO:0048584\_positive\_regulation\_of\_response\_to\_stimulus | UBE2N | 115 | 1 | 1.601739 | -0.328370 | 353 | 347.55 | 0.984561 |
| GO:0051338\_regulation\_of\_transferase\_activity | DUSP16 | 115 | 1 | 1.601739 | -0.328370 | 353 | 347.55 | 0.984561 |
| GO:0006139\_nucleobase\_\_nucleoside\_\_nucleotide\_and\_nucleic\_acid\_metabolic\_process | UBE2N | 1002 | 6 | 1.102994 | -0.327988 | 354 | 347.95 | 0.982910 |
| GO:0006139\_nucleobase\_\_nucleoside\_\_nucleotide\_and\_nucleic\_acid\_metabolic\_process | TRP53BP1 | 1002 | 6 | 1.102994 | -0.327988 | 354 | 347.95 | 0.982910 |
| GO:0006139\_nucleobase\_\_nucleoside\_\_nucleotide\_and\_nucleic\_acid\_metabolic\_process | SALL4 | 1002 | 6 | 1.102994 | -0.327988 | 354 | 347.95 | 0.982910 |
| GO:0006139\_nucleobase\_\_nucleoside\_\_nucleotide\_and\_nucleic\_acid\_metabolic\_process | FOXK1 | 1002 | 6 | 1.102994 | -0.327988 | 354 | 347.95 | 0.982910 |
| GO:0006139\_nucleobase\_\_nucleoside\_\_nucleotide\_and\_nucleic\_acid\_metabolic\_process | SIRT1 | 1002 | 6 | 1.102994 | -0.327988 | 354 | 347.95 | 0.982910 |
| GO:0006139\_nucleobase\_\_nucleoside\_\_nucleotide\_and\_nucleic\_acid\_metabolic\_process | SOD2 | 1002 | 6 | 1.102994 | -0.327988 | 354 | 347.95 | 0.982910 |
| GO:0006519\_cellular\_amino\_acid\_and\_derivative\_metabolic\_process | SOD2 | 118 | 1 | 1.561017 | -0.320292 | 355 | 350.19 | 0.986451 |
| GO:0022403\_cell\_cycle\_phase | RHOU | 119 | 1 | 1.547899 | -0.317661 | 356 | 350.53 | 0.984635 |
| GO:0048468\_cell\_development | PTPRZ1 | 654 | 4 | 1.126606 | -0.315636 | 357 | 351.47 | 0.984510 |
| GO:0048468\_cell\_development | BMPR1B | 654 | 4 | 1.126606 | -0.315636 | 357 | 351.47 | 0.984510 |
| GO:0048468\_cell\_development | SOD2 | 654 | 4 | 1.126606 | -0.315636 | 357 | 351.47 | 0.984510 |
| GO:0048468\_cell\_development | ZFP37 | 654 | 4 | 1.126606 | -0.315636 | 357 | 351.47 | 0.984510 |
| GO:0009308\_amine\_metabolic\_process | GLCE | 124 | 1 | 1.485484 | -0.304942 | 358 | 356.85 | 0.996788 |
| GO:0045893\_positive\_regulation\_of\_transcription\_\_DNA-dependent | SALL4 | 306 | 2 | 1.203922 | -0.299016 | 360 | 358.34 | 0.995389 |
| GO:0045893\_positive\_regulation\_of\_transcription\_\_DNA-dependent | FOXK1 | 306 | 2 | 1.203922 | -0.299016 | 360 | 358.34 | 0.995389 |
| GO:0051254\_positive\_regulation\_of\_RNA\_metabolic\_process | SALL4 | 306 | 2 | 1.203922 | -0.299016 | 360 | 358.34 | 0.995389 |
| GO:0051254\_positive\_regulation\_of\_RNA\_metabolic\_process | FOXK1 | 306 | 2 | 1.203922 | -0.299016 | 360 | 358.34 | 0.995389 |
| GO:0001655\_urogenital\_system\_development | IGF1R | 128 | 1 | 1.439063 | -0.295262 | 361 | 360.08 | 0.997452 |
| GO:0016310\_phosphorylation | IGF1R | 309 | 2 | 1.192233 | -0.294427 | 362 | 360.38 | 0.995525 |
| GO:0016310\_phosphorylation | DUSP16 | 309 | 2 | 1.192233 | -0.294427 | 362 | 360.38 | 0.995525 |
| GO:0050776\_regulation\_of\_immune\_response | UBE2N | 130 | 1 | 1.416923 | -0.290578 | 363 | 362.72 | 0.999229 |
| GO:0009952\_anterior\_posterior\_pattern\_formation | ABI1 | 133 | 1 | 1.384962 | -0.283737 | 364 | 363.54 | 0.998736 |
| GO:0007283\_spermatogenesis | SIRT1 | 134 | 1 | 1.374627 | -0.281504 | 366 | 364.26 | 0.995246 |
| GO:0048232\_male\_gamete\_generation | SIRT1 | 134 | 1 | 1.374627 | -0.281504 | 366 | 364.26 | 0.995246 |
| GO:0001654\_eye\_development | BMPR1B | 136 | 1 | 1.354412 | -0.277109 | 367 | 364.8 | 0.994005 |
| GO:0044237\_cellular\_metabolic\_process | UBE2N | 1974 | 11 | 1.026444 | -0.274888 | 368 | 365.81 | 0.994049 |
| GO:0044237\_cellular\_metabolic\_process | IGF1R | 1974 | 11 | 1.026444 | -0.274888 | 368 | 365.81 | 0.994049 |
| GO:0044237\_cellular\_metabolic\_process | TRP53BP1 | 1974 | 11 | 1.026444 | -0.274888 | 368 | 365.81 | 0.994049 |
| GO:0044237\_cellular\_metabolic\_process | STT3A | 1974 | 11 | 1.026444 | -0.274888 | 368 | 365.81 | 0.994049 |
| GO:0044237\_cellular\_metabolic\_process | SALL4 | 1974 | 11 | 1.026444 | -0.274888 | 368 | 365.81 | 0.994049 |
| GO:0044237\_cellular\_metabolic\_process | FOXK1 | 1974 | 11 | 1.026444 | -0.274888 | 368 | 365.81 | 0.994049 |
| GO:0044237\_cellular\_metabolic\_process | DUSP16 | 1974 | 11 | 1.026444 | -0.274888 | 368 | 365.81 | 0.994049 |
| GO:0044237\_cellular\_metabolic\_process | SIRT1 | 1974 | 11 | 1.026444 | -0.274888 | 368 | 365.81 | 0.994049 |
| GO:0044237\_cellular\_metabolic\_process | GLCE | 1974 | 11 | 1.026444 | -0.274888 | 368 | 365.81 | 0.994049 |
| GO:0044237\_cellular\_metabolic\_process | HDAC6 | 1974 | 11 | 1.026444 | -0.274888 | 368 | 365.81 | 0.994049 |
| GO:0044237\_cellular\_metabolic\_process | SOD2 | 1974 | 11 | 1.026444 | -0.274888 | 368 | 365.81 | 0.994049 |
| GO:0045596\_negative\_regulation\_of\_cell\_differentiation | SOD2 | 144 | 1 | 1.279167 | -0.260404 | 369 | 371.56 | 1.006938 |
| GO:0048522\_positive\_regulation\_of\_cellular\_process | RECQL4 | 895 | 5 | 1.029050 | -0.258338 | 370 | 371.93 | 1.005216 |
| GO:0048522\_positive\_regulation\_of\_cellular\_process | IGF1R | 895 | 5 | 1.029050 | -0.258338 | 370 | 371.93 | 1.005216 |
| GO:0048522\_positive\_regulation\_of\_cellular\_process | SALL4 | 895 | 5 | 1.029050 | -0.258338 | 370 | 371.93 | 1.005216 |
| GO:0048522\_positive\_regulation\_of\_cellular\_process | FOXK1 | 895 | 5 | 1.029050 | -0.258338 | 370 | 371.93 | 1.005216 |
| GO:0048522\_positive\_regulation\_of\_cellular\_process | SOD2 | 895 | 5 | 1.029050 | -0.258338 | 370 | 371.93 | 1.005216 |
| GO:0030900\_forebrain\_development | ATRX | 146 | 1 | 1.261644 | -0.256435 | 371 | 373.1 | 1.005660 |
| GO:0009888\_tissue\_development | IGF1R | 525 | 3 | 1.052571 | -0.255671 | 372 | 373.56 | 1.004194 |
| GO:0009888\_tissue\_development | SALL4 | 525 | 3 | 1.052571 | -0.255671 | 372 | 373.56 | 1.004194 |
| GO:0009888\_tissue\_development | BMPR1B | 525 | 3 | 1.052571 | -0.255671 | 372 | 373.56 | 1.004194 |
| GO:0022603\_regulation\_of\_anatomical\_structure\_morphogenesis | RHOU | 147 | 1 | 1.253061 | -0.254480 | 373 | 373.85 | 1.002279 |
| GO:0045941\_positive\_regulation\_of\_transcription | SALL4 | 338 | 2 | 1.089941 | -0.253798 | 374 | 374.06 | 1.000160 |
| GO:0045941\_positive\_regulation\_of\_transcription | FOXK1 | 338 | 2 | 1.089941 | -0.253798 | 374 | 374.06 | 1.000160 |
| GO:0002684\_positive\_regulation\_of\_immune\_system\_process | UBE2N | 148 | 1 | 1.244595 | -0.252544 | 375 | 375.03 | 1.000080 |
| GO:0006793\_phosphorus\_metabolic\_process | IGF1R | 340 | 2 | 1.083529 | -0.251227 | 377 | 376.21 | 0.997905 |
| GO:0006793\_phosphorus\_metabolic\_process | DUSP16 | 340 | 2 | 1.083529 | -0.251227 | 377 | 376.21 | 0.997905 |
| GO:0006796\_phosphate\_metabolic\_process | IGF1R | 340 | 2 | 1.083529 | -0.251227 | 377 | 376.21 | 0.997905 |
| GO:0006796\_phosphate\_metabolic\_process | DUSP16 | 340 | 2 | 1.083529 | -0.251227 | 377 | 376.21 | 0.997905 |
| GO:0010467\_gene\_expression | UBE2N | 905 | 5 | 1.017680 | -0.249843 | 378 | 376.86 | 0.996984 |
| GO:0010467\_gene\_expression | TRP53BP1 | 905 | 5 | 1.017680 | -0.249843 | 378 | 376.86 | 0.996984 |
| GO:0010467\_gene\_expression | SALL4 | 905 | 5 | 1.017680 | -0.249843 | 378 | 376.86 | 0.996984 |
| GO:0010467\_gene\_expression | FOXK1 | 905 | 5 | 1.017680 | -0.249843 | 378 | 376.86 | 0.996984 |
| GO:0010467\_gene\_expression | SIRT1 | 905 | 5 | 1.017680 | -0.249843 | 378 | 376.86 | 0.996984 |
| GO:0032268\_regulation\_of\_cellular\_protein\_metabolic\_process | HDAC6 | 152 | 1 | 1.211842 | -0.244986 | 379 | 377.79 | 0.996807 |
| GO:0010628\_positive\_regulation\_of\_gene\_expression | SALL4 | 346 | 2 | 1.064740 | -0.243677 | 380 | 378.01 | 0.994763 |
| GO:0010628\_positive\_regulation\_of\_gene\_expression | FOXK1 | 346 | 2 | 1.064740 | -0.243677 | 380 | 378.01 | 0.994763 |
| GO:0008285\_negative\_regulation\_of\_cell\_proliferation | SOD2 | 155 | 1 | 1.188387 | -0.239507 | 381 | 380.12 | 0.997690 |
| GO:0045935\_positive\_regulation\_of\_nucleobase\_\_nucleoside\_\_nucleotide\_and\_nucleic\_acid\_metabolic\_process | SALL4 | 352 | 2 | 1.046591 | -0.236368 | 382 | 380.48 | 0.996021 |
| GO:0045935\_positive\_regulation\_of\_nucleobase\_\_nucleoside\_\_nucleotide\_and\_nucleic\_acid\_metabolic\_process | FOXK1 | 352 | 2 | 1.046591 | -0.236368 | 382 | 380.48 | 0.996021 |
| GO:0008283\_cell\_proliferation | RECQL4 | 544 | 3 | 1.015809 | -0.236195 | 383 | 380.75 | 0.994125 |
| GO:0008283\_cell\_proliferation | PIN1 | 544 | 3 | 1.015809 | -0.236195 | 383 | 380.75 | 0.994125 |
| GO:0008283\_cell\_proliferation | SOD2 | 544 | 3 | 1.015809 | -0.236195 | 383 | 380.75 | 0.994125 |
| GO:0006950\_response\_to\_stress | TRP53BP1 | 549 | 3 | 1.006557 | -0.231313 | 384 | 383.28 | 0.998125 |
| GO:0006950\_response\_to\_stress | PDPK1 | 549 | 3 | 1.006557 | -0.231313 | 384 | 383.28 | 0.998125 |
| GO:0006950\_response\_to\_stress | SOD2 | 549 | 3 | 1.006557 | -0.231313 | 384 | 383.28 | 0.998125 |
| GO:0007626\_locomotory\_behavior | SOD2 | 163 | 1 | 1.130061 | -0.225637 | 385 | 385.97 | 1.002519 |
| GO:0042325\_regulation\_of\_phosphorylation | DUSP16 | 164 | 1 | 1.123171 | -0.223975 | 386 | 386.67 | 1.001736 |
| GO:0019220\_regulation\_of\_phosphate\_metabolic\_process | DUSP16 | 165 | 1 | 1.116364 | -0.222328 | 388 | 387.76 | 0.999381 |
| GO:0051174\_regulation\_of\_phosphorus\_metabolic\_process | DUSP16 | 165 | 1 | 1.116364 | -0.222328 | 388 | 387.76 | 0.999381 |
| GO:0043009\_chordate\_embryonic\_development | SALL4 | 365 | 2 | 1.009315 | -0.221309 | 389 | 388.82 | 0.999537 |
| GO:0043009\_chordate\_embryonic\_development | ABI1 | 365 | 2 | 1.009315 | -0.221309 | 389 | 388.82 | 0.999537 |
| GO:0048856\_anatomical\_structure\_development | ATRX | 1688 | 9 | 0.982109 | -0.220533 | 390 | 389.3 | 0.998205 |
| GO:0048856\_anatomical\_structure\_development | RECQL4 | 1688 | 9 | 0.982109 | -0.220533 | 390 | 389.3 | 0.998205 |
| GO:0048856\_anatomical\_structure\_development | IGF1R | 1688 | 9 | 0.982109 | -0.220533 | 390 | 389.3 | 0.998205 |
| GO:0048856\_anatomical\_structure\_development | SALL4 | 1688 | 9 | 0.982109 | -0.220533 | 390 | 389.3 | 0.998205 |
| GO:0048856\_anatomical\_structure\_development | PTPRZ1 | 1688 | 9 | 0.982109 | -0.220533 | 390 | 389.3 | 0.998205 |
| GO:0048856\_anatomical\_structure\_development | BMPR1B | 1688 | 9 | 0.982109 | -0.220533 | 390 | 389.3 | 0.998205 |
| GO:0048856\_anatomical\_structure\_development | SIRT1 | 1688 | 9 | 0.982109 | -0.220533 | 390 | 389.3 | 0.998205 |
| GO:0048856\_anatomical\_structure\_development | RHOU | 1688 | 9 | 0.982109 | -0.220533 | 390 | 389.3 | 0.998205 |
| GO:0048856\_anatomical\_structure\_development | SOD2 | 1688 | 9 | 0.982109 | -0.220533 | 390 | 389.3 | 0.998205 |
| GO:0009792\_embryonic\_development\_ending\_in\_birth\_or\_egg\_hatching | SALL4 | 368 | 2 | 1.001087 | -0.217978 | 391 | 390.5 | 0.998721 |
| GO:0009792\_embryonic\_development\_ending\_in\_birth\_or\_egg\_hatching | ABI1 | 368 | 2 | 1.001087 | -0.217978 | 391 | 390.5 | 0.998721 |
| GO:0032502\_developmental\_process | ATRX | 2060 | 11 | 0.983592 | -0.217731 | 392 | 390.64 | 0.996531 |
| GO:0032502\_developmental\_process | RECQL4 | 2060 | 11 | 0.983592 | -0.217731 | 392 | 390.64 | 0.996531 |
| GO:0032502\_developmental\_process | IGF1R | 2060 | 11 | 0.983592 | -0.217731 | 392 | 390.64 | 0.996531 |
| GO:0032502\_developmental\_process | SALL4 | 2060 | 11 | 0.983592 | -0.217731 | 392 | 390.64 | 0.996531 |
| GO:0032502\_developmental\_process | PTPRZ1 | 2060 | 11 | 0.983592 | -0.217731 | 392 | 390.64 | 0.996531 |
| GO:0032502\_developmental\_process | ABI1 | 2060 | 11 | 0.983592 | -0.217731 | 392 | 390.64 | 0.996531 |
| GO:0032502\_developmental\_process | BMPR1B | 2060 | 11 | 0.983592 | -0.217731 | 392 | 390.64 | 0.996531 |
| GO:0032502\_developmental\_process | RHOU | 2060 | 11 | 0.983592 | -0.217731 | 392 | 390.64 | 0.996531 |
| GO:0032502\_developmental\_process | SIRT1 | 2060 | 11 | 0.983592 | -0.217731 | 392 | 390.64 | 0.996531 |
| GO:0032502\_developmental\_process | ZFP37 | 2060 | 11 | 0.983592 | -0.217731 | 392 | 390.64 | 0.996531 |
| GO:0032502\_developmental\_process | SOD2 | 2060 | 11 | 0.983592 | -0.217731 | 392 | 390.64 | 0.996531 |
| GO:0010557\_positive\_regulation\_of\_macromolecule\_biosynthetic\_process | SALL4 | 371 | 2 | 0.992992 | -0.214700 | 393 | 391.29 | 0.995649 |
| GO:0010557\_positive\_regulation\_of\_macromolecule\_biosynthetic\_process | FOXK1 | 371 | 2 | 0.992992 | -0.214700 | 393 | 391.29 | 0.995649 |
| GO:0051246\_regulation\_of\_protein\_metabolic\_process | HDAC6 | 170 | 1 | 1.083529 | -0.214317 | 394 | 392.32 | 0.995736 |
| GO:0007600\_sensory\_perception | SOD2 | 172 | 1 | 1.070930 | -0.211213 | 396 | 392.97 | 0.992348 |
| GO:0009611\_response\_to\_wounding | SOD2 | 172 | 1 | 1.070930 | -0.211213 | 396 | 392.97 | 0.992348 |
| GO:0044093\_positive\_regulation\_of\_molecular\_function | UBE2N | 173 | 1 | 1.064740 | -0.209681 | 397 | 394.19 | 0.992922 |
| GO:0006355\_regulation\_of\_transcription\_\_DNA-dependent | TRP53BP1 | 575 | 3 | 0.961043 | -0.207443 | 398 | 395.11 | 0.992739 |
| GO:0006355\_regulation\_of\_transcription\_\_DNA-dependent | SALL4 | 575 | 3 | 0.961043 | -0.207443 | 398 | 395.11 | 0.992739 |
| GO:0006355\_regulation\_of\_transcription\_\_DNA-dependent | FOXK1 | 575 | 3 | 0.961043 | -0.207443 | 398 | 395.11 | 0.992739 |
| GO:0000122\_negative\_regulation\_of\_transcription\_from\_RNA\_polymerase\_II\_promoter | SALL4 | 175 | 1 | 1.052571 | -0.206659 | 399 | 396.26 | 0.993133 |
| GO:0006873\_cellular\_ion\_homeostasis | SOD2 | 176 | 1 | 1.046591 | -0.205168 | 401 | 397.91 | 0.992294 |
| GO:0043066\_negative\_regulation\_of\_apoptosis | SOD2 | 176 | 1 | 1.046591 | -0.205168 | 401 | 397.91 | 0.992294 |
| GO:0009987\_cellular\_process | RECQL4 | 3868 | 21 | 1.000052 | -0.201216 | 402 | 398.7 | 0.991791 |
| GO:0009987\_cellular\_process | FOXK1 | 3868 | 21 | 1.000052 | -0.201216 | 402 | 398.7 | 0.991791 |
| GO:0009987\_cellular\_process | HIST1H1C | 3868 | 21 | 1.000052 | -0.201216 | 402 | 398.7 | 0.991791 |
| GO:0009987\_cellular\_process | PTPRZ1 | 3868 | 21 | 1.000052 | -0.201216 | 402 | 398.7 | 0.991791 |
| GO:0009987\_cellular\_process | ABI1 | 3868 | 21 | 1.000052 | -0.201216 | 402 | 398.7 | 0.991791 |
| GO:0009987\_cellular\_process | SIRT1 | 3868 | 21 | 1.000052 | -0.201216 | 402 | 398.7 | 0.991791 |
| GO:0009987\_cellular\_process | RHOU | 3868 | 21 | 1.000052 | -0.201216 | 402 | 398.7 | 0.991791 |
| GO:0009987\_cellular\_process | GLCE | 3868 | 21 | 1.000052 | -0.201216 | 402 | 398.7 | 0.991791 |
| GO:0009987\_cellular\_process | ZFP37 | 3868 | 21 | 1.000052 | -0.201216 | 402 | 398.7 | 0.991791 |
| GO:0009987\_cellular\_process | SOD2 | 3868 | 21 | 1.000052 | -0.201216 | 402 | 398.7 | 0.991791 |
| GO:0009987\_cellular\_process | PIN1 | 3868 | 21 | 1.000052 | -0.201216 | 402 | 398.7 | 0.991791 |
| GO:0009987\_cellular\_process | UBE2N | 3868 | 21 | 1.000052 | -0.201216 | 402 | 398.7 | 0.991791 |
| GO:0009987\_cellular\_process | NRAS | 3868 | 21 | 1.000052 | -0.201216 | 402 | 398.7 | 0.991791 |
| GO:0009987\_cellular\_process | IGF1R | 3868 | 21 | 1.000052 | -0.201216 | 402 | 398.7 | 0.991791 |
| GO:0009987\_cellular\_process | TRP53BP1 | 3868 | 21 | 1.000052 | -0.201216 | 402 | 398.7 | 0.991791 |
| GO:0009987\_cellular\_process | PDPK1 | 3868 | 21 | 1.000052 | -0.201216 | 402 | 398.7 | 0.991791 |
| GO:0009987\_cellular\_process | STT3A | 3868 | 21 | 1.000052 | -0.201216 | 402 | 398.7 | 0.991791 |
| GO:0009987\_cellular\_process | SALL4 | 3868 | 21 | 1.000052 | -0.201216 | 402 | 398.7 | 0.991791 |
| GO:0009987\_cellular\_process | DUSP16 | 3868 | 21 | 1.000052 | -0.201216 | 402 | 398.7 | 0.991791 |
| GO:0009987\_cellular\_process | BMPR1B | 3868 | 21 | 1.000052 | -0.201216 | 402 | 398.7 | 0.991791 |
| GO:0009987\_cellular\_process | HDAC6 | 3868 | 21 | 1.000052 | -0.201216 | 402 | 398.7 | 0.991791 |
| GO:0043069\_negative\_regulation\_of\_programmed\_cell\_death | SOD2 | 179 | 1 | 1.029050 | -0.200774 | 405 | 400.22 | 0.988198 |
| GO:0048732\_gland\_development | IGF1R | 179 | 1 | 1.029050 | -0.200774 | 405 | 400.22 | 0.988198 |
| GO:0060548\_negative\_regulation\_of\_cell\_death | SOD2 | 179 | 1 | 1.029050 | -0.200774 | 405 | 400.22 | 0.988198 |
| GO:0055082\_cellular\_chemical\_homeostasis | SOD2 | 181 | 1 | 1.017680 | -0.197907 | 406 | 402.13 | 0.990468 |
| GO:0051252\_regulation\_of\_RNA\_metabolic\_process | TRP53BP1 | 590 | 3 | 0.936610 | -0.194760 | 407 | 404.04 | 0.992727 |
| GO:0051252\_regulation\_of\_RNA\_metabolic\_process | SALL4 | 590 | 3 | 0.936610 | -0.194760 | 407 | 404.04 | 0.992727 |
| GO:0051252\_regulation\_of\_RNA\_metabolic\_process | FOXK1 | 590 | 3 | 0.936610 | -0.194760 | 407 | 404.04 | 0.992727 |
| GO:0006351\_transcription\_\_DNA-dependent | TRP53BP1 | 594 | 3 | 0.930303 | -0.191504 | 408 | 405.67 | 0.994289 |
| GO:0006351\_transcription\_\_DNA-dependent | SALL4 | 594 | 3 | 0.930303 | -0.191504 | 408 | 405.67 | 0.994289 |
| GO:0006351\_transcription\_\_DNA-dependent | FOXK1 | 594 | 3 | 0.930303 | -0.191504 | 408 | 405.67 | 0.994289 |
| GO:0006811\_ion\_transport | SLC4A1 | 186 | 1 | 0.990323 | -0.190956 | 411 | 406.87 | 0.989951 |
| GO:0007155\_cell\_adhesion | BMPR1B | 186 | 1 | 0.990323 | -0.190956 | 411 | 406.87 | 0.989951 |
| GO:0022610\_biological\_adhesion | BMPR1B | 186 | 1 | 0.990323 | -0.190956 | 411 | 406.87 | 0.989951 |
| GO:0032774\_RNA\_biosynthetic\_process | TRP53BP1 | 595 | 3 | 0.928739 | -0.190698 | 412 | 407.27 | 0.988519 |
| GO:0032774\_RNA\_biosynthetic\_process | SALL4 | 595 | 3 | 0.928739 | -0.190698 | 412 | 407.27 | 0.988519 |
| GO:0032774\_RNA\_biosynthetic\_process | FOXK1 | 595 | 3 | 0.928739 | -0.190698 | 412 | 407.27 | 0.988519 |
| GO:0048513\_organ\_development | ATRX | 1365 | 7 | 0.944615 | -0.190183 | 413 | 407.38 | 0.986392 |
| GO:0048513\_organ\_development | RECQL4 | 1365 | 7 | 0.944615 | -0.190183 | 413 | 407.38 | 0.986392 |
| GO:0048513\_organ\_development | IGF1R | 1365 | 7 | 0.944615 | -0.190183 | 413 | 407.38 | 0.986392 |
| GO:0048513\_organ\_development | SALL4 | 1365 | 7 | 0.944615 | -0.190183 | 413 | 407.38 | 0.986392 |
| GO:0048513\_organ\_development | BMPR1B | 1365 | 7 | 0.944615 | -0.190183 | 413 | 407.38 | 0.986392 |
| GO:0048513\_organ\_development | SIRT1 | 1365 | 7 | 0.944615 | -0.190183 | 413 | 407.38 | 0.986392 |
| GO:0048513\_organ\_development | SOD2 | 1365 | 7 | 0.944615 | -0.190183 | 413 | 407.38 | 0.986392 |
| GO:0019725\_cellular\_homeostasis | SOD2 | 195 | 1 | 0.944615 | -0.179168 | 414 | 411.45 | 0.993841 |
| GO:0007275\_multicellular\_organismal\_development | ATRX | 1760 | 9 | 0.941932 | -0.178481 | 415 | 411.59 | 0.991783 |
| GO:0007275\_multicellular\_organismal\_development | RECQL4 | 1760 | 9 | 0.941932 | -0.178481 | 415 | 411.59 | 0.991783 |
| GO:0007275\_multicellular\_organismal\_development | IGF1R | 1760 | 9 | 0.941932 | -0.178481 | 415 | 411.59 | 0.991783 |
| GO:0007275\_multicellular\_organismal\_development | SALL4 | 1760 | 9 | 0.941932 | -0.178481 | 415 | 411.59 | 0.991783 |
| GO:0007275\_multicellular\_organismal\_development | PTPRZ1 | 1760 | 9 | 0.941932 | -0.178481 | 415 | 411.59 | 0.991783 |
| GO:0007275\_multicellular\_organismal\_development | ABI1 | 1760 | 9 | 0.941932 | -0.178481 | 415 | 411.59 | 0.991783 |
| GO:0007275\_multicellular\_organismal\_development | BMPR1B | 1760 | 9 | 0.941932 | -0.178481 | 415 | 411.59 | 0.991783 |
| GO:0007275\_multicellular\_organismal\_development | SIRT1 | 1760 | 9 | 0.941932 | -0.178481 | 415 | 411.59 | 0.991783 |
| GO:0007275\_multicellular\_organismal\_development | SOD2 | 1760 | 9 | 0.941932 | -0.178481 | 415 | 411.59 | 0.991783 |
| GO:0050801\_ion\_homeostasis | SOD2 | 197 | 1 | 0.935025 | -0.176668 | 416 | 413.13 | 0.993101 |
| GO:0002009\_morphogenesis\_of\_an\_epithelium | IGF1R | 198 | 1 | 0.930303 | -0.175433 | 418 | 414.52 | 0.991675 |
| GO:0060429\_epithelium\_development | IGF1R | 198 | 1 | 0.930303 | -0.175433 | 418 | 414.52 | 0.991675 |
| GO:0022607\_cellular\_component\_assembly | HIST1H1C | 204 | 1 | 0.902941 | -0.168235 | 419 | 416.76 | 0.994654 |
| GO:0006955\_immune\_response | UBE2N | 205 | 1 | 0.898537 | -0.167070 | 420 | 417.5 | 0.994048 |
| GO:0008284\_positive\_regulation\_of\_cell\_proliferation | RECQL4 | 208 | 1 | 0.885577 | -0.163629 | 421 | 418.57 | 0.994228 |
| GO:0048731\_system\_development | ATRX | 1609 | 8 | 0.915848 | -0.159718 | 422 | 419.07 | 0.993057 |
| GO:0048731\_system\_development | RECQL4 | 1609 | 8 | 0.915848 | -0.159718 | 422 | 419.07 | 0.993057 |
| GO:0048731\_system\_development | IGF1R | 1609 | 8 | 0.915848 | -0.159718 | 422 | 419.07 | 0.993057 |
| GO:0048731\_system\_development | SALL4 | 1609 | 8 | 0.915848 | -0.159718 | 422 | 419.07 | 0.993057 |
| GO:0048731\_system\_development | PTPRZ1 | 1609 | 8 | 0.915848 | -0.159718 | 422 | 419.07 | 0.993057 |
| GO:0048731\_system\_development | BMPR1B | 1609 | 8 | 0.915848 | -0.159718 | 422 | 419.07 | 0.993057 |
| GO:0048731\_system\_development | SIRT1 | 1609 | 8 | 0.915848 | -0.159718 | 422 | 419.07 | 0.993057 |
| GO:0048731\_system\_development | SOD2 | 1609 | 8 | 0.915848 | -0.159718 | 422 | 419.07 | 0.993057 |
| GO:0010604\_positive\_regulation\_of\_macromolecule\_metabolic\_process | SALL4 | 433 | 2 | 0.850808 | -0.157179 | 423 | 419.96 | 0.992813 |
| GO:0010604\_positive\_regulation\_of\_macromolecule\_metabolic\_process | FOXK1 | 433 | 2 | 0.850808 | -0.157179 | 423 | 419.96 | 0.992813 |
| GO:0009887\_organ\_morphogenesis | RECQL4 | 642 | 3 | 0.860748 | -0.156223 | 424 | 420.14 | 0.990896 |
| GO:0009887\_organ\_morphogenesis | IGF1R | 642 | 3 | 0.860748 | -0.156223 | 424 | 420.14 | 0.990896 |
| GO:0009887\_organ\_morphogenesis | BMPR1B | 642 | 3 | 0.860748 | -0.156223 | 424 | 420.14 | 0.990896 |
| GO:0006357\_regulation\_of\_transcription\_from\_RNA\_polymerase\_II\_promoter | SALL4 | 435 | 2 | 0.846897 | -0.155609 | 425 | 420.57 | 0.989576 |
| GO:0006357\_regulation\_of\_transcription\_from\_RNA\_polymerase\_II\_promoter | FOXK1 | 435 | 2 | 0.846897 | -0.155609 | 425 | 420.57 | 0.989576 |
| GO:0048583\_regulation\_of\_response\_to\_stimulus | UBE2N | 217 | 1 | 0.848848 | -0.153792 | 426 | 421.79 | 0.990117 |
| GO:0007423\_sensory\_organ\_development | BMPR1B | 219 | 1 | 0.841096 | -0.151700 | 427 | 422.88 | 0.990351 |
| GO:0001701\_in\_utero\_embryonic\_development | SALL4 | 221 | 1 | 0.833484 | -0.149641 | 428 | 423.79 | 0.990164 |
| GO:0006366\_transcription\_from\_RNA\_polymerase\_II\_promoter | SALL4 | 444 | 2 | 0.829730 | -0.148737 | 429 | 424.27 | 0.988974 |
| GO:0006366\_transcription\_from\_RNA\_polymerase\_II\_promoter | FOXK1 | 444 | 2 | 0.829730 | -0.148737 | 429 | 424.27 | 0.988974 |
| GO:0016070\_RNA\_metabolic\_process | TRP53BP1 | 658 | 3 | 0.839818 | -0.145883 | 430 | 426.02 | 0.990744 |
| GO:0016070\_RNA\_metabolic\_process | SALL4 | 658 | 3 | 0.839818 | -0.145883 | 430 | 426.02 | 0.990744 |
| GO:0016070\_RNA\_metabolic\_process | FOXK1 | 658 | 3 | 0.839818 | -0.145883 | 430 | 426.02 | 0.990744 |
| GO:0002682\_regulation\_of\_immune\_system\_process | UBE2N | 228 | 1 | 0.807895 | -0.142681 | 431 | 428.0 | 0.993039 |
| GO:0007167\_enzyme\_linked\_receptor\_protein\_signaling\_pathway | BMPR1B | 229 | 1 | 0.804367 | -0.141718 | 432 | 428.43 | 0.991736 |
| GO:0050890\_cognition | SOD2 | 233 | 1 | 0.790558 | -0.137936 | 433 | 430.09 | 0.993279 |
| GO:0006468\_protein\_amino\_acid\_phosphorylation | IGF1R | 237 | 1 | 0.777215 | -0.134269 | 435 | 431.79 | 0.992621 |
| GO:0044085\_cellular\_component\_biogenesis | HIST1H1C | 237 | 1 | 0.777215 | -0.134269 | 435 | 431.79 | 0.992621 |
| GO:0030097\_hemopoiesis | SOD2 | 253 | 1 | 0.728063 | -0.120655 | 436 | 437.19 | 1.002729 |
| GO:0048869\_cellular\_developmental\_process | PTPRZ1 | 1113 | 5 | 0.827493 | -0.120094 | 437 | 437.78 | 1.001785 |
| GO:0048869\_cellular\_developmental\_process | BMPR1B | 1113 | 5 | 0.827493 | -0.120094 | 437 | 437.78 | 1.001785 |
| GO:0048869\_cellular\_developmental\_process | RHOU | 1113 | 5 | 0.827493 | -0.120094 | 437 | 437.78 | 1.001785 |
| GO:0048869\_cellular\_developmental\_process | ZFP37 | 1113 | 5 | 0.827493 | -0.120094 | 437 | 437.78 | 1.001785 |
| GO:0048869\_cellular\_developmental\_process | SOD2 | 1113 | 5 | 0.827493 | -0.120094 | 437 | 437.78 | 1.001785 |
| GO:0048878\_chemical\_homeostasis | SOD2 | 254 | 1 | 0.725197 | -0.119857 | 438 | 438.19 | 1.000434 |
| GO:0048729\_tissue\_morphogenesis | IGF1R | 255 | 1 | 0.722353 | -0.119065 | 439 | 438.49 | 0.998838 |
| GO:0009966\_regulation\_of\_signal\_transduction | IGF1R | 256 | 1 | 0.719531 | -0.118278 | 440 | 438.83 | 0.997341 |
| GO:0002376\_immune\_system\_process | UBE2N | 505 | 2 | 0.729505 | -0.109453 | 441 | 442.05 | 1.002381 |
| GO:0002376\_immune\_system\_process | SOD2 | 505 | 2 | 0.729505 | -0.109453 | 441 | 442.05 | 1.002381 |
| GO:0045944\_positive\_regulation\_of\_transcription\_from\_RNA\_polymerase\_II\_promoter | SALL4 | 269 | 1 | 0.684758 | -0.108558 | 442 | 442.61 | 1.001380 |
| GO:0048534\_hemopoietic\_or\_lymphoid\_organ\_development | SOD2 | 277 | 1 | 0.664982 | -0.103014 | 443 | 445.73 | 1.006163 |
| GO:0007610\_behavior | SOD2 | 279 | 1 | 0.660215 | -0.101677 | 444 | 446.39 | 1.005383 |
| GO:0002520\_immune\_system\_development | SOD2 | 295 | 1 | 0.624407 | -0.091631 | 446 | 450.85 | 1.010874 |
| GO:0045595\_regulation\_of\_cell\_differentiation | SOD2 | 295 | 1 | 0.624407 | -0.091631 | 446 | 450.85 | 1.010874 |
| GO:0048598\_embryonic\_morphogenesis | SALL4 | 299 | 1 | 0.616054 | -0.089290 | 447 | 451.34 | 1.009709 |
| GO:0009790\_embryonic\_development | SALL4 | 567 | 2 | 0.649735 | -0.079948 | 448 | 455.65 | 1.017076 |
| GO:0009790\_embryonic\_development | ABI1 | 567 | 2 | 0.649735 | -0.079948 | 448 | 455.65 | 1.017076 |
| GO:0006928\_cell\_motion | BMPR1B | 330 | 1 | 0.558182 | -0.073169 | 451 | 457.3 | 1.013969 |
| GO:0010646\_regulation\_of\_cell\_communication | IGF1R | 330 | 1 | 0.558182 | -0.073169 | 451 | 457.3 | 1.013969 |
| GO:0051674\_localization\_of\_cell | BMPR1B | 330 | 1 | 0.558182 | -0.073169 | 451 | 457.3 | 1.013969 |
| GO:0051093\_negative\_regulation\_of\_developmental\_process | SOD2 | 331 | 1 | 0.556495 | -0.072703 | 452 | 458.12 | 1.013540 |
| GO:0009605\_response\_to\_external\_stimulus | SOD2 | 339 | 1 | 0.543363 | -0.069087 | 453 | 460.11 | 1.015695 |
| GO:0007166\_cell\_surface\_receptor\_linked\_signal\_transduction | UBE2N | 597 | 2 | 0.617085 | -0.068579 | 454 | 460.98 | 1.015374 |
| GO:0007166\_cell\_surface\_receptor\_linked\_signal\_transduction | BMPR1B | 597 | 2 | 0.617085 | -0.068579 | 454 | 460.98 | 1.015374 |
| GO:0030154\_cell\_differentiation | PTPRZ1 | 1060 | 4 | 0.695094 | -0.065045 | 455 | 462.31 | 1.016066 |
| GO:0030154\_cell\_differentiation | BMPR1B | 1060 | 4 | 0.695094 | -0.065045 | 455 | 462.31 | 1.016066 |
| GO:0030154\_cell\_differentiation | ZFP37 | 1060 | 4 | 0.695094 | -0.065045 | 455 | 462.31 | 1.016066 |
| GO:0030154\_cell\_differentiation | SOD2 | 1060 | 4 | 0.695094 | -0.065045 | 455 | 462.31 | 1.016066 |
| GO:0042981\_regulation\_of\_apoptosis | SOD2 | 360 | 1 | 0.511667 | -0.060458 | 456 | 463.68 | 1.016842 |
| GO:0010941\_regulation\_of\_cell\_death | SOD2 | 365 | 1 | 0.504658 | -0.058574 | 458 | 465.19 | 1.015699 |
| GO:0043067\_regulation\_of\_programmed\_cell\_death | SOD2 | 365 | 1 | 0.504658 | -0.058574 | 458 | 465.19 | 1.015699 |
| GO:0050896\_response\_to\_stimulus | UBE2N | 1107 | 4 | 0.665583 | -0.053087 | 459 | 467.48 | 1.018475 |
| GO:0050896\_response\_to\_stimulus | TRP53BP1 | 1107 | 4 | 0.665583 | -0.053087 | 459 | 467.48 | 1.018475 |
| GO:0050896\_response\_to\_stimulus | PDPK1 | 1107 | 4 | 0.665583 | -0.053087 | 459 | 467.48 | 1.018475 |
| GO:0050896\_response\_to\_stimulus | SOD2 | 1107 | 4 | 0.665583 | -0.053087 | 459 | 467.48 | 1.018475 |
| GO:0050877\_neurological\_system\_process | SOD2 | 390 | 1 | 0.472308 | -0.050018 | 460 | 469.25 | 1.020109 |
| GO:0042221\_response\_to\_chemical\_stimulus | SOD2 | 409 | 1 | 0.450367 | -0.044377 | 461 | 471.04 | 1.021779 |
| GO:0042592\_homeostatic\_process | SOD2 | 419 | 1 | 0.439618 | -0.041673 | 462 | 472.46 | 1.022641 |
| GO:0065008\_regulation\_of\_biological\_quality | RHOU | 693 | 2 | 0.531602 | -0.041640 | 463 | 472.59 | 1.020713 |
| GO:0065008\_regulation\_of\_biological\_quality | SOD2 | 693 | 2 | 0.531602 | -0.041640 | 463 | 472.59 | 1.020713 |
| GO:0032501\_multicellular\_organismal\_process | ATRX | 2183 | 9 | 0.759414 | -0.040065 | 464 | 473.16 | 1.019741 |
| GO:0032501\_multicellular\_organismal\_process | RECQL4 | 2183 | 9 | 0.759414 | -0.040065 | 464 | 473.16 | 1.019741 |
| GO:0032501\_multicellular\_organismal\_process | IGF1R | 2183 | 9 | 0.759414 | -0.040065 | 464 | 473.16 | 1.019741 |
| GO:0032501\_multicellular\_organismal\_process | SALL4 | 2183 | 9 | 0.759414 | -0.040065 | 464 | 473.16 | 1.019741 |
| GO:0032501\_multicellular\_organismal\_process | PTPRZ1 | 2183 | 9 | 0.759414 | -0.040065 | 464 | 473.16 | 1.019741 |
| GO:0032501\_multicellular\_organismal\_process | ABI1 | 2183 | 9 | 0.759414 | -0.040065 | 464 | 473.16 | 1.019741 |
| GO:0032501\_multicellular\_organismal\_process | BMPR1B | 2183 | 9 | 0.759414 | -0.040065 | 464 | 473.16 | 1.019741 |
| GO:0032501\_multicellular\_organismal\_process | SIRT1 | 2183 | 9 | 0.759414 | -0.040065 | 464 | 473.16 | 1.019741 |
| GO:0032501\_multicellular\_organismal\_process | SOD2 | 2183 | 9 | 0.759414 | -0.040065 | 464 | 473.16 | 1.019741 |
| GO:0006915\_apoptosis | SOD2 | 427 | 1 | 0.431382 | -0.039629 | 465 | 473.59 | 1.018473 |
| GO:0050793\_regulation\_of\_developmental\_process | RHOU | 703 | 2 | 0.524040 | -0.039499 | 466 | 473.76 | 1.016652 |
| GO:0050793\_regulation\_of\_developmental\_process | SOD2 | 703 | 2 | 0.524040 | -0.039499 | 466 | 473.76 | 1.016652 |
| GO:0012501\_programmed\_cell\_death | SOD2 | 433 | 1 | 0.425404 | -0.038163 | 467 | 474.56 | 1.016188 |
| GO:0008219\_cell\_death | SOD2 | 444 | 1 | 0.414865 | -0.035616 | 468 | 476.34 | 1.017821 |
| GO:0010926\_anatomical\_structure\_formation | HIST1H1C | 447 | 1 | 0.412081 | -0.034951 | 469 | 476.53 | 1.016055 |
| GO:0016265\_death | SOD2 | 450 | 1 | 0.409333 | -0.034299 | 470 | 477.11 | 1.015128 |
| GO:0003008\_system\_process | SOD2 | 516 | 1 | 0.356977 | -0.022655 | 471 | 479.49 | 1.018025 |
| GO:0006810\_transport | SLC4A1 | 718 | 1 | 0.256546 | -0.006241 | 472 | 484.9 | 1.027331 |
| GO:0051234\_establishment\_of\_localization | SLC4A1 | 729 | 1 | 0.252675 | -0.005810 | 473 | 484.97 | 1.025307 |
| GO:0051179\_localization | SLC4A1 | 1058 | 2 | 0.348204 | -0.005341 | 474 | 484.99 | 1.023186 |
| GO:0051179\_localization | BMPR1B | 1058 | 2 | 0.348204 | -0.005341 | 474 | 484.99 | 1.023186 |
| GO:0008150\_biological\_process | FOXK1 | 4605 | 25 | 1.000000 | 0.000000 | 1370 | 1380.55 | 1.007701 |
| GO:0008150\_biological\_process | ABI1 | 4605 | 25 | 1.000000 | 0.000000 | 1370 | 1380.55 | 1.007701 |
| GO:0008150\_biological\_process | RHOU | 4605 | 25 | 1.000000 | 0.000000 | 1370 | 1380.55 | 1.007701 |
| GO:0008150\_biological\_process | PCOLCE | 4605 | 25 | 1.000000 | 0.000000 | 1370 | 1380.55 | 1.007701 |
| GO:0008150\_biological\_process | PIN1 | 4605 | 25 | 1.000000 | 0.000000 | 1370 | 1380.55 | 1.007701 |
| GO:0008150\_biological\_process | IGF1R | 4605 | 25 | 1.000000 | 0.000000 | 1370 | 1380.55 | 1.007701 |
| GO:0008150\_biological\_process | TRP53BP1 | 4605 | 25 | 1.000000 | 0.000000 | 1370 | 1380.55 | 1.007701 |
| GO:0008150\_biological\_process | PDPK1 | 4605 | 25 | 1.000000 | 0.000000 | 1370 | 1380.55 | 1.007701 |
| GO:0008150\_biological\_process | STT3A | 4605 | 25 | 1.000000 | 0.000000 | 1370 | 1380.55 | 1.007701 |
| GO:0008150\_biological\_process | DUSP16 | 4605 | 25 | 1.000000 | 0.000000 | 1370 | 1380.55 | 1.007701 |
| GO:0008150\_biological\_process | SLC4A1 | 4605 | 25 | 1.000000 | 0.000000 | 1370 | 1380.55 | 1.007701 |
| GO:0008150\_biological\_process | RECQL4 | 4605 | 25 | 1.000000 | 0.000000 | 1370 | 1380.55 | 1.007701 |
| GO:0008150\_biological\_process | HIST1H1C | 4605 | 25 | 1.000000 | 0.000000 | 1370 | 1380.55 | 1.007701 |
| GO:0008150\_biological\_process | PTPRZ1 | 4605 | 25 | 1.000000 | 0.000000 | 1370 | 1380.55 | 1.007701 |
| GO:0008150\_biological\_process | CTSS | 4605 | 25 | 1.000000 | 0.000000 | 1370 | 1380.55 | 1.007701 |
| GO:0008150\_biological\_process | SIRT1 | 4605 | 25 | 1.000000 | 0.000000 | 1370 | 1380.55 | 1.007701 |
| GO:0008150\_biological\_process | GLCE | 4605 | 25 | 1.000000 | 0.000000 | 1370 | 1380.55 | 1.007701 |
| GO:0008150\_biological\_process | SOD2 | 4605 | 25 | 1.000000 | 0.000000 | 1370 | 1380.55 | 1.007701 |
| GO:0008150\_biological\_process | ZFP37 | 4605 | 25 | 1.000000 | 0.000000 | 1370 | 1380.55 | 1.007701 |
| GO:0008150\_biological\_process | UBE2N | 4605 | 25 | 1.000000 | 0.000000 | 1370 | 1380.55 | 1.007701 |
| GO:0008150\_biological\_process | ATRX | 4605 | 25 | 1.000000 | 0.000000 | 1370 | 1380.55 | 1.007701 |
| GO:0008150\_biological\_process | NRAS | 4605 | 25 | 1.000000 | 0.000000 | 1370 | 1380.55 | 1.007701 |
| GO:0008150\_biological\_process | SALL4 | 4605 | 25 | 1.000000 | 0.000000 | 1370 | 1380.55 | 1.007701 |
| GO:0008150\_biological\_process | BMPR1B | 4605 | 25 | 1.000000 | 0.000000 | 1370 | 1380.55 | 1.007701 |
| GO:0008150\_biological\_process | HDAC6 | 4605 | 25 | 1.000000 | 0.000000 | 1370 | 1380.55 | 1.007701 |
