# Supplementary material for: RedundancyMiner: De-replication of redundant GO categories in microarray and proteomics analysis
Source: BMC Bioinformatics. 2011 Feb 10;12:52. doi: 10.1186/1471-2105-12-52 (PMC3223614; doi:10.1186/1471-2105-12-52)
Supplement: Additional file 8 — Retinal development HTGM download. compressed package of the results of running HTGM on the retinal development genes list. [file 1471-2105-12-52-S8.ZIP › SCENARIO_2_MODIFIED/total.txt.total.txt.dir/Exp1_BestClusterMap_LEIGS_KM_24.csv.join.20.txt.dir/Exp1_BestClusterMap_LEIGS_KM_24.csv.join.20.txt.change.html]

Category Summary Report for Exp1\_BestClusterMap\_LEIGS\_KM\_24.csv.join.20.txt

# Category Summary Report for Exp1\_BestClusterMap\_LEIGS\_KM\_24.csv.join.20.txt

| HYPERLINKED GO CATEGORY | TOTAL GENES | CHANGED GENES | ENRICHMENT | LOG10(p) | CUMULATIVE NUMBER OF CATEGORIES | CUMULATIVE RANDOMS LOWER BOUND | CUMULATIVE RANDOMS MEAN | CUMULATIVE RANDOMS UPPER BOUND | FALSE DISCOVERY RATE |
| --- | --- | --- | --- | --- | --- | --- | --- | --- | --- |
| GO:0006508\_proteolysis | 76 | 4 | 9.694737 | -3.176313 | 1 | -1.210667 | 0.66 | 2.530667 | 0.660000 |
| GO:0006476\_protein\_amino\_acid\_deacetylation | 9 | 2 | 40.933333 | -3.002047 | 2 | -1.540777 | 1.06 | 3.660777 | 0.530000 |
| GO:0030163\_protein\_catabolic\_process | 101 | 4 | 7.295050 | -2.713343 | 3 | -1.632492 | 1.98 | 5.592492 | 0.660000 |
| GO:0010639\_negative\_regulation\_of\_organelle\_organization | 19 | 2 | 19.389474 | -2.339802 | 4 | -1.566569 | 3.79 | 9.146569 | 0.947500 |
| GO:0043285\_biopolymer\_catabolic\_process | 129 | 4 | 5.711628 | -2.327118 | 5 | -1.564940 | 3.82 | 9.204940 | 0.764000 |
| GO:0003032\_detection\_of\_oxygen | 1 | 1 |  |  |  |  |  |  |  |  |
| GO:0003068\_regulation\_of\_systemic\_arterial\_blood\_pressure\_by\_acetylcholine | 1 | 1 |  |  |  |  |  |  |  |  |
| GO:0003069\_vasodilation\_by\_acetylcholine\_involved\_in\_regulation\_of\_systemic\_arterial\_blood\_pressure | 1 | 1 |  |  |  |  |  |  |  |  |
| GO:0003070\_regulation\_of\_systemic\_arterial\_blood\_pressure\_by\_neurotransmitter | 1 | 1 |  |  |  |  |  |  |  |  |
| GO:0007063\_regulation\_of\_sister\_chromatid\_cohesion | 1 | 1 |  |  |  |  |  |  |  |  |
| GO:0010260\_organ\_senescence | 1 | 1 |  |  |  |  |  |  |  |  |
| GO:0030210\_heparin\_biosynthetic\_process | 1 | 1 |  |  |  |  |  |  |  |  |
| GO:0045875\_negative\_regulation\_of\_sister\_chromatid\_cohesion | 1 | 1 |  |  |  |  |  |  |  |  |
| GO:0048773\_erythrophore\_differentiation | 1 | 1 |  |  |  |  |  |  |  |  |
| GO:0055093\_response\_to\_hyperoxia | 1 | 1 |  |  |  |  |  |  |  |  |
| GO:0009057\_macromolecule\_catabolic\_process | 137 | 4 | 5.378102 | -2.234127 | 6 | -1.464920 | 4.36 | 10.184920 | 0.726667 |
| GO:0001306\_age-dependent\_response\_to\_oxidative\_stress | 2 | 1 |  |  |  |  |  |  |  |  |
| GO:0007571\_age-dependent\_general\_metabolic\_decline | 2 | 1 |  |  |  |  |  |  |  |  |
| GO:0010149\_senescence | 2 | 1 |  |  |  |  |  |  |  |  |
| GO:0016584\_nucleosome\_positioning | 2 | 1 |  |  |  |  |  |  |  |  |
| GO:0030202\_heparin\_metabolic\_process | 2 | 1 |  |  |  |  |  |  |  |  |
| GO:0000303\_response\_to\_superoxide | 3 | 1 |  |  |  |  |  |  |  |  |
| GO:0018196\_peptidyl-asparagine\_modification | 3 | 1 |  |  |  |  |  |  |  |  |
| GO:0018279\_protein\_amino\_acid\_N-linked\_glycosylation\_via\_asparagine | 3 | 1 |  |  |  |  |  |  |  |  |
| GO:0051983\_regulation\_of\_chromosome\_segregation | 3 | 1 |  |  |  |  |  |  |  |  |
| GO:0006511\_ubiquitin-dependent\_protein\_catabolic\_process | 39 | 2 | 9.446154 | -1.731760 | 7 | 0.576615 | 10.68 | 20.783385 | 1.525714 |
| GO:0051129\_negative\_regulation\_of\_cellular\_component\_organization | 40 | 2 | 9.210000 | -1.710919 | 8 | 0.579707 | 11.08 | 21.580293 | 1.385000 |
| GO:0019941\_modification-dependent\_protein\_catabolic\_process | 42 | 2 | 8.771429 | -1.670879 | 11 | 1.204911 | 12.14 | 23.075089 | 1.103636 |
| GO:0043632\_modification-dependent\_macromolecule\_catabolic\_process | 42 | 2 | 8.771429 | -1.670879 | 11 | 1.204911 | 12.14 | 23.075089 | 1.103636 |
| GO:0051603\_proteolysis\_involved\_in\_cellular\_protein\_catabolic\_process | 42 | 2 | 8.771429 | -1.670879 | 11 | 1.204911 | 12.14 | 23.075089 | 1.103636 |
| GO:0000305\_response\_to\_oxygen\_radical | 4 | 1 |  |  |  |  |  |  |  |  |
| GO:0006972\_hyperosmotic\_response | 4 | 1 |  |  |  |  |  |  |  |  |
| GO:0009593\_detection\_of\_chemical\_stimulus | 4 | 1 |  |  |  |  |  |  |  |  |
| GO:0051897\_positive\_regulation\_of\_protein\_kinase\_B\_signaling\_cascade | 4 | 1 |  |  |  |  |  |  |  |  |
| GO:0044257\_cellular\_protein\_catabolic\_process | 44 | 2 | 8.372727 | -1.632858 | 12 | 1.773650 | 13.24 | 24.706350 | 1.103333 |
| GO:0006023\_aminoglycan\_biosynthetic\_process | 5 | 1 | 36.840000 | -1.570848 | 17 | 6.739120 | 21.68 | 36.620880 | 1.275294 |
| GO:0006024\_glycosaminoglycan\_biosynthetic\_process | 5 | 1 | 36.840000 | -1.570848 | 17 | 6.739120 | 21.68 | 36.620880 | 1.275294 |
| GO:0019430\_removal\_of\_superoxide\_radicals | 5 | 1 | 36.840000 | -1.570848 | 17 | 6.739120 | 21.68 | 36.620880 | 1.275294 |
| GO:0042554\_superoxide\_anion\_generation | 5 | 1 | 36.840000 | -1.570848 | 17 | 6.739120 | 21.68 | 36.620880 | 1.275294 |
| GO:0051898\_negative\_regulation\_of\_protein\_kinase\_B\_signaling\_cascade | 5 | 1 | 36.840000 | -1.570848 | 17 | 6.739120 | 21.68 | 36.620880 | 1.275294 |
| GO:0009101\_glycoprotein\_biosynthetic\_process | 48 | 2 | 7.675000 | -1.562169 | 18 | 6.802960 | 22.05 | 37.297040 | 1.225000 |
| GO:0043473\_pigmentation | 49 | 2 | 7.518367 | -1.545502 | 19 | 7.066560 | 22.46 | 37.853440 | 1.182105 |
| GO:0006996\_organelle\_organization | 449 | 6 | 2.461470 | -1.529323 | 20 | 7.224396 | 22.71 | 38.195604 | 1.135500 |
| GO:0051276\_chromosome\_organization | 129 | 3 | 4.283721 | -1.501631 | 21 | 7.587379 | 23.6 | 39.612621 | 1.123810 |
| GO:0014823\_response\_to\_activity | 6 | 1 | 30.700000 | -1.492797 | 25 | 12.089772 | 29.64 | 47.190228 | 1.185600 |
| GO:0015012\_heparan\_sulfate\_proteoglycan\_biosynthetic\_process | 6 | 1 | 30.700000 | -1.492797 | 25 | 12.089772 | 29.64 | 47.190228 | 1.185600 |
| GO:0048147\_negative\_regulation\_of\_fibroblast\_proliferation | 6 | 1 | 30.700000 | -1.492797 | 25 | 12.089772 | 29.64 | 47.190228 | 1.185600 |
| GO:0051881\_regulation\_of\_mitochondrial\_membrane\_potential | 6 | 1 | 30.700000 | -1.492797 | 25 | 12.089772 | 29.64 | 47.190228 | 1.185600 |
| GO:0007265\_Ras\_protein\_signal\_transduction | 54 | 2 | 6.822222 | -1.467420 | 26 | 12.543745 | 30.87 | 49.196255 | 1.187308 |
| GO:0006790\_sulfur\_metabolic\_process | 56 | 2 | 6.578571 | -1.438396 | 27 | 12.998133 | 31.57 | 50.141867 | 1.169259 |
| GO:0000188\_inactivation\_of\_MAPK\_activity | 7 | 1 | 26.314286 | -1.426980 | 39 | 17.743956 | 38.09 | 58.436044 | 0.976667 |
| GO:0003085\_negative\_regulation\_of\_systemic\_arterial\_blood\_pressure | 7 | 1 | 26.314286 | -1.426980 | 39 | 17.743956 | 38.09 | 58.436044 | 0.976667 |
| GO:0007019\_microtubule\_depolymerization | 7 | 1 | 26.314286 | -1.426980 | 39 | 17.743956 | 38.09 | 58.436044 | 0.976667 |
| GO:0007026\_negative\_regulation\_of\_microtubule\_depolymerization | 7 | 1 | 26.314286 | -1.426980 | 39 | 17.743956 | 38.09 | 58.436044 | 0.976667 |
| GO:0007062\_sister\_chromatid\_cohesion | 7 | 1 | 26.314286 | -1.426980 | 39 | 17.743956 | 38.09 | 58.436044 | 0.976667 |
| GO:0010948\_negative\_regulation\_of\_cell\_cycle\_process | 7 | 1 | 26.314286 | -1.426980 | 39 | 17.743956 | 38.09 | 58.436044 | 0.976667 |
| GO:0016575\_histone\_deacetylation | 7 | 1 | 26.314286 | -1.426980 | 39 | 17.743956 | 38.09 | 58.436044 | 0.976667 |
| GO:0030201\_heparan\_sulfate\_proteoglycan\_metabolic\_process | 7 | 1 | 26.314286 | -1.426980 | 39 | 17.743956 | 38.09 | 58.436044 | 0.976667 |
| GO:0031017\_exocrine\_pancreas\_development | 7 | 1 | 26.314286 | -1.426980 | 39 | 17.743956 | 38.09 | 58.436044 | 0.976667 |
| GO:0031114\_regulation\_of\_microtubule\_depolymerization | 7 | 1 | 26.314286 | -1.426980 | 39 | 17.743956 | 38.09 | 58.436044 | 0.976667 |
| GO:0031497\_chromatin\_assembly | 7 | 1 | 26.314286 | -1.426980 | 39 | 17.743956 | 38.09 | 58.436044 | 0.976667 |
| GO:0045599\_negative\_regulation\_of\_fat\_cell\_differentiation | 7 | 1 | 26.314286 | -1.426980 | 39 | 17.743956 | 38.09 | 58.436044 | 0.976667 |
| GO:0033043\_regulation\_of\_organelle\_organization | 58 | 2 | 6.351724 | -1.410499 | 40 | 18.294350 | 38.96 | 59.625650 | 0.974000 |
| GO:0009056\_catabolic\_process | 243 | 4 | 3.032099 | -1.398814 | 41 | 18.396243 | 39.14 | 59.883757 | 0.954634 |
| GO:0031111\_negative\_regulation\_of\_microtubule\_polymerization\_or\_depolymerization | 8 | 1 | 23.025000 | -1.370116 | 44 | 22.591442 | 45.04 | 67.488558 | 1.023636 |
| GO:0034728\_nucleosome\_organization | 8 | 1 | 23.025000 | -1.370116 | 44 | 22.591442 | 45.04 | 67.488558 | 1.023636 |
| GO:0045429\_positive\_regulation\_of\_nitric\_oxide\_biosynthetic\_process | 8 | 1 | 23.025000 | -1.370116 | 44 | 22.591442 | 45.04 | 67.488558 | 1.023636 |
| GO:0001542\_ovulation\_from\_ovarian\_follicle | 9 | 1 | 20.466667 | -1.320091 | 51 | 27.370350 | 52.48 | 77.589650 | 1.029020 |
| GO:0016601\_Rac\_protein\_signal\_transduction | 9 | 1 | 20.466667 | -1.320091 | 51 | 27.370350 | 52.48 | 77.589650 | 1.029020 |
| GO:0030728\_ovulation | 9 | 1 | 20.466667 | -1.320091 | 51 | 27.370350 | 52.48 | 77.589650 | 1.029020 |
| GO:0043242\_negative\_regulation\_of\_protein\_complex\_disassembly | 9 | 1 | 20.466667 | -1.320091 | 51 | 27.370350 | 52.48 | 77.589650 | 1.029020 |
| GO:0043409\_negative\_regulation\_of\_MAPKKK\_cascade | 9 | 1 | 20.466667 | -1.320091 | 51 | 27.370350 | 52.48 | 77.589650 | 1.029020 |
| GO:0045428\_regulation\_of\_nitric\_oxide\_biosynthetic\_process | 9 | 1 | 20.466667 | -1.320091 | 51 | 27.370350 | 52.48 | 77.589650 | 1.029020 |
| GO:0051896\_regulation\_of\_protein\_kinase\_B\_signaling\_cascade | 9 | 1 | 20.466667 | -1.320091 | 51 | 27.370350 | 52.48 | 77.589650 | 1.029020 |
| GO:0034962\_cellular\_biopolymer\_catabolic\_process | 68 | 2 | 5.417647 | -1.285471 | 52 | 27.798760 | 53.61 | 79.421240 | 1.030962 |
| GO:0000209\_protein\_polyubiquitination | 10 | 1 | 18.420000 | -1.275461 | 57 | 32.585766 | 60.08 | 87.574234 | 1.054035 |
| GO:0006801\_superoxide\_metabolic\_process | 10 | 1 | 18.420000 | -1.275461 | 57 | 32.585766 | 60.08 | 87.574234 | 1.054035 |
| GO:0022900\_electron\_transport\_chain | 10 | 1 | 18.420000 | -1.275461 | 57 | 32.585766 | 60.08 | 87.574234 | 1.054035 |
| GO:0022904\_respiratory\_electron\_transport\_chain | 10 | 1 | 18.420000 | -1.275461 | 57 | 32.585766 | 60.08 | 87.574234 | 1.054035 |
| GO:0045776\_negative\_regulation\_of\_blood\_pressure | 10 | 1 | 18.420000 | -1.275461 | 57 | 32.585766 | 60.08 | 87.574234 | 1.054035 |
| GO:0051128\_regulation\_of\_cellular\_component\_organization | 160 | 3 | 3.453750 | -1.266794 | 58 | 32.642838 | 60.43 | 88.217162 | 1.041897 |
| GO:0019538\_protein\_metabolic\_process | 655 | 7 | 1.968550 | -1.265801 | 59 | 32.639371 | 60.48 | 88.320629 | 1.025085 |
| GO:0006281\_DNA\_repair | 71 | 2 | 5.188732 | -1.251966 | 61 | 33.082270 | 61.18 | 89.277730 | 1.002951 |
| GO:0009100\_glycoprotein\_metabolic\_process | 71 | 2 | 5.188732 | -1.251966 | 61 | 33.082270 | 61.18 | 89.277730 | 1.002951 |
| GO:0007264\_small\_GTPase\_mediated\_signal\_transduction | 72 | 2 | 5.116667 | -1.241153 | 62 | 33.603546 | 62.21 | 90.816454 | 1.003387 |
| GO:0000271\_polysaccharide\_biosynthetic\_process | 11 | 1 | 16.745455 | -1.235194 | 70 | 37.178393 | 67.04 | 96.901607 | 0.957714 |
| GO:0006333\_chromatin\_assembly\_or\_disassembly | 11 | 1 | 16.745455 | -1.235194 | 70 | 37.178393 | 67.04 | 96.901607 | 0.957714 |
| GO:0030238\_male\_sex\_determination | 11 | 1 | 16.745455 | -1.235194 | 70 | 37.178393 | 67.04 | 96.901607 | 0.957714 |
| GO:0031110\_regulation\_of\_microtubule\_polymerization\_or\_depolymerization | 11 | 1 | 16.745455 | -1.235194 | 70 | 37.178393 | 67.04 | 96.901607 | 0.957714 |
| GO:0042542\_response\_to\_hydrogen\_peroxide | 11 | 1 | 16.745455 | -1.235194 | 70 | 37.178393 | 67.04 | 96.901607 | 0.957714 |
| GO:0048678\_response\_to\_axon\_injury | 11 | 1 | 16.745455 | -1.235194 | 70 | 37.178393 | 67.04 | 96.901607 | 0.957714 |
| GO:0051092\_positive\_regulation\_of\_NF-kappaB\_transcription\_factor\_activity | 11 | 1 | 16.745455 | -1.235194 | 70 | 37.178393 | 67.04 | 96.901607 | 0.957714 |
| GO:0051494\_negative\_regulation\_of\_cytoskeleton\_organization | 11 | 1 | 16.745455 | -1.235194 | 70 | 37.178393 | 67.04 | 96.901607 | 0.957714 |
| GO:0044265\_cellular\_macromolecule\_catabolic\_process | 75 | 2 | 4.912000 | -1.209714 | 71 | 37.799386 | 68.25 | 98.700614 | 0.961268 |
| GO:0034621\_cellular\_macromolecular\_complex\_subunit\_organization | 76 | 2 | 4.847368 | -1.199552 | 72 | 37.941239 | 68.6 | 99.258761 | 0.952778 |
| GO:0030166\_proteoglycan\_biosynthetic\_process | 12 | 1 | 15.350000 | -1.198531 | 77 | 41.556468 | 73.2 | 104.843532 | 0.950649 |
| GO:0031109\_microtubule\_polymerization\_or\_depolymerization | 12 | 1 | 15.350000 | -1.198531 | 77 | 41.556468 | 73.2 | 104.843532 | 0.950649 |
| GO:0043624\_cellular\_protein\_complex\_disassembly | 12 | 1 | 15.350000 | -1.198531 | 77 | 41.556468 | 73.2 | 104.843532 | 0.950649 |
| GO:0051261\_protein\_depolymerization | 12 | 1 | 15.350000 | -1.198531 | 77 | 41.556468 | 73.2 | 104.843532 | 0.950649 |
| GO:0055114\_oxidation\_reduction | 12 | 1 | 15.350000 | -1.198531 | 77 | 41.556468 | 73.2 | 104.843532 | 0.950649 |
| GO:0001836\_release\_of\_cytochrome\_c\_from\_mitochondria | 13 | 1 | 14.169231 | -1.164893 | 82 | 45.628577 | 79.31 | 112.991423 | 0.967195 |
| GO:0001976\_neurological\_system\_process\_involved\_in\_regulation\_of\_systemic\_arterial\_blood\_pressure | 13 | 1 | 14.169231 | -1.164893 | 82 | 45.628577 | 79.31 | 112.991423 | 0.967195 |
| GO:0031290\_retinal\_ganglion\_cell\_axon\_guidance | 13 | 1 | 14.169231 | -1.164893 | 82 | 45.628577 | 79.31 | 112.991423 | 0.967195 |
| GO:0043241\_protein\_complex\_disassembly | 13 | 1 | 14.169231 | -1.164893 | 82 | 45.628577 | 79.31 | 112.991423 | 0.967195 |
| GO:0043244\_regulation\_of\_protein\_complex\_disassembly | 13 | 1 | 14.169231 | -1.164893 | 82 | 45.628577 | 79.31 | 112.991423 | 0.967195 |
| GO:0001502\_cartilage\_condensation | 14 | 1 | 13.157143 | -1.133832 | 92 | 49.401568 | 84.73 | 120.058432 | 0.920978 |
| GO:0006809\_nitric\_oxide\_biosynthetic\_process | 14 | 1 | 13.157143 | -1.133832 | 92 | 49.401568 | 84.73 | 120.058432 | 0.920978 |
| GO:0006970\_response\_to\_osmotic\_stress | 14 | 1 | 13.157143 | -1.133832 | 92 | 49.401568 | 84.73 | 120.058432 | 0.920978 |
| GO:0007530\_sex\_determination | 14 | 1 | 13.157143 | -1.133832 | 92 | 49.401568 | 84.73 | 120.058432 | 0.920978 |
| GO:0010332\_response\_to\_gamma\_radiation | 14 | 1 | 13.157143 | -1.133832 | 92 | 49.401568 | 84.73 | 120.058432 | 0.920978 |
| GO:0033044\_regulation\_of\_chromosome\_organization | 14 | 1 | 13.157143 | -1.133832 | 92 | 49.401568 | 84.73 | 120.058432 | 0.920978 |
| GO:0034623\_cellular\_macromolecular\_complex\_disassembly | 14 | 1 | 13.157143 | -1.133832 | 92 | 49.401568 | 84.73 | 120.058432 | 0.920978 |
| GO:0043491\_protein\_kinase\_B\_signaling\_cascade | 14 | 1 | 13.157143 | -1.133832 | 92 | 49.401568 | 84.73 | 120.058432 | 0.920978 |
| GO:0045598\_regulation\_of\_fat\_cell\_differentiation | 14 | 1 | 13.157143 | -1.133832 | 92 | 49.401568 | 84.73 | 120.058432 | 0.920978 |
| GO:0046209\_nitric\_oxide\_metabolic\_process | 14 | 1 | 13.157143 | -1.133832 | 92 | 49.401568 | 84.73 | 120.058432 | 0.920978 |
| GO:0006325\_chromatin\_organization | 83 | 2 | 4.438554 | -1.132463 | 93 | 49.771803 | 85.32 | 120.868197 | 0.917419 |
| GO:0043283\_biopolymer\_metabolic\_process | 1490 | 12 | 1.483490 | -1.126677 | 94 | 49.841250 | 85.46 | 121.078750 | 0.909149 |
| GO:0006487\_protein\_amino\_acid\_N-linked\_glycosylation | 15 | 1 | 12.280000 | -1.104991 | 99 | 54.102531 | 90.91 | 127.717469 | 0.918283 |
| GO:0006749\_glutathione\_metabolic\_process | 15 | 1 | 12.280000 | -1.104991 | 99 | 54.102531 | 90.91 | 127.717469 | 0.918283 |
| GO:0048144\_fibroblast\_proliferation | 15 | 1 | 12.280000 | -1.104991 | 99 | 54.102531 | 90.91 | 127.717469 | 0.918283 |
| GO:0048145\_regulation\_of\_fibroblast\_proliferation | 15 | 1 | 12.280000 | -1.104991 | 99 | 54.102531 | 90.91 | 127.717469 | 0.918283 |
| GO:0070507\_regulation\_of\_microtubule\_cytoskeleton\_organization | 15 | 1 | 12.280000 | -1.104991 | 99 | 54.102531 | 90.91 | 127.717469 | 0.918283 |
| GO:0000302\_response\_to\_reactive\_oxygen\_species | 16 | 1 | 11.512500 | -1.078084 | 103 | 57.667590 | 95.95 | 134.232410 | 0.931553 |
| GO:0003044\_regulation\_of\_systemic\_arterial\_blood\_pressure\_mediated\_by\_a\_chemical\_signal | 16 | 1 | 11.512500 | -1.078084 | 103 | 57.667590 | 95.95 | 134.232410 | 0.931553 |
| GO:0006821\_chloride\_transport | 16 | 1 | 11.512500 | -1.078084 | 103 | 57.667590 | 95.95 | 134.232410 | 0.931553 |
| GO:0042311\_vasodilation | 16 | 1 | 11.512500 | -1.078084 | 103 | 57.667590 | 95.95 | 134.232410 | 0.931553 |
| GO:0006323\_DNA\_packaging | 17 | 1 | 10.835294 | -1.052876 | 110 | 61.108619 | 100.79 | 140.471381 | 0.916273 |
| GO:0010741\_negative\_regulation\_of\_protein\_kinase\_cascade | 17 | 1 | 10.835294 | -1.052876 | 110 | 61.108619 | 100.79 | 140.471381 | 0.916273 |
| GO:0043407\_negative\_regulation\_of\_MAP\_kinase\_activity | 17 | 1 | 10.835294 | -1.052876 | 110 | 61.108619 | 100.79 | 140.471381 | 0.916273 |
| GO:0045333\_cellular\_respiration | 17 | 1 | 10.835294 | -1.052876 | 110 | 61.108619 | 100.79 | 140.471381 | 0.916273 |
| GO:0051091\_positive\_regulation\_of\_transcription\_factor\_activity | 17 | 1 | 10.835294 | -1.052876 | 110 | 61.108619 | 100.79 | 140.471381 | 0.916273 |
| GO:0055072\_iron\_ion\_homeostasis | 17 | 1 | 10.835294 | -1.052876 | 110 | 61.108619 | 100.79 | 140.471381 | 0.916273 |
| GO:0090047\_positive\_regulation\_of\_transcription\_regulator\_activity | 17 | 1 | 10.835294 | -1.052876 | 110 | 61.108619 | 100.79 | 140.471381 | 0.916273 |
| GO:0034984\_cellular\_response\_to\_DNA\_damage\_stimulus | 94 | 2 | 3.919149 | -1.039301 | 111 | 61.653293 | 101.92 | 142.186707 | 0.918198 |
| GO:0006022\_aminoglycan\_metabolic\_process | 18 | 1 | 10.233333 | -1.029173 | 115 | 64.518442 | 106.52 | 148.521558 | 0.926261 |
| GO:0030203\_glycosaminoglycan\_metabolic\_process | 18 | 1 | 10.233333 | -1.029173 | 115 | 64.518442 | 106.52 | 148.521558 | 0.926261 |
| GO:0032984\_macromolecular\_complex\_disassembly | 18 | 1 | 10.233333 | -1.029173 | 115 | 64.518442 | 106.52 | 148.521558 | 0.926261 |
| GO:0044272\_sulfur\_compound\_biosynthetic\_process | 18 | 1 | 10.233333 | -1.029173 | 115 | 64.518442 | 106.52 | 148.521558 | 0.926261 |
| GO:0050931\_pigment\_cell\_differentiation | 19 | 1 | 9.694737 | -1.006811 | 116 | 67.752456 | 110.95 | 154.147544 | 0.956466 |
| GO:0010605\_negative\_regulation\_of\_macromolecule\_metabolic\_process | 331 | 4 | 2.225982 | -0.998687 | 117 | 68.181708 | 111.91 | 155.638292 | 0.956496 |
| GO:0031324\_negative\_regulation\_of\_cellular\_metabolic\_process | 332 | 4 | 2.219277 | -0.995007 | 118 | 68.318510 | 112.15 | 155.981490 | 0.950424 |
| GO:0006518\_peptide\_metabolic\_process | 20 | 1 | 9.210000 | -0.985653 | 120 | 70.662633 | 115.32 | 159.977367 | 0.961000 |
| GO:0008360\_regulation\_of\_cell\_shape | 20 | 1 | 9.210000 | -0.985653 | 120 | 70.662633 | 115.32 | 159.977367 | 0.961000 |
| GO:0008637\_apoptotic\_mitochondrial\_changes | 21 | 1 | 8.771429 | -0.965582 | 121 | 73.964221 | 120.0 | 166.035779 | 0.991736 |
| GO:0043170\_macromolecule\_metabolic\_process | 1576 | 12 | 1.402538 | -0.965487 | 122 | 73.997407 | 120.09 | 166.182593 | 0.984344 |
| GO:0048523\_negative\_regulation\_of\_cellular\_process | 774 | 7 | 1.665891 | -0.948841 | 123 | 74.451377 | 121.19 | 167.928623 | 0.985285 |
| GO:0006029\_proteoglycan\_metabolic\_process | 22 | 1 | 8.372727 | -0.946495 | 126 | 78.027692 | 126.24 | 174.452308 | 1.001905 |
| GO:0007059\_chromosome\_segregation | 22 | 1 | 8.372727 | -0.946495 | 126 | 78.027692 | 126.24 | 174.452308 | 1.001905 |
| GO:0032886\_regulation\_of\_microtubule-based\_process | 22 | 1 | 8.372727 | -0.946495 | 126 | 78.027692 | 126.24 | 174.452308 | 1.001905 |
| GO:0009892\_negative\_regulation\_of\_metabolic\_process | 348 | 4 | 2.117241 | -0.938203 | 127 | 78.365651 | 126.88 | 175.394349 | 0.999055 |
| GO:0000082\_G1\_S\_transition\_of\_mitotic\_cell\_cycle | 23 | 1 | 8.008696 | -0.928306 | 131 | 80.365375 | 130.0 | 179.634625 | 0.992366 |
| GO:0003073\_regulation\_of\_systemic\_arterial\_blood\_pressure | 23 | 1 | 8.008696 | -0.928306 | 131 | 80.365375 | 130.0 | 179.634625 | 0.992366 |
| GO:0015698\_inorganic\_anion\_transport | 23 | 1 | 8.008696 | -0.928306 | 131 | 80.365375 | 130.0 | 179.634625 | 0.992366 |
| GO:0043388\_positive\_regulation\_of\_DNA\_binding | 23 | 1 | 8.008696 | -0.928306 | 131 | 80.365375 | 130.0 | 179.634625 | 0.992366 |
| GO:0048705\_skeletal\_system\_morphogenesis | 111 | 2 | 3.318919 | -0.918135 | 132 | 80.716589 | 130.51 | 180.303411 | 0.988712 |
| GO:0043410\_positive\_regulation\_of\_MAPKKK\_cascade | 24 | 1 | 7.675000 | -0.910938 | 134 | 83.529321 | 134.5 | 185.470679 | 1.003731 |
| GO:0051099\_positive\_regulation\_of\_binding | 24 | 1 | 7.675000 | -0.910938 | 134 | 83.529321 | 134.5 | 185.470679 | 1.003731 |
| GO:0006974\_response\_to\_DNA\_damage\_stimulus | 113 | 2 | 3.260177 | -0.905361 | 135 | 83.766859 | 135.06 | 186.353141 | 1.000444 |
| GO:0000165\_MAPKKK\_cascade | 114 | 2 | 3.231579 | -0.899078 | 136 | 83.879513 | 135.33 | 186.780487 | 0.995074 |
| GO:0006302\_double-strand\_break\_repair | 25 | 1 | 7.368000 | -0.894323 | 140 | 86.342023 | 139.02 | 191.697977 | 0.993000 |
| GO:0050852\_T\_cell\_receptor\_signaling\_pathway | 25 | 1 | 7.368000 | -0.894323 | 140 | 86.342023 | 139.02 | 191.697977 | 0.993000 |
| GO:0051090\_regulation\_of\_transcription\_factor\_activity | 25 | 1 | 7.368000 | -0.894323 | 140 | 86.342023 | 139.02 | 191.697977 | 0.993000 |
| GO:0090046\_regulation\_of\_transcription\_regulator\_activity | 25 | 1 | 7.368000 | -0.894323 | 140 | 86.342023 | 139.02 | 191.697977 | 0.993000 |
| GO:0048608\_reproductive\_structure\_development | 116 | 2 | 3.175862 | -0.886710 | 141 | 86.694114 | 139.73 | 192.765886 | 0.990993 |
| GO:0043933\_macromolecular\_complex\_subunit\_organization | 117 | 2 | 3.148718 | -0.880624 | 142 | 87.196899 | 140.46 | 193.723101 | 0.989155 |
| GO:0006800\_oxygen\_and\_reactive\_oxygen\_species\_metabolic\_process | 26 | 1 | 7.084615 | -0.878403 | 144 | 89.458787 | 143.13 | 196.801213 | 0.993958 |
| GO:0010212\_response\_to\_ionizing\_radiation | 26 | 1 | 7.084615 | -0.878403 | 144 | 89.458787 | 143.13 | 196.801213 | 0.993958 |
| GO:0016043\_cellular\_component\_organization | 964 | 8 | 1.528631 | -0.875193 | 145 | 89.533491 | 143.26 | 196.986509 | 0.988000 |
| GO:0007049\_cell\_cycle | 238 | 3 | 2.321849 | -0.865969 | 146 | 90.434380 | 144.49 | 198.545620 | 0.989658 |
| GO:0031016\_pancreas\_development | 27 | 1 | 6.822222 | -0.863126 | 148 | 91.950442 | 146.66 | 201.369558 | 0.990946 |
| GO:0070482\_response\_to\_oxygen\_levels | 27 | 1 | 6.822222 | -0.863126 | 148 | 91.950442 | 146.66 | 201.369558 | 0.990946 |
| GO:0051726\_regulation\_of\_cell\_cycle | 121 | 2 | 3.044628 | -0.856903 | 149 | 92.462387 | 147.36 | 202.257613 | 0.988993 |
| GO:0010564\_regulation\_of\_cell\_cycle\_process | 29 | 1 | 6.351724 | -0.834314 | 152 | 96.788217 | 153.8 | 210.811783 | 1.011842 |
| GO:0048066\_pigmentation\_during\_development | 29 | 1 | 6.351724 | -0.834314 | 152 | 96.788217 | 153.8 | 210.811783 | 1.011842 |
| GO:0060041\_retina\_development\_in\_camera-type\_eye | 29 | 1 | 6.351724 | -0.834314 | 152 | 96.788217 | 153.8 | 210.811783 | 1.011842 |
| GO:0050794\_regulation\_of\_cellular\_process | 2190 | 15 | 1.261644 | -0.831931 | 153 | 96.964272 | 154.11 | 211.255728 | 1.007255 |
| GO:0022411\_cellular\_component\_disassembly | 30 | 1 | 6.140000 | -0.820701 | 155 | 99.909193 | 158.2 | 216.490807 | 1.020645 |
| GO:0060740\_prostate\_gland\_epithelium\_morphogenesis | 30 | 1 | 6.140000 | -0.820701 | 155 | 99.909193 | 158.2 | 216.490807 | 1.020645 |
| GO:0016481\_negative\_regulation\_of\_transcription | 253 | 3 | 2.184190 | -0.808781 | 156 | 100.813275 | 159.63 | 218.446725 | 1.023269 |
| GO:0003018\_vascular\_process\_in\_circulatory\_system | 31 | 1 | 5.941935 | -0.807570 | 163 | 103.871591 | 164.26 | 224.648409 | 1.007730 |
| GO:0006486\_protein\_amino\_acid\_glycosylation | 31 | 1 | 5.941935 | -0.807570 | 163 | 103.871591 | 164.26 | 224.648409 | 1.007730 |
| GO:0035150\_regulation\_of\_tube\_size | 31 | 1 | 5.941935 | -0.807570 | 163 | 103.871591 | 164.26 | 224.648409 | 1.007730 |
| GO:0043413\_biopolymer\_glycosylation | 31 | 1 | 5.941935 | -0.807570 | 163 | 103.871591 | 164.26 | 224.648409 | 1.007730 |
| GO:0050880\_regulation\_of\_blood\_vessel\_size | 31 | 1 | 5.941935 | -0.807570 | 163 | 103.871591 | 164.26 | 224.648409 | 1.007730 |
| GO:0060512\_prostate\_gland\_morphogenesis | 31 | 1 | 5.941935 | -0.807570 | 163 | 103.871591 | 164.26 | 224.648409 | 1.007730 |
| GO:0070085\_glycosylation | 31 | 1 | 5.941935 | -0.807570 | 163 | 103.871591 | 164.26 | 224.648409 | 1.007730 |
| GO:0051493\_regulation\_of\_cytoskeleton\_organization | 32 | 1 | 5.756250 | -0.794890 | 164 | 106.367780 | 167.36 | 228.352220 | 1.020488 |
| GO:0031323\_regulation\_of\_cellular\_metabolic\_process | 1015 | 8 | 1.451823 | -0.778672 | 165 | 108.713796 | 170.78 | 232.846204 | 1.035030 |
| GO:0010629\_negative\_regulation\_of\_gene\_expression | 262 | 3 | 2.109160 | -0.776696 | 167 | 108.927568 | 171.23 | 233.532432 | 1.025329 |
| GO:0048666\_neuron\_development | 262 | 3 | 2.109160 | -0.776696 | 167 | 108.927568 | 171.23 | 233.532432 | 1.025329 |
| GO:0007568\_aging | 34 | 1 | 5.417647 | -0.770776 | 169 | 111.037676 | 174.19 | 237.342324 | 1.030710 |
| GO:0030509\_BMP\_signaling\_pathway | 34 | 1 | 5.417647 | -0.770776 | 169 | 111.037676 | 174.19 | 237.342324 | 1.030710 |
| GO:0048519\_negative\_regulation\_of\_biological\_process | 859 | 7 | 1.501048 | -0.769608 | 170 | 111.181169 | 174.46 | 237.738831 | 1.026235 |
| GO:0001756\_somitogenesis | 35 | 1 | 5.262857 | -0.759292 | 176 | 113.246302 | 177.08 | 240.913698 | 1.006136 |
| GO:0007292\_female\_gamete\_generation | 35 | 1 | 5.262857 | -0.759292 | 176 | 113.246302 | 177.08 | 240.913698 | 1.006136 |
| GO:0016051\_carbohydrate\_biosynthetic\_process | 35 | 1 | 5.262857 | -0.759292 | 176 | 113.246302 | 177.08 | 240.913698 | 1.006136 |
| GO:0016567\_protein\_ubiquitination | 35 | 1 | 5.262857 | -0.759292 | 176 | 113.246302 | 177.08 | 240.913698 | 1.006136 |
| GO:0051325\_interphase | 35 | 1 | 5.262857 | -0.759292 | 176 | 113.246302 | 177.08 | 240.913698 | 1.006136 |
| GO:0051329\_interphase\_of\_mitotic\_cell\_cycle | 35 | 1 | 5.262857 | -0.759292 | 176 | 113.246302 | 177.08 | 240.913698 | 1.006136 |
| GO:0003006\_reproductive\_developmental\_process | 141 | 2 | 2.612766 | -0.751422 | 177 | 113.812389 | 177.85 | 241.887611 | 1.004802 |
| GO:0045934\_negative\_regulation\_of\_nucleobase\_\_nucleoside\_\_nucleotide\_and\_nucleic\_acid\_metabolic\_process | 270 | 3 | 2.046667 | -0.749462 | 178 | 113.969824 | 178.03 | 242.090176 | 1.000169 |
| GO:0001889\_liver\_development | 36 | 1 | 5.116667 | -0.748163 | 183 | 116.389661 | 181.14 | 245.890339 | 0.989836 |
| GO:0006469\_negative\_regulation\_of\_protein\_kinase\_activity | 36 | 1 | 5.116667 | -0.748163 | 183 | 116.389661 | 181.14 | 245.890339 | 0.989836 |
| GO:0022602\_ovulation\_cycle\_process | 36 | 1 | 5.116667 | -0.748163 | 183 | 116.389661 | 181.14 | 245.890339 | 0.989836 |
| GO:0033673\_negative\_regulation\_of\_kinase\_activity | 36 | 1 | 5.116667 | -0.748163 | 183 | 116.389661 | 181.14 | 245.890339 | 0.989836 |
| GO:0050851\_antigen\_receptor-mediated\_signaling\_pathway | 36 | 1 | 5.116667 | -0.748163 | 183 | 116.389661 | 181.14 | 245.890339 | 0.989836 |
| GO:0007242\_intracellular\_signaling\_cascade | 411 | 4 | 1.792701 | -0.747451 | 184 | 116.401110 | 181.23 | 246.058890 | 0.984946 |
| GO:0044267\_cellular\_protein\_metabolic\_process | 559 | 5 | 1.647585 | -0.747414 | 185 | 116.427084 | 181.29 | 246.152916 | 0.979946 |
| GO:0051172\_negative\_regulation\_of\_nitrogen\_compound\_metabolic\_process | 271 | 3 | 2.039114 | -0.746139 | 186 | 116.581836 | 181.47 | 246.358164 | 0.975645 |
| GO:0042698\_ovulation\_cycle | 37 | 1 | 4.978378 | -0.737368 | 188 | 118.446541 | 183.71 | 248.973459 | 0.977181 |
| GO:0051101\_regulation\_of\_DNA\_binding | 37 | 1 | 4.978378 | -0.737368 | 188 | 118.446541 | 183.71 | 248.973459 | 0.977181 |
| GO:0010558\_negative\_regulation\_of\_macromolecule\_biosynthetic\_process | 274 | 3 | 2.016788 | -0.736275 | 189 | 118.809346 | 184.38 | 249.950654 | 0.975556 |
| GO:0005975\_carbohydrate\_metabolic\_process | 146 | 2 | 2.523288 | -0.727993 | 190 | 119.075993 | 184.88 | 250.684007 | 0.973053 |
| GO:0006820\_anion\_transport | 38 | 1 | 4.847368 | -0.726890 | 192 | 121.915558 | 188.34 | 254.764442 | 0.980938 |
| GO:0051348\_negative\_regulation\_of\_transferase\_activity | 38 | 1 | 4.847368 | -0.726890 | 192 | 121.915558 | 188.34 | 254.764442 | 0.980938 |
| GO:0065009\_regulation\_of\_molecular\_function | 279 | 3 | 1.980645 | -0.720179 | 193 | 122.371517 | 189.16 | 255.948483 | 0.980104 |
| GO:0005976\_polysaccharide\_metabolic\_process | 39 | 1 | 4.723077 | -0.716711 | 194 | 124.451633 | 191.98 | 259.508367 | 0.989588 |
| GO:0031327\_negative\_regulation\_of\_cellular\_biosynthetic\_process | 282 | 3 | 1.959574 | -0.710721 | 195 | 125.013963 | 192.71 | 260.406037 | 0.988256 |
| GO:0000902\_cell\_morphogenesis | 283 | 3 | 1.952650 | -0.707600 | 196 | 125.075663 | 192.8 | 260.524337 | 0.983673 |
| GO:0035272\_exocrine\_system\_development | 40 | 1 | 4.605000 | -0.706817 | 197 | 126.901427 | 195.01 | 263.118573 | 0.989898 |
| GO:0009890\_negative\_regulation\_of\_biosynthetic\_process | 284 | 3 | 1.945775 | -0.704496 | 198 | 127.046111 | 195.24 | 263.433889 | 0.986061 |
| GO:0034960\_cellular\_biopolymer\_metabolic\_process | 1395 | 10 | 1.320430 | -0.703527 | 199 | 127.175944 | 195.48 | 263.784056 | 0.982312 |
| GO:0002429\_immune\_response-activating\_cell\_surface\_receptor\_signaling\_pathway | 41 | 1 | 4.492683 | -0.697194 | 203 | 130.152419 | 199.59 | 269.027581 | 0.983202 |
| GO:0006979\_response\_to\_oxidative\_stress | 41 | 1 | 4.492683 | -0.697194 | 203 | 130.152419 | 199.59 | 269.027581 | 0.983202 |
| GO:0008585\_female\_gonad\_development | 41 | 1 | 4.492683 | -0.697194 | 203 | 130.152419 | 199.59 | 269.027581 | 0.983202 |
| GO:0015980\_energy\_derivation\_by\_oxidation\_of\_organic\_compounds | 41 | 1 | 4.492683 | -0.697194 | 203 | 130.152419 | 199.59 | 269.027581 | 0.983202 |
| GO:0034645\_cellular\_macromolecule\_biosynthetic\_process | 901 | 7 | 1.431077 | -0.692796 | 204 | 130.524081 | 200.04 | 269.555919 | 0.980588 |
| GO:0022402\_cell\_cycle\_process | 155 | 2 | 2.376774 | -0.688332 | 205 | 131.056220 | 200.81 | 270.563780 | 0.979561 |
| GO:0010740\_positive\_regulation\_of\_protein\_kinase\_cascade | 42 | 1 | 4.385714 | -0.687829 | 206 | 133.925682 | 204.44 | 274.954318 | 0.992427 |
| GO:0032446\_protein\_modification\_by\_small\_protein\_conjugation | 43 | 1 | 4.283721 | -0.678709 | 207 | 137.428084 | 208.75 | 280.071916 | 1.008454 |
| GO:0009059\_macromolecule\_biosynthetic\_process | 910 | 7 | 1.416923 | -0.677244 | 208 | 137.649186 | 208.94 | 280.230814 | 1.004519 |
| GO:0006464\_protein\_modification\_process | 439 | 4 | 1.678360 | -0.676599 | 209 | 137.793861 | 209.07 | 280.346139 | 1.000335 |
| GO:0007409\_axonogenesis | 158 | 2 | 2.331646 | -0.675776 | 210 | 138.157531 | 209.53 | 280.902469 | 0.997762 |
| GO:0002768\_immune\_response-regulating\_cell\_surface\_receptor\_signaling\_pathway | 44 | 1 | 4.186364 | -0.669823 | 213 | 142.268819 | 214.77 | 287.271181 | 1.008310 |
| GO:0035282\_segmentation | 44 | 1 | 4.186364 | -0.669823 | 213 | 142.268819 | 214.77 | 287.271181 | 1.008310 |
| GO:0046545\_development\_of\_primary\_female\_sexual\_characteristics | 44 | 1 | 4.186364 | -0.669823 | 213 | 142.268819 | 214.77 | 287.271181 | 1.008310 |
| GO:0007165\_signal\_transduction | 915 | 7 | 1.409180 | -0.668736 | 214 | 142.527637 | 215.15 | 287.772363 | 1.005374 |
| GO:0009628\_response\_to\_abiotic\_stimulus | 162 | 2 | 2.274074 | -0.659516 | 215 | 144.253313 | 217.41 | 290.566687 | 1.011209 |
| GO:0019222\_regulation\_of\_metabolic\_process | 1088 | 8 | 1.354412 | -0.656346 | 216 | 144.376243 | 217.77 | 291.163757 | 1.008194 |
| GO:0006732\_coenzyme\_metabolic\_process | 46 | 1 | 4.004348 | -0.652712 | 220 | 145.990751 | 220.04 | 294.089249 | 1.000182 |
| GO:0008217\_regulation\_of\_blood\_pressure | 46 | 1 | 4.004348 | -0.652712 | 220 | 145.990751 | 220.04 | 294.089249 | 1.000182 |
| GO:0030850\_prostate\_gland\_development | 46 | 1 | 4.004348 | -0.652712 | 220 | 145.990751 | 220.04 | 294.089249 | 1.000182 |
| GO:0051098\_regulation\_of\_binding | 46 | 1 | 4.004348 | -0.652712 | 220 | 145.990751 | 220.04 | 294.089249 | 1.000182 |
| GO:0006259\_DNA\_metabolic\_process | 165 | 2 | 2.232727 | -0.647666 | 221 | 146.560438 | 221.01 | 295.459562 | 1.000045 |
| GO:0002757\_immune\_response-activating\_signal\_transduction | 47 | 1 | 3.919149 | -0.644468 | 223 | 148.222483 | 223.45 | 298.677517 | 1.002018 |
| GO:0016570\_histone\_modification | 47 | 1 | 3.919149 | -0.644468 | 223 | 148.222483 | 223.45 | 298.677517 | 1.002018 |
| GO:0032989\_cellular\_component\_morphogenesis | 307 | 3 | 1.800000 | -0.637299 | 224 | 149.033121 | 224.56 | 300.086879 | 1.002500 |
| GO:0051171\_regulation\_of\_nitrogen\_compound\_metabolic\_process | 771 | 6 | 1.433463 | -0.636681 | 225 | 149.150378 | 224.67 | 300.189622 | 0.998533 |
| GO:0032269\_negative\_regulation\_of\_cellular\_protein\_metabolic\_process | 48 | 1 | 3.837500 | -0.636419 | 226 | 150.147111 | 225.94 | 301.732889 | 0.999735 |
| GO:0043412\_biopolymer\_modification | 458 | 4 | 1.608734 | -0.632571 | 227 | 150.476692 | 226.46 | 302.443308 | 0.997621 |
| GO:0044260\_cellular\_macromolecule\_metabolic\_process | 1447 | 10 | 1.272979 | -0.629126 | 228 | 150.889985 | 226.87 | 302.850015 | 0.995044 |
| GO:0046660\_female\_sex\_differentiation | 49 | 1 | 3.759184 | -0.628559 | 229 | 152.686516 | 229.37 | 306.053484 | 1.001616 |
| GO:0048812\_neuron\_projection\_morphogenesis | 170 | 2 | 2.167059 | -0.628544 | 230 | 152.970850 | 229.75 | 306.529150 | 0.998913 |
| GO:0051606\_detection\_of\_stimulus | 50 | 1 | 3.684000 | -0.620878 | 232 | 154.978537 | 232.19 | 309.401463 | 1.000819 |
| GO:0070647\_protein\_modification\_by\_small\_protein\_conjugation\_or\_removal | 50 | 1 | 3.684000 | -0.620878 | 232 | 154.978537 | 232.19 | 309.401463 | 1.000819 |
| GO:0007399\_nervous\_system\_development | 621 | 5 | 1.483092 | -0.617976 | 233 | 155.214487 | 232.49 | 309.765513 | 0.997811 |
| GO:0044248\_cellular\_catabolic\_process | 173 | 2 | 2.129480 | -0.617431 | 235 | 155.603381 | 233.02 | 310.436619 | 0.991574 |
| GO:0048667\_cell\_morphogenesis\_involved\_in\_neuron\_differentiation | 173 | 2 | 2.129480 | -0.617431 | 235 | 155.603381 | 233.02 | 310.436619 | 0.991574 |
| GO:0002764\_immune\_response-regulating\_signal\_transduction | 51 | 1 | 3.611765 | -0.613370 | 238 | 157.067351 | 235.08 | 313.092649 | 0.987731 |
| GO:0016569\_covalent\_chromatin\_modification | 51 | 1 | 3.611765 | -0.613370 | 238 | 157.067351 | 235.08 | 313.092649 | 0.987731 |
| GO:0043408\_regulation\_of\_MAPKKK\_cascade | 51 | 1 | 3.611765 | -0.613370 | 238 | 157.067351 | 235.08 | 313.092649 | 0.987731 |
| GO:0048858\_cell\_projection\_morphogenesis | 176 | 2 | 2.093182 | -0.606576 | 239 | 157.966904 | 236.22 | 314.473096 | 0.988368 |
| GO:0050789\_regulation\_of\_biological\_process | 2357 | 15 | 1.172253 | -0.605940 | 240 | 158.828104 | 237.31 | 315.791896 | 0.988792 |
| GO:0051248\_negative\_regulation\_of\_protein\_metabolic\_process | 53 | 1 | 3.475472 | -0.598847 | 241 | 160.172225 | 239.0 | 317.827775 | 0.991701 |
| GO:0002253\_activation\_of\_immune\_response | 54 | 1 | 3.411111 | -0.591819 | 245 | 162.182336 | 241.69 | 321.197664 | 0.986490 |
| GO:0006091\_generation\_of\_precursor\_metabolites\_and\_energy | 54 | 1 | 3.411111 | -0.591819 | 245 | 162.182336 | 241.69 | 321.197664 | 0.986490 |
| GO:0043405\_regulation\_of\_MAP\_kinase\_activity | 54 | 1 | 3.411111 | -0.591819 | 245 | 162.182336 | 241.69 | 321.197664 | 0.986490 |
| GO:0044271\_nitrogen\_compound\_biosynthetic\_process | 54 | 1 | 3.411111 | -0.591819 | 245 | 162.182336 | 241.69 | 321.197664 | 0.986490 |
| GO:0007605\_sensory\_perception\_of\_sound | 55 | 1 | 3.349091 | -0.584939 | 246 | 164.290222 | 244.12 | 323.949778 | 0.992358 |
| GO:0034961\_cellular\_biopolymer\_biosynthetic\_process | 804 | 6 | 1.374627 | -0.580935 | 247 | 164.498817 | 244.36 | 324.221183 | 0.989312 |
| GO:0032990\_cell\_part\_morphogenesis | 184 | 2 | 2.002174 | -0.578825 | 248 | 165.026125 | 244.95 | 324.873875 | 0.987702 |
| GO:0043284\_biopolymer\_biosynthetic\_process | 807 | 6 | 1.369517 | -0.576093 | 249 | 167.090753 | 247.98 | 328.869247 | 0.995904 |
| GO:0007010\_cytoskeleton\_organization | 185 | 2 | 1.991351 | -0.575473 | 250 | 167.208083 | 248.13 | 329.051917 | 0.992520 |
| GO:0000226\_microtubule\_cytoskeleton\_organization | 57 | 1 | 3.231579 | -0.571602 | 253 | 170.105219 | 251.91 | 333.714781 | 0.995692 |
| GO:0009953\_dorsal\_ventral\_pattern\_formation | 57 | 1 | 3.231579 | -0.571602 | 253 | 170.105219 | 251.91 | 333.714781 | 0.995692 |
| GO:0045444\_fat\_cell\_differentiation | 57 | 1 | 3.231579 | -0.571602 | 253 | 170.105219 | 251.91 | 333.714781 | 0.995692 |
| GO:0031326\_regulation\_of\_cellular\_biosynthetic\_process | 812 | 6 | 1.361084 | -0.568102 | 254 | 170.473541 | 252.31 | 334.146459 | 0.993346 |
| GO:0007276\_gamete\_generation | 188 | 2 | 1.959574 | -0.565565 | 255 | 170.559782 | 252.44 | 334.320218 | 0.989961 |
| GO:0044249\_cellular\_biosynthetic\_process | 1150 | 8 | 1.281391 | -0.565460 | 256 | 170.625543 | 252.57 | 334.514457 | 0.986602 |
| GO:0009889\_regulation\_of\_biosynthetic\_process | 815 | 6 | 1.356074 | -0.563355 | 257 | 171.402819 | 253.38 | 335.357181 | 0.985914 |
| GO:0007005\_mitochondrion\_organization | 61 | 1 | 3.019672 | -0.546488 | 258 | 174.972991 | 257.41 | 339.847009 | 0.997713 |
| GO:0003002\_regionalization | 195 | 2 | 1.889231 | -0.543283 | 260 | 175.899996 | 258.65 | 341.400004 | 0.994808 |
| GO:0007507\_heart\_development | 195 | 2 | 1.889231 | -0.543283 | 260 | 175.899996 | 258.65 | 341.400004 | 0.994808 |
| GO:0022604\_regulation\_of\_cell\_morphogenesis | 62 | 1 | 2.970968 | -0.540509 | 262 | 177.811379 | 260.84 | 343.868621 | 0.995573 |
| GO:0050954\_sensory\_perception\_of\_mechanical\_stimulus | 62 | 1 | 2.970968 | -0.540509 | 262 | 177.811379 | 260.84 | 343.868621 | 0.995573 |
| GO:0033554\_cellular\_response\_to\_stress | 196 | 2 | 1.879592 | -0.540191 | 263 | 177.973222 | 261.02 | 344.066778 | 0.992471 |
| GO:0031175\_neuron\_projection\_development | 197 | 2 | 1.870051 | -0.537122 | 264 | 178.429337 | 261.54 | 344.650663 | 0.990682 |
| GO:0051186\_cofactor\_metabolic\_process | 63 | 1 | 2.923810 | -0.534642 | 266 | 180.191413 | 263.55 | 346.908587 | 0.990789 |
| GO:0051216\_cartilage\_development | 63 | 1 | 2.923810 | -0.534642 | 266 | 180.191413 | 263.55 | 346.908587 | 0.990789 |
| GO:0009058\_biosynthetic\_process | 1175 | 8 | 1.254128 | -0.531875 | 267 | 180.713583 | 264.02 | 347.326417 | 0.988839 |
| GO:0000904\_cell\_morphogenesis\_involved\_in\_differentiation | 199 | 2 | 1.851256 | -0.531049 | 268 | 180.927440 | 264.26 | 347.592560 | 0.986045 |
| GO:0043086\_negative\_regulation\_of\_catalytic\_activity | 65 | 1 | 2.833846 | -0.523231 | 270 | 182.732800 | 266.44 | 350.147200 | 0.986815 |
| GO:0048511\_rhythmic\_process | 65 | 1 | 2.833846 | -0.523231 | 270 | 182.732800 | 266.44 | 350.147200 | 0.986815 |
| GO:0045449\_regulation\_of\_transcription | 676 | 5 | 1.362426 | -0.521407 | 271 | 182.822893 | 266.54 | 350.257107 | 0.983542 |
| GO:0007179\_transforming\_growth\_factor\_beta\_receptor\_signaling\_pathway | 66 | 1 | 2.790909 | -0.517680 | 272 | 184.239792 | 268.21 | 352.180208 | 0.986066 |
| GO:0030182\_neuron\_differentiation | 356 | 3 | 1.552247 | -0.516984 | 273 | 184.385748 | 268.49 | 352.594252 | 0.983480 |
| GO:0007243\_protein\_kinase\_cascade | 205 | 2 | 1.797073 | -0.513340 | 274 | 185.181380 | 269.42 | 353.658620 | 0.983285 |
| GO:0009791\_post-embryonic\_development | 67 | 1 | 2.749254 | -0.512228 | 275 | 186.322781 | 270.72 | 355.117219 | 0.984436 |
| GO:0051173\_positive\_regulation\_of\_nitrogen\_compound\_metabolic\_process | 361 | 3 | 1.530748 | -0.506189 | 276 | 187.593359 | 272.22 | 356.846641 | 0.986304 |
| GO:0044238\_primary\_metabolic\_process | 1905 | 12 | 1.160315 | -0.500570 | 277 | 189.284059 | 274.18 | 359.075941 | 0.989819 |
| GO:0008406\_gonad\_development | 70 | 1 | 2.631429 | -0.496436 | 278 | 191.221130 | 276.3 | 361.378870 | 0.993885 |
| GO:0016568\_chromatin\_modification | 72 | 1 | 2.558333 | -0.486351 | 281 | 195.493777 | 281.18 | 366.866223 | 1.000641 |
| GO:0030879\_mammary\_gland\_development | 72 | 1 | 2.558333 | -0.486351 | 281 | 195.493777 | 281.18 | 366.866223 | 1.000641 |
| GO:0044262\_cellular\_carbohydrate\_metabolic\_process | 72 | 1 | 2.558333 | -0.486351 | 281 | 195.493777 | 281.18 | 366.866223 | 1.000641 |
| GO:0006350\_transcription | 701 | 5 | 1.313837 | -0.482361 | 282 | 195.929142 | 281.68 | 367.430858 | 0.998865 |
| GO:0045892\_negative\_regulation\_of\_transcription\_\_DNA-dependent | 218 | 2 | 1.689908 | -0.477415 | 283 | 197.814875 | 283.89 | 369.965125 | 1.003145 |
| GO:0022414\_reproductive\_process | 376 | 3 | 1.469681 | -0.475236 | 284 | 198.380888 | 284.66 | 370.939112 | 1.002324 |
| GO:0051253\_negative\_regulation\_of\_RNA\_metabolic\_process | 220 | 2 | 1.674545 | -0.472165 | 285 | 198.764548 | 285.08 | 371.395452 | 1.000281 |
| GO:0007281\_germ\_cell\_development | 75 | 1 | 2.456000 | -0.471840 | 286 | 199.671411 | 286.22 | 372.768589 | 1.000769 |
| GO:0000003\_reproduction | 379 | 3 | 1.458047 | -0.469292 | 287 | 200.092161 | 286.69 | 373.287839 | 0.998920 |
| GO:0043687\_post-translational\_protein\_modification | 384 | 3 | 1.439063 | -0.459560 | 288 | 202.142764 | 289.04 | 375.937236 | 1.003611 |
| GO:0030326\_embryonic\_limb\_morphogenesis | 78 | 1 | 2.361538 | -0.458018 | 290 | 203.938446 | 291.28 | 378.621554 | 1.004414 |
| GO:0035113\_embryonic\_appendage\_morphogenesis | 78 | 1 | 2.361538 | -0.458018 | 290 | 203.938446 | 291.28 | 378.621554 | 1.004414 |
| GO:0031328\_positive\_regulation\_of\_cellular\_biosynthetic\_process | 387 | 3 | 1.427907 | -0.453824 | 291 | 204.225650 | 291.57 | 378.914350 | 1.001959 |
| GO:0009891\_positive\_regulation\_of\_biosynthetic\_process | 388 | 3 | 1.424227 | -0.451929 | 292 | 204.828531 | 292.35 | 379.871469 | 1.001199 |
| GO:0019953\_sexual\_reproduction | 228 | 2 | 1.615789 | -0.451852 | 293 | 205.296567 | 293.01 | 380.723433 | 1.000034 |
| GO:0008152\_metabolic\_process | 2133 | 13 | 1.122644 | -0.450411 | 294 | 205.398011 | 293.15 | 380.901989 | 0.997109 |
| GO:0000278\_mitotic\_cell\_cycle | 80 | 1 | 2.302500 | -0.449158 | 296 | 206.988188 | 294.95 | 382.911812 | 0.996453 |
| GO:0044092\_negative\_regulation\_of\_molecular\_function | 80 | 1 | 2.302500 | -0.449158 | 296 | 206.988188 | 294.95 | 382.911812 | 0.996453 |
| GO:0007420\_brain\_development | 231 | 2 | 1.594805 | -0.444507 | 297 | 207.915873 | 295.95 | 383.984127 | 0.996465 |
| GO:0042127\_regulation\_of\_cell\_proliferation | 393 | 3 | 1.406107 | -0.442577 | 298 | 208.223185 | 296.3 | 384.376815 | 0.994295 |
| GO:0007411\_axon\_guidance | 82 | 1 | 2.246341 | -0.440567 | 300 | 209.422295 | 297.97 | 386.517705 | 0.993233 |
| GO:0010627\_regulation\_of\_protein\_kinase\_cascade | 82 | 1 | 2.246341 | -0.440567 | 300 | 209.422295 | 297.97 | 386.517705 | 0.993233 |
| GO:0050790\_regulation\_of\_catalytic\_activity | 233 | 2 | 1.581116 | -0.439690 | 301 | 209.826684 | 298.4 | 386.973316 | 0.991362 |
| GO:0048699\_generation\_of\_neurons | 396 | 3 | 1.395455 | -0.437064 | 302 | 210.149482 | 298.86 | 387.570518 | 0.989603 |
| GO:0006575\_cellular\_amino\_acid\_derivative\_metabolic\_process | 83 | 1 | 2.219277 | -0.436368 | 304 | 211.603600 | 300.55 | 389.496400 | 0.988651 |
| GO:0007017\_microtubule-based\_process | 83 | 1 | 2.219277 | -0.436368 | 304 | 211.603600 | 300.55 | 389.496400 | 0.988651 |
| GO:0001501\_skeletal\_system\_development | 236 | 2 | 1.561017 | -0.432579 | 305 | 212.005785 | 301.08 | 390.154215 | 0.987148 |
| GO:0045137\_development\_of\_primary\_sexual\_characteristics | 84 | 1 | 2.192857 | -0.432231 | 306 | 212.634287 | 301.72 | 390.805713 | 0.986013 |
| GO:0032504\_multicellular\_organism\_reproduction | 86 | 1 | 2.141860 | -0.424137 | 309 | 215.953415 | 305.79 | 395.626585 | 0.989612 |
| GO:0034641\_cellular\_nitrogen\_compound\_metabolic\_process | 86 | 1 | 2.141860 | -0.424137 | 309 | 215.953415 | 305.79 | 395.626585 | 0.989612 |
| GO:0048609\_reproductive\_process\_in\_a\_multicellular\_organism | 86 | 1 | 2.141860 | -0.424137 | 309 | 215.953415 | 305.79 | 395.626585 | 0.989612 |
| GO:0007178\_transmembrane\_receptor\_protein\_serine\_threonine\_kinase\_signaling\_pathway | 87 | 1 | 2.117241 | -0.420178 | 313 | 217.576024 | 307.92 | 398.263976 | 0.983770 |
| GO:0016337\_cell-cell\_adhesion | 87 | 1 | 2.117241 | -0.420178 | 313 | 217.576024 | 307.92 | 398.263976 | 0.983770 |
| GO:0022612\_gland\_morphogenesis | 87 | 1 | 2.117241 | -0.420178 | 313 | 217.576024 | 307.92 | 398.263976 | 0.983770 |
| GO:0050778\_positive\_regulation\_of\_immune\_response | 87 | 1 | 2.117241 | -0.420178 | 313 | 217.576024 | 307.92 | 398.263976 | 0.983770 |
| GO:0010556\_regulation\_of\_macromolecule\_biosynthetic\_process | 745 | 5 | 1.236242 | -0.420112 | 314 | 217.679572 | 308.07 | 398.460428 | 0.981115 |
| GO:0007154\_cell\_communication | 1096 | 7 | 1.176460 | -0.416389 | 315 | 218.153773 | 308.85 | 399.546227 | 0.980476 |
| GO:0080090\_regulation\_of\_primary\_metabolic\_process | 926 | 6 | 1.193521 | -0.410563 | 316 | 219.491583 | 310.24 | 400.988417 | 0.981772 |
| GO:0019219\_regulation\_of\_nucleobase\_\_nucleoside\_\_nucleotide\_and\_nucleic\_acid\_metabolic\_process | 757 | 5 | 1.216645 | -0.404455 | 317 | 222.068202 | 313.36 | 404.651798 | 0.988517 |
| GO:0007389\_pattern\_specification\_process | 250 | 2 | 1.473600 | -0.401140 | 318 | 223.429469 | 314.95 | 406.470531 | 0.990409 |
| GO:0060255\_regulation\_of\_macromolecule\_metabolic\_process | 936 | 6 | 1.180769 | -0.398771 | 319 | 223.911088 | 315.44 | 406.968912 | 0.988840 |
| GO:0035107\_appendage\_morphogenesis | 93 | 1 | 1.980645 | -0.397565 | 322 | 225.028921 | 316.73 | 408.431079 | 0.983634 |
| GO:0035108\_limb\_morphogenesis | 93 | 1 | 1.980645 | -0.397565 | 322 | 225.028921 | 316.73 | 408.431079 | 0.983634 |
| GO:0055066\_di-\_\_tri-valent\_inorganic\_cation\_homeostasis | 93 | 1 | 1.980645 | -0.397565 | 322 | 225.028921 | 316.73 | 408.431079 | 0.983634 |
| GO:0022008\_neurogenesis | 423 | 3 | 1.306383 | -0.390553 | 323 | 228.483917 | 320.83 | 413.176083 | 0.993282 |
| GO:0042391\_regulation\_of\_membrane\_potential | 95 | 1 | 1.938947 | -0.390435 | 324 | 229.053342 | 321.39 | 413.726658 | 0.991944 |
| GO:0048736\_appendage\_development | 96 | 1 | 1.918750 | -0.386941 | 326 | 231.055074 | 323.71 | 416.364926 | 0.992975 |
| GO:0060173\_limb\_development | 96 | 1 | 1.918750 | -0.386941 | 326 | 231.055074 | 323.71 | 416.364926 | 0.992975 |
| GO:0018193\_peptidyl-amino\_acid\_modification | 97 | 1 | 1.898969 | -0.383493 | 327 | 231.846953 | 324.82 | 417.793047 | 0.993333 |
| GO:0007548\_sex\_differentiation | 98 | 1 | 1.879592 | -0.380091 | 330 | 233.266730 | 326.57 | 419.873270 | 0.989606 |
| GO:0009314\_response\_to\_radiation | 98 | 1 | 1.879592 | -0.380091 | 330 | 233.266730 | 326.57 | 419.873270 | 0.989606 |
| GO:0009967\_positive\_regulation\_of\_signal\_transduction | 98 | 1 | 1.879592 | -0.380091 | 330 | 233.266730 | 326.57 | 419.873270 | 0.989606 |
| GO:0010468\_regulation\_of\_gene\_expression | 778 | 5 | 1.183805 | -0.378319 | 331 | 233.311256 | 326.69 | 420.068744 | 0.986979 |
| GO:0030030\_cell\_projection\_organization | 263 | 2 | 1.400760 | -0.374307 | 332 | 236.043657 | 329.57 | 423.096343 | 0.992681 |
| GO:0009653\_anatomical\_structure\_morphogenesis | 958 | 6 | 1.153653 | -0.373851 | 333 | 236.121606 | 329.69 | 423.258394 | 0.990060 |
| GO:0030036\_actin\_cytoskeleton\_organization | 102 | 1 | 1.805882 | -0.366916 | 334 | 237.624275 | 331.3 | 424.975725 | 0.991916 |
| GO:0003013\_circulatory\_system\_process | 103 | 1 | 1.788350 | -0.363727 | 337 | 238.488973 | 332.46 | 426.431027 | 0.986528 |
| GO:0008015\_blood\_circulation | 103 | 1 | 1.788350 | -0.363727 | 337 | 238.488973 | 332.46 | 426.431027 | 0.986528 |
| GO:0009968\_negative\_regulation\_of\_signal\_transduction | 103 | 1 | 1.788350 | -0.363727 | 337 | 238.488973 | 332.46 | 426.431027 | 0.986528 |
| GO:0006807\_nitrogen\_compound\_metabolic\_process | 1147 | 7 | 1.124150 | -0.361894 | 338 | 238.841320 | 332.84 | 426.838680 | 0.984734 |
| GO:0031325\_positive\_regulation\_of\_cellular\_metabolic\_process | 442 | 3 | 1.250226 | -0.360904 | 339 | 239.050540 | 333.04 | 427.029460 | 0.982419 |
| GO:0065007\_biological\_regulation | 2593 | 15 | 1.065561 | -0.360891 | 340 | 239.144315 | 333.17 | 427.195685 | 0.979912 |
| GO:0051716\_cellular\_response\_to\_stimulus | 273 | 2 | 1.349451 | -0.355062 | 341 | 241.689019 | 335.85 | 430.010981 | 0.984897 |
| GO:0045859\_regulation\_of\_protein\_kinase\_activity | 107 | 1 | 1.721495 | -0.351362 | 342 | 243.195807 | 337.38 | 431.564193 | 0.986491 |
| GO:0030029\_actin\_filament-based\_process | 109 | 1 | 1.689908 | -0.345405 | 343 | 245.174665 | 339.58 | 433.985335 | 0.990029 |
| GO:0010647\_positive\_regulation\_of\_cell\_communication | 110 | 1 | 1.674545 | -0.342480 | 347 | 246.864503 | 341.65 | 436.435497 | 0.984582 |
| GO:0010648\_negative\_regulation\_of\_cell\_communication | 110 | 1 | 1.674545 | -0.342480 | 347 | 246.864503 | 341.65 | 436.435497 | 0.984582 |
| GO:0043010\_camera-type\_eye\_development | 110 | 1 | 1.674545 | -0.342480 | 347 | 246.864503 | 341.65 | 436.435497 | 0.984582 |
| GO:0055080\_cation\_homeostasis | 110 | 1 | 1.674545 | -0.342480 | 347 | 246.864503 | 341.65 | 436.435497 | 0.984582 |
| GO:0009893\_positive\_regulation\_of\_metabolic\_process | 458 | 3 | 1.206550 | -0.337715 | 348 | 248.051104 | 342.9 | 437.748896 | 0.985345 |
| GO:0043549\_regulation\_of\_kinase\_activity | 112 | 1 | 1.644643 | -0.336736 | 349 | 248.944668 | 343.9 | 438.855332 | 0.985387 |
| GO:0048518\_positive\_regulation\_of\_biological\_process | 995 | 6 | 1.110754 | -0.334946 | 350 | 249.484070 | 344.58 | 439.675930 | 0.984514 |
| GO:0007417\_central\_nervous\_system\_development | 287 | 2 | 1.283624 | -0.329970 | 351 | 251.346621 | 346.67 | 441.993379 | 0.987664 |
| GO:0048584\_positive\_regulation\_of\_response\_to\_stimulus | 115 | 1 | 1.601739 | -0.328370 | 353 | 252.045135 | 347.55 | 443.054865 | 0.984561 |
| GO:0051338\_regulation\_of\_transferase\_activity | 115 | 1 | 1.601739 | -0.328370 | 353 | 252.045135 | 347.55 | 443.054865 | 0.984561 |
| GO:0006139\_nucleobase\_\_nucleoside\_\_nucleotide\_and\_nucleic\_acid\_metabolic\_process | 1002 | 6 | 1.102994 | -0.327988 | 354 | 252.334569 | 347.95 | 443.565431 | 0.982910 |
| GO:0006519\_cellular\_amino\_acid\_and\_derivative\_metabolic\_process | 118 | 1 | 1.561017 | -0.320292 | 355 | 254.365593 | 350.19 | 446.014407 | 0.986451 |
| GO:0022403\_cell\_cycle\_phase | 119 | 1 | 1.547899 | -0.317661 | 356 | 254.757811 | 350.53 | 446.302189 | 0.984635 |
| GO:0048468\_cell\_development | 654 | 4 | 1.126606 | -0.315636 | 357 | 255.644142 | 351.47 | 447.295858 | 0.984510 |
| GO:0009308\_amine\_metabolic\_process | 124 | 1 | 1.485484 | -0.304942 | 358 | 260.604588 | 356.85 | 453.095412 | 0.996788 |
| GO:0045893\_positive\_regulation\_of\_transcription\_\_DNA-dependent | 306 | 2 | 1.203922 | -0.299016 | 360 | 261.961409 | 358.34 | 454.718591 | 0.995389 |
| GO:0051254\_positive\_regulation\_of\_RNA\_metabolic\_process | 306 | 2 | 1.203922 | -0.299016 | 360 | 261.961409 | 358.34 | 454.718591 | 0.995389 |
| GO:0001655\_urogenital\_system\_development | 128 | 1 | 1.439063 | -0.295262 | 361 | 263.534237 | 360.08 | 456.625763 | 0.997452 |
| GO:0016310\_phosphorylation | 309 | 2 | 1.192233 | -0.294427 | 362 | 263.732062 | 360.38 | 457.027938 | 0.995525 |
| GO:0050776\_regulation\_of\_immune\_response | 130 | 1 | 1.416923 | -0.290578 | 363 | 265.646241 | 362.72 | 459.793759 | 0.999229 |
| GO:0009952\_anterior\_posterior\_pattern\_formation | 133 | 1 | 1.384962 | -0.283737 | 364 | 266.319079 | 363.54 | 460.760921 | 0.998736 |
| GO:0007283\_spermatogenesis | 134 | 1 | 1.374627 | -0.281504 | 366 | 266.998443 | 364.26 | 461.521557 | 0.995246 |
| GO:0048232\_male\_gamete\_generation | 134 | 1 | 1.374627 | -0.281504 | 366 | 266.998443 | 364.26 | 461.521557 | 0.995246 |
| GO:0001654\_eye\_development | 136 | 1 | 1.354412 | -0.277109 | 367 | 267.541623 | 364.8 | 462.058377 | 0.994005 |
| GO:0044237\_cellular\_metabolic\_process | 1974 | 11 | 1.026444 | -0.274888 | 368 | 268.513962 | 365.81 | 463.106038 | 0.994049 |
| GO:0045596\_negative\_regulation\_of\_cell\_differentiation | 144 | 1 | 1.279167 | -0.260404 | 369 | 274.131101 | 371.56 | 468.988899 | 1.006938 |
| GO:0048522\_positive\_regulation\_of\_cellular\_process | 895 | 5 | 1.029050 | -0.258338 | 370 | 274.452082 | 371.93 | 469.407918 | 1.005216 |
| GO:0030900\_forebrain\_development | 146 | 1 | 1.261644 | -0.256435 | 371 | 275.636254 | 373.1 | 470.563746 | 1.005660 |
| GO:0009888\_tissue\_development | 525 | 3 | 1.052571 | -0.255671 | 372 | 276.141780 | 373.56 | 470.978220 | 1.004194 |
| GO:0022603\_regulation\_of\_anatomical\_structure\_morphogenesis | 147 | 1 | 1.253061 | -0.254480 | 373 | 276.395698 | 373.85 | 471.304302 | 1.002279 |
| GO:0045941\_positive\_regulation\_of\_transcription | 338 | 2 | 1.089941 | -0.253798 | 374 | 276.605445 | 374.06 | 471.514555 | 1.000160 |
| GO:0002684\_positive\_regulation\_of\_immune\_system\_process | 148 | 1 | 1.244595 | -0.252544 | 375 | 277.548434 | 375.03 | 472.511566 | 1.000080 |
| GO:0006793\_phosphorus\_metabolic\_process | 340 | 2 | 1.083529 | -0.251227 | 377 | 278.609878 | 376.21 | 473.810122 | 0.997905 |
| GO:0006796\_phosphate\_metabolic\_process | 340 | 2 | 1.083529 | -0.251227 | 377 | 278.609878 | 376.21 | 473.810122 | 0.997905 |
| GO:0010467\_gene\_expression | 905 | 5 | 1.017680 | -0.249843 | 378 | 279.190280 | 376.86 | 474.529720 | 0.996984 |
| GO:0032268\_regulation\_of\_cellular\_protein\_metabolic\_process | 152 | 1 | 1.211842 | -0.244986 | 379 | 280.266494 | 377.79 | 475.313506 | 0.996807 |
| GO:0010628\_positive\_regulation\_of\_gene\_expression | 346 | 2 | 1.064740 | -0.243677 | 380 | 280.468142 | 378.01 | 475.551858 | 0.994763 |
| GO:0008285\_negative\_regulation\_of\_cell\_proliferation | 155 | 1 | 1.188387 | -0.239507 | 381 | 282.654929 | 380.12 | 477.585071 | 0.997690 |
| GO:0045935\_positive\_regulation\_of\_nucleobase\_\_nucleoside\_\_nucleotide\_and\_nucleic\_acid\_metabolic\_process | 352 | 2 | 1.046591 | -0.236368 | 382 | 283.043620 | 380.48 | 477.916380 | 0.996021 |
| GO:0008283\_cell\_proliferation | 544 | 3 | 1.015809 | -0.236195 | 383 | 283.237465 | 380.75 | 478.262535 | 0.994125 |
| GO:0006950\_response\_to\_stress | 549 | 3 | 1.006557 | -0.231313 | 384 | 285.741292 | 383.28 | 480.818708 | 0.998125 |
| GO:0007626\_locomotory\_behavior | 163 | 1 | 1.130061 | -0.225637 | 385 | 288.392840 | 385.97 | 483.547160 | 1.002519 |
| GO:0042325\_regulation\_of\_phosphorylation | 164 | 1 | 1.123171 | -0.223975 | 386 | 289.124563 | 386.67 | 484.215437 | 1.001736 |
| GO:0019220\_regulation\_of\_phosphate\_metabolic\_process | 165 | 1 | 1.116364 | -0.222328 | 388 | 290.127094 | 387.76 | 485.392906 | 0.999381 |
| GO:0051174\_regulation\_of\_phosphorus\_metabolic\_process | 165 | 1 | 1.116364 | -0.222328 | 388 | 290.127094 | 387.76 | 485.392906 | 0.999381 |
| GO:0043009\_chordate\_embryonic\_development | 365 | 2 | 1.009315 | -0.221309 | 389 | 291.122944 | 388.82 | 486.517056 | 0.999537 |
| GO:0048856\_anatomical\_structure\_development | 1688 | 9 | 0.982109 | -0.220533 | 390 | 291.500215 | 389.3 | 487.099785 | 0.998205 |
| GO:0009792\_embryonic\_development\_ending\_in\_birth\_or\_egg\_hatching | 368 | 2 | 1.001087 | -0.217978 | 391 | 292.486756 | 390.5 | 488.513244 | 0.998721 |
| GO:0032502\_developmental\_process | 2060 | 11 | 0.983592 | -0.217731 | 392 | 292.608618 | 390.64 | 488.671382 | 0.996531 |
| GO:0010557\_positive\_regulation\_of\_macromolecule\_biosynthetic\_process | 371 | 2 | 0.992992 | -0.214700 | 393 | 293.366994 | 391.29 | 489.213006 | 0.995649 |
| GO:0051246\_regulation\_of\_protein\_metabolic\_process | 170 | 1 | 1.083529 | -0.214317 | 394 | 294.488266 | 392.32 | 490.151734 | 0.995736 |
| GO:0007600\_sensory\_perception | 172 | 1 | 1.070930 | -0.211213 | 396 | 295.235102 | 392.97 | 490.704898 | 0.992348 |
| GO:0009611\_response\_to\_wounding | 172 | 1 | 1.070930 | -0.211213 | 396 | 295.235102 | 392.97 | 490.704898 | 0.992348 |
| GO:0044093\_positive\_regulation\_of\_molecular\_function | 173 | 1 | 1.064740 | -0.209681 | 397 | 296.439266 | 394.19 | 491.940734 | 0.992922 |
| GO:0006355\_regulation\_of\_transcription\_\_DNA-dependent | 575 | 3 | 0.961043 | -0.207443 | 398 | 297.454463 | 395.11 | 492.765537 | 0.992739 |
| GO:0000122\_negative\_regulation\_of\_transcription\_from\_RNA\_polymerase\_II\_promoter | 175 | 1 | 1.052571 | -0.206659 | 399 | 298.564367 | 396.26 | 493.955633 | 0.993133 |
| GO:0006873\_cellular\_ion\_homeostasis | 176 | 1 | 1.046591 | -0.205168 | 401 | 300.349028 | 397.91 | 495.470972 | 0.992294 |
| GO:0043066\_negative\_regulation\_of\_apoptosis | 176 | 1 | 1.046591 | -0.205168 | 401 | 300.349028 | 397.91 | 495.470972 | 0.992294 |
| GO:0009987\_cellular\_process | 3868 | 21 | 1.000052 | -0.201216 | 402 | 301.170155 | 398.7 | 496.229845 | 0.991791 |
| GO:0043069\_negative\_regulation\_of\_programmed\_cell\_death | 179 | 1 | 1.029050 | -0.200774 | 405 | 302.756556 | 400.22 | 497.683444 | 0.988198 |
| GO:0048732\_gland\_development | 179 | 1 | 1.029050 | -0.200774 | 405 | 302.756556 | 400.22 | 497.683444 | 0.988198 |
| GO:0060548\_negative\_regulation\_of\_cell\_death | 179 | 1 | 1.029050 | -0.200774 | 405 | 302.756556 | 400.22 | 497.683444 | 0.988198 |
| GO:0055082\_cellular\_chemical\_homeostasis | 181 | 1 | 1.017680 | -0.197907 | 406 | 304.598999 | 402.13 | 499.661001 | 0.990468 |
| GO:0051252\_regulation\_of\_RNA\_metabolic\_process | 590 | 3 | 0.936610 | -0.194760 | 407 | 306.446438 | 404.04 | 501.633562 | 0.992727 |
| GO:0006351\_transcription\_\_DNA-dependent | 594 | 3 | 0.930303 | -0.191504 | 408 | 308.241543 | 405.67 | 503.098457 | 0.994289 |
| GO:0006811\_ion\_transport | 186 | 1 | 0.990323 | -0.190956 | 411 | 309.618514 | 406.87 | 504.121486 | 0.989951 |
| GO:0007155\_cell\_adhesion | 186 | 1 | 0.990323 | -0.190956 | 411 | 309.618514 | 406.87 | 504.121486 | 0.989951 |
| GO:0022610\_biological\_adhesion | 186 | 1 | 0.990323 | -0.190956 | 411 | 309.618514 | 406.87 | 504.121486 | 0.989951 |
| GO:0032774\_RNA\_biosynthetic\_process | 595 | 3 | 0.928739 | -0.190698 | 412 | 310.057346 | 407.27 | 504.482654 | 0.988519 |
| GO:0048513\_organ\_development | 1365 | 7 | 0.944615 | -0.190183 | 413 | 310.158210 | 407.38 | 504.601790 | 0.986392 |
| GO:0019725\_cellular\_homeostasis | 195 | 1 | 0.944615 | -0.179168 | 414 | 314.502823 | 411.45 | 508.397177 | 0.993841 |
| GO:0007275\_multicellular\_organismal\_development | 1760 | 9 | 0.941932 | -0.178481 | 415 | 314.614725 | 411.59 | 508.565275 | 0.991783 |
| GO:0050801\_ion\_homeostasis | 197 | 1 | 0.935025 | -0.176668 | 416 | 316.337015 | 413.13 | 509.922985 | 0.993101 |
| GO:0002009\_morphogenesis\_of\_an\_epithelium | 198 | 1 | 0.930303 | -0.175433 | 418 | 317.866137 | 414.52 | 511.173863 | 0.991675 |
| GO:0060429\_epithelium\_development | 198 | 1 | 0.930303 | -0.175433 | 418 | 317.866137 | 414.52 | 511.173863 | 0.991675 |
| GO:0022607\_cellular\_component\_assembly | 204 | 1 | 0.902941 | -0.168235 | 419 | 320.105443 | 416.76 | 513.414557 | 0.994654 |
| GO:0006955\_immune\_response | 205 | 1 | 0.898537 | -0.167070 | 420 | 320.881778 | 417.5 | 514.118222 | 0.994048 |
| GO:0008284\_positive\_regulation\_of\_cell\_proliferation | 208 | 1 | 0.885577 | -0.163629 | 421 | 321.858281 | 418.57 | 515.281719 | 0.994228 |
| GO:0048731\_system\_development | 1609 | 8 | 0.915848 | -0.159718 | 422 | 322.355461 | 419.07 | 515.784539 | 0.993057 |
| GO:0010604\_positive\_regulation\_of\_macromolecule\_metabolic\_process | 433 | 2 | 0.850808 | -0.157179 | 423 | 323.245287 | 419.96 | 516.674713 | 0.992813 |
| GO:0009887\_organ\_morphogenesis | 642 | 3 | 0.860748 | -0.156223 | 424 | 323.409194 | 420.14 | 516.870806 | 0.990896 |
| GO:0006357\_regulation\_of\_transcription\_from\_RNA\_polymerase\_II\_promoter | 435 | 2 | 0.846897 | -0.155609 | 425 | 323.963723 | 420.57 | 517.176277 | 0.989576 |
| GO:0048583\_regulation\_of\_response\_to\_stimulus | 217 | 1 | 0.848848 | -0.153792 | 426 | 325.190410 | 421.79 | 518.389590 | 0.990117 |
| GO:0007423\_sensory\_organ\_development | 219 | 1 | 0.841096 | -0.151700 | 427 | 326.318691 | 422.88 | 519.441309 | 0.990351 |
| GO:0001701\_in\_utero\_embryonic\_development | 221 | 1 | 0.833484 | -0.149641 | 428 | 327.174936 | 423.79 | 520.405064 | 0.990164 |
| GO:0006366\_transcription\_from\_RNA\_polymerase\_II\_promoter | 444 | 2 | 0.829730 | -0.148737 | 429 | 327.608155 | 424.27 | 520.931845 | 0.988974 |
| GO:0016070\_RNA\_metabolic\_process | 658 | 3 | 0.839818 | -0.145883 | 430 | 329.655375 | 426.02 | 522.384625 | 0.990744 |
| GO:0002682\_regulation\_of\_immune\_system\_process | 228 | 1 | 0.807895 | -0.142681 | 431 | 331.899674 | 428.0 | 524.100326 | 0.993039 |
| GO:0007167\_enzyme\_linked\_receptor\_protein\_signaling\_pathway | 229 | 1 | 0.804367 | -0.141718 | 432 | 332.321028 | 428.43 | 524.538972 | 0.991736 |
| GO:0050890\_cognition | 233 | 1 | 0.790558 | -0.137936 | 433 | 334.058535 | 430.09 | 526.121465 | 0.993279 |
| GO:0006468\_protein\_amino\_acid\_phosphorylation | 237 | 1 | 0.777215 | -0.134269 | 435 | 335.724545 | 431.79 | 527.855455 | 0.992621 |
| GO:0044085\_cellular\_component\_biogenesis | 237 | 1 | 0.777215 | -0.134269 | 435 | 335.724545 | 431.79 | 527.855455 | 0.992621 |
| GO:0030097\_hemopoiesis | 253 | 1 | 0.728063 | -0.120655 | 436 | 341.772396 | 437.19 | 532.607604 | 1.002729 |
| GO:0048869\_cellular\_developmental\_process | 1113 | 5 | 0.827493 | -0.120094 | 437 | 342.500869 | 437.78 | 533.059131 | 1.001785 |
| GO:0048878\_chemical\_homeostasis | 254 | 1 | 0.725197 | -0.119857 | 438 | 342.903436 | 438.19 | 533.476564 | 1.000434 |
| GO:0048729\_tissue\_morphogenesis | 255 | 1 | 0.722353 | -0.119065 | 439 | 343.225722 | 438.49 | 533.754278 | 0.998838 |
| GO:0009966\_regulation\_of\_signal\_transduction | 256 | 1 | 0.719531 | -0.118278 | 440 | 343.626332 | 438.83 | 534.033668 | 0.997341 |
| GO:0002376\_immune\_system\_process | 505 | 2 | 0.729505 | -0.109453 | 441 | 347.258642 | 442.05 | 536.841358 | 1.002381 |
| GO:0045944\_positive\_regulation\_of\_transcription\_from\_RNA\_polymerase\_II\_promoter | 269 | 1 | 0.684758 | -0.108558 | 442 | 347.918700 | 442.61 | 537.301300 | 1.001380 |
| GO:0048534\_hemopoietic\_or\_lymphoid\_organ\_development | 277 | 1 | 0.664982 | -0.103014 | 443 | 351.158467 | 445.73 | 540.301533 | 1.006163 |
| GO:0007610\_behavior | 279 | 1 | 0.660215 | -0.101677 | 444 | 351.920520 | 446.39 | 540.859480 | 1.005383 |
| GO:0002520\_immune\_system\_development | 295 | 1 | 0.624407 | -0.091631 | 446 | 357.049182 | 450.85 | 544.650818 | 1.010874 |
| GO:0045595\_regulation\_of\_cell\_differentiation | 295 | 1 | 0.624407 | -0.091631 | 446 | 357.049182 | 450.85 | 544.650818 | 1.010874 |
| GO:0048598\_embryonic\_morphogenesis | 299 | 1 | 0.616054 | -0.089290 | 447 | 357.641233 | 451.34 | 545.038767 | 1.009709 |
| GO:0009790\_embryonic\_development | 567 | 2 | 0.649735 | -0.079948 | 448 | 362.335351 | 455.65 | 548.964649 | 1.017076 |
| GO:0006928\_cell\_motion | 330 | 1 | 0.558182 | -0.073169 | 451 | 364.187762 | 457.3 | 550.412238 | 1.013969 |
| GO:0010646\_regulation\_of\_cell\_communication | 330 | 1 | 0.558182 | -0.073169 | 451 | 364.187762 | 457.3 | 550.412238 | 1.013969 |
| GO:0051674\_localization\_of\_cell | 330 | 1 | 0.558182 | -0.073169 | 451 | 364.187762 | 457.3 | 550.412238 | 1.013969 |
| GO:0051093\_negative\_regulation\_of\_developmental\_process | 331 | 1 | 0.556495 | -0.072703 | 452 | 365.112856 | 458.12 | 551.127144 | 1.013540 |
| GO:0009605\_response\_to\_external\_stimulus | 339 | 1 | 0.543363 | -0.069087 | 453 | 367.540518 | 460.11 | 552.679482 | 1.015695 |
| GO:0007166\_cell\_surface\_receptor\_linked\_signal\_transduction | 597 | 2 | 0.617085 | -0.068579 | 454 | 368.603084 | 460.98 | 553.356916 | 1.015374 |
| GO:0030154\_cell\_differentiation | 1060 | 4 | 0.695094 | -0.065045 | 455 | 370.238915 | 462.31 | 554.381085 | 1.016066 |
| GO:0042981\_regulation\_of\_apoptosis | 360 | 1 | 0.511667 | -0.060458 | 456 | 371.928592 | 463.68 | 555.431408 | 1.016842 |
| GO:0010941\_regulation\_of\_cell\_death | 365 | 1 | 0.504658 | -0.058574 | 458 | 373.769483 | 465.19 | 556.610517 | 1.015699 |
| GO:0043067\_regulation\_of\_programmed\_cell\_death | 365 | 1 | 0.504658 | -0.058574 | 458 | 373.769483 | 465.19 | 556.610517 | 1.015699 |
| GO:0050896\_response\_to\_stimulus | 1107 | 4 | 0.665583 | -0.053087 | 459 | 376.342909 | 467.48 | 558.617091 | 1.018475 |
| GO:0050877\_neurological\_system\_process | 390 | 1 | 0.472308 | -0.050018 | 460 | 378.516583 | 469.25 | 559.983417 | 1.020109 |
| GO:0042221\_response\_to\_chemical\_stimulus | 409 | 1 | 0.450367 | -0.044377 | 461 | 380.535810 | 471.04 | 561.544190 | 1.021779 |
| GO:0042592\_homeostatic\_process | 419 | 1 | 0.439618 | -0.041673 | 462 | 382.350368 | 472.46 | 562.569632 | 1.022641 |
| GO:0065008\_regulation\_of\_biological\_quality | 693 | 2 | 0.531602 | -0.041640 | 463 | 382.518637 | 472.59 | 562.661363 | 1.020713 |
| GO:0032501\_multicellular\_organismal\_process | 2183 | 9 | 0.759414 | -0.040065 | 464 | 383.268851 | 473.16 | 563.051149 | 1.019741 |
| GO:0006915\_apoptosis | 427 | 1 | 0.431382 | -0.039629 | 465 | 383.825426 | 473.59 | 563.354574 | 1.018473 |
| GO:0050793\_regulation\_of\_developmental\_process | 703 | 2 | 0.524040 | -0.039499 | 466 | 384.024911 | 473.76 | 563.495089 | 1.016652 |
| GO:0012501\_programmed\_cell\_death | 433 | 1 | 0.425404 | -0.038163 | 467 | 384.991075 | 474.56 | 564.128925 | 1.016188 |
| GO:0008219\_cell\_death | 444 | 1 | 0.414865 | -0.035616 | 468 | 387.037176 | 476.34 | 565.642824 | 1.017821 |
| GO:0010926\_anatomical\_structure\_formation | 447 | 1 | 0.412081 | -0.034951 | 469 | 387.231109 | 476.53 | 565.828891 | 1.016055 |
| GO:0016265\_death | 450 | 1 | 0.409333 | -0.034299 | 470 | 387.854053 | 477.11 | 566.365947 | 1.015128 |
| GO:0003008\_system\_process | 516 | 1 | 0.356977 | -0.022655 | 471 | 390.903400 | 479.49 | 568.076600 | 1.018025 |
| GO:0006810\_transport | 718 | 1 | 0.256546 | -0.006241 | 472 | 398.077006 | 484.9 | 571.722994 | 1.027331 |
| GO:0051234\_establishment\_of\_localization | 729 | 1 | 0.252675 | -0.005810 | 473 | 398.209741 | 484.97 | 571.730259 | 1.025307 |
| GO:0051179\_localization | 1058 | 2 | 0.348204 | -0.005341 | 474 | 398.243359 | 484.99 | 571.736641 | 1.023186 |
| GO:0006520\_cellular\_amino\_acid\_metabolic\_process | 51 | 0 | 0.000000 | -0.000000 | 481 | 408.868256 | 494.26 | 579.651744 | 1.027568 |
| GO:0006887\_exocytosis | 51 | 0 | 0.000000 | -0.000000 | 481 | 408.868256 | 494.26 | 579.651744 | 1.027568 |
| GO:0007601\_visual\_perception | 51 | 0 | 0.000000 | -0.000000 | 481 | 408.868256 | 494.26 | 579.651744 | 1.027568 |
| GO:0032583\_regulation\_of\_gene-specific\_transcription | 51 | 0 | 0.000000 | -0.000000 | 481 | 408.868256 | 494.26 | 579.651744 | 1.027568 |
| GO:0032880\_regulation\_of\_protein\_localization | 51 | 0 | 0.000000 | -0.000000 | 481 | 408.868256 | 494.26 | 579.651744 | 1.027568 |
| GO:0044106\_cellular\_amine\_metabolic\_process | 51 | 0 | 0.000000 | -0.000000 | 481 | 408.868256 | 494.26 | 579.651744 | 1.027568 |
| GO:0048747\_muscle\_fiber\_development | 51 | 0 | 0.000000 | -0.000000 | 481 | 408.868256 | 494.26 | 579.651744 | 1.027568 |
| GO:0006812\_cation\_transport | 146 | 0 | 0.000000 | -0.000000 | 482 | 410.449272 | 495.6 | 580.750728 | 1.028216 |
| GO:0032940\_secretion\_by\_cell | 149 | 0 | 0.000000 | -0.000000 | 483 | 411.145849 | 496.11 | 581.074151 | 1.027143 |
| GO:0002200\_somatic\_diversification\_of\_immune\_receptors | 34 | 0 | 0.000000 | -0.000000 | 499 | 427.220083 | 511.36 | 595.499917 | 1.024770 |
| GO:0002237\_response\_to\_molecule\_of\_bacterial\_origin | 34 | 0 | 0.000000 | -0.000000 | 499 | 427.220083 | 511.36 | 595.499917 | 1.024770 |
| GO:0002699\_positive\_regulation\_of\_immune\_effector\_process | 34 | 0 | 0.000000 | -0.000000 | 499 | 427.220083 | 511.36 | 595.499917 | 1.024770 |
| GO:0007269\_neurotransmitter\_secretion | 34 | 0 | 0.000000 | -0.000000 | 499 | 427.220083 | 511.36 | 595.499917 | 1.024770 |
| GO:0007338\_single\_fertilization | 34 | 0 | 0.000000 | -0.000000 | 499 | 427.220083 | 511.36 | 595.499917 | 1.024770 |
| GO:0010720\_positive\_regulation\_of\_cell\_development | 34 | 0 | 0.000000 | -0.000000 | 499 | 427.220083 | 511.36 | 595.499917 | 1.024770 |
| GO:0010721\_negative\_regulation\_of\_cell\_development | 34 | 0 | 0.000000 | -0.000000 | 499 | 427.220083 | 511.36 | 595.499917 | 1.024770 |
| GO:0016054\_organic\_acid\_catabolic\_process | 34 | 0 | 0.000000 | -0.000000 | 499 | 427.220083 | 511.36 | 595.499917 | 1.024770 |
| GO:0019882\_antigen\_processing\_and\_presentation | 34 | 0 | 0.000000 | -0.000000 | 499 | 427.220083 | 511.36 | 595.499917 | 1.024770 |
| GO:0045927\_positive\_regulation\_of\_growth | 34 | 0 | 0.000000 | -0.000000 | 499 | 427.220083 | 511.36 | 595.499917 | 1.024770 |
| GO:0046395\_carboxylic\_acid\_catabolic\_process | 34 | 0 | 0.000000 | -0.000000 | 499 | 427.220083 | 511.36 | 595.499917 | 1.024770 |
| GO:0050730\_regulation\_of\_peptidyl-tyrosine\_phosphorylation | 34 | 0 | 0.000000 | -0.000000 | 499 | 427.220083 | 511.36 | 595.499917 | 1.024770 |
| GO:0051047\_positive\_regulation\_of\_secretion | 34 | 0 | 0.000000 | -0.000000 | 499 | 427.220083 | 511.36 | 595.499917 | 1.024770 |
| GO:0051052\_regulation\_of\_DNA\_metabolic\_process | 34 | 0 | 0.000000 | -0.000000 | 499 | 427.220083 | 511.36 | 595.499917 | 1.024770 |
| GO:0060443\_mammary\_gland\_morphogenesis | 34 | 0 | 0.000000 | -0.000000 | 499 | 427.220083 | 511.36 | 595.499917 | 1.024770 |
| GO:0060711\_labyrinthine\_layer\_development | 34 | 0 | 0.000000 | -0.000000 | 499 | 427.220083 | 511.36 | 595.499917 | 1.024770 |
| GO:0000079\_regulation\_of\_cyclin-dependent\_protein\_kinase\_activity | 7 | 0 | 0.000000 | -0.000000 | 669 | 604.649854 | 686.8 | 768.950146 | 1.026607 |
| GO:0001504\_neurotransmitter\_uptake | 7 | 0 | 0.000000 | -0.000000 | 669 | 604.649854 | 686.8 | 768.950146 | 1.026607 |
| GO:0001556\_oocyte\_maturation | 7 | 0 | 0.000000 | -0.000000 | 669 | 604.649854 | 686.8 | 768.950146 | 1.026607 |
| GO:0001573\_ganglioside\_metabolic\_process | 7 | 0 | 0.000000 | -0.000000 | 669 | 604.649854 | 686.8 | 768.950146 | 1.026607 |
| GO:0001736\_establishment\_of\_planar\_polarity | 7 | 0 | 0.000000 | -0.000000 | 669 | 604.649854 | 686.8 | 768.950146 | 1.026607 |
| GO:0001839\_neural\_plate\_morphogenesis | 7 | 0 | 0.000000 | -0.000000 | 669 | 604.649854 | 686.8 | 768.950146 | 1.026607 |
| GO:0001936\_regulation\_of\_endothelial\_cell\_proliferation | 7 | 0 | 0.000000 | -0.000000 | 669 | 604.649854 | 686.8 | 768.950146 | 1.026607 |
| GO:0001967\_suckling\_behavior | 7 | 0 | 0.000000 | -0.000000 | 669 | 604.649854 | 686.8 | 768.950146 | 1.026607 |
| GO:0002011\_morphogenesis\_of\_an\_epithelial\_sheet | 7 | 0 | 0.000000 | -0.000000 | 669 | 604.649854 | 686.8 | 768.950146 | 1.026607 |
| GO:0002052\_positive\_regulation\_of\_neuroblast\_proliferation | 7 | 0 | 0.000000 | -0.000000 | 669 | 604.649854 | 686.8 | 768.950146 | 1.026607 |
| GO:0002063\_chondrocyte\_development | 7 | 0 | 0.000000 | -0.000000 | 669 | 604.649854 | 686.8 | 768.950146 | 1.026607 |
| GO:0002067\_glandular\_epithelial\_cell\_differentiation | 7 | 0 | 0.000000 | -0.000000 | 669 | 604.649854 | 686.8 | 768.950146 | 1.026607 |
| GO:0002076\_osteoblast\_development | 7 | 0 | 0.000000 | -0.000000 | 669 | 604.649854 | 686.8 | 768.950146 | 1.026607 |
| GO:0002087\_regulation\_of\_respiratory\_gaseous\_exchange\_by\_neurological\_system\_process | 7 | 0 | 0.000000 | -0.000000 | 669 | 604.649854 | 686.8 | 768.950146 | 1.026607 |
| GO:0002093\_auditory\_receptor\_cell\_morphogenesis | 7 | 0 | 0.000000 | -0.000000 | 669 | 604.649854 | 686.8 | 768.950146 | 1.026607 |
| GO:0002224\_toll-like\_receptor\_signaling\_pathway | 7 | 0 | 0.000000 | -0.000000 | 669 | 604.649854 | 686.8 | 768.950146 | 1.026607 |
| GO:0002455\_humoral\_immune\_response\_mediated\_by\_circulating\_immunoglobulin | 7 | 0 | 0.000000 | -0.000000 | 669 | 604.649854 | 686.8 | 768.950146 | 1.026607 |
| GO:0002643\_regulation\_of\_tolerance\_induction | 7 | 0 | 0.000000 | -0.000000 | 669 | 604.649854 | 686.8 | 768.950146 | 1.026607 |
| GO:0002645\_positive\_regulation\_of\_tolerance\_induction | 7 | 0 | 0.000000 | -0.000000 | 669 | 604.649854 | 686.8 | 768.950146 | 1.026607 |
| GO:0002714\_positive\_regulation\_of\_B\_cell\_mediated\_immunity | 7 | 0 | 0.000000 | -0.000000 | 669 | 604.649854 | 686.8 | 768.950146 | 1.026607 |
| GO:0002792\_negative\_regulation\_of\_peptide\_secretion | 7 | 0 | 0.000000 | -0.000000 | 669 | 604.649854 | 686.8 | 768.950146 | 1.026607 |
| GO:0002793\_positive\_regulation\_of\_peptide\_secretion | 7 | 0 | 0.000000 | -0.000000 | 669 | 604.649854 | 686.8 | 768.950146 | 1.026607 |
| GO:0002828\_regulation\_of\_T-helper\_2\_type\_immune\_response | 7 | 0 | 0.000000 | -0.000000 | 669 | 604.649854 | 686.8 | 768.950146 | 1.026607 |
| GO:0002863\_positive\_regulation\_of\_inflammatory\_response\_to\_antigenic\_stimulus | 7 | 0 | 0.000000 | -0.000000 | 669 | 604.649854 | 686.8 | 768.950146 | 1.026607 |
| GO:0002891\_positive\_regulation\_of\_immunoglobulin\_mediated\_immune\_response | 7 | 0 | 0.000000 | -0.000000 | 669 | 604.649854 | 686.8 | 768.950146 | 1.026607 |
| GO:0003084\_positive\_regulation\_of\_systemic\_arterial\_blood\_pressure | 7 | 0 | 0.000000 | -0.000000 | 669 | 604.649854 | 686.8 | 768.950146 | 1.026607 |
| GO:0006014\_D-ribose\_metabolic\_process | 7 | 0 | 0.000000 | -0.000000 | 669 | 604.649854 | 686.8 | 768.950146 | 1.026607 |
| GO:0006041\_glucosamine\_metabolic\_process | 7 | 0 | 0.000000 | -0.000000 | 669 | 604.649854 | 686.8 | 768.950146 | 1.026607 |
| GO:0006044\_N-acetylglucosamine\_metabolic\_process | 7 | 0 | 0.000000 | -0.000000 | 669 | 604.649854 | 686.8 | 768.950146 | 1.026607 |
| GO:0006096\_glycolysis | 7 | 0 | 0.000000 | -0.000000 | 669 | 604.649854 | 686.8 | 768.950146 | 1.026607 |
| GO:0006119\_oxidative\_phosphorylation | 7 | 0 | 0.000000 | -0.000000 | 669 | 604.649854 | 686.8 | 768.950146 | 1.026607 |
| GO:0006275\_regulation\_of\_DNA\_replication | 7 | 0 | 0.000000 | -0.000000 | 669 | 604.649854 | 686.8 | 768.950146 | 1.026607 |
| GO:0006298\_mismatch\_repair | 7 | 0 | 0.000000 | -0.000000 | 669 | 604.649854 | 686.8 | 768.950146 | 1.026607 |
| GO:0006352\_transcription\_initiation | 7 | 0 | 0.000000 | -0.000000 | 669 | 604.649854 | 686.8 | 768.950146 | 1.026607 |
| GO:0006401\_RNA\_catabolic\_process | 7 | 0 | 0.000000 | -0.000000 | 669 | 604.649854 | 686.8 | 768.950146 | 1.026607 |
| GO:0006406\_mRNA\_export\_from\_nucleus | 7 | 0 | 0.000000 | -0.000000 | 669 | 604.649854 | 686.8 | 768.950146 | 1.026607 |
| GO:0006505\_GPI\_anchor\_metabolic\_process | 7 | 0 | 0.000000 | -0.000000 | 669 | 604.649854 | 686.8 | 768.950146 | 1.026607 |
| GO:0006516\_glycoprotein\_catabolic\_process | 7 | 0 | 0.000000 | -0.000000 | 669 | 604.649854 | 686.8 | 768.950146 | 1.026607 |
| GO:0006612\_protein\_targeting\_to\_membrane | 7 | 0 | 0.000000 | -0.000000 | 669 | 604.649854 | 686.8 | 768.950146 | 1.026607 |
| GO:0006769\_nicotinamide\_metabolic\_process | 7 | 0 | 0.000000 | -0.000000 | 669 | 604.649854 | 686.8 | 768.950146 | 1.026607 |
| GO:0006783\_heme\_biosynthetic\_process | 7 | 0 | 0.000000 | -0.000000 | 669 | 604.649854 | 686.8 | 768.950146 | 1.026607 |
| GO:0006818\_hydrogen\_transport | 7 | 0 | 0.000000 | -0.000000 | 669 | 604.649854 | 686.8 | 768.950146 | 1.026607 |
| GO:0006878\_cellular\_copper\_ion\_homeostasis | 7 | 0 | 0.000000 | -0.000000 | 669 | 604.649854 | 686.8 | 768.950146 | 1.026607 |
| GO:0006884\_cell\_volume\_homeostasis | 7 | 0 | 0.000000 | -0.000000 | 669 | 604.649854 | 686.8 | 768.950146 | 1.026607 |
| GO:0006949\_syncytium\_formation | 7 | 0 | 0.000000 | -0.000000 | 669 | 604.649854 | 686.8 | 768.950146 | 1.026607 |
| GO:0007034\_vacuolar\_transport | 7 | 0 | 0.000000 | -0.000000 | 669 | 604.649854 | 686.8 | 768.950146 | 1.026607 |
| GO:0007130\_synaptonemal\_complex\_assembly | 7 | 0 | 0.000000 | -0.000000 | 669 | 604.649854 | 686.8 | 768.950146 | 1.026607 |
| GO:0007164\_establishment\_of\_tissue\_polarity | 7 | 0 | 0.000000 | -0.000000 | 669 | 604.649854 | 686.8 | 768.950146 | 1.026607 |
| GO:0007191\_activation\_of\_adenylate\_cyclase\_activity\_by\_dopamine\_receptor\_signaling\_pathway | 7 | 0 | 0.000000 | -0.000000 | 669 | 604.649854 | 686.8 | 768.950146 | 1.026607 |
| GO:0007271\_synaptic\_transmission\_\_cholinergic | 7 | 0 | 0.000000 | -0.000000 | 669 | 604.649854 | 686.8 | 768.950146 | 1.026607 |
| GO:0007413\_axonal\_fasciculation | 7 | 0 | 0.000000 | -0.000000 | 669 | 604.649854 | 686.8 | 768.950146 | 1.026607 |
| GO:0007440\_foregut\_morphogenesis | 7 | 0 | 0.000000 | -0.000000 | 669 | 604.649854 | 686.8 | 768.950146 | 1.026607 |
| GO:0007616\_long-term\_memory | 7 | 0 | 0.000000 | -0.000000 | 669 | 604.649854 | 686.8 | 768.950146 | 1.026607 |
| GO:0008033\_tRNA\_processing | 7 | 0 | 0.000000 | -0.000000 | 669 | 604.649854 | 686.8 | 768.950146 | 1.026607 |
| GO:0008299\_isoprenoid\_biosynthetic\_process | 7 | 0 | 0.000000 | -0.000000 | 669 | 604.649854 | 686.8 | 768.950146 | 1.026607 |
| GO:0008340\_determination\_of\_adult\_lifespan | 7 | 0 | 0.000000 | -0.000000 | 669 | 604.649854 | 686.8 | 768.950146 | 1.026607 |
| GO:0009150\_purine\_ribonucleotide\_metabolic\_process | 7 | 0 | 0.000000 | -0.000000 | 669 | 604.649854 | 686.8 | 768.950146 | 1.026607 |
| GO:0009200\_deoxyribonucleoside\_triphosphate\_metabolic\_process | 7 | 0 | 0.000000 | -0.000000 | 669 | 604.649854 | 686.8 | 768.950146 | 1.026607 |
| GO:0009259\_ribonucleotide\_metabolic\_process | 7 | 0 | 0.000000 | -0.000000 | 669 | 604.649854 | 686.8 | 768.950146 | 1.026607 |
| GO:0009311\_oligosaccharide\_metabolic\_process | 7 | 0 | 0.000000 | -0.000000 | 669 | 604.649854 | 686.8 | 768.950146 | 1.026607 |
| GO:0009394\_2'-deoxyribonucleotide\_metabolic\_process | 7 | 0 | 0.000000 | -0.000000 | 669 | 604.649854 | 686.8 | 768.950146 | 1.026607 |
| GO:0009820\_alkaloid\_metabolic\_process | 7 | 0 | 0.000000 | -0.000000 | 669 | 604.649854 | 686.8 | 768.950146 | 1.026607 |
| GO:0010469\_regulation\_of\_receptor\_activity | 7 | 0 | 0.000000 | -0.000000 | 669 | 604.649854 | 686.8 | 768.950146 | 1.026607 |
| GO:0014047\_glutamate\_secretion | 7 | 0 | 0.000000 | -0.000000 | 669 | 604.649854 | 686.8 | 768.950146 | 1.026607 |
| GO:0014066\_regulation\_of\_phosphoinositide\_3-kinase\_cascade | 7 | 0 | 0.000000 | -0.000000 | 669 | 604.649854 | 686.8 | 768.950146 | 1.026607 |
| GO:0014821\_phasic\_smooth\_muscle\_contraction | 7 | 0 | 0.000000 | -0.000000 | 669 | 604.649854 | 686.8 | 768.950146 | 1.026607 |
| GO:0015697\_quaternary\_ammonium\_group\_transport | 7 | 0 | 0.000000 | -0.000000 | 669 | 604.649854 | 686.8 | 768.950146 | 1.026607 |
| GO:0015813\_L-glutamate\_transport | 7 | 0 | 0.000000 | -0.000000 | 669 | 604.649854 | 686.8 | 768.950146 | 1.026607 |
| GO:0015908\_fatty\_acid\_transport | 7 | 0 | 0.000000 | -0.000000 | 669 | 604.649854 | 686.8 | 768.950146 | 1.026607 |
| GO:0015914\_phospholipid\_transport | 7 | 0 | 0.000000 | -0.000000 | 669 | 604.649854 | 686.8 | 768.950146 | 1.026607 |
| GO:0015992\_proton\_transport | 7 | 0 | 0.000000 | -0.000000 | 669 | 604.649854 | 686.8 | 768.950146 | 1.026607 |
| GO:0016339\_calcium-dependent\_cell-cell\_adhesion | 7 | 0 | 0.000000 | -0.000000 | 669 | 604.649854 | 686.8 | 768.950146 | 1.026607 |
| GO:0019362\_pyridine\_nucleotide\_metabolic\_process | 7 | 0 | 0.000000 | -0.000000 | 669 | 604.649854 | 686.8 | 768.950146 | 1.026607 |
| GO:0019692\_deoxyribose\_phosphate\_metabolic\_process | 7 | 0 | 0.000000 | -0.000000 | 669 | 604.649854 | 686.8 | 768.950146 | 1.026607 |
| GO:0019800\_peptide\_cross-linking\_via\_chondroitin\_4-sulfate\_glycosaminoglycan | 7 | 0 | 0.000000 | -0.000000 | 669 | 604.649854 | 686.8 | 768.950146 | 1.026607 |
| GO:0020027\_hemoglobin\_metabolic\_process | 7 | 0 | 0.000000 | -0.000000 | 669 | 604.649854 | 686.8 | 768.950146 | 1.026607 |
| GO:0021514\_ventral\_spinal\_cord\_interneuron\_differentiation | 7 | 0 | 0.000000 | -0.000000 | 669 | 604.649854 | 686.8 | 768.950146 | 1.026607 |
| GO:0021516\_dorsal\_spinal\_cord\_development | 7 | 0 | 0.000000 | -0.000000 | 669 | 604.649854 | 686.8 | 768.950146 | 1.026607 |
| GO:0021520\_spinal\_cord\_motor\_neuron\_cell\_fate\_specification | 7 | 0 | 0.000000 | -0.000000 | 669 | 604.649854 | 686.8 | 768.950146 | 1.026607 |
| GO:0021521\_ventral\_spinal\_cord\_interneuron\_specification | 7 | 0 | 0.000000 | -0.000000 | 669 | 604.649854 | 686.8 | 768.950146 | 1.026607 |
| GO:0021546\_rhombomere\_development | 7 | 0 | 0.000000 | -0.000000 | 669 | 604.649854 | 686.8 | 768.950146 | 1.026607 |
| GO:0021756\_striatum\_development | 7 | 0 | 0.000000 | -0.000000 | 669 | 604.649854 | 686.8 | 768.950146 | 1.026607 |
| GO:0021884\_forebrain\_neuron\_development | 7 | 0 | 0.000000 | -0.000000 | 669 | 604.649854 | 686.8 | 768.950146 | 1.026607 |
| GO:0021903\_rostrocaudal\_neural\_tube\_patterning | 7 | 0 | 0.000000 | -0.000000 | 669 | 604.649854 | 686.8 | 768.950146 | 1.026607 |
| GO:0021984\_adenohypophysis\_development | 7 | 0 | 0.000000 | -0.000000 | 669 | 604.649854 | 686.8 | 768.950146 | 1.026607 |
| GO:0022407\_regulation\_of\_cell-cell\_adhesion | 7 | 0 | 0.000000 | -0.000000 | 669 | 604.649854 | 686.8 | 768.950146 | 1.026607 |
| GO:0022618\_ribonucleoprotein\_complex\_assembly | 7 | 0 | 0.000000 | -0.000000 | 669 | 604.649854 | 686.8 | 768.950146 | 1.026607 |
| GO:0030104\_water\_homeostasis | 7 | 0 | 0.000000 | -0.000000 | 669 | 604.649854 | 686.8 | 768.950146 | 1.026607 |
| GO:0030432\_peristalsis | 7 | 0 | 0.000000 | -0.000000 | 669 | 604.649854 | 686.8 | 768.950146 | 1.026607 |
| GO:0030517\_negative\_regulation\_of\_axon\_extension | 7 | 0 | 0.000000 | -0.000000 | 669 | 604.649854 | 686.8 | 768.950146 | 1.026607 |
| GO:0030520\_estrogen\_receptor\_signaling\_pathway | 7 | 0 | 0.000000 | -0.000000 | 669 | 604.649854 | 686.8 | 768.950146 | 1.026607 |
| GO:0030521\_androgen\_receptor\_signaling\_pathway | 7 | 0 | 0.000000 | -0.000000 | 669 | 604.649854 | 686.8 | 768.950146 | 1.026607 |
| GO:0030903\_notochord\_development | 7 | 0 | 0.000000 | -0.000000 | 669 | 604.649854 | 686.8 | 768.950146 | 1.026607 |
| GO:0031124\_mRNA\_3'-end\_processing | 7 | 0 | 0.000000 | -0.000000 | 669 | 604.649854 | 686.8 | 768.950146 | 1.026607 |
| GO:0032104\_regulation\_of\_response\_to\_extracellular\_stimulus | 7 | 0 | 0.000000 | -0.000000 | 669 | 604.649854 | 686.8 | 768.950146 | 1.026607 |
| GO:0032107\_regulation\_of\_response\_to\_nutrient\_levels | 7 | 0 | 0.000000 | -0.000000 | 669 | 604.649854 | 686.8 | 768.950146 | 1.026607 |
| GO:0032228\_regulation\_of\_synaptic\_transmission\_\_GABAergic | 7 | 0 | 0.000000 | -0.000000 | 669 | 604.649854 | 686.8 | 768.950146 | 1.026607 |
| GO:0032319\_regulation\_of\_Rho\_GTPase\_activity | 7 | 0 | 0.000000 | -0.000000 | 669 | 604.649854 | 686.8 | 768.950146 | 1.026607 |
| GO:0032387\_negative\_regulation\_of\_intracellular\_transport | 7 | 0 | 0.000000 | -0.000000 | 669 | 604.649854 | 686.8 | 768.950146 | 1.026607 |
| GO:0032507\_maintenance\_of\_protein\_location\_in\_cell | 7 | 0 | 0.000000 | -0.000000 | 669 | 604.649854 | 686.8 | 768.950146 | 1.026607 |
| GO:0033032\_regulation\_of\_myeloid\_cell\_apoptosis | 7 | 0 | 0.000000 | -0.000000 | 669 | 604.649854 | 686.8 | 768.950146 | 1.026607 |
| GO:0033057\_reproductive\_behavior\_in\_a\_multicellular\_organism | 7 | 0 | 0.000000 | -0.000000 | 669 | 604.649854 | 686.8 | 768.950146 | 1.026607 |
| GO:0034599\_cellular\_response\_to\_oxidative\_stress | 7 | 0 | 0.000000 | -0.000000 | 669 | 604.649854 | 686.8 | 768.950146 | 1.026607 |
| GO:0042033\_chemokine\_biosynthetic\_process | 7 | 0 | 0.000000 | -0.000000 | 669 | 604.649854 | 686.8 | 768.950146 | 1.026607 |
| GO:0042133\_neurotransmitter\_metabolic\_process | 7 | 0 | 0.000000 | -0.000000 | 669 | 604.649854 | 686.8 | 768.950146 | 1.026607 |
| GO:0042168\_heme\_metabolic\_process | 7 | 0 | 0.000000 | -0.000000 | 669 | 604.649854 | 686.8 | 768.950146 | 1.026607 |
| GO:0042415\_norepinephrine\_metabolic\_process | 7 | 0 | 0.000000 | -0.000000 | 669 | 604.649854 | 686.8 | 768.950146 | 1.026607 |
| GO:0042438\_melanin\_biosynthetic\_process | 7 | 0 | 0.000000 | -0.000000 | 669 | 604.649854 | 686.8 | 768.950146 | 1.026607 |
| GO:0042503\_tyrosine\_phosphorylation\_of\_Stat3\_protein | 7 | 0 | 0.000000 | -0.000000 | 669 | 604.649854 | 686.8 | 768.950146 | 1.026607 |
| GO:0042572\_retinol\_metabolic\_process | 7 | 0 | 0.000000 | -0.000000 | 669 | 604.649854 | 686.8 | 768.950146 | 1.026607 |
| GO:0043353\_enucleate\_erythrocyte\_differentiation | 7 | 0 | 0.000000 | -0.000000 | 669 | 604.649854 | 686.8 | 768.950146 | 1.026607 |
| GO:0043372\_positive\_regulation\_of\_CD4-positive\_\_alpha\_beta\_T\_cell\_differentiation | 7 | 0 | 0.000000 | -0.000000 | 669 | 604.649854 | 686.8 | 768.950146 | 1.026607 |
| GO:0043449\_cellular\_alkene\_metabolic\_process | 7 | 0 | 0.000000 | -0.000000 | 669 | 604.649854 | 686.8 | 768.950146 | 1.026607 |
| GO:0043507\_positive\_regulation\_of\_JUN\_kinase\_activity | 7 | 0 | 0.000000 | -0.000000 | 669 | 604.649854 | 686.8 | 768.950146 | 1.026607 |
| GO:0043567\_regulation\_of\_insulin-like\_growth\_factor\_receptor\_signaling\_pathway | 7 | 0 | 0.000000 | -0.000000 | 669 | 604.649854 | 686.8 | 768.950146 | 1.026607 |
| GO:0043584\_nose\_development | 7 | 0 | 0.000000 | -0.000000 | 669 | 604.649854 | 686.8 | 768.950146 | 1.026607 |
| GO:0044065\_regulation\_of\_respiratory\_system\_process | 7 | 0 | 0.000000 | -0.000000 | 669 | 604.649854 | 686.8 | 768.950146 | 1.026607 |
| GO:0044275\_cellular\_carbohydrate\_catabolic\_process | 7 | 0 | 0.000000 | -0.000000 | 669 | 604.649854 | 686.8 | 768.950146 | 1.026607 |
| GO:0045059\_positive\_thymic\_T\_cell\_selection | 7 | 0 | 0.000000 | -0.000000 | 669 | 604.649854 | 686.8 | 768.950146 | 1.026607 |
| GO:0045073\_regulation\_of\_chemokine\_biosynthetic\_process | 7 | 0 | 0.000000 | -0.000000 | 669 | 604.649854 | 686.8 | 768.950146 | 1.026607 |
| GO:0045581\_negative\_regulation\_of\_T\_cell\_differentiation | 7 | 0 | 0.000000 | -0.000000 | 669 | 604.649854 | 686.8 | 768.950146 | 1.026607 |
| GO:0045604\_regulation\_of\_epidermal\_cell\_differentiation | 7 | 0 | 0.000000 | -0.000000 | 669 | 604.649854 | 686.8 | 768.950146 | 1.026607 |
| GO:0045668\_negative\_regulation\_of\_osteoblast\_differentiation | 7 | 0 | 0.000000 | -0.000000 | 669 | 604.649854 | 686.8 | 768.950146 | 1.026607 |
| GO:0045823\_positive\_regulation\_of\_heart\_contraction | 7 | 0 | 0.000000 | -0.000000 | 669 | 604.649854 | 686.8 | 768.950146 | 1.026607 |
| GO:0045840\_positive\_regulation\_of\_mitosis | 7 | 0 | 0.000000 | -0.000000 | 669 | 604.649854 | 686.8 | 768.950146 | 1.026607 |
| GO:0045862\_positive\_regulation\_of\_proteolysis | 7 | 0 | 0.000000 | -0.000000 | 669 | 604.649854 | 686.8 | 768.950146 | 1.026607 |
| GO:0045879\_negative\_regulation\_of\_smoothened\_signaling\_pathway | 7 | 0 | 0.000000 | -0.000000 | 669 | 604.649854 | 686.8 | 768.950146 | 1.026607 |
| GO:0045880\_positive\_regulation\_of\_smoothened\_signaling\_pathway | 7 | 0 | 0.000000 | -0.000000 | 669 | 604.649854 | 686.8 | 768.950146 | 1.026607 |
| GO:0045986\_negative\_regulation\_of\_smooth\_muscle\_contraction | 7 | 0 | 0.000000 | -0.000000 | 669 | 604.649854 | 686.8 | 768.950146 | 1.026607 |
| GO:0046496\_nicotinamide\_nucleotide\_metabolic\_process | 7 | 0 | 0.000000 | -0.000000 | 669 | 604.649854 | 686.8 | 768.950146 | 1.026607 |
| GO:0046504\_glycerol\_ether\_biosynthetic\_process | 7 | 0 | 0.000000 | -0.000000 | 669 | 604.649854 | 686.8 | 768.950146 | 1.026607 |
| GO:0046513\_ceramide\_biosynthetic\_process | 7 | 0 | 0.000000 | -0.000000 | 669 | 604.649854 | 686.8 | 768.950146 | 1.026607 |
| GO:0046520\_sphingoid\_biosynthetic\_process | 7 | 0 | 0.000000 | -0.000000 | 669 | 604.649854 | 686.8 | 768.950146 | 1.026607 |
| GO:0046543\_development\_of\_secondary\_female\_sexual\_characteristics | 7 | 0 | 0.000000 | -0.000000 | 669 | 604.649854 | 686.8 | 768.950146 | 1.026607 |
| GO:0046622\_positive\_regulation\_of\_organ\_growth | 7 | 0 | 0.000000 | -0.000000 | 669 | 604.649854 | 686.8 | 768.950146 | 1.026607 |
| GO:0046626\_regulation\_of\_insulin\_receptor\_signaling\_pathway | 7 | 0 | 0.000000 | -0.000000 | 669 | 604.649854 | 686.8 | 768.950146 | 1.026607 |
| GO:0046676\_negative\_regulation\_of\_insulin\_secretion | 7 | 0 | 0.000000 | -0.000000 | 669 | 604.649854 | 686.8 | 768.950146 | 1.026607 |
| GO:0046677\_response\_to\_antibiotic | 7 | 0 | 0.000000 | -0.000000 | 669 | 604.649854 | 686.8 | 768.950146 | 1.026607 |
| GO:0046823\_negative\_regulation\_of\_nucleocytoplasmic\_transport | 7 | 0 | 0.000000 | -0.000000 | 669 | 604.649854 | 686.8 | 768.950146 | 1.026607 |
| GO:0046824\_positive\_regulation\_of\_nucleocytoplasmic\_transport | 7 | 0 | 0.000000 | -0.000000 | 669 | 604.649854 | 686.8 | 768.950146 | 1.026607 |
| GO:0046847\_filopodium\_assembly | 7 | 0 | 0.000000 | -0.000000 | 669 | 604.649854 | 686.8 | 768.950146 | 1.026607 |
| GO:0048148\_behavioral\_response\_to\_cocaine | 7 | 0 | 0.000000 | -0.000000 | 669 | 604.649854 | 686.8 | 768.950146 | 1.026607 |
| GO:0048304\_positive\_regulation\_of\_isotype\_switching\_to\_IgG\_isotypes | 7 | 0 | 0.000000 | -0.000000 | 669 | 604.649854 | 686.8 | 768.950146 | 1.026607 |
| GO:0048486\_parasympathetic\_nervous\_system\_development | 7 | 0 | 0.000000 | -0.000000 | 669 | 604.649854 | 686.8 | 768.950146 | 1.026607 |
| GO:0048537\_mucosal-associated\_lymphoid\_tissue\_development | 7 | 0 | 0.000000 | -0.000000 | 669 | 604.649854 | 686.8 | 768.950146 | 1.026607 |
| GO:0048753\_pigment\_granule\_organization | 7 | 0 | 0.000000 | -0.000000 | 669 | 604.649854 | 686.8 | 768.950146 | 1.026607 |
| GO:0048814\_regulation\_of\_dendrite\_morphogenesis | 7 | 0 | 0.000000 | -0.000000 | 669 | 604.649854 | 686.8 | 768.950146 | 1.026607 |
| GO:0048857\_neural\_nucleus\_development | 7 | 0 | 0.000000 | -0.000000 | 669 | 604.649854 | 686.8 | 768.950146 | 1.026607 |
| GO:0050755\_chemokine\_metabolic\_process | 7 | 0 | 0.000000 | -0.000000 | 669 | 604.649854 | 686.8 | 768.950146 | 1.026607 |
| GO:0050773\_regulation\_of\_dendrite\_development | 7 | 0 | 0.000000 | -0.000000 | 669 | 604.649854 | 686.8 | 768.950146 | 1.026607 |
| GO:0051028\_mRNA\_transport | 7 | 0 | 0.000000 | -0.000000 | 669 | 604.649854 | 686.8 | 768.950146 | 1.026607 |
| GO:0051785\_positive\_regulation\_of\_nuclear\_division | 7 | 0 | 0.000000 | -0.000000 | 669 | 604.649854 | 686.8 | 768.950146 | 1.026607 |
| GO:0051928\_positive\_regulation\_of\_calcium\_ion\_transport | 7 | 0 | 0.000000 | -0.000000 | 669 | 604.649854 | 686.8 | 768.950146 | 1.026607 |
| GO:0055069\_zinc\_ion\_homeostasis | 7 | 0 | 0.000000 | -0.000000 | 669 | 604.649854 | 686.8 | 768.950146 | 1.026607 |
| GO:0055070\_copper\_ion\_homeostasis | 7 | 0 | 0.000000 | -0.000000 | 669 | 604.649854 | 686.8 | 768.950146 | 1.026607 |
| GO:0060037\_pharyngeal\_system\_development | 7 | 0 | 0.000000 | -0.000000 | 669 | 604.649854 | 686.8 | 768.950146 | 1.026607 |
| GO:0060080\_regulation\_of\_inhibitory\_postsynaptic\_membrane\_potential | 7 | 0 | 0.000000 | -0.000000 | 669 | 604.649854 | 686.8 | 768.950146 | 1.026607 |
| GO:0060088\_auditory\_receptor\_cell\_stereocilium\_organization | 7 | 0 | 0.000000 | -0.000000 | 669 | 604.649854 | 686.8 | 768.950146 | 1.026607 |
| GO:0060117\_auditory\_receptor\_cell\_development | 7 | 0 | 0.000000 | -0.000000 | 669 | 604.649854 | 686.8 | 768.950146 | 1.026607 |
| GO:0060441\_branching\_involved\_in\_lung\_morphogenesis | 7 | 0 | 0.000000 | -0.000000 | 669 | 604.649854 | 686.8 | 768.950146 | 1.026607 |
| GO:0060526\_prostate\_glandular\_acinus\_morphogenesis | 7 | 0 | 0.000000 | -0.000000 | 669 | 604.649854 | 686.8 | 768.950146 | 1.026607 |
| GO:0060527\_prostate\_epithelial\_cord\_arborization\_involved\_in\_prostate\_glandular\_acinus\_morphogenesis | 7 | 0 | 0.000000 | -0.000000 | 669 | 604.649854 | 686.8 | 768.950146 | 1.026607 |
| GO:0060579\_ventral\_spinal\_cord\_interneuron\_fate\_commitment | 7 | 0 | 0.000000 | -0.000000 | 669 | 604.649854 | 686.8 | 768.950146 | 1.026607 |
| GO:0060664\_epithelial\_cell\_proliferation\_involved\_in\_salivary\_gland\_morphogenesis | 7 | 0 | 0.000000 | -0.000000 | 669 | 604.649854 | 686.8 | 768.950146 | 1.026607 |
| GO:0060687\_regulation\_of\_branching\_involved\_in\_prostate\_gland\_morphogenesis | 7 | 0 | 0.000000 | -0.000000 | 669 | 604.649854 | 686.8 | 768.950146 | 1.026607 |
| GO:0060770\_negative\_regulation\_of\_epithelial\_cell\_proliferation\_involved\_in\_prostate\_gland\_development | 7 | 0 | 0.000000 | -0.000000 | 669 | 604.649854 | 686.8 | 768.950146 | 1.026607 |
| GO:0060788\_ectodermal\_placode\_formation | 7 | 0 | 0.000000 | -0.000000 | 669 | 604.649854 | 686.8 | 768.950146 | 1.026607 |
| GO:0060795\_cell\_fate\_commitment\_involved\_in\_the\_formation\_of\_primary\_germ\_layers | 7 | 0 | 0.000000 | -0.000000 | 669 | 604.649854 | 686.8 | 768.950146 | 1.026607 |
| GO:0070228\_regulation\_of\_lymphocyte\_apoptosis | 7 | 0 | 0.000000 | -0.000000 | 669 | 604.649854 | 686.8 | 768.950146 | 1.026607 |
| GO:0070646\_protein\_modification\_by\_small\_protein\_removal | 7 | 0 | 0.000000 | -0.000000 | 669 | 604.649854 | 686.8 | 768.950146 | 1.026607 |
| GO:0009607\_response\_to\_biotic\_stimulus | 114 | 0 | 0.000000 | -0.000000 | 670 | 605.854331 | 687.86 | 769.865669 | 1.026657 |
| GO:0001708\_cell\_fate\_specification | 56 | 0 | 0.000000 | -0.000000 | 679 | 614.198598 | 695.18 | 776.161402 | 1.023829 |
| GO:0002683\_negative\_regulation\_of\_immune\_system\_process | 56 | 0 | 0.000000 | -0.000000 | 679 | 614.198598 | 695.18 | 776.161402 | 1.023829 |
| GO:0002703\_regulation\_of\_leukocyte\_mediated\_immunity | 56 | 0 | 0.000000 | -0.000000 | 679 | 614.198598 | 695.18 | 776.161402 | 1.023829 |
| GO:0009187\_cyclic\_nucleotide\_metabolic\_process | 56 | 0 | 0.000000 | -0.000000 | 679 | 614.198598 | 695.18 | 776.161402 | 1.023829 |
| GO:0042089\_cytokine\_biosynthetic\_process | 56 | 0 | 0.000000 | -0.000000 | 679 | 614.198598 | 695.18 | 776.161402 | 1.023829 |
| GO:0042107\_cytokine\_metabolic\_process | 56 | 0 | 0.000000 | -0.000000 | 679 | 614.198598 | 695.18 | 776.161402 | 1.023829 |
| GO:0046486\_glycerolipid\_metabolic\_process | 56 | 0 | 0.000000 | -0.000000 | 679 | 614.198598 | 695.18 | 776.161402 | 1.023829 |
| GO:0050678\_regulation\_of\_epithelial\_cell\_proliferation | 56 | 0 | 0.000000 | -0.000000 | 679 | 614.198598 | 695.18 | 776.161402 | 1.023829 |
| GO:0051321\_meiotic\_cell\_cycle | 56 | 0 | 0.000000 | -0.000000 | 679 | 614.198598 | 695.18 | 776.161402 | 1.023829 |
| GO:0001824\_blastocyst\_development | 40 | 0 | 0.000000 | -0.000000 | 690 | 625.173493 | 705.74 | 786.306507 | 1.022812 |
| GO:0007346\_regulation\_of\_mitotic\_cell\_cycle | 40 | 0 | 0.000000 | -0.000000 | 690 | 625.173493 | 705.74 | 786.306507 | 1.022812 |
| GO:0007599\_hemostasis | 40 | 0 | 0.000000 | -0.000000 | 690 | 625.173493 | 705.74 | 786.306507 | 1.022812 |
| GO:0008203\_cholesterol\_metabolic\_process | 40 | 0 | 0.000000 | -0.000000 | 690 | 625.173493 | 705.74 | 786.306507 | 1.022812 |
| GO:0014031\_mesenchymal\_cell\_development | 40 | 0 | 0.000000 | -0.000000 | 690 | 625.173493 | 705.74 | 786.306507 | 1.022812 |
| GO:0016071\_mRNA\_metabolic\_process | 40 | 0 | 0.000000 | -0.000000 | 690 | 625.173493 | 705.74 | 786.306507 | 1.022812 |
| GO:0016358\_dendrite\_development | 40 | 0 | 0.000000 | -0.000000 | 690 | 625.173493 | 705.74 | 786.306507 | 1.022812 |
| GO:0016485\_protein\_processing | 40 | 0 | 0.000000 | -0.000000 | 690 | 625.173493 | 705.74 | 786.306507 | 1.022812 |
| GO:0017015\_regulation\_of\_transforming\_growth\_factor\_beta\_receptor\_signaling\_pathway | 40 | 0 | 0.000000 | -0.000000 | 690 | 625.173493 | 705.74 | 786.306507 | 1.022812 |
| GO:0019935\_cyclic-nucleotide-mediated\_signaling | 40 | 0 | 0.000000 | -0.000000 | 690 | 625.173493 | 705.74 | 786.306507 | 1.022812 |
| GO:0046850\_regulation\_of\_bone\_remodeling | 40 | 0 | 0.000000 | -0.000000 | 690 | 625.173493 | 705.74 | 786.306507 | 1.022812 |
| GO:0001843\_neural\_tube\_closure | 33 | 0 | 0.000000 | -0.000000 | 705 | 640.388248 | 719.94 | 799.491752 | 1.021191 |
| GO:0002562\_somatic\_diversification\_of\_immune\_receptors\_via\_germline\_recombination\_within\_a\_single\_locus | 33 | 0 | 0.000000 | -0.000000 | 705 | 640.388248 | 719.94 | 799.491752 | 1.021191 |
| GO:0006643\_membrane\_lipid\_metabolic\_process | 33 | 0 | 0.000000 | -0.000000 | 705 | 640.388248 | 719.94 | 799.491752 | 1.021191 |
| GO:0007188\_G-protein\_signaling\_\_coupled\_to\_cAMP\_nucleotide\_second\_messenger | 33 | 0 | 0.000000 | -0.000000 | 705 | 640.388248 | 719.94 | 799.491752 | 1.021191 |
| GO:0007270\_nerve-nerve\_synaptic\_transmission | 33 | 0 | 0.000000 | -0.000000 | 705 | 640.388248 | 719.94 | 799.491752 | 1.021191 |
| GO:0007431\_salivary\_gland\_development | 33 | 0 | 0.000000 | -0.000000 | 705 | 640.388248 | 719.94 | 799.491752 | 1.021191 |
| GO:0007565\_female\_pregnancy | 33 | 0 | 0.000000 | -0.000000 | 705 | 640.388248 | 719.94 | 799.491752 | 1.021191 |
| GO:0008584\_male\_gonad\_development | 33 | 0 | 0.000000 | -0.000000 | 705 | 640.388248 | 719.94 | 799.491752 | 1.021191 |
| GO:0008643\_carbohydrate\_transport | 33 | 0 | 0.000000 | -0.000000 | 705 | 640.388248 | 719.94 | 799.491752 | 1.021191 |
| GO:0016444\_somatic\_cell\_DNA\_recombination | 33 | 0 | 0.000000 | -0.000000 | 705 | 640.388248 | 719.94 | 799.491752 | 1.021191 |
| GO:0021536\_diencephalon\_development | 33 | 0 | 0.000000 | -0.000000 | 705 | 640.388248 | 719.94 | 799.491752 | 1.021191 |
| GO:0021987\_cerebral\_cortex\_development | 33 | 0 | 0.000000 | -0.000000 | 705 | 640.388248 | 719.94 | 799.491752 | 1.021191 |
| GO:0022037\_metencephalon\_development | 33 | 0 | 0.000000 | -0.000000 | 705 | 640.388248 | 719.94 | 799.491752 | 1.021191 |
| GO:0042108\_positive\_regulation\_of\_cytokine\_biosynthetic\_process | 33 | 0 | 0.000000 | -0.000000 | 705 | 640.388248 | 719.94 | 799.491752 | 1.021191 |
| GO:0060606\_tube\_closure | 33 | 0 | 0.000000 | -0.000000 | 705 | 640.388248 | 719.94 | 799.491752 | 1.021191 |
| GO:0040008\_regulation\_of\_growth | 113 | 0 | 0.000000 | -0.000000 | 706 | 641.531994 | 721.02 | 800.508006 | 1.021275 |
| GO:0048589\_developmental\_growth | 75 | 0 | 0.000000 | -0.000000 | 708 | 644.603725 | 723.67 | 802.736275 | 1.022133 |
| GO:0051050\_positive\_regulation\_of\_transport | 75 | 0 | 0.000000 | -0.000000 | 708 | 644.603725 | 723.67 | 802.736275 | 1.022133 |
| GO:0000027\_ribosomal\_large\_subunit\_assembly | 1 | 0 |  |  |  |  |  |  |  |  |
| GO:0000042\_protein\_targeting\_to\_Golgi | 1 | 0 |  |  |  |  |  |  |  |  |
| GO:0000046\_autophagic\_vacuole\_fusion | 1 | 0 |  |  |  |  |  |  |  |  |
| GO:0000050\_urea\_cycle | 1 | 0 |  |  |  |  |  |  |  |  |
| GO:0000054\_ribosome\_export\_from\_nucleus | 1 | 0 |  |  |  |  |  |  |  |  |
| GO:0000055\_ribosomal\_large\_subunit\_export\_from\_nucleus | 1 | 0 |  |  |  |  |  |  |  |  |
| GO:0000056\_ribosomal\_small\_subunit\_export\_from\_nucleus | 1 | 0 |  |  |  |  |  |  |  |  |
| GO:0000072\_M\_phase\_specific\_microtubule\_process | 1 | 0 |  |  |  |  |  |  |  |  |
| GO:0000101\_sulfur\_amino\_acid\_transport | 1 | 0 |  |  |  |  |  |  |  |  |
| GO:0000147\_actin\_cortical\_patch\_assembly | 1 | 0 |  |  |  |  |  |  |  |  |
| GO:0000154\_rRNA\_modification | 1 | 0 |  |  |  |  |  |  |  |  |
| GO:0000183\_chromatin\_silencing\_at\_rDNA | 1 | 0 |  |  |  |  |  |  |  |  |
| GO:0000185\_activation\_of\_MAPKKK\_activity | 1 | 0 |  |  |  |  |  |  |  |  |
| GO:0000238\_zygotene | 1 | 0 |  |  |  |  |  |  |  |  |
| GO:0000255\_allantoin\_metabolic\_process | 1 | 0 |  |  |  |  |  |  |  |  |
| GO:0000266\_mitochondrial\_fission | 1 | 0 |  |  |  |  |  |  |  |  |
| GO:0000273\_lipoic\_acid\_metabolic\_process | 1 | 0 |  |  |  |  |  |  |  |  |
| GO:0000301\_retrograde\_transport\_\_vesicle\_recycling\_within\_Golgi | 1 | 0 |  |  |  |  |  |  |  |  |
| GO:0000394\_RNA\_splicing\_\_via\_endonucleolytic\_cleavage\_and\_ligation | 1 | 0 |  |  |  |  |  |  |  |  |
| GO:0000429\_regulation\_of\_transcription\_from\_RNA\_polymerase\_II\_promoter\_by\_carbon\_catabolites | 1 | 0 |  |  |  |  |  |  |  |  |
| GO:0000430\_regulation\_of\_transcription\_from\_RNA\_polymerase\_II\_promoter\_by\_glucose | 1 | 0 |  |  |  |  |  |  |  |  |
| GO:0000432\_positive\_regulation\_of\_transcription\_from\_RNA\_polymerase\_II\_promoter\_by\_glucose | 1 | 0 |  |  |  |  |  |  |  |  |
| GO:0000436\_positive\_regulation\_of\_transcription\_from\_RNA\_polymerase\_II\_promoter\_by\_carbon\_catabolites | 1 | 0 |  |  |  |  |  |  |  |  |
| GO:0000448\_cleavage\_in\_ITS2\_between\_5.8S\_rRNA\_and\_LSU-rRNA\_of\_tricistronic\_rRNA\_transcript\_(SSU-rRNA\_\_5.8S\_rRNA\_\_LSU-rRNA) | 1 | 0 |  |  |  |  |  |  |  |  |
| GO:0000460\_maturation\_of\_5.8S\_rRNA | 1 | 0 |  |  |  |  |  |  |  |  |
| GO:0000463\_maturation\_of\_LSU-rRNA\_from\_tricistronic\_rRNA\_transcript\_(SSU-rRNA\_\_5.8S\_rRNA\_\_LSU-rRNA) | 1 | 0 |  |  |  |  |  |  |  |  |
| GO:0000466\_maturation\_of\_5.8S\_rRNA\_from\_tricistronic\_rRNA\_transcript\_(SSU-rRNA\_\_5.8S\_rRNA\_\_LSU-rRNA) | 1 | 0 |  |  |  |  |  |  |  |  |
| GO:0000469\_cleavages\_during\_rRNA\_processing | 1 | 0 |  |  |  |  |  |  |  |  |
| GO:0000470\_maturation\_of\_LSU-rRNA | 1 | 0 |  |  |  |  |  |  |  |  |
| GO:0000478\_endonucleolytic\_cleavages\_during\_rRNA\_processing | 1 | 0 |  |  |  |  |  |  |  |  |
| GO:0000479\_endonucleolytic\_cleavage\_of\_tricistronic\_rRNA\_transcript\_(SSU-rRNA\_\_5.8S\_rRNA\_\_LSU-rRNA) | 1 | 0 |  |  |  |  |  |  |  |  |
| GO:0000705\_achiasmate\_meiosis\_I | 1 | 0 |  |  |  |  |  |  |  |  |
| GO:0000966\_RNA\_5'-end\_processing | 1 | 0 |  |  |  |  |  |  |  |  |
| GO:0001300\_chronological\_cell\_aging | 1 | 0 |  |  |  |  |  |  |  |  |
| GO:0001547\_antral\_ovarian\_follicle\_growth | 1 | 0 |  |  |  |  |  |  |  |  |
| GO:0001555\_oocyte\_growth | 1 | 0 |  |  |  |  |  |  |  |  |
| GO:0001560\_regulation\_of\_cell\_growth\_by\_extracellular\_stimulus | 1 | 0 |  |  |  |  |  |  |  |  |
| GO:0001660\_fever | 1 | 0 |  |  |  |  |  |  |  |  |
| GO:0001696\_gastric\_acid\_secretion | 1 | 0 |  |  |  |  |  |  |  |  |
| GO:0001712\_ectodermal\_cell\_fate\_commitment | 1 | 0 |  |  |  |  |  |  |  |  |
| GO:0001714\_endodermal\_cell\_fate\_specification | 1 | 0 |  |  |  |  |  |  |  |  |
| GO:0001762\_beta-alanine\_transport | 1 | 0 |  |  |  |  |  |  |  |  |
| GO:0001766\_membrane\_raft\_polarization | 1 | 0 |  |  |  |  |  |  |  |  |
| GO:0001811\_negative\_regulation\_of\_type\_I\_hypersensitivity | 1 | 0 |  |  |  |  |  |  |  |  |
| GO:0001821\_histamine\_secretion | 1 | 0 |  |  |  |  |  |  |  |  |
| GO:0001826\_inner\_cell\_mass\_cell\_differentiation | 1 | 0 |  |  |  |  |  |  |  |  |
| GO:0001830\_trophectodermal\_cell\_fate\_commitment | 1 | 0 |  |  |  |  |  |  |  |  |
| GO:0001834\_trophectodermal\_cell\_proliferation | 1 | 0 |  |  |  |  |  |  |  |  |
| GO:0001867\_complement\_activation\_\_lectin\_pathway | 1 | 0 |  |  |  |  |  |  |  |  |
| GO:0001880\_Mullerian\_duct\_regression | 1 | 0 |  |  |  |  |  |  |  |  |
| GO:0001887\_selenium\_metabolic\_process | 1 | 0 |  |  |  |  |  |  |  |  |
| GO:0001922\_B-1\_B\_cell\_homeostasis | 1 | 0 |  |  |  |  |  |  |  |  |
| GO:0001923\_B-1\_B\_cell\_differentiation | 1 | 0 |  |  |  |  |  |  |  |  |
| GO:0001941\_postsynaptic\_membrane\_organization | 1 | 0 |  |  |  |  |  |  |  |  |
| GO:0001946\_lymphangiogenesis | 1 | 0 |  |  |  |  |  |  |  |  |
| GO:0001956\_positive\_regulation\_of\_neurotransmitter\_secretion | 1 | 0 |  |  |  |  |  |  |  |  |
| GO:0001961\_positive\_regulation\_of\_cytokine-mediated\_signaling\_pathway | 1 | 0 |  |  |  |  |  |  |  |  |
| GO:0001979\_regulation\_of\_systemic\_arterial\_blood\_pressure\_by\_chemoreceptor\_signaling | 1 | 0 |  |  |  |  |  |  |  |  |
| GO:0001980\_regulation\_of\_systemic\_arterial\_blood\_pressure\_by\_ischemic\_conditions | 1 | 0 |  |  |  |  |  |  |  |  |
| GO:0001984\_vasodilation\_of\_artery\_during\_baroreceptor\_response\_to\_increased\_systemic\_arterial\_blood\_pressure | 1 | 0 |  |  |  |  |  |  |  |  |
| GO:0001985\_negative\_regulation\_of\_heart\_rate\_in\_baroreceptor\_response\_to\_increased\_systemic\_arterial\_blood\_pressure | 1 | 0 |  |  |  |  |  |  |  |  |
| GO:0001987\_vasoconstriction\_of\_artery\_involved\_in\_baroreceptor\_response\_to\_lowering\_of\_systemic\_arterial\_blood\_pressure | 1 | 0 |  |  |  |  |  |  |  |  |
| GO:0001988\_positive\_regulation\_of\_heart\_rate\_in\_baroreceptor\_response\_to\_decreased\_systemic\_arterial\_blood\_pressure | 1 | 0 |  |  |  |  |  |  |  |  |
| GO:0001994\_norepinephrine-epinephrine\_vasoconstriction\_involved\_in\_regulation\_of\_systemic\_arterial\_blood\_pressure | 1 | 0 |  |  |  |  |  |  |  |  |
| GO:0002001\_renin\_secretion\_into\_blood\_stream | 1 | 0 |  |  |  |  |  |  |  |  |
| GO:0002002\_regulation\_of\_angiotensin\_levels\_in\_blood | 1 | 0 |  |  |  |  |  |  |  |  |
| GO:0002003\_angiotensin\_maturation | 1 | 0 |  |  |  |  |  |  |  |  |
| GO:0002007\_detection\_of\_hypoxic\_conditions\_in\_blood\_by\_chemoreceptor\_signaling | 1 | 0 |  |  |  |  |  |  |  |  |
| GO:0002017\_regulation\_of\_blood\_volume\_by\_renal\_aldosterone | 1 | 0 |  |  |  |  |  |  |  |  |
| GO:0002023\_reduction\_of\_food\_intake\_in\_response\_to\_dietary\_excess | 1 | 0 |  |  |  |  |  |  |  |  |
| GO:0002031\_G-protein\_coupled\_receptor\_internalization | 1 | 0 |  |  |  |  |  |  |  |  |
| GO:0002036\_regulation\_of\_L-glutamate\_transport | 1 | 0 |  |  |  |  |  |  |  |  |
| GO:0002040\_sprouting\_angiogenesis | 1 | 0 |  |  |  |  |  |  |  |  |
| GO:0002041\_intussusceptive\_angiogenesis | 1 | 0 |  |  |  |  |  |  |  |  |
| GO:0002068\_glandular\_epithelial\_cell\_development | 1 | 0 |  |  |  |  |  |  |  |  |
| GO:0002069\_columnar\_cuboidal\_epithelial\_cell\_maturation | 1 | 0 |  |  |  |  |  |  |  |  |
| GO:0002071\_glandular\_epithelial\_cell\_maturation | 1 | 0 |  |  |  |  |  |  |  |  |
| GO:0002082\_regulation\_of\_oxidative\_phosphorylation | 1 | 0 |  |  |  |  |  |  |  |  |
| GO:0002084\_protein\_depalmitoylation | 1 | 0 |  |  |  |  |  |  |  |  |
| GO:0002085\_inhibition\_of\_neuroepithelial\_cell\_differentiation | 1 | 0 |  |  |  |  |  |  |  |  |
| GO:0002086\_diaphragm\_contraction | 1 | 0 |  |  |  |  |  |  |  |  |
| GO:0002118\_aggressive\_behavior | 1 | 0 |  |  |  |  |  |  |  |  |
| GO:0002121\_inter-male\_aggressive\_behavior | 1 | 0 |  |  |  |  |  |  |  |  |
| GO:0002124\_territorial\_aggressive\_behavior | 1 | 0 |  |  |  |  |  |  |  |  |
| GO:0002227\_innate\_immune\_response\_in\_mucosa | 1 | 0 |  |  |  |  |  |  |  |  |
| GO:0002232\_leukocyte\_chemotaxis\_during\_inflammatory\_response | 1 | 0 |  |  |  |  |  |  |  |  |
| GO:0002248\_connective\_tissue\_replacement\_during\_inflammatory\_response | 1 | 0 |  |  |  |  |  |  |  |  |
| GO:0002282\_microglial\_cell\_activation\_during\_immune\_response | 1 | 0 |  |  |  |  |  |  |  |  |
| GO:0002287\_alpha-beta\_T\_cell\_activation\_during\_immune\_response | 1 | 0 |  |  |  |  |  |  |  |  |
| GO:0002314\_germinal\_center\_B\_cell\_differentiation | 1 | 0 |  |  |  |  |  |  |  |  |
| GO:0002315\_marginal\_zone\_B\_cell\_differentiation | 1 | 0 |  |  |  |  |  |  |  |  |
| GO:0002316\_follicular\_B\_cell\_differentiation | 1 | 0 |  |  |  |  |  |  |  |  |
| GO:0002317\_plasma\_cell\_differentiation | 1 | 0 |  |  |  |  |  |  |  |  |
| GO:0002349\_histamine\_production\_during\_acute\_inflammatory\_response | 1 | 0 |  |  |  |  |  |  |  |  |
| GO:0002351\_serotonin\_production\_during\_acute\_inflammatory\_response | 1 | 0 |  |  |  |  |  |  |  |  |
| GO:0002355\_detection\_of\_tumor\_cell | 1 | 0 |  |  |  |  |  |  |  |  |
| GO:0002370\_natural\_killer\_cell\_cytokine\_production | 1 | 0 |  |  |  |  |  |  |  |  |
| GO:0002371\_dendritic\_cell\_cytokine\_production | 1 | 0 |  |  |  |  |  |  |  |  |
| GO:0002380\_immunoglobulin\_secretion\_during\_immune\_response | 1 | 0 |  |  |  |  |  |  |  |  |
| GO:0002396\_MHC\_protein\_complex\_assembly | 1 | 0 |  |  |  |  |  |  |  |  |
| GO:0002397\_MHC\_class\_I\_protein\_complex\_assembly | 1 | 0 |  |  |  |  |  |  |  |  |
| GO:0002420\_natural\_killer\_cell\_mediated\_cytotoxicity\_directed\_against\_tumor\_cell\_target | 1 | 0 |  |  |  |  |  |  |  |  |
| GO:0002423\_natural\_killer\_cell\_mediated\_immune\_response\_to\_tumor\_cell | 1 | 0 |  |  |  |  |  |  |  |  |
| GO:0002424\_T\_cell\_mediated\_immune\_response\_to\_tumor\_cell | 1 | 0 |  |  |  |  |  |  |  |  |
| GO:0002426\_immunoglobulin\_production\_in\_mucosal\_tissue | 1 | 0 |  |  |  |  |  |  |  |  |
| GO:0002431\_Fc\_receptor\_mediated\_stimulatory\_signaling\_pathway | 1 | 0 |  |  |  |  |  |  |  |  |
| GO:0002432\_granuloma\_formation | 1 | 0 |  |  |  |  |  |  |  |  |
| GO:0002441\_histamine\_secretion\_during\_acute\_inflammatory\_response | 1 | 0 |  |  |  |  |  |  |  |  |
| GO:0002442\_serotonin\_secretion\_during\_acute\_inflammatory\_response | 1 | 0 |  |  |  |  |  |  |  |  |
| GO:0002457\_T\_cell\_antigen\_processing\_and\_presentation | 1 | 0 |  |  |  |  |  |  |  |  |
| GO:0002458\_peripheral\_T\_cell\_tolerance\_induction | 1 | 0 |  |  |  |  |  |  |  |  |
| GO:0002461\_tolerance\_induction\_dependent\_upon\_immune\_response | 1 | 0 |  |  |  |  |  |  |  |  |
| GO:0002465\_peripheral\_tolerance\_induction | 1 | 0 |  |  |  |  |  |  |  |  |
| GO:0002468\_dendritic\_cell\_antigen\_processing\_and\_presentation | 1 | 0 |  |  |  |  |  |  |  |  |
| GO:0002476\_antigen\_processing\_and\_presentation\_of\_endogenous\_peptide\_antigen\_via\_MHC\_class\_Ib | 1 | 0 |  |  |  |  |  |  |  |  |
| GO:0002479\_antigen\_processing\_and\_presentation\_of\_exogenous\_peptide\_antigen\_via\_MHC\_class\_I\_\_TAP-dependent | 1 | 0 |  |  |  |  |  |  |  |  |
| GO:0002483\_antigen\_processing\_and\_presentation\_of\_endogenous\_peptide\_antigen | 1 | 0 |  |  |  |  |  |  |  |  |
| GO:0002501\_peptide\_antigen\_assembly\_with\_MHC\_protein\_complex | 1 | 0 |  |  |  |  |  |  |  |  |
| GO:0002502\_peptide\_antigen\_assembly\_with\_MHC\_class\_I\_protein\_complex | 1 | 0 |  |  |  |  |  |  |  |  |
| GO:0002508\_central\_tolerance\_induction | 1 | 0 |  |  |  |  |  |  |  |  |
| GO:0002510\_central\_B\_cell\_tolerance\_induction | 1 | 0 |  |  |  |  |  |  |  |  |
| GO:0002545\_chronic\_inflammatory\_response\_to\_non-antigenic\_stimulus | 1 | 0 |  |  |  |  |  |  |  |  |
| GO:0002553\_histamine\_secretion\_by\_mast\_cell | 1 | 0 |  |  |  |  |  |  |  |  |
| GO:0002554\_serotonin\_secretion\_by\_platelet | 1 | 0 |  |  |  |  |  |  |  |  |
| GO:0002572\_pro-T\_cell\_differentiation | 1 | 0 |  |  |  |  |  |  |  |  |
| GO:0002577\_regulation\_of\_antigen\_processing\_and\_presentation | 1 | 0 |  |  |  |  |  |  |  |  |
| GO:0002579\_positive\_regulation\_of\_antigen\_processing\_and\_presentation | 1 | 0 |  |  |  |  |  |  |  |  |
| GO:0002604\_regulation\_of\_dendritic\_cell\_antigen\_processing\_and\_presentation | 1 | 0 |  |  |  |  |  |  |  |  |
| GO:0002606\_positive\_regulation\_of\_dendritic\_cell\_antigen\_processing\_and\_presentation | 1 | 0 |  |  |  |  |  |  |  |  |
| GO:0002635\_negative\_regulation\_of\_germinal\_center\_formation | 1 | 0 |  |  |  |  |  |  |  |  |
| GO:0002646\_regulation\_of\_central\_tolerance\_induction | 1 | 0 |  |  |  |  |  |  |  |  |
| GO:0002648\_positive\_regulation\_of\_central\_tolerance\_induction | 1 | 0 |  |  |  |  |  |  |  |  |
| GO:0002649\_regulation\_of\_tolerance\_induction\_to\_self\_antigen | 1 | 0 |  |  |  |  |  |  |  |  |
| GO:0002651\_positive\_regulation\_of\_tolerance\_induction\_to\_self\_antigen | 1 | 0 |  |  |  |  |  |  |  |  |
| GO:0002652\_regulation\_of\_tolerance\_induction\_dependent\_upon\_immune\_response | 1 | 0 |  |  |  |  |  |  |  |  |
| GO:0002654\_positive\_regulation\_of\_tolerance\_induction\_dependent\_upon\_immune\_response | 1 | 0 |  |  |  |  |  |  |  |  |
| GO:0002658\_regulation\_of\_peripheral\_tolerance\_induction | 1 | 0 |  |  |  |  |  |  |  |  |
| GO:0002660\_positive\_regulation\_of\_peripheral\_tolerance\_induction | 1 | 0 |  |  |  |  |  |  |  |  |
| GO:0002677\_negative\_regulation\_of\_chronic\_inflammatory\_response | 1 | 0 |  |  |  |  |  |  |  |  |
| GO:0002678\_positive\_regulation\_of\_chronic\_inflammatory\_response | 1 | 0 |  |  |  |  |  |  |  |  |
| GO:0002701\_negative\_regulation\_of\_production\_of\_molecular\_mediator\_of\_immune\_response | 1 | 0 |  |  |  |  |  |  |  |  |
| GO:0002719\_negative\_regulation\_of\_cytokine\_production\_during\_immune\_response | 1 | 0 |  |  |  |  |  |  |  |  |
| GO:0002724\_regulation\_of\_T\_cell\_cytokine\_production | 1 | 0 |  |  |  |  |  |  |  |  |
| GO:0002727\_regulation\_of\_natural\_killer\_cell\_cytokine\_production | 1 | 0 |  |  |  |  |  |  |  |  |
| GO:0002729\_positive\_regulation\_of\_natural\_killer\_cell\_cytokine\_production | 1 | 0 |  |  |  |  |  |  |  |  |
| GO:0002730\_regulation\_of\_dendritic\_cell\_cytokine\_production | 1 | 0 |  |  |  |  |  |  |  |  |
| GO:0002756\_MyD88-independent\_toll-like\_receptor\_signaling\_pathway | 1 | 0 |  |  |  |  |  |  |  |  |
| GO:0002767\_immune\_response-inhibiting\_cell\_surface\_receptor\_signaling\_pathway | 1 | 0 |  |  |  |  |  |  |  |  |
| GO:0002769\_natural\_killer\_cell\_inhibitory\_signaling\_pathway | 1 | 0 |  |  |  |  |  |  |  |  |
| GO:0002840\_regulation\_of\_T\_cell\_mediated\_immune\_response\_to\_tumor\_cell | 1 | 0 |  |  |  |  |  |  |  |  |
| GO:0002842\_positive\_regulation\_of\_T\_cell\_mediated\_immune\_response\_to\_tumor\_cell | 1 | 0 |  |  |  |  |  |  |  |  |
| GO:0002849\_regulation\_of\_peripheral\_T\_cell\_tolerance\_induction | 1 | 0 |  |  |  |  |  |  |  |  |
| GO:0002851\_positive\_regulation\_of\_peripheral\_T\_cell\_tolerance\_induction | 1 | 0 |  |  |  |  |  |  |  |  |
| GO:0002855\_regulation\_of\_natural\_killer\_cell\_mediated\_immune\_response\_to\_tumor\_cell | 1 | 0 |  |  |  |  |  |  |  |  |
| GO:0002857\_positive\_regulation\_of\_natural\_killer\_cell\_mediated\_immune\_response\_to\_tumor\_cell | 1 | 0 |  |  |  |  |  |  |  |  |
| GO:0002858\_regulation\_of\_natural\_killer\_cell\_mediated\_cytotoxicity\_directed\_against\_tumor\_cell\_target | 1 | 0 |  |  |  |  |  |  |  |  |
| GO:0002860\_positive\_regulation\_of\_natural\_killer\_cell\_mediated\_cytotoxicity\_directed\_against\_tumor\_cell\_target | 1 | 0 |  |  |  |  |  |  |  |  |
| GO:0002880\_regulation\_of\_chronic\_inflammatory\_response\_to\_non-antigenic\_stimulus | 1 | 0 |  |  |  |  |  |  |  |  |
| GO:0002882\_positive\_regulation\_of\_chronic\_inflammatory\_response\_to\_non-antigenic\_stimulus | 1 | 0 |  |  |  |  |  |  |  |  |
| GO:0002895\_regulation\_of\_central\_B\_cell\_tolerance\_induction | 1 | 0 |  |  |  |  |  |  |  |  |
| GO:0002897\_positive\_regulation\_of\_central\_B\_cell\_tolerance\_induction | 1 | 0 |  |  |  |  |  |  |  |  |
| GO:0002901\_mature\_B\_cell\_apoptosis | 1 | 0 |  |  |  |  |  |  |  |  |
| GO:0002903\_negative\_regulation\_of\_B\_cell\_apoptosis | 1 | 0 |  |  |  |  |  |  |  |  |
| GO:0002905\_regulation\_of\_mature\_B\_cell\_apoptosis | 1 | 0 |  |  |  |  |  |  |  |  |
| GO:0002906\_negative\_regulation\_of\_mature\_B\_cell\_apoptosis | 1 | 0 |  |  |  |  |  |  |  |  |
| GO:0003011\_involuntary\_skeletal\_muscle\_contraction | 1 | 0 |  |  |  |  |  |  |  |  |
| GO:0003027\_regulation\_of\_systemic\_arterial\_blood\_pressure\_by\_carotid\_body\_chemoreceptor\_signaling | 1 | 0 |  |  |  |  |  |  |  |  |
| GO:0003029\_detection\_of\_hypoxic\_conditions\_in\_blood\_by\_carotid\_body\_chemoreceptor\_signaling | 1 | 0 |  |  |  |  |  |  |  |  |
| GO:0003056\_regulation\_of\_vascular\_smooth\_muscle\_contraction | 1 | 0 |  |  |  |  |  |  |  |  |
| GO:0003062\_regulation\_of\_heart\_rate\_by\_chemical\_signal | 1 | 0 |  |  |  |  |  |  |  |  |
| GO:0003065\_positive\_regulation\_of\_heart\_rate\_by\_epinephrine | 1 | 0 |  |  |  |  |  |  |  |  |
| GO:0003097\_renal\_water\_transport | 1 | 0 |  |  |  |  |  |  |  |  |
| GO:0005979\_regulation\_of\_glycogen\_biosynthetic\_process | 1 | 0 |  |  |  |  |  |  |  |  |
| GO:0005984\_disaccharide\_metabolic\_process | 1 | 0 |  |  |  |  |  |  |  |  |
| GO:0005988\_lactose\_metabolic\_process | 1 | 0 |  |  |  |  |  |  |  |  |
| GO:0005989\_lactose\_biosynthetic\_process | 1 | 0 |  |  |  |  |  |  |  |  |
| GO:0005997\_xylulose\_metabolic\_process | 1 | 0 |  |  |  |  |  |  |  |  |
| GO:0006000\_fructose\_metabolic\_process | 1 | 0 |  |  |  |  |  |  |  |  |
| GO:0006002\_fructose\_6-phosphate\_metabolic\_process | 1 | 0 |  |  |  |  |  |  |  |  |
| GO:0006004\_fucose\_metabolic\_process | 1 | 0 |  |  |  |  |  |  |  |  |
| GO:0006013\_mannose\_metabolic\_process | 1 | 0 |  |  |  |  |  |  |  |  |
| GO:0006060\_sorbitol\_metabolic\_process | 1 | 0 |  |  |  |  |  |  |  |  |
| GO:0006064\_glucuronate\_catabolic\_process | 1 | 0 |  |  |  |  |  |  |  |  |
| GO:0006086\_acetyl-CoA\_biosynthetic\_process\_from\_pyruvate | 1 | 0 |  |  |  |  |  |  |  |  |
| GO:0006098\_pentose-phosphate\_shunt | 1 | 0 |  |  |  |  |  |  |  |  |
| GO:0006101\_citrate\_metabolic\_process | 1 | 0 |  |  |  |  |  |  |  |  |
| GO:0006104\_succinyl-CoA\_metabolic\_process | 1 | 0 |  |  |  |  |  |  |  |  |
| GO:0006116\_NADH\_oxidation | 1 | 0 |  |  |  |  |  |  |  |  |
| GO:0006120\_mitochondrial\_electron\_transport\_\_NADH\_to\_ubiquinone | 1 | 0 |  |  |  |  |  |  |  |  |
| GO:0006154\_adenosine\_catabolic\_process | 1 | 0 |  |  |  |  |  |  |  |  |
| GO:0006157\_deoxyadenosine\_catabolic\_process | 1 | 0 |  |  |  |  |  |  |  |  |
| GO:0006167\_AMP\_biosynthetic\_process | 1 | 0 |  |  |  |  |  |  |  |  |
| GO:0006175\_dATP\_biosynthetic\_process | 1 | 0 |  |  |  |  |  |  |  |  |
| GO:0006178\_guanine\_salvage | 1 | 0 |  |  |  |  |  |  |  |  |
| GO:0006196\_AMP\_catabolic\_process | 1 | 0 |  |  |  |  |  |  |  |  |
| GO:0006203\_dGTP\_catabolic\_process | 1 | 0 |  |  |  |  |  |  |  |  |
| GO:0006208\_pyrimidine\_base\_catabolic\_process | 1 | 0 |  |  |  |  |  |  |  |  |
| GO:0006221\_pyrimidine\_nucleotide\_biosynthetic\_process | 1 | 0 |  |  |  |  |  |  |  |  |
| GO:0006235\_dTTP\_biosynthetic\_process | 1 | 0 |  |  |  |  |  |  |  |  |
| GO:0006244\_pyrimidine\_nucleotide\_catabolic\_process | 1 | 0 |  |  |  |  |  |  |  |  |
| GO:0006269\_DNA\_replication\_\_synthesis\_of\_RNA\_primer | 1 | 0 |  |  |  |  |  |  |  |  |
| GO:0006283\_transcription-coupled\_nucleotide-excision\_repair | 1 | 0 |  |  |  |  |  |  |  |  |
| GO:0006296\_nucleotide-excision\_repair\_\_DNA\_incision\_\_5'-to\_lesion | 1 | 0 |  |  |  |  |  |  |  |  |
| GO:0006307\_DNA\_dealkylation | 1 | 0 |  |  |  |  |  |  |  |  |
| GO:0006337\_nucleosome\_disassembly | 1 | 0 |  |  |  |  |  |  |  |  |
| GO:0006344\_maintenance\_of\_chromatin\_silencing | 1 | 0 |  |  |  |  |  |  |  |  |
| GO:0006356\_regulation\_of\_transcription\_from\_RNA\_polymerase\_I\_promoter | 1 | 0 |  |  |  |  |  |  |  |  |
| GO:0006388\_tRNA\_splicing\_\_via\_endonucleolytic\_cleavage\_and\_ligation | 1 | 0 |  |  |  |  |  |  |  |  |
| GO:0006407\_rRNA\_export\_from\_nucleus | 1 | 0 |  |  |  |  |  |  |  |  |
| GO:0006419\_alanyl-tRNA\_aminoacylation | 1 | 0 |  |  |  |  |  |  |  |  |
| GO:0006434\_seryl-tRNA\_aminoacylation | 1 | 0 |  |  |  |  |  |  |  |  |
| GO:0006447\_regulation\_of\_translational\_initiation\_by\_iron | 1 | 0 |  |  |  |  |  |  |  |  |
| GO:0006463\_steroid\_hormone\_receptor\_complex\_assembly | 1 | 0 |  |  |  |  |  |  |  |  |
| GO:0006467\_protein\_thiol-disulfide\_exchange | 1 | 0 |  |  |  |  |  |  |  |  |
| GO:0006474\_N-terminal\_protein\_amino\_acid\_acetylation | 1 | 0 |  |  |  |  |  |  |  |  |
| GO:0006481\_C-terminal\_protein\_amino\_acid\_methylation | 1 | 0 |  |  |  |  |  |  |  |  |
| GO:0006488\_dolichol-linked\_oligosaccharide\_biosynthetic\_process | 1 | 0 |  |  |  |  |  |  |  |  |
| GO:0006494\_protein\_amino\_acid\_terminal\_glycosylation | 1 | 0 |  |  |  |  |  |  |  |  |
| GO:0006496\_protein\_amino\_acid\_terminal\_N-glycosylation | 1 | 0 |  |  |  |  |  |  |  |  |
| GO:0006500\_N-terminal\_protein\_palmitoylation | 1 | 0 |  |  |  |  |  |  |  |  |
| GO:0006507\_GPI\_anchor\_release | 1 | 0 |  |  |  |  |  |  |  |  |
| GO:0006537\_glutamate\_biosynthetic\_process | 1 | 0 |  |  |  |  |  |  |  |  |
| GO:0006544\_glycine\_metabolic\_process | 1 | 0 |  |  |  |  |  |  |  |  |
| GO:0006549\_isoleucine\_metabolic\_process | 1 | 0 |  |  |  |  |  |  |  |  |
| GO:0006553\_lysine\_metabolic\_process | 1 | 0 |  |  |  |  |  |  |  |  |
| GO:0006554\_lysine\_catabolic\_process | 1 | 0 |  |  |  |  |  |  |  |  |
| GO:0006556\_S-adenosylmethionine\_biosynthetic\_process | 1 | 0 |  |  |  |  |  |  |  |  |
| GO:0006559\_L-phenylalanine\_catabolic\_process | 1 | 0 |  |  |  |  |  |  |  |  |
| GO:0006569\_tryptophan\_catabolic\_process | 1 | 0 |  |  |  |  |  |  |  |  |
| GO:0006572\_tyrosine\_catabolic\_process | 1 | 0 |  |  |  |  |  |  |  |  |
| GO:0006573\_valine\_metabolic\_process | 1 | 0 |  |  |  |  |  |  |  |  |
| GO:0006581\_acetylcholine\_catabolic\_process | 1 | 0 |  |  |  |  |  |  |  |  |
| GO:0006585\_dopamine\_biosynthetic\_process\_from\_tyrosine | 1 | 0 |  |  |  |  |  |  |  |  |
| GO:0006590\_thyroid\_hormone\_generation | 1 | 0 |  |  |  |  |  |  |  |  |
| GO:0006591\_ornithine\_metabolic\_process | 1 | 0 |  |  |  |  |  |  |  |  |
| GO:0006596\_polyamine\_biosynthetic\_process | 1 | 0 |  |  |  |  |  |  |  |  |
| GO:0006597\_spermine\_biosynthetic\_process | 1 | 0 |  |  |  |  |  |  |  |  |
| GO:0006601\_creatine\_biosynthetic\_process | 1 | 0 |  |  |  |  |  |  |  |  |
| GO:0006613\_cotranslational\_protein\_targeting\_to\_membrane | 1 | 0 |  |  |  |  |  |  |  |  |
| GO:0006622\_protein\_targeting\_to\_lysosome | 1 | 0 |  |  |  |  |  |  |  |  |
| GO:0006627\_mitochondrial\_protein\_processing\_during\_import | 1 | 0 |  |  |  |  |  |  |  |  |
| GO:0006653\_lecithin\_metabolic\_process | 1 | 0 |  |  |  |  |  |  |  |  |
| GO:0006654\_phosphatidic\_acid\_biosynthetic\_process | 1 | 0 |  |  |  |  |  |  |  |  |
| GO:0006658\_phosphatidylserine\_metabolic\_process | 1 | 0 |  |  |  |  |  |  |  |  |
| GO:0006659\_phosphatidylserine\_biosynthetic\_process | 1 | 0 |  |  |  |  |  |  |  |  |
| GO:0006667\_sphinganine\_metabolic\_process | 1 | 0 |  |  |  |  |  |  |  |  |
| GO:0006668\_sphinganine-1-phosphate\_metabolic\_process | 1 | 0 |  |  |  |  |  |  |  |  |
| GO:0006678\_glucosylceramide\_metabolic\_process | 1 | 0 |  |  |  |  |  |  |  |  |
| GO:0006682\_galactosylceramide\_biosynthetic\_process | 1 | 0 |  |  |  |  |  |  |  |  |
| GO:0006685\_sphingomyelin\_catabolic\_process | 1 | 0 |  |  |  |  |  |  |  |  |
| GO:0006700\_C21-steroid\_hormone\_biosynthetic\_process | 1 | 0 |  |  |  |  |  |  |  |  |
| GO:0006705\_mineralocorticoid\_biosynthetic\_process | 1 | 0 |  |  |  |  |  |  |  |  |
| GO:0006709\_progesterone\_catabolic\_process | 1 | 0 |  |  |  |  |  |  |  |  |
| GO:0006729\_tetrahydrobiopterin\_biosynthetic\_process | 1 | 0 |  |  |  |  |  |  |  |  |
| GO:0006734\_NADH\_metabolic\_process | 1 | 0 |  |  |  |  |  |  |  |  |
| GO:0006740\_NADPH\_regeneration | 1 | 0 |  |  |  |  |  |  |  |  |
| GO:0006741\_NADP\_biosynthetic\_process | 1 | 0 |  |  |  |  |  |  |  |  |
| GO:0006743\_ubiquinone\_metabolic\_process | 1 | 0 |  |  |  |  |  |  |  |  |
| GO:0006744\_ubiquinone\_biosynthetic\_process | 1 | 0 |  |  |  |  |  |  |  |  |
| GO:0006772\_thiamin\_metabolic\_process | 1 | 0 |  |  |  |  |  |  |  |  |
| GO:0006784\_heme\_a\_biosynthetic\_process | 1 | 0 |  |  |  |  |  |  |  |  |
| GO:0006797\_polyphosphate\_metabolic\_process | 1 | 0 |  |  |  |  |  |  |  |  |
| GO:0006798\_polyphosphate\_catabolic\_process | 1 | 0 |  |  |  |  |  |  |  |  |
| GO:0006824\_cobalt\_ion\_transport | 1 | 0 |  |  |  |  |  |  |  |  |
| GO:0006842\_tricarboxylic\_acid\_transport | 1 | 0 |  |  |  |  |  |  |  |  |
| GO:0006844\_acyl\_carnitine\_transport | 1 | 0 |  |  |  |  |  |  |  |  |
| GO:0006855\_multidrug\_transport | 1 | 0 |  |  |  |  |  |  |  |  |
| GO:0006863\_purine\_transport | 1 | 0 |  |  |  |  |  |  |  |  |
| GO:0006890\_retrograde\_vesicle-mediated\_transport\_\_Golgi\_to\_ER | 1 | 0 |  |  |  |  |  |  |  |  |
| GO:0006891\_intra-Golgi\_vesicle-mediated\_transport | 1 | 0 |  |  |  |  |  |  |  |  |
| GO:0006893\_Golgi\_to\_plasma\_membrane\_transport | 1 | 0 |  |  |  |  |  |  |  |  |
| GO:0006895\_Golgi\_to\_endosome\_transport | 1 | 0 |  |  |  |  |  |  |  |  |
| GO:0006896\_Golgi\_to\_vacuole\_transport | 1 | 0 |  |  |  |  |  |  |  |  |
| GO:0006900\_membrane\_budding | 1 | 0 |  |  |  |  |  |  |  |  |
| GO:0006930\_substrate-bound\_cell\_migration\_\_cell\_extension | 1 | 0 |  |  |  |  |  |  |  |  |
| GO:0006931\_substrate-bound\_cell\_migration\_\_cell\_attachment\_to\_substrate | 1 | 0 |  |  |  |  |  |  |  |  |
| GO:0006933\_negative\_regulation\_of\_cell\_adhesion\_involved\_in\_substrate-bound\_cell\_migration | 1 | 0 |  |  |  |  |  |  |  |  |
| GO:0006957\_complement\_activation\_\_alternative\_pathway | 1 | 0 |  |  |  |  |  |  |  |  |
| GO:0006958\_complement\_activation\_\_classical\_pathway | 1 | 0 |  |  |  |  |  |  |  |  |
| GO:0006978\_DNA\_damage\_response\_\_signal\_transduction\_by\_p53\_class\_mediator\_resulting\_in\_transcription\_of\_p21\_class\_mediator | 1 | 0 |  |  |  |  |  |  |  |  |
| GO:0007016\_cytoskeletal\_anchoring\_at\_plasma\_membrane | 1 | 0 |  |  |  |  |  |  |  |  |
| GO:0007021\_tubulin\_complex\_assembly | 1 | 0 |  |  |  |  |  |  |  |  |
| GO:0007052\_mitotic\_spindle\_organization | 1 | 0 |  |  |  |  |  |  |  |  |
| GO:0007056\_spindle\_assembly\_involved\_in\_female\_meiosis | 1 | 0 |  |  |  |  |  |  |  |  |
| GO:0007057\_spindle\_assembly\_involved\_in\_female\_meiosis\_I | 1 | 0 |  |  |  |  |  |  |  |  |
| GO:0007065\_male\_meiosis\_sister\_chromatid\_cohesion | 1 | 0 |  |  |  |  |  |  |  |  |
| GO:0007076\_mitotic\_chromosome\_condensation | 1 | 0 |  |  |  |  |  |  |  |  |
| GO:0007095\_mitotic\_cell\_cycle\_G2\_M\_transition\_DNA\_damage\_checkpoint | 1 | 0 |  |  |  |  |  |  |  |  |
| GO:0007096\_regulation\_of\_exit\_from\_mitosis | 1 | 0 |  |  |  |  |  |  |  |  |
| GO:0007158\_neuron\_adhesion | 1 | 0 |  |  |  |  |  |  |  |  |
| GO:0007168\_receptor\_guanylyl\_cyclase\_signaling\_pathway | 1 | 0 |  |  |  |  |  |  |  |  |
| GO:0007197\_inhibition\_of\_adenylate\_cyclase\_activity\_by\_muscarinic\_acetylcholine\_receptor\_signaling\_pathway | 1 | 0 |  |  |  |  |  |  |  |  |
| GO:0007207\_activation\_of\_phospholipase\_C\_activity\_by\_muscarinic\_acetylcholine\_receptor\_signaling\_pathway | 1 | 0 |  |  |  |  |  |  |  |  |
| GO:0007208\_activation\_of\_phospholipase\_C\_activity\_by\_serotonin\_receptor\_signaling\_pathway | 1 | 0 |  |  |  |  |  |  |  |  |
| GO:0007217\_tachykinin\_receptor\_signaling\_pathway | 1 | 0 |  |  |  |  |  |  |  |  |
| GO:0007221\_positive\_regulation\_of\_transcription\_of\_Notch\_receptor\_target | 1 | 0 |  |  |  |  |  |  |  |  |
| GO:0007223\_Wnt\_receptor\_signaling\_pathway\_\_calcium\_modulating\_pathway | 1 | 0 |  |  |  |  |  |  |  |  |
| GO:0007225\_patched\_ligand\_processing | 1 | 0 |  |  |  |  |  |  |  |  |
| GO:0007227\_signal\_transduction\_downstream\_of\_smoothened | 1 | 0 |  |  |  |  |  |  |  |  |
| GO:0007228\_positive\_regulation\_of\_hh\_target\_transcription\_factor\_activity | 1 | 0 |  |  |  |  |  |  |  |  |
| GO:0007231\_osmosensory\_signaling\_pathway | 1 | 0 |  |  |  |  |  |  |  |  |
| GO:0007284\_spermatogonial\_cell\_division | 1 | 0 |  |  |  |  |  |  |  |  |
| GO:0007290\_spermatid\_nucleus\_elongation | 1 | 0 |  |  |  |  |  |  |  |  |
| GO:0007296\_vitellogenesis | 1 | 0 |  |  |  |  |  |  |  |  |
| GO:0007321\_sperm\_displacement | 1 | 0 |  |  |  |  |  |  |  |  |
| GO:0007380\_specification\_of\_segmental\_identity\_\_head | 1 | 0 |  |  |  |  |  |  |  |  |
| GO:0007382\_specification\_of\_segmental\_identity\_\_maxillary\_segment | 1 | 0 |  |  |  |  |  |  |  |  |
| GO:0007400\_neuroblast\_fate\_determination | 1 | 0 |  |  |  |  |  |  |  |  |
| GO:0007402\_ganglion\_mother\_cell\_fate\_determination | 1 | 0 |  |  |  |  |  |  |  |  |
| GO:0007495\_visceral\_mesoderm-endoderm\_interaction\_involved\_in\_midgut\_development | 1 | 0 |  |  |  |  |  |  |  |  |
| GO:0007497\_posterior\_midgut\_development | 1 | 0 |  |  |  |  |  |  |  |  |
| GO:0007499\_ectoderm\_and\_mesoderm\_interaction | 1 | 0 |  |  |  |  |  |  |  |  |
| GO:0007500\_mesodermal\_cell\_fate\_determination | 1 | 0 |  |  |  |  |  |  |  |  |
| GO:0007509\_mesoderm\_migration | 1 | 0 |  |  |  |  |  |  |  |  |
| GO:0007518\_myoblast\_cell\_fate\_determination | 1 | 0 |  |  |  |  |  |  |  |  |
| GO:0007521\_muscle\_cell\_fate\_determination | 1 | 0 |  |  |  |  |  |  |  |  |
| GO:0007522\_visceral\_muscle\_development | 1 | 0 |  |  |  |  |  |  |  |  |
| GO:0007529\_establishment\_of\_synaptic\_specificity\_at\_neuromuscular\_junction | 1 | 0 |  |  |  |  |  |  |  |  |
| GO:0007538\_primary\_sex\_determination | 1 | 0 |  |  |  |  |  |  |  |  |
| GO:0007542\_primary\_sex\_determination\_\_germ-line | 1 | 0 |  |  |  |  |  |  |  |  |
| GO:0007567\_parturition | 1 | 0 |  |  |  |  |  |  |  |  |
| GO:0007614\_short-term\_memory | 1 | 0 |  |  |  |  |  |  |  |  |
| GO:0007621\_negative\_regulation\_of\_female\_receptivity | 1 | 0 |  |  |  |  |  |  |  |  |
| GO:0008049\_male\_courtship\_behavior | 1 | 0 |  |  |  |  |  |  |  |  |
| GO:0008050\_female\_courtship\_behavior | 1 | 0 |  |  |  |  |  |  |  |  |
| GO:0008052\_sensory\_organ\_boundary\_specification | 1 | 0 |  |  |  |  |  |  |  |  |
| GO:0008054\_cyclin\_catabolic\_process | 1 | 0 |  |  |  |  |  |  |  |  |
| GO:0008057\_eye\_pigment\_granule\_organization | 1 | 0 |  |  |  |  |  |  |  |  |
| GO:0008078\_mesodermal\_cell\_migration | 1 | 0 |  |  |  |  |  |  |  |  |
| GO:0008208\_C21-steroid\_hormone\_catabolic\_process | 1 | 0 |  |  |  |  |  |  |  |  |
| GO:0008216\_spermidine\_metabolic\_process | 1 | 0 |  |  |  |  |  |  |  |  |
| GO:0008292\_acetylcholine\_biosynthetic\_process | 1 | 0 |  |  |  |  |  |  |  |  |
| GO:0008295\_spermidine\_biosynthetic\_process | 1 | 0 |  |  |  |  |  |  |  |  |
| GO:0008300\_isoprenoid\_catabolic\_process | 1 | 0 |  |  |  |  |  |  |  |  |
| GO:0008333\_endosome\_to\_lysosome\_transport | 1 | 0 |  |  |  |  |  |  |  |  |
| GO:0008355\_olfactory\_learning | 1 | 0 |  |  |  |  |  |  |  |  |
| GO:0008611\_ether\_lipid\_biosynthetic\_process | 1 | 0 |  |  |  |  |  |  |  |  |
| GO:0008626\_induction\_of\_apoptosis\_by\_granzyme | 1 | 0 |  |  |  |  |  |  |  |  |
| GO:0008633\_activation\_of\_pro-apoptotic\_gene\_products | 1 | 0 |  |  |  |  |  |  |  |  |
| GO:0008653\_lipopolysaccharide\_metabolic\_process | 1 | 0 |  |  |  |  |  |  |  |  |
| GO:0009068\_aspartate\_family\_amino\_acid\_catabolic\_process | 1 | 0 |  |  |  |  |  |  |  |  |
| GO:0009084\_glutamine\_family\_amino\_acid\_biosynthetic\_process | 1 | 0 |  |  |  |  |  |  |  |  |
| GO:0009088\_threonine\_biosynthetic\_process | 1 | 0 |  |  |  |  |  |  |  |  |
| GO:0009105\_lipoic\_acid\_biosynthetic\_process | 1 | 0 |  |  |  |  |  |  |  |  |
| GO:0009109\_coenzyme\_catabolic\_process | 1 | 0 |  |  |  |  |  |  |  |  |
| GO:0009111\_vitamin\_catabolic\_process | 1 | 0 |  |  |  |  |  |  |  |  |
| GO:0009113\_purine\_base\_biosynthetic\_process | 1 | 0 |  |  |  |  |  |  |  |  |
| GO:0009127\_purine\_nucleoside\_monophosphate\_biosynthetic\_process | 1 | 0 |  |  |  |  |  |  |  |  |
| GO:0009128\_purine\_nucleoside\_monophosphate\_catabolic\_process | 1 | 0 |  |  |  |  |  |  |  |  |
| GO:0009129\_pyrimidine\_nucleoside\_monophosphate\_metabolic\_process | 1 | 0 |  |  |  |  |  |  |  |  |
| GO:0009131\_pyrimidine\_nucleoside\_monophosphate\_catabolic\_process | 1 | 0 |  |  |  |  |  |  |  |  |
| GO:0009133\_nucleoside\_diphosphate\_biosynthetic\_process | 1 | 0 |  |  |  |  |  |  |  |  |
| GO:0009145\_purine\_nucleoside\_triphosphate\_biosynthetic\_process | 1 | 0 |  |  |  |  |  |  |  |  |
| GO:0009147\_pyrimidine\_nucleoside\_triphosphate\_metabolic\_process | 1 | 0 |  |  |  |  |  |  |  |  |
| GO:0009148\_pyrimidine\_nucleoside\_triphosphate\_biosynthetic\_process | 1 | 0 |  |  |  |  |  |  |  |  |
| GO:0009152\_purine\_ribonucleotide\_biosynthetic\_process | 1 | 0 |  |  |  |  |  |  |  |  |
| GO:0009153\_purine\_deoxyribonucleotide\_biosynthetic\_process | 1 | 0 |  |  |  |  |  |  |  |  |
| GO:0009156\_ribonucleoside\_monophosphate\_biosynthetic\_process | 1 | 0 |  |  |  |  |  |  |  |  |
| GO:0009158\_ribonucleoside\_monophosphate\_catabolic\_process | 1 | 0 |  |  |  |  |  |  |  |  |
| GO:0009159\_deoxyribonucleoside\_monophosphate\_catabolic\_process | 1 | 0 |  |  |  |  |  |  |  |  |
| GO:0009162\_deoxyribonucleoside\_monophosphate\_metabolic\_process | 1 | 0 |  |  |  |  |  |  |  |  |
| GO:0009168\_purine\_ribonucleoside\_monophosphate\_biosynthetic\_process | 1 | 0 |  |  |  |  |  |  |  |  |
| GO:0009169\_purine\_ribonucleoside\_monophosphate\_catabolic\_process | 1 | 0 |  |  |  |  |  |  |  |  |
| GO:0009176\_pyrimidine\_deoxyribonucleoside\_monophosphate\_metabolic\_process | 1 | 0 |  |  |  |  |  |  |  |  |
| GO:0009178\_pyrimidine\_deoxyribonucleoside\_monophosphate\_catabolic\_process | 1 | 0 |  |  |  |  |  |  |  |  |
| GO:0009211\_pyrimidine\_deoxyribonucleoside\_triphosphate\_metabolic\_process | 1 | 0 |  |  |  |  |  |  |  |  |
| GO:0009212\_pyrimidine\_deoxyribonucleoside\_triphosphate\_biosynthetic\_process | 1 | 0 |  |  |  |  |  |  |  |  |
| GO:0009216\_purine\_deoxyribonucleoside\_triphosphate\_biosynthetic\_process | 1 | 0 |  |  |  |  |  |  |  |  |
| GO:0009221\_pyrimidine\_deoxyribonucleotide\_biosynthetic\_process | 1 | 0 |  |  |  |  |  |  |  |  |
| GO:0009223\_pyrimidine\_deoxyribonucleotide\_catabolic\_process | 1 | 0 |  |  |  |  |  |  |  |  |
| GO:0009260\_ribonucleotide\_biosynthetic\_process | 1 | 0 |  |  |  |  |  |  |  |  |
| GO:0009405\_pathogenesis | 1 | 0 |  |  |  |  |  |  |  |  |
| GO:0009414\_response\_to\_water\_deprivation | 1 | 0 |  |  |  |  |  |  |  |  |
| GO:0009415\_response\_to\_water | 1 | 0 |  |  |  |  |  |  |  |  |
| GO:0009449\_gamma-aminobutyric\_acid\_biosynthetic\_process | 1 | 0 |  |  |  |  |  |  |  |  |
| GO:0009450\_gamma-aminobutyric\_acid\_catabolic\_process | 1 | 0 |  |  |  |  |  |  |  |  |
| GO:0009589\_detection\_of\_UV | 1 | 0 |  |  |  |  |  |  |  |  |
| GO:0009590\_detection\_of\_gravity | 1 | 0 |  |  |  |  |  |  |  |  |
| GO:0009624\_response\_to\_nematode | 1 | 0 |  |  |  |  |  |  |  |  |
| GO:0009629\_response\_to\_gravity | 1 | 0 |  |  |  |  |  |  |  |  |
| GO:0009648\_photoperiodism | 1 | 0 |  |  |  |  |  |  |  |  |
| GO:0009690\_cytokinin\_metabolic\_process | 1 | 0 |  |  |  |  |  |  |  |  |
| GO:0009691\_cytokinin\_biosynthetic\_process | 1 | 0 |  |  |  |  |  |  |  |  |
| GO:0009786\_regulation\_of\_asymmetric\_cell\_division | 1 | 0 |  |  |  |  |  |  |  |  |
| GO:0009794\_regulation\_of\_mitotic\_cell\_cycle\_\_embryonic | 1 | 0 |  |  |  |  |  |  |  |  |
| GO:0009956\_radial\_pattern\_formation | 1 | 0 |  |  |  |  |  |  |  |  |
| GO:0009957\_epidermal\_cell\_fate\_specification | 1 | 0 |  |  |  |  |  |  |  |  |
| GO:0009992\_cellular\_water\_homeostasis | 1 | 0 |  |  |  |  |  |  |  |  |
| GO:0010032\_meiotic\_chromosome\_condensation | 1 | 0 |  |  |  |  |  |  |  |  |
| GO:0010039\_response\_to\_iron\_ion | 1 | 0 |  |  |  |  |  |  |  |  |
| GO:0010042\_response\_to\_manganese\_ion | 1 | 0 |  |  |  |  |  |  |  |  |
| GO:0010045\_response\_to\_nickel\_ion | 1 | 0 |  |  |  |  |  |  |  |  |
| GO:0010046\_response\_to\_mycotoxin | 1 | 0 |  |  |  |  |  |  |  |  |
| GO:0010107\_potassium\_ion\_import | 1 | 0 |  |  |  |  |  |  |  |  |
| GO:0010155\_regulation\_of\_proton\_transport | 1 | 0 |  |  |  |  |  |  |  |  |
| GO:0010160\_formation\_of\_organ\_boundary | 1 | 0 |  |  |  |  |  |  |  |  |
| GO:0010310\_regulation\_of\_hydrogen\_peroxide\_metabolic\_process | 1 | 0 |  |  |  |  |  |  |  |  |
| GO:0010447\_response\_to\_acidity | 1 | 0 |  |  |  |  |  |  |  |  |
| GO:0010452\_histone\_H3-K36\_methylation | 1 | 0 |  |  |  |  |  |  |  |  |
| GO:0010455\_positive\_regulation\_of\_cell\_fate\_commitment | 1 | 0 |  |  |  |  |  |  |  |  |
| GO:0010470\_regulation\_of\_gastrulation | 1 | 0 |  |  |  |  |  |  |  |  |
| GO:0010508\_positive\_regulation\_of\_autophagy | 1 | 0 |  |  |  |  |  |  |  |  |
| GO:0010519\_negative\_regulation\_of\_phospholipase\_activity | 1 | 0 |  |  |  |  |  |  |  |  |
| GO:0010520\_regulation\_of\_reciprocal\_meiotic\_recombination | 1 | 0 |  |  |  |  |  |  |  |  |
| GO:0010523\_negative\_regulation\_of\_calcium\_ion\_transport\_into\_cytosol | 1 | 0 |  |  |  |  |  |  |  |  |
| GO:0010543\_regulation\_of\_platelet\_activation | 1 | 0 |  |  |  |  |  |  |  |  |
| GO:0010561\_negative\_regulation\_of\_glycoprotein\_biosynthetic\_process | 1 | 0 |  |  |  |  |  |  |  |  |
| GO:0010569\_regulation\_of\_double-strand\_break\_repair\_via\_homologous\_recombination | 1 | 0 |  |  |  |  |  |  |  |  |
| GO:0010572\_positive\_regulation\_of\_platelet\_activation | 1 | 0 |  |  |  |  |  |  |  |  |
| GO:0010594\_regulation\_of\_endothelial\_cell\_migration | 1 | 0 |  |  |  |  |  |  |  |  |
| GO:0010596\_negative\_regulation\_of\_endothelial\_cell\_migration | 1 | 0 |  |  |  |  |  |  |  |  |
| GO:0010611\_regulation\_of\_cardiac\_muscle\_hypertrophy | 1 | 0 |  |  |  |  |  |  |  |  |
| GO:0010612\_regulation\_of\_cardiac\_muscle\_adaptation | 1 | 0 |  |  |  |  |  |  |  |  |
| GO:0010614\_negative\_regulation\_of\_cardiac\_muscle\_hypertrophy | 1 | 0 |  |  |  |  |  |  |  |  |
| GO:0010616\_negative\_regulation\_of\_cardiac\_muscle\_adaptation | 1 | 0 |  |  |  |  |  |  |  |  |
| GO:0010634\_positive\_regulation\_of\_epithelial\_cell\_migration | 1 | 0 |  |  |  |  |  |  |  |  |
| GO:0010656\_negative\_regulation\_of\_muscle\_cell\_apoptosis | 1 | 0 |  |  |  |  |  |  |  |  |
| GO:0010657\_muscle\_cell\_apoptosis | 1 | 0 |  |  |  |  |  |  |  |  |
| GO:0010658\_striated\_muscle\_cell\_apoptosis | 1 | 0 |  |  |  |  |  |  |  |  |
| GO:0010659\_cardiac\_muscle\_cell\_apoptosis | 1 | 0 |  |  |  |  |  |  |  |  |
| GO:0010660\_regulation\_of\_muscle\_cell\_apoptosis | 1 | 0 |  |  |  |  |  |  |  |  |
| GO:0010662\_regulation\_of\_striated\_muscle\_cell\_apoptosis | 1 | 0 |  |  |  |  |  |  |  |  |
| GO:0010664\_negative\_regulation\_of\_striated\_muscle\_cell\_apoptosis | 1 | 0 |  |  |  |  |  |  |  |  |
| GO:0010665\_regulation\_of\_cardiac\_muscle\_cell\_apoptosis | 1 | 0 |  |  |  |  |  |  |  |  |
| GO:0010667\_negative\_regulation\_of\_cardiac\_muscle\_cell\_apoptosis | 1 | 0 |  |  |  |  |  |  |  |  |
| GO:0010668\_ectodermal\_cell\_differentiation | 1 | 0 |  |  |  |  |  |  |  |  |
| GO:0010671\_negative\_regulation\_of\_oxygen\_and\_reactive\_oxygen\_species\_metabolic\_process | 1 | 0 |  |  |  |  |  |  |  |  |
| GO:0010719\_negative\_regulation\_of\_epithelial\_to\_mesenchymal\_transition | 1 | 0 |  |  |  |  |  |  |  |  |
| GO:0010735\_positive\_regulation\_of\_transcription\_via\_serum\_response\_element\_binding | 1 | 0 |  |  |  |  |  |  |  |  |
| GO:0010825\_positive\_regulation\_of\_centrosome\_duplication | 1 | 0 |  |  |  |  |  |  |  |  |
| GO:0010845\_positive\_regulation\_of\_reciprocal\_meiotic\_recombination | 1 | 0 |  |  |  |  |  |  |  |  |
| GO:0010850\_chemoreceptor\_signaling\_pathway\_involved\_in\_regulation\_of\_blood\_pressure | 1 | 0 |  |  |  |  |  |  |  |  |
| GO:0010873\_positive\_regulation\_of\_cholesterol\_esterification | 1 | 0 |  |  |  |  |  |  |  |  |
| GO:0010880\_regulation\_of\_release\_of\_sequestered\_calcium\_ion\_into\_cytosol\_by\_sarcoplasmic\_reticulum | 1 | 0 |  |  |  |  |  |  |  |  |
| GO:0010881\_regulation\_of\_cardiac\_muscle\_contraction\_by\_regulation\_of\_the\_release\_of\_sequestered\_calcium\_ion | 1 | 0 |  |  |  |  |  |  |  |  |
| GO:0010882\_regulation\_of\_cardiac\_muscle\_contraction\_by\_calcium\_ion\_signaling | 1 | 0 |  |  |  |  |  |  |  |  |
| GO:0010890\_positive\_regulation\_of\_sequestering\_of\_triglyceride | 1 | 0 |  |  |  |  |  |  |  |  |
| GO:0010919\_regulation\_of\_inositol\_phosphate\_biosynthetic\_process | 1 | 0 |  |  |  |  |  |  |  |  |
| GO:0010931\_macrophage\_tolerance\_induction | 1 | 0 |  |  |  |  |  |  |  |  |
| GO:0010932\_regulation\_of\_macrophage\_tolerance\_induction | 1 | 0 |  |  |  |  |  |  |  |  |
| GO:0010933\_positive\_regulation\_of\_macrophage\_tolerance\_induction | 1 | 0 |  |  |  |  |  |  |  |  |
| GO:0010934\_macrophage\_cytokine\_production | 1 | 0 |  |  |  |  |  |  |  |  |
| GO:0010935\_regulation\_of\_macrophage\_cytokine\_production | 1 | 0 |  |  |  |  |  |  |  |  |
| GO:0010936\_negative\_regulation\_of\_macrophage\_cytokine\_production | 1 | 0 |  |  |  |  |  |  |  |  |
| GO:0010953\_regulation\_of\_protein\_maturation\_by\_peptide\_bond\_cleavage | 1 | 0 |  |  |  |  |  |  |  |  |
| GO:0010962\_regulation\_of\_glucan\_biosynthetic\_process | 1 | 0 |  |  |  |  |  |  |  |  |
| GO:0010966\_regulation\_of\_phosphate\_transport | 1 | 0 |  |  |  |  |  |  |  |  |
| GO:0014012\_axon\_regeneration\_in\_the\_peripheral\_nervous\_system | 1 | 0 |  |  |  |  |  |  |  |  |
| GO:0014016\_neuroblast\_differentiation | 1 | 0 |  |  |  |  |  |  |  |  |
| GO:0014017\_neuroblast\_fate\_commitment | 1 | 0 |  |  |  |  |  |  |  |  |
| GO:0014041\_regulation\_of\_neuron\_maturation | 1 | 0 |  |  |  |  |  |  |  |  |
| GO:0014042\_positive\_regulation\_of\_neuron\_maturation | 1 | 0 |  |  |  |  |  |  |  |  |
| GO:0014049\_positive\_regulation\_of\_glutamate\_secretion | 1 | 0 |  |  |  |  |  |  |  |  |
| GO:0014061\_regulation\_of\_norepinephrine\_secretion | 1 | 0 |  |  |  |  |  |  |  |  |
| GO:0014071\_response\_to\_cycloalkane | 1 | 0 |  |  |  |  |  |  |  |  |
| GO:0014707\_branchiomeric\_skeletal\_muscle\_development | 1 | 0 |  |  |  |  |  |  |  |  |
| GO:0014738\_regulation\_of\_muscle\_hyperplasia | 1 | 0 |  |  |  |  |  |  |  |  |
| GO:0014740\_negative\_regulation\_of\_muscle\_hyperplasia | 1 | 0 |  |  |  |  |  |  |  |  |
| GO:0014741\_negative\_regulation\_of\_muscle\_hypertrophy | 1 | 0 |  |  |  |  |  |  |  |  |
| GO:0014743\_regulation\_of\_muscle\_hypertrophy | 1 | 0 |  |  |  |  |  |  |  |  |
| GO:0014805\_smooth\_muscle\_adaptation | 1 | 0 |  |  |  |  |  |  |  |  |
| GO:0014806\_smooth\_muscle\_hyperplasia | 1 | 0 |  |  |  |  |  |  |  |  |
| GO:0014807\_regulation\_of\_somitogenesis | 1 | 0 |  |  |  |  |  |  |  |  |
| GO:0014808\_release\_of\_sequestered\_calcium\_ion\_into\_cytosol\_by\_sarcoplasmic\_reticulum | 1 | 0 |  |  |  |  |  |  |  |  |
| GO:0014813\_satellite\_cell\_commitment | 1 | 0 |  |  |  |  |  |  |  |  |
| GO:0014816\_satellite\_cell\_differentiation | 1 | 0 |  |  |  |  |  |  |  |  |
| GO:0014819\_regulation\_of\_skeletal\_muscle\_contraction | 1 | 0 |  |  |  |  |  |  |  |  |
| GO:0014852\_regulation\_of\_skeletal\_muscle\_contraction\_by\_neural\_stimulation\_via\_neuromuscular\_junction | 1 | 0 |  |  |  |  |  |  |  |  |
| GO:0014853\_regulation\_of\_excitatory\_postsynaptic\_membrane\_potential\_involved\_in\_skeletal\_muscle\_contraction | 1 | 0 |  |  |  |  |  |  |  |  |
| GO:0014856\_skeletal\_muscle\_cell\_proliferation | 1 | 0 |  |  |  |  |  |  |  |  |
| GO:0014857\_regulation\_of\_skeletal\_muscle\_cell\_proliferation | 1 | 0 |  |  |  |  |  |  |  |  |
| GO:0014858\_positive\_regulation\_of\_skeletal\_muscle\_cell\_proliferation | 1 | 0 |  |  |  |  |  |  |  |  |
| GO:0014887\_cardiac\_muscle\_adaptation | 1 | 0 |  |  |  |  |  |  |  |  |
| GO:0014889\_muscle\_atrophy | 1 | 0 |  |  |  |  |  |  |  |  |
| GO:0014896\_muscle\_hypertrophy | 1 | 0 |  |  |  |  |  |  |  |  |
| GO:0014897\_striated\_muscle\_hypertrophy | 1 | 0 |  |  |  |  |  |  |  |  |
| GO:0014898\_cardiac\_muscle\_hypertrophy | 1 | 0 |  |  |  |  |  |  |  |  |
| GO:0014900\_muscle\_hyperplasia | 1 | 0 |  |  |  |  |  |  |  |  |
| GO:0014910\_regulation\_of\_smooth\_muscle\_cell\_migration | 1 | 0 |  |  |  |  |  |  |  |  |
| GO:0014911\_positive\_regulation\_of\_smooth\_muscle\_cell\_migration | 1 | 0 |  |  |  |  |  |  |  |  |
| GO:0015014\_heparan\_sulfate\_proteoglycan\_biosynthetic\_process\_\_polysaccharide\_chain\_biosynthetic\_process | 1 | 0 |  |  |  |  |  |  |  |  |
| GO:0015074\_DNA\_integration | 1 | 0 |  |  |  |  |  |  |  |  |
| GO:0015670\_carbon\_dioxide\_transport | 1 | 0 |  |  |  |  |  |  |  |  |
| GO:0015677\_copper\_ion\_import | 1 | 0 |  |  |  |  |  |  |  |  |
| GO:0015680\_intracellular\_copper\_ion\_transport | 1 | 0 |  |  |  |  |  |  |  |  |
| GO:0015684\_ferrous\_iron\_transport | 1 | 0 |  |  |  |  |  |  |  |  |
| GO:0015707\_nitrite\_transport | 1 | 0 |  |  |  |  |  |  |  |  |
| GO:0015724\_formate\_transport | 1 | 0 |  |  |  |  |  |  |  |  |
| GO:0015734\_taurine\_transport | 1 | 0 |  |  |  |  |  |  |  |  |
| GO:0015740\_C4-dicarboxylate\_transport | 1 | 0 |  |  |  |  |  |  |  |  |
| GO:0015744\_succinate\_transport | 1 | 0 |  |  |  |  |  |  |  |  |
| GO:0015746\_citrate\_transport | 1 | 0 |  |  |  |  |  |  |  |  |
| GO:0015747\_urate\_transport | 1 | 0 |  |  |  |  |  |  |  |  |
| GO:0015791\_polyol\_transport | 1 | 0 |  |  |  |  |  |  |  |  |
| GO:0015798\_myo-inositol\_transport | 1 | 0 |  |  |  |  |  |  |  |  |
| GO:0015808\_L-alanine\_transport | 1 | 0 |  |  |  |  |  |  |  |  |
| GO:0015810\_aspartate\_transport | 1 | 0 |  |  |  |  |  |  |  |  |
| GO:0015811\_L-cystine\_transport | 1 | 0 |  |  |  |  |  |  |  |  |
| GO:0015817\_histidine\_transport | 1 | 0 |  |  |  |  |  |  |  |  |
| GO:0015822\_ornithine\_transport | 1 | 0 |  |  |  |  |  |  |  |  |
| GO:0015824\_proline\_transport | 1 | 0 |  |  |  |  |  |  |  |  |
| GO:0015851\_nucleobase\_transport | 1 | 0 |  |  |  |  |  |  |  |  |
| GO:0015864\_pyrimidine\_nucleoside\_transport | 1 | 0 |  |  |  |  |  |  |  |  |
| GO:0015874\_norepinephrine\_transport | 1 | 0 |  |  |  |  |  |  |  |  |
| GO:0015881\_creatine\_transport | 1 | 0 |  |  |  |  |  |  |  |  |
| GO:0015884\_folic\_acid\_transport | 1 | 0 |  |  |  |  |  |  |  |  |
| GO:0015886\_heme\_transport | 1 | 0 |  |  |  |  |  |  |  |  |
| GO:0015888\_thiamin\_transport | 1 | 0 |  |  |  |  |  |  |  |  |
| GO:0015938\_coenzyme\_A\_catabolic\_process | 1 | 0 |  |  |  |  |  |  |  |  |
| GO:0015939\_pantothenate\_metabolic\_process | 1 | 0 |  |  |  |  |  |  |  |  |
| GO:0016073\_snRNA\_metabolic\_process | 1 | 0 |  |  |  |  |  |  |  |  |
| GO:0016074\_snoRNA\_metabolic\_process | 1 | 0 |  |  |  |  |  |  |  |  |
| GO:0016082\_synaptic\_vesicle\_priming | 1 | 0 |  |  |  |  |  |  |  |  |
| GO:0016090\_prenol\_metabolic\_process | 1 | 0 |  |  |  |  |  |  |  |  |
| GO:0016093\_polyprenol\_metabolic\_process | 1 | 0 |  |  |  |  |  |  |  |  |
| GO:0016180\_snRNA\_processing | 1 | 0 |  |  |  |  |  |  |  |  |
| GO:0016239\_positive\_regulation\_of\_macroautophagy | 1 | 0 |  |  |  |  |  |  |  |  |
| GO:0016246\_RNA\_interference | 1 | 0 |  |  |  |  |  |  |  |  |
| GO:0016255\_attachment\_of\_GPI\_anchor\_to\_protein | 1 | 0 |  |  |  |  |  |  |  |  |
| GO:0016333\_morphogenesis\_of\_follicular\_epithelium | 1 | 0 |  |  |  |  |  |  |  |  |
| GO:0016340\_calcium-dependent\_cell-matrix\_adhesion | 1 | 0 |  |  |  |  |  |  |  |  |
| GO:0016344\_meiotic\_chromosome\_movement\_towards\_spindle\_pole | 1 | 0 |  |  |  |  |  |  |  |  |
| GO:0016482\_cytoplasmic\_transport | 1 | 0 |  |  |  |  |  |  |  |  |
| GO:0016553\_base\_conversion\_or\_substitution\_editing | 1 | 0 |  |  |  |  |  |  |  |  |
| GO:0016554\_cytidine\_to\_uridine\_editing | 1 | 0 |  |  |  |  |  |  |  |  |
| GO:0016560\_protein\_import\_into\_peroxisome\_matrix\_\_docking | 1 | 0 |  |  |  |  |  |  |  |  |
| GO:0016578\_histone\_deubiquitination | 1 | 0 |  |  |  |  |  |  |  |  |
| GO:0016598\_protein\_arginylation | 1 | 0 |  |  |  |  |  |  |  |  |
| GO:0017004\_cytochrome\_complex\_assembly | 1 | 0 |  |  |  |  |  |  |  |  |
| GO:0018022\_peptidyl-lysine\_methylation | 1 | 0 |  |  |  |  |  |  |  |  |
| GO:0018023\_peptidyl-lysine\_trimethylation | 1 | 0 |  |  |  |  |  |  |  |  |
| GO:0018120\_peptidyl-arginine\_ADP-ribosylation | 1 | 0 |  |  |  |  |  |  |  |  |
| GO:0018126\_protein\_amino\_acid\_hydroxylation | 1 | 0 |  |  |  |  |  |  |  |  |
| GO:0018146\_keratan\_sulfate\_biosynthetic\_process | 1 | 0 |  |  |  |  |  |  |  |  |
| GO:0018158\_protein\_amino\_acid\_oxidation | 1 | 0 |  |  |  |  |  |  |  |  |
| GO:0018195\_peptidyl-arginine\_modification | 1 | 0 |  |  |  |  |  |  |  |  |
| GO:0018197\_peptidyl-aspartic\_acid\_modification | 1 | 0 |  |  |  |  |  |  |  |  |
| GO:0018282\_metal\_incorporation\_into\_metallo-sulfur\_cluster | 1 | 0 |  |  |  |  |  |  |  |  |
| GO:0018283\_iron\_incorporation\_into\_metallo-sulfur\_cluster | 1 | 0 |  |  |  |  |  |  |  |  |
| GO:0018318\_protein\_amino\_acid\_palmitoylation | 1 | 0 |  |  |  |  |  |  |  |  |
| GO:0018342\_protein\_prenylation | 1 | 0 |  |  |  |  |  |  |  |  |
| GO:0018344\_protein\_geranylgeranylation | 1 | 0 |  |  |  |  |  |  |  |  |
| GO:0018410\_peptide\_or\_protein\_carboxyl-terminal\_blocking | 1 | 0 |  |  |  |  |  |  |  |  |
| GO:0018916\_nitrobenzene\_metabolic\_process | 1 | 0 |  |  |  |  |  |  |  |  |
| GO:0018931\_naphthalene\_metabolic\_process | 1 | 0 |  |  |  |  |  |  |  |  |
| GO:0018992\_germ-line\_sex\_determination | 1 | 0 |  |  |  |  |  |  |  |  |
| GO:0019042\_latent\_virus\_infection | 1 | 0 |  |  |  |  |  |  |  |  |
| GO:0019046\_reactivation\_of\_latent\_virus | 1 | 0 |  |  |  |  |  |  |  |  |
| GO:0019047\_provirus\_integration | 1 | 0 |  |  |  |  |  |  |  |  |
| GO:0019076\_release\_of\_virus\_from\_host | 1 | 0 |  |  |  |  |  |  |  |  |
| GO:0019079\_viral\_genome\_replication | 1 | 0 |  |  |  |  |  |  |  |  |
| GO:0019100\_male\_germ-line\_sex\_determination | 1 | 0 |  |  |  |  |  |  |  |  |
| GO:0019101\_female\_somatic\_sex\_determination | 1 | 0 |  |  |  |  |  |  |  |  |
| GO:0019102\_male\_somatic\_sex\_determination | 1 | 0 |  |  |  |  |  |  |  |  |
| GO:0019255\_glucose\_1-phosphate\_metabolic\_process | 1 | 0 |  |  |  |  |  |  |  |  |
| GO:0019276\_UDP-N-acetylgalactosamine\_metabolic\_process | 1 | 0 |  |  |  |  |  |  |  |  |
| GO:0019344\_cysteine\_biosynthetic\_process | 1 | 0 |  |  |  |  |  |  |  |  |
| GO:0019348\_dolichol\_metabolic\_process | 1 | 0 |  |  |  |  |  |  |  |  |
| GO:0019375\_galactolipid\_biosynthetic\_process | 1 | 0 |  |  |  |  |  |  |  |  |
| GO:0019402\_galactitol\_metabolic\_process | 1 | 0 |  |  |  |  |  |  |  |  |
| GO:0019441\_tryptophan\_catabolic\_process\_to\_kynurenine | 1 | 0 |  |  |  |  |  |  |  |  |
| GO:0019477\_L-lysine\_catabolic\_process | 1 | 0 |  |  |  |  |  |  |  |  |
| GO:0019510\_S-adenosylhomocysteine\_catabolic\_process | 1 | 0 |  |  |  |  |  |  |  |  |
| GO:0019532\_oxalate\_transport | 1 | 0 |  |  |  |  |  |  |  |  |
| GO:0019626\_short-chain\_fatty\_acid\_catabolic\_process | 1 | 0 |  |  |  |  |  |  |  |  |
| GO:0019627\_urea\_metabolic\_process | 1 | 0 |  |  |  |  |  |  |  |  |
| GO:0019676\_ammonia\_assimilation\_cycle | 1 | 0 |  |  |  |  |  |  |  |  |
| GO:0019682\_glyceraldehyde-3-phosphate\_metabolic\_process | 1 | 0 |  |  |  |  |  |  |  |  |
| GO:0019695\_choline\_metabolic\_process | 1 | 0 |  |  |  |  |  |  |  |  |
| GO:0019731\_antibacterial\_humoral\_response | 1 | 0 |  |  |  |  |  |  |  |  |
| GO:0019794\_nonprotein\_amino\_acid\_metabolic\_process | 1 | 0 |  |  |  |  |  |  |  |  |
| GO:0019858\_cytosine\_metabolic\_process | 1 | 0 |  |  |  |  |  |  |  |  |
| GO:0019883\_antigen\_processing\_and\_presentation\_of\_endogenous\_antigen | 1 | 0 |  |  |  |  |  |  |  |  |
| GO:0019889\_pteridine\_metabolic\_process | 1 | 0 |  |  |  |  |  |  |  |  |
| GO:0019896\_axon\_transport\_of\_mitochondrion | 1 | 0 |  |  |  |  |  |  |  |  |
| GO:0021508\_floor\_plate\_formation | 1 | 0 |  |  |  |  |  |  |  |  |
| GO:0021528\_commissural\_neuron\_differentiation\_in\_the\_spinal\_cord | 1 | 0 |  |  |  |  |  |  |  |  |
| GO:0021572\_rhombomere\_6\_development | 1 | 0 |  |  |  |  |  |  |  |  |
| GO:0021577\_hindbrain\_structural\_organization | 1 | 0 |  |  |  |  |  |  |  |  |
| GO:0021586\_pons\_maturation | 1 | 0 |  |  |  |  |  |  |  |  |
| GO:0021589\_cerebellum\_structural\_organization | 1 | 0 |  |  |  |  |  |  |  |  |
| GO:0021590\_cerebellum\_maturation | 1 | 0 |  |  |  |  |  |  |  |  |
| GO:0021592\_fourth\_ventricle\_development | 1 | 0 |  |  |  |  |  |  |  |  |
| GO:0021594\_rhombomere\_formation | 1 | 0 |  |  |  |  |  |  |  |  |
| GO:0021660\_rhombomere\_3\_formation | 1 | 0 |  |  |  |  |  |  |  |  |
| GO:0021664\_rhombomere\_5\_morphogenesis | 1 | 0 |  |  |  |  |  |  |  |  |
| GO:0021666\_rhombomere\_5\_formation | 1 | 0 |  |  |  |  |  |  |  |  |
| GO:0021670\_lateral\_ventricle\_development | 1 | 0 |  |  |  |  |  |  |  |  |
| GO:0021678\_third\_ventricle\_development | 1 | 0 |  |  |  |  |  |  |  |  |
| GO:0021679\_cerebellar\_molecular\_layer\_development | 1 | 0 |  |  |  |  |  |  |  |  |
| GO:0021703\_locus\_ceruleus\_development | 1 | 0 |  |  |  |  |  |  |  |  |
| GO:0021732\_midbrain-hindbrain\_boundary\_maturation | 1 | 0 |  |  |  |  |  |  |  |  |
| GO:0021747\_cochlear\_nucleus\_development | 1 | 0 |  |  |  |  |  |  |  |  |
| GO:0021750\_vestibular\_nucleus\_development | 1 | 0 |  |  |  |  |  |  |  |  |
| GO:0021759\_globus\_pallidus\_development | 1 | 0 |  |  |  |  |  |  |  |  |
| GO:0021768\_nucleus\_accumbens\_development | 1 | 0 |  |  |  |  |  |  |  |  |
| GO:0021771\_lateral\_geniculate\_nucleus\_development | 1 | 0 |  |  |  |  |  |  |  |  |
| GO:0021812\_neuronal-glial\_interaction\_involved\_in\_cerebral\_cortex\_radial\_glia\_guided\_migration | 1 | 0 |  |  |  |  |  |  |  |  |
| GO:0021813\_cell-cell\_adhesion\_involved\_in\_neuronal-glial\_interactions\_involved\_in\_cerebral\_cortex\_radial\_glia\_guided\_migration | 1 | 0 |  |  |  |  |  |  |  |  |
| GO:0021870\_Cajal-Retzius\_cell\_differentiation | 1 | 0 |  |  |  |  |  |  |  |  |
| GO:0021874\_Wnt\_receptor\_signaling\_pathway\_in\_forebrain\_neuroblast\_division | 1 | 0 |  |  |  |  |  |  |  |  |
| GO:0021896\_forebrain\_astrocyte\_differentiation | 1 | 0 |  |  |  |  |  |  |  |  |
| GO:0021897\_forebrain\_astrocyte\_development | 1 | 0 |  |  |  |  |  |  |  |  |
| GO:0021902\_commitment\_of\_a\_neuronal\_cell\_to\_a\_specific\_type\_of\_neuron\_in\_the\_forebrain | 1 | 0 |  |  |  |  |  |  |  |  |
| GO:0021905\_forebrain-midbrain\_boundary\_formation | 1 | 0 |  |  |  |  |  |  |  |  |
| GO:0021914\_negative\_regulation\_of\_smoothened\_signaling\_pathway\_involved\_in\_ventral\_spinal\_cord\_patterning | 1 | 0 |  |  |  |  |  |  |  |  |
| GO:0021917\_somatic\_motor\_neuron\_fate\_commitment | 1 | 0 |  |  |  |  |  |  |  |  |
| GO:0021918\_regulation\_of\_transcription\_from\_RNA\_polymerase\_II\_promoter\_involved\_in\_somatic\_motor\_neuron\_fate\_commitment | 1 | 0 |  |  |  |  |  |  |  |  |
| GO:0021933\_radial\_glia\_guided\_migration\_of\_granule\_cell | 1 | 0 |  |  |  |  |  |  |  |  |
| GO:0021934\_hindbrain\_tangential\_cell\_migration | 1 | 0 |  |  |  |  |  |  |  |  |
| GO:0021935\_granule\_cell\_precursor\_tangential\_migration | 1 | 0 |  |  |  |  |  |  |  |  |
| GO:0021942\_radial\_glia\_guided\_migration\_of\_Purkinje\_cell | 1 | 0 |  |  |  |  |  |  |  |  |
| GO:0021960\_anterior\_commissure\_morphogenesis | 1 | 0 |  |  |  |  |  |  |  |  |
| GO:0021997\_neural\_plate\_axis\_specification | 1 | 0 |  |  |  |  |  |  |  |  |
| GO:0021999\_neural\_plate\_anterior\_posterior\_pattern\_formation | 1 | 0 |  |  |  |  |  |  |  |  |
| GO:0022004\_midbrain-hindbrain\_boundary\_maturation\_during\_brain\_development | 1 | 0 |  |  |  |  |  |  |  |  |
| GO:0022038\_corpus\_callosum\_development | 1 | 0 |  |  |  |  |  |  |  |  |
| GO:0022605\_oogenesis\_stage | 1 | 0 |  |  |  |  |  |  |  |  |
| GO:0030011\_maintenance\_of\_cell\_polarity | 1 | 0 |  |  |  |  |  |  |  |  |
| GO:0030069\_lysogeny | 1 | 0 |  |  |  |  |  |  |  |  |
| GO:0030070\_insulin\_processing | 1 | 0 |  |  |  |  |  |  |  |  |
| GO:0030092\_regulation\_of\_flagellum\_assembly | 1 | 0 |  |  |  |  |  |  |  |  |
| GO:0030103\_vasopressin\_secretion | 1 | 0 |  |  |  |  |  |  |  |  |
| GO:0030194\_positive\_regulation\_of\_blood\_coagulation | 1 | 0 |  |  |  |  |  |  |  |  |
| GO:0030206\_chondroitin\_sulfate\_biosynthetic\_process | 1 | 0 |  |  |  |  |  |  |  |  |
| GO:0030220\_platelet\_formation | 1 | 0 |  |  |  |  |  |  |  |  |
| GO:0030222\_eosinophil\_differentiation | 1 | 0 |  |  |  |  |  |  |  |  |
| GO:0030237\_female\_sex\_determination | 1 | 0 |  |  |  |  |  |  |  |  |
| GO:0030264\_nuclear\_fragmentation\_during\_apoptosis | 1 | 0 |  |  |  |  |  |  |  |  |
| GO:0030322\_stabilization\_of\_membrane\_potential | 1 | 0 |  |  |  |  |  |  |  |  |
| GO:0030327\_prenylated\_protein\_catabolic\_process | 1 | 0 |  |  |  |  |  |  |  |  |
| GO:0030328\_prenylcysteine\_catabolic\_process | 1 | 0 |  |  |  |  |  |  |  |  |
| GO:0030329\_prenylcysteine\_metabolic\_process | 1 | 0 |  |  |  |  |  |  |  |  |
| GO:0030382\_sperm\_mitochondrion\_organization | 1 | 0 |  |  |  |  |  |  |  |  |
| GO:0030389\_fructosamine\_metabolic\_process | 1 | 0 |  |  |  |  |  |  |  |  |
| GO:0030422\_RNA\_interference\_\_production\_of\_siRNA | 1 | 0 |  |  |  |  |  |  |  |  |
| GO:0030449\_regulation\_of\_complement\_activation | 1 | 0 |  |  |  |  |  |  |  |  |
| GO:0030497\_fatty\_acid\_elongation | 1 | 0 |  |  |  |  |  |  |  |  |
| GO:0030575\_nuclear\_body\_organization | 1 | 0 |  |  |  |  |  |  |  |  |
| GO:0030578\_PML\_body\_organization | 1 | 0 |  |  |  |  |  |  |  |  |
| GO:0030853\_negative\_regulation\_of\_granulocyte\_differentiation | 1 | 0 |  |  |  |  |  |  |  |  |
| GO:0030854\_positive\_regulation\_of\_granulocyte\_differentiation | 1 | 0 |  |  |  |  |  |  |  |  |
| GO:0030886\_negative\_regulation\_of\_myeloid\_dendritic\_cell\_activation | 1 | 0 |  |  |  |  |  |  |  |  |
| GO:0030913\_paranodal\_junction\_assembly | 1 | 0 |  |  |  |  |  |  |  |  |
| GO:0031033\_myosin\_filament\_assembly\_or\_disassembly | 1 | 0 |  |  |  |  |  |  |  |  |
| GO:0031034\_myosin\_filament\_assembly | 1 | 0 |  |  |  |  |  |  |  |  |
| GO:0031055\_chromatin\_remodeling\_at\_centromere | 1 | 0 |  |  |  |  |  |  |  |  |
| GO:0031062\_positive\_regulation\_of\_histone\_methylation | 1 | 0 |  |  |  |  |  |  |  |  |
| GO:0031115\_negative\_regulation\_of\_microtubule\_polymerization | 1 | 0 |  |  |  |  |  |  |  |  |
| GO:0031129\_inductive\_cell-cell\_signaling | 1 | 0 |  |  |  |  |  |  |  |  |
| GO:0031284\_positive\_regulation\_of\_guanylate\_cyclase\_activity | 1 | 0 |  |  |  |  |  |  |  |  |
| GO:0031498\_chromatin\_disassembly | 1 | 0 |  |  |  |  |  |  |  |  |
| GO:0031507\_heterochromatin\_formation | 1 | 0 |  |  |  |  |  |  |  |  |
| GO:0031508\_centromeric\_heterochromatin\_formation | 1 | 0 |  |  |  |  |  |  |  |  |
| GO:0031529\_ruffle\_organization | 1 | 0 |  |  |  |  |  |  |  |  |
| GO:0031536\_positive\_regulation\_of\_exit\_from\_mitosis | 1 | 0 |  |  |  |  |  |  |  |  |
| GO:0031572\_G2\_M\_transition\_DNA\_damage\_checkpoint | 1 | 0 |  |  |  |  |  |  |  |  |
| GO:0031576\_G2\_M\_transition\_checkpoint | 1 | 0 |  |  |  |  |  |  |  |  |
| GO:0031580\_membrane\_raft\_distribution | 1 | 0 |  |  |  |  |  |  |  |  |
| GO:0031583\_activation\_of\_phospholipase\_D\_activity\_by\_G-protein\_coupled\_receptor\_protein\_signaling\_pathway | 1 | 0 |  |  |  |  |  |  |  |  |
| GO:0031584\_activation\_of\_phospholipase\_D\_activity | 1 | 0 |  |  |  |  |  |  |  |  |
| GO:0031585\_regulation\_of\_inositol-1\_4\_5-triphosphate\_receptor\_activity | 1 | 0 |  |  |  |  |  |  |  |  |
| GO:0031639\_plasminogen\_activation | 1 | 0 |  |  |  |  |  |  |  |  |
| GO:0031648\_protein\_destabilization | 1 | 0 |  |  |  |  |  |  |  |  |
| GO:0031665\_negative\_regulation\_of\_lipopolysaccharide-mediated\_signaling\_pathway | 1 | 0 |  |  |  |  |  |  |  |  |
| GO:0031914\_negative\_regulation\_of\_synaptic\_plasticity | 1 | 0 |  |  |  |  |  |  |  |  |
| GO:0031944\_negative\_regulation\_of\_glucocorticoid\_metabolic\_process | 1 | 0 |  |  |  |  |  |  |  |  |
| GO:0031947\_negative\_regulation\_of\_glucocorticoid\_biosynthetic\_process | 1 | 0 |  |  |  |  |  |  |  |  |
| GO:0032025\_response\_to\_cobalt\_ion | 1 | 0 |  |  |  |  |  |  |  |  |
| GO:0032026\_response\_to\_magnesium\_ion | 1 | 0 |  |  |  |  |  |  |  |  |
| GO:0032048\_cardiolipin\_metabolic\_process | 1 | 0 |  |  |  |  |  |  |  |  |
| GO:0032066\_nucleolus\_to\_nucleoplasm\_transport | 1 | 0 |  |  |  |  |  |  |  |  |
| GO:0032091\_negative\_regulation\_of\_protein\_binding | 1 | 0 |  |  |  |  |  |  |  |  |
| GO:0032092\_positive\_regulation\_of\_protein\_binding | 1 | 0 |  |  |  |  |  |  |  |  |
| GO:0032097\_positive\_regulation\_of\_response\_to\_food | 1 | 0 |  |  |  |  |  |  |  |  |
| GO:0032100\_positive\_regulation\_of\_appetite | 1 | 0 |  |  |  |  |  |  |  |  |
| GO:0032204\_regulation\_of\_telomere\_maintenance | 1 | 0 |  |  |  |  |  |  |  |  |
| GO:0032206\_positive\_regulation\_of\_telomere\_maintenance | 1 | 0 |  |  |  |  |  |  |  |  |
| GO:0032222\_regulation\_of\_synaptic\_transmission\_\_cholinergic | 1 | 0 |  |  |  |  |  |  |  |  |
| GO:0032224\_positive\_regulation\_of\_synaptic\_transmission\_\_cholinergic | 1 | 0 |  |  |  |  |  |  |  |  |
| GO:0032229\_negative\_regulation\_of\_synaptic\_transmission\_\_GABAergic | 1 | 0 |  |  |  |  |  |  |  |  |
| GO:0032237\_activation\_of\_store-operated\_calcium\_channel\_activity | 1 | 0 |  |  |  |  |  |  |  |  |
| GO:0032239\_regulation\_of\_nucleobase\_\_nucleoside\_\_nucleotide\_and\_nucleic\_acid\_transport | 1 | 0 |  |  |  |  |  |  |  |  |
| GO:0032252\_secretory\_granule\_localization | 1 | 0 |  |  |  |  |  |  |  |  |
| GO:0032274\_gonadotropin\_secretion | 1 | 0 |  |  |  |  |  |  |  |  |
| GO:0032275\_luteinizing\_hormone\_secretion | 1 | 0 |  |  |  |  |  |  |  |  |
| GO:0032287\_myelin\_maintenance\_in\_the\_peripheral\_nervous\_system | 1 | 0 |  |  |  |  |  |  |  |  |
| GO:0032289\_myelin\_formation\_in\_the\_central\_nervous\_system | 1 | 0 |  |  |  |  |  |  |  |  |
| GO:0032303\_regulation\_of\_icosanoid\_secretion | 1 | 0 |  |  |  |  |  |  |  |  |
| GO:0032305\_positive\_regulation\_of\_icosanoid\_secretion | 1 | 0 |  |  |  |  |  |  |  |  |
| GO:0032306\_regulation\_of\_prostaglandin\_secretion | 1 | 0 |  |  |  |  |  |  |  |  |
| GO:0032308\_positive\_regulation\_of\_prostaglandin\_secretion | 1 | 0 |  |  |  |  |  |  |  |  |
| GO:0032310\_prostaglandin\_secretion | 1 | 0 |  |  |  |  |  |  |  |  |
| GO:0032313\_regulation\_of\_Rab\_GTPase\_activity | 1 | 0 |  |  |  |  |  |  |  |  |
| GO:0032314\_regulation\_of\_Rac\_GTPase\_activity | 1 | 0 |  |  |  |  |  |  |  |  |
| GO:0032317\_regulation\_of\_Rap\_GTPase\_activity | 1 | 0 |  |  |  |  |  |  |  |  |
| GO:0032324\_molybdopterin\_cofactor\_biosynthetic\_process | 1 | 0 |  |  |  |  |  |  |  |  |
| GO:0032329\_serine\_transport | 1 | 0 |  |  |  |  |  |  |  |  |
| GO:0032342\_aldosterone\_biosynthetic\_process | 1 | 0 |  |  |  |  |  |  |  |  |
| GO:0032344\_regulation\_of\_aldosterone\_metabolic\_process | 1 | 0 |  |  |  |  |  |  |  |  |
| GO:0032365\_intracellular\_lipid\_transport | 1 | 0 |  |  |  |  |  |  |  |  |
| GO:0032366\_intracellular\_sterol\_transport | 1 | 0 |  |  |  |  |  |  |  |  |
| GO:0032367\_intracellular\_cholesterol\_transport | 1 | 0 |  |  |  |  |  |  |  |  |
| GO:0032370\_positive\_regulation\_of\_lipid\_transport | 1 | 0 |  |  |  |  |  |  |  |  |
| GO:0032410\_negative\_regulation\_of\_transporter\_activity | 1 | 0 |  |  |  |  |  |  |  |  |
| GO:0032413\_negative\_regulation\_of\_ion\_transmembrane\_transporter\_activity | 1 | 0 |  |  |  |  |  |  |  |  |
| GO:0032429\_regulation\_of\_phospholipase\_A2\_activity | 1 | 0 |  |  |  |  |  |  |  |  |
| GO:0032474\_otolith\_morphogenesis | 1 | 0 |  |  |  |  |  |  |  |  |
| GO:0032482\_Rab\_protein\_signal\_transduction | 1 | 0 |  |  |  |  |  |  |  |  |
| GO:0032483\_regulation\_of\_Rab\_protein\_signal\_transduction | 1 | 0 |  |  |  |  |  |  |  |  |
| GO:0032486\_Rap\_protein\_signal\_transduction | 1 | 0 |  |  |  |  |  |  |  |  |
| GO:0032487\_regulation\_of\_Rap\_protein\_signal\_transduction | 1 | 0 |  |  |  |  |  |  |  |  |
| GO:0032594\_protein\_transport\_within\_lipid\_bilayer | 1 | 0 |  |  |  |  |  |  |  |  |
| GO:0032599\_protein\_transport\_out\_of\_membrane\_raft | 1 | 0 |  |  |  |  |  |  |  |  |
| GO:0032600\_chemokine\_receptor\_transport\_out\_of\_membrane\_raft | 1 | 0 |  |  |  |  |  |  |  |  |
| GO:0032607\_interferon-alpha\_production | 1 | 0 |  |  |  |  |  |  |  |  |
| GO:0032621\_interleukin-18\_production | 1 | 0 |  |  |  |  |  |  |  |  |
| GO:0032647\_regulation\_of\_interferon-alpha\_production | 1 | 0 |  |  |  |  |  |  |  |  |
| GO:0032656\_regulation\_of\_interleukin-13\_production | 1 | 0 |  |  |  |  |  |  |  |  |
| GO:0032682\_negative\_regulation\_of\_chemokine\_production | 1 | 0 |  |  |  |  |  |  |  |  |
| GO:0032691\_negative\_regulation\_of\_interleukin-1\_beta\_production | 1 | 0 |  |  |  |  |  |  |  |  |
| GO:0032692\_negative\_regulation\_of\_interleukin-1\_production | 1 | 0 |  |  |  |  |  |  |  |  |
| GO:0032693\_negative\_regulation\_of\_interleukin-10\_production | 1 | 0 |  |  |  |  |  |  |  |  |
| GO:0032696\_negative\_regulation\_of\_interleukin-13\_production | 1 | 0 |  |  |  |  |  |  |  |  |
| GO:0032727\_positive\_regulation\_of\_interferon-alpha\_production | 1 | 0 |  |  |  |  |  |  |  |  |
| GO:0032731\_positive\_regulation\_of\_interleukin-1\_beta\_production | 1 | 0 |  |  |  |  |  |  |  |  |
| GO:0032732\_positive\_regulation\_of\_interleukin-1\_production | 1 | 0 |  |  |  |  |  |  |  |  |
| GO:0032735\_positive\_regulation\_of\_interleukin-12\_production | 1 | 0 |  |  |  |  |  |  |  |  |
| GO:0032764\_negative\_regulation\_of\_mast\_cell\_cytokine\_production | 1 | 0 |  |  |  |  |  |  |  |  |
| GO:0032765\_positive\_regulation\_of\_mast\_cell\_cytokine\_production | 1 | 0 |  |  |  |  |  |  |  |  |
| GO:0032769\_negative\_regulation\_of\_monooxygenase\_activity | 1 | 0 |  |  |  |  |  |  |  |  |
| GO:0032781\_positive\_regulation\_of\_ATPase\_activity | 1 | 0 |  |  |  |  |  |  |  |  |
| GO:0032790\_ribosome\_disassembly | 1 | 0 |  |  |  |  |  |  |  |  |
| GO:0032799\_low-density\_lipoprotein\_receptor\_metabolic\_process | 1 | 0 |  |  |  |  |  |  |  |  |
| GO:0032802\_low-density\_lipoprotein\_receptor\_catabolic\_process | 1 | 0 |  |  |  |  |  |  |  |  |
| GO:0032803\_regulation\_of\_low-density\_lipoprotein\_receptor\_catabolic\_process | 1 | 0 |  |  |  |  |  |  |  |  |
| GO:0032817\_regulation\_of\_natural\_killer\_cell\_proliferation | 1 | 0 |  |  |  |  |  |  |  |  |
| GO:0032819\_positive\_regulation\_of\_natural\_killer\_cell\_proliferation | 1 | 0 |  |  |  |  |  |  |  |  |
| GO:0032836\_glomerular\_basement\_membrane\_development | 1 | 0 |  |  |  |  |  |  |  |  |
| GO:0032855\_positive\_regulation\_of\_Rac\_GTPase\_activity | 1 | 0 |  |  |  |  |  |  |  |  |
| GO:0032863\_activation\_of\_Rac\_GTPase\_activity | 1 | 0 |  |  |  |  |  |  |  |  |
| GO:0032864\_activation\_of\_Cdc42\_GTPase\_activity | 1 | 0 |  |  |  |  |  |  |  |  |
| GO:0032885\_regulation\_of\_polysaccharide\_biosynthetic\_process | 1 | 0 |  |  |  |  |  |  |  |  |
| GO:0032907\_transforming\_growth\_factor-beta3\_production | 1 | 0 |  |  |  |  |  |  |  |  |
| GO:0032910\_regulation\_of\_transforming\_growth\_factor-beta3\_production | 1 | 0 |  |  |  |  |  |  |  |  |
| GO:0032913\_negative\_regulation\_of\_transforming\_growth\_factor-beta3\_production | 1 | 0 |  |  |  |  |  |  |  |  |
| GO:0032924\_activin\_receptor\_signaling\_pathway | 1 | 0 |  |  |  |  |  |  |  |  |
| GO:0032925\_regulation\_of\_activin\_receptor\_signaling\_pathway | 1 | 0 |  |  |  |  |  |  |  |  |
| GO:0032960\_regulation\_of\_inositol\_trisphosphate\_biosynthetic\_process | 1 | 0 |  |  |  |  |  |  |  |  |
| GO:0032962\_positive\_regulation\_of\_inositol\_trisphosphate\_biosynthetic\_process | 1 | 0 |  |  |  |  |  |  |  |  |
| GO:0032964\_collagen\_biosynthetic\_process | 1 | 0 |  |  |  |  |  |  |  |  |
| GO:0032971\_regulation\_of\_muscle\_filament\_sliding | 1 | 0 |  |  |  |  |  |  |  |  |
| GO:0032972\_regulation\_of\_muscle\_filament\_sliding\_speed | 1 | 0 |  |  |  |  |  |  |  |  |
| GO:0032986\_protein-DNA\_complex\_disassembly | 1 | 0 |  |  |  |  |  |  |  |  |
| GO:0032988\_ribonucleoprotein\_complex\_disassembly | 1 | 0 |  |  |  |  |  |  |  |  |
| GO:0033037\_polysaccharide\_localization | 1 | 0 |  |  |  |  |  |  |  |  |
| GO:0033078\_extrathymic\_T\_cell\_differentiation | 1 | 0 |  |  |  |  |  |  |  |  |
| GO:0033085\_negative\_regulation\_of\_T\_cell\_differentiation\_in\_the\_thymus | 1 | 0 |  |  |  |  |  |  |  |  |
| GO:0033087\_negative\_regulation\_of\_immature\_T\_cell\_proliferation | 1 | 0 |  |  |  |  |  |  |  |  |
| GO:0033088\_negative\_regulation\_of\_immature\_T\_cell\_proliferation\_in\_the\_thymus | 1 | 0 |  |  |  |  |  |  |  |  |
| GO:0033108\_mitochondrial\_respiratory\_chain\_complex\_assembly | 1 | 0 |  |  |  |  |  |  |  |  |
| GO:0033127\_regulation\_of\_histone\_phosphorylation | 1 | 0 |  |  |  |  |  |  |  |  |
| GO:0033128\_negative\_regulation\_of\_histone\_phosphorylation | 1 | 0 |  |  |  |  |  |  |  |  |
| GO:0033138\_positive\_regulation\_of\_peptidyl-serine\_phosphorylation | 1 | 0 |  |  |  |  |  |  |  |  |
| GO:0033158\_regulation\_of\_protein\_import\_into\_nucleus\_\_translocation | 1 | 0 |  |  |  |  |  |  |  |  |
| GO:0033160\_positive\_regulation\_of\_protein\_import\_into\_nucleus\_\_translocation | 1 | 0 |  |  |  |  |  |  |  |  |
| GO:0033169\_histone\_H3-K9\_demethylation | 1 | 0 |  |  |  |  |  |  |  |  |
| GO:0033206\_cytokinesis\_after\_meiosis | 1 | 0 |  |  |  |  |  |  |  |  |
| GO:0033240\_positive\_regulation\_of\_cellular\_amine\_metabolic\_process | 1 | 0 |  |  |  |  |  |  |  |  |
| GO:0033313\_meiotic\_cell\_cycle\_checkpoint | 1 | 0 |  |  |  |  |  |  |  |  |
| GO:0033315\_meiotic\_cell\_cycle\_DNA\_replication\_checkpoint | 1 | 0 |  |  |  |  |  |  |  |  |
| GO:0033326\_cerebrospinal\_fluid\_secretion | 1 | 0 |  |  |  |  |  |  |  |  |
| GO:0033366\_protein\_localization\_in\_secretory\_granule | 1 | 0 |  |  |  |  |  |  |  |  |
| GO:0033367\_protein\_localization\_in\_mast\_cell\_secretory\_granule | 1 | 0 |  |  |  |  |  |  |  |  |
| GO:0033368\_protease\_localization\_in\_mast\_cell\_secretory\_granule | 1 | 0 |  |  |  |  |  |  |  |  |
| GO:0033370\_maintenance\_of\_protein\_location\_in\_mast\_cell\_secretory\_granule | 1 | 0 |  |  |  |  |  |  |  |  |
| GO:0033371\_T\_cell\_secretory\_granule\_organization | 1 | 0 |  |  |  |  |  |  |  |  |
| GO:0033373\_maintenance\_of\_protease\_location\_in\_mast\_cell\_secretory\_granule | 1 | 0 |  |  |  |  |  |  |  |  |
| GO:0033374\_protein\_localization\_in\_T\_cell\_secretory\_granule | 1 | 0 |  |  |  |  |  |  |  |  |
| GO:0033375\_protease\_localization\_in\_T\_cell\_secretory\_granule | 1 | 0 |  |  |  |  |  |  |  |  |
| GO:0033377\_maintenance\_of\_protein\_location\_in\_T\_cell\_secretory\_granule | 1 | 0 |  |  |  |  |  |  |  |  |
| GO:0033379\_maintenance\_of\_protease\_location\_in\_T\_cell\_secretory\_granule | 1 | 0 |  |  |  |  |  |  |  |  |
| GO:0033380\_granzyme\_B\_localization\_in\_T\_cell\_secretory\_granule | 1 | 0 |  |  |  |  |  |  |  |  |
| GO:0033382\_maintenance\_of\_granzyme\_B\_location\_in\_T\_cell\_secretory\_granule | 1 | 0 |  |  |  |  |  |  |  |  |
| GO:0033483\_gas\_homeostasis | 1 | 0 |  |  |  |  |  |  |  |  |
| GO:0033484\_nitric\_oxide\_homeostasis | 1 | 0 |  |  |  |  |  |  |  |  |
| GO:0033505\_floor\_plate\_morphogenesis | 1 | 0 |  |  |  |  |  |  |  |  |
| GO:0033522\_histone\_H2A\_ubiquitination | 1 | 0 |  |  |  |  |  |  |  |  |
| GO:0033523\_histone\_H2B\_ubiquitination | 1 | 0 |  |  |  |  |  |  |  |  |
| GO:0033574\_response\_to\_testosterone\_stimulus | 1 | 0 |  |  |  |  |  |  |  |  |
| GO:0033606\_chemokine\_receptor\_transport\_within\_lipid\_bilayer | 1 | 0 |  |  |  |  |  |  |  |  |
| GO:0033628\_regulation\_of\_cell\_adhesion\_mediated\_by\_integrin | 1 | 0 |  |  |  |  |  |  |  |  |
| GO:0033630\_positive\_regulation\_of\_cell\_adhesion\_mediated\_by\_integrin | 1 | 0 |  |  |  |  |  |  |  |  |
| GO:0033632\_regulation\_of\_cell-cell\_adhesion\_mediated\_by\_integrin | 1 | 0 |  |  |  |  |  |  |  |  |
| GO:0033634\_positive\_regulation\_of\_cell-cell\_adhesion\_mediated\_by\_integrin | 1 | 0 |  |  |  |  |  |  |  |  |
| GO:0033683\_nucleotide-excision\_repair\_\_DNA\_incision | 1 | 0 |  |  |  |  |  |  |  |  |
| GO:0033687\_osteoblast\_proliferation | 1 | 0 |  |  |  |  |  |  |  |  |
| GO:0033688\_regulation\_of\_osteoblast\_proliferation | 1 | 0 |  |  |  |  |  |  |  |  |
| GO:0033689\_negative\_regulation\_of\_osteoblast\_proliferation | 1 | 0 |  |  |  |  |  |  |  |  |
| GO:0033750\_ribosome\_localization | 1 | 0 |  |  |  |  |  |  |  |  |
| GO:0033753\_establishment\_of\_ribosome\_localization | 1 | 0 |  |  |  |  |  |  |  |  |
| GO:0033866\_nucleoside\_bisphosphate\_biosynthetic\_process | 1 | 0 |  |  |  |  |  |  |  |  |
| GO:0033875\_ribonucleoside\_bisphosphate\_metabolic\_process | 1 | 0 |  |  |  |  |  |  |  |  |
| GO:0034030\_ribonucleoside\_bisphosphate\_biosynthetic\_process | 1 | 0 |  |  |  |  |  |  |  |  |
| GO:0034032\_purine\_nucleoside\_bisphosphate\_metabolic\_process | 1 | 0 |  |  |  |  |  |  |  |  |
| GO:0034033\_purine\_nucleoside\_bisphosphate\_biosynthetic\_process | 1 | 0 |  |  |  |  |  |  |  |  |
| GO:0034035\_purine\_ribonucleoside\_bisphosphate\_metabolic\_process | 1 | 0 |  |  |  |  |  |  |  |  |
| GO:0034036\_purine\_ribonucleoside\_bisphosphate\_biosynthetic\_process | 1 | 0 |  |  |  |  |  |  |  |  |
| GO:0034067\_protein\_localization\_in\_Golgi\_apparatus | 1 | 0 |  |  |  |  |  |  |  |  |
| GO:0034102\_erythrocyte\_clearance | 1 | 0 |  |  |  |  |  |  |  |  |
| GO:0034106\_regulation\_of\_erythrocyte\_clearance | 1 | 0 |  |  |  |  |  |  |  |  |
| GO:0034107\_negative\_regulation\_of\_erythrocyte\_clearance | 1 | 0 |  |  |  |  |  |  |  |  |
| GO:0034110\_regulation\_of\_homotypic\_cell-cell\_adhesion | 1 | 0 |  |  |  |  |  |  |  |  |
| GO:0034111\_negative\_regulation\_of\_homotypic\_cell-cell\_adhesion | 1 | 0 |  |  |  |  |  |  |  |  |
| GO:0034113\_heterotypic\_cell-cell\_adhesion | 1 | 0 |  |  |  |  |  |  |  |  |
| GO:0034117\_erythrocyte\_aggregation | 1 | 0 |  |  |  |  |  |  |  |  |
| GO:0034118\_regulation\_of\_erythrocyte\_aggregation | 1 | 0 |  |  |  |  |  |  |  |  |
| GO:0034119\_negative\_regulation\_of\_erythrocyte\_aggregation | 1 | 0 |  |  |  |  |  |  |  |  |
| GO:0034121\_regulation\_of\_toll-like\_receptor\_signaling\_pathway | 1 | 0 |  |  |  |  |  |  |  |  |
| GO:0034122\_negative\_regulation\_of\_toll-like\_receptor\_signaling\_pathway | 1 | 0 |  |  |  |  |  |  |  |  |
| GO:0034230\_enkephalin\_processing | 1 | 0 |  |  |  |  |  |  |  |  |
| GO:0034372\_very-low-density\_lipoprotein\_particle\_remodeling | 1 | 0 |  |  |  |  |  |  |  |  |
| GO:0034379\_very-low-density\_lipoprotein\_particle\_assembly | 1 | 0 |  |  |  |  |  |  |  |  |
| GO:0034380\_high-density\_lipoprotein\_particle\_assembly | 1 | 0 |  |  |  |  |  |  |  |  |
| GO:0034394\_protein\_localization\_at\_cell\_surface | 1 | 0 |  |  |  |  |  |  |  |  |
| GO:0034405\_response\_to\_fluid\_shear\_stress | 1 | 0 |  |  |  |  |  |  |  |  |
| GO:0034472\_snRNA\_3'-end\_processing | 1 | 0 |  |  |  |  |  |  |  |  |
| GO:0034474\_U2\_snRNA\_3'-end\_processing | 1 | 0 |  |  |  |  |  |  |  |  |
| GO:0034502\_protein\_localization\_to\_chromosome | 1 | 0 |  |  |  |  |  |  |  |  |
| GO:0034505\_tooth\_mineralization | 1 | 0 |  |  |  |  |  |  |  |  |
| GO:0034508\_centromere\_complex\_assembly | 1 | 0 |  |  |  |  |  |  |  |  |
| GO:0034633\_retinol\_transport | 1 | 0 |  |  |  |  |  |  |  |  |
| GO:0034643\_mitochondrion\_localization\_\_microtubule-mediated | 1 | 0 |  |  |  |  |  |  |  |  |
| GO:0034969\_histone\_arginine\_methylation | 1 | 0 |  |  |  |  |  |  |  |  |
| GO:0034982\_mitochondrial\_protein\_processing | 1 | 0 |  |  |  |  |  |  |  |  |
| GO:0035022\_positive\_regulation\_of\_Rac\_protein\_signal\_transduction | 1 | 0 |  |  |  |  |  |  |  |  |
| GO:0035024\_negative\_regulation\_of\_Rho\_protein\_signal\_transduction | 1 | 0 |  |  |  |  |  |  |  |  |
| GO:0035026\_leading\_edge\_cell\_differentiation | 1 | 0 |  |  |  |  |  |  |  |  |
| GO:0035037\_sperm\_entry | 1 | 0 |  |  |  |  |  |  |  |  |
| GO:0035039\_male\_pronucleus\_formation | 1 | 0 |  |  |  |  |  |  |  |  |
| GO:0035066\_positive\_regulation\_of\_histone\_acetylation | 1 | 0 |  |  |  |  |  |  |  |  |
| GO:0035083\_cilium\_axoneme\_assembly | 1 | 0 |  |  |  |  |  |  |  |  |
| GO:0035090\_maintenance\_of\_apical\_basal\_cell\_polarity | 1 | 0 |  |  |  |  |  |  |  |  |
| GO:0035106\_operant\_conditioning | 1 | 0 |  |  |  |  |  |  |  |  |
| GO:0035172\_hemocyte\_proliferation | 1 | 0 |  |  |  |  |  |  |  |  |
| GO:0035227\_regulation\_of\_glutamate-cysteine\_ligase\_activity | 1 | 0 |  |  |  |  |  |  |  |  |
| GO:0035229\_positive\_regulation\_of\_glutamate-cysteine\_ligase\_activity | 1 | 0 |  |  |  |  |  |  |  |  |
| GO:0035260\_internal\_genitalia\_morphogenesis | 1 | 0 |  |  |  |  |  |  |  |  |
| GO:0035262\_gonad\_morphogenesis | 1 | 0 |  |  |  |  |  |  |  |  |
| GO:0035287\_head\_segmentation | 1 | 0 |  |  |  |  |  |  |  |  |
| GO:0035289\_posterior\_head\_segmentation | 1 | 0 |  |  |  |  |  |  |  |  |
| GO:0035303\_regulation\_of\_dephosphorylation | 1 | 0 |  |  |  |  |  |  |  |  |
| GO:0035304\_regulation\_of\_protein\_amino\_acid\_dephosphorylation | 1 | 0 |  |  |  |  |  |  |  |  |
| GO:0035305\_negative\_regulation\_of\_dephosphorylation | 1 | 0 |  |  |  |  |  |  |  |  |
| GO:0035308\_negative\_regulation\_of\_protein\_amino\_acid\_dephosphorylation | 1 | 0 |  |  |  |  |  |  |  |  |
| GO:0035313\_wound\_healing\_\_spreading\_of\_epidermal\_cells | 1 | 0 |  |  |  |  |  |  |  |  |
| GO:0040013\_negative\_regulation\_of\_locomotion | 1 | 0 |  |  |  |  |  |  |  |  |
| GO:0040019\_positive\_regulation\_of\_embryonic\_development | 1 | 0 |  |  |  |  |  |  |  |  |
| GO:0040032\_post-embryonic\_body\_morphogenesis | 1 | 0 |  |  |  |  |  |  |  |  |
| GO:0040038\_polar\_body\_extrusion\_after\_meiotic\_divisions | 1 | 0 |  |  |  |  |  |  |  |  |
| GO:0042026\_protein\_refolding | 1 | 0 |  |  |  |  |  |  |  |  |
| GO:0042048\_olfactory\_behavior | 1 | 0 |  |  |  |  |  |  |  |  |
| GO:0042059\_negative\_regulation\_of\_epidermal\_growth\_factor\_receptor\_signaling\_pathway | 1 | 0 |  |  |  |  |  |  |  |  |
| GO:0042073\_intraflagellar\_transport | 1 | 0 |  |  |  |  |  |  |  |  |
| GO:0042078\_germ-line\_stem\_cell\_division | 1 | 0 |  |  |  |  |  |  |  |  |
| GO:0042091\_interleukin-10\_biosynthetic\_process | 1 | 0 |  |  |  |  |  |  |  |  |
| GO:0042103\_positive\_regulation\_of\_T\_cell\_homeostatic\_proliferation | 1 | 0 |  |  |  |  |  |  |  |  |
| GO:0042136\_neurotransmitter\_biosynthetic\_process | 1 | 0 |  |  |  |  |  |  |  |  |
| GO:0042137\_sequestering\_of\_neurotransmitter | 1 | 0 |  |  |  |  |  |  |  |  |
| GO:0042138\_meiotic\_DNA\_double-strand\_break\_formation | 1 | 0 |  |  |  |  |  |  |  |  |
| GO:0042178\_xenobiotic\_catabolic\_process | 1 | 0 |  |  |  |  |  |  |  |  |
| GO:0042225\_interleukin-5\_biosynthetic\_process | 1 | 0 |  |  |  |  |  |  |  |  |
| GO:0042231\_interleukin-13\_biosynthetic\_process | 1 | 0 |  |  |  |  |  |  |  |  |
| GO:0042255\_ribosome\_assembly | 1 | 0 |  |  |  |  |  |  |  |  |
| GO:0042257\_ribosomal\_subunit\_assembly | 1 | 0 |  |  |  |  |  |  |  |  |
| GO:0042264\_peptidyl-aspartic\_acid\_hydroxylation | 1 | 0 |  |  |  |  |  |  |  |  |
| GO:0042276\_error-prone\_postreplication\_DNA\_repair | 1 | 0 |  |  |  |  |  |  |  |  |
| GO:0042297\_vocal\_learning | 1 | 0 |  |  |  |  |  |  |  |  |
| GO:0042309\_homoiothermy | 1 | 0 |  |  |  |  |  |  |  |  |
| GO:0042320\_regulation\_of\_circadian\_sleep\_wake\_cycle\_\_REM\_sleep | 1 | 0 |  |  |  |  |  |  |  |  |
| GO:0042339\_keratan\_sulfate\_metabolic\_process | 1 | 0 |  |  |  |  |  |  |  |  |
| GO:0042347\_negative\_regulation\_of\_NF-kappaB\_import\_into\_nucleus | 1 | 0 |  |  |  |  |  |  |  |  |
| GO:0042360\_vitamin\_E\_metabolic\_process | 1 | 0 |  |  |  |  |  |  |  |  |
| GO:0042363\_fat-soluble\_vitamin\_catabolic\_process | 1 | 0 |  |  |  |  |  |  |  |  |
| GO:0042369\_vitamin\_D\_catabolic\_process | 1 | 0 |  |  |  |  |  |  |  |  |
| GO:0042373\_vitamin\_K\_metabolic\_process | 1 | 0 |  |  |  |  |  |  |  |  |
| GO:0042404\_thyroid\_hormone\_catabolic\_process | 1 | 0 |  |  |  |  |  |  |  |  |
| GO:0042414\_epinephrine\_metabolic\_process | 1 | 0 |  |  |  |  |  |  |  |  |
| GO:0042436\_indole\_derivative\_catabolic\_process | 1 | 0 |  |  |  |  |  |  |  |  |
| GO:0042489\_negative\_regulation\_of\_odontogenesis\_of\_dentine-containing\_tooth | 1 | 0 |  |  |  |  |  |  |  |  |
| GO:0042508\_tyrosine\_phosphorylation\_of\_Stat1\_protein | 1 | 0 |  |  |  |  |  |  |  |  |
| GO:0042518\_negative\_regulation\_of\_tyrosine\_phosphorylation\_of\_Stat3\_protein | 1 | 0 |  |  |  |  |  |  |  |  |
| GO:0042524\_negative\_regulation\_of\_tyrosine\_phosphorylation\_of\_Stat5\_protein | 1 | 0 |  |  |  |  |  |  |  |  |
| GO:0042536\_negative\_regulation\_of\_tumor\_necrosis\_factor\_biosynthetic\_process | 1 | 0 |  |  |  |  |  |  |  |  |
| GO:0042538\_hyperosmotic\_salinity\_response | 1 | 0 |  |  |  |  |  |  |  |  |
| GO:0042628\_mating\_plug\_formation | 1 | 0 |  |  |  |  |  |  |  |  |
| GO:0042631\_cellular\_response\_to\_water\_deprivation | 1 | 0 |  |  |  |  |  |  |  |  |
| GO:0042637\_catagen | 1 | 0 |  |  |  |  |  |  |  |  |
| GO:0042660\_positive\_regulation\_of\_cell\_fate\_specification | 1 | 0 |  |  |  |  |  |  |  |  |
| GO:0042663\_regulation\_of\_endodermal\_cell\_fate\_specification | 1 | 0 |  |  |  |  |  |  |  |  |
| GO:0042664\_negative\_regulation\_of\_endodermal\_cell\_fate\_specification | 1 | 0 |  |  |  |  |  |  |  |  |
| GO:0042667\_auditory\_receptor\_cell\_fate\_specification | 1 | 0 |  |  |  |  |  |  |  |  |
| GO:0042694\_muscle\_cell\_fate\_specification | 1 | 0 |  |  |  |  |  |  |  |  |
| GO:0042706\_eye\_photoreceptor\_cell\_fate\_commitment | 1 | 0 |  |  |  |  |  |  |  |  |
| GO:0042713\_sperm\_ejaculation | 1 | 0 |  |  |  |  |  |  |  |  |
| GO:0042723\_thiamin\_and\_derivative\_metabolic\_process | 1 | 0 |  |  |  |  |  |  |  |  |
| GO:0042737\_drug\_catabolic\_process | 1 | 0 |  |  |  |  |  |  |  |  |
| GO:0042738\_exogenous\_drug\_catabolic\_process | 1 | 0 |  |  |  |  |  |  |  |  |
| GO:0042747\_circadian\_sleep\_wake\_cycle\_\_REM\_sleep | 1 | 0 |  |  |  |  |  |  |  |  |
| GO:0042748\_circadian\_sleep\_wake\_cycle\_\_non-REM\_sleep | 1 | 0 |  |  |  |  |  |  |  |  |
| GO:0042772\_DNA\_damage\_response\_\_signal\_transduction\_resulting\_in\_transcription | 1 | 0 |  |  |  |  |  |  |  |  |
| GO:0042790\_transcription\_of\_nuclear\_rRNA\_large\_RNA\_polymerase\_I\_transcript | 1 | 0 |  |  |  |  |  |  |  |  |
| GO:0042839\_D-glucuronate\_metabolic\_process | 1 | 0 |  |  |  |  |  |  |  |  |
| GO:0042840\_D-glucuronate\_catabolic\_process | 1 | 0 |  |  |  |  |  |  |  |  |
| GO:0042891\_antibiotic\_transport | 1 | 0 |  |  |  |  |  |  |  |  |
| GO:0042892\_chloramphenicol\_transport | 1 | 0 |  |  |  |  |  |  |  |  |
| GO:0042940\_D-amino\_acid\_transport | 1 | 0 |  |  |  |  |  |  |  |  |
| GO:0042941\_D-alanine\_transport | 1 | 0 |  |  |  |  |  |  |  |  |
| GO:0042942\_D-serine\_transport | 1 | 0 |  |  |  |  |  |  |  |  |
| GO:0042983\_amyloid\_precursor\_protein\_biosynthetic\_process | 1 | 0 |  |  |  |  |  |  |  |  |
| GO:0042984\_regulation\_of\_amyloid\_precursor\_protein\_biosynthetic\_process | 1 | 0 |  |  |  |  |  |  |  |  |
| GO:0042985\_negative\_regulation\_of\_amyloid\_precursor\_protein\_biosynthetic\_process | 1 | 0 |  |  |  |  |  |  |  |  |
| GO:0042989\_sequestering\_of\_actin\_monomers | 1 | 0 |  |  |  |  |  |  |  |  |
| GO:0043044\_ATP-dependent\_chromatin\_remodeling | 1 | 0 |  |  |  |  |  |  |  |  |
| GO:0043056\_forward\_locomotion | 1 | 0 |  |  |  |  |  |  |  |  |
| GO:0043060\_meiotic\_metaphase\_I\_plate\_congression | 1 | 0 |  |  |  |  |  |  |  |  |
| GO:0043091\_L-arginine\_import | 1 | 0 |  |  |  |  |  |  |  |  |
| GO:0043124\_negative\_regulation\_of\_I-kappaB\_kinase\_NF-kappaB\_cascade | 1 | 0 |  |  |  |  |  |  |  |  |
| GO:0043132\_NAD\_transport | 1 | 0 |  |  |  |  |  |  |  |  |
| GO:0043153\_entrainment\_of\_circadian\_clock\_by\_photoperiod | 1 | 0 |  |  |  |  |  |  |  |  |
| GO:0043171\_peptide\_catabolic\_process | 1 | 0 |  |  |  |  |  |  |  |  |
| GO:0043179\_rhythmic\_excitation | 1 | 0 |  |  |  |  |  |  |  |  |
| GO:0043206\_fibril\_organization | 1 | 0 |  |  |  |  |  |  |  |  |
| GO:0043217\_myelin\_maintenance | 1 | 0 |  |  |  |  |  |  |  |  |
| GO:0043313\_regulation\_of\_neutrophil\_degranulation | 1 | 0 |  |  |  |  |  |  |  |  |
| GO:0043316\_cytotoxic\_T\_cell\_degranulation | 1 | 0 |  |  |  |  |  |  |  |  |
| GO:0043369\_CD4-positive\_or\_CD8-positive\_\_alpha-beta\_T\_cell\_lineage\_commitment | 1 | 0 |  |  |  |  |  |  |  |  |
| GO:0043375\_CD8-positive\_\_alpha-beta\_T\_cell\_lineage\_commitment | 1 | 0 |  |  |  |  |  |  |  |  |
| GO:0043379\_memory\_T\_cell\_differentiation | 1 | 0 |  |  |  |  |  |  |  |  |
| GO:0043380\_regulation\_of\_memory\_T\_cell\_differentiation | 1 | 0 |  |  |  |  |  |  |  |  |
| GO:0043400\_cortisol\_secretion | 1 | 0 |  |  |  |  |  |  |  |  |
| GO:0043415\_positive\_regulation\_of\_skeletal\_muscle\_regeneration | 1 | 0 |  |  |  |  |  |  |  |  |
| GO:0043416\_regulation\_of\_skeletal\_muscle\_regeneration | 1 | 0 |  |  |  |  |  |  |  |  |
| GO:0043437\_butanoic\_acid\_metabolic\_process | 1 | 0 |  |  |  |  |  |  |  |  |
| GO:0043438\_acetoacetic\_acid\_metabolic\_process | 1 | 0 |  |  |  |  |  |  |  |  |
| GO:0043480\_pigment\_accumulation\_in\_tissues | 1 | 0 |  |  |  |  |  |  |  |  |
| GO:0043482\_cellular\_pigment\_accumulation | 1 | 0 |  |  |  |  |  |  |  |  |
| GO:0043486\_histone\_exchange | 1 | 0 |  |  |  |  |  |  |  |  |
| GO:0043496\_regulation\_of\_protein\_homodimerization\_activity | 1 | 0 |  |  |  |  |  |  |  |  |
| GO:0043501\_skeletal\_muscle\_adaptation | 1 | 0 |  |  |  |  |  |  |  |  |
| GO:0043508\_negative\_regulation\_of\_JUN\_kinase\_activity | 1 | 0 |  |  |  |  |  |  |  |  |
| GO:0043517\_positive\_regulation\_of\_DNA\_damage\_response\_\_signal\_transduction\_by\_p53\_class\_mediator | 1 | 0 |  |  |  |  |  |  |  |  |
| GO:0043535\_regulation\_of\_blood\_vessel\_endothelial\_cell\_migration | 1 | 0 |  |  |  |  |  |  |  |  |
| GO:0043537\_negative\_regulation\_of\_blood\_vessel\_endothelial\_cell\_migration | 1 | 0 |  |  |  |  |  |  |  |  |
| GO:0043545\_molybdopterin\_cofactor\_metabolic\_process | 1 | 0 |  |  |  |  |  |  |  |  |
| GO:0043587\_tongue\_morphogenesis | 1 | 0 |  |  |  |  |  |  |  |  |
| GO:0043604\_amide\_biosynthetic\_process | 1 | 0 |  |  |  |  |  |  |  |  |
| GO:0043628\_ncRNA\_3'-end\_processing | 1 | 0 |  |  |  |  |  |  |  |  |
| GO:0044254\_multicellular\_organismal\_protein\_catabolic\_process | 1 | 0 |  |  |  |  |  |  |  |  |
| GO:0044256\_protein\_digestion | 1 | 0 |  |  |  |  |  |  |  |  |
| GO:0044266\_multicellular\_organismal\_macromolecule\_catabolic\_process | 1 | 0 |  |  |  |  |  |  |  |  |
| GO:0045004\_DNA\_replication\_proofreading | 1 | 0 |  |  |  |  |  |  |  |  |
| GO:0045019\_negative\_regulation\_of\_nitric\_oxide\_biosynthetic\_process | 1 | 0 |  |  |  |  |  |  |  |  |
| GO:0045020\_error-prone\_DNA\_repair | 1 | 0 |  |  |  |  |  |  |  |  |
| GO:0045022\_early\_endosome\_to\_late\_endosome\_transport | 1 | 0 |  |  |  |  |  |  |  |  |
| GO:0045062\_extrathymic\_T\_cell\_selection | 1 | 0 |  |  |  |  |  |  |  |  |
| GO:0045069\_regulation\_of\_viral\_genome\_replication | 1 | 0 |  |  |  |  |  |  |  |  |
| GO:0045074\_regulation\_of\_interleukin-10\_biosynthetic\_process | 1 | 0 |  |  |  |  |  |  |  |  |
| GO:0045082\_positive\_regulation\_of\_interleukin-10\_biosynthetic\_process | 1 | 0 |  |  |  |  |  |  |  |  |
| GO:0045083\_negative\_regulation\_of\_interleukin-12\_biosynthetic\_process | 1 | 0 |  |  |  |  |  |  |  |  |
| GO:0045112\_integrin\_biosynthetic\_process | 1 | 0 |  |  |  |  |  |  |  |  |
| GO:0045113\_regulation\_of\_integrin\_biosynthetic\_process | 1 | 0 |  |  |  |  |  |  |  |  |
| GO:0045188\_regulation\_of\_circadian\_sleep\_wake\_cycle\_\_non-REM\_sleep | 1 | 0 |  |  |  |  |  |  |  |  |
| GO:0045210\_FasL\_biosynthetic\_process | 1 | 0 |  |  |  |  |  |  |  |  |
| GO:0045297\_post-mating\_behavior | 1 | 0 |  |  |  |  |  |  |  |  |
| GO:0045299\_otolith\_mineralization | 1 | 0 |  |  |  |  |  |  |  |  |
| GO:0045329\_carnitine\_biosynthetic\_process | 1 | 0 |  |  |  |  |  |  |  |  |
| GO:0045341\_MHC\_class\_I\_biosynthetic\_process | 1 | 0 |  |  |  |  |  |  |  |  |
| GO:0045343\_regulation\_of\_MHC\_class\_I\_biosynthetic\_process | 1 | 0 |  |  |  |  |  |  |  |  |
| GO:0045347\_negative\_regulation\_of\_MHC\_class\_II\_biosynthetic\_process | 1 | 0 |  |  |  |  |  |  |  |  |
| GO:0045405\_regulation\_of\_interleukin-5\_biosynthetic\_process | 1 | 0 |  |  |  |  |  |  |  |  |
| GO:0045407\_positive\_regulation\_of\_interleukin-5\_biosynthetic\_process | 1 | 0 |  |  |  |  |  |  |  |  |
| GO:0045426\_quinone\_cofactor\_biosynthetic\_process | 1 | 0 |  |  |  |  |  |  |  |  |
| GO:0045448\_mitotic\_cell\_cycle\_\_embryonic | 1 | 0 |  |  |  |  |  |  |  |  |
| GO:0045454\_cell\_redox\_homeostasis | 1 | 0 |  |  |  |  |  |  |  |  |
| GO:0045583\_regulation\_of\_cytotoxic\_T\_cell\_differentiation | 1 | 0 |  |  |  |  |  |  |  |  |
| GO:0045585\_positive\_regulation\_of\_cytotoxic\_T\_cell\_differentiation | 1 | 0 |  |  |  |  |  |  |  |  |
| GO:0045601\_regulation\_of\_endothelial\_cell\_differentiation | 1 | 0 |  |  |  |  |  |  |  |  |
| GO:0045602\_negative\_regulation\_of\_endothelial\_cell\_differentiation | 1 | 0 |  |  |  |  |  |  |  |  |
| GO:0045605\_negative\_regulation\_of\_epidermal\_cell\_differentiation | 1 | 0 |  |  |  |  |  |  |  |  |
| GO:0045606\_positive\_regulation\_of\_epidermal\_cell\_differentiation | 1 | 0 |  |  |  |  |  |  |  |  |
| GO:0045609\_positive\_regulation\_of\_auditory\_receptor\_cell\_differentiation | 1 | 0 |  |  |  |  |  |  |  |  |
| GO:0045617\_negative\_regulation\_of\_keratinocyte\_differentiation | 1 | 0 |  |  |  |  |  |  |  |  |
| GO:0045618\_positive\_regulation\_of\_keratinocyte\_differentiation | 1 | 0 |  |  |  |  |  |  |  |  |
| GO:0045626\_negative\_regulation\_of\_T-helper\_1\_cell\_differentiation | 1 | 0 |  |  |  |  |  |  |  |  |
| GO:0045633\_positive\_regulation\_of\_mechanoreceptor\_differentiation | 1 | 0 |  |  |  |  |  |  |  |  |
| GO:0045650\_negative\_regulation\_of\_macrophage\_differentiation | 1 | 0 |  |  |  |  |  |  |  |  |
| GO:0045656\_negative\_regulation\_of\_monocyte\_differentiation | 1 | 0 |  |  |  |  |  |  |  |  |
| GO:0045657\_positive\_regulation\_of\_monocyte\_differentiation | 1 | 0 |  |  |  |  |  |  |  |  |
| GO:0045659\_negative\_regulation\_of\_neutrophil\_differentiation | 1 | 0 |  |  |  |  |  |  |  |  |
| GO:0045660\_positive\_regulation\_of\_neutrophil\_differentiation | 1 | 0 |  |  |  |  |  |  |  |  |
| GO:0045721\_negative\_regulation\_of\_gluconeogenesis | 1 | 0 |  |  |  |  |  |  |  |  |
| GO:0045724\_positive\_regulation\_of\_flagellum\_assembly | 1 | 0 |  |  |  |  |  |  |  |  |
| GO:0045725\_positive\_regulation\_of\_glycogen\_biosynthetic\_process | 1 | 0 |  |  |  |  |  |  |  |  |
| GO:0045740\_positive\_regulation\_of\_DNA\_replication | 1 | 0 |  |  |  |  |  |  |  |  |
| GO:0045759\_negative\_regulation\_of\_action\_potential | 1 | 0 |  |  |  |  |  |  |  |  |
| GO:0045768\_positive\_regulation\_of\_anti-apoptosis | 1 | 0 |  |  |  |  |  |  |  |  |
| GO:0045769\_negative\_regulation\_of\_asymmetric\_cell\_division | 1 | 0 |  |  |  |  |  |  |  |  |
| GO:0045794\_negative\_regulation\_of\_cell\_volume | 1 | 0 |  |  |  |  |  |  |  |  |
| GO:0045815\_positive\_regulation\_of\_gene\_expression\_\_epigenetic | 1 | 0 |  |  |  |  |  |  |  |  |
| GO:0045818\_negative\_regulation\_of\_glycogen\_catabolic\_process | 1 | 0 |  |  |  |  |  |  |  |  |
| GO:0045842\_positive\_regulation\_of\_mitotic\_metaphase\_anaphase\_transition | 1 | 0 |  |  |  |  |  |  |  |  |
| GO:0045898\_regulation\_of\_transcriptional\_preinitiation\_complex\_assembly | 1 | 0 |  |  |  |  |  |  |  |  |
| GO:0045899\_positive\_regulation\_of\_transcriptional\_preinitiation\_complex\_assembly | 1 | 0 |  |  |  |  |  |  |  |  |
| GO:0045906\_negative\_regulation\_of\_vasoconstriction | 1 | 0 |  |  |  |  |  |  |  |  |
| GO:0045908\_negative\_regulation\_of\_vasodilation | 1 | 0 |  |  |  |  |  |  |  |  |
| GO:0045909\_positive\_regulation\_of\_vasodilation | 1 | 0 |  |  |  |  |  |  |  |  |
| GO:0045915\_positive\_regulation\_of\_catecholamine\_metabolic\_process | 1 | 0 |  |  |  |  |  |  |  |  |
| GO:0045920\_negative\_regulation\_of\_exocytosis | 1 | 0 |  |  |  |  |  |  |  |  |
| GO:0045924\_regulation\_of\_female\_receptivity | 1 | 0 |  |  |  |  |  |  |  |  |
| GO:0045947\_negative\_regulation\_of\_translational\_initiation | 1 | 0 |  |  |  |  |  |  |  |  |
| GO:0045955\_negative\_regulation\_of\_calcium\_ion-dependent\_exocytosis | 1 | 0 |  |  |  |  |  |  |  |  |
| GO:0045956\_positive\_regulation\_of\_calcium\_ion-dependent\_exocytosis | 1 | 0 |  |  |  |  |  |  |  |  |
| GO:0045964\_positive\_regulation\_of\_dopamine\_metabolic\_process | 1 | 0 |  |  |  |  |  |  |  |  |
| GO:0045988\_negative\_regulation\_of\_striated\_muscle\_contraction | 1 | 0 |  |  |  |  |  |  |  |  |
| GO:0045989\_positive\_regulation\_of\_striated\_muscle\_contraction | 1 | 0 |  |  |  |  |  |  |  |  |
| GO:0045990\_regulation\_of\_transcription\_by\_carbon\_catabolites | 1 | 0 |  |  |  |  |  |  |  |  |
| GO:0045991\_positive\_regulation\_of\_transcription\_by\_carbon\_catabolites | 1 | 0 |  |  |  |  |  |  |  |  |
| GO:0045994\_positive\_regulation\_of\_translational\_initiation\_by\_iron | 1 | 0 |  |  |  |  |  |  |  |  |
| GO:0046007\_negative\_regulation\_of\_activated\_T\_cell\_proliferation | 1 | 0 |  |  |  |  |  |  |  |  |
| GO:0046014\_negative\_regulation\_of\_T\_cell\_homeostatic\_proliferation | 1 | 0 |  |  |  |  |  |  |  |  |
| GO:0046015\_regulation\_of\_transcription\_by\_glucose | 1 | 0 |  |  |  |  |  |  |  |  |
| GO:0046016\_positive\_regulation\_of\_transcription\_by\_glucose | 1 | 0 |  |  |  |  |  |  |  |  |
| GO:0046031\_ADP\_metabolic\_process | 1 | 0 |  |  |  |  |  |  |  |  |
| GO:0046032\_ADP\_catabolic\_process | 1 | 0 |  |  |  |  |  |  |  |  |
| GO:0046061\_dATP\_catabolic\_process | 1 | 0 |  |  |  |  |  |  |  |  |
| GO:0046075\_dTTP\_metabolic\_process | 1 | 0 |  |  |  |  |  |  |  |  |
| GO:0046078\_dUMP\_metabolic\_process | 1 | 0 |  |  |  |  |  |  |  |  |
| GO:0046079\_dUMP\_catabolic\_process | 1 | 0 |  |  |  |  |  |  |  |  |
| GO:0046086\_adenosine\_biosynthetic\_process | 1 | 0 |  |  |  |  |  |  |  |  |
| GO:0046090\_deoxyadenosine\_metabolic\_process | 1 | 0 |  |  |  |  |  |  |  |  |
| GO:0046098\_guanine\_metabolic\_process | 1 | 0 |  |  |  |  |  |  |  |  |
| GO:0046101\_hypoxanthine\_biosynthetic\_process | 1 | 0 |  |  |  |  |  |  |  |  |
| GO:0046102\_inosine\_metabolic\_process | 1 | 0 |  |  |  |  |  |  |  |  |
| GO:0046103\_inosine\_biosynthetic\_process | 1 | 0 |  |  |  |  |  |  |  |  |
| GO:0046108\_uridine\_metabolic\_process | 1 | 0 |  |  |  |  |  |  |  |  |
| GO:0046110\_xanthine\_metabolic\_process | 1 | 0 |  |  |  |  |  |  |  |  |
| GO:0046111\_xanthine\_biosynthetic\_process | 1 | 0 |  |  |  |  |  |  |  |  |
| GO:0046112\_nucleobase\_biosynthetic\_process | 1 | 0 |  |  |  |  |  |  |  |  |
| GO:0046113\_nucleobase\_catabolic\_process | 1 | 0 |  |  |  |  |  |  |  |  |
| GO:0046121\_deoxyribonucleoside\_catabolic\_process | 1 | 0 |  |  |  |  |  |  |  |  |
| GO:0046122\_purine\_deoxyribonucleoside\_metabolic\_process | 1 | 0 |  |  |  |  |  |  |  |  |
| GO:0046124\_purine\_deoxyribonucleoside\_catabolic\_process | 1 | 0 |  |  |  |  |  |  |  |  |
| GO:0046125\_pyrimidine\_deoxyribonucleoside\_metabolic\_process | 1 | 0 |  |  |  |  |  |  |  |  |
| GO:0046131\_pyrimidine\_ribonucleoside\_metabolic\_process | 1 | 0 |  |  |  |  |  |  |  |  |
| GO:0046160\_heme\_a\_metabolic\_process | 1 | 0 |  |  |  |  |  |  |  |  |
| GO:0046218\_indolalkylamine\_catabolic\_process | 1 | 0 |  |  |  |  |  |  |  |  |
| GO:0046292\_formaldehyde\_metabolic\_process | 1 | 0 |  |  |  |  |  |  |  |  |
| GO:0046294\_formaldehyde\_catabolic\_process | 1 | 0 |  |  |  |  |  |  |  |  |
| GO:0046314\_phosphocreatine\_biosynthetic\_process | 1 | 0 |  |  |  |  |  |  |  |  |
| GO:0046327\_glycerol\_biosynthetic\_process\_from\_pyruvate | 1 | 0 |  |  |  |  |  |  |  |  |
| GO:0046329\_negative\_regulation\_of\_JNK\_cascade | 1 | 0 |  |  |  |  |  |  |  |  |
| GO:0046340\_diacylglycerol\_catabolic\_process | 1 | 0 |  |  |  |  |  |  |  |  |
| GO:0046351\_disaccharide\_biosynthetic\_process | 1 | 0 |  |  |  |  |  |  |  |  |
| GO:0046356\_acetyl-CoA\_catabolic\_process | 1 | 0 |  |  |  |  |  |  |  |  |
| GO:0046358\_butyrate\_biosynthetic\_process | 1 | 0 |  |  |  |  |  |  |  |  |
| GO:0046359\_butyrate\_catabolic\_process | 1 | 0 |  |  |  |  |  |  |  |  |
| GO:0046381\_CMP-N-acetylneuraminate\_metabolic\_process | 1 | 0 |  |  |  |  |  |  |  |  |
| GO:0046415\_urate\_metabolic\_process | 1 | 0 |  |  |  |  |  |  |  |  |
| GO:0046416\_D-amino\_acid\_metabolic\_process | 1 | 0 |  |  |  |  |  |  |  |  |
| GO:0046434\_organophosphate\_catabolic\_process | 1 | 0 |  |  |  |  |  |  |  |  |
| GO:0046437\_D-amino\_acid\_biosynthetic\_process | 1 | 0 |  |  |  |  |  |  |  |  |
| GO:0046440\_L-lysine\_metabolic\_process | 1 | 0 |  |  |  |  |  |  |  |  |
| GO:0046449\_creatinine\_metabolic\_process | 1 | 0 |  |  |  |  |  |  |  |  |
| GO:0046471\_phosphatidylglycerol\_metabolic\_process | 1 | 0 |  |  |  |  |  |  |  |  |
| GO:0046473\_phosphatidic\_acid\_metabolic\_process | 1 | 0 |  |  |  |  |  |  |  |  |
| GO:0046476\_glycosylceramide\_biosynthetic\_process | 1 | 0 |  |  |  |  |  |  |  |  |
| GO:0046477\_glycosylceramide\_catabolic\_process | 1 | 0 |  |  |  |  |  |  |  |  |
| GO:0046485\_ether\_lipid\_metabolic\_process | 1 | 0 |  |  |  |  |  |  |  |  |
| GO:0046487\_glyoxylate\_metabolic\_process | 1 | 0 |  |  |  |  |  |  |  |  |
| GO:0046498\_S-adenosylhomocysteine\_metabolic\_process | 1 | 0 |  |  |  |  |  |  |  |  |
| GO:0046552\_photoreceptor\_cell\_fate\_commitment | 1 | 0 |  |  |  |  |  |  |  |  |
| GO:0046586\_regulation\_of\_calcium-dependent\_cell-cell\_adhesion | 1 | 0 |  |  |  |  |  |  |  |  |
| GO:0046587\_positive\_regulation\_of\_calcium-dependent\_cell-cell\_adhesion | 1 | 0 |  |  |  |  |  |  |  |  |
| GO:0046602\_regulation\_of\_mitotic\_centrosome\_separation | 1 | 0 |  |  |  |  |  |  |  |  |
| GO:0046604\_positive\_regulation\_of\_mitotic\_centrosome\_separation | 1 | 0 |  |  |  |  |  |  |  |  |
| GO:0046607\_positive\_regulation\_of\_centrosome\_cycle | 1 | 0 |  |  |  |  |  |  |  |  |
| GO:0046655\_folic\_acid\_metabolic\_process | 1 | 0 |  |  |  |  |  |  |  |  |
| GO:0046671\_negative\_regulation\_of\_retinal\_cell\_programmed\_cell\_death | 1 | 0 |  |  |  |  |  |  |  |  |
| GO:0046685\_response\_to\_arsenic | 1 | 0 |  |  |  |  |  |  |  |  |
| GO:0046692\_sperm\_competition | 1 | 0 |  |  |  |  |  |  |  |  |
| GO:0046707\_IDP\_metabolic\_process | 1 | 0 |  |  |  |  |  |  |  |  |
| GO:0046709\_IDP\_catabolic\_process | 1 | 0 |  |  |  |  |  |  |  |  |
| GO:0046724\_oxalic\_acid\_secretion | 1 | 0 |  |  |  |  |  |  |  |  |
| GO:0046753\_non-lytic\_viral\_release | 1 | 0 |  |  |  |  |  |  |  |  |
| GO:0046755\_non-lytic\_virus\_budding | 1 | 0 |  |  |  |  |  |  |  |  |
| GO:0046826\_negative\_regulation\_of\_protein\_export\_from\_nucleus | 1 | 0 |  |  |  |  |  |  |  |  |
| GO:0046827\_positive\_regulation\_of\_protein\_export\_from\_nucleus | 1 | 0 |  |  |  |  |  |  |  |  |
| GO:0046831\_regulation\_of\_RNA\_export\_from\_nucleus | 1 | 0 |  |  |  |  |  |  |  |  |
| GO:0046834\_lipid\_phosphorylation | 1 | 0 |  |  |  |  |  |  |  |  |
| GO:0046853\_inositol\_and\_derivative\_phosphorylation | 1 | 0 |  |  |  |  |  |  |  |  |
| GO:0046864\_isoprenoid\_transport | 1 | 0 |  |  |  |  |  |  |  |  |
| GO:0046865\_terpenoid\_transport | 1 | 0 |  |  |  |  |  |  |  |  |
| GO:0046877\_regulation\_of\_saliva\_secretion | 1 | 0 |  |  |  |  |  |  |  |  |
| GO:0046878\_positive\_regulation\_of\_saliva\_secretion | 1 | 0 |  |  |  |  |  |  |  |  |
| GO:0046884\_follicle-stimulating\_hormone\_secretion | 1 | 0 |  |  |  |  |  |  |  |  |
| GO:0046898\_response\_to\_cycloheximide | 1 | 0 |  |  |  |  |  |  |  |  |
| GO:0046929\_negative\_regulation\_of\_neurotransmitter\_secretion | 1 | 0 |  |  |  |  |  |  |  |  |
| GO:0046931\_pore\_complex\_biogenesis | 1 | 0 |  |  |  |  |  |  |  |  |
| GO:0046949\_acyl-CoA\_biosynthetic\_process | 1 | 0 |  |  |  |  |  |  |  |  |
| GO:0046958\_nonassociative\_learning | 1 | 0 |  |  |  |  |  |  |  |  |
| GO:0046960\_sensitization | 1 | 0 |  |  |  |  |  |  |  |  |
| GO:0046986\_negative\_regulation\_of\_hemoglobin\_biosynthetic\_process | 1 | 0 |  |  |  |  |  |  |  |  |
| GO:0047497\_mitochondrion\_transport\_along\_microtubule | 1 | 0 |  |  |  |  |  |  |  |  |
| GO:0048047\_mating\_behavior\_\_sex\_discrimination | 1 | 0 |  |  |  |  |  |  |  |  |
| GO:0048133\_male\_germ-line\_stem\_cell\_division | 1 | 0 |  |  |  |  |  |  |  |  |
| GO:0048137\_spermatocyte\_division | 1 | 0 |  |  |  |  |  |  |  |  |
| GO:0048143\_astrocyte\_activation | 1 | 0 |  |  |  |  |  |  |  |  |
| GO:0048170\_positive\_regulation\_of\_long-term\_neuronal\_synaptic\_plasticity | 1 | 0 |  |  |  |  |  |  |  |  |
| GO:0048199\_vesicle\_targeting\_\_to\_\_from\_or\_within\_Golgi | 1 | 0 |  |  |  |  |  |  |  |  |
| GO:0048241\_epinephrine\_transport | 1 | 0 |  |  |  |  |  |  |  |  |
| GO:0048242\_epinephrine\_secretion | 1 | 0 |  |  |  |  |  |  |  |  |
| GO:0048243\_norepinephrine\_secretion | 1 | 0 |  |  |  |  |  |  |  |  |
| GO:0048247\_lymphocyte\_chemotaxis | 1 | 0 |  |  |  |  |  |  |  |  |
| GO:0048250\_mitochondrial\_iron\_ion\_transport | 1 | 0 |  |  |  |  |  |  |  |  |
| GO:0048259\_regulation\_of\_receptor-mediated\_endocytosis | 1 | 0 |  |  |  |  |  |  |  |  |
| GO:0048260\_positive\_regulation\_of\_receptor-mediated\_endocytosis | 1 | 0 |  |  |  |  |  |  |  |  |
| GO:0048290\_isotype\_switching\_to\_IgA\_isotypes | 1 | 0 |  |  |  |  |  |  |  |  |
| GO:0048296\_regulation\_of\_isotype\_switching\_to\_IgA\_isotypes | 1 | 0 |  |  |  |  |  |  |  |  |
| GO:0048298\_positive\_regulation\_of\_isotype\_switching\_to\_IgA\_isotypes | 1 | 0 |  |  |  |  |  |  |  |  |
| GO:0048319\_axial\_mesoderm\_morphogenesis | 1 | 0 |  |  |  |  |  |  |  |  |
| GO:0048320\_axial\_mesoderm\_formation | 1 | 0 |  |  |  |  |  |  |  |  |
| GO:0048385\_regulation\_of\_retinoic\_acid\_receptor\_signaling\_pathway | 1 | 0 |  |  |  |  |  |  |  |  |
| GO:0048387\_negative\_regulation\_of\_retinoic\_acid\_receptor\_signaling\_pathway | 1 | 0 |  |  |  |  |  |  |  |  |
| GO:0048388\_endosomal\_lumen\_acidification | 1 | 0 |  |  |  |  |  |  |  |  |
| GO:0048389\_intermediate\_mesoderm\_development | 1 | 0 |  |  |  |  |  |  |  |  |
| GO:0048478\_replication\_fork\_protection | 1 | 0 |  |  |  |  |  |  |  |  |
| GO:0048496\_maintenance\_of\_organ\_identity | 1 | 0 |  |  |  |  |  |  |  |  |
| GO:0048525\_negative\_regulation\_of\_viral\_reproduction | 1 | 0 |  |  |  |  |  |  |  |  |
| GO:0048539\_bone\_marrow\_development | 1 | 0 |  |  |  |  |  |  |  |  |
| GO:0048548\_regulation\_of\_pinocytosis | 1 | 0 |  |  |  |  |  |  |  |  |
| GO:0048549\_positive\_regulation\_of\_pinocytosis | 1 | 0 |  |  |  |  |  |  |  |  |
| GO:0048553\_negative\_regulation\_of\_metalloenzyme\_activity | 1 | 0 |  |  |  |  |  |  |  |  |
| GO:0048588\_developmental\_cell\_growth | 1 | 0 |  |  |  |  |  |  |  |  |
| GO:0048601\_oocyte\_morphogenesis | 1 | 0 |  |  |  |  |  |  |  |  |
| GO:0048621\_post-embryonic\_gut\_morphogenesis | 1 | 0 |  |  |  |  |  |  |  |  |
| GO:0048640\_negative\_regulation\_of\_developmental\_growth | 1 | 0 |  |  |  |  |  |  |  |  |
| GO:0048642\_negative\_regulation\_of\_skeletal\_muscle\_tissue\_development | 1 | 0 |  |  |  |  |  |  |  |  |
| GO:0048669\_collateral\_sprouting\_in\_the\_absence\_of\_injury | 1 | 0 |  |  |  |  |  |  |  |  |
| GO:0048680\_positive\_regulation\_of\_axon\_regeneration | 1 | 0 |  |  |  |  |  |  |  |  |
| GO:0048681\_negative\_regulation\_of\_axon\_regeneration | 1 | 0 |  |  |  |  |  |  |  |  |
| GO:0048686\_regulation\_of\_sprouting\_of\_injured\_axon | 1 | 0 |  |  |  |  |  |  |  |  |
| GO:0048687\_positive\_regulation\_of\_sprouting\_of\_injured\_axon | 1 | 0 |  |  |  |  |  |  |  |  |
| GO:0048690\_regulation\_of\_axon\_extension\_involved\_in\_regeneration | 1 | 0 |  |  |  |  |  |  |  |  |
| GO:0048691\_positive\_regulation\_of\_axon\_extension\_involved\_in\_regeneration | 1 | 0 |  |  |  |  |  |  |  |  |
| GO:0048714\_positive\_regulation\_of\_oligodendrocyte\_differentiation | 1 | 0 |  |  |  |  |  |  |  |  |
| GO:0048733\_sebaceous\_gland\_development | 1 | 0 |  |  |  |  |  |  |  |  |
| GO:0048743\_positive\_regulation\_of\_skeletal\_muscle\_fiber\_development | 1 | 0 |  |  |  |  |  |  |  |  |
| GO:0048752\_semicircular\_canal\_morphogenesis | 1 | 0 |  |  |  |  |  |  |  |  |
| GO:0048790\_maintenance\_of\_presynaptic\_active\_zone\_structure | 1 | 0 |  |  |  |  |  |  |  |  |
| GO:0048791\_calcium\_ion-dependent\_exocytosis\_of\_neurotransmitter | 1 | 0 |  |  |  |  |  |  |  |  |
| GO:0048822\_enucleate\_erythrocyte\_development | 1 | 0 |  |  |  |  |  |  |  |  |
| GO:0048866\_stem\_cell\_fate\_specification | 1 | 0 |  |  |  |  |  |  |  |  |
| GO:0048936\_peripheral\_nervous\_system\_neuron\_axonogenesis | 1 | 0 |  |  |  |  |  |  |  |  |
| GO:0050427\_3'-phosphoadenosine\_5'-phosphosulfate\_metabolic\_process | 1 | 0 |  |  |  |  |  |  |  |  |
| GO:0050428\_3'-phosphoadenosine\_5'-phosphosulfate\_biosynthetic\_process | 1 | 0 |  |  |  |  |  |  |  |  |
| GO:0050482\_arachidonic\_acid\_secretion | 1 | 0 |  |  |  |  |  |  |  |  |
| GO:0050667\_homocysteine\_metabolic\_process | 1 | 0 |  |  |  |  |  |  |  |  |
| GO:0050674\_urothelial\_cell\_proliferation | 1 | 0 |  |  |  |  |  |  |  |  |
| GO:0050675\_regulation\_of\_urothelial\_cell\_proliferation | 1 | 0 |  |  |  |  |  |  |  |  |
| GO:0050677\_positive\_regulation\_of\_urothelial\_cell\_proliferation | 1 | 0 |  |  |  |  |  |  |  |  |
| GO:0050691\_regulation\_of\_defense\_response\_to\_virus\_by\_host | 1 | 0 |  |  |  |  |  |  |  |  |
| GO:0050748\_negative\_regulation\_of\_lipoprotein\_metabolic\_process | 1 | 0 |  |  |  |  |  |  |  |  |
| GO:0050757\_thymidylate\_synthase\_biosynthetic\_process | 1 | 0 |  |  |  |  |  |  |  |  |
| GO:0050758\_regulation\_of\_thymidylate\_synthase\_biosynthetic\_process | 1 | 0 |  |  |  |  |  |  |  |  |
| GO:0050760\_negative\_regulation\_of\_thymidylate\_synthase\_biosynthetic\_process | 1 | 0 |  |  |  |  |  |  |  |  |
| GO:0050812\_regulation\_of\_acyl-CoA\_biosynthetic\_process | 1 | 0 |  |  |  |  |  |  |  |  |
| GO:0050832\_defense\_response\_to\_fungus | 1 | 0 |  |  |  |  |  |  |  |  |
| GO:0050861\_positive\_regulation\_of\_B\_cell\_receptor\_signaling\_pathway | 1 | 0 |  |  |  |  |  |  |  |  |
| GO:0050862\_positive\_regulation\_of\_T\_cell\_receptor\_signaling\_pathway | 1 | 0 |  |  |  |  |  |  |  |  |
| GO:0050916\_sensory\_perception\_of\_sweet\_taste | 1 | 0 |  |  |  |  |  |  |  |  |
| GO:0050975\_sensory\_perception\_of\_touch | 1 | 0 |  |  |  |  |  |  |  |  |
| GO:0050995\_negative\_regulation\_of\_lipid\_catabolic\_process | 1 | 0 |  |  |  |  |  |  |  |  |
| GO:0051001\_negative\_regulation\_of\_nitric-oxide\_synthase\_activity | 1 | 0 |  |  |  |  |  |  |  |  |
| GO:0051005\_negative\_regulation\_of\_lipoprotein\_lipase\_activity | 1 | 0 |  |  |  |  |  |  |  |  |
| GO:0051006\_positive\_regulation\_of\_lipoprotein\_lipase\_activity | 1 | 0 |  |  |  |  |  |  |  |  |
| GO:0051016\_barbed-end\_actin\_filament\_capping | 1 | 0 |  |  |  |  |  |  |  |  |
| GO:0051029\_rRNA\_transport | 1 | 0 |  |  |  |  |  |  |  |  |
| GO:0051043\_regulation\_of\_membrane\_protein\_ectodomain\_proteolysis | 1 | 0 |  |  |  |  |  |  |  |  |
| GO:0051044\_positive\_regulation\_of\_membrane\_protein\_ectodomain\_proteolysis | 1 | 0 |  |  |  |  |  |  |  |  |
| GO:0051088\_PMA-inducible\_membrane\_protein\_ectodomain\_proteolysis | 1 | 0 |  |  |  |  |  |  |  |  |
| GO:0051102\_DNA\_ligation\_during\_DNA\_recombination | 1 | 0 |  |  |  |  |  |  |  |  |
| GO:0051103\_DNA\_ligation\_during\_DNA\_repair | 1 | 0 |  |  |  |  |  |  |  |  |
| GO:0051123\_transcriptional\_preinitiation\_complex\_assembly | 1 | 0 |  |  |  |  |  |  |  |  |
| GO:0051125\_regulation\_of\_actin\_nucleation | 1 | 0 |  |  |  |  |  |  |  |  |
| GO:0051127\_positive\_regulation\_of\_actin\_nucleation | 1 | 0 |  |  |  |  |  |  |  |  |
| GO:0051151\_negative\_regulation\_of\_smooth\_muscle\_cell\_differentiation | 1 | 0 |  |  |  |  |  |  |  |  |
| GO:0051154\_negative\_regulation\_of\_striated\_muscle\_cell\_differentiation | 1 | 0 |  |  |  |  |  |  |  |  |
| GO:0051155\_positive\_regulation\_of\_striated\_muscle\_cell\_differentiation | 1 | 0 |  |  |  |  |  |  |  |  |
| GO:0051156\_glucose\_6-phosphate\_metabolic\_process | 1 | 0 |  |  |  |  |  |  |  |  |
| GO:0051187\_cofactor\_catabolic\_process | 1 | 0 |  |  |  |  |  |  |  |  |
| GO:0051189\_prosthetic\_group\_metabolic\_process | 1 | 0 |  |  |  |  |  |  |  |  |
| GO:0051193\_regulation\_of\_cofactor\_metabolic\_process | 1 | 0 |  |  |  |  |  |  |  |  |
| GO:0051196\_regulation\_of\_coenzyme\_metabolic\_process | 1 | 0 |  |  |  |  |  |  |  |  |
| GO:0051255\_spindle\_midzone\_assembly | 1 | 0 |  |  |  |  |  |  |  |  |
| GO:0051257\_spindle\_midzone\_assembly\_involved\_in\_meiosis | 1 | 0 |  |  |  |  |  |  |  |  |
| GO:0051281\_positive\_regulation\_of\_release\_of\_sequestered\_calcium\_ion\_into\_cytosol | 1 | 0 |  |  |  |  |  |  |  |  |
| GO:0051290\_protein\_heterotetramerization | 1 | 0 |  |  |  |  |  |  |  |  |
| GO:0051305\_chromosome\_movement\_towards\_spindle\_pole | 1 | 0 |  |  |  |  |  |  |  |  |
| GO:0051310\_metaphase\_plate\_congression | 1 | 0 |  |  |  |  |  |  |  |  |
| GO:0051311\_meiotic\_metaphase\_plate\_congression | 1 | 0 |  |  |  |  |  |  |  |  |
| GO:0051340\_regulation\_of\_ligase\_activity | 1 | 0 |  |  |  |  |  |  |  |  |
| GO:0051351\_positive\_regulation\_of\_ligase\_activity | 1 | 0 |  |  |  |  |  |  |  |  |
| GO:0051354\_negative\_regulation\_of\_oxidoreductase\_activity | 1 | 0 |  |  |  |  |  |  |  |  |
| GO:0051355\_proprioception\_during\_equilibrioception | 1 | 0 |  |  |  |  |  |  |  |  |
| GO:0051383\_kinetochore\_organization | 1 | 0 |  |  |  |  |  |  |  |  |
| GO:0051386\_regulation\_of\_nerve\_growth\_factor\_receptor\_signaling\_pathway | 1 | 0 |  |  |  |  |  |  |  |  |
| GO:0051409\_response\_to\_nitrosative\_stress | 1 | 0 |  |  |  |  |  |  |  |  |
| GO:0051457\_maintenance\_of\_protein\_location\_in\_nucleus | 1 | 0 |  |  |  |  |  |  |  |  |
| GO:0051462\_regulation\_of\_cortisol\_secretion | 1 | 0 |  |  |  |  |  |  |  |  |
| GO:0051463\_negative\_regulation\_of\_cortisol\_secretion | 1 | 0 |  |  |  |  |  |  |  |  |
| GO:0051481\_reduction\_of\_cytosolic\_calcium\_ion\_concentration | 1 | 0 |  |  |  |  |  |  |  |  |
| GO:0051482\_elevation\_of\_cytosolic\_calcium\_ion\_concentration\_during\_G-protein\_signaling\_\_coupled\_to\_IP3\_second\_messenger\_(phospholipase\_C\_activating) | 1 | 0 |  |  |  |  |  |  |  |  |
| GO:0051542\_elastin\_biosynthetic\_process | 1 | 0 |  |  |  |  |  |  |  |  |
| GO:0051568\_histone\_H3-K4\_methylation | 1 | 0 |  |  |  |  |  |  |  |  |
| GO:0051569\_regulation\_of\_histone\_H3-K4\_methylation | 1 | 0 |  |  |  |  |  |  |  |  |
| GO:0051570\_regulation\_of\_histone\_H3-K9\_methylation | 1 | 0 |  |  |  |  |  |  |  |  |
| GO:0051573\_negative\_regulation\_of\_histone\_H3-K9\_methylation | 1 | 0 |  |  |  |  |  |  |  |  |
| GO:0051580\_regulation\_of\_neurotransmitter\_uptake | 1 | 0 |  |  |  |  |  |  |  |  |
| GO:0051582\_positive\_regulation\_of\_neurotransmitter\_uptake | 1 | 0 |  |  |  |  |  |  |  |  |
| GO:0051584\_regulation\_of\_dopamine\_uptake | 1 | 0 |  |  |  |  |  |  |  |  |
| GO:0051586\_positive\_regulation\_of\_dopamine\_uptake | 1 | 0 |  |  |  |  |  |  |  |  |
| GO:0051589\_negative\_regulation\_of\_neurotransmitter\_transport | 1 | 0 |  |  |  |  |  |  |  |  |
| GO:0051593\_response\_to\_folic\_acid | 1 | 0 |  |  |  |  |  |  |  |  |
| GO:0051615\_histamine\_uptake | 1 | 0 |  |  |  |  |  |  |  |  |
| GO:0051646\_mitochondrion\_localization | 1 | 0 |  |  |  |  |  |  |  |  |
| GO:0051654\_establishment\_of\_mitochondrion\_localization | 1 | 0 |  |  |  |  |  |  |  |  |
| GO:0051661\_maintenance\_of\_centrosome\_location | 1 | 0 |  |  |  |  |  |  |  |  |
| GO:0051665\_membrane\_raft\_localization | 1 | 0 |  |  |  |  |  |  |  |  |
| GO:0051685\_maintenance\_of\_ER\_location | 1 | 0 |  |  |  |  |  |  |  |  |
| GO:0051693\_actin\_filament\_capping | 1 | 0 |  |  |  |  |  |  |  |  |
| GO:0051701\_interaction\_with\_host | 1 | 0 |  |  |  |  |  |  |  |  |
| GO:0051754\_meiotic\_sister\_chromatid\_cohesion\_\_centromeric | 1 | 0 |  |  |  |  |  |  |  |  |
| GO:0051782\_negative\_regulation\_of\_cell\_division | 1 | 0 |  |  |  |  |  |  |  |  |
| GO:0051790\_short-chain\_fatty\_acid\_biosynthetic\_process | 1 | 0 |  |  |  |  |  |  |  |  |
| GO:0051799\_negative\_regulation\_of\_hair\_follicle\_development | 1 | 0 |  |  |  |  |  |  |  |  |
| GO:0051823\_regulation\_of\_synapse\_structural\_plasticity | 1 | 0 |  |  |  |  |  |  |  |  |
| GO:0051865\_protein\_autoubiquitination | 1 | 0 |  |  |  |  |  |  |  |  |
| GO:0051901\_positive\_regulation\_of\_mitochondrial\_depolarization | 1 | 0 |  |  |  |  |  |  |  |  |
| GO:0051917\_regulation\_of\_fibrinolysis | 1 | 0 |  |  |  |  |  |  |  |  |
| GO:0051918\_negative\_regulation\_of\_fibrinolysis | 1 | 0 |  |  |  |  |  |  |  |  |
| GO:0051929\_positive\_regulation\_of\_calcium\_ion\_transport\_via\_voltage-gated\_calcium\_channel\_activity | 1 | 0 |  |  |  |  |  |  |  |  |
| GO:0051933\_amino\_acid\_uptake\_during\_transmission\_of\_nerve\_impulse | 1 | 0 |  |  |  |  |  |  |  |  |
| GO:0051935\_glutamate\_uptake\_during\_transmission\_of\_nerve\_impulse | 1 | 0 |  |  |  |  |  |  |  |  |
| GO:0051940\_regulation\_of\_catecholamine\_uptake\_during\_transmission\_of\_nerve\_impulse | 1 | 0 |  |  |  |  |  |  |  |  |
| GO:0051944\_positive\_regulation\_of\_catecholamine\_uptake\_during\_transmission\_of\_nerve\_impulse | 1 | 0 |  |  |  |  |  |  |  |  |
| GO:0051961\_negative\_regulation\_of\_nervous\_system\_development | 1 | 0 |  |  |  |  |  |  |  |  |
| GO:0051964\_negative\_regulation\_of\_synaptogenesis | 1 | 0 |  |  |  |  |  |  |  |  |
| GO:0051968\_positive\_regulation\_of\_synaptic\_transmission\_\_glutamatergic | 1 | 0 |  |  |  |  |  |  |  |  |
| GO:0051984\_positive\_regulation\_of\_chromosome\_segregation | 1 | 0 |  |  |  |  |  |  |  |  |
| GO:0051987\_positive\_regulation\_of\_attachment\_of\_spindle\_microtubules\_to\_kinetochore | 1 | 0 |  |  |  |  |  |  |  |  |
| GO:0052173\_response\_to\_defenses\_of\_other\_organism\_during\_symbiotic\_interaction | 1 | 0 |  |  |  |  |  |  |  |  |
| GO:0052200\_response\_to\_host\_defenses | 1 | 0 |  |  |  |  |  |  |  |  |
| GO:0052551\_response\_to\_defense-related\_nitric\_oxide\_production\_by\_other\_organism\_during\_symbiotic\_interaction | 1 | 0 |  |  |  |  |  |  |  |  |
| GO:0052564\_response\_to\_immune\_response\_of\_other\_organism\_during\_symbiotic\_interaction | 1 | 0 |  |  |  |  |  |  |  |  |
| GO:0052565\_response\_to\_defense-related\_host\_nitric\_oxide\_production | 1 | 0 |  |  |  |  |  |  |  |  |
| GO:0052572\_response\_to\_host\_immune\_response | 1 | 0 |  |  |  |  |  |  |  |  |
| GO:0055005\_ventricular\_cardiac\_myofibril\_development | 1 | 0 |  |  |  |  |  |  |  |  |
| GO:0055011\_atrial\_cardiac\_muscle\_cell\_differentiation | 1 | 0 |  |  |  |  |  |  |  |  |
| GO:0055014\_atrial\_cardiac\_muscle\_cell\_development | 1 | 0 |  |  |  |  |  |  |  |  |
| GO:0055078\_sodium\_ion\_homeostasis | 1 | 0 |  |  |  |  |  |  |  |  |
| GO:0055089\_fatty\_acid\_homeostasis | 1 | 0 |  |  |  |  |  |  |  |  |
| GO:0060003\_copper\_ion\_export | 1 | 0 |  |  |  |  |  |  |  |  |
| GO:0060005\_vestibular\_reflex | 1 | 0 |  |  |  |  |  |  |  |  |
| GO:0060014\_granulosa\_cell\_differentiation | 1 | 0 |  |  |  |  |  |  |  |  |
| GO:0060018\_astrocyte\_fate\_commitment | 1 | 0 |  |  |  |  |  |  |  |  |
| GO:0060020\_Bergmann\_glial\_cell\_differentiation | 1 | 0 |  |  |  |  |  |  |  |  |
| GO:0060022\_hard\_palate\_development | 1 | 0 |  |  |  |  |  |  |  |  |
| GO:0060034\_notochord\_cell\_differentiation | 1 | 0 |  |  |  |  |  |  |  |  |
| GO:0060035\_notochord\_cell\_development | 1 | 0 |  |  |  |  |  |  |  |  |
| GO:0060046\_regulation\_of\_acrosome\_reaction | 1 | 0 |  |  |  |  |  |  |  |  |
| GO:0060054\_positive\_regulation\_of\_epithelial\_cell\_proliferation\_involved\_in\_wound\_healing | 1 | 0 |  |  |  |  |  |  |  |  |
| GO:0060059\_embryonic\_retina\_morphogenesis\_in\_camera-type\_eye | 1 | 0 |  |  |  |  |  |  |  |  |
| GO:0060061\_Spemann\_organizer\_formation | 1 | 0 |  |  |  |  |  |  |  |  |
| GO:0060064\_Spemann\_organizer\_formation\_at\_the\_anterior\_end\_of\_the\_primitive\_streak | 1 | 0 |  |  |  |  |  |  |  |  |
| GO:0060071\_Wnt\_receptor\_signaling\_pathway\_\_planar\_cell\_polarity\_pathway | 1 | 0 |  |  |  |  |  |  |  |  |
| GO:0060075\_regulation\_of\_resting\_membrane\_potential | 1 | 0 |  |  |  |  |  |  |  |  |
| GO:0060082\_eye\_blink\_reflex | 1 | 0 |  |  |  |  |  |  |  |  |
| GO:0060112\_generation\_of\_ovulation\_cycle\_rhythm | 1 | 0 |  |  |  |  |  |  |  |  |
| GO:0060125\_negative\_regulation\_of\_growth\_hormone\_secretion | 1 | 0 |  |  |  |  |  |  |  |  |
| GO:0060151\_peroxisome\_localization | 1 | 0 |  |  |  |  |  |  |  |  |
| GO:0060152\_microtubule-based\_peroxisome\_localization | 1 | 0 |  |  |  |  |  |  |  |  |
| GO:0060161\_positive\_regulation\_of\_dopamine\_receptor\_signaling\_pathway | 1 | 0 |  |  |  |  |  |  |  |  |
| GO:0060163\_subpallium\_neuron\_fate\_commitment | 1 | 0 |  |  |  |  |  |  |  |  |
| GO:0060165\_regulation\_of\_timing\_of\_subpallium\_neuron\_differentiation | 1 | 0 |  |  |  |  |  |  |  |  |
| GO:0060174\_limb\_bud\_formation | 1 | 0 |  |  |  |  |  |  |  |  |
| GO:0060177\_regulation\_of\_angiotensin\_metabolic\_process | 1 | 0 |  |  |  |  |  |  |  |  |
| GO:0060197\_cloacal\_septation | 1 | 0 |  |  |  |  |  |  |  |  |
| GO:0060215\_primitive\_hemopoiesis | 1 | 0 |  |  |  |  |  |  |  |  |
| GO:0060231\_mesenchymal\_to\_epithelial\_transition | 1 | 0 |  |  |  |  |  |  |  |  |
| GO:0060254\_regulation\_of\_N-terminal\_protein\_palmitoylation | 1 | 0 |  |  |  |  |  |  |  |  |
| GO:0060261\_positive\_regulation\_of\_transcription\_initiation\_from\_RNA\_polymerase\_II\_promoter | 1 | 0 |  |  |  |  |  |  |  |  |
| GO:0060262\_negative\_regulation\_of\_N-terminal\_protein\_palmitoylation | 1 | 0 |  |  |  |  |  |  |  |  |
| GO:0060263\_regulation\_of\_respiratory\_burst | 1 | 0 |  |  |  |  |  |  |  |  |
| GO:0060264\_regulation\_of\_respiratory\_burst\_during\_acute\_inflammatory\_response | 1 | 0 |  |  |  |  |  |  |  |  |
| GO:0060265\_positive\_regulation\_of\_respiratory\_burst\_during\_acute\_inflammatory\_response | 1 | 0 |  |  |  |  |  |  |  |  |
| GO:0060267\_positive\_regulation\_of\_respiratory\_burst | 1 | 0 |  |  |  |  |  |  |  |  |
| GO:0060272\_embryonic\_skeletal\_joint\_morphogenesis | 1 | 0 |  |  |  |  |  |  |  |  |
| GO:0060297\_regulation\_of\_sarcomere\_organization | 1 | 0 |  |  |  |  |  |  |  |  |
| GO:0060298\_positive\_regulation\_of\_sarcomere\_organization | 1 | 0 |  |  |  |  |  |  |  |  |
| GO:0060315\_negative\_regulation\_of\_ryanodine-sensitive\_calcium-release\_channel\_activity | 1 | 0 |  |  |  |  |  |  |  |  |
| GO:0060319\_primitive\_erythrocyte\_differentiation | 1 | 0 |  |  |  |  |  |  |  |  |
| GO:0060371\_regulation\_of\_atrial\_cardiomyocyte\_membrane\_depolarization | 1 | 0 |  |  |  |  |  |  |  |  |
| GO:0060374\_mast\_cell\_differentiation | 1 | 0 |  |  |  |  |  |  |  |  |
| GO:0060375\_regulation\_of\_mast\_cell\_differentiation | 1 | 0 |  |  |  |  |  |  |  |  |
| GO:0060376\_positive\_regulation\_of\_mast\_cell\_differentiation | 1 | 0 |  |  |  |  |  |  |  |  |
| GO:0060390\_regulation\_of\_SMAD\_protein\_nuclear\_translocation | 1 | 0 |  |  |  |  |  |  |  |  |
| GO:0060391\_positive\_regulation\_of\_SMAD\_protein\_nuclear\_translocation | 1 | 0 |  |  |  |  |  |  |  |  |
| GO:0060398\_regulation\_of\_growth\_hormone\_receptor\_signaling\_pathway | 1 | 0 |  |  |  |  |  |  |  |  |
| GO:0060399\_positive\_regulation\_of\_growth\_hormone\_receptor\_signaling\_pathway | 1 | 0 |  |  |  |  |  |  |  |  |
| GO:0060405\_regulation\_of\_penile\_erection | 1 | 0 |  |  |  |  |  |  |  |  |
| GO:0060407\_negative\_regulation\_of\_penile\_erection | 1 | 0 |  |  |  |  |  |  |  |  |
| GO:0060413\_atrial\_septum\_morphogenesis | 1 | 0 |  |  |  |  |  |  |  |  |
| GO:0060414\_aorta\_smooth\_muscle\_tissue\_morphogenesis | 1 | 0 |  |  |  |  |  |  |  |  |
| GO:0060419\_heart\_growth | 1 | 0 |  |  |  |  |  |  |  |  |
| GO:0060420\_regulation\_of\_heart\_growth | 1 | 0 |  |  |  |  |  |  |  |  |
| GO:0060421\_positive\_regulation\_of\_heart\_growth | 1 | 0 |  |  |  |  |  |  |  |  |
| GO:0060431\_primary\_lung\_bud\_formation | 1 | 0 |  |  |  |  |  |  |  |  |
| GO:0060436\_bronchiole\_morphogenesis | 1 | 0 |  |  |  |  |  |  |  |  |
| GO:0060440\_trachea\_formation | 1 | 0 |  |  |  |  |  |  |  |  |
| GO:0060449\_bud\_elongation\_involved\_in\_lung\_branching | 1 | 0 |  |  |  |  |  |  |  |  |
| GO:0060456\_positive\_regulation\_of\_digestive\_system\_process | 1 | 0 |  |  |  |  |  |  |  |  |
| GO:0060461\_right\_lung\_morphogenesis | 1 | 0 |  |  |  |  |  |  |  |  |
| GO:0060481\_lobar\_bronchus\_epithelium\_development | 1 | 0 |  |  |  |  |  |  |  |  |
| GO:0060482\_lobar\_bronchus\_development | 1 | 0 |  |  |  |  |  |  |  |  |
| GO:0060484\_lung-associated\_mesenchyme\_development | 1 | 0 |  |  |  |  |  |  |  |  |
| GO:0060486\_Clara\_cell\_differentiation | 1 | 0 |  |  |  |  |  |  |  |  |
| GO:0060510\_Type\_II\_pneumocyte\_differentiation | 1 | 0 |  |  |  |  |  |  |  |  |
| GO:0060514\_prostate\_induction | 1 | 0 |  |  |  |  |  |  |  |  |
| GO:0060515\_prostate\_field\_specification | 1 | 0 |  |  |  |  |  |  |  |  |
| GO:0060517\_epithelial\_cell\_proliferation\_involved\_in\_prostatic\_bud\_elongation | 1 | 0 |  |  |  |  |  |  |  |  |
| GO:0060520\_activation\_of\_prostate\_induction\_by\_androgen\_receptor\_signaling\_pathway | 1 | 0 |  |  |  |  |  |  |  |  |
| GO:0060535\_trachea\_cartilage\_morphogenesis | 1 | 0 |  |  |  |  |  |  |  |  |
| GO:0060536\_cartilage\_morphogenesis | 1 | 0 |  |  |  |  |  |  |  |  |
| GO:0060563\_neuroepithelial\_cell\_differentiation | 1 | 0 |  |  |  |  |  |  |  |  |
| GO:0060577\_pulmonary\_vein\_morphogenesis | 1 | 0 |  |  |  |  |  |  |  |  |
| GO:0060578\_superior\_vena\_cava\_morphogenesis | 1 | 0 |  |  |  |  |  |  |  |  |
| GO:0060584\_regulation\_of\_prostaglandin-endoperoxide\_synthase\_activity | 1 | 0 |  |  |  |  |  |  |  |  |
| GO:0060585\_positive\_regulation\_of\_prostaglandin-endoperoxidase\_synthase\_activity | 1 | 0 |  |  |  |  |  |  |  |  |
| GO:0060598\_dichotomous\_subdivision\_of\_terminal\_units\_involved\_in\_mammary\_gland\_duct\_morphogenesis | 1 | 0 |  |  |  |  |  |  |  |  |
| GO:0060611\_mammary\_gland\_fat\_development | 1 | 0 |  |  |  |  |  |  |  |  |
| GO:0060618\_nipple\_development | 1 | 0 |  |  |  |  |  |  |  |  |
| GO:0060631\_regulation\_of\_meiosis\_I | 1 | 0 |  |  |  |  |  |  |  |  |
| GO:0060649\_mammary\_gland\_bud\_elongation | 1 | 0 |  |  |  |  |  |  |  |  |
| GO:0060658\_nipple\_morphogenesis | 1 | 0 |  |  |  |  |  |  |  |  |
| GO:0060659\_nipple\_sheath\_formation | 1 | 0 |  |  |  |  |  |  |  |  |
| GO:0060668\_regulation\_of\_branching\_involved\_in\_salivary\_gland\_morphogenesis\_by\_extracellular\_matrix-epithelial\_cell\_signaling | 1 | 0 |  |  |  |  |  |  |  |  |
| GO:0060683\_regulation\_of\_branching\_involved\_in\_salivary\_gland\_morphogenesis\_by\_epithelial-mesenchymal\_signaling | 1 | 0 |  |  |  |  |  |  |  |  |
| GO:0060691\_epithelial\_cell\_maturation\_involved\_in\_salivary\_gland\_development | 1 | 0 |  |  |  |  |  |  |  |  |
| GO:0060709\_glycogen\_cell\_development\_involved\_in\_embryonic\_placenta\_development | 1 | 0 |  |  |  |  |  |  |  |  |
| GO:0060732\_positive\_regulation\_of\_inositol\_phosphate\_biosynthetic\_process | 1 | 0 |  |  |  |  |  |  |  |  |
| GO:0060739\_mesenchymal-epithelial\_cell\_signaling\_involved\_in\_prostate\_gland\_development | 1 | 0 |  |  |  |  |  |  |  |  |
| GO:0060781\_mesenchymal\_cell\_proliferation\_involved\_in\_prostate\_gland\_development | 1 | 0 |  |  |  |  |  |  |  |  |
| GO:0060782\_regulation\_of\_mesenchymal\_cell\_proliferation\_involved\_in\_prostate\_gland\_development | 1 | 0 |  |  |  |  |  |  |  |  |
| GO:0060783\_mesenchymal\_smoothened\_signaling\_pathway\_involved\_in\_prostate\_gland\_development | 1 | 0 |  |  |  |  |  |  |  |  |
| GO:0060872\_semicircular\_canal\_development | 1 | 0 |  |  |  |  |  |  |  |  |
| GO:0060896\_neural\_plate\_pattern\_specification | 1 | 0 |  |  |  |  |  |  |  |  |
| GO:0070091\_glucagon\_secretion | 1 | 0 |  |  |  |  |  |  |  |  |
| GO:0070162\_adiponectin\_secretion | 1 | 0 |  |  |  |  |  |  |  |  |
| GO:0070163\_regulation\_of\_adiponectin\_secretion | 1 | 0 |  |  |  |  |  |  |  |  |
| GO:0070164\_negative\_regulation\_of\_adiponectin\_secretion | 1 | 0 |  |  |  |  |  |  |  |  |
| GO:0070178\_D-serine\_metabolic\_process | 1 | 0 |  |  |  |  |  |  |  |  |
| GO:0070179\_D-serine\_biosynthetic\_process | 1 | 0 |  |  |  |  |  |  |  |  |
| GO:0070296\_sarcoplasmic\_reticulum\_calcium\_ion\_transport | 1 | 0 |  |  |  |  |  |  |  |  |
| GO:0070303\_negative\_regulation\_of\_stress-activated\_protein\_kinase\_signaling\_pathway | 1 | 0 |  |  |  |  |  |  |  |  |
| GO:0070328\_triglyceride\_homeostasis | 1 | 0 |  |  |  |  |  |  |  |  |
| GO:0070365\_hepatocyte\_differentiation | 1 | 0 |  |  |  |  |  |  |  |  |
| GO:0070384\_Harderian\_gland\_development | 1 | 0 |  |  |  |  |  |  |  |  |
| GO:0070391\_response\_to\_lipoteichoic\_acid | 1 | 0 |  |  |  |  |  |  |  |  |
| GO:0070424\_regulation\_of\_nucleotide-binding\_oligomerization\_domain\_containing\_signaling\_pathway | 1 | 0 |  |  |  |  |  |  |  |  |
| GO:0070426\_positive\_regulation\_of\_nucleotide-binding\_oligomerization\_domain\_containing\_signaling\_pathway | 1 | 0 |  |  |  |  |  |  |  |  |
| GO:0070428\_regulation\_of\_nucleotide-binding\_oligomerization\_domain\_containing\_1\_signaling\_pathway | 1 | 0 |  |  |  |  |  |  |  |  |
| GO:0070430\_positive\_regulation\_of\_nucleotide-binding\_oligomerization\_domain\_containing\_1\_signaling\_pathway | 1 | 0 |  |  |  |  |  |  |  |  |
| GO:0070432\_regulation\_of\_nucleotide-binding\_oligomerization\_domain\_containing\_2\_signaling\_pathway | 1 | 0 |  |  |  |  |  |  |  |  |
| GO:0070434\_positive\_regulation\_of\_nucleotide-binding\_oligomerization\_domain\_containing\_2\_signaling\_pathway | 1 | 0 |  |  |  |  |  |  |  |  |
| GO:0070493\_thrombin\_receptor\_signaling\_pathway | 1 | 0 |  |  |  |  |  |  |  |  |
| GO:0070508\_cholesterol\_import | 1 | 0 |  |  |  |  |  |  |  |  |
| GO:0070527\_platelet\_aggregation | 1 | 0 |  |  |  |  |  |  |  |  |
| GO:0070528\_protein\_kinase\_C\_signaling\_cascade | 1 | 0 |  |  |  |  |  |  |  |  |
| GO:0070555\_response\_to\_interleukin-1 | 1 | 0 |  |  |  |  |  |  |  |  |
| GO:0070560\_protein\_secretion\_by\_platelet | 1 | 0 |  |  |  |  |  |  |  |  |
| GO:0070561\_vitamin\_D\_receptor\_signaling\_pathway | 1 | 0 |  |  |  |  |  |  |  |  |
| GO:0070562\_regulation\_of\_vitamin\_D\_receptor\_signaling\_pathway | 1 | 0 |  |  |  |  |  |  |  |  |
| GO:0070571\_negative\_regulation\_of\_neuron\_projection\_regeneration | 1 | 0 |  |  |  |  |  |  |  |  |
| GO:0070572\_positive\_regulation\_of\_neuron\_projection\_regeneration | 1 | 0 |  |  |  |  |  |  |  |  |
| GO:0070613\_regulation\_of\_protein\_processing | 1 | 0 |  |  |  |  |  |  |  |  |
| GO:0070627\_ferrous\_iron\_import | 1 | 0 |  |  |  |  |  |  |  |  |
| GO:0070669\_response\_to\_interleukin-2 | 1 | 0 |  |  |  |  |  |  |  |  |
| GO:0070670\_response\_to\_interleukin-4 | 1 | 0 |  |  |  |  |  |  |  |  |
| GO:0070671\_response\_to\_interleukin-12 | 1 | 0 |  |  |  |  |  |  |  |  |
| GO:0070672\_response\_to\_interleukin-15 | 1 | 0 |  |  |  |  |  |  |  |  |
| GO:0070673\_response\_to\_interleukin-18 | 1 | 0 |  |  |  |  |  |  |  |  |
| GO:0070828\_heterochromatin\_organization | 1 | 0 |  |  |  |  |  |  |  |  |
| GO:0070874\_negative\_regulation\_of\_glycogen\_metabolic\_process | 1 | 0 |  |  |  |  |  |  |  |  |
| GO:0075136\_response\_to\_host | 1 | 0 |  |  |  |  |  |  |  |  |
| GO:0080010\_regulation\_of\_oxygen\_and\_reactive\_oxygen\_species\_metabolic\_process | 1 | 0 |  |  |  |  |  |  |  |  |
| GO:0090032\_negative\_regulation\_of\_steroid\_hormone\_biosynthetic\_process | 1 | 0 |  |  |  |  |  |  |  |  |
| GO:0000187\_activation\_of\_MAPK\_activity | 30 | 0 | 0.000000 | -0.000000 | 730 | 666.444890 | 744.64 | 822.835110 | 1.020055 |
| GO:0002260\_lymphocyte\_homeostasis | 30 | 0 | 0.000000 | -0.000000 | 730 | 666.444890 | 744.64 | 822.835110 | 1.020055 |
| GO:0007219\_Notch\_signaling\_pathway | 30 | 0 | 0.000000 | -0.000000 | 730 | 666.444890 | 744.64 | 822.835110 | 1.020055 |
| GO:0007435\_salivary\_gland\_morphogenesis | 30 | 0 | 0.000000 | -0.000000 | 730 | 666.444890 | 744.64 | 822.835110 | 1.020055 |
| GO:0009266\_response\_to\_temperature\_stimulus | 30 | 0 | 0.000000 | -0.000000 | 730 | 666.444890 | 744.64 | 822.835110 | 1.020055 |
| GO:0014032\_neural\_crest\_cell\_development | 30 | 0 | 0.000000 | -0.000000 | 730 | 666.444890 | 744.64 | 822.835110 | 1.020055 |
| GO:0014033\_neural\_crest\_cell\_differentiation | 30 | 0 | 0.000000 | -0.000000 | 730 | 666.444890 | 744.64 | 822.835110 | 1.020055 |
| GO:0016445\_somatic\_diversification\_of\_immunoglobulins | 30 | 0 | 0.000000 | -0.000000 | 730 | 666.444890 | 744.64 | 822.835110 | 1.020055 |
| GO:0030522\_intracellular\_receptor-mediated\_signaling\_pathway | 30 | 0 | 0.000000 | -0.000000 | 730 | 666.444890 | 744.64 | 822.835110 | 1.020055 |
| GO:0031281\_positive\_regulation\_of\_cyclase\_activity | 30 | 0 | 0.000000 | -0.000000 | 730 | 666.444890 | 744.64 | 822.835110 | 1.020055 |
| GO:0031668\_cellular\_response\_to\_extracellular\_stimulus | 30 | 0 | 0.000000 | -0.000000 | 730 | 666.444890 | 744.64 | 822.835110 | 1.020055 |
| GO:0032102\_negative\_regulation\_of\_response\_to\_external\_stimulus | 30 | 0 | 0.000000 | -0.000000 | 730 | 666.444890 | 744.64 | 822.835110 | 1.020055 |
| GO:0033500\_carbohydrate\_homeostasis | 30 | 0 | 0.000000 | -0.000000 | 730 | 666.444890 | 744.64 | 822.835110 | 1.020055 |
| GO:0035265\_organ\_growth | 30 | 0 | 0.000000 | -0.000000 | 730 | 666.444890 | 744.64 | 822.835110 | 1.020055 |
| GO:0042552\_myelination | 30 | 0 | 0.000000 | -0.000000 | 730 | 666.444890 | 744.64 | 822.835110 | 1.020055 |
| GO:0042593\_glucose\_homeostasis | 30 | 0 | 0.000000 | -0.000000 | 730 | 666.444890 | 744.64 | 822.835110 | 1.020055 |
| GO:0045762\_positive\_regulation\_of\_adenylate\_cyclase\_activity | 30 | 0 | 0.000000 | -0.000000 | 730 | 666.444890 | 744.64 | 822.835110 | 1.020055 |
| GO:0048565\_gut\_development | 30 | 0 | 0.000000 | -0.000000 | 730 | 666.444890 | 744.64 | 822.835110 | 1.020055 |
| GO:0051146\_striated\_muscle\_cell\_differentiation | 30 | 0 | 0.000000 | -0.000000 | 730 | 666.444890 | 744.64 | 822.835110 | 1.020055 |
| GO:0051349\_positive\_regulation\_of\_lyase\_activity | 30 | 0 | 0.000000 | -0.000000 | 730 | 666.444890 | 744.64 | 822.835110 | 1.020055 |
| GO:0052547\_regulation\_of\_peptidase\_activity | 30 | 0 | 0.000000 | -0.000000 | 730 | 666.444890 | 744.64 | 822.835110 | 1.020055 |
| GO:0060021\_palate\_development | 30 | 0 | 0.000000 | -0.000000 | 730 | 666.444890 | 744.64 | 822.835110 | 1.020055 |
| GO:0000060\_protein\_import\_into\_nucleus\_\_translocation | 14 | 0 | 0.000000 | -0.000000 | 785 | 728.271880 | 805.51 | 882.748120 | 1.026127 |
| GO:0000077\_DNA\_damage\_checkpoint | 14 | 0 | 0.000000 | -0.000000 | 785 | 728.271880 | 805.51 | 882.748120 | 1.026127 |
| GO:0001829\_trophectodermal\_cell\_differentiation | 14 | 0 | 0.000000 | -0.000000 | 785 | 728.271880 | 805.51 | 882.748120 | 1.026127 |
| GO:0002027\_regulation\_of\_heart\_rate | 14 | 0 | 0.000000 | -0.000000 | 785 | 728.271880 | 805.51 | 882.748120 | 1.026127 |
| GO:0002262\_myeloid\_cell\_homeostasis | 14 | 0 | 0.000000 | -0.000000 | 785 | 728.271880 | 805.51 | 882.748120 | 1.026127 |
| GO:0002698\_negative\_regulation\_of\_immune\_effector\_process | 14 | 0 | 0.000000 | -0.000000 | 785 | 728.271880 | 805.51 | 882.748120 | 1.026127 |
| GO:0006304\_DNA\_modification | 14 | 0 | 0.000000 | -0.000000 | 785 | 728.271880 | 805.51 | 882.748120 | 1.026127 |
| GO:0006305\_DNA\_alkylation | 14 | 0 | 0.000000 | -0.000000 | 785 | 728.271880 | 805.51 | 882.748120 | 1.026127 |
| GO:0006306\_DNA\_methylation | 14 | 0 | 0.000000 | -0.000000 | 785 | 728.271880 | 805.51 | 882.748120 | 1.026127 |
| GO:0006695\_cholesterol\_biosynthetic\_process | 14 | 0 | 0.000000 | -0.000000 | 785 | 728.271880 | 805.51 | 882.748120 | 1.026127 |
| GO:0006914\_autophagy | 14 | 0 | 0.000000 | -0.000000 | 785 | 728.271880 | 805.51 | 882.748120 | 1.026127 |
| GO:0007157\_heterophilic\_cell\_adhesion | 14 | 0 | 0.000000 | -0.000000 | 785 | 728.271880 | 805.51 | 882.748120 | 1.026127 |
| GO:0007589\_body\_fluid\_secretion | 14 | 0 | 0.000000 | -0.000000 | 785 | 728.271880 | 805.51 | 882.748120 | 1.026127 |
| GO:0008064\_regulation\_of\_actin\_polymerization\_or\_depolymerization | 14 | 0 | 0.000000 | -0.000000 | 785 | 728.271880 | 805.51 | 882.748120 | 1.026127 |
| GO:0008306\_associative\_learning | 14 | 0 | 0.000000 | -0.000000 | 785 | 728.271880 | 805.51 | 882.748120 | 1.026127 |
| GO:0008630\_DNA\_damage\_response\_\_signal\_transduction\_resulting\_in\_induction\_of\_apoptosis | 14 | 0 | 0.000000 | -0.000000 | 785 | 728.271880 | 805.51 | 882.748120 | 1.026127 |
| GO:0009108\_coenzyme\_biosynthetic\_process | 14 | 0 | 0.000000 | -0.000000 | 785 | 728.271880 | 805.51 | 882.748120 | 1.026127 |
| GO:0009267\_cellular\_response\_to\_starvation | 14 | 0 | 0.000000 | -0.000000 | 785 | 728.271880 | 805.51 | 882.748120 | 1.026127 |
| GO:0009895\_negative\_regulation\_of\_catabolic\_process | 14 | 0 | 0.000000 | -0.000000 | 785 | 728.271880 | 805.51 | 882.748120 | 1.026127 |
| GO:0014855\_striated\_muscle\_cell\_proliferation | 14 | 0 | 0.000000 | -0.000000 | 785 | 728.271880 | 805.51 | 882.748120 | 1.026127 |
| GO:0016573\_histone\_acetylation | 14 | 0 | 0.000000 | -0.000000 | 785 | 728.271880 | 805.51 | 882.748120 | 1.026127 |
| GO:0018130\_heterocycle\_biosynthetic\_process | 14 | 0 | 0.000000 | -0.000000 | 785 | 728.271880 | 805.51 | 882.748120 | 1.026127 |
| GO:0019217\_regulation\_of\_fatty\_acid\_metabolic\_process | 14 | 0 | 0.000000 | -0.000000 | 785 | 728.271880 | 805.51 | 882.748120 | 1.026127 |
| GO:0021782\_glial\_cell\_development | 14 | 0 | 0.000000 | -0.000000 | 785 | 728.271880 | 805.51 | 882.748120 | 1.026127 |
| GO:0021904\_dorsal\_ventral\_neural\_tube\_patterning | 14 | 0 | 0.000000 | -0.000000 | 785 | 728.271880 | 805.51 | 882.748120 | 1.026127 |
| GO:0030032\_lamellipodium\_assembly | 14 | 0 | 0.000000 | -0.000000 | 785 | 728.271880 | 805.51 | 882.748120 | 1.026127 |
| GO:0030148\_sphingolipid\_biosynthetic\_process | 14 | 0 | 0.000000 | -0.000000 | 785 | 728.271880 | 805.51 | 882.748120 | 1.026127 |
| GO:0030162\_regulation\_of\_proteolysis | 14 | 0 | 0.000000 | -0.000000 | 785 | 728.271880 | 805.51 | 882.748120 | 1.026127 |
| GO:0030832\_regulation\_of\_actin\_filament\_length | 14 | 0 | 0.000000 | -0.000000 | 785 | 728.271880 | 805.51 | 882.748120 | 1.026127 |
| GO:0031099\_regeneration | 14 | 0 | 0.000000 | -0.000000 | 785 | 728.271880 | 805.51 | 882.748120 | 1.026127 |
| GO:0031346\_positive\_regulation\_of\_cell\_projection\_organization | 14 | 0 | 0.000000 | -0.000000 | 785 | 728.271880 | 805.51 | 882.748120 | 1.026127 |
| GO:0031663\_lipopolysaccharide-mediated\_signaling\_pathway | 14 | 0 | 0.000000 | -0.000000 | 785 | 728.271880 | 805.51 | 882.748120 | 1.026127 |
| GO:0032271\_regulation\_of\_protein\_polymerization | 14 | 0 | 0.000000 | -0.000000 | 785 | 728.271880 | 805.51 | 882.748120 | 1.026127 |
| GO:0034104\_negative\_regulation\_of\_tissue\_remodeling | 14 | 0 | 0.000000 | -0.000000 | 785 | 728.271880 | 805.51 | 882.748120 | 1.026127 |
| GO:0035036\_sperm-egg\_recognition | 14 | 0 | 0.000000 | -0.000000 | 785 | 728.271880 | 805.51 | 882.748120 | 1.026127 |
| GO:0042310\_vasoconstriction | 14 | 0 | 0.000000 | -0.000000 | 785 | 728.271880 | 805.51 | 882.748120 | 1.026127 |
| GO:0042573\_retinoic\_acid\_metabolic\_process | 14 | 0 | 0.000000 | -0.000000 | 785 | 728.271880 | 805.51 | 882.748120 | 1.026127 |
| GO:0043123\_positive\_regulation\_of\_I-kappaB\_kinase\_NF-kappaB\_cascade | 14 | 0 | 0.000000 | -0.000000 | 785 | 728.271880 | 805.51 | 882.748120 | 1.026127 |
| GO:0043254\_regulation\_of\_protein\_complex\_assembly | 14 | 0 | 0.000000 | -0.000000 | 785 | 728.271880 | 805.51 | 882.748120 | 1.026127 |
| GO:0044236\_multicellular\_organismal\_metabolic\_process | 14 | 0 | 0.000000 | -0.000000 | 785 | 728.271880 | 805.51 | 882.748120 | 1.026127 |
| GO:0045061\_thymic\_T\_cell\_selection | 14 | 0 | 0.000000 | -0.000000 | 785 | 728.271880 | 805.51 | 882.748120 | 1.026127 |
| GO:0045453\_bone\_resorption | 14 | 0 | 0.000000 | -0.000000 | 785 | 728.271880 | 805.51 | 882.748120 | 1.026127 |
| GO:0045732\_positive\_regulation\_of\_protein\_catabolic\_process | 14 | 0 | 0.000000 | -0.000000 | 785 | 728.271880 | 805.51 | 882.748120 | 1.026127 |
| GO:0048048\_embryonic\_eye\_morphogenesis | 14 | 0 | 0.000000 | -0.000000 | 785 | 728.271880 | 805.51 | 882.748120 | 1.026127 |
| GO:0048545\_response\_to\_steroid\_hormone\_stimulus | 14 | 0 | 0.000000 | -0.000000 | 785 | 728.271880 | 805.51 | 882.748120 | 1.026127 |
| GO:0048665\_neuron\_fate\_specification | 14 | 0 | 0.000000 | -0.000000 | 785 | 728.271880 | 805.51 | 882.748120 | 1.026127 |
| GO:0048844\_artery\_morphogenesis | 14 | 0 | 0.000000 | -0.000000 | 785 | 728.271880 | 805.51 | 882.748120 | 1.026127 |
| GO:0050810\_regulation\_of\_steroid\_biosynthetic\_process | 14 | 0 | 0.000000 | -0.000000 | 785 | 728.271880 | 805.51 | 882.748120 | 1.026127 |
| GO:0051017\_actin\_filament\_bundle\_formation | 14 | 0 | 0.000000 | -0.000000 | 785 | 728.271880 | 805.51 | 882.748120 | 1.026127 |
| GO:0051053\_negative\_regulation\_of\_DNA\_metabolic\_process | 14 | 0 | 0.000000 | -0.000000 | 785 | 728.271880 | 805.51 | 882.748120 | 1.026127 |
| GO:0051054\_positive\_regulation\_of\_DNA\_metabolic\_process | 14 | 0 | 0.000000 | -0.000000 | 785 | 728.271880 | 805.51 | 882.748120 | 1.026127 |
| GO:0051100\_negative\_regulation\_of\_binding | 14 | 0 | 0.000000 | -0.000000 | 785 | 728.271880 | 805.51 | 882.748120 | 1.026127 |
| GO:0051952\_regulation\_of\_amine\_transport | 14 | 0 | 0.000000 | -0.000000 | 785 | 728.271880 | 805.51 | 882.748120 | 1.026127 |
| GO:0060716\_labyrinthine\_layer\_blood\_vessel\_development | 14 | 0 | 0.000000 | -0.000000 | 785 | 728.271880 | 805.51 | 882.748120 | 1.026127 |
| GO:0060840\_artery\_development | 14 | 0 | 0.000000 | -0.000000 | 785 | 728.271880 | 805.51 | 882.748120 | 1.026127 |
| GO:0002204\_somatic\_recombination\_of\_immunoglobulin\_genes\_during\_immune\_response | 23 | 0 | 0.000000 | -0.000000 | 805 | 751.756817 | 827.8 | 903.843183 | 1.028323 |
| GO:0002208\_somatic\_diversification\_of\_immunoglobulins\_during\_immune\_response | 23 | 0 | 0.000000 | -0.000000 | 805 | 751.756817 | 827.8 | 903.843183 | 1.028323 |
| GO:0002228\_natural\_killer\_cell\_mediated\_immunity | 23 | 0 | 0.000000 | -0.000000 | 805 | 751.756817 | 827.8 | 903.843183 | 1.028323 |
| GO:0002821\_positive\_regulation\_of\_adaptive\_immune\_response | 23 | 0 | 0.000000 | -0.000000 | 805 | 751.756817 | 827.8 | 903.843183 | 1.028323 |
| GO:0002824\_positive\_regulation\_of\_adaptive\_immune\_response\_based\_on\_somatic\_recombination\_of\_immune\_receptors\_built\_from\_immunoglobulin\_superfamily\_domains | 23 | 0 | 0.000000 | -0.000000 | 805 | 751.756817 | 827.8 | 903.843183 | 1.028323 |
| GO:0006397\_mRNA\_processing | 23 | 0 | 0.000000 | -0.000000 | 805 | 751.756817 | 827.8 | 903.843183 | 1.028323 |
| GO:0007018\_microtubule-based\_movement | 23 | 0 | 0.000000 | -0.000000 | 805 | 751.756817 | 827.8 | 903.843183 | 1.028323 |
| GO:0007163\_establishment\_or\_maintenance\_of\_cell\_polarity | 23 | 0 | 0.000000 | -0.000000 | 805 | 751.756817 | 827.8 | 903.843183 | 1.028323 |
| GO:0007584\_response\_to\_nutrient | 23 | 0 | 0.000000 | -0.000000 | 805 | 751.756817 | 827.8 | 903.843183 | 1.028323 |
| GO:0008542\_visual\_learning | 23 | 0 | 0.000000 | -0.000000 | 805 | 751.756817 | 827.8 | 903.843183 | 1.028323 |
| GO:0009954\_proximal\_distal\_pattern\_formation | 23 | 0 | 0.000000 | -0.000000 | 805 | 751.756817 | 827.8 | 903.843183 | 1.028323 |
| GO:0022613\_ribonucleoprotein\_complex\_biogenesis | 23 | 0 | 0.000000 | -0.000000 | 805 | 751.756817 | 827.8 | 903.843183 | 1.028323 |
| GO:0030512\_negative\_regulation\_of\_transforming\_growth\_factor\_beta\_receptor\_signaling\_pathway | 23 | 0 | 0.000000 | -0.000000 | 805 | 751.756817 | 827.8 | 903.843183 | 1.028323 |
| GO:0032635\_interleukin-6\_production | 23 | 0 | 0.000000 | -0.000000 | 805 | 751.756817 | 827.8 | 903.843183 | 1.028323 |
| GO:0032675\_regulation\_of\_interleukin-6\_production | 23 | 0 | 0.000000 | -0.000000 | 805 | 751.756817 | 827.8 | 903.843183 | 1.028323 |
| GO:0042267\_natural\_killer\_cell\_mediated\_cytotoxicity | 23 | 0 | 0.000000 | -0.000000 | 805 | 751.756817 | 827.8 | 903.843183 | 1.028323 |
| GO:0045190\_isotype\_switching | 23 | 0 | 0.000000 | -0.000000 | 805 | 751.756817 | 827.8 | 903.843183 | 1.028323 |
| GO:0051705\_behavioral\_interaction\_between\_organisms | 23 | 0 | 0.000000 | -0.000000 | 805 | 751.756817 | 827.8 | 903.843183 | 1.028323 |
| GO:0060349\_bone\_morphogenesis | 23 | 0 | 0.000000 | -0.000000 | 805 | 751.756817 | 827.8 | 903.843183 | 1.028323 |
| GO:0060445\_branching\_involved\_in\_salivary\_gland\_morphogenesis | 23 | 0 | 0.000000 | -0.000000 | 805 | 751.756817 | 827.8 | 903.843183 | 1.028323 |
| GO:0016044\_membrane\_organization | 140 | 0 | 0.000000 | -0.000000 | 806 | 752.300738 | 828.25 | 904.199262 | 1.027605 |
| GO:0002697\_regulation\_of\_immune\_effector\_process | 68 | 0 | 0.000000 | -0.000000 | 809 | 755.274016 | 831.02 | 906.765984 | 1.027219 |
| GO:0019932\_second-messenger-mediated\_signaling | 68 | 0 | 0.000000 | -0.000000 | 809 | 755.274016 | 831.02 | 906.765984 | 1.027219 |
| GO:0042692\_muscle\_cell\_differentiation | 68 | 0 | 0.000000 | -0.000000 | 809 | 755.274016 | 831.02 | 906.765984 | 1.027219 |
| GO:0001776\_leukocyte\_homeostasis | 41 | 0 | 0.000000 | -0.000000 | 825 | 772.403326 | 847.03 | 921.656674 | 1.026703 |
| GO:0006260\_DNA\_replication | 41 | 0 | 0.000000 | -0.000000 | 825 | 772.403326 | 847.03 | 921.656674 | 1.026703 |
| GO:0006836\_neurotransmitter\_transport | 41 | 0 | 0.000000 | -0.000000 | 825 | 772.403326 | 847.03 | 921.656674 | 1.026703 |
| GO:0006865\_amino\_acid\_transport | 41 | 0 | 0.000000 | -0.000000 | 825 | 772.403326 | 847.03 | 921.656674 | 1.026703 |
| GO:0007254\_JNK\_cascade | 41 | 0 | 0.000000 | -0.000000 | 825 | 772.403326 | 847.03 | 921.656674 | 1.026703 |
| GO:0009894\_regulation\_of\_catabolic\_process | 41 | 0 | 0.000000 | -0.000000 | 825 | 772.403326 | 847.03 | 921.656674 | 1.026703 |
| GO:0010551\_regulation\_of\_specific\_transcription\_from\_RNA\_polymerase\_II\_promoter | 41 | 0 | 0.000000 | -0.000000 | 825 | 772.403326 | 847.03 | 921.656674 | 1.026703 |
| GO:0015833\_peptide\_transport | 41 | 0 | 0.000000 | -0.000000 | 825 | 772.403326 | 847.03 | 921.656674 | 1.026703 |
| GO:0019216\_regulation\_of\_lipid\_metabolic\_process | 41 | 0 | 0.000000 | -0.000000 | 825 | 772.403326 | 847.03 | 921.656674 | 1.026703 |
| GO:0019748\_secondary\_metabolic\_process | 41 | 0 | 0.000000 | -0.000000 | 825 | 772.403326 | 847.03 | 921.656674 | 1.026703 |
| GO:0030817\_regulation\_of\_cAMP\_biosynthetic\_process | 41 | 0 | 0.000000 | -0.000000 | 825 | 772.403326 | 847.03 | 921.656674 | 1.026703 |
| GO:0031344\_regulation\_of\_cell\_projection\_organization | 41 | 0 | 0.000000 | -0.000000 | 825 | 772.403326 | 847.03 | 921.656674 | 1.026703 |
| GO:0032569\_specific\_transcription\_from\_RNA\_polymerase\_II\_promoter | 41 | 0 | 0.000000 | -0.000000 | 825 | 772.403326 | 847.03 | 921.656674 | 1.026703 |
| GO:0032844\_regulation\_of\_homeostatic\_process | 41 | 0 | 0.000000 | -0.000000 | 825 | 772.403326 | 847.03 | 921.656674 | 1.026703 |
| GO:0033077\_T\_cell\_differentiation\_in\_the\_thymus | 41 | 0 | 0.000000 | -0.000000 | 825 | 772.403326 | 847.03 | 921.656674 | 1.026703 |
| GO:0050864\_regulation\_of\_B\_cell\_activation | 41 | 0 | 0.000000 | -0.000000 | 825 | 772.403326 | 847.03 | 921.656674 | 1.026703 |
| GO:0044057\_regulation\_of\_system\_process | 133 | 0 | 0.000000 | -0.000000 | 826 | 773.547040 | 848.02 | 922.492960 | 1.026659 |
| GO:0006954\_inflammatory\_response | 96 | 0 | 0.000000 | -0.000000 | 829 | 777.067661 | 850.84 | 924.612339 | 1.026345 |
| GO:0060249\_anatomical\_structure\_homeostasis | 96 | 0 | 0.000000 | -0.000000 | 829 | 777.067661 | 850.84 | 924.612339 | 1.026345 |
| GO:0070661\_leukocyte\_proliferation | 96 | 0 | 0.000000 | -0.000000 | 829 | 777.067661 | 850.84 | 924.612339 | 1.026345 |
| GO:0015031\_protein\_transport | 175 | 0 | 0.000000 | -0.000000 | 831 | 778.516894 | 851.98 | 925.443106 | 1.025247 |
| GO:0046903\_secretion | 175 | 0 | 0.000000 | -0.000000 | 831 | 778.516894 | 851.98 | 925.443106 | 1.025247 |
| GO:0006140\_regulation\_of\_nucleotide\_metabolic\_process | 47 | 0 | 0.000000 | -0.000000 | 841 | 788.999765 | 861.42 | 933.840235 | 1.024281 |
| GO:0006396\_RNA\_processing | 47 | 0 | 0.000000 | -0.000000 | 841 | 788.999765 | 861.42 | 933.840235 | 1.024281 |
| GO:0030183\_B\_cell\_differentiation | 47 | 0 | 0.000000 | -0.000000 | 841 | 788.999765 | 861.42 | 933.840235 | 1.024281 |
| GO:0030799\_regulation\_of\_cyclic\_nucleotide\_metabolic\_process | 47 | 0 | 0.000000 | -0.000000 | 841 | 788.999765 | 861.42 | 933.840235 | 1.024281 |
| GO:0031667\_response\_to\_nutrient\_levels | 47 | 0 | 0.000000 | -0.000000 | 841 | 788.999765 | 861.42 | 933.840235 | 1.024281 |
| GO:0034754\_cellular\_hormone\_metabolic\_process | 47 | 0 | 0.000000 | -0.000000 | 841 | 788.999765 | 861.42 | 933.840235 | 1.024281 |
| GO:0045087\_innate\_immune\_response | 47 | 0 | 0.000000 | -0.000000 | 841 | 788.999765 | 861.42 | 933.840235 | 1.024281 |
| GO:0045619\_regulation\_of\_lymphocyte\_differentiation | 47 | 0 | 0.000000 | -0.000000 | 841 | 788.999765 | 861.42 | 933.840235 | 1.024281 |
| GO:0048871\_multicellular\_organismal\_homeostasis | 47 | 0 | 0.000000 | -0.000000 | 841 | 788.999765 | 861.42 | 933.840235 | 1.024281 |
| GO:0060627\_regulation\_of\_vesicle-mediated\_transport | 47 | 0 | 0.000000 | -0.000000 | 841 | 788.999765 | 861.42 | 933.840235 | 1.024281 |
| GO:0014706\_striated\_muscle\_tissue\_development | 120 | 0 | 0.000000 | -0.000000 | 842 | 789.555779 | 861.87 | 934.184221 | 1.023599 |
| GO:0009416\_response\_to\_light\_stimulus | 74 | 0 | 0.000000 | -0.000000 | 844 | 791.143394 | 863.3 | 935.456606 | 1.022867 |
| GO:0048771\_tissue\_remodeling | 74 | 0 | 0.000000 | -0.000000 | 844 | 791.143394 | 863.3 | 935.456606 | 1.022867 |
| GO:0000724\_double-strand\_break\_repair\_via\_homologous\_recombination | 10 | 0 | 0.000000 | -0.000000 | 963 | 910.534630 | 980.77 | 1051.005370 | 1.018453 |
| GO:0000725\_recombinational\_repair | 10 | 0 | 0.000000 | -0.000000 | 963 | 910.534630 | 980.77 | 1051.005370 | 1.018453 |
| GO:0001578\_microtubule\_bundle\_formation | 10 | 0 | 0.000000 | -0.000000 | 963 | 910.534630 | 980.77 | 1051.005370 | 1.018453 |
| GO:0001659\_temperature\_homeostasis | 10 | 0 | 0.000000 | -0.000000 | 963 | 910.534630 | 980.77 | 1051.005370 | 1.018453 |
| GO:0001773\_myeloid\_dendritic\_cell\_activation | 10 | 0 | 0.000000 | -0.000000 | 963 | 910.534630 | 980.77 | 1051.005370 | 1.018453 |
| GO:0001832\_blastocyst\_growth | 10 | 0 | 0.000000 | -0.000000 | 963 | 910.534630 | 980.77 | 1051.005370 | 1.018453 |
| GO:0001914\_regulation\_of\_T\_cell\_mediated\_cytotoxicity | 10 | 0 | 0.000000 | -0.000000 | 963 | 910.534630 | 980.77 | 1051.005370 | 1.018453 |
| GO:0001990\_regulation\_of\_systemic\_arterial\_blood\_pressure\_by\_hormone | 10 | 0 | 0.000000 | -0.000000 | 963 | 910.534630 | 980.77 | 1051.005370 | 1.018453 |
| GO:0002070\_epithelial\_cell\_maturation | 10 | 0 | 0.000000 | -0.000000 | 963 | 910.534630 | 980.77 | 1051.005370 | 1.018453 |
| GO:0002673\_regulation\_of\_acute\_inflammatory\_response | 10 | 0 | 0.000000 | -0.000000 | 963 | 910.534630 | 980.77 | 1051.005370 | 1.018453 |
| GO:0002711\_positive\_regulation\_of\_T\_cell\_mediated\_immunity | 10 | 0 | 0.000000 | -0.000000 | 963 | 910.534630 | 980.77 | 1051.005370 | 1.018453 |
| GO:0002762\_negative\_regulation\_of\_myeloid\_leukocyte\_differentiation | 10 | 0 | 0.000000 | -0.000000 | 963 | 910.534630 | 980.77 | 1051.005370 | 1.018453 |
| GO:0006040\_amino\_sugar\_metabolic\_process | 10 | 0 | 0.000000 | -0.000000 | 963 | 910.534630 | 980.77 | 1051.005370 | 1.018453 |
| GO:0006081\_cellular\_aldehyde\_metabolic\_process | 10 | 0 | 0.000000 | -0.000000 | 963 | 910.534630 | 980.77 | 1051.005370 | 1.018453 |
| GO:0006109\_regulation\_of\_carbohydrate\_metabolic\_process | 10 | 0 | 0.000000 | -0.000000 | 963 | 910.534630 | 980.77 | 1051.005370 | 1.018453 |
| GO:0006289\_nucleotide-excision\_repair | 10 | 0 | 0.000000 | -0.000000 | 963 | 910.534630 | 980.77 | 1051.005370 | 1.018453 |
| GO:0006342\_chromatin\_silencing | 10 | 0 | 0.000000 | -0.000000 | 963 | 910.534630 | 980.77 | 1051.005370 | 1.018453 |
| GO:0006405\_RNA\_export\_from\_nucleus | 10 | 0 | 0.000000 | -0.000000 | 963 | 910.534630 | 980.77 | 1051.005370 | 1.018453 |
| GO:0006805\_xenobiotic\_metabolic\_process | 10 | 0 | 0.000000 | -0.000000 | 963 | 910.534630 | 980.77 | 1051.005370 | 1.018453 |
| GO:0006826\_iron\_ion\_transport | 10 | 0 | 0.000000 | -0.000000 | 963 | 910.534630 | 980.77 | 1051.005370 | 1.018453 |
| GO:0006921\_cell\_structure\_disassembly\_during\_apoptosis | 10 | 0 | 0.000000 | -0.000000 | 963 | 910.534630 | 980.77 | 1051.005370 | 1.018453 |
| GO:0006968\_cellular\_defense\_response | 10 | 0 | 0.000000 | -0.000000 | 963 | 910.534630 | 980.77 | 1051.005370 | 1.018453 |
| GO:0007006\_mitochondrial\_membrane\_organization | 10 | 0 | 0.000000 | -0.000000 | 963 | 910.534630 | 980.77 | 1051.005370 | 1.018453 |
| GO:0007044\_cell-substrate\_junction\_assembly | 10 | 0 | 0.000000 | -0.000000 | 963 | 910.534630 | 980.77 | 1051.005370 | 1.018453 |
| GO:0007093\_mitotic\_cell\_cycle\_checkpoint | 10 | 0 | 0.000000 | -0.000000 | 963 | 910.534630 | 980.77 | 1051.005370 | 1.018453 |
| GO:0007172\_signal\_complex\_assembly | 10 | 0 | 0.000000 | -0.000000 | 963 | 910.534630 | 980.77 | 1051.005370 | 1.018453 |
| GO:0007194\_negative\_regulation\_of\_adenylate\_cyclase\_activity | 10 | 0 | 0.000000 | -0.000000 | 963 | 910.534630 | 980.77 | 1051.005370 | 1.018453 |
| GO:0008088\_axon\_cargo\_transport | 10 | 0 | 0.000000 | -0.000000 | 963 | 910.534630 | 980.77 | 1051.005370 | 1.018453 |
| GO:0008206\_bile\_acid\_metabolic\_process | 10 | 0 | 0.000000 | -0.000000 | 963 | 910.534630 | 980.77 | 1051.005370 | 1.018453 |
| GO:0008211\_glucocorticoid\_metabolic\_process | 10 | 0 | 0.000000 | -0.000000 | 963 | 910.534630 | 980.77 | 1051.005370 | 1.018453 |
| GO:0009066\_aspartate\_family\_amino\_acid\_metabolic\_process | 10 | 0 | 0.000000 | -0.000000 | 963 | 910.534630 | 980.77 | 1051.005370 | 1.018453 |
| GO:0009110\_vitamin\_biosynthetic\_process | 10 | 0 | 0.000000 | -0.000000 | 963 | 910.534630 | 980.77 | 1051.005370 | 1.018453 |
| GO:0009620\_response\_to\_fungus | 10 | 0 | 0.000000 | -0.000000 | 963 | 910.534630 | 980.77 | 1051.005370 | 1.018453 |
| GO:0009743\_response\_to\_carbohydrate\_stimulus | 10 | 0 | 0.000000 | -0.000000 | 963 | 910.534630 | 980.77 | 1051.005370 | 1.018453 |
| GO:0009948\_anterior\_posterior\_axis\_specification | 10 | 0 | 0.000000 | -0.000000 | 963 | 910.534630 | 980.77 | 1051.005370 | 1.018453 |
| GO:0010827\_regulation\_of\_glucose\_transport | 10 | 0 | 0.000000 | -0.000000 | 963 | 910.534630 | 980.77 | 1051.005370 | 1.018453 |
| GO:0015718\_monocarboxylic\_acid\_transport | 10 | 0 | 0.000000 | -0.000000 | 963 | 910.534630 | 980.77 | 1051.005370 | 1.018453 |
| GO:0016197\_endosome\_transport | 10 | 0 | 0.000000 | -0.000000 | 963 | 910.534630 | 980.77 | 1051.005370 | 1.018453 |
| GO:0016486\_peptide\_hormone\_processing | 10 | 0 | 0.000000 | -0.000000 | 963 | 910.534630 | 980.77 | 1051.005370 | 1.018453 |
| GO:0017156\_calcium\_ion-dependent\_exocytosis | 10 | 0 | 0.000000 | -0.000000 | 963 | 910.534630 | 980.77 | 1051.005370 | 1.018453 |
| GO:0018149\_peptide\_cross-linking | 10 | 0 | 0.000000 | -0.000000 | 963 | 910.534630 | 980.77 | 1051.005370 | 1.018453 |
| GO:0019321\_pentose\_metabolic\_process | 10 | 0 | 0.000000 | -0.000000 | 963 | 910.534630 | 980.77 | 1051.005370 | 1.018453 |
| GO:0021534\_cell\_proliferation\_in\_hindbrain | 10 | 0 | 0.000000 | -0.000000 | 963 | 910.534630 | 980.77 | 1051.005370 | 1.018453 |
| GO:0021871\_forebrain\_regionalization | 10 | 0 | 0.000000 | -0.000000 | 963 | 910.534630 | 980.77 | 1051.005370 | 1.018453 |
| GO:0021895\_cerebral\_cortex\_neuron\_differentiation | 10 | 0 | 0.000000 | -0.000000 | 963 | 910.534630 | 980.77 | 1051.005370 | 1.018453 |
| GO:0021924\_cell\_proliferation\_in\_the\_external\_granule\_layer | 10 | 0 | 0.000000 | -0.000000 | 963 | 910.534630 | 980.77 | 1051.005370 | 1.018453 |
| GO:0021930\_granule\_cell\_precursor\_proliferation | 10 | 0 | 0.000000 | -0.000000 | 963 | 910.534630 | 980.77 | 1051.005370 | 1.018453 |
| GO:0021952\_central\_nervous\_system\_projection\_neuron\_axonogenesis | 10 | 0 | 0.000000 | -0.000000 | 963 | 910.534630 | 980.77 | 1051.005370 | 1.018453 |
| GO:0030168\_platelet\_activation | 10 | 0 | 0.000000 | -0.000000 | 963 | 910.534630 | 980.77 | 1051.005370 | 1.018453 |
| GO:0030833\_regulation\_of\_actin\_filament\_polymerization | 10 | 0 | 0.000000 | -0.000000 | 963 | 910.534630 | 980.77 | 1051.005370 | 1.018453 |
| GO:0031018\_endocrine\_pancreas\_development | 10 | 0 | 0.000000 | -0.000000 | 963 | 910.534630 | 980.77 | 1051.005370 | 1.018453 |
| GO:0031280\_negative\_regulation\_of\_cyclase\_activity | 10 | 0 | 0.000000 | -0.000000 | 963 | 910.534630 | 980.77 | 1051.005370 | 1.018453 |
| GO:0031331\_positive\_regulation\_of\_cellular\_catabolic\_process | 10 | 0 | 0.000000 | -0.000000 | 963 | 910.534630 | 980.77 | 1051.005370 | 1.018453 |
| GO:0031645\_negative\_regulation\_of\_neurological\_system\_process | 10 | 0 | 0.000000 | -0.000000 | 963 | 910.534630 | 980.77 | 1051.005370 | 1.018453 |
| GO:0032318\_regulation\_of\_Ras\_GTPase\_activity | 10 | 0 | 0.000000 | -0.000000 | 963 | 910.534630 | 980.77 | 1051.005370 | 1.018453 |
| GO:0032602\_chemokine\_production | 10 | 0 | 0.000000 | -0.000000 | 963 | 910.534630 | 980.77 | 1051.005370 | 1.018453 |
| GO:0032633\_interleukin-4\_production | 10 | 0 | 0.000000 | -0.000000 | 963 | 910.534630 | 980.77 | 1051.005370 | 1.018453 |
| GO:0032642\_regulation\_of\_chemokine\_production | 10 | 0 | 0.000000 | -0.000000 | 963 | 910.534630 | 980.77 | 1051.005370 | 1.018453 |
| GO:0032673\_regulation\_of\_interleukin-4\_production | 10 | 0 | 0.000000 | -0.000000 | 963 | 910.534630 | 980.77 | 1051.005370 | 1.018453 |
| GO:0032760\_positive\_regulation\_of\_tumor\_necrosis\_factor\_production | 10 | 0 | 0.000000 | -0.000000 | 963 | 910.534630 | 980.77 | 1051.005370 | 1.018453 |
| GO:0033081\_regulation\_of\_T\_cell\_differentiation\_in\_the\_thymus | 10 | 0 | 0.000000 | -0.000000 | 963 | 910.534630 | 980.77 | 1051.005370 | 1.018453 |
| GO:0034105\_positive\_regulation\_of\_tissue\_remodeling | 10 | 0 | 0.000000 | -0.000000 | 963 | 910.534630 | 980.77 | 1051.005370 | 1.018453 |
| GO:0034637\_cellular\_carbohydrate\_biosynthetic\_process | 10 | 0 | 0.000000 | -0.000000 | 963 | 910.534630 | 980.77 | 1051.005370 | 1.018453 |
| GO:0040015\_negative\_regulation\_of\_multicellular\_organism\_growth | 10 | 0 | 0.000000 | -0.000000 | 963 | 910.534630 | 980.77 | 1051.005370 | 1.018453 |
| GO:0042088\_T-helper\_1\_type\_immune\_response | 10 | 0 | 0.000000 | -0.000000 | 963 | 910.534630 | 980.77 | 1051.005370 | 1.018453 |
| GO:0042116\_macrophage\_activation | 10 | 0 | 0.000000 | -0.000000 | 963 | 910.534630 | 980.77 | 1051.005370 | 1.018453 |
| GO:0042177\_negative\_regulation\_of\_protein\_catabolic\_process | 10 | 0 | 0.000000 | -0.000000 | 963 | 910.534630 | 980.77 | 1051.005370 | 1.018453 |
| GO:0042755\_eating\_behavior | 10 | 0 | 0.000000 | -0.000000 | 963 | 910.534630 | 980.77 | 1051.005370 | 1.018453 |
| GO:0043113\_receptor\_clustering | 10 | 0 | 0.000000 | -0.000000 | 963 | 910.534630 | 980.77 | 1051.005370 | 1.018453 |
| GO:0043330\_response\_to\_exogenous\_dsRNA | 10 | 0 | 0.000000 | -0.000000 | 963 | 910.534630 | 980.77 | 1051.005370 | 1.018453 |
| GO:0043488\_regulation\_of\_mRNA\_stability | 10 | 0 | 0.000000 | -0.000000 | 963 | 910.534630 | 980.77 | 1051.005370 | 1.018453 |
| GO:0043506\_regulation\_of\_JUN\_kinase\_activity | 10 | 0 | 0.000000 | -0.000000 | 963 | 910.534630 | 980.77 | 1051.005370 | 1.018453 |
| GO:0043525\_positive\_regulation\_of\_neuron\_apoptosis | 10 | 0 | 0.000000 | -0.000000 | 963 | 910.534630 | 980.77 | 1051.005370 | 1.018453 |
| GO:0044259\_multicellular\_organismal\_macromolecule\_metabolic\_process | 10 | 0 | 0.000000 | -0.000000 | 963 | 910.534630 | 980.77 | 1051.005370 | 1.018453 |
| GO:0045132\_meiotic\_chromosome\_segregation | 10 | 0 | 0.000000 | -0.000000 | 963 | 910.534630 | 980.77 | 1051.005370 | 1.018453 |
| GO:0045446\_endothelial\_cell\_differentiation | 10 | 0 | 0.000000 | -0.000000 | 963 | 910.534630 | 980.77 | 1051.005370 | 1.018453 |
| GO:0045576\_mast\_cell\_activation | 10 | 0 | 0.000000 | -0.000000 | 963 | 910.534630 | 980.77 | 1051.005370 | 1.018453 |
| GO:0045669\_positive\_regulation\_of\_osteoblast\_differentiation | 10 | 0 | 0.000000 | -0.000000 | 963 | 910.534630 | 980.77 | 1051.005370 | 1.018453 |
| GO:0045777\_positive\_regulation\_of\_blood\_pressure | 10 | 0 | 0.000000 | -0.000000 | 963 | 910.534630 | 980.77 | 1051.005370 | 1.018453 |
| GO:0045814\_negative\_regulation\_of\_gene\_expression\_\_epigenetic | 10 | 0 | 0.000000 | -0.000000 | 963 | 910.534630 | 980.77 | 1051.005370 | 1.018453 |
| GO:0045911\_positive\_regulation\_of\_DNA\_recombination | 10 | 0 | 0.000000 | -0.000000 | 963 | 910.534630 | 980.77 | 1051.005370 | 1.018453 |
| GO:0046887\_positive\_regulation\_of\_hormone\_secretion | 10 | 0 | 0.000000 | -0.000000 | 963 | 910.534630 | 980.77 | 1051.005370 | 1.018453 |
| GO:0048291\_isotype\_switching\_to\_IgG\_isotypes | 10 | 0 | 0.000000 | -0.000000 | 963 | 910.534630 | 980.77 | 1051.005370 | 1.018453 |
| GO:0048302\_regulation\_of\_isotype\_switching\_to\_IgG\_isotypes | 10 | 0 | 0.000000 | -0.000000 | 963 | 910.534630 | 980.77 | 1051.005370 | 1.018453 |
| GO:0048339\_paraxial\_mesoderm\_development | 10 | 0 | 0.000000 | -0.000000 | 963 | 910.534630 | 980.77 | 1051.005370 | 1.018453 |
| GO:0048384\_retinoic\_acid\_receptor\_signaling\_pathway | 10 | 0 | 0.000000 | -0.000000 | 963 | 910.534630 | 980.77 | 1051.005370 | 1.018453 |
| GO:0048596\_embryonic\_camera-type\_eye\_morphogenesis | 10 | 0 | 0.000000 | -0.000000 | 963 | 910.534630 | 980.77 | 1051.005370 | 1.018453 |
| GO:0048641\_regulation\_of\_skeletal\_muscle\_tissue\_development | 10 | 0 | 0.000000 | -0.000000 | 963 | 910.534630 | 980.77 | 1051.005370 | 1.018453 |
| GO:0048738\_cardiac\_muscle\_tissue\_development | 10 | 0 | 0.000000 | -0.000000 | 963 | 910.534630 | 980.77 | 1051.005370 | 1.018453 |
| GO:0050654\_chondroitin\_sulfate\_proteoglycan\_metabolic\_process | 10 | 0 | 0.000000 | -0.000000 | 963 | 910.534630 | 980.77 | 1051.005370 | 1.018453 |
| GO:0050657\_nucleic\_acid\_transport | 10 | 0 | 0.000000 | -0.000000 | 963 | 910.534630 | 980.77 | 1051.005370 | 1.018453 |
| GO:0050658\_RNA\_transport | 10 | 0 | 0.000000 | -0.000000 | 963 | 910.534630 | 980.77 | 1051.005370 | 1.018453 |
| GO:0050663\_cytokine\_secretion | 10 | 0 | 0.000000 | -0.000000 | 963 | 910.534630 | 980.77 | 1051.005370 | 1.018453 |
| GO:0050714\_positive\_regulation\_of\_protein\_secretion | 10 | 0 | 0.000000 | -0.000000 | 963 | 910.534630 | 980.77 | 1051.005370 | 1.018453 |
| GO:0050879\_multicellular\_organismal\_movement | 10 | 0 | 0.000000 | -0.000000 | 963 | 910.534630 | 980.77 | 1051.005370 | 1.018453 |
| GO:0050881\_musculoskeletal\_movement | 10 | 0 | 0.000000 | -0.000000 | 963 | 910.534630 | 980.77 | 1051.005370 | 1.018453 |
| GO:0050886\_endocrine\_process | 10 | 0 | 0.000000 | -0.000000 | 963 | 910.534630 | 980.77 | 1051.005370 | 1.018453 |
| GO:0050892\_intestinal\_absorption | 10 | 0 | 0.000000 | -0.000000 | 963 | 910.534630 | 980.77 | 1051.005370 | 1.018453 |
| GO:0051147\_regulation\_of\_muscle\_cell\_differentiation | 10 | 0 | 0.000000 | -0.000000 | 963 | 910.534630 | 980.77 | 1051.005370 | 1.018453 |
| GO:0051208\_sequestering\_of\_calcium\_ion | 10 | 0 | 0.000000 | -0.000000 | 963 | 910.534630 | 980.77 | 1051.005370 | 1.018453 |
| GO:0051209\_release\_of\_sequestered\_calcium\_ion\_into\_cytosol | 10 | 0 | 0.000000 | -0.000000 | 963 | 910.534630 | 980.77 | 1051.005370 | 1.018453 |
| GO:0051224\_negative\_regulation\_of\_protein\_transport | 10 | 0 | 0.000000 | -0.000000 | 963 | 910.534630 | 980.77 | 1051.005370 | 1.018453 |
| GO:0051236\_establishment\_of\_RNA\_localization | 10 | 0 | 0.000000 | -0.000000 | 963 | 910.534630 | 980.77 | 1051.005370 | 1.018453 |
| GO:0051238\_sequestering\_of\_metal\_ion | 10 | 0 | 0.000000 | -0.000000 | 963 | 910.534630 | 980.77 | 1051.005370 | 1.018453 |
| GO:0051262\_protein\_tetramerization | 10 | 0 | 0.000000 | -0.000000 | 963 | 910.534630 | 980.77 | 1051.005370 | 1.018453 |
| GO:0051282\_regulation\_of\_sequestering\_of\_calcium\_ion | 10 | 0 | 0.000000 | -0.000000 | 963 | 910.534630 | 980.77 | 1051.005370 | 1.018453 |
| GO:0051283\_negative\_regulation\_of\_sequestering\_of\_calcium\_ion | 10 | 0 | 0.000000 | -0.000000 | 963 | 910.534630 | 980.77 | 1051.005370 | 1.018453 |
| GO:0051350\_negative\_regulation\_of\_lyase\_activity | 10 | 0 | 0.000000 | -0.000000 | 963 | 910.534630 | 980.77 | 1051.005370 | 1.018453 |
| GO:0051445\_regulation\_of\_meiotic\_cell\_cycle | 10 | 0 | 0.000000 | -0.000000 | 963 | 910.534630 | 980.77 | 1051.005370 | 1.018453 |
| GO:0051650\_establishment\_of\_vesicle\_localization | 10 | 0 | 0.000000 | -0.000000 | 963 | 910.534630 | 980.77 | 1051.005370 | 1.018453 |
| GO:0051651\_maintenance\_of\_location\_in\_cell | 10 | 0 | 0.000000 | -0.000000 | 963 | 910.534630 | 980.77 | 1051.005370 | 1.018453 |
| GO:0060135\_maternal\_process\_involved\_in\_female\_pregnancy | 10 | 0 | 0.000000 | -0.000000 | 963 | 910.534630 | 980.77 | 1051.005370 | 1.018453 |
| GO:0060216\_definitive\_hemopoiesis | 10 | 0 | 0.000000 | -0.000000 | 963 | 910.534630 | 980.77 | 1051.005370 | 1.018453 |
| GO:0060323\_head\_morphogenesis | 10 | 0 | 0.000000 | -0.000000 | 963 | 910.534630 | 980.77 | 1051.005370 | 1.018453 |
| GO:0060343\_trabecula\_formation | 10 | 0 | 0.000000 | -0.000000 | 963 | 910.534630 | 980.77 | 1051.005370 | 1.018453 |
| GO:0060601\_lateral\_sprouting\_from\_an\_epithelium | 10 | 0 | 0.000000 | -0.000000 | 963 | 910.534630 | 980.77 | 1051.005370 | 1.018453 |
| GO:0060669\_embryonic\_placenta\_morphogenesis | 10 | 0 | 0.000000 | -0.000000 | 963 | 910.534630 | 980.77 | 1051.005370 | 1.018453 |
| GO:0060706\_cell\_differentiation\_involved\_in\_embryonic\_placenta\_development | 10 | 0 | 0.000000 | -0.000000 | 963 | 910.534630 | 980.77 | 1051.005370 | 1.018453 |
| GO:0060768\_regulation\_of\_epithelial\_cell\_proliferation\_involved\_in\_prostate\_gland\_development | 10 | 0 | 0.000000 | -0.000000 | 963 | 910.534630 | 980.77 | 1051.005370 | 1.018453 |
| GO:0006163\_purine\_nucleotide\_metabolic\_process | 73 | 0 | 0.000000 | -0.000000 | 968 | 914.249648 | 984.22 | 1054.190352 | 1.016756 |
| GO:0006936\_muscle\_contraction | 73 | 0 | 0.000000 | -0.000000 | 968 | 914.249648 | 984.22 | 1054.190352 | 1.016756 |
| GO:0048706\_embryonic\_skeletal\_system\_development | 73 | 0 | 0.000000 | -0.000000 | 968 | 914.249648 | 984.22 | 1054.190352 | 1.016756 |
| GO:0051270\_regulation\_of\_cell\_motion | 73 | 0 | 0.000000 | -0.000000 | 968 | 914.249648 | 984.22 | 1054.190352 | 1.016756 |
| GO:0051336\_regulation\_of\_hydrolase\_activity | 73 | 0 | 0.000000 | -0.000000 | 968 | 914.249648 | 984.22 | 1054.190352 | 1.016756 |
| GO:0021700\_developmental\_maturation | 81 | 0 | 0.000000 | -0.000000 | 969 | 914.868725 | 984.76 | 1054.651275 | 1.016264 |
| GO:0000012\_single\_strand\_break\_repair | 2 | 0 |  |  |  |  |  |  |  |  |
| GO:0000019\_regulation\_of\_mitotic\_recombination | 2 | 0 |  |  |  |  |  |  |  |  |
| GO:0000076\_DNA\_replication\_checkpoint | 2 | 0 |  |  |  |  |  |  |  |  |
| GO:0000080\_G1\_phase\_of\_mitotic\_cell\_cycle | 2 | 0 |  |  |  |  |  |  |  |  |
| GO:0000083\_regulation\_of\_transcription\_of\_G1\_S-phase\_of\_mitotic\_cell\_cycle | 2 | 0 |  |  |  |  |  |  |  |  |
| GO:0000085\_G2\_phase\_of\_mitotic\_cell\_cycle | 2 | 0 |  |  |  |  |  |  |  |  |
| GO:0000289\_nuclear-transcribed\_mRNA\_poly(A)\_tail\_shortening | 2 | 0 |  |  |  |  |  |  |  |  |
| GO:0000381\_regulation\_of\_alternative\_nuclear\_mRNA\_splicing\_\_via\_spliceosome | 2 | 0 |  |  |  |  |  |  |  |  |
| GO:0000712\_resolution\_of\_meiotic\_joint\_molecules\_as\_recombinants | 2 | 0 |  |  |  |  |  |  |  |  |
| GO:0000720\_pyrimidine\_dimer\_repair\_by\_nucleotide-excision\_repair | 2 | 0 |  |  |  |  |  |  |  |  |
| GO:0001302\_replicative\_cell\_aging | 2 | 0 |  |  |  |  |  |  |  |  |
| GO:0001514\_selenocysteine\_incorporation | 2 | 0 |  |  |  |  |  |  |  |  |
| GO:0001522\_pseudouridine\_synthesis | 2 | 0 |  |  |  |  |  |  |  |  |
| GO:0001543\_ovarian\_follicle\_rupture | 2 | 0 |  |  |  |  |  |  |  |  |
| GO:0001561\_fatty\_acid\_alpha-oxidation | 2 | 0 |  |  |  |  |  |  |  |  |
| GO:0001675\_acrosome\_assembly | 2 | 0 |  |  |  |  |  |  |  |  |
| GO:0001743\_optic\_placode\_formation | 2 | 0 |  |  |  |  |  |  |  |  |
| GO:0001767\_establishment\_of\_lymphocyte\_polarity | 2 | 0 |  |  |  |  |  |  |  |  |
| GO:0001768\_establishment\_of\_T\_cell\_polarity | 2 | 0 |  |  |  |  |  |  |  |  |
| GO:0001771\_formation\_of\_immunological\_synapse | 2 | 0 |  |  |  |  |  |  |  |  |
| GO:0001774\_microglial\_cell\_activation | 2 | 0 |  |  |  |  |  |  |  |  |
| GO:0001781\_neutrophil\_apoptosis | 2 | 0 |  |  |  |  |  |  |  |  |
| GO:0001787\_natural\_killer\_cell\_proliferation | 2 | 0 |  |  |  |  |  |  |  |  |
| GO:0001788\_antibody-dependent\_cellular\_cytotoxicity | 2 | 0 |  |  |  |  |  |  |  |  |
| GO:0001806\_type\_IV\_hypersensitivity | 2 | 0 |  |  |  |  |  |  |  |  |
| GO:0001807\_regulation\_of\_type\_IV\_hypersensitivity | 2 | 0 |  |  |  |  |  |  |  |  |
| GO:0001808\_negative\_regulation\_of\_type\_IV\_hypersensitivity | 2 | 0 |  |  |  |  |  |  |  |  |
| GO:0001823\_mesonephros\_development | 2 | 0 |  |  |  |  |  |  |  |  |
| GO:0001845\_phagolysosome\_formation | 2 | 0 |  |  |  |  |  |  |  |  |
| GO:0001866\_NK\_T\_cell\_proliferation | 2 | 0 |  |  |  |  |  |  |  |  |
| GO:0001879\_detection\_of\_yeast | 2 | 0 |  |  |  |  |  |  |  |  |
| GO:0001886\_endothelial\_cell\_morphogenesis | 2 | 0 |  |  |  |  |  |  |  |  |
| GO:0001919\_regulation\_of\_receptor\_recycling | 2 | 0 |  |  |  |  |  |  |  |  |
| GO:0001954\_positive\_regulation\_of\_cell-matrix\_adhesion | 2 | 0 |  |  |  |  |  |  |  |  |
| GO:0001977\_renal\_system\_process\_involved\_in\_regulation\_of\_blood\_volume | 2 | 0 |  |  |  |  |  |  |  |  |
| GO:0001982\_baroreceptor\_response\_to\_decreased\_systemic\_arterial\_blood\_pressure | 2 | 0 |  |  |  |  |  |  |  |  |
| GO:0001983\_baroreceptor\_response\_to\_increased\_systemic\_arterial\_blood\_pressure | 2 | 0 |  |  |  |  |  |  |  |  |
| GO:0001992\_regulation\_of\_systemic\_arterial\_blood\_pressure\_by\_vasopressin | 2 | 0 |  |  |  |  |  |  |  |  |
| GO:0001997\_positive\_regulation\_of\_the\_force\_of\_heart\_contraction\_by\_epinephrine-norepinephrine | 2 | 0 |  |  |  |  |  |  |  |  |
| GO:0001998\_angiotensin\_mediated\_vasoconstriction\_involved\_in\_regulation\_of\_systemic\_arterial\_blood\_pressure | 2 | 0 |  |  |  |  |  |  |  |  |
| GO:0001999\_renal\_response\_to\_blood\_flow\_during\_renin-angiotensin\_regulation\_of\_systemic\_arterial\_blood\_pressure | 2 | 0 |  |  |  |  |  |  |  |  |
| GO:0002018\_renin-angiotensin\_regulation\_of\_aldosterone\_production | 2 | 0 |  |  |  |  |  |  |  |  |
| GO:0002019\_regulation\_of\_renal\_output\_by\_angiotensin | 2 | 0 |  |  |  |  |  |  |  |  |
| GO:0002024\_diet\_induced\_thermogenesis | 2 | 0 |  |  |  |  |  |  |  |  |
| GO:0002025\_vasodilation\_by\_norepinephrine-epinephrine\_involved\_in\_regulation\_of\_systemic\_arterial\_blood\_pressure | 2 | 0 |  |  |  |  |  |  |  |  |
| GO:0002029\_desensitization\_of\_G-protein\_coupled\_receptor\_protein\_signaling\_pathway | 2 | 0 |  |  |  |  |  |  |  |  |
| GO:0002033\_vasodilation\_by\_angiotensin\_involved\_in\_regulation\_of\_systemic\_arterial\_blood\_pressure | 2 | 0 |  |  |  |  |  |  |  |  |
| GO:0002066\_columnar\_cuboidal\_epithelial\_cell\_development | 2 | 0 |  |  |  |  |  |  |  |  |
| GO:0002072\_optic\_cup\_morphogenesis\_involved\_in\_camera-type\_eye\_development | 2 | 0 |  |  |  |  |  |  |  |  |
| GO:0002074\_extraocular\_skeletal\_muscle\_development | 2 | 0 |  |  |  |  |  |  |  |  |
| GO:0002138\_retinoic\_acid\_biosynthetic\_process | 2 | 0 |  |  |  |  |  |  |  |  |
| GO:0002223\_stimulatory\_C-type\_lectin\_receptor\_signaling\_pathway | 2 | 0 |  |  |  |  |  |  |  |  |
| GO:0002246\_healing\_during\_inflammatory\_response | 2 | 0 |  |  |  |  |  |  |  |  |
| GO:0002251\_organ\_or\_tissue\_specific\_immune\_response | 2 | 0 |  |  |  |  |  |  |  |  |
| GO:0002266\_follicular\_dendritic\_cell\_activation | 2 | 0 |  |  |  |  |  |  |  |  |
| GO:0002268\_follicular\_dendritic\_cell\_differentiation | 2 | 0 |  |  |  |  |  |  |  |  |
| GO:0002327\_immature\_B\_cell\_differentiation | 2 | 0 |  |  |  |  |  |  |  |  |
| GO:0002329\_pre-B\_cell\_differentiation | 2 | 0 |  |  |  |  |  |  |  |  |
| GO:0002339\_B\_cell\_selection | 2 | 0 |  |  |  |  |  |  |  |  |
| GO:0002352\_B\_cell\_negative\_selection | 2 | 0 |  |  |  |  |  |  |  |  |
| GO:0002358\_B\_cell\_homeostatic\_proliferation | 2 | 0 |  |  |  |  |  |  |  |  |
| GO:0002385\_mucosal\_immune\_response | 2 | 0 |  |  |  |  |  |  |  |  |
| GO:0002514\_B\_cell\_tolerance\_induction | 2 | 0 |  |  |  |  |  |  |  |  |
| GO:0002523\_leukocyte\_migration\_during\_inflammatory\_response | 2 | 0 |  |  |  |  |  |  |  |  |
| GO:0002536\_respiratory\_burst\_during\_acute\_inflammatory\_response | 2 | 0 |  |  |  |  |  |  |  |  |
| GO:0002537\_production\_of\_nitric\_oxide\_during\_acute\_inflammatory\_response | 2 | 0 |  |  |  |  |  |  |  |  |
| GO:0002576\_platelet\_degranulation | 2 | 0 |  |  |  |  |  |  |  |  |
| GO:0002639\_positive\_regulation\_of\_immunoglobulin\_production | 2 | 0 |  |  |  |  |  |  |  |  |
| GO:0002661\_regulation\_of\_B\_cell\_tolerance\_induction | 2 | 0 |  |  |  |  |  |  |  |  |
| GO:0002663\_positive\_regulation\_of\_B\_cell\_tolerance\_induction | 2 | 0 |  |  |  |  |  |  |  |  |
| GO:0002676\_regulation\_of\_chronic\_inflammatory\_response | 2 | 0 |  |  |  |  |  |  |  |  |
| GO:0002679\_respiratory\_burst\_during\_defense\_response | 2 | 0 |  |  |  |  |  |  |  |  |
| GO:0002686\_negative\_regulation\_of\_leukocyte\_migration | 2 | 0 |  |  |  |  |  |  |  |  |
| GO:0002720\_positive\_regulation\_of\_cytokine\_production\_during\_immune\_response | 2 | 0 |  |  |  |  |  |  |  |  |
| GO:0002752\_cell\_surface\_pattern\_recognition\_receptor\_signaling\_pathway | 2 | 0 |  |  |  |  |  |  |  |  |
| GO:0002755\_MyD88-dependent\_toll-like\_receptor\_signaling\_pathway | 2 | 0 |  |  |  |  |  |  |  |  |
| GO:0002765\_immune\_response-inhibiting\_signal\_transduction | 2 | 0 |  |  |  |  |  |  |  |  |
| GO:0002921\_negative\_regulation\_of\_humoral\_immune\_response | 2 | 0 |  |  |  |  |  |  |  |  |
| GO:0002922\_positive\_regulation\_of\_humoral\_immune\_response | 2 | 0 |  |  |  |  |  |  |  |  |
| GO:0002924\_negative\_regulation\_of\_humoral\_immune\_response\_mediated\_by\_circulating\_immunoglobulin | 2 | 0 |  |  |  |  |  |  |  |  |
| GO:0002925\_positive\_regulation\_of\_humoral\_immune\_response\_mediated\_by\_circulating\_immunoglobulin | 2 | 0 |  |  |  |  |  |  |  |  |
| GO:0003057\_regulation\_of\_the\_force\_of\_heart\_contraction\_by\_chemical\_signal | 2 | 0 |  |  |  |  |  |  |  |  |
| GO:0003099\_positive\_regulation\_of\_the\_force\_of\_heart\_contraction\_by\_chemical\_signal | 2 | 0 |  |  |  |  |  |  |  |  |
| GO:0005981\_regulation\_of\_glycogen\_catabolic\_process | 2 | 0 |  |  |  |  |  |  |  |  |
| GO:0006021\_inositol\_biosynthetic\_process | 2 | 0 |  |  |  |  |  |  |  |  |
| GO:0006042\_glucosamine\_biosynthetic\_process | 2 | 0 |  |  |  |  |  |  |  |  |
| GO:0006045\_N-acetylglucosamine\_biosynthetic\_process | 2 | 0 |  |  |  |  |  |  |  |  |
| GO:0006048\_UDP-N-acetylglucosamine\_biosynthetic\_process | 2 | 0 |  |  |  |  |  |  |  |  |
| GO:0006054\_N-acetylneuraminate\_metabolic\_process | 2 | 0 |  |  |  |  |  |  |  |  |
| GO:0006059\_hexitol\_metabolic\_process | 2 | 0 |  |  |  |  |  |  |  |  |
| GO:0006063\_uronic\_acid\_metabolic\_process | 2 | 0 |  |  |  |  |  |  |  |  |
| GO:0006068\_ethanol\_catabolic\_process | 2 | 0 |  |  |  |  |  |  |  |  |
| GO:0006083\_acetate\_metabolic\_process | 2 | 0 |  |  |  |  |  |  |  |  |
| GO:0006089\_lactate\_metabolic\_process | 2 | 0 |  |  |  |  |  |  |  |  |
| GO:0006105\_succinate\_metabolic\_process | 2 | 0 |  |  |  |  |  |  |  |  |
| GO:0006106\_fumarate\_metabolic\_process | 2 | 0 |  |  |  |  |  |  |  |  |
| GO:0006110\_regulation\_of\_glycolysis | 2 | 0 |  |  |  |  |  |  |  |  |
| GO:0006113\_fermentation | 2 | 0 |  |  |  |  |  |  |  |  |
| GO:0006114\_glycerol\_biosynthetic\_process | 2 | 0 |  |  |  |  |  |  |  |  |
| GO:0006122\_mitochondrial\_electron\_transport\_\_ubiquinol\_to\_cytochrome\_c | 2 | 0 |  |  |  |  |  |  |  |  |
| GO:0006152\_purine\_nucleoside\_catabolic\_process | 2 | 0 |  |  |  |  |  |  |  |  |
| GO:0006168\_adenine\_salvage | 2 | 0 |  |  |  |  |  |  |  |  |
| GO:0006200\_ATP\_catabolic\_process | 2 | 0 |  |  |  |  |  |  |  |  |
| GO:0006206\_pyrimidine\_base\_metabolic\_process | 2 | 0 |  |  |  |  |  |  |  |  |
| GO:0006213\_pyrimidine\_nucleoside\_metabolic\_process | 2 | 0 |  |  |  |  |  |  |  |  |
| GO:0006265\_DNA\_topological\_change | 2 | 0 |  |  |  |  |  |  |  |  |
| GO:0006278\_RNA-dependent\_DNA\_replication | 2 | 0 |  |  |  |  |  |  |  |  |
| GO:0006312\_mitotic\_recombination | 2 | 0 |  |  |  |  |  |  |  |  |
| GO:0006398\_histone\_mRNA\_3'-end\_processing | 2 | 0 |  |  |  |  |  |  |  |  |
| GO:0006418\_tRNA\_aminoacylation\_for\_protein\_translation | 2 | 0 |  |  |  |  |  |  |  |  |
| GO:0006451\_translational\_readthrough | 2 | 0 |  |  |  |  |  |  |  |  |
| GO:0006477\_protein\_amino\_acid\_sulfation | 2 | 0 |  |  |  |  |  |  |  |  |
| GO:0006482\_protein\_amino\_acid\_demethylation | 2 | 0 |  |  |  |  |  |  |  |  |
| GO:0006499\_N-terminal\_protein\_myristoylation | 2 | 0 |  |  |  |  |  |  |  |  |
| GO:0006525\_arginine\_metabolic\_process | 2 | 0 |  |  |  |  |  |  |  |  |
| GO:0006527\_arginine\_catabolic\_process | 2 | 0 |  |  |  |  |  |  |  |  |
| GO:0006532\_aspartate\_biosynthetic\_process | 2 | 0 |  |  |  |  |  |  |  |  |
| GO:0006538\_glutamate\_catabolic\_process | 2 | 0 |  |  |  |  |  |  |  |  |
| GO:0006558\_L-phenylalanine\_metabolic\_process | 2 | 0 |  |  |  |  |  |  |  |  |
| GO:0006563\_L-serine\_metabolic\_process | 2 | 0 |  |  |  |  |  |  |  |  |
| GO:0006566\_threonine\_metabolic\_process | 2 | 0 |  |  |  |  |  |  |  |  |
| GO:0006568\_tryptophan\_metabolic\_process | 2 | 0 |  |  |  |  |  |  |  |  |
| GO:0006583\_melanin\_biosynthetic\_process\_from\_tyrosine | 2 | 0 |  |  |  |  |  |  |  |  |
| GO:0006600\_creatine\_metabolic\_process | 2 | 0 |  |  |  |  |  |  |  |  |
| GO:0006603\_phosphocreatine\_metabolic\_process | 2 | 0 |  |  |  |  |  |  |  |  |
| GO:0006610\_ribosomal\_protein\_import\_into\_nucleus | 2 | 0 |  |  |  |  |  |  |  |  |
| GO:0006642\_triglyceride\_mobilization | 2 | 0 |  |  |  |  |  |  |  |  |
| GO:0006649\_phospholipid\_transfer\_to\_membrane | 2 | 0 |  |  |  |  |  |  |  |  |
| GO:0006681\_galactosylceramide\_metabolic\_process | 2 | 0 |  |  |  |  |  |  |  |  |
| GO:0006686\_sphingomyelin\_biosynthetic\_process | 2 | 0 |  |  |  |  |  |  |  |  |
| GO:0006702\_androgen\_biosynthetic\_process | 2 | 0 |  |  |  |  |  |  |  |  |
| GO:0006750\_glutathione\_biosynthetic\_process | 2 | 0 |  |  |  |  |  |  |  |  |
| GO:0006760\_folic\_acid\_and\_derivative\_metabolic\_process | 2 | 0 |  |  |  |  |  |  |  |  |
| GO:0006808\_regulation\_of\_nitrogen\_utilization | 2 | 0 |  |  |  |  |  |  |  |  |
| GO:0006868\_glutamine\_transport | 2 | 0 |  |  |  |  |  |  |  |  |
| GO:0006907\_pinocytosis | 2 | 0 |  |  |  |  |  |  |  |  |
| GO:0006925\_inflammatory\_cell\_apoptosis | 2 | 0 |  |  |  |  |  |  |  |  |
| GO:0006977\_DNA\_damage\_response\_\_signal\_transduction\_by\_p53\_class\_mediator\_resulting\_in\_cell\_cycle\_arrest | 2 | 0 |  |  |  |  |  |  |  |  |
| GO:0006991\_response\_to\_sterol\_depletion | 2 | 0 |  |  |  |  |  |  |  |  |
| GO:0007004\_telomere\_maintenance\_via\_telomerase | 2 | 0 |  |  |  |  |  |  |  |  |
| GO:0007020\_microtubule\_nucleation | 2 | 0 |  |  |  |  |  |  |  |  |
| GO:0007030\_Golgi\_organization | 2 | 0 |  |  |  |  |  |  |  |  |
| GO:0007035\_vacuolar\_acidification | 2 | 0 |  |  |  |  |  |  |  |  |
| GO:0007042\_lysosomal\_lumen\_acidification | 2 | 0 |  |  |  |  |  |  |  |  |
| GO:0007060\_male\_meiosis\_chromosome\_segregation | 2 | 0 |  |  |  |  |  |  |  |  |
| GO:0007089\_traversing\_start\_control\_point\_of\_mitotic\_cell\_cycle | 2 | 0 |  |  |  |  |  |  |  |  |
| GO:0007094\_mitotic\_cell\_cycle\_spindle\_assembly\_checkpoint | 2 | 0 |  |  |  |  |  |  |  |  |
| GO:0007097\_nuclear\_migration | 2 | 0 |  |  |  |  |  |  |  |  |
| GO:0007100\_mitotic\_centrosome\_separation | 2 | 0 |  |  |  |  |  |  |  |  |
| GO:0007132\_meiotic\_metaphase\_I | 2 | 0 |  |  |  |  |  |  |  |  |
| GO:0007171\_activation\_of\_transmembrane\_receptor\_protein\_tyrosine\_kinase\_activity | 2 | 0 |  |  |  |  |  |  |  |  |
| GO:0007182\_common-partner\_SMAD\_protein\_phosphorylation | 2 | 0 |  |  |  |  |  |  |  |  |
| GO:0007185\_transmembrane\_receptor\_protein\_tyrosine\_phosphatase\_signaling\_pathway | 2 | 0 |  |  |  |  |  |  |  |  |
| GO:0007205\_activation\_of\_protein\_kinase\_C\_activity\_by\_G-protein\_coupled\_receptor\_protein\_signaling\_pathway | 2 | 0 |  |  |  |  |  |  |  |  |
| GO:0007210\_serotonin\_receptor\_signaling\_pathway | 2 | 0 |  |  |  |  |  |  |  |  |
| GO:0007220\_Notch\_receptor\_processing | 2 | 0 |  |  |  |  |  |  |  |  |
| GO:0007256\_activation\_of\_JNKK\_activity | 2 | 0 |  |  |  |  |  |  |  |  |
| GO:0007258\_JUN\_phosphorylation | 2 | 0 |  |  |  |  |  |  |  |  |
| GO:0007263\_nitric\_oxide\_mediated\_signal\_transduction | 2 | 0 |  |  |  |  |  |  |  |  |
| GO:0007289\_spermatid\_nucleus\_differentiation | 2 | 0 |  |  |  |  |  |  |  |  |
| GO:0007343\_egg\_activation | 2 | 0 |  |  |  |  |  |  |  |  |
| GO:0007351\_tripartite\_regional\_subdivision | 2 | 0 |  |  |  |  |  |  |  |  |
| GO:0007418\_ventral\_midline\_development | 2 | 0 |  |  |  |  |  |  |  |  |
| GO:0007494\_midgut\_development | 2 | 0 |  |  |  |  |  |  |  |  |
| GO:0007527\_adult\_somatic\_muscle\_development | 2 | 0 |  |  |  |  |  |  |  |  |
| GO:0007549\_dosage\_compensation | 2 | 0 |  |  |  |  |  |  |  |  |
| GO:0007603\_phototransduction\_\_visible\_light | 2 | 0 |  |  |  |  |  |  |  |  |
| GO:0007619\_courtship\_behavior | 2 | 0 |  |  |  |  |  |  |  |  |
| GO:0008065\_establishment\_of\_blood-nerve\_barrier | 2 | 0 |  |  |  |  |  |  |  |  |
| GO:0008089\_anterograde\_axon\_cargo\_transport | 2 | 0 |  |  |  |  |  |  |  |  |
| GO:0008210\_estrogen\_metabolic\_process | 2 | 0 |  |  |  |  |  |  |  |  |
| GO:0008212\_mineralocorticoid\_metabolic\_process | 2 | 0 |  |  |  |  |  |  |  |  |
| GO:0008214\_protein\_amino\_acid\_dealkylation | 2 | 0 |  |  |  |  |  |  |  |  |
| GO:0008228\_opsonization | 2 | 0 |  |  |  |  |  |  |  |  |
| GO:0008272\_sulfate\_transport | 2 | 0 |  |  |  |  |  |  |  |  |
| GO:0008291\_acetylcholine\_metabolic\_process | 2 | 0 |  |  |  |  |  |  |  |  |
| GO:0008298\_intracellular\_mRNA\_localization | 2 | 0 |  |  |  |  |  |  |  |  |
| GO:0008334\_histone\_mRNA\_metabolic\_process | 2 | 0 |  |  |  |  |  |  |  |  |
| GO:0008356\_asymmetric\_cell\_division | 2 | 0 |  |  |  |  |  |  |  |  |
| GO:0008582\_regulation\_of\_synaptic\_growth\_at\_neuromuscular\_junction | 2 | 0 |  |  |  |  |  |  |  |  |
| GO:0008594\_photoreceptor\_cell\_morphogenesis | 2 | 0 |  |  |  |  |  |  |  |  |
| GO:0008595\_determination\_of\_anterior\_posterior\_axis\_\_embryo | 2 | 0 |  |  |  |  |  |  |  |  |
| GO:0008608\_attachment\_of\_spindle\_microtubules\_to\_kinetochore | 2 | 0 |  |  |  |  |  |  |  |  |
| GO:0008616\_queuosine\_biosynthetic\_process | 2 | 0 |  |  |  |  |  |  |  |  |
| GO:0008617\_guanosine\_metabolic\_process | 2 | 0 |  |  |  |  |  |  |  |  |
| GO:0008618\_7-methylguanosine\_metabolic\_process | 2 | 0 |  |  |  |  |  |  |  |  |
| GO:0008634\_negative\_regulation\_of\_survival\_gene\_product\_expression | 2 | 0 |  |  |  |  |  |  |  |  |
| GO:0009048\_dosage\_compensation\_\_by\_inactivation\_of\_X\_chromosome | 2 | 0 |  |  |  |  |  |  |  |  |
| GO:0009070\_serine\_family\_amino\_acid\_biosynthetic\_process | 2 | 0 |  |  |  |  |  |  |  |  |
| GO:0009071\_serine\_family\_amino\_acid\_catabolic\_process | 2 | 0 |  |  |  |  |  |  |  |  |
| GO:0009074\_aromatic\_amino\_acid\_family\_catabolic\_process | 2 | 0 |  |  |  |  |  |  |  |  |
| GO:0009083\_branched\_chain\_family\_amino\_acid\_catabolic\_process | 2 | 0 |  |  |  |  |  |  |  |  |
| GO:0009093\_cysteine\_catabolic\_process | 2 | 0 |  |  |  |  |  |  |  |  |
| GO:0009120\_deoxyribonucleoside\_metabolic\_process | 2 | 0 |  |  |  |  |  |  |  |  |
| GO:0009125\_nucleoside\_monophosphate\_catabolic\_process | 2 | 0 |  |  |  |  |  |  |  |  |
| GO:0009126\_purine\_nucleoside\_monophosphate\_metabolic\_process | 2 | 0 |  |  |  |  |  |  |  |  |
| GO:0009142\_nucleoside\_triphosphate\_biosynthetic\_process | 2 | 0 |  |  |  |  |  |  |  |  |
| GO:0009161\_ribonucleoside\_monophosphate\_metabolic\_process | 2 | 0 |  |  |  |  |  |  |  |  |
| GO:0009164\_nucleoside\_catabolic\_process | 2 | 0 |  |  |  |  |  |  |  |  |
| GO:0009167\_purine\_ribonucleoside\_monophosphate\_metabolic\_process | 2 | 0 |  |  |  |  |  |  |  |  |
| GO:0009202\_deoxyribonucleoside\_triphosphate\_biosynthetic\_process | 2 | 0 |  |  |  |  |  |  |  |  |
| GO:0009203\_ribonucleoside\_triphosphate\_catabolic\_process | 2 | 0 |  |  |  |  |  |  |  |  |
| GO:0009207\_purine\_ribonucleoside\_triphosphate\_catabolic\_process | 2 | 0 |  |  |  |  |  |  |  |  |
| GO:0009219\_pyrimidine\_deoxyribonucleotide\_metabolic\_process | 2 | 0 |  |  |  |  |  |  |  |  |
| GO:0009265\_2'-deoxyribonucleotide\_biosynthetic\_process | 2 | 0 |  |  |  |  |  |  |  |  |
| GO:0009268\_response\_to\_pH | 2 | 0 |  |  |  |  |  |  |  |  |
| GO:0009313\_oligosaccharide\_catabolic\_process | 2 | 0 |  |  |  |  |  |  |  |  |
| GO:0009395\_phospholipid\_catabolic\_process | 2 | 0 |  |  |  |  |  |  |  |  |
| GO:0009435\_NAD\_biosynthetic\_process | 2 | 0 |  |  |  |  |  |  |  |  |
| GO:0009608\_response\_to\_symbiont | 2 | 0 |  |  |  |  |  |  |  |  |
| GO:0009609\_response\_to\_symbiotic\_bacterium | 2 | 0 |  |  |  |  |  |  |  |  |
| GO:0009649\_entrainment\_of\_circadian\_clock | 2 | 0 |  |  |  |  |  |  |  |  |
| GO:0009996\_negative\_regulation\_of\_cell\_fate\_specification | 2 | 0 |  |  |  |  |  |  |  |  |
| GO:0010002\_cardioblast\_differentiation | 2 | 0 |  |  |  |  |  |  |  |  |
| GO:0010225\_response\_to\_UV-C | 2 | 0 |  |  |  |  |  |  |  |  |
| GO:0010389\_regulation\_of\_G2\_M\_transition\_of\_mitotic\_cell\_cycle | 2 | 0 |  |  |  |  |  |  |  |  |
| GO:0010458\_exit\_from\_mitosis | 2 | 0 |  |  |  |  |  |  |  |  |
| GO:0010459\_negative\_regulation\_of\_heart\_rate | 2 | 0 |  |  |  |  |  |  |  |  |
| GO:0010559\_regulation\_of\_glycoprotein\_biosynthetic\_process | 2 | 0 |  |  |  |  |  |  |  |  |
| GO:0010633\_negative\_regulation\_of\_epithelial\_cell\_migration | 2 | 0 |  |  |  |  |  |  |  |  |
| GO:0010677\_negative\_regulation\_of\_cellular\_carbohydrate\_metabolic\_process | 2 | 0 |  |  |  |  |  |  |  |  |
| GO:0010718\_positive\_regulation\_of\_epithelial\_to\_mesenchymal\_transition | 2 | 0 |  |  |  |  |  |  |  |  |
| GO:0010742\_foam\_cell\_differentiation | 2 | 0 |  |  |  |  |  |  |  |  |
| GO:0010743\_regulation\_of\_foam\_cell\_differentiation | 2 | 0 |  |  |  |  |  |  |  |  |
| GO:0010744\_positive\_regulation\_of\_foam\_cell\_differentiation | 2 | 0 |  |  |  |  |  |  |  |  |
| GO:0010765\_positive\_regulation\_of\_sodium\_ion\_transport | 2 | 0 |  |  |  |  |  |  |  |  |
| GO:0010766\_negative\_regulation\_of\_sodium\_ion\_transport | 2 | 0 |  |  |  |  |  |  |  |  |
| GO:0010770\_positive\_regulation\_of\_cell\_morphogenesis\_involved\_in\_differentiation | 2 | 0 |  |  |  |  |  |  |  |  |
| GO:0010771\_negative\_regulation\_of\_cell\_morphogenesis\_involved\_in\_differentiation | 2 | 0 |  |  |  |  |  |  |  |  |
| GO:0010824\_regulation\_of\_centrosome\_duplication | 2 | 0 |  |  |  |  |  |  |  |  |
| GO:0010833\_telomere\_maintenance\_via\_telomere\_lengthening | 2 | 0 |  |  |  |  |  |  |  |  |
| GO:0010862\_positive\_regulation\_of\_pathway-restricted\_SMAD\_protein\_phosphorylation | 2 | 0 |  |  |  |  |  |  |  |  |
| GO:0010872\_regulation\_of\_cholesterol\_esterification | 2 | 0 |  |  |  |  |  |  |  |  |
| GO:0010878\_cholesterol\_storage | 2 | 0 |  |  |  |  |  |  |  |  |
| GO:0010885\_regulation\_of\_cholesterol\_storage | 2 | 0 |  |  |  |  |  |  |  |  |
| GO:0010886\_positive\_regulation\_of\_cholesterol\_storage | 2 | 0 |  |  |  |  |  |  |  |  |
| GO:0010891\_negative\_regulation\_of\_sequestering\_of\_triglyceride | 2 | 0 |  |  |  |  |  |  |  |  |
| GO:0010896\_regulation\_of\_triglyceride\_catabolic\_process | 2 | 0 |  |  |  |  |  |  |  |  |
| GO:0010898\_positive\_regulation\_of\_triglyceride\_catabolic\_process | 2 | 0 |  |  |  |  |  |  |  |  |
| GO:0010907\_positive\_regulation\_of\_glucose\_metabolic\_process | 2 | 0 |  |  |  |  |  |  |  |  |
| GO:0014028\_notochord\_formation | 2 | 0 |  |  |  |  |  |  |  |  |
| GO:0014048\_regulation\_of\_glutamate\_secretion | 2 | 0 |  |  |  |  |  |  |  |  |
| GO:0014052\_regulation\_of\_gamma-aminobutyric\_acid\_secretion | 2 | 0 |  |  |  |  |  |  |  |  |
| GO:0014054\_positive\_regulation\_of\_gamma-aminobutyric\_acid\_secretion | 2 | 0 |  |  |  |  |  |  |  |  |
| GO:0014055\_acetylcholine\_secretion | 2 | 0 |  |  |  |  |  |  |  |  |
| GO:0014056\_regulation\_of\_acetylcholine\_secretion | 2 | 0 |  |  |  |  |  |  |  |  |
| GO:0014067\_negative\_regulation\_of\_phosphoinositide\_3-kinase\_cascade | 2 | 0 |  |  |  |  |  |  |  |  |
| GO:0014745\_negative\_regulation\_of\_muscle\_adaptation | 2 | 0 |  |  |  |  |  |  |  |  |
| GO:0014829\_vascular\_smooth\_muscle\_contraction | 2 | 0 |  |  |  |  |  |  |  |  |
| GO:0014850\_response\_to\_muscle\_activity | 2 | 0 |  |  |  |  |  |  |  |  |
| GO:0014866\_skeletal\_myofibril\_assembly | 2 | 0 |  |  |  |  |  |  |  |  |
| GO:0014888\_striated\_muscle\_adaptation | 2 | 0 |  |  |  |  |  |  |  |  |
| GO:0014916\_regulation\_of\_lung\_blood\_pressure | 2 | 0 |  |  |  |  |  |  |  |  |
| GO:0015671\_oxygen\_transport | 2 | 0 |  |  |  |  |  |  |  |  |
| GO:0015696\_ammonium\_transport | 2 | 0 |  |  |  |  |  |  |  |  |
| GO:0015732\_prostaglandin\_transport | 2 | 0 |  |  |  |  |  |  |  |  |
| GO:0015819\_lysine\_transport | 2 | 0 |  |  |  |  |  |  |  |  |
| GO:0015840\_urea\_transport | 2 | 0 |  |  |  |  |  |  |  |  |
| GO:0015860\_purine\_nucleoside\_transport | 2 | 0 |  |  |  |  |  |  |  |  |
| GO:0015870\_acetylcholine\_transport | 2 | 0 |  |  |  |  |  |  |  |  |
| GO:0015937\_coenzyme\_A\_biosynthetic\_process | 2 | 0 |  |  |  |  |  |  |  |  |
| GO:0016045\_detection\_of\_bacterium | 2 | 0 |  |  |  |  |  |  |  |  |
| GO:0016046\_detection\_of\_fungus | 2 | 0 |  |  |  |  |  |  |  |  |
| GO:0016080\_synaptic\_vesicle\_targeting | 2 | 0 |  |  |  |  |  |  |  |  |
| GO:0016199\_axon\_midline\_choice\_point\_recognition | 2 | 0 |  |  |  |  |  |  |  |  |
| GO:0016226\_iron-sulfur\_cluster\_assembly | 2 | 0 |  |  |  |  |  |  |  |  |
| GO:0016233\_telomere\_capping | 2 | 0 |  |  |  |  |  |  |  |  |
| GO:0016242\_negative\_regulation\_of\_macroautophagy | 2 | 0 |  |  |  |  |  |  |  |  |
| GO:0016441\_posttranscriptional\_gene\_silencing | 2 | 0 |  |  |  |  |  |  |  |  |
| GO:0016540\_protein\_autoprocessing | 2 | 0 |  |  |  |  |  |  |  |  |
| GO:0016558\_protein\_import\_into\_peroxisome\_matrix | 2 | 0 |  |  |  |  |  |  |  |  |
| GO:0016572\_histone\_phosphorylation | 2 | 0 |  |  |  |  |  |  |  |  |
| GO:0016577\_histone\_demethylation | 2 | 0 |  |  |  |  |  |  |  |  |
| GO:0016926\_protein\_desumoylation | 2 | 0 |  |  |  |  |  |  |  |  |
| GO:0017014\_protein\_amino\_acid\_nitrosylation | 2 | 0 |  |  |  |  |  |  |  |  |
| GO:0017144\_drug\_metabolic\_process | 2 | 0 |  |  |  |  |  |  |  |  |
| GO:0018094\_protein\_polyglycylation | 2 | 0 |  |  |  |  |  |  |  |  |
| GO:0018119\_peptidyl-cysteine\_S-nitrosylation | 2 | 0 |  |  |  |  |  |  |  |  |
| GO:0018125\_peptidyl-cysteine\_methylation | 2 | 0 |  |  |  |  |  |  |  |  |
| GO:0018205\_peptidyl-lysine\_modification | 2 | 0 |  |  |  |  |  |  |  |  |
| GO:0018319\_protein\_amino\_acid\_myristoylation | 2 | 0 |  |  |  |  |  |  |  |  |
| GO:0018377\_protein\_myristoylation | 2 | 0 |  |  |  |  |  |  |  |  |
| GO:0018401\_peptidyl-proline\_hydroxylation\_to\_4-hydroxy-L-proline | 2 | 0 |  |  |  |  |  |  |  |  |
| GO:0018993\_somatic\_sex\_determination | 2 | 0 |  |  |  |  |  |  |  |  |
| GO:0019067\_viral\_assembly\_\_maturation\_\_egress\_\_and\_release | 2 | 0 |  |  |  |  |  |  |  |  |
| GO:0019322\_pentose\_biosynthetic\_process | 2 | 0 |  |  |  |  |  |  |  |  |
| GO:0019370\_leukotriene\_biosynthetic\_process | 2 | 0 |  |  |  |  |  |  |  |  |
| GO:0019374\_galactolipid\_metabolic\_process | 2 | 0 |  |  |  |  |  |  |  |  |
| GO:0019401\_alditol\_biosynthetic\_process | 2 | 0 |  |  |  |  |  |  |  |  |
| GO:0019448\_L-cysteine\_catabolic\_process | 2 | 0 |  |  |  |  |  |  |  |  |
| GO:0019452\_L-cysteine\_catabolic\_process\_to\_taurine | 2 | 0 |  |  |  |  |  |  |  |  |
| GO:0019471\_4-hydroxyproline\_metabolic\_process | 2 | 0 |  |  |  |  |  |  |  |  |
| GO:0019511\_peptidyl-proline\_hydroxylation | 2 | 0 |  |  |  |  |  |  |  |  |
| GO:0019550\_glutamate\_catabolic\_process\_to\_aspartate | 2 | 0 |  |  |  |  |  |  |  |  |
| GO:0019551\_glutamate\_catabolic\_process\_to\_2-oxoglutarate | 2 | 0 |  |  |  |  |  |  |  |  |
| GO:0019585\_glucuronate\_metabolic\_process | 2 | 0 |  |  |  |  |  |  |  |  |
| GO:0019730\_antimicrobial\_humoral\_response | 2 | 0 |  |  |  |  |  |  |  |  |
| GO:0019740\_nitrogen\_utilization | 2 | 0 |  |  |  |  |  |  |  |  |
| GO:0019853\_L-ascorbic\_acid\_biosynthetic\_process | 2 | 0 |  |  |  |  |  |  |  |  |
| GO:0021506\_anterior\_neuropore\_closure | 2 | 0 |  |  |  |  |  |  |  |  |
| GO:0021524\_visceral\_motor\_neuron\_differentiation | 2 | 0 |  |  |  |  |  |  |  |  |
| GO:0021526\_medial\_motor\_column\_neuron\_differentiation | 2 | 0 |  |  |  |  |  |  |  |  |
| GO:0021557\_oculomotor\_nerve\_development | 2 | 0 |  |  |  |  |  |  |  |  |
| GO:0021558\_trochlear\_nerve\_development | 2 | 0 |  |  |  |  |  |  |  |  |
| GO:0021562\_vestibulocochlear\_nerve\_development | 2 | 0 |  |  |  |  |  |  |  |  |
| GO:0021568\_rhombomere\_2\_development | 2 | 0 |  |  |  |  |  |  |  |  |
| GO:0021578\_hindbrain\_maturation | 2 | 0 |  |  |  |  |  |  |  |  |
| GO:0021593\_rhombomere\_morphogenesis | 2 | 0 |  |  |  |  |  |  |  |  |
| GO:0021626\_central\_nervous\_system\_maturation | 2 | 0 |  |  |  |  |  |  |  |  |
| GO:0021658\_rhombomere\_3\_morphogenesis | 2 | 0 |  |  |  |  |  |  |  |  |
| GO:0021754\_facial\_nucleus\_development | 2 | 0 |  |  |  |  |  |  |  |  |
| GO:0021775\_smoothened\_signaling\_pathway\_involved\_in\_ventral\_spinal\_cord\_interneuron\_specification | 2 | 0 |  |  |  |  |  |  |  |  |
| GO:0021776\_smoothened\_signaling\_pathway\_involved\_in\_spinal\_cord\_motor\_neuron\_cell\_fate\_specification | 2 | 0 |  |  |  |  |  |  |  |  |
| GO:0021796\_cerebral\_cortex\_regionalization | 2 | 0 |  |  |  |  |  |  |  |  |
| GO:0021831\_embryonic\_olfactory\_bulb\_interneuron\_precursor\_migration | 2 | 0 |  |  |  |  |  |  |  |  |
| GO:0021869\_forebrain\_ventricular\_zone\_progenitor\_cell\_division | 2 | 0 |  |  |  |  |  |  |  |  |
| GO:0021873\_forebrain\_neuroblast\_division | 2 | 0 |  |  |  |  |  |  |  |  |
| GO:0021882\_regulation\_of\_transcription\_from\_RNA\_polymerase\_II\_promoter\_involved\_in\_forebrain\_neuron\_fate\_commitment | 2 | 0 |  |  |  |  |  |  |  |  |
| GO:0021893\_cerebral\_cortex\_GABAergic\_interneuron\_fate\_commitment | 2 | 0 |  |  |  |  |  |  |  |  |
| GO:0021898\_commitment\_of\_multipotent\_stem\_cells\_to\_the\_neuronal\_lineage\_in\_the\_forebrain | 2 | 0 |  |  |  |  |  |  |  |  |
| GO:0021932\_hindbrain\_radial\_glia\_guided\_cell\_migration | 2 | 0 |  |  |  |  |  |  |  |  |
| GO:0021965\_spinal\_cord\_ventral\_commissure\_morphogenesis | 2 | 0 |  |  |  |  |  |  |  |  |
| GO:0021985\_neurohypophysis\_development | 2 | 0 |  |  |  |  |  |  |  |  |
| GO:0021990\_neural\_plate\_formation | 2 | 0 |  |  |  |  |  |  |  |  |
| GO:0021995\_neuropore\_closure | 2 | 0 |  |  |  |  |  |  |  |  |
| GO:0022028\_tangential\_migration\_from\_the\_subventricular\_zone\_to\_the\_olfactory\_bulb | 2 | 0 |  |  |  |  |  |  |  |  |
| GO:0022401\_adaptation\_of\_signaling\_pathway | 2 | 0 |  |  |  |  |  |  |  |  |
| GO:0022408\_negative\_regulation\_of\_cell-cell\_adhesion | 2 | 0 |  |  |  |  |  |  |  |  |
| GO:0022410\_circadian\_sleep\_wake\_cycle\_process | 2 | 0 |  |  |  |  |  |  |  |  |
| GO:0030046\_parallel\_actin\_filament\_bundle\_formation | 2 | 0 |  |  |  |  |  |  |  |  |
| GO:0030049\_muscle\_filament\_sliding | 2 | 0 |  |  |  |  |  |  |  |  |
| GO:0030050\_vesicle\_transport\_along\_actin\_filament | 2 | 0 |  |  |  |  |  |  |  |  |
| GO:0030071\_regulation\_of\_mitotic\_metaphase\_anaphase\_transition | 2 | 0 |  |  |  |  |  |  |  |  |
| GO:0030147\_natriuresis | 2 | 0 |  |  |  |  |  |  |  |  |
| GO:0030174\_regulation\_of\_DNA\_replication\_initiation | 2 | 0 |  |  |  |  |  |  |  |  |
| GO:0030219\_megakaryocyte\_differentiation | 2 | 0 |  |  |  |  |  |  |  |  |
| GO:0030223\_neutrophil\_differentiation | 2 | 0 |  |  |  |  |  |  |  |  |
| GO:0030240\_muscle\_thin\_filament\_assembly | 2 | 0 |  |  |  |  |  |  |  |  |
| GO:0030259\_lipid\_glycosylation | 2 | 0 |  |  |  |  |  |  |  |  |
| GO:0030397\_membrane\_disassembly | 2 | 0 |  |  |  |  |  |  |  |  |
| GO:0030502\_negative\_regulation\_of\_bone\_mineralization | 2 | 0 |  |  |  |  |  |  |  |  |
| GO:0030644\_cellular\_chloride\_ion\_homeostasis | 2 | 0 |  |  |  |  |  |  |  |  |
| GO:0030825\_positive\_regulation\_of\_cGMP\_metabolic\_process | 2 | 0 |  |  |  |  |  |  |  |  |
| GO:0030828\_positive\_regulation\_of\_cGMP\_biosynthetic\_process | 2 | 0 |  |  |  |  |  |  |  |  |
| GO:0030835\_negative\_regulation\_of\_actin\_filament\_depolymerization | 2 | 0 |  |  |  |  |  |  |  |  |
| GO:0030837\_negative\_regulation\_of\_actin\_filament\_polymerization | 2 | 0 |  |  |  |  |  |  |  |  |
| GO:0030852\_regulation\_of\_granulocyte\_differentiation | 2 | 0 |  |  |  |  |  |  |  |  |
| GO:0030885\_regulation\_of\_myeloid\_dendritic\_cell\_activation | 2 | 0 |  |  |  |  |  |  |  |  |
| GO:0030910\_olfactory\_placode\_formation | 2 | 0 |  |  |  |  |  |  |  |  |
| GO:0030948\_negative\_regulation\_of\_vascular\_endothelial\_growth\_factor\_receptor\_signaling\_pathway | 2 | 0 |  |  |  |  |  |  |  |  |
| GO:0030953\_spindle\_astral\_microtubule\_organization | 2 | 0 |  |  |  |  |  |  |  |  |
| GO:0031050\_dsRNA\_fragmentation | 2 | 0 |  |  |  |  |  |  |  |  |
| GO:0031061\_negative\_regulation\_of\_histone\_methylation | 2 | 0 |  |  |  |  |  |  |  |  |
| GO:0031119\_tRNA\_pseudouridine\_synthesis | 2 | 0 |  |  |  |  |  |  |  |  |
| GO:0031163\_metallo-sulfur\_cluster\_assembly | 2 | 0 |  |  |  |  |  |  |  |  |
| GO:0031223\_auditory\_behavior | 2 | 0 |  |  |  |  |  |  |  |  |
| GO:0031296\_B\_cell\_costimulation | 2 | 0 |  |  |  |  |  |  |  |  |
| GO:0031338\_regulation\_of\_vesicle\_fusion | 2 | 0 |  |  |  |  |  |  |  |  |
| GO:0031573\_intra-S\_DNA\_damage\_checkpoint | 2 | 0 |  |  |  |  |  |  |  |  |
| GO:0031577\_spindle\_checkpoint | 2 | 0 |  |  |  |  |  |  |  |  |
| GO:0031629\_synaptic\_vesicle\_fusion\_to\_presynaptic\_membrane | 2 | 0 |  |  |  |  |  |  |  |  |
| GO:0031630\_regulation\_of\_synaptic\_vesicle\_fusion\_to\_presynaptic\_membrane | 2 | 0 |  |  |  |  |  |  |  |  |
| GO:0031664\_regulation\_of\_lipopolysaccharide-mediated\_signaling\_pathway | 2 | 0 |  |  |  |  |  |  |  |  |
| GO:0031670\_cellular\_response\_to\_nutrient | 2 | 0 |  |  |  |  |  |  |  |  |
| GO:0031848\_protection\_from\_non-homologous\_end\_joining\_at\_telomere | 2 | 0 |  |  |  |  |  |  |  |  |
| GO:0031946\_regulation\_of\_glucocorticoid\_biosynthetic\_process | 2 | 0 |  |  |  |  |  |  |  |  |
| GO:0031952\_regulation\_of\_protein\_amino\_acid\_autophosphorylation | 2 | 0 |  |  |  |  |  |  |  |  |
| GO:0031953\_negative\_regulation\_of\_protein\_amino\_acid\_autophosphorylation | 2 | 0 |  |  |  |  |  |  |  |  |
| GO:0031958\_corticosteroid\_receptor\_signaling\_pathway | 2 | 0 |  |  |  |  |  |  |  |  |
| GO:0031987\_locomotion\_involved\_in\_locomotory\_behavior | 2 | 0 |  |  |  |  |  |  |  |  |
| GO:0032096\_negative\_regulation\_of\_response\_to\_food | 2 | 0 |  |  |  |  |  |  |  |  |
| GO:0032099\_negative\_regulation\_of\_appetite | 2 | 0 |  |  |  |  |  |  |  |  |
| GO:0032106\_positive\_regulation\_of\_response\_to\_extracellular\_stimulus | 2 | 0 |  |  |  |  |  |  |  |  |
| GO:0032109\_positive\_regulation\_of\_response\_to\_nutrient\_levels | 2 | 0 |  |  |  |  |  |  |  |  |
| GO:0032226\_positive\_regulation\_of\_synaptic\_transmission\_\_dopaminergic | 2 | 0 |  |  |  |  |  |  |  |  |
| GO:0032230\_positive\_regulation\_of\_synaptic\_transmission\_\_GABAergic | 2 | 0 |  |  |  |  |  |  |  |  |
| GO:0032234\_regulation\_of\_calcium\_ion\_transport\_via\_store-operated\_calcium\_channel\_activity | 2 | 0 |  |  |  |  |  |  |  |  |
| GO:0032236\_positive\_regulation\_of\_calcium\_ion\_transport\_via\_store-operated\_calcium\_channel\_activity | 2 | 0 |  |  |  |  |  |  |  |  |
| GO:0032297\_negative\_regulation\_of\_DNA\_replication\_initiation | 2 | 0 |  |  |  |  |  |  |  |  |
| GO:0032309\_icosanoid\_secretion | 2 | 0 |  |  |  |  |  |  |  |  |
| GO:0032328\_alanine\_transport | 2 | 0 |  |  |  |  |  |  |  |  |
| GO:0032341\_aldosterone\_metabolic\_process | 2 | 0 |  |  |  |  |  |  |  |  |
| GO:0032351\_negative\_regulation\_of\_hormone\_metabolic\_process | 2 | 0 |  |  |  |  |  |  |  |  |
| GO:0032353\_negative\_regulation\_of\_hormone\_biosynthetic\_process | 2 | 0 |  |  |  |  |  |  |  |  |
| GO:0032435\_negative\_regulation\_of\_proteasomal\_ubiquitin-dependent\_protein\_catabolic\_process | 2 | 0 |  |  |  |  |  |  |  |  |
| GO:0032471\_reduction\_of\_endoplasmic\_reticulum\_calcium\_ion\_concentration | 2 | 0 |  |  |  |  |  |  |  |  |
| GO:0032481\_positive\_regulation\_of\_type\_I\_interferon\_production | 2 | 0 |  |  |  |  |  |  |  |  |
| GO:0032488\_Cdc42\_protein\_signal\_transduction | 2 | 0 |  |  |  |  |  |  |  |  |
| GO:0032489\_regulation\_of\_Cdc42\_protein\_signal\_transduction | 2 | 0 |  |  |  |  |  |  |  |  |
| GO:0032495\_response\_to\_muramyl\_dipeptide | 2 | 0 |  |  |  |  |  |  |  |  |
| GO:0032604\_granulocyte\_macrophage\_colony-stimulating\_factor\_production | 2 | 0 |  |  |  |  |  |  |  |  |
| GO:0032616\_interleukin-13\_production | 2 | 0 |  |  |  |  |  |  |  |  |
| GO:0032645\_regulation\_of\_granulocyte\_macrophage\_colony-stimulating\_factor\_production | 2 | 0 |  |  |  |  |  |  |  |  |
| GO:0032672\_regulation\_of\_interleukin-3\_production | 2 | 0 |  |  |  |  |  |  |  |  |
| GO:0032695\_negative\_regulation\_of\_interleukin-12\_production | 2 | 0 |  |  |  |  |  |  |  |  |
| GO:0032714\_negative\_regulation\_of\_interleukin-5\_production | 2 | 0 |  |  |  |  |  |  |  |  |
| GO:0032722\_positive\_regulation\_of\_chemokine\_production | 2 | 0 |  |  |  |  |  |  |  |  |
| GO:0032743\_positive\_regulation\_of\_interleukin-2\_production | 2 | 0 |  |  |  |  |  |  |  |  |
| GO:0032762\_mast\_cell\_cytokine\_production | 2 | 0 |  |  |  |  |  |  |  |  |
| GO:0032763\_regulation\_of\_mast\_cell\_cytokine\_production | 2 | 0 |  |  |  |  |  |  |  |  |
| GO:0032768\_regulation\_of\_monooxygenase\_activity | 2 | 0 |  |  |  |  |  |  |  |  |
| GO:0032788\_saturated\_monocarboxylic\_acid\_metabolic\_process | 2 | 0 |  |  |  |  |  |  |  |  |
| GO:0032789\_unsaturated\_monocarboxylic\_acid\_metabolic\_process | 2 | 0 |  |  |  |  |  |  |  |  |
| GO:0032796\_uropod\_organization | 2 | 0 |  |  |  |  |  |  |  |  |
| GO:0032800\_receptor\_biosynthetic\_process | 2 | 0 |  |  |  |  |  |  |  |  |
| GO:0032801\_receptor\_catabolic\_process | 2 | 0 |  |  |  |  |  |  |  |  |
| GO:0032829\_regulation\_of\_CD4-positive\_\_CD25-positive\_\_alpha-beta\_regulatory\_T\_cell\_differentiation | 2 | 0 |  |  |  |  |  |  |  |  |
| GO:0032831\_positive\_regulation\_of\_CD4-positive\_\_CD25-positive\_\_alpha-beta\_regulatory\_T\_cell\_differentiation | 2 | 0 |  |  |  |  |  |  |  |  |
| GO:0032892\_positive\_regulation\_of\_organic\_acid\_transport | 2 | 0 |  |  |  |  |  |  |  |  |
| GO:0032905\_transforming\_growth\_factor-beta1\_production | 2 | 0 |  |  |  |  |  |  |  |  |
| GO:0032908\_regulation\_of\_transforming\_growth\_factor-beta1\_production | 2 | 0 |  |  |  |  |  |  |  |  |
| GO:0032914\_positive\_regulation\_of\_transforming\_growth\_factor-beta1\_production | 2 | 0 |  |  |  |  |  |  |  |  |
| GO:0032933\_SREBP-mediated\_signaling\_pathway | 2 | 0 |  |  |  |  |  |  |  |  |
| GO:0032957\_inositol\_trisphosphate\_metabolic\_process | 2 | 0 |  |  |  |  |  |  |  |  |
| GO:0032958\_inositol\_phosphate\_biosynthetic\_process | 2 | 0 |  |  |  |  |  |  |  |  |
| GO:0032959\_inositol\_trisphosphate\_biosynthetic\_process | 2 | 0 |  |  |  |  |  |  |  |  |
| GO:0033092\_positive\_regulation\_of\_immature\_T\_cell\_proliferation\_in\_the\_thymus | 2 | 0 |  |  |  |  |  |  |  |  |
| GO:0033119\_negative\_regulation\_of\_RNA\_splicing | 2 | 0 |  |  |  |  |  |  |  |  |
| GO:0033136\_serine\_phosphorylation\_of\_STAT3\_protein | 2 | 0 |  |  |  |  |  |  |  |  |
| GO:0033145\_positive\_regulation\_of\_steroid\_hormone\_receptor\_signaling\_pathway | 2 | 0 |  |  |  |  |  |  |  |  |
| GO:0033147\_negative\_regulation\_of\_estrogen\_receptor\_signaling\_pathway | 2 | 0 |  |  |  |  |  |  |  |  |
| GO:0033148\_positive\_regulation\_of\_estrogen\_receptor\_signaling\_pathway | 2 | 0 |  |  |  |  |  |  |  |  |
| GO:0033194\_response\_to\_hydroperoxide | 2 | 0 |  |  |  |  |  |  |  |  |
| GO:0033275\_actin-myosin\_filament\_sliding | 2 | 0 |  |  |  |  |  |  |  |  |
| GO:0033280\_response\_to\_vitamin\_D | 2 | 0 |  |  |  |  |  |  |  |  |
| GO:0033364\_mast\_cell\_secretory\_granule\_organization | 2 | 0 |  |  |  |  |  |  |  |  |
| GO:0033504\_floor\_plate\_development | 2 | 0 |  |  |  |  |  |  |  |  |
| GO:0033603\_positive\_regulation\_of\_dopamine\_secretion | 2 | 0 |  |  |  |  |  |  |  |  |
| GO:0033605\_positive\_regulation\_of\_catecholamine\_secretion | 2 | 0 |  |  |  |  |  |  |  |  |
| GO:0033622\_integrin\_activation | 2 | 0 |  |  |  |  |  |  |  |  |
| GO:0033623\_regulation\_of\_integrin\_activation | 2 | 0 |  |  |  |  |  |  |  |  |
| GO:0033625\_positive\_regulation\_of\_integrin\_activation | 2 | 0 |  |  |  |  |  |  |  |  |
| GO:0033700\_phospholipid\_efflux | 2 | 0 |  |  |  |  |  |  |  |  |
| GO:0034142\_toll-like\_receptor\_4\_signaling\_pathway | 2 | 0 |  |  |  |  |  |  |  |  |
| GO:0034310\_monohydric\_alcohol\_catabolic\_process | 2 | 0 |  |  |  |  |  |  |  |  |
| GO:0034341\_response\_to\_interferon-gamma | 2 | 0 |  |  |  |  |  |  |  |  |
| GO:0034370\_triglyceride-rich\_lipoprotein\_particle\_remodeling | 2 | 0 |  |  |  |  |  |  |  |  |
| GO:0034374\_low-density\_lipoprotein\_particle\_remodeling | 2 | 0 |  |  |  |  |  |  |  |  |
| GO:0034377\_plasma\_lipoprotein\_particle\_assembly | 2 | 0 |  |  |  |  |  |  |  |  |
| GO:0034384\_high-density\_lipoprotein\_particle\_clearance | 2 | 0 |  |  |  |  |  |  |  |  |
| GO:0034433\_steroid\_esterification | 2 | 0 |  |  |  |  |  |  |  |  |
| GO:0034434\_sterol\_esterification | 2 | 0 |  |  |  |  |  |  |  |  |
| GO:0034435\_cholesterol\_esterification | 2 | 0 |  |  |  |  |  |  |  |  |
| GO:0034453\_microtubule\_anchoring | 2 | 0 |  |  |  |  |  |  |  |  |
| GO:0034644\_cellular\_response\_to\_UV | 2 | 0 |  |  |  |  |  |  |  |  |
| GO:0034755\_iron\_ion\_transmembrane\_transport | 2 | 0 |  |  |  |  |  |  |  |  |
| GO:0034764\_positive\_regulation\_of\_transmembrane\_transport | 2 | 0 |  |  |  |  |  |  |  |  |
| GO:0035021\_negative\_regulation\_of\_Rac\_protein\_signal\_transduction | 2 | 0 |  |  |  |  |  |  |  |  |
| GO:0035054\_embryonic\_heart\_tube\_anterior\_posterior\_pattern\_formation | 2 | 0 |  |  |  |  |  |  |  |  |
| GO:0035092\_sperm\_chromatin\_condensation | 2 | 0 |  |  |  |  |  |  |  |  |
| GO:0035110\_leg\_morphogenesis | 2 | 0 |  |  |  |  |  |  |  |  |
| GO:0035117\_embryonic\_arm\_morphogenesis | 2 | 0 |  |  |  |  |  |  |  |  |
| GO:0035120\_post-embryonic\_appendage\_morphogenesis | 2 | 0 |  |  |  |  |  |  |  |  |
| GO:0035127\_post-embryonic\_limb\_morphogenesis | 2 | 0 |  |  |  |  |  |  |  |  |
| GO:0035129\_post-embryonic\_hindlimb\_morphogenesis | 2 | 0 |  |  |  |  |  |  |  |  |
| GO:0035140\_arm\_morphogenesis | 2 | 0 |  |  |  |  |  |  |  |  |
| GO:0035194\_posttranscriptional\_gene\_silencing\_by\_RNA | 2 | 0 |  |  |  |  |  |  |  |  |
| GO:0035195\_gene\_silencing\_by\_miRNA | 2 | 0 |  |  |  |  |  |  |  |  |
| GO:0035196\_gene\_silencing\_by\_miRNA\_\_production\_of\_miRNAs | 2 | 0 |  |  |  |  |  |  |  |  |
| GO:0035315\_hair\_cell\_differentiation | 2 | 0 |  |  |  |  |  |  |  |  |
| GO:0040009\_regulation\_of\_growth\_rate | 2 | 0 |  |  |  |  |  |  |  |  |
| GO:0040037\_negative\_regulation\_of\_fibroblast\_growth\_factor\_receptor\_signaling\_pathway | 2 | 0 |  |  |  |  |  |  |  |  |
| GO:0042119\_neutrophil\_activation | 2 | 0 |  |  |  |  |  |  |  |  |
| GO:0042147\_retrograde\_transport\_\_endosome\_to\_Golgi | 2 | 0 |  |  |  |  |  |  |  |  |
| GO:0042223\_interleukin-3\_biosynthetic\_process | 2 | 0 |  |  |  |  |  |  |  |  |
| GO:0042249\_establishment\_of\_polarity\_of\_embryonic\_epithelium | 2 | 0 |  |  |  |  |  |  |  |  |
| GO:0042253\_granulocyte\_macrophage\_colony-stimulating\_factor\_biosynthetic\_process | 2 | 0 |  |  |  |  |  |  |  |  |
| GO:0042270\_protection\_from\_natural\_killer\_cell\_mediated\_cytotoxicity | 2 | 0 |  |  |  |  |  |  |  |  |
| GO:0042274\_ribosomal\_small\_subunit\_biogenesis | 2 | 0 |  |  |  |  |  |  |  |  |
| GO:0042312\_regulation\_of\_vasodilation | 2 | 0 |  |  |  |  |  |  |  |  |
| GO:0042346\_positive\_regulation\_of\_NF-kappaB\_import\_into\_nucleus | 2 | 0 |  |  |  |  |  |  |  |  |
| GO:0042396\_phosphagen\_biosynthetic\_process | 2 | 0 |  |  |  |  |  |  |  |  |
| GO:0042454\_ribonucleoside\_catabolic\_process | 2 | 0 |  |  |  |  |  |  |  |  |
| GO:0042482\_positive\_regulation\_of\_odontogenesis | 2 | 0 |  |  |  |  |  |  |  |  |
| GO:0042483\_negative\_regulation\_of\_odontogenesis | 2 | 0 |  |  |  |  |  |  |  |  |
| GO:0042488\_positive\_regulation\_of\_odontogenesis\_of\_dentine-containing\_tooth | 2 | 0 |  |  |  |  |  |  |  |  |
| GO:0042501\_serine\_phosphorylation\_of\_STAT\_protein | 2 | 0 |  |  |  |  |  |  |  |  |
| GO:0042517\_positive\_regulation\_of\_tyrosine\_phosphorylation\_of\_Stat3\_protein | 2 | 0 |  |  |  |  |  |  |  |  |
| GO:0042532\_negative\_regulation\_of\_tyrosine\_phosphorylation\_of\_STAT\_protein | 2 | 0 |  |  |  |  |  |  |  |  |
| GO:0042559\_pteridine\_and\_derivative\_biosynthetic\_process | 2 | 0 |  |  |  |  |  |  |  |  |
| GO:0042730\_fibrinolysis | 2 | 0 |  |  |  |  |  |  |  |  |
| GO:0042749\_regulation\_of\_circadian\_sleep\_wake\_cycle | 2 | 0 |  |  |  |  |  |  |  |  |
| GO:0042886\_amide\_transport | 2 | 0 |  |  |  |  |  |  |  |  |
| GO:0042921\_glucocorticoid\_receptor\_signaling\_pathway | 2 | 0 |  |  |  |  |  |  |  |  |
| GO:0042987\_amyloid\_precursor\_protein\_catabolic\_process | 2 | 0 |  |  |  |  |  |  |  |  |
| GO:0042993\_positive\_regulation\_of\_transcription\_factor\_import\_into\_nucleus | 2 | 0 |  |  |  |  |  |  |  |  |
| GO:0042994\_cytoplasmic\_sequestering\_of\_transcription\_factor | 2 | 0 |  |  |  |  |  |  |  |  |
| GO:0043032\_positive\_regulation\_of\_macrophage\_activation | 2 | 0 |  |  |  |  |  |  |  |  |
| GO:0043038\_amino\_acid\_activation | 2 | 0 |  |  |  |  |  |  |  |  |
| GO:0043039\_tRNA\_aminoacylation | 2 | 0 |  |  |  |  |  |  |  |  |
| GO:0043084\_penile\_erection | 2 | 0 |  |  |  |  |  |  |  |  |
| GO:0043088\_regulation\_of\_Cdc42\_GTPase\_activity | 2 | 0 |  |  |  |  |  |  |  |  |
| GO:0043089\_positive\_regulation\_of\_Cdc42\_GTPase\_activity | 2 | 0 |  |  |  |  |  |  |  |  |
| GO:0043096\_purine\_base\_salvage | 2 | 0 |  |  |  |  |  |  |  |  |
| GO:0043247\_telomere\_maintenance\_in\_response\_to\_DNA\_damage | 2 | 0 |  |  |  |  |  |  |  |  |
| GO:0043297\_apical\_junction\_assembly | 2 | 0 |  |  |  |  |  |  |  |  |
| GO:0043312\_neutrophil\_degranulation | 2 | 0 |  |  |  |  |  |  |  |  |
| GO:0043320\_natural\_killer\_cell\_degranulation | 2 | 0 |  |  |  |  |  |  |  |  |
| GO:0043366\_beta\_selection | 2 | 0 |  |  |  |  |  |  |  |  |
| GO:0043450\_alkene\_biosynthetic\_process | 2 | 0 |  |  |  |  |  |  |  |  |
| GO:0043476\_pigment\_accumulation | 2 | 0 |  |  |  |  |  |  |  |  |
| GO:0043490\_malate-aspartate\_shuttle | 2 | 0 |  |  |  |  |  |  |  |  |
| GO:0043502\_regulation\_of\_muscle\_adaptation | 2 | 0 |  |  |  |  |  |  |  |  |
| GO:0043516\_regulation\_of\_DNA\_damage\_response\_\_signal\_transduction\_by\_p53\_class\_mediator | 2 | 0 |  |  |  |  |  |  |  |  |
| GO:0043568\_positive\_regulation\_of\_insulin-like\_growth\_factor\_receptor\_signaling\_pathway | 2 | 0 |  |  |  |  |  |  |  |  |
| GO:0043589\_skin\_morphogenesis | 2 | 0 |  |  |  |  |  |  |  |  |
| GO:0043618\_regulation\_of\_transcription\_from\_RNA\_polymerase\_II\_promoter\_in\_response\_to\_stress | 2 | 0 |  |  |  |  |  |  |  |  |
| GO:0043619\_regulation\_of\_transcription\_from\_RNA\_polymerase\_II\_promoter\_in\_response\_to\_oxidative\_stress | 2 | 0 |  |  |  |  |  |  |  |  |
| GO:0043620\_regulation\_of\_transcription\_in\_response\_to\_stress | 2 | 0 |  |  |  |  |  |  |  |  |
| GO:0043647\_inositol\_phosphate\_metabolic\_process | 2 | 0 |  |  |  |  |  |  |  |  |
| GO:0043654\_recognition\_of\_apoptotic\_cell | 2 | 0 |  |  |  |  |  |  |  |  |
| GO:0043966\_histone\_H3\_acetylation | 2 | 0 |  |  |  |  |  |  |  |  |
| GO:0043967\_histone\_H4\_acetylation | 2 | 0 |  |  |  |  |  |  |  |  |
| GO:0044070\_regulation\_of\_anion\_transport | 2 | 0 |  |  |  |  |  |  |  |  |
| GO:0044246\_regulation\_of\_multicellular\_organismal\_metabolic\_process | 2 | 0 |  |  |  |  |  |  |  |  |
| GO:0044253\_positive\_regulation\_of\_multicellular\_organismal\_metabolic\_process | 2 | 0 |  |  |  |  |  |  |  |  |
| GO:0044268\_multicellular\_organismal\_protein\_metabolic\_process | 2 | 0 |  |  |  |  |  |  |  |  |
| GO:0045005\_maintenance\_of\_fidelity\_during\_DNA-dependent\_DNA\_replication | 2 | 0 |  |  |  |  |  |  |  |  |
| GO:0045010\_actin\_nucleation | 2 | 0 |  |  |  |  |  |  |  |  |
| GO:0045065\_cytotoxic\_T\_cell\_differentiation | 2 | 0 |  |  |  |  |  |  |  |  |
| GO:0045077\_negative\_regulation\_of\_interferon-gamma\_biosynthetic\_process | 2 | 0 |  |  |  |  |  |  |  |  |
| GO:0045079\_negative\_regulation\_of\_chemokine\_biosynthetic\_process | 2 | 0 |  |  |  |  |  |  |  |  |
| GO:0045116\_protein\_neddylation | 2 | 0 |  |  |  |  |  |  |  |  |
| GO:0045187\_regulation\_of\_circadian\_sleep\_wake\_cycle\_\_sleep | 2 | 0 |  |  |  |  |  |  |  |  |
| GO:0045212\_neurotransmitter\_receptor\_biosynthetic\_process | 2 | 0 |  |  |  |  |  |  |  |  |
| GO:0045399\_regulation\_of\_interleukin-3\_biosynthetic\_process | 2 | 0 |  |  |  |  |  |  |  |  |
| GO:0045401\_positive\_regulation\_of\_interleukin-3\_biosynthetic\_process | 2 | 0 |  |  |  |  |  |  |  |  |
| GO:0045409\_negative\_regulation\_of\_interleukin-6\_biosynthetic\_process | 2 | 0 |  |  |  |  |  |  |  |  |
| GO:0045423\_regulation\_of\_granulocyte\_macrophage\_colony-stimulating\_factor\_biosynthetic\_process | 2 | 0 |  |  |  |  |  |  |  |  |
| GO:0045425\_positive\_regulation\_of\_granulocyte\_macrophage\_colony-stimulating\_factor\_biosynthetic\_process | 2 | 0 |  |  |  |  |  |  |  |  |
| GO:0045475\_locomotor\_rhythm | 2 | 0 |  |  |  |  |  |  |  |  |
| GO:0045578\_negative\_regulation\_of\_B\_cell\_differentiation | 2 | 0 |  |  |  |  |  |  |  |  |
| GO:0045589\_regulation\_of\_regulatory\_T\_cell\_differentiation | 2 | 0 |  |  |  |  |  |  |  |  |
| GO:0045591\_positive\_regulation\_of\_regulatory\_T\_cell\_differentiation | 2 | 0 |  |  |  |  |  |  |  |  |
| GO:0045608\_negative\_regulation\_of\_auditory\_receptor\_cell\_differentiation | 2 | 0 |  |  |  |  |  |  |  |  |
| GO:0045627\_positive\_regulation\_of\_T-helper\_1\_cell\_differentiation | 2 | 0 |  |  |  |  |  |  |  |  |
| GO:0045629\_negative\_regulation\_of\_T-helper\_2\_cell\_differentiation | 2 | 0 |  |  |  |  |  |  |  |  |
| GO:0045630\_positive\_regulation\_of\_T-helper\_2\_cell\_differentiation | 2 | 0 |  |  |  |  |  |  |  |  |
| GO:0045632\_negative\_regulation\_of\_mechanoreceptor\_differentiation | 2 | 0 |  |  |  |  |  |  |  |  |
| GO:0045636\_positive\_regulation\_of\_melanocyte\_differentiation | 2 | 0 |  |  |  |  |  |  |  |  |
| GO:0045655\_regulation\_of\_monocyte\_differentiation | 2 | 0 |  |  |  |  |  |  |  |  |
| GO:0045658\_regulation\_of\_neutrophil\_differentiation | 2 | 0 |  |  |  |  |  |  |  |  |
| GO:0045662\_negative\_regulation\_of\_myoblast\_differentiation | 2 | 0 |  |  |  |  |  |  |  |  |
| GO:0045663\_positive\_regulation\_of\_myoblast\_differentiation | 2 | 0 |  |  |  |  |  |  |  |  |
| GO:0045683\_negative\_regulation\_of\_epidermis\_development | 2 | 0 |  |  |  |  |  |  |  |  |
| GO:0045737\_positive\_regulation\_of\_cyclin-dependent\_protein\_kinase\_activity | 2 | 0 |  |  |  |  |  |  |  |  |
| GO:0045739\_positive\_regulation\_of\_DNA\_repair | 2 | 0 |  |  |  |  |  |  |  |  |
| GO:0045741\_positive\_regulation\_of\_epidermal\_growth\_factor\_receptor\_activity | 2 | 0 |  |  |  |  |  |  |  |  |
| GO:0045743\_positive\_regulation\_of\_fibroblast\_growth\_factor\_receptor\_signaling\_pathway | 2 | 0 |  |  |  |  |  |  |  |  |
| GO:0045749\_negative\_regulation\_of\_S\_phase\_of\_mitotic\_cell\_cycle | 2 | 0 |  |  |  |  |  |  |  |  |
| GO:0045819\_positive\_regulation\_of\_glycogen\_catabolic\_process | 2 | 0 |  |  |  |  |  |  |  |  |
| GO:0045821\_positive\_regulation\_of\_glycolysis | 2 | 0 |  |  |  |  |  |  |  |  |
| GO:0045835\_negative\_regulation\_of\_meiosis | 2 | 0 |  |  |  |  |  |  |  |  |
| GO:0045836\_positive\_regulation\_of\_meiosis | 2 | 0 |  |  |  |  |  |  |  |  |
| GO:0045839\_negative\_regulation\_of\_mitosis | 2 | 0 |  |  |  |  |  |  |  |  |
| GO:0045841\_negative\_regulation\_of\_mitotic\_metaphase\_anaphase\_transition | 2 | 0 |  |  |  |  |  |  |  |  |
| GO:0045872\_positive\_regulation\_of\_rhodopsin\_gene\_expression | 2 | 0 |  |  |  |  |  |  |  |  |
| GO:0045912\_negative\_regulation\_of\_carbohydrate\_metabolic\_process | 2 | 0 |  |  |  |  |  |  |  |  |
| GO:0045948\_positive\_regulation\_of\_translational\_initiation | 2 | 0 |  |  |  |  |  |  |  |  |
| GO:0045950\_negative\_regulation\_of\_mitotic\_recombination | 2 | 0 |  |  |  |  |  |  |  |  |
| GO:0046033\_AMP\_metabolic\_process | 2 | 0 |  |  |  |  |  |  |  |  |
| GO:0046060\_dATP\_metabolic\_process | 2 | 0 |  |  |  |  |  |  |  |  |
| GO:0046070\_dGTP\_metabolic\_process | 2 | 0 |  |  |  |  |  |  |  |  |
| GO:0046083\_adenine\_metabolic\_process | 2 | 0 |  |  |  |  |  |  |  |  |
| GO:0046085\_adenosine\_metabolic\_process | 2 | 0 |  |  |  |  |  |  |  |  |
| GO:0046100\_hypoxanthine\_metabolic\_process | 2 | 0 |  |  |  |  |  |  |  |  |
| GO:0046114\_guanosine\_biosynthetic\_process | 2 | 0 |  |  |  |  |  |  |  |  |
| GO:0046116\_queuosine\_metabolic\_process | 2 | 0 |  |  |  |  |  |  |  |  |
| GO:0046118\_7-methylguanosine\_biosynthetic\_process | 2 | 0 |  |  |  |  |  |  |  |  |
| GO:0046130\_purine\_ribonucleoside\_catabolic\_process | 2 | 0 |  |  |  |  |  |  |  |  |
| GO:0046146\_tetrahydrobiopterin\_metabolic\_process | 2 | 0 |  |  |  |  |  |  |  |  |
| GO:0046185\_aldehyde\_catabolic\_process | 2 | 0 |  |  |  |  |  |  |  |  |
| GO:0046208\_spermine\_catabolic\_process | 2 | 0 |  |  |  |  |  |  |  |  |
| GO:0046349\_amino\_sugar\_biosynthetic\_process | 2 | 0 |  |  |  |  |  |  |  |  |
| GO:0046439\_L-cysteine\_metabolic\_process | 2 | 0 |  |  |  |  |  |  |  |  |
| GO:0046500\_S-adenosylmethionine\_metabolic\_process | 2 | 0 |  |  |  |  |  |  |  |  |
| GO:0046501\_protoporphyrinogen\_IX\_metabolic\_process | 2 | 0 |  |  |  |  |  |  |  |  |
| GO:0046514\_ceramide\_catabolic\_process | 2 | 0 |  |  |  |  |  |  |  |  |
| GO:0046521\_sphingoid\_catabolic\_process | 2 | 0 |  |  |  |  |  |  |  |  |
| GO:0046532\_regulation\_of\_photoreceptor\_cell\_differentiation | 2 | 0 |  |  |  |  |  |  |  |  |
| GO:0046533\_negative\_regulation\_of\_photoreceptor\_cell\_differentiation | 2 | 0 |  |  |  |  |  |  |  |  |
| GO:0046544\_development\_of\_secondary\_male\_sexual\_characteristics | 2 | 0 |  |  |  |  |  |  |  |  |
| GO:0046619\_optic\_placode\_formation\_involved\_in\_camera-type\_eye | 2 | 0 |  |  |  |  |  |  |  |  |
| GO:0046950\_cellular\_ketone\_body\_metabolic\_process | 2 | 0 |  |  |  |  |  |  |  |  |
| GO:0046984\_regulation\_of\_hemoglobin\_biosynthetic\_process | 2 | 0 |  |  |  |  |  |  |  |  |
| GO:0047484\_regulation\_of\_response\_to\_osmotic\_stress | 2 | 0 |  |  |  |  |  |  |  |  |
| GO:0048025\_negative\_regulation\_of\_nuclear\_mRNA\_splicing\_\_via\_spliceosome | 2 | 0 |  |  |  |  |  |  |  |  |
| GO:0048134\_germ-line\_cyst\_formation | 2 | 0 |  |  |  |  |  |  |  |  |
| GO:0048136\_male\_germ-line\_cyst\_formation | 2 | 0 |  |  |  |  |  |  |  |  |
| GO:0048172\_regulation\_of\_short-term\_neuronal\_synaptic\_plasticity | 2 | 0 |  |  |  |  |  |  |  |  |
| GO:0048295\_positive\_regulation\_of\_isotype\_switching\_to\_IgE\_isotypes | 2 | 0 |  |  |  |  |  |  |  |  |
| GO:0048342\_paraxial\_mesodermal\_cell\_differentiation | 2 | 0 |  |  |  |  |  |  |  |  |
| GO:0048343\_paraxial\_mesodermal\_cell\_fate\_commitment | 2 | 0 |  |  |  |  |  |  |  |  |
| GO:0048382\_mesendoderm\_development | 2 | 0 |  |  |  |  |  |  |  |  |
| GO:0048552\_regulation\_of\_metalloenzyme\_activity | 2 | 0 |  |  |  |  |  |  |  |  |
| GO:0048554\_positive\_regulation\_of\_metalloenzyme\_activity | 2 | 0 |  |  |  |  |  |  |  |  |
| GO:0048619\_embryonic\_hindgut\_morphogenesis | 2 | 0 |  |  |  |  |  |  |  |  |
| GO:0048625\_myoblast\_cell\_fate\_commitment | 2 | 0 |  |  |  |  |  |  |  |  |
| GO:0048627\_myoblast\_development | 2 | 0 |  |  |  |  |  |  |  |  |
| GO:0048643\_positive\_regulation\_of\_skeletal\_muscle\_tissue\_development | 2 | 0 |  |  |  |  |  |  |  |  |
| GO:0048661\_positive\_regulation\_of\_smooth\_muscle\_cell\_proliferation | 2 | 0 |  |  |  |  |  |  |  |  |
| GO:0048670\_regulation\_of\_collateral\_sprouting | 2 | 0 |  |  |  |  |  |  |  |  |
| GO:0048671\_negative\_regulation\_of\_collateral\_sprouting | 2 | 0 |  |  |  |  |  |  |  |  |
| GO:0048677\_axon\_extension\_involved\_in\_regeneration | 2 | 0 |  |  |  |  |  |  |  |  |
| GO:0048679\_regulation\_of\_axon\_regeneration | 2 | 0 |  |  |  |  |  |  |  |  |
| GO:0048682\_sprouting\_of\_injured\_axon | 2 | 0 |  |  |  |  |  |  |  |  |
| GO:0048702\_embryonic\_neurocranium\_morphogenesis | 2 | 0 |  |  |  |  |  |  |  |  |
| GO:0048711\_positive\_regulation\_of\_astrocyte\_differentiation | 2 | 0 |  |  |  |  |  |  |  |  |
| GO:0048712\_negative\_regulation\_of\_astrocyte\_differentiation | 2 | 0 |  |  |  |  |  |  |  |  |
| GO:0048739\_cardiac\_muscle\_fiber\_development | 2 | 0 |  |  |  |  |  |  |  |  |
| GO:0048807\_female\_genitalia\_morphogenesis | 2 | 0 |  |  |  |  |  |  |  |  |
| GO:0048808\_male\_genitalia\_morphogenesis | 2 | 0 |  |  |  |  |  |  |  |  |
| GO:0048840\_otolith\_development | 2 | 0 |  |  |  |  |  |  |  |  |
| GO:0048850\_hypophysis\_morphogenesis | 2 | 0 |  |  |  |  |  |  |  |  |
| GO:0048867\_stem\_cell\_fate\_determination | 2 | 0 |  |  |  |  |  |  |  |  |
| GO:0050000\_chromosome\_localization | 2 | 0 |  |  |  |  |  |  |  |  |
| GO:0050686\_negative\_regulation\_of\_mRNA\_processing | 2 | 0 |  |  |  |  |  |  |  |  |
| GO:0050688\_regulation\_of\_defense\_response\_to\_virus | 2 | 0 |  |  |  |  |  |  |  |  |
| GO:0050746\_regulation\_of\_lipoprotein\_metabolic\_process | 2 | 0 |  |  |  |  |  |  |  |  |
| GO:0050779\_RNA\_destabilization | 2 | 0 |  |  |  |  |  |  |  |  |
| GO:0050792\_regulation\_of\_viral\_reproduction | 2 | 0 |  |  |  |  |  |  |  |  |
| GO:0050802\_circadian\_sleep\_wake\_cycle\_\_sleep | 2 | 0 |  |  |  |  |  |  |  |  |
| GO:0050847\_progesterone\_receptor\_signaling\_pathway | 2 | 0 |  |  |  |  |  |  |  |  |
| GO:0050855\_regulation\_of\_B\_cell\_receptor\_signaling\_pathway | 2 | 0 |  |  |  |  |  |  |  |  |
| GO:0050883\_musculoskeletal\_movement\_\_spinal\_reflex\_action | 2 | 0 |  |  |  |  |  |  |  |  |
| GO:0050901\_leukocyte\_tethering\_or\_rolling | 2 | 0 |  |  |  |  |  |  |  |  |
| GO:0050907\_detection\_of\_chemical\_stimulus\_involved\_in\_sensory\_perception | 2 | 0 |  |  |  |  |  |  |  |  |
| GO:0050917\_sensory\_perception\_of\_umami\_taste | 2 | 0 |  |  |  |  |  |  |  |  |
| GO:0050942\_positive\_regulation\_of\_pigment\_cell\_differentiation | 2 | 0 |  |  |  |  |  |  |  |  |
| GO:0050955\_thermoception | 2 | 0 |  |  |  |  |  |  |  |  |
| GO:0050968\_detection\_of\_chemical\_stimulus\_involved\_in\_sensory\_perception\_of\_pain | 2 | 0 |  |  |  |  |  |  |  |  |
| GO:0050973\_detection\_of\_mechanical\_stimulus\_involved\_in\_equilibrioception | 2 | 0 |  |  |  |  |  |  |  |  |
| GO:0050999\_regulation\_of\_nitric-oxide\_synthase\_activity | 2 | 0 |  |  |  |  |  |  |  |  |
| GO:0051004\_regulation\_of\_lipoprotein\_lipase\_activity | 2 | 0 |  |  |  |  |  |  |  |  |
| GO:0051014\_actin\_filament\_severing | 2 | 0 |  |  |  |  |  |  |  |  |
| GO:0051026\_chiasma\_formation | 2 | 0 |  |  |  |  |  |  |  |  |
| GO:0051081\_nuclear\_envelope\_disassembly | 2 | 0 |  |  |  |  |  |  |  |  |
| GO:0051132\_NK\_T\_cell\_activation | 2 | 0 |  |  |  |  |  |  |  |  |
| GO:0051133\_regulation\_of\_NK\_T\_cell\_activation | 2 | 0 |  |  |  |  |  |  |  |  |
| GO:0051135\_positive\_regulation\_of\_NK\_T\_cell\_activation | 2 | 0 |  |  |  |  |  |  |  |  |
| GO:0051150\_regulation\_of\_smooth\_muscle\_cell\_differentiation | 2 | 0 |  |  |  |  |  |  |  |  |
| GO:0051220\_cytoplasmic\_sequestering\_of\_protein | 2 | 0 |  |  |  |  |  |  |  |  |
| GO:0051279\_regulation\_of\_release\_of\_sequestered\_calcium\_ion\_into\_cytosol | 2 | 0 |  |  |  |  |  |  |  |  |
| GO:0051293\_establishment\_of\_spindle\_localization | 2 | 0 |  |  |  |  |  |  |  |  |
| GO:0051295\_establishment\_of\_meiotic\_spindle\_localization | 2 | 0 |  |  |  |  |  |  |  |  |
| GO:0051299\_centrosome\_separation | 2 | 0 |  |  |  |  |  |  |  |  |
| GO:0051303\_establishment\_of\_chromosome\_localization | 2 | 0 |  |  |  |  |  |  |  |  |
| GO:0051304\_chromosome\_separation | 2 | 0 |  |  |  |  |  |  |  |  |
| GO:0051307\_meiotic\_chromosome\_separation | 2 | 0 |  |  |  |  |  |  |  |  |
| GO:0051313\_attachment\_of\_spindle\_microtubules\_to\_chromosome | 2 | 0 |  |  |  |  |  |  |  |  |
| GO:0051318\_G1\_phase | 2 | 0 |  |  |  |  |  |  |  |  |
| GO:0051319\_G2\_phase | 2 | 0 |  |  |  |  |  |  |  |  |
| GO:0051353\_positive\_regulation\_of\_oxidoreductase\_activity | 2 | 0 |  |  |  |  |  |  |  |  |
| GO:0051451\_myoblast\_migration | 2 | 0 |  |  |  |  |  |  |  |  |
| GO:0051489\_regulation\_of\_filopodium\_assembly | 2 | 0 |  |  |  |  |  |  |  |  |
| GO:0051491\_positive\_regulation\_of\_filopodium\_assembly | 2 | 0 |  |  |  |  |  |  |  |  |
| GO:0051541\_elastin\_metabolic\_process | 2 | 0 |  |  |  |  |  |  |  |  |
| GO:0051546\_keratinocyte\_migration | 2 | 0 |  |  |  |  |  |  |  |  |
| GO:0051563\_smooth\_endoplasmic\_reticulum\_calcium\_ion\_homeostasis | 2 | 0 |  |  |  |  |  |  |  |  |
| GO:0051590\_positive\_regulation\_of\_neurotransmitter\_transport | 2 | 0 |  |  |  |  |  |  |  |  |
| GO:0051602\_response\_to\_electrical\_stimulus | 2 | 0 |  |  |  |  |  |  |  |  |
| GO:0051608\_histamine\_transport | 2 | 0 |  |  |  |  |  |  |  |  |
| GO:0051643\_ER\_localization | 2 | 0 |  |  |  |  |  |  |  |  |
| GO:0051653\_spindle\_localization | 2 | 0 |  |  |  |  |  |  |  |  |
| GO:0051657\_maintenance\_of\_organelle\_location | 2 | 0 |  |  |  |  |  |  |  |  |
| GO:0051702\_interaction\_with\_symbiont | 2 | 0 |  |  |  |  |  |  |  |  |
| GO:0051781\_positive\_regulation\_of\_cell\_division | 2 | 0 |  |  |  |  |  |  |  |  |
| GO:0051784\_negative\_regulation\_of\_nuclear\_division | 2 | 0 |  |  |  |  |  |  |  |  |
| GO:0051890\_regulation\_of\_cardioblast\_differentiation | 2 | 0 |  |  |  |  |  |  |  |  |
| GO:0051891\_positive\_regulation\_of\_cardioblast\_differentiation | 2 | 0 |  |  |  |  |  |  |  |  |
| GO:0051923\_sulfation | 2 | 0 |  |  |  |  |  |  |  |  |
| GO:0051938\_L-glutamate\_import | 2 | 0 |  |  |  |  |  |  |  |  |
| GO:0051957\_positive\_regulation\_of\_amino\_acid\_transport | 2 | 0 |  |  |  |  |  |  |  |  |
| GO:0051988\_regulation\_of\_attachment\_of\_spindle\_microtubules\_to\_kinetochore | 2 | 0 |  |  |  |  |  |  |  |  |
| GO:0055057\_neuroblast\_division | 2 | 0 |  |  |  |  |  |  |  |  |
| GO:0055064\_chloride\_ion\_homeostasis | 2 | 0 |  |  |  |  |  |  |  |  |
| GO:0055075\_potassium\_ion\_homeostasis | 2 | 0 |  |  |  |  |  |  |  |  |
| GO:0055090\_acylglycerol\_homeostasis | 2 | 0 |  |  |  |  |  |  |  |  |
| GO:0055091\_phospholipid\_homeostasis | 2 | 0 |  |  |  |  |  |  |  |  |
| GO:0060012\_synaptic\_transmission\_\_glycinergic | 2 | 0 |  |  |  |  |  |  |  |  |
| GO:0060023\_soft\_palate\_development | 2 | 0 |  |  |  |  |  |  |  |  |
| GO:0060032\_notochord\_regression | 2 | 0 |  |  |  |  |  |  |  |  |
| GO:0060039\_pericardium\_development | 2 | 0 |  |  |  |  |  |  |  |  |
| GO:0060044\_negative\_regulation\_of\_cardiac\_muscle\_cell\_proliferation | 2 | 0 |  |  |  |  |  |  |  |  |
| GO:0060060\_post-embryonic\_retina\_morphogenesis\_in\_camera-type\_eye | 2 | 0 |  |  |  |  |  |  |  |  |
| GO:0060083\_smooth\_muscle\_contraction\_involved\_in\_micturition | 2 | 0 |  |  |  |  |  |  |  |  |
| GO:0060124\_positive\_regulation\_of\_growth\_hormone\_secretion | 2 | 0 |  |  |  |  |  |  |  |  |
| GO:0060133\_somatotropin\_secreting\_cell\_development | 2 | 0 |  |  |  |  |  |  |  |  |
| GO:0060155\_platelet\_dense\_granule\_organization | 2 | 0 |  |  |  |  |  |  |  |  |
| GO:0060159\_regulation\_of\_dopamine\_receptor\_signaling\_pathway | 2 | 0 |  |  |  |  |  |  |  |  |
| GO:0060160\_negative\_regulation\_of\_dopamine\_receptor\_signaling\_pathway | 2 | 0 |  |  |  |  |  |  |  |  |
| GO:0060166\_olfactory\_pit\_development | 2 | 0 |  |  |  |  |  |  |  |  |
| GO:0060179\_male\_mating\_behavior | 2 | 0 |  |  |  |  |  |  |  |  |
| GO:0060180\_female\_mating\_behavior | 2 | 0 |  |  |  |  |  |  |  |  |
| GO:0060214\_endocardium\_formation | 2 | 0 |  |  |  |  |  |  |  |  |
| GO:0060218\_hemopoietic\_stem\_cell\_differentiation | 2 | 0 |  |  |  |  |  |  |  |  |
| GO:0060259\_regulation\_of\_feeding\_behavior | 2 | 0 |  |  |  |  |  |  |  |  |
| GO:0060260\_regulation\_of\_transcription\_initiation\_from\_RNA\_polymerase\_II\_promoter | 2 | 0 |  |  |  |  |  |  |  |  |
| GO:0060292\_long\_term\_synaptic\_depression | 2 | 0 |  |  |  |  |  |  |  |  |
| GO:0060318\_definitive\_erythrocyte\_differentiation | 2 | 0 |  |  |  |  |  |  |  |  |
| GO:0060346\_bone\_trabecula\_formation | 2 | 0 |  |  |  |  |  |  |  |  |
| GO:0060363\_cranial\_suture\_morphogenesis | 2 | 0 |  |  |  |  |  |  |  |  |
| GO:0060393\_regulation\_of\_pathway-restricted\_SMAD\_protein\_phosphorylation | 2 | 0 |  |  |  |  |  |  |  |  |
| GO:0060397\_JAK-STAT\_cascade\_involved\_in\_growth\_hormone\_signaling\_pathway | 2 | 0 |  |  |  |  |  |  |  |  |
| GO:0060426\_lung\_vasculature\_development | 2 | 0 |  |  |  |  |  |  |  |  |
| GO:0060430\_lung\_saccule\_development | 2 | 0 |  |  |  |  |  |  |  |  |
| GO:0060434\_bronchus\_morphogenesis | 2 | 0 |  |  |  |  |  |  |  |  |
| GO:0060439\_trachea\_morphogenesis | 2 | 0 |  |  |  |  |  |  |  |  |
| GO:0060458\_right\_lung\_development | 2 | 0 |  |  |  |  |  |  |  |  |
| GO:0060462\_lung\_lobe\_development | 2 | 0 |  |  |  |  |  |  |  |  |
| GO:0060463\_lung\_lobe\_morphogenesis | 2 | 0 |  |  |  |  |  |  |  |  |
| GO:0060479\_lung\_cell\_differentiation | 2 | 0 |  |  |  |  |  |  |  |  |
| GO:0060487\_lung\_epithelial\_cell\_differentiation | 2 | 0 |  |  |  |  |  |  |  |  |
| GO:0060516\_primary\_prostatic\_bud\_elongation | 2 | 0 |  |  |  |  |  |  |  |  |
| GO:0060529\_squamous\_basal\_epithelial\_stem\_cell\_differentiation\_involved\_in\_prostate\_gland\_acinus\_development | 2 | 0 |  |  |  |  |  |  |  |  |
| GO:0060534\_trachea\_cartilage\_development | 2 | 0 |  |  |  |  |  |  |  |  |
| GO:0060599\_lateral\_sprouting\_involved\_in\_mammary\_gland\_duct\_morphogenesis | 2 | 0 |  |  |  |  |  |  |  |  |
| GO:0060612\_adipose\_tissue\_development | 2 | 0 |  |  |  |  |  |  |  |  |
| GO:0060615\_mammary\_gland\_bud\_formation | 2 | 0 |  |  |  |  |  |  |  |  |
| GO:0060667\_branch\_elongation\_involved\_in\_salivary\_gland\_morphogenesis | 2 | 0 |  |  |  |  |  |  |  |  |
| GO:0060690\_epithelial\_cell\_differentiation\_involved\_in\_salivary\_gland\_development | 2 | 0 |  |  |  |  |  |  |  |  |
| GO:0060738\_epithelial-mesenchymal\_signaling\_involved\_in\_prostate\_gland\_development | 2 | 0 |  |  |  |  |  |  |  |  |
| GO:0060741\_prostate\_gland\_stromal\_morphogenesis | 2 | 0 |  |  |  |  |  |  |  |  |
| GO:0060763\_mammary\_duct\_terminal\_end\_bud\_growth | 2 | 0 |  |  |  |  |  |  |  |  |
| GO:0060765\_regulation\_of\_androgen\_receptor\_signaling\_pathway | 2 | 0 |  |  |  |  |  |  |  |  |
| GO:0060766\_negative\_regulation\_of\_androgen\_receptor\_signaling\_pathway | 2 | 0 |  |  |  |  |  |  |  |  |
| GO:0060769\_positive\_regulation\_of\_epithelial\_cell\_proliferation\_involved\_in\_prostate\_gland\_development | 2 | 0 |  |  |  |  |  |  |  |  |
| GO:0065005\_protein-lipid\_complex\_assembly | 2 | 0 |  |  |  |  |  |  |  |  |
| GO:0070076\_histone\_lysine\_demethylation | 2 | 0 |  |  |  |  |  |  |  |  |
| GO:0070168\_negative\_regulation\_of\_biomineral\_formation | 2 | 0 |  |  |  |  |  |  |  |  |
| GO:0070252\_actin-mediated\_cell\_contraction | 2 | 0 |  |  |  |  |  |  |  |  |
| GO:0070256\_negative\_regulation\_of\_mucus\_secretion | 2 | 0 |  |  |  |  |  |  |  |  |
| GO:0070257\_positive\_regulation\_of\_mucus\_secretion | 2 | 0 |  |  |  |  |  |  |  |  |
| GO:0070570\_regulation\_of\_neuron\_projection\_regeneration | 2 | 0 |  |  |  |  |  |  |  |  |
| GO:0070723\_response\_to\_cholesterol | 2 | 0 |  |  |  |  |  |  |  |  |
| GO:0090030\_regulation\_of\_steroid\_hormone\_biosynthetic\_process | 2 | 0 |  |  |  |  |  |  |  |  |
| GO:0006753\_nucleoside\_phosphate\_metabolic\_process | 94 | 0 | 0.000000 | -0.000000 | 974 | 919.018461 | 988.4 | 1057.781539 | 1.014784 |
| GO:0008610\_lipid\_biosynthetic\_process | 94 | 0 | 0.000000 | -0.000000 | 974 | 919.018461 | 988.4 | 1057.781539 | 1.014784 |
| GO:0009117\_nucleotide\_metabolic\_process | 94 | 0 | 0.000000 | -0.000000 | 974 | 919.018461 | 988.4 | 1057.781539 | 1.014784 |
| GO:0032943\_mononuclear\_cell\_proliferation | 94 | 0 | 0.000000 | -0.000000 | 974 | 919.018461 | 988.4 | 1057.781539 | 1.014784 |
| GO:0046651\_lymphocyte\_proliferation | 94 | 0 | 0.000000 | -0.000000 | 974 | 919.018461 | 988.4 | 1057.781539 | 1.014784 |
| GO:0042110\_T\_cell\_activation | 163 | 0 | 0.000000 | -0.000000 | 975 | 920.104804 | 989.25 | 1058.395196 | 1.014615 |
| GO:0001816\_cytokine\_production | 122 | 0 | 0.000000 | -0.000000 | 981 | 923.722814 | 992.26 | 1060.797186 | 1.011478 |
| GO:0002252\_immune\_effector\_process | 122 | 0 | 0.000000 | -0.000000 | 981 | 923.722814 | 992.26 | 1060.797186 | 1.011478 |
| GO:0006886\_intracellular\_protein\_transport | 122 | 0 | 0.000000 | -0.000000 | 981 | 923.722814 | 992.26 | 1060.797186 | 1.011478 |
| GO:0030001\_metal\_ion\_transport | 122 | 0 | 0.000000 | -0.000000 | 981 | 923.722814 | 992.26 | 1060.797186 | 1.011478 |
| GO:0050865\_regulation\_of\_cell\_activation | 122 | 0 | 0.000000 | -0.000000 | 981 | 923.722814 | 992.26 | 1060.797186 | 1.011478 |
| GO:0060284\_regulation\_of\_cell\_development | 122 | 0 | 0.000000 | -0.000000 | 981 | 923.722814 | 992.26 | 1060.797186 | 1.011478 |
| GO:0015674\_di-\_\_tri-valent\_inorganic\_cation\_transport | 79 | 0 | 0.000000 | -0.000000 | 983 | 925.244777 | 993.55 | 1061.855223 | 1.010732 |
| GO:0051046\_regulation\_of\_secretion | 79 | 0 | 0.000000 | -0.000000 | 983 | 925.244777 | 993.55 | 1061.855223 | 1.010732 |
| GO:0060341\_regulation\_of\_cellular\_localization | 97 | 0 | 0.000000 | -0.000000 | 984 | 926.870530 | 994.81 | 1062.749470 | 1.010986 |
| GO:0000375\_RNA\_splicing\_\_via\_transesterification\_reactions | 12 | 0 | 0.000000 | -0.000000 | 1062 | 1006.201945 | 1073.03 | 1139.858055 | 1.010386 |
| GO:0000377\_RNA\_splicing\_\_via\_transesterification\_reactions\_with\_bulged\_adenosine\_as\_nucleophile | 12 | 0 | 0.000000 | -0.000000 | 1062 | 1006.201945 | 1073.03 | 1139.858055 | 1.010386 |
| GO:0000398\_nuclear\_mRNA\_splicing\_\_via\_spliceosome | 12 | 0 | 0.000000 | -0.000000 | 1062 | 1006.201945 | 1073.03 | 1139.858055 | 1.010386 |
| GO:0001662\_behavioral\_fear\_response | 12 | 0 | 0.000000 | -0.000000 | 1062 | 1006.201945 | 1073.03 | 1139.858055 | 1.010386 |
| GO:0001738\_morphogenesis\_of\_a\_polarized\_epithelium | 12 | 0 | 0.000000 | -0.000000 | 1062 | 1006.201945 | 1073.03 | 1139.858055 | 1.010386 |
| GO:0002026\_regulation\_of\_the\_force\_of\_heart\_contraction | 12 | 0 | 0.000000 | -0.000000 | 1062 | 1006.201945 | 1073.03 | 1139.858055 | 1.010386 |
| GO:0002089\_lens\_morphogenesis\_in\_camera-type\_eye | 12 | 0 | 0.000000 | -0.000000 | 1062 | 1006.201945 | 1073.03 | 1139.858055 | 1.010386 |
| GO:0002209\_behavioral\_defense\_response | 12 | 0 | 0.000000 | -0.000000 | 1062 | 1006.201945 | 1073.03 | 1139.858055 | 1.010386 |
| GO:0002218\_activation\_of\_innate\_immune\_response | 12 | 0 | 0.000000 | -0.000000 | 1062 | 1006.201945 | 1073.03 | 1139.858055 | 1.010386 |
| GO:0002244\_hemopoietic\_progenitor\_cell\_differentiation | 12 | 0 | 0.000000 | -0.000000 | 1062 | 1006.201945 | 1073.03 | 1139.858055 | 1.010386 |
| GO:0002437\_inflammatory\_response\_to\_antigenic\_stimulus | 12 | 0 | 0.000000 | -0.000000 | 1062 | 1006.201945 | 1073.03 | 1139.858055 | 1.010386 |
| GO:0002763\_positive\_regulation\_of\_myeloid\_leukocyte\_differentiation | 12 | 0 | 0.000000 | -0.000000 | 1062 | 1006.201945 | 1073.03 | 1139.858055 | 1.010386 |
| GO:0002820\_negative\_regulation\_of\_adaptive\_immune\_response | 12 | 0 | 0.000000 | -0.000000 | 1062 | 1006.201945 | 1073.03 | 1139.858055 | 1.010386 |
| GO:0002823\_negative\_regulation\_of\_adaptive\_immune\_response\_based\_on\_somatic\_recombination\_of\_immune\_receptors\_built\_from\_immunoglobulin\_superfamily\_domains | 12 | 0 | 0.000000 | -0.000000 | 1062 | 1006.201945 | 1073.03 | 1139.858055 | 1.010386 |
| GO:0002861\_regulation\_of\_inflammatory\_response\_to\_antigenic\_stimulus | 12 | 0 | 0.000000 | -0.000000 | 1062 | 1006.201945 | 1073.03 | 1139.858055 | 1.010386 |
| GO:0006094\_gluconeogenesis | 12 | 0 | 0.000000 | -0.000000 | 1062 | 1006.201945 | 1073.03 | 1139.858055 | 1.010386 |
| GO:0006308\_DNA\_catabolic\_process | 12 | 0 | 0.000000 | -0.000000 | 1062 | 1006.201945 | 1073.03 | 1139.858055 | 1.010386 |
| GO:0006403\_RNA\_localization | 12 | 0 | 0.000000 | -0.000000 | 1062 | 1006.201945 | 1073.03 | 1139.858055 | 1.010386 |
| GO:0006413\_translational\_initiation | 12 | 0 | 0.000000 | -0.000000 | 1062 | 1006.201945 | 1073.03 | 1139.858055 | 1.010386 |
| GO:0006446\_regulation\_of\_translational\_initiation | 12 | 0 | 0.000000 | -0.000000 | 1062 | 1006.201945 | 1073.03 | 1139.858055 | 1.010386 |
| GO:0006839\_mitochondrial\_transport | 12 | 0 | 0.000000 | -0.000000 | 1062 | 1006.201945 | 1073.03 | 1139.858055 | 1.010386 |
| GO:0006879\_cellular\_iron\_ion\_homeostasis | 12 | 0 | 0.000000 | -0.000000 | 1062 | 1006.201945 | 1073.03 | 1139.858055 | 1.010386 |
| GO:0007143\_female\_meiosis | 12 | 0 | 0.000000 | -0.000000 | 1062 | 1006.201945 | 1073.03 | 1139.858055 | 1.010386 |
| GO:0007340\_acrosome\_reaction | 12 | 0 | 0.000000 | -0.000000 | 1062 | 1006.201945 | 1073.03 | 1139.858055 | 1.010386 |
| GO:0008038\_neuron\_recognition | 12 | 0 | 0.000000 | -0.000000 | 1062 | 1006.201945 | 1073.03 | 1139.858055 | 1.010386 |
| GO:0008045\_motor\_axon\_guidance | 12 | 0 | 0.000000 | -0.000000 | 1062 | 1006.201945 | 1073.03 | 1139.858055 | 1.010386 |
| GO:0008277\_regulation\_of\_G-protein\_coupled\_receptor\_protein\_signaling\_pathway | 12 | 0 | 0.000000 | -0.000000 | 1062 | 1006.201945 | 1073.03 | 1139.858055 | 1.010386 |
| GO:0009262\_deoxyribonucleotide\_metabolic\_process | 12 | 0 | 0.000000 | -0.000000 | 1062 | 1006.201945 | 1073.03 | 1139.858055 | 1.010386 |
| GO:0009886\_post-embryonic\_morphogenesis | 12 | 0 | 0.000000 | -0.000000 | 1062 | 1006.201945 | 1073.03 | 1139.858055 | 1.010386 |
| GO:0015872\_dopamine\_transport | 12 | 0 | 0.000000 | -0.000000 | 1062 | 1006.201945 | 1073.03 | 1139.858055 | 1.010386 |
| GO:0019319\_hexose\_biosynthetic\_process | 12 | 0 | 0.000000 | -0.000000 | 1062 | 1006.201945 | 1073.03 | 1139.858055 | 1.010386 |
| GO:0021513\_spinal\_cord\_dorsal\_ventral\_patterning | 12 | 0 | 0.000000 | -0.000000 | 1062 | 1006.201945 | 1073.03 | 1139.858055 | 1.010386 |
| GO:0021680\_cerebellar\_Purkinje\_cell\_layer\_development | 12 | 0 | 0.000000 | -0.000000 | 1062 | 1006.201945 | 1073.03 | 1139.858055 | 1.010386 |
| GO:0021697\_cerebellar\_cortex\_formation | 12 | 0 | 0.000000 | -0.000000 | 1062 | 1006.201945 | 1073.03 | 1139.858055 | 1.010386 |
| GO:0030010\_establishment\_of\_cell\_polarity | 12 | 0 | 0.000000 | -0.000000 | 1062 | 1006.201945 | 1073.03 | 1139.858055 | 1.010386 |
| GO:0030225\_macrophage\_differentiation | 12 | 0 | 0.000000 | -0.000000 | 1062 | 1006.201945 | 1073.03 | 1139.858055 | 1.010386 |
| GO:0030239\_myofibril\_assembly | 12 | 0 | 0.000000 | -0.000000 | 1062 | 1006.201945 | 1073.03 | 1139.858055 | 1.010386 |
| GO:0030262\_apoptotic\_nuclear\_changes | 12 | 0 | 0.000000 | -0.000000 | 1062 | 1006.201945 | 1073.03 | 1139.858055 | 1.010386 |
| GO:0030330\_DNA\_damage\_response\_\_signal\_transduction\_by\_p53\_class\_mediator | 12 | 0 | 0.000000 | -0.000000 | 1062 | 1006.201945 | 1073.03 | 1139.858055 | 1.010386 |
| GO:0030514\_negative\_regulation\_of\_BMP\_signaling\_pathway | 12 | 0 | 0.000000 | -0.000000 | 1062 | 1006.201945 | 1073.03 | 1139.858055 | 1.010386 |
| GO:0031056\_regulation\_of\_histone\_modification | 12 | 0 | 0.000000 | -0.000000 | 1062 | 1006.201945 | 1073.03 | 1139.858055 | 1.010386 |
| GO:0032755\_positive\_regulation\_of\_interleukin-6\_production | 12 | 0 | 0.000000 | -0.000000 | 1062 | 1006.201945 | 1073.03 | 1139.858055 | 1.010386 |
| GO:0033598\_mammary\_gland\_epithelial\_cell\_proliferation | 12 | 0 | 0.000000 | -0.000000 | 1062 | 1006.201945 | 1073.03 | 1139.858055 | 1.010386 |
| GO:0035050\_embryonic\_heart\_tube\_development | 12 | 0 | 0.000000 | -0.000000 | 1062 | 1006.201945 | 1073.03 | 1139.858055 | 1.010386 |
| GO:0042278\_purine\_nucleoside\_metabolic\_process | 12 | 0 | 0.000000 | -0.000000 | 1062 | 1006.201945 | 1073.03 | 1139.858055 | 1.010386 |
| GO:0042446\_hormone\_biosynthetic\_process | 12 | 0 | 0.000000 | -0.000000 | 1062 | 1006.201945 | 1073.03 | 1139.858055 | 1.010386 |
| GO:0042743\_hydrogen\_peroxide\_metabolic\_process | 12 | 0 | 0.000000 | -0.000000 | 1062 | 1006.201945 | 1073.03 | 1139.858055 | 1.010386 |
| GO:0043331\_response\_to\_dsRNA | 12 | 0 | 0.000000 | -0.000000 | 1062 | 1006.201945 | 1073.03 | 1139.858055 | 1.010386 |
| GO:0043392\_negative\_regulation\_of\_DNA\_binding | 12 | 0 | 0.000000 | -0.000000 | 1062 | 1006.201945 | 1073.03 | 1139.858055 | 1.010386 |
| GO:0043487\_regulation\_of\_RNA\_stability | 12 | 0 | 0.000000 | -0.000000 | 1062 | 1006.201945 | 1073.03 | 1139.858055 | 1.010386 |
| GO:0045076\_regulation\_of\_interleukin-2\_biosynthetic\_process | 12 | 0 | 0.000000 | -0.000000 | 1062 | 1006.201945 | 1073.03 | 1139.858055 | 1.010386 |
| GO:0045445\_myoblast\_differentiation | 12 | 0 | 0.000000 | -0.000000 | 1062 | 1006.201945 | 1073.03 | 1139.858055 | 1.010386 |
| GO:0045471\_response\_to\_ethanol | 12 | 0 | 0.000000 | -0.000000 | 1062 | 1006.201945 | 1073.03 | 1139.858055 | 1.010386 |
| GO:0045792\_negative\_regulation\_of\_cell\_size | 12 | 0 | 0.000000 | -0.000000 | 1062 | 1006.201945 | 1073.03 | 1139.858055 | 1.010386 |
| GO:0045995\_regulation\_of\_embryonic\_development | 12 | 0 | 0.000000 | -0.000000 | 1062 | 1006.201945 | 1073.03 | 1139.858055 | 1.010386 |
| GO:0046068\_cGMP\_metabolic\_process | 12 | 0 | 0.000000 | -0.000000 | 1062 | 1006.201945 | 1073.03 | 1139.858055 | 1.010386 |
| GO:0046128\_purine\_ribonucleoside\_metabolic\_process | 12 | 0 | 0.000000 | -0.000000 | 1062 | 1006.201945 | 1073.03 | 1139.858055 | 1.010386 |
| GO:0046330\_positive\_regulation\_of\_JNK\_cascade | 12 | 0 | 0.000000 | -0.000000 | 1062 | 1006.201945 | 1073.03 | 1139.858055 | 1.010386 |
| GO:0048169\_regulation\_of\_long-term\_neuronal\_synaptic\_plasticity | 12 | 0 | 0.000000 | -0.000000 | 1062 | 1006.201945 | 1073.03 | 1139.858055 | 1.010386 |
| GO:0048820\_hair\_follicle\_maturation | 12 | 0 | 0.000000 | -0.000000 | 1062 | 1006.201945 | 1073.03 | 1139.858055 | 1.010386 |
| GO:0048821\_erythrocyte\_development | 12 | 0 | 0.000000 | -0.000000 | 1062 | 1006.201945 | 1073.03 | 1139.858055 | 1.010386 |
| GO:0048854\_brain\_morphogenesis | 12 | 0 | 0.000000 | -0.000000 | 1062 | 1006.201945 | 1073.03 | 1139.858055 | 1.010386 |
| GO:0050432\_catecholamine\_secretion | 12 | 0 | 0.000000 | -0.000000 | 1062 | 1006.201945 | 1073.03 | 1139.858055 | 1.010386 |
| GO:0050795\_regulation\_of\_behavior | 12 | 0 | 0.000000 | -0.000000 | 1062 | 1006.201945 | 1073.03 | 1139.858055 | 1.010386 |
| GO:0050803\_regulation\_of\_synapse\_structure\_and\_activity | 12 | 0 | 0.000000 | -0.000000 | 1062 | 1006.201945 | 1073.03 | 1139.858055 | 1.010386 |
| GO:0050848\_regulation\_of\_calcium-mediated\_signaling | 12 | 0 | 0.000000 | -0.000000 | 1062 | 1006.201945 | 1073.03 | 1139.858055 | 1.010386 |
| GO:0050850\_positive\_regulation\_of\_calcium-mediated\_signaling | 12 | 0 | 0.000000 | -0.000000 | 1062 | 1006.201945 | 1073.03 | 1139.858055 | 1.010386 |
| GO:0050853\_B\_cell\_receptor\_signaling\_pathway | 12 | 0 | 0.000000 | -0.000000 | 1062 | 1006.201945 | 1073.03 | 1139.858055 | 1.010386 |
| GO:0050869\_negative\_regulation\_of\_B\_cell\_activation | 12 | 0 | 0.000000 | -0.000000 | 1062 | 1006.201945 | 1073.03 | 1139.858055 | 1.010386 |
| GO:0051145\_smooth\_muscle\_cell\_differentiation | 12 | 0 | 0.000000 | -0.000000 | 1062 | 1006.201945 | 1073.03 | 1139.858055 | 1.010386 |
| GO:0051588\_regulation\_of\_neurotransmitter\_transport | 12 | 0 | 0.000000 | -0.000000 | 1062 | 1006.201945 | 1073.03 | 1139.858055 | 1.010386 |
| GO:0055002\_striated\_muscle\_cell\_development | 12 | 0 | 0.000000 | -0.000000 | 1062 | 1006.201945 | 1073.03 | 1139.858055 | 1.010386 |
| GO:0060042\_retina\_morphogenesis\_in\_camera-type\_eye | 12 | 0 | 0.000000 | -0.000000 | 1062 | 1006.201945 | 1073.03 | 1139.858055 | 1.010386 |
| GO:0060048\_cardiac\_muscle\_contraction | 12 | 0 | 0.000000 | -0.000000 | 1062 | 1006.201945 | 1073.03 | 1139.858055 | 1.010386 |
| GO:0060525\_prostate\_glandular\_acinus\_development | 12 | 0 | 0.000000 | -0.000000 | 1062 | 1006.201945 | 1073.03 | 1139.858055 | 1.010386 |
| GO:0060572\_morphogenesis\_of\_an\_epithelial\_bud | 12 | 0 | 0.000000 | -0.000000 | 1062 | 1006.201945 | 1073.03 | 1139.858055 | 1.010386 |
| GO:0060736\_prostate\_gland\_growth | 12 | 0 | 0.000000 | -0.000000 | 1062 | 1006.201945 | 1073.03 | 1139.858055 | 1.010386 |
| GO:0070304\_positive\_regulation\_of\_stress-activated\_protein\_kinase\_signaling\_pathway | 12 | 0 | 0.000000 | -0.000000 | 1062 | 1006.201945 | 1073.03 | 1139.858055 | 1.010386 |
| GO:0001817\_regulation\_of\_cytokine\_production | 99 | 0 | 0.000000 | -0.000000 | 1066 | 1008.710160 | 1075.33 | 1141.949840 | 1.008752 |
| GO:0007398\_ectoderm\_development | 99 | 0 | 0.000000 | -0.000000 | 1066 | 1008.710160 | 1075.33 | 1141.949840 | 1.008752 |
| GO:0060348\_bone\_development | 99 | 0 | 0.000000 | -0.000000 | 1066 | 1008.710160 | 1075.33 | 1141.949840 | 1.008752 |
| GO:0060562\_epithelial\_tube\_morphogenesis | 99 | 0 | 0.000000 | -0.000000 | 1066 | 1008.710160 | 1075.33 | 1141.949840 | 1.008752 |
| GO:0032787\_monocarboxylic\_acid\_metabolic\_process | 130 | 0 | 0.000000 | -0.000000 | 1068 | 1010.610991 | 1076.89 | 1143.169009 | 1.008324 |
| GO:0045165\_cell\_fate\_commitment | 130 | 0 | 0.000000 | -0.000000 | 1068 | 1010.610991 | 1076.89 | 1143.169009 | 1.008324 |
| GO:0001934\_positive\_regulation\_of\_protein\_amino\_acid\_phosphorylation | 29 | 0 | 0.000000 | -0.000000 | 1087 | 1030.967208 | 1096.3 | 1161.632792 | 1.008556 |
| GO:0006417\_regulation\_of\_translation | 29 | 0 | 0.000000 | -0.000000 | 1087 | 1030.967208 | 1096.3 | 1161.632792 | 1.008556 |
| GO:0006641\_triglyceride\_metabolic\_process | 29 | 0 | 0.000000 | -0.000000 | 1087 | 1030.967208 | 1096.3 | 1161.632792 | 1.008556 |
| GO:0006909\_phagocytosis | 29 | 0 | 0.000000 | -0.000000 | 1087 | 1030.967208 | 1096.3 | 1161.632792 | 1.008556 |
| GO:0007190\_activation\_of\_adenylate\_cyclase\_activity | 29 | 0 | 0.000000 | -0.000000 | 1087 | 1030.967208 | 1096.3 | 1161.632792 | 1.008556 |
| GO:0016447\_somatic\_recombination\_of\_immunoglobulin\_gene\_segments | 29 | 0 | 0.000000 | -0.000000 | 1087 | 1030.967208 | 1096.3 | 1161.632792 | 1.008556 |
| GO:0021761\_limbic\_system\_development | 29 | 0 | 0.000000 | -0.000000 | 1087 | 1030.967208 | 1096.3 | 1161.632792 | 1.008556 |
| GO:0042176\_regulation\_of\_protein\_catabolic\_process | 29 | 0 | 0.000000 | -0.000000 | 1087 | 1030.967208 | 1096.3 | 1161.632792 | 1.008556 |
| GO:0042490\_mechanoreceptor\_differentiation | 29 | 0 | 0.000000 | -0.000000 | 1087 | 1030.967208 | 1096.3 | 1161.632792 | 1.008556 |
| GO:0042770\_DNA\_damage\_response\_\_signal\_transduction | 29 | 0 | 0.000000 | -0.000000 | 1087 | 1030.967208 | 1096.3 | 1161.632792 | 1.008556 |
| GO:0043281\_regulation\_of\_caspase\_activity | 29 | 0 | 0.000000 | -0.000000 | 1087 | 1030.967208 | 1096.3 | 1161.632792 | 1.008556 |
| GO:0044087\_regulation\_of\_cellular\_component\_biogenesis | 29 | 0 | 0.000000 | -0.000000 | 1087 | 1030.967208 | 1096.3 | 1161.632792 | 1.008556 |
| GO:0044270\_nitrogen\_compound\_catabolic\_process | 29 | 0 | 0.000000 | -0.000000 | 1087 | 1030.967208 | 1096.3 | 1161.632792 | 1.008556 |
| GO:0045621\_positive\_regulation\_of\_lymphocyte\_differentiation | 29 | 0 | 0.000000 | -0.000000 | 1087 | 1030.967208 | 1096.3 | 1161.632792 | 1.008556 |
| GO:0046634\_regulation\_of\_alpha-beta\_T\_cell\_activation | 29 | 0 | 0.000000 | -0.000000 | 1087 | 1030.967208 | 1096.3 | 1161.632792 | 1.008556 |
| GO:0050769\_positive\_regulation\_of\_neurogenesis | 29 | 0 | 0.000000 | -0.000000 | 1087 | 1030.967208 | 1096.3 | 1161.632792 | 1.008556 |
| GO:0051301\_cell\_division | 29 | 0 | 0.000000 | -0.000000 | 1087 | 1030.967208 | 1096.3 | 1161.632792 | 1.008556 |
| GO:0052548\_regulation\_of\_endopeptidase\_activity | 29 | 0 | 0.000000 | -0.000000 | 1087 | 1030.967208 | 1096.3 | 1161.632792 | 1.008556 |
| GO:0070302\_regulation\_of\_stress-activated\_protein\_kinase\_signaling\_pathway | 29 | 0 | 0.000000 | -0.000000 | 1087 | 1030.967208 | 1096.3 | 1161.632792 | 1.008556 |
| GO:0000280\_nuclear\_division | 24 | 0 | 0.000000 | -0.000000 | 1114 | 1057.322726 | 1121.43 | 1185.537274 | 1.006670 |
| GO:0001541\_ovarian\_follicle\_development | 24 | 0 | 0.000000 | -0.000000 | 1114 | 1057.322726 | 1121.43 | 1185.537274 | 1.006670 |
| GO:0002381\_immunoglobulin\_production\_during\_immune\_response | 24 | 0 | 0.000000 | -0.000000 | 1114 | 1057.322726 | 1121.43 | 1185.537274 | 1.006670 |
| GO:0006650\_glycerophospholipid\_metabolic\_process | 24 | 0 | 0.000000 | -0.000000 | 1114 | 1057.322726 | 1121.43 | 1185.537274 | 1.006670 |
| GO:0006941\_striated\_muscle\_contraction | 24 | 0 | 0.000000 | -0.000000 | 1114 | 1057.322726 | 1121.43 | 1185.537274 | 1.006670 |
| GO:0006959\_humoral\_immune\_response | 24 | 0 | 0.000000 | -0.000000 | 1114 | 1057.322726 | 1121.43 | 1185.537274 | 1.006670 |
| GO:0007050\_cell\_cycle\_arrest | 24 | 0 | 0.000000 | -0.000000 | 1114 | 1057.322726 | 1121.43 | 1185.537274 | 1.006670 |
| GO:0007067\_mitosis | 24 | 0 | 0.000000 | -0.000000 | 1114 | 1057.322726 | 1121.43 | 1185.537274 | 1.006670 |
| GO:0007204\_elevation\_of\_cytosolic\_calcium\_ion\_concentration | 24 | 0 | 0.000000 | -0.000000 | 1114 | 1057.322726 | 1121.43 | 1185.537274 | 1.006670 |
| GO:0007259\_JAK-STAT\_cascade | 24 | 0 | 0.000000 | -0.000000 | 1114 | 1057.322726 | 1121.43 | 1185.537274 | 1.006670 |
| GO:0007266\_Rho\_protein\_signal\_transduction | 24 | 0 | 0.000000 | -0.000000 | 1114 | 1057.322726 | 1121.43 | 1185.537274 | 1.006670 |
| GO:0007632\_visual\_behavior | 24 | 0 | 0.000000 | -0.000000 | 1114 | 1057.322726 | 1121.43 | 1185.537274 | 1.006670 |
| GO:0008629\_induction\_of\_apoptosis\_by\_intracellular\_signals | 24 | 0 | 0.000000 | -0.000000 | 1114 | 1057.322726 | 1121.43 | 1185.537274 | 1.006670 |
| GO:0009612\_response\_to\_mechanical\_stimulus | 24 | 0 | 0.000000 | -0.000000 | 1114 | 1057.322726 | 1121.43 | 1185.537274 | 1.006670 |
| GO:0014070\_response\_to\_organic\_cyclic\_substance | 24 | 0 | 0.000000 | -0.000000 | 1114 | 1057.322726 | 1121.43 | 1185.537274 | 1.006670 |
| GO:0021515\_cell\_differentiation\_in\_spinal\_cord | 24 | 0 | 0.000000 | -0.000000 | 1114 | 1057.322726 | 1121.43 | 1185.537274 | 1.006670 |
| GO:0032386\_regulation\_of\_intracellular\_transport | 24 | 0 | 0.000000 | -0.000000 | 1114 | 1057.322726 | 1121.43 | 1185.537274 | 1.006670 |
| GO:0042158\_lipoprotein\_biosynthetic\_process | 24 | 0 | 0.000000 | -0.000000 | 1114 | 1057.322726 | 1121.43 | 1185.537274 | 1.006670 |
| GO:0042632\_cholesterol\_homeostasis | 24 | 0 | 0.000000 | -0.000000 | 1114 | 1057.322726 | 1121.43 | 1185.537274 | 1.006670 |
| GO:0043588\_skin\_development | 24 | 0 | 0.000000 | -0.000000 | 1114 | 1057.322726 | 1121.43 | 1185.537274 | 1.006670 |
| GO:0048002\_antigen\_processing\_and\_presentation\_of\_peptide\_antigen | 24 | 0 | 0.000000 | -0.000000 | 1114 | 1057.322726 | 1121.43 | 1185.537274 | 1.006670 |
| GO:0048546\_digestive\_tract\_morphogenesis | 24 | 0 | 0.000000 | -0.000000 | 1114 | 1057.322726 | 1121.43 | 1185.537274 | 1.006670 |
| GO:0050679\_positive\_regulation\_of\_epithelial\_cell\_proliferation | 24 | 0 | 0.000000 | -0.000000 | 1114 | 1057.322726 | 1121.43 | 1185.537274 | 1.006670 |
| GO:0055092\_sterol\_homeostasis | 24 | 0 | 0.000000 | -0.000000 | 1114 | 1057.322726 | 1121.43 | 1185.537274 | 1.006670 |
| GO:0060078\_regulation\_of\_postsynaptic\_membrane\_potential | 24 | 0 | 0.000000 | -0.000000 | 1114 | 1057.322726 | 1121.43 | 1185.537274 | 1.006670 |
| GO:0060113\_inner\_ear\_receptor\_cell\_differentiation | 24 | 0 | 0.000000 | -0.000000 | 1114 | 1057.322726 | 1121.43 | 1185.537274 | 1.006670 |
| GO:0070667\_negative\_regulation\_of\_mast\_cell\_proliferation | 24 | 0 | 0.000000 | -0.000000 | 1114 | 1057.322726 | 1121.43 | 1185.537274 | 1.006670 |
| GO:0043065\_positive\_regulation\_of\_apoptosis | 166 | 0 | 0.000000 | -0.000000 | 1115 | 1057.979056 | 1121.98 | 1185.980944 | 1.006260 |
| GO:0007612\_learning | 46 | 0 | 0.000000 | -0.000000 | 1121 | 1066.479512 | 1129.88 | 1193.280488 | 1.007921 |
| GO:0009581\_detection\_of\_external\_stimulus | 46 | 0 | 0.000000 | -0.000000 | 1121 | 1066.479512 | 1129.88 | 1193.280488 | 1.007921 |
| GO:0019724\_B\_cell\_mediated\_immunity | 46 | 0 | 0.000000 | -0.000000 | 1121 | 1066.479512 | 1129.88 | 1193.280488 | 1.007921 |
| GO:0030218\_erythrocyte\_differentiation | 46 | 0 | 0.000000 | -0.000000 | 1121 | 1066.479512 | 1129.88 | 1193.280488 | 1.007921 |
| GO:0042063\_gliogenesis | 46 | 0 | 0.000000 | -0.000000 | 1121 | 1066.479512 | 1129.88 | 1193.280488 | 1.007921 |
| GO:0046631\_alpha-beta\_T\_cell\_activation | 46 | 0 | 0.000000 | -0.000000 | 1121 | 1066.479512 | 1129.88 | 1193.280488 | 1.007921 |
| GO:0035239\_tube\_morphogenesis | 143 | 0 | 0.000000 | -0.000000 | 1122 | 1066.949057 | 1130.32 | 1193.690943 | 1.007415 |
| GO:0030198\_extracellular\_matrix\_organization | 83 | 0 | 0.000000 | -0.000000 | 1125 | 1071.185626 | 1134.17 | 1197.154374 | 1.008151 |
| GO:0030534\_adult\_behavior | 83 | 0 | 0.000000 | -0.000000 | 1125 | 1071.185626 | 1134.17 | 1197.154374 | 1.008151 |
| GO:0050867\_positive\_regulation\_of\_cell\_activation | 83 | 0 | 0.000000 | -0.000000 | 1125 | 1071.185626 | 1134.17 | 1197.154374 | 1.008151 |
| GO:0003007\_heart\_morphogenesis | 67 | 0 | 0.000000 | -0.000000 | 1129 | 1075.107395 | 1137.68 | 1200.252605 | 1.007688 |
| GO:0031347\_regulation\_of\_defense\_response | 67 | 0 | 0.000000 | -0.000000 | 1129 | 1075.107395 | 1137.68 | 1200.252605 | 1.007688 |
| GO:0042445\_hormone\_metabolic\_process | 67 | 0 | 0.000000 | -0.000000 | 1129 | 1075.107395 | 1137.68 | 1200.252605 | 1.007688 |
| GO:0051247\_positive\_regulation\_of\_protein\_metabolic\_process | 67 | 0 | 0.000000 | -0.000000 | 1129 | 1075.107395 | 1137.68 | 1200.252605 | 1.007688 |
| GO:0001890\_placenta\_development | 77 | 0 | 0.000000 | -0.000000 | 1131 | 1076.713608 | 1139.05 | 1201.386392 | 1.007118 |
| GO:0051241\_negative\_regulation\_of\_multicellular\_organismal\_process | 77 | 0 | 0.000000 | -0.000000 | 1131 | 1076.713608 | 1139.05 | 1201.386392 | 1.007118 |
| GO:0002694\_regulation\_of\_leukocyte\_activation | 121 | 0 | 0.000000 | -0.000000 | 1134 | 1079.244591 | 1141.31 | 1203.375409 | 1.006446 |
| GO:0006917\_induction\_of\_apoptosis | 121 | 0 | 0.000000 | -0.000000 | 1134 | 1079.244591 | 1141.31 | 1203.375409 | 1.006446 |
| GO:0012502\_induction\_of\_programmed\_cell\_death | 121 | 0 | 0.000000 | -0.000000 | 1134 | 1079.244591 | 1141.31 | 1203.375409 | 1.006446 |
| GO:0050767\_regulation\_of\_neurogenesis | 104 | 0 | 0.000000 | -0.000000 | 1136 | 1080.604914 | 1142.53 | 1204.455086 | 1.005748 |
| GO:0055086\_nucleobase\_\_nucleoside\_and\_nucleotide\_metabolic\_process | 104 | 0 | 0.000000 | -0.000000 | 1136 | 1080.604914 | 1142.53 | 1204.455086 | 1.005748 |
| GO:0006576\_biogenic\_amine\_metabolic\_process | 53 | 0 | 0.000000 | -0.000000 | 1143 | 1089.732259 | 1150.84 | 1211.947741 | 1.006859 |
| GO:0006935\_chemotaxis | 53 | 0 | 0.000000 | -0.000000 | 1143 | 1089.732259 | 1150.84 | 1211.947741 | 1.006859 |
| GO:0030031\_cell\_projection\_assembly | 53 | 0 | 0.000000 | -0.000000 | 1143 | 1089.732259 | 1150.84 | 1211.947741 | 1.006859 |
| GO:0042330\_taxis | 53 | 0 | 0.000000 | -0.000000 | 1143 | 1089.732259 | 1150.84 | 1211.947741 | 1.006859 |
| GO:0046942\_carboxylic\_acid\_transport | 53 | 0 | 0.000000 | -0.000000 | 1143 | 1089.732259 | 1150.84 | 1211.947741 | 1.006859 |
| GO:0050905\_neuromuscular\_process | 53 | 0 | 0.000000 | -0.000000 | 1143 | 1089.732259 | 1150.84 | 1211.947741 | 1.006859 |
| GO:0055085\_transmembrane\_transport | 53 | 0 | 0.000000 | -0.000000 | 1143 | 1089.732259 | 1150.84 | 1211.947741 | 1.006859 |
| GO:0006338\_chromatin\_remodeling | 19 | 0 | 0.000000 | -0.000000 | 1175 | 1121.411332 | 1181.77 | 1242.128668 | 1.005762 |
| GO:0006497\_protein\_amino\_acid\_lipidation | 19 | 0 | 0.000000 | -0.000000 | 1175 | 1121.411332 | 1181.77 | 1242.128668 | 1.005762 |
| GO:0006672\_ceramide\_metabolic\_process | 19 | 0 | 0.000000 | -0.000000 | 1175 | 1121.411332 | 1181.77 | 1242.128668 | 1.005762 |
| GO:0006776\_vitamin\_A\_metabolic\_process | 19 | 0 | 0.000000 | -0.000000 | 1175 | 1121.411332 | 1181.77 | 1242.128668 | 1.005762 |
| GO:0007569\_cell\_aging | 19 | 0 | 0.000000 | -0.000000 | 1175 | 1121.411332 | 1181.77 | 1242.128668 | 1.005762 |
| GO:0007595\_lactation | 19 | 0 | 0.000000 | -0.000000 | 1175 | 1121.411332 | 1181.77 | 1242.128668 | 1.005762 |
| GO:0009584\_detection\_of\_visible\_light | 19 | 0 | 0.000000 | -0.000000 | 1175 | 1121.411332 | 1181.77 | 1242.128668 | 1.005762 |
| GO:0009798\_axis\_specification | 19 | 0 | 0.000000 | -0.000000 | 1175 | 1121.411332 | 1181.77 | 1242.128668 | 1.005762 |
| GO:0010952\_positive\_regulation\_of\_peptidase\_activity | 19 | 0 | 0.000000 | -0.000000 | 1175 | 1121.411332 | 1181.77 | 1242.128668 | 1.005762 |
| GO:0019218\_regulation\_of\_steroid\_metabolic\_process | 19 | 0 | 0.000000 | -0.000000 | 1175 | 1121.411332 | 1181.77 | 1242.128668 | 1.005762 |
| GO:0021587\_cerebellum\_morphogenesis | 19 | 0 | 0.000000 | -0.000000 | 1175 | 1121.411332 | 1181.77 | 1242.128668 | 1.005762 |
| GO:0030199\_collagen\_fibril\_organization | 19 | 0 | 0.000000 | -0.000000 | 1175 | 1121.411332 | 1181.77 | 1242.128668 | 1.005762 |
| GO:0030518\_steroid\_hormone\_receptor\_signaling\_pathway | 19 | 0 | 0.000000 | -0.000000 | 1175 | 1121.411332 | 1181.77 | 1242.128668 | 1.005762 |
| GO:0030595\_leukocyte\_chemotaxis | 19 | 0 | 0.000000 | -0.000000 | 1175 | 1121.411332 | 1181.77 | 1242.128668 | 1.005762 |
| GO:0032526\_response\_to\_retinoic\_acid | 19 | 0 | 0.000000 | -0.000000 | 1175 | 1121.411332 | 1181.77 | 1242.128668 | 1.005762 |
| GO:0033002\_muscle\_cell\_proliferation | 19 | 0 | 0.000000 | -0.000000 | 1175 | 1121.411332 | 1181.77 | 1242.128668 | 1.005762 |
| GO:0033189\_response\_to\_vitamin\_A | 19 | 0 | 0.000000 | -0.000000 | 1175 | 1121.411332 | 1181.77 | 1242.128668 | 1.005762 |
| GO:0042462\_eye\_photoreceptor\_cell\_development | 19 | 0 | 0.000000 | -0.000000 | 1175 | 1121.411332 | 1181.77 | 1242.128668 | 1.005762 |
| GO:0042491\_auditory\_receptor\_cell\_differentiation | 19 | 0 | 0.000000 | -0.000000 | 1175 | 1121.411332 | 1181.77 | 1242.128668 | 1.005762 |
| GO:0043280\_positive\_regulation\_of\_caspase\_activity | 19 | 0 | 0.000000 | -0.000000 | 1175 | 1121.411332 | 1181.77 | 1242.128668 | 1.005762 |
| GO:0046165\_alcohol\_biosynthetic\_process | 19 | 0 | 0.000000 | -0.000000 | 1175 | 1121.411332 | 1181.77 | 1242.128668 | 1.005762 |
| GO:0046890\_regulation\_of\_lipid\_biosynthetic\_process | 19 | 0 | 0.000000 | -0.000000 | 1175 | 1121.411332 | 1181.77 | 1242.128668 | 1.005762 |
| GO:0048536\_spleen\_development | 19 | 0 | 0.000000 | -0.000000 | 1175 | 1121.411332 | 1181.77 | 1242.128668 | 1.005762 |
| GO:0048547\_gut\_morphogenesis | 19 | 0 | 0.000000 | -0.000000 | 1175 | 1121.411332 | 1181.77 | 1242.128668 | 1.005762 |
| GO:0048701\_embryonic\_cranial\_skeleton\_morphogenesis | 19 | 0 | 0.000000 | -0.000000 | 1175 | 1121.411332 | 1181.77 | 1242.128668 | 1.005762 |
| GO:0050728\_negative\_regulation\_of\_inflammatory\_response | 19 | 0 | 0.000000 | -0.000000 | 1175 | 1121.411332 | 1181.77 | 1242.128668 | 1.005762 |
| GO:0050908\_detection\_of\_light\_stimulus\_involved\_in\_visual\_perception | 19 | 0 | 0.000000 | -0.000000 | 1175 | 1121.411332 | 1181.77 | 1242.128668 | 1.005762 |
| GO:0050962\_detection\_of\_light\_stimulus\_involved\_in\_sensory\_perception | 19 | 0 | 0.000000 | -0.000000 | 1175 | 1121.411332 | 1181.77 | 1242.128668 | 1.005762 |
| GO:0051056\_regulation\_of\_small\_GTPase\_mediated\_signal\_transduction | 19 | 0 | 0.000000 | -0.000000 | 1175 | 1121.411332 | 1181.77 | 1242.128668 | 1.005762 |
| GO:0060079\_regulation\_of\_excitatory\_postsynaptic\_membrane\_potential | 19 | 0 | 0.000000 | -0.000000 | 1175 | 1121.411332 | 1181.77 | 1242.128668 | 1.005762 |
| GO:0060326\_cell\_chemotaxis | 19 | 0 | 0.000000 | -0.000000 | 1175 | 1121.411332 | 1181.77 | 1242.128668 | 1.005762 |
| GO:0060444\_branching\_involved\_in\_mammary\_gland\_duct\_morphogenesis | 19 | 0 | 0.000000 | -0.000000 | 1175 | 1121.411332 | 1181.77 | 1242.128668 | 1.005762 |
| GO:0000084\_S\_phase\_of\_mitotic\_cell\_cycle | 3 | 0 |  |  |  |  |  |  |  |  |
| GO:0000089\_mitotic\_metaphase | 3 | 0 |  |  |  |  |  |  |  |  |
| GO:0000098\_sulfur\_amino\_acid\_catabolic\_process | 3 | 0 |  |  |  |  |  |  |  |  |
| GO:0000103\_sulfate\_assimilation | 3 | 0 |  |  |  |  |  |  |  |  |
| GO:0000212\_meiotic\_spindle\_organization | 3 | 0 |  |  |  |  |  |  |  |  |
| GO:0000281\_cytokinesis\_after\_mitosis | 3 | 0 |  |  |  |  |  |  |  |  |
| GO:0000320\_re-entry\_into\_mitotic\_cell\_cycle | 3 | 0 |  |  |  |  |  |  |  |  |
| GO:0000380\_alternative\_nuclear\_mRNA\_splicing\_\_via\_spliceosome | 3 | 0 |  |  |  |  |  |  |  |  |
| GO:0001516\_prostaglandin\_biosynthetic\_process | 3 | 0 |  |  |  |  |  |  |  |  |
| GO:0001553\_luteinization | 3 | 0 |  |  |  |  |  |  |  |  |
| GO:0001574\_ganglioside\_biosynthetic\_process | 3 | 0 |  |  |  |  |  |  |  |  |
| GO:0001705\_ectoderm\_formation | 3 | 0 |  |  |  |  |  |  |  |  |
| GO:0001711\_endodermal\_cell\_fate\_commitment | 3 | 0 |  |  |  |  |  |  |  |  |
| GO:0001757\_somite\_specification | 3 | 0 |  |  |  |  |  |  |  |  |
| GO:0001778\_plasma\_membrane\_repair | 3 | 0 |  |  |  |  |  |  |  |  |
| GO:0001780\_neutrophil\_homeostasis | 3 | 0 |  |  |  |  |  |  |  |  |
| GO:0001802\_type\_III\_hypersensitivity | 3 | 0 |  |  |  |  |  |  |  |  |
| GO:0001803\_regulation\_of\_type\_III\_hypersensitivity | 3 | 0 |  |  |  |  |  |  |  |  |
| GO:0001805\_positive\_regulation\_of\_type\_III\_hypersensitivity | 3 | 0 |  |  |  |  |  |  |  |  |
| GO:0001812\_positive\_regulation\_of\_type\_I\_hypersensitivity | 3 | 0 |  |  |  |  |  |  |  |  |
| GO:0001831\_trophectodermal\_cellular\_morphogenesis | 3 | 0 |  |  |  |  |  |  |  |  |
| GO:0001844\_protein\_insertion\_into\_mitochondrial\_membrane\_during\_induction\_of\_apoptosis | 3 | 0 |  |  |  |  |  |  |  |  |
| GO:0001878\_response\_to\_yeast | 3 | 0 |  |  |  |  |  |  |  |  |
| GO:0001895\_retina\_homeostasis | 3 | 0 |  |  |  |  |  |  |  |  |
| GO:0001915\_negative\_regulation\_of\_T\_cell\_mediated\_cytotoxicity | 3 | 0 |  |  |  |  |  |  |  |  |
| GO:0001937\_negative\_regulation\_of\_endothelial\_cell\_proliferation | 3 | 0 |  |  |  |  |  |  |  |  |
| GO:0001953\_negative\_regulation\_of\_cell-matrix\_adhesion | 3 | 0 |  |  |  |  |  |  |  |  |
| GO:0001955\_blood\_vessel\_maturation | 3 | 0 |  |  |  |  |  |  |  |  |
| GO:0001960\_negative\_regulation\_of\_cytokine-mediated\_signaling\_pathway | 3 | 0 |  |  |  |  |  |  |  |  |
| GO:0001973\_adenosine\_receptor\_signaling\_pathway | 3 | 0 |  |  |  |  |  |  |  |  |
| GO:0001996\_positive\_regulation\_of\_heart\_rate\_by\_epinephrine-norepinephrine | 3 | 0 |  |  |  |  |  |  |  |  |
| GO:0002034\_regulation\_of\_blood\_vessel\_size\_by\_renin-angiotensin | 3 | 0 |  |  |  |  |  |  |  |  |
| GO:0002238\_response\_to\_molecule\_of\_fungal\_origin | 3 | 0 |  |  |  |  |  |  |  |  |
| GO:0002275\_myeloid\_cell\_activation\_during\_immune\_response | 3 | 0 |  |  |  |  |  |  |  |  |
| GO:0002281\_macrophage\_activation\_during\_immune\_response | 3 | 0 |  |  |  |  |  |  |  |  |
| GO:0002309\_T\_cell\_proliferation\_during\_immune\_response | 3 | 0 |  |  |  |  |  |  |  |  |
| GO:0002361\_CD4-positive\_\_CD25-positive\_\_alpha-beta\_regulatory\_T\_cell\_differentiation | 3 | 0 |  |  |  |  |  |  |  |  |
| GO:0002369\_T\_cell\_cytokine\_production | 3 | 0 |  |  |  |  |  |  |  |  |
| GO:0002428\_antigen\_processing\_and\_presentation\_of\_peptide\_antigen\_via\_MHC\_class\_Ib | 3 | 0 |  |  |  |  |  |  |  |  |
| GO:0002446\_neutrophil\_mediated\_immunity | 3 | 0 |  |  |  |  |  |  |  |  |
| GO:0002477\_antigen\_processing\_and\_presentation\_of\_exogenous\_peptide\_antigen\_via\_MHC\_class\_Ib | 3 | 0 |  |  |  |  |  |  |  |  |
| GO:0002481\_antigen\_processing\_and\_presentation\_of\_exogenous\_protein\_antigen\_via\_MHC\_class\_Ib\_\_TAP-dependent | 3 | 0 |  |  |  |  |  |  |  |  |
| GO:0002513\_tolerance\_induction\_to\_self\_antigen | 3 | 0 |  |  |  |  |  |  |  |  |
| GO:0002568\_somatic\_diversification\_of\_T\_cell\_receptor\_genes | 3 | 0 |  |  |  |  |  |  |  |  |
| GO:0002674\_negative\_regulation\_of\_acute\_inflammatory\_response | 3 | 0 |  |  |  |  |  |  |  |  |
| GO:0002681\_somatic\_recombination\_of\_T\_cell\_receptor\_gene\_segments | 3 | 0 |  |  |  |  |  |  |  |  |
| GO:0002713\_negative\_regulation\_of\_B\_cell\_mediated\_immunity | 3 | 0 |  |  |  |  |  |  |  |  |
| GO:0002827\_positive\_regulation\_of\_T-helper\_1\_type\_immune\_response | 3 | 0 |  |  |  |  |  |  |  |  |
| GO:0002865\_negative\_regulation\_of\_acute\_inflammatory\_response\_to\_antigenic\_stimulus | 3 | 0 |  |  |  |  |  |  |  |  |
| GO:0002884\_negative\_regulation\_of\_hypersensitivity | 3 | 0 |  |  |  |  |  |  |  |  |
| GO:0002890\_negative\_regulation\_of\_immunoglobulin\_mediated\_immune\_response | 3 | 0 |  |  |  |  |  |  |  |  |
| GO:0002904\_positive\_regulation\_of\_B\_cell\_apoptosis | 3 | 0 |  |  |  |  |  |  |  |  |
| GO:0003009\_skeletal\_muscle\_contraction | 3 | 0 |  |  |  |  |  |  |  |  |
| GO:0003072\_renal\_control\_of\_peripheral\_vascular\_resistance\_involved\_in\_regulation\_of\_systemic\_arterial\_blood\_pressure | 3 | 0 |  |  |  |  |  |  |  |  |
| GO:0006047\_UDP-N-acetylglucosamine\_metabolic\_process | 3 | 0 |  |  |  |  |  |  |  |  |
| GO:0006067\_ethanol\_metabolic\_process | 3 | 0 |  |  |  |  |  |  |  |  |
| GO:0006072\_glycerol-3-phosphate\_metabolic\_process | 3 | 0 |  |  |  |  |  |  |  |  |
| GO:0006103\_2-oxoglutarate\_metabolic\_process | 3 | 0 |  |  |  |  |  |  |  |  |
| GO:0006107\_oxaloacetate\_metabolic\_process | 3 | 0 |  |  |  |  |  |  |  |  |
| GO:0006166\_purine\_ribonucleoside\_salvage | 3 | 0 |  |  |  |  |  |  |  |  |
| GO:0006220\_pyrimidine\_nucleotide\_metabolic\_process | 3 | 0 |  |  |  |  |  |  |  |  |
| GO:0006266\_DNA\_ligation | 3 | 0 |  |  |  |  |  |  |  |  |
| GO:0006282\_regulation\_of\_DNA\_repair | 3 | 0 |  |  |  |  |  |  |  |  |
| GO:0006287\_base-excision\_repair\_\_gap-filling | 3 | 0 |  |  |  |  |  |  |  |  |
| GO:0006301\_postreplication\_repair | 3 | 0 |  |  |  |  |  |  |  |  |
| GO:0006361\_transcription\_initiation\_from\_RNA\_polymerase\_I\_promoter | 3 | 0 |  |  |  |  |  |  |  |  |
| GO:0006367\_transcription\_initiation\_from\_RNA\_polymerase\_II\_promoter | 3 | 0 |  |  |  |  |  |  |  |  |
| GO:0006414\_translational\_elongation | 3 | 0 |  |  |  |  |  |  |  |  |
| GO:0006491\_N-glycan\_processing | 3 | 0 |  |  |  |  |  |  |  |  |
| GO:0006498\_N-terminal\_protein\_lipidation | 3 | 0 |  |  |  |  |  |  |  |  |
| GO:0006531\_aspartate\_metabolic\_process | 3 | 0 |  |  |  |  |  |  |  |  |
| GO:0006598\_polyamine\_catabolic\_process | 3 | 0 |  |  |  |  |  |  |  |  |
| GO:0006620\_posttranslational\_protein\_targeting\_to\_membrane | 3 | 0 |  |  |  |  |  |  |  |  |
| GO:0006625\_protein\_targeting\_to\_peroxisome | 3 | 0 |  |  |  |  |  |  |  |  |
| GO:0006651\_diacylglycerol\_biosynthetic\_process | 3 | 0 |  |  |  |  |  |  |  |  |
| GO:0006670\_sphingosine\_metabolic\_process | 3 | 0 |  |  |  |  |  |  |  |  |
| GO:0006677\_glycosylceramide\_metabolic\_process | 3 | 0 |  |  |  |  |  |  |  |  |
| GO:0006689\_ganglioside\_catabolic\_process | 3 | 0 |  |  |  |  |  |  |  |  |
| GO:0006699\_bile\_acid\_biosynthetic\_process | 3 | 0 |  |  |  |  |  |  |  |  |
| GO:0006791\_sulfur\_utilization | 3 | 0 |  |  |  |  |  |  |  |  |
| GO:0006817\_phosphate\_transport | 3 | 0 |  |  |  |  |  |  |  |  |
| GO:0006825\_copper\_ion\_transport | 3 | 0 |  |  |  |  |  |  |  |  |
| GO:0006828\_manganese\_ion\_transport | 3 | 0 |  |  |  |  |  |  |  |  |
| GO:0006857\_oligopeptide\_transport | 3 | 0 |  |  |  |  |  |  |  |  |
| GO:0006892\_post-Golgi\_vesicle-mediated\_transport | 3 | 0 |  |  |  |  |  |  |  |  |
| GO:0006904\_vesicle\_docking\_during\_exocytosis | 3 | 0 |  |  |  |  |  |  |  |  |
| GO:0006926\_virus-infected\_cell\_apoptosis | 3 | 0 |  |  |  |  |  |  |  |  |
| GO:0006953\_acute-phase\_response | 3 | 0 |  |  |  |  |  |  |  |  |
| GO:0007000\_nucleolus\_organization | 3 | 0 |  |  |  |  |  |  |  |  |
| GO:0007041\_lysosomal\_transport | 3 | 0 |  |  |  |  |  |  |  |  |
| GO:0007043\_cell-cell\_junction\_assembly | 3 | 0 |  |  |  |  |  |  |  |  |
| GO:0007090\_regulation\_of\_S\_phase\_of\_mitotic\_cell\_cycle | 3 | 0 |  |  |  |  |  |  |  |  |
| GO:0007195\_inhibition\_of\_adenylate\_cyclase\_activity\_by\_dopamine\_receptor\_signaling\_pathway | 3 | 0 |  |  |  |  |  |  |  |  |
| GO:0007199\_G-protein\_signaling\_\_coupled\_to\_cGMP\_nucleotide\_second\_messenger | 3 | 0 |  |  |  |  |  |  |  |  |
| GO:0007213\_muscarinic\_acetylcholine\_receptor\_signaling\_pathway | 3 | 0 |  |  |  |  |  |  |  |  |
| GO:0007250\_activation\_of\_NF-kappaB-inducing\_kinase\_activity | 3 | 0 |  |  |  |  |  |  |  |  |
| GO:0007252\_I-kappaB\_phosphorylation | 3 | 0 |  |  |  |  |  |  |  |  |
| GO:0007262\_STAT\_protein\_nuclear\_translocation | 3 | 0 |  |  |  |  |  |  |  |  |
| GO:0007288\_sperm\_axoneme\_assembly | 3 | 0 |  |  |  |  |  |  |  |  |
| GO:0007350\_blastoderm\_segmentation | 3 | 0 |  |  |  |  |  |  |  |  |
| GO:0007403\_glial\_cell\_fate\_determination | 3 | 0 |  |  |  |  |  |  |  |  |
| GO:0007412\_axon\_target\_recognition | 3 | 0 |  |  |  |  |  |  |  |  |
| GO:0007468\_regulation\_of\_rhodopsin\_gene\_expression | 3 | 0 |  |  |  |  |  |  |  |  |
| GO:0007525\_somatic\_muscle\_development | 3 | 0 |  |  |  |  |  |  |  |  |
| GO:0007635\_chemosensory\_behavior | 3 | 0 |  |  |  |  |  |  |  |  |
| GO:0008090\_retrograde\_axon\_cargo\_transport | 3 | 0 |  |  |  |  |  |  |  |  |
| GO:0008347\_glial\_cell\_migration | 3 | 0 |  |  |  |  |  |  |  |  |
| GO:0008635\_activation\_of\_caspase\_activity\_by\_cytochrome\_c | 3 | 0 |  |  |  |  |  |  |  |  |
| GO:0009060\_aerobic\_respiration | 3 | 0 |  |  |  |  |  |  |  |  |
| GO:0009081\_branched\_chain\_family\_amino\_acid\_metabolic\_process | 3 | 0 |  |  |  |  |  |  |  |  |
| GO:0009086\_methionine\_biosynthetic\_process | 3 | 0 |  |  |  |  |  |  |  |  |
| GO:0009135\_purine\_nucleoside\_diphosphate\_metabolic\_process | 3 | 0 |  |  |  |  |  |  |  |  |
| GO:0009137\_purine\_nucleoside\_diphosphate\_catabolic\_process | 3 | 0 |  |  |  |  |  |  |  |  |
| GO:0009155\_purine\_deoxyribonucleotide\_catabolic\_process | 3 | 0 |  |  |  |  |  |  |  |  |
| GO:0009179\_purine\_ribonucleoside\_diphosphate\_metabolic\_process | 3 | 0 |  |  |  |  |  |  |  |  |
| GO:0009181\_purine\_ribonucleoside\_diphosphate\_catabolic\_process | 3 | 0 |  |  |  |  |  |  |  |  |
| GO:0009185\_ribonucleoside\_diphosphate\_metabolic\_process | 3 | 0 |  |  |  |  |  |  |  |  |
| GO:0009191\_ribonucleoside\_diphosphate\_catabolic\_process | 3 | 0 |  |  |  |  |  |  |  |  |
| GO:0009199\_ribonucleoside\_triphosphate\_metabolic\_process | 3 | 0 |  |  |  |  |  |  |  |  |
| GO:0009204\_deoxyribonucleoside\_triphosphate\_catabolic\_process | 3 | 0 |  |  |  |  |  |  |  |  |
| GO:0009205\_purine\_ribonucleoside\_triphosphate\_metabolic\_process | 3 | 0 |  |  |  |  |  |  |  |  |
| GO:0009217\_purine\_deoxyribonucleoside\_triphosphate\_catabolic\_process | 3 | 0 |  |  |  |  |  |  |  |  |
| GO:0009448\_gamma-aminobutyric\_acid\_metabolic\_process | 3 | 0 |  |  |  |  |  |  |  |  |
| GO:0010043\_response\_to\_zinc\_ion | 3 | 0 |  |  |  |  |  |  |  |  |
| GO:0010159\_specification\_of\_organ\_position | 3 | 0 |  |  |  |  |  |  |  |  |
| GO:0010172\_embryonic\_body\_morphogenesis | 3 | 0 |  |  |  |  |  |  |  |  |
| GO:0010216\_maintenance\_of\_DNA\_methylation | 3 | 0 |  |  |  |  |  |  |  |  |
| GO:0010273\_detoxification\_of\_copper\_ion | 3 | 0 |  |  |  |  |  |  |  |  |
| GO:0010454\_negative\_regulation\_of\_cell\_fate\_commitment | 3 | 0 |  |  |  |  |  |  |  |  |
| GO:0010507\_negative\_regulation\_of\_autophagy | 3 | 0 |  |  |  |  |  |  |  |  |
| GO:0010524\_positive\_regulation\_of\_calcium\_ion\_transport\_into\_cytosol | 3 | 0 |  |  |  |  |  |  |  |  |
| GO:0010573\_vascular\_endothelial\_growth\_factor\_production | 3 | 0 |  |  |  |  |  |  |  |  |
| GO:0010574\_regulation\_of\_vascular\_endothelial\_growth\_factor\_production | 3 | 0 |  |  |  |  |  |  |  |  |
| GO:0010575\_positive\_regulation\_vascular\_endothelial\_growth\_factor\_production | 3 | 0 |  |  |  |  |  |  |  |  |
| GO:0010632\_regulation\_of\_epithelial\_cell\_migration | 3 | 0 |  |  |  |  |  |  |  |  |
| GO:0010717\_regulation\_of\_epithelial\_to\_mesenchymal\_transition | 3 | 0 |  |  |  |  |  |  |  |  |
| GO:0010884\_positive\_regulation\_of\_lipid\_storage | 3 | 0 |  |  |  |  |  |  |  |  |
| GO:0010888\_negative\_regulation\_of\_lipid\_storage | 3 | 0 |  |  |  |  |  |  |  |  |
| GO:0010889\_regulation\_of\_sequestering\_of\_triglyceride | 3 | 0 |  |  |  |  |  |  |  |  |
| GO:0010893\_positive\_regulation\_of\_steroid\_biosynthetic\_process | 3 | 0 |  |  |  |  |  |  |  |  |
| GO:0010894\_negative\_regulation\_of\_steroid\_biosynthetic\_process | 3 | 0 |  |  |  |  |  |  |  |  |
| GO:0010998\_regulation\_of\_translational\_initiation\_by\_eIF2\_alpha\_phosphorylation | 3 | 0 |  |  |  |  |  |  |  |  |
| GO:0010999\_regulation\_of\_eIF2\_alpha\_phosphorylation\_by\_heme | 3 | 0 |  |  |  |  |  |  |  |  |
| GO:0014074\_response\_to\_purine | 3 | 0 |  |  |  |  |  |  |  |  |
| GO:0014909\_smooth\_muscle\_cell\_migration | 3 | 0 |  |  |  |  |  |  |  |  |
| GO:0015669\_gas\_transport | 3 | 0 |  |  |  |  |  |  |  |  |
| GO:0015760\_glucose-6-phosphate\_transport | 3 | 0 |  |  |  |  |  |  |  |  |
| GO:0015816\_glycine\_transport | 3 | 0 |  |  |  |  |  |  |  |  |
| GO:0015838\_betaine\_transport | 3 | 0 |  |  |  |  |  |  |  |  |
| GO:0015871\_choline\_transport | 3 | 0 |  |  |  |  |  |  |  |  |
| GO:0015879\_carnitine\_transport | 3 | 0 |  |  |  |  |  |  |  |  |
| GO:0015893\_drug\_transport | 3 | 0 |  |  |  |  |  |  |  |  |
| GO:0015909\_long-chain\_fatty\_acid\_transport | 3 | 0 |  |  |  |  |  |  |  |  |
| GO:0015936\_coenzyme\_A\_metabolic\_process | 3 | 0 |  |  |  |  |  |  |  |  |
| GO:0015988\_energy\_coupled\_proton\_transport\_\_against\_electrochemical\_gradient | 3 | 0 |  |  |  |  |  |  |  |  |
| GO:0015991\_ATP\_hydrolysis\_coupled\_proton\_transport | 3 | 0 |  |  |  |  |  |  |  |  |
| GO:0016241\_regulation\_of\_macroautophagy | 3 | 0 |  |  |  |  |  |  |  |  |
| GO:0016322\_neuron\_remodeling | 3 | 0 |  |  |  |  |  |  |  |  |
| GO:0016556\_mRNA\_modification | 3 | 0 |  |  |  |  |  |  |  |  |
| GO:0016973\_poly(A)+\_mRNA\_export\_from\_nucleus | 3 | 0 |  |  |  |  |  |  |  |  |
| GO:0018208\_peptidyl-proline\_modification | 3 | 0 |  |  |  |  |  |  |  |  |
| GO:0018894\_dibenzo-p-dioxin\_metabolic\_process | 3 | 0 |  |  |  |  |  |  |  |  |
| GO:0019058\_viral\_infectious\_cycle | 3 | 0 |  |  |  |  |  |  |  |  |
| GO:0019230\_proprioception | 3 | 0 |  |  |  |  |  |  |  |  |
| GO:0019236\_response\_to\_pheromone | 3 | 0 |  |  |  |  |  |  |  |  |
| GO:0019359\_nicotinamide\_nucleotide\_biosynthetic\_process | 3 | 0 |  |  |  |  |  |  |  |  |
| GO:0019363\_pyridine\_nucleotide\_biosynthetic\_process | 3 | 0 |  |  |  |  |  |  |  |  |
| GO:0019438\_aromatic\_compound\_biosynthetic\_process | 3 | 0 |  |  |  |  |  |  |  |  |
| GO:0019439\_aromatic\_compound\_catabolic\_process | 3 | 0 |  |  |  |  |  |  |  |  |
| GO:0019605\_butyrate\_metabolic\_process | 3 | 0 |  |  |  |  |  |  |  |  |
| GO:0019614\_catechol\_catabolic\_process | 3 | 0 |  |  |  |  |  |  |  |  |
| GO:0019674\_NAD\_metabolic\_process | 3 | 0 |  |  |  |  |  |  |  |  |
| GO:0019852\_L-ascorbic\_acid\_metabolic\_process | 3 | 0 |  |  |  |  |  |  |  |  |
| GO:0019934\_cGMP-mediated\_signaling | 3 | 0 |  |  |  |  |  |  |  |  |
| GO:0019987\_negative\_regulation\_of\_anti-apoptosis | 3 | 0 |  |  |  |  |  |  |  |  |
| GO:0021527\_spinal\_cord\_association\_neuron\_differentiation | 3 | 0 |  |  |  |  |  |  |  |  |
| GO:0021529\_spinal\_cord\_oligodendrocyte\_cell\_differentiation | 3 | 0 |  |  |  |  |  |  |  |  |
| GO:0021530\_spinal\_cord\_oligodendrocyte\_cell\_fate\_specification | 3 | 0 |  |  |  |  |  |  |  |  |
| GO:0021555\_midbrain-hindbrain\_boundary\_morphogenesis | 3 | 0 |  |  |  |  |  |  |  |  |
| GO:0021563\_glossopharyngeal\_nerve\_development | 3 | 0 |  |  |  |  |  |  |  |  |
| GO:0021570\_rhombomere\_4\_development | 3 | 0 |  |  |  |  |  |  |  |  |
| GO:0021591\_ventricular\_system\_development | 3 | 0 |  |  |  |  |  |  |  |  |
| GO:0021615\_glossopharyngeal\_nerve\_morphogenesis | 3 | 0 |  |  |  |  |  |  |  |  |
| GO:0021794\_thalamus\_development | 3 | 0 |  |  |  |  |  |  |  |  |
| GO:0021797\_forebrain\_anterior\_posterior\_pattern\_formation | 3 | 0 |  |  |  |  |  |  |  |  |
| GO:0021798\_forebrain\_dorsal\_ventral\_pattern\_formation | 3 | 0 |  |  |  |  |  |  |  |  |
| GO:0021800\_cerebral\_cortex\_tangential\_migration | 3 | 0 |  |  |  |  |  |  |  |  |
| GO:0021819\_layer\_formation\_in\_the\_cerebral\_cortex | 3 | 0 |  |  |  |  |  |  |  |  |
| GO:0021859\_pyramidal\_neuron\_differentiation | 3 | 0 |  |  |  |  |  |  |  |  |
| GO:0021860\_pyramidal\_neuron\_development | 3 | 0 |  |  |  |  |  |  |  |  |
| GO:0021889\_olfactory\_bulb\_interneuron\_differentiation | 3 | 0 |  |  |  |  |  |  |  |  |
| GO:0021891\_olfactory\_bulb\_interneuron\_development | 3 | 0 |  |  |  |  |  |  |  |  |
| GO:0021912\_regulation\_of\_transcription\_from\_RNA\_polymerase\_II\_promoter\_involved\_in\_spinal\_cord\_motor\_neuron\_fate\_specification | 3 | 0 |  |  |  |  |  |  |  |  |
| GO:0021979\_hypothalamus\_cell\_differentiation | 3 | 0 |  |  |  |  |  |  |  |  |
| GO:0022010\_myelination\_in\_the\_central\_nervous\_system | 3 | 0 |  |  |  |  |  |  |  |  |
| GO:0022027\_interkinetic\_nuclear\_migration | 3 | 0 |  |  |  |  |  |  |  |  |
| GO:0022406\_membrane\_docking | 3 | 0 |  |  |  |  |  |  |  |  |
| GO:0030033\_microvillus\_assembly | 3 | 0 |  |  |  |  |  |  |  |  |
| GO:0030091\_protein\_repair | 3 | 0 |  |  |  |  |  |  |  |  |
| GO:0030195\_negative\_regulation\_of\_blood\_coagulation | 3 | 0 |  |  |  |  |  |  |  |  |
| GO:0030224\_monocyte\_differentiation | 3 | 0 |  |  |  |  |  |  |  |  |
| GO:0030307\_positive\_regulation\_of\_cell\_growth | 3 | 0 |  |  |  |  |  |  |  |  |
| GO:0030319\_cellular\_di-\_\_tri-valent\_inorganic\_anion\_homeostasis | 3 | 0 |  |  |  |  |  |  |  |  |
| GO:0030320\_cellular\_monovalent\_inorganic\_anion\_homeostasis | 3 | 0 |  |  |  |  |  |  |  |  |
| GO:0030321\_transepithelial\_chloride\_transport | 3 | 0 |  |  |  |  |  |  |  |  |
| GO:0030501\_positive\_regulation\_of\_bone\_mineralization | 3 | 0 |  |  |  |  |  |  |  |  |
| GO:0030513\_positive\_regulation\_of\_BMP\_signaling\_pathway | 3 | 0 |  |  |  |  |  |  |  |  |
| GO:0030538\_embryonic\_genitalia\_morphogenesis | 3 | 0 |  |  |  |  |  |  |  |  |
| GO:0030540\_female\_genitalia\_development | 3 | 0 |  |  |  |  |  |  |  |  |
| GO:0030574\_collagen\_catabolic\_process | 3 | 0 |  |  |  |  |  |  |  |  |
| GO:0030643\_cellular\_phosphate\_ion\_homeostasis | 3 | 0 |  |  |  |  |  |  |  |  |
| GO:0030718\_germ-line\_stem\_cell\_maintenance | 3 | 0 |  |  |  |  |  |  |  |  |
| GO:0030730\_sequestering\_of\_triglyceride | 3 | 0 |  |  |  |  |  |  |  |  |
| GO:0030836\_positive\_regulation\_of\_actin\_filament\_depolymerization | 3 | 0 |  |  |  |  |  |  |  |  |
| GO:0030857\_negative\_regulation\_of\_epithelial\_cell\_differentiation | 3 | 0 |  |  |  |  |  |  |  |  |
| GO:0030916\_otic\_vesicle\_formation | 3 | 0 |  |  |  |  |  |  |  |  |
| GO:0031000\_response\_to\_caffeine | 3 | 0 |  |  |  |  |  |  |  |  |
| GO:0031063\_regulation\_of\_histone\_deacetylation | 3 | 0 |  |  |  |  |  |  |  |  |
| GO:0031065\_positive\_regulation\_of\_histone\_deacetylation | 3 | 0 |  |  |  |  |  |  |  |  |
| GO:0031112\_positive\_regulation\_of\_microtubule\_polymerization\_or\_depolymerization | 3 | 0 |  |  |  |  |  |  |  |  |
| GO:0031116\_positive\_regulation\_of\_microtubule\_polymerization | 3 | 0 |  |  |  |  |  |  |  |  |
| GO:0031133\_regulation\_of\_axon\_diameter | 3 | 0 |  |  |  |  |  |  |  |  |
| GO:0031282\_regulation\_of\_guanylate\_cyclase\_activity | 3 | 0 |  |  |  |  |  |  |  |  |
| GO:0031333\_negative\_regulation\_of\_protein\_complex\_assembly | 3 | 0 |  |  |  |  |  |  |  |  |
| GO:0031397\_negative\_regulation\_of\_protein\_ubiquitination | 3 | 0 |  |  |  |  |  |  |  |  |
| GO:0031398\_positive\_regulation\_of\_protein\_ubiquitination | 3 | 0 |  |  |  |  |  |  |  |  |
| GO:0031503\_protein\_complex\_localization | 3 | 0 |  |  |  |  |  |  |  |  |
| GO:0031571\_G1\_DNA\_damage\_checkpoint | 3 | 0 |  |  |  |  |  |  |  |  |
| GO:0031579\_membrane\_raft\_organization | 3 | 0 |  |  |  |  |  |  |  |  |
| GO:0031638\_zymogen\_activation | 3 | 0 |  |  |  |  |  |  |  |  |
| GO:0031641\_regulation\_of\_myelination | 3 | 0 |  |  |  |  |  |  |  |  |
| GO:0031642\_negative\_regulation\_of\_myelination | 3 | 0 |  |  |  |  |  |  |  |  |
| GO:0031649\_heat\_generation | 3 | 0 |  |  |  |  |  |  |  |  |
| GO:0031943\_regulation\_of\_glucocorticoid\_metabolic\_process | 3 | 0 |  |  |  |  |  |  |  |  |
| GO:0032020\_ISG15-protein\_conjugation | 3 | 0 |  |  |  |  |  |  |  |  |
| GO:0032060\_bleb\_formation | 3 | 0 |  |  |  |  |  |  |  |  |
| GO:0032095\_regulation\_of\_response\_to\_food | 3 | 0 |  |  |  |  |  |  |  |  |
| GO:0032272\_negative\_regulation\_of\_protein\_polymerization | 3 | 0 |  |  |  |  |  |  |  |  |
| GO:0032288\_myelin\_assembly | 3 | 0 |  |  |  |  |  |  |  |  |
| GO:0032291\_ensheathment\_of\_axons\_in\_the\_central\_nervous\_system | 3 | 0 |  |  |  |  |  |  |  |  |
| GO:0032355\_response\_to\_estradiol\_stimulus | 3 | 0 |  |  |  |  |  |  |  |  |
| GO:0032402\_melanosome\_transport | 3 | 0 |  |  |  |  |  |  |  |  |
| GO:0032411\_positive\_regulation\_of\_transporter\_activity | 3 | 0 |  |  |  |  |  |  |  |  |
| GO:0032414\_positive\_regulation\_of\_ion\_transmembrane\_transporter\_activity | 3 | 0 |  |  |  |  |  |  |  |  |
| GO:0032436\_positive\_regulation\_of\_proteasomal\_ubiquitin-dependent\_protein\_catabolic\_process | 3 | 0 |  |  |  |  |  |  |  |  |
| GO:0032528\_microvillus\_organization | 3 | 0 |  |  |  |  |  |  |  |  |
| GO:0032536\_regulation\_of\_cell\_projection\_size | 3 | 0 |  |  |  |  |  |  |  |  |
| GO:0032632\_interleukin-3\_production | 3 | 0 |  |  |  |  |  |  |  |  |
| GO:0032634\_interleukin-5\_production | 3 | 0 |  |  |  |  |  |  |  |  |
| GO:0032674\_regulation\_of\_interleukin-5\_production | 3 | 0 |  |  |  |  |  |  |  |  |
| GO:0032703\_negative\_regulation\_of\_interleukin-2\_production | 3 | 0 |  |  |  |  |  |  |  |  |
| GO:0032753\_positive\_regulation\_of\_interleukin-4\_production | 3 | 0 |  |  |  |  |  |  |  |  |
| GO:0032823\_regulation\_of\_natural\_killer\_cell\_differentiation | 3 | 0 |  |  |  |  |  |  |  |  |
| GO:0032825\_positive\_regulation\_of\_natural\_killer\_cell\_differentiation | 3 | 0 |  |  |  |  |  |  |  |  |
| GO:0032856\_activation\_of\_Ras\_GTPase\_activity | 3 | 0 |  |  |  |  |  |  |  |  |
| GO:0032862\_activation\_of\_Rho\_GTPase\_activity | 3 | 0 |  |  |  |  |  |  |  |  |
| GO:0032874\_positive\_regulation\_of\_stress-activated\_MAPK\_cascade | 3 | 0 |  |  |  |  |  |  |  |  |
| GO:0032881\_regulation\_of\_polysaccharide\_metabolic\_process | 3 | 0 |  |  |  |  |  |  |  |  |
| GO:0032890\_regulation\_of\_organic\_acid\_transport | 3 | 0 |  |  |  |  |  |  |  |  |
| GO:0033058\_directional\_locomotion | 3 | 0 |  |  |  |  |  |  |  |  |
| GO:0033080\_immature\_T\_cell\_proliferation\_in\_the\_thymus | 3 | 0 |  |  |  |  |  |  |  |  |
| GO:0033084\_regulation\_of\_immature\_T\_cell\_proliferation\_in\_the\_thymus | 3 | 0 |  |  |  |  |  |  |  |  |
| GO:0033091\_positive\_regulation\_of\_immature\_T\_cell\_proliferation | 3 | 0 |  |  |  |  |  |  |  |  |
| GO:0033137\_negative\_regulation\_of\_peptidyl-serine\_phosphorylation | 3 | 0 |  |  |  |  |  |  |  |  |
| GO:0033153\_T\_cell\_receptor\_V(D)J\_recombination | 3 | 0 |  |  |  |  |  |  |  |  |
| GO:0033209\_tumor\_necrosis\_factor-mediated\_signaling\_pathway | 3 | 0 |  |  |  |  |  |  |  |  |
| GO:0033261\_regulation\_of\_S\_phase | 3 | 0 |  |  |  |  |  |  |  |  |
| GO:0033600\_negative\_regulation\_of\_mammary\_gland\_epithelial\_cell\_proliferation | 3 | 0 |  |  |  |  |  |  |  |  |
| GO:0033631\_cell-cell\_adhesion\_mediated\_by\_integrin | 3 | 0 |  |  |  |  |  |  |  |  |
| GO:0033993\_response\_to\_lipid | 3 | 0 |  |  |  |  |  |  |  |  |
| GO:0034220\_ion\_transmembrane\_transport | 3 | 0 |  |  |  |  |  |  |  |  |
| GO:0034308\_monohydric\_alcohol\_metabolic\_process | 3 | 0 |  |  |  |  |  |  |  |  |
| GO:0034313\_diol\_catabolic\_process | 3 | 0 |  |  |  |  |  |  |  |  |
| GO:0034331\_cell\_junction\_maintenance | 3 | 0 |  |  |  |  |  |  |  |  |
| GO:0034332\_adherens\_junction\_organization | 3 | 0 |  |  |  |  |  |  |  |  |
| GO:0034375\_high-density\_lipoprotein\_particle\_remodeling | 3 | 0 |  |  |  |  |  |  |  |  |
| GO:0034381\_lipoprotein\_particle\_clearance | 3 | 0 |  |  |  |  |  |  |  |  |
| GO:0034612\_response\_to\_tumor\_necrosis\_factor | 3 | 0 |  |  |  |  |  |  |  |  |
| GO:0034655\_nucleobase\_\_nucleoside\_\_nucleotide\_and\_nucleic\_acid\_catabolic\_process | 3 | 0 |  |  |  |  |  |  |  |  |
| GO:0034656\_nucleobase\_\_nucleoside\_and\_nucleotide\_catabolic\_process | 3 | 0 |  |  |  |  |  |  |  |  |
| GO:0035067\_negative\_regulation\_of\_histone\_acetylation | 3 | 0 |  |  |  |  |  |  |  |  |
| GO:0035084\_flagellar\_axoneme\_assembly | 3 | 0 |  |  |  |  |  |  |  |  |
| GO:0035166\_post-embryonic\_hemopoiesis | 3 | 0 |  |  |  |  |  |  |  |  |
| GO:0035283\_central\_nervous\_system\_segmentation | 3 | 0 |  |  |  |  |  |  |  |  |
| GO:0035284\_brain\_segmentation | 3 | 0 |  |  |  |  |  |  |  |  |
| GO:0042097\_interleukin-4\_biosynthetic\_process | 3 | 0 |  |  |  |  |  |  |  |  |
| GO:0042135\_neurotransmitter\_catabolic\_process | 3 | 0 |  |  |  |  |  |  |  |  |
| GO:0042271\_susceptibility\_to\_natural\_killer\_cell\_mediated\_cytotoxicity | 3 | 0 |  |  |  |  |  |  |  |  |
| GO:0042273\_ribosomal\_large\_subunit\_biogenesis | 3 | 0 |  |  |  |  |  |  |  |  |
| GO:0042375\_quinone\_cofactor\_metabolic\_process | 3 | 0 |  |  |  |  |  |  |  |  |
| GO:0042420\_dopamine\_catabolic\_process | 3 | 0 |  |  |  |  |  |  |  |  |
| GO:0042421\_norepinephrine\_biosynthetic\_process | 3 | 0 |  |  |  |  |  |  |  |  |
| GO:0042424\_catecholamine\_catabolic\_process | 3 | 0 |  |  |  |  |  |  |  |  |
| GO:0042447\_hormone\_catabolic\_process | 3 | 0 |  |  |  |  |  |  |  |  |
| GO:0042448\_progesterone\_metabolic\_process | 3 | 0 |  |  |  |  |  |  |  |  |
| GO:0042523\_positive\_regulation\_of\_tyrosine\_phosphorylation\_of\_Stat5\_protein | 3 | 0 |  |  |  |  |  |  |  |  |
| GO:0042659\_regulation\_of\_cell\_fate\_specification | 3 | 0 |  |  |  |  |  |  |  |  |
| GO:0042668\_auditory\_receptor\_cell\_fate\_determination | 3 | 0 |  |  |  |  |  |  |  |  |
| GO:0042670\_retinal\_cone\_cell\_differentiation | 3 | 0 |  |  |  |  |  |  |  |  |
| GO:0042693\_muscle\_cell\_fate\_commitment | 3 | 0 |  |  |  |  |  |  |  |  |
| GO:0042711\_maternal\_behavior | 3 | 0 |  |  |  |  |  |  |  |  |
| GO:0042745\_circadian\_sleep\_wake\_cycle | 3 | 0 |  |  |  |  |  |  |  |  |
| GO:0042759\_long-chain\_fatty\_acid\_biosynthetic\_process | 3 | 0 |  |  |  |  |  |  |  |  |
| GO:0042787\_protein\_ubiquitination\_during\_ubiquitin-dependent\_protein\_catabolic\_process | 3 | 0 |  |  |  |  |  |  |  |  |
| GO:0043045\_DNA\_methylation\_during\_embryonic\_development | 3 | 0 |  |  |  |  |  |  |  |  |
| GO:0043090\_amino\_acid\_import | 3 | 0 |  |  |  |  |  |  |  |  |
| GO:0043092\_L-amino\_acid\_import | 3 | 0 |  |  |  |  |  |  |  |  |
| GO:0043094\_cellular\_metabolic\_compound\_salvage | 3 | 0 |  |  |  |  |  |  |  |  |
| GO:0043101\_purine\_salvage | 3 | 0 |  |  |  |  |  |  |  |  |
| GO:0043149\_stress\_fiber\_formation | 3 | 0 |  |  |  |  |  |  |  |  |
| GO:0043174\_nucleoside\_salvage | 3 | 0 |  |  |  |  |  |  |  |  |
| GO:0043200\_response\_to\_amino\_acid\_stimulus | 3 | 0 |  |  |  |  |  |  |  |  |
| GO:0043243\_positive\_regulation\_of\_protein\_complex\_disassembly | 3 | 0 |  |  |  |  |  |  |  |  |
| GO:0043249\_erythrocyte\_maturation | 3 | 0 |  |  |  |  |  |  |  |  |
| GO:0043267\_negative\_regulation\_of\_potassium\_ion\_transport | 3 | 0 |  |  |  |  |  |  |  |  |
| GO:0043371\_negative\_regulation\_of\_CD4-positive\_\_alpha\_beta\_T\_cell\_differentiation | 3 | 0 |  |  |  |  |  |  |  |  |
| GO:0043462\_regulation\_of\_ATPase\_activity | 3 | 0 |  |  |  |  |  |  |  |  |
| GO:0043569\_negative\_regulation\_of\_insulin-like\_growth\_factor\_receptor\_signaling\_pathway | 3 | 0 |  |  |  |  |  |  |  |  |
| GO:0043574\_peroxisomal\_transport | 3 | 0 |  |  |  |  |  |  |  |  |
| GO:0043586\_tongue\_development | 3 | 0 |  |  |  |  |  |  |  |  |
| GO:0043900\_regulation\_of\_multi-organism\_process | 3 | 0 |  |  |  |  |  |  |  |  |
| GO:0043954\_cellular\_component\_maintenance | 3 | 0 |  |  |  |  |  |  |  |  |
| GO:0044030\_regulation\_of\_DNA\_methylation | 3 | 0 |  |  |  |  |  |  |  |  |
| GO:0044089\_positive\_regulation\_of\_cellular\_component\_biogenesis | 3 | 0 |  |  |  |  |  |  |  |  |
| GO:0044273\_sulfur\_compound\_catabolic\_process | 3 | 0 |  |  |  |  |  |  |  |  |
| GO:0045047\_protein\_targeting\_to\_ER | 3 | 0 |  |  |  |  |  |  |  |  |
| GO:0045085\_negative\_regulation\_of\_interleukin-2\_biosynthetic\_process | 3 | 0 |  |  |  |  |  |  |  |  |
| GO:0045110\_intermediate\_filament\_bundle\_assembly | 3 | 0 |  |  |  |  |  |  |  |  |
| GO:0045143\_homologous\_chromosome\_segregation | 3 | 0 |  |  |  |  |  |  |  |  |
| GO:0045198\_establishment\_of\_epithelial\_cell\_apical\_basal\_polarity | 3 | 0 |  |  |  |  |  |  |  |  |
| GO:0045217\_cell-cell\_junction\_maintenance | 3 | 0 |  |  |  |  |  |  |  |  |
| GO:0045348\_positive\_regulation\_of\_MHC\_class\_II\_biosynthetic\_process | 3 | 0 |  |  |  |  |  |  |  |  |
| GO:0045402\_regulation\_of\_interleukin-4\_biosynthetic\_process | 3 | 0 |  |  |  |  |  |  |  |  |
| GO:0045404\_positive\_regulation\_of\_interleukin-4\_biosynthetic\_process | 3 | 0 |  |  |  |  |  |  |  |  |
| GO:0045542\_positive\_regulation\_of\_cholesterol\_biosynthetic\_process | 3 | 0 |  |  |  |  |  |  |  |  |
| GO:0045607\_regulation\_of\_auditory\_receptor\_cell\_differentiation | 3 | 0 |  |  |  |  |  |  |  |  |
| GO:0045623\_negative\_regulation\_of\_T-helper\_cell\_differentiation | 3 | 0 |  |  |  |  |  |  |  |  |
| GO:0045625\_regulation\_of\_T-helper\_1\_cell\_differentiation | 3 | 0 |  |  |  |  |  |  |  |  |
| GO:0045631\_regulation\_of\_mechanoreceptor\_differentiation | 3 | 0 |  |  |  |  |  |  |  |  |
| GO:0045717\_negative\_regulation\_of\_fatty\_acid\_biosynthetic\_process | 3 | 0 |  |  |  |  |  |  |  |  |
| GO:0045723\_positive\_regulation\_of\_fatty\_acid\_biosynthetic\_process | 3 | 0 |  |  |  |  |  |  |  |  |
| GO:0045746\_negative\_regulation\_of\_Notch\_signaling\_pathway | 3 | 0 |  |  |  |  |  |  |  |  |
| GO:0045806\_negative\_regulation\_of\_endocytosis | 3 | 0 |  |  |  |  |  |  |  |  |
| GO:0045829\_negative\_regulation\_of\_isotype\_switching | 3 | 0 |  |  |  |  |  |  |  |  |
| GO:0045844\_positive\_regulation\_of\_striated\_muscle\_development | 3 | 0 |  |  |  |  |  |  |  |  |
| GO:0045907\_positive\_regulation\_of\_vasoconstriction | 3 | 0 |  |  |  |  |  |  |  |  |
| GO:0045922\_negative\_regulation\_of\_fatty\_acid\_metabolic\_process | 3 | 0 |  |  |  |  |  |  |  |  |
| GO:0045939\_negative\_regulation\_of\_steroid\_metabolic\_process | 3 | 0 |  |  |  |  |  |  |  |  |
| GO:0046013\_regulation\_of\_T\_cell\_homeostatic\_proliferation | 3 | 0 |  |  |  |  |  |  |  |  |
| GO:0046034\_ATP\_metabolic\_process | 3 | 0 |  |  |  |  |  |  |  |  |
| GO:0046325\_negative\_regulation\_of\_glucose\_import | 3 | 0 |  |  |  |  |  |  |  |  |
| GO:0046426\_negative\_regulation\_of\_JAK-STAT\_cascade | 3 | 0 |  |  |  |  |  |  |  |  |
| GO:0046457\_prostanoid\_biosynthetic\_process | 3 | 0 |  |  |  |  |  |  |  |  |
| GO:0046479\_glycosphingolipid\_catabolic\_process | 3 | 0 |  |  |  |  |  |  |  |  |
| GO:0046488\_phosphatidylinositol\_metabolic\_process | 3 | 0 |  |  |  |  |  |  |  |  |
| GO:0046549\_retinal\_cone\_cell\_development | 3 | 0 |  |  |  |  |  |  |  |  |
| GO:0046605\_regulation\_of\_centrosome\_cycle | 3 | 0 |  |  |  |  |  |  |  |  |
| GO:0046688\_response\_to\_copper\_ion | 3 | 0 |  |  |  |  |  |  |  |  |
| GO:0046717\_acid\_secretion | 3 | 0 |  |  |  |  |  |  |  |  |
| GO:0046825\_regulation\_of\_protein\_export\_from\_nucleus | 3 | 0 |  |  |  |  |  |  |  |  |
| GO:0046885\_regulation\_of\_hormone\_biosynthetic\_process | 3 | 0 |  |  |  |  |  |  |  |  |
| GO:0048003\_antigen\_processing\_and\_presentation\_of\_lipid\_antigen\_via\_MHC\_class\_Ib | 3 | 0 |  |  |  |  |  |  |  |  |
| GO:0048007\_antigen\_processing\_and\_presentation\_\_exogenous\_lipid\_antigen\_via\_MHC\_class\_Ib | 3 | 0 |  |  |  |  |  |  |  |  |
| GO:0048012\_hepatocyte\_growth\_factor\_receptor\_signaling\_pathway | 3 | 0 |  |  |  |  |  |  |  |  |
| GO:0048050\_post-embryonic\_eye\_morphogenesis | 3 | 0 |  |  |  |  |  |  |  |  |
| GO:0048087\_positive\_regulation\_of\_pigmentation\_during\_development | 3 | 0 |  |  |  |  |  |  |  |  |
| GO:0048246\_macrophage\_chemotaxis | 3 | 0 |  |  |  |  |  |  |  |  |
| GO:0048251\_elastic\_fiber\_assembly | 3 | 0 |  |  |  |  |  |  |  |  |
| GO:0048278\_vesicle\_docking | 3 | 0 |  |  |  |  |  |  |  |  |
| GO:0048294\_negative\_regulation\_of\_isotype\_switching\_to\_IgE\_isotypes | 3 | 0 |  |  |  |  |  |  |  |  |
| GO:0048318\_axial\_mesoderm\_development | 3 | 0 |  |  |  |  |  |  |  |  |
| GO:0048597\_post-embryonic\_camera-type\_eye\_morphogenesis | 3 | 0 |  |  |  |  |  |  |  |  |
| GO:0048636\_positive\_regulation\_of\_muscle\_development | 3 | 0 |  |  |  |  |  |  |  |  |
| GO:0048660\_regulation\_of\_smooth\_muscle\_cell\_proliferation | 3 | 0 |  |  |  |  |  |  |  |  |
| GO:0048668\_collateral\_sprouting | 3 | 0 |  |  |  |  |  |  |  |  |
| GO:0048676\_axon\_extension\_involved\_in\_development | 3 | 0 |  |  |  |  |  |  |  |  |
| GO:0048755\_branching\_morphogenesis\_of\_a\_nerve | 3 | 0 |  |  |  |  |  |  |  |  |
| GO:0048845\_venous\_blood\_vessel\_morphogenesis | 3 | 0 |  |  |  |  |  |  |  |  |
| GO:0048852\_diencephalon\_morphogenesis | 3 | 0 |  |  |  |  |  |  |  |  |
| GO:0048859\_formation\_of\_anatomical\_boundary | 3 | 0 |  |  |  |  |  |  |  |  |
| GO:0048865\_stem\_cell\_fate\_commitment | 3 | 0 |  |  |  |  |  |  |  |  |
| GO:0050435\_beta-amyloid\_metabolic\_process | 3 | 0 |  |  |  |  |  |  |  |  |
| GO:0050650\_chondroitin\_sulfate\_proteoglycan\_biosynthetic\_process | 3 | 0 |  |  |  |  |  |  |  |  |
| GO:0050703\_interleukin-1\_alpha\_secretion | 3 | 0 |  |  |  |  |  |  |  |  |
| GO:0050705\_regulation\_of\_interleukin-1\_alpha\_secretion | 3 | 0 |  |  |  |  |  |  |  |  |
| GO:0050709\_negative\_regulation\_of\_protein\_secretion | 3 | 0 |  |  |  |  |  |  |  |  |
| GO:0050710\_negative\_regulation\_of\_cytokine\_secretion | 3 | 0 |  |  |  |  |  |  |  |  |
| GO:0050717\_positive\_regulation\_of\_interleukin-1\_alpha\_secretion | 3 | 0 |  |  |  |  |  |  |  |  |
| GO:0050774\_negative\_regulation\_of\_dendrite\_morphogenesis | 3 | 0 |  |  |  |  |  |  |  |  |
| GO:0050857\_positive\_regulation\_of\_antigen\_receptor-mediated\_signaling\_pathway | 3 | 0 |  |  |  |  |  |  |  |  |
| GO:0050882\_voluntary\_musculoskeletal\_movement | 3 | 0 |  |  |  |  |  |  |  |  |
| GO:0050913\_sensory\_perception\_of\_bitter\_taste | 3 | 0 |  |  |  |  |  |  |  |  |
| GO:0050957\_equilibrioception | 3 | 0 |  |  |  |  |  |  |  |  |
| GO:0050996\_positive\_regulation\_of\_lipid\_catabolic\_process | 3 | 0 |  |  |  |  |  |  |  |  |
| GO:0051149\_positive\_regulation\_of\_muscle\_cell\_differentiation | 3 | 0 |  |  |  |  |  |  |  |  |
| GO:0051153\_regulation\_of\_striated\_muscle\_cell\_differentiation | 3 | 0 |  |  |  |  |  |  |  |  |
| GO:0051204\_protein\_insertion\_into\_mitochondrial\_membrane | 3 | 0 |  |  |  |  |  |  |  |  |
| GO:0051291\_protein\_heterooligomerization | 3 | 0 |  |  |  |  |  |  |  |  |
| GO:0051302\_regulation\_of\_cell\_division | 3 | 0 |  |  |  |  |  |  |  |  |
| GO:0051320\_S\_phase | 3 | 0 |  |  |  |  |  |  |  |  |
| GO:0051450\_myoblast\_proliferation | 3 | 0 |  |  |  |  |  |  |  |  |
| GO:0051583\_dopamine\_uptake | 3 | 0 |  |  |  |  |  |  |  |  |
| GO:0051798\_positive\_regulation\_of\_hair\_follicle\_development | 3 | 0 |  |  |  |  |  |  |  |  |
| GO:0051882\_mitochondrial\_depolarization | 3 | 0 |  |  |  |  |  |  |  |  |
| GO:0051900\_regulation\_of\_mitochondrial\_depolarization | 3 | 0 |  |  |  |  |  |  |  |  |
| GO:0051925\_regulation\_of\_calcium\_ion\_transport\_via\_voltage-gated\_calcium\_channel\_activity | 3 | 0 |  |  |  |  |  |  |  |  |
| GO:0051926\_negative\_regulation\_of\_calcium\_ion\_transport | 3 | 0 |  |  |  |  |  |  |  |  |
| GO:0051930\_regulation\_of\_sensory\_perception\_of\_pain | 3 | 0 |  |  |  |  |  |  |  |  |
| GO:0051931\_regulation\_of\_sensory\_perception | 3 | 0 |  |  |  |  |  |  |  |  |
| GO:0051934\_catecholamine\_uptake\_during\_transmission\_of\_nerve\_impulse | 3 | 0 |  |  |  |  |  |  |  |  |
| GO:0051955\_regulation\_of\_amino\_acid\_transport | 3 | 0 |  |  |  |  |  |  |  |  |
| GO:0051962\_positive\_regulation\_of\_nervous\_system\_development | 3 | 0 |  |  |  |  |  |  |  |  |
| GO:0051965\_positive\_regulation\_of\_synaptogenesis | 3 | 0 |  |  |  |  |  |  |  |  |
| GO:0051967\_negative\_regulation\_of\_synaptic\_transmission\_\_glutamatergic | 3 | 0 |  |  |  |  |  |  |  |  |
| GO:0055061\_di-\_\_tri-valent\_inorganic\_anion\_homeostasis | 3 | 0 |  |  |  |  |  |  |  |  |
| GO:0055062\_phosphate\_ion\_homeostasis | 3 | 0 |  |  |  |  |  |  |  |  |
| GO:0055083\_monovalent\_inorganic\_anion\_homeostasis | 3 | 0 |  |  |  |  |  |  |  |  |
| GO:0055117\_regulation\_of\_cardiac\_muscle\_contraction | 3 | 0 |  |  |  |  |  |  |  |  |
| GO:0060009\_Sertoli\_cell\_development | 3 | 0 |  |  |  |  |  |  |  |  |
| GO:0060024\_rhythmic\_synaptic\_transmission | 3 | 0 |  |  |  |  |  |  |  |  |
| GO:0060033\_anatomical\_structure\_regression | 3 | 0 |  |  |  |  |  |  |  |  |
| GO:0060040\_retinal\_bipolar\_neuron\_differentiation | 3 | 0 |  |  |  |  |  |  |  |  |
| GO:0060055\_angiogenesis\_involved\_in\_wound\_healing | 3 | 0 |  |  |  |  |  |  |  |  |
| GO:0060084\_synaptic\_transmission\_involved\_in\_micturition | 3 | 0 |  |  |  |  |  |  |  |  |
| GO:0060123\_regulation\_of\_growth\_hormone\_secretion | 3 | 0 |  |  |  |  |  |  |  |  |
| GO:0060126\_somatotropin\_secreting\_cell\_differentiation | 3 | 0 |  |  |  |  |  |  |  |  |
| GO:0060192\_negative\_regulation\_of\_lipase\_activity | 3 | 0 |  |  |  |  |  |  |  |  |
| GO:0060219\_camera-type\_eye\_photoreceptor\_cell\_differentiation | 3 | 0 |  |  |  |  |  |  |  |  |
| GO:0060285\_ciliary\_cell\_motility | 3 | 0 |  |  |  |  |  |  |  |  |
| GO:0060294\_cilium\_movement\_involved\_in\_ciliary\_motility | 3 | 0 |  |  |  |  |  |  |  |  |
| GO:0060295\_regulation\_of\_cilium\_movement\_involved\_in\_ciliary\_motility | 3 | 0 |  |  |  |  |  |  |  |  |
| GO:0060296\_regulation\_of\_cilium\_beat\_frequency\_involved\_in\_ciliary\_motility | 3 | 0 |  |  |  |  |  |  |  |  |
| GO:0060314\_regulation\_of\_ryanodine-sensitive\_calcium-release\_channel\_activity | 3 | 0 |  |  |  |  |  |  |  |  |
| GO:0060396\_growth\_hormone\_receptor\_signaling\_pathway | 3 | 0 |  |  |  |  |  |  |  |  |
| GO:0060416\_response\_to\_growth\_hormone\_stimulus | 3 | 0 |  |  |  |  |  |  |  |  |
| GO:0060428\_lung\_epithelium\_development | 3 | 0 |  |  |  |  |  |  |  |  |
| GO:0060433\_bronchus\_development | 3 | 0 |  |  |  |  |  |  |  |  |
| GO:0060435\_bronchiole\_development | 3 | 0 |  |  |  |  |  |  |  |  |
| GO:0060460\_left\_lung\_morphogenesis | 3 | 0 |  |  |  |  |  |  |  |  |
| GO:0060491\_regulation\_of\_cell\_projection\_assembly | 3 | 0 |  |  |  |  |  |  |  |  |
[truncated: 255,448 more chars]
