# Supplementary material for: RedundancyMiner: De-replication of redundant GO categories in microarray and proteomics analysis
Source: BMC Bioinformatics. 2011 Feb 10;12:52. doi: 10.1186/1471-2105-12-52 (PMC3223614; doi:10.1186/1471-2105-12-52)
Supplement: Additional file 8 — Retinal development HTGM download. compressed package of the results of running HTGM on the retinal development genes list. [file 1471-2105-12-52-S8.ZIP › SCENARIO_2_MODIFIED/total.txt.total.txt.dir/Exp1_BestClusterMap_LEIGS_KM_24.csv.join.20.txt.dir/index.html]

Results for Exp1\_BestClusterMap\_LEIGS\_KM\_24.csv.join.20.txt

# Results for Exp1\_BestClusterMap\_LEIGS\_KM\_24.csv.join.20.txt

| Input Files | | | |
| --- | --- | --- | --- |
| Type | File |
| Total File (Text) | total.txt.total.txt |
| Changed File (Text) | Exp1\_BestClusterMap\_LEIGS\_KM\_24.csv.join.20.txt |
|  |
| --- | | | ||| Results files | | | |
| --- | --- | --- | --- |
| Type | Text | Excel | HTML |
| Gene Category Summary (Changed Genes) | Exp1\_BestClusterMap\_LEIGS\_KM\_24.csv.join.20.txt.change | Exp1\_BestClusterMap\_LEIGS\_KM\_24.csv.join.20.txt.change.xls | Exp1\_BestClusterMap\_LEIGS\_KM\_24.csv.join.20.txt.change.html |
| Gene Category Mapping (Changed Genes) | Exp1\_BestClusterMap\_LEIGS\_KM\_24.csv.join.20.txt.change.gce | Exp1\_BestClusterMap\_LEIGS\_KM\_24.csv.join.20.txt.change.gce.xls | Exp1\_BestClusterMap\_LEIGS\_KM\_24.csv.join.20.txt.change.gce.html |
| CIM (Changed) | Exp1\_BestClusterMap\_LEIGS\_KM\_24.csv.join.20.txt.change.gce.CIM |
| --- | | | ||| Navigation | | | |
| --- | --- | --- | --- |
| Up to Parent(Results for Total File) | | | |
