# Supplementary material for: RedundancyMiner: De-replication of redundant GO categories in microarray and proteomics analysis
Source: BMC Bioinformatics. 2011 Feb 10;12:52. doi: 10.1186/1471-2105-12-52 (PMC3223614; doi:10.1186/1471-2105-12-52)
Supplement: Additional file 8 — Retinal development HTGM download. compressed package of the results of running HTGM on the retinal development genes list. [file 1471-2105-12-52-S8.ZIP › SCENARIO_2_MODIFIED/total.txt.total.txt.dir/Exp1_BestClusterMap_LEIGS_KM_24.csv.join.21.txt.dir/Exp1_BestClusterMap_LEIGS_KM_24.csv.join.21.txt.change.gce.CIM.dir/cgi_user_x.html]

**X-axis Names**   
Cluster is based on euclidean distance  
Cluster method is: average  
plclust  
height plot  

|  |
| --- |
| 1.GO:0030030\_cell\_projection\_organization |
| 2.GO:0007411\_axon\_guidance |
| 3.GO:0050769\_positive\_regulation\_of\_neurogenesis |
| 4.GO:0002011\_morphogenesis\_of\_an\_epithelial\_sheet |
| 5.GO:0043583\_ear\_development |
| 6.GO:0048839\_inner\_ear\_development |
| 7.GO:0060113\_inner\_ear\_receptor\_cell\_differentiation |
| 8.GO:0042490\_mechanoreceptor\_differentiation |
| 9.GO:0031122\_cytoplasmic\_microtubule\_organization |
| 10.GO:0048489\_synaptic\_vesicle\_transport |
| 11.GO:0006887\_exocytosis |
