# Supplementary material for: RedundancyMiner: De-replication of redundant GO categories in microarray and proteomics analysis
Source: BMC Bioinformatics. 2011 Feb 10;12:52. doi: 10.1186/1471-2105-12-52 (PMC3223614; doi:10.1186/1471-2105-12-52)
Supplement: Additional file 8 — Retinal development HTGM download. compressed package of the results of running HTGM on the retinal development genes list. [file 1471-2105-12-52-S8.ZIP › SCENARIO_2_MODIFIED/total.txt.total.txt.dir/Exp1_BestClusterMap_LEIGS_KM_24.csv.join.21.txt.dir/Exp1_BestClusterMap_LEIGS_KM_24.csv.join.21.txt.change.gce.CIM.dir/cgi_user_y.html]

**Y-axis Names**   
Cluster is based on euclidean distance  
Cluster method is: average  
plclust  
height plot  

|  |
| --- |
| 1.CHUK |
| 2.SCAMP1 |
| 3.SYTL2 |
| 4.LIN7A |
| 5.CPLX3 |
| 6.SV2B |
| 7.KATNA1 |
| 8.STMN3 |
| 9.PDGFA |
| 10.KIF5C |
| 11.ANK3 |
| 12.EPHA4 |
| 13.EPHB2 |
| 14.NTN1 |
| 15.JAG1 |
| 16.GPR98 |
| 17.HES5 |
| 18.FOXG1 |
| 19.ITGA8 |
