# Supplementary material for: RedundancyMiner: De-replication of redundant GO categories in microarray and proteomics analysis
Source: BMC Bioinformatics. 2011 Feb 10;12:52. doi: 10.1186/1471-2105-12-52 (PMC3223614; doi:10.1186/1471-2105-12-52)
Supplement: Additional file 8 — Retinal development HTGM download. compressed package of the results of running HTGM on the retinal development genes list. [file 1471-2105-12-52-S8.ZIP › SCENARIO_2_MODIFIED/total.txt.total.txt.dir/Exp1_BestClusterMap_LEIGS_KM_24.csv.join.21.txt.dir/Exp1_BestClusterMap_LEIGS_KM_24.csv.join.21.txt.change.gce.html]

Gene Category Report for Exp1\_BestClusterMap\_LEIGS\_KM\_24.csv.join.21.txt

# Gene Category Report for Exp1\_BestClusterMap\_LEIGS\_KM\_24.csv.join.21.txt

| HYPERLINKED GO CATEGORY | HYPERLINKED GENE NAME | TOTAL GENES | CHANGED GENES | ENRICHMENT | LOG10(p) | CUMULATIVE NUMBER OF CATEGORIES | CUMULATIVE RANDOMS MEAN | FALSE DISCOVERY RATE |
| --- | --- | --- | --- | --- | --- | --- | --- | --- |
| GO:0007399\_nervous\_system\_development | DLC1 | 621 | 17 | 2.572710 | -3.898381 | 1 | 0.04 | 0.040000 |
| GO:0007399\_nervous\_system\_development | STMN3 | 621 | 17 | 2.572710 | -3.898381 | 1 | 0.04 | 0.040000 |
| GO:0007399\_nervous\_system\_development | KIF5C | 621 | 17 | 2.572710 | -3.898381 | 1 | 0.04 | 0.040000 |
| GO:0007399\_nervous\_system\_development | JAG1 | 621 | 17 | 2.572710 | -3.898381 | 1 | 0.04 | 0.040000 |
| GO:0007399\_nervous\_system\_development | NTN1 | 621 | 17 | 2.572710 | -3.898381 | 1 | 0.04 | 0.040000 |
| GO:0007399\_nervous\_system\_development | GPR98 | 621 | 17 | 2.572710 | -3.898381 | 1 | 0.04 | 0.040000 |
| GO:0007399\_nervous\_system\_development | APLP1 | 621 | 17 | 2.572710 | -3.898381 | 1 | 0.04 | 0.040000 |
| GO:0007399\_nervous\_system\_development | EPHB2 | 621 | 17 | 2.572710 | -3.898381 | 1 | 0.04 | 0.040000 |
| GO:0007399\_nervous\_system\_development | EPHA4 | 621 | 17 | 2.572710 | -3.898381 | 1 | 0.04 | 0.040000 |
| GO:0007399\_nervous\_system\_development | DKK1 | 621 | 17 | 2.572710 | -3.898381 | 1 | 0.04 | 0.040000 |
| GO:0007399\_nervous\_system\_development | HES5 | 621 | 17 | 2.572710 | -3.898381 | 1 | 0.04 | 0.040000 |
| GO:0007399\_nervous\_system\_development | ANK3 | 621 | 17 | 2.572710 | -3.898381 | 1 | 0.04 | 0.040000 |
| GO:0007399\_nervous\_system\_development | KATNA1 | 621 | 17 | 2.572710 | -3.898381 | 1 | 0.04 | 0.040000 |
| GO:0007399\_nervous\_system\_development | NAB1 | 621 | 17 | 2.572710 | -3.898381 | 1 | 0.04 | 0.040000 |
| GO:0007399\_nervous\_system\_development | FOXG1 | 621 | 17 | 2.572710 | -3.898381 | 1 | 0.04 | 0.040000 |
| GO:0007399\_nervous\_system\_development | POU4F1 | 621 | 17 | 2.572710 | -3.898381 | 1 | 0.04 | 0.040000 |
| GO:0007399\_nervous\_system\_development | LRP2 | 621 | 17 | 2.572710 | -3.898381 | 1 | 0.04 | 0.040000 |
| GO:0007411\_axon\_guidance | EPHA4 | 82 | 6 | 6.876556 | -3.696446 | 2 | 0.07 | 0.035000 |
| GO:0007411\_axon\_guidance | ANK3 | 82 | 6 | 6.876556 | -3.696446 | 2 | 0.07 | 0.035000 |
| GO:0007411\_axon\_guidance | FOXG1 | 82 | 6 | 6.876556 | -3.696446 | 2 | 0.07 | 0.035000 |
| GO:0007411\_axon\_guidance | KIF5C | 82 | 6 | 6.876556 | -3.696446 | 2 | 0.07 | 0.035000 |
| GO:0007411\_axon\_guidance | NTN1 | 82 | 6 | 6.876556 | -3.696446 | 2 | 0.07 | 0.035000 |
| GO:0007411\_axon\_guidance | EPHB2 | 82 | 6 | 6.876556 | -3.696446 | 2 | 0.07 | 0.035000 |
| GO:0022008\_neurogenesis | STMN3 | 423 | 13 | 2.888262 | -3.478792 | 3 | 0.15 | 0.050000 |
| GO:0022008\_neurogenesis | KIF5C | 423 | 13 | 2.888262 | -3.478792 | 3 | 0.15 | 0.050000 |
| GO:0022008\_neurogenesis | JAG1 | 423 | 13 | 2.888262 | -3.478792 | 3 | 0.15 | 0.050000 |
| GO:0022008\_neurogenesis | NTN1 | 423 | 13 | 2.888262 | -3.478792 | 3 | 0.15 | 0.050000 |
| GO:0022008\_neurogenesis | GPR98 | 423 | 13 | 2.888262 | -3.478792 | 3 | 0.15 | 0.050000 |
| GO:0022008\_neurogenesis | EPHB2 | 423 | 13 | 2.888262 | -3.478792 | 3 | 0.15 | 0.050000 |
| GO:0022008\_neurogenesis | EPHA4 | 423 | 13 | 2.888262 | -3.478792 | 3 | 0.15 | 0.050000 |
| GO:0022008\_neurogenesis | HES5 | 423 | 13 | 2.888262 | -3.478792 | 3 | 0.15 | 0.050000 |
| GO:0022008\_neurogenesis | ANK3 | 423 | 13 | 2.888262 | -3.478792 | 3 | 0.15 | 0.050000 |
| GO:0022008\_neurogenesis | KATNA1 | 423 | 13 | 2.888262 | -3.478792 | 3 | 0.15 | 0.050000 |
| GO:0022008\_neurogenesis | NAB1 | 423 | 13 | 2.888262 | -3.478792 | 3 | 0.15 | 0.050000 |
| GO:0022008\_neurogenesis | FOXG1 | 423 | 13 | 2.888262 | -3.478792 | 3 | 0.15 | 0.050000 |
| GO:0022008\_neurogenesis | POU4F1 | 423 | 13 | 2.888262 | -3.478792 | 3 | 0.15 | 0.050000 |
| GO:0030030\_cell\_projection\_organization | EPHA4 | 263 | 10 | 3.573369 | -3.453802 | 4 | 0.15 | 0.037500 |
| GO:0030030\_cell\_projection\_organization | STMN3 | 263 | 10 | 3.573369 | -3.453802 | 4 | 0.15 | 0.037500 |
| GO:0030030\_cell\_projection\_organization | ANK3 | 263 | 10 | 3.573369 | -3.453802 | 4 | 0.15 | 0.037500 |
| GO:0030030\_cell\_projection\_organization | PDGFA | 263 | 10 | 3.573369 | -3.453802 | 4 | 0.15 | 0.037500 |
| GO:0030030\_cell\_projection\_organization | ITGA8 | 263 | 10 | 3.573369 | -3.453802 | 4 | 0.15 | 0.037500 |
| GO:0030030\_cell\_projection\_organization | FOXG1 | 263 | 10 | 3.573369 | -3.453802 | 4 | 0.15 | 0.037500 |
| GO:0030030\_cell\_projection\_organization | KIF5C | 263 | 10 | 3.573369 | -3.453802 | 4 | 0.15 | 0.037500 |
| GO:0030030\_cell\_projection\_organization | NTN1 | 263 | 10 | 3.573369 | -3.453802 | 4 | 0.15 | 0.037500 |
| GO:0030030\_cell\_projection\_organization | GPR98 | 263 | 10 | 3.573369 | -3.453802 | 4 | 0.15 | 0.037500 |
| GO:0030030\_cell\_projection\_organization | EPHB2 | 263 | 10 | 3.573369 | -3.453802 | 4 | 0.15 | 0.037500 |
| GO:0048699\_generation\_of\_neurons | EPHA4 | 396 | 12 | 2.847866 | -3.169871 | 5 | 0.32 | 0.064000 |
| GO:0048699\_generation\_of\_neurons | STMN3 | 396 | 12 | 2.847866 | -3.169871 | 5 | 0.32 | 0.064000 |
| GO:0048699\_generation\_of\_neurons | HES5 | 396 | 12 | 2.847866 | -3.169871 | 5 | 0.32 | 0.064000 |
| GO:0048699\_generation\_of\_neurons | ANK3 | 396 | 12 | 2.847866 | -3.169871 | 5 | 0.32 | 0.064000 |
| GO:0048699\_generation\_of\_neurons | KATNA1 | 396 | 12 | 2.847866 | -3.169871 | 5 | 0.32 | 0.064000 |
| GO:0048699\_generation\_of\_neurons | KIF5C | 396 | 12 | 2.847866 | -3.169871 | 5 | 0.32 | 0.064000 |
| GO:0048699\_generation\_of\_neurons | FOXG1 | 396 | 12 | 2.847866 | -3.169871 | 5 | 0.32 | 0.064000 |
| GO:0048699\_generation\_of\_neurons | POU4F1 | 396 | 12 | 2.847866 | -3.169871 | 5 | 0.32 | 0.064000 |
| GO:0048699\_generation\_of\_neurons | JAG1 | 396 | 12 | 2.847866 | -3.169871 | 5 | 0.32 | 0.064000 |
| GO:0048699\_generation\_of\_neurons | NTN1 | 396 | 12 | 2.847866 | -3.169871 | 5 | 0.32 | 0.064000 |
| GO:0048699\_generation\_of\_neurons | GPR98 | 396 | 12 | 2.847866 | -3.169871 | 5 | 0.32 | 0.064000 |
| GO:0048699\_generation\_of\_neurons | EPHB2 | 396 | 12 | 2.847866 | -3.169871 | 5 | 0.32 | 0.064000 |
| GO:0007275\_multicellular\_organismal\_development | DLC1 | 1760 | 30 | 1.601925 | -3.059568 | 6 | 0.44 | 0.073333 |
| GO:0007275\_multicellular\_organismal\_development | PDGFA | 1760 | 30 | 1.601925 | -3.059568 | 6 | 0.44 | 0.073333 |
| GO:0007275\_multicellular\_organismal\_development | COL3A1 | 1760 | 30 | 1.601925 | -3.059568 | 6 | 0.44 | 0.073333 |
| GO:0007275\_multicellular\_organismal\_development | JAG1 | 1760 | 30 | 1.601925 | -3.059568 | 6 | 0.44 | 0.073333 |
| GO:0007275\_multicellular\_organismal\_development | MMP2 | 1760 | 30 | 1.601925 | -3.059568 | 6 | 0.44 | 0.073333 |
| GO:0007275\_multicellular\_organismal\_development | APLP1 | 1760 | 30 | 1.601925 | -3.059568 | 6 | 0.44 | 0.073333 |
| GO:0007275\_multicellular\_organismal\_development | EPHB2 | 1760 | 30 | 1.601925 | -3.059568 | 6 | 0.44 | 0.073333 |
| GO:0007275\_multicellular\_organismal\_development | HEY1 | 1760 | 30 | 1.601925 | -3.059568 | 6 | 0.44 | 0.073333 |
| GO:0007275\_multicellular\_organismal\_development | XBP1 | 1760 | 30 | 1.601925 | -3.059568 | 6 | 0.44 | 0.073333 |
| GO:0007275\_multicellular\_organismal\_development | ANK3 | 1760 | 30 | 1.601925 | -3.059568 | 6 | 0.44 | 0.073333 |
| GO:0007275\_multicellular\_organismal\_development | KATNA1 | 1760 | 30 | 1.601925 | -3.059568 | 6 | 0.44 | 0.073333 |
| GO:0007275\_multicellular\_organismal\_development | POU4F1 | 1760 | 30 | 1.601925 | -3.059568 | 6 | 0.44 | 0.073333 |
| GO:0007275\_multicellular\_organismal\_development | CHUK | 1760 | 30 | 1.601925 | -3.059568 | 6 | 0.44 | 0.073333 |
| GO:0007275\_multicellular\_organismal\_development | ODZ4 | 1760 | 30 | 1.601925 | -3.059568 | 6 | 0.44 | 0.073333 |
| GO:0007275\_multicellular\_organismal\_development | STMN3 | 1760 | 30 | 1.601925 | -3.059568 | 6 | 0.44 | 0.073333 |
| GO:0007275\_multicellular\_organismal\_development | KIF5C | 1760 | 30 | 1.601925 | -3.059568 | 6 | 0.44 | 0.073333 |
| GO:0007275\_multicellular\_organismal\_development | NTN1 | 1760 | 30 | 1.601925 | -3.059568 | 6 | 0.44 | 0.073333 |
| GO:0007275\_multicellular\_organismal\_development | GPR98 | 1760 | 30 | 1.601925 | -3.059568 | 6 | 0.44 | 0.073333 |
| GO:0007275\_multicellular\_organismal\_development | HBA-A1 | 1760 | 30 | 1.601925 | -3.059568 | 6 | 0.44 | 0.073333 |
| GO:0007275\_multicellular\_organismal\_development | EPHA4 | 1760 | 30 | 1.601925 | -3.059568 | 6 | 0.44 | 0.073333 |
| GO:0007275\_multicellular\_organismal\_development | MAPK1 | 1760 | 30 | 1.601925 | -3.059568 | 6 | 0.44 | 0.073333 |
| GO:0007275\_multicellular\_organismal\_development | DKK1 | 1760 | 30 | 1.601925 | -3.059568 | 6 | 0.44 | 0.073333 |
| GO:0007275\_multicellular\_organismal\_development | HIPK1 | 1760 | 30 | 1.601925 | -3.059568 | 6 | 0.44 | 0.073333 |
| GO:0007275\_multicellular\_organismal\_development | SFRP1 | 1760 | 30 | 1.601925 | -3.059568 | 6 | 0.44 | 0.073333 |
| GO:0007275\_multicellular\_organismal\_development | HES5 | 1760 | 30 | 1.601925 | -3.059568 | 6 | 0.44 | 0.073333 |
| GO:0007275\_multicellular\_organismal\_development | ITGA8 | 1760 | 30 | 1.601925 | -3.059568 | 6 | 0.44 | 0.073333 |
| GO:0007275\_multicellular\_organismal\_development | SP3 | 1760 | 30 | 1.601925 | -3.059568 | 6 | 0.44 | 0.073333 |
| GO:0007275\_multicellular\_organismal\_development | NAB1 | 1760 | 30 | 1.601925 | -3.059568 | 6 | 0.44 | 0.073333 |
| GO:0007275\_multicellular\_organismal\_development | FOXG1 | 1760 | 30 | 1.601925 | -3.059568 | 6 | 0.44 | 0.073333 |
| GO:0007275\_multicellular\_organismal\_development | LRP2 | 1760 | 30 | 1.601925 | -3.059568 | 6 | 0.44 | 0.073333 |
| GO:0048839\_inner\_ear\_development | HES5 | 72 | 5 | 6.526361 | -3.042275 | 7 | 0.45 | 0.064286 |
| GO:0048839\_inner\_ear\_development | ITGA8 | 72 | 5 | 6.526361 | -3.042275 | 7 | 0.45 | 0.064286 |
| GO:0048839\_inner\_ear\_development | FOXG1 | 72 | 5 | 6.526361 | -3.042275 | 7 | 0.45 | 0.064286 |
| GO:0048839\_inner\_ear\_development | JAG1 | 72 | 5 | 6.526361 | -3.042275 | 7 | 0.45 | 0.064286 |
| GO:0048839\_inner\_ear\_development | GPR98 | 72 | 5 | 6.526361 | -3.042275 | 7 | 0.45 | 0.064286 |
| GO:0030182\_neuron\_differentiation | EPHA4 | 356 | 11 | 2.903864 | -2.995982 | 8 | 0.54 | 0.067500 |
| GO:0030182\_neuron\_differentiation | STMN3 | 356 | 11 | 2.903864 | -2.995982 | 8 | 0.54 | 0.067500 |
| GO:0030182\_neuron\_differentiation | HES5 | 356 | 11 | 2.903864 | -2.995982 | 8 | 0.54 | 0.067500 |
| GO:0030182\_neuron\_differentiation | ANK3 | 356 | 11 | 2.903864 | -2.995982 | 8 | 0.54 | 0.067500 |
| GO:0030182\_neuron\_differentiation | KIF5C | 356 | 11 | 2.903864 | -2.995982 | 8 | 0.54 | 0.067500 |
| GO:0030182\_neuron\_differentiation | FOXG1 | 356 | 11 | 2.903864 | -2.995982 | 8 | 0.54 | 0.067500 |
| GO:0030182\_neuron\_differentiation | POU4F1 | 356 | 11 | 2.903864 | -2.995982 | 8 | 0.54 | 0.067500 |
| GO:0030182\_neuron\_differentiation | JAG1 | 356 | 11 | 2.903864 | -2.995982 | 8 | 0.54 | 0.067500 |
| GO:0030182\_neuron\_differentiation | NTN1 | 356 | 11 | 2.903864 | -2.995982 | 8 | 0.54 | 0.067500 |
| GO:0030182\_neuron\_differentiation | GPR98 | 356 | 11 | 2.903864 | -2.995982 | 8 | 0.54 | 0.067500 |
| GO:0030182\_neuron\_differentiation | EPHB2 | 356 | 11 | 2.903864 | -2.995982 | 8 | 0.54 | 0.067500 |
| GO:0048856\_anatomical\_structure\_development | DLC1 | 1688 | 29 | 1.614578 | -2.978962 | 9 | 0.56 | 0.062222 |
| GO:0048856\_anatomical\_structure\_development | PDGFA | 1688 | 29 | 1.614578 | -2.978962 | 9 | 0.56 | 0.062222 |
| GO:0048856\_anatomical\_structure\_development | COL3A1 | 1688 | 29 | 1.614578 | -2.978962 | 9 | 0.56 | 0.062222 |
| GO:0048856\_anatomical\_structure\_development | JAG1 | 1688 | 29 | 1.614578 | -2.978962 | 9 | 0.56 | 0.062222 |
| GO:0048856\_anatomical\_structure\_development | MMP2 | 1688 | 29 | 1.614578 | -2.978962 | 9 | 0.56 | 0.062222 |
| GO:0048856\_anatomical\_structure\_development | EPHB2 | 1688 | 29 | 1.614578 | -2.978962 | 9 | 0.56 | 0.062222 |
| GO:0048856\_anatomical\_structure\_development | APLP1 | 1688 | 29 | 1.614578 | -2.978962 | 9 | 0.56 | 0.062222 |
| GO:0048856\_anatomical\_structure\_development | HEY1 | 1688 | 29 | 1.614578 | -2.978962 | 9 | 0.56 | 0.062222 |
| GO:0048856\_anatomical\_structure\_development | XBP1 | 1688 | 29 | 1.614578 | -2.978962 | 9 | 0.56 | 0.062222 |
| GO:0048856\_anatomical\_structure\_development | ANK3 | 1688 | 29 | 1.614578 | -2.978962 | 9 | 0.56 | 0.062222 |
| GO:0048856\_anatomical\_structure\_development | KATNA1 | 1688 | 29 | 1.614578 | -2.978962 | 9 | 0.56 | 0.062222 |
| GO:0048856\_anatomical\_structure\_development | POU4F1 | 1688 | 29 | 1.614578 | -2.978962 | 9 | 0.56 | 0.062222 |
| GO:0048856\_anatomical\_structure\_development | CHUK | 1688 | 29 | 1.614578 | -2.978962 | 9 | 0.56 | 0.062222 |
| GO:0048856\_anatomical\_structure\_development | ODZ4 | 1688 | 29 | 1.614578 | -2.978962 | 9 | 0.56 | 0.062222 |
| GO:0048856\_anatomical\_structure\_development | STMN3 | 1688 | 29 | 1.614578 | -2.978962 | 9 | 0.56 | 0.062222 |
| GO:0048856\_anatomical\_structure\_development | KIF5C | 1688 | 29 | 1.614578 | -2.978962 | 9 | 0.56 | 0.062222 |
| GO:0048856\_anatomical\_structure\_development | NTN1 | 1688 | 29 | 1.614578 | -2.978962 | 9 | 0.56 | 0.062222 |
| GO:0048856\_anatomical\_structure\_development | GPR98 | 1688 | 29 | 1.614578 | -2.978962 | 9 | 0.56 | 0.062222 |
| GO:0048856\_anatomical\_structure\_development | HBA-A1 | 1688 | 29 | 1.614578 | -2.978962 | 9 | 0.56 | 0.062222 |
| GO:0048856\_anatomical\_structure\_development | MAPK1 | 1688 | 29 | 1.614578 | -2.978962 | 9 | 0.56 | 0.062222 |
| GO:0048856\_anatomical\_structure\_development | EPHA4 | 1688 | 29 | 1.614578 | -2.978962 | 9 | 0.56 | 0.062222 |
| GO:0048856\_anatomical\_structure\_development | DKK1 | 1688 | 29 | 1.614578 | -2.978962 | 9 | 0.56 | 0.062222 |
| GO:0048856\_anatomical\_structure\_development | SFRP1 | 1688 | 29 | 1.614578 | -2.978962 | 9 | 0.56 | 0.062222 |
| GO:0048856\_anatomical\_structure\_development | HES5 | 1688 | 29 | 1.614578 | -2.978962 | 9 | 0.56 | 0.062222 |
| GO:0048856\_anatomical\_structure\_development | ITGA8 | 1688 | 29 | 1.614578 | -2.978962 | 9 | 0.56 | 0.062222 |
| GO:0048856\_anatomical\_structure\_development | SP3 | 1688 | 29 | 1.614578 | -2.978962 | 9 | 0.56 | 0.062222 |
| GO:0048856\_anatomical\_structure\_development | NAB1 | 1688 | 29 | 1.614578 | -2.978962 | 9 | 0.56 | 0.062222 |
| GO:0048856\_anatomical\_structure\_development | FOXG1 | 1688 | 29 | 1.614578 | -2.978962 | 9 | 0.56 | 0.062222 |
| GO:0048856\_anatomical\_structure\_development | LRP2 | 1688 | 29 | 1.614578 | -2.978962 | 9 | 0.56 | 0.062222 |
| GO:0009653\_anatomical\_structure\_morphogenesis | DLC1 | 958 | 20 | 1.961996 | -2.963997 | 10 | 0.56 | 0.056000 |
| GO:0009653\_anatomical\_structure\_morphogenesis | PDGFA | 958 | 20 | 1.961996 | -2.963997 | 10 | 0.56 | 0.056000 |
| GO:0009653\_anatomical\_structure\_morphogenesis | KIF5C | 958 | 20 | 1.961996 | -2.963997 | 10 | 0.56 | 0.056000 |
| GO:0009653\_anatomical\_structure\_morphogenesis | JAG1 | 958 | 20 | 1.961996 | -2.963997 | 10 | 0.56 | 0.056000 |
| GO:0009653\_anatomical\_structure\_morphogenesis | NTN1 | 958 | 20 | 1.961996 | -2.963997 | 10 | 0.56 | 0.056000 |
| GO:0009653\_anatomical\_structure\_morphogenesis | MMP2 | 958 | 20 | 1.961996 | -2.963997 | 10 | 0.56 | 0.056000 |
| GO:0009653\_anatomical\_structure\_morphogenesis | EPHB2 | 958 | 20 | 1.961996 | -2.963997 | 10 | 0.56 | 0.056000 |
| GO:0009653\_anatomical\_structure\_morphogenesis | EPHA4 | 958 | 20 | 1.961996 | -2.963997 | 10 | 0.56 | 0.056000 |
| GO:0009653\_anatomical\_structure\_morphogenesis | MAPK1 | 958 | 20 | 1.961996 | -2.963997 | 10 | 0.56 | 0.056000 |
| GO:0009653\_anatomical\_structure\_morphogenesis | DKK1 | 958 | 20 | 1.961996 | -2.963997 | 10 | 0.56 | 0.056000 |
| GO:0009653\_anatomical\_structure\_morphogenesis | SFRP1 | 958 | 20 | 1.961996 | -2.963997 | 10 | 0.56 | 0.056000 |
| GO:0009653\_anatomical\_structure\_morphogenesis | HEY1 | 958 | 20 | 1.961996 | -2.963997 | 10 | 0.56 | 0.056000 |
| GO:0009653\_anatomical\_structure\_morphogenesis | ANK3 | 958 | 20 | 1.961996 | -2.963997 | 10 | 0.56 | 0.056000 |
| GO:0009653\_anatomical\_structure\_morphogenesis | XBP1 | 958 | 20 | 1.961996 | -2.963997 | 10 | 0.56 | 0.056000 |
| GO:0009653\_anatomical\_structure\_morphogenesis | ITGA8 | 958 | 20 | 1.961996 | -2.963997 | 10 | 0.56 | 0.056000 |
| GO:0009653\_anatomical\_structure\_morphogenesis | SP3 | 958 | 20 | 1.961996 | -2.963997 | 10 | 0.56 | 0.056000 |
| GO:0009653\_anatomical\_structure\_morphogenesis | FOXG1 | 958 | 20 | 1.961996 | -2.963997 | 10 | 0.56 | 0.056000 |
| GO:0009653\_anatomical\_structure\_morphogenesis | NAB1 | 958 | 20 | 1.961996 | -2.963997 | 10 | 0.56 | 0.056000 |
| GO:0009653\_anatomical\_structure\_morphogenesis | CHUK | 958 | 20 | 1.961996 | -2.963997 | 10 | 0.56 | 0.056000 |
| GO:0009653\_anatomical\_structure\_morphogenesis | ODZ4 | 958 | 20 | 1.961996 | -2.963997 | 10 | 0.56 | 0.056000 |
| GO:0031122\_cytoplasmic\_microtubule\_organization | STMN3 | 5 | 2 | 37.591837 | -2.963821 | 11 | 0.86 | 0.078182 |
| GO:0031122\_cytoplasmic\_microtubule\_organization | KATNA1 | 5 | 2 | 37.591837 | -2.963821 | 11 | 0.86 | 0.078182 |
| GO:0048731\_system\_development | DLC1 | 1609 | 28 | 1.635443 | -2.938039 | 12 | 0.92 | 0.076667 |
| GO:0048731\_system\_development | PDGFA | 1609 | 28 | 1.635443 | -2.938039 | 12 | 0.92 | 0.076667 |
| GO:0048731\_system\_development | COL3A1 | 1609 | 28 | 1.635443 | -2.938039 | 12 | 0.92 | 0.076667 |
| GO:0048731\_system\_development | JAG1 | 1609 | 28 | 1.635443 | -2.938039 | 12 | 0.92 | 0.076667 |
| GO:0048731\_system\_development | MMP2 | 1609 | 28 | 1.635443 | -2.938039 | 12 | 0.92 | 0.076667 |
| GO:0048731\_system\_development | APLP1 | 1609 | 28 | 1.635443 | -2.938039 | 12 | 0.92 | 0.076667 |
| GO:0048731\_system\_development | EPHB2 | 1609 | 28 | 1.635443 | -2.938039 | 12 | 0.92 | 0.076667 |
| GO:0048731\_system\_development | HEY1 | 1609 | 28 | 1.635443 | -2.938039 | 12 | 0.92 | 0.076667 |
| GO:0048731\_system\_development | ANK3 | 1609 | 28 | 1.635443 | -2.938039 | 12 | 0.92 | 0.076667 |
| GO:0048731\_system\_development | XBP1 | 1609 | 28 | 1.635443 | -2.938039 | 12 | 0.92 | 0.076667 |
| GO:0048731\_system\_development | KATNA1 | 1609 | 28 | 1.635443 | -2.938039 | 12 | 0.92 | 0.076667 |
| GO:0048731\_system\_development | POU4F1 | 1609 | 28 | 1.635443 | -2.938039 | 12 | 0.92 | 0.076667 |
| GO:0048731\_system\_development | CHUK | 1609 | 28 | 1.635443 | -2.938039 | 12 | 0.92 | 0.076667 |
| GO:0048731\_system\_development | STMN3 | 1609 | 28 | 1.635443 | -2.938039 | 12 | 0.92 | 0.076667 |
| GO:0048731\_system\_development | KIF5C | 1609 | 28 | 1.635443 | -2.938039 | 12 | 0.92 | 0.076667 |
| GO:0048731\_system\_development | NTN1 | 1609 | 28 | 1.635443 | -2.938039 | 12 | 0.92 | 0.076667 |
| GO:0048731\_system\_development | GPR98 | 1609 | 28 | 1.635443 | -2.938039 | 12 | 0.92 | 0.076667 |
| GO:0048731\_system\_development | HBA-A1 | 1609 | 28 | 1.635443 | -2.938039 | 12 | 0.92 | 0.076667 |
| GO:0048731\_system\_development | MAPK1 | 1609 | 28 | 1.635443 | -2.938039 | 12 | 0.92 | 0.076667 |
| GO:0048731\_system\_development | EPHA4 | 1609 | 28 | 1.635443 | -2.938039 | 12 | 0.92 | 0.076667 |
| GO:0048731\_system\_development | DKK1 | 1609 | 28 | 1.635443 | -2.938039 | 12 | 0.92 | 0.076667 |
| GO:0048731\_system\_development | SFRP1 | 1609 | 28 | 1.635443 | -2.938039 | 12 | 0.92 | 0.076667 |
| GO:0048731\_system\_development | HES5 | 1609 | 28 | 1.635443 | -2.938039 | 12 | 0.92 | 0.076667 |
| GO:0048731\_system\_development | ITGA8 | 1609 | 28 | 1.635443 | -2.938039 | 12 | 0.92 | 0.076667 |
| GO:0048731\_system\_development | SP3 | 1609 | 28 | 1.635443 | -2.938039 | 12 | 0.92 | 0.076667 |
| GO:0048731\_system\_development | FOXG1 | 1609 | 28 | 1.635443 | -2.938039 | 12 | 0.92 | 0.076667 |
| GO:0048731\_system\_development | NAB1 | 1609 | 28 | 1.635443 | -2.938039 | 12 | 0.92 | 0.076667 |
| GO:0048731\_system\_development | LRP2 | 1609 | 28 | 1.635443 | -2.938039 | 12 | 0.92 | 0.076667 |
| GO:0032501\_multicellular\_organismal\_process | DLC1 | 2183 | 34 | 1.463722 | -2.833666 | 13 | 1.04 | 0.080000 |
| GO:0032501\_multicellular\_organismal\_process | CPLX3 | 2183 | 34 | 1.463722 | -2.833666 | 13 | 1.04 | 0.080000 |
| GO:0032501\_multicellular\_organismal\_process | PDGFA | 2183 | 34 | 1.463722 | -2.833666 | 13 | 1.04 | 0.080000 |
| GO:0032501\_multicellular\_organismal\_process | COL3A1 | 2183 | 34 | 1.463722 | -2.833666 | 13 | 1.04 | 0.080000 |
| GO:0032501\_multicellular\_organismal\_process | JAG1 | 2183 | 34 | 1.463722 | -2.833666 | 13 | 1.04 | 0.080000 |
| GO:0032501\_multicellular\_organismal\_process | MMP2 | 2183 | 34 | 1.463722 | -2.833666 | 13 | 1.04 | 0.080000 |
| GO:0032501\_multicellular\_organismal\_process | EPHB2 | 2183 | 34 | 1.463722 | -2.833666 | 13 | 1.04 | 0.080000 |
| GO:0032501\_multicellular\_organismal\_process | APLP1 | 2183 | 34 | 1.463722 | -2.833666 | 13 | 1.04 | 0.080000 |
| GO:0032501\_multicellular\_organismal\_process | HEY1 | 2183 | 34 | 1.463722 | -2.833666 | 13 | 1.04 | 0.080000 |
| GO:0032501\_multicellular\_organismal\_process | XBP1 | 2183 | 34 | 1.463722 | -2.833666 | 13 | 1.04 | 0.080000 |
| GO:0032501\_multicellular\_organismal\_process | ANK3 | 2183 | 34 | 1.463722 | -2.833666 | 13 | 1.04 | 0.080000 |
| GO:0032501\_multicellular\_organismal\_process | KATNA1 | 2183 | 34 | 1.463722 | -2.833666 | 13 | 1.04 | 0.080000 |
| GO:0032501\_multicellular\_organismal\_process | POU4F1 | 2183 | 34 | 1.463722 | -2.833666 | 13 | 1.04 | 0.080000 |
| GO:0032501\_multicellular\_organismal\_process | SV2B | 2183 | 34 | 1.463722 | -2.833666 | 13 | 1.04 | 0.080000 |
| GO:0032501\_multicellular\_organismal\_process | CHUK | 2183 | 34 | 1.463722 | -2.833666 | 13 | 1.04 | 0.080000 |
| GO:0032501\_multicellular\_organismal\_process | ODZ4 | 2183 | 34 | 1.463722 | -2.833666 | 13 | 1.04 | 0.080000 |
| GO:0032501\_multicellular\_organismal\_process | STMN3 | 2183 | 34 | 1.463722 | -2.833666 | 13 | 1.04 | 0.080000 |
| GO:0032501\_multicellular\_organismal\_process | KIF5C | 2183 | 34 | 1.463722 | -2.833666 | 13 | 1.04 | 0.080000 |
| GO:0032501\_multicellular\_organismal\_process | NTN1 | 2183 | 34 | 1.463722 | -2.833666 | 13 | 1.04 | 0.080000 |
| GO:0032501\_multicellular\_organismal\_process | GPR98 | 2183 | 34 | 1.463722 | -2.833666 | 13 | 1.04 | 0.080000 |
| GO:0032501\_multicellular\_organismal\_process | LIN7A | 2183 | 34 | 1.463722 | -2.833666 | 13 | 1.04 | 0.080000 |
| GO:0032501\_multicellular\_organismal\_process | HBA-A1 | 2183 | 34 | 1.463722 | -2.833666 | 13 | 1.04 | 0.080000 |
| GO:0032501\_multicellular\_organismal\_process | EPHA4 | 2183 | 34 | 1.463722 | -2.833666 | 13 | 1.04 | 0.080000 |
| GO:0032501\_multicellular\_organismal\_process | MAPK1 | 2183 | 34 | 1.463722 | -2.833666 | 13 | 1.04 | 0.080000 |
| GO:0032501\_multicellular\_organismal\_process | DKK1 | 2183 | 34 | 1.463722 | -2.833666 | 13 | 1.04 | 0.080000 |
| GO:0032501\_multicellular\_organismal\_process | HES5 | 2183 | 34 | 1.463722 | -2.833666 | 13 | 1.04 | 0.080000 |
| GO:0032501\_multicellular\_organismal\_process | SFRP1 | 2183 | 34 | 1.463722 | -2.833666 | 13 | 1.04 | 0.080000 |
| GO:0032501\_multicellular\_organismal\_process | HIPK1 | 2183 | 34 | 1.463722 | -2.833666 | 13 | 1.04 | 0.080000 |
| GO:0032501\_multicellular\_organismal\_process | SP3 | 2183 | 34 | 1.463722 | -2.833666 | 13 | 1.04 | 0.080000 |
| GO:0032501\_multicellular\_organismal\_process | ITGA8 | 2183 | 34 | 1.463722 | -2.833666 | 13 | 1.04 | 0.080000 |
| GO:0032501\_multicellular\_organismal\_process | NAB1 | 2183 | 34 | 1.463722 | -2.833666 | 13 | 1.04 | 0.080000 |
| GO:0032501\_multicellular\_organismal\_process | FOXG1 | 2183 | 34 | 1.463722 | -2.833666 | 13 | 1.04 | 0.080000 |
| GO:0032501\_multicellular\_organismal\_process | SYTL2 | 2183 | 34 | 1.463722 | -2.833666 | 13 | 1.04 | 0.080000 |
| GO:0032501\_multicellular\_organismal\_process | LRP2 | 2183 | 34 | 1.463722 | -2.833666 | 13 | 1.04 | 0.080000 |
| GO:0048489\_synaptic\_vesicle\_transport | CPLX3 | 22 | 3 | 12.815399 | -2.820254 | 14 | 1.08 | 0.077143 |
| GO:0048489\_synaptic\_vesicle\_transport | SV2B | 22 | 3 | 12.815399 | -2.820254 | 14 | 1.08 | 0.077143 |
| GO:0048489\_synaptic\_vesicle\_transport | LIN7A | 22 | 3 | 12.815399 | -2.820254 | 14 | 1.08 | 0.077143 |
| GO:0060113\_inner\_ear\_receptor\_cell\_differentiation | HES5 | 24 | 3 | 11.747449 | -2.708057 | 15 | 1.56 | 0.104000 |
| GO:0060113\_inner\_ear\_receptor\_cell\_differentiation | JAG1 | 24 | 3 | 11.747449 | -2.708057 | 15 | 1.56 | 0.104000 |
| GO:0060113\_inner\_ear\_receptor\_cell\_differentiation | GPR98 | 24 | 3 | 11.747449 | -2.708057 | 15 | 1.56 | 0.104000 |
| GO:0006887\_exocytosis | SCAMP1 | 51 | 4 | 7.370948 | -2.708039 | 16 | 1.56 | 0.097500 |
| GO:0006887\_exocytosis | CPLX3 | 51 | 4 | 7.370948 | -2.708039 | 16 | 1.56 | 0.097500 |
| GO:0006887\_exocytosis | SYTL2 | 51 | 4 | 7.370948 | -2.708039 | 16 | 1.56 | 0.097500 |
| GO:0006887\_exocytosis | SV2B | 51 | 4 | 7.370948 | -2.708039 | 16 | 1.56 | 0.097500 |
| GO:0043583\_ear\_development | HES5 | 87 | 5 | 5.401126 | -2.672415 | 17 | 1.61 | 0.094706 |
| GO:0043583\_ear\_development | ITGA8 | 87 | 5 | 5.401126 | -2.672415 | 17 | 1.61 | 0.094706 |
| GO:0043583\_ear\_development | FOXG1 | 87 | 5 | 5.401126 | -2.672415 | 17 | 1.61 | 0.094706 |
| GO:0043583\_ear\_development | JAG1 | 87 | 5 | 5.401126 | -2.672415 | 17 | 1.61 | 0.094706 |
| GO:0043583\_ear\_development | GPR98 | 87 | 5 | 5.401126 | -2.672415 | 17 | 1.61 | 0.094706 |
| GO:0002011\_morphogenesis\_of\_an\_epithelial\_sheet | JAG1 | 7 | 2 | 26.851312 | -2.647520 | 18 | 2.01 | 0.111667 |
| GO:0002011\_morphogenesis\_of\_an\_epithelial\_sheet | CHUK | 7 | 2 | 26.851312 | -2.647520 | 18 | 2.01 | 0.111667 |
| GO:0048468\_cell\_development | STMN3 | 654 | 15 | 2.155495 | -2.624176 | 19 | 2.04 | 0.107368 |
| GO:0048468\_cell\_development | KIF5C | 654 | 15 | 2.155495 | -2.624176 | 19 | 2.04 | 0.107368 |
| GO:0048468\_cell\_development | JAG1 | 654 | 15 | 2.155495 | -2.624176 | 19 | 2.04 | 0.107368 |
| GO:0048468\_cell\_development | NTN1 | 654 | 15 | 2.155495 | -2.624176 | 19 | 2.04 | 0.107368 |
| GO:0048468\_cell\_development | GPR98 | 654 | 15 | 2.155495 | -2.624176 | 19 | 2.04 | 0.107368 |
| GO:0048468\_cell\_development | EPHB2 | 654 | 15 | 2.155495 | -2.624176 | 19 | 2.04 | 0.107368 |
| GO:0048468\_cell\_development | HBA-A1 | 654 | 15 | 2.155495 | -2.624176 | 19 | 2.04 | 0.107368 |
| GO:0048468\_cell\_development | EPHA4 | 654 | 15 | 2.155495 | -2.624176 | 19 | 2.04 | 0.107368 |
| GO:0048468\_cell\_development | HES5 | 654 | 15 | 2.155495 | -2.624176 | 19 | 2.04 | 0.107368 |
| GO:0048468\_cell\_development | ANK3 | 654 | 15 | 2.155495 | -2.624176 | 19 | 2.04 | 0.107368 |
| GO:0048468\_cell\_development | XBP1 | 654 | 15 | 2.155495 | -2.624176 | 19 | 2.04 | 0.107368 |
| GO:0048468\_cell\_development | KATNA1 | 654 | 15 | 2.155495 | -2.624176 | 19 | 2.04 | 0.107368 |
| GO:0048468\_cell\_development | FOXG1 | 654 | 15 | 2.155495 | -2.624176 | 19 | 2.04 | 0.107368 |
| GO:0048468\_cell\_development | NAB1 | 654 | 15 | 2.155495 | -2.624176 | 19 | 2.04 | 0.107368 |
| GO:0048468\_cell\_development | POU4F1 | 654 | 15 | 2.155495 | -2.624176 | 19 | 2.04 | 0.107368 |
| GO:0042490\_mechanoreceptor\_differentiation | HES5 | 29 | 3 | 9.722027 | -2.467709 | 21 | 2.97 | 0.141429 |
| GO:0042490\_mechanoreceptor\_differentiation | JAG1 | 29 | 3 | 9.722027 | -2.467709 | 21 | 2.97 | 0.141429 |
| GO:0042490\_mechanoreceptor\_differentiation | GPR98 | 29 | 3 | 9.722027 | -2.467709 | 21 | 2.97 | 0.141429 |
| GO:0050769\_positive\_regulation\_of\_neurogenesis | FOXG1 | 29 | 3 | 9.722027 | -2.467709 | 21 | 2.97 | 0.141429 |
| GO:0050769\_positive\_regulation\_of\_neurogenesis | NTN1 | 29 | 3 | 9.722027 | -2.467709 | 21 | 2.97 | 0.141429 |
| GO:0050769\_positive\_regulation\_of\_neurogenesis | EPHB2 | 29 | 3 | 9.722027 | -2.467709 | 21 | 2.97 | 0.141429 |
| GO:0045596\_negative\_regulation\_of\_cell\_differentiation | MAPK1 | 144 | 6 | 3.915816 | -2.410377 | 22 | 3.65 | 0.165909 |
| GO:0045596\_negative\_regulation\_of\_cell\_differentiation | HES5 | 144 | 6 | 3.915816 | -2.410377 | 22 | 3.65 | 0.165909 |
| GO:0045596\_negative\_regulation\_of\_cell\_differentiation | FOXG1 | 144 | 6 | 3.915816 | -2.410377 | 22 | 3.65 | 0.165909 |
| GO:0045596\_negative\_regulation\_of\_cell\_differentiation | JAG1 | 144 | 6 | 3.915816 | -2.410377 | 22 | 3.65 | 0.165909 |
| GO:0045596\_negative\_regulation\_of\_cell\_differentiation | NTN1 | 144 | 6 | 3.915816 | -2.410377 | 22 | 3.65 | 0.165909 |
| GO:0045596\_negative\_regulation\_of\_cell\_differentiation | EPHB2 | 144 | 6 | 3.915816 | -2.410377 | 22 | 3.65 | 0.165909 |
| GO:0031175\_neuron\_projection\_development | EPHA4 | 197 | 7 | 3.339376 | -2.357383 | 23 | 3.89 | 0.169130 |
| GO:0031175\_neuron\_projection\_development | STMN3 | 197 | 7 | 3.339376 | -2.357383 | 23 | 3.89 | 0.169130 |
| GO:0031175\_neuron\_projection\_development | ANK3 | 197 | 7 | 3.339376 | -2.357383 | 23 | 3.89 | 0.169130 |
| GO:0031175\_neuron\_projection\_development | FOXG1 | 197 | 7 | 3.339376 | -2.357383 | 23 | 3.89 | 0.169130 |
| GO:0031175\_neuron\_projection\_development | KIF5C | 197 | 7 | 3.339376 | -2.357383 | 23 | 3.89 | 0.169130 |
| GO:0031175\_neuron\_projection\_development | NTN1 | 197 | 7 | 3.339376 | -2.357383 | 23 | 3.89 | 0.169130 |
| GO:0031175\_neuron\_projection\_development | EPHB2 | 197 | 7 | 3.339376 | -2.357383 | 23 | 3.89 | 0.169130 |
| GO:0007269\_neurotransmitter\_secretion | CPLX3 | 34 | 3 | 8.292317 | -2.269667 | 25 | 4.91 | 0.196400 |
| GO:0007269\_neurotransmitter\_secretion | SV2B | 34 | 3 | 8.292317 | -2.269667 | 25 | 4.91 | 0.196400 |
| GO:0007269\_neurotransmitter\_secretion | LIN7A | 34 | 3 | 8.292317 | -2.269667 | 25 | 4.91 | 0.196400 |
| GO:0010720\_positive\_regulation\_of\_cell\_development | FOXG1 | 34 | 3 | 8.292317 | -2.269667 | 25 | 4.91 | 0.196400 |
| GO:0010720\_positive\_regulation\_of\_cell\_development | NTN1 | 34 | 3 | 8.292317 | -2.269667 | 25 | 4.91 | 0.196400 |
| GO:0010720\_positive\_regulation\_of\_cell\_development | EPHB2 | 34 | 3 | 8.292317 | -2.269667 | 25 | 4.91 | 0.196400 |
| GO:0009887\_organ\_morphogenesis | DLC1 | 642 | 14 | 2.049399 | -2.257305 | 26 | 4.91 | 0.188846 |
| GO:0009887\_organ\_morphogenesis | PDGFA | 642 | 14 | 2.049399 | -2.257305 | 26 | 4.91 | 0.188846 |
| GO:0009887\_organ\_morphogenesis | JAG1 | 642 | 14 | 2.049399 | -2.257305 | 26 | 4.91 | 0.188846 |
| GO:0009887\_organ\_morphogenesis | MMP2 | 642 | 14 | 2.049399 | -2.257305 | 26 | 4.91 | 0.188846 |
| GO:0009887\_organ\_morphogenesis | EPHB2 | 642 | 14 | 2.049399 | -2.257305 | 26 | 4.91 | 0.188846 |
| GO:0009887\_organ\_morphogenesis | MAPK1 | 642 | 14 | 2.049399 | -2.257305 | 26 | 4.91 | 0.188846 |
| GO:0009887\_organ\_morphogenesis | HEY1 | 642 | 14 | 2.049399 | -2.257305 | 26 | 4.91 | 0.188846 |
| GO:0009887\_organ\_morphogenesis | SFRP1 | 642 | 14 | 2.049399 | -2.257305 | 26 | 4.91 | 0.188846 |
| GO:0009887\_organ\_morphogenesis | XBP1 | 642 | 14 | 2.049399 | -2.257305 | 26 | 4.91 | 0.188846 |
| GO:0009887\_organ\_morphogenesis | ITGA8 | 642 | 14 | 2.049399 | -2.257305 | 26 | 4.91 | 0.188846 |
| GO:0009887\_organ\_morphogenesis | SP3 | 642 | 14 | 2.049399 | -2.257305 | 26 | 4.91 | 0.188846 |
| GO:0009887\_organ\_morphogenesis | FOXG1 | 642 | 14 | 2.049399 | -2.257305 | 26 | 4.91 | 0.188846 |
| GO:0009887\_organ\_morphogenesis | NAB1 | 642 | 14 | 2.049399 | -2.257305 | 26 | 4.91 | 0.188846 |
| GO:0009887\_organ\_morphogenesis | CHUK | 642 | 14 | 2.049399 | -2.257305 | 26 | 4.91 | 0.188846 |
| GO:0016079\_synaptic\_vesicle\_exocytosis | CPLX3 | 11 | 2 | 17.087199 | -2.241188 | 27 | 5.48 | 0.202963 |
| GO:0016079\_synaptic\_vesicle\_exocytosis | SV2B | 11 | 2 | 17.087199 | -2.241188 | 27 | 5.48 | 0.202963 |
| GO:0048666\_neuron\_development | EPHA4 | 262 | 8 | 2.869606 | -2.236384 | 28 | 5.49 | 0.196071 |
| GO:0048666\_neuron\_development | STMN3 | 262 | 8 | 2.869606 | -2.236384 | 28 | 5.49 | 0.196071 |
| GO:0048666\_neuron\_development | ANK3 | 262 | 8 | 2.869606 | -2.236384 | 28 | 5.49 | 0.196071 |
| GO:0048666\_neuron\_development | KIF5C | 262 | 8 | 2.869606 | -2.236384 | 28 | 5.49 | 0.196071 |
| GO:0048666\_neuron\_development | FOXG1 | 262 | 8 | 2.869606 | -2.236384 | 28 | 5.49 | 0.196071 |
| GO:0048666\_neuron\_development | NTN1 | 262 | 8 | 2.869606 | -2.236384 | 28 | 5.49 | 0.196071 |
| GO:0048666\_neuron\_development | GPR98 | 262 | 8 | 2.869606 | -2.236384 | 28 | 5.49 | 0.196071 |
| GO:0048666\_neuron\_development | EPHB2 | 262 | 8 | 2.869606 | -2.236384 | 28 | 5.49 | 0.196071 |
| GO:0007409\_axonogenesis | EPHA4 | 158 | 6 | 3.568845 | -2.213376 | 29 | 5.64 | 0.194483 |
| GO:0007409\_axonogenesis | ANK3 | 158 | 6 | 3.568845 | -2.213376 | 29 | 5.64 | 0.194483 |
| GO:0007409\_axonogenesis | FOXG1 | 158 | 6 | 3.568845 | -2.213376 | 29 | 5.64 | 0.194483 |
| GO:0007409\_axonogenesis | KIF5C | 158 | 6 | 3.568845 | -2.213376 | 29 | 5.64 | 0.194483 |
| GO:0007409\_axonogenesis | NTN1 | 158 | 6 | 3.568845 | -2.213376 | 29 | 5.64 | 0.194483 |
| GO:0007409\_axonogenesis | EPHB2 | 158 | 6 | 3.568845 | -2.213376 | 29 | 5.64 | 0.194483 |
| GO:0032502\_developmental\_process | DLC1 | 2060 | 31 | 1.414256 | -2.175414 | 30 | 5.85 | 0.195000 |
| GO:0032502\_developmental\_process | PDGFA | 2060 | 31 | 1.414256 | -2.175414 | 30 | 5.85 | 0.195000 |
| GO:0032502\_developmental\_process | COL3A1 | 2060 | 31 | 1.414256 | -2.175414 | 30 | 5.85 | 0.195000 |
| GO:0032502\_developmental\_process | JAG1 | 2060 | 31 | 1.414256 | -2.175414 | 30 | 5.85 | 0.195000 |
| GO:0032502\_developmental\_process | MMP2 | 2060 | 31 | 1.414256 | -2.175414 | 30 | 5.85 | 0.195000 |
| GO:0032502\_developmental\_process | APLP1 | 2060 | 31 | 1.414256 | -2.175414 | 30 | 5.85 | 0.195000 |
| GO:0032502\_developmental\_process | EPHB2 | 2060 | 31 | 1.414256 | -2.175414 | 30 | 5.85 | 0.195000 |
| GO:0032502\_developmental\_process | HEY1 | 2060 | 31 | 1.414256 | -2.175414 | 30 | 5.85 | 0.195000 |
| GO:0032502\_developmental\_process | XBP1 | 2060 | 31 | 1.414256 | -2.175414 | 30 | 5.85 | 0.195000 |
| GO:0032502\_developmental\_process | ANK3 | 2060 | 31 | 1.414256 | -2.175414 | 30 | 5.85 | 0.195000 |
| GO:0032502\_developmental\_process | KATNA1 | 2060 | 31 | 1.414256 | -2.175414 | 30 | 5.85 | 0.195000 |
| GO:0032502\_developmental\_process | POU4F1 | 2060 | 31 | 1.414256 | -2.175414 | 30 | 5.85 | 0.195000 |
| GO:0032502\_developmental\_process | CHUK | 2060 | 31 | 1.414256 | -2.175414 | 30 | 5.85 | 0.195000 |
| GO:0032502\_developmental\_process | ODZ4 | 2060 | 31 | 1.414256 | -2.175414 | 30 | 5.85 | 0.195000 |
| GO:0032502\_developmental\_process | TM2D1 | 2060 | 31 | 1.414256 | -2.175414 | 30 | 5.85 | 0.195000 |
| GO:0032502\_developmental\_process | STMN3 | 2060 | 31 | 1.414256 | -2.175414 | 30 | 5.85 | 0.195000 |
| GO:0032502\_developmental\_process | KIF5C | 2060 | 31 | 1.414256 | -2.175414 | 30 | 5.85 | 0.195000 |
| GO:0032502\_developmental\_process | NTN1 | 2060 | 31 | 1.414256 | -2.175414 | 30 | 5.85 | 0.195000 |
| GO:0032502\_developmental\_process | GPR98 | 2060 | 31 | 1.414256 | -2.175414 | 30 | 5.85 | 0.195000 |
| GO:0032502\_developmental\_process | HBA-A1 | 2060 | 31 | 1.414256 | -2.175414 | 30 | 5.85 | 0.195000 |
| GO:0032502\_developmental\_process | EPHA4 | 2060 | 31 | 1.414256 | -2.175414 | 30 | 5.85 | 0.195000 |
| GO:0032502\_developmental\_process | MAPK1 | 2060 | 31 | 1.414256 | -2.175414 | 30 | 5.85 | 0.195000 |
| GO:0032502\_developmental\_process | DKK1 | 2060 | 31 | 1.414256 | -2.175414 | 30 | 5.85 | 0.195000 |
| GO:0032502\_developmental\_process | HIPK1 | 2060 | 31 | 1.414256 | -2.175414 | 30 | 5.85 | 0.195000 |
| GO:0032502\_developmental\_process | SFRP1 | 2060 | 31 | 1.414256 | -2.175414 | 30 | 5.85 | 0.195000 |
| GO:0032502\_developmental\_process | HES5 | 2060 | 31 | 1.414256 | -2.175414 | 30 | 5.85 | 0.195000 |
| GO:0032502\_developmental\_process | ITGA8 | 2060 | 31 | 1.414256 | -2.175414 | 30 | 5.85 | 0.195000 |
| GO:0032502\_developmental\_process | SP3 | 2060 | 31 | 1.414256 | -2.175414 | 30 | 5.85 | 0.195000 |
| GO:0032502\_developmental\_process | FOXG1 | 2060 | 31 | 1.414256 | -2.175414 | 30 | 5.85 | 0.195000 |
| GO:0032502\_developmental\_process | NAB1 | 2060 | 31 | 1.414256 | -2.175414 | 30 | 5.85 | 0.195000 |
| GO:0032502\_developmental\_process | LRP2 | 2060 | 31 | 1.414256 | -2.175414 | 30 | 5.85 | 0.195000 |
| GO:0048513\_organ\_development | DLC1 | 1365 | 23 | 1.583539 | -2.120123 | 31 | 6.81 | 0.219677 |
| GO:0048513\_organ\_development | PDGFA | 1365 | 23 | 1.583539 | -2.120123 | 31 | 6.81 | 0.219677 |
| GO:0048513\_organ\_development | COL3A1 | 1365 | 23 | 1.583539 | -2.120123 | 31 | 6.81 | 0.219677 |
| GO:0048513\_organ\_development | JAG1 | 1365 | 23 | 1.583539 | -2.120123 | 31 | 6.81 | 0.219677 |
| GO:0048513\_organ\_development | NTN1 | 1365 | 23 | 1.583539 | -2.120123 | 31 | 6.81 | 0.219677 |
| GO:0048513\_organ\_development | MMP2 | 1365 | 23 | 1.583539 | -2.120123 | 31 | 6.81 | 0.219677 |
| GO:0048513\_organ\_development | GPR98 | 1365 | 23 | 1.583539 | -2.120123 | 31 | 6.81 | 0.219677 |
| GO:0048513\_organ\_development | APLP1 | 1365 | 23 | 1.583539 | -2.120123 | 31 | 6.81 | 0.219677 |
| GO:0048513\_organ\_development | EPHB2 | 1365 | 23 | 1.583539 | -2.120123 | 31 | 6.81 | 0.219677 |
| GO:0048513\_organ\_development | HBA-A1 | 1365 | 23 | 1.583539 | -2.120123 | 31 | 6.81 | 0.219677 |
| GO:0048513\_organ\_development | MAPK1 | 1365 | 23 | 1.583539 | -2.120123 | 31 | 6.81 | 0.219677 |
| GO:0048513\_organ\_development | DKK1 | 1365 | 23 | 1.583539 | -2.120123 | 31 | 6.81 | 0.219677 |
| GO:0048513\_organ\_development | HES5 | 1365 | 23 | 1.583539 | -2.120123 | 31 | 6.81 | 0.219677 |
| GO:0048513\_organ\_development | HEY1 | 1365 | 23 | 1.583539 | -2.120123 | 31 | 6.81 | 0.219677 |
| GO:0048513\_organ\_development | SFRP1 | 1365 | 23 | 1.583539 | -2.120123 | 31 | 6.81 | 0.219677 |
| GO:0048513\_organ\_development | XBP1 | 1365 | 23 | 1.583539 | -2.120123 | 31 | 6.81 | 0.219677 |
| GO:0048513\_organ\_development | ITGA8 | 1365 | 23 | 1.583539 | -2.120123 | 31 | 6.81 | 0.219677 |
| GO:0048513\_organ\_development | SP3 | 1365 | 23 | 1.583539 | -2.120123 | 31 | 6.81 | 0.219677 |
| GO:0048513\_organ\_development | FOXG1 | 1365 | 23 | 1.583539 | -2.120123 | 31 | 6.81 | 0.219677 |
| GO:0048513\_organ\_development | NAB1 | 1365 | 23 | 1.583539 | -2.120123 | 31 | 6.81 | 0.219677 |
| GO:0048513\_organ\_development | POU4F1 | 1365 | 23 | 1.583539 | -2.120123 | 31 | 6.81 | 0.219677 |
| GO:0048513\_organ\_development | LRP2 | 1365 | 23 | 1.583539 | -2.120123 | 31 | 6.81 | 0.219677 |
| GO:0048513\_organ\_development | CHUK | 1365 | 23 | 1.583539 | -2.120123 | 31 | 6.81 | 0.219677 |
| GO:0016043\_cellular\_component\_organization | DLC1 | 964 | 18 | 1.754806 | -2.111540 | 32 | 6.84 | 0.213750 |
| GO:0016043\_cellular\_component\_organization | STMN3 | 964 | 18 | 1.754806 | -2.111540 | 32 | 6.84 | 0.213750 |
| GO:0016043\_cellular\_component\_organization | PDGFA | 964 | 18 | 1.754806 | -2.111540 | 32 | 6.84 | 0.213750 |
| GO:0016043\_cellular\_component\_organization | COL3A1 | 964 | 18 | 1.754806 | -2.111540 | 32 | 6.84 | 0.213750 |
| GO:0016043\_cellular\_component\_organization | KIF5C | 964 | 18 | 1.754806 | -2.111540 | 32 | 6.84 | 0.213750 |
| GO:0016043\_cellular\_component\_organization | NTN1 | 964 | 18 | 1.754806 | -2.111540 | 32 | 6.84 | 0.213750 |
| GO:0016043\_cellular\_component\_organization | GPR98 | 964 | 18 | 1.754806 | -2.111540 | 32 | 6.84 | 0.213750 |
| GO:0016043\_cellular\_component\_organization | APLP1 | 964 | 18 | 1.754806 | -2.111540 | 32 | 6.84 | 0.213750 |
| GO:0016043\_cellular\_component\_organization | EPHB2 | 964 | 18 | 1.754806 | -2.111540 | 32 | 6.84 | 0.213750 |
| GO:0016043\_cellular\_component\_organization | EPHA4 | 964 | 18 | 1.754806 | -2.111540 | 32 | 6.84 | 0.213750 |
| GO:0016043\_cellular\_component\_organization | ANK3 | 964 | 18 | 1.754806 | -2.111540 | 32 | 6.84 | 0.213750 |
| GO:0016043\_cellular\_component\_organization | ITGA8 | 964 | 18 | 1.754806 | -2.111540 | 32 | 6.84 | 0.213750 |
| GO:0016043\_cellular\_component\_organization | KATNA1 | 964 | 18 | 1.754806 | -2.111540 | 32 | 6.84 | 0.213750 |
| GO:0016043\_cellular\_component\_organization | FOXG1 | 964 | 18 | 1.754806 | -2.111540 | 32 | 6.84 | 0.213750 |
| GO:0016043\_cellular\_component\_organization | TUBG1 | 964 | 18 | 1.754806 | -2.111540 | 32 | 6.84 | 0.213750 |
| GO:0016043\_cellular\_component\_organization | LRP2 | 964 | 18 | 1.754806 | -2.111540 | 32 | 6.84 | 0.213750 |
| GO:0016043\_cellular\_component\_organization | MAP3K12 | 964 | 18 | 1.754806 | -2.111540 | 32 | 6.84 | 0.213750 |
| GO:0016043\_cellular\_component\_organization | OLFM1 | 964 | 18 | 1.754806 | -2.111540 | 32 | 6.84 | 0.213750 |
| GO:0007423\_sensory\_organ\_development | HES5 | 219 | 7 | 3.003914 | -2.108026 | 33 | 6.85 | 0.207576 |
| GO:0007423\_sensory\_organ\_development | ITGA8 | 219 | 7 | 3.003914 | -2.108026 | 33 | 6.85 | 0.207576 |
| GO:0007423\_sensory\_organ\_development | SP3 | 219 | 7 | 3.003914 | -2.108026 | 33 | 6.85 | 0.207576 |
| GO:0007423\_sensory\_organ\_development | FOXG1 | 219 | 7 | 3.003914 | -2.108026 | 33 | 6.85 | 0.207576 |
| GO:0007423\_sensory\_organ\_development | JAG1 | 219 | 7 | 3.003914 | -2.108026 | 33 | 6.85 | 0.207576 |
| GO:0007423\_sensory\_organ\_development | GPR98 | 219 | 7 | 3.003914 | -2.108026 | 33 | 6.85 | 0.207576 |
| GO:0007423\_sensory\_organ\_development | EPHB2 | 219 | 7 | 3.003914 | -2.108026 | 33 | 6.85 | 0.207576 |
| GO:0021953\_central\_nervous\_system\_neuron\_differentiation | FOXG1 | 39 | 3 | 7.229199 | -2.101911 | 34 | 6.98 | 0.205294 |
| GO:0021953\_central\_nervous\_system\_neuron\_differentiation | POU4F1 | 39 | 3 | 7.229199 | -2.101911 | 34 | 6.98 | 0.205294 |
| GO:0021953\_central\_nervous\_system\_neuron\_differentiation | EPHB2 | 39 | 3 | 7.229199 | -2.101911 | 34 | 6.98 | 0.205294 |
| GO:0050771\_negative\_regulation\_of\_axonogenesis | NTN1 | 13 | 2 | 14.458399 | -2.095349 | 35 | 7.56 | 0.216000 |
| GO:0050771\_negative\_regulation\_of\_axonogenesis | EPHB2 | 13 | 2 | 14.458399 | -2.095349 | 35 | 7.56 | 0.216000 |
| GO:0048812\_neuron\_projection\_morphogenesis | EPHA4 | 170 | 6 | 3.316927 | -2.061517 | 36 | 7.74 | 0.215000 |
| GO:0048812\_neuron\_projection\_morphogenesis | ANK3 | 170 | 6 | 3.316927 | -2.061517 | 36 | 7.74 | 0.215000 |
| GO:0048812\_neuron\_projection\_morphogenesis | FOXG1 | 170 | 6 | 3.316927 | -2.061517 | 36 | 7.74 | 0.215000 |
| GO:0048812\_neuron\_projection\_morphogenesis | KIF5C | 170 | 6 | 3.316927 | -2.061517 | 36 | 7.74 | 0.215000 |
| GO:0048812\_neuron\_projection\_morphogenesis | NTN1 | 170 | 6 | 3.316927 | -2.061517 | 36 | 7.74 | 0.215000 |
| GO:0048812\_neuron\_projection\_morphogenesis | EPHB2 | 170 | 6 | 3.316927 | -2.061517 | 36 | 7.74 | 0.215000 |
| GO:0006836\_neurotransmitter\_transport | CPLX3 | 41 | 3 | 6.876556 | -2.041504 | 37 | 8.0 | 0.216216 |
| GO:0006836\_neurotransmitter\_transport | SV2B | 41 | 3 | 6.876556 | -2.041504 | 37 | 8.0 | 0.216216 |
| GO:0006836\_neurotransmitter\_transport | LIN7A | 41 | 3 | 6.876556 | -2.041504 | 37 | 8.0 | 0.216216 |
| GO:0030154\_cell\_differentiation | STMN3 | 1060 | 19 | 1.684540 | -2.028725 | 38 | 8.66 | 0.227895 |
| GO:0030154\_cell\_differentiation | KIF5C | 1060 | 19 | 1.684540 | -2.028725 | 38 | 8.66 | 0.227895 |
| GO:0030154\_cell\_differentiation | JAG1 | 1060 | 19 | 1.684540 | -2.028725 | 38 | 8.66 | 0.227895 |
| GO:0030154\_cell\_differentiation | NTN1 | 1060 | 19 | 1.684540 | -2.028725 | 38 | 8.66 | 0.227895 |
| GO:0030154\_cell\_differentiation | GPR98 | 1060 | 19 | 1.684540 | -2.028725 | 38 | 8.66 | 0.227895 |
| GO:0030154\_cell\_differentiation | EPHB2 | 1060 | 19 | 1.684540 | -2.028725 | 38 | 8.66 | 0.227895 |
| GO:0030154\_cell\_differentiation | HBA-A1 | 1060 | 19 | 1.684540 | -2.028725 | 38 | 8.66 | 0.227895 |
| GO:0030154\_cell\_differentiation | MAPK1 | 1060 | 19 | 1.684540 | -2.028725 | 38 | 8.66 | 0.227895 |
| GO:0030154\_cell\_differentiation | EPHA4 | 1060 | 19 | 1.684540 | -2.028725 | 38 | 8.66 | 0.227895 |
| GO:0030154\_cell\_differentiation | HEY1 | 1060 | 19 | 1.684540 | -2.028725 | 38 | 8.66 | 0.227895 |
| GO:0030154\_cell\_differentiation | HES5 | 1060 | 19 | 1.684540 | -2.028725 | 38 | 8.66 | 0.227895 |
| GO:0030154\_cell\_differentiation | XBP1 | 1060 | 19 | 1.684540 | -2.028725 | 38 | 8.66 | 0.227895 |
| GO:0030154\_cell\_differentiation | ANK3 | 1060 | 19 | 1.684540 | -2.028725 | 38 | 8.66 | 0.227895 |
| GO:0030154\_cell\_differentiation | KATNA1 | 1060 | 19 | 1.684540 | -2.028725 | 38 | 8.66 | 0.227895 |
| GO:0030154\_cell\_differentiation | SP3 | 1060 | 19 | 1.684540 | -2.028725 | 38 | 8.66 | 0.227895 |
| GO:0030154\_cell\_differentiation | NAB1 | 1060 | 19 | 1.684540 | -2.028725 | 38 | 8.66 | 0.227895 |
| GO:0030154\_cell\_differentiation | FOXG1 | 1060 | 19 | 1.684540 | -2.028725 | 38 | 8.66 | 0.227895 |
| GO:0030154\_cell\_differentiation | POU4F1 | 1060 | 19 | 1.684540 | -2.028725 | 38 | 8.66 | 0.227895 |
| GO:0030154\_cell\_differentiation | CHUK | 1060 | 19 | 1.684540 | -2.028725 | 38 | 8.66 | 0.227895 |
| GO:0048667\_cell\_morphogenesis\_involved\_in\_neuron\_differentiation | EPHA4 | 173 | 6 | 3.259408 | -2.025718 | 39 | 8.67 | 0.222308 |
| GO:0048667\_cell\_morphogenesis\_involved\_in\_neuron\_differentiation | ANK3 | 173 | 6 | 3.259408 | -2.025718 | 39 | 8.67 | 0.222308 |
| GO:0048667\_cell\_morphogenesis\_involved\_in\_neuron\_differentiation | FOXG1 | 173 | 6 | 3.259408 | -2.025718 | 39 | 8.67 | 0.222308 |
| GO:0048667\_cell\_morphogenesis\_involved\_in\_neuron\_differentiation | KIF5C | 173 | 6 | 3.259408 | -2.025718 | 39 | 8.67 | 0.222308 |
| GO:0048667\_cell\_morphogenesis\_involved\_in\_neuron\_differentiation | NTN1 | 173 | 6 | 3.259408 | -2.025718 | 39 | 8.67 | 0.222308 |
| GO:0048667\_cell\_morphogenesis\_involved\_in\_neuron\_differentiation | EPHB2 | 173 | 6 | 3.259408 | -2.025718 | 39 | 8.67 | 0.222308 |
| GO:0048858\_cell\_projection\_morphogenesis | EPHA4 | 176 | 6 | 3.203850 | -1.990725 | 40 | 8.99 | 0.224750 |
| GO:0048858\_cell\_projection\_morphogenesis | ANK3 | 176 | 6 | 3.203850 | -1.990725 | 40 | 8.99 | 0.224750 |
| GO:0048858\_cell\_projection\_morphogenesis | FOXG1 | 176 | 6 | 3.203850 | -1.990725 | 40 | 8.99 | 0.224750 |
| GO:0048858\_cell\_projection\_morphogenesis | KIF5C | 176 | 6 | 3.203850 | -1.990725 | 40 | 8.99 | 0.224750 |
| GO:0048858\_cell\_projection\_morphogenesis | NTN1 | 176 | 6 | 3.203850 | -1.990725 | 40 | 8.99 | 0.224750 |
| GO:0048858\_cell\_projection\_morphogenesis | EPHB2 | 176 | 6 | 3.203850 | -1.990725 | 40 | 8.99 | 0.224750 |
| GO:0007052\_mitotic\_spindle\_organization | TUBG1 | 1 | 1 |  |  |  |  |  |  |
| GO:0019858\_cytosine\_metabolic\_process | MAPK1 | 1 | 1 |  |  |  |  |  |  |
| GO:0032314\_regulation\_of\_Rac\_GTPase\_activity | STMN3 | 1 | 1 |  |  |  |  |  |  |
| GO:0048170\_positive\_regulation\_of\_long-term\_neuronal\_synaptic\_plasticity | EPHB2 | 1 | 1 |  |  |  |  |  |  |
| GO:0051355\_proprioception\_during\_equilibrioception | POU4F1 | 1 | 1 |  |  |  |  |  |  |
| GO:0060071\_Wnt\_receptor\_signaling\_pathway\_\_planar\_cell\_polarity\_pathway | SFRP1 | 1 | 1 |  |  |  |  |  |  |
| GO:0060683\_regulation\_of\_branching\_involved\_in\_salivary\_gland\_morphogenesis\_by\_epithelial-mesenchymal\_signaling | PDGFA | 1 | 1 |  |  |  |  |  |  |
| GO:0060691\_epithelial\_cell\_maturation\_involved\_in\_salivary\_gland\_development | XBP1 | 1 | 1 |  |  |  |  |  |  |
| GO:0045664\_regulation\_of\_neuron\_differentiation | HES5 | 82 | 4 | 4.584370 | -1.967613 | 41 | 10.05 | 0.245122 |
| GO:0045664\_regulation\_of\_neuron\_differentiation | FOXG1 | 82 | 4 | 4.584370 | -1.967613 | 41 | 10.05 | 0.245122 |
| GO:0045664\_regulation\_of\_neuron\_differentiation | NTN1 | 82 | 4 | 4.584370 | -1.967613 | 41 | 10.05 | 0.245122 |
| GO:0045664\_regulation\_of\_neuron\_differentiation | EPHB2 | 82 | 4 | 4.584370 | -1.967613 | 41 | 10.05 | 0.245122 |
| GO:0031345\_negative\_regulation\_of\_cell\_projection\_organization | NTN1 | 16 | 2 | 11.747449 | -1.917086 | 42 | 11.3 | 0.269048 |
| GO:0031345\_negative\_regulation\_of\_cell\_projection\_organization | EPHB2 | 16 | 2 | 11.747449 | -1.917086 | 42 | 11.3 | 0.269048 |
| GO:0016192\_vesicle-mediated\_transport | SCAMP1 | 184 | 6 | 3.064552 | -1.901142 | 44 | 11.52 | 0.261818 |
| GO:0016192\_vesicle-mediated\_transport | CPLX3 | 184 | 6 | 3.064552 | -1.901142 | 44 | 11.52 | 0.261818 |
| GO:0016192\_vesicle-mediated\_transport | SYTL2 | 184 | 6 | 3.064552 | -1.901142 | 44 | 11.52 | 0.261818 |
| GO:0016192\_vesicle-mediated\_transport | SV2B | 184 | 6 | 3.064552 | -1.901142 | 44 | 11.52 | 0.261818 |
| GO:0016192\_vesicle-mediated\_transport | LRP2 | 184 | 6 | 3.064552 | -1.901142 | 44 | 11.52 | 0.261818 |
| GO:0016192\_vesicle-mediated\_transport | LIN7A | 184 | 6 | 3.064552 | -1.901142 | 44 | 11.52 | 0.261818 |
| GO:0032990\_cell\_part\_morphogenesis | EPHA4 | 184 | 6 | 3.064552 | -1.901142 | 44 | 11.52 | 0.261818 |
| GO:0032990\_cell\_part\_morphogenesis | ANK3 | 184 | 6 | 3.064552 | -1.901142 | 44 | 11.52 | 0.261818 |
| GO:0032990\_cell\_part\_morphogenesis | FOXG1 | 184 | 6 | 3.064552 | -1.901142 | 44 | 11.52 | 0.261818 |
| GO:0032990\_cell\_part\_morphogenesis | KIF5C | 184 | 6 | 3.064552 | -1.901142 | 44 | 11.52 | 0.261818 |
| GO:0032990\_cell\_part\_morphogenesis | NTN1 | 184 | 6 | 3.064552 | -1.901142 | 44 | 11.52 | 0.261818 |
| GO:0032990\_cell\_part\_morphogenesis | EPHB2 | 184 | 6 | 3.064552 | -1.901142 | 44 | 11.52 | 0.261818 |
| GO:0001505\_regulation\_of\_neurotransmitter\_levels | CPLX3 | 48 | 3 | 5.873724 | -1.853858 | 45 | 12.77 | 0.283778 |
| GO:0001505\_regulation\_of\_neurotransmitter\_levels | SV2B | 48 | 3 | 5.873724 | -1.853858 | 45 | 12.77 | 0.283778 |
| GO:0001505\_regulation\_of\_neurotransmitter\_levels | LIN7A | 48 | 3 | 5.873724 | -1.853858 | 45 | 12.77 | 0.283778 |
| GO:0048869\_cellular\_developmental\_process | STMN3 | 1113 | 19 | 1.604324 | -1.799941 | 46 | 14.07 | 0.305870 |
| GO:0048869\_cellular\_developmental\_process | KIF5C | 1113 | 19 | 1.604324 | -1.799941 | 46 | 14.07 | 0.305870 |
| GO:0048869\_cellular\_developmental\_process | JAG1 | 1113 | 19 | 1.604324 | -1.799941 | 46 | 14.07 | 0.305870 |
| GO:0048869\_cellular\_developmental\_process | NTN1 | 1113 | 19 | 1.604324 | -1.799941 | 46 | 14.07 | 0.305870 |
| GO:0048869\_cellular\_developmental\_process | GPR98 | 1113 | 19 | 1.604324 | -1.799941 | 46 | 14.07 | 0.305870 |
| GO:0048869\_cellular\_developmental\_process | EPHB2 | 1113 | 19 | 1.604324 | -1.799941 | 46 | 14.07 | 0.305870 |
| GO:0048869\_cellular\_developmental\_process | HBA-A1 | 1113 | 19 | 1.604324 | -1.799941 | 46 | 14.07 | 0.305870 |
| GO:0048869\_cellular\_developmental\_process | MAPK1 | 1113 | 19 | 1.604324 | -1.799941 | 46 | 14.07 | 0.305870 |
| GO:0048869\_cellular\_developmental\_process | EPHA4 | 1113 | 19 | 1.604324 | -1.799941 | 46 | 14.07 | 0.305870 |
| GO:0048869\_cellular\_developmental\_process | HES5 | 1113 | 19 | 1.604324 | -1.799941 | 46 | 14.07 | 0.305870 |
| GO:0048869\_cellular\_developmental\_process | HEY1 | 1113 | 19 | 1.604324 | -1.799941 | 46 | 14.07 | 0.305870 |
| GO:0048869\_cellular\_developmental\_process | XBP1 | 1113 | 19 | 1.604324 | -1.799941 | 46 | 14.07 | 0.305870 |
| GO:0048869\_cellular\_developmental\_process | ANK3 | 1113 | 19 | 1.604324 | -1.799941 | 46 | 14.07 | 0.305870 |
| GO:0048869\_cellular\_developmental\_process | KATNA1 | 1113 | 19 | 1.604324 | -1.799941 | 46 | 14.07 | 0.305870 |
| GO:0048869\_cellular\_developmental\_process | SP3 | 1113 | 19 | 1.604324 | -1.799941 | 46 | 14.07 | 0.305870 |
| GO:0048869\_cellular\_developmental\_process | NAB1 | 1113 | 19 | 1.604324 | -1.799941 | 46 | 14.07 | 0.305870 |
| GO:0048869\_cellular\_developmental\_process | FOXG1 | 1113 | 19 | 1.604324 | -1.799941 | 46 | 14.07 | 0.305870 |
| GO:0048869\_cellular\_developmental\_process | POU4F1 | 1113 | 19 | 1.604324 | -1.799941 | 46 | 14.07 | 0.305870 |
| GO:0048869\_cellular\_developmental\_process | CHUK | 1113 | 19 | 1.604324 | -1.799941 | 46 | 14.07 | 0.305870 |
| GO:0042491\_auditory\_receptor\_cell\_differentiation | HES5 | 19 | 2 | 9.892589 | -1.772076 | 47 | 15.0 | 0.319149 |
| GO:0042491\_auditory\_receptor\_cell\_differentiation | JAG1 | 19 | 2 | 9.892589 | -1.772076 | 47 | 15.0 | 0.319149 |
| GO:0000904\_cell\_morphogenesis\_involved\_in\_differentiation | EPHA4 | 199 | 6 | 2.833556 | -1.746469 | 48 | 15.44 | 0.321667 |
| GO:0000904\_cell\_morphogenesis\_involved\_in\_differentiation | ANK3 | 199 | 6 | 2.833556 | -1.746469 | 48 | 15.44 | 0.321667 |
| GO:0000904\_cell\_morphogenesis\_involved\_in\_differentiation | FOXG1 | 199 | 6 | 2.833556 | -1.746469 | 48 | 15.44 | 0.321667 |
| GO:0000904\_cell\_morphogenesis\_involved\_in\_differentiation | KIF5C | 199 | 6 | 2.833556 | -1.746469 | 48 | 15.44 | 0.321667 |
| GO:0000904\_cell\_morphogenesis\_involved\_in\_differentiation | NTN1 | 199 | 6 | 2.833556 | -1.746469 | 48 | 15.44 | 0.321667 |
| GO:0000904\_cell\_morphogenesis\_involved\_in\_differentiation | EPHB2 | 199 | 6 | 2.833556 | -1.746469 | 48 | 15.44 | 0.321667 |
| GO:0030900\_forebrain\_development | DLC1 | 146 | 5 | 3.218479 | -1.730831 | 49 | 15.62 | 0.318776 |
| GO:0030900\_forebrain\_development | DKK1 | 146 | 5 | 3.218479 | -1.730831 | 49 | 15.62 | 0.318776 |
| GO:0030900\_forebrain\_development | FOXG1 | 146 | 5 | 3.218479 | -1.730831 | 49 | 15.62 | 0.318776 |
| GO:0030900\_forebrain\_development | LRP2 | 146 | 5 | 3.218479 | -1.730831 | 49 | 15.62 | 0.318776 |
| GO:0030900\_forebrain\_development | APLP1 | 146 | 5 | 3.218479 | -1.730831 | 49 | 15.62 | 0.318776 |
| GO:0032940\_secretion\_by\_cell | SCAMP1 | 149 | 5 | 3.153678 | -1.696353 | 50 | 16.66 | 0.333200 |
| GO:0032940\_secretion\_by\_cell | CPLX3 | 149 | 5 | 3.153678 | -1.696353 | 50 | 16.66 | 0.333200 |
| GO:0032940\_secretion\_by\_cell | SYTL2 | 149 | 5 | 3.153678 | -1.696353 | 50 | 16.66 | 0.333200 |
| GO:0032940\_secretion\_by\_cell | SV2B | 149 | 5 | 3.153678 | -1.696353 | 50 | 16.66 | 0.333200 |
| GO:0032940\_secretion\_by\_cell | LIN7A | 149 | 5 | 3.153678 | -1.696353 | 50 | 16.66 | 0.333200 |
| GO:0006206\_pyrimidine\_base\_metabolic\_process | MAPK1 | 2 | 1 |  |  |  |  |  |  |
| GO:0007020\_microtubule\_nucleation | TUBG1 | 2 | 1 |  |  |  |  |  |  |
| GO:0016199\_axon\_midline\_choice\_point\_recognition | FOXG1 | 2 | 1 |  |  |  |  |  |  |
| GO:0016572\_histone\_phosphorylation | MAP3K12 | 2 | 1 |  |  |  |  |  |  |
| GO:0030219\_megakaryocyte\_differentiation | SP3 | 2 | 1 |  |  |  |  |  |  |
| GO:0035021\_negative\_regulation\_of\_Rac\_protein\_signal\_transduction | STMN3 | 2 | 1 |  |  |  |  |  |  |
| GO:0060346\_bone\_trabecula\_formation | MMP2 | 2 | 1 |  |  |  |  |  |  |
| GO:0060690\_epithelial\_cell\_differentiation\_involved\_in\_salivary\_gland\_development | XBP1 | 2 | 1 |  |  |  |  |  |  |
| GO:0070257\_positive\_regulation\_of\_mucus\_secretion | SYTL2 | 2 | 1 |  |  |  |  |  |  |
| GO:0006928\_cell\_motion | EPHA4 | 330 | 8 | 2.278293 | -1.664418 | 52 | 18.04 | 0.346923 |
| GO:0006928\_cell\_motion | ANK3 | 330 | 8 | 2.278293 | -1.664418 | 52 | 18.04 | 0.346923 |
| GO:0006928\_cell\_motion | KATNA1 | 330 | 8 | 2.278293 | -1.664418 | 52 | 18.04 | 0.346923 |
| GO:0006928\_cell\_motion | FOXG1 | 330 | 8 | 2.278293 | -1.664418 | 52 | 18.04 | 0.346923 |
| GO:0006928\_cell\_motion | KIF5C | 330 | 8 | 2.278293 | -1.664418 | 52 | 18.04 | 0.346923 |
| GO:0006928\_cell\_motion | POU4F1 | 330 | 8 | 2.278293 | -1.664418 | 52 | 18.04 | 0.346923 |
| GO:0006928\_cell\_motion | NTN1 | 330 | 8 | 2.278293 | -1.664418 | 52 | 18.04 | 0.346923 |
| GO:0006928\_cell\_motion | EPHB2 | 330 | 8 | 2.278293 | -1.664418 | 52 | 18.04 | 0.346923 |
| GO:0051674\_localization\_of\_cell | EPHA4 | 330 | 8 | 2.278293 | -1.664418 | 52 | 18.04 | 0.346923 |
| GO:0051674\_localization\_of\_cell | ANK3 | 330 | 8 | 2.278293 | -1.664418 | 52 | 18.04 | 0.346923 |
| GO:0051674\_localization\_of\_cell | KATNA1 | 330 | 8 | 2.278293 | -1.664418 | 52 | 18.04 | 0.346923 |
| GO:0051674\_localization\_of\_cell | FOXG1 | 330 | 8 | 2.278293 | -1.664418 | 52 | 18.04 | 0.346923 |
| GO:0051674\_localization\_of\_cell | KIF5C | 330 | 8 | 2.278293 | -1.664418 | 52 | 18.04 | 0.346923 |
| GO:0051674\_localization\_of\_cell | POU4F1 | 330 | 8 | 2.278293 | -1.664418 | 52 | 18.04 | 0.346923 |
| GO:0051674\_localization\_of\_cell | NTN1 | 330 | 8 | 2.278293 | -1.664418 | 52 | 18.04 | 0.346923 |
| GO:0051674\_localization\_of\_cell | EPHB2 | 330 | 8 | 2.278293 | -1.664418 | 52 | 18.04 | 0.346923 |
| GO:0000226\_microtubule\_cytoskeleton\_organization | STMN3 | 57 | 3 | 4.946294 | -1.654421 | 53 | 18.25 | 0.344340 |
| GO:0000226\_microtubule\_cytoskeleton\_organization | KATNA1 | 57 | 3 | 4.946294 | -1.654421 | 53 | 18.25 | 0.344340 |
| GO:0000226\_microtubule\_cytoskeleton\_organization | TUBG1 | 57 | 3 | 4.946294 | -1.654421 | 53 | 18.25 | 0.344340 |
| GO:0050767\_regulation\_of\_neurogenesis | HES5 | 104 | 4 | 3.614600 | -1.621656 | 54 | 19.66 | 0.364074 |
| GO:0050767\_regulation\_of\_neurogenesis | FOXG1 | 104 | 4 | 3.614600 | -1.621656 | 54 | 19.66 | 0.364074 |
| GO:0050767\_regulation\_of\_neurogenesis | NTN1 | 104 | 4 | 3.614600 | -1.621656 | 54 | 19.66 | 0.364074 |
| GO:0050767\_regulation\_of\_neurogenesis | EPHB2 | 104 | 4 | 3.614600 | -1.621656 | 54 | 19.66 | 0.364074 |
| GO:0030099\_myeloid\_cell\_differentiation | HBA-A1 | 108 | 4 | 3.480726 | -1.568527 | 55 | 21.6 | 0.392727 |
| GO:0030099\_myeloid\_cell\_differentiation | SP3 | 108 | 4 | 3.480726 | -1.568527 | 55 | 21.6 | 0.392727 |
| GO:0030099\_myeloid\_cell\_differentiation | JAG1 | 108 | 4 | 3.480726 | -1.568527 | 55 | 21.6 | 0.392727 |
| GO:0030099\_myeloid\_cell\_differentiation | CHUK | 108 | 4 | 3.480726 | -1.568527 | 55 | 21.6 | 0.392727 |
| GO:0060688\_regulation\_of\_morphogenesis\_of\_a\_branching\_structure | SFRP1 | 25 | 2 | 7.518367 | -1.545502 | 56 | 22.81 | 0.407321 |
| GO:0060688\_regulation\_of\_morphogenesis\_of\_a\_branching\_structure | PDGFA | 25 | 2 | 7.518367 | -1.545502 | 56 | 22.81 | 0.407321 |
| GO:0007417\_central\_nervous\_system\_development | DLC1 | 287 | 7 | 2.292185 | -1.514561 | 57 | 23.37 | 0.410000 |
| GO:0007417\_central\_nervous\_system\_development | DKK1 | 287 | 7 | 2.292185 | -1.514561 | 57 | 23.37 | 0.410000 |
| GO:0007417\_central\_nervous\_system\_development | FOXG1 | 287 | 7 | 2.292185 | -1.514561 | 57 | 23.37 | 0.410000 |
| GO:0007417\_central\_nervous\_system\_development | POU4F1 | 287 | 7 | 2.292185 | -1.514561 | 57 | 23.37 | 0.410000 |
| GO:0007417\_central\_nervous\_system\_development | LRP2 | 287 | 7 | 2.292185 | -1.514561 | 57 | 23.37 | 0.410000 |
| GO:0007417\_central\_nervous\_system\_development | EPHB2 | 287 | 7 | 2.292185 | -1.514561 | 57 | 23.37 | 0.410000 |
| GO:0007417\_central\_nervous\_system\_development | APLP1 | 287 | 7 | 2.292185 | -1.514561 | 57 | 23.37 | 0.410000 |
| GO:0045665\_negative\_regulation\_of\_neuron\_differentiation | HES5 | 26 | 2 | 7.229199 | -1.513657 | 58 | 24.02 | 0.414138 |
| GO:0045665\_negative\_regulation\_of\_neuron\_differentiation | FOXG1 | 26 | 2 | 7.229199 | -1.513657 | 58 | 24.02 | 0.414138 |
| GO:0000212\_meiotic\_spindle\_organization | TUBG1 | 3 | 1 |  |  |  |  |  |  |
| GO:0001955\_blood\_vessel\_maturation | MMP2 | 3 | 1 |  |  |  |  |  |  |
| GO:0007252\_I-kappaB\_phosphorylation | CHUK | 3 | 1 |  |  |  |  |  |  |
| GO:0019230\_proprioception | POU4F1 | 3 | 1 |  |  |  |  |  |  |
| GO:0030224\_monocyte\_differentiation | SP3 | 3 | 1 |  |  |  |  |  |  |
| GO:0030574\_collagen\_catabolic\_process | MMP2 | 3 | 1 |  |  |  |  |  |  |
| GO:0042668\_auditory\_receptor\_cell\_fate\_determination | HES5 | 3 | 1 |  |  |  |  |  |  |
| GO:0050957\_equilibrioception | POU4F1 | 3 | 1 |  |  |  |  |  |  |
| GO:0060684\_epithelial-mesenchymal\_cell\_signaling | PDGFA | 3 | 1 |  |  |  |  |  |  |
| GO:0060689\_cell\_differentiation\_involved\_in\_salivary\_gland\_development | XBP1 | 3 | 1 |  |  |  |  |  |  |
| GO:0007422\_peripheral\_nervous\_system\_development | NAB1 | 27 | 2 | 6.961451 | -1.483149 | 59 | 25.16 | 0.426441 |
| GO:0007422\_peripheral\_nervous\_system\_development | POU4F1 | 27 | 2 | 6.961451 | -1.483149 | 59 | 25.16 | 0.426441 |
| GO:0007420\_brain\_development | DLC1 | 231 | 6 | 2.441028 | -1.464608 | 60 | 25.58 | 0.426333 |
| GO:0007420\_brain\_development | DKK1 | 231 | 6 | 2.441028 | -1.464608 | 60 | 25.58 | 0.426333 |
| GO:0007420\_brain\_development | FOXG1 | 231 | 6 | 2.441028 | -1.464608 | 60 | 25.58 | 0.426333 |
| GO:0007420\_brain\_development | POU4F1 | 231 | 6 | 2.441028 | -1.464608 | 60 | 25.58 | 0.426333 |
| GO:0007420\_brain\_development | LRP2 | 231 | 6 | 2.441028 | -1.464608 | 60 | 25.58 | 0.426333 |
| GO:0007420\_brain\_development | APLP1 | 231 | 6 | 2.441028 | -1.464608 | 60 | 25.58 | 0.426333 |
| GO:0051960\_regulation\_of\_nervous\_system\_development | HES5 | 118 | 4 | 3.185749 | -1.446001 | 61 | 26.7 | 0.437705 |
| GO:0051960\_regulation\_of\_nervous\_system\_development | FOXG1 | 118 | 4 | 3.185749 | -1.446001 | 61 | 26.7 | 0.437705 |
| GO:0051960\_regulation\_of\_nervous\_system\_development | NTN1 | 118 | 4 | 3.185749 | -1.446001 | 61 | 26.7 | 0.437705 |
| GO:0051960\_regulation\_of\_nervous\_system\_development | EPHB2 | 118 | 4 | 3.185749 | -1.446001 | 61 | 26.7 | 0.437705 |
| GO:0046903\_secretion | SCAMP1 | 175 | 5 | 2.685131 | -1.431773 | 62 | 27.15 | 0.437903 |
| GO:0046903\_secretion | CPLX3 | 175 | 5 | 2.685131 | -1.431773 | 62 | 27.15 | 0.437903 |
| GO:0046903\_secretion | SV2B | 175 | 5 | 2.685131 | -1.431773 | 62 | 27.15 | 0.437903 |
| GO:0046903\_secretion | SYTL2 | 175 | 5 | 2.685131 | -1.431773 | 62 | 27.15 | 0.437903 |
| GO:0046903\_secretion | LIN7A | 175 | 5 | 2.685131 | -1.431773 | 62 | 27.15 | 0.437903 |
| GO:0006468\_protein\_amino\_acid\_phosphorylation | MAPK1 | 237 | 6 | 2.379230 | -1.417903 | 63 | 28.68 | 0.455238 |
| GO:0006468\_protein\_amino\_acid\_phosphorylation | HIPK1 | 237 | 6 | 2.379230 | -1.417903 | 63 | 28.68 | 0.455238 |
| GO:0006468\_protein\_amino\_acid\_phosphorylation | PDGFA | 237 | 6 | 2.379230 | -1.417903 | 63 | 28.68 | 0.455238 |
| GO:0006468\_protein\_amino\_acid\_phosphorylation | MAP3K12 | 237 | 6 | 2.379230 | -1.417903 | 63 | 28.68 | 0.455238 |
| GO:0006468\_protein\_amino\_acid\_phosphorylation | CHUK | 237 | 6 | 2.379230 | -1.417903 | 63 | 28.68 | 0.455238 |
| GO:0006468\_protein\_amino\_acid\_phosphorylation | EPHB2 | 237 | 6 | 2.379230 | -1.417903 | 63 | 28.68 | 0.455238 |
| GO:0060284\_regulation\_of\_cell\_development | HES5 | 122 | 4 | 3.081298 | -1.400685 | 64 | 29.23 | 0.456719 |
| GO:0060284\_regulation\_of\_cell\_development | FOXG1 | 122 | 4 | 3.081298 | -1.400685 | 64 | 29.23 | 0.456719 |
| GO:0060284\_regulation\_of\_cell\_development | NTN1 | 122 | 4 | 3.081298 | -1.400685 | 64 | 29.23 | 0.456719 |
| GO:0060284\_regulation\_of\_cell\_development | EPHB2 | 122 | 4 | 3.081298 | -1.400685 | 64 | 29.23 | 0.456719 |
| GO:0048732\_gland\_development | SFRP1 | 179 | 5 | 2.625128 | -1.395810 | 65 | 30.25 | 0.465385 |
| GO:0048732\_gland\_development | XBP1 | 179 | 5 | 2.625128 | -1.395810 | 65 | 30.25 | 0.465385 |
| GO:0048732\_gland\_development | PDGFA | 179 | 5 | 2.625128 | -1.395810 | 65 | 30.25 | 0.465385 |
| GO:0048732\_gland\_development | NTN1 | 179 | 5 | 2.625128 | -1.395810 | 65 | 30.25 | 0.465385 |
| GO:0048732\_gland\_development | CHUK | 179 | 5 | 2.625128 | -1.395810 | 65 | 30.25 | 0.465385 |
| GO:0009912\_auditory\_receptor\_cell\_fate\_commitment | HES5 | 4 | 1 |  |  |  |  |  |  |
| GO:0016198\_axon\_choice\_point\_recognition | FOXG1 | 4 | 1 |  |  |  |  |  |  |
| GO:0021535\_cell\_migration\_in\_hindbrain | POU4F1 | 4 | 1 |  |  |  |  |  |  |
| GO:0021631\_optic\_nerve\_morphogenesis | EPHB2 | 4 | 1 |  |  |  |  |  |  |
| GO:0035020\_regulation\_of\_Rac\_protein\_signal\_transduction | STMN3 | 4 | 1 |  |  |  |  |  |  |
| GO:0044243\_multicellular\_organismal\_catabolic\_process | MMP2 | 4 | 1 |  |  |  |  |  |  |
| GO:0045747\_positive\_regulation\_of\_Notch\_signaling\_pathway | JAG1 | 4 | 1 |  |  |  |  |  |  |
| GO:0060120\_inner\_ear\_receptor\_cell\_fate\_commitment | HES5 | 4 | 1 |  |  |  |  |  |  |
| GO:0070254\_mucus\_secretion | SYTL2 | 4 | 1 |  |  |  |  |  |  |
| GO:0070255\_regulation\_of\_mucus\_secretion | SYTL2 | 4 | 1 |  |  |  |  |  |  |
| GO:0021954\_central\_nervous\_system\_neuron\_development | FOXG1 | 31 | 2 | 6.063199 | -1.372643 | 66 | 32.3 | 0.489394 |
| GO:0021954\_central\_nervous\_system\_neuron\_development | EPHB2 | 31 | 2 | 6.063199 | -1.372643 | 66 | 32.3 | 0.489394 |
| GO:0051094\_positive\_regulation\_of\_developmental\_process | HIPK1 | 308 | 7 | 2.135900 | -1.371296 | 67 | 32.34 | 0.482687 |
| GO:0051094\_positive\_regulation\_of\_developmental\_process | TM2D1 | 308 | 7 | 2.135900 | -1.371296 | 67 | 32.34 | 0.482687 |
| GO:0051094\_positive\_regulation\_of\_developmental\_process | FOXG1 | 308 | 7 | 2.135900 | -1.371296 | 67 | 32.34 | 0.482687 |
| GO:0051094\_positive\_regulation\_of\_developmental\_process | POU4F1 | 308 | 7 | 2.135900 | -1.371296 | 67 | 32.34 | 0.482687 |
| GO:0051094\_positive\_regulation\_of\_developmental\_process | JAG1 | 308 | 7 | 2.135900 | -1.371296 | 67 | 32.34 | 0.482687 |
| GO:0051094\_positive\_regulation\_of\_developmental\_process | NTN1 | 308 | 7 | 2.135900 | -1.371296 | 67 | 32.34 | 0.482687 |
| GO:0051094\_positive\_regulation\_of\_developmental\_process | EPHB2 | 308 | 7 | 2.135900 | -1.371296 | 67 | 32.34 | 0.482687 |
| GO:0043062\_extracellular\_structure\_organization | ANK3 | 125 | 4 | 3.007347 | -1.367954 | 68 | 32.52 | 0.478235 |
| GO:0043062\_extracellular\_structure\_organization | ITGA8 | 125 | 4 | 3.007347 | -1.367954 | 68 | 32.52 | 0.478235 |
| GO:0043062\_extracellular\_structure\_organization | COL3A1 | 125 | 4 | 3.007347 | -1.367954 | 68 | 32.52 | 0.478235 |
| GO:0043062\_extracellular\_structure\_organization | APLP1 | 125 | 4 | 3.007347 | -1.367954 | 68 | 32.52 | 0.478235 |
| GO:0050768\_negative\_regulation\_of\_neurogenesis | NTN1 | 32 | 2 | 5.873724 | -1.347518 | 70 | 34.02 | 0.486000 |
| GO:0050768\_negative\_regulation\_of\_neurogenesis | EPHB2 | 32 | 2 | 5.873724 | -1.347518 | 70 | 34.02 | 0.486000 |
| GO:0050770\_regulation\_of\_axonogenesis | NTN1 | 32 | 2 | 5.873724 | -1.347518 | 70 | 34.02 | 0.486000 |
| GO:0050770\_regulation\_of\_axonogenesis | EPHB2 | 32 | 2 | 5.873724 | -1.347518 | 70 | 34.02 | 0.486000 |
| GO:0007010\_cytoskeleton\_organization | DLC1 | 185 | 5 | 2.539989 | -1.343914 | 71 | 34.12 | 0.480563 |
| GO:0007010\_cytoskeleton\_organization | STMN3 | 185 | 5 | 2.539989 | -1.343914 | 71 | 34.12 | 0.480563 |
| GO:0007010\_cytoskeleton\_organization | PDGFA | 185 | 5 | 2.539989 | -1.343914 | 71 | 34.12 | 0.480563 |
| GO:0007010\_cytoskeleton\_organization | KATNA1 | 185 | 5 | 2.539989 | -1.343914 | 71 | 34.12 | 0.480563 |
| GO:0007010\_cytoskeleton\_organization | TUBG1 | 185 | 5 | 2.539989 | -1.343914 | 71 | 34.12 | 0.480563 |
| GO:0045597\_positive\_regulation\_of\_cell\_differentiation | FOXG1 | 128 | 4 | 2.936862 | -1.336240 | 72 | 34.42 | 0.478056 |
| GO:0045597\_positive\_regulation\_of\_cell\_differentiation | JAG1 | 128 | 4 | 2.936862 | -1.336240 | 72 | 34.42 | 0.478056 |
| GO:0045597\_positive\_regulation\_of\_cell\_differentiation | NTN1 | 128 | 4 | 2.936862 | -1.336240 | 72 | 34.42 | 0.478056 |
| GO:0045597\_positive\_regulation\_of\_cell\_differentiation | EPHB2 | 128 | 4 | 2.936862 | -1.336240 | 72 | 34.42 | 0.478056 |
| GO:0007431\_salivary\_gland\_development | XBP1 | 33 | 2 | 5.695733 | -1.323268 | 73 | 35.58 | 0.487397 |
| GO:0007431\_salivary\_gland\_development | PDGFA | 33 | 2 | 5.695733 | -1.323268 | 73 | 35.58 | 0.487397 |
| GO:0019226\_transmission\_of\_nerve\_impulse | CPLX3 | 189 | 5 | 2.486233 | -1.310613 | 74 | 35.91 | 0.485270 |
| GO:0019226\_transmission\_of\_nerve\_impulse | NAB1 | 189 | 5 | 2.486233 | -1.310613 | 74 | 35.91 | 0.485270 |
| GO:0019226\_transmission\_of\_nerve\_impulse | SV2B | 189 | 5 | 2.486233 | -1.310613 | 74 | 35.91 | 0.485270 |
| GO:0019226\_transmission\_of\_nerve\_impulse | EPHB2 | 189 | 5 | 2.486233 | -1.310613 | 74 | 35.91 | 0.485270 |
| GO:0019226\_transmission\_of\_nerve\_impulse | LIN7A | 189 | 5 | 2.486233 | -1.310613 | 74 | 35.91 | 0.485270 |
| GO:0010721\_negative\_regulation\_of\_cell\_development | NTN1 | 34 | 2 | 5.528211 | -1.299839 | 75 | 37.37 | 0.498267 |
| GO:0010721\_negative\_regulation\_of\_cell\_development | EPHB2 | 34 | 2 | 5.528211 | -1.299839 | 75 | 37.37 | 0.498267 |
| GO:0050877\_neurological\_system\_process | CPLX3 | 390 | 8 | 1.927786 | -1.289732 | 76 | 37.62 | 0.495000 |
| GO:0050877\_neurological\_system\_process | ITGA8 | 390 | 8 | 1.927786 | -1.289732 | 76 | 37.62 | 0.495000 |
| GO:0050877\_neurological\_system\_process | NAB1 | 390 | 8 | 1.927786 | -1.289732 | 76 | 37.62 | 0.495000 |
| GO:0050877\_neurological\_system\_process | POU4F1 | 390 | 8 | 1.927786 | -1.289732 | 76 | 37.62 | 0.495000 |
| GO:0050877\_neurological\_system\_process | SV2B | 390 | 8 | 1.927786 | -1.289732 | 76 | 37.62 | 0.495000 |
| GO:0050877\_neurological\_system\_process | GPR98 | 390 | 8 | 1.927786 | -1.289732 | 76 | 37.62 | 0.495000 |
| GO:0050877\_neurological\_system\_process | EPHB2 | 390 | 8 | 1.927786 | -1.289732 | 76 | 37.62 | 0.495000 |
| GO:0050877\_neurological\_system\_process | LIN7A | 390 | 8 | 1.927786 | -1.289732 | 76 | 37.62 | 0.495000 |
| GO:0001957\_intramembranous\_ossification | MMP2 | 5 | 1 | 18.795918 | -1.283121 | 86 | 51.82 | 0.602558 |
| GO:0006270\_DNA\_replication\_initiation | CCNE2 | 5 | 1 | 18.795918 | -1.283121 | 86 | 51.82 | 0.602558 |
| GO:0006378\_mRNA\_polyadenylation | APLP1 | 5 | 1 | 18.795918 | -1.283121 | 86 | 51.82 | 0.602558 |
| GO:0021554\_optic\_nerve\_development | EPHB2 | 5 | 1 | 18.795918 | -1.283121 | 86 | 51.82 | 0.602558 |
| GO:0030851\_granulocyte\_differentiation | SP3 | 5 | 1 | 18.795918 | -1.283121 | 86 | 51.82 | 0.602558 |
| GO:0043631\_RNA\_polyadenylation | APLP1 | 5 | 1 | 18.795918 | -1.283121 | 86 | 51.82 | 0.602558 |
| GO:0045773\_positive\_regulation\_of\_axon\_extension | NTN1 | 5 | 1 | 18.795918 | -1.283121 | 86 | 51.82 | 0.602558 |
| GO:0048013\_ephrin\_receptor\_signaling\_pathway | EPHB2 | 5 | 1 | 18.795918 | -1.283121 | 86 | 51.82 | 0.602558 |
| GO:0048664\_neuron\_fate\_determination | HES5 | 5 | 1 | 18.795918 | -1.283121 | 86 | 51.82 | 0.602558 |
| GO:0048934\_peripheral\_nervous\_system\_neuron\_differentiation | POU4F1 | 5 | 1 | 18.795918 | -1.283121 | 86 | 51.82 | 0.602558 |
| GO:0021700\_developmental\_maturation | HES5 | 81 | 3 | 3.480726 | -1.266166 | 87 | 52.78 | 0.606667 |
| GO:0021700\_developmental\_maturation | XBP1 | 81 | 3 | 3.480726 | -1.266166 | 87 | 52.78 | 0.606667 |
| GO:0021700\_developmental\_maturation | MMP2 | 81 | 3 | 3.480726 | -1.266166 | 87 | 52.78 | 0.606667 |
| GO:0007154\_cell\_communication | CPLX3 | 1096 | 17 | 1.457713 | -1.255770 | 88 | 53.15 | 0.603977 |
| GO:0007154\_cell\_communication | STMN3 | 1096 | 17 | 1.457713 | -1.255770 | 88 | 53.15 | 0.603977 |
| GO:0007154\_cell\_communication | PDGFA | 1096 | 17 | 1.457713 | -1.255770 | 88 | 53.15 | 0.603977 |
| GO:0007154\_cell\_communication | THOP1 | 1096 | 17 | 1.457713 | -1.255770 | 88 | 53.15 | 0.603977 |
| GO:0007154\_cell\_communication | JAG1 | 1096 | 17 | 1.457713 | -1.255770 | 88 | 53.15 | 0.603977 |
| GO:0007154\_cell\_communication | MAPK10 | 1096 | 17 | 1.457713 | -1.255770 | 88 | 53.15 | 0.603977 |
| GO:0007154\_cell\_communication | LIN7A | 1096 | 17 | 1.457713 | -1.255770 | 88 | 53.15 | 0.603977 |
| GO:0007154\_cell\_communication | EPHB2 | 1096 | 17 | 1.457713 | -1.255770 | 88 | 53.15 | 0.603977 |
| GO:0007154\_cell\_communication | MAPK1 | 1096 | 17 | 1.457713 | -1.255770 | 88 | 53.15 | 0.603977 |
| GO:0007154\_cell\_communication | DKK1 | 1096 | 17 | 1.457713 | -1.255770 | 88 | 53.15 | 0.603977 |
| GO:0007154\_cell\_communication | SFRP1 | 1096 | 17 | 1.457713 | -1.255770 | 88 | 53.15 | 0.603977 |
| GO:0007154\_cell\_communication | HIPK1 | 1096 | 17 | 1.457713 | -1.255770 | 88 | 53.15 | 0.603977 |
| GO:0007154\_cell\_communication | HES5 | 1096 | 17 | 1.457713 | -1.255770 | 88 | 53.15 | 0.603977 |
| GO:0007154\_cell\_communication | ITGA8 | 1096 | 17 | 1.457713 | -1.255770 | 88 | 53.15 | 0.603977 |
| GO:0007154\_cell\_communication | SV2B | 1096 | 17 | 1.457713 | -1.255770 | 88 | 53.15 | 0.603977 |
| GO:0007154\_cell\_communication | GNB4 | 1096 | 17 | 1.457713 | -1.255770 | 88 | 53.15 | 0.603977 |
| GO:0007154\_cell\_communication | CHUK | 1096 | 17 | 1.457713 | -1.255770 | 88 | 53.15 | 0.603977 |
| GO:0007017\_microtubule-based\_process | STMN3 | 83 | 3 | 3.396853 | -1.240332 | 90 | 55.02 | 0.611333 |
| GO:0007017\_microtubule-based\_process | KATNA1 | 83 | 3 | 3.396853 | -1.240332 | 90 | 55.02 | 0.611333 |
| GO:0007017\_microtubule-based\_process | TUBG1 | 83 | 3 | 3.396853 | -1.240332 | 90 | 55.02 | 0.611333 |
| GO:0030198\_extracellular\_matrix\_organization | ITGA8 | 83 | 3 | 3.396853 | -1.240332 | 90 | 55.02 | 0.611333 |
| GO:0030198\_extracellular\_matrix\_organization | COL3A1 | 83 | 3 | 3.396853 | -1.240332 | 90 | 55.02 | 0.611333 |
| GO:0030198\_extracellular\_matrix\_organization | APLP1 | 83 | 3 | 3.396853 | -1.240332 | 90 | 55.02 | 0.611333 |
| GO:0002009\_morphogenesis\_of\_an\_epithelium | DLC1 | 198 | 5 | 2.373222 | -1.239223 | 92 | 55.06 | 0.598478 |
| GO:0002009\_morphogenesis\_of\_an\_epithelium | SFRP1 | 198 | 5 | 2.373222 | -1.239223 | 92 | 55.06 | 0.598478 |
| GO:0002009\_morphogenesis\_of\_an\_epithelium | XBP1 | 198 | 5 | 2.373222 | -1.239223 | 92 | 55.06 | 0.598478 |
| GO:0002009\_morphogenesis\_of\_an\_epithelium | JAG1 | 198 | 5 | 2.373222 | -1.239223 | 92 | 55.06 | 0.598478 |
| GO:0002009\_morphogenesis\_of\_an\_epithelium | CHUK | 198 | 5 | 2.373222 | -1.239223 | 92 | 55.06 | 0.598478 |
| GO:0060429\_epithelium\_development | DLC1 | 198 | 5 | 2.373222 | -1.239223 | 92 | 55.06 | 0.598478 |
| GO:0060429\_epithelium\_development | SFRP1 | 198 | 5 | 2.373222 | -1.239223 | 92 | 55.06 | 0.598478 |
| GO:0060429\_epithelium\_development | XBP1 | 198 | 5 | 2.373222 | -1.239223 | 92 | 55.06 | 0.598478 |
| GO:0060429\_epithelium\_development | JAG1 | 198 | 5 | 2.373222 | -1.239223 | 92 | 55.06 | 0.598478 |
| GO:0060429\_epithelium\_development | CHUK | 198 | 5 | 2.373222 | -1.239223 | 92 | 55.06 | 0.598478 |
| GO:0010646\_regulation\_of\_cell\_communication | CPLX3 | 330 | 7 | 1.993506 | -1.236601 | 93 | 55.2 | 0.593548 |
| GO:0010646\_regulation\_of\_cell\_communication | DKK1 | 330 | 7 | 1.993506 | -1.236601 | 93 | 55.2 | 0.593548 |
| GO:0010646\_regulation\_of\_cell\_communication | STMN3 | 330 | 7 | 1.993506 | -1.236601 | 93 | 55.2 | 0.593548 |
| GO:0010646\_regulation\_of\_cell\_communication | ITGA8 | 330 | 7 | 1.993506 | -1.236601 | 93 | 55.2 | 0.593548 |
| GO:0010646\_regulation\_of\_cell\_communication | JAG1 | 330 | 7 | 1.993506 | -1.236601 | 93 | 55.2 | 0.593548 |
| GO:0010646\_regulation\_of\_cell\_communication | CHUK | 330 | 7 | 1.993506 | -1.236601 | 93 | 55.2 | 0.593548 |
| GO:0010646\_regulation\_of\_cell\_communication | EPHB2 | 330 | 7 | 1.993506 | -1.236601 | 93 | 55.2 | 0.593548 |
| GO:0050793\_regulation\_of\_developmental\_process | MAPK1 | 703 | 12 | 1.604204 | -1.218814 | 94 | 56.47 | 0.600745 |
| GO:0050793\_regulation\_of\_developmental\_process | HES5 | 703 | 12 | 1.604204 | -1.218814 | 94 | 56.47 | 0.600745 |
| GO:0050793\_regulation\_of\_developmental\_process | SFRP1 | 703 | 12 | 1.604204 | -1.218814 | 94 | 56.47 | 0.600745 |
| GO:0050793\_regulation\_of\_developmental\_process | HIPK1 | 703 | 12 | 1.604204 | -1.218814 | 94 | 56.47 | 0.600745 |
| GO:0050793\_regulation\_of\_developmental\_process | TM2D1 | 703 | 12 | 1.604204 | -1.218814 | 94 | 56.47 | 0.600745 |
| GO:0050793\_regulation\_of\_developmental\_process | PDGFA | 703 | 12 | 1.604204 | -1.218814 | 94 | 56.47 | 0.600745 |
| GO:0050793\_regulation\_of\_developmental\_process | NAB1 | 703 | 12 | 1.604204 | -1.218814 | 94 | 56.47 | 0.600745 |
| GO:0050793\_regulation\_of\_developmental\_process | FOXG1 | 703 | 12 | 1.604204 | -1.218814 | 94 | 56.47 | 0.600745 |
| GO:0050793\_regulation\_of\_developmental\_process | POU4F1 | 703 | 12 | 1.604204 | -1.218814 | 94 | 56.47 | 0.600745 |
| GO:0050793\_regulation\_of\_developmental\_process | JAG1 | 703 | 12 | 1.604204 | -1.218814 | 94 | 56.47 | 0.600745 |
| GO:0050793\_regulation\_of\_developmental\_process | NTN1 | 703 | 12 | 1.604204 | -1.218814 | 94 | 56.47 | 0.600745 |
| GO:0050793\_regulation\_of\_developmental\_process | EPHB2 | 703 | 12 | 1.604204 | -1.218814 | 94 | 56.47 | 0.600745 |
| GO:0010975\_regulation\_of\_neuron\_projection\_development | NTN1 | 38 | 2 | 4.946294 | -1.213424 | 95 | 57.69 | 0.607263 |
| GO:0010975\_regulation\_of\_neuron\_projection\_development | EPHB2 | 38 | 2 | 4.946294 | -1.213424 | 95 | 57.69 | 0.607263 |
| GO:0001779\_natural\_killer\_cell\_differentiation | SP3 | 6 | 1 | 15.663265 | -1.206195 | 102 | 68.65 | 0.673039 |
| GO:0009112\_nucleobase\_metabolic\_process | MAPK1 | 6 | 1 | 15.663265 | -1.206195 | 102 | 68.65 | 0.673039 |
| GO:0040023\_establishment\_of\_nucleus\_localization | NTN1 | 6 | 1 | 15.663265 | -1.206195 | 102 | 68.65 | 0.673039 |
| GO:0046580\_negative\_regulation\_of\_Ras\_protein\_signal\_transduction | STMN3 | 6 | 1 | 15.663265 | -1.206195 | 102 | 68.65 | 0.673039 |
| GO:0048041\_focal\_adhesion\_formation | DLC1 | 6 | 1 | 15.663265 | -1.206195 | 102 | 68.65 | 0.673039 |
| GO:0051058\_negative\_regulation\_of\_small\_GTPase\_mediated\_signal\_transduction | STMN3 | 6 | 1 | 15.663265 | -1.206195 | 102 | 68.65 | 0.673039 |
| GO:0060136\_embryonic\_process\_involved\_in\_female\_pregnancy | SP3 | 6 | 1 | 15.663265 | -1.206195 | 102 | 68.65 | 0.673039 |
| GO:0001568\_blood\_vessel\_development | MAPK1 | 203 | 5 | 2.314768 | -1.201549 | 103 | 68.97 | 0.669612 |
| GO:0001568\_blood\_vessel\_development | HEY1 | 203 | 5 | 2.314768 | -1.201549 | 103 | 68.97 | 0.669612 |
| GO:0001568\_blood\_vessel\_development | PDGFA | 203 | 5 | 2.314768 | -1.201549 | 103 | 68.97 | 0.669612 |
| GO:0001568\_blood\_vessel\_development | COL3A1 | 203 | 5 | 2.314768 | -1.201549 | 103 | 68.97 | 0.669612 |
| GO:0001568\_blood\_vessel\_development | MMP2 | 203 | 5 | 2.314768 | -1.201549 | 103 | 68.97 | 0.669612 |
| GO:0003001\_generation\_of\_a\_signal\_involved\_in\_cell-cell\_signaling | CPLX3 | 87 | 3 | 3.240676 | -1.190937 | 104 | 70.66 | 0.679423 |
| GO:0003001\_generation\_of\_a\_signal\_involved\_in\_cell-cell\_signaling | SV2B | 87 | 3 | 3.240676 | -1.190937 | 104 | 70.66 | 0.679423 |
| GO:0003001\_generation\_of\_a\_signal\_involved\_in\_cell-cell\_signaling | LIN7A | 87 | 3 | 3.240676 | -1.190937 | 104 | 70.66 | 0.679423 |
| GO:0001503\_ossification | SP3 | 88 | 3 | 3.203850 | -1.179036 | 105 | 71.21 | 0.678190 |
| GO:0001503\_ossification | NAB1 | 88 | 3 | 3.203850 | -1.179036 | 105 | 71.21 | 0.678190 |
| GO:0001503\_ossification | MMP2 | 88 | 3 | 3.203850 | -1.179036 | 105 | 71.21 | 0.678190 |
| GO:0007346\_regulation\_of\_mitotic\_cell\_cycle | FOXG1 | 40 | 2 | 4.698980 | -1.174060 | 108 | 72.12 | 0.667778 |
| GO:0007346\_regulation\_of\_mitotic\_cell\_cycle | TUBG1 | 40 | 2 | 4.698980 | -1.174060 | 108 | 72.12 | 0.667778 |
| GO:0035272\_exocrine\_system\_development | XBP1 | 40 | 2 | 4.698980 | -1.174060 | 108 | 72.12 | 0.667778 |
| GO:0035272\_exocrine\_system\_development | PDGFA | 40 | 2 | 4.698980 | -1.174060 | 108 | 72.12 | 0.667778 |
| GO:0051129\_negative\_regulation\_of\_cellular\_component\_organization | NTN1 | 40 | 2 | 4.698980 | -1.174060 | 108 | 72.12 | 0.667778 |
| GO:0051129\_negative\_regulation\_of\_cellular\_component\_organization | EPHB2 | 40 | 2 | 4.698980 | -1.174060 | 108 | 72.12 | 0.667778 |
| GO:0051649\_establishment\_of\_localization\_in\_cell | SCAMP1 | 342 | 7 | 1.923559 | -1.169022 | 109 | 72.23 | 0.662661 |
| GO:0051649\_establishment\_of\_localization\_in\_cell | CPLX3 | 342 | 7 | 1.923559 | -1.169022 | 109 | 72.23 | 0.662661 |
| GO:0051649\_establishment\_of\_localization\_in\_cell | SYTL2 | 342 | 7 | 1.923559 | -1.169022 | 109 | 72.23 | 0.662661 |
| GO:0051649\_establishment\_of\_localization\_in\_cell | SV2B | 342 | 7 | 1.923559 | -1.169022 | 109 | 72.23 | 0.662661 |
| GO:0051649\_establishment\_of\_localization\_in\_cell | ARNTL | 342 | 7 | 1.923559 | -1.169022 | 109 | 72.23 | 0.662661 |
| GO:0051649\_establishment\_of\_localization\_in\_cell | NTN1 | 342 | 7 | 1.923559 | -1.169022 | 109 | 72.23 | 0.662661 |
| GO:0051649\_establishment\_of\_localization\_in\_cell | LIN7A | 342 | 7 | 1.923559 | -1.169022 | 109 | 72.23 | 0.662661 |
| GO:0001944\_vasculature\_development | MAPK1 | 208 | 5 | 2.259125 | -1.165204 | 110 | 72.39 | 0.658091 |
| GO:0001944\_vasculature\_development | HEY1 | 208 | 5 | 2.259125 | -1.165204 | 110 | 72.39 | 0.658091 |
| GO:0001944\_vasculature\_development | PDGFA | 208 | 5 | 2.259125 | -1.165204 | 110 | 72.39 | 0.658091 |
| GO:0001944\_vasculature\_development | COL3A1 | 208 | 5 | 2.259125 | -1.165204 | 110 | 72.39 | 0.658091 |
| GO:0001944\_vasculature\_development | MMP2 | 208 | 5 | 2.259125 | -1.165204 | 110 | 72.39 | 0.658091 |
| GO:0022603\_regulation\_of\_anatomical\_structure\_morphogenesis | SFRP1 | 147 | 4 | 2.557268 | -1.156177 | 111 | 72.69 | 0.654865 |
| GO:0022603\_regulation\_of\_anatomical\_structure\_morphogenesis | PDGFA | 147 | 4 | 2.557268 | -1.156177 | 111 | 72.69 | 0.654865 |
| GO:0022603\_regulation\_of\_anatomical\_structure\_morphogenesis | NTN1 | 147 | 4 | 2.557268 | -1.156177 | 111 | 72.69 | 0.654865 |
| GO:0022603\_regulation\_of\_anatomical\_structure\_morphogenesis | EPHB2 | 147 | 4 | 2.557268 | -1.156177 | 111 | 72.69 | 0.654865 |
| GO:0031344\_regulation\_of\_cell\_projection\_organization | NTN1 | 41 | 2 | 4.584370 | -1.155225 | 112 | 74.24 | 0.662857 |
| GO:0031344\_regulation\_of\_cell\_projection\_organization | EPHB2 | 41 | 2 | 4.584370 | -1.155225 | 112 | 74.24 | 0.662857 |
| GO:0001736\_establishment\_of\_planar\_polarity | SFRP1 | 7 | 1 | 13.425656 | -1.141500 | 124 | 86.79 | 0.699919 |
| GO:0001967\_suckling\_behavior | POU4F1 | 7 | 1 | 13.425656 | -1.141500 | 124 | 86.79 | 0.699919 |
| GO:0002052\_positive\_regulation\_of\_neuroblast\_proliferation | FOXG1 | 7 | 1 | 13.425656 | -1.141500 | 124 | 86.79 | 0.699919 |
| GO:0007164\_establishment\_of\_tissue\_polarity | SFRP1 | 7 | 1 | 13.425656 | -1.141500 | 124 | 86.79 | 0.699919 |
| GO:0030517\_negative\_regulation\_of\_axon\_extension | NTN1 | 7 | 1 | 13.425656 | -1.141500 | 124 | 86.79 | 0.699919 |
| GO:0031017\_exocrine\_pancreas\_development | XBP1 | 7 | 1 | 13.425656 | -1.141500 | 124 | 86.79 | 0.699919 |
| GO:0031124\_mRNA\_3'-end\_processing | APLP1 | 7 | 1 | 13.425656 | -1.141500 | 124 | 86.79 | 0.699919 |
| GO:0032319\_regulation\_of\_Rho\_GTPase\_activity | STMN3 | 7 | 1 | 13.425656 | -1.141500 | 124 | 86.79 | 0.699919 |
| GO:0043353\_enucleate\_erythrocyte\_differentiation | SP3 | 7 | 1 | 13.425656 | -1.141500 | 124 | 86.79 | 0.699919 |
| GO:0060526\_prostate\_glandular\_acinus\_morphogenesis | SFRP1 | 7 | 1 | 13.425656 | -1.141500 | 124 | 86.79 | 0.699919 |
| GO:0060527\_prostate\_epithelial\_cord\_arborization\_involved\_in\_prostate\_glandular\_acinus\_morphogenesis | SFRP1 | 7 | 1 | 13.425656 | -1.141500 | 124 | 86.79 | 0.699919 |
| GO:0060687\_regulation\_of\_branching\_involved\_in\_prostate\_gland\_morphogenesis | SFRP1 | 7 | 1 | 13.425656 | -1.141500 | 124 | 86.79 | 0.699919 |
| GO:0010769\_regulation\_of\_cell\_morphogenesis\_involved\_in\_differentiation | NTN1 | 42 | 2 | 4.475219 | -1.136917 | 125 | 88.05 | 0.704400 |
| GO:0010769\_regulation\_of\_cell\_morphogenesis\_involved\_in\_differentiation | EPHB2 | 42 | 2 | 4.475219 | -1.136917 | 125 | 88.05 | 0.704400 |
| GO:0007224\_smoothened\_signaling\_pathway | HES5 | 43 | 2 | 4.371144 | -1.119112 | 126 | 90.24 | 0.716190 |
| GO:0007224\_smoothened\_signaling\_pathway | HIPK1 | 43 | 2 | 4.371144 | -1.119112 | 126 | 90.24 | 0.716190 |
| GO:0051179\_localization | SCAMP1 | 1058 | 16 | 1.421241 | -1.110848 | 127 | 90.8 | 0.714961 |
| GO:0051179\_localization | CPLX3 | 1058 | 16 | 1.421241 | -1.110848 | 127 | 90.8 | 0.714961 |
| GO:0051179\_localization | KIF5C | 1058 | 16 | 1.421241 | -1.110848 | 127 | 90.8 | 0.714961 |
| GO:0051179\_localization | ARNTL | 1058 | 16 | 1.421241 | -1.110848 | 127 | 90.8 | 0.714961 |
| GO:0051179\_localization | NTN1 | 1058 | 16 | 1.421241 | -1.110848 | 127 | 90.8 | 0.714961 |
| GO:0051179\_localization | LIN7A | 1058 | 16 | 1.421241 | -1.110848 | 127 | 90.8 | 0.714961 |
| GO:0051179\_localization | EPHB2 | 1058 | 16 | 1.421241 | -1.110848 | 127 | 90.8 | 0.714961 |
| GO:0051179\_localization | EPHA4 | 1058 | 16 | 1.421241 | -1.110848 | 127 | 90.8 | 0.714961 |
| GO:0051179\_localization | ANK3 | 1058 | 16 | 1.421241 | -1.110848 | 127 | 90.8 | 0.714961 |
| GO:0051179\_localization | KATNA1 | 1058 | 16 | 1.421241 | -1.110848 | 127 | 90.8 | 0.714961 |
| GO:0051179\_localization | FOXG1 | 1058 | 16 | 1.421241 | -1.110848 | 127 | 90.8 | 0.714961 |
| GO:0051179\_localization | POU4F1 | 1058 | 16 | 1.421241 | -1.110848 | 127 | 90.8 | 0.714961 |
| GO:0051179\_localization | SYTL2 | 1058 | 16 | 1.421241 | -1.110848 | 127 | 90.8 | 0.714961 |
| GO:0051179\_localization | SV2B | 1058 | 16 | 1.421241 | -1.110848 | 127 | 90.8 | 0.714961 |
| GO:0051179\_localization | LRP2 | 1058 | 16 | 1.421241 | -1.110848 | 127 | 90.8 | 0.714961 |
| GO:0051179\_localization | YES1 | 1058 | 16 | 1.421241 | -1.110848 | 127 | 90.8 | 0.714961 |
| GO:0000902\_cell\_morphogenesis | EPHA4 | 283 | 6 | 1.992500 | -1.110504 | 128 | 90.84 | 0.709688 |
| GO:0000902\_cell\_morphogenesis | ANK3 | 283 | 6 | 1.992500 | -1.110504 | 128 | 90.84 | 0.709688 |
| GO:0000902\_cell\_morphogenesis | FOXG1 | 283 | 6 | 1.992500 | -1.110504 | 128 | 90.84 | 0.709688 |
| GO:0000902\_cell\_morphogenesis | KIF5C | 283 | 6 | 1.992500 | -1.110504 | 128 | 90.84 | 0.709688 |
| GO:0000902\_cell\_morphogenesis | NTN1 | 283 | 6 | 1.992500 | -1.110504 | 128 | 90.84 | 0.709688 |
| GO:0000902\_cell\_morphogenesis | EPHB2 | 283 | 6 | 1.992500 | -1.110504 | 128 | 90.84 | 0.709688 |
| GO:0048593\_camera-type\_eye\_morphogenesis | SP3 | 44 | 2 | 4.271800 | -1.101787 | 129 | 93.04 | 0.721240 |
| GO:0048593\_camera-type\_eye\_morphogenesis | EPHB2 | 44 | 2 | 4.271800 | -1.101787 | 129 | 93.04 | 0.721240 |
| GO:0007268\_synaptic\_transmission | CPLX3 | 154 | 4 | 2.441028 | -1.097686 | 130 | 93.31 | 0.717769 |
| GO:0007268\_synaptic\_transmission | SV2B | 154 | 4 | 2.441028 | -1.097686 | 130 | 93.31 | 0.717769 |
| GO:0007268\_synaptic\_transmission | EPHB2 | 154 | 4 | 2.441028 | -1.097686 | 130 | 93.31 | 0.717769 |
| GO:0007268\_synaptic\_transmission | LIN7A | 154 | 4 | 2.441028 | -1.097686 | 130 | 93.31 | 0.717769 |
| GO:0008593\_regulation\_of\_Notch\_signaling\_pathway | JAG1 | 8 | 1 | 11.747449 | -1.085756 | 137 | 104.6 | 0.763504 |
| GO:0018107\_peptidyl-threonine\_phosphorylation | MAP3K12 | 8 | 1 | 11.747449 | -1.085756 | 137 | 104.6 | 0.763504 |
| GO:0018210\_peptidyl-threonine\_modification | MAP3K12 | 8 | 1 | 11.747449 | -1.085756 | 137 | 104.6 | 0.763504 |
| GO:0030511\_positive\_regulation\_of\_transforming\_growth\_factor\_beta\_receptor\_signaling\_pathway | ITGA8 | 8 | 1 | 11.747449 | -1.085756 | 137 | 104.6 | 0.763504 |
| GO:0031123\_RNA\_3'-end\_processing | APLP1 | 8 | 1 | 11.747449 | -1.085756 | 137 | 104.6 | 0.763504 |
| GO:0035023\_regulation\_of\_Rho\_protein\_signal\_transduction | STMN3 | 8 | 1 | 11.747449 | -1.085756 | 137 | 104.6 | 0.763504 |
| GO:0042771\_DNA\_damage\_response\_\_signal\_transduction\_by\_p53\_class\_mediator\_resulting\_in\_induction\_of\_apoptosis | HIPK1 | 8 | 1 | 11.747449 | -1.085756 | 137 | 104.6 | 0.763504 |
| GO:0018193\_peptidyl-amino\_acid\_modification | PDGFA | 97 | 3 | 2.906585 | -1.079170 | 138 | 105.29 | 0.762971 |
| GO:0018193\_peptidyl-amino\_acid\_modification | MAP3K12 | 97 | 3 | 2.906585 | -1.079170 | 138 | 105.29 | 0.762971 |
| GO:0018193\_peptidyl-amino\_acid\_modification | EPHB2 | 97 | 3 | 2.906585 | -1.079170 | 138 | 105.29 | 0.762971 |
| GO:0009967\_positive\_regulation\_of\_signal\_transduction | ITGA8 | 98 | 3 | 2.876926 | -1.068815 | 139 | 105.91 | 0.761942 |
| GO:0009967\_positive\_regulation\_of\_signal\_transduction | JAG1 | 98 | 3 | 2.876926 | -1.068815 | 139 | 105.91 | 0.761942 |
| GO:0009967\_positive\_regulation\_of\_signal\_transduction | CHUK | 98 | 3 | 2.876926 | -1.068815 | 139 | 105.91 | 0.761942 |
| GO:0030218\_erythrocyte\_differentiation | HBA-A1 | 46 | 2 | 4.086069 | -1.068485 | 140 | 106.53 | 0.760929 |
| GO:0030218\_erythrocyte\_differentiation | SP3 | 46 | 2 | 4.086069 | -1.068485 | 140 | 106.53 | 0.760929 |
| GO:0051239\_regulation\_of\_multicellular\_organismal\_process | CPLX3 | 587 | 10 | 1.601015 | -1.064675 | 141 | 106.72 | 0.756879 |
| GO:0051239\_regulation\_of\_multicellular\_organismal\_process | SFRP1 | 587 | 10 | 1.601015 | -1.064675 | 141 | 106.72 | 0.756879 |
| GO:0051239\_regulation\_of\_multicellular\_organismal\_process | HES5 | 587 | 10 | 1.601015 | -1.064675 | 141 | 106.72 | 0.756879 |
| GO:0051239\_regulation\_of\_multicellular\_organismal\_process | PDGFA | 587 | 10 | 1.601015 | -1.064675 | 141 | 106.72 | 0.756879 |
| GO:0051239\_regulation\_of\_multicellular\_organismal\_process | FOXG1 | 587 | 10 | 1.601015 | -1.064675 | 141 | 106.72 | 0.756879 |
| GO:0051239\_regulation\_of\_multicellular\_organismal\_process | NAB1 | 587 | 10 | 1.601015 | -1.064675 | 141 | 106.72 | 0.756879 |
| GO:0051239\_regulation\_of\_multicellular\_organismal\_process | SYTL2 | 587 | 10 | 1.601015 | -1.064675 | 141 | 106.72 | 0.756879 |
| GO:0051239\_regulation\_of\_multicellular\_organismal\_process | JAG1 | 587 | 10 | 1.601015 | -1.064675 | 141 | 106.72 | 0.756879 |
| GO:0051239\_regulation\_of\_multicellular\_organismal\_process | NTN1 | 587 | 10 | 1.601015 | -1.064675 | 141 | 106.72 | 0.756879 |
| GO:0051239\_regulation\_of\_multicellular\_organismal\_process | EPHB2 | 587 | 10 | 1.601015 | -1.064675 | 141 | 106.72 | 0.756879 |
| GO:0060348\_bone\_development | SP3 | 99 | 3 | 2.847866 | -1.058598 | 142 | 107.06 | 0.753944 |
| GO:0060348\_bone\_development | NAB1 | 99 | 3 | 2.847866 | -1.058598 | 142 | 107.06 | 0.753944 |
| GO:0060348\_bone\_development | MMP2 | 99 | 3 | 2.847866 | -1.058598 | 142 | 107.06 | 0.753944 |
| GO:0051128\_regulation\_of\_cellular\_component\_organization | STMN3 | 160 | 4 | 2.349490 | -1.050443 | 143 | 108.15 | 0.756294 |
| GO:0051128\_regulation\_of\_cellular\_component\_organization | TUBG1 | 160 | 4 | 2.349490 | -1.050443 | 143 | 108.15 | 0.756294 |
| GO:0051128\_regulation\_of\_cellular\_component\_organization | NTN1 | 160 | 4 | 2.349490 | -1.050443 | 143 | 108.15 | 0.756294 |
| GO:0051128\_regulation\_of\_cellular\_component\_organization | EPHB2 | 160 | 4 | 2.349490 | -1.050443 | 143 | 108.15 | 0.756294 |
| GO:0048518\_positive\_regulation\_of\_biological\_process | FUS | 995 | 15 | 1.416778 | -1.047976 | 144 | 108.45 | 0.753125 |
| GO:0048518\_positive\_regulation\_of\_biological\_process | TM2D1 | 995 | 15 | 1.416778 | -1.047976 | 144 | 108.45 | 0.753125 |
| GO:0048518\_positive\_regulation\_of\_biological\_process | PDGFA | 995 | 15 | 1.416778 | -1.047976 | 144 | 108.45 | 0.753125 |
| GO:0048518\_positive\_regulation\_of\_biological\_process | ARNTL | 995 | 15 | 1.416778 | -1.047976 | 144 | 108.45 | 0.753125 |
| GO:0048518\_positive\_regulation\_of\_biological\_process | JAG1 | 995 | 15 | 1.416778 | -1.047976 | 144 | 108.45 | 0.753125 |
| GO:0048518\_positive\_regulation\_of\_biological\_process | NTN1 | 995 | 15 | 1.416778 | -1.047976 | 144 | 108.45 | 0.753125 |
| GO:0048518\_positive\_regulation\_of\_biological\_process | EPHB2 | 995 | 15 | 1.416778 | -1.047976 | 144 | 108.45 | 0.753125 |
| GO:0048518\_positive\_regulation\_of\_biological\_process | MAPK1 | 995 | 15 | 1.416778 | -1.047976 | 144 | 108.45 | 0.753125 |
| GO:0048518\_positive\_regulation\_of\_biological\_process | HES5 | 995 | 15 | 1.416778 | -1.047976 | 144 | 108.45 | 0.753125 |
| GO:0048518\_positive\_regulation\_of\_biological\_process | HIPK1 | 995 | 15 | 1.416778 | -1.047976 | 144 | 108.45 | 0.753125 |
| GO:0048518\_positive\_regulation\_of\_biological\_process | ITGA8 | 995 | 15 | 1.416778 | -1.047976 | 144 | 108.45 | 0.753125 |
| GO:0048518\_positive\_regulation\_of\_biological\_process | FOXG1 | 995 | 15 | 1.416778 | -1.047976 | 144 | 108.45 | 0.753125 |
| GO:0048518\_positive\_regulation\_of\_biological\_process | SYTL2 | 995 | 15 | 1.416778 | -1.047976 | 144 | 108.45 | 0.753125 |
| GO:0048518\_positive\_regulation\_of\_biological\_process | POU4F1 | 995 | 15 | 1.416778 | -1.047976 | 144 | 108.45 | 0.753125 |
| GO:0048518\_positive\_regulation\_of\_biological\_process | CHUK | 995 | 15 | 1.416778 | -1.047976 | 144 | 108.45 | 0.753125 |
| GO:0045595\_regulation\_of\_cell\_differentiation | MAPK1 | 295 | 6 | 1.911449 | -1.042782 | 145 | 108.7 | 0.749655 |
| GO:0045595\_regulation\_of\_cell\_differentiation | HES5 | 295 | 6 | 1.911449 | -1.042782 | 145 | 108.7 | 0.749655 |
| GO:0045595\_regulation\_of\_cell\_differentiation | FOXG1 | 295 | 6 | 1.911449 | -1.042782 | 145 | 108.7 | 0.749655 |
| GO:0045595\_regulation\_of\_cell\_differentiation | JAG1 | 295 | 6 | 1.911449 | -1.042782 | 145 | 108.7 | 0.749655 |
| GO:0045595\_regulation\_of\_cell\_differentiation | NTN1 | 295 | 6 | 1.911449 | -1.042782 | 145 | 108.7 | 0.749655 |
| GO:0045595\_regulation\_of\_cell\_differentiation | EPHB2 | 295 | 6 | 1.911449 | -1.042782 | 145 | 108.7 | 0.749655 |
| GO:0014037\_Schwann\_cell\_differentiation | NAB1 | 9 | 1 | 10.442177 | -1.036847 | 155 | 120.59 | 0.778000 |
| GO:0016601\_Rac\_protein\_signal\_transduction | STMN3 | 9 | 1 | 10.442177 | -1.036847 | 155 | 120.59 | 0.778000 |
| GO:0032963\_collagen\_metabolic\_process | MMP2 | 9 | 1 | 10.442177 | -1.036847 | 155 | 120.59 | 0.778000 |
| GO:0050884\_neuromuscular\_process\_controlling\_posture | POU4F1 | 9 | 1 | 10.442177 | -1.036847 | 155 | 120.59 | 0.778000 |
| GO:0050910\_detection\_of\_mechanical\_stimulus\_involved\_in\_sensory\_perception\_of\_sound | GPR98 | 9 | 1 | 10.442177 | -1.036847 | 155 | 120.59 | 0.778000 |
| GO:0051647\_nucleus\_localization | NTN1 | 9 | 1 | 10.442177 | -1.036847 | 155 | 120.59 | 0.778000 |
| GO:0060119\_inner\_ear\_receptor\_cell\_development | GPR98 | 9 | 1 | 10.442177 | -1.036847 | 155 | 120.59 | 0.778000 |
| GO:0060122\_inner\_ear\_receptor\_stereocilium\_organization | GPR98 | 9 | 1 | 10.442177 | -1.036847 | 155 | 120.59 | 0.778000 |
| GO:0060325\_face\_morphogenesis | MMP2 | 9 | 1 | 10.442177 | -1.036847 | 155 | 120.59 | 0.778000 |
| GO:0060693\_regulation\_of\_branching\_involved\_in\_salivary\_gland\_morphogenesis | PDGFA | 9 | 1 | 10.442177 | -1.036847 | 155 | 120.59 | 0.778000 |
| GO:0051641\_cellular\_localization | SCAMP1 | 370 | 7 | 1.777992 | -1.025597 | 156 | 121.56 | 0.779231 |
| GO:0051641\_cellular\_localization | CPLX3 | 370 | 7 | 1.777992 | -1.025597 | 156 | 121.56 | 0.779231 |
| GO:0051641\_cellular\_localization | SYTL2 | 370 | 7 | 1.777992 | -1.025597 | 156 | 121.56 | 0.779231 |
| GO:0051641\_cellular\_localization | SV2B | 370 | 7 | 1.777992 | -1.025597 | 156 | 121.56 | 0.779231 |
| GO:0051641\_cellular\_localization | ARNTL | 370 | 7 | 1.777992 | -1.025597 | 156 | 121.56 | 0.779231 |
| GO:0051641\_cellular\_localization | NTN1 | 370 | 7 | 1.777992 | -1.025597 | 156 | 121.56 | 0.779231 |
| GO:0051641\_cellular\_localization | LIN7A | 370 | 7 | 1.777992 | -1.025597 | 156 | 121.56 | 0.779231 |
| GO:0034101\_erythrocyte\_homeostasis | HBA-A1 | 49 | 2 | 3.835902 | -1.021623 | 157 | 122.81 | 0.782229 |
| GO:0034101\_erythrocyte\_homeostasis | SP3 | 49 | 2 | 3.835902 | -1.021623 | 157 | 122.81 | 0.782229 |
| GO:0048598\_embryonic\_morphogenesis | DLC1 | 299 | 6 | 1.885878 | -1.021187 | 158 | 122.88 | 0.777722 |
| GO:0048598\_embryonic\_morphogenesis | DKK1 | 299 | 6 | 1.885878 | -1.021187 | 158 | 122.88 | 0.777722 |
| GO:0048598\_embryonic\_morphogenesis | SP3 | 299 | 6 | 1.885878 | -1.021187 | 158 | 122.88 | 0.777722 |
| GO:0048598\_embryonic\_morphogenesis | ITGA8 | 299 | 6 | 1.885878 | -1.021187 | 158 | 122.88 | 0.777722 |
| GO:0048598\_embryonic\_morphogenesis | FOXG1 | 299 | 6 | 1.885878 | -1.021187 | 158 | 122.88 | 0.777722 |
| GO:0048598\_embryonic\_morphogenesis | ODZ4 | 299 | 6 | 1.885878 | -1.021187 | 158 | 122.88 | 0.777722 |
| GO:0002573\_myeloid\_leukocyte\_differentiation | SP3 | 50 | 2 | 3.759184 | -1.006758 | 159 | 124.58 | 0.783522 |
| GO:0002573\_myeloid\_leukocyte\_differentiation | CHUK | 50 | 2 | 3.759184 | -1.006758 | 159 | 124.58 | 0.783522 |
| GO:0002070\_epithelial\_cell\_maturation | XBP1 | 10 | 1 | 9.397959 | -0.993331 | 169 | 138.17 | 0.817574 |
| GO:0007044\_cell-substrate\_junction\_assembly | DLC1 | 10 | 1 | 9.397959 | -0.993331 | 169 | 138.17 | 0.817574 |
| GO:0021952\_central\_nervous\_system\_projection\_neuron\_axonogenesis | EPHB2 | 10 | 1 | 9.397959 | -0.993331 | 169 | 138.17 | 0.817574 |
| GO:0032318\_regulation\_of\_Ras\_GTPase\_activity | STMN3 | 10 | 1 | 9.397959 | -0.993331 | 169 | 138.17 | 0.817574 |
| GO:0043330\_response\_to\_exogenous\_dsRNA | MAPK1 | 10 | 1 | 9.397959 | -0.993331 | 169 | 138.17 | 0.817574 |
| GO:0044259\_multicellular\_organismal\_macromolecule\_metabolic\_process | MMP2 | 10 | 1 | 9.397959 | -0.993331 | 169 | 138.17 | 0.817574 |
| GO:0048596\_embryonic\_camera-type\_eye\_morphogenesis | SP3 | 10 | 1 | 9.397959 | -0.993331 | 169 | 138.17 | 0.817574 |
| GO:0060216\_definitive\_hemopoiesis | SP3 | 10 | 1 | 9.397959 | -0.993331 | 169 | 138.17 | 0.817574 |
| GO:0060323\_head\_morphogenesis | MMP2 | 10 | 1 | 9.397959 | -0.993331 | 169 | 138.17 | 0.817574 |
| GO:0060343\_trabecula\_formation | MMP2 | 10 | 1 | 9.397959 | -0.993331 | 169 | 138.17 | 0.817574 |
| GO:0050789\_regulation\_of\_biological\_process | FUS | 2357 | 30 | 1.196176 | -0.992707 | 170 | 138.22 | 0.813059 |
| GO:0050789\_regulation\_of\_biological\_process | CPLX3 | 2357 | 30 | 1.196176 | -0.992707 | 170 | 138.22 | 0.813059 |
| GO:0050789\_regulation\_of\_biological\_process | PDGFA | 2357 | 30 | 1.196176 | -0.992707 | 170 | 138.22 | 0.813059 |
| GO:0050789\_regulation\_of\_biological\_process | THOP1 | 2357 | 30 | 1.196176 | -0.992707 | 170 | 138.22 | 0.813059 |
| GO:0050789\_regulation\_of\_biological\_process | JAG1 | 2357 | 30 | 1.196176 | -0.992707 | 170 | 138.22 | 0.813059 |
| GO:0050789\_regulation\_of\_biological\_process | APLP1 | 2357 | 30 | 1.196176 | -0.992707 | 170 | 138.22 | 0.813059 |
| GO:0050789\_regulation\_of\_biological\_process | ZC3H8 | 2357 | 30 | 1.196176 | -0.992707 | 170 | 138.22 | 0.813059 |
| GO:0050789\_regulation\_of\_biological\_process | EPHB2 | 2357 | 30 | 1.196176 | -0.992707 | 170 | 138.22 | 0.813059 |
| GO:0050789\_regulation\_of\_biological\_process | CCNE2 | 2357 | 30 | 1.196176 | -0.992707 | 170 | 138.22 | 0.813059 |
| GO:0050789\_regulation\_of\_biological\_process | POU4F1 | 2357 | 30 | 1.196176 | -0.992707 | 170 | 138.22 | 0.813059 |
| GO:0050789\_regulation\_of\_biological\_process | SV2B | 2357 | 30 | 1.196176 | -0.992707 | 170 | 138.22 | 0.813059 |
| GO:0050789\_regulation\_of\_biological\_process | TUBG1 | 2357 | 30 | 1.196176 | -0.992707 | 170 | 138.22 | 0.813059 |
| GO:0050789\_regulation\_of\_biological\_process | CHUK | 2357 | 30 | 1.196176 | -0.992707 | 170 | 138.22 | 0.813059 |
| GO:0050789\_regulation\_of\_biological\_process | AEBP2 | 2357 | 30 | 1.196176 | -0.992707 | 170 | 138.22 | 0.813059 |
| GO:0050789\_regulation\_of\_biological\_process | TM2D1 | 2357 | 30 | 1.196176 | -0.992707 | 170 | 138.22 | 0.813059 |
| GO:0050789\_regulation\_of\_biological\_process | STMN3 | 2357 | 30 | 1.196176 | -0.992707 | 170 | 138.22 | 0.813059 |
| GO:0050789\_regulation\_of\_biological\_process | MAPK10 | 2357 | 30 | 1.196176 | -0.992707 | 170 | 138.22 | 0.813059 |
| GO:0050789\_regulation\_of\_biological\_process | ARNTL | 2357 | 30 | 1.196176 | -0.992707 | 170 | 138.22 | 0.813059 |
| GO:0050789\_regulation\_of\_biological\_process | NTN1 | 2357 | 30 | 1.196176 | -0.992707 | 170 | 138.22 | 0.813059 |
| GO:0050789\_regulation\_of\_biological\_process | MAPK1 | 2357 | 30 | 1.196176 | -0.992707 | 170 | 138.22 | 0.813059 |
| GO:0050789\_regulation\_of\_biological\_process | DKK1 | 2357 | 30 | 1.196176 | -0.992707 | 170 | 138.22 | 0.813059 |
| GO:0050789\_regulation\_of\_biological\_process | HIPK1 | 2357 | 30 | 1.196176 | -0.992707 | 170 | 138.22 | 0.813059 |
| GO:0050789\_regulation\_of\_biological\_process | HES5 | 2357 | 30 | 1.196176 | -0.992707 | 170 | 138.22 | 0.813059 |
| GO:0050789\_regulation\_of\_biological\_process | SFRP1 | 2357 | 30 | 1.196176 | -0.992707 | 170 | 138.22 | 0.813059 |
| GO:0050789\_regulation\_of\_biological\_process | SP3 | 2357 | 30 | 1.196176 | -0.992707 | 170 | 138.22 | 0.813059 |
| GO:0050789\_regulation\_of\_biological\_process | ITGA8 | 2357 | 30 | 1.196176 | -0.992707 | 170 | 138.22 | 0.813059 |
| GO:0050789\_regulation\_of\_biological\_process | NAB1 | 2357 | 30 | 1.196176 | -0.992707 | 170 | 138.22 | 0.813059 |
| GO:0050789\_regulation\_of\_biological\_process | FOXG1 | 2357 | 30 | 1.196176 | -0.992707 | 170 | 138.22 | 0.813059 |
| GO:0050789\_regulation\_of\_biological\_process | GNB4 | 2357 | 30 | 1.196176 | -0.992707 | 170 | 138.22 | 0.813059 |
| GO:0050789\_regulation\_of\_biological\_process | SYTL2 | 2357 | 30 | 1.196176 | -0.992707 | 170 | 138.22 | 0.813059 |
| GO:0032989\_cellular\_component\_morphogenesis | EPHA4 | 307 | 6 | 1.836735 | -0.979386 | 171 | 139.73 | 0.817135 |
| GO:0032989\_cellular\_component\_morphogenesis | ANK3 | 307 | 6 | 1.836735 | -0.979386 | 171 | 139.73 | 0.817135 |
| GO:0032989\_cellular\_component\_morphogenesis | FOXG1 | 307 | 6 | 1.836735 | -0.979386 | 171 | 139.73 | 0.817135 |
| GO:0032989\_cellular\_component\_morphogenesis | KIF5C | 307 | 6 | 1.836735 | -0.979386 | 171 | 139.73 | 0.817135 |
| GO:0032989\_cellular\_component\_morphogenesis | NTN1 | 307 | 6 | 1.836735 | -0.979386 | 171 | 139.73 | 0.817135 |
| GO:0032989\_cellular\_component\_morphogenesis | EPHB2 | 307 | 6 | 1.836735 | -0.979386 | 171 | 139.73 | 0.817135 |
| GO:0051246\_regulation\_of\_protein\_metabolic\_process | PDGFA | 170 | 4 | 2.211285 | -0.977071 | 172 | 140.4 | 0.816279 |
| GO:0051246\_regulation\_of\_protein\_metabolic\_process | SV2B | 170 | 4 | 2.211285 | -0.977071 | 172 | 140.4 | 0.816279 |
| GO:0051246\_regulation\_of\_protein\_metabolic\_process | ARNTL | 170 | 4 | 2.211285 | -0.977071 | 172 | 140.4 | 0.816279 |
| GO:0051246\_regulation\_of\_protein\_metabolic\_process | APLP1 | 170 | 4 | 2.211285 | -0.977071 | 172 | 140.4 | 0.816279 |
| GO:0016310\_phosphorylation | MAPK1 | 309 | 6 | 1.824846 | -0.969216 | 173 | 140.89 | 0.814393 |
| GO:0016310\_phosphorylation | HIPK1 | 309 | 6 | 1.824846 | -0.969216 | 173 | 140.89 | 0.814393 |
| GO:0016310\_phosphorylation | PDGFA | 309 | 6 | 1.824846 | -0.969216 | 173 | 140.89 | 0.814393 |
| GO:0016310\_phosphorylation | MAP3K12 | 309 | 6 | 1.824846 | -0.969216 | 173 | 140.89 | 0.814393 |
| GO:0016310\_phosphorylation | CHUK | 309 | 6 | 1.824846 | -0.969216 | 173 | 140.89 | 0.814393 |
| GO:0016310\_phosphorylation | EPHB2 | 309 | 6 | 1.824846 | -0.969216 | 173 | 140.89 | 0.814393 |
| GO:0010647\_positive\_regulation\_of\_cell\_communication | ITGA8 | 110 | 3 | 2.563080 | -0.954503 | 174 | 142.8 | 0.820690 |
| GO:0010647\_positive\_regulation\_of\_cell\_communication | JAG1 | 110 | 3 | 2.563080 | -0.954503 | 174 | 142.8 | 0.820690 |
| GO:0010647\_positive\_regulation\_of\_cell\_communication | CHUK | 110 | 3 | 2.563080 | -0.954503 | 174 | 142.8 | 0.820690 |
| GO:0007051\_spindle\_organization | TUBG1 | 11 | 1 | 8.543599 | -0.954175 | 180 | 151.56 | 0.842000 |
| GO:0007088\_regulation\_of\_mitosis | TUBG1 | 11 | 1 | 8.543599 | -0.954175 | 180 | 151.56 | 0.842000 |
| GO:0021602\_cranial\_nerve\_morphogenesis | EPHB2 | 11 | 1 | 8.543599 | -0.954175 | 180 | 151.56 | 0.842000 |
| GO:0046928\_regulation\_of\_neurotransmitter\_secretion | CPLX3 | 11 | 1 | 8.543599 | -0.954175 | 180 | 151.56 | 0.842000 |
| GO:0050772\_positive\_regulation\_of\_axonogenesis | NTN1 | 11 | 1 | 8.543599 | -0.954175 | 180 | 151.56 | 0.842000 |
| GO:0051783\_regulation\_of\_nuclear\_division | TUBG1 | 11 | 1 | 8.543599 | -0.954175 | 180 | 151.56 | 0.842000 |
| GO:0050794\_regulation\_of\_cellular\_process | FUS | 2190 | 28 | 1.201566 | -0.944082 | 181 | 152.6 | 0.843094 |
| GO:0050794\_regulation\_of\_cellular\_process | CPLX3 | 2190 | 28 | 1.201566 | -0.944082 | 181 | 152.6 | 0.843094 |
| GO:0050794\_regulation\_of\_cellular\_process | PDGFA | 2190 | 28 | 1.201566 | -0.944082 | 181 | 152.6 | 0.843094 |
| GO:0050794\_regulation\_of\_cellular\_process | THOP1 | 2190 | 28 | 1.201566 | -0.944082 | 181 | 152.6 | 0.843094 |
| GO:0050794\_regulation\_of\_cellular\_process | JAG1 | 2190 | 28 | 1.201566 | -0.944082 | 181 | 152.6 | 0.843094 |
| GO:0050794\_regulation\_of\_cellular\_process | APLP1 | 2190 | 28 | 1.201566 | -0.944082 | 181 | 152.6 | 0.843094 |
| GO:0050794\_regulation\_of\_cellular\_process | EPHB2 | 2190 | 28 | 1.201566 | -0.944082 | 181 | 152.6 | 0.843094 |
| GO:0050794\_regulation\_of\_cellular\_process | ZC3H8 | 2190 | 28 | 1.201566 | -0.944082 | 181 | 152.6 | 0.843094 |
| GO:0050794\_regulation\_of\_cellular\_process | CCNE2 | 2190 | 28 | 1.201566 | -0.944082 | 181 | 152.6 | 0.843094 |
| GO:0050794\_regulation\_of\_cellular\_process | POU4F1 | 2190 | 28 | 1.201566 | -0.944082 | 181 | 152.6 | 0.843094 |
| GO:0050794\_regulation\_of\_cellular\_process | TUBG1 | 2190 | 28 | 1.201566 | -0.944082 | 181 | 152.6 | 0.843094 |
| GO:0050794\_regulation\_of\_cellular\_process | CHUK | 2190 | 28 | 1.201566 | -0.944082 | 181 | 152.6 | 0.843094 |
| GO:0050794\_regulation\_of\_cellular\_process | AEBP2 | 2190 | 28 | 1.201566 | -0.944082 | 181 | 152.6 | 0.843094 |
| GO:0050794\_regulation\_of\_cellular\_process | STMN3 | 2190 | 28 | 1.201566 | -0.944082 | 181 | 152.6 | 0.843094 |
| GO:0050794\_regulation\_of\_cellular\_process | TM2D1 | 2190 | 28 | 1.201566 | -0.944082 | 181 | 152.6 | 0.843094 |
| GO:0050794\_regulation\_of\_cellular\_process | MAPK10 | 2190 | 28 | 1.201566 | -0.944082 | 181 | 152.6 | 0.843094 |
| GO:0050794\_regulation\_of\_cellular\_process | ARNTL | 2190 | 28 | 1.201566 | -0.944082 | 181 | 152.6 | 0.843094 |
| GO:0050794\_regulation\_of\_cellular\_process | NTN1 | 2190 | 28 | 1.201566 | -0.944082 | 181 | 152.6 | 0.843094 |
| GO:0050794\_regulation\_of\_cellular\_process | MAPK1 | 2190 | 28 | 1.201566 | -0.944082 | 181 | 152.6 | 0.843094 |
| GO:0050794\_regulation\_of\_cellular\_process | DKK1 | 2190 | 28 | 1.201566 | -0.944082 | 181 | 152.6 | 0.843094 |
| GO:0050794\_regulation\_of\_cellular\_process | HES5 | 2190 | 28 | 1.201566 | -0.944082 | 181 | 152.6 | 0.843094 |
| GO:0050794\_regulation\_of\_cellular\_process | HIPK1 | 2190 | 28 | 1.201566 | -0.944082 | 181 | 152.6 | 0.843094 |
| GO:0050794\_regulation\_of\_cellular\_process | SFRP1 | 2190 | 28 | 1.201566 | -0.944082 | 181 | 152.6 | 0.843094 |
| GO:0050794\_regulation\_of\_cellular\_process | ITGA8 | 2190 | 28 | 1.201566 | -0.944082 | 181 | 152.6 | 0.843094 |
| GO:0050794\_regulation\_of\_cellular\_process | SP3 | 2190 | 28 | 1.201566 | -0.944082 | 181 | 152.6 | 0.843094 |
| GO:0050794\_regulation\_of\_cellular\_process | NAB1 | 2190 | 28 | 1.201566 | -0.944082 | 181 | 152.6 | 0.843094 |
| GO:0050794\_regulation\_of\_cellular\_process | FOXG1 | 2190 | 28 | 1.201566 | -0.944082 | 181 | 152.6 | 0.843094 |
| GO:0050794\_regulation\_of\_cellular\_process | GNB4 | 2190 | 28 | 1.201566 | -0.944082 | 181 | 152.6 | 0.843094 |
| GO:0001738\_morphogenesis\_of\_a\_polarized\_epithelium | SFRP1 | 12 | 1 | 7.831633 | -0.918620 | 192 | 164.93 | 0.859010 |
| GO:0008038\_neuron\_recognition | FOXG1 | 12 | 1 | 7.831633 | -0.918620 | 192 | 164.93 | 0.859010 |
| GO:0008045\_motor\_axon\_guidance | KIF5C | 12 | 1 | 7.831633 | -0.918620 | 192 | 164.93 | 0.859010 |
| GO:0030330\_DNA\_damage\_response\_\_signal\_transduction\_by\_p53\_class\_mediator | HIPK1 | 12 | 1 | 7.831633 | -0.918620 | 192 | 164.93 | 0.859010 |
| GO:0033598\_mammary\_gland\_epithelial\_cell\_proliferation | CHUK | 12 | 1 | 7.831633 | -0.918620 | 192 | 164.93 | 0.859010 |
| GO:0043331\_response\_to\_dsRNA | MAPK1 | 12 | 1 | 7.831633 | -0.918620 | 192 | 164.93 | 0.859010 |
| GO:0048169\_regulation\_of\_long-term\_neuronal\_synaptic\_plasticity | EPHB2 | 12 | 1 | 7.831633 | -0.918620 | 192 | 164.93 | 0.859010 |
| GO:0048821\_erythrocyte\_development | HBA-A1 | 12 | 1 | 7.831633 | -0.918620 | 192 | 164.93 | 0.859010 |
| GO:0050853\_B\_cell\_receptor\_signaling\_pathway | MAPK1 | 12 | 1 | 7.831633 | -0.918620 | 192 | 164.93 | 0.859010 |
| GO:0051588\_regulation\_of\_neurotransmitter\_transport | CPLX3 | 12 | 1 | 7.831633 | -0.918620 | 192 | 164.93 | 0.859010 |
| GO:0060525\_prostate\_glandular\_acinus\_development | SFRP1 | 12 | 1 | 7.831633 | -0.918620 | 192 | 164.93 | 0.859010 |
| GO:0001764\_neuron\_migration | KATNA1 | 57 | 2 | 3.297530 | -0.911842 | 197 | 166.48 | 0.845076 |
| GO:0001764\_neuron\_migration | NTN1 | 57 | 2 | 3.297530 | -0.911842 | 197 | 166.48 | 0.845076 |
| GO:0001892\_embryonic\_placenta\_development | MAPK1 | 57 | 2 | 3.297530 | -0.911842 | 197 | 166.48 | 0.845076 |
| GO:0001892\_embryonic\_placenta\_development | SP3 | 57 | 2 | 3.297530 | -0.911842 | 197 | 166.48 | 0.845076 |
| GO:0018108\_peptidyl-tyrosine\_phosphorylation | PDGFA | 57 | 2 | 3.297530 | -0.911842 | 197 | 166.48 | 0.845076 |
| GO:0018108\_peptidyl-tyrosine\_phosphorylation | EPHB2 | 57 | 2 | 3.297530 | -0.911842 | 197 | 166.48 | 0.845076 |
| GO:0018212\_peptidyl-tyrosine\_modification | PDGFA | 57 | 2 | 3.297530 | -0.911842 | 197 | 166.48 | 0.845076 |
| GO:0018212\_peptidyl-tyrosine\_modification | EPHB2 | 57 | 2 | 3.297530 | -0.911842 | 197 | 166.48 | 0.845076 |
| GO:0042472\_inner\_ear\_morphogenesis | ITGA8 | 57 | 2 | 3.297530 | -0.911842 | 197 | 166.48 | 0.845076 |
| GO:0042472\_inner\_ear\_morphogenesis | FOXG1 | 57 | 2 | 3.297530 | -0.911842 | 197 | 166.48 | 0.845076 |
| GO:0030902\_hindbrain\_development | DLC1 | 58 | 2 | 3.240676 | -0.899447 | 200 | 167.95 | 0.839750 |
| GO:0030902\_hindbrain\_development | POU4F1 | 58 | 2 | 3.240676 | -0.899447 | 200 | 167.95 | 0.839750 |
| GO:0033043\_regulation\_of\_organelle\_organization | STMN3 | 58 | 2 | 3.240676 | -0.899447 | 200 | 167.95 | 0.839750 |
| GO:0033043\_regulation\_of\_organelle\_organization | TUBG1 | 58 | 2 | 3.240676 | -0.899447 | 200 | 167.95 | 0.839750 |
| GO:0050804\_regulation\_of\_synaptic\_transmission | CPLX3 | 58 | 2 | 3.240676 | -0.899447 | 200 | 167.95 | 0.839750 |
| GO:0050804\_regulation\_of\_synaptic\_transmission | EPHB2 | 58 | 2 | 3.240676 | -0.899447 | 200 | 167.95 | 0.839750 |
| GO:0007267\_cell-cell\_signaling | CPLX3 | 252 | 5 | 1.864674 | -0.893835 | 201 | 168.41 | 0.837861 |
| GO:0007267\_cell-cell\_signaling | PDGFA | 252 | 5 | 1.864674 | -0.893835 | 201 | 168.41 | 0.837861 |
| GO:0007267\_cell-cell\_signaling | SV2B | 252 | 5 | 1.864674 | -0.893835 | 201 | 168.41 | 0.837861 |
| GO:0007267\_cell-cell\_signaling | EPHB2 | 252 | 5 | 1.864674 | -0.893835 | 201 | 168.41 | 0.837861 |
| GO:0007267\_cell-cell\_signaling | LIN7A | 252 | 5 | 1.864674 | -0.893835 | 201 | 168.41 | 0.837861 |
| GO:0065007\_biological\_regulation | FUS | 2593 | 32 | 1.159794 | -0.891525 | 202 | 168.46 | 0.833960 |
| GO:0065007\_biological\_regulation | CPLX3 | 2593 | 32 | 1.159794 | -0.891525 | 202 | 168.46 | 0.833960 |
| GO:0065007\_biological\_regulation | PDGFA | 2593 | 32 | 1.159794 | -0.891525 | 202 | 168.46 | 0.833960 |
| GO:0065007\_biological\_regulation | THOP1 | 2593 | 32 | 1.159794 | -0.891525 | 202 | 168.46 | 0.833960 |
| GO:0065007\_biological\_regulation | JAG1 | 2593 | 32 | 1.159794 | -0.891525 | 202 | 168.46 | 0.833960 |
| GO:0065007\_biological\_regulation | APLP1 | 2593 | 32 | 1.159794 | -0.891525 | 202 | 168.46 | 0.833960 |
| GO:0065007\_biological\_regulation | ZC3H8 | 2593 | 32 | 1.159794 | -0.891525 | 202 | 168.46 | 0.833960 |
| GO:0065007\_biological\_regulation | EPHB2 | 2593 | 32 | 1.159794 | -0.891525 | 202 | 168.46 | 0.833960 |
| GO:0065007\_biological\_regulation | CCNE2 | 2593 | 32 | 1.159794 | -0.891525 | 202 | 168.46 | 0.833960 |
| GO:0065007\_biological\_regulation | POU4F1 | 2593 | 32 | 1.159794 | -0.891525 | 202 | 168.46 | 0.833960 |
| GO:0065007\_biological\_regulation | SV2B | 2593 | 32 | 1.159794 | -0.891525 | 202 | 168.46 | 0.833960 |
| GO:0065007\_biological\_regulation | TUBG1 | 2593 | 32 | 1.159794 | -0.891525 | 202 | 168.46 | 0.833960 |
| GO:0065007\_biological\_regulation | CHUK | 2593 | 32 | 1.159794 | -0.891525 | 202 | 168.46 | 0.833960 |
| GO:0065007\_biological\_regulation | AEBP2 | 2593 | 32 | 1.159794 | -0.891525 | 202 | 168.46 | 0.833960 |
| GO:0065007\_biological\_regulation | TM2D1 | 2593 | 32 | 1.159794 | -0.891525 | 202 | 168.46 | 0.833960 |
| GO:0065007\_biological\_regulation | STMN3 | 2593 | 32 | 1.159794 | -0.891525 | 202 | 168.46 | 0.833960 |
| GO:0065007\_biological\_regulation | MAPK10 | 2593 | 32 | 1.159794 | -0.891525 | 202 | 168.46 | 0.833960 |
| GO:0065007\_biological\_regulation | ARNTL | 2593 | 32 | 1.159794 | -0.891525 | 202 | 168.46 | 0.833960 |
| GO:0065007\_biological\_regulation | NTN1 | 2593 | 32 | 1.159794 | -0.891525 | 202 | 168.46 | 0.833960 |
| GO:0065007\_biological\_regulation | LIN7A | 2593 | 32 | 1.159794 | -0.891525 | 202 | 168.46 | 0.833960 |
| GO:0065007\_biological\_regulation | HBA-A1 | 2593 | 32 | 1.159794 | -0.891525 | 202 | 168.46 | 0.833960 |
| GO:0065007\_biological\_regulation | MAPK1 | 2593 | 32 | 1.159794 | -0.891525 | 202 | 168.46 | 0.833960 |
| GO:0065007\_biological\_regulation | DKK1 | 2593 | 32 | 1.159794 | -0.891525 | 202 | 168.46 | 0.833960 |
| GO:0065007\_biological\_regulation | HIPK1 | 2593 | 32 | 1.159794 | -0.891525 | 202 | 168.46 | 0.833960 |
| GO:0065007\_biological\_regulation | HES5 | 2593 | 32 | 1.159794 | -0.891525 | 202 | 168.46 | 0.833960 |
| GO:0065007\_biological\_regulation | SFRP1 | 2593 | 32 | 1.159794 | -0.891525 | 202 | 168.46 | 0.833960 |
| GO:0065007\_biological\_regulation | SP3 | 2593 | 32 | 1.159794 | -0.891525 | 202 | 168.46 | 0.833960 |
| GO:0065007\_biological\_regulation | ITGA8 | 2593 | 32 | 1.159794 | -0.891525 | 202 | 168.46 | 0.833960 |
| GO:0065007\_biological\_regulation | NAB1 | 2593 | 32 | 1.159794 | -0.891525 | 202 | 168.46 | 0.833960 |
| GO:0065007\_biological\_regulation | FOXG1 | 2593 | 32 | 1.159794 | -0.891525 | 202 | 168.46 | 0.833960 |
| GO:0065007\_biological\_regulation | GNB4 | 2593 | 32 | 1.159794 | -0.891525 | 202 | 168.46 | 0.833960 |
| GO:0065007\_biological\_regulation | SYTL2 | 2593 | 32 | 1.159794 | -0.891525 | 202 | 168.46 | 0.833960 |
| GO:0016055\_Wnt\_receptor\_signaling\_pathway | DKK1 | 59 | 2 | 3.185749 | -0.887313 | 204 | 169.26 | 0.829706 |
| GO:0016055\_Wnt\_receptor\_signaling\_pathway | SFRP1 | 59 | 2 | 3.185749 | -0.887313 | 204 | 169.26 | 0.829706 |
| GO:0048469\_cell\_maturation | HES5 | 59 | 2 | 3.185749 | -0.887313 | 204 | 169.26 | 0.829706 |
| GO:0048469\_cell\_maturation | XBP1 | 59 | 2 | 3.185749 | -0.887313 | 204 | 169.26 | 0.829706 |
| GO:0001958\_endochondral\_ossification | NAB1 | 13 | 1 | 7.229199 | -0.886087 | 213 | 178.39 | 0.837512 |
| GO:0006898\_receptor-mediated\_endocytosis | LRP2 | 13 | 1 | 7.229199 | -0.886087 | 213 | 178.39 | 0.837512 |
| GO:0018105\_peptidyl-serine\_phosphorylation | MAP3K12 | 13 | 1 | 7.229199 | -0.886087 | 213 | 178.39 | 0.837512 |
| GO:0021955\_central\_nervous\_system\_neuron\_axonogenesis | EPHB2 | 13 | 1 | 7.229199 | -0.886087 | 213 | 178.39 | 0.837512 |
| GO:0030516\_regulation\_of\_axon\_extension | NTN1 | 13 | 1 | 7.229199 | -0.886087 | 213 | 178.39 | 0.837512 |
| GO:0031290\_retinal\_ganglion\_cell\_axon\_guidance | EPHB2 | 13 | 1 | 7.229199 | -0.886087 | 213 | 178.39 | 0.837512 |
| GO:0034329\_cell\_junction\_assembly | DLC1 | 13 | 1 | 7.229199 | -0.886087 | 213 | 178.39 | 0.837512 |
| GO:0045682\_regulation\_of\_epidermis\_development | NAB1 | 13 | 1 | 7.229199 | -0.886087 | 213 | 178.39 | 0.837512 |
| GO:0060324\_face\_development | MMP2 | 13 | 1 | 7.229199 | -0.886087 | 213 | 178.39 | 0.837512 |
| GO:0048729\_tissue\_morphogenesis | DLC1 | 255 | 5 | 1.842737 | -0.878042 | 214 | 179.08 | 0.836822 |
| GO:0048729\_tissue\_morphogenesis | SFRP1 | 255 | 5 | 1.842737 | -0.878042 | 214 | 179.08 | 0.836822 |
| GO:0048729\_tissue\_morphogenesis | XBP1 | 255 | 5 | 1.842737 | -0.878042 | 214 | 179.08 | 0.836822 |
| GO:0048729\_tissue\_morphogenesis | JAG1 | 255 | 5 | 1.842737 | -0.878042 | 214 | 179.08 | 0.836822 |
| GO:0048729\_tissue\_morphogenesis | CHUK | 255 | 5 | 1.842737 | -0.878042 | 214 | 179.08 | 0.836822 |
| GO:0009966\_regulation\_of\_signal\_transduction | DKK1 | 256 | 5 | 1.835539 | -0.872845 | 215 | 179.45 | 0.834651 |
| GO:0009966\_regulation\_of\_signal\_transduction | STMN3 | 256 | 5 | 1.835539 | -0.872845 | 215 | 179.45 | 0.834651 |
| GO:0009966\_regulation\_of\_signal\_transduction | ITGA8 | 256 | 5 | 1.835539 | -0.872845 | 215 | 179.45 | 0.834651 |
| GO:0009966\_regulation\_of\_signal\_transduction | JAG1 | 256 | 5 | 1.835539 | -0.872845 | 215 | 179.45 | 0.834651 |
| GO:0009966\_regulation\_of\_signal\_transduction | CHUK | 256 | 5 | 1.835539 | -0.872845 | 215 | 179.45 | 0.834651 |
| GO:0051093\_negative\_regulation\_of\_developmental\_process | MAPK1 | 331 | 6 | 1.703558 | -0.864239 | 216 | 180.77 | 0.836898 |
| GO:0051093\_negative\_regulation\_of\_developmental\_process | HES5 | 331 | 6 | 1.703558 | -0.864239 | 216 | 180.77 | 0.836898 |
| GO:0051093\_negative\_regulation\_of\_developmental\_process | FOXG1 | 331 | 6 | 1.703558 | -0.864239 | 216 | 180.77 | 0.836898 |
| GO:0051093\_negative\_regulation\_of\_developmental\_process | JAG1 | 331 | 6 | 1.703558 | -0.864239 | 216 | 180.77 | 0.836898 |
| GO:0051093\_negative\_regulation\_of\_developmental\_process | NTN1 | 331 | 6 | 1.703558 | -0.864239 | 216 | 180.77 | 0.836898 |
| GO:0051093\_negative\_regulation\_of\_developmental\_process | EPHB2 | 331 | 6 | 1.703558 | -0.864239 | 216 | 180.77 | 0.836898 |
| GO:0051969\_regulation\_of\_transmission\_of\_nerve\_impulse | CPLX3 | 61 | 2 | 3.081298 | -0.863790 | 217 | 181.37 | 0.835806 |
| GO:0051969\_regulation\_of\_transmission\_of\_nerve\_impulse | EPHB2 | 61 | 2 | 3.081298 | -0.863790 | 217 | 181.37 | 0.835806 |
| GO:0051726\_regulation\_of\_cell\_cycle | CCNE2 | 121 | 3 | 2.330073 | -0.863563 | 218 | 181.74 | 0.833670 |
| GO:0051726\_regulation\_of\_cell\_cycle | FOXG1 | 121 | 3 | 2.330073 | -0.863563 | 218 | 181.74 | 0.833670 |
| GO:0051726\_regulation\_of\_cell\_cycle | TUBG1 | 121 | 3 | 2.330073 | -0.863563 | 218 | 181.74 | 0.833670 |
| GO:0000060\_protein\_import\_into\_nucleus\_\_translocation | ARNTL | 14 | 1 | 6.712828 | -0.856128 | 228 | 190.45 | 0.835307 |
| GO:0001829\_trophectodermal\_cell\_differentiation | SP3 | 14 | 1 | 6.712828 | -0.856128 | 228 | 190.45 | 0.835307 |
| GO:0007589\_body\_fluid\_secretion | SYTL2 | 14 | 1 | 6.712828 | -0.856128 | 228 | 190.45 | 0.835307 |
| GO:0008630\_DNA\_damage\_response\_\_signal\_transduction\_resulting\_in\_induction\_of\_apoptosis | HIPK1 | 14 | 1 | 6.712828 | -0.856128 | 228 | 190.45 | 0.835307 |
| GO:0031346\_positive\_regulation\_of\_cell\_projection\_organization | NTN1 | 14 | 1 | 6.712828 | -0.856128 | 228 | 190.45 | 0.835307 |
| GO:0031663\_lipopolysaccharide-mediated\_signaling\_pathway | MAPK1 | 14 | 1 | 6.712828 | -0.856128 | 228 | 190.45 | 0.835307 |
| GO:0043123\_positive\_regulation\_of\_I-kappaB\_kinase\_NF-kappaB\_cascade | CHUK | 14 | 1 | 6.712828 | -0.856128 | 228 | 190.45 | 0.835307 |
| GO:0044236\_multicellular\_organismal\_metabolic\_process | MMP2 | 14 | 1 | 6.712828 | -0.856128 | 228 | 190.45 | 0.835307 |
| GO:0048048\_embryonic\_eye\_morphogenesis | SP3 | 14 | 1 | 6.712828 | -0.856128 | 228 | 190.45 | 0.835307 |
| GO:0060716\_labyrinthine\_layer\_blood\_vessel\_development | MAPK1 | 14 | 1 | 6.712828 | -0.856128 | 228 | 190.45 | 0.835307 |
| GO:0022604\_regulation\_of\_cell\_morphogenesis | NTN1 | 62 | 2 | 3.031600 | -0.852385 | 229 | 192.33 | 0.839869 |
| GO:0022604\_regulation\_of\_cell\_morphogenesis | EPHB2 | 62 | 2 | 3.031600 | -0.852385 | 229 | 192.33 | 0.839869 |
| GO:0048522\_positive\_regulation\_of\_cellular\_process | FUS | 895 | 13 | 1.365067 | -0.851687 | 230 | 192.36 | 0.836348 |
| GO:0048522\_positive\_regulation\_of\_cellular\_process | TM2D1 | 895 | 13 | 1.365067 | -0.851687 | 230 | 192.36 | 0.836348 |
| GO:0048522\_positive\_regulation\_of\_cellular\_process | PDGFA | 895 | 13 | 1.365067 | -0.851687 | 230 | 192.36 | 0.836348 |
| GO:0048522\_positive\_regulation\_of\_cellular\_process | JAG1 | 895 | 13 | 1.365067 | -0.851687 | 230 | 192.36 | 0.836348 |
| GO:0048522\_positive\_regulation\_of\_cellular\_process | ARNTL | 895 | 13 | 1.365067 | -0.851687 | 230 | 192.36 | 0.836348 |
| GO:0048522\_positive\_regulation\_of\_cellular\_process | NTN1 | 895 | 13 | 1.365067 | -0.851687 | 230 | 192.36 | 0.836348 |
| GO:0048522\_positive\_regulation\_of\_cellular\_process | EPHB2 | 895 | 13 | 1.365067 | -0.851687 | 230 | 192.36 | 0.836348 |
| GO:0048522\_positive\_regulation\_of\_cellular\_process | HIPK1 | 895 | 13 | 1.365067 | -0.851687 | 230 | 192.36 | 0.836348 |
| GO:0048522\_positive\_regulation\_of\_cellular\_process | HES5 | 895 | 13 | 1.365067 | -0.851687 | 230 | 192.36 | 0.836348 |
| GO:0048522\_positive\_regulation\_of\_cellular\_process | ITGA8 | 895 | 13 | 1.365067 | -0.851687 | 230 | 192.36 | 0.836348 |
| GO:0048522\_positive\_regulation\_of\_cellular\_process | FOXG1 | 895 | 13 | 1.365067 | -0.851687 | 230 | 192.36 | 0.836348 |
| GO:0048522\_positive\_regulation\_of\_cellular\_process | POU4F1 | 895 | 13 | 1.365067 | -0.851687 | 230 | 192.36 | 0.836348 |
| GO:0048522\_positive\_regulation\_of\_cellular\_process | CHUK | 895 | 13 | 1.365067 | -0.851687 | 230 | 192.36 | 0.836348 |
| GO:0009790\_embryonic\_development | DLC1 | 567 | 9 | 1.491740 | -0.850271 | 231 | 192.47 | 0.833203 |
| GO:0009790\_embryonic\_development | HBA-A1 | 567 | 9 | 1.491740 | -0.850271 | 231 | 192.47 | 0.833203 |
| GO:0009790\_embryonic\_development | MAPK1 | 567 | 9 | 1.491740 | -0.850271 | 231 | 192.47 | 0.833203 |
| GO:0009790\_embryonic\_development | DKK1 | 567 | 9 | 1.491740 | -0.850271 | 231 | 192.47 | 0.833203 |
| GO:0009790\_embryonic\_development | SFRP1 | 567 | 9 | 1.491740 | -0.850271 | 231 | 192.47 | 0.833203 |
| GO:0009790\_embryonic\_development | ITGA8 | 567 | 9 | 1.491740 | -0.850271 | 231 | 192.47 | 0.833203 |
| GO:0009790\_embryonic\_development | SP3 | 567 | 9 | 1.491740 | -0.850271 | 231 | 192.47 | 0.833203 |
| GO:0009790\_embryonic\_development | FOXG1 | 567 | 9 | 1.491740 | -0.850271 | 231 | 192.47 | 0.833203 |
| GO:0009790\_embryonic\_development | ODZ4 | 567 | 9 | 1.491740 | -0.850271 | 231 | 192.47 | 0.833203 |
| GO:0031644\_regulation\_of\_neurological\_system\_process | CPLX3 | 64 | 2 | 2.936862 | -0.830250 | 232 | 194.98 | 0.840431 |
| GO:0031644\_regulation\_of\_neurological\_system\_process | EPHB2 | 64 | 2 | 2.936862 | -0.830250 | 232 | 194.98 | 0.840431 |
| GO:0010171\_body\_morphogenesis | MMP2 | 15 | 1 | 6.265306 | -0.828387 | 237 | 202.96 | 0.856371 |
| GO:0031076\_embryonic\_camera-type\_eye\_development | SP3 | 15 | 1 | 6.265306 | -0.828387 | 237 | 202.96 | 0.856371 |
| GO:0060322\_head\_development | MMP2 | 15 | 1 | 6.265306 | -0.828387 | 237 | 202.96 | 0.856371 |
| GO:0060442\_branching\_involved\_in\_prostate\_gland\_morphogenesis | SFRP1 | 15 | 1 | 6.265306 | -0.828387 | 237 | 202.96 | 0.856371 |
| GO:0060749\_mammary\_gland\_alveolus\_development | CHUK | 15 | 1 | 6.265306 | -0.828387 | 237 | 202.96 | 0.856371 |
| GO:0006793\_phosphorus\_metabolic\_process | MAPK1 | 340 | 6 | 1.658463 | -0.824685 | 239 | 203.21 | 0.850251 |
| GO:0006793\_phosphorus\_metabolic\_process | HIPK1 | 340 | 6 | 1.658463 | -0.824685 | 239 | 203.21 | 0.850251 |
| GO:0006793\_phosphorus\_metabolic\_process | PDGFA | 340 | 6 | 1.658463 | -0.824685 | 239 | 203.21 | 0.850251 |
| GO:0006793\_phosphorus\_metabolic\_process | MAP3K12 | 340 | 6 | 1.658463 | -0.824685 | 239 | 203.21 | 0.850251 |
| GO:0006793\_phosphorus\_metabolic\_process | CHUK | 340 | 6 | 1.658463 | -0.824685 | 239 | 203.21 | 0.850251 |
| GO:0006793\_phosphorus\_metabolic\_process | EPHB2 | 340 | 6 | 1.658463 | -0.824685 | 239 | 203.21 | 0.850251 |
| GO:0006796\_phosphate\_metabolic\_process | MAPK1 | 340 | 6 | 1.658463 | -0.824685 | 239 | 203.21 | 0.850251 |
| GO:0006796\_phosphate\_metabolic\_process | HIPK1 | 340 | 6 | 1.658463 | -0.824685 | 239 | 203.21 | 0.850251 |
| GO:0006796\_phosphate\_metabolic\_process | PDGFA | 340 | 6 | 1.658463 | -0.824685 | 239 | 203.21 | 0.850251 |
| GO:0006796\_phosphate\_metabolic\_process | MAP3K12 | 340 | 6 | 1.658463 | -0.824685 | 239 | 203.21 | 0.850251 |
| GO:0006796\_phosphate\_metabolic\_process | CHUK | 340 | 6 | 1.658463 | -0.824685 | 239 | 203.21 | 0.850251 |
| GO:0006796\_phosphate\_metabolic\_process | EPHB2 | 340 | 6 | 1.658463 | -0.824685 | 239 | 203.21 | 0.850251 |
| GO:0042471\_ear\_morphogenesis | ITGA8 | 65 | 2 | 2.891680 | -0.819506 | 240 | 204.06 | 0.850250 |
| GO:0042471\_ear\_morphogenesis | FOXG1 | 65 | 2 | 2.891680 | -0.819506 | 240 | 204.06 | 0.850250 |
| GO:0043087\_regulation\_of\_GTPase\_activity | STMN3 | 16 | 1 | 5.873724 | -0.802577 | 244 | 213.74 | 0.875984 |
| GO:0043122\_regulation\_of\_I-kappaB\_kinase\_NF-kappaB\_cascade | CHUK | 16 | 1 | 5.873724 | -0.802577 | 244 | 213.74 | 0.875984 |
| GO:0048286\_lung\_alveolus\_development | PDGFA | 16 | 1 | 5.873724 | -0.802577 | 244 | 213.74 | 0.875984 |
| GO:0050974\_detection\_of\_mechanical\_stimulus\_involved\_in\_sensory\_perception | GPR98 | 16 | 1 | 5.873724 | -0.802577 | 244 | 213.74 | 0.875984 |
| GO:0007165\_signal\_transduction | STMN3 | 915 | 13 | 1.335229 | -0.797558 | 245 | 214.92 | 0.877224 |
| GO:0007165\_signal\_transduction | THOP1 | 915 | 13 | 1.335229 | -0.797558 | 245 | 214.92 | 0.877224 |
| GO:0007165\_signal\_transduction | JAG1 | 915 | 13 | 1.335229 | -0.797558 | 245 | 214.92 | 0.877224 |
| GO:0007165\_signal\_transduction | MAPK10 | 915 | 13 | 1.335229 | -0.797558 | 245 | 214.92 | 0.877224 |
| GO:0007165\_signal\_transduction | EPHB2 | 915 | 13 | 1.335229 | -0.797558 | 245 | 214.92 | 0.877224 |
| GO:0007165\_signal\_transduction | MAPK1 | 915 | 13 | 1.335229 | -0.797558 | 245 | 214.92 | 0.877224 |
| GO:0007165\_signal\_transduction | DKK1 | 915 | 13 | 1.335229 | -0.797558 | 245 | 214.92 | 0.877224 |
| GO:0007165\_signal\_transduction | SFRP1 | 915 | 13 | 1.335229 | -0.797558 | 245 | 214.92 | 0.877224 |
| GO:0007165\_signal\_transduction | HIPK1 | 915 | 13 | 1.335229 | -0.797558 | 245 | 214.92 | 0.877224 |
| GO:0007165\_signal\_transduction | HES5 | 915 | 13 | 1.335229 | -0.797558 | 245 | 214.92 | 0.877224 |
| GO:0007165\_signal\_transduction | ITGA8 | 915 | 13 | 1.335229 | -0.797558 | 245 | 214.92 | 0.877224 |
| GO:0007165\_signal\_transduction | GNB4 | 915 | 13 | 1.335229 | -0.797558 | 245 | 214.92 | 0.877224 |
| GO:0007165\_signal\_transduction | CHUK | 915 | 13 | 1.335229 | -0.797558 | 245 | 214.92 | 0.877224 |
| GO:0006261\_DNA-dependent\_DNA\_replication | CCNE2 | 17 | 1 | 5.528211 | -0.778463 | 250 | 224.52 | 0.898080 |
| GO:0021545\_cranial\_nerve\_development | EPHB2 | 17 | 1 | 5.528211 | -0.778463 | 250 | 224.52 | 0.898080 |
| GO:0030101\_natural\_killer\_cell\_activation | SP3 | 17 | 1 | 5.528211 | -0.778463 | 250 | 224.52 | 0.898080 |
| GO:0048168\_regulation\_of\_neuronal\_synaptic\_plasticity | EPHB2 | 17 | 1 | 5.528211 | -0.778463 | 250 | 224.52 | 0.898080 |
| GO:0060350\_endochondral\_bone\_morphogenesis | NAB1 | 17 | 1 | 5.528211 | -0.778463 | 250 | 224.52 | 0.898080 |
| GO:0007611\_learning\_or\_memory | ITGA8 | 70 | 2 | 2.685131 | -0.768759 | 252 | 226.46 | 0.898651 |
| GO:0007611\_learning\_or\_memory | EPHB2 | 70 | 2 | 2.685131 | -0.768759 | 252 | 226.46 | 0.898651 |
| GO:0048592\_eye\_morphogenesis | SP3 | 70 | 2 | 2.685131 | -0.768759 | 252 | 226.46 | 0.898651 |
| GO:0048592\_eye\_morphogenesis | EPHB2 | 70 | 2 | 2.685131 | -0.768759 | 252 | 226.46 | 0.898651 |
| GO:0007243\_protein\_kinase\_cascade | MAPK1 | 205 | 4 | 1.833748 | -0.763575 | 253 | 226.67 | 0.895929 |
| GO:0007243\_protein\_kinase\_cascade | THOP1 | 205 | 4 | 1.833748 | -0.763575 | 253 | 226.67 | 0.895929 |
| GO:0007243\_protein\_kinase\_cascade | MAPK10 | 205 | 4 | 1.833748 | -0.763575 | 253 | 226.67 | 0.895929 |
| GO:0007243\_protein\_kinase\_cascade | CHUK | 205 | 4 | 1.833748 | -0.763575 | 253 | 226.67 | 0.895929 |
| GO:0001825\_blastocyst\_formation | SP3 | 18 | 1 | 5.221088 | -0.755851 | 260 | 237.21 | 0.912346 |
| GO:0002064\_epithelial\_cell\_development | XBP1 | 18 | 1 | 5.221088 | -0.755851 | 260 | 237.21 | 0.912346 |
| GO:0010553\_negative\_regulation\_of\_specific\_transcription\_from\_RNA\_polymerase\_II\_promoter | HES5 | 18 | 1 | 5.221088 | -0.755851 | 260 | 237.21 | 0.912346 |
| GO:0030178\_negative\_regulation\_of\_Wnt\_receptor\_signaling\_pathway | DKK1 | 18 | 1 | 5.221088 | -0.755851 | 260 | 237.21 | 0.912346 |
| GO:0046578\_regulation\_of\_Ras\_protein\_signal\_transduction | STMN3 | 18 | 1 | 5.221088 | -0.755851 | 260 | 237.21 | 0.912346 |
| GO:0050982\_detection\_of\_mechanical\_stimulus | GPR98 | 18 | 1 | 5.221088 | -0.755851 | 260 | 237.21 | 0.912346 |
| GO:0060674\_placenta\_blood\_vessel\_development | MAPK1 | 18 | 1 | 5.221088 | -0.755851 | 260 | 237.21 | 0.912346 |
| GO:0007166\_cell\_surface\_receptor\_linked\_signal\_transduction | MAPK1 | 597 | 9 | 1.416778 | -0.753362 | 261 | 237.25 | 0.909004 |
| GO:0007166\_cell\_surface\_receptor\_linked\_signal\_transduction | DKK1 | 597 | 9 | 1.416778 | -0.753362 | 261 | 237.25 | 0.909004 |
| GO:0007166\_cell\_surface\_receptor\_linked\_signal\_transduction | HES5 | 597 | 9 | 1.416778 | -0.753362 | 261 | 237.25 | 0.909004 |
| GO:0007166\_cell\_surface\_receptor\_linked\_signal\_transduction | HIPK1 | 597 | 9 | 1.416778 | -0.753362 | 261 | 237.25 | 0.909004 |
| GO:0007166\_cell\_surface\_receptor\_linked\_signal\_transduction | SFRP1 | 597 | 9 | 1.416778 | -0.753362 | 261 | 237.25 | 0.909004 |
| GO:0007166\_cell\_surface\_receptor\_linked\_signal\_transduction | ITGA8 | 597 | 9 | 1.416778 | -0.753362 | 261 | 237.25 | 0.909004 |
| GO:0007166\_cell\_surface\_receptor\_linked\_signal\_transduction | GNB4 | 597 | 9 | 1.416778 | -0.753362 | 261 | 237.25 | 0.909004 |
| GO:0007166\_cell\_surface\_receptor\_linked\_signal\_transduction | JAG1 | 597 | 9 | 1.416778 | -0.753362 | 261 | 237.25 | 0.909004 |
| GO:0007166\_cell\_surface\_receptor\_linked\_signal\_transduction | EPHB2 | 597 | 9 | 1.416778 | -0.753362 | 261 | 237.25 | 0.909004 |
| GO:0003008\_system\_process | CPLX3 | 516 | 8 | 1.457048 | -0.752481 | 262 | 237.36 | 0.905954 |
| GO:0003008\_system\_process | ITGA8 | 516 | 8 | 1.457048 | -0.752481 | 262 | 237.36 | 0.905954 |
| GO:0003008\_system\_process | NAB1 | 516 | 8 | 1.457048 | -0.752481 | 262 | 237.36 | 0.905954 |
| GO:0003008\_system\_process | POU4F1 | 516 | 8 | 1.457048 | -0.752481 | 262 | 237.36 | 0.905954 |
| GO:0003008\_system\_process | SV2B | 516 | 8 | 1.457048 | -0.752481 | 262 | 237.36 | 0.905954 |
| GO:0003008\_system\_process | GPR98 | 516 | 8 | 1.457048 | -0.752481 | 262 | 237.36 | 0.905954 |
| GO:0003008\_system\_process | EPHB2 | 516 | 8 | 1.457048 | -0.752481 | 262 | 237.36 | 0.905954 |
| GO:0003008\_system\_process | LIN7A | 516 | 8 | 1.457048 | -0.752481 | 262 | 237.36 | 0.905954 |
| GO:0030879\_mammary\_gland\_development | NTN1 | 72 | 2 | 2.610544 | -0.749741 | 263 | 239.1 | 0.909125 |
| GO:0030879\_mammary\_gland\_development | CHUK | 72 | 2 | 2.610544 | -0.749741 | 263 | 239.1 | 0.909125 |
| GO:0008284\_positive\_regulation\_of\_cell\_proliferation | HIPK1 | 208 | 4 | 1.807300 | -0.747892 | 264 | 239.45 | 0.907008 |
| GO:0008284\_positive\_regulation\_of\_cell\_proliferation | PDGFA | 208 | 4 | 1.807300 | -0.747892 | 264 | 239.45 | 0.907008 |
| GO:0008284\_positive\_regulation\_of\_cell\_proliferation | FOXG1 | 208 | 4 | 1.807300 | -0.747892 | 264 | 239.45 | 0.907008 |
| GO:0008284\_positive\_regulation\_of\_cell\_proliferation | NTN1 | 208 | 4 | 1.807300 | -0.747892 | 264 | 239.45 | 0.907008 |
| GO:0009987\_cellular\_process | FUS | 3868 | 44 | 1.069054 | -0.742335 | 265 | 239.99 | 0.905623 |
| GO:0009987\_cellular\_process | DLC1 | 3868 | 44 | 1.069054 | -0.742335 | 265 | 239.99 | 0.905623 |
| GO:0009987\_cellular\_process | CPLX3 | 3868 | 44 | 1.069054 | -0.742335 | 265 | 239.99 | 0.905623 |
| GO:0009987\_cellular\_process | PDGFA | 3868 | 44 | 1.069054 | -0.742335 | 265 | 239.99 | 0.905623 |
| GO:0009987\_cellular\_process | THOP1 | 3868 | 44 | 1.069054 | -0.742335 | 265 | 239.99 | 0.905623 |
| GO:0009987\_cellular\_process | COL3A1 | 3868 | 44 | 1.069054 | -0.742335 | 265 | 239.99 | 0.905623 |
| GO:0009987\_cellular\_process | JAG1 | 3868 | 44 | 1.069054 | -0.742335 | 265 | 239.99 | 0.905623 |
| GO:0009987\_cellular\_process | ZC3H8 | 3868 | 44 | 1.069054 | -0.742335 | 265 | 239.99 | 0.905623 |
| GO:0009987\_cellular\_process | APLP1 | 3868 | 44 | 1.069054 | -0.742335 | 265 | 239.99 | 0.905623 |
| GO:0009987\_cellular\_process | EPHB2 | 3868 | 44 | 1.069054 | -0.742335 | 265 | 239.99 | 0.905623 |
| GO:0009987\_cellular\_process | CCNE2 | 3868 | 44 | 1.069054 | -0.742335 | 265 | 239.99 | 0.905623 |
| GO:0009987\_cellular\_process | HEY1 | 3868 | 44 | 1.069054 | -0.742335 | 265 | 239.99 | 0.905623 |
| GO:0009987\_cellular\_process | XBP1 | 3868 | 44 | 1.069054 | -0.742335 | 265 | 239.99 | 0.905623 |
| GO:0009987\_cellular\_process | ANK3 | 3868 | 44 | 1.069054 | -0.742335 | 265 | 239.99 | 0.905623 |
| GO:0009987\_cellular\_process | KATNA1 | 3868 | 44 | 1.069054 | -0.742335 | 265 | 239.99 | 0.905623 |
| GO:0009987\_cellular\_process | SV2B | 3868 | 44 | 1.069054 | -0.742335 | 265 | 239.99 | 0.905623 |
| GO:0009987\_cellular\_process | POU4F1 | 3868 | 44 | 1.069054 | -0.742335 | 265 | 239.99 | 0.905623 |
| GO:0009987\_cellular\_process | TUBG1 | 3868 | 44 | 1.069054 | -0.742335 | 265 | 239.99 | 0.905623 |
| GO:0009987\_cellular\_process | CHUK | 3868 | 44 | 1.069054 | -0.742335 | 265 | 239.99 | 0.905623 |
| GO:0009987\_cellular\_process | SCAMP1 | 3868 | 44 | 1.069054 | -0.742335 | 265 | 239.99 | 0.905623 |
| GO:0009987\_cellular\_process | AEBP2 | 3868 | 44 | 1.069054 | -0.742335 | 265 | 239.99 | 0.905623 |
| GO:0009987\_cellular\_process | TM2D1 | 3868 | 44 | 1.069054 | -0.742335 | 265 | 239.99 | 0.905623 |
| GO:0009987\_cellular\_process | STMN3 | 3868 | 44 | 1.069054 | -0.742335 | 265 | 239.99 | 0.905623 |
| GO:0009987\_cellular\_process | KIF5C | 3868 | 44 | 1.069054 | -0.742335 | 265 | 239.99 | 0.905623 |
| GO:0009987\_cellular\_process | ARNTL | 3868 | 44 | 1.069054 | -0.742335 | 265 | 239.99 | 0.905623 |
| GO:0009987\_cellular\_process | MAPK10 | 3868 | 44 | 1.069054 | -0.742335 | 265 | 239.99 | 0.905623 |
| GO:0009987\_cellular\_process | NTN1 | 3868 | 44 | 1.069054 | -0.742335 | 265 | 239.99 | 0.905623 |
| GO:0009987\_cellular\_process | GPR98 | 3868 | 44 | 1.069054 | -0.742335 | 265 | 239.99 | 0.905623 |
| GO:0009987\_cellular\_process | LIN7A | 3868 | 44 | 1.069054 | -0.742335 | 265 | 239.99 | 0.905623 |
| GO:0009987\_cellular\_process | HBA-A1 | 3868 | 44 | 1.069054 | -0.742335 | 265 | 239.99 | 0.905623 |
| GO:0009987\_cellular\_process | EPHA4 | 3868 | 44 | 1.069054 | -0.742335 | 265 | 239.99 | 0.905623 |
| GO:0009987\_cellular\_process | MAPK1 | 3868 | 44 | 1.069054 | -0.742335 | 265 | 239.99 | 0.905623 |
| GO:0009987\_cellular\_process | DKK1 | 3868 | 44 | 1.069054 | -0.742335 | 265 | 239.99 | 0.905623 |
| GO:0009987\_cellular\_process | HIPK1 | 3868 | 44 | 1.069054 | -0.742335 | 265 | 239.99 | 0.905623 |
| GO:0009987\_cellular\_process | HES5 | 3868 | 44 | 1.069054 | -0.742335 | 265 | 239.99 | 0.905623 |
| GO:0009987\_cellular\_process | SFRP1 | 3868 | 44 | 1.069054 | -0.742335 | 265 | 239.99 | 0.905623 |
| GO:0009987\_cellular\_process | ITGA8 | 3868 | 44 | 1.069054 | -0.742335 | 265 | 239.99 | 0.905623 |
| GO:0009987\_cellular\_process | SP3 | 3868 | 44 | 1.069054 | -0.742335 | 265 | 239.99 | 0.905623 |
| GO:0009987\_cellular\_process | NAB1 | 3868 | 44 | 1.069054 | -0.742335 | 265 | 239.99 | 0.905623 |
| GO:0009987\_cellular\_process | FOXG1 | 3868 | 44 | 1.069054 | -0.742335 | 265 | 239.99 | 0.905623 |
| GO:0009987\_cellular\_process | GNB4 | 3868 | 44 | 1.069054 | -0.742335 | 265 | 239.99 | 0.905623 |
| GO:0009987\_cellular\_process | SYTL2 | 3868 | 44 | 1.069054 | -0.742335 | 265 | 239.99 | 0.905623 |
| GO:0009987\_cellular\_process | LRP2 | 3868 | 44 | 1.069054 | -0.742335 | 265 | 239.99 | 0.905623 |
| GO:0009987\_cellular\_process | MAP3K12 | 3868 | 44 | 1.069054 | -0.742335 | 265 | 239.99 | 0.905623 |
| GO:0007595\_lactation | CHUK | 19 | 1 | 4.946294 | -0.734577 | 268 | 246.8 | 0.920896 |
| GO:0030199\_collagen\_fibril\_organization | COL3A1 | 19 | 1 | 4.946294 | -0.734577 | 268 | 246.8 | 0.920896 |
| GO:0051056\_regulation\_of\_small\_GTPase\_mediated\_signal\_transduction | STMN3 | 19 | 1 | 4.946294 | -0.734577 | 268 | 246.8 | 0.920896 |
| GO:0006518\_peptide\_metabolic\_process | THOP1 | 20 | 1 | 4.698980 | -0.714505 | 272 | 255.09 | 0.937831 |
| GO:0018209\_peptidyl-serine\_modification | MAP3K12 | 20 | 1 | 4.698980 | -0.714505 | 272 | 255.09 | 0.937831 |
| GO:0032582\_negative\_regulation\_of\_gene-specific\_transcription | HES5 | 20 | 1 | 4.698980 | -0.714505 | 272 | 255.09 | 0.937831 |
| GO:0045639\_positive\_regulation\_of\_myeloid\_cell\_differentiation | JAG1 | 20 | 1 | 4.698980 | -0.714505 | 272 | 255.09 | 0.937831 |
| GO:0001890\_placenta\_development | MAPK1 | 77 | 2 | 2.441028 | -0.705050 | 273 | 257.06 | 0.941612 |
| GO:0001890\_placenta\_development | SP3 | 77 | 2 | 2.441028 | -0.705050 | 273 | 257.06 | 0.941612 |
| GO:0001702\_gastrulation\_with\_mouth\_forming\_second | ODZ4 | 21 | 1 | 4.475219 | -0.695516 | 280 | 266.24 | 0.950857 |
| GO:0001709\_cell\_fate\_determination | HES5 | 21 | 1 | 4.475219 | -0.695516 | 280 | 266.24 | 0.950857 |
| GO:0002053\_positive\_regulation\_of\_mesenchymal\_cell\_proliferation | PDGFA | 21 | 1 | 4.475219 | -0.695516 | 280 | 266.24 | 0.950857 |
| GO:0010552\_positive\_regulation\_of\_specific\_transcription\_from\_RNA\_polymerase\_II\_promoter | HES5 | 21 | 1 | 4.475219 | -0.695516 | 280 | 266.24 | 0.950857 |
| GO:0034330\_cell\_junction\_organization | DLC1 | 21 | 1 | 4.475219 | -0.695516 | 280 | 266.24 | 0.950857 |
| GO:0048675\_axon\_extension | NTN1 | 21 | 1 | 4.475219 | -0.695516 | 280 | 266.24 | 0.950857 |
| GO:0051656\_establishment\_of\_organelle\_localization | NTN1 | 21 | 1 | 4.475219 | -0.695516 | 280 | 266.24 | 0.950857 |
| GO:0051046\_regulation\_of\_secretion | CPLX3 | 79 | 2 | 2.379230 | -0.688224 | 281 | 268.0 | 0.953737 |
| GO:0051046\_regulation\_of\_secretion | SYTL2 | 79 | 2 | 2.379230 | -0.688224 | 281 | 268.0 | 0.953737 |
| GO:0000278\_mitotic\_cell\_cycle | FOXG1 | 80 | 2 | 2.349490 | -0.680021 | 282 | 269.67 | 0.956277 |
| GO:0000278\_mitotic\_cell\_cycle | TUBG1 | 80 | 2 | 2.349490 | -0.680021 | 282 | 269.67 | 0.956277 |
| GO:0008624\_induction\_of\_apoptosis\_by\_extracellular\_signals | TM2D1 | 22 | 1 | 4.271800 | -0.677509 | 289 | 278.52 | 0.963737 |
| GO:0010463\_mesenchymal\_cell\_proliferation | PDGFA | 22 | 1 | 4.271800 | -0.677509 | 289 | 278.52 | 0.963737 |
| GO:0010464\_regulation\_of\_mesenchymal\_cell\_proliferation | PDGFA | 22 | 1 | 4.271800 | -0.677509 | 289 | 278.52 | 0.963737 |
| GO:0021575\_hindbrain\_morphogenesis | DLC1 | 22 | 1 | 4.271800 | -0.677509 | 289 | 278.52 | 0.963737 |
| GO:0021675\_nerve\_development | EPHB2 | 22 | 1 | 4.271800 | -0.677509 | 289 | 278.52 | 0.963737 |
| GO:0030316\_osteoclast\_differentiation | CHUK | 22 | 1 | 4.271800 | -0.677509 | 289 | 278.52 | 0.963737 |
| GO:0045787\_positive\_regulation\_of\_cell\_cycle | FOXG1 | 22 | 1 | 4.271800 | -0.677509 | 289 | 278.52 | 0.963737 |
| GO:0006397\_mRNA\_processing | APLP1 | 23 | 1 | 4.086069 | -0.660396 | 294 | 285.17 | 0.969966 |
| GO:0043388\_positive\_regulation\_of\_DNA\_binding | HIPK1 | 23 | 1 | 4.086069 | -0.660396 | 294 | 285.17 | 0.969966 |
| GO:0051705\_behavioral\_interaction\_between\_organisms | POU4F1 | 23 | 1 | 4.086069 | -0.660396 | 294 | 285.17 | 0.969966 |
| GO:0060349\_bone\_morphogenesis | NAB1 | 23 | 1 | 4.086069 | -0.660396 | 294 | 285.17 | 0.969966 |
| GO:0060445\_branching\_involved\_in\_salivary\_gland\_morphogenesis | PDGFA | 23 | 1 | 4.086069 | -0.660396 | 294 | 285.17 | 0.969966 |
| GO:0043687\_post-translational\_protein\_modification | MAPK1 | 384 | 6 | 1.468431 | -0.655640 | 295 | 286.67 | 0.971763 |
| GO:0043687\_post-translational\_protein\_modification | HIPK1 | 384 | 6 | 1.468431 | -0.655640 | 295 | 286.67 | 0.971763 |
| GO:0043687\_post-translational\_protein\_modification | PDGFA | 384 | 6 | 1.468431 | -0.655640 | 295 | 286.67 | 0.971763 |
| GO:0043687\_post-translational\_protein\_modification | MAP3K12 | 384 | 6 | 1.468431 | -0.655640 | 295 | 286.67 | 0.971763 |
| GO:0043687\_post-translational\_protein\_modification | CHUK | 384 | 6 | 1.468431 | -0.655640 | 295 | 286.67 | 0.971763 |
| GO:0043687\_post-translational\_protein\_modification | EPHB2 | 384 | 6 | 1.468431 | -0.655640 | 295 | 286.67 | 0.971763 |
| GO:0034961\_cellular\_biopolymer\_biosynthetic\_process | FUS | 804 | 11 | 1.285790 | -0.647298 | 296 | 288.48 | 0.974595 |
| GO:0034961\_cellular\_biopolymer\_biosynthetic\_process | CCNE2 | 804 | 11 | 1.285790 | -0.647298 | 296 | 288.48 | 0.974595 |
| GO:0034961\_cellular\_biopolymer\_biosynthetic\_process | AEBP2 | 804 | 11 | 1.285790 | -0.647298 | 296 | 288.48 | 0.974595 |
| GO:0034961\_cellular\_biopolymer\_biosynthetic\_process | HIPK1 | 804 | 11 | 1.285790 | -0.647298 | 296 | 288.48 | 0.974595 |
| GO:0034961\_cellular\_biopolymer\_biosynthetic\_process | HES5 | 804 | 11 | 1.285790 | -0.647298 | 296 | 288.48 | 0.974595 |
| GO:0034961\_cellular\_biopolymer\_biosynthetic\_process | SP3 | 804 | 11 | 1.285790 | -0.647298 | 296 | 288.48 | 0.974595 |
| GO:0034961\_cellular\_biopolymer\_biosynthetic\_process | NAB1 | 804 | 11 | 1.285790 | -0.647298 | 296 | 288.48 | 0.974595 |
| GO:0034961\_cellular\_biopolymer\_biosynthetic\_process | POU4F1 | 804 | 11 | 1.285790 | -0.647298 | 296 | 288.48 | 0.974595 |
| GO:0034961\_cellular\_biopolymer\_biosynthetic\_process | ARNTL | 804 | 11 | 1.285790 | -0.647298 | 296 | 288.48 | 0.974595 |
| GO:0034961\_cellular\_biopolymer\_biosynthetic\_process | APLP1 | 804 | 11 | 1.285790 | -0.647298 | 296 | 288.48 | 0.974595 |
| GO:0034961\_cellular\_biopolymer\_biosynthetic\_process | ZC3H8 | 804 | 11 | 1.285790 | -0.647298 | 296 | 288.48 | 0.974595 |
| GO:0000280\_nuclear\_division | TUBG1 | 24 | 1 | 3.915816 | -0.644102 | 303 | 294.49 | 0.971914 |
| GO:0007067\_mitosis | TUBG1 | 24 | 1 | 3.915816 | -0.644102 | 303 | 294.49 | 0.971914 |
| GO:0007266\_Rho\_protein\_signal\_transduction | STMN3 | 24 | 1 | 3.915816 | -0.644102 | 303 | 294.49 | 0.971914 |
| GO:0008629\_induction\_of\_apoptosis\_by\_intracellular\_signals | HIPK1 | 24 | 1 | 3.915816 | -0.644102 | 303 | 294.49 | 0.971914 |
| GO:0009612\_response\_to\_mechanical\_stimulus | GPR98 | 24 | 1 | 3.915816 | -0.644102 | 303 | 294.49 | 0.971914 |
| GO:0043588\_skin\_development | PDGFA | 24 | 1 | 3.915816 | -0.644102 | 303 | 294.49 | 0.971914 |
| GO:0051099\_positive\_regulation\_of\_binding | HIPK1 | 24 | 1 | 3.915816 | -0.644102 | 303 | 294.49 | 0.971914 |
| GO:0043284\_biopolymer\_biosynthetic\_process | FUS | 807 | 11 | 1.281011 | -0.640089 | 304 | 295.51 | 0.972072 |
| GO:0043284\_biopolymer\_biosynthetic\_process | CCNE2 | 807 | 11 | 1.281011 | -0.640089 | 304 | 295.51 | 0.972072 |
| GO:0043284\_biopolymer\_biosynthetic\_process | AEBP2 | 807 | 11 | 1.281011 | -0.640089 | 304 | 295.51 | 0.972072 |
| GO:0043284\_biopolymer\_biosynthetic\_process | HIPK1 | 807 | 11 | 1.281011 | -0.640089 | 304 | 295.51 | 0.972072 |
| GO:0043284\_biopolymer\_biosynthetic\_process | HES5 | 807 | 11 | 1.281011 | -0.640089 | 304 | 295.51 | 0.972072 |
| GO:0043284\_biopolymer\_biosynthetic\_process | SP3 | 807 | 11 | 1.281011 | -0.640089 | 304 | 295.51 | 0.972072 |
| GO:0043284\_biopolymer\_biosynthetic\_process | NAB1 | 807 | 11 | 1.281011 | -0.640089 | 304 | 295.51 | 0.972072 |
| GO:0043284\_biopolymer\_biosynthetic\_process | POU4F1 | 807 | 11 | 1.281011 | -0.640089 | 304 | 295.51 | 0.972072 |
| GO:0043284\_biopolymer\_biosynthetic\_process | ARNTL | 807 | 11 | 1.281011 | -0.640089 | 304 | 295.51 | 0.972072 |
| GO:0043284\_biopolymer\_biosynthetic\_process | APLP1 | 807 | 11 | 1.281011 | -0.640089 | 304 | 295.51 | 0.972072 |
| GO:0043284\_biopolymer\_biosynthetic\_process | ZC3H8 | 807 | 11 | 1.281011 | -0.640089 | 304 | 295.51 | 0.972072 |
| GO:0032504\_multicellular\_organism\_reproduction | SP3 | 86 | 2 | 2.185572 | -0.633523 | 306 | 297.02 | 0.970654 |
| GO:0032504\_multicellular\_organism\_reproduction | CHUK | 86 | 2 | 2.185572 | -0.633523 | 306 | 297.02 | 0.970654 |
| GO:0048609\_reproductive\_process\_in\_a\_multicellular\_organism | SP3 | 86 | 2 | 2.185572 | -0.633523 | 306 | 297.02 | 0.970654 |
| GO:0048609\_reproductive\_process\_in\_a\_multicellular\_organism | CHUK | 86 | 2 | 2.185572 | -0.633523 | 306 | 297.02 | 0.970654 |
| GO:0051704\_multi-organism\_process | MAPK1 | 157 | 3 | 1.795788 | -0.632710 | 307 | 297.11 | 0.967785 |
| GO:0051704\_multi-organism\_process | SP3 | 157 | 3 | 1.795788 | -0.632710 | 307 | 297.11 | 0.967785 |
| GO:0051704\_multi-organism\_process | POU4F1 | 157 | 3 | 1.795788 | -0.632710 | 307 | 297.11 | 0.967785 |
| GO:0050890\_cognition | ITGA8 | 233 | 4 | 1.613384 | -0.630112 | 308 | 297.39 | 0.965552 |
| GO:0050890\_cognition | POU4F1 | 233 | 4 | 1.613384 | -0.630112 | 308 | 297.39 | 0.965552 |
| GO:0050890\_cognition | GPR98 | 233 | 4 | 1.613384 | -0.630112 | 308 | 297.39 | 0.965552 |
| GO:0050890\_cognition | EPHB2 | 233 | 4 | 1.613384 | -0.630112 | 308 | 297.39 | 0.965552 |
| GO:0000087\_M\_phase\_of\_mitotic\_cell\_cycle | TUBG1 | 25 | 1 | 3.759184 | -0.628559 | 312 | 302.55 | 0.969712 |
| GO:0007628\_adult\_walking\_behavior | EPHA4 | 25 | 1 | 3.759184 | -0.628559 | 312 | 302.55 | 0.969712 |
| GO:0048285\_organelle\_fission | TUBG1 | 25 | 1 | 3.759184 | -0.628559 | 312 | 302.55 | 0.969712 |
| GO:0050852\_T\_cell\_receptor\_signaling\_pathway | MAPK1 | 25 | 1 | 3.759184 | -0.628559 | 312 | 302.55 | 0.969712 |
| GO:0022612\_gland\_morphogenesis | SFRP1 | 87 | 2 | 2.160450 | -0.626198 | 313 | 304.47 | 0.972748 |
| GO:0022612\_gland\_morphogenesis | PDGFA | 87 | 2 | 2.160450 | -0.626198 | 313 | 304.47 | 0.972748 |
| GO:0001666\_response\_to\_hypoxia | MMP2 | 26 | 1 | 3.614600 | -0.613707 | 318 | 311.63 | 0.979969 |
| GO:0007405\_neuroblast\_proliferation | FOXG1 | 26 | 1 | 3.614600 | -0.613707 | 318 | 311.63 | 0.979969 |
| GO:0007613\_memory | ITGA8 | 26 | 1 | 3.614600 | -0.613707 | 318 | 311.63 | 0.979969 |
| GO:0007623\_circadian\_rhythm | ARNTL | 26 | 1 | 3.614600 | -0.613707 | 318 | 311.63 | 0.979969 |
| GO:0009636\_response\_to\_toxin | DDC | 26 | 1 | 3.614600 | -0.613707 | 318 | 311.63 | 0.979969 |
| GO:0030324\_lung\_development | PDGFA | 90 | 2 | 2.088435 | -0.604893 | 319 | 313.22 | 0.981881 |
| GO:0030324\_lung\_development | SP3 | 90 | 2 | 2.088435 | -0.604893 | 319 | 313.22 | 0.981881 |
| GO:0031016\_pancreas\_development | XBP1 | 27 | 1 | 3.480726 | -0.599495 | 322 | 317.85 | 0.987112 |
| GO:0032496\_response\_to\_lipopolysaccharide | MAPK1 | 27 | 1 | 3.480726 | -0.599495 | 322 | 317.85 | 0.987112 |
| GO:0070482\_response\_to\_oxygen\_levels | MMP2 | 27 | 1 | 3.480726 | -0.599495 | 322 | 317.85 | 0.987112 |
| GO:0008544\_epidermis\_development | PDGFA | 91 | 2 | 2.065486 | -0.598007 | 323 | 318.44 | 0.985882 |
| GO:0008544\_epidermis\_development | NAB1 | 91 | 2 | 2.065486 | -0.598007 | 323 | 318.44 | 0.985882 |
| GO:0019538\_protein\_metabolic\_process | MAPK1 | 655 | 9 | 1.291323 | -0.593151 | 324 | 319.12 | 0.984938 |
| GO:0019538\_protein\_metabolic\_process | HIPK1 | 655 | 9 | 1.291323 | -0.593151 | 324 | 319.12 | 0.984938 |
| GO:0019538\_protein\_metabolic\_process | PDGFA | 655 | 9 | 1.291323 | -0.593151 | 324 | 319.12 | 0.984938 |
| GO:0019538\_protein\_metabolic\_process | SV2B | 655 | 9 | 1.291323 | -0.593151 | 324 | 319.12 | 0.984938 |
| GO:0019538\_protein\_metabolic\_process | ARNTL | 655 | 9 | 1.291323 | -0.593151 | 324 | 319.12 | 0.984938 |
| GO:0019538\_protein\_metabolic\_process | CHUK | 655 | 9 | 1.291323 | -0.593151 | 324 | 319.12 | 0.984938 |
| GO:0019538\_protein\_metabolic\_process | MAP3K12 | 655 | 9 | 1.291323 | -0.593151 | 324 | 319.12 | 0.984938 |
| GO:0019538\_protein\_metabolic\_process | EPHB2 | 655 | 9 | 1.291323 | -0.593151 | 324 | 319.12 | 0.984938 |
| GO:0019538\_protein\_metabolic\_process | APLP1 | 655 | 9 | 1.291323 | -0.593151 | 324 | 319.12 | 0.984938 |
| GO:0030323\_respiratory\_tube\_development | PDGFA | 92 | 2 | 2.043035 | -0.591224 | 325 | 320.17 | 0.985138 |
| GO:0030323\_respiratory\_tube\_development | SP3 | 92 | 2 | 2.043035 | -0.591224 | 325 | 320.17 | 0.985138 |
| GO:0043065\_positive\_regulation\_of\_apoptosis | HIPK1 | 166 | 3 | 1.698426 | -0.587035 | 326 | 320.61 | 0.983466 |
| GO:0043065\_positive\_regulation\_of\_apoptosis | TM2D1 | 166 | 3 | 1.698426 | -0.587035 | 326 | 320.61 | 0.983466 |
| GO:0043065\_positive\_regulation\_of\_apoptosis | POU4F1 | 166 | 3 | 1.698426 | -0.587035 | 326 | 320.61 | 0.983466 |
| GO:0030073\_insulin\_secretion | CPLX3 | 28 | 1 | 3.356414 | -0.585875 | 329 | 324.69 | 0.986900 |
| GO:0030111\_regulation\_of\_Wnt\_receptor\_signaling\_pathway | DKK1 | 28 | 1 | 3.356414 | -0.585875 | 329 | 324.69 | 0.986900 |
| GO:0043193\_positive\_regulation\_of\_gene-specific\_transcription | HES5 | 28 | 1 | 3.356414 | -0.585875 | 329 | 324.69 | 0.986900 |
| GO:0010556\_regulation\_of\_macromolecule\_biosynthetic\_process | FUS | 745 | 10 | 1.261471 | -0.583263 | 330 | 325.58 | 0.986606 |
| GO:0010556\_regulation\_of\_macromolecule\_biosynthetic\_process | AEBP2 | 745 | 10 | 1.261471 | -0.583263 | 330 | 325.58 | 0.986606 |
| GO:0010556\_regulation\_of\_macromolecule\_biosynthetic\_process | HIPK1 | 745 | 10 | 1.261471 | -0.583263 | 330 | 325.58 | 0.986606 |
| GO:0010556\_regulation\_of\_macromolecule\_biosynthetic\_process | HES5 | 745 | 10 | 1.261471 | -0.583263 | 330 | 325.58 | 0.986606 |
| GO:0010556\_regulation\_of\_macromolecule\_biosynthetic\_process | SP3 | 745 | 10 | 1.261471 | -0.583263 | 330 | 325.58 | 0.986606 |
| GO:0010556\_regulation\_of\_macromolecule\_biosynthetic\_process | NAB1 | 745 | 10 | 1.261471 | -0.583263 | 330 | 325.58 | 0.986606 |
| GO:0010556\_regulation\_of\_macromolecule\_biosynthetic\_process | POU4F1 | 745 | 10 | 1.261471 | -0.583263 | 330 | 325.58 | 0.986606 |
| GO:0010556\_regulation\_of\_macromolecule\_biosynthetic\_process | ARNTL | 745 | 10 | 1.261471 | -0.583263 | 330 | 325.58 | 0.986606 |
| GO:0010556\_regulation\_of\_macromolecule\_biosynthetic\_process | ZC3H8 | 745 | 10 | 1.261471 | -0.583263 | 330 | 325.58 | 0.986606 |
| GO:0010556\_regulation\_of\_macromolecule\_biosynthetic\_process | APLP1 | 745 | 10 | 1.261471 | -0.583263 | 330 | 325.58 | 0.986606 |
| GO:0010942\_positive\_regulation\_of\_cell\_death | HIPK1 | 167 | 3 | 1.688256 | -0.582198 | 332 | 326.12 | 0.982289 |
| GO:0010942\_positive\_regulation\_of\_cell\_death | TM2D1 | 167 | 3 | 1.688256 | -0.582198 | 332 | 326.12 | 0.982289 |
| GO:0010942\_positive\_regulation\_of\_cell\_death | POU4F1 | 167 | 3 | 1.688256 | -0.582198 | 332 | 326.12 | 0.982289 |
| GO:0043068\_positive\_regulation\_of\_programmed\_cell\_death | HIPK1 | 167 | 3 | 1.688256 | -0.582198 | 332 | 326.12 | 0.982289 |
| GO:0043068\_positive\_regulation\_of\_programmed\_cell\_death | TM2D1 | 167 | 3 | 1.688256 | -0.582198 | 332 | 326.12 | 0.982289 |
| GO:0043068\_positive\_regulation\_of\_programmed\_cell\_death | POU4F1 | 167 | 3 | 1.688256 | -0.582198 | 332 | 326.12 | 0.982289 |
| GO:0006417\_regulation\_of\_translation | APLP1 | 29 | 1 | 3.240676 | -0.572805 | 336 | 332.72 | 0.990238 |
| GO:0010564\_regulation\_of\_cell\_cycle\_process | TUBG1 | 29 | 1 | 3.240676 | -0.572805 | 336 | 332.72 | 0.990238 |
| GO:0042176\_regulation\_of\_protein\_catabolic\_process | ARNTL | 29 | 1 | 3.240676 | -0.572805 | 336 | 332.72 | 0.990238 |
| GO:0042770\_DNA\_damage\_response\_\_signal\_transduction | HIPK1 | 29 | 1 | 3.240676 | -0.572805 | 336 | 332.72 | 0.990238 |
| GO:0007242\_intracellular\_signaling\_cascade | MAPK1 | 411 | 6 | 1.371965 | -0.569049 | 337 | 333.57 | 0.989822 |
| GO:0007242\_intracellular\_signaling\_cascade | HIPK1 | 411 | 6 | 1.371965 | -0.569049 | 337 | 333.57 | 0.989822 |
| GO:0007242\_intracellular\_signaling\_cascade | STMN3 | 411 | 6 | 1.371965 | -0.569049 | 337 | 333.57 | 0.989822 |
| GO:0007242\_intracellular\_signaling\_cascade | THOP1 | 411 | 6 | 1.371965 | -0.569049 | 337 | 333.57 | 0.989822 |
| GO:0007242\_intracellular\_signaling\_cascade | MAPK10 | 411 | 6 | 1.371965 | -0.569049 | 337 | 333.57 | 0.989822 |
| GO:0007242\_intracellular\_signaling\_cascade | CHUK | 411 | 6 | 1.371965 | -0.569049 | 337 | 333.57 | 0.989822 |
| GO:0080090\_regulation\_of\_primary\_metabolic\_process | FUS | 926 | 12 | 1.217878 | -0.568490 | 338 | 333.65 | 0.987130 |
| GO:0080090\_regulation\_of\_primary\_metabolic\_process | AEBP2 | 926 | 12 | 1.217878 | -0.568490 | 338 | 333.65 | 0.987130 |
| GO:0080090\_regulation\_of\_primary\_metabolic\_process | HIPK1 | 926 | 12 | 1.217878 | -0.568490 | 338 | 333.65 | 0.987130 |
| GO:0080090\_regulation\_of\_primary\_metabolic\_process | HES5 | 926 | 12 | 1.217878 | -0.568490 | 338 | 333.65 | 0.987130 |
| GO:0080090\_regulation\_of\_primary\_metabolic\_process | PDGFA | 926 | 12 | 1.217878 | -0.568490 | 338 | 333.65 | 0.987130 |
| GO:0080090\_regulation\_of\_primary\_metabolic\_process | SP3 | 926 | 12 | 1.217878 | -0.568490 | 338 | 333.65 | 0.987130 |
| GO:0080090\_regulation\_of\_primary\_metabolic\_process | NAB1 | 926 | 12 | 1.217878 | -0.568490 | 338 | 333.65 | 0.987130 |
| GO:0080090\_regulation\_of\_primary\_metabolic\_process | SV2B | 926 | 12 | 1.217878 | -0.568490 | 338 | 333.65 | 0.987130 |
| GO:0080090\_regulation\_of\_primary\_metabolic\_process | POU4F1 | 926 | 12 | 1.217878 | -0.568490 | 338 | 333.65 | 0.987130 |
| GO:0080090\_regulation\_of\_primary\_metabolic\_process | ARNTL | 926 | 12 | 1.217878 | -0.568490 | 338 | 333.65 | 0.987130 |
| GO:0080090\_regulation\_of\_primary\_metabolic\_process | APLP1 | 926 | 12 | 1.217878 | -0.568490 | 338 | 333.65 | 0.987130 |
| GO:0080090\_regulation\_of\_primary\_metabolic\_process | ZC3H8 | 926 | 12 | 1.217878 | -0.568490 | 338 | 333.65 | 0.987130 |
| GO:0007219\_Notch\_signaling\_pathway | JAG1 | 30 | 1 | 3.132653 | -0.560249 | 343 | 341.5 | 0.995627 |
| GO:0007435\_salivary\_gland\_morphogenesis | PDGFA | 30 | 1 | 3.132653 | -0.560249 | 343 | 341.5 | 0.995627 |
| GO:0042552\_myelination | NAB1 | 30 | 1 | 3.132653 | -0.560249 | 343 | 341.5 | 0.995627 |
| GO:0048565\_gut\_development | COL3A1 | 30 | 1 | 3.132653 | -0.560249 | 343 | 341.5 | 0.995627 |
| GO:0060740\_prostate\_gland\_epithelium\_morphogenesis | SFRP1 | 30 | 1 | 3.132653 | -0.560249 | 343 | 341.5 | 0.995627 |
| GO:0060541\_respiratory\_system\_development | PDGFA | 98 | 2 | 1.917951 | -0.552578 | 344 | 343.87 | 0.999622 |
| GO:0060541\_respiratory\_system\_development | SP3 | 98 | 2 | 1.917951 | -0.552578 | 344 | 343.87 | 0.999622 |
| GO:0030097\_hemopoiesis | HBA-A1 | 253 | 4 | 1.485843 | -0.550196 | 345 | 344.19 | 0.997652 |
| GO:0030097\_hemopoiesis | SP3 | 253 | 4 | 1.485843 | -0.550196 | 345 | 344.19 | 0.997652 |
| GO:0030097\_hemopoiesis | JAG1 | 253 | 4 | 1.485843 | -0.550196 | 345 | 344.19 | 0.997652 |
| GO:0030097\_hemopoiesis | CHUK | 253 | 4 | 1.485843 | -0.550196 | 345 | 344.19 | 0.997652 |
| GO:0008645\_hexose\_transport | YES1 | 31 | 1 | 3.031600 | -0.548172 | 352 | 351.83 | 0.999517 |
| GO:0015749\_monosaccharide\_transport | YES1 | 31 | 1 | 3.031600 | -0.548172 | 352 | 351.83 | 0.999517 |
| GO:0015758\_glucose\_transport | YES1 | 31 | 1 | 3.031600 | -0.548172 | 352 | 351.83 | 0.999517 |
| GO:0048167\_regulation\_of\_synaptic\_plasticity | EPHB2 | 31 | 1 | 3.031600 | -0.548172 | 352 | 351.83 | 0.999517 |
| GO:0048562\_embryonic\_organ\_morphogenesis | SP3 | 31 | 1 | 3.031600 | -0.548172 | 352 | 351.83 | 0.999517 |
| GO:0051640\_organelle\_localization | NTN1 | 31 | 1 | 3.031600 | -0.548172 | 352 | 351.83 | 0.999517 |
| GO:0060512\_prostate\_gland\_morphogenesis | SFRP1 | 31 | 1 | 3.031600 | -0.548172 | 352 | 351.83 | 0.999517 |
| GO:0060255\_regulation\_of\_macromolecule\_metabolic\_process | FUS | 936 | 12 | 1.204867 | -0.547965 | 353 | 351.89 | 0.996856 |
| GO:0060255\_regulation\_of\_macromolecule\_metabolic\_process | AEBP2 | 936 | 12 | 1.204867 | -0.547965 | 353 | 351.89 | 0.996856 |
| GO:0060255\_regulation\_of\_macromolecule\_metabolic\_process | HIPK1 | 936 | 12 | 1.204867 | -0.547965 | 353 | 351.89 | 0.996856 |
| GO:0060255\_regulation\_of\_macromolecule\_metabolic\_process | HES5 | 936 | 12 | 1.204867 | -0.547965 | 353 | 351.89 | 0.996856 |
| GO:0060255\_regulation\_of\_macromolecule\_metabolic\_process | PDGFA | 936 | 12 | 1.204867 | -0.547965 | 353 | 351.89 | 0.996856 |
| GO:0060255\_regulation\_of\_macromolecule\_metabolic\_process | SP3 | 936 | 12 | 1.204867 | -0.547965 | 353 | 351.89 | 0.996856 |
| GO:0060255\_regulation\_of\_macromolecule\_metabolic\_process | NAB1 | 936 | 12 | 1.204867 | -0.547965 | 353 | 351.89 | 0.996856 |
| GO:0060255\_regulation\_of\_macromolecule\_metabolic\_process | SV2B | 936 | 12 | 1.204867 | -0.547965 | 353 | 351.89 | 0.996856 |
| GO:0060255\_regulation\_of\_macromolecule\_metabolic\_process | POU4F1 | 936 | 12 | 1.204867 | -0.547965 | 353 | 351.89 | 0.996856 |
| GO:0060255\_regulation\_of\_macromolecule\_metabolic\_process | ARNTL | 936 | 12 | 1.204867 | -0.547965 | 353 | 351.89 | 0.996856 |
| GO:0060255\_regulation\_of\_macromolecule\_metabolic\_process | ZC3H8 | 936 | 12 | 1.204867 | -0.547965 | 353 | 351.89 | 0.996856 |
| GO:0060255\_regulation\_of\_macromolecule\_metabolic\_process | APLP1 | 936 | 12 | 1.204867 | -0.547965 | 353 | 351.89 | 0.996856 |
| GO:0007398\_ectoderm\_development | PDGFA | 99 | 2 | 1.898578 | -0.546460 | 354 | 352.76 | 0.996497 |
| GO:0007398\_ectoderm\_development | NAB1 | 99 | 2 | 1.898578 | -0.546460 | 354 | 352.76 | 0.996497 |
| GO:0045449\_regulation\_of\_transcription | FUS | 676 | 9 | 1.251208 | -0.542915 | 355 | 353.54 | 0.995887 |
| GO:0045449\_regulation\_of\_transcription | AEBP2 | 676 | 9 | 1.251208 | -0.542915 | 355 | 353.54 | 0.995887 |
| GO:0045449\_regulation\_of\_transcription | HIPK1 | 676 | 9 | 1.251208 | -0.542915 | 355 | 353.54 | 0.995887 |
| GO:0045449\_regulation\_of\_transcription | HES5 | 676 | 9 | 1.251208 | -0.542915 | 355 | 353.54 | 0.995887 |
| GO:0045449\_regulation\_of\_transcription | SP3 | 676 | 9 | 1.251208 | -0.542915 | 355 | 353.54 | 0.995887 |
| GO:0045449\_regulation\_of\_transcription | NAB1 | 676 | 9 | 1.251208 | -0.542915 | 355 | 353.54 | 0.995887 |
| GO:0045449\_regulation\_of\_transcription | POU4F1 | 676 | 9 | 1.251208 | -0.542915 | 355 | 353.54 | 0.995887 |
| GO:0045449\_regulation\_of\_transcription | ARNTL | 676 | 9 | 1.251208 | -0.542915 | 355 | 353.54 | 0.995887 |
| GO:0045449\_regulation\_of\_transcription | ZC3H8 | 676 | 9 | 1.251208 | -0.542915 | 355 | 353.54 | 0.995887 |
| GO:0007249\_I-kappaB\_kinase\_NF-kappaB\_cascade | CHUK | 32 | 1 | 2.936862 | -0.536543 | 361 | 358.5 | 0.993075 |
| GO:0007272\_ensheathment\_of\_neurons | NAB1 | 32 | 1 | 2.936862 | -0.536543 | 361 | 358.5 | 0.993075 |
| GO:0008366\_axon\_ensheathment | NAB1 | 32 | 1 | 2.936862 | -0.536543 | 361 | 358.5 | 0.993075 |
| GO:0050885\_neuromuscular\_process\_controlling\_balance | POU4F1 | 32 | 1 | 2.936862 | -0.536543 | 361 | 358.5 | 0.993075 |
| GO:0051259\_protein\_oligomerization | OLFM1 | 32 | 1 | 2.936862 | -0.536543 | 361 | 358.5 | 0.993075 |
| GO:0051493\_regulation\_of\_cytoskeleton\_organization | STMN3 | 32 | 1 | 2.936862 | -0.536543 | 361 | 358.5 | 0.993075 |
| GO:0030036\_actin\_cytoskeleton\_organization | DLC1 | 102 | 2 | 1.842737 | -0.528619 | 362 | 359.82 | 0.993978 |
| GO:0030036\_actin\_cytoskeleton\_organization | PDGFA | 102 | 2 | 1.842737 | -0.528619 | 362 | 359.82 | 0.993978 |
| GO:0001843\_neural\_tube\_closure | DLC1 | 33 | 1 | 2.847866 | -0.525335 | 366 | 365.14 | 0.997650 |
| GO:0007565\_female\_pregnancy | SP3 | 33 | 1 | 2.847866 | -0.525335 | 366 | 365.14 | 0.997650 |
| GO:0008643\_carbohydrate\_transport | YES1 | 33 | 1 | 2.847866 | -0.525335 | 366 | 365.14 | 0.997650 |
| GO:0060606\_tube\_closure | DLC1 | 33 | 1 | 2.847866 | -0.525335 | 366 | 365.14 | 0.997650 |
| GO:0009968\_negative\_regulation\_of\_signal\_transduction | DKK1 | 103 | 2 | 1.824846 | -0.522838 | 367 | 366.07 | 0.997466 |
| GO:0009968\_negative\_regulation\_of\_signal\_transduction | STMN3 | 103 | 2 | 1.824846 | -0.522838 | 367 | 366.07 | 0.997466 |
| GO:0048523\_negative\_regulation\_of\_cellular\_process | MAPK1 | 774 | 10 | 1.214207 | -0.519136 | 368 | 366.44 | 0.995761 |
| GO:0048523\_negative\_regulation\_of\_cellular\_process | DKK1 | 774 | 10 | 1.214207 | -0.519136 | 368 | 366.44 | 0.995761 |
| GO:0048523\_negative\_regulation\_of\_cellular\_process | HIPK1 | 774 | 10 | 1.214207 | -0.519136 | 368 | 366.44 | 0.995761 |
| GO:0048523\_negative\_regulation\_of\_cellular\_process | HES5 | 774 | 10 | 1.214207 | -0.519136 | 368 | 366.44 | 0.995761 |
| GO:0048523\_negative\_regulation\_of\_cellular\_process | STMN3 | 774 | 10 | 1.214207 | -0.519136 | 368 | 366.44 | 0.995761 |
| GO:0048523\_negative\_regulation\_of\_cellular\_process | FOXG1 | 774 | 10 | 1.214207 | -0.519136 | 368 | 366.44 | 0.995761 |
| GO:0048523\_negative\_regulation\_of\_cellular\_process | JAG1 | 774 | 10 | 1.214207 | -0.519136 | 368 | 366.44 | 0.995761 |
| GO:0048523\_negative\_regulation\_of\_cellular\_process | NTN1 | 774 | 10 | 1.214207 | -0.519136 | 368 | 366.44 | 0.995761 |
| GO:0048523\_negative\_regulation\_of\_cellular\_process | EPHB2 | 774 | 10 | 1.214207 | -0.519136 | 368 | 366.44 | 0.995761 |
| GO:0048523\_negative\_regulation\_of\_cellular\_process | ZC3H8 | 774 | 10 | 1.214207 | -0.519136 | 368 | 366.44 | 0.995761 |
| GO:0002237\_response\_to\_molecule\_of\_bacterial\_origin | MAPK1 | 34 | 1 | 2.764106 | -0.514522 | 372 | 372.59 | 1.001586 |
| GO:0050730\_regulation\_of\_peptidyl-tyrosine\_phosphorylation | PDGFA | 34 | 1 | 2.764106 | -0.514522 | 372 | 372.59 | 1.001586 |
| GO:0051047\_positive\_regulation\_of\_secretion | SYTL2 | 34 | 1 | 2.764106 | -0.514522 | 372 | 372.59 | 1.001586 |
| GO:0060711\_labyrinthine\_layer\_development | MAPK1 | 34 | 1 | 2.764106 | -0.514522 | 372 | 372.59 | 1.001586 |
| GO:0048872\_homeostasis\_of\_number\_of\_cells | HBA-A1 | 105 | 2 | 1.790087 | -0.511514 | 373 | 373.26 | 1.000697 |
| GO:0048872\_homeostasis\_of\_number\_of\_cells | SP3 | 105 | 2 | 1.790087 | -0.511514 | 373 | 373.26 | 1.000697 |
| GO:0010468\_regulation\_of\_gene\_expression | FUS | 778 | 10 | 1.207964 | -0.510773 | 374 | 373.46 | 0.998556 |
| GO:0010468\_regulation\_of\_gene\_expression | AEBP2 | 778 | 10 | 1.207964 | -0.510773 | 374 | 373.46 | 0.998556 |
| GO:0010468\_regulation\_of\_gene\_expression | HIPK1 | 778 | 10 | 1.207964 | -0.510773 | 374 | 373.46 | 0.998556 |
| GO:0010468\_regulation\_of\_gene\_expression | HES5 | 778 | 10 | 1.207964 | -0.510773 | 374 | 373.46 | 0.998556 |
| GO:0010468\_regulation\_of\_gene\_expression | SP3 | 778 | 10 | 1.207964 | -0.510773 | 374 | 373.46 | 0.998556 |
| GO:0010468\_regulation\_of\_gene\_expression | NAB1 | 778 | 10 | 1.207964 | -0.510773 | 374 | 373.46 | 0.998556 |
| GO:0010468\_regulation\_of\_gene\_expression | POU4F1 | 778 | 10 | 1.207964 | -0.510773 | 374 | 373.46 | 0.998556 |
| GO:0010468\_regulation\_of\_gene\_expression | ARNTL | 778 | 10 | 1.207964 | -0.510773 | 374 | 373.46 | 0.998556 |
| GO:0010468\_regulation\_of\_gene\_expression | ZC3H8 | 778 | 10 | 1.207964 | -0.510773 | 374 | 373.46 | 0.998556 |
| GO:0010468\_regulation\_of\_gene\_expression | APLP1 | 778 | 10 | 1.207964 | -0.510773 | 374 | 373.46 | 0.998556 |
| GO:0001756\_somitogenesis | SFRP1 | 35 | 1 | 2.685131 | -0.504082 | 375 | 378.79 | 1.010107 |
| GO:0001889\_liver\_development | SP3 | 36 | 1 | 2.610544 | -0.493992 | 381 | 385.19 | 1.010997 |
| GO:0007631\_feeding\_behavior | POU4F1 | 36 | 1 | 2.610544 | -0.493992 | 381 | 385.19 | 1.010997 |
| GO:0014020\_primary\_neural\_tube\_formation | DLC1 | 36 | 1 | 2.610544 | -0.493992 | 381 | 385.19 | 1.010997 |
| GO:0019228\_regulation\_of\_action\_potential\_in\_neuron | NAB1 | 36 | 1 | 2.610544 | -0.493992 | 381 | 385.19 | 1.010997 |
| GO:0030072\_peptide\_hormone\_secretion | CPLX3 | 36 | 1 | 2.610544 | -0.493992 | 381 | 385.19 | 1.010997 |
| GO:0050851\_antigen\_receptor-mediated\_signaling\_pathway | MAPK1 | 36 | 1 | 2.610544 | -0.493992 | 381 | 385.19 | 1.010997 |
| GO:0009888\_tissue\_development | DLC1 | 525 | 7 | 1.253061 | -0.491052 | 382 | 385.75 | 1.009817 |
| GO:0009888\_tissue\_development | SFRP1 | 525 | 7 | 1.253061 | -0.491052 | 382 | 385.75 | 1.009817 |
| GO:0009888\_tissue\_development | XBP1 | 525 | 7 | 1.253061 | -0.491052 | 382 | 385.75 | 1.009817 |
| GO:0009888\_tissue\_development | PDGFA | 525 | 7 | 1.253061 | -0.491052 | 382 | 385.75 | 1.009817 |
| GO:0009888\_tissue\_development | NAB1 | 525 | 7 | 1.253061 | -0.491052 | 382 | 385.75 | 1.009817 |
| GO:0009888\_tissue\_development | JAG1 | 525 | 7 | 1.253061 | -0.491052 | 382 | 385.75 | 1.009817 |
| GO:0009888\_tissue\_development | CHUK | 525 | 7 | 1.253061 | -0.491052 | 382 | 385.75 | 1.009817 |
| GO:0006464\_protein\_modification\_process | MAPK1 | 439 | 6 | 1.284459 | -0.490711 | 383 | 386.0 | 1.007833 |
| GO:0006464\_protein\_modification\_process | HIPK1 | 439 | 6 | 1.284459 | -0.490711 | 383 | 386.0 | 1.007833 |
| GO:0006464\_protein\_modification\_process | PDGFA | 439 | 6 | 1.284459 | -0.490711 | 383 | 386.0 | 1.007833 |
| GO:0006464\_protein\_modification\_process | CHUK | 439 | 6 | 1.284459 | -0.490711 | 383 | 386.0 | 1.007833 |
| GO:0006464\_protein\_modification\_process | MAP3K12 | 439 | 6 | 1.284459 | -0.490711 | 383 | 386.0 | 1.007833 |
| GO:0006464\_protein\_modification\_process | EPHB2 | 439 | 6 | 1.284459 | -0.490711 | 383 | 386.0 | 1.007833 |
| GO:0030029\_actin\_filament-based\_process | DLC1 | 109 | 2 | 1.724396 | -0.489780 | 384 | 386.68 | 1.006979 |
| GO:0030029\_actin\_filament-based\_process | PDGFA | 109 | 2 | 1.724396 | -0.489780 | 384 | 386.68 | 1.006979 |
| GO:0006350\_transcription | FUS | 701 | 9 | 1.206585 | -0.487897 | 385 | 386.82 | 1.004727 |
| GO:0006350\_transcription | AEBP2 | 701 | 9 | 1.206585 | -0.487897 | 385 | 386.82 | 1.004727 |
| GO:0006350\_transcription | HIPK1 | 701 | 9 | 1.206585 | -0.487897 | 385 | 386.82 | 1.004727 |
| GO:0006350\_transcription | HES5 | 701 | 9 | 1.206585 | -0.487897 | 385 | 386.82 | 1.004727 |
| GO:0006350\_transcription | SP3 | 701 | 9 | 1.206585 | -0.487897 | 385 | 386.82 | 1.004727 |
| GO:0006350\_transcription | NAB1 | 701 | 9 | 1.206585 | -0.487897 | 385 | 386.82 | 1.004727 |
| GO:0006350\_transcription | POU4F1 | 701 | 9 | 1.206585 | -0.487897 | 385 | 386.82 | 1.004727 |
| GO:0006350\_transcription | ARNTL | 701 | 9 | 1.206585 | -0.487897 | 385 | 386.82 | 1.004727 |
| GO:0006350\_transcription | ZC3H8 | 701 | 9 | 1.206585 | -0.487897 | 385 | 386.82 | 1.004727 |
| GO:0010648\_negative\_regulation\_of\_cell\_communication | DKK1 | 110 | 2 | 1.708720 | -0.484527 | 387 | 388.08 | 1.002791 |
| GO:0010648\_negative\_regulation\_of\_cell\_communication | STMN3 | 110 | 2 | 1.708720 | -0.484527 | 387 | 388.08 | 1.002791 |
| GO:0043010\_camera-type\_eye\_development | SP3 | 110 | 2 | 1.708720 | -0.484527 | 387 | 388.08 | 1.002791 |
| GO:0043010\_camera-type\_eye\_development | EPHB2 | 110 | 2 | 1.708720 | -0.484527 | 387 | 388.08 | 1.002791 |
| GO:0002790\_peptide\_secretion | CPLX3 | 37 | 1 | 2.539989 | -0.484234 | 390 | 391.13 | 1.002897 |
| GO:0050906\_detection\_of\_stimulus\_involved\_in\_sensory\_perception | GPR98 | 37 | 1 | 2.539989 | -0.484234 | 390 | 391.13 | 1.002897 |
| GO:0051101\_regulation\_of\_DNA\_binding | HIPK1 | 37 | 1 | 2.539989 | -0.484234 | 390 | 391.13 | 1.002897 |
| GO:0048705\_skeletal\_system\_morphogenesis | NAB1 | 111 | 2 | 1.693326 | -0.479345 | 391 | 392.07 | 1.002737 |
| GO:0048705\_skeletal\_system\_morphogenesis | MMP2 | 111 | 2 | 1.693326 | -0.479345 | 391 | 392.07 | 1.002737 |
| GO:0001570\_vasculogenesis | HEY1 | 38 | 1 | 2.473147 | -0.474789 | 393 | 398.51 | 1.014020 |
| GO:0046777\_protein\_amino\_acid\_autophosphorylation | MAP3K12 | 38 | 1 | 2.473147 | -0.474789 | 393 | 398.51 | 1.014020 |
| GO:0006974\_response\_to\_DNA\_damage\_stimulus | MAPK1 | 113 | 2 | 1.663356 | -0.469185 | 394 | 400.06 | 1.015381 |
| GO:0006974\_response\_to\_DNA\_damage\_stimulus | HIPK1 | 113 | 2 | 1.663356 | -0.469185 | 394 | 400.06 | 1.015381 |
| GO:0048534\_hemopoietic\_or\_lymphoid\_organ\_development | HBA-A1 | 277 | 4 | 1.357106 | -0.467997 | 396 | 400.42 | 1.011162 |
| GO:0048534\_hemopoietic\_or\_lymphoid\_organ\_development | SP3 | 277 | 4 | 1.357106 | -0.467997 | 396 | 400.42 | 1.011162 |
| GO:0048534\_hemopoietic\_or\_lymphoid\_organ\_development | JAG1 | 277 | 4 | 1.357106 | -0.467997 | 396 | 400.42 | 1.011162 |
| GO:0048534\_hemopoietic\_or\_lymphoid\_organ\_development | CHUK | 277 | 4 | 1.357106 | -0.467997 | 396 | 400.42 | 1.011162 |
| GO:0048646\_anatomical\_structure\_formation\_involved\_in\_morphogenesis | DLC1 | 277 | 4 | 1.357106 | -0.467997 | 396 | 400.42 | 1.011162 |
| GO:0048646\_anatomical\_structure\_formation\_involved\_in\_morphogenesis | PDGFA | 277 | 4 | 1.357106 | -0.467997 | 396 | 400.42 | 1.011162 |
| GO:0048646\_anatomical\_structure\_formation\_involved\_in\_morphogenesis | SP3 | 277 | 4 | 1.357106 | -0.467997 | 396 | 400.42 | 1.011162 |
| GO:0048646\_anatomical\_structure\_formation\_involved\_in\_morphogenesis | MMP2 | 277 | 4 | 1.357106 | -0.467997 | 396 | 400.42 | 1.011162 |
| GO:0007160\_cell-matrix\_adhesion | DLC1 | 39 | 1 | 2.409733 | -0.465642 | 401 | 404.98 | 1.009925 |
| GO:0008037\_cell\_recognition | FOXG1 | 39 | 1 | 2.409733 | -0.465642 | 401 | 404.98 | 1.009925 |
| GO:0035148\_tube\_lumen\_formation | DLC1 | 39 | 1 | 2.409733 | -0.465642 | 401 | 404.98 | 1.009925 |
| GO:0042475\_odontogenesis\_of\_dentine-containing\_tooth | CHUK | 39 | 1 | 2.409733 | -0.465642 | 401 | 404.98 | 1.009925 |
| GO:0048663\_neuron\_fate\_commitment | HES5 | 39 | 1 | 2.409733 | -0.465642 | 401 | 404.98 | 1.009925 |
| GO:0006996\_organelle\_organization | DLC1 | 449 | 6 | 1.255852 | -0.465254 | 402 | 405.14 | 1.007811 |
| GO:0006996\_organelle\_organization | STMN3 | 449 | 6 | 1.255852 | -0.465254 | 402 | 405.14 | 1.007811 |
| GO:0006996\_organelle\_organization | PDGFA | 449 | 6 | 1.255852 | -0.465254 | 402 | 405.14 | 1.007811 |
| GO:0006996\_organelle\_organization | KATNA1 | 449 | 6 | 1.255852 | -0.465254 | 402 | 405.14 | 1.007811 |
| GO:0006996\_organelle\_organization | TUBG1 | 449 | 6 | 1.255852 | -0.465254 | 402 | 405.14 | 1.007811 |
| GO:0006996\_organelle\_organization | MAP3K12 | 449 | 6 | 1.255852 | -0.465254 | 402 | 405.14 | 1.007811 |
| GO:0000165\_MAPKKK\_cascade | MAPK1 | 114 | 2 | 1.648765 | -0.464205 | 403 | 405.54 | 1.006303 |
| GO:0000165\_MAPKKK\_cascade | MAPK10 | 114 | 2 | 1.648765 | -0.464205 | 403 | 405.54 | 1.006303 |
| GO:0003002\_regionalization | HIPK1 | 195 | 3 | 1.445840 | -0.463390 | 404 | 406.01 | 1.004975 |
| GO:0003002\_regionalization | SFRP1 | 195 | 3 | 1.445840 | -0.463390 | 404 | 406.01 | 1.004975 |
| GO:0003002\_regionalization | FOXG1 | 195 | 3 | 1.445840 | -0.463390 | 404 | 406.01 | 1.004975 |
| GO:0007610\_behavior | EPHA4 | 279 | 4 | 1.347378 | -0.461740 | 405 | 406.49 | 1.003679 |
| GO:0007610\_behavior | ITGA8 | 279 | 4 | 1.347378 | -0.461740 | 405 | 406.49 | 1.003679 |
| GO:0007610\_behavior | POU4F1 | 279 | 4 | 1.347378 | -0.461740 | 405 | 406.49 | 1.003679 |
| GO:0007610\_behavior | EPHB2 | 279 | 4 | 1.347378 | -0.461740 | 405 | 406.49 | 1.003679 |
| GO:0043009\_chordate\_embryonic\_development | DLC1 | 365 | 5 | 1.287392 | -0.459427 | 406 | 407.23 | 1.003030 |
| GO:0043009\_chordate\_embryonic\_development | HBA-A1 | 365 | 5 | 1.287392 | -0.459427 | 406 | 407.23 | 1.003030 |
| GO:0043009\_chordate\_embryonic\_development | MAPK1 | 365 | 5 | 1.287392 | -0.459427 | 406 | 407.23 | 1.003030 |
| GO:0043009\_chordate\_embryonic\_development | SFRP1 | 365 | 5 | 1.287392 | -0.459427 | 406 | 407.23 | 1.003030 |
| GO:0043009\_chordate\_embryonic\_development | SP3 | 365 | 5 | 1.287392 | -0.459427 | 406 | 407.23 | 1.003030 |
| GO:0001824\_blastocyst\_development | SP3 | 40 | 1 | 2.349490 | -0.456777 | 409 | 411.43 | 1.005941 |
| GO:0016071\_mRNA\_metabolic\_process | APLP1 | 40 | 1 | 2.349490 | -0.456777 | 409 | 411.43 | 1.005941 |
| GO:0017015\_regulation\_of\_transforming\_growth\_factor\_beta\_receptor\_signaling\_pathway | ITGA8 | 40 | 1 | 2.349490 | -0.456777 | 409 | 411.43 | 1.005941 |
| GO:0009792\_embryonic\_development\_ending\_in\_birth\_or\_egg\_hatching | DLC1 | 368 | 5 | 1.276897 | -0.451351 | 410 | 413.69 | 1.009000 |
| GO:0009792\_embryonic\_development\_ending\_in\_birth\_or\_egg\_hatching | HBA-A1 | 368 | 5 | 1.276897 | -0.451351 | 410 | 413.69 | 1.009000 |
| GO:0009792\_embryonic\_development\_ending\_in\_birth\_or\_egg\_hatching | MAPK1 | 368 | 5 | 1.276897 | -0.451351 | 410 | 413.69 | 1.009000 |
| GO:0009792\_embryonic\_development\_ending\_in\_birth\_or\_egg\_hatching | SFRP1 | 368 | 5 | 1.276897 | -0.451351 | 410 | 413.69 | 1.009000 |
| GO:0009792\_embryonic\_development\_ending\_in\_birth\_or\_egg\_hatching | SP3 | 368 | 5 | 1.276897 | -0.451351 | 410 | 413.69 | 1.009000 |
| GO:0002429\_immune\_response-activating\_cell\_surface\_receptor\_signaling\_pathway | MAPK1 | 41 | 1 | 2.292185 | -0.448179 | 417 | 419.85 | 1.006835 |
| GO:0006260\_DNA\_replication | CCNE2 | 41 | 1 | 2.292185 | -0.448179 | 417 | 419.85 | 1.006835 |
| GO:0007254\_JNK\_cascade | MAPK10 | 41 | 1 | 2.292185 | -0.448179 | 417 | 419.85 | 1.006835 |
| GO:0009894\_regulation\_of\_catabolic\_process | ARNTL | 41 | 1 | 2.292185 | -0.448179 | 417 | 419.85 | 1.006835 |
| GO:0010551\_regulation\_of\_specific\_transcription\_from\_RNA\_polymerase\_II\_promoter | HES5 | 41 | 1 | 2.292185 | -0.448179 | 417 | 419.85 | 1.006835 |
| GO:0015833\_peptide\_transport | CPLX3 | 41 | 1 | 2.292185 | -0.448179 | 417 | 419.85 | 1.006835 |
| GO:0032569\_specific\_transcription\_from\_RNA\_polymerase\_II\_promoter | HES5 | 41 | 1 | 2.292185 | -0.448179 | 417 | 419.85 | 1.006835 |
| GO:0034645\_cellular\_macromolecule\_biosynthetic\_process | FUS | 901 | 11 | 1.147365 | -0.445373 | 418 | 420.73 | 1.006531 |
| GO:0034645\_cellular\_macromolecule\_biosynthetic\_process | CCNE2 | 901 | 11 | 1.147365 | -0.445373 | 418 | 420.73 | 1.006531 |
| GO:0034645\_cellular\_macromolecule\_biosynthetic\_process | AEBP2 | 901 | 11 | 1.147365 | -0.445373 | 418 | 420.73 | 1.006531 |
| GO:0034645\_cellular\_macromolecule\_biosynthetic\_process | HIPK1 | 901 | 11 | 1.147365 | -0.445373 | 418 | 420.73 | 1.006531 |
| GO:0034645\_cellular\_macromolecule\_biosynthetic\_process | HES5 | 901 | 11 | 1.147365 | -0.445373 | 418 | 420.73 | 1.006531 |
| GO:0034645\_cellular\_macromolecule\_biosynthetic\_process | SP3 | 901 | 11 | 1.147365 | -0.445373 | 418 | 420.73 | 1.006531 |
| GO:0034645\_cellular\_macromolecule\_biosynthetic\_process | NAB1 | 901 | 11 | 1.147365 | -0.445373 | 418 | 420.73 | 1.006531 |
| GO:0034645\_cellular\_macromolecule\_biosynthetic\_process | POU4F1 | 901 | 11 | 1.147365 | -0.445373 | 418 | 420.73 | 1.006531 |
| GO:0034645\_cellular\_macromolecule\_biosynthetic\_process | ARNTL | 901 | 11 | 1.147365 | -0.445373 | 418 | 420.73 | 1.006531 |
| GO:0034645\_cellular\_macromolecule\_biosynthetic\_process | APLP1 | 901 | 11 | 1.147365 | -0.445373 | 418 | 420.73 | 1.006531 |
| GO:0034645\_cellular\_macromolecule\_biosynthetic\_process | ZC3H8 | 901 | 11 | 1.147365 | -0.445373 | 418 | 420.73 | 1.006531 |
| GO:0031326\_regulation\_of\_cellular\_biosynthetic\_process | FUS | 812 | 10 | 1.157384 | -0.444103 | 419 | 421.42 | 1.005776 |
| GO:0031326\_regulation\_of\_cellular\_biosynthetic\_process | AEBP2 | 812 | 10 | 1.157384 | -0.444103 | 419 | 421.42 | 1.005776 |
| GO:0031326\_regulation\_of\_cellular\_biosynthetic\_process | HIPK1 | 812 | 10 | 1.157384 | -0.444103 | 419 | 421.42 | 1.005776 |
| GO:0031326\_regulation\_of\_cellular\_biosynthetic\_process | HES5 | 812 | 10 | 1.157384 | -0.444103 | 419 | 421.42 | 1.005776 |
| GO:0031326\_regulation\_of\_cellular\_biosynthetic\_process | SP3 | 812 | 10 | 1.157384 | -0.444103 | 419 | 421.42 | 1.005776 |
| GO:0031326\_regulation\_of\_cellular\_biosynthetic\_process | NAB1 | 812 | 10 | 1.157384 | -0.444103 | 419 | 421.42 | 1.005776 |
| GO:0031326\_regulation\_of\_cellular\_biosynthetic\_process | POU4F1 | 812 | 10 | 1.157384 | -0.444103 | 419 | 421.42 | 1.005776 |
| GO:0031326\_regulation\_of\_cellular\_biosynthetic\_process | ARNTL | 812 | 10 | 1.157384 | -0.444103 | 419 | 421.42 | 1.005776 |
| GO:0031326\_regulation\_of\_cellular\_biosynthetic\_process | ZC3H8 | 812 | 10 | 1.157384 | -0.444103 | 419 | 421.42 | 1.005776 |
| GO:0031326\_regulation\_of\_cellular\_biosynthetic\_process | APLP1 | 812 | 10 | 1.157384 | -0.444103 | 419 | 421.42 | 1.005776 |
| GO:0043412\_biopolymer\_modification | MAPK1 | 458 | 6 | 1.231174 | -0.443390 | 420 | 422.04 | 1.004857 |
| GO:0043412\_biopolymer\_modification | HIPK1 | 458 | 6 | 1.231174 | -0.443390 | 420 | 422.04 | 1.004857 |
| GO:0043412\_biopolymer\_modification | PDGFA | 458 | 6 | 1.231174 | -0.443390 | 420 | 422.04 | 1.004857 |
| GO:0043412\_biopolymer\_modification | CHUK | 458 | 6 | 1.231174 | -0.443390 | 420 | 422.04 | 1.004857 |
| GO:0043412\_biopolymer\_modification | MAP3K12 | 458 | 6 | 1.231174 | -0.443390 | 420 | 422.04 | 1.004857 |
| GO:0043412\_biopolymer\_modification | EPHB2 | 458 | 6 | 1.231174 | -0.443390 | 420 | 422.04 | 1.004857 |
| GO:0010740\_positive\_regulation\_of\_protein\_kinase\_cascade | CHUK | 42 | 1 | 2.237609 | -0.439836 | 423 | 428.02 | 1.011868 |
| GO:0042476\_odontogenesis | CHUK | 42 | 1 | 2.237609 | -0.439836 | 423 | 428.02 | 1.011868 |
| GO:0045637\_regulation\_of\_myeloid\_cell\_differentiation | JAG1 | 42 | 1 | 2.237609 | -0.439836 | 423 | 428.02 | 1.011868 |
| GO:0009889\_regulation\_of\_biosynthetic\_process | FUS | 815 | 10 | 1.153124 | -0.438585 | 424 | 428.26 | 1.010047 |
| GO:0009889\_regulation\_of\_biosynthetic\_process | AEBP2 | 815 | 10 | 1.153124 | -0.438585 | 424 | 428.26 | 1.010047 |
| GO:0009889\_regulation\_of\_biosynthetic\_process | HIPK1 | 815 | 10 | 1.153124 | -0.438585 | 424 | 428.26 | 1.010047 |
| GO:0009889\_regulation\_of\_biosynthetic\_process | HES5 | 815 | 10 | 1.153124 | -0.438585 | 424 | 428.26 | 1.010047 |
| GO:0009889\_regulation\_of\_biosynthetic\_process | SP3 | 815 | 10 | 1.153124 | -0.438585 | 424 | 428.26 | 1.010047 |
| GO:0009889\_regulation\_of\_biosynthetic\_process | NAB1 | 815 | 10 | 1.153124 | -0.438585 | 424 | 428.26 | 1.010047 |
| GO:0009889\_regulation\_of\_biosynthetic\_process | POU4F1 | 815 | 10 | 1.153124 | -0.438585 | 424 | 428.26 | 1.010047 |
| GO:0009889\_regulation\_of\_biosynthetic\_process | ARNTL | 815 | 10 | 1.153124 | -0.438585 | 424 | 428.26 | 1.010047 |
| GO:0009889\_regulation\_of\_biosynthetic\_process | APLP1 | 815 | 10 | 1.153124 | -0.438585 | 424 | 428.26 | 1.010047 |
| GO:0009889\_regulation\_of\_biosynthetic\_process | ZC3H8 | 815 | 10 | 1.153124 | -0.438585 | 424 | 428.26 | 1.010047 |
| GO:0051234\_establishment\_of\_localization | SCAMP1 | 729 | 9 | 1.160242 | -0.431971 | 425 | 429.47 | 1.010518 |
| GO:0051234\_establishment\_of\_localization | CPLX3 | 729 | 9 | 1.160242 | -0.431971 | 425 | 429.47 | 1.010518 |
| GO:0051234\_establishment\_of\_localization | SYTL2 | 729 | 9 | 1.160242 | -0.431971 | 425 | 429.47 | 1.010518 |
| GO:0051234\_establishment\_of\_localization | SV2B | 729 | 9 | 1.160242 | -0.431971 | 425 | 429.47 | 1.010518 |
| GO:0051234\_establishment\_of\_localization | ARNTL | 729 | 9 | 1.160242 | -0.431971 | 425 | 429.47 | 1.010518 |
| GO:0051234\_establishment\_of\_localization | YES1 | 729 | 9 | 1.160242 | -0.431971 | 425 | 429.47 | 1.010518 |
| GO:0051234\_establishment\_of\_localization | LRP2 | 729 | 9 | 1.160242 | -0.431971 | 425 | 429.47 | 1.010518 |
| GO:0051234\_establishment\_of\_localization | NTN1 | 729 | 9 | 1.160242 | -0.431971 | 425 | 429.47 | 1.010518 |
| GO:0051234\_establishment\_of\_localization | LIN7A | 729 | 9 | 1.160242 | -0.431971 | 425 | 429.47 | 1.010518 |
| GO:0001508\_regulation\_of\_action\_potential | NAB1 | 43 | 1 | 2.185572 | -0.431736 | 432 | 434.51 | 1.005810 |
| GO:0001841\_neural\_tube\_formation | DLC1 | 43 | 1 | 2.185572 | -0.431736 | 432 | 434.51 | 1.005810 |
| GO:0006766\_vitamin\_metabolic\_process | LRP2 | 43 | 1 | 2.185572 | -0.431736 | 432 | 434.51 | 1.005810 |
| GO:0009582\_detection\_of\_abiotic\_stimulus | GPR98 | 43 | 1 | 2.185572 | -0.431736 | 432 | 434.51 | 1.005810 |
| GO:0010001\_glial\_cell\_differentiation | NAB1 | 43 | 1 | 2.185572 | -0.431736 | 432 | 434.51 | 1.005810 |
| GO:0031098\_stress-activated\_protein\_kinase\_signaling\_pathway | MAPK10 | 43 | 1 | 2.185572 | -0.431736 | 432 | 434.51 | 1.005810 |
| GO:0046879\_hormone\_secretion | CPLX3 | 43 | 1 | 2.185572 | -0.431736 | 432 | 434.51 | 1.005810 |
| GO:0006917\_induction\_of\_apoptosis | HIPK1 | 121 | 2 | 1.553382 | -0.431093 | 434 | 435.57 | 1.003618 |
| GO:0006917\_induction\_of\_apoptosis | TM2D1 | 121 | 2 | 1.553382 | -0.431093 | 434 | 435.57 | 1.003618 |
| GO:0012502\_induction\_of\_programmed\_cell\_death | HIPK1 | 121 | 2 | 1.553382 | -0.431093 | 434 | 435.57 | 1.003618 |
| GO:0012502\_induction\_of\_programmed\_cell\_death | TM2D1 | 121 | 2 | 1.553382 | -0.431093 | 434 | 435.57 | 1.003618 |
| GO:0022607\_cellular\_component\_assembly | DLC1 | 204 | 3 | 1.382053 | -0.431085 | 435 | 435.72 | 1.001655 |
| GO:0022607\_cellular\_component\_assembly | PDGFA | 204 | 3 | 1.382053 | -0.431085 | 435 | 435.72 | 1.001655 |
| GO:0022607\_cellular\_component\_assembly | OLFM1 | 204 | 3 | 1.382053 | -0.431085 | 435 | 435.72 | 1.001655 |
| GO:0009059\_macromolecule\_biosynthetic\_process | CCNE2 | 910 | 11 | 1.136017 | -0.429613 | 436 | 436.03 | 1.000069 |
| GO:0009059\_macromolecule\_biosynthetic\_process | FUS | 910 | 11 | 1.136017 | -0.429613 | 436 | 436.03 | 1.000069 |
| GO:0009059\_macromolecule\_biosynthetic\_process | AEBP2 | 910 | 11 | 1.136017 | -0.429613 | 436 | 436.03 | 1.000069 |
| GO:0009059\_macromolecule\_biosynthetic\_process | HIPK1 | 910 | 11 | 1.136017 | -0.429613 | 436 | 436.03 | 1.000069 |
| GO:0009059\_macromolecule\_biosynthetic\_process | HES5 | 910 | 11 | 1.136017 | -0.429613 | 436 | 436.03 | 1.000069 |
| GO:0009059\_macromolecule\_biosynthetic\_process | SP3 | 910 | 11 | 1.136017 | -0.429613 | 436 | 436.03 | 1.000069 |
| GO:0009059\_macromolecule\_biosynthetic\_process | NAB1 | 910 | 11 | 1.136017 | -0.429613 | 436 | 436.03 | 1.000069 |
| GO:0009059\_macromolecule\_biosynthetic\_process | POU4F1 | 910 | 11 | 1.136017 | -0.429613 | 436 | 436.03 | 1.000069 |
| GO:0009059\_macromolecule\_biosynthetic\_process | ARNTL | 910 | 11 | 1.136017 | -0.429613 | 436 | 436.03 | 1.000069 |
| GO:0009059\_macromolecule\_biosynthetic\_process | ZC3H8 | 910 | 11 | 1.136017 | -0.429613 | 436 | 436.03 | 1.000069 |
| GO:0009059\_macromolecule\_biosynthetic\_process | APLP1 | 910 | 11 | 1.136017 | -0.429613 | 436 | 436.03 | 1.000069 |
| GO:0006139\_nucleobase\_\_nucleoside\_\_nucleotide\_and\_nucleic\_acid\_metabolic\_process | FUS | 1002 | 12 | 1.125504 | -0.426914 | 437 | 436.75 | 0.999428 |
| GO:0006139\_nucleobase\_\_nucleoside\_\_nucleotide\_and\_nucleic\_acid\_metabolic\_process | CCNE2 | 1002 | 12 | 1.125504 | -0.426914 | 437 | 436.75 | 0.999428 |
| GO:0006139\_nucleobase\_\_nucleoside\_\_nucleotide\_and\_nucleic\_acid\_metabolic\_process | MAPK1 | 1002 | 12 | 1.125504 | -0.426914 | 437 | 436.75 | 0.999428 |
| GO:0006139\_nucleobase\_\_nucleoside\_\_nucleotide\_and\_nucleic\_acid\_metabolic\_process | AEBP2 | 1002 | 12 | 1.125504 | -0.426914 | 437 | 436.75 | 0.999428 |
| GO:0006139\_nucleobase\_\_nucleoside\_\_nucleotide\_and\_nucleic\_acid\_metabolic\_process | HIPK1 | 1002 | 12 | 1.125504 | -0.426914 | 437 | 436.75 | 0.999428 |
| GO:0006139\_nucleobase\_\_nucleoside\_\_nucleotide\_and\_nucleic\_acid\_metabolic\_process | HES5 | 1002 | 12 | 1.125504 | -0.426914 | 437 | 436.75 | 0.999428 |
| GO:0006139\_nucleobase\_\_nucleoside\_\_nucleotide\_and\_nucleic\_acid\_metabolic\_process | SP3 | 1002 | 12 | 1.125504 | -0.426914 | 437 | 436.75 | 0.999428 |
| GO:0006139\_nucleobase\_\_nucleoside\_\_nucleotide\_and\_nucleic\_acid\_metabolic\_process | NAB1 | 1002 | 12 | 1.125504 | -0.426914 | 437 | 436.75 | 0.999428 |
| GO:0006139\_nucleobase\_\_nucleoside\_\_nucleotide\_and\_nucleic\_acid\_metabolic\_process | POU4F1 | 1002 | 12 | 1.125504 | -0.426914 | 437 | 436.75 | 0.999428 |
| GO:0006139\_nucleobase\_\_nucleoside\_\_nucleotide\_and\_nucleic\_acid\_metabolic\_process | ARNTL | 1002 | 12 | 1.125504 | -0.426914 | 437 | 436.75 | 0.999428 |
| GO:0006139\_nucleobase\_\_nucleoside\_\_nucleotide\_and\_nucleic\_acid\_metabolic\_process | APLP1 | 1002 | 12 | 1.125504 | -0.426914 | 437 | 436.75 | 0.999428 |
| GO:0006139\_nucleobase\_\_nucleoside\_\_nucleotide\_and\_nucleic\_acid\_metabolic\_process | ZC3H8 | 1002 | 12 | 1.125504 | -0.426914 | 437 | 436.75 | 0.999428 |
| GO:0001942\_hair\_follicle\_development | PDGFA | 44 | 1 | 2.135900 | -0.423867 | 448 | 444.18 | 0.991473 |
| GO:0002768\_immune\_response-regulating\_cell\_surface\_receptor\_signaling\_pathway | MAPK1 | 44 | 1 | 2.135900 | -0.423867 | 448 | 444.18 | 0.991473 |
| GO:0006606\_protein\_import\_into\_nucleus | ARNTL | 44 | 1 | 2.135900 | -0.423867 | 448 | 444.18 | 0.991473 |
| GO:0009914\_hormone\_transport | CPLX3 | 44 | 1 | 2.135900 | -0.423867 | 448 | 444.18 | 0.991473 |
| GO:0022404\_molting\_cycle\_process | PDGFA | 44 | 1 | 2.135900 | -0.423867 | 448 | 444.18 | 0.991473 |
| GO:0022405\_hair\_cycle\_process | PDGFA | 44 | 1 | 2.135900 | -0.423867 | 448 | 444.18 | 0.991473 |
| GO:0035282\_segmentation | SFRP1 | 44 | 1 | 2.135900 | -0.423867 | 448 | 444.18 | 0.991473 |
| GO:0042303\_molting\_cycle | PDGFA | 44 | 1 | 2.135900 | -0.423867 | 448 | 444.18 | 0.991473 |
| GO:0042633\_hair\_cycle | PDGFA | 44 | 1 | 2.135900 | -0.423867 | 448 | 444.18 | 0.991473 |
| GO:0050808\_synapse\_organization | ANK3 | 44 | 1 | 2.135900 | -0.423867 | 448 | 444.18 | 0.991473 |
| GO:0051170\_nuclear\_import | ARNTL | 44 | 1 | 2.135900 | -0.423867 | 448 | 444.18 | 0.991473 |
| GO:0001838\_embryonic\_epithelial\_tube\_formation | DLC1 | 45 | 1 | 2.088435 | -0.416218 | 449 | 447.33 | 0.996281 |
| GO:0044267\_cellular\_protein\_metabolic\_process | MAPK1 | 559 | 7 | 1.176846 | -0.415197 | 450 | 447.51 | 0.994467 |
| GO:0044267\_cellular\_protein\_metabolic\_process | HIPK1 | 559 | 7 | 1.176846 | -0.415197 | 450 | 447.51 | 0.994467 |
| GO:0044267\_cellular\_protein\_metabolic\_process | PDGFA | 559 | 7 | 1.176846 | -0.415197 | 450 | 447.51 | 0.994467 |
| GO:0044267\_cellular\_protein\_metabolic\_process | CHUK | 559 | 7 | 1.176846 | -0.415197 | 450 | 447.51 | 0.994467 |
| GO:0044267\_cellular\_protein\_metabolic\_process | MAP3K12 | 559 | 7 | 1.176846 | -0.415197 | 450 | 447.51 | 0.994467 |
| GO:0044267\_cellular\_protein\_metabolic\_process | EPHB2 | 559 | 7 | 1.176846 | -0.415197 | 450 | 447.51 | 0.994467 |
| GO:0044267\_cellular\_protein\_metabolic\_process | APLP1 | 559 | 7 | 1.176846 | -0.415197 | 450 | 447.51 | 0.994467 |
| GO:0002520\_immune\_system\_development | HBA-A1 | 295 | 4 | 1.274300 | -0.414618 | 451 | 448.13 | 0.993636 |
| GO:0002520\_immune\_system\_development | SP3 | 295 | 4 | 1.274300 | -0.414618 | 451 | 448.13 | 0.993636 |
| GO:0002520\_immune\_system\_development | JAG1 | 295 | 4 | 1.274300 | -0.414618 | 451 | 448.13 | 0.993636 |
| GO:0002520\_immune\_system\_development | CHUK | 295 | 4 | 1.274300 | -0.414618 | 451 | 448.13 | 0.993636 |
| GO:0001763\_morphogenesis\_of\_a\_branching\_structure | SFRP1 | 125 | 2 | 1.503673 | -0.413454 | 452 | 448.7 | 0.992699 |
| GO:0001763\_morphogenesis\_of\_a\_branching\_structure | PDGFA | 125 | 2 | 1.503673 | -0.413454 | 452 | 448.7 | 0.992699 |
| GO:0007612\_learning | EPHB2 | 46 | 1 | 2.043035 | -0.408781 | 457 | 451.97 | 0.988993 |
| GO:0009581\_detection\_of\_external\_stimulus | GPR98 | 46 | 1 | 2.043035 | -0.408781 | 457 | 451.97 | 0.988993 |
| GO:0030850\_prostate\_gland\_development | SFRP1 | 46 | 1 | 2.043035 | -0.408781 | 457 | 451.97 | 0.988993 |
| GO:0042063\_gliogenesis | NAB1 | 46 | 1 | 2.043035 | -0.408781 | 457 | 451.97 | 0.988993 |
| GO:0051098\_regulation\_of\_binding | HIPK1 | 46 | 1 | 2.043035 | -0.408781 | 457 | 451.97 | 0.988993 |
| GO:0035295\_tube\_development | DLC1 | 212 | 3 | 1.329900 | -0.404397 | 458 | 452.43 | 0.987838 |
| GO:0035295\_tube\_development | PDGFA | 212 | 3 | 1.329900 | -0.404397 | 458 | 452.43 | 0.987838 |
| GO:0035295\_tube\_development | SP3 | 212 | 3 | 1.329900 | -0.404397 | 458 | 452.43 | 0.987838 |
| GO:0002757\_immune\_response-activating\_signal\_transduction | MAPK1 | 47 | 1 | 1.999566 | -0.401545 | 462 | 456.95 | 0.989069 |
| GO:0006396\_RNA\_processing | APLP1 | 47 | 1 | 1.999566 | -0.401545 | 462 | 456.95 | 0.989069 |
| GO:0016570\_histone\_modification | MAP3K12 | 47 | 1 | 1.999566 | -0.401545 | 462 | 456.95 | 0.989069 |
| GO:0030183\_B\_cell\_differentiation | SP3 | 47 | 1 | 1.999566 | -0.401545 | 462 | 456.95 | 0.989069 |
| GO:0001655\_urogenital\_system\_development | SFRP1 | 128 | 2 | 1.468431 | -0.400789 | 463 | 457.84 | 0.988855 |
| GO:0001655\_urogenital\_system\_development | ITGA8 | 128 | 2 | 1.468431 | -0.400789 | 463 | 457.84 | 0.988855 |
| GO:0016070\_RNA\_metabolic\_process | FUS | 658 | 8 | 1.142609 | -0.396734 | 464 | 458.28 | 0.987672 |
| GO:0016070\_RNA\_metabolic\_process | AEBP2 | 658 | 8 | 1.142609 | -0.396734 | 464 | 458.28 | 0.987672 |
| GO:0016070\_RNA\_metabolic\_process | HIPK1 | 658 | 8 | 1.142609 | -0.396734 | 464 | 458.28 | 0.987672 |
| GO:0016070\_RNA\_metabolic\_process | HES5 | 658 | 8 | 1.142609 | -0.396734 | 464 | 458.28 | 0.987672 |
| GO:0016070\_RNA\_metabolic\_process | SP3 | 658 | 8 | 1.142609 | -0.396734 | 464 | 458.28 | 0.987672 |
| GO:0016070\_RNA\_metabolic\_process | POU4F1 | 658 | 8 | 1.142609 | -0.396734 | 464 | 458.28 | 0.987672 |
| GO:0016070\_RNA\_metabolic\_process | ARNTL | 658 | 8 | 1.142609 | -0.396734 | 464 | 458.28 | 0.987672 |
| GO:0016070\_RNA\_metabolic\_process | APLP1 | 658 | 8 | 1.142609 | -0.396734 | 464 | 458.28 | 0.987672 |
| GO:0034504\_protein\_localization\_in\_nucleus | ARNTL | 48 | 1 | 1.957908 | -0.394502 | 465 | 461.16 | 0.991742 |
| GO:0043170\_macromolecule\_metabolic\_process | FUS | 1576 | 18 | 1.073371 | -0.390570 | 466 | 462.36 | 0.992189 |
| GO:0043170\_macromolecule\_metabolic\_process | AEBP2 | 1576 | 18 | 1.073371 | -0.390570 | 466 | 462.36 | 0.992189 |
| GO:0043170\_macromolecule\_metabolic\_process | PDGFA | 1576 | 18 | 1.073371 | -0.390570 | 466 | 462.36 | 0.992189 |
| GO:0043170\_macromolecule\_metabolic\_process | ARNTL | 1576 | 18 | 1.073371 | -0.390570 | 466 | 462.36 | 0.992189 |
| GO:0043170\_macromolecule\_metabolic\_process | MMP2 | 1576 | 18 | 1.073371 | -0.390570 | 466 | 462.36 | 0.992189 |
| GO:0043170\_macromolecule\_metabolic\_process | EPHB2 | 1576 | 18 | 1.073371 | -0.390570 | 466 | 462.36 | 0.992189 |
| GO:0043170\_macromolecule\_metabolic\_process | APLP1 | 1576 | 18 | 1.073371 | -0.390570 | 466 | 462.36 | 0.992189 |
| GO:0043170\_macromolecule\_metabolic\_process | ZC3H8 | 1576 | 18 | 1.073371 | -0.390570 | 466 | 462.36 | 0.992189 |
| GO:0043170\_macromolecule\_metabolic\_process | CCNE2 | 1576 | 18 | 1.073371 | -0.390570 | 466 | 462.36 | 0.992189 |
| GO:0043170\_macromolecule\_metabolic\_process | MAPK1 | 1576 | 18 | 1.073371 | -0.390570 | 466 | 462.36 | 0.992189 |
| GO:0043170\_macromolecule\_metabolic\_process | HES5 | 1576 | 18 | 1.073371 | -0.390570 | 466 | 462.36 | 0.992189 |
| GO:0043170\_macromolecule\_metabolic\_process | HIPK1 | 1576 | 18 | 1.073371 | -0.390570 | 466 | 462.36 | 0.992189 |
| GO:0043170\_macromolecule\_metabolic\_process | SP3 | 1576 | 18 | 1.073371 | -0.390570 | 466 | 462.36 | 0.992189 |
| GO:0043170\_macromolecule\_metabolic\_process | NAB1 | 1576 | 18 | 1.073371 | -0.390570 | 466 | 462.36 | 0.992189 |
| GO:0043170\_macromolecule\_metabolic\_process | SV2B | 1576 | 18 | 1.073371 | -0.390570 | 466 | 462.36 | 0.992189 |
| GO:0043170\_macromolecule\_metabolic\_process | POU4F1 | 1576 | 18 | 1.073371 | -0.390570 | 466 | 462.36 | 0.992189 |
| GO:0043170\_macromolecule\_metabolic\_process | CHUK | 1576 | 18 | 1.073371 | -0.390570 | 466 | 462.36 | 0.992189 |
| GO:0043170\_macromolecule\_metabolic\_process | MAP3K12 | 1576 | 18 | 1.073371 | -0.390570 | 466 | 462.36 | 0.992189 |
| GO:0006725\_cellular\_aromatic\_compound\_metabolic\_process | MAPK1 | 49 | 1 | 1.917951 | -0.387643 | 467 | 466.93 | 0.999850 |
| GO:0034960\_cellular\_biopolymer\_metabolic\_process | FUS | 1395 | 16 | 1.077902 | -0.385836 | 468 | 467.23 | 0.998355 |
| GO:0034960\_cellular\_biopolymer\_metabolic\_process | AEBP2 | 1395 | 16 | 1.077902 | -0.385836 | 468 | 467.23 | 0.998355 |
| GO:0034960\_cellular\_biopolymer\_metabolic\_process | PDGFA | 1395 | 16 | 1.077902 | -0.385836 | 468 | 467.23 | 0.998355 |
| GO:0034960\_cellular\_biopolymer\_metabolic\_process | ARNTL | 1395 | 16 | 1.077902 | -0.385836 | 468 | 467.23 | 0.998355 |
| GO:0034960\_cellular\_biopolymer\_metabolic\_process | EPHB2 | 1395 | 16 | 1.077902 | -0.385836 | 468 | 467.23 | 0.998355 |
| GO:0034960\_cellular\_biopolymer\_metabolic\_process | APLP1 | 1395 | 16 | 1.077902 | -0.385836 | 468 | 467.23 | 0.998355 |
| GO:0034960\_cellular\_biopolymer\_metabolic\_process | ZC3H8 | 1395 | 16 | 1.077902 | -0.385836 | 468 | 467.23 | 0.998355 |
| GO:0034960\_cellular\_biopolymer\_metabolic\_process | CCNE2 | 1395 | 16 | 1.077902 | -0.385836 | 468 | 467.23 | 0.998355 |
| GO:0034960\_cellular\_biopolymer\_metabolic\_process | MAPK1 | 1395 | 16 | 1.077902 | -0.385836 | 468 | 467.23 | 0.998355 |
| GO:0034960\_cellular\_biopolymer\_metabolic\_process | HES5 | 1395 | 16 | 1.077902 | -0.385836 | 468 | 467.23 | 0.998355 |
| GO:0034960\_cellular\_biopolymer\_metabolic\_process | HIPK1 | 1395 | 16 | 1.077902 | -0.385836 | 468 | 467.23 | 0.998355 |
| GO:0034960\_cellular\_biopolymer\_metabolic\_process | SP3 | 1395 | 16 | 1.077902 | -0.385836 | 468 | 467.23 | 0.998355 |
| GO:0034960\_cellular\_biopolymer\_metabolic\_process | NAB1 | 1395 | 16 | 1.077902 | -0.385836 | 468 | 467.23 | 0.998355 |
| GO:0034960\_cellular\_biopolymer\_metabolic\_process | POU4F1 | 1395 | 16 | 1.077902 | -0.385836 | 468 | 467.23 | 0.998355 |
| GO:0034960\_cellular\_biopolymer\_metabolic\_process | CHUK | 1395 | 16 | 1.077902 | -0.385836 | 468 | 467.23 | 0.998355 |
| GO:0034960\_cellular\_biopolymer\_metabolic\_process | MAP3K12 | 1395 | 16 | 1.077902 | -0.385836 | 468 | 467.23 | 0.998355 |
| GO:0006355\_regulation\_of\_transcription\_\_DNA-dependent | FUS | 575 | 7 | 1.144099 | -0.383243 | 469 | 468.05 | 0.997974 |
| GO:0006355\_regulation\_of\_transcription\_\_DNA-dependent | AEBP2 | 575 | 7 | 1.144099 | -0.383243 | 469 | 468.05 | 0.997974 |
| GO:0006355\_regulation\_of\_transcription\_\_DNA-dependent | HIPK1 | 575 | 7 | 1.144099 | -0.383243 | 469 | 468.05 | 0.997974 |
| GO:0006355\_regulation\_of\_transcription\_\_DNA-dependent | HES5 | 575 | 7 | 1.144099 | -0.383243 | 469 | 468.05 | 0.997974 |
| GO:0006355\_regulation\_of\_transcription\_\_DNA-dependent | SP3 | 575 | 7 | 1.144099 | -0.383243 | 469 | 468.05 | 0.997974 |
| GO:0006355\_regulation\_of\_transcription\_\_DNA-dependent | POU4F1 | 575 | 7 | 1.144099 | -0.383243 | 469 | 468.05 | 0.997974 |
| GO:0006355\_regulation\_of\_transcription\_\_DNA-dependent | ARNTL | 575 | 7 | 1.144099 | -0.383243 | 469 | 468.05 | 0.997974 |
| GO:0043283\_biopolymer\_metabolic\_process | FUS | 1490 | 17 | 1.072250 | -0.382049 | 470 | 468.83 | 0.997511 |
| GO:0043283\_biopolymer\_metabolic\_process | AEBP2 | 1490 | 17 | 1.072250 | -0.382049 | 470 | 468.83 | 0.997511 |
| GO:0043283\_biopolymer\_metabolic\_process | PDGFA | 1490 | 17 | 1.072250 | -0.382049 | 470 | 468.83 | 0.997511 |
| GO:0043283\_biopolymer\_metabolic\_process | ARNTL | 1490 | 17 | 1.072250 | -0.382049 | 470 | 468.83 | 0.997511 |
| GO:0043283\_biopolymer\_metabolic\_process | ZC3H8 | 1490 | 17 | 1.072250 | -0.382049 | 470 | 468.83 | 0.997511 |
| GO:0043283\_biopolymer\_metabolic\_process | APLP1 | 1490 | 17 | 1.072250 | -0.382049 | 470 | 468.83 | 0.997511 |
| GO:0043283\_biopolymer\_metabolic\_process | EPHB2 | 1490 | 17 | 1.072250 | -0.382049 | 470 | 468.83 | 0.997511 |
| GO:0043283\_biopolymer\_metabolic\_process | CCNE2 | 1490 | 17 | 1.072250 | -0.382049 | 470 | 468.83 | 0.997511 |
| GO:0043283\_biopolymer\_metabolic\_process | MAPK1 | 1490 | 17 | 1.072250 | -0.382049 | 470 | 468.83 | 0.997511 |
| GO:0043283\_biopolymer\_metabolic\_process | HIPK1 | 1490 | 17 | 1.072250 | -0.382049 | 470 | 468.83 | 0.997511 |
| GO:0043283\_biopolymer\_metabolic\_process | HES5 | 1490 | 17 | 1.072250 | -0.382049 | 470 | 468.83 | 0.997511 |
| GO:0043283\_biopolymer\_metabolic\_process | SP3 | 1490 | 17 | 1.072250 | -0.382049 | 470 | 468.83 | 0.997511 |
| GO:0043283\_biopolymer\_metabolic\_process | NAB1 | 1490 | 17 | 1.072250 | -0.382049 | 470 | 468.83 | 0.997511 |
| GO:0043283\_biopolymer\_metabolic\_process | SV2B | 1490 | 17 | 1.072250 | -0.382049 | 470 | 468.83 | 0.997511 |
| GO:0043283\_biopolymer\_metabolic\_process | POU4F1 | 1490 | 17 | 1.072250 | -0.382049 | 470 | 468.83 | 0.997511 |
| GO:0043283\_biopolymer\_metabolic\_process | MAP3K12 | 1490 | 17 | 1.072250 | -0.382049 | 470 | 468.83 | 0.997511 |
| GO:0043283\_biopolymer\_metabolic\_process | CHUK | 1490 | 17 | 1.072250 | -0.382049 | 470 | 468.83 | 0.997511 |
| GO:0019219\_regulation\_of\_nucleobase\_\_nucleoside\_\_nucleotide\_and\_nucleic\_acid\_metabolic\_process | FUS | 757 | 9 | 1.117327 | -0.381556 | 471 | 469.03 | 0.995817 |
| GO:0019219\_regulation\_of\_nucleobase\_\_nucleoside\_\_nucleotide\_and\_nucleic\_acid\_metabolic\_process | AEBP2 | 757 | 9 | 1.117327 | -0.381556 | 471 | 469.03 | 0.995817 |
| GO:0019219\_regulation\_of\_nucleobase\_\_nucleoside\_\_nucleotide\_and\_nucleic\_acid\_metabolic\_process | HIPK1 | 757 | 9 | 1.117327 | -0.381556 | 471 | 469.03 | 0.995817 |
| GO:0019219\_regulation\_of\_nucleobase\_\_nucleoside\_\_nucleotide\_and\_nucleic\_acid\_metabolic\_process | HES5 | 757 | 9 | 1.117327 | -0.381556 | 471 | 469.03 | 0.995817 |
| GO:0019219\_regulation\_of\_nucleobase\_\_nucleoside\_\_nucleotide\_and\_nucleic\_acid\_metabolic\_process | SP3 | 757 | 9 | 1.117327 | -0.381556 | 471 | 469.03 | 0.995817 |
| GO:0019219\_regulation\_of\_nucleobase\_\_nucleoside\_\_nucleotide\_and\_nucleic\_acid\_metabolic\_process | NAB1 | 757 | 9 | 1.117327 | -0.381556 | 471 | 469.03 | 0.995817 |
| GO:0019219\_regulation\_of\_nucleobase\_\_nucleoside\_\_nucleotide\_and\_nucleic\_acid\_metabolic\_process | POU4F1 | 757 | 9 | 1.117327 | -0.381556 | 471 | 469.03 | 0.995817 |
| GO:0019219\_regulation\_of\_nucleobase\_\_nucleoside\_\_nucleotide\_and\_nucleic\_acid\_metabolic\_process | ARNTL | 757 | 9 | 1.117327 | -0.381556 | 471 | 469.03 | 0.995817 |
| GO:0019219\_regulation\_of\_nucleobase\_\_nucleoside\_\_nucleotide\_and\_nucleic\_acid\_metabolic\_process | ZC3H8 | 757 | 9 | 1.117327 | -0.381556 | 471 | 469.03 | 0.995817 |
| GO:0001656\_metanephros\_development | ITGA8 | 50 | 1 | 1.879592 | -0.380962 | 474 | 471.44 | 0.994599 |
| GO:0017038\_protein\_import | ARNTL | 50 | 1 | 1.879592 | -0.380962 | 474 | 471.44 | 0.994599 |
| GO:0051606\_detection\_of\_stimulus | GPR98 | 50 | 1 | 1.879592 | -0.380962 | 474 | 471.44 | 0.994599 |
| GO:0009952\_anterior\_posterior\_pattern\_formation | HIPK1 | 133 | 2 | 1.413227 | -0.380686 | 476 | 472.04 | 0.991681 |
| GO:0009952\_anterior\_posterior\_pattern\_formation | SFRP1 | 133 | 2 | 1.413227 | -0.380686 | 476 | 472.04 | 0.991681 |
| GO:0044057\_regulation\_of\_system\_process | CPLX3 | 133 | 2 | 1.413227 | -0.380686 | 476 | 472.04 | 0.991681 |
| GO:0044057\_regulation\_of\_system\_process | EPHB2 | 133 | 2 | 1.413227 | -0.380686 | 476 | 472.04 | 0.991681 |
| GO:0001701\_in\_utero\_embryonic\_development | HBA-A1 | 221 | 3 | 1.275741 | -0.376457 | 477 | 473.4 | 0.992453 |
| GO:0001701\_in\_utero\_embryonic\_development | MAPK1 | 221 | 3 | 1.275741 | -0.376457 | 477 | 473.4 | 0.992453 |
| GO:0001701\_in\_utero\_embryonic\_development | SP3 | 221 | 3 | 1.275741 | -0.376457 | 477 | 473.4 | 0.992453 |
| GO:0002764\_immune\_response-regulating\_signal\_transduction | MAPK1 | 51 | 1 | 1.842737 | -0.374451 | 481 | 476.82 | 0.991310 |
| GO:0007601\_visual\_perception | GPR98 | 51 | 1 | 1.842737 | -0.374451 | 481 | 476.82 | 0.991310 |
| GO:0016569\_covalent\_chromatin\_modification | MAP3K12 | 51 | 1 | 1.842737 | -0.374451 | 481 | 476.82 | 0.991310 |
| GO:0032583\_regulation\_of\_gene-specific\_transcription | HES5 | 51 | 1 | 1.842737 | -0.374451 | 481 | 476.82 | 0.991310 |
| GO:0001654\_eye\_development | SP3 | 136 | 2 | 1.382053 | -0.369192 | 482 | 477.06 | 0.989751 |
| GO:0001654\_eye\_development | EPHB2 | 136 | 2 | 1.382053 | -0.369192 | 482 | 477.06 | 0.989751 |
| GO:0010608\_posttranscriptional\_regulation\_of\_gene\_expression | APLP1 | 52 | 1 | 1.807300 | -0.368104 | 484 | 478.61 | 0.988864 |
| GO:0050953\_sensory\_perception\_of\_light\_stimulus | GPR98 | 52 | 1 | 1.807300 | -0.368104 | 484 | 478.61 | 0.988864 |
| GO:0048519\_negative\_regulation\_of\_biological\_process | MAPK1 | 859 | 10 | 1.094058 | -0.363936 | 485 | 479.54 | 0.988742 |
| GO:0048519\_negative\_regulation\_of\_biological\_process | DKK1 | 859 | 10 | 1.094058 | -0.363936 | 485 | 479.54 | 0.988742 |
| GO:0048519\_negative\_regulation\_of\_biological\_process | STMN3 | 859 | 10 | 1.094058 | -0.363936 | 485 | 479.54 | 0.988742 |
| GO:0048519\_negative\_regulation\_of\_biological\_process | HIPK1 | 859 | 10 | 1.094058 | -0.363936 | 485 | 479.54 | 0.988742 |
| GO:0048519\_negative\_regulation\_of\_biological\_process | HES5 | 859 | 10 | 1.094058 | -0.363936 | 485 | 479.54 | 0.988742 |
| GO:0048519\_negative\_regulation\_of\_biological\_process | FOXG1 | 859 | 10 | 1.094058 | -0.363936 | 485 | 479.54 | 0.988742 |
| GO:0048519\_negative\_regulation\_of\_biological\_process | JAG1 | 859 | 10 | 1.094058 | -0.363936 | 485 | 479.54 | 0.988742 |
| GO:0048519\_negative\_regulation\_of\_biological\_process | NTN1 | 859 | 10 | 1.094058 | -0.363936 | 485 | 479.54 | 0.988742 |
| GO:0048519\_negative\_regulation\_of\_biological\_process | EPHB2 | 859 | 10 | 1.094058 | -0.363936 | 485 | 479.54 | 0.988742 |
| GO:0048519\_negative\_regulation\_of\_biological\_process | ZC3H8 | 859 | 10 | 1.094058 | -0.363936 | 485 | 479.54 | 0.988742 |
| GO:0030031\_cell\_projection\_assembly | PDGFA | 53 | 1 | 1.773200 | -0.361913 | 487 | 482.05 | 0.989836 |
| GO:0050905\_neuromuscular\_process | POU4F1 | 53 | 1 | 1.773200 | -0.361913 | 487 | 482.05 | 0.989836 |
| GO:0051171\_regulation\_of\_nitrogen\_compound\_metabolic\_process | FUS | 771 | 9 | 1.097038 | -0.358265 | 488 | 482.4 | 0.988525 |
| GO:0051171\_regulation\_of\_nitrogen\_compound\_metabolic\_process | AEBP2 | 771 | 9 | 1.097038 | -0.358265 | 488 | 482.4 | 0.988525 |
| GO:0051171\_regulation\_of\_nitrogen\_compound\_metabolic\_process | HIPK1 | 771 | 9 | 1.097038 | -0.358265 | 488 | 482.4 | 0.988525 |
| GO:0051171\_regulation\_of\_nitrogen\_compound\_metabolic\_process | HES5 | 771 | 9 | 1.097038 | -0.358265 | 488 | 482.4 | 0.988525 |
| GO:0051171\_regulation\_of\_nitrogen\_compound\_metabolic\_process | SP3 | 771 | 9 | 1.097038 | -0.358265 | 488 | 482.4 | 0.988525 |
| GO:0051171\_regulation\_of\_nitrogen\_compound\_metabolic\_process | NAB1 | 771 | 9 | 1.097038 | -0.358265 | 488 | 482.4 | 0.988525 |
| GO:0051171\_regulation\_of\_nitrogen\_compound\_metabolic\_process | POU4F1 | 771 | 9 | 1.097038 | -0.358265 | 488 | 482.4 | 0.988525 |
| GO:0051171\_regulation\_of\_nitrogen\_compound\_metabolic\_process | ARNTL | 771 | 9 | 1.097038 | -0.358265 | 488 | 482.4 | 0.988525 |
| GO:0051171\_regulation\_of\_nitrogen\_compound\_metabolic\_process | ZC3H8 | 771 | 9 | 1.097038 | -0.358265 | 488 | 482.4 | 0.988525 |
| GO:0002253\_activation\_of\_immune\_response | MAPK1 | 54 | 1 | 1.740363 | -0.355873 | 491 | 486.85 | 0.991548 |
| GO:0006412\_translation | APLP1 | 54 | 1 | 1.740363 | -0.355873 | 491 | 486.85 | 0.991548 |
| GO:0007265\_Ras\_protein\_signal\_transduction | STMN3 | 54 | 1 | 1.740363 | -0.355873 | 491 | 486.85 | 0.991548 |
| GO:0051252\_regulation\_of\_RNA\_metabolic\_process | FUS | 590 | 7 | 1.115012 | -0.355272 | 492 | 487.0 | 0.989837 |
| GO:0051252\_regulation\_of\_RNA\_metabolic\_process | AEBP2 | 590 | 7 | 1.115012 | -0.355272 | 492 | 487.0 | 0.989837 |
| GO:0051252\_regulation\_of\_RNA\_metabolic\_process | HIPK1 | 590 | 7 | 1.115012 | -0.355272 | 492 | 487.0 | 0.989837 |
| GO:0051252\_regulation\_of\_RNA\_metabolic\_process | HES5 | 590 | 7 | 1.115012 | -0.355272 | 492 | 487.0 | 0.989837 |
| GO:0051252\_regulation\_of\_RNA\_metabolic\_process | SP3 | 590 | 7 | 1.115012 | -0.355272 | 492 | 487.0 | 0.989837 |
| GO:0051252\_regulation\_of\_RNA\_metabolic\_process | POU4F1 | 590 | 7 | 1.115012 | -0.355272 | 492 | 487.0 | 0.989837 |
| GO:0051252\_regulation\_of\_RNA\_metabolic\_process | ARNTL | 590 | 7 | 1.115012 | -0.355272 | 492 | 487.0 | 0.989837 |
| GO:0007605\_sensory\_perception\_of\_sound | GPR98 | 55 | 1 | 1.708720 | -0.349978 | 494 | 490.74 | 0.993401 |
| GO:0048568\_embryonic\_organ\_development | SP3 | 55 | 1 | 1.708720 | -0.349978 | 494 | 490.74 | 0.993401 |
| GO:0006351\_transcription\_\_DNA-dependent | FUS | 594 | 7 | 1.107504 | -0.348123 | 495 | 491.08 | 0.992081 |
| GO:0006351\_transcription\_\_DNA-dependent | AEBP2 | 594 | 7 | 1.107504 | -0.348123 | 495 | 491.08 | 0.992081 |
| GO:0006351\_transcription\_\_DNA-dependent | HIPK1 | 594 | 7 | 1.107504 | -0.348123 | 495 | 491.08 | 0.992081 |
| GO:0006351\_transcription\_\_DNA-dependent | HES5 | 594 | 7 | 1.107504 | -0.348123 | 495 | 491.08 | 0.992081 |
| GO:0006351\_transcription\_\_DNA-dependent | SP3 | 594 | 7 | 1.107504 | -0.348123 | 495 | 491.08 | 0.992081 |
| GO:0006351\_transcription\_\_DNA-dependent | POU4F1 | 594 | 7 | 1.107504 | -0.348123 | 495 | 491.08 | 0.992081 |
| GO:0006351\_transcription\_\_DNA-dependent | ARNTL | 594 | 7 | 1.107504 | -0.348123 | 495 | 491.08 | 0.992081 |
| GO:0032774\_RNA\_biosynthetic\_process | FUS | 595 | 7 | 1.105642 | -0.346355 | 496 | 491.82 | 0.991573 |
| GO:0032774\_RNA\_biosynthetic\_process | AEBP2 | 595 | 7 | 1.105642 | -0.346355 | 496 | 491.82 | 0.991573 |
| GO:0032774\_RNA\_biosynthetic\_process | HIPK1 | 595 | 7 | 1.105642 | -0.346355 | 496 | 491.82 | 0.991573 |
| GO:0032774\_RNA\_biosynthetic\_process | HES5 | 595 | 7 | 1.105642 | -0.346355 | 496 | 491.82 | 0.991573 |
| GO:0032774\_RNA\_biosynthetic\_process | SP3 | 595 | 7 | 1.105642 | -0.346355 | 496 | 491.82 | 0.991573 |
| GO:0032774\_RNA\_biosynthetic\_process | POU4F1 | 595 | 7 | 1.105642 | -0.346355 | 496 | 491.82 | 0.991573 |
| GO:0032774\_RNA\_biosynthetic\_process | ARNTL | 595 | 7 | 1.105642 | -0.346355 | 496 | 491.82 | 0.991573 |
| GO:0051321\_meiotic\_cell\_cycle | TUBG1 | 56 | 1 | 1.678207 | -0.344224 | 497 | 495.48 | 0.996942 |
| GO:0016477\_cell\_migration | KATNA1 | 234 | 3 | 1.204867 | -0.339620 | 498 | 497.62 | 0.999237 |
| GO:0016477\_cell\_migration | POU4F1 | 234 | 3 | 1.204867 | -0.339620 | 498 | 497.62 | 0.999237 |
| GO:0016477\_cell\_migration | NTN1 | 234 | 3 | 1.204867 | -0.339620 | 498 | 497.62 | 0.999237 |
| GO:0008344\_adult\_locomotory\_behavior | EPHA4 | 57 | 1 | 1.648765 | -0.338604 | 502 | 501.73 | 0.999462 |
| GO:0009953\_dorsal\_ventral\_pattern\_formation | FOXG1 | 57 | 1 | 1.648765 | -0.338604 | 502 | 501.73 | 0.999462 |
| GO:0033365\_protein\_localization\_in\_organelle | ARNTL | 57 | 1 | 1.648765 | -0.338604 | 502 | 501.73 | 0.999462 |
| GO:0050878\_regulation\_of\_body\_fluid\_levels | SYTL2 | 57 | 1 | 1.648765 | -0.338604 | 502 | 501.73 | 0.999462 |
| GO:0001501\_skeletal\_system\_development | SP3 | 236 | 3 | 1.194656 | -0.334295 | 503 | 502.14 | 0.998290 |
| GO:0001501\_skeletal\_system\_development | NAB1 | 236 | 3 | 1.194656 | -0.334295 | 503 | 502.14 | 0.998290 |
| GO:0001501\_skeletal\_system\_development | MMP2 | 236 | 3 | 1.194656 | -0.334295 | 503 | 502.14 | 0.998290 |
| GO:0044085\_cellular\_component\_biogenesis | DLC1 | 237 | 3 | 1.189615 | -0.331664 | 504 | 504.98 | 1.001944 |
| GO:0044085\_cellular\_component\_biogenesis | PDGFA | 237 | 3 | 1.189615 | -0.331664 | 504 | 504.98 | 1.001944 |
| GO:0044085\_cellular\_component\_biogenesis | OLFM1 | 237 | 3 | 1.189615 | -0.331664 | 504 | 504.98 | 1.001944 |
| GO:0007049\_cell\_cycle | CCNE2 | 238 | 3 | 1.184617 | -0.329055 | 505 | 505.82 | 1.001624 |
| GO:0007049\_cell\_cycle | FOXG1 | 238 | 3 | 1.184617 | -0.329055 | 505 | 505.82 | 1.001624 |
| GO:0007049\_cell\_cycle | TUBG1 | 238 | 3 | 1.184617 | -0.329055 | 505 | 505.82 | 1.001624 |
| GO:0030334\_regulation\_of\_cell\_migration | NTN1 | 59 | 1 | 1.592874 | -0.327749 | 506 | 507.73 | 1.003419 |
| GO:0044260\_cellular\_macromolecule\_metabolic\_process | FUS | 1447 | 16 | 1.039166 | -0.318972 | 507 | 511.1 | 1.008087 |
| GO:0044260\_cellular\_macromolecule\_metabolic\_process | AEBP2 | 1447 | 16 | 1.039166 | -0.318972 | 507 | 511.1 | 1.008087 |
| GO:0044260\_cellular\_macromolecule\_metabolic\_process | PDGFA | 1447 | 16 | 1.039166 | -0.318972 | 507 | 511.1 | 1.008087 |
| GO:0044260\_cellular\_macromolecule\_metabolic\_process | ARNTL | 1447 | 16 | 1.039166 | -0.318972 | 507 | 511.1 | 1.008087 |
| GO:0044260\_cellular\_macromolecule\_metabolic\_process | EPHB2 | 1447 | 16 | 1.039166 | -0.318972 | 507 | 511.1 | 1.008087 |
| GO:0044260\_cellular\_macromolecule\_metabolic\_process | ZC3H8 | 1447 | 16 | 1.039166 | -0.318972 | 507 | 511.1 | 1.008087 |
| GO:0044260\_cellular\_macromolecule\_metabolic\_process | APLP1 | 1447 | 16 | 1.039166 | -0.318972 | 507 | 511.1 | 1.008087 |
| GO:0044260\_cellular\_macromolecule\_metabolic\_process | CCNE2 | 1447 | 16 | 1.039166 | -0.318972 | 507 | 511.1 | 1.008087 |
| GO:0044260\_cellular\_macromolecule\_metabolic\_process | MAPK1 | 1447 | 16 | 1.039166 | -0.318972 | 507 | 511.1 | 1.008087 |
| GO:0044260\_cellular\_macromolecule\_metabolic\_process | HES5 | 1447 | 16 | 1.039166 | -0.318972 | 507 | 511.1 | 1.008087 |
| GO:0044260\_cellular\_macromolecule\_metabolic\_process | HIPK1 | 1447 | 16 | 1.039166 | -0.318972 | 507 | 511.1 | 1.008087 |
| GO:0044260\_cellular\_macromolecule\_metabolic\_process | SP3 | 1447 | 16 | 1.039166 | -0.318972 | 507 | 511.1 | 1.008087 |
| GO:0044260\_cellular\_macromolecule\_metabolic\_process | NAB1 | 1447 | 16 | 1.039166 | -0.318972 | 507 | 511.1 | 1.008087 |
| GO:0044260\_cellular\_macromolecule\_metabolic\_process | POU4F1 | 1447 | 16 | 1.039166 | -0.318972 | 507 | 511.1 | 1.008087 |
| GO:0044260\_cellular\_macromolecule\_metabolic\_process | CHUK | 1447 | 16 | 1.039166 | -0.318972 | 507 | 511.1 | 1.008087 |
| GO:0044260\_cellular\_macromolecule\_metabolic\_process | MAP3K12 | 1447 | 16 | 1.039166 | -0.318972 | 507 | 511.1 | 1.008087 |
| GO:0032268\_regulation\_of\_cellular\_protein\_metabolic\_process | PDGFA | 152 | 2 | 1.236574 | -0.314250 | 508 | 513.31 | 1.010453 |
| GO:0032268\_regulation\_of\_cellular\_protein\_metabolic\_process | APLP1 | 152 | 2 | 1.236574 | -0.314250 | 508 | 513.31 | 1.010453 |
| GO:0030855\_epithelial\_cell\_differentiation | XBP1 | 62 | 1 | 1.515800 | -0.312366 | 510 | 516.51 | 1.012765 |
| GO:0050954\_sensory\_perception\_of\_mechanical\_stimulus | GPR98 | 62 | 1 | 1.515800 | -0.312366 | 510 | 516.51 | 1.012765 |
| GO:0007369\_gastrulation | ODZ4 | 63 | 1 | 1.491740 | -0.307462 | 511 | 519.98 | 1.017573 |
| GO:0032879\_regulation\_of\_localization | CPLX3 | 248 | 3 | 1.136850 | -0.304095 | 512 | 521.99 | 1.019512 |
| GO:0032879\_regulation\_of\_localization | SYTL2 | 248 | 3 | 1.136850 | -0.304095 | 512 | 521.99 | 1.019512 |
| GO:0032879\_regulation\_of\_localization | NTN1 | 248 | 3 | 1.136850 | -0.304095 | 512 | 521.99 | 1.019512 |
| GO:0019222\_regulation\_of\_metabolic\_process | FUS | 1088 | 12 | 1.036540 | -0.302239 | 513 | 524.61 | 1.022632 |
| GO:0019222\_regulation\_of\_metabolic\_process | AEBP2 | 1088 | 12 | 1.036540 | -0.302239 | 513 | 524.61 | 1.022632 |
| GO:0019222\_regulation\_of\_metabolic\_process | HIPK1 | 1088 | 12 | 1.036540 | -0.302239 | 513 | 524.61 | 1.022632 |
| GO:0019222\_regulation\_of\_metabolic\_process | HES5 | 1088 | 12 | 1.036540 | -0.302239 | 513 | 524.61 | 1.022632 |
| GO:0019222\_regulation\_of\_metabolic\_process | PDGFA | 1088 | 12 | 1.036540 | -0.302239 | 513 | 524.61 | 1.022632 |
| GO:0019222\_regulation\_of\_metabolic\_process | SP3 | 1088 | 12 | 1.036540 | -0.302239 | 513 | 524.61 | 1.022632 |
| GO:0019222\_regulation\_of\_metabolic\_process | NAB1 | 1088 | 12 | 1.036540 | -0.302239 | 513 | 524.61 | 1.022632 |
| GO:0019222\_regulation\_of\_metabolic\_process | SV2B | 1088 | 12 | 1.036540 | -0.302239 | 513 | 524.61 | 1.022632 |
| GO:0019222\_regulation\_of\_metabolic\_process | POU4F1 | 1088 | 12 | 1.036540 | -0.302239 | 513 | 524.61 | 1.022632 |
| GO:0019222\_regulation\_of\_metabolic\_process | ARNTL | 1088 | 12 | 1.036540 | -0.302239 | 513 | 524.61 | 1.022632 |
| GO:0019222\_regulation\_of\_metabolic\_process | APLP1 | 1088 | 12 | 1.036540 | -0.302239 | 513 | 524.61 | 1.022632 |
| GO:0019222\_regulation\_of\_metabolic\_process | ZC3H8 | 1088 | 12 | 1.036540 | -0.302239 | 513 | 524.61 | 1.022632 |
| GO:0007389\_pattern\_specification\_process | HIPK1 | 250 | 3 | 1.127755 | -0.299339 | 514 | 525.14 | 1.021673 |
| GO:0007389\_pattern\_specification\_process | SFRP1 | 250 | 3 | 1.127755 | -0.299339 | 514 | 525.14 | 1.021673 |
| GO:0007389\_pattern\_specification\_process | FOXG1 | 250 | 3 | 1.127755 | -0.299339 | 514 | 525.14 | 1.021673 |
| GO:0048511\_rhythmic\_process | ARNTL | 65 | 1 | 1.445840 | -0.297967 | 515 | 526.57 | 1.022466 |
| GO:0006810\_transport | SCAMP1 | 718 | 8 | 1.047126 | -0.297545 | 516 | 526.65 | 1.020640 |
| GO:0006810\_transport | CPLX3 | 718 | 8 | 1.047126 | -0.297545 | 516 | 526.65 | 1.020640 |
| GO:0006810\_transport | SYTL2 | 718 | 8 | 1.047126 | -0.297545 | 516 | 526.65 | 1.020640 |
| GO:0006810\_transport | SV2B | 718 | 8 | 1.047126 | -0.297545 | 516 | 526.65 | 1.020640 |
| GO:0006810\_transport | ARNTL | 718 | 8 | 1.047126 | -0.297545 | 516 | 526.65 | 1.020640 |
| GO:0006810\_transport | YES1 | 718 | 8 | 1.047126 | -0.297545 | 516 | 526.65 | 1.020640 |
| GO:0006810\_transport | LRP2 | 718 | 8 | 1.047126 | -0.297545 | 516 | 526.65 | 1.020640 |
| GO:0006810\_transport | LIN7A | 718 | 8 | 1.047126 | -0.297545 | 516 | 526.65 | 1.020640 |
| GO:0010467\_gene\_expression | FUS | 905 | 10 | 1.038449 | -0.297401 | 517 | 526.8 | 1.018956 |
| GO:0010467\_gene\_expression | AEBP2 | 905 | 10 | 1.038449 | -0.297401 | 517 | 526.8 | 1.018956 |
| GO:0010467\_gene\_expression | HIPK1 | 905 | 10 | 1.038449 | -0.297401 | 517 | 526.8 | 1.018956 |
| GO:0010467\_gene\_expression | HES5 | 905 | 10 | 1.038449 | -0.297401 | 517 | 526.8 | 1.018956 |
| GO:0010467\_gene\_expression | SP3 | 905 | 10 | 1.038449 | -0.297401 | 517 | 526.8 | 1.018956 |
| GO:0010467\_gene\_expression | NAB1 | 905 | 10 | 1.038449 | -0.297401 | 517 | 526.8 | 1.018956 |
| GO:0010467\_gene\_expression | POU4F1 | 905 | 10 | 1.038449 | -0.297401 | 517 | 526.8 | 1.018956 |
| GO:0010467\_gene\_expression | ARNTL | 905 | 10 | 1.038449 | -0.297401 | 517 | 526.8 | 1.018956 |
| GO:0010467\_gene\_expression | APLP1 | 905 | 10 | 1.038449 | -0.297401 | 517 | 526.8 | 1.018956 |
| GO:0010467\_gene\_expression | ZC3H8 | 905 | 10 | 1.038449 | -0.297401 | 517 | 526.8 | 1.018956 |
| GO:0008104\_protein\_localization | KATNA1 | 251 | 3 | 1.123262 | -0.296990 | 518 | 527.09 | 1.017548 |
| GO:0008104\_protein\_localization | SV2B | 251 | 3 | 1.123262 | -0.296990 | 518 | 527.09 | 1.017548 |
| GO:0008104\_protein\_localization | ARNTL | 251 | 3 | 1.123262 | -0.296990 | 518 | 527.09 | 1.017548 |
| GO:0048514\_blood\_vessel\_morphogenesis | HEY1 | 158 | 2 | 1.189615 | -0.296086 | 519 | 528.27 | 1.017861 |
| GO:0048514\_blood\_vessel\_morphogenesis | PDGFA | 158 | 2 | 1.189615 | -0.296086 | 519 | 528.27 | 1.017861 |
| GO:0007179\_transforming\_growth\_factor\_beta\_receptor\_signaling\_pathway | ITGA8 | 66 | 1 | 1.423933 | -0.293370 | 522 | 530.6 | 1.016475 |
| GO:0031589\_cell-substrate\_adhesion | DLC1 | 66 | 1 | 1.423933 | -0.293370 | 522 | 530.6 | 1.016475 |
| GO:0051130\_positive\_regulation\_of\_cellular\_component\_organization | NTN1 | 66 | 1 | 1.423933 | -0.293370 | 522 | 530.6 | 1.016475 |
| GO:0016481\_negative\_regulation\_of\_transcription | HIPK1 | 253 | 3 | 1.114383 | -0.292348 | 523 | 531.48 | 1.016214 |
| GO:0016481\_negative\_regulation\_of\_transcription | HES5 | 253 | 3 | 1.114383 | -0.292348 | 523 | 531.48 | 1.016214 |
| GO:0016481\_negative\_regulation\_of\_transcription | ZC3H8 | 253 | 3 | 1.114383 | -0.292348 | 523 | 531.48 | 1.016214 |
| GO:0003007\_heart\_morphogenesis | DLC1 | 67 | 1 | 1.402680 | -0.288869 | 524 | 534.54 | 1.020115 |
| GO:0002521\_leukocyte\_differentiation | SP3 | 161 | 2 | 1.167448 | -0.287446 | 525 | 535.04 | 1.019124 |
| GO:0002521\_leukocyte\_differentiation | CHUK | 161 | 2 | 1.167448 | -0.287446 | 525 | 535.04 | 1.019124 |
| GO:0048870\_cell\_motility | KATNA1 | 257 | 3 | 1.097038 | -0.283283 | 526 | 537.9 | 1.022624 |
| GO:0048870\_cell\_motility | POU4F1 | 257 | 3 | 1.097038 | -0.283283 | 526 | 537.9 | 1.022624 |
| GO:0048870\_cell\_motility | NTN1 | 257 | 3 | 1.097038 | -0.283283 | 526 | 537.9 | 1.022624 |
| GO:0010926\_anatomical\_structure\_formation | DLC1 | 447 | 5 | 1.051226 | -0.281045 | 527 | 539.24 | 1.023226 |
| GO:0010926\_anatomical\_structure\_formation | PDGFA | 447 | 5 | 1.051226 | -0.281045 | 527 | 539.24 | 1.023226 |
| GO:0010926\_anatomical\_structure\_formation | SP3 | 447 | 5 | 1.051226 | -0.281045 | 527 | 539.24 | 1.023226 |
| GO:0010926\_anatomical\_structure\_formation | MMP2 | 447 | 5 | 1.051226 | -0.281045 | 527 | 539.24 | 1.023226 |
| GO:0010926\_anatomical\_structure\_formation | OLFM1 | 447 | 5 | 1.051226 | -0.281045 | 527 | 539.24 | 1.023226 |
| GO:0001932\_regulation\_of\_protein\_amino\_acid\_phosphorylation | PDGFA | 69 | 1 | 1.362023 | -0.280143 | 528 | 541.11 | 1.024830 |
| GO:0008283\_cell\_proliferation | HIPK1 | 544 | 6 | 1.036540 | -0.276817 | 529 | 542.01 | 1.024594 |
| GO:0008283\_cell\_proliferation | PDGFA | 544 | 6 | 1.036540 | -0.276817 | 529 | 542.01 | 1.024594 |
| GO:0008283\_cell\_proliferation | FOXG1 | 544 | 6 | 1.036540 | -0.276817 | 529 | 542.01 | 1.024594 |
| GO:0008283\_cell\_proliferation | LRP2 | 544 | 6 | 1.036540 | -0.276817 | 529 | 542.01 | 1.024594 |
| GO:0008283\_cell\_proliferation | NTN1 | 544 | 6 | 1.036540 | -0.276817 | 529 | 542.01 | 1.024594 |
| GO:0008283\_cell\_proliferation | CHUK | 544 | 6 | 1.036540 | -0.276817 | 529 | 542.01 | 1.024594 |
| GO:0031323\_regulation\_of\_cellular\_metabolic\_process | FUS | 1015 | 11 | 1.018498 | -0.276780 | 530 | 542.17 | 1.022962 |
| GO:0031323\_regulation\_of\_cellular\_metabolic\_process | AEBP2 | 1015 | 11 | 1.018498 | -0.276780 | 530 | 542.17 | 1.022962 |
| GO:0031323\_regulation\_of\_cellular\_metabolic\_process | HIPK1 | 1015 | 11 | 1.018498 | -0.276780 | 530 | 542.17 | 1.022962 |
| GO:0031323\_regulation\_of\_cellular\_metabolic\_process | HES5 | 1015 | 11 | 1.018498 | -0.276780 | 530 | 542.17 | 1.022962 |
| GO:0031323\_regulation\_of\_cellular\_metabolic\_process | PDGFA | 1015 | 11 | 1.018498 | -0.276780 | 530 | 542.17 | 1.022962 |
| GO:0031323\_regulation\_of\_cellular\_metabolic\_process | SP3 | 1015 | 11 | 1.018498 | -0.276780 | 530 | 542.17 | 1.022962 |
| GO:0031323\_regulation\_of\_cellular\_metabolic\_process | NAB1 | 1015 | 11 | 1.018498 | -0.276780 | 530 | 542.17 | 1.022962 |
| GO:0031323\_regulation\_of\_cellular\_metabolic\_process | POU4F1 | 1015 | 11 | 1.018498 | -0.276780 | 530 | 542.17 | 1.022962 |
| GO:0031323\_regulation\_of\_cellular\_metabolic\_process | ARNTL | 1015 | 11 | 1.018498 | -0.276780 | 530 | 542.17 | 1.022962 |
| GO:0031323\_regulation\_of\_cellular\_metabolic\_process | APLP1 | 1015 | 11 | 1.018498 | -0.276780 | 530 | 542.17 | 1.022962 |
| GO:0031323\_regulation\_of\_cellular\_metabolic\_process | ZC3H8 | 1015 | 11 | 1.018498 | -0.276780 | 530 | 542.17 | 1.022962 |
| GO:0009617\_response\_to\_bacterium | MAPK1 | 70 | 1 | 1.342566 | -0.275913 | 531 | 545.0 | 1.026365 |
| GO:0010629\_negative\_regulation\_of\_gene\_expression | HIPK1 | 262 | 3 | 1.076102 | -0.272350 | 532 | 546.26 | 1.026805 |
| GO:0010629\_negative\_regulation\_of\_gene\_expression | HES5 | 262 | 3 | 1.076102 | -0.272350 | 532 | 546.26 | 1.026805 |
| GO:0010629\_negative\_regulation\_of\_gene\_expression | ZC3H8 | 262 | 3 | 1.076102 | -0.272350 | 532 | 546.26 | 1.026805 |
| GO:0006913\_nucleocytoplasmic\_transport | ARNTL | 71 | 1 | 1.323656 | -0.271768 | 534 | 548.34 | 1.026854 |
| GO:0016331\_morphogenesis\_of\_embryonic\_epithelium | DLC1 | 71 | 1 | 1.323656 | -0.271768 | 534 | 548.34 | 1.026854 |
| GO:0051049\_regulation\_of\_transport | CPLX3 | 167 | 2 | 1.125504 | -0.270990 | 535 | 549.48 | 1.027065 |
| GO:0051049\_regulation\_of\_transport | SYTL2 | 167 | 2 | 1.125504 | -0.270990 | 535 | 549.48 | 1.027065 |
| GO:0007264\_small\_GTPase\_mediated\_signal\_transduction | STMN3 | 72 | 1 | 1.305272 | -0.267705 | 541 | 554.76 | 1.025434 |
| GO:0016568\_chromatin\_modification | MAP3K12 | 72 | 1 | 1.305272 | -0.267705 | 541 | 554.76 | 1.025434 |
| GO:0021915\_neural\_tube\_development | DLC1 | 72 | 1 | 1.305272 | -0.267705 | 541 | 554.76 | 1.025434 |
| GO:0040012\_regulation\_of\_locomotion | NTN1 | 72 | 1 | 1.305272 | -0.267705 | 541 | 554.76 | 1.025434 |
| GO:0050673\_epithelial\_cell\_proliferation | CHUK | 72 | 1 | 1.305272 | -0.267705 | 541 | 554.76 | 1.025434 |
| GO:0051169\_nuclear\_transport | ARNTL | 72 | 1 | 1.305272 | -0.267705 | 541 | 554.76 | 1.025434 |
| GO:0048706\_embryonic\_skeletal\_system\_development | SP3 | 73 | 1 | 1.287392 | -0.263722 | 544 | 557.19 | 1.024246 |
| GO:0051270\_regulation\_of\_cell\_motion | NTN1 | 73 | 1 | 1.287392 | -0.263722 | 544 | 557.19 | 1.024246 |
| GO:0051336\_regulation\_of\_hydrolase\_activity | STMN3 | 73 | 1 | 1.287392 | -0.263722 | 544 | 557.19 | 1.024246 |
| GO:0007600\_sensory\_perception | POU4F1 | 172 | 2 | 1.092786 | -0.258066 | 545 | 560.9 | 1.029174 |
| GO:0007600\_sensory\_perception | GPR98 | 172 | 2 | 1.092786 | -0.258066 | 545 | 560.9 | 1.029174 |
| GO:0045944\_positive\_regulation\_of\_transcription\_from\_RNA\_polymerase\_II\_promoter | FUS | 269 | 3 | 1.048100 | -0.257753 | 546 | 561.16 | 1.027766 |
| GO:0045944\_positive\_regulation\_of\_transcription\_from\_RNA\_polymerase\_II\_promoter | HES5 | 269 | 3 | 1.048100 | -0.257753 | 546 | 561.16 | 1.027766 |
| GO:0045944\_positive\_regulation\_of\_transcription\_from\_RNA\_polymerase\_II\_promoter | ARNTL | 269 | 3 | 1.048100 | -0.257753 | 546 | 561.16 | 1.027766 |
| GO:0051050\_positive\_regulation\_of\_transport | SYTL2 | 75 | 1 | 1.253061 | -0.255988 | 547 | 562.55 | 1.028428 |
| GO:0045934\_negative\_regulation\_of\_nucleobase\_\_nucleoside\_\_nucleotide\_and\_nucleic\_acid\_metabolic\_process | HIPK1 | 270 | 3 | 1.044218 | -0.255733 | 548 | 562.86 | 1.027117 |
| GO:0045934\_negative\_regulation\_of\_nucleobase\_\_nucleoside\_\_nucleotide\_and\_nucleic\_acid\_metabolic\_process | HES5 | 270 | 3 | 1.044218 | -0.255733 | 548 | 562.86 | 1.027117 |
| GO:0045934\_negative\_regulation\_of\_nucleobase\_\_nucleoside\_\_nucleotide\_and\_nucleic\_acid\_metabolic\_process | ZC3H8 | 270 | 3 | 1.044218 | -0.255733 | 548 | 562.86 | 1.027117 |
| GO:0051172\_negative\_regulation\_of\_nitrogen\_compound\_metabolic\_process | HIPK1 | 271 | 3 | 1.040364 | -0.253728 | 549 | 564.54 | 1.028306 |
| GO:0051172\_negative\_regulation\_of\_nitrogen\_compound\_metabolic\_process | HES5 | 271 | 3 | 1.040364 | -0.253728 | 549 | 564.54 | 1.028306 |
| GO:0051172\_negative\_regulation\_of\_nitrogen\_compound\_metabolic\_process | ZC3H8 | 271 | 3 | 1.040364 | -0.253728 | 549 | 564.54 | 1.028306 |
| GO:0000122\_negative\_regulation\_of\_transcription\_from\_RNA\_polymerase\_II\_promoter | HIPK1 | 175 | 2 | 1.074052 | -0.250635 | 551 | 567.0 | 1.029038 |
| GO:0000122\_negative\_regulation\_of\_transcription\_from\_RNA\_polymerase\_II\_promoter | HES5 | 175 | 2 | 1.074052 | -0.250635 | 551 | 567.0 | 1.029038 |
| GO:0015031\_protein\_transport | SV2B | 175 | 2 | 1.074052 | -0.250635 | 551 | 567.0 | 1.029038 |
| GO:0015031\_protein\_transport | ARNTL | 175 | 2 | 1.074052 | -0.250635 | 551 | 567.0 | 1.029038 |
| GO:0010558\_negative\_regulation\_of\_macromolecule\_biosynthetic\_process | HIPK1 | 274 | 3 | 1.028974 | -0.247809 | 553 | 570.05 | 1.030832 |
| GO:0010558\_negative\_regulation\_of\_macromolecule\_biosynthetic\_process | HES5 | 274 | 3 | 1.028974 | -0.247809 | 553 | 570.05 | 1.030832 |
| GO:0010558\_negative\_regulation\_of\_macromolecule\_biosynthetic\_process | ZC3H8 | 274 | 3 | 1.028974 | -0.247809 | 553 | 570.05 | 1.030832 |
| GO:0033036\_macromolecule\_localization | KATNA1 | 274 | 3 | 1.028974 | -0.247809 | 553 | 570.05 | 1.030832 |
| GO:0033036\_macromolecule\_localization | SV2B | 274 | 3 | 1.028974 | -0.247809 | 553 | 570.05 | 1.030832 |
| GO:0033036\_macromolecule\_localization | ARNTL | 274 | 3 | 1.028974 | -0.247809 | 553 | 570.05 | 1.030832 |
| GO:0006461\_protein\_complex\_assembly | OLFM1 | 78 | 1 | 1.204867 | -0.244933 | 557 | 573.19 | 1.029066 |
| GO:0030326\_embryonic\_limb\_morphogenesis | DKK1 | 78 | 1 | 1.204867 | -0.244933 | 557 | 573.19 | 1.029066 |
| GO:0035113\_embryonic\_appendage\_morphogenesis | DKK1 | 78 | 1 | 1.204867 | -0.244933 | 557 | 573.19 | 1.029066 |
| GO:0070271\_protein\_complex\_biogenesis | OLFM1 | 78 | 1 | 1.204867 | -0.244933 | 557 | 573.19 | 1.029066 |
| GO:0045184\_establishment\_of\_protein\_localization | SV2B | 180 | 2 | 1.044218 | -0.238760 | 558 | 575.81 | 1.031918 |
| GO:0045184\_establishment\_of\_protein\_localization | ARNTL | 180 | 2 | 1.044218 | -0.238760 | 558 | 575.81 | 1.031918 |
| GO:0006807\_nitrogen\_compound\_metabolic\_process | FUS | 1147 | 12 | 0.983222 | -0.234948 | 559 | 579.58 | 1.036816 |
| GO:0006807\_nitrogen\_compound\_metabolic\_process | CCNE2 | 1147 | 12 | 0.983222 | -0.234948 | 559 | 579.58 | 1.036816 |
| GO:0006807\_nitrogen\_compound\_metabolic\_process | MAPK1 | 1147 | 12 | 0.983222 | -0.234948 | 559 | 579.58 | 1.036816 |
| GO:0006807\_nitrogen\_compound\_metabolic\_process | AEBP2 | 1147 | 12 | 0.983222 | -0.234948 | 559 | 579.58 | 1.036816 |
| GO:0006807\_nitrogen\_compound\_metabolic\_process | HIPK1 | 1147 | 12 | 0.983222 | -0.234948 | 559 | 579.58 | 1.036816 |
| GO:0006807\_nitrogen\_compound\_metabolic\_process | HES5 | 1147 | 12 | 0.983222 | -0.234948 | 559 | 579.58 | 1.036816 |
| GO:0006807\_nitrogen\_compound\_metabolic\_process | SP3 | 1147 | 12 | 0.983222 | -0.234948 | 559 | 579.58 | 1.036816 |
| GO:0006807\_nitrogen\_compound\_metabolic\_process | NAB1 | 1147 | 12 | 0.983222 | -0.234948 | 559 | 579.58 | 1.036816 |
| GO:0006807\_nitrogen\_compound\_metabolic\_process | POU4F1 | 1147 | 12 | 0.983222 | -0.234948 | 559 | 579.58 | 1.036816 |
| GO:0006807\_nitrogen\_compound\_metabolic\_process | ARNTL | 1147 | 12 | 0.983222 | -0.234948 | 559 | 579.58 | 1.036816 |
| GO:0006807\_nitrogen\_compound\_metabolic\_process | APLP1 | 1147 | 12 | 0.983222 | -0.234948 | 559 | 579.58 | 1.036816 |
| GO:0006807\_nitrogen\_compound\_metabolic\_process | ZC3H8 | 1147 | 12 | 0.983222 | -0.234948 | 559 | 579.58 | 1.036816 |
| GO:0031327\_negative\_regulation\_of\_cellular\_biosynthetic\_process | HIPK1 | 282 | 3 | 0.999783 | -0.232686 | 560 | 580.73 | 1.037018 |
| GO:0031327\_negative\_regulation\_of\_cellular\_biosynthetic\_process | HES5 | 282 | 3 | 0.999783 | -0.232686 | 560 | 580.73 | 1.037018 |
| GO:0031327\_negative\_regulation\_of\_cellular\_biosynthetic\_process | ZC3H8 | 282 | 3 | 0.999783 | -0.232686 | 560 | 580.73 | 1.037018 |
| GO:0010627\_regulation\_of\_protein\_kinase\_cascade | CHUK | 82 | 1 | 1.146093 | -0.231134 | 561 | 583.46 | 1.040036 |
| GO:0009890\_negative\_regulation\_of\_biosynthetic\_process | HIPK1 | 284 | 3 | 0.992742 | -0.229051 | 562 | 584.52 | 1.040071 |
| GO:0009890\_negative\_regulation\_of\_biosynthetic\_process | HES5 | 284 | 3 | 0.992742 | -0.229051 | 562 | 584.52 | 1.040071 |
| GO:0009890\_negative\_regulation\_of\_biosynthetic\_process | ZC3H8 | 284 | 3 | 0.992742 | -0.229051 | 562 | 584.52 | 1.040071 |
| GO:0006325\_chromatin\_organization | MAP3K12 | 83 | 1 | 1.132284 | -0.227840 | 564 | 586.95 | 1.040691 |
| GO:0030534\_adult\_behavior | EPHA4 | 83 | 1 | 1.132284 | -0.227840 | 564 | 586.95 | 1.040691 |
| GO:0007155\_cell\_adhesion | DLC1 | 186 | 2 | 1.010533 | -0.225304 | 566 | 588.87 | 1.040406 |
| GO:0007155\_cell\_adhesion | HES5 | 186 | 2 | 1.010533 | -0.225304 | 566 | 588.87 | 1.040406 |
| GO:0022610\_biological\_adhesion | DLC1 | 186 | 2 | 1.010533 | -0.225304 | 566 | 588.87 | 1.040406 |
| GO:0022610\_biological\_adhesion | HES5 | 186 | 2 | 1.010533 | -0.225304 | 566 | 588.87 | 1.040406 |
| GO:0000279\_M\_phase | TUBG1 | 85 | 1 | 1.105642 | -0.221430 | 567 | 591.73 | 1.043616 |
| GO:0006605\_protein\_targeting | ARNTL | 86 | 1 | 1.092786 | -0.218310 | 570 | 594.64 | 1.043228 |
| GO:0006897\_endocytosis | LRP2 | 86 | 1 | 1.092786 | -0.218310 | 570 | 594.64 | 1.043228 |
| GO:0010324\_membrane\_invagination | LRP2 | 86 | 1 | 1.092786 | -0.218310 | 570 | 594.64 | 1.043228 |
| GO:0001822\_kidney\_development | ITGA8 | 87 | 1 | 1.080225 | -0.215245 | 573 | 597.45 | 1.042670 |
| GO:0007178\_transmembrane\_receptor\_protein\_serine\_threonine\_kinase\_signaling\_pathway | ITGA8 | 87 | 1 | 1.080225 | -0.215245 | 573 | 597.45 | 1.042670 |
| GO:0050778\_positive\_regulation\_of\_immune\_response | MAPK1 | 87 | 1 | 1.080225 | -0.215245 | 573 | 597.45 | 1.042670 |
| GO:0042127\_regulation\_of\_cell\_proliferation | HIPK1 | 393 | 4 | 0.956535 | -0.213109 | 574 | 597.65 | 1.041202 |
| GO:0042127\_regulation\_of\_cell\_proliferation | PDGFA | 393 | 4 | 0.956535 | -0.213109 | 574 | 597.65 | 1.041202 |
| GO:0042127\_regulation\_of\_cell\_proliferation | FOXG1 | 393 | 4 | 0.956535 | -0.213109 | 574 | 597.65 | 1.041202 |
| GO:0042127\_regulation\_of\_cell\_proliferation | NTN1 | 393 | 4 | 0.956535 | -0.213109 | 574 | 597.65 | 1.041202 |
| GO:0040011\_locomotion | KATNA1 | 295 | 3 | 0.955725 | -0.210037 | 575 | 599.69 | 1.042939 |
| GO:0040011\_locomotion | POU4F1 | 295 | 3 | 0.955725 | -0.210037 | 575 | 599.69 | 1.042939 |
| GO:0040011\_locomotion | NTN1 | 295 | 3 | 0.955725 | -0.210037 | 575 | 599.69 | 1.042939 |
| GO:0065008\_regulation\_of\_biological\_quality | HBA-A1 | 693 | 7 | 0.949289 | -0.206719 | 576 | 601.07 | 1.043524 |
| GO:0065008\_regulation\_of\_biological\_quality | CPLX3 | 693 | 7 | 0.949289 | -0.206719 | 576 | 601.07 | 1.043524 |
| GO:0065008\_regulation\_of\_biological\_quality | SP3 | 693 | 7 | 0.949289 | -0.206719 | 576 | 601.07 | 1.043524 |
| GO:0065008\_regulation\_of\_biological\_quality | NAB1 | 693 | 7 | 0.949289 | -0.206719 | 576 | 601.07 | 1.043524 |
| GO:0065008\_regulation\_of\_biological\_quality | SYTL2 | 693 | 7 | 0.949289 | -0.206719 | 576 | 601.07 | 1.043524 |
| GO:0065008\_regulation\_of\_biological\_quality | SV2B | 693 | 7 | 0.949289 | -0.206719 | 576 | 601.07 | 1.043524 |
| GO:0065008\_regulation\_of\_biological\_quality | LIN7A | 693 | 7 | 0.949289 | -0.206719 | 576 | 601.07 | 1.043524 |
| GO:0042113\_B\_cell\_activation | SP3 | 90 | 1 | 1.044218 | -0.206367 | 577 | 603.39 | 1.045737 |
| GO:0033554\_cellular\_response\_to\_stress | HIPK1 | 196 | 2 | 0.958975 | -0.204643 | 578 | 603.67 | 1.044412 |
| GO:0033554\_cellular\_response\_to\_stress | MAPK10 | 196 | 2 | 0.958975 | -0.204643 | 578 | 603.67 | 1.044412 |
| GO:0031399\_regulation\_of\_protein\_modification\_process | PDGFA | 91 | 1 | 1.032743 | -0.203510 | 579 | 605.39 | 1.045579 |
| GO:0030217\_T\_cell\_differentiation | SP3 | 92 | 1 | 1.021517 | -0.200701 | 580 | 607.54 | 1.047483 |
| GO:0035107\_appendage\_morphogenesis | DKK1 | 93 | 1 | 1.010533 | -0.197940 | 583 | 609.85 | 1.046055 |
| GO:0035108\_limb\_morphogenesis | DKK1 | 93 | 1 | 1.010533 | -0.197940 | 583 | 609.85 | 1.046055 |
| GO:0065003\_macromolecular\_complex\_assembly | OLFM1 | 93 | 1 | 1.010533 | -0.197940 | 583 | 609.85 | 1.046055 |
| GO:0002376\_immune\_system\_process | HBA-A1 | 505 | 5 | 0.930491 | -0.196194 | 584 | 610.18 | 1.044829 |
| GO:0002376\_immune\_system\_process | MAPK1 | 505 | 5 | 0.930491 | -0.196194 | 584 | 610.18 | 1.044829 |
| GO:0002376\_immune\_system\_process | SP3 | 505 | 5 | 0.930491 | -0.196194 | 584 | 610.18 | 1.044829 |
| GO:0002376\_immune\_system\_process | JAG1 | 505 | 5 | 0.930491 | -0.196194 | 584 | 610.18 | 1.044829 |
| GO:0002376\_immune\_system\_process | CHUK | 505 | 5 | 0.930491 | -0.196194 | 584 | 610.18 | 1.044829 |
| GO:0034984\_cellular\_response\_to\_DNA\_damage\_stimulus | HIPK1 | 94 | 1 | 0.999783 | -0.195225 | 585 | 612.85 | 1.047607 |
| GO:0045893\_positive\_regulation\_of\_transcription\_\_DNA-dependent | FUS | 306 | 3 | 0.921369 | -0.192575 | 587 | 613.52 | 1.045179 |
| GO:0045893\_positive\_regulation\_of\_transcription\_\_DNA-dependent | HES5 | 306 | 3 | 0.921369 | -0.192575 | 587 | 613.52 | 1.045179 |
| GO:0045893\_positive\_regulation\_of\_transcription\_\_DNA-dependent | ARNTL | 306 | 3 | 0.921369 | -0.192575 | 587 | 613.52 | 1.045179 |
| GO:0051254\_positive\_regulation\_of\_RNA\_metabolic\_process | FUS | 306 | 3 | 0.921369 | -0.192575 | 587 | 613.52 | 1.045179 |
| GO:0051254\_positive\_regulation\_of\_RNA\_metabolic\_process | HES5 | 306 | 3 | 0.921369 | -0.192575 | 587 | 613.52 | 1.045179 |
| GO:0051254\_positive\_regulation\_of\_RNA\_metabolic\_process | ARNTL | 306 | 3 | 0.921369 | -0.192575 | 587 | 613.52 | 1.045179 |
| GO:0042391\_regulation\_of\_membrane\_potential | NAB1 | 95 | 1 | 0.989259 | -0.192556 | 589 | 614.2 | 1.042784 |
| GO:0051707\_response\_to\_other\_organism | MAPK1 | 95 | 1 | 0.989259 | -0.192556 | 589 | 614.2 | 1.042784 |
| GO:0048736\_appendage\_development | DKK1 | 96 | 1 | 0.978954 | -0.189931 | 591 | 617.2 | 1.044332 |
| GO:0060173\_limb\_development | DKK1 | 96 | 1 | 0.978954 | -0.189931 | 591 | 617.2 | 1.044332 |
| GO:0060341\_regulation\_of\_cellular\_localization | CPLX3 | 97 | 1 | 0.968862 | -0.187350 | 592 | 620.11 | 1.047483 |
| GO:0060562\_epithelial\_tube\_morphogenesis | DLC1 | 99 | 1 | 0.949289 | -0.182313 | 593 | 624.78 | 1.053592 |
| GO:0001525\_angiogenesis | PDGFA | 100 | 1 | 0.939796 | -0.179857 | 594 | 625.78 | 1.053502 |
| GO:0030163\_protein\_catabolic\_process | ARNTL | 101 | 1 | 0.930491 | -0.177440 | 595 | 626.94 | 1.053681 |
| GO:0055086\_nucleobase\_\_nucleoside\_and\_nucleotide\_metabolic\_process | MAPK1 | 104 | 1 | 0.903650 | -0.170420 | 596 | 630.31 | 1.057567 |
| GO:0010817\_regulation\_of\_hormone\_levels | CPLX3 | 106 | 1 | 0.886600 | -0.165923 | 597 | 632.48 | 1.059430 |
| GO:0045892\_negative\_regulation\_of\_transcription\_\_DNA-dependent | HIPK1 | 218 | 2 | 0.862198 | -0.165886 | 598 | 632.77 | 1.058144 |
| GO:0045892\_negative\_regulation\_of\_transcription\_\_DNA-dependent | HES5 | 218 | 2 | 0.862198 | -0.165886 | 598 | 632.77 | 1.058144 |
| GO:0051253\_negative\_regulation\_of\_RNA\_metabolic\_process | HIPK1 | 220 | 2 | 0.854360 | -0.162763 | 599 | 633.78 | 1.058063 |
| GO:0051253\_negative\_regulation\_of\_RNA\_metabolic\_process | HES5 | 220 | 2 | 0.854360 | -0.162763 | 599 | 633.78 | 1.058063 |
| GO:0051240\_positive\_regulation\_of\_multicellular\_organismal\_process | SYTL2 | 108 | 1 | 0.870181 | -0.161567 | 600 | 634.52 | 1.057533 |
| GO:0006357\_regulation\_of\_transcription\_from\_RNA\_polymerase\_II\_promoter | FUS | 435 | 4 | 0.864180 | -0.159030 | 601 | 636.7 | 1.059401 |
| GO:0006357\_regulation\_of\_transcription\_from\_RNA\_polymerase\_II\_promoter | HIPK1 | 435 | 4 | 0.864180 | -0.159030 | 601 | 636.7 | 1.059401 |
| GO:0006357\_regulation\_of\_transcription\_from\_RNA\_polymerase\_II\_promoter | HES5 | 435 | 4 | 0.864180 | -0.159030 | 601 | 636.7 | 1.059401 |
| GO:0006357\_regulation\_of\_transcription\_from\_RNA\_polymerase\_II\_promoter | ARNTL | 435 | 4 | 0.864180 | -0.159030 | 601 | 636.7 | 1.059401 |
| GO:0010605\_negative\_regulation\_of\_macromolecule\_metabolic\_process | HIPK1 | 331 | 3 | 0.851779 | -0.157977 | 602 | 637.39 | 1.058787 |
| GO:0010605\_negative\_regulation\_of\_macromolecule\_metabolic\_process | HES5 | 331 | 3 | 0.851779 | -0.157977 | 602 | 637.39 | 1.058787 |
| GO:0010605\_negative\_regulation\_of\_macromolecule\_metabolic\_process | ZC3H8 | 331 | 3 | 0.851779 | -0.157977 | 602 | 637.39 | 1.058787 |
| GO:0031324\_negative\_regulation\_of\_cellular\_metabolic\_process | HIPK1 | 332 | 3 | 0.849213 | -0.156726 | 603 | 639.51 | 1.060547 |
| GO:0031324\_negative\_regulation\_of\_cellular\_metabolic\_process | HES5 | 332 | 3 | 0.849213 | -0.156726 | 603 | 639.51 | 1.060547 |
| GO:0031324\_negative\_regulation\_of\_cellular\_metabolic\_process | ZC3H8 | 332 | 3 | 0.849213 | -0.156726 | 603 | 639.51 | 1.060547 |
| GO:0002682\_regulation\_of\_immune\_system\_process | MAPK1 | 228 | 2 | 0.824382 | -0.150864 | 604 | 643.02 | 1.064603 |
| GO:0002682\_regulation\_of\_immune\_system\_process | JAG1 | 228 | 2 | 0.824382 | -0.150864 | 604 | 643.02 | 1.064603 |
| GO:0007167\_enzyme\_linked\_receptor\_protein\_signaling\_pathway | ITGA8 | 229 | 2 | 0.820782 | -0.149441 | 605 | 643.49 | 1.063620 |
| GO:0007167\_enzyme\_linked\_receptor\_protein\_signaling\_pathway | EPHB2 | 229 | 2 | 0.820782 | -0.149441 | 605 | 643.49 | 1.063620 |
| GO:0045941\_positive\_regulation\_of\_transcription | FUS | 338 | 3 | 0.834138 | -0.149419 | 606 | 643.74 | 1.062277 |
| GO:0045941\_positive\_regulation\_of\_transcription | HES5 | 338 | 3 | 0.834138 | -0.149419 | 606 | 643.74 | 1.062277 |
| GO:0045941\_positive\_regulation\_of\_transcription | ARNTL | 338 | 3 | 0.834138 | -0.149419 | 606 | 643.74 | 1.062277 |
| GO:0009607\_response\_to\_biotic\_stimulus | MAPK1 | 114 | 1 | 0.824382 | -0.149284 | 607 | 644.54 | 1.061845 |
| GO:0006366\_transcription\_from\_RNA\_polymerase\_II\_promoter | FUS | 444 | 4 | 0.846663 | -0.149239 | 608 | 644.97 | 1.060806 |
| GO:0006366\_transcription\_from\_RNA\_polymerase\_II\_promoter | HIPK1 | 444 | 4 | 0.846663 | -0.149239 | 608 | 644.97 | 1.060806 |
| GO:0006366\_transcription\_from\_RNA\_polymerase\_II\_promoter | HES5 | 444 | 4 | 0.846663 | -0.149239 | 608 | 644.97 | 1.060806 |
| GO:0006366\_transcription\_from\_RNA\_polymerase\_II\_promoter | ARNTL | 444 | 4 | 0.846663 | -0.149239 | 608 | 644.97 | 1.060806 |
| GO:0044249\_cellular\_biosynthetic\_process | CCNE2 | 1150 | 11 | 0.898935 | -0.147990 | 609 | 645.49 | 1.059918 |
| GO:0044249\_cellular\_biosynthetic\_process | FUS | 1150 | 11 | 0.898935 | -0.147990 | 609 | 645.49 | 1.059918 |
| GO:0044249\_cellular\_biosynthetic\_process | AEBP2 | 1150 | 11 | 0.898935 | -0.147990 | 609 | 645.49 | 1.059918 |
| GO:0044249\_cellular\_biosynthetic\_process | HIPK1 | 1150 | 11 | 0.898935 | -0.147990 | 609 | 645.49 | 1.059918 |
| GO:0044249\_cellular\_biosynthetic\_process | HES5 | 1150 | 11 | 0.898935 | -0.147990 | 609 | 645.49 | 1.059918 |
| GO:0044249\_cellular\_biosynthetic\_process | SP3 | 1150 | 11 | 0.898935 | -0.147990 | 609 | 645.49 | 1.059918 |
| GO:0044249\_cellular\_biosynthetic\_process | NAB1 | 1150 | 11 | 0.898935 | -0.147990 | 609 | 645.49 | 1.059918 |
| GO:0044249\_cellular\_biosynthetic\_process | POU4F1 | 1150 | 11 | 0.898935 | -0.147990 | 609 | 645.49 | 1.059918 |
| GO:0044249\_cellular\_biosynthetic\_process | ARNTL | 1150 | 11 | 0.898935 | -0.147990 | 609 | 645.49 | 1.059918 |
| GO:0044249\_cellular\_biosynthetic\_process | ZC3H8 | 1150 | 11 | 0.898935 | -0.147990 | 609 | 645.49 | 1.059918 |
| GO:0044249\_cellular\_biosynthetic\_process | APLP1 | 1150 | 11 | 0.898935 | -0.147990 | 609 | 645.49 | 1.059918 |
| GO:0048584\_positive\_regulation\_of\_response\_to\_stimulus | MAPK1 | 115 | 1 | 0.817214 | -0.147344 | 610 | 646.21 | 1.059361 |
| GO:0046483\_heterocycle\_metabolic\_process | MAPK1 | 116 | 1 | 0.810169 | -0.145434 | 612 | 648.43 | 1.059526 |
| GO:0048608\_reproductive\_structure\_development | SFRP1 | 116 | 1 | 0.810169 | -0.145434 | 612 | 648.43 | 1.059526 |
| GO:0043933\_macromolecular\_complex\_subunit\_organization | OLFM1 | 117 | 1 | 0.803244 | -0.143552 | 613 | 650.16 | 1.060620 |
| GO:0010628\_positive\_regulation\_of\_gene\_expression | FUS | 346 | 3 | 0.814852 | -0.140183 | 614 | 652.08 | 1.062020 |
| GO:0010628\_positive\_regulation\_of\_gene\_expression | HES5 | 346 | 3 | 0.814852 | -0.140183 | 614 | 652.08 | 1.062020 |
| GO:0010628\_positive\_regulation\_of\_gene\_expression | ARNTL | 346 | 3 | 0.814852 | -0.140183 | 614 | 652.08 | 1.062020 |
| GO:0022403\_cell\_cycle\_phase | TUBG1 | 119 | 1 | 0.789744 | -0.139873 | 615 | 652.4 | 1.060813 |
| GO:0009892\_negative\_regulation\_of\_metabolic\_process | HIPK1 | 348 | 3 | 0.810169 | -0.137961 | 616 | 654.13 | 1.061899 |
| GO:0009892\_negative\_regulation\_of\_metabolic\_process | HES5 | 348 | 3 | 0.810169 | -0.137961 | 616 | 654.13 | 1.061899 |
| GO:0009892\_negative\_regulation\_of\_metabolic\_process | ZC3H8 | 348 | 3 | 0.810169 | -0.137961 | 616 | 654.13 | 1.061899 |
| GO:0006886\_intracellular\_protein\_transport | ARNTL | 122 | 1 | 0.770325 | -0.134556 | 617 | 658.8 | 1.067747 |
| GO:0045935\_positive\_regulation\_of\_nucleobase\_\_nucleoside\_\_nucleotide\_and\_nucleic\_acid\_metabolic\_process | FUS | 352 | 3 | 0.800962 | -0.133618 | 618 | 659.03 | 1.066392 |
| GO:0045935\_positive\_regulation\_of\_nucleobase\_\_nucleoside\_\_nucleotide\_and\_nucleic\_acid\_metabolic\_process | HES5 | 352 | 3 | 0.800962 | -0.133618 | 618 | 659.03 | 1.066392 |
| GO:0045935\_positive\_regulation\_of\_nucleobase\_\_nucleoside\_\_nucleotide\_and\_nucleic\_acid\_metabolic\_process | ARNTL | 352 | 3 | 0.800962 | -0.133618 | 618 | 659.03 | 1.066392 |
| GO:0030098\_lymphocyte\_differentiation | SP3 | 124 | 1 | 0.757900 | -0.131140 | 619 | 660.17 | 1.066511 |
| GO:0009058\_biosynthetic\_process | CCNE2 | 1175 | 11 | 0.879809 | -0.130659 | 620 | 660.66 | 1.065581 |
| GO:0009058\_biosynthetic\_process | FUS | 1175 | 11 | 0.879809 | -0.130659 | 620 | 660.66 | 1.065581 |
| GO:0009058\_biosynthetic\_process | AEBP2 | 1175 | 11 | 0.879809 | -0.130659 | 620 | 660.66 | 1.065581 |
| GO:0009058\_biosynthetic\_process | HIPK1 | 1175 | 11 | 0.879809 | -0.130659 | 620 | 660.66 | 1.065581 |
| GO:0009058\_biosynthetic\_process | HES5 | 1175 | 11 | 0.879809 | -0.130659 | 620 | 660.66 | 1.065581 |
| GO:0009058\_biosynthetic\_process | SP3 | 1175 | 11 | 0.879809 | -0.130659 | 620 | 660.66 | 1.065581 |
| GO:0009058\_biosynthetic\_process | NAB1 | 1175 | 11 | 0.879809 | -0.130659 | 620 | 660.66 | 1.065581 |
| GO:0009058\_biosynthetic\_process | POU4F1 | 1175 | 11 | 0.879809 | -0.130659 | 620 | 660.66 | 1.065581 |
| GO:0009058\_biosynthetic\_process | ARNTL | 1175 | 11 | 0.879809 | -0.130659 | 620 | 660.66 | 1.065581 |
| GO:0009058\_biosynthetic\_process | ZC3H8 | 1175 | 11 | 0.879809 | -0.130659 | 620 | 660.66 | 1.065581 |
| GO:0009058\_biosynthetic\_process | APLP1 | 1175 | 11 | 0.879809 | -0.130659 | 620 | 660.66 | 1.065581 |
| GO:0042981\_regulation\_of\_apoptosis | HIPK1 | 360 | 3 | 0.783163 | -0.125319 | 621 | 662.15 | 1.066264 |
| GO:0042981\_regulation\_of\_apoptosis | TM2D1 | 360 | 3 | 0.783163 | -0.125319 | 621 | 662.15 | 1.066264 |
| GO:0042981\_regulation\_of\_apoptosis | POU4F1 | 360 | 3 | 0.783163 | -0.125319 | 621 | 662.15 | 1.066264 |
| GO:0051173\_positive\_regulation\_of\_nitrogen\_compound\_metabolic\_process | FUS | 361 | 3 | 0.780994 | -0.124317 | 622 | 664.28 | 1.067974 |
| GO:0051173\_positive\_regulation\_of\_nitrogen\_compound\_metabolic\_process | HES5 | 361 | 3 | 0.780994 | -0.124317 | 622 | 664.28 | 1.067974 |
| GO:0051173\_positive\_regulation\_of\_nitrogen\_compound\_metabolic\_process | ARNTL | 361 | 3 | 0.780994 | -0.124317 | 622 | 664.28 | 1.067974 |
| GO:0043285\_biopolymer\_catabolic\_process | ARNTL | 129 | 1 | 0.728524 | -0.123023 | 624 | 665.02 | 1.065737 |
| GO:0051276\_chromosome\_organization | MAP3K12 | 129 | 1 | 0.728524 | -0.123023 | 624 | 665.02 | 1.065737 |
| GO:0045165\_cell\_fate\_commitment | HES5 | 130 | 1 | 0.722920 | -0.121469 | 626 | 666.67 | 1.064968 |
| GO:0050776\_regulation\_of\_immune\_response | MAPK1 | 130 | 1 | 0.722920 | -0.121469 | 626 | 666.67 | 1.064968 |
| GO:0010941\_regulation\_of\_cell\_death | HIPK1 | 365 | 3 | 0.772435 | -0.120384 | 628 | 667.5 | 1.062898 |
| GO:0010941\_regulation\_of\_cell\_death | TM2D1 | 365 | 3 | 0.772435 | -0.120384 | 628 | 667.5 | 1.062898 |
| GO:0010941\_regulation\_of\_cell\_death | POU4F1 | 365 | 3 | 0.772435 | -0.120384 | 628 | 667.5 | 1.062898 |
| GO:0043067\_regulation\_of\_programmed\_cell\_death | HIPK1 | 365 | 3 | 0.772435 | -0.120384 | 628 | 667.5 | 1.062898 |
| GO:0043067\_regulation\_of\_programmed\_cell\_death | TM2D1 | 365 | 3 | 0.772435 | -0.120384 | 628 | 667.5 | 1.062898 |
| GO:0043067\_regulation\_of\_programmed\_cell\_death | POU4F1 | 365 | 3 | 0.772435 | -0.120384 | 628 | 667.5 | 1.062898 |
| GO:0010557\_positive\_regulation\_of\_macromolecule\_biosynthetic\_process | FUS | 371 | 3 | 0.759943 | -0.114706 | 629 | 671.43 | 1.067456 |
| GO:0010557\_positive\_regulation\_of\_macromolecule\_biosynthetic\_process | HES5 | 371 | 3 | 0.759943 | -0.114706 | 629 | 671.43 | 1.067456 |
| GO:0010557\_positive\_regulation\_of\_macromolecule\_biosynthetic\_process | ARNTL | 371 | 3 | 0.759943 | -0.114706 | 629 | 671.43 | 1.067456 |
| GO:0009057\_macromolecule\_catabolic\_process | ARNTL | 137 | 1 | 0.685982 | -0.111189 | 630 | 672.83 | 1.067984 |
| GO:0050896\_response\_to\_stimulus | DDC | 1107 | 10 | 0.848957 | -0.111009 | 631 | 672.93 | 1.066450 |
| GO:0050896\_response\_to\_stimulus | EPHA4 | 1107 | 10 | 0.848957 | -0.111009 | 631 | 672.93 | 1.066450 |
| GO:0050896\_response\_to\_stimulus | MAPK1 | 1107 | 10 | 0.848957 | -0.111009 | 631 | 672.93 | 1.066450 |
| GO:0050896\_response\_to\_stimulus | HIPK1 | 1107 | 10 | 0.848957 | -0.111009 | 631 | 672.93 | 1.066450 |
| GO:0050896\_response\_to\_stimulus | ITGA8 | 1107 | 10 | 0.848957 | -0.111009 | 631 | 672.93 | 1.066450 |
| GO:0050896\_response\_to\_stimulus | POU4F1 | 1107 | 10 | 0.848957 | -0.111009 | 631 | 672.93 | 1.066450 |
| GO:0050896\_response\_to\_stimulus | MAPK10 | 1107 | 10 | 0.848957 | -0.111009 | 631 | 672.93 | 1.066450 |
| GO:0050896\_response\_to\_stimulus | MMP2 | 1107 | 10 | 0.848957 | -0.111009 | 631 | 672.93 | 1.066450 |
| GO:0050896\_response\_to\_stimulus | GPR98 | 1107 | 10 | 0.848957 | -0.111009 | 631 | 672.93 | 1.066450 |
| GO:0050896\_response\_to\_stimulus | EPHB2 | 1107 | 10 | 0.848957 | -0.111009 | 631 | 672.93 | 1.066450 |
| GO:0022414\_reproductive\_process | SFRP1 | 376 | 3 | 0.749837 | -0.110169 | 632 | 673.25 | 1.065269 |
| GO:0022414\_reproductive\_process | SP3 | 376 | 3 | 0.749837 | -0.110169 | 632 | 673.25 | 1.065269 |
| GO:0022414\_reproductive\_process | CHUK | 376 | 3 | 0.749837 | -0.110169 | 632 | 673.25 | 1.065269 |
| GO:0007169\_transmembrane\_receptor\_protein\_tyrosine\_kinase\_signaling\_pathway | EPHB2 | 139 | 1 | 0.676112 | -0.108433 | 634 | 675.34 | 1.065205 |
| GO:0034613\_cellular\_protein\_localization | ARNTL | 139 | 1 | 0.676112 | -0.108433 | 634 | 675.34 | 1.065205 |
| GO:0000003\_reproduction | SFRP1 | 379 | 3 | 0.743902 | -0.107529 | 635 | 676.3 | 1.065039 |
| GO:0000003\_reproduction | SP3 | 379 | 3 | 0.743902 | -0.107529 | 635 | 676.3 | 1.065039 |
| GO:0000003\_reproduction | CHUK | 379 | 3 | 0.743902 | -0.107529 | 635 | 676.3 | 1.065039 |
| GO:0016044\_membrane\_organization | LRP2 | 140 | 1 | 0.671283 | -0.107084 | 636 | 676.95 | 1.064387 |
| GO:0003006\_reproductive\_developmental\_process | SFRP1 | 141 | 1 | 0.666522 | -0.105753 | 638 | 677.98 | 1.062665 |
| GO:0070727\_cellular\_macromolecule\_localization | ARNTL | 141 | 1 | 0.666522 | -0.105753 | 638 | 677.98 | 1.062665 |
| GO:0035239\_tube\_morphogenesis | DLC1 | 143 | 1 | 0.657200 | -0.103146 | 639 | 678.78 | 1.062254 |
| GO:0007186\_G-protein\_coupled\_receptor\_protein\_signaling\_pathway | GNB4 | 144 | 1 | 0.652636 | -0.101870 | 640 | 679.97 | 1.062453 |
| GO:0031328\_positive\_regulation\_of\_cellular\_biosynthetic\_process | FUS | 387 | 3 | 0.728524 | -0.100779 | 641 | 680.36 | 1.061404 |
| GO:0031328\_positive\_regulation\_of\_cellular\_biosynthetic\_process | HES5 | 387 | 3 | 0.728524 | -0.100779 | 641 | 680.36 | 1.061404 |
| GO:0031328\_positive\_regulation\_of\_cellular\_biosynthetic\_process | ARNTL | 387 | 3 | 0.728524 | -0.100779 | 641 | 680.36 | 1.061404 |
| GO:0009891\_positive\_regulation\_of\_biosynthetic\_process | FUS | 388 | 3 | 0.726646 | -0.099964 | 642 | 680.74 | 1.060343 |
| GO:0009891\_positive\_regulation\_of\_biosynthetic\_process | HES5 | 388 | 3 | 0.726646 | -0.099964 | 642 | 680.74 | 1.060343 |
| GO:0009891\_positive\_regulation\_of\_biosynthetic\_process | ARNTL | 388 | 3 | 0.726646 | -0.099964 | 642 | 680.74 | 1.060343 |
| GO:0051716\_cellular\_response\_to\_stimulus | HIPK1 | 273 | 2 | 0.688495 | -0.098570 | 643 | 681.92 | 1.060529 |
| GO:0051716\_cellular\_response\_to\_stimulus | MAPK10 | 273 | 2 | 0.688495 | -0.098570 | 643 | 681.92 | 1.060529 |
| GO:0002684\_positive\_regulation\_of\_immune\_system\_process | MAPK1 | 148 | 1 | 0.634997 | -0.096934 | 644 | 683.69 | 1.061630 |
| GO:0065009\_regulation\_of\_molecular\_function | STMN3 | 279 | 2 | 0.673689 | -0.093132 | 645 | 685.13 | 1.062217 |
| GO:0065009\_regulation\_of\_molecular\_function | HIPK1 | 279 | 2 | 0.673689 | -0.093132 | 645 | 685.13 | 1.062217 |
| GO:0022402\_cell\_cycle\_process | TUBG1 | 155 | 1 | 0.606320 | -0.088916 | 646 | 687.73 | 1.064598 |
| GO:0008152\_metabolic\_process | FUS | 2133 | 20 | 0.881196 | -0.085566 | 647 | 689.96 | 1.066399 |
| GO:0008152\_metabolic\_process | AEBP2 | 2133 | 20 | 0.881196 | -0.085566 | 647 | 689.96 | 1.066399 |
| GO:0008152\_metabolic\_process | PDGFA | 2133 | 20 | 0.881196 | -0.085566 | 647 | 689.96 | 1.066399 |
| GO:0008152\_metabolic\_process | THOP1 | 2133 | 20 | 0.881196 | -0.085566 | 647 | 689.96 | 1.066399 |
| GO:0008152\_metabolic\_process | ARNTL | 2133 | 20 | 0.881196 | -0.085566 | 647 | 689.96 | 1.066399 |
| GO:0008152\_metabolic\_process | MMP2 | 2133 | 20 | 0.881196 | -0.085566 | 647 | 689.96 | 1.066399 |
| GO:0008152\_metabolic\_process | EPHB2 | 2133 | 20 | 0.881196 | -0.085566 | 647 | 689.96 | 1.066399 |
| GO:0008152\_metabolic\_process | ZC3H8 | 2133 | 20 | 0.881196 | -0.085566 | 647 | 689.96 | 1.066399 |
| GO:0008152\_metabolic\_process | APLP1 | 2133 | 20 | 0.881196 | -0.085566 | 647 | 689.96 | 1.066399 |
| GO:0008152\_metabolic\_process | CCNE2 | 2133 | 20 | 0.881196 | -0.085566 | 647 | 689.96 | 1.066399 |
| GO:0008152\_metabolic\_process | MAPK1 | 2133 | 20 | 0.881196 | -0.085566 | 647 | 689.96 | 1.066399 |
| GO:0008152\_metabolic\_process | HES5 | 2133 | 20 | 0.881196 | -0.085566 | 647 | 689.96 | 1.066399 |
| GO:0008152\_metabolic\_process | HIPK1 | 2133 | 20 | 0.881196 | -0.085566 | 647 | 689.96 | 1.066399 |
| GO:0008152\_metabolic\_process | SP3 | 2133 | 20 | 0.881196 | -0.085566 | 647 | 689.96 | 1.066399 |
| GO:0008152\_metabolic\_process | NAB1 | 2133 | 20 | 0.881196 | -0.085566 | 647 | 689.96 | 1.066399 |
| GO:0008152\_metabolic\_process | SV2B | 2133 | 20 | 0.881196 | -0.085566 | 647 | 689.96 | 1.066399 |
| GO:0008152\_metabolic\_process | POU4F1 | 2133 | 20 | 0.881196 | -0.085566 | 647 | 689.96 | 1.066399 |
| GO:0008152\_metabolic\_process | LRP2 | 2133 | 20 | 0.881196 | -0.085566 | 647 | 689.96 | 1.066399 |
| GO:0008152\_metabolic\_process | CHUK | 2133 | 20 | 0.881196 | -0.085566 | 647 | 689.96 | 1.066399 |
| GO:0008152\_metabolic\_process | MAP3K12 | 2133 | 20 | 0.881196 | -0.085566 | 647 | 689.96 | 1.066399 |
| GO:0042221\_response\_to\_chemical\_stimulus | MAPK1 | 409 | 3 | 0.689337 | -0.084217 | 648 | 690.2 | 1.065123 |
| GO:0042221\_response\_to\_chemical\_stimulus | DDC | 409 | 3 | 0.689337 | -0.084217 | 648 | 690.2 | 1.065123 |
| GO:0042221\_response\_to\_chemical\_stimulus | MMP2 | 409 | 3 | 0.689337 | -0.084217 | 648 | 690.2 | 1.065123 |
| GO:0009628\_response\_to\_abiotic\_stimulus | GPR98 | 162 | 1 | 0.580121 | -0.081614 | 649 | 691.84 | 1.066009 |
| GO:0007626\_locomotory\_behavior | EPHA4 | 163 | 1 | 0.576562 | -0.080625 | 651 | 692.53 | 1.063794 |
| GO:0042110\_T\_cell\_activation | SP3 | 163 | 1 | 0.576562 | -0.080625 | 651 | 692.53 | 1.063794 |
| GO:0042325\_regulation\_of\_phosphorylation | PDGFA | 164 | 1 | 0.573046 | -0.079649 | 652 | 693.37 | 1.063451 |
| GO:0006259\_DNA\_metabolic\_process | CCNE2 | 165 | 1 | 0.569573 | -0.078686 | 655 | 694.38 | 1.060122 |
| GO:0019220\_regulation\_of\_phosphate\_metabolic\_process | PDGFA | 165 | 1 | 0.569573 | -0.078686 | 655 | 694.38 | 1.060122 |
| GO:0051174\_regulation\_of\_phosphorus\_metabolic\_process | PDGFA | 165 | 1 | 0.569573 | -0.078686 | 655 | 694.38 | 1.060122 |
| GO:0042592\_homeostatic\_process | HBA-A1 | 419 | 3 | 0.672885 | -0.077568 | 656 | 695.11 | 1.059619 |
| GO:0042592\_homeostatic\_process | SP3 | 419 | 3 | 0.672885 | -0.077568 | 656 | 695.11 | 1.059619 |
| GO:0042592\_homeostatic\_process | NAB1 | 419 | 3 | 0.672885 | -0.077568 | 656 | 695.11 | 1.059619 |
| GO:0044237\_cellular\_metabolic\_process | FUS | 1974 | 18 | 0.856957 | -0.072881 | 657 | 697.64 | 1.061857 |
| GO:0044237\_cellular\_metabolic\_process | AEBP2 | 1974 | 18 | 0.856957 | -0.072881 | 657 | 697.64 | 1.061857 |
| GO:0044237\_cellular\_metabolic\_process | PDGFA | 1974 | 18 | 0.856957 | -0.072881 | 657 | 697.64 | 1.061857 |
| GO:0044237\_cellular\_metabolic\_process | THOP1 | 1974 | 18 | 0.856957 | -0.072881 | 657 | 697.64 | 1.061857 |
| GO:0044237\_cellular\_metabolic\_process | ARNTL | 1974 | 18 | 0.856957 | -0.072881 | 657 | 697.64 | 1.061857 |
| GO:0044237\_cellular\_metabolic\_process | EPHB2 | 1974 | 18 | 0.856957 | -0.072881 | 657 | 697.64 | 1.061857 |
| GO:0044237\_cellular\_metabolic\_process | ZC3H8 | 1974 | 18 | 0.856957 | -0.072881 | 657 | 697.64 | 1.061857 |
| GO:0044237\_cellular\_metabolic\_process | APLP1 | 1974 | 18 | 0.856957 | -0.072881 | 657 | 697.64 | 1.061857 |
| GO:0044237\_cellular\_metabolic\_process | CCNE2 | 1974 | 18 | 0.856957 | -0.072881 | 657 | 697.64 | 1.061857 |
| GO:0044237\_cellular\_metabolic\_process | MAPK1 | 1974 | 18 | 0.856957 | -0.072881 | 657 | 697.64 | 1.061857 |
| GO:0044237\_cellular\_metabolic\_process | HES5 | 1974 | 18 | 0.856957 | -0.072881 | 657 | 697.64 | 1.061857 |
| GO:0044237\_cellular\_metabolic\_process | HIPK1 | 1974 | 18 | 0.856957 | -0.072881 | 657 | 697.64 | 1.061857 |
| GO:0044237\_cellular\_metabolic\_process | SP3 | 1974 | 18 | 0.856957 | -0.072881 | 657 | 697.64 | 1.061857 |
| GO:0044237\_cellular\_metabolic\_process | NAB1 | 1974 | 18 | 0.856957 | -0.072881 | 657 | 697.64 | 1.061857 |
| GO:0044237\_cellular\_metabolic\_process | POU4F1 | 1974 | 18 | 0.856957 | -0.072881 | 657 | 697.64 | 1.061857 |
| GO:0044237\_cellular\_metabolic\_process | LRP2 | 1974 | 18 | 0.856957 | -0.072881 | 657 | 697.64 | 1.061857 |
| GO:0044237\_cellular\_metabolic\_process | MAP3K12 | 1974 | 18 | 0.856957 | -0.072881 | 657 | 697.64 | 1.061857 |
| GO:0044237\_cellular\_metabolic\_process | CHUK | 1974 | 18 | 0.856957 | -0.072881 | 657 | 697.64 | 1.061857 |
| GO:0006915\_apoptosis | HIPK1 | 427 | 3 | 0.660278 | -0.072607 | 658 | 697.84 | 1.060547 |
| GO:0006915\_apoptosis | TM2D1 | 427 | 3 | 0.660278 | -0.072607 | 658 | 697.84 | 1.060547 |
| GO:0006915\_apoptosis | POU4F1 | 427 | 3 | 0.660278 | -0.072607 | 658 | 697.84 | 1.060547 |
| GO:0044093\_positive\_regulation\_of\_molecular\_function | HIPK1 | 173 | 1 | 0.543235 | -0.071413 | 659 | 700.27 | 1.062625 |
| GO:0006950\_response\_to\_stress | MAPK1 | 549 | 4 | 0.684733 | -0.069291 | 660 | 702.18 | 1.063909 |
| GO:0006950\_response\_to\_stress | HIPK1 | 549 | 4 | 0.684733 | -0.069291 | 660 | 702.18 | 1.063909 |
| GO:0006950\_response\_to\_stress | MAPK10 | 549 | 4 | 0.684733 | -0.069291 | 660 | 702.18 | 1.063909 |
| GO:0006950\_response\_to\_stress | MMP2 | 549 | 4 | 0.684733 | -0.069291 | 660 | 702.18 | 1.063909 |
| GO:0010604\_positive\_regulation\_of\_macromolecule\_metabolic\_process | FUS | 433 | 3 | 0.651129 | -0.069084 | 662 | 702.66 | 1.061420 |
| GO:0010604\_positive\_regulation\_of\_macromolecule\_metabolic\_process | HES5 | 433 | 3 | 0.651129 | -0.069084 | 662 | 702.66 | 1.061420 |
| GO:0010604\_positive\_regulation\_of\_macromolecule\_metabolic\_process | ARNTL | 433 | 3 | 0.651129 | -0.069084 | 662 | 702.66 | 1.061420 |
| GO:0012501\_programmed\_cell\_death | HIPK1 | 433 | 3 | 0.651129 | -0.069084 | 662 | 702.66 | 1.061420 |
| GO:0012501\_programmed\_cell\_death | TM2D1 | 433 | 3 | 0.651129 | -0.069084 | 662 | 702.66 | 1.061420 |
| GO:0012501\_programmed\_cell\_death | POU4F1 | 433 | 3 | 0.651129 | -0.069084 | 662 | 702.66 | 1.061420 |
| GO:0006873\_cellular\_ion\_homeostasis | NAB1 | 176 | 1 | 0.533975 | -0.068874 | 663 | 703.59 | 1.061222 |
| GO:0055082\_cellular\_chemical\_homeostasis | NAB1 | 181 | 1 | 0.519224 | -0.064852 | 664 | 706.38 | 1.063825 |
| GO:0031325\_positive\_regulation\_of\_cellular\_metabolic\_process | FUS | 442 | 3 | 0.637871 | -0.064097 | 665 | 706.7 | 1.062707 |
| GO:0031325\_positive\_regulation\_of\_cellular\_metabolic\_process | HES5 | 442 | 3 | 0.637871 | -0.064097 | 665 | 706.7 | 1.062707 |
| GO:0031325\_positive\_regulation\_of\_cellular\_metabolic\_process | ARNTL | 442 | 3 | 0.637871 | -0.064097 | 665 | 706.7 | 1.062707 |
| GO:0044238\_primary\_metabolic\_process | FUS | 1905 | 17 | 0.838663 | -0.063130 | 666 | 707.38 | 1.062132 |
| GO:0044238\_primary\_metabolic\_process | AEBP2 | 1905 | 17 | 0.838663 | -0.063130 | 666 | 707.38 | 1.062132 |
| GO:0044238\_primary\_metabolic\_process | PDGFA | 1905 | 17 | 0.838663 | -0.063130 | 666 | 707.38 | 1.062132 |
| GO:0044238\_primary\_metabolic\_process | ARNTL | 1905 | 17 | 0.838663 | -0.063130 | 666 | 707.38 | 1.062132 |
| GO:0044238\_primary\_metabolic\_process | EPHB2 | 1905 | 17 | 0.838663 | -0.063130 | 666 | 707.38 | 1.062132 |
| GO:0044238\_primary\_metabolic\_process | ZC3H8 | 1905 | 17 | 0.838663 | -0.063130 | 666 | 707.38 | 1.062132 |
| GO:0044238\_primary\_metabolic\_process | APLP1 | 1905 | 17 | 0.838663 | -0.063130 | 666 | 707.38 | 1.062132 |
| GO:0044238\_primary\_metabolic\_process | CCNE2 | 1905 | 17 | 0.838663 | -0.063130 | 666 | 707.38 | 1.062132 |
| GO:0044238\_primary\_metabolic\_process | MAPK1 | 1905 | 17 | 0.838663 | -0.063130 | 666 | 707.38 | 1.062132 |
| GO:0044238\_primary\_metabolic\_process | HES5 | 1905 | 17 | 0.838663 | -0.063130 | 666 | 707.38 | 1.062132 |
| GO:0044238\_primary\_metabolic\_process | HIPK1 | 1905 | 17 | 0.838663 | -0.063130 | 666 | 707.38 | 1.062132 |
| GO:0044238\_primary\_metabolic\_process | SP3 | 1905 | 17 | 0.838663 | -0.063130 | 666 | 707.38 | 1.062132 |
| GO:0044238\_primary\_metabolic\_process | NAB1 | 1905 | 17 | 0.838663 | -0.063130 | 666 | 707.38 | 1.062132 |
| GO:0044238\_primary\_metabolic\_process | SV2B | 1905 | 17 | 0.838663 | -0.063130 | 666 | 707.38 | 1.062132 |
| GO:0044238\_primary\_metabolic\_process | POU4F1 | 1905 | 17 | 0.838663 | -0.063130 | 666 | 707.38 | 1.062132 |
| GO:0044238\_primary\_metabolic\_process | CHUK | 1905 | 17 | 0.838663 | -0.063130 | 666 | 707.38 | 1.062132 |
| GO:0044238\_primary\_metabolic\_process | MAP3K12 | 1905 | 17 | 0.838663 | -0.063130 | 666 | 707.38 | 1.062132 |
| GO:0008219\_cell\_death | HIPK1 | 444 | 3 | 0.634997 | -0.063036 | 667 | 707.69 | 1.061004 |
| GO:0008219\_cell\_death | TM2D1 | 444 | 3 | 0.634997 | -0.063036 | 667 | 707.69 | 1.061004 |
| GO:0008219\_cell\_death | POU4F1 | 444 | 3 | 0.634997 | -0.063036 | 667 | 707.69 | 1.061004 |
| GO:0016265\_death | HIPK1 | 450 | 3 | 0.626531 | -0.059949 | 668 | 710.69 | 1.063907 |
| GO:0016265\_death | TM2D1 | 450 | 3 | 0.626531 | -0.059949 | 668 | 710.69 | 1.063907 |
| GO:0016265\_death | POU4F1 | 450 | 3 | 0.626531 | -0.059949 | 668 | 710.69 | 1.063907 |
| GO:0009893\_positive\_regulation\_of\_metabolic\_process | FUS | 458 | 3 | 0.615587 | -0.056054 | 669 | 712.92 | 1.065650 |
| GO:0009893\_positive\_regulation\_of\_metabolic\_process | HES5 | 458 | 3 | 0.615587 | -0.056054 | 669 | 712.92 | 1.065650 |
| GO:0009893\_positive\_regulation\_of\_metabolic\_process | ARNTL | 458 | 3 | 0.615587 | -0.056054 | 669 | 712.92 | 1.065650 |
| GO:0046907\_intracellular\_transport | ARNTL | 194 | 1 | 0.484431 | -0.055513 | 670 | 713.28 | 1.064597 |
| GO:0007507\_heart\_development | DLC1 | 195 | 1 | 0.481947 | -0.054856 | 672 | 714.23 | 1.062842 |
| GO:0019725\_cellular\_homeostasis | NAB1 | 195 | 1 | 0.481947 | -0.054856 | 672 | 714.23 | 1.062842 |
| GO:0050801\_ion\_homeostasis | NAB1 | 197 | 1 | 0.477054 | -0.053565 | 673 | 715.19 | 1.062689 |
| GO:0006955\_immune\_response | MAPK1 | 205 | 1 | 0.458437 | -0.048712 | 674 | 718.74 | 1.066380 |
| GO:0010033\_response\_to\_organic\_substance | MAPK1 | 216 | 1 | 0.435091 | -0.042772 | 675 | 721.18 | 1.068415 |
| GO:0048583\_regulation\_of\_response\_to\_stimulus | MAPK1 | 217 | 1 | 0.433086 | -0.042270 | 676 | 721.84 | 1.067811 |
| GO:0046649\_lymphocyte\_activation | SP3 | 228 | 1 | 0.412191 | -0.037136 | 677 | 725.15 | 1.071123 |
| GO:0050790\_regulation\_of\_catalytic\_activity | STMN3 | 233 | 1 | 0.403346 | -0.035019 | 678 | 726.39 | 1.071372 |
| GO:0009056\_catabolic\_process | ARNTL | 243 | 1 | 0.386747 | -0.031145 | 679 | 728.86 | 1.073432 |
| GO:0045321\_leukocyte\_activation | SP3 | 248 | 1 | 0.378950 | -0.029374 | 680 | 729.79 | 1.073221 |
| GO:0048878\_chemical\_homeostasis | NAB1 | 254 | 1 | 0.369998 | -0.027384 | 681 | 731.01 | 1.073436 |
| GO:0001775\_cell\_activation | SP3 | 262 | 1 | 0.358701 | -0.024940 | 682 | 732.74 | 1.074399 |
| GO:0009605\_response\_to\_external\_stimulus | GPR98 | 339 | 1 | 0.277226 | -0.010156 | 683 | 740.67 | 1.084436 |
| GO:0008150\_biological\_process | DLC1 | 4605 | 49 | 1.000000 | 0.000000 | 2716 | 2717.44 | 1.000530 |
| GO:0008150\_biological\_process | PDGFA | 4605 | 49 | 1.000000 | 0.000000 | 2716 | 2717.44 | 1.000530 |
| GO:0008150\_biological\_process | THOP1 | 4605 | 49 | 1.000000 | 0.000000 | 2716 | 2717.44 | 1.000530 |
| GO:0008150\_biological\_process | JAG1 | 4605 | 49 | 1.000000 | 0.000000 | 2716 | 2717.44 | 1.000530 |
| GO:0008150\_biological\_process | MMP2 | 4605 | 49 | 1.000000 | 0.000000 | 2716 | 2717.44 | 1.000530 |
| GO:0008150\_biological\_process | CCNE2 | 4605 | 49 | 1.000000 | 0.000000 | 2716 | 2717.44 | 1.000530 |
| GO:0008150\_biological\_process | HEY1 | 4605 | 49 | 1.000000 | 0.000000 | 2716 | 2717.44 | 1.000530 |
| GO:0008150\_biological\_process | ANK3 | 4605 | 49 | 1.000000 | 0.000000 | 2716 | 2717.44 | 1.000530 |
| GO:0008150\_biological\_process | SV2B | 4605 | 49 | 1.000000 | 0.000000 | 2716 | 2717.44 | 1.000530 |
| GO:0008150\_biological\_process | POU4F1 | 4605 | 49 | 1.000000 | 0.000000 | 2716 | 2717.44 | 1.000530 |
| GO:0008150\_biological\_process | TUBG1 | 4605 | 49 | 1.000000 | 0.000000 | 2716 | 2717.44 | 1.000530 |
| GO:0008150\_biological\_process | CHUK | 4605 | 49 | 1.000000 | 0.000000 | 2716 | 2717.44 | 1.000530 |
| GO:0008150\_biological\_process | ODZ4 | 4605 | 49 | 1.000000 | 0.000000 | 2716 | 2717.44 | 1.000530 |
| GO:0008150\_biological\_process | SCAMP1 | 4605 | 49 | 1.000000 | 0.000000 | 2716 | 2717.44 | 1.000530 |
| GO:0008150\_biological\_process | DDC | 4605 | 49 | 1.000000 | 0.000000 | 2716 | 2717.44 | 1.000530 |
| GO:0008150\_biological\_process | STMN3 | 4605 | 49 | 1.000000 | 0.000000 | 2716 | 2717.44 | 1.000530 |
| GO:0008150\_biological\_process | KIF5C | 4605 | 49 | 1.000000 | 0.000000 | 2716 | 2717.44 | 1.000530 |
| GO:0008150\_biological\_process | ARNTL | 4605 | 49 | 1.000000 | 0.000000 | 2716 | 2717.44 | 1.000530 |
| GO:0008150\_biological\_process | GPR98 | 4605 | 49 | 1.000000 | 0.000000 | 2716 | 2717.44 | 1.000530 |
| GO:0008150\_biological\_process | MAPK1 | 4605 | 49 | 1.000000 | 0.000000 | 2716 | 2717.44 | 1.000530 |
| GO:0008150\_biological\_process | HES5 | 4605 | 49 | 1.000000 | 0.000000 | 2716 | 2717.44 | 1.000530 |
| GO:0008150\_biological\_process | HIPK1 | 4605 | 49 | 1.000000 | 0.000000 | 2716 | 2717.44 | 1.000530 |
| GO:0008150\_biological\_process | NAB1 | 4605 | 49 | 1.000000 | 0.000000 | 2716 | 2717.44 | 1.000530 |
| GO:0008150\_biological\_process | FOXG1 | 4605 | 49 | 1.000000 | 0.000000 | 2716 | 2717.44 | 1.000530 |
| GO:0008150\_biological\_process | GNB4 | 4605 | 49 | 1.000000 | 0.000000 | 2716 | 2717.44 | 1.000530 |
| GO:0008150\_biological\_process | MAP3K12 | 4605 | 49 | 1.000000 | 0.000000 | 2716 | 2717.44 | 1.000530 |
| GO:0008150\_biological\_process | FUS | 4605 | 49 | 1.000000 | 0.000000 | 2716 | 2717.44 | 1.000530 |
| GO:0008150\_biological\_process | CPLX3 | 4605 | 49 | 1.000000 | 0.000000 | 2716 | 2717.44 | 1.000530 |
| GO:0008150\_biological\_process | COL3A1 | 4605 | 49 | 1.000000 | 0.000000 | 2716 | 2717.44 | 1.000530 |
| GO:0008150\_biological\_process | EPHB2 | 4605 | 49 | 1.000000 | 0.000000 | 2716 | 2717.44 | 1.000530 |
| GO:0008150\_biological\_process | ZC3H8 | 4605 | 49 | 1.000000 | 0.000000 | 2716 | 2717.44 | 1.000530 |
| GO:0008150\_biological\_process | APLP1 | 4605 | 49 | 1.000000 | 0.000000 | 2716 | 2717.44 | 1.000530 |
| GO:0008150\_biological\_process | XBP1 | 4605 | 49 | 1.000000 | 0.000000 | 2716 | 2717.44 | 1.000530 |
| GO:0008150\_biological\_process | KATNA1 | 4605 | 49 | 1.000000 | 0.000000 | 2716 | 2717.44 | 1.000530 |
| GO:0008150\_biological\_process | YES1 | 4605 | 49 | 1.000000 | 0.000000 | 2716 | 2717.44 | 1.000530 |
| GO:0008150\_biological\_process | OLFM1 | 4605 | 49 | 1.000000 | 0.000000 | 2716 | 2717.44 | 1.000530 |
| GO:0008150\_biological\_process | AEBP2 | 4605 | 49 | 1.000000 | 0.000000 | 2716 | 2717.44 | 1.000530 |
| GO:0008150\_biological\_process | TM2D1 | 4605 | 49 | 1.000000 | 0.000000 | 2716 | 2717.44 | 1.000530 |
| GO:0008150\_biological\_process | MAPK10 | 4605 | 49 | 1.000000 | 0.000000 | 2716 | 2717.44 | 1.000530 |
| GO:0008150\_biological\_process | NTN1 | 4605 | 49 | 1.000000 | 0.000000 | 2716 | 2717.44 | 1.000530 |
| GO:0008150\_biological\_process | LIN7A | 4605 | 49 | 1.000000 | 0.000000 | 2716 | 2717.44 | 1.000530 |
| GO:0008150\_biological\_process | HBA-A1 | 4605 | 49 | 1.000000 | 0.000000 | 2716 | 2717.44 | 1.000530 |
| GO:0008150\_biological\_process | EPHA4 | 4605 | 49 | 1.000000 | 0.000000 | 2716 | 2717.44 | 1.000530 |
| GO:0008150\_biological\_process | DKK1 | 4605 | 49 | 1.000000 | 0.000000 | 2716 | 2717.44 | 1.000530 |
| GO:0008150\_biological\_process | SFRP1 | 4605 | 49 | 1.000000 | 0.000000 | 2716 | 2717.44 | 1.000530 |
| GO:0008150\_biological\_process | SP3 | 4605 | 49 | 1.000000 | 0.000000 | 2716 | 2717.44 | 1.000530 |
| GO:0008150\_biological\_process | ITGA8 | 4605 | 49 | 1.000000 | 0.000000 | 2716 | 2717.44 | 1.000530 |
| GO:0008150\_biological\_process | SYTL2 | 4605 | 49 | 1.000000 | 0.000000 | 2716 | 2717.44 | 1.000530 |
| GO:0008150\_biological\_process | LRP2 | 4605 | 49 | 1.000000 | 0.000000 | 2716 | 2717.44 | 1.000530 |
