# Supplementary material for: RedundancyMiner: De-replication of redundant GO categories in microarray and proteomics analysis
Source: BMC Bioinformatics. 2011 Feb 10;12:52. doi: 10.1186/1471-2105-12-52 (PMC3223614; doi:10.1186/1471-2105-12-52)
Supplement: Additional file 8 — Retinal development HTGM download. compressed package of the results of running HTGM on the retinal development genes list. [file 1471-2105-12-52-S8.ZIP › SCENARIO_2_MODIFIED/total.txt.total.txt.dir/Exp1_BestClusterMap_LEIGS_KM_24.csv.join.21.txt.dir/Exp1_BestClusterMap_LEIGS_KM_24.csv.join.21.txt.change.html]

Category Summary Report for Exp1\_BestClusterMap\_LEIGS\_KM\_24.csv.join.21.txt

# Category Summary Report for Exp1\_BestClusterMap\_LEIGS\_KM\_24.csv.join.21.txt

| HYPERLINKED GO CATEGORY | TOTAL GENES | CHANGED GENES | ENRICHMENT | LOG10(p) | CUMULATIVE NUMBER OF CATEGORIES | CUMULATIVE RANDOMS LOWER BOUND | CUMULATIVE RANDOMS MEAN | CUMULATIVE RANDOMS UPPER BOUND | FALSE DISCOVERY RATE |
| --- | --- | --- | --- | --- | --- | --- | --- | --- | --- |
| GO:0007399\_nervous\_system\_development | 621 | 17 | 2.572710 | -3.898381 | 1 | -0.275268 | 0.04 | 0.355268 | 0.040000 |
| GO:0007411\_axon\_guidance | 82 | 6 | 6.876556 | -3.696446 | 2 | -0.312839 | 0.07 | 0.452839 | 0.035000 |
| GO:0022008\_neurogenesis | 423 | 13 | 2.888262 | -3.478792 | 3 | -0.369810 | 0.15 | 0.669810 | 0.050000 |
| GO:0030030\_cell\_projection\_organization | 263 | 10 | 3.573369 | -3.453802 | 4 | -0.369810 | 0.15 | 0.669810 | 0.037500 |
| GO:0048699\_generation\_of\_neurons | 396 | 12 | 2.847866 | -3.169871 | 5 | -0.482521 | 0.32 | 1.122521 | 0.064000 |
| GO:0007275\_multicellular\_organismal\_development | 1760 | 30 | 1.601925 | -3.059568 | 6 | -0.568249 | 0.44 | 1.448249 | 0.073333 |
| GO:0048839\_inner\_ear\_development | 72 | 5 | 6.526361 | -3.042275 | 7 | -0.558800 | 0.45 | 1.458800 | 0.064286 |
| GO:0030182\_neuron\_differentiation | 356 | 11 | 2.903864 | -2.995982 | 8 | -0.669558 | 0.54 | 1.749558 | 0.067500 |
| GO:0048856\_anatomical\_structure\_development | 1688 | 29 | 1.614578 | -2.978962 | 9 | -0.729585 | 0.56 | 1.849585 | 0.062222 |
| GO:0009653\_anatomical\_structure\_morphogenesis | 958 | 20 | 1.961996 | -2.963997 | 10 | -0.729585 | 0.56 | 1.849585 | 0.056000 |
| GO:0031122\_cytoplasmic\_microtubule\_organization | 5 | 2 | 37.591837 | -2.963821 | 11 | -0.589626 | 0.86 | 2.309626 | 0.078182 |
| GO:0048731\_system\_development | 1609 | 28 | 1.635443 | -2.938039 | 12 | -0.615308 | 0.92 | 2.455308 | 0.076667 |
| GO:0032501\_multicellular\_organismal\_process | 2183 | 34 | 1.463722 | -2.833666 | 13 | -0.740478 | 1.04 | 2.820478 | 0.080000 |
| GO:0048489\_synaptic\_vesicle\_transport | 22 | 3 | 12.815399 | -2.820254 | 14 | -0.721683 | 1.08 | 2.881683 | 0.077143 |
| GO:0060113\_inner\_ear\_receptor\_cell\_differentiation | 24 | 3 | 11.747449 | -2.708057 | 15 | -0.661656 | 1.56 | 3.781656 | 0.104000 |
| GO:0006887\_exocytosis | 51 | 4 | 7.370948 | -2.708039 | 16 | -0.661656 | 1.56 | 3.781656 | 0.097500 |
| GO:0043583\_ear\_development | 87 | 5 | 5.401126 | -2.672415 | 17 | -0.668113 | 1.61 | 3.888113 | 0.094706 |
| GO:0002011\_morphogenesis\_of\_an\_epithelial\_sheet | 7 | 2 | 26.851312 | -2.647520 | 18 | -0.506591 | 2.01 | 4.526591 | 0.111667 |
| GO:0048468\_cell\_development | 654 | 15 | 2.155495 | -2.624176 | 19 | -0.502249 | 2.04 | 4.582249 | 0.107368 |
| GO:0042490\_mechanoreceptor\_differentiation | 29 | 3 | 9.722027 | -2.467709 | 21 | -0.187339 | 2.97 | 6.127339 | 0.141429 |
| GO:0050769\_positive\_regulation\_of\_neurogenesis | 29 | 3 | 9.722027 | -2.467709 | 21 | -0.187339 | 2.97 | 6.127339 | 0.141429 |
| GO:0045596\_negative\_regulation\_of\_cell\_differentiation | 144 | 6 | 3.915816 | -2.410377 | 22 | 0.095589 | 3.65 | 7.204411 | 0.165909 |
| GO:0031175\_neuron\_projection\_development | 197 | 7 | 3.339376 | -2.357383 | 23 | 0.200279 | 3.89 | 7.579721 | 0.169130 |
| GO:0007269\_neurotransmitter\_secretion | 34 | 3 | 8.292317 | -2.269667 | 25 | 0.600989 | 4.91 | 9.219011 | 0.196400 |
| GO:0010720\_positive\_regulation\_of\_cell\_development | 34 | 3 | 8.292317 | -2.269667 | 25 | 0.600989 | 4.91 | 9.219011 | 0.196400 |
| GO:0009887\_organ\_morphogenesis | 642 | 14 | 2.049399 | -2.257305 | 26 | 0.600989 | 4.91 | 9.219011 | 0.188846 |
| GO:0016079\_synaptic\_vesicle\_exocytosis | 11 | 2 | 17.087199 | -2.241188 | 27 | 0.849229 | 5.48 | 10.110771 | 0.202963 |
| GO:0048666\_neuron\_development | 262 | 8 | 2.869606 | -2.236384 | 28 | 0.854836 | 5.49 | 10.125164 | 0.196071 |
| GO:0007409\_axonogenesis | 158 | 6 | 3.568845 | -2.213376 | 29 | 0.933417 | 5.64 | 10.346583 | 0.194483 |
| GO:0032502\_developmental\_process | 2060 | 31 | 1.414256 | -2.175414 | 30 | 0.993733 | 5.85 | 10.706267 | 0.195000 |
| GO:0048513\_organ\_development | 1365 | 23 | 1.583539 | -2.120123 | 31 | 1.042282 | 6.81 | 12.577718 | 0.219677 |
| GO:0016043\_cellular\_component\_organization | 964 | 18 | 1.754806 | -2.111540 | 32 | 1.065238 | 6.84 | 12.614762 | 0.213750 |
| GO:0007423\_sensory\_organ\_development | 219 | 7 | 3.003914 | -2.108026 | 33 | 1.082843 | 6.85 | 12.617157 | 0.207576 |
| GO:0021953\_central\_nervous\_system\_neuron\_differentiation | 39 | 3 | 7.229199 | -2.101911 | 34 | 1.126606 | 6.98 | 12.833394 | 0.205294 |
| GO:0050771\_negative\_regulation\_of\_axonogenesis | 13 | 2 | 14.458399 | -2.095349 | 35 | 1.209433 | 7.56 | 13.910567 | 0.216000 |
| GO:0048812\_neuron\_projection\_morphogenesis | 170 | 6 | 3.316927 | -2.061517 | 36 | 1.248949 | 7.74 | 14.231051 | 0.215000 |
| GO:0006836\_neurotransmitter\_transport | 41 | 3 | 6.876556 | -2.041504 | 37 | 1.395747 | 8.0 | 14.604253 | 0.216216 |
| GO:0030154\_cell\_differentiation | 1060 | 19 | 1.684540 | -2.028725 | 38 | 1.499192 | 8.66 | 15.820808 | 0.227895 |
| GO:0048667\_cell\_morphogenesis\_involved\_in\_neuron\_differentiation | 173 | 6 | 3.259408 | -2.025718 | 39 | 1.513658 | 8.67 | 15.826342 | 0.222308 |
| GO:0048858\_cell\_projection\_morphogenesis | 176 | 6 | 3.203850 | -1.990725 | 40 | 1.543904 | 8.99 | 16.436096 | 0.224750 |
| GO:0007052\_mitotic\_spindle\_organization | 1 | 1 |  |  |  |  |  |  |  |  |
| GO:0019858\_cytosine\_metabolic\_process | 1 | 1 |  |  |  |  |  |  |  |  |
| GO:0032314\_regulation\_of\_Rac\_GTPase\_activity | 1 | 1 |  |  |  |  |  |  |  |  |
| GO:0048170\_positive\_regulation\_of\_long-term\_neuronal\_synaptic\_plasticity | 1 | 1 |  |  |  |  |  |  |  |  |
| GO:0051355\_proprioception\_during\_equilibrioception | 1 | 1 |  |  |  |  |  |  |  |  |
| GO:0060071\_Wnt\_receptor\_signaling\_pathway\_\_planar\_cell\_polarity\_pathway | 1 | 1 |  |  |  |  |  |  |  |  |
| GO:0060683\_regulation\_of\_branching\_involved\_in\_salivary\_gland\_morphogenesis\_by\_epithelial-mesenchymal\_signaling | 1 | 1 |  |  |  |  |  |  |  |  |
| GO:0060691\_epithelial\_cell\_maturation\_involved\_in\_salivary\_gland\_development | 1 | 1 |  |  |  |  |  |  |  |  |
| GO:0045664\_regulation\_of\_neuron\_differentiation | 82 | 4 | 4.584370 | -1.967613 | 41 | 2.350630 | 10.05 | 17.749370 | 0.245122 |
| GO:0031345\_negative\_regulation\_of\_cell\_projection\_organization | 16 | 2 | 11.747449 | -1.917086 | 42 | 2.953344 | 11.3 | 19.646656 | 0.269048 |
| GO:0016192\_vesicle-mediated\_transport | 184 | 6 | 3.064552 | -1.901142 | 44 | 3.069933 | 11.52 | 19.970067 | 0.261818 |
| GO:0032990\_cell\_part\_morphogenesis | 184 | 6 | 3.064552 | -1.901142 | 44 | 3.069933 | 11.52 | 19.970067 | 0.261818 |
| GO:0001505\_regulation\_of\_neurotransmitter\_levels | 48 | 3 | 5.873724 | -1.853858 | 45 | 3.591792 | 12.77 | 21.948208 | 0.283778 |
| GO:0048869\_cellular\_developmental\_process | 1113 | 19 | 1.604324 | -1.799941 | 46 | 4.306892 | 14.07 | 23.833108 | 0.305870 |
| GO:0042491\_auditory\_receptor\_cell\_differentiation | 19 | 2 | 9.892589 | -1.772076 | 47 | 4.942589 | 15.0 | 25.057411 | 0.319149 |
| GO:0000904\_cell\_morphogenesis\_involved\_in\_differentiation | 199 | 6 | 2.833556 | -1.746469 | 48 | 5.018267 | 15.44 | 25.861733 | 0.321667 |
| GO:0030900\_forebrain\_development | 146 | 5 | 3.218479 | -1.730831 | 49 | 5.156229 | 15.62 | 26.083771 | 0.318776 |
| GO:0032940\_secretion\_by\_cell | 149 | 5 | 3.153678 | -1.696353 | 50 | 5.743217 | 16.66 | 27.576783 | 0.333200 |
| GO:0006206\_pyrimidine\_base\_metabolic\_process | 2 | 1 |  |  |  |  |  |  |  |  |
| GO:0007020\_microtubule\_nucleation | 2 | 1 |  |  |  |  |  |  |  |  |
| GO:0016199\_axon\_midline\_choice\_point\_recognition | 2 | 1 |  |  |  |  |  |  |  |  |
| GO:0016572\_histone\_phosphorylation | 2 | 1 |  |  |  |  |  |  |  |  |
| GO:0030219\_megakaryocyte\_differentiation | 2 | 1 |  |  |  |  |  |  |  |  |
| GO:0035021\_negative\_regulation\_of\_Rac\_protein\_signal\_transduction | 2 | 1 |  |  |  |  |  |  |  |  |
| GO:0060346\_bone\_trabecula\_formation | 2 | 1 |  |  |  |  |  |  |  |  |
| GO:0060690\_epithelial\_cell\_differentiation\_involved\_in\_salivary\_gland\_development | 2 | 1 |  |  |  |  |  |  |  |  |
| GO:0070257\_positive\_regulation\_of\_mucus\_secretion | 2 | 1 |  |  |  |  |  |  |  |  |
| GO:0006928\_cell\_motion | 330 | 8 | 2.278293 | -1.664418 | 52 | 6.318553 | 18.04 | 29.761447 | 0.346923 |
| GO:0051674\_localization\_of\_cell | 330 | 8 | 2.278293 | -1.664418 | 52 | 6.318553 | 18.04 | 29.761447 | 0.346923 |
| GO:0000226\_microtubule\_cytoskeleton\_organization | 57 | 3 | 4.946294 | -1.654421 | 53 | 6.421789 | 18.25 | 30.078211 | 0.344340 |
| GO:0050767\_regulation\_of\_neurogenesis | 104 | 4 | 3.614600 | -1.621656 | 54 | 7.396710 | 19.66 | 31.923290 | 0.364074 |
| GO:0030099\_myeloid\_cell\_differentiation | 108 | 4 | 3.480726 | -1.568527 | 55 | 8.225065 | 21.6 | 34.974935 | 0.392727 |
| GO:0060688\_regulation\_of\_morphogenesis\_of\_a\_branching\_structure | 25 | 2 | 7.518367 | -1.545502 | 56 | 8.929780 | 22.81 | 36.690220 | 0.407321 |
| GO:0007417\_central\_nervous\_system\_development | 287 | 7 | 2.292185 | -1.514561 | 57 | 9.141238 | 23.37 | 37.598762 | 0.410000 |
| GO:0045665\_negative\_regulation\_of\_neuron\_differentiation | 26 | 2 | 7.229199 | -1.513657 | 58 | 9.501478 | 24.02 | 38.538522 | 0.414138 |
| GO:0000212\_meiotic\_spindle\_organization | 3 | 1 |  |  |  |  |  |  |  |  |
| GO:0001955\_blood\_vessel\_maturation | 3 | 1 |  |  |  |  |  |  |  |  |
| GO:0007252\_I-kappaB\_phosphorylation | 3 | 1 |  |  |  |  |  |  |  |  |
| GO:0019230\_proprioception | 3 | 1 |  |  |  |  |  |  |  |  |
| GO:0030224\_monocyte\_differentiation | 3 | 1 |  |  |  |  |  |  |  |  |
| GO:0030574\_collagen\_catabolic\_process | 3 | 1 |  |  |  |  |  |  |  |  |
| GO:0042668\_auditory\_receptor\_cell\_fate\_determination | 3 | 1 |  |  |  |  |  |  |  |  |
| GO:0050957\_equilibrioception | 3 | 1 |  |  |  |  |  |  |  |  |
| GO:0060684\_epithelial-mesenchymal\_cell\_signaling | 3 | 1 |  |  |  |  |  |  |  |  |
| GO:0060689\_cell\_differentiation\_involved\_in\_salivary\_gland\_development | 3 | 1 |  |  |  |  |  |  |  |  |
| GO:0007422\_peripheral\_nervous\_system\_development | 27 | 2 | 6.961451 | -1.483149 | 59 | 10.259170 | 25.16 | 40.060830 | 0.426441 |
| GO:0007420\_brain\_development | 231 | 6 | 2.441028 | -1.464608 | 60 | 10.470180 | 25.58 | 40.689820 | 0.426333 |
| GO:0051960\_regulation\_of\_nervous\_system\_development | 118 | 4 | 3.185749 | -1.446001 | 61 | 10.946533 | 26.7 | 42.453467 | 0.437705 |
| GO:0046903\_secretion | 175 | 5 | 2.685131 | -1.431773 | 62 | 11.157341 | 27.15 | 43.142659 | 0.437903 |
| GO:0006468\_protein\_amino\_acid\_phosphorylation | 237 | 6 | 2.379230 | -1.417903 | 63 | 12.149722 | 28.68 | 45.210278 | 0.455238 |
| GO:0060284\_regulation\_of\_cell\_development | 122 | 4 | 3.081298 | -1.400685 | 64 | 12.434826 | 29.23 | 46.025174 | 0.456719 |
| GO:0048732\_gland\_development | 179 | 5 | 2.625128 | -1.395810 | 65 | 13.158225 | 30.25 | 47.341775 | 0.465385 |
| GO:0009912\_auditory\_receptor\_cell\_fate\_commitment | 4 | 1 |  |  |  |  |  |  |  |  |
| GO:0016198\_axon\_choice\_point\_recognition | 4 | 1 |  |  |  |  |  |  |  |  |
| GO:0021535\_cell\_migration\_in\_hindbrain | 4 | 1 |  |  |  |  |  |  |  |  |
| GO:0021631\_optic\_nerve\_morphogenesis | 4 | 1 |  |  |  |  |  |  |  |  |
| GO:0035020\_regulation\_of\_Rac\_protein\_signal\_transduction | 4 | 1 |  |  |  |  |  |  |  |  |
| GO:0044243\_multicellular\_organismal\_catabolic\_process | 4 | 1 |  |  |  |  |  |  |  |  |
| GO:0045747\_positive\_regulation\_of\_Notch\_signaling\_pathway | 4 | 1 |  |  |  |  |  |  |  |  |
| GO:0060120\_inner\_ear\_receptor\_cell\_fate\_commitment | 4 | 1 |  |  |  |  |  |  |  |  |
| GO:0070254\_mucus\_secretion | 4 | 1 |  |  |  |  |  |  |  |  |
| GO:0070255\_regulation\_of\_mucus\_secretion | 4 | 1 |  |  |  |  |  |  |  |  |
| GO:0021954\_central\_nervous\_system\_neuron\_development | 31 | 2 | 6.063199 | -1.372643 | 66 | 14.178915 | 32.3 | 50.421085 | 0.489394 |
| GO:0051094\_positive\_regulation\_of\_developmental\_process | 308 | 7 | 2.135900 | -1.371296 | 67 | 14.195117 | 32.34 | 50.484883 | 0.482687 |
| GO:0043062\_extracellular\_structure\_organization | 125 | 4 | 3.007347 | -1.367954 | 68 | 14.255678 | 32.52 | 50.784322 | 0.478235 |
| GO:0050768\_negative\_regulation\_of\_neurogenesis | 32 | 2 | 5.873724 | -1.347518 | 70 | 15.094852 | 34.02 | 52.945148 | 0.486000 |
| GO:0050770\_regulation\_of\_axonogenesis | 32 | 2 | 5.873724 | -1.347518 | 70 | 15.094852 | 34.02 | 52.945148 | 0.486000 |
| GO:0007010\_cytoskeleton\_organization | 185 | 5 | 2.539989 | -1.343914 | 71 | 15.152574 | 34.12 | 53.087426 | 0.480563 |
| GO:0045597\_positive\_regulation\_of\_cell\_differentiation | 128 | 4 | 2.936862 | -1.336240 | 72 | 15.310438 | 34.42 | 53.529562 | 0.478056 |
| GO:0007431\_salivary\_gland\_development | 33 | 2 | 5.695733 | -1.323268 | 73 | 15.991207 | 35.58 | 55.168793 | 0.487397 |
| GO:0019226\_transmission\_of\_nerve\_impulse | 189 | 5 | 2.486233 | -1.310613 | 74 | 16.048309 | 35.91 | 55.771691 | 0.485270 |
| GO:0010721\_negative\_regulation\_of\_cell\_development | 34 | 2 | 5.528211 | -1.299839 | 75 | 16.790195 | 37.37 | 57.949805 | 0.498267 |
| GO:0050877\_neurological\_system\_process | 390 | 8 | 1.927786 | -1.289732 | 76 | 16.934876 | 37.62 | 58.305124 | 0.495000 |
| GO:0001957\_intramembranous\_ossification | 5 | 1 | 18.795918 | -1.283121 | 86 | 28.487801 | 51.82 | 75.152199 | 0.602558 |
| GO:0006270\_DNA\_replication\_initiation | 5 | 1 | 18.795918 | -1.283121 | 86 | 28.487801 | 51.82 | 75.152199 | 0.602558 |
| GO:0006378\_mRNA\_polyadenylation | 5 | 1 | 18.795918 | -1.283121 | 86 | 28.487801 | 51.82 | 75.152199 | 0.602558 |
| GO:0021554\_optic\_nerve\_development | 5 | 1 | 18.795918 | -1.283121 | 86 | 28.487801 | 51.82 | 75.152199 | 0.602558 |
| GO:0030851\_granulocyte\_differentiation | 5 | 1 | 18.795918 | -1.283121 | 86 | 28.487801 | 51.82 | 75.152199 | 0.602558 |
| GO:0043631\_RNA\_polyadenylation | 5 | 1 | 18.795918 | -1.283121 | 86 | 28.487801 | 51.82 | 75.152199 | 0.602558 |
| GO:0045773\_positive\_regulation\_of\_axon\_extension | 5 | 1 | 18.795918 | -1.283121 | 86 | 28.487801 | 51.82 | 75.152199 | 0.602558 |
| GO:0048013\_ephrin\_receptor\_signaling\_pathway | 5 | 1 | 18.795918 | -1.283121 | 86 | 28.487801 | 51.82 | 75.152199 | 0.602558 |
| GO:0048664\_neuron\_fate\_determination | 5 | 1 | 18.795918 | -1.283121 | 86 | 28.487801 | 51.82 | 75.152199 | 0.602558 |
| GO:0048934\_peripheral\_nervous\_system\_neuron\_differentiation | 5 | 1 | 18.795918 | -1.283121 | 86 | 28.487801 | 51.82 | 75.152199 | 0.602558 |
| GO:0021700\_developmental\_maturation | 81 | 3 | 3.480726 | -1.266166 | 87 | 29.240401 | 52.78 | 76.319599 | 0.606667 |
| GO:0007154\_cell\_communication | 1096 | 17 | 1.457713 | -1.255770 | 88 | 29.342958 | 53.15 | 76.957042 | 0.603977 |
| GO:0007017\_microtubule-based\_process | 83 | 3 | 3.396853 | -1.240332 | 90 | 30.718867 | 55.02 | 79.321133 | 0.611333 |
| GO:0030198\_extracellular\_matrix\_organization | 83 | 3 | 3.396853 | -1.240332 | 90 | 30.718867 | 55.02 | 79.321133 | 0.611333 |
| GO:0002009\_morphogenesis\_of\_an\_epithelium | 198 | 5 | 2.373222 | -1.239223 | 92 | 30.752284 | 55.06 | 79.367716 | 0.598478 |
| GO:0060429\_epithelium\_development | 198 | 5 | 2.373222 | -1.239223 | 92 | 30.752284 | 55.06 | 79.367716 | 0.598478 |
| GO:0010646\_regulation\_of\_cell\_communication | 330 | 7 | 1.993506 | -1.236601 | 93 | 30.828299 | 55.2 | 79.571701 | 0.593548 |
| GO:0050793\_regulation\_of\_developmental\_process | 703 | 12 | 1.604204 | -1.218814 | 94 | 31.672861 | 56.47 | 81.267139 | 0.600745 |
| GO:0010975\_regulation\_of\_neuron\_projection\_development | 38 | 2 | 4.946294 | -1.213424 | 95 | 32.663269 | 57.69 | 82.716731 | 0.607263 |
| GO:0001779\_natural\_killer\_cell\_differentiation | 6 | 1 | 15.663265 | -1.206195 | 102 | 41.809515 | 68.65 | 95.490485 | 0.673039 |
| GO:0009112\_nucleobase\_metabolic\_process | 6 | 1 | 15.663265 | -1.206195 | 102 | 41.809515 | 68.65 | 95.490485 | 0.673039 |
| GO:0040023\_establishment\_of\_nucleus\_localization | 6 | 1 | 15.663265 | -1.206195 | 102 | 41.809515 | 68.65 | 95.490485 | 0.673039 |
| GO:0046580\_negative\_regulation\_of\_Ras\_protein\_signal\_transduction | 6 | 1 | 15.663265 | -1.206195 | 102 | 41.809515 | 68.65 | 95.490485 | 0.673039 |
| GO:0048041\_focal\_adhesion\_formation | 6 | 1 | 15.663265 | -1.206195 | 102 | 41.809515 | 68.65 | 95.490485 | 0.673039 |
| GO:0051058\_negative\_regulation\_of\_small\_GTPase\_mediated\_signal\_transduction | 6 | 1 | 15.663265 | -1.206195 | 102 | 41.809515 | 68.65 | 95.490485 | 0.673039 |
| GO:0060136\_embryonic\_process\_involved\_in\_female\_pregnancy | 6 | 1 | 15.663265 | -1.206195 | 102 | 41.809515 | 68.65 | 95.490485 | 0.673039 |
| GO:0001568\_blood\_vessel\_development | 203 | 5 | 2.314768 | -1.201549 | 103 | 42.152451 | 68.97 | 95.787549 | 0.669612 |
| GO:0003001\_generation\_of\_a\_signal\_involved\_in\_cell-cell\_signaling | 87 | 3 | 3.240676 | -1.190937 | 104 | 43.267890 | 70.66 | 98.052110 | 0.679423 |
| GO:0001503\_ossification | 88 | 3 | 3.203850 | -1.179036 | 105 | 43.573362 | 71.21 | 98.846638 | 0.678190 |
| GO:0007346\_regulation\_of\_mitotic\_cell\_cycle | 40 | 2 | 4.698980 | -1.174060 | 108 | 44.157081 | 72.12 | 100.082919 | 0.667778 |
| GO:0035272\_exocrine\_system\_development | 40 | 2 | 4.698980 | -1.174060 | 108 | 44.157081 | 72.12 | 100.082919 | 0.667778 |
| GO:0051129\_negative\_regulation\_of\_cellular\_component\_organization | 40 | 2 | 4.698980 | -1.174060 | 108 | 44.157081 | 72.12 | 100.082919 | 0.667778 |
| GO:0051649\_establishment\_of\_localization\_in\_cell | 342 | 7 | 1.923559 | -1.169022 | 109 | 44.252067 | 72.23 | 100.207933 | 0.662661 |
| GO:0001944\_vasculature\_development | 208 | 5 | 2.259125 | -1.165204 | 110 | 44.304670 | 72.39 | 100.475330 | 0.658091 |
| GO:0022603\_regulation\_of\_anatomical\_structure\_morphogenesis | 147 | 4 | 2.557268 | -1.156177 | 111 | 44.542590 | 72.69 | 100.837410 | 0.654865 |
| GO:0031344\_regulation\_of\_cell\_projection\_organization | 41 | 2 | 4.584370 | -1.155225 | 112 | 45.544060 | 74.24 | 102.935940 | 0.662857 |
| GO:0001736\_establishment\_of\_planar\_polarity | 7 | 1 | 13.425656 | -1.141500 | 124 | 55.528866 | 86.79 | 118.051134 | 0.699919 |
| GO:0001967\_suckling\_behavior | 7 | 1 | 13.425656 | -1.141500 | 124 | 55.528866 | 86.79 | 118.051134 | 0.699919 |
| GO:0002052\_positive\_regulation\_of\_neuroblast\_proliferation | 7 | 1 | 13.425656 | -1.141500 | 124 | 55.528866 | 86.79 | 118.051134 | 0.699919 |
| GO:0007164\_establishment\_of\_tissue\_polarity | 7 | 1 | 13.425656 | -1.141500 | 124 | 55.528866 | 86.79 | 118.051134 | 0.699919 |
| GO:0030517\_negative\_regulation\_of\_axon\_extension | 7 | 1 | 13.425656 | -1.141500 | 124 | 55.528866 | 86.79 | 118.051134 | 0.699919 |
| GO:0031017\_exocrine\_pancreas\_development | 7 | 1 | 13.425656 | -1.141500 | 124 | 55.528866 | 86.79 | 118.051134 | 0.699919 |
| GO:0031124\_mRNA\_3'-end\_processing | 7 | 1 | 13.425656 | -1.141500 | 124 | 55.528866 | 86.79 | 118.051134 | 0.699919 |
| GO:0032319\_regulation\_of\_Rho\_GTPase\_activity | 7 | 1 | 13.425656 | -1.141500 | 124 | 55.528866 | 86.79 | 118.051134 | 0.699919 |
| GO:0043353\_enucleate\_erythrocyte\_differentiation | 7 | 1 | 13.425656 | -1.141500 | 124 | 55.528866 | 86.79 | 118.051134 | 0.699919 |
| GO:0060526\_prostate\_glandular\_acinus\_morphogenesis | 7 | 1 | 13.425656 | -1.141500 | 124 | 55.528866 | 86.79 | 118.051134 | 0.699919 |
| GO:0060527\_prostate\_epithelial\_cord\_arborization\_involved\_in\_prostate\_glandular\_acinus\_morphogenesis | 7 | 1 | 13.425656 | -1.141500 | 124 | 55.528866 | 86.79 | 118.051134 | 0.699919 |
| GO:0060687\_regulation\_of\_branching\_involved\_in\_prostate\_gland\_morphogenesis | 7 | 1 | 13.425656 | -1.141500 | 124 | 55.528866 | 86.79 | 118.051134 | 0.699919 |
| GO:0010769\_regulation\_of\_cell\_morphogenesis\_involved\_in\_differentiation | 42 | 2 | 4.475219 | -1.136917 | 125 | 56.543267 | 88.05 | 119.556733 | 0.704400 |
| GO:0007224\_smoothened\_signaling\_pathway | 43 | 2 | 4.371144 | -1.119112 | 126 | 57.931162 | 90.24 | 122.548838 | 0.716190 |
| GO:0051179\_localization | 1058 | 16 | 1.421241 | -1.110848 | 127 | 58.215688 | 90.8 | 123.384312 | 0.714961 |
| GO:0000902\_cell\_morphogenesis | 283 | 6 | 1.992500 | -1.110504 | 128 | 58.256395 | 90.84 | 123.423605 | 0.709688 |
| GO:0048593\_camera-type\_eye\_morphogenesis | 44 | 2 | 4.271800 | -1.101787 | 129 | 59.795597 | 93.04 | 126.284403 | 0.721240 |
| GO:0007268\_synaptic\_transmission | 154 | 4 | 2.441028 | -1.097686 | 130 | 59.899576 | 93.31 | 126.720424 | 0.717769 |
| GO:0008593\_regulation\_of\_Notch\_signaling\_pathway | 8 | 1 | 11.747449 | -1.085756 | 137 | 68.639865 | 104.6 | 140.560135 | 0.763504 |
| GO:0018107\_peptidyl-threonine\_phosphorylation | 8 | 1 | 11.747449 | -1.085756 | 137 | 68.639865 | 104.6 | 140.560135 | 0.763504 |
| GO:0018210\_peptidyl-threonine\_modification | 8 | 1 | 11.747449 | -1.085756 | 137 | 68.639865 | 104.6 | 140.560135 | 0.763504 |
| GO:0030511\_positive\_regulation\_of\_transforming\_growth\_factor\_beta\_receptor\_signaling\_pathway | 8 | 1 | 11.747449 | -1.085756 | 137 | 68.639865 | 104.6 | 140.560135 | 0.763504 |
| GO:0031123\_RNA\_3'-end\_processing | 8 | 1 | 11.747449 | -1.085756 | 137 | 68.639865 | 104.6 | 140.560135 | 0.763504 |
| GO:0035023\_regulation\_of\_Rho\_protein\_signal\_transduction | 8 | 1 | 11.747449 | -1.085756 | 137 | 68.639865 | 104.6 | 140.560135 | 0.763504 |
| GO:0042771\_DNA\_damage\_response\_\_signal\_transduction\_by\_p53\_class\_mediator\_resulting\_in\_induction\_of\_apoptosis | 8 | 1 | 11.747449 | -1.085756 | 137 | 68.639865 | 104.6 | 140.560135 | 0.763504 |
| GO:0018193\_peptidyl-amino\_acid\_modification | 97 | 3 | 2.906585 | -1.079170 | 138 | 69.002624 | 105.29 | 141.577376 | 0.762971 |
| GO:0009967\_positive\_regulation\_of\_signal\_transduction | 98 | 3 | 2.876926 | -1.068815 | 139 | 69.259013 | 105.91 | 142.560987 | 0.761942 |
| GO:0030218\_erythrocyte\_differentiation | 46 | 2 | 4.086069 | -1.068485 | 140 | 69.617202 | 106.53 | 143.442798 | 0.760929 |
| GO:0051239\_regulation\_of\_multicellular\_organismal\_process | 587 | 10 | 1.601015 | -1.064675 | 141 | 69.766281 | 106.72 | 143.673719 | 0.756879 |
| GO:0060348\_bone\_development | 99 | 3 | 2.847866 | -1.058598 | 142 | 70.091594 | 107.06 | 144.028406 | 0.753944 |
| GO:0051128\_regulation\_of\_cellular\_component\_organization | 160 | 4 | 2.349490 | -1.050443 | 143 | 70.890231 | 108.15 | 145.409769 | 0.756294 |
| GO:0048518\_positive\_regulation\_of\_biological\_process | 995 | 15 | 1.416778 | -1.047976 | 144 | 71.036295 | 108.45 | 145.863705 | 0.753125 |
| GO:0045595\_regulation\_of\_cell\_differentiation | 295 | 6 | 1.911449 | -1.042782 | 145 | 71.179231 | 108.7 | 146.220769 | 0.749655 |
| GO:0014037\_Schwann\_cell\_differentiation | 9 | 1 | 10.442177 | -1.036847 | 155 | 80.467688 | 120.59 | 160.712312 | 0.778000 |
| GO:0016601\_Rac\_protein\_signal\_transduction | 9 | 1 | 10.442177 | -1.036847 | 155 | 80.467688 | 120.59 | 160.712312 | 0.778000 |
| GO:0032963\_collagen\_metabolic\_process | 9 | 1 | 10.442177 | -1.036847 | 155 | 80.467688 | 120.59 | 160.712312 | 0.778000 |
| GO:0050884\_neuromuscular\_process\_controlling\_posture | 9 | 1 | 10.442177 | -1.036847 | 155 | 80.467688 | 120.59 | 160.712312 | 0.778000 |
| GO:0050910\_detection\_of\_mechanical\_stimulus\_involved\_in\_sensory\_perception\_of\_sound | 9 | 1 | 10.442177 | -1.036847 | 155 | 80.467688 | 120.59 | 160.712312 | 0.778000 |
| GO:0051647\_nucleus\_localization | 9 | 1 | 10.442177 | -1.036847 | 155 | 80.467688 | 120.59 | 160.712312 | 0.778000 |
| GO:0060119\_inner\_ear\_receptor\_cell\_development | 9 | 1 | 10.442177 | -1.036847 | 155 | 80.467688 | 120.59 | 160.712312 | 0.778000 |
| GO:0060122\_inner\_ear\_receptor\_stereocilium\_organization | 9 | 1 | 10.442177 | -1.036847 | 155 | 80.467688 | 120.59 | 160.712312 | 0.778000 |
| GO:0060325\_face\_morphogenesis | 9 | 1 | 10.442177 | -1.036847 | 155 | 80.467688 | 120.59 | 160.712312 | 0.778000 |
| GO:0060693\_regulation\_of\_branching\_involved\_in\_salivary\_gland\_morphogenesis | 9 | 1 | 10.442177 | -1.036847 | 155 | 80.467688 | 120.59 | 160.712312 | 0.778000 |
| GO:0051641\_cellular\_localization | 370 | 7 | 1.777992 | -1.025597 | 156 | 80.851050 | 121.56 | 162.268950 | 0.779231 |
| GO:0034101\_erythrocyte\_homeostasis | 49 | 2 | 3.835902 | -1.021623 | 157 | 81.671973 | 122.81 | 163.948027 | 0.782229 |
| GO:0048598\_embryonic\_morphogenesis | 299 | 6 | 1.885878 | -1.021187 | 158 | 81.690544 | 122.88 | 164.069456 | 0.777722 |
| GO:0002573\_myeloid\_leukocyte\_differentiation | 50 | 2 | 3.759184 | -1.006758 | 159 | 82.670000 | 124.58 | 166.490000 | 0.783522 |
| GO:0002070\_epithelial\_cell\_maturation | 10 | 1 | 9.397959 | -0.993331 | 169 | 93.606108 | 138.17 | 182.733892 | 0.817574 |
| GO:0007044\_cell-substrate\_junction\_assembly | 10 | 1 | 9.397959 | -0.993331 | 169 | 93.606108 | 138.17 | 182.733892 | 0.817574 |
| GO:0021952\_central\_nervous\_system\_projection\_neuron\_axonogenesis | 10 | 1 | 9.397959 | -0.993331 | 169 | 93.606108 | 138.17 | 182.733892 | 0.817574 |
| GO:0032318\_regulation\_of\_Ras\_GTPase\_activity | 10 | 1 | 9.397959 | -0.993331 | 169 | 93.606108 | 138.17 | 182.733892 | 0.817574 |
| GO:0043330\_response\_to\_exogenous\_dsRNA | 10 | 1 | 9.397959 | -0.993331 | 169 | 93.606108 | 138.17 | 182.733892 | 0.817574 |
| GO:0044259\_multicellular\_organismal\_macromolecule\_metabolic\_process | 10 | 1 | 9.397959 | -0.993331 | 169 | 93.606108 | 138.17 | 182.733892 | 0.817574 |
| GO:0048596\_embryonic\_camera-type\_eye\_morphogenesis | 10 | 1 | 9.397959 | -0.993331 | 169 | 93.606108 | 138.17 | 182.733892 | 0.817574 |
| GO:0060216\_definitive\_hemopoiesis | 10 | 1 | 9.397959 | -0.993331 | 169 | 93.606108 | 138.17 | 182.733892 | 0.817574 |
| GO:0060323\_head\_morphogenesis | 10 | 1 | 9.397959 | -0.993331 | 169 | 93.606108 | 138.17 | 182.733892 | 0.817574 |
| GO:0060343\_trabecula\_formation | 10 | 1 | 9.397959 | -0.993331 | 169 | 93.606108 | 138.17 | 182.733892 | 0.817574 |
| GO:0050789\_regulation\_of\_biological\_process | 2357 | 30 | 1.196176 | -0.992707 | 170 | 93.626079 | 138.22 | 182.813921 | 0.813059 |
| GO:0032989\_cellular\_component\_morphogenesis | 307 | 6 | 1.836735 | -0.979386 | 171 | 94.793040 | 139.73 | 184.666960 | 0.817135 |
| GO:0051246\_regulation\_of\_protein\_metabolic\_process | 170 | 4 | 2.211285 | -0.977071 | 172 | 95.252878 | 140.4 | 185.547122 | 0.816279 |
| GO:0016310\_phosphorylation | 309 | 6 | 1.824846 | -0.969216 | 173 | 95.475435 | 140.89 | 186.304565 | 0.814393 |
| GO:0010647\_positive\_regulation\_of\_cell\_communication | 110 | 3 | 2.563080 | -0.954503 | 174 | 96.705455 | 142.8 | 188.894545 | 0.820690 |
| GO:0007051\_spindle\_organization | 11 | 1 | 8.543599 | -0.954175 | 180 | 103.616995 | 151.56 | 199.503005 | 0.842000 |
| GO:0007088\_regulation\_of\_mitosis | 11 | 1 | 8.543599 | -0.954175 | 180 | 103.616995 | 151.56 | 199.503005 | 0.842000 |
| GO:0021602\_cranial\_nerve\_morphogenesis | 11 | 1 | 8.543599 | -0.954175 | 180 | 103.616995 | 151.56 | 199.503005 | 0.842000 |
| GO:0046928\_regulation\_of\_neurotransmitter\_secretion | 11 | 1 | 8.543599 | -0.954175 | 180 | 103.616995 | 151.56 | 199.503005 | 0.842000 |
| GO:0050772\_positive\_regulation\_of\_axonogenesis | 11 | 1 | 8.543599 | -0.954175 | 180 | 103.616995 | 151.56 | 199.503005 | 0.842000 |
| GO:0051783\_regulation\_of\_nuclear\_division | 11 | 1 | 8.543599 | -0.954175 | 180 | 103.616995 | 151.56 | 199.503005 | 0.842000 |
| GO:0050794\_regulation\_of\_cellular\_process | 2190 | 28 | 1.201566 | -0.944082 | 181 | 104.416009 | 152.6 | 200.783991 | 0.843094 |
| GO:0001738\_morphogenesis\_of\_a\_polarized\_epithelium | 12 | 1 | 7.831633 | -0.918620 | 192 | 114.402829 | 164.93 | 215.457171 | 0.859010 |
| GO:0008038\_neuron\_recognition | 12 | 1 | 7.831633 | -0.918620 | 192 | 114.402829 | 164.93 | 215.457171 | 0.859010 |
| GO:0008045\_motor\_axon\_guidance | 12 | 1 | 7.831633 | -0.918620 | 192 | 114.402829 | 164.93 | 215.457171 | 0.859010 |
| GO:0030330\_DNA\_damage\_response\_\_signal\_transduction\_by\_p53\_class\_mediator | 12 | 1 | 7.831633 | -0.918620 | 192 | 114.402829 | 164.93 | 215.457171 | 0.859010 |
| GO:0033598\_mammary\_gland\_epithelial\_cell\_proliferation | 12 | 1 | 7.831633 | -0.918620 | 192 | 114.402829 | 164.93 | 215.457171 | 0.859010 |
| GO:0043331\_response\_to\_dsRNA | 12 | 1 | 7.831633 | -0.918620 | 192 | 114.402829 | 164.93 | 215.457171 | 0.859010 |
| GO:0048169\_regulation\_of\_long-term\_neuronal\_synaptic\_plasticity | 12 | 1 | 7.831633 | -0.918620 | 192 | 114.402829 | 164.93 | 215.457171 | 0.859010 |
| GO:0048821\_erythrocyte\_development | 12 | 1 | 7.831633 | -0.918620 | 192 | 114.402829 | 164.93 | 215.457171 | 0.859010 |
| GO:0050853\_B\_cell\_receptor\_signaling\_pathway | 12 | 1 | 7.831633 | -0.918620 | 192 | 114.402829 | 164.93 | 215.457171 | 0.859010 |
| GO:0051588\_regulation\_of\_neurotransmitter\_transport | 12 | 1 | 7.831633 | -0.918620 | 192 | 114.402829 | 164.93 | 215.457171 | 0.859010 |
| GO:0060525\_prostate\_glandular\_acinus\_development | 12 | 1 | 7.831633 | -0.918620 | 192 | 114.402829 | 164.93 | 215.457171 | 0.859010 |
| GO:0001764\_neuron\_migration | 57 | 2 | 3.297530 | -0.911842 | 197 | 115.647441 | 166.48 | 217.312559 | 0.845076 |
| GO:0001892\_embryonic\_placenta\_development | 57 | 2 | 3.297530 | -0.911842 | 197 | 115.647441 | 166.48 | 217.312559 | 0.845076 |
| GO:0018108\_peptidyl-tyrosine\_phosphorylation | 57 | 2 | 3.297530 | -0.911842 | 197 | 115.647441 | 166.48 | 217.312559 | 0.845076 |
| GO:0018212\_peptidyl-tyrosine\_modification | 57 | 2 | 3.297530 | -0.911842 | 197 | 115.647441 | 166.48 | 217.312559 | 0.845076 |
| GO:0042472\_inner\_ear\_morphogenesis | 57 | 2 | 3.297530 | -0.911842 | 197 | 115.647441 | 166.48 | 217.312559 | 0.845076 |
| GO:0030902\_hindbrain\_development | 58 | 2 | 3.240676 | -0.899447 | 200 | 116.506911 | 167.95 | 219.393089 | 0.839750 |
| GO:0033043\_regulation\_of\_organelle\_organization | 58 | 2 | 3.240676 | -0.899447 | 200 | 116.506911 | 167.95 | 219.393089 | 0.839750 |
| GO:0050804\_regulation\_of\_synaptic\_transmission | 58 | 2 | 3.240676 | -0.899447 | 200 | 116.506911 | 167.95 | 219.393089 | 0.839750 |
| GO:0007267\_cell-cell\_signaling | 252 | 5 | 1.864674 | -0.893835 | 201 | 116.870059 | 168.41 | 219.949941 | 0.837861 |
| GO:0065007\_biological\_regulation | 2593 | 32 | 1.159794 | -0.891525 | 202 | 116.929599 | 168.46 | 219.990401 | 0.833960 |
| GO:0016055\_Wnt\_receptor\_signaling\_pathway | 59 | 2 | 3.185749 | -0.887313 | 204 | 117.443771 | 169.26 | 221.076229 | 0.829706 |
| GO:0048469\_cell\_maturation | 59 | 2 | 3.185749 | -0.887313 | 204 | 117.443771 | 169.26 | 221.076229 | 0.829706 |
| GO:0001958\_endochondral\_ossification | 13 | 1 | 7.229199 | -0.886087 | 213 | 124.759032 | 178.39 | 232.020968 | 0.837512 |
| GO:0006898\_receptor-mediated\_endocytosis | 13 | 1 | 7.229199 | -0.886087 | 213 | 124.759032 | 178.39 | 232.020968 | 0.837512 |
| GO:0018105\_peptidyl-serine\_phosphorylation | 13 | 1 | 7.229199 | -0.886087 | 213 | 124.759032 | 178.39 | 232.020968 | 0.837512 |
| GO:0021955\_central\_nervous\_system\_neuron\_axonogenesis | 13 | 1 | 7.229199 | -0.886087 | 213 | 124.759032 | 178.39 | 232.020968 | 0.837512 |
| GO:0030516\_regulation\_of\_axon\_extension | 13 | 1 | 7.229199 | -0.886087 | 213 | 124.759032 | 178.39 | 232.020968 | 0.837512 |
| GO:0031290\_retinal\_ganglion\_cell\_axon\_guidance | 13 | 1 | 7.229199 | -0.886087 | 213 | 124.759032 | 178.39 | 232.020968 | 0.837512 |
| GO:0034329\_cell\_junction\_assembly | 13 | 1 | 7.229199 | -0.886087 | 213 | 124.759032 | 178.39 | 232.020968 | 0.837512 |
| GO:0045682\_regulation\_of\_epidermis\_development | 13 | 1 | 7.229199 | -0.886087 | 213 | 124.759032 | 178.39 | 232.020968 | 0.837512 |
| GO:0060324\_face\_development | 13 | 1 | 7.229199 | -0.886087 | 213 | 124.759032 | 178.39 | 232.020968 | 0.837512 |
| GO:0048729\_tissue\_morphogenesis | 255 | 5 | 1.842737 | -0.878042 | 214 | 125.228600 | 179.08 | 232.931400 | 0.836822 |
| GO:0009966\_regulation\_of\_signal\_transduction | 256 | 5 | 1.835539 | -0.872845 | 215 | 125.499710 | 179.45 | 233.400290 | 0.834651 |
| GO:0051093\_negative\_regulation\_of\_developmental\_process | 331 | 6 | 1.703558 | -0.864239 | 216 | 126.429165 | 180.77 | 235.110835 | 0.836898 |
| GO:0051969\_regulation\_of\_transmission\_of\_nerve\_impulse | 61 | 2 | 3.081298 | -0.863790 | 217 | 126.823818 | 181.37 | 235.916182 | 0.835806 |
| GO:0051726\_regulation\_of\_cell\_cycle | 121 | 3 | 2.330073 | -0.863563 | 218 | 127.077284 | 181.74 | 236.402716 | 0.833670 |
| GO:0000060\_protein\_import\_into\_nucleus\_\_translocation | 14 | 1 | 6.712828 | -0.856128 | 228 | 133.500550 | 190.45 | 247.399450 | 0.835307 |
| GO:0001829\_trophectodermal\_cell\_differentiation | 14 | 1 | 6.712828 | -0.856128 | 228 | 133.500550 | 190.45 | 247.399450 | 0.835307 |
| GO:0007589\_body\_fluid\_secretion | 14 | 1 | 6.712828 | -0.856128 | 228 | 133.500550 | 190.45 | 247.399450 | 0.835307 |
| GO:0008630\_DNA\_damage\_response\_\_signal\_transduction\_resulting\_in\_induction\_of\_apoptosis | 14 | 1 | 6.712828 | -0.856128 | 228 | 133.500550 | 190.45 | 247.399450 | 0.835307 |
| GO:0031346\_positive\_regulation\_of\_cell\_projection\_organization | 14 | 1 | 6.712828 | -0.856128 | 228 | 133.500550 | 190.45 | 247.399450 | 0.835307 |
| GO:0031663\_lipopolysaccharide-mediated\_signaling\_pathway | 14 | 1 | 6.712828 | -0.856128 | 228 | 133.500550 | 190.45 | 247.399450 | 0.835307 |
| GO:0043123\_positive\_regulation\_of\_I-kappaB\_kinase\_NF-kappaB\_cascade | 14 | 1 | 6.712828 | -0.856128 | 228 | 133.500550 | 190.45 | 247.399450 | 0.835307 |
| GO:0044236\_multicellular\_organismal\_metabolic\_process | 14 | 1 | 6.712828 | -0.856128 | 228 | 133.500550 | 190.45 | 247.399450 | 0.835307 |
| GO:0048048\_embryonic\_eye\_morphogenesis | 14 | 1 | 6.712828 | -0.856128 | 228 | 133.500550 | 190.45 | 247.399450 | 0.835307 |
| GO:0060716\_labyrinthine\_layer\_blood\_vessel\_development | 14 | 1 | 6.712828 | -0.856128 | 228 | 133.500550 | 190.45 | 247.399450 | 0.835307 |
| GO:0022604\_regulation\_of\_cell\_morphogenesis | 62 | 2 | 3.031600 | -0.852385 | 229 | 134.852371 | 192.33 | 249.807629 | 0.839869 |
| GO:0048522\_positive\_regulation\_of\_cellular\_process | 895 | 13 | 1.365067 | -0.851687 | 230 | 134.866826 | 192.36 | 249.853174 | 0.836348 |
| GO:0009790\_embryonic\_development | 567 | 9 | 1.491740 | -0.850271 | 231 | 134.908359 | 192.47 | 250.031641 | 0.833203 |
| GO:0031644\_regulation\_of\_neurological\_system\_process | 64 | 2 | 2.936862 | -0.830250 | 232 | 136.788748 | 194.98 | 253.171252 | 0.840431 |
| GO:0010171\_body\_morphogenesis | 15 | 1 | 6.265306 | -0.828387 | 237 | 143.170653 | 202.96 | 262.749347 | 0.856371 |
| GO:0031076\_embryonic\_camera-type\_eye\_development | 15 | 1 | 6.265306 | -0.828387 | 237 | 143.170653 | 202.96 | 262.749347 | 0.856371 |
| GO:0060322\_head\_development | 15 | 1 | 6.265306 | -0.828387 | 237 | 143.170653 | 202.96 | 262.749347 | 0.856371 |
| GO:0060442\_branching\_involved\_in\_prostate\_gland\_morphogenesis | 15 | 1 | 6.265306 | -0.828387 | 237 | 143.170653 | 202.96 | 262.749347 | 0.856371 |
| GO:0060749\_mammary\_gland\_alveolus\_development | 15 | 1 | 6.265306 | -0.828387 | 237 | 143.170653 | 202.96 | 262.749347 | 0.856371 |
| GO:0006793\_phosphorus\_metabolic\_process | 340 | 6 | 1.658463 | -0.824685 | 239 | 143.325718 | 203.21 | 263.094282 | 0.850251 |
| GO:0006796\_phosphate\_metabolic\_process | 340 | 6 | 1.658463 | -0.824685 | 239 | 143.325718 | 203.21 | 263.094282 | 0.850251 |
| GO:0042471\_ear\_morphogenesis | 65 | 2 | 2.891680 | -0.819506 | 240 | 144.078720 | 204.06 | 264.041280 | 0.850250 |
| GO:0043087\_regulation\_of\_GTPase\_activity | 16 | 1 | 5.873724 | -0.802577 | 244 | 151.865474 | 213.74 | 275.614526 | 0.875984 |
| GO:0043122\_regulation\_of\_I-kappaB\_kinase\_NF-kappaB\_cascade | 16 | 1 | 5.873724 | -0.802577 | 244 | 151.865474 | 213.74 | 275.614526 | 0.875984 |
| GO:0048286\_lung\_alveolus\_development | 16 | 1 | 5.873724 | -0.802577 | 244 | 151.865474 | 213.74 | 275.614526 | 0.875984 |
| GO:0050974\_detection\_of\_mechanical\_stimulus\_involved\_in\_sensory\_perception | 16 | 1 | 5.873724 | -0.802577 | 244 | 151.865474 | 213.74 | 275.614526 | 0.875984 |
| GO:0007165\_signal\_transduction | 915 | 13 | 1.335229 | -0.797558 | 245 | 152.704558 | 214.92 | 277.135442 | 0.877224 |
| GO:0006261\_DNA-dependent\_DNA\_replication | 17 | 1 | 5.528211 | -0.778463 | 250 | 160.461554 | 224.52 | 288.578446 | 0.898080 |
| GO:0021545\_cranial\_nerve\_development | 17 | 1 | 5.528211 | -0.778463 | 250 | 160.461554 | 224.52 | 288.578446 | 0.898080 |
| GO:0030101\_natural\_killer\_cell\_activation | 17 | 1 | 5.528211 | -0.778463 | 250 | 160.461554 | 224.52 | 288.578446 | 0.898080 |
| GO:0048168\_regulation\_of\_neuronal\_synaptic\_plasticity | 17 | 1 | 5.528211 | -0.778463 | 250 | 160.461554 | 224.52 | 288.578446 | 0.898080 |
| GO:0060350\_endochondral\_bone\_morphogenesis | 17 | 1 | 5.528211 | -0.778463 | 250 | 160.461554 | 224.52 | 288.578446 | 0.898080 |
| GO:0007611\_learning\_or\_memory | 70 | 2 | 2.685131 | -0.768759 | 252 | 161.805140 | 226.46 | 291.114860 | 0.898651 |
| GO:0048592\_eye\_morphogenesis | 70 | 2 | 2.685131 | -0.768759 | 252 | 161.805140 | 226.46 | 291.114860 | 0.898651 |
| GO:0007243\_protein\_kinase\_cascade | 205 | 4 | 1.833748 | -0.763575 | 253 | 161.909516 | 226.67 | 291.430484 | 0.895929 |
| GO:0001825\_blastocyst\_formation | 18 | 1 | 5.221088 | -0.755851 | 260 | 170.258745 | 237.21 | 304.161255 | 0.912346 |
| GO:0002064\_epithelial\_cell\_development | 18 | 1 | 5.221088 | -0.755851 | 260 | 170.258745 | 237.21 | 304.161255 | 0.912346 |
| GO:0010553\_negative\_regulation\_of\_specific\_transcription\_from\_RNA\_polymerase\_II\_promoter | 18 | 1 | 5.221088 | -0.755851 | 260 | 170.258745 | 237.21 | 304.161255 | 0.912346 |
| GO:0030178\_negative\_regulation\_of\_Wnt\_receptor\_signaling\_pathway | 18 | 1 | 5.221088 | -0.755851 | 260 | 170.258745 | 237.21 | 304.161255 | 0.912346 |
| GO:0046578\_regulation\_of\_Ras\_protein\_signal\_transduction | 18 | 1 | 5.221088 | -0.755851 | 260 | 170.258745 | 237.21 | 304.161255 | 0.912346 |
| GO:0050982\_detection\_of\_mechanical\_stimulus | 18 | 1 | 5.221088 | -0.755851 | 260 | 170.258745 | 237.21 | 304.161255 | 0.912346 |
| GO:0060674\_placenta\_blood\_vessel\_development | 18 | 1 | 5.221088 | -0.755851 | 260 | 170.258745 | 237.21 | 304.161255 | 0.912346 |
| GO:0007166\_cell\_surface\_receptor\_linked\_signal\_transduction | 597 | 9 | 1.416778 | -0.753362 | 261 | 170.262081 | 237.25 | 304.237919 | 0.909004 |
| GO:0003008\_system\_process | 516 | 8 | 1.457048 | -0.752481 | 262 | 170.324728 | 237.36 | 304.395272 | 0.905954 |
| GO:0030879\_mammary\_gland\_development | 72 | 2 | 2.610544 | -0.749741 | 263 | 171.585916 | 239.1 | 306.614084 | 0.909125 |
| GO:0008284\_positive\_regulation\_of\_cell\_proliferation | 208 | 4 | 1.807300 | -0.747892 | 264 | 171.871183 | 239.45 | 307.028817 | 0.907008 |
| GO:0009987\_cellular\_process | 3868 | 44 | 1.069054 | -0.742335 | 265 | 172.322438 | 239.99 | 307.657562 | 0.905623 |
| GO:0007595\_lactation | 19 | 1 | 4.946294 | -0.734577 | 268 | 177.758803 | 246.8 | 315.841197 | 0.920896 |
| GO:0030199\_collagen\_fibril\_organization | 19 | 1 | 4.946294 | -0.734577 | 268 | 177.758803 | 246.8 | 315.841197 | 0.920896 |
| GO:0051056\_regulation\_of\_small\_GTPase\_mediated\_signal\_transduction | 19 | 1 | 4.946294 | -0.734577 | 268 | 177.758803 | 246.8 | 315.841197 | 0.920896 |
| GO:0006518\_peptide\_metabolic\_process | 20 | 1 | 4.698980 | -0.714505 | 272 | 184.222633 | 255.09 | 325.957367 | 0.937831 |
| GO:0018209\_peptidyl-serine\_modification | 20 | 1 | 4.698980 | -0.714505 | 272 | 184.222633 | 255.09 | 325.957367 | 0.937831 |
| GO:0032582\_negative\_regulation\_of\_gene-specific\_transcription | 20 | 1 | 4.698980 | -0.714505 | 272 | 184.222633 | 255.09 | 325.957367 | 0.937831 |
| GO:0045639\_positive\_regulation\_of\_myeloid\_cell\_differentiation | 20 | 1 | 4.698980 | -0.714505 | 272 | 184.222633 | 255.09 | 325.957367 | 0.937831 |
| GO:0001890\_placenta\_development | 77 | 2 | 2.441028 | -0.705050 | 273 | 185.622266 | 257.06 | 328.497734 | 0.941612 |
| GO:0001702\_gastrulation\_with\_mouth\_forming\_second | 21 | 1 | 4.475219 | -0.695516 | 280 | 193.564977 | 266.24 | 338.915023 | 0.950857 |
| GO:0001709\_cell\_fate\_determination | 21 | 1 | 4.475219 | -0.695516 | 280 | 193.564977 | 266.24 | 338.915023 | 0.950857 |
| GO:0002053\_positive\_regulation\_of\_mesenchymal\_cell\_proliferation | 21 | 1 | 4.475219 | -0.695516 | 280 | 193.564977 | 266.24 | 338.915023 | 0.950857 |
| GO:0010552\_positive\_regulation\_of\_specific\_transcription\_from\_RNA\_polymerase\_II\_promoter | 21 | 1 | 4.475219 | -0.695516 | 280 | 193.564977 | 266.24 | 338.915023 | 0.950857 |
| GO:0034330\_cell\_junction\_organization | 21 | 1 | 4.475219 | -0.695516 | 280 | 193.564977 | 266.24 | 338.915023 | 0.950857 |
| GO:0048675\_axon\_extension | 21 | 1 | 4.475219 | -0.695516 | 280 | 193.564977 | 266.24 | 338.915023 | 0.950857 |
| GO:0051656\_establishment\_of\_organelle\_localization | 21 | 1 | 4.475219 | -0.695516 | 280 | 193.564977 | 266.24 | 338.915023 | 0.950857 |
| GO:0051046\_regulation\_of\_secretion | 79 | 2 | 2.379230 | -0.688224 | 281 | 195.006158 | 268.0 | 340.993842 | 0.953737 |
| GO:0000278\_mitotic\_cell\_cycle | 80 | 2 | 2.349490 | -0.680021 | 282 | 196.231363 | 269.67 | 343.108637 | 0.956277 |
| GO:0008624\_induction\_of\_apoptosis\_by\_extracellular\_signals | 22 | 1 | 4.271800 | -0.677509 | 289 | 203.206181 | 278.52 | 353.833819 | 0.963737 |
| GO:0010463\_mesenchymal\_cell\_proliferation | 22 | 1 | 4.271800 | -0.677509 | 289 | 203.206181 | 278.52 | 353.833819 | 0.963737 |
| GO:0010464\_regulation\_of\_mesenchymal\_cell\_proliferation | 22 | 1 | 4.271800 | -0.677509 | 289 | 203.206181 | 278.52 | 353.833819 | 0.963737 |
| GO:0021575\_hindbrain\_morphogenesis | 22 | 1 | 4.271800 | -0.677509 | 289 | 203.206181 | 278.52 | 353.833819 | 0.963737 |
| GO:0021675\_nerve\_development | 22 | 1 | 4.271800 | -0.677509 | 289 | 203.206181 | 278.52 | 353.833819 | 0.963737 |
| GO:0030316\_osteoclast\_differentiation | 22 | 1 | 4.271800 | -0.677509 | 289 | 203.206181 | 278.52 | 353.833819 | 0.963737 |
| GO:0045787\_positive\_regulation\_of\_cell\_cycle | 22 | 1 | 4.271800 | -0.677509 | 289 | 203.206181 | 278.52 | 353.833819 | 0.963737 |
| GO:0006397\_mRNA\_processing | 23 | 1 | 4.086069 | -0.660396 | 294 | 208.379447 | 285.17 | 361.960553 | 0.969966 |
| GO:0043388\_positive\_regulation\_of\_DNA\_binding | 23 | 1 | 4.086069 | -0.660396 | 294 | 208.379447 | 285.17 | 361.960553 | 0.969966 |
| GO:0051705\_behavioral\_interaction\_between\_organisms | 23 | 1 | 4.086069 | -0.660396 | 294 | 208.379447 | 285.17 | 361.960553 | 0.969966 |
| GO:0060349\_bone\_morphogenesis | 23 | 1 | 4.086069 | -0.660396 | 294 | 208.379447 | 285.17 | 361.960553 | 0.969966 |
| GO:0060445\_branching\_involved\_in\_salivary\_gland\_morphogenesis | 23 | 1 | 4.086069 | -0.660396 | 294 | 208.379447 | 285.17 | 361.960553 | 0.969966 |
| GO:0043687\_post-translational\_protein\_modification | 384 | 6 | 1.468431 | -0.655640 | 295 | 209.754192 | 286.67 | 363.585808 | 0.971763 |
| GO:0034961\_cellular\_biopolymer\_biosynthetic\_process | 804 | 11 | 1.285790 | -0.647298 | 296 | 211.188805 | 288.48 | 365.771195 | 0.974595 |
| GO:0000280\_nuclear\_division | 24 | 1 | 3.915816 | -0.644102 | 303 | 215.931588 | 294.49 | 373.048412 | 0.971914 |
| GO:0007067\_mitosis | 24 | 1 | 3.915816 | -0.644102 | 303 | 215.931588 | 294.49 | 373.048412 | 0.971914 |
| GO:0007266\_Rho\_protein\_signal\_transduction | 24 | 1 | 3.915816 | -0.644102 | 303 | 215.931588 | 294.49 | 373.048412 | 0.971914 |
| GO:0008629\_induction\_of\_apoptosis\_by\_intracellular\_signals | 24 | 1 | 3.915816 | -0.644102 | 303 | 215.931588 | 294.49 | 373.048412 | 0.971914 |
| GO:0009612\_response\_to\_mechanical\_stimulus | 24 | 1 | 3.915816 | -0.644102 | 303 | 215.931588 | 294.49 | 373.048412 | 0.971914 |
| GO:0043588\_skin\_development | 24 | 1 | 3.915816 | -0.644102 | 303 | 215.931588 | 294.49 | 373.048412 | 0.971914 |
| GO:0051099\_positive\_regulation\_of\_binding | 24 | 1 | 3.915816 | -0.644102 | 303 | 215.931588 | 294.49 | 373.048412 | 0.971914 |
| GO:0043284\_biopolymer\_biosynthetic\_process | 807 | 11 | 1.281011 | -0.640089 | 304 | 216.754465 | 295.51 | 374.265535 | 0.972072 |
| GO:0032504\_multicellular\_organism\_reproduction | 86 | 2 | 2.185572 | -0.633523 | 306 | 217.833992 | 297.02 | 376.206008 | 0.970654 |
| GO:0048609\_reproductive\_process\_in\_a\_multicellular\_organism | 86 | 2 | 2.185572 | -0.633523 | 306 | 217.833992 | 297.02 | 376.206008 | 0.970654 |
| GO:0051704\_multi-organism\_process | 157 | 3 | 1.795788 | -0.632710 | 307 | 217.895180 | 297.11 | 376.324820 | 0.967785 |
| GO:0050890\_cognition | 233 | 4 | 1.613384 | -0.630112 | 308 | 218.194819 | 297.39 | 376.585181 | 0.965552 |
| GO:0000087\_M\_phase\_of\_mitotic\_cell\_cycle | 25 | 1 | 3.759184 | -0.628559 | 312 | 221.960082 | 302.55 | 383.139918 | 0.969712 |
| GO:0007628\_adult\_walking\_behavior | 25 | 1 | 3.759184 | -0.628559 | 312 | 221.960082 | 302.55 | 383.139918 | 0.969712 |
| GO:0048285\_organelle\_fission | 25 | 1 | 3.759184 | -0.628559 | 312 | 221.960082 | 302.55 | 383.139918 | 0.969712 |
| GO:0050852\_T\_cell\_receptor\_signaling\_pathway | 25 | 1 | 3.759184 | -0.628559 | 312 | 221.960082 | 302.55 | 383.139918 | 0.969712 |
| GO:0022612\_gland\_morphogenesis | 87 | 2 | 2.160450 | -0.626198 | 313 | 223.414656 | 304.47 | 385.525344 | 0.972748 |
| GO:0001666\_response\_to\_hypoxia | 26 | 1 | 3.614600 | -0.613707 | 318 | 229.468831 | 311.63 | 393.791169 | 0.979969 |
| GO:0007405\_neuroblast\_proliferation | 26 | 1 | 3.614600 | -0.613707 | 318 | 229.468831 | 311.63 | 393.791169 | 0.979969 |
| GO:0007613\_memory | 26 | 1 | 3.614600 | -0.613707 | 318 | 229.468831 | 311.63 | 393.791169 | 0.979969 |
| GO:0007623\_circadian\_rhythm | 26 | 1 | 3.614600 | -0.613707 | 318 | 229.468831 | 311.63 | 393.791169 | 0.979969 |
| GO:0009636\_response\_to\_toxin | 26 | 1 | 3.614600 | -0.613707 | 318 | 229.468831 | 311.63 | 393.791169 | 0.979969 |
| GO:0030324\_lung\_development | 90 | 2 | 2.088435 | -0.604893 | 319 | 230.751447 | 313.22 | 395.688553 | 0.981881 |
| GO:0031016\_pancreas\_development | 27 | 1 | 3.480726 | -0.599495 | 322 | 234.656618 | 317.85 | 401.043382 | 0.987112 |
| GO:0032496\_response\_to\_lipopolysaccharide | 27 | 1 | 3.480726 | -0.599495 | 322 | 234.656618 | 317.85 | 401.043382 | 0.987112 |
| GO:0070482\_response\_to\_oxygen\_levels | 27 | 1 | 3.480726 | -0.599495 | 322 | 234.656618 | 317.85 | 401.043382 | 0.987112 |
| GO:0008544\_epidermis\_development | 91 | 2 | 2.065486 | -0.598007 | 323 | 235.045196 | 318.44 | 401.834804 | 0.985882 |
| GO:0019538\_protein\_metabolic\_process | 655 | 9 | 1.291323 | -0.593151 | 324 | 235.571033 | 319.12 | 402.668967 | 0.984938 |
| GO:0030323\_respiratory\_tube\_development | 92 | 2 | 2.043035 | -0.591224 | 325 | 236.387688 | 320.17 | 403.952312 | 0.985138 |
| GO:0043065\_positive\_regulation\_of\_apoptosis | 166 | 3 | 1.698426 | -0.587035 | 326 | 236.727941 | 320.61 | 404.492059 | 0.983466 |
| GO:0030073\_insulin\_secretion | 28 | 1 | 3.356414 | -0.585875 | 329 | 240.232050 | 324.69 | 409.147950 | 0.986900 |
| GO:0030111\_regulation\_of\_Wnt\_receptor\_signaling\_pathway | 28 | 1 | 3.356414 | -0.585875 | 329 | 240.232050 | 324.69 | 409.147950 | 0.986900 |
| GO:0043193\_positive\_regulation\_of\_gene-specific\_transcription | 28 | 1 | 3.356414 | -0.585875 | 329 | 240.232050 | 324.69 | 409.147950 | 0.986900 |
| GO:0010556\_regulation\_of\_macromolecule\_biosynthetic\_process | 745 | 10 | 1.261471 | -0.583263 | 330 | 241.002359 | 325.58 | 410.157641 | 0.986606 |
| GO:0010942\_positive\_regulation\_of\_cell\_death | 167 | 3 | 1.688256 | -0.582198 | 332 | 241.297038 | 326.12 | 410.942962 | 0.982289 |
| GO:0043068\_positive\_regulation\_of\_programmed\_cell\_death | 167 | 3 | 1.688256 | -0.582198 | 332 | 241.297038 | 326.12 | 410.942962 | 0.982289 |
| GO:0006417\_regulation\_of\_translation | 29 | 1 | 3.240676 | -0.572805 | 336 | 247.191922 | 332.72 | 418.248078 | 0.990238 |
| GO:0010564\_regulation\_of\_cell\_cycle\_process | 29 | 1 | 3.240676 | -0.572805 | 336 | 247.191922 | 332.72 | 418.248078 | 0.990238 |
| GO:0042176\_regulation\_of\_protein\_catabolic\_process | 29 | 1 | 3.240676 | -0.572805 | 336 | 247.191922 | 332.72 | 418.248078 | 0.990238 |
| GO:0042770\_DNA\_damage\_response\_\_signal\_transduction | 29 | 1 | 3.240676 | -0.572805 | 336 | 247.191922 | 332.72 | 418.248078 | 0.990238 |
| GO:0007242\_intracellular\_signaling\_cascade | 411 | 6 | 1.371965 | -0.569049 | 337 | 247.812501 | 333.57 | 419.327499 | 0.989822 |
| GO:0080090\_regulation\_of\_primary\_metabolic\_process | 926 | 12 | 1.217878 | -0.568490 | 338 | 247.883889 | 333.65 | 419.416111 | 0.987130 |
| GO:0007219\_Notch\_signaling\_pathway | 30 | 1 | 3.132653 | -0.560249 | 343 | 254.413894 | 341.5 | 428.586106 | 0.995627 |
| GO:0007435\_salivary\_gland\_morphogenesis | 30 | 1 | 3.132653 | -0.560249 | 343 | 254.413894 | 341.5 | 428.586106 | 0.995627 |
| GO:0042552\_myelination | 30 | 1 | 3.132653 | -0.560249 | 343 | 254.413894 | 341.5 | 428.586106 | 0.995627 |
| GO:0048565\_gut\_development | 30 | 1 | 3.132653 | -0.560249 | 343 | 254.413894 | 341.5 | 428.586106 | 0.995627 |
| GO:0060740\_prostate\_gland\_epithelium\_morphogenesis | 30 | 1 | 3.132653 | -0.560249 | 343 | 254.413894 | 341.5 | 428.586106 | 0.995627 |
| GO:0060541\_respiratory\_system\_development | 98 | 2 | 1.917951 | -0.552578 | 344 | 256.531965 | 343.87 | 431.208035 | 0.999622 |
| GO:0030097\_hemopoiesis | 253 | 4 | 1.485843 | -0.550196 | 345 | 256.851729 | 344.19 | 431.528271 | 0.997652 |
| GO:0008645\_hexose\_transport | 31 | 1 | 3.031600 | -0.548172 | 352 | 262.693168 | 351.83 | 440.966832 | 0.999517 |
| GO:0015749\_monosaccharide\_transport | 31 | 1 | 3.031600 | -0.548172 | 352 | 262.693168 | 351.83 | 440.966832 | 0.999517 |
| GO:0015758\_glucose\_transport | 31 | 1 | 3.031600 | -0.548172 | 352 | 262.693168 | 351.83 | 440.966832 | 0.999517 |
| GO:0048167\_regulation\_of\_synaptic\_plasticity | 31 | 1 | 3.031600 | -0.548172 | 352 | 262.693168 | 351.83 | 440.966832 | 0.999517 |
| GO:0048562\_embryonic\_organ\_morphogenesis | 31 | 1 | 3.031600 | -0.548172 | 352 | 262.693168 | 351.83 | 440.966832 | 0.999517 |
| GO:0051640\_organelle\_localization | 31 | 1 | 3.031600 | -0.548172 | 352 | 262.693168 | 351.83 | 440.966832 | 0.999517 |
| GO:0060512\_prostate\_gland\_morphogenesis | 31 | 1 | 3.031600 | -0.548172 | 352 | 262.693168 | 351.83 | 440.966832 | 0.999517 |
| GO:0060255\_regulation\_of\_macromolecule\_metabolic\_process | 936 | 12 | 1.204867 | -0.547965 | 353 | 262.736190 | 351.89 | 441.043810 | 0.996856 |
| GO:0007398\_ectoderm\_development | 99 | 2 | 1.898578 | -0.546460 | 354 | 263.339631 | 352.76 | 442.180369 | 0.996497 |
| GO:0045449\_regulation\_of\_transcription | 676 | 9 | 1.251208 | -0.542915 | 355 | 263.970387 | 353.54 | 443.109613 | 0.995887 |
| GO:0007249\_I-kappaB\_kinase\_NF-kappaB\_cascade | 32 | 1 | 2.936862 | -0.536543 | 361 | 268.568208 | 358.5 | 448.431792 | 0.993075 |
| GO:0007272\_ensheathment\_of\_neurons | 32 | 1 | 2.936862 | -0.536543 | 361 | 268.568208 | 358.5 | 448.431792 | 0.993075 |
| GO:0008366\_axon\_ensheathment | 32 | 1 | 2.936862 | -0.536543 | 361 | 268.568208 | 358.5 | 448.431792 | 0.993075 |
| GO:0050885\_neuromuscular\_process\_controlling\_balance | 32 | 1 | 2.936862 | -0.536543 | 361 | 268.568208 | 358.5 | 448.431792 | 0.993075 |
| GO:0051259\_protein\_oligomerization | 32 | 1 | 2.936862 | -0.536543 | 361 | 268.568208 | 358.5 | 448.431792 | 0.993075 |
| GO:0051493\_regulation\_of\_cytoskeleton\_organization | 32 | 1 | 2.936862 | -0.536543 | 361 | 268.568208 | 358.5 | 448.431792 | 0.993075 |
| GO:0030036\_actin\_cytoskeleton\_organization | 102 | 2 | 1.842737 | -0.528619 | 362 | 269.641123 | 359.82 | 449.998877 | 0.993978 |
| GO:0001843\_neural\_tube\_closure | 33 | 1 | 2.847866 | -0.525335 | 366 | 274.360767 | 365.14 | 455.919233 | 0.997650 |
| GO:0007565\_female\_pregnancy | 33 | 1 | 2.847866 | -0.525335 | 366 | 274.360767 | 365.14 | 455.919233 | 0.997650 |
| GO:0008643\_carbohydrate\_transport | 33 | 1 | 2.847866 | -0.525335 | 366 | 274.360767 | 365.14 | 455.919233 | 0.997650 |
| GO:0060606\_tube\_closure | 33 | 1 | 2.847866 | -0.525335 | 366 | 274.360767 | 365.14 | 455.919233 | 0.997650 |
| GO:0009968\_negative\_regulation\_of\_signal\_transduction | 103 | 2 | 1.824846 | -0.522838 | 367 | 274.889166 | 366.07 | 457.250834 | 0.997466 |
| GO:0048523\_negative\_regulation\_of\_cellular\_process | 774 | 10 | 1.214207 | -0.519136 | 368 | 275.143688 | 366.44 | 457.736312 | 0.995761 |
| GO:0002237\_response\_to\_molecule\_of\_bacterial\_origin | 34 | 1 | 2.764106 | -0.514522 | 372 | 280.213072 | 372.59 | 464.966928 | 1.001586 |
| GO:0050730\_regulation\_of\_peptidyl-tyrosine\_phosphorylation | 34 | 1 | 2.764106 | -0.514522 | 372 | 280.213072 | 372.59 | 464.966928 | 1.001586 |
| GO:0051047\_positive\_regulation\_of\_secretion | 34 | 1 | 2.764106 | -0.514522 | 372 | 280.213072 | 372.59 | 464.966928 | 1.001586 |
| GO:0060711\_labyrinthine\_layer\_development | 34 | 1 | 2.764106 | -0.514522 | 372 | 280.213072 | 372.59 | 464.966928 | 1.001586 |
| GO:0048872\_homeostasis\_of\_number\_of\_cells | 105 | 2 | 1.790087 | -0.511514 | 373 | 280.713684 | 373.26 | 465.806316 | 1.000697 |
| GO:0010468\_regulation\_of\_gene\_expression | 778 | 10 | 1.207964 | -0.510773 | 374 | 280.853477 | 373.46 | 466.066523 | 0.998556 |
| GO:0001756\_somitogenesis | 35 | 1 | 2.685131 | -0.504082 | 375 | 285.680713 | 378.79 | 471.899287 | 1.010107 |
| GO:0001889\_liver\_development | 36 | 1 | 2.610544 | -0.493992 | 381 | 291.307988 | 385.19 | 479.072012 | 1.010997 |
| GO:0007631\_feeding\_behavior | 36 | 1 | 2.610544 | -0.493992 | 381 | 291.307988 | 385.19 | 479.072012 | 1.010997 |
| GO:0014020\_primary\_neural\_tube\_formation | 36 | 1 | 2.610544 | -0.493992 | 381 | 291.307988 | 385.19 | 479.072012 | 1.010997 |
| GO:0019228\_regulation\_of\_action\_potential\_in\_neuron | 36 | 1 | 2.610544 | -0.493992 | 381 | 291.307988 | 385.19 | 479.072012 | 1.010997 |
| GO:0030072\_peptide\_hormone\_secretion | 36 | 1 | 2.610544 | -0.493992 | 381 | 291.307988 | 385.19 | 479.072012 | 1.010997 |
| GO:0050851\_antigen\_receptor-mediated\_signaling\_pathway | 36 | 1 | 2.610544 | -0.493992 | 381 | 291.307988 | 385.19 | 479.072012 | 1.010997 |
| GO:0009888\_tissue\_development | 525 | 7 | 1.253061 | -0.491052 | 382 | 291.810693 | 385.75 | 479.689307 | 1.009817 |
| GO:0006464\_protein\_modification\_process | 439 | 6 | 1.284459 | -0.490711 | 383 | 292.023106 | 386.0 | 479.976894 | 1.007833 |
| GO:0030029\_actin\_filament-based\_process | 109 | 2 | 1.724396 | -0.489780 | 384 | 292.712901 | 386.68 | 480.647099 | 1.006979 |
| GO:0006350\_transcription | 701 | 9 | 1.206585 | -0.487897 | 385 | 292.867037 | 386.82 | 480.772963 | 1.004727 |
| GO:0010648\_negative\_regulation\_of\_cell\_communication | 110 | 2 | 1.708720 | -0.484527 | 387 | 294.040821 | 388.08 | 482.119179 | 1.002791 |
| GO:0043010\_camera-type\_eye\_development | 110 | 2 | 1.708720 | -0.484527 | 387 | 294.040821 | 388.08 | 482.119179 | 1.002791 |
| GO:0002790\_peptide\_secretion | 37 | 1 | 2.539989 | -0.484234 | 390 | 296.615648 | 391.13 | 485.644352 | 1.002897 |
| GO:0050906\_detection\_of\_stimulus\_involved\_in\_sensory\_perception | 37 | 1 | 2.539989 | -0.484234 | 390 | 296.615648 | 391.13 | 485.644352 | 1.002897 |
| GO:0051101\_regulation\_of\_DNA\_binding | 37 | 1 | 2.539989 | -0.484234 | 390 | 296.615648 | 391.13 | 485.644352 | 1.002897 |
| GO:0048705\_skeletal\_system\_morphogenesis | 111 | 2 | 1.693326 | -0.479345 | 391 | 297.369808 | 392.07 | 486.770192 | 1.002737 |
| GO:0001570\_vasculogenesis | 38 | 1 | 2.473147 | -0.474789 | 393 | 302.612486 | 398.51 | 494.407514 | 1.014020 |
| GO:0046777\_protein\_amino\_acid\_autophosphorylation | 38 | 1 | 2.473147 | -0.474789 | 393 | 302.612486 | 398.51 | 494.407514 | 1.014020 |
| GO:0006974\_response\_to\_DNA\_damage\_stimulus | 113 | 2 | 1.663356 | -0.469185 | 394 | 303.924593 | 400.06 | 496.195407 | 1.015381 |
| GO:0048534\_hemopoietic\_or\_lymphoid\_organ\_development | 277 | 4 | 1.357106 | -0.467997 | 396 | 304.229513 | 400.42 | 496.610487 | 1.011162 |
| GO:0048646\_anatomical\_structure\_formation\_involved\_in\_morphogenesis | 277 | 4 | 1.357106 | -0.467997 | 396 | 304.229513 | 400.42 | 496.610487 | 1.011162 |
| GO:0007160\_cell-matrix\_adhesion | 39 | 1 | 2.409733 | -0.465642 | 401 | 308.385776 | 404.98 | 501.574224 | 1.009925 |
| GO:0008037\_cell\_recognition | 39 | 1 | 2.409733 | -0.465642 | 401 | 308.385776 | 404.98 | 501.574224 | 1.009925 |
| GO:0035148\_tube\_lumen\_formation | 39 | 1 | 2.409733 | -0.465642 | 401 | 308.385776 | 404.98 | 501.574224 | 1.009925 |
| GO:0042475\_odontogenesis\_of\_dentine-containing\_tooth | 39 | 1 | 2.409733 | -0.465642 | 401 | 308.385776 | 404.98 | 501.574224 | 1.009925 |
| GO:0048663\_neuron\_fate\_commitment | 39 | 1 | 2.409733 | -0.465642 | 401 | 308.385776 | 404.98 | 501.574224 | 1.009925 |
| GO:0006996\_organelle\_organization | 449 | 6 | 1.255852 | -0.465254 | 402 | 308.563028 | 405.14 | 501.716972 | 1.007811 |
| GO:0000165\_MAPKKK\_cascade | 114 | 2 | 1.648765 | -0.464205 | 403 | 308.933183 | 405.54 | 502.146817 | 1.006303 |
| GO:0003002\_regionalization | 195 | 3 | 1.445840 | -0.463390 | 404 | 309.476916 | 406.01 | 502.543084 | 1.004975 |
| GO:0007610\_behavior | 279 | 4 | 1.347378 | -0.461740 | 405 | 309.859025 | 406.49 | 503.120975 | 1.003679 |
| GO:0043009\_chordate\_embryonic\_development | 365 | 5 | 1.287392 | -0.459427 | 406 | 310.392548 | 407.23 | 504.067452 | 1.003030 |
| GO:0001824\_blastocyst\_development | 40 | 1 | 2.349490 | -0.456777 | 409 | 314.355910 | 411.43 | 508.504090 | 1.005941 |
| GO:0016071\_mRNA\_metabolic\_process | 40 | 1 | 2.349490 | -0.456777 | 409 | 314.355910 | 411.43 | 508.504090 | 1.005941 |
| GO:0017015\_regulation\_of\_transforming\_growth\_factor\_beta\_receptor\_signaling\_pathway | 40 | 1 | 2.349490 | -0.456777 | 409 | 314.355910 | 411.43 | 508.504090 | 1.005941 |
| GO:0009792\_embryonic\_development\_ending\_in\_birth\_or\_egg\_hatching | 368 | 5 | 1.276897 | -0.451351 | 410 | 316.036656 | 413.69 | 511.343344 | 1.009000 |
| GO:0002429\_immune\_response-activating\_cell\_surface\_receptor\_signaling\_pathway | 41 | 1 | 2.292185 | -0.448179 | 417 | 321.402108 | 419.85 | 518.297892 | 1.006835 |
| GO:0006260\_DNA\_replication | 41 | 1 | 2.292185 | -0.448179 | 417 | 321.402108 | 419.85 | 518.297892 | 1.006835 |
| GO:0007254\_JNK\_cascade | 41 | 1 | 2.292185 | -0.448179 | 417 | 321.402108 | 419.85 | 518.297892 | 1.006835 |
| GO:0009894\_regulation\_of\_catabolic\_process | 41 | 1 | 2.292185 | -0.448179 | 417 | 321.402108 | 419.85 | 518.297892 | 1.006835 |
| GO:0010551\_regulation\_of\_specific\_transcription\_from\_RNA\_polymerase\_II\_promoter | 41 | 1 | 2.292185 | -0.448179 | 417 | 321.402108 | 419.85 | 518.297892 | 1.006835 |
| GO:0015833\_peptide\_transport | 41 | 1 | 2.292185 | -0.448179 | 417 | 321.402108 | 419.85 | 518.297892 | 1.006835 |
| GO:0032569\_specific\_transcription\_from\_RNA\_polymerase\_II\_promoter | 41 | 1 | 2.292185 | -0.448179 | 417 | 321.402108 | 419.85 | 518.297892 | 1.006835 |
| GO:0034645\_cellular\_macromolecule\_biosynthetic\_process | 901 | 11 | 1.147365 | -0.445373 | 418 | 322.093349 | 420.73 | 519.366651 | 1.006531 |
| GO:0031326\_regulation\_of\_cellular\_biosynthetic\_process | 812 | 10 | 1.157384 | -0.444103 | 419 | 322.762529 | 421.42 | 520.077471 | 1.005776 |
| GO:0043412\_biopolymer\_modification | 458 | 6 | 1.231174 | -0.443390 | 420 | 323.385320 | 422.04 | 520.694680 | 1.004857 |
| GO:0010740\_positive\_regulation\_of\_protein\_kinase\_cascade | 42 | 1 | 2.237609 | -0.439836 | 423 | 329.285177 | 428.02 | 526.754823 | 1.011868 |
| GO:0042476\_odontogenesis | 42 | 1 | 2.237609 | -0.439836 | 423 | 329.285177 | 428.02 | 526.754823 | 1.011868 |
| GO:0045637\_regulation\_of\_myeloid\_cell\_differentiation | 42 | 1 | 2.237609 | -0.439836 | 423 | 329.285177 | 428.02 | 526.754823 | 1.011868 |
| GO:0009889\_regulation\_of\_biosynthetic\_process | 815 | 10 | 1.153124 | -0.438585 | 424 | 329.510381 | 428.26 | 527.009619 | 1.010047 |
| GO:0051234\_establishment\_of\_localization | 729 | 9 | 1.160242 | -0.431971 | 425 | 330.460312 | 429.47 | 528.479688 | 1.010518 |
| GO:0001508\_regulation\_of\_action\_potential | 43 | 1 | 2.185572 | -0.431736 | 432 | 335.166041 | 434.51 | 533.853959 | 1.005810 |
| GO:0001841\_neural\_tube\_formation | 43 | 1 | 2.185572 | -0.431736 | 432 | 335.166041 | 434.51 | 533.853959 | 1.005810 |
| GO:0006766\_vitamin\_metabolic\_process | 43 | 1 | 2.185572 | -0.431736 | 432 | 335.166041 | 434.51 | 533.853959 | 1.005810 |
| GO:0009582\_detection\_of\_abiotic\_stimulus | 43 | 1 | 2.185572 | -0.431736 | 432 | 335.166041 | 434.51 | 533.853959 | 1.005810 |
| GO:0010001\_glial\_cell\_differentiation | 43 | 1 | 2.185572 | -0.431736 | 432 | 335.166041 | 434.51 | 533.853959 | 1.005810 |
| GO:0031098\_stress-activated\_protein\_kinase\_signaling\_pathway | 43 | 1 | 2.185572 | -0.431736 | 432 | 335.166041 | 434.51 | 533.853959 | 1.005810 |
| GO:0046879\_hormone\_secretion | 43 | 1 | 2.185572 | -0.431736 | 432 | 335.166041 | 434.51 | 533.853959 | 1.005810 |
| GO:0006917\_induction\_of\_apoptosis | 121 | 2 | 1.553382 | -0.431093 | 434 | 335.922616 | 435.57 | 535.217384 | 1.003618 |
| GO:0012502\_induction\_of\_programmed\_cell\_death | 121 | 2 | 1.553382 | -0.431093 | 434 | 335.922616 | 435.57 | 535.217384 | 1.003618 |
| GO:0022607\_cellular\_component\_assembly | 204 | 3 | 1.382053 | -0.431085 | 435 | 336.015175 | 435.72 | 535.424825 | 1.001655 |
| GO:0009059\_macromolecule\_biosynthetic\_process | 910 | 11 | 1.136017 | -0.429613 | 436 | 336.351582 | 436.03 | 535.708418 | 1.000069 |
| GO:0006139\_nucleobase\_\_nucleoside\_\_nucleotide\_and\_nucleic\_acid\_metabolic\_process | 1002 | 12 | 1.125504 | -0.426914 | 437 | 336.941459 | 436.75 | 536.558541 | 0.999428 |
| GO:0001942\_hair\_follicle\_development | 44 | 1 | 2.135900 | -0.423867 | 448 | 343.699902 | 444.18 | 544.660098 | 0.991473 |
| GO:0002768\_immune\_response-regulating\_cell\_surface\_receptor\_signaling\_pathway | 44 | 1 | 2.135900 | -0.423867 | 448 | 343.699902 | 444.18 | 544.660098 | 0.991473 |
| GO:0006606\_protein\_import\_into\_nucleus | 44 | 1 | 2.135900 | -0.423867 | 448 | 343.699902 | 444.18 | 544.660098 | 0.991473 |
| GO:0009914\_hormone\_transport | 44 | 1 | 2.135900 | -0.423867 | 448 | 343.699902 | 444.18 | 544.660098 | 0.991473 |
| GO:0022404\_molting\_cycle\_process | 44 | 1 | 2.135900 | -0.423867 | 448 | 343.699902 | 444.18 | 544.660098 | 0.991473 |
| GO:0022405\_hair\_cycle\_process | 44 | 1 | 2.135900 | -0.423867 | 448 | 343.699902 | 444.18 | 544.660098 | 0.991473 |
| GO:0035282\_segmentation | 44 | 1 | 2.135900 | -0.423867 | 448 | 343.699902 | 444.18 | 544.660098 | 0.991473 |
| GO:0042303\_molting\_cycle | 44 | 1 | 2.135900 | -0.423867 | 448 | 343.699902 | 444.18 | 544.660098 | 0.991473 |
| GO:0042633\_hair\_cycle | 44 | 1 | 2.135900 | -0.423867 | 448 | 343.699902 | 444.18 | 544.660098 | 0.991473 |
| GO:0050808\_synapse\_organization | 44 | 1 | 2.135900 | -0.423867 | 448 | 343.699902 | 444.18 | 544.660098 | 0.991473 |
| GO:0051170\_nuclear\_import | 44 | 1 | 2.135900 | -0.423867 | 448 | 343.699902 | 444.18 | 544.660098 | 0.991473 |
| GO:0001838\_embryonic\_epithelial\_tube\_formation | 45 | 1 | 2.088435 | -0.416218 | 449 | 346.352849 | 447.33 | 548.307151 | 0.996281 |
| GO:0044267\_cellular\_protein\_metabolic\_process | 559 | 7 | 1.176846 | -0.415197 | 450 | 346.507600 | 447.51 | 548.512400 | 0.994467 |
| GO:0002520\_immune\_system\_development | 295 | 4 | 1.274300 | -0.414618 | 451 | 346.980181 | 448.13 | 549.279819 | 0.993636 |
| GO:0001763\_morphogenesis\_of\_a\_branching\_structure | 125 | 2 | 1.503673 | -0.413454 | 452 | 347.531424 | 448.7 | 549.868576 | 0.992699 |
| GO:0007612\_learning | 46 | 1 | 2.043035 | -0.408781 | 457 | 350.410598 | 451.97 | 553.529402 | 0.988993 |
| GO:0009581\_detection\_of\_external\_stimulus | 46 | 1 | 2.043035 | -0.408781 | 457 | 350.410598 | 451.97 | 553.529402 | 0.988993 |
| GO:0030850\_prostate\_gland\_development | 46 | 1 | 2.043035 | -0.408781 | 457 | 350.410598 | 451.97 | 553.529402 | 0.988993 |
| GO:0042063\_gliogenesis | 46 | 1 | 2.043035 | -0.408781 | 457 | 350.410598 | 451.97 | 553.529402 | 0.988993 |
| GO:0051098\_regulation\_of\_binding | 46 | 1 | 2.043035 | -0.408781 | 457 | 350.410598 | 451.97 | 553.529402 | 0.988993 |
| GO:0035295\_tube\_development | 212 | 3 | 1.329900 | -0.404397 | 458 | 350.743489 | 452.43 | 554.116511 | 0.987838 |
| GO:0002757\_immune\_response-activating\_signal\_transduction | 47 | 1 | 1.999566 | -0.401545 | 462 | 354.971554 | 456.95 | 558.928446 | 0.989069 |
| GO:0006396\_RNA\_processing | 47 | 1 | 1.999566 | -0.401545 | 462 | 354.971554 | 456.95 | 558.928446 | 0.989069 |
| GO:0016570\_histone\_modification | 47 | 1 | 1.999566 | -0.401545 | 462 | 354.971554 | 456.95 | 558.928446 | 0.989069 |
| GO:0030183\_B\_cell\_differentiation | 47 | 1 | 1.999566 | -0.401545 | 462 | 354.971554 | 456.95 | 558.928446 | 0.989069 |
| GO:0001655\_urogenital\_system\_development | 128 | 2 | 1.468431 | -0.400789 | 463 | 355.693272 | 457.84 | 559.986728 | 0.988855 |
| GO:0016070\_RNA\_metabolic\_process | 658 | 8 | 1.142609 | -0.396734 | 464 | 356.000221 | 458.28 | 560.559779 | 0.987672 |
| GO:0034504\_protein\_localization\_in\_nucleus | 48 | 1 | 1.957908 | -0.394502 | 465 | 358.691111 | 461.16 | 563.628889 | 0.991742 |
| GO:0043170\_macromolecule\_metabolic\_process | 1576 | 18 | 1.073371 | -0.390570 | 466 | 359.692692 | 462.36 | 565.027308 | 0.992189 |
| GO:0006725\_cellular\_aromatic\_compound\_metabolic\_process | 49 | 1 | 1.917951 | -0.387643 | 467 | 363.779501 | 466.93 | 570.080499 | 0.999850 |
| GO:0034960\_cellular\_biopolymer\_metabolic\_process | 1395 | 16 | 1.077902 | -0.385836 | 468 | 364.097854 | 467.23 | 570.362146 | 0.998355 |
| GO:0006355\_regulation\_of\_transcription\_\_DNA-dependent | 575 | 7 | 1.144099 | -0.383243 | 469 | 364.905561 | 468.05 | 571.194439 | 0.997974 |
| GO:0043283\_biopolymer\_metabolic\_process | 1490 | 17 | 1.072250 | -0.382049 | 470 | 365.663854 | 468.83 | 571.996146 | 0.997511 |
| GO:0019219\_regulation\_of\_nucleobase\_\_nucleoside\_\_nucleotide\_and\_nucleic\_acid\_metabolic\_process | 757 | 9 | 1.117327 | -0.381556 | 471 | 365.923655 | 469.03 | 572.136345 | 0.995817 |
| GO:0001656\_metanephros\_development | 50 | 1 | 1.879592 | -0.380962 | 474 | 368.277941 | 471.44 | 574.602059 | 0.994599 |
| GO:0017038\_protein\_import | 50 | 1 | 1.879592 | -0.380962 | 474 | 368.277941 | 471.44 | 574.602059 | 0.994599 |
| GO:0051606\_detection\_of\_stimulus | 50 | 1 | 1.879592 | -0.380962 | 474 | 368.277941 | 471.44 | 574.602059 | 0.994599 |
| GO:0009952\_anterior\_posterior\_pattern\_formation | 133 | 2 | 1.413227 | -0.380686 | 476 | 368.848316 | 472.04 | 575.231684 | 0.991681 |
| GO:0044057\_regulation\_of\_system\_process | 133 | 2 | 1.413227 | -0.380686 | 476 | 368.848316 | 472.04 | 575.231684 | 0.991681 |
| GO:0001701\_in\_utero\_embryonic\_development | 221 | 3 | 1.275741 | -0.376457 | 477 | 370.128758 | 473.4 | 576.671242 | 0.992453 |
| GO:0002764\_immune\_response-regulating\_signal\_transduction | 51 | 1 | 1.842737 | -0.374451 | 481 | 373.183165 | 476.82 | 580.456835 | 0.991310 |
| GO:0007601\_visual\_perception | 51 | 1 | 1.842737 | -0.374451 | 481 | 373.183165 | 476.82 | 580.456835 | 0.991310 |
| GO:0016569\_covalent\_chromatin\_modification | 51 | 1 | 1.842737 | -0.374451 | 481 | 373.183165 | 476.82 | 580.456835 | 0.991310 |
| GO:0032583\_regulation\_of\_gene-specific\_transcription | 51 | 1 | 1.842737 | -0.374451 | 481 | 373.183165 | 476.82 | 580.456835 | 0.991310 |
| GO:0001654\_eye\_development | 136 | 2 | 1.382053 | -0.369192 | 482 | 373.336317 | 477.06 | 580.783683 | 0.989751 |
| GO:0010608\_posttranscriptional\_regulation\_of\_gene\_expression | 52 | 1 | 1.807300 | -0.368104 | 484 | 374.888063 | 478.61 | 582.331937 | 0.988864 |
| GO:0050953\_sensory\_perception\_of\_light\_stimulus | 52 | 1 | 1.807300 | -0.368104 | 484 | 374.888063 | 478.61 | 582.331937 | 0.988864 |
| GO:0048519\_negative\_regulation\_of\_biological\_process | 859 | 10 | 1.094058 | -0.363936 | 485 | 375.741300 | 479.54 | 583.338700 | 0.988742 |
| GO:0030031\_cell\_projection\_assembly | 53 | 1 | 1.773200 | -0.361913 | 487 | 377.803170 | 482.05 | 586.296830 | 0.989836 |
| GO:0050905\_neuromuscular\_process | 53 | 1 | 1.773200 | -0.361913 | 487 | 377.803170 | 482.05 | 586.296830 | 0.989836 |
| GO:0051171\_regulation\_of\_nitrogen\_compound\_metabolic\_process | 771 | 9 | 1.097038 | -0.358265 | 488 | 378.162897 | 482.4 | 586.637103 | 0.988525 |
| GO:0002253\_activation\_of\_immune\_response | 54 | 1 | 1.740363 | -0.355873 | 491 | 382.234168 | 486.85 | 591.465832 | 0.991548 |
| GO:0006412\_translation | 54 | 1 | 1.740363 | -0.355873 | 491 | 382.234168 | 486.85 | 591.465832 | 0.991548 |
| GO:0007265\_Ras\_protein\_signal\_transduction | 54 | 1 | 1.740363 | -0.355873 | 491 | 382.234168 | 486.85 | 591.465832 | 0.991548 |
| GO:0051252\_regulation\_of\_RNA\_metabolic\_process | 590 | 7 | 1.115012 | -0.355272 | 492 | 382.404483 | 487.0 | 591.595517 | 0.989837 |
| GO:0007605\_sensory\_perception\_of\_sound | 55 | 1 | 1.708720 | -0.349978 | 494 | 385.705458 | 490.74 | 595.774542 | 0.993401 |
| GO:0048568\_embryonic\_organ\_development | 55 | 1 | 1.708720 | -0.349978 | 494 | 385.705458 | 490.74 | 595.774542 | 0.993401 |
| GO:0006351\_transcription\_\_DNA-dependent | 594 | 7 | 1.107504 | -0.348123 | 495 | 385.987094 | 491.08 | 596.172906 | 0.992081 |
| GO:0032774\_RNA\_biosynthetic\_process | 595 | 7 | 1.105642 | -0.346355 | 496 | 386.789905 | 491.82 | 596.850095 | 0.991573 |
| GO:0051321\_meiotic\_cell\_cycle | 56 | 1 | 1.678207 | -0.344224 | 497 | 390.081240 | 495.48 | 600.878760 | 0.996942 |
| GO:0016477\_cell\_migration | 234 | 3 | 1.204867 | -0.339620 | 498 | 391.850403 | 497.62 | 603.389597 | 0.999237 |
| GO:0008344\_adult\_locomotory\_behavior | 57 | 1 | 1.648765 | -0.338604 | 502 | 395.655712 | 501.73 | 607.804288 | 0.999462 |
| GO:0009953\_dorsal\_ventral\_pattern\_formation | 57 | 1 | 1.648765 | -0.338604 | 502 | 395.655712 | 501.73 | 607.804288 | 0.999462 |
| GO:0033365\_protein\_localization\_in\_organelle | 57 | 1 | 1.648765 | -0.338604 | 502 | 395.655712 | 501.73 | 607.804288 | 0.999462 |
| GO:0050878\_regulation\_of\_body\_fluid\_levels | 57 | 1 | 1.648765 | -0.338604 | 502 | 395.655712 | 501.73 | 607.804288 | 0.999462 |
| GO:0001501\_skeletal\_system\_development | 236 | 3 | 1.194656 | -0.334295 | 503 | 396.061411 | 502.14 | 608.218589 | 0.998290 |
| GO:0044085\_cellular\_component\_biogenesis | 237 | 3 | 1.189615 | -0.331664 | 504 | 398.546545 | 504.98 | 611.413455 | 1.001944 |
| GO:0007049\_cell\_cycle | 238 | 3 | 1.184617 | -0.329055 | 505 | 399.297429 | 505.82 | 612.342571 | 1.001624 |
| GO:0030334\_regulation\_of\_cell\_migration | 59 | 1 | 1.592874 | -0.327749 | 506 | 401.189653 | 507.73 | 614.270347 | 1.003419 |
| GO:0044260\_cellular\_macromolecule\_metabolic\_process | 1447 | 16 | 1.039166 | -0.318972 | 507 | 404.097640 | 511.1 | 618.102360 | 1.008087 |
| GO:0032268\_regulation\_of\_cellular\_protein\_metabolic\_process | 152 | 2 | 1.236574 | -0.314250 | 508 | 405.953641 | 513.31 | 620.666359 | 1.010453 |
| GO:0030855\_epithelial\_cell\_differentiation | 62 | 1 | 1.515800 | -0.312366 | 510 | 409.207398 | 516.51 | 623.812602 | 1.012765 |
| GO:0050954\_sensory\_perception\_of\_mechanical\_stimulus | 62 | 1 | 1.515800 | -0.312366 | 510 | 409.207398 | 516.51 | 623.812602 | 1.012765 |
| GO:0007369\_gastrulation | 63 | 1 | 1.491740 | -0.307462 | 511 | 412.692415 | 519.98 | 627.267585 | 1.017573 |
| GO:0032879\_regulation\_of\_localization | 248 | 3 | 1.136850 | -0.304095 | 512 | 414.575435 | 521.99 | 629.404565 | 1.019512 |
| GO:0019222\_regulation\_of\_metabolic\_process | 1088 | 12 | 1.036540 | -0.302239 | 513 | 416.872956 | 524.61 | 632.347044 | 1.022632 |
| GO:0007389\_pattern\_specification\_process | 250 | 3 | 1.127755 | -0.299339 | 514 | 417.401538 | 525.14 | 632.878462 | 1.021673 |
| GO:0048511\_rhythmic\_process | 65 | 1 | 1.445840 | -0.297967 | 515 | 418.796364 | 526.57 | 634.343636 | 1.022466 |
| GO:0006810\_transport | 718 | 8 | 1.047126 | -0.297545 | 516 | 418.843554 | 526.65 | 634.456446 | 1.020640 |
| GO:0010467\_gene\_expression | 905 | 10 | 1.038449 | -0.297401 | 517 | 419.034636 | 526.8 | 634.565364 | 1.018956 |
| GO:0008104\_protein\_localization | 251 | 3 | 1.123262 | -0.296990 | 518 | 419.231030 | 527.09 | 634.948970 | 1.017548 |
| GO:0048514\_blood\_vessel\_morphogenesis | 158 | 2 | 1.189615 | -0.296086 | 519 | 420.330262 | 528.27 | 636.209738 | 1.017861 |
| GO:0007179\_transforming\_growth\_factor\_beta\_receptor\_signaling\_pathway | 66 | 1 | 1.423933 | -0.293370 | 522 | 422.380153 | 530.6 | 638.819847 | 1.016475 |
| GO:0031589\_cell-substrate\_adhesion | 66 | 1 | 1.423933 | -0.293370 | 522 | 422.380153 | 530.6 | 638.819847 | 1.016475 |
| GO:0051130\_positive\_regulation\_of\_cellular\_component\_organization | 66 | 1 | 1.423933 | -0.293370 | 522 | 422.380153 | 530.6 | 638.819847 | 1.016475 |
| GO:0016481\_negative\_regulation\_of\_transcription | 253 | 3 | 1.114383 | -0.292348 | 523 | 423.093255 | 531.48 | 639.866745 | 1.016214 |
| GO:0003007\_heart\_morphogenesis | 67 | 1 | 1.402680 | -0.288869 | 524 | 426.020075 | 534.54 | 643.059925 | 1.020115 |
| GO:0002521\_leukocyte\_differentiation | 161 | 2 | 1.167448 | -0.287446 | 525 | 426.443358 | 535.04 | 643.636642 | 1.019124 |
| GO:0048870\_cell\_motility | 257 | 3 | 1.097038 | -0.283283 | 526 | 429.072317 | 537.9 | 646.727683 | 1.022624 |
| GO:0010926\_anatomical\_structure\_formation | 447 | 5 | 1.051226 | -0.281045 | 527 | 430.490717 | 539.24 | 647.989283 | 1.023226 |
| GO:0001932\_regulation\_of\_protein\_amino\_acid\_phosphorylation | 69 | 1 | 1.362023 | -0.280143 | 528 | 432.158162 | 541.11 | 650.061838 | 1.024830 |
| GO:0008283\_cell\_proliferation | 544 | 6 | 1.036540 | -0.276817 | 529 | 433.044479 | 542.01 | 650.975521 | 1.024594 |
| GO:0031323\_regulation\_of\_cellular\_metabolic\_process | 1015 | 11 | 1.018498 | -0.276780 | 530 | 433.225472 | 542.17 | 651.114528 | 1.022962 |
| GO:0009617\_response\_to\_bacterium | 70 | 1 | 1.342566 | -0.275913 | 531 | 436.007553 | 545.0 | 653.992447 | 1.026365 |
| GO:0010629\_negative\_regulation\_of\_gene\_expression | 262 | 3 | 1.076102 | -0.272350 | 532 | 437.123942 | 546.26 | 655.396058 | 1.026805 |
| GO:0006913\_nucleocytoplasmic\_transport | 71 | 1 | 1.323656 | -0.271768 | 534 | 439.047212 | 548.34 | 657.632788 | 1.026854 |
| GO:0016331\_morphogenesis\_of\_embryonic\_epithelium | 71 | 1 | 1.323656 | -0.271768 | 534 | 439.047212 | 548.34 | 657.632788 | 1.026854 |
| GO:0051049\_regulation\_of\_transport | 167 | 2 | 1.125504 | -0.270990 | 535 | 440.262630 | 549.48 | 658.697370 | 1.027065 |
| GO:0007264\_small\_GTPase\_mediated\_signal\_transduction | 72 | 1 | 1.305272 | -0.267705 | 541 | 445.639353 | 554.76 | 663.880647 | 1.025434 |
| GO:0016568\_chromatin\_modification | 72 | 1 | 1.305272 | -0.267705 | 541 | 445.639353 | 554.76 | 663.880647 | 1.025434 |
| GO:0021915\_neural\_tube\_development | 72 | 1 | 1.305272 | -0.267705 | 541 | 445.639353 | 554.76 | 663.880647 | 1.025434 |
| GO:0040012\_regulation\_of\_locomotion | 72 | 1 | 1.305272 | -0.267705 | 541 | 445.639353 | 554.76 | 663.880647 | 1.025434 |
| GO:0050673\_epithelial\_cell\_proliferation | 72 | 1 | 1.305272 | -0.267705 | 541 | 445.639353 | 554.76 | 663.880647 | 1.025434 |
| GO:0051169\_nuclear\_transport | 72 | 1 | 1.305272 | -0.267705 | 541 | 445.639353 | 554.76 | 663.880647 | 1.025434 |
| GO:0048706\_embryonic\_skeletal\_system\_development | 73 | 1 | 1.287392 | -0.263722 | 544 | 448.208054 | 557.19 | 666.171946 | 1.024246 |
| GO:0051270\_regulation\_of\_cell\_motion | 73 | 1 | 1.287392 | -0.263722 | 544 | 448.208054 | 557.19 | 666.171946 | 1.024246 |
| GO:0051336\_regulation\_of\_hydrolase\_activity | 73 | 1 | 1.287392 | -0.263722 | 544 | 448.208054 | 557.19 | 666.171946 | 1.024246 |
| GO:0007600\_sensory\_perception | 172 | 2 | 1.092786 | -0.258066 | 545 | 452.178551 | 560.9 | 669.621449 | 1.029174 |
| GO:0045944\_positive\_regulation\_of\_transcription\_from\_RNA\_polymerase\_II\_promoter | 269 | 3 | 1.048100 | -0.257753 | 546 | 452.498658 | 561.16 | 669.821342 | 1.027766 |
| GO:0051050\_positive\_regulation\_of\_transport | 75 | 1 | 1.253061 | -0.255988 | 547 | 453.957129 | 562.55 | 671.142871 | 1.028428 |
| GO:0045934\_negative\_regulation\_of\_nucleobase\_\_nucleoside\_\_nucleotide\_and\_nucleic\_acid\_metabolic\_process | 270 | 3 | 1.044218 | -0.255733 | 548 | 454.225591 | 562.86 | 671.494409 | 1.027117 |
| GO:0051172\_negative\_regulation\_of\_nitrogen\_compound\_metabolic\_process | 271 | 3 | 1.040364 | -0.253728 | 549 | 455.872086 | 564.54 | 673.207914 | 1.028306 |
| GO:0000122\_negative\_regulation\_of\_transcription\_from\_RNA\_polymerase\_II\_promoter | 175 | 2 | 1.074052 | -0.250635 | 551 | 458.557025 | 567.0 | 675.442975 | 1.029038 |
| GO:0015031\_protein\_transport | 175 | 2 | 1.074052 | -0.250635 | 551 | 458.557025 | 567.0 | 675.442975 | 1.029038 |
| GO:0010558\_negative\_regulation\_of\_macromolecule\_biosynthetic\_process | 274 | 3 | 1.028974 | -0.247809 | 553 | 461.486806 | 570.05 | 678.613194 | 1.030832 |
| GO:0033036\_macromolecule\_localization | 274 | 3 | 1.028974 | -0.247809 | 553 | 461.486806 | 570.05 | 678.613194 | 1.030832 |
| GO:0006461\_protein\_complex\_assembly | 78 | 1 | 1.204867 | -0.244933 | 557 | 464.650504 | 573.19 | 681.729496 | 1.029066 |
| GO:0030326\_embryonic\_limb\_morphogenesis | 78 | 1 | 1.204867 | -0.244933 | 557 | 464.650504 | 573.19 | 681.729496 | 1.029066 |
| GO:0035113\_embryonic\_appendage\_morphogenesis | 78 | 1 | 1.204867 | -0.244933 | 557 | 464.650504 | 573.19 | 681.729496 | 1.029066 |
| GO:0070271\_protein\_complex\_biogenesis | 78 | 1 | 1.204867 | -0.244933 | 557 | 464.650504 | 573.19 | 681.729496 | 1.029066 |
| GO:0045184\_establishment\_of\_protein\_localization | 180 | 2 | 1.044218 | -0.238760 | 558 | 467.150612 | 575.81 | 684.469388 | 1.031918 |
| GO:0006807\_nitrogen\_compound\_metabolic\_process | 1147 | 12 | 0.983222 | -0.234948 | 559 | 470.969847 | 579.58 | 688.190153 | 1.036816 |
| GO:0031327\_negative\_regulation\_of\_cellular\_biosynthetic\_process | 282 | 3 | 0.999783 | -0.232686 | 560 | 472.204264 | 580.73 | 689.255736 | 1.037018 |
| GO:0010627\_regulation\_of\_protein\_kinase\_cascade | 82 | 1 | 1.146093 | -0.231134 | 561 | 475.187670 | 583.46 | 691.732330 | 1.040036 |
| GO:0009890\_negative\_regulation\_of\_biosynthetic\_process | 284 | 3 | 0.992742 | -0.229051 | 562 | 476.300388 | 584.52 | 692.739612 | 1.040071 |
| GO:0006325\_chromatin\_organization | 83 | 1 | 1.132284 | -0.227840 | 564 | 478.682246 | 586.95 | 695.217754 | 1.040691 |
| GO:0030534\_adult\_behavior | 83 | 1 | 1.132284 | -0.227840 | 564 | 478.682246 | 586.95 | 695.217754 | 1.040691 |
| GO:0007155\_cell\_adhesion | 186 | 2 | 1.010533 | -0.225304 | 566 | 480.637118 | 588.87 | 697.102882 | 1.040406 |
| GO:0022610\_biological\_adhesion | 186 | 2 | 1.010533 | -0.225304 | 566 | 480.637118 | 588.87 | 697.102882 | 1.040406 |
| GO:0000279\_M\_phase | 85 | 1 | 1.105642 | -0.221430 | 567 | 483.206404 | 591.73 | 700.253596 | 1.043616 |
| GO:0006605\_protein\_targeting | 86 | 1 | 1.092786 | -0.218310 | 570 | 485.826331 | 594.64 | 703.453669 | 1.043228 |
| GO:0006897\_endocytosis | 86 | 1 | 1.092786 | -0.218310 | 570 | 485.826331 | 594.64 | 703.453669 | 1.043228 |
| GO:0010324\_membrane\_invagination | 86 | 1 | 1.092786 | -0.218310 | 570 | 485.826331 | 594.64 | 703.453669 | 1.043228 |
| GO:0001822\_kidney\_development | 87 | 1 | 1.080225 | -0.215245 | 573 | 488.824764 | 597.45 | 706.075236 | 1.042670 |
| GO:0007178\_transmembrane\_receptor\_protein\_serine\_threonine\_kinase\_signaling\_pathway | 87 | 1 | 1.080225 | -0.215245 | 573 | 488.824764 | 597.45 | 706.075236 | 1.042670 |
| GO:0050778\_positive\_regulation\_of\_immune\_response | 87 | 1 | 1.080225 | -0.215245 | 573 | 488.824764 | 597.45 | 706.075236 | 1.042670 |
| GO:0042127\_regulation\_of\_cell\_proliferation | 393 | 4 | 0.956535 | -0.213109 | 574 | 489.032389 | 597.65 | 706.267611 | 1.041202 |
| GO:0040011\_locomotion | 295 | 3 | 0.955725 | -0.210037 | 575 | 490.883556 | 599.69 | 708.496444 | 1.042939 |
| GO:0065008\_regulation\_of\_biological\_quality | 693 | 7 | 0.949289 | -0.206719 | 576 | 492.465328 | 601.07 | 709.674672 | 1.043524 |
| GO:0042113\_B\_cell\_activation | 90 | 1 | 1.044218 | -0.206367 | 577 | 494.669722 | 603.39 | 712.110278 | 1.045737 |
| GO:0033554\_cellular\_response\_to\_stress | 196 | 2 | 0.958975 | -0.204643 | 578 | 494.913014 | 603.67 | 712.426986 | 1.044412 |
| GO:0031399\_regulation\_of\_protein\_modification\_process | 91 | 1 | 1.032743 | -0.203510 | 579 | 496.746584 | 605.39 | 714.033416 | 1.045579 |
| GO:0030217\_T\_cell\_differentiation | 92 | 1 | 1.021517 | -0.200701 | 580 | 499.100339 | 607.54 | 715.979661 | 1.047483 |
| GO:0035107\_appendage\_morphogenesis | 93 | 1 | 1.010533 | -0.197940 | 583 | 501.444721 | 609.85 | 718.255279 | 1.046055 |
| GO:0035108\_limb\_morphogenesis | 93 | 1 | 1.010533 | -0.197940 | 583 | 501.444721 | 609.85 | 718.255279 | 1.046055 |
| GO:0065003\_macromolecular\_complex\_assembly | 93 | 1 | 1.010533 | -0.197940 | 583 | 501.444721 | 609.85 | 718.255279 | 1.046055 |
| GO:0002376\_immune\_system\_process | 505 | 5 | 0.930491 | -0.196194 | 584 | 501.753478 | 610.18 | 718.606522 | 1.044829 |
| GO:0034984\_cellular\_response\_to\_DNA\_damage\_stimulus | 94 | 1 | 0.999783 | -0.195225 | 585 | 504.593815 | 612.85 | 721.106185 | 1.047607 |
| GO:0045893\_positive\_regulation\_of\_transcription\_\_DNA-dependent | 306 | 3 | 0.921369 | -0.192575 | 587 | 505.291801 | 613.52 | 721.748199 | 1.045179 |
| GO:0051254\_positive\_regulation\_of\_RNA\_metabolic\_process | 306 | 3 | 0.921369 | -0.192575 | 587 | 505.291801 | 613.52 | 721.748199 | 1.045179 |
| GO:0042391\_regulation\_of\_membrane\_potential | 95 | 1 | 0.989259 | -0.192556 | 589 | 505.969233 | 614.2 | 722.430767 | 1.042784 |
| GO:0051707\_response\_to\_other\_organism | 95 | 1 | 0.989259 | -0.192556 | 589 | 505.969233 | 614.2 | 722.430767 | 1.042784 |
| GO:0048736\_appendage\_development | 96 | 1 | 0.978954 | -0.189931 | 591 | 509.043361 | 617.2 | 725.356639 | 1.044332 |
| GO:0060173\_limb\_development | 96 | 1 | 0.978954 | -0.189931 | 591 | 509.043361 | 617.2 | 725.356639 | 1.044332 |
| GO:0060341\_regulation\_of\_cellular\_localization | 97 | 1 | 0.968862 | -0.187350 | 592 | 512.047925 | 620.11 | 728.172075 | 1.047483 |
| GO:0060562\_epithelial\_tube\_morphogenesis | 99 | 1 | 0.949289 | -0.182313 | 593 | 516.856479 | 624.78 | 732.703521 | 1.053592 |
| GO:0001525\_angiogenesis | 100 | 1 | 0.939796 | -0.179857 | 594 | 517.966602 | 625.78 | 733.593398 | 1.053502 |
| GO:0030163\_protein\_catabolic\_process | 101 | 1 | 0.930491 | -0.177440 | 595 | 519.183557 | 626.94 | 734.696443 | 1.053681 |
| GO:0055086\_nucleobase\_\_nucleoside\_and\_nucleotide\_metabolic\_process | 104 | 1 | 0.903650 | -0.170420 | 596 | 522.810910 | 630.31 | 737.809090 | 1.057567 |
| GO:0010817\_regulation\_of\_hormone\_levels | 106 | 1 | 0.886600 | -0.165923 | 597 | 525.073336 | 632.48 | 739.886664 | 1.059430 |
| GO:0045892\_negative\_regulation\_of\_transcription\_\_DNA-dependent | 218 | 2 | 0.862198 | -0.165886 | 598 | 525.375245 | 632.77 | 740.164755 | 1.058144 |
| GO:0051253\_negative\_regulation\_of\_RNA\_metabolic\_process | 220 | 2 | 0.854360 | -0.162763 | 599 | 526.417725 | 633.78 | 741.142275 | 1.058063 |
| GO:0051240\_positive\_regulation\_of\_multicellular\_organismal\_process | 108 | 1 | 0.870181 | -0.161567 | 600 | 527.110044 | 634.52 | 741.929956 | 1.057533 |
| GO:0006357\_regulation\_of\_transcription\_from\_RNA\_polymerase\_II\_promoter | 435 | 4 | 0.864180 | -0.159030 | 601 | 529.470095 | 636.7 | 743.929905 | 1.059401 |
| GO:0010605\_negative\_regulation\_of\_macromolecule\_metabolic\_process | 331 | 3 | 0.851779 | -0.157977 | 602 | 530.153652 | 637.39 | 744.626348 | 1.058787 |
| GO:0031324\_negative\_regulation\_of\_cellular\_metabolic\_process | 332 | 3 | 0.849213 | -0.156726 | 603 | 532.272089 | 639.51 | 746.747911 | 1.060547 |
| GO:0002682\_regulation\_of\_immune\_system\_process | 228 | 2 | 0.824382 | -0.150864 | 604 | 535.740795 | 643.02 | 750.299205 | 1.064603 |
| GO:0007167\_enzyme\_linked\_receptor\_protein\_signaling\_pathway | 229 | 2 | 0.820782 | -0.149441 | 605 | 536.196530 | 643.49 | 750.783470 | 1.063620 |
| GO:0045941\_positive\_regulation\_of\_transcription | 338 | 3 | 0.834138 | -0.149419 | 606 | 536.471940 | 643.74 | 751.008060 | 1.062277 |
| GO:0009607\_response\_to\_biotic\_stimulus | 114 | 1 | 0.824382 | -0.149284 | 607 | 537.274784 | 644.54 | 751.805216 | 1.061845 |
| GO:0006366\_transcription\_from\_RNA\_polymerase\_II\_promoter | 444 | 4 | 0.846663 | -0.149239 | 608 | 537.769305 | 644.97 | 752.170695 | 1.060806 |
| GO:0044249\_cellular\_biosynthetic\_process | 1150 | 11 | 0.898935 | -0.147990 | 609 | 538.279691 | 645.49 | 752.700309 | 1.059918 |
| GO:0048584\_positive\_regulation\_of\_response\_to\_stimulus | 115 | 1 | 0.817214 | -0.147344 | 610 | 539.062853 | 646.21 | 753.357147 | 1.059361 |
| GO:0046483\_heterocycle\_metabolic\_process | 116 | 1 | 0.810169 | -0.145434 | 612 | 541.450321 | 648.43 | 755.409679 | 1.059526 |
| GO:0048608\_reproductive\_structure\_development | 116 | 1 | 0.810169 | -0.145434 | 612 | 541.450321 | 648.43 | 755.409679 | 1.059526 |
| GO:0043933\_macromolecular\_complex\_subunit\_organization | 117 | 1 | 0.803244 | -0.143552 | 613 | 543.394069 | 650.16 | 756.925931 | 1.060620 |
| GO:0010628\_positive\_regulation\_of\_gene\_expression | 346 | 3 | 0.814852 | -0.140183 | 614 | 545.537015 | 652.08 | 758.622985 | 1.062020 |
| GO:0022403\_cell\_cycle\_phase | 119 | 1 | 0.789744 | -0.139873 | 615 | 545.805706 | 652.4 | 758.994294 | 1.060813 |
| GO:0009892\_negative\_regulation\_of\_metabolic\_process | 348 | 3 | 0.810169 | -0.137961 | 616 | 547.676932 | 654.13 | 760.583068 | 1.061899 |
| GO:0006886\_intracellular\_protein\_transport | 122 | 1 | 0.770325 | -0.134556 | 617 | 552.730554 | 658.8 | 764.869446 | 1.067747 |
| GO:0045935\_positive\_regulation\_of\_nucleobase\_\_nucleoside\_\_nucleotide\_and\_nucleic\_acid\_metabolic\_process | 352 | 3 | 0.800962 | -0.133618 | 618 | 552.994323 | 659.03 | 765.065677 | 1.066392 |
| GO:0030098\_lymphocyte\_differentiation | 124 | 1 | 0.757900 | -0.131140 | 619 | 554.259703 | 660.17 | 766.080297 | 1.066511 |
| GO:0009058\_biosynthetic\_process | 1175 | 11 | 0.879809 | -0.130659 | 620 | 554.733094 | 660.66 | 766.586906 | 1.065581 |
| GO:0042981\_regulation\_of\_apoptosis | 360 | 3 | 0.783163 | -0.125319 | 621 | 556.435751 | 662.15 | 767.864249 | 1.066264 |
| GO:0051173\_positive\_regulation\_of\_nitrogen\_compound\_metabolic\_process | 361 | 3 | 0.780994 | -0.124317 | 622 | 558.926235 | 664.28 | 769.633765 | 1.067974 |
| GO:0043285\_biopolymer\_catabolic\_process | 129 | 1 | 0.728524 | -0.123023 | 624 | 559.882574 | 665.02 | 770.157426 | 1.065737 |
| GO:0051276\_chromosome\_organization | 129 | 1 | 0.728524 | -0.123023 | 624 | 559.882574 | 665.02 | 770.157426 | 1.065737 |
| GO:0045165\_cell\_fate\_commitment | 130 | 1 | 0.722920 | -0.121469 | 626 | 561.622723 | 666.67 | 771.717277 | 1.064968 |
| GO:0050776\_regulation\_of\_immune\_response | 130 | 1 | 0.722920 | -0.121469 | 626 | 561.622723 | 666.67 | 771.717277 | 1.064968 |
| GO:0010941\_regulation\_of\_cell\_death | 365 | 3 | 0.772435 | -0.120384 | 628 | 562.515874 | 667.5 | 772.484126 | 1.062898 |
| GO:0043067\_regulation\_of\_programmed\_cell\_death | 365 | 3 | 0.772435 | -0.120384 | 628 | 562.515874 | 667.5 | 772.484126 | 1.062898 |
| GO:0010557\_positive\_regulation\_of\_macromolecule\_biosynthetic\_process | 371 | 3 | 0.759943 | -0.114706 | 629 | 566.787728 | 671.43 | 776.072272 | 1.067456 |
| GO:0009057\_macromolecule\_catabolic\_process | 137 | 1 | 0.685982 | -0.111189 | 630 | 568.467863 | 672.83 | 777.192137 | 1.067984 |
| GO:0050896\_response\_to\_stimulus | 1107 | 10 | 0.848957 | -0.111009 | 631 | 568.591366 | 672.93 | 777.268634 | 1.066450 |
| GO:0022414\_reproductive\_process | 376 | 3 | 0.749837 | -0.110169 | 632 | 568.899641 | 673.25 | 777.600359 | 1.065269 |
| GO:0007169\_transmembrane\_receptor\_protein\_tyrosine\_kinase\_signaling\_pathway | 139 | 1 | 0.676112 | -0.108433 | 634 | 571.241346 | 675.34 | 779.438654 | 1.065205 |
| GO:0034613\_cellular\_protein\_localization | 139 | 1 | 0.676112 | -0.108433 | 634 | 571.241346 | 675.34 | 779.438654 | 1.065205 |
| GO:0000003\_reproduction | 379 | 3 | 0.743902 | -0.107529 | 635 | 572.161834 | 676.3 | 780.438166 | 1.065039 |
| GO:0016044\_membrane\_organization | 140 | 1 | 0.671283 | -0.107084 | 636 | 572.952197 | 676.95 | 780.947803 | 1.064387 |
| GO:0003006\_reproductive\_developmental\_process | 141 | 1 | 0.666522 | -0.105753 | 638 | 574.090881 | 677.98 | 781.869119 | 1.062665 |
| GO:0070727\_cellular\_macromolecule\_localization | 141 | 1 | 0.666522 | -0.105753 | 638 | 574.090881 | 677.98 | 781.869119 | 1.062665 |
| GO:0035239\_tube\_morphogenesis | 143 | 1 | 0.657200 | -0.103146 | 639 | 575.013695 | 678.78 | 782.546305 | 1.062254 |
| GO:0007186\_G-protein\_coupled\_receptor\_protein\_signaling\_pathway | 144 | 1 | 0.652636 | -0.101870 | 640 | 576.425694 | 679.97 | 783.514306 | 1.062453 |
| GO:0031328\_positive\_regulation\_of\_cellular\_biosynthetic\_process | 387 | 3 | 0.728524 | -0.100779 | 641 | 576.816370 | 680.36 | 783.903630 | 1.061404 |
| GO:0009891\_positive\_regulation\_of\_biosynthetic\_process | 388 | 3 | 0.726646 | -0.099964 | 642 | 577.196263 | 680.74 | 784.283737 | 1.060343 |
| GO:0051716\_cellular\_response\_to\_stimulus | 273 | 2 | 0.688495 | -0.098570 | 643 | 578.469462 | 681.92 | 785.370538 | 1.060529 |
| GO:0002684\_positive\_regulation\_of\_immune\_system\_process | 148 | 1 | 0.634997 | -0.096934 | 644 | 580.335193 | 683.69 | 787.044807 | 1.061630 |
| GO:0065009\_regulation\_of\_molecular\_function | 279 | 2 | 0.673689 | -0.093132 | 645 | 582.219462 | 685.13 | 788.040538 | 1.062217 |
| GO:0022402\_cell\_cycle\_process | 155 | 1 | 0.606320 | -0.088916 | 646 | 585.135195 | 687.73 | 790.324805 | 1.064598 |
| GO:0008152\_metabolic\_process | 2133 | 20 | 0.881196 | -0.085566 | 647 | 587.624651 | 689.96 | 792.295349 | 1.066399 |
| GO:0042221\_response\_to\_chemical\_stimulus | 409 | 3 | 0.689337 | -0.084217 | 648 | 587.952825 | 690.2 | 792.447175 | 1.065123 |
| GO:0009628\_response\_to\_abiotic\_stimulus | 162 | 1 | 0.580121 | -0.081614 | 649 | 590.083138 | 691.84 | 793.596862 | 1.066009 |
| GO:0007626\_locomotory\_behavior | 163 | 1 | 0.576562 | -0.080625 | 651 | 590.912033 | 692.53 | 794.147967 | 1.063794 |
| GO:0042110\_T\_cell\_activation | 163 | 1 | 0.576562 | -0.080625 | 651 | 590.912033 | 692.53 | 794.147967 | 1.063794 |
| GO:0042325\_regulation\_of\_phosphorylation | 164 | 1 | 0.573046 | -0.079649 | 652 | 591.899135 | 693.37 | 794.840865 | 1.063451 |
| GO:0006259\_DNA\_metabolic\_process | 165 | 1 | 0.569573 | -0.078686 | 655 | 592.833396 | 694.38 | 795.926604 | 1.060122 |
| GO:0019220\_regulation\_of\_phosphate\_metabolic\_process | 165 | 1 | 0.569573 | -0.078686 | 655 | 592.833396 | 694.38 | 795.926604 | 1.060122 |
| GO:0051174\_regulation\_of\_phosphorus\_metabolic\_process | 165 | 1 | 0.569573 | -0.078686 | 655 | 592.833396 | 694.38 | 795.926604 | 1.060122 |
| GO:0042592\_homeostatic\_process | 419 | 3 | 0.672885 | -0.077568 | 656 | 593.699255 | 695.11 | 796.520745 | 1.059619 |
| GO:0044237\_cellular\_metabolic\_process | 1974 | 18 | 0.856957 | -0.072881 | 657 | 596.511866 | 697.64 | 798.768134 | 1.061857 |
| GO:0006915\_apoptosis | 427 | 3 | 0.660278 | -0.072607 | 658 | 596.728229 | 697.84 | 798.951771 | 1.060547 |
| GO:0044093\_positive\_regulation\_of\_molecular\_function | 173 | 1 | 0.543235 | -0.071413 | 659 | 599.409675 | 700.27 | 801.130325 | 1.062625 |
| GO:0006950\_response\_to\_stress | 549 | 4 | 0.684733 | -0.069291 | 660 | 601.577835 | 702.18 | 802.782165 | 1.063909 |
| GO:0010604\_positive\_regulation\_of\_macromolecule\_metabolic\_process | 433 | 3 | 0.651129 | -0.069084 | 662 | 602.139715 | 702.66 | 803.180285 | 1.061420 |
| GO:0012501\_programmed\_cell\_death | 433 | 3 | 0.651129 | -0.069084 | 662 | 602.139715 | 702.66 | 803.180285 | 1.061420 |
| GO:0006873\_cellular\_ion\_homeostasis | 176 | 1 | 0.533975 | -0.068874 | 663 | 603.148440 | 703.59 | 804.031560 | 1.061222 |
| GO:0055082\_cellular\_chemical\_homeostasis | 181 | 1 | 0.519224 | -0.064852 | 664 | 606.236993 | 706.38 | 806.523007 | 1.063825 |
| GO:0031325\_positive\_regulation\_of\_cellular\_metabolic\_process | 442 | 3 | 0.637871 | -0.064097 | 665 | 606.626340 | 706.7 | 806.773660 | 1.062707 |
| GO:0044238\_primary\_metabolic\_process | 1905 | 17 | 0.838663 | -0.063130 | 666 | 607.310047 | 707.38 | 807.449953 | 1.062132 |
| GO:0008219\_cell\_death | 444 | 3 | 0.634997 | -0.063036 | 667 | 607.648524 | 707.69 | 807.731476 | 1.061004 |
| GO:0016265\_death | 450 | 3 | 0.626531 | -0.059949 | 668 | 610.974678 | 710.69 | 810.405322 | 1.063907 |
| GO:0009893\_positive\_regulation\_of\_metabolic\_process | 458 | 3 | 0.615587 | -0.056054 | 669 | 613.615888 | 712.92 | 812.224112 | 1.065650 |
| GO:0046907\_intracellular\_transport | 194 | 1 | 0.484431 | -0.055513 | 670 | 614.039238 | 713.28 | 812.520762 | 1.064597 |
| GO:0007507\_heart\_development | 195 | 1 | 0.481947 | -0.054856 | 672 | 615.212824 | 714.23 | 813.247176 | 1.062842 |
| GO:0019725\_cellular\_homeostasis | 195 | 1 | 0.481947 | -0.054856 | 672 | 615.212824 | 714.23 | 813.247176 | 1.062842 |
| GO:0050801\_ion\_homeostasis | 197 | 1 | 0.477054 | -0.053565 | 673 | 616.316068 | 715.19 | 814.063932 | 1.062689 |
| GO:0006955\_immune\_response | 205 | 1 | 0.458437 | -0.048712 | 674 | 620.490409 | 718.74 | 816.989591 | 1.066380 |
| GO:0010033\_response\_to\_organic\_substance | 216 | 1 | 0.435091 | -0.042772 | 675 | 623.301248 | 721.18 | 819.058752 | 1.068415 |
| GO:0048583\_regulation\_of\_response\_to\_stimulus | 217 | 1 | 0.433086 | -0.042270 | 676 | 624.180447 | 721.84 | 819.499553 | 1.067811 |
| GO:0046649\_lymphocyte\_activation | 228 | 1 | 0.412191 | -0.037136 | 677 | 628.394620 | 725.15 | 821.905380 | 1.071123 |
| GO:0050790\_regulation\_of\_catalytic\_activity | 233 | 1 | 0.403346 | -0.035019 | 678 | 629.867967 | 726.39 | 822.912033 | 1.071372 |
| GO:0009056\_catabolic\_process | 243 | 1 | 0.386747 | -0.031145 | 679 | 632.993508 | 728.86 | 824.726492 | 1.073432 |
| GO:0045321\_leukocyte\_activation | 248 | 1 | 0.378950 | -0.029374 | 680 | 634.236610 | 729.79 | 825.343390 | 1.073221 |
| GO:0048878\_chemical\_homeostasis | 254 | 1 | 0.369998 | -0.027384 | 681 | 635.803314 | 731.01 | 826.216686 | 1.073436 |
| GO:0001775\_cell\_activation | 262 | 1 | 0.358701 | -0.024940 | 682 | 638.141367 | 732.74 | 827.338633 | 1.074399 |
| GO:0009605\_response\_to\_external\_stimulus | 339 | 1 | 0.277226 | -0.010156 | 683 | 648.489122 | 740.67 | 832.850878 | 1.084436 |
| GO:0000910\_cytokinesis | 8 | 0 | 0.000000 | -0.000000 | 808 | 780.610597 | 867.79 | 954.969403 | 1.073998 |
| GO:0001783\_B\_cell\_apoptosis | 8 | 0 | 0.000000 | -0.000000 | 808 | 780.610597 | 867.79 | 954.969403 | 1.073998 |
| GO:0001833\_inner\_cell\_mass\_cell\_proliferation | 8 | 0 | 0.000000 | -0.000000 | 808 | 780.610597 | 867.79 | 954.969403 | 1.073998 |
| GO:0001840\_neural\_plate\_development | 8 | 0 | 0.000000 | -0.000000 | 808 | 780.610597 | 867.79 | 954.969403 | 1.073998 |
| GO:0001893\_maternal\_placenta\_development | 8 | 0 | 0.000000 | -0.000000 | 808 | 780.610597 | 867.79 | 954.969403 | 1.073998 |
| GO:0001911\_negative\_regulation\_of\_leukocyte\_mediated\_cytotoxicity | 8 | 0 | 0.000000 | -0.000000 | 808 | 780.610597 | 867.79 | 954.969403 | 1.073998 |
| GO:0001916\_positive\_regulation\_of\_T\_cell\_mediated\_cytotoxicity | 8 | 0 | 0.000000 | -0.000000 | 808 | 780.610597 | 867.79 | 954.969403 | 1.073998 |
| GO:0002065\_columnar\_cuboidal\_epithelial\_cell\_differentiation | 8 | 0 | 0.000000 | -0.000000 | 808 | 780.610597 | 867.79 | 954.969403 | 1.073998 |
| GO:0002320\_lymphoid\_progenitor\_cell\_differentiation | 8 | 0 | 0.000000 | -0.000000 | 808 | 780.610597 | 867.79 | 954.969403 | 1.073998 |
| GO:0002438\_acute\_inflammatory\_response\_to\_antigenic\_stimulus | 8 | 0 | 0.000000 | -0.000000 | 808 | 780.610597 | 867.79 | 954.969403 | 1.073998 |
| GO:0002524\_hypersensitivity | 8 | 0 | 0.000000 | -0.000000 | 808 | 780.610597 | 867.79 | 954.969403 | 1.073998 |
| GO:0002566\_somatic\_diversification\_of\_immune\_receptors\_via\_somatic\_mutation | 8 | 0 | 0.000000 | -0.000000 | 808 | 780.610597 | 867.79 | 954.969403 | 1.073998 |
| GO:0002864\_regulation\_of\_acute\_inflammatory\_response\_to\_antigenic\_stimulus | 8 | 0 | 0.000000 | -0.000000 | 808 | 780.610597 | 867.79 | 954.969403 | 1.073998 |
| GO:0002883\_regulation\_of\_hypersensitivity | 8 | 0 | 0.000000 | -0.000000 | 808 | 780.610597 | 867.79 | 954.969403 | 1.073998 |
| GO:0003081\_regulation\_of\_systemic\_arterial\_blood\_pressure\_by\_renin-angiotensin | 8 | 0 | 0.000000 | -0.000000 | 808 | 780.610597 | 867.79 | 954.969403 | 1.073998 |
| GO:0006020\_inositol\_metabolic\_process | 8 | 0 | 0.000000 | -0.000000 | 808 | 780.610597 | 867.79 | 954.969403 | 1.073998 |
| GO:0006195\_purine\_nucleotide\_catabolic\_process | 8 | 0 | 0.000000 | -0.000000 | 808 | 780.610597 | 867.79 | 954.969403 | 1.073998 |
| GO:0006284\_base-excision\_repair | 8 | 0 | 0.000000 | -0.000000 | 808 | 780.610597 | 867.79 | 954.969403 | 1.073998 |
| GO:0006349\_genetic\_imprinting | 8 | 0 | 0.000000 | -0.000000 | 808 | 780.610597 | 867.79 | 954.969403 | 1.073998 |
| GO:0006360\_transcription\_from\_RNA\_polymerase\_I\_promoter | 8 | 0 | 0.000000 | -0.000000 | 808 | 780.610597 | 867.79 | 954.969403 | 1.073998 |
| GO:0006399\_tRNA\_metabolic\_process | 8 | 0 | 0.000000 | -0.000000 | 808 | 780.610597 | 867.79 | 954.969403 | 1.073998 |
| GO:0006458\_'de\_novo'\_protein\_folding | 8 | 0 | 0.000000 | -0.000000 | 808 | 780.610597 | 867.79 | 954.969403 | 1.073998 |
| GO:0006493\_protein\_amino\_acid\_O-linked\_glycosylation | 8 | 0 | 0.000000 | -0.000000 | 808 | 780.610597 | 867.79 | 954.969403 | 1.073998 |
| GO:0006582\_melanin\_metabolic\_process | 8 | 0 | 0.000000 | -0.000000 | 808 | 780.610597 | 867.79 | 954.969403 | 1.073998 |
| GO:0006733\_oxidoreduction\_coenzyme\_metabolic\_process | 8 | 0 | 0.000000 | -0.000000 | 808 | 780.610597 | 867.79 | 954.969403 | 1.073998 |
| GO:0006829\_zinc\_ion\_transport | 8 | 0 | 0.000000 | -0.000000 | 808 | 780.610597 | 867.79 | 954.969403 | 1.073998 |
| GO:0007009\_plasma\_membrane\_organization | 8 | 0 | 0.000000 | -0.000000 | 808 | 780.610597 | 867.79 | 954.969403 | 1.073998 |
| GO:0007098\_centrosome\_cycle | 8 | 0 | 0.000000 | -0.000000 | 808 | 780.610597 | 867.79 | 954.969403 | 1.073998 |
| GO:0007131\_reciprocal\_meiotic\_recombination | 8 | 0 | 0.000000 | -0.000000 | 808 | 780.610597 | 867.79 | 954.969403 | 1.073998 |
| GO:0007141\_male\_meiosis\_I | 8 | 0 | 0.000000 | -0.000000 | 808 | 780.610597 | 867.79 | 954.969403 | 1.073998 |
| GO:0007625\_grooming\_behavior | 8 | 0 | 0.000000 | -0.000000 | 808 | 780.610597 | 867.79 | 954.969403 | 1.073998 |
| GO:0008105\_asymmetric\_protein\_localization | 8 | 0 | 0.000000 | -0.000000 | 808 | 780.610597 | 867.79 | 954.969403 | 1.073998 |
| GO:0009072\_aromatic\_amino\_acid\_family\_metabolic\_process | 8 | 0 | 0.000000 | -0.000000 | 808 | 780.610597 | 867.79 | 954.969403 | 1.073998 |
| GO:0009144\_purine\_nucleoside\_triphosphate\_metabolic\_process | 8 | 0 | 0.000000 | -0.000000 | 808 | 780.610597 | 867.79 | 954.969403 | 1.073998 |
| GO:0009746\_response\_to\_hexose\_stimulus | 8 | 0 | 0.000000 | -0.000000 | 808 | 780.610597 | 867.79 | 954.969403 | 1.073998 |
| GO:0009749\_response\_to\_glucose\_stimulus | 8 | 0 | 0.000000 | -0.000000 | 808 | 780.610597 | 867.79 | 954.969403 | 1.073998 |
| GO:0014014\_negative\_regulation\_of\_gliogenesis | 8 | 0 | 0.000000 | -0.000000 | 808 | 780.610597 | 867.79 | 954.969403 | 1.073998 |
| GO:0014046\_dopamine\_secretion | 8 | 0 | 0.000000 | -0.000000 | 808 | 780.610597 | 867.79 | 954.969403 | 1.073998 |
| GO:0014059\_regulation\_of\_dopamine\_secretion | 8 | 0 | 0.000000 | -0.000000 | 808 | 780.610597 | 867.79 | 954.969403 | 1.073998 |
| GO:0014065\_phosphoinositide\_3-kinase\_cascade | 8 | 0 | 0.000000 | -0.000000 | 808 | 780.610597 | 867.79 | 954.969403 | 1.073998 |
| GO:0015800\_acidic\_amino\_acid\_transport | 8 | 0 | 0.000000 | -0.000000 | 808 | 780.610597 | 867.79 | 954.969403 | 1.073998 |
| GO:0015804\_neutral\_amino\_acid\_transport | 8 | 0 | 0.000000 | -0.000000 | 808 | 780.610597 | 867.79 | 954.969403 | 1.073998 |
| GO:0016236\_macroautophagy | 8 | 0 | 0.000000 | -0.000000 | 808 | 780.610597 | 867.79 | 954.969403 | 1.073998 |
| GO:0016446\_somatic\_hypermutation\_of\_immunoglobulin\_genes | 8 | 0 | 0.000000 | -0.000000 | 808 | 780.610597 | 867.79 | 954.969403 | 1.073998 |
| GO:0018345\_protein\_palmitoylation | 8 | 0 | 0.000000 | -0.000000 | 808 | 780.610597 | 867.79 | 954.969403 | 1.073998 |
| GO:0019229\_regulation\_of\_vasoconstriction | 8 | 0 | 0.000000 | -0.000000 | 808 | 780.610597 | 867.79 | 954.969403 | 1.073998 |
| GO:0019400\_alditol\_metabolic\_process | 8 | 0 | 0.000000 | -0.000000 | 808 | 780.610597 | 867.79 | 954.969403 | 1.073998 |
| GO:0021692\_cerebellar\_Purkinje\_cell\_layer\_morphogenesis | 8 | 0 | 0.000000 | -0.000000 | 808 | 780.610597 | 867.79 | 954.969403 | 1.073998 |
| GO:0021694\_cerebellar\_Purkinje\_cell\_layer\_formation | 8 | 0 | 0.000000 | -0.000000 | 808 | 780.610597 | 867.79 | 954.969403 | 1.073998 |
| GO:0021702\_cerebellar\_Purkinje\_cell\_differentiation | 8 | 0 | 0.000000 | -0.000000 | 808 | 780.610597 | 867.79 | 954.969403 | 1.073998 |
| GO:0021781\_glial\_cell\_fate\_commitment | 8 | 0 | 0.000000 | -0.000000 | 808 | 780.610597 | 867.79 | 954.969403 | 1.073998 |
| GO:0021799\_cerebral\_cortex\_radially\_oriented\_cell\_migration | 8 | 0 | 0.000000 | -0.000000 | 808 | 780.610597 | 867.79 | 954.969403 | 1.073998 |
| GO:0022898\_regulation\_of\_transmembrane\_transporter\_activity | 8 | 0 | 0.000000 | -0.000000 | 808 | 780.610597 | 867.79 | 954.969403 | 1.073998 |
| GO:0030035\_microspike\_assembly | 8 | 0 | 0.000000 | -0.000000 | 808 | 780.610597 | 867.79 | 954.969403 | 1.073998 |
| GO:0030193\_regulation\_of\_blood\_coagulation | 8 | 0 | 0.000000 | -0.000000 | 808 | 780.610597 | 867.79 | 954.969403 | 1.073998 |
| GO:0030204\_chondroitin\_sulfate\_metabolic\_process | 8 | 0 | 0.000000 | -0.000000 | 808 | 780.610597 | 867.79 | 954.969403 | 1.073998 |
| GO:0030500\_regulation\_of\_bone\_mineralization | 8 | 0 | 0.000000 | -0.000000 | 808 | 780.610597 | 867.79 | 954.969403 | 1.073998 |
| GO:0031102\_neuron\_projection\_regeneration | 8 | 0 | 0.000000 | -0.000000 | 808 | 780.610597 | 867.79 | 954.969403 | 1.073998 |
| GO:0031103\_axon\_regeneration | 8 | 0 | 0.000000 | -0.000000 | 808 | 780.610597 | 867.79 | 954.969403 | 1.073998 |
| GO:0031111\_negative\_regulation\_of\_microtubule\_polymerization\_or\_depolymerization | 8 | 0 | 0.000000 | -0.000000 | 808 | 780.610597 | 867.79 | 954.969403 | 1.073998 |
| GO:0031294\_lymphocyte\_costimulation | 8 | 0 | 0.000000 | -0.000000 | 808 | 780.610597 | 867.79 | 954.969403 | 1.073998 |
| GO:0031295\_T\_cell\_costimulation | 8 | 0 | 0.000000 | -0.000000 | 808 | 780.610597 | 867.79 | 954.969403 | 1.073998 |
| GO:0031334\_positive\_regulation\_of\_protein\_complex\_assembly | 8 | 0 | 0.000000 | -0.000000 | 808 | 780.610597 | 867.79 | 954.969403 | 1.073998 |
| GO:0031342\_negative\_regulation\_of\_cell\_killing | 8 | 0 | 0.000000 | -0.000000 | 808 | 780.610597 | 867.79 | 954.969403 | 1.073998 |
| GO:0031396\_regulation\_of\_protein\_ubiquitination | 8 | 0 | 0.000000 | -0.000000 | 808 | 780.610597 | 867.79 | 954.969403 | 1.073998 |
| GO:0032094\_response\_to\_food | 8 | 0 | 0.000000 | -0.000000 | 808 | 780.610597 | 867.79 | 954.969403 | 1.073998 |
| GO:0032273\_positive\_regulation\_of\_protein\_polymerization | 8 | 0 | 0.000000 | -0.000000 | 808 | 780.610597 | 867.79 | 954.969403 | 1.073998 |
| GO:0032409\_regulation\_of\_transporter\_activity | 8 | 0 | 0.000000 | -0.000000 | 808 | 780.610597 | 867.79 | 954.969403 | 1.073998 |
| GO:0032412\_regulation\_of\_ion\_transmembrane\_transporter\_activity | 8 | 0 | 0.000000 | -0.000000 | 808 | 780.610597 | 867.79 | 954.969403 | 1.073998 |
| GO:0032613\_interleukin-10\_production | 8 | 0 | 0.000000 | -0.000000 | 808 | 780.610597 | 867.79 | 954.969403 | 1.073998 |
| GO:0033198\_response\_to\_ATP | 8 | 0 | 0.000000 | -0.000000 | 808 | 780.610597 | 867.79 | 954.969403 | 1.073998 |
| GO:0034284\_response\_to\_monosaccharide\_stimulus | 8 | 0 | 0.000000 | -0.000000 | 808 | 780.610597 | 867.79 | 954.969403 | 1.073998 |
| GO:0034728\_nucleosome\_organization | 8 | 0 | 0.000000 | -0.000000 | 808 | 780.610597 | 867.79 | 954.969403 | 1.073998 |
| GO:0035112\_genitalia\_morphogenesis | 8 | 0 | 0.000000 | -0.000000 | 808 | 780.610597 | 867.79 | 954.969403 | 1.073998 |
| GO:0040017\_positive\_regulation\_of\_locomotion | 8 | 0 | 0.000000 | -0.000000 | 808 | 780.610597 | 867.79 | 954.969403 | 1.073998 |
| GO:0040034\_regulation\_of\_development\_\_heterochronic | 8 | 0 | 0.000000 | -0.000000 | 808 | 780.610597 | 867.79 | 954.969403 | 1.073998 |
| GO:0042074\_cell\_migration\_involved\_in\_gastrulation | 8 | 0 | 0.000000 | -0.000000 | 808 | 780.610597 | 867.79 | 954.969403 | 1.073998 |
| GO:0042090\_interleukin-12\_biosynthetic\_process | 8 | 0 | 0.000000 | -0.000000 | 808 | 780.610597 | 867.79 | 954.969403 | 1.073998 |
| GO:0042092\_T-helper\_2\_type\_immune\_response | 8 | 0 | 0.000000 | -0.000000 | 808 | 780.610597 | 867.79 | 954.969403 | 1.073998 |
| GO:0042095\_interferon-gamma\_biosynthetic\_process | 8 | 0 | 0.000000 | -0.000000 | 808 | 780.610597 | 867.79 | 954.969403 | 1.073998 |
| GO:0042104\_positive\_regulation\_of\_activated\_T\_cell\_proliferation | 8 | 0 | 0.000000 | -0.000000 | 808 | 780.610597 | 867.79 | 954.969403 | 1.073998 |
| GO:0042226\_interleukin-6\_biosynthetic\_process | 8 | 0 | 0.000000 | -0.000000 | 808 | 780.610597 | 867.79 | 954.969403 | 1.073998 |
| GO:0042304\_regulation\_of\_fatty\_acid\_biosynthetic\_process | 8 | 0 | 0.000000 | -0.000000 | 808 | 780.610597 | 867.79 | 954.969403 | 1.073998 |
| GO:0042423\_catecholamine\_biosynthetic\_process | 8 | 0 | 0.000000 | -0.000000 | 808 | 780.610597 | 867.79 | 954.969403 | 1.073998 |
| GO:0042990\_regulation\_of\_transcription\_factor\_import\_into\_nucleus | 8 | 0 | 0.000000 | -0.000000 | 808 | 780.610597 | 867.79 | 954.969403 | 1.073998 |
| GO:0042991\_transcription\_factor\_import\_into\_nucleus | 8 | 0 | 0.000000 | -0.000000 | 808 | 780.610597 | 867.79 | 954.969403 | 1.073998 |
| GO:0043011\_myeloid\_dendritic\_cell\_differentiation | 8 | 0 | 0.000000 | -0.000000 | 808 | 780.610597 | 867.79 | 954.969403 | 1.073998 |
| GO:0043368\_positive\_T\_cell\_selection | 8 | 0 | 0.000000 | -0.000000 | 808 | 780.610597 | 867.79 | 954.969403 | 1.073998 |
| GO:0043370\_regulation\_of\_CD4-positive\_\_alpha\_beta\_T\_cell\_differentiation | 8 | 0 | 0.000000 | -0.000000 | 808 | 780.610597 | 867.79 | 954.969403 | 1.073998 |
| GO:0043542\_endothelial\_cell\_migration | 8 | 0 | 0.000000 | -0.000000 | 808 | 780.610597 | 867.79 | 954.969403 | 1.073998 |
| GO:0043616\_keratinocyte\_proliferation | 8 | 0 | 0.000000 | -0.000000 | 808 | 780.610597 | 867.79 | 954.969403 | 1.073998 |
| GO:0045075\_regulation\_of\_interleukin-12\_biosynthetic\_process | 8 | 0 | 0.000000 | -0.000000 | 808 | 780.610597 | 867.79 | 954.969403 | 1.073998 |
| GO:0045086\_positive\_regulation\_of\_interleukin-2\_biosynthetic\_process | 8 | 0 | 0.000000 | -0.000000 | 808 | 780.610597 | 867.79 | 954.969403 | 1.073998 |
| GO:0045351\_type\_I\_interferon\_biosynthetic\_process | 8 | 0 | 0.000000 | -0.000000 | 808 | 780.610597 | 867.79 | 954.969403 | 1.073998 |
| GO:0045408\_regulation\_of\_interleukin-6\_biosynthetic\_process | 8 | 0 | 0.000000 | -0.000000 | 808 | 780.610597 | 867.79 | 954.969403 | 1.073998 |
| GO:0045429\_positive\_regulation\_of\_nitric\_oxide\_biosynthetic\_process | 8 | 0 | 0.000000 | -0.000000 | 808 | 780.610597 | 867.79 | 954.969403 | 1.073998 |
| GO:0045494\_photoreceptor\_cell\_maintenance | 8 | 0 | 0.000000 | -0.000000 | 808 | 780.610597 | 867.79 | 954.969403 | 1.073998 |
| GO:0045686\_negative\_regulation\_of\_glial\_cell\_differentiation | 8 | 0 | 0.000000 | -0.000000 | 808 | 780.610597 | 867.79 | 954.969403 | 1.073998 |
| GO:0045910\_negative\_regulation\_of\_DNA\_recombination | 8 | 0 | 0.000000 | -0.000000 | 808 | 780.610597 | 867.79 | 954.969403 | 1.073998 |
| GO:0045921\_positive\_regulation\_of\_exocytosis | 8 | 0 | 0.000000 | -0.000000 | 808 | 780.610597 | 867.79 | 954.969403 | 1.073998 |
| GO:0045932\_negative\_regulation\_of\_muscle\_contraction | 8 | 0 | 0.000000 | -0.000000 | 808 | 780.610597 | 867.79 | 954.969403 | 1.073998 |
| GO:0046470\_phosphatidylcholine\_metabolic\_process | 8 | 0 | 0.000000 | -0.000000 | 808 | 780.610597 | 867.79 | 954.969403 | 1.073998 |
| GO:0048266\_behavioral\_response\_to\_pain | 8 | 0 | 0.000000 | -0.000000 | 808 | 780.610597 | 867.79 | 954.969403 | 1.073998 |
| GO:0048505\_regulation\_of\_timing\_of\_cell\_differentiation | 8 | 0 | 0.000000 | -0.000000 | 808 | 780.610597 | 867.79 | 954.969403 | 1.073998 |
| GO:0048520\_positive\_regulation\_of\_behavior | 8 | 0 | 0.000000 | -0.000000 | 808 | 780.610597 | 867.79 | 954.969403 | 1.073998 |
| GO:0048557\_embryonic\_digestive\_tract\_morphogenesis | 8 | 0 | 0.000000 | -0.000000 | 808 | 780.610597 | 867.79 | 954.969403 | 1.073998 |
| GO:0048638\_regulation\_of\_developmental\_growth | 8 | 0 | 0.000000 | -0.000000 | 808 | 780.610597 | 867.79 | 954.969403 | 1.073998 |
| GO:0048742\_regulation\_of\_skeletal\_muscle\_fiber\_development | 8 | 0 | 0.000000 | -0.000000 | 808 | 780.610597 | 867.79 | 954.969403 | 1.073998 |
| GO:0050707\_regulation\_of\_cytokine\_secretion | 8 | 0 | 0.000000 | -0.000000 | 808 | 780.610597 | 867.79 | 954.969403 | 1.073998 |
| GO:0050909\_sensory\_perception\_of\_taste | 8 | 0 | 0.000000 | -0.000000 | 808 | 780.610597 | 867.79 | 954.969403 | 1.073998 |
| GO:0050920\_regulation\_of\_chemotaxis | 8 | 0 | 0.000000 | -0.000000 | 808 | 780.610597 | 867.79 | 954.969403 | 1.073998 |
| GO:0050921\_positive\_regulation\_of\_chemotaxis | 8 | 0 | 0.000000 | -0.000000 | 808 | 780.610597 | 867.79 | 954.969403 | 1.073998 |
| GO:0050926\_regulation\_of\_positive\_chemotaxis | 8 | 0 | 0.000000 | -0.000000 | 808 | 780.610597 | 867.79 | 954.969403 | 1.073998 |
| GO:0050927\_positive\_regulation\_of\_positive\_chemotaxis | 8 | 0 | 0.000000 | -0.000000 | 808 | 780.610597 | 867.79 | 954.969403 | 1.073998 |
| GO:0050930\_induction\_of\_positive\_chemotaxis | 8 | 0 | 0.000000 | -0.000000 | 808 | 780.610597 | 867.79 | 954.969403 | 1.073998 |
| GO:0051084\_'de\_novo'\_posttranslational\_protein\_folding | 8 | 0 | 0.000000 | -0.000000 | 808 | 780.610597 | 867.79 | 954.969403 | 1.073998 |
| GO:0051181\_cofactor\_transport | 8 | 0 | 0.000000 | -0.000000 | 808 | 780.610597 | 867.79 | 954.969403 | 1.073998 |
| GO:0060043\_regulation\_of\_cardiac\_muscle\_cell\_proliferation | 8 | 0 | 0.000000 | -0.000000 | 808 | 780.610597 | 867.79 | 954.969403 | 1.073998 |
| GO:0060347\_heart\_trabecula\_formation | 8 | 0 | 0.000000 | -0.000000 | 808 | 780.610597 | 867.79 | 954.969403 | 1.073998 |
| GO:0060670\_branching\_involved\_in\_embryonic\_placenta\_morphogenesis | 8 | 0 | 0.000000 | -0.000000 | 808 | 780.610597 | 867.79 | 954.969403 | 1.073998 |
| GO:0060712\_spongiotrophoblast\_layer\_development | 8 | 0 | 0.000000 | -0.000000 | 808 | 780.610597 | 867.79 | 954.969403 | 1.073998 |
| GO:0070167\_regulation\_of\_biomineral\_formation | 8 | 0 | 0.000000 | -0.000000 | 808 | 780.610597 | 867.79 | 954.969403 | 1.073998 |
| GO:0070193\_synaptonemal\_complex\_organization | 8 | 0 | 0.000000 | -0.000000 | 808 | 780.610597 | 867.79 | 954.969403 | 1.073998 |
| GO:0070231\_T\_cell\_apoptosis | 8 | 0 | 0.000000 | -0.000000 | 808 | 780.610597 | 867.79 | 954.969403 | 1.073998 |
| GO:0070584\_mitochondrion\_morphogenesis | 8 | 0 | 0.000000 | -0.000000 | 808 | 780.610597 | 867.79 | 954.969403 | 1.073998 |
| GO:0001708\_cell\_fate\_specification | 56 | 0 | 0.000000 | -0.000000 | 817 | 786.754711 | 873.42 | 960.085289 | 1.069058 |
| GO:0002683\_negative\_regulation\_of\_immune\_system\_process | 56 | 0 | 0.000000 | -0.000000 | 817 | 786.754711 | 873.42 | 960.085289 | 1.069058 |
| GO:0002703\_regulation\_of\_leukocyte\_mediated\_immunity | 56 | 0 | 0.000000 | -0.000000 | 817 | 786.754711 | 873.42 | 960.085289 | 1.069058 |
| GO:0006790\_sulfur\_metabolic\_process | 56 | 0 | 0.000000 | -0.000000 | 817 | 786.754711 | 873.42 | 960.085289 | 1.069058 |
| GO:0009187\_cyclic\_nucleotide\_metabolic\_process | 56 | 0 | 0.000000 | -0.000000 | 817 | 786.754711 | 873.42 | 960.085289 | 1.069058 |
| GO:0042089\_cytokine\_biosynthetic\_process | 56 | 0 | 0.000000 | -0.000000 | 817 | 786.754711 | 873.42 | 960.085289 | 1.069058 |
| GO:0042107\_cytokine\_metabolic\_process | 56 | 0 | 0.000000 | -0.000000 | 817 | 786.754711 | 873.42 | 960.085289 | 1.069058 |
| GO:0046486\_glycerolipid\_metabolic\_process | 56 | 0 | 0.000000 | -0.000000 | 817 | 786.754711 | 873.42 | 960.085289 | 1.069058 |
| GO:0050678\_regulation\_of\_epithelial\_cell\_proliferation | 56 | 0 | 0.000000 | -0.000000 | 817 | 786.754711 | 873.42 | 960.085289 | 1.069058 |
| GO:0000038\_very-long-chain\_fatty\_acid\_metabolic\_process | 6 | 0 | 0.000000 | -0.000000 | 997 | 965.208136 | 1049.32 | 1133.431864 | 1.052477 |
| GO:0000245\_spliceosome\_assembly | 6 | 0 | 0.000000 | -0.000000 | 997 | 965.208136 | 1049.32 | 1133.431864 | 1.052477 |
| GO:0000768\_syncytium\_formation\_by\_plasma\_membrane\_fusion | 6 | 0 | 0.000000 | -0.000000 | 997 | 965.208136 | 1049.32 | 1133.431864 | 1.052477 |
| GO:0001710\_mesodermal\_cell\_fate\_commitment | 6 | 0 | 0.000000 | -0.000000 | 997 | 965.208136 | 1049.32 | 1133.431864 | 1.052477 |
| GO:0001885\_endothelial\_cell\_development | 6 | 0 | 0.000000 | -0.000000 | 997 | 965.208136 | 1049.32 | 1133.431864 | 1.052477 |
| GO:0002016\_regulation\_of\_blood\_volume\_by\_renin-angiotensin | 6 | 0 | 0.000000 | -0.000000 | 997 | 965.208136 | 1049.32 | 1133.431864 | 1.052477 |
| GO:0002335\_mature\_B\_cell\_differentiation | 6 | 0 | 0.000000 | -0.000000 | 997 | 965.208136 | 1049.32 | 1133.431864 | 1.052477 |
| GO:0002360\_T\_cell\_lineage\_commitment | 6 | 0 | 0.000000 | -0.000000 | 997 | 965.208136 | 1049.32 | 1133.431864 | 1.052477 |
| GO:0002367\_cytokine\_production\_during\_immune\_response | 6 | 0 | 0.000000 | -0.000000 | 997 | 965.208136 | 1049.32 | 1133.431864 | 1.052477 |
| GO:0002474\_antigen\_processing\_and\_presentation\_of\_peptide\_antigen\_via\_MHC\_class\_I | 6 | 0 | 0.000000 | -0.000000 | 997 | 965.208136 | 1049.32 | 1133.431864 | 1.052477 |
| GO:0002475\_antigen\_processing\_and\_presentation\_via\_MHC\_class\_Ib | 6 | 0 | 0.000000 | -0.000000 | 997 | 965.208136 | 1049.32 | 1133.431864 | 1.052477 |
| GO:0002532\_production\_of\_molecular\_mediator\_of\_acute\_inflammatory\_response | 6 | 0 | 0.000000 | -0.000000 | 997 | 965.208136 | 1049.32 | 1133.431864 | 1.052477 |
| GO:0002541\_activation\_of\_plasma\_proteins\_involved\_in\_acute\_inflammatory\_response | 6 | 0 | 0.000000 | -0.000000 | 997 | 965.208136 | 1049.32 | 1133.431864 | 1.052477 |
| GO:0002675\_positive\_regulation\_of\_acute\_inflammatory\_response | 6 | 0 | 0.000000 | -0.000000 | 997 | 965.208136 | 1049.32 | 1133.431864 | 1.052477 |
| GO:0002685\_regulation\_of\_leukocyte\_migration | 6 | 0 | 0.000000 | -0.000000 | 997 | 965.208136 | 1049.32 | 1133.431864 | 1.052477 |
| GO:0002831\_regulation\_of\_response\_to\_biotic\_stimulus | 6 | 0 | 0.000000 | -0.000000 | 997 | 965.208136 | 1049.32 | 1133.431864 | 1.052477 |
| GO:0002920\_regulation\_of\_humoral\_immune\_response | 6 | 0 | 0.000000 | -0.000000 | 997 | 965.208136 | 1049.32 | 1133.431864 | 1.052477 |
| GO:0006071\_glycerol\_metabolic\_process | 6 | 0 | 0.000000 | -0.000000 | 997 | 965.208136 | 1049.32 | 1133.431864 | 1.052477 |
| GO:0006084\_acetyl-CoA\_metabolic\_process | 6 | 0 | 0.000000 | -0.000000 | 997 | 965.208136 | 1049.32 | 1133.431864 | 1.052477 |
| GO:0006264\_mitochondrial\_DNA\_replication | 6 | 0 | 0.000000 | -0.000000 | 997 | 965.208136 | 1049.32 | 1133.431864 | 1.052477 |
| GO:0006402\_mRNA\_catabolic\_process | 6 | 0 | 0.000000 | -0.000000 | 997 | 965.208136 | 1049.32 | 1133.431864 | 1.052477 |
| GO:0006471\_protein\_amino\_acid\_ADP-ribosylation | 6 | 0 | 0.000000 | -0.000000 | 997 | 965.208136 | 1049.32 | 1133.431864 | 1.052477 |
| GO:0006536\_glutamate\_metabolic\_process | 6 | 0 | 0.000000 | -0.000000 | 997 | 965.208136 | 1049.32 | 1133.431864 | 1.052477 |
| GO:0006656\_phosphatidylcholine\_biosynthetic\_process | 6 | 0 | 0.000000 | -0.000000 | 997 | 965.208136 | 1049.32 | 1133.431864 | 1.052477 |
| GO:0006692\_prostanoid\_metabolic\_process | 6 | 0 | 0.000000 | -0.000000 | 997 | 965.208136 | 1049.32 | 1133.431864 | 1.052477 |
| GO:0006693\_prostaglandin\_metabolic\_process | 6 | 0 | 0.000000 | -0.000000 | 997 | 965.208136 | 1049.32 | 1133.431864 | 1.052477 |
| GO:0006706\_steroid\_catabolic\_process | 6 | 0 | 0.000000 | -0.000000 | 997 | 965.208136 | 1049.32 | 1133.431864 | 1.052477 |
| GO:0006752\_group\_transfer\_coenzyme\_metabolic\_process | 6 | 0 | 0.000000 | -0.000000 | 997 | 965.208136 | 1049.32 | 1133.431864 | 1.052477 |
| GO:0006882\_cellular\_zinc\_ion\_homeostasis | 6 | 0 | 0.000000 | -0.000000 | 997 | 965.208136 | 1049.32 | 1133.431864 | 1.052477 |
| GO:0006942\_regulation\_of\_striated\_muscle\_contraction | 6 | 0 | 0.000000 | -0.000000 | 997 | 965.208136 | 1049.32 | 1133.431864 | 1.052477 |
| GO:0006956\_complement\_activation | 6 | 0 | 0.000000 | -0.000000 | 997 | 965.208136 | 1049.32 | 1133.431864 | 1.052477 |
| GO:0006998\_nuclear\_envelope\_organization | 6 | 0 | 0.000000 | -0.000000 | 997 | 965.208136 | 1049.32 | 1133.431864 | 1.052477 |
| GO:0007032\_endosome\_organization | 6 | 0 | 0.000000 | -0.000000 | 997 | 965.208136 | 1049.32 | 1133.431864 | 1.052477 |
| GO:0007176\_regulation\_of\_epidermal\_growth\_factor\_receptor\_activity | 6 | 0 | 0.000000 | -0.000000 | 997 | 965.208136 | 1049.32 | 1133.431864 | 1.052477 |
| GO:0007214\_gamma-aminobutyric\_acid\_signaling\_pathway | 6 | 0 | 0.000000 | -0.000000 | 997 | 965.208136 | 1049.32 | 1133.431864 | 1.052477 |
| GO:0007257\_activation\_of\_JUN\_kinase\_activity | 6 | 0 | 0.000000 | -0.000000 | 997 | 965.208136 | 1049.32 | 1133.431864 | 1.052477 |
| GO:0007341\_penetration\_of\_zona\_pellucida | 6 | 0 | 0.000000 | -0.000000 | 997 | 965.208136 | 1049.32 | 1133.431864 | 1.052477 |
| GO:0007406\_negative\_regulation\_of\_neuroblast\_proliferation | 6 | 0 | 0.000000 | -0.000000 | 997 | 965.208136 | 1049.32 | 1133.431864 | 1.052477 |
| GO:0007442\_hindgut\_morphogenesis | 6 | 0 | 0.000000 | -0.000000 | 997 | 965.208136 | 1049.32 | 1133.431864 | 1.052477 |
| GO:0007520\_myoblast\_fusion | 6 | 0 | 0.000000 | -0.000000 | 997 | 965.208136 | 1049.32 | 1133.431864 | 1.052477 |
| GO:0007620\_copulation | 6 | 0 | 0.000000 | -0.000000 | 997 | 965.208136 | 1049.32 | 1133.431864 | 1.052477 |
| GO:0008156\_negative\_regulation\_of\_DNA\_replication | 6 | 0 | 0.000000 | -0.000000 | 997 | 965.208136 | 1049.32 | 1133.431864 | 1.052477 |
| GO:0008209\_androgen\_metabolic\_process | 6 | 0 | 0.000000 | -0.000000 | 997 | 965.208136 | 1049.32 | 1133.431864 | 1.052477 |
| GO:0008625\_induction\_of\_apoptosis\_via\_death\_domain\_receptors | 6 | 0 | 0.000000 | -0.000000 | 997 | 965.208136 | 1049.32 | 1133.431864 | 1.052477 |
| GO:0009067\_aspartate\_family\_amino\_acid\_biosynthetic\_process | 6 | 0 | 0.000000 | -0.000000 | 997 | 965.208136 | 1049.32 | 1133.431864 | 1.052477 |
| GO:0009069\_serine\_family\_amino\_acid\_metabolic\_process | 6 | 0 | 0.000000 | -0.000000 | 997 | 965.208136 | 1049.32 | 1133.431864 | 1.052477 |
| GO:0009143\_nucleoside\_triphosphate\_catabolic\_process | 6 | 0 | 0.000000 | -0.000000 | 997 | 965.208136 | 1049.32 | 1133.431864 | 1.052477 |
| GO:0009247\_glycolipid\_biosynthetic\_process | 6 | 0 | 0.000000 | -0.000000 | 997 | 965.208136 | 1049.32 | 1133.431864 | 1.052477 |
| GO:0009650\_UV\_protection | 6 | 0 | 0.000000 | -0.000000 | 997 | 965.208136 | 1049.32 | 1133.431864 | 1.052477 |
| GO:0009651\_response\_to\_salt\_stress | 6 | 0 | 0.000000 | -0.000000 | 997 | 965.208136 | 1049.32 | 1133.431864 | 1.052477 |
| GO:0010466\_negative\_regulation\_of\_peptidase\_activity | 6 | 0 | 0.000000 | -0.000000 | 997 | 965.208136 | 1049.32 | 1133.431864 | 1.052477 |
| GO:0010883\_regulation\_of\_lipid\_storage | 6 | 0 | 0.000000 | -0.000000 | 997 | 965.208136 | 1049.32 | 1133.431864 | 1.052477 |
| GO:0010906\_regulation\_of\_glucose\_metabolic\_process | 6 | 0 | 0.000000 | -0.000000 | 997 | 965.208136 | 1049.32 | 1133.431864 | 1.052477 |
| GO:0014003\_oligodendrocyte\_development | 6 | 0 | 0.000000 | -0.000000 | 997 | 965.208136 | 1049.32 | 1133.431864 | 1.052477 |
| GO:0014051\_gamma-aminobutyric\_acid\_secretion | 6 | 0 | 0.000000 | -0.000000 | 997 | 965.208136 | 1049.32 | 1133.431864 | 1.052477 |
| GO:0014072\_response\_to\_isoquinoline\_alkaloid | 6 | 0 | 0.000000 | -0.000000 | 997 | 965.208136 | 1049.32 | 1133.431864 | 1.052477 |
| GO:0014812\_muscle\_cell\_migration | 6 | 0 | 0.000000 | -0.000000 | 997 | 965.208136 | 1049.32 | 1133.431864 | 1.052477 |
| GO:0014823\_response\_to\_activity | 6 | 0 | 0.000000 | -0.000000 | 997 | 965.208136 | 1049.32 | 1133.431864 | 1.052477 |
| GO:0015012\_heparan\_sulfate\_proteoglycan\_biosynthetic\_process | 6 | 0 | 0.000000 | -0.000000 | 997 | 965.208136 | 1049.32 | 1133.431864 | 1.052477 |
| GO:0015812\_gamma-aminobutyric\_acid\_transport | 6 | 0 | 0.000000 | -0.000000 | 997 | 965.208136 | 1049.32 | 1133.431864 | 1.052477 |
| GO:0016032\_viral\_reproduction | 6 | 0 | 0.000000 | -0.000000 | 997 | 965.208136 | 1049.32 | 1133.431864 | 1.052477 |
| GO:0016574\_histone\_ubiquitination | 6 | 0 | 0.000000 | -0.000000 | 997 | 965.208136 | 1049.32 | 1133.431864 | 1.052477 |
| GO:0016925\_protein\_sumoylation | 6 | 0 | 0.000000 | -0.000000 | 997 | 965.208136 | 1049.32 | 1133.431864 | 1.052477 |
| GO:0019433\_triglyceride\_catabolic\_process | 6 | 0 | 0.000000 | -0.000000 | 997 | 965.208136 | 1049.32 | 1133.431864 | 1.052477 |
| GO:0019835\_cytolysis | 6 | 0 | 0.000000 | -0.000000 | 997 | 965.208136 | 1049.32 | 1133.431864 | 1.052477 |
| GO:0021548\_pons\_development | 6 | 0 | 0.000000 | -0.000000 | 997 | 965.208136 | 1049.32 | 1133.431864 | 1.052477 |
| GO:0021783\_preganglionic\_parasympathetic\_nervous\_system\_development | 6 | 0 | 0.000000 | -0.000000 | 997 | 965.208136 | 1049.32 | 1133.431864 | 1.052477 |
| GO:0021892\_cerebral\_cortex\_GABAergic\_interneuron\_differentiation | 6 | 0 | 0.000000 | -0.000000 | 997 | 965.208136 | 1049.32 | 1133.431864 | 1.052477 |
| GO:0021937\_Purkinje\_cell-granule\_cell\_precursor\_cell\_signaling\_involved\_in\_regulation\_of\_granule\_cell\_precursor\_cell\_proliferation | 6 | 0 | 0.000000 | -0.000000 | 997 | 965.208136 | 1049.32 | 1133.431864 | 1.052477 |
| GO:0022409\_positive\_regulation\_of\_cell-cell\_adhesion | 6 | 0 | 0.000000 | -0.000000 | 997 | 965.208136 | 1049.32 | 1133.431864 | 1.052477 |
| GO:0030002\_cellular\_anion\_homeostasis | 6 | 0 | 0.000000 | -0.000000 | 997 | 965.208136 | 1049.32 | 1133.431864 | 1.052477 |
| GO:0030149\_sphingolipid\_catabolic\_process | 6 | 0 | 0.000000 | -0.000000 | 997 | 965.208136 | 1049.32 | 1133.431864 | 1.052477 |
| GO:0030252\_growth\_hormone\_secretion | 6 | 0 | 0.000000 | -0.000000 | 997 | 965.208136 | 1049.32 | 1133.431864 | 1.052477 |
| GO:0030865\_cortical\_cytoskeleton\_organization | 6 | 0 | 0.000000 | -0.000000 | 997 | 965.208136 | 1049.32 | 1133.431864 | 1.052477 |
| GO:0030947\_regulation\_of\_vascular\_endothelial\_growth\_factor\_receptor\_signaling\_pathway | 6 | 0 | 0.000000 | -0.000000 | 997 | 965.208136 | 1049.32 | 1133.431864 | 1.052477 |
| GO:0031077\_post-embryonic\_camera-type\_eye\_development | 6 | 0 | 0.000000 | -0.000000 | 997 | 965.208136 | 1049.32 | 1133.431864 | 1.052477 |
| GO:0031330\_negative\_regulation\_of\_cellular\_catabolic\_process | 6 | 0 | 0.000000 | -0.000000 | 997 | 965.208136 | 1049.32 | 1133.431864 | 1.052477 |
| GO:0031575\_G1\_S\_transition\_checkpoint | 6 | 0 | 0.000000 | -0.000000 | 997 | 965.208136 | 1049.32 | 1133.431864 | 1.052477 |
| GO:0031960\_response\_to\_corticosteroid\_stimulus | 6 | 0 | 0.000000 | -0.000000 | 997 | 965.208136 | 1049.32 | 1133.431864 | 1.052477 |
| GO:0032042\_mitochondrial\_DNA\_metabolic\_process | 6 | 0 | 0.000000 | -0.000000 | 997 | 965.208136 | 1049.32 | 1133.431864 | 1.052477 |
| GO:0032331\_negative\_regulation\_of\_chondrocyte\_differentiation | 6 | 0 | 0.000000 | -0.000000 | 997 | 965.208136 | 1049.32 | 1133.431864 | 1.052477 |
| GO:0032392\_DNA\_geometric\_change | 6 | 0 | 0.000000 | -0.000000 | 997 | 965.208136 | 1049.32 | 1133.431864 | 1.052477 |
| GO:0032438\_melanosome\_organization | 6 | 0 | 0.000000 | -0.000000 | 997 | 965.208136 | 1049.32 | 1133.431864 | 1.052477 |
| GO:0032469\_endoplasmic\_reticulum\_calcium\_ion\_homeostasis | 6 | 0 | 0.000000 | -0.000000 | 997 | 965.208136 | 1049.32 | 1133.431864 | 1.052477 |
| GO:0032653\_regulation\_of\_interleukin-10\_production | 6 | 0 | 0.000000 | -0.000000 | 997 | 965.208136 | 1049.32 | 1133.431864 | 1.052477 |
| GO:0033238\_regulation\_of\_cellular\_amine\_metabolic\_process | 6 | 0 | 0.000000 | -0.000000 | 997 | 965.208136 | 1049.32 | 1133.431864 | 1.052477 |
| GO:0034968\_histone\_lysine\_methylation | 6 | 0 | 0.000000 | -0.000000 | 997 | 965.208136 | 1049.32 | 1133.431864 | 1.052477 |
| GO:0035019\_somatic\_stem\_cell\_maintenance | 6 | 0 | 0.000000 | -0.000000 | 997 | 965.208136 | 1049.32 | 1133.431864 | 1.052477 |
| GO:0035094\_response\_to\_nicotine | 6 | 0 | 0.000000 | -0.000000 | 997 | 965.208136 | 1049.32 | 1133.431864 | 1.052477 |
| GO:0035121\_tail\_morphogenesis | 6 | 0 | 0.000000 | -0.000000 | 997 | 965.208136 | 1049.32 | 1133.431864 | 1.052477 |
| GO:0040016\_embryonic\_cleavage | 6 | 0 | 0.000000 | -0.000000 | 997 | 965.208136 | 1049.32 | 1133.431864 | 1.052477 |
| GO:0040036\_regulation\_of\_fibroblast\_growth\_factor\_receptor\_signaling\_pathway | 6 | 0 | 0.000000 | -0.000000 | 997 | 965.208136 | 1049.32 | 1133.431864 | 1.052477 |
| GO:0042053\_regulation\_of\_dopamine\_metabolic\_process | 6 | 0 | 0.000000 | -0.000000 | 997 | 965.208136 | 1049.32 | 1133.431864 | 1.052477 |
| GO:0042069\_regulation\_of\_catecholamine\_metabolic\_process | 6 | 0 | 0.000000 | -0.000000 | 997 | 965.208136 | 1049.32 | 1133.431864 | 1.052477 |
| GO:0042246\_tissue\_regeneration | 6 | 0 | 0.000000 | -0.000000 | 997 | 965.208136 | 1049.32 | 1133.431864 | 1.052477 |
| GO:0042307\_positive\_regulation\_of\_protein\_import\_into\_nucleus | 6 | 0 | 0.000000 | -0.000000 | 997 | 965.208136 | 1049.32 | 1133.431864 | 1.052477 |
| GO:0042308\_negative\_regulation\_of\_protein\_import\_into\_nucleus | 6 | 0 | 0.000000 | -0.000000 | 997 | 965.208136 | 1049.32 | 1133.431864 | 1.052477 |
| GO:0042403\_thyroid\_hormone\_metabolic\_process | 6 | 0 | 0.000000 | -0.000000 | 997 | 965.208136 | 1049.32 | 1133.431864 | 1.052477 |
| GO:0042481\_regulation\_of\_odontogenesis | 6 | 0 | 0.000000 | -0.000000 | 997 | 965.208136 | 1049.32 | 1133.431864 | 1.052477 |
| GO:0042492\_gamma-delta\_T\_cell\_differentiation | 6 | 0 | 0.000000 | -0.000000 | 997 | 965.208136 | 1049.32 | 1133.431864 | 1.052477 |
| GO:0042953\_lipoprotein\_transport | 6 | 0 | 0.000000 | -0.000000 | 997 | 965.208136 | 1049.32 | 1133.431864 | 1.052477 |
| GO:0043064\_flagellum\_organization | 6 | 0 | 0.000000 | -0.000000 | 997 | 965.208136 | 1049.32 | 1133.431864 | 1.052477 |
| GO:0043154\_negative\_regulation\_of\_caspase\_activity | 6 | 0 | 0.000000 | -0.000000 | 997 | 965.208136 | 1049.32 | 1133.431864 | 1.052477 |
| GO:0043255\_regulation\_of\_carbohydrate\_biosynthetic\_process | 6 | 0 | 0.000000 | -0.000000 | 997 | 965.208136 | 1049.32 | 1133.431864 | 1.052477 |
| GO:0043271\_negative\_regulation\_of\_ion\_transport | 6 | 0 | 0.000000 | -0.000000 | 997 | 965.208136 | 1049.32 | 1133.431864 | 1.052477 |
| GO:0043278\_response\_to\_morphine | 6 | 0 | 0.000000 | -0.000000 | 997 | 965.208136 | 1049.32 | 1133.431864 | 1.052477 |
| GO:0043300\_regulation\_of\_leukocyte\_degranulation | 6 | 0 | 0.000000 | -0.000000 | 997 | 965.208136 | 1049.32 | 1133.431864 | 1.052477 |
| GO:0043467\_regulation\_of\_generation\_of\_precursor\_metabolites\_and\_energy | 6 | 0 | 0.000000 | -0.000000 | 997 | 965.208136 | 1049.32 | 1133.431864 | 1.052477 |
| GO:0043547\_positive\_regulation\_of\_GTPase\_activity | 6 | 0 | 0.000000 | -0.000000 | 997 | 965.208136 | 1049.32 | 1133.431864 | 1.052477 |
| GO:0043627\_response\_to\_estrogen\_stimulus | 6 | 0 | 0.000000 | -0.000000 | 997 | 965.208136 | 1049.32 | 1133.431864 | 1.052477 |
| GO:0044269\_glycerol\_ether\_catabolic\_process | 6 | 0 | 0.000000 | -0.000000 | 997 | 965.208136 | 1049.32 | 1133.431864 | 1.052477 |
| GO:0045072\_regulation\_of\_interferon-gamma\_biosynthetic\_process | 6 | 0 | 0.000000 | -0.000000 | 997 | 965.208136 | 1049.32 | 1133.431864 | 1.052477 |
| GO:0045084\_positive\_regulation\_of\_interleukin-12\_biosynthetic\_process | 6 | 0 | 0.000000 | -0.000000 | 997 | 965.208136 | 1049.32 | 1133.431864 | 1.052477 |
| GO:0045124\_regulation\_of\_bone\_resorption | 6 | 0 | 0.000000 | -0.000000 | 997 | 965.208136 | 1049.32 | 1133.431864 | 1.052477 |
| GO:0045176\_apical\_protein\_localization | 6 | 0 | 0.000000 | -0.000000 | 997 | 965.208136 | 1049.32 | 1133.431864 | 1.052477 |
| GO:0045540\_regulation\_of\_cholesterol\_biosynthetic\_process | 6 | 0 | 0.000000 | -0.000000 | 997 | 965.208136 | 1049.32 | 1133.431864 | 1.052477 |
| GO:0045579\_positive\_regulation\_of\_B\_cell\_differentiation | 6 | 0 | 0.000000 | -0.000000 | 997 | 965.208136 | 1049.32 | 1133.431864 | 1.052477 |
| GO:0045649\_regulation\_of\_macrophage\_differentiation | 6 | 0 | 0.000000 | -0.000000 | 997 | 965.208136 | 1049.32 | 1133.431864 | 1.052477 |
| GO:0045727\_positive\_regulation\_of\_translation | 6 | 0 | 0.000000 | -0.000000 | 997 | 965.208136 | 1049.32 | 1133.431864 | 1.052477 |
| GO:0045778\_positive\_regulation\_of\_ossification | 6 | 0 | 0.000000 | -0.000000 | 997 | 965.208136 | 1049.32 | 1133.431864 | 1.052477 |
| GO:0045822\_negative\_regulation\_of\_heart\_contraction | 6 | 0 | 0.000000 | -0.000000 | 997 | 965.208136 | 1049.32 | 1133.431864 | 1.052477 |
| GO:0045824\_negative\_regulation\_of\_innate\_immune\_response | 6 | 0 | 0.000000 | -0.000000 | 997 | 965.208136 | 1049.32 | 1133.431864 | 1.052477 |
| GO:0045833\_negative\_regulation\_of\_lipid\_metabolic\_process | 6 | 0 | 0.000000 | -0.000000 | 997 | 965.208136 | 1049.32 | 1133.431864 | 1.052477 |
| GO:0045843\_negative\_regulation\_of\_striated\_muscle\_development | 6 | 0 | 0.000000 | -0.000000 | 997 | 965.208136 | 1049.32 | 1133.431864 | 1.052477 |
| GO:0045861\_negative\_regulation\_of\_proteolysis | 6 | 0 | 0.000000 | -0.000000 | 997 | 965.208136 | 1049.32 | 1133.431864 | 1.052477 |
| GO:0045913\_positive\_regulation\_of\_carbohydrate\_metabolic\_process | 6 | 0 | 0.000000 | -0.000000 | 997 | 965.208136 | 1049.32 | 1133.431864 | 1.052477 |
| GO:0045931\_positive\_regulation\_of\_mitotic\_cell\_cycle | 6 | 0 | 0.000000 | -0.000000 | 997 | 965.208136 | 1049.32 | 1133.431864 | 1.052477 |
| GO:0045933\_positive\_regulation\_of\_muscle\_contraction | 6 | 0 | 0.000000 | -0.000000 | 997 | 965.208136 | 1049.32 | 1133.431864 | 1.052477 |
| GO:0046427\_positive\_regulation\_of\_JAK-STAT\_cascade | 6 | 0 | 0.000000 | -0.000000 | 997 | 965.208136 | 1049.32 | 1133.431864 | 1.052477 |
| GO:0046460\_neutral\_lipid\_biosynthetic\_process | 6 | 0 | 0.000000 | -0.000000 | 997 | 965.208136 | 1049.32 | 1133.431864 | 1.052477 |
| GO:0046461\_neutral\_lipid\_catabolic\_process | 6 | 0 | 0.000000 | -0.000000 | 997 | 965.208136 | 1049.32 | 1133.431864 | 1.052477 |
| GO:0046463\_acylglycerol\_biosynthetic\_process | 6 | 0 | 0.000000 | -0.000000 | 997 | 965.208136 | 1049.32 | 1133.431864 | 1.052477 |
| GO:0046464\_acylglycerol\_catabolic\_process | 6 | 0 | 0.000000 | -0.000000 | 997 | 965.208136 | 1049.32 | 1133.431864 | 1.052477 |
| GO:0046466\_membrane\_lipid\_catabolic\_process | 6 | 0 | 0.000000 | -0.000000 | 997 | 965.208136 | 1049.32 | 1133.431864 | 1.052477 |
| GO:0046503\_glycerolipid\_catabolic\_process | 6 | 0 | 0.000000 | -0.000000 | 997 | 965.208136 | 1049.32 | 1133.431864 | 1.052477 |
| GO:0046627\_negative\_regulation\_of\_insulin\_receptor\_signaling\_pathway | 6 | 0 | 0.000000 | -0.000000 | 997 | 965.208136 | 1049.32 | 1133.431864 | 1.052477 |
| GO:0046629\_gamma-delta\_T\_cell\_activation | 6 | 0 | 0.000000 | -0.000000 | 997 | 965.208136 | 1049.32 | 1133.431864 | 1.052477 |
| GO:0046666\_retinal\_cell\_programmed\_cell\_death | 6 | 0 | 0.000000 | -0.000000 | 997 | 965.208136 | 1049.32 | 1133.431864 | 1.052477 |
| GO:0046852\_positive\_regulation\_of\_bone\_remodeling | 6 | 0 | 0.000000 | -0.000000 | 997 | 965.208136 | 1049.32 | 1133.431864 | 1.052477 |
| GO:0046889\_positive\_regulation\_of\_lipid\_biosynthetic\_process | 6 | 0 | 0.000000 | -0.000000 | 997 | 965.208136 | 1049.32 | 1133.431864 | 1.052477 |
| GO:0048103\_somatic\_stem\_cell\_division | 6 | 0 | 0.000000 | -0.000000 | 997 | 965.208136 | 1049.32 | 1133.431864 | 1.052477 |
| GO:0048147\_negative\_regulation\_of\_fibroblast\_proliferation | 6 | 0 | 0.000000 | -0.000000 | 997 | 965.208136 | 1049.32 | 1133.431864 | 1.052477 |
| GO:0048333\_mesodermal\_cell\_differentiation | 6 | 0 | 0.000000 | -0.000000 | 997 | 965.208136 | 1049.32 | 1133.431864 | 1.052477 |
| GO:0048340\_paraxial\_mesoderm\_morphogenesis | 6 | 0 | 0.000000 | -0.000000 | 997 | 965.208136 | 1049.32 | 1133.431864 | 1.052477 |
| GO:0048541\_Peyer's\_patch\_development | 6 | 0 | 0.000000 | -0.000000 | 997 | 965.208136 | 1049.32 | 1133.431864 | 1.052477 |
| GO:0048563\_post-embryonic\_organ\_morphogenesis | 6 | 0 | 0.000000 | -0.000000 | 997 | 965.208136 | 1049.32 | 1133.431864 | 1.052477 |
| GO:0048617\_embryonic\_foregut\_morphogenesis | 6 | 0 | 0.000000 | -0.000000 | 997 | 965.208136 | 1049.32 | 1133.431864 | 1.052477 |
| GO:0048635\_negative\_regulation\_of\_muscle\_development | 6 | 0 | 0.000000 | -0.000000 | 997 | 965.208136 | 1049.32 | 1133.431864 | 1.052477 |
| GO:0048644\_muscle\_organ\_morphogenesis | 6 | 0 | 0.000000 | -0.000000 | 997 | 965.208136 | 1049.32 | 1133.431864 | 1.052477 |
| GO:0048703\_embryonic\_viscerocranium\_morphogenesis | 6 | 0 | 0.000000 | -0.000000 | 997 | 965.208136 | 1049.32 | 1133.431864 | 1.052477 |
| GO:0048713\_regulation\_of\_oligodendrocyte\_differentiation | 6 | 0 | 0.000000 | -0.000000 | 997 | 965.208136 | 1049.32 | 1133.431864 | 1.052477 |
| GO:0048853\_forebrain\_morphogenesis | 6 | 0 | 0.000000 | -0.000000 | 997 | 965.208136 | 1049.32 | 1133.431864 | 1.052477 |
| GO:0050684\_regulation\_of\_mRNA\_processing | 6 | 0 | 0.000000 | -0.000000 | 997 | 965.208136 | 1049.32 | 1133.431864 | 1.052477 |
| GO:0050732\_negative\_regulation\_of\_peptidyl-tyrosine\_phosphorylation | 6 | 0 | 0.000000 | -0.000000 | 997 | 965.208136 | 1049.32 | 1133.431864 | 1.052477 |
| GO:0050805\_negative\_regulation\_of\_synaptic\_transmission | 6 | 0 | 0.000000 | -0.000000 | 997 | 965.208136 | 1049.32 | 1133.431864 | 1.052477 |
| GO:0050821\_protein\_stabilization | 6 | 0 | 0.000000 | -0.000000 | 997 | 965.208136 | 1049.32 | 1133.431864 | 1.052477 |
| GO:0050829\_defense\_response\_to\_Gram-negative\_bacterium | 6 | 0 | 0.000000 | -0.000000 | 997 | 965.208136 | 1049.32 | 1133.431864 | 1.052477 |
| GO:0050872\_white\_fat\_cell\_differentiation | 6 | 0 | 0.000000 | -0.000000 | 997 | 965.208136 | 1049.32 | 1133.431864 | 1.052477 |
| GO:0050951\_sensory\_perception\_of\_temperature\_stimulus | 6 | 0 | 0.000000 | -0.000000 | 997 | 965.208136 | 1049.32 | 1133.431864 | 1.052477 |
| GO:0050966\_detection\_of\_mechanical\_stimulus\_involved\_in\_sensory\_perception\_of\_pain | 6 | 0 | 0.000000 | -0.000000 | 997 | 965.208136 | 1049.32 | 1133.431864 | 1.052477 |
| GO:0051085\_chaperone\_mediated\_protein\_folding\_requiring\_cofactor | 6 | 0 | 0.000000 | -0.000000 | 997 | 965.208136 | 1049.32 | 1133.431864 | 1.052477 |
| GO:0051180\_vitamin\_transport | 6 | 0 | 0.000000 | -0.000000 | 997 | 965.208136 | 1049.32 | 1133.431864 | 1.052477 |
| GO:0051384\_response\_to\_glucocorticoid\_stimulus | 6 | 0 | 0.000000 | -0.000000 | 997 | 965.208136 | 1049.32 | 1133.431864 | 1.052477 |
| GO:0051592\_response\_to\_calcium\_ion | 6 | 0 | 0.000000 | -0.000000 | 997 | 965.208136 | 1049.32 | 1133.431864 | 1.052477 |
| GO:0051875\_pigment\_granule\_localization | 6 | 0 | 0.000000 | -0.000000 | 997 | 965.208136 | 1049.32 | 1133.431864 | 1.052477 |
| GO:0051881\_regulation\_of\_mitochondrial\_membrane\_potential | 6 | 0 | 0.000000 | -0.000000 | 997 | 965.208136 | 1049.32 | 1133.431864 | 1.052477 |
| GO:0051970\_negative\_regulation\_of\_transmission\_of\_nerve\_impulse | 6 | 0 | 0.000000 | -0.000000 | 997 | 965.208136 | 1049.32 | 1133.431864 | 1.052477 |
| GO:0055081\_anion\_homeostasis | 6 | 0 | 0.000000 | -0.000000 | 997 | 965.208136 | 1049.32 | 1133.431864 | 1.052477 |
| GO:0060013\_righting\_reflex | 6 | 0 | 0.000000 | -0.000000 | 997 | 965.208136 | 1049.32 | 1133.431864 | 1.052477 |
| GO:0060017\_parathyroid\_gland\_development | 6 | 0 | 0.000000 | -0.000000 | 997 | 965.208136 | 1049.32 | 1133.431864 | 1.052477 |
| GO:0060056\_mammary\_gland\_involution | 6 | 0 | 0.000000 | -0.000000 | 997 | 965.208136 | 1049.32 | 1133.431864 | 1.052477 |
| GO:0060068\_vagina\_development | 6 | 0 | 0.000000 | -0.000000 | 997 | 965.208136 | 1049.32 | 1133.431864 | 1.052477 |
| GO:0060134\_prepulse\_inhibition | 6 | 0 | 0.000000 | -0.000000 | 997 | 965.208136 | 1049.32 | 1133.431864 | 1.052477 |
| GO:0060271\_cilium\_morphogenesis | 6 | 0 | 0.000000 | -0.000000 | 997 | 965.208136 | 1049.32 | 1133.431864 | 1.052477 |
| GO:0060389\_pathway-restricted\_SMAD\_protein\_phosphorylation | 6 | 0 | 0.000000 | -0.000000 | 997 | 965.208136 | 1049.32 | 1133.431864 | 1.052477 |
| GO:0060411\_heart\_septum\_morphogenesis | 6 | 0 | 0.000000 | -0.000000 | 997 | 965.208136 | 1049.32 | 1133.431864 | 1.052477 |
| GO:0060638\_mesenchymal-epithelial\_cell\_signaling | 6 | 0 | 0.000000 | -0.000000 | 997 | 965.208136 | 1049.32 | 1133.431864 | 1.052477 |
| GO:0060685\_regulation\_of\_prostatic\_bud\_formation | 6 | 0 | 0.000000 | -0.000000 | 997 | 965.208136 | 1049.32 | 1133.431864 | 1.052477 |
| GO:0060710\_chorio-allantoic\_fusion | 6 | 0 | 0.000000 | -0.000000 | 997 | 965.208136 | 1049.32 | 1133.431864 | 1.052477 |
| GO:0065004\_protein-DNA\_complex\_assembly | 6 | 0 | 0.000000 | -0.000000 | 997 | 965.208136 | 1049.32 | 1133.431864 | 1.052477 |
| GO:0002562\_somatic\_diversification\_of\_immune\_receptors\_via\_germline\_recombination\_within\_a\_single\_locus | 33 | 0 | 0.000000 | -0.000000 | 1007 | 976.061167 | 1059.54 | 1143.018833 | 1.052175 |
| GO:0006643\_membrane\_lipid\_metabolic\_process | 33 | 0 | 0.000000 | -0.000000 | 1007 | 976.061167 | 1059.54 | 1143.018833 | 1.052175 |
| GO:0007188\_G-protein\_signaling\_\_coupled\_to\_cAMP\_nucleotide\_second\_messenger | 33 | 0 | 0.000000 | -0.000000 | 1007 | 976.061167 | 1059.54 | 1143.018833 | 1.052175 |
| GO:0007270\_nerve-nerve\_synaptic\_transmission | 33 | 0 | 0.000000 | -0.000000 | 1007 | 976.061167 | 1059.54 | 1143.018833 | 1.052175 |
| GO:0008584\_male\_gonad\_development | 33 | 0 | 0.000000 | -0.000000 | 1007 | 976.061167 | 1059.54 | 1143.018833 | 1.052175 |
| GO:0016444\_somatic\_cell\_DNA\_recombination | 33 | 0 | 0.000000 | -0.000000 | 1007 | 976.061167 | 1059.54 | 1143.018833 | 1.052175 |
| GO:0021536\_diencephalon\_development | 33 | 0 | 0.000000 | -0.000000 | 1007 | 976.061167 | 1059.54 | 1143.018833 | 1.052175 |
| GO:0021987\_cerebral\_cortex\_development | 33 | 0 | 0.000000 | -0.000000 | 1007 | 976.061167 | 1059.54 | 1143.018833 | 1.052175 |
| GO:0022037\_metencephalon\_development | 33 | 0 | 0.000000 | -0.000000 | 1007 | 976.061167 | 1059.54 | 1143.018833 | 1.052175 |
| GO:0042108\_positive\_regulation\_of\_cytokine\_biosynthetic\_process | 33 | 0 | 0.000000 | -0.000000 | 1007 | 976.061167 | 1059.54 | 1143.018833 | 1.052175 |
| GO:0007281\_germ\_cell\_development | 75 | 0 | 0.000000 | -0.000000 | 1010 | 978.025559 | 1061.26 | 1144.494441 | 1.050752 |
| GO:0044265\_cellular\_macromolecule\_catabolic\_process | 75 | 0 | 0.000000 | -0.000000 | 1010 | 978.025559 | 1061.26 | 1144.494441 | 1.050752 |
| GO:0048589\_developmental\_growth | 75 | 0 | 0.000000 | -0.000000 | 1010 | 978.025559 | 1061.26 | 1144.494441 | 1.050752 |
| GO:0002200\_somatic\_diversification\_of\_immune\_receptors | 34 | 0 | 0.000000 | -0.000000 | 1021 | 991.949966 | 1073.9 | 1155.850034 | 1.051812 |
| GO:0002699\_positive\_regulation\_of\_immune\_effector\_process | 34 | 0 | 0.000000 | -0.000000 | 1021 | 991.949966 | 1073.9 | 1155.850034 | 1.051812 |
| GO:0007338\_single\_fertilization | 34 | 0 | 0.000000 | -0.000000 | 1021 | 991.949966 | 1073.9 | 1155.850034 | 1.051812 |
| GO:0007568\_aging | 34 | 0 | 0.000000 | -0.000000 | 1021 | 991.949966 | 1073.9 | 1155.850034 | 1.051812 |
| GO:0016054\_organic\_acid\_catabolic\_process | 34 | 0 | 0.000000 | -0.000000 | 1021 | 991.949966 | 1073.9 | 1155.850034 | 1.051812 |
| GO:0019882\_antigen\_processing\_and\_presentation | 34 | 0 | 0.000000 | -0.000000 | 1021 | 991.949966 | 1073.9 | 1155.850034 | 1.051812 |
| GO:0030509\_BMP\_signaling\_pathway | 34 | 0 | 0.000000 | -0.000000 | 1021 | 991.949966 | 1073.9 | 1155.850034 | 1.051812 |
| GO:0045927\_positive\_regulation\_of\_growth | 34 | 0 | 0.000000 | -0.000000 | 1021 | 991.949966 | 1073.9 | 1155.850034 | 1.051812 |
| GO:0046395\_carboxylic\_acid\_catabolic\_process | 34 | 0 | 0.000000 | -0.000000 | 1021 | 991.949966 | 1073.9 | 1155.850034 | 1.051812 |
| GO:0051052\_regulation\_of\_DNA\_metabolic\_process | 34 | 0 | 0.000000 | -0.000000 | 1021 | 991.949966 | 1073.9 | 1155.850034 | 1.051812 |
| GO:0060443\_mammary\_gland\_morphogenesis | 34 | 0 | 0.000000 | -0.000000 | 1021 | 991.949966 | 1073.9 | 1155.850034 | 1.051812 |
| GO:0001759\_induction\_of\_an\_organ | 15 | 0 | 0.000000 | -0.000000 | 1072 | 1041.304884 | 1121.26 | 1201.215116 | 1.045951 |
| GO:0001782\_B\_cell\_homeostasis | 15 | 0 | 0.000000 | -0.000000 | 1072 | 1041.304884 | 1121.26 | 1201.215116 | 1.045951 |
| GO:0001964\_startle\_response | 15 | 0 | 0.000000 | -0.000000 | 1072 | 1041.304884 | 1121.26 | 1201.215116 | 1.045951 |
| GO:0002286\_T\_cell\_activation\_during\_immune\_response | 15 | 0 | 0.000000 | -0.000000 | 1072 | 1041.304884 | 1121.26 | 1201.215116 | 1.045951 |
| GO:0002495\_antigen\_processing\_and\_presentation\_of\_peptide\_antigen\_via\_MHC\_class\_II | 15 | 0 | 0.000000 | -0.000000 | 1072 | 1041.304884 | 1121.26 | 1201.215116 | 1.045951 |
| GO:0002504\_antigen\_processing\_and\_presentation\_of\_peptide\_or\_polysaccharide\_antigen\_via\_MHC\_class\_II | 15 | 0 | 0.000000 | -0.000000 | 1072 | 1041.304884 | 1121.26 | 1201.215116 | 1.045951 |
| GO:0002709\_regulation\_of\_T\_cell\_mediated\_immunity | 15 | 0 | 0.000000 | -0.000000 | 1072 | 1041.304884 | 1121.26 | 1201.215116 | 1.045951 |
| GO:0006473\_protein\_amino\_acid\_acetylation | 15 | 0 | 0.000000 | -0.000000 | 1072 | 1041.304884 | 1121.26 | 1201.215116 | 1.045951 |
| GO:0006487\_protein\_amino\_acid\_N-linked\_glycosylation | 15 | 0 | 0.000000 | -0.000000 | 1072 | 1041.304884 | 1121.26 | 1201.215116 | 1.045951 |
| GO:0006749\_glutathione\_metabolic\_process | 15 | 0 | 0.000000 | -0.000000 | 1072 | 1041.304884 | 1121.26 | 1201.215116 | 1.045951 |
| GO:0006885\_regulation\_of\_pH | 15 | 0 | 0.000000 | -0.000000 | 1072 | 1041.304884 | 1121.26 | 1201.215116 | 1.045951 |
| GO:0007040\_lysosome\_organization | 15 | 0 | 0.000000 | -0.000000 | 1072 | 1041.304884 | 1121.26 | 1201.215116 | 1.045951 |
| GO:0007173\_epidermal\_growth\_factor\_receptor\_signaling\_pathway | 15 | 0 | 0.000000 | -0.000000 | 1072 | 1041.304884 | 1121.26 | 1201.215116 | 1.045951 |
| GO:0007200\_activation\_of\_phospholipase\_C\_activity\_by\_G-protein\_coupled\_receptor\_protein\_signaling\_pathway\_coupled\_to\_IP3\_second\_messenger | 15 | 0 | 0.000000 | -0.000000 | 1072 | 1041.304884 | 1121.26 | 1201.215116 | 1.045951 |
| GO:0007202\_activation\_of\_phospholipase\_C\_activity | 15 | 0 | 0.000000 | -0.000000 | 1072 | 1041.304884 | 1121.26 | 1201.215116 | 1.045951 |
| GO:0007218\_neuropeptide\_signaling\_pathway | 15 | 0 | 0.000000 | -0.000000 | 1072 | 1041.304884 | 1121.26 | 1201.215116 | 1.045951 |
| GO:0007588\_excretion | 15 | 0 | 0.000000 | -0.000000 | 1072 | 1041.304884 | 1121.26 | 1201.215116 | 1.045951 |
| GO:0007618\_mating | 15 | 0 | 0.000000 | -0.000000 | 1072 | 1041.304884 | 1121.26 | 1201.215116 | 1.045951 |
| GO:0008543\_fibroblast\_growth\_factor\_receptor\_signaling\_pathway | 15 | 0 | 0.000000 | -0.000000 | 1072 | 1041.304884 | 1121.26 | 1201.215116 | 1.045951 |
| GO:0009062\_fatty\_acid\_catabolic\_process | 15 | 0 | 0.000000 | -0.000000 | 1072 | 1041.304884 | 1121.26 | 1201.215116 | 1.045951 |
| GO:0009116\_nucleoside\_metabolic\_process | 15 | 0 | 0.000000 | -0.000000 | 1072 | 1041.304884 | 1121.26 | 1201.215116 | 1.045951 |
| GO:0010092\_specification\_of\_organ\_identity | 15 | 0 | 0.000000 | -0.000000 | 1072 | 1041.304884 | 1121.26 | 1201.215116 | 1.045951 |
| GO:0010518\_positive\_regulation\_of\_phospholipase\_activity | 15 | 0 | 0.000000 | -0.000000 | 1072 | 1041.304884 | 1121.26 | 1201.215116 | 1.045951 |
| GO:0010863\_positive\_regulation\_of\_phospholipase\_C\_activity | 15 | 0 | 0.000000 | -0.000000 | 1072 | 1041.304884 | 1121.26 | 1201.215116 | 1.045951 |
| GO:0015931\_nucleobase\_\_nucleoside\_\_nucleotide\_and\_nucleic\_acid\_transport | 15 | 0 | 0.000000 | -0.000000 | 1072 | 1041.304884 | 1121.26 | 1201.215116 | 1.045951 |
| GO:0019886\_antigen\_processing\_and\_presentation\_of\_exogenous\_peptide\_antigen\_via\_MHC\_class\_II | 15 | 0 | 0.000000 | -0.000000 | 1072 | 1041.304884 | 1121.26 | 1201.215116 | 1.045951 |
| GO:0021795\_cerebral\_cortex\_cell\_migration | 15 | 0 | 0.000000 | -0.000000 | 1072 | 1041.304884 | 1121.26 | 1201.215116 | 1.045951 |
| GO:0021872\_generation\_of\_neurons\_in\_the\_forebrain | 15 | 0 | 0.000000 | -0.000000 | 1072 | 1041.304884 | 1121.26 | 1201.215116 | 1.045951 |
| GO:0022600\_digestive\_system\_process | 15 | 0 | 0.000000 | -0.000000 | 1072 | 1041.304884 | 1121.26 | 1201.215116 | 1.045951 |
| GO:0030041\_actin\_filament\_polymerization | 15 | 0 | 0.000000 | -0.000000 | 1072 | 1041.304884 | 1121.26 | 1201.215116 | 1.045951 |
| GO:0031069\_hair\_follicle\_morphogenesis | 15 | 0 | 0.000000 | -0.000000 | 1072 | 1041.304884 | 1121.26 | 1201.215116 | 1.045951 |
| GO:0031329\_regulation\_of\_cellular\_catabolic\_process | 15 | 0 | 0.000000 | -0.000000 | 1072 | 1041.304884 | 1121.26 | 1201.215116 | 1.045951 |
| GO:0035116\_embryonic\_hindlimb\_morphogenesis | 15 | 0 | 0.000000 | -0.000000 | 1072 | 1041.304884 | 1121.26 | 1201.215116 | 1.045951 |
| GO:0035249\_synaptic\_transmission\_\_glutamatergic | 15 | 0 | 0.000000 | -0.000000 | 1072 | 1041.304884 | 1121.26 | 1201.215116 | 1.045951 |
| GO:0042306\_regulation\_of\_protein\_import\_into\_nucleus | 15 | 0 | 0.000000 | -0.000000 | 1072 | 1041.304884 | 1121.26 | 1201.215116 | 1.045951 |
| GO:0045666\_positive\_regulation\_of\_neuron\_differentiation | 15 | 0 | 0.000000 | -0.000000 | 1072 | 1041.304884 | 1121.26 | 1201.215116 | 1.045951 |
| GO:0046164\_alcohol\_catabolic\_process | 15 | 0 | 0.000000 | -0.000000 | 1072 | 1041.304884 | 1121.26 | 1201.215116 | 1.045951 |
| GO:0046638\_positive\_regulation\_of\_alpha-beta\_T\_cell\_differentiation | 15 | 0 | 0.000000 | -0.000000 | 1072 | 1041.304884 | 1121.26 | 1201.215116 | 1.045951 |
| GO:0048008\_platelet-derived\_growth\_factor\_receptor\_signaling\_pathway | 15 | 0 | 0.000000 | -0.000000 | 1072 | 1041.304884 | 1121.26 | 1201.215116 | 1.045951 |
| GO:0048010\_vascular\_endothelial\_growth\_factor\_receptor\_signaling\_pathway | 15 | 0 | 0.000000 | -0.000000 | 1072 | 1041.304884 | 1121.26 | 1201.215116 | 1.045951 |
| GO:0048144\_fibroblast\_proliferation | 15 | 0 | 0.000000 | -0.000000 | 1072 | 1041.304884 | 1121.26 | 1201.215116 | 1.045951 |
| GO:0048145\_regulation\_of\_fibroblast\_proliferation | 15 | 0 | 0.000000 | -0.000000 | 1072 | 1041.304884 | 1121.26 | 1201.215116 | 1.045951 |
| GO:0048610\_reproductive\_cellular\_process | 15 | 0 | 0.000000 | -0.000000 | 1072 | 1041.304884 | 1121.26 | 1201.215116 | 1.045951 |
| GO:0048709\_oligodendrocyte\_differentiation | 15 | 0 | 0.000000 | -0.000000 | 1072 | 1041.304884 | 1121.26 | 1201.215116 | 1.045951 |
| GO:0050729\_positive\_regulation\_of\_inflammatory\_response | 15 | 0 | 0.000000 | -0.000000 | 1072 | 1041.304884 | 1121.26 | 1201.215116 | 1.045951 |
| GO:0050796\_regulation\_of\_insulin\_secretion | 15 | 0 | 0.000000 | -0.000000 | 1072 | 1041.304884 | 1121.26 | 1201.215116 | 1.045951 |
| GO:0050798\_activated\_T\_cell\_proliferation | 15 | 0 | 0.000000 | -0.000000 | 1072 | 1041.304884 | 1121.26 | 1201.215116 | 1.045951 |
| GO:0055010\_ventricular\_cardiac\_muscle\_morphogenesis | 15 | 0 | 0.000000 | -0.000000 | 1072 | 1041.304884 | 1121.26 | 1201.215116 | 1.045951 |
| GO:0060425\_lung\_morphogenesis | 15 | 0 | 0.000000 | -0.000000 | 1072 | 1041.304884 | 1121.26 | 1201.215116 | 1.045951 |
| GO:0070227\_lymphocyte\_apoptosis | 15 | 0 | 0.000000 | -0.000000 | 1072 | 1041.304884 | 1121.26 | 1201.215116 | 1.045951 |
| GO:0070507\_regulation\_of\_microtubule\_cytoskeleton\_organization | 15 | 0 | 0.000000 | -0.000000 | 1072 | 1041.304884 | 1121.26 | 1201.215116 | 1.045951 |
| GO:0006520\_cellular\_amino\_acid\_metabolic\_process | 51 | 0 | 0.000000 | -0.000000 | 1077 | 1047.393728 | 1126.7 | 1206.006272 | 1.046147 |
| GO:0032880\_regulation\_of\_protein\_localization | 51 | 0 | 0.000000 | -0.000000 | 1077 | 1047.393728 | 1126.7 | 1206.006272 | 1.046147 |
| GO:0043408\_regulation\_of\_MAPKKK\_cascade | 51 | 0 | 0.000000 | -0.000000 | 1077 | 1047.393728 | 1126.7 | 1206.006272 | 1.046147 |
| GO:0044106\_cellular\_amine\_metabolic\_process | 51 | 0 | 0.000000 | -0.000000 | 1077 | 1047.393728 | 1126.7 | 1206.006272 | 1.046147 |
| GO:0048747\_muscle\_fiber\_development | 51 | 0 | 0.000000 | -0.000000 | 1077 | 1047.393728 | 1126.7 | 1206.006272 | 1.046147 |
| GO:0005996\_monosaccharide\_metabolic\_process | 69 | 0 | 0.000000 | -0.000000 | 1081 | 1050.429095 | 1129.11 | 1207.790905 | 1.044505 |
| GO:0006816\_calcium\_ion\_transport | 69 | 0 | 0.000000 | -0.000000 | 1081 | 1050.429095 | 1129.11 | 1207.790905 | 1.044505 |
| GO:0032101\_regulation\_of\_response\_to\_external\_stimulus | 69 | 0 | 0.000000 | -0.000000 | 1081 | 1050.429095 | 1129.11 | 1207.790905 | 1.044505 |
| GO:0055065\_metal\_ion\_homeostasis | 69 | 0 | 0.000000 | -0.000000 | 1081 | 1050.429095 | 1129.11 | 1207.790905 | 1.044505 |
| GO:0015674\_di-\_\_tri-valent\_inorganic\_cation\_transport | 79 | 0 | 0.000000 | -0.000000 | 1082 | 1051.577621 | 1129.95 | 1208.322379 | 1.044316 |
| GO:0000077\_DNA\_damage\_checkpoint | 14 | 0 | 0.000000 | -0.000000 | 1137 | 1109.603463 | 1185.73 | 1261.856537 | 1.042858 |
| GO:0001502\_cartilage\_condensation | 14 | 0 | 0.000000 | -0.000000 | 1137 | 1109.603463 | 1185.73 | 1261.856537 | 1.042858 |
| GO:0002027\_regulation\_of\_heart\_rate | 14 | 0 | 0.000000 | -0.000000 | 1137 | 1109.603463 | 1185.73 | 1261.856537 | 1.042858 |
| GO:0002262\_myeloid\_cell\_homeostasis | 14 | 0 | 0.000000 | -0.000000 | 1137 | 1109.603463 | 1185.73 | 1261.856537 | 1.042858 |
| GO:0002698\_negative\_regulation\_of\_immune\_effector\_process | 14 | 0 | 0.000000 | -0.000000 | 1137 | 1109.603463 | 1185.73 | 1261.856537 | 1.042858 |
| GO:0006304\_DNA\_modification | 14 | 0 | 0.000000 | -0.000000 | 1137 | 1109.603463 | 1185.73 | 1261.856537 | 1.042858 |
| GO:0006305\_DNA\_alkylation | 14 | 0 | 0.000000 | -0.000000 | 1137 | 1109.603463 | 1185.73 | 1261.856537 | 1.042858 |
| GO:0006306\_DNA\_methylation | 14 | 0 | 0.000000 | -0.000000 | 1137 | 1109.603463 | 1185.73 | 1261.856537 | 1.042858 |
| GO:0006695\_cholesterol\_biosynthetic\_process | 14 | 0 | 0.000000 | -0.000000 | 1137 | 1109.603463 | 1185.73 | 1261.856537 | 1.042858 |
| GO:0006809\_nitric\_oxide\_biosynthetic\_process | 14 | 0 | 0.000000 | -0.000000 | 1137 | 1109.603463 | 1185.73 | 1261.856537 | 1.042858 |
| GO:0006914\_autophagy | 14 | 0 | 0.000000 | -0.000000 | 1137 | 1109.603463 | 1185.73 | 1261.856537 | 1.042858 |
| GO:0006970\_response\_to\_osmotic\_stress | 14 | 0 | 0.000000 | -0.000000 | 1137 | 1109.603463 | 1185.73 | 1261.856537 | 1.042858 |
| GO:0007157\_heterophilic\_cell\_adhesion | 14 | 0 | 0.000000 | -0.000000 | 1137 | 1109.603463 | 1185.73 | 1261.856537 | 1.042858 |
| GO:0007530\_sex\_determination | 14 | 0 | 0.000000 | -0.000000 | 1137 | 1109.603463 | 1185.73 | 1261.856537 | 1.042858 |
| GO:0008064\_regulation\_of\_actin\_polymerization\_or\_depolymerization | 14 | 0 | 0.000000 | -0.000000 | 1137 | 1109.603463 | 1185.73 | 1261.856537 | 1.042858 |
| GO:0008306\_associative\_learning | 14 | 0 | 0.000000 | -0.000000 | 1137 | 1109.603463 | 1185.73 | 1261.856537 | 1.042858 |
| GO:0009108\_coenzyme\_biosynthetic\_process | 14 | 0 | 0.000000 | -0.000000 | 1137 | 1109.603463 | 1185.73 | 1261.856537 | 1.042858 |
| GO:0009267\_cellular\_response\_to\_starvation | 14 | 0 | 0.000000 | -0.000000 | 1137 | 1109.603463 | 1185.73 | 1261.856537 | 1.042858 |
| GO:0009895\_negative\_regulation\_of\_catabolic\_process | 14 | 0 | 0.000000 | -0.000000 | 1137 | 1109.603463 | 1185.73 | 1261.856537 | 1.042858 |
| GO:0010332\_response\_to\_gamma\_radiation | 14 | 0 | 0.000000 | -0.000000 | 1137 | 1109.603463 | 1185.73 | 1261.856537 | 1.042858 |
| GO:0014855\_striated\_muscle\_cell\_proliferation | 14 | 0 | 0.000000 | -0.000000 | 1137 | 1109.603463 | 1185.73 | 1261.856537 | 1.042858 |
| GO:0016573\_histone\_acetylation | 14 | 0 | 0.000000 | -0.000000 | 1137 | 1109.603463 | 1185.73 | 1261.856537 | 1.042858 |
| GO:0018130\_heterocycle\_biosynthetic\_process | 14 | 0 | 0.000000 | -0.000000 | 1137 | 1109.603463 | 1185.73 | 1261.856537 | 1.042858 |
| GO:0019217\_regulation\_of\_fatty\_acid\_metabolic\_process | 14 | 0 | 0.000000 | -0.000000 | 1137 | 1109.603463 | 1185.73 | 1261.856537 | 1.042858 |
| GO:0021782\_glial\_cell\_development | 14 | 0 | 0.000000 | -0.000000 | 1137 | 1109.603463 | 1185.73 | 1261.856537 | 1.042858 |
| GO:0021904\_dorsal\_ventral\_neural\_tube\_patterning | 14 | 0 | 0.000000 | -0.000000 | 1137 | 1109.603463 | 1185.73 | 1261.856537 | 1.042858 |
| GO:0030032\_lamellipodium\_assembly | 14 | 0 | 0.000000 | -0.000000 | 1137 | 1109.603463 | 1185.73 | 1261.856537 | 1.042858 |
| GO:0030148\_sphingolipid\_biosynthetic\_process | 14 | 0 | 0.000000 | -0.000000 | 1137 | 1109.603463 | 1185.73 | 1261.856537 | 1.042858 |
| GO:0030162\_regulation\_of\_proteolysis | 14 | 0 | 0.000000 | -0.000000 | 1137 | 1109.603463 | 1185.73 | 1261.856537 | 1.042858 |
| GO:0030832\_regulation\_of\_actin\_filament\_length | 14 | 0 | 0.000000 | -0.000000 | 1137 | 1109.603463 | 1185.73 | 1261.856537 | 1.042858 |
| GO:0031099\_regeneration | 14 | 0 | 0.000000 | -0.000000 | 1137 | 1109.603463 | 1185.73 | 1261.856537 | 1.042858 |
| GO:0032271\_regulation\_of\_protein\_polymerization | 14 | 0 | 0.000000 | -0.000000 | 1137 | 1109.603463 | 1185.73 | 1261.856537 | 1.042858 |
| GO:0033044\_regulation\_of\_chromosome\_organization | 14 | 0 | 0.000000 | -0.000000 | 1137 | 1109.603463 | 1185.73 | 1261.856537 | 1.042858 |
| GO:0034104\_negative\_regulation\_of\_tissue\_remodeling | 14 | 0 | 0.000000 | -0.000000 | 1137 | 1109.603463 | 1185.73 | 1261.856537 | 1.042858 |
| GO:0034623\_cellular\_macromolecular\_complex\_disassembly | 14 | 0 | 0.000000 | -0.000000 | 1137 | 1109.603463 | 1185.73 | 1261.856537 | 1.042858 |
| GO:0035036\_sperm-egg\_recognition | 14 | 0 | 0.000000 | -0.000000 | 1137 | 1109.603463 | 1185.73 | 1261.856537 | 1.042858 |
| GO:0042310\_vasoconstriction | 14 | 0 | 0.000000 | -0.000000 | 1137 | 1109.603463 | 1185.73 | 1261.856537 | 1.042858 |
| GO:0042573\_retinoic\_acid\_metabolic\_process | 14 | 0 | 0.000000 | -0.000000 | 1137 | 1109.603463 | 1185.73 | 1261.856537 | 1.042858 |
| GO:0043254\_regulation\_of\_protein\_complex\_assembly | 14 | 0 | 0.000000 | -0.000000 | 1137 | 1109.603463 | 1185.73 | 1261.856537 | 1.042858 |
| GO:0043491\_protein\_kinase\_B\_signaling\_cascade | 14 | 0 | 0.000000 | -0.000000 | 1137 | 1109.603463 | 1185.73 | 1261.856537 | 1.042858 |
| GO:0045061\_thymic\_T\_cell\_selection | 14 | 0 | 0.000000 | -0.000000 | 1137 | 1109.603463 | 1185.73 | 1261.856537 | 1.042858 |
| GO:0045453\_bone\_resorption | 14 | 0 | 0.000000 | -0.000000 | 1137 | 1109.603463 | 1185.73 | 1261.856537 | 1.042858 |
| GO:0045598\_regulation\_of\_fat\_cell\_differentiation | 14 | 0 | 0.000000 | -0.000000 | 1137 | 1109.603463 | 1185.73 | 1261.856537 | 1.042858 |
| GO:0045732\_positive\_regulation\_of\_protein\_catabolic\_process | 14 | 0 | 0.000000 | -0.000000 | 1137 | 1109.603463 | 1185.73 | 1261.856537 | 1.042858 |
| GO:0046209\_nitric\_oxide\_metabolic\_process | 14 | 0 | 0.000000 | -0.000000 | 1137 | 1109.603463 | 1185.73 | 1261.856537 | 1.042858 |
| GO:0048545\_response\_to\_steroid\_hormone\_stimulus | 14 | 0 | 0.000000 | -0.000000 | 1137 | 1109.603463 | 1185.73 | 1261.856537 | 1.042858 |
| GO:0048665\_neuron\_fate\_specification | 14 | 0 | 0.000000 | -0.000000 | 1137 | 1109.603463 | 1185.73 | 1261.856537 | 1.042858 |
| GO:0048844\_artery\_morphogenesis | 14 | 0 | 0.000000 | -0.000000 | 1137 | 1109.603463 | 1185.73 | 1261.856537 | 1.042858 |
| GO:0050810\_regulation\_of\_steroid\_biosynthetic\_process | 14 | 0 | 0.000000 | -0.000000 | 1137 | 1109.603463 | 1185.73 | 1261.856537 | 1.042858 |
| GO:0051017\_actin\_filament\_bundle\_formation | 14 | 0 | 0.000000 | -0.000000 | 1137 | 1109.603463 | 1185.73 | 1261.856537 | 1.042858 |
| GO:0051053\_negative\_regulation\_of\_DNA\_metabolic\_process | 14 | 0 | 0.000000 | -0.000000 | 1137 | 1109.603463 | 1185.73 | 1261.856537 | 1.042858 |
| GO:0051054\_positive\_regulation\_of\_DNA\_metabolic\_process | 14 | 0 | 0.000000 | -0.000000 | 1137 | 1109.603463 | 1185.73 | 1261.856537 | 1.042858 |
| GO:0051100\_negative\_regulation\_of\_binding | 14 | 0 | 0.000000 | -0.000000 | 1137 | 1109.603463 | 1185.73 | 1261.856537 | 1.042858 |
| GO:0051952\_regulation\_of\_amine\_transport | 14 | 0 | 0.000000 | -0.000000 | 1137 | 1109.603463 | 1185.73 | 1261.856537 | 1.042858 |
| GO:0060840\_artery\_development | 14 | 0 | 0.000000 | -0.000000 | 1137 | 1109.603463 | 1185.73 | 1261.856537 | 1.042858 |
| GO:0001776\_leukocyte\_homeostasis | 41 | 0 | 0.000000 | -0.000000 | 1148 | 1123.857740 | 1198.78 | 1273.702260 | 1.044233 |
| GO:0006865\_amino\_acid\_transport | 41 | 0 | 0.000000 | -0.000000 | 1148 | 1123.857740 | 1198.78 | 1273.702260 | 1.044233 |
| GO:0006979\_response\_to\_oxidative\_stress | 41 | 0 | 0.000000 | -0.000000 | 1148 | 1123.857740 | 1198.78 | 1273.702260 | 1.044233 |
| GO:0008585\_female\_gonad\_development | 41 | 0 | 0.000000 | -0.000000 | 1148 | 1123.857740 | 1198.78 | 1273.702260 | 1.044233 |
| GO:0015980\_energy\_derivation\_by\_oxidation\_of\_organic\_compounds | 41 | 0 | 0.000000 | -0.000000 | 1148 | 1123.857740 | 1198.78 | 1273.702260 | 1.044233 |
| GO:0019216\_regulation\_of\_lipid\_metabolic\_process | 41 | 0 | 0.000000 | -0.000000 | 1148 | 1123.857740 | 1198.78 | 1273.702260 | 1.044233 |
| GO:0019748\_secondary\_metabolic\_process | 41 | 0 | 0.000000 | -0.000000 | 1148 | 1123.857740 | 1198.78 | 1273.702260 | 1.044233 |
| GO:0030817\_regulation\_of\_cAMP\_biosynthetic\_process | 41 | 0 | 0.000000 | -0.000000 | 1148 | 1123.857740 | 1198.78 | 1273.702260 | 1.044233 |
| GO:0032844\_regulation\_of\_homeostatic\_process | 41 | 0 | 0.000000 | -0.000000 | 1148 | 1123.857740 | 1198.78 | 1273.702260 | 1.044233 |
| GO:0033077\_T\_cell\_differentiation\_in\_the\_thymus | 41 | 0 | 0.000000 | -0.000000 | 1148 | 1123.857740 | 1198.78 | 1273.702260 | 1.044233 |
| GO:0050864\_regulation\_of\_B\_cell\_activation | 41 | 0 | 0.000000 | -0.000000 | 1148 | 1123.857740 | 1198.78 | 1273.702260 | 1.044233 |
| GO:0000302\_response\_to\_reactive\_oxygen\_species | 16 | 0 | 0.000000 | -0.000000 | 1191 | 1165.597588 | 1239.18 | 1312.762412 | 1.040453 |
| GO:0001933\_negative\_regulation\_of\_protein\_amino\_acid\_phosphorylation | 16 | 0 | 0.000000 | -0.000000 | 1191 | 1165.597588 | 1239.18 | 1312.762412 | 1.040453 |
| GO:0003044\_regulation\_of\_systemic\_arterial\_blood\_pressure\_mediated\_by\_a\_chemical\_signal | 16 | 0 | 0.000000 | -0.000000 | 1191 | 1165.597588 | 1239.18 | 1312.762412 | 1.040453 |
| GO:0006664\_glycolipid\_metabolic\_process | 16 | 0 | 0.000000 | -0.000000 | 1191 | 1165.597588 | 1239.18 | 1312.762412 | 1.040453 |
| GO:0006821\_chloride\_transport | 16 | 0 | 0.000000 | -0.000000 | 1191 | 1165.597588 | 1239.18 | 1312.762412 | 1.040453 |
| GO:0007033\_vacuole\_organization | 16 | 0 | 0.000000 | -0.000000 | 1191 | 1165.597588 | 1239.18 | 1312.762412 | 1.040453 |
| GO:0007156\_homophilic\_cell\_adhesion | 16 | 0 | 0.000000 | -0.000000 | 1191 | 1165.597588 | 1239.18 | 1312.762412 | 1.040453 |
| GO:0007602\_phototransduction | 16 | 0 | 0.000000 | -0.000000 | 1191 | 1165.597588 | 1239.18 | 1312.762412 | 1.040453 |
| GO:0008654\_phospholipid\_biosynthetic\_process | 16 | 0 | 0.000000 | -0.000000 | 1191 | 1165.597588 | 1239.18 | 1312.762412 | 1.040453 |
| GO:0009988\_cell-cell\_recognition | 16 | 0 | 0.000000 | -0.000000 | 1191 | 1165.597588 | 1239.18 | 1312.762412 | 1.040453 |
| GO:0010038\_response\_to\_metal\_ion | 16 | 0 | 0.000000 | -0.000000 | 1191 | 1165.597588 | 1239.18 | 1312.762412 | 1.040453 |
| GO:0010243\_response\_to\_organic\_nitrogen | 16 | 0 | 0.000000 | -0.000000 | 1191 | 1165.597588 | 1239.18 | 1312.762412 | 1.040453 |
| GO:0010876\_lipid\_localization | 16 | 0 | 0.000000 | -0.000000 | 1191 | 1165.597588 | 1239.18 | 1312.762412 | 1.040453 |
| GO:0014075\_response\_to\_amine\_stimulus | 16 | 0 | 0.000000 | -0.000000 | 1191 | 1165.597588 | 1239.18 | 1312.762412 | 1.040453 |
| GO:0016126\_sterol\_biosynthetic\_process | 16 | 0 | 0.000000 | -0.000000 | 1191 | 1165.597588 | 1239.18 | 1312.762412 | 1.040453 |
| GO:0019722\_calcium-mediated\_signaling | 16 | 0 | 0.000000 | -0.000000 | 1191 | 1165.597588 | 1239.18 | 1312.762412 | 1.040453 |
| GO:0019751\_polyol\_metabolic\_process | 16 | 0 | 0.000000 | -0.000000 | 1191 | 1165.597588 | 1239.18 | 1312.762412 | 1.040453 |
| GO:0019915\_lipid\_storage | 16 | 0 | 0.000000 | -0.000000 | 1191 | 1165.597588 | 1239.18 | 1312.762412 | 1.040453 |
| GO:0021522\_spinal\_cord\_motor\_neuron\_differentiation | 16 | 0 | 0.000000 | -0.000000 | 1191 | 1165.597588 | 1239.18 | 1312.762412 | 1.040453 |
| GO:0021696\_cerebellar\_cortex\_morphogenesis | 16 | 0 | 0.000000 | -0.000000 | 1191 | 1165.597588 | 1239.18 | 1312.762412 | 1.040453 |
| GO:0030890\_positive\_regulation\_of\_B\_cell\_proliferation | 16 | 0 | 0.000000 | -0.000000 | 1191 | 1165.597588 | 1239.18 | 1312.762412 | 1.040453 |
| GO:0031570\_DNA\_integrity\_checkpoint | 16 | 0 | 0.000000 | -0.000000 | 1191 | 1165.597588 | 1239.18 | 1312.762412 | 1.040453 |
| GO:0031669\_cellular\_response\_to\_nutrient\_levels | 16 | 0 | 0.000000 | -0.000000 | 1191 | 1165.597588 | 1239.18 | 1312.762412 | 1.040453 |
| GO:0032663\_regulation\_of\_interleukin-2\_production | 16 | 0 | 0.000000 | -0.000000 | 1191 | 1165.597588 | 1239.18 | 1312.762412 | 1.040453 |
| GO:0032956\_regulation\_of\_actin\_cytoskeleton\_organization | 16 | 0 | 0.000000 | -0.000000 | 1191 | 1165.597588 | 1239.18 | 1312.762412 | 1.040453 |
| GO:0034976\_response\_to\_endoplasmic\_reticulum\_stress | 16 | 0 | 0.000000 | -0.000000 | 1191 | 1165.597588 | 1239.18 | 1312.762412 | 1.040453 |
| GO:0042311\_vasodilation | 16 | 0 | 0.000000 | -0.000000 | 1191 | 1165.597588 | 1239.18 | 1312.762412 | 1.040453 |
| GO:0042594\_response\_to\_starvation | 16 | 0 | 0.000000 | -0.000000 | 1191 | 1165.597588 | 1239.18 | 1312.762412 | 1.040453 |
| GO:0042596\_fear\_response | 16 | 0 | 0.000000 | -0.000000 | 1191 | 1165.597588 | 1239.18 | 1312.762412 | 1.040453 |
| GO:0043367\_CD4-positive\_\_alpha\_beta\_T\_cell\_differentiation | 16 | 0 | 0.000000 | -0.000000 | 1191 | 1165.597588 | 1239.18 | 1312.762412 | 1.040453 |
| GO:0045104\_intermediate\_filament\_cytoskeleton\_organization | 16 | 0 | 0.000000 | -0.000000 | 1191 | 1165.597588 | 1239.18 | 1312.762412 | 1.040453 |
| GO:0046148\_pigment\_biosynthetic\_process | 16 | 0 | 0.000000 | -0.000000 | 1191 | 1165.597588 | 1239.18 | 1312.762412 | 1.040453 |
| GO:0046364\_monosaccharide\_biosynthetic\_process | 16 | 0 | 0.000000 | -0.000000 | 1191 | 1165.597588 | 1239.18 | 1312.762412 | 1.040453 |
| GO:0046467\_membrane\_lipid\_biosynthetic\_process | 16 | 0 | 0.000000 | -0.000000 | 1191 | 1165.597588 | 1239.18 | 1312.762412 | 1.040453 |
| GO:0046633\_alpha-beta\_T\_cell\_proliferation | 16 | 0 | 0.000000 | -0.000000 | 1191 | 1165.597588 | 1239.18 | 1312.762412 | 1.040453 |
| GO:0046700\_heterocycle\_catabolic\_process | 16 | 0 | 0.000000 | -0.000000 | 1191 | 1165.597588 | 1239.18 | 1312.762412 | 1.040453 |
| GO:0048015\_phosphoinositide-mediated\_signaling | 16 | 0 | 0.000000 | -0.000000 | 1191 | 1165.597588 | 1239.18 | 1312.762412 | 1.040453 |
| GO:0048483\_autonomic\_nervous\_system\_development | 16 | 0 | 0.000000 | -0.000000 | 1191 | 1165.597588 | 1239.18 | 1312.762412 | 1.040453 |
| GO:0051048\_negative\_regulation\_of\_secretion | 16 | 0 | 0.000000 | -0.000000 | 1191 | 1165.597588 | 1239.18 | 1312.762412 | 1.040453 |
| GO:0051937\_catecholamine\_transport | 16 | 0 | 0.000000 | -0.000000 | 1191 | 1165.597588 | 1239.18 | 1312.762412 | 1.040453 |
| GO:0055007\_cardiac\_muscle\_cell\_differentiation | 16 | 0 | 0.000000 | -0.000000 | 1191 | 1165.597588 | 1239.18 | 1312.762412 | 1.040453 |
| GO:0060193\_positive\_regulation\_of\_lipase\_activity | 16 | 0 | 0.000000 | -0.000000 | 1191 | 1165.597588 | 1239.18 | 1312.762412 | 1.040453 |
| GO:0060713\_labyrinthine\_layer\_morphogenesis | 16 | 0 | 0.000000 | -0.000000 | 1191 | 1165.597588 | 1239.18 | 1312.762412 | 1.040453 |
| GO:0006576\_biogenic\_amine\_metabolic\_process | 53 | 0 | 0.000000 | -0.000000 | 1197 | 1170.654871 | 1243.78 | 1316.905129 | 1.039081 |
| GO:0006935\_chemotaxis | 53 | 0 | 0.000000 | -0.000000 | 1197 | 1170.654871 | 1243.78 | 1316.905129 | 1.039081 |
| GO:0042330\_taxis | 53 | 0 | 0.000000 | -0.000000 | 1197 | 1170.654871 | 1243.78 | 1316.905129 | 1.039081 |
| GO:0046942\_carboxylic\_acid\_transport | 53 | 0 | 0.000000 | -0.000000 | 1197 | 1170.654871 | 1243.78 | 1316.905129 | 1.039081 |
| GO:0051248\_negative\_regulation\_of\_protein\_metabolic\_process | 53 | 0 | 0.000000 | -0.000000 | 1197 | 1170.654871 | 1243.78 | 1316.905129 | 1.039081 |
| GO:0055085\_transmembrane\_transport | 53 | 0 | 0.000000 | -0.000000 | 1197 | 1170.654871 | 1243.78 | 1316.905129 | 1.039081 |
| GO:0009416\_response\_to\_light\_stimulus | 74 | 0 | 0.000000 | -0.000000 | 1199 | 1171.552785 | 1244.55 | 1317.547215 | 1.037990 |
| GO:0048771\_tissue\_remodeling | 74 | 0 | 0.000000 | -0.000000 | 1199 | 1171.552785 | 1244.55 | 1317.547215 | 1.037990 |
| GO:0002697\_regulation\_of\_immune\_effector\_process | 68 | 0 | 0.000000 | -0.000000 | 1203 | 1173.687851 | 1246.44 | 1319.192149 | 1.036110 |
| GO:0019932\_second-messenger-mediated\_signaling | 68 | 0 | 0.000000 | -0.000000 | 1203 | 1173.687851 | 1246.44 | 1319.192149 | 1.036110 |
| GO:0034962\_cellular\_biopolymer\_catabolic\_process | 68 | 0 | 0.000000 | -0.000000 | 1203 | 1173.687851 | 1246.44 | 1319.192149 | 1.036110 |
| GO:0042692\_muscle\_cell\_differentiation | 68 | 0 | 0.000000 | -0.000000 | 1203 | 1173.687851 | 1246.44 | 1319.192149 | 1.036110 |
| GO:0002250\_adaptive\_immune\_response | 80 | 0 | 0.000000 | -0.000000 | 1207 | 1176.026535 | 1248.48 | 1320.933465 | 1.034366 |
| GO:0002460\_adaptive\_immune\_response\_based\_on\_somatic\_recombination\_of\_immune\_receptors\_built\_from\_immunoglobulin\_superfamily\_domains | 80 | 0 | 0.000000 | -0.000000 | 1207 | 1176.026535 | 1248.48 | 1320.933465 | 1.034366 |
| GO:0006631\_fatty\_acid\_metabolic\_process | 80 | 0 | 0.000000 | -0.000000 | 1207 | 1176.026535 | 1248.48 | 1320.933465 | 1.034366 |
| GO:0044092\_negative\_regulation\_of\_molecular\_function | 80 | 0 | 0.000000 | -0.000000 | 1207 | 1176.026535 | 1248.48 | 1320.933465 | 1.034366 |
| GO:0001894\_tissue\_homeostasis | 43 | 0 | 0.000000 | -0.000000 | 1217 | 1188.300317 | 1259.86 | 1331.419683 | 1.035218 |
| GO:0002819\_regulation\_of\_adaptive\_immune\_response | 43 | 0 | 0.000000 | -0.000000 | 1217 | 1188.300317 | 1259.86 | 1331.419683 | 1.035218 |
| GO:0002822\_regulation\_of\_adaptive\_immune\_response\_based\_on\_somatic\_recombination\_of\_immune\_receptors\_built\_from\_immunoglobulin\_superfamily\_domains | 43 | 0 | 0.000000 | -0.000000 | 1217 | 1188.300317 | 1259.86 | 1331.419683 | 1.035218 |
| GO:0019637\_organophosphate\_metabolic\_process | 43 | 0 | 0.000000 | -0.000000 | 1217 | 1188.300317 | 1259.86 | 1331.419683 | 1.035218 |
| GO:0030814\_regulation\_of\_cAMP\_metabolic\_process | 43 | 0 | 0.000000 | -0.000000 | 1217 | 1188.300317 | 1259.86 | 1331.419683 | 1.035218 |
| GO:0032446\_protein\_modification\_by\_small\_protein\_conjugation | 43 | 0 | 0.000000 | -0.000000 | 1217 | 1188.300317 | 1259.86 | 1331.419683 | 1.035218 |
| GO:0032868\_response\_to\_insulin\_stimulus | 43 | 0 | 0.000000 | -0.000000 | 1217 | 1188.300317 | 1259.86 | 1331.419683 | 1.035218 |
| GO:0048762\_mesenchymal\_cell\_differentiation | 43 | 0 | 0.000000 | -0.000000 | 1217 | 1188.300317 | 1259.86 | 1331.419683 | 1.035218 |
| GO:0051604\_protein\_maturation | 43 | 0 | 0.000000 | -0.000000 | 1217 | 1188.300317 | 1259.86 | 1331.419683 | 1.035218 |
| GO:0051789\_response\_to\_protein\_stimulus | 43 | 0 | 0.000000 | -0.000000 | 1217 | 1188.300317 | 1259.86 | 1331.419683 | 1.035218 |
| GO:0002440\_production\_of\_molecular\_mediator\_of\_immune\_response | 49 | 0 | 0.000000 | -0.000000 | 1227 | 1196.034910 | 1266.94 | 1337.845090 | 1.032551 |
| GO:0003015\_heart\_process | 49 | 0 | 0.000000 | -0.000000 | 1227 | 1196.034910 | 1266.94 | 1337.845090 | 1.032551 |
| GO:0007606\_sensory\_perception\_of\_chemical\_stimulus | 49 | 0 | 0.000000 | -0.000000 | 1227 | 1196.034910 | 1266.94 | 1337.845090 | 1.032551 |
| GO:0021543\_pallium\_development | 49 | 0 | 0.000000 | -0.000000 | 1227 | 1196.034910 | 1266.94 | 1337.845090 | 1.032551 |
| GO:0042035\_regulation\_of\_cytokine\_biosynthetic\_process | 49 | 0 | 0.000000 | -0.000000 | 1227 | 1196.034910 | 1266.94 | 1337.845090 | 1.032551 |
| GO:0043473\_pigmentation | 49 | 0 | 0.000000 | -0.000000 | 1227 | 1196.034910 | 1266.94 | 1337.845090 | 1.032551 |
| GO:0046660\_female\_sex\_differentiation | 49 | 0 | 0.000000 | -0.000000 | 1227 | 1196.034910 | 1266.94 | 1337.845090 | 1.032551 |
| GO:0046661\_male\_sex\_differentiation | 49 | 0 | 0.000000 | -0.000000 | 1227 | 1196.034910 | 1266.94 | 1337.845090 | 1.032551 |
| GO:0048741\_skeletal\_muscle\_fiber\_development | 49 | 0 | 0.000000 | -0.000000 | 1227 | 1196.034910 | 1266.94 | 1337.845090 | 1.032551 |
| GO:0060047\_heart\_contraction | 49 | 0 | 0.000000 | -0.000000 | 1227 | 1196.034910 | 1266.94 | 1337.845090 | 1.032551 |
| GO:0000209\_protein\_polyubiquitination | 10 | 0 | 0.000000 | -0.000000 | 1341 | 1309.727787 | 1377.58 | 1445.432213 | 1.027278 |
| GO:0000724\_double-strand\_break\_repair\_via\_homologous\_recombination | 10 | 0 | 0.000000 | -0.000000 | 1341 | 1309.727787 | 1377.58 | 1445.432213 | 1.027278 |
| GO:0000725\_recombinational\_repair | 10 | 0 | 0.000000 | -0.000000 | 1341 | 1309.727787 | 1377.58 | 1445.432213 | 1.027278 |
| GO:0001578\_microtubule\_bundle\_formation | 10 | 0 | 0.000000 | -0.000000 | 1341 | 1309.727787 | 1377.58 | 1445.432213 | 1.027278 |
| GO:0001659\_temperature\_homeostasis | 10 | 0 | 0.000000 | -0.000000 | 1341 | 1309.727787 | 1377.58 | 1445.432213 | 1.027278 |
| GO:0001773\_myeloid\_dendritic\_cell\_activation | 10 | 0 | 0.000000 | -0.000000 | 1341 | 1309.727787 | 1377.58 | 1445.432213 | 1.027278 |
| GO:0001832\_blastocyst\_growth | 10 | 0 | 0.000000 | -0.000000 | 1341 | 1309.727787 | 1377.58 | 1445.432213 | 1.027278 |
| GO:0001914\_regulation\_of\_T\_cell\_mediated\_cytotoxicity | 10 | 0 | 0.000000 | -0.000000 | 1341 | 1309.727787 | 1377.58 | 1445.432213 | 1.027278 |
| GO:0001990\_regulation\_of\_systemic\_arterial\_blood\_pressure\_by\_hormone | 10 | 0 | 0.000000 | -0.000000 | 1341 | 1309.727787 | 1377.58 | 1445.432213 | 1.027278 |
| GO:0002673\_regulation\_of\_acute\_inflammatory\_response | 10 | 0 | 0.000000 | -0.000000 | 1341 | 1309.727787 | 1377.58 | 1445.432213 | 1.027278 |
| GO:0002711\_positive\_regulation\_of\_T\_cell\_mediated\_immunity | 10 | 0 | 0.000000 | -0.000000 | 1341 | 1309.727787 | 1377.58 | 1445.432213 | 1.027278 |
| GO:0002762\_negative\_regulation\_of\_myeloid\_leukocyte\_differentiation | 10 | 0 | 0.000000 | -0.000000 | 1341 | 1309.727787 | 1377.58 | 1445.432213 | 1.027278 |
| GO:0006040\_amino\_sugar\_metabolic\_process | 10 | 0 | 0.000000 | -0.000000 | 1341 | 1309.727787 | 1377.58 | 1445.432213 | 1.027278 |
| GO:0006081\_cellular\_aldehyde\_metabolic\_process | 10 | 0 | 0.000000 | -0.000000 | 1341 | 1309.727787 | 1377.58 | 1445.432213 | 1.027278 |
| GO:0006109\_regulation\_of\_carbohydrate\_metabolic\_process | 10 | 0 | 0.000000 | -0.000000 | 1341 | 1309.727787 | 1377.58 | 1445.432213 | 1.027278 |
| GO:0006289\_nucleotide-excision\_repair | 10 | 0 | 0.000000 | -0.000000 | 1341 | 1309.727787 | 1377.58 | 1445.432213 | 1.027278 |
| GO:0006342\_chromatin\_silencing | 10 | 0 | 0.000000 | -0.000000 | 1341 | 1309.727787 | 1377.58 | 1445.432213 | 1.027278 |
| GO:0006405\_RNA\_export\_from\_nucleus | 10 | 0 | 0.000000 | -0.000000 | 1341 | 1309.727787 | 1377.58 | 1445.432213 | 1.027278 |
| GO:0006801\_superoxide\_metabolic\_process | 10 | 0 | 0.000000 | -0.000000 | 1341 | 1309.727787 | 1377.58 | 1445.432213 | 1.027278 |
| GO:0006805\_xenobiotic\_metabolic\_process | 10 | 0 | 0.000000 | -0.000000 | 1341 | 1309.727787 | 1377.58 | 1445.432213 | 1.027278 |
| GO:0006826\_iron\_ion\_transport | 10 | 0 | 0.000000 | -0.000000 | 1341 | 1309.727787 | 1377.58 | 1445.432213 | 1.027278 |
| GO:0006921\_cell\_structure\_disassembly\_during\_apoptosis | 10 | 0 | 0.000000 | -0.000000 | 1341 | 1309.727787 | 1377.58 | 1445.432213 | 1.027278 |
| GO:0006968\_cellular\_defense\_response | 10 | 0 | 0.000000 | -0.000000 | 1341 | 1309.727787 | 1377.58 | 1445.432213 | 1.027278 |
| GO:0007006\_mitochondrial\_membrane\_organization | 10 | 0 | 0.000000 | -0.000000 | 1341 | 1309.727787 | 1377.58 | 1445.432213 | 1.027278 |
| GO:0007093\_mitotic\_cell\_cycle\_checkpoint | 10 | 0 | 0.000000 | -0.000000 | 1341 | 1309.727787 | 1377.58 | 1445.432213 | 1.027278 |
| GO:0007172\_signal\_complex\_assembly | 10 | 0 | 0.000000 | -0.000000 | 1341 | 1309.727787 | 1377.58 | 1445.432213 | 1.027278 |
| GO:0007194\_negative\_regulation\_of\_adenylate\_cyclase\_activity | 10 | 0 | 0.000000 | -0.000000 | 1341 | 1309.727787 | 1377.58 | 1445.432213 | 1.027278 |
| GO:0008088\_axon\_cargo\_transport | 10 | 0 | 0.000000 | -0.000000 | 1341 | 1309.727787 | 1377.58 | 1445.432213 | 1.027278 |
| GO:0008206\_bile\_acid\_metabolic\_process | 10 | 0 | 0.000000 | -0.000000 | 1341 | 1309.727787 | 1377.58 | 1445.432213 | 1.027278 |
| GO:0008211\_glucocorticoid\_metabolic\_process | 10 | 0 | 0.000000 | -0.000000 | 1341 | 1309.727787 | 1377.58 | 1445.432213 | 1.027278 |
| GO:0009066\_aspartate\_family\_amino\_acid\_metabolic\_process | 10 | 0 | 0.000000 | -0.000000 | 1341 | 1309.727787 | 1377.58 | 1445.432213 | 1.027278 |
| GO:0009110\_vitamin\_biosynthetic\_process | 10 | 0 | 0.000000 | -0.000000 | 1341 | 1309.727787 | 1377.58 | 1445.432213 | 1.027278 |
| GO:0009620\_response\_to\_fungus | 10 | 0 | 0.000000 | -0.000000 | 1341 | 1309.727787 | 1377.58 | 1445.432213 | 1.027278 |
| GO:0009743\_response\_to\_carbohydrate\_stimulus | 10 | 0 | 0.000000 | -0.000000 | 1341 | 1309.727787 | 1377.58 | 1445.432213 | 1.027278 |
| GO:0009948\_anterior\_posterior\_axis\_specification | 10 | 0 | 0.000000 | -0.000000 | 1341 | 1309.727787 | 1377.58 | 1445.432213 | 1.027278 |
| GO:0010827\_regulation\_of\_glucose\_transport | 10 | 0 | 0.000000 | -0.000000 | 1341 | 1309.727787 | 1377.58 | 1445.432213 | 1.027278 |
| GO:0015718\_monocarboxylic\_acid\_transport | 10 | 0 | 0.000000 | -0.000000 | 1341 | 1309.727787 | 1377.58 | 1445.432213 | 1.027278 |
| GO:0016197\_endosome\_transport | 10 | 0 | 0.000000 | -0.000000 | 1341 | 1309.727787 | 1377.58 | 1445.432213 | 1.027278 |
| GO:0016486\_peptide\_hormone\_processing | 10 | 0 | 0.000000 | -0.000000 | 1341 | 1309.727787 | 1377.58 | 1445.432213 | 1.027278 |
| GO:0017156\_calcium\_ion-dependent\_exocytosis | 10 | 0 | 0.000000 | -0.000000 | 1341 | 1309.727787 | 1377.58 | 1445.432213 | 1.027278 |
| GO:0018149\_peptide\_cross-linking | 10 | 0 | 0.000000 | -0.000000 | 1341 | 1309.727787 | 1377.58 | 1445.432213 | 1.027278 |
| GO:0019321\_pentose\_metabolic\_process | 10 | 0 | 0.000000 | -0.000000 | 1341 | 1309.727787 | 1377.58 | 1445.432213 | 1.027278 |
| GO:0021534\_cell\_proliferation\_in\_hindbrain | 10 | 0 | 0.000000 | -0.000000 | 1341 | 1309.727787 | 1377.58 | 1445.432213 | 1.027278 |
| GO:0021871\_forebrain\_regionalization | 10 | 0 | 0.000000 | -0.000000 | 1341 | 1309.727787 | 1377.58 | 1445.432213 | 1.027278 |
| GO:0021895\_cerebral\_cortex\_neuron\_differentiation | 10 | 0 | 0.000000 | -0.000000 | 1341 | 1309.727787 | 1377.58 | 1445.432213 | 1.027278 |
| GO:0021924\_cell\_proliferation\_in\_the\_external\_granule\_layer | 10 | 0 | 0.000000 | -0.000000 | 1341 | 1309.727787 | 1377.58 | 1445.432213 | 1.027278 |
| GO:0021930\_granule\_cell\_precursor\_proliferation | 10 | 0 | 0.000000 | -0.000000 | 1341 | 1309.727787 | 1377.58 | 1445.432213 | 1.027278 |
| GO:0022900\_electron\_transport\_chain | 10 | 0 | 0.000000 | -0.000000 | 1341 | 1309.727787 | 1377.58 | 1445.432213 | 1.027278 |
| GO:0022904\_respiratory\_electron\_transport\_chain | 10 | 0 | 0.000000 | -0.000000 | 1341 | 1309.727787 | 1377.58 | 1445.432213 | 1.027278 |
| GO:0030168\_platelet\_activation | 10 | 0 | 0.000000 | -0.000000 | 1341 | 1309.727787 | 1377.58 | 1445.432213 | 1.027278 |
| GO:0030833\_regulation\_of\_actin\_filament\_polymerization | 10 | 0 | 0.000000 | -0.000000 | 1341 | 1309.727787 | 1377.58 | 1445.432213 | 1.027278 |
| GO:0031018\_endocrine\_pancreas\_development | 10 | 0 | 0.000000 | -0.000000 | 1341 | 1309.727787 | 1377.58 | 1445.432213 | 1.027278 |
| GO:0031280\_negative\_regulation\_of\_cyclase\_activity | 10 | 0 | 0.000000 | -0.000000 | 1341 | 1309.727787 | 1377.58 | 1445.432213 | 1.027278 |
| GO:0031331\_positive\_regulation\_of\_cellular\_catabolic\_process | 10 | 0 | 0.000000 | -0.000000 | 1341 | 1309.727787 | 1377.58 | 1445.432213 | 1.027278 |
| GO:0031645\_negative\_regulation\_of\_neurological\_system\_process | 10 | 0 | 0.000000 | -0.000000 | 1341 | 1309.727787 | 1377.58 | 1445.432213 | 1.027278 |
| GO:0032602\_chemokine\_production | 10 | 0 | 0.000000 | -0.000000 | 1341 | 1309.727787 | 1377.58 | 1445.432213 | 1.027278 |
| GO:0032633\_interleukin-4\_production | 10 | 0 | 0.000000 | -0.000000 | 1341 | 1309.727787 | 1377.58 | 1445.432213 | 1.027278 |
| GO:0032642\_regulation\_of\_chemokine\_production | 10 | 0 | 0.000000 | -0.000000 | 1341 | 1309.727787 | 1377.58 | 1445.432213 | 1.027278 |
| GO:0032673\_regulation\_of\_interleukin-4\_production | 10 | 0 | 0.000000 | -0.000000 | 1341 | 1309.727787 | 1377.58 | 1445.432213 | 1.027278 |
| GO:0032760\_positive\_regulation\_of\_tumor\_necrosis\_factor\_production | 10 | 0 | 0.000000 | -0.000000 | 1341 | 1309.727787 | 1377.58 | 1445.432213 | 1.027278 |
| GO:0033081\_regulation\_of\_T\_cell\_differentiation\_in\_the\_thymus | 10 | 0 | 0.000000 | -0.000000 | 1341 | 1309.727787 | 1377.58 | 1445.432213 | 1.027278 |
| GO:0034105\_positive\_regulation\_of\_tissue\_remodeling | 10 | 0 | 0.000000 | -0.000000 | 1341 | 1309.727787 | 1377.58 | 1445.432213 | 1.027278 |
| GO:0034637\_cellular\_carbohydrate\_biosynthetic\_process | 10 | 0 | 0.000000 | -0.000000 | 1341 | 1309.727787 | 1377.58 | 1445.432213 | 1.027278 |
| GO:0040015\_negative\_regulation\_of\_multicellular\_organism\_growth | 10 | 0 | 0.000000 | -0.000000 | 1341 | 1309.727787 | 1377.58 | 1445.432213 | 1.027278 |
| GO:0042088\_T-helper\_1\_type\_immune\_response | 10 | 0 | 0.000000 | -0.000000 | 1341 | 1309.727787 | 1377.58 | 1445.432213 | 1.027278 |
| GO:0042116\_macrophage\_activation | 10 | 0 | 0.000000 | -0.000000 | 1341 | 1309.727787 | 1377.58 | 1445.432213 | 1.027278 |
| GO:0042177\_negative\_regulation\_of\_protein\_catabolic\_process | 10 | 0 | 0.000000 | -0.000000 | 1341 | 1309.727787 | 1377.58 | 1445.432213 | 1.027278 |
| GO:0042755\_eating\_behavior | 10 | 0 | 0.000000 | -0.000000 | 1341 | 1309.727787 | 1377.58 | 1445.432213 | 1.027278 |
| GO:0043113\_receptor\_clustering | 10 | 0 | 0.000000 | -0.000000 | 1341 | 1309.727787 | 1377.58 | 1445.432213 | 1.027278 |
| GO:0043488\_regulation\_of\_mRNA\_stability | 10 | 0 | 0.000000 | -0.000000 | 1341 | 1309.727787 | 1377.58 | 1445.432213 | 1.027278 |
| GO:0043506\_regulation\_of\_JUN\_kinase\_activity | 10 | 0 | 0.000000 | -0.000000 | 1341 | 1309.727787 | 1377.58 | 1445.432213 | 1.027278 |
| GO:0043525\_positive\_regulation\_of\_neuron\_apoptosis | 10 | 0 | 0.000000 | -0.000000 | 1341 | 1309.727787 | 1377.58 | 1445.432213 | 1.027278 |
| GO:0045132\_meiotic\_chromosome\_segregation | 10 | 0 | 0.000000 | -0.000000 | 1341 | 1309.727787 | 1377.58 | 1445.432213 | 1.027278 |
| GO:0045446\_endothelial\_cell\_differentiation | 10 | 0 | 0.000000 | -0.000000 | 1341 | 1309.727787 | 1377.58 | 1445.432213 | 1.027278 |
| GO:0045576\_mast\_cell\_activation | 10 | 0 | 0.000000 | -0.000000 | 1341 | 1309.727787 | 1377.58 | 1445.432213 | 1.027278 |
| GO:0045669\_positive\_regulation\_of\_osteoblast\_differentiation | 10 | 0 | 0.000000 | -0.000000 | 1341 | 1309.727787 | 1377.58 | 1445.432213 | 1.027278 |
| GO:0045776\_negative\_regulation\_of\_blood\_pressure | 10 | 0 | 0.000000 | -0.000000 | 1341 | 1309.727787 | 1377.58 | 1445.432213 | 1.027278 |
| GO:0045777\_positive\_regulation\_of\_blood\_pressure | 10 | 0 | 0.000000 | -0.000000 | 1341 | 1309.727787 | 1377.58 | 1445.432213 | 1.027278 |
| GO:0045814\_negative\_regulation\_of\_gene\_expression\_\_epigenetic | 10 | 0 | 0.000000 | -0.000000 | 1341 | 1309.727787 | 1377.58 | 1445.432213 | 1.027278 |
| GO:0045911\_positive\_regulation\_of\_DNA\_recombination | 10 | 0 | 0.000000 | -0.000000 | 1341 | 1309.727787 | 1377.58 | 1445.432213 | 1.027278 |
| GO:0046887\_positive\_regulation\_of\_hormone\_secretion | 10 | 0 | 0.000000 | -0.000000 | 1341 | 1309.727787 | 1377.58 | 1445.432213 | 1.027278 |
| GO:0048291\_isotype\_switching\_to\_IgG\_isotypes | 10 | 0 | 0.000000 | -0.000000 | 1341 | 1309.727787 | 1377.58 | 1445.432213 | 1.027278 |
| GO:0048302\_regulation\_of\_isotype\_switching\_to\_IgG\_isotypes | 10 | 0 | 0.000000 | -0.000000 | 1341 | 1309.727787 | 1377.58 | 1445.432213 | 1.027278 |
| GO:0048339\_paraxial\_mesoderm\_development | 10 | 0 | 0.000000 | -0.000000 | 1341 | 1309.727787 | 1377.58 | 1445.432213 | 1.027278 |
| GO:0048384\_retinoic\_acid\_receptor\_signaling\_pathway | 10 | 0 | 0.000000 | -0.000000 | 1341 | 1309.727787 | 1377.58 | 1445.432213 | 1.027278 |
| GO:0048641\_regulation\_of\_skeletal\_muscle\_tissue\_development | 10 | 0 | 0.000000 | -0.000000 | 1341 | 1309.727787 | 1377.58 | 1445.432213 | 1.027278 |
| GO:0048738\_cardiac\_muscle\_tissue\_development | 10 | 0 | 0.000000 | -0.000000 | 1341 | 1309.727787 | 1377.58 | 1445.432213 | 1.027278 |
| GO:0050654\_chondroitin\_sulfate\_proteoglycan\_metabolic\_process | 10 | 0 | 0.000000 | -0.000000 | 1341 | 1309.727787 | 1377.58 | 1445.432213 | 1.027278 |
| GO:0050657\_nucleic\_acid\_transport | 10 | 0 | 0.000000 | -0.000000 | 1341 | 1309.727787 | 1377.58 | 1445.432213 | 1.027278 |
| GO:0050658\_RNA\_transport | 10 | 0 | 0.000000 | -0.000000 | 1341 | 1309.727787 | 1377.58 | 1445.432213 | 1.027278 |
| GO:0050663\_cytokine\_secretion | 10 | 0 | 0.000000 | -0.000000 | 1341 | 1309.727787 | 1377.58 | 1445.432213 | 1.027278 |
| GO:0050714\_positive\_regulation\_of\_protein\_secretion | 10 | 0 | 0.000000 | -0.000000 | 1341 | 1309.727787 | 1377.58 | 1445.432213 | 1.027278 |
| GO:0050879\_multicellular\_organismal\_movement | 10 | 0 | 0.000000 | -0.000000 | 1341 | 1309.727787 | 1377.58 | 1445.432213 | 1.027278 |
| GO:0050881\_musculoskeletal\_movement | 10 | 0 | 0.000000 | -0.000000 | 1341 | 1309.727787 | 1377.58 | 1445.432213 | 1.027278 |
| GO:0050886\_endocrine\_process | 10 | 0 | 0.000000 | -0.000000 | 1341 | 1309.727787 | 1377.58 | 1445.432213 | 1.027278 |
| GO:0050892\_intestinal\_absorption | 10 | 0 | 0.000000 | -0.000000 | 1341 | 1309.727787 | 1377.58 | 1445.432213 | 1.027278 |
| GO:0051147\_regulation\_of\_muscle\_cell\_differentiation | 10 | 0 | 0.000000 | -0.000000 | 1341 | 1309.727787 | 1377.58 | 1445.432213 | 1.027278 |
| GO:0051208\_sequestering\_of\_calcium\_ion | 10 | 0 | 0.000000 | -0.000000 | 1341 | 1309.727787 | 1377.58 | 1445.432213 | 1.027278 |
| GO:0051209\_release\_of\_sequestered\_calcium\_ion\_into\_cytosol | 10 | 0 | 0.000000 | -0.000000 | 1341 | 1309.727787 | 1377.58 | 1445.432213 | 1.027278 |
| GO:0051224\_negative\_regulation\_of\_protein\_transport | 10 | 0 | 0.000000 | -0.000000 | 1341 | 1309.727787 | 1377.58 | 1445.432213 | 1.027278 |
| GO:0051236\_establishment\_of\_RNA\_localization | 10 | 0 | 0.000000 | -0.000000 | 1341 | 1309.727787 | 1377.58 | 1445.432213 | 1.027278 |
| GO:0051238\_sequestering\_of\_metal\_ion | 10 | 0 | 0.000000 | -0.000000 | 1341 | 1309.727787 | 1377.58 | 1445.432213 | 1.027278 |
| GO:0051262\_protein\_tetramerization | 10 | 0 | 0.000000 | -0.000000 | 1341 | 1309.727787 | 1377.58 | 1445.432213 | 1.027278 |
| GO:0051282\_regulation\_of\_sequestering\_of\_calcium\_ion | 10 | 0 | 0.000000 | -0.000000 | 1341 | 1309.727787 | 1377.58 | 1445.432213 | 1.027278 |
| GO:0051283\_negative\_regulation\_of\_sequestering\_of\_calcium\_ion | 10 | 0 | 0.000000 | -0.000000 | 1341 | 1309.727787 | 1377.58 | 1445.432213 | 1.027278 |
| GO:0051350\_negative\_regulation\_of\_lyase\_activity | 10 | 0 | 0.000000 | -0.000000 | 1341 | 1309.727787 | 1377.58 | 1445.432213 | 1.027278 |
| GO:0051445\_regulation\_of\_meiotic\_cell\_cycle | 10 | 0 | 0.000000 | -0.000000 | 1341 | 1309.727787 | 1377.58 | 1445.432213 | 1.027278 |
| GO:0051650\_establishment\_of\_vesicle\_localization | 10 | 0 | 0.000000 | -0.000000 | 1341 | 1309.727787 | 1377.58 | 1445.432213 | 1.027278 |
| GO:0051651\_maintenance\_of\_location\_in\_cell | 10 | 0 | 0.000000 | -0.000000 | 1341 | 1309.727787 | 1377.58 | 1445.432213 | 1.027278 |
| GO:0060135\_maternal\_process\_involved\_in\_female\_pregnancy | 10 | 0 | 0.000000 | -0.000000 | 1341 | 1309.727787 | 1377.58 | 1445.432213 | 1.027278 |
| GO:0060601\_lateral\_sprouting\_from\_an\_epithelium | 10 | 0 | 0.000000 | -0.000000 | 1341 | 1309.727787 | 1377.58 | 1445.432213 | 1.027278 |
| GO:0060669\_embryonic\_placenta\_morphogenesis | 10 | 0 | 0.000000 | -0.000000 | 1341 | 1309.727787 | 1377.58 | 1445.432213 | 1.027278 |
| GO:0060706\_cell\_differentiation\_involved\_in\_embryonic\_placenta\_development | 10 | 0 | 0.000000 | -0.000000 | 1341 | 1309.727787 | 1377.58 | 1445.432213 | 1.027278 |
| GO:0060768\_regulation\_of\_epithelial\_cell\_proliferation\_involved\_in\_prostate\_gland\_development | 10 | 0 | 0.000000 | -0.000000 | 1341 | 1309.727787 | 1377.58 | 1445.432213 | 1.027278 |
| GO:0007015\_actin\_filament\_organization | 50 | 0 | 0.000000 | -0.000000 | 1345 | 1314.927622 | 1382.34 | 1449.752378 | 1.027762 |
| GO:0009190\_cyclic\_nucleotide\_biosynthetic\_process | 50 | 0 | 0.000000 | -0.000000 | 1345 | 1314.927622 | 1382.34 | 1449.752378 | 1.027762 |
| GO:0042129\_regulation\_of\_T\_cell\_proliferation | 50 | 0 | 0.000000 | -0.000000 | 1345 | 1314.927622 | 1382.34 | 1449.752378 | 1.027762 |
| GO:0070647\_protein\_modification\_by\_small\_protein\_conjugation\_or\_removal | 50 | 0 | 0.000000 | -0.000000 | 1345 | 1314.927622 | 1382.34 | 1449.752378 | 1.027762 |
| GO:0000082\_G1\_S\_transition\_of\_mitotic\_cell\_cycle | 23 | 0 | 0.000000 | -0.000000 | 1364 | 1335.015019 | 1400.95 | 1466.884981 | 1.027089 |
| GO:0002204\_somatic\_recombination\_of\_immunoglobulin\_genes\_during\_immune\_response | 23 | 0 | 0.000000 | -0.000000 | 1364 | 1335.015019 | 1400.95 | 1466.884981 | 1.027089 |
| GO:0002208\_somatic\_diversification\_of\_immunoglobulins\_during\_immune\_response | 23 | 0 | 0.000000 | -0.000000 | 1364 | 1335.015019 | 1400.95 | 1466.884981 | 1.027089 |
| GO:0002228\_natural\_killer\_cell\_mediated\_immunity | 23 | 0 | 0.000000 | -0.000000 | 1364 | 1335.015019 | 1400.95 | 1466.884981 | 1.027089 |
| GO:0002821\_positive\_regulation\_of\_adaptive\_immune\_response | 23 | 0 | 0.000000 | -0.000000 | 1364 | 1335.015019 | 1400.95 | 1466.884981 | 1.027089 |
| GO:0002824\_positive\_regulation\_of\_adaptive\_immune\_response\_based\_on\_somatic\_recombination\_of\_immune\_receptors\_built\_from\_immunoglobulin\_superfamily\_domains | 23 | 0 | 0.000000 | -0.000000 | 1364 | 1335.015019 | 1400.95 | 1466.884981 | 1.027089 |
| GO:0003073\_regulation\_of\_systemic\_arterial\_blood\_pressure | 23 | 0 | 0.000000 | -0.000000 | 1364 | 1335.015019 | 1400.95 | 1466.884981 | 1.027089 |
| GO:0007018\_microtubule-based\_movement | 23 | 0 | 0.000000 | -0.000000 | 1364 | 1335.015019 | 1400.95 | 1466.884981 | 1.027089 |
| GO:0007163\_establishment\_or\_maintenance\_of\_cell\_polarity | 23 | 0 | 0.000000 | -0.000000 | 1364 | 1335.015019 | 1400.95 | 1466.884981 | 1.027089 |
| GO:0007584\_response\_to\_nutrient | 23 | 0 | 0.000000 | -0.000000 | 1364 | 1335.015019 | 1400.95 | 1466.884981 | 1.027089 |
| GO:0008542\_visual\_learning | 23 | 0 | 0.000000 | -0.000000 | 1364 | 1335.015019 | 1400.95 | 1466.884981 | 1.027089 |
| GO:0009954\_proximal\_distal\_pattern\_formation | 23 | 0 | 0.000000 | -0.000000 | 1364 | 1335.015019 | 1400.95 | 1466.884981 | 1.027089 |
| GO:0015698\_inorganic\_anion\_transport | 23 | 0 | 0.000000 | -0.000000 | 1364 | 1335.015019 | 1400.95 | 1466.884981 | 1.027089 |
| GO:0022613\_ribonucleoprotein\_complex\_biogenesis | 23 | 0 | 0.000000 | -0.000000 | 1364 | 1335.015019 | 1400.95 | 1466.884981 | 1.027089 |
| GO:0030512\_negative\_regulation\_of\_transforming\_growth\_factor\_beta\_receptor\_signaling\_pathway | 23 | 0 | 0.000000 | -0.000000 | 1364 | 1335.015019 | 1400.95 | 1466.884981 | 1.027089 |
| GO:0032635\_interleukin-6\_production | 23 | 0 | 0.000000 | -0.000000 | 1364 | 1335.015019 | 1400.95 | 1466.884981 | 1.027089 |
| GO:0032675\_regulation\_of\_interleukin-6\_production | 23 | 0 | 0.000000 | -0.000000 | 1364 | 1335.015019 | 1400.95 | 1466.884981 | 1.027089 |
| GO:0042267\_natural\_killer\_cell\_mediated\_cytotoxicity | 23 | 0 | 0.000000 | -0.000000 | 1364 | 1335.015019 | 1400.95 | 1466.884981 | 1.027089 |
| GO:0045190\_isotype\_switching | 23 | 0 | 0.000000 | -0.000000 | 1364 | 1335.015019 | 1400.95 | 1466.884981 | 1.027089 |
| GO:0030003\_cellular\_cation\_homeostasis | 90 | 0 | 0.000000 | -0.000000 | 1366 | 1336.856105 | 1402.52 | 1468.183895 | 1.026735 |
| GO:0035264\_multicellular\_organism\_growth | 90 | 0 | 0.000000 | -0.000000 | 1366 | 1336.856105 | 1402.52 | 1468.183895 | 1.026735 |
| GO:0000002\_mitochondrial\_genome\_maintenance | 9 | 0 | 0.000000 | -0.000000 | 1476 | 1447.676740 | 1511.08 | 1574.483260 | 1.023767 |
| GO:0000186\_activation\_of\_MAPKK\_activity | 9 | 0 | 0.000000 | -0.000000 | 1476 | 1447.676740 | 1511.08 | 1574.483260 | 1.023767 |
| GO:0001539\_ciliary\_or\_flagellar\_motility | 9 | 0 | 0.000000 | -0.000000 | 1476 | 1447.676740 | 1511.08 | 1574.483260 | 1.023767 |
| GO:0001542\_ovulation\_from\_ovarian\_follicle | 9 | 0 | 0.000000 | -0.000000 | 1476 | 1447.676740 | 1511.08 | 1574.483260 | 1.023767 |
| GO:0001667\_ameboidal\_cell\_migration | 9 | 0 | 0.000000 | -0.000000 | 1476 | 1447.676740 | 1511.08 | 1574.483260 | 1.023767 |
| GO:0001676\_long-chain\_fatty\_acid\_metabolic\_process | 9 | 0 | 0.000000 | -0.000000 | 1476 | 1447.676740 | 1511.08 | 1574.483260 | 1.023767 |
| GO:0001935\_endothelial\_cell\_proliferation | 9 | 0 | 0.000000 | -0.000000 | 1476 | 1447.676740 | 1511.08 | 1574.483260 | 1.023767 |
| GO:0002021\_response\_to\_dietary\_excess | 9 | 0 | 0.000000 | -0.000000 | 1476 | 1447.676740 | 1511.08 | 1574.483260 | 1.023767 |
| GO:0002028\_regulation\_of\_sodium\_ion\_transport | 9 | 0 | 0.000000 | -0.000000 | 1476 | 1447.676740 | 1511.08 | 1574.483260 | 1.023767 |
| GO:0002221\_pattern\_recognition\_receptor\_signaling\_pathway | 9 | 0 | 0.000000 | -0.000000 | 1476 | 1447.676740 | 1511.08 | 1574.483260 | 1.023767 |
| GO:0002292\_T\_cell\_differentiation\_during\_immune\_response | 9 | 0 | 0.000000 | -0.000000 | 1476 | 1447.676740 | 1511.08 | 1574.483260 | 1.023767 |
| GO:0002293\_alpha-beta\_T\_cell\_differentiation\_during\_immune\_response | 9 | 0 | 0.000000 | -0.000000 | 1476 | 1447.676740 | 1511.08 | 1574.483260 | 1.023767 |
| GO:0002294\_CD4-positive\_\_alpha-beta\_T\_cell\_differentiation\_during\_immune\_response | 9 | 0 | 0.000000 | -0.000000 | 1476 | 1447.676740 | 1511.08 | 1574.483260 | 1.023767 |
| GO:0002507\_tolerance\_induction | 9 | 0 | 0.000000 | -0.000000 | 1476 | 1447.676740 | 1511.08 | 1574.483260 | 1.023767 |
| GO:0002886\_regulation\_of\_myeloid\_leukocyte\_mediated\_immunity | 9 | 0 | 0.000000 | -0.000000 | 1476 | 1447.676740 | 1511.08 | 1574.483260 | 1.023767 |
| GO:0006007\_glucose\_catabolic\_process | 9 | 0 | 0.000000 | -0.000000 | 1476 | 1447.676740 | 1511.08 | 1574.483260 | 1.023767 |
| GO:0006182\_cGMP\_biosynthetic\_process | 9 | 0 | 0.000000 | -0.000000 | 1476 | 1447.676740 | 1511.08 | 1574.483260 | 1.023767 |
| GO:0006309\_DNA\_fragmentation\_involved\_in\_apoptosis | 9 | 0 | 0.000000 | -0.000000 | 1476 | 1447.676740 | 1511.08 | 1574.483260 | 1.023767 |
| GO:0006364\_rRNA\_processing | 9 | 0 | 0.000000 | -0.000000 | 1476 | 1447.676740 | 1511.08 | 1574.483260 | 1.023767 |
| GO:0006476\_protein\_amino\_acid\_deacetylation | 9 | 0 | 0.000000 | -0.000000 | 1476 | 1447.676740 | 1511.08 | 1574.483260 | 1.023767 |
| GO:0006595\_polyamine\_metabolic\_process | 9 | 0 | 0.000000 | -0.000000 | 1476 | 1447.676740 | 1511.08 | 1574.483260 | 1.023767 |
| GO:0006611\_protein\_export\_from\_nucleus | 9 | 0 | 0.000000 | -0.000000 | 1476 | 1447.676740 | 1511.08 | 1574.483260 | 1.023767 |
| GO:0006910\_phagocytosis\_\_recognition | 9 | 0 | 0.000000 | -0.000000 | 1476 | 1447.676740 | 1511.08 | 1574.483260 | 1.023767 |
| GO:0006911\_phagocytosis\_\_engulfment | 9 | 0 | 0.000000 | -0.000000 | 1476 | 1447.676740 | 1511.08 | 1574.483260 | 1.023767 |
| GO:0007128\_meiotic\_prophase\_I | 9 | 0 | 0.000000 | -0.000000 | 1476 | 1447.676740 | 1511.08 | 1574.483260 | 1.023767 |
| GO:0007193\_inhibition\_of\_adenylate\_cyclase\_activity\_by\_G-protein\_signaling | 9 | 0 | 0.000000 | -0.000000 | 1476 | 1447.676740 | 1511.08 | 1574.483260 | 1.023767 |
| GO:0007379\_segment\_specification | 9 | 0 | 0.000000 | -0.000000 | 1476 | 1447.676740 | 1511.08 | 1574.483260 | 1.023767 |
| GO:0007617\_mating\_behavior | 9 | 0 | 0.000000 | -0.000000 | 1476 | 1447.676740 | 1511.08 | 1574.483260 | 1.023767 |
| GO:0009451\_RNA\_modification | 9 | 0 | 0.000000 | -0.000000 | 1476 | 1447.676740 | 1511.08 | 1574.483260 | 1.023767 |
| GO:0010165\_response\_to\_X-ray | 9 | 0 | 0.000000 | -0.000000 | 1476 | 1447.676740 | 1511.08 | 1574.483260 | 1.023767 |
| GO:0010675\_regulation\_of\_cellular\_carbohydrate\_metabolic\_process | 9 | 0 | 0.000000 | -0.000000 | 1476 | 1447.676740 | 1511.08 | 1574.483260 | 1.023767 |
| GO:0014073\_response\_to\_tropane | 9 | 0 | 0.000000 | -0.000000 | 1476 | 1447.676740 | 1511.08 | 1574.483260 | 1.023767 |
| GO:0015695\_organic\_cation\_transport | 9 | 0 | 0.000000 | -0.000000 | 1476 | 1447.676740 | 1511.08 | 1574.483260 | 1.023767 |
| GO:0016072\_rRNA\_metabolic\_process | 9 | 0 | 0.000000 | -0.000000 | 1476 | 1447.676740 | 1511.08 | 1574.483260 | 1.023767 |
| GO:0017145\_stem\_cell\_division | 9 | 0 | 0.000000 | -0.000000 | 1476 | 1447.676740 | 1511.08 | 1574.483260 | 1.023767 |
| GO:0019320\_hexose\_catabolic\_process | 9 | 0 | 0.000000 | -0.000000 | 1476 | 1447.676740 | 1511.08 | 1574.483260 | 1.023767 |
| GO:0021544\_subpallium\_development | 9 | 0 | 0.000000 | -0.000000 | 1476 | 1447.676740 | 1511.08 | 1574.483260 | 1.023767 |
| GO:0021936\_regulation\_of\_granule\_cell\_precursor\_proliferation | 9 | 0 | 0.000000 | -0.000000 | 1476 | 1447.676740 | 1511.08 | 1574.483260 | 1.023767 |
| GO:0021940\_positive\_regulation\_of\_granule\_cell\_precursor\_proliferation | 9 | 0 | 0.000000 | -0.000000 | 1476 | 1447.676740 | 1511.08 | 1574.483260 | 1.023767 |
| GO:0030048\_actin\_filament-based\_movement | 9 | 0 | 0.000000 | -0.000000 | 1476 | 1447.676740 | 1511.08 | 1574.483260 | 1.023767 |
| GO:0030279\_negative\_regulation\_of\_ossification | 9 | 0 | 0.000000 | -0.000000 | 1476 | 1447.676740 | 1511.08 | 1574.483260 | 1.023767 |
| GO:0030325\_adrenal\_gland\_development | 9 | 0 | 0.000000 | -0.000000 | 1476 | 1447.676740 | 1511.08 | 1574.483260 | 1.023767 |
| GO:0030728\_ovulation | 9 | 0 | 0.000000 | -0.000000 | 1476 | 1447.676740 | 1511.08 | 1574.483260 | 1.023767 |
| GO:0031023\_microtubule\_organizing\_center\_organization | 9 | 0 | 0.000000 | -0.000000 | 1476 | 1447.676740 | 1511.08 | 1574.483260 | 1.023767 |
| GO:0032388\_positive\_regulation\_of\_intracellular\_transport | 9 | 0 | 0.000000 | -0.000000 | 1476 | 1447.676740 | 1511.08 | 1574.483260 | 1.023767 |
| GO:0032606\_type\_I\_interferon\_production | 9 | 0 | 0.000000 | -0.000000 | 1476 | 1447.676740 | 1511.08 | 1574.483260 | 1.023767 |
| GO:0032814\_regulation\_of\_natural\_killer\_cell\_activation | 9 | 0 | 0.000000 | -0.000000 | 1476 | 1447.676740 | 1511.08 | 1574.483260 | 1.023767 |
| GO:0032816\_positive\_regulation\_of\_natural\_killer\_cell\_activation | 9 | 0 | 0.000000 | -0.000000 | 1476 | 1447.676740 | 1511.08 | 1574.483260 | 1.023767 |
| GO:0033028\_myeloid\_cell\_apoptosis | 9 | 0 | 0.000000 | -0.000000 | 1476 | 1447.676740 | 1511.08 | 1574.483260 | 1.023767 |
| GO:0033143\_regulation\_of\_steroid\_hormone\_receptor\_signaling\_pathway | 9 | 0 | 0.000000 | -0.000000 | 1476 | 1447.676740 | 1511.08 | 1574.483260 | 1.023767 |
| GO:0033151\_V(D)J\_recombination | 9 | 0 | 0.000000 | -0.000000 | 1476 | 1447.676740 | 1511.08 | 1574.483260 | 1.023767 |
| GO:0033344\_cholesterol\_efflux | 9 | 0 | 0.000000 | -0.000000 | 1476 | 1447.676740 | 1511.08 | 1574.483260 | 1.023767 |
| GO:0034605\_cellular\_response\_to\_heat | 9 | 0 | 0.000000 | -0.000000 | 1476 | 1447.676740 | 1511.08 | 1574.483260 | 1.023767 |
| GO:0035088\_establishment\_or\_maintenance\_of\_apical\_basal\_cell\_polarity | 9 | 0 | 0.000000 | -0.000000 | 1476 | 1447.676740 | 1511.08 | 1574.483260 | 1.023767 |
| GO:0035162\_embryonic\_hemopoiesis | 9 | 0 | 0.000000 | -0.000000 | 1476 | 1447.676740 | 1511.08 | 1574.483260 | 1.023767 |
| GO:0040020\_regulation\_of\_meiosis | 9 | 0 | 0.000000 | -0.000000 | 1476 | 1447.676740 | 1511.08 | 1574.483260 | 1.023767 |
| GO:0042058\_regulation\_of\_epidermal\_growth\_factor\_receptor\_signaling\_pathway | 9 | 0 | 0.000000 | -0.000000 | 1476 | 1447.676740 | 1511.08 | 1574.483260 | 1.023767 |
| GO:0042093\_T-helper\_cell\_differentiation | 9 | 0 | 0.000000 | -0.000000 | 1476 | 1447.676740 | 1511.08 | 1574.483260 | 1.023767 |
| GO:0042220\_response\_to\_cocaine | 9 | 0 | 0.000000 | -0.000000 | 1476 | 1447.676740 | 1511.08 | 1574.483260 | 1.023767 |
| GO:0042402\_biogenic\_amine\_catabolic\_process | 9 | 0 | 0.000000 | -0.000000 | 1476 | 1447.676740 | 1511.08 | 1574.483260 | 1.023767 |
| GO:0042509\_regulation\_of\_tyrosine\_phosphorylation\_of\_STAT\_protein | 9 | 0 | 0.000000 | -0.000000 | 1476 | 1447.676740 | 1511.08 | 1574.483260 | 1.023767 |
| GO:0042640\_anagen | 9 | 0 | 0.000000 | -0.000000 | 1476 | 1447.676740 | 1511.08 | 1574.483260 | 1.023767 |
| GO:0043242\_negative\_regulation\_of\_protein\_complex\_disassembly | 9 | 0 | 0.000000 | -0.000000 | 1476 | 1447.676740 | 1511.08 | 1574.483260 | 1.023767 |
| GO:0043299\_leukocyte\_degranulation | 9 | 0 | 0.000000 | -0.000000 | 1476 | 1447.676740 | 1511.08 | 1574.483260 | 1.023767 |
| GO:0043383\_negative\_T\_cell\_selection | 9 | 0 | 0.000000 | -0.000000 | 1476 | 1447.676740 | 1511.08 | 1574.483260 | 1.023767 |
| GO:0043409\_negative\_regulation\_of\_MAPKKK\_cascade | 9 | 0 | 0.000000 | -0.000000 | 1476 | 1447.676740 | 1511.08 | 1574.483260 | 1.023767 |
| GO:0043433\_negative\_regulation\_of\_transcription\_factor\_activity | 9 | 0 | 0.000000 | -0.000000 | 1476 | 1447.676740 | 1511.08 | 1574.483260 | 1.023767 |
| GO:0043603\_cellular\_amide\_metabolic\_process | 9 | 0 | 0.000000 | -0.000000 | 1476 | 1447.676740 | 1511.08 | 1574.483260 | 1.023767 |
| GO:0045060\_negative\_thymic\_T\_cell\_selection | 9 | 0 | 0.000000 | -0.000000 | 1476 | 1447.676740 | 1511.08 | 1574.483260 | 1.023767 |
| GO:0045109\_intermediate\_filament\_organization | 9 | 0 | 0.000000 | -0.000000 | 1476 | 1447.676740 | 1511.08 | 1574.483260 | 1.023767 |
| GO:0045136\_development\_of\_secondary\_sexual\_characteristics | 9 | 0 | 0.000000 | -0.000000 | 1476 | 1447.676740 | 1511.08 | 1574.483260 | 1.023767 |
| GO:0045185\_maintenance\_of\_protein\_location | 9 | 0 | 0.000000 | -0.000000 | 1476 | 1447.676740 | 1511.08 | 1574.483260 | 1.023767 |
| GO:0045214\_sarcomere\_organization | 9 | 0 | 0.000000 | -0.000000 | 1476 | 1447.676740 | 1511.08 | 1574.483260 | 1.023767 |
| GO:0045428\_regulation\_of\_nitric\_oxide\_biosynthetic\_process | 9 | 0 | 0.000000 | -0.000000 | 1476 | 1447.676740 | 1511.08 | 1574.483260 | 1.023767 |
| GO:0045620\_negative\_regulation\_of\_lymphocyte\_differentiation | 9 | 0 | 0.000000 | -0.000000 | 1476 | 1447.676740 | 1511.08 | 1574.483260 | 1.023767 |
| GO:0045646\_regulation\_of\_erythrocyte\_differentiation | 9 | 0 | 0.000000 | -0.000000 | 1476 | 1447.676740 | 1511.08 | 1574.483260 | 1.023767 |
| GO:0045671\_negative\_regulation\_of\_osteoclast\_differentiation | 9 | 0 | 0.000000 | -0.000000 | 1476 | 1447.676740 | 1511.08 | 1574.483260 | 1.023767 |
| GO:0045766\_positive\_regulation\_of\_angiogenesis | 9 | 0 | 0.000000 | -0.000000 | 1476 | 1447.676740 | 1511.08 | 1574.483260 | 1.023767 |
| GO:0045830\_positive\_regulation\_of\_isotype\_switching | 9 | 0 | 0.000000 | -0.000000 | 1476 | 1447.676740 | 1511.08 | 1574.483260 | 1.023767 |
| GO:0045884\_regulation\_of\_survival\_gene\_product\_expression | 9 | 0 | 0.000000 | -0.000000 | 1476 | 1447.676740 | 1511.08 | 1574.483260 | 1.023767 |
| GO:0046006\_regulation\_of\_activated\_T\_cell\_proliferation | 9 | 0 | 0.000000 | -0.000000 | 1476 | 1447.676740 | 1511.08 | 1574.483260 | 1.023767 |
| GO:0046324\_regulation\_of\_glucose\_import | 9 | 0 | 0.000000 | -0.000000 | 1476 | 1447.676740 | 1511.08 | 1574.483260 | 1.023767 |
| GO:0046365\_monosaccharide\_catabolic\_process | 9 | 0 | 0.000000 | -0.000000 | 1476 | 1447.676740 | 1511.08 | 1574.483260 | 1.023767 |
| GO:0046636\_negative\_regulation\_of\_alpha-beta\_T\_cell\_activation | 9 | 0 | 0.000000 | -0.000000 | 1476 | 1447.676740 | 1511.08 | 1574.483260 | 1.023767 |
| GO:0046641\_positive\_regulation\_of\_alpha-beta\_T\_cell\_proliferation | 9 | 0 | 0.000000 | -0.000000 | 1476 | 1447.676740 | 1511.08 | 1574.483260 | 1.023767 |
| GO:0046888\_negative\_regulation\_of\_hormone\_secretion | 9 | 0 | 0.000000 | -0.000000 | 1476 | 1447.676740 | 1511.08 | 1574.483260 | 1.023767 |
| GO:0048070\_regulation\_of\_pigmentation\_during\_development | 9 | 0 | 0.000000 | -0.000000 | 1476 | 1447.676740 | 1511.08 | 1574.483260 | 1.023767 |
| GO:0048146\_positive\_regulation\_of\_fibroblast\_proliferation | 9 | 0 | 0.000000 | -0.000000 | 1476 | 1447.676740 | 1511.08 | 1574.483260 | 1.023767 |
| GO:0048284\_organelle\_fusion | 9 | 0 | 0.000000 | -0.000000 | 1476 | 1447.676740 | 1511.08 | 1574.483260 | 1.023767 |
| GO:0048488\_synaptic\_vesicle\_endocytosis | 9 | 0 | 0.000000 | -0.000000 | 1476 | 1447.676740 | 1511.08 | 1574.483260 | 1.023767 |
| GO:0048569\_post-embryonic\_organ\_development | 9 | 0 | 0.000000 | -0.000000 | 1476 | 1447.676740 | 1511.08 | 1574.483260 | 1.023767 |
| GO:0048708\_astrocyte\_differentiation | 9 | 0 | 0.000000 | -0.000000 | 1476 | 1447.676740 | 1511.08 | 1574.483260 | 1.023767 |
| GO:0050433\_regulation\_of\_catecholamine\_secretion | 9 | 0 | 0.000000 | -0.000000 | 1476 | 1447.676740 | 1511.08 | 1574.483260 | 1.023767 |
| GO:0050856\_regulation\_of\_T\_cell\_receptor\_signaling\_pathway | 9 | 0 | 0.000000 | -0.000000 | 1476 | 1447.676740 | 1511.08 | 1574.483260 | 1.023767 |
| GO:0050918\_positive\_chemotaxis | 9 | 0 | 0.000000 | -0.000000 | 1476 | 1447.676740 | 1511.08 | 1574.483260 | 1.023767 |
| GO:0051023\_regulation\_of\_immunoglobulin\_secretion | 9 | 0 | 0.000000 | -0.000000 | 1476 | 1447.676740 | 1511.08 | 1574.483260 | 1.023767 |
| GO:0051297\_centrosome\_organization | 9 | 0 | 0.000000 | -0.000000 | 1476 | 1447.676740 | 1511.08 | 1574.483260 | 1.023767 |
| GO:0051324\_prophase | 9 | 0 | 0.000000 | -0.000000 | 1476 | 1447.676740 | 1511.08 | 1574.483260 | 1.023767 |
| GO:0051607\_defense\_response\_to\_virus | 9 | 0 | 0.000000 | -0.000000 | 1476 | 1447.676740 | 1511.08 | 1574.483260 | 1.023767 |
| GO:0051896\_regulation\_of\_protein\_kinase\_B\_signaling\_cascade | 9 | 0 | 0.000000 | -0.000000 | 1476 | 1447.676740 | 1511.08 | 1574.483260 | 1.023767 |
| GO:0051932\_synaptic\_transmission\_\_GABAergic | 9 | 0 | 0.000000 | -0.000000 | 1476 | 1447.676740 | 1511.08 | 1574.483260 | 1.023767 |
| GO:0051963\_regulation\_of\_synaptogenesis | 9 | 0 | 0.000000 | -0.000000 | 1476 | 1447.676740 | 1511.08 | 1574.483260 | 1.023767 |
| GO:0055012\_ventricular\_cardiac\_muscle\_cell\_differentiation | 9 | 0 | 0.000000 | -0.000000 | 1476 | 1447.676740 | 1511.08 | 1574.483260 | 1.023767 |
| GO:0055013\_cardiac\_muscle\_cell\_development | 9 | 0 | 0.000000 | -0.000000 | 1476 | 1447.676740 | 1511.08 | 1574.483260 | 1.023767 |
| GO:0060052\_neurofilament\_cytoskeleton\_organization | 9 | 0 | 0.000000 | -0.000000 | 1476 | 1447.676740 | 1511.08 | 1574.483260 | 1.023767 |
| GO:0060081\_membrane\_hyperpolarization | 9 | 0 | 0.000000 | -0.000000 | 1476 | 1447.676740 | 1511.08 | 1574.483260 | 1.023767 |
| GO:0060513\_prostatic\_bud\_formation | 9 | 0 | 0.000000 | -0.000000 | 1476 | 1447.676740 | 1511.08 | 1574.483260 | 1.023767 |
| GO:0060602\_branch\_elongation\_of\_an\_epithelium | 9 | 0 | 0.000000 | -0.000000 | 1476 | 1447.676740 | 1511.08 | 1574.483260 | 1.023767 |
| GO:0070306\_lens\_fiber\_cell\_differentiation | 9 | 0 | 0.000000 | -0.000000 | 1476 | 1447.676740 | 1511.08 | 1574.483260 | 1.023767 |
| GO:0090048\_negative\_regulation\_of\_transcription\_regulator\_activity | 9 | 0 | 0.000000 | -0.000000 | 1476 | 1447.676740 | 1511.08 | 1574.483260 | 1.023767 |
| GO:0001541\_ovarian\_follicle\_development | 24 | 0 | 0.000000 | -0.000000 | 1497 | 1471.692975 | 1533.63 | 1595.567025 | 1.024469 |
| GO:0002381\_immunoglobulin\_production\_during\_immune\_response | 24 | 0 | 0.000000 | -0.000000 | 1497 | 1471.692975 | 1533.63 | 1595.567025 | 1.024469 |
| GO:0006650\_glycerophospholipid\_metabolic\_process | 24 | 0 | 0.000000 | -0.000000 | 1497 | 1471.692975 | 1533.63 | 1595.567025 | 1.024469 |
| GO:0006941\_striated\_muscle\_contraction | 24 | 0 | 0.000000 | -0.000000 | 1497 | 1471.692975 | 1533.63 | 1595.567025 | 1.024469 |
| GO:0006959\_humoral\_immune\_response | 24 | 0 | 0.000000 | -0.000000 | 1497 | 1471.692975 | 1533.63 | 1595.567025 | 1.024469 |
| GO:0007050\_cell\_cycle\_arrest | 24 | 0 | 0.000000 | -0.000000 | 1497 | 1471.692975 | 1533.63 | 1595.567025 | 1.024469 |
| GO:0007204\_elevation\_of\_cytosolic\_calcium\_ion\_concentration | 24 | 0 | 0.000000 | -0.000000 | 1497 | 1471.692975 | 1533.63 | 1595.567025 | 1.024469 |
| GO:0007259\_JAK-STAT\_cascade | 24 | 0 | 0.000000 | -0.000000 | 1497 | 1471.692975 | 1533.63 | 1595.567025 | 1.024469 |
| GO:0007632\_visual\_behavior | 24 | 0 | 0.000000 | -0.000000 | 1497 | 1471.692975 | 1533.63 | 1595.567025 | 1.024469 |
| GO:0014070\_response\_to\_organic\_cyclic\_substance | 24 | 0 | 0.000000 | -0.000000 | 1497 | 1471.692975 | 1533.63 | 1595.567025 | 1.024469 |
| GO:0021515\_cell\_differentiation\_in\_spinal\_cord | 24 | 0 | 0.000000 | -0.000000 | 1497 | 1471.692975 | 1533.63 | 1595.567025 | 1.024469 |
| GO:0032386\_regulation\_of\_intracellular\_transport | 24 | 0 | 0.000000 | -0.000000 | 1497 | 1471.692975 | 1533.63 | 1595.567025 | 1.024469 |
| GO:0042158\_lipoprotein\_biosynthetic\_process | 24 | 0 | 0.000000 | -0.000000 | 1497 | 1471.692975 | 1533.63 | 1595.567025 | 1.024469 |
| GO:0042632\_cholesterol\_homeostasis | 24 | 0 | 0.000000 | -0.000000 | 1497 | 1471.692975 | 1533.63 | 1595.567025 | 1.024469 |
| GO:0043410\_positive\_regulation\_of\_MAPKKK\_cascade | 24 | 0 | 0.000000 | -0.000000 | 1497 | 1471.692975 | 1533.63 | 1595.567025 | 1.024469 |
| GO:0048002\_antigen\_processing\_and\_presentation\_of\_peptide\_antigen | 24 | 0 | 0.000000 | -0.000000 | 1497 | 1471.692975 | 1533.63 | 1595.567025 | 1.024469 |
| GO:0048546\_digestive\_tract\_morphogenesis | 24 | 0 | 0.000000 | -0.000000 | 1497 | 1471.692975 | 1533.63 | 1595.567025 | 1.024469 |
| GO:0050679\_positive\_regulation\_of\_epithelial\_cell\_proliferation | 24 | 0 | 0.000000 | -0.000000 | 1497 | 1471.692975 | 1533.63 | 1595.567025 | 1.024469 |
| GO:0055092\_sterol\_homeostasis | 24 | 0 | 0.000000 | -0.000000 | 1497 | 1471.692975 | 1533.63 | 1595.567025 | 1.024469 |
| GO:0060078\_regulation\_of\_postsynaptic\_membrane\_potential | 24 | 0 | 0.000000 | -0.000000 | 1497 | 1471.692975 | 1533.63 | 1595.567025 | 1.024469 |
| GO:0070667\_negative\_regulation\_of\_mast\_cell\_proliferation | 24 | 0 | 0.000000 | -0.000000 | 1497 | 1471.692975 | 1533.63 | 1595.567025 | 1.024469 |
| GO:0048754\_branching\_morphogenesis\_of\_a\_tube | 88 | 0 | 0.000000 | -0.000000 | 1499 | 1473.183129 | 1534.73 | 1596.276871 | 1.023836 |
| GO:0050863\_regulation\_of\_T\_cell\_activation | 88 | 0 | 0.000000 | -0.000000 | 1499 | 1473.183129 | 1534.73 | 1596.276871 | 1.023836 |
| GO:0006163\_purine\_nucleotide\_metabolic\_process | 73 | 0 | 0.000000 | -0.000000 | 1501 | 1475.508778 | 1536.89 | 1598.271222 | 1.023911 |
| GO:0006936\_muscle\_contraction | 73 | 0 | 0.000000 | -0.000000 | 1501 | 1475.508778 | 1536.89 | 1598.271222 | 1.023911 |
| GO:0009165\_nucleotide\_biosynthetic\_process | 63 | 0 | 0.000000 | -0.000000 | 1506 | 1478.635628 | 1539.9 | 1601.164372 | 1.022510 |
| GO:0051186\_cofactor\_metabolic\_process | 63 | 0 | 0.000000 | -0.000000 | 1506 | 1478.635628 | 1539.9 | 1601.164372 | 1.022510 |
| GO:0051216\_cartilage\_development | 63 | 0 | 0.000000 | -0.000000 | 1506 | 1478.635628 | 1539.9 | 1601.164372 | 1.022510 |
| GO:0070662\_mast\_cell\_proliferation | 63 | 0 | 0.000000 | -0.000000 | 1506 | 1478.635628 | 1539.9 | 1601.164372 | 1.022510 |
| GO:0070666\_regulation\_of\_mast\_cell\_proliferation | 63 | 0 | 0.000000 | -0.000000 | 1506 | 1478.635628 | 1539.9 | 1601.164372 | 1.022510 |
| GO:0000723\_telomere\_maintenance | 13 | 0 | 0.000000 | -0.000000 | 1571 | 1546.328955 | 1605.38 | 1664.431045 | 1.021884 |
| GO:0001836\_release\_of\_cytochrome\_c\_from\_mitochondria | 13 | 0 | 0.000000 | -0.000000 | 1571 | 1546.328955 | 1605.38 | 1664.431045 | 1.021884 |
| GO:0001975\_response\_to\_amphetamine | 13 | 0 | 0.000000 | -0.000000 | 1571 | 1546.328955 | 1605.38 | 1664.431045 | 1.021884 |
| GO:0001976\_neurological\_system\_process\_involved\_in\_regulation\_of\_systemic\_arterial\_blood\_pressure | 13 | 0 | 0.000000 | -0.000000 | 1571 | 1546.328955 | 1605.38 | 1664.431045 | 1.021884 |
| GO:0002704\_negative\_regulation\_of\_leukocyte\_mediated\_immunity | 13 | 0 | 0.000000 | -0.000000 | 1571 | 1546.328955 | 1605.38 | 1664.431045 | 1.021884 |
| GO:0002707\_negative\_regulation\_of\_lymphocyte\_mediated\_immunity | 13 | 0 | 0.000000 | -0.000000 | 1571 | 1546.328955 | 1605.38 | 1664.431045 | 1.021884 |
| GO:0002717\_positive\_regulation\_of\_natural\_killer\_cell\_mediated\_immunity | 13 | 0 | 0.000000 | -0.000000 | 1571 | 1546.328955 | 1605.38 | 1664.431045 | 1.021884 |
| GO:0003016\_respiratory\_system\_process | 13 | 0 | 0.000000 | -0.000000 | 1571 | 1546.328955 | 1605.38 | 1664.431045 | 1.021884 |
| GO:0006090\_pyruvate\_metabolic\_process | 13 | 0 | 0.000000 | -0.000000 | 1571 | 1546.328955 | 1605.38 | 1664.431045 | 1.021884 |
| GO:0006687\_glycosphingolipid\_metabolic\_process | 13 | 0 | 0.000000 | -0.000000 | 1571 | 1546.328955 | 1605.38 | 1664.431045 | 1.021884 |
| GO:0006778\_porphyrin\_metabolic\_process | 13 | 0 | 0.000000 | -0.000000 | 1571 | 1546.328955 | 1605.38 | 1664.431045 | 1.021884 |
| GO:0006833\_water\_transport | 13 | 0 | 0.000000 | -0.000000 | 1571 | 1546.328955 | 1605.38 | 1664.431045 | 1.021884 |
| GO:0006986\_response\_to\_unfolded\_protein | 13 | 0 | 0.000000 | -0.000000 | 1571 | 1546.328955 | 1605.38 | 1664.431045 | 1.021884 |
| GO:0007129\_synapsis | 13 | 0 | 0.000000 | -0.000000 | 1571 | 1546.328955 | 1605.38 | 1664.431045 | 1.021884 |
| GO:0007212\_dopamine\_receptor\_signaling\_pathway | 13 | 0 | 0.000000 | -0.000000 | 1571 | 1546.328955 | 1605.38 | 1664.431045 | 1.021884 |
| GO:0007274\_neuromuscular\_synaptic\_transmission | 13 | 0 | 0.000000 | -0.000000 | 1571 | 1546.328955 | 1605.38 | 1664.431045 | 1.021884 |
| GO:0007339\_binding\_of\_sperm\_to\_zona\_pellucida | 13 | 0 | 0.000000 | -0.000000 | 1571 | 1546.328955 | 1605.38 | 1664.431045 | 1.021884 |
| GO:0007439\_ectodermal\_gut\_development | 13 | 0 | 0.000000 | -0.000000 | 1571 | 1546.328955 | 1605.38 | 1664.431045 | 1.021884 |
| GO:0007512\_adult\_heart\_development | 13 | 0 | 0.000000 | -0.000000 | 1571 | 1546.328955 | 1605.38 | 1664.431045 | 1.021884 |
| GO:0007566\_embryo\_implantation | 13 | 0 | 0.000000 | -0.000000 | 1571 | 1546.328955 | 1605.38 | 1664.431045 | 1.021884 |
| GO:0009119\_ribonucleoside\_metabolic\_process | 13 | 0 | 0.000000 | -0.000000 | 1571 | 1546.328955 | 1605.38 | 1664.431045 | 1.021884 |
| GO:0009410\_response\_to\_xenobiotic\_stimulus | 13 | 0 | 0.000000 | -0.000000 | 1571 | 1546.328955 | 1605.38 | 1664.431045 | 1.021884 |
| GO:0009994\_oocyte\_differentiation | 13 | 0 | 0.000000 | -0.000000 | 1571 | 1546.328955 | 1605.38 | 1664.431045 | 1.021884 |
| GO:0010623\_developmental\_programmed\_cell\_death | 13 | 0 | 0.000000 | -0.000000 | 1571 | 1546.328955 | 1605.38 | 1664.431045 | 1.021884 |
| GO:0010970\_microtubule-based\_transport | 13 | 0 | 0.000000 | -0.000000 | 1571 | 1546.328955 | 1605.38 | 1664.431045 | 1.021884 |
| GO:0016525\_negative\_regulation\_of\_angiogenesis | 13 | 0 | 0.000000 | -0.000000 | 1571 | 1546.328955 | 1605.38 | 1664.431045 | 1.021884 |
| GO:0019098\_reproductive\_behavior | 13 | 0 | 0.000000 | -0.000000 | 1571 | 1546.328955 | 1605.38 | 1664.431045 | 1.021884 |
| GO:0021511\_spinal\_cord\_patterning | 13 | 0 | 0.000000 | -0.000000 | 1571 | 1546.328955 | 1605.38 | 1664.431045 | 1.021884 |
| GO:0021533\_cell\_differentiation\_in\_hindbrain | 13 | 0 | 0.000000 | -0.000000 | 1571 | 1546.328955 | 1605.38 | 1664.431045 | 1.021884 |
| GO:0021879\_forebrain\_neuron\_differentiation | 13 | 0 | 0.000000 | -0.000000 | 1571 | 1546.328955 | 1605.38 | 1664.431045 | 1.021884 |
| GO:0030384\_phosphoinositide\_metabolic\_process | 13 | 0 | 0.000000 | -0.000000 | 1571 | 1546.328955 | 1605.38 | 1664.431045 | 1.021884 |
| GO:0030539\_male\_genitalia\_development | 13 | 0 | 0.000000 | -0.000000 | 1571 | 1546.328955 | 1605.38 | 1664.431045 | 1.021884 |
| GO:0031032\_actomyosin\_structure\_organization | 13 | 0 | 0.000000 | -0.000000 | 1571 | 1546.328955 | 1605.38 | 1664.431045 | 1.021884 |
| GO:0032200\_telomere\_organization | 13 | 0 | 0.000000 | -0.000000 | 1571 | 1546.328955 | 1605.38 | 1664.431045 | 1.021884 |
| GO:0032330\_regulation\_of\_chondrocyte\_differentiation | 13 | 0 | 0.000000 | -0.000000 | 1571 | 1546.328955 | 1605.38 | 1664.431045 | 1.021884 |
| GO:0032615\_interleukin-12\_production | 13 | 0 | 0.000000 | -0.000000 | 1571 | 1546.328955 | 1605.38 | 1664.431045 | 1.021884 |
| GO:0032729\_positive\_regulation\_of\_interferon-gamma\_production | 13 | 0 | 0.000000 | -0.000000 | 1571 | 1546.328955 | 1605.38 | 1664.431045 | 1.021884 |
| GO:0033013\_tetrapyrrole\_metabolic\_process | 13 | 0 | 0.000000 | -0.000000 | 1571 | 1546.328955 | 1605.38 | 1664.431045 | 1.021884 |
| GO:0042044\_fluid\_transport | 13 | 0 | 0.000000 | -0.000000 | 1571 | 1546.328955 | 1605.38 | 1664.431045 | 1.021884 |
| GO:0042094\_interleukin-2\_biosynthetic\_process | 13 | 0 | 0.000000 | -0.000000 | 1571 | 1546.328955 | 1605.38 | 1664.431045 | 1.021884 |
| GO:0042474\_middle\_ear\_morphogenesis | 13 | 0 | 0.000000 | -0.000000 | 1571 | 1546.328955 | 1605.38 | 1664.431045 | 1.021884 |
| GO:0043241\_protein\_complex\_disassembly | 13 | 0 | 0.000000 | -0.000000 | 1571 | 1546.328955 | 1605.38 | 1664.431045 | 1.021884 |
| GO:0043244\_regulation\_of\_protein\_complex\_disassembly | 13 | 0 | 0.000000 | -0.000000 | 1571 | 1546.328955 | 1605.38 | 1664.431045 | 1.021884 |
| GO:0045191\_regulation\_of\_isotype\_switching | 13 | 0 | 0.000000 | -0.000000 | 1571 | 1546.328955 | 1605.38 | 1664.431045 | 1.021884 |
| GO:0045577\_regulation\_of\_B\_cell\_differentiation | 13 | 0 | 0.000000 | -0.000000 | 1571 | 1546.328955 | 1605.38 | 1664.431045 | 1.021884 |
| GO:0045954\_positive\_regulation\_of\_natural\_killer\_cell\_mediated\_cytotoxicity | 13 | 0 | 0.000000 | -0.000000 | 1571 | 1546.328955 | 1605.38 | 1664.431045 | 1.021884 |
| GO:0046474\_glycerophospholipid\_biosynthetic\_process | 13 | 0 | 0.000000 | -0.000000 | 1571 | 1546.328955 | 1605.38 | 1664.431045 | 1.021884 |
| GO:0046640\_regulation\_of\_alpha-beta\_T\_cell\_proliferation | 13 | 0 | 0.000000 | -0.000000 | 1571 | 1546.328955 | 1605.38 | 1664.431045 | 1.021884 |
| GO:0046851\_negative\_regulation\_of\_bone\_remodeling | 13 | 0 | 0.000000 | -0.000000 | 1571 | 1546.328955 | 1605.38 | 1664.431045 | 1.021884 |
| GO:0048305\_immunoglobulin\_secretion | 13 | 0 | 0.000000 | -0.000000 | 1571 | 1546.328955 | 1605.38 | 1664.431045 | 1.021884 |
| GO:0048566\_embryonic\_gut\_development | 13 | 0 | 0.000000 | -0.000000 | 1571 | 1546.328955 | 1605.38 | 1664.431045 | 1.021884 |
| GO:0048567\_ectodermal\_gut\_morphogenesis | 13 | 0 | 0.000000 | -0.000000 | 1571 | 1546.328955 | 1605.38 | 1664.431045 | 1.021884 |
| GO:0048599\_oocyte\_development | 13 | 0 | 0.000000 | -0.000000 | 1571 | 1546.328955 | 1605.38 | 1664.431045 | 1.021884 |
| GO:0050764\_regulation\_of\_phagocytosis | 13 | 0 | 0.000000 | -0.000000 | 1571 | 1546.328955 | 1605.38 | 1664.431045 | 1.021884 |
| GO:0050766\_positive\_regulation\_of\_phagocytosis | 13 | 0 | 0.000000 | -0.000000 | 1571 | 1546.328955 | 1605.38 | 1664.431045 | 1.021884 |
| GO:0050818\_regulation\_of\_coagulation | 13 | 0 | 0.000000 | -0.000000 | 1571 | 1546.328955 | 1605.38 | 1664.431045 | 1.021884 |
| GO:0051346\_negative\_regulation\_of\_hydrolase\_activity | 13 | 0 | 0.000000 | -0.000000 | 1571 | 1546.328955 | 1605.38 | 1664.431045 | 1.021884 |
| GO:0051495\_positive\_regulation\_of\_cytoskeleton\_organization | 13 | 0 | 0.000000 | -0.000000 | 1571 | 1546.328955 | 1605.38 | 1664.431045 | 1.021884 |
| GO:0060038\_cardiac\_muscle\_cell\_proliferation | 13 | 0 | 0.000000 | -0.000000 | 1571 | 1546.328955 | 1605.38 | 1664.431045 | 1.021884 |
| GO:0060070\_Wnt\_receptor\_signaling\_pathway\_through\_beta-catenin | 13 | 0 | 0.000000 | -0.000000 | 1571 | 1546.328955 | 1605.38 | 1664.431045 | 1.021884 |
| GO:0060401\_cytosolic\_calcium\_ion\_transport | 13 | 0 | 0.000000 | -0.000000 | 1571 | 1546.328955 | 1605.38 | 1664.431045 | 1.021884 |
| GO:0060402\_calcium\_ion\_transport\_into\_cytosol | 13 | 0 | 0.000000 | -0.000000 | 1571 | 1546.328955 | 1605.38 | 1664.431045 | 1.021884 |
| GO:0060560\_developmental\_growth\_involved\_in\_morphogenesis | 13 | 0 | 0.000000 | -0.000000 | 1571 | 1546.328955 | 1605.38 | 1664.431045 | 1.021884 |
| GO:0060742\_epithelial\_cell\_differentiation\_involved\_in\_prostate\_gland\_development | 13 | 0 | 0.000000 | -0.000000 | 1571 | 1546.328955 | 1605.38 | 1664.431045 | 1.021884 |
| GO:0070192\_chromosome\_organization\_involved\_in\_meiosis | 13 | 0 | 0.000000 | -0.000000 | 1571 | 1546.328955 | 1605.38 | 1664.431045 | 1.021884 |
| GO:0007599\_hemostasis | 40 | 0 | 0.000000 | -0.000000 | 1578 | 1555.180184 | 1613.8 | 1672.419816 | 1.022687 |
| GO:0008203\_cholesterol\_metabolic\_process | 40 | 0 | 0.000000 | -0.000000 | 1578 | 1555.180184 | 1613.8 | 1672.419816 | 1.022687 |
| GO:0014031\_mesenchymal\_cell\_development | 40 | 0 | 0.000000 | -0.000000 | 1578 | 1555.180184 | 1613.8 | 1672.419816 | 1.022687 |
| GO:0016358\_dendrite\_development | 40 | 0 | 0.000000 | -0.000000 | 1578 | 1555.180184 | 1613.8 | 1672.419816 | 1.022687 |
| GO:0016485\_protein\_processing | 40 | 0 | 0.000000 | -0.000000 | 1578 | 1555.180184 | 1613.8 | 1672.419816 | 1.022687 |
| GO:0019935\_cyclic-nucleotide-mediated\_signaling | 40 | 0 | 0.000000 | -0.000000 | 1578 | 1555.180184 | 1613.8 | 1672.419816 | 1.022687 |
| GO:0046850\_regulation\_of\_bone\_remodeling | 40 | 0 | 0.000000 | -0.000000 | 1578 | 1555.180184 | 1613.8 | 1672.419816 | 1.022687 |
| GO:0051241\_negative\_regulation\_of\_multicellular\_organismal\_process | 77 | 0 | 0.000000 | -0.000000 | 1579 | 1556.356900 | 1614.8 | 1673.243100 | 1.022673 |
| GO:0034622\_cellular\_macromolecular\_complex\_assembly | 58 | 0 | 0.000000 | -0.000000 | 1580 | 1559.362856 | 1617.39 | 1675.417144 | 1.023665 |
| GO:0009791\_post-embryonic\_development | 67 | 0 | 0.000000 | -0.000000 | 1584 | 1562.257354 | 1619.88 | 1677.502646 | 1.022652 |
| GO:0031347\_regulation\_of\_defense\_response | 67 | 0 | 0.000000 | -0.000000 | 1584 | 1562.257354 | 1619.88 | 1677.502646 | 1.022652 |
| GO:0042445\_hormone\_metabolic\_process | 67 | 0 | 0.000000 | -0.000000 | 1584 | 1562.257354 | 1619.88 | 1677.502646 | 1.022652 |
| GO:0051247\_positive\_regulation\_of\_protein\_metabolic\_process | 67 | 0 | 0.000000 | -0.000000 | 1584 | 1562.257354 | 1619.88 | 1677.502646 | 1.022652 |
| GO:0002706\_regulation\_of\_lymphocyte\_mediated\_immunity | 52 | 0 | 0.000000 | -0.000000 | 1587 | 1565.401756 | 1622.87 | 1680.338244 | 1.022602 |
| GO:0009124\_nucleoside\_monophosphate\_biosynthetic\_process | 52 | 0 | 0.000000 | -0.000000 | 1587 | 1565.401756 | 1622.87 | 1680.338244 | 1.022602 |
| GO:0048585\_negative\_regulation\_of\_response\_to\_stimulus | 52 | 0 | 0.000000 | -0.000000 | 1587 | 1565.401756 | 1622.87 | 1680.338244 | 1.022602 |
| GO:0006006\_glucose\_metabolic\_process | 42 | 0 | 0.000000 | -0.000000 | 1602 | 1578.173582 | 1635.12 | 1692.066418 | 1.020674 |
| GO:0006171\_cAMP\_biosynthetic\_process | 42 | 0 | 0.000000 | -0.000000 | 1602 | 1578.173582 | 1635.12 | 1692.066418 | 1.020674 |
| GO:0008361\_regulation\_of\_cell\_size | 42 | 0 | 0.000000 | -0.000000 | 1602 | 1578.173582 | 1635.12 | 1692.066418 | 1.020674 |
| GO:0015672\_monovalent\_inorganic\_cation\_transport | 42 | 0 | 0.000000 | -0.000000 | 1602 | 1578.173582 | 1635.12 | 1692.066418 | 1.020674 |
| GO:0016125\_sterol\_metabolic\_process | 42 | 0 | 0.000000 | -0.000000 | 1602 | 1578.173582 | 1635.12 | 1692.066418 | 1.020674 |
| GO:0019221\_cytokine-mediated\_signaling\_pathway | 42 | 0 | 0.000000 | -0.000000 | 1602 | 1578.173582 | 1635.12 | 1692.066418 | 1.020674 |
| GO:0019941\_modification-dependent\_protein\_catabolic\_process | 42 | 0 | 0.000000 | -0.000000 | 1602 | 1578.173582 | 1635.12 | 1692.066418 | 1.020674 |
| GO:0032946\_positive\_regulation\_of\_mononuclear\_cell\_proliferation | 42 | 0 | 0.000000 | -0.000000 | 1602 | 1578.173582 | 1635.12 | 1692.066418 | 1.020674 |
| GO:0043632\_modification-dependent\_macromolecule\_catabolic\_process | 42 | 0 | 0.000000 | -0.000000 | 1602 | 1578.173582 | 1635.12 | 1692.066418 | 1.020674 |
| GO:0048515\_spermatid\_differentiation | 42 | 0 | 0.000000 | -0.000000 | 1602 | 1578.173582 | 1635.12 | 1692.066418 | 1.020674 |
| GO:0050671\_positive\_regulation\_of\_lymphocyte\_proliferation | 42 | 0 | 0.000000 | -0.000000 | 1602 | 1578.173582 | 1635.12 | 1692.066418 | 1.020674 |
| GO:0050817\_coagulation | 42 | 0 | 0.000000 | -0.000000 | 1602 | 1578.173582 | 1635.12 | 1692.066418 | 1.020674 |
| GO:0051345\_positive\_regulation\_of\_hydrolase\_activity | 42 | 0 | 0.000000 | -0.000000 | 1602 | 1578.173582 | 1635.12 | 1692.066418 | 1.020674 |
| GO:0051603\_proteolysis\_involved\_in\_cellular\_protein\_catabolic\_process | 42 | 0 | 0.000000 | -0.000000 | 1602 | 1578.173582 | 1635.12 | 1692.066418 | 1.020674 |
| GO:0080135\_regulation\_of\_cellular\_response\_to\_stress | 42 | 0 | 0.000000 | -0.000000 | 1602 | 1578.173582 | 1635.12 | 1692.066418 | 1.020674 |
| GO:0001934\_positive\_regulation\_of\_protein\_amino\_acid\_phosphorylation | 29 | 0 | 0.000000 | -0.000000 | 1618 | 1595.002672 | 1651.17 | 1707.337328 | 1.020501 |
| GO:0006641\_triglyceride\_metabolic\_process | 29 | 0 | 0.000000 | -0.000000 | 1618 | 1595.002672 | 1651.17 | 1707.337328 | 1.020501 |
| GO:0006909\_phagocytosis | 29 | 0 | 0.000000 | -0.000000 | 1618 | 1595.002672 | 1651.17 | 1707.337328 | 1.020501 |
| GO:0007190\_activation\_of\_adenylate\_cyclase\_activity | 29 | 0 | 0.000000 | -0.000000 | 1618 | 1595.002672 | 1651.17 | 1707.337328 | 1.020501 |
| GO:0016447\_somatic\_recombination\_of\_immunoglobulin\_gene\_segments | 29 | 0 | 0.000000 | -0.000000 | 1618 | 1595.002672 | 1651.17 | 1707.337328 | 1.020501 |
| GO:0021761\_limbic\_system\_development | 29 | 0 | 0.000000 | -0.000000 | 1618 | 1595.002672 | 1651.17 | 1707.337328 | 1.020501 |
| GO:0043281\_regulation\_of\_caspase\_activity | 29 | 0 | 0.000000 | -0.000000 | 1618 | 1595.002672 | 1651.17 | 1707.337328 | 1.020501 |
| GO:0044087\_regulation\_of\_cellular\_component\_biogenesis | 29 | 0 | 0.000000 | -0.000000 | 1618 | 1595.002672 | 1651.17 | 1707.337328 | 1.020501 |
| GO:0044270\_nitrogen\_compound\_catabolic\_process | 29 | 0 | 0.000000 | -0.000000 | 1618 | 1595.002672 | 1651.17 | 1707.337328 | 1.020501 |
| GO:0045621\_positive\_regulation\_of\_lymphocyte\_differentiation | 29 | 0 | 0.000000 | -0.000000 | 1618 | 1595.002672 | 1651.17 | 1707.337328 | 1.020501 |
| GO:0046634\_regulation\_of\_alpha-beta\_T\_cell\_activation | 29 | 0 | 0.000000 | -0.000000 | 1618 | 1595.002672 | 1651.17 | 1707.337328 | 1.020501 |
| GO:0048066\_pigmentation\_during\_development | 29 | 0 | 0.000000 | -0.000000 | 1618 | 1595.002672 | 1651.17 | 1707.337328 | 1.020501 |
| GO:0051301\_cell\_division | 29 | 0 | 0.000000 | -0.000000 | 1618 | 1595.002672 | 1651.17 | 1707.337328 | 1.020501 |
| GO:0052548\_regulation\_of\_endopeptidase\_activity | 29 | 0 | 0.000000 | -0.000000 | 1618 | 1595.002672 | 1651.17 | 1707.337328 | 1.020501 |
| GO:0060041\_retina\_development\_in\_camera-type\_eye | 29 | 0 | 0.000000 | -0.000000 | 1618 | 1595.002672 | 1651.17 | 1707.337328 | 1.020501 |
| GO:0070302\_regulation\_of\_stress-activated\_protein\_kinase\_signaling\_pathway | 29 | 0 | 0.000000 | -0.000000 | 1618 | 1595.002672 | 1651.17 | 1707.337328 | 1.020501 |
| GO:0000084\_S\_phase\_of\_mitotic\_cell\_cycle | 3 | 0 |  |  |  |  |  |  |  |  |
| GO:0000089\_mitotic\_metaphase | 3 | 0 |  |  |  |  |  |  |  |  |
| GO:0000098\_sulfur\_amino\_acid\_catabolic\_process | 3 | 0 |  |  |  |  |  |  |  |  |
| GO:0000103\_sulfate\_assimilation | 3 | 0 |  |  |  |  |  |  |  |  |
| GO:0000281\_cytokinesis\_after\_mitosis | 3 | 0 |  |  |  |  |  |  |  |  |
| GO:0000303\_response\_to\_superoxide | 3 | 0 |  |  |  |  |  |  |  |  |
| GO:0000320\_re-entry\_into\_mitotic\_cell\_cycle | 3 | 0 |  |  |  |  |  |  |  |  |
| GO:0000380\_alternative\_nuclear\_mRNA\_splicing\_\_via\_spliceosome | 3 | 0 |  |  |  |  |  |  |  |  |
| GO:0001516\_prostaglandin\_biosynthetic\_process | 3 | 0 |  |  |  |  |  |  |  |  |
| GO:0001553\_luteinization | 3 | 0 |  |  |  |  |  |  |  |  |
| GO:0001574\_ganglioside\_biosynthetic\_process | 3 | 0 |  |  |  |  |  |  |  |  |
| GO:0001705\_ectoderm\_formation | 3 | 0 |  |  |  |  |  |  |  |  |
| GO:0001711\_endodermal\_cell\_fate\_commitment | 3 | 0 |  |  |  |  |  |  |  |  |
| GO:0001757\_somite\_specification | 3 | 0 |  |  |  |  |  |  |  |  |
| GO:0001778\_plasma\_membrane\_repair | 3 | 0 |  |  |  |  |  |  |  |  |
| GO:0001780\_neutrophil\_homeostasis | 3 | 0 |  |  |  |  |  |  |  |  |
| GO:0001802\_type\_III\_hypersensitivity | 3 | 0 |  |  |  |  |  |  |  |  |
| GO:0001803\_regulation\_of\_type\_III\_hypersensitivity | 3 | 0 |  |  |  |  |  |  |  |  |
| GO:0001805\_positive\_regulation\_of\_type\_III\_hypersensitivity | 3 | 0 |  |  |  |  |  |  |  |  |
| GO:0001812\_positive\_regulation\_of\_type\_I\_hypersensitivity | 3 | 0 |  |  |  |  |  |  |  |  |
| GO:0001831\_trophectodermal\_cellular\_morphogenesis | 3 | 0 |  |  |  |  |  |  |  |  |
| GO:0001844\_protein\_insertion\_into\_mitochondrial\_membrane\_during\_induction\_of\_apoptosis | 3 | 0 |  |  |  |  |  |  |  |  |
| GO:0001878\_response\_to\_yeast | 3 | 0 |  |  |  |  |  |  |  |  |
| GO:0001895\_retina\_homeostasis | 3 | 0 |  |  |  |  |  |  |  |  |
| GO:0001915\_negative\_regulation\_of\_T\_cell\_mediated\_cytotoxicity | 3 | 0 |  |  |  |  |  |  |  |  |
| GO:0001937\_negative\_regulation\_of\_endothelial\_cell\_proliferation | 3 | 0 |  |  |  |  |  |  |  |  |
| GO:0001953\_negative\_regulation\_of\_cell-matrix\_adhesion | 3 | 0 |  |  |  |  |  |  |  |  |
| GO:0001960\_negative\_regulation\_of\_cytokine-mediated\_signaling\_pathway | 3 | 0 |  |  |  |  |  |  |  |  |
| GO:0001973\_adenosine\_receptor\_signaling\_pathway | 3 | 0 |  |  |  |  |  |  |  |  |
| GO:0001996\_positive\_regulation\_of\_heart\_rate\_by\_epinephrine-norepinephrine | 3 | 0 |  |  |  |  |  |  |  |  |
| GO:0002034\_regulation\_of\_blood\_vessel\_size\_by\_renin-angiotensin | 3 | 0 |  |  |  |  |  |  |  |  |
| GO:0002238\_response\_to\_molecule\_of\_fungal\_origin | 3 | 0 |  |  |  |  |  |  |  |  |
| GO:0002275\_myeloid\_cell\_activation\_during\_immune\_response | 3 | 0 |  |  |  |  |  |  |  |  |
| GO:0002281\_macrophage\_activation\_during\_immune\_response | 3 | 0 |  |  |  |  |  |  |  |  |
| GO:0002309\_T\_cell\_proliferation\_during\_immune\_response | 3 | 0 |  |  |  |  |  |  |  |  |
| GO:0002361\_CD4-positive\_\_CD25-positive\_\_alpha-beta\_regulatory\_T\_cell\_differentiation | 3 | 0 |  |  |  |  |  |  |  |  |
| GO:0002369\_T\_cell\_cytokine\_production | 3 | 0 |  |  |  |  |  |  |  |  |
| GO:0002428\_antigen\_processing\_and\_presentation\_of\_peptide\_antigen\_via\_MHC\_class\_Ib | 3 | 0 |  |  |  |  |  |  |  |  |
| GO:0002446\_neutrophil\_mediated\_immunity | 3 | 0 |  |  |  |  |  |  |  |  |
| GO:0002477\_antigen\_processing\_and\_presentation\_of\_exogenous\_peptide\_antigen\_via\_MHC\_class\_Ib | 3 | 0 |  |  |  |  |  |  |  |  |
| GO:0002481\_antigen\_processing\_and\_presentation\_of\_exogenous\_protein\_antigen\_via\_MHC\_class\_Ib\_\_TAP-dependent | 3 | 0 |  |  |  |  |  |  |  |  |
| GO:0002513\_tolerance\_induction\_to\_self\_antigen | 3 | 0 |  |  |  |  |  |  |  |  |
| GO:0002568\_somatic\_diversification\_of\_T\_cell\_receptor\_genes | 3 | 0 |  |  |  |  |  |  |  |  |
| GO:0002674\_negative\_regulation\_of\_acute\_inflammatory\_response | 3 | 0 |  |  |  |  |  |  |  |  |
| GO:0002681\_somatic\_recombination\_of\_T\_cell\_receptor\_gene\_segments | 3 | 0 |  |  |  |  |  |  |  |  |
| GO:0002713\_negative\_regulation\_of\_B\_cell\_mediated\_immunity | 3 | 0 |  |  |  |  |  |  |  |  |
| GO:0002827\_positive\_regulation\_of\_T-helper\_1\_type\_immune\_response | 3 | 0 |  |  |  |  |  |  |  |  |
| GO:0002865\_negative\_regulation\_of\_acute\_inflammatory\_response\_to\_antigenic\_stimulus | 3 | 0 |  |  |  |  |  |  |  |  |
| GO:0002884\_negative\_regulation\_of\_hypersensitivity | 3 | 0 |  |  |  |  |  |  |  |  |
| GO:0002890\_negative\_regulation\_of\_immunoglobulin\_mediated\_immune\_response | 3 | 0 |  |  |  |  |  |  |  |  |
| GO:0002904\_positive\_regulation\_of\_B\_cell\_apoptosis | 3 | 0 |  |  |  |  |  |  |  |  |
| GO:0003009\_skeletal\_muscle\_contraction | 3 | 0 |  |  |  |  |  |  |  |  |
| GO:0003072\_renal\_control\_of\_peripheral\_vascular\_resistance\_involved\_in\_regulation\_of\_systemic\_arterial\_blood\_pressure | 3 | 0 |  |  |  |  |  |  |  |  |
| GO:0006047\_UDP-N-acetylglucosamine\_metabolic\_process | 3 | 0 |  |  |  |  |  |  |  |  |
| GO:0006067\_ethanol\_metabolic\_process | 3 | 0 |  |  |  |  |  |  |  |  |
| GO:0006072\_glycerol-3-phosphate\_metabolic\_process | 3 | 0 |  |  |  |  |  |  |  |  |
| GO:0006103\_2-oxoglutarate\_metabolic\_process | 3 | 0 |  |  |  |  |  |  |  |  |
| GO:0006107\_oxaloacetate\_metabolic\_process | 3 | 0 |  |  |  |  |  |  |  |  |
| GO:0006166\_purine\_ribonucleoside\_salvage | 3 | 0 |  |  |  |  |  |  |  |  |
| GO:0006220\_pyrimidine\_nucleotide\_metabolic\_process | 3 | 0 |  |  |  |  |  |  |  |  |
| GO:0006266\_DNA\_ligation | 3 | 0 |  |  |  |  |  |  |  |  |
| GO:0006282\_regulation\_of\_DNA\_repair | 3 | 0 |  |  |  |  |  |  |  |  |
| GO:0006287\_base-excision\_repair\_\_gap-filling | 3 | 0 |  |  |  |  |  |  |  |  |
| GO:0006301\_postreplication\_repair | 3 | 0 |  |  |  |  |  |  |  |  |
| GO:0006361\_transcription\_initiation\_from\_RNA\_polymerase\_I\_promoter | 3 | 0 |  |  |  |  |  |  |  |  |
| GO:0006367\_transcription\_initiation\_from\_RNA\_polymerase\_II\_promoter | 3 | 0 |  |  |  |  |  |  |  |  |
| GO:0006414\_translational\_elongation | 3 | 0 |  |  |  |  |  |  |  |  |
| GO:0006491\_N-glycan\_processing | 3 | 0 |  |  |  |  |  |  |  |  |
| GO:0006498\_N-terminal\_protein\_lipidation | 3 | 0 |  |  |  |  |  |  |  |  |
| GO:0006531\_aspartate\_metabolic\_process | 3 | 0 |  |  |  |  |  |  |  |  |
| GO:0006598\_polyamine\_catabolic\_process | 3 | 0 |  |  |  |  |  |  |  |  |
| GO:0006620\_posttranslational\_protein\_targeting\_to\_membrane | 3 | 0 |  |  |  |  |  |  |  |  |
| GO:0006625\_protein\_targeting\_to\_peroxisome | 3 | 0 |  |  |  |  |  |  |  |  |
| GO:0006651\_diacylglycerol\_biosynthetic\_process | 3 | 0 |  |  |  |  |  |  |  |  |
| GO:0006670\_sphingosine\_metabolic\_process | 3 | 0 |  |  |  |  |  |  |  |  |
| GO:0006677\_glycosylceramide\_metabolic\_process | 3 | 0 |  |  |  |  |  |  |  |  |
| GO:0006689\_ganglioside\_catabolic\_process | 3 | 0 |  |  |  |  |  |  |  |  |
| GO:0006699\_bile\_acid\_biosynthetic\_process | 3 | 0 |  |  |  |  |  |  |  |  |
| GO:0006791\_sulfur\_utilization | 3 | 0 |  |  |  |  |  |  |  |  |
| GO:0006817\_phosphate\_transport | 3 | 0 |  |  |  |  |  |  |  |  |
| GO:0006825\_copper\_ion\_transport | 3 | 0 |  |  |  |  |  |  |  |  |
| GO:0006828\_manganese\_ion\_transport | 3 | 0 |  |  |  |  |  |  |  |  |
| GO:0006857\_oligopeptide\_transport | 3 | 0 |  |  |  |  |  |  |  |  |
| GO:0006892\_post-Golgi\_vesicle-mediated\_transport | 3 | 0 |  |  |  |  |  |  |  |  |
| GO:0006904\_vesicle\_docking\_during\_exocytosis | 3 | 0 |  |  |  |  |  |  |  |  |
| GO:0006926\_virus-infected\_cell\_apoptosis | 3 | 0 |  |  |  |  |  |  |  |  |
| GO:0006953\_acute-phase\_response | 3 | 0 |  |  |  |  |  |  |  |  |
| GO:0007000\_nucleolus\_organization | 3 | 0 |  |  |  |  |  |  |  |  |
| GO:0007041\_lysosomal\_transport | 3 | 0 |  |  |  |  |  |  |  |  |
| GO:0007043\_cell-cell\_junction\_assembly | 3 | 0 |  |  |  |  |  |  |  |  |
| GO:0007090\_regulation\_of\_S\_phase\_of\_mitotic\_cell\_cycle | 3 | 0 |  |  |  |  |  |  |  |  |
| GO:0007195\_inhibition\_of\_adenylate\_cyclase\_activity\_by\_dopamine\_receptor\_signaling\_pathway | 3 | 0 |  |  |  |  |  |  |  |  |
| GO:0007199\_G-protein\_signaling\_\_coupled\_to\_cGMP\_nucleotide\_second\_messenger | 3 | 0 |  |  |  |  |  |  |  |  |
| GO:0007213\_muscarinic\_acetylcholine\_receptor\_signaling\_pathway | 3 | 0 |  |  |  |  |  |  |  |  |
| GO:0007250\_activation\_of\_NF-kappaB-inducing\_kinase\_activity | 3 | 0 |  |  |  |  |  |  |  |  |
| GO:0007262\_STAT\_protein\_nuclear\_translocation | 3 | 0 |  |  |  |  |  |  |  |  |
| GO:0007288\_sperm\_axoneme\_assembly | 3 | 0 |  |  |  |  |  |  |  |  |
| GO:0007350\_blastoderm\_segmentation | 3 | 0 |  |  |  |  |  |  |  |  |
| GO:0007403\_glial\_cell\_fate\_determination | 3 | 0 |  |  |  |  |  |  |  |  |
| GO:0007412\_axon\_target\_recognition | 3 | 0 |  |  |  |  |  |  |  |  |
| GO:0007468\_regulation\_of\_rhodopsin\_gene\_expression | 3 | 0 |  |  |  |  |  |  |  |  |
| GO:0007525\_somatic\_muscle\_development | 3 | 0 |  |  |  |  |  |  |  |  |
| GO:0007635\_chemosensory\_behavior | 3 | 0 |  |  |  |  |  |  |  |  |
| GO:0008090\_retrograde\_axon\_cargo\_transport | 3 | 0 |  |  |  |  |  |  |  |  |
| GO:0008347\_glial\_cell\_migration | 3 | 0 |  |  |  |  |  |  |  |  |
| GO:0008635\_activation\_of\_caspase\_activity\_by\_cytochrome\_c | 3 | 0 |  |  |  |  |  |  |  |  |
| GO:0009060\_aerobic\_respiration | 3 | 0 |  |  |  |  |  |  |  |  |
| GO:0009081\_branched\_chain\_family\_amino\_acid\_metabolic\_process | 3 | 0 |  |  |  |  |  |  |  |  |
| GO:0009086\_methionine\_biosynthetic\_process | 3 | 0 |  |  |  |  |  |  |  |  |
| GO:0009135\_purine\_nucleoside\_diphosphate\_metabolic\_process | 3 | 0 |  |  |  |  |  |  |  |  |
| GO:0009137\_purine\_nucleoside\_diphosphate\_catabolic\_process | 3 | 0 |  |  |  |  |  |  |  |  |
| GO:0009155\_purine\_deoxyribonucleotide\_catabolic\_process | 3 | 0 |  |  |  |  |  |  |  |  |
| GO:0009179\_purine\_ribonucleoside\_diphosphate\_metabolic\_process | 3 | 0 |  |  |  |  |  |  |  |  |
| GO:0009181\_purine\_ribonucleoside\_diphosphate\_catabolic\_process | 3 | 0 |  |  |  |  |  |  |  |  |
| GO:0009185\_ribonucleoside\_diphosphate\_metabolic\_process | 3 | 0 |  |  |  |  |  |  |  |  |
| GO:0009191\_ribonucleoside\_diphosphate\_catabolic\_process | 3 | 0 |  |  |  |  |  |  |  |  |
| GO:0009199\_ribonucleoside\_triphosphate\_metabolic\_process | 3 | 0 |  |  |  |  |  |  |  |  |
| GO:0009204\_deoxyribonucleoside\_triphosphate\_catabolic\_process | 3 | 0 |  |  |  |  |  |  |  |  |
| GO:0009205\_purine\_ribonucleoside\_triphosphate\_metabolic\_process | 3 | 0 |  |  |  |  |  |  |  |  |
| GO:0009217\_purine\_deoxyribonucleoside\_triphosphate\_catabolic\_process | 3 | 0 |  |  |  |  |  |  |  |  |
| GO:0009448\_gamma-aminobutyric\_acid\_metabolic\_process | 3 | 0 |  |  |  |  |  |  |  |  |
| GO:0010043\_response\_to\_zinc\_ion | 3 | 0 |  |  |  |  |  |  |  |  |
| GO:0010159\_specification\_of\_organ\_position | 3 | 0 |  |  |  |  |  |  |  |  |
| GO:0010172\_embryonic\_body\_morphogenesis | 3 | 0 |  |  |  |  |  |  |  |  |
| GO:0010216\_maintenance\_of\_DNA\_methylation | 3 | 0 |  |  |  |  |  |  |  |  |
| GO:0010273\_detoxification\_of\_copper\_ion | 3 | 0 |  |  |  |  |  |  |  |  |
| GO:0010454\_negative\_regulation\_of\_cell\_fate\_commitment | 3 | 0 |  |  |  |  |  |  |  |  |
| GO:0010507\_negative\_regulation\_of\_autophagy | 3 | 0 |  |  |  |  |  |  |  |  |
| GO:0010524\_positive\_regulation\_of\_calcium\_ion\_transport\_into\_cytosol | 3 | 0 |  |  |  |  |  |  |  |  |
| GO:0010573\_vascular\_endothelial\_growth\_factor\_production | 3 | 0 |  |  |  |  |  |  |  |  |
| GO:0010574\_regulation\_of\_vascular\_endothelial\_growth\_factor\_production | 3 | 0 |  |  |  |  |  |  |  |  |
| GO:0010575\_positive\_regulation\_vascular\_endothelial\_growth\_factor\_production | 3 | 0 |  |  |  |  |  |  |  |  |
| GO:0010632\_regulation\_of\_epithelial\_cell\_migration | 3 | 0 |  |  |  |  |  |  |  |  |
| GO:0010717\_regulation\_of\_epithelial\_to\_mesenchymal\_transition | 3 | 0 |  |  |  |  |  |  |  |  |
| GO:0010884\_positive\_regulation\_of\_lipid\_storage | 3 | 0 |  |  |  |  |  |  |  |  |
| GO:0010888\_negative\_regulation\_of\_lipid\_storage | 3 | 0 |  |  |  |  |  |  |  |  |
| GO:0010889\_regulation\_of\_sequestering\_of\_triglyceride | 3 | 0 |  |  |  |  |  |  |  |  |
| GO:0010893\_positive\_regulation\_of\_steroid\_biosynthetic\_process | 3 | 0 |  |  |  |  |  |  |  |  |
| GO:0010894\_negative\_regulation\_of\_steroid\_biosynthetic\_process | 3 | 0 |  |  |  |  |  |  |  |  |
| GO:0010998\_regulation\_of\_translational\_initiation\_by\_eIF2\_alpha\_phosphorylation | 3 | 0 |  |  |  |  |  |  |  |  |
| GO:0010999\_regulation\_of\_eIF2\_alpha\_phosphorylation\_by\_heme | 3 | 0 |  |  |  |  |  |  |  |  |
| GO:0014074\_response\_to\_purine | 3 | 0 |  |  |  |  |  |  |  |  |
| GO:0014909\_smooth\_muscle\_cell\_migration | 3 | 0 |  |  |  |  |  |  |  |  |
| GO:0015669\_gas\_transport | 3 | 0 |  |  |  |  |  |  |  |  |
| GO:0015760\_glucose-6-phosphate\_transport | 3 | 0 |  |  |  |  |  |  |  |  |
| GO:0015816\_glycine\_transport | 3 | 0 |  |  |  |  |  |  |  |  |
| GO:0015838\_betaine\_transport | 3 | 0 |  |  |  |  |  |  |  |  |
| GO:0015871\_choline\_transport | 3 | 0 |  |  |  |  |  |  |  |  |
| GO:0015879\_carnitine\_transport | 3 | 0 |  |  |  |  |  |  |  |  |
| GO:0015893\_drug\_transport | 3 | 0 |  |  |  |  |  |  |  |  |
| GO:0015909\_long-chain\_fatty\_acid\_transport | 3 | 0 |  |  |  |  |  |  |  |  |
| GO:0015936\_coenzyme\_A\_metabolic\_process | 3 | 0 |  |  |  |  |  |  |  |  |
| GO:0015988\_energy\_coupled\_proton\_transport\_\_against\_electrochemical\_gradient | 3 | 0 |  |  |  |  |  |  |  |  |
| GO:0015991\_ATP\_hydrolysis\_coupled\_proton\_transport | 3 | 0 |  |  |  |  |  |  |  |  |
| GO:0016241\_regulation\_of\_macroautophagy | 3 | 0 |  |  |  |  |  |  |  |  |
| GO:0016322\_neuron\_remodeling | 3 | 0 |  |  |  |  |  |  |  |  |
| GO:0016556\_mRNA\_modification | 3 | 0 |  |  |  |  |  |  |  |  |
| GO:0016973\_poly(A)+\_mRNA\_export\_from\_nucleus | 3 | 0 |  |  |  |  |  |  |  |  |
| GO:0018196\_peptidyl-asparagine\_modification | 3 | 0 |  |  |  |  |  |  |  |  |
| GO:0018208\_peptidyl-proline\_modification | 3 | 0 |  |  |  |  |  |  |  |  |
| GO:0018279\_protein\_amino\_acid\_N-linked\_glycosylation\_via\_asparagine | 3 | 0 |  |  |  |  |  |  |  |  |
| GO:0018894\_dibenzo-p-dioxin\_metabolic\_process | 3 | 0 |  |  |  |  |  |  |  |  |
| GO:0019058\_viral\_infectious\_cycle | 3 | 0 |  |  |  |  |  |  |  |  |
| GO:0019236\_response\_to\_pheromone | 3 | 0 |  |  |  |  |  |  |  |  |
| GO:0019359\_nicotinamide\_nucleotide\_biosynthetic\_process | 3 | 0 |  |  |  |  |  |  |  |  |
| GO:0019363\_pyridine\_nucleotide\_biosynthetic\_process | 3 | 0 |  |  |  |  |  |  |  |  |
| GO:0019438\_aromatic\_compound\_biosynthetic\_process | 3 | 0 |  |  |  |  |  |  |  |  |
| GO:0019439\_aromatic\_compound\_catabolic\_process | 3 | 0 |  |  |  |  |  |  |  |  |
| GO:0019605\_butyrate\_metabolic\_process | 3 | 0 |  |  |  |  |  |  |  |  |
| GO:0019614\_catechol\_catabolic\_process | 3 | 0 |  |  |  |  |  |  |  |  |
| GO:0019674\_NAD\_metabolic\_process | 3 | 0 |  |  |  |  |  |  |  |  |
| GO:0019852\_L-ascorbic\_acid\_metabolic\_process | 3 | 0 |  |  |  |  |  |  |  |  |
| GO:0019934\_cGMP-mediated\_signaling | 3 | 0 |  |  |  |  |  |  |  |  |
| GO:0019987\_negative\_regulation\_of\_anti-apoptosis | 3 | 0 |  |  |  |  |  |  |  |  |
| GO:0021527\_spinal\_cord\_association\_neuron\_differentiation | 3 | 0 |  |  |  |  |  |  |  |  |
| GO:0021529\_spinal\_cord\_oligodendrocyte\_cell\_differentiation | 3 | 0 |  |  |  |  |  |  |  |  |
| GO:0021530\_spinal\_cord\_oligodendrocyte\_cell\_fate\_specification | 3 | 0 |  |  |  |  |  |  |  |  |
| GO:0021555\_midbrain-hindbrain\_boundary\_morphogenesis | 3 | 0 |  |  |  |  |  |  |  |  |
| GO:0021563\_glossopharyngeal\_nerve\_development | 3 | 0 |  |  |  |  |  |  |  |  |
| GO:0021570\_rhombomere\_4\_development | 3 | 0 |  |  |  |  |  |  |  |  |
| GO:0021591\_ventricular\_system\_development | 3 | 0 |  |  |  |  |  |  |  |  |
| GO:0021615\_glossopharyngeal\_nerve\_morphogenesis | 3 | 0 |  |  |  |  |  |  |  |  |
| GO:0021794\_thalamus\_development | 3 | 0 |  |  |  |  |  |  |  |  |
| GO:0021797\_forebrain\_anterior\_posterior\_pattern\_formation | 3 | 0 |  |  |  |  |  |  |  |  |
| GO:0021798\_forebrain\_dorsal\_ventral\_pattern\_formation | 3 | 0 |  |  |  |  |  |  |  |  |
| GO:0021800\_cerebral\_cortex\_tangential\_migration | 3 | 0 |  |  |  |  |  |  |  |  |
| GO:0021819\_layer\_formation\_in\_the\_cerebral\_cortex | 3 | 0 |  |  |  |  |  |  |  |  |
| GO:0021859\_pyramidal\_neuron\_differentiation | 3 | 0 |  |  |  |  |  |  |  |  |
| GO:0021860\_pyramidal\_neuron\_development | 3 | 0 |  |  |  |  |  |  |  |  |
| GO:0021889\_olfactory\_bulb\_interneuron\_differentiation | 3 | 0 |  |  |  |  |  |  |  |  |
| GO:0021891\_olfactory\_bulb\_interneuron\_development | 3 | 0 |  |  |  |  |  |  |  |  |
| GO:0021912\_regulation\_of\_transcription\_from\_RNA\_polymerase\_II\_promoter\_involved\_in\_spinal\_cord\_motor\_neuron\_fate\_specification | 3 | 0 |  |  |  |  |  |  |  |  |
| GO:0021979\_hypothalamus\_cell\_differentiation | 3 | 0 |  |  |  |  |  |  |  |  |
| GO:0022010\_myelination\_in\_the\_central\_nervous\_system | 3 | 0 |  |  |  |  |  |  |  |  |
| GO:0022027\_interkinetic\_nuclear\_migration | 3 | 0 |  |  |  |  |  |  |  |  |
| GO:0022406\_membrane\_docking | 3 | 0 |  |  |  |  |  |  |  |  |
| GO:0030033\_microvillus\_assembly | 3 | 0 |  |  |  |  |  |  |  |  |
| GO:0030091\_protein\_repair | 3 | 0 |  |  |  |  |  |  |  |  |
| GO:0030195\_negative\_regulation\_of\_blood\_coagulation | 3 | 0 |  |  |  |  |  |  |  |  |
| GO:0030307\_positive\_regulation\_of\_cell\_growth | 3 | 0 |  |  |  |  |  |  |  |  |
| GO:0030319\_cellular\_di-\_\_tri-valent\_inorganic\_anion\_homeostasis | 3 | 0 |  |  |  |  |  |  |  |  |
| GO:0030320\_cellular\_monovalent\_inorganic\_anion\_homeostasis | 3 | 0 |  |  |  |  |  |  |  |  |
| GO:0030321\_transepithelial\_chloride\_transport | 3 | 0 |  |  |  |  |  |  |  |  |
| GO:0030501\_positive\_regulation\_of\_bone\_mineralization | 3 | 0 |  |  |  |  |  |  |  |  |
| GO:0030513\_positive\_regulation\_of\_BMP\_signaling\_pathway | 3 | 0 |  |  |  |  |  |  |  |  |
| GO:0030538\_embryonic\_genitalia\_morphogenesis | 3 | 0 |  |  |  |  |  |  |  |  |
| GO:0030540\_female\_genitalia\_development | 3 | 0 |  |  |  |  |  |  |  |  |
| GO:0030643\_cellular\_phosphate\_ion\_homeostasis | 3 | 0 |  |  |  |  |  |  |  |  |
| GO:0030718\_germ-line\_stem\_cell\_maintenance | 3 | 0 |  |  |  |  |  |  |  |  |
| GO:0030730\_sequestering\_of\_triglyceride | 3 | 0 |  |  |  |  |  |  |  |  |
| GO:0030836\_positive\_regulation\_of\_actin\_filament\_depolymerization | 3 | 0 |  |  |  |  |  |  |  |  |
| GO:0030857\_negative\_regulation\_of\_epithelial\_cell\_differentiation | 3 | 0 |  |  |  |  |  |  |  |  |
| GO:0030916\_otic\_vesicle\_formation | 3 | 0 |  |  |  |  |  |  |  |  |
| GO:0031000\_response\_to\_caffeine | 3 | 0 |  |  |  |  |  |  |  |  |
| GO:0031063\_regulation\_of\_histone\_deacetylation | 3 | 0 |  |  |  |  |  |  |  |  |
| GO:0031065\_positive\_regulation\_of\_histone\_deacetylation | 3 | 0 |  |  |  |  |  |  |  |  |
| GO:0031112\_positive\_regulation\_of\_microtubule\_polymerization\_or\_depolymerization | 3 | 0 |  |  |  |  |  |  |  |  |
| GO:0031116\_positive\_regulation\_of\_microtubule\_polymerization | 3 | 0 |  |  |  |  |  |  |  |  |
| GO:0031133\_regulation\_of\_axon\_diameter | 3 | 0 |  |  |  |  |  |  |  |  |
| GO:0031282\_regulation\_of\_guanylate\_cyclase\_activity | 3 | 0 |  |  |  |  |  |  |  |  |
| GO:0031333\_negative\_regulation\_of\_protein\_complex\_assembly | 3 | 0 |  |  |  |  |  |  |  |  |
| GO:0031397\_negative\_regulation\_of\_protein\_ubiquitination | 3 | 0 |  |  |  |  |  |  |  |  |
| GO:0031398\_positive\_regulation\_of\_protein\_ubiquitination | 3 | 0 |  |  |  |  |  |  |  |  |
| GO:0031503\_protein\_complex\_localization | 3 | 0 |  |  |  |  |  |  |  |  |
| GO:0031571\_G1\_DNA\_damage\_checkpoint | 3 | 0 |  |  |  |  |  |  |  |  |
| GO:0031579\_membrane\_raft\_organization | 3 | 0 |  |  |  |  |  |  |  |  |
| GO:0031638\_zymogen\_activation | 3 | 0 |  |  |  |  |  |  |  |  |
| GO:0031641\_regulation\_of\_myelination | 3 | 0 |  |  |  |  |  |  |  |  |
| GO:0031642\_negative\_regulation\_of\_myelination | 3 | 0 |  |  |  |  |  |  |  |  |
| GO:0031649\_heat\_generation | 3 | 0 |  |  |  |  |  |  |  |  |
| GO:0031943\_regulation\_of\_glucocorticoid\_metabolic\_process | 3 | 0 |  |  |  |  |  |  |  |  |
| GO:0032020\_ISG15-protein\_conjugation | 3 | 0 |  |  |  |  |  |  |  |  |
| GO:0032060\_bleb\_formation | 3 | 0 |  |  |  |  |  |  |  |  |
| GO:0032095\_regulation\_of\_response\_to\_food | 3 | 0 |  |  |  |  |  |  |  |  |
| GO:0032272\_negative\_regulation\_of\_protein\_polymerization | 3 | 0 |  |  |  |  |  |  |  |  |
| GO:0032288\_myelin\_assembly | 3 | 0 |  |  |  |  |  |  |  |  |
| GO:0032291\_ensheathment\_of\_axons\_in\_the\_central\_nervous\_system | 3 | 0 |  |  |  |  |  |  |  |  |
| GO:0032355\_response\_to\_estradiol\_stimulus | 3 | 0 |  |  |  |  |  |  |  |  |
| GO:0032402\_melanosome\_transport | 3 | 0 |  |  |  |  |  |  |  |  |
| GO:0032411\_positive\_regulation\_of\_transporter\_activity | 3 | 0 |  |  |  |  |  |  |  |  |
| GO:0032414\_positive\_regulation\_of\_ion\_transmembrane\_transporter\_activity | 3 | 0 |  |  |  |  |  |  |  |  |
| GO:0032436\_positive\_regulation\_of\_proteasomal\_ubiquitin-dependent\_protein\_catabolic\_process | 3 | 0 |  |  |  |  |  |  |  |  |
| GO:0032528\_microvillus\_organization | 3 | 0 |  |  |  |  |  |  |  |  |
| GO:0032536\_regulation\_of\_cell\_projection\_size | 3 | 0 |  |  |  |  |  |  |  |  |
| GO:0032632\_interleukin-3\_production | 3 | 0 |  |  |  |  |  |  |  |  |
| GO:0032634\_interleukin-5\_production | 3 | 0 |  |  |  |  |  |  |  |  |
| GO:0032674\_regulation\_of\_interleukin-5\_production | 3 | 0 |  |  |  |  |  |  |  |  |
| GO:0032703\_negative\_regulation\_of\_interleukin-2\_production | 3 | 0 |  |  |  |  |  |  |  |  |
| GO:0032753\_positive\_regulation\_of\_interleukin-4\_production | 3 | 0 |  |  |  |  |  |  |  |  |
| GO:0032823\_regulation\_of\_natural\_killer\_cell\_differentiation | 3 | 0 |  |  |  |  |  |  |  |  |
| GO:0032825\_positive\_regulation\_of\_natural\_killer\_cell\_differentiation | 3 | 0 |  |  |  |  |  |  |  |  |
| GO:0032856\_activation\_of\_Ras\_GTPase\_activity | 3 | 0 |  |  |  |  |  |  |  |  |
| GO:0032862\_activation\_of\_Rho\_GTPase\_activity | 3 | 0 |  |  |  |  |  |  |  |  |
| GO:0032874\_positive\_regulation\_of\_stress-activated\_MAPK\_cascade | 3 | 0 |  |  |  |  |  |  |  |  |
| GO:0032881\_regulation\_of\_polysaccharide\_metabolic\_process | 3 | 0 |  |  |  |  |  |  |  |  |
| GO:0032890\_regulation\_of\_organic\_acid\_transport | 3 | 0 |  |  |  |  |  |  |  |  |
| GO:0033058\_directional\_locomotion | 3 | 0 |  |  |  |  |  |  |  |  |
| GO:0033080\_immature\_T\_cell\_proliferation\_in\_the\_thymus | 3 | 0 |  |  |  |  |  |  |  |  |
| GO:0033084\_regulation\_of\_immature\_T\_cell\_proliferation\_in\_the\_thymus | 3 | 0 |  |  |  |  |  |  |  |  |
| GO:0033091\_positive\_regulation\_of\_immature\_T\_cell\_proliferation | 3 | 0 |  |  |  |  |  |  |  |  |
| GO:0033137\_negative\_regulation\_of\_peptidyl-serine\_phosphorylation | 3 | 0 |  |  |  |  |  |  |  |  |
| GO:0033153\_T\_cell\_receptor\_V(D)J\_recombination | 3 | 0 |  |  |  |  |  |  |  |  |
| GO:0033209\_tumor\_necrosis\_factor-mediated\_signaling\_pathway | 3 | 0 |  |  |  |  |  |  |  |  |
| GO:0033261\_regulation\_of\_S\_phase | 3 | 0 |  |  |  |  |  |  |  |  |
| GO:0033600\_negative\_regulation\_of\_mammary\_gland\_epithelial\_cell\_proliferation | 3 | 0 |  |  |  |  |  |  |  |  |
| GO:0033631\_cell-cell\_adhesion\_mediated\_by\_integrin | 3 | 0 |  |  |  |  |  |  |  |  |
| GO:0033993\_response\_to\_lipid | 3 | 0 |  |  |  |  |  |  |  |  |
| GO:0034220\_ion\_transmembrane\_transport | 3 | 0 |  |  |  |  |  |  |  |  |
| GO:0034308\_monohydric\_alcohol\_metabolic\_process | 3 | 0 |  |  |  |  |  |  |  |  |
| GO:0034313\_diol\_catabolic\_process | 3 | 0 |  |  |  |  |  |  |  |  |
| GO:0034331\_cell\_junction\_maintenance | 3 | 0 |  |  |  |  |  |  |  |  |
| GO:0034332\_adherens\_junction\_organization | 3 | 0 |  |  |  |  |  |  |  |  |
| GO:0034375\_high-density\_lipoprotein\_particle\_remodeling | 3 | 0 |  |  |  |  |  |  |  |  |
| GO:0034381\_lipoprotein\_particle\_clearance | 3 | 0 |  |  |  |  |  |  |  |  |
| GO:0034612\_response\_to\_tumor\_necrosis\_factor | 3 | 0 |  |  |  |  |  |  |  |  |
| GO:0034655\_nucleobase\_\_nucleoside\_\_nucleotide\_and\_nucleic\_acid\_catabolic\_process | 3 | 0 |  |  |  |  |  |  |  |  |
| GO:0034656\_nucleobase\_\_nucleoside\_and\_nucleotide\_catabolic\_process | 3 | 0 |  |  |  |  |  |  |  |  |
| GO:0035067\_negative\_regulation\_of\_histone\_acetylation | 3 | 0 |  |  |  |  |  |  |  |  |
| GO:0035084\_flagellar\_axoneme\_assembly | 3 | 0 |  |  |  |  |  |  |  |  |
| GO:0035166\_post-embryonic\_hemopoiesis | 3 | 0 |  |  |  |  |  |  |  |  |
| GO:0035283\_central\_nervous\_system\_segmentation | 3 | 0 |  |  |  |  |  |  |  |  |
| GO:0035284\_brain\_segmentation | 3 | 0 |  |  |  |  |  |  |  |  |
| GO:0042097\_interleukin-4\_biosynthetic\_process | 3 | 0 |  |  |  |  |  |  |  |  |
| GO:0042135\_neurotransmitter\_catabolic\_process | 3 | 0 |  |  |  |  |  |  |  |  |
| GO:0042271\_susceptibility\_to\_natural\_killer\_cell\_mediated\_cytotoxicity | 3 | 0 |  |  |  |  |  |  |  |  |
| GO:0042273\_ribosomal\_large\_subunit\_biogenesis | 3 | 0 |  |  |  |  |  |  |  |  |
| GO:0042375\_quinone\_cofactor\_metabolic\_process | 3 | 0 |  |  |  |  |  |  |  |  |
| GO:0042420\_dopamine\_catabolic\_process | 3 | 0 |  |  |  |  |  |  |  |  |
| GO:0042421\_norepinephrine\_biosynthetic\_process | 3 | 0 |  |  |  |  |  |  |  |  |
| GO:0042424\_catecholamine\_catabolic\_process | 3 | 0 |  |  |  |  |  |  |  |  |
| GO:0042447\_hormone\_catabolic\_process | 3 | 0 |  |  |  |  |  |  |  |  |
| GO:0042448\_progesterone\_metabolic\_process | 3 | 0 |  |  |  |  |  |  |  |  |
| GO:0042523\_positive\_regulation\_of\_tyrosine\_phosphorylation\_of\_Stat5\_protein | 3 | 0 |  |  |  |  |  |  |  |  |
| GO:0042659\_regulation\_of\_cell\_fate\_specification | 3 | 0 |  |  |  |  |  |  |  |  |
| GO:0042670\_retinal\_cone\_cell\_differentiation | 3 | 0 |  |  |  |  |  |  |  |  |
| GO:0042693\_muscle\_cell\_fate\_commitment | 3 | 0 |  |  |  |  |  |  |  |  |
| GO:0042711\_maternal\_behavior | 3 | 0 |  |  |  |  |  |  |  |  |
| GO:0042745\_circadian\_sleep\_wake\_cycle | 3 | 0 |  |  |  |  |  |  |  |  |
| GO:0042759\_long-chain\_fatty\_acid\_biosynthetic\_process | 3 | 0 |  |  |  |  |  |  |  |  |
| GO:0042787\_protein\_ubiquitination\_during\_ubiquitin-dependent\_protein\_catabolic\_process | 3 | 0 |  |  |  |  |  |  |  |  |
| GO:0043045\_DNA\_methylation\_during\_embryonic\_development | 3 | 0 |  |  |  |  |  |  |  |  |
| GO:0043090\_amino\_acid\_import | 3 | 0 |  |  |  |  |  |  |  |  |
| GO:0043092\_L-amino\_acid\_import | 3 | 0 |  |  |  |  |  |  |  |  |
| GO:0043094\_cellular\_metabolic\_compound\_salvage | 3 | 0 |  |  |  |  |  |  |  |  |
| GO:0043101\_purine\_salvage | 3 | 0 |  |  |  |  |  |  |  |  |
| GO:0043149\_stress\_fiber\_formation | 3 | 0 |  |  |  |  |  |  |  |  |
| GO:0043174\_nucleoside\_salvage | 3 | 0 |  |  |  |  |  |  |  |  |
| GO:0043200\_response\_to\_amino\_acid\_stimulus | 3 | 0 |  |  |  |  |  |  |  |  |
| GO:0043243\_positive\_regulation\_of\_protein\_complex\_disassembly | 3 | 0 |  |  |  |  |  |  |  |  |
| GO:0043249\_erythrocyte\_maturation | 3 | 0 |  |  |  |  |  |  |  |  |
| GO:0043267\_negative\_regulation\_of\_potassium\_ion\_transport | 3 | 0 |  |  |  |  |  |  |  |  |
| GO:0043371\_negative\_regulation\_of\_CD4-positive\_\_alpha\_beta\_T\_cell\_differentiation | 3 | 0 |  |  |  |  |  |  |  |  |
| GO:0043462\_regulation\_of\_ATPase\_activity | 3 | 0 |  |  |  |  |  |  |  |  |
| GO:0043569\_negative\_regulation\_of\_insulin-like\_growth\_factor\_receptor\_signaling\_pathway | 3 | 0 |  |  |  |  |  |  |  |  |
| GO:0043574\_peroxisomal\_transport | 3 | 0 |  |  |  |  |  |  |  |  |
| GO:0043586\_tongue\_development | 3 | 0 |  |  |  |  |  |  |  |  |
| GO:0043900\_regulation\_of\_multi-organism\_process | 3 | 0 |  |  |  |  |  |  |  |  |
| GO:0043954\_cellular\_component\_maintenance | 3 | 0 |  |  |  |  |  |  |  |  |
| GO:0044030\_regulation\_of\_DNA\_methylation | 3 | 0 |  |  |  |  |  |  |  |  |
| GO:0044089\_positive\_regulation\_of\_cellular\_component\_biogenesis | 3 | 0 |  |  |  |  |  |  |  |  |
| GO:0044273\_sulfur\_compound\_catabolic\_process | 3 | 0 |  |  |  |  |  |  |  |  |
| GO:0045047\_protein\_targeting\_to\_ER | 3 | 0 |  |  |  |  |  |  |  |  |
| GO:0045085\_negative\_regulation\_of\_interleukin-2\_biosynthetic\_process | 3 | 0 |  |  |  |  |  |  |  |  |
| GO:0045110\_intermediate\_filament\_bundle\_assembly | 3 | 0 |  |  |  |  |  |  |  |  |
| GO:0045143\_homologous\_chromosome\_segregation | 3 | 0 |  |  |  |  |  |  |  |  |
| GO:0045198\_establishment\_of\_epithelial\_cell\_apical\_basal\_polarity | 3 | 0 |  |  |  |  |  |  |  |  |
| GO:0045217\_cell-cell\_junction\_maintenance | 3 | 0 |  |  |  |  |  |  |  |  |
| GO:0045348\_positive\_regulation\_of\_MHC\_class\_II\_biosynthetic\_process | 3 | 0 |  |  |  |  |  |  |  |  |
| GO:0045402\_regulation\_of\_interleukin-4\_biosynthetic\_process | 3 | 0 |  |  |  |  |  |  |  |  |
| GO:0045404\_positive\_regulation\_of\_interleukin-4\_biosynthetic\_process | 3 | 0 |  |  |  |  |  |  |  |  |
| GO:0045542\_positive\_regulation\_of\_cholesterol\_biosynthetic\_process | 3 | 0 |  |  |  |  |  |  |  |  |
| GO:0045607\_regulation\_of\_auditory\_receptor\_cell\_differentiation | 3 | 0 |  |  |  |  |  |  |  |  |
| GO:0045623\_negative\_regulation\_of\_T-helper\_cell\_differentiation | 3 | 0 |  |  |  |  |  |  |  |  |
| GO:0045625\_regulation\_of\_T-helper\_1\_cell\_differentiation | 3 | 0 |  |  |  |  |  |  |  |  |
| GO:0045631\_regulation\_of\_mechanoreceptor\_differentiation | 3 | 0 |  |  |  |  |  |  |  |  |
| GO:0045717\_negative\_regulation\_of\_fatty\_acid\_biosynthetic\_process | 3 | 0 |  |  |  |  |  |  |  |  |
| GO:0045723\_positive\_regulation\_of\_fatty\_acid\_biosynthetic\_process | 3 | 0 |  |  |  |  |  |  |  |  |
| GO:0045746\_negative\_regulation\_of\_Notch\_signaling\_pathway | 3 | 0 |  |  |  |  |  |  |  |  |
| GO:0045806\_negative\_regulation\_of\_endocytosis | 3 | 0 |  |  |  |  |  |  |  |  |
| GO:0045829\_negative\_regulation\_of\_isotype\_switching | 3 | 0 |  |  |  |  |  |  |  |  |
| GO:0045844\_positive\_regulation\_of\_striated\_muscle\_development | 3 | 0 |  |  |  |  |  |  |  |  |
| GO:0045907\_positive\_regulation\_of\_vasoconstriction | 3 | 0 |  |  |  |  |  |  |  |  |
| GO:0045922\_negative\_regulation\_of\_fatty\_acid\_metabolic\_process | 3 | 0 |  |  |  |  |  |  |  |  |
| GO:0045939\_negative\_regulation\_of\_steroid\_metabolic\_process | 3 | 0 |  |  |  |  |  |  |  |  |
| GO:0046013\_regulation\_of\_T\_cell\_homeostatic\_proliferation | 3 | 0 |  |  |  |  |  |  |  |  |
| GO:0046034\_ATP\_metabolic\_process | 3 | 0 |  |  |  |  |  |  |  |  |
| GO:0046325\_negative\_regulation\_of\_glucose\_import | 3 | 0 |  |  |  |  |  |  |  |  |
| GO:0046426\_negative\_regulation\_of\_JAK-STAT\_cascade | 3 | 0 |  |  |  |  |  |  |  |  |
| GO:0046457\_prostanoid\_biosynthetic\_process | 3 | 0 |  |  |  |  |  |  |  |  |
| GO:0046479\_glycosphingolipid\_catabolic\_process | 3 | 0 |  |  |  |  |  |  |  |  |
| GO:0046488\_phosphatidylinositol\_metabolic\_process | 3 | 0 |  |  |  |  |  |  |  |  |
| GO:0046549\_retinal\_cone\_cell\_development | 3 | 0 |  |  |  |  |  |  |  |  |
| GO:0046605\_regulation\_of\_centrosome\_cycle | 3 | 0 |  |  |  |  |  |  |  |  |
| GO:0046688\_response\_to\_copper\_ion | 3 | 0 |  |  |  |  |  |  |  |  |
| GO:0046717\_acid\_secretion | 3 | 0 |  |  |  |  |  |  |  |  |
| GO:0046825\_regulation\_of\_protein\_export\_from\_nucleus | 3 | 0 |  |  |  |  |  |  |  |  |
| GO:0046885\_regulation\_of\_hormone\_biosynthetic\_process | 3 | 0 |  |  |  |  |  |  |  |  |
| GO:0048003\_antigen\_processing\_and\_presentation\_of\_lipid\_antigen\_via\_MHC\_class\_Ib | 3 | 0 |  |  |  |  |  |  |  |  |
| GO:0048007\_antigen\_processing\_and\_presentation\_\_exogenous\_lipid\_antigen\_via\_MHC\_class\_Ib | 3 | 0 |  |  |  |  |  |  |  |  |
| GO:0048012\_hepatocyte\_growth\_factor\_receptor\_signaling\_pathway | 3 | 0 |  |  |  |  |  |  |  |  |
| GO:0048050\_post-embryonic\_eye\_morphogenesis | 3 | 0 |  |  |  |  |  |  |  |  |
| GO:0048087\_positive\_regulation\_of\_pigmentation\_during\_development | 3 | 0 |  |  |  |  |  |  |  |  |
| GO:0048246\_macrophage\_chemotaxis | 3 | 0 |  |  |  |  |  |  |  |  |
| GO:0048251\_elastic\_fiber\_assembly | 3 | 0 |  |  |  |  |  |  |  |  |
| GO:0048278\_vesicle\_docking | 3 | 0 |  |  |  |  |  |  |  |  |
| GO:0048294\_negative\_regulation\_of\_isotype\_switching\_to\_IgE\_isotypes | 3 | 0 |  |  |  |  |  |  |  |  |
| GO:0048318\_axial\_mesoderm\_development | 3 | 0 |  |  |  |  |  |  |  |  |
| GO:0048597\_post-embryonic\_camera-type\_eye\_morphogenesis | 3 | 0 |  |  |  |  |  |  |  |  |
| GO:0048636\_positive\_regulation\_of\_muscle\_development | 3 | 0 |  |  |  |  |  |  |  |  |
| GO:0048660\_regulation\_of\_smooth\_muscle\_cell\_proliferation | 3 | 0 |  |  |  |  |  |  |  |  |
| GO:0048668\_collateral\_sprouting | 3 | 0 |  |  |  |  |  |  |  |  |
| GO:0048676\_axon\_extension\_involved\_in\_development | 3 | 0 |  |  |  |  |  |  |  |  |
| GO:0048755\_branching\_morphogenesis\_of\_a\_nerve | 3 | 0 |  |  |  |  |  |  |  |  |
| GO:0048845\_venous\_blood\_vessel\_morphogenesis | 3 | 0 |  |  |  |  |  |  |  |  |
| GO:0048852\_diencephalon\_morphogenesis | 3 | 0 |  |  |  |  |  |  |  |  |
| GO:0048859\_formation\_of\_anatomical\_boundary | 3 | 0 |  |  |  |  |  |  |  |  |
| GO:0048865\_stem\_cell\_fate\_commitment | 3 | 0 |  |  |  |  |  |  |  |  |
| GO:0050435\_beta-amyloid\_metabolic\_process | 3 | 0 |  |  |  |  |  |  |  |  |
| GO:0050650\_chondroitin\_sulfate\_proteoglycan\_biosynthetic\_process | 3 | 0 |  |  |  |  |  |  |  |  |
| GO:0050703\_interleukin-1\_alpha\_secretion | 3 | 0 |  |  |  |  |  |  |  |  |
| GO:0050705\_regulation\_of\_interleukin-1\_alpha\_secretion | 3 | 0 |  |  |  |  |  |  |  |  |
| GO:0050709\_negative\_regulation\_of\_protein\_secretion | 3 | 0 |  |  |  |  |  |  |  |  |
| GO:0050710\_negative\_regulation\_of\_cytokine\_secretion | 3 | 0 |  |  |  |  |  |  |  |  |
| GO:0050717\_positive\_regulation\_of\_interleukin-1\_alpha\_secretion | 3 | 0 |  |  |  |  |  |  |  |  |
| GO:0050774\_negative\_regulation\_of\_dendrite\_morphogenesis | 3 | 0 |  |  |  |  |  |  |  |  |
| GO:0050857\_positive\_regulation\_of\_antigen\_receptor-mediated\_signaling\_pathway | 3 | 0 |  |  |  |  |  |  |  |  |
| GO:0050882\_voluntary\_musculoskeletal\_movement | 3 | 0 |  |  |  |  |  |  |  |  |
| GO:0050913\_sensory\_perception\_of\_bitter\_taste | 3 | 0 |  |  |  |  |  |  |  |  |
| GO:0050996\_positive\_regulation\_of\_lipid\_catabolic\_process | 3 | 0 |  |  |  |  |  |  |  |  |
| GO:0051149\_positive\_regulation\_of\_muscle\_cell\_differentiation | 3 | 0 |  |  |  |  |  |  |  |  |
| GO:0051153\_regulation\_of\_striated\_muscle\_cell\_differentiation | 3 | 0 |  |  |  |  |  |  |  |  |
| GO:0051204\_protein\_insertion\_into\_mitochondrial\_membrane | 3 | 0 |  |  |  |  |  |  |  |  |
| GO:0051291\_protein\_heterooligomerization | 3 | 0 |  |  |  |  |  |  |  |  |
| GO:0051302\_regulation\_of\_cell\_division | 3 | 0 |  |  |  |  |  |  |  |  |
| GO:0051320\_S\_phase | 3 | 0 |  |  |  |  |  |  |  |  |
| GO:0051450\_myoblast\_proliferation | 3 | 0 |  |  |  |  |  |  |  |  |
| GO:0051583\_dopamine\_uptake | 3 | 0 |  |  |  |  |  |  |  |  |
| GO:0051798\_positive\_regulation\_of\_hair\_follicle\_development | 3 | 0 |  |  |  |  |  |  |  |  |
| GO:0051882\_mitochondrial\_depolarization | 3 | 0 |  |  |  |  |  |  |  |  |
| GO:0051900\_regulation\_of\_mitochondrial\_depolarization | 3 | 0 |  |  |  |  |  |  |  |  |
| GO:0051925\_regulation\_of\_calcium\_ion\_transport\_via\_voltage-gated\_calcium\_channel\_activity | 3 | 0 |  |  |  |  |  |  |  |  |
| GO:0051926\_negative\_regulation\_of\_calcium\_ion\_transport | 3 | 0 |  |  |  |  |  |  |  |  |
| GO:0051930\_regulation\_of\_sensory\_perception\_of\_pain | 3 | 0 |  |  |  |  |  |  |  |  |
| GO:0051931\_regulation\_of\_sensory\_perception | 3 | 0 |  |  |  |  |  |  |  |  |
| GO:0051934\_catecholamine\_uptake\_during\_transmission\_of\_nerve\_impulse | 3 | 0 |  |  |  |  |  |  |  |  |
| GO:0051955\_regulation\_of\_amino\_acid\_transport | 3 | 0 |  |  |  |  |  |  |  |  |
| GO:0051962\_positive\_regulation\_of\_nervous\_system\_development | 3 | 0 |  |  |  |  |  |  |  |  |
| GO:0051965\_positive\_regulation\_of\_synaptogenesis | 3 | 0 |  |  |  |  |  |  |  |  |
| GO:0051967\_negative\_regulation\_of\_synaptic\_transmission\_\_glutamatergic | 3 | 0 |  |  |  |  |  |  |  |  |
| GO:0051983\_regulation\_of\_chromosome\_segregation | 3 | 0 |  |  |  |  |  |  |  |  |
| GO:0055061\_di-\_\_tri-valent\_inorganic\_anion\_homeostasis | 3 | 0 |  |  |  |  |  |  |  |  |
| GO:0055062\_phosphate\_ion\_homeostasis | 3 | 0 |  |  |  |  |  |  |  |  |
| GO:0055083\_monovalent\_inorganic\_anion\_homeostasis | 3 | 0 |  |  |  |  |  |  |  |  |
| GO:0055117\_regulation\_of\_cardiac\_muscle\_contraction | 3 | 0 |  |  |  |  |  |  |  |  |
| GO:0060009\_Sertoli\_cell\_development | 3 | 0 |  |  |  |  |  |  |  |  |
| GO:0060024\_rhythmic\_synaptic\_transmission | 3 | 0 |  |  |  |  |  |  |  |  |
| GO:0060033\_anatomical\_structure\_regression | 3 | 0 |  |  |  |  |  |  |  |  |
| GO:0060040\_retinal\_bipolar\_neuron\_differentiation | 3 | 0 |  |  |  |  |  |  |  |  |
| GO:0060055\_angiogenesis\_involved\_in\_wound\_healing | 3 | 0 |  |  |  |  |  |  |  |  |
| GO:0060084\_synaptic\_transmission\_involved\_in\_micturition | 3 | 0 |  |  |  |  |  |  |  |  |
| GO:0060123\_regulation\_of\_growth\_hormone\_secretion | 3 | 0 |  |  |  |  |  |  |  |  |
| GO:0060126\_somatotropin\_secreting\_cell\_differentiation | 3 | 0 |  |  |  |  |  |  |  |  |
| GO:0060192\_negative\_regulation\_of\_lipase\_activity | 3 | 0 |  |  |  |  |  |  |  |  |
| GO:0060219\_camera-type\_eye\_photoreceptor\_cell\_differentiation | 3 | 0 |  |  |  |  |  |  |  |  |
| GO:0060285\_ciliary\_cell\_motility | 3 | 0 |  |  |  |  |  |  |  |  |
| GO:0060294\_cilium\_movement\_involved\_in\_ciliary\_motility | 3 | 0 |  |  |  |  |  |  |  |  |
| GO:0060295\_regulation\_of\_cilium\_movement\_involved\_in\_ciliary\_motility | 3 | 0 |  |  |  |  |  |  |  |  |
| GO:0060296\_regulation\_of\_cilium\_beat\_frequency\_involved\_in\_ciliary\_motility | 3 | 0 |  |  |  |  |  |  |  |  |
| GO:0060314\_regulation\_of\_ryanodine-sensitive\_calcium-release\_channel\_activity | 3 | 0 |  |  |  |  |  |  |  |  |
| GO:0060396\_growth\_hormone\_receptor\_signaling\_pathway | 3 | 0 |  |  |  |  |  |  |  |  |
| GO:0060416\_response\_to\_growth\_hormone\_stimulus | 3 | 0 |  |  |  |  |  |  |  |  |
| GO:0060428\_lung\_epithelium\_development | 3 | 0 |  |  |  |  |  |  |  |  |
| GO:0060433\_bronchus\_development | 3 | 0 |  |  |  |  |  |  |  |  |
| GO:0060435\_bronchiole\_development | 3 | 0 |  |  |  |  |  |  |  |  |
| GO:0060460\_left\_lung\_morphogenesis | 3 | 0 |  |  |  |  |  |  |  |  |
| GO:0060491\_regulation\_of\_cell\_projection\_assembly | 3 | 0 |  |  |  |  |  |  |  |  |
| GO:0060523\_prostate\_epithelial\_cord\_elongation | 3 | 0 |  |  |  |  |  |  |  |  |
| GO:0060586\_multicellular\_organismal\_iron\_ion\_homeostasis | 3 | 0 |  |  |  |  |  |  |  |  |
| GO:0060596\_mammary\_placode\_formation | 3 | 0 |  |  |  |  |  |  |  |  |
| GO:0060632\_regulation\_of\_microtubule-based\_movement | 3 | 0 |  |  |  |  |  |  |  |  |
| GO:0060648\_mammary\_gland\_bud\_morphogenesis | 3 | 0 |  |  |  |  |  |  |  |  |
| GO:0060686\_negative\_regulation\_of\_prostatic\_bud\_formation | 3 | 0 |  |  |  |  |  |  |  |  |
| GO:0060708\_spongiotrophoblast\_differentiation | 3 | 0 |  |  |  |  |  |  |  |  |
| GO:0060746\_parental\_behavior | 3 | 0 |  |  |  |  |  |  |  |  |
| GO:0060748\_tertiary\_branching\_involved\_in\_mammary\_gland\_duct\_morphogenesis | 3 | 0 |  |  |  |  |  |  |  |  |
| GO:0060750\_epithelial\_cell\_proliferation\_involved\_in\_mammary\_gland\_duct\_elongation | 3 | 0 |  |  |  |  |  |  |  |  |
| GO:0060841\_venous\_blood\_vessel\_development | 3 | 0 |  |  |  |  |  |  |  |  |
| GO:0070102\_interleukin-6-mediated\_signaling\_pathway | 3 | 0 |  |  |  |  |  |  |  |  |
| GO:0070169\_positive\_regulation\_of\_biomineral\_formation | 3 | 0 |  |  |  |  |  |  |  |  |
| GO:0070206\_protein\_trimerization | 3 | 0 |  |  |  |  |  |  |  |  |
| GO:0070207\_protein\_homotrimerization | 3 | 0 |  |  |  |  |  |  |  |  |
| GO:0070229\_negative\_regulation\_of\_lymphocyte\_apoptosis | 3 | 0 |  |  |  |  |  |  |  |  |
| GO:0070230\_positive\_regulation\_of\_lymphocyte\_apoptosis | 3 | 0 |  |  |  |  |  |  |  |  |
| GO:0070232\_regulation\_of\_T\_cell\_apoptosis | 3 | 0 |  |  |  |  |  |  |  |  |
| GO:0070233\_negative\_regulation\_of\_T\_cell\_apoptosis | 3 | 0 |  |  |  |  |  |  |  |  |
| GO:0070242\_thymocyte\_apoptosis | 3 | 0 |  |  |  |  |  |  |  |  |
| GO:0070243\_regulation\_of\_thymocyte\_apoptosis | 3 | 0 |  |  |  |  |  |  |  |  |
| GO:0070244\_negative\_regulation\_of\_thymocyte\_apoptosis | 3 | 0 |  |  |  |  |  |  |  |  |
| GO:0070307\_lens\_fiber\_cell\_development | 3 | 0 |  |  |  |  |  |  |  |  |
| GO:0070309\_lens\_fiber\_cell\_morphogenesis | 3 | 0 |  |  |  |  |  |  |  |  |
| GO:0070423\_nucleotide-binding\_oligomerization\_domain\_containing\_signaling\_pathway | 3 | 0 |  |  |  |  |  |  |  |  |
| GO:0070427\_nucleotide-binding\_oligomerization\_domain\_containing\_1\_signaling\_pathway | 3 | 0 |  |  |  |  |  |  |  |  |
| GO:0070431\_nucleotide-binding\_oligomerization\_domain\_containing\_2\_signaling\_pathway | 3 | 0 |  |  |  |  |  |  |  |  |
| GO:0070633\_transepithelial\_transport | 3 | 0 |  |  |  |  |  |  |  |  |
| GO:0070846\_Hsp90\_deacetylation | 3 | 0 |  |  |  |  |  |  |  |  |
| GO:0070873\_regulation\_of\_glycogen\_metabolic\_process | 3 | 0 |  |  |  |  |  |  |  |  |
| GO:0070875\_positive\_regulation\_of\_glycogen\_metabolic\_process | 3 | 0 |  |  |  |  |  |  |  |  |
| GO:0006140\_regulation\_of\_nucleotide\_metabolic\_process | 47 | 0 | 0.000000 | -0.000000 | 1626 | 1603.008926 | 1658.48 | 1713.951074 | 1.019975 |
| GO:0030799\_regulation\_of\_cyclic\_nucleotide\_metabolic\_process | 47 | 0 | 0.000000 | -0.000000 | 1626 | 1603.008926 | 1658.48 | 1713.951074 | 1.019975 |
| GO:0031667\_response\_to\_nutrient\_levels | 47 | 0 | 0.000000 | -0.000000 | 1626 | 1603.008926 | 1658.48 | 1713.951074 | 1.019975 |
| GO:0034754\_cellular\_hormone\_metabolic\_process | 47 | 0 | 0.000000 | -0.000000 | 1626 | 1603.008926 | 1658.48 | 1713.951074 | 1.019975 |
| GO:0045087\_innate\_immune\_response | 47 | 0 | 0.000000 | -0.000000 | 1626 | 1603.008926 | 1658.48 | 1713.951074 | 1.019975 |
| GO:0045619\_regulation\_of\_lymphocyte\_differentiation | 47 | 0 | 0.000000 | -0.000000 | 1626 | 1603.008926 | 1658.48 | 1713.951074 | 1.019975 |
| GO:0048871\_multicellular\_organismal\_homeostasis | 47 | 0 | 0.000000 | -0.000000 | 1626 | 1603.008926 | 1658.48 | 1713.951074 | 1.019975 |
| GO:0060627\_regulation\_of\_vesicle-mediated\_transport | 47 | 0 | 0.000000 | -0.000000 | 1626 | 1603.008926 | 1658.48 | 1713.951074 | 1.019975 |
| GO:0007519\_skeletal\_muscle\_tissue\_development | 78 | 0 | 0.000000 | -0.000000 | 1629 | 1606.302451 | 1661.27 | 1716.237549 | 1.019810 |
| GO:0051251\_positive\_regulation\_of\_lymphocyte\_activation | 78 | 0 | 0.000000 | -0.000000 | 1629 | 1606.302451 | 1661.27 | 1716.237549 | 1.019810 |
| GO:0060538\_skeletal\_muscle\_organ\_development | 78 | 0 | 0.000000 | -0.000000 | 1629 | 1606.302451 | 1661.27 | 1716.237549 | 1.019810 |
| GO:0000041\_transition\_metal\_ion\_transport | 22 | 0 | 0.000000 | -0.000000 | 1667 | 1644.428298 | 1697.64 | 1750.851702 | 1.018380 |
| GO:0001523\_retinoid\_metabolic\_process | 22 | 0 | 0.000000 | -0.000000 | 1667 | 1644.428298 | 1697.64 | 1750.851702 | 1.018380 |
| GO:0001558\_regulation\_of\_cell\_growth | 22 | 0 | 0.000000 | -0.000000 | 1667 | 1644.428298 | 1697.64 | 1750.851702 | 1.018380 |
| GO:0001569\_patterning\_of\_blood\_vessels | 22 | 0 | 0.000000 | -0.000000 | 1667 | 1644.428298 | 1697.64 | 1750.851702 | 1.018380 |
| GO:0001947\_heart\_looping | 22 | 0 | 0.000000 | -0.000000 | 1667 | 1644.428298 | 1697.64 | 1750.851702 | 1.018380 |
| GO:0002478\_antigen\_processing\_and\_presentation\_of\_exogenous\_peptide\_antigen | 22 | 0 | 0.000000 | -0.000000 | 1667 | 1644.428298 | 1697.64 | 1750.851702 | 1.018380 |
| GO:0002637\_regulation\_of\_immunoglobulin\_production | 22 | 0 | 0.000000 | -0.000000 | 1667 | 1644.428298 | 1697.64 | 1750.851702 | 1.018380 |
| GO:0002712\_regulation\_of\_B\_cell\_mediated\_immunity | 22 | 0 | 0.000000 | -0.000000 | 1667 | 1644.428298 | 1697.64 | 1750.851702 | 1.018380 |
| GO:0002889\_regulation\_of\_immunoglobulin\_mediated\_immune\_response | 22 | 0 | 0.000000 | -0.000000 | 1667 | 1644.428298 | 1697.64 | 1750.851702 | 1.018380 |
| GO:0006029\_proteoglycan\_metabolic\_process | 22 | 0 | 0.000000 | -0.000000 | 1667 | 1644.428298 | 1697.64 | 1750.851702 | 1.018380 |
| GO:0006112\_energy\_reserve\_metabolic\_process | 22 | 0 | 0.000000 | -0.000000 | 1667 | 1644.428298 | 1697.64 | 1750.851702 | 1.018380 |
| GO:0006721\_terpenoid\_metabolic\_process | 22 | 0 | 0.000000 | -0.000000 | 1667 | 1644.428298 | 1697.64 | 1750.851702 | 1.018380 |
| GO:0007059\_chromosome\_segregation | 22 | 0 | 0.000000 | -0.000000 | 1667 | 1644.428298 | 1697.64 | 1750.851702 | 1.018380 |
| GO:0009309\_amine\_biosynthetic\_process | 22 | 0 | 0.000000 | -0.000000 | 1667 | 1644.428298 | 1697.64 | 1750.851702 | 1.018380 |
| GO:0009896\_positive\_regulation\_of\_catabolic\_process | 22 | 0 | 0.000000 | -0.000000 | 1667 | 1644.428298 | 1697.64 | 1750.851702 | 1.018380 |
| GO:0015918\_sterol\_transport | 22 | 0 | 0.000000 | -0.000000 | 1667 | 1644.428298 | 1697.64 | 1750.851702 | 1.018380 |
| GO:0016101\_diterpenoid\_metabolic\_process | 22 | 0 | 0.000000 | -0.000000 | 1667 | 1644.428298 | 1697.64 | 1750.851702 | 1.018380 |
| GO:0021766\_hippocampus\_development | 22 | 0 | 0.000000 | -0.000000 | 1667 | 1644.428298 | 1697.64 | 1750.851702 | 1.018380 |
| GO:0030258\_lipid\_modification | 22 | 0 | 0.000000 | -0.000000 | 1667 | 1644.428298 | 1697.64 | 1750.851702 | 1.018380 |
| GO:0030301\_cholesterol\_transport | 22 | 0 | 0.000000 | -0.000000 | 1667 | 1644.428298 | 1697.64 | 1750.851702 | 1.018380 |
| GO:0030335\_positive\_regulation\_of\_cell\_migration | 22 | 0 | 0.000000 | -0.000000 | 1667 | 1644.428298 | 1697.64 | 1750.851702 | 1.018380 |
| GO:0030705\_cytoskeleton-dependent\_intracellular\_transport | 22 | 0 | 0.000000 | -0.000000 | 1667 | 1644.428298 | 1697.64 | 1750.851702 | 1.018380 |
| GO:0032649\_regulation\_of\_interferon-gamma\_production | 22 | 0 | 0.000000 | -0.000000 | 1667 | 1644.428298 | 1697.64 | 1750.851702 | 1.018380 |
| GO:0032886\_regulation\_of\_microtubule-based\_process | 22 | 0 | 0.000000 | -0.000000 | 1667 | 1644.428298 | 1697.64 | 1750.851702 | 1.018380 |
| GO:0033273\_response\_to\_vitamin | 22 | 0 | 0.000000 | -0.000000 | 1667 | 1644.428298 | 1697.64 | 1750.851702 | 1.018380 |
| GO:0034097\_response\_to\_cytokine\_stimulus | 22 | 0 | 0.000000 | -0.000000 | 1667 | 1644.428298 | 1697.64 | 1750.851702 | 1.018380 |
| GO:0034660\_ncRNA\_metabolic\_process | 22 | 0 | 0.000000 | -0.000000 | 1667 | 1644.428298 | 1697.64 | 1750.851702 | 1.018380 |
| GO:0040018\_positive\_regulation\_of\_multicellular\_organism\_growth | 22 | 0 | 0.000000 | -0.000000 | 1667 | 1644.428298 | 1697.64 | 1750.851702 | 1.018380 |
| GO:0042130\_negative\_regulation\_of\_T\_cell\_proliferation | 22 | 0 | 0.000000 | -0.000000 | 1667 | 1644.428298 | 1697.64 | 1750.851702 | 1.018380 |
| GO:0042461\_photoreceptor\_cell\_development | 22 | 0 | 0.000000 | -0.000000 | 1667 | 1644.428298 | 1697.64 | 1750.851702 | 1.018380 |
| GO:0042733\_embryonic\_digit\_morphogenesis | 22 | 0 | 0.000000 | -0.000000 | 1667 | 1644.428298 | 1697.64 | 1750.851702 | 1.018380 |
| GO:0043112\_receptor\_metabolic\_process | 22 | 0 | 0.000000 | -0.000000 | 1667 | 1644.428298 | 1697.64 | 1750.851702 | 1.018380 |
| GO:0044264\_cellular\_polysaccharide\_metabolic\_process | 22 | 0 | 0.000000 | -0.000000 | 1667 | 1644.428298 | 1697.64 | 1750.851702 | 1.018380 |
| GO:0046635\_positive\_regulation\_of\_alpha-beta\_T\_cell\_activation | 22 | 0 | 0.000000 | -0.000000 | 1667 | 1644.428298 | 1697.64 | 1750.851702 | 1.018380 |
| GO:0046883\_regulation\_of\_hormone\_secretion | 22 | 0 | 0.000000 | -0.000000 | 1667 | 1644.428298 | 1697.64 | 1750.851702 | 1.018380 |
| GO:0048477\_oogenesis | 22 | 0 | 0.000000 | -0.000000 | 1667 | 1644.428298 | 1697.64 | 1750.851702 | 1.018380 |
| GO:0048864\_stem\_cell\_development | 22 | 0 | 0.000000 | -0.000000 | 1667 | 1644.428298 | 1697.64 | 1750.851702 | 1.018380 |
| GO:0051260\_protein\_homooligomerization | 22 | 0 | 0.000000 | -0.000000 | 1667 | 1644.428298 | 1697.64 | 1750.851702 | 1.018380 |
| GO:0000027\_ribosomal\_large\_subunit\_assembly | 1 | 0 |  |  |  |  |  |  |  |  |
| GO:0000042\_protein\_targeting\_to\_Golgi | 1 | 0 |  |  |  |  |  |  |  |  |
| GO:0000046\_autophagic\_vacuole\_fusion | 1 | 0 |  |  |  |  |  |  |  |  |
| GO:0000050\_urea\_cycle | 1 | 0 |  |  |  |  |  |  |  |  |
| GO:0000054\_ribosome\_export\_from\_nucleus | 1 | 0 |  |  |  |  |  |  |  |  |
| GO:0000055\_ribosomal\_large\_subunit\_export\_from\_nucleus | 1 | 0 |  |  |  |  |  |  |  |  |
| GO:0000056\_ribosomal\_small\_subunit\_export\_from\_nucleus | 1 | 0 |  |  |  |  |  |  |  |  |
| GO:0000072\_M\_phase\_specific\_microtubule\_process | 1 | 0 |  |  |  |  |  |  |  |  |
| GO:0000101\_sulfur\_amino\_acid\_transport | 1 | 0 |  |  |  |  |  |  |  |  |
| GO:0000147\_actin\_cortical\_patch\_assembly | 1 | 0 |  |  |  |  |  |  |  |  |
| GO:0000154\_rRNA\_modification | 1 | 0 |  |  |  |  |  |  |  |  |
| GO:0000183\_chromatin\_silencing\_at\_rDNA | 1 | 0 |  |  |  |  |  |  |  |  |
| GO:0000185\_activation\_of\_MAPKKK\_activity | 1 | 0 |  |  |  |  |  |  |  |  |
| GO:0000238\_zygotene | 1 | 0 |  |  |  |  |  |  |  |  |
| GO:0000255\_allantoin\_metabolic\_process | 1 | 0 |  |  |  |  |  |  |  |  |
| GO:0000266\_mitochondrial\_fission | 1 | 0 |  |  |  |  |  |  |  |  |
| GO:0000273\_lipoic\_acid\_metabolic\_process | 1 | 0 |  |  |  |  |  |  |  |  |
| GO:0000301\_retrograde\_transport\_\_vesicle\_recycling\_within\_Golgi | 1 | 0 |  |  |  |  |  |  |  |  |
| GO:0000394\_RNA\_splicing\_\_via\_endonucleolytic\_cleavage\_and\_ligation | 1 | 0 |  |  |  |  |  |  |  |  |
| GO:0000429\_regulation\_of\_transcription\_from\_RNA\_polymerase\_II\_promoter\_by\_carbon\_catabolites | 1 | 0 |  |  |  |  |  |  |  |  |
| GO:0000430\_regulation\_of\_transcription\_from\_RNA\_polymerase\_II\_promoter\_by\_glucose | 1 | 0 |  |  |  |  |  |  |  |  |
| GO:0000432\_positive\_regulation\_of\_transcription\_from\_RNA\_polymerase\_II\_promoter\_by\_glucose | 1 | 0 |  |  |  |  |  |  |  |  |
| GO:0000436\_positive\_regulation\_of\_transcription\_from\_RNA\_polymerase\_II\_promoter\_by\_carbon\_catabolites | 1 | 0 |  |  |  |  |  |  |  |  |
| GO:0000448\_cleavage\_in\_ITS2\_between\_5.8S\_rRNA\_and\_LSU-rRNA\_of\_tricistronic\_rRNA\_transcript\_(SSU-rRNA\_\_5.8S\_rRNA\_\_LSU-rRNA) | 1 | 0 |  |  |  |  |  |  |  |  |
| GO:0000460\_maturation\_of\_5.8S\_rRNA | 1 | 0 |  |  |  |  |  |  |  |  |
| GO:0000463\_maturation\_of\_LSU-rRNA\_from\_tricistronic\_rRNA\_transcript\_(SSU-rRNA\_\_5.8S\_rRNA\_\_LSU-rRNA) | 1 | 0 |  |  |  |  |  |  |  |  |
| GO:0000466\_maturation\_of\_5.8S\_rRNA\_from\_tricistronic\_rRNA\_transcript\_(SSU-rRNA\_\_5.8S\_rRNA\_\_LSU-rRNA) | 1 | 0 |  |  |  |  |  |  |  |  |
| GO:0000469\_cleavages\_during\_rRNA\_processing | 1 | 0 |  |  |  |  |  |  |  |  |
| GO:0000470\_maturation\_of\_LSU-rRNA | 1 | 0 |  |  |  |  |  |  |  |  |
| GO:0000478\_endonucleolytic\_cleavages\_during\_rRNA\_processing | 1 | 0 |  |  |  |  |  |  |  |  |
| GO:0000479\_endonucleolytic\_cleavage\_of\_tricistronic\_rRNA\_transcript\_(SSU-rRNA\_\_5.8S\_rRNA\_\_LSU-rRNA) | 1 | 0 |  |  |  |  |  |  |  |  |
| GO:0000705\_achiasmate\_meiosis\_I | 1 | 0 |  |  |  |  |  |  |  |  |
| GO:0000966\_RNA\_5'-end\_processing | 1 | 0 |  |  |  |  |  |  |  |  |
| GO:0001300\_chronological\_cell\_aging | 1 | 0 |  |  |  |  |  |  |  |  |
| GO:0001547\_antral\_ovarian\_follicle\_growth | 1 | 0 |  |  |  |  |  |  |  |  |
| GO:0001555\_oocyte\_growth | 1 | 0 |  |  |  |  |  |  |  |  |
| GO:0001560\_regulation\_of\_cell\_growth\_by\_extracellular\_stimulus | 1 | 0 |  |  |  |  |  |  |  |  |
| GO:0001660\_fever | 1 | 0 |  |  |  |  |  |  |  |  |
| GO:0001696\_gastric\_acid\_secretion | 1 | 0 |  |  |  |  |  |  |  |  |
| GO:0001712\_ectodermal\_cell\_fate\_commitment | 1 | 0 |  |  |  |  |  |  |  |  |
| GO:0001714\_endodermal\_cell\_fate\_specification | 1 | 0 |  |  |  |  |  |  |  |  |
| GO:0001762\_beta-alanine\_transport | 1 | 0 |  |  |  |  |  |  |  |  |
| GO:0001766\_membrane\_raft\_polarization | 1 | 0 |  |  |  |  |  |  |  |  |
| GO:0001811\_negative\_regulation\_of\_type\_I\_hypersensitivity | 1 | 0 |  |  |  |  |  |  |  |  |
| GO:0001821\_histamine\_secretion | 1 | 0 |  |  |  |  |  |  |  |  |
| GO:0001826\_inner\_cell\_mass\_cell\_differentiation | 1 | 0 |  |  |  |  |  |  |  |  |
| GO:0001830\_trophectodermal\_cell\_fate\_commitment | 1 | 0 |  |  |  |  |  |  |  |  |
| GO:0001834\_trophectodermal\_cell\_proliferation | 1 | 0 |  |  |  |  |  |  |  |  |
| GO:0001867\_complement\_activation\_\_lectin\_pathway | 1 | 0 |  |  |  |  |  |  |  |  |
| GO:0001880\_Mullerian\_duct\_regression | 1 | 0 |  |  |  |  |  |  |  |  |
| GO:0001887\_selenium\_metabolic\_process | 1 | 0 |  |  |  |  |  |  |  |  |
| GO:0001922\_B-1\_B\_cell\_homeostasis | 1 | 0 |  |  |  |  |  |  |  |  |
| GO:0001923\_B-1\_B\_cell\_differentiation | 1 | 0 |  |  |  |  |  |  |  |  |
| GO:0001941\_postsynaptic\_membrane\_organization | 1 | 0 |  |  |  |  |  |  |  |  |
| GO:0001946\_lymphangiogenesis | 1 | 0 |  |  |  |  |  |  |  |  |
| GO:0001956\_positive\_regulation\_of\_neurotransmitter\_secretion | 1 | 0 |  |  |  |  |  |  |  |  |
| GO:0001961\_positive\_regulation\_of\_cytokine-mediated\_signaling\_pathway | 1 | 0 |  |  |  |  |  |  |  |  |
| GO:0001979\_regulation\_of\_systemic\_arterial\_blood\_pressure\_by\_chemoreceptor\_signaling | 1 | 0 |  |  |  |  |  |  |  |  |
| GO:0001980\_regulation\_of\_systemic\_arterial\_blood\_pressure\_by\_ischemic\_conditions | 1 | 0 |  |  |  |  |  |  |  |  |
| GO:0001984\_vasodilation\_of\_artery\_during\_baroreceptor\_response\_to\_increased\_systemic\_arterial\_blood\_pressure | 1 | 0 |  |  |  |  |  |  |  |  |
| GO:0001985\_negative\_regulation\_of\_heart\_rate\_in\_baroreceptor\_response\_to\_increased\_systemic\_arterial\_blood\_pressure | 1 | 0 |  |  |  |  |  |  |  |  |
| GO:0001987\_vasoconstriction\_of\_artery\_involved\_in\_baroreceptor\_response\_to\_lowering\_of\_systemic\_arterial\_blood\_pressure | 1 | 0 |  |  |  |  |  |  |  |  |
| GO:0001988\_positive\_regulation\_of\_heart\_rate\_in\_baroreceptor\_response\_to\_decreased\_systemic\_arterial\_blood\_pressure | 1 | 0 |  |  |  |  |  |  |  |  |
| GO:0001994\_norepinephrine-epinephrine\_vasoconstriction\_involved\_in\_regulation\_of\_systemic\_arterial\_blood\_pressure | 1 | 0 |  |  |  |  |  |  |  |  |
| GO:0002001\_renin\_secretion\_into\_blood\_stream | 1 | 0 |  |  |  |  |  |  |  |  |
| GO:0002002\_regulation\_of\_angiotensin\_levels\_in\_blood | 1 | 0 |  |  |  |  |  |  |  |  |
| GO:0002003\_angiotensin\_maturation | 1 | 0 |  |  |  |  |  |  |  |  |
| GO:0002007\_detection\_of\_hypoxic\_conditions\_in\_blood\_by\_chemoreceptor\_signaling | 1 | 0 |  |  |  |  |  |  |  |  |
| GO:0002017\_regulation\_of\_blood\_volume\_by\_renal\_aldosterone | 1 | 0 |  |  |  |  |  |  |  |  |
| GO:0002023\_reduction\_of\_food\_intake\_in\_response\_to\_dietary\_excess | 1 | 0 |  |  |  |  |  |  |  |  |
| GO:0002031\_G-protein\_coupled\_receptor\_internalization | 1 | 0 |  |  |  |  |  |  |  |  |
| GO:0002036\_regulation\_of\_L-glutamate\_transport | 1 | 0 |  |  |  |  |  |  |  |  |
| GO:0002040\_sprouting\_angiogenesis | 1 | 0 |  |  |  |  |  |  |  |  |
| GO:0002041\_intussusceptive\_angiogenesis | 1 | 0 |  |  |  |  |  |  |  |  |
| GO:0002068\_glandular\_epithelial\_cell\_development | 1 | 0 |  |  |  |  |  |  |  |  |
| GO:0002069\_columnar\_cuboidal\_epithelial\_cell\_maturation | 1 | 0 |  |  |  |  |  |  |  |  |
| GO:0002071\_glandular\_epithelial\_cell\_maturation | 1 | 0 |  |  |  |  |  |  |  |  |
| GO:0002082\_regulation\_of\_oxidative\_phosphorylation | 1 | 0 |  |  |  |  |  |  |  |  |
| GO:0002084\_protein\_depalmitoylation | 1 | 0 |  |  |  |  |  |  |  |  |
| GO:0002085\_inhibition\_of\_neuroepithelial\_cell\_differentiation | 1 | 0 |  |  |  |  |  |  |  |  |
| GO:0002086\_diaphragm\_contraction | 1 | 0 |  |  |  |  |  |  |  |  |
| GO:0002118\_aggressive\_behavior | 1 | 0 |  |  |  |  |  |  |  |  |
| GO:0002121\_inter-male\_aggressive\_behavior | 1 | 0 |  |  |  |  |  |  |  |  |
| GO:0002124\_territorial\_aggressive\_behavior | 1 | 0 |  |  |  |  |  |  |  |  |
| GO:0002227\_innate\_immune\_response\_in\_mucosa | 1 | 0 |  |  |  |  |  |  |  |  |
| GO:0002232\_leukocyte\_chemotaxis\_during\_inflammatory\_response | 1 | 0 |  |  |  |  |  |  |  |  |
| GO:0002248\_connective\_tissue\_replacement\_during\_inflammatory\_response | 1 | 0 |  |  |  |  |  |  |  |  |
| GO:0002282\_microglial\_cell\_activation\_during\_immune\_response | 1 | 0 |  |  |  |  |  |  |  |  |
| GO:0002287\_alpha-beta\_T\_cell\_activation\_during\_immune\_response | 1 | 0 |  |  |  |  |  |  |  |  |
| GO:0002314\_germinal\_center\_B\_cell\_differentiation | 1 | 0 |  |  |  |  |  |  |  |  |
| GO:0002315\_marginal\_zone\_B\_cell\_differentiation | 1 | 0 |  |  |  |  |  |  |  |  |
| GO:0002316\_follicular\_B\_cell\_differentiation | 1 | 0 |  |  |  |  |  |  |  |  |
| GO:0002317\_plasma\_cell\_differentiation | 1 | 0 |  |  |  |  |  |  |  |  |
| GO:0002349\_histamine\_production\_during\_acute\_inflammatory\_response | 1 | 0 |  |  |  |  |  |  |  |  |
| GO:0002351\_serotonin\_production\_during\_acute\_inflammatory\_response | 1 | 0 |  |  |  |  |  |  |  |  |
| GO:0002355\_detection\_of\_tumor\_cell | 1 | 0 |  |  |  |  |  |  |  |  |
| GO:0002370\_natural\_killer\_cell\_cytokine\_production | 1 | 0 |  |  |  |  |  |  |  |  |
| GO:0002371\_dendritic\_cell\_cytokine\_production | 1 | 0 |  |  |  |  |  |  |  |  |
| GO:0002380\_immunoglobulin\_secretion\_during\_immune\_response | 1 | 0 |  |  |  |  |  |  |  |  |
| GO:0002396\_MHC\_protein\_complex\_assembly | 1 | 0 |  |  |  |  |  |  |  |  |
| GO:0002397\_MHC\_class\_I\_protein\_complex\_assembly | 1 | 0 |  |  |  |  |  |  |  |  |
| GO:0002420\_natural\_killer\_cell\_mediated\_cytotoxicity\_directed\_against\_tumor\_cell\_target | 1 | 0 |  |  |  |  |  |  |  |  |
| GO:0002423\_natural\_killer\_cell\_mediated\_immune\_response\_to\_tumor\_cell | 1 | 0 |  |  |  |  |  |  |  |  |
| GO:0002424\_T\_cell\_mediated\_immune\_response\_to\_tumor\_cell | 1 | 0 |  |  |  |  |  |  |  |  |
| GO:0002426\_immunoglobulin\_production\_in\_mucosal\_tissue | 1 | 0 |  |  |  |  |  |  |  |  |
| GO:0002431\_Fc\_receptor\_mediated\_stimulatory\_signaling\_pathway | 1 | 0 |  |  |  |  |  |  |  |  |
| GO:0002432\_granuloma\_formation | 1 | 0 |  |  |  |  |  |  |  |  |
| GO:0002441\_histamine\_secretion\_during\_acute\_inflammatory\_response | 1 | 0 |  |  |  |  |  |  |  |  |
| GO:0002442\_serotonin\_secretion\_during\_acute\_inflammatory\_response | 1 | 0 |  |  |  |  |  |  |  |  |
| GO:0002457\_T\_cell\_antigen\_processing\_and\_presentation | 1 | 0 |  |  |  |  |  |  |  |  |
| GO:0002458\_peripheral\_T\_cell\_tolerance\_induction | 1 | 0 |  |  |  |  |  |  |  |  |
| GO:0002461\_tolerance\_induction\_dependent\_upon\_immune\_response | 1 | 0 |  |  |  |  |  |  |  |  |
| GO:0002465\_peripheral\_tolerance\_induction | 1 | 0 |  |  |  |  |  |  |  |  |
| GO:0002468\_dendritic\_cell\_antigen\_processing\_and\_presentation | 1 | 0 |  |  |  |  |  |  |  |  |
| GO:0002476\_antigen\_processing\_and\_presentation\_of\_endogenous\_peptide\_antigen\_via\_MHC\_class\_Ib | 1 | 0 |  |  |  |  |  |  |  |  |
| GO:0002479\_antigen\_processing\_and\_presentation\_of\_exogenous\_peptide\_antigen\_via\_MHC\_class\_I\_\_TAP-dependent | 1 | 0 |  |  |  |  |  |  |  |  |
| GO:0002483\_antigen\_processing\_and\_presentation\_of\_endogenous\_peptide\_antigen | 1 | 0 |  |  |  |  |  |  |  |  |
| GO:0002501\_peptide\_antigen\_assembly\_with\_MHC\_protein\_complex | 1 | 0 |  |  |  |  |  |  |  |  |
| GO:0002502\_peptide\_antigen\_assembly\_with\_MHC\_class\_I\_protein\_complex | 1 | 0 |  |  |  |  |  |  |  |  |
| GO:0002508\_central\_tolerance\_induction | 1 | 0 |  |  |  |  |  |  |  |  |
| GO:0002510\_central\_B\_cell\_tolerance\_induction | 1 | 0 |  |  |  |  |  |  |  |  |
| GO:0002545\_chronic\_inflammatory\_response\_to\_non-antigenic\_stimulus | 1 | 0 |  |  |  |  |  |  |  |  |
| GO:0002553\_histamine\_secretion\_by\_mast\_cell | 1 | 0 |  |  |  |  |  |  |  |  |
| GO:0002554\_serotonin\_secretion\_by\_platelet | 1 | 0 |  |  |  |  |  |  |  |  |
| GO:0002572\_pro-T\_cell\_differentiation | 1 | 0 |  |  |  |  |  |  |  |  |
| GO:0002577\_regulation\_of\_antigen\_processing\_and\_presentation | 1 | 0 |  |  |  |  |  |  |  |  |
| GO:0002579\_positive\_regulation\_of\_antigen\_processing\_and\_presentation | 1 | 0 |  |  |  |  |  |  |  |  |
| GO:0002604\_regulation\_of\_dendritic\_cell\_antigen\_processing\_and\_presentation | 1 | 0 |  |  |  |  |  |  |  |  |
| GO:0002606\_positive\_regulation\_of\_dendritic\_cell\_antigen\_processing\_and\_presentation | 1 | 0 |  |  |  |  |  |  |  |  |
| GO:0002635\_negative\_regulation\_of\_germinal\_center\_formation | 1 | 0 |  |  |  |  |  |  |  |  |
| GO:0002646\_regulation\_of\_central\_tolerance\_induction | 1 | 0 |  |  |  |  |  |  |  |  |
| GO:0002648\_positive\_regulation\_of\_central\_tolerance\_induction | 1 | 0 |  |  |  |  |  |  |  |  |
| GO:0002649\_regulation\_of\_tolerance\_induction\_to\_self\_antigen | 1 | 0 |  |  |  |  |  |  |  |  |
| GO:0002651\_positive\_regulation\_of\_tolerance\_induction\_to\_self\_antigen | 1 | 0 |  |  |  |  |  |  |  |  |
| GO:0002652\_regulation\_of\_tolerance\_induction\_dependent\_upon\_immune\_response | 1 | 0 |  |  |  |  |  |  |  |  |
| GO:0002654\_positive\_regulation\_of\_tolerance\_induction\_dependent\_upon\_immune\_response | 1 | 0 |  |  |  |  |  |  |  |  |
| GO:0002658\_regulation\_of\_peripheral\_tolerance\_induction | 1 | 0 |  |  |  |  |  |  |  |  |
| GO:0002660\_positive\_regulation\_of\_peripheral\_tolerance\_induction | 1 | 0 |  |  |  |  |  |  |  |  |
| GO:0002677\_negative\_regulation\_of\_chronic\_inflammatory\_response | 1 | 0 |  |  |  |  |  |  |  |  |
| GO:0002678\_positive\_regulation\_of\_chronic\_inflammatory\_response | 1 | 0 |  |  |  |  |  |  |  |  |
| GO:0002701\_negative\_regulation\_of\_production\_of\_molecular\_mediator\_of\_immune\_response | 1 | 0 |  |  |  |  |  |  |  |  |
| GO:0002719\_negative\_regulation\_of\_cytokine\_production\_during\_immune\_response | 1 | 0 |  |  |  |  |  |  |  |  |
| GO:0002724\_regulation\_of\_T\_cell\_cytokine\_production | 1 | 0 |  |  |  |  |  |  |  |  |
| GO:0002727\_regulation\_of\_natural\_killer\_cell\_cytokine\_production | 1 | 0 |  |  |  |  |  |  |  |  |
| GO:0002729\_positive\_regulation\_of\_natural\_killer\_cell\_cytokine\_production | 1 | 0 |  |  |  |  |  |  |  |  |
| GO:0002730\_regulation\_of\_dendritic\_cell\_cytokine\_production | 1 | 0 |  |  |  |  |  |  |  |  |
| GO:0002756\_MyD88-independent\_toll-like\_receptor\_signaling\_pathway | 1 | 0 |  |  |  |  |  |  |  |  |
| GO:0002767\_immune\_response-inhibiting\_cell\_surface\_receptor\_signaling\_pathway | 1 | 0 |  |  |  |  |  |  |  |  |
| GO:0002769\_natural\_killer\_cell\_inhibitory\_signaling\_pathway | 1 | 0 |  |  |  |  |  |  |  |  |
| GO:0002840\_regulation\_of\_T\_cell\_mediated\_immune\_response\_to\_tumor\_cell | 1 | 0 |  |  |  |  |  |  |  |  |
| GO:0002842\_positive\_regulation\_of\_T\_cell\_mediated\_immune\_response\_to\_tumor\_cell | 1 | 0 |  |  |  |  |  |  |  |  |
| GO:0002849\_regulation\_of\_peripheral\_T\_cell\_tolerance\_induction | 1 | 0 |  |  |  |  |  |  |  |  |
| GO:0002851\_positive\_regulation\_of\_peripheral\_T\_cell\_tolerance\_induction | 1 | 0 |  |  |  |  |  |  |  |  |
| GO:0002855\_regulation\_of\_natural\_killer\_cell\_mediated\_immune\_response\_to\_tumor\_cell | 1 | 0 |  |  |  |  |  |  |  |  |
| GO:0002857\_positive\_regulation\_of\_natural\_killer\_cell\_mediated\_immune\_response\_to\_tumor\_cell | 1 | 0 |  |  |  |  |  |  |  |  |
| GO:0002858\_regulation\_of\_natural\_killer\_cell\_mediated\_cytotoxicity\_directed\_against\_tumor\_cell\_target | 1 | 0 |  |  |  |  |  |  |  |  |
| GO:0002860\_positive\_regulation\_of\_natural\_killer\_cell\_mediated\_cytotoxicity\_directed\_against\_tumor\_cell\_target | 1 | 0 |  |  |  |  |  |  |  |  |
| GO:0002880\_regulation\_of\_chronic\_inflammatory\_response\_to\_non-antigenic\_stimulus | 1 | 0 |  |  |  |  |  |  |  |  |
| GO:0002882\_positive\_regulation\_of\_chronic\_inflammatory\_response\_to\_non-antigenic\_stimulus | 1 | 0 |  |  |  |  |  |  |  |  |
| GO:0002895\_regulation\_of\_central\_B\_cell\_tolerance\_induction | 1 | 0 |  |  |  |  |  |  |  |  |
| GO:0002897\_positive\_regulation\_of\_central\_B\_cell\_tolerance\_induction | 1 | 0 |  |  |  |  |  |  |  |  |
| GO:0002901\_mature\_B\_cell\_apoptosis | 1 | 0 |  |  |  |  |  |  |  |  |
| GO:0002903\_negative\_regulation\_of\_B\_cell\_apoptosis | 1 | 0 |  |  |  |  |  |  |  |  |
| GO:0002905\_regulation\_of\_mature\_B\_cell\_apoptosis | 1 | 0 |  |  |  |  |  |  |  |  |
| GO:0002906\_negative\_regulation\_of\_mature\_B\_cell\_apoptosis | 1 | 0 |  |  |  |  |  |  |  |  |
| GO:0003011\_involuntary\_skeletal\_muscle\_contraction | 1 | 0 |  |  |  |  |  |  |  |  |
| GO:0003027\_regulation\_of\_systemic\_arterial\_blood\_pressure\_by\_carotid\_body\_chemoreceptor\_signaling | 1 | 0 |  |  |  |  |  |  |  |  |
| GO:0003029\_detection\_of\_hypoxic\_conditions\_in\_blood\_by\_carotid\_body\_chemoreceptor\_signaling | 1 | 0 |  |  |  |  |  |  |  |  |
| GO:0003032\_detection\_of\_oxygen | 1 | 0 |  |  |  |  |  |  |  |  |
| GO:0003056\_regulation\_of\_vascular\_smooth\_muscle\_contraction | 1 | 0 |  |  |  |  |  |  |  |  |
| GO:0003062\_regulation\_of\_heart\_rate\_by\_chemical\_signal | 1 | 0 |  |  |  |  |  |  |  |  |
| GO:0003065\_positive\_regulation\_of\_heart\_rate\_by\_epinephrine | 1 | 0 |  |  |  |  |  |  |  |  |
| GO:0003068\_regulation\_of\_systemic\_arterial\_blood\_pressure\_by\_acetylcholine | 1 | 0 |  |  |  |  |  |  |  |  |
| GO:0003069\_vasodilation\_by\_acetylcholine\_involved\_in\_regulation\_of\_systemic\_arterial\_blood\_pressure | 1 | 0 |  |  |  |  |  |  |  |  |
| GO:0003070\_regulation\_of\_systemic\_arterial\_blood\_pressure\_by\_neurotransmitter | 1 | 0 |  |  |  |  |  |  |  |  |
| GO:0003097\_renal\_water\_transport | 1 | 0 |  |  |  |  |  |  |  |  |
| GO:0005979\_regulation\_of\_glycogen\_biosynthetic\_process | 1 | 0 |  |  |  |  |  |  |  |  |
| GO:0005984\_disaccharide\_metabolic\_process | 1 | 0 |  |  |  |  |  |  |  |  |
| GO:0005988\_lactose\_metabolic\_process | 1 | 0 |  |  |  |  |  |  |  |  |
| GO:0005989\_lactose\_biosynthetic\_process | 1 | 0 |  |  |  |  |  |  |  |  |
| GO:0005997\_xylulose\_metabolic\_process | 1 | 0 |  |  |  |  |  |  |  |  |
| GO:0006000\_fructose\_metabolic\_process | 1 | 0 |  |  |  |  |  |  |  |  |
| GO:0006002\_fructose\_6-phosphate\_metabolic\_process | 1 | 0 |  |  |  |  |  |  |  |  |
| GO:0006004\_fucose\_metabolic\_process | 1 | 0 |  |  |  |  |  |  |  |  |
| GO:0006013\_mannose\_metabolic\_process | 1 | 0 |  |  |  |  |  |  |  |  |
| GO:0006060\_sorbitol\_metabolic\_process | 1 | 0 |  |  |  |  |  |  |  |  |
| GO:0006064\_glucuronate\_catabolic\_process | 1 | 0 |  |  |  |  |  |  |  |  |
| GO:0006086\_acetyl-CoA\_biosynthetic\_process\_from\_pyruvate | 1 | 0 |  |  |  |  |  |  |  |  |
| GO:0006098\_pentose-phosphate\_shunt | 1 | 0 |  |  |  |  |  |  |  |  |
| GO:0006101\_citrate\_metabolic\_process | 1 | 0 |  |  |  |  |  |  |  |  |
| GO:0006104\_succinyl-CoA\_metabolic\_process | 1 | 0 |  |  |  |  |  |  |  |  |
| GO:0006116\_NADH\_oxidation | 1 | 0 |  |  |  |  |  |  |  |  |
| GO:0006120\_mitochondrial\_electron\_transport\_\_NADH\_to\_ubiquinone | 1 | 0 |  |  |  |  |  |  |  |  |
| GO:0006154\_adenosine\_catabolic\_process | 1 | 0 |  |  |  |  |  |  |  |  |
| GO:0006157\_deoxyadenosine\_catabolic\_process | 1 | 0 |  |  |  |  |  |  |  |  |
| GO:0006167\_AMP\_biosynthetic\_process | 1 | 0 |  |  |  |  |  |  |  |  |
| GO:0006175\_dATP\_biosynthetic\_process | 1 | 0 |  |  |  |  |  |  |  |  |
| GO:0006178\_guanine\_salvage | 1 | 0 |  |  |  |  |  |  |  |  |
| GO:0006196\_AMP\_catabolic\_process | 1 | 0 |  |  |  |  |  |  |  |  |
| GO:0006203\_dGTP\_catabolic\_process | 1 | 0 |  |  |  |  |  |  |  |  |
| GO:0006208\_pyrimidine\_base\_catabolic\_process | 1 | 0 |  |  |  |  |  |  |  |  |
| GO:0006221\_pyrimidine\_nucleotide\_biosynthetic\_process | 1 | 0 |  |  |  |  |  |  |  |  |
| GO:0006235\_dTTP\_biosynthetic\_process | 1 | 0 |  |  |  |  |  |  |  |  |
| GO:0006244\_pyrimidine\_nucleotide\_catabolic\_process | 1 | 0 |  |  |  |  |  |  |  |  |
| GO:0006269\_DNA\_replication\_\_synthesis\_of\_RNA\_primer | 1 | 0 |  |  |  |  |  |  |  |  |
| GO:0006283\_transcription-coupled\_nucleotide-excision\_repair | 1 | 0 |  |  |  |  |  |  |  |  |
| GO:0006296\_nucleotide-excision\_repair\_\_DNA\_incision\_\_5'-to\_lesion | 1 | 0 |  |  |  |  |  |  |  |  |
| GO:0006307\_DNA\_dealkylation | 1 | 0 |  |  |  |  |  |  |  |  |
| GO:0006337\_nucleosome\_disassembly | 1 | 0 |  |  |  |  |  |  |  |  |
| GO:0006344\_maintenance\_of\_chromatin\_silencing | 1 | 0 |  |  |  |  |  |  |  |  |
| GO:0006356\_regulation\_of\_transcription\_from\_RNA\_polymerase\_I\_promoter | 1 | 0 |  |  |  |  |  |  |  |  |
| GO:0006388\_tRNA\_splicing\_\_via\_endonucleolytic\_cleavage\_and\_ligation | 1 | 0 |  |  |  |  |  |  |  |  |
| GO:0006407\_rRNA\_export\_from\_nucleus | 1 | 0 |  |  |  |  |  |  |  |  |
| GO:0006419\_alanyl-tRNA\_aminoacylation | 1 | 0 |  |  |  |  |  |  |  |  |
| GO:0006434\_seryl-tRNA\_aminoacylation | 1 | 0 |  |  |  |  |  |  |  |  |
| GO:0006447\_regulation\_of\_translational\_initiation\_by\_iron | 1 | 0 |  |  |  |  |  |  |  |  |
| GO:0006463\_steroid\_hormone\_receptor\_complex\_assembly | 1 | 0 |  |  |  |  |  |  |  |  |
| GO:0006467\_protein\_thiol-disulfide\_exchange | 1 | 0 |  |  |  |  |  |  |  |  |
| GO:0006474\_N-terminal\_protein\_amino\_acid\_acetylation | 1 | 0 |  |  |  |  |  |  |  |  |
| GO:0006481\_C-terminal\_protein\_amino\_acid\_methylation | 1 | 0 |  |  |  |  |  |  |  |  |
| GO:0006488\_dolichol-linked\_oligosaccharide\_biosynthetic\_process | 1 | 0 |  |  |  |  |  |  |  |  |
| GO:0006494\_protein\_amino\_acid\_terminal\_glycosylation | 1 | 0 |  |  |  |  |  |  |  |  |
| GO:0006496\_protein\_amino\_acid\_terminal\_N-glycosylation | 1 | 0 |  |  |  |  |  |  |  |  |
| GO:0006500\_N-terminal\_protein\_palmitoylation | 1 | 0 |  |  |  |  |  |  |  |  |
| GO:0006507\_GPI\_anchor\_release | 1 | 0 |  |  |  |  |  |  |  |  |
| GO:0006537\_glutamate\_biosynthetic\_process | 1 | 0 |  |  |  |  |  |  |  |  |
| GO:0006544\_glycine\_metabolic\_process | 1 | 0 |  |  |  |  |  |  |  |  |
| GO:0006549\_isoleucine\_metabolic\_process | 1 | 0 |  |  |  |  |  |  |  |  |
| GO:0006553\_lysine\_metabolic\_process | 1 | 0 |  |  |  |  |  |  |  |  |
| GO:0006554\_lysine\_catabolic\_process | 1 | 0 |  |  |  |  |  |  |  |  |
| GO:0006556\_S-adenosylmethionine\_biosynthetic\_process | 1 | 0 |  |  |  |  |  |  |  |  |
| GO:0006559\_L-phenylalanine\_catabolic\_process | 1 | 0 |  |  |  |  |  |  |  |  |
| GO:0006569\_tryptophan\_catabolic\_process | 1 | 0 |  |  |  |  |  |  |  |  |
| GO:0006572\_tyrosine\_catabolic\_process | 1 | 0 |  |  |  |  |  |  |  |  |
| GO:0006573\_valine\_metabolic\_process | 1 | 0 |  |  |  |  |  |  |  |  |
| GO:0006581\_acetylcholine\_catabolic\_process | 1 | 0 |  |  |  |  |  |  |  |  |
| GO:0006585\_dopamine\_biosynthetic\_process\_from\_tyrosine | 1 | 0 |  |  |  |  |  |  |  |  |
| GO:0006590\_thyroid\_hormone\_generation | 1 | 0 |  |  |  |  |  |  |  |  |
| GO:0006591\_ornithine\_metabolic\_process | 1 | 0 |  |  |  |  |  |  |  |  |
| GO:0006596\_polyamine\_biosynthetic\_process | 1 | 0 |  |  |  |  |  |  |  |  |
| GO:0006597\_spermine\_biosynthetic\_process | 1 | 0 |  |  |  |  |  |  |  |  |
| GO:0006601\_creatine\_biosynthetic\_process | 1 | 0 |  |  |  |  |  |  |  |  |
| GO:0006613\_cotranslational\_protein\_targeting\_to\_membrane | 1 | 0 |  |  |  |  |  |  |  |  |
| GO:0006622\_protein\_targeting\_to\_lysosome | 1 | 0 |  |  |  |  |  |  |  |  |
| GO:0006627\_mitochondrial\_protein\_processing\_during\_import | 1 | 0 |  |  |  |  |  |  |  |  |
| GO:0006653\_lecithin\_metabolic\_process | 1 | 0 |  |  |  |  |  |  |  |  |
| GO:0006654\_phosphatidic\_acid\_biosynthetic\_process | 1 | 0 |  |  |  |  |  |  |  |  |
| GO:0006658\_phosphatidylserine\_metabolic\_process | 1 | 0 |  |  |  |  |  |  |  |  |
| GO:0006659\_phosphatidylserine\_biosynthetic\_process | 1 | 0 |  |  |  |  |  |  |  |  |
| GO:0006667\_sphinganine\_metabolic\_process | 1 | 0 |  |  |  |  |  |  |  |  |
| GO:0006668\_sphinganine-1-phosphate\_metabolic\_process | 1 | 0 |  |  |  |  |  |  |  |  |
| GO:0006678\_glucosylceramide\_metabolic\_process | 1 | 0 |  |  |  |  |  |  |  |  |
| GO:0006682\_galactosylceramide\_biosynthetic\_process | 1 | 0 |  |  |  |  |  |  |  |  |
| GO:0006685\_sphingomyelin\_catabolic\_process | 1 | 0 |  |  |  |  |  |  |  |  |
| GO:0006700\_C21-steroid\_hormone\_biosynthetic\_process | 1 | 0 |  |  |  |  |  |  |  |  |
| GO:0006705\_mineralocorticoid\_biosynthetic\_process | 1 | 0 |  |  |  |  |  |  |  |  |
| GO:0006709\_progesterone\_catabolic\_process | 1 | 0 |  |  |  |  |  |  |  |  |
| GO:0006729\_tetrahydrobiopterin\_biosynthetic\_process | 1 | 0 |  |  |  |  |  |  |  |  |
| GO:0006734\_NADH\_metabolic\_process | 1 | 0 |  |  |  |  |  |  |  |  |
| GO:0006740\_NADPH\_regeneration | 1 | 0 |  |  |  |  |  |  |  |  |
| GO:0006741\_NADP\_biosynthetic\_process | 1 | 0 |  |  |  |  |  |  |  |  |
| GO:0006743\_ubiquinone\_metabolic\_process | 1 | 0 |  |  |  |  |  |  |  |  |
| GO:0006744\_ubiquinone\_biosynthetic\_process | 1 | 0 |  |  |  |  |  |  |  |  |
| GO:0006772\_thiamin\_metabolic\_process | 1 | 0 |  |  |  |  |  |  |  |  |
| GO:0006784\_heme\_a\_biosynthetic\_process | 1 | 0 |  |  |  |  |  |  |  |  |
| GO:0006797\_polyphosphate\_metabolic\_process | 1 | 0 |  |  |  |  |  |  |  |  |
| GO:0006798\_polyphosphate\_catabolic\_process | 1 | 0 |  |  |  |  |  |  |  |  |
| GO:0006824\_cobalt\_ion\_transport | 1 | 0 |  |  |  |  |  |  |  |  |
| GO:0006842\_tricarboxylic\_acid\_transport | 1 | 0 |  |  |  |  |  |  |  |  |
| GO:0006844\_acyl\_carnitine\_transport | 1 | 0 |  |  |  |  |  |  |  |  |
| GO:0006855\_multidrug\_transport | 1 | 0 |  |  |  |  |  |  |  |  |
| GO:0006863\_purine\_transport | 1 | 0 |  |  |  |  |  |  |  |  |
| GO:0006890\_retrograde\_vesicle-mediated\_transport\_\_Golgi\_to\_ER | 1 | 0 |  |  |  |  |  |  |  |  |
| GO:0006891\_intra-Golgi\_vesicle-mediated\_transport | 1 | 0 |  |  |  |  |  |  |  |  |
| GO:0006893\_Golgi\_to\_plasma\_membrane\_transport | 1 | 0 |  |  |  |  |  |  |  |  |
| GO:0006895\_Golgi\_to\_endosome\_transport | 1 | 0 |  |  |  |  |  |  |  |  |
| GO:0006896\_Golgi\_to\_vacuole\_transport | 1 | 0 |  |  |  |  |  |  |  |  |
| GO:0006900\_membrane\_budding | 1 | 0 |  |  |  |  |  |  |  |  |
| GO:0006930\_substrate-bound\_cell\_migration\_\_cell\_extension | 1 | 0 |  |  |  |  |  |  |  |  |
| GO:0006931\_substrate-bound\_cell\_migration\_\_cell\_attachment\_to\_substrate | 1 | 0 |  |  |  |  |  |  |  |  |
| GO:0006933\_negative\_regulation\_of\_cell\_adhesion\_involved\_in\_substrate-bound\_cell\_migration | 1 | 0 |  |  |  |  |  |  |  |  |
| GO:0006957\_complement\_activation\_\_alternative\_pathway | 1 | 0 |  |  |  |  |  |  |  |  |
| GO:0006958\_complement\_activation\_\_classical\_pathway | 1 | 0 |  |  |  |  |  |  |  |  |
| GO:0006978\_DNA\_damage\_response\_\_signal\_transduction\_by\_p53\_class\_mediator\_resulting\_in\_transcription\_of\_p21\_class\_mediator | 1 | 0 |  |  |  |  |  |  |  |  |
| GO:0007016\_cytoskeletal\_anchoring\_at\_plasma\_membrane | 1 | 0 |  |  |  |  |  |  |  |  |
| GO:0007021\_tubulin\_complex\_assembly | 1 | 0 |  |  |  |  |  |  |  |  |
| GO:0007056\_spindle\_assembly\_involved\_in\_female\_meiosis | 1 | 0 |  |  |  |  |  |  |  |  |
| GO:0007057\_spindle\_assembly\_involved\_in\_female\_meiosis\_I | 1 | 0 |  |  |  |  |  |  |  |  |
| GO:0007063\_regulation\_of\_sister\_chromatid\_cohesion | 1 | 0 |  |  |  |  |  |  |  |  |
| GO:0007065\_male\_meiosis\_sister\_chromatid\_cohesion | 1 | 0 |  |  |  |  |  |  |  |  |
| GO:0007076\_mitotic\_chromosome\_condensation | 1 | 0 |  |  |  |  |  |  |  |  |
| GO:0007095\_mitotic\_cell\_cycle\_G2\_M\_transition\_DNA\_damage\_checkpoint | 1 | 0 |  |  |  |  |  |  |  |  |
| GO:0007096\_regulation\_of\_exit\_from\_mitosis | 1 | 0 |  |  |  |  |  |  |  |  |
| GO:0007158\_neuron\_adhesion | 1 | 0 |  |  |  |  |  |  |  |  |
| GO:0007168\_receptor\_guanylyl\_cyclase\_signaling\_pathway | 1 | 0 |  |  |  |  |  |  |  |  |
| GO:0007197\_inhibition\_of\_adenylate\_cyclase\_activity\_by\_muscarinic\_acetylcholine\_receptor\_signaling\_pathway | 1 | 0 |  |  |  |  |  |  |  |  |
| GO:0007207\_activation\_of\_phospholipase\_C\_activity\_by\_muscarinic\_acetylcholine\_receptor\_signaling\_pathway | 1 | 0 |  |  |  |  |  |  |  |  |
| GO:0007208\_activation\_of\_phospholipase\_C\_activity\_by\_serotonin\_receptor\_signaling\_pathway | 1 | 0 |  |  |  |  |  |  |  |  |
| GO:0007217\_tachykinin\_receptor\_signaling\_pathway | 1 | 0 |  |  |  |  |  |  |  |  |
| GO:0007221\_positive\_regulation\_of\_transcription\_of\_Notch\_receptor\_target | 1 | 0 |  |  |  |  |  |  |  |  |
| GO:0007223\_Wnt\_receptor\_signaling\_pathway\_\_calcium\_modulating\_pathway | 1 | 0 |  |  |  |  |  |  |  |  |
| GO:0007225\_patched\_ligand\_processing | 1 | 0 |  |  |  |  |  |  |  |  |
| GO:0007227\_signal\_transduction\_downstream\_of\_smoothened | 1 | 0 |  |  |  |  |  |  |  |  |
| GO:0007228\_positive\_regulation\_of\_hh\_target\_transcription\_factor\_activity | 1 | 0 |  |  |  |  |  |  |  |  |
| GO:0007231\_osmosensory\_signaling\_pathway | 1 | 0 |  |  |  |  |  |  |  |  |
| GO:0007284\_spermatogonial\_cell\_division | 1 | 0 |  |  |  |  |  |  |  |  |
| GO:0007290\_spermatid\_nucleus\_elongation | 1 | 0 |  |  |  |  |  |  |  |  |
| GO:0007296\_vitellogenesis | 1 | 0 |  |  |  |  |  |  |  |  |
| GO:0007321\_sperm\_displacement | 1 | 0 |  |  |  |  |  |  |  |  |
| GO:0007380\_specification\_of\_segmental\_identity\_\_head | 1 | 0 |  |  |  |  |  |  |  |  |
| GO:0007382\_specification\_of\_segmental\_identity\_\_maxillary\_segment | 1 | 0 |  |  |  |  |  |  |  |  |
| GO:0007400\_neuroblast\_fate\_determination | 1 | 0 |  |  |  |  |  |  |  |  |
| GO:0007402\_ganglion\_mother\_cell\_fate\_determination | 1 | 0 |  |  |  |  |  |  |  |  |
| GO:0007495\_visceral\_mesoderm-endoderm\_interaction\_involved\_in\_midgut\_development | 1 | 0 |  |  |  |  |  |  |  |  |
| GO:0007497\_posterior\_midgut\_development | 1 | 0 |  |  |  |  |  |  |  |  |
| GO:0007499\_ectoderm\_and\_mesoderm\_interaction | 1 | 0 |  |  |  |  |  |  |  |  |
| GO:0007500\_mesodermal\_cell\_fate\_determination | 1 | 0 |  |  |  |  |  |  |  |  |
| GO:0007509\_mesoderm\_migration | 1 | 0 |  |  |  |  |  |  |  |  |
| GO:0007518\_myoblast\_cell\_fate\_determination | 1 | 0 |  |  |  |  |  |  |  |  |
| GO:0007521\_muscle\_cell\_fate\_determination | 1 | 0 |  |  |  |  |  |  |  |  |
| GO:0007522\_visceral\_muscle\_development | 1 | 0 |  |  |  |  |  |  |  |  |
| GO:0007529\_establishment\_of\_synaptic\_specificity\_at\_neuromuscular\_junction | 1 | 0 |  |  |  |  |  |  |  |  |
| GO:0007538\_primary\_sex\_determination | 1 | 0 |  |  |  |  |  |  |  |  |
| GO:0007542\_primary\_sex\_determination\_\_germ-line | 1 | 0 |  |  |  |  |  |  |  |  |
| GO:0007567\_parturition | 1 | 0 |  |  |  |  |  |  |  |  |
| GO:0007614\_short-term\_memory | 1 | 0 |  |  |  |  |  |  |  |  |
| GO:0007621\_negative\_regulation\_of\_female\_receptivity | 1 | 0 |  |  |  |  |  |  |  |  |
| GO:0008049\_male\_courtship\_behavior | 1 | 0 |  |  |  |  |  |  |  |  |
| GO:0008050\_female\_courtship\_behavior | 1 | 0 |  |  |  |  |  |  |  |  |
| GO:0008052\_sensory\_organ\_boundary\_specification | 1 | 0 |  |  |  |  |  |  |  |  |
| GO:0008054\_cyclin\_catabolic\_process | 1 | 0 |  |  |  |  |  |  |  |  |
| GO:0008057\_eye\_pigment\_granule\_organization | 1 | 0 |  |  |  |  |  |  |  |  |
| GO:0008078\_mesodermal\_cell\_migration | 1 | 0 |  |  |  |  |  |  |  |  |
| GO:0008208\_C21-steroid\_hormone\_catabolic\_process | 1 | 0 |  |  |  |  |  |  |  |  |
| GO:0008216\_spermidine\_metabolic\_process | 1 | 0 |  |  |  |  |  |  |  |  |
| GO:0008292\_acetylcholine\_biosynthetic\_process | 1 | 0 |  |  |  |  |  |  |  |  |
| GO:0008295\_spermidine\_biosynthetic\_process | 1 | 0 |  |  |  |  |  |  |  |  |
| GO:0008300\_isoprenoid\_catabolic\_process | 1 | 0 |  |  |  |  |  |  |  |  |
| GO:0008333\_endosome\_to\_lysosome\_transport | 1 | 0 |  |  |  |  |  |  |  |  |
| GO:0008355\_olfactory\_learning | 1 | 0 |  |  |  |  |  |  |  |  |
| GO:0008611\_ether\_lipid\_biosynthetic\_process | 1 | 0 |  |  |  |  |  |  |  |  |
| GO:0008626\_induction\_of\_apoptosis\_by\_granzyme | 1 | 0 |  |  |  |  |  |  |  |  |
| GO:0008633\_activation\_of\_pro-apoptotic\_gene\_products | 1 | 0 |  |  |  |  |  |  |  |  |
| GO:0008653\_lipopolysaccharide\_metabolic\_process | 1 | 0 |  |  |  |  |  |  |  |  |
| GO:0009068\_aspartate\_family\_amino\_acid\_catabolic\_process | 1 | 0 |  |  |  |  |  |  |  |  |
| GO:0009084\_glutamine\_family\_amino\_acid\_biosynthetic\_process | 1 | 0 |  |  |  |  |  |  |  |  |
| GO:0009088\_threonine\_biosynthetic\_process | 1 | 0 |  |  |  |  |  |  |  |  |
| GO:0009105\_lipoic\_acid\_biosynthetic\_process | 1 | 0 |  |  |  |  |  |  |  |  |
| GO:0009109\_coenzyme\_catabolic\_process | 1 | 0 |  |  |  |  |  |  |  |  |
| GO:0009111\_vitamin\_catabolic\_process | 1 | 0 |  |  |  |  |  |  |  |  |
| GO:0009113\_purine\_base\_biosynthetic\_process | 1 | 0 |  |  |  |  |  |  |  |  |
| GO:0009127\_purine\_nucleoside\_monophosphate\_biosynthetic\_process | 1 | 0 |  |  |  |  |  |  |  |  |
| GO:0009128\_purine\_nucleoside\_monophosphate\_catabolic\_process | 1 | 0 |  |  |  |  |  |  |  |  |
| GO:0009129\_pyrimidine\_nucleoside\_monophosphate\_metabolic\_process | 1 | 0 |  |  |  |  |  |  |  |  |
| GO:0009131\_pyrimidine\_nucleoside\_monophosphate\_catabolic\_process | 1 | 0 |  |  |  |  |  |  |  |  |
| GO:0009133\_nucleoside\_diphosphate\_biosynthetic\_process | 1 | 0 |  |  |  |  |  |  |  |  |
| GO:0009145\_purine\_nucleoside\_triphosphate\_biosynthetic\_process | 1 | 0 |  |  |  |  |  |  |  |  |
| GO:0009147\_pyrimidine\_nucleoside\_triphosphate\_metabolic\_process | 1 | 0 |  |  |  |  |  |  |  |  |
| GO:0009148\_pyrimidine\_nucleoside\_triphosphate\_biosynthetic\_process | 1 | 0 |  |  |  |  |  |  |  |  |
| GO:0009152\_purine\_ribonucleotide\_biosynthetic\_process | 1 | 0 |  |  |  |  |  |  |  |  |
| GO:0009153\_purine\_deoxyribonucleotide\_biosynthetic\_process | 1 | 0 |  |  |  |  |  |  |  |  |
| GO:0009156\_ribonucleoside\_monophosphate\_biosynthetic\_process | 1 | 0 |  |  |  |  |  |  |  |  |
| GO:0009158\_ribonucleoside\_monophosphate\_catabolic\_process | 1 | 0 |  |  |  |  |  |  |  |  |
| GO:0009159\_deoxyribonucleoside\_monophosphate\_catabolic\_process | 1 | 0 |  |  |  |  |  |  |  |  |
| GO:0009162\_deoxyribonucleoside\_monophosphate\_metabolic\_process | 1 | 0 |  |  |  |  |  |  |  |  |
| GO:0009168\_purine\_ribonucleoside\_monophosphate\_biosynthetic\_process | 1 | 0 |  |  |  |  |  |  |  |  |
| GO:0009169\_purine\_ribonucleoside\_monophosphate\_catabolic\_process | 1 | 0 |  |  |  |  |  |  |  |  |
| GO:0009176\_pyrimidine\_deoxyribonucleoside\_monophosphate\_metabolic\_process | 1 | 0 |  |  |  |  |  |  |  |  |
| GO:0009178\_pyrimidine\_deoxyribonucleoside\_monophosphate\_catabolic\_process | 1 | 0 |  |  |  |  |  |  |  |  |
| GO:0009211\_pyrimidine\_deoxyribonucleoside\_triphosphate\_metabolic\_process | 1 | 0 |  |  |  |  |  |  |  |  |
| GO:0009212\_pyrimidine\_deoxyribonucleoside\_triphosphate\_biosynthetic\_process | 1 | 0 |  |  |  |  |  |  |  |  |
| GO:0009216\_purine\_deoxyribonucleoside\_triphosphate\_biosynthetic\_process | 1 | 0 |  |  |  |  |  |  |  |  |
| GO:0009221\_pyrimidine\_deoxyribonucleotide\_biosynthetic\_process | 1 | 0 |  |  |  |  |  |  |  |  |
| GO:0009223\_pyrimidine\_deoxyribonucleotide\_catabolic\_process | 1 | 0 |  |  |  |  |  |  |  |  |
| GO:0009260\_ribonucleotide\_biosynthetic\_process | 1 | 0 |  |  |  |  |  |  |  |  |
| GO:0009405\_pathogenesis | 1 | 0 |  |  |  |  |  |  |  |  |
| GO:0009414\_response\_to\_water\_deprivation | 1 | 0 |  |  |  |  |  |  |  |  |
| GO:0009415\_response\_to\_water | 1 | 0 |  |  |  |  |  |  |  |  |
| GO:0009449\_gamma-aminobutyric\_acid\_biosynthetic\_process | 1 | 0 |  |  |  |  |  |  |  |  |
| GO:0009450\_gamma-aminobutyric\_acid\_catabolic\_process | 1 | 0 |  |  |  |  |  |  |  |  |
| GO:0009589\_detection\_of\_UV | 1 | 0 |  |  |  |  |  |  |  |  |
| GO:0009590\_detection\_of\_gravity | 1 | 0 |  |  |  |  |  |  |  |  |
| GO:0009624\_response\_to\_nematode | 1 | 0 |  |  |  |  |  |  |  |  |
| GO:0009629\_response\_to\_gravity | 1 | 0 |  |  |  |  |  |  |  |  |
| GO:0009648\_photoperiodism | 1 | 0 |  |  |  |  |  |  |  |  |
| GO:0009690\_cytokinin\_metabolic\_process | 1 | 0 |  |  |  |  |  |  |  |  |
| GO:0009691\_cytokinin\_biosynthetic\_process | 1 | 0 |  |  |  |  |  |  |  |  |
| GO:0009786\_regulation\_of\_asymmetric\_cell\_division | 1 | 0 |  |  |  |  |  |  |  |  |
| GO:0009794\_regulation\_of\_mitotic\_cell\_cycle\_\_embryonic | 1 | 0 |  |  |  |  |  |  |  |  |
| GO:0009956\_radial\_pattern\_formation | 1 | 0 |  |  |  |  |  |  |  |  |
| GO:0009957\_epidermal\_cell\_fate\_specification | 1 | 0 |  |  |  |  |  |  |  |  |
| GO:0009992\_cellular\_water\_homeostasis | 1 | 0 |  |  |  |  |  |  |  |  |
| GO:0010032\_meiotic\_chromosome\_condensation | 1 | 0 |  |  |  |  |  |  |  |  |
| GO:0010039\_response\_to\_iron\_ion | 1 | 0 |  |  |  |  |  |  |  |  |
| GO:0010042\_response\_to\_manganese\_ion | 1 | 0 |  |  |  |  |  |  |  |  |
| GO:0010045\_response\_to\_nickel\_ion | 1 | 0 |  |  |  |  |  |  |  |  |
| GO:0010046\_response\_to\_mycotoxin | 1 | 0 |  |  |  |  |  |  |  |  |
| GO:0010107\_potassium\_ion\_import | 1 | 0 |  |  |  |  |  |  |  |  |
| GO:0010155\_regulation\_of\_proton\_transport | 1 | 0 |  |  |  |  |  |  |  |  |
| GO:0010160\_formation\_of\_organ\_boundary | 1 | 0 |  |  |  |  |  |  |  |  |
| GO:0010260\_organ\_senescence | 1 | 0 |  |  |  |  |  |  |  |  |
| GO:0010310\_regulation\_of\_hydrogen\_peroxide\_metabolic\_process | 1 | 0 |  |  |  |  |  |  |  |  |
| GO:0010447\_response\_to\_acidity | 1 | 0 |  |  |  |  |  |  |  |  |
| GO:0010452\_histone\_H3-K36\_methylation | 1 | 0 |  |  |  |  |  |  |  |  |
| GO:0010455\_positive\_regulation\_of\_cell\_fate\_commitment | 1 | 0 |  |  |  |  |  |  |  |  |
| GO:0010470\_regulation\_of\_gastrulation | 1 | 0 |  |  |  |  |  |  |  |  |
| GO:0010508\_positive\_regulation\_of\_autophagy | 1 | 0 |  |  |  |  |  |  |  |  |
| GO:0010519\_negative\_regulation\_of\_phospholipase\_activity | 1 | 0 |  |  |  |  |  |  |  |  |
| GO:0010520\_regulation\_of\_reciprocal\_meiotic\_recombination | 1 | 0 |  |  |  |  |  |  |  |  |
| GO:0010523\_negative\_regulation\_of\_calcium\_ion\_transport\_into\_cytosol | 1 | 0 |  |  |  |  |  |  |  |  |
| GO:0010543\_regulation\_of\_platelet\_activation | 1 | 0 |  |  |  |  |  |  |  |  |
| GO:0010561\_negative\_regulation\_of\_glycoprotein\_biosynthetic\_process | 1 | 0 |  |  |  |  |  |  |  |  |
| GO:0010569\_regulation\_of\_double-strand\_break\_repair\_via\_homologous\_recombination | 1 | 0 |  |  |  |  |  |  |  |  |
| GO:0010572\_positive\_regulation\_of\_platelet\_activation | 1 | 0 |  |  |  |  |  |  |  |  |
| GO:0010594\_regulation\_of\_endothelial\_cell\_migration | 1 | 0 |  |  |  |  |  |  |  |  |
| GO:0010596\_negative\_regulation\_of\_endothelial\_cell\_migration | 1 | 0 |  |  |  |  |  |  |  |  |
| GO:0010611\_regulation\_of\_cardiac\_muscle\_hypertrophy | 1 | 0 |  |  |  |  |  |  |  |  |
| GO:0010612\_regulation\_of\_cardiac\_muscle\_adaptation | 1 | 0 |  |  |  |  |  |  |  |  |
| GO:0010614\_negative\_regulation\_of\_cardiac\_muscle\_hypertrophy | 1 | 0 |  |  |  |  |  |  |  |  |
| GO:0010616\_negative\_regulation\_of\_cardiac\_muscle\_adaptation | 1 | 0 |  |  |  |  |  |  |  |  |
| GO:0010634\_positive\_regulation\_of\_epithelial\_cell\_migration | 1 | 0 |  |  |  |  |  |  |  |  |
| GO:0010656\_negative\_regulation\_of\_muscle\_cell\_apoptosis | 1 | 0 |  |  |  |  |  |  |  |  |
| GO:0010657\_muscle\_cell\_apoptosis | 1 | 0 |  |  |  |  |  |  |  |  |
| GO:0010658\_striated\_muscle\_cell\_apoptosis | 1 | 0 |  |  |  |  |  |  |  |  |
| GO:0010659\_cardiac\_muscle\_cell\_apoptosis | 1 | 0 |  |  |  |  |  |  |  |  |
| GO:0010660\_regulation\_of\_muscle\_cell\_apoptosis | 1 | 0 |  |  |  |  |  |  |  |  |
| GO:0010662\_regulation\_of\_striated\_muscle\_cell\_apoptosis | 1 | 0 |  |  |  |  |  |  |  |  |
| GO:0010664\_negative\_regulation\_of\_striated\_muscle\_cell\_apoptosis | 1 | 0 |  |  |  |  |  |  |  |  |
| GO:0010665\_regulation\_of\_cardiac\_muscle\_cell\_apoptosis | 1 | 0 |  |  |  |  |  |  |  |  |
| GO:0010667\_negative\_regulation\_of\_cardiac\_muscle\_cell\_apoptosis | 1 | 0 |  |  |  |  |  |  |  |  |
| GO:0010668\_ectodermal\_cell\_differentiation | 1 | 0 |  |  |  |  |  |  |  |  |
| GO:0010671\_negative\_regulation\_of\_oxygen\_and\_reactive\_oxygen\_species\_metabolic\_process | 1 | 0 |  |  |  |  |  |  |  |  |
| GO:0010719\_negative\_regulation\_of\_epithelial\_to\_mesenchymal\_transition | 1 | 0 |  |  |  |  |  |  |  |  |
| GO:0010735\_positive\_regulation\_of\_transcription\_via\_serum\_response\_element\_binding | 1 | 0 |  |  |  |  |  |  |  |  |
| GO:0010825\_positive\_regulation\_of\_centrosome\_duplication | 1 | 0 |  |  |  |  |  |  |  |  |
| GO:0010845\_positive\_regulation\_of\_reciprocal\_meiotic\_recombination | 1 | 0 |  |  |  |  |  |  |  |  |
| GO:0010850\_chemoreceptor\_signaling\_pathway\_involved\_in\_regulation\_of\_blood\_pressure | 1 | 0 |  |  |  |  |  |  |  |  |
| GO:0010873\_positive\_regulation\_of\_cholesterol\_esterification | 1 | 0 |  |  |  |  |  |  |  |  |
| GO:0010880\_regulation\_of\_release\_of\_sequestered\_calcium\_ion\_into\_cytosol\_by\_sarcoplasmic\_reticulum | 1 | 0 |  |  |  |  |  |  |  |  |
| GO:0010881\_regulation\_of\_cardiac\_muscle\_contraction\_by\_regulation\_of\_the\_release\_of\_sequestered\_calcium\_ion | 1 | 0 |  |  |  |  |  |  |  |  |
| GO:0010882\_regulation\_of\_cardiac\_muscle\_contraction\_by\_calcium\_ion\_signaling | 1 | 0 |  |  |  |  |  |  |  |  |
| GO:0010890\_positive\_regulation\_of\_sequestering\_of\_triglyceride | 1 | 0 |  |  |  |  |  |  |  |  |
| GO:0010919\_regulation\_of\_inositol\_phosphate\_biosynthetic\_process | 1 | 0 |  |  |  |  |  |  |  |  |
| GO:0010931\_macrophage\_tolerance\_induction | 1 | 0 |  |  |  |  |  |  |  |  |
| GO:0010932\_regulation\_of\_macrophage\_tolerance\_induction | 1 | 0 |  |  |  |  |  |  |  |  |
| GO:0010933\_positive\_regulation\_of\_macrophage\_tolerance\_induction | 1 | 0 |  |  |  |  |  |  |  |  |
| GO:0010934\_macrophage\_cytokine\_production | 1 | 0 |  |  |  |  |  |  |  |  |
| GO:0010935\_regulation\_of\_macrophage\_cytokine\_production | 1 | 0 |  |  |  |  |  |  |  |  |
| GO:0010936\_negative\_regulation\_of\_macrophage\_cytokine\_production | 1 | 0 |  |  |  |  |  |  |  |  |
| GO:0010953\_regulation\_of\_protein\_maturation\_by\_peptide\_bond\_cleavage | 1 | 0 |  |  |  |  |  |  |  |  |
| GO:0010962\_regulation\_of\_glucan\_biosynthetic\_process | 1 | 0 |  |  |  |  |  |  |  |  |
| GO:0010966\_regulation\_of\_phosphate\_transport | 1 | 0 |  |  |  |  |  |  |  |  |
| GO:0014012\_axon\_regeneration\_in\_the\_peripheral\_nervous\_system | 1 | 0 |  |  |  |  |  |  |  |  |
| GO:0014016\_neuroblast\_differentiation | 1 | 0 |  |  |  |  |  |  |  |  |
| GO:0014017\_neuroblast\_fate\_commitment | 1 | 0 |  |  |  |  |  |  |  |  |
| GO:0014041\_regulation\_of\_neuron\_maturation | 1 | 0 |  |  |  |  |  |  |  |  |
| GO:0014042\_positive\_regulation\_of\_neuron\_maturation | 1 | 0 |  |  |  |  |  |  |  |  |
| GO:0014049\_positive\_regulation\_of\_glutamate\_secretion | 1 | 0 |  |  |  |  |  |  |  |  |
| GO:0014061\_regulation\_of\_norepinephrine\_secretion | 1 | 0 |  |  |  |  |  |  |  |  |
| GO:0014071\_response\_to\_cycloalkane | 1 | 0 |  |  |  |  |  |  |  |  |
| GO:0014707\_branchiomeric\_skeletal\_muscle\_development | 1 | 0 |  |  |  |  |  |  |  |  |
| GO:0014738\_regulation\_of\_muscle\_hyperplasia | 1 | 0 |  |  |  |  |  |  |  |  |
| GO:0014740\_negative\_regulation\_of\_muscle\_hyperplasia | 1 | 0 |  |  |  |  |  |  |  |  |
| GO:0014741\_negative\_regulation\_of\_muscle\_hypertrophy | 1 | 0 |  |  |  |  |  |  |  |  |
| GO:0014743\_regulation\_of\_muscle\_hypertrophy | 1 | 0 |  |  |  |  |  |  |  |  |
| GO:0014805\_smooth\_muscle\_adaptation | 1 | 0 |  |  |  |  |  |  |  |  |
| GO:0014806\_smooth\_muscle\_hyperplasia | 1 | 0 |  |  |  |  |  |  |  |  |
| GO:0014807\_regulation\_of\_somitogenesis | 1 | 0 |  |  |  |  |  |  |  |  |
| GO:0014808\_release\_of\_sequestered\_calcium\_ion\_into\_cytosol\_by\_sarcoplasmic\_reticulum | 1 | 0 |  |  |  |  |  |  |  |  |
| GO:0014813\_satellite\_cell\_commitment | 1 | 0 |  |  |  |  |  |  |  |  |
| GO:0014816\_satellite\_cell\_differentiation | 1 | 0 |  |  |  |  |  |  |  |  |
| GO:0014819\_regulation\_of\_skeletal\_muscle\_contraction | 1 | 0 |  |  |  |  |  |  |  |  |
| GO:0014852\_regulation\_of\_skeletal\_muscle\_contraction\_by\_neural\_stimulation\_via\_neuromuscular\_junction | 1 | 0 |  |  |  |  |  |  |  |  |
| GO:0014853\_regulation\_of\_excitatory\_postsynaptic\_membrane\_potential\_involved\_in\_skeletal\_muscle\_contraction | 1 | 0 |  |  |  |  |  |  |  |  |
| GO:0014856\_skeletal\_muscle\_cell\_proliferation | 1 | 0 |  |  |  |  |  |  |  |  |
| GO:0014857\_regulation\_of\_skeletal\_muscle\_cell\_proliferation | 1 | 0 |  |  |  |  |  |  |  |  |
| GO:0014858\_positive\_regulation\_of\_skeletal\_muscle\_cell\_proliferation | 1 | 0 |  |  |  |  |  |  |  |  |
| GO:0014887\_cardiac\_muscle\_adaptation | 1 | 0 |  |  |  |  |  |  |  |  |
| GO:0014889\_muscle\_atrophy | 1 | 0 |  |  |  |  |  |  |  |  |
| GO:0014896\_muscle\_hypertrophy | 1 | 0 |  |  |  |  |  |  |  |  |
| GO:0014897\_striated\_muscle\_hypertrophy | 1 | 0 |  |  |  |  |  |  |  |  |
| GO:0014898\_cardiac\_muscle\_hypertrophy | 1 | 0 |  |  |  |  |  |  |  |  |
| GO:0014900\_muscle\_hyperplasia | 1 | 0 |  |  |  |  |  |  |  |  |
| GO:0014910\_regulation\_of\_smooth\_muscle\_cell\_migration | 1 | 0 |  |  |  |  |  |  |  |  |
| GO:0014911\_positive\_regulation\_of\_smooth\_muscle\_cell\_migration | 1 | 0 |  |  |  |  |  |  |  |  |
| GO:0015014\_heparan\_sulfate\_proteoglycan\_biosynthetic\_process\_\_polysaccharide\_chain\_biosynthetic\_process | 1 | 0 |  |  |  |  |  |  |  |  |
| GO:0015074\_DNA\_integration | 1 | 0 |  |  |  |  |  |  |  |  |
| GO:0015670\_carbon\_dioxide\_transport | 1 | 0 |  |  |  |  |  |  |  |  |
| GO:0015677\_copper\_ion\_import | 1 | 0 |  |  |  |  |  |  |  |  |
| GO:0015680\_intracellular\_copper\_ion\_transport | 1 | 0 |  |  |  |  |  |  |  |  |
| GO:0015684\_ferrous\_iron\_transport | 1 | 0 |  |  |  |  |  |  |  |  |
| GO:0015707\_nitrite\_transport | 1 | 0 |  |  |  |  |  |  |  |  |
| GO:0015724\_formate\_transport | 1 | 0 |  |  |  |  |  |  |  |  |
| GO:0015734\_taurine\_transport | 1 | 0 |  |  |  |  |  |  |  |  |
| GO:0015740\_C4-dicarboxylate\_transport | 1 | 0 |  |  |  |  |  |  |  |  |
| GO:0015744\_succinate\_transport | 1 | 0 |  |  |  |  |  |  |  |  |
| GO:0015746\_citrate\_transport | 1 | 0 |  |  |  |  |  |  |  |  |
| GO:0015747\_urate\_transport | 1 | 0 |  |  |  |  |  |  |  |  |
| GO:0015791\_polyol\_transport | 1 | 0 |  |  |  |  |  |  |  |  |
| GO:0015798\_myo-inositol\_transport | 1 | 0 |  |  |  |  |  |  |  |  |
| GO:0015808\_L-alanine\_transport | 1 | 0 |  |  |  |  |  |  |  |  |
| GO:0015810\_aspartate\_transport | 1 | 0 |  |  |  |  |  |  |  |  |
| GO:0015811\_L-cystine\_transport | 1 | 0 |  |  |  |  |  |  |  |  |
| GO:0015817\_histidine\_transport | 1 | 0 |  |  |  |  |  |  |  |  |
| GO:0015822\_ornithine\_transport | 1 | 0 |  |  |  |  |  |  |  |  |
| GO:0015824\_proline\_transport | 1 | 0 |  |  |  |  |  |  |  |  |
| GO:0015851\_nucleobase\_transport | 1 | 0 |  |  |  |  |  |  |  |  |
| GO:0015864\_pyrimidine\_nucleoside\_transport | 1 | 0 |  |  |  |  |  |  |  |  |
| GO:0015874\_norepinephrine\_transport | 1 | 0 |  |  |  |  |  |  |  |  |
| GO:0015881\_creatine\_transport | 1 | 0 |  |  |  |  |  |  |  |  |
| GO:0015884\_folic\_acid\_transport | 1 | 0 |  |  |  |  |  |  |  |  |
| GO:0015886\_heme\_transport | 1 | 0 |  |  |  |  |  |  |  |  |
| GO:0015888\_thiamin\_transport | 1 | 0 |  |  |  |  |  |  |  |  |
| GO:0015938\_coenzyme\_A\_catabolic\_process | 1 | 0 |  |  |  |  |  |  |  |  |
| GO:0015939\_pantothenate\_metabolic\_process | 1 | 0 |  |  |  |  |  |  |  |  |
| GO:0016073\_snRNA\_metabolic\_process | 1 | 0 |  |  |  |  |  |  |  |  |
| GO:0016074\_snoRNA\_metabolic\_process | 1 | 0 |  |  |  |  |  |  |  |  |
| GO:0016082\_synaptic\_vesicle\_priming | 1 | 0 |  |  |  |  |  |  |  |  |
| GO:0016090\_prenol\_metabolic\_process | 1 | 0 |  |  |  |  |  |  |  |  |
| GO:0016093\_polyprenol\_metabolic\_process | 1 | 0 |  |  |  |  |  |  |  |  |
| GO:0016180\_snRNA\_processing | 1 | 0 |  |  |  |  |  |  |  |  |
| GO:0016239\_positive\_regulation\_of\_macroautophagy | 1 | 0 |  |  |  |  |  |  |  |  |
| GO:0016246\_RNA\_interference | 1 | 0 |  |  |  |  |  |  |  |  |
| GO:0016255\_attachment\_of\_GPI\_anchor\_to\_protein | 1 | 0 |  |  |  |  |  |  |  |  |
| GO:0016333\_morphogenesis\_of\_follicular\_epithelium | 1 | 0 |  |  |  |  |  |  |  |  |
| GO:0016340\_calcium-dependent\_cell-matrix\_adhesion | 1 | 0 |  |  |  |  |  |  |  |  |
| GO:0016344\_meiotic\_chromosome\_movement\_towards\_spindle\_pole | 1 | 0 |  |  |  |  |  |  |  |  |
| GO:0016482\_cytoplasmic\_transport | 1 | 0 |  |  |  |  |  |  |  |  |
| GO:0016553\_base\_conversion\_or\_substitution\_editing | 1 | 0 |  |  |  |  |  |  |  |  |
| GO:0016554\_cytidine\_to\_uridine\_editing | 1 | 0 |  |  |  |  |  |  |  |  |
| GO:0016560\_protein\_import\_into\_peroxisome\_matrix\_\_docking | 1 | 0 |  |  |  |  |  |  |  |  |
| GO:0016578\_histone\_deubiquitination | 1 | 0 |  |  |  |  |  |  |  |  |
| GO:0016598\_protein\_arginylation | 1 | 0 |  |  |  |  |  |  |  |  |
| GO:0017004\_cytochrome\_complex\_assembly | 1 | 0 |  |  |  |  |  |  |  |  |
| GO:0018022\_peptidyl-lysine\_methylation | 1 | 0 |  |  |  |  |  |  |  |  |
| GO:0018023\_peptidyl-lysine\_trimethylation | 1 | 0 |  |  |  |  |  |  |  |  |
| GO:0018120\_peptidyl-arginine\_ADP-ribosylation | 1 | 0 |  |  |  |  |  |  |  |  |
| GO:0018126\_protein\_amino\_acid\_hydroxylation | 1 | 0 |  |  |  |  |  |  |  |  |
| GO:0018146\_keratan\_sulfate\_biosynthetic\_process | 1 | 0 |  |  |  |  |  |  |  |  |
| GO:0018158\_protein\_amino\_acid\_oxidation | 1 | 0 |  |  |  |  |  |  |  |  |
| GO:0018195\_peptidyl-arginine\_modification | 1 | 0 |  |  |  |  |  |  |  |  |
| GO:0018197\_peptidyl-aspartic\_acid\_modification | 1 | 0 |  |  |  |  |  |  |  |  |
| GO:0018282\_metal\_incorporation\_into\_metallo-sulfur\_cluster | 1 | 0 |  |  |  |  |  |  |  |  |
| GO:0018283\_iron\_incorporation\_into\_metallo-sulfur\_cluster | 1 | 0 |  |  |  |  |  |  |  |  |
| GO:0018318\_protein\_amino\_acid\_palmitoylation | 1 | 0 |  |  |  |  |  |  |  |  |
| GO:0018342\_protein\_prenylation | 1 | 0 |  |  |  |  |  |  |  |  |
| GO:0018344\_protein\_geranylgeranylation | 1 | 0 |  |  |  |  |  |  |  |  |
| GO:0018410\_peptide\_or\_protein\_carboxyl-terminal\_blocking | 1 | 0 |  |  |  |  |  |  |  |  |
| GO:0018916\_nitrobenzene\_metabolic\_process | 1 | 0 |  |  |  |  |  |  |  |  |
| GO:0018931\_naphthalene\_metabolic\_process | 1 | 0 |  |  |  |  |  |  |  |  |
| GO:0018992\_germ-line\_sex\_determination | 1 | 0 |  |  |  |  |  |  |  |  |
| GO:0019042\_latent\_virus\_infection | 1 | 0 |  |  |  |  |  |  |  |  |
| GO:0019046\_reactivation\_of\_latent\_virus | 1 | 0 |  |  |  |  |  |  |  |  |
| GO:0019047\_provirus\_integration | 1 | 0 |  |  |  |  |  |  |  |  |
| GO:0019076\_release\_of\_virus\_from\_host | 1 | 0 |  |  |  |  |  |  |  |  |
| GO:0019079\_viral\_genome\_replication | 1 | 0 |  |  |  |  |  |  |  |  |
| GO:0019100\_male\_germ-line\_sex\_determination | 1 | 0 |  |  |  |  |  |  |  |  |
| GO:0019101\_female\_somatic\_sex\_determination | 1 | 0 |  |  |  |  |  |  |  |  |
| GO:0019102\_male\_somatic\_sex\_determination | 1 | 0 |  |  |  |  |  |  |  |  |
| GO:0019255\_glucose\_1-phosphate\_metabolic\_process | 1 | 0 |  |  |  |  |  |  |  |  |
| GO:0019276\_UDP-N-acetylgalactosamine\_metabolic\_process | 1 | 0 |  |  |  |  |  |  |  |  |
| GO:0019344\_cysteine\_biosynthetic\_process | 1 | 0 |  |  |  |  |  |  |  |  |
| GO:0019348\_dolichol\_metabolic\_process | 1 | 0 |  |  |  |  |  |  |  |  |
| GO:0019375\_galactolipid\_biosynthetic\_process | 1 | 0 |  |  |  |  |  |  |  |  |
| GO:0019402\_galactitol\_metabolic\_process | 1 | 0 |  |  |  |  |  |  |  |  |
| GO:0019441\_tryptophan\_catabolic\_process\_to\_kynurenine | 1 | 0 |  |  |  |  |  |  |  |  |
| GO:0019477\_L-lysine\_catabolic\_process | 1 | 0 |  |  |  |  |  |  |  |  |
| GO:0019510\_S-adenosylhomocysteine\_catabolic\_process | 1 | 0 |  |  |  |  |  |  |  |  |
| GO:0019532\_oxalate\_transport | 1 | 0 |  |  |  |  |  |  |  |  |
| GO:0019626\_short-chain\_fatty\_acid\_catabolic\_process | 1 | 0 |  |  |  |  |  |  |  |  |
| GO:0019627\_urea\_metabolic\_process | 1 | 0 |  |  |  |  |  |  |  |  |
| GO:0019676\_ammonia\_assimilation\_cycle | 1 | 0 |  |  |  |  |  |  |  |  |
| GO:0019682\_glyceraldehyde-3-phosphate\_metabolic\_process | 1 | 0 |  |  |  |  |  |  |  |  |
| GO:0019695\_choline\_metabolic\_process | 1 | 0 |  |  |  |  |  |  |  |  |
| GO:0019731\_antibacterial\_humoral\_response | 1 | 0 |  |  |  |  |  |  |  |  |
| GO:0019794\_nonprotein\_amino\_acid\_metabolic\_process | 1 | 0 |  |  |  |  |  |  |  |  |
| GO:0019883\_antigen\_processing\_and\_presentation\_of\_endogenous\_antigen | 1 | 0 |  |  |  |  |  |  |  |  |
| GO:0019889\_pteridine\_metabolic\_process | 1 | 0 |  |  |  |  |  |  |  |  |
| GO:0019896\_axon\_transport\_of\_mitochondrion | 1 | 0 |  |  |  |  |  |  |  |  |
| GO:0021508\_floor\_plate\_formation | 1 | 0 |  |  |  |  |  |  |  |  |
| GO:0021528\_commissural\_neuron\_differentiation\_in\_the\_spinal\_cord | 1 | 0 |  |  |  |  |  |  |  |  |
| GO:0021572\_rhombomere\_6\_development | 1 | 0 |  |  |  |  |  |  |  |  |
| GO:0021577\_hindbrain\_structural\_organization | 1 | 0 |  |  |  |  |  |  |  |  |
| GO:0021586\_pons\_maturation | 1 | 0 |  |  |  |  |  |  |  |  |
| GO:0021589\_cerebellum\_structural\_organization | 1 | 0 |  |  |  |  |  |  |  |  |
| GO:0021590\_cerebellum\_maturation | 1 | 0 |  |  |  |  |  |  |  |  |
| GO:0021592\_fourth\_ventricle\_development | 1 | 0 |  |  |  |  |  |  |  |  |
| GO:0021594\_rhombomere\_formation | 1 | 0 |  |  |  |  |  |  |  |  |
| GO:0021660\_rhombomere\_3\_formation | 1 | 0 |  |  |  |  |  |  |  |  |
| GO:0021664\_rhombomere\_5\_morphogenesis | 1 | 0 |  |  |  |  |  |  |  |  |
| GO:0021666\_rhombomere\_5\_formation | 1 | 0 |  |  |  |  |  |  |  |  |
| GO:0021670\_lateral\_ventricle\_development | 1 | 0 |  |  |  |  |  |  |  |  |
| GO:0021678\_third\_ventricle\_development | 1 | 0 |  |  |  |  |  |  |  |  |
| GO:0021679\_cerebellar\_molecular\_layer\_development | 1 | 0 |  |  |  |  |  |  |  |  |
| GO:0021703\_locus\_ceruleus\_development | 1 | 0 |  |  |  |  |  |  |  |  |
| GO:0021732\_midbrain-hindbrain\_boundary\_maturation | 1 | 0 |  |  |  |  |  |  |  |  |
| GO:0021747\_cochlear\_nucleus\_development | 1 | 0 |  |  |  |  |  |  |  |  |
| GO:0021750\_vestibular\_nucleus\_development | 1 | 0 |  |  |  |  |  |  |  |  |
| GO:0021759\_globus\_pallidus\_development | 1 | 0 |  |  |  |  |  |  |  |  |
| GO:0021768\_nucleus\_accumbens\_development | 1 | 0 |  |  |  |  |  |  |  |  |
| GO:0021771\_lateral\_geniculate\_nucleus\_development | 1 | 0 |  |  |  |  |  |  |  |  |
| GO:0021812\_neuronal-glial\_interaction\_involved\_in\_cerebral\_cortex\_radial\_glia\_guided\_migration | 1 | 0 |  |  |  |  |  |  |  |  |
| GO:0021813\_cell-cell\_adhesion\_involved\_in\_neuronal-glial\_interactions\_involved\_in\_cerebral\_cortex\_radial\_glia\_guided\_migration | 1 | 0 |  |  |  |  |  |  |  |  |
| GO:0021870\_Cajal-Retzius\_cell\_differentiation | 1 | 0 |  |  |  |  |  |  |  |  |
| GO:0021874\_Wnt\_receptor\_signaling\_pathway\_in\_forebrain\_neuroblast\_division | 1 | 0 |  |  |  |  |  |  |  |  |
| GO:0021896\_forebrain\_astrocyte\_differentiation | 1 | 0 |  |  |  |  |  |  |  |  |
| GO:0021897\_forebrain\_astrocyte\_development | 1 | 0 |  |  |  |  |  |  |  |  |
| GO:0021902\_commitment\_of\_a\_neuronal\_cell\_to\_a\_specific\_type\_of\_neuron\_in\_the\_forebrain | 1 | 0 |  |  |  |  |  |  |  |  |
| GO:0021905\_forebrain-midbrain\_boundary\_formation | 1 | 0 |  |  |  |  |  |  |  |  |
| GO:0021914\_negative\_regulation\_of\_smoothened\_signaling\_pathway\_involved\_in\_ventral\_spinal\_cord\_patterning | 1 | 0 |  |  |  |  |  |  |  |  |
| GO:0021917\_somatic\_motor\_neuron\_fate\_commitment | 1 | 0 |  |  |  |  |  |  |  |  |
| GO:0021918\_regulation\_of\_transcription\_from\_RNA\_polymerase\_II\_promoter\_involved\_in\_somatic\_motor\_neuron\_fate\_commitment | 1 | 0 |  |  |  |  |  |  |  |  |
| GO:0021933\_radial\_glia\_guided\_migration\_of\_granule\_cell | 1 | 0 |  |  |  |  |  |  |  |  |
| GO:0021934\_hindbrain\_tangential\_cell\_migration | 1 | 0 |  |  |  |  |  |  |  |  |
| GO:0021935\_granule\_cell\_precursor\_tangential\_migration | 1 | 0 |  |  |  |  |  |  |  |  |
| GO:0021942\_radial\_glia\_guided\_migration\_of\_Purkinje\_cell | 1 | 0 |  |  |  |  |  |  |  |  |
| GO:0021960\_anterior\_commissure\_morphogenesis | 1 | 0 |  |  |  |  |  |  |  |  |
| GO:0021997\_neural\_plate\_axis\_specification | 1 | 0 |  |  |  |  |  |  |  |  |
| GO:0021999\_neural\_plate\_anterior\_posterior\_pattern\_formation | 1 | 0 |  |  |  |  |  |  |  |  |
| GO:0022004\_midbrain-hindbrain\_boundary\_maturation\_during\_brain\_development | 1 | 0 |  |  |  |  |  |  |  |  |
| GO:0022038\_corpus\_callosum\_development | 1 | 0 |  |  |  |  |  |  |  |  |
| GO:0022605\_oogenesis\_stage | 1 | 0 |  |  |  |  |  |  |  |  |
| GO:0030011\_maintenance\_of\_cell\_polarity | 1 | 0 |  |  |  |  |  |  |  |  |
| GO:0030069\_lysogeny | 1 | 0 |  |  |  |  |  |  |  |  |
| GO:0030070\_insulin\_processing | 1 | 0 |  |  |  |  |  |  |  |  |
| GO:0030092\_regulation\_of\_flagellum\_assembly | 1 | 0 |  |  |  |  |  |  |  |  |
| GO:0030103\_vasopressin\_secretion | 1 | 0 |  |  |  |  |  |  |  |  |
| GO:0030194\_positive\_regulation\_of\_blood\_coagulation | 1 | 0 |  |  |  |  |  |  |  |  |
| GO:0030206\_chondroitin\_sulfate\_biosynthetic\_process | 1 | 0 |  |  |  |  |  |  |  |  |
| GO:0030210\_heparin\_biosynthetic\_process | 1 | 0 |  |  |  |  |  |  |  |  |
| GO:0030220\_platelet\_formation | 1 | 0 |  |  |  |  |  |  |  |  |
| GO:0030222\_eosinophil\_differentiation | 1 | 0 |  |  |  |  |  |  |  |  |
| GO:0030237\_female\_sex\_determination | 1 | 0 |  |  |  |  |  |  |  |  |
| GO:0030264\_nuclear\_fragmentation\_during\_apoptosis | 1 | 0 |  |  |  |  |  |  |  |  |
| GO:0030322\_stabilization\_of\_membrane\_potential | 1 | 0 |  |  |  |  |  |  |  |  |
| GO:0030327\_prenylated\_protein\_catabolic\_process | 1 | 0 |  |  |  |  |  |  |  |  |
| GO:0030328\_prenylcysteine\_catabolic\_process | 1 | 0 |  |  |  |  |  |  |  |  |
| GO:0030329\_prenylcysteine\_metabolic\_process | 1 | 0 |  |  |  |  |  |  |  |  |
| GO:0030382\_sperm\_mitochondrion\_organization | 1 | 0 |  |  |  |  |  |  |  |  |
| GO:0030389\_fructosamine\_metabolic\_process | 1 | 0 |  |  |  |  |  |  |  |  |
| GO:0030422\_RNA\_interference\_\_production\_of\_siRNA | 1 | 0 |  |  |  |  |  |  |  |  |
| GO:0030449\_regulation\_of\_complement\_activation | 1 | 0 |  |  |  |  |  |  |  |  |
| GO:0030497\_fatty\_acid\_elongation | 1 | 0 |  |  |  |  |  |  |  |  |
| GO:0030575\_nuclear\_body\_organization | 1 | 0 |  |  |  |  |  |  |  |  |
| GO:0030578\_PML\_body\_organization | 1 | 0 |  |  |  |  |  |  |  |  |
| GO:0030853\_negative\_regulation\_of\_granulocyte\_differentiation | 1 | 0 |  |  |  |  |  |  |  |  |
| GO:0030854\_positive\_regulation\_of\_granulocyte\_differentiation | 1 | 0 |  |  |  |  |  |  |  |  |
| GO:0030886\_negative\_regulation\_of\_myeloid\_dendritic\_cell\_activation | 1 | 0 |  |  |  |  |  |  |  |  |
| GO:0030913\_paranodal\_junction\_assembly | 1 | 0 |  |  |  |  |  |  |  |  |
| GO:0031033\_myosin\_filament\_assembly\_or\_disassembly | 1 | 0 |  |  |  |  |  |  |  |  |
| GO:0031034\_myosin\_filament\_assembly | 1 | 0 |  |  |  |  |  |  |  |  |
| GO:0031055\_chromatin\_remodeling\_at\_centromere | 1 | 0 |  |  |  |  |  |  |  |  |
| GO:0031062\_positive\_regulation\_of\_histone\_methylation | 1 | 0 |  |  |  |  |  |  |  |  |
| GO:0031115\_negative\_regulation\_of\_microtubule\_polymerization | 1 | 0 |  |  |  |  |  |  |  |  |
| GO:0031129\_inductive\_cell-cell\_signaling | 1 | 0 |  |  |  |  |  |  |  |  |
| GO:0031284\_positive\_regulation\_of\_guanylate\_cyclase\_activity | 1 | 0 |  |  |  |  |  |  |  |  |
| GO:0031498\_chromatin\_disassembly | 1 | 0 |  |  |  |  |  |  |  |  |
| GO:0031507\_heterochromatin\_formation | 1 | 0 |  |  |  |  |  |  |  |  |
| GO:0031508\_centromeric\_heterochromatin\_formation | 1 | 0 |  |  |  |  |  |  |  |  |
| GO:0031529\_ruffle\_organization | 1 | 0 |  |  |  |  |  |  |  |  |
| GO:0031536\_positive\_regulation\_of\_exit\_from\_mitosis | 1 | 0 |  |  |  |  |  |  |  |  |
| GO:0031572\_G2\_M\_transition\_DNA\_damage\_checkpoint | 1 | 0 |  |  |  |  |  |  |  |  |
| GO:0031576\_G2\_M\_transition\_checkpoint | 1 | 0 |  |  |  |  |  |  |  |  |
| GO:0031580\_membrane\_raft\_distribution | 1 | 0 |  |  |  |  |  |  |  |  |
| GO:0031583\_activation\_of\_phospholipase\_D\_activity\_by\_G-protein\_coupled\_receptor\_protein\_signaling\_pathway | 1 | 0 |  |  |  |  |  |  |  |  |
| GO:0031584\_activation\_of\_phospholipase\_D\_activity | 1 | 0 |  |  |  |  |  |  |  |  |
| GO:0031585\_regulation\_of\_inositol-1\_4\_5-triphosphate\_receptor\_activity | 1 | 0 |  |  |  |  |  |  |  |  |
| GO:0031639\_plasminogen\_activation | 1 | 0 |  |  |  |  |  |  |  |  |
| GO:0031648\_protein\_destabilization | 1 | 0 |  |  |  |  |  |  |  |  |
| GO:0031665\_negative\_regulation\_of\_lipopolysaccharide-mediated\_signaling\_pathway | 1 | 0 |  |  |  |  |  |  |  |  |
| GO:0031914\_negative\_regulation\_of\_synaptic\_plasticity | 1 | 0 |  |  |  |  |  |  |  |  |
| GO:0031944\_negative\_regulation\_of\_glucocorticoid\_metabolic\_process | 1 | 0 |  |  |  |  |  |  |  |  |
| GO:0031947\_negative\_regulation\_of\_glucocorticoid\_biosynthetic\_process | 1 | 0 |  |  |  |  |  |  |  |  |
| GO:0032025\_response\_to\_cobalt\_ion | 1 | 0 |  |  |  |  |  |  |  |  |
| GO:0032026\_response\_to\_magnesium\_ion | 1 | 0 |  |  |  |  |  |  |  |  |
| GO:0032048\_cardiolipin\_metabolic\_process | 1 | 0 |  |  |  |  |  |  |  |  |
| GO:0032066\_nucleolus\_to\_nucleoplasm\_transport | 1 | 0 |  |  |  |  |  |  |  |  |
| GO:0032091\_negative\_regulation\_of\_protein\_binding | 1 | 0 |  |  |  |  |  |  |  |  |
| GO:0032092\_positive\_regulation\_of\_protein\_binding | 1 | 0 |  |  |  |  |  |  |  |  |
| GO:0032097\_positive\_regulation\_of\_response\_to\_food | 1 | 0 |  |  |  |  |  |  |  |  |
| GO:0032100\_positive\_regulation\_of\_appetite | 1 | 0 |  |  |  |  |  |  |  |  |
| GO:0032204\_regulation\_of\_telomere\_maintenance | 1 | 0 |  |  |  |  |  |  |  |  |
| GO:0032206\_positive\_regulation\_of\_telomere\_maintenance | 1 | 0 |  |  |  |  |  |  |  |  |
| GO:0032222\_regulation\_of\_synaptic\_transmission\_\_cholinergic | 1 | 0 |  |  |  |  |  |  |  |  |
| GO:0032224\_positive\_regulation\_of\_synaptic\_transmission\_\_cholinergic | 1 | 0 |  |  |  |  |  |  |  |  |
| GO:0032229\_negative\_regulation\_of\_synaptic\_transmission\_\_GABAergic | 1 | 0 |  |  |  |  |  |  |  |  |
| GO:0032237\_activation\_of\_store-operated\_calcium\_channel\_activity | 1 | 0 |  |  |  |  |  |  |  |  |
| GO:0032239\_regulation\_of\_nucleobase\_\_nucleoside\_\_nucleotide\_and\_nucleic\_acid\_transport | 1 | 0 |  |  |  |  |  |  |  |  |
| GO:0032252\_secretory\_granule\_localization | 1 | 0 |  |  |  |  |  |  |  |  |
| GO:0032274\_gonadotropin\_secretion | 1 | 0 |  |  |  |  |  |  |  |  |
| GO:0032275\_luteinizing\_hormone\_secretion | 1 | 0 |  |  |  |  |  |  |  |  |
| GO:0032287\_myelin\_maintenance\_in\_the\_peripheral\_nervous\_system | 1 | 0 |  |  |  |  |  |  |  |  |
| GO:0032289\_myelin\_formation\_in\_the\_central\_nervous\_system | 1 | 0 |  |  |  |  |  |  |  |  |
| GO:0032303\_regulation\_of\_icosanoid\_secretion | 1 | 0 |  |  |  |  |  |  |  |  |
| GO:0032305\_positive\_regulation\_of\_icosanoid\_secretion | 1 | 0 |  |  |  |  |  |  |  |  |
| GO:0032306\_regulation\_of\_prostaglandin\_secretion | 1 | 0 |  |  |  |  |  |  |  |  |
| GO:0032308\_positive\_regulation\_of\_prostaglandin\_secretion | 1 | 0 |  |  |  |  |  |  |  |  |
| GO:0032310\_prostaglandin\_secretion | 1 | 0 |  |  |  |  |  |  |  |  |
| GO:0032313\_regulation\_of\_Rab\_GTPase\_activity | 1 | 0 |  |  |  |  |  |  |  |  |
| GO:0032317\_regulation\_of\_Rap\_GTPase\_activity | 1 | 0 |  |  |  |  |  |  |  |  |
| GO:0032324\_molybdopterin\_cofactor\_biosynthetic\_process | 1 | 0 |  |  |  |  |  |  |  |  |
| GO:0032329\_serine\_transport | 1 | 0 |  |  |  |  |  |  |  |  |
| GO:0032342\_aldosterone\_biosynthetic\_process | 1 | 0 |  |  |  |  |  |  |  |  |
| GO:0032344\_regulation\_of\_aldosterone\_metabolic\_process | 1 | 0 |  |  |  |  |  |  |  |  |
| GO:0032365\_intracellular\_lipid\_transport | 1 | 0 |  |  |  |  |  |  |  |  |
| GO:0032366\_intracellular\_sterol\_transport | 1 | 0 |  |  |  |  |  |  |  |  |
| GO:0032367\_intracellular\_cholesterol\_transport | 1 | 0 |  |  |  |  |  |  |  |  |
| GO:0032370\_positive\_regulation\_of\_lipid\_transport | 1 | 0 |  |  |  |  |  |  |  |  |
| GO:0032410\_negative\_regulation\_of\_transporter\_activity | 1 | 0 |  |  |  |  |  |  |  |  |
| GO:0032413\_negative\_regulation\_of\_ion\_transmembrane\_transporter\_activity | 1 | 0 |  |  |  |  |  |  |  |  |
| GO:0032429\_regulation\_of\_phospholipase\_A2\_activity | 1 | 0 |  |  |  |  |  |  |  |  |
| GO:0032474\_otolith\_morphogenesis | 1 | 0 |  |  |  |  |  |  |  |  |
| GO:0032482\_Rab\_protein\_signal\_transduction | 1 | 0 |  |  |  |  |  |  |  |  |
| GO:0032483\_regulation\_of\_Rab\_protein\_signal\_transduction | 1 | 0 |  |  |  |  |  |  |  |  |
| GO:0032486\_Rap\_protein\_signal\_transduction | 1 | 0 |  |  |  |  |  |  |  |  |
| GO:0032487\_regulation\_of\_Rap\_protein\_signal\_transduction | 1 | 0 |  |  |  |  |  |  |  |  |
| GO:0032594\_protein\_transport\_within\_lipid\_bilayer | 1 | 0 |  |  |  |  |  |  |  |  |
| GO:0032599\_protein\_transport\_out\_of\_membrane\_raft | 1 | 0 |  |  |  |  |  |  |  |  |
| GO:0032600\_chemokine\_receptor\_transport\_out\_of\_membrane\_raft | 1 | 0 |  |  |  |  |  |  |  |  |
| GO:0032607\_interferon-alpha\_production | 1 | 0 |  |  |  |  |  |  |  |  |
| GO:0032621\_interleukin-18\_production | 1 | 0 |  |  |  |  |  |  |  |  |
| GO:0032647\_regulation\_of\_interferon-alpha\_production | 1 | 0 |  |  |  |  |  |  |  |  |
| GO:0032656\_regulation\_of\_interleukin-13\_production | 1 | 0 |  |  |  |  |  |  |  |  |
| GO:0032682\_negative\_regulation\_of\_chemokine\_production | 1 | 0 |  |  |  |  |  |  |  |  |
| GO:0032691\_negative\_regulation\_of\_interleukin-1\_beta\_production | 1 | 0 |  |  |  |  |  |  |  |  |
| GO:0032692\_negative\_regulation\_of\_interleukin-1\_production | 1 | 0 |  |  |  |  |  |  |  |  |
| GO:0032693\_negative\_regulation\_of\_interleukin-10\_production | 1 | 0 |  |  |  |  |  |  |  |  |
| GO:0032696\_negative\_regulation\_of\_interleukin-13\_production | 1 | 0 |  |  |  |  |  |  |  |  |
| GO:0032727\_positive\_regulation\_of\_interferon-alpha\_production | 1 | 0 |  |  |  |  |  |  |  |  |
| GO:0032731\_positive\_regulation\_of\_interleukin-1\_beta\_production | 1 | 0 |  |  |  |  |  |  |  |  |
| GO:0032732\_positive\_regulation\_of\_interleukin-1\_production | 1 | 0 |  |  |  |  |  |  |  |  |
| GO:0032735\_positive\_regulation\_of\_interleukin-12\_production | 1 | 0 |  |  |  |  |  |  |  |  |
| GO:0032764\_negative\_regulation\_of\_mast\_cell\_cytokine\_production | 1 | 0 |  |  |  |  |  |  |  |  |
| GO:0032765\_positive\_regulation\_of\_mast\_cell\_cytokine\_production | 1 | 0 |  |  |  |  |  |  |  |  |
| GO:0032769\_negative\_regulation\_of\_monooxygenase\_activity | 1 | 0 |  |  |  |  |  |  |  |  |
| GO:0032781\_positive\_regulation\_of\_ATPase\_activity | 1 | 0 |  |  |  |  |  |  |  |  |
| GO:0032790\_ribosome\_disassembly | 1 | 0 |  |  |  |  |  |  |  |  |
| GO:0032799\_low-density\_lipoprotein\_receptor\_metabolic\_process | 1 | 0 |  |  |  |  |  |  |  |  |
| GO:0032802\_low-density\_lipoprotein\_receptor\_catabolic\_process | 1 | 0 |  |  |  |  |  |  |  |  |
| GO:0032803\_regulation\_of\_low-density\_lipoprotein\_receptor\_catabolic\_process | 1 | 0 |  |  |  |  |  |  |  |  |
| GO:0032817\_regulation\_of\_natural\_killer\_cell\_proliferation | 1 | 0 |  |  |  |  |  |  |  |  |
| GO:0032819\_positive\_regulation\_of\_natural\_killer\_cell\_proliferation | 1 | 0 |  |  |  |  |  |  |  |  |
| GO:0032836\_glomerular\_basement\_membrane\_development | 1 | 0 |  |  |  |  |  |  |  |  |
| GO:0032855\_positive\_regulation\_of\_Rac\_GTPase\_activity | 1 | 0 |  |  |  |  |  |  |  |  |
| GO:0032863\_activation\_of\_Rac\_GTPase\_activity | 1 | 0 |  |  |  |  |  |  |  |  |
| GO:0032864\_activation\_of\_Cdc42\_GTPase\_activity | 1 | 0 |  |  |  |  |  |  |  |  |
| GO:0032885\_regulation\_of\_polysaccharide\_biosynthetic\_process | 1 | 0 |  |  |  |  |  |  |  |  |
| GO:0032907\_transforming\_growth\_factor-beta3\_production | 1 | 0 |  |  |  |  |  |  |  |  |
| GO:0032910\_regulation\_of\_transforming\_growth\_factor-beta3\_production | 1 | 0 |  |  |  |  |  |  |  |  |
| GO:0032913\_negative\_regulation\_of\_transforming\_growth\_factor-beta3\_production | 1 | 0 |  |  |  |  |  |  |  |  |
| GO:0032924\_activin\_receptor\_signaling\_pathway | 1 | 0 |  |  |  |  |  |  |  |  |
| GO:0032925\_regulation\_of\_activin\_receptor\_signaling\_pathway | 1 | 0 |  |  |  |  |  |  |  |  |
| GO:0032960\_regulation\_of\_inositol\_trisphosphate\_biosynthetic\_process | 1 | 0 |  |  |  |  |  |  |  |  |
| GO:0032962\_positive\_regulation\_of\_inositol\_trisphosphate\_biosynthetic\_process | 1 | 0 |  |  |  |  |  |  |  |  |
| GO:0032964\_collagen\_biosynthetic\_process | 1 | 0 |  |  |  |  |  |  |  |  |
| GO:0032971\_regulation\_of\_muscle\_filament\_sliding | 1 | 0 |  |  |  |  |  |  |  |  |
| GO:0032972\_regulation\_of\_muscle\_filament\_sliding\_speed | 1 | 0 |  |  |  |  |  |  |  |  |
| GO:0032986\_protein-DNA\_complex\_disassembly | 1 | 0 |  |  |  |  |  |  |  |  |
| GO:0032988\_ribonucleoprotein\_complex\_disassembly | 1 | 0 |  |  |  |  |  |  |  |  |
| GO:0033037\_polysaccharide\_localization | 1 | 0 |  |  |  |  |  |  |  |  |
| GO:0033078\_extrathymic\_T\_cell\_differentiation | 1 | 0 |  |  |  |  |  |  |  |  |
| GO:0033085\_negative\_regulation\_of\_T\_cell\_differentiation\_in\_the\_thymus | 1 | 0 |  |  |  |  |  |  |  |  |
| GO:0033087\_negative\_regulation\_of\_immature\_T\_cell\_proliferation | 1 | 0 |  |  |  |  |  |  |  |  |
| GO:0033088\_negative\_regulation\_of\_immature\_T\_cell\_proliferation\_in\_the\_thymus | 1 | 0 |  |  |  |  |  |  |  |  |
| GO:0033108\_mitochondrial\_respiratory\_chain\_complex\_assembly | 1 | 0 |  |  |  |  |  |  |  |  |
| GO:0033127\_regulation\_of\_histone\_phosphorylation | 1 | 0 |  |  |  |  |  |  |  |  |
| GO:0033128\_negative\_regulation\_of\_histone\_phosphorylation | 1 | 0 |  |  |  |  |  |  |  |  |
| GO:0033138\_positive\_regulation\_of\_peptidyl-serine\_phosphorylation | 1 | 0 |  |  |  |  |  |  |  |  |
| GO:0033158\_regulation\_of\_protein\_import\_into\_nucleus\_\_translocation | 1 | 0 |  |  |  |  |  |  |  |  |
| GO:0033160\_positive\_regulation\_of\_protein\_import\_into\_nucleus\_\_translocation | 1 | 0 |  |  |  |  |  |  |  |  |
| GO:0033169\_histone\_H3-K9\_demethylation | 1 | 0 |  |  |  |  |  |  |  |  |
| GO:0033206\_cytokinesis\_after\_meiosis | 1 | 0 |  |  |  |  |  |  |  |  |
| GO:0033240\_positive\_regulation\_of\_cellular\_amine\_metabolic\_process | 1 | 0 |  |  |  |  |  |  |  |  |
| GO:0033313\_meiotic\_cell\_cycle\_checkpoint | 1 | 0 |  |  |  |  |  |  |  |  |
| GO:0033315\_meiotic\_cell\_cycle\_DNA\_replication\_checkpoint | 1 | 0 |  |  |  |  |  |  |  |  |
| GO:0033326\_cerebrospinal\_fluid\_secretion | 1 | 0 |  |  |  |  |  |  |  |  |
| GO:0033366\_protein\_localization\_in\_secretory\_granule | 1 | 0 |  |  |  |  |  |  |  |  |
| GO:0033367\_protein\_localization\_in\_mast\_cell\_secretory\_granule | 1 | 0 |  |  |  |  |  |  |  |  |
| GO:0033368\_protease\_localization\_in\_mast\_cell\_secretory\_granule | 1 | 0 |  |  |  |  |  |  |  |  |
| GO:0033370\_maintenance\_of\_protein\_location\_in\_mast\_cell\_secretory\_granule | 1 | 0 |  |  |  |  |  |  |  |  |
| GO:0033371\_T\_cell\_secretory\_granule\_organization | 1 | 0 |  |  |  |  |  |  |  |  |
| GO:0033373\_maintenance\_of\_protease\_location\_in\_mast\_cell\_secretory\_granule | 1 | 0 |  |  |  |  |  |  |  |  |
| GO:0033374\_protein\_localization\_in\_T\_cell\_secretory\_granule | 1 | 0 |  |  |  |  |  |  |  |  |
| GO:0033375\_protease\_localization\_in\_T\_cell\_secretory\_granule | 1 | 0 |  |  |  |  |  |  |  |  |
| GO:0033377\_maintenance\_of\_protein\_location\_in\_T\_cell\_secretory\_granule | 1 | 0 |  |  |  |  |  |  |  |  |
| GO:0033379\_maintenance\_of\_protease\_location\_in\_T\_cell\_secretory\_granule | 1 | 0 |  |  |  |  |  |  |  |  |
| GO:0033380\_granzyme\_B\_localization\_in\_T\_cell\_secretory\_granule | 1 | 0 |  |  |  |  |  |  |  |  |
| GO:0033382\_maintenance\_of\_granzyme\_B\_location\_in\_T\_cell\_secretory\_granule | 1 | 0 |  |  |  |  |  |  |  |  |
| GO:0033483\_gas\_homeostasis | 1 | 0 |  |  |  |  |  |  |  |  |
| GO:0033484\_nitric\_oxide\_homeostasis | 1 | 0 |  |  |  |  |  |  |  |  |
| GO:0033505\_floor\_plate\_morphogenesis | 1 | 0 |  |  |  |  |  |  |  |  |
| GO:0033522\_histone\_H2A\_ubiquitination | 1 | 0 |  |  |  |  |  |  |  |  |
| GO:0033523\_histone\_H2B\_ubiquitination | 1 | 0 |  |  |  |  |  |  |  |  |
| GO:0033574\_response\_to\_testosterone\_stimulus | 1 | 0 |  |  |  |  |  |  |  |  |
| GO:0033606\_chemokine\_receptor\_transport\_within\_lipid\_bilayer | 1 | 0 |  |  |  |  |  |  |  |  |
| GO:0033628\_regulation\_of\_cell\_adhesion\_mediated\_by\_integrin | 1 | 0 |  |  |  |  |  |  |  |  |
| GO:0033630\_positive\_regulation\_of\_cell\_adhesion\_mediated\_by\_integrin | 1 | 0 |  |  |  |  |  |  |  |  |
| GO:0033632\_regulation\_of\_cell-cell\_adhesion\_mediated\_by\_integrin | 1 | 0 |  |  |  |  |  |  |  |  |
| GO:0033634\_positive\_regulation\_of\_cell-cell\_adhesion\_mediated\_by\_integrin | 1 | 0 |  |  |  |  |  |  |  |  |
| GO:0033683\_nucleotide-excision\_repair\_\_DNA\_incision | 1 | 0 |  |  |  |  |  |  |  |  |
| GO:0033687\_osteoblast\_proliferation | 1 | 0 |  |  |  |  |  |  |  |  |
| GO:0033688\_regulation\_of\_osteoblast\_proliferation | 1 | 0 |  |  |  |  |  |  |  |  |
| GO:0033689\_negative\_regulation\_of\_osteoblast\_proliferation | 1 | 0 |  |  |  |  |  |  |  |  |
| GO:0033750\_ribosome\_localization | 1 | 0 |  |  |  |  |  |  |  |  |
| GO:0033753\_establishment\_of\_ribosome\_localization | 1 | 0 |  |  |  |  |  |  |  |  |
| GO:0033866\_nucleoside\_bisphosphate\_biosynthetic\_process | 1 | 0 |  |  |  |  |  |  |  |  |
| GO:0033875\_ribonucleoside\_bisphosphate\_metabolic\_process | 1 | 0 |  |  |  |  |  |  |  |  |
| GO:0034030\_ribonucleoside\_bisphosphate\_biosynthetic\_process | 1 | 0 |  |  |  |  |  |  |  |  |
| GO:0034032\_purine\_nucleoside\_bisphosphate\_metabolic\_process | 1 | 0 |  |  |  |  |  |  |  |  |
| GO:0034033\_purine\_nucleoside\_bisphosphate\_biosynthetic\_process | 1 | 0 |  |  |  |  |  |  |  |  |
| GO:0034035\_purine\_ribonucleoside\_bisphosphate\_metabolic\_process | 1 | 0 |  |  |  |  |  |  |  |  |
| GO:0034036\_purine\_ribonucleoside\_bisphosphate\_biosynthetic\_process | 1 | 0 |  |  |  |  |  |  |  |  |
| GO:0034067\_protein\_localization\_in\_Golgi\_apparatus | 1 | 0 |  |  |  |  |  |  |  |  |
| GO:0034102\_erythrocyte\_clearance | 1 | 0 |  |  |  |  |  |  |  |  |
| GO:0034106\_regulation\_of\_erythrocyte\_clearance | 1 | 0 |  |  |  |  |  |  |  |  |
| GO:0034107\_negative\_regulation\_of\_erythrocyte\_clearance | 1 | 0 |  |  |  |  |  |  |  |  |
| GO:0034110\_regulation\_of\_homotypic\_cell-cell\_adhesion | 1 | 0 |  |  |  |  |  |  |  |  |
| GO:0034111\_negative\_regulation\_of\_homotypic\_cell-cell\_adhesion | 1 | 0 |  |  |  |  |  |  |  |  |
| GO:0034113\_heterotypic\_cell-cell\_adhesion | 1 | 0 |  |  |  |  |  |  |  |  |
| GO:0034117\_erythrocyte\_aggregation | 1 | 0 |  |  |  |  |  |  |  |  |
| GO:0034118\_regulation\_of\_erythrocyte\_aggregation | 1 | 0 |  |  |  |  |  |  |  |  |
| GO:0034119\_negative\_regulation\_of\_erythrocyte\_aggregation | 1 | 0 |  |  |  |  |  |  |  |  |
| GO:0034121\_regulation\_of\_toll-like\_receptor\_signaling\_pathway | 1 | 0 |  |  |  |  |  |  |  |  |
| GO:0034122\_negative\_regulation\_of\_toll-like\_receptor\_signaling\_pathway | 1 | 0 |  |  |  |  |  |  |  |  |
| GO:0034230\_enkephalin\_processing | 1 | 0 |  |  |  |  |  |  |  |  |
| GO:0034372\_very-low-density\_lipoprotein\_particle\_remodeling | 1 | 0 |  |  |  |  |  |  |  |  |
| GO:0034379\_very-low-density\_lipoprotein\_particle\_assembly | 1 | 0 |  |  |  |  |  |  |  |  |
| GO:0034380\_high-density\_lipoprotein\_particle\_assembly | 1 | 0 |  |  |  |  |  |  |  |  |
| GO:0034394\_protein\_localization\_at\_cell\_surface | 1 | 0 |  |  |  |  |  |  |  |  |
| GO:0034405\_response\_to\_fluid\_shear\_stress | 1 | 0 |  |  |  |  |  |  |  |  |
| GO:0034472\_snRNA\_3'-end\_processing | 1 | 0 |  |  |  |  |  |  |  |  |
| GO:0034474\_U2\_snRNA\_3'-end\_processing | 1 | 0 |  |  |  |  |  |  |  |  |
| GO:0034502\_protein\_localization\_to\_chromosome | 1 | 0 |  |  |  |  |  |  |  |  |
| GO:0034505\_tooth\_mineralization | 1 | 0 |  |  |  |  |  |  |  |  |
| GO:0034508\_centromere\_complex\_assembly | 1 | 0 |  |  |  |  |  |  |  |  |
| GO:0034633\_retinol\_transport | 1 | 0 |  |  |  |  |  |  |  |  |
| GO:0034643\_mitochondrion\_localization\_\_microtubule-mediated | 1 | 0 |  |  |  |  |  |  |  |  |
| GO:0034969\_histone\_arginine\_methylation | 1 | 0 |  |  |  |  |  |  |  |  |
| GO:0034982\_mitochondrial\_protein\_processing | 1 | 0 |  |  |  |  |  |  |  |  |
| GO:0035022\_positive\_regulation\_of\_Rac\_protein\_signal\_transduction | 1 | 0 |  |  |  |  |  |  |  |  |
| GO:0035024\_negative\_regulation\_of\_Rho\_protein\_signal\_transduction | 1 | 0 |  |  |  |  |  |  |  |  |
| GO:0035026\_leading\_edge\_cell\_differentiation | 1 | 0 |  |  |  |  |  |  |  |  |
| GO:0035037\_sperm\_entry | 1 | 0 |  |  |  |  |  |  |  |  |
| GO:0035039\_male\_pronucleus\_formation | 1 | 0 |  |  |  |  |  |  |  |  |
| GO:0035066\_positive\_regulation\_of\_histone\_acetylation | 1 | 0 |  |  |  |  |  |  |  |  |
| GO:0035083\_cilium\_axoneme\_assembly | 1 | 0 |  |  |  |  |  |  |  |  |
| GO:0035090\_maintenance\_of\_apical\_basal\_cell\_polarity | 1 | 0 |  |  |  |  |  |  |  |  |
| GO:0035106\_operant\_conditioning | 1 | 0 |  |  |  |  |  |  |  |  |
| GO:0035172\_hemocyte\_proliferation | 1 | 0 |  |  |  |  |  |  |  |  |
| GO:0035227\_regulation\_of\_glutamate-cysteine\_ligase\_activity | 1 | 0 |  |  |  |  |  |  |  |  |
| GO:0035229\_positive\_regulation\_of\_glutamate-cysteine\_ligase\_activity | 1 | 0 |  |  |  |  |  |  |  |  |
| GO:0035260\_internal\_genitalia\_morphogenesis | 1 | 0 |  |  |  |  |  |  |  |  |
| GO:0035262\_gonad\_morphogenesis | 1 | 0 |  |  |  |  |  |  |  |  |
| GO:0035287\_head\_segmentation | 1 | 0 |  |  |  |  |  |  |  |  |
| GO:0035289\_posterior\_head\_segmentation | 1 | 0 |  |  |  |  |  |  |  |  |
| GO:0035303\_regulation\_of\_dephosphorylation | 1 | 0 |  |  |  |  |  |  |  |  |
| GO:0035304\_regulation\_of\_protein\_amino\_acid\_dephosphorylation | 1 | 0 |  |  |  |  |  |  |  |  |
| GO:0035305\_negative\_regulation\_of\_dephosphorylation | 1 | 0 |  |  |  |  |  |  |  |  |
| GO:0035308\_negative\_regulation\_of\_protein\_amino\_acid\_dephosphorylation | 1 | 0 |  |  |  |  |  |  |  |  |
| GO:0035313\_wound\_healing\_\_spreading\_of\_epidermal\_cells | 1 | 0 |  |  |  |  |  |  |  |  |
| GO:0040013\_negative\_regulation\_of\_locomotion | 1 | 0 |  |  |  |  |  |  |  |  |
| GO:0040019\_positive\_regulation\_of\_embryonic\_development | 1 | 0 |  |  |  |  |  |  |  |  |
| GO:0040032\_post-embryonic\_body\_morphogenesis | 1 | 0 |  |  |  |  |  |  |  |  |
| GO:0040038\_polar\_body\_extrusion\_after\_meiotic\_divisions | 1 | 0 |  |  |  |  |  |  |  |  |
| GO:0042026\_protein\_refolding | 1 | 0 |  |  |  |  |  |  |  |  |
| GO:0042048\_olfactory\_behavior | 1 | 0 |  |  |  |  |  |  |  |  |
| GO:0042059\_negative\_regulation\_of\_epidermal\_growth\_factor\_receptor\_signaling\_pathway | 1 | 0 |  |  |  |  |  |  |  |  |
| GO:0042073\_intraflagellar\_transport | 1 | 0 |  |  |  |  |  |  |  |  |
| GO:0042078\_germ-line\_stem\_cell\_division | 1 | 0 |  |  |  |  |  |  |  |  |
| GO:0042091\_interleukin-10\_biosynthetic\_process | 1 | 0 |  |  |  |  |  |  |  |  |
| GO:0042103\_positive\_regulation\_of\_T\_cell\_homeostatic\_proliferation | 1 | 0 |  |  |  |  |  |  |  |  |
| GO:0042136\_neurotransmitter\_biosynthetic\_process | 1 | 0 |  |  |  |  |  |  |  |  |
| GO:0042137\_sequestering\_of\_neurotransmitter | 1 | 0 |  |  |  |  |  |  |  |  |
| GO:0042138\_meiotic\_DNA\_double-strand\_break\_formation | 1 | 0 |  |  |  |  |  |  |  |  |
| GO:0042178\_xenobiotic\_catabolic\_process | 1 | 0 |  |  |  |  |  |  |  |  |
| GO:0042225\_interleukin-5\_biosynthetic\_process | 1 | 0 |  |  |  |  |  |  |  |  |
| GO:0042231\_interleukin-13\_biosynthetic\_process | 1 | 0 |  |  |  |  |  |  |  |  |
| GO:0042255\_ribosome\_assembly | 1 | 0 |  |  |  |  |  |  |  |  |
| GO:0042257\_ribosomal\_subunit\_assembly | 1 | 0 |  |  |  |  |  |  |  |  |
| GO:0042264\_peptidyl-aspartic\_acid\_hydroxylation | 1 | 0 |  |  |  |  |  |  |  |  |
| GO:0042276\_error-prone\_postreplication\_DNA\_repair | 1 | 0 |  |  |  |  |  |  |  |  |
| GO:0042297\_vocal\_learning | 1 | 0 |  |  |  |  |  |  |  |  |
| GO:0042309\_homoiothermy | 1 | 0 |  |  |  |  |  |  |  |  |
| GO:0042320\_regulation\_of\_circadian\_sleep\_wake\_cycle\_\_REM\_sleep | 1 | 0 |  |  |  |  |  |  |  |  |
| GO:0042339\_keratan\_sulfate\_metabolic\_process | 1 | 0 |  |  |  |  |  |  |  |  |
| GO:0042347\_negative\_regulation\_of\_NF-kappaB\_import\_into\_nucleus | 1 | 0 |  |  |  |  |  |  |  |  |
| GO:0042360\_vitamin\_E\_metabolic\_process | 1 | 0 |  |  |  |  |  |  |  |  |
| GO:0042363\_fat-soluble\_vitamin\_catabolic\_process | 1 | 0 |  |  |  |  |  |  |  |  |
| GO:0042369\_vitamin\_D\_catabolic\_process | 1 | 0 |  |  |  |  |  |  |  |  |
| GO:0042373\_vitamin\_K\_metabolic\_process | 1 | 0 |  |  |  |  |  |  |  |  |
| GO:0042404\_thyroid\_hormone\_catabolic\_process | 1 | 0 |  |  |  |  |  |  |  |  |
| GO:0042414\_epinephrine\_metabolic\_process | 1 | 0 |  |  |  |  |  |  |  |  |
| GO:0042436\_indole\_derivative\_catabolic\_process | 1 | 0 |  |  |  |  |  |  |  |  |
| GO:0042489\_negative\_regulation\_of\_odontogenesis\_of\_dentine-containing\_tooth | 1 | 0 |  |  |  |  |  |  |  |  |
| GO:0042508\_tyrosine\_phosphorylation\_of\_Stat1\_protein | 1 | 0 |  |  |  |  |  |  |  |  |
| GO:0042518\_negative\_regulation\_of\_tyrosine\_phosphorylation\_of\_Stat3\_protein | 1 | 0 |  |  |  |  |  |  |  |  |
| GO:0042524\_negative\_regulation\_of\_tyrosine\_phosphorylation\_of\_Stat5\_protein | 1 | 0 |  |  |  |  |  |  |  |  |
| GO:0042536\_negative\_regulation\_of\_tumor\_necrosis\_factor\_biosynthetic\_process | 1 | 0 |  |  |  |  |  |  |  |  |
| GO:0042538\_hyperosmotic\_salinity\_response | 1 | 0 |  |  |  |  |  |  |  |  |
| GO:0042628\_mating\_plug\_formation | 1 | 0 |  |  |  |  |  |  |  |  |
| GO:0042631\_cellular\_response\_to\_water\_deprivation | 1 | 0 |  |  |  |  |  |  |  |  |
| GO:0042637\_catagen | 1 | 0 |  |  |  |  |  |  |  |  |
| GO:0042660\_positive\_regulation\_of\_cell\_fate\_specification | 1 | 0 |  |  |  |  |  |  |  |  |
| GO:0042663\_regulation\_of\_endodermal\_cell\_fate\_specification | 1 | 0 |  |  |  |  |  |  |  |  |
| GO:0042664\_negative\_regulation\_of\_endodermal\_cell\_fate\_specification | 1 | 0 |  |  |  |  |  |  |  |  |
| GO:0042667\_auditory\_receptor\_cell\_fate\_specification | 1 | 0 |  |  |  |  |  |  |  |  |
| GO:0042694\_muscle\_cell\_fate\_specification | 1 | 0 |  |  |  |  |  |  |  |  |
| GO:0042706\_eye\_photoreceptor\_cell\_fate\_commitment | 1 | 0 |  |  |  |  |  |  |  |  |
| GO:0042713\_sperm\_ejaculation | 1 | 0 |  |  |  |  |  |  |  |  |
| GO:0042723\_thiamin\_and\_derivative\_metabolic\_process | 1 | 0 |  |  |  |  |  |  |  |  |
| GO:0042737\_drug\_catabolic\_process | 1 | 0 |  |  |  |  |  |  |  |  |
| GO:0042738\_exogenous\_drug\_catabolic\_process | 1 | 0 |  |  |  |  |  |  |  |  |
| GO:0042747\_circadian\_sleep\_wake\_cycle\_\_REM\_sleep | 1 | 0 |  |  |  |  |  |  |  |  |
| GO:0042748\_circadian\_sleep\_wake\_cycle\_\_non-REM\_sleep | 1 | 0 |  |  |  |  |  |  |  |  |
| GO:0042772\_DNA\_damage\_response\_\_signal\_transduction\_resulting\_in\_transcription | 1 | 0 |  |  |  |  |  |  |  |  |
| GO:0042790\_transcription\_of\_nuclear\_rRNA\_large\_RNA\_polymerase\_I\_transcript | 1 | 0 |  |  |  |  |  |  |  |  |
| GO:0042839\_D-glucuronate\_metabolic\_process | 1 | 0 |  |  |  |  |  |  |  |  |
| GO:0042840\_D-glucuronate\_catabolic\_process | 1 | 0 |  |  |  |  |  |  |  |  |
| GO:0042891\_antibiotic\_transport | 1 | 0 |  |  |  |  |  |  |  |  |
| GO:0042892\_chloramphenicol\_transport | 1 | 0 |  |  |  |  |  |  |  |  |
| GO:0042940\_D-amino\_acid\_transport | 1 | 0 |  |  |  |  |  |  |  |  |
| GO:0042941\_D-alanine\_transport | 1 | 0 |  |  |  |  |  |  |  |  |
| GO:0042942\_D-serine\_transport | 1 | 0 |  |  |  |  |  |  |  |  |
| GO:0042983\_amyloid\_precursor\_protein\_biosynthetic\_process | 1 | 0 |  |  |  |  |  |  |  |  |
| GO:0042984\_regulation\_of\_amyloid\_precursor\_protein\_biosynthetic\_process | 1 | 0 |  |  |  |  |  |  |  |  |
| GO:0042985\_negative\_regulation\_of\_amyloid\_precursor\_protein\_biosynthetic\_process | 1 | 0 |  |  |  |  |  |  |  |  |
| GO:0042989\_sequestering\_of\_actin\_monomers | 1 | 0 |  |  |  |  |  |  |  |  |
| GO:0043044\_ATP-dependent\_chromatin\_remodeling | 1 | 0 |  |  |  |  |  |  |  |  |
| GO:0043056\_forward\_locomotion | 1 | 0 |  |  |  |  |  |  |  |  |
| GO:0043060\_meiotic\_metaphase\_I\_plate\_congression | 1 | 0 |  |  |  |  |  |  |  |  |
| GO:0043091\_L-arginine\_import | 1 | 0 |  |  |  |  |  |  |  |  |
| GO:0043124\_negative\_regulation\_of\_I-kappaB\_kinase\_NF-kappaB\_cascade | 1 | 0 |  |  |  |  |  |  |  |  |
| GO:0043132\_NAD\_transport | 1 | 0 |  |  |  |  |  |  |  |  |
| GO:0043153\_entrainment\_of\_circadian\_clock\_by\_photoperiod | 1 | 0 |  |  |  |  |  |  |  |  |
| GO:0043171\_peptide\_catabolic\_process | 1 | 0 |  |  |  |  |  |  |  |  |
| GO:0043179\_rhythmic\_excitation | 1 | 0 |  |  |  |  |  |  |  |  |
| GO:0043206\_fibril\_organization | 1 | 0 |  |  |  |  |  |  |  |  |
| GO:0043217\_myelin\_maintenance | 1 | 0 |  |  |  |  |  |  |  |  |
| GO:0043313\_regulation\_of\_neutrophil\_degranulation | 1 | 0 |  |  |  |  |  |  |  |  |
| GO:0043316\_cytotoxic\_T\_cell\_degranulation | 1 | 0 |  |  |  |  |  |  |  |  |
| GO:0043369\_CD4-positive\_or\_CD8-positive\_\_alpha-beta\_T\_cell\_lineage\_commitment | 1 | 0 |  |  |  |  |  |  |  |  |
| GO:0043375\_CD8-positive\_\_alpha-beta\_T\_cell\_lineage\_commitment | 1 | 0 |  |  |  |  |  |  |  |  |
| GO:0043379\_memory\_T\_cell\_differentiation | 1 | 0 |  |  |  |  |  |  |  |  |
| GO:0043380\_regulation\_of\_memory\_T\_cell\_differentiation | 1 | 0 |  |  |  |  |  |  |  |  |
| GO:0043400\_cortisol\_secretion | 1 | 0 |  |  |  |  |  |  |  |  |
| GO:0043415\_positive\_regulation\_of\_skeletal\_muscle\_regeneration | 1 | 0 |  |  |  |  |  |  |  |  |
| GO:0043416\_regulation\_of\_skeletal\_muscle\_regeneration | 1 | 0 |  |  |  |  |  |  |  |  |
| GO:0043437\_butanoic\_acid\_metabolic\_process | 1 | 0 |  |  |  |  |  |  |  |  |
| GO:0043438\_acetoacetic\_acid\_metabolic\_process | 1 | 0 |  |  |  |  |  |  |  |  |
| GO:0043480\_pigment\_accumulation\_in\_tissues | 1 | 0 |  |  |  |  |  |  |  |  |
| GO:0043482\_cellular\_pigment\_accumulation | 1 | 0 |  |  |  |  |  |  |  |  |
| GO:0043486\_histone\_exchange | 1 | 0 |  |  |  |  |  |  |  |  |
| GO:0043496\_regulation\_of\_protein\_homodimerization\_activity | 1 | 0 |  |  |  |  |  |  |  |  |
| GO:0043501\_skeletal\_muscle\_adaptation | 1 | 0 |  |  |  |  |  |  |  |  |
| GO:0043508\_negative\_regulation\_of\_JUN\_kinase\_activity | 1 | 0 |  |  |  |  |  |  |  |  |
| GO:0043517\_positive\_regulation\_of\_DNA\_damage\_response\_\_signal\_transduction\_by\_p53\_class\_mediator | 1 | 0 |  |  |  |  |  |  |  |  |
| GO:0043535\_regulation\_of\_blood\_vessel\_endothelial\_cell\_migration | 1 | 0 |  |  |  |  |  |  |  |  |
| GO:0043537\_negative\_regulation\_of\_blood\_vessel\_endothelial\_cell\_migration | 1 | 0 |  |  |  |  |  |  |  |  |
| GO:0043545\_molybdopterin\_cofactor\_metabolic\_process | 1 | 0 |  |  |  |  |  |  |  |  |
| GO:0043587\_tongue\_morphogenesis | 1 | 0 |  |  |  |  |  |  |  |  |
| GO:0043604\_amide\_biosynthetic\_process | 1 | 0 |  |  |  |  |  |  |  |  |
| GO:0043628\_ncRNA\_3'-end\_processing | 1 | 0 |  |  |  |  |  |  |  |  |
| GO:0044254\_multicellular\_organismal\_protein\_catabolic\_process | 1 | 0 |  |  |  |  |  |  |  |  |
| GO:0044256\_protein\_digestion | 1 | 0 |  |  |  |  |  |  |  |  |
| GO:0044266\_multicellular\_organismal\_macromolecule\_catabolic\_process | 1 | 0 |  |  |  |  |  |  |  |  |
| GO:0045004\_DNA\_replication\_proofreading | 1 | 0 |  |  |  |  |  |  |  |  |
| GO:0045019\_negative\_regulation\_of\_nitric\_oxide\_biosynthetic\_process | 1 | 0 |  |  |  |  |  |  |  |  |
| GO:0045020\_error-prone\_DNA\_repair | 1 | 0 |  |  |  |  |  |  |  |  |
| GO:0045022\_early\_endosome\_to\_late\_endosome\_transport | 1 | 0 |  |  |  |  |  |  |  |  |
| GO:0045062\_extrathymic\_T\_cell\_selection | 1 | 0 |  |  |  |  |  |  |  |  |
| GO:0045069\_regulation\_of\_viral\_genome\_replication | 1 | 0 |  |  |  |  |  |  |  |  |
| GO:0045074\_regulation\_of\_interleukin-10\_biosynthetic\_process | 1 | 0 |  |  |  |  |  |  |  |  |
| GO:0045082\_positive\_regulation\_of\_interleukin-10\_biosynthetic\_process | 1 | 0 |  |  |  |  |  |  |  |  |
| GO:0045083\_negative\_regulation\_of\_interleukin-12\_biosynthetic\_process | 1 | 0 |  |  |  |  |  |  |  |  |
| GO:0045112\_integrin\_biosynthetic\_process | 1 | 0 |  |  |  |  |  |  |  |  |
| GO:0045113\_regulation\_of\_integrin\_biosynthetic\_process | 1 | 0 |  |  |  |  |  |  |  |  |
| GO:0045188\_regulation\_of\_circadian\_sleep\_wake\_cycle\_\_non-REM\_sleep | 1 | 0 |  |  |  |  |  |  |  |  |
| GO:0045210\_FasL\_biosynthetic\_process | 1 | 0 |  |  |  |  |  |  |  |  |
| GO:0045297\_post-mating\_behavior | 1 | 0 |  |  |  |  |  |  |  |  |
| GO:0045299\_otolith\_mineralization | 1 | 0 |  |  |  |  |  |  |  |  |
| GO:0045329\_carnitine\_biosynthetic\_process | 1 | 0 |  |  |  |  |  |  |  |  |
| GO:0045341\_MHC\_class\_I\_biosynthetic\_process | 1 | 0 |  |  |  |  |  |  |  |  |
| GO:0045343\_regulation\_of\_MHC\_class\_I\_biosynthetic\_process | 1 | 0 |  |  |  |  |  |  |  |  |
| GO:0045347\_negative\_regulation\_of\_MHC\_class\_II\_biosynthetic\_process | 1 | 0 |  |  |  |  |  |  |  |  |
| GO:0045405\_regulation\_of\_interleukin-5\_biosynthetic\_process | 1 | 0 |  |  |  |  |  |  |  |  |
| GO:0045407\_positive\_regulation\_of\_interleukin-5\_biosynthetic\_process | 1 | 0 |  |  |  |  |  |  |  |  |
| GO:0045426\_quinone\_cofactor\_biosynthetic\_process | 1 | 0 |  |  |  |  |  |  |  |  |
| GO:0045448\_mitotic\_cell\_cycle\_\_embryonic | 1 | 0 |  |  |  |  |  |  |  |  |
| GO:0045454\_cell\_redox\_homeostasis | 1 | 0 |  |  |  |  |  |  |  |  |
| GO:0045583\_regulation\_of\_cytotoxic\_T\_cell\_differentiation | 1 | 0 |  |  |  |  |  |  |  |  |
| GO:0045585\_positive\_regulation\_of\_cytotoxic\_T\_cell\_differentiation | 1 | 0 |  |  |  |  |  |  |  |  |
| GO:0045601\_regulation\_of\_endothelial\_cell\_differentiation | 1 | 0 |  |  |  |  |  |  |  |  |
| GO:0045602\_negative\_regulation\_of\_endothelial\_cell\_differentiation | 1 | 0 |  |  |  |  |  |  |  |  |
| GO:0045605\_negative\_regulation\_of\_epidermal\_cell\_differentiation | 1 | 0 |  |  |  |  |  |  |  |  |
| GO:0045606\_positive\_regulation\_of\_epidermal\_cell\_differentiation | 1 | 0 |  |  |  |  |  |  |  |  |
| GO:0045609\_positive\_regulation\_of\_auditory\_receptor\_cell\_differentiation | 1 | 0 |  |  |  |  |  |  |  |  |
| GO:0045617\_negative\_regulation\_of\_keratinocyte\_differentiation | 1 | 0 |  |  |  |  |  |  |  |  |
| GO:0045618\_positive\_regulation\_of\_keratinocyte\_differentiation | 1 | 0 |  |  |  |  |  |  |  |  |
| GO:0045626\_negative\_regulation\_of\_T-helper\_1\_cell\_differentiation | 1 | 0 |  |  |  |  |  |  |  |  |
| GO:0045633\_positive\_regulation\_of\_mechanoreceptor\_differentiation | 1 | 0 |  |  |  |  |  |  |  |  |
| GO:0045650\_negative\_regulation\_of\_macrophage\_differentiation | 1 | 0 |  |  |  |  |  |  |  |  |
| GO:0045656\_negative\_regulation\_of\_monocyte\_differentiation | 1 | 0 |  |  |  |  |  |  |  |  |
| GO:0045657\_positive\_regulation\_of\_monocyte\_differentiation | 1 | 0 |  |  |  |  |  |  |  |  |
| GO:0045659\_negative\_regulation\_of\_neutrophil\_differentiation | 1 | 0 |  |  |  |  |  |  |  |  |
| GO:0045660\_positive\_regulation\_of\_neutrophil\_differentiation | 1 | 0 |  |  |  |  |  |  |  |  |
| GO:0045721\_negative\_regulation\_of\_gluconeogenesis | 1 | 0 |  |  |  |  |  |  |  |  |
| GO:0045724\_positive\_regulation\_of\_flagellum\_assembly | 1 | 0 |  |  |  |  |  |  |  |  |
| GO:0045725\_positive\_regulation\_of\_glycogen\_biosynthetic\_process | 1 | 0 |  |  |  |  |  |  |  |  |
| GO:0045740\_positive\_regulation\_of\_DNA\_replication | 1 | 0 |  |  |  |  |  |  |  |  |
| GO:0045759\_negative\_regulation\_of\_action\_potential | 1 | 0 |  |  |  |  |  |  |  |  |
| GO:0045768\_positive\_regulation\_of\_anti-apoptosis | 1 | 0 |  |  |  |  |  |  |  |  |
| GO:0045769\_negative\_regulation\_of\_asymmetric\_cell\_division | 1 | 0 |  |  |  |  |  |  |  |  |
| GO:0045794\_negative\_regulation\_of\_cell\_volume | 1 | 0 |  |  |  |  |  |  |  |  |
| GO:0045815\_positive\_regulation\_of\_gene\_expression\_\_epigenetic | 1 | 0 |  |  |  |  |  |  |  |  |
| GO:0045818\_negative\_regulation\_of\_glycogen\_catabolic\_process | 1 | 0 |  |  |  |  |  |  |  |  |
| GO:0045842\_positive\_regulation\_of\_mitotic\_metaphase\_anaphase\_transition | 1 | 0 |  |  |  |  |  |  |  |  |
| GO:0045875\_negative\_regulation\_of\_sister\_chromatid\_cohesion | 1 | 0 |  |  |  |  |  |  |  |  |
| GO:0045898\_regulation\_of\_transcriptional\_preinitiation\_complex\_assembly | 1 | 0 |  |  |  |  |  |  |  |  |
| GO:0045899\_positive\_regulation\_of\_transcriptional\_preinitiation\_complex\_assembly | 1 | 0 |  |  |  |  |  |  |  |  |
| GO:0045906\_negative\_regulation\_of\_vasoconstriction | 1 | 0 |  |  |  |  |  |  |  |  |
| GO:0045908\_negative\_regulation\_of\_vasodilation | 1 | 0 |  |  |  |  |  |  |  |  |
| GO:0045909\_positive\_regulation\_of\_vasodilation | 1 | 0 |  |  |  |  |  |  |  |  |
| GO:0045915\_positive\_regulation\_of\_catecholamine\_metabolic\_process | 1 | 0 |  |  |  |  |  |  |  |  |
| GO:0045920\_negative\_regulation\_of\_exocytosis | 1 | 0 |  |  |  |  |  |  |  |  |
| GO:0045924\_regulation\_of\_female\_receptivity | 1 | 0 |  |  |  |  |  |  |  |  |
| GO:0045947\_negative\_regulation\_of\_translational\_initiation | 1 | 0 |  |  |  |  |  |  |  |  |
| GO:0045955\_negative\_regulation\_of\_calcium\_ion-dependent\_exocytosis | 1 | 0 |  |  |  |  |  |  |  |  |
| GO:0045956\_positive\_regulation\_of\_calcium\_ion-dependent\_exocytosis | 1 | 0 |  |  |  |  |  |  |  |  |
| GO:0045964\_positive\_regulation\_of\_dopamine\_metabolic\_process | 1 | 0 |  |  |  |  |  |  |  |  |
| GO:0045988\_negative\_regulation\_of\_striated\_muscle\_contraction | 1 | 0 |  |  |  |  |  |  |  |  |
| GO:0045989\_positive\_regulation\_of\_striated\_muscle\_contraction | 1 | 0 |  |  |  |  |  |  |  |  |
| GO:0045990\_regulation\_of\_transcription\_by\_carbon\_catabolites | 1 | 0 |  |  |  |  |  |  |  |  |
| GO:0045991\_positive\_regulation\_of\_transcription\_by\_carbon\_catabolites | 1 | 0 |  |  |  |  |  |  |  |  |
| GO:0045994\_positive\_regulation\_of\_translational\_initiation\_by\_iron | 1 | 0 |  |  |  |  |  |  |  |  |
| GO:0046007\_negative\_regulation\_of\_activated\_T\_cell\_proliferation | 1 | 0 |  |  |  |  |  |  |  |  |
| GO:0046014\_negative\_regulation\_of\_T\_cell\_homeostatic\_proliferation | 1 | 0 |  |  |  |  |  |  |  |  |
| GO:0046015\_regulation\_of\_transcription\_by\_glucose | 1 | 0 |  |  |  |  |  |  |  |  |
| GO:0046016\_positive\_regulation\_of\_transcription\_by\_glucose | 1 | 0 |  |  |  |  |  |  |  |  |
| GO:0046031\_ADP\_metabolic\_process | 1 | 0 |  |  |  |  |  |  |  |  |
| GO:0046032\_ADP\_catabolic\_process | 1 | 0 |  |  |  |  |  |  |  |  |
| GO:0046061\_dATP\_catabolic\_process | 1 | 0 |  |  |  |  |  |  |  |  |
| GO:0046075\_dTTP\_metabolic\_process | 1 | 0 |  |  |  |  |  |  |  |  |
| GO:0046078\_dUMP\_metabolic\_process | 1 | 0 |  |  |  |  |  |  |  |  |
| GO:0046079\_dUMP\_catabolic\_process | 1 | 0 |  |  |  |  |  |  |  |  |
| GO:0046086\_adenosine\_biosynthetic\_process | 1 | 0 |  |  |  |  |  |  |  |  |
| GO:0046090\_deoxyadenosine\_metabolic\_process | 1 | 0 |  |  |  |  |  |  |  |  |
| GO:0046098\_guanine\_metabolic\_process | 1 | 0 |  |  |  |  |  |  |  |  |
| GO:0046101\_hypoxanthine\_biosynthetic\_process | 1 | 0 |  |  |  |  |  |  |  |  |
| GO:0046102\_inosine\_metabolic\_process | 1 | 0 |  |  |  |  |  |  |  |  |
| GO:0046103\_inosine\_biosynthetic\_process | 1 | 0 |  |  |  |  |  |  |  |  |
| GO:0046108\_uridine\_metabolic\_process | 1 | 0 |  |  |  |  |  |  |  |  |
| GO:0046110\_xanthine\_metabolic\_process | 1 | 0 |  |  |  |  |  |  |  |  |
| GO:0046111\_xanthine\_biosynthetic\_process | 1 | 0 |  |  |  |  |  |  |  |  |
| GO:0046112\_nucleobase\_biosynthetic\_process | 1 | 0 |  |  |  |  |  |  |  |  |
| GO:0046113\_nucleobase\_catabolic\_process | 1 | 0 |  |  |  |  |  |  |  |  |
| GO:0046121\_deoxyribonucleoside\_catabolic\_process | 1 | 0 |  |  |  |  |  |  |  |  |
| GO:0046122\_purine\_deoxyribonucleoside\_metabolic\_process | 1 | 0 |  |  |  |  |  |  |  |  |
| GO:0046124\_purine\_deoxyribonucleoside\_catabolic\_process | 1 | 0 |  |  |  |  |  |  |  |  |
| GO:0046125\_pyrimidine\_deoxyribonucleoside\_metabolic\_process | 1 | 0 |  |  |  |  |  |  |  |  |
| GO:0046131\_pyrimidine\_ribonucleoside\_metabolic\_process | 1 | 0 |  |  |  |  |  |  |  |  |
| GO:0046160\_heme\_a\_metabolic\_process | 1 | 0 |  |  |  |  |  |  |  |  |
| GO:0046218\_indolalkylamine\_catabolic\_process | 1 | 0 |  |  |  |  |  |  |  |  |
| GO:0046292\_formaldehyde\_metabolic\_process | 1 | 0 |  |  |  |  |  |  |  |  |
| GO:0046294\_formaldehyde\_catabolic\_process | 1 | 0 |  |  |  |  |  |  |  |  |
| GO:0046314\_phosphocreatine\_biosynthetic\_process | 1 | 0 |  |  |  |  |  |  |  |  |
| GO:0046327\_glycerol\_biosynthetic\_process\_from\_pyruvate | 1 | 0 |  |  |  |  |  |  |  |  |
| GO:0046329\_negative\_regulation\_of\_JNK\_cascade | 1 | 0 |  |  |  |  |  |  |  |  |
| GO:0046340\_diacylglycerol\_catabolic\_process | 1 | 0 |  |  |  |  |  |  |  |  |
| GO:0046351\_disaccharide\_biosynthetic\_process | 1 | 0 |  |  |  |  |  |  |  |  |
| GO:0046356\_acetyl-CoA\_catabolic\_process | 1 | 0 |  |  |  |  |  |  |  |  |
| GO:0046358\_butyrate\_biosynthetic\_process | 1 | 0 |  |  |  |  |  |  |  |  |
| GO:0046359\_butyrate\_catabolic\_process | 1 | 0 |  |  |  |  |  |  |  |  |
| GO:0046381\_CMP-N-acetylneuraminate\_metabolic\_process | 1 | 0 |  |  |  |  |  |  |  |  |
| GO:0046415\_urate\_metabolic\_process | 1 | 0 |  |  |  |  |  |  |  |  |
| GO:0046416\_D-amino\_acid\_metabolic\_process | 1 | 0 |  |  |  |  |  |  |  |  |
| GO:0046434\_organophosphate\_catabolic\_process | 1 | 0 |  |  |  |  |  |  |  |  |
| GO:0046437\_D-amino\_acid\_biosynthetic\_process | 1 | 0 |  |  |  |  |  |  |  |  |
| GO:0046440\_L-lysine\_metabolic\_process | 1 | 0 |  |  |  |  |  |  |  |  |
| GO:0046449\_creatinine\_metabolic\_process | 1 | 0 |  |  |  |  |  |  |  |  |
| GO:0046471\_phosphatidylglycerol\_metabolic\_process | 1 | 0 |  |  |  |  |  |  |  |  |
| GO:0046473\_phosphatidic\_acid\_metabolic\_process | 1 | 0 |  |  |  |  |  |  |  |  |
| GO:0046476\_glycosylceramide\_biosynthetic\_process | 1 | 0 |  |  |  |  |  |  |  |  |
| GO:0046477\_glycosylceramide\_catabolic\_process | 1 | 0 |  |  |  |  |  |  |  |  |
| GO:0046485\_ether\_lipid\_metabolic\_process | 1 | 0 |  |  |  |  |  |  |  |  |
| GO:0046487\_glyoxylate\_metabolic\_process | 1 | 0 |  |  |  |  |  |  |  |  |
| GO:0046498\_S-adenosylhomocysteine\_metabolic\_process | 1 | 0 |  |  |  |  |  |  |  |  |
| GO:0046552\_photoreceptor\_cell\_fate\_commitment | 1 | 0 |  |  |  |  |  |  |  |  |
| GO:0046586\_regulation\_of\_calcium-dependent\_cell-cell\_adhesion | 1 | 0 |  |  |  |  |  |  |  |  |
| GO:0046587\_positive\_regulation\_of\_calcium-dependent\_cell-cell\_adhesion | 1 | 0 |  |  |  |  |  |  |  |  |
| GO:0046602\_regulation\_of\_mitotic\_centrosome\_separation | 1 | 0 |  |  |  |  |  |  |  |  |
| GO:0046604\_positive\_regulation\_of\_mitotic\_centrosome\_separation | 1 | 0 |  |  |  |  |  |  |  |  |
| GO:0046607\_positive\_regulation\_of\_centrosome\_cycle | 1 | 0 |  |  |  |  |  |  |  |  |
| GO:0046655\_folic\_acid\_metabolic\_process | 1 | 0 |  |  |  |  |  |  |  |  |
| GO:0046671\_negative\_regulation\_of\_retinal\_cell\_programmed\_cell\_death | 1 | 0 |  |  |  |  |  |  |  |  |
| GO:0046685\_response\_to\_arsenic | 1 | 0 |  |  |  |  |  |  |  |  |
| GO:0046692\_sperm\_competition | 1 | 0 |  |  |  |  |  |  |  |  |
| GO:0046707\_IDP\_metabolic\_process | 1 | 0 |  |  |  |  |  |  |  |  |
| GO:0046709\_IDP\_catabolic\_process | 1 | 0 |  |  |  |  |  |  |  |  |
| GO:0046724\_oxalic\_acid\_secretion | 1 | 0 |  |  |  |  |  |  |  |  |
| GO:0046753\_non-lytic\_viral\_release | 1 | 0 |  |  |  |  |  |  |  |  |
| GO:0046755\_non-lytic\_virus\_budding | 1 | 0 |  |  |  |  |  |  |  |  |
| GO:0046826\_negative\_regulation\_of\_protein\_export\_from\_nucleus | 1 | 0 |  |  |  |  |  |  |  |  |
| GO:0046827\_positive\_regulation\_of\_protein\_export\_from\_nucleus | 1 | 0 |  |  |  |  |  |  |  |  |
| GO:0046831\_regulation\_of\_RNA\_export\_from\_nucleus | 1 | 0 |  |  |  |  |  |  |  |  |
| GO:0046834\_lipid\_phosphorylation | 1 | 0 |  |  |  |  |  |  |  |  |
| GO:0046853\_inositol\_and\_derivative\_phosphorylation | 1 | 0 |  |  |  |  |  |  |  |  |
| GO:0046864\_isoprenoid\_transport | 1 | 0 |  |  |  |  |  |  |  |  |
| GO:0046865\_terpenoid\_transport | 1 | 0 |  |  |  |  |  |  |  |  |
| GO:0046877\_regulation\_of\_saliva\_secretion | 1 | 0 |  |  |  |  |  |  |  |  |
| GO:0046878\_positive\_regulation\_of\_saliva\_secretion | 1 | 0 |  |  |  |  |  |  |  |  |
| GO:0046884\_follicle-stimulating\_hormone\_secretion | 1 | 0 |  |  |  |  |  |  |  |  |
| GO:0046898\_response\_to\_cycloheximide | 1 | 0 |  |  |  |  |  |  |  |  |
| GO:0046929\_negative\_regulation\_of\_neurotransmitter\_secretion | 1 | 0 |  |  |  |  |  |  |  |  |
| GO:0046931\_pore\_complex\_biogenesis | 1 | 0 |  |  |  |  |  |  |  |  |
| GO:0046949\_acyl-CoA\_biosynthetic\_process | 1 | 0 |  |  |  |  |  |  |  |  |
| GO:0046958\_nonassociative\_learning | 1 | 0 |  |  |  |  |  |  |  |  |
| GO:0046960\_sensitization | 1 | 0 |  |  |  |  |  |  |  |  |
| GO:0046986\_negative\_regulation\_of\_hemoglobin\_biosynthetic\_process | 1 | 0 |  |  |  |  |  |  |  |  |
| GO:0047497\_mitochondrion\_transport\_along\_microtubule | 1 | 0 |  |  |  |  |  |  |  |  |
| GO:0048047\_mating\_behavior\_\_sex\_discrimination | 1 | 0 |  |  |  |  |  |  |  |  |
| GO:0048133\_male\_germ-line\_stem\_cell\_division | 1 | 0 |  |  |  |  |  |  |  |  |
| GO:0048137\_spermatocyte\_division | 1 | 0 |  |  |  |  |  |  |  |  |
| GO:0048143\_astrocyte\_activation | 1 | 0 |  |  |  |  |  |  |  |  |
| GO:0048199\_vesicle\_targeting\_\_to\_\_from\_or\_within\_Golgi | 1 | 0 |  |  |  |  |  |  |  |  |
| GO:0048241\_epinephrine\_transport | 1 | 0 |  |  |  |  |  |  |  |  |
| GO:0048242\_epinephrine\_secretion | 1 | 0 |  |  |  |  |  |  |  |  |
| GO:0048243\_norepinephrine\_secretion | 1 | 0 |  |  |  |  |  |  |  |  |
| GO:0048247\_lymphocyte\_chemotaxis | 1 | 0 |  |  |  |  |  |  |  |  |
| GO:0048250\_mitochondrial\_iron\_ion\_transport | 1 | 0 |  |  |  |  |  |  |  |  |
| GO:0048259\_regulation\_of\_receptor-mediated\_endocytosis | 1 | 0 |  |  |  |  |  |  |  |  |
| GO:0048260\_positive\_regulation\_of\_receptor-mediated\_endocytosis | 1 | 0 |  |  |  |  |  |  |  |  |
| GO:0048290\_isotype\_switching\_to\_IgA\_isotypes | 1 | 0 |  |  |  |  |  |  |  |  |
| GO:0048296\_regulation\_of\_isotype\_switching\_to\_IgA\_isotypes | 1 | 0 |  |  |  |  |  |  |  |  |
| GO:0048298\_positive\_regulation\_of\_isotype\_switching\_to\_IgA\_isotypes | 1 | 0 |  |  |  |  |  |  |  |  |
| GO:0048319\_axial\_mesoderm\_morphogenesis | 1 | 0 |  |  |  |  |  |  |  |  |
| GO:0048320\_axial\_mesoderm\_formation | 1 | 0 |  |  |  |  |  |  |  |  |
| GO:0048385\_regulation\_of\_retinoic\_acid\_receptor\_signaling\_pathway | 1 | 0 |  |  |  |  |  |  |  |  |
| GO:0048387\_negative\_regulation\_of\_retinoic\_acid\_receptor\_signaling\_pathway | 1 | 0 |  |  |  |  |  |  |  |  |
| GO:0048388\_endosomal\_lumen\_acidification | 1 | 0 |  |  |  |  |  |  |  |  |
| GO:0048389\_intermediate\_mesoderm\_development | 1 | 0 |  |  |  |  |  |  |  |  |
| GO:0048478\_replication\_fork\_protection | 1 | 0 |  |  |  |  |  |  |  |  |
| GO:0048496\_maintenance\_of\_organ\_identity | 1 | 0 |  |  |  |  |  |  |  |  |
| GO:0048525\_negative\_regulation\_of\_viral\_reproduction | 1 | 0 |  |  |  |  |  |  |  |  |
| GO:0048539\_bone\_marrow\_development | 1 | 0 |  |  |  |  |  |  |  |  |
| GO:0048548\_regulation\_of\_pinocytosis | 1 | 0 |  |  |  |  |  |  |  |  |
| GO:0048549\_positive\_regulation\_of\_pinocytosis | 1 | 0 |  |  |  |  |  |  |  |  |
| GO:0048553\_negative\_regulation\_of\_metalloenzyme\_activity | 1 | 0 |  |  |  |  |  |  |  |  |
| GO:0048588\_developmental\_cell\_growth | 1 | 0 |  |  |  |  |  |  |  |  |
| GO:0048601\_oocyte\_morphogenesis | 1 | 0 |  |  |  |  |  |  |  |  |
| GO:0048621\_post-embryonic\_gut\_morphogenesis | 1 | 0 |  |  |  |  |  |  |  |  |
| GO:0048640\_negative\_regulation\_of\_developmental\_growth | 1 | 0 |  |  |  |  |  |  |  |  |
| GO:0048642\_negative\_regulation\_of\_skeletal\_muscle\_tissue\_development | 1 | 0 |  |  |  |  |  |  |  |  |
| GO:0048669\_collateral\_sprouting\_in\_the\_absence\_of\_injury | 1 | 0 |  |  |  |  |  |  |  |  |
| GO:0048680\_positive\_regulation\_of\_axon\_regeneration | 1 | 0 |  |  |  |  |  |  |  |  |
| GO:0048681\_negative\_regulation\_of\_axon\_regeneration | 1 | 0 |  |  |  |  |  |  |  |  |
| GO:0048686\_regulation\_of\_sprouting\_of\_injured\_axon | 1 | 0 |  |  |  |  |  |  |  |  |
| GO:0048687\_positive\_regulation\_of\_sprouting\_of\_injured\_axon | 1 | 0 |  |  |  |  |  |  |  |  |
| GO:0048690\_regulation\_of\_axon\_extension\_involved\_in\_regeneration | 1 | 0 |  |  |  |  |  |  |  |  |
| GO:0048691\_positive\_regulation\_of\_axon\_extension\_involved\_in\_regeneration | 1 | 0 |  |  |  |  |  |  |  |  |
| GO:0048714\_positive\_regulation\_of\_oligodendrocyte\_differentiation | 1 | 0 |  |  |  |  |  |  |  |  |
| GO:0048733\_sebaceous\_gland\_development | 1 | 0 |  |  |  |  |  |  |  |  |
| GO:0048743\_positive\_regulation\_of\_skeletal\_muscle\_fiber\_development | 1 | 0 |  |  |  |  |  |  |  |  |
| GO:0048752\_semicircular\_canal\_morphogenesis | 1 | 0 |  |  |  |  |  |  |  |  |
| GO:0048773\_erythrophore\_differentiation | 1 | 0 |  |  |  |  |  |  |  |  |
| GO:0048790\_maintenance\_of\_presynaptic\_active\_zone\_structure | 1 | 0 |  |  |  |  |  |  |  |  |
| GO:0048791\_calcium\_ion-dependent\_exocytosis\_of\_neurotransmitter | 1 | 0 |  |  |  |  |  |  |  |  |
| GO:0048822\_enucleate\_erythrocyte\_development | 1 | 0 |  |  |  |  |  |  |  |  |
| GO:0048866\_stem\_cell\_fate\_specification | 1 | 0 |  |  |  |  |  |  |  |  |
| GO:0048936\_peripheral\_nervous\_system\_neuron\_axonogenesis | 1 | 0 |  |  |  |  |  |  |  |  |
| GO:0050427\_3'-phosphoadenosine\_5'-phosphosulfate\_metabolic\_process | 1 | 0 |  |  |  |  |  |  |  |  |
| GO:0050428\_3'-phosphoadenosine\_5'-phosphosulfate\_biosynthetic\_process | 1 | 0 |  |  |  |  |  |  |  |  |
| GO:0050482\_arachidonic\_acid\_secretion | 1 | 0 |  |  |  |  |  |  |  |  |
| GO:0050667\_homocysteine\_metabolic\_process | 1 | 0 |  |  |  |  |  |  |  |  |
| GO:0050674\_urothelial\_cell\_proliferation | 1 | 0 |  |  |  |  |  |  |  |  |
| GO:0050675\_regulation\_of\_urothelial\_cell\_proliferation | 1 | 0 |  |  |  |  |  |  |  |  |
| GO:0050677\_positive\_regulation\_of\_urothelial\_cell\_proliferation | 1 | 0 |  |  |  |  |  |  |  |  |
| GO:0050691\_regulation\_of\_defense\_response\_to\_virus\_by\_host | 1 | 0 |  |  |  |  |  |  |  |  |
| GO:0050748\_negative\_regulation\_of\_lipoprotein\_metabolic\_process | 1 | 0 |  |  |  |  |  |  |  |  |
| GO:0050757\_thymidylate\_synthase\_biosynthetic\_process | 1 | 0 |  |  |  |  |  |  |  |  |
| GO:0050758\_regulation\_of\_thymidylate\_synthase\_biosynthetic\_process | 1 | 0 |  |  |  |  |  |  |  |  |
| GO:0050760\_negative\_regulation\_of\_thymidylate\_synthase\_biosynthetic\_process | 1 | 0 |  |  |  |  |  |  |  |  |
| GO:0050812\_regulation\_of\_acyl-CoA\_biosynthetic\_process | 1 | 0 |  |  |  |  |  |  |  |  |
| GO:0050832\_defense\_response\_to\_fungus | 1 | 0 |  |  |  |  |  |  |  |  |
| GO:0050861\_positive\_regulation\_of\_B\_cell\_receptor\_signaling\_pathway | 1 | 0 |  |  |  |  |  |  |  |  |
| GO:0050862\_positive\_regulation\_of\_T\_cell\_receptor\_signaling\_pathway | 1 | 0 |  |  |  |  |  |  |  |  |
| GO:0050916\_sensory\_perception\_of\_sweet\_taste | 1 | 0 |  |  |  |  |  |  |  |  |
| GO:0050975\_sensory\_perception\_of\_touch | 1 | 0 |  |  |  |  |  |  |  |  |
| GO:0050995\_negative\_regulation\_of\_lipid\_catabolic\_process | 1 | 0 |  |  |  |  |  |  |  |  |
| GO:0051001\_negative\_regulation\_of\_nitric-oxide\_synthase\_activity | 1 | 0 |  |  |  |  |  |  |  |  |
| GO:0051005\_negative\_regulation\_of\_lipoprotein\_lipase\_activity | 1 | 0 |  |  |  |  |  |  |  |  |
| GO:0051006\_positive\_regulation\_of\_lipoprotein\_lipase\_activity | 1 | 0 |  |  |  |  |  |  |  |  |
| GO:0051016\_barbed-end\_actin\_filament\_capping | 1 | 0 |  |  |  |  |  |  |  |  |
| GO:0051029\_rRNA\_transport | 1 | 0 |  |  |  |  |  |  |  |  |
| GO:0051043\_regulation\_of\_membrane\_protein\_ectodomain\_proteolysis | 1 | 0 |  |  |  |  |  |  |  |  |
| GO:0051044\_positive\_regulation\_of\_membrane\_protein\_ectodomain\_proteolysis | 1 | 0 |  |  |  |  |  |  |  |  |
| GO:0051088\_PMA-inducible\_membrane\_protein\_ectodomain\_proteolysis | 1 | 0 |  |  |  |  |  |  |  |  |
| GO:0051102\_DNA\_ligation\_during\_DNA\_recombination | 1 | 0 |  |  |  |  |  |  |  |  |
| GO:0051103\_DNA\_ligation\_during\_DNA\_repair | 1 | 0 |  |  |  |  |  |  |  |  |
| GO:0051123\_transcriptional\_preinitiation\_complex\_assembly | 1 | 0 |  |  |  |  |  |  |  |  |
| GO:0051125\_regulation\_of\_actin\_nucleation | 1 | 0 |  |  |  |  |  |  |  |  |
| GO:0051127\_positive\_regulation\_of\_actin\_nucleation | 1 | 0 |  |  |  |  |  |  |  |  |
| GO:0051151\_negative\_regulation\_of\_smooth\_muscle\_cell\_differentiation | 1 | 0 |  |  |  |  |  |  |  |  |
| GO:0051154\_negative\_regulation\_of\_striated\_muscle\_cell\_differentiation | 1 | 0 |  |  |  |  |  |  |  |  |
| GO:0051155\_positive\_regulation\_of\_striated\_muscle\_cell\_differentiation | 1 | 0 |  |  |  |  |  |  |  |  |
| GO:0051156\_glucose\_6-phosphate\_metabolic\_process | 1 | 0 |  |  |  |  |  |  |  |  |
| GO:0051187\_cofactor\_catabolic\_process | 1 | 0 |  |  |  |  |  |  |  |  |
| GO:0051189\_prosthetic\_group\_metabolic\_process | 1 | 0 |  |  |  |  |  |  |  |  |
| GO:0051193\_regulation\_of\_cofactor\_metabolic\_process | 1 | 0 |  |  |  |  |  |  |  |  |
| GO:0051196\_regulation\_of\_coenzyme\_metabolic\_process | 1 | 0 |  |  |  |  |  |  |  |  |
| GO:0051255\_spindle\_midzone\_assembly | 1 | 0 |  |  |  |  |  |  |  |  |
| GO:0051257\_spindle\_midzone\_assembly\_involved\_in\_meiosis | 1 | 0 |  |  |  |  |  |  |  |  |
| GO:0051281\_positive\_regulation\_of\_release\_of\_sequestered\_calcium\_ion\_into\_cytosol | 1 | 0 |  |  |  |  |  |  |  |  |
| GO:0051290\_protein\_heterotetramerization | 1 | 0 |  |  |  |  |  |  |  |  |
| GO:0051305\_chromosome\_movement\_towards\_spindle\_pole | 1 | 0 |  |  |  |  |  |  |  |  |
| GO:0051310\_metaphase\_plate\_congression | 1 | 0 |  |  |  |  |  |  |  |  |
| GO:0051311\_meiotic\_metaphase\_plate\_congression | 1 | 0 |  |  |  |  |  |  |  |  |
| GO:0051340\_regulation\_of\_ligase\_activity | 1 | 0 |  |  |  |  |  |  |  |  |
| GO:0051351\_positive\_regulation\_of\_ligase\_activity | 1 | 0 |  |  |  |  |  |  |  |  |
| GO:0051354\_negative\_regulation\_of\_oxidoreductase\_activity | 1 | 0 |  |  |  |  |  |  |  |  |
| GO:0051383\_kinetochore\_organization | 1 | 0 |  |  |  |  |  |  |  |  |
| GO:0051386\_regulation\_of\_nerve\_growth\_factor\_receptor\_signaling\_pathway | 1 | 0 |  |  |  |  |  |  |  |  |
| GO:0051409\_response\_to\_nitrosative\_stress | 1 | 0 |  |  |  |  |  |  |  |  |
| GO:0051457\_maintenance\_of\_protein\_location\_in\_nucleus | 1 | 0 |  |  |  |  |  |  |  |  |
| GO:0051462\_regulation\_of\_cortisol\_secretion | 1 | 0 |  |  |  |  |  |  |  |  |
| GO:0051463\_negative\_regulation\_of\_cortisol\_secretion | 1 | 0 |  |  |  |  |  |  |  |  |
| GO:0051481\_reduction\_of\_cytosolic\_calcium\_ion\_concentration | 1 | 0 |  |  |  |  |  |  |  |  |
| GO:0051482\_elevation\_of\_cytosolic\_calcium\_ion\_concentration\_during\_G-protein\_signaling\_\_coupled\_to\_IP3\_second\_messenger\_(phospholipase\_C\_activating) | 1 | 0 |  |  |  |  |  |  |  |  |
| GO:0051542\_elastin\_biosynthetic\_process | 1 | 0 |  |  |  |  |  |  |  |  |
| GO:0051568\_histone\_H3-K4\_methylation | 1 | 0 |  |  |  |  |  |  |  |  |
| GO:0051569\_regulation\_of\_histone\_H3-K4\_methylation | 1 | 0 |  |  |  |  |  |  |  |  |
| GO:0051570\_regulation\_of\_histone\_H3-K9\_methylation | 1 | 0 |  |  |  |  |  |  |  |  |
| GO:0051573\_negative\_regulation\_of\_histone\_H3-K9\_methylation | 1 | 0 |  |  |  |  |  |  |  |  |
| GO:0051580\_regulation\_of\_neurotransmitter\_uptake | 1 | 0 |  |  |  |  |  |  |  |  |
| GO:0051582\_positive\_regulation\_of\_neurotransmitter\_uptake | 1 | 0 |  |  |  |  |  |  |  |  |
| GO:0051584\_regulation\_of\_dopamine\_uptake | 1 | 0 |  |  |  |  |  |  |  |  |
| GO:0051586\_positive\_regulation\_of\_dopamine\_uptake | 1 | 0 |  |  |  |  |  |  |  |  |
| GO:0051589\_negative\_regulation\_of\_neurotransmitter\_transport | 1 | 0 |  |  |  |  |  |  |  |  |
| GO:0051593\_response\_to\_folic\_acid | 1 | 0 |  |  |  |  |  |  |  |  |
| GO:0051615\_histamine\_uptake | 1 | 0 |  |  |  |  |  |  |  |  |
| GO:0051646\_mitochondrion\_localization | 1 | 0 |  |  |  |  |  |  |  |  |
| GO:0051654\_establishment\_of\_mitochondrion\_localization | 1 | 0 |  |  |  |  |  |  |  |  |
| GO:0051661\_maintenance\_of\_centrosome\_location | 1 | 0 |  |  |  |  |  |  |  |  |
| GO:0051665\_membrane\_raft\_localization | 1 | 0 |  |  |  |  |  |  |  |  |
| GO:0051685\_maintenance\_of\_ER\_location | 1 | 0 |  |  |  |  |  |  |  |  |
| GO:0051693\_actin\_filament\_capping | 1 | 0 |  |  |  |  |  |  |  |  |
| GO:0051701\_interaction\_with\_host | 1 | 0 |  |  |  |  |  |  |  |  |
| GO:0051754\_meiotic\_sister\_chromatid\_cohesion\_\_centromeric | 1 | 0 |  |  |  |  |  |  |  |  |
| GO:0051782\_negative\_regulation\_of\_cell\_division | 1 | 0 |  |  |  |  |  |  |  |  |
| GO:0051790\_short-chain\_fatty\_acid\_biosynthetic\_process | 1 | 0 |  |  |  |  |  |  |  |  |
| GO:0051799\_negative\_regulation\_of\_hair\_follicle\_development | 1 | 0 |  |  |  |  |  |  |  |  |
| GO:0051823\_regulation\_of\_synapse\_structural\_plasticity | 1 | 0 |  |  |  |  |  |  |  |  |
| GO:0051865\_protein\_autoubiquitination | 1 | 0 |  |  |  |  |  |  |  |  |
| GO:0051901\_positive\_regulation\_of\_mitochondrial\_depolarization | 1 | 0 |  |  |  |  |  |  |  |  |
| GO:0051917\_regulation\_of\_fibrinolysis | 1 | 0 |  |  |  |  |  |  |  |  |
| GO:0051918\_negative\_regulation\_of\_fibrinolysis | 1 | 0 |  |  |  |  |  |  |  |  |
| GO:0051929\_positive\_regulation\_of\_calcium\_ion\_transport\_via\_voltage-gated\_calcium\_channel\_activity | 1 | 0 |  |  |  |  |  |  |  |  |
| GO:0051933\_amino\_acid\_uptake\_during\_transmission\_of\_nerve\_impulse | 1 | 0 |  |  |  |  |  |  |  |  |
| GO:0051935\_glutamate\_uptake\_during\_transmission\_of\_nerve\_impulse | 1 | 0 |  |  |  |  |  |  |  |  |
| GO:0051940\_regulation\_of\_catecholamine\_uptake\_during\_transmission\_of\_nerve\_impulse | 1 | 0 |  |  |  |  |  |  |  |  |
| GO:0051944\_positive\_regulation\_of\_catecholamine\_uptake\_during\_transmission\_of\_nerve\_impulse | 1 | 0 |  |  |  |  |  |  |  |  |
| GO:0051961\_negative\_regulation\_of\_nervous\_system\_development | 1 | 0 |  |  |  |  |  |  |  |  |
| GO:0051964\_negative\_regulation\_of\_synaptogenesis | 1 | 0 |  |  |  |  |  |  |  |  |
| GO:0051968\_positive\_regulation\_of\_synaptic\_transmission\_\_glutamatergic | 1 | 0 |  |  |  |  |  |  |  |  |
| GO:0051984\_positive\_regulation\_of\_chromosome\_segregation | 1 | 0 |  |  |  |  |  |  |  |  |
| GO:0051987\_positive\_regulation\_of\_attachment\_of\_spindle\_microtubules\_to\_kinetochore | 1 | 0 |  |  |  |  |  |  |  |  |
| GO:0052173\_response\_to\_defenses\_of\_other\_organism\_during\_symbiotic\_interaction | 1 | 0 |  |  |  |  |  |  |  |  |
| GO:0052200\_response\_to\_host\_defenses | 1 | 0 |  |  |  |  |  |  |  |  |
| GO:0052551\_response\_to\_defense-related\_nitric\_oxide\_production\_by\_other\_organism\_during\_symbiotic\_interaction | 1 | 0 |  |  |  |  |  |  |  |  |
| GO:0052564\_response\_to\_immune\_response\_of\_other\_organism\_during\_symbiotic\_interaction | 1 | 0 |  |  |  |  |  |  |  |  |
| GO:0052565\_response\_to\_defense-related\_host\_nitric\_oxide\_production | 1 | 0 |  |  |  |  |  |  |  |  |
| GO:0052572\_response\_to\_host\_immune\_response | 1 | 0 |  |  |  |  |  |  |  |  |
| GO:0055005\_ventricular\_cardiac\_myofibril\_development | 1 | 0 |  |  |  |  |  |  |  |  |
| GO:0055011\_atrial\_cardiac\_muscle\_cell\_differentiation | 1 | 0 |  |  |  |  |  |  |  |  |
| GO:0055014\_atrial\_cardiac\_muscle\_cell\_development | 1 | 0 |  |  |  |  |  |  |  |  |
| GO:0055078\_sodium\_ion\_homeostasis | 1 | 0 |  |  |  |  |  |  |  |  |
| GO:0055089\_fatty\_acid\_homeostasis | 1 | 0 |  |  |  |  |  |  |  |  |
| GO:0055093\_response\_to\_hyperoxia | 1 | 0 |  |  |  |  |  |  |  |  |
| GO:0060003\_copper\_ion\_export | 1 | 0 |  |  |  |  |  |  |  |  |
| GO:0060005\_vestibular\_reflex | 1 | 0 |  |  |  |  |  |  |  |  |
| GO:0060014\_granulosa\_cell\_differentiation | 1 | 0 |  |  |  |  |  |  |  |  |
| GO:0060018\_astrocyte\_fate\_commitment | 1 | 0 |  |  |  |  |  |  |  |  |
| GO:0060020\_Bergmann\_glial\_cell\_differentiation | 1 | 0 |  |  |  |  |  |  |  |  |
| GO:0060022\_hard\_palate\_development | 1 | 0 |  |  |  |  |  |  |  |  |
| GO:0060034\_notochord\_cell\_differentiation | 1 | 0 |  |  |  |  |  |  |  |  |
| GO:0060035\_notochord\_cell\_development | 1 | 0 |  |  |  |  |  |  |  |  |
| GO:0060046\_regulation\_of\_acrosome\_reaction | 1 | 0 |  |  |  |  |  |  |  |  |
| GO:0060054\_positive\_regulation\_of\_epithelial\_cell\_proliferation\_involved\_in\_wound\_healing | 1 | 0 |  |  |  |  |  |  |  |  |
| GO:0060059\_embryonic\_retina\_morphogenesis\_in\_camera-type\_eye | 1 | 0 |  |  |  |  |  |  |  |  |
| GO:0060061\_Spemann\_organizer\_formation | 1 | 0 |  |  |  |  |  |  |  |  |
| GO:0060064\_Spemann\_organizer\_formation\_at\_the\_anterior\_end\_of\_the\_primitive\_streak | 1 | 0 |  |  |  |  |  |  |  |  |
| GO:0060075\_regulation\_of\_resting\_membrane\_potential | 1 | 0 |  |  |  |  |  |  |  |  |
| GO:0060082\_eye\_blink\_reflex | 1 | 0 |  |  |  |  |  |  |  |  |
| GO:0060112\_generation\_of\_ovulation\_cycle\_rhythm | 1 | 0 |  |  |  |  |  |  |  |  |
| GO:0060125\_negative\_regulation\_of\_growth\_hormone\_secretion | 1 | 0 |  |  |  |  |  |  |  |  |
| GO:0060151\_peroxisome\_localization | 1 | 0 |  |  |  |  |  |  |  |  |
| GO:0060152\_microtubule-based\_peroxisome\_localization | 1 | 0 |  |  |  |  |  |  |  |  |
| GO:0060161\_positive\_regulation\_of\_dopamine\_receptor\_signaling\_pathway | 1 | 0 |  |  |  |  |  |  |  |  |
| GO:0060163\_subpallium\_neuron\_fate\_commitment | 1 | 0 |  |  |  |  |  |  |  |  |
| GO:0060165\_regulation\_of\_timing\_of\_subpallium\_neuron\_differentiation | 1 | 0 |  |  |  |  |  |  |  |  |
| GO:0060174\_limb\_bud\_formation | 1 | 0 |  |  |  |  |  |  |  |  |
| GO:0060177\_regulation\_of\_angiotensin\_metabolic\_process | 1 | 0 |  |  |  |  |  |  |  |  |
| GO:0060197\_cloacal\_septation | 1 | 0 |  |  |  |  |  |  |  |  |
| GO:0060215\_primitive\_hemopoiesis | 1 | 0 |  |  |  |  |  |  |  |  |
| GO:0060231\_mesenchymal\_to\_epithelial\_transition | 1 | 0 |  |  |  |  |  |  |  |  |
| GO:0060254\_regulation\_of\_N-terminal\_protein\_palmitoylation | 1 | 0 |  |  |  |  |  |  |  |  |
| GO:0060261\_positive\_regulation\_of\_transcription\_initiation\_from\_RNA\_polymerase\_II\_promoter | 1 | 0 |  |  |  |  |  |  |  |  |
| GO:0060262\_negative\_regulation\_of\_N-terminal\_protein\_palmitoylation | 1 | 0 |  |  |  |  |  |  |  |  |
| GO:0060263\_regulation\_of\_respiratory\_burst | 1 | 0 |  |  |  |  |  |  |  |  |
| GO:0060264\_regulation\_of\_respiratory\_burst\_during\_acute\_inflammatory\_response | 1 | 0 |  |  |  |  |  |  |  |  |
| GO:0060265\_positive\_regulation\_of\_respiratory\_burst\_during\_acute\_inflammatory\_response | 1 | 0 |  |  |  |  |  |  |  |  |
| GO:0060267\_positive\_regulation\_of\_respiratory\_burst | 1 | 0 |  |  |  |  |  |  |  |  |
| GO:0060272\_embryonic\_skeletal\_joint\_morphogenesis | 1 | 0 |  |  |  |  |  |  |  |  |
| GO:0060297\_regulation\_of\_sarcomere\_organization | 1 | 0 |  |  |  |  |  |  |  |  |
| GO:0060298\_positive\_regulation\_of\_sarcomere\_organization | 1 | 0 |  |  |  |  |  |  |  |  |
| GO:0060315\_negative\_regulation\_of\_ryanodine-sensitive\_calcium-release\_channel\_activity | 1 | 0 |  |  |  |  |  |  |  |  |
| GO:0060319\_primitive\_erythrocyte\_differentiation | 1 | 0 |  |  |  |  |  |  |  |  |
| GO:0060371\_regulation\_of\_atrial\_cardiomyocyte\_membrane\_depolarization | 1 | 0 |  |  |  |  |  |  |  |  |
| GO:0060374\_mast\_cell\_differentiation | 1 | 0 |  |  |  |  |  |  |  |  |
| GO:0060375\_regulation\_of\_mast\_cell\_differentiation | 1 | 0 |  |  |  |  |  |  |  |  |
| GO:0060376\_positive\_regulation\_of\_mast\_cell\_differentiation | 1 | 0 |  |  |  |  |  |  |  |  |
| GO:0060390\_regulation\_of\_SMAD\_protein\_nuclear\_translocation | 1 | 0 |  |  |  |  |  |  |  |  |
| GO:0060391\_positive\_regulation\_of\_SMAD\_protein\_nuclear\_translocation | 1 | 0 |  |  |  |  |  |  |  |  |
| GO:0060398\_regulation\_of\_growth\_hormone\_receptor\_signaling\_pathway | 1 | 0 |  |  |  |  |  |  |  |  |
| GO:0060399\_positive\_regulation\_of\_growth\_hormone\_receptor\_signaling\_pathway | 1 | 0 |  |  |  |  |  |  |  |  |
| GO:0060405\_regulation\_of\_penile\_erection | 1 | 0 |  |  |  |  |  |  |  |  |
| GO:0060407\_negative\_regulation\_of\_penile\_erection | 1 | 0 |  |  |  |  |  |  |  |  |
| GO:0060413\_atrial\_septum\_morphogenesis | 1 | 0 |  |  |  |  |  |  |  |  |
| GO:0060414\_aorta\_smooth\_muscle\_tissue\_morphogenesis | 1 | 0 |  |  |  |  |  |  |  |  |
| GO:0060419\_heart\_growth | 1 | 0 |  |  |  |  |  |  |  |  |
| GO:0060420\_regulation\_of\_heart\_growth | 1 | 0 |  |  |  |  |  |  |  |  |
| GO:0060421\_positive\_regulation\_of\_heart\_growth | 1 | 0 |  |  |  |  |  |  |  |  |
| GO:0060431\_primary\_lung\_bud\_formation | 1 | 0 |  |  |  |  |  |  |  |  |
| GO:0060436\_bronchiole\_morphogenesis | 1 | 0 |  |  |  |  |  |  |  |  |
| GO:0060440\_trachea\_formation | 1 | 0 |  |  |  |  |  |  |  |  |
| GO:0060449\_bud\_elongation\_involved\_in\_lung\_branching | 1 | 0 |  |  |  |  |  |  |  |  |
| GO:0060456\_positive\_regulation\_of\_digestive\_system\_process | 1 | 0 |  |  |  |  |  |  |  |  |
| GO:0060461\_right\_lung\_morphogenesis | 1 | 0 |  |  |  |  |  |  |  |  |
| GO:0060481\_lobar\_bronchus\_epithelium\_development | 1 | 0 |  |  |  |  |  |  |  |  |
| GO:0060482\_lobar\_bronchus\_development | 1 | 0 |  |  |  |  |  |  |  |  |
| GO:0060484\_lung-associated\_mesenchyme\_development | 1 | 0 |  |  |  |  |  |  |  |  |
| GO:0060486\_Clara\_cell\_differentiation | 1 | 0 |  |  |  |  |  |  |  |  |
| GO:0060510\_Type\_II\_pneumocyte\_differentiation | 1 | 0 |  |  |  |  |  |  |  |  |
| GO:0060514\_prostate\_induction | 1 | 0 |  |  |  |  |  |  |  |  |
| GO:0060515\_prostate\_field\_specification | 1 | 0 |  |  |  |  |  |  |  |  |
| GO:0060517\_epithelial\_cell\_proliferation\_involved\_in\_prostatic\_bud\_elongation | 1 | 0 |  |  |  |  |  |  |  |  |
| GO:0060520\_activation\_of\_prostate\_induction\_by\_androgen\_receptor\_signaling\_pathway | 1 | 0 |  |  |  |  |  |  |  |  |
| GO:0060535\_trachea\_cartilage\_morphogenesis | 1 | 0 |  |  |  |  |  |  |  |  |
| GO:0060536\_cartilage\_morphogenesis | 1 | 0 |  |  |  |  |  |  |  |  |
[truncated: 256,971 more chars]
