# Supplementary material for: RedundancyMiner: De-replication of redundant GO categories in microarray and proteomics analysis
Source: BMC Bioinformatics. 2011 Feb 10;12:52. doi: 10.1186/1471-2105-12-52 (PMC3223614; doi:10.1186/1471-2105-12-52)
Supplement: Additional file 8 — Retinal development HTGM download. compressed package of the results of running HTGM on the retinal development genes list. [file 1471-2105-12-52-S8.ZIP › SCENARIO_2_MODIFIED/total.txt.total.txt.dir/Exp1_BestClusterMap_LEIGS_KM_24.csv.join.22.txt.dir/Exp1_BestClusterMap_LEIGS_KM_24.csv.join.22.txt.change.gce.CIM.dir/cgi_user_x.html]

**X-axis Names**   
Cluster is based on euclidean distance  
Cluster method is: average  
plclust  
height plot  

|  |
| --- |
| 1.GO:0007417\_central\_nervous\_system\_development |
| 2.GO:0040011\_locomotion |
| 3.GO:0016477\_cell\_migration |
| 4.GO:0048870\_cell\_motility |
| 5.GO:0048666\_neuron\_development |
| 6.GO:0030030\_cell\_projection\_organization |
| 7.GO:0031175\_neuron\_projection\_development |
| 8.GO:0048812\_neuron\_projection\_morphogenesis |
| 9.GO:0048858\_cell\_projection\_morphogenesis |
| 10.GO:0048667\_cell\_morphogenesis\_involved\_in\_neuron\_differentiation |
| 11.GO:0007409\_axonogenesis |
| 12.GO:0007155\_cell\_adhesion |
| 13.GO:0022610\_biological\_adhesion |
| 14.GO:0045860\_positive\_regulation\_of\_protein\_kinase\_activity |
| 15.GO:0019220\_regulation\_of\_phosphate\_metabolic\_process |
| 16.GO:0051174\_regulation\_of\_phosphorus\_metabolic\_process |
| 17.GO:0042325\_regulation\_of\_phosphorylation |
| 18.GO:0007179\_transforming\_growth\_factor\_beta\_receptor\_signaling\_pathway |
| 19.GO:0048592\_eye\_morphogenesis |
| 20.GO:0001654\_eye\_development |
| 21.GO:0007423\_sensory\_organ\_development |
| 22.GO:0001569\_patterning\_of\_blood\_vessels |
| 23.GO:0042461\_photoreceptor\_cell\_development |
| 24.GO:0001754\_eye\_photoreceptor\_cell\_differentiation |
| 25.GO:0042462\_eye\_photoreceptor\_cell\_development |
| 26.GO:0046530\_photoreceptor\_cell\_differentiation |
| 27.GO:0010463\_mesenchymal\_cell\_proliferation |
| 28.GO:0002053\_positive\_regulation\_of\_mesenchymal\_cell\_proliferation |
| 29.GO:0010464\_regulation\_of\_mesenchymal\_cell\_proliferation |
| 30.GO:0040012\_regulation\_of\_locomotion |
| 31.GO:0030335\_positive\_regulation\_of\_cell\_migration |
| 32.GO:0051272\_positive\_regulation\_of\_cell\_motion |
| 33.GO:0030334\_regulation\_of\_cell\_migration |
| 34.GO:0051270\_regulation\_of\_cell\_motion |
| 35.GO:0010721\_negative\_regulation\_of\_cell\_development |
| 36.GO:0050768\_negative\_regulation\_of\_neurogenesis |
| 37.GO:0045664\_regulation\_of\_neuron\_differentiation |
| 38.GO:0050767\_regulation\_of\_neurogenesis |
| 39.GO:0014047\_glutamate\_secretion |
| 40.GO:0007006\_mitochondrial\_membrane\_organization |
| 41.GO:0042493\_response\_to\_drug |
| 42.GO:0051402\_neuron\_apoptosis |
| 43.GO:0043523\_regulation\_of\_neuron\_apoptosis |
| 44.GO:0043524\_negative\_regulation\_of\_neuron\_apoptosis |
| 45.GO:0046394\_carboxylic\_acid\_biosynthetic\_process |
| 46.GO:0016053\_organic\_acid\_biosynthetic\_process |
| 47.GO:0042116\_macrophage\_activation |
| 48.GO:0006637\_acyl-CoA\_metabolic\_process |
| 49.GO:0001657\_ureteric\_bud\_development |
| 50.GO:0001656\_metanephros\_development |
| 51.GO:0048469\_cell\_maturation |
| 52.GO:0000082\_G1\_S\_transition\_of\_mitotic\_cell\_cycle |
| 53.GO:0001952\_regulation\_of\_cell-matrix\_adhesion |
| 54.GO:0031103\_axon\_regeneration |
| 55.GO:0048678\_response\_to\_axon\_injury |
| 56.GO:0031102\_neuron\_projection\_regeneration |
| 57.GO:0010623\_developmental\_programmed\_cell\_death |
| 58.GO:0046666\_retinal\_cell\_programmed\_cell\_death |
| 59.GO:0042551\_neuron\_maturation |
| 60.GO:0048066\_pigmentation\_during\_development |
| 61.GO:0030318\_melanocyte\_differentiation |
| 62.GO:0048070\_regulation\_of\_pigmentation\_during\_development |
| 63.GO:0050931\_pigment\_cell\_differentiation |
| 64.GO:0051384\_response\_to\_glucocorticoid\_stimulus |
| 65.GO:0031960\_response\_to\_corticosteroid\_stimulus |
| 66.GO:0042327\_positive\_regulation\_of\_phosphorylation |
| 67.GO:0045937\_positive\_regulation\_of\_phosphate\_metabolic\_process |
| 68.GO:0001934\_positive\_regulation\_of\_protein\_amino\_acid\_phosphorylation |
| 69.GO:0010562\_positive\_regulation\_of\_phosphorus\_metabolic\_process |
| 70.GO:0031401\_positive\_regulation\_of\_protein\_modification\_process |
| 71.GO:0033032\_regulation\_of\_myeloid\_cell\_apoptosis |
| 72.GO:0033028\_myeloid\_cell\_apoptosis |
| 73.GO:0033033\_negative\_regulation\_of\_myeloid\_cell\_apoptosis |
