# Supplementary material for: RedundancyMiner: De-replication of redundant GO categories in microarray and proteomics analysis
Source: BMC Bioinformatics. 2011 Feb 10;12:52. doi: 10.1186/1471-2105-12-52 (PMC3223614; doi:10.1186/1471-2105-12-52)
Supplement: Additional file 8 — Retinal development HTGM download. compressed package of the results of running HTGM on the retinal development genes list. [file 1471-2105-12-52-S8.ZIP › SCENARIO_2_MODIFIED/total.txt.total.txt.dir/Exp1_BestClusterMap_LEIGS_KM_24.csv.join.22.txt.dir/Exp1_BestClusterMap_LEIGS_KM_24.csv.join.22.txt.change.gce.CIM.dir/cgi_user_y.html]

**Y-axis Names**   
Cluster is based on euclidean distance  
Cluster method is: average  
plclust  
height plot  

|  |
| --- |
| 1.BCL2 |
| 2.HMGB1 |
| 3.KITL |
| 4.BDNF |
| 5.SNCA |
| 6.ONECUT2 |
| 7.IRS2 |
| 8.PITX2 |
| 9.VEGFA |
| 10.NTRK2 |
| 11.RORB |
| 12.DAB1 |
| 13.GNAQ |
| 14.ID4 |
| 15.CDC25B |
| 16.SLC11A1 |
| 17.NR2F2 |
| 18.SALL1 |
| 19.SPRY1 |
| 20.EFHD1 |
| 21.TGFBI |
| 22.GPNMB |
| 23.PKP2 |
| 24.ALDH18A1 |
| 25.SCD1 |
| 26.TNFRSF21 |
| 27.MYB |
| 28.TOB1 |
| 29.LTBP1 |
| 30.LTBP3 |
| 31.ACOT10 |
| 32.FABP7 |
| 33.NEFL |
| 34.RNF6 |
| 35.EPHB1 |
| 36.NRCAM |
| 37.SEMA5A |
