# Supplementary material for: RedundancyMiner: De-replication of redundant GO categories in microarray and proteomics analysis
Source: BMC Bioinformatics. 2011 Feb 10;12:52. doi: 10.1186/1471-2105-12-52 (PMC3223614; doi:10.1186/1471-2105-12-52)
Supplement: Additional file 8 — Retinal development HTGM download. compressed package of the results of running HTGM on the retinal development genes list. [file 1471-2105-12-52-S8.ZIP › SCENARIO_2_MODIFIED/total.txt.total.txt.dir/Exp1_BestClusterMap_LEIGS_KM_24.csv.join.22.txt.dir/Exp1_BestClusterMap_LEIGS_KM_24.csv.join.22.txt.change.gce.html]

Gene Category Report for Exp1\_BestClusterMap\_LEIGS\_KM\_24.csv.join.22.txt

# Gene Category Report for Exp1\_BestClusterMap\_LEIGS\_KM\_24.csv.join.22.txt

| HYPERLINKED GO CATEGORY | HYPERLINKED GENE NAME | TOTAL GENES | CHANGED GENES | ENRICHMENT | LOG10(p) | CUMULATIVE NUMBER OF CATEGORIES | CUMULATIVE RANDOMS MEAN | FALSE DISCOVERY RATE |
| --- | --- | --- | --- | --- | --- | --- | --- | --- |
| GO:0045634\_regulation\_of\_melanocyte\_differentiation | GNAQ | 4 | 3 |  |  |  |  |  |  |
| GO:0045634\_regulation\_of\_melanocyte\_differentiation | BCL2 | 4 | 3 |  |  |  |  |  |  |
| GO:0045634\_regulation\_of\_melanocyte\_differentiation | KITL | 4 | 3 |  |  |  |  |  |  |
| GO:0050932\_regulation\_of\_pigment\_cell\_differentiation | GNAQ | 4 | 3 |  |  |  |  |  |  |
| GO:0050932\_regulation\_of\_pigment\_cell\_differentiation | BCL2 | 4 | 3 |  |  |  |  |  |  |
| GO:0050932\_regulation\_of\_pigment\_cell\_differentiation | KITL | 4 | 3 |  |  |  |  |  |  |
| GO:0022008\_neurogenesis | ONECUT2 | 423 | 17 | 2.985015 | -4.587653 | 1 | 0.02 | 0.020000 |
| GO:0022008\_neurogenesis | RORB | 423 | 17 | 2.985015 | -4.587653 | 1 | 0.02 | 0.020000 |
| GO:0022008\_neurogenesis | EPHB1 | 423 | 17 | 2.985015 | -4.587653 | 1 | 0.02 | 0.020000 |
| GO:0022008\_neurogenesis | NRCAM | 423 | 17 | 2.985015 | -4.587653 | 1 | 0.02 | 0.020000 |
| GO:0022008\_neurogenesis | SEMA5A | 423 | 17 | 2.985015 | -4.587653 | 1 | 0.02 | 0.020000 |
| GO:0022008\_neurogenesis | EFHD1 | 423 | 17 | 2.985015 | -4.587653 | 1 | 0.02 | 0.020000 |
| GO:0022008\_neurogenesis | BDNF | 423 | 17 | 2.985015 | -4.587653 | 1 | 0.02 | 0.020000 |
| GO:0022008\_neurogenesis | RNF6 | 423 | 17 | 2.985015 | -4.587653 | 1 | 0.02 | 0.020000 |
| GO:0022008\_neurogenesis | DAB1 | 423 | 17 | 2.985015 | -4.587653 | 1 | 0.02 | 0.020000 |
| GO:0022008\_neurogenesis | GNAQ | 423 | 17 | 2.985015 | -4.587653 | 1 | 0.02 | 0.020000 |
| GO:0022008\_neurogenesis | BCL2 | 423 | 17 | 2.985015 | -4.587653 | 1 | 0.02 | 0.020000 |
| GO:0022008\_neurogenesis | NTRK2 | 423 | 17 | 2.985015 | -4.587653 | 1 | 0.02 | 0.020000 |
| GO:0022008\_neurogenesis | VEGFA | 423 | 17 | 2.985015 | -4.587653 | 1 | 0.02 | 0.020000 |
| GO:0022008\_neurogenesis | ID4 | 423 | 17 | 2.985015 | -4.587653 | 1 | 0.02 | 0.020000 |
| GO:0022008\_neurogenesis | FABP7 | 423 | 17 | 2.985015 | -4.587653 | 1 | 0.02 | 0.020000 |
| GO:0022008\_neurogenesis | NR2F2 | 423 | 17 | 2.985015 | -4.587653 | 1 | 0.02 | 0.020000 |
| GO:0022008\_neurogenesis | NEFL | 423 | 17 | 2.985015 | -4.587653 | 1 | 0.02 | 0.020000 |
| GO:0048666\_neuron\_development | ONECUT2 | 262 | 13 | 3.685361 | -4.485241 | 2 | 0.02 | 0.010000 |
| GO:0048666\_neuron\_development | RORB | 262 | 13 | 3.685361 | -4.485241 | 2 | 0.02 | 0.010000 |
| GO:0048666\_neuron\_development | EPHB1 | 262 | 13 | 3.685361 | -4.485241 | 2 | 0.02 | 0.010000 |
| GO:0048666\_neuron\_development | SEMA5A | 262 | 13 | 3.685361 | -4.485241 | 2 | 0.02 | 0.010000 |
| GO:0048666\_neuron\_development | NRCAM | 262 | 13 | 3.685361 | -4.485241 | 2 | 0.02 | 0.010000 |
| GO:0048666\_neuron\_development | EFHD1 | 262 | 13 | 3.685361 | -4.485241 | 2 | 0.02 | 0.010000 |
| GO:0048666\_neuron\_development | RNF6 | 262 | 13 | 3.685361 | -4.485241 | 2 | 0.02 | 0.010000 |
| GO:0048666\_neuron\_development | BDNF | 262 | 13 | 3.685361 | -4.485241 | 2 | 0.02 | 0.010000 |
| GO:0048666\_neuron\_development | GNAQ | 262 | 13 | 3.685361 | -4.485241 | 2 | 0.02 | 0.010000 |
| GO:0048666\_neuron\_development | BCL2 | 262 | 13 | 3.685361 | -4.485241 | 2 | 0.02 | 0.010000 |
| GO:0048666\_neuron\_development | NTRK2 | 262 | 13 | 3.685361 | -4.485241 | 2 | 0.02 | 0.010000 |
| GO:0048666\_neuron\_development | VEGFA | 262 | 13 | 3.685361 | -4.485241 | 2 | 0.02 | 0.010000 |
| GO:0048666\_neuron\_development | NEFL | 262 | 13 | 3.685361 | -4.485241 | 2 | 0.02 | 0.010000 |
| GO:0048699\_generation\_of\_neurons | ONECUT2 | 396 | 16 | 3.000978 | -4.349756 | 3 | 0.03 | 0.010000 |
| GO:0048699\_generation\_of\_neurons | RORB | 396 | 16 | 3.000978 | -4.349756 | 3 | 0.03 | 0.010000 |
| GO:0048699\_generation\_of\_neurons | EPHB1 | 396 | 16 | 3.000978 | -4.349756 | 3 | 0.03 | 0.010000 |
| GO:0048699\_generation\_of\_neurons | NRCAM | 396 | 16 | 3.000978 | -4.349756 | 3 | 0.03 | 0.010000 |
| GO:0048699\_generation\_of\_neurons | SEMA5A | 396 | 16 | 3.000978 | -4.349756 | 3 | 0.03 | 0.010000 |
| GO:0048699\_generation\_of\_neurons | EFHD1 | 396 | 16 | 3.000978 | -4.349756 | 3 | 0.03 | 0.010000 |
| GO:0048699\_generation\_of\_neurons | BDNF | 396 | 16 | 3.000978 | -4.349756 | 3 | 0.03 | 0.010000 |
| GO:0048699\_generation\_of\_neurons | RNF6 | 396 | 16 | 3.000978 | -4.349756 | 3 | 0.03 | 0.010000 |
| GO:0048699\_generation\_of\_neurons | DAB1 | 396 | 16 | 3.000978 | -4.349756 | 3 | 0.03 | 0.010000 |
| GO:0048699\_generation\_of\_neurons | GNAQ | 396 | 16 | 3.000978 | -4.349756 | 3 | 0.03 | 0.010000 |
| GO:0048699\_generation\_of\_neurons | BCL2 | 396 | 16 | 3.000978 | -4.349756 | 3 | 0.03 | 0.010000 |
| GO:0048699\_generation\_of\_neurons | NTRK2 | 396 | 16 | 3.000978 | -4.349756 | 3 | 0.03 | 0.010000 |
| GO:0048699\_generation\_of\_neurons | VEGFA | 396 | 16 | 3.000978 | -4.349756 | 3 | 0.03 | 0.010000 |
| GO:0048699\_generation\_of\_neurons | ID4 | 396 | 16 | 3.000978 | -4.349756 | 3 | 0.03 | 0.010000 |
| GO:0048699\_generation\_of\_neurons | NR2F2 | 396 | 16 | 3.000978 | -4.349756 | 3 | 0.03 | 0.010000 |
| GO:0048699\_generation\_of\_neurons | NEFL | 396 | 16 | 3.000978 | -4.349756 | 3 | 0.03 | 0.010000 |
| GO:0014047\_glutamate\_secretion | BDNF | 7 | 3 | 31.831797 | -4.106238 | 4 | 0.08 | 0.020000 |
| GO:0014047\_glutamate\_secretion | NTRK2 | 7 | 3 | 31.831797 | -4.106238 | 4 | 0.08 | 0.020000 |
| GO:0014047\_glutamate\_secretion | SNCA | 7 | 3 | 31.831797 | -4.106238 | 4 | 0.08 | 0.020000 |
| GO:0045636\_positive\_regulation\_of\_melanocyte\_differentiation | BCL2 | 2 | 2 |  |  |  |  |  |  |
| GO:0045636\_positive\_regulation\_of\_melanocyte\_differentiation | KITL | 2 | 2 |  |  |  |  |  |  |
| GO:0050942\_positive\_regulation\_of\_pigment\_cell\_differentiation | BCL2 | 2 | 2 |  |  |  |  |  |  |
| GO:0050942\_positive\_regulation\_of\_pigment\_cell\_differentiation | KITL | 2 | 2 |  |  |  |  |  |  |
| GO:0030335\_positive\_regulation\_of\_cell\_migration | HMGB1 | 22 | 4 | 13.504399 | -3.740480 | 5 | 0.2 | 0.040000 |
| GO:0030335\_positive\_regulation\_of\_cell\_migration | IRS2 | 22 | 4 | 13.504399 | -3.740480 | 5 | 0.2 | 0.040000 |
| GO:0030335\_positive\_regulation\_of\_cell\_migration | BCL2 | 22 | 4 | 13.504399 | -3.740480 | 5 | 0.2 | 0.040000 |
| GO:0030335\_positive\_regulation\_of\_cell\_migration | ONECUT2 | 22 | 4 | 13.504399 | -3.740480 | 5 | 0.2 | 0.040000 |
| GO:0048070\_regulation\_of\_pigmentation\_during\_development | GNAQ | 9 | 3 | 24.758065 | -3.734400 | 6 | 0.2 | 0.033333 |
| GO:0048070\_regulation\_of\_pigmentation\_during\_development | BCL2 | 9 | 3 | 24.758065 | -3.734400 | 6 | 0.2 | 0.033333 |
| GO:0048070\_regulation\_of\_pigmentation\_during\_development | KITL | 9 | 3 | 24.758065 | -3.734400 | 6 | 0.2 | 0.033333 |
| GO:0030182\_neuron\_differentiation | ONECUT2 | 356 | 14 | 2.920895 | -3.700699 | 7 | 0.21 | 0.030000 |
| GO:0030182\_neuron\_differentiation | RORB | 356 | 14 | 2.920895 | -3.700699 | 7 | 0.21 | 0.030000 |
| GO:0030182\_neuron\_differentiation | EPHB1 | 356 | 14 | 2.920895 | -3.700699 | 7 | 0.21 | 0.030000 |
| GO:0030182\_neuron\_differentiation | SEMA5A | 356 | 14 | 2.920895 | -3.700699 | 7 | 0.21 | 0.030000 |
| GO:0030182\_neuron\_differentiation | NRCAM | 356 | 14 | 2.920895 | -3.700699 | 7 | 0.21 | 0.030000 |
| GO:0030182\_neuron\_differentiation | EFHD1 | 356 | 14 | 2.920895 | -3.700699 | 7 | 0.21 | 0.030000 |
| GO:0030182\_neuron\_differentiation | BDNF | 356 | 14 | 2.920895 | -3.700699 | 7 | 0.21 | 0.030000 |
| GO:0030182\_neuron\_differentiation | RNF6 | 356 | 14 | 2.920895 | -3.700699 | 7 | 0.21 | 0.030000 |
| GO:0030182\_neuron\_differentiation | GNAQ | 356 | 14 | 2.920895 | -3.700699 | 7 | 0.21 | 0.030000 |
| GO:0030182\_neuron\_differentiation | BCL2 | 356 | 14 | 2.920895 | -3.700699 | 7 | 0.21 | 0.030000 |
| GO:0030182\_neuron\_differentiation | NTRK2 | 356 | 14 | 2.920895 | -3.700699 | 7 | 0.21 | 0.030000 |
| GO:0030182\_neuron\_differentiation | VEGFA | 356 | 14 | 2.920895 | -3.700699 | 7 | 0.21 | 0.030000 |
| GO:0030182\_neuron\_differentiation | ID4 | 356 | 14 | 2.920895 | -3.700699 | 7 | 0.21 | 0.030000 |
| GO:0030182\_neuron\_differentiation | NEFL | 356 | 14 | 2.920895 | -3.700699 | 7 | 0.21 | 0.030000 |
| GO:0048592\_eye\_morphogenesis | BDNF | 70 | 6 | 6.366359 | -3.505509 | 8 | 0.27 | 0.033750 |
| GO:0048592\_eye\_morphogenesis | BCL2 | 70 | 6 | 6.366359 | -3.505509 | 8 | 0.27 | 0.033750 |
| GO:0048592\_eye\_morphogenesis | VEGFA | 70 | 6 | 6.366359 | -3.505509 | 8 | 0.27 | 0.033750 |
| GO:0048592\_eye\_morphogenesis | NTRK2 | 70 | 6 | 6.366359 | -3.505509 | 8 | 0.27 | 0.033750 |
| GO:0048592\_eye\_morphogenesis | RORB | 70 | 6 | 6.366359 | -3.505509 | 8 | 0.27 | 0.033750 |
| GO:0048592\_eye\_morphogenesis | EPHB1 | 70 | 6 | 6.366359 | -3.505509 | 8 | 0.27 | 0.033750 |
| GO:0007399\_nervous\_system\_development | IRS2 | 621 | 19 | 2.272479 | -3.480218 | 9 | 0.27 | 0.030000 |
| GO:0007399\_nervous\_system\_development | ONECUT2 | 621 | 19 | 2.272479 | -3.480218 | 9 | 0.27 | 0.030000 |
| GO:0007399\_nervous\_system\_development | RORB | 621 | 19 | 2.272479 | -3.480218 | 9 | 0.27 | 0.030000 |
| GO:0007399\_nervous\_system\_development | EPHB1 | 621 | 19 | 2.272479 | -3.480218 | 9 | 0.27 | 0.030000 |
| GO:0007399\_nervous\_system\_development | SEMA5A | 621 | 19 | 2.272479 | -3.480218 | 9 | 0.27 | 0.030000 |
| GO:0007399\_nervous\_system\_development | NRCAM | 621 | 19 | 2.272479 | -3.480218 | 9 | 0.27 | 0.030000 |
| GO:0007399\_nervous\_system\_development | EFHD1 | 621 | 19 | 2.272479 | -3.480218 | 9 | 0.27 | 0.030000 |
| GO:0007399\_nervous\_system\_development | RNF6 | 621 | 19 | 2.272479 | -3.480218 | 9 | 0.27 | 0.030000 |
| GO:0007399\_nervous\_system\_development | BDNF | 621 | 19 | 2.272479 | -3.480218 | 9 | 0.27 | 0.030000 |
| GO:0007399\_nervous\_system\_development | DAB1 | 621 | 19 | 2.272479 | -3.480218 | 9 | 0.27 | 0.030000 |
| GO:0007399\_nervous\_system\_development | GNAQ | 621 | 19 | 2.272479 | -3.480218 | 9 | 0.27 | 0.030000 |
| GO:0007399\_nervous\_system\_development | BCL2 | 621 | 19 | 2.272479 | -3.480218 | 9 | 0.27 | 0.030000 |
| GO:0007399\_nervous\_system\_development | VEGFA | 621 | 19 | 2.272479 | -3.480218 | 9 | 0.27 | 0.030000 |
| GO:0007399\_nervous\_system\_development | NTRK2 | 621 | 19 | 2.272479 | -3.480218 | 9 | 0.27 | 0.030000 |
| GO:0007399\_nervous\_system\_development | ID4 | 621 | 19 | 2.272479 | -3.480218 | 9 | 0.27 | 0.030000 |
| GO:0007399\_nervous\_system\_development | FABP7 | 621 | 19 | 2.272479 | -3.480218 | 9 | 0.27 | 0.030000 |
| GO:0007399\_nervous\_system\_development | NR2F2 | 621 | 19 | 2.272479 | -3.480218 | 9 | 0.27 | 0.030000 |
| GO:0007399\_nervous\_system\_development | NEFL | 621 | 19 | 2.272479 | -3.480218 | 9 | 0.27 | 0.030000 |
| GO:0007399\_nervous\_system\_development | PITX2 | 621 | 19 | 2.272479 | -3.480218 | 9 | 0.27 | 0.030000 |
| GO:0006928\_cell\_motion | HMGB1 | 330 | 13 | 2.925953 | -3.461063 | 11 | 0.27 | 0.024545 |
| GO:0006928\_cell\_motion | IRS2 | 330 | 13 | 2.925953 | -3.461063 | 11 | 0.27 | 0.024545 |
| GO:0006928\_cell\_motion | ONECUT2 | 330 | 13 | 2.925953 | -3.461063 | 11 | 0.27 | 0.024545 |
| GO:0006928\_cell\_motion | KITL | 330 | 13 | 2.925953 | -3.461063 | 11 | 0.27 | 0.024545 |
| GO:0006928\_cell\_motion | EPHB1 | 330 | 13 | 2.925953 | -3.461063 | 11 | 0.27 | 0.024545 |
| GO:0006928\_cell\_motion | SEMA5A | 330 | 13 | 2.925953 | -3.461063 | 11 | 0.27 | 0.024545 |
| GO:0006928\_cell\_motion | NRCAM | 330 | 13 | 2.925953 | -3.461063 | 11 | 0.27 | 0.024545 |
| GO:0006928\_cell\_motion | BDNF | 330 | 13 | 2.925953 | -3.461063 | 11 | 0.27 | 0.024545 |
| GO:0006928\_cell\_motion | DAB1 | 330 | 13 | 2.925953 | -3.461063 | 11 | 0.27 | 0.024545 |
| GO:0006928\_cell\_motion | BCL2 | 330 | 13 | 2.925953 | -3.461063 | 11 | 0.27 | 0.024545 |
| GO:0006928\_cell\_motion | VEGFA | 330 | 13 | 2.925953 | -3.461063 | 11 | 0.27 | 0.024545 |
| GO:0006928\_cell\_motion | NR2F2 | 330 | 13 | 2.925953 | -3.461063 | 11 | 0.27 | 0.024545 |
| GO:0006928\_cell\_motion | PITX2 | 330 | 13 | 2.925953 | -3.461063 | 11 | 0.27 | 0.024545 |
| GO:0051674\_localization\_of\_cell | HMGB1 | 330 | 13 | 2.925953 | -3.461063 | 11 | 0.27 | 0.024545 |
| GO:0051674\_localization\_of\_cell | IRS2 | 330 | 13 | 2.925953 | -3.461063 | 11 | 0.27 | 0.024545 |
| GO:0051674\_localization\_of\_cell | ONECUT2 | 330 | 13 | 2.925953 | -3.461063 | 11 | 0.27 | 0.024545 |
| GO:0051674\_localization\_of\_cell | KITL | 330 | 13 | 2.925953 | -3.461063 | 11 | 0.27 | 0.024545 |
| GO:0051674\_localization\_of\_cell | EPHB1 | 330 | 13 | 2.925953 | -3.461063 | 11 | 0.27 | 0.024545 |
| GO:0051674\_localization\_of\_cell | SEMA5A | 330 | 13 | 2.925953 | -3.461063 | 11 | 0.27 | 0.024545 |
| GO:0051674\_localization\_of\_cell | NRCAM | 330 | 13 | 2.925953 | -3.461063 | 11 | 0.27 | 0.024545 |
| GO:0051674\_localization\_of\_cell | BDNF | 330 | 13 | 2.925953 | -3.461063 | 11 | 0.27 | 0.024545 |
| GO:0051674\_localization\_of\_cell | DAB1 | 330 | 13 | 2.925953 | -3.461063 | 11 | 0.27 | 0.024545 |
| GO:0051674\_localization\_of\_cell | BCL2 | 330 | 13 | 2.925953 | -3.461063 | 11 | 0.27 | 0.024545 |
| GO:0051674\_localization\_of\_cell | VEGFA | 330 | 13 | 2.925953 | -3.461063 | 11 | 0.27 | 0.024545 |
| GO:0051674\_localization\_of\_cell | NR2F2 | 330 | 13 | 2.925953 | -3.461063 | 11 | 0.27 | 0.024545 |
| GO:0051674\_localization\_of\_cell | PITX2 | 330 | 13 | 2.925953 | -3.461063 | 11 | 0.27 | 0.024545 |
| GO:0040012\_regulation\_of\_locomotion | HMGB1 | 72 | 6 | 6.189516 | -3.438460 | 12 | 0.32 | 0.026667 |
| GO:0040012\_regulation\_of\_locomotion | IRS2 | 72 | 6 | 6.189516 | -3.438460 | 12 | 0.32 | 0.026667 |
| GO:0040012\_regulation\_of\_locomotion | BCL2 | 72 | 6 | 6.189516 | -3.438460 | 12 | 0.32 | 0.026667 |
| GO:0040012\_regulation\_of\_locomotion | SNCA | 72 | 6 | 6.189516 | -3.438460 | 12 | 0.32 | 0.026667 |
| GO:0040012\_regulation\_of\_locomotion | ONECUT2 | 72 | 6 | 6.189516 | -3.438460 | 12 | 0.32 | 0.026667 |
| GO:0040012\_regulation\_of\_locomotion | PITX2 | 72 | 6 | 6.189516 | -3.438460 | 12 | 0.32 | 0.026667 |
| GO:0051272\_positive\_regulation\_of\_cell\_motion | HMGB1 | 27 | 4 | 11.003584 | -3.382314 | 13 | 0.32 | 0.024615 |
| GO:0051272\_positive\_regulation\_of\_cell\_motion | IRS2 | 27 | 4 | 11.003584 | -3.382314 | 13 | 0.32 | 0.024615 |
| GO:0051272\_positive\_regulation\_of\_cell\_motion | BCL2 | 27 | 4 | 11.003584 | -3.382314 | 13 | 0.32 | 0.024615 |
| GO:0051272\_positive\_regulation\_of\_cell\_motion | ONECUT2 | 27 | 4 | 11.003584 | -3.382314 | 13 | 0.32 | 0.024615 |
| GO:0001654\_eye\_development | HMGB1 | 136 | 8 | 4.369070 | -3.382301 | 14 | 0.32 | 0.022857 |
| GO:0001654\_eye\_development | BDNF | 136 | 8 | 4.369070 | -3.382301 | 14 | 0.32 | 0.022857 |
| GO:0001654\_eye\_development | BCL2 | 136 | 8 | 4.369070 | -3.382301 | 14 | 0.32 | 0.022857 |
| GO:0001654\_eye\_development | NTRK2 | 136 | 8 | 4.369070 | -3.382301 | 14 | 0.32 | 0.022857 |
| GO:0001654\_eye\_development | VEGFA | 136 | 8 | 4.369070 | -3.382301 | 14 | 0.32 | 0.022857 |
| GO:0001654\_eye\_development | RORB | 136 | 8 | 4.369070 | -3.382301 | 14 | 0.32 | 0.022857 |
| GO:0001654\_eye\_development | EPHB1 | 136 | 8 | 4.369070 | -3.382301 | 14 | 0.32 | 0.022857 |
| GO:0001654\_eye\_development | PITX2 | 136 | 8 | 4.369070 | -3.382301 | 14 | 0.32 | 0.022857 |
| GO:0048087\_positive\_regulation\_of\_pigmentation\_during\_development | BCL2 | 3 | 2 |  |  |  |  |  |  |
| GO:0048087\_positive\_regulation\_of\_pigmentation\_during\_development | KITL | 3 | 2 |  |  |  |  |  |  |
| GO:0010623\_developmental\_programmed\_cell\_death | BDNF | 13 | 3 | 17.140199 | -3.219037 | 15 | 0.44 | 0.029333 |
| GO:0010623\_developmental\_programmed\_cell\_death | BCL2 | 13 | 3 | 17.140199 | -3.219037 | 15 | 0.44 | 0.029333 |
| GO:0010623\_developmental\_programmed\_cell\_death | KITL | 13 | 3 | 17.140199 | -3.219037 | 15 | 0.44 | 0.029333 |
| GO:0048468\_cell\_development | ONECUT2 | 654 | 19 | 2.157813 | -3.190461 | 16 | 0.47 | 0.029375 |
| GO:0048468\_cell\_development | RORB | 654 | 19 | 2.157813 | -3.190461 | 16 | 0.47 | 0.029375 |
| GO:0048468\_cell\_development | KITL | 654 | 19 | 2.157813 | -3.190461 | 16 | 0.47 | 0.029375 |
| GO:0048468\_cell\_development | EPHB1 | 654 | 19 | 2.157813 | -3.190461 | 16 | 0.47 | 0.029375 |
| GO:0048468\_cell\_development | CDC25B | 654 | 19 | 2.157813 | -3.190461 | 16 | 0.47 | 0.029375 |
| GO:0048468\_cell\_development | SEMA5A | 654 | 19 | 2.157813 | -3.190461 | 16 | 0.47 | 0.029375 |
| GO:0048468\_cell\_development | NRCAM | 654 | 19 | 2.157813 | -3.190461 | 16 | 0.47 | 0.029375 |
| GO:0048468\_cell\_development | EFHD1 | 654 | 19 | 2.157813 | -3.190461 | 16 | 0.47 | 0.029375 |
| GO:0048468\_cell\_development | RNF6 | 654 | 19 | 2.157813 | -3.190461 | 16 | 0.47 | 0.029375 |
| GO:0048468\_cell\_development | BDNF | 654 | 19 | 2.157813 | -3.190461 | 16 | 0.47 | 0.029375 |
| GO:0048468\_cell\_development | DAB1 | 654 | 19 | 2.157813 | -3.190461 | 16 | 0.47 | 0.029375 |
| GO:0048468\_cell\_development | GNAQ | 654 | 19 | 2.157813 | -3.190461 | 16 | 0.47 | 0.029375 |
| GO:0048468\_cell\_development | BCL2 | 654 | 19 | 2.157813 | -3.190461 | 16 | 0.47 | 0.029375 |
| GO:0048468\_cell\_development | VEGFA | 654 | 19 | 2.157813 | -3.190461 | 16 | 0.47 | 0.029375 |
| GO:0048468\_cell\_development | NTRK2 | 654 | 19 | 2.157813 | -3.190461 | 16 | 0.47 | 0.029375 |
| GO:0048468\_cell\_development | ID4 | 654 | 19 | 2.157813 | -3.190461 | 16 | 0.47 | 0.029375 |
| GO:0048468\_cell\_development | FABP7 | 654 | 19 | 2.157813 | -3.190461 | 16 | 0.47 | 0.029375 |
| GO:0048468\_cell\_development | NR2F2 | 654 | 19 | 2.157813 | -3.190461 | 16 | 0.47 | 0.029375 |
| GO:0048468\_cell\_development | NEFL | 654 | 19 | 2.157813 | -3.190461 | 16 | 0.47 | 0.029375 |
| GO:0046668\_regulation\_of\_retinal\_cell\_programmed\_cell\_death | BDNF | 4 | 2 |  |  |  |  |  |  |
| GO:0046668\_regulation\_of\_retinal\_cell\_programmed\_cell\_death | BCL2 | 4 | 2 |  |  |  |  |  |  |
| GO:0030334\_regulation\_of\_cell\_migration | HMGB1 | 59 | 5 | 6.294423 | -2.968128 | 17 | 0.74 | 0.043529 |
| GO:0030334\_regulation\_of\_cell\_migration | IRS2 | 59 | 5 | 6.294423 | -2.968128 | 17 | 0.74 | 0.043529 |
| GO:0030334\_regulation\_of\_cell\_migration | BCL2 | 59 | 5 | 6.294423 | -2.968128 | 17 | 0.74 | 0.043529 |
| GO:0030334\_regulation\_of\_cell\_migration | ONECUT2 | 59 | 5 | 6.294423 | -2.968128 | 17 | 0.74 | 0.043529 |
| GO:0030334\_regulation\_of\_cell\_migration | PITX2 | 59 | 5 | 6.294423 | -2.968128 | 17 | 0.74 | 0.043529 |
| GO:0001657\_ureteric\_bud\_development | SPRY1 | 38 | 4 | 7.818336 | -2.806512 | 18 | 1.07 | 0.059444 |
| GO:0001657\_ureteric\_bud\_development | BDNF | 38 | 4 | 7.818336 | -2.806512 | 18 | 1.07 | 0.059444 |
| GO:0001657\_ureteric\_bud\_development | SALL1 | 38 | 4 | 7.818336 | -2.806512 | 18 | 1.07 | 0.059444 |
| GO:0001657\_ureteric\_bud\_development | BCL2 | 38 | 4 | 7.818336 | -2.806512 | 18 | 1.07 | 0.059444 |
| GO:0030318\_melanocyte\_differentiation | GNAQ | 18 | 3 | 12.379032 | -2.784571 | 19 | 1.1 | 0.057895 |
| GO:0030318\_melanocyte\_differentiation | BCL2 | 18 | 3 | 12.379032 | -2.784571 | 19 | 1.1 | 0.057895 |
| GO:0030318\_melanocyte\_differentiation | KITL | 18 | 3 | 12.379032 | -2.784571 | 19 | 1.1 | 0.057895 |
| GO:0040011\_locomotion | HMGB1 | 295 | 11 | 2.769546 | -2.780667 | 20 | 1.11 | 0.055500 |
| GO:0040011\_locomotion | IRS2 | 295 | 11 | 2.769546 | -2.780667 | 20 | 1.11 | 0.055500 |
| GO:0040011\_locomotion | DAB1 | 295 | 11 | 2.769546 | -2.780667 | 20 | 1.11 | 0.055500 |
| GO:0040011\_locomotion | BCL2 | 295 | 11 | 2.769546 | -2.780667 | 20 | 1.11 | 0.055500 |
| GO:0040011\_locomotion | VEGFA | 295 | 11 | 2.769546 | -2.780667 | 20 | 1.11 | 0.055500 |
| GO:0040011\_locomotion | SNCA | 295 | 11 | 2.769546 | -2.780667 | 20 | 1.11 | 0.055500 |
| GO:0040011\_locomotion | ONECUT2 | 295 | 11 | 2.769546 | -2.780667 | 20 | 1.11 | 0.055500 |
| GO:0040011\_locomotion | NR2F2 | 295 | 11 | 2.769546 | -2.780667 | 20 | 1.11 | 0.055500 |
| GO:0040011\_locomotion | NEFL | 295 | 11 | 2.769546 | -2.780667 | 20 | 1.11 | 0.055500 |
| GO:0040011\_locomotion | KITL | 295 | 11 | 2.769546 | -2.780667 | 20 | 1.11 | 0.055500 |
| GO:0040011\_locomotion | PITX2 | 295 | 11 | 2.769546 | -2.780667 | 20 | 1.11 | 0.055500 |
| GO:0043524\_negative\_regulation\_of\_neuron\_apoptosis | BDNF | 39 | 4 | 7.617866 | -2.763878 | 21 | 1.13 | 0.053810 |
| GO:0043524\_negative\_regulation\_of\_neuron\_apoptosis | BCL2 | 39 | 4 | 7.617866 | -2.763878 | 21 | 1.13 | 0.053810 |
| GO:0043524\_negative\_regulation\_of\_neuron\_apoptosis | SNCA | 39 | 4 | 7.617866 | -2.763878 | 21 | 1.13 | 0.053810 |
| GO:0043524\_negative\_regulation\_of\_neuron\_apoptosis | NEFL | 39 | 4 | 7.617866 | -2.763878 | 21 | 1.13 | 0.053810 |
| GO:0033033\_negative\_regulation\_of\_myeloid\_cell\_apoptosis | BCL2 | 5 | 2 | 29.709677 | -2.760004 | 22 | 1.61 | 0.073182 |
| GO:0033033\_negative\_regulation\_of\_myeloid\_cell\_apoptosis | KITL | 5 | 2 | 29.709677 | -2.760004 | 22 | 1.61 | 0.073182 |
| GO:0051402\_neuron\_apoptosis | TNFRSF21 | 66 | 5 | 5.626833 | -2.747771 | 23 | 1.62 | 0.070435 |
| GO:0051402\_neuron\_apoptosis | BDNF | 66 | 5 | 5.626833 | -2.747771 | 23 | 1.62 | 0.070435 |
| GO:0051402\_neuron\_apoptosis | BCL2 | 66 | 5 | 5.626833 | -2.747771 | 23 | 1.62 | 0.070435 |
| GO:0051402\_neuron\_apoptosis | SNCA | 66 | 5 | 5.626833 | -2.747771 | 23 | 1.62 | 0.070435 |
| GO:0051402\_neuron\_apoptosis | NEFL | 66 | 5 | 5.626833 | -2.747771 | 23 | 1.62 | 0.070435 |
| GO:0042462\_eye\_photoreceptor\_cell\_development | VEGFA | 19 | 3 | 11.727504 | -2.714103 | 25 | 1.76 | 0.070400 |
| GO:0042462\_eye\_photoreceptor\_cell\_development | NTRK2 | 19 | 3 | 11.727504 | -2.714103 | 25 | 1.76 | 0.070400 |
| GO:0042462\_eye\_photoreceptor\_cell\_development | RORB | 19 | 3 | 11.727504 | -2.714103 | 25 | 1.76 | 0.070400 |
| GO:0050931\_pigment\_cell\_differentiation | GNAQ | 19 | 3 | 11.727504 | -2.714103 | 25 | 1.76 | 0.070400 |
| GO:0050931\_pigment\_cell\_differentiation | BCL2 | 19 | 3 | 11.727504 | -2.714103 | 25 | 1.76 | 0.070400 |
| GO:0050931\_pigment\_cell\_differentiation | KITL | 19 | 3 | 11.727504 | -2.714103 | 25 | 1.76 | 0.070400 |
| GO:0031960\_response\_to\_corticosteroid\_stimulus | HMGB1 | 6 | 2 | 24.758065 | -2.587693 | 28 | 2.61 | 0.093214 |
| GO:0031960\_response\_to\_corticosteroid\_stimulus | BCL2 | 6 | 2 | 24.758065 | -2.587693 | 28 | 2.61 | 0.093214 |
| GO:0046666\_retinal\_cell\_programmed\_cell\_death | BDNF | 6 | 2 | 24.758065 | -2.587693 | 28 | 2.61 | 0.093214 |
| GO:0046666\_retinal\_cell\_programmed\_cell\_death | BCL2 | 6 | 2 | 24.758065 | -2.587693 | 28 | 2.61 | 0.093214 |
| GO:0051384\_response\_to\_glucocorticoid\_stimulus | HMGB1 | 6 | 2 | 24.758065 | -2.587693 | 28 | 2.61 | 0.093214 |
| GO:0051384\_response\_to\_glucocorticoid\_stimulus | BCL2 | 6 | 2 | 24.758065 | -2.587693 | 28 | 2.61 | 0.093214 |
| GO:0001754\_eye\_photoreceptor\_cell\_differentiation | VEGFA | 21 | 3 | 10.610599 | -2.584899 | 30 | 2.7 | 0.090000 |
| GO:0001754\_eye\_photoreceptor\_cell\_differentiation | NTRK2 | 21 | 3 | 10.610599 | -2.584899 | 30 | 2.7 | 0.090000 |
| GO:0001754\_eye\_photoreceptor\_cell\_differentiation | RORB | 21 | 3 | 10.610599 | -2.584899 | 30 | 2.7 | 0.090000 |
| GO:0002053\_positive\_regulation\_of\_mesenchymal\_cell\_proliferation | HMGB1 | 21 | 3 | 10.610599 | -2.584899 | 30 | 2.7 | 0.090000 |
| GO:0002053\_positive\_regulation\_of\_mesenchymal\_cell\_proliferation | IRS2 | 21 | 3 | 10.610599 | -2.584899 | 30 | 2.7 | 0.090000 |
| GO:0002053\_positive\_regulation\_of\_mesenchymal\_cell\_proliferation | VEGFA | 21 | 3 | 10.610599 | -2.584899 | 30 | 2.7 | 0.090000 |
| GO:0051270\_regulation\_of\_cell\_motion | HMGB1 | 73 | 5 | 5.087274 | -2.553550 | 31 | 2.81 | 0.090645 |
| GO:0051270\_regulation\_of\_cell\_motion | IRS2 | 73 | 5 | 5.087274 | -2.553550 | 31 | 2.81 | 0.090645 |
| GO:0051270\_regulation\_of\_cell\_motion | BCL2 | 73 | 5 | 5.087274 | -2.553550 | 31 | 2.81 | 0.090645 |
| GO:0051270\_regulation\_of\_cell\_motion | ONECUT2 | 73 | 5 | 5.087274 | -2.553550 | 31 | 2.81 | 0.090645 |
| GO:0051270\_regulation\_of\_cell\_motion | PITX2 | 73 | 5 | 5.087274 | -2.553550 | 31 | 2.81 | 0.090645 |
| GO:0001569\_patterning\_of\_blood\_vessels | SEMA5A | 22 | 3 | 10.128299 | -2.525389 | 35 | 3.04 | 0.086857 |
| GO:0001569\_patterning\_of\_blood\_vessels | VEGFA | 22 | 3 | 10.128299 | -2.525389 | 35 | 3.04 | 0.086857 |
| GO:0001569\_patterning\_of\_blood\_vessels | PITX2 | 22 | 3 | 10.128299 | -2.525389 | 35 | 3.04 | 0.086857 |
| GO:0010463\_mesenchymal\_cell\_proliferation | HMGB1 | 22 | 3 | 10.128299 | -2.525389 | 35 | 3.04 | 0.086857 |
| GO:0010463\_mesenchymal\_cell\_proliferation | IRS2 | 22 | 3 | 10.128299 | -2.525389 | 35 | 3.04 | 0.086857 |
| GO:0010463\_mesenchymal\_cell\_proliferation | VEGFA | 22 | 3 | 10.128299 | -2.525389 | 35 | 3.04 | 0.086857 |
| GO:0010464\_regulation\_of\_mesenchymal\_cell\_proliferation | HMGB1 | 22 | 3 | 10.128299 | -2.525389 | 35 | 3.04 | 0.086857 |
| GO:0010464\_regulation\_of\_mesenchymal\_cell\_proliferation | IRS2 | 22 | 3 | 10.128299 | -2.525389 | 35 | 3.04 | 0.086857 |
| GO:0010464\_regulation\_of\_mesenchymal\_cell\_proliferation | VEGFA | 22 | 3 | 10.128299 | -2.525389 | 35 | 3.04 | 0.086857 |
| GO:0042461\_photoreceptor\_cell\_development | VEGFA | 22 | 3 | 10.128299 | -2.525389 | 35 | 3.04 | 0.086857 |
| GO:0042461\_photoreceptor\_cell\_development | NTRK2 | 22 | 3 | 10.128299 | -2.525389 | 35 | 3.04 | 0.086857 |
| GO:0042461\_photoreceptor\_cell\_development | RORB | 22 | 3 | 10.128299 | -2.525389 | 35 | 3.04 | 0.086857 |
| GO:0048869\_cellular\_developmental\_process | HMGB1 | 1113 | 25 | 1.668333 | -2.474393 | 36 | 3.18 | 0.088333 |
| GO:0048869\_cellular\_developmental\_process | PARD3 | 1113 | 25 | 1.668333 | -2.474393 | 36 | 3.18 | 0.088333 |
| GO:0048869\_cellular\_developmental\_process | ONECUT2 | 1113 | 25 | 1.668333 | -2.474393 | 36 | 3.18 | 0.088333 |
| GO:0048869\_cellular\_developmental\_process | RORB | 1113 | 25 | 1.668333 | -2.474393 | 36 | 3.18 | 0.088333 |
| GO:0048869\_cellular\_developmental\_process | EPHB1 | 1113 | 25 | 1.668333 | -2.474393 | 36 | 3.18 | 0.088333 |
| GO:0048869\_cellular\_developmental\_process | SEMA5A | 1113 | 25 | 1.668333 | -2.474393 | 36 | 3.18 | 0.088333 |
| GO:0048869\_cellular\_developmental\_process | NRCAM | 1113 | 25 | 1.668333 | -2.474393 | 36 | 3.18 | 0.088333 |
| GO:0048869\_cellular\_developmental\_process | EFHD1 | 1113 | 25 | 1.668333 | -2.474393 | 36 | 3.18 | 0.088333 |
| GO:0048869\_cellular\_developmental\_process | BDNF | 1113 | 25 | 1.668333 | -2.474393 | 36 | 3.18 | 0.088333 |
| GO:0048869\_cellular\_developmental\_process | SPRY1 | 1113 | 25 | 1.668333 | -2.474393 | 36 | 3.18 | 0.088333 |
| GO:0048869\_cellular\_developmental\_process | DAB1 | 1113 | 25 | 1.668333 | -2.474393 | 36 | 3.18 | 0.088333 |
| GO:0048869\_cellular\_developmental\_process | BCL2 | 1113 | 25 | 1.668333 | -2.474393 | 36 | 3.18 | 0.088333 |
| GO:0048869\_cellular\_developmental\_process | NR2F2 | 1113 | 25 | 1.668333 | -2.474393 | 36 | 3.18 | 0.088333 |
| GO:0048869\_cellular\_developmental\_process | NEFL | 1113 | 25 | 1.668333 | -2.474393 | 36 | 3.18 | 0.088333 |
| GO:0048869\_cellular\_developmental\_process | SCD1 | 1113 | 25 | 1.668333 | -2.474393 | 36 | 3.18 | 0.088333 |
| GO:0048869\_cellular\_developmental\_process | KITL | 1113 | 25 | 1.668333 | -2.474393 | 36 | 3.18 | 0.088333 |
| GO:0048869\_cellular\_developmental\_process | CDC25B | 1113 | 25 | 1.668333 | -2.474393 | 36 | 3.18 | 0.088333 |
| GO:0048869\_cellular\_developmental\_process | RNF6 | 1113 | 25 | 1.668333 | -2.474393 | 36 | 3.18 | 0.088333 |
| GO:0048869\_cellular\_developmental\_process | GNAQ | 1113 | 25 | 1.668333 | -2.474393 | 36 | 3.18 | 0.088333 |
| GO:0048869\_cellular\_developmental\_process | SALL1 | 1113 | 25 | 1.668333 | -2.474393 | 36 | 3.18 | 0.088333 |
| GO:0048869\_cellular\_developmental\_process | VEGFA | 1113 | 25 | 1.668333 | -2.474393 | 36 | 3.18 | 0.088333 |
| GO:0048869\_cellular\_developmental\_process | NTRK2 | 1113 | 25 | 1.668333 | -2.474393 | 36 | 3.18 | 0.088333 |
| GO:0048869\_cellular\_developmental\_process | ID4 | 1113 | 25 | 1.668333 | -2.474393 | 36 | 3.18 | 0.088333 |
| GO:0048869\_cellular\_developmental\_process | FABP7 | 1113 | 25 | 1.668333 | -2.474393 | 36 | 3.18 | 0.088333 |
| GO:0048869\_cellular\_developmental\_process | TOB1 | 1113 | 25 | 1.668333 | -2.474393 | 36 | 3.18 | 0.088333 |
| GO:0000082\_G1\_S\_transition\_of\_mitotic\_cell\_cycle | BCL2 | 23 | 3 | 9.687938 | -2.468848 | 37 | 3.28 | 0.088649 |
| GO:0000082\_G1\_S\_transition\_of\_mitotic\_cell\_cycle | ID4 | 23 | 3 | 9.687938 | -2.468848 | 37 | 3.28 | 0.088649 |
| GO:0000082\_G1\_S\_transition\_of\_mitotic\_cell\_cycle | MYB | 23 | 3 | 9.687938 | -2.468848 | 37 | 3.28 | 0.088649 |
| GO:0033032\_regulation\_of\_myeloid\_cell\_apoptosis | BCL2 | 7 | 2 | 21.221198 | -2.445341 | 38 | 4.1 | 0.107895 |
| GO:0033032\_regulation\_of\_myeloid\_cell\_apoptosis | KITL | 7 | 2 | 21.221198 | -2.445341 | 38 | 4.1 | 0.107895 |
| GO:0016477\_cell\_migration | HMGB1 | 234 | 9 | 2.856700 | -2.431034 | 39 | 4.1 | 0.105128 |
| GO:0016477\_cell\_migration | IRS2 | 234 | 9 | 2.856700 | -2.431034 | 39 | 4.1 | 0.105128 |
| GO:0016477\_cell\_migration | DAB1 | 234 | 9 | 2.856700 | -2.431034 | 39 | 4.1 | 0.105128 |
| GO:0016477\_cell\_migration | BCL2 | 234 | 9 | 2.856700 | -2.431034 | 39 | 4.1 | 0.105128 |
| GO:0016477\_cell\_migration | VEGFA | 234 | 9 | 2.856700 | -2.431034 | 39 | 4.1 | 0.105128 |
| GO:0016477\_cell\_migration | ONECUT2 | 234 | 9 | 2.856700 | -2.431034 | 39 | 4.1 | 0.105128 |
| GO:0016477\_cell\_migration | NR2F2 | 234 | 9 | 2.856700 | -2.431034 | 39 | 4.1 | 0.105128 |
| GO:0016477\_cell\_migration | KITL | 234 | 9 | 2.856700 | -2.431034 | 39 | 4.1 | 0.105128 |
| GO:0016477\_cell\_migration | PITX2 | 234 | 9 | 2.856700 | -2.431034 | 39 | 4.1 | 0.105128 |
| GO:0051179\_localization | HMGB1 | 1058 | 24 | 1.684859 | -2.429746 | 40 | 4.11 | 0.102750 |
| GO:0051179\_localization | IRS2 | 1058 | 24 | 1.684859 | -2.429746 | 40 | 4.11 | 0.102750 |
| GO:0051179\_localization | PFKL | 1058 | 24 | 1.684859 | -2.429746 | 40 | 4.11 | 0.102750 |
| GO:0051179\_localization | SNCA | 1058 | 24 | 1.684859 | -2.429746 | 40 | 4.11 | 0.102750 |
| GO:0051179\_localization | ONECUT2 | 1058 | 24 | 1.684859 | -2.429746 | 40 | 4.11 | 0.102750 |
| GO:0051179\_localization | KITL | 1058 | 24 | 1.684859 | -2.429746 | 40 | 4.11 | 0.102750 |
| GO:0051179\_localization | EPHB1 | 1058 | 24 | 1.684859 | -2.429746 | 40 | 4.11 | 0.102750 |
| GO:0051179\_localization | SEMA5A | 1058 | 24 | 1.684859 | -2.429746 | 40 | 4.11 | 0.102750 |
| GO:0051179\_localization | NRCAM | 1058 | 24 | 1.684859 | -2.429746 | 40 | 4.11 | 0.102750 |
| GO:0051179\_localization | CADPS | 1058 | 24 | 1.684859 | -2.429746 | 40 | 4.11 | 0.102750 |
| GO:0051179\_localization | SLC11A1 | 1058 | 24 | 1.684859 | -2.429746 | 40 | 4.11 | 0.102750 |
| GO:0051179\_localization | BDNF | 1058 | 24 | 1.684859 | -2.429746 | 40 | 4.11 | 0.102750 |
| GO:0051179\_localization | DAB1 | 1058 | 24 | 1.684859 | -2.429746 | 40 | 4.11 | 0.102750 |
| GO:0051179\_localization | BCL2 | 1058 | 24 | 1.684859 | -2.429746 | 40 | 4.11 | 0.102750 |
| GO:0051179\_localization | VEGFA | 1058 | 24 | 1.684859 | -2.429746 | 40 | 4.11 | 0.102750 |
| GO:0051179\_localization | NTRK2 | 1058 | 24 | 1.684859 | -2.429746 | 40 | 4.11 | 0.102750 |
| GO:0051179\_localization | CHRNB4 | 1058 | 24 | 1.684859 | -2.429746 | 40 | 4.11 | 0.102750 |
| GO:0051179\_localization | STXBP3A | 1058 | 24 | 1.684859 | -2.429746 | 40 | 4.11 | 0.102750 |
| GO:0051179\_localization | NR2F2 | 1058 | 24 | 1.684859 | -2.429746 | 40 | 4.11 | 0.102750 |
| GO:0051179\_localization | MYB | 1058 | 24 | 1.684859 | -2.429746 | 40 | 4.11 | 0.102750 |
| GO:0051179\_localization | SLC4A4 | 1058 | 24 | 1.684859 | -2.429746 | 40 | 4.11 | 0.102750 |
| GO:0051179\_localization | EHD1 | 1058 | 24 | 1.684859 | -2.429746 | 40 | 4.11 | 0.102750 |
| GO:0051179\_localization | PITX2 | 1058 | 24 | 1.684859 | -2.429746 | 40 | 4.11 | 0.102750 |
| GO:0051179\_localization | TOB1 | 1058 | 24 | 1.684859 | -2.429746 | 40 | 4.11 | 0.102750 |
| GO:0030154\_cell\_differentiation | SCD1 | 1060 | 24 | 1.681680 | -2.418439 | 41 | 4.18 | 0.101951 |
| GO:0030154\_cell\_differentiation | HMGB1 | 1060 | 24 | 1.681680 | -2.418439 | 41 | 4.18 | 0.101951 |
| GO:0030154\_cell\_differentiation | ONECUT2 | 1060 | 24 | 1.681680 | -2.418439 | 41 | 4.18 | 0.101951 |
| GO:0030154\_cell\_differentiation | RORB | 1060 | 24 | 1.681680 | -2.418439 | 41 | 4.18 | 0.101951 |
| GO:0030154\_cell\_differentiation | KITL | 1060 | 24 | 1.681680 | -2.418439 | 41 | 4.18 | 0.101951 |
| GO:0030154\_cell\_differentiation | EPHB1 | 1060 | 24 | 1.681680 | -2.418439 | 41 | 4.18 | 0.101951 |
| GO:0030154\_cell\_differentiation | CDC25B | 1060 | 24 | 1.681680 | -2.418439 | 41 | 4.18 | 0.101951 |
| GO:0030154\_cell\_differentiation | NRCAM | 1060 | 24 | 1.681680 | -2.418439 | 41 | 4.18 | 0.101951 |
| GO:0030154\_cell\_differentiation | SEMA5A | 1060 | 24 | 1.681680 | -2.418439 | 41 | 4.18 | 0.101951 |
| GO:0030154\_cell\_differentiation | EFHD1 | 1060 | 24 | 1.681680 | -2.418439 | 41 | 4.18 | 0.101951 |
| GO:0030154\_cell\_differentiation | SPRY1 | 1060 | 24 | 1.681680 | -2.418439 | 41 | 4.18 | 0.101951 |
| GO:0030154\_cell\_differentiation | BDNF | 1060 | 24 | 1.681680 | -2.418439 | 41 | 4.18 | 0.101951 |
| GO:0030154\_cell\_differentiation | RNF6 | 1060 | 24 | 1.681680 | -2.418439 | 41 | 4.18 | 0.101951 |
| GO:0030154\_cell\_differentiation | DAB1 | 1060 | 24 | 1.681680 | -2.418439 | 41 | 4.18 | 0.101951 |
| GO:0030154\_cell\_differentiation | GNAQ | 1060 | 24 | 1.681680 | -2.418439 | 41 | 4.18 | 0.101951 |
| GO:0030154\_cell\_differentiation | SALL1 | 1060 | 24 | 1.681680 | -2.418439 | 41 | 4.18 | 0.101951 |
| GO:0030154\_cell\_differentiation | BCL2 | 1060 | 24 | 1.681680 | -2.418439 | 41 | 4.18 | 0.101951 |
| GO:0030154\_cell\_differentiation | NTRK2 | 1060 | 24 | 1.681680 | -2.418439 | 41 | 4.18 | 0.101951 |
| GO:0030154\_cell\_differentiation | VEGFA | 1060 | 24 | 1.681680 | -2.418439 | 41 | 4.18 | 0.101951 |
| GO:0030154\_cell\_differentiation | ID4 | 1060 | 24 | 1.681680 | -2.418439 | 41 | 4.18 | 0.101951 |
| GO:0030154\_cell\_differentiation | FABP7 | 1060 | 24 | 1.681680 | -2.418439 | 41 | 4.18 | 0.101951 |
| GO:0030154\_cell\_differentiation | NR2F2 | 1060 | 24 | 1.681680 | -2.418439 | 41 | 4.18 | 0.101951 |
| GO:0030154\_cell\_differentiation | NEFL | 1060 | 24 | 1.681680 | -2.418439 | 41 | 4.18 | 0.101951 |
| GO:0030154\_cell\_differentiation | TOB1 | 1060 | 24 | 1.681680 | -2.418439 | 41 | 4.18 | 0.101951 |
| GO:0001656\_metanephros\_development | SPRY1 | 50 | 4 | 5.941935 | -2.364623 | 42 | 4.47 | 0.106429 |
| GO:0001656\_metanephros\_development | BDNF | 50 | 4 | 5.941935 | -2.364623 | 42 | 4.47 | 0.106429 |
| GO:0001656\_metanephros\_development | SALL1 | 50 | 4 | 5.941935 | -2.364623 | 42 | 4.47 | 0.106429 |
| GO:0001656\_metanephros\_development | BCL2 | 50 | 4 | 5.941935 | -2.364623 | 42 | 4.47 | 0.106429 |
| GO:0007417\_central\_nervous\_system\_development | NRCAM | 287 | 10 | 2.587951 | -2.347184 | 43 | 4.51 | 0.104884 |
| GO:0007417\_central\_nervous\_system\_development | IRS2 | 287 | 10 | 2.587951 | -2.347184 | 43 | 4.51 | 0.104884 |
| GO:0007417\_central\_nervous\_system\_development | DAB1 | 287 | 10 | 2.587951 | -2.347184 | 43 | 4.51 | 0.104884 |
| GO:0007417\_central\_nervous\_system\_development | GNAQ | 287 | 10 | 2.587951 | -2.347184 | 43 | 4.51 | 0.104884 |
| GO:0007417\_central\_nervous\_system\_development | BCL2 | 287 | 10 | 2.587951 | -2.347184 | 43 | 4.51 | 0.104884 |
| GO:0007417\_central\_nervous\_system\_development | ID4 | 287 | 10 | 2.587951 | -2.347184 | 43 | 4.51 | 0.104884 |
| GO:0007417\_central\_nervous\_system\_development | NR2F2 | 287 | 10 | 2.587951 | -2.347184 | 43 | 4.51 | 0.104884 |
| GO:0007417\_central\_nervous\_system\_development | FABP7 | 287 | 10 | 2.587951 | -2.347184 | 43 | 4.51 | 0.104884 |
| GO:0007417\_central\_nervous\_system\_development | EPHB1 | 287 | 10 | 2.587951 | -2.347184 | 43 | 4.51 | 0.104884 |
| GO:0007417\_central\_nervous\_system\_development | PITX2 | 287 | 10 | 2.587951 | -2.347184 | 43 | 4.51 | 0.104884 |
| GO:0031175\_neuron\_projection\_development | NRCAM | 197 | 8 | 3.016211 | -2.345987 | 44 | 4.52 | 0.102727 |
| GO:0031175\_neuron\_projection\_development | SEMA5A | 197 | 8 | 3.016211 | -2.345987 | 44 | 4.52 | 0.102727 |
| GO:0031175\_neuron\_projection\_development | EFHD1 | 197 | 8 | 3.016211 | -2.345987 | 44 | 4.52 | 0.102727 |
| GO:0031175\_neuron\_projection\_development | BDNF | 197 | 8 | 3.016211 | -2.345987 | 44 | 4.52 | 0.102727 |
| GO:0031175\_neuron\_projection\_development | RNF6 | 197 | 8 | 3.016211 | -2.345987 | 44 | 4.52 | 0.102727 |
| GO:0031175\_neuron\_projection\_development | BCL2 | 197 | 8 | 3.016211 | -2.345987 | 44 | 4.52 | 0.102727 |
| GO:0031175\_neuron\_projection\_development | NEFL | 197 | 8 | 3.016211 | -2.345987 | 44 | 4.52 | 0.102727 |
| GO:0031175\_neuron\_projection\_development | EPHB1 | 197 | 8 | 3.016211 | -2.345987 | 44 | 4.52 | 0.102727 |
| GO:0045664\_regulation\_of\_neuron\_differentiation | BDNF | 82 | 5 | 4.528914 | -2.334448 | 45 | 4.52 | 0.100444 |
| GO:0045664\_regulation\_of\_neuron\_differentiation | RNF6 | 82 | 5 | 4.528914 | -2.334448 | 45 | 4.52 | 0.100444 |
| GO:0045664\_regulation\_of\_neuron\_differentiation | BCL2 | 82 | 5 | 4.528914 | -2.334448 | 45 | 4.52 | 0.100444 |
| GO:0045664\_regulation\_of\_neuron\_differentiation | ID4 | 82 | 5 | 4.528914 | -2.334448 | 45 | 4.52 | 0.100444 |
| GO:0045664\_regulation\_of\_neuron\_differentiation | NEFL | 82 | 5 | 4.528914 | -2.334448 | 45 | 4.52 | 0.100444 |
| GO:0031102\_neuron\_projection\_regeneration | BCL2 | 8 | 2 | 18.568548 | -2.324175 | 47 | 5.26 | 0.111915 |
| GO:0031102\_neuron\_projection\_regeneration | NEFL | 8 | 2 | 18.568548 | -2.324175 | 47 | 5.26 | 0.111915 |
| GO:0031103\_axon\_regeneration | BCL2 | 8 | 2 | 18.568548 | -2.324175 | 47 | 5.26 | 0.111915 |
| GO:0031103\_axon\_regeneration | NEFL | 8 | 2 | 18.568548 | -2.324175 | 47 | 5.26 | 0.111915 |
| GO:0046530\_photoreceptor\_cell\_differentiation | VEGFA | 26 | 3 | 8.570099 | -2.314551 | 48 | 5.36 | 0.111667 |
| GO:0046530\_photoreceptor\_cell\_differentiation | NTRK2 | 26 | 3 | 8.570099 | -2.314551 | 48 | 5.36 | 0.111667 |
| GO:0046530\_photoreceptor\_cell\_differentiation | RORB | 26 | 3 | 8.570099 | -2.314551 | 48 | 5.36 | 0.111667 |
| GO:0007409\_axonogenesis | NRCAM | 158 | 7 | 3.290629 | -2.306897 | 49 | 5.38 | 0.109796 |
| GO:0007409\_axonogenesis | SEMA5A | 158 | 7 | 3.290629 | -2.306897 | 49 | 5.38 | 0.109796 |
| GO:0007409\_axonogenesis | RNF6 | 158 | 7 | 3.290629 | -2.306897 | 49 | 5.38 | 0.109796 |
| GO:0007409\_axonogenesis | BDNF | 158 | 7 | 3.290629 | -2.306897 | 49 | 5.38 | 0.109796 |
| GO:0007409\_axonogenesis | BCL2 | 158 | 7 | 3.290629 | -2.306897 | 49 | 5.38 | 0.109796 |
| GO:0007409\_axonogenesis | NEFL | 158 | 7 | 3.290629 | -2.306897 | 49 | 5.38 | 0.109796 |
| GO:0007409\_axonogenesis | EPHB1 | 158 | 7 | 3.290629 | -2.306897 | 49 | 5.38 | 0.109796 |
| GO:0033028\_myeloid\_cell\_apoptosis | BCL2 | 9 | 2 | 16.505376 | -2.218799 | 50 | 6.57 | 0.131400 |
| GO:0033028\_myeloid\_cell\_apoptosis | KITL | 9 | 2 | 16.505376 | -2.218799 | 50 | 6.57 | 0.131400 |
| GO:0042325\_regulation\_of\_phosphorylation | SLC11A1 | 164 | 7 | 3.170240 | -2.218789 | 51 | 6.57 | 0.128824 |
| GO:0042325\_regulation\_of\_phosphorylation | HMGB1 | 164 | 7 | 3.170240 | -2.218789 | 51 | 6.57 | 0.128824 |
| GO:0042325\_regulation\_of\_phosphorylation | SPRY1 | 164 | 7 | 3.170240 | -2.218789 | 51 | 6.57 | 0.128824 |
| GO:0042325\_regulation\_of\_phosphorylation | DAB1 | 164 | 7 | 3.170240 | -2.218789 | 51 | 6.57 | 0.128824 |
| GO:0042325\_regulation\_of\_phosphorylation | BCL2 | 164 | 7 | 3.170240 | -2.218789 | 51 | 6.57 | 0.128824 |
| GO:0042325\_regulation\_of\_phosphorylation | KITL | 164 | 7 | 3.170240 | -2.218789 | 51 | 6.57 | 0.128824 |
| GO:0042325\_regulation\_of\_phosphorylation | CDC25B | 164 | 7 | 3.170240 | -2.218789 | 51 | 6.57 | 0.128824 |
| GO:0019220\_regulation\_of\_phosphate\_metabolic\_process | SLC11A1 | 165 | 7 | 3.151026 | -2.204523 | 53 | 6.64 | 0.125283 |
| GO:0019220\_regulation\_of\_phosphate\_metabolic\_process | HMGB1 | 165 | 7 | 3.151026 | -2.204523 | 53 | 6.64 | 0.125283 |
| GO:0019220\_regulation\_of\_phosphate\_metabolic\_process | SPRY1 | 165 | 7 | 3.151026 | -2.204523 | 53 | 6.64 | 0.125283 |
| GO:0019220\_regulation\_of\_phosphate\_metabolic\_process | DAB1 | 165 | 7 | 3.151026 | -2.204523 | 53 | 6.64 | 0.125283 |
| GO:0019220\_regulation\_of\_phosphate\_metabolic\_process | BCL2 | 165 | 7 | 3.151026 | -2.204523 | 53 | 6.64 | 0.125283 |
| GO:0019220\_regulation\_of\_phosphate\_metabolic\_process | KITL | 165 | 7 | 3.151026 | -2.204523 | 53 | 6.64 | 0.125283 |
| GO:0019220\_regulation\_of\_phosphate\_metabolic\_process | CDC25B | 165 | 7 | 3.151026 | -2.204523 | 53 | 6.64 | 0.125283 |
| GO:0051174\_regulation\_of\_phosphorus\_metabolic\_process | SLC11A1 | 165 | 7 | 3.151026 | -2.204523 | 53 | 6.64 | 0.125283 |
| GO:0051174\_regulation\_of\_phosphorus\_metabolic\_process | HMGB1 | 165 | 7 | 3.151026 | -2.204523 | 53 | 6.64 | 0.125283 |
| GO:0051174\_regulation\_of\_phosphorus\_metabolic\_process | SPRY1 | 165 | 7 | 3.151026 | -2.204523 | 53 | 6.64 | 0.125283 |
| GO:0051174\_regulation\_of\_phosphorus\_metabolic\_process | DAB1 | 165 | 7 | 3.151026 | -2.204523 | 53 | 6.64 | 0.125283 |
| GO:0051174\_regulation\_of\_phosphorus\_metabolic\_process | BCL2 | 165 | 7 | 3.151026 | -2.204523 | 53 | 6.64 | 0.125283 |
| GO:0051174\_regulation\_of\_phosphorus\_metabolic\_process | KITL | 165 | 7 | 3.151026 | -2.204523 | 53 | 6.64 | 0.125283 |
| GO:0051174\_regulation\_of\_phosphorus\_metabolic\_process | CDC25B | 165 | 7 | 3.151026 | -2.204523 | 53 | 6.64 | 0.125283 |
| GO:0001934\_positive\_regulation\_of\_protein\_amino\_acid\_phosphorylation | HMGB1 | 29 | 3 | 7.683537 | -2.179193 | 55 | 6.83 | 0.124182 |
| GO:0001934\_positive\_regulation\_of\_protein\_amino\_acid\_phosphorylation | BCL2 | 29 | 3 | 7.683537 | -2.179193 | 55 | 6.83 | 0.124182 |
| GO:0001934\_positive\_regulation\_of\_protein\_amino\_acid\_phosphorylation | KITL | 29 | 3 | 7.683537 | -2.179193 | 55 | 6.83 | 0.124182 |
| GO:0048066\_pigmentation\_during\_development | GNAQ | 29 | 3 | 7.683537 | -2.179193 | 55 | 6.83 | 0.124182 |
| GO:0048066\_pigmentation\_during\_development | BCL2 | 29 | 3 | 7.683537 | -2.179193 | 55 | 6.83 | 0.124182 |
| GO:0048066\_pigmentation\_during\_development | KITL | 29 | 3 | 7.683537 | -2.179193 | 55 | 6.83 | 0.124182 |
| GO:0048870\_cell\_motility | HMGB1 | 257 | 9 | 2.601042 | -2.164158 | 56 | 6.85 | 0.122321 |
| GO:0048870\_cell\_motility | IRS2 | 257 | 9 | 2.601042 | -2.164158 | 56 | 6.85 | 0.122321 |
| GO:0048870\_cell\_motility | DAB1 | 257 | 9 | 2.601042 | -2.164158 | 56 | 6.85 | 0.122321 |
| GO:0048870\_cell\_motility | BCL2 | 257 | 9 | 2.601042 | -2.164158 | 56 | 6.85 | 0.122321 |
| GO:0048870\_cell\_motility | VEGFA | 257 | 9 | 2.601042 | -2.164158 | 56 | 6.85 | 0.122321 |
| GO:0048870\_cell\_motility | ONECUT2 | 257 | 9 | 2.601042 | -2.164158 | 56 | 6.85 | 0.122321 |
| GO:0048870\_cell\_motility | NR2F2 | 257 | 9 | 2.601042 | -2.164158 | 56 | 6.85 | 0.122321 |
| GO:0048870\_cell\_motility | KITL | 257 | 9 | 2.601042 | -2.164158 | 56 | 6.85 | 0.122321 |
| GO:0048870\_cell\_motility | PITX2 | 257 | 9 | 2.601042 | -2.164158 | 56 | 6.85 | 0.122321 |
| GO:0043523\_regulation\_of\_neuron\_apoptosis | BDNF | 57 | 4 | 5.212224 | -2.160705 | 57 | 6.96 | 0.122105 |
| GO:0043523\_regulation\_of\_neuron\_apoptosis | BCL2 | 57 | 4 | 5.212224 | -2.160705 | 57 | 6.96 | 0.122105 |
| GO:0043523\_regulation\_of\_neuron\_apoptosis | SNCA | 57 | 4 | 5.212224 | -2.160705 | 57 | 6.96 | 0.122105 |
| GO:0043523\_regulation\_of\_neuron\_apoptosis | NEFL | 57 | 4 | 5.212224 | -2.160705 | 57 | 6.96 | 0.122105 |
| GO:0048812\_neuron\_projection\_morphogenesis | NRCAM | 170 | 7 | 3.058349 | -2.134899 | 58 | 7.17 | 0.123621 |
| GO:0048812\_neuron\_projection\_morphogenesis | SEMA5A | 170 | 7 | 3.058349 | -2.134899 | 58 | 7.17 | 0.123621 |
| GO:0048812\_neuron\_projection\_morphogenesis | RNF6 | 170 | 7 | 3.058349 | -2.134899 | 58 | 7.17 | 0.123621 |
| GO:0048812\_neuron\_projection\_morphogenesis | BDNF | 170 | 7 | 3.058349 | -2.134899 | 58 | 7.17 | 0.123621 |
| GO:0048812\_neuron\_projection\_morphogenesis | BCL2 | 170 | 7 | 3.058349 | -2.134899 | 58 | 7.17 | 0.123621 |
| GO:0048812\_neuron\_projection\_morphogenesis | NEFL | 170 | 7 | 3.058349 | -2.134899 | 58 | 7.17 | 0.123621 |
| GO:0048812\_neuron\_projection\_morphogenesis | EPHB1 | 170 | 7 | 3.058349 | -2.134899 | 58 | 7.17 | 0.123621 |
| GO:0007006\_mitochondrial\_membrane\_organization | BCL2 | 10 | 2 | 14.854839 | -2.125655 | 60 | 8.31 | 0.138500 |
| GO:0007006\_mitochondrial\_membrane\_organization | SNCA | 10 | 2 | 14.854839 | -2.125655 | 60 | 8.31 | 0.138500 |
| GO:0042116\_macrophage\_activation | SLC11A1 | 10 | 2 | 14.854839 | -2.125655 | 60 | 8.31 | 0.138500 |
| GO:0042116\_macrophage\_activation | SNCA | 10 | 2 | 14.854839 | -2.125655 | 60 | 8.31 | 0.138500 |
| GO:0048469\_cell\_maturation | GNAQ | 59 | 4 | 5.035539 | -2.107854 | 61 | 8.45 | 0.138525 |
| GO:0048469\_cell\_maturation | BCL2 | 59 | 4 | 5.035539 | -2.107854 | 61 | 8.45 | 0.138525 |
| GO:0048469\_cell\_maturation | VEGFA | 59 | 4 | 5.035539 | -2.107854 | 61 | 8.45 | 0.138525 |
| GO:0048469\_cell\_maturation | CDC25B | 59 | 4 | 5.035539 | -2.107854 | 61 | 8.45 | 0.138525 |
| GO:0030030\_cell\_projection\_organization | NRCAM | 263 | 9 | 2.541702 | -2.100116 | 62 | 8.5 | 0.137097 |
| GO:0030030\_cell\_projection\_organization | SEMA5A | 263 | 9 | 2.541702 | -2.100116 | 62 | 8.5 | 0.137097 |
| GO:0030030\_cell\_projection\_organization | EFHD1 | 263 | 9 | 2.541702 | -2.100116 | 62 | 8.5 | 0.137097 |
| GO:0030030\_cell\_projection\_organization | BDNF | 263 | 9 | 2.541702 | -2.100116 | 62 | 8.5 | 0.137097 |
| GO:0030030\_cell\_projection\_organization | RNF6 | 263 | 9 | 2.541702 | -2.100116 | 62 | 8.5 | 0.137097 |
| GO:0030030\_cell\_projection\_organization | BCL2 | 263 | 9 | 2.541702 | -2.100116 | 62 | 8.5 | 0.137097 |
| GO:0030030\_cell\_projection\_organization | ONECUT2 | 263 | 9 | 2.541702 | -2.100116 | 62 | 8.5 | 0.137097 |
| GO:0030030\_cell\_projection\_organization | NEFL | 263 | 9 | 2.541702 | -2.100116 | 62 | 8.5 | 0.137097 |
| GO:0030030\_cell\_projection\_organization | EPHB1 | 263 | 9 | 2.541702 | -2.100116 | 62 | 8.5 | 0.137097 |
| GO:0010562\_positive\_regulation\_of\_phosphorus\_metabolic\_process | HMGB1 | 31 | 3 | 7.187825 | -2.097513 | 65 | 8.7 | 0.133846 |
| GO:0010562\_positive\_regulation\_of\_phosphorus\_metabolic\_process | BCL2 | 31 | 3 | 7.187825 | -2.097513 | 65 | 8.7 | 0.133846 |
| GO:0010562\_positive\_regulation\_of\_phosphorus\_metabolic\_process | KITL | 31 | 3 | 7.187825 | -2.097513 | 65 | 8.7 | 0.133846 |
| GO:0042327\_positive\_regulation\_of\_phosphorylation | HMGB1 | 31 | 3 | 7.187825 | -2.097513 | 65 | 8.7 | 0.133846 |
| GO:0042327\_positive\_regulation\_of\_phosphorylation | BCL2 | 31 | 3 | 7.187825 | -2.097513 | 65 | 8.7 | 0.133846 |
| GO:0042327\_positive\_regulation\_of\_phosphorylation | KITL | 31 | 3 | 7.187825 | -2.097513 | 65 | 8.7 | 0.133846 |
| GO:0045937\_positive\_regulation\_of\_phosphate\_metabolic\_process | HMGB1 | 31 | 3 | 7.187825 | -2.097513 | 65 | 8.7 | 0.133846 |
| GO:0045937\_positive\_regulation\_of\_phosphate\_metabolic\_process | BCL2 | 31 | 3 | 7.187825 | -2.097513 | 65 | 8.7 | 0.133846 |
| GO:0045937\_positive\_regulation\_of\_phosphate\_metabolic\_process | KITL | 31 | 3 | 7.187825 | -2.097513 | 65 | 8.7 | 0.133846 |
| GO:0048667\_cell\_morphogenesis\_involved\_in\_neuron\_differentiation | NRCAM | 173 | 7 | 3.005314 | -2.094441 | 66 | 8.71 | 0.131970 |
| GO:0048667\_cell\_morphogenesis\_involved\_in\_neuron\_differentiation | SEMA5A | 173 | 7 | 3.005314 | -2.094441 | 66 | 8.71 | 0.131970 |
| GO:0048667\_cell\_morphogenesis\_involved\_in\_neuron\_differentiation | RNF6 | 173 | 7 | 3.005314 | -2.094441 | 66 | 8.71 | 0.131970 |
| GO:0048667\_cell\_morphogenesis\_involved\_in\_neuron\_differentiation | BDNF | 173 | 7 | 3.005314 | -2.094441 | 66 | 8.71 | 0.131970 |
| GO:0048667\_cell\_morphogenesis\_involved\_in\_neuron\_differentiation | BCL2 | 173 | 7 | 3.005314 | -2.094441 | 66 | 8.71 | 0.131970 |
| GO:0048667\_cell\_morphogenesis\_involved\_in\_neuron\_differentiation | NEFL | 173 | 7 | 3.005314 | -2.094441 | 66 | 8.71 | 0.131970 |
| GO:0048667\_cell\_morphogenesis\_involved\_in\_neuron\_differentiation | EPHB1 | 173 | 7 | 3.005314 | -2.094441 | 66 | 8.71 | 0.131970 |
| GO:0007423\_sensory\_organ\_development | HMGB1 | 219 | 8 | 2.713213 | -2.072663 | 67 | 8.81 | 0.131493 |
| GO:0007423\_sensory\_organ\_development | BDNF | 219 | 8 | 2.713213 | -2.072663 | 67 | 8.81 | 0.131493 |
| GO:0007423\_sensory\_organ\_development | BCL2 | 219 | 8 | 2.713213 | -2.072663 | 67 | 8.81 | 0.131493 |
| GO:0007423\_sensory\_organ\_development | NTRK2 | 219 | 8 | 2.713213 | -2.072663 | 67 | 8.81 | 0.131493 |
| GO:0007423\_sensory\_organ\_development | VEGFA | 219 | 8 | 2.713213 | -2.072663 | 67 | 8.81 | 0.131493 |
| GO:0007423\_sensory\_organ\_development | RORB | 219 | 8 | 2.713213 | -2.072663 | 67 | 8.81 | 0.131493 |
| GO:0007423\_sensory\_organ\_development | EPHB1 | 219 | 8 | 2.713213 | -2.072663 | 67 | 8.81 | 0.131493 |
| GO:0007423\_sensory\_organ\_development | PITX2 | 219 | 8 | 2.713213 | -2.072663 | 67 | 8.81 | 0.131493 |
| GO:0050768\_negative\_regulation\_of\_neurogenesis | RNF6 | 32 | 3 | 6.963206 | -2.058898 | 68 | 8.94 | 0.131471 |
| GO:0050768\_negative\_regulation\_of\_neurogenesis | BDNF | 32 | 3 | 6.963206 | -2.058898 | 68 | 8.94 | 0.131471 |
| GO:0050768\_negative\_regulation\_of\_neurogenesis | ID4 | 32 | 3 | 6.963206 | -2.058898 | 68 | 8.94 | 0.131471 |
| GO:0048858\_cell\_projection\_morphogenesis | NRCAM | 176 | 7 | 2.954087 | -2.054927 | 69 | 9.06 | 0.131304 |
| GO:0048858\_cell\_projection\_morphogenesis | SEMA5A | 176 | 7 | 2.954087 | -2.054927 | 69 | 9.06 | 0.131304 |
| GO:0048858\_cell\_projection\_morphogenesis | RNF6 | 176 | 7 | 2.954087 | -2.054927 | 69 | 9.06 | 0.131304 |
| GO:0048858\_cell\_projection\_morphogenesis | BDNF | 176 | 7 | 2.954087 | -2.054927 | 69 | 9.06 | 0.131304 |
| GO:0048858\_cell\_projection\_morphogenesis | BCL2 | 176 | 7 | 2.954087 | -2.054927 | 69 | 9.06 | 0.131304 |
| GO:0048858\_cell\_projection\_morphogenesis | NEFL | 176 | 7 | 2.954087 | -2.054927 | 69 | 9.06 | 0.131304 |
| GO:0048858\_cell\_projection\_morphogenesis | EPHB1 | 176 | 7 | 2.954087 | -2.054927 | 69 | 9.06 | 0.131304 |
| GO:0001952\_regulation\_of\_cell-matrix\_adhesion | BCL2 | 11 | 2 | 13.504399 | -2.042266 | 73 | 9.81 | 0.134384 |
| GO:0001952\_regulation\_of\_cell-matrix\_adhesion | ONECUT2 | 11 | 2 | 13.504399 | -2.042266 | 73 | 9.81 | 0.134384 |
| GO:0006637\_acyl-CoA\_metabolic\_process | ACOT10 | 11 | 2 | 13.504399 | -2.042266 | 73 | 9.81 | 0.134384 |
| GO:0006637\_acyl-CoA\_metabolic\_process | SNCA | 11 | 2 | 13.504399 | -2.042266 | 73 | 9.81 | 0.134384 |
| GO:0042551\_neuron\_maturation | GNAQ | 11 | 2 | 13.504399 | -2.042266 | 73 | 9.81 | 0.134384 |
| GO:0042551\_neuron\_maturation | BCL2 | 11 | 2 | 13.504399 | -2.042266 | 73 | 9.81 | 0.134384 |
| GO:0048678\_response\_to\_axon\_injury | BCL2 | 11 | 2 | 13.504399 | -2.042266 | 73 | 9.81 | 0.134384 |
| GO:0048678\_response\_to\_axon\_injury | NEFL | 11 | 2 | 13.504399 | -2.042266 | 73 | 9.81 | 0.134384 |
| GO:0010721\_negative\_regulation\_of\_cell\_development | RNF6 | 34 | 3 | 6.553605 | -1.985656 | 74 | 10.69 | 0.144459 |
| GO:0010721\_negative\_regulation\_of\_cell\_development | BDNF | 34 | 3 | 6.553605 | -1.985656 | 74 | 10.69 | 0.144459 |
| GO:0010721\_negative\_regulation\_of\_cell\_development | ID4 | 34 | 3 | 6.553605 | -1.985656 | 74 | 10.69 | 0.144459 |
| GO:0050432\_catecholamine\_secretion | CADPS | 12 | 2 | 12.379032 | -1.966843 | 75 | 11.66 | 0.155467 |
| GO:0050432\_catecholamine\_secretion | SNCA | 12 | 2 | 12.379032 | -1.966843 | 75 | 11.66 | 0.155467 |
| GO:0032990\_cell\_part\_morphogenesis | NRCAM | 184 | 7 | 2.825649 | -1.953933 | 76 | 11.8 | 0.155263 |
| GO:0032990\_cell\_part\_morphogenesis | SEMA5A | 184 | 7 | 2.825649 | -1.953933 | 76 | 11.8 | 0.155263 |
| GO:0032990\_cell\_part\_morphogenesis | RNF6 | 184 | 7 | 2.825649 | -1.953933 | 76 | 11.8 | 0.155263 |
| GO:0032990\_cell\_part\_morphogenesis | BDNF | 184 | 7 | 2.825649 | -1.953933 | 76 | 11.8 | 0.155263 |
| GO:0032990\_cell\_part\_morphogenesis | BCL2 | 184 | 7 | 2.825649 | -1.953933 | 76 | 11.8 | 0.155263 |
| GO:0032990\_cell\_part\_morphogenesis | NEFL | 184 | 7 | 2.825649 | -1.953933 | 76 | 11.8 | 0.155263 |
| GO:0032990\_cell\_part\_morphogenesis | EPHB1 | 184 | 7 | 2.825649 | -1.953933 | 76 | 11.8 | 0.155263 |
| GO:0051325\_interphase | BCL2 | 35 | 3 | 6.366359 | -1.950868 | 78 | 11.97 | 0.153462 |
| GO:0051325\_interphase | ID4 | 35 | 3 | 6.366359 | -1.950868 | 78 | 11.97 | 0.153462 |
| GO:0051325\_interphase | MYB | 35 | 3 | 6.366359 | -1.950868 | 78 | 11.97 | 0.153462 |
| GO:0051329\_interphase\_of\_mitotic\_cell\_cycle | BCL2 | 35 | 3 | 6.366359 | -1.950868 | 78 | 11.97 | 0.153462 |
| GO:0051329\_interphase\_of\_mitotic\_cell\_cycle | ID4 | 35 | 3 | 6.366359 | -1.950868 | 78 | 11.97 | 0.153462 |
| GO:0051329\_interphase\_of\_mitotic\_cell\_cycle | MYB | 35 | 3 | 6.366359 | -1.950868 | 78 | 11.97 | 0.153462 |
| GO:0007420\_brain\_development | IRS2 | 231 | 8 | 2.572266 | -1.939363 | 79 | 12.03 | 0.152278 |
| GO:0007420\_brain\_development | DAB1 | 231 | 8 | 2.572266 | -1.939363 | 79 | 12.03 | 0.152278 |
| GO:0007420\_brain\_development | GNAQ | 231 | 8 | 2.572266 | -1.939363 | 79 | 12.03 | 0.152278 |
| GO:0007420\_brain\_development | BCL2 | 231 | 8 | 2.572266 | -1.939363 | 79 | 12.03 | 0.152278 |
| GO:0007420\_brain\_development | ID4 | 231 | 8 | 2.572266 | -1.939363 | 79 | 12.03 | 0.152278 |
| GO:0007420\_brain\_development | NR2F2 | 231 | 8 | 2.572266 | -1.939363 | 79 | 12.03 | 0.152278 |
| GO:0007420\_brain\_development | FABP7 | 231 | 8 | 2.572266 | -1.939363 | 79 | 12.03 | 0.152278 |
| GO:0007420\_brain\_development | PITX2 | 231 | 8 | 2.572266 | -1.939363 | 79 | 12.03 | 0.152278 |
| GO:0007179\_transforming\_growth\_factor\_beta\_receptor\_signaling\_pathway | LTBP1 | 66 | 4 | 4.501466 | -1.938528 | 81 | 12.09 | 0.149259 |
| GO:0007179\_transforming\_growth\_factor\_beta\_receptor\_signaling\_pathway | LTBP3 | 66 | 4 | 4.501466 | -1.938528 | 81 | 12.09 | 0.149259 |
| GO:0007179\_transforming\_growth\_factor\_beta\_receptor\_signaling\_pathway | ONECUT2 | 66 | 4 | 4.501466 | -1.938528 | 81 | 12.09 | 0.149259 |
| GO:0007179\_transforming\_growth\_factor\_beta\_receptor\_signaling\_pathway | TOB1 | 66 | 4 | 4.501466 | -1.938528 | 81 | 12.09 | 0.149259 |
| GO:0045860\_positive\_regulation\_of\_protein\_kinase\_activity | SLC11A1 | 66 | 4 | 4.501466 | -1.938528 | 81 | 12.09 | 0.149259 |
| GO:0045860\_positive\_regulation\_of\_protein\_kinase\_activity | DAB1 | 66 | 4 | 4.501466 | -1.938528 | 81 | 12.09 | 0.149259 |
| GO:0045860\_positive\_regulation\_of\_protein\_kinase\_activity | KITL | 66 | 4 | 4.501466 | -1.938528 | 81 | 12.09 | 0.149259 |
| GO:0045860\_positive\_regulation\_of\_protein\_kinase\_activity | CDC25B | 66 | 4 | 4.501466 | -1.938528 | 81 | 12.09 | 0.149259 |
| GO:0019222\_regulation\_of\_metabolic\_process | HMGB1 | 1088 | 23 | 1.570135 | -1.938309 | 82 | 12.1 | 0.147561 |
| GO:0019222\_regulation\_of\_metabolic\_process | SNCA | 1088 | 23 | 1.570135 | -1.938309 | 82 | 12.1 | 0.147561 |
| GO:0019222\_regulation\_of\_metabolic\_process | ONECUT2 | 1088 | 23 | 1.570135 | -1.938309 | 82 | 12.1 | 0.147561 |
| GO:0019222\_regulation\_of\_metabolic\_process | RORB | 1088 | 23 | 1.570135 | -1.938309 | 82 | 12.1 | 0.147561 |
| GO:0019222\_regulation\_of\_metabolic\_process | KITL | 1088 | 23 | 1.570135 | -1.938309 | 82 | 12.1 | 0.147561 |
| GO:0019222\_regulation\_of\_metabolic\_process | AHR | 1088 | 23 | 1.570135 | -1.938309 | 82 | 12.1 | 0.147561 |
| GO:0019222\_regulation\_of\_metabolic\_process | CDC25B | 1088 | 23 | 1.570135 | -1.938309 | 82 | 12.1 | 0.147561 |
| GO:0019222\_regulation\_of\_metabolic\_process | SUZ12 | 1088 | 23 | 1.570135 | -1.938309 | 82 | 12.1 | 0.147561 |
| GO:0019222\_regulation\_of\_metabolic\_process | SLC11A1 | 1088 | 23 | 1.570135 | -1.938309 | 82 | 12.1 | 0.147561 |
| GO:0019222\_regulation\_of\_metabolic\_process | BDNF | 1088 | 23 | 1.570135 | -1.938309 | 82 | 12.1 | 0.147561 |
| GO:0019222\_regulation\_of\_metabolic\_process | SPRY1 | 1088 | 23 | 1.570135 | -1.938309 | 82 | 12.1 | 0.147561 |
| GO:0019222\_regulation\_of\_metabolic\_process | RNF6 | 1088 | 23 | 1.570135 | -1.938309 | 82 | 12.1 | 0.147561 |
| GO:0019222\_regulation\_of\_metabolic\_process | DAB1 | 1088 | 23 | 1.570135 | -1.938309 | 82 | 12.1 | 0.147561 |
| GO:0019222\_regulation\_of\_metabolic\_process | GNAQ | 1088 | 23 | 1.570135 | -1.938309 | 82 | 12.1 | 0.147561 |
| GO:0019222\_regulation\_of\_metabolic\_process | BCL2 | 1088 | 23 | 1.570135 | -1.938309 | 82 | 12.1 | 0.147561 |
| GO:0019222\_regulation\_of\_metabolic\_process | NTRK2 | 1088 | 23 | 1.570135 | -1.938309 | 82 | 12.1 | 0.147561 |
| GO:0019222\_regulation\_of\_metabolic\_process | GARNL1 | 1088 | 23 | 1.570135 | -1.938309 | 82 | 12.1 | 0.147561 |
| GO:0019222\_regulation\_of\_metabolic\_process | NFAT5 | 1088 | 23 | 1.570135 | -1.938309 | 82 | 12.1 | 0.147561 |
| GO:0019222\_regulation\_of\_metabolic\_process | ACSL4 | 1088 | 23 | 1.570135 | -1.938309 | 82 | 12.1 | 0.147561 |
| GO:0019222\_regulation\_of\_metabolic\_process | NR2F2 | 1088 | 23 | 1.570135 | -1.938309 | 82 | 12.1 | 0.147561 |
| GO:0019222\_regulation\_of\_metabolic\_process | MYB | 1088 | 23 | 1.570135 | -1.938309 | 82 | 12.1 | 0.147561 |
| GO:0019222\_regulation\_of\_metabolic\_process | ETV3 | 1088 | 23 | 1.570135 | -1.938309 | 82 | 12.1 | 0.147561 |
| GO:0019222\_regulation\_of\_metabolic\_process | PITX2 | 1088 | 23 | 1.570135 | -1.938309 | 82 | 12.1 | 0.147561 |
| GO:0007155\_cell\_adhesion | NRCAM | 186 | 7 | 2.795265 | -1.929631 | 84 | 12.17 | 0.144881 |
| GO:0007155\_cell\_adhesion | DAB1 | 186 | 7 | 2.795265 | -1.929631 | 84 | 12.17 | 0.144881 |
| GO:0007155\_cell\_adhesion | PKP2 | 186 | 7 | 2.795265 | -1.929631 | 84 | 12.17 | 0.144881 |
| GO:0007155\_cell\_adhesion | BCL2 | 186 | 7 | 2.795265 | -1.929631 | 84 | 12.17 | 0.144881 |
| GO:0007155\_cell\_adhesion | TGFBI | 186 | 7 | 2.795265 | -1.929631 | 84 | 12.17 | 0.144881 |
| GO:0007155\_cell\_adhesion | ONECUT2 | 186 | 7 | 2.795265 | -1.929631 | 84 | 12.17 | 0.144881 |
| GO:0007155\_cell\_adhesion | GPNMB | 186 | 7 | 2.795265 | -1.929631 | 84 | 12.17 | 0.144881 |
| GO:0022610\_biological\_adhesion | NRCAM | 186 | 7 | 2.795265 | -1.929631 | 84 | 12.17 | 0.144881 |
| GO:0022610\_biological\_adhesion | DAB1 | 186 | 7 | 2.795265 | -1.929631 | 84 | 12.17 | 0.144881 |
| GO:0022610\_biological\_adhesion | PKP2 | 186 | 7 | 2.795265 | -1.929631 | 84 | 12.17 | 0.144881 |
| GO:0022610\_biological\_adhesion | BCL2 | 186 | 7 | 2.795265 | -1.929631 | 84 | 12.17 | 0.144881 |
| GO:0022610\_biological\_adhesion | TGFBI | 186 | 7 | 2.795265 | -1.929631 | 84 | 12.17 | 0.144881 |
| GO:0022610\_biological\_adhesion | ONECUT2 | 186 | 7 | 2.795265 | -1.929631 | 84 | 12.17 | 0.144881 |
| GO:0022610\_biological\_adhesion | GPNMB | 186 | 7 | 2.795265 | -1.929631 | 84 | 12.17 | 0.144881 |
| GO:0050767\_regulation\_of\_neurogenesis | BDNF | 104 | 5 | 3.570875 | -1.904616 | 85 | 12.68 | 0.149176 |
| GO:0050767\_regulation\_of\_neurogenesis | RNF6 | 104 | 5 | 3.570875 | -1.904616 | 85 | 12.68 | 0.149176 |
| GO:0050767\_regulation\_of\_neurogenesis | BCL2 | 104 | 5 | 3.570875 | -1.904616 | 85 | 12.68 | 0.149176 |
| GO:0050767\_regulation\_of\_neurogenesis | ID4 | 104 | 5 | 3.570875 | -1.904616 | 85 | 12.68 | 0.149176 |
| GO:0050767\_regulation\_of\_neurogenesis | NEFL | 104 | 5 | 3.570875 | -1.904616 | 85 | 12.68 | 0.149176 |
| GO:0009994\_oocyte\_differentiation | BCL2 | 13 | 2 | 11.426799 | -1.898047 | 87 | 13.49 | 0.155057 |
| GO:0009994\_oocyte\_differentiation | CDC25B | 13 | 2 | 11.426799 | -1.898047 | 87 | 13.49 | 0.155057 |
| GO:0048599\_oocyte\_development | BCL2 | 13 | 2 | 11.426799 | -1.898047 | 87 | 13.49 | 0.155057 |
| GO:0048599\_oocyte\_development | CDC25B | 13 | 2 | 11.426799 | -1.898047 | 87 | 13.49 | 0.155057 |
| GO:0030900\_forebrain\_development | DAB1 | 146 | 6 | 3.052364 | -1.883615 | 88 | 13.7 | 0.155682 |
| GO:0030900\_forebrain\_development | GNAQ | 146 | 6 | 3.052364 | -1.883615 | 88 | 13.7 | 0.155682 |
| GO:0030900\_forebrain\_development | ID4 | 146 | 6 | 3.052364 | -1.883615 | 88 | 13.7 | 0.155682 |
| GO:0030900\_forebrain\_development | NR2F2 | 146 | 6 | 3.052364 | -1.883615 | 88 | 13.7 | 0.155682 |
| GO:0030900\_forebrain\_development | FABP7 | 146 | 6 | 3.052364 | -1.883615 | 88 | 13.7 | 0.155682 |
| GO:0030900\_forebrain\_development | PITX2 | 146 | 6 | 3.052364 | -1.883615 | 88 | 13.7 | 0.155682 |
| GO:0001956\_positive\_regulation\_of\_neurotransmitter\_secretion | SNCA | 1 | 1 |  |  |  |  |  |  |
| GO:0002468\_dendritic\_cell\_antigen\_processing\_and\_presentation | SLC11A1 | 1 | 1 |  |  |  |  |  |  |
| GO:0002577\_regulation\_of\_antigen\_processing\_and\_presentation | SLC11A1 | 1 | 1 |  |  |  |  |  |  |
| GO:0002579\_positive\_regulation\_of\_antigen\_processing\_and\_presentation | SLC11A1 | 1 | 1 |  |  |  |  |  |  |
| GO:0002604\_regulation\_of\_dendritic\_cell\_antigen\_processing\_and\_presentation | SLC11A1 | 1 | 1 |  |  |  |  |  |  |
| GO:0002606\_positive\_regulation\_of\_dendritic\_cell\_antigen\_processing\_and\_presentation | SLC11A1 | 1 | 1 |  |  |  |  |  |  |
| GO:0006000\_fructose\_metabolic\_process | PFKL | 1 | 1 |  |  |  |  |  |  |
| GO:0006002\_fructose\_6-phosphate\_metabolic\_process | PFKL | 1 | 1 |  |  |  |  |  |  |
| GO:0009956\_radial\_pattern\_formation | NR2F2 | 1 | 1 |  |  |  |  |  |  |
| GO:0010523\_negative\_regulation\_of\_calcium\_ion\_transport\_into\_cytosol | BCL2 | 1 | 1 |  |  |  |  |  |  |
| GO:0014012\_axon\_regeneration\_in\_the\_peripheral\_nervous\_system | NEFL | 1 | 1 |  |  |  |  |  |  |
| GO:0014041\_regulation\_of\_neuron\_maturation | BCL2 | 1 | 1 |  |  |  |  |  |  |
| GO:0014042\_positive\_regulation\_of\_neuron\_maturation | BCL2 | 1 | 1 |  |  |  |  |  |  |
| GO:0014910\_regulation\_of\_smooth\_muscle\_cell\_migration | BCL2 | 1 | 1 |  |  |  |  |  |  |
| GO:0014911\_positive\_regulation\_of\_smooth\_muscle\_cell\_migration | BCL2 | 1 | 1 |  |  |  |  |  |  |
| GO:0015707\_nitrite\_transport | SLC11A1 | 1 | 1 |  |  |  |  |  |  |
| GO:0019079\_viral\_genome\_replication | BCL2 | 1 | 1 |  |  |  |  |  |  |
| GO:0021577\_hindbrain\_structural\_organization | DAB1 | 1 | 1 |  |  |  |  |  |  |
| GO:0021589\_cerebellum\_structural\_organization | DAB1 | 1 | 1 |  |  |  |  |  |  |
| GO:0021747\_cochlear\_nucleus\_development | BCL2 | 1 | 1 |  |  |  |  |  |  |
| GO:0021812\_neuronal-glial\_interaction\_involved\_in\_cerebral\_cortex\_radial\_glia\_guided\_migration | DAB1 | 1 | 1 |  |  |  |  |  |  |
| GO:0021813\_cell-cell\_adhesion\_involved\_in\_neuronal-glial\_interactions\_involved\_in\_cerebral\_cortex\_radial\_glia\_guided\_migration | DAB1 | 1 | 1 |  |  |  |  |  |  |
| GO:0021942\_radial\_glia\_guided\_migration\_of\_Purkinje\_cell | DAB1 | 1 | 1 |  |  |  |  |  |  |
| GO:0031129\_inductive\_cell-cell\_signaling | SALL1 | 1 | 1 |  |  |  |  |  |  |
| GO:0033138\_positive\_regulation\_of\_peptidyl-serine\_phosphorylation | BCL2 | 1 | 1 |  |  |  |  |  |  |
| GO:0033687\_osteoblast\_proliferation | BCL2 | 1 | 1 |  |  |  |  |  |  |
| GO:0033688\_regulation\_of\_osteoblast\_proliferation | BCL2 | 1 | 1 |  |  |  |  |  |  |
| GO:0033689\_negative\_regulation\_of\_osteoblast\_proliferation | BCL2 | 1 | 1 |  |  |  |  |  |  |
| GO:0043091\_L-arginine\_import | SLC11A1 | 1 | 1 |  |  |  |  |  |  |
| GO:0043369\_CD4-positive\_or\_CD8-positive\_\_alpha-beta\_T\_cell\_lineage\_commitment | BCL2 | 1 | 1 |  |  |  |  |  |  |
| GO:0043375\_CD8-positive\_\_alpha-beta\_T\_cell\_lineage\_commitment | BCL2 | 1 | 1 |  |  |  |  |  |  |
| GO:0045069\_regulation\_of\_viral\_genome\_replication | BCL2 | 1 | 1 |  |  |  |  |  |  |
| GO:0045898\_regulation\_of\_transcriptional\_preinitiation\_complex\_assembly | AHR | 1 | 1 |  |  |  |  |  |  |
| GO:0045899\_positive\_regulation\_of\_transcriptional\_preinitiation\_complex\_assembly | AHR | 1 | 1 |  |  |  |  |  |  |
| GO:0046671\_negative\_regulation\_of\_retinal\_cell\_programmed\_cell\_death | BCL2 | 1 | 1 |  |  |  |  |  |  |
| GO:0046949\_acyl-CoA\_biosynthetic\_process | SNCA | 1 | 1 |  |  |  |  |  |  |
| GO:0048743\_positive\_regulation\_of\_skeletal\_muscle\_fiber\_development | BCL2 | 1 | 1 |  |  |  |  |  |  |
| GO:0050812\_regulation\_of\_acyl-CoA\_biosynthetic\_process | SNCA | 1 | 1 |  |  |  |  |  |  |
| GO:0051123\_transcriptional\_preinitiation\_complex\_assembly | AHR | 1 | 1 |  |  |  |  |  |  |
| GO:0051193\_regulation\_of\_cofactor\_metabolic\_process | SNCA | 1 | 1 |  |  |  |  |  |  |
| GO:0051196\_regulation\_of\_coenzyme\_metabolic\_process | SNCA | 1 | 1 |  |  |  |  |  |  |
| GO:0060215\_primitive\_hemopoiesis | VEGFA | 1 | 1 |  |  |  |  |  |  |
| GO:0060319\_primitive\_erythrocyte\_differentiation | VEGFA | 1 | 1 |  |  |  |  |  |  |
| GO:0060577\_pulmonary\_vein\_morphogenesis | PITX2 | 1 | 1 |  |  |  |  |  |  |
| GO:0060578\_superior\_vena\_cava\_morphogenesis | PITX2 | 1 | 1 |  |  |  |  |  |  |
| GO:0043085\_positive\_regulation\_of\_catalytic\_activity | SLC11A1 | 148 | 6 | 3.011116 | -1.856559 | 89 | 13.86 | 0.155730 |
| GO:0043085\_positive\_regulation\_of\_catalytic\_activity | DAB1 | 148 | 6 | 3.011116 | -1.856559 | 89 | 13.86 | 0.155730 |
| GO:0043085\_positive\_regulation\_of\_catalytic\_activity | GNAQ | 148 | 6 | 3.011116 | -1.856559 | 89 | 13.86 | 0.155730 |
| GO:0043085\_positive\_regulation\_of\_catalytic\_activity | BCL2 | 148 | 6 | 3.011116 | -1.856559 | 89 | 13.86 | 0.155730 |
| GO:0043085\_positive\_regulation\_of\_catalytic\_activity | KITL | 148 | 6 | 3.011116 | -1.856559 | 89 | 13.86 | 0.155730 |
| GO:0043085\_positive\_regulation\_of\_catalytic\_activity | CDC25B | 148 | 6 | 3.011116 | -1.856559 | 89 | 13.86 | 0.155730 |
| GO:0045859\_regulation\_of\_protein\_kinase\_activity | SLC11A1 | 107 | 5 | 3.470757 | -1.854967 | 90 | 13.86 | 0.154000 |
| GO:0045859\_regulation\_of\_protein\_kinase\_activity | SPRY1 | 107 | 5 | 3.470757 | -1.854967 | 90 | 13.86 | 0.154000 |
| GO:0045859\_regulation\_of\_protein\_kinase\_activity | DAB1 | 107 | 5 | 3.470757 | -1.854967 | 90 | 13.86 | 0.154000 |
| GO:0045859\_regulation\_of\_protein\_kinase\_activity | KITL | 107 | 5 | 3.470757 | -1.854967 | 90 | 13.86 | 0.154000 |
| GO:0045859\_regulation\_of\_protein\_kinase\_activity | CDC25B | 107 | 5 | 3.470757 | -1.854967 | 90 | 13.86 | 0.154000 |
| GO:0016053\_organic\_acid\_biosynthetic\_process | SCD1 | 38 | 3 | 5.863752 | -1.853019 | 94 | 14.02 | 0.149149 |
| GO:0016053\_organic\_acid\_biosynthetic\_process | ALDH18A1 | 38 | 3 | 5.863752 | -1.853019 | 94 | 14.02 | 0.149149 |
| GO:0016053\_organic\_acid\_biosynthetic\_process | SNCA | 38 | 3 | 5.863752 | -1.853019 | 94 | 14.02 | 0.149149 |
| GO:0031401\_positive\_regulation\_of\_protein\_modification\_process | HMGB1 | 38 | 3 | 5.863752 | -1.853019 | 94 | 14.02 | 0.149149 |
| GO:0031401\_positive\_regulation\_of\_protein\_modification\_process | BCL2 | 38 | 3 | 5.863752 | -1.853019 | 94 | 14.02 | 0.149149 |
| GO:0031401\_positive\_regulation\_of\_protein\_modification\_process | KITL | 38 | 3 | 5.863752 | -1.853019 | 94 | 14.02 | 0.149149 |
| GO:0042493\_response\_to\_drug | BDNF | 38 | 3 | 5.863752 | -1.853019 | 94 | 14.02 | 0.149149 |
| GO:0042493\_response\_to\_drug | BCL2 | 38 | 3 | 5.863752 | -1.853019 | 94 | 14.02 | 0.149149 |
| GO:0042493\_response\_to\_drug | SNCA | 38 | 3 | 5.863752 | -1.853019 | 94 | 14.02 | 0.149149 |
| GO:0046394\_carboxylic\_acid\_biosynthetic\_process | SCD1 | 38 | 3 | 5.863752 | -1.853019 | 94 | 14.02 | 0.149149 |
| GO:0046394\_carboxylic\_acid\_biosynthetic\_process | ALDH18A1 | 38 | 3 | 5.863752 | -1.853019 | 94 | 14.02 | 0.149149 |
| GO:0046394\_carboxylic\_acid\_biosynthetic\_process | SNCA | 38 | 3 | 5.863752 | -1.853019 | 94 | 14.02 | 0.149149 |
| GO:0000060\_protein\_import\_into\_nucleus\_\_translocation | SLC11A1 | 14 | 2 | 10.610599 | -1.834851 | 98 | 15.07 | 0.153776 |
| GO:0000060\_protein\_import\_into\_nucleus\_\_translocation | TOB1 | 14 | 2 | 10.610599 | -1.834851 | 98 | 15.07 | 0.153776 |
| GO:0019217\_regulation\_of\_fatty\_acid\_metabolic\_process | SNCA | 14 | 2 | 10.610599 | -1.834851 | 98 | 15.07 | 0.153776 |
| GO:0019217\_regulation\_of\_fatty\_acid\_metabolic\_process | ACSL4 | 14 | 2 | 10.610599 | -1.834851 | 98 | 15.07 | 0.153776 |
| GO:0031099\_regeneration | BCL2 | 14 | 2 | 10.610599 | -1.834851 | 98 | 15.07 | 0.153776 |
| GO:0031099\_regeneration | NEFL | 14 | 2 | 10.610599 | -1.834851 | 98 | 15.07 | 0.153776 |
| GO:0048545\_response\_to\_steroid\_hormone\_stimulus | HMGB1 | 14 | 2 | 10.610599 | -1.834851 | 98 | 15.07 | 0.153776 |
| GO:0048545\_response\_to\_steroid\_hormone\_stimulus | BCL2 | 14 | 2 | 10.610599 | -1.834851 | 98 | 15.07 | 0.153776 |
| GO:0033674\_positive\_regulation\_of\_kinase\_activity | SLC11A1 | 71 | 4 | 4.184462 | -1.830398 | 99 | 15.11 | 0.152626 |
| GO:0033674\_positive\_regulation\_of\_kinase\_activity | DAB1 | 71 | 4 | 4.184462 | -1.830398 | 99 | 15.11 | 0.152626 |
| GO:0033674\_positive\_regulation\_of\_kinase\_activity | KITL | 71 | 4 | 4.184462 | -1.830398 | 99 | 15.11 | 0.152626 |
| GO:0033674\_positive\_regulation\_of\_kinase\_activity | CDC25B | 71 | 4 | 4.184462 | -1.830398 | 99 | 15.11 | 0.152626 |
| GO:0051347\_positive\_regulation\_of\_transferase\_activity | SLC11A1 | 72 | 4 | 4.126344 | -1.809891 | 100 | 15.67 | 0.156700 |
| GO:0051347\_positive\_regulation\_of\_transferase\_activity | DAB1 | 72 | 4 | 4.126344 | -1.809891 | 100 | 15.67 | 0.156700 |
| GO:0051347\_positive\_regulation\_of\_transferase\_activity | KITL | 72 | 4 | 4.126344 | -1.809891 | 100 | 15.67 | 0.156700 |
| GO:0051347\_positive\_regulation\_of\_transferase\_activity | CDC25B | 72 | 4 | 4.126344 | -1.809891 | 100 | 15.67 | 0.156700 |
| GO:0045595\_regulation\_of\_cell\_differentiation | HMGB1 | 295 | 9 | 2.265992 | -1.791857 | 101 | 16.02 | 0.158614 |
| GO:0045595\_regulation\_of\_cell\_differentiation | RNF6 | 295 | 9 | 2.265992 | -1.791857 | 101 | 16.02 | 0.158614 |
| GO:0045595\_regulation\_of\_cell\_differentiation | BDNF | 295 | 9 | 2.265992 | -1.791857 | 101 | 16.02 | 0.158614 |
| GO:0045595\_regulation\_of\_cell\_differentiation | GNAQ | 295 | 9 | 2.265992 | -1.791857 | 101 | 16.02 | 0.158614 |
| GO:0045595\_regulation\_of\_cell\_differentiation | BCL2 | 295 | 9 | 2.265992 | -1.791857 | 101 | 16.02 | 0.158614 |
| GO:0045595\_regulation\_of\_cell\_differentiation | ID4 | 295 | 9 | 2.265992 | -1.791857 | 101 | 16.02 | 0.158614 |
| GO:0045595\_regulation\_of\_cell\_differentiation | KITL | 295 | 9 | 2.265992 | -1.791857 | 101 | 16.02 | 0.158614 |
| GO:0045595\_regulation\_of\_cell\_differentiation | NEFL | 295 | 9 | 2.265992 | -1.791857 | 101 | 16.02 | 0.158614 |
| GO:0045595\_regulation\_of\_cell\_differentiation | TOB1 | 295 | 9 | 2.265992 | -1.791857 | 101 | 16.02 | 0.158614 |
| GO:0000904\_cell\_morphogenesis\_involved\_in\_differentiation | NRCAM | 199 | 7 | 2.612660 | -1.780153 | 102 | 16.15 | 0.158333 |
| GO:0000904\_cell\_morphogenesis\_involved\_in\_differentiation | SEMA5A | 199 | 7 | 2.612660 | -1.780153 | 102 | 16.15 | 0.158333 |
| GO:0000904\_cell\_morphogenesis\_involved\_in\_differentiation | BDNF | 199 | 7 | 2.612660 | -1.780153 | 102 | 16.15 | 0.158333 |
| GO:0000904\_cell\_morphogenesis\_involved\_in\_differentiation | RNF6 | 199 | 7 | 2.612660 | -1.780153 | 102 | 16.15 | 0.158333 |
| GO:0000904\_cell\_morphogenesis\_involved\_in\_differentiation | BCL2 | 199 | 7 | 2.612660 | -1.780153 | 102 | 16.15 | 0.158333 |
| GO:0000904\_cell\_morphogenesis\_involved\_in\_differentiation | NEFL | 199 | 7 | 2.612660 | -1.780153 | 102 | 16.15 | 0.158333 |
| GO:0000904\_cell\_morphogenesis\_involved\_in\_differentiation | EPHB1 | 199 | 7 | 2.612660 | -1.780153 | 102 | 16.15 | 0.158333 |
| GO:0006885\_regulation\_of\_pH | SLC11A1 | 15 | 2 | 9.903226 | -1.776451 | 103 | 17.07 | 0.165728 |
| GO:0006885\_regulation\_of\_pH | SLC4A4 | 15 | 2 | 9.903226 | -1.776451 | 103 | 17.07 | 0.165728 |
| GO:0043549\_regulation\_of\_kinase\_activity | SLC11A1 | 112 | 5 | 3.315812 | -1.776096 | 104 | 17.09 | 0.164327 |
| GO:0043549\_regulation\_of\_kinase\_activity | SPRY1 | 112 | 5 | 3.315812 | -1.776096 | 104 | 17.09 | 0.164327 |
| GO:0043549\_regulation\_of\_kinase\_activity | DAB1 | 112 | 5 | 3.315812 | -1.776096 | 104 | 17.09 | 0.164327 |
| GO:0043549\_regulation\_of\_kinase\_activity | KITL | 112 | 5 | 3.315812 | -1.776096 | 104 | 17.09 | 0.164327 |
| GO:0043549\_regulation\_of\_kinase\_activity | CDC25B | 112 | 5 | 3.315812 | -1.776096 | 104 | 17.09 | 0.164327 |
| GO:0032879\_regulation\_of\_localization | HMGB1 | 248 | 8 | 2.395942 | -1.766834 | 105 | 17.15 | 0.163333 |
| GO:0032879\_regulation\_of\_localization | SLC11A1 | 248 | 8 | 2.395942 | -1.766834 | 105 | 17.15 | 0.163333 |
| GO:0032879\_regulation\_of\_localization | IRS2 | 248 | 8 | 2.395942 | -1.766834 | 105 | 17.15 | 0.163333 |
| GO:0032879\_regulation\_of\_localization | PFKL | 248 | 8 | 2.395942 | -1.766834 | 105 | 17.15 | 0.163333 |
| GO:0032879\_regulation\_of\_localization | BCL2 | 248 | 8 | 2.395942 | -1.766834 | 105 | 17.15 | 0.163333 |
| GO:0032879\_regulation\_of\_localization | ONECUT2 | 248 | 8 | 2.395942 | -1.766834 | 105 | 17.15 | 0.163333 |
| GO:0032879\_regulation\_of\_localization | SNCA | 248 | 8 | 2.395942 | -1.766834 | 105 | 17.15 | 0.163333 |
| GO:0032879\_regulation\_of\_localization | PITX2 | 248 | 8 | 2.395942 | -1.766834 | 105 | 17.15 | 0.163333 |
| GO:0051338\_regulation\_of\_transferase\_activity | SLC11A1 | 115 | 5 | 3.229313 | -1.730944 | 106 | 18.08 | 0.170566 |
| GO:0051338\_regulation\_of\_transferase\_activity | SPRY1 | 115 | 5 | 3.229313 | -1.730944 | 106 | 18.08 | 0.170566 |
| GO:0051338\_regulation\_of\_transferase\_activity | DAB1 | 115 | 5 | 3.229313 | -1.730944 | 106 | 18.08 | 0.170566 |
| GO:0051338\_regulation\_of\_transferase\_activity | KITL | 115 | 5 | 3.229313 | -1.730944 | 106 | 18.08 | 0.170566 |
| GO:0051338\_regulation\_of\_transferase\_activity | CDC25B | 115 | 5 | 3.229313 | -1.730944 | 106 | 18.08 | 0.170566 |
| GO:0042596\_fear\_response | BDNF | 16 | 2 | 9.284274 | -1.722202 | 108 | 18.94 | 0.175370 |
| GO:0042596\_fear\_response | BCL2 | 16 | 2 | 9.284274 | -1.722202 | 108 | 18.94 | 0.175370 |
| GO:0051937\_catecholamine\_transport | CADPS | 16 | 2 | 9.284274 | -1.722202 | 108 | 18.94 | 0.175370 |
| GO:0051937\_catecholamine\_transport | SNCA | 16 | 2 | 9.284274 | -1.722202 | 108 | 18.94 | 0.175370 |
| GO:0048731\_system\_development | HMGB1 | 1609 | 30 | 1.384851 | -1.714370 | 109 | 19.04 | 0.174679 |
| GO:0048731\_system\_development | LTBP3 | 1609 | 30 | 1.384851 | -1.714370 | 109 | 19.04 | 0.174679 |
| GO:0048731\_system\_development | ONECUT2 | 1609 | 30 | 1.384851 | -1.714370 | 109 | 19.04 | 0.174679 |
| GO:0048731\_system\_development | RORB | 1609 | 30 | 1.384851 | -1.714370 | 109 | 19.04 | 0.174679 |
| GO:0048731\_system\_development | EPHB1 | 1609 | 30 | 1.384851 | -1.714370 | 109 | 19.04 | 0.174679 |
| GO:0048731\_system\_development | SEMA5A | 1609 | 30 | 1.384851 | -1.714370 | 109 | 19.04 | 0.174679 |
| GO:0048731\_system\_development | NRCAM | 1609 | 30 | 1.384851 | -1.714370 | 109 | 19.04 | 0.174679 |
| GO:0048731\_system\_development | EFHD1 | 1609 | 30 | 1.384851 | -1.714370 | 109 | 19.04 | 0.174679 |
| GO:0048731\_system\_development | SPRY1 | 1609 | 30 | 1.384851 | -1.714370 | 109 | 19.04 | 0.174679 |
| GO:0048731\_system\_development | BDNF | 1609 | 30 | 1.384851 | -1.714370 | 109 | 19.04 | 0.174679 |
| GO:0048731\_system\_development | DAB1 | 1609 | 30 | 1.384851 | -1.714370 | 109 | 19.04 | 0.174679 |
| GO:0048731\_system\_development | BCL2 | 1609 | 30 | 1.384851 | -1.714370 | 109 | 19.04 | 0.174679 |
| GO:0048731\_system\_development | MYB | 1609 | 30 | 1.384851 | -1.714370 | 109 | 19.04 | 0.174679 |
| GO:0048731\_system\_development | NR2F2 | 1609 | 30 | 1.384851 | -1.714370 | 109 | 19.04 | 0.174679 |
| GO:0048731\_system\_development | NEFL | 1609 | 30 | 1.384851 | -1.714370 | 109 | 19.04 | 0.174679 |
| GO:0048731\_system\_development | MAP2K5 | 1609 | 30 | 1.384851 | -1.714370 | 109 | 19.04 | 0.174679 |
| GO:0048731\_system\_development | PITX2 | 1609 | 30 | 1.384851 | -1.714370 | 109 | 19.04 | 0.174679 |
| GO:0048731\_system\_development | IRS2 | 1609 | 30 | 1.384851 | -1.714370 | 109 | 19.04 | 0.174679 |
| GO:0048731\_system\_development | COL13A1 | 1609 | 30 | 1.384851 | -1.714370 | 109 | 19.04 | 0.174679 |
| GO:0048731\_system\_development | KITL | 1609 | 30 | 1.384851 | -1.714370 | 109 | 19.04 | 0.174679 |
| GO:0048731\_system\_development | AHR | 1609 | 30 | 1.384851 | -1.714370 | 109 | 19.04 | 0.174679 |
| GO:0048731\_system\_development | RNF6 | 1609 | 30 | 1.384851 | -1.714370 | 109 | 19.04 | 0.174679 |
| GO:0048731\_system\_development | GNAQ | 1609 | 30 | 1.384851 | -1.714370 | 109 | 19.04 | 0.174679 |
| GO:0048731\_system\_development | PKP2 | 1609 | 30 | 1.384851 | -1.714370 | 109 | 19.04 | 0.174679 |
| GO:0048731\_system\_development | SALL1 | 1609 | 30 | 1.384851 | -1.714370 | 109 | 19.04 | 0.174679 |
| GO:0048731\_system\_development | VEGFA | 1609 | 30 | 1.384851 | -1.714370 | 109 | 19.04 | 0.174679 |
| GO:0048731\_system\_development | NTRK2 | 1609 | 30 | 1.384851 | -1.714370 | 109 | 19.04 | 0.174679 |
| GO:0048731\_system\_development | ID4 | 1609 | 30 | 1.384851 | -1.714370 | 109 | 19.04 | 0.174679 |
| GO:0048731\_system\_development | FABP7 | 1609 | 30 | 1.384851 | -1.714370 | 109 | 19.04 | 0.174679 |
| GO:0048731\_system\_development | TOB1 | 1609 | 30 | 1.384851 | -1.714370 | 109 | 19.04 | 0.174679 |
| GO:0051960\_regulation\_of\_nervous\_system\_development | BDNF | 118 | 5 | 3.147212 | -1.687314 | 110 | 19.62 | 0.178364 |
| GO:0051960\_regulation\_of\_nervous\_system\_development | RNF6 | 118 | 5 | 3.147212 | -1.687314 | 110 | 19.62 | 0.178364 |
| GO:0051960\_regulation\_of\_nervous\_system\_development | BCL2 | 118 | 5 | 3.147212 | -1.687314 | 110 | 19.62 | 0.178364 |
| GO:0051960\_regulation\_of\_nervous\_system\_development | ID4 | 118 | 5 | 3.147212 | -1.687314 | 110 | 19.62 | 0.178364 |
| GO:0051960\_regulation\_of\_nervous\_system\_development | NEFL | 118 | 5 | 3.147212 | -1.687314 | 110 | 19.62 | 0.178364 |
| GO:0008284\_positive\_regulation\_of\_cell\_proliferation | SUZ12 | 208 | 7 | 2.499612 | -1.684590 | 111 | 19.62 | 0.176757 |
| GO:0008284\_positive\_regulation\_of\_cell\_proliferation | HMGB1 | 208 | 7 | 2.499612 | -1.684590 | 111 | 19.62 | 0.176757 |
| GO:0008284\_positive\_regulation\_of\_cell\_proliferation | IRS2 | 208 | 7 | 2.499612 | -1.684590 | 111 | 19.62 | 0.176757 |
| GO:0008284\_positive\_regulation\_of\_cell\_proliferation | BCL2 | 208 | 7 | 2.499612 | -1.684590 | 111 | 19.62 | 0.176757 |
| GO:0008284\_positive\_regulation\_of\_cell\_proliferation | VEGFA | 208 | 7 | 2.499612 | -1.684590 | 111 | 19.62 | 0.176757 |
| GO:0008284\_positive\_regulation\_of\_cell\_proliferation | ID4 | 208 | 7 | 2.499612 | -1.684590 | 111 | 19.62 | 0.176757 |
| GO:0008284\_positive\_regulation\_of\_cell\_proliferation | KITL | 208 | 7 | 2.499612 | -1.684590 | 111 | 19.62 | 0.176757 |
| GO:0048856\_anatomical\_structure\_development | HMGB1 | 1688 | 31 | 1.364040 | -1.679779 | 112 | 19.99 | 0.178482 |
| GO:0048856\_anatomical\_structure\_development | PARD3 | 1688 | 31 | 1.364040 | -1.679779 | 112 | 19.99 | 0.178482 |
| GO:0048856\_anatomical\_structure\_development | LTBP3 | 1688 | 31 | 1.364040 | -1.679779 | 112 | 19.99 | 0.178482 |
| GO:0048856\_anatomical\_structure\_development | ONECUT2 | 1688 | 31 | 1.364040 | -1.679779 | 112 | 19.99 | 0.178482 |
| GO:0048856\_anatomical\_structure\_development | RORB | 1688 | 31 | 1.364040 | -1.679779 | 112 | 19.99 | 0.178482 |
| GO:0048856\_anatomical\_structure\_development | EPHB1 | 1688 | 31 | 1.364040 | -1.679779 | 112 | 19.99 | 0.178482 |
| GO:0048856\_anatomical\_structure\_development | SEMA5A | 1688 | 31 | 1.364040 | -1.679779 | 112 | 19.99 | 0.178482 |
| GO:0048856\_anatomical\_structure\_development | NRCAM | 1688 | 31 | 1.364040 | -1.679779 | 112 | 19.99 | 0.178482 |
| GO:0048856\_anatomical\_structure\_development | EFHD1 | 1688 | 31 | 1.364040 | -1.679779 | 112 | 19.99 | 0.178482 |
| GO:0048856\_anatomical\_structure\_development | SPRY1 | 1688 | 31 | 1.364040 | -1.679779 | 112 | 19.99 | 0.178482 |
| GO:0048856\_anatomical\_structure\_development | BDNF | 1688 | 31 | 1.364040 | -1.679779 | 112 | 19.99 | 0.178482 |
| GO:0048856\_anatomical\_structure\_development | DAB1 | 1688 | 31 | 1.364040 | -1.679779 | 112 | 19.99 | 0.178482 |
| GO:0048856\_anatomical\_structure\_development | BCL2 | 1688 | 31 | 1.364040 | -1.679779 | 112 | 19.99 | 0.178482 |
| GO:0048856\_anatomical\_structure\_development | MYB | 1688 | 31 | 1.364040 | -1.679779 | 112 | 19.99 | 0.178482 |
| GO:0048856\_anatomical\_structure\_development | NR2F2 | 1688 | 31 | 1.364040 | -1.679779 | 112 | 19.99 | 0.178482 |
| GO:0048856\_anatomical\_structure\_development | NEFL | 1688 | 31 | 1.364040 | -1.679779 | 112 | 19.99 | 0.178482 |
| GO:0048856\_anatomical\_structure\_development | PITX2 | 1688 | 31 | 1.364040 | -1.679779 | 112 | 19.99 | 0.178482 |
| GO:0048856\_anatomical\_structure\_development | MAP2K5 | 1688 | 31 | 1.364040 | -1.679779 | 112 | 19.99 | 0.178482 |
| GO:0048856\_anatomical\_structure\_development | IRS2 | 1688 | 31 | 1.364040 | -1.679779 | 112 | 19.99 | 0.178482 |
| GO:0048856\_anatomical\_structure\_development | COL13A1 | 1688 | 31 | 1.364040 | -1.679779 | 112 | 19.99 | 0.178482 |
| GO:0048856\_anatomical\_structure\_development | KITL | 1688 | 31 | 1.364040 | -1.679779 | 112 | 19.99 | 0.178482 |
| GO:0048856\_anatomical\_structure\_development | AHR | 1688 | 31 | 1.364040 | -1.679779 | 112 | 19.99 | 0.178482 |
| GO:0048856\_anatomical\_structure\_development | RNF6 | 1688 | 31 | 1.364040 | -1.679779 | 112 | 19.99 | 0.178482 |
| GO:0048856\_anatomical\_structure\_development | GNAQ | 1688 | 31 | 1.364040 | -1.679779 | 112 | 19.99 | 0.178482 |
| GO:0048856\_anatomical\_structure\_development | PKP2 | 1688 | 31 | 1.364040 | -1.679779 | 112 | 19.99 | 0.178482 |
| GO:0048856\_anatomical\_structure\_development | SALL1 | 1688 | 31 | 1.364040 | -1.679779 | 112 | 19.99 | 0.178482 |
| GO:0048856\_anatomical\_structure\_development | NTRK2 | 1688 | 31 | 1.364040 | -1.679779 | 112 | 19.99 | 0.178482 |
| GO:0048856\_anatomical\_structure\_development | VEGFA | 1688 | 31 | 1.364040 | -1.679779 | 112 | 19.99 | 0.178482 |
| GO:0048856\_anatomical\_structure\_development | ID4 | 1688 | 31 | 1.364040 | -1.679779 | 112 | 19.99 | 0.178482 |
| GO:0048856\_anatomical\_structure\_development | FABP7 | 1688 | 31 | 1.364040 | -1.679779 | 112 | 19.99 | 0.178482 |
| GO:0048856\_anatomical\_structure\_development | TOB1 | 1688 | 31 | 1.364040 | -1.679779 | 112 | 19.99 | 0.178482 |
| GO:0031323\_regulation\_of\_cellular\_metabolic\_process | HMGB1 | 1015 | 21 | 1.536707 | -1.676938 | 113 | 20.02 | 0.177168 |
| GO:0031323\_regulation\_of\_cellular\_metabolic\_process | SNCA | 1015 | 21 | 1.536707 | -1.676938 | 113 | 20.02 | 0.177168 |
| GO:0031323\_regulation\_of\_cellular\_metabolic\_process | ONECUT2 | 1015 | 21 | 1.536707 | -1.676938 | 113 | 20.02 | 0.177168 |
| GO:0031323\_regulation\_of\_cellular\_metabolic\_process | RORB | 1015 | 21 | 1.536707 | -1.676938 | 113 | 20.02 | 0.177168 |
| GO:0031323\_regulation\_of\_cellular\_metabolic\_process | KITL | 1015 | 21 | 1.536707 | -1.676938 | 113 | 20.02 | 0.177168 |
| GO:0031323\_regulation\_of\_cellular\_metabolic\_process | AHR | 1015 | 21 | 1.536707 | -1.676938 | 113 | 20.02 | 0.177168 |
| GO:0031323\_regulation\_of\_cellular\_metabolic\_process | CDC25B | 1015 | 21 | 1.536707 | -1.676938 | 113 | 20.02 | 0.177168 |
| GO:0031323\_regulation\_of\_cellular\_metabolic\_process | SUZ12 | 1015 | 21 | 1.536707 | -1.676938 | 113 | 20.02 | 0.177168 |
| GO:0031323\_regulation\_of\_cellular\_metabolic\_process | SLC11A1 | 1015 | 21 | 1.536707 | -1.676938 | 113 | 20.02 | 0.177168 |
| GO:0031323\_regulation\_of\_cellular\_metabolic\_process | SPRY1 | 1015 | 21 | 1.536707 | -1.676938 | 113 | 20.02 | 0.177168 |
| GO:0031323\_regulation\_of\_cellular\_metabolic\_process | RNF6 | 1015 | 21 | 1.536707 | -1.676938 | 113 | 20.02 | 0.177168 |
| GO:0031323\_regulation\_of\_cellular\_metabolic\_process | DAB1 | 1015 | 21 | 1.536707 | -1.676938 | 113 | 20.02 | 0.177168 |
| GO:0031323\_regulation\_of\_cellular\_metabolic\_process | GNAQ | 1015 | 21 | 1.536707 | -1.676938 | 113 | 20.02 | 0.177168 |
| GO:0031323\_regulation\_of\_cellular\_metabolic\_process | BCL2 | 1015 | 21 | 1.536707 | -1.676938 | 113 | 20.02 | 0.177168 |
| GO:0031323\_regulation\_of\_cellular\_metabolic\_process | GARNL1 | 1015 | 21 | 1.536707 | -1.676938 | 113 | 20.02 | 0.177168 |
| GO:0031323\_regulation\_of\_cellular\_metabolic\_process | NFAT5 | 1015 | 21 | 1.536707 | -1.676938 | 113 | 20.02 | 0.177168 |
| GO:0031323\_regulation\_of\_cellular\_metabolic\_process | MYB | 1015 | 21 | 1.536707 | -1.676938 | 113 | 20.02 | 0.177168 |
| GO:0031323\_regulation\_of\_cellular\_metabolic\_process | ACSL4 | 1015 | 21 | 1.536707 | -1.676938 | 113 | 20.02 | 0.177168 |
| GO:0031323\_regulation\_of\_cellular\_metabolic\_process | NR2F2 | 1015 | 21 | 1.536707 | -1.676938 | 113 | 20.02 | 0.177168 |
| GO:0031323\_regulation\_of\_cellular\_metabolic\_process | ETV3 | 1015 | 21 | 1.536707 | -1.676938 | 113 | 20.02 | 0.177168 |
| GO:0031323\_regulation\_of\_cellular\_metabolic\_process | PITX2 | 1015 | 21 | 1.536707 | -1.676938 | 113 | 20.02 | 0.177168 |
| GO:0010565\_regulation\_of\_cellular\_ketone\_metabolic\_process | SNCA | 17 | 2 | 8.738140 | -1.671584 | 116 | 20.94 | 0.180517 |
| GO:0010565\_regulation\_of\_cellular\_ketone\_metabolic\_process | ACSL4 | 17 | 2 | 8.738140 | -1.671584 | 116 | 20.94 | 0.180517 |
| GO:0016052\_carbohydrate\_catabolic\_process | HMGB1 | 17 | 2 | 8.738140 | -1.671584 | 116 | 20.94 | 0.180517 |
| GO:0016052\_carbohydrate\_catabolic\_process | PFKL | 17 | 2 | 8.738140 | -1.671584 | 116 | 20.94 | 0.180517 |
| GO:0055067\_monovalent\_inorganic\_cation\_homeostasis | SLC11A1 | 17 | 2 | 8.738140 | -1.671584 | 116 | 20.94 | 0.180517 |
| GO:0055067\_monovalent\_inorganic\_cation\_homeostasis | SLC4A4 | 17 | 2 | 8.738140 | -1.671584 | 116 | 20.94 | 0.180517 |
| GO:0000278\_mitotic\_cell\_cycle | HMGB1 | 80 | 4 | 3.713710 | -1.657608 | 118 | 21.12 | 0.178983 |
| GO:0000278\_mitotic\_cell\_cycle | BCL2 | 80 | 4 | 3.713710 | -1.657608 | 118 | 21.12 | 0.178983 |
| GO:0000278\_mitotic\_cell\_cycle | ID4 | 80 | 4 | 3.713710 | -1.657608 | 118 | 21.12 | 0.178983 |
| GO:0000278\_mitotic\_cell\_cycle | MYB | 80 | 4 | 3.713710 | -1.657608 | 118 | 21.12 | 0.178983 |
| GO:0006631\_fatty\_acid\_metabolic\_process | SCD1 | 80 | 4 | 3.713710 | -1.657608 | 118 | 21.12 | 0.178983 |
| GO:0006631\_fatty\_acid\_metabolic\_process | ACOT10 | 80 | 4 | 3.713710 | -1.657608 | 118 | 21.12 | 0.178983 |
| GO:0006631\_fatty\_acid\_metabolic\_process | SNCA | 80 | 4 | 3.713710 | -1.657608 | 118 | 21.12 | 0.178983 |
| GO:0006631\_fatty\_acid\_metabolic\_process | ACSL4 | 80 | 4 | 3.713710 | -1.657608 | 118 | 21.12 | 0.178983 |
| GO:0021700\_developmental\_maturation | GNAQ | 81 | 4 | 3.667861 | -1.639917 | 119 | 21.43 | 0.180084 |
| GO:0021700\_developmental\_maturation | BCL2 | 81 | 4 | 3.667861 | -1.639917 | 119 | 21.43 | 0.180084 |
| GO:0021700\_developmental\_maturation | VEGFA | 81 | 4 | 3.667861 | -1.639917 | 119 | 21.43 | 0.180084 |
| GO:0021700\_developmental\_maturation | CDC25B | 81 | 4 | 3.667861 | -1.639917 | 119 | 21.43 | 0.180084 |
| GO:0060284\_regulation\_of\_cell\_development | RNF6 | 122 | 5 | 3.044024 | -1.631376 | 120 | 21.61 | 0.180083 |
| GO:0060284\_regulation\_of\_cell\_development | BDNF | 122 | 5 | 3.044024 | -1.631376 | 120 | 21.61 | 0.180083 |
| GO:0060284\_regulation\_of\_cell\_development | BCL2 | 122 | 5 | 3.044024 | -1.631376 | 120 | 21.61 | 0.180083 |
| GO:0060284\_regulation\_of\_cell\_development | ID4 | 122 | 5 | 3.044024 | -1.631376 | 120 | 21.61 | 0.180083 |
| GO:0060284\_regulation\_of\_cell\_development | NEFL | 122 | 5 | 3.044024 | -1.631376 | 120 | 21.61 | 0.180083 |
| GO:0003014\_renal\_system\_process | BCL2 | 18 | 2 | 8.252688 | -1.624168 | 121 | 23.12 | 0.191074 |
| GO:0003014\_renal\_system\_process | CHRNB4 | 18 | 2 | 8.252688 | -1.624168 | 121 | 23.12 | 0.191074 |
| GO:0007411\_axon\_guidance | NRCAM | 82 | 4 | 3.623131 | -1.622499 | 122 | 23.21 | 0.190246 |
| GO:0007411\_axon\_guidance | SEMA5A | 82 | 4 | 3.623131 | -1.622499 | 122 | 23.21 | 0.190246 |
| GO:0007411\_axon\_guidance | BDNF | 82 | 4 | 3.623131 | -1.622499 | 122 | 23.21 | 0.190246 |
| GO:0007411\_axon\_guidance | EPHB1 | 82 | 4 | 3.623131 | -1.622499 | 122 | 23.21 | 0.190246 |
| GO:0048871\_multicellular\_organismal\_homeostasis | SLC11A1 | 47 | 3 | 4.740906 | -1.606248 | 123 | 23.53 | 0.191301 |
| GO:0048871\_multicellular\_organismal\_homeostasis | BCL2 | 47 | 3 | 4.740906 | -1.606248 | 123 | 23.53 | 0.191301 |
| GO:0048871\_multicellular\_organismal\_homeostasis | VEGFA | 47 | 3 | 4.740906 | -1.606248 | 123 | 23.53 | 0.191301 |
| GO:0010033\_response\_to\_organic\_substance | HMGB1 | 216 | 7 | 2.407034 | -1.604560 | 124 | 23.69 | 0.191048 |
| GO:0010033\_response\_to\_organic\_substance | SLC11A1 | 216 | 7 | 2.407034 | -1.604560 | 124 | 23.69 | 0.191048 |
| GO:0010033\_response\_to\_organic\_substance | IRS2 | 216 | 7 | 2.407034 | -1.604560 | 124 | 23.69 | 0.191048 |
| GO:0010033\_response\_to\_organic\_substance | PFKL | 216 | 7 | 2.407034 | -1.604560 | 124 | 23.69 | 0.191048 |
| GO:0010033\_response\_to\_organic\_substance | BCL2 | 216 | 7 | 2.407034 | -1.604560 | 124 | 23.69 | 0.191048 |
| GO:0010033\_response\_to\_organic\_substance | CHRNB4 | 216 | 7 | 2.407034 | -1.604560 | 124 | 23.69 | 0.191048 |
| GO:0010033\_response\_to\_organic\_substance | ABAT | 216 | 7 | 2.407034 | -1.604560 | 124 | 23.69 | 0.191048 |
| GO:0001763\_morphogenesis\_of\_a\_branching\_structure | SEMA5A | 125 | 5 | 2.970968 | -1.591005 | 125 | 23.84 | 0.190720 |
| GO:0001763\_morphogenesis\_of\_a\_branching\_structure | COL13A1 | 125 | 5 | 2.970968 | -1.591005 | 125 | 23.84 | 0.190720 |
| GO:0001763\_morphogenesis\_of\_a\_branching\_structure | BCL2 | 125 | 5 | 2.970968 | -1.591005 | 125 | 23.84 | 0.190720 |
| GO:0001763\_morphogenesis\_of\_a\_branching\_structure | VEGFA | 125 | 5 | 2.970968 | -1.591005 | 125 | 23.84 | 0.190720 |
| GO:0001763\_morphogenesis\_of\_a\_branching\_structure | PITX2 | 125 | 5 | 2.970968 | -1.591005 | 125 | 23.84 | 0.190720 |
| GO:0001774\_microglial\_cell\_activation | SNCA | 2 | 1 |  |  |  |  |  |  |
| GO:0002074\_extraocular\_skeletal\_muscle\_development | PITX2 | 2 | 1 |  |  |  |  |  |  |
| GO:0005981\_regulation\_of\_glycogen\_catabolic\_process | HMGB1 | 2 | 1 |  |  |  |  |  |  |
| GO:0006808\_regulation\_of\_nitrogen\_utilization | BCL2 | 2 | 1 |  |  |  |  |  |  |
| GO:0007035\_vacuolar\_acidification | SLC11A1 | 2 | 1 |  |  |  |  |  |  |
| GO:0010559\_regulation\_of\_glycoprotein\_biosynthetic\_process | BCL2 | 2 | 1 |  |  |  |  |  |  |
| GO:0014048\_regulation\_of\_glutamate\_secretion | SNCA | 2 | 1 |  |  |  |  |  |  |
| GO:0019740\_nitrogen\_utilization | BCL2 | 2 | 1 |  |  |  |  |  |  |
| GO:0021932\_hindbrain\_radial\_glia\_guided\_cell\_migration | DAB1 | 2 | 1 |  |  |  |  |  |  |
| GO:0034341\_response\_to\_interferon-gamma | SLC11A1 | 2 | 1 |  |  |  |  |  |  |
| GO:0045819\_positive\_regulation\_of\_glycogen\_catabolic\_process | HMGB1 | 2 | 1 |  |  |  |  |  |  |
| GO:0048643\_positive\_regulation\_of\_skeletal\_muscle\_tissue\_development | BCL2 | 2 | 1 |  |  |  |  |  |  |
| GO:0048712\_negative\_regulation\_of\_astrocyte\_differentiation | ID4 | 2 | 1 |  |  |  |  |  |  |
| GO:0050792\_regulation\_of\_viral\_reproduction | BCL2 | 2 | 1 |  |  |  |  |  |  |
| GO:0051590\_positive\_regulation\_of\_neurotransmitter\_transport | SNCA | 2 | 1 |  |  |  |  |  |  |
| GO:0060260\_regulation\_of\_transcription\_initiation\_from\_RNA\_polymerase\_II\_promoter | AHR | 2 | 1 |  |  |  |  |  |  |
| GO:0043473\_pigmentation | GNAQ | 49 | 3 | 4.547400 | -1.558980 | 126 | 25.43 | 0.201825 |
| GO:0043473\_pigmentation | BCL2 | 49 | 3 | 4.547400 | -1.558980 | 126 | 25.43 | 0.201825 |
| GO:0043473\_pigmentation | KITL | 49 | 3 | 4.547400 | -1.558980 | 126 | 25.43 | 0.201825 |
| GO:0044093\_positive\_regulation\_of\_molecular\_function | SLC11A1 | 173 | 6 | 2.575984 | -1.555817 | 127 | 25.54 | 0.201102 |
| GO:0044093\_positive\_regulation\_of\_molecular\_function | DAB1 | 173 | 6 | 2.575984 | -1.555817 | 127 | 25.54 | 0.201102 |
| GO:0044093\_positive\_regulation\_of\_molecular\_function | GNAQ | 173 | 6 | 2.575984 | -1.555817 | 127 | 25.54 | 0.201102 |
| GO:0044093\_positive\_regulation\_of\_molecular\_function | BCL2 | 173 | 6 | 2.575984 | -1.555817 | 127 | 25.54 | 0.201102 |
| GO:0044093\_positive\_regulation\_of\_molecular\_function | KITL | 173 | 6 | 2.575984 | -1.555817 | 127 | 25.54 | 0.201102 |
| GO:0044093\_positive\_regulation\_of\_molecular\_function | CDC25B | 173 | 6 | 2.575984 | -1.555817 | 127 | 25.54 | 0.201102 |
| GO:0001655\_urogenital\_system\_development | SPRY1 | 128 | 5 | 2.901336 | -1.551918 | 129 | 25.79 | 0.199922 |
| GO:0001655\_urogenital\_system\_development | BDNF | 128 | 5 | 2.901336 | -1.551918 | 129 | 25.79 | 0.199922 |
| GO:0001655\_urogenital\_system\_development | SALL1 | 128 | 5 | 2.901336 | -1.551918 | 129 | 25.79 | 0.199922 |
| GO:0001655\_urogenital\_system\_development | BCL2 | 128 | 5 | 2.901336 | -1.551918 | 129 | 25.79 | 0.199922 |
| GO:0001655\_urogenital\_system\_development | AHR | 128 | 5 | 2.901336 | -1.551918 | 129 | 25.79 | 0.199922 |
| GO:0045597\_positive\_regulation\_of\_cell\_differentiation | HMGB1 | 128 | 5 | 2.901336 | -1.551918 | 129 | 25.79 | 0.199922 |
| GO:0045597\_positive\_regulation\_of\_cell\_differentiation | BDNF | 128 | 5 | 2.901336 | -1.551918 | 129 | 25.79 | 0.199922 |
| GO:0045597\_positive\_regulation\_of\_cell\_differentiation | BCL2 | 128 | 5 | 2.901336 | -1.551918 | 129 | 25.79 | 0.199922 |
| GO:0045597\_positive\_regulation\_of\_cell\_differentiation | KITL | 128 | 5 | 2.901336 | -1.551918 | 129 | 25.79 | 0.199922 |
| GO:0045597\_positive\_regulation\_of\_cell\_differentiation | NEFL | 128 | 5 | 2.901336 | -1.551918 | 129 | 25.79 | 0.199922 |
| GO:0001822\_kidney\_development | SPRY1 | 87 | 4 | 3.414905 | -1.539285 | 133 | 25.98 | 0.195338 |
| GO:0001822\_kidney\_development | BDNF | 87 | 4 | 3.414905 | -1.539285 | 133 | 25.98 | 0.195338 |
| GO:0001822\_kidney\_development | SALL1 | 87 | 4 | 3.414905 | -1.539285 | 133 | 25.98 | 0.195338 |
| GO:0001822\_kidney\_development | BCL2 | 87 | 4 | 3.414905 | -1.539285 | 133 | 25.98 | 0.195338 |
| GO:0003001\_generation\_of\_a\_signal\_involved\_in\_cell-cell\_signaling | BDNF | 87 | 4 | 3.414905 | -1.539285 | 133 | 25.98 | 0.195338 |
| GO:0003001\_generation\_of\_a\_signal\_involved\_in\_cell-cell\_signaling | PFKL | 87 | 4 | 3.414905 | -1.539285 | 133 | 25.98 | 0.195338 |
| GO:0003001\_generation\_of\_a\_signal\_involved\_in\_cell-cell\_signaling | NTRK2 | 87 | 4 | 3.414905 | -1.539285 | 133 | 25.98 | 0.195338 |
| GO:0003001\_generation\_of\_a\_signal\_involved\_in\_cell-cell\_signaling | SNCA | 87 | 4 | 3.414905 | -1.539285 | 133 | 25.98 | 0.195338 |
| GO:0007178\_transmembrane\_receptor\_protein\_serine\_threonine\_kinase\_signaling\_pathway | LTBP1 | 87 | 4 | 3.414905 | -1.539285 | 133 | 25.98 | 0.195338 |
| GO:0007178\_transmembrane\_receptor\_protein\_serine\_threonine\_kinase\_signaling\_pathway | LTBP3 | 87 | 4 | 3.414905 | -1.539285 | 133 | 25.98 | 0.195338 |
| GO:0007178\_transmembrane\_receptor\_protein\_serine\_threonine\_kinase\_signaling\_pathway | ONECUT2 | 87 | 4 | 3.414905 | -1.539285 | 133 | 25.98 | 0.195338 |
| GO:0007178\_transmembrane\_receptor\_protein\_serine\_threonine\_kinase\_signaling\_pathway | TOB1 | 87 | 4 | 3.414905 | -1.539285 | 133 | 25.98 | 0.195338 |
| GO:0016337\_cell-cell\_adhesion | NRCAM | 87 | 4 | 3.414905 | -1.539285 | 133 | 25.98 | 0.195338 |
| GO:0016337\_cell-cell\_adhesion | DAB1 | 87 | 4 | 3.414905 | -1.539285 | 133 | 25.98 | 0.195338 |
| GO:0016337\_cell-cell\_adhesion | PKP2 | 87 | 4 | 3.414905 | -1.539285 | 133 | 25.98 | 0.195338 |
| GO:0016337\_cell-cell\_adhesion | BCL2 | 87 | 4 | 3.414905 | -1.539285 | 133 | 25.98 | 0.195338 |
| GO:0031128\_developmental\_induction | SPRY1 | 20 | 2 | 7.427419 | -1.537567 | 136 | 26.76 | 0.196765 |
| GO:0031128\_developmental\_induction | SALL1 | 20 | 2 | 7.427419 | -1.537567 | 136 | 26.76 | 0.196765 |
| GO:0045168\_cell-cell\_signaling\_involved\_in\_cell\_fate\_specification | SPRY1 | 20 | 2 | 7.427419 | -1.537567 | 136 | 26.76 | 0.196765 |
| GO:0045168\_cell-cell\_signaling\_involved\_in\_cell\_fate\_specification | SALL1 | 20 | 2 | 7.427419 | -1.537567 | 136 | 26.76 | 0.196765 |
| GO:0045639\_positive\_regulation\_of\_myeloid\_cell\_differentiation | HMGB1 | 20 | 2 | 7.427419 | -1.537567 | 136 | 26.76 | 0.196765 |
| GO:0045639\_positive\_regulation\_of\_myeloid\_cell\_differentiation | KITL | 20 | 2 | 7.427419 | -1.537567 | 136 | 26.76 | 0.196765 |
| GO:0046903\_secretion | CADPS | 175 | 6 | 2.546544 | -1.534406 | 137 | 27.01 | 0.197153 |
| GO:0046903\_secretion | BDNF | 175 | 6 | 2.546544 | -1.534406 | 137 | 27.01 | 0.197153 |
| GO:0046903\_secretion | PFKL | 175 | 6 | 2.546544 | -1.534406 | 137 | 27.01 | 0.197153 |
| GO:0046903\_secretion | NTRK2 | 175 | 6 | 2.546544 | -1.534406 | 137 | 27.01 | 0.197153 |
| GO:0046903\_secretion | SNCA | 175 | 6 | 2.546544 | -1.534406 | 137 | 27.01 | 0.197153 |
| GO:0046903\_secretion | CHRNB4 | 175 | 6 | 2.546544 | -1.534406 | 137 | 27.01 | 0.197153 |
| GO:0043066\_negative\_regulation\_of\_apoptosis | BDNF | 176 | 6 | 2.532075 | -1.523831 | 138 | 27.17 | 0.196884 |
| GO:0043066\_negative\_regulation\_of\_apoptosis | BCL2 | 176 | 6 | 2.532075 | -1.523831 | 138 | 27.17 | 0.196884 |
| GO:0043066\_negative\_regulation\_of\_apoptosis | SNCA | 176 | 6 | 2.532075 | -1.523831 | 138 | 27.17 | 0.196884 |
| GO:0043066\_negative\_regulation\_of\_apoptosis | VEGFA | 176 | 6 | 2.532075 | -1.523831 | 138 | 27.17 | 0.196884 |
| GO:0043066\_negative\_regulation\_of\_apoptosis | NEFL | 176 | 6 | 2.532075 | -1.523831 | 138 | 27.17 | 0.196884 |
| GO:0043066\_negative\_regulation\_of\_apoptosis | KITL | 176 | 6 | 2.532075 | -1.523831 | 138 | 27.17 | 0.196884 |
| GO:0048754\_branching\_morphogenesis\_of\_a\_tube | SEMA5A | 88 | 4 | 3.376100 | -1.523374 | 139 | 27.24 | 0.195971 |
| GO:0048754\_branching\_morphogenesis\_of\_a\_tube | BCL2 | 88 | 4 | 3.376100 | -1.523374 | 139 | 27.24 | 0.195971 |
| GO:0048754\_branching\_morphogenesis\_of\_a\_tube | VEGFA | 88 | 4 | 3.376100 | -1.523374 | 139 | 27.24 | 0.195971 |
| GO:0048754\_branching\_morphogenesis\_of\_a\_tube | PITX2 | 88 | 4 | 3.376100 | -1.523374 | 139 | 27.24 | 0.195971 |
| GO:0051093\_negative\_regulation\_of\_developmental\_process | BDNF | 331 | 9 | 2.019540 | -1.501420 | 140 | 27.68 | 0.197714 |
| GO:0051093\_negative\_regulation\_of\_developmental\_process | RNF6 | 331 | 9 | 2.019540 | -1.501420 | 140 | 27.68 | 0.197714 |
| GO:0051093\_negative\_regulation\_of\_developmental\_process | BCL2 | 331 | 9 | 2.019540 | -1.501420 | 140 | 27.68 | 0.197714 |
| GO:0051093\_negative\_regulation\_of\_developmental\_process | SNCA | 331 | 9 | 2.019540 | -1.501420 | 140 | 27.68 | 0.197714 |
| GO:0051093\_negative\_regulation\_of\_developmental\_process | VEGFA | 331 | 9 | 2.019540 | -1.501420 | 140 | 27.68 | 0.197714 |
| GO:0051093\_negative\_regulation\_of\_developmental\_process | ID4 | 331 | 9 | 2.019540 | -1.501420 | 140 | 27.68 | 0.197714 |
| GO:0051093\_negative\_regulation\_of\_developmental\_process | NEFL | 331 | 9 | 2.019540 | -1.501420 | 140 | 27.68 | 0.197714 |
| GO:0051093\_negative\_regulation\_of\_developmental\_process | KITL | 331 | 9 | 2.019540 | -1.501420 | 140 | 27.68 | 0.197714 |
| GO:0051093\_negative\_regulation\_of\_developmental\_process | TOB1 | 331 | 9 | 2.019540 | -1.501420 | 140 | 27.68 | 0.197714 |
| GO:0006633\_fatty\_acid\_biosynthetic\_process | SCD1 | 21 | 2 | 7.073733 | -1.497826 | 143 | 28.94 | 0.202378 |
| GO:0006633\_fatty\_acid\_biosynthetic\_process | SNCA | 21 | 2 | 7.073733 | -1.497826 | 143 | 28.94 | 0.202378 |
| GO:0015844\_monoamine\_transport | CADPS | 21 | 2 | 7.073733 | -1.497826 | 143 | 28.94 | 0.202378 |
| GO:0015844\_monoamine\_transport | SNCA | 21 | 2 | 7.073733 | -1.497826 | 143 | 28.94 | 0.202378 |
| GO:0043279\_response\_to\_alkaloid | CHRNB4 | 21 | 2 | 7.073733 | -1.497826 | 143 | 28.94 | 0.202378 |
| GO:0043279\_response\_to\_alkaloid | ABAT | 21 | 2 | 7.073733 | -1.497826 | 143 | 28.94 | 0.202378 |
| GO:0007610\_behavior | HMGB1 | 279 | 8 | 2.129726 | -1.493925 | 144 | 28.99 | 0.201319 |
| GO:0007610\_behavior | BDNF | 279 | 8 | 2.129726 | -1.493925 | 144 | 28.99 | 0.201319 |
| GO:0007610\_behavior | GNAQ | 279 | 8 | 2.129726 | -1.493925 | 144 | 28.99 | 0.201319 |
| GO:0007610\_behavior | BCL2 | 279 | 8 | 2.129726 | -1.493925 | 144 | 28.99 | 0.201319 |
| GO:0007610\_behavior | NTRK2 | 279 | 8 | 2.129726 | -1.493925 | 144 | 28.99 | 0.201319 |
| GO:0007610\_behavior | SNCA | 279 | 8 | 2.129726 | -1.493925 | 144 | 28.99 | 0.201319 |
| GO:0007610\_behavior | CHRNB4 | 279 | 8 | 2.129726 | -1.493925 | 144 | 28.99 | 0.201319 |
| GO:0007610\_behavior | ABAT | 279 | 8 | 2.129726 | -1.493925 | 144 | 28.99 | 0.201319 |
| GO:0043069\_negative\_regulation\_of\_programmed\_cell\_death | BDNF | 179 | 6 | 2.489638 | -1.492618 | 146 | 29.02 | 0.198767 |
| GO:0043069\_negative\_regulation\_of\_programmed\_cell\_death | BCL2 | 179 | 6 | 2.489638 | -1.492618 | 146 | 29.02 | 0.198767 |
| GO:0043069\_negative\_regulation\_of\_programmed\_cell\_death | SNCA | 179 | 6 | 2.489638 | -1.492618 | 146 | 29.02 | 0.198767 |
| GO:0043069\_negative\_regulation\_of\_programmed\_cell\_death | VEGFA | 179 | 6 | 2.489638 | -1.492618 | 146 | 29.02 | 0.198767 |
| GO:0043069\_negative\_regulation\_of\_programmed\_cell\_death | NEFL | 179 | 6 | 2.489638 | -1.492618 | 146 | 29.02 | 0.198767 |
| GO:0043069\_negative\_regulation\_of\_programmed\_cell\_death | KITL | 179 | 6 | 2.489638 | -1.492618 | 146 | 29.02 | 0.198767 |
| GO:0060548\_negative\_regulation\_of\_cell\_death | BDNF | 179 | 6 | 2.489638 | -1.492618 | 146 | 29.02 | 0.198767 |
| GO:0060548\_negative\_regulation\_of\_cell\_death | BCL2 | 179 | 6 | 2.489638 | -1.492618 | 146 | 29.02 | 0.198767 |
| GO:0060548\_negative\_regulation\_of\_cell\_death | SNCA | 179 | 6 | 2.489638 | -1.492618 | 146 | 29.02 | 0.198767 |
| GO:0060548\_negative\_regulation\_of\_cell\_death | VEGFA | 179 | 6 | 2.489638 | -1.492618 | 146 | 29.02 | 0.198767 |
| GO:0060548\_negative\_regulation\_of\_cell\_death | NEFL | 179 | 6 | 2.489638 | -1.492618 | 146 | 29.02 | 0.198767 |
| GO:0060548\_negative\_regulation\_of\_cell\_death | KITL | 179 | 6 | 2.489638 | -1.492618 | 146 | 29.02 | 0.198767 |
| GO:0032501\_multicellular\_organismal\_process | HMGB1 | 2183 | 37 | 1.258885 | -1.464563 | 147 | 29.99 | 0.204014 |
| GO:0032501\_multicellular\_organismal\_process | LTBP3 | 2183 | 37 | 1.258885 | -1.464563 | 147 | 29.99 | 0.204014 |
| GO:0032501\_multicellular\_organismal\_process | ONECUT2 | 2183 | 37 | 1.258885 | -1.464563 | 147 | 29.99 | 0.204014 |
| GO:0032501\_multicellular\_organismal\_process | SNCA | 2183 | 37 | 1.258885 | -1.464563 | 147 | 29.99 | 0.204014 |
| GO:0032501\_multicellular\_organismal\_process | RORB | 2183 | 37 | 1.258885 | -1.464563 | 147 | 29.99 | 0.204014 |
| GO:0032501\_multicellular\_organismal\_process | EPHB1 | 2183 | 37 | 1.258885 | -1.464563 | 147 | 29.99 | 0.204014 |
| GO:0032501\_multicellular\_organismal\_process | NRCAM | 2183 | 37 | 1.258885 | -1.464563 | 147 | 29.99 | 0.204014 |
| GO:0032501\_multicellular\_organismal\_process | SEMA5A | 2183 | 37 | 1.258885 | -1.464563 | 147 | 29.99 | 0.204014 |
| GO:0032501\_multicellular\_organismal\_process | EFHD1 | 2183 | 37 | 1.258885 | -1.464563 | 147 | 29.99 | 0.204014 |
| GO:0032501\_multicellular\_organismal\_process | SLC11A1 | 2183 | 37 | 1.258885 | -1.464563 | 147 | 29.99 | 0.204014 |
| GO:0032501\_multicellular\_organismal\_process | BDNF | 2183 | 37 | 1.258885 | -1.464563 | 147 | 29.99 | 0.204014 |
| GO:0032501\_multicellular\_organismal\_process | SPRY1 | 2183 | 37 | 1.258885 | -1.464563 | 147 | 29.99 | 0.204014 |
| GO:0032501\_multicellular\_organismal\_process | DAB1 | 2183 | 37 | 1.258885 | -1.464563 | 147 | 29.99 | 0.204014 |
| GO:0032501\_multicellular\_organismal\_process | MORC3 | 2183 | 37 | 1.258885 | -1.464563 | 147 | 29.99 | 0.204014 |
| GO:0032501\_multicellular\_organismal\_process | BCL2 | 2183 | 37 | 1.258885 | -1.464563 | 147 | 29.99 | 0.204014 |
| GO:0032501\_multicellular\_organismal\_process | NFAT5 | 2183 | 37 | 1.258885 | -1.464563 | 147 | 29.99 | 0.204014 |
| GO:0032501\_multicellular\_organismal\_process | MYB | 2183 | 37 | 1.258885 | -1.464563 | 147 | 29.99 | 0.204014 |
| GO:0032501\_multicellular\_organismal\_process | NR2F2 | 2183 | 37 | 1.258885 | -1.464563 | 147 | 29.99 | 0.204014 |
| GO:0032501\_multicellular\_organismal\_process | NEFL | 2183 | 37 | 1.258885 | -1.464563 | 147 | 29.99 | 0.204014 |
| GO:0032501\_multicellular\_organismal\_process | MAP2K5 | 2183 | 37 | 1.258885 | -1.464563 | 147 | 29.99 | 0.204014 |
| GO:0032501\_multicellular\_organismal\_process | PITX2 | 2183 | 37 | 1.258885 | -1.464563 | 147 | 29.99 | 0.204014 |
| GO:0032501\_multicellular\_organismal\_process | IRS2 | 2183 | 37 | 1.258885 | -1.464563 | 147 | 29.99 | 0.204014 |
| GO:0032501\_multicellular\_organismal\_process | COL13A1 | 2183 | 37 | 1.258885 | -1.464563 | 147 | 29.99 | 0.204014 |
| GO:0032501\_multicellular\_organismal\_process | KITL | 2183 | 37 | 1.258885 | -1.464563 | 147 | 29.99 | 0.204014 |
| GO:0032501\_multicellular\_organismal\_process | AHR | 2183 | 37 | 1.258885 | -1.464563 | 147 | 29.99 | 0.204014 |
| GO:0032501\_multicellular\_organismal\_process | RNF6 | 2183 | 37 | 1.258885 | -1.464563 | 147 | 29.99 | 0.204014 |
| GO:0032501\_multicellular\_organismal\_process | GNAQ | 2183 | 37 | 1.258885 | -1.464563 | 147 | 29.99 | 0.204014 |
| GO:0032501\_multicellular\_organismal\_process | PKP2 | 2183 | 37 | 1.258885 | -1.464563 | 147 | 29.99 | 0.204014 |
| GO:0032501\_multicellular\_organismal\_process | SALL1 | 2183 | 37 | 1.258885 | -1.464563 | 147 | 29.99 | 0.204014 |
| GO:0032501\_multicellular\_organismal\_process | NTRK2 | 2183 | 37 | 1.258885 | -1.464563 | 147 | 29.99 | 0.204014 |
| GO:0032501\_multicellular\_organismal\_process | VEGFA | 2183 | 37 | 1.258885 | -1.464563 | 147 | 29.99 | 0.204014 |
| GO:0032501\_multicellular\_organismal\_process | SP4 | 2183 | 37 | 1.258885 | -1.464563 | 147 | 29.99 | 0.204014 |
| GO:0032501\_multicellular\_organismal\_process | CHRNB4 | 2183 | 37 | 1.258885 | -1.464563 | 147 | 29.99 | 0.204014 |
| GO:0032501\_multicellular\_organismal\_process | ABAT | 2183 | 37 | 1.258885 | -1.464563 | 147 | 29.99 | 0.204014 |
| GO:0032501\_multicellular\_organismal\_process | ID4 | 2183 | 37 | 1.258885 | -1.464563 | 147 | 29.99 | 0.204014 |
| GO:0032501\_multicellular\_organismal\_process | FABP7 | 2183 | 37 | 1.258885 | -1.464563 | 147 | 29.99 | 0.204014 |
| GO:0032501\_multicellular\_organismal\_process | TOB1 | 2183 | 37 | 1.258885 | -1.464563 | 147 | 29.99 | 0.204014 |
| GO:0000902\_cell\_morphogenesis | NRCAM | 283 | 8 | 2.099624 | -1.462129 | 148 | 30.03 | 0.202905 |
| GO:0000902\_cell\_morphogenesis | SEMA5A | 283 | 8 | 2.099624 | -1.462129 | 148 | 30.03 | 0.202905 |
| GO:0000902\_cell\_morphogenesis | PARD3 | 283 | 8 | 2.099624 | -1.462129 | 148 | 30.03 | 0.202905 |
| GO:0000902\_cell\_morphogenesis | BDNF | 283 | 8 | 2.099624 | -1.462129 | 148 | 30.03 | 0.202905 |
| GO:0000902\_cell\_morphogenesis | RNF6 | 283 | 8 | 2.099624 | -1.462129 | 148 | 30.03 | 0.202905 |
| GO:0000902\_cell\_morphogenesis | BCL2 | 283 | 8 | 2.099624 | -1.462129 | 148 | 30.03 | 0.202905 |
| GO:0000902\_cell\_morphogenesis | NEFL | 283 | 8 | 2.099624 | -1.462129 | 148 | 30.03 | 0.202905 |
| GO:0000902\_cell\_morphogenesis | EPHB1 | 283 | 8 | 2.099624 | -1.462129 | 148 | 30.03 | 0.202905 |
| GO:0009309\_amine\_biosynthetic\_process | ALDH18A1 | 22 | 2 | 6.752199 | -1.460155 | 152 | 31.59 | 0.207829 |
| GO:0009309\_amine\_biosynthetic\_process | SNCA | 22 | 2 | 6.752199 | -1.460155 | 152 | 31.59 | 0.207829 |
| GO:0021675\_nerve\_development | BDNF | 22 | 2 | 6.752199 | -1.460155 | 152 | 31.59 | 0.207829 |
| GO:0021675\_nerve\_development | EPHB1 | 22 | 2 | 6.752199 | -1.460155 | 152 | 31.59 | 0.207829 |
| GO:0034097\_response\_to\_cytokine\_stimulus | SLC11A1 | 22 | 2 | 6.752199 | -1.460155 | 152 | 31.59 | 0.207829 |
| GO:0034097\_response\_to\_cytokine\_stimulus | BCL2 | 22 | 2 | 6.752199 | -1.460155 | 152 | 31.59 | 0.207829 |
| GO:0048477\_oogenesis | BCL2 | 22 | 2 | 6.752199 | -1.460155 | 152 | 31.59 | 0.207829 |
| GO:0048477\_oogenesis | CDC25B | 22 | 2 | 6.752199 | -1.460155 | 152 | 31.59 | 0.207829 |
| GO:0006091\_generation\_of\_precursor\_metabolites\_and\_energy | HMGB1 | 54 | 3 | 4.126344 | -1.450297 | 153 | 31.95 | 0.208824 |
| GO:0006091\_generation\_of\_precursor\_metabolites\_and\_energy | PFKL | 54 | 3 | 4.126344 | -1.450297 | 153 | 31.95 | 0.208824 |
| GO:0006091\_generation\_of\_precursor\_metabolites\_and\_energy | SNCA | 54 | 3 | 4.126344 | -1.450297 | 153 | 31.95 | 0.208824 |
| GO:0050790\_regulation\_of\_catalytic\_activity | SLC11A1 | 233 | 7 | 2.231414 | -1.448276 | 154 | 31.99 | 0.207727 |
| GO:0050790\_regulation\_of\_catalytic\_activity | SPRY1 | 233 | 7 | 2.231414 | -1.448276 | 154 | 31.99 | 0.207727 |
| GO:0050790\_regulation\_of\_catalytic\_activity | DAB1 | 233 | 7 | 2.231414 | -1.448276 | 154 | 31.99 | 0.207727 |
| GO:0050790\_regulation\_of\_catalytic\_activity | GNAQ | 233 | 7 | 2.231414 | -1.448276 | 154 | 31.99 | 0.207727 |
| GO:0050790\_regulation\_of\_catalytic\_activity | BCL2 | 233 | 7 | 2.231414 | -1.448276 | 154 | 31.99 | 0.207727 |
| GO:0050790\_regulation\_of\_catalytic\_activity | KITL | 233 | 7 | 2.231414 | -1.448276 | 154 | 31.99 | 0.207727 |
| GO:0050790\_regulation\_of\_catalytic\_activity | CDC25B | 233 | 7 | 2.231414 | -1.448276 | 154 | 31.99 | 0.207727 |
| GO:0006793\_phosphorus\_metabolic\_process | SLC11A1 | 340 | 9 | 1.966082 | -1.436646 | 156 | 32.41 | 0.207756 |
| GO:0006793\_phosphorus\_metabolic\_process | HMGB1 | 340 | 9 | 1.966082 | -1.436646 | 156 | 32.41 | 0.207756 |
| GO:0006793\_phosphorus\_metabolic\_process | SPRY1 | 340 | 9 | 1.966082 | -1.436646 | 156 | 32.41 | 0.207756 |
| GO:0006793\_phosphorus\_metabolic\_process | DAB1 | 340 | 9 | 1.966082 | -1.436646 | 156 | 32.41 | 0.207756 |
| GO:0006793\_phosphorus\_metabolic\_process | BCL2 | 340 | 9 | 1.966082 | -1.436646 | 156 | 32.41 | 0.207756 |
| GO:0006793\_phosphorus\_metabolic\_process | SNCA | 340 | 9 | 1.966082 | -1.436646 | 156 | 32.41 | 0.207756 |
| GO:0006793\_phosphorus\_metabolic\_process | PPM1A | 340 | 9 | 1.966082 | -1.436646 | 156 | 32.41 | 0.207756 |
| GO:0006793\_phosphorus\_metabolic\_process | KITL | 340 | 9 | 1.966082 | -1.436646 | 156 | 32.41 | 0.207756 |
| GO:0006793\_phosphorus\_metabolic\_process | CDC25B | 340 | 9 | 1.966082 | -1.436646 | 156 | 32.41 | 0.207756 |
| GO:0006796\_phosphate\_metabolic\_process | SLC11A1 | 340 | 9 | 1.966082 | -1.436646 | 156 | 32.41 | 0.207756 |
| GO:0006796\_phosphate\_metabolic\_process | HMGB1 | 340 | 9 | 1.966082 | -1.436646 | 156 | 32.41 | 0.207756 |
| GO:0006796\_phosphate\_metabolic\_process | SPRY1 | 340 | 9 | 1.966082 | -1.436646 | 156 | 32.41 | 0.207756 |
| GO:0006796\_phosphate\_metabolic\_process | DAB1 | 340 | 9 | 1.966082 | -1.436646 | 156 | 32.41 | 0.207756 |
| GO:0006796\_phosphate\_metabolic\_process | BCL2 | 340 | 9 | 1.966082 | -1.436646 | 156 | 32.41 | 0.207756 |
| GO:0006796\_phosphate\_metabolic\_process | SNCA | 340 | 9 | 1.966082 | -1.436646 | 156 | 32.41 | 0.207756 |
| GO:0006796\_phosphate\_metabolic\_process | PPM1A | 340 | 9 | 1.966082 | -1.436646 | 156 | 32.41 | 0.207756 |
| GO:0006796\_phosphate\_metabolic\_process | KITL | 340 | 9 | 1.966082 | -1.436646 | 156 | 32.41 | 0.207756 |
| GO:0006796\_phosphate\_metabolic\_process | CDC25B | 340 | 9 | 1.966082 | -1.436646 | 156 | 32.41 | 0.207756 |
| GO:0015698\_inorganic\_anion\_transport | SLC11A1 | 23 | 2 | 6.458626 | -1.424364 | 158 | 33.64 | 0.212911 |
| GO:0015698\_inorganic\_anion\_transport | SLC4A4 | 23 | 2 | 6.458626 | -1.424364 | 158 | 33.64 | 0.212911 |
| GO:0030512\_negative\_regulation\_of\_transforming\_growth\_factor\_beta\_receptor\_signaling\_pathway | ONECUT2 | 23 | 2 | 6.458626 | -1.424364 | 158 | 33.64 | 0.212911 |
| GO:0030512\_negative\_regulation\_of\_transforming\_growth\_factor\_beta\_receptor\_signaling\_pathway | TOB1 | 23 | 2 | 6.458626 | -1.424364 | 158 | 33.64 | 0.212911 |
| GO:0051649\_establishment\_of\_localization\_in\_cell | CADPS | 342 | 9 | 1.954584 | -1.422637 | 159 | 33.81 | 0.212642 |
| GO:0051649\_establishment\_of\_localization\_in\_cell | SLC11A1 | 342 | 9 | 1.954584 | -1.422637 | 159 | 33.81 | 0.212642 |
| GO:0051649\_establishment\_of\_localization\_in\_cell | BDNF | 342 | 9 | 1.954584 | -1.422637 | 159 | 33.81 | 0.212642 |
| GO:0051649\_establishment\_of\_localization\_in\_cell | PFKL | 342 | 9 | 1.954584 | -1.422637 | 159 | 33.81 | 0.212642 |
| GO:0051649\_establishment\_of\_localization\_in\_cell | BCL2 | 342 | 9 | 1.954584 | -1.422637 | 159 | 33.81 | 0.212642 |
| GO:0051649\_establishment\_of\_localization\_in\_cell | NTRK2 | 342 | 9 | 1.954584 | -1.422637 | 159 | 33.81 | 0.212642 |
| GO:0051649\_establishment\_of\_localization\_in\_cell | SNCA | 342 | 9 | 1.954584 | -1.422637 | 159 | 33.81 | 0.212642 |
| GO:0051649\_establishment\_of\_localization\_in\_cell | EHD1 | 342 | 9 | 1.954584 | -1.422637 | 159 | 33.81 | 0.212642 |
| GO:0051649\_establishment\_of\_localization\_in\_cell | TOB1 | 342 | 9 | 1.954584 | -1.422637 | 159 | 33.81 | 0.212642 |
| GO:0007275\_multicellular\_organismal\_development | HMGB1 | 1760 | 31 | 1.308239 | -1.419898 | 160 | 33.85 | 0.211563 |
| GO:0007275\_multicellular\_organismal\_development | LTBP3 | 1760 | 31 | 1.308239 | -1.419898 | 160 | 33.85 | 0.211563 |
| GO:0007275\_multicellular\_organismal\_development | ONECUT2 | 1760 | 31 | 1.308239 | -1.419898 | 160 | 33.85 | 0.211563 |
| GO:0007275\_multicellular\_organismal\_development | RORB | 1760 | 31 | 1.308239 | -1.419898 | 160 | 33.85 | 0.211563 |
| GO:0007275\_multicellular\_organismal\_development | EPHB1 | 1760 | 31 | 1.308239 | -1.419898 | 160 | 33.85 | 0.211563 |
| GO:0007275\_multicellular\_organismal\_development | SEMA5A | 1760 | 31 | 1.308239 | -1.419898 | 160 | 33.85 | 0.211563 |
| GO:0007275\_multicellular\_organismal\_development | NRCAM | 1760 | 31 | 1.308239 | -1.419898 | 160 | 33.85 | 0.211563 |
| GO:0007275\_multicellular\_organismal\_development | EFHD1 | 1760 | 31 | 1.308239 | -1.419898 | 160 | 33.85 | 0.211563 |
| GO:0007275\_multicellular\_organismal\_development | SPRY1 | 1760 | 31 | 1.308239 | -1.419898 | 160 | 33.85 | 0.211563 |
| GO:0007275\_multicellular\_organismal\_development | BDNF | 1760 | 31 | 1.308239 | -1.419898 | 160 | 33.85 | 0.211563 |
| GO:0007275\_multicellular\_organismal\_development | DAB1 | 1760 | 31 | 1.308239 | -1.419898 | 160 | 33.85 | 0.211563 |
| GO:0007275\_multicellular\_organismal\_development | MORC3 | 1760 | 31 | 1.308239 | -1.419898 | 160 | 33.85 | 0.211563 |
| GO:0007275\_multicellular\_organismal\_development | BCL2 | 1760 | 31 | 1.308239 | -1.419898 | 160 | 33.85 | 0.211563 |
| GO:0007275\_multicellular\_organismal\_development | NR2F2 | 1760 | 31 | 1.308239 | -1.419898 | 160 | 33.85 | 0.211563 |
| GO:0007275\_multicellular\_organismal\_development | MYB | 1760 | 31 | 1.308239 | -1.419898 | 160 | 33.85 | 0.211563 |
| GO:0007275\_multicellular\_organismal\_development | NEFL | 1760 | 31 | 1.308239 | -1.419898 | 160 | 33.85 | 0.211563 |
| GO:0007275\_multicellular\_organismal\_development | MAP2K5 | 1760 | 31 | 1.308239 | -1.419898 | 160 | 33.85 | 0.211563 |
| GO:0007275\_multicellular\_organismal\_development | PITX2 | 1760 | 31 | 1.308239 | -1.419898 | 160 | 33.85 | 0.211563 |
| GO:0007275\_multicellular\_organismal\_development | IRS2 | 1760 | 31 | 1.308239 | -1.419898 | 160 | 33.85 | 0.211563 |
| GO:0007275\_multicellular\_organismal\_development | COL13A1 | 1760 | 31 | 1.308239 | -1.419898 | 160 | 33.85 | 0.211563 |
| GO:0007275\_multicellular\_organismal\_development | KITL | 1760 | 31 | 1.308239 | -1.419898 | 160 | 33.85 | 0.211563 |
| GO:0007275\_multicellular\_organismal\_development | AHR | 1760 | 31 | 1.308239 | -1.419898 | 160 | 33.85 | 0.211563 |
| GO:0007275\_multicellular\_organismal\_development | RNF6 | 1760 | 31 | 1.308239 | -1.419898 | 160 | 33.85 | 0.211563 |
| GO:0007275\_multicellular\_organismal\_development | GNAQ | 1760 | 31 | 1.308239 | -1.419898 | 160 | 33.85 | 0.211563 |
| GO:0007275\_multicellular\_organismal\_development | PKP2 | 1760 | 31 | 1.308239 | -1.419898 | 160 | 33.85 | 0.211563 |
| GO:0007275\_multicellular\_organismal\_development | SALL1 | 1760 | 31 | 1.308239 | -1.419898 | 160 | 33.85 | 0.211563 |
| GO:0007275\_multicellular\_organismal\_development | VEGFA | 1760 | 31 | 1.308239 | -1.419898 | 160 | 33.85 | 0.211563 |
| GO:0007275\_multicellular\_organismal\_development | NTRK2 | 1760 | 31 | 1.308239 | -1.419898 | 160 | 33.85 | 0.211563 |
| GO:0007275\_multicellular\_organismal\_development | ID4 | 1760 | 31 | 1.308239 | -1.419898 | 160 | 33.85 | 0.211563 |
| GO:0007275\_multicellular\_organismal\_development | FABP7 | 1760 | 31 | 1.308239 | -1.419898 | 160 | 33.85 | 0.211563 |
| GO:0007275\_multicellular\_organismal\_development | TOB1 | 1760 | 31 | 1.308239 | -1.419898 | 160 | 33.85 | 0.211563 |
| GO:0042391\_regulation\_of\_membrane\_potential | GNAQ | 95 | 4 | 3.127334 | -1.418173 | 161 | 33.93 | 0.210745 |
| GO:0042391\_regulation\_of\_membrane\_potential | BCL2 | 95 | 4 | 3.127334 | -1.418173 | 161 | 33.93 | 0.210745 |
| GO:0042391\_regulation\_of\_membrane\_potential | SNCA | 95 | 4 | 3.127334 | -1.418173 | 161 | 33.93 | 0.210745 |
| GO:0042391\_regulation\_of\_membrane\_potential | CHRNB4 | 95 | 4 | 3.127334 | -1.418173 | 161 | 33.93 | 0.210745 |
| GO:0002309\_T\_cell\_proliferation\_during\_immune\_response | SLC11A1 | 3 | 1 |  |  |  |  |  |  |
| GO:0002369\_T\_cell\_cytokine\_production | SLC11A1 | 3 | 1 |  |  |  |  |  |  |
| GO:0002827\_positive\_regulation\_of\_T-helper\_1\_type\_immune\_response | SLC11A1 | 3 | 1 |  |  |  |  |  |  |
| GO:0006367\_transcription\_initiation\_from\_RNA\_polymerase\_II\_promoter | AHR | 3 | 1 |  |  |  |  |  |  |
| GO:0006828\_manganese\_ion\_transport | SLC11A1 | 3 | 1 |  |  |  |  |  |  |
| GO:0007412\_axon\_target\_recognition | BDNF | 3 | 1 |  |  |  |  |  |  |
| GO:0014909\_smooth\_muscle\_cell\_migration | BCL2 | 3 | 1 |  |  |  |  |  |  |
| GO:0016322\_neuron\_remodeling | GNAQ | 3 | 1 |  |  |  |  |  |  |
| GO:0019058\_viral\_infectious\_cycle | BCL2 | 3 | 1 |  |  |  |  |  |  |
| GO:0031133\_regulation\_of\_axon\_diameter | NEFL | 3 | 1 |  |  |  |  |  |  |
| GO:0032536\_regulation\_of\_cell\_projection\_size | NEFL | 3 | 1 |  |  |  |  |  |  |
| GO:0032632\_interleukin-3\_production | SLC11A1 | 3 | 1 |  |  |  |  |  |  |
| GO:0032881\_regulation\_of\_polysaccharide\_metabolic\_process | HMGB1 | 3 | 1 |  |  |  |  |  |  |
| GO:0043090\_amino\_acid\_import | SLC11A1 | 3 | 1 |  |  |  |  |  |  |
| GO:0043092\_L-amino\_acid\_import | SLC11A1 | 3 | 1 |  |  |  |  |  |  |
| GO:0045110\_intermediate\_filament\_bundle\_assembly | NEFL | 3 | 1 |  |  |  |  |  |  |
| GO:0045844\_positive\_regulation\_of\_striated\_muscle\_development | BCL2 | 3 | 1 |  |  |  |  |  |  |
| GO:0048636\_positive\_regulation\_of\_muscle\_development | BCL2 | 3 | 1 |  |  |  |  |  |  |
| GO:0048845\_venous\_blood\_vessel\_morphogenesis | PITX2 | 3 | 1 |  |  |  |  |  |  |
| GO:0051926\_negative\_regulation\_of\_calcium\_ion\_transport | BCL2 | 3 | 1 |  |  |  |  |  |  |
| GO:0060084\_synaptic\_transmission\_involved\_in\_micturition | CHRNB4 | 3 | 1 |  |  |  |  |  |  |
| GO:0060460\_left\_lung\_morphogenesis | PITX2 | 3 | 1 |  |  |  |  |  |  |
| GO:0060586\_multicellular\_organismal\_iron\_ion\_homeostasis | SLC11A1 | 3 | 1 |  |  |  |  |  |  |
| GO:0060841\_venous\_blood\_vessel\_development | PITX2 | 3 | 1 |  |  |  |  |  |  |
| GO:0070873\_regulation\_of\_glycogen\_metabolic\_process | HMGB1 | 3 | 1 |  |  |  |  |  |  |
| GO:0070875\_positive\_regulation\_of\_glycogen\_metabolic\_process | HMGB1 | 3 | 1 |  |  |  |  |  |  |
| GO:0001541\_ovarian\_follicle\_development | BCL2 | 24 | 2 | 6.189516 | -1.390289 | 163 | 36.42 | 0.223436 |
| GO:0001541\_ovarian\_follicle\_development | VEGFA | 24 | 2 | 6.189516 | -1.390289 | 163 | 36.42 | 0.223436 |
| GO:0014070\_response\_to\_organic\_cyclic\_substance | CHRNB4 | 24 | 2 | 6.189516 | -1.390289 | 163 | 36.42 | 0.223436 |
| GO:0014070\_response\_to\_organic\_cyclic\_substance | ABAT | 24 | 2 | 6.189516 | -1.390289 | 163 | 36.42 | 0.223436 |
| GO:0032502\_developmental\_process | HMGB1 | 2060 | 35 | 1.261940 | -1.383669 | 164 | 36.51 | 0.222622 |
| GO:0032502\_developmental\_process | PARD3 | 2060 | 35 | 1.261940 | -1.383669 | 164 | 36.51 | 0.222622 |
| GO:0032502\_developmental\_process | LTBP3 | 2060 | 35 | 1.261940 | -1.383669 | 164 | 36.51 | 0.222622 |
| GO:0032502\_developmental\_process | SNCA | 2060 | 35 | 1.261940 | -1.383669 | 164 | 36.51 | 0.222622 |
| GO:0032502\_developmental\_process | ONECUT2 | 2060 | 35 | 1.261940 | -1.383669 | 164 | 36.51 | 0.222622 |
| GO:0032502\_developmental\_process | RORB | 2060 | 35 | 1.261940 | -1.383669 | 164 | 36.51 | 0.222622 |
| GO:0032502\_developmental\_process | EPHB1 | 2060 | 35 | 1.261940 | -1.383669 | 164 | 36.51 | 0.222622 |
| GO:0032502\_developmental\_process | SEMA5A | 2060 | 35 | 1.261940 | -1.383669 | 164 | 36.51 | 0.222622 |
| GO:0032502\_developmental\_process | NRCAM | 2060 | 35 | 1.261940 | -1.383669 | 164 | 36.51 | 0.222622 |
| GO:0032502\_developmental\_process | EFHD1 | 2060 | 35 | 1.261940 | -1.383669 | 164 | 36.51 | 0.222622 |
| GO:0032502\_developmental\_process | SPRY1 | 2060 | 35 | 1.261940 | -1.383669 | 164 | 36.51 | 0.222622 |
| GO:0032502\_developmental\_process | BDNF | 2060 | 35 | 1.261940 | -1.383669 | 164 | 36.51 | 0.222622 |
| GO:0032502\_developmental\_process | DAB1 | 2060 | 35 | 1.261940 | -1.383669 | 164 | 36.51 | 0.222622 |
| GO:0032502\_developmental\_process | MORC3 | 2060 | 35 | 1.261940 | -1.383669 | 164 | 36.51 | 0.222622 |
| GO:0032502\_developmental\_process | BCL2 | 2060 | 35 | 1.261940 | -1.383669 | 164 | 36.51 | 0.222622 |
| GO:0032502\_developmental\_process | NR2F2 | 2060 | 35 | 1.261940 | -1.383669 | 164 | 36.51 | 0.222622 |
| GO:0032502\_developmental\_process | MYB | 2060 | 35 | 1.261940 | -1.383669 | 164 | 36.51 | 0.222622 |
| GO:0032502\_developmental\_process | NEFL | 2060 | 35 | 1.261940 | -1.383669 | 164 | 36.51 | 0.222622 |
| GO:0032502\_developmental\_process | MAP2K5 | 2060 | 35 | 1.261940 | -1.383669 | 164 | 36.51 | 0.222622 |
| GO:0032502\_developmental\_process | PITX2 | 2060 | 35 | 1.261940 | -1.383669 | 164 | 36.51 | 0.222622 |
| GO:0032502\_developmental\_process | SCD1 | 2060 | 35 | 1.261940 | -1.383669 | 164 | 36.51 | 0.222622 |
| GO:0032502\_developmental\_process | IRS2 | 2060 | 35 | 1.261940 | -1.383669 | 164 | 36.51 | 0.222622 |
| GO:0032502\_developmental\_process | COL13A1 | 2060 | 35 | 1.261940 | -1.383669 | 164 | 36.51 | 0.222622 |
| GO:0032502\_developmental\_process | KITL | 2060 | 35 | 1.261940 | -1.383669 | 164 | 36.51 | 0.222622 |
| GO:0032502\_developmental\_process | AHR | 2060 | 35 | 1.261940 | -1.383669 | 164 | 36.51 | 0.222622 |
| GO:0032502\_developmental\_process | CDC25B | 2060 | 35 | 1.261940 | -1.383669 | 164 | 36.51 | 0.222622 |
| GO:0032502\_developmental\_process | RNF6 | 2060 | 35 | 1.261940 | -1.383669 | 164 | 36.51 | 0.222622 |
| GO:0032502\_developmental\_process | GNAQ | 2060 | 35 | 1.261940 | -1.383669 | 164 | 36.51 | 0.222622 |
| GO:0032502\_developmental\_process | PKP2 | 2060 | 35 | 1.261940 | -1.383669 | 164 | 36.51 | 0.222622 |
| GO:0032502\_developmental\_process | SALL1 | 2060 | 35 | 1.261940 | -1.383669 | 164 | 36.51 | 0.222622 |
| GO:0032502\_developmental\_process | VEGFA | 2060 | 35 | 1.261940 | -1.383669 | 164 | 36.51 | 0.222622 |
| GO:0032502\_developmental\_process | NTRK2 | 2060 | 35 | 1.261940 | -1.383669 | 164 | 36.51 | 0.222622 |
| GO:0032502\_developmental\_process | ID4 | 2060 | 35 | 1.261940 | -1.383669 | 164 | 36.51 | 0.222622 |
| GO:0032502\_developmental\_process | FABP7 | 2060 | 35 | 1.261940 | -1.383669 | 164 | 36.51 | 0.222622 |
| GO:0032502\_developmental\_process | TOB1 | 2060 | 35 | 1.261940 | -1.383669 | 164 | 36.51 | 0.222622 |
| GO:0055123\_digestive\_system\_development | BCL2 | 25 | 2 | 5.941935 | -1.357787 | 165 | 38.28 | 0.232000 |
| GO:0055123\_digestive\_system\_development | PITX2 | 25 | 2 | 5.941935 | -1.357787 | 165 | 38.28 | 0.232000 |
| GO:0042221\_response\_to\_chemical\_stimulus | SLC11A1 | 409 | 10 | 1.815995 | -1.349992 | 166 | 38.52 | 0.232048 |
| GO:0042221\_response\_to\_chemical\_stimulus | HMGB1 | 409 | 10 | 1.815995 | -1.349992 | 166 | 38.52 | 0.232048 |
| GO:0042221\_response\_to\_chemical\_stimulus | IRS2 | 409 | 10 | 1.815995 | -1.349992 | 166 | 38.52 | 0.232048 |
| GO:0042221\_response\_to\_chemical\_stimulus | BDNF | 409 | 10 | 1.815995 | -1.349992 | 166 | 38.52 | 0.232048 |
| GO:0042221\_response\_to\_chemical\_stimulus | PFKL | 409 | 10 | 1.815995 | -1.349992 | 166 | 38.52 | 0.232048 |
| GO:0042221\_response\_to\_chemical\_stimulus | BCL2 | 409 | 10 | 1.815995 | -1.349992 | 166 | 38.52 | 0.232048 |
| GO:0042221\_response\_to\_chemical\_stimulus | SNCA | 409 | 10 | 1.815995 | -1.349992 | 166 | 38.52 | 0.232048 |
| GO:0042221\_response\_to\_chemical\_stimulus | CHRNB4 | 409 | 10 | 1.815995 | -1.349992 | 166 | 38.52 | 0.232048 |
| GO:0042221\_response\_to\_chemical\_stimulus | ABAT | 409 | 10 | 1.815995 | -1.349992 | 166 | 38.52 | 0.232048 |
| GO:0042221\_response\_to\_chemical\_stimulus | AHR | 409 | 10 | 1.815995 | -1.349992 | 166 | 38.52 | 0.232048 |
| GO:0033554\_cellular\_response\_to\_stress | PAXIP1 | 196 | 6 | 2.273700 | -1.329141 | 167 | 39.24 | 0.234970 |
| GO:0033554\_cellular\_response\_to\_stress | DCLRE1A | 196 | 6 | 2.273700 | -1.329141 | 167 | 39.24 | 0.234970 |
| GO:0033554\_cellular\_response\_to\_stress | BCL2 | 196 | 6 | 2.273700 | -1.329141 | 167 | 39.24 | 0.234970 |
| GO:0033554\_cellular\_response\_to\_stress | SNCA | 196 | 6 | 2.273700 | -1.329141 | 167 | 39.24 | 0.234970 |
| GO:0033554\_cellular\_response\_to\_stress | NEFL | 196 | 6 | 2.273700 | -1.329141 | 167 | 39.24 | 0.234970 |
| GO:0033554\_cellular\_response\_to\_stress | FEN1 | 196 | 6 | 2.273700 | -1.329141 | 167 | 39.24 | 0.234970 |
| GO:0007405\_neuroblast\_proliferation | BDNF | 26 | 2 | 5.713400 | -1.326731 | 168 | 40.46 | 0.240833 |
| GO:0007405\_neuroblast\_proliferation | ID4 | 26 | 2 | 5.713400 | -1.326731 | 168 | 40.46 | 0.240833 |
| GO:0050801\_ion\_homeostasis | SLC11A1 | 197 | 6 | 2.262158 | -1.320181 | 169 | 40.62 | 0.240355 |
| GO:0050801\_ion\_homeostasis | GNAQ | 197 | 6 | 2.262158 | -1.320181 | 169 | 40.62 | 0.240355 |
| GO:0050801\_ion\_homeostasis | BCL2 | 197 | 6 | 2.262158 | -1.320181 | 169 | 40.62 | 0.240355 |
| GO:0050801\_ion\_homeostasis | SNCA | 197 | 6 | 2.262158 | -1.320181 | 169 | 40.62 | 0.240355 |
| GO:0050801\_ion\_homeostasis | CHRNB4 | 197 | 6 | 2.262158 | -1.320181 | 169 | 40.62 | 0.240355 |
| GO:0050801\_ion\_homeostasis | SLC4A4 | 197 | 6 | 2.262158 | -1.320181 | 169 | 40.62 | 0.240355 |
| GO:0032270\_positive\_regulation\_of\_cellular\_protein\_metabolic\_process | HMGB1 | 61 | 3 | 3.652829 | -1.317173 | 170 | 40.91 | 0.240647 |
| GO:0032270\_positive\_regulation\_of\_cellular\_protein\_metabolic\_process | BCL2 | 61 | 3 | 3.652829 | -1.317173 | 170 | 40.91 | 0.240647 |
| GO:0032270\_positive\_regulation\_of\_cellular\_protein\_metabolic\_process | KITL | 61 | 3 | 3.652829 | -1.317173 | 170 | 40.91 | 0.240647 |
| GO:0032940\_secretion\_by\_cell | CADPS | 149 | 5 | 2.492423 | -1.309598 | 171 | 41.23 | 0.241111 |
| GO:0032940\_secretion\_by\_cell | BDNF | 149 | 5 | 2.492423 | -1.309598 | 171 | 41.23 | 0.241111 |
| GO:0032940\_secretion\_by\_cell | PFKL | 149 | 5 | 2.492423 | -1.309598 | 171 | 41.23 | 0.241111 |
| GO:0032940\_secretion\_by\_cell | NTRK2 | 149 | 5 | 2.492423 | -1.309598 | 171 | 41.23 | 0.241111 |
| GO:0032940\_secretion\_by\_cell | SNCA | 149 | 5 | 2.492423 | -1.309598 | 171 | 41.23 | 0.241111 |
| GO:0006351\_transcription\_\_DNA-dependent | HMGB1 | 594 | 13 | 1.625529 | -1.305197 | 172 | 41.35 | 0.240407 |
| GO:0006351\_transcription\_\_DNA-dependent | LCORL | 594 | 13 | 1.625529 | -1.305197 | 172 | 41.35 | 0.240407 |
| GO:0006351\_transcription\_\_DNA-dependent | ONECUT2 | 594 | 13 | 1.625529 | -1.305197 | 172 | 41.35 | 0.240407 |
| GO:0006351\_transcription\_\_DNA-dependent | RORB | 594 | 13 | 1.625529 | -1.305197 | 172 | 41.35 | 0.240407 |
| GO:0006351\_transcription\_\_DNA-dependent | AHR | 594 | 13 | 1.625529 | -1.305197 | 172 | 41.35 | 0.240407 |
| GO:0006351\_transcription\_\_DNA-dependent | SUZ12 | 594 | 13 | 1.625529 | -1.305197 | 172 | 41.35 | 0.240407 |
| GO:0006351\_transcription\_\_DNA-dependent | SLC11A1 | 594 | 13 | 1.625529 | -1.305197 | 172 | 41.35 | 0.240407 |
| GO:0006351\_transcription\_\_DNA-dependent | RNF6 | 594 | 13 | 1.625529 | -1.305197 | 172 | 41.35 | 0.240407 |
| GO:0006351\_transcription\_\_DNA-dependent | NFAT5 | 594 | 13 | 1.625529 | -1.305197 | 172 | 41.35 | 0.240407 |
| GO:0006351\_transcription\_\_DNA-dependent | NR2F2 | 594 | 13 | 1.625529 | -1.305197 | 172 | 41.35 | 0.240407 |
| GO:0006351\_transcription\_\_DNA-dependent | MYB | 594 | 13 | 1.625529 | -1.305197 | 172 | 41.35 | 0.240407 |
| GO:0006351\_transcription\_\_DNA-dependent | ETV3 | 594 | 13 | 1.625529 | -1.305197 | 172 | 41.35 | 0.240407 |
| GO:0006351\_transcription\_\_DNA-dependent | PITX2 | 594 | 13 | 1.625529 | -1.305197 | 172 | 41.35 | 0.240407 |
| GO:0032774\_RNA\_biosynthetic\_process | HMGB1 | 595 | 13 | 1.622798 | -1.300232 | 173 | 41.51 | 0.239942 |
| GO:0032774\_RNA\_biosynthetic\_process | LCORL | 595 | 13 | 1.622798 | -1.300232 | 173 | 41.51 | 0.239942 |
| GO:0032774\_RNA\_biosynthetic\_process | ONECUT2 | 595 | 13 | 1.622798 | -1.300232 | 173 | 41.51 | 0.239942 |
| GO:0032774\_RNA\_biosynthetic\_process | RORB | 595 | 13 | 1.622798 | -1.300232 | 173 | 41.51 | 0.239942 |
| GO:0032774\_RNA\_biosynthetic\_process | AHR | 595 | 13 | 1.622798 | -1.300232 | 173 | 41.51 | 0.239942 |
| GO:0032774\_RNA\_biosynthetic\_process | SUZ12 | 595 | 13 | 1.622798 | -1.300232 | 173 | 41.51 | 0.239942 |
| GO:0032774\_RNA\_biosynthetic\_process | SLC11A1 | 595 | 13 | 1.622798 | -1.300232 | 173 | 41.51 | 0.239942 |
| GO:0032774\_RNA\_biosynthetic\_process | RNF6 | 595 | 13 | 1.622798 | -1.300232 | 173 | 41.51 | 0.239942 |
| GO:0032774\_RNA\_biosynthetic\_process | NFAT5 | 595 | 13 | 1.622798 | -1.300232 | 173 | 41.51 | 0.239942 |
| GO:0032774\_RNA\_biosynthetic\_process | NR2F2 | 595 | 13 | 1.622798 | -1.300232 | 173 | 41.51 | 0.239942 |
| GO:0032774\_RNA\_biosynthetic\_process | MYB | 595 | 13 | 1.622798 | -1.300232 | 173 | 41.51 | 0.239942 |
| GO:0032774\_RNA\_biosynthetic\_process | ETV3 | 595 | 13 | 1.622798 | -1.300232 | 173 | 41.51 | 0.239942 |
| GO:0032774\_RNA\_biosynthetic\_process | PITX2 | 595 | 13 | 1.622798 | -1.300232 | 173 | 41.51 | 0.239942 |
| GO:0006916\_anti-apoptosis | BDNF | 62 | 3 | 3.593913 | -1.299700 | 175 | 41.76 | 0.238629 |
| GO:0006916\_anti-apoptosis | BCL2 | 62 | 3 | 3.593913 | -1.299700 | 175 | 41.76 | 0.238629 |
| GO:0006916\_anti-apoptosis | VEGFA | 62 | 3 | 3.593913 | -1.299700 | 175 | 41.76 | 0.238629 |
| GO:0030155\_regulation\_of\_cell\_adhesion | DAB1 | 62 | 3 | 3.593913 | -1.299700 | 175 | 41.76 | 0.238629 |
| GO:0030155\_regulation\_of\_cell\_adhesion | BCL2 | 62 | 3 | 3.593913 | -1.299700 | 175 | 41.76 | 0.238629 |
| GO:0030155\_regulation\_of\_cell\_adhesion | ONECUT2 | 62 | 3 | 3.593913 | -1.299700 | 175 | 41.76 | 0.238629 |
| GO:0016050\_vesicle\_organization | CADPS | 27 | 2 | 5.501792 | -1.297009 | 176 | 42.5 | 0.241477 |
| GO:0016050\_vesicle\_organization | BCL2 | 27 | 2 | 5.501792 | -1.297009 | 176 | 42.5 | 0.241477 |
| GO:0007267\_cell-cell\_signaling | SPRY1 | 252 | 7 | 2.063172 | -1.293046 | 177 | 42.66 | 0.241017 |
| GO:0007267\_cell-cell\_signaling | BDNF | 252 | 7 | 2.063172 | -1.293046 | 177 | 42.66 | 0.241017 |
| GO:0007267\_cell-cell\_signaling | PFKL | 252 | 7 | 2.063172 | -1.293046 | 177 | 42.66 | 0.241017 |
| GO:0007267\_cell-cell\_signaling | SALL1 | 252 | 7 | 2.063172 | -1.293046 | 177 | 42.66 | 0.241017 |
| GO:0007267\_cell-cell\_signaling | NTRK2 | 252 | 7 | 2.063172 | -1.293046 | 177 | 42.66 | 0.241017 |
| GO:0007267\_cell-cell\_signaling | SNCA | 252 | 7 | 2.063172 | -1.293046 | 177 | 42.66 | 0.241017 |
| GO:0007267\_cell-cell\_signaling | CHRNB4 | 252 | 7 | 2.063172 | -1.293046 | 177 | 42.66 | 0.241017 |
| GO:0045893\_positive\_regulation\_of\_transcription\_\_DNA-dependent | SLC11A1 | 306 | 8 | 1.941809 | -1.292432 | 179 | 42.72 | 0.238659 |
| GO:0045893\_positive\_regulation\_of\_transcription\_\_DNA-dependent | HMGB1 | 306 | 8 | 1.941809 | -1.292432 | 179 | 42.72 | 0.238659 |
| GO:0045893\_positive\_regulation\_of\_transcription\_\_DNA-dependent | RNF6 | 306 | 8 | 1.941809 | -1.292432 | 179 | 42.72 | 0.238659 |
| GO:0045893\_positive\_regulation\_of\_transcription\_\_DNA-dependent | ONECUT2 | 306 | 8 | 1.941809 | -1.292432 | 179 | 42.72 | 0.238659 |
| GO:0045893\_positive\_regulation\_of\_transcription\_\_DNA-dependent | NFAT5 | 306 | 8 | 1.941809 | -1.292432 | 179 | 42.72 | 0.238659 |
| GO:0045893\_positive\_regulation\_of\_transcription\_\_DNA-dependent | RORB | 306 | 8 | 1.941809 | -1.292432 | 179 | 42.72 | 0.238659 |
| GO:0045893\_positive\_regulation\_of\_transcription\_\_DNA-dependent | AHR | 306 | 8 | 1.941809 | -1.292432 | 179 | 42.72 | 0.238659 |
| GO:0045893\_positive\_regulation\_of\_transcription\_\_DNA-dependent | PITX2 | 306 | 8 | 1.941809 | -1.292432 | 179 | 42.72 | 0.238659 |
| GO:0051254\_positive\_regulation\_of\_RNA\_metabolic\_process | SLC11A1 | 306 | 8 | 1.941809 | -1.292432 | 179 | 42.72 | 0.238659 |
| GO:0051254\_positive\_regulation\_of\_RNA\_metabolic\_process | HMGB1 | 306 | 8 | 1.941809 | -1.292432 | 179 | 42.72 | 0.238659 |
| GO:0051254\_positive\_regulation\_of\_RNA\_metabolic\_process | RNF6 | 306 | 8 | 1.941809 | -1.292432 | 179 | 42.72 | 0.238659 |
| GO:0051254\_positive\_regulation\_of\_RNA\_metabolic\_process | ONECUT2 | 306 | 8 | 1.941809 | -1.292432 | 179 | 42.72 | 0.238659 |
| GO:0051254\_positive\_regulation\_of\_RNA\_metabolic\_process | NFAT5 | 306 | 8 | 1.941809 | -1.292432 | 179 | 42.72 | 0.238659 |
| GO:0051254\_positive\_regulation\_of\_RNA\_metabolic\_process | RORB | 306 | 8 | 1.941809 | -1.292432 | 179 | 42.72 | 0.238659 |
| GO:0051254\_positive\_regulation\_of\_RNA\_metabolic\_process | AHR | 306 | 8 | 1.941809 | -1.292432 | 179 | 42.72 | 0.238659 |
| GO:0051254\_positive\_regulation\_of\_RNA\_metabolic\_process | PITX2 | 306 | 8 | 1.941809 | -1.292432 | 179 | 42.72 | 0.238659 |
| GO:0032989\_cellular\_component\_morphogenesis | NRCAM | 307 | 8 | 1.935484 | -1.285529 | 180 | 42.86 | 0.238111 |
| GO:0032989\_cellular\_component\_morphogenesis | SEMA5A | 307 | 8 | 1.935484 | -1.285529 | 180 | 42.86 | 0.238111 |
| GO:0032989\_cellular\_component\_morphogenesis | PARD3 | 307 | 8 | 1.935484 | -1.285529 | 180 | 42.86 | 0.238111 |
| GO:0032989\_cellular\_component\_morphogenesis | BDNF | 307 | 8 | 1.935484 | -1.285529 | 180 | 42.86 | 0.238111 |
| GO:0032989\_cellular\_component\_morphogenesis | RNF6 | 307 | 8 | 1.935484 | -1.285529 | 180 | 42.86 | 0.238111 |
| GO:0032989\_cellular\_component\_morphogenesis | BCL2 | 307 | 8 | 1.935484 | -1.285529 | 180 | 42.86 | 0.238111 |
| GO:0032989\_cellular\_component\_morphogenesis | NEFL | 307 | 8 | 1.935484 | -1.285529 | 180 | 42.86 | 0.238111 |
| GO:0032989\_cellular\_component\_morphogenesis | EPHB1 | 307 | 8 | 1.935484 | -1.285529 | 180 | 42.86 | 0.238111 |
| GO:0048878\_chemical\_homeostasis | SLC11A1 | 254 | 7 | 2.046927 | -1.277775 | 181 | 43.26 | 0.239006 |
| GO:0048878\_chemical\_homeostasis | GNAQ | 254 | 7 | 2.046927 | -1.277775 | 181 | 43.26 | 0.239006 |
| GO:0048878\_chemical\_homeostasis | BCL2 | 254 | 7 | 2.046927 | -1.277775 | 181 | 43.26 | 0.239006 |
| GO:0048878\_chemical\_homeostasis | VEGFA | 254 | 7 | 2.046927 | -1.277775 | 181 | 43.26 | 0.239006 |
| GO:0048878\_chemical\_homeostasis | SNCA | 254 | 7 | 2.046927 | -1.277775 | 181 | 43.26 | 0.239006 |
| GO:0048878\_chemical\_homeostasis | CHRNB4 | 254 | 7 | 2.046927 | -1.277775 | 181 | 43.26 | 0.239006 |
| GO:0048878\_chemical\_homeostasis | SLC4A4 | 254 | 7 | 2.046927 | -1.277775 | 181 | 43.26 | 0.239006 |
| GO:0001938\_positive\_regulation\_of\_endothelial\_cell\_proliferation | VEGFA | 4 | 1 |  |  |  |  |  |  |
| GO:0002326\_B\_cell\_lineage\_commitment | BCL2 | 4 | 1 |  |  |  |  |  |  |
| GO:0007144\_female\_meiosis\_I | CDC25B | 4 | 1 |  |  |  |  |  |  |
| GO:0007184\_SMAD\_protein\_nuclear\_translocation | TOB1 | 4 | 1 |  |  |  |  |  |  |
| GO:0010224\_response\_to\_UV-B | BCL2 | 4 | 1 |  |  |  |  |  |  |
| GO:0015701\_bicarbonate\_transport | SLC4A4 | 4 | 1 |  |  |  |  |  |  |
| GO:0015809\_arginine\_transport | SLC11A1 | 4 | 1 |  |  |  |  |  |  |
| GO:0021535\_cell\_migration\_in\_hindbrain | DAB1 | 4 | 1 |  |  |  |  |  |  |
| GO:0021631\_optic\_nerve\_morphogenesis | EPHB1 | 4 | 1 |  |  |  |  |  |  |
| GO:0021801\_cerebral\_cortex\_radial\_glia\_guided\_migration | DAB1 | 4 | 1 |  |  |  |  |  |  |
| GO:0032835\_glomerulus\_development | BCL2 | 4 | 1 |  |  |  |  |  |  |
| GO:0033026\_negative\_regulation\_of\_mast\_cell\_apoptosis | KITL | 4 | 1 |  |  |  |  |  |  |
| GO:0033135\_regulation\_of\_peptidyl-serine\_phosphorylation | BCL2 | 4 | 1 |  |  |  |  |  |  |
| GO:0042773\_ATP\_synthesis\_coupled\_electron\_transport | SNCA | 4 | 1 |  |  |  |  |  |  |
| GO:0042775\_mitochondrial\_ATP\_synthesis\_coupled\_electron\_transport | SNCA | 4 | 1 |  |  |  |  |  |  |
| GO:0042832\_defense\_response\_to\_protozoan | SLC11A1 | 4 | 1 |  |  |  |  |  |  |
| GO:0043129\_surfactant\_homeostasis | VEGFA | 4 | 1 |  |  |  |  |  |  |
| GO:0043374\_CD8-positive\_\_alpha-beta\_T\_cell\_differentiation | BCL2 | 4 | 1 |  |  |  |  |  |  |
| GO:0043470\_regulation\_of\_carbohydrate\_catabolic\_process | HMGB1 | 4 | 1 |  |  |  |  |  |  |
| GO:0043471\_regulation\_of\_cellular\_carbohydrate\_catabolic\_process | HMGB1 | 4 | 1 |  |  |  |  |  |  |
| GO:0045930\_negative\_regulation\_of\_mitotic\_cell\_cycle | BCL2 | 4 | 1 |  |  |  |  |  |  |
| GO:0046548\_retinal\_rod\_cell\_development | NTRK2 | 4 | 1 |  |  |  |  |  |  |
| GO:0046579\_positive\_regulation\_of\_Ras\_protein\_signal\_transduction | KITL | 4 | 1 |  |  |  |  |  |  |
| GO:0046902\_regulation\_of\_mitochondrial\_membrane\_permeability | BCL2 | 4 | 1 |  |  |  |  |  |  |
| GO:0048710\_regulation\_of\_astrocyte\_differentiation | ID4 | 4 | 1 |  |  |  |  |  |  |
| GO:0048875\_chemical\_homeostasis\_within\_a\_tissue | VEGFA | 4 | 1 |  |  |  |  |  |  |
| GO:0048935\_peripheral\_nervous\_system\_neuron\_development | ONECUT2 | 4 | 1 |  |  |  |  |  |  |
| GO:0051452\_intracellular\_pH\_reduction | SLC11A1 | 4 | 1 |  |  |  |  |  |  |
| GO:0055009\_atrial\_cardiac\_muscle\_morphogenesis | PITX2 | 4 | 1 |  |  |  |  |  |  |
| GO:0060158\_activation\_of\_phospholipase\_C\_activity\_by\_dopamine\_receptor\_signaling\_pathway | GNAQ | 4 | 1 |  |  |  |  |  |  |
| GO:0060291\_long-term\_synaptic\_potentiation | SNCA | 4 | 1 |  |  |  |  |  |  |
| GO:0060459\_left\_lung\_development | PITX2 | 4 | 1 |  |  |  |  |  |  |
| GO:0070059\_apoptosis\_in\_response\_to\_endoplasmic\_reticulum\_stress | BCL2 | 4 | 1 |  |  |  |  |  |  |
| GO:0016310\_phosphorylation | SLC11A1 | 309 | 8 | 1.922956 | -1.271835 | 182 | 43.34 | 0.238132 |
| GO:0016310\_phosphorylation | HMGB1 | 309 | 8 | 1.922956 | -1.271835 | 182 | 43.34 | 0.238132 |
| GO:0016310\_phosphorylation | SPRY1 | 309 | 8 | 1.922956 | -1.271835 | 182 | 43.34 | 0.238132 |
| GO:0016310\_phosphorylation | DAB1 | 309 | 8 | 1.922956 | -1.271835 | 182 | 43.34 | 0.238132 |
| GO:0016310\_phosphorylation | BCL2 | 309 | 8 | 1.922956 | -1.271835 | 182 | 43.34 | 0.238132 |
| GO:0016310\_phosphorylation | SNCA | 309 | 8 | 1.922956 | -1.271835 | 182 | 43.34 | 0.238132 |
| GO:0016310\_phosphorylation | KITL | 309 | 8 | 1.922956 | -1.271835 | 182 | 43.34 | 0.238132 |
| GO:0016310\_phosphorylation | CDC25B | 309 | 8 | 1.922956 | -1.271835 | 182 | 43.34 | 0.238132 |
| GO:0006470\_protein\_amino\_acid\_dephosphorylation | BCL2 | 28 | 2 | 5.305300 | -1.268523 | 184 | 44.34 | 0.240978 |
| GO:0006470\_protein\_amino\_acid\_dephosphorylation | PPM1A | 28 | 2 | 5.305300 | -1.268523 | 184 | 44.34 | 0.240978 |
| GO:0043193\_positive\_regulation\_of\_gene-specific\_transcription | HMGB1 | 28 | 2 | 5.305300 | -1.268523 | 184 | 44.34 | 0.240978 |
| GO:0043193\_positive\_regulation\_of\_gene-specific\_transcription | RORB | 28 | 2 | 5.305300 | -1.268523 | 184 | 44.34 | 0.240978 |
| GO:0048511\_rhythmic\_process | CSNK1E | 65 | 3 | 3.428040 | -1.249324 | 185 | 45.14 | 0.244000 |
| GO:0048511\_rhythmic\_process | BCL2 | 65 | 3 | 3.428040 | -1.249324 | 185 | 45.14 | 0.244000 |
| GO:0048511\_rhythmic\_process | VEGFA | 65 | 3 | 3.428040 | -1.249324 | 185 | 45.14 | 0.244000 |
| GO:0042490\_mechanoreceptor\_differentiation | BDNF | 29 | 2 | 5.122358 | -1.241184 | 186 | 46.46 | 0.249785 |
| GO:0042490\_mechanoreceptor\_differentiation | NTRK2 | 29 | 2 | 5.122358 | -1.241184 | 186 | 46.46 | 0.249785 |
| GO:0048519\_negative\_regulation\_of\_biological\_process | PFKL | 859 | 17 | 1.469920 | -1.240875 | 187 | 46.48 | 0.248556 |
| GO:0048519\_negative\_regulation\_of\_biological\_process | SNCA | 859 | 17 | 1.469920 | -1.240875 | 187 | 46.48 | 0.248556 |
| GO:0048519\_negative\_regulation\_of\_biological\_process | ONECUT2 | 859 | 17 | 1.469920 | -1.240875 | 187 | 46.48 | 0.248556 |
| GO:0048519\_negative\_regulation\_of\_biological\_process | KITL | 859 | 17 | 1.469920 | -1.240875 | 187 | 46.48 | 0.248556 |
| GO:0048519\_negative\_regulation\_of\_biological\_process | SUZ12 | 859 | 17 | 1.469920 | -1.240875 | 187 | 46.48 | 0.248556 |
| GO:0048519\_negative\_regulation\_of\_biological\_process | SLC11A1 | 859 | 17 | 1.469920 | -1.240875 | 187 | 46.48 | 0.248556 |
| GO:0048519\_negative\_regulation\_of\_biological\_process | BDNF | 859 | 17 | 1.469920 | -1.240875 | 187 | 46.48 | 0.248556 |
| GO:0048519\_negative\_regulation\_of\_biological\_process | RNF6 | 859 | 17 | 1.469920 | -1.240875 | 187 | 46.48 | 0.248556 |
| GO:0048519\_negative\_regulation\_of\_biological\_process | DAB1 | 859 | 17 | 1.469920 | -1.240875 | 187 | 46.48 | 0.248556 |
| GO:0048519\_negative\_regulation\_of\_biological\_process | BCL2 | 859 | 17 | 1.469920 | -1.240875 | 187 | 46.48 | 0.248556 |
| GO:0048519\_negative\_regulation\_of\_biological\_process | VEGFA | 859 | 17 | 1.469920 | -1.240875 | 187 | 46.48 | 0.248556 |
| GO:0048519\_negative\_regulation\_of\_biological\_process | ID4 | 859 | 17 | 1.469920 | -1.240875 | 187 | 46.48 | 0.248556 |
| GO:0048519\_negative\_regulation\_of\_biological\_process | FABP7 | 859 | 17 | 1.469920 | -1.240875 | 187 | 46.48 | 0.248556 |
| GO:0048519\_negative\_regulation\_of\_biological\_process | NR2F2 | 859 | 17 | 1.469920 | -1.240875 | 187 | 46.48 | 0.248556 |
| GO:0048519\_negative\_regulation\_of\_biological\_process | NEFL | 859 | 17 | 1.469920 | -1.240875 | 187 | 46.48 | 0.248556 |
| GO:0048519\_negative\_regulation\_of\_biological\_process | ETV3 | 859 | 17 | 1.469920 | -1.240875 | 187 | 46.48 | 0.248556 |
| GO:0048519\_negative\_regulation\_of\_biological\_process | TOB1 | 859 | 17 | 1.469920 | -1.240875 | 187 | 46.48 | 0.248556 |
| GO:0051641\_cellular\_localization | CADPS | 370 | 9 | 1.806670 | -1.240121 | 188 | 46.52 | 0.247447 |
| GO:0051641\_cellular\_localization | SLC11A1 | 370 | 9 | 1.806670 | -1.240121 | 188 | 46.52 | 0.247447 |
| GO:0051641\_cellular\_localization | BDNF | 370 | 9 | 1.806670 | -1.240121 | 188 | 46.52 | 0.247447 |
| GO:0051641\_cellular\_localization | PFKL | 370 | 9 | 1.806670 | -1.240121 | 188 | 46.52 | 0.247447 |
| GO:0051641\_cellular\_localization | BCL2 | 370 | 9 | 1.806670 | -1.240121 | 188 | 46.52 | 0.247447 |
| GO:0051641\_cellular\_localization | NTRK2 | 370 | 9 | 1.806670 | -1.240121 | 188 | 46.52 | 0.247447 |
| GO:0051641\_cellular\_localization | SNCA | 370 | 9 | 1.806670 | -1.240121 | 188 | 46.52 | 0.247447 |
| GO:0051641\_cellular\_localization | EHD1 | 370 | 9 | 1.806670 | -1.240121 | 188 | 46.52 | 0.247447 |
| GO:0051641\_cellular\_localization | TOB1 | 370 | 9 | 1.806670 | -1.240121 | 188 | 46.52 | 0.247447 |
| GO:0015837\_amine\_transport | CADPS | 66 | 3 | 3.376100 | -1.233178 | 190 | 46.88 | 0.246737 |
| GO:0015837\_amine\_transport | SLC11A1 | 66 | 3 | 3.376100 | -1.233178 | 190 | 46.88 | 0.246737 |
| GO:0015837\_amine\_transport | SNCA | 66 | 3 | 3.376100 | -1.233178 | 190 | 46.88 | 0.246737 |
| GO:0051130\_positive\_regulation\_of\_cellular\_component\_organization | SLC11A1 | 66 | 3 | 3.376100 | -1.233178 | 190 | 46.88 | 0.246737 |
| GO:0051130\_positive\_regulation\_of\_cellular\_component\_organization | NEFL | 66 | 3 | 3.376100 | -1.233178 | 190 | 46.88 | 0.246737 |
| GO:0051130\_positive\_regulation\_of\_cellular\_component\_organization | AHR | 66 | 3 | 3.376100 | -1.233178 | 190 | 46.88 | 0.246737 |
| GO:0080090\_regulation\_of\_primary\_metabolic\_process | HMGB1 | 926 | 18 | 1.443775 | -1.231926 | 191 | 46.91 | 0.245602 |
| GO:0080090\_regulation\_of\_primary\_metabolic\_process | SNCA | 926 | 18 | 1.443775 | -1.231926 | 191 | 46.91 | 0.245602 |
| GO:0080090\_regulation\_of\_primary\_metabolic\_process | ONECUT2 | 926 | 18 | 1.443775 | -1.231926 | 191 | 46.91 | 0.245602 |
| GO:0080090\_regulation\_of\_primary\_metabolic\_process | RORB | 926 | 18 | 1.443775 | -1.231926 | 191 | 46.91 | 0.245602 |
| GO:0080090\_regulation\_of\_primary\_metabolic\_process | KITL | 926 | 18 | 1.443775 | -1.231926 | 191 | 46.91 | 0.245602 |
| GO:0080090\_regulation\_of\_primary\_metabolic\_process | AHR | 926 | 18 | 1.443775 | -1.231926 | 191 | 46.91 | 0.245602 |
| GO:0080090\_regulation\_of\_primary\_metabolic\_process | SUZ12 | 926 | 18 | 1.443775 | -1.231926 | 191 | 46.91 | 0.245602 |
| GO:0080090\_regulation\_of\_primary\_metabolic\_process | SLC11A1 | 926 | 18 | 1.443775 | -1.231926 | 191 | 46.91 | 0.245602 |
| GO:0080090\_regulation\_of\_primary\_metabolic\_process | RNF6 | 926 | 18 | 1.443775 | -1.231926 | 191 | 46.91 | 0.245602 |
| GO:0080090\_regulation\_of\_primary\_metabolic\_process | GNAQ | 926 | 18 | 1.443775 | -1.231926 | 191 | 46.91 | 0.245602 |
| GO:0080090\_regulation\_of\_primary\_metabolic\_process | BCL2 | 926 | 18 | 1.443775 | -1.231926 | 191 | 46.91 | 0.245602 |
| GO:0080090\_regulation\_of\_primary\_metabolic\_process | GARNL1 | 926 | 18 | 1.443775 | -1.231926 | 191 | 46.91 | 0.245602 |
| GO:0080090\_regulation\_of\_primary\_metabolic\_process | NFAT5 | 926 | 18 | 1.443775 | -1.231926 | 191 | 46.91 | 0.245602 |
| GO:0080090\_regulation\_of\_primary\_metabolic\_process | MYB | 926 | 18 | 1.443775 | -1.231926 | 191 | 46.91 | 0.245602 |
| GO:0080090\_regulation\_of\_primary\_metabolic\_process | ACSL4 | 926 | 18 | 1.443775 | -1.231926 | 191 | 46.91 | 0.245602 |
| GO:0080090\_regulation\_of\_primary\_metabolic\_process | NR2F2 | 926 | 18 | 1.443775 | -1.231926 | 191 | 46.91 | 0.245602 |
| GO:0080090\_regulation\_of\_primary\_metabolic\_process | ETV3 | 926 | 18 | 1.443775 | -1.231926 | 191 | 46.91 | 0.245602 |
| GO:0080090\_regulation\_of\_primary\_metabolic\_process | PITX2 | 926 | 18 | 1.443775 | -1.231926 | 191 | 46.91 | 0.245602 |
| GO:0048514\_blood\_vessel\_morphogenesis | SEMA5A | 158 | 5 | 2.350449 | -1.220018 | 192 | 47.38 | 0.246771 |
| GO:0048514\_blood\_vessel\_morphogenesis | NTRK2 | 158 | 5 | 2.350449 | -1.220018 | 192 | 47.38 | 0.246771 |
| GO:0048514\_blood\_vessel\_morphogenesis | VEGFA | 158 | 5 | 2.350449 | -1.220018 | 192 | 47.38 | 0.246771 |
| GO:0048514\_blood\_vessel\_morphogenesis | NR2F2 | 158 | 5 | 2.350449 | -1.220018 | 192 | 47.38 | 0.246771 |
| GO:0048514\_blood\_vessel\_morphogenesis | PITX2 | 158 | 5 | 2.350449 | -1.220018 | 192 | 47.38 | 0.246771 |
| GO:0009791\_post-embryonic\_development | GNAQ | 67 | 3 | 3.325710 | -1.217337 | 194 | 47.75 | 0.246134 |
| GO:0009791\_post-embryonic\_development | MORC3 | 67 | 3 | 3.325710 | -1.217337 | 194 | 47.75 | 0.246134 |
| GO:0009791\_post-embryonic\_development | BCL2 | 67 | 3 | 3.325710 | -1.217337 | 194 | 47.75 | 0.246134 |
| GO:0051247\_positive\_regulation\_of\_protein\_metabolic\_process | HMGB1 | 67 | 3 | 3.325710 | -1.217337 | 194 | 47.75 | 0.246134 |
| GO:0051247\_positive\_regulation\_of\_protein\_metabolic\_process | BCL2 | 67 | 3 | 3.325710 | -1.217337 | 194 | 47.75 | 0.246134 |
| GO:0051247\_positive\_regulation\_of\_protein\_metabolic\_process | KITL | 67 | 3 | 3.325710 | -1.217337 | 194 | 47.75 | 0.246134 |
| GO:0048565\_gut\_development | BCL2 | 30 | 2 | 4.951613 | -1.214912 | 195 | 49.15 | 0.252051 |
| GO:0048565\_gut\_development | MYB | 30 | 2 | 4.951613 | -1.214912 | 195 | 49.15 | 0.252051 |
| GO:0010604\_positive\_regulation\_of\_macromolecule\_metabolic\_process | SLC11A1 | 433 | 10 | 1.715339 | -1.210277 | 196 | 49.26 | 0.251327 |
| GO:0010604\_positive\_regulation\_of\_macromolecule\_metabolic\_process | HMGB1 | 433 | 10 | 1.715339 | -1.210277 | 196 | 49.26 | 0.251327 |
| GO:0010604\_positive\_regulation\_of\_macromolecule\_metabolic\_process | RNF6 | 433 | 10 | 1.715339 | -1.210277 | 196 | 49.26 | 0.251327 |
| GO:0010604\_positive\_regulation\_of\_macromolecule\_metabolic\_process | BCL2 | 433 | 10 | 1.715339 | -1.210277 | 196 | 49.26 | 0.251327 |
| GO:0010604\_positive\_regulation\_of\_macromolecule\_metabolic\_process | ONECUT2 | 433 | 10 | 1.715339 | -1.210277 | 196 | 49.26 | 0.251327 |
| GO:0010604\_positive\_regulation\_of\_macromolecule\_metabolic\_process | NFAT5 | 433 | 10 | 1.715339 | -1.210277 | 196 | 49.26 | 0.251327 |
| GO:0010604\_positive\_regulation\_of\_macromolecule\_metabolic\_process | RORB | 433 | 10 | 1.715339 | -1.210277 | 196 | 49.26 | 0.251327 |
| GO:0010604\_positive\_regulation\_of\_macromolecule\_metabolic\_process | KITL | 433 | 10 | 1.715339 | -1.210277 | 196 | 49.26 | 0.251327 |
| GO:0010604\_positive\_regulation\_of\_macromolecule\_metabolic\_process | AHR | 433 | 10 | 1.715339 | -1.210277 | 196 | 49.26 | 0.251327 |
| GO:0010604\_positive\_regulation\_of\_macromolecule\_metabolic\_process | PITX2 | 433 | 10 | 1.715339 | -1.210277 | 196 | 49.26 | 0.251327 |
| GO:0016311\_dephosphorylation | BCL2 | 31 | 2 | 4.791883 | -1.189635 | 200 | 51.55 | 0.257750 |
| GO:0016311\_dephosphorylation | PPM1A | 31 | 2 | 4.791883 | -1.189635 | 200 | 51.55 | 0.257750 |
| GO:0021954\_central\_nervous\_system\_neuron\_development | GNAQ | 31 | 2 | 4.791883 | -1.189635 | 200 | 51.55 | 0.257750 |
| GO:0021954\_central\_nervous\_system\_neuron\_development | EPHB1 | 31 | 2 | 4.791883 | -1.189635 | 200 | 51.55 | 0.257750 |
| GO:0033555\_multicellular\_organismal\_response\_to\_stress | BDNF | 31 | 2 | 4.791883 | -1.189635 | 200 | 51.55 | 0.257750 |
| GO:0033555\_multicellular\_organismal\_response\_to\_stress | BCL2 | 31 | 2 | 4.791883 | -1.189635 | 200 | 51.55 | 0.257750 |
| GO:0048167\_regulation\_of\_synaptic\_plasticity | BDNF | 31 | 2 | 4.791883 | -1.189635 | 200 | 51.55 | 0.257750 |
| GO:0048167\_regulation\_of\_synaptic\_plasticity | SNCA | 31 | 2 | 4.791883 | -1.189635 | 200 | 51.55 | 0.257750 |
| GO:0001932\_regulation\_of\_protein\_amino\_acid\_phosphorylation | HMGB1 | 69 | 3 | 3.229313 | -1.186537 | 201 | 51.98 | 0.258607 |
| GO:0001932\_regulation\_of\_protein\_amino\_acid\_phosphorylation | BCL2 | 69 | 3 | 3.229313 | -1.186537 | 201 | 51.98 | 0.258607 |
| GO:0001932\_regulation\_of\_protein\_amino\_acid\_phosphorylation | KITL | 69 | 3 | 3.229313 | -1.186537 | 201 | 51.98 | 0.258607 |
| GO:0000272\_polysaccharide\_catabolic\_process | HMGB1 | 5 | 1 | 14.854839 | -1.183378 | 233 | 70.14 | 0.301030 |
| GO:0001562\_response\_to\_protozoan | SLC11A1 | 5 | 1 | 14.854839 | -1.183378 | 233 | 70.14 | 0.301030 |
| GO:0002825\_regulation\_of\_T-helper\_1\_type\_immune\_response | SLC11A1 | 5 | 1 | 14.854839 | -1.183378 | 233 | 70.14 | 0.301030 |
| GO:0005980\_glycogen\_catabolic\_process | HMGB1 | 5 | 1 | 14.854839 | -1.183378 | 233 | 70.14 | 0.301030 |
| GO:0009251\_glucan\_catabolic\_process | HMGB1 | 5 | 1 | 14.854839 | -1.183378 | 233 | 70.14 | 0.301030 |
| GO:0010522\_regulation\_of\_calcium\_ion\_transport\_into\_cytosol | BCL2 | 5 | 1 | 14.854839 | -1.183378 | 233 | 70.14 | 0.301030 |
| GO:0010676\_positive\_regulation\_of\_cellular\_carbohydrate\_metabolic\_process | HMGB1 | 5 | 1 | 14.854839 | -1.183378 | 233 | 70.14 | 0.301030 |
| GO:0015802\_basic\_amino\_acid\_transport | SLC11A1 | 5 | 1 | 14.854839 | -1.183378 | 233 | 70.14 | 0.301030 |
| GO:0019227\_neuronal\_action\_potential\_propagation | NRCAM | 5 | 1 | 14.854839 | -1.183378 | 233 | 70.14 | 0.301030 |
| GO:0021554\_optic\_nerve\_development | EPHB1 | 5 | 1 | 14.854839 | -1.183378 | 233 | 70.14 | 0.301030 |
| GO:0022415\_viral\_reproductive\_process | BCL2 | 5 | 1 | 14.854839 | -1.183378 | 233 | 70.14 | 0.301030 |
| GO:0030004\_cellular\_monovalent\_inorganic\_cation\_homeostasis | SLC11A1 | 5 | 1 | 14.854839 | -1.183378 | 233 | 70.14 | 0.301030 |
| GO:0030641\_regulation\_of\_cellular\_pH | SLC11A1 | 5 | 1 | 14.854839 | -1.183378 | 233 | 70.14 | 0.301030 |
| GO:0032845\_negative\_regulation\_of\_homeostatic\_process | BCL2 | 5 | 1 | 14.854839 | -1.183378 | 233 | 70.14 | 0.301030 |
| GO:0033023\_mast\_cell\_homeostasis | KITL | 5 | 1 | 14.854839 | -1.183378 | 233 | 70.14 | 0.301030 |
| GO:0033024\_mast\_cell\_apoptosis | KITL | 5 | 1 | 14.854839 | -1.183378 | 233 | 70.14 | 0.301030 |
| GO:0033025\_regulation\_of\_mast\_cell\_apoptosis | KITL | 5 | 1 | 14.854839 | -1.183378 | 233 | 70.14 | 0.301030 |
| GO:0035095\_behavioral\_response\_to\_nicotine | CHRNB4 | 5 | 1 | 14.854839 | -1.183378 | 233 | 70.14 | 0.301030 |
| GO:0035234\_germ\_cell\_programmed\_cell\_death | KITL | 5 | 1 | 14.854839 | -1.183378 | 233 | 70.14 | 0.301030 |
| GO:0042416\_dopamine\_biosynthetic\_process | SNCA | 5 | 1 | 14.854839 | -1.183378 | 233 | 70.14 | 0.301030 |
| GO:0043030\_regulation\_of\_macrophage\_activation | SNCA | 5 | 1 | 14.854839 | -1.183378 | 233 | 70.14 | 0.301030 |
| GO:0043489\_RNA\_stabilization | SLC11A1 | 5 | 1 | 14.854839 | -1.183378 | 233 | 70.14 | 0.301030 |
| GO:0044247\_cellular\_polysaccharide\_catabolic\_process | HMGB1 | 5 | 1 | 14.854839 | -1.183378 | 233 | 70.14 | 0.301030 |
| GO:0045342\_MHC\_class\_II\_biosynthetic\_process | SLC11A1 | 5 | 1 | 14.854839 | -1.183378 | 233 | 70.14 | 0.301030 |
| GO:0045730\_respiratory\_burst | SLC11A1 | 5 | 1 | 14.854839 | -1.183378 | 233 | 70.14 | 0.301030 |
| GO:0045851\_pH\_reduction | SLC11A1 | 5 | 1 | 14.854839 | -1.183378 | 233 | 70.14 | 0.301030 |
| GO:0048255\_mRNA\_stabilization | SLC11A1 | 5 | 1 | 14.854839 | -1.183378 | 233 | 70.14 | 0.301030 |
| GO:0048532\_anatomical\_structure\_arrangement | DAB1 | 5 | 1 | 14.854839 | -1.183378 | 233 | 70.14 | 0.301030 |
| GO:0048934\_peripheral\_nervous\_system\_neuron\_differentiation | ONECUT2 | 5 | 1 | 14.854839 | -1.183378 | 233 | 70.14 | 0.301030 |
| GO:0051057\_positive\_regulation\_of\_small\_GTPase\_mediated\_signal\_transduction | KITL | 5 | 1 | 14.854839 | -1.183378 | 233 | 70.14 | 0.301030 |
| GO:0051453\_regulation\_of\_intracellular\_pH | SLC11A1 | 5 | 1 | 14.854839 | -1.183378 | 233 | 70.14 | 0.301030 |
| GO:0060073\_micturition | CHRNB4 | 5 | 1 | 14.854839 | -1.183378 | 233 | 70.14 | 0.301030 |
| GO:0031326\_regulation\_of\_cellular\_biosynthetic\_process | HMGB1 | 812 | 16 | 1.463531 | -1.170900 | 234 | 70.8 | 0.302564 |
| GO:0031326\_regulation\_of\_cellular\_biosynthetic\_process | SNCA | 812 | 16 | 1.463531 | -1.170900 | 234 | 70.8 | 0.302564 |
| GO:0031326\_regulation\_of\_cellular\_biosynthetic\_process | ONECUT2 | 812 | 16 | 1.463531 | -1.170900 | 234 | 70.8 | 0.302564 |
| GO:0031326\_regulation\_of\_cellular\_biosynthetic\_process | RORB | 812 | 16 | 1.463531 | -1.170900 | 234 | 70.8 | 0.302564 |
| GO:0031326\_regulation\_of\_cellular\_biosynthetic\_process | AHR | 812 | 16 | 1.463531 | -1.170900 | 234 | 70.8 | 0.302564 |
| GO:0031326\_regulation\_of\_cellular\_biosynthetic\_process | SUZ12 | 812 | 16 | 1.463531 | -1.170900 | 234 | 70.8 | 0.302564 |
| GO:0031326\_regulation\_of\_cellular\_biosynthetic\_process | SLC11A1 | 812 | 16 | 1.463531 | -1.170900 | 234 | 70.8 | 0.302564 |
| GO:0031326\_regulation\_of\_cellular\_biosynthetic\_process | RNF6 | 812 | 16 | 1.463531 | -1.170900 | 234 | 70.8 | 0.302564 |
| GO:0031326\_regulation\_of\_cellular\_biosynthetic\_process | GNAQ | 812 | 16 | 1.463531 | -1.170900 | 234 | 70.8 | 0.302564 |
| GO:0031326\_regulation\_of\_cellular\_biosynthetic\_process | BCL2 | 812 | 16 | 1.463531 | -1.170900 | 234 | 70.8 | 0.302564 |
| GO:0031326\_regulation\_of\_cellular\_biosynthetic\_process | GARNL1 | 812 | 16 | 1.463531 | -1.170900 | 234 | 70.8 | 0.302564 |
| GO:0031326\_regulation\_of\_cellular\_biosynthetic\_process | NFAT5 | 812 | 16 | 1.463531 | -1.170900 | 234 | 70.8 | 0.302564 |
| GO:0031326\_regulation\_of\_cellular\_biosynthetic\_process | MYB | 812 | 16 | 1.463531 | -1.170900 | 234 | 70.8 | 0.302564 |
| GO:0031326\_regulation\_of\_cellular\_biosynthetic\_process | NR2F2 | 812 | 16 | 1.463531 | -1.170900 | 234 | 70.8 | 0.302564 |
| GO:0031326\_regulation\_of\_cellular\_biosynthetic\_process | ETV3 | 812 | 16 | 1.463531 | -1.170900 | 234 | 70.8 | 0.302564 |
| GO:0031326\_regulation\_of\_cellular\_biosynthetic\_process | PITX2 | 812 | 16 | 1.463531 | -1.170900 | 234 | 70.8 | 0.302564 |
| GO:0002274\_myeloid\_leukocyte\_activation | SLC11A1 | 32 | 2 | 4.642137 | -1.165290 | 236 | 72.05 | 0.305297 |
| GO:0002274\_myeloid\_leukocyte\_activation | SNCA | 32 | 2 | 4.642137 | -1.165290 | 236 | 72.05 | 0.305297 |
| GO:0050770\_regulation\_of\_axonogenesis | RNF6 | 32 | 2 | 4.642137 | -1.165290 | 236 | 72.05 | 0.305297 |
| GO:0050770\_regulation\_of\_axonogenesis | NEFL | 32 | 2 | 4.642137 | -1.165290 | 236 | 72.05 | 0.305297 |
| GO:0031325\_positive\_regulation\_of\_cellular\_metabolic\_process | SLC11A1 | 442 | 10 | 1.680412 | -1.161473 | 237 | 72.26 | 0.304895 |
| GO:0031325\_positive\_regulation\_of\_cellular\_metabolic\_process | HMGB1 | 442 | 10 | 1.680412 | -1.161473 | 237 | 72.26 | 0.304895 |
| GO:0031325\_positive\_regulation\_of\_cellular\_metabolic\_process | RNF6 | 442 | 10 | 1.680412 | -1.161473 | 237 | 72.26 | 0.304895 |
| GO:0031325\_positive\_regulation\_of\_cellular\_metabolic\_process | BCL2 | 442 | 10 | 1.680412 | -1.161473 | 237 | 72.26 | 0.304895 |
| GO:0031325\_positive\_regulation\_of\_cellular\_metabolic\_process | ONECUT2 | 442 | 10 | 1.680412 | -1.161473 | 237 | 72.26 | 0.304895 |
| GO:0031325\_positive\_regulation\_of\_cellular\_metabolic\_process | NFAT5 | 442 | 10 | 1.680412 | -1.161473 | 237 | 72.26 | 0.304895 |
| GO:0031325\_positive\_regulation\_of\_cellular\_metabolic\_process | RORB | 442 | 10 | 1.680412 | -1.161473 | 237 | 72.26 | 0.304895 |
| GO:0031325\_positive\_regulation\_of\_cellular\_metabolic\_process | KITL | 442 | 10 | 1.680412 | -1.161473 | 237 | 72.26 | 0.304895 |
| GO:0031325\_positive\_regulation\_of\_cellular\_metabolic\_process | AHR | 442 | 10 | 1.680412 | -1.161473 | 237 | 72.26 | 0.304895 |
| GO:0031325\_positive\_regulation\_of\_cellular\_metabolic\_process | PITX2 | 442 | 10 | 1.680412 | -1.161473 | 237 | 72.26 | 0.304895 |
| GO:0009889\_regulation\_of\_biosynthetic\_process | HMGB1 | 815 | 16 | 1.458144 | -1.158930 | 238 | 72.32 | 0.303866 |
| GO:0009889\_regulation\_of\_biosynthetic\_process | SNCA | 815 | 16 | 1.458144 | -1.158930 | 238 | 72.32 | 0.303866 |
| GO:0009889\_regulation\_of\_biosynthetic\_process | ONECUT2 | 815 | 16 | 1.458144 | -1.158930 | 238 | 72.32 | 0.303866 |
| GO:0009889\_regulation\_of\_biosynthetic\_process | RORB | 815 | 16 | 1.458144 | -1.158930 | 238 | 72.32 | 0.303866 |
| GO:0009889\_regulation\_of\_biosynthetic\_process | AHR | 815 | 16 | 1.458144 | -1.158930 | 238 | 72.32 | 0.303866 |
| GO:0009889\_regulation\_of\_biosynthetic\_process | SUZ12 | 815 | 16 | 1.458144 | -1.158930 | 238 | 72.32 | 0.303866 |
| GO:0009889\_regulation\_of\_biosynthetic\_process | SLC11A1 | 815 | 16 | 1.458144 | -1.158930 | 238 | 72.32 | 0.303866 |
| GO:0009889\_regulation\_of\_biosynthetic\_process | RNF6 | 815 | 16 | 1.458144 | -1.158930 | 238 | 72.32 | 0.303866 |
| GO:0009889\_regulation\_of\_biosynthetic\_process | GNAQ | 815 | 16 | 1.458144 | -1.158930 | 238 | 72.32 | 0.303866 |
| GO:0009889\_regulation\_of\_biosynthetic\_process | BCL2 | 815 | 16 | 1.458144 | -1.158930 | 238 | 72.32 | 0.303866 |
| GO:0009889\_regulation\_of\_biosynthetic\_process | GARNL1 | 815 | 16 | 1.458144 | -1.158930 | 238 | 72.32 | 0.303866 |
| GO:0009889\_regulation\_of\_biosynthetic\_process | NFAT5 | 815 | 16 | 1.458144 | -1.158930 | 238 | 72.32 | 0.303866 |
| GO:0009889\_regulation\_of\_biosynthetic\_process | NR2F2 | 815 | 16 | 1.458144 | -1.158930 | 238 | 72.32 | 0.303866 |
| GO:0009889\_regulation\_of\_biosynthetic\_process | MYB | 815 | 16 | 1.458144 | -1.158930 | 238 | 72.32 | 0.303866 |
| GO:0009889\_regulation\_of\_biosynthetic\_process | ETV3 | 815 | 16 | 1.458144 | -1.158930 | 238 | 72.32 | 0.303866 |
| GO:0009889\_regulation\_of\_biosynthetic\_process | PITX2 | 815 | 16 | 1.458144 | -1.158930 | 238 | 72.32 | 0.303866 |
| GO:0006807\_nitrogen\_compound\_metabolic\_process | HMGB1 | 1147 | 21 | 1.359859 | -1.150901 | 239 | 73.03 | 0.305565 |
| GO:0006807\_nitrogen\_compound\_metabolic\_process | ALDH18A1 | 1147 | 21 | 1.359859 | -1.150901 | 239 | 73.03 | 0.305565 |
| GO:0006807\_nitrogen\_compound\_metabolic\_process | LCORL | 1147 | 21 | 1.359859 | -1.150901 | 239 | 73.03 | 0.305565 |
| GO:0006807\_nitrogen\_compound\_metabolic\_process | SNCA | 1147 | 21 | 1.359859 | -1.150901 | 239 | 73.03 | 0.305565 |
| GO:0006807\_nitrogen\_compound\_metabolic\_process | ONECUT2 | 1147 | 21 | 1.359859 | -1.150901 | 239 | 73.03 | 0.305565 |
| GO:0006807\_nitrogen\_compound\_metabolic\_process | RORB | 1147 | 21 | 1.359859 | -1.150901 | 239 | 73.03 | 0.305565 |
| GO:0006807\_nitrogen\_compound\_metabolic\_process | AHR | 1147 | 21 | 1.359859 | -1.150901 | 239 | 73.03 | 0.305565 |
| GO:0006807\_nitrogen\_compound\_metabolic\_process | SUZ12 | 1147 | 21 | 1.359859 | -1.150901 | 239 | 73.03 | 0.305565 |
| GO:0006807\_nitrogen\_compound\_metabolic\_process | SLC11A1 | 1147 | 21 | 1.359859 | -1.150901 | 239 | 73.03 | 0.305565 |
| GO:0006807\_nitrogen\_compound\_metabolic\_process | DCLRE1A | 1147 | 21 | 1.359859 | -1.150901 | 239 | 73.03 | 0.305565 |
| GO:0006807\_nitrogen\_compound\_metabolic\_process | RNF6 | 1147 | 21 | 1.359859 | -1.150901 | 239 | 73.03 | 0.305565 |
| GO:0006807\_nitrogen\_compound\_metabolic\_process | GNAQ | 1147 | 21 | 1.359859 | -1.150901 | 239 | 73.03 | 0.305565 |
| GO:0006807\_nitrogen\_compound\_metabolic\_process | BCL2 | 1147 | 21 | 1.359859 | -1.150901 | 239 | 73.03 | 0.305565 |
| GO:0006807\_nitrogen\_compound\_metabolic\_process | GARNL1 | 1147 | 21 | 1.359859 | -1.150901 | 239 | 73.03 | 0.305565 |
| GO:0006807\_nitrogen\_compound\_metabolic\_process | FABP3 | 1147 | 21 | 1.359859 | -1.150901 | 239 | 73.03 | 0.305565 |
| GO:0006807\_nitrogen\_compound\_metabolic\_process | NFAT5 | 1147 | 21 | 1.359859 | -1.150901 | 239 | 73.03 | 0.305565 |
| GO:0006807\_nitrogen\_compound\_metabolic\_process | MYB | 1147 | 21 | 1.359859 | -1.150901 | 239 | 73.03 | 0.305565 |
| GO:0006807\_nitrogen\_compound\_metabolic\_process | NR2F2 | 1147 | 21 | 1.359859 | -1.150901 | 239 | 73.03 | 0.305565 |
| GO:0006807\_nitrogen\_compound\_metabolic\_process | FEN1 | 1147 | 21 | 1.359859 | -1.150901 | 239 | 73.03 | 0.305565 |
| GO:0006807\_nitrogen\_compound\_metabolic\_process | ETV3 | 1147 | 21 | 1.359859 | -1.150901 | 239 | 73.03 | 0.305565 |
| GO:0006807\_nitrogen\_compound\_metabolic\_process | PITX2 | 1147 | 21 | 1.359859 | -1.150901 | 239 | 73.03 | 0.305565 |
| GO:0006366\_transcription\_from\_RNA\_polymerase\_II\_promoter | SUZ12 | 444 | 10 | 1.672842 | -1.150879 | 240 | 73.08 | 0.304500 |
| GO:0006366\_transcription\_from\_RNA\_polymerase\_II\_promoter | SLC11A1 | 444 | 10 | 1.672842 | -1.150879 | 240 | 73.08 | 0.304500 |
| GO:0006366\_transcription\_from\_RNA\_polymerase\_II\_promoter | HMGB1 | 444 | 10 | 1.672842 | -1.150879 | 240 | 73.08 | 0.304500 |
| GO:0006366\_transcription\_from\_RNA\_polymerase\_II\_promoter | LCORL | 444 | 10 | 1.672842 | -1.150879 | 240 | 73.08 | 0.304500 |
| GO:0006366\_transcription\_from\_RNA\_polymerase\_II\_promoter | ONECUT2 | 444 | 10 | 1.672842 | -1.150879 | 240 | 73.08 | 0.304500 |
| GO:0006366\_transcription\_from\_RNA\_polymerase\_II\_promoter | NFAT5 | 444 | 10 | 1.672842 | -1.150879 | 240 | 73.08 | 0.304500 |
| GO:0006366\_transcription\_from\_RNA\_polymerase\_II\_promoter | NR2F2 | 444 | 10 | 1.672842 | -1.150879 | 240 | 73.08 | 0.304500 |
| GO:0006366\_transcription\_from\_RNA\_polymerase\_II\_promoter | AHR | 444 | 10 | 1.672842 | -1.150879 | 240 | 73.08 | 0.304500 |
| GO:0006366\_transcription\_from\_RNA\_polymerase\_II\_promoter | PITX2 | 444 | 10 | 1.672842 | -1.150879 | 240 | 73.08 | 0.304500 |
| GO:0006366\_transcription\_from\_RNA\_polymerase\_II\_promoter | ETV3 | 444 | 10 | 1.672842 | -1.150879 | 240 | 73.08 | 0.304500 |
| GO:0022037\_metencephalon\_development | DAB1 | 33 | 2 | 4.501466 | -1.141817 | 241 | 74.76 | 0.310207 |
| GO:0022037\_metencephalon\_development | BCL2 | 33 | 2 | 4.501466 | -1.141817 | 241 | 74.76 | 0.310207 |
| GO:0051716\_cellular\_response\_to\_stimulus | IRS2 | 273 | 7 | 1.904467 | -1.141798 | 242 | 74.86 | 0.309339 |
| GO:0051716\_cellular\_response\_to\_stimulus | PAXIP1 | 273 | 7 | 1.904467 | -1.141798 | 242 | 74.86 | 0.309339 |
| GO:0051716\_cellular\_response\_to\_stimulus | DCLRE1A | 273 | 7 | 1.904467 | -1.141798 | 242 | 74.86 | 0.309339 |
| GO:0051716\_cellular\_response\_to\_stimulus | BCL2 | 273 | 7 | 1.904467 | -1.141798 | 242 | 74.86 | 0.309339 |
| GO:0051716\_cellular\_response\_to\_stimulus | SNCA | 273 | 7 | 1.904467 | -1.141798 | 242 | 74.86 | 0.309339 |
| GO:0051716\_cellular\_response\_to\_stimulus | NEFL | 273 | 7 | 1.904467 | -1.141798 | 242 | 74.86 | 0.309339 |
| GO:0051716\_cellular\_response\_to\_stimulus | FEN1 | 273 | 7 | 1.904467 | -1.141798 | 242 | 74.86 | 0.309339 |
| GO:0044249\_cellular\_biosynthetic\_process | SCD1 | 1150 | 21 | 1.356311 | -1.140632 | 243 | 74.94 | 0.308395 |
| GO:0044249\_cellular\_biosynthetic\_process | HMGB1 | 1150 | 21 | 1.356311 | -1.140632 | 243 | 74.94 | 0.308395 |
| GO:0044249\_cellular\_biosynthetic\_process | LCORL | 1150 | 21 | 1.356311 | -1.140632 | 243 | 74.94 | 0.308395 |
| GO:0044249\_cellular\_biosynthetic\_process | ALDH18A1 | 1150 | 21 | 1.356311 | -1.140632 | 243 | 74.94 | 0.308395 |
| GO:0044249\_cellular\_biosynthetic\_process | SNCA | 1150 | 21 | 1.356311 | -1.140632 | 243 | 74.94 | 0.308395 |
| GO:0044249\_cellular\_biosynthetic\_process | ONECUT2 | 1150 | 21 | 1.356311 | -1.140632 | 243 | 74.94 | 0.308395 |
| GO:0044249\_cellular\_biosynthetic\_process | RORB | 1150 | 21 | 1.356311 | -1.140632 | 243 | 74.94 | 0.308395 |
| GO:0044249\_cellular\_biosynthetic\_process | AHR | 1150 | 21 | 1.356311 | -1.140632 | 243 | 74.94 | 0.308395 |
| GO:0044249\_cellular\_biosynthetic\_process | SUZ12 | 1150 | 21 | 1.356311 | -1.140632 | 243 | 74.94 | 0.308395 |
| GO:0044249\_cellular\_biosynthetic\_process | SLC11A1 | 1150 | 21 | 1.356311 | -1.140632 | 243 | 74.94 | 0.308395 |
| GO:0044249\_cellular\_biosynthetic\_process | RNF6 | 1150 | 21 | 1.356311 | -1.140632 | 243 | 74.94 | 0.308395 |
| GO:0044249\_cellular\_biosynthetic\_process | GNAQ | 1150 | 21 | 1.356311 | -1.140632 | 243 | 74.94 | 0.308395 |
| GO:0044249\_cellular\_biosynthetic\_process | BCL2 | 1150 | 21 | 1.356311 | -1.140632 | 243 | 74.94 | 0.308395 |
| GO:0044249\_cellular\_biosynthetic\_process | GARNL1 | 1150 | 21 | 1.356311 | -1.140632 | 243 | 74.94 | 0.308395 |
| GO:0044249\_cellular\_biosynthetic\_process | NFAT5 | 1150 | 21 | 1.356311 | -1.140632 | 243 | 74.94 | 0.308395 |
| GO:0044249\_cellular\_biosynthetic\_process | FABP3 | 1150 | 21 | 1.356311 | -1.140632 | 243 | 74.94 | 0.308395 |
| GO:0044249\_cellular\_biosynthetic\_process | MYB | 1150 | 21 | 1.356311 | -1.140632 | 243 | 74.94 | 0.308395 |
| GO:0044249\_cellular\_biosynthetic\_process | NR2F2 | 1150 | 21 | 1.356311 | -1.140632 | 243 | 74.94 | 0.308395 |
| GO:0044249\_cellular\_biosynthetic\_process | FEN1 | 1150 | 21 | 1.356311 | -1.140632 | 243 | 74.94 | 0.308395 |
| GO:0044249\_cellular\_biosynthetic\_process | PITX2 | 1150 | 21 | 1.356311 | -1.140632 | 243 | 74.94 | 0.308395 |
| GO:0044249\_cellular\_biosynthetic\_process | ETV3 | 1150 | 21 | 1.356311 | -1.140632 | 243 | 74.94 | 0.308395 |
| GO:0022403\_cell\_cycle\_phase | BCL2 | 119 | 4 | 2.496612 | -1.123468 | 244 | 76.15 | 0.312090 |
| GO:0022403\_cell\_cycle\_phase | ID4 | 119 | 4 | 2.496612 | -1.123468 | 244 | 76.15 | 0.312090 |
| GO:0022403\_cell\_cycle\_phase | MYB | 119 | 4 | 2.496612 | -1.123468 | 244 | 76.15 | 0.312090 |
| GO:0022403\_cell\_cycle\_phase | CDC25B | 119 | 4 | 2.496612 | -1.123468 | 244 | 76.15 | 0.312090 |
| GO:0010720\_positive\_regulation\_of\_cell\_development | BCL2 | 34 | 2 | 4.369070 | -1.119164 | 245 | 77.13 | 0.314816 |
| GO:0010720\_positive\_regulation\_of\_cell\_development | NEFL | 34 | 2 | 4.369070 | -1.119164 | 245 | 77.13 | 0.314816 |
| GO:0009653\_anatomical\_structure\_morphogenesis | PARD3 | 958 | 18 | 1.395549 | -1.111652 | 246 | 77.49 | 0.315000 |
| GO:0009653\_anatomical\_structure\_morphogenesis | COL13A1 | 958 | 18 | 1.395549 | -1.111652 | 246 | 77.49 | 0.315000 |
| GO:0009653\_anatomical\_structure\_morphogenesis | ONECUT2 | 958 | 18 | 1.395549 | -1.111652 | 246 | 77.49 | 0.315000 |
| GO:0009653\_anatomical\_structure\_morphogenesis | RORB | 958 | 18 | 1.395549 | -1.111652 | 246 | 77.49 | 0.315000 |
| GO:0009653\_anatomical\_structure\_morphogenesis | EPHB1 | 958 | 18 | 1.395549 | -1.111652 | 246 | 77.49 | 0.315000 |
| GO:0009653\_anatomical\_structure\_morphogenesis | NRCAM | 958 | 18 | 1.395549 | -1.111652 | 246 | 77.49 | 0.315000 |
| GO:0009653\_anatomical\_structure\_morphogenesis | SEMA5A | 958 | 18 | 1.395549 | -1.111652 | 246 | 77.49 | 0.315000 |
| GO:0009653\_anatomical\_structure\_morphogenesis | RNF6 | 958 | 18 | 1.395549 | -1.111652 | 246 | 77.49 | 0.315000 |
| GO:0009653\_anatomical\_structure\_morphogenesis | BDNF | 958 | 18 | 1.395549 | -1.111652 | 246 | 77.49 | 0.315000 |
| GO:0009653\_anatomical\_structure\_morphogenesis | SPRY1 | 958 | 18 | 1.395549 | -1.111652 | 246 | 77.49 | 0.315000 |
| GO:0009653\_anatomical\_structure\_morphogenesis | DAB1 | 958 | 18 | 1.395549 | -1.111652 | 246 | 77.49 | 0.315000 |
| GO:0009653\_anatomical\_structure\_morphogenesis | GNAQ | 958 | 18 | 1.395549 | -1.111652 | 246 | 77.49 | 0.315000 |
| GO:0009653\_anatomical\_structure\_morphogenesis | BCL2 | 958 | 18 | 1.395549 | -1.111652 | 246 | 77.49 | 0.315000 |
| GO:0009653\_anatomical\_structure\_morphogenesis | VEGFA | 958 | 18 | 1.395549 | -1.111652 | 246 | 77.49 | 0.315000 |
| GO:0009653\_anatomical\_structure\_morphogenesis | NTRK2 | 958 | 18 | 1.395549 | -1.111652 | 246 | 77.49 | 0.315000 |
| GO:0009653\_anatomical\_structure\_morphogenesis | NR2F2 | 958 | 18 | 1.395549 | -1.111652 | 246 | 77.49 | 0.315000 |
| GO:0009653\_anatomical\_structure\_morphogenesis | NEFL | 958 | 18 | 1.395549 | -1.111652 | 246 | 77.49 | 0.315000 |
| GO:0009653\_anatomical\_structure\_morphogenesis | PITX2 | 958 | 18 | 1.395549 | -1.111652 | 246 | 77.49 | 0.315000 |
| GO:0048513\_organ\_development | HMGB1 | 1365 | 24 | 1.305920 | -1.107505 | 247 | 77.6 | 0.314170 |
| GO:0048513\_organ\_development | IRS2 | 1365 | 24 | 1.305920 | -1.107505 | 247 | 77.6 | 0.314170 |
| GO:0048513\_organ\_development | COL13A1 | 1365 | 24 | 1.305920 | -1.107505 | 247 | 77.6 | 0.314170 |
| GO:0048513\_organ\_development | ONECUT2 | 1365 | 24 | 1.305920 | -1.107505 | 247 | 77.6 | 0.314170 |
| GO:0048513\_organ\_development | RORB | 1365 | 24 | 1.305920 | -1.107505 | 247 | 77.6 | 0.314170 |
| GO:0048513\_organ\_development | KITL | 1365 | 24 | 1.305920 | -1.107505 | 247 | 77.6 | 0.314170 |
| GO:0048513\_organ\_development | AHR | 1365 | 24 | 1.305920 | -1.107505 | 247 | 77.6 | 0.314170 |
| GO:0048513\_organ\_development | EPHB1 | 1365 | 24 | 1.305920 | -1.107505 | 247 | 77.6 | 0.314170 |
| GO:0048513\_organ\_development | SEMA5A | 1365 | 24 | 1.305920 | -1.107505 | 247 | 77.6 | 0.314170 |
| GO:0048513\_organ\_development | BDNF | 1365 | 24 | 1.305920 | -1.107505 | 247 | 77.6 | 0.314170 |
| GO:0048513\_organ\_development | SPRY1 | 1365 | 24 | 1.305920 | -1.107505 | 247 | 77.6 | 0.314170 |
| GO:0048513\_organ\_development | DAB1 | 1365 | 24 | 1.305920 | -1.107505 | 247 | 77.6 | 0.314170 |
| GO:0048513\_organ\_development | GNAQ | 1365 | 24 | 1.305920 | -1.107505 | 247 | 77.6 | 0.314170 |
| GO:0048513\_organ\_development | PKP2 | 1365 | 24 | 1.305920 | -1.107505 | 247 | 77.6 | 0.314170 |
| GO:0048513\_organ\_development | BCL2 | 1365 | 24 | 1.305920 | -1.107505 | 247 | 77.6 | 0.314170 |
| GO:0048513\_organ\_development | SALL1 | 1365 | 24 | 1.305920 | -1.107505 | 247 | 77.6 | 0.314170 |
| GO:0048513\_organ\_development | VEGFA | 1365 | 24 | 1.305920 | -1.107505 | 247 | 77.6 | 0.314170 |
| GO:0048513\_organ\_development | NTRK2 | 1365 | 24 | 1.305920 | -1.107505 | 247 | 77.6 | 0.314170 |
| GO:0048513\_organ\_development | ID4 | 1365 | 24 | 1.305920 | -1.107505 | 247 | 77.6 | 0.314170 |
| GO:0048513\_organ\_development | MYB | 1365 | 24 | 1.305920 | -1.107505 | 247 | 77.6 | 0.314170 |
| GO:0048513\_organ\_development | FABP7 | 1365 | 24 | 1.305920 | -1.107505 | 247 | 77.6 | 0.314170 |
| GO:0048513\_organ\_development | NR2F2 | 1365 | 24 | 1.305920 | -1.107505 | 247 | 77.6 | 0.314170 |
| GO:0048513\_organ\_development | MAP2K5 | 1365 | 24 | 1.305920 | -1.107505 | 247 | 77.6 | 0.314170 |
| GO:0048513\_organ\_development | PITX2 | 1365 | 24 | 1.305920 | -1.107505 | 247 | 77.6 | 0.314170 |
| GO:0042127\_regulation\_of\_cell\_proliferation | SUZ12 | 393 | 9 | 1.700936 | -1.107396 | 248 | 77.64 | 0.313065 |
| GO:0042127\_regulation\_of\_cell\_proliferation | HMGB1 | 393 | 9 | 1.700936 | -1.107396 | 248 | 77.64 | 0.313065 |
| GO:0042127\_regulation\_of\_cell\_proliferation | BDNF | 393 | 9 | 1.700936 | -1.107396 | 248 | 77.64 | 0.313065 |
| GO:0042127\_regulation\_of\_cell\_proliferation | IRS2 | 393 | 9 | 1.700936 | -1.107396 | 248 | 77.64 | 0.313065 |
| GO:0042127\_regulation\_of\_cell\_proliferation | BCL2 | 393 | 9 | 1.700936 | -1.107396 | 248 | 77.64 | 0.313065 |
| GO:0042127\_regulation\_of\_cell\_proliferation | VEGFA | 393 | 9 | 1.700936 | -1.107396 | 248 | 77.64 | 0.313065 |
| GO:0042127\_regulation\_of\_cell\_proliferation | ID4 | 393 | 9 | 1.700936 | -1.107396 | 248 | 77.64 | 0.313065 |
| GO:0042127\_regulation\_of\_cell\_proliferation | KITL | 393 | 9 | 1.700936 | -1.107396 | 248 | 77.64 | 0.313065 |
| GO:0042127\_regulation\_of\_cell\_proliferation | PITX2 | 393 | 9 | 1.700936 | -1.107396 | 248 | 77.64 | 0.313065 |
| GO:0002360\_T\_cell\_lineage\_commitment | BCL2 | 6 | 1 | 12.379032 | -1.107060 | 269 | 90.91 | 0.337955 |
| GO:0002367\_cytokine\_production\_during\_immune\_response | SLC11A1 | 6 | 1 | 12.379032 | -1.107060 | 269 | 90.91 | 0.337955 |
| GO:0006656\_phosphatidylcholine\_biosynthetic\_process | FABP3 | 6 | 1 | 12.379032 | -1.107060 | 269 | 90.91 | 0.337955 |
| GO:0007406\_negative\_regulation\_of\_neuroblast\_proliferation | BDNF | 6 | 1 | 12.379032 | -1.107060 | 269 | 90.91 | 0.337955 |
| GO:0014812\_muscle\_cell\_migration | BCL2 | 6 | 1 | 12.379032 | -1.107060 | 269 | 90.91 | 0.337955 |
| GO:0016032\_viral\_reproduction | BCL2 | 6 | 1 | 12.379032 | -1.107060 | 269 | 90.91 | 0.337955 |
| GO:0016574\_histone\_ubiquitination | SUZ12 | 6 | 1 | 12.379032 | -1.107060 | 269 | 90.91 | 0.337955 |
| GO:0021548\_pons\_development | BCL2 | 6 | 1 | 12.379032 | -1.107060 | 269 | 90.91 | 0.337955 |
| GO:0032392\_DNA\_geometric\_change | HMGB1 | 6 | 1 | 12.379032 | -1.107060 | 269 | 90.91 | 0.337955 |
| GO:0032469\_endoplasmic\_reticulum\_calcium\_ion\_homeostasis | BCL2 | 6 | 1 | 12.379032 | -1.107060 | 269 | 90.91 | 0.337955 |
| GO:0035094\_response\_to\_nicotine | CHRNB4 | 6 | 1 | 12.379032 | -1.107060 | 269 | 90.91 | 0.337955 |
| GO:0043271\_negative\_regulation\_of\_ion\_transport | BCL2 | 6 | 1 | 12.379032 | -1.107060 | 269 | 90.91 | 0.337955 |
| GO:0043467\_regulation\_of\_generation\_of\_precursor\_metabolites\_and\_energy | HMGB1 | 6 | 1 | 12.379032 | -1.107060 | 269 | 90.91 | 0.337955 |
| GO:0045913\_positive\_regulation\_of\_carbohydrate\_metabolic\_process | HMGB1 | 6 | 1 | 12.379032 | -1.107060 | 269 | 90.91 | 0.337955 |
| GO:0045931\_positive\_regulation\_of\_mitotic\_cell\_cycle | HMGB1 | 6 | 1 | 12.379032 | -1.107060 | 269 | 90.91 | 0.337955 |
| GO:0048041\_focal\_adhesion\_formation | BCL2 | 6 | 1 | 12.379032 | -1.107060 | 269 | 90.91 | 0.337955 |
| GO:0050829\_defense\_response\_to\_Gram-negative\_bacterium | SLC11A1 | 6 | 1 | 12.379032 | -1.107060 | 269 | 90.91 | 0.337955 |
| GO:0050872\_white\_fat\_cell\_differentiation | SCD1 | 6 | 1 | 12.379032 | -1.107060 | 269 | 90.91 | 0.337955 |
| GO:0051881\_regulation\_of\_mitochondrial\_membrane\_potential | BCL2 | 6 | 1 | 12.379032 | -1.107060 | 269 | 90.91 | 0.337955 |
| GO:0060134\_prepulse\_inhibition | FABP7 | 6 | 1 | 12.379032 | -1.107060 | 269 | 90.91 | 0.337955 |
| GO:0065004\_protein-DNA\_complex\_assembly | AHR | 6 | 1 | 12.379032 | -1.107060 | 269 | 90.91 | 0.337955 |
| GO:0006355\_regulation\_of\_transcription\_\_DNA-dependent | SUZ12 | 575 | 12 | 1.550070 | -1.104381 | 270 | 90.97 | 0.336926 |
| GO:0006355\_regulation\_of\_transcription\_\_DNA-dependent | SLC11A1 | 575 | 12 | 1.550070 | -1.104381 | 270 | 90.97 | 0.336926 |
| GO:0006355\_regulation\_of\_transcription\_\_DNA-dependent | HMGB1 | 575 | 12 | 1.550070 | -1.104381 | 270 | 90.97 | 0.336926 |
| GO:0006355\_regulation\_of\_transcription\_\_DNA-dependent | RNF6 | 575 | 12 | 1.550070 | -1.104381 | 270 | 90.97 | 0.336926 |
| GO:0006355\_regulation\_of\_transcription\_\_DNA-dependent | ONECUT2 | 575 | 12 | 1.550070 | -1.104381 | 270 | 90.97 | 0.336926 |
| GO:0006355\_regulation\_of\_transcription\_\_DNA-dependent | NFAT5 | 575 | 12 | 1.550070 | -1.104381 | 270 | 90.97 | 0.336926 |
| GO:0006355\_regulation\_of\_transcription\_\_DNA-dependent | RORB | 575 | 12 | 1.550070 | -1.104381 | 270 | 90.97 | 0.336926 |
| GO:0006355\_regulation\_of\_transcription\_\_DNA-dependent | MYB | 575 | 12 | 1.550070 | -1.104381 | 270 | 90.97 | 0.336926 |
| GO:0006355\_regulation\_of\_transcription\_\_DNA-dependent | NR2F2 | 575 | 12 | 1.550070 | -1.104381 | 270 | 90.97 | 0.336926 |
| GO:0006355\_regulation\_of\_transcription\_\_DNA-dependent | AHR | 575 | 12 | 1.550070 | -1.104381 | 270 | 90.97 | 0.336926 |
| GO:0006355\_regulation\_of\_transcription\_\_DNA-dependent | PITX2 | 575 | 12 | 1.550070 | -1.104381 | 270 | 90.97 | 0.336926 |
| GO:0006355\_regulation\_of\_transcription\_\_DNA-dependent | ETV3 | 575 | 12 | 1.550070 | -1.104381 | 270 | 90.97 | 0.336926 |
| GO:0006350\_transcription | HMGB1 | 701 | 14 | 1.483365 | -1.102438 | 271 | 91.14 | 0.336310 |
| GO:0006350\_transcription | LCORL | 701 | 14 | 1.483365 | -1.102438 | 271 | 91.14 | 0.336310 |
| GO:0006350\_transcription | ONECUT2 | 701 | 14 | 1.483365 | -1.102438 | 271 | 91.14 | 0.336310 |
| GO:0006350\_transcription | RORB | 701 | 14 | 1.483365 | -1.102438 | 271 | 91.14 | 0.336310 |
| GO:0006350\_transcription | AHR | 701 | 14 | 1.483365 | -1.102438 | 271 | 91.14 | 0.336310 |
| GO:0006350\_transcription | SUZ12 | 701 | 14 | 1.483365 | -1.102438 | 271 | 91.14 | 0.336310 |
| GO:0006350\_transcription | SLC11A1 | 701 | 14 | 1.483365 | -1.102438 | 271 | 91.14 | 0.336310 |
| GO:0006350\_transcription | RNF6 | 701 | 14 | 1.483365 | -1.102438 | 271 | 91.14 | 0.336310 |
| GO:0006350\_transcription | GARNL1 | 701 | 14 | 1.483365 | -1.102438 | 271 | 91.14 | 0.336310 |
| GO:0006350\_transcription | NFAT5 | 701 | 14 | 1.483365 | -1.102438 | 271 | 91.14 | 0.336310 |
| GO:0006350\_transcription | NR2F2 | 701 | 14 | 1.483365 | -1.102438 | 271 | 91.14 | 0.336310 |
| GO:0006350\_transcription | MYB | 701 | 14 | 1.483365 | -1.102438 | 271 | 91.14 | 0.336310 |
| GO:0006350\_transcription | ETV3 | 701 | 14 | 1.483365 | -1.102438 | 271 | 91.14 | 0.336310 |
| GO:0006350\_transcription | PITX2 | 701 | 14 | 1.483365 | -1.102438 | 271 | 91.14 | 0.336310 |
| GO:0065009\_regulation\_of\_molecular\_function | SLC11A1 | 279 | 7 | 1.863510 | -1.102046 | 272 | 91.31 | 0.335699 |
| GO:0065009\_regulation\_of\_molecular\_function | SPRY1 | 279 | 7 | 1.863510 | -1.102046 | 272 | 91.31 | 0.335699 |
| GO:0065009\_regulation\_of\_molecular\_function | DAB1 | 279 | 7 | 1.863510 | -1.102046 | 272 | 91.31 | 0.335699 |
| GO:0065009\_regulation\_of\_molecular\_function | GNAQ | 279 | 7 | 1.863510 | -1.102046 | 272 | 91.31 | 0.335699 |
| GO:0065009\_regulation\_of\_molecular\_function | BCL2 | 279 | 7 | 1.863510 | -1.102046 | 272 | 91.31 | 0.335699 |
| GO:0065009\_regulation\_of\_molecular\_function | KITL | 279 | 7 | 1.863510 | -1.102046 | 272 | 91.31 | 0.335699 |
| GO:0065009\_regulation\_of\_molecular\_function | CDC25B | 279 | 7 | 1.863510 | -1.102046 | 272 | 91.31 | 0.335699 |
| GO:0048522\_positive\_regulation\_of\_cellular\_process | HMGB1 | 895 | 17 | 1.410795 | -1.101460 | 273 | 91.35 | 0.334615 |
| GO:0048522\_positive\_regulation\_of\_cellular\_process | IRS2 | 895 | 17 | 1.410795 | -1.101460 | 273 | 91.35 | 0.334615 |
| GO:0048522\_positive\_regulation\_of\_cellular\_process | SNCA | 895 | 17 | 1.410795 | -1.101460 | 273 | 91.35 | 0.334615 |
| GO:0048522\_positive\_regulation\_of\_cellular\_process | ONECUT2 | 895 | 17 | 1.410795 | -1.101460 | 273 | 91.35 | 0.334615 |
| GO:0048522\_positive\_regulation\_of\_cellular\_process | RORB | 895 | 17 | 1.410795 | -1.101460 | 273 | 91.35 | 0.334615 |
| GO:0048522\_positive\_regulation\_of\_cellular\_process | KITL | 895 | 17 | 1.410795 | -1.101460 | 273 | 91.35 | 0.334615 |
| GO:0048522\_positive\_regulation\_of\_cellular\_process | AHR | 895 | 17 | 1.410795 | -1.101460 | 273 | 91.35 | 0.334615 |
| GO:0048522\_positive\_regulation\_of\_cellular\_process | SUZ12 | 895 | 17 | 1.410795 | -1.101460 | 273 | 91.35 | 0.334615 |
| GO:0048522\_positive\_regulation\_of\_cellular\_process | SLC11A1 | 895 | 17 | 1.410795 | -1.101460 | 273 | 91.35 | 0.334615 |
| GO:0048522\_positive\_regulation\_of\_cellular\_process | BDNF | 895 | 17 | 1.410795 | -1.101460 | 273 | 91.35 | 0.334615 |
| GO:0048522\_positive\_regulation\_of\_cellular\_process | RNF6 | 895 | 17 | 1.410795 | -1.101460 | 273 | 91.35 | 0.334615 |
| GO:0048522\_positive\_regulation\_of\_cellular\_process | BCL2 | 895 | 17 | 1.410795 | -1.101460 | 273 | 91.35 | 0.334615 |
| GO:0048522\_positive\_regulation\_of\_cellular\_process | VEGFA | 895 | 17 | 1.410795 | -1.101460 | 273 | 91.35 | 0.334615 |
| GO:0048522\_positive\_regulation\_of\_cellular\_process | NFAT5 | 895 | 17 | 1.410795 | -1.101460 | 273 | 91.35 | 0.334615 |
| GO:0048522\_positive\_regulation\_of\_cellular\_process | ID4 | 895 | 17 | 1.410795 | -1.101460 | 273 | 91.35 | 0.334615 |
| GO:0048522\_positive\_regulation\_of\_cellular\_process | NEFL | 895 | 17 | 1.410795 | -1.101460 | 273 | 91.35 | 0.334615 |
| GO:0048522\_positive\_regulation\_of\_cellular\_process | PITX2 | 895 | 17 | 1.410795 | -1.101460 | 273 | 91.35 | 0.334615 |
| GO:0007281\_germ\_cell\_development | BCL2 | 75 | 3 | 2.970968 | -1.100596 | 274 | 91.52 | 0.334015 |
| GO:0007281\_germ\_cell\_development | KITL | 75 | 3 | 2.970968 | -1.100596 | 274 | 91.52 | 0.334015 |
| GO:0007281\_germ\_cell\_development | CDC25B | 75 | 3 | 2.970968 | -1.100596 | 274 | 91.52 | 0.334015 |
| GO:0007292\_female\_gamete\_generation | BCL2 | 35 | 2 | 4.244240 | -1.097281 | 277 | 92.42 | 0.333646 |
| GO:0007292\_female\_gamete\_generation | CDC25B | 35 | 2 | 4.244240 | -1.097281 | 277 | 92.42 | 0.333646 |
| GO:0010810\_regulation\_of\_cell-substrate\_adhesion | BCL2 | 35 | 2 | 4.244240 | -1.097281 | 277 | 92.42 | 0.333646 |
| GO:0010810\_regulation\_of\_cell-substrate\_adhesion | ONECUT2 | 35 | 2 | 4.244240 | -1.097281 | 277 | 92.42 | 0.333646 |
| GO:0051051\_negative\_regulation\_of\_transport | PFKL | 35 | 2 | 4.244240 | -1.097281 | 277 | 92.42 | 0.333646 |
| GO:0051051\_negative\_regulation\_of\_transport | BCL2 | 35 | 2 | 4.244240 | -1.097281 | 277 | 92.42 | 0.333646 |
| GO:0030001\_metal\_ion\_transport | SLC11A1 | 122 | 4 | 2.435219 | -1.092378 | 278 | 93.08 | 0.334820 |
| GO:0030001\_metal\_ion\_transport | BCL2 | 122 | 4 | 2.435219 | -1.092378 | 278 | 93.08 | 0.334820 |
| GO:0030001\_metal\_ion\_transport | MYB | 122 | 4 | 2.435219 | -1.092378 | 278 | 93.08 | 0.334820 |
| GO:0030001\_metal\_ion\_transport | SLC4A4 | 122 | 4 | 2.435219 | -1.092378 | 278 | 93.08 | 0.334820 |
| GO:0045941\_positive\_regulation\_of\_transcription | SLC11A1 | 338 | 8 | 1.757969 | -1.088919 | 279 | 93.22 | 0.334122 |
| GO:0045941\_positive\_regulation\_of\_transcription | HMGB1 | 338 | 8 | 1.757969 | -1.088919 | 279 | 93.22 | 0.334122 |
| GO:0045941\_positive\_regulation\_of\_transcription | RNF6 | 338 | 8 | 1.757969 | -1.088919 | 279 | 93.22 | 0.334122 |
| GO:0045941\_positive\_regulation\_of\_transcription | ONECUT2 | 338 | 8 | 1.757969 | -1.088919 | 279 | 93.22 | 0.334122 |
| GO:0045941\_positive\_regulation\_of\_transcription | NFAT5 | 338 | 8 | 1.757969 | -1.088919 | 279 | 93.22 | 0.334122 |
| GO:0045941\_positive\_regulation\_of\_transcription | RORB | 338 | 8 | 1.757969 | -1.088919 | 279 | 93.22 | 0.334122 |
| GO:0045941\_positive\_regulation\_of\_transcription | AHR | 338 | 8 | 1.757969 | -1.088919 | 279 | 93.22 | 0.334122 |
| GO:0045941\_positive\_regulation\_of\_transcription | PITX2 | 338 | 8 | 1.757969 | -1.088919 | 279 | 93.22 | 0.334122 |
| GO:0009725\_response\_to\_hormone\_stimulus | HMGB1 | 76 | 3 | 2.931876 | -1.087137 | 280 | 93.39 | 0.333536 |
| GO:0009725\_response\_to\_hormone\_stimulus | IRS2 | 76 | 3 | 2.931876 | -1.087137 | 280 | 93.39 | 0.333536 |
| GO:0009725\_response\_to\_hormone\_stimulus | BCL2 | 76 | 3 | 2.931876 | -1.087137 | 280 | 93.39 | 0.333536 |
| GO:0009893\_positive\_regulation\_of\_metabolic\_process | SLC11A1 | 458 | 10 | 1.621707 | -1.079181 | 281 | 93.85 | 0.333986 |
| GO:0009893\_positive\_regulation\_of\_metabolic\_process | HMGB1 | 458 | 10 | 1.621707 | -1.079181 | 281 | 93.85 | 0.333986 |
| GO:0009893\_positive\_regulation\_of\_metabolic\_process | RNF6 | 458 | 10 | 1.621707 | -1.079181 | 281 | 93.85 | 0.333986 |
| GO:0009893\_positive\_regulation\_of\_metabolic\_process | BCL2 | 458 | 10 | 1.621707 | -1.079181 | 281 | 93.85 | 0.333986 |
| GO:0009893\_positive\_regulation\_of\_metabolic\_process | ONECUT2 | 458 | 10 | 1.621707 | -1.079181 | 281 | 93.85 | 0.333986 |
| GO:0009893\_positive\_regulation\_of\_metabolic\_process | NFAT5 | 458 | 10 | 1.621707 | -1.079181 | 281 | 93.85 | 0.333986 |
| GO:0009893\_positive\_regulation\_of\_metabolic\_process | RORB | 458 | 10 | 1.621707 | -1.079181 | 281 | 93.85 | 0.333986 |
| GO:0009893\_positive\_regulation\_of\_metabolic\_process | KITL | 458 | 10 | 1.621707 | -1.079181 | 281 | 93.85 | 0.333986 |
| GO:0009893\_positive\_regulation\_of\_metabolic\_process | AHR | 458 | 10 | 1.621707 | -1.079181 | 281 | 93.85 | 0.333986 |
| GO:0009893\_positive\_regulation\_of\_metabolic\_process | PITX2 | 458 | 10 | 1.621707 | -1.079181 | 281 | 93.85 | 0.333986 |
| GO:0051171\_regulation\_of\_nitrogen\_compound\_metabolic\_process | HMGB1 | 771 | 15 | 1.445023 | -1.078583 | 282 | 93.9 | 0.332979 |
| GO:0051171\_regulation\_of\_nitrogen\_compound\_metabolic\_process | ONECUT2 | 771 | 15 | 1.445023 | -1.078583 | 282 | 93.9 | 0.332979 |
| GO:0051171\_regulation\_of\_nitrogen\_compound\_metabolic\_process | RORB | 771 | 15 | 1.445023 | -1.078583 | 282 | 93.9 | 0.332979 |
| GO:0051171\_regulation\_of\_nitrogen\_compound\_metabolic\_process | AHR | 771 | 15 | 1.445023 | -1.078583 | 282 | 93.9 | 0.332979 |
| GO:0051171\_regulation\_of\_nitrogen\_compound\_metabolic\_process | SUZ12 | 771 | 15 | 1.445023 | -1.078583 | 282 | 93.9 | 0.332979 |
| GO:0051171\_regulation\_of\_nitrogen\_compound\_metabolic\_process | SLC11A1 | 771 | 15 | 1.445023 | -1.078583 | 282 | 93.9 | 0.332979 |
| GO:0051171\_regulation\_of\_nitrogen\_compound\_metabolic\_process | RNF6 | 771 | 15 | 1.445023 | -1.078583 | 282 | 93.9 | 0.332979 |
| GO:0051171\_regulation\_of\_nitrogen\_compound\_metabolic\_process | GNAQ | 771 | 15 | 1.445023 | -1.078583 | 282 | 93.9 | 0.332979 |
| GO:0051171\_regulation\_of\_nitrogen\_compound\_metabolic\_process | BCL2 | 771 | 15 | 1.445023 | -1.078583 | 282 | 93.9 | 0.332979 |
| GO:0051171\_regulation\_of\_nitrogen\_compound\_metabolic\_process | GARNL1 | 771 | 15 | 1.445023 | -1.078583 | 282 | 93.9 | 0.332979 |
| GO:0051171\_regulation\_of\_nitrogen\_compound\_metabolic\_process | NFAT5 | 771 | 15 | 1.445023 | -1.078583 | 282 | 93.9 | 0.332979 |
| GO:0051171\_regulation\_of\_nitrogen\_compound\_metabolic\_process | MYB | 771 | 15 | 1.445023 | -1.078583 | 282 | 93.9 | 0.332979 |
| GO:0051171\_regulation\_of\_nitrogen\_compound\_metabolic\_process | NR2F2 | 771 | 15 | 1.445023 | -1.078583 | 282 | 93.9 | 0.332979 |
| GO:0051171\_regulation\_of\_nitrogen\_compound\_metabolic\_process | PITX2 | 771 | 15 | 1.445023 | -1.078583 | 282 | 93.9 | 0.332979 |
| GO:0051171\_regulation\_of\_nitrogen\_compound\_metabolic\_process | ETV3 | 771 | 15 | 1.445023 | -1.078583 | 282 | 93.9 | 0.332979 |
| GO:0007631\_feeding\_behavior | BDNF | 36 | 2 | 4.126344 | -1.076126 | 285 | 95.24 | 0.334175 |
| GO:0007631\_feeding\_behavior | NTRK2 | 36 | 2 | 4.126344 | -1.076126 | 285 | 95.24 | 0.334175 |
| GO:0022602\_ovulation\_cycle\_process | BCL2 | 36 | 2 | 4.126344 | -1.076126 | 285 | 95.24 | 0.334175 |
| GO:0022602\_ovulation\_cycle\_process | VEGFA | 36 | 2 | 4.126344 | -1.076126 | 285 | 95.24 | 0.334175 |
| GO:0030278\_regulation\_of\_ossification | BCL2 | 36 | 2 | 4.126344 | -1.076126 | 285 | 95.24 | 0.334175 |
| GO:0030278\_regulation\_of\_ossification | TOB1 | 36 | 2 | 4.126344 | -1.076126 | 285 | 95.24 | 0.334175 |
| GO:0048523\_negative\_regulation\_of\_cellular\_process | PFKL | 774 | 15 | 1.439422 | -1.066981 | 286 | 95.89 | 0.335280 |
| GO:0048523\_negative\_regulation\_of\_cellular\_process | SNCA | 774 | 15 | 1.439422 | -1.066981 | 286 | 95.89 | 0.335280 |
| GO:0048523\_negative\_regulation\_of\_cellular\_process | ONECUT2 | 774 | 15 | 1.439422 | -1.066981 | 286 | 95.89 | 0.335280 |
| GO:0048523\_negative\_regulation\_of\_cellular\_process | KITL | 774 | 15 | 1.439422 | -1.066981 | 286 | 95.89 | 0.335280 |
| GO:0048523\_negative\_regulation\_of\_cellular\_process | SUZ12 | 774 | 15 | 1.439422 | -1.066981 | 286 | 95.89 | 0.335280 |
| GO:0048523\_negative\_regulation\_of\_cellular\_process | BDNF | 774 | 15 | 1.439422 | -1.066981 | 286 | 95.89 | 0.335280 |
| GO:0048523\_negative\_regulation\_of\_cellular\_process | RNF6 | 774 | 15 | 1.439422 | -1.066981 | 286 | 95.89 | 0.335280 |
| GO:0048523\_negative\_regulation\_of\_cellular\_process | DAB1 | 774 | 15 | 1.439422 | -1.066981 | 286 | 95.89 | 0.335280 |
| GO:0048523\_negative\_regulation\_of\_cellular\_process | BCL2 | 774 | 15 | 1.439422 | -1.066981 | 286 | 95.89 | 0.335280 |
| GO:0048523\_negative\_regulation\_of\_cellular\_process | VEGFA | 774 | 15 | 1.439422 | -1.066981 | 286 | 95.89 | 0.335280 |
| GO:0048523\_negative\_regulation\_of\_cellular\_process | ID4 | 774 | 15 | 1.439422 | -1.066981 | 286 | 95.89 | 0.335280 |
| GO:0048523\_negative\_regulation\_of\_cellular\_process | NR2F2 | 774 | 15 | 1.439422 | -1.066981 | 286 | 95.89 | 0.335280 |
| GO:0048523\_negative\_regulation\_of\_cellular\_process | NEFL | 774 | 15 | 1.439422 | -1.066981 | 286 | 95.89 | 0.335280 |
| GO:0048523\_negative\_regulation\_of\_cellular\_process | TOB1 | 774 | 15 | 1.439422 | -1.066981 | 286 | 95.89 | 0.335280 |
| GO:0048523\_negative\_regulation\_of\_cellular\_process | ETV3 | 774 | 15 | 1.439422 | -1.066981 | 286 | 95.89 | 0.335280 |
| GO:0007167\_enzyme\_linked\_receptor\_protein\_signaling\_pathway | IRS2 | 229 | 6 | 1.946049 | -1.065954 | 287 | 95.94 | 0.334286 |
| GO:0007167\_enzyme\_linked\_receptor\_protein\_signaling\_pathway | LTBP1 | 229 | 6 | 1.946049 | -1.065954 | 287 | 95.94 | 0.334286 |
| GO:0007167\_enzyme\_linked\_receptor\_protein\_signaling\_pathway | LTBP3 | 229 | 6 | 1.946049 | -1.065954 | 287 | 95.94 | 0.334286 |
| GO:0007167\_enzyme\_linked\_receptor\_protein\_signaling\_pathway | ONECUT2 | 229 | 6 | 1.946049 | -1.065954 | 287 | 95.94 | 0.334286 |
| GO:0007167\_enzyme\_linked\_receptor\_protein\_signaling\_pathway | VEGFA | 229 | 6 | 1.946049 | -1.065954 | 287 | 95.94 | 0.334286 |
| GO:0007167\_enzyme\_linked\_receptor\_protein\_signaling\_pathway | TOB1 | 229 | 6 | 1.946049 | -1.065954 | 287 | 95.94 | 0.334286 |
| GO:0006873\_cellular\_ion\_homeostasis | SLC11A1 | 176 | 5 | 2.110062 | -1.061512 | 288 | 96.25 | 0.334201 |
| GO:0006873\_cellular\_ion\_homeostasis | GNAQ | 176 | 5 | 2.110062 | -1.061512 | 288 | 96.25 | 0.334201 |
| GO:0006873\_cellular\_ion\_homeostasis | BCL2 | 176 | 5 | 2.110062 | -1.061512 | 288 | 96.25 | 0.334201 |
| GO:0006873\_cellular\_ion\_homeostasis | SNCA | 176 | 5 | 2.110062 | -1.061512 | 288 | 96.25 | 0.334201 |
| GO:0006873\_cellular\_ion\_homeostasis | CHRNB4 | 176 | 5 | 2.110062 | -1.061512 | 288 | 96.25 | 0.334201 |
| GO:0007519\_skeletal\_muscle\_tissue\_development | BCL2 | 78 | 3 | 2.856700 | -1.060900 | 290 | 96.68 | 0.333379 |
| GO:0007519\_skeletal\_muscle\_tissue\_development | NR2F2 | 78 | 3 | 2.856700 | -1.060900 | 290 | 96.68 | 0.333379 |
| GO:0007519\_skeletal\_muscle\_tissue\_development | PITX2 | 78 | 3 | 2.856700 | -1.060900 | 290 | 96.68 | 0.333379 |
| GO:0060538\_skeletal\_muscle\_organ\_development | BCL2 | 78 | 3 | 2.856700 | -1.060900 | 290 | 96.68 | 0.333379 |
| GO:0060538\_skeletal\_muscle\_organ\_development | NR2F2 | 78 | 3 | 2.856700 | -1.060900 | 290 | 96.68 | 0.333379 |
| GO:0060538\_skeletal\_muscle\_organ\_development | PITX2 | 78 | 3 | 2.856700 | -1.060900 | 290 | 96.68 | 0.333379 |
| GO:0009058\_biosynthetic\_process | SCD1 | 1175 | 21 | 1.327454 | -1.057708 | 291 | 96.75 | 0.332474 |
| GO:0009058\_biosynthetic\_process | HMGB1 | 1175 | 21 | 1.327454 | -1.057708 | 291 | 96.75 | 0.332474 |
| GO:0009058\_biosynthetic\_process | ALDH18A1 | 1175 | 21 | 1.327454 | -1.057708 | 291 | 96.75 | 0.332474 |
| GO:0009058\_biosynthetic\_process | LCORL | 1175 | 21 | 1.327454 | -1.057708 | 291 | 96.75 | 0.332474 |
| GO:0009058\_biosynthetic\_process | SNCA | 1175 | 21 | 1.327454 | -1.057708 | 291 | 96.75 | 0.332474 |
| GO:0009058\_biosynthetic\_process | ONECUT2 | 1175 | 21 | 1.327454 | -1.057708 | 291 | 96.75 | 0.332474 |
| GO:0009058\_biosynthetic\_process | RORB | 1175 | 21 | 1.327454 | -1.057708 | 291 | 96.75 | 0.332474 |
| GO:0009058\_biosynthetic\_process | AHR | 1175 | 21 | 1.327454 | -1.057708 | 291 | 96.75 | 0.332474 |
| GO:0009058\_biosynthetic\_process | SUZ12 | 1175 | 21 | 1.327454 | -1.057708 | 291 | 96.75 | 0.332474 |
| GO:0009058\_biosynthetic\_process | SLC11A1 | 1175 | 21 | 1.327454 | -1.057708 | 291 | 96.75 | 0.332474 |
| GO:0009058\_biosynthetic\_process | RNF6 | 1175 | 21 | 1.327454 | -1.057708 | 291 | 96.75 | 0.332474 |
| GO:0009058\_biosynthetic\_process | GNAQ | 1175 | 21 | 1.327454 | -1.057708 | 291 | 96.75 | 0.332474 |
| GO:0009058\_biosynthetic\_process | BCL2 | 1175 | 21 | 1.327454 | -1.057708 | 291 | 96.75 | 0.332474 |
| GO:0009058\_biosynthetic\_process | GARNL1 | 1175 | 21 | 1.327454 | -1.057708 | 291 | 96.75 | 0.332474 |
| GO:0009058\_biosynthetic\_process | FABP3 | 1175 | 21 | 1.327454 | -1.057708 | 291 | 96.75 | 0.332474 |
| GO:0009058\_biosynthetic\_process | NFAT5 | 1175 | 21 | 1.327454 | -1.057708 | 291 | 96.75 | 0.332474 |
| GO:0009058\_biosynthetic\_process | MYB | 1175 | 21 | 1.327454 | -1.057708 | 291 | 96.75 | 0.332474 |
| GO:0009058\_biosynthetic\_process | NR2F2 | 1175 | 21 | 1.327454 | -1.057708 | 291 | 96.75 | 0.332474 |
| GO:0009058\_biosynthetic\_process | FEN1 | 1175 | 21 | 1.327454 | -1.057708 | 291 | 96.75 | 0.332474 |
| GO:0009058\_biosynthetic\_process | ETV3 | 1175 | 21 | 1.327454 | -1.057708 | 291 | 96.75 | 0.332474 |
| GO:0009058\_biosynthetic\_process | PITX2 | 1175 | 21 | 1.327454 | -1.057708 | 291 | 96.75 | 0.332474 |
| GO:0042698\_ovulation\_cycle | BCL2 | 37 | 2 | 4.014821 | -1.055656 | 292 | 97.77 | 0.334829 |
| GO:0042698\_ovulation\_cycle | VEGFA | 37 | 2 | 4.014821 | -1.055656 | 292 | 97.77 | 0.334829 |
| GO:0051239\_regulation\_of\_multicellular\_organismal\_process | HMGB1 | 587 | 12 | 1.518382 | -1.051295 | 293 | 97.86 | 0.333993 |
| GO:0051239\_regulation\_of\_multicellular\_organismal\_process | SLC11A1 | 587 | 12 | 1.518382 | -1.051295 | 293 | 97.86 | 0.333993 |
| GO:0051239\_regulation\_of\_multicellular\_organismal\_process | RNF6 | 587 | 12 | 1.518382 | -1.051295 | 293 | 97.86 | 0.333993 |
| GO:0051239\_regulation\_of\_multicellular\_organismal\_process | BDNF | 587 | 12 | 1.518382 | -1.051295 | 293 | 97.86 | 0.333993 |
| GO:0051239\_regulation\_of\_multicellular\_organismal\_process | BCL2 | 587 | 12 | 1.518382 | -1.051295 | 293 | 97.86 | 0.333993 |
| GO:0051239\_regulation\_of\_multicellular\_organismal\_process | SNCA | 587 | 12 | 1.518382 | -1.051295 | 293 | 97.86 | 0.333993 |
| GO:0051239\_regulation\_of\_multicellular\_organismal\_process | SP4 | 587 | 12 | 1.518382 | -1.051295 | 293 | 97.86 | 0.333993 |
| GO:0051239\_regulation\_of\_multicellular\_organismal\_process | CHRNB4 | 587 | 12 | 1.518382 | -1.051295 | 293 | 97.86 | 0.333993 |
| GO:0051239\_regulation\_of\_multicellular\_organismal\_process | ID4 | 587 | 12 | 1.518382 | -1.051295 | 293 | 97.86 | 0.333993 |
| GO:0051239\_regulation\_of\_multicellular\_organismal\_process | NEFL | 587 | 12 | 1.518382 | -1.051295 | 293 | 97.86 | 0.333993 |
| GO:0051239\_regulation\_of\_multicellular\_organismal\_process | KITL | 587 | 12 | 1.518382 | -1.051295 | 293 | 97.86 | 0.333993 |
| GO:0051239\_regulation\_of\_multicellular\_organismal\_process | TOB1 | 587 | 12 | 1.518382 | -1.051295 | 293 | 97.86 | 0.333993 |
| GO:0015674\_di-\_\_tri-valent\_inorganic\_cation\_transport | SLC11A1 | 79 | 3 | 2.820539 | -1.048110 | 294 | 98.09 | 0.333639 |
| GO:0015674\_di-\_\_tri-valent\_inorganic\_cation\_transport | BCL2 | 79 | 3 | 2.820539 | -1.048110 | 294 | 98.09 | 0.333639 |
| GO:0015674\_di-\_\_tri-valent\_inorganic\_cation\_transport | MYB | 79 | 3 | 2.820539 | -1.048110 | 294 | 98.09 | 0.333639 |
| GO:0010628\_positive\_regulation\_of\_gene\_expression | SLC11A1 | 346 | 8 | 1.717322 | -1.043199 | 295 | 98.17 | 0.332780 |
| GO:0010628\_positive\_regulation\_of\_gene\_expression | HMGB1 | 346 | 8 | 1.717322 | -1.043199 | 295 | 98.17 | 0.332780 |
| GO:0010628\_positive\_regulation\_of\_gene\_expression | RNF6 | 346 | 8 | 1.717322 | -1.043199 | 295 | 98.17 | 0.332780 |
| GO:0010628\_positive\_regulation\_of\_gene\_expression | ONECUT2 | 346 | 8 | 1.717322 | -1.043199 | 295 | 98.17 | 0.332780 |
| GO:0010628\_positive\_regulation\_of\_gene\_expression | NFAT5 | 346 | 8 | 1.717322 | -1.043199 | 295 | 98.17 | 0.332780 |
| GO:0010628\_positive\_regulation\_of\_gene\_expression | RORB | 346 | 8 | 1.717322 | -1.043199 | 295 | 98.17 | 0.332780 |
| GO:0010628\_positive\_regulation\_of\_gene\_expression | AHR | 346 | 8 | 1.717322 | -1.043199 | 295 | 98.17 | 0.332780 |
| GO:0010628\_positive\_regulation\_of\_gene\_expression | PITX2 | 346 | 8 | 1.717322 | -1.043199 | 295 | 98.17 | 0.332780 |
| GO:0001556\_oocyte\_maturation | CDC25B | 7 | 1 | 10.610599 | -1.042969 | 310 | 113.73 | 0.366871 |
| GO:0001936\_regulation\_of\_endothelial\_cell\_proliferation | VEGFA | 7 | 1 | 10.610599 | -1.042969 | 310 | 113.73 | 0.366871 |
| GO:0002792\_negative\_regulation\_of\_peptide\_secretion | PFKL | 7 | 1 | 10.610599 | -1.042969 | 310 | 113.73 | 0.366871 |
| GO:0006096\_glycolysis | PFKL | 7 | 1 | 10.610599 | -1.042969 | 310 | 113.73 | 0.366871 |
| GO:0006119\_oxidative\_phosphorylation | SNCA | 7 | 1 | 10.610599 | -1.042969 | 310 | 113.73 | 0.366871 |
| GO:0006352\_transcription\_initiation | AHR | 7 | 1 | 10.610599 | -1.042969 | 310 | 113.73 | 0.366871 |
| GO:0021884\_forebrain\_neuron\_development | GNAQ | 7 | 1 | 10.610599 | -1.042969 | 310 | 113.73 | 0.366871 |
| GO:0030517\_negative\_regulation\_of\_axon\_extension | RNF6 | 7 | 1 | 10.610599 | -1.042969 | 310 | 113.73 | 0.366871 |
| GO:0034599\_cellular\_response\_to\_oxidative\_stress | SNCA | 7 | 1 | 10.610599 | -1.042969 | 310 | 113.73 | 0.366871 |
| GO:0044275\_cellular\_carbohydrate\_catabolic\_process | HMGB1 | 7 | 1 | 10.610599 | -1.042969 | 310 | 113.73 | 0.366871 |
| GO:0045668\_negative\_regulation\_of\_osteoblast\_differentiation | TOB1 | 7 | 1 | 10.610599 | -1.042969 | 310 | 113.73 | 0.366871 |
| GO:0046676\_negative\_regulation\_of\_insulin\_secretion | PFKL | 7 | 1 | 10.610599 | -1.042969 | 310 | 113.73 | 0.366871 |
| GO:0048148\_behavioral\_response\_to\_cocaine | ABAT | 7 | 1 | 10.610599 | -1.042969 | 310 | 113.73 | 0.366871 |
| GO:0048753\_pigment\_granule\_organization | BCL2 | 7 | 1 | 10.610599 | -1.042969 | 310 | 113.73 | 0.366871 |
| GO:0048857\_neural\_nucleus\_development | BCL2 | 7 | 1 | 10.610599 | -1.042969 | 310 | 113.73 | 0.366871 |
| GO:0051252\_regulation\_of\_RNA\_metabolic\_process | SUZ12 | 590 | 12 | 1.510662 | -1.038376 | 311 | 113.84 | 0.366045 |
| GO:0051252\_regulation\_of\_RNA\_metabolic\_process | SLC11A1 | 590 | 12 | 1.510662 | -1.038376 | 311 | 113.84 | 0.366045 |
| GO:0051252\_regulation\_of\_RNA\_metabolic\_process | HMGB1 | 590 | 12 | 1.510662 | -1.038376 | 311 | 113.84 | 0.366045 |
| GO:0051252\_regulation\_of\_RNA\_metabolic\_process | RNF6 | 590 | 12 | 1.510662 | -1.038376 | 311 | 113.84 | 0.366045 |
| GO:0051252\_regulation\_of\_RNA\_metabolic\_process | ONECUT2 | 590 | 12 | 1.510662 | -1.038376 | 311 | 113.84 | 0.366045 |
| GO:0051252\_regulation\_of\_RNA\_metabolic\_process | NFAT5 | 590 | 12 | 1.510662 | -1.038376 | 311 | 113.84 | 0.366045 |
| GO:0051252\_regulation\_of\_RNA\_metabolic\_process | RORB | 590 | 12 | 1.510662 | -1.038376 | 311 | 113.84 | 0.366045 |
| GO:0051252\_regulation\_of\_RNA\_metabolic\_process | MYB | 590 | 12 | 1.510662 | -1.038376 | 311 | 113.84 | 0.366045 |
| GO:0051252\_regulation\_of\_RNA\_metabolic\_process | NR2F2 | 590 | 12 | 1.510662 | -1.038376 | 311 | 113.84 | 0.366045 |
| GO:0051252\_regulation\_of\_RNA\_metabolic\_process | AHR | 590 | 12 | 1.510662 | -1.038376 | 311 | 113.84 | 0.366045 |
| GO:0051252\_regulation\_of\_RNA\_metabolic\_process | PITX2 | 590 | 12 | 1.510662 | -1.038376 | 311 | 113.84 | 0.366045 |
| GO:0051252\_regulation\_of\_RNA\_metabolic\_process | ETV3 | 590 | 12 | 1.510662 | -1.038376 | 311 | 113.84 | 0.366045 |
| GO:0048732\_gland\_development | IRS2 | 179 | 5 | 2.074698 | -1.037455 | 312 | 114.05 | 0.365545 |
| GO:0048732\_gland\_development | BCL2 | 179 | 5 | 2.074698 | -1.037455 | 312 | 114.05 | 0.365545 |
| GO:0048732\_gland\_development | VEGFA | 179 | 5 | 2.074698 | -1.037455 | 312 | 114.05 | 0.365545 |
| GO:0048732\_gland\_development | AHR | 179 | 5 | 2.074698 | -1.037455 | 312 | 114.05 | 0.365545 |
| GO:0048732\_gland\_development | PITX2 | 179 | 5 | 2.074698 | -1.037455 | 312 | 114.05 | 0.365545 |
| GO:0006820\_anion\_transport | SLC11A1 | 38 | 2 | 3.909168 | -1.035835 | 314 | 115.61 | 0.368185 |
| GO:0006820\_anion\_transport | SLC4A4 | 38 | 2 | 3.909168 | -1.035835 | 314 | 115.61 | 0.368185 |
| GO:0010975\_regulation\_of\_neuron\_projection\_development | RNF6 | 38 | 2 | 3.909168 | -1.035835 | 314 | 115.61 | 0.368185 |
| GO:0010975\_regulation\_of\_neuron\_projection\_development | NEFL | 38 | 2 | 3.909168 | -1.035835 | 314 | 115.61 | 0.368185 |
| GO:0019752\_carboxylic\_acid\_metabolic\_process | SCD1 | 181 | 5 | 2.051773 | -1.021758 | 317 | 116.96 | 0.368959 |
| GO:0019752\_carboxylic\_acid\_metabolic\_process | ALDH18A1 | 181 | 5 | 2.051773 | -1.021758 | 317 | 116.96 | 0.368959 |
| GO:0019752\_carboxylic\_acid\_metabolic\_process | ACOT10 | 181 | 5 | 2.051773 | -1.021758 | 317 | 116.96 | 0.368959 |
| GO:0019752\_carboxylic\_acid\_metabolic\_process | SNCA | 181 | 5 | 2.051773 | -1.021758 | 317 | 116.96 | 0.368959 |
| GO:0019752\_carboxylic\_acid\_metabolic\_process | ACSL4 | 181 | 5 | 2.051773 | -1.021758 | 317 | 116.96 | 0.368959 |
| GO:0043436\_oxoacid\_metabolic\_process | SCD1 | 181 | 5 | 2.051773 | -1.021758 | 317 | 116.96 | 0.368959 |
| GO:0043436\_oxoacid\_metabolic\_process | ALDH18A1 | 181 | 5 | 2.051773 | -1.021758 | 317 | 116.96 | 0.368959 |
| GO:0043436\_oxoacid\_metabolic\_process | ACOT10 | 181 | 5 | 2.051773 | -1.021758 | 317 | 116.96 | 0.368959 |
| GO:0043436\_oxoacid\_metabolic\_process | SNCA | 181 | 5 | 2.051773 | -1.021758 | 317 | 116.96 | 0.368959 |
| GO:0043436\_oxoacid\_metabolic\_process | ACSL4 | 181 | 5 | 2.051773 | -1.021758 | 317 | 116.96 | 0.368959 |
| GO:0055082\_cellular\_chemical\_homeostasis | SLC11A1 | 181 | 5 | 2.051773 | -1.021758 | 317 | 116.96 | 0.368959 |
| GO:0055082\_cellular\_chemical\_homeostasis | GNAQ | 181 | 5 | 2.051773 | -1.021758 | 317 | 116.96 | 0.368959 |
| GO:0055082\_cellular\_chemical\_homeostasis | BCL2 | 181 | 5 | 2.051773 | -1.021758 | 317 | 116.96 | 0.368959 |
| GO:0055082\_cellular\_chemical\_homeostasis | SNCA | 181 | 5 | 2.051773 | -1.021758 | 317 | 116.96 | 0.368959 |
| GO:0055082\_cellular\_chemical\_homeostasis | CHRNB4 | 181 | 5 | 2.051773 | -1.021758 | 317 | 116.96 | 0.368959 |
| GO:0016070\_RNA\_metabolic\_process | HMGB1 | 658 | 13 | 1.467423 | -1.018549 | 318 | 117.13 | 0.368333 |
| GO:0016070\_RNA\_metabolic\_process | LCORL | 658 | 13 | 1.467423 | -1.018549 | 318 | 117.13 | 0.368333 |
| GO:0016070\_RNA\_metabolic\_process | ONECUT2 | 658 | 13 | 1.467423 | -1.018549 | 318 | 117.13 | 0.368333 |
| GO:0016070\_RNA\_metabolic\_process | RORB | 658 | 13 | 1.467423 | -1.018549 | 318 | 117.13 | 0.368333 |
| GO:0016070\_RNA\_metabolic\_process | AHR | 658 | 13 | 1.467423 | -1.018549 | 318 | 117.13 | 0.368333 |
| GO:0016070\_RNA\_metabolic\_process | SUZ12 | 658 | 13 | 1.467423 | -1.018549 | 318 | 117.13 | 0.368333 |
| GO:0016070\_RNA\_metabolic\_process | SLC11A1 | 658 | 13 | 1.467423 | -1.018549 | 318 | 117.13 | 0.368333 |
| GO:0016070\_RNA\_metabolic\_process | RNF6 | 658 | 13 | 1.467423 | -1.018549 | 318 | 117.13 | 0.368333 |
| GO:0016070\_RNA\_metabolic\_process | NFAT5 | 658 | 13 | 1.467423 | -1.018549 | 318 | 117.13 | 0.368333 |
| GO:0016070\_RNA\_metabolic\_process | NR2F2 | 658 | 13 | 1.467423 | -1.018549 | 318 | 117.13 | 0.368333 |
| GO:0016070\_RNA\_metabolic\_process | MYB | 658 | 13 | 1.467423 | -1.018549 | 318 | 117.13 | 0.368333 |
| GO:0016070\_RNA\_metabolic\_process | ETV3 | 658 | 13 | 1.467423 | -1.018549 | 318 | 117.13 | 0.368333 |
| GO:0016070\_RNA\_metabolic\_process | PITX2 | 658 | 13 | 1.467423 | -1.018549 | 318 | 117.13 | 0.368333 |
| GO:0006644\_phospholipid\_metabolic\_process | SNCA | 39 | 2 | 3.808933 | -1.016628 | 321 | 118.68 | 0.369720 |
| GO:0006644\_phospholipid\_metabolic\_process | FABP3 | 39 | 2 | 3.808933 | -1.016628 | 321 | 118.68 | 0.369720 |
| GO:0007160\_cell-matrix\_adhesion | BCL2 | 39 | 2 | 3.808933 | -1.016628 | 321 | 118.68 | 0.369720 |
| GO:0007160\_cell-matrix\_adhesion | ONECUT2 | 39 | 2 | 3.808933 | -1.016628 | 321 | 118.68 | 0.369720 |
| GO:0021953\_central\_nervous\_system\_neuron\_differentiation | GNAQ | 39 | 2 | 3.808933 | -1.016628 | 321 | 118.68 | 0.369720 |
| GO:0021953\_central\_nervous\_system\_neuron\_differentiation | EPHB1 | 39 | 2 | 3.808933 | -1.016628 | 321 | 118.68 | 0.369720 |
| GO:0032787\_monocarboxylic\_acid\_metabolic\_process | SCD1 | 130 | 4 | 2.285360 | -1.014518 | 323 | 118.91 | 0.368142 |
| GO:0032787\_monocarboxylic\_acid\_metabolic\_process | ACOT10 | 130 | 4 | 2.285360 | -1.014518 | 323 | 118.91 | 0.368142 |
| GO:0032787\_monocarboxylic\_acid\_metabolic\_process | SNCA | 130 | 4 | 2.285360 | -1.014518 | 323 | 118.91 | 0.368142 |
| GO:0032787\_monocarboxylic\_acid\_metabolic\_process | ACSL4 | 130 | 4 | 2.285360 | -1.014518 | 323 | 118.91 | 0.368142 |
| GO:0045165\_cell\_fate\_commitment | SPRY1 | 130 | 4 | 2.285360 | -1.014518 | 323 | 118.91 | 0.368142 |
| GO:0045165\_cell\_fate\_commitment | BCL2 | 130 | 4 | 2.285360 | -1.014518 | 323 | 118.91 | 0.368142 |
| GO:0045165\_cell\_fate\_commitment | SALL1 | 130 | 4 | 2.285360 | -1.014518 | 323 | 118.91 | 0.368142 |
| GO:0045165\_cell\_fate\_commitment | ONECUT2 | 130 | 4 | 2.285360 | -1.014518 | 323 | 118.91 | 0.368142 |
| GO:0006082\_organic\_acid\_metabolic\_process | SCD1 | 182 | 5 | 2.040500 | -1.014009 | 324 | 118.98 | 0.367222 |
| GO:0006082\_organic\_acid\_metabolic\_process | ALDH18A1 | 182 | 5 | 2.040500 | -1.014009 | 324 | 118.98 | 0.367222 |
| GO:0006082\_organic\_acid\_metabolic\_process | ACOT10 | 182 | 5 | 2.040500 | -1.014009 | 324 | 118.98 | 0.367222 |
| GO:0006082\_organic\_acid\_metabolic\_process | SNCA | 182 | 5 | 2.040500 | -1.014009 | 324 | 118.98 | 0.367222 |
| GO:0006082\_organic\_acid\_metabolic\_process | ACSL4 | 182 | 5 | 2.040500 | -1.014009 | 324 | 118.98 | 0.367222 |
| GO:0045935\_positive\_regulation\_of\_nucleobase\_\_nucleoside\_\_nucleotide\_and\_nucleic\_acid\_metabolic\_process | SLC11A1 | 352 | 8 | 1.688050 | -1.010139 | 325 | 119.59 | 0.367969 |
| GO:0045935\_positive\_regulation\_of\_nucleobase\_\_nucleoside\_\_nucleotide\_and\_nucleic\_acid\_metabolic\_process | HMGB1 | 352 | 8 | 1.688050 | -1.010139 | 325 | 119.59 | 0.367969 |
| GO:0045935\_positive\_regulation\_of\_nucleobase\_\_nucleoside\_\_nucleotide\_and\_nucleic\_acid\_metabolic\_process | RNF6 | 352 | 8 | 1.688050 | -1.010139 | 325 | 119.59 | 0.367969 |
| GO:0045935\_positive\_regulation\_of\_nucleobase\_\_nucleoside\_\_nucleotide\_and\_nucleic\_acid\_metabolic\_process | ONECUT2 | 352 | 8 | 1.688050 | -1.010139 | 325 | 119.59 | 0.367969 |
| GO:0045935\_positive\_regulation\_of\_nucleobase\_\_nucleoside\_\_nucleotide\_and\_nucleic\_acid\_metabolic\_process | NFAT5 | 352 | 8 | 1.688050 | -1.010139 | 325 | 119.59 | 0.367969 |
| GO:0045935\_positive\_regulation\_of\_nucleobase\_\_nucleoside\_\_nucleotide\_and\_nucleic\_acid\_metabolic\_process | RORB | 352 | 8 | 1.688050 | -1.010139 | 325 | 119.59 | 0.367969 |
| GO:0045935\_positive\_regulation\_of\_nucleobase\_\_nucleoside\_\_nucleotide\_and\_nucleic\_acid\_metabolic\_process | AHR | 352 | 8 | 1.688050 | -1.010139 | 325 | 119.59 | 0.367969 |
| GO:0045935\_positive\_regulation\_of\_nucleobase\_\_nucleoside\_\_nucleotide\_and\_nucleic\_acid\_metabolic\_process | PITX2 | 352 | 8 | 1.688050 | -1.010139 | 325 | 119.59 | 0.367969 |
| GO:0042180\_cellular\_ketone\_metabolic\_process | SCD1 | 183 | 5 | 2.029350 | -1.006327 | 326 | 119.8 | 0.367485 |
| GO:0042180\_cellular\_ketone\_metabolic\_process | ALDH18A1 | 183 | 5 | 2.029350 | -1.006327 | 326 | 119.8 | 0.367485 |
| GO:0042180\_cellular\_ketone\_metabolic\_process | ACOT10 | 183 | 5 | 2.029350 | -1.006327 | 326 | 119.8 | 0.367485 |
| GO:0042180\_cellular\_ketone\_metabolic\_process | SNCA | 183 | 5 | 2.029350 | -1.006327 | 326 | 119.8 | 0.367485 |
| GO:0042180\_cellular\_ketone\_metabolic\_process | ACSL4 | 183 | 5 | 2.029350 | -1.006327 | 326 | 119.8 | 0.367485 |
| GO:0007049\_cell\_cycle | HMGB1 | 238 | 6 | 1.872459 | -1.004376 | 327 | 119.91 | 0.366697 |
| GO:0007049\_cell\_cycle | BCL2 | 238 | 6 | 1.872459 | -1.004376 | 327 | 119.91 | 0.366697 |
| GO:0007049\_cell\_cycle | ID4 | 238 | 6 | 1.872459 | -1.004376 | 327 | 119.91 | 0.366697 |
| GO:0007049\_cell\_cycle | MYB | 238 | 6 | 1.872459 | -1.004376 | 327 | 119.91 | 0.366697 |
| GO:0007049\_cell\_cycle | AHR | 238 | 6 | 1.872459 | -1.004376 | 327 | 119.91 | 0.366697 |
| GO:0007049\_cell\_cycle | CDC25B | 238 | 6 | 1.872459 | -1.004376 | 327 | 119.91 | 0.366697 |
| GO:0030534\_adult\_behavior | SNCA | 83 | 3 | 2.684609 | -0.999013 | 328 | 120.54 | 0.367500 |
| GO:0030534\_adult\_behavior | CHRNB4 | 83 | 3 | 2.684609 | -0.999013 | 328 | 120.54 | 0.367500 |
| GO:0030534\_adult\_behavior | ABAT | 83 | 3 | 2.684609 | -0.999013 | 328 | 120.54 | 0.367500 |
| GO:0007346\_regulation\_of\_mitotic\_cell\_cycle | HMGB1 | 40 | 2 | 3.713710 | -0.998004 | 332 | 121.98 | 0.367410 |
| GO:0007346\_regulation\_of\_mitotic\_cell\_cycle | BCL2 | 40 | 2 | 3.713710 | -0.998004 | 332 | 121.98 | 0.367410 |
| GO:0014031\_mesenchymal\_cell\_development | BCL2 | 40 | 2 | 3.713710 | -0.998004 | 332 | 121.98 | 0.367410 |
| GO:0014031\_mesenchymal\_cell\_development | KITL | 40 | 2 | 3.713710 | -0.998004 | 332 | 121.98 | 0.367410 |
| GO:0017015\_regulation\_of\_transforming\_growth\_factor\_beta\_receptor\_signaling\_pathway | ONECUT2 | 40 | 2 | 3.713710 | -0.998004 | 332 | 121.98 | 0.367410 |
| GO:0017015\_regulation\_of\_transforming\_growth\_factor\_beta\_receptor\_signaling\_pathway | TOB1 | 40 | 2 | 3.713710 | -0.998004 | 332 | 121.98 | 0.367410 |
| GO:0046850\_regulation\_of\_bone\_remodeling | BCL2 | 40 | 2 | 3.713710 | -0.998004 | 332 | 121.98 | 0.367410 |
| GO:0046850\_regulation\_of\_bone\_remodeling | TOB1 | 40 | 2 | 3.713710 | -0.998004 | 332 | 121.98 | 0.367410 |
| GO:0002320\_lymphoid\_progenitor\_cell\_differentiation | BCL2 | 8 | 1 | 9.284274 | -0.987828 | 356 | 135.34 | 0.380169 |
| GO:0006582\_melanin\_metabolic\_process | BCL2 | 8 | 1 | 9.284274 | -0.987828 | 356 | 135.34 | 0.380169 |
| GO:0009746\_response\_to\_hexose\_stimulus | PFKL | 8 | 1 | 9.284274 | -0.987828 | 356 | 135.34 | 0.380169 |
| GO:0009749\_response\_to\_glucose\_stimulus | PFKL | 8 | 1 | 9.284274 | -0.987828 | 356 | 135.34 | 0.380169 |
| GO:0014014\_negative\_regulation\_of\_gliogenesis | ID4 | 8 | 1 | 9.284274 | -0.987828 | 356 | 135.34 | 0.380169 |
| GO:0014046\_dopamine\_secretion | SNCA | 8 | 1 | 9.284274 | -0.987828 | 356 | 135.34 | 0.380169 |
| GO:0014059\_regulation\_of\_dopamine\_secretion | SNCA | 8 | 1 | 9.284274 | -0.987828 | 356 | 135.34 | 0.380169 |
| GO:0018107\_peptidyl-threonine\_phosphorylation | BCL2 | 8 | 1 | 9.284274 | -0.987828 | 356 | 135.34 | 0.380169 |
| GO:0018210\_peptidyl-threonine\_modification | BCL2 | 8 | 1 | 9.284274 | -0.987828 | 356 | 135.34 | 0.380169 |
| GO:0021799\_cerebral\_cortex\_radially\_oriented\_cell\_migration | DAB1 | 8 | 1 | 9.284274 | -0.987828 | 356 | 135.34 | 0.380169 |
| GO:0034284\_response\_to\_monosaccharide\_stimulus | PFKL | 8 | 1 | 9.284274 | -0.987828 | 356 | 135.34 | 0.380169 |
| GO:0040017\_positive\_regulation\_of\_locomotion | HMGB1 | 8 | 1 | 9.284274 | -0.987828 | 356 | 135.34 | 0.380169 |
| GO:0042304\_regulation\_of\_fatty\_acid\_biosynthetic\_process | SNCA | 8 | 1 | 9.284274 | -0.987828 | 356 | 135.34 | 0.380169 |
| GO:0042423\_catecholamine\_biosynthetic\_process | SNCA | 8 | 1 | 9.284274 | -0.987828 | 356 | 135.34 | 0.380169 |
| GO:0043368\_positive\_T\_cell\_selection | BCL2 | 8 | 1 | 9.284274 | -0.987828 | 356 | 135.34 | 0.380169 |
| GO:0045686\_negative\_regulation\_of\_glial\_cell\_differentiation | ID4 | 8 | 1 | 9.284274 | -0.987828 | 356 | 135.34 | 0.380169 |
| GO:0046470\_phosphatidylcholine\_metabolic\_process | FABP3 | 8 | 1 | 9.284274 | -0.987828 | 356 | 135.34 | 0.380169 |
| GO:0048520\_positive\_regulation\_of\_behavior | HMGB1 | 8 | 1 | 9.284274 | -0.987828 | 356 | 135.34 | 0.380169 |
| GO:0048742\_regulation\_of\_skeletal\_muscle\_fiber\_development | BCL2 | 8 | 1 | 9.284274 | -0.987828 | 356 | 135.34 | 0.380169 |
| GO:0050920\_regulation\_of\_chemotaxis | HMGB1 | 8 | 1 | 9.284274 | -0.987828 | 356 | 135.34 | 0.380169 |
| GO:0050921\_positive\_regulation\_of\_chemotaxis | HMGB1 | 8 | 1 | 9.284274 | -0.987828 | 356 | 135.34 | 0.380169 |
| GO:0050926\_regulation\_of\_positive\_chemotaxis | HMGB1 | 8 | 1 | 9.284274 | -0.987828 | 356 | 135.34 | 0.380169 |
| GO:0050927\_positive\_regulation\_of\_positive\_chemotaxis | HMGB1 | 8 | 1 | 9.284274 | -0.987828 | 356 | 135.34 | 0.380169 |
| GO:0050930\_induction\_of\_positive\_chemotaxis | HMGB1 | 8 | 1 | 9.284274 | -0.987828 | 356 | 135.34 | 0.380169 |
| GO:0044057\_regulation\_of\_system\_process | BDNF | 133 | 4 | 2.233810 | -0.987074 | 357 | 135.7 | 0.380112 |
| GO:0044057\_regulation\_of\_system\_process | SNCA | 133 | 4 | 2.233810 | -0.987074 | 357 | 135.7 | 0.380112 |
| GO:0044057\_regulation\_of\_system\_process | SP4 | 133 | 4 | 2.233810 | -0.987074 | 357 | 135.7 | 0.380112 |
| GO:0044057\_regulation\_of\_system\_process | CHRNB4 | 133 | 4 | 2.233810 | -0.987074 | 357 | 135.7 | 0.380112 |
| GO:0001776\_leukocyte\_homeostasis | BCL2 | 41 | 2 | 3.623131 | -0.979934 | 364 | 137.7 | 0.378297 |
| GO:0001776\_leukocyte\_homeostasis | KITL | 41 | 2 | 3.623131 | -0.979934 | 364 | 137.7 | 0.378297 |
| GO:0006979\_response\_to\_oxidative\_stress | BCL2 | 41 | 2 | 3.623131 | -0.979934 | 364 | 137.7 | 0.378297 |
| GO:0006979\_response\_to\_oxidative\_stress | SNCA | 41 | 2 | 3.623131 | -0.979934 | 364 | 137.7 | 0.378297 |
| GO:0008585\_female\_gonad\_development | BCL2 | 41 | 2 | 3.623131 | -0.979934 | 364 | 137.7 | 0.378297 |
| GO:0008585\_female\_gonad\_development | VEGFA | 41 | 2 | 3.623131 | -0.979934 | 364 | 137.7 | 0.378297 |
| GO:0015980\_energy\_derivation\_by\_oxidation\_of\_organic\_compounds | HMGB1 | 41 | 2 | 3.623131 | -0.979934 | 364 | 137.7 | 0.378297 |
| GO:0015980\_energy\_derivation\_by\_oxidation\_of\_organic\_compounds | SNCA | 41 | 2 | 3.623131 | -0.979934 | 364 | 137.7 | 0.378297 |
| GO:0019216\_regulation\_of\_lipid\_metabolic\_process | SNCA | 41 | 2 | 3.623131 | -0.979934 | 364 | 137.7 | 0.378297 |
| GO:0019216\_regulation\_of\_lipid\_metabolic\_process | ACSL4 | 41 | 2 | 3.623131 | -0.979934 | 364 | 137.7 | 0.378297 |
| GO:0031344\_regulation\_of\_cell\_projection\_organization | RNF6 | 41 | 2 | 3.623131 | -0.979934 | 364 | 137.7 | 0.378297 |
| GO:0031344\_regulation\_of\_cell\_projection\_organization | NEFL | 41 | 2 | 3.623131 | -0.979934 | 364 | 137.7 | 0.378297 |
| GO:0032844\_regulation\_of\_homeostatic\_process | BCL2 | 41 | 2 | 3.623131 | -0.979934 | 364 | 137.7 | 0.378297 |
| GO:0032844\_regulation\_of\_homeostatic\_process | KITL | 41 | 2 | 3.623131 | -0.979934 | 364 | 137.7 | 0.378297 |
| GO:0008283\_cell\_proliferation | SUZ12 | 544 | 11 | 1.501868 | -0.967077 | 365 | 138.45 | 0.379315 |
| GO:0008283\_cell\_proliferation | SLC11A1 | 544 | 11 | 1.501868 | -0.967077 | 365 | 138.45 | 0.379315 |
| GO:0008283\_cell\_proliferation | HMGB1 | 544 | 11 | 1.501868 | -0.967077 | 365 | 138.45 | 0.379315 |
| GO:0008283\_cell\_proliferation | IRS2 | 544 | 11 | 1.501868 | -0.967077 | 365 | 138.45 | 0.379315 |
| GO:0008283\_cell\_proliferation | BDNF | 544 | 11 | 1.501868 | -0.967077 | 365 | 138.45 | 0.379315 |
| GO:0008283\_cell\_proliferation | BCL2 | 544 | 11 | 1.501868 | -0.967077 | 365 | 138.45 | 0.379315 |
| GO:0008283\_cell\_proliferation | VEGFA | 544 | 11 | 1.501868 | -0.967077 | 365 | 138.45 | 0.379315 |
| GO:0008283\_cell\_proliferation | ID4 | 544 | 11 | 1.501868 | -0.967077 | 365 | 138.45 | 0.379315 |
| GO:0008283\_cell\_proliferation | FABP7 | 544 | 11 | 1.501868 | -0.967077 | 365 | 138.45 | 0.379315 |
| GO:0008283\_cell\_proliferation | KITL | 544 | 11 | 1.501868 | -0.967077 | 365 | 138.45 | 0.379315 |
| GO:0008283\_cell\_proliferation | PITX2 | 544 | 11 | 1.501868 | -0.967077 | 365 | 138.45 | 0.379315 |
| GO:0006897\_endocytosis | SLC11A1 | 86 | 3 | 2.590960 | -0.964217 | 367 | 138.87 | 0.378392 |
| GO:0006897\_endocytosis | SNCA | 86 | 3 | 2.590960 | -0.964217 | 367 | 138.87 | 0.378392 |
| GO:0006897\_endocytosis | EHD1 | 86 | 3 | 2.590960 | -0.964217 | 367 | 138.87 | 0.378392 |
| GO:0010324\_membrane\_invagination | SLC11A1 | 86 | 3 | 2.590960 | -0.964217 | 367 | 138.87 | 0.378392 |
| GO:0010324\_membrane\_invagination | SNCA | 86 | 3 | 2.590960 | -0.964217 | 367 | 138.87 | 0.378392 |
| GO:0010324\_membrane\_invagination | EHD1 | 86 | 3 | 2.590960 | -0.964217 | 367 | 138.87 | 0.378392 |
| GO:0051173\_positive\_regulation\_of\_nitrogen\_compound\_metabolic\_process | SLC11A1 | 361 | 8 | 1.645966 | -0.962435 | 368 | 138.9 | 0.377446 |
| GO:0051173\_positive\_regulation\_of\_nitrogen\_compound\_metabolic\_process | HMGB1 | 361 | 8 | 1.645966 | -0.962435 | 368 | 138.9 | 0.377446 |
| GO:0051173\_positive\_regulation\_of\_nitrogen\_compound\_metabolic\_process | RNF6 | 361 | 8 | 1.645966 | -0.962435 | 368 | 138.9 | 0.377446 |
| GO:0051173\_positive\_regulation\_of\_nitrogen\_compound\_metabolic\_process | ONECUT2 | 361 | 8 | 1.645966 | -0.962435 | 368 | 138.9 | 0.377446 |
| GO:0051173\_positive\_regulation\_of\_nitrogen\_compound\_metabolic\_process | NFAT5 | 361 | 8 | 1.645966 | -0.962435 | 368 | 138.9 | 0.377446 |
| GO:0051173\_positive\_regulation\_of\_nitrogen\_compound\_metabolic\_process | RORB | 361 | 8 | 1.645966 | -0.962435 | 368 | 138.9 | 0.377446 |
| GO:0051173\_positive\_regulation\_of\_nitrogen\_compound\_metabolic\_process | AHR | 361 | 8 | 1.645966 | -0.962435 | 368 | 138.9 | 0.377446 |
| GO:0051173\_positive\_regulation\_of\_nitrogen\_compound\_metabolic\_process | PITX2 | 361 | 8 | 1.645966 | -0.962435 | 368 | 138.9 | 0.377446 |
| GO:0010769\_regulation\_of\_cell\_morphogenesis\_involved\_in\_differentiation | RNF6 | 42 | 2 | 3.536866 | -0.962389 | 370 | 140.59 | 0.379973 |
| GO:0010769\_regulation\_of\_cell\_morphogenesis\_involved\_in\_differentiation | NEFL | 42 | 2 | 3.536866 | -0.962389 | 370 | 140.59 | 0.379973 |
| GO:0045637\_regulation\_of\_myeloid\_cell\_differentiation | HMGB1 | 42 | 2 | 3.536866 | -0.962389 | 370 | 140.59 | 0.379973 |
| GO:0045637\_regulation\_of\_myeloid\_cell\_differentiation | KITL | 42 | 2 | 3.536866 | -0.962389 | 370 | 140.59 | 0.379973 |
| GO:0034961\_cellular\_biopolymer\_biosynthetic\_process | HMGB1 | 804 | 15 | 1.385713 | -0.956464 | 371 | 140.94 | 0.379892 |
| GO:0034961\_cellular\_biopolymer\_biosynthetic\_process | LCORL | 804 | 15 | 1.385713 | -0.956464 | 371 | 140.94 | 0.379892 |
| GO:0034961\_cellular\_biopolymer\_biosynthetic\_process | ONECUT2 | 804 | 15 | 1.385713 | -0.956464 | 371 | 140.94 | 0.379892 |
| GO:0034961\_cellular\_biopolymer\_biosynthetic\_process | RORB | 804 | 15 | 1.385713 | -0.956464 | 371 | 140.94 | 0.379892 |
| GO:0034961\_cellular\_biopolymer\_biosynthetic\_process | AHR | 804 | 15 | 1.385713 | -0.956464 | 371 | 140.94 | 0.379892 |
| GO:0034961\_cellular\_biopolymer\_biosynthetic\_process | SUZ12 | 804 | 15 | 1.385713 | -0.956464 | 371 | 140.94 | 0.379892 |
| GO:0034961\_cellular\_biopolymer\_biosynthetic\_process | SLC11A1 | 804 | 15 | 1.385713 | -0.956464 | 371 | 140.94 | 0.379892 |
| GO:0034961\_cellular\_biopolymer\_biosynthetic\_process | RNF6 | 804 | 15 | 1.385713 | -0.956464 | 371 | 140.94 | 0.379892 |
| GO:0034961\_cellular\_biopolymer\_biosynthetic\_process | GARNL1 | 804 | 15 | 1.385713 | -0.956464 | 371 | 140.94 | 0.379892 |
| GO:0034961\_cellular\_biopolymer\_biosynthetic\_process | NFAT5 | 804 | 15 | 1.385713 | -0.956464 | 371 | 140.94 | 0.379892 |
| GO:0034961\_cellular\_biopolymer\_biosynthetic\_process | NR2F2 | 804 | 15 | 1.385713 | -0.956464 | 371 | 140.94 | 0.379892 |
| GO:0034961\_cellular\_biopolymer\_biosynthetic\_process | MYB | 804 | 15 | 1.385713 | -0.956464 | 371 | 140.94 | 0.379892 |
| GO:0034961\_cellular\_biopolymer\_biosynthetic\_process | FEN1 | 804 | 15 | 1.385713 | -0.956464 | 371 | 140.94 | 0.379892 |
| GO:0034961\_cellular\_biopolymer\_biosynthetic\_process | ETV3 | 804 | 15 | 1.385713 | -0.956464 | 371 | 140.94 | 0.379892 |
| GO:0034961\_cellular\_biopolymer\_biosynthetic\_process | PITX2 | 804 | 15 | 1.385713 | -0.956464 | 371 | 140.94 | 0.379892 |
| GO:0045449\_regulation\_of\_transcription | HMGB1 | 676 | 13 | 1.428350 | -0.948354 | 372 | 141.81 | 0.381210 |
| GO:0045449\_regulation\_of\_transcription | ONECUT2 | 676 | 13 | 1.428350 | -0.948354 | 372 | 141.81 | 0.381210 |
| GO:0045449\_regulation\_of\_transcription | RORB | 676 | 13 | 1.428350 | -0.948354 | 372 | 141.81 | 0.381210 |
| GO:0045449\_regulation\_of\_transcription | AHR | 676 | 13 | 1.428350 | -0.948354 | 372 | 141.81 | 0.381210 |
| GO:0045449\_regulation\_of\_transcription | SUZ12 | 676 | 13 | 1.428350 | -0.948354 | 372 | 141.81 | 0.381210 |
| GO:0045449\_regulation\_of\_transcription | SLC11A1 | 676 | 13 | 1.428350 | -0.948354 | 372 | 141.81 | 0.381210 |
| GO:0045449\_regulation\_of\_transcription | RNF6 | 676 | 13 | 1.428350 | -0.948354 | 372 | 141.81 | 0.381210 |
| GO:0045449\_regulation\_of\_transcription | GARNL1 | 676 | 13 | 1.428350 | -0.948354 | 372 | 141.81 | 0.381210 |
| GO:0045449\_regulation\_of\_transcription | NFAT5 | 676 | 13 | 1.428350 | -0.948354 | 372 | 141.81 | 0.381210 |
| GO:0045449\_regulation\_of\_transcription | NR2F2 | 676 | 13 | 1.428350 | -0.948354 | 372 | 141.81 | 0.381210 |
| GO:0045449\_regulation\_of\_transcription | MYB | 676 | 13 | 1.428350 | -0.948354 | 372 | 141.81 | 0.381210 |
| GO:0045449\_regulation\_of\_transcription | ETV3 | 676 | 13 | 1.428350 | -0.948354 | 372 | 141.81 | 0.381210 |
| GO:0045449\_regulation\_of\_transcription | PITX2 | 676 | 13 | 1.428350 | -0.948354 | 372 | 141.81 | 0.381210 |
| GO:0043284\_biopolymer\_biosynthetic\_process | HMGB1 | 807 | 15 | 1.380561 | -0.945945 | 373 | 141.92 | 0.380483 |
| GO:0043284\_biopolymer\_biosynthetic\_process | LCORL | 807 | 15 | 1.380561 | -0.945945 | 373 | 141.92 | 0.380483 |
| GO:0043284\_biopolymer\_biosynthetic\_process | ONECUT2 | 807 | 15 | 1.380561 | -0.945945 | 373 | 141.92 | 0.380483 |
| GO:0043284\_biopolymer\_biosynthetic\_process | RORB | 807 | 15 | 1.380561 | -0.945945 | 373 | 141.92 | 0.380483 |
| GO:0043284\_biopolymer\_biosynthetic\_process | AHR | 807 | 15 | 1.380561 | -0.945945 | 373 | 141.92 | 0.380483 |
| GO:0043284\_biopolymer\_biosynthetic\_process | SUZ12 | 807 | 15 | 1.380561 | -0.945945 | 373 | 141.92 | 0.380483 |
| GO:0043284\_biopolymer\_biosynthetic\_process | SLC11A1 | 807 | 15 | 1.380561 | -0.945945 | 373 | 141.92 | 0.380483 |
| GO:0043284\_biopolymer\_biosynthetic\_process | RNF6 | 807 | 15 | 1.380561 | -0.945945 | 373 | 141.92 | 0.380483 |
| GO:0043284\_biopolymer\_biosynthetic\_process | GARNL1 | 807 | 15 | 1.380561 | -0.945945 | 373 | 141.92 | 0.380483 |
| GO:0043284\_biopolymer\_biosynthetic\_process | NFAT5 | 807 | 15 | 1.380561 | -0.945945 | 373 | 141.92 | 0.380483 |
| GO:0043284\_biopolymer\_biosynthetic\_process | NR2F2 | 807 | 15 | 1.380561 | -0.945945 | 373 | 141.92 | 0.380483 |
| GO:0043284\_biopolymer\_biosynthetic\_process | MYB | 807 | 15 | 1.380561 | -0.945945 | 373 | 141.92 | 0.380483 |
| GO:0043284\_biopolymer\_biosynthetic\_process | FEN1 | 807 | 15 | 1.380561 | -0.945945 | 373 | 141.92 | 0.380483 |
| GO:0043284\_biopolymer\_biosynthetic\_process | ETV3 | 807 | 15 | 1.380561 | -0.945945 | 373 | 141.92 | 0.380483 |
| GO:0043284\_biopolymer\_biosynthetic\_process | PITX2 | 807 | 15 | 1.380561 | -0.945945 | 373 | 141.92 | 0.380483 |
| GO:0001508\_regulation\_of\_action\_potential | GNAQ | 43 | 2 | 3.454614 | -0.945345 | 377 | 143.73 | 0.381247 |
| GO:0001508\_regulation\_of\_action\_potential | CHRNB4 | 43 | 2 | 3.454614 | -0.945345 | 377 | 143.73 | 0.381247 |
| GO:0001894\_tissue\_homeostasis | BCL2 | 43 | 2 | 3.454614 | -0.945345 | 377 | 143.73 | 0.381247 |
| GO:0001894\_tissue\_homeostasis | VEGFA | 43 | 2 | 3.454614 | -0.945345 | 377 | 143.73 | 0.381247 |
| GO:0019637\_organophosphate\_metabolic\_process | SNCA | 43 | 2 | 3.454614 | -0.945345 | 377 | 143.73 | 0.381247 |
| GO:0019637\_organophosphate\_metabolic\_process | FABP3 | 43 | 2 | 3.454614 | -0.945345 | 377 | 143.73 | 0.381247 |
| GO:0048762\_mesenchymal\_cell\_differentiation | BCL2 | 43 | 2 | 3.454614 | -0.945345 | 377 | 143.73 | 0.381247 |
| GO:0048762\_mesenchymal\_cell\_differentiation | KITL | 43 | 2 | 3.454614 | -0.945345 | 377 | 143.73 | 0.381247 |
| GO:0001503\_ossification | COL13A1 | 88 | 3 | 2.532075 | -0.941915 | 378 | 144.28 | 0.381693 |
| GO:0001503\_ossification | BCL2 | 88 | 3 | 2.532075 | -0.941915 | 378 | 144.28 | 0.381693 |
| GO:0001503\_ossification | TOB1 | 88 | 3 | 2.532075 | -0.941915 | 378 | 144.28 | 0.381693 |
| GO:0001935\_endothelial\_cell\_proliferation | VEGFA | 9 | 1 | 8.252688 | -0.939520 | 394 | 156.93 | 0.398299 |
| GO:0006007\_glucose\_catabolic\_process | PFKL | 9 | 1 | 8.252688 | -0.939520 | 394 | 156.93 | 0.398299 |
| GO:0010675\_regulation\_of\_cellular\_carbohydrate\_metabolic\_process | HMGB1 | 9 | 1 | 8.252688 | -0.939520 | 394 | 156.93 | 0.398299 |
| GO:0014073\_response\_to\_tropane | ABAT | 9 | 1 | 8.252688 | -0.939520 | 394 | 156.93 | 0.398299 |
| GO:0019320\_hexose\_catabolic\_process | PFKL | 9 | 1 | 8.252688 | -0.939520 | 394 | 156.93 | 0.398299 |
| GO:0030279\_negative\_regulation\_of\_ossification | BCL2 | 9 | 1 | 8.252688 | -0.939520 | 394 | 156.93 | 0.398299 |
| GO:0035162\_embryonic\_hemopoiesis | VEGFA | 9 | 1 | 8.252688 | -0.939520 | 394 | 156.93 | 0.398299 |
| GO:0042220\_response\_to\_cocaine | ABAT | 9 | 1 | 8.252688 | -0.939520 | 394 | 156.93 | 0.398299 |
| GO:0045109\_intermediate\_filament\_organization | NEFL | 9 | 1 | 8.252688 | -0.939520 | 394 | 156.93 | 0.398299 |
| GO:0046365\_monosaccharide\_catabolic\_process | PFKL | 9 | 1 | 8.252688 | -0.939520 | 394 | 156.93 | 0.398299 |
| GO:0046888\_negative\_regulation\_of\_hormone\_secretion | PFKL | 9 | 1 | 8.252688 | -0.939520 | 394 | 156.93 | 0.398299 |
| GO:0048488\_synaptic\_vesicle\_endocytosis | SNCA | 9 | 1 | 8.252688 | -0.939520 | 394 | 156.93 | 0.398299 |
| GO:0048708\_astrocyte\_differentiation | ID4 | 9 | 1 | 8.252688 | -0.939520 | 394 | 156.93 | 0.398299 |
| GO:0050433\_regulation\_of\_catecholamine\_secretion | SNCA | 9 | 1 | 8.252688 | -0.939520 | 394 | 156.93 | 0.398299 |
| GO:0050918\_positive\_chemotaxis | HMGB1 | 9 | 1 | 8.252688 | -0.939520 | 394 | 156.93 | 0.398299 |
| GO:0060052\_neurofilament\_cytoskeleton\_organization | NEFL | 9 | 1 | 8.252688 | -0.939520 | 394 | 156.93 | 0.398299 |
| GO:0010556\_regulation\_of\_macromolecule\_biosynthetic\_process | HMGB1 | 745 | 14 | 1.395757 | -0.933347 | 395 | 157.23 | 0.398051 |
| GO:0010556\_regulation\_of\_macromolecule\_biosynthetic\_process | ONECUT2 | 745 | 14 | 1.395757 | -0.933347 | 395 | 157.23 | 0.398051 |
| GO:0010556\_regulation\_of\_macromolecule\_biosynthetic\_process | RORB | 745 | 14 | 1.395757 | -0.933347 | 395 | 157.23 | 0.398051 |
| GO:0010556\_regulation\_of\_macromolecule\_biosynthetic\_process | AHR | 745 | 14 | 1.395757 | -0.933347 | 395 | 157.23 | 0.398051 |
| GO:0010556\_regulation\_of\_macromolecule\_biosynthetic\_process | SUZ12 | 745 | 14 | 1.395757 | -0.933347 | 395 | 157.23 | 0.398051 |
| GO:0010556\_regulation\_of\_macromolecule\_biosynthetic\_process | SLC11A1 | 745 | 14 | 1.395757 | -0.933347 | 395 | 157.23 | 0.398051 |
| GO:0010556\_regulation\_of\_macromolecule\_biosynthetic\_process | RNF6 | 745 | 14 | 1.395757 | -0.933347 | 395 | 157.23 | 0.398051 |
| GO:0010556\_regulation\_of\_macromolecule\_biosynthetic\_process | BCL2 | 745 | 14 | 1.395757 | -0.933347 | 395 | 157.23 | 0.398051 |
| GO:0010556\_regulation\_of\_macromolecule\_biosynthetic\_process | GARNL1 | 745 | 14 | 1.395757 | -0.933347 | 395 | 157.23 | 0.398051 |
| GO:0010556\_regulation\_of\_macromolecule\_biosynthetic\_process | NFAT5 | 745 | 14 | 1.395757 | -0.933347 | 395 | 157.23 | 0.398051 |
| GO:0010556\_regulation\_of\_macromolecule\_biosynthetic\_process | NR2F2 | 745 | 14 | 1.395757 | -0.933347 | 395 | 157.23 | 0.398051 |
| GO:0010556\_regulation\_of\_macromolecule\_biosynthetic\_process | MYB | 745 | 14 | 1.395757 | -0.933347 | 395 | 157.23 | 0.398051 |
| GO:0010556\_regulation\_of\_macromolecule\_biosynthetic\_process | PITX2 | 745 | 14 | 1.395757 | -0.933347 | 395 | 157.23 | 0.398051 |
| GO:0010556\_regulation\_of\_macromolecule\_biosynthetic\_process | ETV3 | 745 | 14 | 1.395757 | -0.933347 | 395 | 157.23 | 0.398051 |
| GO:0006606\_protein\_import\_into\_nucleus | SLC11A1 | 44 | 2 | 3.376100 | -0.928778 | 400 | 159.49 | 0.398725 |
| GO:0006606\_protein\_import\_into\_nucleus | TOB1 | 44 | 2 | 3.376100 | -0.928778 | 400 | 159.49 | 0.398725 |
| GO:0046545\_development\_of\_primary\_female\_sexual\_characteristics | BCL2 | 44 | 2 | 3.376100 | -0.928778 | 400 | 159.49 | 0.398725 |
| GO:0046545\_development\_of\_primary\_female\_sexual\_characteristics | VEGFA | 44 | 2 | 3.376100 | -0.928778 | 400 | 159.49 | 0.398725 |
| GO:0048593\_camera-type\_eye\_morphogenesis | VEGFA | 44 | 2 | 3.376100 | -0.928778 | 400 | 159.49 | 0.398725 |
| GO:0048593\_camera-type\_eye\_morphogenesis | EPHB1 | 44 | 2 | 3.376100 | -0.928778 | 400 | 159.49 | 0.398725 |
| GO:0051170\_nuclear\_import | SLC11A1 | 44 | 2 | 3.376100 | -0.928778 | 400 | 159.49 | 0.398725 |
| GO:0051170\_nuclear\_import | TOB1 | 44 | 2 | 3.376100 | -0.928778 | 400 | 159.49 | 0.398725 |
| GO:0060485\_mesenchyme\_development | BCL2 | 44 | 2 | 3.376100 | -0.928778 | 400 | 159.49 | 0.398725 |
| GO:0060485\_mesenchyme\_development | KITL | 44 | 2 | 3.376100 | -0.928778 | 400 | 159.49 | 0.398725 |
| GO:0016044\_membrane\_organization | SLC11A1 | 140 | 4 | 2.122120 | -0.926421 | 401 | 159.79 | 0.398479 |
| GO:0016044\_membrane\_organization | BCL2 | 140 | 4 | 2.122120 | -0.926421 | 401 | 159.79 | 0.398479 |
| GO:0016044\_membrane\_organization | SNCA | 140 | 4 | 2.122120 | -0.926421 | 401 | 159.79 | 0.398479 |
| GO:0016044\_membrane\_organization | EHD1 | 140 | 4 | 2.122120 | -0.926421 | 401 | 159.79 | 0.398479 |
| GO:0030324\_lung\_development | HMGB1 | 90 | 3 | 2.475806 | -0.920291 | 402 | 160.38 | 0.398955 |
| GO:0030324\_lung\_development | VEGFA | 90 | 3 | 2.475806 | -0.920291 | 402 | 160.38 | 0.398955 |
| GO:0030324\_lung\_development | PITX2 | 90 | 3 | 2.475806 | -0.920291 | 402 | 160.38 | 0.398955 |
| GO:0019725\_cellular\_homeostasis | SLC11A1 | 195 | 5 | 1.904467 | -0.918998 | 403 | 160.69 | 0.398734 |
| GO:0019725\_cellular\_homeostasis | GNAQ | 195 | 5 | 1.904467 | -0.918998 | 403 | 160.69 | 0.398734 |
| GO:0019725\_cellular\_homeostasis | BCL2 | 195 | 5 | 1.904467 | -0.918998 | 403 | 160.69 | 0.398734 |
| GO:0019725\_cellular\_homeostasis | SNCA | 195 | 5 | 1.904467 | -0.918998 | 403 | 160.69 | 0.398734 |
| GO:0019725\_cellular\_homeostasis | CHRNB4 | 195 | 5 | 1.904467 | -0.918998 | 403 | 160.69 | 0.398734 |
| GO:0034103\_regulation\_of\_tissue\_remodeling | BCL2 | 45 | 2 | 3.301075 | -0.912666 | 404 | 161.71 | 0.400272 |
| GO:0034103\_regulation\_of\_tissue\_remodeling | TOB1 | 45 | 2 | 3.301075 | -0.912666 | 404 | 161.71 | 0.400272 |
| GO:0010557\_positive\_regulation\_of\_macromolecule\_biosynthetic\_process | SLC11A1 | 371 | 8 | 1.601600 | -0.911960 | 405 | 161.86 | 0.399654 |
| GO:0010557\_positive\_regulation\_of\_macromolecule\_biosynthetic\_process | HMGB1 | 371 | 8 | 1.601600 | -0.911960 | 405 | 161.86 | 0.399654 |
| GO:0010557\_positive\_regulation\_of\_macromolecule\_biosynthetic\_process | RNF6 | 371 | 8 | 1.601600 | -0.911960 | 405 | 161.86 | 0.399654 |
| GO:0010557\_positive\_regulation\_of\_macromolecule\_biosynthetic\_process | ONECUT2 | 371 | 8 | 1.601600 | -0.911960 | 405 | 161.86 | 0.399654 |
| GO:0010557\_positive\_regulation\_of\_macromolecule\_biosynthetic\_process | NFAT5 | 371 | 8 | 1.601600 | -0.911960 | 405 | 161.86 | 0.399654 |
| GO:0010557\_positive\_regulation\_of\_macromolecule\_biosynthetic\_process | RORB | 371 | 8 | 1.601600 | -0.911960 | 405 | 161.86 | 0.399654 |
| GO:0010557\_positive\_regulation\_of\_macromolecule\_biosynthetic\_process | AHR | 371 | 8 | 1.601600 | -0.911960 | 405 | 161.86 | 0.399654 |
| GO:0010557\_positive\_regulation\_of\_macromolecule\_biosynthetic\_process | PITX2 | 371 | 8 | 1.601600 | -0.911960 | 405 | 161.86 | 0.399654 |
| GO:0031399\_regulation\_of\_protein\_modification\_process | HMGB1 | 91 | 3 | 2.448600 | -0.909724 | 406 | 162.26 | 0.399655 |
| GO:0031399\_regulation\_of\_protein\_modification\_process | BCL2 | 91 | 3 | 2.448600 | -0.909724 | 406 | 162.26 | 0.399655 |
| GO:0031399\_regulation\_of\_protein\_modification\_process | KITL | 91 | 3 | 2.448600 | -0.909724 | 406 | 162.26 | 0.399655 |
| GO:0035239\_tube\_morphogenesis | SEMA5A | 143 | 4 | 2.077600 | -0.901784 | 407 | 162.9 | 0.400246 |
| GO:0035239\_tube\_morphogenesis | BCL2 | 143 | 4 | 2.077600 | -0.901784 | 407 | 162.9 | 0.400246 |
| GO:0035239\_tube\_morphogenesis | VEGFA | 143 | 4 | 2.077600 | -0.901784 | 407 | 162.9 | 0.400246 |
| GO:0035239\_tube\_morphogenesis | PITX2 | 143 | 4 | 2.077600 | -0.901784 | 407 | 162.9 | 0.400246 |
| GO:0009719\_response\_to\_endogenous\_stimulus | HMGB1 | 92 | 3 | 2.421985 | -0.899315 | 409 | 163.11 | 0.398802 |
| GO:0009719\_response\_to\_endogenous\_stimulus | IRS2 | 92 | 3 | 2.421985 | -0.899315 | 409 | 163.11 | 0.398802 |
| GO:0009719\_response\_to\_endogenous\_stimulus | BCL2 | 92 | 3 | 2.421985 | -0.899315 | 409 | 163.11 | 0.398802 |
| GO:0030323\_respiratory\_tube\_development | HMGB1 | 92 | 3 | 2.421985 | -0.899315 | 409 | 163.11 | 0.398802 |
| GO:0030323\_respiratory\_tube\_development | VEGFA | 92 | 3 | 2.421985 | -0.899315 | 409 | 163.11 | 0.398802 |
| GO:0030323\_respiratory\_tube\_development | PITX2 | 92 | 3 | 2.421985 | -0.899315 | 409 | 163.11 | 0.398802 |
| GO:0006357\_regulation\_of\_transcription\_from\_RNA\_polymerase\_II\_promoter | SUZ12 | 435 | 9 | 1.536707 | -0.898892 | 410 | 163.2 | 0.398049 |
| GO:0006357\_regulation\_of\_transcription\_from\_RNA\_polymerase\_II\_promoter | SLC11A1 | 435 | 9 | 1.536707 | -0.898892 | 410 | 163.2 | 0.398049 |
| GO:0006357\_regulation\_of\_transcription\_from\_RNA\_polymerase\_II\_promoter | HMGB1 | 435 | 9 | 1.536707 | -0.898892 | 410 | 163.2 | 0.398049 |
| GO:0006357\_regulation\_of\_transcription\_from\_RNA\_polymerase\_II\_promoter | ONECUT2 | 435 | 9 | 1.536707 | -0.898892 | 410 | 163.2 | 0.398049 |
| GO:0006357\_regulation\_of\_transcription\_from\_RNA\_polymerase\_II\_promoter | NFAT5 | 435 | 9 | 1.536707 | -0.898892 | 410 | 163.2 | 0.398049 |
| GO:0006357\_regulation\_of\_transcription\_from\_RNA\_polymerase\_II\_promoter | NR2F2 | 435 | 9 | 1.536707 | -0.898892 | 410 | 163.2 | 0.398049 |
| GO:0006357\_regulation\_of\_transcription\_from\_RNA\_polymerase\_II\_promoter | AHR | 435 | 9 | 1.536707 | -0.898892 | 410 | 163.2 | 0.398049 |
| GO:0006357\_regulation\_of\_transcription\_from\_RNA\_polymerase\_II\_promoter | PITX2 | 435 | 9 | 1.536707 | -0.898892 | 410 | 163.2 | 0.398049 |
| GO:0006357\_regulation\_of\_transcription\_from\_RNA\_polymerase\_II\_promoter | ETV3 | 435 | 9 | 1.536707 | -0.898892 | 410 | 163.2 | 0.398049 |
| GO:0006732\_coenzyme\_metabolic\_process | ACOT10 | 46 | 2 | 3.229313 | -0.896990 | 411 | 164.34 | 0.399854 |
| GO:0006732\_coenzyme\_metabolic\_process | SNCA | 46 | 2 | 3.229313 | -0.896990 | 411 | 164.34 | 0.399854 |
| GO:0006109\_regulation\_of\_carbohydrate\_metabolic\_process | HMGB1 | 10 | 1 | 7.427419 | -0.896601 | 427 | 178.91 | 0.418993 |
| GO:0006289\_nucleotide-excision\_repair | DCLRE1A | 10 | 1 | 7.427419 | -0.896601 | 427 | 178.91 | 0.418993 |
| GO:0006805\_xenobiotic\_metabolic\_process | AHR | 10 | 1 | 7.427419 | -0.896601 | 427 | 178.91 | 0.418993 |
| GO:0006826\_iron\_ion\_transport | SLC11A1 | 10 | 1 | 7.427419 | -0.896601 | 427 | 178.91 | 0.418993 |
| GO:0007044\_cell-substrate\_junction\_assembly | BCL2 | 10 | 1 | 7.427419 | -0.896601 | 427 | 178.91 | 0.418993 |
| GO:0009743\_response\_to\_carbohydrate\_stimulus | PFKL | 10 | 1 | 7.427419 | -0.896601 | 427 | 178.91 | 0.418993 |
| GO:0016197\_endosome\_transport | EHD1 | 10 | 1 | 7.427419 | -0.896601 | 427 | 178.91 | 0.418993 |
| GO:0021895\_cerebral\_cortex\_neuron\_differentiation | ID4 | 10 | 1 | 7.427419 | -0.896601 | 427 | 178.91 | 0.418993 |
| GO:0021952\_central\_nervous\_system\_projection\_neuron\_axonogenesis | EPHB1 | 10 | 1 | 7.427419 | -0.896601 | 427 | 178.91 | 0.418993 |
| GO:0022900\_electron\_transport\_chain | SNCA | 10 | 1 | 7.427419 | -0.896601 | 427 | 178.91 | 0.418993 |
| GO:0022904\_respiratory\_electron\_transport\_chain | SNCA | 10 | 1 | 7.427419 | -0.896601 | 427 | 178.91 | 0.418993 |
| GO:0031018\_endocrine\_pancreas\_development | ONECUT2 | 10 | 1 | 7.427419 | -0.896601 | 427 | 178.91 | 0.418993 |
| GO:0031331\_positive\_regulation\_of\_cellular\_catabolic\_process | HMGB1 | 10 | 1 | 7.427419 | -0.896601 | 427 | 178.91 | 0.418993 |
| GO:0042088\_T-helper\_1\_type\_immune\_response | SLC11A1 | 10 | 1 | 7.427419 | -0.896601 | 427 | 178.91 | 0.418993 |
| GO:0043488\_regulation\_of\_mRNA\_stability | SLC11A1 | 10 | 1 | 7.427419 | -0.896601 | 427 | 178.91 | 0.418993 |
| GO:0048641\_regulation\_of\_skeletal\_muscle\_tissue\_development | BCL2 | 10 | 1 | 7.427419 | -0.896601 | 427 | 178.91 | 0.418993 |
| GO:0045596\_negative\_regulation\_of\_cell\_differentiation | RNF6 | 144 | 4 | 2.063172 | -0.893743 | 428 | 179.16 | 0.418598 |
| GO:0045596\_negative\_regulation\_of\_cell\_differentiation | BDNF | 144 | 4 | 2.063172 | -0.893743 | 428 | 179.16 | 0.418598 |
| GO:0045596\_negative\_regulation\_of\_cell\_differentiation | ID4 | 144 | 4 | 2.063172 | -0.893743 | 428 | 179.16 | 0.418598 |
| GO:0045596\_negative\_regulation\_of\_cell\_differentiation | TOB1 | 144 | 4 | 2.063172 | -0.893743 | 428 | 179.16 | 0.418598 |
| GO:0019219\_regulation\_of\_nucleobase\_\_nucleoside\_\_nucleotide\_and\_nucleic\_acid\_metabolic\_process | HMGB1 | 757 | 14 | 1.373631 | -0.891156 | 429 | 179.34 | 0.418042 |
| GO:0019219\_regulation\_of\_nucleobase\_\_nucleoside\_\_nucleotide\_and\_nucleic\_acid\_metabolic\_process | ONECUT2 | 757 | 14 | 1.373631 | -0.891156 | 429 | 179.34 | 0.418042 |
| GO:0019219\_regulation\_of\_nucleobase\_\_nucleoside\_\_nucleotide\_and\_nucleic\_acid\_metabolic\_process | RORB | 757 | 14 | 1.373631 | -0.891156 | 429 | 179.34 | 0.418042 |
| GO:0019219\_regulation\_of\_nucleobase\_\_nucleoside\_\_nucleotide\_and\_nucleic\_acid\_metabolic\_process | AHR | 757 | 14 | 1.373631 | -0.891156 | 429 | 179.34 | 0.418042 |
| GO:0019219\_regulation\_of\_nucleobase\_\_nucleoside\_\_nucleotide\_and\_nucleic\_acid\_metabolic\_process | SUZ12 | 757 | 14 | 1.373631 | -0.891156 | 429 | 179.34 | 0.418042 |
| GO:0019219\_regulation\_of\_nucleobase\_\_nucleoside\_\_nucleotide\_and\_nucleic\_acid\_metabolic\_process | SLC11A1 | 757 | 14 | 1.373631 | -0.891156 | 429 | 179.34 | 0.418042 |
| GO:0019219\_regulation\_of\_nucleobase\_\_nucleoside\_\_nucleotide\_and\_nucleic\_acid\_metabolic\_process | RNF6 | 757 | 14 | 1.373631 | -0.891156 | 429 | 179.34 | 0.418042 |
| GO:0019219\_regulation\_of\_nucleobase\_\_nucleoside\_\_nucleotide\_and\_nucleic\_acid\_metabolic\_process | GNAQ | 757 | 14 | 1.373631 | -0.891156 | 429 | 179.34 | 0.418042 |
| GO:0019219\_regulation\_of\_nucleobase\_\_nucleoside\_\_nucleotide\_and\_nucleic\_acid\_metabolic\_process | GARNL1 | 757 | 14 | 1.373631 | -0.891156 | 429 | 179.34 | 0.418042 |
| GO:0019219\_regulation\_of\_nucleobase\_\_nucleoside\_\_nucleotide\_and\_nucleic\_acid\_metabolic\_process | NFAT5 | 757 | 14 | 1.373631 | -0.891156 | 429 | 179.34 | 0.418042 |
| GO:0019219\_regulation\_of\_nucleobase\_\_nucleoside\_\_nucleotide\_and\_nucleic\_acid\_metabolic\_process | NR2F2 | 757 | 14 | 1.373631 | -0.891156 | 429 | 179.34 | 0.418042 |
| GO:0019219\_regulation\_of\_nucleobase\_\_nucleoside\_\_nucleotide\_and\_nucleic\_acid\_metabolic\_process | MYB | 757 | 14 | 1.373631 | -0.891156 | 429 | 179.34 | 0.418042 |
| GO:0019219\_regulation\_of\_nucleobase\_\_nucleoside\_\_nucleotide\_and\_nucleic\_acid\_metabolic\_process | PITX2 | 757 | 14 | 1.373631 | -0.891156 | 429 | 179.34 | 0.418042 |
| GO:0019219\_regulation\_of\_nucleobase\_\_nucleoside\_\_nucleotide\_and\_nucleic\_acid\_metabolic\_process | ETV3 | 757 | 14 | 1.373631 | -0.891156 | 429 | 179.34 | 0.418042 |
| GO:0008610\_lipid\_biosynthetic\_process | SCD1 | 94 | 3 | 2.370453 | -0.878958 | 431 | 182.02 | 0.422320 |
| GO:0008610\_lipid\_biosynthetic\_process | SNCA | 94 | 3 | 2.370453 | -0.878958 | 431 | 182.02 | 0.422320 |
| GO:0008610\_lipid\_biosynthetic\_process | FABP3 | 94 | 3 | 2.370453 | -0.878958 | 431 | 182.02 | 0.422320 |
| GO:0034984\_cellular\_response\_to\_DNA\_damage\_stimulus | PAXIP1 | 94 | 3 | 2.370453 | -0.878958 | 431 | 182.02 | 0.422320 |
| GO:0034984\_cellular\_response\_to\_DNA\_damage\_stimulus | DCLRE1A | 94 | 3 | 2.370453 | -0.878958 | 431 | 182.02 | 0.422320 |
| GO:0034984\_cellular\_response\_to\_DNA\_damage\_stimulus | FEN1 | 94 | 3 | 2.370453 | -0.878958 | 431 | 182.02 | 0.422320 |
| GO:0006812\_cation\_transport | SLC11A1 | 146 | 4 | 2.034909 | -0.877911 | 432 | 182.2 | 0.421759 |
| GO:0006812\_cation\_transport | BCL2 | 146 | 4 | 2.034909 | -0.877911 | 432 | 182.2 | 0.421759 |
| GO:0006812\_cation\_transport | MYB | 146 | 4 | 2.034909 | -0.877911 | 432 | 182.2 | 0.421759 |
| GO:0006812\_cation\_transport | SLC4A4 | 146 | 4 | 2.034909 | -0.877911 | 432 | 182.2 | 0.421759 |
| GO:0022603\_regulation\_of\_anatomical\_structure\_morphogenesis | BDNF | 147 | 4 | 2.021066 | -0.870117 | 433 | 182.58 | 0.421663 |
| GO:0022603\_regulation\_of\_anatomical\_structure\_morphogenesis | RNF6 | 147 | 4 | 2.021066 | -0.870117 | 433 | 182.58 | 0.421663 |
| GO:0022603\_regulation\_of\_anatomical\_structure\_morphogenesis | BCL2 | 147 | 4 | 2.021066 | -0.870117 | 433 | 182.58 | 0.421663 |
| GO:0022603\_regulation\_of\_anatomical\_structure\_morphogenesis | NEFL | 147 | 4 | 2.021066 | -0.870117 | 433 | 182.58 | 0.421663 |
| GO:0016043\_cellular\_component\_organization | PARD3 | 964 | 17 | 1.309815 | -0.869028 | 434 | 182.63 | 0.420806 |
| GO:0016043\_cellular\_component\_organization | SNCA | 964 | 17 | 1.309815 | -0.869028 | 434 | 182.63 | 0.420806 |
| GO:0016043\_cellular\_component\_organization | ONECUT2 | 964 | 17 | 1.309815 | -0.869028 | 434 | 182.63 | 0.420806 |
| GO:0016043\_cellular\_component\_organization | AHR | 964 | 17 | 1.309815 | -0.869028 | 434 | 182.63 | 0.420806 |
| GO:0016043\_cellular\_component\_organization | EPHB1 | 964 | 17 | 1.309815 | -0.869028 | 434 | 182.63 | 0.420806 |
| GO:0016043\_cellular\_component\_organization | NRCAM | 964 | 17 | 1.309815 | -0.869028 | 434 | 182.63 | 0.420806 |
| GO:0016043\_cellular\_component\_organization | SUZ12 | 964 | 17 | 1.309815 | -0.869028 | 434 | 182.63 | 0.420806 |
| GO:0016043\_cellular\_component\_organization | CADPS | 964 | 17 | 1.309815 | -0.869028 | 434 | 182.63 | 0.420806 |
| GO:0016043\_cellular\_component\_organization | SEMA5A | 964 | 17 | 1.309815 | -0.869028 | 434 | 182.63 | 0.420806 |
| GO:0016043\_cellular\_component\_organization | EFHD1 | 964 | 17 | 1.309815 | -0.869028 | 434 | 182.63 | 0.420806 |
| GO:0016043\_cellular\_component\_organization | SLC11A1 | 964 | 17 | 1.309815 | -0.869028 | 434 | 182.63 | 0.420806 |
| GO:0016043\_cellular\_component\_organization | RNF6 | 964 | 17 | 1.309815 | -0.869028 | 434 | 182.63 | 0.420806 |
| GO:0016043\_cellular\_component\_organization | BDNF | 964 | 17 | 1.309815 | -0.869028 | 434 | 182.63 | 0.420806 |
| GO:0016043\_cellular\_component\_organization | BCL2 | 964 | 17 | 1.309815 | -0.869028 | 434 | 182.63 | 0.420806 |
| GO:0016043\_cellular\_component\_organization | TGFBI | 964 | 17 | 1.309815 | -0.869028 | 434 | 182.63 | 0.420806 |
| GO:0016043\_cellular\_component\_organization | EHD1 | 964 | 17 | 1.309815 | -0.869028 | 434 | 182.63 | 0.420806 |
| GO:0016043\_cellular\_component\_organization | NEFL | 964 | 17 | 1.309815 | -0.869028 | 434 | 182.63 | 0.420806 |
| GO:0034504\_protein\_localization\_in\_nucleus | SLC11A1 | 48 | 2 | 3.094758 | -0.866866 | 436 | 183.55 | 0.420986 |
| GO:0034504\_protein\_localization\_in\_nucleus | TOB1 | 48 | 2 | 3.094758 | -0.866866 | 436 | 183.55 | 0.420986 |
| GO:0046849\_bone\_remodeling | BCL2 | 48 | 2 | 3.094758 | -0.866866 | 436 | 183.55 | 0.420986 |
| GO:0046849\_bone\_remodeling | TOB1 | 48 | 2 | 3.094758 | -0.866866 | 436 | 183.55 | 0.420986 |
| GO:0001568\_blood\_vessel\_development | SEMA5A | 203 | 5 | 1.829414 | -0.865402 | 437 | 183.64 | 0.420229 |
| GO:0001568\_blood\_vessel\_development | NTRK2 | 203 | 5 | 1.829414 | -0.865402 | 437 | 183.64 | 0.420229 |
| GO:0001568\_blood\_vessel\_development | VEGFA | 203 | 5 | 1.829414 | -0.865402 | 437 | 183.64 | 0.420229 |
| GO:0001568\_blood\_vessel\_development | NR2F2 | 203 | 5 | 1.829414 | -0.865402 | 437 | 183.64 | 0.420229 |
| GO:0001568\_blood\_vessel\_development | PITX2 | 203 | 5 | 1.829414 | -0.865402 | 437 | 183.64 | 0.420229 |
| GO:0001101\_response\_to\_acid | BCL2 | 11 | 1 | 6.752199 | -0.858040 | 458 | 195.55 | 0.426965 |
| GO:0001963\_synaptic\_transmission\_\_dopaminergic | SNCA | 11 | 1 | 6.752199 | -0.858040 | 458 | 195.55 | 0.426965 |
| GO:0007162\_negative\_regulation\_of\_cell\_adhesion | DAB1 | 11 | 1 | 6.752199 | -0.858040 | 458 | 195.55 | 0.426965 |
| GO:0007215\_glutamate\_signaling\_pathway | GNAQ | 11 | 1 | 6.752199 | -0.858040 | 458 | 195.55 | 0.426965 |
| GO:0008354\_germ\_cell\_migration | KITL | 11 | 1 | 6.752199 | -0.858040 | 458 | 195.55 | 0.426965 |
| GO:0008652\_cellular\_amino\_acid\_biosynthetic\_process | ALDH18A1 | 11 | 1 | 6.752199 | -0.858040 | 458 | 195.55 | 0.426965 |
| GO:0014013\_regulation\_of\_gliogenesis | ID4 | 11 | 1 | 6.752199 | -0.858040 | 458 | 195.55 | 0.426965 |
| GO:0021602\_cranial\_nerve\_morphogenesis | EPHB1 | 11 | 1 | 6.752199 | -0.858040 | 458 | 195.55 | 0.426965 |
| GO:0021846\_cell\_proliferation\_in\_forebrain | FABP7 | 11 | 1 | 6.752199 | -0.858040 | 458 | 195.55 | 0.426965 |
| GO:0030308\_negative\_regulation\_of\_cell\_growth | BCL2 | 11 | 1 | 6.752199 | -0.858040 | 458 | 195.55 | 0.426965 |
| GO:0031646\_positive\_regulation\_of\_neurological\_system\_process | SNCA | 11 | 1 | 6.752199 | -0.858040 | 458 | 195.55 | 0.426965 |
| GO:0031647\_regulation\_of\_protein\_stability | BCL2 | 11 | 1 | 6.752199 | -0.858040 | 458 | 195.55 | 0.426965 |
| GO:0033059\_cellular\_pigmentation | BCL2 | 11 | 1 | 6.752199 | -0.858040 | 458 | 195.55 | 0.426965 |
| GO:0042401\_biogenic\_amine\_biosynthetic\_process | SNCA | 11 | 1 | 6.752199 | -0.858040 | 458 | 195.55 | 0.426965 |
| GO:0042439\_ethanolamine\_and\_derivative\_metabolic\_process | FABP3 | 11 | 1 | 6.752199 | -0.858040 | 458 | 195.55 | 0.426965 |
| GO:0042542\_response\_to\_hydrogen\_peroxide | BCL2 | 11 | 1 | 6.752199 | -0.858040 | 458 | 195.55 | 0.426965 |
| GO:0045685\_regulation\_of\_glial\_cell\_differentiation | ID4 | 11 | 1 | 6.752199 | -0.858040 | 458 | 195.55 | 0.426965 |
| GO:0046928\_regulation\_of\_neurotransmitter\_secretion | SNCA | 11 | 1 | 6.752199 | -0.858040 | 458 | 195.55 | 0.426965 |
| GO:0050772\_positive\_regulation\_of\_axonogenesis | NEFL | 11 | 1 | 6.752199 | -0.858040 | 458 | 195.55 | 0.426965 |
| GO:0050806\_positive\_regulation\_of\_synaptic\_transmission | SNCA | 11 | 1 | 6.752199 | -0.858040 | 458 | 195.55 | 0.426965 |
| GO:0051971\_positive\_regulation\_of\_transmission\_of\_nerve\_impulse | SNCA | 11 | 1 | 6.752199 | -0.858040 | 458 | 195.55 | 0.426965 |
| GO:0034645\_cellular\_macromolecule\_biosynthetic\_process | HMGB1 | 901 | 16 | 1.318965 | -0.855385 | 459 | 195.91 | 0.426819 |
| GO:0034645\_cellular\_macromolecule\_biosynthetic\_process | LCORL | 901 | 16 | 1.318965 | -0.855385 | 459 | 195.91 | 0.426819 |
| GO:0034645\_cellular\_macromolecule\_biosynthetic\_process | ONECUT2 | 901 | 16 | 1.318965 | -0.855385 | 459 | 195.91 | 0.426819 |
| GO:0034645\_cellular\_macromolecule\_biosynthetic\_process | RORB | 901 | 16 | 1.318965 | -0.855385 | 459 | 195.91 | 0.426819 |
| GO:0034645\_cellular\_macromolecule\_biosynthetic\_process | AHR | 901 | 16 | 1.318965 | -0.855385 | 459 | 195.91 | 0.426819 |
| GO:0034645\_cellular\_macromolecule\_biosynthetic\_process | SUZ12 | 901 | 16 | 1.318965 | -0.855385 | 459 | 195.91 | 0.426819 |
| GO:0034645\_cellular\_macromolecule\_biosynthetic\_process | SLC11A1 | 901 | 16 | 1.318965 | -0.855385 | 459 | 195.91 | 0.426819 |
| GO:0034645\_cellular\_macromolecule\_biosynthetic\_process | RNF6 | 901 | 16 | 1.318965 | -0.855385 | 459 | 195.91 | 0.426819 |
| GO:0034645\_cellular\_macromolecule\_biosynthetic\_process | BCL2 | 901 | 16 | 1.318965 | -0.855385 | 459 | 195.91 | 0.426819 |
| GO:0034645\_cellular\_macromolecule\_biosynthetic\_process | GARNL1 | 901 | 16 | 1.318965 | -0.855385 | 459 | 195.91 | 0.426819 |
| GO:0034645\_cellular\_macromolecule\_biosynthetic\_process | NFAT5 | 901 | 16 | 1.318965 | -0.855385 | 459 | 195.91 | 0.426819 |
| GO:0034645\_cellular\_macromolecule\_biosynthetic\_process | MYB | 901 | 16 | 1.318965 | -0.855385 | 459 | 195.91 | 0.426819 |
| GO:0034645\_cellular\_macromolecule\_biosynthetic\_process | NR2F2 | 901 | 16 | 1.318965 | -0.855385 | 459 | 195.91 | 0.426819 |
| GO:0034645\_cellular\_macromolecule\_biosynthetic\_process | FEN1 | 901 | 16 | 1.318965 | -0.855385 | 459 | 195.91 | 0.426819 |
| GO:0034645\_cellular\_macromolecule\_biosynthetic\_process | ETV3 | 901 | 16 | 1.318965 | -0.855385 | 459 | 195.91 | 0.426819 |
| GO:0034645\_cellular\_macromolecule\_biosynthetic\_process | PITX2 | 901 | 16 | 1.318965 | -0.855385 | 459 | 195.91 | 0.426819 |
| GO:0021543\_pallium\_development | DAB1 | 49 | 2 | 3.031600 | -0.852385 | 461 | 197.41 | 0.428221 |
| GO:0021543\_pallium\_development | ID4 | 49 | 2 | 3.031600 | -0.852385 | 461 | 197.41 | 0.428221 |
| GO:0046660\_female\_sex\_differentiation | BCL2 | 49 | 2 | 3.031600 | -0.852385 | 461 | 197.41 | 0.428221 |
| GO:0046660\_female\_sex\_differentiation | VEGFA | 49 | 2 | 3.031600 | -0.852385 | 461 | 197.41 | 0.428221 |
| GO:0060541\_respiratory\_system\_development | HMGB1 | 98 | 3 | 2.273700 | -0.839994 | 462 | 198.77 | 0.430238 |
| GO:0060541\_respiratory\_system\_development | VEGFA | 98 | 3 | 2.273700 | -0.839994 | 462 | 198.77 | 0.430238 |
| GO:0060541\_respiratory\_system\_development | PITX2 | 98 | 3 | 2.273700 | -0.839994 | 462 | 198.77 | 0.430238 |
| GO:0017038\_protein\_import | SLC11A1 | 50 | 2 | 2.970968 | -0.838269 | 463 | 199.7 | 0.431317 |
| GO:0017038\_protein\_import | TOB1 | 50 | 2 | 2.970968 | -0.838269 | 463 | 199.7 | 0.431317 |
| GO:0031328\_positive\_regulation\_of\_cellular\_biosynthetic\_process | SLC11A1 | 387 | 8 | 1.535384 | -0.836376 | 464 | 199.85 | 0.430711 |
| GO:0031328\_positive\_regulation\_of\_cellular\_biosynthetic\_process | HMGB1 | 387 | 8 | 1.535384 | -0.836376 | 464 | 199.85 | 0.430711 |
| GO:0031328\_positive\_regulation\_of\_cellular\_biosynthetic\_process | RNF6 | 387 | 8 | 1.535384 | -0.836376 | 464 | 199.85 | 0.430711 |
| GO:0031328\_positive\_regulation\_of\_cellular\_biosynthetic\_process | ONECUT2 | 387 | 8 | 1.535384 | -0.836376 | 464 | 199.85 | 0.430711 |
| GO:0031328\_positive\_regulation\_of\_cellular\_biosynthetic\_process | NFAT5 | 387 | 8 | 1.535384 | -0.836376 | 464 | 199.85 | 0.430711 |
| GO:0031328\_positive\_regulation\_of\_cellular\_biosynthetic\_process | RORB | 387 | 8 | 1.535384 | -0.836376 | 464 | 199.85 | 0.430711 |
| GO:0031328\_positive\_regulation\_of\_cellular\_biosynthetic\_process | AHR | 387 | 8 | 1.535384 | -0.836376 | 464 | 199.85 | 0.430711 |
| GO:0031328\_positive\_regulation\_of\_cellular\_biosynthetic\_process | PITX2 | 387 | 8 | 1.535384 | -0.836376 | 464 | 199.85 | 0.430711 |
| GO:0009887\_organ\_morphogenesis | SEMA5A | 642 | 12 | 1.388303 | -0.835139 | 465 | 199.91 | 0.429914 |
| GO:0009887\_organ\_morphogenesis | SPRY1 | 642 | 12 | 1.388303 | -0.835139 | 465 | 199.91 | 0.429914 |
| GO:0009887\_organ\_morphogenesis | BDNF | 642 | 12 | 1.388303 | -0.835139 | 465 | 199.91 | 0.429914 |
| GO:0009887\_organ\_morphogenesis | COL13A1 | 642 | 12 | 1.388303 | -0.835139 | 465 | 199.91 | 0.429914 |
| GO:0009887\_organ\_morphogenesis | BCL2 | 642 | 12 | 1.388303 | -0.835139 | 465 | 199.91 | 0.429914 |
| GO:0009887\_organ\_morphogenesis | ONECUT2 | 642 | 12 | 1.388303 | -0.835139 | 465 | 199.91 | 0.429914 |
| GO:0009887\_organ\_morphogenesis | NTRK2 | 642 | 12 | 1.388303 | -0.835139 | 465 | 199.91 | 0.429914 |
| GO:0009887\_organ\_morphogenesis | VEGFA | 642 | 12 | 1.388303 | -0.835139 | 465 | 199.91 | 0.429914 |
| GO:0009887\_organ\_morphogenesis | RORB | 642 | 12 | 1.388303 | -0.835139 | 465 | 199.91 | 0.429914 |
| GO:0009887\_organ\_morphogenesis | NR2F2 | 642 | 12 | 1.388303 | -0.835139 | 465 | 199.91 | 0.429914 |
| GO:0009887\_organ\_morphogenesis | EPHB1 | 642 | 12 | 1.388303 | -0.835139 | 465 | 199.91 | 0.429914 |
| GO:0009887\_organ\_morphogenesis | PITX2 | 642 | 12 | 1.388303 | -0.835139 | 465 | 199.91 | 0.429914 |
| GO:0001944\_vasculature\_development | SEMA5A | 208 | 5 | 1.785437 | -0.833622 | 466 | 200.25 | 0.429721 |
| GO:0001944\_vasculature\_development | NTRK2 | 208 | 5 | 1.785437 | -0.833622 | 466 | 200.25 | 0.429721 |
| GO:0001944\_vasculature\_development | VEGFA | 208 | 5 | 1.785437 | -0.833622 | 466 | 200.25 | 0.429721 |
| GO:0001944\_vasculature\_development | NR2F2 | 208 | 5 | 1.785437 | -0.833622 | 466 | 200.25 | 0.429721 |
| GO:0001944\_vasculature\_development | PITX2 | 208 | 5 | 1.785437 | -0.833622 | 466 | 200.25 | 0.429721 |
| GO:0009891\_positive\_regulation\_of\_biosynthetic\_process | SLC11A1 | 388 | 8 | 1.531427 | -0.831854 | 467 | 200.47 | 0.429272 |
| GO:0009891\_positive\_regulation\_of\_biosynthetic\_process | HMGB1 | 388 | 8 | 1.531427 | -0.831854 | 467 | 200.47 | 0.429272 |
| GO:0009891\_positive\_regulation\_of\_biosynthetic\_process | RNF6 | 388 | 8 | 1.531427 | -0.831854 | 467 | 200.47 | 0.429272 |
| GO:0009891\_positive\_regulation\_of\_biosynthetic\_process | ONECUT2 | 388 | 8 | 1.531427 | -0.831854 | 467 | 200.47 | 0.429272 |
| GO:0009891\_positive\_regulation\_of\_biosynthetic\_process | NFAT5 | 388 | 8 | 1.531427 | -0.831854 | 467 | 200.47 | 0.429272 |
| GO:0009891\_positive\_regulation\_of\_biosynthetic\_process | RORB | 388 | 8 | 1.531427 | -0.831854 | 467 | 200.47 | 0.429272 |
| GO:0009891\_positive\_regulation\_of\_biosynthetic\_process | AHR | 388 | 8 | 1.531427 | -0.831854 | 467 | 200.47 | 0.429272 |
| GO:0009891\_positive\_regulation\_of\_biosynthetic\_process | PITX2 | 388 | 8 | 1.531427 | -0.831854 | 467 | 200.47 | 0.429272 |
| GO:0060348\_bone\_development | COL13A1 | 99 | 3 | 2.250733 | -0.830599 | 468 | 200.94 | 0.429359 |
| GO:0060348\_bone\_development | BCL2 | 99 | 3 | 2.250733 | -0.830599 | 468 | 200.94 | 0.429359 |
| GO:0060348\_bone\_development | TOB1 | 99 | 3 | 2.250733 | -0.830599 | 468 | 200.94 | 0.429359 |
| GO:0009059\_macromolecule\_biosynthetic\_process | HMGB1 | 910 | 16 | 1.305920 | -0.827706 | 469 | 201.02 | 0.428614 |
| GO:0009059\_macromolecule\_biosynthetic\_process | LCORL | 910 | 16 | 1.305920 | -0.827706 | 469 | 201.02 | 0.428614 |
| GO:0009059\_macromolecule\_biosynthetic\_process | ONECUT2 | 910 | 16 | 1.305920 | -0.827706 | 469 | 201.02 | 0.428614 |
| GO:0009059\_macromolecule\_biosynthetic\_process | RORB | 910 | 16 | 1.305920 | -0.827706 | 469 | 201.02 | 0.428614 |
| GO:0009059\_macromolecule\_biosynthetic\_process | AHR | 910 | 16 | 1.305920 | -0.827706 | 469 | 201.02 | 0.428614 |
| GO:0009059\_macromolecule\_biosynthetic\_process | SUZ12 | 910 | 16 | 1.305920 | -0.827706 | 469 | 201.02 | 0.428614 |
| GO:0009059\_macromolecule\_biosynthetic\_process | SLC11A1 | 910 | 16 | 1.305920 | -0.827706 | 469 | 201.02 | 0.428614 |
| GO:0009059\_macromolecule\_biosynthetic\_process | RNF6 | 910 | 16 | 1.305920 | -0.827706 | 469 | 201.02 | 0.428614 |
| GO:0009059\_macromolecule\_biosynthetic\_process | BCL2 | 910 | 16 | 1.305920 | -0.827706 | 469 | 201.02 | 0.428614 |
| GO:0009059\_macromolecule\_biosynthetic\_process | GARNL1 | 910 | 16 | 1.305920 | -0.827706 | 469 | 201.02 | 0.428614 |
| GO:0009059\_macromolecule\_biosynthetic\_process | NFAT5 | 910 | 16 | 1.305920 | -0.827706 | 469 | 201.02 | 0.428614 |
| GO:0009059\_macromolecule\_biosynthetic\_process | NR2F2 | 910 | 16 | 1.305920 | -0.827706 | 469 | 201.02 | 0.428614 |
| GO:0009059\_macromolecule\_biosynthetic\_process | MYB | 910 | 16 | 1.305920 | -0.827706 | 469 | 201.02 | 0.428614 |
| GO:0009059\_macromolecule\_biosynthetic\_process | FEN1 | 910 | 16 | 1.305920 | -0.827706 | 469 | 201.02 | 0.428614 |
| GO:0009059\_macromolecule\_biosynthetic\_process | PITX2 | 910 | 16 | 1.305920 | -0.827706 | 469 | 201.02 | 0.428614 |
| GO:0009059\_macromolecule\_biosynthetic\_process | ETV3 | 910 | 16 | 1.305920 | -0.827706 | 469 | 201.02 | 0.428614 |
| GO:0032583\_regulation\_of\_gene-specific\_transcription | HMGB1 | 51 | 2 | 2.912713 | -0.824503 | 470 | 202.08 | 0.429957 |
| GO:0032583\_regulation\_of\_gene-specific\_transcription | RORB | 51 | 2 | 2.912713 | -0.824503 | 470 | 202.08 | 0.429957 |
| GO:0001662\_behavioral\_fear\_response | BCL2 | 12 | 1 | 6.189516 | -0.823078 | 486 | 213.27 | 0.438827 |
| GO:0002209\_behavioral\_defense\_response | BCL2 | 12 | 1 | 6.189516 | -0.823078 | 486 | 213.27 | 0.438827 |
| GO:0002244\_hemopoietic\_progenitor\_cell\_differentiation | BCL2 | 12 | 1 | 6.189516 | -0.823078 | 486 | 213.27 | 0.438827 |
| GO:0002763\_positive\_regulation\_of\_myeloid\_leukocyte\_differentiation | KITL | 12 | 1 | 6.189516 | -0.823078 | 486 | 213.27 | 0.438827 |
| GO:0006839\_mitochondrial\_transport | BCL2 | 12 | 1 | 6.189516 | -0.823078 | 486 | 213.27 | 0.438827 |
| GO:0006879\_cellular\_iron\_ion\_homeostasis | SLC11A1 | 12 | 1 | 6.189516 | -0.823078 | 486 | 213.27 | 0.438827 |
| GO:0007143\_female\_meiosis | CDC25B | 12 | 1 | 6.189516 | -0.823078 | 486 | 213.27 | 0.438827 |
| GO:0008038\_neuron\_recognition | BDNF | 12 | 1 | 6.189516 | -0.823078 | 486 | 213.27 | 0.438827 |
| GO:0015872\_dopamine\_transport | SNCA | 12 | 1 | 6.189516 | -0.823078 | 486 | 213.27 | 0.438827 |
| GO:0030514\_negative\_regulation\_of\_BMP\_signaling\_pathway | TOB1 | 12 | 1 | 6.189516 | -0.823078 | 486 | 213.27 | 0.438827 |
| GO:0043487\_regulation\_of\_RNA\_stability | SLC11A1 | 12 | 1 | 6.189516 | -0.823078 | 486 | 213.27 | 0.438827 |
| GO:0045792\_negative\_regulation\_of\_cell\_size | BCL2 | 12 | 1 | 6.189516 | -0.823078 | 486 | 213.27 | 0.438827 |
| GO:0048169\_regulation\_of\_long-term\_neuronal\_synaptic\_plasticity | SNCA | 12 | 1 | 6.189516 | -0.823078 | 486 | 213.27 | 0.438827 |
| GO:0050795\_regulation\_of\_behavior | HMGB1 | 12 | 1 | 6.189516 | -0.823078 | 486 | 213.27 | 0.438827 |
| GO:0051588\_regulation\_of\_neurotransmitter\_transport | SNCA | 12 | 1 | 6.189516 | -0.823078 | 486 | 213.27 | 0.438827 |
| GO:0055114\_oxidation\_reduction | SNCA | 12 | 1 | 6.189516 | -0.823078 | 486 | 213.27 | 0.438827 |
| GO:0001525\_angiogenesis | SEMA5A | 100 | 3 | 2.228226 | -0.821338 | 487 | 213.52 | 0.438439 |
| GO:0001525\_angiogenesis | VEGFA | 100 | 3 | 2.228226 | -0.821338 | 487 | 213.52 | 0.438439 |
| GO:0001525\_angiogenesis | PITX2 | 100 | 3 | 2.228226 | -0.821338 | 487 | 213.52 | 0.438439 |
| GO:0010468\_regulation\_of\_gene\_expression | HMGB1 | 778 | 14 | 1.336554 | -0.821083 | 488 | 213.58 | 0.437664 |
| GO:0010468\_regulation\_of\_gene\_expression | ONECUT2 | 778 | 14 | 1.336554 | -0.821083 | 488 | 213.58 | 0.437664 |
| GO:0010468\_regulation\_of\_gene\_expression | RORB | 778 | 14 | 1.336554 | -0.821083 | 488 | 213.58 | 0.437664 |
| GO:0010468\_regulation\_of\_gene\_expression | AHR | 778 | 14 | 1.336554 | -0.821083 | 488 | 213.58 | 0.437664 |
| GO:0010468\_regulation\_of\_gene\_expression | SUZ12 | 778 | 14 | 1.336554 | -0.821083 | 488 | 213.58 | 0.437664 |
| GO:0010468\_regulation\_of\_gene\_expression | SLC11A1 | 778 | 14 | 1.336554 | -0.821083 | 488 | 213.58 | 0.437664 |
| GO:0010468\_regulation\_of\_gene\_expression | RNF6 | 778 | 14 | 1.336554 | -0.821083 | 488 | 213.58 | 0.437664 |
| GO:0010468\_regulation\_of\_gene\_expression | BCL2 | 778 | 14 | 1.336554 | -0.821083 | 488 | 213.58 | 0.437664 |
| GO:0010468\_regulation\_of\_gene\_expression | GARNL1 | 778 | 14 | 1.336554 | -0.821083 | 488 | 213.58 | 0.437664 |
| GO:0010468\_regulation\_of\_gene\_expression | NFAT5 | 778 | 14 | 1.336554 | -0.821083 | 488 | 213.58 | 0.437664 |
| GO:0010468\_regulation\_of\_gene\_expression | NR2F2 | 778 | 14 | 1.336554 | -0.821083 | 488 | 213.58 | 0.437664 |
| GO:0010468\_regulation\_of\_gene\_expression | MYB | 778 | 14 | 1.336554 | -0.821083 | 488 | 213.58 | 0.437664 |
| GO:0010468\_regulation\_of\_gene\_expression | PITX2 | 778 | 14 | 1.336554 | -0.821083 | 488 | 213.58 | 0.437664 |
| GO:0010468\_regulation\_of\_gene\_expression | ETV3 | 778 | 14 | 1.336554 | -0.821083 | 488 | 213.58 | 0.437664 |
| GO:0045944\_positive\_regulation\_of\_transcription\_from\_RNA\_polymerase\_II\_promoter | SLC11A1 | 269 | 6 | 1.656673 | -0.819139 | 489 | 213.65 | 0.436912 |
| GO:0045944\_positive\_regulation\_of\_transcription\_from\_RNA\_polymerase\_II\_promoter | HMGB1 | 269 | 6 | 1.656673 | -0.819139 | 489 | 213.65 | 0.436912 |
| GO:0045944\_positive\_regulation\_of\_transcription\_from\_RNA\_polymerase\_II\_promoter | ONECUT2 | 269 | 6 | 1.656673 | -0.819139 | 489 | 213.65 | 0.436912 |
| GO:0045944\_positive\_regulation\_of\_transcription\_from\_RNA\_polymerase\_II\_promoter | NFAT5 | 269 | 6 | 1.656673 | -0.819139 | 489 | 213.65 | 0.436912 |
| GO:0045944\_positive\_regulation\_of\_transcription\_from\_RNA\_polymerase\_II\_promoter | AHR | 269 | 6 | 1.656673 | -0.819139 | 489 | 213.65 | 0.436912 |
| GO:0045944\_positive\_regulation\_of\_transcription\_from\_RNA\_polymerase\_II\_promoter | PITX2 | 269 | 6 | 1.656673 | -0.819139 | 489 | 213.65 | 0.436912 |
| GO:0010608\_posttranscriptional\_regulation\_of\_gene\_expression | SLC11A1 | 52 | 2 | 2.856700 | -0.811075 | 490 | 214.99 | 0.438755 |
| GO:0010608\_posttranscriptional\_regulation\_of\_gene\_expression | BCL2 | 52 | 2 | 2.856700 | -0.811075 | 490 | 214.99 | 0.438755 |
| GO:0022402\_cell\_cycle\_process | BCL2 | 155 | 4 | 1.916753 | -0.810559 | 491 | 215.38 | 0.438656 |
| GO:0022402\_cell\_cycle\_process | ID4 | 155 | 4 | 1.916753 | -0.810559 | 491 | 215.38 | 0.438656 |
| GO:0022402\_cell\_cycle\_process | MYB | 155 | 4 | 1.916753 | -0.810559 | 491 | 215.38 | 0.438656 |
| GO:0022402\_cell\_cycle\_process | CDC25B | 155 | 4 | 1.916753 | -0.810559 | 491 | 215.38 | 0.438656 |
| GO:0035295\_tube\_development | SEMA5A | 212 | 5 | 1.751750 | -0.809096 | 492 | 215.52 | 0.438049 |
| GO:0035295\_tube\_development | HMGB1 | 212 | 5 | 1.751750 | -0.809096 | 492 | 215.52 | 0.438049 |
| GO:0035295\_tube\_development | BCL2 | 212 | 5 | 1.751750 | -0.809096 | 492 | 215.52 | 0.438049 |
| GO:0035295\_tube\_development | VEGFA | 212 | 5 | 1.751750 | -0.809096 | 492 | 215.52 | 0.438049 |
| GO:0035295\_tube\_development | PITX2 | 212 | 5 | 1.751750 | -0.809096 | 492 | 215.52 | 0.438049 |
| GO:0006810\_transport | PFKL | 718 | 13 | 1.344797 | -0.800161 | 493 | 216.16 | 0.438458 |
| GO:0006810\_transport | SNCA | 718 | 13 | 1.344797 | -0.800161 | 493 | 216.16 | 0.438458 |
| GO:0006810\_transport | CADPS | 718 | 13 | 1.344797 | -0.800161 | 493 | 216.16 | 0.438458 |
| GO:0006810\_transport | SLC11A1 | 718 | 13 | 1.344797 | -0.800161 | 493 | 216.16 | 0.438458 |
| GO:0006810\_transport | BDNF | 718 | 13 | 1.344797 | -0.800161 | 493 | 216.16 | 0.438458 |
| GO:0006810\_transport | BCL2 | 718 | 13 | 1.344797 | -0.800161 | 493 | 216.16 | 0.438458 |
| GO:0006810\_transport | NTRK2 | 718 | 13 | 1.344797 | -0.800161 | 493 | 216.16 | 0.438458 |
| GO:0006810\_transport | CHRNB4 | 718 | 13 | 1.344797 | -0.800161 | 493 | 216.16 | 0.438458 |
| GO:0006810\_transport | STXBP3A | 718 | 13 | 1.344797 | -0.800161 | 493 | 216.16 | 0.438458 |
| GO:0006810\_transport | EHD1 | 718 | 13 | 1.344797 | -0.800161 | 493 | 216.16 | 0.438458 |
| GO:0006810\_transport | SLC4A4 | 718 | 13 | 1.344797 | -0.800161 | 493 | 216.16 | 0.438458 |
| GO:0006810\_transport | MYB | 718 | 13 | 1.344797 | -0.800161 | 493 | 216.16 | 0.438458 |
| GO:0006810\_transport | TOB1 | 718 | 13 | 1.344797 | -0.800161 | 493 | 216.16 | 0.438458 |
| GO:0006576\_biogenic\_amine\_metabolic\_process | SNCA | 53 | 2 | 2.802800 | -0.797970 | 495 | 217.2 | 0.438788 |
| GO:0006576\_biogenic\_amine\_metabolic\_process | FABP3 | 53 | 2 | 2.802800 | -0.797970 | 495 | 217.2 | 0.438788 |
| GO:0050905\_neuromuscular\_process | FABP7 | 53 | 2 | 2.802800 | -0.797970 | 495 | 217.2 | 0.438788 |
| GO:0050905\_neuromuscular\_process | NEFL | 53 | 2 | 2.802800 | -0.797970 | 495 | 217.2 | 0.438788 |
| GO:0001836\_release\_of\_cytochrome\_c\_from\_mitochondria | BCL2 | 13 | 1 | 5.713400 | -0.791136 | 515 | 229.39 | 0.445417 |
| GO:0001958\_endochondral\_ossification | COL13A1 | 13 | 1 | 5.713400 | -0.791136 | 515 | 229.39 | 0.445417 |
| GO:0007212\_dopamine\_receptor\_signaling\_pathway | GNAQ | 13 | 1 | 5.713400 | -0.791136 | 515 | 229.39 | 0.445417 |
| GO:0007274\_neuromuscular\_synaptic\_transmission | CHRNB4 | 13 | 1 | 5.713400 | -0.791136 | 515 | 229.39 | 0.445417 |
| GO:0009410\_response\_to\_xenobiotic\_stimulus | AHR | 13 | 1 | 5.713400 | -0.791136 | 515 | 229.39 | 0.445417 |
| GO:0018105\_peptidyl-serine\_phosphorylation | BCL2 | 13 | 1 | 5.713400 | -0.791136 | 515 | 229.39 | 0.445417 |
| GO:0021879\_forebrain\_neuron\_differentiation | GNAQ | 13 | 1 | 5.713400 | -0.791136 | 515 | 229.39 | 0.445417 |
| GO:0021955\_central\_nervous\_system\_neuron\_axonogenesis | EPHB1 | 13 | 1 | 5.713400 | -0.791136 | 515 | 229.39 | 0.445417 |
| GO:0030516\_regulation\_of\_axon\_extension | RNF6 | 13 | 1 | 5.713400 | -0.791136 | 515 | 229.39 | 0.445417 |
| GO:0031290\_retinal\_ganglion\_cell\_axon\_guidance | EPHB1 | 13 | 1 | 5.713400 | -0.791136 | 515 | 229.39 | 0.445417 |
| GO:0032729\_positive\_regulation\_of\_interferon-gamma\_production | SLC11A1 | 13 | 1 | 5.713400 | -0.791136 | 515 | 229.39 | 0.445417 |
| GO:0034329\_cell\_junction\_assembly | BCL2 | 13 | 1 | 5.713400 | -0.791136 | 515 | 229.39 | 0.445417 |
| GO:0046474\_glycerophospholipid\_biosynthetic\_process | FABP3 | 13 | 1 | 5.713400 | -0.791136 | 515 | 229.39 | 0.445417 |
| GO:0046851\_negative\_regulation\_of\_bone\_remodeling | BCL2 | 13 | 1 | 5.713400 | -0.791136 | 515 | 229.39 | 0.445417 |
| GO:0048566\_embryonic\_gut\_development | MYB | 13 | 1 | 5.713400 | -0.791136 | 515 | 229.39 | 0.445417 |
| GO:0050764\_regulation\_of\_phagocytosis | SLC11A1 | 13 | 1 | 5.713400 | -0.791136 | 515 | 229.39 | 0.445417 |
| GO:0050766\_positive\_regulation\_of\_phagocytosis | SLC11A1 | 13 | 1 | 5.713400 | -0.791136 | 515 | 229.39 | 0.445417 |
| GO:0050771\_negative\_regulation\_of\_axonogenesis | RNF6 | 13 | 1 | 5.713400 | -0.791136 | 515 | 229.39 | 0.445417 |
| GO:0060401\_cytosolic\_calcium\_ion\_transport | BCL2 | 13 | 1 | 5.713400 | -0.791136 | 515 | 229.39 | 0.445417 |
| GO:0060402\_calcium\_ion\_transport\_into\_cytosol | BCL2 | 13 | 1 | 5.713400 | -0.791136 | 515 | 229.39 | 0.445417 |
| GO:0043405\_regulation\_of\_MAP\_kinase\_activity | SPRY1 | 54 | 2 | 2.750896 | -0.785176 | 517 | 231.22 | 0.447234 |
| GO:0043405\_regulation\_of\_MAP\_kinase\_activity | KITL | 54 | 2 | 2.750896 | -0.785176 | 517 | 231.22 | 0.447234 |
| GO:0044271\_nitrogen\_compound\_biosynthetic\_process | ALDH18A1 | 54 | 2 | 2.750896 | -0.785176 | 517 | 231.22 | 0.447234 |
| GO:0044271\_nitrogen\_compound\_biosynthetic\_process | SNCA | 54 | 2 | 2.750896 | -0.785176 | 517 | 231.22 | 0.447234 |
| GO:0048518\_positive\_regulation\_of\_biological\_process | HMGB1 | 995 | 17 | 1.269006 | -0.778047 | 518 | 231.79 | 0.447471 |
| GO:0048518\_positive\_regulation\_of\_biological\_process | IRS2 | 995 | 17 | 1.269006 | -0.778047 | 518 | 231.79 | 0.447471 |
| GO:0048518\_positive\_regulation\_of\_biological\_process | ONECUT2 | 995 | 17 | 1.269006 | -0.778047 | 518 | 231.79 | 0.447471 |
| GO:0048518\_positive\_regulation\_of\_biological\_process | SNCA | 995 | 17 | 1.269006 | -0.778047 | 518 | 231.79 | 0.447471 |
| GO:0048518\_positive\_regulation\_of\_biological\_process | RORB | 995 | 17 | 1.269006 | -0.778047 | 518 | 231.79 | 0.447471 |
| GO:0048518\_positive\_regulation\_of\_biological\_process | KITL | 995 | 17 | 1.269006 | -0.778047 | 518 | 231.79 | 0.447471 |
| GO:0048518\_positive\_regulation\_of\_biological\_process | AHR | 995 | 17 | 1.269006 | -0.778047 | 518 | 231.79 | 0.447471 |
| GO:0048518\_positive\_regulation\_of\_biological\_process | SUZ12 | 995 | 17 | 1.269006 | -0.778047 | 518 | 231.79 | 0.447471 |
| GO:0048518\_positive\_regulation\_of\_biological\_process | SLC11A1 | 995 | 17 | 1.269006 | -0.778047 | 518 | 231.79 | 0.447471 |
| GO:0048518\_positive\_regulation\_of\_biological\_process | BDNF | 995 | 17 | 1.269006 | -0.778047 | 518 | 231.79 | 0.447471 |
| GO:0048518\_positive\_regulation\_of\_biological\_process | RNF6 | 995 | 17 | 1.269006 | -0.778047 | 518 | 231.79 | 0.447471 |
| GO:0048518\_positive\_regulation\_of\_biological\_process | BCL2 | 995 | 17 | 1.269006 | -0.778047 | 518 | 231.79 | 0.447471 |
| GO:0048518\_positive\_regulation\_of\_biological\_process | VEGFA | 995 | 17 | 1.269006 | -0.778047 | 518 | 231.79 | 0.447471 |
| GO:0048518\_positive\_regulation\_of\_biological\_process | NFAT5 | 995 | 17 | 1.269006 | -0.778047 | 518 | 231.79 | 0.447471 |
| GO:0048518\_positive\_regulation\_of\_biological\_process | ID4 | 995 | 17 | 1.269006 | -0.778047 | 518 | 231.79 | 0.447471 |
| GO:0048518\_positive\_regulation\_of\_biological\_process | NEFL | 995 | 17 | 1.269006 | -0.778047 | 518 | 231.79 | 0.447471 |
| GO:0048518\_positive\_regulation\_of\_biological\_process | PITX2 | 995 | 17 | 1.269006 | -0.778047 | 518 | 231.79 | 0.447471 |
| GO:0048872\_homeostasis\_of\_number\_of\_cells | BCL2 | 105 | 3 | 2.122120 | -0.776931 | 519 | 232.22 | 0.447437 |
| GO:0048872\_homeostasis\_of\_number\_of\_cells | VEGFA | 105 | 3 | 2.122120 | -0.776931 | 519 | 232.22 | 0.447437 |
| GO:0048872\_homeostasis\_of\_number\_of\_cells | KITL | 105 | 3 | 2.122120 | -0.776931 | 519 | 232.22 | 0.447437 |
| GO:0051128\_regulation\_of\_cellular\_component\_organization | SLC11A1 | 160 | 4 | 1.856855 | -0.775700 | 520 | 232.34 | 0.446808 |
| GO:0051128\_regulation\_of\_cellular\_component\_organization | RNF6 | 160 | 4 | 1.856855 | -0.775700 | 520 | 232.34 | 0.446808 |
| GO:0051128\_regulation\_of\_cellular\_component\_organization | NEFL | 160 | 4 | 1.856855 | -0.775700 | 520 | 232.34 | 0.446808 |
| GO:0051128\_regulation\_of\_cellular\_component\_organization | AHR | 160 | 4 | 1.856855 | -0.775700 | 520 | 232.34 | 0.446808 |
| GO:0048568\_embryonic\_organ\_development | VEGFA | 55 | 2 | 2.700880 | -0.772682 | 521 | 233.44 | 0.448061 |
| GO:0048568\_embryonic\_organ\_development | MYB | 55 | 2 | 2.700880 | -0.772682 | 521 | 233.44 | 0.448061 |
| GO:0051234\_establishment\_of\_localization | PFKL | 729 | 13 | 1.324506 | -0.764703 | 522 | 234.45 | 0.449138 |
| GO:0051234\_establishment\_of\_localization | SNCA | 729 | 13 | 1.324506 | -0.764703 | 522 | 234.45 | 0.449138 |
| GO:0051234\_establishment\_of\_localization | CADPS | 729 | 13 | 1.324506 | -0.764703 | 522 | 234.45 | 0.449138 |
| GO:0051234\_establishment\_of\_localization | SLC11A1 | 729 | 13 | 1.324506 | -0.764703 | 522 | 234.45 | 0.449138 |
| GO:0051234\_establishment\_of\_localization | BDNF | 729 | 13 | 1.324506 | -0.764703 | 522 | 234.45 | 0.449138 |
| GO:0051234\_establishment\_of\_localization | BCL2 | 729 | 13 | 1.324506 | -0.764703 | 522 | 234.45 | 0.449138 |
| GO:0051234\_establishment\_of\_localization | NTRK2 | 729 | 13 | 1.324506 | -0.764703 | 522 | 234.45 | 0.449138 |
| GO:0051234\_establishment\_of\_localization | CHRNB4 | 729 | 13 | 1.324506 | -0.764703 | 522 | 234.45 | 0.449138 |
| GO:0051234\_establishment\_of\_localization | STXBP3A | 729 | 13 | 1.324506 | -0.764703 | 522 | 234.45 | 0.449138 |
| GO:0051234\_establishment\_of\_localization | EHD1 | 729 | 13 | 1.324506 | -0.764703 | 522 | 234.45 | 0.449138 |
| GO:0051234\_establishment\_of\_localization | SLC4A4 | 729 | 13 | 1.324506 | -0.764703 | 522 | 234.45 | 0.449138 |
| GO:0051234\_establishment\_of\_localization | MYB | 729 | 13 | 1.324506 | -0.764703 | 522 | 234.45 | 0.449138 |
| GO:0051234\_establishment\_of\_localization | TOB1 | 729 | 13 | 1.324506 | -0.764703 | 522 | 234.45 | 0.449138 |
| GO:0002262\_myeloid\_cell\_homeostasis | KITL | 14 | 1 | 5.305300 | -0.761765 | 528 | 245.42 | 0.464811 |
| GO:0009108\_coenzyme\_biosynthetic\_process | SNCA | 14 | 1 | 5.305300 | -0.761765 | 528 | 245.42 | 0.464811 |
| GO:0010332\_response\_to\_gamma\_radiation | BCL2 | 14 | 1 | 5.305300 | -0.761765 | 528 | 245.42 | 0.464811 |
| GO:0031346\_positive\_regulation\_of\_cell\_projection\_organization | NEFL | 14 | 1 | 5.305300 | -0.761765 | 528 | 245.42 | 0.464811 |
| GO:0034104\_negative\_regulation\_of\_tissue\_remodeling | BCL2 | 14 | 1 | 5.305300 | -0.761765 | 528 | 245.42 | 0.464811 |
| GO:0051952\_regulation\_of\_amine\_transport | SNCA | 14 | 1 | 5.305300 | -0.761765 | 528 | 245.42 | 0.464811 |
| GO:0001708\_cell\_fate\_specification | SPRY1 | 56 | 2 | 2.652650 | -0.760476 | 529 | 246.88 | 0.466692 |
| GO:0001708\_cell\_fate\_specification | SALL1 | 56 | 2 | 2.652650 | -0.760476 | 529 | 246.88 | 0.466692 |
| GO:0006139\_nucleobase\_\_nucleoside\_\_nucleotide\_and\_nucleic\_acid\_metabolic\_process | HMGB1 | 1002 | 17 | 1.260141 | -0.758569 | 530 | 247.15 | 0.466321 |
| GO:0006139\_nucleobase\_\_nucleoside\_\_nucleotide\_and\_nucleic\_acid\_metabolic\_process | LCORL | 1002 | 17 | 1.260141 | -0.758569 | 530 | 247.15 | 0.466321 |
| GO:0006139\_nucleobase\_\_nucleoside\_\_nucleotide\_and\_nucleic\_acid\_metabolic\_process | ONECUT2 | 1002 | 17 | 1.260141 | -0.758569 | 530 | 247.15 | 0.466321 |
| GO:0006139\_nucleobase\_\_nucleoside\_\_nucleotide\_and\_nucleic\_acid\_metabolic\_process | RORB | 1002 | 17 | 1.260141 | -0.758569 | 530 | 247.15 | 0.466321 |
| GO:0006139\_nucleobase\_\_nucleoside\_\_nucleotide\_and\_nucleic\_acid\_metabolic\_process | AHR | 1002 | 17 | 1.260141 | -0.758569 | 530 | 247.15 | 0.466321 |
| GO:0006139\_nucleobase\_\_nucleoside\_\_nucleotide\_and\_nucleic\_acid\_metabolic\_process | SUZ12 | 1002 | 17 | 1.260141 | -0.758569 | 530 | 247.15 | 0.466321 |
| GO:0006139\_nucleobase\_\_nucleoside\_\_nucleotide\_and\_nucleic\_acid\_metabolic\_process | SLC11A1 | 1002 | 17 | 1.260141 | -0.758569 | 530 | 247.15 | 0.466321 |
| GO:0006139\_nucleobase\_\_nucleoside\_\_nucleotide\_and\_nucleic\_acid\_metabolic\_process | DCLRE1A | 1002 | 17 | 1.260141 | -0.758569 | 530 | 247.15 | 0.466321 |
| GO:0006139\_nucleobase\_\_nucleoside\_\_nucleotide\_and\_nucleic\_acid\_metabolic\_process | RNF6 | 1002 | 17 | 1.260141 | -0.758569 | 530 | 247.15 | 0.466321 |
| GO:0006139\_nucleobase\_\_nucleoside\_\_nucleotide\_and\_nucleic\_acid\_metabolic\_process | GNAQ | 1002 | 17 | 1.260141 | -0.758569 | 530 | 247.15 | 0.466321 |
| GO:0006139\_nucleobase\_\_nucleoside\_\_nucleotide\_and\_nucleic\_acid\_metabolic\_process | GARNL1 | 1002 | 17 | 1.260141 | -0.758569 | 530 | 247.15 | 0.466321 |
| GO:0006139\_nucleobase\_\_nucleoside\_\_nucleotide\_and\_nucleic\_acid\_metabolic\_process | NFAT5 | 1002 | 17 | 1.260141 | -0.758569 | 530 | 247.15 | 0.466321 |
| GO:0006139\_nucleobase\_\_nucleoside\_\_nucleotide\_and\_nucleic\_acid\_metabolic\_process | MYB | 1002 | 17 | 1.260141 | -0.758569 | 530 | 247.15 | 0.466321 |
| GO:0006139\_nucleobase\_\_nucleoside\_\_nucleotide\_and\_nucleic\_acid\_metabolic\_process | NR2F2 | 1002 | 17 | 1.260141 | -0.758569 | 530 | 247.15 | 0.466321 |
| GO:0006139\_nucleobase\_\_nucleoside\_\_nucleotide\_and\_nucleic\_acid\_metabolic\_process | FEN1 | 1002 | 17 | 1.260141 | -0.758569 | 530 | 247.15 | 0.466321 |
| GO:0006139\_nucleobase\_\_nucleoside\_\_nucleotide\_and\_nucleic\_acid\_metabolic\_process | ETV3 | 1002 | 17 | 1.260141 | -0.758569 | 530 | 247.15 | 0.466321 |
| GO:0006139\_nucleobase\_\_nucleoside\_\_nucleotide\_and\_nucleic\_acid\_metabolic\_process | PITX2 | 1002 | 17 | 1.260141 | -0.758569 | 530 | 247.15 | 0.466321 |
| GO:0030099\_myeloid\_cell\_differentiation | HMGB1 | 108 | 3 | 2.063172 | -0.751719 | 532 | 247.75 | 0.465695 |
| GO:0030099\_myeloid\_cell\_differentiation | VEGFA | 108 | 3 | 2.063172 | -0.751719 | 532 | 247.75 | 0.465695 |
| GO:0030099\_myeloid\_cell\_differentiation | KITL | 108 | 3 | 2.063172 | -0.751719 | 532 | 247.75 | 0.465695 |
| GO:0051240\_positive\_regulation\_of\_multicellular\_organismal\_process | SLC11A1 | 108 | 3 | 2.063172 | -0.751719 | 532 | 247.75 | 0.465695 |
| GO:0051240\_positive\_regulation\_of\_multicellular\_organismal\_process | BCL2 | 108 | 3 | 2.063172 | -0.751719 | 532 | 247.75 | 0.465695 |
| GO:0051240\_positive\_regulation\_of\_multicellular\_organismal\_process | SNCA | 108 | 3 | 2.063172 | -0.751719 | 532 | 247.75 | 0.465695 |
| GO:0001764\_neuron\_migration | DAB1 | 57 | 2 | 2.606112 | -0.748548 | 534 | 249.77 | 0.467734 |
| GO:0001764\_neuron\_migration | NR2F2 | 57 | 2 | 2.606112 | -0.748548 | 534 | 249.77 | 0.467734 |
| GO:0033365\_protein\_localization\_in\_organelle | SLC11A1 | 57 | 2 | 2.606112 | -0.748548 | 534 | 249.77 | 0.467734 |
| GO:0033365\_protein\_localization\_in\_organelle | TOB1 | 57 | 2 | 2.606112 | -0.748548 | 534 | 249.77 | 0.467734 |
| GO:0030902\_hindbrain\_development | DAB1 | 58 | 2 | 2.561179 | -0.736888 | 536 | 251.39 | 0.469011 |
| GO:0030902\_hindbrain\_development | BCL2 | 58 | 2 | 2.561179 | -0.736888 | 536 | 251.39 | 0.469011 |
| GO:0050804\_regulation\_of\_synaptic\_transmission | BDNF | 58 | 2 | 2.561179 | -0.736888 | 536 | 251.39 | 0.469011 |
| GO:0050804\_regulation\_of\_synaptic\_transmission | SNCA | 58 | 2 | 2.561179 | -0.736888 | 536 | 251.39 | 0.469011 |
| GO:0043010\_camera-type\_eye\_development | VEGFA | 110 | 3 | 2.025660 | -0.735469 | 538 | 252.02 | 0.468439 |
| GO:0043010\_camera-type\_eye\_development | EPHB1 | 110 | 3 | 2.025660 | -0.735469 | 538 | 252.02 | 0.468439 |
| GO:0043010\_camera-type\_eye\_development | PITX2 | 110 | 3 | 2.025660 | -0.735469 | 538 | 252.02 | 0.468439 |
| GO:0055080\_cation\_homeostasis | SLC11A1 | 110 | 3 | 2.025660 | -0.735469 | 538 | 252.02 | 0.468439 |
| GO:0055080\_cation\_homeostasis | BCL2 | 110 | 3 | 2.025660 | -0.735469 | 538 | 252.02 | 0.468439 |
| GO:0055080\_cation\_homeostasis | SLC4A4 | 110 | 3 | 2.025660 | -0.735469 | 538 | 252.02 | 0.468439 |
| GO:0001759\_induction\_of\_an\_organ | SPRY1 | 15 | 1 | 4.951613 | -0.734610 | 559 | 261.53 | 0.467853 |
| GO:0001782\_B\_cell\_homeostasis | BCL2 | 15 | 1 | 4.951613 | -0.734610 | 559 | 261.53 | 0.467853 |
| GO:0001964\_startle\_response | FABP7 | 15 | 1 | 4.951613 | -0.734610 | 559 | 261.53 | 0.467853 |
| GO:0002286\_T\_cell\_activation\_during\_immune\_response | SLC11A1 | 15 | 1 | 4.951613 | -0.734610 | 559 | 261.53 | 0.467853 |
| GO:0007200\_activation\_of\_phospholipase\_C\_activity\_by\_G-protein\_coupled\_receptor\_protein\_signaling\_pathway\_coupled\_to\_IP3\_second\_messenger | GNAQ | 15 | 1 | 4.951613 | -0.734610 | 559 | 261.53 | 0.467853 |
| GO:0007202\_activation\_of\_phospholipase\_C\_activity | GNAQ | 15 | 1 | 4.951613 | -0.734610 | 559 | 261.53 | 0.467853 |
| GO:0007588\_excretion | CHRNB4 | 15 | 1 | 4.951613 | -0.734610 | 559 | 261.53 | 0.467853 |
| GO:0010092\_specification\_of\_organ\_identity | SPRY1 | 15 | 1 | 4.951613 | -0.734610 | 559 | 261.53 | 0.467853 |
| GO:0010518\_positive\_regulation\_of\_phospholipase\_activity | GNAQ | 15 | 1 | 4.951613 | -0.734610 | 559 | 261.53 | 0.467853 |
| GO:0010863\_positive\_regulation\_of\_phospholipase\_C\_activity | GNAQ | 15 | 1 | 4.951613 | -0.734610 | 559 | 261.53 | 0.467853 |
| GO:0021795\_cerebral\_cortex\_cell\_migration | DAB1 | 15 | 1 | 4.951613 | -0.734610 | 559 | 261.53 | 0.467853 |
| GO:0021872\_generation\_of\_neurons\_in\_the\_forebrain | GNAQ | 15 | 1 | 4.951613 | -0.734610 | 559 | 261.53 | 0.467853 |
| GO:0031069\_hair\_follicle\_morphogenesis | BCL2 | 15 | 1 | 4.951613 | -0.734610 | 559 | 261.53 | 0.467853 |
| GO:0031076\_embryonic\_camera-type\_eye\_development | PITX2 | 15 | 1 | 4.951613 | -0.734610 | 559 | 261.53 | 0.467853 |
| GO:0031329\_regulation\_of\_cellular\_catabolic\_process | HMGB1 | 15 | 1 | 4.951613 | -0.734610 | 559 | 261.53 | 0.467853 |
| GO:0045666\_positive\_regulation\_of\_neuron\_differentiation | BDNF | 15 | 1 | 4.951613 | -0.734610 | 559 | 261.53 | 0.467853 |
| GO:0046164\_alcohol\_catabolic\_process | PFKL | 15 | 1 | 4.951613 | -0.734610 | 559 | 261.53 | 0.467853 |
| GO:0048010\_vascular\_endothelial\_growth\_factor\_receptor\_signaling\_pathway | VEGFA | 15 | 1 | 4.951613 | -0.734610 | 559 | 261.53 | 0.467853 |
| GO:0050796\_regulation\_of\_insulin\_secretion | PFKL | 15 | 1 | 4.951613 | -0.734610 | 559 | 261.53 | 0.467853 |
| GO:0060425\_lung\_morphogenesis | PITX2 | 15 | 1 | 4.951613 | -0.734610 | 559 | 261.53 | 0.467853 |
| GO:0060749\_mammary\_gland\_alveolus\_development | VEGFA | 15 | 1 | 4.951613 | -0.734610 | 559 | 261.53 | 0.467853 |
| GO:0051049\_regulation\_of\_transport | SLC11A1 | 167 | 4 | 1.779023 | -0.729692 | 560 | 262.05 | 0.467946 |
| GO:0051049\_regulation\_of\_transport | PFKL | 167 | 4 | 1.779023 | -0.729692 | 560 | 262.05 | 0.467946 |
| GO:0051049\_regulation\_of\_transport | BCL2 | 167 | 4 | 1.779023 | -0.729692 | 560 | 262.05 | 0.467946 |
| GO:0051049\_regulation\_of\_transport | SNCA | 167 | 4 | 1.779023 | -0.729692 | 560 | 262.05 | 0.467946 |
| GO:0035270\_endocrine\_system\_development | ONECUT2 | 59 | 2 | 2.517769 | -0.725488 | 561 | 263.15 | 0.469073 |
| GO:0035270\_endocrine\_system\_development | PITX2 | 59 | 2 | 2.517769 | -0.725488 | 561 | 263.15 | 0.469073 |
| GO:0006974\_response\_to\_DNA\_damage\_stimulus | PAXIP1 | 113 | 3 | 1.971881 | -0.711891 | 562 | 264.76 | 0.471103 |
| GO:0006974\_response\_to\_DNA\_damage\_stimulus | DCLRE1A | 113 | 3 | 1.971881 | -0.711891 | 562 | 264.76 | 0.471103 |
| GO:0006974\_response\_to\_DNA\_damage\_stimulus | FEN1 | 113 | 3 | 1.971881 | -0.711891 | 562 | 264.76 | 0.471103 |
| GO:0000302\_response\_to\_reactive\_oxygen\_species | BCL2 | 16 | 1 | 4.642137 | -0.709384 | 569 | 273.81 | 0.481213 |
| GO:0008654\_phospholipid\_biosynthetic\_process | FABP3 | 16 | 1 | 4.642137 | -0.709384 | 569 | 273.81 | 0.481213 |
| GO:0031345\_negative\_regulation\_of\_cell\_projection\_organization | RNF6 | 16 | 1 | 4.642137 | -0.709384 | 569 | 273.81 | 0.481213 |
| GO:0045104\_intermediate\_filament\_cytoskeleton\_organization | NEFL | 16 | 1 | 4.642137 | -0.709384 | 569 | 273.81 | 0.481213 |
| GO:0048015\_phosphoinositide-mediated\_signaling | GNAQ | 16 | 1 | 4.642137 | -0.709384 | 569 | 273.81 | 0.481213 |
| GO:0051048\_negative\_regulation\_of\_secretion | PFKL | 16 | 1 | 4.642137 | -0.709384 | 569 | 273.81 | 0.481213 |
| GO:0060193\_positive\_regulation\_of\_lipase\_activity | GNAQ | 16 | 1 | 4.642137 | -0.709384 | 569 | 273.81 | 0.481213 |
| GO:0007005\_mitochondrion\_organization | BCL2 | 61 | 2 | 2.435219 | -0.703427 | 571 | 274.91 | 0.481454 |
| GO:0007005\_mitochondrion\_organization | SNCA | 61 | 2 | 2.435219 | -0.703427 | 571 | 274.91 | 0.481454 |
| GO:0051969\_regulation\_of\_transmission\_of\_nerve\_impulse | BDNF | 61 | 2 | 2.435219 | -0.703427 | 571 | 274.91 | 0.481454 |
| GO:0051969\_regulation\_of\_transmission\_of\_nerve\_impulse | SNCA | 61 | 2 | 2.435219 | -0.703427 | 571 | 274.91 | 0.481454 |
| GO:0042592\_homeostatic\_process | SLC11A1 | 419 | 8 | 1.418123 | -0.702398 | 572 | 275.16 | 0.481049 |
| GO:0042592\_homeostatic\_process | GNAQ | 419 | 8 | 1.418123 | -0.702398 | 572 | 275.16 | 0.481049 |
| GO:0042592\_homeostatic\_process | BCL2 | 419 | 8 | 1.418123 | -0.702398 | 572 | 275.16 | 0.481049 |
| GO:0042592\_homeostatic\_process | VEGFA | 419 | 8 | 1.418123 | -0.702398 | 572 | 275.16 | 0.481049 |
| GO:0042592\_homeostatic\_process | SNCA | 419 | 8 | 1.418123 | -0.702398 | 572 | 275.16 | 0.481049 |
| GO:0042592\_homeostatic\_process | CHRNB4 | 419 | 8 | 1.418123 | -0.702398 | 572 | 275.16 | 0.481049 |
| GO:0042592\_homeostatic\_process | SLC4A4 | 419 | 8 | 1.418123 | -0.702398 | 572 | 275.16 | 0.481049 |
| GO:0042592\_homeostatic\_process | KITL | 419 | 8 | 1.418123 | -0.702398 | 572 | 275.16 | 0.481049 |
| GO:0009987\_cellular\_process | LTBP1 | 3868 | 55 | 1.056122 | -0.694332 | 573 | 275.84 | 0.481396 |
| GO:0009987\_cellular\_process | LTBP3 | 3868 | 55 | 1.056122 | -0.694332 | 573 | 275.84 | 0.481396 |
| GO:0009987\_cellular\_process | SNCA | 3868 | 55 | 1.056122 | -0.694332 | 573 | 275.84 | 0.481396 |
| GO:0009987\_cellular\_process | RORB | 3868 | 55 | 1.056122 | -0.694332 | 573 | 275.84 | 0.481396 |
| GO:0009987\_cellular\_process | NRCAM | 3868 | 55 | 1.056122 | -0.694332 | 573 | 275.84 | 0.481396 |
| GO:0009987\_cellular\_process | EFHD1 | 3868 | 55 | 1.056122 | -0.694332 | 573 | 275.84 | 0.481396 |
| GO:0009987\_cellular\_process | BDNF | 3868 | 55 | 1.056122 | -0.694332 | 573 | 275.84 | 0.481396 |
| GO:0009987\_cellular\_process | SPRY1 | 3868 | 55 | 1.056122 | -0.694332 | 573 | 275.84 | 0.481396 |
| GO:0009987\_cellular\_process | DAB1 | 3868 | 55 | 1.056122 | -0.694332 | 573 | 275.84 | 0.481396 |
| GO:0009987\_cellular\_process | ACOT10 | 3868 | 55 | 1.056122 | -0.694332 | 573 | 275.84 | 0.481396 |
| GO:0009987\_cellular\_process | GARNL1 | 3868 | 55 | 1.056122 | -0.694332 | 573 | 275.84 | 0.481396 |
| GO:0009987\_cellular\_process | TGFBI | 3868 | 55 | 1.056122 | -0.694332 | 573 | 275.84 | 0.481396 |
| GO:0009987\_cellular\_process | NR2F2 | 3868 | 55 | 1.056122 | -0.694332 | 573 | 275.84 | 0.481396 |
| GO:0009987\_cellular\_process | MYB | 3868 | 55 | 1.056122 | -0.694332 | 573 | 275.84 | 0.481396 |
| GO:0009987\_cellular\_process | PITX2 | 3868 | 55 | 1.056122 | -0.694332 | 573 | 275.84 | 0.481396 |
| GO:0009987\_cellular\_process | MAP2K5 | 3868 | 55 | 1.056122 | -0.694332 | 573 | 275.84 | 0.481396 |
| GO:0009987\_cellular\_process | IRS2 | 3868 | 55 | 1.056122 | -0.694332 | 573 | 275.84 | 0.481396 |
| GO:0009987\_cellular\_process | PFKL | 3868 | 55 | 1.056122 | -0.694332 | 573 | 275.84 | 0.481396 |
| GO:0009987\_cellular\_process | AHR | 3868 | 55 | 1.056122 | -0.694332 | 573 | 275.84 | 0.481396 |
| GO:0009987\_cellular\_process | SUZ12 | 3868 | 55 | 1.056122 | -0.694332 | 573 | 275.84 | 0.481396 |
| GO:0009987\_cellular\_process | DCLRE1A | 3868 | 55 | 1.056122 | -0.694332 | 573 | 275.84 | 0.481396 |
| GO:0009987\_cellular\_process | GNAQ | 3868 | 55 | 1.056122 | -0.694332 | 573 | 275.84 | 0.481396 |
| GO:0009987\_cellular\_process | VEGFA | 3868 | 55 | 1.056122 | -0.694332 | 573 | 275.84 | 0.481396 |
| GO:0009987\_cellular\_process | HMGB1 | 3868 | 55 | 1.056122 | -0.694332 | 573 | 275.84 | 0.481396 |
| GO:0009987\_cellular\_process | TNFRSF21 | 3868 | 55 | 1.056122 | -0.694332 | 573 | 275.84 | 0.481396 |
| GO:0009987\_cellular\_process | PARD3 | 3868 | 55 | 1.056122 | -0.694332 | 573 | 275.84 | 0.481396 |
| GO:0009987\_cellular\_process | LCORL | 3868 | 55 | 1.056122 | -0.694332 | 573 | 275.84 | 0.481396 |
| GO:0009987\_cellular\_process | ALDH18A1 | 3868 | 55 | 1.056122 | -0.694332 | 573 | 275.84 | 0.481396 |
| GO:0009987\_cellular\_process | ONECUT2 | 3868 | 55 | 1.056122 | -0.694332 | 573 | 275.84 | 0.481396 |
| GO:0009987\_cellular\_process | PPM1A | 3868 | 55 | 1.056122 | -0.694332 | 573 | 275.84 | 0.481396 |
| GO:0009987\_cellular\_process | EPHB1 | 3868 | 55 | 1.056122 | -0.694332 | 573 | 275.84 | 0.481396 |
| GO:0009987\_cellular\_process | SEMA5A | 3868 | 55 | 1.056122 | -0.694332 | 573 | 275.84 | 0.481396 |
| GO:0009987\_cellular\_process | SLC11A1 | 3868 | 55 | 1.056122 | -0.694332 | 573 | 275.84 | 0.481396 |
| GO:0009987\_cellular\_process | BCL2 | 3868 | 55 | 1.056122 | -0.694332 | 573 | 275.84 | 0.481396 |
| GO:0009987\_cellular\_process | NFAT5 | 3868 | 55 | 1.056122 | -0.694332 | 573 | 275.84 | 0.481396 |
| GO:0009987\_cellular\_process | GPNMB | 3868 | 55 | 1.056122 | -0.694332 | 573 | 275.84 | 0.481396 |
| GO:0009987\_cellular\_process | EHD1 | 3868 | 55 | 1.056122 | -0.694332 | 573 | 275.84 | 0.481396 |
| GO:0009987\_cellular\_process | ACSL4 | 3868 | 55 | 1.056122 | -0.694332 | 573 | 275.84 | 0.481396 |
| GO:0009987\_cellular\_process | NEFL | 3868 | 55 | 1.056122 | -0.694332 | 573 | 275.84 | 0.481396 |
| GO:0009987\_cellular\_process | FEN1 | 3868 | 55 | 1.056122 | -0.694332 | 573 | 275.84 | 0.481396 |
| GO:0009987\_cellular\_process | ETV3 | 3868 | 55 | 1.056122 | -0.694332 | 573 | 275.84 | 0.481396 |
| GO:0009987\_cellular\_process | SCD1 | 3868 | 55 | 1.056122 | -0.694332 | 573 | 275.84 | 0.481396 |
| GO:0009987\_cellular\_process | KITL | 3868 | 55 | 1.056122 | -0.694332 | 573 | 275.84 | 0.481396 |
| GO:0009987\_cellular\_process | CDC25B | 3868 | 55 | 1.056122 | -0.694332 | 573 | 275.84 | 0.481396 |
| GO:0009987\_cellular\_process | CADPS | 3868 | 55 | 1.056122 | -0.694332 | 573 | 275.84 | 0.481396 |
| GO:0009987\_cellular\_process | PAXIP1 | 3868 | 55 | 1.056122 | -0.694332 | 573 | 275.84 | 0.481396 |
| GO:0009987\_cellular\_process | RNF6 | 3868 | 55 | 1.056122 | -0.694332 | 573 | 275.84 | 0.481396 |
| GO:0009987\_cellular\_process | PKP2 | 3868 | 55 | 1.056122 | -0.694332 | 573 | 275.84 | 0.481396 |
| GO:0009987\_cellular\_process | SALL1 | 3868 | 55 | 1.056122 | -0.694332 | 573 | 275.84 | 0.481396 |
| GO:0009987\_cellular\_process | NTRK2 | 3868 | 55 | 1.056122 | -0.694332 | 573 | 275.84 | 0.481396 |
| GO:0009987\_cellular\_process | FABP3 | 3868 | 55 | 1.056122 | -0.694332 | 573 | 275.84 | 0.481396 |
| GO:0009987\_cellular\_process | CHRNB4 | 3868 | 55 | 1.056122 | -0.694332 | 573 | 275.84 | 0.481396 |
| GO:0009987\_cellular\_process | ID4 | 3868 | 55 | 1.056122 | -0.694332 | 573 | 275.84 | 0.481396 |
| GO:0009987\_cellular\_process | FABP7 | 3868 | 55 | 1.056122 | -0.694332 | 573 | 275.84 | 0.481396 |
| GO:0009987\_cellular\_process | TOB1 | 3868 | 55 | 1.056122 | -0.694332 | 573 | 275.84 | 0.481396 |
| GO:0007154\_cell\_communication | IRS2 | 1096 | 18 | 1.219832 | -0.693258 | 574 | 275.91 | 0.480679 |
| GO:0007154\_cell\_communication | LTBP1 | 1096 | 18 | 1.219832 | -0.693258 | 574 | 275.91 | 0.480679 |
| GO:0007154\_cell\_communication | PFKL | 1096 | 18 | 1.219832 | -0.693258 | 574 | 275.91 | 0.480679 |
| GO:0007154\_cell\_communication | LTBP3 | 1096 | 18 | 1.219832 | -0.693258 | 574 | 275.91 | 0.480679 |
| GO:0007154\_cell\_communication | ONECUT2 | 1096 | 18 | 1.219832 | -0.693258 | 574 | 275.91 | 0.480679 |
| GO:0007154\_cell\_communication | SNCA | 1096 | 18 | 1.219832 | -0.693258 | 574 | 275.91 | 0.480679 |
| GO:0007154\_cell\_communication | KITL | 1096 | 18 | 1.219832 | -0.693258 | 574 | 275.91 | 0.480679 |
| GO:0007154\_cell\_communication | SPRY1 | 1096 | 18 | 1.219832 | -0.693258 | 574 | 275.91 | 0.480679 |
| GO:0007154\_cell\_communication | BDNF | 1096 | 18 | 1.219832 | -0.693258 | 574 | 275.91 | 0.480679 |
| GO:0007154\_cell\_communication | DAB1 | 1096 | 18 | 1.219832 | -0.693258 | 574 | 275.91 | 0.480679 |
| GO:0007154\_cell\_communication | GNAQ | 1096 | 18 | 1.219832 | -0.693258 | 574 | 275.91 | 0.480679 |
| GO:0007154\_cell\_communication | SALL1 | 1096 | 18 | 1.219832 | -0.693258 | 574 | 275.91 | 0.480679 |
| GO:0007154\_cell\_communication | NTRK2 | 1096 | 18 | 1.219832 | -0.693258 | 574 | 275.91 | 0.480679 |
| GO:0007154\_cell\_communication | VEGFA | 1096 | 18 | 1.219832 | -0.693258 | 574 | 275.91 | 0.480679 |
| GO:0007154\_cell\_communication | CHRNB4 | 1096 | 18 | 1.219832 | -0.693258 | 574 | 275.91 | 0.480679 |
| GO:0007154\_cell\_communication | PITX2 | 1096 | 18 | 1.219832 | -0.693258 | 574 | 275.91 | 0.480679 |
| GO:0007154\_cell\_communication | TOB1 | 1096 | 18 | 1.219832 | -0.693258 | 574 | 275.91 | 0.480679 |
| GO:0007154\_cell\_communication | MAP2K5 | 1096 | 18 | 1.219832 | -0.693258 | 574 | 275.91 | 0.480679 |
| GO:0021537\_telencephalon\_development | DAB1 | 62 | 2 | 2.395942 | -0.692750 | 577 | 277.36 | 0.480693 |
| GO:0021537\_telencephalon\_development | ID4 | 62 | 2 | 2.395942 | -0.692750 | 577 | 277.36 | 0.480693 |
| GO:0022604\_regulation\_of\_cell\_morphogenesis | RNF6 | 62 | 2 | 2.395942 | -0.692750 | 577 | 277.36 | 0.480693 |
| GO:0022604\_regulation\_of\_cell\_morphogenesis | NEFL | 62 | 2 | 2.395942 | -0.692750 | 577 | 277.36 | 0.480693 |
| GO:0030855\_epithelial\_cell\_differentiation | VEGFA | 62 | 2 | 2.395942 | -0.692750 | 577 | 277.36 | 0.480693 |
| GO:0030855\_epithelial\_cell\_differentiation | ONECUT2 | 62 | 2 | 2.395942 | -0.692750 | 577 | 277.36 | 0.480693 |
| GO:0048608\_reproductive\_structure\_development | BCL2 | 116 | 3 | 1.920884 | -0.689222 | 578 | 278.66 | 0.482111 |
| GO:0048608\_reproductive\_structure\_development | VEGFA | 116 | 3 | 1.920884 | -0.689222 | 578 | 278.66 | 0.482111 |
| GO:0048608\_reproductive\_structure\_development | AHR | 116 | 3 | 1.920884 | -0.689222 | 578 | 278.66 | 0.482111 |
| GO:0002791\_regulation\_of\_peptide\_secretion | PFKL | 17 | 1 | 4.369070 | -0.685851 | 596 | 287.42 | 0.482248 |
| GO:0010517\_regulation\_of\_phospholipase\_activity | GNAQ | 17 | 1 | 4.369070 | -0.685851 | 596 | 287.42 | 0.482248 |
| GO:0015807\_L-amino\_acid\_transport | SLC11A1 | 17 | 1 | 4.369070 | -0.685851 | 596 | 287.42 | 0.482248 |
| GO:0021545\_cranial\_nerve\_development | EPHB1 | 17 | 1 | 4.369070 | -0.685851 | 596 | 287.42 | 0.482248 |
| GO:0022029\_telencephalon\_cell\_migration | DAB1 | 17 | 1 | 4.369070 | -0.685851 | 596 | 287.42 | 0.482248 |
| GO:0032535\_regulation\_of\_cellular\_component\_size | NEFL | 17 | 1 | 4.369070 | -0.685851 | 596 | 287.42 | 0.482248 |
| GO:0042384\_cilium\_assembly | ONECUT2 | 17 | 1 | 4.369070 | -0.685851 | 596 | 287.42 | 0.482248 |
| GO:0042398\_cellular\_amino\_acid\_derivative\_biosynthetic\_process | SNCA | 17 | 1 | 4.369070 | -0.685851 | 596 | 287.42 | 0.482248 |
| GO:0042417\_dopamine\_metabolic\_process | SNCA | 17 | 1 | 4.369070 | -0.685851 | 596 | 287.42 | 0.482248 |
| GO:0042440\_pigment\_metabolic\_process | BCL2 | 17 | 1 | 4.369070 | -0.685851 | 596 | 287.42 | 0.482248 |
| GO:0043407\_negative\_regulation\_of\_MAP\_kinase\_activity | SPRY1 | 17 | 1 | 4.369070 | -0.685851 | 596 | 287.42 | 0.482248 |
| GO:0045333\_cellular\_respiration | SNCA | 17 | 1 | 4.369070 | -0.685851 | 596 | 287.42 | 0.482248 |
| GO:0045667\_regulation\_of\_osteoblast\_differentiation | TOB1 | 17 | 1 | 4.369070 | -0.685851 | 596 | 287.42 | 0.482248 |
| GO:0045786\_negative\_regulation\_of\_cell\_cycle | BCL2 | 17 | 1 | 4.369070 | -0.685851 | 596 | 287.42 | 0.482248 |
| GO:0048168\_regulation\_of\_neuronal\_synaptic\_plasticity | SNCA | 17 | 1 | 4.369070 | -0.685851 | 596 | 287.42 | 0.482248 |
| GO:0048873\_homeostasis\_of\_number\_of\_cells\_within\_a\_tissue | BCL2 | 17 | 1 | 4.369070 | -0.685851 | 596 | 287.42 | 0.482248 |
| GO:0055072\_iron\_ion\_homeostasis | SLC11A1 | 17 | 1 | 4.369070 | -0.685851 | 596 | 287.42 | 0.482248 |
| GO:0060350\_endochondral\_bone\_morphogenesis | COL13A1 | 17 | 1 | 4.369070 | -0.685851 | 596 | 287.42 | 0.482248 |
| GO:0051186\_cofactor\_metabolic\_process | ACOT10 | 63 | 2 | 2.357911 | -0.682299 | 597 | 288.52 | 0.483283 |
| GO:0051186\_cofactor\_metabolic\_process | SNCA | 63 | 2 | 2.357911 | -0.682299 | 597 | 288.52 | 0.483283 |
| GO:0001501\_skeletal\_system\_development | GNAQ | 236 | 5 | 1.573606 | -0.676994 | 598 | 289.26 | 0.483712 |
| GO:0001501\_skeletal\_system\_development | LTBP3 | 236 | 5 | 1.573606 | -0.676994 | 598 | 289.26 | 0.483712 |
| GO:0001501\_skeletal\_system\_development | COL13A1 | 236 | 5 | 1.573606 | -0.676994 | 598 | 289.26 | 0.483712 |
| GO:0001501\_skeletal\_system\_development | BCL2 | 236 | 5 | 1.573606 | -0.676994 | 598 | 289.26 | 0.483712 |
| GO:0001501\_skeletal\_system\_development | TOB1 | 236 | 5 | 1.573606 | -0.676994 | 598 | 289.26 | 0.483712 |
| GO:0006519\_cellular\_amino\_acid\_and\_derivative\_metabolic\_process | ALDH18A1 | 118 | 3 | 1.888327 | -0.674590 | 599 | 290.01 | 0.484157 |
| GO:0006519\_cellular\_amino\_acid\_and\_derivative\_metabolic\_process | SNCA | 118 | 3 | 1.888327 | -0.674590 | 599 | 290.01 | 0.484157 |
| GO:0006519\_cellular\_amino\_acid\_and\_derivative\_metabolic\_process | FABP3 | 118 | 3 | 1.888327 | -0.674590 | 599 | 290.01 | 0.484157 |
| GO:0006915\_apoptosis | TNFRSF21 | 427 | 8 | 1.391554 | -0.672140 | 600 | 290.19 | 0.483650 |
| GO:0006915\_apoptosis | BDNF | 427 | 8 | 1.391554 | -0.672140 | 600 | 290.19 | 0.483650 |
| GO:0006915\_apoptosis | BCL2 | 427 | 8 | 1.391554 | -0.672140 | 600 | 290.19 | 0.483650 |
| GO:0006915\_apoptosis | SNCA | 427 | 8 | 1.391554 | -0.672140 | 600 | 290.19 | 0.483650 |
| GO:0006915\_apoptosis | VEGFA | 427 | 8 | 1.391554 | -0.672140 | 600 | 290.19 | 0.483650 |
| GO:0006915\_apoptosis | NEFL | 427 | 8 | 1.391554 | -0.672140 | 600 | 290.19 | 0.483650 |
| GO:0006915\_apoptosis | KITL | 427 | 8 | 1.391554 | -0.672140 | 600 | 290.19 | 0.483650 |
| GO:0006915\_apoptosis | AHR | 427 | 8 | 1.391554 | -0.672140 | 600 | 290.19 | 0.483650 |
| GO:0031644\_regulation\_of\_neurological\_system\_process | BDNF | 64 | 2 | 2.321069 | -0.672067 | 601 | 291.11 | 0.484376 |
| GO:0031644\_regulation\_of\_neurological\_system\_process | SNCA | 64 | 2 | 2.321069 | -0.672067 | 601 | 291.11 | 0.484376 |
| GO:0001818\_negative\_regulation\_of\_cytokine\_production | SLC11A1 | 18 | 1 | 4.126344 | -0.663817 | 620 | 302.18 | 0.487387 |
| GO:0002064\_epithelial\_cell\_development | ONECUT2 | 18 | 1 | 4.126344 | -0.663817 | 620 | 302.18 | 0.487387 |
| GO:0002285\_lymphocyte\_activation\_during\_immune\_response | SLC11A1 | 18 | 1 | 4.126344 | -0.663817 | 620 | 302.18 | 0.487387 |
| GO:0006940\_regulation\_of\_smooth\_muscle\_contraction | CHRNB4 | 18 | 1 | 4.126344 | -0.663817 | 620 | 302.18 | 0.487387 |
| GO:0015711\_organic\_anion\_transport | SLC4A4 | 18 | 1 | 4.126344 | -0.663817 | 620 | 302.18 | 0.487387 |
| GO:0021885\_forebrain\_cell\_migration | DAB1 | 18 | 1 | 4.126344 | -0.663817 | 620 | 302.18 | 0.487387 |
| GO:0030336\_negative\_regulation\_of\_cell\_migration | BCL2 | 18 | 1 | 4.126344 | -0.663817 | 620 | 302.18 | 0.487387 |
| GO:0030510\_regulation\_of\_BMP\_signaling\_pathway | TOB1 | 18 | 1 | 4.126344 | -0.663817 | 620 | 302.18 | 0.487387 |
| GO:0032623\_interleukin-2\_production | SLC11A1 | 18 | 1 | 4.126344 | -0.663817 | 620 | 302.18 | 0.487387 |
| GO:0043029\_T\_cell\_homeostasis | BCL2 | 18 | 1 | 4.126344 | -0.663817 | 620 | 302.18 | 0.487387 |
| GO:0045058\_T\_cell\_selection | BCL2 | 18 | 1 | 4.126344 | -0.663817 | 620 | 302.18 | 0.487387 |
| GO:0045103\_intermediate\_filament-based\_process | NEFL | 18 | 1 | 4.126344 | -0.663817 | 620 | 302.18 | 0.487387 |
| GO:0045807\_positive\_regulation\_of\_endocytosis | SLC11A1 | 18 | 1 | 4.126344 | -0.663817 | 620 | 302.18 | 0.487387 |
| GO:0046578\_regulation\_of\_Ras\_protein\_signal\_transduction | KITL | 18 | 1 | 4.126344 | -0.663817 | 620 | 302.18 | 0.487387 |
| GO:0048730\_epidermis\_morphogenesis | BCL2 | 18 | 1 | 4.126344 | -0.663817 | 620 | 302.18 | 0.487387 |
| GO:0050731\_positive\_regulation\_of\_peptidyl-tyrosine\_phosphorylation | KITL | 18 | 1 | 4.126344 | -0.663817 | 620 | 302.18 | 0.487387 |
| GO:0051924\_regulation\_of\_calcium\_ion\_transport | BCL2 | 18 | 1 | 4.126344 | -0.663817 | 620 | 302.18 | 0.487387 |
| GO:0055008\_cardiac\_muscle\_tissue\_morphogenesis | PITX2 | 18 | 1 | 4.126344 | -0.663817 | 620 | 302.18 | 0.487387 |
| GO:0060415\_muscle\_tissue\_morphogenesis | PITX2 | 18 | 1 | 4.126344 | -0.663817 | 620 | 302.18 | 0.487387 |
| GO:0014706\_striated\_muscle\_tissue\_development | BCL2 | 120 | 3 | 1.856855 | -0.660328 | 621 | 303.16 | 0.488180 |
| GO:0014706\_striated\_muscle\_tissue\_development | NR2F2 | 120 | 3 | 1.856855 | -0.660328 | 621 | 303.16 | 0.488180 |
| GO:0014706\_striated\_muscle\_tissue\_development | PITX2 | 120 | 3 | 1.856855 | -0.660328 | 621 | 303.16 | 0.488180 |
| GO:0031589\_cell-substrate\_adhesion | BCL2 | 66 | 2 | 2.250733 | -0.652229 | 622 | 305.56 | 0.491254 |
| GO:0031589\_cell-substrate\_adhesion | ONECUT2 | 66 | 2 | 2.250733 | -0.652229 | 622 | 305.56 | 0.491254 |
| GO:0012501\_programmed\_cell\_death | TNFRSF21 | 433 | 8 | 1.372271 | -0.650231 | 623 | 306.17 | 0.491445 |
| GO:0012501\_programmed\_cell\_death | BDNF | 433 | 8 | 1.372271 | -0.650231 | 623 | 306.17 | 0.491445 |
| GO:0012501\_programmed\_cell\_death | BCL2 | 433 | 8 | 1.372271 | -0.650231 | 623 | 306.17 | 0.491445 |
| GO:0012501\_programmed\_cell\_death | SNCA | 433 | 8 | 1.372271 | -0.650231 | 623 | 306.17 | 0.491445 |
| GO:0012501\_programmed\_cell\_death | VEGFA | 433 | 8 | 1.372271 | -0.650231 | 623 | 306.17 | 0.491445 |
| GO:0012501\_programmed\_cell\_death | NEFL | 433 | 8 | 1.372271 | -0.650231 | 623 | 306.17 | 0.491445 |
| GO:0012501\_programmed\_cell\_death | KITL | 433 | 8 | 1.372271 | -0.650231 | 623 | 306.17 | 0.491445 |
| GO:0012501\_programmed\_cell\_death | AHR | 433 | 8 | 1.372271 | -0.650231 | 623 | 306.17 | 0.491445 |
| GO:0010467\_gene\_expression | HMGB1 | 905 | 15 | 1.231064 | -0.650025 | 624 | 306.22 | 0.490737 |
| GO:0010467\_gene\_expression | LCORL | 905 | 15 | 1.231064 | -0.650025 | 624 | 306.22 | 0.490737 |
| GO:0010467\_gene\_expression | ONECUT2 | 905 | 15 | 1.231064 | -0.650025 | 624 | 306.22 | 0.490737 |
| GO:0010467\_gene\_expression | RORB | 905 | 15 | 1.231064 | -0.650025 | 624 | 306.22 | 0.490737 |
| GO:0010467\_gene\_expression | AHR | 905 | 15 | 1.231064 | -0.650025 | 624 | 306.22 | 0.490737 |
| GO:0010467\_gene\_expression | SUZ12 | 905 | 15 | 1.231064 | -0.650025 | 624 | 306.22 | 0.490737 |
| GO:0010467\_gene\_expression | SLC11A1 | 905 | 15 | 1.231064 | -0.650025 | 624 | 306.22 | 0.490737 |
| GO:0010467\_gene\_expression | RNF6 | 905 | 15 | 1.231064 | -0.650025 | 624 | 306.22 | 0.490737 |
| GO:0010467\_gene\_expression | BCL2 | 905 | 15 | 1.231064 | -0.650025 | 624 | 306.22 | 0.490737 |
| GO:0010467\_gene\_expression | GARNL1 | 905 | 15 | 1.231064 | -0.650025 | 624 | 306.22 | 0.490737 |
| GO:0010467\_gene\_expression | NFAT5 | 905 | 15 | 1.231064 | -0.650025 | 624 | 306.22 | 0.490737 |
| GO:0010467\_gene\_expression | NR2F2 | 905 | 15 | 1.231064 | -0.650025 | 624 | 306.22 | 0.490737 |
| GO:0010467\_gene\_expression | MYB | 905 | 15 | 1.231064 | -0.650025 | 624 | 306.22 | 0.490737 |
| GO:0010467\_gene\_expression | ETV3 | 905 | 15 | 1.231064 | -0.650025 | 624 | 306.22 | 0.490737 |
| GO:0010467\_gene\_expression | PITX2 | 905 | 15 | 1.231064 | -0.650025 | 624 | 306.22 | 0.490737 |
| GO:0044237\_cellular\_metabolic\_process | HMGB1 | 1974 | 30 | 1.128787 | -0.648933 | 625 | 306.3 | 0.490080 |
| GO:0044237\_cellular\_metabolic\_process | ALDH18A1 | 1974 | 30 | 1.128787 | -0.648933 | 625 | 306.3 | 0.490080 |
| GO:0044237\_cellular\_metabolic\_process | LCORL | 1974 | 30 | 1.128787 | -0.648933 | 625 | 306.3 | 0.490080 |
| GO:0044237\_cellular\_metabolic\_process | SNCA | 1974 | 30 | 1.128787 | -0.648933 | 625 | 306.3 | 0.490080 |
| GO:0044237\_cellular\_metabolic\_process | ONECUT2 | 1974 | 30 | 1.128787 | -0.648933 | 625 | 306.3 | 0.490080 |
| GO:0044237\_cellular\_metabolic\_process | PPM1A | 1974 | 30 | 1.128787 | -0.648933 | 625 | 306.3 | 0.490080 |
| GO:0044237\_cellular\_metabolic\_process | RORB | 1974 | 30 | 1.128787 | -0.648933 | 625 | 306.3 | 0.490080 |
| GO:0044237\_cellular\_metabolic\_process | SLC11A1 | 1974 | 30 | 1.128787 | -0.648933 | 625 | 306.3 | 0.490080 |
| GO:0044237\_cellular\_metabolic\_process | SPRY1 | 1974 | 30 | 1.128787 | -0.648933 | 625 | 306.3 | 0.490080 |
| GO:0044237\_cellular\_metabolic\_process | DAB1 | 1974 | 30 | 1.128787 | -0.648933 | 625 | 306.3 | 0.490080 |
| GO:0044237\_cellular\_metabolic\_process | ACOT10 | 1974 | 30 | 1.128787 | -0.648933 | 625 | 306.3 | 0.490080 |
| GO:0044237\_cellular\_metabolic\_process | BCL2 | 1974 | 30 | 1.128787 | -0.648933 | 625 | 306.3 | 0.490080 |
| GO:0044237\_cellular\_metabolic\_process | GARNL1 | 1974 | 30 | 1.128787 | -0.648933 | 625 | 306.3 | 0.490080 |
| GO:0044237\_cellular\_metabolic\_process | NFAT5 | 1974 | 30 | 1.128787 | -0.648933 | 625 | 306.3 | 0.490080 |
| GO:0044237\_cellular\_metabolic\_process | NR2F2 | 1974 | 30 | 1.128787 | -0.648933 | 625 | 306.3 | 0.490080 |
| GO:0044237\_cellular\_metabolic\_process | ACSL4 | 1974 | 30 | 1.128787 | -0.648933 | 625 | 306.3 | 0.490080 |
| GO:0044237\_cellular\_metabolic\_process | MYB | 1974 | 30 | 1.128787 | -0.648933 | 625 | 306.3 | 0.490080 |
| GO:0044237\_cellular\_metabolic\_process | FEN1 | 1974 | 30 | 1.128787 | -0.648933 | 625 | 306.3 | 0.490080 |
| GO:0044237\_cellular\_metabolic\_process | ETV3 | 1974 | 30 | 1.128787 | -0.648933 | 625 | 306.3 | 0.490080 |
| GO:0044237\_cellular\_metabolic\_process | PITX2 | 1974 | 30 | 1.128787 | -0.648933 | 625 | 306.3 | 0.490080 |
| GO:0044237\_cellular\_metabolic\_process | SCD1 | 1974 | 30 | 1.128787 | -0.648933 | 625 | 306.3 | 0.490080 |
| GO:0044237\_cellular\_metabolic\_process | PFKL | 1974 | 30 | 1.128787 | -0.648933 | 625 | 306.3 | 0.490080 |
| GO:0044237\_cellular\_metabolic\_process | KITL | 1974 | 30 | 1.128787 | -0.648933 | 625 | 306.3 | 0.490080 |
| GO:0044237\_cellular\_metabolic\_process | AHR | 1974 | 30 | 1.128787 | -0.648933 | 625 | 306.3 | 0.490080 |
| GO:0044237\_cellular\_metabolic\_process | CDC25B | 1974 | 30 | 1.128787 | -0.648933 | 625 | 306.3 | 0.490080 |
| GO:0044237\_cellular\_metabolic\_process | SUZ12 | 1974 | 30 | 1.128787 | -0.648933 | 625 | 306.3 | 0.490080 |
| GO:0044237\_cellular\_metabolic\_process | DCLRE1A | 1974 | 30 | 1.128787 | -0.648933 | 625 | 306.3 | 0.490080 |
| GO:0044237\_cellular\_metabolic\_process | RNF6 | 1974 | 30 | 1.128787 | -0.648933 | 625 | 306.3 | 0.490080 |
| GO:0044237\_cellular\_metabolic\_process | GNAQ | 1974 | 30 | 1.128787 | -0.648933 | 625 | 306.3 | 0.490080 |
| GO:0044237\_cellular\_metabolic\_process | FABP3 | 1974 | 30 | 1.128787 | -0.648933 | 625 | 306.3 | 0.490080 |
| GO:0007569\_cell\_aging | BCL2 | 19 | 1 | 3.909168 | -0.643120 | 633 | 314.6 | 0.496998 |
| GO:0007595\_lactation | VEGFA | 19 | 1 | 3.909168 | -0.643120 | 633 | 314.6 | 0.496998 |
| GO:0021587\_cerebellum\_morphogenesis | DAB1 | 19 | 1 | 3.909168 | -0.643120 | 633 | 314.6 | 0.496998 |
| GO:0046890\_regulation\_of\_lipid\_biosynthetic\_process | SNCA | 19 | 1 | 3.909168 | -0.643120 | 633 | 314.6 | 0.496998 |
| GO:0048536\_spleen\_development | BCL2 | 19 | 1 | 3.909168 | -0.643120 | 633 | 314.6 | 0.496998 |
| GO:0048547\_gut\_morphogenesis | BCL2 | 19 | 1 | 3.909168 | -0.643120 | 633 | 314.6 | 0.496998 |
| GO:0051056\_regulation\_of\_small\_GTPase\_mediated\_signal\_transduction | KITL | 19 | 1 | 3.909168 | -0.643120 | 633 | 314.6 | 0.496998 |
| GO:0060079\_regulation\_of\_excitatory\_postsynaptic\_membrane\_potential | SNCA | 19 | 1 | 3.909168 | -0.643120 | 633 | 314.6 | 0.496998 |
| GO:0034962\_cellular\_biopolymer\_catabolic\_process | HMGB1 | 68 | 2 | 2.184535 | -0.633185 | 634 | 317.36 | 0.500568 |
| GO:0034962\_cellular\_biopolymer\_catabolic\_process | RNF6 | 68 | 2 | 2.184535 | -0.633185 | 634 | 317.36 | 0.500568 |
| GO:0009308\_amine\_metabolic\_process | ALDH18A1 | 124 | 3 | 1.796956 | -0.632859 | 635 | 317.64 | 0.500220 |
| GO:0009308\_amine\_metabolic\_process | SNCA | 124 | 3 | 1.796956 | -0.632859 | 635 | 317.64 | 0.500220 |
| GO:0009308\_amine\_metabolic\_process | FABP3 | 124 | 3 | 1.796956 | -0.632859 | 635 | 317.64 | 0.500220 |
| GO:0016192\_vesicle-mediated\_transport | CADPS | 184 | 4 | 1.614656 | -0.629987 | 636 | 317.96 | 0.499937 |
| GO:0016192\_vesicle-mediated\_transport | SLC11A1 | 184 | 4 | 1.614656 | -0.629987 | 636 | 317.96 | 0.499937 |
| GO:0016192\_vesicle-mediated\_transport | SNCA | 184 | 4 | 1.614656 | -0.629987 | 636 | 317.96 | 0.499937 |
| GO:0016192\_vesicle-mediated\_transport | EHD1 | 184 | 4 | 1.614656 | -0.629987 | 636 | 317.96 | 0.499937 |
| GO:0008152\_metabolic\_process | HMGB1 | 2133 | 32 | 1.114287 | -0.624377 | 637 | 318.98 | 0.500754 |
| GO:0008152\_metabolic\_process | LCORL | 2133 | 32 | 1.114287 | -0.624377 | 637 | 318.98 | 0.500754 |
| GO:0008152\_metabolic\_process | ALDH18A1 | 2133 | 32 | 1.114287 | -0.624377 | 637 | 318.98 | 0.500754 |
| GO:0008152\_metabolic\_process | ONECUT2 | 2133 | 32 | 1.114287 | -0.624377 | 637 | 318.98 | 0.500754 |
| GO:0008152\_metabolic\_process | SNCA | 2133 | 32 | 1.114287 | -0.624377 | 637 | 318.98 | 0.500754 |
| GO:0008152\_metabolic\_process | PPM1A | 2133 | 32 | 1.114287 | -0.624377 | 637 | 318.98 | 0.500754 |
| GO:0008152\_metabolic\_process | RORB | 2133 | 32 | 1.114287 | -0.624377 | 637 | 318.98 | 0.500754 |
| GO:0008152\_metabolic\_process | SLC11A1 | 2133 | 32 | 1.114287 | -0.624377 | 637 | 318.98 | 0.500754 |
| GO:0008152\_metabolic\_process | BDNF | 2133 | 32 | 1.114287 | -0.624377 | 637 | 318.98 | 0.500754 |
| GO:0008152\_metabolic\_process | SPRY1 | 2133 | 32 | 1.114287 | -0.624377 | 637 | 318.98 | 0.500754 |
| GO:0008152\_metabolic\_process | DAB1 | 2133 | 32 | 1.114287 | -0.624377 | 637 | 318.98 | 0.500754 |
| GO:0008152\_metabolic\_process | ACOT10 | 2133 | 32 | 1.114287 | -0.624377 | 637 | 318.98 | 0.500754 |
| GO:0008152\_metabolic\_process | BCL2 | 2133 | 32 | 1.114287 | -0.624377 | 637 | 318.98 | 0.500754 |
| GO:0008152\_metabolic\_process | GARNL1 | 2133 | 32 | 1.114287 | -0.624377 | 637 | 318.98 | 0.500754 |
| GO:0008152\_metabolic\_process | NFAT5 | 2133 | 32 | 1.114287 | -0.624377 | 637 | 318.98 | 0.500754 |
| GO:0008152\_metabolic\_process | NR2F2 | 2133 | 32 | 1.114287 | -0.624377 | 637 | 318.98 | 0.500754 |
| GO:0008152\_metabolic\_process | MYB | 2133 | 32 | 1.114287 | -0.624377 | 637 | 318.98 | 0.500754 |
| GO:0008152\_metabolic\_process | ACSL4 | 2133 | 32 | 1.114287 | -0.624377 | 637 | 318.98 | 0.500754 |
| GO:0008152\_metabolic\_process | FEN1 | 2133 | 32 | 1.114287 | -0.624377 | 637 | 318.98 | 0.500754 |
| GO:0008152\_metabolic\_process | PITX2 | 2133 | 32 | 1.114287 | -0.624377 | 637 | 318.98 | 0.500754 |
| GO:0008152\_metabolic\_process | ETV3 | 2133 | 32 | 1.114287 | -0.624377 | 637 | 318.98 | 0.500754 |
| GO:0008152\_metabolic\_process | SCD1 | 2133 | 32 | 1.114287 | -0.624377 | 637 | 318.98 | 0.500754 |
| GO:0008152\_metabolic\_process | PFKL | 2133 | 32 | 1.114287 | -0.624377 | 637 | 318.98 | 0.500754 |
| GO:0008152\_metabolic\_process | KITL | 2133 | 32 | 1.114287 | -0.624377 | 637 | 318.98 | 0.500754 |
| GO:0008152\_metabolic\_process | AHR | 2133 | 32 | 1.114287 | -0.624377 | 637 | 318.98 | 0.500754 |
| GO:0008152\_metabolic\_process | CDC25B | 2133 | 32 | 1.114287 | -0.624377 | 637 | 318.98 | 0.500754 |
| GO:0008152\_metabolic\_process | SUZ12 | 2133 | 32 | 1.114287 | -0.624377 | 637 | 318.98 | 0.500754 |
| GO:0008152\_metabolic\_process | DCLRE1A | 2133 | 32 | 1.114287 | -0.624377 | 637 | 318.98 | 0.500754 |
| GO:0008152\_metabolic\_process | RNF6 | 2133 | 32 | 1.114287 | -0.624377 | 637 | 318.98 | 0.500754 |
| GO:0008152\_metabolic\_process | GNAQ | 2133 | 32 | 1.114287 | -0.624377 | 637 | 318.98 | 0.500754 |
| GO:0008152\_metabolic\_process | NTRK2 | 2133 | 32 | 1.114287 | -0.624377 | 637 | 318.98 | 0.500754 |
| GO:0008152\_metabolic\_process | FABP3 | 2133 | 32 | 1.114287 | -0.624377 | 637 | 318.98 | 0.500754 |
| GO:0006816\_calcium\_ion\_transport | BCL2 | 69 | 2 | 2.152875 | -0.623945 | 639 | 319.84 | 0.500532 |
| GO:0006816\_calcium\_ion\_transport | MYB | 69 | 2 | 2.152875 | -0.623945 | 639 | 319.84 | 0.500532 |
| GO:0032101\_regulation\_of\_response\_to\_external\_stimulus | HMGB1 | 69 | 2 | 2.152875 | -0.623945 | 639 | 319.84 | 0.500532 |
| GO:0032101\_regulation\_of\_response\_to\_external\_stimulus | FABP7 | 69 | 2 | 2.152875 | -0.623945 | 639 | 319.84 | 0.500532 |
| GO:0005977\_glycogen\_metabolic\_process | HMGB1 | 20 | 1 | 3.713710 | -0.623621 | 650 | 325.9 | 0.501385 |
| GO:0006073\_cellular\_glucan\_metabolic\_process | HMGB1 | 20 | 1 | 3.713710 | -0.623621 | 650 | 325.9 | 0.501385 |
| GO:0006584\_catecholamine\_metabolic\_process | SNCA | 20 | 1 | 3.713710 | -0.623621 | 650 | 325.9 | 0.501385 |
| GO:0009712\_catechol\_metabolic\_process | SNCA | 20 | 1 | 3.713710 | -0.623621 | 650 | 325.9 | 0.501385 |
| GO:0016571\_histone\_methylation | SUZ12 | 20 | 1 | 3.713710 | -0.623621 | 650 | 325.9 | 0.501385 |
| GO:0018209\_peptidyl-serine\_modification | BCL2 | 20 | 1 | 3.713710 | -0.623621 | 650 | 325.9 | 0.501385 |
| GO:0018958\_phenol\_metabolic\_process | SNCA | 20 | 1 | 3.713710 | -0.623621 | 650 | 325.9 | 0.501385 |
| GO:0034311\_diol\_metabolic\_process | SNCA | 20 | 1 | 3.713710 | -0.623621 | 650 | 325.9 | 0.501385 |
| GO:0044042\_glucan\_metabolic\_process | HMGB1 | 20 | 1 | 3.713710 | -0.623621 | 650 | 325.9 | 0.501385 |
| GO:0045017\_glycerolipid\_biosynthetic\_process | FABP3 | 20 | 1 | 3.713710 | -0.623621 | 650 | 325.9 | 0.501385 |
| GO:0060191\_regulation\_of\_lipase\_activity | GNAQ | 20 | 1 | 3.713710 | -0.623621 | 650 | 325.9 | 0.501385 |
| GO:0050789\_regulation\_of\_biological\_process | HMGB1 | 2357 | 35 | 1.102926 | -0.619966 | 651 | 326.27 | 0.501183 |
| GO:0050789\_regulation\_of\_biological\_process | LTBP1 | 2357 | 35 | 1.102926 | -0.619966 | 651 | 326.27 | 0.501183 |
| GO:0050789\_regulation\_of\_biological\_process | LTBP3 | 2357 | 35 | 1.102926 | -0.619966 | 651 | 326.27 | 0.501183 |
| GO:0050789\_regulation\_of\_biological\_process | ONECUT2 | 2357 | 35 | 1.102926 | -0.619966 | 651 | 326.27 | 0.501183 |
| GO:0050789\_regulation\_of\_biological\_process | SNCA | 2357 | 35 | 1.102926 | -0.619966 | 651 | 326.27 | 0.501183 |
| GO:0050789\_regulation\_of\_biological\_process | RORB | 2357 | 35 | 1.102926 | -0.619966 | 651 | 326.27 | 0.501183 |
| GO:0050789\_regulation\_of\_biological\_process | SLC11A1 | 2357 | 35 | 1.102926 | -0.619966 | 651 | 326.27 | 0.501183 |
| GO:0050789\_regulation\_of\_biological\_process | SPRY1 | 2357 | 35 | 1.102926 | -0.619966 | 651 | 326.27 | 0.501183 |
| GO:0050789\_regulation\_of\_biological\_process | BDNF | 2357 | 35 | 1.102926 | -0.619966 | 651 | 326.27 | 0.501183 |
| GO:0050789\_regulation\_of\_biological\_process | DAB1 | 2357 | 35 | 1.102926 | -0.619966 | 651 | 326.27 | 0.501183 |
| GO:0050789\_regulation\_of\_biological\_process | BCL2 | 2357 | 35 | 1.102926 | -0.619966 | 651 | 326.27 | 0.501183 |
| GO:0050789\_regulation\_of\_biological\_process | GARNL1 | 2357 | 35 | 1.102926 | -0.619966 | 651 | 326.27 | 0.501183 |
| GO:0050789\_regulation\_of\_biological\_process | NFAT5 | 2357 | 35 | 1.102926 | -0.619966 | 651 | 326.27 | 0.501183 |
| GO:0050789\_regulation\_of\_biological\_process | NR2F2 | 2357 | 35 | 1.102926 | -0.619966 | 651 | 326.27 | 0.501183 |
| GO:0050789\_regulation\_of\_biological\_process | MYB | 2357 | 35 | 1.102926 | -0.619966 | 651 | 326.27 | 0.501183 |
| GO:0050789\_regulation\_of\_biological\_process | ACSL4 | 2357 | 35 | 1.102926 | -0.619966 | 651 | 326.27 | 0.501183 |
| GO:0050789\_regulation\_of\_biological\_process | NEFL | 2357 | 35 | 1.102926 | -0.619966 | 651 | 326.27 | 0.501183 |
| GO:0050789\_regulation\_of\_biological\_process | MAP2K5 | 2357 | 35 | 1.102926 | -0.619966 | 651 | 326.27 | 0.501183 |
| GO:0050789\_regulation\_of\_biological\_process | PITX2 | 2357 | 35 | 1.102926 | -0.619966 | 651 | 326.27 | 0.501183 |
| GO:0050789\_regulation\_of\_biological\_process | ETV3 | 2357 | 35 | 1.102926 | -0.619966 | 651 | 326.27 | 0.501183 |
| GO:0050789\_regulation\_of\_biological\_process | IRS2 | 2357 | 35 | 1.102926 | -0.619966 | 651 | 326.27 | 0.501183 |
| GO:0050789\_regulation\_of\_biological\_process | PFKL | 2357 | 35 | 1.102926 | -0.619966 | 651 | 326.27 | 0.501183 |
| GO:0050789\_regulation\_of\_biological\_process | KITL | 2357 | 35 | 1.102926 | -0.619966 | 651 | 326.27 | 0.501183 |
| GO:0050789\_regulation\_of\_biological\_process | AHR | 2357 | 35 | 1.102926 | -0.619966 | 651 | 326.27 | 0.501183 |
| GO:0050789\_regulation\_of\_biological\_process | CDC25B | 2357 | 35 | 1.102926 | -0.619966 | 651 | 326.27 | 0.501183 |
| GO:0050789\_regulation\_of\_biological\_process | SUZ12 | 2357 | 35 | 1.102926 | -0.619966 | 651 | 326.27 | 0.501183 |
| GO:0050789\_regulation\_of\_biological\_process | RNF6 | 2357 | 35 | 1.102926 | -0.619966 | 651 | 326.27 | 0.501183 |
| GO:0050789\_regulation\_of\_biological\_process | GNAQ | 2357 | 35 | 1.102926 | -0.619966 | 651 | 326.27 | 0.501183 |
| GO:0050789\_regulation\_of\_biological\_process | SP4 | 2357 | 35 | 1.102926 | -0.619966 | 651 | 326.27 | 0.501183 |
| GO:0050789\_regulation\_of\_biological\_process | VEGFA | 2357 | 35 | 1.102926 | -0.619966 | 651 | 326.27 | 0.501183 |
| GO:0050789\_regulation\_of\_biological\_process | NTRK2 | 2357 | 35 | 1.102926 | -0.619966 | 651 | 326.27 | 0.501183 |
| GO:0050789\_regulation\_of\_biological\_process | CHRNB4 | 2357 | 35 | 1.102926 | -0.619966 | 651 | 326.27 | 0.501183 |
| GO:0050789\_regulation\_of\_biological\_process | ID4 | 2357 | 35 | 1.102926 | -0.619966 | 651 | 326.27 | 0.501183 |
| GO:0050789\_regulation\_of\_biological\_process | FABP7 | 2357 | 35 | 1.102926 | -0.619966 | 651 | 326.27 | 0.501183 |
| GO:0050789\_regulation\_of\_biological\_process | TOB1 | 2357 | 35 | 1.102926 | -0.619966 | 651 | 326.27 | 0.501183 |
| GO:0006811\_ion\_transport | SLC11A1 | 186 | 4 | 1.597294 | -0.619265 | 652 | 326.85 | 0.501304 |
| GO:0006811\_ion\_transport | BCL2 | 186 | 4 | 1.597294 | -0.619265 | 652 | 326.85 | 0.501304 |
| GO:0006811\_ion\_transport | MYB | 186 | 4 | 1.597294 | -0.619265 | 652 | 326.85 | 0.501304 |
| GO:0006811\_ion\_transport | SLC4A4 | 186 | 4 | 1.597294 | -0.619265 | 652 | 326.85 | 0.501304 |
| GO:0008406\_gonad\_development | BCL2 | 70 | 2 | 2.122120 | -0.614887 | 654 | 327.92 | 0.501407 |
| GO:0008406\_gonad\_development | VEGFA | 70 | 2 | 2.122120 | -0.614887 | 654 | 327.92 | 0.501407 |
| GO:0070838\_divalent\_metal\_ion\_transport | BCL2 | 70 | 2 | 2.122120 | -0.614887 | 654 | 327.92 | 0.501407 |
| GO:0070838\_divalent\_metal\_ion\_transport | MYB | 70 | 2 | 2.122120 | -0.614887 | 654 | 327.92 | 0.501407 |
| GO:0008219\_cell\_death | TNFRSF21 | 444 | 8 | 1.338274 | -0.611735 | 655 | 328.21 | 0.501084 |
| GO:0008219\_cell\_death | BDNF | 444 | 8 | 1.338274 | -0.611735 | 655 | 328.21 | 0.501084 |
| GO:0008219\_cell\_death | BCL2 | 444 | 8 | 1.338274 | -0.611735 | 655 | 328.21 | 0.501084 |
| GO:0008219\_cell\_death | SNCA | 444 | 8 | 1.338274 | -0.611735 | 655 | 328.21 | 0.501084 |
| GO:0008219\_cell\_death | VEGFA | 444 | 8 | 1.338274 | -0.611735 | 655 | 328.21 | 0.501084 |
| GO:0008219\_cell\_death | NEFL | 444 | 8 | 1.338274 | -0.611735 | 655 | 328.21 | 0.501084 |
| GO:0008219\_cell\_death | KITL | 444 | 8 | 1.338274 | -0.611735 | 655 | 328.21 | 0.501084 |
| GO:0008219\_cell\_death | AHR | 444 | 8 | 1.338274 | -0.611735 | 655 | 328.21 | 0.501084 |
| GO:0007389\_pattern\_specification\_process | SEMA5A | 250 | 5 | 1.485484 | -0.610391 | 656 | 328.44 | 0.500671 |
| GO:0007389\_pattern\_specification\_process | SPRY1 | 250 | 5 | 1.485484 | -0.610391 | 656 | 328.44 | 0.500671 |
| GO:0007389\_pattern\_specification\_process | VEGFA | 250 | 5 | 1.485484 | -0.610391 | 656 | 328.44 | 0.500671 |
| GO:0007389\_pattern\_specification\_process | NR2F2 | 250 | 5 | 1.485484 | -0.610391 | 656 | 328.44 | 0.500671 |
| GO:0007389\_pattern\_specification\_process | PITX2 | 250 | 5 | 1.485484 | -0.610391 | 656 | 328.44 | 0.500671 |
| GO:0060537\_muscle\_tissue\_development | BCL2 | 128 | 3 | 1.740801 | -0.606721 | 657 | 329.09 | 0.500898 |
| GO:0060537\_muscle\_tissue\_development | NR2F2 | 128 | 3 | 1.740801 | -0.606721 | 657 | 329.09 | 0.500898 |
| GO:0060537\_muscle\_tissue\_development | PITX2 | 128 | 3 | 1.740801 | -0.606721 | 657 | 329.09 | 0.500898 |
| GO:0006281\_DNA\_repair | DCLRE1A | 71 | 2 | 2.092231 | -0.606004 | 659 | 330.03 | 0.500804 |
| GO:0006281\_DNA\_repair | FEN1 | 71 | 2 | 2.092231 | -0.606004 | 659 | 330.03 | 0.500804 |
| GO:0006913\_nucleocytoplasmic\_transport | SLC11A1 | 71 | 2 | 2.092231 | -0.606004 | 659 | 330.03 | 0.500804 |
| GO:0006913\_nucleocytoplasmic\_transport | TOB1 | 71 | 2 | 2.092231 | -0.606004 | 659 | 330.03 | 0.500804 |
| GO:0001755\_neural\_crest\_cell\_migration | KITL | 21 | 1 | 3.536866 | -0.605203 | 676 | 338.54 | 0.500799 |
| GO:0002263\_cell\_activation\_during\_immune\_response | SLC11A1 | 21 | 1 | 3.536866 | -0.605203 | 676 | 338.54 | 0.500799 |
| GO:0002366\_leukocyte\_activation\_during\_immune\_response | SLC11A1 | 21 | 1 | 3.536866 | -0.605203 | 676 | 338.54 | 0.500799 |
| GO:0002456\_T\_cell\_mediated\_immunity | SLC11A1 | 21 | 1 | 3.536866 | -0.605203 | 676 | 338.54 | 0.500799 |
| GO:0006814\_sodium\_ion\_transport | SLC4A4 | 21 | 1 | 3.536866 | -0.605203 | 676 | 338.54 | 0.500799 |
| GO:0007189\_activation\_of\_adenylate\_cyclase\_activity\_by\_G-protein\_signaling\_pathway | GNAQ | 21 | 1 | 3.536866 | -0.605203 | 676 | 338.54 | 0.500799 |
| GO:0008637\_apoptotic\_mitochondrial\_changes | BCL2 | 21 | 1 | 3.536866 | -0.605203 | 676 | 338.54 | 0.500799 |
| GO:0010552\_positive\_regulation\_of\_specific\_transcription\_from\_RNA\_polymerase\_II\_promoter | HMGB1 | 21 | 1 | 3.536866 | -0.605203 | 676 | 338.54 | 0.500799 |
| GO:0010578\_regulation\_of\_adenylate\_cyclase\_activity\_involved\_in\_G-protein\_signaling | GNAQ | 21 | 1 | 3.536866 | -0.605203 | 676 | 338.54 | 0.500799 |
| GO:0010579\_positive\_regulation\_of\_adenylate\_cyclase\_activity\_by\_G-protein\_signaling\_pathway | GNAQ | 21 | 1 | 3.536866 | -0.605203 | 676 | 338.54 | 0.500799 |
| GO:0016202\_regulation\_of\_striated\_muscle\_tissue\_development | BCL2 | 21 | 1 | 3.536866 | -0.605203 | 676 | 338.54 | 0.500799 |
| GO:0032147\_activation\_of\_protein\_kinase\_activity | SLC11A1 | 21 | 1 | 3.536866 | -0.605203 | 676 | 338.54 | 0.500799 |
| GO:0034330\_cell\_junction\_organization | BCL2 | 21 | 1 | 3.536866 | -0.605203 | 676 | 338.54 | 0.500799 |
| GO:0048538\_thymus\_development | BCL2 | 21 | 1 | 3.536866 | -0.605203 | 676 | 338.54 | 0.500799 |
| GO:0048634\_regulation\_of\_muscle\_development | BCL2 | 21 | 1 | 3.536866 | -0.605203 | 676 | 338.54 | 0.500799 |
| GO:0048675\_axon\_extension | RNF6 | 21 | 1 | 3.536866 | -0.605203 | 676 | 338.54 | 0.500799 |
| GO:0051271\_negative\_regulation\_of\_cell\_motion | BCL2 | 21 | 1 | 3.536866 | -0.605203 | 676 | 338.54 | 0.500799 |
| GO:0019226\_transmission\_of\_nerve\_impulse | NRCAM | 189 | 4 | 1.571941 | -0.603546 | 677 | 338.63 | 0.500192 |
| GO:0019226\_transmission\_of\_nerve\_impulse | BDNF | 189 | 4 | 1.571941 | -0.603546 | 677 | 338.63 | 0.500192 |
| GO:0019226\_transmission\_of\_nerve\_impulse | SNCA | 189 | 4 | 1.571941 | -0.603546 | 677 | 338.63 | 0.500192 |
| GO:0019226\_transmission\_of\_nerve\_impulse | CHRNB4 | 189 | 4 | 1.571941 | -0.603546 | 677 | 338.63 | 0.500192 |
| GO:0010926\_anatomical\_structure\_formation | SEMA5A | 447 | 8 | 1.329292 | -0.601599 | 678 | 338.79 | 0.499690 |
| GO:0010926\_anatomical\_structure\_formation | SPRY1 | 447 | 8 | 1.329292 | -0.601599 | 678 | 338.79 | 0.499690 |
| GO:0010926\_anatomical\_structure\_formation | BCL2 | 447 | 8 | 1.329292 | -0.601599 | 678 | 338.79 | 0.499690 |
| GO:0010926\_anatomical\_structure\_formation | VEGFA | 447 | 8 | 1.329292 | -0.601599 | 678 | 338.79 | 0.499690 |
| GO:0010926\_anatomical\_structure\_formation | ONECUT2 | 447 | 8 | 1.329292 | -0.601599 | 678 | 338.79 | 0.499690 |
| GO:0010926\_anatomical\_structure\_formation | NEFL | 447 | 8 | 1.329292 | -0.601599 | 678 | 338.79 | 0.499690 |
| GO:0010926\_anatomical\_structure\_formation | AHR | 447 | 8 | 1.329292 | -0.601599 | 678 | 338.79 | 0.499690 |
| GO:0010926\_anatomical\_structure\_formation | PITX2 | 447 | 8 | 1.329292 | -0.601599 | 678 | 338.79 | 0.499690 |
| GO:0007264\_small\_GTPase\_mediated\_signal\_transduction | DAB1 | 72 | 2 | 2.063172 | -0.597292 | 681 | 341.11 | 0.500896 |
| GO:0007264\_small\_GTPase\_mediated\_signal\_transduction | KITL | 72 | 2 | 2.063172 | -0.597292 | 681 | 341.11 | 0.500896 |
| GO:0030879\_mammary\_gland\_development | IRS2 | 72 | 2 | 2.063172 | -0.597292 | 681 | 341.11 | 0.500896 |
| GO:0030879\_mammary\_gland\_development | VEGFA | 72 | 2 | 2.063172 | -0.597292 | 681 | 341.11 | 0.500896 |
| GO:0051169\_nuclear\_transport | SLC11A1 | 72 | 2 | 2.063172 | -0.597292 | 681 | 341.11 | 0.500896 |
| GO:0051169\_nuclear\_transport | TOB1 | 72 | 2 | 2.063172 | -0.597292 | 681 | 341.11 | 0.500896 |
| GO:0016265\_death | TNFRSF21 | 450 | 8 | 1.320430 | -0.591615 | 682 | 342.25 | 0.501833 |
| GO:0016265\_death | BDNF | 450 | 8 | 1.320430 | -0.591615 | 682 | 342.25 | 0.501833 |
| GO:0016265\_death | BCL2 | 450 | 8 | 1.320430 | -0.591615 | 682 | 342.25 | 0.501833 |
| GO:0016265\_death | SNCA | 450 | 8 | 1.320430 | -0.591615 | 682 | 342.25 | 0.501833 |
| GO:0016265\_death | VEGFA | 450 | 8 | 1.320430 | -0.591615 | 682 | 342.25 | 0.501833 |
| GO:0016265\_death | NEFL | 450 | 8 | 1.320430 | -0.591615 | 682 | 342.25 | 0.501833 |
| GO:0016265\_death | KITL | 450 | 8 | 1.320430 | -0.591615 | 682 | 342.25 | 0.501833 |
| GO:0016265\_death | AHR | 450 | 8 | 1.320430 | -0.591615 | 682 | 342.25 | 0.501833 |
| GO:0000041\_transition\_metal\_ion\_transport | SLC11A1 | 22 | 1 | 3.376100 | -0.587766 | 695 | 353.67 | 0.508878 |
| GO:0001558\_regulation\_of\_cell\_growth | BCL2 | 22 | 1 | 3.376100 | -0.587766 | 695 | 353.67 | 0.508878 |
| GO:0006112\_energy\_reserve\_metabolic\_process | HMGB1 | 22 | 1 | 3.376100 | -0.587766 | 695 | 353.67 | 0.508878 |
| GO:0009896\_positive\_regulation\_of\_catabolic\_process | HMGB1 | 22 | 1 | 3.376100 | -0.587766 | 695 | 353.67 | 0.508878 |
| GO:0021575\_hindbrain\_morphogenesis | DAB1 | 22 | 1 | 3.376100 | -0.587766 | 695 | 353.67 | 0.508878 |
| GO:0021766\_hippocampus\_development | ID4 | 22 | 1 | 3.376100 | -0.587766 | 695 | 353.67 | 0.508878 |
| GO:0032649\_regulation\_of\_interferon-gamma\_production | SLC11A1 | 22 | 1 | 3.376100 | -0.587766 | 695 | 353.67 | 0.508878 |
| GO:0040018\_positive\_regulation\_of\_multicellular\_organism\_growth | BCL2 | 22 | 1 | 3.376100 | -0.587766 | 695 | 353.67 | 0.508878 |
| GO:0042733\_embryonic\_digit\_morphogenesis | GNAQ | 22 | 1 | 3.376100 | -0.587766 | 695 | 353.67 | 0.508878 |
| GO:0044264\_cellular\_polysaccharide\_metabolic\_process | HMGB1 | 22 | 1 | 3.376100 | -0.587766 | 695 | 353.67 | 0.508878 |
| GO:0045787\_positive\_regulation\_of\_cell\_cycle | HMGB1 | 22 | 1 | 3.376100 | -0.587766 | 695 | 353.67 | 0.508878 |
| GO:0046883\_regulation\_of\_hormone\_secretion | PFKL | 22 | 1 | 3.376100 | -0.587766 | 695 | 353.67 | 0.508878 |
| GO:0048489\_synaptic\_vesicle\_transport | SNCA | 22 | 1 | 3.376100 | -0.587766 | 695 | 353.67 | 0.508878 |
| GO:0048771\_tissue\_remodeling | BCL2 | 74 | 2 | 2.007411 | -0.580360 | 696 | 354.56 | 0.509425 |
| GO:0048771\_tissue\_remodeling | TOB1 | 74 | 2 | 2.007411 | -0.580360 | 696 | 354.56 | 0.509425 |
| GO:0046907\_intracellular\_transport | SLC11A1 | 194 | 4 | 1.531427 | -0.578283 | 697 | 354.88 | 0.509154 |
| GO:0046907\_intracellular\_transport | BCL2 | 194 | 4 | 1.531427 | -0.578283 | 697 | 354.88 | 0.509154 |
| GO:0046907\_intracellular\_transport | EHD1 | 194 | 4 | 1.531427 | -0.578283 | 697 | 354.88 | 0.509154 |
| GO:0046907\_intracellular\_transport | TOB1 | 194 | 4 | 1.531427 | -0.578283 | 697 | 354.88 | 0.509154 |
| GO:0060255\_regulation\_of\_macromolecule\_metabolic\_process | HMGB1 | 936 | 15 | 1.190292 | -0.573673 | 698 | 355.84 | 0.509799 |
| GO:0060255\_regulation\_of\_macromolecule\_metabolic\_process | ONECUT2 | 936 | 15 | 1.190292 | -0.573673 | 698 | 355.84 | 0.509799 |
| GO:0060255\_regulation\_of\_macromolecule\_metabolic\_process | RORB | 936 | 15 | 1.190292 | -0.573673 | 698 | 355.84 | 0.509799 |
| GO:0060255\_regulation\_of\_macromolecule\_metabolic\_process | KITL | 936 | 15 | 1.190292 | -0.573673 | 698 | 355.84 | 0.509799 |
| GO:0060255\_regulation\_of\_macromolecule\_metabolic\_process | AHR | 936 | 15 | 1.190292 | -0.573673 | 698 | 355.84 | 0.509799 |
| GO:0060255\_regulation\_of\_macromolecule\_metabolic\_process | SUZ12 | 936 | 15 | 1.190292 | -0.573673 | 698 | 355.84 | 0.509799 |
| GO:0060255\_regulation\_of\_macromolecule\_metabolic\_process | SLC11A1 | 936 | 15 | 1.190292 | -0.573673 | 698 | 355.84 | 0.509799 |
| GO:0060255\_regulation\_of\_macromolecule\_metabolic\_process | RNF6 | 936 | 15 | 1.190292 | -0.573673 | 698 | 355.84 | 0.509799 |
| GO:0060255\_regulation\_of\_macromolecule\_metabolic\_process | BCL2 | 936 | 15 | 1.190292 | -0.573673 | 698 | 355.84 | 0.509799 |
| GO:0060255\_regulation\_of\_macromolecule\_metabolic\_process | GARNL1 | 936 | 15 | 1.190292 | -0.573673 | 698 | 355.84 | 0.509799 |
| GO:0060255\_regulation\_of\_macromolecule\_metabolic\_process | NFAT5 | 936 | 15 | 1.190292 | -0.573673 | 698 | 355.84 | 0.509799 |
| GO:0060255\_regulation\_of\_macromolecule\_metabolic\_process | NR2F2 | 936 | 15 | 1.190292 | -0.573673 | 698 | 355.84 | 0.509799 |
| GO:0060255\_regulation\_of\_macromolecule\_metabolic\_process | MYB | 936 | 15 | 1.190292 | -0.573673 | 698 | 355.84 | 0.509799 |
| GO:0060255\_regulation\_of\_macromolecule\_metabolic\_process | PITX2 | 936 | 15 | 1.190292 | -0.573673 | 698 | 355.84 | 0.509799 |
| GO:0060255\_regulation\_of\_macromolecule\_metabolic\_process | ETV3 | 936 | 15 | 1.190292 | -0.573673 | 698 | 355.84 | 0.509799 |
| GO:0007507\_heart\_development | GNAQ | 195 | 4 | 1.523573 | -0.573366 | 699 | 356.26 | 0.509671 |
| GO:0007507\_heart\_development | PKP2 | 195 | 4 | 1.523573 | -0.573366 | 699 | 356.26 | 0.509671 |
| GO:0007507\_heart\_development | MAP2K5 | 195 | 4 | 1.523573 | -0.573366 | 699 | 356.26 | 0.509671 |
| GO:0007507\_heart\_development | PITX2 | 195 | 4 | 1.523573 | -0.573366 | 699 | 356.26 | 0.509671 |
| GO:0044265\_cellular\_macromolecule\_catabolic\_process | HMGB1 | 75 | 2 | 1.980645 | -0.572132 | 701 | 356.97 | 0.509230 |
| GO:0044265\_cellular\_macromolecule\_catabolic\_process | RNF6 | 75 | 2 | 1.980645 | -0.572132 | 701 | 356.97 | 0.509230 |
| GO:0051050\_positive\_regulation\_of\_transport | SLC11A1 | 75 | 2 | 1.980645 | -0.572132 | 701 | 356.97 | 0.509230 |
| GO:0051050\_positive\_regulation\_of\_transport | SNCA | 75 | 2 | 1.980645 | -0.572132 | 701 | 356.97 | 0.509230 |
| GO:0002821\_positive\_regulation\_of\_adaptive\_immune\_response | SLC11A1 | 23 | 1 | 3.229313 | -0.571220 | 705 | 362.71 | 0.514482 |
| GO:0002824\_positive\_regulation\_of\_adaptive\_immune\_response\_based\_on\_somatic\_recombination\_of\_immune\_receptors\_built\_from\_immunoglobulin\_superfamily\_domains | SLC11A1 | 23 | 1 | 3.229313 | -0.571220 | 705 | 362.71 | 0.514482 |
| GO:0007163\_establishment\_or\_maintenance\_of\_cell\_polarity | PARD3 | 23 | 1 | 3.229313 | -0.571220 | 705 | 362.71 | 0.514482 |
| GO:0060349\_bone\_morphogenesis | COL13A1 | 23 | 1 | 3.229313 | -0.571220 | 705 | 362.71 | 0.514482 |
| GO:0051241\_negative\_regulation\_of\_multicellular\_organismal\_process | SLC11A1 | 77 | 2 | 1.929200 | -0.556127 | 706 | 366.59 | 0.519249 |
| GO:0051241\_negative\_regulation\_of\_multicellular\_organismal\_process | BCL2 | 77 | 2 | 1.929200 | -0.556127 | 706 | 366.59 | 0.519249 |
| GO:0006650\_glycerophospholipid\_metabolic\_process | FABP3 | 24 | 1 | 3.094758 | -0.555490 | 712 | 373.4 | 0.524438 |
| GO:0007204\_elevation\_of\_cytosolic\_calcium\_ion\_concentration | BCL2 | 24 | 1 | 3.094758 | -0.555490 | 712 | 373.4 | 0.524438 |
| GO:0048002\_antigen\_processing\_and\_presentation\_of\_peptide\_antigen | SLC11A1 | 24 | 1 | 3.094758 | -0.555490 | 712 | 373.4 | 0.524438 |
| GO:0048546\_digestive\_tract\_morphogenesis | BCL2 | 24 | 1 | 3.094758 | -0.555490 | 712 | 373.4 | 0.524438 |
| GO:0050679\_positive\_regulation\_of\_epithelial\_cell\_proliferation | VEGFA | 24 | 1 | 3.094758 | -0.555490 | 712 | 373.4 | 0.524438 |
| GO:0060078\_regulation\_of\_postsynaptic\_membrane\_potential | SNCA | 24 | 1 | 3.094758 | -0.555490 | 712 | 373.4 | 0.524438 |
| GO:0044255\_cellular\_lipid\_metabolic\_process | SCD1 | 264 | 5 | 1.406708 | -0.550376 | 713 | 374.16 | 0.524769 |
| GO:0044255\_cellular\_lipid\_metabolic\_process | ACOT10 | 264 | 5 | 1.406708 | -0.550376 | 713 | 374.16 | 0.524769 |
| GO:0044255\_cellular\_lipid\_metabolic\_process | SNCA | 264 | 5 | 1.406708 | -0.550376 | 713 | 374.16 | 0.524769 |
| GO:0044255\_cellular\_lipid\_metabolic\_process | FABP3 | 264 | 5 | 1.406708 | -0.550376 | 713 | 374.16 | 0.524769 |
| GO:0044255\_cellular\_lipid\_metabolic\_process | ACSL4 | 264 | 5 | 1.406708 | -0.550376 | 713 | 374.16 | 0.524769 |
| GO:0010646\_regulation\_of\_cell\_communication | BDNF | 330 | 6 | 1.350440 | -0.548293 | 714 | 376.54 | 0.527367 |
| GO:0010646\_regulation\_of\_cell\_communication | PFKL | 330 | 6 | 1.350440 | -0.548293 | 714 | 376.54 | 0.527367 |
| GO:0010646\_regulation\_of\_cell\_communication | SNCA | 330 | 6 | 1.350440 | -0.548293 | 714 | 376.54 | 0.527367 |
| GO:0010646\_regulation\_of\_cell\_communication | ONECUT2 | 330 | 6 | 1.350440 | -0.548293 | 714 | 376.54 | 0.527367 |
| GO:0010646\_regulation\_of\_cell\_communication | KITL | 330 | 6 | 1.350440 | -0.548293 | 714 | 376.54 | 0.527367 |
| GO:0010646\_regulation\_of\_cell\_communication | TOB1 | 330 | 6 | 1.350440 | -0.548293 | 714 | 376.54 | 0.527367 |
| GO:0051046\_regulation\_of\_secretion | PFKL | 79 | 2 | 1.880359 | -0.540700 | 715 | 377.67 | 0.528210 |
| GO:0051046\_regulation\_of\_secretion | SNCA | 79 | 2 | 1.880359 | -0.540700 | 715 | 377.67 | 0.528210 |
| GO:0021983\_pituitary\_gland\_development | PITX2 | 25 | 1 | 2.970968 | -0.540509 | 717 | 383.44 | 0.534784 |
| GO:0032103\_positive\_regulation\_of\_response\_to\_external\_stimulus | HMGB1 | 25 | 1 | 2.970968 | -0.540509 | 717 | 383.44 | 0.534784 |
| GO:0022607\_cellular\_component\_assembly | BCL2 | 204 | 4 | 1.456357 | -0.531042 | 718 | 385.26 | 0.536574 |
| GO:0022607\_cellular\_component\_assembly | ONECUT2 | 204 | 4 | 1.456357 | -0.531042 | 718 | 385.26 | 0.536574 |
| GO:0022607\_cellular\_component\_assembly | NEFL | 204 | 4 | 1.456357 | -0.531042 | 718 | 385.26 | 0.536574 |
| GO:0022607\_cellular\_component\_assembly | AHR | 204 | 4 | 1.456357 | -0.531042 | 718 | 385.26 | 0.536574 |
| GO:0003006\_reproductive\_developmental\_process | BCL2 | 141 | 3 | 1.580302 | -0.529977 | 719 | 385.67 | 0.536398 |
| GO:0003006\_reproductive\_developmental\_process | VEGFA | 141 | 3 | 1.580302 | -0.529977 | 719 | 385.67 | 0.536398 |
| GO:0003006\_reproductive\_developmental\_process | AHR | 141 | 3 | 1.580302 | -0.529977 | 719 | 385.67 | 0.536398 |
| GO:0001658\_branching\_involved\_in\_ureteric\_bud\_morphogenesis | BCL2 | 26 | 1 | 2.856700 | -0.526217 | 731 | 392.75 | 0.537278 |
| GO:0006800\_oxygen\_and\_reactive\_oxygen\_species\_metabolic\_process | BCL2 | 26 | 1 | 2.856700 | -0.526217 | 731 | 392.75 | 0.537278 |
| GO:0007623\_circadian\_rhythm | CSNK1E | 26 | 1 | 2.856700 | -0.526217 | 731 | 392.75 | 0.537278 |
| GO:0009636\_response\_to\_toxin | BCL2 | 26 | 1 | 2.856700 | -0.526217 | 731 | 392.75 | 0.537278 |
| GO:0010212\_response\_to\_ionizing\_radiation | BCL2 | 26 | 1 | 2.856700 | -0.526217 | 731 | 392.75 | 0.537278 |
| GO:0010959\_regulation\_of\_metal\_ion\_transport | BCL2 | 26 | 1 | 2.856700 | -0.526217 | 731 | 392.75 | 0.537278 |
| GO:0032609\_interferon-gamma\_production | SLC11A1 | 26 | 1 | 2.856700 | -0.526217 | 731 | 392.75 | 0.537278 |
| GO:0045665\_negative\_regulation\_of\_neuron\_differentiation | ID4 | 26 | 1 | 2.856700 | -0.526217 | 731 | 392.75 | 0.537278 |
| GO:0048645\_organ\_formation | SPRY1 | 26 | 1 | 2.856700 | -0.526217 | 731 | 392.75 | 0.537278 |
| GO:0050873\_brown\_fat\_cell\_differentiation | SCD1 | 26 | 1 | 2.856700 | -0.526217 | 731 | 392.75 | 0.537278 |
| GO:0051480\_cytosolic\_calcium\_ion\_homeostasis | BCL2 | 26 | 1 | 2.856700 | -0.526217 | 731 | 392.75 | 0.537278 |
| GO:0060675\_ureteric\_bud\_morphogenesis | BCL2 | 26 | 1 | 2.856700 | -0.526217 | 731 | 392.75 | 0.537278 |
| GO:0002761\_regulation\_of\_myeloid\_leukocyte\_differentiation | KITL | 27 | 1 | 2.750896 | -0.512562 | 740 | 400.8 | 0.541622 |
| GO:0006479\_protein\_amino\_acid\_methylation | SUZ12 | 27 | 1 | 2.750896 | -0.512562 | 740 | 400.8 | 0.541622 |
| GO:0007422\_peripheral\_nervous\_system\_development | ONECUT2 | 27 | 1 | 2.750896 | -0.512562 | 740 | 400.8 | 0.541622 |
| GO:0008213\_protein\_amino\_acid\_alkylation | SUZ12 | 27 | 1 | 2.750896 | -0.512562 | 740 | 400.8 | 0.541622 |
| GO:0008286\_insulin\_receptor\_signaling\_pathway | IRS2 | 27 | 1 | 2.750896 | -0.512562 | 740 | 400.8 | 0.541622 |
| GO:0009411\_response\_to\_UV | BCL2 | 27 | 1 | 2.750896 | -0.512562 | 740 | 400.8 | 0.541622 |
| GO:0030100\_regulation\_of\_endocytosis | SLC11A1 | 27 | 1 | 2.750896 | -0.512562 | 740 | 400.8 | 0.541622 |
| GO:0031016\_pancreas\_development | ONECUT2 | 27 | 1 | 2.750896 | -0.512562 | 740 | 400.8 | 0.541622 |
| GO:0032496\_response\_to\_lipopolysaccharide | SLC11A1 | 27 | 1 | 2.750896 | -0.512562 | 740 | 400.8 | 0.541622 |
| GO:0006575\_cellular\_amino\_acid\_derivative\_metabolic\_process | SNCA | 83 | 2 | 1.789740 | -0.511457 | 741 | 402.19 | 0.542767 |
| GO:0006575\_cellular\_amino\_acid\_derivative\_metabolic\_process | FABP3 | 83 | 2 | 1.789740 | -0.511457 | 741 | 402.19 | 0.542767 |
| GO:0050896\_response\_to\_stimulus | HMGB1 | 1107 | 17 | 1.140615 | -0.508982 | 742 | 402.67 | 0.542682 |
| GO:0050896\_response\_to\_stimulus | IRS2 | 1107 | 17 | 1.140615 | -0.508982 | 742 | 402.67 | 0.542682 |
| GO:0050896\_response\_to\_stimulus | PFKL | 1107 | 17 | 1.140615 | -0.508982 | 742 | 402.67 | 0.542682 |
| GO:0050896\_response\_to\_stimulus | SNCA | 1107 | 17 | 1.140615 | -0.508982 | 742 | 402.67 | 0.542682 |
| GO:0050896\_response\_to\_stimulus | AHR | 1107 | 17 | 1.140615 | -0.508982 | 742 | 402.67 | 0.542682 |
| GO:0050896\_response\_to\_stimulus | SLC11A1 | 1107 | 17 | 1.140615 | -0.508982 | 742 | 402.67 | 0.542682 |
| GO:0050896\_response\_to\_stimulus | DCLRE1A | 1107 | 17 | 1.140615 | -0.508982 | 742 | 402.67 | 0.542682 |
| GO:0050896\_response\_to\_stimulus | PAXIP1 | 1107 | 17 | 1.140615 | -0.508982 | 742 | 402.67 | 0.542682 |
| GO:0050896\_response\_to\_stimulus | BDNF | 1107 | 17 | 1.140615 | -0.508982 | 742 | 402.67 | 0.542682 |
| GO:0050896\_response\_to\_stimulus | GNAQ | 1107 | 17 | 1.140615 | -0.508982 | 742 | 402.67 | 0.542682 |
| GO:0050896\_response\_to\_stimulus | BCL2 | 1107 | 17 | 1.140615 | -0.508982 | 742 | 402.67 | 0.542682 |
| GO:0050896\_response\_to\_stimulus | NTRK2 | 1107 | 17 | 1.140615 | -0.508982 | 742 | 402.67 | 0.542682 |
| GO:0050896\_response\_to\_stimulus | CHRNB4 | 1107 | 17 | 1.140615 | -0.508982 | 742 | 402.67 | 0.542682 |
| GO:0050896\_response\_to\_stimulus | ABAT | 1107 | 17 | 1.140615 | -0.508982 | 742 | 402.67 | 0.542682 |
| GO:0050896\_response\_to\_stimulus | FABP7 | 1107 | 17 | 1.140615 | -0.508982 | 742 | 402.67 | 0.542682 |
| GO:0050896\_response\_to\_stimulus | NEFL | 1107 | 17 | 1.140615 | -0.508982 | 742 | 402.67 | 0.542682 |
| GO:0050896\_response\_to\_stimulus | FEN1 | 1107 | 17 | 1.140615 | -0.508982 | 742 | 402.67 | 0.542682 |
| GO:0030005\_cellular\_di-\_\_tri-valent\_inorganic\_cation\_homeostasis | SLC11A1 | 84 | 2 | 1.768433 | -0.504463 | 744 | 403.58 | 0.542446 |
| GO:0030005\_cellular\_di-\_\_tri-valent\_inorganic\_cation\_homeostasis | BCL2 | 84 | 2 | 1.768433 | -0.504463 | 744 | 403.58 | 0.542446 |
| GO:0045137\_development\_of\_primary\_sexual\_characteristics | BCL2 | 84 | 2 | 1.768433 | -0.504463 | 744 | 403.58 | 0.542446 |
| GO:0045137\_development\_of\_primary\_sexual\_characteristics | VEGFA | 84 | 2 | 1.768433 | -0.504463 | 744 | 403.58 | 0.542446 |
| GO:0021549\_cerebellum\_development | DAB1 | 28 | 1 | 2.652650 | -0.499496 | 748 | 409.77 | 0.547821 |
| GO:0030073\_insulin\_secretion | PFKL | 28 | 1 | 2.652650 | -0.499496 | 748 | 409.77 | 0.547821 |
| GO:0045926\_negative\_regulation\_of\_growth | BCL2 | 28 | 1 | 2.652650 | -0.499496 | 748 | 409.77 | 0.547821 |
| GO:0051188\_cofactor\_biosynthetic\_process | SNCA | 28 | 1 | 2.652650 | -0.499496 | 748 | 409.77 | 0.547821 |
| GO:0006950\_response\_to\_stress | SLC11A1 | 549 | 9 | 1.217610 | -0.499139 | 749 | 409.88 | 0.547236 |
| GO:0006950\_response\_to\_stress | BDNF | 549 | 9 | 1.217610 | -0.499139 | 749 | 409.88 | 0.547236 |
| GO:0006950\_response\_to\_stress | PAXIP1 | 549 | 9 | 1.217610 | -0.499139 | 749 | 409.88 | 0.547236 |
| GO:0006950\_response\_to\_stress | DCLRE1A | 549 | 9 | 1.217610 | -0.499139 | 749 | 409.88 | 0.547236 |
| GO:0006950\_response\_to\_stress | BCL2 | 549 | 9 | 1.217610 | -0.499139 | 749 | 409.88 | 0.547236 |
| GO:0006950\_response\_to\_stress | SNCA | 549 | 9 | 1.217610 | -0.499139 | 749 | 409.88 | 0.547236 |
| GO:0006950\_response\_to\_stress | NEFL | 549 | 9 | 1.217610 | -0.499139 | 749 | 409.88 | 0.547236 |
| GO:0006950\_response\_to\_stress | AHR | 549 | 9 | 1.217610 | -0.499139 | 749 | 409.88 | 0.547236 |
| GO:0006950\_response\_to\_stress | FEN1 | 549 | 9 | 1.217610 | -0.499139 | 749 | 409.88 | 0.547236 |
| GO:0070887\_cellular\_response\_to\_chemical\_stimulus | IRS2 | 85 | 2 | 1.747628 | -0.497590 | 750 | 410.8 | 0.547733 |
| GO:0070887\_cellular\_response\_to\_chemical\_stimulus | SNCA | 85 | 2 | 1.747628 | -0.497590 | 750 | 410.8 | 0.547733 |
| GO:0006605\_protein\_targeting | SLC11A1 | 86 | 2 | 1.727307 | -0.490833 | 754 | 413.48 | 0.548382 |
| GO:0006605\_protein\_targeting | TOB1 | 86 | 2 | 1.727307 | -0.490833 | 754 | 413.48 | 0.548382 |
| GO:0032504\_multicellular\_organism\_reproduction | BCL2 | 86 | 2 | 1.727307 | -0.490833 | 754 | 413.48 | 0.548382 |
| GO:0032504\_multicellular\_organism\_reproduction | VEGFA | 86 | 2 | 1.727307 | -0.490833 | 754 | 413.48 | 0.548382 |
| GO:0034641\_cellular\_nitrogen\_compound\_metabolic\_process | ALDH18A1 | 86 | 2 | 1.727307 | -0.490833 | 754 | 413.48 | 0.548382 |
| GO:0034641\_cellular\_nitrogen\_compound\_metabolic\_process | SNCA | 86 | 2 | 1.727307 | -0.490833 | 754 | 413.48 | 0.548382 |
| GO:0048609\_reproductive\_process\_in\_a\_multicellular\_organism | BCL2 | 86 | 2 | 1.727307 | -0.490833 | 754 | 413.48 | 0.548382 |
| GO:0048609\_reproductive\_process\_in\_a\_multicellular\_organism | VEGFA | 86 | 2 | 1.727307 | -0.490833 | 754 | 413.48 | 0.548382 |
| GO:0006909\_phagocytosis | SLC11A1 | 29 | 1 | 2.561179 | -0.486980 | 759 | 420.12 | 0.553518 |
| GO:0007190\_activation\_of\_adenylate\_cyclase\_activity | GNAQ | 29 | 1 | 2.561179 | -0.486980 | 759 | 420.12 | 0.553518 |
| GO:0021761\_limbic\_system\_development | ID4 | 29 | 1 | 2.561179 | -0.486980 | 759 | 420.12 | 0.553518 |
| GO:0044087\_regulation\_of\_cellular\_component\_biogenesis | AHR | 29 | 1 | 2.561179 | -0.486980 | 759 | 420.12 | 0.553518 |
| GO:0050769\_positive\_regulation\_of\_neurogenesis | NEFL | 29 | 1 | 2.561179 | -0.486980 | 759 | 420.12 | 0.553518 |
| GO:0043583\_ear\_development | BDNF | 87 | 2 | 1.707453 | -0.484191 | 760 | 422.0 | 0.555263 |
| GO:0043583\_ear\_development | BCL2 | 87 | 2 | 1.707453 | -0.484191 | 760 | 422.0 | 0.555263 |
| GO:0002260\_lymphocyte\_homeostasis | BCL2 | 30 | 1 | 2.475806 | -0.474973 | 768 | 429.76 | 0.559583 |
| GO:0014032\_neural\_crest\_cell\_development | KITL | 30 | 1 | 2.475806 | -0.474973 | 768 | 429.76 | 0.559583 |
| GO:0014033\_neural\_crest\_cell\_differentiation | KITL | 30 | 1 | 2.475806 | -0.474973 | 768 | 429.76 | 0.559583 |
| GO:0031281\_positive\_regulation\_of\_cyclase\_activity | GNAQ | 30 | 1 | 2.475806 | -0.474973 | 768 | 429.76 | 0.559583 |
| GO:0032102\_negative\_regulation\_of\_response\_to\_external\_stimulus | FABP7 | 30 | 1 | 2.475806 | -0.474973 | 768 | 429.76 | 0.559583 |
| GO:0035265\_organ\_growth | BCL2 | 30 | 1 | 2.475806 | -0.474973 | 768 | 429.76 | 0.559583 |
| GO:0045762\_positive\_regulation\_of\_adenylate\_cyclase\_activity | GNAQ | 30 | 1 | 2.475806 | -0.474973 | 768 | 429.76 | 0.559583 |
| GO:0051349\_positive\_regulation\_of\_lyase\_activity | GNAQ | 30 | 1 | 2.475806 | -0.474973 | 768 | 429.76 | 0.559583 |
| GO:0032268\_regulation\_of\_cellular\_protein\_metabolic\_process | HMGB1 | 152 | 3 | 1.465938 | -0.473520 | 769 | 430.19 | 0.559415 |
| GO:0032268\_regulation\_of\_cellular\_protein\_metabolic\_process | BCL2 | 152 | 3 | 1.465938 | -0.473520 | 769 | 430.19 | 0.559415 |
| GO:0032268\_regulation\_of\_cellular\_protein\_metabolic\_process | KITL | 152 | 3 | 1.465938 | -0.473520 | 769 | 430.19 | 0.559415 |
| GO:0006629\_lipid\_metabolic\_process | SCD1 | 285 | 5 | 1.303056 | -0.471119 | 770 | 430.62 | 0.559247 |
| GO:0006629\_lipid\_metabolic\_process | ACOT10 | 285 | 5 | 1.303056 | -0.471119 | 770 | 430.62 | 0.559247 |
| GO:0006629\_lipid\_metabolic\_process | SNCA | 285 | 5 | 1.303056 | -0.471119 | 770 | 430.62 | 0.559247 |
| GO:0006629\_lipid\_metabolic\_process | FABP3 | 285 | 5 | 1.303056 | -0.471119 | 770 | 430.62 | 0.559247 |
| GO:0006629\_lipid\_metabolic\_process | ACSL4 | 285 | 5 | 1.303056 | -0.471119 | 770 | 430.62 | 0.559247 |
| GO:0007517\_muscle\_organ\_development | BCL2 | 153 | 3 | 1.456357 | -0.468727 | 771 | 430.91 | 0.558898 |
| GO:0007517\_muscle\_organ\_development | NR2F2 | 153 | 3 | 1.456357 | -0.468727 | 771 | 430.91 | 0.558898 |
| GO:0007517\_muscle\_organ\_development | PITX2 | 153 | 3 | 1.456357 | -0.468727 | 771 | 430.91 | 0.558898 |
| GO:0030003\_cellular\_cation\_homeostasis | SLC11A1 | 90 | 2 | 1.650538 | -0.464926 | 772 | 432.39 | 0.560091 |
| GO:0030003\_cellular\_cation\_homeostasis | BCL2 | 90 | 2 | 1.650538 | -0.464926 | 772 | 432.39 | 0.560091 |
| GO:0007268\_synaptic\_transmission | BDNF | 154 | 3 | 1.446900 | -0.463988 | 773 | 432.87 | 0.559987 |
| GO:0007268\_synaptic\_transmission | SNCA | 154 | 3 | 1.446900 | -0.463988 | 773 | 432.87 | 0.559987 |
| GO:0007268\_synaptic\_transmission | CHRNB4 | 154 | 3 | 1.446900 | -0.463988 | 773 | 432.87 | 0.559987 |
| GO:0050793\_regulation\_of\_developmental\_process | HMGB1 | 703 | 11 | 1.162185 | -0.463782 | 774 | 432.97 | 0.559393 |
| GO:0050793\_regulation\_of\_developmental\_process | RNF6 | 703 | 11 | 1.162185 | -0.463782 | 774 | 432.97 | 0.559393 |
| GO:0050793\_regulation\_of\_developmental\_process | BDNF | 703 | 11 | 1.162185 | -0.463782 | 774 | 432.97 | 0.559393 |
| GO:0050793\_regulation\_of\_developmental\_process | GNAQ | 703 | 11 | 1.162185 | -0.463782 | 774 | 432.97 | 0.559393 |
| GO:0050793\_regulation\_of\_developmental\_process | BCL2 | 703 | 11 | 1.162185 | -0.463782 | 774 | 432.97 | 0.559393 |
| GO:0050793\_regulation\_of\_developmental\_process | SNCA | 703 | 11 | 1.162185 | -0.463782 | 774 | 432.97 | 0.559393 |
| GO:0050793\_regulation\_of\_developmental\_process | VEGFA | 703 | 11 | 1.162185 | -0.463782 | 774 | 432.97 | 0.559393 |
| GO:0050793\_regulation\_of\_developmental\_process | ID4 | 703 | 11 | 1.162185 | -0.463782 | 774 | 432.97 | 0.559393 |
| GO:0050793\_regulation\_of\_developmental\_process | NEFL | 703 | 11 | 1.162185 | -0.463782 | 774 | 432.97 | 0.559393 |
| GO:0050793\_regulation\_of\_developmental\_process | KITL | 703 | 11 | 1.162185 | -0.463782 | 774 | 432.97 | 0.559393 |
| GO:0050793\_regulation\_of\_developmental\_process | TOB1 | 703 | 11 | 1.162185 | -0.463782 | 774 | 432.97 | 0.559393 |
| GO:0006939\_smooth\_muscle\_contraction | CHRNB4 | 31 | 1 | 2.395942 | -0.463444 | 782 | 442.44 | 0.565780 |
| GO:0008645\_hexose\_transport | STXBP3A | 31 | 1 | 2.395942 | -0.463444 | 782 | 442.44 | 0.565780 |
| GO:0015749\_monosaccharide\_transport | STXBP3A | 31 | 1 | 2.395942 | -0.463444 | 782 | 442.44 | 0.565780 |
| GO:0015758\_glucose\_transport | STXBP3A | 31 | 1 | 2.395942 | -0.463444 | 782 | 442.44 | 0.565780 |
| GO:0016049\_cell\_growth | BCL2 | 31 | 1 | 2.395942 | -0.463444 | 782 | 442.44 | 0.565780 |
| GO:0043269\_regulation\_of\_ion\_transport | BCL2 | 31 | 1 | 2.395942 | -0.463444 | 782 | 442.44 | 0.565780 |
| GO:0046632\_alpha-beta\_T\_cell\_differentiation | BCL2 | 31 | 1 | 2.395942 | -0.463444 | 782 | 442.44 | 0.565780 |
| GO:0051899\_membrane\_depolarization | SNCA | 31 | 1 | 2.395942 | -0.463444 | 782 | 442.44 | 0.565780 |
| GO:0006638\_neutral\_lipid\_metabolic\_process | SNCA | 32 | 1 | 2.321069 | -0.452360 | 785 | 449.49 | 0.572599 |
| GO:0006937\_regulation\_of\_muscle\_contraction | CHRNB4 | 32 | 1 | 2.321069 | -0.452360 | 785 | 449.49 | 0.572599 |
| GO:0050885\_neuromuscular\_process\_controlling\_balance | NEFL | 32 | 1 | 2.321069 | -0.452360 | 785 | 449.49 | 0.572599 |
| GO:0042981\_regulation\_of\_apoptosis | BDNF | 360 | 6 | 1.237903 | -0.448921 | 786 | 449.75 | 0.572201 |
| GO:0042981\_regulation\_of\_apoptosis | BCL2 | 360 | 6 | 1.237903 | -0.448921 | 786 | 449.75 | 0.572201 |
| GO:0042981\_regulation\_of\_apoptosis | SNCA | 360 | 6 | 1.237903 | -0.448921 | 786 | 449.75 | 0.572201 |
| GO:0042981\_regulation\_of\_apoptosis | VEGFA | 360 | 6 | 1.237903 | -0.448921 | 786 | 449.75 | 0.572201 |
| GO:0042981\_regulation\_of\_apoptosis | NEFL | 360 | 6 | 1.237903 | -0.448921 | 786 | 449.75 | 0.572201 |
| GO:0042981\_regulation\_of\_apoptosis | KITL | 360 | 6 | 1.237903 | -0.448921 | 786 | 449.75 | 0.572201 |
| GO:0055066\_di-\_\_tri-valent\_inorganic\_cation\_homeostasis | SLC11A1 | 93 | 2 | 1.597294 | -0.446598 | 787 | 450.87 | 0.572897 |
| GO:0055066\_di-\_\_tri-valent\_inorganic\_cation\_homeostasis | BCL2 | 93 | 2 | 1.597294 | -0.446598 | 787 | 450.87 | 0.572897 |
| GO:0006066\_alcohol\_metabolic\_process | PFKL | 158 | 3 | 1.410269 | -0.445547 | 788 | 451.71 | 0.573236 |
| GO:0006066\_alcohol\_metabolic\_process | SNCA | 158 | 3 | 1.410269 | -0.445547 | 788 | 451.71 | 0.573236 |
| GO:0006066\_alcohol\_metabolic\_process | FABP3 | 158 | 3 | 1.410269 | -0.445547 | 788 | 451.71 | 0.573236 |
| GO:0007188\_G-protein\_signaling\_\_coupled\_to\_cAMP\_nucleotide\_second\_messenger | GNAQ | 33 | 1 | 2.250733 | -0.441695 | 794 | 456.17 | 0.574521 |
| GO:0007270\_nerve-nerve\_synaptic\_transmission | SNCA | 33 | 1 | 2.250733 | -0.441695 | 794 | 456.17 | 0.574521 |
| GO:0008584\_male\_gonad\_development | BCL2 | 33 | 1 | 2.250733 | -0.441695 | 794 | 456.17 | 0.574521 |
| GO:0008643\_carbohydrate\_transport | STXBP3A | 33 | 1 | 2.250733 | -0.441695 | 794 | 456.17 | 0.574521 |
| GO:0021536\_diencephalon\_development | PITX2 | 33 | 1 | 2.250733 | -0.441695 | 794 | 456.17 | 0.574521 |
| GO:0021987\_cerebral\_cortex\_development | DAB1 | 33 | 1 | 2.250733 | -0.441695 | 794 | 456.17 | 0.574521 |
| GO:0010941\_regulation\_of\_cell\_death | BDNF | 365 | 6 | 1.220946 | -0.434094 | 796 | 459.51 | 0.577274 |
| GO:0010941\_regulation\_of\_cell\_death | BCL2 | 365 | 6 | 1.220946 | -0.434094 | 796 | 459.51 | 0.577274 |
| GO:0010941\_regulation\_of\_cell\_death | SNCA | 365 | 6 | 1.220946 | -0.434094 | 796 | 459.51 | 0.577274 |
| GO:0010941\_regulation\_of\_cell\_death | VEGFA | 365 | 6 | 1.220946 | -0.434094 | 796 | 459.51 | 0.577274 |
| GO:0010941\_regulation\_of\_cell\_death | NEFL | 365 | 6 | 1.220946 | -0.434094 | 796 | 459.51 | 0.577274 |
| GO:0010941\_regulation\_of\_cell\_death | KITL | 365 | 6 | 1.220946 | -0.434094 | 796 | 459.51 | 0.577274 |
| GO:0043067\_regulation\_of\_programmed\_cell\_death | BDNF | 365 | 6 | 1.220946 | -0.434094 | 796 | 459.51 | 0.577274 |
| GO:0043067\_regulation\_of\_programmed\_cell\_death | BCL2 | 365 | 6 | 1.220946 | -0.434094 | 796 | 459.51 | 0.577274 |
| GO:0043067\_regulation\_of\_programmed\_cell\_death | SNCA | 365 | 6 | 1.220946 | -0.434094 | 796 | 459.51 | 0.577274 |
| GO:0043067\_regulation\_of\_programmed\_cell\_death | VEGFA | 365 | 6 | 1.220946 | -0.434094 | 796 | 459.51 | 0.577274 |
| GO:0043067\_regulation\_of\_programmed\_cell\_death | NEFL | 365 | 6 | 1.220946 | -0.434094 | 796 | 459.51 | 0.577274 |
| GO:0043067\_regulation\_of\_programmed\_cell\_death | KITL | 365 | 6 | 1.220946 | -0.434094 | 796 | 459.51 | 0.577274 |
| GO:0002682\_regulation\_of\_immune\_system\_process | HMGB1 | 228 | 4 | 1.303056 | -0.433252 | 797 | 460.26 | 0.577491 |
| GO:0002682\_regulation\_of\_immune\_system\_process | SLC11A1 | 228 | 4 | 1.303056 | -0.433252 | 797 | 460.26 | 0.577491 |
| GO:0002682\_regulation\_of\_immune\_system\_process | SNCA | 228 | 4 | 1.303056 | -0.433252 | 797 | 460.26 | 0.577491 |
| GO:0002682\_regulation\_of\_immune\_system\_process | KITL | 228 | 4 | 1.303056 | -0.433252 | 797 | 460.26 | 0.577491 |
| GO:0002237\_response\_to\_molecule\_of\_bacterial\_origin | SLC11A1 | 34 | 1 | 2.184535 | -0.431423 | 805 | 466.18 | 0.579106 |
| GO:0007269\_neurotransmitter\_secretion | SNCA | 34 | 1 | 2.184535 | -0.431423 | 805 | 466.18 | 0.579106 |
| GO:0007568\_aging | BCL2 | 34 | 1 | 2.184535 | -0.431423 | 805 | 466.18 | 0.579106 |
| GO:0019882\_antigen\_processing\_and\_presentation | SLC11A1 | 34 | 1 | 2.184535 | -0.431423 | 805 | 466.18 | 0.579106 |
| GO:0030509\_BMP\_signaling\_pathway | TOB1 | 34 | 1 | 2.184535 | -0.431423 | 805 | 466.18 | 0.579106 |
| GO:0045927\_positive\_regulation\_of\_growth | BCL2 | 34 | 1 | 2.184535 | -0.431423 | 805 | 466.18 | 0.579106 |
| GO:0050730\_regulation\_of\_peptidyl-tyrosine\_phosphorylation | KITL | 34 | 1 | 2.184535 | -0.431423 | 805 | 466.18 | 0.579106 |
| GO:0051047\_positive\_regulation\_of\_secretion | SNCA | 34 | 1 | 2.184535 | -0.431423 | 805 | 466.18 | 0.579106 |
| GO:0048736\_appendage\_development | GNAQ | 96 | 2 | 1.547379 | -0.429144 | 809 | 467.9 | 0.578368 |
| GO:0048736\_appendage\_development | NR2F2 | 96 | 2 | 1.547379 | -0.429144 | 809 | 467.9 | 0.578368 |
| GO:0060173\_limb\_development | GNAQ | 96 | 2 | 1.547379 | -0.429144 | 809 | 467.9 | 0.578368 |
| GO:0060173\_limb\_development | NR2F2 | 96 | 2 | 1.547379 | -0.429144 | 809 | 467.9 | 0.578368 |
| GO:0060249\_anatomical\_structure\_homeostasis | BCL2 | 96 | 2 | 1.547379 | -0.429144 | 809 | 467.9 | 0.578368 |
| GO:0060249\_anatomical\_structure\_homeostasis | VEGFA | 96 | 2 | 1.547379 | -0.429144 | 809 | 467.9 | 0.578368 |
| GO:0070661\_leukocyte\_proliferation | SLC11A1 | 96 | 2 | 1.547379 | -0.429144 | 809 | 467.9 | 0.578368 |
| GO:0070661\_leukocyte\_proliferation | KITL | 96 | 2 | 1.547379 | -0.429144 | 809 | 467.9 | 0.578368 |
| GO:0007626\_locomotory\_behavior | HMGB1 | 163 | 3 | 1.367010 | -0.423607 | 810 | 469.0 | 0.579012 |
| GO:0007626\_locomotory\_behavior | SNCA | 163 | 3 | 1.367010 | -0.423607 | 810 | 469.0 | 0.579012 |
| GO:0007626\_locomotory\_behavior | CHRNB4 | 163 | 3 | 1.367010 | -0.423607 | 810 | 469.0 | 0.579012 |
| GO:0018193\_peptidyl-amino\_acid\_modification | BCL2 | 97 | 2 | 1.531427 | -0.423511 | 812 | 469.57 | 0.578288 |
| GO:0018193\_peptidyl-amino\_acid\_modification | KITL | 97 | 2 | 1.531427 | -0.423511 | 812 | 469.57 | 0.578288 |
| GO:0060341\_regulation\_of\_cellular\_localization | PFKL | 97 | 2 | 1.531427 | -0.423511 | 812 | 469.57 | 0.578288 |
| GO:0060341\_regulation\_of\_cellular\_localization | SNCA | 97 | 2 | 1.531427 | -0.423511 | 812 | 469.57 | 0.578288 |
| GO:0016567\_protein\_ubiquitination | SUZ12 | 35 | 1 | 2.122120 | -0.421520 | 814 | 474.21 | 0.582568 |
| GO:0043406\_positive\_regulation\_of\_MAP\_kinase\_activity | KITL | 35 | 1 | 2.122120 | -0.421520 | 814 | 474.21 | 0.582568 |
| GO:0007548\_sex\_differentiation | BCL2 | 98 | 2 | 1.515800 | -0.417967 | 815 | 475.38 | 0.583288 |
| GO:0007548\_sex\_differentiation | VEGFA | 98 | 2 | 1.515800 | -0.417967 | 815 | 475.38 | 0.583288 |
| GO:0006259\_DNA\_metabolic\_process | HMGB1 | 165 | 3 | 1.350440 | -0.415160 | 816 | 476.76 | 0.584265 |
| GO:0006259\_DNA\_metabolic\_process | DCLRE1A | 165 | 3 | 1.350440 | -0.415160 | 816 | 476.76 | 0.584265 |
| GO:0006259\_DNA\_metabolic\_process | FEN1 | 165 | 3 | 1.350440 | -0.415160 | 816 | 476.76 | 0.584265 |
| GO:0001819\_positive\_regulation\_of\_cytokine\_production | SLC11A1 | 36 | 1 | 2.063172 | -0.411966 | 824 | 483.2 | 0.586408 |
| GO:0001889\_liver\_development | ONECUT2 | 36 | 1 | 2.063172 | -0.411966 | 824 | 483.2 | 0.586408 |
| GO:0006469\_negative\_regulation\_of\_protein\_kinase\_activity | SPRY1 | 36 | 1 | 2.063172 | -0.411966 | 824 | 483.2 | 0.586408 |
| GO:0007187\_G-protein\_signaling\_\_coupled\_to\_cyclic\_nucleotide\_second\_messenger | GNAQ | 36 | 1 | 2.063172 | -0.411966 | 824 | 483.2 | 0.586408 |
| GO:0007368\_determination\_of\_left\_right\_symmetry | PITX2 | 36 | 1 | 2.063172 | -0.411966 | 824 | 483.2 | 0.586408 |
| GO:0030072\_peptide\_hormone\_secretion | PFKL | 36 | 1 | 2.063172 | -0.411966 | 824 | 483.2 | 0.586408 |
| GO:0033673\_negative\_regulation\_of\_kinase\_activity | SPRY1 | 36 | 1 | 2.063172 | -0.411966 | 824 | 483.2 | 0.586408 |
| GO:0042742\_defense\_response\_to\_bacterium | SLC11A1 | 36 | 1 | 2.063172 | -0.411966 | 824 | 483.2 | 0.586408 |
| GO:0003008\_system\_process | NRCAM | 516 | 8 | 1.151538 | -0.406064 | 825 | 485.42 | 0.588388 |
| GO:0003008\_system\_process | BDNF | 516 | 8 | 1.151538 | -0.406064 | 825 | 485.42 | 0.588388 |
| GO:0003008\_system\_process | BCL2 | 516 | 8 | 1.151538 | -0.406064 | 825 | 485.42 | 0.588388 |
| GO:0003008\_system\_process | SNCA | 516 | 8 | 1.151538 | -0.406064 | 825 | 485.42 | 0.588388 |
| GO:0003008\_system\_process | SP4 | 516 | 8 | 1.151538 | -0.406064 | 825 | 485.42 | 0.588388 |
| GO:0003008\_system\_process | CHRNB4 | 516 | 8 | 1.151538 | -0.406064 | 825 | 485.42 | 0.588388 |
| GO:0003008\_system\_process | FABP7 | 516 | 8 | 1.151538 | -0.406064 | 825 | 485.42 | 0.588388 |
| GO:0003008\_system\_process | NEFL | 516 | 8 | 1.151538 | -0.406064 | 825 | 485.42 | 0.588388 |
| GO:0002790\_peptide\_secretion | PFKL | 37 | 1 | 2.007411 | -0.402742 | 831 | 489.52 | 0.589073 |
| GO:0009799\_determination\_of\_symmetry | PITX2 | 37 | 1 | 2.007411 | -0.402742 | 831 | 489.52 | 0.589073 |
| GO:0009855\_determination\_of\_bilateral\_symmetry | PITX2 | 37 | 1 | 2.007411 | -0.402742 | 831 | 489.52 | 0.589073 |
| GO:0019933\_cAMP-mediated\_signaling | GNAQ | 37 | 1 | 2.007411 | -0.402742 | 831 | 489.52 | 0.589073 |
| GO:0032869\_cellular\_response\_to\_insulin\_stimulus | IRS2 | 37 | 1 | 2.007411 | -0.402742 | 831 | 489.52 | 0.589073 |
| GO:0045761\_regulation\_of\_adenylate\_cyclase\_activity | GNAQ | 37 | 1 | 2.007411 | -0.402742 | 831 | 489.52 | 0.589073 |
| GO:0044085\_cellular\_component\_biogenesis | BCL2 | 237 | 4 | 1.253573 | -0.401480 | 832 | 490.29 | 0.589291 |
| GO:0044085\_cellular\_component\_biogenesis | ONECUT2 | 237 | 4 | 1.253573 | -0.401480 | 832 | 490.29 | 0.589291 |
| GO:0044085\_cellular\_component\_biogenesis | NEFL | 237 | 4 | 1.253573 | -0.401480 | 832 | 490.29 | 0.589291 |
| GO:0044085\_cellular\_component\_biogenesis | AHR | 237 | 4 | 1.253573 | -0.401480 | 832 | 490.29 | 0.589291 |
| GO:0050794\_regulation\_of\_cellular\_process | HMGB1 | 2190 | 31 | 1.051370 | -0.401314 | 833 | 490.38 | 0.588691 |
| GO:0050794\_regulation\_of\_cellular\_process | LTBP1 | 2190 | 31 | 1.051370 | -0.401314 | 833 | 490.38 | 0.588691 |
| GO:0050794\_regulation\_of\_cellular\_process | LTBP3 | 2190 | 31 | 1.051370 | -0.401314 | 833 | 490.38 | 0.588691 |
| GO:0050794\_regulation\_of\_cellular\_process | ONECUT2 | 2190 | 31 | 1.051370 | -0.401314 | 833 | 490.38 | 0.588691 |
| GO:0050794\_regulation\_of\_cellular\_process | SNCA | 2190 | 31 | 1.051370 | -0.401314 | 833 | 490.38 | 0.588691 |
| GO:0050794\_regulation\_of\_cellular\_process | RORB | 2190 | 31 | 1.051370 | -0.401314 | 833 | 490.38 | 0.588691 |
| GO:0050794\_regulation\_of\_cellular\_process | SLC11A1 | 2190 | 31 | 1.051370 | -0.401314 | 833 | 490.38 | 0.588691 |
| GO:0050794\_regulation\_of\_cellular\_process | SPRY1 | 2190 | 31 | 1.051370 | -0.401314 | 833 | 490.38 | 0.588691 |
| GO:0050794\_regulation\_of\_cellular\_process | BDNF | 2190 | 31 | 1.051370 | -0.401314 | 833 | 490.38 | 0.588691 |
| GO:0050794\_regulation\_of\_cellular\_process | DAB1 | 2190 | 31 | 1.051370 | -0.401314 | 833 | 490.38 | 0.588691 |
| GO:0050794\_regulation\_of\_cellular\_process | BCL2 | 2190 | 31 | 1.051370 | -0.401314 | 833 | 490.38 | 0.588691 |
| GO:0050794\_regulation\_of\_cellular\_process | GARNL1 | 2190 | 31 | 1.051370 | -0.401314 | 833 | 490.38 | 0.588691 |
| GO:0050794\_regulation\_of\_cellular\_process | NFAT5 | 2190 | 31 | 1.051370 | -0.401314 | 833 | 490.38 | 0.588691 |
| GO:0050794\_regulation\_of\_cellular\_process | MYB | 2190 | 31 | 1.051370 | -0.401314 | 833 | 490.38 | 0.588691 |
| GO:0050794\_regulation\_of\_cellular\_process | ACSL4 | 2190 | 31 | 1.051370 | -0.401314 | 833 | 490.38 | 0.588691 |
| GO:0050794\_regulation\_of\_cellular\_process | NR2F2 | 2190 | 31 | 1.051370 | -0.401314 | 833 | 490.38 | 0.588691 |
| GO:0050794\_regulation\_of\_cellular\_process | NEFL | 2190 | 31 | 1.051370 | -0.401314 | 833 | 490.38 | 0.588691 |
| GO:0050794\_regulation\_of\_cellular\_process | ETV3 | 2190 | 31 | 1.051370 | -0.401314 | 833 | 490.38 | 0.588691 |
| GO:0050794\_regulation\_of\_cellular\_process | MAP2K5 | 2190 | 31 | 1.051370 | -0.401314 | 833 | 490.38 | 0.588691 |
| GO:0050794\_regulation\_of\_cellular\_process | PITX2 | 2190 | 31 | 1.051370 | -0.401314 | 833 | 490.38 | 0.588691 |
| GO:0050794\_regulation\_of\_cellular\_process | IRS2 | 2190 | 31 | 1.051370 | -0.401314 | 833 | 490.38 | 0.588691 |
| GO:0050794\_regulation\_of\_cellular\_process | PFKL | 2190 | 31 | 1.051370 | -0.401314 | 833 | 490.38 | 0.588691 |
| GO:0050794\_regulation\_of\_cellular\_process | KITL | 2190 | 31 | 1.051370 | -0.401314 | 833 | 490.38 | 0.588691 |
| GO:0050794\_regulation\_of\_cellular\_process | AHR | 2190 | 31 | 1.051370 | -0.401314 | 833 | 490.38 | 0.588691 |
| GO:0050794\_regulation\_of\_cellular\_process | CDC25B | 2190 | 31 | 1.051370 | -0.401314 | 833 | 490.38 | 0.588691 |
| GO:0050794\_regulation\_of\_cellular\_process | SUZ12 | 2190 | 31 | 1.051370 | -0.401314 | 833 | 490.38 | 0.588691 |
| GO:0050794\_regulation\_of\_cellular\_process | RNF6 | 2190 | 31 | 1.051370 | -0.401314 | 833 | 490.38 | 0.588691 |
| GO:0050794\_regulation\_of\_cellular\_process | GNAQ | 2190 | 31 | 1.051370 | -0.401314 | 833 | 490.38 | 0.588691 |
| GO:0050794\_regulation\_of\_cellular\_process | VEGFA | 2190 | 31 | 1.051370 | -0.401314 | 833 | 490.38 | 0.588691 |
| GO:0050794\_regulation\_of\_cellular\_process | ID4 | 2190 | 31 | 1.051370 | -0.401314 | 833 | 490.38 | 0.588691 |
| GO:0050794\_regulation\_of\_cellular\_process | TOB1 | 2190 | 31 | 1.051370 | -0.401314 | 833 | 490.38 | 0.588691 |
| GO:0051094\_positive\_regulation\_of\_developmental\_process | HMGB1 | 308 | 5 | 1.205750 | -0.397011 | 834 | 491.2 | 0.588969 |
| GO:0051094\_positive\_regulation\_of\_developmental\_process | BDNF | 308 | 5 | 1.205750 | -0.397011 | 834 | 491.2 | 0.588969 |
| GO:0051094\_positive\_regulation\_of\_developmental\_process | BCL2 | 308 | 5 | 1.205750 | -0.397011 | 834 | 491.2 | 0.588969 |
| GO:0051094\_positive\_regulation\_of\_developmental\_process | KITL | 308 | 5 | 1.205750 | -0.397011 | 834 | 491.2 | 0.588969 |
| GO:0051094\_positive\_regulation\_of\_developmental\_process | NEFL | 308 | 5 | 1.205750 | -0.397011 | 834 | 491.2 | 0.588969 |
| GO:0051246\_regulation\_of\_protein\_metabolic\_process | HMGB1 | 170 | 3 | 1.310721 | -0.394822 | 835 | 492.45 | 0.589760 |
| GO:0051246\_regulation\_of\_protein\_metabolic\_process | BCL2 | 170 | 3 | 1.310721 | -0.394822 | 835 | 492.45 | 0.589760 |
| GO:0051246\_regulation\_of\_protein\_metabolic\_process | KITL | 170 | 3 | 1.310721 | -0.394822 | 835 | 492.45 | 0.589760 |
| GO:0001570\_vasculogenesis | NTRK2 | 38 | 1 | 1.954584 | -0.393828 | 841 | 499.37 | 0.593781 |
| GO:0001649\_osteoblast\_differentiation | TOB1 | 38 | 1 | 1.954584 | -0.393828 | 841 | 499.37 | 0.593781 |
| GO:0008016\_regulation\_of\_heart\_contraction | SP4 | 38 | 1 | 1.954584 | -0.393828 | 841 | 499.37 | 0.593781 |
| GO:0032259\_methylation | SUZ12 | 38 | 1 | 1.954584 | -0.393828 | 841 | 499.37 | 0.593781 |
| GO:0043414\_biopolymer\_methylation | SUZ12 | 38 | 1 | 1.954584 | -0.393828 | 841 | 499.37 | 0.593781 |
| GO:0051348\_negative\_regulation\_of\_transferase\_activity | SPRY1 | 38 | 1 | 1.954584 | -0.393828 | 841 | 499.37 | 0.593781 |
| GO:0009968\_negative\_regulation\_of\_signal\_transduction | ONECUT2 | 103 | 2 | 1.442217 | -0.391513 | 842 | 500.33 | 0.594216 |
| GO:0009968\_negative\_regulation\_of\_signal\_transduction | TOB1 | 103 | 2 | 1.442217 | -0.391513 | 842 | 500.33 | 0.594216 |
| GO:0044238\_primary\_metabolic\_process | HMGB1 | 1905 | 27 | 1.052705 | -0.387331 | 843 | 500.74 | 0.593998 |
| GO:0044238\_primary\_metabolic\_process | LCORL | 1905 | 27 | 1.052705 | -0.387331 | 843 | 500.74 | 0.593998 |
| GO:0044238\_primary\_metabolic\_process | ALDH18A1 | 1905 | 27 | 1.052705 | -0.387331 | 843 | 500.74 | 0.593998 |
| GO:0044238\_primary\_metabolic\_process | ONECUT2 | 1905 | 27 | 1.052705 | -0.387331 | 843 | 500.74 | 0.593998 |
| GO:0044238\_primary\_metabolic\_process | SNCA | 1905 | 27 | 1.052705 | -0.387331 | 843 | 500.74 | 0.593998 |
| GO:0044238\_primary\_metabolic\_process | PPM1A | 1905 | 27 | 1.052705 | -0.387331 | 843 | 500.74 | 0.593998 |
| GO:0044238\_primary\_metabolic\_process | RORB | 1905 | 27 | 1.052705 | -0.387331 | 843 | 500.74 | 0.593998 |
| GO:0044238\_primary\_metabolic\_process | SLC11A1 | 1905 | 27 | 1.052705 | -0.387331 | 843 | 500.74 | 0.593998 |
| GO:0044238\_primary\_metabolic\_process | ACOT10 | 1905 | 27 | 1.052705 | -0.387331 | 843 | 500.74 | 0.593998 |
| GO:0044238\_primary\_metabolic\_process | BCL2 | 1905 | 27 | 1.052705 | -0.387331 | 843 | 500.74 | 0.593998 |
| GO:0044238\_primary\_metabolic\_process | GARNL1 | 1905 | 27 | 1.052705 | -0.387331 | 843 | 500.74 | 0.593998 |
| GO:0044238\_primary\_metabolic\_process | NFAT5 | 1905 | 27 | 1.052705 | -0.387331 | 843 | 500.74 | 0.593998 |
| GO:0044238\_primary\_metabolic\_process | NR2F2 | 1905 | 27 | 1.052705 | -0.387331 | 843 | 500.74 | 0.593998 |
| GO:0044238\_primary\_metabolic\_process | MYB | 1905 | 27 | 1.052705 | -0.387331 | 843 | 500.74 | 0.593998 |
| GO:0044238\_primary\_metabolic\_process | ACSL4 | 1905 | 27 | 1.052705 | -0.387331 | 843 | 500.74 | 0.593998 |
| GO:0044238\_primary\_metabolic\_process | FEN1 | 1905 | 27 | 1.052705 | -0.387331 | 843 | 500.74 | 0.593998 |
| GO:0044238\_primary\_metabolic\_process | PITX2 | 1905 | 27 | 1.052705 | -0.387331 | 843 | 500.74 | 0.593998 |
| GO:0044238\_primary\_metabolic\_process | ETV3 | 1905 | 27 | 1.052705 | -0.387331 | 843 | 500.74 | 0.593998 |
| GO:0044238\_primary\_metabolic\_process | SCD1 | 1905 | 27 | 1.052705 | -0.387331 | 843 | 500.74 | 0.593998 |
| GO:0044238\_primary\_metabolic\_process | PFKL | 1905 | 27 | 1.052705 | -0.387331 | 843 | 500.74 | 0.593998 |
| GO:0044238\_primary\_metabolic\_process | KITL | 1905 | 27 | 1.052705 | -0.387331 | 843 | 500.74 | 0.593998 |
| GO:0044238\_primary\_metabolic\_process | AHR | 1905 | 27 | 1.052705 | -0.387331 | 843 | 500.74 | 0.593998 |
| GO:0044238\_primary\_metabolic\_process | SUZ12 | 1905 | 27 | 1.052705 | -0.387331 | 843 | 500.74 | 0.593998 |
| GO:0044238\_primary\_metabolic\_process | DCLRE1A | 1905 | 27 | 1.052705 | -0.387331 | 843 | 500.74 | 0.593998 |
| GO:0044238\_primary\_metabolic\_process | RNF6 | 1905 | 27 | 1.052705 | -0.387331 | 843 | 500.74 | 0.593998 |
| GO:0044238\_primary\_metabolic\_process | GNAQ | 1905 | 27 | 1.052705 | -0.387331 | 843 | 500.74 | 0.593998 |
| GO:0044238\_primary\_metabolic\_process | FABP3 | 1905 | 27 | 1.052705 | -0.387331 | 843 | 500.74 | 0.593998 |
| GO:0009611\_response\_to\_wounding | SLC11A1 | 172 | 3 | 1.295480 | -0.386987 | 844 | 501.12 | 0.593744 |
| GO:0009611\_response\_to\_wounding | BCL2 | 172 | 3 | 1.295480 | -0.386987 | 844 | 501.12 | 0.593744 |
| GO:0009611\_response\_to\_wounding | NEFL | 172 | 3 | 1.295480 | -0.386987 | 844 | 501.12 | 0.593744 |
| GO:0005976\_polysaccharide\_metabolic\_process | HMGB1 | 39 | 1 | 1.904467 | -0.385209 | 850 | 506.62 | 0.596024 |
| GO:0006511\_ubiquitin-dependent\_protein\_catabolic\_process | RNF6 | 39 | 1 | 1.904467 | -0.385209 | 850 | 506.62 | 0.596024 |
| GO:0006730\_one-carbon\_metabolic\_process | SUZ12 | 39 | 1 | 1.904467 | -0.385209 | 850 | 506.62 | 0.596024 |
| GO:0008037\_cell\_recognition | BDNF | 39 | 1 | 1.904467 | -0.385209 | 850 | 506.62 | 0.596024 |
| GO:0031279\_regulation\_of\_cyclase\_activity | GNAQ | 39 | 1 | 1.904467 | -0.385209 | 850 | 506.62 | 0.596024 |
| GO:0051339\_regulation\_of\_lyase\_activity | GNAQ | 39 | 1 | 1.904467 | -0.385209 | 850 | 506.62 | 0.596024 |
| GO:0016071\_mRNA\_metabolic\_process | SLC11A1 | 40 | 1 | 1.856855 | -0.376870 | 854 | 513.07 | 0.600785 |
| GO:0016358\_dendrite\_development | BDNF | 40 | 1 | 1.856855 | -0.376870 | 854 | 513.07 | 0.600785 |
| GO:0019935\_cyclic-nucleotide-mediated\_signaling | GNAQ | 40 | 1 | 1.856855 | -0.376870 | 854 | 513.07 | 0.600785 |
| GO:0051129\_negative\_regulation\_of\_cellular\_component\_organization | RNF6 | 40 | 1 | 1.856855 | -0.376870 | 854 | 513.07 | 0.600785 |
| GO:0000122\_negative\_regulation\_of\_transcription\_from\_RNA\_polymerase\_II\_promoter | SUZ12 | 175 | 3 | 1.273272 | -0.375542 | 855 | 514.15 | 0.601345 |
| GO:0000122\_negative\_regulation\_of\_transcription\_from\_RNA\_polymerase\_II\_promoter | NR2F2 | 175 | 3 | 1.273272 | -0.375542 | 855 | 514.15 | 0.601345 |
| GO:0000122\_negative\_regulation\_of\_transcription\_from\_RNA\_polymerase\_II\_promoter | ETV3 | 175 | 3 | 1.273272 | -0.375542 | 855 | 514.15 | 0.601345 |
| GO:0006260\_DNA\_replication | FEN1 | 41 | 1 | 1.811566 | -0.368796 | 865 | 522.58 | 0.604139 |
| GO:0006836\_neurotransmitter\_transport | SNCA | 41 | 1 | 1.811566 | -0.368796 | 865 | 522.58 | 0.604139 |
| GO:0006865\_amino\_acid\_transport | SLC11A1 | 41 | 1 | 1.811566 | -0.368796 | 865 | 522.58 | 0.604139 |
| GO:0009894\_regulation\_of\_catabolic\_process | HMGB1 | 41 | 1 | 1.811566 | -0.368796 | 865 | 522.58 | 0.604139 |
| GO:0010551\_regulation\_of\_specific\_transcription\_from\_RNA\_polymerase\_II\_promoter | HMGB1 | 41 | 1 | 1.811566 | -0.368796 | 865 | 522.58 | 0.604139 |
| GO:0015833\_peptide\_transport | PFKL | 41 | 1 | 1.811566 | -0.368796 | 865 | 522.58 | 0.604139 |
| GO:0019748\_secondary\_metabolic\_process | BCL2 | 41 | 1 | 1.811566 | -0.368796 | 865 | 522.58 | 0.604139 |
| GO:0030817\_regulation\_of\_cAMP\_biosynthetic\_process | GNAQ | 41 | 1 | 1.811566 | -0.368796 | 865 | 522.58 | 0.604139 |
| GO:0032569\_specific\_transcription\_from\_RNA\_polymerase\_II\_promoter | HMGB1 | 41 | 1 | 1.811566 | -0.368796 | 865 | 522.58 | 0.604139 |
| GO:0033077\_T\_cell\_differentiation\_in\_the\_thymus | BCL2 | 41 | 1 | 1.811566 | -0.368796 | 865 | 522.58 | 0.604139 |
| GO:0050877\_neurological\_system\_process | NRCAM | 390 | 6 | 1.142680 | -0.366498 | 866 | 523.4 | 0.604388 |
| GO:0050877\_neurological\_system\_process | BDNF | 390 | 6 | 1.142680 | -0.366498 | 866 | 523.4 | 0.604388 |
| GO:0050877\_neurological\_system\_process | SNCA | 390 | 6 | 1.142680 | -0.366498 | 866 | 523.4 | 0.604388 |
| GO:0050877\_neurological\_system\_process | CHRNB4 | 390 | 6 | 1.142680 | -0.366498 | 866 | 523.4 | 0.604388 |
| GO:0050877\_neurological\_system\_process | FABP7 | 390 | 6 | 1.142680 | -0.366498 | 866 | 523.4 | 0.604388 |
| GO:0050877\_neurological\_system\_process | NEFL | 390 | 6 | 1.142680 | -0.366498 | 866 | 523.4 | 0.604388 |
| GO:0006006\_glucose\_metabolic\_process | PFKL | 42 | 1 | 1.768433 | -0.360975 | 875 | 530.26 | 0.606011 |
| GO:0006171\_cAMP\_biosynthetic\_process | GNAQ | 42 | 1 | 1.768433 | -0.360975 | 875 | 530.26 | 0.606011 |
| GO:0008361\_regulation\_of\_cell\_size | BCL2 | 42 | 1 | 1.768433 | -0.360975 | 875 | 530.26 | 0.606011 |
| GO:0015672\_monovalent\_inorganic\_cation\_transport | SLC4A4 | 42 | 1 | 1.768433 | -0.360975 | 875 | 530.26 | 0.606011 |
| GO:0019941\_modification-dependent\_protein\_catabolic\_process | RNF6 | 42 | 1 | 1.768433 | -0.360975 | 875 | 530.26 | 0.606011 |
| GO:0042476\_odontogenesis | PITX2 | 42 | 1 | 1.768433 | -0.360975 | 875 | 530.26 | 0.606011 |
| GO:0043632\_modification-dependent\_macromolecule\_catabolic\_process | RNF6 | 42 | 1 | 1.768433 | -0.360975 | 875 | 530.26 | 0.606011 |
| GO:0051345\_positive\_regulation\_of\_hydrolase\_activity | GNAQ | 42 | 1 | 1.768433 | -0.360975 | 875 | 530.26 | 0.606011 |
| GO:0051603\_proteolysis\_involved\_in\_cellular\_protein\_catabolic\_process | RNF6 | 42 | 1 | 1.768433 | -0.360975 | 875 | 530.26 | 0.606011 |
| GO:0010647\_positive\_regulation\_of\_cell\_communication | SNCA | 110 | 2 | 1.350440 | -0.357725 | 877 | 532.32 | 0.606978 |
| GO:0010647\_positive\_regulation\_of\_cell\_communication | KITL | 110 | 2 | 1.350440 | -0.357725 | 877 | 532.32 | 0.606978 |
| GO:0010648\_negative\_regulation\_of\_cell\_communication | ONECUT2 | 110 | 2 | 1.350440 | -0.357725 | 877 | 532.32 | 0.606978 |
| GO:0010648\_negative\_regulation\_of\_cell\_communication | TOB1 | 110 | 2 | 1.350440 | -0.357725 | 877 | 532.32 | 0.606978 |
| GO:0008104\_protein\_localization | NRCAM | 251 | 4 | 1.183652 | -0.356614 | 878 | 532.92 | 0.606970 |
| GO:0008104\_protein\_localization | SLC11A1 | 251 | 4 | 1.183652 | -0.356614 | 878 | 532.92 | 0.606970 |
| GO:0008104\_protein\_localization | BCL2 | 251 | 4 | 1.183652 | -0.356614 | 878 | 532.92 | 0.606970 |
| GO:0008104\_protein\_localization | TOB1 | 251 | 4 | 1.183652 | -0.356614 | 878 | 532.92 | 0.606970 |
| GO:0065007\_biological\_regulation | HMGB1 | 2593 | 36 | 1.031188 | -0.354769 | 879 | 533.21 | 0.606610 |
| GO:0065007\_biological\_regulation | LTBP1 | 2593 | 36 | 1.031188 | -0.354769 | 879 | 533.21 | 0.606610 |
| GO:0065007\_biological\_regulation | LTBP3 | 2593 | 36 | 1.031188 | -0.354769 | 879 | 533.21 | 0.606610 |
| GO:0065007\_biological\_regulation | ONECUT2 | 2593 | 36 | 1.031188 | -0.354769 | 879 | 533.21 | 0.606610 |
| GO:0065007\_biological\_regulation | SNCA | 2593 | 36 | 1.031188 | -0.354769 | 879 | 533.21 | 0.606610 |
| GO:0065007\_biological\_regulation | RORB | 2593 | 36 | 1.031188 | -0.354769 | 879 | 533.21 | 0.606610 |
| GO:0065007\_biological\_regulation | SLC11A1 | 2593 | 36 | 1.031188 | -0.354769 | 879 | 533.21 | 0.606610 |
| GO:0065007\_biological\_regulation | SPRY1 | 2593 | 36 | 1.031188 | -0.354769 | 879 | 533.21 | 0.606610 |
| GO:0065007\_biological\_regulation | BDNF | 2593 | 36 | 1.031188 | -0.354769 | 879 | 533.21 | 0.606610 |
| GO:0065007\_biological\_regulation | DAB1 | 2593 | 36 | 1.031188 | -0.354769 | 879 | 533.21 | 0.606610 |
| GO:0065007\_biological\_regulation | BCL2 | 2593 | 36 | 1.031188 | -0.354769 | 879 | 533.21 | 0.606610 |
| GO:0065007\_biological\_regulation | GARNL1 | 2593 | 36 | 1.031188 | -0.354769 | 879 | 533.21 | 0.606610 |
| GO:0065007\_biological\_regulation | NFAT5 | 2593 | 36 | 1.031188 | -0.354769 | 879 | 533.21 | 0.606610 |
| GO:0065007\_biological\_regulation | SLC4A4 | 2593 | 36 | 1.031188 | -0.354769 | 879 | 533.21 | 0.606610 |
| GO:0065007\_biological\_regulation | NR2F2 | 2593 | 36 | 1.031188 | -0.354769 | 879 | 533.21 | 0.606610 |
| GO:0065007\_biological\_regulation | MYB | 2593 | 36 | 1.031188 | -0.354769 | 879 | 533.21 | 0.606610 |
| GO:0065007\_biological\_regulation | ACSL4 | 2593 | 36 | 1.031188 | -0.354769 | 879 | 533.21 | 0.606610 |
| GO:0065007\_biological\_regulation | NEFL | 2593 | 36 | 1.031188 | -0.354769 | 879 | 533.21 | 0.606610 |
| GO:0065007\_biological\_regulation | MAP2K5 | 2593 | 36 | 1.031188 | -0.354769 | 879 | 533.21 | 0.606610 |
| GO:0065007\_biological\_regulation | PITX2 | 2593 | 36 | 1.031188 | -0.354769 | 879 | 533.21 | 0.606610 |
| GO:0065007\_biological\_regulation | ETV3 | 2593 | 36 | 1.031188 | -0.354769 | 879 | 533.21 | 0.606610 |
| GO:0065007\_biological\_regulation | IRS2 | 2593 | 36 | 1.031188 | -0.354769 | 879 | 533.21 | 0.606610 |
| GO:0065007\_biological\_regulation | PFKL | 2593 | 36 | 1.031188 | -0.354769 | 879 | 533.21 | 0.606610 |
| GO:0065007\_biological\_regulation | KITL | 2593 | 36 | 1.031188 | -0.354769 | 879 | 533.21 | 0.606610 |
| GO:0065007\_biological\_regulation | AHR | 2593 | 36 | 1.031188 | -0.354769 | 879 | 533.21 | 0.606610 |
| GO:0065007\_biological\_regulation | CDC25B | 2593 | 36 | 1.031188 | -0.354769 | 879 | 533.21 | 0.606610 |
| GO:0065007\_biological\_regulation | SUZ12 | 2593 | 36 | 1.031188 | -0.354769 | 879 | 533.21 | 0.606610 |
| GO:0065007\_biological\_regulation | RNF6 | 2593 | 36 | 1.031188 | -0.354769 | 879 | 533.21 | 0.606610 |
| GO:0065007\_biological\_regulation | GNAQ | 2593 | 36 | 1.031188 | -0.354769 | 879 | 533.21 | 0.606610 |
| GO:0065007\_biological\_regulation | SP4 | 2593 | 36 | 1.031188 | -0.354769 | 879 | 533.21 | 0.606610 |
| GO:0065007\_biological\_regulation | VEGFA | 2593 | 36 | 1.031188 | -0.354769 | 879 | 533.21 | 0.606610 |
| GO:0065007\_biological\_regulation | NTRK2 | 2593 | 36 | 1.031188 | -0.354769 | 879 | 533.21 | 0.606610 |
| GO:0065007\_biological\_regulation | CHRNB4 | 2593 | 36 | 1.031188 | -0.354769 | 879 | 533.21 | 0.606610 |
| GO:0065007\_biological\_regulation | ID4 | 2593 | 36 | 1.031188 | -0.354769 | 879 | 533.21 | 0.606610 |
| GO:0065007\_biological\_regulation | FABP7 | 2593 | 36 | 1.031188 | -0.354769 | 879 | 533.21 | 0.606610 |
| GO:0065007\_biological\_regulation | TOB1 | 2593 | 36 | 1.031188 | -0.354769 | 879 | 533.21 | 0.606610 |
| GO:0002819\_regulation\_of\_adaptive\_immune\_response | SLC11A1 | 43 | 1 | 1.727307 | -0.353394 | 887 | 540.02 | 0.608816 |
| GO:0002822\_regulation\_of\_adaptive\_immune\_response\_based\_on\_somatic\_recombination\_of\_immune\_receptors\_built\_from\_immunoglobulin\_superfamily\_domains | SLC11A1 | 43 | 1 | 1.727307 | -0.353394 | 887 | 540.02 | 0.608816 |
| GO:0010001\_glial\_cell\_differentiation | ID4 | 43 | 1 | 1.727307 | -0.353394 | 887 | 540.02 | 0.608816 |
| GO:0030814\_regulation\_of\_cAMP\_metabolic\_process | GNAQ | 43 | 1 | 1.727307 | -0.353394 | 887 | 540.02 | 0.608816 |
| GO:0032446\_protein\_modification\_by\_small\_protein\_conjugation | SUZ12 | 43 | 1 | 1.727307 | -0.353394 | 887 | 540.02 | 0.608816 |
| GO:0032868\_response\_to\_insulin\_stimulus | IRS2 | 43 | 1 | 1.727307 | -0.353394 | 887 | 540.02 | 0.608816 |
| GO:0046879\_hormone\_secretion | PFKL | 43 | 1 | 1.727307 | -0.353394 | 887 | 540.02 | 0.608816 |
| GO:0051789\_response\_to\_protein\_stimulus | BCL2 | 43 | 1 | 1.727307 | -0.353394 | 887 | 540.02 | 0.608816 |
| GO:0030097\_hemopoiesis | HMGB1 | 253 | 4 | 1.174296 | -0.350623 | 888 | 540.84 | 0.609054 |
| GO:0030097\_hemopoiesis | BCL2 | 253 | 4 | 1.174296 | -0.350623 | 888 | 540.84 | 0.609054 |
| GO:0030097\_hemopoiesis | VEGFA | 253 | 4 | 1.174296 | -0.350623 | 888 | 540.84 | 0.609054 |
| GO:0030097\_hemopoiesis | KITL | 253 | 4 | 1.174296 | -0.350623 | 888 | 540.84 | 0.609054 |
| GO:0001942\_hair\_follicle\_development | BCL2 | 44 | 1 | 1.688050 | -0.346041 | 899 | 549.28 | 0.610990 |
| GO:0009914\_hormone\_transport | PFKL | 44 | 1 | 1.688050 | -0.346041 | 899 | 549.28 | 0.610990 |
| GO:0022404\_molting\_cycle\_process | BCL2 | 44 | 1 | 1.688050 | -0.346041 | 899 | 549.28 | 0.610990 |
| GO:0022405\_hair\_cycle\_process | BCL2 | 44 | 1 | 1.688050 | -0.346041 | 899 | 549.28 | 0.610990 |
| GO:0030802\_regulation\_of\_cyclic\_nucleotide\_biosynthetic\_process | GNAQ | 44 | 1 | 1.688050 | -0.346041 | 899 | 549.28 | 0.610990 |
| GO:0030808\_regulation\_of\_nucleotide\_biosynthetic\_process | GNAQ | 44 | 1 | 1.688050 | -0.346041 | 899 | 549.28 | 0.610990 |
| GO:0042303\_molting\_cycle | BCL2 | 44 | 1 | 1.688050 | -0.346041 | 899 | 549.28 | 0.610990 |
| GO:0042633\_hair\_cycle | BCL2 | 44 | 1 | 1.688050 | -0.346041 | 899 | 549.28 | 0.610990 |
| GO:0044257\_cellular\_protein\_catabolic\_process | RNF6 | 44 | 1 | 1.688050 | -0.346041 | 899 | 549.28 | 0.610990 |
| GO:0070665\_positive\_regulation\_of\_leukocyte\_proliferation | KITL | 44 | 1 | 1.688050 | -0.346041 | 899 | 549.28 | 0.610990 |
| GO:0070668\_positive\_regulation\_of\_mast\_cell\_proliferation | KITL | 44 | 1 | 1.688050 | -0.346041 | 899 | 549.28 | 0.610990 |
| GO:0048729\_tissue\_morphogenesis | BCL2 | 255 | 4 | 1.165085 | -0.344731 | 900 | 549.56 | 0.610622 |
| GO:0048729\_tissue\_morphogenesis | VEGFA | 255 | 4 | 1.165085 | -0.344731 | 900 | 549.56 | 0.610622 |
| GO:0048729\_tissue\_morphogenesis | ONECUT2 | 255 | 4 | 1.165085 | -0.344731 | 900 | 549.56 | 0.610622 |
| GO:0048729\_tissue\_morphogenesis | PITX2 | 255 | 4 | 1.165085 | -0.344731 | 900 | 549.56 | 0.610622 |
| GO:0032870\_cellular\_response\_to\_hormone\_stimulus | IRS2 | 45 | 1 | 1.650538 | -0.338907 | 903 | 554.22 | 0.613754 |
| GO:0046058\_cAMP\_metabolic\_process | GNAQ | 45 | 1 | 1.650538 | -0.338907 | 903 | 554.22 | 0.613754 |
| GO:0046546\_development\_of\_primary\_male\_sexual\_characteristics | BCL2 | 45 | 1 | 1.650538 | -0.338907 | 903 | 554.22 | 0.613754 |
| GO:0065008\_regulation\_of\_biological\_quality | SLC11A1 | 693 | 10 | 1.071778 | -0.337630 | 904 | 554.46 | 0.613341 |
| GO:0065008\_regulation\_of\_biological\_quality | GNAQ | 693 | 10 | 1.071778 | -0.337630 | 904 | 554.46 | 0.613341 |
| GO:0065008\_regulation\_of\_biological\_quality | PFKL | 693 | 10 | 1.071778 | -0.337630 | 904 | 554.46 | 0.613341 |
| GO:0065008\_regulation\_of\_biological\_quality | BCL2 | 693 | 10 | 1.071778 | -0.337630 | 904 | 554.46 | 0.613341 |
| GO:0065008\_regulation\_of\_biological\_quality | VEGFA | 693 | 10 | 1.071778 | -0.337630 | 904 | 554.46 | 0.613341 |
| GO:0065008\_regulation\_of\_biological\_quality | SNCA | 693 | 10 | 1.071778 | -0.337630 | 904 | 554.46 | 0.613341 |
| GO:0065008\_regulation\_of\_biological\_quality | CHRNB4 | 693 | 10 | 1.071778 | -0.337630 | 904 | 554.46 | 0.613341 |
| GO:0065008\_regulation\_of\_biological\_quality | SLC4A4 | 693 | 10 | 1.071778 | -0.337630 | 904 | 554.46 | 0.613341 |
| GO:0065008\_regulation\_of\_biological\_quality | KITL | 693 | 10 | 1.071778 | -0.337630 | 904 | 554.46 | 0.613341 |
| GO:0065008\_regulation\_of\_biological\_quality | NEFL | 693 | 10 | 1.071778 | -0.337630 | 904 | 554.46 | 0.613341 |
| GO:0048584\_positive\_regulation\_of\_response\_to\_stimulus | SLC11A1 | 115 | 2 | 1.291725 | -0.335660 | 905 | 556.3 | 0.614696 |
| GO:0048584\_positive\_regulation\_of\_response\_to\_stimulus | HMGB1 | 115 | 2 | 1.291725 | -0.335660 | 905 | 556.3 | 0.614696 |
| GO:0030218\_erythrocyte\_differentiation | VEGFA | 46 | 1 | 1.614656 | -0.331982 | 909 | 560.79 | 0.616931 |
| GO:0030850\_prostate\_gland\_development | AHR | 46 | 1 | 1.614656 | -0.331982 | 909 | 560.79 | 0.616931 |
| GO:0042063\_gliogenesis | ID4 | 46 | 1 | 1.614656 | -0.331982 | 909 | 560.79 | 0.616931 |
| GO:0046631\_alpha-beta\_T\_cell\_activation | BCL2 | 46 | 1 | 1.614656 | -0.331982 | 909 | 560.79 | 0.616931 |
| GO:0007276\_gamete\_generation | BCL2 | 188 | 3 | 1.185226 | -0.329905 | 910 | 561.93 | 0.617505 |
| GO:0007276\_gamete\_generation | KITL | 188 | 3 | 1.185226 | -0.329905 | 910 | 561.93 | 0.617505 |
| GO:0007276\_gamete\_generation | CDC25B | 188 | 3 | 1.185226 | -0.329905 | 910 | 561.93 | 0.617505 |
| GO:0006140\_regulation\_of\_nucleotide\_metabolic\_process | GNAQ | 47 | 1 | 1.580302 | -0.325255 | 916 | 566.53 | 0.618483 |
| GO:0016570\_histone\_modification | SUZ12 | 47 | 1 | 1.580302 | -0.325255 | 916 | 566.53 | 0.618483 |
| GO:0030183\_B\_cell\_differentiation | BCL2 | 47 | 1 | 1.580302 | -0.325255 | 916 | 566.53 | 0.618483 |
| GO:0030799\_regulation\_of\_cyclic\_nucleotide\_metabolic\_process | GNAQ | 47 | 1 | 1.580302 | -0.325255 | 916 | 566.53 | 0.618483 |
| GO:0045087\_innate\_immune\_response | SLC11A1 | 47 | 1 | 1.580302 | -0.325255 | 916 | 566.53 | 0.618483 |
| GO:0060627\_regulation\_of\_vesicle-mediated\_transport | SLC11A1 | 47 | 1 | 1.580302 | -0.325255 | 916 | 566.53 | 0.618483 |
| GO:0001505\_regulation\_of\_neurotransmitter\_levels | SNCA | 48 | 1 | 1.547379 | -0.318719 | 920 | 571.42 | 0.621109 |
| GO:0007498\_mesoderm\_development | VEGFA | 48 | 1 | 1.547379 | -0.318719 | 920 | 571.42 | 0.621109 |
| GO:0009101\_glycoprotein\_biosynthetic\_process | BCL2 | 48 | 1 | 1.547379 | -0.318719 | 920 | 571.42 | 0.621109 |
| GO:0019318\_hexose\_metabolic\_process | PFKL | 48 | 1 | 1.547379 | -0.318719 | 920 | 571.42 | 0.621109 |
| GO:0009605\_response\_to\_external\_stimulus | HMGB1 | 339 | 5 | 1.095490 | -0.314495 | 921 | 572.87 | 0.622009 |
| GO:0009605\_response\_to\_external\_stimulus | SLC11A1 | 339 | 5 | 1.095490 | -0.314495 | 921 | 572.87 | 0.622009 |
| GO:0009605\_response\_to\_external\_stimulus | BCL2 | 339 | 5 | 1.095490 | -0.314495 | 921 | 572.87 | 0.622009 |
| GO:0009605\_response\_to\_external\_stimulus | FABP7 | 339 | 5 | 1.095490 | -0.314495 | 921 | 572.87 | 0.622009 |
| GO:0009605\_response\_to\_external\_stimulus | NEFL | 339 | 5 | 1.095490 | -0.314495 | 921 | 572.87 | 0.622009 |
| GO:0002440\_production\_of\_molecular\_mediator\_of\_immune\_response | SLC11A1 | 49 | 1 | 1.515800 | -0.312366 | 928 | 577.12 | 0.621897 |
| GO:0003015\_heart\_process | SP4 | 49 | 1 | 1.515800 | -0.312366 | 928 | 577.12 | 0.621897 |
| GO:0006725\_cellular\_aromatic\_compound\_metabolic\_process | SNCA | 49 | 1 | 1.515800 | -0.312366 | 928 | 577.12 | 0.621897 |
| GO:0034101\_erythrocyte\_homeostasis | VEGFA | 49 | 1 | 1.515800 | -0.312366 | 928 | 577.12 | 0.621897 |
| GO:0046661\_male\_sex\_differentiation | BCL2 | 49 | 1 | 1.515800 | -0.312366 | 928 | 577.12 | 0.621897 |
| GO:0048741\_skeletal\_muscle\_fiber\_development | BCL2 | 49 | 1 | 1.515800 | -0.312366 | 928 | 577.12 | 0.621897 |
| GO:0060047\_heart\_contraction | SP4 | 49 | 1 | 1.515800 | -0.312366 | 928 | 577.12 | 0.621897 |
| GO:0051726\_regulation\_of\_cell\_cycle | HMGB1 | 121 | 2 | 1.227673 | -0.311208 | 929 | 578.98 | 0.623229 |
| GO:0051726\_regulation\_of\_cell\_cycle | BCL2 | 121 | 2 | 1.227673 | -0.311208 | 929 | 578.98 | 0.623229 |
| GO:0001816\_cytokine\_production | SLC11A1 | 122 | 2 | 1.217610 | -0.307333 | 931 | 581.97 | 0.625102 |
| GO:0001816\_cytokine\_production | NFAT5 | 122 | 2 | 1.217610 | -0.307333 | 931 | 581.97 | 0.625102 |
| GO:0006886\_intracellular\_protein\_transport | SLC11A1 | 122 | 2 | 1.217610 | -0.307333 | 931 | 581.97 | 0.625102 |
| GO:0006886\_intracellular\_protein\_transport | TOB1 | 122 | 2 | 1.217610 | -0.307333 | 931 | 581.97 | 0.625102 |
| GO:0002573\_myeloid\_leukocyte\_differentiation | KITL | 50 | 1 | 1.485484 | -0.306188 | 935 | 584.83 | 0.625487 |
| GO:0007015\_actin\_filament\_organization | BCL2 | 50 | 1 | 1.485484 | -0.306188 | 935 | 584.83 | 0.625487 |
| GO:0009190\_cyclic\_nucleotide\_biosynthetic\_process | GNAQ | 50 | 1 | 1.485484 | -0.306188 | 935 | 584.83 | 0.625487 |
| GO:0070647\_protein\_modification\_by\_small\_protein\_conjugation\_or\_removal | SUZ12 | 50 | 1 | 1.485484 | -0.306188 | 935 | 584.83 | 0.625487 |
| GO:0006520\_cellular\_amino\_acid\_metabolic\_process | ALDH18A1 | 51 | 1 | 1.456357 | -0.300177 | 941 | 590.51 | 0.627535 |
| GO:0006887\_exocytosis | CADPS | 51 | 1 | 1.456357 | -0.300177 | 941 | 590.51 | 0.627535 |
| GO:0016569\_covalent\_chromatin\_modification | SUZ12 | 51 | 1 | 1.456357 | -0.300177 | 941 | 590.51 | 0.627535 |
| GO:0032880\_regulation\_of\_protein\_localization | BCL2 | 51 | 1 | 1.456357 | -0.300177 | 941 | 590.51 | 0.627535 |
| GO:0044106\_cellular\_amine\_metabolic\_process | ALDH18A1 | 51 | 1 | 1.456357 | -0.300177 | 941 | 590.51 | 0.627535 |
| GO:0048747\_muscle\_fiber\_development | BCL2 | 51 | 1 | 1.456357 | -0.300177 | 941 | 590.51 | 0.627535 |
| GO:0002009\_morphogenesis\_of\_an\_epithelium | BCL2 | 198 | 3 | 1.125367 | -0.298739 | 943 | 591.68 | 0.627444 |
| GO:0002009\_morphogenesis\_of\_an\_epithelium | VEGFA | 198 | 3 | 1.125367 | -0.298739 | 943 | 591.68 | 0.627444 |
| GO:0002009\_morphogenesis\_of\_an\_epithelium | ONECUT2 | 198 | 3 | 1.125367 | -0.298739 | 943 | 591.68 | 0.627444 |
| GO:0060429\_epithelium\_development | BCL2 | 198 | 3 | 1.125367 | -0.298739 | 943 | 591.68 | 0.627444 |
| GO:0060429\_epithelium\_development | VEGFA | 198 | 3 | 1.125367 | -0.298739 | 943 | 591.68 | 0.627444 |
| GO:0060429\_epithelium\_development | ONECUT2 | 198 | 3 | 1.125367 | -0.298739 | 943 | 591.68 | 0.627444 |
| GO:0009124\_nucleoside\_monophosphate\_biosynthetic\_process | GNAQ | 52 | 1 | 1.428350 | -0.294327 | 945 | 594.91 | 0.629534 |
| GO:0048585\_negative\_regulation\_of\_response\_to\_stimulus | FABP7 | 52 | 1 | 1.428350 | -0.294327 | 945 | 594.91 | 0.629534 |
| GO:0033036\_macromolecule\_localization | NRCAM | 274 | 4 | 1.084295 | -0.293368 | 946 | 595.63 | 0.629630 |
| GO:0033036\_macromolecule\_localization | SLC11A1 | 274 | 4 | 1.084295 | -0.293368 | 946 | 595.63 | 0.629630 |
| GO:0033036\_macromolecule\_localization | BCL2 | 274 | 4 | 1.084295 | -0.293368 | 946 | 595.63 | 0.629630 |
| GO:0033036\_macromolecule\_localization | TOB1 | 274 | 4 | 1.084295 | -0.293368 | 946 | 595.63 | 0.629630 |
| GO:0006935\_chemotaxis | HMGB1 | 53 | 1 | 1.401400 | -0.288632 | 951 | 598.86 | 0.629716 |
| GO:0030031\_cell\_projection\_assembly | ONECUT2 | 53 | 1 | 1.401400 | -0.288632 | 951 | 598.86 | 0.629716 |
| GO:0042330\_taxis | HMGB1 | 53 | 1 | 1.401400 | -0.288632 | 951 | 598.86 | 0.629716 |
| GO:0046942\_carboxylic\_acid\_transport | SLC11A1 | 53 | 1 | 1.401400 | -0.288632 | 951 | 598.86 | 0.629716 |
| GO:0055085\_transmembrane\_transport | SLC11A1 | 53 | 1 | 1.401400 | -0.288632 | 951 | 598.86 | 0.629716 |
| GO:0048534\_hemopoietic\_or\_lymphoid\_organ\_development | HMGB1 | 277 | 4 | 1.072552 | -0.285968 | 953 | 599.38 | 0.628940 |
| GO:0048534\_hemopoietic\_or\_lymphoid\_organ\_development | BCL2 | 277 | 4 | 1.072552 | -0.285968 | 953 | 599.38 | 0.628940 |
| GO:0048534\_hemopoietic\_or\_lymphoid\_organ\_development | VEGFA | 277 | 4 | 1.072552 | -0.285968 | 953 | 599.38 | 0.628940 |
| GO:0048534\_hemopoietic\_or\_lymphoid\_organ\_development | KITL | 277 | 4 | 1.072552 | -0.285968 | 953 | 599.38 | 0.628940 |
| GO:0048646\_anatomical\_structure\_formation\_involved\_in\_morphogenesis | SEMA5A | 277 | 4 | 1.072552 | -0.285968 | 953 | 599.38 | 0.628940 |
| GO:0048646\_anatomical\_structure\_formation\_involved\_in\_morphogenesis | SPRY1 | 277 | 4 | 1.072552 | -0.285968 | 953 | 599.38 | 0.628940 |
| GO:0048646\_anatomical\_structure\_formation\_involved\_in\_morphogenesis | VEGFA | 277 | 4 | 1.072552 | -0.285968 | 953 | 599.38 | 0.628940 |
| GO:0048646\_anatomical\_structure\_formation\_involved\_in\_morphogenesis | PITX2 | 277 | 4 | 1.072552 | -0.285968 | 953 | 599.38 | 0.628940 |
| GO:0006164\_purine\_nucleotide\_biosynthetic\_process | GNAQ | 54 | 1 | 1.375448 | -0.283085 | 956 | 604.68 | 0.632510 |
| GO:0007265\_Ras\_protein\_signal\_transduction | KITL | 54 | 1 | 1.375448 | -0.283085 | 956 | 604.68 | 0.632510 |
| GO:0015849\_organic\_acid\_transport | SLC11A1 | 54 | 1 | 1.375448 | -0.283085 | 956 | 604.68 | 0.632510 |
| GO:0043285\_biopolymer\_catabolic\_process | HMGB1 | 129 | 2 | 1.151538 | -0.281670 | 957 | 605.32 | 0.632518 |
| GO:0043285\_biopolymer\_catabolic\_process | RNF6 | 129 | 2 | 1.151538 | -0.281670 | 957 | 605.32 | 0.632518 |
| GO:0034960\_cellular\_biopolymer\_metabolic\_process | HMGB1 | 1395 | 19 | 1.011620 | -0.280589 | 958 | 606.08 | 0.632651 |
| GO:0034960\_cellular\_biopolymer\_metabolic\_process | LCORL | 1395 | 19 | 1.011620 | -0.280589 | 958 | 606.08 | 0.632651 |
| GO:0034960\_cellular\_biopolymer\_metabolic\_process | ONECUT2 | 1395 | 19 | 1.011620 | -0.280589 | 958 | 606.08 | 0.632651 |
| GO:0034960\_cellular\_biopolymer\_metabolic\_process | PPM1A | 1395 | 19 | 1.011620 | -0.280589 | 958 | 606.08 | 0.632651 |
| GO:0034960\_cellular\_biopolymer\_metabolic\_process | RORB | 1395 | 19 | 1.011620 | -0.280589 | 958 | 606.08 | 0.632651 |
| GO:0034960\_cellular\_biopolymer\_metabolic\_process | KITL | 1395 | 19 | 1.011620 | -0.280589 | 958 | 606.08 | 0.632651 |
| GO:0034960\_cellular\_biopolymer\_metabolic\_process | AHR | 1395 | 19 | 1.011620 | -0.280589 | 958 | 606.08 | 0.632651 |
| GO:0034960\_cellular\_biopolymer\_metabolic\_process | SUZ12 | 1395 | 19 | 1.011620 | -0.280589 | 958 | 606.08 | 0.632651 |
| GO:0034960\_cellular\_biopolymer\_metabolic\_process | SLC11A1 | 1395 | 19 | 1.011620 | -0.280589 | 958 | 606.08 | 0.632651 |
| GO:0034960\_cellular\_biopolymer\_metabolic\_process | DCLRE1A | 1395 | 19 | 1.011620 | -0.280589 | 958 | 606.08 | 0.632651 |
| GO:0034960\_cellular\_biopolymer\_metabolic\_process | RNF6 | 1395 | 19 | 1.011620 | -0.280589 | 958 | 606.08 | 0.632651 |
| GO:0034960\_cellular\_biopolymer\_metabolic\_process | BCL2 | 1395 | 19 | 1.011620 | -0.280589 | 958 | 606.08 | 0.632651 |
| GO:0034960\_cellular\_biopolymer\_metabolic\_process | GARNL1 | 1395 | 19 | 1.011620 | -0.280589 | 958 | 606.08 | 0.632651 |
| GO:0034960\_cellular\_biopolymer\_metabolic\_process | NFAT5 | 1395 | 19 | 1.011620 | -0.280589 | 958 | 606.08 | 0.632651 |
| GO:0034960\_cellular\_biopolymer\_metabolic\_process | MYB | 1395 | 19 | 1.011620 | -0.280589 | 958 | 606.08 | 0.632651 |
| GO:0034960\_cellular\_biopolymer\_metabolic\_process | NR2F2 | 1395 | 19 | 1.011620 | -0.280589 | 958 | 606.08 | 0.632651 |
| GO:0034960\_cellular\_biopolymer\_metabolic\_process | FEN1 | 1395 | 19 | 1.011620 | -0.280589 | 958 | 606.08 | 0.632651 |
| GO:0034960\_cellular\_biopolymer\_metabolic\_process | ETV3 | 1395 | 19 | 1.011620 | -0.280589 | 958 | 606.08 | 0.632651 |
| GO:0034960\_cellular\_biopolymer\_metabolic\_process | PITX2 | 1395 | 19 | 1.011620 | -0.280589 | 958 | 606.08 | 0.632651 |
| GO:0007126\_meiosis | CDC25B | 55 | 1 | 1.350440 | -0.277681 | 961 | 610.47 | 0.635245 |
| GO:0043434\_response\_to\_peptide\_hormone\_stimulus | IRS2 | 55 | 1 | 1.350440 | -0.277681 | 961 | 610.47 | 0.635245 |
| GO:0051327\_M\_phase\_of\_meiotic\_cell\_cycle | CDC25B | 55 | 1 | 1.350440 | -0.277681 | 961 | 610.47 | 0.635245 |
| GO:0009187\_cyclic\_nucleotide\_metabolic\_process | GNAQ | 56 | 1 | 1.326325 | -0.272415 | 965 | 615.2 | 0.637513 |
| GO:0046486\_glycerolipid\_metabolic\_process | FABP3 | 56 | 1 | 1.326325 | -0.272415 | 965 | 615.2 | 0.637513 |
| GO:0050678\_regulation\_of\_epithelial\_cell\_proliferation | VEGFA | 56 | 1 | 1.326325 | -0.272415 | 965 | 615.2 | 0.637513 |
| GO:0051321\_meiotic\_cell\_cycle | CDC25B | 56 | 1 | 1.326325 | -0.272415 | 965 | 615.2 | 0.637513 |
| GO:0000226\_microtubule\_cytoskeleton\_organization | NEFL | 57 | 1 | 1.303056 | -0.267281 | 970 | 622.17 | 0.641412 |
| GO:0008344\_adult\_locomotory\_behavior | SNCA | 57 | 1 | 1.303056 | -0.267281 | 970 | 622.17 | 0.641412 |
| GO:0018108\_peptidyl-tyrosine\_phosphorylation | KITL | 57 | 1 | 1.303056 | -0.267281 | 970 | 622.17 | 0.641412 |
| GO:0018212\_peptidyl-tyrosine\_modification | KITL | 57 | 1 | 1.303056 | -0.267281 | 970 | 622.17 | 0.641412 |
| GO:0045444\_fat\_cell\_differentiation | SCD1 | 57 | 1 | 1.303056 | -0.267281 | 970 | 622.17 | 0.641412 |
| GO:0034622\_cellular\_macromolecular\_complex\_assembly | AHR | 58 | 1 | 1.280590 | -0.262275 | 971 | 624.93 | 0.643594 |
| GO:0016055\_Wnt\_receptor\_signaling\_pathway | PITX2 | 59 | 1 | 1.258885 | -0.257392 | 972 | 628.47 | 0.646574 |
| GO:0009057\_macromolecule\_catabolic\_process | HMGB1 | 137 | 2 | 1.084295 | -0.255211 | 973 | 629.02 | 0.646475 |
| GO:0009057\_macromolecule\_catabolic\_process | RNF6 | 137 | 2 | 1.084295 | -0.255211 | 973 | 629.02 | 0.646475 |
| GO:0009123\_nucleoside\_monophosphate\_metabolic\_process | GNAQ | 60 | 1 | 1.237903 | -0.252627 | 974 | 629.84 | 0.646653 |
| GO:0007166\_cell\_surface\_receptor\_linked\_signal\_transduction | IRS2 | 597 | 8 | 0.995299 | -0.249242 | 975 | 631.06 | 0.647241 |
| GO:0007166\_cell\_surface\_receptor\_linked\_signal\_transduction | LTBP1 | 597 | 8 | 0.995299 | -0.249242 | 975 | 631.06 | 0.647241 |
| GO:0007166\_cell\_surface\_receptor\_linked\_signal\_transduction | GNAQ | 597 | 8 | 0.995299 | -0.249242 | 975 | 631.06 | 0.647241 |
| GO:0007166\_cell\_surface\_receptor\_linked\_signal\_transduction | LTBP3 | 597 | 8 | 0.995299 | -0.249242 | 975 | 631.06 | 0.647241 |
| GO:0007166\_cell\_surface\_receptor\_linked\_signal\_transduction | ONECUT2 | 597 | 8 | 0.995299 | -0.249242 | 975 | 631.06 | 0.647241 |
| GO:0007166\_cell\_surface\_receptor\_linked\_signal\_transduction | VEGFA | 597 | 8 | 0.995299 | -0.249242 | 975 | 631.06 | 0.647241 |
| GO:0007166\_cell\_surface\_receptor\_linked\_signal\_transduction | TOB1 | 597 | 8 | 0.995299 | -0.249242 | 975 | 631.06 | 0.647241 |
| GO:0007166\_cell\_surface\_receptor\_linked\_signal\_transduction | PITX2 | 597 | 8 | 0.995299 | -0.249242 | 975 | 631.06 | 0.647241 |
| GO:0007169\_transmembrane\_receptor\_protein\_tyrosine\_kinase\_signaling\_pathway | IRS2 | 139 | 2 | 1.068693 | -0.249031 | 977 | 631.66 | 0.646530 |
| GO:0007169\_transmembrane\_receptor\_protein\_tyrosine\_kinase\_signaling\_pathway | VEGFA | 139 | 2 | 1.068693 | -0.249031 | 977 | 631.66 | 0.646530 |
| GO:0034613\_cellular\_protein\_localization | SLC11A1 | 139 | 2 | 1.068693 | -0.249031 | 977 | 631.66 | 0.646530 |
| GO:0034613\_cellular\_protein\_localization | TOB1 | 139 | 2 | 1.068693 | -0.249031 | 977 | 631.66 | 0.646530 |
| GO:0006874\_cellular\_calcium\_ion\_homeostasis | BCL2 | 61 | 1 | 1.217610 | -0.247977 | 978 | 633.51 | 0.647761 |
| GO:0048583\_regulation\_of\_response\_to\_stimulus | SLC11A1 | 217 | 3 | 1.026832 | -0.247541 | 979 | 633.93 | 0.647528 |
| GO:0048583\_regulation\_of\_response\_to\_stimulus | HMGB1 | 217 | 3 | 1.026832 | -0.247541 | 979 | 633.93 | 0.647528 |
| GO:0048583\_regulation\_of\_response\_to\_stimulus | FABP7 | 217 | 3 | 1.026832 | -0.247541 | 979 | 633.93 | 0.647528 |
[truncated: 42,849 more chars]
