# Supplementary material for: RedundancyMiner: De-replication of redundant GO categories in microarray and proteomics analysis
Source: BMC Bioinformatics. 2011 Feb 10;12:52. doi: 10.1186/1471-2105-12-52 (PMC3223614; doi:10.1186/1471-2105-12-52)
Supplement: Additional file 8 — Retinal development HTGM download. compressed package of the results of running HTGM on the retinal development genes list. [file 1471-2105-12-52-S8.ZIP › SCENARIO_2_MODIFIED/total.txt.total.txt.dir/Exp1_BestClusterMap_LEIGS_KM_24.csv.join.22.txt.dir/Exp1_BestClusterMap_LEIGS_KM_24.csv.join.22.txt.change.html]

Category Summary Report for Exp1\_BestClusterMap\_LEIGS\_KM\_24.csv.join.22.txt

# Category Summary Report for Exp1\_BestClusterMap\_LEIGS\_KM\_24.csv.join.22.txt

| HYPERLINKED GO CATEGORY | TOTAL GENES | CHANGED GENES | ENRICHMENT | LOG10(p) | CUMULATIVE NUMBER OF CATEGORIES | CUMULATIVE RANDOMS LOWER BOUND | CUMULATIVE RANDOMS MEAN | CUMULATIVE RANDOMS UPPER BOUND | FALSE DISCOVERY RATE |
| --- | --- | --- | --- | --- | --- | --- | --- | --- | --- |
| GO:0045634\_regulation\_of\_melanocyte\_differentiation | 4 | 3 |  |  |  |  |  |  |  |  |
| GO:0050932\_regulation\_of\_pigment\_cell\_differentiation | 4 | 3 |  |  |  |  |  |  |  |  |
| GO:0022008\_neurogenesis | 423 | 17 | 2.985015 | -4.587653 | 1 | -0.180000 | 0.02 | 0.220000 | 0.020000 |
| GO:0048666\_neuron\_development | 262 | 13 | 3.685361 | -4.485241 | 2 | -0.180000 | 0.02 | 0.220000 | 0.010000 |
| GO:0048699\_generation\_of\_neurons | 396 | 16 | 3.000978 | -4.349756 | 3 | -0.192702 | 0.03 | 0.252702 | 0.010000 |
| GO:0014047\_glutamate\_secretion | 7 | 3 | 31.831797 | -4.106238 | 4 | -0.313893 | 0.08 | 0.473893 | 0.020000 |
| GO:0045636\_positive\_regulation\_of\_melanocyte\_differentiation | 2 | 2 |  |  |  |  |  |  |  |  |
| GO:0050942\_positive\_regulation\_of\_pigment\_cell\_differentiation | 2 | 2 |  |  |  |  |  |  |  |  |
| GO:0030335\_positive\_regulation\_of\_cell\_migration | 22 | 4 | 13.504399 | -3.740480 | 5 | -0.524743 | 0.2 | 0.924743 | 0.040000 |
| GO:0048070\_regulation\_of\_pigmentation\_during\_development | 9 | 3 | 24.758065 | -3.734400 | 6 | -0.524743 | 0.2 | 0.924743 | 0.033333 |
| GO:0030182\_neuron\_differentiation | 356 | 14 | 2.920895 | -3.700699 | 7 | -0.532573 | 0.21 | 0.952573 | 0.030000 |
| GO:0048592\_eye\_morphogenesis | 70 | 6 | 6.366359 | -3.505509 | 8 | -0.557006 | 0.27 | 1.097006 | 0.033750 |
| GO:0007399\_nervous\_system\_development | 621 | 19 | 2.272479 | -3.480218 | 9 | -0.557006 | 0.27 | 1.097006 | 0.030000 |
| GO:0006928\_cell\_motion | 330 | 13 | 2.925953 | -3.461063 | 11 | -0.557006 | 0.27 | 1.097006 | 0.024545 |
| GO:0051674\_localization\_of\_cell | 330 | 13 | 2.925953 | -3.461063 | 11 | -0.557006 | 0.27 | 1.097006 | 0.024545 |
| GO:0040012\_regulation\_of\_locomotion | 72 | 6 | 6.189516 | -3.438460 | 12 | -0.577584 | 0.32 | 1.217584 | 0.026667 |
| GO:0051272\_positive\_regulation\_of\_cell\_motion | 27 | 4 | 11.003584 | -3.382314 | 13 | -0.577584 | 0.32 | 1.217584 | 0.024615 |
| GO:0001654\_eye\_development | 136 | 8 | 4.369070 | -3.382301 | 14 | -0.577584 | 0.32 | 1.217584 | 0.022857 |
| GO:0048087\_positive\_regulation\_of\_pigmentation\_during\_development | 3 | 2 |  |  |  |  |  |  |  |  |
| GO:0010623\_developmental\_programmed\_cell\_death | 13 | 3 | 17.140199 | -3.219037 | 15 | -0.636095 | 0.44 | 1.516095 | 0.029333 |
| GO:0048468\_cell\_development | 654 | 19 | 2.157813 | -3.190461 | 16 | -0.679923 | 0.47 | 1.619923 | 0.029375 |
| GO:0046668\_regulation\_of\_retinal\_cell\_programmed\_cell\_death | 4 | 2 |  |  |  |  |  |  |  |  |
| GO:0030334\_regulation\_of\_cell\_migration | 59 | 5 | 6.294423 | -2.968128 | 17 | -0.720732 | 0.74 | 2.200732 | 0.043529 |
| GO:0001657\_ureteric\_bud\_development | 38 | 4 | 7.818336 | -2.806512 | 18 | -0.890494 | 1.07 | 3.030494 | 0.059444 |
| GO:0030318\_melanocyte\_differentiation | 18 | 3 | 12.379032 | -2.784571 | 19 | -0.861756 | 1.1 | 3.061756 | 0.057895 |
| GO:0040011\_locomotion | 295 | 11 | 2.769546 | -2.780667 | 20 | -0.889470 | 1.11 | 3.109470 | 0.055500 |
| GO:0043524\_negative\_regulation\_of\_neuron\_apoptosis | 39 | 4 | 7.617866 | -2.763878 | 21 | -0.918182 | 1.13 | 3.178182 | 0.053810 |
| GO:0033033\_negative\_regulation\_of\_myeloid\_cell\_apoptosis | 5 | 2 | 29.709677 | -2.760004 | 22 | -0.654772 | 1.61 | 3.874772 | 0.073182 |
| GO:0051402\_neuron\_apoptosis | 66 | 5 | 5.626833 | -2.747771 | 23 | -0.662033 | 1.62 | 3.902033 | 0.070435 |
| GO:0042462\_eye\_photoreceptor\_cell\_development | 19 | 3 | 11.727504 | -2.714103 | 25 | -0.593248 | 1.76 | 4.113248 | 0.070400 |
| GO:0050931\_pigment\_cell\_differentiation | 19 | 3 | 11.727504 | -2.714103 | 25 | -0.593248 | 1.76 | 4.113248 | 0.070400 |
| GO:0031960\_response\_to\_corticosteroid\_stimulus | 6 | 2 | 24.758065 | -2.587693 | 28 | -0.497160 | 2.61 | 5.717160 | 0.093214 |
| GO:0046666\_retinal\_cell\_programmed\_cell\_death | 6 | 2 | 24.758065 | -2.587693 | 28 | -0.497160 | 2.61 | 5.717160 | 0.093214 |
| GO:0051384\_response\_to\_glucocorticoid\_stimulus | 6 | 2 | 24.758065 | -2.587693 | 28 | -0.497160 | 2.61 | 5.717160 | 0.093214 |
| GO:0001754\_eye\_photoreceptor\_cell\_differentiation | 21 | 3 | 10.610599 | -2.584899 | 30 | -0.489313 | 2.7 | 5.889313 | 0.090000 |
| GO:0002053\_positive\_regulation\_of\_mesenchymal\_cell\_proliferation | 21 | 3 | 10.610599 | -2.584899 | 30 | -0.489313 | 2.7 | 5.889313 | 0.090000 |
| GO:0051270\_regulation\_of\_cell\_motion | 73 | 5 | 5.087274 | -2.553550 | 31 | -0.579511 | 2.81 | 6.199511 | 0.090645 |
| GO:0001569\_patterning\_of\_blood\_vessels | 22 | 3 | 10.128299 | -2.525389 | 35 | -0.636076 | 3.04 | 6.716076 | 0.086857 |
| GO:0010463\_mesenchymal\_cell\_proliferation | 22 | 3 | 10.128299 | -2.525389 | 35 | -0.636076 | 3.04 | 6.716076 | 0.086857 |
| GO:0010464\_regulation\_of\_mesenchymal\_cell\_proliferation | 22 | 3 | 10.128299 | -2.525389 | 35 | -0.636076 | 3.04 | 6.716076 | 0.086857 |
| GO:0042461\_photoreceptor\_cell\_development | 22 | 3 | 10.128299 | -2.525389 | 35 | -0.636076 | 3.04 | 6.716076 | 0.086857 |
| GO:0048869\_cellular\_developmental\_process | 1113 | 25 | 1.668333 | -2.474393 | 36 | -0.565381 | 3.18 | 6.925381 | 0.088333 |
| GO:0000082\_G1\_S\_transition\_of\_mitotic\_cell\_cycle | 23 | 3 | 9.687938 | -2.468848 | 37 | -0.560455 | 3.28 | 7.120455 | 0.088649 |
| GO:0033032\_regulation\_of\_myeloid\_cell\_apoptosis | 7 | 2 | 21.221198 | -2.445341 | 38 | -0.103173 | 4.1 | 8.303173 | 0.107895 |
| GO:0016477\_cell\_migration | 234 | 9 | 2.856700 | -2.431034 | 39 | -0.103173 | 4.1 | 8.303173 | 0.105128 |
| GO:0051179\_localization | 1058 | 24 | 1.684859 | -2.429746 | 40 | -0.113300 | 4.11 | 8.333300 | 0.102750 |
| GO:0030154\_cell\_differentiation | 1060 | 24 | 1.681680 | -2.418439 | 41 | -0.169341 | 4.18 | 8.529341 | 0.101951 |
| GO:0001656\_metanephros\_development | 50 | 4 | 5.941935 | -2.364623 | 42 | -0.162897 | 4.47 | 9.102897 | 0.106429 |
| GO:0007417\_central\_nervous\_system\_development | 287 | 10 | 2.587951 | -2.347184 | 43 | -0.138221 | 4.51 | 9.158221 | 0.104884 |
| GO:0031175\_neuron\_projection\_development | 197 | 8 | 3.016211 | -2.345987 | 44 | -0.147706 | 4.52 | 9.187706 | 0.102727 |
| GO:0045664\_regulation\_of\_neuron\_differentiation | 82 | 5 | 4.528914 | -2.334448 | 45 | -0.147706 | 4.52 | 9.187706 | 0.100444 |
| GO:0031102\_neuron\_projection\_regeneration | 8 | 2 | 18.568548 | -2.324175 | 47 | 0.185564 | 5.26 | 10.334436 | 0.111915 |
| GO:0031103\_axon\_regeneration | 8 | 2 | 18.568548 | -2.324175 | 47 | 0.185564 | 5.26 | 10.334436 | 0.111915 |
| GO:0046530\_photoreceptor\_cell\_differentiation | 26 | 3 | 8.570099 | -2.314551 | 48 | 0.248081 | 5.36 | 10.471919 | 0.111667 |
| GO:0007409\_axonogenesis | 158 | 7 | 3.290629 | -2.306897 | 49 | 0.281416 | 5.38 | 10.478584 | 0.109796 |
| GO:0033028\_myeloid\_cell\_apoptosis | 9 | 2 | 16.505376 | -2.218799 | 50 | 0.405168 | 6.57 | 12.734832 | 0.131400 |
| GO:0042325\_regulation\_of\_phosphorylation | 164 | 7 | 3.170240 | -2.218789 | 51 | 0.405168 | 6.57 | 12.734832 | 0.128824 |
| GO:0019220\_regulation\_of\_phosphate\_metabolic\_process | 165 | 7 | 3.151026 | -2.204523 | 53 | 0.455105 | 6.64 | 12.824895 | 0.125283 |
| GO:0051174\_regulation\_of\_phosphorus\_metabolic\_process | 165 | 7 | 3.151026 | -2.204523 | 53 | 0.455105 | 6.64 | 12.824895 | 0.125283 |
| GO:0001934\_positive\_regulation\_of\_protein\_amino\_acid\_phosphorylation | 29 | 3 | 7.683537 | -2.179193 | 55 | 0.503760 | 6.83 | 13.156240 | 0.124182 |
| GO:0048066\_pigmentation\_during\_development | 29 | 3 | 7.683537 | -2.179193 | 55 | 0.503760 | 6.83 | 13.156240 | 0.124182 |
| GO:0048870\_cell\_motility | 257 | 9 | 2.601042 | -2.164158 | 56 | 0.497756 | 6.85 | 13.202244 | 0.122321 |
| GO:0043523\_regulation\_of\_neuron\_apoptosis | 57 | 4 | 5.212224 | -2.160705 | 57 | 0.560947 | 6.96 | 13.359053 | 0.122105 |
| GO:0048812\_neuron\_projection\_morphogenesis | 170 | 7 | 3.058349 | -2.134899 | 58 | 0.667390 | 7.17 | 13.672610 | 0.123621 |
| GO:0007006\_mitochondrial\_membrane\_organization | 10 | 2 | 14.854839 | -2.125655 | 60 | 1.262262 | 8.31 | 15.357738 | 0.138500 |
| GO:0042116\_macrophage\_activation | 10 | 2 | 14.854839 | -2.125655 | 60 | 1.262262 | 8.31 | 15.357738 | 0.138500 |
| GO:0048469\_cell\_maturation | 59 | 4 | 5.035539 | -2.107854 | 61 | 1.258008 | 8.45 | 15.641992 | 0.138525 |
| GO:0030030\_cell\_projection\_organization | 263 | 9 | 2.541702 | -2.100116 | 62 | 1.211570 | 8.5 | 15.788430 | 0.137097 |
| GO:0010562\_positive\_regulation\_of\_phosphorus\_metabolic\_process | 31 | 3 | 7.187825 | -2.097513 | 65 | 1.346721 | 8.7 | 16.053279 | 0.133846 |
| GO:0042327\_positive\_regulation\_of\_phosphorylation | 31 | 3 | 7.187825 | -2.097513 | 65 | 1.346721 | 8.7 | 16.053279 | 0.133846 |
| GO:0045937\_positive\_regulation\_of\_phosphate\_metabolic\_process | 31 | 3 | 7.187825 | -2.097513 | 65 | 1.346721 | 8.7 | 16.053279 | 0.133846 |
| GO:0048667\_cell\_morphogenesis\_involved\_in\_neuron\_differentiation | 173 | 7 | 3.005314 | -2.094441 | 66 | 1.326843 | 8.71 | 16.093157 | 0.131970 |
| GO:0007423\_sensory\_organ\_development | 219 | 8 | 2.713213 | -2.072663 | 67 | 1.328447 | 8.81 | 16.291553 | 0.131493 |
| GO:0050768\_negative\_regulation\_of\_neurogenesis | 32 | 3 | 6.963206 | -2.058898 | 68 | 1.319162 | 8.94 | 16.560838 | 0.131471 |
| GO:0048858\_cell\_projection\_morphogenesis | 176 | 7 | 2.954087 | -2.054927 | 69 | 1.416663 | 9.06 | 16.703337 | 0.131304 |
| GO:0001952\_regulation\_of\_cell-matrix\_adhesion | 11 | 2 | 13.504399 | -2.042266 | 73 | 1.604686 | 9.81 | 18.015314 | 0.134384 |
| GO:0006637\_acyl-CoA\_metabolic\_process | 11 | 2 | 13.504399 | -2.042266 | 73 | 1.604686 | 9.81 | 18.015314 | 0.134384 |
| GO:0042551\_neuron\_maturation | 11 | 2 | 13.504399 | -2.042266 | 73 | 1.604686 | 9.81 | 18.015314 | 0.134384 |
| GO:0048678\_response\_to\_axon\_injury | 11 | 2 | 13.504399 | -2.042266 | 73 | 1.604686 | 9.81 | 18.015314 | 0.134384 |
| GO:0010721\_negative\_regulation\_of\_cell\_development | 34 | 3 | 6.553605 | -1.985656 | 74 | 1.772086 | 10.69 | 19.607914 | 0.144459 |
| GO:0050432\_catecholamine\_secretion | 12 | 2 | 12.379032 | -1.966843 | 75 | 2.277855 | 11.66 | 21.042145 | 0.155467 |
| GO:0032990\_cell\_part\_morphogenesis | 184 | 7 | 2.825649 | -1.953933 | 76 | 2.330218 | 11.8 | 21.269782 | 0.155263 |
| GO:0051325\_interphase | 35 | 3 | 6.366359 | -1.950868 | 78 | 2.474169 | 11.97 | 21.465831 | 0.153462 |
| GO:0051329\_interphase\_of\_mitotic\_cell\_cycle | 35 | 3 | 6.366359 | -1.950868 | 78 | 2.474169 | 11.97 | 21.465831 | 0.153462 |
| GO:0007420\_brain\_development | 231 | 8 | 2.572266 | -1.939363 | 79 | 2.439964 | 12.03 | 21.620036 | 0.152278 |
| GO:0007179\_transforming\_growth\_factor\_beta\_receptor\_signaling\_pathway | 66 | 4 | 4.501466 | -1.938528 | 81 | 2.501397 | 12.09 | 21.678603 | 0.149259 |
| GO:0045860\_positive\_regulation\_of\_protein\_kinase\_activity | 66 | 4 | 4.501466 | -1.938528 | 81 | 2.501397 | 12.09 | 21.678603 | 0.149259 |
| GO:0019222\_regulation\_of\_metabolic\_process | 1088 | 23 | 1.570135 | -1.938309 | 82 | 2.473123 | 12.1 | 21.726877 | 0.147561 |
| GO:0007155\_cell\_adhesion | 186 | 7 | 2.795265 | -1.929631 | 84 | 2.524721 | 12.17 | 21.815279 | 0.144881 |
| GO:0022610\_biological\_adhesion | 186 | 7 | 2.795265 | -1.929631 | 84 | 2.524721 | 12.17 | 21.815279 | 0.144881 |
| GO:0050767\_regulation\_of\_neurogenesis | 104 | 5 | 3.570875 | -1.904616 | 85 | 2.718579 | 12.68 | 22.641421 | 0.149176 |
| GO:0009994\_oocyte\_differentiation | 13 | 2 | 11.426799 | -1.898047 | 87 | 3.249957 | 13.49 | 23.730043 | 0.155057 |
| GO:0048599\_oocyte\_development | 13 | 2 | 11.426799 | -1.898047 | 87 | 3.249957 | 13.49 | 23.730043 | 0.155057 |
| GO:0030900\_forebrain\_development | 146 | 6 | 3.052364 | -1.883615 | 88 | 3.412713 | 13.7 | 23.987287 | 0.155682 |
| GO:0001956\_positive\_regulation\_of\_neurotransmitter\_secretion | 1 | 1 |  |  |  |  |  |  |  |  |
| GO:0002468\_dendritic\_cell\_antigen\_processing\_and\_presentation | 1 | 1 |  |  |  |  |  |  |  |  |
| GO:0002577\_regulation\_of\_antigen\_processing\_and\_presentation | 1 | 1 |  |  |  |  |  |  |  |  |
| GO:0002579\_positive\_regulation\_of\_antigen\_processing\_and\_presentation | 1 | 1 |  |  |  |  |  |  |  |  |
| GO:0002604\_regulation\_of\_dendritic\_cell\_antigen\_processing\_and\_presentation | 1 | 1 |  |  |  |  |  |  |  |  |
| GO:0002606\_positive\_regulation\_of\_dendritic\_cell\_antigen\_processing\_and\_presentation | 1 | 1 |  |  |  |  |  |  |  |  |
| GO:0006000\_fructose\_metabolic\_process | 1 | 1 |  |  |  |  |  |  |  |  |
| GO:0006002\_fructose\_6-phosphate\_metabolic\_process | 1 | 1 |  |  |  |  |  |  |  |  |
| GO:0009956\_radial\_pattern\_formation | 1 | 1 |  |  |  |  |  |  |  |  |
| GO:0010523\_negative\_regulation\_of\_calcium\_ion\_transport\_into\_cytosol | 1 | 1 |  |  |  |  |  |  |  |  |
| GO:0014012\_axon\_regeneration\_in\_the\_peripheral\_nervous\_system | 1 | 1 |  |  |  |  |  |  |  |  |
| GO:0014041\_regulation\_of\_neuron\_maturation | 1 | 1 |  |  |  |  |  |  |  |  |
| GO:0014042\_positive\_regulation\_of\_neuron\_maturation | 1 | 1 |  |  |  |  |  |  |  |  |
| GO:0014910\_regulation\_of\_smooth\_muscle\_cell\_migration | 1 | 1 |  |  |  |  |  |  |  |  |
| GO:0014911\_positive\_regulation\_of\_smooth\_muscle\_cell\_migration | 1 | 1 |  |  |  |  |  |  |  |  |
| GO:0015707\_nitrite\_transport | 1 | 1 |  |  |  |  |  |  |  |  |
| GO:0019079\_viral\_genome\_replication | 1 | 1 |  |  |  |  |  |  |  |  |
| GO:0021577\_hindbrain\_structural\_organization | 1 | 1 |  |  |  |  |  |  |  |  |
| GO:0021589\_cerebellum\_structural\_organization | 1 | 1 |  |  |  |  |  |  |  |  |
| GO:0021747\_cochlear\_nucleus\_development | 1 | 1 |  |  |  |  |  |  |  |  |
| GO:0021812\_neuronal-glial\_interaction\_involved\_in\_cerebral\_cortex\_radial\_glia\_guided\_migration | 1 | 1 |  |  |  |  |  |  |  |  |
| GO:0021813\_cell-cell\_adhesion\_involved\_in\_neuronal-glial\_interactions\_involved\_in\_cerebral\_cortex\_radial\_glia\_guided\_migration | 1 | 1 |  |  |  |  |  |  |  |  |
| GO:0021942\_radial\_glia\_guided\_migration\_of\_Purkinje\_cell | 1 | 1 |  |  |  |  |  |  |  |  |
| GO:0031129\_inductive\_cell-cell\_signaling | 1 | 1 |  |  |  |  |  |  |  |  |
| GO:0033138\_positive\_regulation\_of\_peptidyl-serine\_phosphorylation | 1 | 1 |  |  |  |  |  |  |  |  |
| GO:0033687\_osteoblast\_proliferation | 1 | 1 |  |  |  |  |  |  |  |  |
| GO:0033688\_regulation\_of\_osteoblast\_proliferation | 1 | 1 |  |  |  |  |  |  |  |  |
| GO:0033689\_negative\_regulation\_of\_osteoblast\_proliferation | 1 | 1 |  |  |  |  |  |  |  |  |
| GO:0043091\_L-arginine\_import | 1 | 1 |  |  |  |  |  |  |  |  |
| GO:0043369\_CD4-positive\_or\_CD8-positive\_\_alpha-beta\_T\_cell\_lineage\_commitment | 1 | 1 |  |  |  |  |  |  |  |  |
| GO:0043375\_CD8-positive\_\_alpha-beta\_T\_cell\_lineage\_commitment | 1 | 1 |  |  |  |  |  |  |  |  |
| GO:0045069\_regulation\_of\_viral\_genome\_replication | 1 | 1 |  |  |  |  |  |  |  |  |
| GO:0045898\_regulation\_of\_transcriptional\_preinitiation\_complex\_assembly | 1 | 1 |  |  |  |  |  |  |  |  |
| GO:0045899\_positive\_regulation\_of\_transcriptional\_preinitiation\_complex\_assembly | 1 | 1 |  |  |  |  |  |  |  |  |
| GO:0046671\_negative\_regulation\_of\_retinal\_cell\_programmed\_cell\_death | 1 | 1 |  |  |  |  |  |  |  |  |
| GO:0046949\_acyl-CoA\_biosynthetic\_process | 1 | 1 |  |  |  |  |  |  |  |  |
| GO:0048743\_positive\_regulation\_of\_skeletal\_muscle\_fiber\_development | 1 | 1 |  |  |  |  |  |  |  |  |
| GO:0050812\_regulation\_of\_acyl-CoA\_biosynthetic\_process | 1 | 1 |  |  |  |  |  |  |  |  |
| GO:0051123\_transcriptional\_preinitiation\_complex\_assembly | 1 | 1 |  |  |  |  |  |  |  |  |
| GO:0051193\_regulation\_of\_cofactor\_metabolic\_process | 1 | 1 |  |  |  |  |  |  |  |  |
| GO:0051196\_regulation\_of\_coenzyme\_metabolic\_process | 1 | 1 |  |  |  |  |  |  |  |  |
| GO:0060215\_primitive\_hemopoiesis | 1 | 1 |  |  |  |  |  |  |  |  |
| GO:0060319\_primitive\_erythrocyte\_differentiation | 1 | 1 |  |  |  |  |  |  |  |  |
| GO:0060577\_pulmonary\_vein\_morphogenesis | 1 | 1 |  |  |  |  |  |  |  |  |
| GO:0060578\_superior\_vena\_cava\_morphogenesis | 1 | 1 |  |  |  |  |  |  |  |  |
| GO:0043085\_positive\_regulation\_of\_catalytic\_activity | 148 | 6 | 3.011116 | -1.856559 | 89 | 3.476427 | 13.86 | 24.243573 | 0.155730 |
| GO:0045859\_regulation\_of\_protein\_kinase\_activity | 107 | 5 | 3.470757 | -1.854967 | 90 | 3.476427 | 13.86 | 24.243573 | 0.154000 |
| GO:0016053\_organic\_acid\_biosynthetic\_process | 38 | 3 | 5.863752 | -1.853019 | 94 | 3.522304 | 14.02 | 24.517696 | 0.149149 |
| GO:0031401\_positive\_regulation\_of\_protein\_modification\_process | 38 | 3 | 5.863752 | -1.853019 | 94 | 3.522304 | 14.02 | 24.517696 | 0.149149 |
| GO:0042493\_response\_to\_drug | 38 | 3 | 5.863752 | -1.853019 | 94 | 3.522304 | 14.02 | 24.517696 | 0.149149 |
| GO:0046394\_carboxylic\_acid\_biosynthetic\_process | 38 | 3 | 5.863752 | -1.853019 | 94 | 3.522304 | 14.02 | 24.517696 | 0.149149 |
| GO:0000060\_protein\_import\_into\_nucleus\_\_translocation | 14 | 2 | 10.610599 | -1.834851 | 98 | 4.313508 | 15.07 | 25.826492 | 0.153776 |
| GO:0019217\_regulation\_of\_fatty\_acid\_metabolic\_process | 14 | 2 | 10.610599 | -1.834851 | 98 | 4.313508 | 15.07 | 25.826492 | 0.153776 |
| GO:0031099\_regeneration | 14 | 2 | 10.610599 | -1.834851 | 98 | 4.313508 | 15.07 | 25.826492 | 0.153776 |
| GO:0048545\_response\_to\_steroid\_hormone\_stimulus | 14 | 2 | 10.610599 | -1.834851 | 98 | 4.313508 | 15.07 | 25.826492 | 0.153776 |
| GO:0033674\_positive\_regulation\_of\_kinase\_activity | 71 | 4 | 4.184462 | -1.830398 | 99 | 4.343521 | 15.11 | 25.876479 | 0.152626 |
| GO:0051347\_positive\_regulation\_of\_transferase\_activity | 72 | 4 | 4.126344 | -1.809891 | 100 | 4.702597 | 15.67 | 26.637403 | 0.156700 |
| GO:0045595\_regulation\_of\_cell\_differentiation | 295 | 9 | 2.265992 | -1.791857 | 101 | 4.888118 | 16.02 | 27.151882 | 0.158614 |
| GO:0000904\_cell\_morphogenesis\_involved\_in\_differentiation | 199 | 7 | 2.612660 | -1.780153 | 102 | 4.977907 | 16.15 | 27.322093 | 0.158333 |
| GO:0006885\_regulation\_of\_pH | 15 | 2 | 9.903226 | -1.776451 | 103 | 5.446917 | 17.07 | 28.693083 | 0.165728 |
| GO:0043549\_regulation\_of\_kinase\_activity | 112 | 5 | 3.315812 | -1.776096 | 104 | 5.483580 | 17.09 | 28.696420 | 0.164327 |
| GO:0032879\_regulation\_of\_localization | 248 | 8 | 2.395942 | -1.766834 | 105 | 5.474787 | 17.15 | 28.825213 | 0.163333 |
| GO:0051338\_regulation\_of\_transferase\_activity | 115 | 5 | 3.229313 | -1.730944 | 106 | 5.741606 | 18.08 | 30.418394 | 0.170566 |
| GO:0042596\_fear\_response | 16 | 2 | 9.284274 | -1.722202 | 108 | 6.231282 | 18.94 | 31.648718 | 0.175370 |
| GO:0051937\_catecholamine\_transport | 16 | 2 | 9.284274 | -1.722202 | 108 | 6.231282 | 18.94 | 31.648718 | 0.175370 |
| GO:0048731\_system\_development | 1609 | 30 | 1.384851 | -1.714370 | 109 | 6.213323 | 19.04 | 31.866677 | 0.174679 |
| GO:0051960\_regulation\_of\_nervous\_system\_development | 118 | 5 | 3.147212 | -1.687314 | 110 | 6.285318 | 19.62 | 32.954682 | 0.178364 |
| GO:0008284\_positive\_regulation\_of\_cell\_proliferation | 208 | 7 | 2.499612 | -1.684590 | 111 | 6.285318 | 19.62 | 32.954682 | 0.176757 |
| GO:0048856\_anatomical\_structure\_development | 1688 | 31 | 1.364040 | -1.679779 | 112 | 6.407112 | 19.99 | 33.572888 | 0.178482 |
| GO:0031323\_regulation\_of\_cellular\_metabolic\_process | 1015 | 21 | 1.536707 | -1.676938 | 113 | 6.430060 | 20.02 | 33.609940 | 0.177168 |
| GO:0010565\_regulation\_of\_cellular\_ketone\_metabolic\_process | 17 | 2 | 8.738140 | -1.671584 | 116 | 6.943738 | 20.94 | 34.936262 | 0.180517 |
| GO:0016052\_carbohydrate\_catabolic\_process | 17 | 2 | 8.738140 | -1.671584 | 116 | 6.943738 | 20.94 | 34.936262 | 0.180517 |
| GO:0055067\_monovalent\_inorganic\_cation\_homeostasis | 17 | 2 | 8.738140 | -1.671584 | 116 | 6.943738 | 20.94 | 34.936262 | 0.180517 |
| GO:0000278\_mitotic\_cell\_cycle | 80 | 4 | 3.713710 | -1.657608 | 118 | 6.946985 | 21.12 | 35.293015 | 0.178983 |
| GO:0006631\_fatty\_acid\_metabolic\_process | 80 | 4 | 3.713710 | -1.657608 | 118 | 6.946985 | 21.12 | 35.293015 | 0.178983 |
| GO:0021700\_developmental\_maturation | 81 | 4 | 3.667861 | -1.639917 | 119 | 7.041273 | 21.43 | 35.818727 | 0.180084 |
| GO:0060284\_regulation\_of\_cell\_development | 122 | 5 | 3.044024 | -1.631376 | 120 | 7.068596 | 21.61 | 36.151404 | 0.180083 |
| GO:0003014\_renal\_system\_process | 18 | 2 | 8.252688 | -1.624168 | 121 | 7.841918 | 23.12 | 38.398082 | 0.191074 |
| GO:0007411\_axon\_guidance | 82 | 4 | 3.623131 | -1.622499 | 122 | 7.887016 | 23.21 | 38.532984 | 0.190246 |
| GO:0048871\_multicellular\_organismal\_homeostasis | 47 | 3 | 4.740906 | -1.606248 | 123 | 7.914380 | 23.53 | 39.145620 | 0.191301 |
| GO:0010033\_response\_to\_organic\_substance | 216 | 7 | 2.407034 | -1.604560 | 124 | 8.049018 | 23.69 | 39.330982 | 0.191048 |
| GO:0001763\_morphogenesis\_of\_a\_branching\_structure | 125 | 5 | 2.970968 | -1.591005 | 125 | 8.076136 | 23.84 | 39.603864 | 0.190720 |
| GO:0001774\_microglial\_cell\_activation | 2 | 1 |  |  |  |  |  |  |  |  |
| GO:0002074\_extraocular\_skeletal\_muscle\_development | 2 | 1 |  |  |  |  |  |  |  |  |
| GO:0005981\_regulation\_of\_glycogen\_catabolic\_process | 2 | 1 |  |  |  |  |  |  |  |  |
| GO:0006808\_regulation\_of\_nitrogen\_utilization | 2 | 1 |  |  |  |  |  |  |  |  |
| GO:0007035\_vacuolar\_acidification | 2 | 1 |  |  |  |  |  |  |  |  |
| GO:0010559\_regulation\_of\_glycoprotein\_biosynthetic\_process | 2 | 1 |  |  |  |  |  |  |  |  |
| GO:0014048\_regulation\_of\_glutamate\_secretion | 2 | 1 |  |  |  |  |  |  |  |  |
| GO:0019740\_nitrogen\_utilization | 2 | 1 |  |  |  |  |  |  |  |  |
| GO:0021932\_hindbrain\_radial\_glia\_guided\_cell\_migration | 2 | 1 |  |  |  |  |  |  |  |  |
| GO:0034341\_response\_to\_interferon-gamma | 2 | 1 |  |  |  |  |  |  |  |  |
| GO:0045819\_positive\_regulation\_of\_glycogen\_catabolic\_process | 2 | 1 |  |  |  |  |  |  |  |  |
| GO:0048643\_positive\_regulation\_of\_skeletal\_muscle\_tissue\_development | 2 | 1 |  |  |  |  |  |  |  |  |
| GO:0048712\_negative\_regulation\_of\_astrocyte\_differentiation | 2 | 1 |  |  |  |  |  |  |  |  |
| GO:0050792\_regulation\_of\_viral\_reproduction | 2 | 1 |  |  |  |  |  |  |  |  |
| GO:0051590\_positive\_regulation\_of\_neurotransmitter\_transport | 2 | 1 |  |  |  |  |  |  |  |  |
| GO:0060260\_regulation\_of\_transcription\_initiation\_from\_RNA\_polymerase\_II\_promoter | 2 | 1 |  |  |  |  |  |  |  |  |
| GO:0043473\_pigmentation | 49 | 3 | 4.547400 | -1.558980 | 126 | 8.698457 | 25.43 | 42.161543 | 0.201825 |
| GO:0044093\_positive\_regulation\_of\_molecular\_function | 173 | 6 | 2.575984 | -1.555817 | 127 | 8.689247 | 25.54 | 42.390753 | 0.201102 |
| GO:0001655\_urogenital\_system\_development | 128 | 5 | 2.901336 | -1.551918 | 129 | 8.862170 | 25.79 | 42.717830 | 0.199922 |
| GO:0045597\_positive\_regulation\_of\_cell\_differentiation | 128 | 5 | 2.901336 | -1.551918 | 129 | 8.862170 | 25.79 | 42.717830 | 0.199922 |
| GO:0001822\_kidney\_development | 87 | 4 | 3.414905 | -1.539285 | 133 | 8.948846 | 25.98 | 43.011154 | 0.195338 |
| GO:0003001\_generation\_of\_a\_signal\_involved\_in\_cell-cell\_signaling | 87 | 4 | 3.414905 | -1.539285 | 133 | 8.948846 | 25.98 | 43.011154 | 0.195338 |
| GO:0007178\_transmembrane\_receptor\_protein\_serine\_threonine\_kinase\_signaling\_pathway | 87 | 4 | 3.414905 | -1.539285 | 133 | 8.948846 | 25.98 | 43.011154 | 0.195338 |
| GO:0016337\_cell-cell\_adhesion | 87 | 4 | 3.414905 | -1.539285 | 133 | 8.948846 | 25.98 | 43.011154 | 0.195338 |
| GO:0031128\_developmental\_induction | 20 | 2 | 7.427419 | -1.537567 | 136 | 9.418440 | 26.76 | 44.101560 | 0.196765 |
| GO:0045168\_cell-cell\_signaling\_involved\_in\_cell\_fate\_specification | 20 | 2 | 7.427419 | -1.537567 | 136 | 9.418440 | 26.76 | 44.101560 | 0.196765 |
| GO:0045639\_positive\_regulation\_of\_myeloid\_cell\_differentiation | 20 | 2 | 7.427419 | -1.537567 | 136 | 9.418440 | 26.76 | 44.101560 | 0.196765 |
| GO:0046903\_secretion | 175 | 6 | 2.546544 | -1.534406 | 137 | 9.559640 | 27.01 | 44.460360 | 0.197153 |
| GO:0043066\_negative\_regulation\_of\_apoptosis | 176 | 6 | 2.532075 | -1.523831 | 138 | 9.520177 | 27.17 | 44.819823 | 0.196884 |
| GO:0048754\_branching\_morphogenesis\_of\_a\_tube | 88 | 4 | 3.376100 | -1.523374 | 139 | 9.567834 | 27.24 | 44.912166 | 0.195971 |
| GO:0051093\_negative\_regulation\_of\_developmental\_process | 331 | 9 | 2.019540 | -1.501420 | 140 | 9.639148 | 27.68 | 45.720852 | 0.197714 |
| GO:0006633\_fatty\_acid\_biosynthetic\_process | 21 | 2 | 7.073733 | -1.497826 | 143 | 10.408116 | 28.94 | 47.471884 | 0.202378 |
| GO:0015844\_monoamine\_transport | 21 | 2 | 7.073733 | -1.497826 | 143 | 10.408116 | 28.94 | 47.471884 | 0.202378 |
| GO:0043279\_response\_to\_alkaloid | 21 | 2 | 7.073733 | -1.497826 | 143 | 10.408116 | 28.94 | 47.471884 | 0.202378 |
| GO:0007610\_behavior | 279 | 8 | 2.129726 | -1.493925 | 144 | 10.398976 | 28.99 | 47.581024 | 0.201319 |
| GO:0043069\_negative\_regulation\_of\_programmed\_cell\_death | 179 | 6 | 2.489638 | -1.492618 | 146 | 10.357132 | 29.02 | 47.682868 | 0.198767 |
| GO:0060548\_negative\_regulation\_of\_cell\_death | 179 | 6 | 2.489638 | -1.492618 | 146 | 10.357132 | 29.02 | 47.682868 | 0.198767 |
| GO:0032501\_multicellular\_organismal\_process | 2183 | 37 | 1.258885 | -1.464563 | 147 | 10.602148 | 29.99 | 49.377852 | 0.204014 |
| GO:0000902\_cell\_morphogenesis | 283 | 8 | 2.099624 | -1.462129 | 148 | 10.617698 | 30.03 | 49.442302 | 0.202905 |
| GO:0009309\_amine\_biosynthetic\_process | 22 | 2 | 6.752199 | -1.460155 | 152 | 11.449536 | 31.59 | 51.730464 | 0.207829 |
| GO:0021675\_nerve\_development | 22 | 2 | 6.752199 | -1.460155 | 152 | 11.449536 | 31.59 | 51.730464 | 0.207829 |
| GO:0034097\_response\_to\_cytokine\_stimulus | 22 | 2 | 6.752199 | -1.460155 | 152 | 11.449536 | 31.59 | 51.730464 | 0.207829 |
| GO:0048477\_oogenesis | 22 | 2 | 6.752199 | -1.460155 | 152 | 11.449536 | 31.59 | 51.730464 | 0.207829 |
| GO:0006091\_generation\_of\_precursor\_metabolites\_and\_energy | 54 | 3 | 4.126344 | -1.450297 | 153 | 11.613748 | 31.95 | 52.286252 | 0.208824 |
| GO:0050790\_regulation\_of\_catalytic\_activity | 233 | 7 | 2.231414 | -1.448276 | 154 | 11.668595 | 31.99 | 52.311405 | 0.207727 |
| GO:0006793\_phosphorus\_metabolic\_process | 340 | 9 | 1.966082 | -1.436646 | 156 | 11.915066 | 32.41 | 52.904934 | 0.207756 |
| GO:0006796\_phosphate\_metabolic\_process | 340 | 9 | 1.966082 | -1.436646 | 156 | 11.915066 | 32.41 | 52.904934 | 0.207756 |
| GO:0015698\_inorganic\_anion\_transport | 23 | 2 | 6.458626 | -1.424364 | 158 | 12.421788 | 33.64 | 54.858212 | 0.212911 |
| GO:0030512\_negative\_regulation\_of\_transforming\_growth\_factor\_beta\_receptor\_signaling\_pathway | 23 | 2 | 6.458626 | -1.424364 | 158 | 12.421788 | 33.64 | 54.858212 | 0.212911 |
| GO:0051649\_establishment\_of\_localization\_in\_cell | 342 | 9 | 1.954584 | -1.422637 | 159 | 12.487230 | 33.81 | 55.132770 | 0.212642 |
| GO:0007275\_multicellular\_organismal\_development | 1760 | 31 | 1.308239 | -1.419898 | 160 | 12.517910 | 33.85 | 55.182090 | 0.211563 |
| GO:0042391\_regulation\_of\_membrane\_potential | 95 | 4 | 3.127334 | -1.418173 | 161 | 12.524226 | 33.93 | 55.335774 | 0.210745 |
| GO:0002309\_T\_cell\_proliferation\_during\_immune\_response | 3 | 1 |  |  |  |  |  |  |  |  |
| GO:0002369\_T\_cell\_cytokine\_production | 3 | 1 |  |  |  |  |  |  |  |  |
| GO:0002827\_positive\_regulation\_of\_T-helper\_1\_type\_immune\_response | 3 | 1 |  |  |  |  |  |  |  |  |
| GO:0006367\_transcription\_initiation\_from\_RNA\_polymerase\_II\_promoter | 3 | 1 |  |  |  |  |  |  |  |  |
| GO:0006828\_manganese\_ion\_transport | 3 | 1 |  |  |  |  |  |  |  |  |
| GO:0007412\_axon\_target\_recognition | 3 | 1 |  |  |  |  |  |  |  |  |
| GO:0014909\_smooth\_muscle\_cell\_migration | 3 | 1 |  |  |  |  |  |  |  |  |
| GO:0016322\_neuron\_remodeling | 3 | 1 |  |  |  |  |  |  |  |  |
| GO:0019058\_viral\_infectious\_cycle | 3 | 1 |  |  |  |  |  |  |  |  |
| GO:0031133\_regulation\_of\_axon\_diameter | 3 | 1 |  |  |  |  |  |  |  |  |
| GO:0032536\_regulation\_of\_cell\_projection\_size | 3 | 1 |  |  |  |  |  |  |  |  |
| GO:0032632\_interleukin-3\_production | 3 | 1 |  |  |  |  |  |  |  |  |
| GO:0032881\_regulation\_of\_polysaccharide\_metabolic\_process | 3 | 1 |  |  |  |  |  |  |  |  |
| GO:0043090\_amino\_acid\_import | 3 | 1 |  |  |  |  |  |  |  |  |
| GO:0043092\_L-amino\_acid\_import | 3 | 1 |  |  |  |  |  |  |  |  |
| GO:0045110\_intermediate\_filament\_bundle\_assembly | 3 | 1 |  |  |  |  |  |  |  |  |
| GO:0045844\_positive\_regulation\_of\_striated\_muscle\_development | 3 | 1 |  |  |  |  |  |  |  |  |
| GO:0048636\_positive\_regulation\_of\_muscle\_development | 3 | 1 |  |  |  |  |  |  |  |  |
| GO:0048845\_venous\_blood\_vessel\_morphogenesis | 3 | 1 |  |  |  |  |  |  |  |  |
| GO:0051926\_negative\_regulation\_of\_calcium\_ion\_transport | 3 | 1 |  |  |  |  |  |  |  |  |
| GO:0060084\_synaptic\_transmission\_involved\_in\_micturition | 3 | 1 |  |  |  |  |  |  |  |  |
| GO:0060460\_left\_lung\_morphogenesis | 3 | 1 |  |  |  |  |  |  |  |  |
| GO:0060586\_multicellular\_organismal\_iron\_ion\_homeostasis | 3 | 1 |  |  |  |  |  |  |  |  |
| GO:0060841\_venous\_blood\_vessel\_development | 3 | 1 |  |  |  |  |  |  |  |  |
| GO:0070873\_regulation\_of\_glycogen\_metabolic\_process | 3 | 1 |  |  |  |  |  |  |  |  |
| GO:0070875\_positive\_regulation\_of\_glycogen\_metabolic\_process | 3 | 1 |  |  |  |  |  |  |  |  |
| GO:0001541\_ovarian\_follicle\_development | 24 | 2 | 6.189516 | -1.390289 | 163 | 13.934009 | 36.42 | 58.905991 | 0.223436 |
| GO:0014070\_response\_to\_organic\_cyclic\_substance | 24 | 2 | 6.189516 | -1.390289 | 163 | 13.934009 | 36.42 | 58.905991 | 0.223436 |
| GO:0032502\_developmental\_process | 2060 | 35 | 1.261940 | -1.383669 | 164 | 13.991547 | 36.51 | 59.028453 | 0.222622 |
| GO:0055123\_digestive\_system\_development | 25 | 2 | 5.941935 | -1.357787 | 165 | 14.579819 | 38.28 | 61.980181 | 0.232000 |
| GO:0042221\_response\_to\_chemical\_stimulus | 409 | 10 | 1.815995 | -1.349992 | 166 | 14.785152 | 38.52 | 62.254848 | 0.232048 |
| GO:0033554\_cellular\_response\_to\_stress | 196 | 6 | 2.273700 | -1.329141 | 167 | 15.286290 | 39.24 | 63.193710 | 0.234970 |
| GO:0007405\_neuroblast\_proliferation | 26 | 2 | 5.713400 | -1.326731 | 168 | 16.120059 | 40.46 | 64.799941 | 0.240833 |
| GO:0050801\_ion\_homeostasis | 197 | 6 | 2.262158 | -1.320181 | 169 | 16.184647 | 40.62 | 65.055353 | 0.240355 |
| GO:0032270\_positive\_regulation\_of\_cellular\_protein\_metabolic\_process | 61 | 3 | 3.652829 | -1.317173 | 170 | 16.429913 | 40.91 | 65.390087 | 0.240647 |
| GO:0032940\_secretion\_by\_cell | 149 | 5 | 2.492423 | -1.309598 | 171 | 16.537199 | 41.23 | 65.922801 | 0.241111 |
| GO:0006351\_transcription\_\_DNA-dependent | 594 | 13 | 1.625529 | -1.305197 | 172 | 16.569601 | 41.35 | 66.130399 | 0.240407 |
| GO:0032774\_RNA\_biosynthetic\_process | 595 | 13 | 1.622798 | -1.300232 | 173 | 16.653850 | 41.51 | 66.366150 | 0.239942 |
| GO:0006916\_anti-apoptosis | 62 | 3 | 3.593913 | -1.299700 | 175 | 16.797353 | 41.76 | 66.722647 | 0.238629 |
| GO:0030155\_regulation\_of\_cell\_adhesion | 62 | 3 | 3.593913 | -1.299700 | 175 | 16.797353 | 41.76 | 66.722647 | 0.238629 |
| GO:0016050\_vesicle\_organization | 27 | 2 | 5.501792 | -1.297009 | 176 | 17.039593 | 42.5 | 67.960407 | 0.241477 |
| GO:0007267\_cell-cell\_signaling | 252 | 7 | 2.063172 | -1.293046 | 177 | 17.140263 | 42.66 | 68.179737 | 0.241017 |
| GO:0045893\_positive\_regulation\_of\_transcription\_\_DNA-dependent | 306 | 8 | 1.941809 | -1.292432 | 179 | 17.172627 | 42.72 | 68.267373 | 0.238659 |
| GO:0051254\_positive\_regulation\_of\_RNA\_metabolic\_process | 306 | 8 | 1.941809 | -1.292432 | 179 | 17.172627 | 42.72 | 68.267373 | 0.238659 |
| GO:0032989\_cellular\_component\_morphogenesis | 307 | 8 | 1.935484 | -1.285529 | 180 | 17.268011 | 42.86 | 68.451989 | 0.238111 |
| GO:0048878\_chemical\_homeostasis | 254 | 7 | 2.046927 | -1.277775 | 181 | 17.418752 | 43.26 | 69.101248 | 0.239006 |
| GO:0001938\_positive\_regulation\_of\_endothelial\_cell\_proliferation | 4 | 1 |  |  |  |  |  |  |  |  |
| GO:0002326\_B\_cell\_lineage\_commitment | 4 | 1 |  |  |  |  |  |  |  |  |
| GO:0007144\_female\_meiosis\_I | 4 | 1 |  |  |  |  |  |  |  |  |
| GO:0007184\_SMAD\_protein\_nuclear\_translocation | 4 | 1 |  |  |  |  |  |  |  |  |
| GO:0010224\_response\_to\_UV-B | 4 | 1 |  |  |  |  |  |  |  |  |
| GO:0015701\_bicarbonate\_transport | 4 | 1 |  |  |  |  |  |  |  |  |
| GO:0015809\_arginine\_transport | 4 | 1 |  |  |  |  |  |  |  |  |
| GO:0021535\_cell\_migration\_in\_hindbrain | 4 | 1 |  |  |  |  |  |  |  |  |
| GO:0021631\_optic\_nerve\_morphogenesis | 4 | 1 |  |  |  |  |  |  |  |  |
| GO:0021801\_cerebral\_cortex\_radial\_glia\_guided\_migration | 4 | 1 |  |  |  |  |  |  |  |  |
| GO:0032835\_glomerulus\_development | 4 | 1 |  |  |  |  |  |  |  |  |
| GO:0033026\_negative\_regulation\_of\_mast\_cell\_apoptosis | 4 | 1 |  |  |  |  |  |  |  |  |
| GO:0033135\_regulation\_of\_peptidyl-serine\_phosphorylation | 4 | 1 |  |  |  |  |  |  |  |  |
| GO:0042773\_ATP\_synthesis\_coupled\_electron\_transport | 4 | 1 |  |  |  |  |  |  |  |  |
| GO:0042775\_mitochondrial\_ATP\_synthesis\_coupled\_electron\_transport | 4 | 1 |  |  |  |  |  |  |  |  |
| GO:0042832\_defense\_response\_to\_protozoan | 4 | 1 |  |  |  |  |  |  |  |  |
| GO:0043129\_surfactant\_homeostasis | 4 | 1 |  |  |  |  |  |  |  |  |
| GO:0043374\_CD8-positive\_\_alpha-beta\_T\_cell\_differentiation | 4 | 1 |  |  |  |  |  |  |  |  |
| GO:0043470\_regulation\_of\_carbohydrate\_catabolic\_process | 4 | 1 |  |  |  |  |  |  |  |  |
| GO:0043471\_regulation\_of\_cellular\_carbohydrate\_catabolic\_process | 4 | 1 |  |  |  |  |  |  |  |  |
| GO:0045930\_negative\_regulation\_of\_mitotic\_cell\_cycle | 4 | 1 |  |  |  |  |  |  |  |  |
| GO:0046548\_retinal\_rod\_cell\_development | 4 | 1 |  |  |  |  |  |  |  |  |
| GO:0046579\_positive\_regulation\_of\_Ras\_protein\_signal\_transduction | 4 | 1 |  |  |  |  |  |  |  |  |
| GO:0046902\_regulation\_of\_mitochondrial\_membrane\_permeability | 4 | 1 |  |  |  |  |  |  |  |  |
| GO:0048710\_regulation\_of\_astrocyte\_differentiation | 4 | 1 |  |  |  |  |  |  |  |  |
| GO:0048875\_chemical\_homeostasis\_within\_a\_tissue | 4 | 1 |  |  |  |  |  |  |  |  |
| GO:0048935\_peripheral\_nervous\_system\_neuron\_development | 4 | 1 |  |  |  |  |  |  |  |  |
| GO:0051452\_intracellular\_pH\_reduction | 4 | 1 |  |  |  |  |  |  |  |  |
| GO:0055009\_atrial\_cardiac\_muscle\_morphogenesis | 4 | 1 |  |  |  |  |  |  |  |  |
| GO:0060158\_activation\_of\_phospholipase\_C\_activity\_by\_dopamine\_receptor\_signaling\_pathway | 4 | 1 |  |  |  |  |  |  |  |  |
| GO:0060291\_long-term\_synaptic\_potentiation | 4 | 1 |  |  |  |  |  |  |  |  |
| GO:0060459\_left\_lung\_development | 4 | 1 |  |  |  |  |  |  |  |  |
| GO:0070059\_apoptosis\_in\_response\_to\_endoplasmic\_reticulum\_stress | 4 | 1 |  |  |  |  |  |  |  |  |
| GO:0016310\_phosphorylation | 309 | 8 | 1.922956 | -1.271835 | 182 | 17.427084 | 43.34 | 69.252916 | 0.238132 |
| GO:0006470\_protein\_amino\_acid\_dephosphorylation | 28 | 2 | 5.305300 | -1.268523 | 184 | 17.996419 | 44.34 | 70.683581 | 0.240978 |
| GO:0043193\_positive\_regulation\_of\_gene-specific\_transcription | 28 | 2 | 5.305300 | -1.268523 | 184 | 17.996419 | 44.34 | 70.683581 | 0.240978 |
| GO:0048511\_rhythmic\_process | 65 | 3 | 3.428040 | -1.249324 | 185 | 18.487724 | 45.14 | 71.792276 | 0.244000 |
| GO:0042490\_mechanoreceptor\_differentiation | 29 | 2 | 5.122358 | -1.241184 | 186 | 19.564234 | 46.46 | 73.355766 | 0.249785 |
| GO:0048519\_negative\_regulation\_of\_biological\_process | 859 | 17 | 1.469920 | -1.240875 | 187 | 19.589094 | 46.48 | 73.370906 | 0.248556 |
| GO:0051641\_cellular\_localization | 370 | 9 | 1.806670 | -1.240121 | 188 | 19.572059 | 46.52 | 73.467941 | 0.247447 |
| GO:0015837\_amine\_transport | 66 | 3 | 3.376100 | -1.233178 | 190 | 19.938882 | 46.88 | 73.821118 | 0.246737 |
| GO:0051130\_positive\_regulation\_of\_cellular\_component\_organization | 66 | 3 | 3.376100 | -1.233178 | 190 | 19.938882 | 46.88 | 73.821118 | 0.246737 |
| GO:0080090\_regulation\_of\_primary\_metabolic\_process | 926 | 18 | 1.443775 | -1.231926 | 191 | 19.967077 | 46.91 | 73.852923 | 0.245602 |
| GO:0048514\_blood\_vessel\_morphogenesis | 158 | 5 | 2.350449 | -1.220018 | 192 | 20.231408 | 47.38 | 74.528592 | 0.246771 |
| GO:0009791\_post-embryonic\_development | 67 | 3 | 3.325710 | -1.217337 | 194 | 20.513522 | 47.75 | 74.986478 | 0.246134 |
| GO:0051247\_positive\_regulation\_of\_protein\_metabolic\_process | 67 | 3 | 3.325710 | -1.217337 | 194 | 20.513522 | 47.75 | 74.986478 | 0.246134 |
| GO:0048565\_gut\_development | 30 | 2 | 4.951613 | -1.214912 | 195 | 21.623199 | 49.15 | 76.676801 | 0.252051 |
| GO:0010604\_positive\_regulation\_of\_macromolecule\_metabolic\_process | 433 | 10 | 1.715339 | -1.210277 | 196 | 21.752932 | 49.26 | 76.767068 | 0.251327 |
| GO:0016311\_dephosphorylation | 31 | 2 | 4.791883 | -1.189635 | 200 | 23.211721 | 51.55 | 79.888279 | 0.257750 |
| GO:0021954\_central\_nervous\_system\_neuron\_development | 31 | 2 | 4.791883 | -1.189635 | 200 | 23.211721 | 51.55 | 79.888279 | 0.257750 |
| GO:0033555\_multicellular\_organismal\_response\_to\_stress | 31 | 2 | 4.791883 | -1.189635 | 200 | 23.211721 | 51.55 | 79.888279 | 0.257750 |
| GO:0048167\_regulation\_of\_synaptic\_plasticity | 31 | 2 | 4.791883 | -1.189635 | 200 | 23.211721 | 51.55 | 79.888279 | 0.257750 |
| GO:0001932\_regulation\_of\_protein\_amino\_acid\_phosphorylation | 69 | 3 | 3.229313 | -1.186537 | 201 | 23.411111 | 51.98 | 80.548889 | 0.258607 |
| GO:0000272\_polysaccharide\_catabolic\_process | 5 | 1 | 14.854839 | -1.183378 | 233 | 38.498696 | 70.14 | 101.781304 | 0.301030 |
| GO:0001562\_response\_to\_protozoan | 5 | 1 | 14.854839 | -1.183378 | 233 | 38.498696 | 70.14 | 101.781304 | 0.301030 |
| GO:0002825\_regulation\_of\_T-helper\_1\_type\_immune\_response | 5 | 1 | 14.854839 | -1.183378 | 233 | 38.498696 | 70.14 | 101.781304 | 0.301030 |
| GO:0005980\_glycogen\_catabolic\_process | 5 | 1 | 14.854839 | -1.183378 | 233 | 38.498696 | 70.14 | 101.781304 | 0.301030 |
| GO:0009251\_glucan\_catabolic\_process | 5 | 1 | 14.854839 | -1.183378 | 233 | 38.498696 | 70.14 | 101.781304 | 0.301030 |
| GO:0010522\_regulation\_of\_calcium\_ion\_transport\_into\_cytosol | 5 | 1 | 14.854839 | -1.183378 | 233 | 38.498696 | 70.14 | 101.781304 | 0.301030 |
| GO:0010676\_positive\_regulation\_of\_cellular\_carbohydrate\_metabolic\_process | 5 | 1 | 14.854839 | -1.183378 | 233 | 38.498696 | 70.14 | 101.781304 | 0.301030 |
| GO:0015802\_basic\_amino\_acid\_transport | 5 | 1 | 14.854839 | -1.183378 | 233 | 38.498696 | 70.14 | 101.781304 | 0.301030 |
| GO:0019227\_neuronal\_action\_potential\_propagation | 5 | 1 | 14.854839 | -1.183378 | 233 | 38.498696 | 70.14 | 101.781304 | 0.301030 |
| GO:0021554\_optic\_nerve\_development | 5 | 1 | 14.854839 | -1.183378 | 233 | 38.498696 | 70.14 | 101.781304 | 0.301030 |
| GO:0022415\_viral\_reproductive\_process | 5 | 1 | 14.854839 | -1.183378 | 233 | 38.498696 | 70.14 | 101.781304 | 0.301030 |
| GO:0030004\_cellular\_monovalent\_inorganic\_cation\_homeostasis | 5 | 1 | 14.854839 | -1.183378 | 233 | 38.498696 | 70.14 | 101.781304 | 0.301030 |
| GO:0030641\_regulation\_of\_cellular\_pH | 5 | 1 | 14.854839 | -1.183378 | 233 | 38.498696 | 70.14 | 101.781304 | 0.301030 |
| GO:0032845\_negative\_regulation\_of\_homeostatic\_process | 5 | 1 | 14.854839 | -1.183378 | 233 | 38.498696 | 70.14 | 101.781304 | 0.301030 |
| GO:0033023\_mast\_cell\_homeostasis | 5 | 1 | 14.854839 | -1.183378 | 233 | 38.498696 | 70.14 | 101.781304 | 0.301030 |
| GO:0033024\_mast\_cell\_apoptosis | 5 | 1 | 14.854839 | -1.183378 | 233 | 38.498696 | 70.14 | 101.781304 | 0.301030 |
| GO:0033025\_regulation\_of\_mast\_cell\_apoptosis | 5 | 1 | 14.854839 | -1.183378 | 233 | 38.498696 | 70.14 | 101.781304 | 0.301030 |
| GO:0035095\_behavioral\_response\_to\_nicotine | 5 | 1 | 14.854839 | -1.183378 | 233 | 38.498696 | 70.14 | 101.781304 | 0.301030 |
| GO:0035234\_germ\_cell\_programmed\_cell\_death | 5 | 1 | 14.854839 | -1.183378 | 233 | 38.498696 | 70.14 | 101.781304 | 0.301030 |
| GO:0042416\_dopamine\_biosynthetic\_process | 5 | 1 | 14.854839 | -1.183378 | 233 | 38.498696 | 70.14 | 101.781304 | 0.301030 |
| GO:0043030\_regulation\_of\_macrophage\_activation | 5 | 1 | 14.854839 | -1.183378 | 233 | 38.498696 | 70.14 | 101.781304 | 0.301030 |
| GO:0043489\_RNA\_stabilization | 5 | 1 | 14.854839 | -1.183378 | 233 | 38.498696 | 70.14 | 101.781304 | 0.301030 |
| GO:0044247\_cellular\_polysaccharide\_catabolic\_process | 5 | 1 | 14.854839 | -1.183378 | 233 | 38.498696 | 70.14 | 101.781304 | 0.301030 |
| GO:0045342\_MHC\_class\_II\_biosynthetic\_process | 5 | 1 | 14.854839 | -1.183378 | 233 | 38.498696 | 70.14 | 101.781304 | 0.301030 |
| GO:0045730\_respiratory\_burst | 5 | 1 | 14.854839 | -1.183378 | 233 | 38.498696 | 70.14 | 101.781304 | 0.301030 |
| GO:0045851\_pH\_reduction | 5 | 1 | 14.854839 | -1.183378 | 233 | 38.498696 | 70.14 | 101.781304 | 0.301030 |
| GO:0048255\_mRNA\_stabilization | 5 | 1 | 14.854839 | -1.183378 | 233 | 38.498696 | 70.14 | 101.781304 | 0.301030 |
| GO:0048532\_anatomical\_structure\_arrangement | 5 | 1 | 14.854839 | -1.183378 | 233 | 38.498696 | 70.14 | 101.781304 | 0.301030 |
| GO:0048934\_peripheral\_nervous\_system\_neuron\_differentiation | 5 | 1 | 14.854839 | -1.183378 | 233 | 38.498696 | 70.14 | 101.781304 | 0.301030 |
| GO:0051057\_positive\_regulation\_of\_small\_GTPase\_mediated\_signal\_transduction | 5 | 1 | 14.854839 | -1.183378 | 233 | 38.498696 | 70.14 | 101.781304 | 0.301030 |
| GO:0051453\_regulation\_of\_intracellular\_pH | 5 | 1 | 14.854839 | -1.183378 | 233 | 38.498696 | 70.14 | 101.781304 | 0.301030 |
| GO:0060073\_micturition | 5 | 1 | 14.854839 | -1.183378 | 233 | 38.498696 | 70.14 | 101.781304 | 0.301030 |
| GO:0031326\_regulation\_of\_cellular\_biosynthetic\_process | 812 | 16 | 1.463531 | -1.170900 | 234 | 38.996627 | 70.8 | 102.603373 | 0.302564 |
| GO:0002274\_myeloid\_leukocyte\_activation | 32 | 2 | 4.642137 | -1.165290 | 236 | 39.740457 | 72.05 | 104.359543 | 0.305297 |
| GO:0050770\_regulation\_of\_axonogenesis | 32 | 2 | 4.642137 | -1.165290 | 236 | 39.740457 | 72.05 | 104.359543 | 0.305297 |
| GO:0031325\_positive\_regulation\_of\_cellular\_metabolic\_process | 442 | 10 | 1.680412 | -1.161473 | 237 | 39.835225 | 72.26 | 104.684775 | 0.304895 |
| GO:0009889\_regulation\_of\_biosynthetic\_process | 815 | 16 | 1.458144 | -1.158930 | 238 | 39.869609 | 72.32 | 104.770391 | 0.303866 |
| GO:0006807\_nitrogen\_compound\_metabolic\_process | 1147 | 21 | 1.359859 | -1.150901 | 239 | 40.331359 | 73.03 | 105.728641 | 0.305565 |
| GO:0006366\_transcription\_from\_RNA\_polymerase\_II\_promoter | 444 | 10 | 1.672842 | -1.150879 | 240 | 40.337762 | 73.08 | 105.822238 | 0.304500 |
| GO:0022037\_metencephalon\_development | 33 | 2 | 4.501466 | -1.141817 | 241 | 41.744203 | 74.76 | 107.775797 | 0.310207 |
| GO:0051716\_cellular\_response\_to\_stimulus | 273 | 7 | 1.904467 | -1.141798 | 242 | 41.876680 | 74.86 | 107.843320 | 0.309339 |
| GO:0044249\_cellular\_biosynthetic\_process | 1150 | 21 | 1.356311 | -1.140632 | 243 | 41.846676 | 74.94 | 108.033324 | 0.308395 |
| GO:0022403\_cell\_cycle\_phase | 119 | 4 | 2.496612 | -1.123468 | 244 | 42.494474 | 76.15 | 109.805526 | 0.312090 |
| GO:0010720\_positive\_regulation\_of\_cell\_development | 34 | 2 | 4.369070 | -1.119164 | 245 | 43.132776 | 77.13 | 111.127224 | 0.314816 |
| GO:0009653\_anatomical\_structure\_morphogenesis | 958 | 18 | 1.395549 | -1.111652 | 246 | 43.315612 | 77.49 | 111.664388 | 0.315000 |
| GO:0048513\_organ\_development | 1365 | 24 | 1.305920 | -1.107505 | 247 | 43.335137 | 77.6 | 111.864863 | 0.314170 |
| GO:0042127\_regulation\_of\_cell\_proliferation | 393 | 9 | 1.700936 | -1.107396 | 248 | 43.382060 | 77.64 | 111.897940 | 0.313065 |
| GO:0002360\_T\_cell\_lineage\_commitment | 6 | 1 | 12.379032 | -1.107060 | 269 | 54.474061 | 90.91 | 127.345939 | 0.337955 |
| GO:0002367\_cytokine\_production\_during\_immune\_response | 6 | 1 | 12.379032 | -1.107060 | 269 | 54.474061 | 90.91 | 127.345939 | 0.337955 |
| GO:0006656\_phosphatidylcholine\_biosynthetic\_process | 6 | 1 | 12.379032 | -1.107060 | 269 | 54.474061 | 90.91 | 127.345939 | 0.337955 |
| GO:0007406\_negative\_regulation\_of\_neuroblast\_proliferation | 6 | 1 | 12.379032 | -1.107060 | 269 | 54.474061 | 90.91 | 127.345939 | 0.337955 |
| GO:0014812\_muscle\_cell\_migration | 6 | 1 | 12.379032 | -1.107060 | 269 | 54.474061 | 90.91 | 127.345939 | 0.337955 |
| GO:0016032\_viral\_reproduction | 6 | 1 | 12.379032 | -1.107060 | 269 | 54.474061 | 90.91 | 127.345939 | 0.337955 |
| GO:0016574\_histone\_ubiquitination | 6 | 1 | 12.379032 | -1.107060 | 269 | 54.474061 | 90.91 | 127.345939 | 0.337955 |
| GO:0021548\_pons\_development | 6 | 1 | 12.379032 | -1.107060 | 269 | 54.474061 | 90.91 | 127.345939 | 0.337955 |
| GO:0032392\_DNA\_geometric\_change | 6 | 1 | 12.379032 | -1.107060 | 269 | 54.474061 | 90.91 | 127.345939 | 0.337955 |
| GO:0032469\_endoplasmic\_reticulum\_calcium\_ion\_homeostasis | 6 | 1 | 12.379032 | -1.107060 | 269 | 54.474061 | 90.91 | 127.345939 | 0.337955 |
| GO:0035094\_response\_to\_nicotine | 6 | 1 | 12.379032 | -1.107060 | 269 | 54.474061 | 90.91 | 127.345939 | 0.337955 |
| GO:0043271\_negative\_regulation\_of\_ion\_transport | 6 | 1 | 12.379032 | -1.107060 | 269 | 54.474061 | 90.91 | 127.345939 | 0.337955 |
| GO:0043467\_regulation\_of\_generation\_of\_precursor\_metabolites\_and\_energy | 6 | 1 | 12.379032 | -1.107060 | 269 | 54.474061 | 90.91 | 127.345939 | 0.337955 |
| GO:0045913\_positive\_regulation\_of\_carbohydrate\_metabolic\_process | 6 | 1 | 12.379032 | -1.107060 | 269 | 54.474061 | 90.91 | 127.345939 | 0.337955 |
| GO:0045931\_positive\_regulation\_of\_mitotic\_cell\_cycle | 6 | 1 | 12.379032 | -1.107060 | 269 | 54.474061 | 90.91 | 127.345939 | 0.337955 |
| GO:0048041\_focal\_adhesion\_formation | 6 | 1 | 12.379032 | -1.107060 | 269 | 54.474061 | 90.91 | 127.345939 | 0.337955 |
| GO:0050829\_defense\_response\_to\_Gram-negative\_bacterium | 6 | 1 | 12.379032 | -1.107060 | 269 | 54.474061 | 90.91 | 127.345939 | 0.337955 |
| GO:0050872\_white\_fat\_cell\_differentiation | 6 | 1 | 12.379032 | -1.107060 | 269 | 54.474061 | 90.91 | 127.345939 | 0.337955 |
| GO:0051881\_regulation\_of\_mitochondrial\_membrane\_potential | 6 | 1 | 12.379032 | -1.107060 | 269 | 54.474061 | 90.91 | 127.345939 | 0.337955 |
| GO:0060134\_prepulse\_inhibition | 6 | 1 | 12.379032 | -1.107060 | 269 | 54.474061 | 90.91 | 127.345939 | 0.337955 |
| GO:0065004\_protein-DNA\_complex\_assembly | 6 | 1 | 12.379032 | -1.107060 | 269 | 54.474061 | 90.91 | 127.345939 | 0.337955 |
| GO:0006355\_regulation\_of\_transcription\_\_DNA-dependent | 575 | 12 | 1.550070 | -1.104381 | 270 | 54.502371 | 90.97 | 127.437629 | 0.336926 |
| GO:0006350\_transcription | 701 | 14 | 1.483365 | -1.102438 | 271 | 54.608563 | 91.14 | 127.671437 | 0.336310 |
| GO:0065009\_regulation\_of\_molecular\_function | 279 | 7 | 1.863510 | -1.102046 | 272 | 54.775888 | 91.31 | 127.844112 | 0.335699 |
| GO:0048522\_positive\_regulation\_of\_cellular\_process | 895 | 17 | 1.410795 | -1.101460 | 273 | 54.756581 | 91.35 | 127.943419 | 0.334615 |
| GO:0007281\_germ\_cell\_development | 75 | 3 | 2.970968 | -1.100596 | 274 | 54.922688 | 91.52 | 128.117312 | 0.334015 |
| GO:0007292\_female\_gamete\_generation | 35 | 2 | 4.244240 | -1.097281 | 277 | 55.711434 | 92.42 | 129.128566 | 0.333646 |
| GO:0010810\_regulation\_of\_cell-substrate\_adhesion | 35 | 2 | 4.244240 | -1.097281 | 277 | 55.711434 | 92.42 | 129.128566 | 0.333646 |
| GO:0051051\_negative\_regulation\_of\_transport | 35 | 2 | 4.244240 | -1.097281 | 277 | 55.711434 | 92.42 | 129.128566 | 0.333646 |
| GO:0030001\_metal\_ion\_transport | 122 | 4 | 2.435219 | -1.092378 | 278 | 56.031934 | 93.08 | 130.128066 | 0.334820 |
| GO:0045941\_positive\_regulation\_of\_transcription | 338 | 8 | 1.757969 | -1.088919 | 279 | 56.161057 | 93.22 | 130.278943 | 0.334122 |
| GO:0009725\_response\_to\_hormone\_stimulus | 76 | 3 | 2.931876 | -1.087137 | 280 | 56.280581 | 93.39 | 130.499419 | 0.333536 |
| GO:0009893\_positive\_regulation\_of\_metabolic\_process | 458 | 10 | 1.621707 | -1.079181 | 281 | 56.576407 | 93.85 | 131.123593 | 0.333986 |
| GO:0051171\_regulation\_of\_nitrogen\_compound\_metabolic\_process | 771 | 15 | 1.445023 | -1.078583 | 282 | 56.616890 | 93.9 | 131.183110 | 0.332979 |
| GO:0007631\_feeding\_behavior | 36 | 2 | 4.126344 | -1.076126 | 285 | 57.354175 | 95.24 | 133.125825 | 0.334175 |
| GO:0022602\_ovulation\_cycle\_process | 36 | 2 | 4.126344 | -1.076126 | 285 | 57.354175 | 95.24 | 133.125825 | 0.334175 |
| GO:0030278\_regulation\_of\_ossification | 36 | 2 | 4.126344 | -1.076126 | 285 | 57.354175 | 95.24 | 133.125825 | 0.334175 |
| GO:0048523\_negative\_regulation\_of\_cellular\_process | 774 | 15 | 1.439422 | -1.066981 | 286 | 57.634903 | 95.89 | 134.145097 | 0.335280 |
| GO:0007167\_enzyme\_linked\_receptor\_protein\_signaling\_pathway | 229 | 6 | 1.946049 | -1.065954 | 287 | 57.660903 | 95.94 | 134.219097 | 0.334286 |
| GO:0006873\_cellular\_ion\_homeostasis | 176 | 5 | 2.110062 | -1.061512 | 288 | 57.868245 | 96.25 | 134.631755 | 0.334201 |
| GO:0007519\_skeletal\_muscle\_tissue\_development | 78 | 3 | 2.856700 | -1.060900 | 290 | 58.095741 | 96.68 | 135.264259 | 0.333379 |
| GO:0060538\_skeletal\_muscle\_organ\_development | 78 | 3 | 2.856700 | -1.060900 | 290 | 58.095741 | 96.68 | 135.264259 | 0.333379 |
| GO:0009058\_biosynthetic\_process | 1175 | 21 | 1.327454 | -1.057708 | 291 | 58.074354 | 96.75 | 135.425646 | 0.332474 |
| GO:0042698\_ovulation\_cycle | 37 | 2 | 4.014821 | -1.055656 | 292 | 58.795039 | 97.77 | 136.744961 | 0.334829 |
| GO:0051239\_regulation\_of\_multicellular\_organismal\_process | 587 | 12 | 1.518382 | -1.051295 | 293 | 58.805255 | 97.86 | 136.914745 | 0.333993 |
| GO:0015674\_di-\_\_tri-valent\_inorganic\_cation\_transport | 79 | 3 | 2.820539 | -1.048110 | 294 | 58.963400 | 98.09 | 137.216600 | 0.333639 |
| GO:0010628\_positive\_regulation\_of\_gene\_expression | 346 | 8 | 1.717322 | -1.043199 | 295 | 58.961916 | 98.17 | 137.378084 | 0.332780 |
| GO:0001556\_oocyte\_maturation | 7 | 1 | 10.610599 | -1.042969 | 310 | 71.199713 | 113.73 | 156.260287 | 0.366871 |
| GO:0001936\_regulation\_of\_endothelial\_cell\_proliferation | 7 | 1 | 10.610599 | -1.042969 | 310 | 71.199713 | 113.73 | 156.260287 | 0.366871 |
| GO:0002792\_negative\_regulation\_of\_peptide\_secretion | 7 | 1 | 10.610599 | -1.042969 | 310 | 71.199713 | 113.73 | 156.260287 | 0.366871 |
| GO:0006096\_glycolysis | 7 | 1 | 10.610599 | -1.042969 | 310 | 71.199713 | 113.73 | 156.260287 | 0.366871 |
| GO:0006119\_oxidative\_phosphorylation | 7 | 1 | 10.610599 | -1.042969 | 310 | 71.199713 | 113.73 | 156.260287 | 0.366871 |
| GO:0006352\_transcription\_initiation | 7 | 1 | 10.610599 | -1.042969 | 310 | 71.199713 | 113.73 | 156.260287 | 0.366871 |
| GO:0021884\_forebrain\_neuron\_development | 7 | 1 | 10.610599 | -1.042969 | 310 | 71.199713 | 113.73 | 156.260287 | 0.366871 |
| GO:0030517\_negative\_regulation\_of\_axon\_extension | 7 | 1 | 10.610599 | -1.042969 | 310 | 71.199713 | 113.73 | 156.260287 | 0.366871 |
| GO:0034599\_cellular\_response\_to\_oxidative\_stress | 7 | 1 | 10.610599 | -1.042969 | 310 | 71.199713 | 113.73 | 156.260287 | 0.366871 |
| GO:0044275\_cellular\_carbohydrate\_catabolic\_process | 7 | 1 | 10.610599 | -1.042969 | 310 | 71.199713 | 113.73 | 156.260287 | 0.366871 |
| GO:0045668\_negative\_regulation\_of\_osteoblast\_differentiation | 7 | 1 | 10.610599 | -1.042969 | 310 | 71.199713 | 113.73 | 156.260287 | 0.366871 |
| GO:0046676\_negative\_regulation\_of\_insulin\_secretion | 7 | 1 | 10.610599 | -1.042969 | 310 | 71.199713 | 113.73 | 156.260287 | 0.366871 |
| GO:0048148\_behavioral\_response\_to\_cocaine | 7 | 1 | 10.610599 | -1.042969 | 310 | 71.199713 | 113.73 | 156.260287 | 0.366871 |
| GO:0048753\_pigment\_granule\_organization | 7 | 1 | 10.610599 | -1.042969 | 310 | 71.199713 | 113.73 | 156.260287 | 0.366871 |
| GO:0048857\_neural\_nucleus\_development | 7 | 1 | 10.610599 | -1.042969 | 310 | 71.199713 | 113.73 | 156.260287 | 0.366871 |
| GO:0051252\_regulation\_of\_RNA\_metabolic\_process | 590 | 12 | 1.510662 | -1.038376 | 311 | 71.253494 | 113.84 | 156.426506 | 0.366045 |
| GO:0048732\_gland\_development | 179 | 5 | 2.074698 | -1.037455 | 312 | 71.355318 | 114.05 | 156.744682 | 0.365545 |
| GO:0006820\_anion\_transport | 38 | 2 | 3.909168 | -1.035835 | 314 | 72.261309 | 115.61 | 158.958691 | 0.368185 |
| GO:0010975\_regulation\_of\_neuron\_projection\_development | 38 | 2 | 3.909168 | -1.035835 | 314 | 72.261309 | 115.61 | 158.958691 | 0.368185 |
| GO:0019752\_carboxylic\_acid\_metabolic\_process | 181 | 5 | 2.051773 | -1.021758 | 317 | 73.308737 | 116.96 | 160.611263 | 0.368959 |
| GO:0043436\_oxoacid\_metabolic\_process | 181 | 5 | 2.051773 | -1.021758 | 317 | 73.308737 | 116.96 | 160.611263 | 0.368959 |
| GO:0055082\_cellular\_chemical\_homeostasis | 181 | 5 | 2.051773 | -1.021758 | 317 | 73.308737 | 116.96 | 160.611263 | 0.368959 |
| GO:0016070\_RNA\_metabolic\_process | 658 | 13 | 1.467423 | -1.018549 | 318 | 73.374330 | 117.13 | 160.885670 | 0.368333 |
| GO:0006644\_phospholipid\_metabolic\_process | 39 | 2 | 3.808933 | -1.016628 | 321 | 74.700234 | 118.68 | 162.659766 | 0.369720 |
| GO:0007160\_cell-matrix\_adhesion | 39 | 2 | 3.808933 | -1.016628 | 321 | 74.700234 | 118.68 | 162.659766 | 0.369720 |
| GO:0021953\_central\_nervous\_system\_neuron\_differentiation | 39 | 2 | 3.808933 | -1.016628 | 321 | 74.700234 | 118.68 | 162.659766 | 0.369720 |
| GO:0032787\_monocarboxylic\_acid\_metabolic\_process | 130 | 4 | 2.285360 | -1.014518 | 323 | 74.833598 | 118.91 | 162.986402 | 0.368142 |
| GO:0045165\_cell\_fate\_commitment | 130 | 4 | 2.285360 | -1.014518 | 323 | 74.833598 | 118.91 | 162.986402 | 0.368142 |
| GO:0006082\_organic\_acid\_metabolic\_process | 182 | 5 | 2.040500 | -1.014009 | 324 | 74.912334 | 118.98 | 163.047666 | 0.367222 |
| GO:0045935\_positive\_regulation\_of\_nucleobase\_\_nucleoside\_\_nucleotide\_and\_nucleic\_acid\_metabolic\_process | 352 | 8 | 1.688050 | -1.010139 | 325 | 75.362376 | 119.59 | 163.817624 | 0.367969 |
| GO:0042180\_cellular\_ketone\_metabolic\_process | 183 | 5 | 2.029350 | -1.006327 | 326 | 75.464274 | 119.8 | 164.135726 | 0.367485 |
| GO:0007049\_cell\_cycle | 238 | 6 | 1.872459 | -1.004376 | 327 | 75.582455 | 119.91 | 164.237545 | 0.366697 |
| GO:0030534\_adult\_behavior | 83 | 3 | 2.684609 | -0.999013 | 328 | 76.055655 | 120.54 | 165.024345 | 0.367500 |
| GO:0007346\_regulation\_of\_mitotic\_cell\_cycle | 40 | 2 | 3.713710 | -0.998004 | 332 | 77.040764 | 121.98 | 166.919236 | 0.367410 |
| GO:0014031\_mesenchymal\_cell\_development | 40 | 2 | 3.713710 | -0.998004 | 332 | 77.040764 | 121.98 | 166.919236 | 0.367410 |
| GO:0017015\_regulation\_of\_transforming\_growth\_factor\_beta\_receptor\_signaling\_pathway | 40 | 2 | 3.713710 | -0.998004 | 332 | 77.040764 | 121.98 | 166.919236 | 0.367410 |
| GO:0046850\_regulation\_of\_bone\_remodeling | 40 | 2 | 3.713710 | -0.998004 | 332 | 77.040764 | 121.98 | 166.919236 | 0.367410 |
| GO:0002320\_lymphoid\_progenitor\_cell\_differentiation | 8 | 1 | 9.284274 | -0.987828 | 356 | 87.123690 | 135.34 | 183.556310 | 0.380169 |
| GO:0006582\_melanin\_metabolic\_process | 8 | 1 | 9.284274 | -0.987828 | 356 | 87.123690 | 135.34 | 183.556310 | 0.380169 |
| GO:0009746\_response\_to\_hexose\_stimulus | 8 | 1 | 9.284274 | -0.987828 | 356 | 87.123690 | 135.34 | 183.556310 | 0.380169 |
| GO:0009749\_response\_to\_glucose\_stimulus | 8 | 1 | 9.284274 | -0.987828 | 356 | 87.123690 | 135.34 | 183.556310 | 0.380169 |
| GO:0014014\_negative\_regulation\_of\_gliogenesis | 8 | 1 | 9.284274 | -0.987828 | 356 | 87.123690 | 135.34 | 183.556310 | 0.380169 |
| GO:0014046\_dopamine\_secretion | 8 | 1 | 9.284274 | -0.987828 | 356 | 87.123690 | 135.34 | 183.556310 | 0.380169 |
| GO:0014059\_regulation\_of\_dopamine\_secretion | 8 | 1 | 9.284274 | -0.987828 | 356 | 87.123690 | 135.34 | 183.556310 | 0.380169 |
| GO:0018107\_peptidyl-threonine\_phosphorylation | 8 | 1 | 9.284274 | -0.987828 | 356 | 87.123690 | 135.34 | 183.556310 | 0.380169 |
| GO:0018210\_peptidyl-threonine\_modification | 8 | 1 | 9.284274 | -0.987828 | 356 | 87.123690 | 135.34 | 183.556310 | 0.380169 |
| GO:0021799\_cerebral\_cortex\_radially\_oriented\_cell\_migration | 8 | 1 | 9.284274 | -0.987828 | 356 | 87.123690 | 135.34 | 183.556310 | 0.380169 |
| GO:0034284\_response\_to\_monosaccharide\_stimulus | 8 | 1 | 9.284274 | -0.987828 | 356 | 87.123690 | 135.34 | 183.556310 | 0.380169 |
| GO:0040017\_positive\_regulation\_of\_locomotion | 8 | 1 | 9.284274 | -0.987828 | 356 | 87.123690 | 135.34 | 183.556310 | 0.380169 |
| GO:0042304\_regulation\_of\_fatty\_acid\_biosynthetic\_process | 8 | 1 | 9.284274 | -0.987828 | 356 | 87.123690 | 135.34 | 183.556310 | 0.380169 |
| GO:0042423\_catecholamine\_biosynthetic\_process | 8 | 1 | 9.284274 | -0.987828 | 356 | 87.123690 | 135.34 | 183.556310 | 0.380169 |
| GO:0043368\_positive\_T\_cell\_selection | 8 | 1 | 9.284274 | -0.987828 | 356 | 87.123690 | 135.34 | 183.556310 | 0.380169 |
| GO:0045686\_negative\_regulation\_of\_glial\_cell\_differentiation | 8 | 1 | 9.284274 | -0.987828 | 356 | 87.123690 | 135.34 | 183.556310 | 0.380169 |
| GO:0046470\_phosphatidylcholine\_metabolic\_process | 8 | 1 | 9.284274 | -0.987828 | 356 | 87.123690 | 135.34 | 183.556310 | 0.380169 |
| GO:0048520\_positive\_regulation\_of\_behavior | 8 | 1 | 9.284274 | -0.987828 | 356 | 87.123690 | 135.34 | 183.556310 | 0.380169 |
| GO:0048742\_regulation\_of\_skeletal\_muscle\_fiber\_development | 8 | 1 | 9.284274 | -0.987828 | 356 | 87.123690 | 135.34 | 183.556310 | 0.380169 |
| GO:0050920\_regulation\_of\_chemotaxis | 8 | 1 | 9.284274 | -0.987828 | 356 | 87.123690 | 135.34 | 183.556310 | 0.380169 |
| GO:0050921\_positive\_regulation\_of\_chemotaxis | 8 | 1 | 9.284274 | -0.987828 | 356 | 87.123690 | 135.34 | 183.556310 | 0.380169 |
| GO:0050926\_regulation\_of\_positive\_chemotaxis | 8 | 1 | 9.284274 | -0.987828 | 356 | 87.123690 | 135.34 | 183.556310 | 0.380169 |
| GO:0050927\_positive\_regulation\_of\_positive\_chemotaxis | 8 | 1 | 9.284274 | -0.987828 | 356 | 87.123690 | 135.34 | 183.556310 | 0.380169 |
| GO:0050930\_induction\_of\_positive\_chemotaxis | 8 | 1 | 9.284274 | -0.987828 | 356 | 87.123690 | 135.34 | 183.556310 | 0.380169 |
| GO:0044057\_regulation\_of\_system\_process | 133 | 4 | 2.233810 | -0.987074 | 357 | 87.463106 | 135.7 | 183.936894 | 0.380112 |
| GO:0001776\_leukocyte\_homeostasis | 41 | 2 | 3.623131 | -0.979934 | 364 | 89.096991 | 137.7 | 186.303009 | 0.378297 |
| GO:0006979\_response\_to\_oxidative\_stress | 41 | 2 | 3.623131 | -0.979934 | 364 | 89.096991 | 137.7 | 186.303009 | 0.378297 |
| GO:0008585\_female\_gonad\_development | 41 | 2 | 3.623131 | -0.979934 | 364 | 89.096991 | 137.7 | 186.303009 | 0.378297 |
| GO:0015980\_energy\_derivation\_by\_oxidation\_of\_organic\_compounds | 41 | 2 | 3.623131 | -0.979934 | 364 | 89.096991 | 137.7 | 186.303009 | 0.378297 |
| GO:0019216\_regulation\_of\_lipid\_metabolic\_process | 41 | 2 | 3.623131 | -0.979934 | 364 | 89.096991 | 137.7 | 186.303009 | 0.378297 |
| GO:0031344\_regulation\_of\_cell\_projection\_organization | 41 | 2 | 3.623131 | -0.979934 | 364 | 89.096991 | 137.7 | 186.303009 | 0.378297 |
| GO:0032844\_regulation\_of\_homeostatic\_process | 41 | 2 | 3.623131 | -0.979934 | 364 | 89.096991 | 137.7 | 186.303009 | 0.378297 |
| GO:0008283\_cell\_proliferation | 544 | 11 | 1.501868 | -0.967077 | 365 | 89.600535 | 138.45 | 187.299465 | 0.379315 |
| GO:0006897\_endocytosis | 86 | 3 | 2.590960 | -0.964217 | 367 | 89.848534 | 138.87 | 187.891466 | 0.378392 |
| GO:0010324\_membrane\_invagination | 86 | 3 | 2.590960 | -0.964217 | 367 | 89.848534 | 138.87 | 187.891466 | 0.378392 |
| GO:0051173\_positive\_regulation\_of\_nitrogen\_compound\_metabolic\_process | 361 | 8 | 1.645966 | -0.962435 | 368 | 89.842314 | 138.9 | 187.957686 | 0.377446 |
| GO:0010769\_regulation\_of\_cell\_morphogenesis\_involved\_in\_differentiation | 42 | 2 | 3.536866 | -0.962389 | 370 | 90.989761 | 140.59 | 190.190239 | 0.379973 |
| GO:0045637\_regulation\_of\_myeloid\_cell\_differentiation | 42 | 2 | 3.536866 | -0.962389 | 370 | 90.989761 | 140.59 | 190.190239 | 0.379973 |
| GO:0034961\_cellular\_biopolymer\_biosynthetic\_process | 804 | 15 | 1.385713 | -0.956464 | 371 | 91.198482 | 140.94 | 190.681518 | 0.379892 |
| GO:0045449\_regulation\_of\_transcription | 676 | 13 | 1.428350 | -0.948354 | 372 | 91.700283 | 141.81 | 191.919717 | 0.381210 |
| GO:0043284\_biopolymer\_biosynthetic\_process | 807 | 15 | 1.380561 | -0.945945 | 373 | 91.786707 | 141.92 | 192.053293 | 0.380483 |
| GO:0001508\_regulation\_of\_action\_potential | 43 | 2 | 3.454614 | -0.945345 | 377 | 93.416467 | 143.73 | 194.043533 | 0.381247 |
| GO:0001894\_tissue\_homeostasis | 43 | 2 | 3.454614 | -0.945345 | 377 | 93.416467 | 143.73 | 194.043533 | 0.381247 |
| GO:0019637\_organophosphate\_metabolic\_process | 43 | 2 | 3.454614 | -0.945345 | 377 | 93.416467 | 143.73 | 194.043533 | 0.381247 |
| GO:0048762\_mesenchymal\_cell\_differentiation | 43 | 2 | 3.454614 | -0.945345 | 377 | 93.416467 | 143.73 | 194.043533 | 0.381247 |
| GO:0001503\_ossification | 88 | 3 | 2.532075 | -0.941915 | 378 | 93.770059 | 144.28 | 194.789941 | 0.381693 |
| GO:0001935\_endothelial\_cell\_proliferation | 9 | 1 | 8.252688 | -0.939520 | 394 | 104.349277 | 156.93 | 209.510723 | 0.398299 |
| GO:0006007\_glucose\_catabolic\_process | 9 | 1 | 8.252688 | -0.939520 | 394 | 104.349277 | 156.93 | 209.510723 | 0.398299 |
| GO:0010675\_regulation\_of\_cellular\_carbohydrate\_metabolic\_process | 9 | 1 | 8.252688 | -0.939520 | 394 | 104.349277 | 156.93 | 209.510723 | 0.398299 |
| GO:0014073\_response\_to\_tropane | 9 | 1 | 8.252688 | -0.939520 | 394 | 104.349277 | 156.93 | 209.510723 | 0.398299 |
| GO:0019320\_hexose\_catabolic\_process | 9 | 1 | 8.252688 | -0.939520 | 394 | 104.349277 | 156.93 | 209.510723 | 0.398299 |
| GO:0030279\_negative\_regulation\_of\_ossification | 9 | 1 | 8.252688 | -0.939520 | 394 | 104.349277 | 156.93 | 209.510723 | 0.398299 |
| GO:0035162\_embryonic\_hemopoiesis | 9 | 1 | 8.252688 | -0.939520 | 394 | 104.349277 | 156.93 | 209.510723 | 0.398299 |
| GO:0042220\_response\_to\_cocaine | 9 | 1 | 8.252688 | -0.939520 | 394 | 104.349277 | 156.93 | 209.510723 | 0.398299 |
| GO:0045109\_intermediate\_filament\_organization | 9 | 1 | 8.252688 | -0.939520 | 394 | 104.349277 | 156.93 | 209.510723 | 0.398299 |
| GO:0046365\_monosaccharide\_catabolic\_process | 9 | 1 | 8.252688 | -0.939520 | 394 | 104.349277 | 156.93 | 209.510723 | 0.398299 |
| GO:0046888\_negative\_regulation\_of\_hormone\_secretion | 9 | 1 | 8.252688 | -0.939520 | 394 | 104.349277 | 156.93 | 209.510723 | 0.398299 |
| GO:0048488\_synaptic\_vesicle\_endocytosis | 9 | 1 | 8.252688 | -0.939520 | 394 | 104.349277 | 156.93 | 209.510723 | 0.398299 |
| GO:0048708\_astrocyte\_differentiation | 9 | 1 | 8.252688 | -0.939520 | 394 | 104.349277 | 156.93 | 209.510723 | 0.398299 |
| GO:0050433\_regulation\_of\_catecholamine\_secretion | 9 | 1 | 8.252688 | -0.939520 | 394 | 104.349277 | 156.93 | 209.510723 | 0.398299 |
| GO:0050918\_positive\_chemotaxis | 9 | 1 | 8.252688 | -0.939520 | 394 | 104.349277 | 156.93 | 209.510723 | 0.398299 |
| GO:0060052\_neurofilament\_cytoskeleton\_organization | 9 | 1 | 8.252688 | -0.939520 | 394 | 104.349277 | 156.93 | 209.510723 | 0.398299 |
| GO:0010556\_regulation\_of\_macromolecule\_biosynthetic\_process | 745 | 14 | 1.395757 | -0.933347 | 395 | 104.645512 | 157.23 | 209.814488 | 0.398051 |
| GO:0006606\_protein\_import\_into\_nucleus | 44 | 2 | 3.376100 | -0.928778 | 400 | 106.417057 | 159.49 | 212.562943 | 0.398725 |
| GO:0046545\_development\_of\_primary\_female\_sexual\_characteristics | 44 | 2 | 3.376100 | -0.928778 | 400 | 106.417057 | 159.49 | 212.562943 | 0.398725 |
| GO:0048593\_camera-type\_eye\_morphogenesis | 44 | 2 | 3.376100 | -0.928778 | 400 | 106.417057 | 159.49 | 212.562943 | 0.398725 |
| GO:0051170\_nuclear\_import | 44 | 2 | 3.376100 | -0.928778 | 400 | 106.417057 | 159.49 | 212.562943 | 0.398725 |
| GO:0060485\_mesenchyme\_development | 44 | 2 | 3.376100 | -0.928778 | 400 | 106.417057 | 159.49 | 212.562943 | 0.398725 |
| GO:0016044\_membrane\_organization | 140 | 4 | 2.122120 | -0.926421 | 401 | 106.687413 | 159.79 | 212.892587 | 0.398479 |
| GO:0030324\_lung\_development | 90 | 3 | 2.475806 | -0.920291 | 402 | 107.033074 | 160.38 | 213.726926 | 0.398955 |
| GO:0019725\_cellular\_homeostasis | 195 | 5 | 1.904467 | -0.918998 | 403 | 107.200891 | 160.69 | 214.179109 | 0.398734 |
| GO:0034103\_regulation\_of\_tissue\_remodeling | 45 | 2 | 3.301075 | -0.912666 | 404 | 107.804303 | 161.71 | 215.615697 | 0.400272 |
| GO:0010557\_positive\_regulation\_of\_macromolecule\_biosynthetic\_process | 371 | 8 | 1.601600 | -0.911960 | 405 | 107.903598 | 161.86 | 215.816402 | 0.399654 |
| GO:0031399\_regulation\_of\_protein\_modification\_process | 91 | 3 | 2.448600 | -0.909724 | 406 | 108.201554 | 162.26 | 216.318446 | 0.399655 |
| GO:0035239\_tube\_morphogenesis | 143 | 4 | 2.077600 | -0.901784 | 407 | 108.598094 | 162.9 | 217.201906 | 0.400246 |
| GO:0009719\_response\_to\_endogenous\_stimulus | 92 | 3 | 2.421985 | -0.899315 | 409 | 108.781241 | 163.11 | 217.438759 | 0.398802 |
| GO:0030323\_respiratory\_tube\_development | 92 | 3 | 2.421985 | -0.899315 | 409 | 108.781241 | 163.11 | 217.438759 | 0.398802 |
| GO:0006357\_regulation\_of\_transcription\_from\_RNA\_polymerase\_II\_promoter | 435 | 9 | 1.536707 | -0.898892 | 410 | 108.859510 | 163.2 | 217.540490 | 0.398049 |
| GO:0006732\_coenzyme\_metabolic\_process | 46 | 2 | 3.229313 | -0.896990 | 411 | 109.775198 | 164.34 | 218.904802 | 0.399854 |
| GO:0006109\_regulation\_of\_carbohydrate\_metabolic\_process | 10 | 1 | 7.427419 | -0.896601 | 427 | 121.469808 | 178.91 | 236.350192 | 0.418993 |
| GO:0006289\_nucleotide-excision\_repair | 10 | 1 | 7.427419 | -0.896601 | 427 | 121.469808 | 178.91 | 236.350192 | 0.418993 |
| GO:0006805\_xenobiotic\_metabolic\_process | 10 | 1 | 7.427419 | -0.896601 | 427 | 121.469808 | 178.91 | 236.350192 | 0.418993 |
| GO:0006826\_iron\_ion\_transport | 10 | 1 | 7.427419 | -0.896601 | 427 | 121.469808 | 178.91 | 236.350192 | 0.418993 |
| GO:0007044\_cell-substrate\_junction\_assembly | 10 | 1 | 7.427419 | -0.896601 | 427 | 121.469808 | 178.91 | 236.350192 | 0.418993 |
| GO:0009743\_response\_to\_carbohydrate\_stimulus | 10 | 1 | 7.427419 | -0.896601 | 427 | 121.469808 | 178.91 | 236.350192 | 0.418993 |
| GO:0016197\_endosome\_transport | 10 | 1 | 7.427419 | -0.896601 | 427 | 121.469808 | 178.91 | 236.350192 | 0.418993 |
| GO:0021895\_cerebral\_cortex\_neuron\_differentiation | 10 | 1 | 7.427419 | -0.896601 | 427 | 121.469808 | 178.91 | 236.350192 | 0.418993 |
| GO:0021952\_central\_nervous\_system\_projection\_neuron\_axonogenesis | 10 | 1 | 7.427419 | -0.896601 | 427 | 121.469808 | 178.91 | 236.350192 | 0.418993 |
| GO:0022900\_electron\_transport\_chain | 10 | 1 | 7.427419 | -0.896601 | 427 | 121.469808 | 178.91 | 236.350192 | 0.418993 |
| GO:0022904\_respiratory\_electron\_transport\_chain | 10 | 1 | 7.427419 | -0.896601 | 427 | 121.469808 | 178.91 | 236.350192 | 0.418993 |
| GO:0031018\_endocrine\_pancreas\_development | 10 | 1 | 7.427419 | -0.896601 | 427 | 121.469808 | 178.91 | 236.350192 | 0.418993 |
| GO:0031331\_positive\_regulation\_of\_cellular\_catabolic\_process | 10 | 1 | 7.427419 | -0.896601 | 427 | 121.469808 | 178.91 | 236.350192 | 0.418993 |
| GO:0042088\_T-helper\_1\_type\_immune\_response | 10 | 1 | 7.427419 | -0.896601 | 427 | 121.469808 | 178.91 | 236.350192 | 0.418993 |
| GO:0043488\_regulation\_of\_mRNA\_stability | 10 | 1 | 7.427419 | -0.896601 | 427 | 121.469808 | 178.91 | 236.350192 | 0.418993 |
| GO:0048641\_regulation\_of\_skeletal\_muscle\_tissue\_development | 10 | 1 | 7.427419 | -0.896601 | 427 | 121.469808 | 178.91 | 236.350192 | 0.418993 |
| GO:0045596\_negative\_regulation\_of\_cell\_differentiation | 144 | 4 | 2.063172 | -0.893743 | 428 | 121.660115 | 179.16 | 236.659885 | 0.418598 |
| GO:0019219\_regulation\_of\_nucleobase\_\_nucleoside\_\_nucleotide\_and\_nucleic\_acid\_metabolic\_process | 757 | 14 | 1.373631 | -0.891156 | 429 | 121.726306 | 179.34 | 236.953694 | 0.418042 |
| GO:0008610\_lipid\_biosynthetic\_process | 94 | 3 | 2.370453 | -0.878958 | 431 | 123.416387 | 182.02 | 240.623613 | 0.422320 |
| GO:0034984\_cellular\_response\_to\_DNA\_damage\_stimulus | 94 | 3 | 2.370453 | -0.878958 | 431 | 123.416387 | 182.02 | 240.623613 | 0.422320 |
| GO:0006812\_cation\_transport | 146 | 4 | 2.034909 | -0.877911 | 432 | 123.533506 | 182.2 | 240.866494 | 0.421759 |
| GO:0022603\_regulation\_of\_anatomical\_structure\_morphogenesis | 147 | 4 | 2.021066 | -0.870117 | 433 | 123.838969 | 182.58 | 241.321031 | 0.421663 |
| GO:0016043\_cellular\_component\_organization | 964 | 17 | 1.309815 | -0.869028 | 434 | 123.882526 | 182.63 | 241.377474 | 0.420806 |
| GO:0034504\_protein\_localization\_in\_nucleus | 48 | 2 | 3.094758 | -0.866866 | 436 | 124.418000 | 183.55 | 242.682000 | 0.420986 |
| GO:0046849\_bone\_remodeling | 48 | 2 | 3.094758 | -0.866866 | 436 | 124.418000 | 183.55 | 242.682000 | 0.420986 |
| GO:0001568\_blood\_vessel\_development | 203 | 5 | 1.829414 | -0.865402 | 437 | 124.489700 | 183.64 | 242.790300 | 0.420229 |
| GO:0001101\_response\_to\_acid | 11 | 1 | 6.752199 | -0.858040 | 458 | 134.662394 | 195.55 | 256.437606 | 0.426965 |
| GO:0001963\_synaptic\_transmission\_\_dopaminergic | 11 | 1 | 6.752199 | -0.858040 | 458 | 134.662394 | 195.55 | 256.437606 | 0.426965 |
| GO:0007162\_negative\_regulation\_of\_cell\_adhesion | 11 | 1 | 6.752199 | -0.858040 | 458 | 134.662394 | 195.55 | 256.437606 | 0.426965 |
| GO:0007215\_glutamate\_signaling\_pathway | 11 | 1 | 6.752199 | -0.858040 | 458 | 134.662394 | 195.55 | 256.437606 | 0.426965 |
| GO:0008354\_germ\_cell\_migration | 11 | 1 | 6.752199 | -0.858040 | 458 | 134.662394 | 195.55 | 256.437606 | 0.426965 |
| GO:0008652\_cellular\_amino\_acid\_biosynthetic\_process | 11 | 1 | 6.752199 | -0.858040 | 458 | 134.662394 | 195.55 | 256.437606 | 0.426965 |
| GO:0014013\_regulation\_of\_gliogenesis | 11 | 1 | 6.752199 | -0.858040 | 458 | 134.662394 | 195.55 | 256.437606 | 0.426965 |
| GO:0021602\_cranial\_nerve\_morphogenesis | 11 | 1 | 6.752199 | -0.858040 | 458 | 134.662394 | 195.55 | 256.437606 | 0.426965 |
| GO:0021846\_cell\_proliferation\_in\_forebrain | 11 | 1 | 6.752199 | -0.858040 | 458 | 134.662394 | 195.55 | 256.437606 | 0.426965 |
| GO:0030308\_negative\_regulation\_of\_cell\_growth | 11 | 1 | 6.752199 | -0.858040 | 458 | 134.662394 | 195.55 | 256.437606 | 0.426965 |
| GO:0031646\_positive\_regulation\_of\_neurological\_system\_process | 11 | 1 | 6.752199 | -0.858040 | 458 | 134.662394 | 195.55 | 256.437606 | 0.426965 |
| GO:0031647\_regulation\_of\_protein\_stability | 11 | 1 | 6.752199 | -0.858040 | 458 | 134.662394 | 195.55 | 256.437606 | 0.426965 |
| GO:0033059\_cellular\_pigmentation | 11 | 1 | 6.752199 | -0.858040 | 458 | 134.662394 | 195.55 | 256.437606 | 0.426965 |
| GO:0042401\_biogenic\_amine\_biosynthetic\_process | 11 | 1 | 6.752199 | -0.858040 | 458 | 134.662394 | 195.55 | 256.437606 | 0.426965 |
| GO:0042439\_ethanolamine\_and\_derivative\_metabolic\_process | 11 | 1 | 6.752199 | -0.858040 | 458 | 134.662394 | 195.55 | 256.437606 | 0.426965 |
| GO:0042542\_response\_to\_hydrogen\_peroxide | 11 | 1 | 6.752199 | -0.858040 | 458 | 134.662394 | 195.55 | 256.437606 | 0.426965 |
| GO:0045685\_regulation\_of\_glial\_cell\_differentiation | 11 | 1 | 6.752199 | -0.858040 | 458 | 134.662394 | 195.55 | 256.437606 | 0.426965 |
| GO:0046928\_regulation\_of\_neurotransmitter\_secretion | 11 | 1 | 6.752199 | -0.858040 | 458 | 134.662394 | 195.55 | 256.437606 | 0.426965 |
| GO:0050772\_positive\_regulation\_of\_axonogenesis | 11 | 1 | 6.752199 | -0.858040 | 458 | 134.662394 | 195.55 | 256.437606 | 0.426965 |
| GO:0050806\_positive\_regulation\_of\_synaptic\_transmission | 11 | 1 | 6.752199 | -0.858040 | 458 | 134.662394 | 195.55 | 256.437606 | 0.426965 |
| GO:0051971\_positive\_regulation\_of\_transmission\_of\_nerve\_impulse | 11 | 1 | 6.752199 | -0.858040 | 458 | 134.662394 | 195.55 | 256.437606 | 0.426965 |
| GO:0034645\_cellular\_macromolecule\_biosynthetic\_process | 901 | 16 | 1.318965 | -0.855385 | 459 | 134.939052 | 195.91 | 256.880948 | 0.426819 |
| GO:0021543\_pallium\_development | 49 | 2 | 3.031600 | -0.852385 | 461 | 135.766336 | 197.41 | 259.053664 | 0.428221 |
| GO:0046660\_female\_sex\_differentiation | 49 | 2 | 3.031600 | -0.852385 | 461 | 135.766336 | 197.41 | 259.053664 | 0.428221 |
| GO:0060541\_respiratory\_system\_development | 98 | 3 | 2.273700 | -0.839994 | 462 | 136.819734 | 198.77 | 260.720266 | 0.430238 |
| GO:0017038\_protein\_import | 50 | 2 | 2.970968 | -0.838269 | 463 | 137.597039 | 199.7 | 261.802961 | 0.431317 |
| GO:0031328\_positive\_regulation\_of\_cellular\_biosynthetic\_process | 387 | 8 | 1.535384 | -0.836376 | 464 | 137.680734 | 199.85 | 262.019266 | 0.430711 |
| GO:0009887\_organ\_morphogenesis | 642 | 12 | 1.388303 | -0.835139 | 465 | 137.719986 | 199.91 | 262.100014 | 0.429914 |
| GO:0001944\_vasculature\_development | 208 | 5 | 1.785437 | -0.833622 | 466 | 137.893356 | 200.25 | 262.606644 | 0.429721 |
| GO:0009891\_positive\_regulation\_of\_biosynthetic\_process | 388 | 8 | 1.531427 | -0.831854 | 467 | 137.942357 | 200.47 | 262.997643 | 0.429272 |
| GO:0060348\_bone\_development | 99 | 3 | 2.250733 | -0.830599 | 468 | 138.272073 | 200.94 | 263.607927 | 0.429359 |
| GO:0009059\_macromolecule\_biosynthetic\_process | 910 | 16 | 1.305920 | -0.827706 | 469 | 138.343666 | 201.02 | 263.696334 | 0.428614 |
| GO:0032583\_regulation\_of\_gene-specific\_transcription | 51 | 2 | 2.912713 | -0.824503 | 470 | 139.091837 | 202.08 | 265.068163 | 0.429957 |
| GO:0001662\_behavioral\_fear\_response | 12 | 1 | 6.189516 | -0.823078 | 486 | 148.820685 | 213.27 | 277.719315 | 0.438827 |
| GO:0002209\_behavioral\_defense\_response | 12 | 1 | 6.189516 | -0.823078 | 486 | 148.820685 | 213.27 | 277.719315 | 0.438827 |
| GO:0002244\_hemopoietic\_progenitor\_cell\_differentiation | 12 | 1 | 6.189516 | -0.823078 | 486 | 148.820685 | 213.27 | 277.719315 | 0.438827 |
| GO:0002763\_positive\_regulation\_of\_myeloid\_leukocyte\_differentiation | 12 | 1 | 6.189516 | -0.823078 | 486 | 148.820685 | 213.27 | 277.719315 | 0.438827 |
| GO:0006839\_mitochondrial\_transport | 12 | 1 | 6.189516 | -0.823078 | 486 | 148.820685 | 213.27 | 277.719315 | 0.438827 |
| GO:0006879\_cellular\_iron\_ion\_homeostasis | 12 | 1 | 6.189516 | -0.823078 | 486 | 148.820685 | 213.27 | 277.719315 | 0.438827 |
| GO:0007143\_female\_meiosis | 12 | 1 | 6.189516 | -0.823078 | 486 | 148.820685 | 213.27 | 277.719315 | 0.438827 |
| GO:0008038\_neuron\_recognition | 12 | 1 | 6.189516 | -0.823078 | 486 | 148.820685 | 213.27 | 277.719315 | 0.438827 |
| GO:0015872\_dopamine\_transport | 12 | 1 | 6.189516 | -0.823078 | 486 | 148.820685 | 213.27 | 277.719315 | 0.438827 |
| GO:0030514\_negative\_regulation\_of\_BMP\_signaling\_pathway | 12 | 1 | 6.189516 | -0.823078 | 486 | 148.820685 | 213.27 | 277.719315 | 0.438827 |
| GO:0043487\_regulation\_of\_RNA\_stability | 12 | 1 | 6.189516 | -0.823078 | 486 | 148.820685 | 213.27 | 277.719315 | 0.438827 |
| GO:0045792\_negative\_regulation\_of\_cell\_size | 12 | 1 | 6.189516 | -0.823078 | 486 | 148.820685 | 213.27 | 277.719315 | 0.438827 |
| GO:0048169\_regulation\_of\_long-term\_neuronal\_synaptic\_plasticity | 12 | 1 | 6.189516 | -0.823078 | 486 | 148.820685 | 213.27 | 277.719315 | 0.438827 |
| GO:0050795\_regulation\_of\_behavior | 12 | 1 | 6.189516 | -0.823078 | 486 | 148.820685 | 213.27 | 277.719315 | 0.438827 |
| GO:0051588\_regulation\_of\_neurotransmitter\_transport | 12 | 1 | 6.189516 | -0.823078 | 486 | 148.820685 | 213.27 | 277.719315 | 0.438827 |
| GO:0055114\_oxidation\_reduction | 12 | 1 | 6.189516 | -0.823078 | 486 | 148.820685 | 213.27 | 277.719315 | 0.438827 |
| GO:0001525\_angiogenesis | 100 | 3 | 2.228226 | -0.821338 | 487 | 148.992427 | 213.52 | 278.047573 | 0.438439 |
| GO:0010468\_regulation\_of\_gene\_expression | 778 | 14 | 1.336554 | -0.821083 | 488 | 149.032284 | 213.58 | 278.127716 | 0.437664 |
| GO:0045944\_positive\_regulation\_of\_transcription\_from\_RNA\_polymerase\_II\_promoter | 269 | 6 | 1.656673 | -0.819139 | 489 | 149.053916 | 213.65 | 278.246084 | 0.436912 |
| GO:0010608\_posttranscriptional\_regulation\_of\_gene\_expression | 52 | 2 | 2.856700 | -0.811075 | 490 | 150.321694 | 214.99 | 279.658306 | 0.438755 |
| GO:0022402\_cell\_cycle\_process | 155 | 4 | 1.916753 | -0.810559 | 491 | 150.586968 | 215.38 | 280.173032 | 0.438656 |
| GO:0035295\_tube\_development | 212 | 5 | 1.751750 | -0.809096 | 492 | 150.683223 | 215.52 | 280.356777 | 0.438049 |
| GO:0006810\_transport | 718 | 13 | 1.344797 | -0.800161 | 493 | 151.148778 | 216.16 | 281.171222 | 0.438458 |
| GO:0006576\_biogenic\_amine\_metabolic\_process | 53 | 2 | 2.802800 | -0.797970 | 495 | 151.830653 | 217.2 | 282.569347 | 0.438788 |
| GO:0050905\_neuromuscular\_process | 53 | 2 | 2.802800 | -0.797970 | 495 | 151.830653 | 217.2 | 282.569347 | 0.438788 |
| GO:0001836\_release\_of\_cytochrome\_c\_from\_mitochondria | 13 | 1 | 5.713400 | -0.791136 | 515 | 161.788689 | 229.39 | 296.991311 | 0.445417 |
| GO:0001958\_endochondral\_ossification | 13 | 1 | 5.713400 | -0.791136 | 515 | 161.788689 | 229.39 | 296.991311 | 0.445417 |
| GO:0007212\_dopamine\_receptor\_signaling\_pathway | 13 | 1 | 5.713400 | -0.791136 | 515 | 161.788689 | 229.39 | 296.991311 | 0.445417 |
| GO:0007274\_neuromuscular\_synaptic\_transmission | 13 | 1 | 5.713400 | -0.791136 | 515 | 161.788689 | 229.39 | 296.991311 | 0.445417 |
| GO:0009410\_response\_to\_xenobiotic\_stimulus | 13 | 1 | 5.713400 | -0.791136 | 515 | 161.788689 | 229.39 | 296.991311 | 0.445417 |
| GO:0018105\_peptidyl-serine\_phosphorylation | 13 | 1 | 5.713400 | -0.791136 | 515 | 161.788689 | 229.39 | 296.991311 | 0.445417 |
| GO:0021879\_forebrain\_neuron\_differentiation | 13 | 1 | 5.713400 | -0.791136 | 515 | 161.788689 | 229.39 | 296.991311 | 0.445417 |
| GO:0021955\_central\_nervous\_system\_neuron\_axonogenesis | 13 | 1 | 5.713400 | -0.791136 | 515 | 161.788689 | 229.39 | 296.991311 | 0.445417 |
| GO:0030516\_regulation\_of\_axon\_extension | 13 | 1 | 5.713400 | -0.791136 | 515 | 161.788689 | 229.39 | 296.991311 | 0.445417 |
| GO:0031290\_retinal\_ganglion\_cell\_axon\_guidance | 13 | 1 | 5.713400 | -0.791136 | 515 | 161.788689 | 229.39 | 296.991311 | 0.445417 |
| GO:0032729\_positive\_regulation\_of\_interferon-gamma\_production | 13 | 1 | 5.713400 | -0.791136 | 515 | 161.788689 | 229.39 | 296.991311 | 0.445417 |
| GO:0034329\_cell\_junction\_assembly | 13 | 1 | 5.713400 | -0.791136 | 515 | 161.788689 | 229.39 | 296.991311 | 0.445417 |
| GO:0046474\_glycerophospholipid\_biosynthetic\_process | 13 | 1 | 5.713400 | -0.791136 | 515 | 161.788689 | 229.39 | 296.991311 | 0.445417 |
| GO:0046851\_negative\_regulation\_of\_bone\_remodeling | 13 | 1 | 5.713400 | -0.791136 | 515 | 161.788689 | 229.39 | 296.991311 | 0.445417 |
| GO:0048566\_embryonic\_gut\_development | 13 | 1 | 5.713400 | -0.791136 | 515 | 161.788689 | 229.39 | 296.991311 | 0.445417 |
| GO:0050764\_regulation\_of\_phagocytosis | 13 | 1 | 5.713400 | -0.791136 | 515 | 161.788689 | 229.39 | 296.991311 | 0.445417 |
| GO:0050766\_positive\_regulation\_of\_phagocytosis | 13 | 1 | 5.713400 | -0.791136 | 515 | 161.788689 | 229.39 | 296.991311 | 0.445417 |
| GO:0050771\_negative\_regulation\_of\_axonogenesis | 13 | 1 | 5.713400 | -0.791136 | 515 | 161.788689 | 229.39 | 296.991311 | 0.445417 |
| GO:0060401\_cytosolic\_calcium\_ion\_transport | 13 | 1 | 5.713400 | -0.791136 | 515 | 161.788689 | 229.39 | 296.991311 | 0.445417 |
| GO:0060402\_calcium\_ion\_transport\_into\_cytosol | 13 | 1 | 5.713400 | -0.791136 | 515 | 161.788689 | 229.39 | 296.991311 | 0.445417 |
| GO:0043405\_regulation\_of\_MAP\_kinase\_activity | 54 | 2 | 2.750896 | -0.785176 | 517 | 163.298093 | 231.22 | 299.141907 | 0.447234 |
| GO:0044271\_nitrogen\_compound\_biosynthetic\_process | 54 | 2 | 2.750896 | -0.785176 | 517 | 163.298093 | 231.22 | 299.141907 | 0.447234 |
| GO:0048518\_positive\_regulation\_of\_biological\_process | 995 | 17 | 1.269006 | -0.778047 | 518 | 163.664255 | 231.79 | 299.915745 | 0.447471 |
| GO:0048872\_homeostasis\_of\_number\_of\_cells | 105 | 3 | 2.122120 | -0.776931 | 519 | 163.896559 | 232.22 | 300.543441 | 0.447437 |
| GO:0051128\_regulation\_of\_cellular\_component\_organization | 160 | 4 | 1.856855 | -0.775700 | 520 | 164.014543 | 232.34 | 300.665457 | 0.446808 |
| GO:0048568\_embryonic\_organ\_development | 55 | 2 | 2.700880 | -0.772682 | 521 | 164.957116 | 233.44 | 301.922884 | 0.448061 |
| GO:0051234\_establishment\_of\_localization | 729 | 13 | 1.324506 | -0.764703 | 522 | 165.735928 | 234.45 | 303.164072 | 0.449138 |
| GO:0002262\_myeloid\_cell\_homeostasis | 14 | 1 | 5.305300 | -0.761765 | 528 | 175.098203 | 245.42 | 315.741797 | 0.464811 |
| GO:0009108\_coenzyme\_biosynthetic\_process | 14 | 1 | 5.305300 | -0.761765 | 528 | 175.098203 | 245.42 | 315.741797 | 0.464811 |
| GO:0010332\_response\_to\_gamma\_radiation | 14 | 1 | 5.305300 | -0.761765 | 528 | 175.098203 | 245.42 | 315.741797 | 0.464811 |
| GO:0031346\_positive\_regulation\_of\_cell\_projection\_organization | 14 | 1 | 5.305300 | -0.761765 | 528 | 175.098203 | 245.42 | 315.741797 | 0.464811 |
| GO:0034104\_negative\_regulation\_of\_tissue\_remodeling | 14 | 1 | 5.305300 | -0.761765 | 528 | 175.098203 | 245.42 | 315.741797 | 0.464811 |
| GO:0051952\_regulation\_of\_amine\_transport | 14 | 1 | 5.305300 | -0.761765 | 528 | 175.098203 | 245.42 | 315.741797 | 0.464811 |
| GO:0001708\_cell\_fate\_specification | 56 | 2 | 2.652650 | -0.760476 | 529 | 175.943796 | 246.88 | 317.816204 | 0.466692 |
| GO:0006139\_nucleobase\_\_nucleoside\_\_nucleotide\_and\_nucleic\_acid\_metabolic\_process | 1002 | 17 | 1.260141 | -0.758569 | 530 | 176.122850 | 247.15 | 318.177150 | 0.466321 |
| GO:0030099\_myeloid\_cell\_differentiation | 108 | 3 | 2.063172 | -0.751719 | 532 | 176.501626 | 247.75 | 318.998374 | 0.465695 |
| GO:0051240\_positive\_regulation\_of\_multicellular\_organismal\_process | 108 | 3 | 2.063172 | -0.751719 | 532 | 176.501626 | 247.75 | 318.998374 | 0.465695 |
| GO:0001764\_neuron\_migration | 57 | 2 | 2.606112 | -0.748548 | 534 | 178.256654 | 249.77 | 321.283346 | 0.467734 |
| GO:0033365\_protein\_localization\_in\_organelle | 57 | 2 | 2.606112 | -0.748548 | 534 | 178.256654 | 249.77 | 321.283346 | 0.467734 |
| GO:0030902\_hindbrain\_development | 58 | 2 | 2.561179 | -0.736888 | 536 | 179.315979 | 251.39 | 323.464021 | 0.469011 |
| GO:0050804\_regulation\_of\_synaptic\_transmission | 58 | 2 | 2.561179 | -0.736888 | 536 | 179.315979 | 251.39 | 323.464021 | 0.469011 |
| GO:0043010\_camera-type\_eye\_development | 110 | 3 | 2.025660 | -0.735469 | 538 | 179.742200 | 252.02 | 324.297800 | 0.468439 |
| GO:0055080\_cation\_homeostasis | 110 | 3 | 2.025660 | -0.735469 | 538 | 179.742200 | 252.02 | 324.297800 | 0.468439 |
| GO:0001759\_induction\_of\_an\_organ | 15 | 1 | 4.951613 | -0.734610 | 559 | 188.094884 | 261.53 | 334.965116 | 0.467853 |
| GO:0001782\_B\_cell\_homeostasis | 15 | 1 | 4.951613 | -0.734610 | 559 | 188.094884 | 261.53 | 334.965116 | 0.467853 |
| GO:0001964\_startle\_response | 15 | 1 | 4.951613 | -0.734610 | 559 | 188.094884 | 261.53 | 334.965116 | 0.467853 |
| GO:0002286\_T\_cell\_activation\_during\_immune\_response | 15 | 1 | 4.951613 | -0.734610 | 559 | 188.094884 | 261.53 | 334.965116 | 0.467853 |
| GO:0007200\_activation\_of\_phospholipase\_C\_activity\_by\_G-protein\_coupled\_receptor\_protein\_signaling\_pathway\_coupled\_to\_IP3\_second\_messenger | 15 | 1 | 4.951613 | -0.734610 | 559 | 188.094884 | 261.53 | 334.965116 | 0.467853 |
| GO:0007202\_activation\_of\_phospholipase\_C\_activity | 15 | 1 | 4.951613 | -0.734610 | 559 | 188.094884 | 261.53 | 334.965116 | 0.467853 |
| GO:0007588\_excretion | 15 | 1 | 4.951613 | -0.734610 | 559 | 188.094884 | 261.53 | 334.965116 | 0.467853 |
| GO:0010092\_specification\_of\_organ\_identity | 15 | 1 | 4.951613 | -0.734610 | 559 | 188.094884 | 261.53 | 334.965116 | 0.467853 |
| GO:0010518\_positive\_regulation\_of\_phospholipase\_activity | 15 | 1 | 4.951613 | -0.734610 | 559 | 188.094884 | 261.53 | 334.965116 | 0.467853 |
| GO:0010863\_positive\_regulation\_of\_phospholipase\_C\_activity | 15 | 1 | 4.951613 | -0.734610 | 559 | 188.094884 | 261.53 | 334.965116 | 0.467853 |
| GO:0021795\_cerebral\_cortex\_cell\_migration | 15 | 1 | 4.951613 | -0.734610 | 559 | 188.094884 | 261.53 | 334.965116 | 0.467853 |
| GO:0021872\_generation\_of\_neurons\_in\_the\_forebrain | 15 | 1 | 4.951613 | -0.734610 | 559 | 188.094884 | 261.53 | 334.965116 | 0.467853 |
| GO:0031069\_hair\_follicle\_morphogenesis | 15 | 1 | 4.951613 | -0.734610 | 559 | 188.094884 | 261.53 | 334.965116 | 0.467853 |
| GO:0031076\_embryonic\_camera-type\_eye\_development | 15 | 1 | 4.951613 | -0.734610 | 559 | 188.094884 | 261.53 | 334.965116 | 0.467853 |
| GO:0031329\_regulation\_of\_cellular\_catabolic\_process | 15 | 1 | 4.951613 | -0.734610 | 559 | 188.094884 | 261.53 | 334.965116 | 0.467853 |
| GO:0045666\_positive\_regulation\_of\_neuron\_differentiation | 15 | 1 | 4.951613 | -0.734610 | 559 | 188.094884 | 261.53 | 334.965116 | 0.467853 |
| GO:0046164\_alcohol\_catabolic\_process | 15 | 1 | 4.951613 | -0.734610 | 559 | 188.094884 | 261.53 | 334.965116 | 0.467853 |
| GO:0048010\_vascular\_endothelial\_growth\_factor\_receptor\_signaling\_pathway | 15 | 1 | 4.951613 | -0.734610 | 559 | 188.094884 | 261.53 | 334.965116 | 0.467853 |
| GO:0050796\_regulation\_of\_insulin\_secretion | 15 | 1 | 4.951613 | -0.734610 | 559 | 188.094884 | 261.53 | 334.965116 | 0.467853 |
| GO:0060425\_lung\_morphogenesis | 15 | 1 | 4.951613 | -0.734610 | 559 | 188.094884 | 261.53 | 334.965116 | 0.467853 |
| GO:0060749\_mammary\_gland\_alveolus\_development | 15 | 1 | 4.951613 | -0.734610 | 559 | 188.094884 | 261.53 | 334.965116 | 0.467853 |
| GO:0051049\_regulation\_of\_transport | 167 | 4 | 1.779023 | -0.729692 | 560 | 188.458392 | 262.05 | 335.641608 | 0.467946 |
| GO:0035270\_endocrine\_system\_development | 59 | 2 | 2.517769 | -0.725488 | 561 | 189.192139 | 263.15 | 337.107861 | 0.469073 |
| GO:0006974\_response\_to\_DNA\_damage\_stimulus | 113 | 3 | 1.971881 | -0.711891 | 562 | 190.305198 | 264.76 | 339.214802 | 0.471103 |
| GO:0000302\_response\_to\_reactive\_oxygen\_species | 16 | 1 | 4.642137 | -0.709384 | 569 | 198.428051 | 273.81 | 349.191949 | 0.481213 |
| GO:0008654\_phospholipid\_biosynthetic\_process | 16 | 1 | 4.642137 | -0.709384 | 569 | 198.428051 | 273.81 | 349.191949 | 0.481213 |
| GO:0031345\_negative\_regulation\_of\_cell\_projection\_organization | 16 | 1 | 4.642137 | -0.709384 | 569 | 198.428051 | 273.81 | 349.191949 | 0.481213 |
| GO:0045104\_intermediate\_filament\_cytoskeleton\_organization | 16 | 1 | 4.642137 | -0.709384 | 569 | 198.428051 | 273.81 | 349.191949 | 0.481213 |
| GO:0048015\_phosphoinositide-mediated\_signaling | 16 | 1 | 4.642137 | -0.709384 | 569 | 198.428051 | 273.81 | 349.191949 | 0.481213 |
| GO:0051048\_negative\_regulation\_of\_secretion | 16 | 1 | 4.642137 | -0.709384 | 569 | 198.428051 | 273.81 | 349.191949 | 0.481213 |
| GO:0060193\_positive\_regulation\_of\_lipase\_activity | 16 | 1 | 4.642137 | -0.709384 | 569 | 198.428051 | 273.81 | 349.191949 | 0.481213 |
| GO:0007005\_mitochondrion\_organization | 61 | 2 | 2.435219 | -0.703427 | 571 | 199.242987 | 274.91 | 350.577013 | 0.481454 |
| GO:0051969\_regulation\_of\_transmission\_of\_nerve\_impulse | 61 | 2 | 2.435219 | -0.703427 | 571 | 199.242987 | 274.91 | 350.577013 | 0.481454 |
| GO:0042592\_homeostatic\_process | 419 | 8 | 1.418123 | -0.702398 | 572 | 199.289068 | 275.16 | 351.030932 | 0.481049 |
| GO:0009987\_cellular\_process | 3868 | 55 | 1.056122 | -0.694332 | 573 | 199.690928 | 275.84 | 351.989072 | 0.481396 |
| GO:0007154\_cell\_communication | 1096 | 18 | 1.219832 | -0.693258 | 574 | 199.704524 | 275.91 | 352.115476 | 0.480679 |
| GO:0021537\_telencephalon\_development | 62 | 2 | 2.395942 | -0.692750 | 577 | 200.642294 | 277.36 | 354.077706 | 0.480693 |
| GO:0022604\_regulation\_of\_cell\_morphogenesis | 62 | 2 | 2.395942 | -0.692750 | 577 | 200.642294 | 277.36 | 354.077706 | 0.480693 |
| GO:0030855\_epithelial\_cell\_differentiation | 62 | 2 | 2.395942 | -0.692750 | 577 | 200.642294 | 277.36 | 354.077706 | 0.480693 |
| GO:0048608\_reproductive\_structure\_development | 116 | 3 | 1.920884 | -0.689222 | 578 | 201.501470 | 278.66 | 355.818530 | 0.482111 |
| GO:0002791\_regulation\_of\_peptide\_secretion | 17 | 1 | 4.369070 | -0.685851 | 596 | 209.345162 | 287.42 | 365.494838 | 0.482248 |
| GO:0010517\_regulation\_of\_phospholipase\_activity | 17 | 1 | 4.369070 | -0.685851 | 596 | 209.345162 | 287.42 | 365.494838 | 0.482248 |
| GO:0015807\_L-amino\_acid\_transport | 17 | 1 | 4.369070 | -0.685851 | 596 | 209.345162 | 287.42 | 365.494838 | 0.482248 |
| GO:0021545\_cranial\_nerve\_development | 17 | 1 | 4.369070 | -0.685851 | 596 | 209.345162 | 287.42 | 365.494838 | 0.482248 |
| GO:0022029\_telencephalon\_cell\_migration | 17 | 1 | 4.369070 | -0.685851 | 596 | 209.345162 | 287.42 | 365.494838 | 0.482248 |
| GO:0032535\_regulation\_of\_cellular\_component\_size | 17 | 1 | 4.369070 | -0.685851 | 596 | 209.345162 | 287.42 | 365.494838 | 0.482248 |
| GO:0042384\_cilium\_assembly | 17 | 1 | 4.369070 | -0.685851 | 596 | 209.345162 | 287.42 | 365.494838 | 0.482248 |
| GO:0042398\_cellular\_amino\_acid\_derivative\_biosynthetic\_process | 17 | 1 | 4.369070 | -0.685851 | 596 | 209.345162 | 287.42 | 365.494838 | 0.482248 |
| GO:0042417\_dopamine\_metabolic\_process | 17 | 1 | 4.369070 | -0.685851 | 596 | 209.345162 | 287.42 | 365.494838 | 0.482248 |
| GO:0042440\_pigment\_metabolic\_process | 17 | 1 | 4.369070 | -0.685851 | 596 | 209.345162 | 287.42 | 365.494838 | 0.482248 |
| GO:0043407\_negative\_regulation\_of\_MAP\_kinase\_activity | 17 | 1 | 4.369070 | -0.685851 | 596 | 209.345162 | 287.42 | 365.494838 | 0.482248 |
| GO:0045333\_cellular\_respiration | 17 | 1 | 4.369070 | -0.685851 | 596 | 209.345162 | 287.42 | 365.494838 | 0.482248 |
| GO:0045667\_regulation\_of\_osteoblast\_differentiation | 17 | 1 | 4.369070 | -0.685851 | 596 | 209.345162 | 287.42 | 365.494838 | 0.482248 |
| GO:0045786\_negative\_regulation\_of\_cell\_cycle | 17 | 1 | 4.369070 | -0.685851 | 596 | 209.345162 | 287.42 | 365.494838 | 0.482248 |
| GO:0048168\_regulation\_of\_neuronal\_synaptic\_plasticity | 17 | 1 | 4.369070 | -0.685851 | 596 | 209.345162 | 287.42 | 365.494838 | 0.482248 |
| GO:0048873\_homeostasis\_of\_number\_of\_cells\_within\_a\_tissue | 17 | 1 | 4.369070 | -0.685851 | 596 | 209.345162 | 287.42 | 365.494838 | 0.482248 |
| GO:0055072\_iron\_ion\_homeostasis | 17 | 1 | 4.369070 | -0.685851 | 596 | 209.345162 | 287.42 | 365.494838 | 0.482248 |
| GO:0060350\_endochondral\_bone\_morphogenesis | 17 | 1 | 4.369070 | -0.685851 | 596 | 209.345162 | 287.42 | 365.494838 | 0.482248 |
| GO:0051186\_cofactor\_metabolic\_process | 63 | 2 | 2.357911 | -0.682299 | 597 | 210.201500 | 288.52 | 366.838500 | 0.483283 |
| GO:0001501\_skeletal\_system\_development | 236 | 5 | 1.573606 | -0.676994 | 598 | 210.709158 | 289.26 | 367.810842 | 0.483712 |
| GO:0006519\_cellular\_amino\_acid\_and\_derivative\_metabolic\_process | 118 | 3 | 1.888327 | -0.674590 | 599 | 211.232408 | 290.01 | 368.787592 | 0.484157 |
| GO:0006915\_apoptosis | 427 | 8 | 1.391554 | -0.672140 | 600 | 211.414306 | 290.19 | 368.965694 | 0.483650 |
| GO:0031644\_regulation\_of\_neurological\_system\_process | 64 | 2 | 2.321069 | -0.672067 | 601 | 211.988320 | 291.11 | 370.231680 | 0.484376 |
| GO:0001818\_negative\_regulation\_of\_cytokine\_production | 18 | 1 | 4.126344 | -0.663817 | 620 | 221.150466 | 302.18 | 383.209534 | 0.487387 |
| GO:0002064\_epithelial\_cell\_development | 18 | 1 | 4.126344 | -0.663817 | 620 | 221.150466 | 302.18 | 383.209534 | 0.487387 |
| GO:0002285\_lymphocyte\_activation\_during\_immune\_response | 18 | 1 | 4.126344 | -0.663817 | 620 | 221.150466 | 302.18 | 383.209534 | 0.487387 |
| GO:0006940\_regulation\_of\_smooth\_muscle\_contraction | 18 | 1 | 4.126344 | -0.663817 | 620 | 221.150466 | 302.18 | 383.209534 | 0.487387 |
| GO:0015711\_organic\_anion\_transport | 18 | 1 | 4.126344 | -0.663817 | 620 | 221.150466 | 302.18 | 383.209534 | 0.487387 |
| GO:0021885\_forebrain\_cell\_migration | 18 | 1 | 4.126344 | -0.663817 | 620 | 221.150466 | 302.18 | 383.209534 | 0.487387 |
| GO:0030336\_negative\_regulation\_of\_cell\_migration | 18 | 1 | 4.126344 | -0.663817 | 620 | 221.150466 | 302.18 | 383.209534 | 0.487387 |
| GO:0030510\_regulation\_of\_BMP\_signaling\_pathway | 18 | 1 | 4.126344 | -0.663817 | 620 | 221.150466 | 302.18 | 383.209534 | 0.487387 |
| GO:0032623\_interleukin-2\_production | 18 | 1 | 4.126344 | -0.663817 | 620 | 221.150466 | 302.18 | 383.209534 | 0.487387 |
| GO:0043029\_T\_cell\_homeostasis | 18 | 1 | 4.126344 | -0.663817 | 620 | 221.150466 | 302.18 | 383.209534 | 0.487387 |
| GO:0045058\_T\_cell\_selection | 18 | 1 | 4.126344 | -0.663817 | 620 | 221.150466 | 302.18 | 383.209534 | 0.487387 |
| GO:0045103\_intermediate\_filament-based\_process | 18 | 1 | 4.126344 | -0.663817 | 620 | 221.150466 | 302.18 | 383.209534 | 0.487387 |
| GO:0045807\_positive\_regulation\_of\_endocytosis | 18 | 1 | 4.126344 | -0.663817 | 620 | 221.150466 | 302.18 | 383.209534 | 0.487387 |
| GO:0046578\_regulation\_of\_Ras\_protein\_signal\_transduction | 18 | 1 | 4.126344 | -0.663817 | 620 | 221.150466 | 302.18 | 383.209534 | 0.487387 |
| GO:0048730\_epidermis\_morphogenesis | 18 | 1 | 4.126344 | -0.663817 | 620 | 221.150466 | 302.18 | 383.209534 | 0.487387 |
| GO:0050731\_positive\_regulation\_of\_peptidyl-tyrosine\_phosphorylation | 18 | 1 | 4.126344 | -0.663817 | 620 | 221.150466 | 302.18 | 383.209534 | 0.487387 |
| GO:0051924\_regulation\_of\_calcium\_ion\_transport | 18 | 1 | 4.126344 | -0.663817 | 620 | 221.150466 | 302.18 | 383.209534 | 0.487387 |
| GO:0055008\_cardiac\_muscle\_tissue\_morphogenesis | 18 | 1 | 4.126344 | -0.663817 | 620 | 221.150466 | 302.18 | 383.209534 | 0.487387 |
| GO:0060415\_muscle\_tissue\_morphogenesis | 18 | 1 | 4.126344 | -0.663817 | 620 | 221.150466 | 302.18 | 383.209534 | 0.487387 |
| GO:0014706\_striated\_muscle\_tissue\_development | 120 | 3 | 1.856855 | -0.660328 | 621 | 222.003620 | 303.16 | 384.316380 | 0.488180 |
| GO:0031589\_cell-substrate\_adhesion | 66 | 2 | 2.250733 | -0.652229 | 622 | 223.802126 | 305.56 | 387.317874 | 0.491254 |
| GO:0012501\_programmed\_cell\_death | 433 | 8 | 1.372271 | -0.650231 | 623 | 224.250841 | 306.17 | 388.089159 | 0.491445 |
| GO:0010467\_gene\_expression | 905 | 15 | 1.231064 | -0.650025 | 624 | 224.306449 | 306.22 | 388.133551 | 0.490737 |
| GO:0044237\_cellular\_metabolic\_process | 1974 | 30 | 1.128787 | -0.648933 | 625 | 224.398544 | 306.3 | 388.201456 | 0.490080 |
| GO:0007569\_cell\_aging | 19 | 1 | 3.909168 | -0.643120 | 633 | 232.042276 | 314.6 | 397.157724 | 0.496998 |
| GO:0007595\_lactation | 19 | 1 | 3.909168 | -0.643120 | 633 | 232.042276 | 314.6 | 397.157724 | 0.496998 |
| GO:0021587\_cerebellum\_morphogenesis | 19 | 1 | 3.909168 | -0.643120 | 633 | 232.042276 | 314.6 | 397.157724 | 0.496998 |
| GO:0046890\_regulation\_of\_lipid\_biosynthetic\_process | 19 | 1 | 3.909168 | -0.643120 | 633 | 232.042276 | 314.6 | 397.157724 | 0.496998 |
| GO:0048536\_spleen\_development | 19 | 1 | 3.909168 | -0.643120 | 633 | 232.042276 | 314.6 | 397.157724 | 0.496998 |
| GO:0048547\_gut\_morphogenesis | 19 | 1 | 3.909168 | -0.643120 | 633 | 232.042276 | 314.6 | 397.157724 | 0.496998 |
| GO:0051056\_regulation\_of\_small\_GTPase\_mediated\_signal\_transduction | 19 | 1 | 3.909168 | -0.643120 | 633 | 232.042276 | 314.6 | 397.157724 | 0.496998 |
| GO:0060079\_regulation\_of\_excitatory\_postsynaptic\_membrane\_potential | 19 | 1 | 3.909168 | -0.643120 | 633 | 232.042276 | 314.6 | 397.157724 | 0.496998 |
| GO:0034962\_cellular\_biopolymer\_catabolic\_process | 68 | 2 | 2.184535 | -0.633185 | 634 | 234.012059 | 317.36 | 400.707941 | 0.500568 |
| GO:0009308\_amine\_metabolic\_process | 124 | 3 | 1.796956 | -0.632859 | 635 | 234.122927 | 317.64 | 401.157073 | 0.500220 |
| GO:0016192\_vesicle-mediated\_transport | 184 | 4 | 1.614656 | -0.629987 | 636 | 234.384966 | 317.96 | 401.535034 | 0.499937 |
| GO:0008152\_metabolic\_process | 2133 | 32 | 1.114287 | -0.624377 | 637 | 235.104433 | 318.98 | 402.855567 | 0.500754 |
| GO:0006816\_calcium\_ion\_transport | 69 | 2 | 2.152875 | -0.623945 | 639 | 235.684814 | 319.84 | 403.995186 | 0.500532 |
| GO:0032101\_regulation\_of\_response\_to\_external\_stimulus | 69 | 2 | 2.152875 | -0.623945 | 639 | 235.684814 | 319.84 | 403.995186 | 0.500532 |
| GO:0005977\_glycogen\_metabolic\_process | 20 | 1 | 3.713710 | -0.623621 | 650 | 240.727626 | 325.9 | 411.072374 | 0.501385 |
| GO:0006073\_cellular\_glucan\_metabolic\_process | 20 | 1 | 3.713710 | -0.623621 | 650 | 240.727626 | 325.9 | 411.072374 | 0.501385 |
| GO:0006584\_catecholamine\_metabolic\_process | 20 | 1 | 3.713710 | -0.623621 | 650 | 240.727626 | 325.9 | 411.072374 | 0.501385 |
| GO:0009712\_catechol\_metabolic\_process | 20 | 1 | 3.713710 | -0.623621 | 650 | 240.727626 | 325.9 | 411.072374 | 0.501385 |
| GO:0016571\_histone\_methylation | 20 | 1 | 3.713710 | -0.623621 | 650 | 240.727626 | 325.9 | 411.072374 | 0.501385 |
| GO:0018209\_peptidyl-serine\_modification | 20 | 1 | 3.713710 | -0.623621 | 650 | 240.727626 | 325.9 | 411.072374 | 0.501385 |
| GO:0018958\_phenol\_metabolic\_process | 20 | 1 | 3.713710 | -0.623621 | 650 | 240.727626 | 325.9 | 411.072374 | 0.501385 |
| GO:0034311\_diol\_metabolic\_process | 20 | 1 | 3.713710 | -0.623621 | 650 | 240.727626 | 325.9 | 411.072374 | 0.501385 |
| GO:0044042\_glucan\_metabolic\_process | 20 | 1 | 3.713710 | -0.623621 | 650 | 240.727626 | 325.9 | 411.072374 | 0.501385 |
| GO:0045017\_glycerolipid\_biosynthetic\_process | 20 | 1 | 3.713710 | -0.623621 | 650 | 240.727626 | 325.9 | 411.072374 | 0.501385 |
| GO:0060191\_regulation\_of\_lipase\_activity | 20 | 1 | 3.713710 | -0.623621 | 650 | 240.727626 | 325.9 | 411.072374 | 0.501385 |
| GO:0050789\_regulation\_of\_biological\_process | 2357 | 35 | 1.102926 | -0.619966 | 651 | 241.051581 | 326.27 | 411.488419 | 0.501183 |
| GO:0006811\_ion\_transport | 186 | 4 | 1.597294 | -0.619265 | 652 | 241.484905 | 326.85 | 412.215095 | 0.501304 |
| GO:0008406\_gonad\_development | 70 | 2 | 2.122120 | -0.614887 | 654 | 242.422918 | 327.92 | 413.417082 | 0.501407 |
| GO:0070838\_divalent\_metal\_ion\_transport | 70 | 2 | 2.122120 | -0.614887 | 654 | 242.422918 | 327.92 | 413.417082 | 0.501407 |
| GO:0008219\_cell\_death | 444 | 8 | 1.338274 | -0.611735 | 655 | 242.626879 | 328.21 | 413.793121 | 0.501084 |
| GO:0007389\_pattern\_specification\_process | 250 | 5 | 1.485484 | -0.610391 | 656 | 242.817582 | 328.44 | 414.062418 | 0.500671 |
| GO:0060537\_muscle\_tissue\_development | 128 | 3 | 1.740801 | -0.606721 | 657 | 243.423853 | 329.09 | 414.756147 | 0.500898 |
| GO:0006281\_DNA\_repair | 71 | 2 | 2.092231 | -0.606004 | 659 | 244.253634 | 330.03 | 415.806366 | 0.500804 |
| GO:0006913\_nucleocytoplasmic\_transport | 71 | 2 | 2.092231 | -0.606004 | 659 | 244.253634 | 330.03 | 415.806366 | 0.500804 |
| GO:0001755\_neural\_crest\_cell\_migration | 21 | 1 | 3.536866 | -0.605203 | 676 | 250.885442 | 338.54 | 426.194558 | 0.500799 |
| GO:0002263\_cell\_activation\_during\_immune\_response | 21 | 1 | 3.536866 | -0.605203 | 676 | 250.885442 | 338.54 | 426.194558 | 0.500799 |
| GO:0002366\_leukocyte\_activation\_during\_immune\_response | 21 | 1 | 3.536866 | -0.605203 | 676 | 250.885442 | 338.54 | 426.194558 | 0.500799 |
| GO:0002456\_T\_cell\_mediated\_immunity | 21 | 1 | 3.536866 | -0.605203 | 676 | 250.885442 | 338.54 | 426.194558 | 0.500799 |
| GO:0006814\_sodium\_ion\_transport | 21 | 1 | 3.536866 | -0.605203 | 676 | 250.885442 | 338.54 | 426.194558 | 0.500799 |
| GO:0007189\_activation\_of\_adenylate\_cyclase\_activity\_by\_G-protein\_signaling\_pathway | 21 | 1 | 3.536866 | -0.605203 | 676 | 250.885442 | 338.54 | 426.194558 | 0.500799 |
| GO:0008637\_apoptotic\_mitochondrial\_changes | 21 | 1 | 3.536866 | -0.605203 | 676 | 250.885442 | 338.54 | 426.194558 | 0.500799 |
| GO:0010552\_positive\_regulation\_of\_specific\_transcription\_from\_RNA\_polymerase\_II\_promoter | 21 | 1 | 3.536866 | -0.605203 | 676 | 250.885442 | 338.54 | 426.194558 | 0.500799 |
| GO:0010578\_regulation\_of\_adenylate\_cyclase\_activity\_involved\_in\_G-protein\_signaling | 21 | 1 | 3.536866 | -0.605203 | 676 | 250.885442 | 338.54 | 426.194558 | 0.500799 |
| GO:0010579\_positive\_regulation\_of\_adenylate\_cyclase\_activity\_by\_G-protein\_signaling\_pathway | 21 | 1 | 3.536866 | -0.605203 | 676 | 250.885442 | 338.54 | 426.194558 | 0.500799 |
| GO:0016202\_regulation\_of\_striated\_muscle\_tissue\_development | 21 | 1 | 3.536866 | -0.605203 | 676 | 250.885442 | 338.54 | 426.194558 | 0.500799 |
| GO:0032147\_activation\_of\_protein\_kinase\_activity | 21 | 1 | 3.536866 | -0.605203 | 676 | 250.885442 | 338.54 | 426.194558 | 0.500799 |
| GO:0034330\_cell\_junction\_organization | 21 | 1 | 3.536866 | -0.605203 | 676 | 250.885442 | 338.54 | 426.194558 | 0.500799 |
| GO:0048538\_thymus\_development | 21 | 1 | 3.536866 | -0.605203 | 676 | 250.885442 | 338.54 | 426.194558 | 0.500799 |
| GO:0048634\_regulation\_of\_muscle\_development | 21 | 1 | 3.536866 | -0.605203 | 676 | 250.885442 | 338.54 | 426.194558 | 0.500799 |
| GO:0048675\_axon\_extension | 21 | 1 | 3.536866 | -0.605203 | 676 | 250.885442 | 338.54 | 426.194558 | 0.500799 |
| GO:0051271\_negative\_regulation\_of\_cell\_motion | 21 | 1 | 3.536866 | -0.605203 | 676 | 250.885442 | 338.54 | 426.194558 | 0.500799 |
| GO:0019226\_transmission\_of\_nerve\_impulse | 189 | 4 | 1.571941 | -0.603546 | 677 | 250.967348 | 338.63 | 426.292652 | 0.500192 |
| GO:0010926\_anatomical\_structure\_formation | 447 | 8 | 1.329292 | -0.601599 | 678 | 251.128888 | 338.79 | 426.451112 | 0.499690 |
| GO:0007264\_small\_GTPase\_mediated\_signal\_transduction | 72 | 2 | 2.063172 | -0.597292 | 681 | 252.807044 | 341.11 | 429.412956 | 0.500896 |
| GO:0030879\_mammary\_gland\_development | 72 | 2 | 2.063172 | -0.597292 | 681 | 252.807044 | 341.11 | 429.412956 | 0.500896 |
| GO:0051169\_nuclear\_transport | 72 | 2 | 2.063172 | -0.597292 | 681 | 252.807044 | 341.11 | 429.412956 | 0.500896 |
| GO:0016265\_death | 450 | 8 | 1.320430 | -0.591615 | 682 | 253.780708 | 342.25 | 430.719292 | 0.501833 |
| GO:0000041\_transition\_metal\_ion\_transport | 22 | 1 | 3.376100 | -0.587766 | 695 | 263.666402 | 353.67 | 443.673598 | 0.508878 |
| GO:0001558\_regulation\_of\_cell\_growth | 22 | 1 | 3.376100 | -0.587766 | 695 | 263.666402 | 353.67 | 443.673598 | 0.508878 |
| GO:0006112\_energy\_reserve\_metabolic\_process | 22 | 1 | 3.376100 | -0.587766 | 695 | 263.666402 | 353.67 | 443.673598 | 0.508878 |
| GO:0009896\_positive\_regulation\_of\_catabolic\_process | 22 | 1 | 3.376100 | -0.587766 | 695 | 263.666402 | 353.67 | 443.673598 | 0.508878 |
| GO:0021575\_hindbrain\_morphogenesis | 22 | 1 | 3.376100 | -0.587766 | 695 | 263.666402 | 353.67 | 443.673598 | 0.508878 |
| GO:0021766\_hippocampus\_development | 22 | 1 | 3.376100 | -0.587766 | 695 | 263.666402 | 353.67 | 443.673598 | 0.508878 |
| GO:0032649\_regulation\_of\_interferon-gamma\_production | 22 | 1 | 3.376100 | -0.587766 | 695 | 263.666402 | 353.67 | 443.673598 | 0.508878 |
| GO:0040018\_positive\_regulation\_of\_multicellular\_organism\_growth | 22 | 1 | 3.376100 | -0.587766 | 695 | 263.666402 | 353.67 | 443.673598 | 0.508878 |
| GO:0042733\_embryonic\_digit\_morphogenesis | 22 | 1 | 3.376100 | -0.587766 | 695 | 263.666402 | 353.67 | 443.673598 | 0.508878 |
| GO:0044264\_cellular\_polysaccharide\_metabolic\_process | 22 | 1 | 3.376100 | -0.587766 | 695 | 263.666402 | 353.67 | 443.673598 | 0.508878 |
| GO:0045787\_positive\_regulation\_of\_cell\_cycle | 22 | 1 | 3.376100 | -0.587766 | 695 | 263.666402 | 353.67 | 443.673598 | 0.508878 |
| GO:0046883\_regulation\_of\_hormone\_secretion | 22 | 1 | 3.376100 | -0.587766 | 695 | 263.666402 | 353.67 | 443.673598 | 0.508878 |
| GO:0048489\_synaptic\_vesicle\_transport | 22 | 1 | 3.376100 | -0.587766 | 695 | 263.666402 | 353.67 | 443.673598 | 0.508878 |
| GO:0048771\_tissue\_remodeling | 74 | 2 | 2.007411 | -0.580360 | 696 | 264.263259 | 354.56 | 444.856741 | 0.509425 |
| GO:0046907\_intracellular\_transport | 194 | 4 | 1.531427 | -0.578283 | 697 | 264.594227 | 354.88 | 445.165773 | 0.509154 |
| GO:0060255\_regulation\_of\_macromolecule\_metabolic\_process | 936 | 15 | 1.190292 | -0.573673 | 698 | 265.250938 | 355.84 | 446.429062 | 0.509799 |
| GO:0007507\_heart\_development | 195 | 4 | 1.523573 | -0.573366 | 699 | 265.580899 | 356.26 | 446.939101 | 0.509671 |
| GO:0044265\_cellular\_macromolecule\_catabolic\_process | 75 | 2 | 1.980645 | -0.572132 | 701 | 266.126539 | 356.97 | 447.813461 | 0.509230 |
| GO:0051050\_positive\_regulation\_of\_transport | 75 | 2 | 1.980645 | -0.572132 | 701 | 266.126539 | 356.97 | 447.813461 | 0.509230 |
| GO:0002821\_positive\_regulation\_of\_adaptive\_immune\_response | 23 | 1 | 3.229313 | -0.571220 | 705 | 271.221711 | 362.71 | 454.198289 | 0.514482 |
| GO:0002824\_positive\_regulation\_of\_adaptive\_immune\_response\_based\_on\_somatic\_recombination\_of\_immune\_receptors\_built\_from\_immunoglobulin\_superfamily\_domains | 23 | 1 | 3.229313 | -0.571220 | 705 | 271.221711 | 362.71 | 454.198289 | 0.514482 |
| GO:0007163\_establishment\_or\_maintenance\_of\_cell\_polarity | 23 | 1 | 3.229313 | -0.571220 | 705 | 271.221711 | 362.71 | 454.198289 | 0.514482 |
| GO:0060349\_bone\_morphogenesis | 23 | 1 | 3.229313 | -0.571220 | 705 | 271.221711 | 362.71 | 454.198289 | 0.514482 |
| GO:0051241\_negative\_regulation\_of\_multicellular\_organismal\_process | 77 | 2 | 1.929200 | -0.556127 | 706 | 274.148908 | 366.59 | 459.031092 | 0.519249 |
| GO:0006650\_glycerophospholipid\_metabolic\_process | 24 | 1 | 3.094758 | -0.555490 | 712 | 280.187741 | 373.4 | 466.612259 | 0.524438 |
| GO:0007204\_elevation\_of\_cytosolic\_calcium\_ion\_concentration | 24 | 1 | 3.094758 | -0.555490 | 712 | 280.187741 | 373.4 | 466.612259 | 0.524438 |
| GO:0048002\_antigen\_processing\_and\_presentation\_of\_peptide\_antigen | 24 | 1 | 3.094758 | -0.555490 | 712 | 280.187741 | 373.4 | 466.612259 | 0.524438 |
| GO:0048546\_digestive\_tract\_morphogenesis | 24 | 1 | 3.094758 | -0.555490 | 712 | 280.187741 | 373.4 | 466.612259 | 0.524438 |
| GO:0050679\_positive\_regulation\_of\_epithelial\_cell\_proliferation | 24 | 1 | 3.094758 | -0.555490 | 712 | 280.187741 | 373.4 | 466.612259 | 0.524438 |
| GO:0060078\_regulation\_of\_postsynaptic\_membrane\_potential | 24 | 1 | 3.094758 | -0.555490 | 712 | 280.187741 | 373.4 | 466.612259 | 0.524438 |
| GO:0044255\_cellular\_lipid\_metabolic\_process | 264 | 5 | 1.406708 | -0.550376 | 713 | 280.793911 | 374.16 | 467.526089 | 0.524769 |
| GO:0010646\_regulation\_of\_cell\_communication | 330 | 6 | 1.350440 | -0.548293 | 714 | 282.730347 | 376.54 | 470.349653 | 0.527367 |
| GO:0051046\_regulation\_of\_secretion | 79 | 2 | 1.880359 | -0.540700 | 715 | 283.632821 | 377.67 | 471.707179 | 0.528210 |
| GO:0021983\_pituitary\_gland\_development | 25 | 1 | 2.970968 | -0.540509 | 717 | 288.673480 | 383.44 | 478.206520 | 0.534784 |
| GO:0032103\_positive\_regulation\_of\_response\_to\_external\_stimulus | 25 | 1 | 2.970968 | -0.540509 | 717 | 288.673480 | 383.44 | 478.206520 | 0.534784 |
| GO:0022607\_cellular\_component\_assembly | 204 | 4 | 1.456357 | -0.531042 | 718 | 290.123031 | 385.26 | 480.396969 | 0.536574 |
| GO:0003006\_reproductive\_developmental\_process | 141 | 3 | 1.580302 | -0.529977 | 719 | 290.449994 | 385.67 | 480.890006 | 0.536398 |
| GO:0001658\_branching\_involved\_in\_ureteric\_bud\_morphogenesis | 26 | 1 | 2.856700 | -0.526217 | 731 | 296.571360 | 392.75 | 488.928640 | 0.537278 |
| GO:0006800\_oxygen\_and\_reactive\_oxygen\_species\_metabolic\_process | 26 | 1 | 2.856700 | -0.526217 | 731 | 296.571360 | 392.75 | 488.928640 | 0.537278 |
| GO:0007623\_circadian\_rhythm | 26 | 1 | 2.856700 | -0.526217 | 731 | 296.571360 | 392.75 | 488.928640 | 0.537278 |
| GO:0009636\_response\_to\_toxin | 26 | 1 | 2.856700 | -0.526217 | 731 | 296.571360 | 392.75 | 488.928640 | 0.537278 |
| GO:0010212\_response\_to\_ionizing\_radiation | 26 | 1 | 2.856700 | -0.526217 | 731 | 296.571360 | 392.75 | 488.928640 | 0.537278 |
| GO:0010959\_regulation\_of\_metal\_ion\_transport | 26 | 1 | 2.856700 | -0.526217 | 731 | 296.571360 | 392.75 | 488.928640 | 0.537278 |
| GO:0032609\_interferon-gamma\_production | 26 | 1 | 2.856700 | -0.526217 | 731 | 296.571360 | 392.75 | 488.928640 | 0.537278 |
| GO:0045665\_negative\_regulation\_of\_neuron\_differentiation | 26 | 1 | 2.856700 | -0.526217 | 731 | 296.571360 | 392.75 | 488.928640 | 0.537278 |
| GO:0048645\_organ\_formation | 26 | 1 | 2.856700 | -0.526217 | 731 | 296.571360 | 392.75 | 488.928640 | 0.537278 |
| GO:0050873\_brown\_fat\_cell\_differentiation | 26 | 1 | 2.856700 | -0.526217 | 731 | 296.571360 | 392.75 | 488.928640 | 0.537278 |
| GO:0051480\_cytosolic\_calcium\_ion\_homeostasis | 26 | 1 | 2.856700 | -0.526217 | 731 | 296.571360 | 392.75 | 488.928640 | 0.537278 |
| GO:0060675\_ureteric\_bud\_morphogenesis | 26 | 1 | 2.856700 | -0.526217 | 731 | 296.571360 | 392.75 | 488.928640 | 0.537278 |
| GO:0002761\_regulation\_of\_myeloid\_leukocyte\_differentiation | 27 | 1 | 2.750896 | -0.512562 | 740 | 303.375593 | 400.8 | 498.224407 | 0.541622 |
| GO:0006479\_protein\_amino\_acid\_methylation | 27 | 1 | 2.750896 | -0.512562 | 740 | 303.375593 | 400.8 | 498.224407 | 0.541622 |
| GO:0007422\_peripheral\_nervous\_system\_development | 27 | 1 | 2.750896 | -0.512562 | 740 | 303.375593 | 400.8 | 498.224407 | 0.541622 |
| GO:0008213\_protein\_amino\_acid\_alkylation | 27 | 1 | 2.750896 | -0.512562 | 740 | 303.375593 | 400.8 | 498.224407 | 0.541622 |
| GO:0008286\_insulin\_receptor\_signaling\_pathway | 27 | 1 | 2.750896 | -0.512562 | 740 | 303.375593 | 400.8 | 498.224407 | 0.541622 |
| GO:0009411\_response\_to\_UV | 27 | 1 | 2.750896 | -0.512562 | 740 | 303.375593 | 400.8 | 498.224407 | 0.541622 |
| GO:0030100\_regulation\_of\_endocytosis | 27 | 1 | 2.750896 | -0.512562 | 740 | 303.375593 | 400.8 | 498.224407 | 0.541622 |
| GO:0031016\_pancreas\_development | 27 | 1 | 2.750896 | -0.512562 | 740 | 303.375593 | 400.8 | 498.224407 | 0.541622 |
| GO:0032496\_response\_to\_lipopolysaccharide | 27 | 1 | 2.750896 | -0.512562 | 740 | 303.375593 | 400.8 | 498.224407 | 0.541622 |
| GO:0006575\_cellular\_amino\_acid\_derivative\_metabolic\_process | 83 | 2 | 1.789740 | -0.511457 | 741 | 304.498288 | 402.19 | 499.881712 | 0.542767 |
| GO:0050896\_response\_to\_stimulus | 1107 | 17 | 1.140615 | -0.508982 | 742 | 304.944549 | 402.67 | 500.395451 | 0.542682 |
| GO:0030005\_cellular\_di-\_\_tri-valent\_inorganic\_cation\_homeostasis | 84 | 2 | 1.768433 | -0.504463 | 744 | 305.758767 | 403.58 | 501.401233 | 0.542446 |
| GO:0045137\_development\_of\_primary\_sexual\_characteristics | 84 | 2 | 1.768433 | -0.504463 | 744 | 305.758767 | 403.58 | 501.401233 | 0.542446 |
| GO:0021549\_cerebellum\_development | 28 | 1 | 2.652650 | -0.499496 | 748 | 311.106931 | 409.77 | 508.433069 | 0.547821 |
| GO:0030073\_insulin\_secretion | 28 | 1 | 2.652650 | -0.499496 | 748 | 311.106931 | 409.77 | 508.433069 | 0.547821 |
| GO:0045926\_negative\_regulation\_of\_growth | 28 | 1 | 2.652650 | -0.499496 | 748 | 311.106931 | 409.77 | 508.433069 | 0.547821 |
| GO:0051188\_cofactor\_biosynthetic\_process | 28 | 1 | 2.652650 | -0.499496 | 748 | 311.106931 | 409.77 | 508.433069 | 0.547821 |
| GO:0006950\_response\_to\_stress | 549 | 9 | 1.217610 | -0.499139 | 749 | 311.145044 | 409.88 | 508.614956 | 0.547236 |
| GO:0070887\_cellular\_response\_to\_chemical\_stimulus | 85 | 2 | 1.747628 | -0.497590 | 750 | 311.947595 | 410.8 | 509.652405 | 0.547733 |
| GO:0006605\_protein\_targeting | 86 | 2 | 1.727307 | -0.490833 | 754 | 314.068044 | 413.48 | 512.891956 | 0.548382 |
| GO:0032504\_multicellular\_organism\_reproduction | 86 | 2 | 1.727307 | -0.490833 | 754 | 314.068044 | 413.48 | 512.891956 | 0.548382 |
| GO:0034641\_cellular\_nitrogen\_compound\_metabolic\_process | 86 | 2 | 1.727307 | -0.490833 | 754 | 314.068044 | 413.48 | 512.891956 | 0.548382 |
| GO:0048609\_reproductive\_process\_in\_a\_multicellular\_organism | 86 | 2 | 1.727307 | -0.490833 | 754 | 314.068044 | 413.48 | 512.891956 | 0.548382 |
| GO:0006909\_phagocytosis | 29 | 1 | 2.561179 | -0.486980 | 759 | 319.504392 | 420.12 | 520.735608 | 0.553518 |
| GO:0007190\_activation\_of\_adenylate\_cyclase\_activity | 29 | 1 | 2.561179 | -0.486980 | 759 | 319.504392 | 420.12 | 520.735608 | 0.553518 |
| GO:0021761\_limbic\_system\_development | 29 | 1 | 2.561179 | -0.486980 | 759 | 319.504392 | 420.12 | 520.735608 | 0.553518 |
| GO:0044087\_regulation\_of\_cellular\_component\_biogenesis | 29 | 1 | 2.561179 | -0.486980 | 759 | 319.504392 | 420.12 | 520.735608 | 0.553518 |
| GO:0050769\_positive\_regulation\_of\_neurogenesis | 29 | 1 | 2.561179 | -0.486980 | 759 | 319.504392 | 420.12 | 520.735608 | 0.553518 |
| GO:0043583\_ear\_development | 87 | 2 | 1.707453 | -0.484191 | 760 | 321.290497 | 422.0 | 522.709503 | 0.555263 |
| GO:0002260\_lymphocyte\_homeostasis | 30 | 1 | 2.475806 | -0.474973 | 768 | 327.679311 | 429.76 | 531.840689 | 0.559583 |
| GO:0014032\_neural\_crest\_cell\_development | 30 | 1 | 2.475806 | -0.474973 | 768 | 327.679311 | 429.76 | 531.840689 | 0.559583 |
| GO:0014033\_neural\_crest\_cell\_differentiation | 30 | 1 | 2.475806 | -0.474973 | 768 | 327.679311 | 429.76 | 531.840689 | 0.559583 |
| GO:0031281\_positive\_regulation\_of\_cyclase\_activity | 30 | 1 | 2.475806 | -0.474973 | 768 | 327.679311 | 429.76 | 531.840689 | 0.559583 |
| GO:0032102\_negative\_regulation\_of\_response\_to\_external\_stimulus | 30 | 1 | 2.475806 | -0.474973 | 768 | 327.679311 | 429.76 | 531.840689 | 0.559583 |
| GO:0035265\_organ\_growth | 30 | 1 | 2.475806 | -0.474973 | 768 | 327.679311 | 429.76 | 531.840689 | 0.559583 |
| GO:0045762\_positive\_regulation\_of\_adenylate\_cyclase\_activity | 30 | 1 | 2.475806 | -0.474973 | 768 | 327.679311 | 429.76 | 531.840689 | 0.559583 |
| GO:0051349\_positive\_regulation\_of\_lyase\_activity | 30 | 1 | 2.475806 | -0.474973 | 768 | 327.679311 | 429.76 | 531.840689 | 0.559583 |
| GO:0032268\_regulation\_of\_cellular\_protein\_metabolic\_process | 152 | 3 | 1.465938 | -0.473520 | 769 | 328.059494 | 430.19 | 532.320506 | 0.559415 |
| GO:0006629\_lipid\_metabolic\_process | 285 | 5 | 1.303056 | -0.471119 | 770 | 328.427195 | 430.62 | 532.812805 | 0.559247 |
| GO:0007517\_muscle\_organ\_development | 153 | 3 | 1.456357 | -0.468727 | 771 | 328.680006 | 430.91 | 533.139994 | 0.558898 |
| GO:0030003\_cellular\_cation\_homeostasis | 90 | 2 | 1.650538 | -0.464926 | 772 | 329.858517 | 432.39 | 534.921483 | 0.560091 |
| GO:0007268\_synaptic\_transmission | 154 | 3 | 1.446900 | -0.463988 | 773 | 330.262416 | 432.87 | 535.477584 | 0.559987 |
| GO:0050793\_regulation\_of\_developmental\_process | 703 | 11 | 1.162185 | -0.463782 | 774 | 330.381142 | 432.97 | 535.558858 | 0.559393 |
| GO:0006939\_smooth\_muscle\_contraction | 31 | 1 | 2.395942 | -0.463444 | 782 | 338.526543 | 442.44 | 546.353457 | 0.565780 |
| GO:0008645\_hexose\_transport | 31 | 1 | 2.395942 | -0.463444 | 782 | 338.526543 | 442.44 | 546.353457 | 0.565780 |
| GO:0015749\_monosaccharide\_transport | 31 | 1 | 2.395942 | -0.463444 | 782 | 338.526543 | 442.44 | 546.353457 | 0.565780 |
| GO:0015758\_glucose\_transport | 31 | 1 | 2.395942 | -0.463444 | 782 | 338.526543 | 442.44 | 546.353457 | 0.565780 |
| GO:0016049\_cell\_growth | 31 | 1 | 2.395942 | -0.463444 | 782 | 338.526543 | 442.44 | 546.353457 | 0.565780 |
| GO:0043269\_regulation\_of\_ion\_transport | 31 | 1 | 2.395942 | -0.463444 | 782 | 338.526543 | 442.44 | 546.353457 | 0.565780 |
| GO:0046632\_alpha-beta\_T\_cell\_differentiation | 31 | 1 | 2.395942 | -0.463444 | 782 | 338.526543 | 442.44 | 546.353457 | 0.565780 |
| GO:0051899\_membrane\_depolarization | 31 | 1 | 2.395942 | -0.463444 | 782 | 338.526543 | 442.44 | 546.353457 | 0.565780 |
| GO:0006638\_neutral\_lipid\_metabolic\_process | 32 | 1 | 2.321069 | -0.452360 | 785 | 345.078277 | 449.49 | 553.901723 | 0.572599 |
| GO:0006937\_regulation\_of\_muscle\_contraction | 32 | 1 | 2.321069 | -0.452360 | 785 | 345.078277 | 449.49 | 553.901723 | 0.572599 |
| GO:0050885\_neuromuscular\_process\_controlling\_balance | 32 | 1 | 2.321069 | -0.452360 | 785 | 345.078277 | 449.49 | 553.901723 | 0.572599 |
| GO:0042981\_regulation\_of\_apoptosis | 360 | 6 | 1.237903 | -0.448921 | 786 | 345.265757 | 449.75 | 554.234243 | 0.572201 |
| GO:0055066\_di-\_\_tri-valent\_inorganic\_cation\_homeostasis | 93 | 2 | 1.597294 | -0.446598 | 787 | 346.131687 | 450.87 | 555.608313 | 0.572897 |
| GO:0006066\_alcohol\_metabolic\_process | 158 | 3 | 1.410269 | -0.445547 | 788 | 346.812812 | 451.71 | 556.607188 | 0.573236 |
| GO:0007188\_G-protein\_signaling\_\_coupled\_to\_cAMP\_nucleotide\_second\_messenger | 33 | 1 | 2.250733 | -0.441695 | 794 | 350.689469 | 456.17 | 561.650531 | 0.574521 |
| GO:0007270\_nerve-nerve\_synaptic\_transmission | 33 | 1 | 2.250733 | -0.441695 | 794 | 350.689469 | 456.17 | 561.650531 | 0.574521 |
| GO:0008584\_male\_gonad\_development | 33 | 1 | 2.250733 | -0.441695 | 794 | 350.689469 | 456.17 | 561.650531 | 0.574521 |
| GO:0008643\_carbohydrate\_transport | 33 | 1 | 2.250733 | -0.441695 | 794 | 350.689469 | 456.17 | 561.650531 | 0.574521 |
| GO:0021536\_diencephalon\_development | 33 | 1 | 2.250733 | -0.441695 | 794 | 350.689469 | 456.17 | 561.650531 | 0.574521 |
| GO:0021987\_cerebral\_cortex\_development | 33 | 1 | 2.250733 | -0.441695 | 794 | 350.689469 | 456.17 | 561.650531 | 0.574521 |
| GO:0010941\_regulation\_of\_cell\_death | 365 | 6 | 1.220946 | -0.434094 | 796 | 353.971315 | 459.51 | 565.048685 | 0.577274 |
| GO:0043067\_regulation\_of\_programmed\_cell\_death | 365 | 6 | 1.220946 | -0.434094 | 796 | 353.971315 | 459.51 | 565.048685 | 0.577274 |
| GO:0002682\_regulation\_of\_immune\_system\_process | 228 | 4 | 1.303056 | -0.433252 | 797 | 354.687427 | 460.26 | 565.832573 | 0.577491 |
| GO:0002237\_response\_to\_molecule\_of\_bacterial\_origin | 34 | 1 | 2.184535 | -0.431423 | 805 | 360.038739 | 466.18 | 572.321261 | 0.579106 |
| GO:0007269\_neurotransmitter\_secretion | 34 | 1 | 2.184535 | -0.431423 | 805 | 360.038739 | 466.18 | 572.321261 | 0.579106 |
| GO:0007568\_aging | 34 | 1 | 2.184535 | -0.431423 | 805 | 360.038739 | 466.18 | 572.321261 | 0.579106 |
| GO:0019882\_antigen\_processing\_and\_presentation | 34 | 1 | 2.184535 | -0.431423 | 805 | 360.038739 | 466.18 | 572.321261 | 0.579106 |
| GO:0030509\_BMP\_signaling\_pathway | 34 | 1 | 2.184535 | -0.431423 | 805 | 360.038739 | 466.18 | 572.321261 | 0.579106 |
| GO:0045927\_positive\_regulation\_of\_growth | 34 | 1 | 2.184535 | -0.431423 | 805 | 360.038739 | 466.18 | 572.321261 | 0.579106 |
| GO:0050730\_regulation\_of\_peptidyl-tyrosine\_phosphorylation | 34 | 1 | 2.184535 | -0.431423 | 805 | 360.038739 | 466.18 | 572.321261 | 0.579106 |
| GO:0051047\_positive\_regulation\_of\_secretion | 34 | 1 | 2.184535 | -0.431423 | 805 | 360.038739 | 466.18 | 572.321261 | 0.579106 |
| GO:0048736\_appendage\_development | 96 | 2 | 1.547379 | -0.429144 | 809 | 361.404636 | 467.9 | 574.395364 | 0.578368 |
| GO:0060173\_limb\_development | 96 | 2 | 1.547379 | -0.429144 | 809 | 361.404636 | 467.9 | 574.395364 | 0.578368 |
| GO:0060249\_anatomical\_structure\_homeostasis | 96 | 2 | 1.547379 | -0.429144 | 809 | 361.404636 | 467.9 | 574.395364 | 0.578368 |
| GO:0070661\_leukocyte\_proliferation | 96 | 2 | 1.547379 | -0.429144 | 809 | 361.404636 | 467.9 | 574.395364 | 0.578368 |
| GO:0007626\_locomotory\_behavior | 163 | 3 | 1.367010 | -0.423607 | 810 | 362.308431 | 469.0 | 575.691569 | 0.579012 |
| GO:0018193\_peptidyl-amino\_acid\_modification | 97 | 2 | 1.531427 | -0.423511 | 812 | 362.815088 | 469.57 | 576.324912 | 0.578288 |
| GO:0060341\_regulation\_of\_cellular\_localization | 97 | 2 | 1.531427 | -0.423511 | 812 | 362.815088 | 469.57 | 576.324912 | 0.578288 |
| GO:0016567\_protein\_ubiquitination | 35 | 1 | 2.122120 | -0.421520 | 814 | 367.111792 | 474.21 | 581.308208 | 0.582568 |
| GO:0043406\_positive\_regulation\_of\_MAP\_kinase\_activity | 35 | 1 | 2.122120 | -0.421520 | 814 | 367.111792 | 474.21 | 581.308208 | 0.582568 |
| GO:0007548\_sex\_differentiation | 98 | 2 | 1.515800 | -0.417967 | 815 | 368.209148 | 475.38 | 582.550852 | 0.583288 |
| GO:0006259\_DNA\_metabolic\_process | 165 | 3 | 1.350440 | -0.415160 | 816 | 369.500085 | 476.76 | 584.019915 | 0.584265 |
| GO:0001819\_positive\_regulation\_of\_cytokine\_production | 36 | 1 | 2.063172 | -0.411966 | 824 | 375.176340 | 483.2 | 591.223660 | 0.586408 |
| GO:0001889\_liver\_development | 36 | 1 | 2.063172 | -0.411966 | 824 | 375.176340 | 483.2 | 591.223660 | 0.586408 |
| GO:0006469\_negative\_regulation\_of\_protein\_kinase\_activity | 36 | 1 | 2.063172 | -0.411966 | 824 | 375.176340 | 483.2 | 591.223660 | 0.586408 |
| GO:0007187\_G-protein\_signaling\_\_coupled\_to\_cyclic\_nucleotide\_second\_messenger | 36 | 1 | 2.063172 | -0.411966 | 824 | 375.176340 | 483.2 | 591.223660 | 0.586408 |
| GO:0007368\_determination\_of\_left\_right\_symmetry | 36 | 1 | 2.063172 | -0.411966 | 824 | 375.176340 | 483.2 | 591.223660 | 0.586408 |
| GO:0030072\_peptide\_hormone\_secretion | 36 | 1 | 2.063172 | -0.411966 | 824 | 375.176340 | 483.2 | 591.223660 | 0.586408 |
| GO:0033673\_negative\_regulation\_of\_kinase\_activity | 36 | 1 | 2.063172 | -0.411966 | 824 | 375.176340 | 483.2 | 591.223660 | 0.586408 |
| GO:0042742\_defense\_response\_to\_bacterium | 36 | 1 | 2.063172 | -0.411966 | 824 | 375.176340 | 483.2 | 591.223660 | 0.586408 |
| GO:0003008\_system\_process | 516 | 8 | 1.151538 | -0.406064 | 825 | 376.963876 | 485.42 | 593.876124 | 0.588388 |
| GO:0002790\_peptide\_secretion | 37 | 1 | 2.007411 | -0.402742 | 831 | 380.685450 | 489.52 | 598.354550 | 0.589073 |
| GO:0009799\_determination\_of\_symmetry | 37 | 1 | 2.007411 | -0.402742 | 831 | 380.685450 | 489.52 | 598.354550 | 0.589073 |
| GO:0009855\_determination\_of\_bilateral\_symmetry | 37 | 1 | 2.007411 | -0.402742 | 831 | 380.685450 | 489.52 | 598.354550 | 0.589073 |
| GO:0019933\_cAMP-mediated\_signaling | 37 | 1 | 2.007411 | -0.402742 | 831 | 380.685450 | 489.52 | 598.354550 | 0.589073 |
| GO:0032869\_cellular\_response\_to\_insulin\_stimulus | 37 | 1 | 2.007411 | -0.402742 | 831 | 380.685450 | 489.52 | 598.354550 | 0.589073 |
| GO:0045761\_regulation\_of\_adenylate\_cyclase\_activity | 37 | 1 | 2.007411 | -0.402742 | 831 | 380.685450 | 489.52 | 598.354550 | 0.589073 |
| GO:0044085\_cellular\_component\_biogenesis | 237 | 4 | 1.253573 | -0.401480 | 832 | 381.342575 | 490.29 | 599.237425 | 0.589291 |
| GO:0050794\_regulation\_of\_cellular\_process | 2190 | 31 | 1.051370 | -0.401314 | 833 | 381.435127 | 490.38 | 599.324873 | 0.588691 |
| GO:0051094\_positive\_regulation\_of\_developmental\_process | 308 | 5 | 1.205750 | -0.397011 | 834 | 382.124361 | 491.2 | 600.275639 | 0.588969 |
| GO:0051246\_regulation\_of\_protein\_metabolic\_process | 170 | 3 | 1.310721 | -0.394822 | 835 | 383.383402 | 492.45 | 601.516598 | 0.589760 |
| GO:0001570\_vasculogenesis | 38 | 1 | 1.954584 | -0.393828 | 841 | 389.542803 | 499.37 | 609.197197 | 0.593781 |
| GO:0001649\_osteoblast\_differentiation | 38 | 1 | 1.954584 | -0.393828 | 841 | 389.542803 | 499.37 | 609.197197 | 0.593781 |
| GO:0008016\_regulation\_of\_heart\_contraction | 38 | 1 | 1.954584 | -0.393828 | 841 | 389.542803 | 499.37 | 609.197197 | 0.593781 |
| GO:0032259\_methylation | 38 | 1 | 1.954584 | -0.393828 | 841 | 389.542803 | 499.37 | 609.197197 | 0.593781 |
| GO:0043414\_biopolymer\_methylation | 38 | 1 | 1.954584 | -0.393828 | 841 | 389.542803 | 499.37 | 609.197197 | 0.593781 |
| GO:0051348\_negative\_regulation\_of\_transferase\_activity | 38 | 1 | 1.954584 | -0.393828 | 841 | 389.542803 | 499.37 | 609.197197 | 0.593781 |
| GO:0009968\_negative\_regulation\_of\_signal\_transduction | 103 | 2 | 1.442217 | -0.391513 | 842 | 390.420206 | 500.33 | 610.239794 | 0.594216 |
| GO:0044238\_primary\_metabolic\_process | 1905 | 27 | 1.052705 | -0.387331 | 843 | 390.810949 | 500.74 | 610.669051 | 0.593998 |
| GO:0009611\_response\_to\_wounding | 172 | 3 | 1.295480 | -0.386987 | 844 | 391.120893 | 501.12 | 611.119107 | 0.593744 |
| GO:0005976\_polysaccharide\_metabolic\_process | 39 | 1 | 1.904467 | -0.385209 | 850 | 396.079840 | 506.62 | 617.160160 | 0.596024 |
| GO:0006511\_ubiquitin-dependent\_protein\_catabolic\_process | 39 | 1 | 1.904467 | -0.385209 | 850 | 396.079840 | 506.62 | 617.160160 | 0.596024 |
| GO:0006730\_one-carbon\_metabolic\_process | 39 | 1 | 1.904467 | -0.385209 | 850 | 396.079840 | 506.62 | 617.160160 | 0.596024 |
| GO:0008037\_cell\_recognition | 39 | 1 | 1.904467 | -0.385209 | 850 | 396.079840 | 506.62 | 617.160160 | 0.596024 |
| GO:0031279\_regulation\_of\_cyclase\_activity | 39 | 1 | 1.904467 | -0.385209 | 850 | 396.079840 | 506.62 | 617.160160 | 0.596024 |
| GO:0051339\_regulation\_of\_lyase\_activity | 39 | 1 | 1.904467 | -0.385209 | 850 | 396.079840 | 506.62 | 617.160160 | 0.596024 |
| GO:0016071\_mRNA\_metabolic\_process | 40 | 1 | 1.856855 | -0.376870 | 854 | 402.290553 | 513.07 | 623.849447 | 0.600785 |
| GO:0016358\_dendrite\_development | 40 | 1 | 1.856855 | -0.376870 | 854 | 402.290553 | 513.07 | 623.849447 | 0.600785 |
| GO:0019935\_cyclic-nucleotide-mediated\_signaling | 40 | 1 | 1.856855 | -0.376870 | 854 | 402.290553 | 513.07 | 623.849447 | 0.600785 |
| GO:0051129\_negative\_regulation\_of\_cellular\_component\_organization | 40 | 1 | 1.856855 | -0.376870 | 854 | 402.290553 | 513.07 | 623.849447 | 0.600785 |
| GO:0000122\_negative\_regulation\_of\_transcription\_from\_RNA\_polymerase\_II\_promoter | 175 | 3 | 1.273272 | -0.375542 | 855 | 403.260905 | 514.15 | 625.039095 | 0.601345 |
| GO:0006260\_DNA\_replication | 41 | 1 | 1.811566 | -0.368796 | 865 | 410.681310 | 522.58 | 634.478690 | 0.604139 |
| GO:0006836\_neurotransmitter\_transport | 41 | 1 | 1.811566 | -0.368796 | 865 | 410.681310 | 522.58 | 634.478690 | 0.604139 |
| GO:0006865\_amino\_acid\_transport | 41 | 1 | 1.811566 | -0.368796 | 865 | 410.681310 | 522.58 | 634.478690 | 0.604139 |
| GO:0009894\_regulation\_of\_catabolic\_process | 41 | 1 | 1.811566 | -0.368796 | 865 | 410.681310 | 522.58 | 634.478690 | 0.604139 |
| GO:0010551\_regulation\_of\_specific\_transcription\_from\_RNA\_polymerase\_II\_promoter | 41 | 1 | 1.811566 | -0.368796 | 865 | 410.681310 | 522.58 | 634.478690 | 0.604139 |
| GO:0015833\_peptide\_transport | 41 | 1 | 1.811566 | -0.368796 | 865 | 410.681310 | 522.58 | 634.478690 | 0.604139 |
| GO:0019748\_secondary\_metabolic\_process | 41 | 1 | 1.811566 | -0.368796 | 865 | 410.681310 | 522.58 | 634.478690 | 0.604139 |
| GO:0030817\_regulation\_of\_cAMP\_biosynthetic\_process | 41 | 1 | 1.811566 | -0.368796 | 865 | 410.681310 | 522.58 | 634.478690 | 0.604139 |
| GO:0032569\_specific\_transcription\_from\_RNA\_polymerase\_II\_promoter | 41 | 1 | 1.811566 | -0.368796 | 865 | 410.681310 | 522.58 | 634.478690 | 0.604139 |
| GO:0033077\_T\_cell\_differentiation\_in\_the\_thymus | 41 | 1 | 1.811566 | -0.368796 | 865 | 410.681310 | 522.58 | 634.478690 | 0.604139 |
| GO:0050877\_neurological\_system\_process | 390 | 6 | 1.142680 | -0.366498 | 866 | 411.485440 | 523.4 | 635.314560 | 0.604388 |
| GO:0006006\_glucose\_metabolic\_process | 42 | 1 | 1.768433 | -0.360975 | 875 | 418.036952 | 530.26 | 642.483048 | 0.606011 |
| GO:0006171\_cAMP\_biosynthetic\_process | 42 | 1 | 1.768433 | -0.360975 | 875 | 418.036952 | 530.26 | 642.483048 | 0.606011 |
| GO:0008361\_regulation\_of\_cell\_size | 42 | 1 | 1.768433 | -0.360975 | 875 | 418.036952 | 530.26 | 642.483048 | 0.606011 |
| GO:0015672\_monovalent\_inorganic\_cation\_transport | 42 | 1 | 1.768433 | -0.360975 | 875 | 418.036952 | 530.26 | 642.483048 | 0.606011 |
| GO:0019941\_modification-dependent\_protein\_catabolic\_process | 42 | 1 | 1.768433 | -0.360975 | 875 | 418.036952 | 530.26 | 642.483048 | 0.606011 |
| GO:0042476\_odontogenesis | 42 | 1 | 1.768433 | -0.360975 | 875 | 418.036952 | 530.26 | 642.483048 | 0.606011 |
| GO:0043632\_modification-dependent\_macromolecule\_catabolic\_process | 42 | 1 | 1.768433 | -0.360975 | 875 | 418.036952 | 530.26 | 642.483048 | 0.606011 |
| GO:0051345\_positive\_regulation\_of\_hydrolase\_activity | 42 | 1 | 1.768433 | -0.360975 | 875 | 418.036952 | 530.26 | 642.483048 | 0.606011 |
| GO:0051603\_proteolysis\_involved\_in\_cellular\_protein\_catabolic\_process | 42 | 1 | 1.768433 | -0.360975 | 875 | 418.036952 | 530.26 | 642.483048 | 0.606011 |
| GO:0010647\_positive\_regulation\_of\_cell\_communication | 110 | 2 | 1.350440 | -0.357725 | 877 | 419.764660 | 532.32 | 644.875340 | 0.606978 |
| GO:0010648\_negative\_regulation\_of\_cell\_communication | 110 | 2 | 1.350440 | -0.357725 | 877 | 419.764660 | 532.32 | 644.875340 | 0.606978 |
| GO:0008104\_protein\_localization | 251 | 4 | 1.183652 | -0.356614 | 878 | 420.333721 | 532.92 | 645.506279 | 0.606970 |
| GO:0065007\_biological\_regulation | 2593 | 36 | 1.031188 | -0.354769 | 879 | 420.625998 | 533.21 | 645.794002 | 0.606610 |
| GO:0002819\_regulation\_of\_adaptive\_immune\_response | 43 | 1 | 1.727307 | -0.353394 | 887 | 426.968570 | 540.02 | 653.071430 | 0.608816 |
| GO:0002822\_regulation\_of\_adaptive\_immune\_response\_based\_on\_somatic\_recombination\_of\_immune\_receptors\_built\_from\_immunoglobulin\_superfamily\_domains | 43 | 1 | 1.727307 | -0.353394 | 887 | 426.968570 | 540.02 | 653.071430 | 0.608816 |
| GO:0010001\_glial\_cell\_differentiation | 43 | 1 | 1.727307 | -0.353394 | 887 | 426.968570 | 540.02 | 653.071430 | 0.608816 |
| GO:0030814\_regulation\_of\_cAMP\_metabolic\_process | 43 | 1 | 1.727307 | -0.353394 | 887 | 426.968570 | 540.02 | 653.071430 | 0.608816 |
| GO:0032446\_protein\_modification\_by\_small\_protein\_conjugation | 43 | 1 | 1.727307 | -0.353394 | 887 | 426.968570 | 540.02 | 653.071430 | 0.608816 |
| GO:0032868\_response\_to\_insulin\_stimulus | 43 | 1 | 1.727307 | -0.353394 | 887 | 426.968570 | 540.02 | 653.071430 | 0.608816 |
| GO:0046879\_hormone\_secretion | 43 | 1 | 1.727307 | -0.353394 | 887 | 426.968570 | 540.02 | 653.071430 | 0.608816 |
| GO:0051789\_response\_to\_protein\_stimulus | 43 | 1 | 1.727307 | -0.353394 | 887 | 426.968570 | 540.02 | 653.071430 | 0.608816 |
| GO:0030097\_hemopoiesis | 253 | 4 | 1.174296 | -0.350623 | 888 | 427.742141 | 540.84 | 653.937859 | 0.609054 |
| GO:0001942\_hair\_follicle\_development | 44 | 1 | 1.688050 | -0.346041 | 899 | 435.427883 | 549.28 | 663.132117 | 0.610990 |
| GO:0009914\_hormone\_transport | 44 | 1 | 1.688050 | -0.346041 | 899 | 435.427883 | 549.28 | 663.132117 | 0.610990 |
| GO:0022404\_molting\_cycle\_process | 44 | 1 | 1.688050 | -0.346041 | 899 | 435.427883 | 549.28 | 663.132117 | 0.610990 |
| GO:0022405\_hair\_cycle\_process | 44 | 1 | 1.688050 | -0.346041 | 899 | 435.427883 | 549.28 | 663.132117 | 0.610990 |
| GO:0030802\_regulation\_of\_cyclic\_nucleotide\_biosynthetic\_process | 44 | 1 | 1.688050 | -0.346041 | 899 | 435.427883 | 549.28 | 663.132117 | 0.610990 |
| GO:0030808\_regulation\_of\_nucleotide\_biosynthetic\_process | 44 | 1 | 1.688050 | -0.346041 | 899 | 435.427883 | 549.28 | 663.132117 | 0.610990 |
| GO:0042303\_molting\_cycle | 44 | 1 | 1.688050 | -0.346041 | 899 | 435.427883 | 549.28 | 663.132117 | 0.610990 |
| GO:0042633\_hair\_cycle | 44 | 1 | 1.688050 | -0.346041 | 899 | 435.427883 | 549.28 | 663.132117 | 0.610990 |
| GO:0044257\_cellular\_protein\_catabolic\_process | 44 | 1 | 1.688050 | -0.346041 | 899 | 435.427883 | 549.28 | 663.132117 | 0.610990 |
| GO:0070665\_positive\_regulation\_of\_leukocyte\_proliferation | 44 | 1 | 1.688050 | -0.346041 | 899 | 435.427883 | 549.28 | 663.132117 | 0.610990 |
| GO:0070668\_positive\_regulation\_of\_mast\_cell\_proliferation | 44 | 1 | 1.688050 | -0.346041 | 899 | 435.427883 | 549.28 | 663.132117 | 0.610990 |
| GO:0048729\_tissue\_morphogenesis | 255 | 4 | 1.165085 | -0.344731 | 900 | 435.666880 | 549.56 | 663.453120 | 0.610622 |
| GO:0032870\_cellular\_response\_to\_hormone\_stimulus | 45 | 1 | 1.650538 | -0.338907 | 903 | 439.988392 | 554.22 | 668.451608 | 0.613754 |
| GO:0046058\_cAMP\_metabolic\_process | 45 | 1 | 1.650538 | -0.338907 | 903 | 439.988392 | 554.22 | 668.451608 | 0.613754 |
| GO:0046546\_development\_of\_primary\_male\_sexual\_characteristics | 45 | 1 | 1.650538 | -0.338907 | 903 | 439.988392 | 554.22 | 668.451608 | 0.613754 |
| GO:0065008\_regulation\_of\_biological\_quality | 693 | 10 | 1.071778 | -0.337630 | 904 | 440.222747 | 554.46 | 668.697253 | 0.613341 |
| GO:0048584\_positive\_regulation\_of\_response\_to\_stimulus | 115 | 2 | 1.291725 | -0.335660 | 905 | 441.865289 | 556.3 | 670.734711 | 0.614696 |
| GO:0030218\_erythrocyte\_differentiation | 46 | 1 | 1.614656 | -0.331982 | 909 | 445.950517 | 560.79 | 675.629483 | 0.616931 |
| GO:0030850\_prostate\_gland\_development | 46 | 1 | 1.614656 | -0.331982 | 909 | 445.950517 | 560.79 | 675.629483 | 0.616931 |
| GO:0042063\_gliogenesis | 46 | 1 | 1.614656 | -0.331982 | 909 | 445.950517 | 560.79 | 675.629483 | 0.616931 |
| GO:0046631\_alpha-beta\_T\_cell\_activation | 46 | 1 | 1.614656 | -0.331982 | 909 | 445.950517 | 560.79 | 675.629483 | 0.616931 |
| GO:0007276\_gamete\_generation | 188 | 3 | 1.185226 | -0.329905 | 910 | 447.020527 | 561.93 | 676.839473 | 0.617505 |
| GO:0006140\_regulation\_of\_nucleotide\_metabolic\_process | 47 | 1 | 1.580302 | -0.325255 | 916 | 451.075303 | 566.53 | 681.984697 | 0.618483 |
| GO:0016570\_histone\_modification | 47 | 1 | 1.580302 | -0.325255 | 916 | 451.075303 | 566.53 | 681.984697 | 0.618483 |
| GO:0030183\_B\_cell\_differentiation | 47 | 1 | 1.580302 | -0.325255 | 916 | 451.075303 | 566.53 | 681.984697 | 0.618483 |
| GO:0030799\_regulation\_of\_cyclic\_nucleotide\_metabolic\_process | 47 | 1 | 1.580302 | -0.325255 | 916 | 451.075303 | 566.53 | 681.984697 | 0.618483 |
| GO:0045087\_innate\_immune\_response | 47 | 1 | 1.580302 | -0.325255 | 916 | 451.075303 | 566.53 | 681.984697 | 0.618483 |
| GO:0060627\_regulation\_of\_vesicle-mediated\_transport | 47 | 1 | 1.580302 | -0.325255 | 916 | 451.075303 | 566.53 | 681.984697 | 0.618483 |
| GO:0001505\_regulation\_of\_neurotransmitter\_levels | 48 | 1 | 1.547379 | -0.318719 | 920 | 455.816605 | 571.42 | 687.023395 | 0.621109 |
| GO:0007498\_mesoderm\_development | 48 | 1 | 1.547379 | -0.318719 | 920 | 455.816605 | 571.42 | 687.023395 | 0.621109 |
| GO:0009101\_glycoprotein\_biosynthetic\_process | 48 | 1 | 1.547379 | -0.318719 | 920 | 455.816605 | 571.42 | 687.023395 | 0.621109 |
| GO:0019318\_hexose\_metabolic\_process | 48 | 1 | 1.547379 | -0.318719 | 920 | 455.816605 | 571.42 | 687.023395 | 0.621109 |
| GO:0009605\_response\_to\_external\_stimulus | 339 | 5 | 1.095490 | -0.314495 | 921 | 457.078420 | 572.87 | 688.661580 | 0.622009 |
| GO:0002440\_production\_of\_molecular\_mediator\_of\_immune\_response | 49 | 1 | 1.515800 | -0.312366 | 928 | 461.036150 | 577.12 | 693.203850 | 0.621897 |
| GO:0003015\_heart\_process | 49 | 1 | 1.515800 | -0.312366 | 928 | 461.036150 | 577.12 | 693.203850 | 0.621897 |
| GO:0006725\_cellular\_aromatic\_compound\_metabolic\_process | 49 | 1 | 1.515800 | -0.312366 | 928 | 461.036150 | 577.12 | 693.203850 | 0.621897 |
| GO:0034101\_erythrocyte\_homeostasis | 49 | 1 | 1.515800 | -0.312366 | 928 | 461.036150 | 577.12 | 693.203850 | 0.621897 |
| GO:0046661\_male\_sex\_differentiation | 49 | 1 | 1.515800 | -0.312366 | 928 | 461.036150 | 577.12 | 693.203850 | 0.621897 |
| GO:0048741\_skeletal\_muscle\_fiber\_development | 49 | 1 | 1.515800 | -0.312366 | 928 | 461.036150 | 577.12 | 693.203850 | 0.621897 |
| GO:0060047\_heart\_contraction | 49 | 1 | 1.515800 | -0.312366 | 928 | 461.036150 | 577.12 | 693.203850 | 0.621897 |
| GO:0051726\_regulation\_of\_cell\_cycle | 121 | 2 | 1.227673 | -0.311208 | 929 | 462.639245 | 578.98 | 695.320755 | 0.623229 |
| GO:0001816\_cytokine\_production | 122 | 2 | 1.217610 | -0.307333 | 931 | 465.407063 | 581.97 | 698.532937 | 0.625102 |
| GO:0006886\_intracellular\_protein\_transport | 122 | 2 | 1.217610 | -0.307333 | 931 | 465.407063 | 581.97 | 698.532937 | 0.625102 |
| GO:0002573\_myeloid\_leukocyte\_differentiation | 50 | 1 | 1.485484 | -0.306188 | 935 | 468.227156 | 584.83 | 701.432844 | 0.625487 |
| GO:0007015\_actin\_filament\_organization | 50 | 1 | 1.485484 | -0.306188 | 935 | 468.227156 | 584.83 | 701.432844 | 0.625487 |
| GO:0009190\_cyclic\_nucleotide\_biosynthetic\_process | 50 | 1 | 1.485484 | -0.306188 | 935 | 468.227156 | 584.83 | 701.432844 | 0.625487 |
| GO:0070647\_protein\_modification\_by\_small\_protein\_conjugation\_or\_removal | 50 | 1 | 1.485484 | -0.306188 | 935 | 468.227156 | 584.83 | 701.432844 | 0.625487 |
| GO:0006520\_cellular\_amino\_acid\_metabolic\_process | 51 | 1 | 1.456357 | -0.300177 | 941 | 473.671728 | 590.51 | 707.348272 | 0.627535 |
| GO:0006887\_exocytosis | 51 | 1 | 1.456357 | -0.300177 | 941 | 473.671728 | 590.51 | 707.348272 | 0.627535 |
| GO:0016569\_covalent\_chromatin\_modification | 51 | 1 | 1.456357 | -0.300177 | 941 | 473.671728 | 590.51 | 707.348272 | 0.627535 |
| GO:0032880\_regulation\_of\_protein\_localization | 51 | 1 | 1.456357 | -0.300177 | 941 | 473.671728 | 590.51 | 707.348272 | 0.627535 |
| GO:0044106\_cellular\_amine\_metabolic\_process | 51 | 1 | 1.456357 | -0.300177 | 941 | 473.671728 | 590.51 | 707.348272 | 0.627535 |
| GO:0048747\_muscle\_fiber\_development | 51 | 1 | 1.456357 | -0.300177 | 941 | 473.671728 | 590.51 | 707.348272 | 0.627535 |
| GO:0002009\_morphogenesis\_of\_an\_epithelium | 198 | 3 | 1.125367 | -0.298739 | 943 | 474.888908 | 591.68 | 708.471092 | 0.627444 |
| GO:0060429\_epithelium\_development | 198 | 3 | 1.125367 | -0.298739 | 943 | 474.888908 | 591.68 | 708.471092 | 0.627444 |
| GO:0009124\_nucleoside\_monophosphate\_biosynthetic\_process | 52 | 1 | 1.428350 | -0.294327 | 945 | 478.109981 | 594.91 | 711.710019 | 0.629534 |
| GO:0048585\_negative\_regulation\_of\_response\_to\_stimulus | 52 | 1 | 1.428350 | -0.294327 | 945 | 478.109981 | 594.91 | 711.710019 | 0.629534 |
| GO:0033036\_macromolecule\_localization | 274 | 4 | 1.084295 | -0.293368 | 946 | 478.880103 | 595.63 | 712.379897 | 0.629630 |
| GO:0006935\_chemotaxis | 53 | 1 | 1.401400 | -0.288632 | 951 | 481.940186 | 598.86 | 715.779814 | 0.629716 |
| GO:0030031\_cell\_projection\_assembly | 53 | 1 | 1.401400 | -0.288632 | 951 | 481.940186 | 598.86 | 715.779814 | 0.629716 |
| GO:0042330\_taxis | 53 | 1 | 1.401400 | -0.288632 | 951 | 481.940186 | 598.86 | 715.779814 | 0.629716 |
| GO:0046942\_carboxylic\_acid\_transport | 53 | 1 | 1.401400 | -0.288632 | 951 | 481.940186 | 598.86 | 715.779814 | 0.629716 |
| GO:0055085\_transmembrane\_transport | 53 | 1 | 1.401400 | -0.288632 | 951 | 481.940186 | 598.86 | 715.779814 | 0.629716 |
| GO:0048534\_hemopoietic\_or\_lymphoid\_organ\_development | 277 | 4 | 1.072552 | -0.285968 | 953 | 482.368580 | 599.38 | 716.391420 | 0.628940 |
| GO:0048646\_anatomical\_structure\_formation\_involved\_in\_morphogenesis | 277 | 4 | 1.072552 | -0.285968 | 953 | 482.368580 | 599.38 | 716.391420 | 0.628940 |
| GO:0006164\_purine\_nucleotide\_biosynthetic\_process | 54 | 1 | 1.375448 | -0.283085 | 956 | 487.122975 | 604.68 | 722.237025 | 0.632510 |
| GO:0007265\_Ras\_protein\_signal\_transduction | 54 | 1 | 1.375448 | -0.283085 | 956 | 487.122975 | 604.68 | 722.237025 | 0.632510 |
| GO:0015849\_organic\_acid\_transport | 54 | 1 | 1.375448 | -0.283085 | 956 | 487.122975 | 604.68 | 722.237025 | 0.632510 |
| GO:0043285\_biopolymer\_catabolic\_process | 129 | 2 | 1.151538 | -0.281670 | 957 | 487.718560 | 605.32 | 722.921440 | 0.632518 |
| GO:0034960\_cellular\_biopolymer\_metabolic\_process | 1395 | 19 | 1.011620 | -0.280589 | 958 | 488.509073 | 606.08 | 723.650927 | 0.632651 |
| GO:0007126\_meiosis | 55 | 1 | 1.350440 | -0.277681 | 961 | 492.509858 | 610.47 | 728.430142 | 0.635245 |
| GO:0043434\_response\_to\_peptide\_hormone\_stimulus | 55 | 1 | 1.350440 | -0.277681 | 961 | 492.509858 | 610.47 | 728.430142 | 0.635245 |
| GO:0051327\_M\_phase\_of\_meiotic\_cell\_cycle | 55 | 1 | 1.350440 | -0.277681 | 961 | 492.509858 | 610.47 | 728.430142 | 0.635245 |
| GO:0009187\_cyclic\_nucleotide\_metabolic\_process | 56 | 1 | 1.326325 | -0.272415 | 965 | 497.176462 | 615.2 | 733.223538 | 0.637513 |
| GO:0046486\_glycerolipid\_metabolic\_process | 56 | 1 | 1.326325 | -0.272415 | 965 | 497.176462 | 615.2 | 733.223538 | 0.637513 |
| GO:0050678\_regulation\_of\_epithelial\_cell\_proliferation | 56 | 1 | 1.326325 | -0.272415 | 965 | 497.176462 | 615.2 | 733.223538 | 0.637513 |
| GO:0051321\_meiotic\_cell\_cycle | 56 | 1 | 1.326325 | -0.272415 | 965 | 497.176462 | 615.2 | 733.223538 | 0.637513 |
| GO:0000226\_microtubule\_cytoskeleton\_organization | 57 | 1 | 1.303056 | -0.267281 | 970 | 503.926538 | 622.17 | 740.413462 | 0.641412 |
| GO:0008344\_adult\_locomotory\_behavior | 57 | 1 | 1.303056 | -0.267281 | 970 | 503.926538 | 622.17 | 740.413462 | 0.641412 |
| GO:0018108\_peptidyl-tyrosine\_phosphorylation | 57 | 1 | 1.303056 | -0.267281 | 970 | 503.926538 | 622.17 | 740.413462 | 0.641412 |
| GO:0018212\_peptidyl-tyrosine\_modification | 57 | 1 | 1.303056 | -0.267281 | 970 | 503.926538 | 622.17 | 740.413462 | 0.641412 |
| GO:0045444\_fat\_cell\_differentiation | 57 | 1 | 1.303056 | -0.267281 | 970 | 503.926538 | 622.17 | 740.413462 | 0.641412 |
| GO:0034622\_cellular\_macromolecular\_complex\_assembly | 58 | 1 | 1.280590 | -0.262275 | 971 | 507.081591 | 624.93 | 742.778409 | 0.643594 |
| GO:0016055\_Wnt\_receptor\_signaling\_pathway | 59 | 1 | 1.258885 | -0.257392 | 972 | 510.408002 | 628.47 | 746.531998 | 0.646574 |
| GO:0009057\_macromolecule\_catabolic\_process | 137 | 2 | 1.084295 | -0.255211 | 973 | 510.881323 | 629.02 | 747.158677 | 0.646475 |
| GO:0009123\_nucleoside\_monophosphate\_metabolic\_process | 60 | 1 | 1.237903 | -0.252627 | 974 | 511.679373 | 629.84 | 748.000627 | 0.646653 |
| GO:0007166\_cell\_surface\_receptor\_linked\_signal\_transduction | 597 | 8 | 0.995299 | -0.249242 | 975 | 512.833815 | 631.06 | 749.286185 | 0.647241 |
| GO:0007169\_transmembrane\_receptor\_protein\_tyrosine\_kinase\_signaling\_pathway | 139 | 2 | 1.068693 | -0.249031 | 977 | 513.433098 | 631.66 | 749.886902 | 0.646530 |
| GO:0034613\_cellular\_protein\_localization | 139 | 2 | 1.068693 | -0.249031 | 977 | 513.433098 | 631.66 | 749.886902 | 0.646530 |
| GO:0006874\_cellular\_calcium\_ion\_homeostasis | 61 | 1 | 1.217610 | -0.247977 | 978 | 515.170948 | 633.51 | 751.849052 | 0.647761 |
| GO:0048583\_regulation\_of\_response\_to\_stimulus | 217 | 3 | 1.026832 | -0.247541 | 979 | 515.519120 | 633.93 | 752.340880 | 0.647528 |
| GO:0002520\_immune\_system\_development | 295 | 4 | 1.007108 | -0.245198 | 980 | 516.799293 | 635.35 | 753.900707 | 0.648316 |
| GO:0045892\_negative\_regulation\_of\_transcription\_\_DNA-dependent | 218 | 3 | 1.022122 | -0.245106 | 981 | 516.998733 | 635.56 | 754.121267 | 0.647870 |
| GO:0040014\_regulation\_of\_multicellular\_organism\_growth | 62 | 1 | 1.197971 | -0.243438 | 982 | 520.389377 | 639.1 | 757.810623 | 0.650815 |
| GO:0070727\_cellular\_macromolecule\_localization | 141 | 2 | 1.053535 | -0.243013 | 983 | 521.322787 | 640.11 | 758.897213 | 0.651180 |
| GO:0051253\_negative\_regulation\_of\_RNA\_metabolic\_process | 220 | 3 | 1.012830 | -0.240307 | 984 | 521.932716 | 640.77 | 759.607284 | 0.651189 |
| GO:0009165\_nucleotide\_biosynthetic\_process | 63 | 1 | 1.178955 | -0.239006 | 987 | 524.179083 | 643.04 | 761.900917 | 0.651510 |
| GO:0070662\_mast\_cell\_proliferation | 63 | 1 | 1.178955 | -0.239006 | 987 | 524.179083 | 643.04 | 761.900917 | 0.651510 |
| GO:0070666\_regulation\_of\_mast\_cell\_proliferation | 63 | 1 | 1.178955 | -0.239006 | 987 | 524.179083 | 643.04 | 761.900917 | 0.651510 |
| GO:0022414\_reproductive\_process | 376 | 5 | 0.987689 | -0.236984 | 988 | 524.985519 | 643.91 | 762.834481 | 0.651731 |
| GO:0006875\_cellular\_metal\_ion\_homeostasis | 64 | 1 | 1.160534 | -0.234678 | 992 | 527.008349 | 645.81 | 764.611651 | 0.651018 |
| GO:0042060\_wound\_healing | 64 | 1 | 1.160534 | -0.234678 | 992 | 527.008349 | 645.81 | 764.611651 | 0.651018 |
| GO:0055074\_calcium\_ion\_homeostasis | 64 | 1 | 1.160534 | -0.234678 | 992 | 527.008349 | 645.81 | 764.611651 | 0.651018 |
| GO:0070663\_regulation\_of\_leukocyte\_proliferation | 64 | 1 | 1.160534 | -0.234678 | 992 | 527.008349 | 645.81 | 764.611651 | 0.651018 |
| GO:0000003\_reproduction | 379 | 5 | 0.979871 | -0.231545 | 993 | 527.986325 | 646.85 | 765.713675 | 0.651410 |
| GO:0043086\_negative\_regulation\_of\_catalytic\_activity | 65 | 1 | 1.142680 | -0.230449 | 994 | 528.898998 | 647.85 | 766.801002 | 0.651761 |
| GO:0005975\_carbohydrate\_metabolic\_process | 146 | 2 | 1.017455 | -0.228646 | 995 | 529.996597 | 648.98 | 767.963403 | 0.652241 |
| GO:0043687\_post-translational\_protein\_modification | 384 | 5 | 0.967112 | -0.222732 | 996 | 533.822234 | 653.05 | 772.277766 | 0.655673 |
| GO:0003007\_heart\_morphogenesis | 67 | 1 | 1.108570 | -0.222279 | 997 | 535.458898 | 654.88 | 774.301102 | 0.656851 |
| GO:0019953\_sexual\_reproduction | 228 | 3 | 0.977292 | -0.222033 | 998 | 536.081552 | 655.48 | 774.878448 | 0.656794 |
| GO:0044260\_cellular\_macromolecule\_metabolic\_process | 1447 | 19 | 0.975266 | -0.221522 | 999 | 536.214308 | 655.61 | 775.005692 | 0.656266 |
| GO:0019932\_second-messenger-mediated\_signaling | 68 | 1 | 1.092268 | -0.218331 | 1000 | 539.152354 | 658.42 | 777.687646 | 0.658420 |
| GO:0005996\_monosaccharide\_metabolic\_process | 69 | 1 | 1.076438 | -0.214472 | 1002 | 542.580620 | 661.69 | 780.799380 | 0.660369 |
| GO:0055065\_metal\_ion\_homeostasis | 69 | 1 | 1.076438 | -0.214472 | 1002 | 542.580620 | 661.69 | 780.799380 | 0.660369 |
| GO:0007611\_learning\_or\_memory | 70 | 1 | 1.061060 | -0.210697 | 1004 | 546.113012 | 665.13 | 784.146988 | 0.662480 |
| GO:0009617\_response\_to\_bacterium | 70 | 1 | 1.061060 | -0.210697 | 1004 | 546.113012 | 665.13 | 784.146988 | 0.662480 |
| GO:0009100\_glycoprotein\_metabolic\_process | 71 | 1 | 1.046115 | -0.207006 | 1005 | 549.856717 | 668.85 | 787.843283 | 0.665522 |
| GO:0008285\_negative\_regulation\_of\_cell\_proliferation | 155 | 2 | 0.958377 | -0.205031 | 1006 | 550.927485 | 669.97 | 789.012515 | 0.665974 |
| GO:0016568\_chromatin\_modification | 72 | 1 | 1.031586 | -0.203394 | 1011 | 555.627389 | 674.59 | 793.552611 | 0.667250 |
| GO:0042098\_T\_cell\_proliferation | 72 | 1 | 1.031586 | -0.203394 | 1011 | 555.627389 | 674.59 | 793.552611 | 0.667250 |
| GO:0044262\_cellular\_carbohydrate\_metabolic\_process | 72 | 1 | 1.031586 | -0.203394 | 1011 | 555.627389 | 674.59 | 793.552611 | 0.667250 |
| GO:0048839\_inner\_ear\_development | 72 | 1 | 1.031586 | -0.203394 | 1011 | 555.627389 | 674.59 | 793.552611 | 0.667250 |
| GO:0050673\_epithelial\_cell\_proliferation | 72 | 1 | 1.031586 | -0.203394 | 1011 | 555.627389 | 674.59 | 793.552611 | 0.667250 |
| GO:0006468\_protein\_amino\_acid\_phosphorylation | 237 | 3 | 0.940180 | -0.203119 | 1012 | 556.408677 | 675.25 | 794.091323 | 0.667243 |
| GO:0006163\_purine\_nucleotide\_metabolic\_process | 73 | 1 | 1.017455 | -0.199860 | 1015 | 558.931423 | 677.95 | 796.968577 | 0.667931 |
| GO:0006936\_muscle\_contraction | 73 | 1 | 1.017455 | -0.199860 | 1015 | 558.931423 | 677.95 | 796.968577 | 0.667931 |
| GO:0051336\_regulation\_of\_hydrolase\_activity | 73 | 1 | 1.017455 | -0.199860 | 1015 | 558.931423 | 677.95 | 796.968577 | 0.667931 |
| GO:0009416\_response\_to\_light\_stimulus | 74 | 1 | 1.003705 | -0.196402 | 1016 | 560.542831 | 679.48 | 798.417169 | 0.668780 |
| GO:0048589\_developmental\_growth | 75 | 1 | 0.990323 | -0.193017 | 1017 | 562.458454 | 681.56 | 800.661546 | 0.670167 |
| GO:0009056\_catabolic\_process | 243 | 3 | 0.916965 | -0.191401 | 1018 | 562.869654 | 681.95 | 801.030346 | 0.669892 |
| GO:0002521\_leukocyte\_differentiation | 161 | 2 | 0.922661 | -0.190737 | 1019 | 563.123237 | 682.18 | 801.236763 | 0.669460 |
| GO:0003012\_muscle\_system\_process | 76 | 1 | 0.977292 | -0.189704 | 1022 | 564.579409 | 683.62 | 802.660591 | 0.668904 |
| GO:0006508\_proteolysis | 76 | 1 | 0.977292 | -0.189704 | 1022 | 564.579409 | 683.62 | 802.660591 | 0.668904 |
| GO:0034621\_cellular\_macromolecular\_complex\_subunit\_organization | 76 | 1 | 0.977292 | -0.189704 | 1022 | 564.579409 | 683.62 | 802.660591 | 0.668904 |
| GO:0042110\_T\_cell\_activation | 163 | 2 | 0.911340 | -0.186209 | 1023 | 566.539533 | 685.37 | 804.200467 | 0.669961 |
| GO:0030326\_embryonic\_limb\_morphogenesis | 78 | 1 | 0.952233 | -0.183282 | 1025 | 569.486221 | 688.17 | 806.853779 | 0.671385 |
| GO:0035113\_embryonic\_appendage\_morphogenesis | 78 | 1 | 0.952233 | -0.183282 | 1025 | 569.486221 | 688.17 | 806.853779 | 0.671385 |
| GO:0045321\_leukocyte\_activation | 248 | 3 | 0.898478 | -0.182146 | 1026 | 570.349348 | 688.91 | 807.470652 | 0.671452 |
| GO:0043283\_biopolymer\_metabolic\_process | 1490 | 19 | 0.947121 | -0.180301 | 1027 | 571.656979 | 690.31 | 808.963021 | 0.672162 |
| GO:0002250\_adaptive\_immune\_response | 80 | 1 | 0.928427 | -0.177122 | 1030 | 576.900994 | 695.66 | 814.419006 | 0.675398 |
| GO:0002460\_adaptive\_immune\_response\_based\_on\_somatic\_recombination\_of\_immune\_receptors\_built\_from\_immunoglobulin\_superfamily\_domains | 80 | 1 | 0.928427 | -0.177122 | 1030 | 576.900994 | 695.66 | 814.419006 | 0.675398 |
| GO:0044092\_negative\_regulation\_of\_molecular\_function | 80 | 1 | 0.928427 | -0.177122 | 1030 | 576.900994 | 695.66 | 814.419006 | 0.675398 |
| GO:0016481\_negative\_regulation\_of\_transcription | 253 | 3 | 0.880722 | -0.173328 | 1031 | 578.723903 | 697.47 | 816.216097 | 0.676499 |
| GO:0006325\_chromatin\_organization | 83 | 1 | 0.894870 | -0.168343 | 1034 | 584.825510 | 703.33 | 821.834490 | 0.680203 |
| GO:0007017\_microtubule-based\_process | 83 | 1 | 0.894870 | -0.168343 | 1034 | 584.825510 | 703.33 | 821.834490 | 0.680203 |
| GO:0030198\_extracellular\_matrix\_organization | 83 | 1 | 0.894870 | -0.168343 | 1034 | 584.825510 | 703.33 | 821.834490 | 0.680203 |
| GO:0009966\_regulation\_of\_signal\_transduction | 256 | 3 | 0.870401 | -0.168239 | 1035 | 585.028044 | 703.56 | 822.091956 | 0.679768 |
| GO:0044248\_cellular\_catabolic\_process | 173 | 2 | 0.858661 | -0.165196 | 1036 | 588.421393 | 706.66 | 824.898607 | 0.682104 |
| GO:0000279\_M\_phase | 85 | 1 | 0.873814 | -0.162778 | 1038 | 590.255739 | 708.29 | 826.324261 | 0.682360 |
| GO:0002449\_lymphocyte\_mediated\_immunity | 85 | 1 | 0.873814 | -0.162778 | 1038 | 590.255739 | 708.29 | 826.324261 | 0.682360 |
| GO:0002376\_immune\_system\_process | 505 | 6 | 0.882466 | -0.161975 | 1039 | 590.361489 | 708.45 | 826.538511 | 0.681858 |
| GO:0015031\_protein\_transport | 175 | 2 | 0.848848 | -0.161298 | 1040 | 591.166303 | 709.31 | 827.453697 | 0.682029 |
| GO:0001775\_cell\_activation | 262 | 3 | 0.850468 | -0.158492 | 1042 | 596.527305 | 713.99 | 831.452695 | 0.685211 |
| GO:0010629\_negative\_regulation\_of\_gene\_expression | 262 | 3 | 0.850468 | -0.158492 | 1042 | 596.527305 | 713.99 | 831.452695 | 0.685211 |
| GO:0022612\_gland\_morphogenesis | 87 | 1 | 0.853726 | -0.157430 | 1044 | 599.715361 | 717.14 | 834.564639 | 0.686916 |
| GO:0050778\_positive\_regulation\_of\_immune\_response | 87 | 1 | 0.853726 | -0.157430 | 1044 | 599.715361 | 717.14 | 834.564639 | 0.686916 |
| GO:0045184\_establishment\_of\_protein\_localization | 180 | 2 | 0.825269 | -0.151962 | 1045 | 603.499083 | 720.44 | 837.380917 | 0.689416 |
| GO:0035264\_multicellular\_organism\_growth | 90 | 1 | 0.825269 | -0.149791 | 1047 | 606.917948 | 723.8 | 840.682052 | 0.691309 |
| GO:0042113\_B\_cell\_activation | 90 | 1 | 0.825269 | -0.149791 | 1047 | 606.917948 | 723.8 | 840.682052 | 0.691309 |
| GO:0002443\_leukocyte\_mediated\_immunity | 91 | 1 | 0.816200 | -0.147342 | 1049 | 609.953597 | 726.69 | 843.426403 | 0.692745 |
| GO:0008544\_epidermis\_development | 91 | 1 | 0.816200 | -0.147342 | 1049 | 609.953597 | 726.69 | 843.426403 | 0.692745 |
| GO:0007165\_signal\_transduction | 915 | 11 | 0.892914 | -0.147110 | 1050 | 610.121742 | 726.89 | 843.658258 | 0.692276 |
| GO:0045934\_negative\_regulation\_of\_nucleobase\_\_nucleoside\_\_nucleotide\_and\_nucleic\_acid\_metabolic\_process | 270 | 3 | 0.825269 | -0.146344 | 1051 | 610.700001 | 727.46 | 844.219999 | 0.692160 |
| GO:0030217\_T\_cell\_differentiation | 92 | 1 | 0.807328 | -0.144939 | 1052 | 612.532575 | 729.28 | 846.027425 | 0.693232 |
| GO:0051172\_negative\_regulation\_of\_nitrogen\_compound\_metabolic\_process | 271 | 3 | 0.822224 | -0.144891 | 1053 | 613.665616 | 730.13 | 846.594384 | 0.693381 |
| GO:0006464\_protein\_modification\_process | 439 | 5 | 0.845948 | -0.144029 | 1054 | 614.078186 | 730.42 | 846.761814 | 0.692998 |
| GO:0007010\_cytoskeleton\_organization | 185 | 2 | 0.802964 | -0.143182 | 1055 | 614.439841 | 730.79 | 847.140159 | 0.692692 |
| GO:0035107\_appendage\_morphogenesis | 93 | 1 | 0.798647 | -0.142582 | 1058 | 616.069726 | 732.23 | 848.390274 | 0.692089 |
| GO:0035108\_limb\_morphogenesis | 93 | 1 | 0.798647 | -0.142582 | 1058 | 616.069726 | 732.23 | 848.390274 | 0.692089 |
| GO:0065003\_macromolecular\_complex\_assembly | 93 | 1 | 0.798647 | -0.142582 | 1058 | 616.069726 | 732.23 | 848.390274 | 0.692089 |
| GO:0010558\_negative\_regulation\_of\_macromolecule\_biosynthetic\_process | 274 | 3 | 0.813221 | -0.140613 | 1059 | 617.654848 | 733.68 | 849.705152 | 0.692805 |
| GO:0006753\_nucleoside\_phosphate\_metabolic\_process | 94 | 1 | 0.790151 | -0.140269 | 1063 | 619.902216 | 735.99 | 852.077784 | 0.692371 |
| GO:0009117\_nucleotide\_metabolic\_process | 94 | 1 | 0.790151 | -0.140269 | 1063 | 619.902216 | 735.99 | 852.077784 | 0.692371 |
| GO:0032943\_mononuclear\_cell\_proliferation | 94 | 1 | 0.790151 | -0.140269 | 1063 | 619.902216 | 735.99 | 852.077784 | 0.692371 |
| GO:0046651\_lymphocyte\_proliferation | 94 | 1 | 0.790151 | -0.140269 | 1063 | 619.902216 | 735.99 | 852.077784 | 0.692371 |
| GO:0006952\_defense\_response | 187 | 2 | 0.794376 | -0.139816 | 1064 | 620.266670 | 736.31 | 852.353330 | 0.692021 |
| GO:0009888\_tissue\_development | 525 | 6 | 0.848848 | -0.139439 | 1065 | 620.517533 | 736.51 | 852.502467 | 0.691559 |
| GO:0051707\_response\_to\_other\_organism | 95 | 1 | 0.781834 | -0.137999 | 1066 | 622.108001 | 737.91 | 853.711999 | 0.692223 |
| GO:0006954\_inflammatory\_response | 96 | 1 | 0.773690 | -0.135772 | 1067 | 625.154625 | 740.5 | 855.845375 | 0.694002 |
| GO:0006996\_organelle\_organization | 449 | 5 | 0.827107 | -0.132792 | 1068 | 628.222979 | 743.22 | 858.217021 | 0.695899 |
| GO:0009314\_response\_to\_radiation | 98 | 1 | 0.757900 | -0.131440 | 1070 | 630.030263 | 745.0 | 859.969737 | 0.696262 |
| GO:0009967\_positive\_regulation\_of\_signal\_transduction | 98 | 1 | 0.757900 | -0.131440 | 1070 | 630.030263 | 745.0 | 859.969737 | 0.696262 |
| GO:0031327\_negative\_regulation\_of\_cellular\_biosynthetic\_process | 282 | 3 | 0.790151 | -0.129790 | 1071 | 630.280835 | 745.21 | 860.139165 | 0.695808 |
| GO:0001817\_regulation\_of\_cytokine\_production | 99 | 1 | 0.750244 | -0.129333 | 1074 | 632.041827 | 746.84 | 861.638173 | 0.695382 |
| GO:0007398\_ectoderm\_development | 99 | 1 | 0.750244 | -0.129333 | 1074 | 632.041827 | 746.84 | 861.638173 | 0.695382 |
| GO:0060562\_epithelial\_tube\_morphogenesis | 99 | 1 | 0.750244 | -0.129333 | 1074 | 632.041827 | 746.84 | 861.638173 | 0.695382 |
| GO:0009890\_negative\_regulation\_of\_biosynthetic\_process | 284 | 3 | 0.784587 | -0.127212 | 1075 | 633.961244 | 748.43 | 862.898756 | 0.696214 |
| GO:0003002\_regionalization | 195 | 2 | 0.761787 | -0.127142 | 1076 | 634.697112 | 749.03 | 863.362888 | 0.696125 |
| GO:0030163\_protein\_catabolic\_process | 101 | 1 | 0.735388 | -0.125235 | 1077 | 635.878283 | 750.32 | 864.761717 | 0.696676 |
| GO:0043412\_biopolymer\_modification | 458 | 5 | 0.810854 | -0.123361 | 1078 | 637.254496 | 751.55 | 865.845504 | 0.697171 |
| GO:0030036\_actin\_cytoskeleton\_organization | 102 | 1 | 0.728178 | -0.123241 | 1079 | 637.520613 | 751.86 | 866.199387 | 0.696812 |
| GO:0003013\_circulatory\_system\_process | 103 | 1 | 0.721109 | -0.121283 | 1081 | 639.262988 | 753.47 | 867.677012 | 0.697012 |
| GO:0008015\_blood\_circulation | 103 | 1 | 0.721109 | -0.121283 | 1081 | 639.262988 | 753.47 | 867.677012 | 0.697012 |
| GO:0055086\_nucleobase\_\_nucleoside\_and\_nucleotide\_metabolic\_process | 104 | 1 | 0.714175 | -0.119361 | 1082 | 640.664794 | 754.71 | 868.755206 | 0.697514 |
| GO:0043170\_macromolecule\_metabolic\_process | 1576 | 19 | 0.895438 | -0.115772 | 1083 | 641.615452 | 755.56 | 869.504548 | 0.697655 |
| GO:0010817\_regulation\_of\_hormone\_levels | 106 | 1 | 0.700700 | -0.115618 | 1084 | 642.161021 | 756.1 | 870.038979 | 0.697509 |
| GO:0007243\_protein\_kinase\_cascade | 205 | 2 | 0.724626 | -0.112921 | 1085 | 644.337917 | 758.19 | 872.042083 | 0.698793 |
| GO:0030029\_actin\_filament-based\_process | 109 | 1 | 0.681415 | -0.110249 | 1086 | 645.711268 | 759.59 | 873.468732 | 0.699438 |
| GO:0044267\_cellular\_protein\_metabolic\_process | 559 | 6 | 0.797219 | -0.107424 | 1087 | 649.140564 | 762.76 | 876.379436 | 0.701711 |
| GO:0048705\_skeletal\_system\_morphogenesis | 111 | 1 | 0.669137 | -0.106824 | 1088 | 649.379097 | 763.07 | 876.760903 | 0.701351 |
| GO:0040008\_regulation\_of\_growth | 113 | 1 | 0.657294 | -0.103518 | 1089 | 651.781746 | 765.3 | 878.818254 | 0.702755 |
| GO:0000165\_MAPKKK\_cascade | 114 | 1 | 0.651528 | -0.101907 | 1091 | 652.733639 | 766.26 | 879.786361 | 0.702346 |
| GO:0009607\_response\_to\_biotic\_stimulus | 114 | 1 | 0.651528 | -0.101907 | 1091 | 652.733639 | 766.26 | 879.786361 | 0.702346 |
| GO:0046483\_heterocycle\_metabolic\_process | 116 | 1 | 0.640295 | -0.098769 | 1092 | 657.338746 | 770.29 | 883.241254 | 0.705394 |
| GO:0043933\_macromolecular\_complex\_subunit\_organization | 117 | 1 | 0.634822 | -0.097240 | 1093 | 659.048547 | 771.81 | 884.571453 | 0.706139 |
| GO:0001701\_in\_utero\_embryonic\_development | 221 | 2 | 0.672165 | -0.093413 | 1094 | 661.939167 | 774.46 | 886.980833 | 0.707916 |
| GO:0002694\_regulation\_of\_leukocyte\_activation | 121 | 1 | 0.613836 | -0.091379 | 1095 | 663.847836 | 776.28 | 888.712164 | 0.708932 |
| GO:0002252\_immune\_effector\_process | 122 | 1 | 0.608805 | -0.089975 | 1097 | 666.011088 | 778.22 | 890.428912 | 0.709407 |
| GO:0050865\_regulation\_of\_cell\_activation | 122 | 1 | 0.608805 | -0.089975 | 1097 | 666.011088 | 778.22 | 890.428912 | 0.709407 |
| GO:0007242\_intracellular\_signaling\_cascade | 411 | 4 | 0.722863 | -0.087771 | 1098 | 666.665654 | 778.74 | 890.814346 | 0.709235 |
| GO:0030098\_lymphocyte\_differentiation | 124 | 1 | 0.598985 | -0.087237 | 1099 | 667.630547 | 779.54 | 891.449453 | 0.709318 |
| GO:0046649\_lymphocyte\_activation | 228 | 2 | 0.651528 | -0.085972 | 1100 | 668.697973 | 780.39 | 892.082027 | 0.709445 |
| GO:0043062\_extracellular\_structure\_organization | 125 | 1 | 0.594194 | -0.085903 | 1101 | 669.472546 | 781.13 | 892.787454 | 0.709473 |
| GO:0051276\_chromosome\_organization | 129 | 1 | 0.575769 | -0.080781 | 1102 | 673.262286 | 784.32 | 895.377714 | 0.711724 |
| GO:0050776\_regulation\_of\_immune\_response | 130 | 1 | 0.571340 | -0.079553 | 1103 | 675.325788 | 786.2 | 897.074212 | 0.712783 |
| GO:0010605\_negative\_regulation\_of\_macromolecule\_metabolic\_process | 331 | 3 | 0.673180 | -0.078960 | 1104 | 675.795294 | 786.57 | 897.344706 | 0.712473 |
| GO:0031324\_negative\_regulation\_of\_cellular\_metabolic\_process | 332 | 3 | 0.671152 | -0.078153 | 1105 | 676.640999 | 787.25 | 897.859001 | 0.712443 |
| GO:0009952\_anterior\_posterior\_pattern\_formation | 133 | 1 | 0.558453 | -0.075988 | 1106 | 678.218812 | 788.64 | 899.061188 | 0.713056 |
| GO:0009892\_negative\_regulation\_of\_metabolic\_process | 348 | 3 | 0.640295 | -0.066251 | 1107 | 686.628607 | 795.65 | 904.671393 | 0.718744 |
| GO:0007186\_G-protein\_coupled\_receptor\_protein\_signaling\_pathway | 144 | 1 | 0.515793 | -0.064312 | 1108 | 689.755753 | 798.05 | 906.344247 | 0.720262 |
| GO:0002684\_positive\_regulation\_of\_immune\_system\_process | 148 | 1 | 0.501853 | -0.060553 | 1109 | 694.752279 | 802.16 | 909.567721 | 0.723318 |
| GO:0051704\_multi-organism\_process | 157 | 1 | 0.473084 | -0.052918 | 1110 | 701.038092 | 807.22 | 913.401908 | 0.727225 |
| GO:0019538\_protein\_metabolic\_process | 655 | 6 | 0.680374 | -0.049141 | 1111 | 704.765388 | 810.5 | 916.234612 | 0.729523 |
| GO:0009628\_response\_to\_abiotic\_stimulus | 162 | 1 | 0.458483 | -0.049118 | 1112 | 704.947154 | 810.69 | 916.432846 | 0.729038 |
| GO:0006955\_immune\_response | 205 | 1 | 0.362313 | -0.026073 | 1113 | 730.007877 | 830.79 | 931.572123 | 0.746442 |
| GO:0040007\_growth | 217 | 1 | 0.342277 | -0.021881 | 1114 | 734.449491 | 834.29 | 934.130509 | 0.748914 |
| GO:0009790\_embryonic\_development | 567 | 4 | 0.523980 | -0.019070 | 1115 | 736.647775 | 835.94 | 935.232225 | 0.749722 |
| GO:0050890\_cognition | 233 | 1 | 0.318773 | -0.017328 | 1116 | 738.257767 | 837.25 | 936.242233 | 0.750224 |
| GO:0043009\_chordate\_embryonic\_development | 365 | 2 | 0.406982 | -0.016372 | 1117 | 739.374632 | 838.02 | 936.665368 | 0.750242 |
| GO:0009792\_embryonic\_development\_ending\_in\_birth\_or\_egg\_hatching | 368 | 2 | 0.403664 | -0.015772 | 1118 | 740.123150 | 838.53 | 936.936850 | 0.750027 |
| GO:0048598\_embryonic\_morphogenesis | 299 | 1 | 0.248409 | -0.006621 | 1119 | 749.291365 | 845.38 | 941.468635 | 0.755478 |
| GO:0002683\_negative\_regulation\_of\_immune\_system\_process | 56 | 0 | 0.000000 | -0.000000 | 1124 | 760.794655 | 853.78 | 946.765345 | 0.759591 |
| GO:0002703\_regulation\_of\_leukocyte\_mediated\_immunity | 56 | 0 | 0.000000 | -0.000000 | 1124 | 760.794655 | 853.78 | 946.765345 | 0.759591 |
| GO:0006790\_sulfur\_metabolic\_process | 56 | 0 | 0.000000 | -0.000000 | 1124 | 760.794655 | 853.78 | 946.765345 | 0.759591 |
| GO:0042089\_cytokine\_biosynthetic\_process | 56 | 0 | 0.000000 | -0.000000 | 1124 | 760.794655 | 853.78 | 946.765345 | 0.759591 |
| GO:0042107\_cytokine\_metabolic\_process | 56 | 0 | 0.000000 | -0.000000 | 1124 | 760.794655 | 853.78 | 946.765345 | 0.759591 |
| GO:0000077\_DNA\_damage\_checkpoint | 14 | 0 | 0.000000 | -0.000000 | 1179 | 816.175779 | 907.33 | 998.484221 | 0.769576 |
| GO:0001502\_cartilage\_condensation | 14 | 0 | 0.000000 | -0.000000 | 1179 | 816.175779 | 907.33 | 998.484221 | 0.769576 |
| GO:0001829\_trophectodermal\_cell\_differentiation | 14 | 0 | 0.000000 | -0.000000 | 1179 | 816.175779 | 907.33 | 998.484221 | 0.769576 |
| GO:0002027\_regulation\_of\_heart\_rate | 14 | 0 | 0.000000 | -0.000000 | 1179 | 816.175779 | 907.33 | 998.484221 | 0.769576 |
| GO:0002698\_negative\_regulation\_of\_immune\_effector\_process | 14 | 0 | 0.000000 | -0.000000 | 1179 | 816.175779 | 907.33 | 998.484221 | 0.769576 |
| GO:0006304\_DNA\_modification | 14 | 0 | 0.000000 | -0.000000 | 1179 | 816.175779 | 907.33 | 998.484221 | 0.769576 |
| GO:0006305\_DNA\_alkylation | 14 | 0 | 0.000000 | -0.000000 | 1179 | 816.175779 | 907.33 | 998.484221 | 0.769576 |
| GO:0006306\_DNA\_methylation | 14 | 0 | 0.000000 | -0.000000 | 1179 | 816.175779 | 907.33 | 998.484221 | 0.769576 |
| GO:0006695\_cholesterol\_biosynthetic\_process | 14 | 0 | 0.000000 | -0.000000 | 1179 | 816.175779 | 907.33 | 998.484221 | 0.769576 |
| GO:0006809\_nitric\_oxide\_biosynthetic\_process | 14 | 0 | 0.000000 | -0.000000 | 1179 | 816.175779 | 907.33 | 998.484221 | 0.769576 |
| GO:0006914\_autophagy | 14 | 0 | 0.000000 | -0.000000 | 1179 | 816.175779 | 907.33 | 998.484221 | 0.769576 |
| GO:0006970\_response\_to\_osmotic\_stress | 14 | 0 | 0.000000 | -0.000000 | 1179 | 816.175779 | 907.33 | 998.484221 | 0.769576 |
| GO:0007157\_heterophilic\_cell\_adhesion | 14 | 0 | 0.000000 | -0.000000 | 1179 | 816.175779 | 907.33 | 998.484221 | 0.769576 |
| GO:0007530\_sex\_determination | 14 | 0 | 0.000000 | -0.000000 | 1179 | 816.175779 | 907.33 | 998.484221 | 0.769576 |
| GO:0007589\_body\_fluid\_secretion | 14 | 0 | 0.000000 | -0.000000 | 1179 | 816.175779 | 907.33 | 998.484221 | 0.769576 |
| GO:0008064\_regulation\_of\_actin\_polymerization\_or\_depolymerization | 14 | 0 | 0.000000 | -0.000000 | 1179 | 816.175779 | 907.33 | 998.484221 | 0.769576 |
| GO:0008306\_associative\_learning | 14 | 0 | 0.000000 | -0.000000 | 1179 | 816.175779 | 907.33 | 998.484221 | 0.769576 |
| GO:0008630\_DNA\_damage\_response\_\_signal\_transduction\_resulting\_in\_induction\_of\_apoptosis | 14 | 0 | 0.000000 | -0.000000 | 1179 | 816.175779 | 907.33 | 998.484221 | 0.769576 |
| GO:0009267\_cellular\_response\_to\_starvation | 14 | 0 | 0.000000 | -0.000000 | 1179 | 816.175779 | 907.33 | 998.484221 | 0.769576 |
| GO:0009895\_negative\_regulation\_of\_catabolic\_process | 14 | 0 | 0.000000 | -0.000000 | 1179 | 816.175779 | 907.33 | 998.484221 | 0.769576 |
| GO:0014855\_striated\_muscle\_cell\_proliferation | 14 | 0 | 0.000000 | -0.000000 | 1179 | 816.175779 | 907.33 | 998.484221 | 0.769576 |
| GO:0016573\_histone\_acetylation | 14 | 0 | 0.000000 | -0.000000 | 1179 | 816.175779 | 907.33 | 998.484221 | 0.769576 |
| GO:0018130\_heterocycle\_biosynthetic\_process | 14 | 0 | 0.000000 | -0.000000 | 1179 | 816.175779 | 907.33 | 998.484221 | 0.769576 |
| GO:0021782\_glial\_cell\_development | 14 | 0 | 0.000000 | -0.000000 | 1179 | 816.175779 | 907.33 | 998.484221 | 0.769576 |
| GO:0021904\_dorsal\_ventral\_neural\_tube\_patterning | 14 | 0 | 0.000000 | -0.000000 | 1179 | 816.175779 | 907.33 | 998.484221 | 0.769576 |
| GO:0030032\_lamellipodium\_assembly | 14 | 0 | 0.000000 | -0.000000 | 1179 | 816.175779 | 907.33 | 998.484221 | 0.769576 |
| GO:0030148\_sphingolipid\_biosynthetic\_process | 14 | 0 | 0.000000 | -0.000000 | 1179 | 816.175779 | 907.33 | 998.484221 | 0.769576 |
| GO:0030162\_regulation\_of\_proteolysis | 14 | 0 | 0.000000 | -0.000000 | 1179 | 816.175779 | 907.33 | 998.484221 | 0.769576 |
| GO:0030832\_regulation\_of\_actin\_filament\_length | 14 | 0 | 0.000000 | -0.000000 | 1179 | 816.175779 | 907.33 | 998.484221 | 0.769576 |
| GO:0031663\_lipopolysaccharide-mediated\_signaling\_pathway | 14 | 0 | 0.000000 | -0.000000 | 1179 | 816.175779 | 907.33 | 998.484221 | 0.769576 |
| GO:0032271\_regulation\_of\_protein\_polymerization | 14 | 0 | 0.000000 | -0.000000 | 1179 | 816.175779 | 907.33 | 998.484221 | 0.769576 |
| GO:0033044\_regulation\_of\_chromosome\_organization | 14 | 0 | 0.000000 | -0.000000 | 1179 | 816.175779 | 907.33 | 998.484221 | 0.769576 |
| GO:0034623\_cellular\_macromolecular\_complex\_disassembly | 14 | 0 | 0.000000 | -0.000000 | 1179 | 816.175779 | 907.33 | 998.484221 | 0.769576 |
| GO:0035036\_sperm-egg\_recognition | 14 | 0 | 0.000000 | -0.000000 | 1179 | 816.175779 | 907.33 | 998.484221 | 0.769576 |
| GO:0042310\_vasoconstriction | 14 | 0 | 0.000000 | -0.000000 | 1179 | 816.175779 | 907.33 | 998.484221 | 0.769576 |
| GO:0042573\_retinoic\_acid\_metabolic\_process | 14 | 0 | 0.000000 | -0.000000 | 1179 | 816.175779 | 907.33 | 998.484221 | 0.769576 |
| GO:0043123\_positive\_regulation\_of\_I-kappaB\_kinase\_NF-kappaB\_cascade | 14 | 0 | 0.000000 | -0.000000 | 1179 | 816.175779 | 907.33 | 998.484221 | 0.769576 |
| GO:0043254\_regulation\_of\_protein\_complex\_assembly | 14 | 0 | 0.000000 | -0.000000 | 1179 | 816.175779 | 907.33 | 998.484221 | 0.769576 |
| GO:0043491\_protein\_kinase\_B\_signaling\_cascade | 14 | 0 | 0.000000 | -0.000000 | 1179 | 816.175779 | 907.33 | 998.484221 | 0.769576 |
| GO:0044236\_multicellular\_organismal\_metabolic\_process | 14 | 0 | 0.000000 | -0.000000 | 1179 | 816.175779 | 907.33 | 998.484221 | 0.769576 |
| GO:0045061\_thymic\_T\_cell\_selection | 14 | 0 | 0.000000 | -0.000000 | 1179 | 816.175779 | 907.33 | 998.484221 | 0.769576 |
| GO:0045453\_bone\_resorption | 14 | 0 | 0.000000 | -0.000000 | 1179 | 816.175779 | 907.33 | 998.484221 | 0.769576 |
| GO:0045598\_regulation\_of\_fat\_cell\_differentiation | 14 | 0 | 0.000000 | -0.000000 | 1179 | 816.175779 | 907.33 | 998.484221 | 0.769576 |
| GO:0045732\_positive\_regulation\_of\_protein\_catabolic\_process | 14 | 0 | 0.000000 | -0.000000 | 1179 | 816.175779 | 907.33 | 998.484221 | 0.769576 |
| GO:0046209\_nitric\_oxide\_metabolic\_process | 14 | 0 | 0.000000 | -0.000000 | 1179 | 816.175779 | 907.33 | 998.484221 | 0.769576 |
| GO:0048048\_embryonic\_eye\_morphogenesis | 14 | 0 | 0.000000 | -0.000000 | 1179 | 816.175779 | 907.33 | 998.484221 | 0.769576 |
| GO:0048665\_neuron\_fate\_specification | 14 | 0 | 0.000000 | -0.000000 | 1179 | 816.175779 | 907.33 | 998.484221 | 0.769576 |
| GO:0048844\_artery\_morphogenesis | 14 | 0 | 0.000000 | -0.000000 | 1179 | 816.175779 | 907.33 | 998.484221 | 0.769576 |
| GO:0050810\_regulation\_of\_steroid\_biosynthetic\_process | 14 | 0 | 0.000000 | -0.000000 | 1179 | 816.175779 | 907.33 | 998.484221 | 0.769576 |
| GO:0051017\_actin\_filament\_bundle\_formation | 14 | 0 | 0.000000 | -0.000000 | 1179 | 816.175779 | 907.33 | 998.484221 | 0.769576 |
| GO:0051053\_negative\_regulation\_of\_DNA\_metabolic\_process | 14 | 0 | 0.000000 | -0.000000 | 1179 | 816.175779 | 907.33 | 998.484221 | 0.769576 |
| GO:0051054\_positive\_regulation\_of\_DNA\_metabolic\_process | 14 | 0 | 0.000000 | -0.000000 | 1179 | 816.175779 | 907.33 | 998.484221 | 0.769576 |
| GO:0051100\_negative\_regulation\_of\_binding | 14 | 0 | 0.000000 | -0.000000 | 1179 | 816.175779 | 907.33 | 998.484221 | 0.769576 |
| GO:0060716\_labyrinthine\_layer\_blood\_vessel\_development | 14 | 0 | 0.000000 | -0.000000 | 1179 | 816.175779 | 907.33 | 998.484221 | 0.769576 |
| GO:0060840\_artery\_development | 14 | 0 | 0.000000 | -0.000000 | 1179 | 816.175779 | 907.33 | 998.484221 | 0.769576 |
| GO:0000002\_mitochondrial\_genome\_maintenance | 9 | 0 | 0.000000 | -0.000000 | 1281 | 925.600601 | 1014.4 | 1103.199399 | 0.791881 |
| GO:0000186\_activation\_of\_MAPKK\_activity | 9 | 0 | 0.000000 | -0.000000 | 1281 | 925.600601 | 1014.4 | 1103.199399 | 0.791881 |
| GO:0001539\_ciliary\_or\_flagellar\_motility | 9 | 0 | 0.000000 | -0.000000 | 1281 | 925.600601 | 1014.4 | 1103.199399 | 0.791881 |
| GO:0001542\_ovulation\_from\_ovarian\_follicle | 9 | 0 | 0.000000 | -0.000000 | 1281 | 925.600601 | 1014.4 | 1103.199399 | 0.791881 |
| GO:0001667\_ameboidal\_cell\_migration | 9 | 0 | 0.000000 | -0.000000 | 1281 | 925.600601 | 1014.4 | 1103.199399 | 0.791881 |
| GO:0001676\_long-chain\_fatty\_acid\_metabolic\_process | 9 | 0 | 0.000000 | -0.000000 | 1281 | 925.600601 | 1014.4 | 1103.199399 | 0.791881 |
| GO:0002021\_response\_to\_dietary\_excess | 9 | 0 | 0.000000 | -0.000000 | 1281 | 925.600601 | 1014.4 | 1103.199399 | 0.791881 |
| GO:0002028\_regulation\_of\_sodium\_ion\_transport | 9 | 0 | 0.000000 | -0.000000 | 1281 | 925.600601 | 1014.4 | 1103.199399 | 0.791881 |
| GO:0002221\_pattern\_recognition\_receptor\_signaling\_pathway | 9 | 0 | 0.000000 | -0.000000 | 1281 | 925.600601 | 1014.4 | 1103.199399 | 0.791881 |
| GO:0002292\_T\_cell\_differentiation\_during\_immune\_response | 9 | 0 | 0.000000 | -0.000000 | 1281 | 925.600601 | 1014.4 | 1103.199399 | 0.791881 |
| GO:0002293\_alpha-beta\_T\_cell\_differentiation\_during\_immune\_response | 9 | 0 | 0.000000 | -0.000000 | 1281 | 925.600601 | 1014.4 | 1103.199399 | 0.791881 |
| GO:0002294\_CD4-positive\_\_alpha-beta\_T\_cell\_differentiation\_during\_immune\_response | 9 | 0 | 0.000000 | -0.000000 | 1281 | 925.600601 | 1014.4 | 1103.199399 | 0.791881 |
| GO:0002507\_tolerance\_induction | 9 | 0 | 0.000000 | -0.000000 | 1281 | 925.600601 | 1014.4 | 1103.199399 | 0.791881 |
| GO:0002886\_regulation\_of\_myeloid\_leukocyte\_mediated\_immunity | 9 | 0 | 0.000000 | -0.000000 | 1281 | 925.600601 | 1014.4 | 1103.199399 | 0.791881 |
| GO:0006182\_cGMP\_biosynthetic\_process | 9 | 0 | 0.000000 | -0.000000 | 1281 | 925.600601 | 1014.4 | 1103.199399 | 0.791881 |
| GO:0006309\_DNA\_fragmentation\_involved\_in\_apoptosis | 9 | 0 | 0.000000 | -0.000000 | 1281 | 925.600601 | 1014.4 | 1103.199399 | 0.791881 |
| GO:0006364\_rRNA\_processing | 9 | 0 | 0.000000 | -0.000000 | 1281 | 925.600601 | 1014.4 | 1103.199399 | 0.791881 |
| GO:0006476\_protein\_amino\_acid\_deacetylation | 9 | 0 | 0.000000 | -0.000000 | 1281 | 925.600601 | 1014.4 | 1103.199399 | 0.791881 |
| GO:0006595\_polyamine\_metabolic\_process | 9 | 0 | 0.000000 | -0.000000 | 1281 | 925.600601 | 1014.4 | 1103.199399 | 0.791881 |
| GO:0006611\_protein\_export\_from\_nucleus | 9 | 0 | 0.000000 | -0.000000 | 1281 | 925.600601 | 1014.4 | 1103.199399 | 0.791881 |
| GO:0006910\_phagocytosis\_\_recognition | 9 | 0 | 0.000000 | -0.000000 | 1281 | 925.600601 | 1014.4 | 1103.199399 | 0.791881 |
| GO:0006911\_phagocytosis\_\_engulfment | 9 | 0 | 0.000000 | -0.000000 | 1281 | 925.600601 | 1014.4 | 1103.199399 | 0.791881 |
| GO:0007128\_meiotic\_prophase\_I | 9 | 0 | 0.000000 | -0.000000 | 1281 | 925.600601 | 1014.4 | 1103.199399 | 0.791881 |
| GO:0007193\_inhibition\_of\_adenylate\_cyclase\_activity\_by\_G-protein\_signaling | 9 | 0 | 0.000000 | -0.000000 | 1281 | 925.600601 | 1014.4 | 1103.199399 | 0.791881 |
| GO:0007379\_segment\_specification | 9 | 0 | 0.000000 | -0.000000 | 1281 | 925.600601 | 1014.4 | 1103.199399 | 0.791881 |
| GO:0007617\_mating\_behavior | 9 | 0 | 0.000000 | -0.000000 | 1281 | 925.600601 | 1014.4 | 1103.199399 | 0.791881 |
| GO:0009451\_RNA\_modification | 9 | 0 | 0.000000 | -0.000000 | 1281 | 925.600601 | 1014.4 | 1103.199399 | 0.791881 |
| GO:0010165\_response\_to\_X-ray | 9 | 0 | 0.000000 | -0.000000 | 1281 | 925.600601 | 1014.4 | 1103.199399 | 0.791881 |
| GO:0014037\_Schwann\_cell\_differentiation | 9 | 0 | 0.000000 | -0.000000 | 1281 | 925.600601 | 1014.4 | 1103.199399 | 0.791881 |
| GO:0015695\_organic\_cation\_transport | 9 | 0 | 0.000000 | -0.000000 | 1281 | 925.600601 | 1014.4 | 1103.199399 | 0.791881 |
| GO:0016072\_rRNA\_metabolic\_process | 9 | 0 | 0.000000 | -0.000000 | 1281 | 925.600601 | 1014.4 | 1103.199399 | 0.791881 |
| GO:0016601\_Rac\_protein\_signal\_transduction | 9 | 0 | 0.000000 | -0.000000 | 1281 | 925.600601 | 1014.4 | 1103.199399 | 0.791881 |
| GO:0017145\_stem\_cell\_division | 9 | 0 | 0.000000 | -0.000000 | 1281 | 925.600601 | 1014.4 | 1103.199399 | 0.791881 |
| GO:0021544\_subpallium\_development | 9 | 0 | 0.000000 | -0.000000 | 1281 | 925.600601 | 1014.4 | 1103.199399 | 0.791881 |
| GO:0021936\_regulation\_of\_granule\_cell\_precursor\_proliferation | 9 | 0 | 0.000000 | -0.000000 | 1281 | 925.600601 | 1014.4 | 1103.199399 | 0.791881 |
| GO:0021940\_positive\_regulation\_of\_granule\_cell\_precursor\_proliferation | 9 | 0 | 0.000000 | -0.000000 | 1281 | 925.600601 | 1014.4 | 1103.199399 | 0.791881 |
| GO:0030048\_actin\_filament-based\_movement | 9 | 0 | 0.000000 | -0.000000 | 1281 | 925.600601 | 1014.4 | 1103.199399 | 0.791881 |
| GO:0030325\_adrenal\_gland\_development | 9 | 0 | 0.000000 | -0.000000 | 1281 | 925.600601 | 1014.4 | 1103.199399 | 0.791881 |
| GO:0030728\_ovulation | 9 | 0 | 0.000000 | -0.000000 | 1281 | 925.600601 | 1014.4 | 1103.199399 | 0.791881 |
| GO:0031023\_microtubule\_organizing\_center\_organization | 9 | 0 | 0.000000 | -0.000000 | 1281 | 925.600601 | 1014.4 | 1103.199399 | 0.791881 |
| GO:0032388\_positive\_regulation\_of\_intracellular\_transport | 9 | 0 | 0.000000 | -0.000000 | 1281 | 925.600601 | 1014.4 | 1103.199399 | 0.791881 |
| GO:0032606\_type\_I\_interferon\_production | 9 | 0 | 0.000000 | -0.000000 | 1281 | 925.600601 | 1014.4 | 1103.199399 | 0.791881 |
| GO:0032814\_regulation\_of\_natural\_killer\_cell\_activation | 9 | 0 | 0.000000 | -0.000000 | 1281 | 925.600601 | 1014.4 | 1103.199399 | 0.791881 |
| GO:0032816\_positive\_regulation\_of\_natural\_killer\_cell\_activation | 9 | 0 | 0.000000 | -0.000000 | 1281 | 925.600601 | 1014.4 | 1103.199399 | 0.791881 |
| GO:0032963\_collagen\_metabolic\_process | 9 | 0 | 0.000000 | -0.000000 | 1281 | 925.600601 | 1014.4 | 1103.199399 | 0.791881 |
| GO:0033143\_regulation\_of\_steroid\_hormone\_receptor\_signaling\_pathway | 9 | 0 | 0.000000 | -0.000000 | 1281 | 925.600601 | 1014.4 | 1103.199399 | 0.791881 |
| GO:0033151\_V(D)J\_recombination | 9 | 0 | 0.000000 | -0.000000 | 1281 | 925.600601 | 1014.4 | 1103.199399 | 0.791881 |
| GO:0033344\_cholesterol\_efflux | 9 | 0 | 0.000000 | -0.000000 | 1281 | 925.600601 | 1014.4 | 1103.199399 | 0.791881 |
| GO:0034605\_cellular\_response\_to\_heat | 9 | 0 | 0.000000 | -0.000000 | 1281 | 925.600601 | 1014.4 | 1103.199399 | 0.791881 |
| GO:0035088\_establishment\_or\_maintenance\_of\_apical\_basal\_cell\_polarity | 9 | 0 | 0.000000 | -0.000000 | 1281 | 925.600601 | 1014.4 | 1103.199399 | 0.791881 |
| GO:0040020\_regulation\_of\_meiosis | 9 | 0 | 0.000000 | -0.000000 | 1281 | 925.600601 | 1014.4 | 1103.199399 | 0.791881 |
| GO:0042058\_regulation\_of\_epidermal\_growth\_factor\_receptor\_signaling\_pathway | 9 | 0 | 0.000000 | -0.000000 | 1281 | 925.600601 | 1014.4 | 1103.199399 | 0.791881 |
| GO:0042093\_T-helper\_cell\_differentiation | 9 | 0 | 0.000000 | -0.000000 | 1281 | 925.600601 | 1014.4 | 1103.199399 | 0.791881 |
| GO:0042402\_biogenic\_amine\_catabolic\_process | 9 | 0 | 0.000000 | -0.000000 | 1281 | 925.600601 | 1014.4 | 1103.199399 | 0.791881 |
| GO:0042509\_regulation\_of\_tyrosine\_phosphorylation\_of\_STAT\_protein | 9 | 0 | 0.000000 | -0.000000 | 1281 | 925.600601 | 1014.4 | 1103.199399 | 0.791881 |
| GO:0042640\_anagen | 9 | 0 | 0.000000 | -0.000000 | 1281 | 925.600601 | 1014.4 | 1103.199399 | 0.791881 |
| GO:0043242\_negative\_regulation\_of\_protein\_complex\_disassembly | 9 | 0 | 0.000000 | -0.000000 | 1281 | 925.600601 | 1014.4 | 1103.199399 | 0.791881 |
| GO:0043299\_leukocyte\_degranulation | 9 | 0 | 0.000000 | -0.000000 | 1281 | 925.600601 | 1014.4 | 1103.199399 | 0.791881 |
| GO:0043383\_negative\_T\_cell\_selection | 9 | 0 | 0.000000 | -0.000000 | 1281 | 925.600601 | 1014.4 | 1103.199399 | 0.791881 |
| GO:0043409\_negative\_regulation\_of\_MAPKKK\_cascade | 9 | 0 | 0.000000 | -0.000000 | 1281 | 925.600601 | 1014.4 | 1103.199399 | 0.791881 |
| GO:0043433\_negative\_regulation\_of\_transcription\_factor\_activity | 9 | 0 | 0.000000 | -0.000000 | 1281 | 925.600601 | 1014.4 | 1103.199399 | 0.791881 |
| GO:0043603\_cellular\_amide\_metabolic\_process | 9 | 0 | 0.000000 | -0.000000 | 1281 | 925.600601 | 1014.4 | 1103.199399 | 0.791881 |
| GO:0045060\_negative\_thymic\_T\_cell\_selection | 9 | 0 | 0.000000 | -0.000000 | 1281 | 925.600601 | 1014.4 | 1103.199399 | 0.791881 |
| GO:0045136\_development\_of\_secondary\_sexual\_characteristics | 9 | 0 | 0.000000 | -0.000000 | 1281 | 925.600601 | 1014.4 | 1103.199399 | 0.791881 |
| GO:0045185\_maintenance\_of\_protein\_location | 9 | 0 | 0.000000 | -0.000000 | 1281 | 925.600601 | 1014.4 | 1103.199399 | 0.791881 |
| GO:0045214\_sarcomere\_organization | 9 | 0 | 0.000000 | -0.000000 | 1281 | 925.600601 | 1014.4 | 1103.199399 | 0.791881 |
| GO:0045428\_regulation\_of\_nitric\_oxide\_biosynthetic\_process | 9 | 0 | 0.000000 | -0.000000 | 1281 | 925.600601 | 1014.4 | 1103.199399 | 0.791881 |
| GO:0045620\_negative\_regulation\_of\_lymphocyte\_differentiation | 9 | 0 | 0.000000 | -0.000000 | 1281 | 925.600601 | 1014.4 | 1103.199399 | 0.791881 |
| GO:0045646\_regulation\_of\_erythrocyte\_differentiation | 9 | 0 | 0.000000 | -0.000000 | 1281 | 925.600601 | 1014.4 | 1103.199399 | 0.791881 |
| GO:0045671\_negative\_regulation\_of\_osteoclast\_differentiation | 9 | 0 | 0.000000 | -0.000000 | 1281 | 925.600601 | 1014.4 | 1103.199399 | 0.791881 |
| GO:0045766\_positive\_regulation\_of\_angiogenesis | 9 | 0 | 0.000000 | -0.000000 | 1281 | 925.600601 | 1014.4 | 1103.199399 | 0.791881 |
| GO:0045830\_positive\_regulation\_of\_isotype\_switching | 9 | 0 | 0.000000 | -0.000000 | 1281 | 925.600601 | 1014.4 | 1103.199399 | 0.791881 |
| GO:0045884\_regulation\_of\_survival\_gene\_product\_expression | 9 | 0 | 0.000000 | -0.000000 | 1281 | 925.600601 | 1014.4 | 1103.199399 | 0.791881 |
| GO:0046006\_regulation\_of\_activated\_T\_cell\_proliferation | 9 | 0 | 0.000000 | -0.000000 | 1281 | 925.600601 | 1014.4 | 1103.199399 | 0.791881 |
| GO:0046324\_regulation\_of\_glucose\_import | 9 | 0 | 0.000000 | -0.000000 | 1281 | 925.600601 | 1014.4 | 1103.199399 | 0.791881 |
| GO:0046636\_negative\_regulation\_of\_alpha-beta\_T\_cell\_activation | 9 | 0 | 0.000000 | -0.000000 | 1281 | 925.600601 | 1014.4 | 1103.199399 | 0.791881 |
| GO:0046641\_positive\_regulation\_of\_alpha-beta\_T\_cell\_proliferation | 9 | 0 | 0.000000 | -0.000000 | 1281 | 925.600601 | 1014.4 | 1103.199399 | 0.791881 |
| GO:0048146\_positive\_regulation\_of\_fibroblast\_proliferation | 9 | 0 | 0.000000 | -0.000000 | 1281 | 925.600601 | 1014.4 | 1103.199399 | 0.791881 |
| GO:0048284\_organelle\_fusion | 9 | 0 | 0.000000 | -0.000000 | 1281 | 925.600601 | 1014.4 | 1103.199399 | 0.791881 |
| GO:0048569\_post-embryonic\_organ\_development | 9 | 0 | 0.000000 | -0.000000 | 1281 | 925.600601 | 1014.4 | 1103.199399 | 0.791881 |
| GO:0050856\_regulation\_of\_T\_cell\_receptor\_signaling\_pathway | 9 | 0 | 0.000000 | -0.000000 | 1281 | 925.600601 | 1014.4 | 1103.199399 | 0.791881 |
| GO:0050884\_neuromuscular\_process\_controlling\_posture | 9 | 0 | 0.000000 | -0.000000 | 1281 | 925.600601 | 1014.4 | 1103.199399 | 0.791881 |
| GO:0050910\_detection\_of\_mechanical\_stimulus\_involved\_in\_sensory\_perception\_of\_sound | 9 | 0 | 0.000000 | -0.000000 | 1281 | 925.600601 | 1014.4 | 1103.199399 | 0.791881 |
| GO:0051023\_regulation\_of\_immunoglobulin\_secretion | 9 | 0 | 0.000000 | -0.000000 | 1281 | 925.600601 | 1014.4 | 1103.199399 | 0.791881 |
| GO:0051297\_centrosome\_organization | 9 | 0 | 0.000000 | -0.000000 | 1281 | 925.600601 | 1014.4 | 1103.199399 | 0.791881 |
| GO:0051324\_prophase | 9 | 0 | 0.000000 | -0.000000 | 1281 | 925.600601 | 1014.4 | 1103.199399 | 0.791881 |
| GO:0051607\_defense\_response\_to\_virus | 9 | 0 | 0.000000 | -0.000000 | 1281 | 925.600601 | 1014.4 | 1103.199399 | 0.791881 |
| GO:0051647\_nucleus\_localization | 9 | 0 | 0.000000 | -0.000000 | 1281 | 925.600601 | 1014.4 | 1103.199399 | 0.791881 |
| GO:0051896\_regulation\_of\_protein\_kinase\_B\_signaling\_cascade | 9 | 0 | 0.000000 | -0.000000 | 1281 | 925.600601 | 1014.4 | 1103.199399 | 0.791881 |
| GO:0051932\_synaptic\_transmission\_\_GABAergic | 9 | 0 | 0.000000 | -0.000000 | 1281 | 925.600601 | 1014.4 | 1103.199399 | 0.791881 |
| GO:0051963\_regulation\_of\_synaptogenesis | 9 | 0 | 0.000000 | -0.000000 | 1281 | 925.600601 | 1014.4 | 1103.199399 | 0.791881 |
| GO:0055012\_ventricular\_cardiac\_muscle\_cell\_differentiation | 9 | 0 | 0.000000 | -0.000000 | 1281 | 925.600601 | 1014.4 | 1103.199399 | 0.791881 |
| GO:0055013\_cardiac\_muscle\_cell\_development | 9 | 0 | 0.000000 | -0.000000 | 1281 | 925.600601 | 1014.4 | 1103.199399 | 0.791881 |
| GO:0060081\_membrane\_hyperpolarization | 9 | 0 | 0.000000 | -0.000000 | 1281 | 925.600601 | 1014.4 | 1103.199399 | 0.791881 |
| GO:0060119\_inner\_ear\_receptor\_cell\_development | 9 | 0 | 0.000000 | -0.000000 | 1281 | 925.600601 | 1014.4 | 1103.199399 | 0.791881 |
| GO:0060122\_inner\_ear\_receptor\_stereocilium\_organization | 9 | 0 | 0.000000 | -0.000000 | 1281 | 925.600601 | 1014.4 | 1103.199399 | 0.791881 |
| GO:0060325\_face\_morphogenesis | 9 | 0 | 0.000000 | -0.000000 | 1281 | 925.600601 | 1014.4 | 1103.199399 | 0.791881 |
| GO:0060513\_prostatic\_bud\_formation | 9 | 0 | 0.000000 | -0.000000 | 1281 | 925.600601 | 1014.4 | 1103.199399 | 0.791881 |
| GO:0060602\_branch\_elongation\_of\_an\_epithelium | 9 | 0 | 0.000000 | -0.000000 | 1281 | 925.600601 | 1014.4 | 1103.199399 | 0.791881 |
| GO:0060693\_regulation\_of\_branching\_involved\_in\_salivary\_gland\_morphogenesis | 9 | 0 | 0.000000 | -0.000000 | 1281 | 925.600601 | 1014.4 | 1103.199399 | 0.791881 |
| GO:0070306\_lens\_fiber\_cell\_differentiation | 9 | 0 | 0.000000 | -0.000000 | 1281 | 925.600601 | 1014.4 | 1103.199399 | 0.791881 |
| GO:0090048\_negative\_regulation\_of\_transcription\_regulator\_activity | 9 | 0 | 0.000000 | -0.000000 | 1281 | 925.600601 | 1014.4 | 1103.199399 | 0.791881 |
| GO:0002429\_immune\_response-activating\_cell\_surface\_receptor\_signaling\_pathway | 41 | 0 | 0.000000 | -0.000000 | 1284 | 938.919854 | 1026.14 | 1113.360146 | 0.799174 |
| GO:0007254\_JNK\_cascade | 41 | 0 | 0.000000 | -0.000000 | 1284 | 938.919854 | 1026.14 | 1113.360146 | 0.799174 |
| GO:0050864\_regulation\_of\_B\_cell\_activation | 41 | 0 | 0.000000 | -0.000000 | 1284 | 938.919854 | 1026.14 | 1113.360146 | 0.799174 |
| GO:0001843\_neural\_tube\_closure | 33 | 0 | 0.000000 | -0.000000 | 1292 | 949.483286 | 1035.78 | 1122.076714 | 0.801687 |
| GO:0002562\_somatic\_diversification\_of\_immune\_receptors\_via\_germline\_recombination\_within\_a\_single\_locus | 33 | 0 | 0.000000 | -0.000000 | 1292 | 949.483286 | 1035.78 | 1122.076714 | 0.801687 |
| GO:0006643\_membrane\_lipid\_metabolic\_process | 33 | 0 | 0.000000 | -0.000000 | 1292 | 949.483286 | 1035.78 | 1122.076714 | 0.801687 |
| GO:0007431\_salivary\_gland\_development | 33 | 0 | 0.000000 | -0.000000 | 1292 | 949.483286 | 1035.78 | 1122.076714 | 0.801687 |
| GO:0007565\_female\_pregnancy | 33 | 0 | 0.000000 | -0.000000 | 1292 | 949.483286 | 1035.78 | 1122.076714 | 0.801687 |
| GO:0016444\_somatic\_cell\_DNA\_recombination | 33 | 0 | 0.000000 | -0.000000 | 1292 | 949.483286 | 1035.78 | 1122.076714 | 0.801687 |
| GO:0042108\_positive\_regulation\_of\_cytokine\_biosynthetic\_process | 33 | 0 | 0.000000 | -0.000000 | 1292 | 949.483286 | 1035.78 | 1122.076714 | 0.801687 |
| GO:0060606\_tube\_closure | 33 | 0 | 0.000000 | -0.000000 | 1292 | 949.483286 | 1035.78 | 1122.076714 | 0.801687 |
| GO:0000280\_nuclear\_division | 24 | 0 | 0.000000 | -0.000000 | 1313 | 972.178002 | 1056.83 | 1141.481998 | 0.804897 |
| GO:0002381\_immunoglobulin\_production\_during\_immune\_response | 24 | 0 | 0.000000 | -0.000000 | 1313 | 972.178002 | 1056.83 | 1141.481998 | 0.804897 |
| GO:0006941\_striated\_muscle\_contraction | 24 | 0 | 0.000000 | -0.000000 | 1313 | 972.178002 | 1056.83 | 1141.481998 | 0.804897 |
| GO:0006959\_humoral\_immune\_response | 24 | 0 | 0.000000 | -0.000000 | 1313 | 972.178002 | 1056.83 | 1141.481998 | 0.804897 |
| GO:0007050\_cell\_cycle\_arrest | 24 | 0 | 0.000000 | -0.000000 | 1313 | 972.178002 | 1056.83 | 1141.481998 | 0.804897 |
| GO:0007067\_mitosis | 24 | 0 | 0.000000 | -0.000000 | 1313 | 972.178002 | 1056.83 | 1141.481998 | 0.804897 |
| GO:0007259\_JAK-STAT\_cascade | 24 | 0 | 0.000000 | -0.000000 | 1313 | 972.178002 | 1056.83 | 1141.481998 | 0.804897 |
| GO:0007266\_Rho\_protein\_signal\_transduction | 24 | 0 | 0.000000 | -0.000000 | 1313 | 972.178002 | 1056.83 | 1141.481998 | 0.804897 |
| GO:0007632\_visual\_behavior | 24 | 0 | 0.000000 | -0.000000 | 1313 | 972.178002 | 1056.83 | 1141.481998 | 0.804897 |
| GO:0008629\_induction\_of\_apoptosis\_by\_intracellular\_signals | 24 | 0 | 0.000000 | -0.000000 | 1313 | 972.178002 | 1056.83 | 1141.481998 | 0.804897 |
| GO:0009612\_response\_to\_mechanical\_stimulus | 24 | 0 | 0.000000 | -0.000000 | 1313 | 972.178002 | 1056.83 | 1141.481998 | 0.804897 |
| GO:0021515\_cell\_differentiation\_in\_spinal\_cord | 24 | 0 | 0.000000 | -0.000000 | 1313 | 972.178002 | 1056.83 | 1141.481998 | 0.804897 |
| GO:0032386\_regulation\_of\_intracellular\_transport | 24 | 0 | 0.000000 | -0.000000 | 1313 | 972.178002 | 1056.83 | 1141.481998 | 0.804897 |
| GO:0042158\_lipoprotein\_biosynthetic\_process | 24 | 0 | 0.000000 | -0.000000 | 1313 | 972.178002 | 1056.83 | 1141.481998 | 0.804897 |
| GO:0042632\_cholesterol\_homeostasis | 24 | 0 | 0.000000 | -0.000000 | 1313 | 972.178002 | 1056.83 | 1141.481998 | 0.804897 |
| GO:0043410\_positive\_regulation\_of\_MAPKKK\_cascade | 24 | 0 | 0.000000 | -0.000000 | 1313 | 972.178002 | 1056.83 | 1141.481998 | 0.804897 |
| GO:0043588\_skin\_development | 24 | 0 | 0.000000 | -0.000000 | 1313 | 972.178002 | 1056.83 | 1141.481998 | 0.804897 |
| GO:0051099\_positive\_regulation\_of\_binding | 24 | 0 | 0.000000 | -0.000000 | 1313 | 972.178002 | 1056.83 | 1141.481998 | 0.804897 |
| GO:0055092\_sterol\_homeostasis | 24 | 0 | 0.000000 | -0.000000 | 1313 | 972.178002 | 1056.83 | 1141.481998 | 0.804897 |
| GO:0060113\_inner\_ear\_receptor\_cell\_differentiation | 24 | 0 | 0.000000 | -0.000000 | 1313 | 972.178002 | 1056.83 | 1141.481998 | 0.804897 |
| GO:0070667\_negative\_regulation\_of\_mast\_cell\_proliferation | 24 | 0 | 0.000000 | -0.000000 | 1313 | 972.178002 | 1056.83 | 1141.481998 | 0.804897 |
| GO:0006417\_regulation\_of\_translation | 29 | 0 | 0.000000 | -0.000000 | 1327 | 988.129519 | 1071.48 | 1154.830481 | 0.807445 |
| GO:0006641\_triglyceride\_metabolic\_process | 29 | 0 | 0.000000 | -0.000000 | 1327 | 988.129519 | 1071.48 | 1154.830481 | 0.807445 |
| GO:0010564\_regulation\_of\_cell\_cycle\_process | 29 | 0 | 0.000000 | -0.000000 | 1327 | 988.129519 | 1071.48 | 1154.830481 | 0.807445 |
| GO:0016447\_somatic\_recombination\_of\_immunoglobulin\_gene\_segments | 29 | 0 | 0.000000 | -0.000000 | 1327 | 988.129519 | 1071.48 | 1154.830481 | 0.807445 |
| GO:0042176\_regulation\_of\_protein\_catabolic\_process | 29 | 0 | 0.000000 | -0.000000 | 1327 | 988.129519 | 1071.48 | 1154.830481 | 0.807445 |
| GO:0042770\_DNA\_damage\_response\_\_signal\_transduction | 29 | 0 | 0.000000 | -0.000000 | 1327 | 988.129519 | 1071.48 | 1154.830481 | 0.807445 |
| GO:0043281\_regulation\_of\_caspase\_activity | 29 | 0 | 0.000000 | -0.000000 | 1327 | 988.129519 | 1071.48 | 1154.830481 | 0.807445 |
| GO:0044270\_nitrogen\_compound\_catabolic\_process | 29 | 0 | 0.000000 | -0.000000 | 1327 | 988.129519 | 1071.48 | 1154.830481 | 0.807445 |
| GO:0045621\_positive\_regulation\_of\_lymphocyte\_differentiation | 29 | 0 | 0.000000 | -0.000000 | 1327 | 988.129519 | 1071.48 | 1154.830481 | 0.807445 |
| GO:0046634\_regulation\_of\_alpha-beta\_T\_cell\_activation | 29 | 0 | 0.000000 | -0.000000 | 1327 | 988.129519 | 1071.48 | 1154.830481 | 0.807445 |
| GO:0051301\_cell\_division | 29 | 0 | 0.000000 | -0.000000 | 1327 | 988.129519 | 1071.48 | 1154.830481 | 0.807445 |
| GO:0052548\_regulation\_of\_endopeptidase\_activity | 29 | 0 | 0.000000 | -0.000000 | 1327 | 988.129519 | 1071.48 | 1154.830481 | 0.807445 |
| GO:0060041\_retina\_development\_in\_camera-type\_eye | 29 | 0 | 0.000000 | -0.000000 | 1327 | 988.129519 | 1071.48 | 1154.830481 | 0.807445 |
| GO:0070302\_regulation\_of\_stress-activated\_protein\_kinase\_signaling\_pathway | 29 | 0 | 0.000000 | -0.000000 | 1327 | 988.129519 | 1071.48 | 1154.830481 | 0.807445 |
| GO:0042129\_regulation\_of\_T\_cell\_proliferation | 50 | 0 | 0.000000 | -0.000000 | 1329 | 992.431922 | 1075.47 | 1158.508078 | 0.809233 |
| GO:0051606\_detection\_of\_stimulus | 50 | 0 | 0.000000 | -0.000000 | 1329 | 992.431922 | 1075.47 | 1158.508078 | 0.809233 |
| GO:0002764\_immune\_response-regulating\_signal\_transduction | 51 | 0 | 0.000000 | -0.000000 | 1332 | 997.984608 | 1080.46 | 1162.935392 | 0.811156 |
| GO:0007601\_visual\_perception | 51 | 0 | 0.000000 | -0.000000 | 1332 | 997.984608 | 1080.46 | 1162.935392 | 0.811156 |
| GO:0043408\_regulation\_of\_MAPKKK\_cascade | 51 | 0 | 0.000000 | -0.000000 | 1332 | 997.984608 | 1080.46 | 1162.935392 | 0.811156 |
| GO:0000086\_G2\_M\_transition\_of\_mitotic\_cell\_cycle | 4 | 0 |  |  |  |  |  |  |  |  |
| GO:0000305\_response\_to\_oxygen\_radical | 4 | 0 |  |  |  |  |  |  |  |  |
| GO:0001661\_conditioned\_taste\_aversion | 4 | 0 |  |  |  |  |  |  |  |  |
| GO:0001678\_cellular\_glucose\_homeostasis | 4 | 0 |  |  |  |  |  |  |  |  |
| GO:0001777\_T\_cell\_homeostatic\_proliferation | 4 | 0 |  |  |  |  |  |  |  |  |
| GO:0001794\_type\_IIa\_hypersensitivity | 4 | 0 |  |  |  |  |  |  |  |  |
| GO:0001796\_regulation\_of\_type\_IIa\_hypersensitivity | 4 | 0 |  |  |  |  |  |  |  |  |
| GO:0001798\_positive\_regulation\_of\_type\_IIa\_hypersensitivity | 4 | 0 |  |  |  |  |  |  |  |  |
| GO:0001810\_regulation\_of\_type\_I\_hypersensitivity | 4 | 0 |  |  |  |  |  |  |  |  |
| GO:0001820\_serotonin\_secretion | 4 | 0 |  |  |  |  |  |  |  |  |
| GO:0001835\_blastocyst\_hatching | 4 | 0 |  |  |  |  |  |  |  |  |
| GO:0001842\_neural\_fold\_formation | 4 | 0 |  |  |  |  |  |  |  |  |
| GO:0001881\_receptor\_recycling | 4 | 0 |  |  |  |  |  |  |  |  |
| GO:0001978\_regulation\_of\_systemic\_arterial\_blood\_pressure\_by\_carotid\_sinus\_baroreceptor\_feedback | 4 | 0 |  |  |  |  |  |  |  |  |
| GO:0002035\_brain\_renin-angiotensin\_system | 4 | 0 |  |  |  |  |  |  |  |  |
| GO:0002051\_osteoblast\_fate\_commitment | 4 | 0 |  |  |  |  |  |  |  |  |
| GO:0002220\_innate\_immune\_response\_activating\_cell\_surface\_receptor\_signaling\_pathway | 4 | 0 |  |  |  |  |  |  |  |  |
| GO:0002249\_lymphocyte\_anergy | 4 | 0 |  |  |  |  |  |  |  |  |
| GO:0002312\_B\_cell\_activation\_during\_immune\_response | 4 | 0 |  |  |  |  |  |  |  |  |
| GO:0002313\_mature\_B\_cell\_differentiation\_during\_immune\_response | 4 | 0 |  |  |  |  |  |  |  |  |
| GO:0002318\_myeloid\_progenitor\_cell\_differentiation | 4 | 0 |  |  |  |  |  |  |  |  |
| GO:0002347\_response\_to\_tumor\_cell | 4 | 0 |  |  |  |  |  |  |  |  |
| GO:0002418\_immune\_response\_to\_tumor\_cell | 4 | 0 |  |  |  |  |  |  |  |  |
| GO:0002445\_type\_II\_hypersensitivity | 4 | 0 |  |  |  |  |  |  |  |  |
| GO:0002544\_chronic\_inflammatory\_response | 4 | 0 |  |  |  |  |  |  |  |  |
| GO:0002636\_positive\_regulation\_of\_germinal\_center\_formation | 4 | 0 |  |  |  |  |  |  |  |  |
| GO:0002667\_regulation\_of\_T\_cell\_anergy | 4 | 0 |  |  |  |  |  |  |  |  |
| GO:0002669\_positive\_regulation\_of\_T\_cell\_anergy | 4 | 0 |  |  |  |  |  |  |  |  |
| GO:0002687\_positive\_regulation\_of\_leukocyte\_migration | 4 | 0 |  |  |  |  |  |  |  |  |
| GO:0002702\_positive\_regulation\_of\_production\_of\_molecular\_mediator\_of\_immune\_response | 4 | 0 |  |  |  |  |  |  |  |  |
| GO:0002718\_regulation\_of\_cytokine\_production\_during\_immune\_response | 4 | 0 |  |  |  |  |  |  |  |  |
| GO:0002829\_negative\_regulation\_of\_T-helper\_2\_type\_immune\_response | 4 | 0 |  |  |  |  |  |  |  |  |
| GO:0002833\_positive\_regulation\_of\_response\_to\_biotic\_stimulus | 4 | 0 |  |  |  |  |  |  |  |  |
| GO:0002834\_regulation\_of\_response\_to\_tumor\_cell | 4 | 0 |  |  |  |  |  |  |  |  |
| GO:0002836\_positive\_regulation\_of\_response\_to\_tumor\_cell | 4 | 0 |  |  |  |  |  |  |  |  |
| GO:0002837\_regulation\_of\_immune\_response\_to\_tumor\_cell | 4 | 0 |  |  |  |  |  |  |  |  |
| GO:0002839\_positive\_regulation\_of\_immune\_response\_to\_tumor\_cell | 4 | 0 |  |  |  |  |  |  |  |  |
| GO:0002870\_T\_cell\_anergy | 4 | 0 |  |  |  |  |  |  |  |  |
| GO:0002888\_positive\_regulation\_of\_myeloid\_leukocyte\_mediated\_immunity | 4 | 0 |  |  |  |  |  |  |  |  |
| GO:0002892\_regulation\_of\_type\_II\_hypersensitivity | 4 | 0 |  |  |  |  |  |  |  |  |
| GO:0002894\_positive\_regulation\_of\_type\_II\_hypersensitivity | 4 | 0 |  |  |  |  |  |  |  |  |
| GO:0002911\_regulation\_of\_lymphocyte\_anergy | 4 | 0 |  |  |  |  |  |  |  |  |
| GO:0002913\_positive\_regulation\_of\_lymphocyte\_anergy | 4 | 0 |  |  |  |  |  |  |  |  |
| GO:0002923\_regulation\_of\_humoral\_immune\_response\_mediated\_by\_circulating\_immunoglobulin | 4 | 0 |  |  |  |  |  |  |  |  |
| GO:0003025\_regulation\_of\_systemic\_arterial\_blood\_pressure\_by\_baroreceptor\_feedback | 4 | 0 |  |  |  |  |  |  |  |  |
| GO:0003091\_renal\_water\_homeostasis | 4 | 0 |  |  |  |  |  |  |  |  |
| GO:0005978\_glycogen\_biosynthetic\_process | 4 | 0 |  |  |  |  |  |  |  |  |
| GO:0006012\_galactose\_metabolic\_process | 4 | 0 |  |  |  |  |  |  |  |  |
| GO:0006085\_acetyl-CoA\_biosynthetic\_process | 4 | 0 |  |  |  |  |  |  |  |  |
| GO:0006111\_regulation\_of\_gluconeogenesis | 4 | 0 |  |  |  |  |  |  |  |  |
| GO:0006144\_purine\_base\_metabolic\_process | 4 | 0 |  |  |  |  |  |  |  |  |
| GO:0006290\_pyrimidine\_dimer\_repair | 4 | 0 |  |  |  |  |  |  |  |  |
| GO:0006334\_nucleosome\_assembly | 4 | 0 |  |  |  |  |  |  |  |  |
| GO:0006534\_cysteine\_metabolic\_process | 4 | 0 |  |  |  |  |  |  |  |  |
| GO:0006547\_histidine\_metabolic\_process | 4 | 0 |  |  |  |  |  |  |  |  |
| GO:0006548\_histidine\_catabolic\_process | 4 | 0 |  |  |  |  |  |  |  |  |
| GO:0006555\_methionine\_metabolic\_process | 4 | 0 |  |  |  |  |  |  |  |  |
| GO:0006599\_phosphagen\_metabolic\_process | 4 | 0 |  |  |  |  |  |  |  |  |
| GO:0006623\_protein\_targeting\_to\_vacuole | 4 | 0 |  |  |  |  |  |  |  |  |
| GO:0006626\_protein\_targeting\_to\_mitochondrion | 4 | 0 |  |  |  |  |  |  |  |  |
| GO:0006684\_sphingomyelin\_metabolic\_process | 4 | 0 |  |  |  |  |  |  |  |  |
| GO:0006688\_glycosphingolipid\_biosynthetic\_process | 4 | 0 |  |  |  |  |  |  |  |  |
| GO:0006707\_cholesterol\_catabolic\_process | 4 | 0 |  |  |  |  |  |  |  |  |
| GO:0006739\_NADP\_metabolic\_process | 4 | 0 |  |  |  |  |  |  |  |  |
| GO:0006835\_dicarboxylic\_acid\_transport | 4 | 0 |  |  |  |  |  |  |  |  |
| GO:0006837\_serotonin\_transport | 4 | 0 |  |  |  |  |  |  |  |  |
| GO:0006888\_ER\_to\_Golgi\_vesicle-mediated\_transport | 4 | 0 |  |  |  |  |  |  |  |  |
| GO:0006906\_vesicle\_fusion | 4 | 0 |  |  |  |  |  |  |  |  |
| GO:0006927\_transformed\_cell\_apoptosis | 4 | 0 |  |  |  |  |  |  |  |  |
| GO:0006972\_hyperosmotic\_response | 4 | 0 |  |  |  |  |  |  |  |  |
| GO:0007028\_cytoplasm\_organization | 4 | 0 |  |  |  |  |  |  |  |  |
| GO:0007031\_peroxisome\_organization | 4 | 0 |  |  |  |  |  |  |  |  |
| GO:0007066\_female\_meiosis\_sister\_chromatid\_cohesion | 4 | 0 |  |  |  |  |  |  |  |  |
| GO:0007216\_metabotropic\_glutamate\_receptor\_signaling\_pathway | 4 | 0 |  |  |  |  |  |  |  |  |
| GO:0007342\_fusion\_of\_sperm\_to\_egg\_plasma\_membrane | 4 | 0 |  |  |  |  |  |  |  |  |
| GO:0007386\_compartment\_specification | 4 | 0 |  |  |  |  |  |  |  |  |
| GO:0008053\_mitochondrial\_fusion | 4 | 0 |  |  |  |  |  |  |  |  |
| GO:0008207\_C21-steroid\_hormone\_metabolic\_process | 4 | 0 |  |  |  |  |  |  |  |  |
| GO:0008215\_spermine\_metabolic\_process | 4 | 0 |  |  |  |  |  |  |  |  |
| GO:0009065\_glutamine\_family\_amino\_acid\_catabolic\_process | 4 | 0 |  |  |  |  |  |  |  |  |
| GO:0009075\_histidine\_family\_amino\_acid\_metabolic\_process | 4 | 0 |  |  |  |  |  |  |  |  |
| GO:0009077\_histidine\_family\_amino\_acid\_catabolic\_process | 4 | 0 |  |  |  |  |  |  |  |  |
| GO:0009134\_nucleoside\_diphosphate\_catabolic\_process | 4 | 0 |  |  |  |  |  |  |  |  |
| GO:0009163\_nucleoside\_biosynthetic\_process | 4 | 0 |  |  |  |  |  |  |  |  |
| GO:0009225\_nucleotide-sugar\_metabolic\_process | 4 | 0 |  |  |  |  |  |  |  |  |
| GO:0009250\_glucan\_biosynthetic\_process | 4 | 0 |  |  |  |  |  |  |  |  |
| GO:0009404\_toxin\_metabolic\_process | 4 | 0 |  |  |  |  |  |  |  |  |
| GO:0009593\_detection\_of\_chemical\_stimulus | 4 | 0 |  |  |  |  |  |  |  |  |
| GO:0009595\_detection\_of\_biotic\_stimulus | 4 | 0 |  |  |  |  |  |  |  |  |
| GO:0009755\_hormone-mediated\_signaling | 4 | 0 |  |  |  |  |  |  |  |  |
| GO:0009912\_auditory\_receptor\_cell\_fate\_commitment | 4 | 0 |  |  |  |  |  |  |  |  |
| GO:0010453\_regulation\_of\_cell\_fate\_commitment | 4 | 0 |  |  |  |  |  |  |  |  |
| GO:0010506\_regulation\_of\_autophagy | 4 | 0 |  |  |  |  |  |  |  |  |
| GO:0010631\_epithelial\_cell\_migration | 4 | 0 |  |  |  |  |  |  |  |  |
| GO:0010812\_negative\_regulation\_of\_cell-substrate\_adhesion | 4 | 0 |  |  |  |  |  |  |  |  |
| GO:0010829\_negative\_regulation\_of\_glucose\_transport | 4 | 0 |  |  |  |  |  |  |  |  |
| GO:0014002\_astrocyte\_development | 4 | 0 |  |  |  |  |  |  |  |  |
| GO:0014832\_urinary\_bladder\_smooth\_muscle\_contraction | 4 | 0 |  |  |  |  |  |  |  |  |
| GO:0014848\_urinary\_tract\_smooth\_muscle\_contraction | 4 | 0 |  |  |  |  |  |  |  |  |
| GO:0015850\_organic\_alcohol\_transport | 4 | 0 |  |  |  |  |  |  |  |  |
| GO:0015858\_nucleoside\_transport | 4 | 0 |  |  |  |  |  |  |  |  |
| GO:0016068\_type\_I\_hypersensitivity | 4 | 0 |  |  |  |  |  |  |  |  |
| GO:0016127\_sterol\_catabolic\_process | 4 | 0 |  |  |  |  |  |  |  |  |
| GO:0016198\_axon\_choice\_point\_recognition | 4 | 0 |  |  |  |  |  |  |  |  |
| GO:0016338\_calcium-independent\_cell-cell\_adhesion | 4 | 0 |  |  |  |  |  |  |  |  |
| GO:0018198\_peptidyl-cysteine\_modification | 4 | 0 |  |  |  |  |  |  |  |  |
| GO:0018409\_peptide\_or\_protein\_amino-terminal\_blocking | 4 | 0 |  |  |  |  |  |  |  |  |
| GO:0019377\_glycolipid\_catabolic\_process | 4 | 0 |  |  |  |  |  |  |  |  |
| GO:0019432\_triglyceride\_biosynthetic\_process | 4 | 0 |  |  |  |  |  |  |  |  |
| GO:0019530\_taurine\_metabolic\_process | 4 | 0 |  |  |  |  |  |  |  |  |
| GO:0021523\_somatic\_motor\_neuron\_differentiation | 4 | 0 |  |  |  |  |  |  |  |  |
| GO:0021542\_dentate\_gyrus\_development | 4 | 0 |  |  |  |  |  |  |  |  |
| GO:0021561\_facial\_nerve\_development | 4 | 0 |  |  |  |  |  |  |  |  |
| GO:0021569\_rhombomere\_3\_development | 4 | 0 |  |  |  |  |  |  |  |  |
| GO:0021571\_rhombomere\_5\_development | 4 | 0 |  |  |  |  |  |  |  |  |
| GO:0021604\_cranial\_nerve\_structural\_organization | 4 | 0 |  |  |  |  |  |  |  |  |
| GO:0021610\_facial\_nerve\_morphogenesis | 4 | 0 |  |  |  |  |  |  |  |  |
| GO:0021612\_facial\_nerve\_structural\_organization | 4 | 0 |  |  |  |  |  |  |  |  |
| GO:0021681\_cerebellar\_granular\_layer\_development | 4 | 0 |  |  |  |  |  |  |  |  |
| GO:0021683\_cerebellar\_granular\_layer\_morphogenesis | 4 | 0 |  |  |  |  |  |  |  |  |
| GO:0021684\_cerebellar\_granular\_layer\_formation | 4 | 0 |  |  |  |  |  |  |  |  |
| GO:0021707\_cerebellar\_granule\_cell\_differentiation | 4 | 0 |  |  |  |  |  |  |  |  |
| GO:0021778\_oligodendrocyte\_cell\_fate\_specification | 4 | 0 |  |  |  |  |  |  |  |  |
| GO:0021779\_oligodendrocyte\_cell\_fate\_commitment | 4 | 0 |  |  |  |  |  |  |  |  |
| GO:0021780\_glial\_cell\_fate\_specification | 4 | 0 |  |  |  |  |  |  |  |  |
| GO:0021830\_interneuron\_migration\_from\_the\_subpallium\_to\_the\_cortex | 4 | 0 |  |  |  |  |  |  |  |  |
| GO:0021853\_cerebral\_cortex\_GABAergic\_interneuron\_migration | 4 | 0 |  |  |  |  |  |  |  |  |
| GO:0021877\_forebrain\_neuron\_fate\_commitment | 4 | 0 |  |  |  |  |  |  |  |  |
| GO:0021894\_cerebral\_cortex\_GABAergic\_interneuron\_development | 4 | 0 |  |  |  |  |  |  |  |  |
| GO:0021910\_smoothened\_signaling\_pathway\_involved\_in\_ventral\_spinal\_cord\_patterning | 4 | 0 |  |  |  |  |  |  |  |  |
| GO:0021913\_regulation\_of\_transcription\_from\_RNA\_polymerase\_II\_promoter\_involved\_in\_ventral\_spinal\_cord\_interneuron\_specification | 4 | 0 |  |  |  |  |  |  |  |  |
| GO:0021938\_smoothened\_signaling\_pathway\_involved\_in\_regulation\_of\_granule\_cell\_precursor\_cell\_proliferation | 4 | 0 |  |  |  |  |  |  |  |  |
| GO:0021978\_telencephalon\_regionalization | 4 | 0 |  |  |  |  |  |  |  |  |
| GO:0022011\_myelination\_in\_the\_peripheral\_nervous\_system | 4 | 0 |  |  |  |  |  |  |  |  |
| GO:0030146\_diuresis | 4 | 0 |  |  |  |  |  |  |  |  |
| GO:0030300\_regulation\_of\_intestinal\_cholesterol\_absorption | 4 | 0 |  |  |  |  |  |  |  |  |
| GO:0030800\_negative\_regulation\_of\_cyclic\_nucleotide\_metabolic\_process | 4 | 0 |  |  |  |  |  |  |  |  |
| GO:0030803\_negative\_regulation\_of\_cyclic\_nucleotide\_biosynthetic\_process | 4 | 0 |  |  |  |  |  |  |  |  |
| GO:0030809\_negative\_regulation\_of\_nucleotide\_biosynthetic\_process | 4 | 0 |  |  |  |  |  |  |  |  |
| GO:0030815\_negative\_regulation\_of\_cAMP\_metabolic\_process | 4 | 0 |  |  |  |  |  |  |  |  |
| GO:0030816\_positive\_regulation\_of\_cAMP\_metabolic\_process | 4 | 0 |  |  |  |  |  |  |  |  |
| GO:0030818\_negative\_regulation\_of\_cAMP\_biosynthetic\_process | 4 | 0 |  |  |  |  |  |  |  |  |
| GO:0030819\_positive\_regulation\_of\_cAMP\_biosynthetic\_process | 4 | 0 |  |  |  |  |  |  |  |  |
| GO:0030826\_regulation\_of\_cGMP\_biosynthetic\_process | 4 | 0 |  |  |  |  |  |  |  |  |
| GO:0030858\_positive\_regulation\_of\_epithelial\_cell\_differentiation | 4 | 0 |  |  |  |  |  |  |  |  |
| GO:0030859\_polarized\_epithelial\_cell\_differentiation | 4 | 0 |  |  |  |  |  |  |  |  |
| GO:0030949\_positive\_regulation\_of\_vascular\_endothelial\_growth\_factor\_receptor\_signaling\_pathway | 4 | 0 |  |  |  |  |  |  |  |  |
| GO:0031113\_regulation\_of\_microtubule\_polymerization | 4 | 0 |  |  |  |  |  |  |  |  |
| GO:0031365\_N-terminal\_protein\_amino\_acid\_modification | 4 | 0 |  |  |  |  |  |  |  |  |
| GO:0031424\_keratinization | 4 | 0 |  |  |  |  |  |  |  |  |
| GO:0031557\_induction\_of\_programmed\_cell\_death\_in\_response\_to\_chemical\_stimulus | 4 | 0 |  |  |  |  |  |  |  |  |
| GO:0031558\_induction\_of\_apoptosis\_in\_response\_to\_chemical\_stimulus | 4 | 0 |  |  |  |  |  |  |  |  |
| GO:0031623\_receptor\_internalization | 4 | 0 |  |  |  |  |  |  |  |  |
| GO:0032088\_negative\_regulation\_of\_NF-kappaB\_transcription\_factor\_activity | 4 | 0 |  |  |  |  |  |  |  |  |
| GO:0032098\_regulation\_of\_appetite | 4 | 0 |  |  |  |  |  |  |  |  |
| GO:0032105\_negative\_regulation\_of\_response\_to\_extracellular\_stimulus | 4 | 0 |  |  |  |  |  |  |  |  |
| GO:0032108\_negative\_regulation\_of\_response\_to\_nutrient\_levels | 4 | 0 |  |  |  |  |  |  |  |  |
| GO:0032225\_regulation\_of\_synaptic\_transmission\_\_dopaminergic | 4 | 0 |  |  |  |  |  |  |  |  |
| GO:0032292\_ensheathment\_of\_axons\_in\_the\_peripheral\_nervous\_system | 4 | 0 |  |  |  |  |  |  |  |  |
| GO:0032321\_positive\_regulation\_of\_Rho\_GTPase\_activity | 4 | 0 |  |  |  |  |  |  |  |  |
| GO:0032371\_regulation\_of\_sterol\_transport | 4 | 0 |  |  |  |  |  |  |  |  |
| GO:0032374\_regulation\_of\_cholesterol\_transport | 4 | 0 |  |  |  |  |  |  |  |  |
| GO:0032401\_establishment\_of\_melanosome\_localization | 4 | 0 |  |  |  |  |  |  |  |  |
| GO:0032608\_interferon-beta\_production | 4 | 0 |  |  |  |  |  |  |  |  |
| GO:0032611\_interleukin-1\_beta\_production | 4 | 0 |  |  |  |  |  |  |  |  |
| GO:0032612\_interleukin-1\_production | 4 | 0 |  |  |  |  |  |  |  |  |
| GO:0032648\_regulation\_of\_interferon-beta\_production | 4 | 0 |  |  |  |  |  |  |  |  |
| GO:0032651\_regulation\_of\_interleukin-1\_beta\_production | 4 | 0 |  |  |  |  |  |  |  |  |
| GO:0032652\_regulation\_of\_interleukin-1\_production | 4 | 0 |  |  |  |  |  |  |  |  |
| GO:0032689\_negative\_regulation\_of\_interferon-gamma\_production | 4 | 0 |  |  |  |  |  |  |  |  |
| GO:0032713\_negative\_regulation\_of\_interleukin-4\_production | 4 | 0 |  |  |  |  |  |  |  |  |
| GO:0032715\_negative\_regulation\_of\_interleukin-6\_production | 4 | 0 |  |  |  |  |  |  |  |  |
| GO:0032733\_positive\_regulation\_of\_interleukin-10\_production | 4 | 0 |  |  |  |  |  |  |  |  |
| GO:0032808\_lacrimal\_gland\_development | 4 | 0 |  |  |  |  |  |  |  |  |
| GO:0032872\_regulation\_of\_stress-activated\_MAPK\_cascade | 4 | 0 |  |  |  |  |  |  |  |  |
| GO:0032922\_circadian\_regulation\_of\_gene\_expression | 4 | 0 |  |  |  |  |  |  |  |  |
| GO:0033079\_immature\_T\_cell\_proliferation | 4 | 0 |  |  |  |  |  |  |  |  |
| GO:0033083\_regulation\_of\_immature\_T\_cell\_proliferation | 4 | 0 |  |  |  |  |  |  |  |  |
| GO:0033089\_positive\_regulation\_of\_T\_cell\_differentiation\_in\_the\_thymus | 4 | 0 |  |  |  |  |  |  |  |  |
| GO:0033299\_secretion\_of\_lysosomal\_enzymes | 4 | 0 |  |  |  |  |  |  |  |  |
| GO:0033327\_Leydig\_cell\_differentiation | 4 | 0 |  |  |  |  |  |  |  |  |
| GO:0033363\_secretory\_granule\_organization | 4 | 0 |  |  |  |  |  |  |  |  |
| GO:0033599\_regulation\_of\_mammary\_gland\_epithelial\_cell\_proliferation | 4 | 0 |  |  |  |  |  |  |  |  |
| GO:0033865\_nucleoside\_bisphosphate\_metabolic\_process | 4 | 0 |  |  |  |  |  |  |  |  |
| GO:0034204\_lipid\_translocation | 4 | 0 |  |  |  |  |  |  |  |  |
| GO:0034404\_nucleobase\_\_nucleoside\_and\_nucleotide\_biosynthetic\_process | 4 | 0 |  |  |  |  |  |  |  |  |
| GO:0034587\_piRNA\_metabolic\_process | 4 | 0 |  |  |  |  |  |  |  |  |
| GO:0034614\_cellular\_response\_to\_reactive\_oxygen\_species | 4 | 0 |  |  |  |  |  |  |  |  |
| GO:0034654\_nucleobase\_\_nucleoside\_\_nucleotide\_and\_nucleic\_acid\_biosynthetic\_process | 4 | 0 |  |  |  |  |  |  |  |  |
| GO:0035020\_regulation\_of\_Rac\_protein\_signal\_transduction | 4 | 0 |  |  |  |  |  |  |  |  |
| GO:0035082\_axoneme\_assembly | 4 | 0 |  |  |  |  |  |  |  |  |
| GO:0035188\_hatching | 4 | 0 |  |  |  |  |  |  |  |  |
| GO:0035235\_ionotropic\_glutamate\_receptor\_signaling\_pathway | 4 | 0 |  |  |  |  |  |  |  |  |
| GO:0042345\_regulation\_of\_NF-kappaB\_import\_into\_nucleus | 4 | 0 |  |  |  |  |  |  |  |  |
| GO:0042348\_NF-kappaB\_import\_into\_nucleus | 4 | 0 |  |  |  |  |  |  |  |  |
| GO:0042359\_vitamin\_D\_metabolic\_process | 4 | 0 |  |  |  |  |  |  |  |  |
| GO:0042428\_serotonin\_metabolic\_process | 4 | 0 |  |  |  |  |  |  |  |  |
| GO:0042451\_purine\_nucleoside\_biosynthetic\_process | 4 | 0 |  |  |  |  |  |  |  |  |
| GO:0042455\_ribonucleoside\_biosynthetic\_process | 4 | 0 |  |  |  |  |  |  |  |  |
| GO:0042473\_outer\_ear\_morphogenesis | 4 | 0 |  |  |  |  |  |  |  |  |
| GO:0042522\_regulation\_of\_tyrosine\_phosphorylation\_of\_Stat5\_protein | 4 | 0 |  |  |  |  |  |  |  |  |
| GO:0042535\_positive\_regulation\_of\_tumor\_necrosis\_factor\_biosynthetic\_process | 4 | 0 |  |  |  |  |  |  |  |  |
| GO:0042541\_hemoglobin\_biosynthetic\_process | 4 | 0 |  |  |  |  |  |  |  |  |
| GO:0042558\_pteridine\_and\_derivative\_metabolic\_process | 4 | 0 |  |  |  |  |  |  |  |  |
| GO:0042634\_regulation\_of\_hair\_cycle | 4 | 0 |  |  |  |  |  |  |  |  |
| GO:0042744\_hydrogen\_peroxide\_catabolic\_process | 4 | 0 |  |  |  |  |  |  |  |  |
| GO:0042982\_amyloid\_precursor\_protein\_metabolic\_process | 4 | 0 |  |  |  |  |  |  |  |  |
| GO:0042992\_negative\_regulation\_of\_transcription\_factor\_import\_into\_nucleus | 4 | 0 |  |  |  |  |  |  |  |  |
| GO:0043043\_peptide\_biosynthetic\_process | 4 | 0 |  |  |  |  |  |  |  |  |
| GO:0043484\_regulation\_of\_RNA\_splicing | 4 | 0 |  |  |  |  |  |  |  |  |
| GO:0043500\_muscle\_adaptation | 4 | 0 |  |  |  |  |  |  |  |  |
| GO:0043534\_blood\_vessel\_endothelial\_cell\_migration | 4 | 0 |  |  |  |  |  |  |  |  |
| GO:0043691\_reverse\_cholesterol\_transport | 4 | 0 |  |  |  |  |  |  |  |  |
| GO:0044243\_multicellular\_organismal\_catabolic\_process | 4 | 0 |  |  |  |  |  |  |  |  |
| GO:0044403\_symbiosis\_\_encompassing\_mutualism\_through\_parasitism | 4 | 0 |  |  |  |  |  |  |  |  |
| GO:0044419\_interspecies\_interaction\_between\_organisms | 4 | 0 |  |  |  |  |  |  |  |  |
| GO:0045066\_regulatory\_T\_cell\_differentiation | 4 | 0 |  |  |  |  |  |  |  |  |
| GO:0045078\_positive\_regulation\_of\_interferon-gamma\_biosynthetic\_process | 4 | 0 |  |  |  |  |  |  |  |  |
| GO:0045332\_phospholipid\_translocation | 4 | 0 |  |  |  |  |  |  |  |  |
| GO:0045346\_regulation\_of\_MHC\_class\_II\_biosynthetic\_process | 4 | 0 |  |  |  |  |  |  |  |  |
| GO:0045350\_interferon-beta\_biosynthetic\_process | 4 | 0 |  |  |  |  |  |  |  |  |
| GO:0045357\_regulation\_of\_interferon-beta\_biosynthetic\_process | 4 | 0 |  |  |  |  |  |  |  |  |
| GO:0045359\_positive\_regulation\_of\_interferon-beta\_biosynthetic\_process | 4 | 0 |  |  |  |  |  |  |  |  |
| GO:0045600\_positive\_regulation\_of\_fat\_cell\_differentiation | 4 | 0 |  |  |  |  |  |  |  |  |
| GO:0045616\_regulation\_of\_keratinocyte\_differentiation | 4 | 0 |  |  |  |  |  |  |  |  |
| GO:0045624\_positive\_regulation\_of\_T-helper\_cell\_differentiation | 4 | 0 |  |  |  |  |  |  |  |  |
| GO:0045628\_regulation\_of\_T-helper\_2\_cell\_differentiation | 4 | 0 |  |  |  |  |  |  |  |  |
| GO:0045647\_negative\_regulation\_of\_erythrocyte\_differentiation | 4 | 0 |  |  |  |  |  |  |  |  |
| GO:0045672\_positive\_regulation\_of\_osteoclast\_differentiation | 4 | 0 |  |  |  |  |  |  |  |  |
| GO:0045684\_positive\_regulation\_of\_epidermis\_development | 4 | 0 |  |  |  |  |  |  |  |  |
| GO:0045736\_negative\_regulation\_of\_cyclin-dependent\_protein\_kinase\_activity | 4 | 0 |  |  |  |  |  |  |  |  |
| GO:0045742\_positive\_regulation\_of\_epidermal\_growth\_factor\_receptor\_signaling\_pathway | 4 | 0 |  |  |  |  |  |  |  |  |
| GO:0045747\_positive\_regulation\_of\_Notch\_signaling\_pathway | 4 | 0 |  |  |  |  |  |  |  |  |
| GO:0045767\_regulation\_of\_anti-apoptosis | 4 | 0 |  |  |  |  |  |  |  |  |
| GO:0045779\_negative\_regulation\_of\_bone\_resorption | 4 | 0 |  |  |  |  |  |  |  |  |
| GO:0045923\_positive\_regulation\_of\_fatty\_acid\_metabolic\_process | 4 | 0 |  |  |  |  |  |  |  |  |
| GO:0045940\_positive\_regulation\_of\_steroid\_metabolic\_process | 4 | 0 |  |  |  |  |  |  |  |  |
| GO:0045980\_negative\_regulation\_of\_nucleotide\_metabolic\_process | 4 | 0 |  |  |  |  |  |  |  |  |
| GO:0046129\_purine\_ribonucleoside\_biosynthetic\_process | 4 | 0 |  |  |  |  |  |  |  |  |
| GO:0046173\_polyol\_biosynthetic\_process | 4 | 0 |  |  |  |  |  |  |  |  |
| GO:0046541\_saliva\_secretion | 4 | 0 |  |  |  |  |  |  |  |  |
| GO:0046639\_negative\_regulation\_of\_alpha-beta\_T\_cell\_differentiation | 4 | 0 |  |  |  |  |  |  |  |  |
| GO:0046642\_negative\_regulation\_of\_alpha-beta\_T\_cell\_proliferation | 4 | 0 |  |  |  |  |  |  |  |  |
| GO:0046686\_response\_to\_cadmium\_ion | 4 | 0 |  |  |  |  |  |  |  |  |
| GO:0046835\_carbohydrate\_phosphorylation | 4 | 0 |  |  |  |  |  |  |  |  |
| GO:0047496\_vesicle\_transport\_along\_microtubule | 4 | 0 |  |  |  |  |  |  |  |  |
| GO:0048011\_nerve\_growth\_factor\_receptor\_signaling\_pathway | 4 | 0 |  |  |  |  |  |  |  |  |
| GO:0048024\_regulation\_of\_nuclear\_mRNA\_splicing\_\_via\_spliceosome | 4 | 0 |  |  |  |  |  |  |  |  |
| GO:0048240\_sperm\_capacitation | 4 | 0 |  |  |  |  |  |  |  |  |
| GO:0048341\_paraxial\_mesoderm\_formation | 4 | 0 |  |  |  |  |  |  |  |  |
| GO:0048484\_enteric\_nervous\_system\_development | 4 | 0 |  |  |  |  |  |  |  |  |
| GO:0048512\_circadian\_behavior | 4 | 0 |  |  |  |  |  |  |  |  |
| GO:0048558\_embryonic\_gut\_morphogenesis | 4 | 0 |  |  |  |  |  |  |  |  |
| GO:0048639\_positive\_regulation\_of\_developmental\_growth | 4 | 0 |  |  |  |  |  |  |  |  |
| GO:0048841\_regulation\_of\_axon\_extension\_involved\_in\_axon\_guidance | 4 | 0 |  |  |  |  |  |  |  |  |
| GO:0048843\_negative\_regulation\_of\_axon\_extension\_involved\_in\_axon\_guidance | 4 | 0 |  |  |  |  |  |  |  |  |
| GO:0048846\_axon\_extension\_involved\_in\_axon\_guidance | 4 | 0 |  |  |  |  |  |  |  |  |
| GO:0050702\_interleukin-1\_beta\_secretion | 4 | 0 |  |  |  |  |  |  |  |  |
| GO:0050704\_regulation\_of\_interleukin-1\_secretion | 4 | 0 |  |  |  |  |  |  |  |  |
| GO:0050706\_regulation\_of\_interleukin-1\_beta\_secretion | 4 | 0 |  |  |  |  |  |  |  |  |
| GO:0050716\_positive\_regulation\_of\_interleukin-1\_secretion | 4 | 0 |  |  |  |  |  |  |  |  |
| GO:0050718\_positive\_regulation\_of\_interleukin-1\_beta\_secretion | 4 | 0 |  |  |  |  |  |  |  |  |
| GO:0050820\_positive\_regulation\_of\_coagulation | 4 | 0 |  |  |  |  |  |  |  |  |
| GO:0050891\_multicellular\_organismal\_water\_homeostasis | 4 | 0 |  |  |  |  |  |  |  |  |
| GO:0050919\_negative\_chemotaxis | 4 | 0 |  |  |  |  |  |  |  |  |
| GO:0050961\_detection\_of\_temperature\_stimulus\_involved\_in\_sensory\_perception | 4 | 0 |  |  |  |  |  |  |  |  |
| GO:0050965\_detection\_of\_temperature\_stimulus\_involved\_in\_sensory\_perception\_of\_pain | 4 | 0 |  |  |  |  |  |  |  |  |
| GO:0050994\_regulation\_of\_lipid\_catabolic\_process | 4 | 0 |  |  |  |  |  |  |  |  |
| GO:0051024\_positive\_regulation\_of\_immunoglobulin\_secretion | 4 | 0 |  |  |  |  |  |  |  |  |
| GO:0051055\_negative\_regulation\_of\_lipid\_biosynthetic\_process | 4 | 0 |  |  |  |  |  |  |  |  |
| GO:0051124\_synaptic\_growth\_at\_neuromuscular\_junction | 4 | 0 |  |  |  |  |  |  |  |  |
| GO:0051148\_negative\_regulation\_of\_muscle\_cell\_differentiation | 4 | 0 |  |  |  |  |  |  |  |  |
| GO:0051205\_protein\_insertion\_into\_membrane | 4 | 0 |  |  |  |  |  |  |  |  |
| GO:0051225\_spindle\_assembly | 4 | 0 |  |  |  |  |  |  |  |  |
| GO:0051341\_regulation\_of\_oxidoreductase\_activity | 4 | 0 |  |  |  |  |  |  |  |  |
| GO:0051567\_histone\_H3-K9\_methylation | 4 | 0 |  |  |  |  |  |  |  |  |
| GO:0051642\_centrosome\_localization | 4 | 0 |  |  |  |  |  |  |  |  |
| GO:0051797\_regulation\_of\_hair\_follicle\_development | 4 | 0 |  |  |  |  |  |  |  |  |
| GO:0051897\_positive\_regulation\_of\_protein\_kinase\_B\_signaling\_cascade | 4 | 0 |  |  |  |  |  |  |  |  |
| GO:0051904\_pigment\_granule\_transport | 4 | 0 |  |  |  |  |  |  |  |  |
| GO:0060008\_Sertoli\_cell\_differentiation | 4 | 0 |  |  |  |  |  |  |  |  |
| GO:0060011\_Sertoli\_cell\_proliferation | 4 | 0 |  |  |  |  |  |  |  |  |
| GO:0060057\_apoptosis\_involved\_in\_mammary\_gland\_involution | 4 | 0 |  |  |  |  |  |  |  |  |
| GO:0060058\_positive\_regulation\_of\_apoptosis\_involved\_in\_mammary\_gland\_involution | 4 | 0 |  |  |  |  |  |  |  |  |
| GO:0060065\_uterus\_development | 4 | 0 |  |  |  |  |  |  |  |  |
| GO:0060087\_relaxation\_of\_vascular\_smooth\_muscle | 4 | 0 |  |  |  |  |  |  |  |  |
| GO:0060120\_inner\_ear\_receptor\_cell\_fate\_commitment | 4 | 0 |  |  |  |  |  |  |  |  |
| GO:0060157\_urinary\_bladder\_development | 4 | 0 |  |  |  |  |  |  |  |  |
| GO:0060164\_regulation\_of\_timing\_of\_neuron\_differentiation | 4 | 0 |  |  |  |  |  |  |  |  |
| GO:0060235\_lens\_induction\_in\_camera-type\_eye | 4 | 0 |  |  |  |  |  |  |  |  |
| GO:0060412\_ventricular\_septum\_morphogenesis | 4 | 0 |  |  |  |  |  |  |  |  |
| GO:0060528\_secretory\_columnal\_luminar\_epithelial\_cell\_differentiation\_involved\_in\_prostate\_glandular\_acinus\_development | 4 | 0 |  |  |  |  |  |  |  |  |
| GO:0060561\_apoptosis\_involved\_in\_morphogenesis | 4 | 0 |  |  |  |  |  |  |  |  |
| GO:0060592\_mammary\_gland\_formation | 4 | 0 |  |  |  |  |  |  |  |  |
| GO:0060644\_mammary\_gland\_epithelial\_cell\_differentiation | 4 | 0 |  |  |  |  |  |  |  |  |
| GO:0060666\_dichotomous\_subdivision\_of\_terminal\_units\_involved\_in\_salivary\_gland\_branching | 4 | 0 |  |  |  |  |  |  |  |  |
| GO:0060737\_prostate\_gland\_morphogenetic\_growth | 4 | 0 |  |  |  |  |  |  |  |  |
| GO:0060743\_epithelial\_cell\_maturation\_involved\_in\_prostate\_gland\_development | 4 | 0 |  |  |  |  |  |  |  |  |
| GO:0060751\_mammary\_gland\_duct\_branch\_elongation | 4 | 0 |  |  |  |  |  |  |  |  |
| GO:0060900\_embryonic\_camera-type\_eye\_formation | 4 | 0 |  |  |  |  |  |  |  |  |
| GO:0070254\_mucus\_secretion | 4 | 0 |  |  |  |  |  |  |  |  |
| GO:0070255\_regulation\_of\_mucus\_secretion | 4 | 0 |  |  |  |  |  |  |  |  |
| GO:0070301\_cellular\_response\_to\_hydrogen\_peroxide | 4 | 0 |  |  |  |  |  |  |  |  |
| GO:0070585\_protein\_localization\_in\_mitochondrion | 4 | 0 |  |  |  |  |  |  |  |  |
| GO:0002495\_antigen\_processing\_and\_presentation\_of\_peptide\_antigen\_via\_MHC\_class\_II | 15 | 0 | 0.000000 | -0.000000 | 1366 | 1045.181835 | 1126.0 | 1206.818165 | 0.824305 |
| GO:0002504\_antigen\_processing\_and\_presentation\_of\_peptide\_or\_polysaccharide\_antigen\_via\_MHC\_class\_II | 15 | 0 | 0.000000 | -0.000000 | 1366 | 1045.181835 | 1126.0 | 1206.818165 | 0.824305 |
| GO:0002709\_regulation\_of\_T\_cell\_mediated\_immunity | 15 | 0 | 0.000000 | -0.000000 | 1366 | 1045.181835 | 1126.0 | 1206.818165 | 0.824305 |
| GO:0006473\_protein\_amino\_acid\_acetylation | 15 | 0 | 0.000000 | -0.000000 | 1366 | 1045.181835 | 1126.0 | 1206.818165 | 0.824305 |
| GO:0006487\_protein\_amino\_acid\_N-linked\_glycosylation | 15 | 0 | 0.000000 | -0.000000 | 1366 | 1045.181835 | 1126.0 | 1206.818165 | 0.824305 |
| GO:0006749\_glutathione\_metabolic\_process | 15 | 0 | 0.000000 | -0.000000 | 1366 | 1045.181835 | 1126.0 | 1206.818165 | 0.824305 |
| GO:0007040\_lysosome\_organization | 15 | 0 | 0.000000 | -0.000000 | 1366 | 1045.181835 | 1126.0 | 1206.818165 | 0.824305 |
| GO:0007173\_epidermal\_growth\_factor\_receptor\_signaling\_pathway | 15 | 0 | 0.000000 | -0.000000 | 1366 | 1045.181835 | 1126.0 | 1206.818165 | 0.824305 |
| GO:0007218\_neuropeptide\_signaling\_pathway | 15 | 0 | 0.000000 | -0.000000 | 1366 | 1045.181835 | 1126.0 | 1206.818165 | 0.824305 |
| GO:0007618\_mating | 15 | 0 | 0.000000 | -0.000000 | 1366 | 1045.181835 | 1126.0 | 1206.818165 | 0.824305 |
| GO:0008543\_fibroblast\_growth\_factor\_receptor\_signaling\_pathway | 15 | 0 | 0.000000 | -0.000000 | 1366 | 1045.181835 | 1126.0 | 1206.818165 | 0.824305 |
| GO:0009062\_fatty\_acid\_catabolic\_process | 15 | 0 | 0.000000 | -0.000000 | 1366 | 1045.181835 | 1126.0 | 1206.818165 | 0.824305 |
| GO:0009116\_nucleoside\_metabolic\_process | 15 | 0 | 0.000000 | -0.000000 | 1366 | 1045.181835 | 1126.0 | 1206.818165 | 0.824305 |
| GO:0010171\_body\_morphogenesis | 15 | 0 | 0.000000 | -0.000000 | 1366 | 1045.181835 | 1126.0 | 1206.818165 | 0.824305 |
| GO:0015931\_nucleobase\_\_nucleoside\_\_nucleotide\_and\_nucleic\_acid\_transport | 15 | 0 | 0.000000 | -0.000000 | 1366 | 1045.181835 | 1126.0 | 1206.818165 | 0.824305 |
| GO:0019886\_antigen\_processing\_and\_presentation\_of\_exogenous\_peptide\_antigen\_via\_MHC\_class\_II | 15 | 0 | 0.000000 | -0.000000 | 1366 | 1045.181835 | 1126.0 | 1206.818165 | 0.824305 |
| GO:0022600\_digestive\_system\_process | 15 | 0 | 0.000000 | -0.000000 | 1366 | 1045.181835 | 1126.0 | 1206.818165 | 0.824305 |
| GO:0030041\_actin\_filament\_polymerization | 15 | 0 | 0.000000 | -0.000000 | 1366 | 1045.181835 | 1126.0 | 1206.818165 | 0.824305 |
| GO:0035116\_embryonic\_hindlimb\_morphogenesis | 15 | 0 | 0.000000 | -0.000000 | 1366 | 1045.181835 | 1126.0 | 1206.818165 | 0.824305 |
| GO:0035249\_synaptic\_transmission\_\_glutamatergic | 15 | 0 | 0.000000 | -0.000000 | 1366 | 1045.181835 | 1126.0 | 1206.818165 | 0.824305 |
| GO:0042306\_regulation\_of\_protein\_import\_into\_nucleus | 15 | 0 | 0.000000 | -0.000000 | 1366 | 1045.181835 | 1126.0 | 1206.818165 | 0.824305 |
| GO:0046638\_positive\_regulation\_of\_alpha-beta\_T\_cell\_differentiation | 15 | 0 | 0.000000 | -0.000000 | 1366 | 1045.181835 | 1126.0 | 1206.818165 | 0.824305 |
| GO:0048008\_platelet-derived\_growth\_factor\_receptor\_signaling\_pathway | 15 | 0 | 0.000000 | -0.000000 | 1366 | 1045.181835 | 1126.0 | 1206.818165 | 0.824305 |
| GO:0048144\_fibroblast\_proliferation | 15 | 0 | 0.000000 | -0.000000 | 1366 | 1045.181835 | 1126.0 | 1206.818165 | 0.824305 |
| GO:0048145\_regulation\_of\_fibroblast\_proliferation | 15 | 0 | 0.000000 | -0.000000 | 1366 | 1045.181835 | 1126.0 | 1206.818165 | 0.824305 |
| GO:0048610\_reproductive\_cellular\_process | 15 | 0 | 0.000000 | -0.000000 | 1366 | 1045.181835 | 1126.0 | 1206.818165 | 0.824305 |
| GO:0048709\_oligodendrocyte\_differentiation | 15 | 0 | 0.000000 | -0.000000 | 1366 | 1045.181835 | 1126.0 | 1206.818165 | 0.824305 |
| GO:0050729\_positive\_regulation\_of\_inflammatory\_response | 15 | 0 | 0.000000 | -0.000000 | 1366 | 1045.181835 | 1126.0 | 1206.818165 | 0.824305 |
| GO:0050798\_activated\_T\_cell\_proliferation | 15 | 0 | 0.000000 | -0.000000 | 1366 | 1045.181835 | 1126.0 | 1206.818165 | 0.824305 |
| GO:0055010\_ventricular\_cardiac\_muscle\_morphogenesis | 15 | 0 | 0.000000 | -0.000000 | 1366 | 1045.181835 | 1126.0 | 1206.818165 | 0.824305 |
| GO:0060322\_head\_development | 15 | 0 | 0.000000 | -0.000000 | 1366 | 1045.181835 | 1126.0 | 1206.818165 | 0.824305 |
| GO:0060442\_branching\_involved\_in\_prostate\_gland\_morphogenesis | 15 | 0 | 0.000000 | -0.000000 | 1366 | 1045.181835 | 1126.0 | 1206.818165 | 0.824305 |
| GO:0070227\_lymphocyte\_apoptosis | 15 | 0 | 0.000000 | -0.000000 | 1366 | 1045.181835 | 1126.0 | 1206.818165 | 0.824305 |
| GO:0070507\_regulation\_of\_microtubule\_cytoskeleton\_organization | 15 | 0 | 0.000000 | -0.000000 | 1366 | 1045.181835 | 1126.0 | 1206.818165 | 0.824305 |
| GO:0000910\_cytokinesis | 8 | 0 | 0.000000 | -0.000000 | 1472 | 1166.907699 | 1244.36 | 1321.812301 | 0.845353 |
| GO:0001783\_B\_cell\_apoptosis | 8 | 0 | 0.000000 | -0.000000 | 1472 | 1166.907699 | 1244.36 | 1321.812301 | 0.845353 |
| GO:0001833\_inner\_cell\_mass\_cell\_proliferation | 8 | 0 | 0.000000 | -0.000000 | 1472 | 1166.907699 | 1244.36 | 1321.812301 | 0.845353 |
| GO:0001840\_neural\_plate\_development | 8 | 0 | 0.000000 | -0.000000 | 1472 | 1166.907699 | 1244.36 | 1321.812301 | 0.845353 |
| GO:0001893\_maternal\_placenta\_development | 8 | 0 | 0.000000 | -0.000000 | 1472 | 1166.907699 | 1244.36 | 1321.812301 | 0.845353 |
| GO:0001911\_negative\_regulation\_of\_leukocyte\_mediated\_cytotoxicity | 8 | 0 | 0.000000 | -0.000000 | 1472 | 1166.907699 | 1244.36 | 1321.812301 | 0.845353 |
| GO:0001916\_positive\_regulation\_of\_T\_cell\_mediated\_cytotoxicity | 8 | 0 | 0.000000 | -0.000000 | 1472 | 1166.907699 | 1244.36 | 1321.812301 | 0.845353 |
| GO:0002065\_columnar\_cuboidal\_epithelial\_cell\_differentiation | 8 | 0 | 0.000000 | -0.000000 | 1472 | 1166.907699 | 1244.36 | 1321.812301 | 0.845353 |
| GO:0002438\_acute\_inflammatory\_response\_to\_antigenic\_stimulus | 8 | 0 | 0.000000 | -0.000000 | 1472 | 1166.907699 | 1244.36 | 1321.812301 | 0.845353 |
| GO:0002524\_hypersensitivity | 8 | 0 | 0.000000 | -0.000000 | 1472 | 1166.907699 | 1244.36 | 1321.812301 | 0.845353 |
| GO:0002566\_somatic\_diversification\_of\_immune\_receptors\_via\_somatic\_mutation | 8 | 0 | 0.000000 | -0.000000 | 1472 | 1166.907699 | 1244.36 | 1321.812301 | 0.845353 |
| GO:0002864\_regulation\_of\_acute\_inflammatory\_response\_to\_antigenic\_stimulus | 8 | 0 | 0.000000 | -0.000000 | 1472 | 1166.907699 | 1244.36 | 1321.812301 | 0.845353 |
| GO:0002883\_regulation\_of\_hypersensitivity | 8 | 0 | 0.000000 | -0.000000 | 1472 | 1166.907699 | 1244.36 | 1321.812301 | 0.845353 |
| GO:0003081\_regulation\_of\_systemic\_arterial\_blood\_pressure\_by\_renin-angiotensin | 8 | 0 | 0.000000 | -0.000000 | 1472 | 1166.907699 | 1244.36 | 1321.812301 | 0.845353 |
| GO:0006020\_inositol\_metabolic\_process | 8 | 0 | 0.000000 | -0.000000 | 1472 | 1166.907699 | 1244.36 | 1321.812301 | 0.845353 |
| GO:0006195\_purine\_nucleotide\_catabolic\_process | 8 | 0 | 0.000000 | -0.000000 | 1472 | 1166.907699 | 1244.36 | 1321.812301 | 0.845353 |
| GO:0006284\_base-excision\_repair | 8 | 0 | 0.000000 | -0.000000 | 1472 | 1166.907699 | 1244.36 | 1321.812301 | 0.845353 |
| GO:0006349\_genetic\_imprinting | 8 | 0 | 0.000000 | -0.000000 | 1472 | 1166.907699 | 1244.36 | 1321.812301 | 0.845353 |
| GO:0006360\_transcription\_from\_RNA\_polymerase\_I\_promoter | 8 | 0 | 0.000000 | -0.000000 | 1472 | 1166.907699 | 1244.36 | 1321.812301 | 0.845353 |
| GO:0006399\_tRNA\_metabolic\_process | 8 | 0 | 0.000000 | -0.000000 | 1472 | 1166.907699 | 1244.36 | 1321.812301 | 0.845353 |
| GO:0006458\_'de\_novo'\_protein\_folding | 8 | 0 | 0.000000 | -0.000000 | 1472 | 1166.907699 | 1244.36 | 1321.812301 | 0.845353 |
| GO:0006493\_protein\_amino\_acid\_O-linked\_glycosylation | 8 | 0 | 0.000000 | -0.000000 | 1472 | 1166.907699 | 1244.36 | 1321.812301 | 0.845353 |
| GO:0006733\_oxidoreduction\_coenzyme\_metabolic\_process | 8 | 0 | 0.000000 | -0.000000 | 1472 | 1166.907699 | 1244.36 | 1321.812301 | 0.845353 |
| GO:0006829\_zinc\_ion\_transport | 8 | 0 | 0.000000 | -0.000000 | 1472 | 1166.907699 | 1244.36 | 1321.812301 | 0.845353 |
| GO:0007009\_plasma\_membrane\_organization | 8 | 0 | 0.000000 | -0.000000 | 1472 | 1166.907699 | 1244.36 | 1321.812301 | 0.845353 |
| GO:0007098\_centrosome\_cycle | 8 | 0 | 0.000000 | -0.000000 | 1472 | 1166.907699 | 1244.36 | 1321.812301 | 0.845353 |
| GO:0007131\_reciprocal\_meiotic\_recombination | 8 | 0 | 0.000000 | -0.000000 | 1472 | 1166.907699 | 1244.36 | 1321.812301 | 0.845353 |
| GO:0007141\_male\_meiosis\_I | 8 | 0 | 0.000000 | -0.000000 | 1472 | 1166.907699 | 1244.36 | 1321.812301 | 0.845353 |
| GO:0007625\_grooming\_behavior | 8 | 0 | 0.000000 | -0.000000 | 1472 | 1166.907699 | 1244.36 | 1321.812301 | 0.845353 |
| GO:0008105\_asymmetric\_protein\_localization | 8 | 0 | 0.000000 | -0.000000 | 1472 | 1166.907699 | 1244.36 | 1321.812301 | 0.845353 |
| GO:0008593\_regulation\_of\_Notch\_signaling\_pathway | 8 | 0 | 0.000000 | -0.000000 | 1472 | 1166.907699 | 1244.36 | 1321.812301 | 0.845353 |
| GO:0009072\_aromatic\_amino\_acid\_family\_metabolic\_process | 8 | 0 | 0.000000 | -0.000000 | 1472 | 1166.907699 | 1244.36 | 1321.812301 | 0.845353 |
| GO:0009144\_purine\_nucleoside\_triphosphate\_metabolic\_process | 8 | 0 | 0.000000 | -0.000000 | 1472 | 1166.907699 | 1244.36 | 1321.812301 | 0.845353 |
| GO:0014065\_phosphoinositide\_3-kinase\_cascade | 8 | 0 | 0.000000 | -0.000000 | 1472 | 1166.907699 | 1244.36 | 1321.812301 | 0.845353 |
| GO:0015800\_acidic\_amino\_acid\_transport | 8 | 0 | 0.000000 | -0.000000 | 1472 | 1166.907699 | 1244.36 | 1321.812301 | 0.845353 |
| GO:0015804\_neutral\_amino\_acid\_transport | 8 | 0 | 0.000000 | -0.000000 | 1472 | 1166.907699 | 1244.36 | 1321.812301 | 0.845353 |
| GO:0016236\_macroautophagy | 8 | 0 | 0.000000 | -0.000000 | 1472 | 1166.907699 | 1244.36 | 1321.812301 | 0.845353 |
| GO:0016446\_somatic\_hypermutation\_of\_immunoglobulin\_genes | 8 | 0 | 0.000000 | -0.000000 | 1472 | 1166.907699 | 1244.36 | 1321.812301 | 0.845353 |
| GO:0018345\_protein\_palmitoylation | 8 | 0 | 0.000000 | -0.000000 | 1472 | 1166.907699 | 1244.36 | 1321.812301 | 0.845353 |
| GO:0019229\_regulation\_of\_vasoconstriction | 8 | 0 | 0.000000 | -0.000000 | 1472 | 1166.907699 | 1244.36 | 1321.812301 | 0.845353 |
| GO:0019400\_alditol\_metabolic\_process | 8 | 0 | 0.000000 | -0.000000 | 1472 | 1166.907699 | 1244.36 | 1321.812301 | 0.845353 |
| GO:0021692\_cerebellar\_Purkinje\_cell\_layer\_morphogenesis | 8 | 0 | 0.000000 | -0.000000 | 1472 | 1166.907699 | 1244.36 | 1321.812301 | 0.845353 |
| GO:0021694\_cerebellar\_Purkinje\_cell\_layer\_formation | 8 | 0 | 0.000000 | -0.000000 | 1472 | 1166.907699 | 1244.36 | 1321.812301 | 0.845353 |
| GO:0021702\_cerebellar\_Purkinje\_cell\_differentiation | 8 | 0 | 0.000000 | -0.000000 | 1472 | 1166.907699 | 1244.36 | 1321.812301 | 0.845353 |
| GO:0021781\_glial\_cell\_fate\_commitment | 8 | 0 | 0.000000 | -0.000000 | 1472 | 1166.907699 | 1244.36 | 1321.812301 | 0.845353 |
| GO:0022898\_regulation\_of\_transmembrane\_transporter\_activity | 8 | 0 | 0.000000 | -0.000000 | 1472 | 1166.907699 | 1244.36 | 1321.812301 | 0.845353 |
| GO:0030035\_microspike\_assembly | 8 | 0 | 0.000000 | -0.000000 | 1472 | 1166.907699 | 1244.36 | 1321.812301 | 0.845353 |
| GO:0030193\_regulation\_of\_blood\_coagulation | 8 | 0 | 0.000000 | -0.000000 | 1472 | 1166.907699 | 1244.36 | 1321.812301 | 0.845353 |
| GO:0030204\_chondroitin\_sulfate\_metabolic\_process | 8 | 0 | 0.000000 | -0.000000 | 1472 | 1166.907699 | 1244.36 | 1321.812301 | 0.845353 |
| GO:0030500\_regulation\_of\_bone\_mineralization | 8 | 0 | 0.000000 | -0.000000 | 1472 | 1166.907699 | 1244.36 | 1321.812301 | 0.845353 |
| GO:0030511\_positive\_regulation\_of\_transforming\_growth\_factor\_beta\_receptor\_signaling\_pathway | 8 | 0 | 0.000000 | -0.000000 | 1472 | 1166.907699 | 1244.36 | 1321.812301 | 0.845353 |
| GO:0031111\_negative\_regulation\_of\_microtubule\_polymerization\_or\_depolymerization | 8 | 0 | 0.000000 | -0.000000 | 1472 | 1166.907699 | 1244.36 | 1321.812301 | 0.845353 |
| GO:0031123\_RNA\_3'-end\_processing | 8 | 0 | 0.000000 | -0.000000 | 1472 | 1166.907699 | 1244.36 | 1321.812301 | 0.845353 |
| GO:0031294\_lymphocyte\_costimulation | 8 | 0 | 0.000000 | -0.000000 | 1472 | 1166.907699 | 1244.36 | 1321.812301 | 0.845353 |
| GO:0031295\_T\_cell\_costimulation | 8 | 0 | 0.000000 | -0.000000 | 1472 | 1166.907699 | 1244.36 | 1321.812301 | 0.845353 |
| GO:0031334\_positive\_regulation\_of\_protein\_complex\_assembly | 8 | 0 | 0.000000 | -0.000000 | 1472 | 1166.907699 | 1244.36 | 1321.812301 | 0.845353 |
| GO:0031342\_negative\_regulation\_of\_cell\_killing | 8 | 0 | 0.000000 | -0.000000 | 1472 | 1166.907699 | 1244.36 | 1321.812301 | 0.845353 |
| GO:0031396\_regulation\_of\_protein\_ubiquitination | 8 | 0 | 0.000000 | -0.000000 | 1472 | 1166.907699 | 1244.36 | 1321.812301 | 0.845353 |
| GO:0032094\_response\_to\_food | 8 | 0 | 0.000000 | -0.000000 | 1472 | 1166.907699 | 1244.36 | 1321.812301 | 0.845353 |
| GO:0032273\_positive\_regulation\_of\_protein\_polymerization | 8 | 0 | 0.000000 | -0.000000 | 1472 | 1166.907699 | 1244.36 | 1321.812301 | 0.845353 |
| GO:0032409\_regulation\_of\_transporter\_activity | 8 | 0 | 0.000000 | -0.000000 | 1472 | 1166.907699 | 1244.36 | 1321.812301 | 0.845353 |
| GO:0032412\_regulation\_of\_ion\_transmembrane\_transporter\_activity | 8 | 0 | 0.000000 | -0.000000 | 1472 | 1166.907699 | 1244.36 | 1321.812301 | 0.845353 |
| GO:0032613\_interleukin-10\_production | 8 | 0 | 0.000000 | -0.000000 | 1472 | 1166.907699 | 1244.36 | 1321.812301 | 0.845353 |
| GO:0033198\_response\_to\_ATP | 8 | 0 | 0.000000 | -0.000000 | 1472 | 1166.907699 | 1244.36 | 1321.812301 | 0.845353 |
| GO:0034728\_nucleosome\_organization | 8 | 0 | 0.000000 | -0.000000 | 1472 | 1166.907699 | 1244.36 | 1321.812301 | 0.845353 |
| GO:0035023\_regulation\_of\_Rho\_protein\_signal\_transduction | 8 | 0 | 0.000000 | -0.000000 | 1472 | 1166.907699 | 1244.36 | 1321.812301 | 0.845353 |
| GO:0035112\_genitalia\_morphogenesis | 8 | 0 | 0.000000 | -0.000000 | 1472 | 1166.907699 | 1244.36 | 1321.812301 | 0.845353 |
| GO:0040034\_regulation\_of\_development\_\_heterochronic | 8 | 0 | 0.000000 | -0.000000 | 1472 | 1166.907699 | 1244.36 | 1321.812301 | 0.845353 |
| GO:0042074\_cell\_migration\_involved\_in\_gastrulation | 8 | 0 | 0.000000 | -0.000000 | 1472 | 1166.907699 | 1244.36 | 1321.812301 | 0.845353 |
| GO:0042090\_interleukin-12\_biosynthetic\_process | 8 | 0 | 0.000000 | -0.000000 | 1472 | 1166.907699 | 1244.36 | 1321.812301 | 0.845353 |
| GO:0042092\_T-helper\_2\_type\_immune\_response | 8 | 0 | 0.000000 | -0.000000 | 1472 | 1166.907699 | 1244.36 | 1321.812301 | 0.845353 |
| GO:0042095\_interferon-gamma\_biosynthetic\_process | 8 | 0 | 0.000000 | -0.000000 | 1472 | 1166.907699 | 1244.36 | 1321.812301 | 0.845353 |
| GO:0042104\_positive\_regulation\_of\_activated\_T\_cell\_proliferation | 8 | 0 | 0.000000 | -0.000000 | 1472 | 1166.907699 | 1244.36 | 1321.812301 | 0.845353 |
| GO:0042226\_interleukin-6\_biosynthetic\_process | 8 | 0 | 0.000000 | -0.000000 | 1472 | 1166.907699 | 1244.36 | 1321.812301 | 0.845353 |
| GO:0042771\_DNA\_damage\_response\_\_signal\_transduction\_by\_p53\_class\_mediator\_resulting\_in\_induction\_of\_apoptosis | 8 | 0 | 0.000000 | -0.000000 | 1472 | 1166.907699 | 1244.36 | 1321.812301 | 0.845353 |
| GO:0042990\_regulation\_of\_transcription\_factor\_import\_into\_nucleus | 8 | 0 | 0.000000 | -0.000000 | 1472 | 1166.907699 | 1244.36 | 1321.812301 | 0.845353 |
| GO:0042991\_transcription\_factor\_import\_into\_nucleus | 8 | 0 | 0.000000 | -0.000000 | 1472 | 1166.907699 | 1244.36 | 1321.812301 | 0.845353 |
| GO:0043011\_myeloid\_dendritic\_cell\_differentiation | 8 | 0 | 0.000000 | -0.000000 | 1472 | 1166.907699 | 1244.36 | 1321.812301 | 0.845353 |
| GO:0043370\_regulation\_of\_CD4-positive\_\_alpha\_beta\_T\_cell\_differentiation | 8 | 0 | 0.000000 | -0.000000 | 1472 | 1166.907699 | 1244.36 | 1321.812301 | 0.845353 |
| GO:0043542\_endothelial\_cell\_migration | 8 | 0 | 0.000000 | -0.000000 | 1472 | 1166.907699 | 1244.36 | 1321.812301 | 0.845353 |
| GO:0043616\_keratinocyte\_proliferation | 8 | 0 | 0.000000 | -0.000000 | 1472 | 1166.907699 | 1244.36 | 1321.812301 | 0.845353 |
| GO:0045075\_regulation\_of\_interleukin-12\_biosynthetic\_process | 8 | 0 | 0.000000 | -0.000000 | 1472 | 1166.907699 | 1244.36 | 1321.812301 | 0.845353 |
| GO:0045086\_positive\_regulation\_of\_interleukin-2\_biosynthetic\_process | 8 | 0 | 0.000000 | -0.000000 | 1472 | 1166.907699 | 1244.36 | 1321.812301 | 0.845353 |
| GO:0045351\_type\_I\_interferon\_biosynthetic\_process | 8 | 0 | 0.000000 | -0.000000 | 1472 | 1166.907699 | 1244.36 | 1321.812301 | 0.845353 |
| GO:0045408\_regulation\_of\_interleukin-6\_biosynthetic\_process | 8 | 0 | 0.000000 | -0.000000 | 1472 | 1166.907699 | 1244.36 | 1321.812301 | 0.845353 |
| GO:0045429\_positive\_regulation\_of\_nitric\_oxide\_biosynthetic\_process | 8 | 0 | 0.000000 | -0.000000 | 1472 | 1166.907699 | 1244.36 | 1321.812301 | 0.845353 |
| GO:0045494\_photoreceptor\_cell\_maintenance | 8 | 0 | 0.000000 | -0.000000 | 1472 | 1166.907699 | 1244.36 | 1321.812301 | 0.845353 |
| GO:0045910\_negative\_regulation\_of\_DNA\_recombination | 8 | 0 | 0.000000 | -0.000000 | 1472 | 1166.907699 | 1244.36 | 1321.812301 | 0.845353 |
| GO:0045921\_positive\_regulation\_of\_exocytosis | 8 | 0 | 0.000000 | -0.000000 | 1472 | 1166.907699 | 1244.36 | 1321.812301 | 0.845353 |
| GO:0045932\_negative\_regulation\_of\_muscle\_contraction | 8 | 0 | 0.000000 | -0.000000 | 1472 | 1166.907699 | 1244.36 | 1321.812301 | 0.845353 |
| GO:0048266\_behavioral\_response\_to\_pain | 8 | 0 | 0.000000 | -0.000000 | 1472 | 1166.907699 | 1244.36 | 1321.812301 | 0.845353 |
| GO:0048505\_regulation\_of\_timing\_of\_cell\_differentiation | 8 | 0 | 0.000000 | -0.000000 | 1472 | 1166.907699 | 1244.36 | 1321.812301 | 0.845353 |
| GO:0048557\_embryonic\_digestive\_tract\_morphogenesis | 8 | 0 | 0.000000 | -0.000000 | 1472 | 1166.907699 | 1244.36 | 1321.812301 | 0.845353 |
| GO:0048638\_regulation\_of\_developmental\_growth | 8 | 0 | 0.000000 | -0.000000 | 1472 | 1166.907699 | 1244.36 | 1321.812301 | 0.845353 |
| GO:0050707\_regulation\_of\_cytokine\_secretion | 8 | 0 | 0.000000 | -0.000000 | 1472 | 1166.907699 | 1244.36 | 1321.812301 | 0.845353 |
| GO:0050909\_sensory\_perception\_of\_taste | 8 | 0 | 0.000000 | -0.000000 | 1472 | 1166.907699 | 1244.36 | 1321.812301 | 0.845353 |
| GO:0051084\_'de\_novo'\_posttranslational\_protein\_folding | 8 | 0 | 0.000000 | -0.000000 | 1472 | 1166.907699 | 1244.36 | 1321.812301 | 0.845353 |
| GO:0051181\_cofactor\_transport | 8 | 0 | 0.000000 | -0.000000 | 1472 | 1166.907699 | 1244.36 | 1321.812301 | 0.845353 |
| GO:0060043\_regulation\_of\_cardiac\_muscle\_cell\_proliferation | 8 | 0 | 0.000000 | -0.000000 | 1472 | 1166.907699 | 1244.36 | 1321.812301 | 0.845353 |
| GO:0060347\_heart\_trabecula\_formation | 8 | 0 | 0.000000 | -0.000000 | 1472 | 1166.907699 | 1244.36 | 1321.812301 | 0.845353 |
| GO:0060670\_branching\_involved\_in\_embryonic\_placenta\_morphogenesis | 8 | 0 | 0.000000 | -0.000000 | 1472 | 1166.907699 | 1244.36 | 1321.812301 | 0.845353 |
| GO:0060712\_spongiotrophoblast\_layer\_development | 8 | 0 | 0.000000 | -0.000000 | 1472 | 1166.907699 | 1244.36 | 1321.812301 | 0.845353 |
| GO:0070167\_regulation\_of\_biomineral\_formation | 8 | 0 | 0.000000 | -0.000000 | 1472 | 1166.907699 | 1244.36 | 1321.812301 | 0.845353 |
| GO:0070193\_synaptonemal\_complex\_organization | 8 | 0 | 0.000000 | -0.000000 | 1472 | 1166.907699 | 1244.36 | 1321.812301 | 0.845353 |
| GO:0070231\_T\_cell\_apoptosis | 8 | 0 | 0.000000 | -0.000000 | 1472 | 1166.907699 | 1244.36 | 1321.812301 | 0.845353 |
| GO:0070584\_mitochondrion\_morphogenesis | 8 | 0 | 0.000000 | -0.000000 | 1472 | 1166.907699 | 1244.36 | 1321.812301 | 0.845353 |
| GO:0001824\_blastocyst\_development | 40 | 0 | 0.000000 | -0.000000 | 1477 | 1174.893020 | 1251.78 | 1328.666980 | 0.847515 |
| GO:0007599\_hemostasis | 40 | 0 | 0.000000 | -0.000000 | 1477 | 1174.893020 | 1251.78 | 1328.666980 | 0.847515 |
| GO:0008203\_cholesterol\_metabolic\_process | 40 | 0 | 0.000000 | -0.000000 | 1477 | 1174.893020 | 1251.78 | 1328.666980 | 0.847515 |
| GO:0016485\_protein\_processing | 40 | 0 | 0.000000 | -0.000000 | 1477 | 1174.893020 | 1251.78 | 1328.666980 | 0.847515 |
| GO:0035272\_exocrine\_system\_development | 40 | 0 | 0.000000 | -0.000000 | 1477 | 1174.893020 | 1251.78 | 1328.666980 | 0.847515 |
| GO:0000027\_ribosomal\_large\_subunit\_assembly | 1 | 0 |  |  |  |  |  |  |  |  |
| GO:0000042\_protein\_targeting\_to\_Golgi | 1 | 0 |  |  |  |  |  |  |  |  |
| GO:0000046\_autophagic\_vacuole\_fusion | 1 | 0 |  |  |  |  |  |  |  |  |
| GO:0000050\_urea\_cycle | 1 | 0 |  |  |  |  |  |  |  |  |
| GO:0000054\_ribosome\_export\_from\_nucleus | 1 | 0 |  |  |  |  |  |  |  |  |
| GO:0000055\_ribosomal\_large\_subunit\_export\_from\_nucleus | 1 | 0 |  |  |  |  |  |  |  |  |
| GO:0000056\_ribosomal\_small\_subunit\_export\_from\_nucleus | 1 | 0 |  |  |  |  |  |  |  |  |
| GO:0000072\_M\_phase\_specific\_microtubule\_process | 1 | 0 |  |  |  |  |  |  |  |  |
| GO:0000101\_sulfur\_amino\_acid\_transport | 1 | 0 |  |  |  |  |  |  |  |  |
| GO:0000147\_actin\_cortical\_patch\_assembly | 1 | 0 |  |  |  |  |  |  |  |  |
| GO:0000154\_rRNA\_modification | 1 | 0 |  |  |  |  |  |  |  |  |
| GO:0000183\_chromatin\_silencing\_at\_rDNA | 1 | 0 |  |  |  |  |  |  |  |  |
| GO:0000185\_activation\_of\_MAPKKK\_activity | 1 | 0 |  |  |  |  |  |  |  |  |
| GO:0000238\_zygotene | 1 | 0 |  |  |  |  |  |  |  |  |
| GO:0000255\_allantoin\_metabolic\_process | 1 | 0 |  |  |  |  |  |  |  |  |
| GO:0000266\_mitochondrial\_fission | 1 | 0 |  |  |  |  |  |  |  |  |
| GO:0000273\_lipoic\_acid\_metabolic\_process | 1 | 0 |  |  |  |  |  |  |  |  |
| GO:0000301\_retrograde\_transport\_\_vesicle\_recycling\_within\_Golgi | 1 | 0 |  |  |  |  |  |  |  |  |
| GO:0000394\_RNA\_splicing\_\_via\_endonucleolytic\_cleavage\_and\_ligation | 1 | 0 |  |  |  |  |  |  |  |  |
| GO:0000429\_regulation\_of\_transcription\_from\_RNA\_polymerase\_II\_promoter\_by\_carbon\_catabolites | 1 | 0 |  |  |  |  |  |  |  |  |
| GO:0000430\_regulation\_of\_transcription\_from\_RNA\_polymerase\_II\_promoter\_by\_glucose | 1 | 0 |  |  |  |  |  |  |  |  |
| GO:0000432\_positive\_regulation\_of\_transcription\_from\_RNA\_polymerase\_II\_promoter\_by\_glucose | 1 | 0 |  |  |  |  |  |  |  |  |
| GO:0000436\_positive\_regulation\_of\_transcription\_from\_RNA\_polymerase\_II\_promoter\_by\_carbon\_catabolites | 1 | 0 |  |  |  |  |  |  |  |  |
| GO:0000448\_cleavage\_in\_ITS2\_between\_5.8S\_rRNA\_and\_LSU-rRNA\_of\_tricistronic\_rRNA\_transcript\_(SSU-rRNA\_\_5.8S\_rRNA\_\_LSU-rRNA) | 1 | 0 |  |  |  |  |  |  |  |  |
| GO:0000460\_maturation\_of\_5.8S\_rRNA | 1 | 0 |  |  |  |  |  |  |  |  |
| GO:0000463\_maturation\_of\_LSU-rRNA\_from\_tricistronic\_rRNA\_transcript\_(SSU-rRNA\_\_5.8S\_rRNA\_\_LSU-rRNA) | 1 | 0 |  |  |  |  |  |  |  |  |
| GO:0000466\_maturation\_of\_5.8S\_rRNA\_from\_tricistronic\_rRNA\_transcript\_(SSU-rRNA\_\_5.8S\_rRNA\_\_LSU-rRNA) | 1 | 0 |  |  |  |  |  |  |  |  |
| GO:0000469\_cleavages\_during\_rRNA\_processing | 1 | 0 |  |  |  |  |  |  |  |  |
| GO:0000470\_maturation\_of\_LSU-rRNA | 1 | 0 |  |  |  |  |  |  |  |  |
| GO:0000478\_endonucleolytic\_cleavages\_during\_rRNA\_processing | 1 | 0 |  |  |  |  |  |  |  |  |
| GO:0000479\_endonucleolytic\_cleavage\_of\_tricistronic\_rRNA\_transcript\_(SSU-rRNA\_\_5.8S\_rRNA\_\_LSU-rRNA) | 1 | 0 |  |  |  |  |  |  |  |  |
| GO:0000705\_achiasmate\_meiosis\_I | 1 | 0 |  |  |  |  |  |  |  |  |
| GO:0000966\_RNA\_5'-end\_processing | 1 | 0 |  |  |  |  |  |  |  |  |
| GO:0001300\_chronological\_cell\_aging | 1 | 0 |  |  |  |  |  |  |  |  |
| GO:0001547\_antral\_ovarian\_follicle\_growth | 1 | 0 |  |  |  |  |  |  |  |  |
| GO:0001555\_oocyte\_growth | 1 | 0 |  |  |  |  |  |  |  |  |
| GO:0001560\_regulation\_of\_cell\_growth\_by\_extracellular\_stimulus | 1 | 0 |  |  |  |  |  |  |  |  |
| GO:0001660\_fever | 1 | 0 |  |  |  |  |  |  |  |  |
| GO:0001696\_gastric\_acid\_secretion | 1 | 0 |  |  |  |  |  |  |  |  |
| GO:0001712\_ectodermal\_cell\_fate\_commitment | 1 | 0 |  |  |  |  |  |  |  |  |
| GO:0001714\_endodermal\_cell\_fate\_specification | 1 | 0 |  |  |  |  |  |  |  |  |
| GO:0001762\_beta-alanine\_transport | 1 | 0 |  |  |  |  |  |  |  |  |
| GO:0001766\_membrane\_raft\_polarization | 1 | 0 |  |  |  |  |  |  |  |  |
| GO:0001811\_negative\_regulation\_of\_type\_I\_hypersensitivity | 1 | 0 |  |  |  |  |  |  |  |  |
| GO:0001821\_histamine\_secretion | 1 | 0 |  |  |  |  |  |  |  |  |
| GO:0001826\_inner\_cell\_mass\_cell\_differentiation | 1 | 0 |  |  |  |  |  |  |  |  |
| GO:0001830\_trophectodermal\_cell\_fate\_commitment | 1 | 0 |  |  |  |  |  |  |  |  |
| GO:0001834\_trophectodermal\_cell\_proliferation | 1 | 0 |  |  |  |  |  |  |  |  |
| GO:0001867\_complement\_activation\_\_lectin\_pathway | 1 | 0 |  |  |  |  |  |  |  |  |
| GO:0001880\_Mullerian\_duct\_regression | 1 | 0 |  |  |  |  |  |  |  |  |
| GO:0001887\_selenium\_metabolic\_process | 1 | 0 |  |  |  |  |  |  |  |  |
| GO:0001922\_B-1\_B\_cell\_homeostasis | 1 | 0 |  |  |  |  |  |  |  |  |
| GO:0001923\_B-1\_B\_cell\_differentiation | 1 | 0 |  |  |  |  |  |  |  |  |
| GO:0001941\_postsynaptic\_membrane\_organization | 1 | 0 |  |  |  |  |  |  |  |  |
| GO:0001946\_lymphangiogenesis | 1 | 0 |  |  |  |  |  |  |  |  |
| GO:0001961\_positive\_regulation\_of\_cytokine-mediated\_signaling\_pathway | 1 | 0 |  |  |  |  |  |  |  |  |
| GO:0001979\_regulation\_of\_systemic\_arterial\_blood\_pressure\_by\_chemoreceptor\_signaling | 1 | 0 |  |  |  |  |  |  |  |  |
| GO:0001980\_regulation\_of\_systemic\_arterial\_blood\_pressure\_by\_ischemic\_conditions | 1 | 0 |  |  |  |  |  |  |  |  |
| GO:0001984\_vasodilation\_of\_artery\_during\_baroreceptor\_response\_to\_increased\_systemic\_arterial\_blood\_pressure | 1 | 0 |  |  |  |  |  |  |  |  |
| GO:0001985\_negative\_regulation\_of\_heart\_rate\_in\_baroreceptor\_response\_to\_increased\_systemic\_arterial\_blood\_pressure | 1 | 0 |  |  |  |  |  |  |  |  |
| GO:0001987\_vasoconstriction\_of\_artery\_involved\_in\_baroreceptor\_response\_to\_lowering\_of\_systemic\_arterial\_blood\_pressure | 1 | 0 |  |  |  |  |  |  |  |  |
| GO:0001988\_positive\_regulation\_of\_heart\_rate\_in\_baroreceptor\_response\_to\_decreased\_systemic\_arterial\_blood\_pressure | 1 | 0 |  |  |  |  |  |  |  |  |
| GO:0001994\_norepinephrine-epinephrine\_vasoconstriction\_involved\_in\_regulation\_of\_systemic\_arterial\_blood\_pressure | 1 | 0 |  |  |  |  |  |  |  |  |
| GO:0002001\_renin\_secretion\_into\_blood\_stream | 1 | 0 |  |  |  |  |  |  |  |  |
| GO:0002002\_regulation\_of\_angiotensin\_levels\_in\_blood | 1 | 0 |  |  |  |  |  |  |  |  |
| GO:0002003\_angiotensin\_maturation | 1 | 0 |  |  |  |  |  |  |  |  |
| GO:0002007\_detection\_of\_hypoxic\_conditions\_in\_blood\_by\_chemoreceptor\_signaling | 1 | 0 |  |  |  |  |  |  |  |  |
| GO:0002017\_regulation\_of\_blood\_volume\_by\_renal\_aldosterone | 1 | 0 |  |  |  |  |  |  |  |  |
| GO:0002023\_reduction\_of\_food\_intake\_in\_response\_to\_dietary\_excess | 1 | 0 |  |  |  |  |  |  |  |  |
| GO:0002031\_G-protein\_coupled\_receptor\_internalization | 1 | 0 |  |  |  |  |  |  |  |  |
| GO:0002036\_regulation\_of\_L-glutamate\_transport | 1 | 0 |  |  |  |  |  |  |  |  |
| GO:0002040\_sprouting\_angiogenesis | 1 | 0 |  |  |  |  |  |  |  |  |
| GO:0002041\_intussusceptive\_angiogenesis | 1 | 0 |  |  |  |  |  |  |  |  |
| GO:0002068\_glandular\_epithelial\_cell\_development | 1 | 0 |  |  |  |  |  |  |  |  |
| GO:0002069\_columnar\_cuboidal\_epithelial\_cell\_maturation | 1 | 0 |  |  |  |  |  |  |  |  |
| GO:0002071\_glandular\_epithelial\_cell\_maturation | 1 | 0 |  |  |  |  |  |  |  |  |
| GO:0002082\_regulation\_of\_oxidative\_phosphorylation | 1 | 0 |  |  |  |  |  |  |  |  |
| GO:0002084\_protein\_depalmitoylation | 1 | 0 |  |  |  |  |  |  |  |  |
| GO:0002085\_inhibition\_of\_neuroepithelial\_cell\_differentiation | 1 | 0 |  |  |  |  |  |  |  |  |
| GO:0002086\_diaphragm\_contraction | 1 | 0 |  |  |  |  |  |  |  |  |
| GO:0002118\_aggressive\_behavior | 1 | 0 |  |  |  |  |  |  |  |  |
| GO:0002121\_inter-male\_aggressive\_behavior | 1 | 0 |  |  |  |  |  |  |  |  |
| GO:0002124\_territorial\_aggressive\_behavior | 1 | 0 |  |  |  |  |  |  |  |  |
| GO:0002227\_innate\_immune\_response\_in\_mucosa | 1 | 0 |  |  |  |  |  |  |  |  |
| GO:0002232\_leukocyte\_chemotaxis\_during\_inflammatory\_response | 1 | 0 |  |  |  |  |  |  |  |  |
| GO:0002248\_connective\_tissue\_replacement\_during\_inflammatory\_response | 1 | 0 |  |  |  |  |  |  |  |  |
| GO:0002282\_microglial\_cell\_activation\_during\_immune\_response | 1 | 0 |  |  |  |  |  |  |  |  |
| GO:0002287\_alpha-beta\_T\_cell\_activation\_during\_immune\_response | 1 | 0 |  |  |  |  |  |  |  |  |
| GO:0002314\_germinal\_center\_B\_cell\_differentiation | 1 | 0 |  |  |  |  |  |  |  |  |
| GO:0002315\_marginal\_zone\_B\_cell\_differentiation | 1 | 0 |  |  |  |  |  |  |  |  |
| GO:0002316\_follicular\_B\_cell\_differentiation | 1 | 0 |  |  |  |  |  |  |  |  |
| GO:0002317\_plasma\_cell\_differentiation | 1 | 0 |  |  |  |  |  |  |  |  |
| GO:0002349\_histamine\_production\_during\_acute\_inflammatory\_response | 1 | 0 |  |  |  |  |  |  |  |  |
| GO:0002351\_serotonin\_production\_during\_acute\_inflammatory\_response | 1 | 0 |  |  |  |  |  |  |  |  |
| GO:0002355\_detection\_of\_tumor\_cell | 1 | 0 |  |  |  |  |  |  |  |  |
| GO:0002370\_natural\_killer\_cell\_cytokine\_production | 1 | 0 |  |  |  |  |  |  |  |  |
| GO:0002371\_dendritic\_cell\_cytokine\_production | 1 | 0 |  |  |  |  |  |  |  |  |
| GO:0002380\_immunoglobulin\_secretion\_during\_immune\_response | 1 | 0 |  |  |  |  |  |  |  |  |
| GO:0002396\_MHC\_protein\_complex\_assembly | 1 | 0 |  |  |  |  |  |  |  |  |
| GO:0002397\_MHC\_class\_I\_protein\_complex\_assembly | 1 | 0 |  |  |  |  |  |  |  |  |
| GO:0002420\_natural\_killer\_cell\_mediated\_cytotoxicity\_directed\_against\_tumor\_cell\_target | 1 | 0 |  |  |  |  |  |  |  |  |
| GO:0002423\_natural\_killer\_cell\_mediated\_immune\_response\_to\_tumor\_cell | 1 | 0 |  |  |  |  |  |  |  |  |
| GO:0002424\_T\_cell\_mediated\_immune\_response\_to\_tumor\_cell | 1 | 0 |  |  |  |  |  |  |  |  |
| GO:0002426\_immunoglobulin\_production\_in\_mucosal\_tissue | 1 | 0 |  |  |  |  |  |  |  |  |
| GO:0002431\_Fc\_receptor\_mediated\_stimulatory\_signaling\_pathway | 1 | 0 |  |  |  |  |  |  |  |  |
| GO:0002432\_granuloma\_formation | 1 | 0 |  |  |  |  |  |  |  |  |
| GO:0002441\_histamine\_secretion\_during\_acute\_inflammatory\_response | 1 | 0 |  |  |  |  |  |  |  |  |
| GO:0002442\_serotonin\_secretion\_during\_acute\_inflammatory\_response | 1 | 0 |  |  |  |  |  |  |  |  |
| GO:0002457\_T\_cell\_antigen\_processing\_and\_presentation | 1 | 0 |  |  |  |  |  |  |  |  |
| GO:0002458\_peripheral\_T\_cell\_tolerance\_induction | 1 | 0 |  |  |  |  |  |  |  |  |
| GO:0002461\_tolerance\_induction\_dependent\_upon\_immune\_response | 1 | 0 |  |  |  |  |  |  |  |  |
| GO:0002465\_peripheral\_tolerance\_induction | 1 | 0 |  |  |  |  |  |  |  |  |
| GO:0002476\_antigen\_processing\_and\_presentation\_of\_endogenous\_peptide\_antigen\_via\_MHC\_class\_Ib | 1 | 0 |  |  |  |  |  |  |  |  |
| GO:0002479\_antigen\_processing\_and\_presentation\_of\_exogenous\_peptide\_antigen\_via\_MHC\_class\_I\_\_TAP-dependent | 1 | 0 |  |  |  |  |  |  |  |  |
| GO:0002483\_antigen\_processing\_and\_presentation\_of\_endogenous\_peptide\_antigen | 1 | 0 |  |  |  |  |  |  |  |  |
| GO:0002501\_peptide\_antigen\_assembly\_with\_MHC\_protein\_complex | 1 | 0 |  |  |  |  |  |  |  |  |
| GO:0002502\_peptide\_antigen\_assembly\_with\_MHC\_class\_I\_protein\_complex | 1 | 0 |  |  |  |  |  |  |  |  |
| GO:0002508\_central\_tolerance\_induction | 1 | 0 |  |  |  |  |  |  |  |  |
| GO:0002510\_central\_B\_cell\_tolerance\_induction | 1 | 0 |  |  |  |  |  |  |  |  |
| GO:0002545\_chronic\_inflammatory\_response\_to\_non-antigenic\_stimulus | 1 | 0 |  |  |  |  |  |  |  |  |
| GO:0002553\_histamine\_secretion\_by\_mast\_cell | 1 | 0 |  |  |  |  |  |  |  |  |
| GO:0002554\_serotonin\_secretion\_by\_platelet | 1 | 0 |  |  |  |  |  |  |  |  |
| GO:0002572\_pro-T\_cell\_differentiation | 1 | 0 |  |  |  |  |  |  |  |  |
| GO:0002635\_negative\_regulation\_of\_germinal\_center\_formation | 1 | 0 |  |  |  |  |  |  |  |  |
| GO:0002646\_regulation\_of\_central\_tolerance\_induction | 1 | 0 |  |  |  |  |  |  |  |  |
| GO:0002648\_positive\_regulation\_of\_central\_tolerance\_induction | 1 | 0 |  |  |  |  |  |  |  |  |
| GO:0002649\_regulation\_of\_tolerance\_induction\_to\_self\_antigen | 1 | 0 |  |  |  |  |  |  |  |  |
| GO:0002651\_positive\_regulation\_of\_tolerance\_induction\_to\_self\_antigen | 1 | 0 |  |  |  |  |  |  |  |  |
| GO:0002652\_regulation\_of\_tolerance\_induction\_dependent\_upon\_immune\_response | 1 | 0 |  |  |  |  |  |  |  |  |
| GO:0002654\_positive\_regulation\_of\_tolerance\_induction\_dependent\_upon\_immune\_response | 1 | 0 |  |  |  |  |  |  |  |  |
| GO:0002658\_regulation\_of\_peripheral\_tolerance\_induction | 1 | 0 |  |  |  |  |  |  |  |  |
| GO:0002660\_positive\_regulation\_of\_peripheral\_tolerance\_induction | 1 | 0 |  |  |  |  |  |  |  |  |
| GO:0002677\_negative\_regulation\_of\_chronic\_inflammatory\_response | 1 | 0 |  |  |  |  |  |  |  |  |
| GO:0002678\_positive\_regulation\_of\_chronic\_inflammatory\_response | 1 | 0 |  |  |  |  |  |  |  |  |
| GO:0002701\_negative\_regulation\_of\_production\_of\_molecular\_mediator\_of\_immune\_response | 1 | 0 |  |  |  |  |  |  |  |  |
| GO:0002719\_negative\_regulation\_of\_cytokine\_production\_during\_immune\_response | 1 | 0 |  |  |  |  |  |  |  |  |
| GO:0002724\_regulation\_of\_T\_cell\_cytokine\_production | 1 | 0 |  |  |  |  |  |  |  |  |
| GO:0002727\_regulation\_of\_natural\_killer\_cell\_cytokine\_production | 1 | 0 |  |  |  |  |  |  |  |  |
| GO:0002729\_positive\_regulation\_of\_natural\_killer\_cell\_cytokine\_production | 1 | 0 |  |  |  |  |  |  |  |  |
| GO:0002730\_regulation\_of\_dendritic\_cell\_cytokine\_production | 1 | 0 |  |  |  |  |  |  |  |  |
| GO:0002756\_MyD88-independent\_toll-like\_receptor\_signaling\_pathway | 1 | 0 |  |  |  |  |  |  |  |  |
| GO:0002767\_immune\_response-inhibiting\_cell\_surface\_receptor\_signaling\_pathway | 1 | 0 |  |  |  |  |  |  |  |  |
| GO:0002769\_natural\_killer\_cell\_inhibitory\_signaling\_pathway | 1 | 0 |  |  |  |  |  |  |  |  |
| GO:0002840\_regulation\_of\_T\_cell\_mediated\_immune\_response\_to\_tumor\_cell | 1 | 0 |  |  |  |  |  |  |  |  |
| GO:0002842\_positive\_regulation\_of\_T\_cell\_mediated\_immune\_response\_to\_tumor\_cell | 1 | 0 |  |  |  |  |  |  |  |  |
| GO:0002849\_regulation\_of\_peripheral\_T\_cell\_tolerance\_induction | 1 | 0 |  |  |  |  |  |  |  |  |
| GO:0002851\_positive\_regulation\_of\_peripheral\_T\_cell\_tolerance\_induction | 1 | 0 |  |  |  |  |  |  |  |  |
| GO:0002855\_regulation\_of\_natural\_killer\_cell\_mediated\_immune\_response\_to\_tumor\_cell | 1 | 0 |  |  |  |  |  |  |  |  |
| GO:0002857\_positive\_regulation\_of\_natural\_killer\_cell\_mediated\_immune\_response\_to\_tumor\_cell | 1 | 0 |  |  |  |  |  |  |  |  |
| GO:0002858\_regulation\_of\_natural\_killer\_cell\_mediated\_cytotoxicity\_directed\_against\_tumor\_cell\_target | 1 | 0 |  |  |  |  |  |  |  |  |
| GO:0002860\_positive\_regulation\_of\_natural\_killer\_cell\_mediated\_cytotoxicity\_directed\_against\_tumor\_cell\_target | 1 | 0 |  |  |  |  |  |  |  |  |
| GO:0002880\_regulation\_of\_chronic\_inflammatory\_response\_to\_non-antigenic\_stimulus | 1 | 0 |  |  |  |  |  |  |  |  |
| GO:0002882\_positive\_regulation\_of\_chronic\_inflammatory\_response\_to\_non-antigenic\_stimulus | 1 | 0 |  |  |  |  |  |  |  |  |
| GO:0002895\_regulation\_of\_central\_B\_cell\_tolerance\_induction | 1 | 0 |  |  |  |  |  |  |  |  |
| GO:0002897\_positive\_regulation\_of\_central\_B\_cell\_tolerance\_induction | 1 | 0 |  |  |  |  |  |  |  |  |
| GO:0002901\_mature\_B\_cell\_apoptosis | 1 | 0 |  |  |  |  |  |  |  |  |
| GO:0002903\_negative\_regulation\_of\_B\_cell\_apoptosis | 1 | 0 |  |  |  |  |  |  |  |  |
| GO:0002905\_regulation\_of\_mature\_B\_cell\_apoptosis | 1 | 0 |  |  |  |  |  |  |  |  |
| GO:0002906\_negative\_regulation\_of\_mature\_B\_cell\_apoptosis | 1 | 0 |  |  |  |  |  |  |  |  |
| GO:0003011\_involuntary\_skeletal\_muscle\_contraction | 1 | 0 |  |  |  |  |  |  |  |  |
| GO:0003027\_regulation\_of\_systemic\_arterial\_blood\_pressure\_by\_carotid\_body\_chemoreceptor\_signaling | 1 | 0 |  |  |  |  |  |  |  |  |
| GO:0003029\_detection\_of\_hypoxic\_conditions\_in\_blood\_by\_carotid\_body\_chemoreceptor\_signaling | 1 | 0 |  |  |  |  |  |  |  |  |
| GO:0003032\_detection\_of\_oxygen | 1 | 0 |  |  |  |  |  |  |  |  |
| GO:0003056\_regulation\_of\_vascular\_smooth\_muscle\_contraction | 1 | 0 |  |  |  |  |  |  |  |  |
| GO:0003062\_regulation\_of\_heart\_rate\_by\_chemical\_signal | 1 | 0 |  |  |  |  |  |  |  |  |
| GO:0003065\_positive\_regulation\_of\_heart\_rate\_by\_epinephrine | 1 | 0 |  |  |  |  |  |  |  |  |
| GO:0003068\_regulation\_of\_systemic\_arterial\_blood\_pressure\_by\_acetylcholine | 1 | 0 |  |  |  |  |  |  |  |  |
| GO:0003069\_vasodilation\_by\_acetylcholine\_involved\_in\_regulation\_of\_systemic\_arterial\_blood\_pressure | 1 | 0 |  |  |  |  |  |  |  |  |
| GO:0003070\_regulation\_of\_systemic\_arterial\_blood\_pressure\_by\_neurotransmitter | 1 | 0 |  |  |  |  |  |  |  |  |
| GO:0003097\_renal\_water\_transport | 1 | 0 |  |  |  |  |  |  |  |  |
| GO:0005979\_regulation\_of\_glycogen\_biosynthetic\_process | 1 | 0 |  |  |  |  |  |  |  |  |
| GO:0005984\_disaccharide\_metabolic\_process | 1 | 0 |  |  |  |  |  |  |  |  |
| GO:0005988\_lactose\_metabolic\_process | 1 | 0 |  |  |  |  |  |  |  |  |
| GO:0005989\_lactose\_biosynthetic\_process | 1 | 0 |  |  |  |  |  |  |  |  |
| GO:0005997\_xylulose\_metabolic\_process | 1 | 0 |  |  |  |  |  |  |  |  |
| GO:0006004\_fucose\_metabolic\_process | 1 | 0 |  |  |  |  |  |  |  |  |
| GO:0006013\_mannose\_metabolic\_process | 1 | 0 |  |  |  |  |  |  |  |  |
| GO:0006060\_sorbitol\_metabolic\_process | 1 | 0 |  |  |  |  |  |  |  |  |
| GO:0006064\_glucuronate\_catabolic\_process | 1 | 0 |  |  |  |  |  |  |  |  |
| GO:0006086\_acetyl-CoA\_biosynthetic\_process\_from\_pyruvate | 1 | 0 |  |  |  |  |  |  |  |  |
| GO:0006098\_pentose-phosphate\_shunt | 1 | 0 |  |  |  |  |  |  |  |  |
| GO:0006101\_citrate\_metabolic\_process | 1 | 0 |  |  |  |  |  |  |  |  |
| GO:0006104\_succinyl-CoA\_metabolic\_process | 1 | 0 |  |  |  |  |  |  |  |  |
| GO:0006116\_NADH\_oxidation | 1 | 0 |  |  |  |  |  |  |  |  |
| GO:0006120\_mitochondrial\_electron\_transport\_\_NADH\_to\_ubiquinone | 1 | 0 |  |  |  |  |  |  |  |  |
| GO:0006154\_adenosine\_catabolic\_process | 1 | 0 |  |  |  |  |  |  |  |  |
| GO:0006157\_deoxyadenosine\_catabolic\_process | 1 | 0 |  |  |  |  |  |  |  |  |
| GO:0006167\_AMP\_biosynthetic\_process | 1 | 0 |  |  |  |  |  |  |  |  |
| GO:0006175\_dATP\_biosynthetic\_process | 1 | 0 |  |  |  |  |  |  |  |  |
| GO:0006178\_guanine\_salvage | 1 | 0 |  |  |  |  |  |  |  |  |
| GO:0006196\_AMP\_catabolic\_process | 1 | 0 |  |  |  |  |  |  |  |  |
| GO:0006203\_dGTP\_catabolic\_process | 1 | 0 |  |  |  |  |  |  |  |  |
| GO:0006208\_pyrimidine\_base\_catabolic\_process | 1 | 0 |  |  |  |  |  |  |  |  |
| GO:0006221\_pyrimidine\_nucleotide\_biosynthetic\_process | 1 | 0 |  |  |  |  |  |  |  |  |
| GO:0006235\_dTTP\_biosynthetic\_process | 1 | 0 |  |  |  |  |  |  |  |  |
| GO:0006244\_pyrimidine\_nucleotide\_catabolic\_process | 1 | 0 |  |  |  |  |  |  |  |  |
| GO:0006269\_DNA\_replication\_\_synthesis\_of\_RNA\_primer | 1 | 0 |  |  |  |  |  |  |  |  |
| GO:0006283\_transcription-coupled\_nucleotide-excision\_repair | 1 | 0 |  |  |  |  |  |  |  |  |
| GO:0006296\_nucleotide-excision\_repair\_\_DNA\_incision\_\_5'-to\_lesion | 1 | 0 |  |  |  |  |  |  |  |  |
| GO:0006307\_DNA\_dealkylation | 1 | 0 |  |  |  |  |  |  |  |  |
| GO:0006337\_nucleosome\_disassembly | 1 | 0 |  |  |  |  |  |  |  |  |
| GO:0006344\_maintenance\_of\_chromatin\_silencing | 1 | 0 |  |  |  |  |  |  |  |  |
| GO:0006356\_regulation\_of\_transcription\_from\_RNA\_polymerase\_I\_promoter | 1 | 0 |  |  |  |  |  |  |  |  |
| GO:0006388\_tRNA\_splicing\_\_via\_endonucleolytic\_cleavage\_and\_ligation | 1 | 0 |  |  |  |  |  |  |  |  |
| GO:0006407\_rRNA\_export\_from\_nucleus | 1 | 0 |  |  |  |  |  |  |  |  |
| GO:0006419\_alanyl-tRNA\_aminoacylation | 1 | 0 |  |  |  |  |  |  |  |  |
| GO:0006434\_seryl-tRNA\_aminoacylation | 1 | 0 |  |  |  |  |  |  |  |  |
| GO:0006447\_regulation\_of\_translational\_initiation\_by\_iron | 1 | 0 |  |  |  |  |  |  |  |  |
| GO:0006463\_steroid\_hormone\_receptor\_complex\_assembly | 1 | 0 |  |  |  |  |  |  |  |  |
| GO:0006467\_protein\_thiol-disulfide\_exchange | 1 | 0 |  |  |  |  |  |  |  |  |
| GO:0006474\_N-terminal\_protein\_amino\_acid\_acetylation | 1 | 0 |  |  |  |  |  |  |  |  |
| GO:0006481\_C-terminal\_protein\_amino\_acid\_methylation | 1 | 0 |  |  |  |  |  |  |  |  |
| GO:0006488\_dolichol-linked\_oligosaccharide\_biosynthetic\_process | 1 | 0 |  |  |  |  |  |  |  |  |
| GO:0006494\_protein\_amino\_acid\_terminal\_glycosylation | 1 | 0 |  |  |  |  |  |  |  |  |
| GO:0006496\_protein\_amino\_acid\_terminal\_N-glycosylation | 1 | 0 |  |  |  |  |  |  |  |  |
| GO:0006500\_N-terminal\_protein\_palmitoylation | 1 | 0 |  |  |  |  |  |  |  |  |
| GO:0006507\_GPI\_anchor\_release | 1 | 0 |  |  |  |  |  |  |  |  |
| GO:0006537\_glutamate\_biosynthetic\_process | 1 | 0 |  |  |  |  |  |  |  |  |
| GO:0006544\_glycine\_metabolic\_process | 1 | 0 |  |  |  |  |  |  |  |  |
| GO:0006549\_isoleucine\_metabolic\_process | 1 | 0 |  |  |  |  |  |  |  |  |
| GO:0006553\_lysine\_metabolic\_process | 1 | 0 |  |  |  |  |  |  |  |  |
| GO:0006554\_lysine\_catabolic\_process | 1 | 0 |  |  |  |  |  |  |  |  |
| GO:0006556\_S-adenosylmethionine\_biosynthetic\_process | 1 | 0 |  |  |  |  |  |  |  |  |
| GO:0006559\_L-phenylalanine\_catabolic\_process | 1 | 0 |  |  |  |  |  |  |  |  |
| GO:0006569\_tryptophan\_catabolic\_process | 1 | 0 |  |  |  |  |  |  |  |  |
| GO:0006572\_tyrosine\_catabolic\_process | 1 | 0 |  |  |  |  |  |  |  |  |
| GO:0006573\_valine\_metabolic\_process | 1 | 0 |  |  |  |  |  |  |  |  |
| GO:0006581\_acetylcholine\_catabolic\_process | 1 | 0 |  |  |  |  |  |  |  |  |
| GO:0006585\_dopamine\_biosynthetic\_process\_from\_tyrosine | 1 | 0 |  |  |  |  |  |  |  |  |
| GO:0006590\_thyroid\_hormone\_generation | 1 | 0 |  |  |  |  |  |  |  |  |
| GO:0006591\_ornithine\_metabolic\_process | 1 | 0 |  |  |  |  |  |  |  |  |
| GO:0006596\_polyamine\_biosynthetic\_process | 1 | 0 |  |  |  |  |  |  |  |  |
| GO:0006597\_spermine\_biosynthetic\_process | 1 | 0 |  |  |  |  |  |  |  |  |
| GO:0006601\_creatine\_biosynthetic\_process | 1 | 0 |  |  |  |  |  |  |  |  |
| GO:0006613\_cotranslational\_protein\_targeting\_to\_membrane | 1 | 0 |  |  |  |  |  |  |  |  |
| GO:0006622\_protein\_targeting\_to\_lysosome | 1 | 0 |  |  |  |  |  |  |  |  |
| GO:0006627\_mitochondrial\_protein\_processing\_during\_import | 1 | 0 |  |  |  |  |  |  |  |  |
| GO:0006653\_lecithin\_metabolic\_process | 1 | 0 |  |  |  |  |  |  |  |  |
| GO:0006654\_phosphatidic\_acid\_biosynthetic\_process | 1 | 0 |  |  |  |  |  |  |  |  |
| GO:0006658\_phosphatidylserine\_metabolic\_process | 1 | 0 |  |  |  |  |  |  |  |  |
| GO:0006659\_phosphatidylserine\_biosynthetic\_process | 1 | 0 |  |  |  |  |  |  |  |  |
| GO:0006667\_sphinganine\_metabolic\_process | 1 | 0 |  |  |  |  |  |  |  |  |
| GO:0006668\_sphinganine-1-phosphate\_metabolic\_process | 1 | 0 |  |  |  |  |  |  |  |  |
| GO:0006678\_glucosylceramide\_metabolic\_process | 1 | 0 |  |  |  |  |  |  |  |  |
| GO:0006682\_galactosylceramide\_biosynthetic\_process | 1 | 0 |  |  |  |  |  |  |  |  |
| GO:0006685\_sphingomyelin\_catabolic\_process | 1 | 0 |  |  |  |  |  |  |  |  |
| GO:0006700\_C21-steroid\_hormone\_biosynthetic\_process | 1 | 0 |  |  |  |  |  |  |  |  |
| GO:0006705\_mineralocorticoid\_biosynthetic\_process | 1 | 0 |  |  |  |  |  |  |  |  |
| GO:0006709\_progesterone\_catabolic\_process | 1 | 0 |  |  |  |  |  |  |  |  |
| GO:0006729\_tetrahydrobiopterin\_biosynthetic\_process | 1 | 0 |  |  |  |  |  |  |  |  |
| GO:0006734\_NADH\_metabolic\_process | 1 | 0 |  |  |  |  |  |  |  |  |
| GO:0006740\_NADPH\_regeneration | 1 | 0 |  |  |  |  |  |  |  |  |
| GO:0006741\_NADP\_biosynthetic\_process | 1 | 0 |  |  |  |  |  |  |  |  |
| GO:0006743\_ubiquinone\_metabolic\_process | 1 | 0 |  |  |  |  |  |  |  |  |
| GO:0006744\_ubiquinone\_biosynthetic\_process | 1 | 0 |  |  |  |  |  |  |  |  |
| GO:0006772\_thiamin\_metabolic\_process | 1 | 0 |  |  |  |  |  |  |  |  |
| GO:0006784\_heme\_a\_biosynthetic\_process | 1 | 0 |  |  |  |  |  |  |  |  |
| GO:0006797\_polyphosphate\_metabolic\_process | 1 | 0 |  |  |  |  |  |  |  |  |
| GO:0006798\_polyphosphate\_catabolic\_process | 1 | 0 |  |  |  |  |  |  |  |  |
| GO:0006824\_cobalt\_ion\_transport | 1 | 0 |  |  |  |  |  |  |  |  |
| GO:0006842\_tricarboxylic\_acid\_transport | 1 | 0 |  |  |  |  |  |  |  |  |
| GO:0006844\_acyl\_carnitine\_transport | 1 | 0 |  |  |  |  |  |  |  |  |
| GO:0006855\_multidrug\_transport | 1 | 0 |  |  |  |  |  |  |  |  |
| GO:0006863\_purine\_transport | 1 | 0 |  |  |  |  |  |  |  |  |
| GO:0006890\_retrograde\_vesicle-mediated\_transport\_\_Golgi\_to\_ER | 1 | 0 |  |  |  |  |  |  |  |  |
| GO:0006891\_intra-Golgi\_vesicle-mediated\_transport | 1 | 0 |  |  |  |  |  |  |  |  |
| GO:0006893\_Golgi\_to\_plasma\_membrane\_transport | 1 | 0 |  |  |  |  |  |  |  |  |
| GO:0006895\_Golgi\_to\_endosome\_transport | 1 | 0 |  |  |  |  |  |  |  |  |
| GO:0006896\_Golgi\_to\_vacuole\_transport | 1 | 0 |  |  |  |  |  |  |  |  |
| GO:0006900\_membrane\_budding | 1 | 0 |  |  |  |  |  |  |  |  |
| GO:0006930\_substrate-bound\_cell\_migration\_\_cell\_extension | 1 | 0 |  |  |  |  |  |  |  |  |
| GO:0006931\_substrate-bound\_cell\_migration\_\_cell\_attachment\_to\_substrate | 1 | 0 |  |  |  |  |  |  |  |  |
| GO:0006933\_negative\_regulation\_of\_cell\_adhesion\_involved\_in\_substrate-bound\_cell\_migration | 1 | 0 |  |  |  |  |  |  |  |  |
| GO:0006957\_complement\_activation\_\_alternative\_pathway | 1 | 0 |  |  |  |  |  |  |  |  |
| GO:0006958\_complement\_activation\_\_classical\_pathway | 1 | 0 |  |  |  |  |  |  |  |  |
| GO:0006978\_DNA\_damage\_response\_\_signal\_transduction\_by\_p53\_class\_mediator\_resulting\_in\_transcription\_of\_p21\_class\_mediator | 1 | 0 |  |  |  |  |  |  |  |  |
| GO:0007016\_cytoskeletal\_anchoring\_at\_plasma\_membrane | 1 | 0 |  |  |  |  |  |  |  |  |
| GO:0007021\_tubulin\_complex\_assembly | 1 | 0 |  |  |  |  |  |  |  |  |
| GO:0007052\_mitotic\_spindle\_organization | 1 | 0 |  |  |  |  |  |  |  |  |
| GO:0007056\_spindle\_assembly\_involved\_in\_female\_meiosis | 1 | 0 |  |  |  |  |  |  |  |  |
| GO:0007057\_spindle\_assembly\_involved\_in\_female\_meiosis\_I | 1 | 0 |  |  |  |  |  |  |  |  |
| GO:0007063\_regulation\_of\_sister\_chromatid\_cohesion | 1 | 0 |  |  |  |  |  |  |  |  |
| GO:0007065\_male\_meiosis\_sister\_chromatid\_cohesion | 1 | 0 |  |  |  |  |  |  |  |  |
| GO:0007076\_mitotic\_chromosome\_condensation | 1 | 0 |  |  |  |  |  |  |  |  |
| GO:0007095\_mitotic\_cell\_cycle\_G2\_M\_transition\_DNA\_damage\_checkpoint | 1 | 0 |  |  |  |  |  |  |  |  |
| GO:0007096\_regulation\_of\_exit\_from\_mitosis | 1 | 0 |  |  |  |  |  |  |  |  |
| GO:0007158\_neuron\_adhesion | 1 | 0 |  |  |  |  |  |  |  |  |
| GO:0007168\_receptor\_guanylyl\_cyclase\_signaling\_pathway | 1 | 0 |  |  |  |  |  |  |  |  |
| GO:0007197\_inhibition\_of\_adenylate\_cyclase\_activity\_by\_muscarinic\_acetylcholine\_receptor\_signaling\_pathway | 1 | 0 |  |  |  |  |  |  |  |  |
| GO:0007207\_activation\_of\_phospholipase\_C\_activity\_by\_muscarinic\_acetylcholine\_receptor\_signaling\_pathway | 1 | 0 |  |  |  |  |  |  |  |  |
| GO:0007208\_activation\_of\_phospholipase\_C\_activity\_by\_serotonin\_receptor\_signaling\_pathway | 1 | 0 |  |  |  |  |  |  |  |  |
| GO:0007217\_tachykinin\_receptor\_signaling\_pathway | 1 | 0 |  |  |  |  |  |  |  |  |
| GO:0007221\_positive\_regulation\_of\_transcription\_of\_Notch\_receptor\_target | 1 | 0 |  |  |  |  |  |  |  |  |
| GO:0007223\_Wnt\_receptor\_signaling\_pathway\_\_calcium\_modulating\_pathway | 1 | 0 |  |  |  |  |  |  |  |  |
| GO:0007225\_patched\_ligand\_processing | 1 | 0 |  |  |  |  |  |  |  |  |
| GO:0007227\_signal\_transduction\_downstream\_of\_smoothened | 1 | 0 |  |  |  |  |  |  |  |  |
| GO:0007228\_positive\_regulation\_of\_hh\_target\_transcription\_factor\_activity | 1 | 0 |  |  |  |  |  |  |  |  |
| GO:0007231\_osmosensory\_signaling\_pathway | 1 | 0 |  |  |  |  |  |  |  |  |
| GO:0007284\_spermatogonial\_cell\_division | 1 | 0 |  |  |  |  |  |  |  |  |
| GO:0007290\_spermatid\_nucleus\_elongation | 1 | 0 |  |  |  |  |  |  |  |  |
| GO:0007296\_vitellogenesis | 1 | 0 |  |  |  |  |  |  |  |  |
| GO:0007321\_sperm\_displacement | 1 | 0 |  |  |  |  |  |  |  |  |
| GO:0007380\_specification\_of\_segmental\_identity\_\_head | 1 | 0 |  |  |  |  |  |  |  |  |
| GO:0007382\_specification\_of\_segmental\_identity\_\_maxillary\_segment | 1 | 0 |  |  |  |  |  |  |  |  |
| GO:0007400\_neuroblast\_fate\_determination | 1 | 0 |  |  |  |  |  |  |  |  |
| GO:0007402\_ganglion\_mother\_cell\_fate\_determination | 1 | 0 |  |  |  |  |  |  |  |  |
| GO:0007495\_visceral\_mesoderm-endoderm\_interaction\_involved\_in\_midgut\_development | 1 | 0 |  |  |  |  |  |  |  |  |
| GO:0007497\_posterior\_midgut\_development | 1 | 0 |  |  |  |  |  |  |  |  |
| GO:0007499\_ectoderm\_and\_mesoderm\_interaction | 1 | 0 |  |  |  |  |  |  |  |  |
| GO:0007500\_mesodermal\_cell\_fate\_determination | 1 | 0 |  |  |  |  |  |  |  |  |
| GO:0007509\_mesoderm\_migration | 1 | 0 |  |  |  |  |  |  |  |  |
| GO:0007518\_myoblast\_cell\_fate\_determination | 1 | 0 |  |  |  |  |  |  |  |  |
| GO:0007521\_muscle\_cell\_fate\_determination | 1 | 0 |  |  |  |  |  |  |  |  |
| GO:0007522\_visceral\_muscle\_development | 1 | 0 |  |  |  |  |  |  |  |  |
| GO:0007529\_establishment\_of\_synaptic\_specificity\_at\_neuromuscular\_junction | 1 | 0 |  |  |  |  |  |  |  |  |
| GO:0007538\_primary\_sex\_determination | 1 | 0 |  |  |  |  |  |  |  |  |
| GO:0007542\_primary\_sex\_determination\_\_germ-line | 1 | 0 |  |  |  |  |  |  |  |  |
| GO:0007567\_parturition | 1 | 0 |  |  |  |  |  |  |  |  |
| GO:0007614\_short-term\_memory | 1 | 0 |  |  |  |  |  |  |  |  |
| GO:0007621\_negative\_regulation\_of\_female\_receptivity | 1 | 0 |  |  |  |  |  |  |  |  |
| GO:0008049\_male\_courtship\_behavior | 1 | 0 |  |  |  |  |  |  |  |  |
| GO:0008050\_female\_courtship\_behavior | 1 | 0 |  |  |  |  |  |  |  |  |
| GO:0008052\_sensory\_organ\_boundary\_specification | 1 | 0 |  |  |  |  |  |  |  |  |
| GO:0008054\_cyclin\_catabolic\_process | 1 | 0 |  |  |  |  |  |  |  |  |
| GO:0008057\_eye\_pigment\_granule\_organization | 1 | 0 |  |  |  |  |  |  |  |  |
| GO:0008078\_mesodermal\_cell\_migration | 1 | 0 |  |  |  |  |  |  |  |  |
| GO:0008208\_C21-steroid\_hormone\_catabolic\_process | 1 | 0 |  |  |  |  |  |  |  |  |
| GO:0008216\_spermidine\_metabolic\_process | 1 | 0 |  |  |  |  |  |  |  |  |
| GO:0008292\_acetylcholine\_biosynthetic\_process | 1 | 0 |  |  |  |  |  |  |  |  |
| GO:0008295\_spermidine\_biosynthetic\_process | 1 | 0 |  |  |  |  |  |  |  |  |
| GO:0008300\_isoprenoid\_catabolic\_process | 1 | 0 |  |  |  |  |  |  |  |  |
| GO:0008333\_endosome\_to\_lysosome\_transport | 1 | 0 |  |  |  |  |  |  |  |  |
| GO:0008355\_olfactory\_learning | 1 | 0 |  |  |  |  |  |  |  |  |
| GO:0008611\_ether\_lipid\_biosynthetic\_process | 1 | 0 |  |  |  |  |  |  |  |  |
| GO:0008626\_induction\_of\_apoptosis\_by\_granzyme | 1 | 0 |  |  |  |  |  |  |  |  |
| GO:0008633\_activation\_of\_pro-apoptotic\_gene\_products | 1 | 0 |  |  |  |  |  |  |  |  |
| GO:0008653\_lipopolysaccharide\_metabolic\_process | 1 | 0 |  |  |  |  |  |  |  |  |
| GO:0009068\_aspartate\_family\_amino\_acid\_catabolic\_process | 1 | 0 |  |  |  |  |  |  |  |  |
| GO:0009084\_glutamine\_family\_amino\_acid\_biosynthetic\_process | 1 | 0 |  |  |  |  |  |  |  |  |
| GO:0009088\_threonine\_biosynthetic\_process | 1 | 0 |  |  |  |  |  |  |  |  |
| GO:0009105\_lipoic\_acid\_biosynthetic\_process | 1 | 0 |  |  |  |  |  |  |  |  |
| GO:0009109\_coenzyme\_catabolic\_process | 1 | 0 |  |  |  |  |  |  |  |  |
| GO:0009111\_vitamin\_catabolic\_process | 1 | 0 |  |  |  |  |  |  |  |  |
| GO:0009113\_purine\_base\_biosynthetic\_process | 1 | 0 |  |  |  |  |  |  |  |  |
| GO:0009127\_purine\_nucleoside\_monophosphate\_biosynthetic\_process | 1 | 0 |  |  |  |  |  |  |  |  |
| GO:0009128\_purine\_nucleoside\_monophosphate\_catabolic\_process | 1 | 0 |  |  |  |  |  |  |  |  |
| GO:0009129\_pyrimidine\_nucleoside\_monophosphate\_metabolic\_process | 1 | 0 |  |  |  |  |  |  |  |  |
| GO:0009131\_pyrimidine\_nucleoside\_monophosphate\_catabolic\_process | 1 | 0 |  |  |  |  |  |  |  |  |
| GO:0009133\_nucleoside\_diphosphate\_biosynthetic\_process | 1 | 0 |  |  |  |  |  |  |  |  |
| GO:0009145\_purine\_nucleoside\_triphosphate\_biosynthetic\_process | 1 | 0 |  |  |  |  |  |  |  |  |
| GO:0009147\_pyrimidine\_nucleoside\_triphosphate\_metabolic\_process | 1 | 0 |  |  |  |  |  |  |  |  |
| GO:0009148\_pyrimidine\_nucleoside\_triphosphate\_biosynthetic\_process | 1 | 0 |  |  |  |  |  |  |  |  |
| GO:0009152\_purine\_ribonucleotide\_biosynthetic\_process | 1 | 0 |  |  |  |  |  |  |  |  |
| GO:0009153\_purine\_deoxyribonucleotide\_biosynthetic\_process | 1 | 0 |  |  |  |  |  |  |  |  |
| GO:0009156\_ribonucleoside\_monophosphate\_biosynthetic\_process | 1 | 0 |  |  |  |  |  |  |  |  |
| GO:0009158\_ribonucleoside\_monophosphate\_catabolic\_process | 1 | 0 |  |  |  |  |  |  |  |  |
| GO:0009159\_deoxyribonucleoside\_monophosphate\_catabolic\_process | 1 | 0 |  |  |  |  |  |  |  |  |
| GO:0009162\_deoxyribonucleoside\_monophosphate\_metabolic\_process | 1 | 0 |  |  |  |  |  |  |  |  |
| GO:0009168\_purine\_ribonucleoside\_monophosphate\_biosynthetic\_process | 1 | 0 |  |  |  |  |  |  |  |  |
| GO:0009169\_purine\_ribonucleoside\_monophosphate\_catabolic\_process | 1 | 0 |  |  |  |  |  |  |  |  |
| GO:0009176\_pyrimidine\_deoxyribonucleoside\_monophosphate\_metabolic\_process | 1 | 0 |  |  |  |  |  |  |  |  |
| GO:0009178\_pyrimidine\_deoxyribonucleoside\_monophosphate\_catabolic\_process | 1 | 0 |  |  |  |  |  |  |  |  |
| GO:0009211\_pyrimidine\_deoxyribonucleoside\_triphosphate\_metabolic\_process | 1 | 0 |  |  |  |  |  |  |  |  |
| GO:0009212\_pyrimidine\_deoxyribonucleoside\_triphosphate\_biosynthetic\_process | 1 | 0 |  |  |  |  |  |  |  |  |
| GO:0009216\_purine\_deoxyribonucleoside\_triphosphate\_biosynthetic\_process | 1 | 0 |  |  |  |  |  |  |  |  |
| GO:0009221\_pyrimidine\_deoxyribonucleotide\_biosynthetic\_process | 1 | 0 |  |  |  |  |  |  |  |  |
| GO:0009223\_pyrimidine\_deoxyribonucleotide\_catabolic\_process | 1 | 0 |  |  |  |  |  |  |  |  |
| GO:0009260\_ribonucleotide\_biosynthetic\_process | 1 | 0 |  |  |  |  |  |  |  |  |
| GO:0009405\_pathogenesis | 1 | 0 |  |  |  |  |  |  |  |  |
| GO:0009414\_response\_to\_water\_deprivation | 1 | 0 |  |  |  |  |  |  |  |  |
| GO:0009415\_response\_to\_water | 1 | 0 |  |  |  |  |  |  |  |  |
| GO:0009449\_gamma-aminobutyric\_acid\_biosynthetic\_process | 1 | 0 |  |  |  |  |  |  |  |  |
| GO:0009450\_gamma-aminobutyric\_acid\_catabolic\_process | 1 | 0 |  |  |  |  |  |  |  |  |
| GO:0009589\_detection\_of\_UV | 1 | 0 |  |  |  |  |  |  |  |  |
| GO:0009590\_detection\_of\_gravity | 1 | 0 |  |  |  |  |  |  |  |  |
| GO:0009624\_response\_to\_nematode | 1 | 0 |  |  |  |  |  |  |  |  |
| GO:0009629\_response\_to\_gravity | 1 | 0 |  |  |  |  |  |  |  |  |
| GO:0009648\_photoperiodism | 1 | 0 |  |  |  |  |  |  |  |  |
| GO:0009690\_cytokinin\_metabolic\_process | 1 | 0 |  |  |  |  |  |  |  |  |
| GO:0009691\_cytokinin\_biosynthetic\_process | 1 | 0 |  |  |  |  |  |  |  |  |
| GO:0009786\_regulation\_of\_asymmetric\_cell\_division | 1 | 0 |  |  |  |  |  |  |  |  |
| GO:0009794\_regulation\_of\_mitotic\_cell\_cycle\_\_embryonic | 1 | 0 |  |  |  |  |  |  |  |  |
| GO:0009957\_epidermal\_cell\_fate\_specification | 1 | 0 |  |  |  |  |  |  |  |  |
| GO:0009992\_cellular\_water\_homeostasis | 1 | 0 |  |  |  |  |  |  |  |  |
| GO:0010032\_meiotic\_chromosome\_condensation | 1 | 0 |  |  |  |  |  |  |  |  |
| GO:0010039\_response\_to\_iron\_ion | 1 | 0 |  |  |  |  |  |  |  |  |
| GO:0010042\_response\_to\_manganese\_ion | 1 | 0 |  |  |  |  |  |  |  |  |
| GO:0010045\_response\_to\_nickel\_ion | 1 | 0 |  |  |  |  |  |  |  |  |
| GO:0010046\_response\_to\_mycotoxin | 1 | 0 |  |  |  |  |  |  |  |  |
| GO:0010107\_potassium\_ion\_import | 1 | 0 |  |  |  |  |  |  |  |  |
| GO:0010155\_regulation\_of\_proton\_transport | 1 | 0 |  |  |  |  |  |  |  |  |
| GO:0010160\_formation\_of\_organ\_boundary | 1 | 0 |  |  |  |  |  |  |  |  |
| GO:0010260\_organ\_senescence | 1 | 0 |  |  |  |  |  |  |  |  |
| GO:0010310\_regulation\_of\_hydrogen\_peroxide\_metabolic\_process | 1 | 0 |  |  |  |  |  |  |  |  |
| GO:0010447\_response\_to\_acidity | 1 | 0 |  |  |  |  |  |  |  |  |
| GO:0010452\_histone\_H3-K36\_methylation | 1 | 0 |  |  |  |  |  |  |  |  |
| GO:0010455\_positive\_regulation\_of\_cell\_fate\_commitment | 1 | 0 |  |  |  |  |  |  |  |  |
| GO:0010470\_regulation\_of\_gastrulation | 1 | 0 |  |  |  |  |  |  |  |  |
| GO:0010508\_positive\_regulation\_of\_autophagy | 1 | 0 |  |  |  |  |  |  |  |  |
| GO:0010519\_negative\_regulation\_of\_phospholipase\_activity | 1 | 0 |  |  |  |  |  |  |  |  |
| GO:0010520\_regulation\_of\_reciprocal\_meiotic\_recombination | 1 | 0 |  |  |  |  |  |  |  |  |
| GO:0010543\_regulation\_of\_platelet\_activation | 1 | 0 |  |  |  |  |  |  |  |  |
| GO:0010561\_negative\_regulation\_of\_glycoprotein\_biosynthetic\_process | 1 | 0 |  |  |  |  |  |  |  |  |
| GO:0010569\_regulation\_of\_double-strand\_break\_repair\_via\_homologous\_recombination | 1 | 0 |  |  |  |  |  |  |  |  |
| GO:0010572\_positive\_regulation\_of\_platelet\_activation | 1 | 0 |  |  |  |  |  |  |  |  |
| GO:0010594\_regulation\_of\_endothelial\_cell\_migration | 1 | 0 |  |  |  |  |  |  |  |  |
| GO:0010596\_negative\_regulation\_of\_endothelial\_cell\_migration | 1 | 0 |  |  |  |  |  |  |  |  |
| GO:0010611\_regulation\_of\_cardiac\_muscle\_hypertrophy | 1 | 0 |  |  |  |  |  |  |  |  |
| GO:0010612\_regulation\_of\_cardiac\_muscle\_adaptation | 1 | 0 |  |  |  |  |  |  |  |  |
| GO:0010614\_negative\_regulation\_of\_cardiac\_muscle\_hypertrophy | 1 | 0 |  |  |  |  |  |  |  |  |
| GO:0010616\_negative\_regulation\_of\_cardiac\_muscle\_adaptation | 1 | 0 |  |  |  |  |  |  |  |  |
| GO:0010634\_positive\_regulation\_of\_epithelial\_cell\_migration | 1 | 0 |  |  |  |  |  |  |  |  |
| GO:0010656\_negative\_regulation\_of\_muscle\_cell\_apoptosis | 1 | 0 |  |  |  |  |  |  |  |  |
| GO:0010657\_muscle\_cell\_apoptosis | 1 | 0 |  |  |  |  |  |  |  |  |
| GO:0010658\_striated\_muscle\_cell\_apoptosis | 1 | 0 |  |  |  |  |  |  |  |  |
| GO:0010659\_cardiac\_muscle\_cell\_apoptosis | 1 | 0 |  |  |  |  |  |  |  |  |
| GO:0010660\_regulation\_of\_muscle\_cell\_apoptosis | 1 | 0 |  |  |  |  |  |  |  |  |
| GO:0010662\_regulation\_of\_striated\_muscle\_cell\_apoptosis | 1 | 0 |  |  |  |  |  |  |  |  |
| GO:0010664\_negative\_regulation\_of\_striated\_muscle\_cell\_apoptosis | 1 | 0 |  |  |  |  |  |  |  |  |
| GO:0010665\_regulation\_of\_cardiac\_muscle\_cell\_apoptosis | 1 | 0 |  |  |  |  |  |  |  |  |
| GO:0010667\_negative\_regulation\_of\_cardiac\_muscle\_cell\_apoptosis | 1 | 0 |  |  |  |  |  |  |  |  |
| GO:0010668\_ectodermal\_cell\_differentiation | 1 | 0 |  |  |  |  |  |  |  |  |
| GO:0010671\_negative\_regulation\_of\_oxygen\_and\_reactive\_oxygen\_species\_metabolic\_process | 1 | 0 |  |  |  |  |  |  |  |  |
| GO:0010719\_negative\_regulation\_of\_epithelial\_to\_mesenchymal\_transition | 1 | 0 |  |  |  |  |  |  |  |  |
| GO:0010735\_positive\_regulation\_of\_transcription\_via\_serum\_response\_element\_binding | 1 | 0 |  |  |  |  |  |  |  |  |
| GO:0010825\_positive\_regulation\_of\_centrosome\_duplication | 1 | 0 |  |  |  |  |  |  |  |  |
| GO:0010845\_positive\_regulation\_of\_reciprocal\_meiotic\_recombination | 1 | 0 |  |  |  |  |  |  |  |  |
| GO:0010850\_chemoreceptor\_signaling\_pathway\_involved\_in\_regulation\_of\_blood\_pressure | 1 | 0 |  |  |  |  |  |  |  |  |
| GO:0010873\_positive\_regulation\_of\_cholesterol\_esterification | 1 | 0 |  |  |  |  |  |  |  |  |
| GO:0010880\_regulation\_of\_release\_of\_sequestered\_calcium\_ion\_into\_cytosol\_by\_sarcoplasmic\_reticulum | 1 | 0 |  |  |  |  |  |  |  |  |
| GO:0010881\_regulation\_of\_cardiac\_muscle\_contraction\_by\_regulation\_of\_the\_release\_of\_sequestered\_calcium\_ion | 1 | 0 |  |  |  |  |  |  |  |  |
| GO:0010882\_regulation\_of\_cardiac\_muscle\_contraction\_by\_calcium\_ion\_signaling | 1 | 0 |  |  |  |  |  |  |  |  |
| GO:0010890\_positive\_regulation\_of\_sequestering\_of\_triglyceride | 1 | 0 |  |  |  |  |  |  |  |  |
| GO:0010919\_regulation\_of\_inositol\_phosphate\_biosynthetic\_process | 1 | 0 |  |  |  |  |  |  |  |  |
| GO:0010931\_macrophage\_tolerance\_induction | 1 | 0 |  |  |  |  |  |  |  |  |
| GO:0010932\_regulation\_of\_macrophage\_tolerance\_induction | 1 | 0 |  |  |  |  |  |  |  |  |
| GO:0010933\_positive\_regulation\_of\_macrophage\_tolerance\_induction | 1 | 0 |  |  |  |  |  |  |  |  |
| GO:0010934\_macrophage\_cytokine\_production | 1 | 0 |  |  |  |  |  |  |  |  |
| GO:0010935\_regulation\_of\_macrophage\_cytokine\_production | 1 | 0 |  |  |  |  |  |  |  |  |
| GO:0010936\_negative\_regulation\_of\_macrophage\_cytokine\_production | 1 | 0 |  |  |  |  |  |  |  |  |
| GO:0010953\_regulation\_of\_protein\_maturation\_by\_peptide\_bond\_cleavage | 1 | 0 |  |  |  |  |  |  |  |  |
| GO:0010962\_regulation\_of\_glucan\_biosynthetic\_process | 1 | 0 |  |  |  |  |  |  |  |  |
| GO:0010966\_regulation\_of\_phosphate\_transport | 1 | 0 |  |  |  |  |  |  |  |  |
| GO:0014016\_neuroblast\_differentiation | 1 | 0 |  |  |  |  |  |  |  |  |
| GO:0014017\_neuroblast\_fate\_commitment | 1 | 0 |  |  |  |  |  |  |  |  |
| GO:0014049\_positive\_regulation\_of\_glutamate\_secretion | 1 | 0 |  |  |  |  |  |  |  |  |
| GO:0014061\_regulation\_of\_norepinephrine\_secretion | 1 | 0 |  |  |  |  |  |  |  |  |
| GO:0014071\_response\_to\_cycloalkane | 1 | 0 |  |  |  |  |  |  |  |  |
| GO:0014707\_branchiomeric\_skeletal\_muscle\_development | 1 | 0 |  |  |  |  |  |  |  |  |
| GO:0014738\_regulation\_of\_muscle\_hyperplasia | 1 | 0 |  |  |  |  |  |  |  |  |
| GO:0014740\_negative\_regulation\_of\_muscle\_hyperplasia | 1 | 0 |  |  |  |  |  |  |  |  |
| GO:0014741\_negative\_regulation\_of\_muscle\_hypertrophy | 1 | 0 |  |  |  |  |  |  |  |  |
| GO:0014743\_regulation\_of\_muscle\_hypertrophy | 1 | 0 |  |  |  |  |  |  |  |  |
| GO:0014805\_smooth\_muscle\_adaptation | 1 | 0 |  |  |  |  |  |  |  |  |
| GO:0014806\_smooth\_muscle\_hyperplasia | 1 | 0 |  |  |  |  |  |  |  |  |
| GO:0014807\_regulation\_of\_somitogenesis | 1 | 0 |  |  |  |  |  |  |  |  |
| GO:0014808\_release\_of\_sequestered\_calcium\_ion\_into\_cytosol\_by\_sarcoplasmic\_reticulum | 1 | 0 |  |  |  |  |  |  |  |  |
| GO:0014813\_satellite\_cell\_commitment | 1 | 0 |  |  |  |  |  |  |  |  |
| GO:0014816\_satellite\_cell\_differentiation | 1 | 0 |  |  |  |  |  |  |  |  |
| GO:0014819\_regulation\_of\_skeletal\_muscle\_contraction | 1 | 0 |  |  |  |  |  |  |  |  |
| GO:0014852\_regulation\_of\_skeletal\_muscle\_contraction\_by\_neural\_stimulation\_via\_neuromuscular\_junction | 1 | 0 |  |  |  |  |  |  |  |  |
| GO:0014853\_regulation\_of\_excitatory\_postsynaptic\_membrane\_potential\_involved\_in\_skeletal\_muscle\_contraction | 1 | 0 |  |  |  |  |  |  |  |  |
| GO:0014856\_skeletal\_muscle\_cell\_proliferation | 1 | 0 |  |  |  |  |  |  |  |  |
| GO:0014857\_regulation\_of\_skeletal\_muscle\_cell\_proliferation | 1 | 0 |  |  |  |  |  |  |  |  |
| GO:0014858\_positive\_regulation\_of\_skeletal\_muscle\_cell\_proliferation | 1 | 0 |  |  |  |  |  |  |  |  |
| GO:0014887\_cardiac\_muscle\_adaptation | 1 | 0 |  |  |  |  |  |  |  |  |
| GO:0014889\_muscle\_atrophy | 1 | 0 |  |  |  |  |  |  |  |  |
| GO:0014896\_muscle\_hypertrophy | 1 | 0 |  |  |  |  |  |  |  |  |
| GO:0014897\_striated\_muscle\_hypertrophy | 1 | 0 |  |  |  |  |  |  |  |  |
| GO:0014898\_cardiac\_muscle\_hypertrophy | 1 | 0 |  |  |  |  |  |  |  |  |
| GO:0014900\_muscle\_hyperplasia | 1 | 0 |  |  |  |  |  |  |  |  |
| GO:0015014\_heparan\_sulfate\_proteoglycan\_biosynthetic\_process\_\_polysaccharide\_chain\_biosynthetic\_process | 1 | 0 |  |  |  |  |  |  |  |  |
| GO:0015074\_DNA\_integration | 1 | 0 |  |  |  |  |  |  |  |  |
| GO:0015670\_carbon\_dioxide\_transport | 1 | 0 |  |  |  |  |  |  |  |  |
| GO:0015677\_copper\_ion\_import | 1 | 0 |  |  |  |  |  |  |  |  |
| GO:0015680\_intracellular\_copper\_ion\_transport | 1 | 0 |  |  |  |  |  |  |  |  |
| GO:0015684\_ferrous\_iron\_transport | 1 | 0 |  |  |  |  |  |  |  |  |
| GO:0015724\_formate\_transport | 1 | 0 |  |  |  |  |  |  |  |  |
| GO:0015734\_taurine\_transport | 1 | 0 |  |  |  |  |  |  |  |  |
| GO:0015740\_C4-dicarboxylate\_transport | 1 | 0 |  |  |  |  |  |  |  |  |
| GO:0015744\_succinate\_transport | 1 | 0 |  |  |  |  |  |  |  |  |
| GO:0015746\_citrate\_transport | 1 | 0 |  |  |  |  |  |  |  |  |
| GO:0015747\_urate\_transport | 1 | 0 |  |  |  |  |  |  |  |  |
| GO:0015791\_polyol\_transport | 1 | 0 |  |  |  |  |  |  |  |  |
| GO:0015798\_myo-inositol\_transport | 1 | 0 |  |  |  |  |  |  |  |  |
| GO:0015808\_L-alanine\_transport | 1 | 0 |  |  |  |  |  |  |  |  |
| GO:0015810\_aspartate\_transport | 1 | 0 |  |  |  |  |  |  |  |  |
| GO:0015811\_L-cystine\_transport | 1 | 0 |  |  |  |  |  |  |  |  |
| GO:0015817\_histidine\_transport | 1 | 0 |  |  |  |  |  |  |  |  |
| GO:0015822\_ornithine\_transport | 1 | 0 |  |  |  |  |  |  |  |  |
| GO:0015824\_proline\_transport | 1 | 0 |  |  |  |  |  |  |  |  |
| GO:0015851\_nucleobase\_transport | 1 | 0 |  |  |  |  |  |  |  |  |
| GO:0015864\_pyrimidine\_nucleoside\_transport | 1 | 0 |  |  |  |  |  |  |  |  |
| GO:0015874\_norepinephrine\_transport | 1 | 0 |  |  |  |  |  |  |  |  |
| GO:0015881\_creatine\_transport | 1 | 0 |  |  |  |  |  |  |  |  |
| GO:0015884\_folic\_acid\_transport | 1 | 0 |  |  |  |  |  |  |  |  |
| GO:0015886\_heme\_transport | 1 | 0 |  |  |  |  |  |  |  |  |
| GO:0015888\_thiamin\_transport | 1 | 0 |  |  |  |  |  |  |  |  |
| GO:0015938\_coenzyme\_A\_catabolic\_process | 1 | 0 |  |  |  |  |  |  |  |  |
| GO:0015939\_pantothenate\_metabolic\_process | 1 | 0 |  |  |  |  |  |  |  |  |
| GO:0016073\_snRNA\_metabolic\_process | 1 | 0 |  |  |  |  |  |  |  |  |
| GO:0016074\_snoRNA\_metabolic\_process | 1 | 0 |  |  |  |  |  |  |  |  |
| GO:0016082\_synaptic\_vesicle\_priming | 1 | 0 |  |  |  |  |  |  |  |  |
| GO:0016090\_prenol\_metabolic\_process | 1 | 0 |  |  |  |  |  |  |  |  |
| GO:0016093\_polyprenol\_metabolic\_process | 1 | 0 |  |  |  |  |  |  |  |  |
| GO:0016180\_snRNA\_processing | 1 | 0 |  |  |  |  |  |  |  |  |
| GO:0016239\_positive\_regulation\_of\_macroautophagy | 1 | 0 |  |  |  |  |  |  |  |  |
| GO:0016246\_RNA\_interference | 1 | 0 |  |  |  |  |  |  |  |  |
| GO:0016255\_attachment\_of\_GPI\_anchor\_to\_protein | 1 | 0 |  |  |  |  |  |  |  |  |
| GO:0016333\_morphogenesis\_of\_follicular\_epithelium | 1 | 0 |  |  |  |  |  |  |  |  |
| GO:0016340\_calcium-dependent\_cell-matrix\_adhesion | 1 | 0 |  |  |  |  |  |  |  |  |
| GO:0016344\_meiotic\_chromosome\_movement\_towards\_spindle\_pole | 1 | 0 |  |  |  |  |  |  |  |  |
| GO:0016482\_cytoplasmic\_transport | 1 | 0 |  |  |  |  |  |  |  |  |
| GO:0016553\_base\_conversion\_or\_substitution\_editing | 1 | 0 |  |  |  |  |  |  |  |  |
| GO:0016554\_cytidine\_to\_uridine\_editing | 1 | 0 |  |  |  |  |  |  |  |  |
| GO:0016560\_protein\_import\_into\_peroxisome\_matrix\_\_docking | 1 | 0 |  |  |  |  |  |  |  |  |
| GO:0016578\_histone\_deubiquitination | 1 | 0 |  |  |  |  |  |  |  |  |
| GO:0016598\_protein\_arginylation | 1 | 0 |  |  |  |  |  |  |  |  |
| GO:0017004\_cytochrome\_complex\_assembly | 1 | 0 |  |  |  |  |  |  |  |  |
| GO:0018022\_peptidyl-lysine\_methylation | 1 | 0 |  |  |  |  |  |  |  |  |
| GO:0018023\_peptidyl-lysine\_trimethylation | 1 | 0 |  |  |  |  |  |  |  |  |
| GO:0018120\_peptidyl-arginine\_ADP-ribosylation | 1 | 0 |  |  |  |  |  |  |  |  |
| GO:0018126\_protein\_amino\_acid\_hydroxylation | 1 | 0 |  |  |  |  |  |  |  |  |
| GO:0018146\_keratan\_sulfate\_biosynthetic\_process | 1 | 0 |  |  |  |  |  |  |  |  |
| GO:0018158\_protein\_amino\_acid\_oxidation | 1 | 0 |  |  |  |  |  |  |  |  |
| GO:0018195\_peptidyl-arginine\_modification | 1 | 0 |  |  |  |  |  |  |  |  |
| GO:0018197\_peptidyl-aspartic\_acid\_modification | 1 | 0 |  |  |  |  |  |  |  |  |
| GO:0018282\_metal\_incorporation\_into\_metallo-sulfur\_cluster | 1 | 0 |  |  |  |  |  |  |  |  |
| GO:0018283\_iron\_incorporation\_into\_metallo-sulfur\_cluster | 1 | 0 |  |  |  |  |  |  |  |  |
| GO:0018318\_protein\_amino\_acid\_palmitoylation | 1 | 0 |  |  |  |  |  |  |  |  |
| GO:0018342\_protein\_prenylation | 1 | 0 |  |  |  |  |  |  |  |  |
| GO:0018344\_protein\_geranylgeranylation | 1 | 0 |  |  |  |  |  |  |  |  |
| GO:0018410\_peptide\_or\_protein\_carboxyl-terminal\_blocking | 1 | 0 |  |  |  |  |  |  |  |  |
| GO:0018916\_nitrobenzene\_metabolic\_process | 1 | 0 |  |  |  |  |  |  |  |  |
| GO:0018931\_naphthalene\_metabolic\_process | 1 | 0 |  |  |  |  |  |  |  |  |
| GO:0018992\_germ-line\_sex\_determination | 1 | 0 |  |  |  |  |  |  |  |  |
| GO:0019042\_latent\_virus\_infection | 1 | 0 |  |  |  |  |  |  |  |  |
| GO:0019046\_reactivation\_of\_latent\_virus | 1 | 0 |  |  |  |  |  |  |  |  |
| GO:0019047\_provirus\_integration | 1 | 0 |  |  |  |  |  |  |  |  |
| GO:0019076\_release\_of\_virus\_from\_host | 1 | 0 |  |  |  |  |  |  |  |  |
| GO:0019100\_male\_germ-line\_sex\_determination | 1 | 0 |  |  |  |  |  |  |  |  |
| GO:0019101\_female\_somatic\_sex\_determination | 1 | 0 |  |  |  |  |  |  |  |  |
| GO:0019102\_male\_somatic\_sex\_determination | 1 | 0 |  |  |  |  |  |  |  |  |
| GO:0019255\_glucose\_1-phosphate\_metabolic\_process | 1 | 0 |  |  |  |  |  |  |  |  |
| GO:0019276\_UDP-N-acetylgalactosamine\_metabolic\_process | 1 | 0 |  |  |  |  |  |  |  |  |
| GO:0019344\_cysteine\_biosynthetic\_process | 1 | 0 |  |  |  |  |  |  |  |  |
| GO:0019348\_dolichol\_metabolic\_process | 1 | 0 |  |  |  |  |  |  |  |  |
| GO:0019375\_galactolipid\_biosynthetic\_process | 1 | 0 |  |  |  |  |  |  |  |  |
| GO:0019402\_galactitol\_metabolic\_process | 1 | 0 |  |  |  |  |  |  |  |  |
| GO:0019441\_tryptophan\_catabolic\_process\_to\_kynurenine | 1 | 0 |  |  |  |  |  |  |  |  |
| GO:0019477\_L-lysine\_catabolic\_process | 1 | 0 |  |  |  |  |  |  |  |  |
| GO:0019510\_S-adenosylhomocysteine\_catabolic\_process | 1 | 0 |  |  |  |  |  |  |  |  |
| GO:0019532\_oxalate\_transport | 1 | 0 |  |  |  |  |  |  |  |  |
| GO:0019626\_short-chain\_fatty\_acid\_catabolic\_process | 1 | 0 |  |  |  |  |  |  |  |  |
| GO:0019627\_urea\_metabolic\_process | 1 | 0 |  |  |  |  |  |  |  |  |
| GO:0019676\_ammonia\_assimilation\_cycle | 1 | 0 |  |  |  |  |  |  |  |  |
| GO:0019682\_glyceraldehyde-3-phosphate\_metabolic\_process | 1 | 0 |  |  |  |  |  |  |  |  |
| GO:0019695\_choline\_metabolic\_process | 1 | 0 |  |  |  |  |  |  |  |  |
| GO:0019731\_antibacterial\_humoral\_response | 1 | 0 |  |  |  |  |  |  |  |  |
| GO:0019794\_nonprotein\_amino\_acid\_metabolic\_process | 1 | 0 |  |  |  |  |  |  |  |  |
| GO:0019858\_cytosine\_metabolic\_process | 1 | 0 |  |  |  |  |  |  |  |  |
| GO:0019883\_antigen\_processing\_and\_presentation\_of\_endogenous\_antigen | 1 | 0 |  |  |  |  |  |  |  |  |
| GO:0019889\_pteridine\_metabolic\_process | 1 | 0 |  |  |  |  |  |  |  |  |
| GO:0019896\_axon\_transport\_of\_mitochondrion | 1 | 0 |  |  |  |  |  |  |  |  |
| GO:0021508\_floor\_plate\_formation | 1 | 0 |  |  |  |  |  |  |  |  |
| GO:0021528\_commissural\_neuron\_differentiation\_in\_the\_spinal\_cord | 1 | 0 |  |  |  |  |  |  |  |  |
| GO:0021572\_rhombomere\_6\_development | 1 | 0 |  |  |  |  |  |  |  |  |
| GO:0021586\_pons\_maturation | 1 | 0 |  |  |  |  |  |  |  |  |
| GO:0021590\_cerebellum\_maturation | 1 | 0 |  |  |  |  |  |  |  |  |
| GO:0021592\_fourth\_ventricle\_development | 1 | 0 |  |  |  |  |  |  |  |  |
| GO:0021594\_rhombomere\_formation | 1 | 0 |  |  |  |  |  |  |  |  |
| GO:0021660\_rhombomere\_3\_formation | 1 | 0 |  |  |  |  |  |  |  |  |
| GO:0021664\_rhombomere\_5\_morphogenesis | 1 | 0 |  |  |  |  |  |  |  |  |
| GO:0021666\_rhombomere\_5\_formation | 1 | 0 |  |  |  |  |  |  |  |  |
| GO:0021670\_lateral\_ventricle\_development | 1 | 0 |  |  |  |  |  |  |  |  |
| GO:0021678\_third\_ventricle\_development | 1 | 0 |  |  |  |  |  |  |  |  |
| GO:0021679\_cerebellar\_molecular\_layer\_development | 1 | 0 |  |  |  |  |  |  |  |  |
| GO:0021703\_locus\_ceruleus\_development | 1 | 0 |  |  |  |  |  |  |  |  |
| GO:0021732\_midbrain-hindbrain\_boundary\_maturation | 1 | 0 |  |  |  |  |  |  |  |  |
| GO:0021750\_vestibular\_nucleus\_development | 1 | 0 |  |  |  |  |  |  |  |  |
| GO:0021759\_globus\_pallidus\_development | 1 | 0 |  |  |  |  |  |  |  |  |
| GO:0021768\_nucleus\_accumbens\_development | 1 | 0 |  |  |  |  |  |  |  |  |
| GO:0021771\_lateral\_geniculate\_nucleus\_development | 1 | 0 |  |  |  |  |  |  |  |  |
| GO:0021870\_Cajal-Retzius\_cell\_differentiation | 1 | 0 |  |  |  |  |  |  |  |  |
| GO:0021874\_Wnt\_receptor\_signaling\_pathway\_in\_forebrain\_neuroblast\_division | 1 | 0 |  |  |  |  |  |  |  |  |
| GO:0021896\_forebrain\_astrocyte\_differentiation | 1 | 0 |  |  |  |  |  |  |  |  |
| GO:0021897\_forebrain\_astrocyte\_development | 1 | 0 |  |  |  |  |  |  |  |  |
| GO:0021902\_commitment\_of\_a\_neuronal\_cell\_to\_a\_specific\_type\_of\_neuron\_in\_the\_forebrain | 1 | 0 |  |  |  |  |  |  |  |  |
| GO:0021905\_forebrain-midbrain\_boundary\_formation | 1 | 0 |  |  |  |  |  |  |  |  |
| GO:0021914\_negative\_regulation\_of\_smoothened\_signaling\_pathway\_involved\_in\_ventral\_spinal\_cord\_patterning | 1 | 0 |  |  |  |  |  |  |  |  |
| GO:0021917\_somatic\_motor\_neuron\_fate\_commitment | 1 | 0 |  |  |  |  |  |  |  |  |
| GO:0021918\_regulation\_of\_transcription\_from\_RNA\_polymerase\_II\_promoter\_involved\_in\_somatic\_motor\_neuron\_fate\_commitment | 1 | 0 |  |  |  |  |  |  |  |  |
| GO:0021933\_radial\_glia\_guided\_migration\_of\_granule\_cell | 1 | 0 |  |  |  |  |  |  |  |  |
| GO:0021934\_hindbrain\_tangential\_cell\_migration | 1 | 0 |  |  |  |  |  |  |  |  |
| GO:0021935\_granule\_cell\_precursor\_tangential\_migration | 1 | 0 |  |  |  |  |  |  |  |  |
| GO:0021960\_anterior\_commissure\_morphogenesis | 1 | 0 |  |  |  |  |  |  |  |  |
| GO:0021997\_neural\_plate\_axis\_specification | 1 | 0 |  |  |  |  |  |  |  |  |
| GO:0021999\_neural\_plate\_anterior\_posterior\_pattern\_formation | 1 | 0 |  |  |  |  |  |  |  |  |
| GO:0022004\_midbrain-hindbrain\_boundary\_maturation\_during\_brain\_development | 1 | 0 |  |  |  |  |  |  |  |  |
| GO:0022038\_corpus\_callosum\_development | 1 | 0 |  |  |  |  |  |  |  |  |
| GO:0022605\_oogenesis\_stage | 1 | 0 |  |  |  |  |  |  |  |  |
| GO:0030011\_maintenance\_of\_cell\_polarity | 1 | 0 |  |  |  |  |  |  |  |  |
| GO:0030069\_lysogeny | 1 | 0 |  |  |  |  |  |  |  |  |
| GO:0030070\_insulin\_processing | 1 | 0 |  |  |  |  |  |  |  |  |
| GO:0030092\_regulation\_of\_flagellum\_assembly | 1 | 0 |  |  |  |  |  |  |  |  |
| GO:0030103\_vasopressin\_secretion | 1 | 0 |  |  |  |  |  |  |  |  |
| GO:0030194\_positive\_regulation\_of\_blood\_coagulation | 1 | 0 |  |  |  |  |  |  |  |  |
| GO:0030206\_chondroitin\_sulfate\_biosynthetic\_process | 1 | 0 |  |  |  |  |  |  |  |  |
| GO:0030210\_heparin\_biosynthetic\_process | 1 | 0 |  |  |  |  |  |  |  |  |
| GO:0030220\_platelet\_formation | 1 | 0 |  |  |  |  |  |  |  |  |
| GO:0030222\_eosinophil\_differentiation | 1 | 0 |  |  |  |  |  |  |  |  |
| GO:0030237\_female\_sex\_determination | 1 | 0 |  |  |  |  |  |  |  |  |
| GO:0030264\_nuclear\_fragmentation\_during\_apoptosis | 1 | 0 |  |  |  |  |  |  |  |  |
| GO:0030322\_stabilization\_of\_membrane\_potential | 1 | 0 |  |  |  |  |  |  |  |  |
| GO:0030327\_prenylated\_protein\_catabolic\_process | 1 | 0 |  |  |  |  |  |  |  |  |
| GO:0030328\_prenylcysteine\_catabolic\_process | 1 | 0 |  |  |  |  |  |  |  |  |
| GO:0030329\_prenylcysteine\_metabolic\_process | 1 | 0 |  |  |  |  |  |  |  |  |
| GO:0030382\_sperm\_mitochondrion\_organization | 1 | 0 |  |  |  |  |  |  |  |  |
| GO:0030389\_fructosamine\_metabolic\_process | 1 | 0 |  |  |  |  |  |  |  |  |
| GO:0030422\_RNA\_interference\_\_production\_of\_siRNA | 1 | 0 |  |  |  |  |  |  |  |  |
| GO:0030449\_regulation\_of\_complement\_activation | 1 | 0 |  |  |  |  |  |  |  |  |
| GO:0030497\_fatty\_acid\_elongation | 1 | 0 |  |  |  |  |  |  |  |  |
| GO:0030575\_nuclear\_body\_organization | 1 | 0 |  |  |  |  |  |  |  |  |
| GO:0030578\_PML\_body\_organization | 1 | 0 |  |  |  |  |  |  |  |  |
| GO:0030853\_negative\_regulation\_of\_granulocyte\_differentiation | 1 | 0 |  |  |  |  |  |  |  |  |
| GO:0030854\_positive\_regulation\_of\_granulocyte\_differentiation | 1 | 0 |  |  |  |  |  |  |  |  |
| GO:0030886\_negative\_regulation\_of\_myeloid\_dendritic\_cell\_activation | 1 | 0 |  |  |  |  |  |  |  |  |
| GO:0030913\_paranodal\_junction\_assembly | 1 | 0 |  |  |  |  |  |  |  |  |
| GO:0031033\_myosin\_filament\_assembly\_or\_disassembly | 1 | 0 |  |  |  |  |  |  |  |  |
| GO:0031034\_myosin\_filament\_assembly | 1 | 0 |  |  |  |  |  |  |  |  |
| GO:0031055\_chromatin\_remodeling\_at\_centromere | 1 | 0 |  |  |  |  |  |  |  |  |
| GO:0031062\_positive\_regulation\_of\_histone\_methylation | 1 | 0 |  |  |  |  |  |  |  |  |
| GO:0031115\_negative\_regulation\_of\_microtubule\_polymerization | 1 | 0 |  |  |  |  |  |  |  |  |
| GO:0031284\_positive\_regulation\_of\_guanylate\_cyclase\_activity | 1 | 0 |  |  |  |  |  |  |  |  |
| GO:0031498\_chromatin\_disassembly | 1 | 0 |  |  |  |  |  |  |  |  |
| GO:0031507\_heterochromatin\_formation | 1 | 0 |  |  |  |  |  |  |  |  |
| GO:0031508\_centromeric\_heterochromatin\_formation | 1 | 0 |  |  |  |  |  |  |  |  |
| GO:0031529\_ruffle\_organization | 1 | 0 |  |  |  |  |  |  |  |  |
| GO:0031536\_positive\_regulation\_of\_exit\_from\_mitosis | 1 | 0 |  |  |  |  |  |  |  |  |
| GO:0031572\_G2\_M\_transition\_DNA\_damage\_checkpoint | 1 | 0 |  |  |  |  |  |  |  |  |
| GO:0031576\_G2\_M\_transition\_checkpoint | 1 | 0 |  |  |  |  |  |  |  |  |
| GO:0031580\_membrane\_raft\_distribution | 1 | 0 |  |  |  |  |  |  |  |  |
| GO:0031583\_activation\_of\_phospholipase\_D\_activity\_by\_G-protein\_coupled\_receptor\_protein\_signaling\_pathway | 1 | 0 |  |  |  |  |  |  |  |  |
| GO:0031584\_activation\_of\_phospholipase\_D\_activity | 1 | 0 |  |  |  |  |  |  |  |  |
| GO:0031585\_regulation\_of\_inositol-1\_4\_5-triphosphate\_receptor\_activity | 1 | 0 |  |  |  |  |  |  |  |  |
| GO:0031639\_plasminogen\_activation | 1 | 0 |  |  |  |  |  |  |  |  |
| GO:0031648\_protein\_destabilization | 1 | 0 |  |  |  |  |  |  |  |  |
| GO:0031665\_negative\_regulation\_of\_lipopolysaccharide-mediated\_signaling\_pathway | 1 | 0 |  |  |  |  |  |  |  |  |
| GO:0031914\_negative\_regulation\_of\_synaptic\_plasticity | 1 | 0 |  |  |  |  |  |  |  |  |
| GO:0031944\_negative\_regulation\_of\_glucocorticoid\_metabolic\_process | 1 | 0 |  |  |  |  |  |  |  |  |
| GO:0031947\_negative\_regulation\_of\_glucocorticoid\_biosynthetic\_process | 1 | 0 |  |  |  |  |  |  |  |  |
| GO:0032025\_response\_to\_cobalt\_ion | 1 | 0 |  |  |  |  |  |  |  |  |
| GO:0032026\_response\_to\_magnesium\_ion | 1 | 0 |  |  |  |  |  |  |  |  |
| GO:0032048\_cardiolipin\_metabolic\_process | 1 | 0 |  |  |  |  |  |  |  |  |
| GO:0032066\_nucleolus\_to\_nucleoplasm\_transport | 1 | 0 |  |  |  |  |  |  |  |  |
| GO:0032091\_negative\_regulation\_of\_protein\_binding | 1 | 0 |  |  |  |  |  |  |  |  |
| GO:0032092\_positive\_regulation\_of\_protein\_binding | 1 | 0 |  |  |  |  |  |  |  |  |
| GO:0032097\_positive\_regulation\_of\_response\_to\_food | 1 | 0 |  |  |  |  |  |  |  |  |
| GO:0032100\_positive\_regulation\_of\_appetite | 1 | 0 |  |  |  |  |  |  |  |  |
| GO:0032204\_regulation\_of\_telomere\_maintenance | 1 | 0 |  |  |  |  |  |  |  |  |
| GO:0032206\_positive\_regulation\_of\_telomere\_maintenance | 1 | 0 |  |  |  |  |  |  |  |  |
| GO:0032222\_regulation\_of\_synaptic\_transmission\_\_cholinergic | 1 | 0 |  |  |  |  |  |  |  |  |
| GO:0032224\_positive\_regulation\_of\_synaptic\_transmission\_\_cholinergic | 1 | 0 |  |  |  |  |  |  |  |  |
| GO:0032229\_negative\_regulation\_of\_synaptic\_transmission\_\_GABAergic | 1 | 0 |  |  |  |  |  |  |  |  |
| GO:0032237\_activation\_of\_store-operated\_calcium\_channel\_activity | 1 | 0 |  |  |  |  |  |  |  |  |
| GO:0032239\_regulation\_of\_nucleobase\_\_nucleoside\_\_nucleotide\_and\_nucleic\_acid\_transport | 1 | 0 |  |  |  |  |  |  |  |  |
| GO:0032252\_secretory\_granule\_localization | 1 | 0 |  |  |  |  |  |  |  |  |
| GO:0032274\_gonadotropin\_secretion | 1 | 0 |  |  |  |  |  |  |  |  |
| GO:0032275\_luteinizing\_hormone\_secretion | 1 | 0 |  |  |  |  |  |  |  |  |
| GO:0032287\_myelin\_maintenance\_in\_the\_peripheral\_nervous\_system | 1 | 0 |  |  |  |  |  |  |  |  |
| GO:0032289\_myelin\_formation\_in\_the\_central\_nervous\_system | 1 | 0 |  |  |  |  |  |  |  |  |
| GO:0032303\_regulation\_of\_icosanoid\_secretion | 1 | 0 |  |  |  |  |  |  |  |  |
| GO:0032305\_positive\_regulation\_of\_icosanoid\_secretion | 1 | 0 |  |  |  |  |  |  |  |  |
| GO:0032306\_regulation\_of\_prostaglandin\_secretion | 1 | 0 |  |  |  |  |  |  |  |  |
| GO:0032308\_positive\_regulation\_of\_prostaglandin\_secretion | 1 | 0 |  |  |  |  |  |  |  |  |
| GO:0032310\_prostaglandin\_secretion | 1 | 0 |  |  |  |  |  |  |  |  |
| GO:0032313\_regulation\_of\_Rab\_GTPase\_activity | 1 | 0 |  |  |  |  |  |  |  |  |
| GO:0032314\_regulation\_of\_Rac\_GTPase\_activity | 1 | 0 |  |  |  |  |  |  |  |  |
| GO:0032317\_regulation\_of\_Rap\_GTPase\_activity | 1 | 0 |  |  |  |  |  |  |  |  |
| GO:0032324\_molybdopterin\_cofactor\_biosynthetic\_process | 1 | 0 |  |  |  |  |  |  |  |  |
| GO:0032329\_serine\_transport | 1 | 0 |  |  |  |  |  |  |  |  |
| GO:0032342\_aldosterone\_biosynthetic\_process | 1 | 0 |  |  |  |  |  |  |  |  |
| GO:0032344\_regulation\_of\_aldosterone\_metabolic\_process | 1 | 0 |  |  |  |  |  |  |  |  |
| GO:0032365\_intracellular\_lipid\_transport | 1 | 0 |  |  |  |  |  |  |  |  |
| GO:0032366\_intracellular\_sterol\_transport | 1 | 0 |  |  |  |  |  |  |  |  |
| GO:0032367\_intracellular\_cholesterol\_transport | 1 | 0 |  |  |  |  |  |  |  |  |
| GO:0032370\_positive\_regulation\_of\_lipid\_transport | 1 | 0 |  |  |  |  |  |  |  |  |
| GO:0032410\_negative\_regulation\_of\_transporter\_activity | 1 | 0 |  |  |  |  |  |  |  |  |
| GO:0032413\_negative\_regulation\_of\_ion\_transmembrane\_transporter\_activity | 1 | 0 |  |  |  |  |  |  |  |  |
| GO:0032429\_regulation\_of\_phospholipase\_A2\_activity | 1 | 0 |  |  |  |  |  |  |  |  |
| GO:0032474\_otolith\_morphogenesis | 1 | 0 |  |  |  |  |  |  |  |  |
| GO:0032482\_Rab\_protein\_signal\_transduction | 1 | 0 |  |  |  |  |  |  |  |  |
| GO:0032483\_regulation\_of\_Rab\_protein\_signal\_transduction | 1 | 0 |  |  |  |  |  |  |  |  |
| GO:0032486\_Rap\_protein\_signal\_transduction | 1 | 0 |  |  |  |  |  |  |  |  |
| GO:0032487\_regulation\_of\_Rap\_protein\_signal\_transduction | 1 | 0 |  |  |  |  |  |  |  |  |
| GO:0032594\_protein\_transport\_within\_lipid\_bilayer | 1 | 0 |  |  |  |  |  |  |  |  |
| GO:0032599\_protein\_transport\_out\_of\_membrane\_raft | 1 | 0 |  |  |  |  |  |  |  |  |
| GO:0032600\_chemokine\_receptor\_transport\_out\_of\_membrane\_raft | 1 | 0 |  |  |  |  |  |  |  |  |
| GO:0032607\_interferon-alpha\_production | 1 | 0 |  |  |  |  |  |  |  |  |
| GO:0032621\_interleukin-18\_production | 1 | 0 |  |  |  |  |  |  |  |  |
| GO:0032647\_regulation\_of\_interferon-alpha\_production | 1 | 0 |  |  |  |  |  |  |  |  |
| GO:0032656\_regulation\_of\_interleukin-13\_production | 1 | 0 |  |  |  |  |  |  |  |  |
| GO:0032682\_negative\_regulation\_of\_chemokine\_production | 1 | 0 |  |  |  |  |  |  |  |  |
| GO:0032691\_negative\_regulation\_of\_interleukin-1\_beta\_production | 1 | 0 |  |  |  |  |  |  |  |  |
| GO:0032692\_negative\_regulation\_of\_interleukin-1\_production | 1 | 0 |  |  |  |  |  |  |  |  |
| GO:0032693\_negative\_regulation\_of\_interleukin-10\_production | 1 | 0 |  |  |  |  |  |  |  |  |
| GO:0032696\_negative\_regulation\_of\_interleukin-13\_production | 1 | 0 |  |  |  |  |  |  |  |  |
| GO:0032727\_positive\_regulation\_of\_interferon-alpha\_production | 1 | 0 |  |  |  |  |  |  |  |  |
| GO:0032731\_positive\_regulation\_of\_interleukin-1\_beta\_production | 1 | 0 |  |  |  |  |  |  |  |  |
| GO:0032732\_positive\_regulation\_of\_interleukin-1\_production | 1 | 0 |  |  |  |  |  |  |  |  |
| GO:0032735\_positive\_regulation\_of\_interleukin-12\_production | 1 | 0 |  |  |  |  |  |  |  |  |
| GO:0032764\_negative\_regulation\_of\_mast\_cell\_cytokine\_production | 1 | 0 |  |  |  |  |  |  |  |  |
| GO:0032765\_positive\_regulation\_of\_mast\_cell\_cytokine\_production | 1 | 0 |  |  |  |  |  |  |  |  |
| GO:0032769\_negative\_regulation\_of\_monooxygenase\_activity | 1 | 0 |  |  |  |  |  |  |  |  |
| GO:0032781\_positive\_regulation\_of\_ATPase\_activity | 1 | 0 |  |  |  |  |  |  |  |  |
| GO:0032790\_ribosome\_disassembly | 1 | 0 |  |  |  |  |  |  |  |  |
| GO:0032799\_low-density\_lipoprotein\_receptor\_metabolic\_process | 1 | 0 |  |  |  |  |  |  |  |  |
| GO:0032802\_low-density\_lipoprotein\_receptor\_catabolic\_process | 1 | 0 |  |  |  |  |  |  |  |  |
| GO:0032803\_regulation\_of\_low-density\_lipoprotein\_receptor\_catabolic\_process | 1 | 0 |  |  |  |  |  |  |  |  |
| GO:0032817\_regulation\_of\_natural\_killer\_cell\_proliferation | 1 | 0 |  |  |  |  |  |  |  |  |
| GO:0032819\_positive\_regulation\_of\_natural\_killer\_cell\_proliferation | 1 | 0 |  |  |  |  |  |  |  |  |
| GO:0032836\_glomerular\_basement\_membrane\_development | 1 | 0 |  |  |  |  |  |  |  |  |
| GO:0032855\_positive\_regulation\_of\_Rac\_GTPase\_activity | 1 | 0 |  |  |  |  |  |  |  |  |
| GO:0032863\_activation\_of\_Rac\_GTPase\_activity | 1 | 0 |  |  |  |  |  |  |  |  |
| GO:0032864\_activation\_of\_Cdc42\_GTPase\_activity | 1 | 0 |  |  |  |  |  |  |  |  |
| GO:0032885\_regulation\_of\_polysaccharide\_biosynthetic\_process | 1 | 0 |  |  |  |  |  |  |  |  |
| GO:0032907\_transforming\_growth\_factor-beta3\_production | 1 | 0 |  |  |  |  |  |  |  |  |
| GO:0032910\_regulation\_of\_transforming\_growth\_factor-beta3\_production | 1 | 0 |  |  |  |  |  |  |  |  |
| GO:0032913\_negative\_regulation\_of\_transforming\_growth\_factor-beta3\_production | 1 | 0 |  |  |  |  |  |  |  |  |
| GO:0032924\_activin\_receptor\_signaling\_pathway | 1 | 0 |  |  |  |  |  |  |  |  |
| GO:0032925\_regulation\_of\_activin\_receptor\_signaling\_pathway | 1 | 0 |  |  |  |  |  |  |  |  |
| GO:0032960\_regulation\_of\_inositol\_trisphosphate\_biosynthetic\_process | 1 | 0 |  |  |  |  |  |  |  |  |
| GO:0032962\_positive\_regulation\_of\_inositol\_trisphosphate\_biosynthetic\_process | 1 | 0 |  |  |  |  |  |  |  |  |
| GO:0032964\_collagen\_biosynthetic\_process | 1 | 0 |  |  |  |  |  |  |  |  |
| GO:0032971\_regulation\_of\_muscle\_filament\_sliding | 1 | 0 |  |  |  |  |  |  |  |  |
| GO:0032972\_regulation\_of\_muscle\_filament\_sliding\_speed | 1 | 0 |  |  |  |  |  |  |  |  |
| GO:0032986\_protein-DNA\_complex\_disassembly | 1 | 0 |  |  |  |  |  |  |  |  |
| GO:0032988\_ribonucleoprotein\_complex\_disassembly | 1 | 0 |  |  |  |  |  |  |  |  |
| GO:0033037\_polysaccharide\_localization | 1 | 0 |  |  |  |  |  |  |  |  |
| GO:0033078\_extrathymic\_T\_cell\_differentiation | 1 | 0 |  |  |  |  |  |  |  |  |
| GO:0033085\_negative\_regulation\_of\_T\_cell\_differentiation\_in\_the\_thymus | 1 | 0 |  |  |  |  |  |  |  |  |
| GO:0033087\_negative\_regulation\_of\_immature\_T\_cell\_proliferation | 1 | 0 |  |  |  |  |  |  |  |  |
| GO:0033088\_negative\_regulation\_of\_immature\_T\_cell\_proliferation\_in\_the\_thymus | 1 | 0 |  |  |  |  |  |  |  |  |
| GO:0033108\_mitochondrial\_respiratory\_chain\_complex\_assembly | 1 | 0 |  |  |  |  |  |  |  |  |
| GO:0033127\_regulation\_of\_histone\_phosphorylation | 1 | 0 |  |  |  |  |  |  |  |  |
| GO:0033128\_negative\_regulation\_of\_histone\_phosphorylation | 1 | 0 |  |  |  |  |  |  |  |  |
| GO:0033158\_regulation\_of\_protein\_import\_into\_nucleus\_\_translocation | 1 | 0 |  |  |  |  |  |  |  |  |
| GO:0033160\_positive\_regulation\_of\_protein\_import\_into\_nucleus\_\_translocation | 1 | 0 |  |  |  |  |  |  |  |  |
| GO:0033169\_histone\_H3-K9\_demethylation | 1 | 0 |  |  |  |  |  |  |  |  |
| GO:0033206\_cytokinesis\_after\_meiosis | 1 | 0 |  |  |  |  |  |  |  |  |
| GO:0033240\_positive\_regulation\_of\_cellular\_amine\_metabolic\_process | 1 | 0 |  |  |  |  |  |  |  |  |
| GO:0033313\_meiotic\_cell\_cycle\_checkpoint | 1 | 0 |  |  |  |  |  |  |  |  |
| GO:0033315\_meiotic\_cell\_cycle\_DNA\_replication\_checkpoint | 1 | 0 |  |  |  |  |  |  |  |  |
| GO:0033326\_cerebrospinal\_fluid\_secretion | 1 | 0 |  |  |  |  |  |  |  |  |
| GO:0033366\_protein\_localization\_in\_secretory\_granule | 1 | 0 |  |  |  |  |  |  |  |  |
| GO:0033367\_protein\_localization\_in\_mast\_cell\_secretory\_granule | 1 | 0 |  |  |  |  |  |  |  |  |
| GO:0033368\_protease\_localization\_in\_mast\_cell\_secretory\_granule | 1 | 0 |  |  |  |  |  |  |  |  |
| GO:0033370\_maintenance\_of\_protein\_location\_in\_mast\_cell\_secretory\_granule | 1 | 0 |  |  |  |  |  |  |  |  |
| GO:0033371\_T\_cell\_secretory\_granule\_organization | 1 | 0 |  |  |  |  |  |  |  |  |
| GO:0033373\_maintenance\_of\_protease\_location\_in\_mast\_cell\_secretory\_granule | 1 | 0 |  |  |  |  |  |  |  |  |
| GO:0033374\_protein\_localization\_in\_T\_cell\_secretory\_granule | 1 | 0 |  |  |  |  |  |  |  |  |
| GO:0033375\_protease\_localization\_in\_T\_cell\_secretory\_granule | 1 | 0 |  |  |  |  |  |  |  |  |
| GO:0033377\_maintenance\_of\_protein\_location\_in\_T\_cell\_secretory\_granule | 1 | 0 |  |  |  |  |  |  |  |  |
| GO:0033379\_maintenance\_of\_protease\_location\_in\_T\_cell\_secretory\_granule | 1 | 0 |  |  |  |  |  |  |  |  |
| GO:0033380\_granzyme\_B\_localization\_in\_T\_cell\_secretory\_granule | 1 | 0 |  |  |  |  |  |  |  |  |
| GO:0033382\_maintenance\_of\_granzyme\_B\_location\_in\_T\_cell\_secretory\_granule | 1 | 0 |  |  |  |  |  |  |  |  |
| GO:0033483\_gas\_homeostasis | 1 | 0 |  |  |  |  |  |  |  |  |
| GO:0033484\_nitric\_oxide\_homeostasis | 1 | 0 |  |  |  |  |  |  |  |  |
| GO:0033505\_floor\_plate\_morphogenesis | 1 | 0 |  |  |  |  |  |  |  |  |
| GO:0033522\_histone\_H2A\_ubiquitination | 1 | 0 |  |  |  |  |  |  |  |  |
| GO:0033523\_histone\_H2B\_ubiquitination | 1 | 0 |  |  |  |  |  |  |  |  |
| GO:0033574\_response\_to\_testosterone\_stimulus | 1 | 0 |  |  |  |  |  |  |  |  |
| GO:0033606\_chemokine\_receptor\_transport\_within\_lipid\_bilayer | 1 | 0 |  |  |  |  |  |  |  |  |
| GO:0033628\_regulation\_of\_cell\_adhesion\_mediated\_by\_integrin | 1 | 0 |  |  |  |  |  |  |  |  |
| GO:0033630\_positive\_regulation\_of\_cell\_adhesion\_mediated\_by\_integrin | 1 | 0 |  |  |  |  |  |  |  |  |
| GO:0033632\_regulation\_of\_cell-cell\_adhesion\_mediated\_by\_integrin | 1 | 0 |  |  |  |  |  |  |  |  |
| GO:0033634\_positive\_regulation\_of\_cell-cell\_adhesion\_mediated\_by\_integrin | 1 | 0 |  |  |  |  |  |  |  |  |
| GO:0033683\_nucleotide-excision\_repair\_\_DNA\_incision | 1 | 0 |  |  |  |  |  |  |  |  |
| GO:0033750\_ribosome\_localization | 1 | 0 |  |  |  |  |  |  |  |  |
| GO:0033753\_establishment\_of\_ribosome\_localization | 1 | 0 |  |  |  |  |  |  |  |  |
| GO:0033866\_nucleoside\_bisphosphate\_biosynthetic\_process | 1 | 0 |  |  |  |  |  |  |  |  |
| GO:0033875\_ribonucleoside\_bisphosphate\_metabolic\_process | 1 | 0 |  |  |  |  |  |  |  |  |
| GO:0034030\_ribonucleoside\_bisphosphate\_biosynthetic\_process | 1 | 0 |  |  |  |  |  |  |  |  |
| GO:0034032\_purine\_nucleoside\_bisphosphate\_metabolic\_process | 1 | 0 |  |  |  |  |  |  |  |  |
| GO:0034033\_purine\_nucleoside\_bisphosphate\_biosynthetic\_process | 1 | 0 |  |  |  |  |  |  |  |  |
| GO:0034035\_purine\_ribonucleoside\_bisphosphate\_metabolic\_process | 1 | 0 |  |  |  |  |  |  |  |  |
| GO:0034036\_purine\_ribonucleoside\_bisphosphate\_biosynthetic\_process | 1 | 0 |  |  |  |  |  |  |  |  |
| GO:0034067\_protein\_localization\_in\_Golgi\_apparatus | 1 | 0 |  |  |  |  |  |  |  |  |
| GO:0034102\_erythrocyte\_clearance | 1 | 0 |  |  |  |  |  |  |  |  |
| GO:0034106\_regulation\_of\_erythrocyte\_clearance | 1 | 0 |  |  |  |  |  |  |  |  |
| GO:0034107\_negative\_regulation\_of\_erythrocyte\_clearance | 1 | 0 |  |  |  |  |  |  |  |  |
| GO:0034110\_regulation\_of\_homotypic\_cell-cell\_adhesion | 1 | 0 |  |  |  |  |  |  |  |  |
| GO:0034111\_negative\_regulation\_of\_homotypic\_cell-cell\_adhesion | 1 | 0 |  |  |  |  |  |  |  |  |
| GO:0034113\_heterotypic\_cell-cell\_adhesion | 1 | 0 |  |  |  |  |  |  |  |  |
| GO:0034117\_erythrocyte\_aggregation | 1 | 0 |  |  |  |  |  |  |  |  |
| GO:0034118\_regulation\_of\_erythrocyte\_aggregation | 1 | 0 |  |  |  |  |  |  |  |  |
| GO:0034119\_negative\_regulation\_of\_erythrocyte\_aggregation | 1 | 0 |  |  |  |  |  |  |  |  |
| GO:0034121\_regulation\_of\_toll-like\_receptor\_signaling\_pathway | 1 | 0 |  |  |  |  |  |  |  |  |
| GO:0034122\_negative\_regulation\_of\_toll-like\_receptor\_signaling\_pathway | 1 | 0 |  |  |  |  |  |  |  |  |
| GO:0034230\_enkephalin\_processing | 1 | 0 |  |  |  |  |  |  |  |  |
| GO:0034372\_very-low-density\_lipoprotein\_particle\_remodeling | 1 | 0 |  |  |  |  |  |  |  |  |
| GO:0034379\_very-low-density\_lipoprotein\_particle\_assembly | 1 | 0 |  |  |  |  |  |  |  |  |
| GO:0034380\_high-density\_lipoprotein\_particle\_assembly | 1 | 0 |  |  |  |  |  |  |  |  |
| GO:0034394\_protein\_localization\_at\_cell\_surface | 1 | 0 |  |  |  |  |  |  |  |  |
| GO:0034405\_response\_to\_fluid\_shear\_stress | 1 | 0 |  |  |  |  |  |  |  |  |
| GO:0034472\_snRNA\_3'-end\_processing | 1 | 0 |  |  |  |  |  |  |  |  |
| GO:0034474\_U2\_snRNA\_3'-end\_processing | 1 | 0 |  |  |  |  |  |  |  |  |
| GO:0034502\_protein\_localization\_to\_chromosome | 1 | 0 |  |  |  |  |  |  |  |  |
| GO:0034505\_tooth\_mineralization | 1 | 0 |  |  |  |  |  |  |  |  |
| GO:0034508\_centromere\_complex\_assembly | 1 | 0 |  |  |  |  |  |  |  |  |
| GO:0034633\_retinol\_transport | 1 | 0 |  |  |  |  |  |  |  |  |
| GO:0034643\_mitochondrion\_localization\_\_microtubule-mediated | 1 | 0 |  |  |  |  |  |  |  |  |
| GO:0034969\_histone\_arginine\_methylation | 1 | 0 |  |  |  |  |  |  |  |  |
| GO:0034982\_mitochondrial\_protein\_processing | 1 | 0 |  |  |  |  |  |  |  |  |
| GO:0035022\_positive\_regulation\_of\_Rac\_protein\_signal\_transduction | 1 | 0 |  |  |  |  |  |  |  |  |
| GO:0035024\_negative\_regulation\_of\_Rho\_protein\_signal\_transduction | 1 | 0 |  |  |  |  |  |  |  |  |
| GO:0035026\_leading\_edge\_cell\_differentiation | 1 | 0 |  |  |  |  |  |  |  |  |
| GO:0035037\_sperm\_entry | 1 | 0 |  |  |  |  |  |  |  |  |
| GO:0035039\_male\_pronucleus\_formation | 1 | 0 |  |  |  |  |  |  |  |  |
| GO:0035066\_positive\_regulation\_of\_histone\_acetylation | 1 | 0 |  |  |  |  |  |  |  |  |
| GO:0035083\_cilium\_axoneme\_assembly | 1 | 0 |  |  |  |  |  |  |  |  |
| GO:0035090\_maintenance\_of\_apical\_basal\_cell\_polarity | 1 | 0 |  |  |  |  |  |  |  |  |
| GO:0035106\_operant\_conditioning | 1 | 0 |  |  |  |  |  |  |  |  |
| GO:0035172\_hemocyte\_proliferation | 1 | 0 |  |  |  |  |  |  |  |  |
| GO:0035227\_regulation\_of\_glutamate-cysteine\_ligase\_activity | 1 | 0 |  |  |  |  |  |  |  |  |
| GO:0035229\_positive\_regulation\_of\_glutamate-cysteine\_ligase\_activity | 1 | 0 |  |  |  |  |  |  |  |  |
| GO:0035260\_internal\_genitalia\_morphogenesis | 1 | 0 |  |  |  |  |  |  |  |  |
| GO:0035262\_gonad\_morphogenesis | 1 | 0 |  |  |  |  |  |  |  |  |
| GO:0035287\_head\_segmentation | 1 | 0 |  |  |  |  |  |  |  |  |
| GO:0035289\_posterior\_head\_segmentation | 1 | 0 |  |  |  |  |  |  |  |  |
| GO:0035303\_regulation\_of\_dephosphorylation | 1 | 0 |  |  |  |  |  |  |  |  |
| GO:0035304\_regulation\_of\_protein\_amino\_acid\_dephosphorylation | 1 | 0 |  |  |  |  |  |  |  |  |
| GO:0035305\_negative\_regulation\_of\_dephosphorylation | 1 | 0 |  |  |  |  |  |  |  |  |
| GO:0035308\_negative\_regulation\_of\_protein\_amino\_acid\_dephosphorylation | 1 | 0 |  |  |  |  |  |  |  |  |
| GO:0035313\_wound\_healing\_\_spreading\_of\_epidermal\_cells | 1 | 0 |  |  |  |  |  |  |  |  |
| GO:0040013\_negative\_regulation\_of\_locomotion | 1 | 0 |  |  |  |  |  |  |  |  |
| GO:0040019\_positive\_regulation\_of\_embryonic\_development | 1 | 0 |  |  |  |  |  |  |  |  |
| GO:0040032\_post-embryonic\_body\_morphogenesis | 1 | 0 |  |  |  |  |  |  |  |  |
| GO:0040038\_polar\_body\_extrusion\_after\_meiotic\_divisions | 1 | 0 |  |  |  |  |  |  |  |  |
| GO:0042026\_protein\_refolding | 1 | 0 |  |  |  |  |  |  |  |  |
| GO:0042048\_olfactory\_behavior | 1 | 0 |  |  |  |  |  |  |  |  |
| GO:0042059\_negative\_regulation\_of\_epidermal\_growth\_factor\_receptor\_signaling\_pathway | 1 | 0 |  |  |  |  |  |  |  |  |
| GO:0042073\_intraflagellar\_transport | 1 | 0 |  |  |  |  |  |  |  |  |
| GO:0042078\_germ-line\_stem\_cell\_division | 1 | 0 |  |  |  |  |  |  |  |  |
| GO:0042091\_interleukin-10\_biosynthetic\_process | 1 | 0 |  |  |  |  |  |  |  |  |
| GO:0042103\_positive\_regulation\_of\_T\_cell\_homeostatic\_proliferation | 1 | 0 |  |  |  |  |  |  |  |  |
| GO:0042136\_neurotransmitter\_biosynthetic\_process | 1 | 0 |  |  |  |  |  |  |  |  |
| GO:0042137\_sequestering\_of\_neurotransmitter | 1 | 0 |  |  |  |  |  |  |  |  |
| GO:0042138\_meiotic\_DNA\_double-strand\_break\_formation | 1 | 0 |  |  |  |  |  |  |  |  |
| GO:0042178\_xenobiotic\_catabolic\_process | 1 | 0 |  |  |  |  |  |  |  |  |
| GO:0042225\_interleukin-5\_biosynthetic\_process | 1 | 0 |  |  |  |  |  |  |  |  |
| GO:0042231\_interleukin-13\_biosynthetic\_process | 1 | 0 |  |  |  |  |  |  |  |  |
| GO:0042255\_ribosome\_assembly | 1 | 0 |  |  |  |  |  |  |  |  |
| GO:0042257\_ribosomal\_subunit\_assembly | 1 | 0 |  |  |  |  |  |  |  |  |
| GO:0042264\_peptidyl-aspartic\_acid\_hydroxylation | 1 | 0 |  |  |  |  |  |  |  |  |
| GO:0042276\_error-prone\_postreplication\_DNA\_repair | 1 | 0 |  |  |  |  |  |  |  |  |
| GO:0042297\_vocal\_learning | 1 | 0 |  |  |  |  |  |  |  |  |
| GO:0042309\_homoiothermy | 1 | 0 |  |  |  |  |  |  |  |  |
| GO:0042320\_regulation\_of\_circadian\_sleep\_wake\_cycle\_\_REM\_sleep | 1 | 0 |  |  |  |  |  |  |  |  |
| GO:0042339\_keratan\_sulfate\_metabolic\_process | 1 | 0 |  |  |  |  |  |  |  |  |
| GO:0042347\_negative\_regulation\_of\_NF-kappaB\_import\_into\_nucleus | 1 | 0 |  |  |  |  |  |  |  |  |
| GO:0042360\_vitamin\_E\_metabolic\_process | 1 | 0 |  |  |  |  |  |  |  |  |
| GO:0042363\_fat-soluble\_vitamin\_catabolic\_process | 1 | 0 |  |  |  |  |  |  |  |  |
| GO:0042369\_vitamin\_D\_catabolic\_process | 1 | 0 |  |  |  |  |  |  |  |  |
| GO:0042373\_vitamin\_K\_metabolic\_process | 1 | 0 |  |  |  |  |  |  |  |  |
| GO:0042404\_thyroid\_hormone\_catabolic\_process | 1 | 0 |  |  |  |  |  |  |  |  |
| GO:0042414\_epinephrine\_metabolic\_process | 1 | 0 |  |  |  |  |  |  |  |  |
| GO:0042436\_indole\_derivative\_catabolic\_process | 1 | 0 |  |  |  |  |  |  |  |  |
| GO:0042489\_negative\_regulation\_of\_odontogenesis\_of\_dentine-containing\_tooth | 1 | 0 |  |  |  |  |  |  |  |  |
| GO:0042508\_tyrosine\_phosphorylation\_of\_Stat1\_protein | 1 | 0 |  |  |  |  |  |  |  |  |
| GO:0042518\_negative\_regulation\_of\_tyrosine\_phosphorylation\_of\_Stat3\_protein | 1 | 0 |  |  |  |  |  |  |  |  |
| GO:0042524\_negative\_regulation\_of\_tyrosine\_phosphorylation\_of\_Stat5\_protein | 1 | 0 |  |  |  |  |  |  |  |  |
| GO:0042536\_negative\_regulation\_of\_tumor\_necrosis\_factor\_biosynthetic\_process | 1 | 0 |  |  |  |  |  |  |  |  |
| GO:0042538\_hyperosmotic\_salinity\_response | 1 | 0 |  |  |  |  |  |  |  |  |
| GO:0042628\_mating\_plug\_formation | 1 | 0 |  |  |  |  |  |  |  |  |
| GO:0042631\_cellular\_response\_to\_water\_deprivation | 1 | 0 |  |  |  |  |  |  |  |  |
| GO:0042637\_catagen | 1 | 0 |  |  |  |  |  |  |  |  |
| GO:0042660\_positive\_regulation\_of\_cell\_fate\_specification | 1 | 0 |  |  |  |  |  |  |  |  |
| GO:0042663\_regulation\_of\_endodermal\_cell\_fate\_specification | 1 | 0 |  |  |  |  |  |  |  |  |
| GO:0042664\_negative\_regulation\_of\_endodermal\_cell\_fate\_specification | 1 | 0 |  |  |  |  |  |  |  |  |
| GO:0042667\_auditory\_receptor\_cell\_fate\_specification | 1 | 0 |  |  |  |  |  |  |  |  |
| GO:0042694\_muscle\_cell\_fate\_specification | 1 | 0 |  |  |  |  |  |  |  |  |
| GO:0042706\_eye\_photoreceptor\_cell\_fate\_commitment | 1 | 0 |  |  |  |  |  |  |  |  |
| GO:0042713\_sperm\_ejaculation | 1 | 0 |  |  |  |  |  |  |  |  |
| GO:0042723\_thiamin\_and\_derivative\_metabolic\_process | 1 | 0 |  |  |  |  |  |  |  |  |
| GO:0042737\_drug\_catabolic\_process | 1 | 0 |  |  |  |  |  |  |  |  |
| GO:0042738\_exogenous\_drug\_catabolic\_process | 1 | 0 |  |  |  |  |  |  |  |  |
| GO:0042747\_circadian\_sleep\_wake\_cycle\_\_REM\_sleep | 1 | 0 |  |  |  |  |  |  |  |  |
| GO:0042748\_circadian\_sleep\_wake\_cycle\_\_non-REM\_sleep | 1 | 0 |  |  |  |  |  |  |  |  |
| GO:0042772\_DNA\_damage\_response\_\_signal\_transduction\_resulting\_in\_transcription | 1 | 0 |  |  |  |  |  |  |  |  |
| GO:0042790\_transcription\_of\_nuclear\_rRNA\_large\_RNA\_polymerase\_I\_transcript | 1 | 0 |  |  |  |  |  |  |  |  |
| GO:0042839\_D-glucuronate\_metabolic\_process | 1 | 0 |  |  |  |  |  |  |  |  |
| GO:0042840\_D-glucuronate\_catabolic\_process | 1 | 0 |  |  |  |  |  |  |  |  |
| GO:0042891\_antibiotic\_transport | 1 | 0 |  |  |  |  |  |  |  |  |
| GO:0042892\_chloramphenicol\_transport | 1 | 0 |  |  |  |  |  |  |  |  |
| GO:0042940\_D-amino\_acid\_transport | 1 | 0 |  |  |  |  |  |  |  |  |
| GO:0042941\_D-alanine\_transport | 1 | 0 |  |  |  |  |  |  |  |  |
| GO:0042942\_D-serine\_transport | 1 | 0 |  |  |  |  |  |  |  |  |
| GO:0042983\_amyloid\_precursor\_protein\_biosynthetic\_process | 1 | 0 |  |  |  |  |  |  |  |  |
| GO:0042984\_regulation\_of\_amyloid\_precursor\_protein\_biosynthetic\_process | 1 | 0 |  |  |  |  |  |  |  |  |
| GO:0042985\_negative\_regulation\_of\_amyloid\_precursor\_protein\_biosynthetic\_process | 1 | 0 |  |  |  |  |  |  |  |  |
| GO:0042989\_sequestering\_of\_actin\_monomers | 1 | 0 |  |  |  |  |  |  |  |  |
| GO:0043044\_ATP-dependent\_chromatin\_remodeling | 1 | 0 |  |  |  |  |  |  |  |  |
| GO:0043056\_forward\_locomotion | 1 | 0 |  |  |  |  |  |  |  |  |
| GO:0043060\_meiotic\_metaphase\_I\_plate\_congression | 1 | 0 |  |  |  |  |  |  |  |  |
| GO:0043124\_negative\_regulation\_of\_I-kappaB\_kinase\_NF-kappaB\_cascade | 1 | 0 |  |  |  |  |  |  |  |  |
| GO:0043132\_NAD\_transport | 1 | 0 |  |  |  |  |  |  |  |  |
| GO:0043153\_entrainment\_of\_circadian\_clock\_by\_photoperiod | 1 | 0 |  |  |  |  |  |  |  |  |
| GO:0043171\_peptide\_catabolic\_process | 1 | 0 |  |  |  |  |  |  |  |  |
| GO:0043179\_rhythmic\_excitation | 1 | 0 |  |  |  |  |  |  |  |  |
| GO:0043206\_fibril\_organization | 1 | 0 |  |  |  |  |  |  |  |  |
| GO:0043217\_myelin\_maintenance | 1 | 0 |  |  |  |  |  |  |  |  |
| GO:0043313\_regulation\_of\_neutrophil\_degranulation | 1 | 0 |  |  |  |  |  |  |  |  |
| GO:0043316\_cytotoxic\_T\_cell\_degranulation | 1 | 0 |  |  |  |  |  |  |  |  |
| GO:0043379\_memory\_T\_cell\_differentiation | 1 | 0 |  |  |  |  |  |  |  |  |
| GO:0043380\_regulation\_of\_memory\_T\_cell\_differentiation | 1 | 0 |  |  |  |  |  |  |  |  |
| GO:0043400\_cortisol\_secretion | 1 | 0 |  |  |  |  |  |  |  |  |
| GO:0043415\_positive\_regulation\_of\_skeletal\_muscle\_regeneration | 1 | 0 |  |  |  |  |  |  |  |  |
| GO:0043416\_regulation\_of\_skeletal\_muscle\_regeneration | 1 | 0 |  |  |  |  |  |  |  |  |
| GO:0043437\_butanoic\_acid\_metabolic\_process | 1 | 0 |  |  |  |  |  |  |  |  |
| GO:0043438\_acetoacetic\_acid\_metabolic\_process | 1 | 0 |  |  |  |  |  |  |  |  |
| GO:0043480\_pigment\_accumulation\_in\_tissues | 1 | 0 |  |  |  |  |  |  |  |  |
| GO:0043482\_cellular\_pigment\_accumulation | 1 | 0 |  |  |  |  |  |  |  |  |
| GO:0043486\_histone\_exchange | 1 | 0 |  |  |  |  |  |  |  |  |
| GO:0043496\_regulation\_of\_protein\_homodimerization\_activity | 1 | 0 |  |  |  |  |  |  |  |  |
| GO:0043501\_skeletal\_muscle\_adaptation | 1 | 0 |  |  |  |  |  |  |  |  |
| GO:0043508\_negative\_regulation\_of\_JUN\_kinase\_activity | 1 | 0 |  |  |  |  |  |  |  |  |
| GO:0043517\_positive\_regulation\_of\_DNA\_damage\_response\_\_signal\_transduction\_by\_p53\_class\_mediator | 1 | 0 |  |  |  |  |  |  |  |  |
| GO:0043535\_regulation\_of\_blood\_vessel\_endothelial\_cell\_migration | 1 | 0 |  |  |  |  |  |  |  |  |
| GO:0043537\_negative\_regulation\_of\_blood\_vessel\_endothelial\_cell\_migration | 1 | 0 |  |  |  |  |  |  |  |  |
| GO:0043545\_molybdopterin\_cofactor\_metabolic\_process | 1 | 0 |  |  |  |  |  |  |  |  |
| GO:0043587\_tongue\_morphogenesis | 1 | 0 |  |  |  |  |  |  |  |  |
| GO:0043604\_amide\_biosynthetic\_process | 1 | 0 |  |  |  |  |  |  |  |  |
| GO:0043628\_ncRNA\_3'-end\_processing | 1 | 0 |  |  |  |  |  |  |  |  |
| GO:0044254\_multicellular\_organismal\_protein\_catabolic\_process | 1 | 0 |  |  |  |  |  |  |  |  |
| GO:0044256\_protein\_digestion | 1 | 0 |  |  |  |  |  |  |  |  |
| GO:0044266\_multicellular\_organismal\_macromolecule\_catabolic\_process | 1 | 0 |  |  |  |  |  |  |  |  |
| GO:0045004\_DNA\_replication\_proofreading | 1 | 0 |  |  |  |  |  |  |  |  |
| GO:0045019\_negative\_regulation\_of\_nitric\_oxide\_biosynthetic\_process | 1 | 0 |  |  |  |  |  |  |  |  |
| GO:0045020\_error-prone\_DNA\_repair | 1 | 0 |  |  |  |  |  |  |  |  |
| GO:0045022\_early\_endosome\_to\_late\_endosome\_transport | 1 | 0 |  |  |  |  |  |  |  |  |
| GO:0045062\_extrathymic\_T\_cell\_selection | 1 | 0 |  |  |  |  |  |  |  |  |
| GO:0045074\_regulation\_of\_interleukin-10\_biosynthetic\_process | 1 | 0 |  |  |  |  |  |  |  |  |
| GO:0045082\_positive\_regulation\_of\_interleukin-10\_biosynthetic\_process | 1 | 0 |  |  |  |  |  |  |  |  |
| GO:0045083\_negative\_regulation\_of\_interleukin-12\_biosynthetic\_process | 1 | 0 |  |  |  |  |  |  |  |  |
| GO:0045112\_integrin\_biosynthetic\_process | 1 | 0 |  |  |  |  |  |  |  |  |
| GO:0045113\_regulation\_of\_integrin\_biosynthetic\_process | 1 | 0 |  |  |  |  |  |  |  |  |
| GO:0045188\_regulation\_of\_circadian\_sleep\_wake\_cycle\_\_non-REM\_sleep | 1 | 0 |  |  |  |  |  |  |  |  |
| GO:0045210\_FasL\_biosynthetic\_process | 1 | 0 |  |  |  |  |  |  |  |  |
| GO:0045297\_post-mating\_behavior | 1 | 0 |  |  |  |  |  |  |  |  |
| GO:0045299\_otolith\_mineralization | 1 | 0 |  |  |  |  |  |  |  |  |
| GO:0045329\_carnitine\_biosynthetic\_process | 1 | 0 |  |  |  |  |  |  |  |  |
| GO:0045341\_MHC\_class\_I\_biosynthetic\_process | 1 | 0 |  |  |  |  |  |  |  |  |
| GO:0045343\_regulation\_of\_MHC\_class\_I\_biosynthetic\_process | 1 | 0 |  |  |  |  |  |  |  |  |
| GO:0045347\_negative\_regulation\_of\_MHC\_class\_II\_biosynthetic\_process | 1 | 0 |  |  |  |  |  |  |  |  |
| GO:0045405\_regulation\_of\_interleukin-5\_biosynthetic\_process | 1 | 0 |  |  |  |  |  |  |  |  |
| GO:0045407\_positive\_regulation\_of\_interleukin-5\_biosynthetic\_process | 1 | 0 |  |  |  |  |  |  |  |  |
| GO:0045426\_quinone\_cofactor\_biosynthetic\_process | 1 | 0 |  |  |  |  |  |  |  |  |
| GO:0045448\_mitotic\_cell\_cycle\_\_embryonic | 1 | 0 |  |  |  |  |  |  |  |  |
| GO:0045454\_cell\_redox\_homeostasis | 1 | 0 |  |  |  |  |  |  |  |  |
| GO:0045583\_regulation\_of\_cytotoxic\_T\_cell\_differentiation | 1 | 0 |  |  |  |  |  |  |  |  |
| GO:0045585\_positive\_regulation\_of\_cytotoxic\_T\_cell\_differentiation | 1 | 0 |  |  |  |  |  |  |  |  |
| GO:0045601\_regulation\_of\_endothelial\_cell\_differentiation | 1 | 0 |  |  |  |  |  |  |  |  |
| GO:0045602\_negative\_regulation\_of\_endothelial\_cell\_differentiation | 1 | 0 |  |  |  |  |  |  |  |  |
| GO:0045605\_negative\_regulation\_of\_epidermal\_cell\_differentiation | 1 | 0 |  |  |  |  |  |  |  |  |
| GO:0045606\_positive\_regulation\_of\_epidermal\_cell\_differentiation | 1 | 0 |  |  |  |  |  |  |  |  |
| GO:0045609\_positive\_regulation\_of\_auditory\_receptor\_cell\_differentiation | 1 | 0 |  |  |  |  |  |  |  |  |
| GO:0045617\_negative\_regulation\_of\_keratinocyte\_differentiation | 1 | 0 |  |  |  |  |  |  |  |  |
| GO:0045618\_positive\_regulation\_of\_keratinocyte\_differentiation | 1 | 0 |  |  |  |  |  |  |  |  |
| GO:0045626\_negative\_regulation\_of\_T-helper\_1\_cell\_differentiation | 1 | 0 |  |  |  |  |  |  |  |  |
| GO:0045633\_positive\_regulation\_of\_mechanoreceptor\_differentiation | 1 | 0 |  |  |  |  |  |  |  |  |
| GO:0045650\_negative\_regulation\_of\_macrophage\_differentiation | 1 | 0 |  |  |  |  |  |  |  |  |
| GO:0045656\_negative\_regulation\_of\_monocyte\_differentiation | 1 | 0 |  |  |  |  |  |  |  |  |
| GO:0045657\_positive\_regulation\_of\_monocyte\_differentiation | 1 | 0 |  |  |  |  |  |  |  |  |
| GO:0045659\_negative\_regulation\_of\_neutrophil\_differentiation | 1 | 0 |  |  |  |  |  |  |  |  |
| GO:0045660\_positive\_regulation\_of\_neutrophil\_differentiation | 1 | 0 |  |  |  |  |  |  |  |  |
| GO:0045721\_negative\_regulation\_of\_gluconeogenesis | 1 | 0 |  |  |  |  |  |  |  |  |
| GO:0045724\_positive\_regulation\_of\_flagellum\_assembly | 1 | 0 |  |  |  |  |  |  |  |  |
| GO:0045725\_positive\_regulation\_of\_glycogen\_biosynthetic\_process | 1 | 0 |  |  |  |  |  |  |  |  |
| GO:0045740\_positive\_regulation\_of\_DNA\_replication | 1 | 0 |  |  |  |  |  |  |  |  |
| GO:0045759\_negative\_regulation\_of\_action\_potential | 1 | 0 |  |  |  |  |  |  |  |  |
| GO:0045768\_positive\_regulation\_of\_anti-apoptosis | 1 | 0 |  |  |  |  |  |  |  |  |
| GO:0045769\_negative\_regulation\_of\_asymmetric\_cell\_division | 1 | 0 |  |  |  |  |  |  |  |  |
| GO:0045794\_negative\_regulation\_of\_cell\_volume | 1 | 0 |  |  |  |  |  |  |  |  |
| GO:0045815\_positive\_regulation\_of\_gene\_expression\_\_epigenetic | 1 | 0 |  |  |  |  |  |  |  |  |
| GO:0045818\_negative\_regulation\_of\_glycogen\_catabolic\_process | 1 | 0 |  |  |  |  |  |  |  |  |
| GO:0045842\_positive\_regulation\_of\_mitotic\_metaphase\_anaphase\_transition | 1 | 0 |  |  |  |  |  |  |  |  |
| GO:0045875\_negative\_regulation\_of\_sister\_chromatid\_cohesion | 1 | 0 |  |  |  |  |  |  |  |  |
| GO:0045906\_negative\_regulation\_of\_vasoconstriction | 1 | 0 |  |  |  |  |  |  |  |  |
| GO:0045908\_negative\_regulation\_of\_vasodilation | 1 | 0 |  |  |  |  |  |  |  |  |
| GO:0045909\_positive\_regulation\_of\_vasodilation | 1 | 0 |  |  |  |  |  |  |  |  |
| GO:0045915\_positive\_regulation\_of\_catecholamine\_metabolic\_process | 1 | 0 |  |  |  |  |  |  |  |  |
| GO:0045920\_negative\_regulation\_of\_exocytosis | 1 | 0 |  |  |  |  |  |  |  |  |
| GO:0045924\_regulation\_of\_female\_receptivity | 1 | 0 |  |  |  |  |  |  |  |  |
| GO:0045947\_negative\_regulation\_of\_translational\_initiation | 1 | 0 |  |  |  |  |  |  |  |  |
| GO:0045955\_negative\_regulation\_of\_calcium\_ion-dependent\_exocytosis | 1 | 0 |  |  |  |  |  |  |  |  |
| GO:0045956\_positive\_regulation\_of\_calcium\_ion-dependent\_exocytosis | 1 | 0 |  |  |  |  |  |  |  |  |
| GO:0045964\_positive\_regulation\_of\_dopamine\_metabolic\_process | 1 | 0 |  |  |  |  |  |  |  |  |
| GO:0045988\_negative\_regulation\_of\_striated\_muscle\_contraction | 1 | 0 |  |  |  |  |  |  |  |  |
| GO:0045989\_positive\_regulation\_of\_striated\_muscle\_contraction | 1 | 0 |  |  |  |  |  |  |  |  |
| GO:0045990\_regulation\_of\_transcription\_by\_carbon\_catabolites | 1 | 0 |  |  |  |  |  |  |  |  |
| GO:0045991\_positive\_regulation\_of\_transcription\_by\_carbon\_catabolites | 1 | 0 |  |  |  |  |  |  |  |  |
| GO:0045994\_positive\_regulation\_of\_translational\_initiation\_by\_iron | 1 | 0 |  |  |  |  |  |  |  |  |
| GO:0046007\_negative\_regulation\_of\_activated\_T\_cell\_proliferation | 1 | 0 |  |  |  |  |  |  |  |  |
| GO:0046014\_negative\_regulation\_of\_T\_cell\_homeostatic\_proliferation | 1 | 0 |  |  |  |  |  |  |  |  |
| GO:0046015\_regulation\_of\_transcription\_by\_glucose | 1 | 0 |  |  |  |  |  |  |  |  |
| GO:0046016\_positive\_regulation\_of\_transcription\_by\_glucose | 1 | 0 |  |  |  |  |  |  |  |  |
| GO:0046031\_ADP\_metabolic\_process | 1 | 0 |  |  |  |  |  |  |  |  |
| GO:0046032\_ADP\_catabolic\_process | 1 | 0 |  |  |  |  |  |  |  |  |
| GO:0046061\_dATP\_catabolic\_process | 1 | 0 |  |  |  |  |  |  |  |  |
| GO:0046075\_dTTP\_metabolic\_process | 1 | 0 |  |  |  |  |  |  |  |  |
| GO:0046078\_dUMP\_metabolic\_process | 1 | 0 |  |  |  |  |  |  |  |  |
| GO:0046079\_dUMP\_catabolic\_process | 1 | 0 |  |  |  |  |  |  |  |  |
| GO:0046086\_adenosine\_biosynthetic\_process | 1 | 0 |  |  |  |  |  |  |  |  |
| GO:0046090\_deoxyadenosine\_metabolic\_process | 1 | 0 |  |  |  |  |  |  |  |  |
| GO:0046098\_guanine\_metabolic\_process | 1 | 0 |  |  |  |  |  |  |  |  |
| GO:0046101\_hypoxanthine\_biosynthetic\_process | 1 | 0 |  |  |  |  |  |  |  |  |
| GO:0046102\_inosine\_metabolic\_process | 1 | 0 |  |  |  |  |  |  |  |  |
| GO:0046103\_inosine\_biosynthetic\_process | 1 | 0 |  |  |  |  |  |  |  |  |
| GO:0046108\_uridine\_metabolic\_process | 1 | 0 |  |  |  |  |  |  |  |  |
| GO:0046110\_xanthine\_metabolic\_process | 1 | 0 |  |  |  |  |  |  |  |  |
| GO:0046111\_xanthine\_biosynthetic\_process | 1 | 0 |  |  |  |  |  |  |  |  |
| GO:0046112\_nucleobase\_biosynthetic\_process | 1 | 0 |  |  |  |  |  |  |  |  |
| GO:0046113\_nucleobase\_catabolic\_process | 1 | 0 |  |  |  |  |  |  |  |  |
| GO:0046121\_deoxyribonucleoside\_catabolic\_process | 1 | 0 |  |  |  |  |  |  |  |  |
| GO:0046122\_purine\_deoxyribonucleoside\_metabolic\_process | 1 | 0 |  |  |  |  |  |  |  |  |
| GO:0046124\_purine\_deoxyribonucleoside\_catabolic\_process | 1 | 0 |  |  |  |  |  |  |  |  |
| GO:0046125\_pyrimidine\_deoxyribonucleoside\_metabolic\_process | 1 | 0 |  |  |  |  |  |  |  |  |
| GO:0046131\_pyrimidine\_ribonucleoside\_metabolic\_process | 1 | 0 |  |  |  |  |  |  |  |  |
| GO:0046160\_heme\_a\_metabolic\_process | 1 | 0 |  |  |  |  |  |  |  |  |
| GO:0046218\_indolalkylamine\_catabolic\_process | 1 | 0 |  |  |  |  |  |  |  |  |
| GO:0046292\_formaldehyde\_metabolic\_process | 1 | 0 |  |  |  |  |  |  |  |  |
| GO:0046294\_formaldehyde\_catabolic\_process | 1 | 0 |  |  |  |  |  |  |  |  |
| GO:0046314\_phosphocreatine\_biosynthetic\_process | 1 | 0 |  |  |  |  |  |  |  |  |
| GO:0046327\_glycerol\_biosynthetic\_process\_from\_pyruvate | 1 | 0 |  |  |  |  |  |  |  |  |
| GO:0046329\_negative\_regulation\_of\_JNK\_cascade | 1 | 0 |  |  |  |  |  |  |  |  |
| GO:0046340\_diacylglycerol\_catabolic\_process | 1 | 0 |  |  |  |  |  |  |  |  |
| GO:0046351\_disaccharide\_biosynthetic\_process | 1 | 0 |  |  |  |  |  |  |  |  |
| GO:0046356\_acetyl-CoA\_catabolic\_process | 1 | 0 |  |  |  |  |  |  |  |  |
| GO:0046358\_butyrate\_biosynthetic\_process | 1 | 0 |  |  |  |  |  |  |  |  |
| GO:0046359\_butyrate\_catabolic\_process | 1 | 0 |  |  |  |  |  |  |  |  |
| GO:0046381\_CMP-N-acetylneuraminate\_metabolic\_process | 1 | 0 |  |  |  |  |  |  |  |  |
| GO:0046415\_urate\_metabolic\_process | 1 | 0 |  |  |  |  |  |  |  |  |
| GO:0046416\_D-amino\_acid\_metabolic\_process | 1 | 0 |  |  |  |  |  |  |  |  |
| GO:0046434\_organophosphate\_catabolic\_process | 1 | 0 |  |  |  |  |  |  |  |  |
| GO:0046437\_D-amino\_acid\_biosynthetic\_process | 1 | 0 |  |  |  |  |  |  |  |  |
| GO:0046440\_L-lysine\_metabolic\_process | 1 | 0 |  |  |  |  |  |  |  |  |
| GO:0046449\_creatinine\_metabolic\_process | 1 | 0 |  |  |  |  |  |  |  |  |
| GO:0046471\_phosphatidylglycerol\_metabolic\_process | 1 | 0 |  |  |  |  |  |  |  |  |
| GO:0046473\_phosphatidic\_acid\_metabolic\_process | 1 | 0 |  |  |  |  |  |  |  |  |
| GO:0046476\_glycosylceramide\_biosynthetic\_process | 1 | 0 |  |  |  |  |  |  |  |  |
| GO:0046477\_glycosylceramide\_catabolic\_process | 1 | 0 |  |  |  |  |  |  |  |  |
| GO:0046485\_ether\_lipid\_metabolic\_process | 1 | 0 |  |  |  |  |  |  |  |  |
| GO:0046487\_glyoxylate\_metabolic\_process | 1 | 0 |  |  |  |  |  |  |  |  |
| GO:0046498\_S-adenosylhomocysteine\_metabolic\_process | 1 | 0 |  |  |  |  |  |  |  |  |
| GO:0046552\_photoreceptor\_cell\_fate\_commitment | 1 | 0 |  |  |  |  |  |  |  |  |
| GO:0046586\_regulation\_of\_calcium-dependent\_cell-cell\_adhesion | 1 | 0 |  |  |  |  |  |  |  |  |
| GO:0046587\_positive\_regulation\_of\_calcium-dependent\_cell-cell\_adhesion | 1 | 0 |  |  |  |  |  |  |  |  |
| GO:0046602\_regulation\_of\_mitotic\_centrosome\_separation | 1 | 0 |  |  |  |  |  |  |  |  |
| GO:0046604\_positive\_regulation\_of\_mitotic\_centrosome\_separation | 1 | 0 |  |  |  |  |  |  |  |  |
| GO:0046607\_positive\_regulation\_of\_centrosome\_cycle | 1 | 0 |  |  |  |  |  |  |  |  |
| GO:0046655\_folic\_acid\_metabolic\_process | 1 | 0 |  |  |  |  |  |  |  |  |
| GO:0046685\_response\_to\_arsenic | 1 | 0 |  |  |  |  |  |  |  |  |
| GO:0046692\_sperm\_competition | 1 | 0 |  |  |  |  |  |  |  |  |
| GO:0046707\_IDP\_metabolic\_process | 1 | 0 |  |  |  |  |  |  |  |  |
| GO:0046709\_IDP\_catabolic\_process | 1 | 0 |  |  |  |  |  |  |  |  |
| GO:0046724\_oxalic\_acid\_secretion | 1 | 0 |  |  |  |  |  |  |  |  |
| GO:0046753\_non-lytic\_viral\_release | 1 | 0 |  |  |  |  |  |  |  |  |
| GO:0046755\_non-lytic\_virus\_budding | 1 | 0 |  |  |  |  |  |  |  |  |
| GO:0046826\_negative\_regulation\_of\_protein\_export\_from\_nucleus | 1 | 0 |  |  |  |  |  |  |  |  |
| GO:0046827\_positive\_regulation\_of\_protein\_export\_from\_nucleus | 1 | 0 |  |  |  |  |  |  |  |  |
| GO:0046831\_regulation\_of\_RNA\_export\_from\_nucleus | 1 | 0 |  |  |  |  |  |  |  |  |
| GO:0046834\_lipid\_phosphorylation | 1 | 0 |  |  |  |  |  |  |  |  |
| GO:0046853\_inositol\_and\_derivative\_phosphorylation | 1 | 0 |  |  |  |  |  |  |  |  |
| GO:0046864\_isoprenoid\_transport | 1 | 0 |  |  |  |  |  |  |  |  |
| GO:0046865\_terpenoid\_transport | 1 | 0 |  |  |  |  |  |  |  |  |
| GO:0046877\_regulation\_of\_saliva\_secretion | 1 | 0 |  |  |  |  |  |  |  |  |
| GO:0046878\_positive\_regulation\_of\_saliva\_secretion | 1 | 0 |  |  |  |  |  |  |  |  |
| GO:0046884\_follicle-stimulating\_hormone\_secretion | 1 | 0 |  |  |  |  |  |  |  |  |
| GO:0046898\_response\_to\_cycloheximide | 1 | 0 |  |  |  |  |  |  |  |  |
| GO:0046929\_negative\_regulation\_of\_neurotransmitter\_secretion | 1 | 0 |  |  |  |  |  |  |  |  |
| GO:0046931\_pore\_complex\_biogenesis | 1 | 0 |  |  |  |  |  |  |  |  |
| GO:0046958\_nonassociative\_learning | 1 | 0 |  |  |  |  |  |  |  |  |
| GO:0046960\_sensitization | 1 | 0 |  |  |  |  |  |  |  |  |
| GO:0046986\_negative\_regulation\_of\_hemoglobin\_biosynthetic\_process | 1 | 0 |  |  |  |  |  |  |  |  |
| GO:0047497\_mitochondrion\_transport\_along\_microtubule | 1 | 0 |  |  |  |  |  |  |  |  |
| GO:0048047\_mating\_behavior\_\_sex\_discrimination | 1 | 0 |  |  |  |  |  |  |  |  |
| GO:0048133\_male\_germ-line\_stem\_cell\_division | 1 | 0 |  |  |  |  |  |  |  |  |
| GO:0048137\_spermatocyte\_division | 1 | 0 |  |  |  |  |  |  |  |  |
| GO:0048143\_astrocyte\_activation | 1 | 0 |  |  |  |  |  |  |  |  |
| GO:0048170\_positive\_regulation\_of\_long-term\_neuronal\_synaptic\_plasticity | 1 | 0 |  |  |  |  |  |  |  |  |
| GO:0048199\_vesicle\_targeting\_\_to\_\_from\_or\_within\_Golgi | 1 | 0 |  |  |  |  |  |  |  |  |
| GO:0048241\_epinephrine\_transport | 1 | 0 |  |  |  |  |  |  |  |  |
| GO:0048242\_epinephrine\_secretion | 1 | 0 |  |  |  |  |  |  |  |  |
| GO:0048243\_norepinephrine\_secretion | 1 | 0 |  |  |  |  |  |  |  |  |
| GO:0048247\_lymphocyte\_chemotaxis | 1 | 0 |  |  |  |  |  |  |  |  |
| GO:0048250\_mitochondrial\_iron\_ion\_transport | 1 | 0 |  |  |  |  |  |  |  |  |
| GO:0048259\_regulation\_of\_receptor-mediated\_endocytosis | 1 | 0 |  |  |  |  |  |  |  |  |
| GO:0048260\_positive\_regulation\_of\_receptor-mediated\_endocytosis | 1 | 0 |  |  |  |  |  |  |  |  |
| GO:0048290\_isotype\_switching\_to\_IgA\_isotypes | 1 | 0 |  |  |  |  |  |  |  |  |
| GO:0048296\_regulation\_of\_isotype\_switching\_to\_IgA\_isotypes | 1 | 0 |  |  |  |  |  |  |  |  |
| GO:0048298\_positive\_regulation\_of\_isotype\_switching\_to\_IgA\_isotypes | 1 | 0 |  |  |  |  |  |  |  |  |
| GO:0048319\_axial\_mesoderm\_morphogenesis | 1 | 0 |  |  |  |  |  |  |  |  |
| GO:0048320\_axial\_mesoderm\_formation | 1 | 0 |  |  |  |  |  |  |  |  |
| GO:0048385\_regulation\_of\_retinoic\_acid\_receptor\_signaling\_pathway | 1 | 0 |  |  |  |  |  |  |  |  |
| GO:0048387\_negative\_regulation\_of\_retinoic\_acid\_receptor\_signaling\_pathway | 1 | 0 |  |  |  |  |  |  |  |  |
| GO:0048388\_endosomal\_lumen\_acidification | 1 | 0 |  |  |  |  |  |  |  |  |
| GO:0048389\_intermediate\_mesoderm\_development | 1 | 0 |  |  |  |  |  |  |  |  |
| GO:0048478\_replication\_fork\_protection | 1 | 0 |  |  |  |  |  |  |  |  |
| GO:0048496\_maintenance\_of\_organ\_identity | 1 | 0 |  |  |  |  |  |  |  |  |
| GO:0048525\_negative\_regulation\_of\_viral\_reproduction | 1 | 0 |  |  |  |  |  |  |  |  |
| GO:0048539\_bone\_marrow\_development | 1 | 0 |  |  |  |  |  |  |  |  |
| GO:0048548\_regulation\_of\_pinocytosis | 1 | 0 |  |  |  |  |  |  |  |  |
| GO:0048549\_positive\_regulation\_of\_pinocytosis | 1 | 0 |  |  |  |  |  |  |  |  |
| GO:0048553\_negative\_regulation\_of\_metalloenzyme\_activity | 1 | 0 |  |  |  |  |  |  |  |  |
| GO:0048588\_developmental\_cell\_growth | 1 | 0 |  |  |  |  |  |  |  |  |
| GO:0048601\_oocyte\_morphogenesis | 1 | 0 |  |  |  |  |  |  |  |  |
| GO:0048621\_post-embryonic\_gut\_morphogenesis | 1 | 0 |  |  |  |  |  |  |  |  |
| GO:0048640\_negative\_regulation\_of\_developmental\_growth | 1 | 0 |  |  |  |  |  |  |  |  |
| GO:0048642\_negative\_regulation\_of\_skeletal\_muscle\_tissue\_development | 1 | 0 |  |  |  |  |  |  |  |  |
| GO:0048669\_collateral\_sprouting\_in\_the\_absence\_of\_injury | 1 | 0 |  |  |  |  |  |  |  |  |
| GO:0048680\_positive\_regulation\_of\_axon\_regeneration | 1 | 0 |  |  |  |  |  |  |  |  |
| GO:0048681\_negative\_regulation\_of\_axon\_regeneration | 1 | 0 |  |  |  |  |  |  |  |  |
| GO:0048686\_regulation\_of\_sprouting\_of\_injured\_axon | 1 | 0 |  |  |  |  |  |  |  |  |
| GO:0048687\_positive\_regulation\_of\_sprouting\_of\_injured\_axon | 1 | 0 |  |  |  |  |  |  |  |  |
| GO:0048690\_regulation\_of\_axon\_extension\_involved\_in\_regeneration | 1 | 0 |  |  |  |  |  |  |  |  |
| GO:0048691\_positive\_regulation\_of\_axon\_extension\_involved\_in\_regeneration | 1 | 0 |  |  |  |  |  |  |  |  |
| GO:0048714\_positive\_regulation\_of\_oligodendrocyte\_differentiation | 1 | 0 |  |  |  |  |  |  |  |  |
| GO:0048733\_sebaceous\_gland\_development | 1 | 0 |  |  |  |  |  |  |  |  |
| GO:0048752\_semicircular\_canal\_morphogenesis | 1 | 0 |  |  |  |  |  |  |  |  |
| GO:0048773\_erythrophore\_differentiation | 1 | 0 |  |  |  |  |  |  |  |  |
| GO:0048790\_maintenance\_of\_presynaptic\_active\_zone\_structure | 1 | 0 |  |  |  |  |  |  |  |  |
| GO:0048791\_calcium\_ion-dependent\_exocytosis\_of\_neurotransmitter | 1 | 0 |  |  |  |  |  |  |  |  |
| GO:0048822\_enucleate\_erythrocyte\_development | 1 | 0 |  |  |  |  |  |  |  |  |
| GO:0048866\_stem\_cell\_fate\_specification | 1 | 0 |  |  |  |  |  |  |  |  |
| GO:0048936\_peripheral\_nervous\_system\_neuron\_axonogenesis | 1 | 0 |  |  |  |  |  |  |  |  |
| GO:0050427\_3'-phosphoadenosine\_5'-phosphosulfate\_metabolic\_process | 1 | 0 |  |  |  |  |  |  |  |  |
| GO:0050428\_3'-phosphoadenosine\_5'-phosphosulfate\_biosynthetic\_process | 1 | 0 |  |  |  |  |  |  |  |  |
| GO:0050482\_arachidonic\_acid\_secretion | 1 | 0 |  |  |  |  |  |  |  |  |
| GO:0050667\_homocysteine\_metabolic\_process | 1 | 0 |  |  |  |  |  |  |  |  |
| GO:0050674\_urothelial\_cell\_proliferation | 1 | 0 |  |  |  |  |  |  |  |  |
| GO:0050675\_regulation\_of\_urothelial\_cell\_proliferation | 1 | 0 |  |  |  |  |  |  |  |  |
| GO:0050677\_positive\_regulation\_of\_urothelial\_cell\_proliferation | 1 | 0 |  |  |  |  |  |  |  |  |
| GO:0050691\_regulation\_of\_defense\_response\_to\_virus\_by\_host | 1 | 0 |  |  |  |  |  |  |  |  |
| GO:0050748\_negative\_regulation\_of\_lipoprotein\_metabolic\_process | 1 | 0 |  |  |  |  |  |  |  |  |
| GO:0050757\_thymidylate\_synthase\_biosynthetic\_process | 1 | 0 |  |  |  |  |  |  |  |  |
| GO:0050758\_regulation\_of\_thymidylate\_synthase\_biosynthetic\_process | 1 | 0 |  |  |  |  |  |  |  |  |
| GO:0050760\_negative\_regulation\_of\_thymidylate\_synthase\_biosynthetic\_process | 1 | 0 |  |  |  |  |  |  |  |  |
| GO:0050832\_defense\_response\_to\_fungus | 1 | 0 |  |  |  |  |  |  |  |  |
| GO:0050861\_positive\_regulation\_of\_B\_cell\_receptor\_signaling\_pathway | 1 | 0 |  |  |  |  |  |  |  |  |
| GO:0050862\_positive\_regulation\_of\_T\_cell\_receptor\_signaling\_pathway | 1 | 0 |  |  |  |  |  |  |  |  |
| GO:0050916\_sensory\_perception\_of\_sweet\_taste | 1 | 0 |  |  |  |  |  |  |  |  |
| GO:0050975\_sensory\_perception\_of\_touch | 1 | 0 |  |  |  |  |  |  |  |  |
| GO:0050995\_negative\_regulation\_of\_lipid\_catabolic\_process | 1 | 0 |  |  |  |  |  |  |  |  |
| GO:0051001\_negative\_regulation\_of\_nitric-oxide\_synthase\_activity | 1 | 0 |  |  |  |  |  |  |  |  |
| GO:0051005\_negative\_regulation\_of\_lipoprotein\_lipase\_activity | 1 | 0 |  |  |  |  |  |  |  |  |
| GO:0051006\_positive\_regulation\_of\_lipoprotein\_lipase\_activity | 1 | 0 |  |  |  |  |  |  |  |  |
| GO:0051016\_barbed-end\_actin\_filament\_capping | 1 | 0 |  |  |  |  |  |  |  |  |
| GO:0051029\_rRNA\_transport | 1 | 0 |  |  |  |  |  |  |  |  |
| GO:0051043\_regulation\_of\_membrane\_protein\_ectodomain\_proteolysis | 1 | 0 |  |  |  |  |  |  |  |  |
| GO:0051044\_positive\_regulation\_of\_membrane\_protein\_ectodomain\_proteolysis | 1 | 0 |  |  |  |  |  |  |  |  |
| GO:0051088\_PMA-inducible\_membrane\_protein\_ectodomain\_proteolysis | 1 | 0 |  |  |  |  |  |  |  |  |
| GO:0051102\_DNA\_ligation\_during\_DNA\_recombination | 1 | 0 |  |  |  |  |  |  |  |  |
| GO:0051103\_DNA\_ligation\_during\_DNA\_repair | 1 | 0 |  |  |  |  |  |  |  |  |
| GO:0051125\_regulation\_of\_actin\_nucleation | 1 | 0 |  |  |  |  |  |  |  |  |
| GO:0051127\_positive\_regulation\_of\_actin\_nucleation | 1 | 0 |  |  |  |  |  |  |  |  |
| GO:0051151\_negative\_regulation\_of\_smooth\_muscle\_cell\_differentiation | 1 | 0 |  |  |  |  |  |  |  |  |
| GO:0051154\_negative\_regulation\_of\_striated\_muscle\_cell\_differentiation | 1 | 0 |  |  |  |  |  |  |  |  |
| GO:0051155\_positive\_regulation\_of\_striated\_muscle\_cell\_differentiation | 1 | 0 |  |  |  |  |  |  |  |  |
| GO:0051156\_glucose\_6-phosphate\_metabolic\_process | 1 | 0 |  |  |  |  |  |  |  |  |
| GO:0051187\_cofactor\_catabolic\_process | 1 | 0 |  |  |  |  |  |  |  |  |
| GO:0051189\_prosthetic\_group\_metabolic\_process | 1 | 0 |  |  |  |  |  |  |  |  |
| GO:0051255\_spindle\_midzone\_assembly | 1 | 0 |  |  |  |  |  |  |  |  |
| GO:0051257\_spindle\_midzone\_assembly\_involved\_in\_meiosis | 1 | 0 |  |  |  |  |  |  |  |  |
| GO:0051281\_positive\_regulation\_of\_release\_of\_sequestered\_calcium\_ion\_into\_cytosol | 1 | 0 |  |  |  |  |  |  |  |  |
| GO:0051290\_protein\_heterotetramerization | 1 | 0 |  |  |  |  |  |  |  |  |
| GO:0051305\_chromosome\_movement\_towards\_spindle\_pole | 1 | 0 |  |  |  |  |  |  |  |  |
| GO:0051310\_metaphase\_plate\_congression | 1 | 0 |  |  |  |  |  |  |  |  |
| GO:0051311\_meiotic\_metaphase\_plate\_congression | 1 | 0 |  |  |  |  |  |  |  |  |
| GO:0051340\_regulation\_of\_ligase\_activity | 1 | 0 |  |  |  |  |  |  |  |  |
| GO:0051351\_positive\_regulation\_of\_ligase\_activity | 1 | 0 |  |  |  |  |  |  |  |  |
| GO:0051354\_negative\_regulation\_of\_oxidoreductase\_activity | 1 | 0 |  |  |  |  |  |  |  |  |
| GO:0051355\_proprioception\_during\_equilibrioception | 1 | 0 |  |  |  |  |  |  |  |  |
| GO:0051383\_kinetochore\_organization | 1 | 0 |  |  |  |  |  |  |  |  |
| GO:0051386\_regulation\_of\_nerve\_growth\_factor\_receptor\_signaling\_pathway | 1 | 0 |  |  |  |  |  |  |  |  |
| GO:0051409\_response\_to\_nitrosative\_stress | 1 | 0 |  |  |  |  |  |  |  |  |
| GO:0051457\_maintenance\_of\_protein\_location\_in\_nucleus | 1 | 0 |  |  |  |  |  |  |  |  |
| GO:0051462\_regulation\_of\_cortisol\_secretion | 1 | 0 |  |  |  |  |  |  |  |  |
| GO:0051463\_negative\_regulation\_of\_cortisol\_secretion | 1 | 0 |  |  |  |  |  |  |  |  |
| GO:0051481\_reduction\_of\_cytosolic\_calcium\_ion\_concentration | 1 | 0 |  |  |  |  |  |  |  |  |
| GO:0051482\_elevation\_of\_cytosolic\_calcium\_ion\_concentration\_during\_G-protein\_signaling\_\_coupled\_to\_IP3\_second\_messenger\_(phospholipase\_C\_activating) | 1 | 0 |  |  |  |  |  |  |  |  |
| GO:0051542\_elastin\_biosynthetic\_process | 1 | 0 |  |  |  |  |  |  |  |  |
| GO:0051568\_histone\_H3-K4\_methylation | 1 | 0 |  |  |  |  |  |  |  |  |
| GO:0051569\_regulation\_of\_histone\_H3-K4\_methylation | 1 | 0 |  |  |  |  |  |  |  |  |
| GO:0051570\_regulation\_of\_histone\_H3-K9\_methylation | 1 | 0 |  |  |  |  |  |  |  |  |
| GO:0051573\_negative\_regulation\_of\_histone\_H3-K9\_methylation | 1 | 0 |  |  |  |  |  |  |  |  |
| GO:0051580\_regulation\_of\_neurotransmitter\_uptake | 1 | 0 |  |  |  |  |  |  |  |  |
| GO:0051582\_positive\_regulation\_of\_neurotransmitter\_uptake | 1 | 0 |  |  |  |  |  |  |  |  |
| GO:0051584\_regulation\_of\_dopamine\_uptake | 1 | 0 |  |  |  |  |  |  |  |  |
| GO:0051586\_positive\_regulation\_of\_dopamine\_uptake | 1 | 0 |  |  |  |  |  |  |  |  |
| GO:0051589\_negative\_regulation\_of\_neurotransmitter\_transport | 1 | 0 |  |  |  |  |  |  |  |  |
| GO:0051593\_response\_to\_folic\_acid | 1 | 0 |  |  |  |  |  |  |  |  |
| GO:0051615\_histamine\_uptake | 1 | 0 |  |  |  |  |  |  |  |  |
| GO:0051646\_mitochondrion\_localization | 1 | 0 |  |  |  |  |  |  |  |  |
| GO:0051654\_establishment\_of\_mitochondrion\_localization | 1 | 0 |  |  |  |  |  |  |  |  |
| GO:0051661\_maintenance\_of\_centrosome\_location | 1 | 0 |  |  |  |  |  |  |  |  |
| GO:0051665\_membrane\_raft\_localization | 1 | 0 |  |  |  |  |  |  |  |  |
| GO:0051685\_maintenance\_of\_ER\_location | 1 | 0 |  |  |  |  |  |  |  |  |
| GO:0051693\_actin\_filament\_capping | 1 | 0 |  |  |  |  |  |  |  |  |
| GO:0051701\_interaction\_with\_host | 1 | 0 |  |  |  |  |  |  |  |  |
| GO:0051754\_meiotic\_sister\_chromatid\_cohesion\_\_centromeric | 1 | 0 |  |  |  |  |  |  |  |  |
| GO:0051782\_negative\_regulation\_of\_cell\_division | 1 | 0 |  |  |  |  |  |  |  |  |
| GO:0051790\_short-chain\_fatty\_acid\_biosynthetic\_process | 1 | 0 |  |  |  |  |  |  |  |  |
| GO:0051799\_negative\_regulation\_of\_hair\_follicle\_development | 1 | 0 |  |  |  |  |  |  |  |  |
| GO:0051823\_regulation\_of\_synapse\_structural\_plasticity | 1 | 0 |  |  |  |  |  |  |  |  |
| GO:0051865\_protein\_autoubiquitination | 1 | 0 |  |  |  |  |  |  |  |  |
| GO:0051901\_positive\_regulation\_of\_mitochondrial\_depolarization | 1 | 0 |  |  |  |  |  |  |  |  |
| GO:0051917\_regulation\_of\_fibrinolysis | 1 | 0 |  |  |  |  |  |  |  |  |
| GO:0051918\_negative\_regulation\_of\_fibrinolysis | 1 | 0 |  |  |  |  |  |  |  |  |
| GO:0051929\_positive\_regulation\_of\_calcium\_ion\_transport\_via\_voltage-gated\_calcium\_channel\_activity | 1 | 0 |  |  |  |  |  |  |  |  |
| GO:0051933\_amino\_acid\_uptake\_during\_transmission\_of\_nerve\_impulse | 1 | 0 |  |  |  |  |  |  |  |  |
| GO:0051935\_glutamate\_uptake\_during\_transmission\_of\_nerve\_impulse | 1 | 0 |  |  |  |  |  |  |  |  |
| GO:0051940\_regulation\_of\_catecholamine\_uptake\_during\_transmission\_of\_nerve\_impulse | 1 | 0 |  |  |  |  |  |  |  |  |
| GO:0051944\_positive\_regulation\_of\_catecholamine\_uptake\_during\_transmission\_of\_nerve\_impulse | 1 | 0 |  |  |  |  |  |  |  |  |
| GO:0051961\_negative\_regulation\_of\_nervous\_system\_development | 1 | 0 |  |  |  |  |  |  |  |  |
| GO:0051964\_negative\_regulation\_of\_synaptogenesis | 1 | 0 |  |  |  |  |  |  |  |  |
| GO:0051968\_positive\_regulation\_of\_synaptic\_transmission\_\_glutamatergic | 1 | 0 |  |  |  |  |  |  |  |  |
| GO:0051984\_positive\_regulation\_of\_chromosome\_segregation | 1 | 0 |  |  |  |  |  |  |  |  |
| GO:0051987\_positive\_regulation\_of\_attachment\_of\_spindle\_microtubules\_to\_kinetochore | 1 | 0 |  |  |  |  |  |  |  |  |
| GO:0052173\_response\_to\_defenses\_of\_other\_organism\_during\_symbiotic\_interaction | 1 | 0 |  |  |  |  |  |  |  |  |
| GO:0052200\_response\_to\_host\_defenses | 1 | 0 |  |  |  |  |  |  |  |  |
| GO:0052551\_response\_to\_defense-related\_nitric\_oxide\_production\_by\_other\_organism\_during\_symbiotic\_interaction | 1 | 0 |  |  |  |  |  |  |  |  |
| GO:0052564\_response\_to\_immune\_response\_of\_other\_organism\_during\_symbiotic\_interaction | 1 | 0 |  |  |  |  |  |  |  |  |
| GO:0052565\_response\_to\_defense-related\_host\_nitric\_oxide\_production | 1 | 0 |  |  |  |  |  |  |  |  |
| GO:0052572\_response\_to\_host\_immune\_response | 1 | 0 |  |  |  |  |  |  |  |  |
| GO:0055005\_ventricular\_cardiac\_myofibril\_development | 1 | 0 |  |  |  |  |  |  |  |  |
| GO:0055011\_atrial\_cardiac\_muscle\_cell\_differentiation | 1 | 0 |  |  |  |  |  |  |  |  |
| GO:0055014\_atrial\_cardiac\_muscle\_cell\_development | 1 | 0 |  |  |  |  |  |  |  |  |
| GO:0055078\_sodium\_ion\_homeostasis | 1 | 0 |  |  |  |  |  |  |  |  |
| GO:0055089\_fatty\_acid\_homeostasis | 1 | 0 |  |  |  |  |  |  |  |  |
| GO:0055093\_response\_to\_hyperoxia | 1 | 0 |  |  |  |  |  |  |  |  |
| GO:0060003\_copper\_ion\_export | 1 | 0 |  |  |  |  |  |  |  |  |
| GO:0060005\_vestibular\_reflex | 1 | 0 |  |  |  |  |  |  |  |  |
| GO:0060014\_granulosa\_cell\_differentiation | 1 | 0 |  |  |  |  |  |  |  |  |
| GO:0060018\_astrocyte\_fate\_commitment | 1 | 0 |  |  |  |  |  |  |  |  |
| GO:0060020\_Bergmann\_glial\_cell\_differentiation | 1 | 0 |  |  |  |  |  |  |  |  |
| GO:0060022\_hard\_palate\_development | 1 | 0 |  |  |  |  |  |  |  |  |
| GO:0060034\_notochord\_cell\_differentiation | 1 | 0 |  |  |  |  |  |  |  |  |
| GO:0060035\_notochord\_cell\_development | 1 | 0 |  |  |  |  |  |  |  |  |
| GO:0060046\_regulation\_of\_acrosome\_reaction | 1 | 0 |  |  |  |  |  |  |  |  |
| GO:0060054\_positive\_regulation\_of\_epithelial\_cell\_proliferation\_involved\_in\_wound\_healing | 1 | 0 |  |  |  |  |  |  |  |  |
| GO:0060059\_embryonic\_retina\_morphogenesis\_in\_camera-type\_eye | 1 | 0 |  |  |  |  |  |  |  |  |
| GO:0060061\_Spemann\_organizer\_formation | 1 | 0 |  |  |  |  |  |  |  |  |
| GO:0060064\_Spemann\_organizer\_formation\_at\_the\_anterior\_end\_of\_the\_primitive\_streak | 1 | 0 |  |  |  |  |  |  |  |  |
| GO:0060071\_Wnt\_receptor\_signaling\_pathway\_\_planar\_cell\_polarity\_pathway | 1 | 0 |  |  |  |  |  |  |  |  |
| GO:0060075\_regulation\_of\_resting\_membrane\_potential | 1 | 0 |  |  |  |  |  |  |  |  |
| GO:0060082\_eye\_blink\_reflex | 1 | 0 |  |  |  |  |  |  |  |  |
| GO:0060112\_generation\_of\_ovulation\_cycle\_rhythm | 1 | 0 |  |  |  |  |  |  |  |  |
| GO:0060125\_negative\_regulation\_of\_growth\_hormone\_secretion | 1 | 0 |  |  |  |  |  |  |  |  |
| GO:0060151\_peroxisome\_localization | 1 | 0 |  |  |  |  |  |  |  |  |
| GO:0060152\_microtubule-based\_peroxisome\_localization | 1 | 0 |  |  |  |  |  |  |  |  |
| GO:0060161\_positive\_regulation\_of\_dopamine\_receptor\_signaling\_pathway | 1 | 0 |  |  |  |  |  |  |  |  |
| GO:0060163\_subpallium\_neuron\_fate\_commitment | 1 | 0 |  |  |  |  |  |  |  |  |
| GO:0060165\_regulation\_of\_timing\_of\_subpallium\_neuron\_differentiation | 1 | 0 |  |  |  |  |  |  |  |  |
| GO:0060174\_limb\_bud\_formation | 1 | 0 |  |  |  |  |  |  |  |  |
| GO:0060177\_regulation\_of\_angiotensin\_metabolic\_process | 1 | 0 |  |  |  |  |  |  |  |  |
| GO:0060197\_cloacal\_septation | 1 | 0 |  |  |  |  |  |  |  |  |
| GO:0060231\_mesenchymal\_to\_epithelial\_transition | 1 | 0 |  |  |  |  |  |  |  |  |
| GO:0060254\_regulation\_of\_N-terminal\_protein\_palmitoylation | 1 | 0 |  |  |  |  |  |  |  |  |
| GO:0060261\_positive\_regulation\_of\_transcription\_initiation\_from\_RNA\_polymerase\_II\_promoter | 1 | 0 |  |  |  |  |  |  |  |  |
| GO:0060262\_negative\_regulation\_of\_N-terminal\_protein\_palmitoylation | 1 | 0 |  |  |  |  |  |  |  |  |
| GO:0060263\_regulation\_of\_respiratory\_burst | 1 | 0 |  |  |  |  |  |  |  |  |
| GO:0060264\_regulation\_of\_respiratory\_burst\_during\_acute\_inflammatory\_response | 1 | 0 |  |  |  |  |  |  |  |  |
| GO:0060265\_positive\_regulation\_of\_respiratory\_burst\_during\_acute\_inflammatory\_response | 1 | 0 |  |  |  |  |  |  |  |  |
| GO:0060267\_positive\_regulation\_of\_respiratory\_burst | 1 | 0 |  |  |  |  |  |  |  |  |
| GO:0060272\_embryonic\_skeletal\_joint\_morphogenesis | 1 | 0 |  |  |  |  |  |  |  |  |
| GO:0060297\_regulation\_of\_sarcomere\_organization | 1 | 0 |  |  |  |  |  |  |  |  |
| GO:0060298\_positive\_regulation\_of\_sarcomere\_organization | 1 | 0 |  |  |  |  |  |  |  |  |
| GO:0060315\_negative\_regulation\_of\_ryanodine-sensitive\_calcium-release\_channel\_activity | 1 | 0 |  |  |  |  |  |  |  |  |
| GO:0060371\_regulation\_of\_atrial\_cardiomyocyte\_membrane\_depolarization | 1 | 0 |  |  |  |  |  |  |  |  |
| GO:0060374\_mast\_cell\_differentiation | 1 | 0 |  |  |  |  |  |  |  |  |
| GO:0060375\_regulation\_of\_mast\_cell\_differentiation | 1 | 0 |  |  |  |  |  |  |  |  |
| GO:0060376\_positive\_regulation\_of\_mast\_cell\_differentiation | 1 | 0 |  |  |  |  |  |  |  |  |
| GO:0060390\_regulation\_of\_SMAD\_protein\_nuclear\_translocation | 1 | 0 |  |  |  |  |  |  |  |  |
| GO:0060391\_positive\_regulation\_of\_SMAD\_protein\_nuclear\_translocation | 1 | 0 |  |  |  |  |  |  |  |  |
| GO:0060398\_regulation\_of\_growth\_hormone\_receptor\_signaling\_pathway | 1 | 0 |  |  |  |  |  |  |  |  |
| GO:0060399\_positive\_regulation\_of\_growth\_hormone\_receptor\_signaling\_pathway | 1 | 0 |  |  |  |  |  |  |  |  |
| GO:0060405\_regulation\_of\_penile\_erection | 1 | 0 |  |  |  |  |  |  |  |  |
| GO:0060407\_negative\_regulation\_of\_penile\_erection | 1 | 0 |  |  |  |  |  |  |  |  |
| GO:0060413\_atrial\_septum\_morphogenesis | 1 | 0 |  |  |  |  |  |  |  |  |
| GO:0060414\_aorta\_smooth\_muscle\_tissue\_morphogenesis | 1 | 0 |  |  |  |  |  |  |  |  |
| GO:0060419\_heart\_growth | 1 | 0 |  |  |  |  |  |  |  |  |
| GO:0060420\_regulation\_of\_heart\_growth | 1 | 0 |  |  |  |  |  |  |  |  |
| GO:0060421\_positive\_regulation\_of\_heart\_growth | 1 | 0 |  |  |  |  |  |  |  |  |
| GO:0060431\_primary\_lung\_bud\_formation | 1 | 0 |  |  |  |  |  |  |  |  |
| GO:0060436\_bronchiole\_morphogenesis | 1 | 0 |  |  |  |  |  |  |  |  |
| GO:0060440\_trachea\_formation | 1 | 0 |  |  |  |  |  |  |  |  |
| GO:0060449\_bud\_elongation\_involved\_in\_lung\_branching | 1 | 0 |  |  |  |  |  |  |  |  |
| GO:0060456\_positive\_regulation\_of\_digestive\_system\_process | 1 | 0 |  |  |  |  |  |  |  |  |
| GO:0060461\_right\_lung\_morphogenesis | 1 | 0 |  |  |  |  |  |  |  |  |
| GO:0060481\_lobar\_bronchus\_epithelium\_development | 1 | 0 |  |  |  |  |  |  |  |  |
| GO:0060482\_lobar\_bronchus\_development | 1 | 0 |  |  |  |  |  |  |  |  |
| GO:0060484\_lung-associated\_mesenchyme\_development | 1 | 0 |  |  |  |  |  |  |  |  |
| GO:0060486\_Clara\_cell\_differentiation | 1 | 0 |  |  |  |  |  |  |  |  |
| GO:0060510\_Type\_II\_pneumocyte\_differentiation | 1 | 0 |  |  |  |  |  |  |  |  |
| GO:0060514\_prostate\_induction | 1 | 0 |  |  |  |  |  |  |  |  |
| GO:0060515\_prostate\_field\_specification | 1 | 0 |  |  |  |  |  |  |  |  |
| GO:0060517\_epithelial\_cell\_proliferation\_involved\_in\_prostatic\_bud\_elongation | 1 | 0 |  |  |  |  |  |  |  |  |
| GO:0060520\_activation\_of\_prostate\_induction\_by\_androgen\_receptor\_signaling\_pathway | 1 | 0 |  |  |  |  |  |  |  |  |
| GO:0060535\_trachea\_cartilage\_morphogenesis | 1 | 0 |  |  |  |  |  |  |  |  |
| GO:0060536\_cartilage\_morphogenesis | 1 | 0 |  |  |  |  |  |  |  |  |
| GO:0060563\_neuroepithelial\_cell\_differentiation | 1 | 0 |  |  |  |  |  |  |  |  |
| GO:0060584\_regulation\_of\_prostaglandin-endoperoxide\_synthase\_activity | 1 | 0 |  |  |  |  |  |  |  |  |
| GO:0060585\_positive\_regulation\_of\_prostaglandin-endoperoxidase\_synthase\_activity | 1 | 0 |  |  |  |  |  |  |  |  |
| GO:0060598\_dichotomous\_subdivision\_of\_terminal\_units\_involved\_in\_mammary\_gland\_duct\_morphogenesis | 1 | 0 |  |  |  |  |  |  |  |  |
| GO:0060611\_mammary\_gland\_fat\_development | 1 | 0 |  |  |  |  |  |  |  |  |
| GO:0060618\_nipple\_development | 1 | 0 |  |  |  |  |  |  |  |  |
| GO:0060631\_regulation\_of\_meiosis\_I | 1 | 0 |  |  |  |  |  |  |  |  |
| GO:0060649\_mammary\_gland\_bud\_elongation | 1 | 0 |  |  |  |  |  |  |  |  |
| GO:0060658\_nipple\_morphogenesis | 1 | 0 |  |  |  |  |  |  |  |  |
| GO:0060659\_nipple\_sheath\_formation | 1 | 0 |  |  |  |  |  |  |  |  |
| GO:0060668\_regulation\_of\_branching\_involved\_in\_salivary\_gland\_morphogenesis\_by\_extracellular\_matrix-epithelial\_cell\_signaling | 1 | 0 |  |  |  |  |  |  |  |  |
| GO:0060683\_regulation\_of\_branching\_involved\_in\_salivary\_gland\_morphogenesis\_by\_epithelial-mesenchymal\_signaling | 1 | 0 |  |  |  |  |  |  |  |  |
| GO:0060691\_epithelial\_cell\_maturation\_involved\_in\_salivary\_gland\_development | 1 | 0 |  |  |  |  |  |  |  |  |
| GO:0060709\_glycogen\_cell\_development\_involved\_in\_embryonic\_placenta\_development | 1 | 0 |  |  |  |  |  |  |  |  |
| GO:0060732\_positive\_regulation\_of\_inositol\_phosphate\_biosynthetic\_process | 1 | 0 |  |  |  |  |  |  |  |  |
| GO:0060739\_mesenchymal-epithelial\_cell\_signaling\_involved\_in\_prostate\_gland\_development | 1 | 0 |  |  |  |  |  |  |  |  |
| GO:0060781\_mesenchymal\_cell\_proliferation\_involved\_in\_prostate\_gland\_development | 1 | 0 |  |  |  |  |  |  |  |  |
| GO:0060782\_regulation\_of\_mesenchymal\_cell\_proliferation\_involved\_in\_prostate\_gland\_development | 1 | 0 |  |  |  |  |  |  |  |  |
| GO:0060783\_mesenchymal\_smoothened\_signaling\_pathway\_involved\_in\_prostate\_gland\_development | 1 | 0 |  |  |  |  |  |  |  |  |
| GO:0060872\_semicircular\_canal\_development | 1 | 0 |  |  |  |  |  |  |  |  |
| GO:0060896\_neural\_plate\_pattern\_specification | 1 | 0 |  |  |  |  |  |  |  |  |
| GO:0070091\_glucagon\_secretion | 1 | 0 |  |  |  |  |  |  |  |  |
| GO:0070162\_adiponectin\_secretion | 1 | 0 |  |  |  |  |  |  |  |  |
| GO:0070163\_regulation\_of\_adiponectin\_secretion | 1 | 0 |  |  |  |  |  |  |  |  |
| GO:0070164\_negative\_regulation\_of\_adiponectin\_secretion | 1 | 0 |  |  |  |  |  |  |  |  |
| GO:0070178\_D-serine\_metabolic\_process | 1 | 0 |  |  |  |  |  |  |  |  |
| GO:0070179\_D-serine\_biosynthetic\_process | 1 | 0 |  |  |  |  |  |  |  |  |
| GO:0070296\_sarcoplasmic\_reticulum\_calcium\_ion\_transport | 1 | 0 |  |  |  |  |  |  |  |  |
| GO:0070303\_negative\_regulation\_of\_stress-activated\_protein\_kinase\_signaling\_pathway | 1 | 0 |  |  |  |  |  |  |  |  |
| GO:0070328\_triglyceride\_homeostasis | 1 | 0 |  |  |  |  |  |  |  |  |
| GO:0070365\_hepatocyte\_differentiation | 1 | 0 |  |  |  |  |  |  |  |  |
| GO:0070384\_Harderian\_gland\_development | 1 | 0 |  |  |  |  |  |  |  |  |
| GO:0070391\_response\_to\_lipoteichoic\_acid | 1 | 0 |  |  |  |  |  |  |  |  |
| GO:0070424\_regulation\_of\_nucleotide-binding\_oligomerization\_domain\_containing\_signaling\_pathway | 1 | 0 |  |  |  |  |  |  |  |  |
| GO:0070426\_positive\_regulation\_of\_nucleotide-binding\_oligomerization\_domain\_containing\_signaling\_pathway | 1 | 0 |  |  |  |  |  |  |  |  |
| GO:0070428\_regulation\_of\_nucleotide-binding\_oligomerization\_domain\_containing\_1\_signaling\_pathway | 1 | 0 |  |  |  |  |  |  |  |  |
| GO:0070430\_positive\_regulation\_of\_nucleotide-binding\_oligomerization\_domain\_containing\_1\_signaling\_pathway | 1 | 0 |  |  |  |  |  |  |  |  |
| GO:0070432\_regulation\_of\_nucleotide-binding\_oligomerization\_domain\_containing\_2\_signaling\_pathway | 1 | 0 |  |  |  |  |  |  |  |  |
| GO:0070434\_positive\_regulation\_of\_nucleotide-binding\_oligomerization\_domain\_containing\_2\_signaling\_pathway | 1 | 0 |  |  |  |  |  |  |  |  |
| GO:0070493\_thrombin\_receptor\_signaling\_pathway | 1 | 0 |  |  |  |  |  |  |  |  |
| GO:0070508\_cholesterol\_import | 1 | 0 |  |  |  |  |  |  |  |  |
| GO:0070527\_platelet\_aggregation | 1 | 0 |  |  |  |  |  |  |  |  |
| GO:0070528\_protein\_kinase\_C\_signaling\_cascade | 1 | 0 |  |  |  |  |  |  |  |  |
| GO:0070555\_response\_to\_interleukin-1 | 1 | 0 |  |  |  |  |  |  |  |  |
| GO:0070560\_protein\_secretion\_by\_platelet | 1 | 0 |  |  |  |  |  |  |  |  |
| GO:0070561\_vitamin\_D\_receptor\_signaling\_pathway | 1 | 0 |  |  |  |  |  |  |  |  |
| GO:0070562\_regulation\_of\_vitamin\_D\_receptor\_signaling\_pathway | 1 | 0 |  |  |  |  |  |  |  |  |
| GO:0070571\_negative\_regulation\_of\_neuron\_projection\_regeneration | 1 | 0 |  |  |  |  |  |  |  |  |
| GO:0070572\_positive\_regulation\_of\_neuron\_projection\_regeneration | 1 | 0 |  |  |  |  |  |  |  |  |
| GO:0070613\_regulation\_of\_protein\_processing | 1 | 0 |  |  |  |  |  |  |  |  |
| GO:0070627\_ferrous\_iron\_import | 1 | 0 |  |  |  |  |  |  |  |  |
| GO:0070669\_response\_to\_interleukin-2 | 1 | 0 |  |  |  |  |  |  |  |  |
| GO:0070670\_response\_to\_interleukin-4 | 1 | 0 |  |  |  |  |  |  |  |  |
| GO:0070671\_response\_to\_interleukin-12 | 1 | 0 |  |  |  |  |  |  |  |  |
| GO:0070672\_response\_to\_interleukin-15 | 1 | 0 |  |  |  |  |  |  |  |  |
| GO:0070673\_response\_to\_interleukin-18 | 1 | 0 |  |  |  |  |  |  |  |  |
| GO:0070828\_heterochromatin\_organization | 1 | 0 |  |  |  |  |  |  |  |  |
| GO:0070874\_negative\_regulation\_of\_glycogen\_metabolic\_process | 1 | 0 |  |  |  |  |  |  |  |  |
| GO:0075136\_response\_to\_host | 1 | 0 |  |  |  |  |  |  |  |  |
| GO:0080010\_regulation\_of\_oxygen\_and\_reactive\_oxygen\_species\_metabolic\_process | 1 | 0 |  |  |  |  |  |  |  |  |
| GO:0090032\_negative\_regulation\_of\_steroid\_hormone\_biosynthetic\_process | 1 | 0 |  |  |  |  |  |  |  |  |
| GO:0000012\_single\_strand\_break\_repair | 2 | 0 |  |  |  |  |  |  |  |  |
| GO:0000019\_regulation\_of\_mitotic\_recombination | 2 | 0 |  |  |  |  |  |  |  |  |
| GO:0000076\_DNA\_replication\_checkpoint | 2 | 0 |  |  |  |  |  |  |  |  |
| GO:0000080\_G1\_phase\_of\_mitotic\_cell\_cycle | 2 | 0 |  |  |  |  |  |  |  |  |
| GO:0000083\_regulation\_of\_transcription\_of\_G1\_S-phase\_of\_mitotic\_cell\_cycle | 2 | 0 |  |  |  |  |  |  |  |  |
| GO:0000085\_G2\_phase\_of\_mitotic\_cell\_cycle | 2 | 0 |  |  |  |  |  |  |  |  |
| GO:0000289\_nuclear-transcribed\_mRNA\_poly(A)\_tail\_shortening | 2 | 0 |  |  |  |  |  |  |  |  |
| GO:0000381\_regulation\_of\_alternative\_nuclear\_mRNA\_splicing\_\_via\_spliceosome | 2 | 0 |  |  |  |  |  |  |  |  |
| GO:0000712\_resolution\_of\_meiotic\_joint\_molecules\_as\_recombinants | 2 | 0 |  |  |  |  |  |  |  |  |
| GO:0000720\_pyrimidine\_dimer\_repair\_by\_nucleotide-excision\_repair | 2 | 0 |  |  |  |  |  |  |  |  |
| GO:0001302\_replicative\_cell\_aging | 2 | 0 |  |  |  |  |  |  |  |  |
| GO:0001306\_age-dependent\_response\_to\_oxidative\_stress | 2 | 0 |  |  |  |  |  |  |  |  |
| GO:0001514\_selenocysteine\_incorporation | 2 | 0 |  |  |  |  |  |  |  |  |
| GO:0001522\_pseudouridine\_synthesis | 2 | 0 |  |  |  |  |  |  |  |  |
| GO:0001543\_ovarian\_follicle\_rupture | 2 | 0 |  |  |  |  |  |  |  |  |
| GO:0001561\_fatty\_acid\_alpha-oxidation | 2 | 0 |  |  |  |  |  |  |  |  |
| GO:0001675\_acrosome\_assembly | 2 | 0 |  |  |  |  |  |  |  |  |
| GO:0001743\_optic\_placode\_formation | 2 | 0 |  |  |  |  |  |  |  |  |
| GO:0001767\_establishment\_of\_lymphocyte\_polarity | 2 | 0 |  |  |  |  |  |  |  |  |
| GO:0001768\_establishment\_of\_T\_cell\_polarity | 2 | 0 |  |  |  |  |  |  |  |  |
| GO:0001771\_formation\_of\_immunological\_synapse | 2 | 0 |  |  |  |  |  |  |  |  |
| GO:0001781\_neutrophil\_apoptosis | 2 | 0 |  |  |  |  |  |  |  |  |
| GO:0001787\_natural\_killer\_cell\_proliferation | 2 | 0 |  |  |  |  |  |  |  |  |
| GO:0001788\_antibody-dependent\_cellular\_cytotoxicity | 2 | 0 |  |  |  |  |  |  |  |  |
| GO:0001806\_type\_IV\_hypersensitivity | 2 | 0 |  |  |  |  |  |  |  |  |
| GO:0001807\_regulation\_of\_type\_IV\_hypersensitivity | 2 | 0 |  |  |  |  |  |  |  |  |
| GO:0001808\_negative\_regulation\_of\_type\_IV\_hypersensitivity | 2 | 0 |  |  |  |  |  |  |  |  |
| GO:0001823\_mesonephros\_development | 2 | 0 |  |  |  |  |  |  |  |  |
| GO:0001845\_phagolysosome\_formation | 2 | 0 |  |  |  |  |  |  |  |  |
| GO:0001866\_NK\_T\_cell\_proliferation | 2 | 0 |  |  |  |  |  |  |  |  |
| GO:0001879\_detection\_of\_yeast | 2 | 0 |  |  |  |  |  |  |  |  |
| GO:0001886\_endothelial\_cell\_morphogenesis | 2 | 0 |  |  |  |  |  |  |  |  |
| GO:0001919\_regulation\_of\_receptor\_recycling | 2 | 0 |  |  |  |  |  |  |  |  |
| GO:0001954\_positive\_regulation\_of\_cell-matrix\_adhesion | 2 | 0 |  |  |  |  |  |  |  |  |
| GO:0001977\_renal\_system\_process\_involved\_in\_regulation\_of\_blood\_volume | 2 | 0 |  |  |  |  |  |  |  |  |
| GO:0001982\_baroreceptor\_response\_to\_decreased\_systemic\_arterial\_blood\_pressure | 2 | 0 |  |  |  |  |  |  |  |  |
| GO:0001983\_baroreceptor\_response\_to\_increased\_systemic\_arterial\_blood\_pressure | 2 | 0 |  |  |  |  |  |  |  |  |
| GO:0001992\_regulation\_of\_systemic\_arterial\_blood\_pressure\_by\_vasopressin | 2 | 0 |  |  |  |  |  |  |  |  |
| GO:0001997\_positive\_regulation\_of\_the\_force\_of\_heart\_contraction\_by\_epinephrine-norepinephrine | 2 | 0 |  |  |  |  |  |  |  |  |
| GO:0001998\_angiotensin\_mediated\_vasoconstriction\_involved\_in\_regulation\_of\_systemic\_arterial\_blood\_pressure | 2 | 0 |  |  |  |  |  |  |  |  |
| GO:0001999\_renal\_response\_to\_blood\_flow\_during\_renin-angiotensin\_regulation\_of\_systemic\_arterial\_blood\_pressure | 2 | 0 |  |  |  |  |  |  |  |  |
| GO:0002018\_renin-angiotensin\_regulation\_of\_aldosterone\_production | 2 | 0 |  |  |  |  |  |  |  |  |
| GO:0002019\_regulation\_of\_renal\_output\_by\_angiotensin | 2 | 0 |  |  |  |  |  |  |  |  |
| GO:0002024\_diet\_induced\_thermogenesis | 2 | 0 |  |  |  |  |  |  |  |  |
| GO:0002025\_vasodilation\_by\_norepinephrine-epinephrine\_involved\_in\_regulation\_of\_systemic\_arterial\_blood\_pressure | 2 | 0 |  |  |  |  |  |  |  |  |
| GO:0002029\_desensitization\_of\_G-protein\_coupled\_receptor\_protein\_signaling\_pathway | 2 | 0 |  |  |  |  |  |  |  |  |
| GO:0002033\_vasodilation\_by\_angiotensin\_involved\_in\_regulation\_of\_systemic\_arterial\_blood\_pressure | 2 | 0 |  |  |  |  |  |  |  |  |
| GO:0002066\_columnar\_cuboidal\_epithelial\_cell\_development | 2 | 0 |  |  |  |  |  |  |  |  |
| GO:0002072\_optic\_cup\_morphogenesis\_involved\_in\_camera-type\_eye\_development | 2 | 0 |  |  |  |  |  |  |  |  |
| GO:0002138\_retinoic\_acid\_biosynthetic\_process | 2 | 0 |  |  |  |  |  |  |  |  |
| GO:0002223\_stimulatory\_C-type\_lectin\_receptor\_signaling\_pathway | 2 | 0 |  |  |  |  |  |  |  |  |
| GO:0002246\_healing\_during\_inflammatory\_response | 2 | 0 |  |  |  |  |  |  |  |  |
| GO:0002251\_organ\_or\_tissue\_specific\_immune\_response | 2 | 0 |  |  |  |  |  |  |  |  |
| GO:0002266\_follicular\_dendritic\_cell\_activation | 2 | 0 |  |  |  |  |  |  |  |  |
| GO:0002268\_follicular\_dendritic\_cell\_differentiation | 2 | 0 |  |  |  |  |  |  |  |  |
| GO:0002327\_immature\_B\_cell\_differentiation | 2 | 0 |  |  |  |  |  |  |  |  |
| GO:0002329\_pre-B\_cell\_differentiation | 2 | 0 |  |  |  |  |  |  |  |  |
| GO:0002339\_B\_cell\_selection | 2 | 0 |  |  |  |  |  |  |  |  |
| GO:0002352\_B\_cell\_negative\_selection | 2 | 0 |  |  |  |  |  |  |  |  |
| GO:0002358\_B\_cell\_homeostatic\_proliferation | 2 | 0 |  |  |  |  |  |  |  |  |
| GO:0002385\_mucosal\_immune\_response | 2 | 0 |  |  |  |  |  |  |  |  |
| GO:0002514\_B\_cell\_tolerance\_induction | 2 | 0 |  |  |  |  |  |  |  |  |
| GO:0002523\_leukocyte\_migration\_during\_inflammatory\_response | 2 | 0 |  |  |  |  |  |  |  |  |
| GO:0002536\_respiratory\_burst\_during\_acute\_inflammatory\_response | 2 | 0 |  |  |  |  |  |  |  |  |
| GO:0002537\_production\_of\_nitric\_oxide\_during\_acute\_inflammatory\_response | 2 | 0 |  |  |  |  |  |  |  |  |
| GO:0002576\_platelet\_degranulation | 2 | 0 |  |  |  |  |  |  |  |  |
| GO:0002639\_positive\_regulation\_of\_immunoglobulin\_production | 2 | 0 |  |  |  |  |  |  |  |  |
| GO:0002661\_regulation\_of\_B\_cell\_tolerance\_induction | 2 | 0 |  |  |  |  |  |  |  |  |
| GO:0002663\_positive\_regulation\_of\_B\_cell\_tolerance\_induction | 2 | 0 |  |  |  |  |  |  |  |  |
| GO:0002676\_regulation\_of\_chronic\_inflammatory\_response | 2 | 0 |  |  |  |  |  |  |  |  |
| GO:0002679\_respiratory\_burst\_during\_defense\_response | 2 | 0 |  |  |  |  |  |  |  |  |
| GO:0002686\_negative\_regulation\_of\_leukocyte\_migration | 2 | 0 |  |  |  |  |  |  |  |  |
| GO:0002720\_positive\_regulation\_of\_cytokine\_production\_during\_immune\_response | 2 | 0 |  |  |  |  |  |  |  |  |
| GO:0002752\_cell\_surface\_pattern\_recognition\_receptor\_signaling\_pathway | 2 | 0 |  |  |  |  |  |  |  |  |
| GO:0002755\_MyD88-dependent\_toll-like\_receptor\_signaling\_pathway | 2 | 0 |  |  |  |  |  |  |  |  |
| GO:0002765\_immune\_response-inhibiting\_signal\_transduction | 2 | 0 |  |  |  |  |  |  |  |  |
| GO:0002921\_negative\_regulation\_of\_humoral\_immune\_response | 2 | 0 |  |  |  |  |  |  |  |  |
| GO:0002922\_positive\_regulation\_of\_humoral\_immune\_response | 2 | 0 |  |  |  |  |  |  |  |  |
| GO:0002924\_negative\_regulation\_of\_humoral\_immune\_response\_mediated\_by\_circulating\_immunoglobulin | 2 | 0 |  |  |  |  |  |  |  |  |
| GO:0002925\_positive\_regulation\_of\_humoral\_immune\_response\_mediated\_by\_circulating\_immunoglobulin | 2 | 0 |  |  |  |  |  |  |  |  |
| GO:0003057\_regulation\_of\_the\_force\_of\_heart\_contraction\_by\_chemical\_signal | 2 | 0 |  |  |  |  |  |  |  |  |
| GO:0003099\_positive\_regulation\_of\_the\_force\_of\_heart\_contraction\_by\_chemical\_signal | 2 | 0 |  |  |  |  |  |  |  |  |
| GO:0006021\_inositol\_biosynthetic\_process | 2 | 0 |  |  |  |  |  |  |  |  |
| GO:0006042\_glucosamine\_biosynthetic\_process | 2 | 0 |  |  |  |  |  |  |  |  |
| GO:0006045\_N-acetylglucosamine\_biosynthetic\_process | 2 | 0 |  |  |  |  |  |  |  |  |
| GO:0006048\_UDP-N-acetylglucosamine\_biosynthetic\_process | 2 | 0 |  |  |  |  |  |  |  |  |
| GO:0006054\_N-acetylneuraminate\_metabolic\_process | 2 | 0 |  |  |  |  |  |  |  |  |
| GO:0006059\_hexitol\_metabolic\_process | 2 | 0 |  |  |  |  |  |  |  |  |
| GO:0006063\_uronic\_acid\_metabolic\_process | 2 | 0 |  |  |  |  |  |  |  |  |
| GO:0006068\_ethanol\_catabolic\_process | 2 | 0 |  |  |  |  |  |  |  |  |
| GO:0006083\_acetate\_metabolic\_process | 2 | 0 |  |  |  |  |  |  |  |  |
| GO:0006089\_lactate\_metabolic\_process | 2 | 0 |  |  |  |  |  |  |  |  |
| GO:0006105\_succinate\_metabolic\_process | 2 | 0 |  |  |  |  |  |  |  |  |
| GO:0006106\_fumarate\_metabolic\_process | 2 | 0 |  |  |  |  |  |  |  |  |
| GO:0006110\_regulation\_of\_glycolysis | 2 | 0 |  |  |  |  |  |  |  |  |
| GO:0006113\_fermentation | 2 | 0 |  |  |  |  |  |  |  |  |
| GO:0006114\_glycerol\_biosynthetic\_process | 2 | 0 |  |  |  |  |  |  |  |  |
| GO:0006122\_mitochondrial\_electron\_transport\_\_ubiquinol\_to\_cytochrome\_c | 2 | 0 |  |  |  |  |  |  |  |  |
| GO:0006152\_purine\_nucleoside\_catabolic\_process | 2 | 0 |  |  |  |  |  |  |  |  |
| GO:0006168\_adenine\_salvage | 2 | 0 |  |  |  |  |  |  |  |  |
| GO:0006200\_ATP\_catabolic\_process | 2 | 0 |  |  |  |  |  |  |  |  |
| GO:0006206\_pyrimidine\_base\_metabolic\_process | 2 | 0 |  |  |  |  |  |  |  |  |
| GO:0006213\_pyrimidine\_nucleoside\_metabolic\_process | 2 | 0 |  |  |  |  |  |  |  |  |
| GO:0006265\_DNA\_topological\_change | 2 | 0 |  |  |  |  |  |  |  |  |
| GO:0006278\_RNA-dependent\_DNA\_replication | 2 | 0 |  |  |  |  |  |  |  |  |
| GO:0006312\_mitotic\_recombination | 2 | 0 |  |  |  |  |  |  |  |  |
| GO:0006398\_histone\_mRNA\_3'-end\_processing | 2 | 0 |  |  |  |  |  |  |  |  |
| GO:0006418\_tRNA\_aminoacylation\_for\_protein\_translation | 2 | 0 |  |  |  |  |  |  |  |  |
| GO:0006451\_translational\_readthrough | 2 | 0 |  |  |  |  |  |  |  |  |
| GO:0006477\_protein\_amino\_acid\_sulfation | 2 | 0 |  |  |  |  |  |  |  |  |
| GO:0006482\_protein\_amino\_acid\_demethylation | 2 | 0 |  |  |  |  |  |  |  |  |
| GO:0006499\_N-terminal\_protein\_myristoylation | 2 | 0 |  |  |  |  |  |  |  |  |
| GO:0006525\_arginine\_metabolic\_process | 2 | 0 |  |  |  |  |  |  |  |  |
| GO:0006527\_arginine\_catabolic\_process | 2 | 0 |  |  |  |  |  |  |  |  |
| GO:0006532\_aspartate\_biosynthetic\_process | 2 | 0 |  |  |  |  |  |  |  |  |
| GO:0006538\_glutamate\_catabolic\_process | 2 | 0 |  |  |  |  |  |  |  |  |
| GO:0006558\_L-phenylalanine\_metabolic\_process | 2 | 0 |  |  |  |  |  |  |  |  |
| GO:0006563\_L-serine\_metabolic\_process | 2 | 0 |  |  |  |  |  |  |  |  |
| GO:0006566\_threonine\_metabolic\_process | 2 | 0 |  |  |  |  |  |  |  |  |
| GO:0006568\_tryptophan\_metabolic\_process | 2 | 0 |  |  |  |  |  |  |  |  |
| GO:0006583\_melanin\_biosynthetic\_process\_from\_tyrosine | 2 | 0 |  |  |  |  |  |  |  |  |
| GO:0006600\_creatine\_metabolic\_process | 2 | 0 |  |  |  |  |  |  |  |  |
| GO:0006603\_phosphocreatine\_metabolic\_process | 2 | 0 |  |  |  |  |  |  |  |  |
| GO:0006610\_ribosomal\_protein\_import\_into\_nucleus | 2 | 0 |  |  |  |  |  |  |  |  |
| GO:0006642\_triglyceride\_mobilization | 2 | 0 |  |  |  |  |  |  |  |  |
| GO:0006649\_phospholipid\_transfer\_to\_membrane | 2 | 0 |  |  |  |  |  |  |  |  |
| GO:0006681\_galactosylceramide\_metabolic\_process | 2 | 0 |  |  |  |  |  |  |  |  |
| GO:0006686\_sphingomyelin\_biosynthetic\_process | 2 | 0 |  |  |  |  |  |  |  |  |
| GO:0006702\_androgen\_biosynthetic\_process | 2 | 0 |  |  |  |  |  |  |  |  |
| GO:0006750\_glutathione\_biosynthetic\_process | 2 | 0 |  |  |  |  |  |  |  |  |
| GO:0006760\_folic\_acid\_and\_derivative\_metabolic\_process | 2 | 0 |  |  |  |  |  |  |  |  |
| GO:0006868\_glutamine\_transport | 2 | 0 |  |  |  |  |  |  |  |  |
| GO:0006907\_pinocytosis | 2 | 0 |  |  |  |  |  |  |  |  |
| GO:0006925\_inflammatory\_cell\_apoptosis | 2 | 0 |  |  |  |  |  |  |  |  |
| GO:0006977\_DNA\_damage\_response\_\_signal\_transduction\_by\_p53\_class\_mediator\_resulting\_in\_cell\_cycle\_arrest | 2 | 0 |  |  |  |  |  |  |  |  |
| GO:0006991\_response\_to\_sterol\_depletion | 2 | 0 |  |  |  |  |  |  |  |  |
| GO:0007004\_telomere\_maintenance\_via\_telomerase | 2 | 0 |  |  |  |  |  |  |  |  |
| GO:0007020\_microtubule\_nucleation | 2 | 0 |  |  |  |  |  |  |  |  |
| GO:0007030\_Golgi\_organization | 2 | 0 |  |  |  |  |  |  |  |  |
| GO:0007042\_lysosomal\_lumen\_acidification | 2 | 0 |  |  |  |  |  |  |  |  |
| GO:0007060\_male\_meiosis\_chromosome\_segregation | 2 | 0 |  |  |  |  |  |  |  |  |
| GO:0007089\_traversing\_start\_control\_point\_of\_mitotic\_cell\_cycle | 2 | 0 |  |  |  |  |  |  |  |  |
| GO:0007094\_mitotic\_cell\_cycle\_spindle\_assembly\_checkpoint | 2 | 0 |  |  |  |  |  |  |  |  |
| GO:0007097\_nuclear\_migration | 2 | 0 |  |  |  |  |  |  |  |  |
| GO:0007100\_mitotic\_centrosome\_separation | 2 | 0 |  |  |  |  |  |  |  |  |
| GO:0007132\_meiotic\_metaphase\_I | 2 | 0 |  |  |  |  |  |  |  |  |
| GO:0007171\_activation\_of\_transmembrane\_receptor\_protein\_tyrosine\_kinase\_activity | 2 | 0 |  |  |  |  |  |  |  |  |
| GO:0007182\_common-partner\_SMAD\_protein\_phosphorylation | 2 | 0 |  |  |  |  |  |  |  |  |
| GO:0007185\_transmembrane\_receptor\_protein\_tyrosine\_phosphatase\_signaling\_pathway | 2 | 0 |  |  |  |  |  |  |  |  |
| GO:0007205\_activation\_of\_protein\_kinase\_C\_activity\_by\_G-protein\_coupled\_receptor\_protein\_signaling\_pathway | 2 | 0 |  |  |  |  |  |  |  |  |
| GO:0007210\_serotonin\_receptor\_signaling\_pathway | 2 | 0 |  |  |  |  |  |  |  |  |
| GO:0007220\_Notch\_receptor\_processing | 2 | 0 |  |  |  |  |  |  |  |  |
| GO:0007256\_activation\_of\_JNKK\_activity | 2 | 0 |  |  |  |  |  |  |  |  |
| GO:0007258\_JUN\_phosphorylation | 2 | 0 |  |  |  |  |  |  |  |  |
| GO:0007263\_nitric\_oxide\_mediated\_signal\_transduction | 2 | 0 |  |  |  |  |  |  |  |  |
| GO:0007289\_spermatid\_nucleus\_differentiation | 2 | 0 |  |  |  |  |  |  |  |  |
| GO:0007343\_egg\_activation | 2 | 0 |  |  |  |  |  |  |  |  |
| GO:0007351\_tripartite\_regional\_subdivision | 2 | 0 |  |  |  |  |  |  |  |  |
| GO:0007418\_ventral\_midline\_development | 2 | 0 |  |  |  |  |  |  |  |  |
| GO:0007494\_midgut\_development | 2 | 0 |  |  |  |  |  |  |  |  |
| GO:0007527\_adult\_somatic\_muscle\_development | 2 | 0 |  |  |  |  |  |  |  |  |
| GO:0007549\_dosage\_compensation | 2 | 0 |  |  |  |  |  |  |  |  |
| GO:0007571\_age-dependent\_general\_metabolic\_decline | 2 | 0 |  |  |  |  |  |  |  |  |
| GO:0007603\_phototransduction\_\_visible\_light | 2 | 0 |  |  |  |  |  |  |  |  |
| GO:0007619\_courtship\_behavior | 2 | 0 |  |  |  |  |  |  |  |  |
| GO:0008065\_establishment\_of\_blood-nerve\_barrier | 2 | 0 |  |  |  |  |  |  |  |  |
| GO:0008089\_anterograde\_axon\_cargo\_transport | 2 | 0 |  |  |  |  |  |  |  |  |
| GO:0008210\_estrogen\_metabolic\_process | 2 | 0 |  |  |  |  |  |  |  |  |
| GO:0008212\_mineralocorticoid\_metabolic\_process | 2 | 0 |  |  |  |  |  |  |  |  |
| GO:0008214\_protein\_amino\_acid\_dealkylation | 2 | 0 |  |  |  |  |  |  |  |  |
| GO:0008228\_opsonization | 2 | 0 |  |  |  |  |  |  |  |  |
| GO:0008272\_sulfate\_transport | 2 | 0 |  |  |  |  |  |  |  |  |
| GO:0008291\_acetylcholine\_metabolic\_process | 2 | 0 |  |  |  |  |  |  |  |  |
| GO:0008298\_intracellular\_mRNA\_localization | 2 | 0 |  |  |  |  |  |  |  |  |
| GO:0008334\_histone\_mRNA\_metabolic\_process | 2 | 0 |  |  |  |  |  |  |  |  |
| GO:0008356\_asymmetric\_cell\_division | 2 | 0 |  |  |  |  |  |  |  |  |
| GO:0008582\_regulation\_of\_synaptic\_growth\_at\_neuromuscular\_junction | 2 | 0 |  |  |  |  |  |  |  |  |
| GO:0008594\_photoreceptor\_cell\_morphogenesis | 2 | 0 |  |  |  |  |  |  |  |  |
| GO:0008595\_determination\_of\_anterior\_posterior\_axis\_\_embryo | 2 | 0 |  |  |  |  |  |  |  |  |
| GO:0008608\_attachment\_of\_spindle\_microtubules\_to\_kinetochore | 2 | 0 |  |  |  |  |  |  |  |  |
| GO:0008616\_queuosine\_biosynthetic\_process | 2 | 0 |  |  |  |  |  |  |  |  |
| GO:0008617\_guanosine\_metabolic\_process | 2 | 0 |  |  |  |  |  |  |  |  |
| GO:0008618\_7-methylguanosine\_metabolic\_process | 2 | 0 |  |  |  |  |  |  |  |  |
| GO:0008634\_negative\_regulation\_of\_survival\_gene\_product\_expression | 2 | 0 |  |  |  |  |  |  |  |  |
| GO:0009048\_dosage\_compensation\_\_by\_inactivation\_of\_X\_chromosome | 2 | 0 |  |  |  |  |  |  |  |  |
| GO:0009070\_serine\_family\_amino\_acid\_biosynthetic\_process | 2 | 0 |  |  |  |  |  |  |  |  |
| GO:0009071\_serine\_family\_amino\_acid\_catabolic\_process | 2 | 0 |  |  |  |  |  |  |  |  |
| GO:0009074\_aromatic\_amino\_acid\_family\_catabolic\_process | 2 | 0 |  |  |  |  |  |  |  |  |
| GO:0009083\_branched\_chain\_family\_amino\_acid\_catabolic\_process | 2 | 0 |  |  |  |  |  |  |  |  |
| GO:0009093\_cysteine\_catabolic\_process | 2 | 0 |  |  |  |  |  |  |  |  |
| GO:0009120\_deoxyribonucleoside\_metabolic\_process | 2 | 0 |  |  |  |  |  |  |  |  |
| GO:0009125\_nucleoside\_monophosphate\_catabolic\_process | 2 | 0 |  |  |  |  |  |  |  |  |
| GO:0009126\_purine\_nucleoside\_monophosphate\_metabolic\_process | 2 | 0 |  |  |  |  |  |  |  |  |
| GO:0009142\_nucleoside\_triphosphate\_biosynthetic\_process | 2 | 0 |  |  |  |  |  |  |  |  |
| GO:0009161\_ribonucleoside\_monophosphate\_metabolic\_process | 2 | 0 |  |  |  |  |  |  |  |  |
| GO:0009164\_nucleoside\_catabolic\_process | 2 | 0 |  |  |  |  |  |  |  |  |
| GO:0009167\_purine\_ribonucleoside\_monophosphate\_metabolic\_process | 2 | 0 |  |  |  |  |  |  |  |  |
| GO:0009202\_deoxyribonucleoside\_triphosphate\_biosynthetic\_process | 2 | 0 |  |  |  |  |  |  |  |  |
| GO:0009203\_ribonucleoside\_triphosphate\_catabolic\_process | 2 | 0 |  |  |  |  |  |  |  |  |
| GO:0009207\_purine\_ribonucleoside\_triphosphate\_catabolic\_process | 2 | 0 |  |  |  |  |  |  |  |  |
| GO:0009219\_pyrimidine\_deoxyribonucleotide\_metabolic\_process | 2 | 0 |  |  |  |  |  |  |  |  |
| GO:0009265\_2'-deoxyribonucleotide\_biosynthetic\_process | 2 | 0 |  |  |  |  |  |  |  |  |
| GO:0009268\_response\_to\_pH | 2 | 0 |  |  |  |  |  |  |  |  |
| GO:0009313\_oligosaccharide\_catabolic\_process | 2 | 0 |  |  |  |  |  |  |  |  |
| GO:0009395\_phospholipid\_catabolic\_process | 2 | 0 |  |  |  |  |  |  |  |  |
| GO:0009435\_NAD\_biosynthetic\_process | 2 | 0 |  |  |  |  |  |  |  |  |
| GO:0009608\_response\_to\_symbiont | 2 | 0 |  |  |  |  |  |  |  |  |
| GO:0009609\_response\_to\_symbiotic\_bacterium | 2 | 0 |  |  |  |  |  |  |  |  |
| GO:0009649\_entrainment\_of\_circadian\_clock | 2 | 0 |  |  |  |  |  |  |  |  |
| GO:0009996\_negative\_regulation\_of\_cell\_fate\_specification | 2 | 0 |  |  |  |  |  |  |  |  |
| GO:0010002\_cardioblast\_differentiation | 2 | 0 |  |  |  |  |  |  |  |  |
| GO:0010149\_senescence | 2 | 0 |  |  |  |  |  |  |  |  |
| GO:0010225\_response\_to\_UV-C | 2 | 0 |  |  |  |  |  |  |  |  |
| GO:0010389\_regulation\_of\_G2\_M\_transition\_of\_mitotic\_cell\_cycle | 2 | 0 |  |  |  |  |  |  |  |  |
| GO:0010458\_exit\_from\_mitosis | 2 | 0 |  |  |  |  |  |  |  |  |
| GO:0010459\_negative\_regulation\_of\_heart\_rate | 2 | 0 |  |  |  |  |  |  |  |  |
| GO:0010633\_negative\_regulation\_of\_epithelial\_cell\_migration | 2 | 0 |  |  |  |  |  |  |  |  |
| GO:0010677\_negative\_regulation\_of\_cellular\_carbohydrate\_metabolic\_process | 2 | 0 |  |  |  |  |  |  |  |  |
| GO:0010718\_positive\_regulation\_of\_epithelial\_to\_mesenchymal\_transition | 2 | 0 |  |  |  |  |  |  |  |  |
| GO:0010742\_foam\_cell\_differentiation | 2 | 0 |  |  |  |  |  |  |  |  |
| GO:0010743\_regulation\_of\_foam\_cell\_differentiation | 2 | 0 |  |  |  |  |  |  |  |  |
| GO:0010744\_positive\_regulation\_of\_foam\_cell\_differentiation | 2 | 0 |  |  |  |  |  |  |  |  |
| GO:0010765\_positive\_regulation\_of\_sodium\_ion\_transport | 2 | 0 |  |  |  |  |  |  |  |  |
| GO:0010766\_negative\_regulation\_of\_sodium\_ion\_transport | 2 | 0 |  |  |  |  |  |  |  |  |
| GO:0010770\_positive\_regulation\_of\_cell\_morphogenesis\_involved\_in\_differentiation | 2 | 0 |  |  |  |  |  |  |  |  |
| GO:0010771\_negative\_regulation\_of\_cell\_morphogenesis\_involved\_in\_differentiation | 2 | 0 |  |  |  |  |  |  |  |  |
| GO:0010824\_regulation\_of\_centrosome\_duplication | 2 | 0 |  |  |  |  |  |  |  |  |
| GO:0010833\_telomere\_maintenance\_via\_telomere\_lengthening | 2 | 0 |  |  |  |  |  |  |  |  |
| GO:0010862\_positive\_regulation\_of\_pathway-restricted\_SMAD\_protein\_phosphorylation | 2 | 0 |  |  |  |  |  |  |  |  |
| GO:0010872\_regulation\_of\_cholesterol\_esterification | 2 | 0 |  |  |  |  |  |  |  |  |
| GO:0010878\_cholesterol\_storage | 2 | 0 |  |  |  |  |  |  |  |  |
| GO:0010885\_regulation\_of\_cholesterol\_storage | 2 | 0 |  |  |  |  |  |  |  |  |
| GO:0010886\_positive\_regulation\_of\_cholesterol\_storage | 2 | 0 |  |  |  |  |  |  |  |  |
| GO:0010891\_negative\_regulation\_of\_sequestering\_of\_triglyceride | 2 | 0 |  |  |  |  |  |  |  |  |
| GO:0010896\_regulation\_of\_triglyceride\_catabolic\_process | 2 | 0 |  |  |  |  |  |  |  |  |
| GO:0010898\_positive\_regulation\_of\_triglyceride\_catabolic\_process | 2 | 0 |  |  |  |  |  |  |  |  |
| GO:0010907\_positive\_regulation\_of\_glucose\_metabolic\_process | 2 | 0 |  |  |  |  |  |  |  |  |
| GO:0014028\_notochord\_formation | 2 | 0 |  |  |  |  |  |  |  |  |
| GO:0014052\_regulation\_of\_gamma-aminobutyric\_acid\_secretion | 2 | 0 |  |  |  |  |  |  |  |  |
| GO:0014054\_positive\_regulation\_of\_gamma-aminobutyric\_acid\_secretion | 2 | 0 |  |  |  |  |  |  |  |  |
| GO:0014055\_acetylcholine\_secretion | 2 | 0 |  |  |  |  |  |  |  |  |
| GO:0014056\_regulation\_of\_acetylcholine\_secretion | 2 | 0 |  |  |  |  |  |  |  |  |
| GO:0014067\_negative\_regulation\_of\_phosphoinositide\_3-kinase\_cascade | 2 | 0 |  |  |  |  |  |  |  |  |
| GO:0014745\_negative\_regulation\_of\_muscle\_adaptation | 2 | 0 |  |  |  |  |  |  |  |  |
| GO:0014829\_vascular\_smooth\_muscle\_contraction | 2 | 0 |  |  |  |  |  |  |  |  |
| GO:0014850\_response\_to\_muscle\_activity | 2 | 0 |  |  |  |  |  |  |  |  |
| GO:0014866\_skeletal\_myofibril\_assembly | 2 | 0 |  |  |  |  |  |  |  |  |
| GO:0014888\_striated\_muscle\_adaptation | 2 | 0 |  |  |  |  |  |  |  |  |
| GO:0014916\_regulation\_of\_lung\_blood\_pressure | 2 | 0 |  |  |  |  |  |  |  |  |
| GO:0015671\_oxygen\_transport | 2 | 0 |  |  |  |  |  |  |  |  |
| GO:0015696\_ammonium\_transport | 2 | 0 |  |  |  |  |  |  |  |  |
| GO:0015732\_prostaglandin\_transport | 2 | 0 |  |  |  |  |  |  |  |  |
| GO:0015819\_lysine\_transport | 2 | 0 |  |  |  |  |  |  |  |  |
| GO:0015840\_urea\_transport | 2 | 0 |  |  |  |  |  |  |  |  |
| GO:0015860\_purine\_nucleoside\_transport | 2 | 0 |  |  |  |  |  |  |  |  |
| GO:0015870\_acetylcholine\_transport | 2 | 0 |  |  |  |  |  |  |  |  |
| GO:0015937\_coenzyme\_A\_biosynthetic\_process | 2 | 0 |  |  |  |  |  |  |  |  |
| GO:0016045\_detection\_of\_bacterium | 2 | 0 |  |  |  |  |  |  |  |  |
| GO:0016046\_detection\_of\_fungus | 2 | 0 |  |  |  |  |  |  |  |  |
| GO:0016080\_synaptic\_vesicle\_targeting | 2 | 0 |  |  |  |  |  |  |  |  |
| GO:0016199\_axon\_midline\_choice\_point\_recognition | 2 | 0 |  |  |  |  |  |  |  |  |
| GO:0016226\_iron-sulfur\_cluster\_assembly | 2 | 0 |  |  |  |  |  |  |  |  |
| GO:0016233\_telomere\_capping | 2 | 0 |  |  |  |  |  |  |  |  |
| GO:0016242\_negative\_regulation\_of\_macroautophagy | 2 | 0 |  |  |  |  |  |  |  |  |
| GO:0016441\_posttranscriptional\_gene\_silencing | 2 | 0 |  |  |  |  |  |  |  |  |
| GO:0016540\_protein\_autoprocessing | 2 | 0 |  |  |  |  |  |  |  |  |
| GO:0016558\_protein\_import\_into\_peroxisome\_matrix | 2 | 0 |  |  |  |  |  |  |  |  |
| GO:0016572\_histone\_phosphorylation | 2 | 0 |  |  |  |  |  |  |  |  |
| GO:0016577\_histone\_demethylation | 2 | 0 |  |  |  |  |  |  |  |  |
| GO:0016584\_nucleosome\_positioning | 2 | 0 |  |  |  |  |  |  |  |  |
| GO:0016926\_protein\_desumoylation | 2 | 0 |  |  |  |  |  |  |  |  |
| GO:0017014\_protein\_amino\_acid\_nitrosylation | 2 | 0 |  |  |  |  |  |  |  |  |
| GO:0017144\_drug\_metabolic\_process | 2 | 0 |  |  |  |  |  |  |  |  |
| GO:0018094\_protein\_polyglycylation | 2 | 0 |  |  |  |  |  |  |  |  |
| GO:0018119\_peptidyl-cysteine\_S-nitrosylation | 2 | 0 |  |  |  |  |  |  |  |  |
| GO:0018125\_peptidyl-cysteine\_methylation | 2 | 0 |  |  |  |  |  |  |  |  |
| GO:0018205\_peptidyl-lysine\_modification | 2 | 0 |  |  |  |  |  |  |  |  |
| GO:0018319\_protein\_amino\_acid\_myristoylation | 2 | 0 |  |  |  |  |  |  |  |  |
| GO:0018377\_protein\_myristoylation | 2 | 0 |  |  |  |  |  |  |  |  |
| GO:0018401\_peptidyl-proline\_hydroxylation\_to\_4-hydroxy-L-proline | 2 | 0 |  |  |  |  |  |  |  |  |
| GO:0018993\_somatic\_sex\_determination | 2 | 0 |  |  |  |  |  |  |  |  |
| GO:0019067\_viral\_assembly\_\_maturation\_\_egress\_\_and\_release | 2 | 0 |  |  |  |  |  |  |  |  |
| GO:0019322\_pentose\_biosynthetic\_process | 2 | 0 |  |  |  |  |  |  |  |  |
| GO:0019370\_leukotriene\_biosynthetic\_process | 2 | 0 |  |  |  |  |  |  |  |  |
| GO:0019374\_galactolipid\_metabolic\_process | 2 | 0 |  |  |  |  |  |  |  |  |
| GO:0019401\_alditol\_biosynthetic\_process | 2 | 0 |  |  |  |  |  |  |  |  |
| GO:0019448\_L-cysteine\_catabolic\_process | 2 | 0 |  |  |  |  |  |  |  |  |
| GO:0019452\_L-cysteine\_catabolic\_process\_to\_taurine | 2 | 0 |  |  |  |  |  |  |  |  |
| GO:0019471\_4-hydroxyproline\_metabolic\_process | 2 | 0 |  |  |  |  |  |  |  |  |
| GO:0019511\_peptidyl-proline\_hydroxylation | 2 | 0 |  |  |  |  |  |  |  |  |
| GO:0019550\_glutamate\_catabolic\_process\_to\_aspartate | 2 | 0 |  |  |  |  |  |  |  |  |
| GO:0019551\_glutamate\_catabolic\_process\_to\_2-oxoglutarate | 2 | 0 |  |  |  |  |  |  |  |  |
| GO:0019585\_glucuronate\_metabolic\_process | 2 | 0 |  |  |  |  |  |  |  |  |
| GO:0019730\_antimicrobial\_humoral\_response | 2 | 0 |  |  |  |  |  |  |  |  |
| GO:0019853\_L-ascorbic\_acid\_biosynthetic\_process | 2 | 0 |  |  |  |  |  |  |  |  |
| GO:0021506\_anterior\_neuropore\_closure | 2 | 0 |  |  |  |  |  |  |  |  |
| GO:0021524\_visceral\_motor\_neuron\_differentiation | 2 | 0 |  |  |  |  |  |  |  |  |
| GO:0021526\_medial\_motor\_column\_neuron\_differentiation | 2 | 0 |  |  |  |  |  |  |  |  |
| GO:0021557\_oculomotor\_nerve\_development | 2 | 0 |  |  |  |  |  |  |  |  |
| GO:0021558\_trochlear\_nerve\_development | 2 | 0 |  |  |  |  |  |  |  |  |
| GO:0021562\_vestibulocochlear\_nerve\_development | 2 | 0 |  |  |  |  |  |  |  |  |
| GO:0021568\_rhombomere\_2\_development | 2 | 0 |  |  |  |  |  |  |  |  |
| GO:0021578\_hindbrain\_maturation | 2 | 0 |  |  |  |  |  |  |  |  |
| GO:0021593\_rhombomere\_morphogenesis | 2 | 0 |  |  |  |  |  |  |  |  |
| GO:0021626\_central\_nervous\_system\_maturation | 2 | 0 |  |  |  |  |  |  |  |  |
| GO:0021658\_rhombomere\_3\_morphogenesis | 2 | 0 |  |  |  |  |  |  |  |  |
| GO:0021754\_facial\_nucleus\_development | 2 | 0 |  |  |  |  |  |  |  |  |
| GO:0021775\_smoothened\_signaling\_pathway\_involved\_in\_ventral\_spinal\_cord\_interneuron\_specification | 2 | 0 |  |  |  |  |  |  |  |  |
| GO:0021776\_smoothened\_signaling\_pathway\_involved\_in\_spinal\_cord\_motor\_neuron\_cell\_fate\_specification | 2 | 0 |  |  |  |  |  |  |  |  |
| GO:0021796\_cerebral\_cortex\_regionalization | 2 | 0 |  |  |  |  |  |  |  |  |
| GO:0021831\_embryonic\_olfactory\_bulb\_interneuron\_precursor\_migration | 2 | 0 |  |  |  |  |  |  |  |  |
| GO:0021869\_forebrain\_ventricular\_zone\_progenitor\_cell\_division | 2 | 0 |  |  |  |  |  |  |  |  |
| GO:0021873\_forebrain\_neuroblast\_division | 2 | 0 |  |  |  |  |  |  |  |  |
| GO:0021882\_regulation\_of\_transcription\_from\_RNA\_polymerase\_II\_promoter\_involved\_in\_forebrain\_neuron\_fate\_commitment | 2 | 0 |  |  |  |  |  |  |  |  |
| GO:0021893\_cerebral\_cortex\_GABAergic\_interneuron\_fate\_commitment | 2 | 0 |  |  |  |  |  |  |  |  |
| GO:0021898\_commitment\_of\_multipotent\_stem\_cells\_to\_the\_neuronal\_lineage\_in\_the\_forebrain | 2 | 0 |  |  |  |  |  |  |  |  |
| GO:0021965\_spinal\_cord\_ventral\_commissure\_morphogenesis | 2 | 0 |  |  |  |  |  |  |  |  |
| GO:0021985\_neurohypophysis\_development | 2 | 0 |  |  |  |  |  |  |  |  |
| GO:0021990\_neural\_plate\_formation | 2 | 0 |  |  |  |  |  |  |  |  |
| GO:0021995\_neuropore\_closure | 2 | 0 |  |  |  |  |  |  |  |  |
| GO:0022028\_tangential\_migration\_from\_the\_subventricular\_zone\_to\_the\_olfactory\_bulb | 2 | 0 |  |  |  |  |  |  |  |  |
| GO:0022401\_adaptation\_of\_signaling\_pathway | 2 | 0 |  |  |  |  |  |  |  |  |
| GO:0022408\_negative\_regulation\_of\_cell-cell\_adhesion | 2 | 0 |  |  |  |  |  |  |  |  |
| GO:0022410\_circadian\_sleep\_wake\_cycle\_process | 2 | 0 |  |  |  |  |  |  |  |  |
| GO:0030046\_parallel\_actin\_filament\_bundle\_formation | 2 | 0 |  |  |  |  |  |  |  |  |
| GO:0030049\_muscle\_filament\_sliding | 2 | 0 |  |  |  |  |  |  |  |  |
| GO:0030050\_vesicle\_transport\_along\_actin\_filament | 2 | 0 |  |  |  |  |  |  |  |  |
| GO:0030071\_regulation\_of\_mitotic\_metaphase\_anaphase\_transition | 2 | 0 |  |  |  |  |  |  |  |  |
| GO:0030147\_natriuresis | 2 | 0 |  |  |  |  |  |  |  |  |
| GO:0030174\_regulation\_of\_DNA\_replication\_initiation | 2 | 0 |  |  |  |  |  |  |  |  |
| GO:0030202\_heparin\_metabolic\_process | 2 | 0 |  |  |  |  |  |  |  |  |
| GO:0030219\_megakaryocyte\_differentiation | 2 | 0 |  |  |  |  |  |  |  |  |
| GO:0030223\_neutrophil\_differentiation | 2 | 0 |  |  |  |  |  |  |  |  |
| GO:0030240\_muscle\_thin\_filament\_assembly | 2 | 0 |  |  |  |  |  |  |  |  |
| GO:0030259\_lipid\_glycosylation | 2 | 0 |  |  |  |  |  |  |  |  |
| GO:0030397\_membrane\_disassembly | 2 | 0 |  |  |  |  |  |  |  |  |
| GO:0030502\_negative\_regulation\_of\_bone\_mineralization | 2 | 0 |  |  |  |  |  |  |  |  |
| GO:0030644\_cellular\_chloride\_ion\_homeostasis | 2 | 0 |  |  |  |  |  |  |  |  |
| GO:0030825\_positive\_regulation\_of\_cGMP\_metabolic\_process | 2 | 0 |  |  |  |  |  |  |  |  |
| GO:0030828\_positive\_regulation\_of\_cGMP\_biosynthetic\_process | 2 | 0 |  |  |  |  |  |  |  |  |
| GO:0030835\_negative\_regulation\_of\_actin\_filament\_depolymerization | 2 | 0 |  |  |  |  |  |  |  |  |
| GO:0030837\_negative\_regulation\_of\_actin\_filament\_polymerization | 2 | 0 |  |  |  |  |  |  |  |  |
| GO:0030852\_regulation\_of\_granulocyte\_differentiation | 2 | 0 |  |  |  |  |  |  |  |  |
| GO:0030885\_regulation\_of\_myeloid\_dendritic\_cell\_activation | 2 | 0 |  |  |  |  |  |  |  |  |
| GO:0030910\_olfactory\_placode\_formation | 2 | 0 |  |  |  |  |  |  |  |  |
| GO:0030948\_negative\_regulation\_of\_vascular\_endothelial\_growth\_factor\_receptor\_signaling\_pathway | 2 | 0 |  |  |  |  |  |  |  |  |
| GO:0030953\_spindle\_astral\_microtubule\_organization | 2 | 0 |  |  |  |  |  |  |  |  |
| GO:0031050\_dsRNA\_fragmentation | 2 | 0 |  |  |  |  |  |  |  |  |
| GO:0031061\_negative\_regulation\_of\_histone\_methylation | 2 | 0 |  |  |  |  |  |  |  |  |
| GO:0031119\_tRNA\_pseudouridine\_synthesis | 2 | 0 |  |  |  |  |  |  |  |  |
| GO:0031163\_metallo-sulfur\_cluster\_assembly | 2 | 0 |  |  |  |  |  |  |  |  |
| GO:0031223\_auditory\_behavior | 2 | 0 |  |  |  |  |  |  |  |  |
| GO:0031296\_B\_cell\_costimulation | 2 | 0 |  |  |  |  |  |  |  |  |
| GO:0031338\_regulation\_of\_vesicle\_fusion | 2 | 0 |  |  |  |  |  |  |  |  |
| GO:0031573\_intra-S\_DNA\_damage\_checkpoint | 2 | 0 |  |  |  |  |  |  |  |  |
| GO:0031577\_spindle\_checkpoint | 2 | 0 |  |  |  |  |  |  |  |  |
| GO:0031629\_synaptic\_vesicle\_fusion\_to\_presynaptic\_membrane | 2 | 0 |  |  |  |  |  |  |  |  |
| GO:0031630\_regulation\_of\_synaptic\_vesicle\_fusion\_to\_presynaptic\_membrane | 2 | 0 |  |  |  |  |  |  |  |  |
| GO:0031664\_regulation\_of\_lipopolysaccharide-mediated\_signaling\_pathway | 2 | 0 |  |  |  |  |  |  |  |  |
| GO:0031670\_cellular\_response\_to\_nutrient | 2 | 0 |  |  |  |  |  |  |  |  |
| GO:0031848\_protection\_from\_non-homologous\_end\_joining\_at\_telomere | 2 | 0 |  |  |  |  |  |  |  |  |
| GO:0031946\_regulation\_of\_glucocorticoid\_biosynthetic\_process | 2 | 0 |  |  |  |  |  |  |  |  |
| GO:0031952\_regulation\_of\_protein\_amino\_acid\_autophosphorylation | 2 | 0 |  |  |  |  |  |  |  |  |
| GO:0031953\_negative\_regulation\_of\_protein\_amino\_acid\_autophosphorylation | 2 | 0 |  |  |  |  |  |  |  |  |
| GO:0031958\_corticosteroid\_receptor\_signaling\_pathway | 2 | 0 |  |  |  |  |  |  |  |  |
| GO:0031987\_locomotion\_involved\_in\_locomotory\_behavior | 2 | 0 |  |  |  |  |  |  |  |  |
| GO:0032096\_negative\_regulation\_of\_response\_to\_food | 2 | 0 |  |  |  |  |  |  |  |  |
| GO:0032099\_negative\_regulation\_of\_appetite | 2 | 0 |  |  |  |  |  |  |  |  |
| GO:0032106\_positive\_regulation\_of\_response\_to\_extracellular\_stimulus | 2 | 0 |  |  |  |  |  |  |  |  |
[truncated: 254,138 more chars]
