# Supplementary material for: RedundancyMiner: De-replication of redundant GO categories in microarray and proteomics analysis
Source: BMC Bioinformatics. 2011 Feb 10;12:52. doi: 10.1186/1471-2105-12-52 (PMC3223614; doi:10.1186/1471-2105-12-52)
Supplement: Additional file 8 — Retinal development HTGM download. compressed package of the results of running HTGM on the retinal development genes list. [file 1471-2105-12-52-S8.ZIP › SCENARIO_2_MODIFIED/total.txt.total.txt.dir/Exp1_BestClusterMap_LEIGS_KM_24.csv.join.23.txt.dir/Exp1_BestClusterMap_LEIGS_KM_24.csv.join.23.txt.change.gce.html]

Gene Category Report for Exp1\_BestClusterMap\_LEIGS\_KM\_24.csv.join.23.txt

# Gene Category Report for Exp1\_BestClusterMap\_LEIGS\_KM\_24.csv.join.23.txt

| HYPERLINKED GO CATEGORY | HYPERLINKED GENE NAME | TOTAL GENES | CHANGED GENES | ENRICHMENT | LOG10(p) | CUMULATIVE NUMBER OF CATEGORIES | CUMULATIVE RANDOMS MEAN | FALSE DISCOVERY RATE |
| --- | --- | --- | --- | --- | --- | --- | --- | --- |
| GO:0009950\_dorsal\_ventral\_axis\_specification | PAX6 | 5 | 2 | 65.785714 | -3.452757 | 1 | 0.26 | 0.260000 |
| GO:0009950\_dorsal\_ventral\_axis\_specification | BMPR1A | 5 | 2 | 65.785714 | -3.452757 | 1 | 0.26 | 0.260000 |
| GO:0048641\_regulation\_of\_skeletal\_muscle\_tissue\_development | AGRN | 10 | 2 | 32.892857 | -2.807724 | 2 | 1.39 | 0.695000 |
| GO:0048641\_regulation\_of\_skeletal\_muscle\_tissue\_development | CSDA | 10 | 2 | 32.892857 | -2.807724 | 2 | 1.39 | 0.695000 |
| GO:0030856\_regulation\_of\_epithelial\_cell\_differentiation | CCND1 | 11 | 2 | 29.902597 | -2.722208 | 3 | 1.63 | 0.543333 |
| GO:0030856\_regulation\_of\_epithelial\_cell\_differentiation | PAX6 | 11 | 2 | 29.902597 | -2.722208 | 3 | 1.63 | 0.543333 |
| GO:0006986\_response\_to\_unfolded\_protein | CCND1 | 13 | 2 | 25.302198 | -2.573744 | 4 | 2.28 | 0.570000 |
| GO:0006986\_response\_to\_unfolded\_protein | HSP90AA1 | 13 | 2 | 25.302198 | -2.573744 | 4 | 2.28 | 0.570000 |
| GO:0009798\_axis\_specification | PAX6 | 19 | 2 | 17.312030 | -2.242631 | 5 | 4.75 | 0.950000 |
| GO:0009798\_axis\_specification | BMPR1A | 19 | 2 | 17.312030 | -2.242631 | 5 | 4.75 | 0.950000 |
| GO:0001880\_Mullerian\_duct\_regression | BMPR1A | 1 | 1 |  |  |  |  |  |  |
| GO:0006175\_dATP\_biosynthetic\_process | ADK | 1 | 1 |  |  |  |  |  |  |
| GO:0009145\_purine\_nucleoside\_triphosphate\_biosynthetic\_process | ADK | 1 | 1 |  |  |  |  |  |  |
| GO:0009153\_purine\_deoxyribonucleotide\_biosynthetic\_process | ADK | 1 | 1 |  |  |  |  |  |  |
| GO:0009216\_purine\_deoxyribonucleoside\_triphosphate\_biosynthetic\_process | ADK | 1 | 1 |  |  |  |  |  |  |
| GO:0021902\_commitment\_of\_a\_neuronal\_cell\_to\_a\_specific\_type\_of\_neuron\_in\_the\_forebrain | PAX6 | 1 | 1 |  |  |  |  |  |  |
| GO:0021905\_forebrain-midbrain\_boundary\_formation | PAX6 | 1 | 1 |  |  |  |  |  |  |
| GO:0021917\_somatic\_motor\_neuron\_fate\_commitment | PAX6 | 1 | 1 |  |  |  |  |  |  |
| GO:0021918\_regulation\_of\_transcription\_from\_RNA\_polymerase\_II\_promoter\_involved\_in\_somatic\_motor\_neuron\_fate\_commitment | PAX6 | 1 | 1 |  |  |  |  |  |  |
| GO:0035303\_regulation\_of\_dephosphorylation | YWHAE | 1 | 1 |  |  |  |  |  |  |
| GO:0035304\_regulation\_of\_protein\_amino\_acid\_dephosphorylation | YWHAE | 1 | 1 |  |  |  |  |  |  |
| GO:0035305\_negative\_regulation\_of\_dephosphorylation | YWHAE | 1 | 1 |  |  |  |  |  |  |
| GO:0035308\_negative\_regulation\_of\_protein\_amino\_acid\_dephosphorylation | YWHAE | 1 | 1 |  |  |  |  |  |  |
| GO:0042026\_protein\_refolding | HSP90AA1 | 1 | 1 |  |  |  |  |  |  |
| GO:0042059\_negative\_regulation\_of\_epidermal\_growth\_factor\_receptor\_signaling\_pathway | NUP62 | 1 | 1 |  |  |  |  |  |  |
| GO:0045583\_regulation\_of\_cytotoxic\_T\_cell\_differentiation | HSP90AA1 | 1 | 1 |  |  |  |  |  |  |
| GO:0045585\_positive\_regulation\_of\_cytotoxic\_T\_cell\_differentiation | HSP90AA1 | 1 | 1 |  |  |  |  |  |  |
| GO:0048642\_negative\_regulation\_of\_skeletal\_muscle\_tissue\_development | CSDA | 1 | 1 |  |  |  |  |  |  |
| GO:0016202\_regulation\_of\_striated\_muscle\_tissue\_development | AGRN | 21 | 2 | 15.663265 | -2.156667 | 7 | 5.53 | 0.790000 |
| GO:0016202\_regulation\_of\_striated\_muscle\_tissue\_development | CSDA | 21 | 2 | 15.663265 | -2.156667 | 7 | 5.53 | 0.790000 |
| GO:0048634\_regulation\_of\_muscle\_development | AGRN | 21 | 2 | 15.663265 | -2.156667 | 7 | 5.53 | 0.790000 |
| GO:0048634\_regulation\_of\_muscle\_development | CSDA | 21 | 2 | 15.663265 | -2.156667 | 7 | 5.53 | 0.790000 |
| GO:0009952\_anterior\_posterior\_pattern\_formation | SFRP2 | 133 | 4 | 4.946294 | -2.099692 | 8 | 5.99 | 0.748750 |
| GO:0009952\_anterior\_posterior\_pattern\_formation | PAX6 | 133 | 4 | 4.946294 | -2.099692 | 8 | 5.99 | 0.748750 |
| GO:0009952\_anterior\_posterior\_pattern\_formation | ZIC3 | 133 | 4 | 4.946294 | -2.099692 | 8 | 5.99 | 0.748750 |
| GO:0009952\_anterior\_posterior\_pattern\_formation | BMPR1A | 133 | 4 | 4.946294 | -2.099692 | 8 | 5.99 | 0.748750 |
| GO:0050673\_epithelial\_cell\_proliferation | CCND1 | 72 | 3 | 6.852679 | -2.042262 | 9 | 6.78 | 0.753333 |
| GO:0050673\_epithelial\_cell\_proliferation | PAX6 | 72 | 3 | 6.852679 | -2.042262 | 9 | 6.78 | 0.753333 |
| GO:0050673\_epithelial\_cell\_proliferation | BMPR1A | 72 | 3 | 6.852679 | -2.042262 | 9 | 6.78 | 0.753333 |
| GO:0021983\_pituitary\_gland\_development | PAX6 | 25 | 2 | 13.157143 | -2.008277 | 10 | 7.19 | 0.719000 |
| GO:0021983\_pituitary\_gland\_development | BMPR1A | 25 | 2 | 13.157143 | -2.008277 | 10 | 7.19 | 0.719000 |
| GO:0007519\_skeletal\_muscle\_tissue\_development | NEO1 | 78 | 3 | 6.325549 | -1.947103 | 12 | 7.77 | 0.647500 |
| GO:0007519\_skeletal\_muscle\_tissue\_development | AGRN | 78 | 3 | 6.325549 | -1.947103 | 12 | 7.77 | 0.647500 |
| GO:0007519\_skeletal\_muscle\_tissue\_development | CSDA | 78 | 3 | 6.325549 | -1.947103 | 12 | 7.77 | 0.647500 |
| GO:0060538\_skeletal\_muscle\_organ\_development | NEO1 | 78 | 3 | 6.325549 | -1.947103 | 12 | 7.77 | 0.647500 |
| GO:0060538\_skeletal\_muscle\_organ\_development | AGRN | 78 | 3 | 6.325549 | -1.947103 | 12 | 7.77 | 0.647500 |
| GO:0060538\_skeletal\_muscle\_organ\_development | CSDA | 78 | 3 | 6.325549 | -1.947103 | 12 | 7.77 | 0.647500 |
| GO:0008582\_regulation\_of\_synaptic\_growth\_at\_neuromuscular\_junction | AGRN | 2 | 1 |  |  |  |  |  |  |
| GO:0009142\_nucleoside\_triphosphate\_biosynthetic\_process | ADK | 2 | 1 |  |  |  |  |  |  |
| GO:0009202\_deoxyribonucleoside\_triphosphate\_biosynthetic\_process | ADK | 2 | 1 |  |  |  |  |  |  |
| GO:0009265\_2'-deoxyribonucleotide\_biosynthetic\_process | ADK | 2 | 1 |  |  |  |  |  |  |
| GO:0045065\_cytotoxic\_T\_cell\_differentiation | HSP90AA1 | 2 | 1 |  |  |  |  |  |  |
| GO:0046060\_dATP\_metabolic\_process | ADK | 2 | 1 |  |  |  |  |  |  |
| GO:0048382\_mesendoderm\_development | BMPR1A | 2 | 1 |  |  |  |  |  |  |
| GO:0031668\_cellular\_response\_to\_extracellular\_stimulus | LDHA | 30 | 2 | 10.964286 | -1.855036 | 13 | 9.51 | 0.731538 |
| GO:0031668\_cellular\_response\_to\_extracellular\_stimulus | SFRP2 | 30 | 2 | 10.964286 | -1.855036 | 13 | 9.51 | 0.731538 |
| GO:0021536\_diencephalon\_development | PAX6 | 33 | 2 | 9.967532 | -1.775761 | 14 | 11.01 | 0.786429 |
| GO:0021536\_diencephalon\_development | BMPR1A | 33 | 2 | 9.967532 | -1.775761 | 14 | 11.01 | 0.786429 |
| GO:0000320\_re-entry\_into\_mitotic\_cell\_cycle | CCND1 | 3 | 1 |  |  |  |  |  |  |
| GO:0006166\_purine\_ribonucleoside\_salvage | ADK | 3 | 1 |  |  |  |  |  |  |
| GO:0021797\_forebrain\_anterior\_posterior\_pattern\_formation | PAX6 | 3 | 1 |  |  |  |  |  |  |
| GO:0021798\_forebrain\_dorsal\_ventral\_pattern\_formation | PAX6 | 3 | 1 |  |  |  |  |  |  |
| GO:0021912\_regulation\_of\_transcription\_from\_RNA\_polymerase\_II\_promoter\_involved\_in\_spinal\_cord\_motor\_neuron\_fate\_specification | PAX6 | 3 | 1 |  |  |  |  |  |  |
| GO:0030033\_microvillus\_assembly | RDX | 3 | 1 |  |  |  |  |  |  |
| GO:0030857\_negative\_regulation\_of\_epithelial\_cell\_differentiation | CCND1 | 3 | 1 |  |  |  |  |  |  |
| GO:0032528\_microvillus\_organization | RDX | 3 | 1 |  |  |  |  |  |  |
| GO:0043094\_cellular\_metabolic\_compound\_salvage | ADK | 3 | 1 |  |  |  |  |  |  |
| GO:0043101\_purine\_salvage | ADK | 3 | 1 |  |  |  |  |  |  |
| GO:0043174\_nucleoside\_salvage | ADK | 3 | 1 |  |  |  |  |  |  |
| GO:0060033\_anatomical\_structure\_regression | BMPR1A | 3 | 1 |  |  |  |  |  |  |
| GO:0021523\_somatic\_motor\_neuron\_differentiation | PAX6 | 4 | 1 |  |  |  |  |  |  |
| GO:0021778\_oligodendrocyte\_cell\_fate\_specification | PAX6 | 4 | 1 |  |  |  |  |  |  |
| GO:0021779\_oligodendrocyte\_cell\_fate\_commitment | PAX6 | 4 | 1 |  |  |  |  |  |  |
| GO:0021780\_glial\_cell\_fate\_specification | PAX6 | 4 | 1 |  |  |  |  |  |  |
| GO:0021877\_forebrain\_neuron\_fate\_commitment | PAX6 | 4 | 1 |  |  |  |  |  |  |
| GO:0021913\_regulation\_of\_transcription\_from\_RNA\_polymerase\_II\_promoter\_involved\_in\_ventral\_spinal\_cord\_interneuron\_specification | PAX6 | 4 | 1 |  |  |  |  |  |  |
| GO:0030858\_positive\_regulation\_of\_epithelial\_cell\_differentiation | PAX6 | 4 | 1 |  |  |  |  |  |  |
| GO:0032808\_lacrimal\_gland\_development | PAX6 | 4 | 1 |  |  |  |  |  |  |
| GO:0051124\_synaptic\_growth\_at\_neuromuscular\_junction | AGRN | 4 | 1 |  |  |  |  |  |  |
| GO:0051789\_response\_to\_protein\_stimulus | CCND1 | 43 | 2 | 7.649502 | -1.558905 | 15 | 16.98 | 1.132000 |
| GO:0051789\_response\_to\_protein\_stimulus | HSP90AA1 | 43 | 2 | 7.649502 | -1.558905 | 15 | 16.98 | 1.132000 |
| GO:0003002\_regionalization | SFRP2 | 195 | 4 | 3.373626 | -1.540950 | 16 | 17.22 | 1.076250 |
| GO:0003002\_regionalization | PAX6 | 195 | 4 | 3.373626 | -1.540950 | 16 | 17.22 | 1.076250 |
| GO:0003002\_regionalization | ZIC3 | 195 | 4 | 3.373626 | -1.540950 | 16 | 17.22 | 1.076250 |
| GO:0003002\_regionalization | BMPR1A | 195 | 4 | 3.373626 | -1.540950 | 16 | 17.22 | 1.076250 |
| GO:0006270\_DNA\_replication\_initiation | CCNE1 | 5 | 1 | 32.892857 | -1.522196 | 22 | 26.8 | 1.218182 |
| GO:0009151\_purine\_deoxyribonucleotide\_metabolic\_process | ADK | 5 | 1 | 32.892857 | -1.522196 | 22 | 26.8 | 1.218182 |
| GO:0009215\_purine\_deoxyribonucleoside\_triphosphate\_metabolic\_process | ADK | 5 | 1 | 32.892857 | -1.522196 | 22 | 26.8 | 1.218182 |
| GO:0009263\_deoxyribonucleotide\_biosynthetic\_process | ADK | 5 | 1 | 32.892857 | -1.522196 | 22 | 26.8 | 1.218182 |
| GO:0030866\_cortical\_actin\_cytoskeleton\_organization | RHOQ | 5 | 1 | 32.892857 | -1.522196 | 22 | 26.8 | 1.218182 |
| GO:0045213\_neurotransmitter\_receptor\_metabolic\_process | AGRN | 5 | 1 | 32.892857 | -1.522196 | 22 | 26.8 | 1.218182 |
| GO:0040008\_regulation\_of\_growth | EI24 | 113 | 3 | 4.366308 | -1.520257 | 23 | 27.07 | 1.176957 |
| GO:0040008\_regulation\_of\_growth | AGRN | 113 | 3 | 4.366308 | -1.520257 | 23 | 27.07 | 1.176957 |
| GO:0040008\_regulation\_of\_growth | CSDA | 113 | 3 | 4.366308 | -1.520257 | 23 | 27.07 | 1.176957 |
| GO:0045595\_regulation\_of\_cell\_differentiation | CCND1 | 295 | 5 | 2.787530 | -1.518123 | 24 | 27.14 | 1.130833 |
| GO:0045595\_regulation\_of\_cell\_differentiation | HSP90AA1 | 295 | 5 | 2.787530 | -1.518123 | 24 | 27.14 | 1.130833 |
| GO:0045595\_regulation\_of\_cell\_differentiation | PAX6 | 295 | 5 | 2.787530 | -1.518123 | 24 | 27.14 | 1.130833 |
| GO:0045595\_regulation\_of\_cell\_differentiation | AGRN | 295 | 5 | 2.787530 | -1.518123 | 24 | 27.14 | 1.130833 |
| GO:0045595\_regulation\_of\_cell\_differentiation | BMPR1A | 295 | 5 | 2.787530 | -1.518123 | 24 | 27.14 | 1.130833 |
| GO:0051960\_regulation\_of\_nervous\_system\_development | PAX6 | 118 | 3 | 4.181295 | -1.472093 | 25 | 28.42 | 1.136800 |
| GO:0051960\_regulation\_of\_nervous\_system\_development | AGRN | 118 | 3 | 4.181295 | -1.472093 | 25 | 28.42 | 1.136800 |
| GO:0051960\_regulation\_of\_nervous\_system\_development | BMPR1A | 118 | 3 | 4.181295 | -1.472093 | 25 | 28.42 | 1.136800 |
| GO:0021543\_pallium\_development | PAX6 | 49 | 2 | 6.712828 | -1.453877 | 28 | 29.22 | 1.043571 |
| GO:0021543\_pallium\_development | YWHAE | 49 | 2 | 6.712828 | -1.453877 | 28 | 29.22 | 1.043571 |
| GO:0046661\_male\_sex\_differentiation | CSDA | 49 | 2 | 6.712828 | -1.453877 | 28 | 29.22 | 1.043571 |
| GO:0046661\_male\_sex\_differentiation | BMPR1A | 49 | 2 | 6.712828 | -1.453877 | 28 | 29.22 | 1.043571 |
| GO:0048741\_skeletal\_muscle\_fiber\_development | NEO1 | 49 | 2 | 6.712828 | -1.453877 | 28 | 29.22 | 1.043571 |
| GO:0048741\_skeletal\_muscle\_fiber\_development | AGRN | 49 | 2 | 6.712828 | -1.453877 | 28 | 29.22 | 1.043571 |
| GO:0014706\_striated\_muscle\_tissue\_development | NEO1 | 120 | 3 | 4.111607 | -1.453504 | 29 | 29.24 | 1.008276 |
| GO:0014706\_striated\_muscle\_tissue\_development | AGRN | 120 | 3 | 4.111607 | -1.453504 | 29 | 29.24 | 1.008276 |
| GO:0014706\_striated\_muscle\_tissue\_development | CSDA | 120 | 3 | 4.111607 | -1.453504 | 29 | 29.24 | 1.008276 |
| GO:0000768\_syncytium\_formation\_by\_plasma\_membrane\_fusion | NEO1 | 6 | 1 | 27.410714 | -1.444286 | 38 | 36.01 | 0.947632 |
| GO:0007032\_endosome\_organization | STX6 | 6 | 1 | 27.410714 | -1.444286 | 38 | 36.01 | 0.947632 |
| GO:0007520\_myoblast\_fusion | NEO1 | 6 | 1 | 27.410714 | -1.444286 | 38 | 36.01 | 0.947632 |
| GO:0030865\_cortical\_cytoskeleton\_organization | RHOQ | 6 | 1 | 27.410714 | -1.444286 | 38 | 36.01 | 0.947632 |
| GO:0045176\_apical\_protein\_localization | RDX | 6 | 1 | 27.410714 | -1.444286 | 38 | 36.01 | 0.947632 |
| GO:0045843\_negative\_regulation\_of\_striated\_muscle\_development | CSDA | 6 | 1 | 27.410714 | -1.444286 | 38 | 36.01 | 0.947632 |
| GO:0046580\_negative\_regulation\_of\_Ras\_protein\_signal\_transduction | NUP62 | 6 | 1 | 27.410714 | -1.444286 | 38 | 36.01 | 0.947632 |
| GO:0048635\_negative\_regulation\_of\_muscle\_development | CSDA | 6 | 1 | 27.410714 | -1.444286 | 38 | 36.01 | 0.947632 |
| GO:0051058\_negative\_regulation\_of\_small\_GTPase\_mediated\_signal\_transduction | NUP62 | 6 | 1 | 27.410714 | -1.444286 | 38 | 36.01 | 0.947632 |
| GO:0060284\_regulation\_of\_cell\_development | PAX6 | 122 | 3 | 4.044204 | -1.435282 | 39 | 36.54 | 0.936923 |
| GO:0060284\_regulation\_of\_cell\_development | AGRN | 122 | 3 | 4.044204 | -1.435282 | 39 | 36.54 | 0.936923 |
| GO:0060284\_regulation\_of\_cell\_development | BMPR1A | 122 | 3 | 4.044204 | -1.435282 | 39 | 36.54 | 0.936923 |
| GO:0048747\_muscle\_fiber\_development | NEO1 | 51 | 2 | 6.449580 | -1.422001 | 40 | 36.96 | 0.924000 |
| GO:0048747\_muscle\_fiber\_development | AGRN | 51 | 2 | 6.449580 | -1.422001 | 40 | 36.96 | 0.924000 |
| GO:0040007\_growth | EI24 | 217 | 4 | 3.031600 | -1.393656 | 41 | 37.48 | 0.914146 |
| GO:0040007\_growth | AGRN | 217 | 4 | 3.031600 | -1.393656 | 41 | 37.48 | 0.914146 |
| GO:0040007\_growth | CSDA | 217 | 4 | 3.031600 | -1.393656 | 41 | 37.48 | 0.914146 |
| GO:0040007\_growth | BMPR1A | 217 | 4 | 3.031600 | -1.393656 | 41 | 37.48 | 0.914146 |
| GO:0060537\_muscle\_tissue\_development | NEO1 | 128 | 3 | 3.854632 | -1.382699 | 42 | 37.87 | 0.901667 |
| GO:0060537\_muscle\_tissue\_development | AGRN | 128 | 3 | 3.854632 | -1.382699 | 42 | 37.87 | 0.901667 |
| GO:0060537\_muscle\_tissue\_development | CSDA | 128 | 3 | 3.854632 | -1.382699 | 42 | 37.87 | 0.901667 |
| GO:0002052\_positive\_regulation\_of\_neuroblast\_proliferation | PAX6 | 7 | 1 | 23.494898 | -1.378609 | 55 | 45.42 | 0.825818 |
| GO:0006014\_D-ribose\_metabolic\_process | ADK | 7 | 1 | 23.494898 | -1.378609 | 55 | 45.42 | 0.825818 |
| GO:0006949\_syncytium\_formation | NEO1 | 7 | 1 | 23.494898 | -1.378609 | 55 | 45.42 | 0.825818 |
| GO:0009200\_deoxyribonucleoside\_triphosphate\_metabolic\_process | ADK | 7 | 1 | 23.494898 | -1.378609 | 55 | 45.42 | 0.825818 |
| GO:0009394\_2'-deoxyribonucleotide\_metabolic\_process | ADK | 7 | 1 | 23.494898 | -1.378609 | 55 | 45.42 | 0.825818 |
| GO:0019692\_deoxyribose\_phosphate\_metabolic\_process | ADK | 7 | 1 | 23.494898 | -1.378609 | 55 | 45.42 | 0.825818 |
| GO:0021514\_ventral\_spinal\_cord\_interneuron\_differentiation | PAX6 | 7 | 1 | 23.494898 | -1.378609 | 55 | 45.42 | 0.825818 |
| GO:0021520\_spinal\_cord\_motor\_neuron\_cell\_fate\_specification | PAX6 | 7 | 1 | 23.494898 | -1.378609 | 55 | 45.42 | 0.825818 |
| GO:0021521\_ventral\_spinal\_cord\_interneuron\_specification | PAX6 | 7 | 1 | 23.494898 | -1.378609 | 55 | 45.42 | 0.825818 |
| GO:0021903\_rostrocaudal\_neural\_tube\_patterning | PAX6 | 7 | 1 | 23.494898 | -1.378609 | 55 | 45.42 | 0.825818 |
| GO:0030521\_androgen\_receptor\_signaling\_pathway | DNAJA1 | 7 | 1 | 23.494898 | -1.378609 | 55 | 45.42 | 0.825818 |
| GO:0046622\_positive\_regulation\_of\_organ\_growth | CSDA | 7 | 1 | 23.494898 | -1.378609 | 55 | 45.42 | 0.825818 |
| GO:0060579\_ventral\_spinal\_cord\_interneuron\_fate\_commitment | PAX6 | 7 | 1 | 23.494898 | -1.378609 | 55 | 45.42 | 0.825818 |
| GO:0050678\_regulation\_of\_epithelial\_cell\_proliferation | PAX6 | 56 | 2 | 5.873724 | -1.348046 | 56 | 46.96 | 0.838571 |
| GO:0050678\_regulation\_of\_epithelial\_cell\_proliferation | BMPR1A | 56 | 2 | 5.873724 | -1.348046 | 56 | 46.96 | 0.838571 |
| GO:0001764\_neuron\_migration | PAX6 | 57 | 2 | 5.770677 | -1.334143 | 58 | 47.77 | 0.823621 |
| GO:0001764\_neuron\_migration | YWHAE | 57 | 2 | 5.770677 | -1.334143 | 58 | 47.77 | 0.823621 |
| GO:0009953\_dorsal\_ventral\_pattern\_formation | PAX6 | 57 | 2 | 5.770677 | -1.334143 | 58 | 47.77 | 0.823621 |
| GO:0009953\_dorsal\_ventral\_pattern\_formation | BMPR1A | 57 | 2 | 5.770677 | -1.334143 | 58 | 47.77 | 0.823621 |
| GO:0007283\_spermatogenesis | GPX4 | 134 | 3 | 3.682036 | -1.333019 | 60 | 47.81 | 0.796833 |
| GO:0007283\_spermatogenesis | DNAJA1 | 134 | 3 | 3.682036 | -1.333019 | 60 | 47.81 | 0.796833 |
| GO:0007283\_spermatogenesis | CSDA | 134 | 3 | 3.682036 | -1.333019 | 60 | 47.81 | 0.796833 |
| GO:0048232\_male\_gamete\_generation | GPX4 | 134 | 3 | 3.682036 | -1.333019 | 60 | 47.81 | 0.796833 |
| GO:0048232\_male\_gamete\_generation | DNAJA1 | 134 | 3 | 3.682036 | -1.333019 | 60 | 47.81 | 0.796833 |
| GO:0048232\_male\_gamete\_generation | CSDA | 134 | 3 | 3.682036 | -1.333019 | 60 | 47.81 | 0.796833 |
| GO:0009987\_cellular\_process | LDHA | 3868 | 27 | 1.148019 | -1.323317 | 61 | 48.06 | 0.787869 |
| GO:0009987\_cellular\_process | PAX6 | 3868 | 27 | 1.148019 | -1.323317 | 61 | 48.06 | 0.787869 |
| GO:0009987\_cellular\_process | RHOQ | 3868 | 27 | 1.148019 | -1.323317 | 61 | 48.06 | 0.787869 |
| GO:0009987\_cellular\_process | RDX | 3868 | 27 | 1.148019 | -1.323317 | 61 | 48.06 | 0.787869 |
| GO:0009987\_cellular\_process | NEO1 | 3868 | 27 | 1.148019 | -1.323317 | 61 | 48.06 | 0.787869 |
| GO:0009987\_cellular\_process | ZIC3 | 3868 | 27 | 1.148019 | -1.323317 | 61 | 48.06 | 0.787869 |
| GO:0009987\_cellular\_process | CCNE1 | 3868 | 27 | 1.148019 | -1.323317 | 61 | 48.06 | 0.787869 |
| GO:0009987\_cellular\_process | UBE2D3 | 3868 | 27 | 1.148019 | -1.323317 | 61 | 48.06 | 0.787869 |
| GO:0009987\_cellular\_process | GPX4 | 3868 | 27 | 1.148019 | -1.323317 | 61 | 48.06 | 0.787869 |
| GO:0009987\_cellular\_process | DNAJA1 | 3868 | 27 | 1.148019 | -1.323317 | 61 | 48.06 | 0.787869 |
| GO:0009987\_cellular\_process | AGRN | 3868 | 27 | 1.148019 | -1.323317 | 61 | 48.06 | 0.787869 |
| GO:0009987\_cellular\_process | STX6 | 3868 | 27 | 1.148019 | -1.323317 | 61 | 48.06 | 0.787869 |
| GO:0009987\_cellular\_process | HSP90AA1 | 3868 | 27 | 1.148019 | -1.323317 | 61 | 48.06 | 0.787869 |
| GO:0009987\_cellular\_process | CSDA | 3868 | 27 | 1.148019 | -1.323317 | 61 | 48.06 | 0.787869 |
| GO:0009987\_cellular\_process | YWHAE | 3868 | 27 | 1.148019 | -1.323317 | 61 | 48.06 | 0.787869 |
| GO:0009987\_cellular\_process | CCND1 | 3868 | 27 | 1.148019 | -1.323317 | 61 | 48.06 | 0.787869 |
| GO:0009987\_cellular\_process | EI24 | 3868 | 27 | 1.148019 | -1.323317 | 61 | 48.06 | 0.787869 |
| GO:0009987\_cellular\_process | ATF4 | 3868 | 27 | 1.148019 | -1.323317 | 61 | 48.06 | 0.787869 |
| GO:0009987\_cellular\_process | NUP62 | 3868 | 27 | 1.148019 | -1.323317 | 61 | 48.06 | 0.787869 |
| GO:0009987\_cellular\_process | SFRP2 | 3868 | 27 | 1.148019 | -1.323317 | 61 | 48.06 | 0.787869 |
| GO:0009987\_cellular\_process | ADK | 3868 | 27 | 1.148019 | -1.323317 | 61 | 48.06 | 0.787869 |
| GO:0009987\_cellular\_process | TMSB4X | 3868 | 27 | 1.148019 | -1.323317 | 61 | 48.06 | 0.787869 |
| GO:0009987\_cellular\_process | RAP1B | 3868 | 27 | 1.148019 | -1.323317 | 61 | 48.06 | 0.787869 |
| GO:0009987\_cellular\_process | SMC1A | 3868 | 27 | 1.148019 | -1.323317 | 61 | 48.06 | 0.787869 |
| GO:0009987\_cellular\_process | MYBBP1A | 3868 | 27 | 1.148019 | -1.323317 | 61 | 48.06 | 0.787869 |
| GO:0009987\_cellular\_process | CALM1 | 3868 | 27 | 1.148019 | -1.323317 | 61 | 48.06 | 0.787869 |
| GO:0009987\_cellular\_process | BMPR1A | 3868 | 27 | 1.148019 | -1.323317 | 61 | 48.06 | 0.787869 |
| GO:0006020\_inositol\_metabolic\_process | IMPA1 | 8 | 1 | 20.558036 | -1.321886 | 71 | 54.25 | 0.764085 |
| GO:0007009\_plasma\_membrane\_organization | AGRN | 8 | 1 | 20.558036 | -1.321886 | 71 | 54.25 | 0.764085 |
| GO:0008105\_asymmetric\_protein\_localization | RDX | 8 | 1 | 20.558036 | -1.321886 | 71 | 54.25 | 0.764085 |
| GO:0009144\_purine\_nucleoside\_triphosphate\_metabolic\_process | ADK | 8 | 1 | 20.558036 | -1.321886 | 71 | 54.25 | 0.764085 |
| GO:0021781\_glial\_cell\_fate\_commitment | PAX6 | 8 | 1 | 20.558036 | -1.321886 | 71 | 54.25 | 0.764085 |
| GO:0040034\_regulation\_of\_development\_\_heterochronic | PAX6 | 8 | 1 | 20.558036 | -1.321886 | 71 | 54.25 | 0.764085 |
| GO:0045429\_positive\_regulation\_of\_nitric\_oxide\_biosynthetic\_process | HSP90AA1 | 8 | 1 | 20.558036 | -1.321886 | 71 | 54.25 | 0.764085 |
| GO:0048505\_regulation\_of\_timing\_of\_cell\_differentiation | PAX6 | 8 | 1 | 20.558036 | -1.321886 | 71 | 54.25 | 0.764085 |
| GO:0048638\_regulation\_of\_developmental\_growth | AGRN | 8 | 1 | 20.558036 | -1.321886 | 71 | 54.25 | 0.764085 |
| GO:0048742\_regulation\_of\_skeletal\_muscle\_fiber\_development | AGRN | 8 | 1 | 20.558036 | -1.321886 | 71 | 54.25 | 0.764085 |
| GO:0030334\_regulation\_of\_cell\_migration | PAX6 | 59 | 2 | 5.575061 | -1.307143 | 73 | 54.92 | 0.752329 |
| GO:0030334\_regulation\_of\_cell\_migration | TMSB4X | 59 | 2 | 5.575061 | -1.307143 | 73 | 54.92 | 0.752329 |
| GO:0035270\_endocrine\_system\_development | PAX6 | 59 | 2 | 5.575061 | -1.307143 | 73 | 54.92 | 0.752329 |
| GO:0035270\_endocrine\_system\_development | BMPR1A | 59 | 2 | 5.575061 | -1.307143 | 73 | 54.92 | 0.752329 |
| GO:0009991\_response\_to\_extracellular\_stimulus | LDHA | 61 | 2 | 5.392272 | -1.281157 | 74 | 55.86 | 0.754865 |
| GO:0009991\_response\_to\_extracellular\_stimulus | SFRP2 | 61 | 2 | 5.392272 | -1.281157 | 74 | 55.86 | 0.754865 |
| GO:0050793\_regulation\_of\_developmental\_process | CCND1 | 703 | 8 | 1.871571 | -1.279392 | 75 | 55.92 | 0.745600 |
| GO:0050793\_regulation\_of\_developmental\_process | EI24 | 703 | 8 | 1.871571 | -1.279392 | 75 | 55.92 | 0.745600 |
| GO:0050793\_regulation\_of\_developmental\_process | HSP90AA1 | 703 | 8 | 1.871571 | -1.279392 | 75 | 55.92 | 0.745600 |
| GO:0050793\_regulation\_of\_developmental\_process | PAX6 | 703 | 8 | 1.871571 | -1.279392 | 75 | 55.92 | 0.745600 |
| GO:0050793\_regulation\_of\_developmental\_process | RHOQ | 703 | 8 | 1.871571 | -1.279392 | 75 | 55.92 | 0.745600 |
| GO:0050793\_regulation\_of\_developmental\_process | AGRN | 703 | 8 | 1.871571 | -1.279392 | 75 | 55.92 | 0.745600 |
| GO:0050793\_regulation\_of\_developmental\_process | CSDA | 703 | 8 | 1.871571 | -1.279392 | 75 | 55.92 | 0.745600 |
| GO:0050793\_regulation\_of\_developmental\_process | BMPR1A | 703 | 8 | 1.871571 | -1.279392 | 75 | 55.92 | 0.745600 |
| GO:0042058\_regulation\_of\_epidermal\_growth\_factor\_receptor\_signaling\_pathway | NUP62 | 9 | 1 | 18.273810 | -1.272002 | 78 | 61.98 | 0.794615 |
| GO:0045428\_regulation\_of\_nitric\_oxide\_biosynthetic\_process | HSP90AA1 | 9 | 1 | 18.273810 | -1.272002 | 78 | 61.98 | 0.794615 |
| GO:0051963\_regulation\_of\_synaptogenesis | AGRN | 9 | 1 | 18.273810 | -1.272002 | 78 | 61.98 | 0.794615 |
| GO:0007049\_cell\_cycle | CCNE1 | 238 | 4 | 2.764106 | -1.270037 | 79 | 62.05 | 0.785443 |
| GO:0007049\_cell\_cycle | CCND1 | 238 | 4 | 2.764106 | -1.270037 | 79 | 62.05 | 0.785443 |
| GO:0007049\_cell\_cycle | SMC1A | 238 | 4 | 2.764106 | -1.270037 | 79 | 62.05 | 0.785443 |
| GO:0007049\_cell\_cycle | CALM1 | 238 | 4 | 2.764106 | -1.270037 | 79 | 62.05 | 0.785443 |
| GO:0021537\_telencephalon\_development | PAX6 | 62 | 2 | 5.305300 | -1.268523 | 81 | 62.49 | 0.771481 |
| GO:0021537\_telencephalon\_development | YWHAE | 62 | 2 | 5.305300 | -1.268523 | 81 | 62.49 | 0.771481 |
| GO:0030855\_epithelial\_cell\_differentiation | CCND1 | 62 | 2 | 5.305300 | -1.268523 | 81 | 62.49 | 0.771481 |
| GO:0030855\_epithelial\_cell\_differentiation | PAX6 | 62 | 2 | 5.305300 | -1.268523 | 81 | 62.49 | 0.771481 |
| GO:0045596\_negative\_regulation\_of\_cell\_differentiation | CCND1 | 144 | 3 | 3.426339 | -1.255982 | 82 | 62.98 | 0.768049 |
| GO:0045596\_negative\_regulation\_of\_cell\_differentiation | PAX6 | 144 | 3 | 3.426339 | -1.255982 | 82 | 62.98 | 0.768049 |
| GO:0045596\_negative\_regulation\_of\_cell\_differentiation | BMPR1A | 144 | 3 | 3.426339 | -1.255982 | 82 | 62.98 | 0.768049 |
| GO:0050794\_regulation\_of\_cellular\_process | HSP90AA1 | 2190 | 18 | 1.351761 | -1.252876 | 83 | 63.08 | 0.760000 |
| GO:0050794\_regulation\_of\_cellular\_process | PAX6 | 2190 | 18 | 1.351761 | -1.252876 | 83 | 63.08 | 0.760000 |
| GO:0050794\_regulation\_of\_cellular\_process | RHOQ | 2190 | 18 | 1.351761 | -1.252876 | 83 | 63.08 | 0.760000 |
| GO:0050794\_regulation\_of\_cellular\_process | NEO1 | 2190 | 18 | 1.351761 | -1.252876 | 83 | 63.08 | 0.760000 |
| GO:0050794\_regulation\_of\_cellular\_process | CSDA | 2190 | 18 | 1.351761 | -1.252876 | 83 | 63.08 | 0.760000 |
| GO:0050794\_regulation\_of\_cellular\_process | YWHAE | 2190 | 18 | 1.351761 | -1.252876 | 83 | 63.08 | 0.760000 |
| GO:0050794\_regulation\_of\_cellular\_process | ZIC3 | 2190 | 18 | 1.351761 | -1.252876 | 83 | 63.08 | 0.760000 |
| GO:0050794\_regulation\_of\_cellular\_process | CCNE1 | 2190 | 18 | 1.351761 | -1.252876 | 83 | 63.08 | 0.760000 |
| GO:0050794\_regulation\_of\_cellular\_process | EI24 | 2190 | 18 | 1.351761 | -1.252876 | 83 | 63.08 | 0.760000 |
| GO:0050794\_regulation\_of\_cellular\_process | CCND1 | 2190 | 18 | 1.351761 | -1.252876 | 83 | 63.08 | 0.760000 |
| GO:0050794\_regulation\_of\_cellular\_process | ATF4 | 2190 | 18 | 1.351761 | -1.252876 | 83 | 63.08 | 0.760000 |
| GO:0050794\_regulation\_of\_cellular\_process | NUP62 | 2190 | 18 | 1.351761 | -1.252876 | 83 | 63.08 | 0.760000 |
| GO:0050794\_regulation\_of\_cellular\_process | DNAJA1 | 2190 | 18 | 1.351761 | -1.252876 | 83 | 63.08 | 0.760000 |
| GO:0050794\_regulation\_of\_cellular\_process | TMSB4X | 2190 | 18 | 1.351761 | -1.252876 | 83 | 63.08 | 0.760000 |
| GO:0050794\_regulation\_of\_cellular\_process | RAP1B | 2190 | 18 | 1.351761 | -1.252876 | 83 | 63.08 | 0.760000 |
| GO:0050794\_regulation\_of\_cellular\_process | AGRN | 2190 | 18 | 1.351761 | -1.252876 | 83 | 63.08 | 0.760000 |
| GO:0050794\_regulation\_of\_cellular\_process | MYBBP1A | 2190 | 18 | 1.351761 | -1.252876 | 83 | 63.08 | 0.760000 |
| GO:0050794\_regulation\_of\_cellular\_process | BMPR1A | 2190 | 18 | 1.351761 | -1.252876 | 83 | 63.08 | 0.760000 |
| GO:0030900\_forebrain\_development | PAX6 | 146 | 3 | 3.379403 | -1.241366 | 84 | 63.65 | 0.757738 |
| GO:0030900\_forebrain\_development | YWHAE | 146 | 3 | 3.379403 | -1.241366 | 84 | 63.65 | 0.757738 |
| GO:0030900\_forebrain\_development | BMPR1A | 146 | 3 | 3.379403 | -1.241366 | 84 | 63.65 | 0.757738 |
| GO:0022603\_regulation\_of\_anatomical\_structure\_morphogenesis | CCND1 | 147 | 3 | 3.356414 | -1.234151 | 85 | 63.8 | 0.750588 |
| GO:0022603\_regulation\_of\_anatomical\_structure\_morphogenesis | PAX6 | 147 | 3 | 3.356414 | -1.234151 | 85 | 63.8 | 0.750588 |
| GO:0022603\_regulation\_of\_anatomical\_structure\_morphogenesis | RHOQ | 147 | 3 | 3.356414 | -1.234151 | 85 | 63.8 | 0.750588 |
| GO:0007172\_signal\_complex\_assembly | AGRN | 10 | 1 | 16.446429 | -1.227511 | 91 | 70.84 | 0.778462 |
| GO:0019321\_pentose\_metabolic\_process | ADK | 10 | 1 | 16.446429 | -1.227511 | 91 | 70.84 | 0.778462 |
| GO:0021871\_forebrain\_regionalization | PAX6 | 10 | 1 | 16.446429 | -1.227511 | 91 | 70.84 | 0.778462 |
| GO:0022900\_electron\_transport\_chain | MYBBP1A | 10 | 1 | 16.446429 | -1.227511 | 91 | 70.84 | 0.778462 |
| GO:0022904\_respiratory\_electron\_transport\_chain | MYBBP1A | 10 | 1 | 16.446429 | -1.227511 | 91 | 70.84 | 0.778462 |
| GO:0043113\_receptor\_clustering | AGRN | 10 | 1 | 16.446429 | -1.227511 | 91 | 70.84 | 0.778462 |
| GO:0003007\_heart\_morphogenesis | ZIC3 | 67 | 2 | 4.909382 | -1.208650 | 92 | 71.87 | 0.781196 |
| GO:0003007\_heart\_morphogenesis | BMPR1A | 67 | 2 | 4.909382 | -1.208650 | 92 | 71.87 | 0.781196 |
| GO:0007389\_pattern\_specification\_process | SFRP2 | 250 | 4 | 2.631429 | -1.205701 | 93 | 71.93 | 0.773441 |
| GO:0007389\_pattern\_specification\_process | PAX6 | 250 | 4 | 2.631429 | -1.205701 | 93 | 71.93 | 0.773441 |
| GO:0007389\_pattern\_specification\_process | ZIC3 | 250 | 4 | 2.631429 | -1.205701 | 93 | 71.93 | 0.773441 |
| GO:0007389\_pattern\_specification\_process | BMPR1A | 250 | 4 | 2.631429 | -1.205701 | 93 | 71.93 | 0.773441 |
| GO:0008104\_protein\_localization | PAX6 | 251 | 4 | 2.620945 | -1.200527 | 94 | 72.06 | 0.766596 |
| GO:0008104\_protein\_localization | RHOQ | 251 | 4 | 2.620945 | -1.200527 | 94 | 72.06 | 0.766596 |
| GO:0008104\_protein\_localization | RDX | 251 | 4 | 2.620945 | -1.200527 | 94 | 72.06 | 0.766596 |
| GO:0008104\_protein\_localization | YWHAE | 251 | 4 | 2.620945 | -1.200527 | 94 | 72.06 | 0.766596 |
| GO:0051173\_positive\_regulation\_of\_nitrogen\_compound\_metabolic\_process | ATF4 | 361 | 5 | 2.277899 | -1.198089 | 95 | 72.15 | 0.759474 |
| GO:0051173\_positive\_regulation\_of\_nitrogen\_compound\_metabolic\_process | HSP90AA1 | 361 | 5 | 2.277899 | -1.198089 | 95 | 72.15 | 0.759474 |
| GO:0051173\_positive\_regulation\_of\_nitrogen\_compound\_metabolic\_process | PAX6 | 361 | 5 | 2.277899 | -1.198089 | 95 | 72.15 | 0.759474 |
| GO:0051173\_positive\_regulation\_of\_nitrogen\_compound\_metabolic\_process | AGRN | 361 | 5 | 2.277899 | -1.198089 | 95 | 72.15 | 0.759474 |
| GO:0051173\_positive\_regulation\_of\_nitrogen\_compound\_metabolic\_process | ZIC3 | 361 | 5 | 2.277899 | -1.198089 | 95 | 72.15 | 0.759474 |
| GO:0007517\_muscle\_organ\_development | NEO1 | 153 | 3 | 3.224790 | -1.192110 | 96 | 72.49 | 0.755104 |
| GO:0007517\_muscle\_organ\_development | AGRN | 153 | 3 | 3.224790 | -1.192110 | 96 | 72.49 | 0.755104 |
| GO:0007517\_muscle\_organ\_development | CSDA | 153 | 3 | 3.224790 | -1.192110 | 96 | 72.49 | 0.755104 |
| GO:0009141\_nucleoside\_triphosphate\_metabolic\_process | ADK | 11 | 1 | 14.951299 | -1.187384 | 103 | 77.57 | 0.753107 |
| GO:0014902\_myotube\_differentiation | NEO1 | 11 | 1 | 14.951299 | -1.187384 | 103 | 77.57 | 0.753107 |
| GO:0030308\_negative\_regulation\_of\_cell\_growth | EI24 | 11 | 1 | 14.951299 | -1.187384 | 103 | 77.57 | 0.753107 |
| GO:0030968\_endoplasmic\_reticulum\_unfolded\_protein\_response | CCND1 | 11 | 1 | 14.951299 | -1.187384 | 103 | 77.57 | 0.753107 |
| GO:0034620\_cellular\_response\_to\_unfolded\_protein | CCND1 | 11 | 1 | 14.951299 | -1.187384 | 103 | 77.57 | 0.753107 |
| GO:0045026\_plasma\_membrane\_fusion | NEO1 | 11 | 1 | 14.951299 | -1.187384 | 103 | 77.57 | 0.753107 |
| GO:0050807\_regulation\_of\_synapse\_organization | AGRN | 11 | 1 | 14.951299 | -1.187384 | 103 | 77.57 | 0.753107 |
| GO:0005996\_monosaccharide\_metabolic\_process | ATF4 | 69 | 2 | 4.767081 | -1.186117 | 104 | 77.87 | 0.748750 |
| GO:0005996\_monosaccharide\_metabolic\_process | ADK | 69 | 2 | 4.767081 | -1.186117 | 104 | 77.87 | 0.748750 |
| GO:0048870\_cell\_motility | DNAJA1 | 257 | 4 | 2.559755 | -1.170060 | 105 | 78.79 | 0.750381 |
| GO:0048870\_cell\_motility | PAX6 | 257 | 4 | 2.559755 | -1.170060 | 105 | 78.79 | 0.750381 |
| GO:0048870\_cell\_motility | TMSB4X | 257 | 4 | 2.559755 | -1.170060 | 105 | 78.79 | 0.750381 |
| GO:0048870\_cell\_motility | YWHAE | 257 | 4 | 2.559755 | -1.170060 | 105 | 78.79 | 0.750381 |
| GO:0006066\_alcohol\_metabolic\_process | ATF4 | 158 | 3 | 3.122740 | -1.158627 | 106 | 79.5 | 0.750000 |
| GO:0006066\_alcohol\_metabolic\_process | IMPA1 | 158 | 3 | 3.122740 | -1.158627 | 106 | 79.5 | 0.750000 |
| GO:0006066\_alcohol\_metabolic\_process | ADK | 158 | 3 | 3.122740 | -1.158627 | 106 | 79.5 | 0.750000 |
| GO:0007264\_small\_GTPase\_mediated\_signal\_transduction | NUP62 | 72 | 2 | 4.568452 | -1.153688 | 108 | 80.2 | 0.742593 |
| GO:0007264\_small\_GTPase\_mediated\_signal\_transduction | RAP1B | 72 | 2 | 4.568452 | -1.153688 | 108 | 80.2 | 0.742593 |
| GO:0040012\_regulation\_of\_locomotion | PAX6 | 72 | 2 | 4.568452 | -1.153688 | 108 | 80.2 | 0.742593 |
| GO:0040012\_regulation\_of\_locomotion | TMSB4X | 72 | 2 | 4.568452 | -1.153688 | 108 | 80.2 | 0.742593 |
| GO:0006094\_gluconeogenesis | ATF4 | 12 | 1 | 13.705357 | -1.150860 | 118 | 85.7 | 0.726271 |
| GO:0009262\_deoxyribonucleotide\_metabolic\_process | ADK | 12 | 1 | 13.705357 | -1.150860 | 118 | 85.7 | 0.726271 |
| GO:0019319\_hexose\_biosynthetic\_process | ATF4 | 12 | 1 | 13.705357 | -1.150860 | 118 | 85.7 | 0.726271 |
| GO:0021513\_spinal\_cord\_dorsal\_ventral\_patterning | PAX6 | 12 | 1 | 13.705357 | -1.150860 | 118 | 85.7 | 0.726271 |
| GO:0033598\_mammary\_gland\_epithelial\_cell\_proliferation | CCND1 | 12 | 1 | 13.705357 | -1.150860 | 118 | 85.7 | 0.726271 |
| GO:0042278\_purine\_nucleoside\_metabolic\_process | ADK | 12 | 1 | 13.705357 | -1.150860 | 118 | 85.7 | 0.726271 |
| GO:0045792\_negative\_regulation\_of\_cell\_size | EI24 | 12 | 1 | 13.705357 | -1.150860 | 118 | 85.7 | 0.726271 |
| GO:0046128\_purine\_ribonucleoside\_metabolic\_process | ADK | 12 | 1 | 13.705357 | -1.150860 | 118 | 85.7 | 0.726271 |
| GO:0050803\_regulation\_of\_synapse\_structure\_and\_activity | AGRN | 12 | 1 | 13.705357 | -1.150860 | 118 | 85.7 | 0.726271 |
| GO:0055114\_oxidation\_reduction | MYBBP1A | 12 | 1 | 13.705357 | -1.150860 | 118 | 85.7 | 0.726271 |
| GO:0051270\_regulation\_of\_cell\_motion | PAX6 | 73 | 2 | 4.505871 | -1.143224 | 119 | 86.25 | 0.724790 |
| GO:0051270\_regulation\_of\_cell\_motion | TMSB4X | 73 | 2 | 4.505871 | -1.143224 | 119 | 86.25 | 0.724790 |
| GO:0022414\_reproductive\_process | CCND1 | 376 | 5 | 2.187025 | -1.136723 | 120 | 86.4 | 0.720000 |
| GO:0022414\_reproductive\_process | GPX4 | 376 | 5 | 2.187025 | -1.136723 | 120 | 86.4 | 0.720000 |
| GO:0022414\_reproductive\_process | DNAJA1 | 376 | 5 | 2.187025 | -1.136723 | 120 | 86.4 | 0.720000 |
| GO:0022414\_reproductive\_process | CSDA | 376 | 5 | 2.187025 | -1.136723 | 120 | 86.4 | 0.720000 |
| GO:0022414\_reproductive\_process | BMPR1A | 376 | 5 | 2.187025 | -1.136723 | 120 | 86.4 | 0.720000 |
| GO:0000003\_reproduction | CCND1 | 379 | 5 | 2.169714 | -1.124877 | 121 | 86.83 | 0.717603 |
| GO:0000003\_reproduction | GPX4 | 379 | 5 | 2.169714 | -1.124877 | 121 | 86.83 | 0.717603 |
| GO:0000003\_reproduction | DNAJA1 | 379 | 5 | 2.169714 | -1.124877 | 121 | 86.83 | 0.717603 |
| GO:0000003\_reproduction | CSDA | 379 | 5 | 2.169714 | -1.124877 | 121 | 86.83 | 0.717603 |
| GO:0000003\_reproduction | BMPR1A | 379 | 5 | 2.169714 | -1.124877 | 121 | 86.83 | 0.717603 |
| GO:0048589\_developmental\_growth | AGRN | 75 | 2 | 4.385714 | -1.122785 | 122 | 87.1 | 0.713934 |
| GO:0048589\_developmental\_growth | BMPR1A | 75 | 2 | 4.385714 | -1.122785 | 122 | 87.1 | 0.713934 |
| GO:0006090\_pyruvate\_metabolic\_process | ATF4 | 13 | 1 | 12.651099 | -1.117362 | 127 | 92.85 | 0.731102 |
| GO:0009119\_ribonucleoside\_metabolic\_process | ADK | 13 | 1 | 12.651099 | -1.117362 | 127 | 92.85 | 0.731102 |
| GO:0021511\_spinal\_cord\_patterning | PAX6 | 13 | 1 | 12.651099 | -1.117362 | 127 | 92.85 | 0.731102 |
| GO:0021879\_forebrain\_neuron\_differentiation | PAX6 | 13 | 1 | 12.651099 | -1.117362 | 127 | 92.85 | 0.731102 |
| GO:0060070\_Wnt\_receptor\_signaling\_pathway\_through\_beta-catenin | CCND1 | 13 | 1 | 12.651099 | -1.117362 | 127 | 92.85 | 0.731102 |
| GO:0045944\_positive\_regulation\_of\_transcription\_from\_RNA\_polymerase\_II\_promoter | ATF4 | 269 | 4 | 2.445566 | -1.111944 | 128 | 93.53 | 0.730703 |
| GO:0045944\_positive\_regulation\_of\_transcription\_from\_RNA\_polymerase\_II\_promoter | PAX6 | 269 | 4 | 2.445566 | -1.111944 | 128 | 93.53 | 0.730703 |
| GO:0045944\_positive\_regulation\_of\_transcription\_from\_RNA\_polymerase\_II\_promoter | AGRN | 269 | 4 | 2.445566 | -1.111944 | 128 | 93.53 | 0.730703 |
| GO:0045944\_positive\_regulation\_of\_transcription\_from\_RNA\_polymerase\_II\_promoter | ZIC3 | 269 | 4 | 2.445566 | -1.111944 | 128 | 93.53 | 0.730703 |
| GO:0031328\_positive\_regulation\_of\_cellular\_biosynthetic\_process | ATF4 | 387 | 5 | 2.124862 | -1.093955 | 129 | 94.27 | 0.730775 |
| GO:0031328\_positive\_regulation\_of\_cellular\_biosynthetic\_process | HSP90AA1 | 387 | 5 | 2.124862 | -1.093955 | 129 | 94.27 | 0.730775 |
| GO:0031328\_positive\_regulation\_of\_cellular\_biosynthetic\_process | PAX6 | 387 | 5 | 2.124862 | -1.093955 | 129 | 94.27 | 0.730775 |
| GO:0031328\_positive\_regulation\_of\_cellular\_biosynthetic\_process | AGRN | 387 | 5 | 2.124862 | -1.093955 | 129 | 94.27 | 0.730775 |
| GO:0031328\_positive\_regulation\_of\_cellular\_biosynthetic\_process | ZIC3 | 387 | 5 | 2.124862 | -1.093955 | 129 | 94.27 | 0.730775 |
| GO:0051716\_cellular\_response\_to\_stimulus | CCND1 | 273 | 4 | 2.409733 | -1.093361 | 130 | 94.33 | 0.725615 |
| GO:0051716\_cellular\_response\_to\_stimulus | LDHA | 273 | 4 | 2.409733 | -1.093361 | 130 | 94.33 | 0.725615 |
| GO:0051716\_cellular\_response\_to\_stimulus | SFRP2 | 273 | 4 | 2.409733 | -1.093361 | 130 | 94.33 | 0.725615 |
| GO:0051716\_cellular\_response\_to\_stimulus | RHOQ | 273 | 4 | 2.409733 | -1.093361 | 130 | 94.33 | 0.725615 |
| GO:0009891\_positive\_regulation\_of\_biosynthetic\_process | ATF4 | 388 | 5 | 2.119385 | -1.090156 | 131 | 94.97 | 0.724962 |
| GO:0009891\_positive\_regulation\_of\_biosynthetic\_process | HSP90AA1 | 388 | 5 | 2.119385 | -1.090156 | 131 | 94.97 | 0.724962 |
| GO:0009891\_positive\_regulation\_of\_biosynthetic\_process | PAX6 | 388 | 5 | 2.119385 | -1.090156 | 131 | 94.97 | 0.724962 |
| GO:0009891\_positive\_regulation\_of\_biosynthetic\_process | AGRN | 388 | 5 | 2.119385 | -1.090156 | 131 | 94.97 | 0.724962 |
| GO:0009891\_positive\_regulation\_of\_biosynthetic\_process | ZIC3 | 388 | 5 | 2.119385 | -1.090156 | 131 | 94.97 | 0.724962 |
| GO:0033036\_macromolecule\_localization | PAX6 | 274 | 4 | 2.400938 | -1.088774 | 132 | 95.13 | 0.720682 |
| GO:0033036\_macromolecule\_localization | RHOQ | 274 | 4 | 2.400938 | -1.088774 | 132 | 95.13 | 0.720682 |
| GO:0033036\_macromolecule\_localization | RDX | 274 | 4 | 2.400938 | -1.088774 | 132 | 95.13 | 0.720682 |
| GO:0033036\_macromolecule\_localization | YWHAE | 274 | 4 | 2.400938 | -1.088774 | 132 | 95.13 | 0.720682 |
| GO:0006809\_nitric\_oxide\_biosynthetic\_process | HSP90AA1 | 14 | 1 | 11.747449 | -1.086439 | 135 | 99.55 | 0.737407 |
| GO:0046209\_nitric\_oxide\_metabolic\_process | HSP90AA1 | 14 | 1 | 11.747449 | -1.086439 | 135 | 99.55 | 0.737407 |
| GO:0048665\_neuron\_fate\_specification | PAX6 | 14 | 1 | 11.747449 | -1.086439 | 135 | 99.55 | 0.737407 |
| GO:0048523\_negative\_regulation\_of\_cellular\_process | EI24 | 774 | 8 | 1.699889 | -1.074655 | 136 | 100.24 | 0.737059 |
| GO:0048523\_negative\_regulation\_of\_cellular\_process | CCND1 | 774 | 8 | 1.699889 | -1.074655 | 136 | 100.24 | 0.737059 |
| GO:0048523\_negative\_regulation\_of\_cellular\_process | NUP62 | 774 | 8 | 1.699889 | -1.074655 | 136 | 100.24 | 0.737059 |
| GO:0048523\_negative\_regulation\_of\_cellular\_process | PAX6 | 774 | 8 | 1.699889 | -1.074655 | 136 | 100.24 | 0.737059 |
| GO:0048523\_negative\_regulation\_of\_cellular\_process | CSDA | 774 | 8 | 1.699889 | -1.074655 | 136 | 100.24 | 0.737059 |
| GO:0048523\_negative\_regulation\_of\_cellular\_process | MYBBP1A | 774 | 8 | 1.699889 | -1.074655 | 136 | 100.24 | 0.737059 |
| GO:0048523\_negative\_regulation\_of\_cellular\_process | YWHAE | 774 | 8 | 1.699889 | -1.074655 | 136 | 100.24 | 0.737059 |
| GO:0048523\_negative\_regulation\_of\_cellular\_process | BMPR1A | 774 | 8 | 1.699889 | -1.074655 | 136 | 100.24 | 0.737059 |
| GO:0007173\_epidermal\_growth\_factor\_receptor\_signaling\_pathway | NUP62 | 15 | 1 | 10.964286 | -1.057737 | 141 | 106.18 | 0.753050 |
| GO:0009116\_nucleoside\_metabolic\_process | ADK | 15 | 1 | 10.964286 | -1.057737 | 141 | 106.18 | 0.753050 |
| GO:0021872\_generation\_of\_neurons\_in\_the\_forebrain | PAX6 | 15 | 1 | 10.964286 | -1.057737 | 141 | 106.18 | 0.753050 |
| GO:0048709\_oligodendrocyte\_differentiation | PAX6 | 15 | 1 | 10.964286 | -1.057737 | 141 | 106.18 | 0.753050 |
| GO:0060749\_mammary\_gland\_alveolus\_development | CCND1 | 15 | 1 | 10.964286 | -1.057737 | 141 | 106.18 | 0.753050 |
| GO:0009888\_tissue\_development | CCND1 | 525 | 6 | 1.879592 | -1.039964 | 142 | 107.96 | 0.760282 |
| GO:0009888\_tissue\_development | PAX6 | 525 | 6 | 1.879592 | -1.039964 | 142 | 107.96 | 0.760282 |
| GO:0009888\_tissue\_development | NEO1 | 525 | 6 | 1.879592 | -1.039964 | 142 | 107.96 | 0.760282 |
| GO:0009888\_tissue\_development | AGRN | 525 | 6 | 1.879592 | -1.039964 | 142 | 107.96 | 0.760282 |
| GO:0009888\_tissue\_development | CSDA | 525 | 6 | 1.879592 | -1.039964 | 142 | 107.96 | 0.760282 |
| GO:0009888\_tissue\_development | BMPR1A | 525 | 6 | 1.879592 | -1.039964 | 142 | 107.96 | 0.760282 |
| GO:0048732\_gland\_development | CCND1 | 179 | 3 | 2.756385 | -1.031459 | 143 | 108.59 | 0.759371 |
| GO:0048732\_gland\_development | PAX6 | 179 | 3 | 2.756385 | -1.031459 | 143 | 108.59 | 0.759371 |
| GO:0048732\_gland\_development | BMPR1A | 179 | 3 | 2.756385 | -1.031459 | 143 | 108.59 | 0.759371 |
| GO:0019751\_polyol\_metabolic\_process | IMPA1 | 16 | 1 | 10.279018 | -1.030969 | 147 | 112.69 | 0.766599 |
| GO:0021522\_spinal\_cord\_motor\_neuron\_differentiation | PAX6 | 16 | 1 | 10.279018 | -1.030969 | 147 | 112.69 | 0.766599 |
| GO:0034976\_response\_to\_endoplasmic\_reticulum\_stress | CCND1 | 16 | 1 | 10.279018 | -1.030969 | 147 | 112.69 | 0.766599 |
| GO:0046364\_monosaccharide\_biosynthetic\_process | ATF4 | 16 | 1 | 10.279018 | -1.030969 | 147 | 112.69 | 0.766599 |
| GO:0070887\_cellular\_response\_to\_chemical\_stimulus | CCND1 | 85 | 2 | 3.869748 | -1.029338 | 148 | 112.94 | 0.763108 |
| GO:0070887\_cellular\_response\_to\_chemical\_stimulus | RHOQ | 85 | 2 | 3.869748 | -1.029338 | 148 | 112.94 | 0.763108 |
| GO:0006261\_DNA-dependent\_DNA\_replication | CCNE1 | 17 | 1 | 9.674370 | -1.005899 | 153 | 119.08 | 0.778301 |
| GO:0006984\_ER-nuclear\_signaling\_pathway | CCND1 | 17 | 1 | 9.674370 | -1.005899 | 153 | 119.08 | 0.778301 |
| GO:0030317\_sperm\_motility | DNAJA1 | 17 | 1 | 9.674370 | -1.005899 | 153 | 119.08 | 0.778301 |
| GO:0043407\_negative\_regulation\_of\_MAP\_kinase\_activity | NUP62 | 17 | 1 | 9.674370 | -1.005899 | 153 | 119.08 | 0.778301 |
| GO:0045333\_cellular\_respiration | MYBBP1A | 17 | 1 | 9.674370 | -1.005899 | 153 | 119.08 | 0.778301 |
| GO:0040011\_locomotion | DNAJA1 | 295 | 4 | 2.230024 | -0.997570 | 154 | 120.0 | 0.779221 |
| GO:0040011\_locomotion | PAX6 | 295 | 4 | 2.230024 | -0.997570 | 154 | 120.0 | 0.779221 |
| GO:0040011\_locomotion | TMSB4X | 295 | 4 | 2.230024 | -0.997570 | 154 | 120.0 | 0.779221 |
| GO:0040011\_locomotion | YWHAE | 295 | 4 | 2.230024 | -0.997570 | 154 | 120.0 | 0.779221 |
| GO:0007276\_gamete\_generation | GPX4 | 188 | 3 | 2.624430 | -0.982749 | 155 | 120.88 | 0.779871 |
| GO:0007276\_gamete\_generation | DNAJA1 | 188 | 3 | 2.624430 | -0.982749 | 155 | 120.88 | 0.779871 |
| GO:0007276\_gamete\_generation | CSDA | 188 | 3 | 2.624430 | -0.982749 | 155 | 120.88 | 0.779871 |
| GO:0006457\_protein\_folding | HSP90AA1 | 18 | 1 | 9.136905 | -0.982334 | 162 | 125.92 | 0.777284 |
| GO:0010498\_proteasomal\_protein\_catabolic\_process | UBE2D3 | 18 | 1 | 9.136905 | -0.982334 | 162 | 125.92 | 0.777284 |
| GO:0021517\_ventral\_spinal\_cord\_development | PAX6 | 18 | 1 | 9.136905 | -0.982334 | 162 | 125.92 | 0.777284 |
| GO:0030178\_negative\_regulation\_of\_Wnt\_receptor\_signaling\_pathway | CCND1 | 18 | 1 | 9.136905 | -0.982334 | 162 | 125.92 | 0.777284 |
| GO:0043161\_proteasomal\_ubiquitin-dependent\_protein\_catabolic\_process | UBE2D3 | 18 | 1 | 9.136905 | -0.982334 | 162 | 125.92 | 0.777284 |
| GO:0046578\_regulation\_of\_Ras\_protein\_signal\_transduction | NUP62 | 18 | 1 | 9.136905 | -0.982334 | 162 | 125.92 | 0.777284 |
| GO:0046620\_regulation\_of\_organ\_growth | CSDA | 18 | 1 | 9.136905 | -0.982334 | 162 | 125.92 | 0.777284 |
| GO:0007569\_cell\_aging | NUP62 | 19 | 1 | 8.656015 | -0.960110 | 168 | 131.23 | 0.781131 |
| GO:0007595\_lactation | CCND1 | 19 | 1 | 8.656015 | -0.960110 | 168 | 131.23 | 0.781131 |
| GO:0030518\_steroid\_hormone\_receptor\_signaling\_pathway | DNAJA1 | 19 | 1 | 8.656015 | -0.960110 | 168 | 131.23 | 0.781131 |
| GO:0042462\_eye\_photoreceptor\_cell\_development | PAX6 | 19 | 1 | 8.656015 | -0.960110 | 168 | 131.23 | 0.781131 |
| GO:0046165\_alcohol\_biosynthetic\_process | ATF4 | 19 | 1 | 8.656015 | -0.960110 | 168 | 131.23 | 0.781131 |
| GO:0051056\_regulation\_of\_small\_GTPase\_mediated\_signal\_transduction | NUP62 | 19 | 1 | 8.656015 | -0.960110 | 168 | 131.23 | 0.781131 |
| GO:0045893\_positive\_regulation\_of\_transcription\_\_DNA-dependent | ATF4 | 306 | 4 | 2.149860 | -0.953407 | 170 | 132.13 | 0.777235 |
| GO:0045893\_positive\_regulation\_of\_transcription\_\_DNA-dependent | PAX6 | 306 | 4 | 2.149860 | -0.953407 | 170 | 132.13 | 0.777235 |
| GO:0045893\_positive\_regulation\_of\_transcription\_\_DNA-dependent | AGRN | 306 | 4 | 2.149860 | -0.953407 | 170 | 132.13 | 0.777235 |
| GO:0045893\_positive\_regulation\_of\_transcription\_\_DNA-dependent | ZIC3 | 306 | 4 | 2.149860 | -0.953407 | 170 | 132.13 | 0.777235 |
| GO:0051254\_positive\_regulation\_of\_RNA\_metabolic\_process | ATF4 | 306 | 4 | 2.149860 | -0.953407 | 170 | 132.13 | 0.777235 |
| GO:0051254\_positive\_regulation\_of\_RNA\_metabolic\_process | PAX6 | 306 | 4 | 2.149860 | -0.953407 | 170 | 132.13 | 0.777235 |
| GO:0051254\_positive\_regulation\_of\_RNA\_metabolic\_process | AGRN | 306 | 4 | 2.149860 | -0.953407 | 170 | 132.13 | 0.777235 |
| GO:0051254\_positive\_regulation\_of\_RNA\_metabolic\_process | ZIC3 | 306 | 4 | 2.149860 | -0.953407 | 170 | 132.13 | 0.777235 |
| GO:0050789\_regulation\_of\_biological\_process | HSP90AA1 | 2357 | 18 | 1.255985 | -0.941772 | 171 | 133.09 | 0.778304 |
| GO:0050789\_regulation\_of\_biological\_process | PAX6 | 2357 | 18 | 1.255985 | -0.941772 | 171 | 133.09 | 0.778304 |
| GO:0050789\_regulation\_of\_biological\_process | RHOQ | 2357 | 18 | 1.255985 | -0.941772 | 171 | 133.09 | 0.778304 |
| GO:0050789\_regulation\_of\_biological\_process | NEO1 | 2357 | 18 | 1.255985 | -0.941772 | 171 | 133.09 | 0.778304 |
| GO:0050789\_regulation\_of\_biological\_process | CSDA | 2357 | 18 | 1.255985 | -0.941772 | 171 | 133.09 | 0.778304 |
| GO:0050789\_regulation\_of\_biological\_process | YWHAE | 2357 | 18 | 1.255985 | -0.941772 | 171 | 133.09 | 0.778304 |
| GO:0050789\_regulation\_of\_biological\_process | ZIC3 | 2357 | 18 | 1.255985 | -0.941772 | 171 | 133.09 | 0.778304 |
| GO:0050789\_regulation\_of\_biological\_process | CCNE1 | 2357 | 18 | 1.255985 | -0.941772 | 171 | 133.09 | 0.778304 |
| GO:0050789\_regulation\_of\_biological\_process | CCND1 | 2357 | 18 | 1.255985 | -0.941772 | 171 | 133.09 | 0.778304 |
| GO:0050789\_regulation\_of\_biological\_process | ATF4 | 2357 | 18 | 1.255985 | -0.941772 | 171 | 133.09 | 0.778304 |
| GO:0050789\_regulation\_of\_biological\_process | EI24 | 2357 | 18 | 1.255985 | -0.941772 | 171 | 133.09 | 0.778304 |
| GO:0050789\_regulation\_of\_biological\_process | NUP62 | 2357 | 18 | 1.255985 | -0.941772 | 171 | 133.09 | 0.778304 |
| GO:0050789\_regulation\_of\_biological\_process | DNAJA1 | 2357 | 18 | 1.255985 | -0.941772 | 171 | 133.09 | 0.778304 |
| GO:0050789\_regulation\_of\_biological\_process | RAP1B | 2357 | 18 | 1.255985 | -0.941772 | 171 | 133.09 | 0.778304 |
| GO:0050789\_regulation\_of\_biological\_process | TMSB4X | 2357 | 18 | 1.255985 | -0.941772 | 171 | 133.09 | 0.778304 |
| GO:0050789\_regulation\_of\_biological\_process | AGRN | 2357 | 18 | 1.255985 | -0.941772 | 171 | 133.09 | 0.778304 |
| GO:0050789\_regulation\_of\_biological\_process | MYBBP1A | 2357 | 18 | 1.255985 | -0.941772 | 171 | 133.09 | 0.778304 |
| GO:0050789\_regulation\_of\_biological\_process | BMPR1A | 2357 | 18 | 1.255985 | -0.941772 | 171 | 133.09 | 0.778304 |
| GO:0007416\_synaptogenesis | AGRN | 20 | 1 | 8.223214 | -0.939089 | 174 | 137.1 | 0.787931 |
| GO:0007528\_neuromuscular\_junction\_development | AGRN | 20 | 1 | 8.223214 | -0.939089 | 174 | 137.1 | 0.787931 |
| GO:0008360\_regulation\_of\_cell\_shape | RHOQ | 20 | 1 | 8.223214 | -0.939089 | 174 | 137.1 | 0.787931 |
| GO:0007548\_sex\_differentiation | CSDA | 98 | 2 | 3.356414 | -0.925694 | 175 | 138.6 | 0.792000 |
| GO:0007548\_sex\_differentiation | BMPR1A | 98 | 2 | 3.356414 | -0.925694 | 175 | 138.6 | 0.792000 |
| GO:0001709\_cell\_fate\_determination | PAX6 | 21 | 1 | 7.831633 | -0.919155 | 184 | 143.15 | 0.777989 |
| GO:0001754\_eye\_photoreceptor\_cell\_differentiation | PAX6 | 21 | 1 | 7.831633 | -0.919155 | 184 | 143.15 | 0.777989 |
| GO:0002053\_positive\_regulation\_of\_mesenchymal\_cell\_proliferation | BMPR1A | 21 | 1 | 7.831633 | -0.919155 | 184 | 143.15 | 0.777989 |
| GO:0006944\_membrane\_fusion | NEO1 | 21 | 1 | 7.831633 | -0.919155 | 184 | 143.15 | 0.777989 |
| GO:0010563\_negative\_regulation\_of\_phosphorus\_metabolic\_process | YWHAE | 21 | 1 | 7.831633 | -0.919155 | 184 | 143.15 | 0.777989 |
| GO:0019827\_stem\_cell\_maintenance | BMPR1A | 21 | 1 | 7.831633 | -0.919155 | 184 | 143.15 | 0.777989 |
| GO:0021532\_neural\_tube\_patterning | PAX6 | 21 | 1 | 7.831633 | -0.919155 | 184 | 143.15 | 0.777989 |
| GO:0030216\_keratinocyte\_differentiation | PAX6 | 21 | 1 | 7.831633 | -0.919155 | 184 | 143.15 | 0.777989 |
| GO:0045936\_negative\_regulation\_of\_phosphate\_metabolic\_process | YWHAE | 21 | 1 | 7.831633 | -0.919155 | 184 | 143.15 | 0.777989 |
| GO:0007398\_ectoderm\_development | PAX6 | 99 | 2 | 3.322511 | -0.918416 | 185 | 143.54 | 0.775892 |
| GO:0007398\_ectoderm\_development | BMPR1A | 99 | 2 | 3.322511 | -0.918416 | 185 | 143.54 | 0.775892 |
| GO:0031325\_positive\_regulation\_of\_cellular\_metabolic\_process | ATF4 | 442 | 5 | 1.860456 | -0.904817 | 186 | 144.05 | 0.774462 |
| GO:0031325\_positive\_regulation\_of\_cellular\_metabolic\_process | HSP90AA1 | 442 | 5 | 1.860456 | -0.904817 | 186 | 144.05 | 0.774462 |
| GO:0031325\_positive\_regulation\_of\_cellular\_metabolic\_process | PAX6 | 442 | 5 | 1.860456 | -0.904817 | 186 | 144.05 | 0.774462 |
| GO:0031325\_positive\_regulation\_of\_cellular\_metabolic\_process | AGRN | 442 | 5 | 1.860456 | -0.904817 | 186 | 144.05 | 0.774462 |
| GO:0031325\_positive\_regulation\_of\_cellular\_metabolic\_process | ZIC3 | 442 | 5 | 1.860456 | -0.904817 | 186 | 144.05 | 0.774462 |
| GO:0001558\_regulation\_of\_cell\_growth | EI24 | 22 | 1 | 7.475649 | -0.900206 | 194 | 149.8 | 0.772165 |
| GO:0001947\_heart\_looping | ZIC3 | 22 | 1 | 7.475649 | -0.900206 | 194 | 149.8 | 0.772165 |
| GO:0010463\_mesenchymal\_cell\_proliferation | BMPR1A | 22 | 1 | 7.475649 | -0.900206 | 194 | 149.8 | 0.772165 |
| GO:0010464\_regulation\_of\_mesenchymal\_cell\_proliferation | BMPR1A | 22 | 1 | 7.475649 | -0.900206 | 194 | 149.8 | 0.772165 |
| GO:0021766\_hippocampus\_development | YWHAE | 22 | 1 | 7.475649 | -0.900206 | 194 | 149.8 | 0.772165 |
| GO:0042461\_photoreceptor\_cell\_development | PAX6 | 22 | 1 | 7.475649 | -0.900206 | 194 | 149.8 | 0.772165 |
| GO:0043112\_receptor\_metabolic\_process | AGRN | 22 | 1 | 7.475649 | -0.900206 | 194 | 149.8 | 0.772165 |
| GO:0048864\_stem\_cell\_development | BMPR1A | 22 | 1 | 7.475649 | -0.900206 | 194 | 149.8 | 0.772165 |
| GO:0009968\_negative\_regulation\_of\_signal\_transduction | CCND1 | 103 | 2 | 3.193481 | -0.890172 | 195 | 150.7 | 0.772821 |
| GO:0009968\_negative\_regulation\_of\_signal\_transduction | NUP62 | 103 | 2 | 3.193481 | -0.890172 | 195 | 150.7 | 0.772821 |
| GO:0050767\_regulation\_of\_neurogenesis | PAX6 | 104 | 2 | 3.162775 | -0.883320 | 196 | 151.25 | 0.771684 |
| GO:0050767\_regulation\_of\_neurogenesis | BMPR1A | 104 | 2 | 3.162775 | -0.883320 | 196 | 151.25 | 0.771684 |
| GO:0043388\_positive\_regulation\_of\_DNA\_binding | CALM1 | 23 | 1 | 7.150621 | -0.882153 | 197 | 154.18 | 0.782640 |
| GO:0048519\_negative\_regulation\_of\_biological\_process | CCND1 | 859 | 8 | 1.531681 | -0.869154 | 198 | 154.55 | 0.780556 |
| GO:0048519\_negative\_regulation\_of\_biological\_process | EI24 | 859 | 8 | 1.531681 | -0.869154 | 198 | 154.55 | 0.780556 |
| GO:0048519\_negative\_regulation\_of\_biological\_process | NUP62 | 859 | 8 | 1.531681 | -0.869154 | 198 | 154.55 | 0.780556 |
| GO:0048519\_negative\_regulation\_of\_biological\_process | PAX6 | 859 | 8 | 1.531681 | -0.869154 | 198 | 154.55 | 0.780556 |
| GO:0048519\_negative\_regulation\_of\_biological\_process | CSDA | 859 | 8 | 1.531681 | -0.869154 | 198 | 154.55 | 0.780556 |
| GO:0048519\_negative\_regulation\_of\_biological\_process | YWHAE | 859 | 8 | 1.531681 | -0.869154 | 198 | 154.55 | 0.780556 |
| GO:0048519\_negative\_regulation\_of\_biological\_process | MYBBP1A | 859 | 8 | 1.531681 | -0.869154 | 198 | 154.55 | 0.780556 |
| GO:0048519\_negative\_regulation\_of\_biological\_process | BMPR1A | 859 | 8 | 1.531681 | -0.869154 | 198 | 154.55 | 0.780556 |
| GO:0021515\_cell\_differentiation\_in\_spinal\_cord | PAX6 | 24 | 1 | 6.852679 | -0.864921 | 201 | 158.71 | 0.789602 |
| GO:0050679\_positive\_regulation\_of\_epithelial\_cell\_proliferation | BMPR1A | 24 | 1 | 6.852679 | -0.864921 | 201 | 158.71 | 0.789602 |
| GO:0051099\_positive\_regulation\_of\_binding | CALM1 | 24 | 1 | 6.852679 | -0.864921 | 201 | 158.71 | 0.789602 |
| GO:0006928\_cell\_motion | DNAJA1 | 330 | 4 | 1.993506 | -0.864642 | 203 | 159.07 | 0.783596 |
| GO:0006928\_cell\_motion | PAX6 | 330 | 4 | 1.993506 | -0.864642 | 203 | 159.07 | 0.783596 |
| GO:0006928\_cell\_motion | TMSB4X | 330 | 4 | 1.993506 | -0.864642 | 203 | 159.07 | 0.783596 |
| GO:0006928\_cell\_motion | YWHAE | 330 | 4 | 1.993506 | -0.864642 | 203 | 159.07 | 0.783596 |
| GO:0051674\_localization\_of\_cell | DNAJA1 | 330 | 4 | 1.993506 | -0.864642 | 203 | 159.07 | 0.783596 |
| GO:0051674\_localization\_of\_cell | PAX6 | 330 | 4 | 1.993506 | -0.864642 | 203 | 159.07 | 0.783596 |
| GO:0051674\_localization\_of\_cell | TMSB4X | 330 | 4 | 1.993506 | -0.864642 | 203 | 159.07 | 0.783596 |
| GO:0051674\_localization\_of\_cell | YWHAE | 330 | 4 | 1.993506 | -0.864642 | 203 | 159.07 | 0.783596 |
| GO:0051239\_regulation\_of\_multicellular\_organismal\_process | CCND1 | 587 | 6 | 1.681066 | -0.862615 | 204 | 159.28 | 0.780784 |
| GO:0051239\_regulation\_of\_multicellular\_organismal\_process | HSP90AA1 | 587 | 6 | 1.681066 | -0.862615 | 204 | 159.28 | 0.780784 |
| GO:0051239\_regulation\_of\_multicellular\_organismal\_process | PAX6 | 587 | 6 | 1.681066 | -0.862615 | 204 | 159.28 | 0.780784 |
| GO:0051239\_regulation\_of\_multicellular\_organismal\_process | AGRN | 587 | 6 | 1.681066 | -0.862615 | 204 | 159.28 | 0.780784 |
| GO:0051239\_regulation\_of\_multicellular\_organismal\_process | CSDA | 587 | 6 | 1.681066 | -0.862615 | 204 | 159.28 | 0.780784 |
| GO:0051239\_regulation\_of\_multicellular\_organismal\_process | BMPR1A | 587 | 6 | 1.681066 | -0.862615 | 204 | 159.28 | 0.780784 |
| GO:0051093\_negative\_regulation\_of\_developmental\_process | CCND1 | 331 | 4 | 1.987484 | -0.861153 | 205 | 159.49 | 0.778000 |
| GO:0051093\_negative\_regulation\_of\_developmental\_process | PAX6 | 331 | 4 | 1.987484 | -0.861153 | 205 | 159.49 | 0.778000 |
| GO:0051093\_negative\_regulation\_of\_developmental\_process | CSDA | 331 | 4 | 1.987484 | -0.861153 | 205 | 159.49 | 0.778000 |
| GO:0051093\_negative\_regulation\_of\_developmental\_process | BMPR1A | 331 | 4 | 1.987484 | -0.861153 | 205 | 159.49 | 0.778000 |
| GO:0009893\_positive\_regulation\_of\_metabolic\_process | ATF4 | 458 | 5 | 1.795462 | -0.856557 | 206 | 160.04 | 0.776893 |
| GO:0009893\_positive\_regulation\_of\_metabolic\_process | HSP90AA1 | 458 | 5 | 1.795462 | -0.856557 | 206 | 160.04 | 0.776893 |
| GO:0009893\_positive\_regulation\_of\_metabolic\_process | PAX6 | 458 | 5 | 1.795462 | -0.856557 | 206 | 160.04 | 0.776893 |
| GO:0009893\_positive\_regulation\_of\_metabolic\_process | AGRN | 458 | 5 | 1.795462 | -0.856557 | 206 | 160.04 | 0.776893 |
| GO:0009893\_positive\_regulation\_of\_metabolic\_process | ZIC3 | 458 | 5 | 1.795462 | -0.856557 | 206 | 160.04 | 0.776893 |
| GO:0010033\_response\_to\_organic\_substance | CCND1 | 216 | 3 | 2.284226 | -0.849178 | 207 | 160.34 | 0.774589 |
| GO:0010033\_response\_to\_organic\_substance | HSP90AA1 | 216 | 3 | 2.284226 | -0.849178 | 207 | 160.34 | 0.774589 |
| GO:0010033\_response\_to\_organic\_substance | RHOQ | 216 | 3 | 2.284226 | -0.849178 | 207 | 160.34 | 0.774589 |
| GO:0007492\_endoderm\_development | BMPR1A | 25 | 1 | 6.578571 | -0.848443 | 210 | 163.4 | 0.778095 |
| GO:0031400\_negative\_regulation\_of\_protein\_modification\_process | YWHAE | 25 | 1 | 6.578571 | -0.848443 | 210 | 163.4 | 0.778095 |
| GO:0035137\_hindlimb\_morphogenesis | BMPR1A | 25 | 1 | 6.578571 | -0.848443 | 210 | 163.4 | 0.778095 |
| GO:0010648\_negative\_regulation\_of\_cell\_communication | CCND1 | 110 | 2 | 2.990260 | -0.843848 | 211 | 164.38 | 0.779052 |
| GO:0010648\_negative\_regulation\_of\_cell\_communication | NUP62 | 110 | 2 | 2.990260 | -0.843848 | 211 | 164.38 | 0.779052 |
| GO:0031323\_regulation\_of\_cellular\_metabolic\_process | ATF4 | 1015 | 9 | 1.458304 | -0.841765 | 212 | 164.5 | 0.775943 |
| GO:0031323\_regulation\_of\_cellular\_metabolic\_process | HSP90AA1 | 1015 | 9 | 1.458304 | -0.841765 | 212 | 164.5 | 0.775943 |
| GO:0031323\_regulation\_of\_cellular\_metabolic\_process | NUP62 | 1015 | 9 | 1.458304 | -0.841765 | 212 | 164.5 | 0.775943 |
| GO:0031323\_regulation\_of\_cellular\_metabolic\_process | PAX6 | 1015 | 9 | 1.458304 | -0.841765 | 212 | 164.5 | 0.775943 |
| GO:0031323\_regulation\_of\_cellular\_metabolic\_process | NEO1 | 1015 | 9 | 1.458304 | -0.841765 | 212 | 164.5 | 0.775943 |
| GO:0031323\_regulation\_of\_cellular\_metabolic\_process | AGRN | 1015 | 9 | 1.458304 | -0.841765 | 212 | 164.5 | 0.775943 |
| GO:0031323\_regulation\_of\_cellular\_metabolic\_process | YWHAE | 1015 | 9 | 1.458304 | -0.841765 | 212 | 164.5 | 0.775943 |
| GO:0031323\_regulation\_of\_cellular\_metabolic\_process | MYBBP1A | 1015 | 9 | 1.458304 | -0.841765 | 212 | 164.5 | 0.775943 |
| GO:0031323\_regulation\_of\_cellular\_metabolic\_process | ZIC3 | 1015 | 9 | 1.458304 | -0.841765 | 212 | 164.5 | 0.775943 |
| GO:0045941\_positive\_regulation\_of\_transcription | ATF4 | 338 | 4 | 1.946323 | -0.837164 | 213 | 164.82 | 0.773803 |
| GO:0045941\_positive\_regulation\_of\_transcription | PAX6 | 338 | 4 | 1.946323 | -0.837164 | 213 | 164.82 | 0.773803 |
| GO:0045941\_positive\_regulation\_of\_transcription | AGRN | 338 | 4 | 1.946323 | -0.837164 | 213 | 164.82 | 0.773803 |
| GO:0045941\_positive\_regulation\_of\_transcription | ZIC3 | 338 | 4 | 1.946323 | -0.837164 | 213 | 164.82 | 0.773803 |
| GO:0007405\_neuroblast\_proliferation | PAX6 | 26 | 1 | 6.325549 | -0.832659 | 217 | 168.47 | 0.776359 |
| GO:0045665\_negative\_regulation\_of\_neuron\_differentiation | PAX6 | 26 | 1 | 6.325549 | -0.832659 | 217 | 168.47 | 0.776359 |
| GO:0046530\_photoreceptor\_cell\_differentiation | PAX6 | 26 | 1 | 6.325549 | -0.832659 | 217 | 168.47 | 0.776359 |
| GO:0050680\_negative\_regulation\_of\_epithelial\_cell\_proliferation | PAX6 | 26 | 1 | 6.325549 | -0.832659 | 217 | 168.47 | 0.776359 |
| GO:0006793\_phosphorus\_metabolic\_process | CCNE1 | 340 | 4 | 1.934874 | -0.830447 | 219 | 169.06 | 0.771963 |
| GO:0006793\_phosphorus\_metabolic\_process | CCND1 | 340 | 4 | 1.934874 | -0.830447 | 219 | 169.06 | 0.771963 |
| GO:0006793\_phosphorus\_metabolic\_process | NUP62 | 340 | 4 | 1.934874 | -0.830447 | 219 | 169.06 | 0.771963 |
| GO:0006793\_phosphorus\_metabolic\_process | YWHAE | 340 | 4 | 1.934874 | -0.830447 | 219 | 169.06 | 0.771963 |
| GO:0006796\_phosphate\_metabolic\_process | CCNE1 | 340 | 4 | 1.934874 | -0.830447 | 219 | 169.06 | 0.771963 |
| GO:0006796\_phosphate\_metabolic\_process | CCND1 | 340 | 4 | 1.934874 | -0.830447 | 219 | 169.06 | 0.771963 |
| GO:0006796\_phosphate\_metabolic\_process | NUP62 | 340 | 4 | 1.934874 | -0.830447 | 219 | 169.06 | 0.771963 |
| GO:0006796\_phosphate\_metabolic\_process | YWHAE | 340 | 4 | 1.934874 | -0.830447 | 219 | 169.06 | 0.771963 |
| GO:0065007\_biological\_regulation | HSP90AA1 | 2593 | 19 | 1.205099 | -0.830148 | 220 | 169.08 | 0.768545 |
| GO:0065007\_biological\_regulation | PAX6 | 2593 | 19 | 1.205099 | -0.830148 | 220 | 169.08 | 0.768545 |
| GO:0065007\_biological\_regulation | RHOQ | 2593 | 19 | 1.205099 | -0.830148 | 220 | 169.08 | 0.768545 |
| GO:0065007\_biological\_regulation | NEO1 | 2593 | 19 | 1.205099 | -0.830148 | 220 | 169.08 | 0.768545 |
| GO:0065007\_biological\_regulation | CSDA | 2593 | 19 | 1.205099 | -0.830148 | 220 | 169.08 | 0.768545 |
| GO:0065007\_biological\_regulation | YWHAE | 2593 | 19 | 1.205099 | -0.830148 | 220 | 169.08 | 0.768545 |
| GO:0065007\_biological\_regulation | ZIC3 | 2593 | 19 | 1.205099 | -0.830148 | 220 | 169.08 | 0.768545 |
| GO:0065007\_biological\_regulation | CCNE1 | 2593 | 19 | 1.205099 | -0.830148 | 220 | 169.08 | 0.768545 |
| GO:0065007\_biological\_regulation | CCND1 | 2593 | 19 | 1.205099 | -0.830148 | 220 | 169.08 | 0.768545 |
| GO:0065007\_biological\_regulation | ATF4 | 2593 | 19 | 1.205099 | -0.830148 | 220 | 169.08 | 0.768545 |
| GO:0065007\_biological\_regulation | EI24 | 2593 | 19 | 1.205099 | -0.830148 | 220 | 169.08 | 0.768545 |
| GO:0065007\_biological\_regulation | NUP62 | 2593 | 19 | 1.205099 | -0.830148 | 220 | 169.08 | 0.768545 |
| GO:0065007\_biological\_regulation | DNAJA1 | 2593 | 19 | 1.205099 | -0.830148 | 220 | 169.08 | 0.768545 |
| GO:0065007\_biological\_regulation | RAP1B | 2593 | 19 | 1.205099 | -0.830148 | 220 | 169.08 | 0.768545 |
| GO:0065007\_biological\_regulation | TMSB4X | 2593 | 19 | 1.205099 | -0.830148 | 220 | 169.08 | 0.768545 |
| GO:0065007\_biological\_regulation | AGRN | 2593 | 19 | 1.205099 | -0.830148 | 220 | 169.08 | 0.768545 |
| GO:0065007\_biological\_regulation | MYBBP1A | 2593 | 19 | 1.205099 | -0.830148 | 220 | 169.08 | 0.768545 |
| GO:0065007\_biological\_regulation | CALM1 | 2593 | 19 | 1.205099 | -0.830148 | 220 | 169.08 | 0.768545 |
| GO:0065007\_biological\_regulation | BMPR1A | 2593 | 19 | 1.205099 | -0.830148 | 220 | 169.08 | 0.768545 |
| GO:0009607\_response\_to\_biotic\_stimulus | CCND1 | 114 | 2 | 2.885338 | -0.818989 | 221 | 169.78 | 0.768235 |
| GO:0009607\_response\_to\_biotic\_stimulus | HSP90AA1 | 114 | 2 | 2.885338 | -0.818989 | 221 | 169.78 | 0.768235 |
| GO:0009913\_epidermal\_cell\_differentiation | PAX6 | 27 | 1 | 6.091270 | -0.817517 | 223 | 172.19 | 0.772152 |
| GO:0045582\_positive\_regulation\_of\_T\_cell\_differentiation | HSP90AA1 | 27 | 1 | 6.091270 | -0.817517 | 223 | 172.19 | 0.772152 |
| GO:0010628\_positive\_regulation\_of\_gene\_expression | ATF4 | 346 | 4 | 1.901321 | -0.810655 | 224 | 172.67 | 0.770848 |
| GO:0010628\_positive\_regulation\_of\_gene\_expression | PAX6 | 346 | 4 | 1.901321 | -0.810655 | 224 | 172.67 | 0.770848 |
| GO:0010628\_positive\_regulation\_of\_gene\_expression | AGRN | 346 | 4 | 1.901321 | -0.810655 | 224 | 172.67 | 0.770848 |
| GO:0010628\_positive\_regulation\_of\_gene\_expression | ZIC3 | 346 | 4 | 1.901321 | -0.810655 | 224 | 172.67 | 0.770848 |
| GO:0002088\_lens\_development\_in\_camera-type\_eye | PAX6 | 28 | 1 | 5.873724 | -0.802970 | 229 | 175.72 | 0.767336 |
| GO:0006470\_protein\_amino\_acid\_dephosphorylation | YWHAE | 28 | 1 | 5.873724 | -0.802970 | 229 | 175.72 | 0.767336 |
| GO:0030111\_regulation\_of\_Wnt\_receptor\_signaling\_pathway | CCND1 | 28 | 1 | 5.873724 | -0.802970 | 229 | 175.72 | 0.767336 |
| GO:0045926\_negative\_regulation\_of\_growth | EI24 | 28 | 1 | 5.873724 | -0.802970 | 229 | 175.72 | 0.767336 |
| GO:0048863\_stem\_cell\_differentiation | BMPR1A | 28 | 1 | 5.873724 | -0.802970 | 229 | 175.72 | 0.767336 |
| GO:0019953\_sexual\_reproduction | GPX4 | 228 | 3 | 2.164004 | -0.798997 | 230 | 176.19 | 0.766043 |
| GO:0019953\_sexual\_reproduction | DNAJA1 | 228 | 3 | 2.164004 | -0.798997 | 230 | 176.19 | 0.766043 |
| GO:0019953\_sexual\_reproduction | CSDA | 228 | 3 | 2.164004 | -0.798997 | 230 | 176.19 | 0.766043 |
| GO:0007167\_enzyme\_linked\_receptor\_protein\_signaling\_pathway | NUP62 | 229 | 3 | 2.154554 | -0.794983 | 231 | 176.63 | 0.764632 |
| GO:0007167\_enzyme\_linked\_receptor\_protein\_signaling\_pathway | AGRN | 229 | 3 | 2.154554 | -0.794983 | 231 | 176.63 | 0.764632 |
| GO:0007167\_enzyme\_linked\_receptor\_protein\_signaling\_pathway | BMPR1A | 229 | 3 | 2.154554 | -0.794983 | 231 | 176.63 | 0.764632 |
| GO:0045935\_positive\_regulation\_of\_nucleobase\_\_nucleoside\_\_nucleotide\_and\_nucleic\_acid\_metabolic\_process | ATF4 | 352 | 4 | 1.868912 | -0.791382 | 232 | 176.87 | 0.762371 |
| GO:0045935\_positive\_regulation\_of\_nucleobase\_\_nucleoside\_\_nucleotide\_and\_nucleic\_acid\_metabolic\_process | PAX6 | 352 | 4 | 1.868912 | -0.791382 | 232 | 176.87 | 0.762371 |
| GO:0045935\_positive\_regulation\_of\_nucleobase\_\_nucleoside\_\_nucleotide\_and\_nucleic\_acid\_metabolic\_process | AGRN | 352 | 4 | 1.868912 | -0.791382 | 232 | 176.87 | 0.762371 |
| GO:0045935\_positive\_regulation\_of\_nucleobase\_\_nucleoside\_\_nucleotide\_and\_nucleic\_acid\_metabolic\_process | ZIC3 | 352 | 4 | 1.868912 | -0.791382 | 232 | 176.87 | 0.762371 |
| GO:0021761\_limbic\_system\_development | YWHAE | 29 | 1 | 5.671182 | -0.788977 | 236 | 180.12 | 0.763220 |
| GO:0044087\_regulation\_of\_cellular\_component\_biogenesis | AGRN | 29 | 1 | 5.671182 | -0.788977 | 236 | 180.12 | 0.763220 |
| GO:0045621\_positive\_regulation\_of\_lymphocyte\_differentiation | HSP90AA1 | 29 | 1 | 5.671182 | -0.788977 | 236 | 180.12 | 0.763220 |
| GO:0050769\_positive\_regulation\_of\_neurogenesis | PAX6 | 29 | 1 | 5.671182 | -0.788977 | 236 | 180.12 | 0.763220 |
| GO:0007420\_brain\_development | PAX6 | 231 | 3 | 2.135900 | -0.787029 | 237 | 180.23 | 0.760464 |
| GO:0007420\_brain\_development | YWHAE | 231 | 3 | 2.135900 | -0.787029 | 237 | 180.23 | 0.760464 |
| GO:0007420\_brain\_development | BMPR1A | 231 | 3 | 2.135900 | -0.787029 | 237 | 180.23 | 0.760464 |
| GO:0051726\_regulation\_of\_cell\_cycle | CCNE1 | 121 | 2 | 2.718418 | -0.778015 | 238 | 181.58 | 0.762941 |
| GO:0051726\_regulation\_of\_cell\_cycle | CCND1 | 121 | 2 | 2.718418 | -0.778015 | 238 | 181.58 | 0.762941 |
| GO:0007435\_salivary\_gland\_morphogenesis | PAX6 | 30 | 1 | 5.482143 | -0.775499 | 242 | 185.36 | 0.765950 |
| GO:0030522\_intracellular\_receptor-mediated\_signaling\_pathway | DNAJA1 | 30 | 1 | 5.482143 | -0.775499 | 242 | 185.36 | 0.765950 |
| GO:0035265\_organ\_growth | CSDA | 30 | 1 | 5.482143 | -0.775499 | 242 | 185.36 | 0.765950 |
| GO:0060021\_palate\_development | BMPR1A | 30 | 1 | 5.482143 | -0.775499 | 242 | 185.36 | 0.765950 |
| GO:0016477\_cell\_migration | PAX6 | 234 | 3 | 2.108516 | -0.775279 | 243 | 185.54 | 0.763539 |
| GO:0016477\_cell\_migration | TMSB4X | 234 | 3 | 2.108516 | -0.775279 | 243 | 185.54 | 0.763539 |
| GO:0016477\_cell\_migration | YWHAE | 234 | 3 | 2.108516 | -0.775279 | 243 | 185.54 | 0.763539 |
| GO:0016049\_cell\_growth | EI24 | 31 | 1 | 5.305300 | -0.762502 | 245 | 192.27 | 0.784776 |
| GO:0016311\_dephosphorylation | YWHAE | 31 | 1 | 5.305300 | -0.762502 | 245 | 192.27 | 0.784776 |
| GO:0051171\_regulation\_of\_nitrogen\_compound\_metabolic\_process | ATF4 | 771 | 7 | 1.493191 | -0.755007 | 246 | 193.08 | 0.784878 |
| GO:0051171\_regulation\_of\_nitrogen\_compound\_metabolic\_process | HSP90AA1 | 771 | 7 | 1.493191 | -0.755007 | 246 | 193.08 | 0.784878 |
| GO:0051171\_regulation\_of\_nitrogen\_compound\_metabolic\_process | PAX6 | 771 | 7 | 1.493191 | -0.755007 | 246 | 193.08 | 0.784878 |
| GO:0051171\_regulation\_of\_nitrogen\_compound\_metabolic\_process | NEO1 | 771 | 7 | 1.493191 | -0.755007 | 246 | 193.08 | 0.784878 |
| GO:0051171\_regulation\_of\_nitrogen\_compound\_metabolic\_process | AGRN | 771 | 7 | 1.493191 | -0.755007 | 246 | 193.08 | 0.784878 |
| GO:0051171\_regulation\_of\_nitrogen\_compound\_metabolic\_process | MYBBP1A | 771 | 7 | 1.493191 | -0.755007 | 246 | 193.08 | 0.784878 |
| GO:0051171\_regulation\_of\_nitrogen\_compound\_metabolic\_process | ZIC3 | 771 | 7 | 1.493191 | -0.755007 | 246 | 193.08 | 0.784878 |
| GO:0043009\_chordate\_embryonic\_development | SFRP2 | 365 | 4 | 1.802348 | -0.751324 | 247 | 193.45 | 0.783198 |
| GO:0043009\_chordate\_embryonic\_development | PAX6 | 365 | 4 | 1.802348 | -0.751324 | 247 | 193.45 | 0.783198 |
| GO:0043009\_chordate\_embryonic\_development | CSDA | 365 | 4 | 1.802348 | -0.751324 | 247 | 193.45 | 0.783198 |
| GO:0043009\_chordate\_embryonic\_development | BMPR1A | 365 | 4 | 1.802348 | -0.751324 | 247 | 193.45 | 0.783198 |
| GO:0001707\_mesoderm\_formation | BMPR1A | 32 | 1 | 5.139509 | -0.749957 | 250 | 195.86 | 0.783440 |
| GO:0048332\_mesoderm\_morphogenesis | BMPR1A | 32 | 1 | 5.139509 | -0.749957 | 250 | 195.86 | 0.783440 |
| GO:0050768\_negative\_regulation\_of\_neurogenesis | BMPR1A | 32 | 1 | 5.139509 | -0.749957 | 250 | 195.86 | 0.783440 |
| GO:0009792\_embryonic\_development\_ending\_in\_birth\_or\_egg\_hatching | SFRP2 | 368 | 4 | 1.787655 | -0.742397 | 251 | 196.11 | 0.781315 |
| GO:0009792\_embryonic\_development\_ending\_in\_birth\_or\_egg\_hatching | PAX6 | 368 | 4 | 1.787655 | -0.742397 | 251 | 196.11 | 0.781315 |
| GO:0009792\_embryonic\_development\_ending\_in\_birth\_or\_egg\_hatching | CSDA | 368 | 4 | 1.787655 | -0.742397 | 251 | 196.11 | 0.781315 |
| GO:0009792\_embryonic\_development\_ending\_in\_birth\_or\_egg\_hatching | BMPR1A | 368 | 4 | 1.787655 | -0.742397 | 251 | 196.11 | 0.781315 |
| GO:0045597\_positive\_regulation\_of\_cell\_differentiation | HSP90AA1 | 128 | 2 | 2.569754 | -0.739947 | 252 | 196.78 | 0.780873 |
| GO:0045597\_positive\_regulation\_of\_cell\_differentiation | PAX6 | 128 | 2 | 2.569754 | -0.739947 | 252 | 196.78 | 0.780873 |
| GO:0007431\_salivary\_gland\_development | PAX6 | 33 | 1 | 4.983766 | -0.737835 | 255 | 199.21 | 0.781216 |
| GO:0008584\_male\_gonad\_development | CSDA | 33 | 1 | 4.983766 | -0.737835 | 255 | 199.21 | 0.781216 |
| GO:0021987\_cerebral\_cortex\_development | YWHAE | 33 | 1 | 4.983766 | -0.737835 | 255 | 199.21 | 0.781216 |
| GO:0010557\_positive\_regulation\_of\_macromolecule\_biosynthetic\_process | ATF4 | 371 | 4 | 1.773200 | -0.733584 | 256 | 199.56 | 0.779531 |
| GO:0010557\_positive\_regulation\_of\_macromolecule\_biosynthetic\_process | PAX6 | 371 | 4 | 1.773200 | -0.733584 | 256 | 199.56 | 0.779531 |
| GO:0010557\_positive\_regulation\_of\_macromolecule\_biosynthetic\_process | AGRN | 371 | 4 | 1.773200 | -0.733584 | 256 | 199.56 | 0.779531 |
| GO:0010557\_positive\_regulation\_of\_macromolecule\_biosynthetic\_process | ZIC3 | 371 | 4 | 1.773200 | -0.733584 | 256 | 199.56 | 0.779531 |
| GO:0007568\_aging | NUP62 | 34 | 1 | 4.837185 | -0.726111 | 261 | 203.18 | 0.778467 |
| GO:0010720\_positive\_regulation\_of\_cell\_development | PAX6 | 34 | 1 | 4.837185 | -0.726111 | 261 | 203.18 | 0.778467 |
| GO:0010721\_negative\_regulation\_of\_cell\_development | BMPR1A | 34 | 1 | 4.837185 | -0.726111 | 261 | 203.18 | 0.778467 |
| GO:0030509\_BMP\_signaling\_pathway | BMPR1A | 34 | 1 | 4.837185 | -0.726111 | 261 | 203.18 | 0.778467 |
| GO:0045927\_positive\_regulation\_of\_growth | CSDA | 34 | 1 | 4.837185 | -0.726111 | 261 | 203.18 | 0.778467 |
| GO:0032879\_regulation\_of\_localization | PAX6 | 248 | 3 | 1.989487 | -0.723170 | 262 | 203.44 | 0.776489 |
| GO:0032879\_regulation\_of\_localization | RHOQ | 248 | 3 | 1.989487 | -0.723170 | 262 | 203.44 | 0.776489 |
| GO:0032879\_regulation\_of\_localization | TMSB4X | 248 | 3 | 1.989487 | -0.723170 | 262 | 203.44 | 0.776489 |
| GO:0001756\_somitogenesis | SFRP2 | 35 | 1 | 4.698980 | -0.714761 | 264 | 205.75 | 0.779356 |
| GO:0016051\_carbohydrate\_biosynthetic\_process | ATF4 | 35 | 1 | 4.698980 | -0.714761 | 264 | 205.75 | 0.779356 |
| GO:0019222\_regulation\_of\_metabolic\_process | ATF4 | 1088 | 9 | 1.360458 | -0.705867 | 265 | 206.76 | 0.780226 |
| GO:0019222\_regulation\_of\_metabolic\_process | HSP90AA1 | 1088 | 9 | 1.360458 | -0.705867 | 265 | 206.76 | 0.780226 |
| GO:0019222\_regulation\_of\_metabolic\_process | NUP62 | 1088 | 9 | 1.360458 | -0.705867 | 265 | 206.76 | 0.780226 |
| GO:0019222\_regulation\_of\_metabolic\_process | PAX6 | 1088 | 9 | 1.360458 | -0.705867 | 265 | 206.76 | 0.780226 |
| GO:0019222\_regulation\_of\_metabolic\_process | NEO1 | 1088 | 9 | 1.360458 | -0.705867 | 265 | 206.76 | 0.780226 |
| GO:0019222\_regulation\_of\_metabolic\_process | AGRN | 1088 | 9 | 1.360458 | -0.705867 | 265 | 206.76 | 0.780226 |
| GO:0019222\_regulation\_of\_metabolic\_process | YWHAE | 1088 | 9 | 1.360458 | -0.705867 | 265 | 206.76 | 0.780226 |
| GO:0019222\_regulation\_of\_metabolic\_process | MYBBP1A | 1088 | 9 | 1.360458 | -0.705867 | 265 | 206.76 | 0.780226 |
| GO:0019222\_regulation\_of\_metabolic\_process | ZIC3 | 1088 | 9 | 1.360458 | -0.705867 | 265 | 206.76 | 0.780226 |
| GO:0001704\_formation\_of\_primary\_germ\_layer | BMPR1A | 36 | 1 | 4.568452 | -0.703766 | 270 | 210.34 | 0.779037 |
| GO:0006469\_negative\_regulation\_of\_protein\_kinase\_activity | NUP62 | 36 | 1 | 4.568452 | -0.703766 | 270 | 210.34 | 0.779037 |
| GO:0007368\_determination\_of\_left\_right\_symmetry | ZIC3 | 36 | 1 | 4.568452 | -0.703766 | 270 | 210.34 | 0.779037 |
| GO:0021510\_spinal\_cord\_development | PAX6 | 36 | 1 | 4.568452 | -0.703766 | 270 | 210.34 | 0.779037 |
| GO:0033673\_negative\_regulation\_of\_kinase\_activity | NUP62 | 36 | 1 | 4.568452 | -0.703766 | 270 | 210.34 | 0.779037 |
| GO:0048729\_tissue\_morphogenesis | CCND1 | 255 | 3 | 1.934874 | -0.698689 | 271 | 211.07 | 0.778856 |
| GO:0048729\_tissue\_morphogenesis | PAX6 | 255 | 3 | 1.934874 | -0.698689 | 271 | 211.07 | 0.778856 |
| GO:0048729\_tissue\_morphogenesis | BMPR1A | 255 | 3 | 1.934874 | -0.698689 | 271 | 211.07 | 0.778856 |
| GO:0034960\_cellular\_biopolymer\_metabolic\_process | CCNE1 | 1395 | 11 | 1.296851 | -0.698310 | 272 | 211.11 | 0.776140 |
| GO:0034960\_cellular\_biopolymer\_metabolic\_process | CCND1 | 1395 | 11 | 1.296851 | -0.698310 | 272 | 211.11 | 0.776140 |
| GO:0034960\_cellular\_biopolymer\_metabolic\_process | UBE2D3 | 1395 | 11 | 1.296851 | -0.698310 | 272 | 211.11 | 0.776140 |
| GO:0034960\_cellular\_biopolymer\_metabolic\_process | ATF4 | 1395 | 11 | 1.296851 | -0.698310 | 272 | 211.11 | 0.776140 |
| GO:0034960\_cellular\_biopolymer\_metabolic\_process | HSP90AA1 | 1395 | 11 | 1.296851 | -0.698310 | 272 | 211.11 | 0.776140 |
| GO:0034960\_cellular\_biopolymer\_metabolic\_process | PAX6 | 1395 | 11 | 1.296851 | -0.698310 | 272 | 211.11 | 0.776140 |
| GO:0034960\_cellular\_biopolymer\_metabolic\_process | NEO1 | 1395 | 11 | 1.296851 | -0.698310 | 272 | 211.11 | 0.776140 |
| GO:0034960\_cellular\_biopolymer\_metabolic\_process | AGRN | 1395 | 11 | 1.296851 | -0.698310 | 272 | 211.11 | 0.776140 |
| GO:0034960\_cellular\_biopolymer\_metabolic\_process | YWHAE | 1395 | 11 | 1.296851 | -0.698310 | 272 | 211.11 | 0.776140 |
| GO:0034960\_cellular\_biopolymer\_metabolic\_process | MYBBP1A | 1395 | 11 | 1.296851 | -0.698310 | 272 | 211.11 | 0.776140 |
| GO:0034960\_cellular\_biopolymer\_metabolic\_process | ZIC3 | 1395 | 11 | 1.296851 | -0.698310 | 272 | 211.11 | 0.776140 |
| GO:0009799\_determination\_of\_symmetry | ZIC3 | 37 | 1 | 4.444981 | -0.693104 | 276 | 213.73 | 0.774384 |
| GO:0009855\_determination\_of\_bilateral\_symmetry | ZIC3 | 37 | 1 | 4.444981 | -0.693104 | 276 | 213.73 | 0.774384 |
| GO:0032869\_cellular\_response\_to\_insulin\_stimulus | RHOQ | 37 | 1 | 4.444981 | -0.693104 | 276 | 213.73 | 0.774384 |
| GO:0051101\_regulation\_of\_DNA\_binding | CALM1 | 37 | 1 | 4.444981 | -0.693104 | 276 | 213.73 | 0.774384 |
| GO:0007154\_cell\_communication | LDHA | 1096 | 9 | 1.350528 | -0.692150 | 277 | 213.83 | 0.771949 |
| GO:0007154\_cell\_communication | CCND1 | 1096 | 9 | 1.350528 | -0.692150 | 277 | 213.83 | 0.771949 |
| GO:0007154\_cell\_communication | NUP62 | 1096 | 9 | 1.350528 | -0.692150 | 277 | 213.83 | 0.771949 |
| GO:0007154\_cell\_communication | SFRP2 | 1096 | 9 | 1.350528 | -0.692150 | 277 | 213.83 | 0.771949 |
| GO:0007154\_cell\_communication | DNAJA1 | 1096 | 9 | 1.350528 | -0.692150 | 277 | 213.83 | 0.771949 |
| GO:0007154\_cell\_communication | PAX6 | 1096 | 9 | 1.350528 | -0.692150 | 277 | 213.83 | 0.771949 |
| GO:0007154\_cell\_communication | RAP1B | 1096 | 9 | 1.350528 | -0.692150 | 277 | 213.83 | 0.771949 |
| GO:0007154\_cell\_communication | AGRN | 1096 | 9 | 1.350528 | -0.692150 | 277 | 213.83 | 0.771949 |
| GO:0007154\_cell\_communication | BMPR1A | 1096 | 9 | 1.350528 | -0.692150 | 277 | 213.83 | 0.771949 |
| GO:0034961\_cellular\_biopolymer\_biosynthetic\_process | CCNE1 | 804 | 7 | 1.431903 | -0.688760 | 278 | 214.27 | 0.770755 |
| GO:0034961\_cellular\_biopolymer\_biosynthetic\_process | ATF4 | 804 | 7 | 1.431903 | -0.688760 | 278 | 214.27 | 0.770755 |
| GO:0034961\_cellular\_biopolymer\_biosynthetic\_process | PAX6 | 804 | 7 | 1.431903 | -0.688760 | 278 | 214.27 | 0.770755 |
| GO:0034961\_cellular\_biopolymer\_biosynthetic\_process | NEO1 | 804 | 7 | 1.431903 | -0.688760 | 278 | 214.27 | 0.770755 |
| GO:0034961\_cellular\_biopolymer\_biosynthetic\_process | AGRN | 804 | 7 | 1.431903 | -0.688760 | 278 | 214.27 | 0.770755 |
| GO:0034961\_cellular\_biopolymer\_biosynthetic\_process | MYBBP1A | 804 | 7 | 1.431903 | -0.688760 | 278 | 214.27 | 0.770755 |
| GO:0034961\_cellular\_biopolymer\_biosynthetic\_process | ZIC3 | 804 | 7 | 1.431903 | -0.688760 | 278 | 214.27 | 0.770755 |
| GO:0007169\_transmembrane\_receptor\_protein\_tyrosine\_kinase\_signaling\_pathway | NUP62 | 139 | 2 | 2.366393 | -0.685254 | 280 | 215.05 | 0.768036 |
| GO:0007169\_transmembrane\_receptor\_protein\_tyrosine\_kinase\_signaling\_pathway | AGRN | 139 | 2 | 2.366393 | -0.685254 | 280 | 215.05 | 0.768036 |
| GO:0034613\_cellular\_protein\_localization | PAX6 | 139 | 2 | 2.366393 | -0.685254 | 280 | 215.05 | 0.768036 |
| GO:0034613\_cellular\_protein\_localization | YWHAE | 139 | 2 | 2.366393 | -0.685254 | 280 | 215.05 | 0.768036 |
| GO:0043284\_biopolymer\_biosynthetic\_process | CCNE1 | 807 | 7 | 1.426580 | -0.683003 | 281 | 215.17 | 0.765730 |
| GO:0043284\_biopolymer\_biosynthetic\_process | ATF4 | 807 | 7 | 1.426580 | -0.683003 | 281 | 215.17 | 0.765730 |
| GO:0043284\_biopolymer\_biosynthetic\_process | PAX6 | 807 | 7 | 1.426580 | -0.683003 | 281 | 215.17 | 0.765730 |
| GO:0043284\_biopolymer\_biosynthetic\_process | NEO1 | 807 | 7 | 1.426580 | -0.683003 | 281 | 215.17 | 0.765730 |
| GO:0043284\_biopolymer\_biosynthetic\_process | AGRN | 807 | 7 | 1.426580 | -0.683003 | 281 | 215.17 | 0.765730 |
| GO:0043284\_biopolymer\_biosynthetic\_process | MYBBP1A | 807 | 7 | 1.426580 | -0.683003 | 281 | 215.17 | 0.765730 |
| GO:0043284\_biopolymer\_biosynthetic\_process | ZIC3 | 807 | 7 | 1.426580 | -0.683003 | 281 | 215.17 | 0.765730 |
| GO:0042493\_response\_to\_drug | EI24 | 38 | 1 | 4.328008 | -0.682758 | 284 | 218.77 | 0.770317 |
| GO:0045580\_regulation\_of\_T\_cell\_differentiation | HSP90AA1 | 38 | 1 | 4.328008 | -0.682758 | 284 | 218.77 | 0.770317 |
| GO:0051348\_negative\_regulation\_of\_transferase\_activity | NUP62 | 38 | 1 | 4.328008 | -0.682758 | 284 | 218.77 | 0.770317 |
| GO:0016044\_membrane\_organization | AGRN | 140 | 2 | 2.349490 | -0.680564 | 285 | 219.01 | 0.768456 |
| GO:0016044\_membrane\_organization | NEO1 | 140 | 2 | 2.349490 | -0.680564 | 285 | 219.01 | 0.768456 |
| GO:0003006\_reproductive\_developmental\_process | CSDA | 141 | 2 | 2.332827 | -0.675918 | 287 | 219.5 | 0.764808 |
| GO:0003006\_reproductive\_developmental\_process | BMPR1A | 141 | 2 | 2.332827 | -0.675918 | 287 | 219.5 | 0.764808 |
| GO:0070727\_cellular\_macromolecule\_localization | PAX6 | 141 | 2 | 2.332827 | -0.675918 | 287 | 219.5 | 0.764808 |
| GO:0070727\_cellular\_macromolecule\_localization | YWHAE | 141 | 2 | 2.332827 | -0.675918 | 287 | 219.5 | 0.764808 |
| GO:0031326\_regulation\_of\_cellular\_biosynthetic\_process | ATF4 | 812 | 7 | 1.417796 | -0.673503 | 288 | 220.2 | 0.764583 |
| GO:0031326\_regulation\_of\_cellular\_biosynthetic\_process | HSP90AA1 | 812 | 7 | 1.417796 | -0.673503 | 288 | 220.2 | 0.764583 |
| GO:0031326\_regulation\_of\_cellular\_biosynthetic\_process | PAX6 | 812 | 7 | 1.417796 | -0.673503 | 288 | 220.2 | 0.764583 |
| GO:0031326\_regulation\_of\_cellular\_biosynthetic\_process | NEO1 | 812 | 7 | 1.417796 | -0.673503 | 288 | 220.2 | 0.764583 |
| GO:0031326\_regulation\_of\_cellular\_biosynthetic\_process | AGRN | 812 | 7 | 1.417796 | -0.673503 | 288 | 220.2 | 0.764583 |
| GO:0031326\_regulation\_of\_cellular\_biosynthetic\_process | MYBBP1A | 812 | 7 | 1.417796 | -0.673503 | 288 | 220.2 | 0.764583 |
| GO:0031326\_regulation\_of\_cellular\_biosynthetic\_process | ZIC3 | 812 | 7 | 1.417796 | -0.673503 | 288 | 220.2 | 0.764583 |
| GO:0006511\_ubiquitin-dependent\_protein\_catabolic\_process | UBE2D3 | 39 | 1 | 4.217033 | -0.672713 | 292 | 223.06 | 0.763904 |
| GO:0042475\_odontogenesis\_of\_dentine-containing\_tooth | BMPR1A | 39 | 1 | 4.217033 | -0.672713 | 292 | 223.06 | 0.763904 |
| GO:0048663\_neuron\_fate\_commitment | PAX6 | 39 | 1 | 4.217033 | -0.672713 | 292 | 223.06 | 0.763904 |
| GO:0070201\_regulation\_of\_establishment\_of\_protein\_localization | RHOQ | 39 | 1 | 4.217033 | -0.672713 | 292 | 223.06 | 0.763904 |
| GO:0009889\_regulation\_of\_biosynthetic\_process | ATF4 | 815 | 7 | 1.412577 | -0.667859 | 293 | 223.53 | 0.762901 |
| GO:0009889\_regulation\_of\_biosynthetic\_process | HSP90AA1 | 815 | 7 | 1.412577 | -0.667859 | 293 | 223.53 | 0.762901 |
| GO:0009889\_regulation\_of\_biosynthetic\_process | PAX6 | 815 | 7 | 1.412577 | -0.667859 | 293 | 223.53 | 0.762901 |
| GO:0009889\_regulation\_of\_biosynthetic\_process | NEO1 | 815 | 7 | 1.412577 | -0.667859 | 293 | 223.53 | 0.762901 |
| GO:0009889\_regulation\_of\_biosynthetic\_process | AGRN | 815 | 7 | 1.412577 | -0.667859 | 293 | 223.53 | 0.762901 |
| GO:0009889\_regulation\_of\_biosynthetic\_process | MYBBP1A | 815 | 7 | 1.412577 | -0.667859 | 293 | 223.53 | 0.762901 |
| GO:0009889\_regulation\_of\_biosynthetic\_process | ZIC3 | 815 | 7 | 1.412577 | -0.667859 | 293 | 223.53 | 0.762901 |
| GO:0035272\_exocrine\_system\_development | PAX6 | 40 | 1 | 4.111607 | -0.662952 | 294 | 226.16 | 0.769252 |
| GO:0045449\_regulation\_of\_transcription | ATF4 | 676 | 6 | 1.459742 | -0.658228 | 295 | 226.7 | 0.768475 |
| GO:0045449\_regulation\_of\_transcription | PAX6 | 676 | 6 | 1.459742 | -0.658228 | 295 | 226.7 | 0.768475 |
| GO:0045449\_regulation\_of\_transcription | NEO1 | 676 | 6 | 1.459742 | -0.658228 | 295 | 226.7 | 0.768475 |
| GO:0045449\_regulation\_of\_transcription | AGRN | 676 | 6 | 1.459742 | -0.658228 | 295 | 226.7 | 0.768475 |
| GO:0045449\_regulation\_of\_transcription | MYBBP1A | 676 | 6 | 1.459742 | -0.658228 | 295 | 226.7 | 0.768475 |
| GO:0045449\_regulation\_of\_transcription | ZIC3 | 676 | 6 | 1.459742 | -0.658228 | 295 | 226.7 | 0.768475 |
| GO:0006260\_DNA\_replication | CCNE1 | 41 | 1 | 4.011324 | -0.653461 | 299 | 230.65 | 0.771405 |
| GO:0010551\_regulation\_of\_specific\_transcription\_from\_RNA\_polymerase\_II\_promoter | PAX6 | 41 | 1 | 4.011324 | -0.653461 | 299 | 230.65 | 0.771405 |
| GO:0015980\_energy\_derivation\_by\_oxidation\_of\_organic\_compounds | MYBBP1A | 41 | 1 | 4.011324 | -0.653461 | 299 | 230.65 | 0.771405 |
| GO:0032569\_specific\_transcription\_from\_RNA\_polymerase\_II\_promoter | PAX6 | 41 | 1 | 4.011324 | -0.653461 | 299 | 230.65 | 0.771405 |
| GO:0005975\_carbohydrate\_metabolic\_process | ATF4 | 146 | 2 | 2.252935 | -0.653324 | 300 | 231.13 | 0.770433 |
| GO:0005975\_carbohydrate\_metabolic\_process | ADK | 146 | 2 | 2.252935 | -0.653324 | 300 | 231.13 | 0.770433 |
| GO:0006006\_glucose\_metabolic\_process | ATF4 | 42 | 1 | 3.915816 | -0.644228 | 306 | 235.6 | 0.769935 |
| GO:0008361\_regulation\_of\_cell\_size | EI24 | 42 | 1 | 3.915816 | -0.644228 | 306 | 235.6 | 0.769935 |
| GO:0019941\_modification-dependent\_protein\_catabolic\_process | UBE2D3 | 42 | 1 | 3.915816 | -0.644228 | 306 | 235.6 | 0.769935 |
| GO:0042476\_odontogenesis | BMPR1A | 42 | 1 | 3.915816 | -0.644228 | 306 | 235.6 | 0.769935 |
| GO:0043632\_modification-dependent\_macromolecule\_catabolic\_process | UBE2D3 | 42 | 1 | 3.915816 | -0.644228 | 306 | 235.6 | 0.769935 |
| GO:0051603\_proteolysis\_involved\_in\_cellular\_protein\_catabolic\_process | UBE2D3 | 42 | 1 | 3.915816 | -0.644228 | 306 | 235.6 | 0.769935 |
| GO:0008283\_cell\_proliferation | CCND1 | 544 | 5 | 1.511620 | -0.638509 | 307 | 236.11 | 0.769088 |
| GO:0008283\_cell\_proliferation | NUP62 | 544 | 5 | 1.511620 | -0.638509 | 307 | 236.11 | 0.769088 |
| GO:0008283\_cell\_proliferation | PAX6 | 544 | 5 | 1.511620 | -0.638509 | 307 | 236.11 | 0.769088 |
| GO:0008283\_cell\_proliferation | RAP1B | 544 | 5 | 1.511620 | -0.638509 | 307 | 236.11 | 0.769088 |
| GO:0008283\_cell\_proliferation | BMPR1A | 544 | 5 | 1.511620 | -0.638509 | 307 | 236.11 | 0.769088 |
| GO:0007224\_smoothened\_signaling\_pathway | PAX6 | 43 | 1 | 3.824751 | -0.635239 | 310 | 239.86 | 0.773742 |
| GO:0010001\_glial\_cell\_differentiation | PAX6 | 43 | 1 | 3.824751 | -0.635239 | 310 | 239.86 | 0.773742 |
| GO:0032868\_response\_to\_insulin\_stimulus | RHOQ | 43 | 1 | 3.824751 | -0.635239 | 310 | 239.86 | 0.773742 |
| GO:0042221\_response\_to\_chemical\_stimulus | CCND1 | 409 | 4 | 1.608453 | -0.631110 | 311 | 240.06 | 0.771897 |
| GO:0042221\_response\_to\_chemical\_stimulus | EI24 | 409 | 4 | 1.608453 | -0.631110 | 311 | 240.06 | 0.771897 |
| GO:0042221\_response\_to\_chemical\_stimulus | HSP90AA1 | 409 | 4 | 1.608453 | -0.631110 | 311 | 240.06 | 0.771897 |
| GO:0042221\_response\_to\_chemical\_stimulus | RHOQ | 409 | 4 | 1.608453 | -0.631110 | 311 | 240.06 | 0.771897 |
| GO:0035282\_segmentation | SFRP2 | 44 | 1 | 3.737825 | -0.626485 | 314 | 244.94 | 0.780064 |
| GO:0044257\_cellular\_protein\_catabolic\_process | UBE2D3 | 44 | 1 | 3.737825 | -0.626485 | 314 | 244.94 | 0.780064 |
| GO:0050808\_synapse\_organization | AGRN | 44 | 1 | 3.737825 | -0.626485 | 314 | 244.94 | 0.780064 |
| GO:0007242\_intracellular\_signaling\_cascade | CCND1 | 411 | 4 | 1.600626 | -0.626152 | 315 | 245.09 | 0.778063 |
| GO:0007242\_intracellular\_signaling\_cascade | NUP62 | 411 | 4 | 1.600626 | -0.626152 | 315 | 245.09 | 0.778063 |
| GO:0007242\_intracellular\_signaling\_cascade | DNAJA1 | 411 | 4 | 1.600626 | -0.626152 | 315 | 245.09 | 0.778063 |
| GO:0007242\_intracellular\_signaling\_cascade | RAP1B | 411 | 4 | 1.600626 | -0.626152 | 315 | 245.09 | 0.778063 |
| GO:0044260\_cellular\_macromolecule\_metabolic\_process | CCNE1 | 1447 | 11 | 1.250247 | -0.620268 | 316 | 245.95 | 0.778323 |
| GO:0044260\_cellular\_macromolecule\_metabolic\_process | CCND1 | 1447 | 11 | 1.250247 | -0.620268 | 316 | 245.95 | 0.778323 |
| GO:0044260\_cellular\_macromolecule\_metabolic\_process | UBE2D3 | 1447 | 11 | 1.250247 | -0.620268 | 316 | 245.95 | 0.778323 |
| GO:0044260\_cellular\_macromolecule\_metabolic\_process | ATF4 | 1447 | 11 | 1.250247 | -0.620268 | 316 | 245.95 | 0.778323 |
| GO:0044260\_cellular\_macromolecule\_metabolic\_process | HSP90AA1 | 1447 | 11 | 1.250247 | -0.620268 | 316 | 245.95 | 0.778323 |
| GO:0044260\_cellular\_macromolecule\_metabolic\_process | PAX6 | 1447 | 11 | 1.250247 | -0.620268 | 316 | 245.95 | 0.778323 |
| GO:0044260\_cellular\_macromolecule\_metabolic\_process | NEO1 | 1447 | 11 | 1.250247 | -0.620268 | 316 | 245.95 | 0.778323 |
| GO:0044260\_cellular\_macromolecule\_metabolic\_process | AGRN | 1447 | 11 | 1.250247 | -0.620268 | 316 | 245.95 | 0.778323 |
| GO:0044260\_cellular\_macromolecule\_metabolic\_process | YWHAE | 1447 | 11 | 1.250247 | -0.620268 | 316 | 245.95 | 0.778323 |
| GO:0044260\_cellular\_macromolecule\_metabolic\_process | MYBBP1A | 1447 | 11 | 1.250247 | -0.620268 | 316 | 245.95 | 0.778323 |
| GO:0044260\_cellular\_macromolecule\_metabolic\_process | ZIC3 | 1447 | 11 | 1.250247 | -0.620268 | 316 | 245.95 | 0.778323 |
| GO:0032870\_cellular\_response\_to\_hormone\_stimulus | RHOQ | 45 | 1 | 3.654762 | -0.617954 | 319 | 247.41 | 0.775580 |
| GO:0043623\_cellular\_protein\_complex\_assembly | AGRN | 45 | 1 | 3.654762 | -0.617954 | 319 | 247.41 | 0.775580 |
| GO:0046546\_development\_of\_primary\_male\_sexual\_characteristics | CSDA | 45 | 1 | 3.654762 | -0.617954 | 319 | 247.41 | 0.775580 |
| GO:0008285\_negative\_regulation\_of\_cell\_proliferation | NUP62 | 155 | 2 | 2.122120 | -0.615154 | 321 | 247.83 | 0.772056 |
| GO:0008285\_negative\_regulation\_of\_cell\_proliferation | PAX6 | 155 | 2 | 2.122120 | -0.615154 | 321 | 247.83 | 0.772056 |
| GO:0022402\_cell\_cycle\_process | CCND1 | 155 | 2 | 2.122120 | -0.615154 | 321 | 247.83 | 0.772056 |
| GO:0022402\_cell\_cycle\_process | SMC1A | 155 | 2 | 2.122120 | -0.615154 | 321 | 247.83 | 0.772056 |
| GO:0048518\_positive\_regulation\_of\_biological\_process | EI24 | 995 | 8 | 1.322326 | -0.611481 | 322 | 248.15 | 0.770652 |
| GO:0048518\_positive\_regulation\_of\_biological\_process | ATF4 | 995 | 8 | 1.322326 | -0.611481 | 322 | 248.15 | 0.770652 |
| GO:0048518\_positive\_regulation\_of\_biological\_process | HSP90AA1 | 995 | 8 | 1.322326 | -0.611481 | 322 | 248.15 | 0.770652 |
| GO:0048518\_positive\_regulation\_of\_biological\_process | PAX6 | 995 | 8 | 1.322326 | -0.611481 | 322 | 248.15 | 0.770652 |
| GO:0048518\_positive\_regulation\_of\_biological\_process | AGRN | 995 | 8 | 1.322326 | -0.611481 | 322 | 248.15 | 0.770652 |
| GO:0048518\_positive\_regulation\_of\_biological\_process | CSDA | 995 | 8 | 1.322326 | -0.611481 | 322 | 248.15 | 0.770652 |
| GO:0048518\_positive\_regulation\_of\_biological\_process | ZIC3 | 995 | 8 | 1.322326 | -0.611481 | 322 | 248.15 | 0.770652 |
| GO:0048518\_positive\_regulation\_of\_biological\_process | BMPR1A | 995 | 8 | 1.322326 | -0.611481 | 322 | 248.15 | 0.770652 |
| GO:0006807\_nitrogen\_compound\_metabolic\_process | CCNE1 | 1147 | 9 | 1.290478 | -0.609745 | 323 | 248.42 | 0.769102 |
| GO:0006807\_nitrogen\_compound\_metabolic\_process | ATF4 | 1147 | 9 | 1.290478 | -0.609745 | 323 | 248.42 | 0.769102 |
| GO:0006807\_nitrogen\_compound\_metabolic\_process | HSP90AA1 | 1147 | 9 | 1.290478 | -0.609745 | 323 | 248.42 | 0.769102 |
| GO:0006807\_nitrogen\_compound\_metabolic\_process | ADK | 1147 | 9 | 1.290478 | -0.609745 | 323 | 248.42 | 0.769102 |
| GO:0006807\_nitrogen\_compound\_metabolic\_process | PAX6 | 1147 | 9 | 1.290478 | -0.609745 | 323 | 248.42 | 0.769102 |
| GO:0006807\_nitrogen\_compound\_metabolic\_process | NEO1 | 1147 | 9 | 1.290478 | -0.609745 | 323 | 248.42 | 0.769102 |
| GO:0006807\_nitrogen\_compound\_metabolic\_process | AGRN | 1147 | 9 | 1.290478 | -0.609745 | 323 | 248.42 | 0.769102 |
| GO:0006807\_nitrogen\_compound\_metabolic\_process | MYBBP1A | 1147 | 9 | 1.290478 | -0.609745 | 323 | 248.42 | 0.769102 |
| GO:0006807\_nitrogen\_compound\_metabolic\_process | ZIC3 | 1147 | 9 | 1.290478 | -0.609745 | 323 | 248.42 | 0.769102 |
| GO:0042063\_gliogenesis | PAX6 | 46 | 1 | 3.575311 | -0.609636 | 325 | 250.45 | 0.770615 |
| GO:0051098\_regulation\_of\_binding | CALM1 | 46 | 1 | 3.575311 | -0.609636 | 325 | 250.45 | 0.770615 |
| GO:0006350\_transcription | ATF4 | 701 | 6 | 1.407683 | -0.609558 | 326 | 250.58 | 0.768650 |
| GO:0006350\_transcription | PAX6 | 701 | 6 | 1.407683 | -0.609558 | 326 | 250.58 | 0.768650 |
| GO:0006350\_transcription | NEO1 | 701 | 6 | 1.407683 | -0.609558 | 326 | 250.58 | 0.768650 |
| GO:0006350\_transcription | AGRN | 701 | 6 | 1.407683 | -0.609558 | 326 | 250.58 | 0.768650 |
| GO:0006350\_transcription | MYBBP1A | 701 | 6 | 1.407683 | -0.609558 | 326 | 250.58 | 0.768650 |
| GO:0006350\_transcription | ZIC3 | 701 | 6 | 1.407683 | -0.609558 | 326 | 250.58 | 0.768650 |
| GO:0044267\_cellular\_protein\_metabolic\_process | CCNE1 | 559 | 5 | 1.471058 | -0.606553 | 327 | 251.18 | 0.768135 |
| GO:0044267\_cellular\_protein\_metabolic\_process | UBE2D3 | 559 | 5 | 1.471058 | -0.606553 | 327 | 251.18 | 0.768135 |
| GO:0044267\_cellular\_protein\_metabolic\_process | CCND1 | 559 | 5 | 1.471058 | -0.606553 | 327 | 251.18 | 0.768135 |
| GO:0044267\_cellular\_protein\_metabolic\_process | HSP90AA1 | 559 | 5 | 1.471058 | -0.606553 | 327 | 251.18 | 0.768135 |
| GO:0044267\_cellular\_protein\_metabolic\_process | YWHAE | 559 | 5 | 1.471058 | -0.606553 | 327 | 251.18 | 0.768135 |
| GO:0044249\_cellular\_biosynthetic\_process | CCNE1 | 1150 | 9 | 1.287112 | -0.605159 | 328 | 251.45 | 0.766616 |
| GO:0044249\_cellular\_biosynthetic\_process | ATF4 | 1150 | 9 | 1.287112 | -0.605159 | 328 | 251.45 | 0.766616 |
| GO:0044249\_cellular\_biosynthetic\_process | HSP90AA1 | 1150 | 9 | 1.287112 | -0.605159 | 328 | 251.45 | 0.766616 |
| GO:0044249\_cellular\_biosynthetic\_process | ADK | 1150 | 9 | 1.287112 | -0.605159 | 328 | 251.45 | 0.766616 |
| GO:0044249\_cellular\_biosynthetic\_process | PAX6 | 1150 | 9 | 1.287112 | -0.605159 | 328 | 251.45 | 0.766616 |
| GO:0044249\_cellular\_biosynthetic\_process | NEO1 | 1150 | 9 | 1.287112 | -0.605159 | 328 | 251.45 | 0.766616 |
| GO:0044249\_cellular\_biosynthetic\_process | AGRN | 1150 | 9 | 1.287112 | -0.605159 | 328 | 251.45 | 0.766616 |
| GO:0044249\_cellular\_biosynthetic\_process | MYBBP1A | 1150 | 9 | 1.287112 | -0.605159 | 328 | 251.45 | 0.766616 |
| GO:0044249\_cellular\_biosynthetic\_process | ZIC3 | 1150 | 9 | 1.287112 | -0.605159 | 328 | 251.45 | 0.766616 |
| GO:0045619\_regulation\_of\_lymphocyte\_differentiation | HSP90AA1 | 47 | 1 | 3.499240 | -0.601523 | 329 | 254.58 | 0.773799 |
| GO:0006139\_nucleobase\_\_nucleoside\_\_nucleotide\_and\_nucleic\_acid\_metabolic\_process | CCNE1 | 1002 | 8 | 1.313088 | -0.600211 | 330 | 254.69 | 0.771788 |
| GO:0006139\_nucleobase\_\_nucleoside\_\_nucleotide\_and\_nucleic\_acid\_metabolic\_process | ATF4 | 1002 | 8 | 1.313088 | -0.600211 | 330 | 254.69 | 0.771788 |
| GO:0006139\_nucleobase\_\_nucleoside\_\_nucleotide\_and\_nucleic\_acid\_metabolic\_process | ADK | 1002 | 8 | 1.313088 | -0.600211 | 330 | 254.69 | 0.771788 |
| GO:0006139\_nucleobase\_\_nucleoside\_\_nucleotide\_and\_nucleic\_acid\_metabolic\_process | PAX6 | 1002 | 8 | 1.313088 | -0.600211 | 330 | 254.69 | 0.771788 |
| GO:0006139\_nucleobase\_\_nucleoside\_\_nucleotide\_and\_nucleic\_acid\_metabolic\_process | NEO1 | 1002 | 8 | 1.313088 | -0.600211 | 330 | 254.69 | 0.771788 |
| GO:0006139\_nucleobase\_\_nucleoside\_\_nucleotide\_and\_nucleic\_acid\_metabolic\_process | AGRN | 1002 | 8 | 1.313088 | -0.600211 | 330 | 254.69 | 0.771788 |
| GO:0006139\_nucleobase\_\_nucleoside\_\_nucleotide\_and\_nucleic\_acid\_metabolic\_process | MYBBP1A | 1002 | 8 | 1.313088 | -0.600211 | 330 | 254.69 | 0.771788 |
| GO:0006139\_nucleobase\_\_nucleoside\_\_nucleotide\_and\_nucleic\_acid\_metabolic\_process | ZIC3 | 1002 | 8 | 1.313088 | -0.600211 | 330 | 254.69 | 0.771788 |
| GO:0007417\_central\_nervous\_system\_development | PAX6 | 287 | 3 | 1.719139 | -0.598378 | 331 | 254.83 | 0.769879 |
| GO:0007417\_central\_nervous\_system\_development | YWHAE | 287 | 3 | 1.719139 | -0.598378 | 331 | 254.83 | 0.769879 |
| GO:0007417\_central\_nervous\_system\_development | BMPR1A | 287 | 3 | 1.719139 | -0.598378 | 331 | 254.83 | 0.769879 |
| GO:0051128\_regulation\_of\_cellular\_component\_organization | RHOQ | 160 | 2 | 2.055804 | -0.595222 | 332 | 255.17 | 0.768584 |
| GO:0051128\_regulation\_of\_cellular\_component\_organization | AGRN | 160 | 2 | 2.055804 | -0.595222 | 332 | 255.17 | 0.768584 |
| GO:0007498\_mesoderm\_development | BMPR1A | 48 | 1 | 3.426339 | -0.593605 | 335 | 256.79 | 0.766537 |
| GO:0019318\_hexose\_metabolic\_process | ATF4 | 48 | 1 | 3.426339 | -0.593605 | 335 | 256.79 | 0.766537 |
| GO:0032269\_negative\_regulation\_of\_cellular\_protein\_metabolic\_process | YWHAE | 48 | 1 | 3.426339 | -0.593605 | 335 | 256.79 | 0.766537 |
| GO:0019220\_regulation\_of\_phosphate\_metabolic\_process | NUP62 | 165 | 2 | 1.993506 | -0.576128 | 337 | 263.46 | 0.781780 |
| GO:0019220\_regulation\_of\_phosphate\_metabolic\_process | YWHAE | 165 | 2 | 1.993506 | -0.576128 | 337 | 263.46 | 0.781780 |
| GO:0051174\_regulation\_of\_phosphorus\_metabolic\_process | NUP62 | 165 | 2 | 1.993506 | -0.576128 | 337 | 263.46 | 0.781780 |
| GO:0051174\_regulation\_of\_phosphorus\_metabolic\_process | YWHAE | 165 | 2 | 1.993506 | -0.576128 | 337 | 263.46 | 0.781780 |
| GO:0010604\_positive\_regulation\_of\_macromolecule\_metabolic\_process | ATF4 | 433 | 4 | 1.519301 | -0.574200 | 338 | 264.08 | 0.781302 |
| GO:0010604\_positive\_regulation\_of\_macromolecule\_metabolic\_process | PAX6 | 433 | 4 | 1.519301 | -0.574200 | 338 | 264.08 | 0.781302 |
| GO:0010604\_positive\_regulation\_of\_macromolecule\_metabolic\_process | AGRN | 433 | 4 | 1.519301 | -0.574200 | 338 | 264.08 | 0.781302 |
| GO:0010604\_positive\_regulation\_of\_macromolecule\_metabolic\_process | ZIC3 | 433 | 4 | 1.519301 | -0.574200 | 338 | 264.08 | 0.781302 |
| GO:0032583\_regulation\_of\_gene-specific\_transcription | PAX6 | 51 | 1 | 3.224790 | -0.570946 | 340 | 266.66 | 0.784294 |
| GO:0032880\_regulation\_of\_protein\_localization | RHOQ | 51 | 1 | 3.224790 | -0.570946 | 340 | 266.66 | 0.784294 |
| GO:0006357\_regulation\_of\_transcription\_from\_RNA\_polymerase\_II\_promoter | ATF4 | 435 | 4 | 1.512315 | -0.569702 | 341 | 266.78 | 0.782346 |
| GO:0006357\_regulation\_of\_transcription\_from\_RNA\_polymerase\_II\_promoter | PAX6 | 435 | 4 | 1.512315 | -0.569702 | 341 | 266.78 | 0.782346 |
| GO:0006357\_regulation\_of\_transcription\_from\_RNA\_polymerase\_II\_promoter | AGRN | 435 | 4 | 1.512315 | -0.569702 | 341 | 266.78 | 0.782346 |
| GO:0006357\_regulation\_of\_transcription\_from\_RNA\_polymerase\_II\_promoter | ZIC3 | 435 | 4 | 1.512315 | -0.569702 | 341 | 266.78 | 0.782346 |
| GO:0009058\_biosynthetic\_process | CCNE1 | 1175 | 9 | 1.259726 | -0.568015 | 342 | 267.39 | 0.781842 |
| GO:0009058\_biosynthetic\_process | ATF4 | 1175 | 9 | 1.259726 | -0.568015 | 342 | 267.39 | 0.781842 |
| GO:0009058\_biosynthetic\_process | HSP90AA1 | 1175 | 9 | 1.259726 | -0.568015 | 342 | 267.39 | 0.781842 |
| GO:0009058\_biosynthetic\_process | ADK | 1175 | 9 | 1.259726 | -0.568015 | 342 | 267.39 | 0.781842 |
| GO:0009058\_biosynthetic\_process | PAX6 | 1175 | 9 | 1.259726 | -0.568015 | 342 | 267.39 | 0.781842 |
| GO:0009058\_biosynthetic\_process | NEO1 | 1175 | 9 | 1.259726 | -0.568015 | 342 | 267.39 | 0.781842 |
| GO:0009058\_biosynthetic\_process | AGRN | 1175 | 9 | 1.259726 | -0.568015 | 342 | 267.39 | 0.781842 |
| GO:0009058\_biosynthetic\_process | MYBBP1A | 1175 | 9 | 1.259726 | -0.568015 | 342 | 267.39 | 0.781842 |
| GO:0009058\_biosynthetic\_process | ZIC3 | 1175 | 9 | 1.259726 | -0.568015 | 342 | 267.39 | 0.781842 |
| GO:0043283\_biopolymer\_metabolic\_process | CCNE1 | 1490 | 11 | 1.214166 | -0.560920 | 343 | 268.81 | 0.783703 |
| GO:0043283\_biopolymer\_metabolic\_process | ATF4 | 1490 | 11 | 1.214166 | -0.560920 | 343 | 268.81 | 0.783703 |
| GO:0043283\_biopolymer\_metabolic\_process | CCND1 | 1490 | 11 | 1.214166 | -0.560920 | 343 | 268.81 | 0.783703 |
| GO:0043283\_biopolymer\_metabolic\_process | UBE2D3 | 1490 | 11 | 1.214166 | -0.560920 | 343 | 268.81 | 0.783703 |
| GO:0043283\_biopolymer\_metabolic\_process | HSP90AA1 | 1490 | 11 | 1.214166 | -0.560920 | 343 | 268.81 | 0.783703 |
| GO:0043283\_biopolymer\_metabolic\_process | PAX6 | 1490 | 11 | 1.214166 | -0.560920 | 343 | 268.81 | 0.783703 |
| GO:0043283\_biopolymer\_metabolic\_process | NEO1 | 1490 | 11 | 1.214166 | -0.560920 | 343 | 268.81 | 0.783703 |
| GO:0043283\_biopolymer\_metabolic\_process | AGRN | 1490 | 11 | 1.214166 | -0.560920 | 343 | 268.81 | 0.783703 |
| GO:0043283\_biopolymer\_metabolic\_process | MYBBP1A | 1490 | 11 | 1.214166 | -0.560920 | 343 | 268.81 | 0.783703 |
| GO:0043283\_biopolymer\_metabolic\_process | YWHAE | 1490 | 11 | 1.214166 | -0.560920 | 343 | 268.81 | 0.783703 |
| GO:0043283\_biopolymer\_metabolic\_process | ZIC3 | 1490 | 11 | 1.214166 | -0.560920 | 343 | 268.81 | 0.783703 |
| GO:0030031\_cell\_projection\_assembly | RDX | 53 | 1 | 3.103100 | -0.556682 | 345 | 271.35 | 0.786522 |
| GO:0051248\_negative\_regulation\_of\_protein\_metabolic\_process | YWHAE | 53 | 1 | 3.103100 | -0.556682 | 345 | 271.35 | 0.786522 |
| GO:0006366\_transcription\_from\_RNA\_polymerase\_II\_promoter | ATF4 | 444 | 4 | 1.481660 | -0.549901 | 346 | 272.4 | 0.787283 |
| GO:0006366\_transcription\_from\_RNA\_polymerase\_II\_promoter | PAX6 | 444 | 4 | 1.481660 | -0.549901 | 346 | 272.4 | 0.787283 |
| GO:0006366\_transcription\_from\_RNA\_polymerase\_II\_promoter | AGRN | 444 | 4 | 1.481660 | -0.549901 | 346 | 272.4 | 0.787283 |
| GO:0006366\_transcription\_from\_RNA\_polymerase\_II\_promoter | ZIC3 | 444 | 4 | 1.481660 | -0.549901 | 346 | 272.4 | 0.787283 |
| GO:0006091\_generation\_of\_precursor\_metabolites\_and\_energy | MYBBP1A | 54 | 1 | 3.045635 | -0.549783 | 352 | 274.74 | 0.780511 |
| GO:0006164\_purine\_nucleotide\_biosynthetic\_process | ADK | 54 | 1 | 3.045635 | -0.549783 | 352 | 274.74 | 0.780511 |
| GO:0007265\_Ras\_protein\_signal\_transduction | NUP62 | 54 | 1 | 3.045635 | -0.549783 | 352 | 274.74 | 0.780511 |
| GO:0009566\_fertilization | CSDA | 54 | 1 | 3.045635 | -0.549783 | 352 | 274.74 | 0.780511 |
| GO:0043405\_regulation\_of\_MAP\_kinase\_activity | NUP62 | 54 | 1 | 3.045635 | -0.549783 | 352 | 274.74 | 0.780511 |
| GO:0044271\_nitrogen\_compound\_biosynthetic\_process | HSP90AA1 | 54 | 1 | 3.045635 | -0.549783 | 352 | 274.74 | 0.780511 |
| GO:0010926\_anatomical\_structure\_formation | RDX | 447 | 4 | 1.471716 | -0.543457 | 353 | 276.04 | 0.781983 |
| GO:0010926\_anatomical\_structure\_formation | NEO1 | 447 | 4 | 1.471716 | -0.543457 | 353 | 276.04 | 0.781983 |
| GO:0010926\_anatomical\_structure\_formation | AGRN | 447 | 4 | 1.471716 | -0.543457 | 353 | 276.04 | 0.781983 |
| GO:0010926\_anatomical\_structure\_formation | BMPR1A | 447 | 4 | 1.471716 | -0.543457 | 353 | 276.04 | 0.781983 |
| GO:0007126\_meiosis | SMC1A | 55 | 1 | 2.990260 | -0.543032 | 357 | 277.67 | 0.777787 |
| GO:0043434\_response\_to\_peptide\_hormone\_stimulus | RHOQ | 55 | 1 | 2.990260 | -0.543032 | 357 | 277.67 | 0.777787 |
| GO:0048568\_embryonic\_organ\_development | BMPR1A | 55 | 1 | 2.990260 | -0.543032 | 357 | 277.67 | 0.777787 |
| GO:0051327\_M\_phase\_of\_meiotic\_cell\_cycle | SMC1A | 55 | 1 | 2.990260 | -0.543032 | 357 | 277.67 | 0.777787 |
| GO:0051094\_positive\_regulation\_of\_developmental\_process | EI24 | 308 | 3 | 1.601925 | -0.541455 | 358 | 277.85 | 0.776117 |
| GO:0051094\_positive\_regulation\_of\_developmental\_process | HSP90AA1 | 308 | 3 | 1.601925 | -0.541455 | 358 | 277.85 | 0.776117 |
| GO:0051094\_positive\_regulation\_of\_developmental\_process | PAX6 | 308 | 3 | 1.601925 | -0.541455 | 358 | 277.85 | 0.776117 |
| GO:0016310\_phosphorylation | CCNE1 | 309 | 3 | 1.596741 | -0.538898 | 359 | 278.73 | 0.776407 |
| GO:0016310\_phosphorylation | CCND1 | 309 | 3 | 1.596741 | -0.538898 | 359 | 278.73 | 0.776407 |
| GO:0016310\_phosphorylation | NUP62 | 309 | 3 | 1.596741 | -0.538898 | 359 | 278.73 | 0.776407 |
| GO:0001708\_cell\_fate\_specification | PAX6 | 56 | 1 | 2.936862 | -0.536423 | 361 | 282.18 | 0.781662 |
| GO:0051321\_meiotic\_cell\_cycle | SMC1A | 56 | 1 | 2.936862 | -0.536423 | 361 | 282.18 | 0.781662 |
| GO:0007166\_cell\_surface\_receptor\_linked\_signal\_transduction | CCND1 | 597 | 5 | 1.377423 | -0.532323 | 362 | 282.54 | 0.780497 |
| GO:0007166\_cell\_surface\_receptor\_linked\_signal\_transduction | NUP62 | 597 | 5 | 1.377423 | -0.532323 | 362 | 282.54 | 0.780497 |
| GO:0007166\_cell\_surface\_receptor\_linked\_signal\_transduction | PAX6 | 597 | 5 | 1.377423 | -0.532323 | 362 | 282.54 | 0.780497 |
| GO:0007166\_cell\_surface\_receptor\_linked\_signal\_transduction | AGRN | 597 | 5 | 1.377423 | -0.532323 | 362 | 282.54 | 0.780497 |
| GO:0007166\_cell\_surface\_receptor\_linked\_signal\_transduction | BMPR1A | 597 | 5 | 1.377423 | -0.532323 | 362 | 282.54 | 0.780497 |
| GO:0048522\_positive\_regulation\_of\_cellular\_process | ATF4 | 895 | 7 | 1.286313 | -0.531863 | 363 | 282.6 | 0.778512 |
| GO:0048522\_positive\_regulation\_of\_cellular\_process | EI24 | 895 | 7 | 1.286313 | -0.531863 | 363 | 282.6 | 0.778512 |
| GO:0048522\_positive\_regulation\_of\_cellular\_process | HSP90AA1 | 895 | 7 | 1.286313 | -0.531863 | 363 | 282.6 | 0.778512 |
| GO:0048522\_positive\_regulation\_of\_cellular\_process | PAX6 | 895 | 7 | 1.286313 | -0.531863 | 363 | 282.6 | 0.778512 |
| GO:0048522\_positive\_regulation\_of\_cellular\_process | AGRN | 895 | 7 | 1.286313 | -0.531863 | 363 | 282.6 | 0.778512 |
| GO:0048522\_positive\_regulation\_of\_cellular\_process | ZIC3 | 895 | 7 | 1.286313 | -0.531863 | 363 | 282.6 | 0.778512 |
| GO:0048522\_positive\_regulation\_of\_cellular\_process | BMPR1A | 895 | 7 | 1.286313 | -0.531863 | 363 | 282.6 | 0.778512 |
| GO:0010556\_regulation\_of\_macromolecule\_biosynthetic\_process | ATF4 | 745 | 6 | 1.324545 | -0.531800 | 364 | 282.76 | 0.776813 |
| GO:0010556\_regulation\_of\_macromolecule\_biosynthetic\_process | PAX6 | 745 | 6 | 1.324545 | -0.531800 | 364 | 282.76 | 0.776813 |
| GO:0010556\_regulation\_of\_macromolecule\_biosynthetic\_process | NEO1 | 745 | 6 | 1.324545 | -0.531800 | 364 | 282.76 | 0.776813 |
| GO:0010556\_regulation\_of\_macromolecule\_biosynthetic\_process | AGRN | 745 | 6 | 1.324545 | -0.531800 | 364 | 282.76 | 0.776813 |
| GO:0010556\_regulation\_of\_macromolecule\_biosynthetic\_process | MYBBP1A | 745 | 6 | 1.324545 | -0.531800 | 364 | 282.76 | 0.776813 |
| GO:0010556\_regulation\_of\_macromolecule\_biosynthetic\_process | ZIC3 | 745 | 6 | 1.324545 | -0.531800 | 364 | 282.76 | 0.776813 |
| GO:0033365\_protein\_localization\_in\_organelle | PAX6 | 57 | 1 | 2.885338 | -0.529952 | 366 | 285.78 | 0.780820 |
| GO:0045444\_fat\_cell\_differentiation | CCND1 | 57 | 1 | 2.885338 | -0.529952 | 366 | 285.78 | 0.780820 |
| GO:0034622\_cellular\_macromolecular\_complex\_assembly | AGRN | 58 | 1 | 2.835591 | -0.523613 | 367 | 287.07 | 0.782207 |
| GO:0045184\_establishment\_of\_protein\_localization | RHOQ | 180 | 2 | 1.827381 | -0.523375 | 368 | 287.19 | 0.780408 |
| GO:0045184\_establishment\_of\_protein\_localization | YWHAE | 180 | 2 | 1.827381 | -0.523375 | 368 | 287.19 | 0.780408 |
| GO:0034645\_cellular\_macromolecule\_biosynthetic\_process | CCNE1 | 901 | 7 | 1.277747 | -0.522711 | 369 | 287.34 | 0.778699 |
| GO:0034645\_cellular\_macromolecule\_biosynthetic\_process | ATF4 | 901 | 7 | 1.277747 | -0.522711 | 369 | 287.34 | 0.778699 |
| GO:0034645\_cellular\_macromolecule\_biosynthetic\_process | PAX6 | 901 | 7 | 1.277747 | -0.522711 | 369 | 287.34 | 0.778699 |
| GO:0034645\_cellular\_macromolecule\_biosynthetic\_process | NEO1 | 901 | 7 | 1.277747 | -0.522711 | 369 | 287.34 | 0.778699 |
| GO:0034645\_cellular\_macromolecule\_biosynthetic\_process | AGRN | 901 | 7 | 1.277747 | -0.522711 | 369 | 287.34 | 0.778699 |
| GO:0034645\_cellular\_macromolecule\_biosynthetic\_process | MYBBP1A | 901 | 7 | 1.277747 | -0.522711 | 369 | 287.34 | 0.778699 |
| GO:0034645\_cellular\_macromolecule\_biosynthetic\_process | ZIC3 | 901 | 7 | 1.277747 | -0.522711 | 369 | 287.34 | 0.778699 |
| GO:0016055\_Wnt\_receptor\_signaling\_pathway | CCND1 | 59 | 1 | 2.787530 | -0.517403 | 371 | 289.71 | 0.780889 |
| GO:0050870\_positive\_regulation\_of\_T\_cell\_activation | HSP90AA1 | 59 | 1 | 2.787530 | -0.517403 | 371 | 289.71 | 0.780889 |
| GO:0019219\_regulation\_of\_nucleobase\_\_nucleoside\_\_nucleotide\_and\_nucleic\_acid\_metabolic\_process | ATF4 | 757 | 6 | 1.303548 | -0.512208 | 372 | 290.66 | 0.781344 |
| GO:0019219\_regulation\_of\_nucleobase\_\_nucleoside\_\_nucleotide\_and\_nucleic\_acid\_metabolic\_process | PAX6 | 757 | 6 | 1.303548 | -0.512208 | 372 | 290.66 | 0.781344 |
| GO:0019219\_regulation\_of\_nucleobase\_\_nucleoside\_\_nucleotide\_and\_nucleic\_acid\_metabolic\_process | NEO1 | 757 | 6 | 1.303548 | -0.512208 | 372 | 290.66 | 0.781344 |
| GO:0019219\_regulation\_of\_nucleobase\_\_nucleoside\_\_nucleotide\_and\_nucleic\_acid\_metabolic\_process | AGRN | 757 | 6 | 1.303548 | -0.512208 | 372 | 290.66 | 0.781344 |
| GO:0019219\_regulation\_of\_nucleobase\_\_nucleoside\_\_nucleotide\_and\_nucleic\_acid\_metabolic\_process | MYBBP1A | 757 | 6 | 1.303548 | -0.512208 | 372 | 290.66 | 0.781344 |
| GO:0019219\_regulation\_of\_nucleobase\_\_nucleoside\_\_nucleotide\_and\_nucleic\_acid\_metabolic\_process | ZIC3 | 757 | 6 | 1.303548 | -0.512208 | 372 | 290.66 | 0.781344 |
| GO:0009059\_macromolecule\_biosynthetic\_process | CCNE1 | 910 | 7 | 1.265110 | -0.509238 | 373 | 291.54 | 0.781609 |
| GO:0009059\_macromolecule\_biosynthetic\_process | ATF4 | 910 | 7 | 1.265110 | -0.509238 | 373 | 291.54 | 0.781609 |
| GO:0009059\_macromolecule\_biosynthetic\_process | PAX6 | 910 | 7 | 1.265110 | -0.509238 | 373 | 291.54 | 0.781609 |
| GO:0009059\_macromolecule\_biosynthetic\_process | NEO1 | 910 | 7 | 1.265110 | -0.509238 | 373 | 291.54 | 0.781609 |
| GO:0009059\_macromolecule\_biosynthetic\_process | AGRN | 910 | 7 | 1.265110 | -0.509238 | 373 | 291.54 | 0.781609 |
| GO:0009059\_macromolecule\_biosynthetic\_process | MYBBP1A | 910 | 7 | 1.265110 | -0.509238 | 373 | 291.54 | 0.781609 |
| GO:0009059\_macromolecule\_biosynthetic\_process | ZIC3 | 910 | 7 | 1.265110 | -0.509238 | 373 | 291.54 | 0.781609 |
| GO:0007165\_signal\_transduction | CCND1 | 915 | 7 | 1.258197 | -0.501882 | 374 | 293.84 | 0.785668 |
| GO:0007165\_signal\_transduction | NUP62 | 915 | 7 | 1.258197 | -0.501882 | 374 | 293.84 | 0.785668 |
| GO:0007165\_signal\_transduction | DNAJA1 | 915 | 7 | 1.258197 | -0.501882 | 374 | 293.84 | 0.785668 |
| GO:0007165\_signal\_transduction | PAX6 | 915 | 7 | 1.258197 | -0.501882 | 374 | 293.84 | 0.785668 |
| GO:0007165\_signal\_transduction | RAP1B | 915 | 7 | 1.258197 | -0.501882 | 374 | 293.84 | 0.785668 |
| GO:0007165\_signal\_transduction | AGRN | 915 | 7 | 1.258197 | -0.501882 | 374 | 293.84 | 0.785668 |
| GO:0007165\_signal\_transduction | BMPR1A | 915 | 7 | 1.258197 | -0.501882 | 374 | 293.84 | 0.785668 |
| GO:0022604\_regulation\_of\_cell\_morphogenesis | RHOQ | 62 | 1 | 2.652650 | -0.499496 | 375 | 296.36 | 0.790293 |
| GO:0007369\_gastrulation | BMPR1A | 63 | 1 | 2.610544 | -0.493757 | 378 | 298.39 | 0.789392 |
| GO:0009165\_nucleotide\_biosynthetic\_process | ADK | 63 | 1 | 2.610544 | -0.493757 | 378 | 298.39 | 0.789392 |
| GO:0051216\_cartilage\_development | BMPR1A | 63 | 1 | 2.610544 | -0.493757 | 378 | 298.39 | 0.789392 |
| GO:0080090\_regulation\_of\_primary\_metabolic\_process | ATF4 | 926 | 7 | 1.243251 | -0.486019 | 379 | 301.08 | 0.794406 |
| GO:0080090\_regulation\_of\_primary\_metabolic\_process | PAX6 | 926 | 7 | 1.243251 | -0.486019 | 379 | 301.08 | 0.794406 |
| GO:0080090\_regulation\_of\_primary\_metabolic\_process | NEO1 | 926 | 7 | 1.243251 | -0.486019 | 379 | 301.08 | 0.794406 |
| GO:0080090\_regulation\_of\_primary\_metabolic\_process | AGRN | 926 | 7 | 1.243251 | -0.486019 | 379 | 301.08 | 0.794406 |
| GO:0080090\_regulation\_of\_primary\_metabolic\_process | YWHAE | 926 | 7 | 1.243251 | -0.486019 | 379 | 301.08 | 0.794406 |
| GO:0080090\_regulation\_of\_primary\_metabolic\_process | MYBBP1A | 926 | 7 | 1.243251 | -0.486019 | 379 | 301.08 | 0.794406 |
| GO:0080090\_regulation\_of\_primary\_metabolic\_process | ZIC3 | 926 | 7 | 1.243251 | -0.486019 | 379 | 301.08 | 0.794406 |
| GO:0043086\_negative\_regulation\_of\_catalytic\_activity | NUP62 | 65 | 1 | 2.530220 | -0.482598 | 380 | 302.58 | 0.796263 |
| GO:0046907\_intracellular\_transport | MYBBP1A | 194 | 2 | 1.695508 | -0.479509 | 381 | 302.98 | 0.795223 |
| GO:0046907\_intracellular\_transport | YWHAE | 194 | 2 | 1.695508 | -0.479509 | 381 | 302.98 | 0.795223 |
| GO:0010468\_regulation\_of\_gene\_expression | ATF4 | 778 | 6 | 1.268362 | -0.479468 | 382 | 303.12 | 0.793508 |
| GO:0010468\_regulation\_of\_gene\_expression | PAX6 | 778 | 6 | 1.268362 | -0.479468 | 382 | 303.12 | 0.793508 |
| GO:0010468\_regulation\_of\_gene\_expression | NEO1 | 778 | 6 | 1.268362 | -0.479468 | 382 | 303.12 | 0.793508 |
| GO:0010468\_regulation\_of\_gene\_expression | AGRN | 778 | 6 | 1.268362 | -0.479468 | 382 | 303.12 | 0.793508 |
| GO:0010468\_regulation\_of\_gene\_expression | MYBBP1A | 778 | 6 | 1.268362 | -0.479468 | 382 | 303.12 | 0.793508 |
| GO:0010468\_regulation\_of\_gene\_expression | ZIC3 | 778 | 6 | 1.268362 | -0.479468 | 382 | 303.12 | 0.793508 |
| GO:0007507\_heart\_development | ZIC3 | 195 | 2 | 1.686813 | -0.476552 | 383 | 305.27 | 0.797050 |
| GO:0007507\_heart\_development | BMPR1A | 195 | 2 | 1.686813 | -0.476552 | 383 | 305.27 | 0.797050 |
| GO:0060255\_regulation\_of\_macromolecule\_metabolic\_process | ATF4 | 936 | 7 | 1.229968 | -0.471972 | 384 | 305.74 | 0.796198 |
| GO:0060255\_regulation\_of\_macromolecule\_metabolic\_process | PAX6 | 936 | 7 | 1.229968 | -0.471972 | 384 | 305.74 | 0.796198 |
| GO:0060255\_regulation\_of\_macromolecule\_metabolic\_process | NEO1 | 936 | 7 | 1.229968 | -0.471972 | 384 | 305.74 | 0.796198 |
| GO:0060255\_regulation\_of\_macromolecule\_metabolic\_process | AGRN | 936 | 7 | 1.229968 | -0.471972 | 384 | 305.74 | 0.796198 |
| GO:0060255\_regulation\_of\_macromolecule\_metabolic\_process | YWHAE | 936 | 7 | 1.229968 | -0.471972 | 384 | 305.74 | 0.796198 |
| GO:0060255\_regulation\_of\_macromolecule\_metabolic\_process | MYBBP1A | 936 | 7 | 1.229968 | -0.471972 | 384 | 305.74 | 0.796198 |
| GO:0060255\_regulation\_of\_macromolecule\_metabolic\_process | ZIC3 | 936 | 7 | 1.229968 | -0.471972 | 384 | 305.74 | 0.796198 |
| GO:0002009\_morphogenesis\_of\_an\_epithelium | CCND1 | 198 | 2 | 1.661255 | -0.467816 | 386 | 308.11 | 0.798212 |
| GO:0002009\_morphogenesis\_of\_an\_epithelium | PAX6 | 198 | 2 | 1.661255 | -0.467816 | 386 | 308.11 | 0.798212 |
| GO:0060429\_epithelium\_development | CCND1 | 198 | 2 | 1.661255 | -0.467816 | 386 | 308.11 | 0.798212 |
| GO:0060429\_epithelium\_development | PAX6 | 198 | 2 | 1.661255 | -0.467816 | 386 | 308.11 | 0.798212 |
| GO:0034962\_cellular\_biopolymer\_catabolic\_process | UBE2D3 | 68 | 1 | 2.418592 | -0.466617 | 388 | 309.25 | 0.797036 |
| GO:0042692\_muscle\_cell\_differentiation | NEO1 | 68 | 1 | 2.418592 | -0.466617 | 388 | 309.25 | 0.797036 |
| GO:0008406\_gonad\_development | CSDA | 70 | 1 | 2.349490 | -0.456431 | 390 | 313.13 | 0.802897 |
| GO:0048592\_eye\_morphogenesis | PAX6 | 70 | 1 | 2.349490 | -0.456431 | 390 | 313.13 | 0.802897 |
| GO:0043170\_macromolecule\_metabolic\_process | CCNE1 | 1576 | 11 | 1.147911 | -0.455211 | 391 | 313.34 | 0.801381 |
| GO:0043170\_macromolecule\_metabolic\_process | CCND1 | 1576 | 11 | 1.147911 | -0.455211 | 391 | 313.34 | 0.801381 |
| GO:0043170\_macromolecule\_metabolic\_process | ATF4 | 1576 | 11 | 1.147911 | -0.455211 | 391 | 313.34 | 0.801381 |
| GO:0043170\_macromolecule\_metabolic\_process | UBE2D3 | 1576 | 11 | 1.147911 | -0.455211 | 391 | 313.34 | 0.801381 |
| GO:0043170\_macromolecule\_metabolic\_process | HSP90AA1 | 1576 | 11 | 1.147911 | -0.455211 | 391 | 313.34 | 0.801381 |
| GO:0043170\_macromolecule\_metabolic\_process | PAX6 | 1576 | 11 | 1.147911 | -0.455211 | 391 | 313.34 | 0.801381 |
| GO:0043170\_macromolecule\_metabolic\_process | NEO1 | 1576 | 11 | 1.147911 | -0.455211 | 391 | 313.34 | 0.801381 |
| GO:0043170\_macromolecule\_metabolic\_process | AGRN | 1576 | 11 | 1.147911 | -0.455211 | 391 | 313.34 | 0.801381 |
| GO:0043170\_macromolecule\_metabolic\_process | YWHAE | 1576 | 11 | 1.147911 | -0.455211 | 391 | 313.34 | 0.801381 |
| GO:0043170\_macromolecule\_metabolic\_process | MYBBP1A | 1576 | 11 | 1.147911 | -0.455211 | 391 | 313.34 | 0.801381 |
| GO:0043170\_macromolecule\_metabolic\_process | ZIC3 | 1576 | 11 | 1.147911 | -0.455211 | 391 | 313.34 | 0.801381 |
| GO:0006913\_nucleocytoplasmic\_transport | MYBBP1A | 71 | 1 | 2.316398 | -0.451471 | 392 | 315.28 | 0.804286 |
| GO:0022607\_cellular\_component\_assembly | RDX | 204 | 2 | 1.612395 | -0.450918 | 393 | 315.49 | 0.802774 |
| GO:0022607\_cellular\_component\_assembly | AGRN | 204 | 2 | 1.612395 | -0.450918 | 393 | 315.49 | 0.802774 |
| GO:0021915\_neural\_tube\_development | PAX6 | 72 | 1 | 2.284226 | -0.446596 | 396 | 319.17 | 0.805985 |
| GO:0030879\_mammary\_gland\_development | CCND1 | 72 | 1 | 2.284226 | -0.446596 | 396 | 319.17 | 0.805985 |
| GO:0051169\_nuclear\_transport | MYBBP1A | 72 | 1 | 2.284226 | -0.446596 | 396 | 319.17 | 0.805985 |
| GO:0048869\_cellular\_developmental\_process | CCND1 | 1113 | 8 | 1.182133 | -0.443306 | 397 | 319.45 | 0.804660 |
| GO:0048869\_cellular\_developmental\_process | HSP90AA1 | 1113 | 8 | 1.182133 | -0.443306 | 397 | 319.45 | 0.804660 |
| GO:0048869\_cellular\_developmental\_process | PAX6 | 1113 | 8 | 1.182133 | -0.443306 | 397 | 319.45 | 0.804660 |
| GO:0048869\_cellular\_developmental\_process | RHOQ | 1113 | 8 | 1.182133 | -0.443306 | 397 | 319.45 | 0.804660 |
| GO:0048869\_cellular\_developmental\_process | AGRN | 1113 | 8 | 1.182133 | -0.443306 | 397 | 319.45 | 0.804660 |
| GO:0048869\_cellular\_developmental\_process | NEO1 | 1113 | 8 | 1.182133 | -0.443306 | 397 | 319.45 | 0.804660 |
| GO:0048869\_cellular\_developmental\_process | YWHAE | 1113 | 8 | 1.182133 | -0.443306 | 397 | 319.45 | 0.804660 |
| GO:0048869\_cellular\_developmental\_process | BMPR1A | 1113 | 8 | 1.182133 | -0.443306 | 397 | 319.45 | 0.804660 |
| GO:0006163\_purine\_nucleotide\_metabolic\_process | ADK | 73 | 1 | 2.252935 | -0.441803 | 398 | 321.04 | 0.806633 |
| GO:0008284\_positive\_regulation\_of\_cell\_proliferation | PAX6 | 208 | 2 | 1.581387 | -0.440061 | 399 | 321.77 | 0.806441 |
| GO:0008284\_positive\_regulation\_of\_cell\_proliferation | BMPR1A | 208 | 2 | 1.581387 | -0.440061 | 399 | 321.77 | 0.806441 |
| GO:0048468\_cell\_development | PAX6 | 654 | 5 | 1.257372 | -0.436845 | 400 | 322.71 | 0.806775 |
| GO:0048468\_cell\_development | NEO1 | 654 | 5 | 1.257372 | -0.436845 | 400 | 322.71 | 0.806775 |
| GO:0048468\_cell\_development | AGRN | 654 | 5 | 1.257372 | -0.436845 | 400 | 322.71 | 0.806775 |
| GO:0048468\_cell\_development | YWHAE | 654 | 5 | 1.257372 | -0.436845 | 400 | 322.71 | 0.806775 |
| GO:0048468\_cell\_development | BMPR1A | 654 | 5 | 1.257372 | -0.436845 | 400 | 322.71 | 0.806775 |
| GO:0019538\_protein\_metabolic\_process | CCNE1 | 655 | 5 | 1.255453 | -0.435322 | 401 | 322.84 | 0.805087 |
| GO:0019538\_protein\_metabolic\_process | CCND1 | 655 | 5 | 1.255453 | -0.435322 | 401 | 322.84 | 0.805087 |
| GO:0019538\_protein\_metabolic\_process | UBE2D3 | 655 | 5 | 1.255453 | -0.435322 | 401 | 322.84 | 0.805087 |
| GO:0019538\_protein\_metabolic\_process | HSP90AA1 | 655 | 5 | 1.255453 | -0.435322 | 401 | 322.84 | 0.805087 |
| GO:0019538\_protein\_metabolic\_process | YWHAE | 655 | 5 | 1.255453 | -0.435322 | 401 | 322.84 | 0.805087 |
| GO:0016043\_cellular\_component\_organization | STX6 | 964 | 7 | 1.194243 | -0.434462 | 402 | 322.93 | 0.803308 |
| GO:0016043\_cellular\_component\_organization | GPX4 | 964 | 7 | 1.194243 | -0.434462 | 402 | 322.93 | 0.803308 |
| GO:0016043\_cellular\_component\_organization | PAX6 | 964 | 7 | 1.194243 | -0.434462 | 402 | 322.93 | 0.803308 |
| GO:0016043\_cellular\_component\_organization | RHOQ | 964 | 7 | 1.194243 | -0.434462 | 402 | 322.93 | 0.803308 |
| GO:0016043\_cellular\_component\_organization | RDX | 964 | 7 | 1.194243 | -0.434462 | 402 | 322.93 | 0.803308 |
| GO:0016043\_cellular\_component\_organization | AGRN | 964 | 7 | 1.194243 | -0.434462 | 402 | 322.93 | 0.803308 |
| GO:0016043\_cellular\_component\_organization | NEO1 | 964 | 7 | 1.194243 | -0.434462 | 402 | 322.93 | 0.803308 |
| GO:0044265\_cellular\_macromolecule\_catabolic\_process | UBE2D3 | 75 | 1 | 2.192857 | -0.432458 | 403 | 324.13 | 0.804293 |
| GO:0006508\_proteolysis | UBE2D3 | 76 | 1 | 2.164004 | -0.427900 | 406 | 326.09 | 0.803177 |
| GO:0009725\_response\_to\_hormone\_stimulus | RHOQ | 76 | 1 | 2.164004 | -0.427900 | 406 | 326.09 | 0.803177 |
| GO:0034621\_cellular\_macromolecular\_complex\_subunit\_organization | AGRN | 76 | 1 | 2.164004 | -0.427900 | 406 | 326.09 | 0.803177 |
| GO:0006461\_protein\_complex\_assembly | AGRN | 78 | 1 | 2.108516 | -0.419005 | 409 | 329.39 | 0.805355 |
| GO:0051251\_positive\_regulation\_of\_lymphocyte\_activation | HSP90AA1 | 78 | 1 | 2.108516 | -0.419005 | 409 | 329.39 | 0.805355 |
| GO:0070271\_protein\_complex\_biogenesis | AGRN | 78 | 1 | 2.108516 | -0.419005 | 409 | 329.39 | 0.805355 |
| GO:0044092\_negative\_regulation\_of\_molecular\_function | NUP62 | 80 | 1 | 2.055804 | -0.410391 | 410 | 333.21 | 0.812707 |
| GO:0001701\_in\_utero\_embryonic\_development | CSDA | 221 | 2 | 1.488365 | -0.406864 | 411 | 333.92 | 0.812457 |
| GO:0001701\_in\_utero\_embryonic\_development | BMPR1A | 221 | 2 | 1.488365 | -0.406864 | 411 | 333.92 | 0.812457 |
| GO:0051641\_cellular\_localization | PAX6 | 370 | 3 | 1.333494 | -0.405122 | 412 | 334.6 | 0.812136 |
| GO:0051641\_cellular\_localization | MYBBP1A | 370 | 3 | 1.333494 | -0.405122 | 412 | 334.6 | 0.812136 |
| GO:0051641\_cellular\_localization | YWHAE | 370 | 3 | 1.333494 | -0.405122 | 412 | 334.6 | 0.812136 |
| GO:0002696\_positive\_regulation\_of\_leukocyte\_activation | HSP90AA1 | 82 | 1 | 2.005662 | -0.402044 | 415 | 336.61 | 0.811108 |
| GO:0007411\_axon\_guidance | PAX6 | 82 | 1 | 2.005662 | -0.402044 | 415 | 336.61 | 0.811108 |
| GO:0045664\_regulation\_of\_neuron\_differentiation | PAX6 | 82 | 1 | 2.005662 | -0.402044 | 415 | 336.61 | 0.811108 |
| GO:0006325\_chromatin\_organization | GPX4 | 83 | 1 | 1.981497 | -0.397966 | 417 | 338.65 | 0.812110 |
| GO:0050867\_positive\_regulation\_of\_cell\_activation | HSP90AA1 | 83 | 1 | 1.981497 | -0.397966 | 417 | 338.65 | 0.812110 |
| GO:0045137\_development\_of\_primary\_sexual\_characteristics | CSDA | 84 | 1 | 1.957908 | -0.393951 | 418 | 339.53 | 0.812273 |
| GO:0000279\_M\_phase | SMC1A | 85 | 1 | 1.934874 | -0.389996 | 419 | 341.19 | 0.814296 |
| GO:0006605\_protein\_targeting | YWHAE | 86 | 1 | 1.912375 | -0.386100 | 423 | 343.92 | 0.813050 |
| GO:0032504\_multicellular\_organism\_reproduction | CCND1 | 86 | 1 | 1.912375 | -0.386100 | 423 | 343.92 | 0.813050 |
| GO:0034641\_cellular\_nitrogen\_compound\_metabolic\_process | HSP90AA1 | 86 | 1 | 1.912375 | -0.386100 | 423 | 343.92 | 0.813050 |
| GO:0048609\_reproductive\_process\_in\_a\_multicellular\_organism | CCND1 | 86 | 1 | 1.912375 | -0.386100 | 423 | 343.92 | 0.813050 |
| GO:0007178\_transmembrane\_receptor\_protein\_serine\_threonine\_kinase\_signaling\_pathway | BMPR1A | 87 | 1 | 1.890394 | -0.382261 | 425 | 346.45 | 0.815176 |
| GO:0022612\_gland\_morphogenesis | PAX6 | 87 | 1 | 1.890394 | -0.382261 | 425 | 346.45 | 0.815176 |
| GO:0043687\_post-translational\_protein\_modification | CCNE1 | 384 | 3 | 1.284877 | -0.379680 | 426 | 346.76 | 0.813991 |
| GO:0043687\_post-translational\_protein\_modification | CCND1 | 384 | 3 | 1.284877 | -0.379680 | 426 | 346.76 | 0.813991 |
| GO:0043687\_post-translational\_protein\_modification | YWHAE | 384 | 3 | 1.284877 | -0.379680 | 426 | 346.76 | 0.813991 |
| GO:0050863\_regulation\_of\_T\_cell\_activation | HSP90AA1 | 88 | 1 | 1.868912 | -0.378479 | 427 | 348.07 | 0.815152 |
| GO:0008152\_metabolic\_process | IMPA1 | 2133 | 14 | 1.079466 | -0.378253 | 428 | 348.19 | 0.813528 |
| GO:0008152\_metabolic\_process | HSP90AA1 | 2133 | 14 | 1.079466 | -0.378253 | 428 | 348.19 | 0.813528 |
| GO:0008152\_metabolic\_process | PAX6 | 2133 | 14 | 1.079466 | -0.378253 | 428 | 348.19 | 0.813528 |
| GO:0008152\_metabolic\_process | NEO1 | 2133 | 14 | 1.079466 | -0.378253 | 428 | 348.19 | 0.813528 |
| GO:0008152\_metabolic\_process | YWHAE | 2133 | 14 | 1.079466 | -0.378253 | 428 | 348.19 | 0.813528 |
| GO:0008152\_metabolic\_process | ZIC3 | 2133 | 14 | 1.079466 | -0.378253 | 428 | 348.19 | 0.813528 |
| GO:0008152\_metabolic\_process | CCNE1 | 2133 | 14 | 1.079466 | -0.378253 | 428 | 348.19 | 0.813528 |
| GO:0008152\_metabolic\_process | CCND1 | 2133 | 14 | 1.079466 | -0.378253 | 428 | 348.19 | 0.813528 |
| GO:0008152\_metabolic\_process | ATF4 | 2133 | 14 | 1.079466 | -0.378253 | 428 | 348.19 | 0.813528 |
| GO:0008152\_metabolic\_process | UBE2D3 | 2133 | 14 | 1.079466 | -0.378253 | 428 | 348.19 | 0.813528 |
| GO:0008152\_metabolic\_process | NUP62 | 2133 | 14 | 1.079466 | -0.378253 | 428 | 348.19 | 0.813528 |
| GO:0008152\_metabolic\_process | ADK | 2133 | 14 | 1.079466 | -0.378253 | 428 | 348.19 | 0.813528 |
| GO:0008152\_metabolic\_process | AGRN | 2133 | 14 | 1.079466 | -0.378253 | 428 | 348.19 | 0.813528 |
| GO:0008152\_metabolic\_process | MYBBP1A | 2133 | 14 | 1.079466 | -0.378253 | 428 | 348.19 | 0.813528 |
| GO:0044237\_cellular\_metabolic\_process | HSP90AA1 | 1974 | 13 | 1.083098 | -0.375483 | 429 | 348.81 | 0.813077 |
| GO:0044237\_cellular\_metabolic\_process | PAX6 | 1974 | 13 | 1.083098 | -0.375483 | 429 | 348.81 | 0.813077 |
| GO:0044237\_cellular\_metabolic\_process | NEO1 | 1974 | 13 | 1.083098 | -0.375483 | 429 | 348.81 | 0.813077 |
| GO:0044237\_cellular\_metabolic\_process | YWHAE | 1974 | 13 | 1.083098 | -0.375483 | 429 | 348.81 | 0.813077 |
| GO:0044237\_cellular\_metabolic\_process | ZIC3 | 1974 | 13 | 1.083098 | -0.375483 | 429 | 348.81 | 0.813077 |
| GO:0044237\_cellular\_metabolic\_process | CCNE1 | 1974 | 13 | 1.083098 | -0.375483 | 429 | 348.81 | 0.813077 |
| GO:0044237\_cellular\_metabolic\_process | ATF4 | 1974 | 13 | 1.083098 | -0.375483 | 429 | 348.81 | 0.813077 |
| GO:0044237\_cellular\_metabolic\_process | CCND1 | 1974 | 13 | 1.083098 | -0.375483 | 429 | 348.81 | 0.813077 |
| GO:0044237\_cellular\_metabolic\_process | UBE2D3 | 1974 | 13 | 1.083098 | -0.375483 | 429 | 348.81 | 0.813077 |
| GO:0044237\_cellular\_metabolic\_process | NUP62 | 1974 | 13 | 1.083098 | -0.375483 | 429 | 348.81 | 0.813077 |
| GO:0044237\_cellular\_metabolic\_process | ADK | 1974 | 13 | 1.083098 | -0.375483 | 429 | 348.81 | 0.813077 |
| GO:0044237\_cellular\_metabolic\_process | AGRN | 1974 | 13 | 1.083098 | -0.375483 | 429 | 348.81 | 0.813077 |
| GO:0044237\_cellular\_metabolic\_process | MYBBP1A | 1974 | 13 | 1.083098 | -0.375483 | 429 | 348.81 | 0.813077 |
| GO:0030324\_lung\_development | BMPR1A | 90 | 1 | 1.827381 | -0.371079 | 430 | 351.04 | 0.816372 |
| GO:0006468\_protein\_amino\_acid\_phosphorylation | CCNE1 | 237 | 2 | 1.387884 | -0.369949 | 432 | 351.81 | 0.814375 |
| GO:0006468\_protein\_amino\_acid\_phosphorylation | CCND1 | 237 | 2 | 1.387884 | -0.369949 | 432 | 351.81 | 0.814375 |
| GO:0044085\_cellular\_component\_biogenesis | RDX | 237 | 2 | 1.387884 | -0.369949 | 432 | 351.81 | 0.814375 |
| GO:0044085\_cellular\_component\_biogenesis | AGRN | 237 | 2 | 1.387884 | -0.369949 | 432 | 351.81 | 0.814375 |
| GO:0008544\_epidermis\_development | PAX6 | 91 | 1 | 1.807300 | -0.367458 | 434 | 353.44 | 0.814378 |
| GO:0031399\_regulation\_of\_protein\_modification\_process | YWHAE | 91 | 1 | 1.807300 | -0.367458 | 434 | 353.44 | 0.814378 |
| GO:0042127\_regulation\_of\_cell\_proliferation | NUP62 | 393 | 3 | 1.255453 | -0.364200 | 435 | 353.68 | 0.813057 |
| GO:0042127\_regulation\_of\_cell\_proliferation | PAX6 | 393 | 3 | 1.255453 | -0.364200 | 435 | 353.68 | 0.813057 |
| GO:0042127\_regulation\_of\_cell\_proliferation | BMPR1A | 393 | 3 | 1.255453 | -0.364200 | 435 | 353.68 | 0.813057 |
| GO:0009719\_response\_to\_endogenous\_stimulus | RHOQ | 92 | 1 | 1.787655 | -0.363888 | 438 | 354.62 | 0.809635 |
| GO:0030217\_T\_cell\_differentiation | HSP90AA1 | 92 | 1 | 1.787655 | -0.363888 | 438 | 354.62 | 0.809635 |
| GO:0030323\_respiratory\_tube\_development | BMPR1A | 92 | 1 | 1.787655 | -0.363888 | 438 | 354.62 | 0.809635 |
| GO:0035107\_appendage\_morphogenesis | BMPR1A | 93 | 1 | 1.768433 | -0.360369 | 441 | 356.07 | 0.807415 |
| GO:0035108\_limb\_morphogenesis | BMPR1A | 93 | 1 | 1.768433 | -0.360369 | 441 | 356.07 | 0.807415 |
| GO:0065003\_macromolecular\_complex\_assembly | AGRN | 93 | 1 | 1.768433 | -0.360369 | 441 | 356.07 | 0.807415 |
| GO:0048699\_generation\_of\_neurons | PAX6 | 396 | 3 | 1.245942 | -0.359185 | 442 | 356.34 | 0.806199 |
| GO:0048699\_generation\_of\_neurons | YWHAE | 396 | 3 | 1.245942 | -0.359185 | 442 | 356.34 | 0.806199 |
| GO:0048699\_generation\_of\_neurons | BMPR1A | 396 | 3 | 1.245942 | -0.359185 | 442 | 356.34 | 0.806199 |
| GO:0006753\_nucleoside\_phosphate\_metabolic\_process | ADK | 94 | 1 | 1.749620 | -0.356898 | 444 | 358.37 | 0.807140 |
| GO:0009117\_nucleotide\_metabolic\_process | ADK | 94 | 1 | 1.749620 | -0.356898 | 444 | 358.37 | 0.807140 |
| GO:0048736\_appendage\_development | BMPR1A | 96 | 1 | 1.713170 | -0.350101 | 447 | 360.6 | 0.806711 |
| GO:0060173\_limb\_development | BMPR1A | 96 | 1 | 1.713170 | -0.350101 | 447 | 360.6 | 0.806711 |
| GO:0060249\_anatomical\_structure\_homeostasis | BMPR1A | 96 | 1 | 1.713170 | -0.350101 | 447 | 360.6 | 0.806711 |
| GO:0060541\_respiratory\_system\_development | BMPR1A | 98 | 1 | 1.678207 | -0.343487 | 448 | 363.16 | 0.810625 |
| GO:0048513\_organ\_development | CCND1 | 1365 | 9 | 1.084380 | -0.341212 | 449 | 363.51 | 0.809599 |
| GO:0048513\_organ\_development | HSP90AA1 | 1365 | 9 | 1.084380 | -0.341212 | 449 | 363.51 | 0.809599 |
| GO:0048513\_organ\_development | PAX6 | 1365 | 9 | 1.084380 | -0.341212 | 449 | 363.51 | 0.809599 |
| GO:0048513\_organ\_development | NEO1 | 1365 | 9 | 1.084380 | -0.341212 | 449 | 363.51 | 0.809599 |
| GO:0048513\_organ\_development | AGRN | 1365 | 9 | 1.084380 | -0.341212 | 449 | 363.51 | 0.809599 |
| GO:0048513\_organ\_development | CSDA | 1365 | 9 | 1.084380 | -0.341212 | 449 | 363.51 | 0.809599 |
| GO:0048513\_organ\_development | YWHAE | 1365 | 9 | 1.084380 | -0.341212 | 449 | 363.51 | 0.809599 |
| GO:0048513\_organ\_development | ZIC3 | 1365 | 9 | 1.084380 | -0.341212 | 449 | 363.51 | 0.809599 |
| GO:0048513\_organ\_development | BMPR1A | 1365 | 9 | 1.084380 | -0.341212 | 449 | 363.51 | 0.809599 |
| GO:0009790\_embryonic\_development | SFRP2 | 567 | 4 | 1.160242 | -0.338611 | 450 | 365.54 | 0.812311 |
| GO:0009790\_embryonic\_development | PAX6 | 567 | 4 | 1.160242 | -0.338611 | 450 | 365.54 | 0.812311 |
| GO:0009790\_embryonic\_development | CSDA | 567 | 4 | 1.160242 | -0.338611 | 450 | 365.54 | 0.812311 |
| GO:0009790\_embryonic\_development | BMPR1A | 567 | 4 | 1.160242 | -0.338611 | 450 | 365.54 | 0.812311 |
| GO:0030163\_protein\_catabolic\_process | UBE2D3 | 101 | 1 | 1.628359 | -0.333895 | 451 | 367.55 | 0.814967 |
| GO:0009966\_regulation\_of\_signal\_transduction | CCND1 | 256 | 2 | 1.284877 | -0.330997 | 452 | 368.11 | 0.814403 |
| GO:0009966\_regulation\_of\_signal\_transduction | NUP62 | 256 | 2 | 1.284877 | -0.330997 | 452 | 368.11 | 0.814403 |
| GO:0030036\_actin\_cytoskeleton\_organization | RHOQ | 102 | 1 | 1.612395 | -0.330782 | 453 | 368.48 | 0.813422 |
| GO:0006355\_regulation\_of\_transcription\_\_DNA-dependent | ATF4 | 575 | 4 | 1.144099 | -0.327998 | 454 | 369.06 | 0.812907 |
| GO:0006355\_regulation\_of\_transcription\_\_DNA-dependent | PAX6 | 575 | 4 | 1.144099 | -0.327998 | 454 | 369.06 | 0.812907 |
| GO:0006355\_regulation\_of\_transcription\_\_DNA-dependent | AGRN | 575 | 4 | 1.144099 | -0.327998 | 454 | 369.06 | 0.812907 |
| GO:0006355\_regulation\_of\_transcription\_\_DNA-dependent | ZIC3 | 575 | 4 | 1.144099 | -0.327998 | 454 | 369.06 | 0.812907 |
| GO:0051179\_localization | DNAJA1 | 1058 | 7 | 1.088138 | -0.326333 | 455 | 370.48 | 0.814242 |
| GO:0051179\_localization | PAX6 | 1058 | 7 | 1.088138 | -0.326333 | 455 | 370.48 | 0.814242 |
| GO:0051179\_localization | RHOQ | 1058 | 7 | 1.088138 | -0.326333 | 455 | 370.48 | 0.814242 |
| GO:0051179\_localization | RDX | 1058 | 7 | 1.088138 | -0.326333 | 455 | 370.48 | 0.814242 |
| GO:0051179\_localization | TMSB4X | 1058 | 7 | 1.088138 | -0.326333 | 455 | 370.48 | 0.814242 |
| GO:0051179\_localization | MYBBP1A | 1058 | 7 | 1.088138 | -0.326333 | 455 | 370.48 | 0.814242 |
| GO:0051179\_localization | YWHAE | 1058 | 7 | 1.088138 | -0.326333 | 455 | 370.48 | 0.814242 |
| GO:0055086\_nucleobase\_\_nucleoside\_and\_nucleotide\_metabolic\_process | ADK | 104 | 1 | 1.581387 | -0.324677 | 456 | 371.21 | 0.814057 |
| GO:0030154\_cell\_differentiation | CCND1 | 1060 | 7 | 1.086085 | -0.324304 | 457 | 371.35 | 0.812582 |
| GO:0030154\_cell\_differentiation | HSP90AA1 | 1060 | 7 | 1.086085 | -0.324304 | 457 | 371.35 | 0.812582 |
| GO:0030154\_cell\_differentiation | PAX6 | 1060 | 7 | 1.086085 | -0.324304 | 457 | 371.35 | 0.812582 |
| GO:0030154\_cell\_differentiation | AGRN | 1060 | 7 | 1.086085 | -0.324304 | 457 | 371.35 | 0.812582 |
| GO:0030154\_cell\_differentiation | NEO1 | 1060 | 7 | 1.086085 | -0.324304 | 457 | 371.35 | 0.812582 |
| GO:0030154\_cell\_differentiation | YWHAE | 1060 | 7 | 1.086085 | -0.324304 | 457 | 371.35 | 0.812582 |
| GO:0030154\_cell\_differentiation | BMPR1A | 1060 | 7 | 1.086085 | -0.324304 | 457 | 371.35 | 0.812582 |
| GO:0010467\_gene\_expression | ATF4 | 905 | 6 | 1.090371 | -0.317830 | 458 | 373.64 | 0.815808 |
| GO:0010467\_gene\_expression | PAX6 | 905 | 6 | 1.090371 | -0.317830 | 458 | 373.64 | 0.815808 |
| GO:0010467\_gene\_expression | NEO1 | 905 | 6 | 1.090371 | -0.317830 | 458 | 373.64 | 0.815808 |
| GO:0010467\_gene\_expression | AGRN | 905 | 6 | 1.090371 | -0.317830 | 458 | 373.64 | 0.815808 |
| GO:0010467\_gene\_expression | MYBBP1A | 905 | 6 | 1.090371 | -0.317830 | 458 | 373.64 | 0.815808 |
| GO:0010467\_gene\_expression | ZIC3 | 905 | 6 | 1.090371 | -0.317830 | 458 | 373.64 | 0.815808 |
| GO:0030030\_cell\_projection\_organization | PAX6 | 263 | 2 | 1.250679 | -0.317827 | 459 | 373.9 | 0.814597 |
| GO:0030030\_cell\_projection\_organization | RDX | 263 | 2 | 1.250679 | -0.317827 | 459 | 373.9 | 0.814597 |
| GO:0022008\_neurogenesis | PAX6 | 423 | 3 | 1.166413 | -0.317086 | 460 | 374.14 | 0.813348 |
| GO:0022008\_neurogenesis | YWHAE | 423 | 3 | 1.166413 | -0.317086 | 460 | 374.14 | 0.813348 |
| GO:0022008\_neurogenesis | BMPR1A | 423 | 3 | 1.166413 | -0.317086 | 460 | 374.14 | 0.813348 |
| GO:0045859\_regulation\_of\_protein\_kinase\_activity | NUP62 | 107 | 1 | 1.537049 | -0.315810 | 461 | 374.89 | 0.813210 |
| GO:0030029\_actin\_filament-based\_process | RHOQ | 109 | 1 | 1.508847 | -0.310084 | 462 | 376.81 | 0.815606 |
| GO:0051252\_regulation\_of\_RNA\_metabolic\_process | ATF4 | 590 | 4 | 1.115012 | -0.308938 | 463 | 377.01 | 0.814276 |
| GO:0051252\_regulation\_of\_RNA\_metabolic\_process | PAX6 | 590 | 4 | 1.115012 | -0.308938 | 463 | 377.01 | 0.814276 |
| GO:0051252\_regulation\_of\_RNA\_metabolic\_process | AGRN | 590 | 4 | 1.115012 | -0.308938 | 463 | 377.01 | 0.814276 |
| GO:0051252\_regulation\_of\_RNA\_metabolic\_process | ZIC3 | 590 | 4 | 1.115012 | -0.308938 | 463 | 377.01 | 0.814276 |
| GO:0043010\_camera-type\_eye\_development | PAX6 | 110 | 1 | 1.495130 | -0.307274 | 464 | 378.48 | 0.815690 |
| GO:0006351\_transcription\_\_DNA-dependent | ATF4 | 594 | 4 | 1.107504 | -0.304034 | 465 | 379.54 | 0.816215 |
| GO:0006351\_transcription\_\_DNA-dependent | PAX6 | 594 | 4 | 1.107504 | -0.304034 | 465 | 379.54 | 0.816215 |
| GO:0006351\_transcription\_\_DNA-dependent | AGRN | 594 | 4 | 1.107504 | -0.304034 | 465 | 379.54 | 0.816215 |
| GO:0006351\_transcription\_\_DNA-dependent | ZIC3 | 594 | 4 | 1.107504 | -0.304034 | 465 | 379.54 | 0.816215 |
| GO:0032774\_RNA\_biosynthetic\_process | ATF4 | 595 | 4 | 1.105642 | -0.302819 | 466 | 380.14 | 0.815751 |
| GO:0032774\_RNA\_biosynthetic\_process | PAX6 | 595 | 4 | 1.105642 | -0.302819 | 466 | 380.14 | 0.815751 |
| GO:0032774\_RNA\_biosynthetic\_process | AGRN | 595 | 4 | 1.105642 | -0.302819 | 466 | 380.14 | 0.815751 |
| GO:0032774\_RNA\_biosynthetic\_process | ZIC3 | 595 | 4 | 1.105642 | -0.302819 | 466 | 380.14 | 0.815751 |
| GO:0043549\_regulation\_of\_kinase\_activity | NUP62 | 112 | 1 | 1.468431 | -0.301759 | 468 | 381.22 | 0.814573 |
| GO:0051249\_regulation\_of\_lymphocyte\_activation | HSP90AA1 | 112 | 1 | 1.468431 | -0.301759 | 468 | 381.22 | 0.814573 |
| GO:0032502\_developmental\_process | HSP90AA1 | 2060 | 13 | 1.037881 | -0.299998 | 469 | 382.16 | 0.814840 |
| GO:0032502\_developmental\_process | PAX6 | 2060 | 13 | 1.037881 | -0.299998 | 469 | 382.16 | 0.814840 |
| GO:0032502\_developmental\_process | RHOQ | 2060 | 13 | 1.037881 | -0.299998 | 469 | 382.16 | 0.814840 |
| GO:0032502\_developmental\_process | NEO1 | 2060 | 13 | 1.037881 | -0.299998 | 469 | 382.16 | 0.814840 |
| GO:0032502\_developmental\_process | CSDA | 2060 | 13 | 1.037881 | -0.299998 | 469 | 382.16 | 0.814840 |
| GO:0032502\_developmental\_process | YWHAE | 2060 | 13 | 1.037881 | -0.299998 | 469 | 382.16 | 0.814840 |
| GO:0032502\_developmental\_process | ZIC3 | 2060 | 13 | 1.037881 | -0.299998 | 469 | 382.16 | 0.814840 |
| GO:0032502\_developmental\_process | CCND1 | 2060 | 13 | 1.037881 | -0.299998 | 469 | 382.16 | 0.814840 |
| GO:0032502\_developmental\_process | EI24 | 2060 | 13 | 1.037881 | -0.299998 | 469 | 382.16 | 0.814840 |
| GO:0032502\_developmental\_process | NUP62 | 2060 | 13 | 1.037881 | -0.299998 | 469 | 382.16 | 0.814840 |
| GO:0032502\_developmental\_process | SFRP2 | 2060 | 13 | 1.037881 | -0.299998 | 469 | 382.16 | 0.814840 |
| GO:0032502\_developmental\_process | AGRN | 2060 | 13 | 1.037881 | -0.299998 | 469 | 382.16 | 0.814840 |
| GO:0032502\_developmental\_process | BMPR1A | 2060 | 13 | 1.037881 | -0.299998 | 469 | 382.16 | 0.814840 |
| GO:0006464\_protein\_modification\_process | CCNE1 | 439 | 3 | 1.123902 | -0.294516 | 470 | 384.64 | 0.818383 |
| GO:0006464\_protein\_modification\_process | CCND1 | 439 | 3 | 1.123902 | -0.294516 | 470 | 384.64 | 0.818383 |
| GO:0006464\_protein\_modification\_process | YWHAE | 439 | 3 | 1.123902 | -0.294516 | 470 | 384.64 | 0.818383 |
| GO:0044238\_primary\_metabolic\_process | CCNE1 | 1905 | 12 | 1.035996 | -0.293896 | 471 | 384.8 | 0.816985 |
| GO:0044238\_primary\_metabolic\_process | CCND1 | 1905 | 12 | 1.035996 | -0.293896 | 471 | 384.8 | 0.816985 |
| GO:0044238\_primary\_metabolic\_process | UBE2D3 | 1905 | 12 | 1.035996 | -0.293896 | 471 | 384.8 | 0.816985 |
| GO:0044238\_primary\_metabolic\_process | ATF4 | 1905 | 12 | 1.035996 | -0.293896 | 471 | 384.8 | 0.816985 |
| GO:0044238\_primary\_metabolic\_process | HSP90AA1 | 1905 | 12 | 1.035996 | -0.293896 | 471 | 384.8 | 0.816985 |
| GO:0044238\_primary\_metabolic\_process | ADK | 1905 | 12 | 1.035996 | -0.293896 | 471 | 384.8 | 0.816985 |
| GO:0044238\_primary\_metabolic\_process | PAX6 | 1905 | 12 | 1.035996 | -0.293896 | 471 | 384.8 | 0.816985 |
| GO:0044238\_primary\_metabolic\_process | NEO1 | 1905 | 12 | 1.035996 | -0.293896 | 471 | 384.8 | 0.816985 |
| GO:0044238\_primary\_metabolic\_process | AGRN | 1905 | 12 | 1.035996 | -0.293896 | 471 | 384.8 | 0.816985 |
| GO:0044238\_primary\_metabolic\_process | YWHAE | 1905 | 12 | 1.035996 | -0.293896 | 471 | 384.8 | 0.816985 |
| GO:0044238\_primary\_metabolic\_process | MYBBP1A | 1905 | 12 | 1.035996 | -0.293896 | 471 | 384.8 | 0.816985 |
| GO:0044238\_primary\_metabolic\_process | ZIC3 | 1905 | 12 | 1.035996 | -0.293896 | 471 | 384.8 | 0.816985 |
| GO:0051338\_regulation\_of\_transferase\_activity | NUP62 | 115 | 1 | 1.430124 | -0.293736 | 472 | 385.53 | 0.816801 |
| GO:0048646\_anatomical\_structure\_formation\_involved\_in\_morphogenesis | NEO1 | 277 | 2 | 1.187468 | -0.293196 | 473 | 386.11 | 0.816300 |
| GO:0048646\_anatomical\_structure\_formation\_involved\_in\_morphogenesis | BMPR1A | 277 | 2 | 1.187468 | -0.293196 | 473 | 386.11 | 0.816300 |
| GO:0046483\_heterocycle\_metabolic\_process | ADK | 116 | 1 | 1.417796 | -0.291125 | 475 | 387.24 | 0.815242 |
| GO:0048608\_reproductive\_structure\_development | CSDA | 116 | 1 | 1.417796 | -0.291125 | 475 | 387.24 | 0.815242 |
| GO:0065009\_regulation\_of\_molecular\_function | NUP62 | 279 | 2 | 1.178955 | -0.289852 | 476 | 388.0 | 0.815126 |
| GO:0065009\_regulation\_of\_molecular\_function | CALM1 | 279 | 2 | 1.178955 | -0.289852 | 476 | 388.0 | 0.815126 |
| GO:0043933\_macromolecular\_complex\_subunit\_organization | AGRN | 117 | 1 | 1.405678 | -0.288546 | 477 | 388.45 | 0.814361 |
| GO:0022403\_cell\_cycle\_phase | SMC1A | 119 | 1 | 1.382053 | -0.283479 | 478 | 391.06 | 0.818117 |
| GO:0000902\_cell\_morphogenesis | PAX6 | 283 | 2 | 1.162292 | -0.283288 | 479 | 391.33 | 0.816973 |
| GO:0000902\_cell\_morphogenesis | RHOQ | 283 | 2 | 1.162292 | -0.283288 | 479 | 391.33 | 0.816973 |
| GO:0006996\_organelle\_organization | STX6 | 449 | 3 | 1.098871 | -0.281229 | 480 | 391.89 | 0.816437 |
| GO:0006996\_organelle\_organization | GPX4 | 449 | 3 | 1.098871 | -0.281229 | 480 | 391.89 | 0.816437 |
| GO:0006996\_organelle\_organization | RHOQ | 449 | 3 | 1.098871 | -0.281229 | 480 | 391.89 | 0.816437 |
| GO:0002694\_regulation\_of\_leukocyte\_activation | HSP90AA1 | 121 | 1 | 1.359209 | -0.278531 | 483 | 394.55 | 0.816874 |
| GO:0006917\_induction\_of\_apoptosis | EI24 | 121 | 1 | 1.359209 | -0.278531 | 483 | 394.55 | 0.816874 |
| GO:0012502\_induction\_of\_programmed\_cell\_death | EI24 | 121 | 1 | 1.359209 | -0.278531 | 483 | 394.55 | 0.816874 |
| GO:0006886\_intracellular\_protein\_transport | YWHAE | 122 | 1 | 1.348068 | -0.276100 | 485 | 397.24 | 0.819052 |
| GO:0050865\_regulation\_of\_cell\_activation | HSP90AA1 | 122 | 1 | 1.348068 | -0.276100 | 485 | 397.24 | 0.819052 |
| GO:0007399\_nervous\_system\_development | PAX6 | 621 | 4 | 1.059351 | -0.272780 | 486 | 397.55 | 0.818004 |
| GO:0007399\_nervous\_system\_development | AGRN | 621 | 4 | 1.059351 | -0.272780 | 486 | 397.55 | 0.818004 |
| GO:0007399\_nervous\_system\_development | YWHAE | 621 | 4 | 1.059351 | -0.272780 | 486 | 397.55 | 0.818004 |
| GO:0007399\_nervous\_system\_development | BMPR1A | 621 | 4 | 1.059351 | -0.272780 | 486 | 397.55 | 0.818004 |
| GO:0030098\_lymphocyte\_differentiation | HSP90AA1 | 124 | 1 | 1.326325 | -0.271321 | 487 | 398.34 | 0.817947 |
| GO:0043412\_biopolymer\_modification | CCNE1 | 458 | 3 | 1.077277 | -0.269777 | 488 | 398.97 | 0.817561 |
| GO:0043412\_biopolymer\_modification | CCND1 | 458 | 3 | 1.077277 | -0.269777 | 488 | 398.97 | 0.817561 |
| GO:0043412\_biopolymer\_modification | YWHAE | 458 | 3 | 1.077277 | -0.269777 | 488 | 398.97 | 0.817561 |
| GO:0043062\_extracellular\_structure\_organization | AGRN | 125 | 1 | 1.315714 | -0.268972 | 489 | 399.6 | 0.817178 |
| GO:0009653\_anatomical\_structure\_morphogenesis | CCND1 | 958 | 6 | 1.030048 | -0.265839 | 490 | 400.01 | 0.816347 |
| GO:0009653\_anatomical\_structure\_morphogenesis | PAX6 | 958 | 6 | 1.030048 | -0.265839 | 490 | 400.01 | 0.816347 |
| GO:0009653\_anatomical\_structure\_morphogenesis | RHOQ | 958 | 6 | 1.030048 | -0.265839 | 490 | 400.01 | 0.816347 |
| GO:0009653\_anatomical\_structure\_morphogenesis | NEO1 | 958 | 6 | 1.030048 | -0.265839 | 490 | 400.01 | 0.816347 |
| GO:0009653\_anatomical\_structure\_morphogenesis | ZIC3 | 958 | 6 | 1.030048 | -0.265839 | 490 | 400.01 | 0.816347 |
| GO:0009653\_anatomical\_structure\_morphogenesis | BMPR1A | 958 | 6 | 1.030048 | -0.265839 | 490 | 400.01 | 0.816347 |
| GO:0043285\_biopolymer\_catabolic\_process | UBE2D3 | 129 | 1 | 1.274917 | -0.259841 | 492 | 402.99 | 0.819085 |
| GO:0051276\_chromosome\_organization | GPX4 | 129 | 1 | 1.274917 | -0.259841 | 492 | 402.99 | 0.819085 |
| GO:0032787\_monocarboxylic\_acid\_metabolic\_process | ATF4 | 130 | 1 | 1.265110 | -0.257621 | 494 | 404.33 | 0.818482 |
| GO:0045165\_cell\_fate\_commitment | PAX6 | 130 | 1 | 1.265110 | -0.257621 | 494 | 404.33 | 0.818482 |
| GO:0009887\_organ\_morphogenesis | CCND1 | 642 | 4 | 1.024700 | -0.250558 | 495 | 405.7 | 0.819596 |
| GO:0009887\_organ\_morphogenesis | PAX6 | 642 | 4 | 1.024700 | -0.250558 | 495 | 405.7 | 0.819596 |
| GO:0009887\_organ\_morphogenesis | ZIC3 | 642 | 4 | 1.024700 | -0.250558 | 495 | 405.7 | 0.819596 |
| GO:0009887\_organ\_morphogenesis | BMPR1A | 642 | 4 | 1.024700 | -0.250558 | 495 | 405.7 | 0.819596 |
| GO:0032989\_cellular\_component\_morphogenesis | PAX6 | 307 | 2 | 1.071429 | -0.247131 | 496 | 407.52 | 0.821613 |
| GO:0032989\_cellular\_component\_morphogenesis | RHOQ | 307 | 2 | 1.071429 | -0.247131 | 496 | 407.52 | 0.821613 |
| GO:0001654\_eye\_development | PAX6 | 136 | 1 | 1.209296 | -0.244807 | 497 | 408.68 | 0.822294 |
| GO:0009057\_macromolecule\_catabolic\_process | UBE2D3 | 137 | 1 | 1.200469 | -0.242752 | 498 | 409.46 | 0.822209 |
| GO:0016070\_RNA\_metabolic\_process | ATF4 | 658 | 4 | 0.999783 | -0.234759 | 499 | 411.46 | 0.824569 |
| GO:0016070\_RNA\_metabolic\_process | PAX6 | 658 | 4 | 0.999783 | -0.234759 | 499 | 411.46 | 0.824569 |
| GO:0016070\_RNA\_metabolic\_process | AGRN | 658 | 4 | 0.999783 | -0.234759 | 499 | 411.46 | 0.824569 |
| GO:0016070\_RNA\_metabolic\_process | ZIC3 | 658 | 4 | 0.999783 | -0.234759 | 499 | 411.46 | 0.824569 |
| GO:0002684\_positive\_regulation\_of\_immune\_system\_process | HSP90AA1 | 148 | 1 | 1.111245 | -0.221526 | 500 | 416.12 | 0.832240 |
| GO:0010646\_regulation\_of\_cell\_communication | CCND1 | 330 | 2 | 0.996753 | -0.217063 | 501 | 417.72 | 0.833772 |
| GO:0010646\_regulation\_of\_cell\_communication | NUP62 | 330 | 2 | 0.996753 | -0.217063 | 501 | 417.72 | 0.833772 |
| GO:0010605\_negative\_regulation\_of\_macromolecule\_metabolic\_process | YWHAE | 331 | 2 | 0.993742 | -0.215847 | 502 | 418.47 | 0.833606 |
| GO:0010605\_negative\_regulation\_of\_macromolecule\_metabolic\_process | MYBBP1A | 331 | 2 | 0.993742 | -0.215847 | 502 | 418.47 | 0.833606 |
| GO:0031324\_negative\_regulation\_of\_cellular\_metabolic\_process | MYBBP1A | 332 | 2 | 0.990749 | -0.214637 | 503 | 418.67 | 0.832346 |
| GO:0031324\_negative\_regulation\_of\_cellular\_metabolic\_process | YWHAE | 332 | 2 | 0.990749 | -0.214637 | 503 | 418.67 | 0.832346 |
| GO:0032268\_regulation\_of\_cellular\_protein\_metabolic\_process | YWHAE | 152 | 1 | 1.082002 | -0.214389 | 504 | 419.03 | 0.831409 |
| GO:0048856\_anatomical\_structure\_development | CCND1 | 1688 | 10 | 0.974314 | -0.213549 | 505 | 419.16 | 0.830020 |
| GO:0048856\_anatomical\_structure\_development | HSP90AA1 | 1688 | 10 | 0.974314 | -0.213549 | 505 | 419.16 | 0.830020 |
| GO:0048856\_anatomical\_structure\_development | PAX6 | 1688 | 10 | 0.974314 | -0.213549 | 505 | 419.16 | 0.830020 |
| GO:0048856\_anatomical\_structure\_development | RHOQ | 1688 | 10 | 0.974314 | -0.213549 | 505 | 419.16 | 0.830020 |
| GO:0048856\_anatomical\_structure\_development | AGRN | 1688 | 10 | 0.974314 | -0.213549 | 505 | 419.16 | 0.830020 |
| GO:0048856\_anatomical\_structure\_development | NEO1 | 1688 | 10 | 0.974314 | -0.213549 | 505 | 419.16 | 0.830020 |
| GO:0048856\_anatomical\_structure\_development | CSDA | 1688 | 10 | 0.974314 | -0.213549 | 505 | 419.16 | 0.830020 |
| GO:0048856\_anatomical\_structure\_development | YWHAE | 1688 | 10 | 0.974314 | -0.213549 | 505 | 419.16 | 0.830020 |
| GO:0048856\_anatomical\_structure\_development | ZIC3 | 1688 | 10 | 0.974314 | -0.213549 | 505 | 419.16 | 0.830020 |
| GO:0048856\_anatomical\_structure\_development | BMPR1A | 1688 | 10 | 0.974314 | -0.213549 | 505 | 419.16 | 0.830020 |
| GO:0007268\_synaptic\_transmission | AGRN | 154 | 1 | 1.067950 | -0.210927 | 506 | 420.07 | 0.830178 |
| GO:0009605\_response\_to\_external\_stimulus | LDHA | 339 | 2 | 0.970291 | -0.206365 | 507 | 421.71 | 0.831775 |
| GO:0009605\_response\_to\_external\_stimulus | SFRP2 | 339 | 2 | 0.970291 | -0.206365 | 507 | 421.71 | 0.831775 |
| GO:0007409\_axonogenesis | PAX6 | 158 | 1 | 1.040913 | -0.204208 | 508 | 424.19 | 0.835020 |
| GO:0065008\_regulation\_of\_biological\_quality | EI24 | 693 | 4 | 0.949289 | -0.203322 | 509 | 424.39 | 0.833772 |
| GO:0065008\_regulation\_of\_biological\_quality | RHOQ | 693 | 4 | 0.949289 | -0.203322 | 509 | 424.39 | 0.833772 |
| GO:0065008\_regulation\_of\_biological\_quality | AGRN | 693 | 4 | 0.949289 | -0.203322 | 509 | 424.39 | 0.833772 |
| GO:0065008\_regulation\_of\_biological\_quality | BMPR1A | 693 | 4 | 0.949289 | -0.203322 | 509 | 424.39 | 0.833772 |
| GO:0051649\_establishment\_of\_localization\_in\_cell | MYBBP1A | 342 | 2 | 0.961779 | -0.202923 | 510 | 424.65 | 0.832647 |
| GO:0051649\_establishment\_of\_localization\_in\_cell | YWHAE | 342 | 2 | 0.961779 | -0.202923 | 510 | 424.65 | 0.832647 |
| GO:0002521\_leukocyte\_differentiation | HSP90AA1 | 161 | 1 | 1.021517 | -0.199341 | 511 | 425.42 | 0.832524 |
| GO:0009892\_negative\_regulation\_of\_metabolic\_process | YWHAE | 348 | 2 | 0.945197 | -0.196214 | 512 | 426.8 | 0.833594 |
| GO:0009892\_negative\_regulation\_of\_metabolic\_process | MYBBP1A | 348 | 2 | 0.945197 | -0.196214 | 512 | 426.8 | 0.833594 |
| GO:0042110\_T\_cell\_activation | HSP90AA1 | 163 | 1 | 1.008983 | -0.196174 | 513 | 427.58 | 0.833489 |
| GO:0042325\_regulation\_of\_phosphorylation | NUP62 | 164 | 1 | 1.002831 | -0.194614 | 514 | 428.15 | 0.832977 |
| GO:0006259\_DNA\_metabolic\_process | CCNE1 | 165 | 1 | 0.996753 | -0.193068 | 515 | 429.24 | 0.833476 |
| GO:0043065\_positive\_regulation\_of\_apoptosis | EI24 | 166 | 1 | 0.990749 | -0.191538 | 516 | 429.94 | 0.833217 |
| GO:0010942\_positive\_regulation\_of\_cell\_death | EI24 | 167 | 1 | 0.984816 | -0.190022 | 518 | 431.04 | 0.832124 |
| GO:0043068\_positive\_regulation\_of\_programmed\_cell\_death | EI24 | 167 | 1 | 0.984816 | -0.190022 | 518 | 431.04 | 0.832124 |
| GO:0048812\_neuron\_projection\_morphogenesis | PAX6 | 170 | 1 | 0.967437 | -0.185561 | 520 | 432.7 | 0.832115 |
| GO:0051246\_regulation\_of\_protein\_metabolic\_process | YWHAE | 170 | 1 | 0.967437 | -0.185561 | 520 | 432.7 | 0.832115 |
| GO:0042981\_regulation\_of\_apoptosis | EI24 | 360 | 2 | 0.913690 | -0.183474 | 521 | 432.96 | 0.831017 |
| GO:0042981\_regulation\_of\_apoptosis | CSDA | 360 | 2 | 0.913690 | -0.183474 | 521 | 432.96 | 0.831017 |
| GO:0044093\_positive\_regulation\_of\_molecular\_function | CALM1 | 173 | 1 | 0.950661 | -0.181224 | 524 | 435.42 | 0.830954 |
| GO:0044248\_cellular\_catabolic\_process | UBE2D3 | 173 | 1 | 0.950661 | -0.181224 | 524 | 435.42 | 0.830954 |
| GO:0048667\_cell\_morphogenesis\_involved\_in\_neuron\_differentiation | PAX6 | 173 | 1 | 0.950661 | -0.181224 | 524 | 435.42 | 0.830954 |
| GO:0010941\_regulation\_of\_cell\_death | EI24 | 365 | 2 | 0.901174 | -0.178419 | 526 | 436.8 | 0.830418 |
| GO:0010941\_regulation\_of\_cell\_death | CSDA | 365 | 2 | 0.901174 | -0.178419 | 526 | 436.8 | 0.830418 |
| GO:0043067\_regulation\_of\_programmed\_cell\_death | EI24 | 365 | 2 | 0.901174 | -0.178419 | 526 | 436.8 | 0.830418 |
| GO:0043067\_regulation\_of\_programmed\_cell\_death | CSDA | 365 | 2 | 0.901174 | -0.178419 | 526 | 436.8 | 0.830418 |
| GO:0015031\_protein\_transport | YWHAE | 175 | 1 | 0.939796 | -0.178400 | 527 | 437.94 | 0.831006 |
| GO:0043066\_negative\_regulation\_of\_apoptosis | CSDA | 176 | 1 | 0.934456 | -0.177008 | 529 | 439.25 | 0.830340 |
| GO:0048858\_cell\_projection\_morphogenesis | PAX6 | 176 | 1 | 0.934456 | -0.177008 | 529 | 439.25 | 0.830340 |
| GO:0043069\_negative\_regulation\_of\_programmed\_cell\_death | CSDA | 179 | 1 | 0.918795 | -0.172908 | 531 | 441.63 | 0.831695 |
| GO:0060548\_negative\_regulation\_of\_cell\_death | CSDA | 179 | 1 | 0.918795 | -0.172908 | 531 | 441.63 | 0.831695 |
| GO:0007275\_multicellular\_organismal\_development | CCND1 | 1760 | 10 | 0.934456 | -0.170325 | 532 | 442.61 | 0.831974 |
| GO:0007275\_multicellular\_organismal\_development | HSP90AA1 | 1760 | 10 | 0.934456 | -0.170325 | 532 | 442.61 | 0.831974 |
| GO:0007275\_multicellular\_organismal\_development | SFRP2 | 1760 | 10 | 0.934456 | -0.170325 | 532 | 442.61 | 0.831974 |
| GO:0007275\_multicellular\_organismal\_development | PAX6 | 1760 | 10 | 0.934456 | -0.170325 | 532 | 442.61 | 0.831974 |
| GO:0007275\_multicellular\_organismal\_development | NEO1 | 1760 | 10 | 0.934456 | -0.170325 | 532 | 442.61 | 0.831974 |
| GO:0007275\_multicellular\_organismal\_development | AGRN | 1760 | 10 | 0.934456 | -0.170325 | 532 | 442.61 | 0.831974 |
| GO:0007275\_multicellular\_organismal\_development | CSDA | 1760 | 10 | 0.934456 | -0.170325 | 532 | 442.61 | 0.831974 |
| GO:0007275\_multicellular\_organismal\_development | YWHAE | 1760 | 10 | 0.934456 | -0.170325 | 532 | 442.61 | 0.831974 |
| GO:0007275\_multicellular\_organismal\_development | ZIC3 | 1760 | 10 | 0.934456 | -0.170325 | 532 | 442.61 | 0.831974 |
| GO:0007275\_multicellular\_organismal\_development | BMPR1A | 1760 | 10 | 0.934456 | -0.170325 | 532 | 442.61 | 0.831974 |
| GO:0019752\_carboxylic\_acid\_metabolic\_process | ATF4 | 181 | 1 | 0.908642 | -0.170238 | 534 | 443.8 | 0.831086 |
| GO:0043436\_oxoacid\_metabolic\_process | ATF4 | 181 | 1 | 0.908642 | -0.170238 | 534 | 443.8 | 0.831086 |
| GO:0006082\_organic\_acid\_metabolic\_process | ATF4 | 182 | 1 | 0.903650 | -0.168920 | 535 | 444.22 | 0.830318 |
| GO:0042180\_cellular\_ketone\_metabolic\_process | ATF4 | 183 | 1 | 0.898712 | -0.167615 | 536 | 445.63 | 0.831399 |
| GO:0032990\_cell\_part\_morphogenesis | PAX6 | 184 | 1 | 0.893828 | -0.166322 | 537 | 446.44 | 0.831359 |
| GO:0007010\_cytoskeleton\_organization | RHOQ | 185 | 1 | 0.888996 | -0.165040 | 538 | 446.83 | 0.830539 |
| GO:0048731\_system\_development | CCND1 | 1609 | 9 | 0.919937 | -0.161574 | 539 | 449.58 | 0.834100 |
| GO:0048731\_system\_development | HSP90AA1 | 1609 | 9 | 0.919937 | -0.161574 | 539 | 449.58 | 0.834100 |
| GO:0048731\_system\_development | PAX6 | 1609 | 9 | 0.919937 | -0.161574 | 539 | 449.58 | 0.834100 |
| GO:0048731\_system\_development | NEO1 | 1609 | 9 | 0.919937 | -0.161574 | 539 | 449.58 | 0.834100 |
| GO:0048731\_system\_development | AGRN | 1609 | 9 | 0.919937 | -0.161574 | 539 | 449.58 | 0.834100 |
| GO:0048731\_system\_development | CSDA | 1609 | 9 | 0.919937 | -0.161574 | 539 | 449.58 | 0.834100 |
| GO:0048731\_system\_development | YWHAE | 1609 | 9 | 0.919937 | -0.161574 | 539 | 449.58 | 0.834100 |
| GO:0048731\_system\_development | ZIC3 | 1609 | 9 | 0.919937 | -0.161574 | 539 | 449.58 | 0.834100 |
| GO:0048731\_system\_development | BMPR1A | 1609 | 9 | 0.919937 | -0.161574 | 539 | 449.58 | 0.834100 |
| GO:0019226\_transmission\_of\_nerve\_impulse | AGRN | 189 | 1 | 0.870181 | -0.160028 | 540 | 450.83 | 0.834870 |
| GO:0050896\_response\_to\_stimulus | EI24 | 1107 | 6 | 0.891405 | -0.156522 | 541 | 451.67 | 0.834880 |
| GO:0050896\_response\_to\_stimulus | LDHA | 1107 | 6 | 0.891405 | -0.156522 | 541 | 451.67 | 0.834880 |
| GO:0050896\_response\_to\_stimulus | CCND1 | 1107 | 6 | 0.891405 | -0.156522 | 541 | 451.67 | 0.834880 |
| GO:0050896\_response\_to\_stimulus | HSP90AA1 | 1107 | 6 | 0.891405 | -0.156522 | 541 | 451.67 | 0.834880 |
| GO:0050896\_response\_to\_stimulus | SFRP2 | 1107 | 6 | 0.891405 | -0.156522 | 541 | 451.67 | 0.834880 |
| GO:0050896\_response\_to\_stimulus | RHOQ | 1107 | 6 | 0.891405 | -0.156522 | 541 | 451.67 | 0.834880 |
| GO:0033554\_cellular\_response\_to\_stress | CCND1 | 196 | 1 | 0.839103 | -0.151677 | 542 | 454.78 | 0.839077 |
| GO:0031175\_neuron\_projection\_development | PAX6 | 197 | 1 | 0.834844 | -0.150526 | 543 | 455.48 | 0.838821 |
| GO:0000904\_cell\_morphogenesis\_involved\_in\_differentiation | PAX6 | 199 | 1 | 0.826454 | -0.148253 | 544 | 457.14 | 0.840331 |
| GO:0035295\_tube\_development | BMPR1A | 212 | 1 | 0.775775 | -0.134404 | 545 | 463.27 | 0.850037 |
| GO:0007423\_sensory\_organ\_development | PAX6 | 219 | 1 | 0.750978 | -0.127558 | 546 | 465.57 | 0.852692 |
| GO:0006915\_apoptosis | EI24 | 427 | 2 | 0.770325 | -0.126263 | 547 | 466.49 | 0.852815 |
| GO:0006915\_apoptosis | CSDA | 427 | 2 | 0.770325 | -0.126263 | 547 | 466.49 | 0.852815 |
| GO:0012501\_programmed\_cell\_death | EI24 | 433 | 2 | 0.759650 | -0.122107 | 548 | 468.4 | 0.854745 |
| GO:0012501\_programmed\_cell\_death | CSDA | 433 | 2 | 0.759650 | -0.122107 | 548 | 468.4 | 0.854745 |
| GO:0002682\_regulation\_of\_immune\_system\_process | HSP90AA1 | 228 | 1 | 0.721335 | -0.119325 | 550 | 470.35 | 0.855182 |
| GO:0046649\_lymphocyte\_activation | HSP90AA1 | 228 | 1 | 0.721335 | -0.119325 | 550 | 470.35 | 0.855182 |
| GO:0050790\_regulation\_of\_catalytic\_activity | NUP62 | 233 | 1 | 0.705855 | -0.115008 | 551 | 472.15 | 0.856897 |
| GO:0008219\_cell\_death | EI24 | 444 | 2 | 0.740830 | -0.114834 | 552 | 472.69 | 0.856322 |
| GO:0008219\_cell\_death | CSDA | 444 | 2 | 0.740830 | -0.114834 | 552 | 472.69 | 0.856322 |
| GO:0001501\_skeletal\_system\_development | BMPR1A | 236 | 1 | 0.696883 | -0.112501 | 553 | 473.86 | 0.856890 |
| GO:0016265\_death | EI24 | 450 | 2 | 0.730952 | -0.111048 | 554 | 475.25 | 0.857852 |
| GO:0016265\_death | CSDA | 450 | 2 | 0.730952 | -0.111048 | 554 | 475.25 | 0.857852 |
| GO:0009056\_catabolic\_process | UBE2D3 | 243 | 1 | 0.676808 | -0.106882 | 555 | 476.36 | 0.858306 |
| GO:0045321\_leukocyte\_activation | HSP90AA1 | 248 | 1 | 0.663162 | -0.103057 | 556 | 478.2 | 0.860072 |
| GO:0007267\_cell-cell\_signaling | AGRN | 252 | 1 | 0.652636 | -0.100105 | 557 | 479.44 | 0.860754 |
| GO:0016481\_negative\_regulation\_of\_transcription | MYBBP1A | 253 | 1 | 0.650056 | -0.099382 | 559 | 480.4 | 0.859392 |
| GO:0030097\_hemopoiesis | HSP90AA1 | 253 | 1 | 0.650056 | -0.099382 | 559 | 480.4 | 0.859392 |
| GO:0001775\_cell\_activation | HSP90AA1 | 262 | 1 | 0.627726 | -0.093119 | 562 | 483.27 | 0.859911 |
| GO:0010629\_negative\_regulation\_of\_gene\_expression | MYBBP1A | 262 | 1 | 0.627726 | -0.093119 | 562 | 483.27 | 0.859911 |
| GO:0048666\_neuron\_development | PAX6 | 262 | 1 | 0.627726 | -0.093119 | 562 | 483.27 | 0.859911 |
| GO:0045934\_negative\_regulation\_of\_nucleobase\_\_nucleoside\_\_nucleotide\_and\_nucleic\_acid\_metabolic\_process | MYBBP1A | 270 | 1 | 0.609127 | -0.087909 | 563 | 484.79 | 0.861083 |
| GO:0051172\_negative\_regulation\_of\_nitrogen\_compound\_metabolic\_process | MYBBP1A | 271 | 1 | 0.606879 | -0.087280 | 564 | 485.32 | 0.860496 |
| GO:0010558\_negative\_regulation\_of\_macromolecule\_biosynthetic\_process | MYBBP1A | 274 | 1 | 0.600235 | -0.085422 | 565 | 486.29 | 0.860690 |
| GO:0048534\_hemopoietic\_or\_lymphoid\_organ\_development | HSP90AA1 | 277 | 1 | 0.593734 | -0.083606 | 566 | 487.54 | 0.861378 |
| GO:0031327\_negative\_regulation\_of\_cellular\_biosynthetic\_process | MYBBP1A | 282 | 1 | 0.583207 | -0.080671 | 567 | 489.34 | 0.863034 |
| GO:0009890\_negative\_regulation\_of\_biosynthetic\_process | MYBBP1A | 284 | 1 | 0.579100 | -0.079528 | 568 | 490.25 | 0.863116 |
| GO:0051234\_establishment\_of\_localization | RHOQ | 729 | 3 | 0.676808 | -0.074031 | 569 | 491.97 | 0.864622 |
| GO:0051234\_establishment\_of\_localization | MYBBP1A | 729 | 3 | 0.676808 | -0.074031 | 569 | 491.97 | 0.864622 |
| GO:0051234\_establishment\_of\_localization | YWHAE | 729 | 3 | 0.676808 | -0.074031 | 569 | 491.97 | 0.864622 |
| GO:0002520\_immune\_system\_development | HSP90AA1 | 295 | 1 | 0.557506 | -0.073539 | 570 | 493.09 | 0.865070 |
| GO:0048598\_embryonic\_morphogenesis | BMPR1A | 299 | 1 | 0.550048 | -0.071481 | 571 | 493.59 | 0.864431 |
| GO:0006950\_response\_to\_stress | CCND1 | 549 | 2 | 0.599141 | -0.063574 | 572 | 497.0 | 0.868881 |
| GO:0006950\_response\_to\_stress | HSP90AA1 | 549 | 2 | 0.599141 | -0.063574 | 572 | 497.0 | 0.868881 |
| GO:0030182\_neuron\_differentiation | PAX6 | 356 | 1 | 0.461978 | -0.047874 | 573 | 505.27 | 0.881798 |
| GO:0050877\_neurological\_system\_process | AGRN | 390 | 1 | 0.421703 | -0.037768 | 574 | 509.91 | 0.888345 |
| GO:0032501\_multicellular\_organismal\_process | CCND1 | 2183 | 10 | 0.753387 | -0.033796 | 575 | 510.87 | 0.888470 |
| GO:0032501\_multicellular\_organismal\_process | HSP90AA1 | 2183 | 10 | 0.753387 | -0.033796 | 575 | 510.87 | 0.888470 |
| GO:0032501\_multicellular\_organismal\_process | SFRP2 | 2183 | 10 | 0.753387 | -0.033796 | 575 | 510.87 | 0.888470 |
| GO:0032501\_multicellular\_organismal\_process | PAX6 | 2183 | 10 | 0.753387 | -0.033796 | 575 | 510.87 | 0.888470 |
| GO:0032501\_multicellular\_organismal\_process | NEO1 | 2183 | 10 | 0.753387 | -0.033796 | 575 | 510.87 | 0.888470 |
| GO:0032501\_multicellular\_organismal\_process | AGRN | 2183 | 10 | 0.753387 | -0.033796 | 575 | 510.87 | 0.888470 |
| GO:0032501\_multicellular\_organismal\_process | CSDA | 2183 | 10 | 0.753387 | -0.033796 | 575 | 510.87 | 0.888470 |
| GO:0032501\_multicellular\_organismal\_process | YWHAE | 2183 | 10 | 0.753387 | -0.033796 | 575 | 510.87 | 0.888470 |
| GO:0032501\_multicellular\_organismal\_process | ZIC3 | 2183 | 10 | 0.753387 | -0.033796 | 575 | 510.87 | 0.888470 |
| GO:0032501\_multicellular\_organismal\_process | BMPR1A | 2183 | 10 | 0.753387 | -0.033796 | 575 | 510.87 | 0.888470 |
| GO:0042592\_homeostatic\_process | BMPR1A | 419 | 1 | 0.392516 | -0.030867 | 576 | 511.86 | 0.888646 |
| GO:0006810\_transport | MYBBP1A | 718 | 2 | 0.458118 | -0.023698 | 577 | 515.72 | 0.893795 |
| GO:0006810\_transport | YWHAE | 718 | 2 | 0.458118 | -0.023698 | 577 | 515.72 | 0.893795 |
| GO:0002376\_immune\_system\_process | HSP90AA1 | 505 | 1 | 0.325672 | -0.016957 | 578 | 517.57 | 0.895450 |
| GO:0003008\_system\_process | AGRN | 516 | 1 | 0.318729 | -0.015702 | 579 | 518.0 | 0.894646 |
| GO:0008150\_biological\_process | LDHA | 4605 | 28 | 1.000000 | 0.000000 | 1778 | 1741.41 | 0.979421 |
| GO:0008150\_biological\_process | IMPA1 | 4605 | 28 | 1.000000 | 0.000000 | 1778 | 1741.41 | 0.979421 |
| GO:0008150\_biological\_process | PAX6 | 4605 | 28 | 1.000000 | 0.000000 | 1778 | 1741.41 | 0.979421 |
| GO:0008150\_biological\_process | RHOQ | 4605 | 28 | 1.000000 | 0.000000 | 1778 | 1741.41 | 0.979421 |
| GO:0008150\_biological\_process | RDX | 4605 | 28 | 1.000000 | 0.000000 | 1778 | 1741.41 | 0.979421 |
| GO:0008150\_biological\_process | NEO1 | 4605 | 28 | 1.000000 | 0.000000 | 1778 | 1741.41 | 0.979421 |
| GO:0008150\_biological\_process | ZIC3 | 4605 | 28 | 1.000000 | 0.000000 | 1778 | 1741.41 | 0.979421 |
| GO:0008150\_biological\_process | CCNE1 | 4605 | 28 | 1.000000 | 0.000000 | 1778 | 1741.41 | 0.979421 |
| GO:0008150\_biological\_process | UBE2D3 | 4605 | 28 | 1.000000 | 0.000000 | 1778 | 1741.41 | 0.979421 |
| GO:0008150\_biological\_process | GPX4 | 4605 | 28 | 1.000000 | 0.000000 | 1778 | 1741.41 | 0.979421 |
| GO:0008150\_biological\_process | DNAJA1 | 4605 | 28 | 1.000000 | 0.000000 | 1778 | 1741.41 | 0.979421 |
| GO:0008150\_biological\_process | AGRN | 4605 | 28 | 1.000000 | 0.000000 | 1778 | 1741.41 | 0.979421 |
| GO:0008150\_biological\_process | STX6 | 4605 | 28 | 1.000000 | 0.000000 | 1778 | 1741.41 | 0.979421 |
| GO:0008150\_biological\_process | HSP90AA1 | 4605 | 28 | 1.000000 | 0.000000 | 1778 | 1741.41 | 0.979421 |
| GO:0008150\_biological\_process | CSDA | 4605 | 28 | 1.000000 | 0.000000 | 1778 | 1741.41 | 0.979421 |
| GO:0008150\_biological\_process | YWHAE | 4605 | 28 | 1.000000 | 0.000000 | 1778 | 1741.41 | 0.979421 |
| GO:0008150\_biological\_process | ATF4 | 4605 | 28 | 1.000000 | 0.000000 | 1778 | 1741.41 | 0.979421 |
| GO:0008150\_biological\_process | CCND1 | 4605 | 28 | 1.000000 | 0.000000 | 1778 | 1741.41 | 0.979421 |
| GO:0008150\_biological\_process | EI24 | 4605 | 28 | 1.000000 | 0.000000 | 1778 | 1741.41 | 0.979421 |
| GO:0008150\_biological\_process | NUP62 | 4605 | 28 | 1.000000 | 0.000000 | 1778 | 1741.41 | 0.979421 |
| GO:0008150\_biological\_process | SFRP2 | 4605 | 28 | 1.000000 | 0.000000 | 1778 | 1741.41 | 0.979421 |
| GO:0008150\_biological\_process | ADK | 4605 | 28 | 1.000000 | 0.000000 | 1778 | 1741.41 | 0.979421 |
| GO:0008150\_biological\_process | TMSB4X | 4605 | 28 | 1.000000 | 0.000000 | 1778 | 1741.41 | 0.979421 |
| GO:0008150\_biological\_process | RAP1B | 4605 | 28 | 1.000000 | 0.000000 | 1778 | 1741.41 | 0.979421 |
| GO:0008150\_biological\_process | SMC1A | 4605 | 28 | 1.000000 | 0.000000 | 1778 | 1741.41 | 0.979421 |
| GO:0008150\_biological\_process | MYBBP1A | 4605 | 28 | 1.000000 | 0.000000 | 1778 | 1741.41 | 0.979421 |
| GO:0008150\_biological\_process | BMPR1A | 4605 | 28 | 1.000000 | 0.000000 | 1778 | 1741.41 | 0.979421 |
| GO:0008150\_biological\_process | CALM1 | 4605 | 28 | 1.000000 | 0.000000 | 1778 | 1741.41 | 0.979421 |
