# Supplementary material for: RedundancyMiner: De-replication of redundant GO categories in microarray and proteomics analysis
Source: BMC Bioinformatics. 2011 Feb 10;12:52. doi: 10.1186/1471-2105-12-52 (PMC3223614; doi:10.1186/1471-2105-12-52)
Supplement: Additional file 8 — Retinal development HTGM download. compressed package of the results of running HTGM on the retinal development genes list. [file 1471-2105-12-52-S8.ZIP › SCENARIO_2_MODIFIED/total.txt.total.txt.dir/Exp1_BestClusterMap_LEIGS_KM_24.csv.join.23.txt.dir/Exp1_BestClusterMap_LEIGS_KM_24.csv.join.23.txt.change.html]

Category Summary Report for Exp1\_BestClusterMap\_LEIGS\_KM\_24.csv.join.23.txt

# Category Summary Report for Exp1\_BestClusterMap\_LEIGS\_KM\_24.csv.join.23.txt

| HYPERLINKED GO CATEGORY | TOTAL GENES | CHANGED GENES | ENRICHMENT | LOG10(p) | CUMULATIVE NUMBER OF CATEGORIES | CUMULATIVE RANDOMS LOWER BOUND | CUMULATIVE RANDOMS MEAN | CUMULATIVE RANDOMS UPPER BOUND | FALSE DISCOVERY RATE |
| --- | --- | --- | --- | --- | --- | --- | --- | --- | --- |
| GO:0009950\_dorsal\_ventral\_axis\_specification | 5 | 2 | 65.785714 | -3.452757 | 1 | -0.445247 | 0.26 | 0.965247 | 0.260000 |
| GO:0048641\_regulation\_of\_skeletal\_muscle\_tissue\_development | 10 | 2 | 32.892857 | -2.807724 | 2 | -0.703255 | 1.39 | 3.483255 | 0.695000 |
| GO:0030856\_regulation\_of\_epithelial\_cell\_differentiation | 11 | 2 | 29.902597 | -2.722208 | 3 | -0.647048 | 1.63 | 3.907048 | 0.543333 |
| GO:0006986\_response\_to\_unfolded\_protein | 13 | 2 | 25.302198 | -2.573744 | 4 | -0.494633 | 2.28 | 5.054633 | 0.570000 |
| GO:0009798\_axis\_specification | 19 | 2 | 17.312030 | -2.242631 | 5 | 0.169905 | 4.75 | 9.330095 | 0.950000 |
| GO:0001880\_Mullerian\_duct\_regression | 1 | 1 |  |  |  |  |  |  |  |  |
| GO:0006175\_dATP\_biosynthetic\_process | 1 | 1 |  |  |  |  |  |  |  |  |
| GO:0009145\_purine\_nucleoside\_triphosphate\_biosynthetic\_process | 1 | 1 |  |  |  |  |  |  |  |  |
| GO:0009153\_purine\_deoxyribonucleotide\_biosynthetic\_process | 1 | 1 |  |  |  |  |  |  |  |  |
| GO:0009216\_purine\_deoxyribonucleoside\_triphosphate\_biosynthetic\_process | 1 | 1 |  |  |  |  |  |  |  |  |
| GO:0021902\_commitment\_of\_a\_neuronal\_cell\_to\_a\_specific\_type\_of\_neuron\_in\_the\_forebrain | 1 | 1 |  |  |  |  |  |  |  |  |
| GO:0021905\_forebrain-midbrain\_boundary\_formation | 1 | 1 |  |  |  |  |  |  |  |  |
| GO:0021917\_somatic\_motor\_neuron\_fate\_commitment | 1 | 1 |  |  |  |  |  |  |  |  |
| GO:0021918\_regulation\_of\_transcription\_from\_RNA\_polymerase\_II\_promoter\_involved\_in\_somatic\_motor\_neuron\_fate\_commitment | 1 | 1 |  |  |  |  |  |  |  |  |
| GO:0035303\_regulation\_of\_dephosphorylation | 1 | 1 |  |  |  |  |  |  |  |  |
| GO:0035304\_regulation\_of\_protein\_amino\_acid\_dephosphorylation | 1 | 1 |  |  |  |  |  |  |  |  |
| GO:0035305\_negative\_regulation\_of\_dephosphorylation | 1 | 1 |  |  |  |  |  |  |  |  |
| GO:0035308\_negative\_regulation\_of\_protein\_amino\_acid\_dephosphorylation | 1 | 1 |  |  |  |  |  |  |  |  |
| GO:0042026\_protein\_refolding | 1 | 1 |  |  |  |  |  |  |  |  |
| GO:0042059\_negative\_regulation\_of\_epidermal\_growth\_factor\_receptor\_signaling\_pathway | 1 | 1 |  |  |  |  |  |  |  |  |
| GO:0045583\_regulation\_of\_cytotoxic\_T\_cell\_differentiation | 1 | 1 |  |  |  |  |  |  |  |  |
| GO:0045585\_positive\_regulation\_of\_cytotoxic\_T\_cell\_differentiation | 1 | 1 |  |  |  |  |  |  |  |  |
| GO:0048642\_negative\_regulation\_of\_skeletal\_muscle\_tissue\_development | 1 | 1 |  |  |  |  |  |  |  |  |
| GO:0016202\_regulation\_of\_striated\_muscle\_tissue\_development | 21 | 2 | 15.663265 | -2.156667 | 7 | 0.400456 | 5.53 | 10.659544 | 0.790000 |
| GO:0048634\_regulation\_of\_muscle\_development | 21 | 2 | 15.663265 | -2.156667 | 7 | 0.400456 | 5.53 | 10.659544 | 0.790000 |
| GO:0009952\_anterior\_posterior\_pattern\_formation | 133 | 4 | 4.946294 | -2.099692 | 8 | 0.595474 | 5.99 | 11.384526 | 0.748750 |
| GO:0050673\_epithelial\_cell\_proliferation | 72 | 3 | 6.852679 | -2.042262 | 9 | 0.949776 | 6.78 | 12.610224 | 0.753333 |
| GO:0021983\_pituitary\_gland\_development | 25 | 2 | 13.157143 | -2.008277 | 10 | 1.060596 | 7.19 | 13.319404 | 0.719000 |
| GO:0007519\_skeletal\_muscle\_tissue\_development | 78 | 3 | 6.325549 | -1.947103 | 12 | 1.185882 | 7.77 | 14.354118 | 0.647500 |
| GO:0060538\_skeletal\_muscle\_organ\_development | 78 | 3 | 6.325549 | -1.947103 | 12 | 1.185882 | 7.77 | 14.354118 | 0.647500 |
| GO:0008582\_regulation\_of\_synaptic\_growth\_at\_neuromuscular\_junction | 2 | 1 |  |  |  |  |  |  |  |  |
| GO:0009142\_nucleoside\_triphosphate\_biosynthetic\_process | 2 | 1 |  |  |  |  |  |  |  |  |
| GO:0009202\_deoxyribonucleoside\_triphosphate\_biosynthetic\_process | 2 | 1 |  |  |  |  |  |  |  |  |
| GO:0009265\_2'-deoxyribonucleotide\_biosynthetic\_process | 2 | 1 |  |  |  |  |  |  |  |  |
| GO:0045065\_cytotoxic\_T\_cell\_differentiation | 2 | 1 |  |  |  |  |  |  |  |  |
| GO:0046060\_dATP\_metabolic\_process | 2 | 1 |  |  |  |  |  |  |  |  |
| GO:0048382\_mesendoderm\_development | 2 | 1 |  |  |  |  |  |  |  |  |
| GO:0031668\_cellular\_response\_to\_extracellular\_stimulus | 30 | 2 | 10.964286 | -1.855036 | 13 | 1.340712 | 9.51 | 17.679288 | 0.731538 |
| GO:0021536\_diencephalon\_development | 33 | 2 | 9.967532 | -1.775761 | 14 | 1.757651 | 11.01 | 20.262349 | 0.786429 |
| GO:0000320\_re-entry\_into\_mitotic\_cell\_cycle | 3 | 1 |  |  |  |  |  |  |  |  |
| GO:0006166\_purine\_ribonucleoside\_salvage | 3 | 1 |  |  |  |  |  |  |  |  |
| GO:0021797\_forebrain\_anterior\_posterior\_pattern\_formation | 3 | 1 |  |  |  |  |  |  |  |  |
| GO:0021798\_forebrain\_dorsal\_ventral\_pattern\_formation | 3 | 1 |  |  |  |  |  |  |  |  |
| GO:0021912\_regulation\_of\_transcription\_from\_RNA\_polymerase\_II\_promoter\_involved\_in\_spinal\_cord\_motor\_neuron\_fate\_specification | 3 | 1 |  |  |  |  |  |  |  |  |
| GO:0030033\_microvillus\_assembly | 3 | 1 |  |  |  |  |  |  |  |  |
| GO:0030857\_negative\_regulation\_of\_epithelial\_cell\_differentiation | 3 | 1 |  |  |  |  |  |  |  |  |
| GO:0032528\_microvillus\_organization | 3 | 1 |  |  |  |  |  |  |  |  |
| GO:0043094\_cellular\_metabolic\_compound\_salvage | 3 | 1 |  |  |  |  |  |  |  |  |
| GO:0043101\_purine\_salvage | 3 | 1 |  |  |  |  |  |  |  |  |
| GO:0043174\_nucleoside\_salvage | 3 | 1 |  |  |  |  |  |  |  |  |
| GO:0060033\_anatomical\_structure\_regression | 3 | 1 |  |  |  |  |  |  |  |  |
| GO:0021523\_somatic\_motor\_neuron\_differentiation | 4 | 1 |  |  |  |  |  |  |  |  |
| GO:0021778\_oligodendrocyte\_cell\_fate\_specification | 4 | 1 |  |  |  |  |  |  |  |  |
| GO:0021779\_oligodendrocyte\_cell\_fate\_commitment | 4 | 1 |  |  |  |  |  |  |  |  |
| GO:0021780\_glial\_cell\_fate\_specification | 4 | 1 |  |  |  |  |  |  |  |  |
| GO:0021877\_forebrain\_neuron\_fate\_commitment | 4 | 1 |  |  |  |  |  |  |  |  |
| GO:0021913\_regulation\_of\_transcription\_from\_RNA\_polymerase\_II\_promoter\_involved\_in\_ventral\_spinal\_cord\_interneuron\_specification | 4 | 1 |  |  |  |  |  |  |  |  |
| GO:0030858\_positive\_regulation\_of\_epithelial\_cell\_differentiation | 4 | 1 |  |  |  |  |  |  |  |  |
| GO:0032808\_lacrimal\_gland\_development | 4 | 1 |  |  |  |  |  |  |  |  |
| GO:0051124\_synaptic\_growth\_at\_neuromuscular\_junction | 4 | 1 |  |  |  |  |  |  |  |  |
| GO:0051789\_response\_to\_protein\_stimulus | 43 | 2 | 7.649502 | -1.558905 | 15 | 3.183514 | 16.98 | 30.776486 | 1.132000 |
| GO:0003002\_regionalization | 195 | 4 | 3.373626 | -1.540950 | 16 | 3.282492 | 17.22 | 31.157508 | 1.076250 |
| GO:0006270\_DNA\_replication\_initiation | 5 | 1 | 32.892857 | -1.522196 | 22 | 9.097635 | 26.8 | 44.502365 | 1.218182 |
| GO:0009151\_purine\_deoxyribonucleotide\_metabolic\_process | 5 | 1 | 32.892857 | -1.522196 | 22 | 9.097635 | 26.8 | 44.502365 | 1.218182 |
| GO:0009215\_purine\_deoxyribonucleoside\_triphosphate\_metabolic\_process | 5 | 1 | 32.892857 | -1.522196 | 22 | 9.097635 | 26.8 | 44.502365 | 1.218182 |
| GO:0009263\_deoxyribonucleotide\_biosynthetic\_process | 5 | 1 | 32.892857 | -1.522196 | 22 | 9.097635 | 26.8 | 44.502365 | 1.218182 |
| GO:0030866\_cortical\_actin\_cytoskeleton\_organization | 5 | 1 | 32.892857 | -1.522196 | 22 | 9.097635 | 26.8 | 44.502365 | 1.218182 |
| GO:0045213\_neurotransmitter\_receptor\_metabolic\_process | 5 | 1 | 32.892857 | -1.522196 | 22 | 9.097635 | 26.8 | 44.502365 | 1.218182 |
| GO:0040008\_regulation\_of\_growth | 113 | 3 | 4.366308 | -1.520257 | 23 | 9.106370 | 27.07 | 45.033630 | 1.176957 |
| GO:0045595\_regulation\_of\_cell\_differentiation | 295 | 5 | 2.787530 | -1.518123 | 24 | 9.108031 | 27.14 | 45.171969 | 1.130833 |
| GO:0051960\_regulation\_of\_nervous\_system\_development | 118 | 3 | 4.181295 | -1.472093 | 25 | 9.464880 | 28.42 | 47.375120 | 1.136800 |
| GO:0021543\_pallium\_development | 49 | 2 | 6.712828 | -1.453877 | 28 | 9.755668 | 29.22 | 48.684332 | 1.043571 |
| GO:0046661\_male\_sex\_differentiation | 49 | 2 | 6.712828 | -1.453877 | 28 | 9.755668 | 29.22 | 48.684332 | 1.043571 |
| GO:0048741\_skeletal\_muscle\_fiber\_development | 49 | 2 | 6.712828 | -1.453877 | 28 | 9.755668 | 29.22 | 48.684332 | 1.043571 |
| GO:0014706\_striated\_muscle\_tissue\_development | 120 | 3 | 4.111607 | -1.453504 | 29 | 9.779021 | 29.24 | 48.700979 | 1.008276 |
| GO:0000768\_syncytium\_formation\_by\_plasma\_membrane\_fusion | 6 | 1 | 27.410714 | -1.444286 | 38 | 14.076448 | 36.01 | 57.943552 | 0.947632 |
| GO:0007032\_endosome\_organization | 6 | 1 | 27.410714 | -1.444286 | 38 | 14.076448 | 36.01 | 57.943552 | 0.947632 |
| GO:0007520\_myoblast\_fusion | 6 | 1 | 27.410714 | -1.444286 | 38 | 14.076448 | 36.01 | 57.943552 | 0.947632 |
| GO:0030865\_cortical\_cytoskeleton\_organization | 6 | 1 | 27.410714 | -1.444286 | 38 | 14.076448 | 36.01 | 57.943552 | 0.947632 |
| GO:0045176\_apical\_protein\_localization | 6 | 1 | 27.410714 | -1.444286 | 38 | 14.076448 | 36.01 | 57.943552 | 0.947632 |
| GO:0045843\_negative\_regulation\_of\_striated\_muscle\_development | 6 | 1 | 27.410714 | -1.444286 | 38 | 14.076448 | 36.01 | 57.943552 | 0.947632 |
| GO:0046580\_negative\_regulation\_of\_Ras\_protein\_signal\_transduction | 6 | 1 | 27.410714 | -1.444286 | 38 | 14.076448 | 36.01 | 57.943552 | 0.947632 |
| GO:0048635\_negative\_regulation\_of\_muscle\_development | 6 | 1 | 27.410714 | -1.444286 | 38 | 14.076448 | 36.01 | 57.943552 | 0.947632 |
| GO:0051058\_negative\_regulation\_of\_small\_GTPase\_mediated\_signal\_transduction | 6 | 1 | 27.410714 | -1.444286 | 38 | 14.076448 | 36.01 | 57.943552 | 0.947632 |
| GO:0060284\_regulation\_of\_cell\_development | 122 | 3 | 4.044204 | -1.435282 | 39 | 14.090767 | 36.54 | 58.989233 | 0.936923 |
| GO:0048747\_muscle\_fiber\_development | 51 | 2 | 6.449580 | -1.422001 | 40 | 14.296934 | 36.96 | 59.623066 | 0.924000 |
| GO:0040007\_growth | 217 | 4 | 3.031600 | -1.393656 | 41 | 14.368291 | 37.48 | 60.591709 | 0.914146 |
| GO:0060537\_muscle\_tissue\_development | 128 | 3 | 3.854632 | -1.382699 | 42 | 14.654429 | 37.87 | 61.085571 | 0.901667 |
| GO:0002052\_positive\_regulation\_of\_neuroblast\_proliferation | 7 | 1 | 23.494898 | -1.378609 | 55 | 20.269070 | 45.42 | 70.570930 | 0.825818 |
| GO:0006014\_D-ribose\_metabolic\_process | 7 | 1 | 23.494898 | -1.378609 | 55 | 20.269070 | 45.42 | 70.570930 | 0.825818 |
| GO:0006949\_syncytium\_formation | 7 | 1 | 23.494898 | -1.378609 | 55 | 20.269070 | 45.42 | 70.570930 | 0.825818 |
| GO:0009200\_deoxyribonucleoside\_triphosphate\_metabolic\_process | 7 | 1 | 23.494898 | -1.378609 | 55 | 20.269070 | 45.42 | 70.570930 | 0.825818 |
| GO:0009394\_2'-deoxyribonucleotide\_metabolic\_process | 7 | 1 | 23.494898 | -1.378609 | 55 | 20.269070 | 45.42 | 70.570930 | 0.825818 |
| GO:0019692\_deoxyribose\_phosphate\_metabolic\_process | 7 | 1 | 23.494898 | -1.378609 | 55 | 20.269070 | 45.42 | 70.570930 | 0.825818 |
| GO:0021514\_ventral\_spinal\_cord\_interneuron\_differentiation | 7 | 1 | 23.494898 | -1.378609 | 55 | 20.269070 | 45.42 | 70.570930 | 0.825818 |
| GO:0021520\_spinal\_cord\_motor\_neuron\_cell\_fate\_specification | 7 | 1 | 23.494898 | -1.378609 | 55 | 20.269070 | 45.42 | 70.570930 | 0.825818 |
| GO:0021521\_ventral\_spinal\_cord\_interneuron\_specification | 7 | 1 | 23.494898 | -1.378609 | 55 | 20.269070 | 45.42 | 70.570930 | 0.825818 |
| GO:0021903\_rostrocaudal\_neural\_tube\_patterning | 7 | 1 | 23.494898 | -1.378609 | 55 | 20.269070 | 45.42 | 70.570930 | 0.825818 |
| GO:0030521\_androgen\_receptor\_signaling\_pathway | 7 | 1 | 23.494898 | -1.378609 | 55 | 20.269070 | 45.42 | 70.570930 | 0.825818 |
| GO:0046622\_positive\_regulation\_of\_organ\_growth | 7 | 1 | 23.494898 | -1.378609 | 55 | 20.269070 | 45.42 | 70.570930 | 0.825818 |
| GO:0060579\_ventral\_spinal\_cord\_interneuron\_fate\_commitment | 7 | 1 | 23.494898 | -1.378609 | 55 | 20.269070 | 45.42 | 70.570930 | 0.825818 |
| GO:0050678\_regulation\_of\_epithelial\_cell\_proliferation | 56 | 2 | 5.873724 | -1.348046 | 56 | 20.830207 | 46.96 | 73.089793 | 0.838571 |
| GO:0001764\_neuron\_migration | 57 | 2 | 5.770677 | -1.334143 | 58 | 21.352660 | 47.77 | 74.187340 | 0.823621 |
| GO:0009953\_dorsal\_ventral\_pattern\_formation | 57 | 2 | 5.770677 | -1.334143 | 58 | 21.352660 | 47.77 | 74.187340 | 0.823621 |
| GO:0007283\_spermatogenesis | 134 | 3 | 3.682036 | -1.333019 | 60 | 21.416821 | 47.81 | 74.203179 | 0.796833 |
| GO:0048232\_male\_gamete\_generation | 134 | 3 | 3.682036 | -1.333019 | 60 | 21.416821 | 47.81 | 74.203179 | 0.796833 |
| GO:0009987\_cellular\_process | 3868 | 27 | 1.148019 | -1.323317 | 61 | 21.466988 | 48.06 | 74.653012 | 0.787869 |
| GO:0006020\_inositol\_metabolic\_process | 8 | 1 | 20.558036 | -1.321886 | 71 | 26.019214 | 54.25 | 82.480786 | 0.764085 |
| GO:0007009\_plasma\_membrane\_organization | 8 | 1 | 20.558036 | -1.321886 | 71 | 26.019214 | 54.25 | 82.480786 | 0.764085 |
| GO:0008105\_asymmetric\_protein\_localization | 8 | 1 | 20.558036 | -1.321886 | 71 | 26.019214 | 54.25 | 82.480786 | 0.764085 |
| GO:0009144\_purine\_nucleoside\_triphosphate\_metabolic\_process | 8 | 1 | 20.558036 | -1.321886 | 71 | 26.019214 | 54.25 | 82.480786 | 0.764085 |
| GO:0021781\_glial\_cell\_fate\_commitment | 8 | 1 | 20.558036 | -1.321886 | 71 | 26.019214 | 54.25 | 82.480786 | 0.764085 |
| GO:0040034\_regulation\_of\_development\_\_heterochronic | 8 | 1 | 20.558036 | -1.321886 | 71 | 26.019214 | 54.25 | 82.480786 | 0.764085 |
| GO:0045429\_positive\_regulation\_of\_nitric\_oxide\_biosynthetic\_process | 8 | 1 | 20.558036 | -1.321886 | 71 | 26.019214 | 54.25 | 82.480786 | 0.764085 |
| GO:0048505\_regulation\_of\_timing\_of\_cell\_differentiation | 8 | 1 | 20.558036 | -1.321886 | 71 | 26.019214 | 54.25 | 82.480786 | 0.764085 |
| GO:0048638\_regulation\_of\_developmental\_growth | 8 | 1 | 20.558036 | -1.321886 | 71 | 26.019214 | 54.25 | 82.480786 | 0.764085 |
| GO:0048742\_regulation\_of\_skeletal\_muscle\_fiber\_development | 8 | 1 | 20.558036 | -1.321886 | 71 | 26.019214 | 54.25 | 82.480786 | 0.764085 |
| GO:0030334\_regulation\_of\_cell\_migration | 59 | 2 | 5.575061 | -1.307143 | 73 | 26.407136 | 54.92 | 83.432864 | 0.752329 |
| GO:0035270\_endocrine\_system\_development | 59 | 2 | 5.575061 | -1.307143 | 73 | 26.407136 | 54.92 | 83.432864 | 0.752329 |
| GO:0009991\_response\_to\_extracellular\_stimulus | 61 | 2 | 5.392272 | -1.281157 | 74 | 26.573184 | 55.86 | 85.146816 | 0.754865 |
| GO:0050793\_regulation\_of\_developmental\_process | 703 | 8 | 1.871571 | -1.279392 | 75 | 26.568188 | 55.92 | 85.271812 | 0.745600 |
| GO:0042058\_regulation\_of\_epidermal\_growth\_factor\_receptor\_signaling\_pathway | 9 | 1 | 18.273810 | -1.272002 | 78 | 30.668280 | 61.98 | 93.291720 | 0.794615 |
| GO:0045428\_regulation\_of\_nitric\_oxide\_biosynthetic\_process | 9 | 1 | 18.273810 | -1.272002 | 78 | 30.668280 | 61.98 | 93.291720 | 0.794615 |
| GO:0051963\_regulation\_of\_synaptogenesis | 9 | 1 | 18.273810 | -1.272002 | 78 | 30.668280 | 61.98 | 93.291720 | 0.794615 |
| GO:0007049\_cell\_cycle | 238 | 4 | 2.764106 | -1.270037 | 79 | 30.738152 | 62.05 | 93.361848 | 0.785443 |
| GO:0021537\_telencephalon\_development | 62 | 2 | 5.305300 | -1.268523 | 81 | 31.042595 | 62.49 | 93.937405 | 0.771481 |
| GO:0030855\_epithelial\_cell\_differentiation | 62 | 2 | 5.305300 | -1.268523 | 81 | 31.042595 | 62.49 | 93.937405 | 0.771481 |
| GO:0045596\_negative\_regulation\_of\_cell\_differentiation | 144 | 3 | 3.426339 | -1.255982 | 82 | 31.216359 | 62.98 | 94.743641 | 0.768049 |
| GO:0050794\_regulation\_of\_cellular\_process | 2190 | 18 | 1.351761 | -1.252876 | 83 | 31.239907 | 63.08 | 94.920093 | 0.760000 |
| GO:0030900\_forebrain\_development | 146 | 3 | 3.379403 | -1.241366 | 84 | 31.594374 | 63.65 | 95.705626 | 0.757738 |
| GO:0022603\_regulation\_of\_anatomical\_structure\_morphogenesis | 147 | 3 | 3.356414 | -1.234151 | 85 | 31.654497 | 63.8 | 95.945503 | 0.750588 |
| GO:0007172\_signal\_complex\_assembly | 10 | 1 | 16.446429 | -1.227511 | 91 | 36.800890 | 70.84 | 104.879110 | 0.778462 |
| GO:0019321\_pentose\_metabolic\_process | 10 | 1 | 16.446429 | -1.227511 | 91 | 36.800890 | 70.84 | 104.879110 | 0.778462 |
| GO:0021871\_forebrain\_regionalization | 10 | 1 | 16.446429 | -1.227511 | 91 | 36.800890 | 70.84 | 104.879110 | 0.778462 |
| GO:0022900\_electron\_transport\_chain | 10 | 1 | 16.446429 | -1.227511 | 91 | 36.800890 | 70.84 | 104.879110 | 0.778462 |
| GO:0022904\_respiratory\_electron\_transport\_chain | 10 | 1 | 16.446429 | -1.227511 | 91 | 36.800890 | 70.84 | 104.879110 | 0.778462 |
| GO:0043113\_receptor\_clustering | 10 | 1 | 16.446429 | -1.227511 | 91 | 36.800890 | 70.84 | 104.879110 | 0.778462 |
| GO:0003007\_heart\_morphogenesis | 67 | 2 | 4.909382 | -1.208650 | 92 | 37.093178 | 71.87 | 106.646822 | 0.781196 |
| GO:0007389\_pattern\_specification\_process | 250 | 4 | 2.631429 | -1.205701 | 93 | 37.121649 | 71.93 | 106.738351 | 0.773441 |
| GO:0008104\_protein\_localization | 251 | 4 | 2.620945 | -1.200527 | 94 | 37.179303 | 72.06 | 106.940697 | 0.766596 |
| GO:0051173\_positive\_regulation\_of\_nitrogen\_compound\_metabolic\_process | 361 | 5 | 2.277899 | -1.198089 | 95 | 37.224864 | 72.15 | 107.075136 | 0.759474 |
| GO:0007517\_muscle\_organ\_development | 153 | 3 | 3.224790 | -1.192110 | 96 | 37.373888 | 72.49 | 107.606112 | 0.755104 |
| GO:0009141\_nucleoside\_triphosphate\_metabolic\_process | 11 | 1 | 14.951299 | -1.187384 | 103 | 41.004781 | 77.57 | 114.135219 | 0.753107 |
| GO:0014902\_myotube\_differentiation | 11 | 1 | 14.951299 | -1.187384 | 103 | 41.004781 | 77.57 | 114.135219 | 0.753107 |
| GO:0030308\_negative\_regulation\_of\_cell\_growth | 11 | 1 | 14.951299 | -1.187384 | 103 | 41.004781 | 77.57 | 114.135219 | 0.753107 |
| GO:0030968\_endoplasmic\_reticulum\_unfolded\_protein\_response | 11 | 1 | 14.951299 | -1.187384 | 103 | 41.004781 | 77.57 | 114.135219 | 0.753107 |
| GO:0034620\_cellular\_response\_to\_unfolded\_protein | 11 | 1 | 14.951299 | -1.187384 | 103 | 41.004781 | 77.57 | 114.135219 | 0.753107 |
| GO:0045026\_plasma\_membrane\_fusion | 11 | 1 | 14.951299 | -1.187384 | 103 | 41.004781 | 77.57 | 114.135219 | 0.753107 |
| GO:0050807\_regulation\_of\_synapse\_organization | 11 | 1 | 14.951299 | -1.187384 | 103 | 41.004781 | 77.57 | 114.135219 | 0.753107 |
| GO:0005996\_monosaccharide\_metabolic\_process | 69 | 2 | 4.767081 | -1.186117 | 104 | 41.101916 | 77.87 | 114.638084 | 0.748750 |
| GO:0048870\_cell\_motility | 257 | 4 | 2.559755 | -1.170060 | 105 | 41.447118 | 78.79 | 116.132882 | 0.750381 |
| GO:0006066\_alcohol\_metabolic\_process | 158 | 3 | 3.122740 | -1.158627 | 106 | 41.608208 | 79.5 | 117.391792 | 0.750000 |
| GO:0007264\_small\_GTPase\_mediated\_signal\_transduction | 72 | 2 | 4.568452 | -1.153688 | 108 | 41.866798 | 80.2 | 118.533202 | 0.742593 |
| GO:0040012\_regulation\_of\_locomotion | 72 | 2 | 4.568452 | -1.153688 | 108 | 41.866798 | 80.2 | 118.533202 | 0.742593 |
| GO:0006094\_gluconeogenesis | 12 | 1 | 13.705357 | -1.150860 | 118 | 46.213524 | 85.7 | 125.186476 | 0.726271 |
| GO:0009262\_deoxyribonucleotide\_metabolic\_process | 12 | 1 | 13.705357 | -1.150860 | 118 | 46.213524 | 85.7 | 125.186476 | 0.726271 |
| GO:0019319\_hexose\_biosynthetic\_process | 12 | 1 | 13.705357 | -1.150860 | 118 | 46.213524 | 85.7 | 125.186476 | 0.726271 |
| GO:0021513\_spinal\_cord\_dorsal\_ventral\_patterning | 12 | 1 | 13.705357 | -1.150860 | 118 | 46.213524 | 85.7 | 125.186476 | 0.726271 |
| GO:0033598\_mammary\_gland\_epithelial\_cell\_proliferation | 12 | 1 | 13.705357 | -1.150860 | 118 | 46.213524 | 85.7 | 125.186476 | 0.726271 |
| GO:0042278\_purine\_nucleoside\_metabolic\_process | 12 | 1 | 13.705357 | -1.150860 | 118 | 46.213524 | 85.7 | 125.186476 | 0.726271 |
| GO:0045792\_negative\_regulation\_of\_cell\_size | 12 | 1 | 13.705357 | -1.150860 | 118 | 46.213524 | 85.7 | 125.186476 | 0.726271 |
| GO:0046128\_purine\_ribonucleoside\_metabolic\_process | 12 | 1 | 13.705357 | -1.150860 | 118 | 46.213524 | 85.7 | 125.186476 | 0.726271 |
| GO:0050803\_regulation\_of\_synapse\_structure\_and\_activity | 12 | 1 | 13.705357 | -1.150860 | 118 | 46.213524 | 85.7 | 125.186476 | 0.726271 |
| GO:0055114\_oxidation\_reduction | 12 | 1 | 13.705357 | -1.150860 | 118 | 46.213524 | 85.7 | 125.186476 | 0.726271 |
| GO:0051270\_regulation\_of\_cell\_motion | 73 | 2 | 4.505871 | -1.143224 | 119 | 46.570380 | 86.25 | 125.929620 | 0.724790 |
| GO:0022414\_reproductive\_process | 376 | 5 | 2.187025 | -1.136723 | 120 | 46.670358 | 86.4 | 126.129642 | 0.720000 |
| GO:0000003\_reproduction | 379 | 5 | 2.169714 | -1.124877 | 121 | 46.843120 | 86.83 | 126.816880 | 0.717603 |
| GO:0048589\_developmental\_growth | 75 | 2 | 4.385714 | -1.122785 | 122 | 46.960219 | 87.1 | 127.239781 | 0.713934 |
| GO:0006090\_pyruvate\_metabolic\_process | 13 | 1 | 12.651099 | -1.117362 | 127 | 50.981187 | 92.85 | 134.718813 | 0.731102 |
| GO:0009119\_ribonucleoside\_metabolic\_process | 13 | 1 | 12.651099 | -1.117362 | 127 | 50.981187 | 92.85 | 134.718813 | 0.731102 |
| GO:0021511\_spinal\_cord\_patterning | 13 | 1 | 12.651099 | -1.117362 | 127 | 50.981187 | 92.85 | 134.718813 | 0.731102 |
| GO:0021879\_forebrain\_neuron\_differentiation | 13 | 1 | 12.651099 | -1.117362 | 127 | 50.981187 | 92.85 | 134.718813 | 0.731102 |
| GO:0060070\_Wnt\_receptor\_signaling\_pathway\_through\_beta-catenin | 13 | 1 | 12.651099 | -1.117362 | 127 | 50.981187 | 92.85 | 134.718813 | 0.731102 |
| GO:0045944\_positive\_regulation\_of\_transcription\_from\_RNA\_polymerase\_II\_promoter | 269 | 4 | 2.445566 | -1.111944 | 128 | 51.269037 | 93.53 | 135.790963 | 0.730703 |
| GO:0031328\_positive\_regulation\_of\_cellular\_biosynthetic\_process | 387 | 5 | 2.124862 | -1.093955 | 129 | 51.784386 | 94.27 | 136.755614 | 0.730775 |
| GO:0051716\_cellular\_response\_to\_stimulus | 273 | 4 | 2.409733 | -1.093361 | 130 | 51.808691 | 94.33 | 136.851309 | 0.725615 |
| GO:0009891\_positive\_regulation\_of\_biosynthetic\_process | 388 | 5 | 2.119385 | -1.090156 | 131 | 52.225881 | 94.97 | 137.714119 | 0.724962 |
| GO:0033036\_macromolecule\_localization | 274 | 4 | 2.400938 | -1.088774 | 132 | 52.321841 | 95.13 | 137.938159 | 0.720682 |
| GO:0006809\_nitric\_oxide\_biosynthetic\_process | 14 | 1 | 11.747449 | -1.086439 | 135 | 55.406209 | 99.55 | 143.693791 | 0.737407 |
| GO:0046209\_nitric\_oxide\_metabolic\_process | 14 | 1 | 11.747449 | -1.086439 | 135 | 55.406209 | 99.55 | 143.693791 | 0.737407 |
| GO:0048665\_neuron\_fate\_specification | 14 | 1 | 11.747449 | -1.086439 | 135 | 55.406209 | 99.55 | 143.693791 | 0.737407 |
| GO:0048523\_negative\_regulation\_of\_cellular\_process | 774 | 8 | 1.699889 | -1.074655 | 136 | 55.570140 | 100.24 | 144.909860 | 0.737059 |
| GO:0007173\_epidermal\_growth\_factor\_receptor\_signaling\_pathway | 15 | 1 | 10.964286 | -1.057737 | 141 | 59.850617 | 106.18 | 152.509383 | 0.753050 |
| GO:0009116\_nucleoside\_metabolic\_process | 15 | 1 | 10.964286 | -1.057737 | 141 | 59.850617 | 106.18 | 152.509383 | 0.753050 |
| GO:0021872\_generation\_of\_neurons\_in\_the\_forebrain | 15 | 1 | 10.964286 | -1.057737 | 141 | 59.850617 | 106.18 | 152.509383 | 0.753050 |
| GO:0048709\_oligodendrocyte\_differentiation | 15 | 1 | 10.964286 | -1.057737 | 141 | 59.850617 | 106.18 | 152.509383 | 0.753050 |
| GO:0060749\_mammary\_gland\_alveolus\_development | 15 | 1 | 10.964286 | -1.057737 | 141 | 59.850617 | 106.18 | 152.509383 | 0.753050 |
| GO:0009888\_tissue\_development | 525 | 6 | 1.879592 | -1.039964 | 142 | 60.872521 | 107.96 | 155.047479 | 0.760282 |
| GO:0048732\_gland\_development | 179 | 3 | 2.756385 | -1.031459 | 143 | 61.200342 | 108.59 | 155.979658 | 0.759371 |
| GO:0019751\_polyol\_metabolic\_process | 16 | 1 | 10.279018 | -1.030969 | 147 | 64.056201 | 112.69 | 161.323799 | 0.766599 |
| GO:0021522\_spinal\_cord\_motor\_neuron\_differentiation | 16 | 1 | 10.279018 | -1.030969 | 147 | 64.056201 | 112.69 | 161.323799 | 0.766599 |
| GO:0034976\_response\_to\_endoplasmic\_reticulum\_stress | 16 | 1 | 10.279018 | -1.030969 | 147 | 64.056201 | 112.69 | 161.323799 | 0.766599 |
| GO:0046364\_monosaccharide\_biosynthetic\_process | 16 | 1 | 10.279018 | -1.030969 | 147 | 64.056201 | 112.69 | 161.323799 | 0.766599 |
| GO:0070887\_cellular\_response\_to\_chemical\_stimulus | 85 | 2 | 3.869748 | -1.029338 | 148 | 64.171153 | 112.94 | 161.708847 | 0.763108 |
| GO:0006261\_DNA-dependent\_DNA\_replication | 17 | 1 | 9.674370 | -1.005899 | 153 | 69.157498 | 119.08 | 169.002502 | 0.778301 |
| GO:0006984\_ER-nuclear\_signaling\_pathway | 17 | 1 | 9.674370 | -1.005899 | 153 | 69.157498 | 119.08 | 169.002502 | 0.778301 |
| GO:0030317\_sperm\_motility | 17 | 1 | 9.674370 | -1.005899 | 153 | 69.157498 | 119.08 | 169.002502 | 0.778301 |
| GO:0043407\_negative\_regulation\_of\_MAP\_kinase\_activity | 17 | 1 | 9.674370 | -1.005899 | 153 | 69.157498 | 119.08 | 169.002502 | 0.778301 |
| GO:0045333\_cellular\_respiration | 17 | 1 | 9.674370 | -1.005899 | 153 | 69.157498 | 119.08 | 169.002502 | 0.778301 |
| GO:0040011\_locomotion | 295 | 4 | 2.230024 | -0.997570 | 154 | 69.810058 | 120.0 | 170.189942 | 0.779221 |
| GO:0007276\_gamete\_generation | 188 | 3 | 2.624430 | -0.982749 | 155 | 70.133396 | 120.88 | 171.626604 | 0.779871 |
| GO:0006457\_protein\_folding | 18 | 1 | 9.136905 | -0.982334 | 162 | 73.882585 | 125.92 | 177.957415 | 0.777284 |
| GO:0010498\_proteasomal\_protein\_catabolic\_process | 18 | 1 | 9.136905 | -0.982334 | 162 | 73.882585 | 125.92 | 177.957415 | 0.777284 |
| GO:0021517\_ventral\_spinal\_cord\_development | 18 | 1 | 9.136905 | -0.982334 | 162 | 73.882585 | 125.92 | 177.957415 | 0.777284 |
| GO:0030178\_negative\_regulation\_of\_Wnt\_receptor\_signaling\_pathway | 18 | 1 | 9.136905 | -0.982334 | 162 | 73.882585 | 125.92 | 177.957415 | 0.777284 |
| GO:0043161\_proteasomal\_ubiquitin-dependent\_protein\_catabolic\_process | 18 | 1 | 9.136905 | -0.982334 | 162 | 73.882585 | 125.92 | 177.957415 | 0.777284 |
| GO:0046578\_regulation\_of\_Ras\_protein\_signal\_transduction | 18 | 1 | 9.136905 | -0.982334 | 162 | 73.882585 | 125.92 | 177.957415 | 0.777284 |
| GO:0046620\_regulation\_of\_organ\_growth | 18 | 1 | 9.136905 | -0.982334 | 162 | 73.882585 | 125.92 | 177.957415 | 0.777284 |
| GO:0007569\_cell\_aging | 19 | 1 | 8.656015 | -0.960110 | 168 | 77.363938 | 131.23 | 185.096062 | 0.781131 |
| GO:0007595\_lactation | 19 | 1 | 8.656015 | -0.960110 | 168 | 77.363938 | 131.23 | 185.096062 | 0.781131 |
| GO:0030518\_steroid\_hormone\_receptor\_signaling\_pathway | 19 | 1 | 8.656015 | -0.960110 | 168 | 77.363938 | 131.23 | 185.096062 | 0.781131 |
| GO:0042462\_eye\_photoreceptor\_cell\_development | 19 | 1 | 8.656015 | -0.960110 | 168 | 77.363938 | 131.23 | 185.096062 | 0.781131 |
| GO:0046165\_alcohol\_biosynthetic\_process | 19 | 1 | 8.656015 | -0.960110 | 168 | 77.363938 | 131.23 | 185.096062 | 0.781131 |
| GO:0051056\_regulation\_of\_small\_GTPase\_mediated\_signal\_transduction | 19 | 1 | 8.656015 | -0.960110 | 168 | 77.363938 | 131.23 | 185.096062 | 0.781131 |
| GO:0045893\_positive\_regulation\_of\_transcription\_\_DNA-dependent | 306 | 4 | 2.149860 | -0.953407 | 170 | 77.903082 | 132.13 | 186.356918 | 0.777235 |
| GO:0051254\_positive\_regulation\_of\_RNA\_metabolic\_process | 306 | 4 | 2.149860 | -0.953407 | 170 | 77.903082 | 132.13 | 186.356918 | 0.777235 |
| GO:0050789\_regulation\_of\_biological\_process | 2357 | 18 | 1.255985 | -0.941772 | 171 | 78.478406 | 133.09 | 187.701594 | 0.778304 |
| GO:0007416\_synaptogenesis | 20 | 1 | 8.223214 | -0.939089 | 174 | 81.578687 | 137.1 | 192.621313 | 0.787931 |
| GO:0007528\_neuromuscular\_junction\_development | 20 | 1 | 8.223214 | -0.939089 | 174 | 81.578687 | 137.1 | 192.621313 | 0.787931 |
| GO:0008360\_regulation\_of\_cell\_shape | 20 | 1 | 8.223214 | -0.939089 | 174 | 81.578687 | 137.1 | 192.621313 | 0.787931 |
| GO:0007548\_sex\_differentiation | 98 | 2 | 3.356414 | -0.925694 | 175 | 82.216035 | 138.6 | 194.983965 | 0.792000 |
| GO:0001709\_cell\_fate\_determination | 21 | 1 | 7.831633 | -0.919155 | 184 | 85.237775 | 143.15 | 201.062225 | 0.777989 |
| GO:0001754\_eye\_photoreceptor\_cell\_differentiation | 21 | 1 | 7.831633 | -0.919155 | 184 | 85.237775 | 143.15 | 201.062225 | 0.777989 |
| GO:0002053\_positive\_regulation\_of\_mesenchymal\_cell\_proliferation | 21 | 1 | 7.831633 | -0.919155 | 184 | 85.237775 | 143.15 | 201.062225 | 0.777989 |
| GO:0006944\_membrane\_fusion | 21 | 1 | 7.831633 | -0.919155 | 184 | 85.237775 | 143.15 | 201.062225 | 0.777989 |
| GO:0010563\_negative\_regulation\_of\_phosphorus\_metabolic\_process | 21 | 1 | 7.831633 | -0.919155 | 184 | 85.237775 | 143.15 | 201.062225 | 0.777989 |
| GO:0019827\_stem\_cell\_maintenance | 21 | 1 | 7.831633 | -0.919155 | 184 | 85.237775 | 143.15 | 201.062225 | 0.777989 |
| GO:0021532\_neural\_tube\_patterning | 21 | 1 | 7.831633 | -0.919155 | 184 | 85.237775 | 143.15 | 201.062225 | 0.777989 |
| GO:0030216\_keratinocyte\_differentiation | 21 | 1 | 7.831633 | -0.919155 | 184 | 85.237775 | 143.15 | 201.062225 | 0.777989 |
| GO:0045936\_negative\_regulation\_of\_phosphate\_metabolic\_process | 21 | 1 | 7.831633 | -0.919155 | 184 | 85.237775 | 143.15 | 201.062225 | 0.777989 |
| GO:0007398\_ectoderm\_development | 99 | 2 | 3.322511 | -0.918416 | 185 | 85.453262 | 143.54 | 201.626738 | 0.775892 |
| GO:0031325\_positive\_regulation\_of\_cellular\_metabolic\_process | 442 | 5 | 1.860456 | -0.904817 | 186 | 85.802119 | 144.05 | 202.297881 | 0.774462 |
| GO:0001558\_regulation\_of\_cell\_growth | 22 | 1 | 7.475649 | -0.900206 | 194 | 90.185582 | 149.8 | 209.414418 | 0.772165 |
| GO:0001947\_heart\_looping | 22 | 1 | 7.475649 | -0.900206 | 194 | 90.185582 | 149.8 | 209.414418 | 0.772165 |
| GO:0010463\_mesenchymal\_cell\_proliferation | 22 | 1 | 7.475649 | -0.900206 | 194 | 90.185582 | 149.8 | 209.414418 | 0.772165 |
| GO:0010464\_regulation\_of\_mesenchymal\_cell\_proliferation | 22 | 1 | 7.475649 | -0.900206 | 194 | 90.185582 | 149.8 | 209.414418 | 0.772165 |
| GO:0021766\_hippocampus\_development | 22 | 1 | 7.475649 | -0.900206 | 194 | 90.185582 | 149.8 | 209.414418 | 0.772165 |
| GO:0042461\_photoreceptor\_cell\_development | 22 | 1 | 7.475649 | -0.900206 | 194 | 90.185582 | 149.8 | 209.414418 | 0.772165 |
| GO:0043112\_receptor\_metabolic\_process | 22 | 1 | 7.475649 | -0.900206 | 194 | 90.185582 | 149.8 | 209.414418 | 0.772165 |
| GO:0048864\_stem\_cell\_development | 22 | 1 | 7.475649 | -0.900206 | 194 | 90.185582 | 149.8 | 209.414418 | 0.772165 |
| GO:0009968\_negative\_regulation\_of\_signal\_transduction | 103 | 2 | 3.193481 | -0.890172 | 195 | 90.689311 | 150.7 | 210.710689 | 0.772821 |
| GO:0050767\_regulation\_of\_neurogenesis | 104 | 2 | 3.162775 | -0.883320 | 196 | 90.940968 | 151.25 | 211.559032 | 0.771684 |
| GO:0043388\_positive\_regulation\_of\_DNA\_binding | 23 | 1 | 7.150621 | -0.882153 | 197 | 93.127716 | 154.18 | 215.232284 | 0.782640 |
| GO:0048519\_negative\_regulation\_of\_biological\_process | 859 | 8 | 1.531681 | -0.869154 | 198 | 93.361704 | 154.55 | 215.738296 | 0.780556 |
| GO:0021515\_cell\_differentiation\_in\_spinal\_cord | 24 | 1 | 6.852679 | -0.864921 | 201 | 96.273884 | 158.71 | 221.146116 | 0.789602 |
| GO:0050679\_positive\_regulation\_of\_epithelial\_cell\_proliferation | 24 | 1 | 6.852679 | -0.864921 | 201 | 96.273884 | 158.71 | 221.146116 | 0.789602 |
| GO:0051099\_positive\_regulation\_of\_binding | 24 | 1 | 6.852679 | -0.864921 | 201 | 96.273884 | 158.71 | 221.146116 | 0.789602 |
| GO:0006928\_cell\_motion | 330 | 4 | 1.993506 | -0.864642 | 203 | 96.379280 | 159.07 | 221.760720 | 0.783596 |
| GO:0051674\_localization\_of\_cell | 330 | 4 | 1.993506 | -0.864642 | 203 | 96.379280 | 159.07 | 221.760720 | 0.783596 |
| GO:0051239\_regulation\_of\_multicellular\_organismal\_process | 587 | 6 | 1.681066 | -0.862615 | 204 | 96.504131 | 159.28 | 222.055869 | 0.780784 |
| GO:0051093\_negative\_regulation\_of\_developmental\_process | 331 | 4 | 1.987484 | -0.861153 | 205 | 96.641377 | 159.49 | 222.338623 | 0.778000 |
| GO:0009893\_positive\_regulation\_of\_metabolic\_process | 458 | 5 | 1.795462 | -0.856557 | 206 | 96.939484 | 160.04 | 223.140516 | 0.776893 |
| GO:0010033\_response\_to\_organic\_substance | 216 | 3 | 2.284226 | -0.849178 | 207 | 97.193670 | 160.34 | 223.486330 | 0.774589 |
| GO:0007492\_endoderm\_development | 25 | 1 | 6.578571 | -0.848443 | 210 | 99.367180 | 163.4 | 227.432820 | 0.778095 |
| GO:0031400\_negative\_regulation\_of\_protein\_modification\_process | 25 | 1 | 6.578571 | -0.848443 | 210 | 99.367180 | 163.4 | 227.432820 | 0.778095 |
| GO:0035137\_hindlimb\_morphogenesis | 25 | 1 | 6.578571 | -0.848443 | 210 | 99.367180 | 163.4 | 227.432820 | 0.778095 |
| GO:0010648\_negative\_regulation\_of\_cell\_communication | 110 | 2 | 2.990260 | -0.843848 | 211 | 99.867723 | 164.38 | 228.892277 | 0.779052 |
| GO:0031323\_regulation\_of\_cellular\_metabolic\_process | 1015 | 9 | 1.458304 | -0.841765 | 212 | 99.945505 | 164.5 | 229.054495 | 0.775943 |
| GO:0045941\_positive\_regulation\_of\_transcription | 338 | 4 | 1.946323 | -0.837164 | 213 | 100.199057 | 164.82 | 229.440943 | 0.773803 |
| GO:0007405\_neuroblast\_proliferation | 26 | 1 | 6.325549 | -0.832659 | 217 | 102.408282 | 168.47 | 234.531718 | 0.776359 |
| GO:0045665\_negative\_regulation\_of\_neuron\_differentiation | 26 | 1 | 6.325549 | -0.832659 | 217 | 102.408282 | 168.47 | 234.531718 | 0.776359 |
| GO:0046530\_photoreceptor\_cell\_differentiation | 26 | 1 | 6.325549 | -0.832659 | 217 | 102.408282 | 168.47 | 234.531718 | 0.776359 |
| GO:0050680\_negative\_regulation\_of\_epithelial\_cell\_proliferation | 26 | 1 | 6.325549 | -0.832659 | 217 | 102.408282 | 168.47 | 234.531718 | 0.776359 |
| GO:0006793\_phosphorus\_metabolic\_process | 340 | 4 | 1.934874 | -0.830447 | 219 | 102.749006 | 169.06 | 235.370994 | 0.771963 |
| GO:0006796\_phosphate\_metabolic\_process | 340 | 4 | 1.934874 | -0.830447 | 219 | 102.749006 | 169.06 | 235.370994 | 0.771963 |
| GO:0065007\_biological\_regulation | 2593 | 19 | 1.205099 | -0.830148 | 220 | 102.752121 | 169.08 | 235.407879 | 0.768545 |
| GO:0009607\_response\_to\_biotic\_stimulus | 114 | 2 | 2.885338 | -0.818989 | 221 | 103.345620 | 169.78 | 236.214380 | 0.768235 |
| GO:0009913\_epidermal\_cell\_differentiation | 27 | 1 | 6.091270 | -0.817517 | 223 | 105.227068 | 172.19 | 239.152932 | 0.772152 |
| GO:0045582\_positive\_regulation\_of\_T\_cell\_differentiation | 27 | 1 | 6.091270 | -0.817517 | 223 | 105.227068 | 172.19 | 239.152932 | 0.772152 |
| GO:0010628\_positive\_regulation\_of\_gene\_expression | 346 | 4 | 1.901321 | -0.810655 | 224 | 105.510149 | 172.67 | 239.829851 | 0.770848 |
| GO:0002088\_lens\_development\_in\_camera-type\_eye | 28 | 1 | 5.873724 | -0.802970 | 229 | 107.623651 | 175.72 | 243.816349 | 0.767336 |
| GO:0006470\_protein\_amino\_acid\_dephosphorylation | 28 | 1 | 5.873724 | -0.802970 | 229 | 107.623651 | 175.72 | 243.816349 | 0.767336 |
| GO:0030111\_regulation\_of\_Wnt\_receptor\_signaling\_pathway | 28 | 1 | 5.873724 | -0.802970 | 229 | 107.623651 | 175.72 | 243.816349 | 0.767336 |
| GO:0045926\_negative\_regulation\_of\_growth | 28 | 1 | 5.873724 | -0.802970 | 229 | 107.623651 | 175.72 | 243.816349 | 0.767336 |
| GO:0048863\_stem\_cell\_differentiation | 28 | 1 | 5.873724 | -0.802970 | 229 | 107.623651 | 175.72 | 243.816349 | 0.767336 |
| GO:0019953\_sexual\_reproduction | 228 | 3 | 2.164004 | -0.798997 | 230 | 107.933695 | 176.19 | 244.446305 | 0.766043 |
| GO:0007167\_enzyme\_linked\_receptor\_protein\_signaling\_pathway | 229 | 3 | 2.154554 | -0.794983 | 231 | 108.295018 | 176.63 | 244.964982 | 0.764632 |
| GO:0045935\_positive\_regulation\_of\_nucleobase\_\_nucleoside\_\_nucleotide\_and\_nucleic\_acid\_metabolic\_process | 352 | 4 | 1.868912 | -0.791382 | 232 | 108.490392 | 176.87 | 245.249608 | 0.762371 |
| GO:0021761\_limbic\_system\_development | 29 | 1 | 5.671182 | -0.788977 | 236 | 111.009887 | 180.12 | 249.230113 | 0.763220 |
| GO:0044087\_regulation\_of\_cellular\_component\_biogenesis | 29 | 1 | 5.671182 | -0.788977 | 236 | 111.009887 | 180.12 | 249.230113 | 0.763220 |
| GO:0045621\_positive\_regulation\_of\_lymphocyte\_differentiation | 29 | 1 | 5.671182 | -0.788977 | 236 | 111.009887 | 180.12 | 249.230113 | 0.763220 |
| GO:0050769\_positive\_regulation\_of\_neurogenesis | 29 | 1 | 5.671182 | -0.788977 | 236 | 111.009887 | 180.12 | 249.230113 | 0.763220 |
| GO:0007420\_brain\_development | 231 | 3 | 2.135900 | -0.787029 | 237 | 111.060781 | 180.23 | 249.399219 | 0.760464 |
| GO:0051726\_regulation\_of\_cell\_cycle | 121 | 2 | 2.718418 | -0.778015 | 238 | 112.006526 | 181.58 | 251.153474 | 0.762941 |
| GO:0007435\_salivary\_gland\_morphogenesis | 30 | 1 | 5.482143 | -0.775499 | 242 | 114.741732 | 185.36 | 255.978268 | 0.765950 |
| GO:0030522\_intracellular\_receptor-mediated\_signaling\_pathway | 30 | 1 | 5.482143 | -0.775499 | 242 | 114.741732 | 185.36 | 255.978268 | 0.765950 |
| GO:0035265\_organ\_growth | 30 | 1 | 5.482143 | -0.775499 | 242 | 114.741732 | 185.36 | 255.978268 | 0.765950 |
| GO:0060021\_palate\_development | 30 | 1 | 5.482143 | -0.775499 | 242 | 114.741732 | 185.36 | 255.978268 | 0.765950 |
| GO:0016477\_cell\_migration | 234 | 3 | 2.108516 | -0.775279 | 243 | 114.823691 | 185.54 | 256.256309 | 0.763539 |
| GO:0016049\_cell\_growth | 31 | 1 | 5.305300 | -0.762502 | 245 | 119.736642 | 192.27 | 264.803358 | 0.784776 |
| GO:0016311\_dephosphorylation | 31 | 1 | 5.305300 | -0.762502 | 245 | 119.736642 | 192.27 | 264.803358 | 0.784776 |
| GO:0051171\_regulation\_of\_nitrogen\_compound\_metabolic\_process | 771 | 7 | 1.493191 | -0.755007 | 246 | 120.181332 | 193.08 | 265.978668 | 0.784878 |
| GO:0043009\_chordate\_embryonic\_development | 365 | 4 | 1.802348 | -0.751324 | 247 | 120.301697 | 193.45 | 266.598303 | 0.783198 |
| GO:0001707\_mesoderm\_formation | 32 | 1 | 5.139509 | -0.749957 | 250 | 122.164473 | 195.86 | 269.555527 | 0.783440 |
| GO:0048332\_mesoderm\_morphogenesis | 32 | 1 | 5.139509 | -0.749957 | 250 | 122.164473 | 195.86 | 269.555527 | 0.783440 |
| GO:0050768\_negative\_regulation\_of\_neurogenesis | 32 | 1 | 5.139509 | -0.749957 | 250 | 122.164473 | 195.86 | 269.555527 | 0.783440 |
| GO:0009792\_embryonic\_development\_ending\_in\_birth\_or\_egg\_hatching | 368 | 4 | 1.787655 | -0.742397 | 251 | 122.286173 | 196.11 | 269.933827 | 0.781315 |
| GO:0045597\_positive\_regulation\_of\_cell\_differentiation | 128 | 2 | 2.569754 | -0.739947 | 252 | 122.685250 | 196.78 | 270.874750 | 0.780873 |
| GO:0007431\_salivary\_gland\_development | 33 | 1 | 4.983766 | -0.737835 | 255 | 124.232792 | 199.21 | 274.187208 | 0.781216 |
| GO:0008584\_male\_gonad\_development | 33 | 1 | 4.983766 | -0.737835 | 255 | 124.232792 | 199.21 | 274.187208 | 0.781216 |
| GO:0021987\_cerebral\_cortex\_development | 33 | 1 | 4.983766 | -0.737835 | 255 | 124.232792 | 199.21 | 274.187208 | 0.781216 |
| GO:0010557\_positive\_regulation\_of\_macromolecule\_biosynthetic\_process | 371 | 4 | 1.773200 | -0.733584 | 256 | 124.408124 | 199.56 | 274.711876 | 0.779531 |
| GO:0007568\_aging | 34 | 1 | 4.837185 | -0.726111 | 261 | 126.890628 | 203.18 | 279.469372 | 0.778467 |
| GO:0010720\_positive\_regulation\_of\_cell\_development | 34 | 1 | 4.837185 | -0.726111 | 261 | 126.890628 | 203.18 | 279.469372 | 0.778467 |
| GO:0010721\_negative\_regulation\_of\_cell\_development | 34 | 1 | 4.837185 | -0.726111 | 261 | 126.890628 | 203.18 | 279.469372 | 0.778467 |
| GO:0030509\_BMP\_signaling\_pathway | 34 | 1 | 4.837185 | -0.726111 | 261 | 126.890628 | 203.18 | 279.469372 | 0.778467 |
| GO:0045927\_positive\_regulation\_of\_growth | 34 | 1 | 4.837185 | -0.726111 | 261 | 126.890628 | 203.18 | 279.469372 | 0.778467 |
| GO:0032879\_regulation\_of\_localization | 248 | 3 | 1.989487 | -0.723170 | 262 | 127.002487 | 203.44 | 279.877513 | 0.776489 |
| GO:0001756\_somitogenesis | 35 | 1 | 4.698980 | -0.714761 | 264 | 128.712376 | 205.75 | 282.787624 | 0.779356 |
| GO:0016051\_carbohydrate\_biosynthetic\_process | 35 | 1 | 4.698980 | -0.714761 | 264 | 128.712376 | 205.75 | 282.787624 | 0.779356 |
| GO:0019222\_regulation\_of\_metabolic\_process | 1088 | 9 | 1.360458 | -0.705867 | 265 | 129.445201 | 206.76 | 284.074799 | 0.780226 |
| GO:0001704\_formation\_of\_primary\_germ\_layer | 36 | 1 | 4.568452 | -0.703766 | 270 | 131.770695 | 210.34 | 288.909305 | 0.779037 |
| GO:0006469\_negative\_regulation\_of\_protein\_kinase\_activity | 36 | 1 | 4.568452 | -0.703766 | 270 | 131.770695 | 210.34 | 288.909305 | 0.779037 |
| GO:0007368\_determination\_of\_left\_right\_symmetry | 36 | 1 | 4.568452 | -0.703766 | 270 | 131.770695 | 210.34 | 288.909305 | 0.779037 |
| GO:0021510\_spinal\_cord\_development | 36 | 1 | 4.568452 | -0.703766 | 270 | 131.770695 | 210.34 | 288.909305 | 0.779037 |
| GO:0033673\_negative\_regulation\_of\_kinase\_activity | 36 | 1 | 4.568452 | -0.703766 | 270 | 131.770695 | 210.34 | 288.909305 | 0.779037 |
| GO:0048729\_tissue\_morphogenesis | 255 | 3 | 1.934874 | -0.698689 | 271 | 132.373517 | 211.07 | 289.766483 | 0.778856 |
| GO:0034960\_cellular\_biopolymer\_metabolic\_process | 1395 | 11 | 1.296851 | -0.698310 | 272 | 132.406376 | 211.11 | 289.813624 | 0.776140 |
| GO:0009799\_determination\_of\_symmetry | 37 | 1 | 4.444981 | -0.693104 | 276 | 133.900528 | 213.73 | 293.559472 | 0.774384 |
| GO:0009855\_determination\_of\_bilateral\_symmetry | 37 | 1 | 4.444981 | -0.693104 | 276 | 133.900528 | 213.73 | 293.559472 | 0.774384 |
| GO:0032869\_cellular\_response\_to\_insulin\_stimulus | 37 | 1 | 4.444981 | -0.693104 | 276 | 133.900528 | 213.73 | 293.559472 | 0.774384 |
| GO:0051101\_regulation\_of\_DNA\_binding | 37 | 1 | 4.444981 | -0.693104 | 276 | 133.900528 | 213.73 | 293.559472 | 0.774384 |
| GO:0007154\_cell\_communication | 1096 | 9 | 1.350528 | -0.692150 | 277 | 133.975454 | 213.83 | 293.684546 | 0.771949 |
| GO:0034961\_cellular\_biopolymer\_biosynthetic\_process | 804 | 7 | 1.431903 | -0.688760 | 278 | 134.310054 | 214.27 | 294.229946 | 0.770755 |
| GO:0007169\_transmembrane\_receptor\_protein\_tyrosine\_kinase\_signaling\_pathway | 139 | 2 | 2.366393 | -0.685254 | 280 | 134.817714 | 215.05 | 295.282286 | 0.768036 |
| GO:0034613\_cellular\_protein\_localization | 139 | 2 | 2.366393 | -0.685254 | 280 | 134.817714 | 215.05 | 295.282286 | 0.768036 |
| GO:0043284\_biopolymer\_biosynthetic\_process | 807 | 7 | 1.426580 | -0.683003 | 281 | 134.938384 | 215.17 | 295.401616 | 0.765730 |
| GO:0042493\_response\_to\_drug | 38 | 1 | 4.328008 | -0.682758 | 284 | 137.929609 | 218.77 | 299.610391 | 0.770317 |
| GO:0045580\_regulation\_of\_T\_cell\_differentiation | 38 | 1 | 4.328008 | -0.682758 | 284 | 137.929609 | 218.77 | 299.610391 | 0.770317 |
| GO:0051348\_negative\_regulation\_of\_transferase\_activity | 38 | 1 | 4.328008 | -0.682758 | 284 | 137.929609 | 218.77 | 299.610391 | 0.770317 |
| GO:0016044\_membrane\_organization | 140 | 2 | 2.349490 | -0.680564 | 285 | 138.021848 | 219.01 | 299.998152 | 0.768456 |
| GO:0003006\_reproductive\_developmental\_process | 141 | 2 | 2.332827 | -0.675918 | 287 | 138.348626 | 219.5 | 300.651374 | 0.764808 |
| GO:0070727\_cellular\_macromolecule\_localization | 141 | 2 | 2.332827 | -0.675918 | 287 | 138.348626 | 219.5 | 300.651374 | 0.764808 |
| GO:0031326\_regulation\_of\_cellular\_biosynthetic\_process | 812 | 7 | 1.417796 | -0.673503 | 288 | 138.821225 | 220.2 | 301.578775 | 0.764583 |
| GO:0006511\_ubiquitin-dependent\_protein\_catabolic\_process | 39 | 1 | 4.217033 | -0.672713 | 292 | 141.146666 | 223.06 | 304.973334 | 0.763904 |
| GO:0042475\_odontogenesis\_of\_dentine-containing\_tooth | 39 | 1 | 4.217033 | -0.672713 | 292 | 141.146666 | 223.06 | 304.973334 | 0.763904 |
| GO:0048663\_neuron\_fate\_commitment | 39 | 1 | 4.217033 | -0.672713 | 292 | 141.146666 | 223.06 | 304.973334 | 0.763904 |
| GO:0070201\_regulation\_of\_establishment\_of\_protein\_localization | 39 | 1 | 4.217033 | -0.672713 | 292 | 141.146666 | 223.06 | 304.973334 | 0.763904 |
| GO:0009889\_regulation\_of\_biosynthetic\_process | 815 | 7 | 1.412577 | -0.667859 | 293 | 141.515779 | 223.53 | 305.544221 | 0.762901 |
| GO:0035272\_exocrine\_system\_development | 40 | 1 | 4.111607 | -0.662952 | 294 | 143.537245 | 226.16 | 308.782755 | 0.769252 |
| GO:0045449\_regulation\_of\_transcription | 676 | 6 | 1.459742 | -0.658228 | 295 | 144.056369 | 226.7 | 309.343631 | 0.768475 |
| GO:0006260\_DNA\_replication | 41 | 1 | 4.011324 | -0.653461 | 299 | 147.055213 | 230.65 | 314.244787 | 0.771405 |
| GO:0010551\_regulation\_of\_specific\_transcription\_from\_RNA\_polymerase\_II\_promoter | 41 | 1 | 4.011324 | -0.653461 | 299 | 147.055213 | 230.65 | 314.244787 | 0.771405 |
| GO:0015980\_energy\_derivation\_by\_oxidation\_of\_organic\_compounds | 41 | 1 | 4.011324 | -0.653461 | 299 | 147.055213 | 230.65 | 314.244787 | 0.771405 |
| GO:0032569\_specific\_transcription\_from\_RNA\_polymerase\_II\_promoter | 41 | 1 | 4.011324 | -0.653461 | 299 | 147.055213 | 230.65 | 314.244787 | 0.771405 |
| GO:0005975\_carbohydrate\_metabolic\_process | 146 | 2 | 2.252935 | -0.653324 | 300 | 147.389461 | 231.13 | 314.870539 | 0.770433 |
| GO:0006006\_glucose\_metabolic\_process | 42 | 1 | 3.915816 | -0.644228 | 306 | 150.725761 | 235.6 | 320.474239 | 0.769935 |
| GO:0008361\_regulation\_of\_cell\_size | 42 | 1 | 3.915816 | -0.644228 | 306 | 150.725761 | 235.6 | 320.474239 | 0.769935 |
| GO:0019941\_modification-dependent\_protein\_catabolic\_process | 42 | 1 | 3.915816 | -0.644228 | 306 | 150.725761 | 235.6 | 320.474239 | 0.769935 |
| GO:0042476\_odontogenesis | 42 | 1 | 3.915816 | -0.644228 | 306 | 150.725761 | 235.6 | 320.474239 | 0.769935 |
| GO:0043632\_modification-dependent\_macromolecule\_catabolic\_process | 42 | 1 | 3.915816 | -0.644228 | 306 | 150.725761 | 235.6 | 320.474239 | 0.769935 |
| GO:0051603\_proteolysis\_involved\_in\_cellular\_protein\_catabolic\_process | 42 | 1 | 3.915816 | -0.644228 | 306 | 150.725761 | 235.6 | 320.474239 | 0.769935 |
| GO:0008283\_cell\_proliferation | 544 | 5 | 1.511620 | -0.638509 | 307 | 151.119460 | 236.11 | 321.100540 | 0.769088 |
| GO:0007224\_smoothened\_signaling\_pathway | 43 | 1 | 3.824751 | -0.635239 | 310 | 153.858353 | 239.86 | 325.861647 | 0.773742 |
| GO:0010001\_glial\_cell\_differentiation | 43 | 1 | 3.824751 | -0.635239 | 310 | 153.858353 | 239.86 | 325.861647 | 0.773742 |
| GO:0032868\_response\_to\_insulin\_stimulus | 43 | 1 | 3.824751 | -0.635239 | 310 | 153.858353 | 239.86 | 325.861647 | 0.773742 |
| GO:0042221\_response\_to\_chemical\_stimulus | 409 | 4 | 1.608453 | -0.631110 | 311 | 154.026671 | 240.06 | 326.093329 | 0.771897 |
| GO:0035282\_segmentation | 44 | 1 | 3.737825 | -0.626485 | 314 | 157.715476 | 244.94 | 332.164524 | 0.780064 |
| GO:0044257\_cellular\_protein\_catabolic\_process | 44 | 1 | 3.737825 | -0.626485 | 314 | 157.715476 | 244.94 | 332.164524 | 0.780064 |
| GO:0050808\_synapse\_organization | 44 | 1 | 3.737825 | -0.626485 | 314 | 157.715476 | 244.94 | 332.164524 | 0.780064 |
| GO:0007242\_intracellular\_signaling\_cascade | 411 | 4 | 1.600626 | -0.626152 | 315 | 157.834530 | 245.09 | 332.345470 | 0.778063 |
| GO:0044260\_cellular\_macromolecule\_metabolic\_process | 1447 | 11 | 1.250247 | -0.620268 | 316 | 158.365885 | 245.95 | 333.534115 | 0.778323 |
| GO:0032870\_cellular\_response\_to\_hormone\_stimulus | 45 | 1 | 3.654762 | -0.617954 | 319 | 159.533814 | 247.41 | 335.286186 | 0.775580 |
| GO:0043623\_cellular\_protein\_complex\_assembly | 45 | 1 | 3.654762 | -0.617954 | 319 | 159.533814 | 247.41 | 335.286186 | 0.775580 |
| GO:0046546\_development\_of\_primary\_male\_sexual\_characteristics | 45 | 1 | 3.654762 | -0.617954 | 319 | 159.533814 | 247.41 | 335.286186 | 0.775580 |
| GO:0008285\_negative\_regulation\_of\_cell\_proliferation | 155 | 2 | 2.122120 | -0.615154 | 321 | 159.849509 | 247.83 | 335.810491 | 0.772056 |
| GO:0022402\_cell\_cycle\_process | 155 | 2 | 2.122120 | -0.615154 | 321 | 159.849509 | 247.83 | 335.810491 | 0.772056 |
| GO:0048518\_positive\_regulation\_of\_biological\_process | 995 | 8 | 1.322326 | -0.611481 | 322 | 160.076296 | 248.15 | 336.223704 | 0.770652 |
| GO:0006807\_nitrogen\_compound\_metabolic\_process | 1147 | 9 | 1.290478 | -0.609745 | 323 | 160.307448 | 248.42 | 336.532552 | 0.769102 |
| GO:0042063\_gliogenesis | 46 | 1 | 3.575311 | -0.609636 | 325 | 161.845285 | 250.45 | 339.054715 | 0.770615 |
| GO:0051098\_regulation\_of\_binding | 46 | 1 | 3.575311 | -0.609636 | 325 | 161.845285 | 250.45 | 339.054715 | 0.770615 |
| GO:0006350\_transcription | 701 | 6 | 1.407683 | -0.609558 | 326 | 161.994462 | 250.58 | 339.165538 | 0.768650 |
| GO:0044267\_cellular\_protein\_metabolic\_process | 559 | 5 | 1.471058 | -0.606553 | 327 | 162.413210 | 251.18 | 339.946790 | 0.768135 |
| GO:0044249\_cellular\_biosynthetic\_process | 1150 | 9 | 1.287112 | -0.605159 | 328 | 162.580174 | 251.45 | 340.319826 | 0.766616 |
| GO:0045619\_regulation\_of\_lymphocyte\_differentiation | 47 | 1 | 3.499240 | -0.601523 | 329 | 164.758833 | 254.58 | 344.401167 | 0.773799 |
| GO:0006139\_nucleobase\_\_nucleoside\_\_nucleotide\_and\_nucleic\_acid\_metabolic\_process | 1002 | 8 | 1.313088 | -0.600211 | 330 | 164.886320 | 254.69 | 344.493680 | 0.771788 |
| GO:0007417\_central\_nervous\_system\_development | 287 | 3 | 1.719139 | -0.598378 | 331 | 164.942747 | 254.83 | 344.717253 | 0.769879 |
| GO:0051128\_regulation\_of\_cellular\_component\_organization | 160 | 2 | 2.055804 | -0.595222 | 332 | 165.227700 | 255.17 | 345.112300 | 0.768584 |
| GO:0007498\_mesoderm\_development | 48 | 1 | 3.426339 | -0.593605 | 335 | 166.524030 | 256.79 | 347.055970 | 0.766537 |
| GO:0019318\_hexose\_metabolic\_process | 48 | 1 | 3.426339 | -0.593605 | 335 | 166.524030 | 256.79 | 347.055970 | 0.766537 |
| GO:0032269\_negative\_regulation\_of\_cellular\_protein\_metabolic\_process | 48 | 1 | 3.426339 | -0.593605 | 335 | 166.524030 | 256.79 | 347.055970 | 0.766537 |
| GO:0019220\_regulation\_of\_phosphate\_metabolic\_process | 165 | 2 | 1.993506 | -0.576128 | 337 | 171.492678 | 263.46 | 355.427322 | 0.781780 |
| GO:0051174\_regulation\_of\_phosphorus\_metabolic\_process | 165 | 2 | 1.993506 | -0.576128 | 337 | 171.492678 | 263.46 | 355.427322 | 0.781780 |
| GO:0010604\_positive\_regulation\_of\_macromolecule\_metabolic\_process | 433 | 4 | 1.519301 | -0.574200 | 338 | 171.782427 | 264.08 | 356.377573 | 0.781302 |
| GO:0032583\_regulation\_of\_gene-specific\_transcription | 51 | 1 | 3.224790 | -0.570946 | 340 | 173.935338 | 266.66 | 359.384662 | 0.784294 |
| GO:0032880\_regulation\_of\_protein\_localization | 51 | 1 | 3.224790 | -0.570946 | 340 | 173.935338 | 266.66 | 359.384662 | 0.784294 |
| GO:0006357\_regulation\_of\_transcription\_from\_RNA\_polymerase\_II\_promoter | 435 | 4 | 1.512315 | -0.569702 | 341 | 174.063469 | 266.78 | 359.496531 | 0.782346 |
| GO:0009058\_biosynthetic\_process | 1175 | 9 | 1.259726 | -0.568015 | 342 | 174.513532 | 267.39 | 360.266468 | 0.781842 |
| GO:0043283\_biopolymer\_metabolic\_process | 1490 | 11 | 1.214166 | -0.560920 | 343 | 175.512096 | 268.81 | 362.107904 | 0.783703 |
| GO:0030031\_cell\_projection\_assembly | 53 | 1 | 3.103100 | -0.556682 | 345 | 177.721314 | 271.35 | 364.978686 | 0.786522 |
| GO:0051248\_negative\_regulation\_of\_protein\_metabolic\_process | 53 | 1 | 3.103100 | -0.556682 | 345 | 177.721314 | 271.35 | 364.978686 | 0.786522 |
| GO:0006366\_transcription\_from\_RNA\_polymerase\_II\_promoter | 444 | 4 | 1.481660 | -0.549901 | 346 | 178.434608 | 272.4 | 366.365392 | 0.787283 |
| GO:0006091\_generation\_of\_precursor\_metabolites\_and\_energy | 54 | 1 | 3.045635 | -0.549783 | 352 | 180.371325 | 274.74 | 369.108675 | 0.780511 |
| GO:0006164\_purine\_nucleotide\_biosynthetic\_process | 54 | 1 | 3.045635 | -0.549783 | 352 | 180.371325 | 274.74 | 369.108675 | 0.780511 |
| GO:0007265\_Ras\_protein\_signal\_transduction | 54 | 1 | 3.045635 | -0.549783 | 352 | 180.371325 | 274.74 | 369.108675 | 0.780511 |
| GO:0009566\_fertilization | 54 | 1 | 3.045635 | -0.549783 | 352 | 180.371325 | 274.74 | 369.108675 | 0.780511 |
| GO:0043405\_regulation\_of\_MAP\_kinase\_activity | 54 | 1 | 3.045635 | -0.549783 | 352 | 180.371325 | 274.74 | 369.108675 | 0.780511 |
| GO:0044271\_nitrogen\_compound\_biosynthetic\_process | 54 | 1 | 3.045635 | -0.549783 | 352 | 180.371325 | 274.74 | 369.108675 | 0.780511 |
| GO:0010926\_anatomical\_structure\_formation | 447 | 4 | 1.471716 | -0.543457 | 353 | 181.325019 | 276.04 | 370.754981 | 0.781983 |
| GO:0007126\_meiosis | 55 | 1 | 2.990260 | -0.543032 | 357 | 182.840961 | 277.67 | 372.499039 | 0.777787 |
| GO:0043434\_response\_to\_peptide\_hormone\_stimulus | 55 | 1 | 2.990260 | -0.543032 | 357 | 182.840961 | 277.67 | 372.499039 | 0.777787 |
| GO:0048568\_embryonic\_organ\_development | 55 | 1 | 2.990260 | -0.543032 | 357 | 182.840961 | 277.67 | 372.499039 | 0.777787 |
| GO:0051327\_M\_phase\_of\_meiotic\_cell\_cycle | 55 | 1 | 2.990260 | -0.543032 | 357 | 182.840961 | 277.67 | 372.499039 | 0.777787 |
| GO:0051094\_positive\_regulation\_of\_developmental\_process | 308 | 3 | 1.601925 | -0.541455 | 358 | 183.063011 | 277.85 | 372.636989 | 0.776117 |
| GO:0016310\_phosphorylation | 309 | 3 | 1.596741 | -0.538898 | 359 | 183.801334 | 278.73 | 373.658666 | 0.776407 |
| GO:0001708\_cell\_fate\_specification | 56 | 1 | 2.936862 | -0.536423 | 361 | 186.469749 | 282.18 | 377.890251 | 0.781662 |
| GO:0051321\_meiotic\_cell\_cycle | 56 | 1 | 2.936862 | -0.536423 | 361 | 186.469749 | 282.18 | 377.890251 | 0.781662 |
| GO:0007166\_cell\_surface\_receptor\_linked\_signal\_transduction | 597 | 5 | 1.377423 | -0.532323 | 362 | 186.652501 | 282.54 | 378.427499 | 0.780497 |
| GO:0048522\_positive\_regulation\_of\_cellular\_process | 895 | 7 | 1.286313 | -0.531863 | 363 | 186.672839 | 282.6 | 378.527161 | 0.778512 |
| GO:0010556\_regulation\_of\_macromolecule\_biosynthetic\_process | 745 | 6 | 1.324545 | -0.531800 | 364 | 186.867371 | 282.76 | 378.652629 | 0.776813 |
| GO:0033365\_protein\_localization\_in\_organelle | 57 | 1 | 2.885338 | -0.529952 | 366 | 189.523128 | 285.78 | 382.036872 | 0.780820 |
| GO:0045444\_fat\_cell\_differentiation | 57 | 1 | 2.885338 | -0.529952 | 366 | 189.523128 | 285.78 | 382.036872 | 0.780820 |
| GO:0034622\_cellular\_macromolecular\_complex\_assembly | 58 | 1 | 2.835591 | -0.523613 | 367 | 190.617441 | 287.07 | 383.522559 | 0.782207 |
| GO:0045184\_establishment\_of\_protein\_localization | 180 | 2 | 1.827381 | -0.523375 | 368 | 190.760228 | 287.19 | 383.619772 | 0.780408 |
| GO:0034645\_cellular\_macromolecule\_biosynthetic\_process | 901 | 7 | 1.277747 | -0.522711 | 369 | 190.888283 | 287.34 | 383.791717 | 0.778699 |
| GO:0016055\_Wnt\_receptor\_signaling\_pathway | 59 | 1 | 2.787530 | -0.517403 | 371 | 192.552698 | 289.71 | 386.867302 | 0.780889 |
| GO:0050870\_positive\_regulation\_of\_T\_cell\_activation | 59 | 1 | 2.787530 | -0.517403 | 371 | 192.552698 | 289.71 | 386.867302 | 0.780889 |
| GO:0019219\_regulation\_of\_nucleobase\_\_nucleoside\_\_nucleotide\_and\_nucleic\_acid\_metabolic\_process | 757 | 6 | 1.303548 | -0.512208 | 372 | 193.286905 | 290.66 | 388.033095 | 0.781344 |
| GO:0009059\_macromolecule\_biosynthetic\_process | 910 | 7 | 1.265110 | -0.509238 | 373 | 194.040721 | 291.54 | 389.039279 | 0.781609 |
| GO:0007165\_signal\_transduction | 915 | 7 | 1.258197 | -0.501882 | 374 | 195.801076 | 293.84 | 391.878924 | 0.785668 |
| GO:0022604\_regulation\_of\_cell\_morphogenesis | 62 | 1 | 2.652650 | -0.499496 | 375 | 197.595151 | 296.36 | 395.124849 | 0.790293 |
| GO:0007369\_gastrulation | 63 | 1 | 2.610544 | -0.493757 | 378 | 199.261994 | 298.39 | 397.518006 | 0.789392 |
| GO:0009165\_nucleotide\_biosynthetic\_process | 63 | 1 | 2.610544 | -0.493757 | 378 | 199.261994 | 298.39 | 397.518006 | 0.789392 |
| GO:0051216\_cartilage\_development | 63 | 1 | 2.610544 | -0.493757 | 378 | 199.261994 | 298.39 | 397.518006 | 0.789392 |
| GO:0080090\_regulation\_of\_primary\_metabolic\_process | 926 | 7 | 1.243251 | -0.486019 | 379 | 201.215781 | 301.08 | 400.944219 | 0.794406 |
| GO:0043086\_negative\_regulation\_of\_catalytic\_activity | 65 | 1 | 2.530220 | -0.482598 | 380 | 202.442400 | 302.58 | 402.717600 | 0.796263 |
| GO:0046907\_intracellular\_transport | 194 | 2 | 1.695508 | -0.479509 | 381 | 202.770625 | 302.98 | 403.189375 | 0.795223 |
| GO:0010468\_regulation\_of\_gene\_expression | 778 | 6 | 1.268362 | -0.479468 | 382 | 202.948704 | 303.12 | 403.291296 | 0.793508 |
| GO:0007507\_heart\_development | 195 | 2 | 1.686813 | -0.476552 | 383 | 204.706351 | 305.27 | 405.833649 | 0.797050 |
| GO:0060255\_regulation\_of\_macromolecule\_metabolic\_process | 936 | 7 | 1.229968 | -0.471972 | 384 | 205.142329 | 305.74 | 406.337671 | 0.796198 |
| GO:0002009\_morphogenesis\_of\_an\_epithelium | 198 | 2 | 1.661255 | -0.467816 | 386 | 207.083261 | 308.11 | 409.136739 | 0.798212 |
| GO:0060429\_epithelium\_development | 198 | 2 | 1.661255 | -0.467816 | 386 | 207.083261 | 308.11 | 409.136739 | 0.798212 |
| GO:0034962\_cellular\_biopolymer\_catabolic\_process | 68 | 1 | 2.418592 | -0.466617 | 388 | 207.965684 | 309.25 | 410.534316 | 0.797036 |
| GO:0042692\_muscle\_cell\_differentiation | 68 | 1 | 2.418592 | -0.466617 | 388 | 207.965684 | 309.25 | 410.534316 | 0.797036 |
| GO:0008406\_gonad\_development | 70 | 1 | 2.349490 | -0.456431 | 390 | 210.999696 | 313.13 | 415.260304 | 0.802897 |
| GO:0048592\_eye\_morphogenesis | 70 | 1 | 2.349490 | -0.456431 | 390 | 210.999696 | 313.13 | 415.260304 | 0.802897 |
| GO:0043170\_macromolecule\_metabolic\_process | 1576 | 11 | 1.147911 | -0.455211 | 391 | 211.173546 | 313.34 | 415.506454 | 0.801381 |
| GO:0006913\_nucleocytoplasmic\_transport | 71 | 1 | 2.316398 | -0.451471 | 392 | 212.942069 | 315.28 | 417.617931 | 0.804286 |
| GO:0022607\_cellular\_component\_assembly | 204 | 2 | 1.612395 | -0.450918 | 393 | 213.170191 | 315.49 | 417.809809 | 0.802774 |
| GO:0021915\_neural\_tube\_development | 72 | 1 | 2.284226 | -0.446596 | 396 | 216.197899 | 319.17 | 422.142101 | 0.805985 |
| GO:0030879\_mammary\_gland\_development | 72 | 1 | 2.284226 | -0.446596 | 396 | 216.197899 | 319.17 | 422.142101 | 0.805985 |
| GO:0051169\_nuclear\_transport | 72 | 1 | 2.284226 | -0.446596 | 396 | 216.197899 | 319.17 | 422.142101 | 0.805985 |
| GO:0048869\_cellular\_developmental\_process | 1113 | 8 | 1.182133 | -0.443306 | 397 | 216.482282 | 319.45 | 422.417718 | 0.804660 |
| GO:0006163\_purine\_nucleotide\_metabolic\_process | 73 | 1 | 2.252935 | -0.441803 | 398 | 217.707457 | 321.04 | 424.372543 | 0.806633 |
| GO:0008284\_positive\_regulation\_of\_cell\_proliferation | 208 | 2 | 1.581387 | -0.440061 | 399 | 218.300505 | 321.77 | 425.239495 | 0.806441 |
| GO:0048468\_cell\_development | 654 | 5 | 1.257372 | -0.436845 | 400 | 219.026401 | 322.71 | 426.393599 | 0.806775 |
| GO:0019538\_protein\_metabolic\_process | 655 | 5 | 1.255453 | -0.435322 | 401 | 219.075142 | 322.84 | 426.604858 | 0.805087 |
| GO:0016043\_cellular\_component\_organization | 964 | 7 | 1.194243 | -0.434462 | 402 | 219.117012 | 322.93 | 426.742988 | 0.803308 |
| GO:0044265\_cellular\_macromolecule\_catabolic\_process | 75 | 1 | 2.192857 | -0.432458 | 403 | 220.045375 | 324.13 | 428.214625 | 0.804293 |
| GO:0006508\_proteolysis | 76 | 1 | 2.164004 | -0.427900 | 406 | 221.848721 | 326.09 | 430.331279 | 0.803177 |
| GO:0009725\_response\_to\_hormone\_stimulus | 76 | 1 | 2.164004 | -0.427900 | 406 | 221.848721 | 326.09 | 430.331279 | 0.803177 |
| GO:0034621\_cellular\_macromolecular\_complex\_subunit\_organization | 76 | 1 | 2.164004 | -0.427900 | 406 | 221.848721 | 326.09 | 430.331279 | 0.803177 |
| GO:0006461\_protein\_complex\_assembly | 78 | 1 | 2.108516 | -0.419005 | 409 | 224.768132 | 329.39 | 434.011868 | 0.805355 |
| GO:0051251\_positive\_regulation\_of\_lymphocyte\_activation | 78 | 1 | 2.108516 | -0.419005 | 409 | 224.768132 | 329.39 | 434.011868 | 0.805355 |
| GO:0070271\_protein\_complex\_biogenesis | 78 | 1 | 2.108516 | -0.419005 | 409 | 224.768132 | 329.39 | 434.011868 | 0.805355 |
| GO:0044092\_negative\_regulation\_of\_molecular\_function | 80 | 1 | 2.055804 | -0.410391 | 410 | 227.799087 | 333.21 | 438.620913 | 0.812707 |
| GO:0001701\_in\_utero\_embryonic\_development | 221 | 2 | 1.488365 | -0.406864 | 411 | 228.340629 | 333.92 | 439.499371 | 0.812457 |
| GO:0051641\_cellular\_localization | 370 | 3 | 1.333494 | -0.405122 | 412 | 228.848815 | 334.6 | 440.351185 | 0.812136 |
| GO:0002696\_positive\_regulation\_of\_leukocyte\_activation | 82 | 1 | 2.005662 | -0.402044 | 415 | 230.620588 | 336.61 | 442.599412 | 0.811108 |
| GO:0007411\_axon\_guidance | 82 | 1 | 2.005662 | -0.402044 | 415 | 230.620588 | 336.61 | 442.599412 | 0.811108 |
| GO:0045664\_regulation\_of\_neuron\_differentiation | 82 | 1 | 2.005662 | -0.402044 | 415 | 230.620588 | 336.61 | 442.599412 | 0.811108 |
| GO:0006325\_chromatin\_organization | 83 | 1 | 1.981497 | -0.397966 | 417 | 232.548145 | 338.65 | 444.751855 | 0.812110 |
| GO:0050867\_positive\_regulation\_of\_cell\_activation | 83 | 1 | 1.981497 | -0.397966 | 417 | 232.548145 | 338.65 | 444.751855 | 0.812110 |
| GO:0045137\_development\_of\_primary\_sexual\_characteristics | 84 | 1 | 1.957908 | -0.393951 | 418 | 233.181093 | 339.53 | 445.878907 | 0.812273 |
| GO:0000279\_M\_phase | 85 | 1 | 1.934874 | -0.389996 | 419 | 234.546850 | 341.19 | 447.833150 | 0.814296 |
| GO:0006605\_protein\_targeting | 86 | 1 | 1.912375 | -0.386100 | 423 | 237.164291 | 343.92 | 450.675709 | 0.813050 |
| GO:0032504\_multicellular\_organism\_reproduction | 86 | 1 | 1.912375 | -0.386100 | 423 | 237.164291 | 343.92 | 450.675709 | 0.813050 |
| GO:0034641\_cellular\_nitrogen\_compound\_metabolic\_process | 86 | 1 | 1.912375 | -0.386100 | 423 | 237.164291 | 343.92 | 450.675709 | 0.813050 |
| GO:0048609\_reproductive\_process\_in\_a\_multicellular\_organism | 86 | 1 | 1.912375 | -0.386100 | 423 | 237.164291 | 343.92 | 450.675709 | 0.813050 |
| GO:0007178\_transmembrane\_receptor\_protein\_serine\_threonine\_kinase\_signaling\_pathway | 87 | 1 | 1.890394 | -0.382261 | 425 | 239.019837 | 346.45 | 453.880163 | 0.815176 |
| GO:0022612\_gland\_morphogenesis | 87 | 1 | 1.890394 | -0.382261 | 425 | 239.019837 | 346.45 | 453.880163 | 0.815176 |
| GO:0043687\_post-translational\_protein\_modification | 384 | 3 | 1.284877 | -0.379680 | 426 | 239.343872 | 346.76 | 454.176128 | 0.813991 |
| GO:0050863\_regulation\_of\_T\_cell\_activation | 88 | 1 | 1.868912 | -0.378479 | 427 | 240.407484 | 348.07 | 455.732516 | 0.815152 |
| GO:0008152\_metabolic\_process | 2133 | 14 | 1.079466 | -0.378253 | 428 | 240.547429 | 348.19 | 455.832571 | 0.813528 |
| GO:0044237\_cellular\_metabolic\_process | 1974 | 13 | 1.083098 | -0.375483 | 429 | 241.092572 | 348.81 | 456.527428 | 0.813077 |
| GO:0030324\_lung\_development | 90 | 1 | 1.827381 | -0.371079 | 430 | 242.769956 | 351.04 | 459.310044 | 0.816372 |
| GO:0006468\_protein\_amino\_acid\_phosphorylation | 237 | 2 | 1.387884 | -0.369949 | 432 | 243.345818 | 351.81 | 460.274182 | 0.814375 |
| GO:0044085\_cellular\_component\_biogenesis | 237 | 2 | 1.387884 | -0.369949 | 432 | 243.345818 | 351.81 | 460.274182 | 0.814375 |
| GO:0008544\_epidermis\_development | 91 | 1 | 1.807300 | -0.367458 | 434 | 244.806958 | 353.44 | 462.073042 | 0.814378 |
| GO:0031399\_regulation\_of\_protein\_modification\_process | 91 | 1 | 1.807300 | -0.367458 | 434 | 244.806958 | 353.44 | 462.073042 | 0.814378 |
| GO:0042127\_regulation\_of\_cell\_proliferation | 393 | 3 | 1.255453 | -0.364200 | 435 | 244.972824 | 353.68 | 462.387176 | 0.813057 |
| GO:0009719\_response\_to\_endogenous\_stimulus | 92 | 1 | 1.787655 | -0.363888 | 438 | 245.785608 | 354.62 | 463.454392 | 0.809635 |
| GO:0030217\_T\_cell\_differentiation | 92 | 1 | 1.787655 | -0.363888 | 438 | 245.785608 | 354.62 | 463.454392 | 0.809635 |
| GO:0030323\_respiratory\_tube\_development | 92 | 1 | 1.787655 | -0.363888 | 438 | 245.785608 | 354.62 | 463.454392 | 0.809635 |
| GO:0035107\_appendage\_morphogenesis | 93 | 1 | 1.768433 | -0.360369 | 441 | 247.229624 | 356.07 | 464.910376 | 0.807415 |
| GO:0035108\_limb\_morphogenesis | 93 | 1 | 1.768433 | -0.360369 | 441 | 247.229624 | 356.07 | 464.910376 | 0.807415 |
| GO:0065003\_macromolecular\_complex\_assembly | 93 | 1 | 1.768433 | -0.360369 | 441 | 247.229624 | 356.07 | 464.910376 | 0.807415 |
| GO:0048699\_generation\_of\_neurons | 396 | 3 | 1.245942 | -0.359185 | 442 | 247.494338 | 356.34 | 465.185662 | 0.806199 |
| GO:0006753\_nucleoside\_phosphate\_metabolic\_process | 94 | 1 | 1.749620 | -0.356898 | 444 | 249.203400 | 358.37 | 467.536600 | 0.807140 |
| GO:0009117\_nucleotide\_metabolic\_process | 94 | 1 | 1.749620 | -0.356898 | 444 | 249.203400 | 358.37 | 467.536600 | 0.807140 |
| GO:0048736\_appendage\_development | 96 | 1 | 1.713170 | -0.350101 | 447 | 251.268145 | 360.6 | 469.931855 | 0.806711 |
| GO:0060173\_limb\_development | 96 | 1 | 1.713170 | -0.350101 | 447 | 251.268145 | 360.6 | 469.931855 | 0.806711 |
| GO:0060249\_anatomical\_structure\_homeostasis | 96 | 1 | 1.713170 | -0.350101 | 447 | 251.268145 | 360.6 | 469.931855 | 0.806711 |
| GO:0060541\_respiratory\_system\_development | 98 | 1 | 1.678207 | -0.343487 | 448 | 253.528227 | 363.16 | 472.791773 | 0.810625 |
| GO:0048513\_organ\_development | 1365 | 9 | 1.084380 | -0.341212 | 449 | 253.863323 | 363.51 | 473.156677 | 0.809599 |
| GO:0009790\_embryonic\_development | 567 | 4 | 1.160242 | -0.338611 | 450 | 255.646441 | 365.54 | 475.433559 | 0.812311 |
| GO:0030163\_protein\_catabolic\_process | 101 | 1 | 1.628359 | -0.333895 | 451 | 257.472032 | 367.55 | 477.627968 | 0.814967 |
| GO:0009966\_regulation\_of\_signal\_transduction | 256 | 2 | 1.284877 | -0.330997 | 452 | 257.938243 | 368.11 | 478.281757 | 0.814403 |
| GO:0030036\_actin\_cytoskeleton\_organization | 102 | 1 | 1.612395 | -0.330782 | 453 | 258.269872 | 368.48 | 478.690128 | 0.813422 |
| GO:0006355\_regulation\_of\_transcription\_\_DNA-dependent | 575 | 4 | 1.144099 | -0.327998 | 454 | 258.943182 | 369.06 | 479.176818 | 0.812907 |
| GO:0051179\_localization | 1058 | 7 | 1.088138 | -0.326333 | 455 | 260.359820 | 370.48 | 480.600180 | 0.814242 |
| GO:0055086\_nucleobase\_\_nucleoside\_and\_nucleotide\_metabolic\_process | 104 | 1 | 1.581387 | -0.324677 | 456 | 260.972305 | 371.21 | 481.447695 | 0.814057 |
| GO:0030154\_cell\_differentiation | 1060 | 7 | 1.086085 | -0.324304 | 457 | 261.123844 | 371.35 | 481.576156 | 0.812582 |
| GO:0010467\_gene\_expression | 905 | 6 | 1.090371 | -0.317830 | 458 | 263.118507 | 373.64 | 484.161493 | 0.815808 |
| GO:0030030\_cell\_projection\_organization | 263 | 2 | 1.250679 | -0.317827 | 459 | 263.410319 | 373.9 | 484.389681 | 0.814597 |
| GO:0022008\_neurogenesis | 423 | 3 | 1.166413 | -0.317086 | 460 | 263.643140 | 374.14 | 484.636860 | 0.813348 |
| GO:0045859\_regulation\_of\_protein\_kinase\_activity | 107 | 1 | 1.537049 | -0.315810 | 461 | 264.310453 | 374.89 | 485.469547 | 0.813210 |
| GO:0030029\_actin\_filament-based\_process | 109 | 1 | 1.508847 | -0.310084 | 462 | 266.063160 | 376.81 | 487.556840 | 0.815606 |
| GO:0051252\_regulation\_of\_RNA\_metabolic\_process | 590 | 4 | 1.115012 | -0.308938 | 463 | 266.334161 | 377.01 | 487.685839 | 0.814276 |
| GO:0043010\_camera-type\_eye\_development | 110 | 1 | 1.495130 | -0.307274 | 464 | 267.760089 | 378.48 | 489.199911 | 0.815690 |
| GO:0006351\_transcription\_\_DNA-dependent | 594 | 4 | 1.107504 | -0.304034 | 465 | 268.822741 | 379.54 | 490.257259 | 0.816215 |
| GO:0032774\_RNA\_biosynthetic\_process | 595 | 4 | 1.105642 | -0.302819 | 466 | 269.463657 | 380.14 | 490.816343 | 0.815751 |
| GO:0043549\_regulation\_of\_kinase\_activity | 112 | 1 | 1.468431 | -0.301759 | 468 | 270.534662 | 381.22 | 491.905338 | 0.814573 |
| GO:0051249\_regulation\_of\_lymphocyte\_activation | 112 | 1 | 1.468431 | -0.301759 | 468 | 270.534662 | 381.22 | 491.905338 | 0.814573 |
| GO:0032502\_developmental\_process | 2060 | 13 | 1.037881 | -0.299998 | 469 | 271.456125 | 382.16 | 492.863875 | 0.814840 |
| GO:0006464\_protein\_modification\_process | 439 | 3 | 1.123902 | -0.294516 | 470 | 273.753807 | 384.64 | 495.526193 | 0.818383 |
| GO:0044238\_primary\_metabolic\_process | 1905 | 12 | 1.035996 | -0.293896 | 471 | 273.912397 | 384.8 | 495.687603 | 0.816985 |
| GO:0051338\_regulation\_of\_transferase\_activity | 115 | 1 | 1.430124 | -0.293736 | 472 | 274.526364 | 385.53 | 496.533636 | 0.816801 |
| GO:0048646\_anatomical\_structure\_formation\_involved\_in\_morphogenesis | 277 | 2 | 1.187468 | -0.293196 | 473 | 275.017910 | 386.11 | 497.202090 | 0.816300 |
| GO:0046483\_heterocycle\_metabolic\_process | 116 | 1 | 1.417796 | -0.291125 | 475 | 276.042830 | 387.24 | 498.437170 | 0.815242 |
| GO:0048608\_reproductive\_structure\_development | 116 | 1 | 1.417796 | -0.291125 | 475 | 276.042830 | 387.24 | 498.437170 | 0.815242 |
| GO:0065009\_regulation\_of\_molecular\_function | 279 | 2 | 1.178955 | -0.289852 | 476 | 276.777773 | 388.0 | 499.222227 | 0.815126 |
| GO:0043933\_macromolecular\_complex\_subunit\_organization | 117 | 1 | 1.405678 | -0.288546 | 477 | 277.241362 | 388.45 | 499.658638 | 0.814361 |
| GO:0022403\_cell\_cycle\_phase | 119 | 1 | 1.382053 | -0.283479 | 478 | 279.829707 | 391.06 | 502.290293 | 0.818117 |
| GO:0000902\_cell\_morphogenesis | 283 | 2 | 1.162292 | -0.283288 | 479 | 280.103863 | 391.33 | 502.556137 | 0.816973 |
| GO:0006996\_organelle\_organization | 449 | 3 | 1.098871 | -0.281229 | 480 | 280.640631 | 391.89 | 503.139369 | 0.816437 |
| GO:0002694\_regulation\_of\_leukocyte\_activation | 121 | 1 | 1.359209 | -0.278531 | 483 | 283.143077 | 394.55 | 505.956923 | 0.816874 |
| GO:0006917\_induction\_of\_apoptosis | 121 | 1 | 1.359209 | -0.278531 | 483 | 283.143077 | 394.55 | 505.956923 | 0.816874 |
| GO:0012502\_induction\_of\_programmed\_cell\_death | 121 | 1 | 1.359209 | -0.278531 | 483 | 283.143077 | 394.55 | 505.956923 | 0.816874 |
| GO:0006886\_intracellular\_protein\_transport | 122 | 1 | 1.348068 | -0.276100 | 485 | 285.724986 | 397.24 | 508.755014 | 0.819052 |
| GO:0050865\_regulation\_of\_cell\_activation | 122 | 1 | 1.348068 | -0.276100 | 485 | 285.724986 | 397.24 | 508.755014 | 0.819052 |
| GO:0007399\_nervous\_system\_development | 621 | 4 | 1.059351 | -0.272780 | 486 | 285.982892 | 397.55 | 509.117108 | 0.818004 |
| GO:0030098\_lymphocyte\_differentiation | 124 | 1 | 1.326325 | -0.271321 | 487 | 286.643241 | 398.34 | 510.036759 | 0.817947 |
| GO:0043412\_biopolymer\_modification | 458 | 3 | 1.077277 | -0.269777 | 488 | 287.279459 | 398.97 | 510.660541 | 0.817561 |
| GO:0043062\_extracellular\_structure\_organization | 125 | 1 | 1.315714 | -0.268972 | 489 | 287.875953 | 399.6 | 511.324047 | 0.817178 |
| GO:0009653\_anatomical\_structure\_morphogenesis | 958 | 6 | 1.030048 | -0.265839 | 490 | 288.366674 | 400.01 | 511.653326 | 0.816347 |
| GO:0043285\_biopolymer\_catabolic\_process | 129 | 1 | 1.274917 | -0.259841 | 492 | 291.042998 | 402.99 | 514.937002 | 0.819085 |
| GO:0051276\_chromosome\_organization | 129 | 1 | 1.274917 | -0.259841 | 492 | 291.042998 | 402.99 | 514.937002 | 0.819085 |
| GO:0032787\_monocarboxylic\_acid\_metabolic\_process | 130 | 1 | 1.265110 | -0.257621 | 494 | 292.225606 | 404.33 | 516.434394 | 0.818482 |
| GO:0045165\_cell\_fate\_commitment | 130 | 1 | 1.265110 | -0.257621 | 494 | 292.225606 | 404.33 | 516.434394 | 0.818482 |
| GO:0009887\_organ\_morphogenesis | 642 | 4 | 1.024700 | -0.250558 | 495 | 293.624493 | 405.7 | 517.775507 | 0.819596 |
| GO:0032989\_cellular\_component\_morphogenesis | 307 | 2 | 1.071429 | -0.247131 | 496 | 295.138392 | 407.52 | 519.901608 | 0.821613 |
| GO:0001654\_eye\_development | 136 | 1 | 1.209296 | -0.244807 | 497 | 296.166218 | 408.68 | 521.193782 | 0.822294 |
| GO:0009057\_macromolecule\_catabolic\_process | 137 | 1 | 1.200469 | -0.242752 | 498 | 296.959816 | 409.46 | 521.960184 | 0.822209 |
| GO:0016070\_RNA\_metabolic\_process | 658 | 4 | 0.999783 | -0.234759 | 499 | 299.356024 | 411.46 | 523.563976 | 0.824569 |
| GO:0002684\_positive\_regulation\_of\_immune\_system\_process | 148 | 1 | 1.111245 | -0.221526 | 500 | 303.829227 | 416.12 | 528.410773 | 0.832240 |
| GO:0010646\_regulation\_of\_cell\_communication | 330 | 2 | 0.996753 | -0.217063 | 501 | 305.501501 | 417.72 | 529.938499 | 0.833772 |
| GO:0010605\_negative\_regulation\_of\_macromolecule\_metabolic\_process | 331 | 2 | 0.993742 | -0.215847 | 502 | 306.175523 | 418.47 | 530.764477 | 0.833606 |
| GO:0031324\_negative\_regulation\_of\_cellular\_metabolic\_process | 332 | 2 | 0.990749 | -0.214637 | 503 | 306.347947 | 418.67 | 530.992053 | 0.832346 |
| GO:0032268\_regulation\_of\_cellular\_protein\_metabolic\_process | 152 | 1 | 1.082002 | -0.214389 | 504 | 306.607146 | 419.03 | 531.452854 | 0.831409 |
| GO:0048856\_anatomical\_structure\_development | 1688 | 10 | 0.974314 | -0.213549 | 505 | 306.754644 | 419.16 | 531.565356 | 0.830020 |
| GO:0007268\_synaptic\_transmission | 154 | 1 | 1.067950 | -0.210927 | 506 | 307.540295 | 420.07 | 532.599705 | 0.830178 |
| GO:0009605\_response\_to\_external\_stimulus | 339 | 2 | 0.970291 | -0.206365 | 507 | 309.147264 | 421.71 | 534.272736 | 0.831775 |
| GO:0007409\_axonogenesis | 158 | 1 | 1.040913 | -0.204208 | 508 | 311.544073 | 424.19 | 536.835927 | 0.835020 |
| GO:0065008\_regulation\_of\_biological\_quality | 693 | 4 | 0.949289 | -0.203322 | 509 | 311.763515 | 424.39 | 537.016485 | 0.833772 |
| GO:0051649\_establishment\_of\_localization\_in\_cell | 342 | 2 | 0.961779 | -0.202923 | 510 | 312.043204 | 424.65 | 537.256796 | 0.832647 |
| GO:0002521\_leukocyte\_differentiation | 161 | 1 | 1.021517 | -0.199341 | 511 | 312.730189 | 425.42 | 538.109811 | 0.832524 |
| GO:0009892\_negative\_regulation\_of\_metabolic\_process | 348 | 2 | 0.945197 | -0.196214 | 512 | 314.093803 | 426.8 | 539.506197 | 0.833594 |
| GO:0042110\_T\_cell\_activation | 163 | 1 | 1.008983 | -0.196174 | 513 | 314.876117 | 427.58 | 540.283883 | 0.833489 |
| GO:0042325\_regulation\_of\_phosphorylation | 164 | 1 | 1.002831 | -0.194614 | 514 | 315.482314 | 428.15 | 540.817686 | 0.832977 |
| GO:0006259\_DNA\_metabolic\_process | 165 | 1 | 0.996753 | -0.193068 | 515 | 316.544906 | 429.24 | 541.935094 | 0.833476 |
| GO:0043065\_positive\_regulation\_of\_apoptosis | 166 | 1 | 0.990749 | -0.191538 | 516 | 317.174773 | 429.94 | 542.705227 | 0.833217 |
| GO:0010942\_positive\_regulation\_of\_cell\_death | 167 | 1 | 0.984816 | -0.190022 | 518 | 318.251925 | 431.04 | 543.828075 | 0.832124 |
| GO:0043068\_positive\_regulation\_of\_programmed\_cell\_death | 167 | 1 | 0.984816 | -0.190022 | 518 | 318.251925 | 431.04 | 543.828075 | 0.832124 |
| GO:0048812\_neuron\_projection\_morphogenesis | 170 | 1 | 0.967437 | -0.185561 | 520 | 320.063506 | 432.7 | 545.336494 | 0.832115 |
| GO:0051246\_regulation\_of\_protein\_metabolic\_process | 170 | 1 | 0.967437 | -0.185561 | 520 | 320.063506 | 432.7 | 545.336494 | 0.832115 |
| GO:0042981\_regulation\_of\_apoptosis | 360 | 2 | 0.913690 | -0.183474 | 521 | 320.381415 | 432.96 | 545.538585 | 0.831017 |
| GO:0044093\_positive\_regulation\_of\_molecular\_function | 173 | 1 | 0.950661 | -0.181224 | 524 | 322.964830 | 435.42 | 547.875170 | 0.830954 |
| GO:0044248\_cellular\_catabolic\_process | 173 | 1 | 0.950661 | -0.181224 | 524 | 322.964830 | 435.42 | 547.875170 | 0.830954 |
| GO:0048667\_cell\_morphogenesis\_involved\_in\_neuron\_differentiation | 173 | 1 | 0.950661 | -0.181224 | 524 | 322.964830 | 435.42 | 547.875170 | 0.830954 |
| GO:0010941\_regulation\_of\_cell\_death | 365 | 2 | 0.901174 | -0.178419 | 526 | 324.589700 | 436.8 | 549.010300 | 0.830418 |
| GO:0043067\_regulation\_of\_programmed\_cell\_death | 365 | 2 | 0.901174 | -0.178419 | 526 | 324.589700 | 436.8 | 549.010300 | 0.830418 |
| GO:0015031\_protein\_transport | 175 | 1 | 0.939796 | -0.178400 | 527 | 325.779777 | 437.94 | 550.100223 | 0.831006 |
| GO:0043066\_negative\_regulation\_of\_apoptosis | 176 | 1 | 0.934456 | -0.177008 | 529 | 327.140352 | 439.25 | 551.359648 | 0.830340 |
| GO:0048858\_cell\_projection\_morphogenesis | 176 | 1 | 0.934456 | -0.177008 | 529 | 327.140352 | 439.25 | 551.359648 | 0.830340 |
| GO:0043069\_negative\_regulation\_of\_programmed\_cell\_death | 179 | 1 | 0.918795 | -0.172908 | 531 | 329.524111 | 441.63 | 553.735889 | 0.831695 |
| GO:0060548\_negative\_regulation\_of\_cell\_death | 179 | 1 | 0.918795 | -0.172908 | 531 | 329.524111 | 441.63 | 553.735889 | 0.831695 |
| GO:0007275\_multicellular\_organismal\_development | 1760 | 10 | 0.934456 | -0.170325 | 532 | 330.515082 | 442.61 | 554.704918 | 0.831974 |
| GO:0019752\_carboxylic\_acid\_metabolic\_process | 181 | 1 | 0.908642 | -0.170238 | 534 | 331.604374 | 443.8 | 555.995626 | 0.831086 |
| GO:0043436\_oxoacid\_metabolic\_process | 181 | 1 | 0.908642 | -0.170238 | 534 | 331.604374 | 443.8 | 555.995626 | 0.831086 |
| GO:0006082\_organic\_acid\_metabolic\_process | 182 | 1 | 0.903650 | -0.168920 | 535 | 331.974816 | 444.22 | 556.465184 | 0.830318 |
| GO:0042180\_cellular\_ketone\_metabolic\_process | 183 | 1 | 0.898712 | -0.167615 | 536 | 333.321653 | 445.63 | 557.938347 | 0.831399 |
| GO:0032990\_cell\_part\_morphogenesis | 184 | 1 | 0.893828 | -0.166322 | 537 | 334.097961 | 446.44 | 558.782039 | 0.831359 |
| GO:0007010\_cytoskeleton\_organization | 185 | 1 | 0.888996 | -0.165040 | 538 | 334.483579 | 446.83 | 559.176421 | 0.830539 |
| GO:0048731\_system\_development | 1609 | 9 | 0.919937 | -0.161574 | 539 | 337.334402 | 449.58 | 561.825598 | 0.834100 |
| GO:0019226\_transmission\_of\_nerve\_impulse | 189 | 1 | 0.870181 | -0.160028 | 540 | 338.318447 | 450.83 | 563.341553 | 0.834870 |
| GO:0050896\_response\_to\_stimulus | 1107 | 6 | 0.891405 | -0.156522 | 541 | 339.096876 | 451.67 | 564.243124 | 0.834880 |
| GO:0033554\_cellular\_response\_to\_stress | 196 | 1 | 0.839103 | -0.151677 | 542 | 342.294169 | 454.78 | 567.265831 | 0.839077 |
| GO:0031175\_neuron\_projection\_development | 197 | 1 | 0.834844 | -0.150526 | 543 | 342.969935 | 455.48 | 567.990065 | 0.838821 |
| GO:0000904\_cell\_morphogenesis\_involved\_in\_differentiation | 199 | 1 | 0.826454 | -0.148253 | 544 | 344.541758 | 457.14 | 569.738242 | 0.840331 |
| GO:0035295\_tube\_development | 212 | 1 | 0.775775 | -0.134404 | 545 | 351.085461 | 463.27 | 575.454539 | 0.850037 |
| GO:0007423\_sensory\_organ\_development | 219 | 1 | 0.750978 | -0.127558 | 546 | 353.738213 | 465.57 | 577.401787 | 0.852692 |
| GO:0006915\_apoptosis | 427 | 2 | 0.770325 | -0.126263 | 547 | 354.844865 | 466.49 | 578.135135 | 0.852815 |
| GO:0012501\_programmed\_cell\_death | 433 | 2 | 0.759650 | -0.122107 | 548 | 357.100604 | 468.4 | 579.699396 | 0.854745 |
| GO:0002682\_regulation\_of\_immune\_system\_process | 228 | 1 | 0.721335 | -0.119325 | 550 | 359.132552 | 470.35 | 581.567448 | 0.855182 |
| GO:0046649\_lymphocyte\_activation | 228 | 1 | 0.721335 | -0.119325 | 550 | 359.132552 | 470.35 | 581.567448 | 0.855182 |
| GO:0050790\_regulation\_of\_catalytic\_activity | 233 | 1 | 0.705855 | -0.115008 | 551 | 360.892598 | 472.15 | 583.407402 | 0.856897 |
| GO:0008219\_cell\_death | 444 | 2 | 0.740830 | -0.114834 | 552 | 361.440286 | 472.69 | 583.939714 | 0.856322 |
| GO:0001501\_skeletal\_system\_development | 236 | 1 | 0.696883 | -0.112501 | 553 | 362.746170 | 473.86 | 584.973830 | 0.856890 |
| GO:0016265\_death | 450 | 2 | 0.730952 | -0.111048 | 554 | 364.246462 | 475.25 | 586.253538 | 0.857852 |
| GO:0009056\_catabolic\_process | 243 | 1 | 0.676808 | -0.106882 | 555 | 365.447939 | 476.36 | 587.272061 | 0.858306 |
| GO:0045321\_leukocyte\_activation | 248 | 1 | 0.663162 | -0.103057 | 556 | 367.442370 | 478.2 | 588.957630 | 0.860072 |
| GO:0007267\_cell-cell\_signaling | 252 | 1 | 0.652636 | -0.100105 | 557 | 368.618520 | 479.44 | 590.261480 | 0.860754 |
| GO:0016481\_negative\_regulation\_of\_transcription | 253 | 1 | 0.650056 | -0.099382 | 559 | 369.727674 | 480.4 | 591.072326 | 0.859392 |
| GO:0030097\_hemopoiesis | 253 | 1 | 0.650056 | -0.099382 | 559 | 369.727674 | 480.4 | 591.072326 | 0.859392 |
| GO:0001775\_cell\_activation | 262 | 1 | 0.627726 | -0.093119 | 562 | 373.102648 | 483.27 | 593.437352 | 0.859911 |
| GO:0010629\_negative\_regulation\_of\_gene\_expression | 262 | 1 | 0.627726 | -0.093119 | 562 | 373.102648 | 483.27 | 593.437352 | 0.859911 |
| GO:0048666\_neuron\_development | 262 | 1 | 0.627726 | -0.093119 | 562 | 373.102648 | 483.27 | 593.437352 | 0.859911 |
| GO:0045934\_negative\_regulation\_of\_nucleobase\_\_nucleoside\_\_nucleotide\_and\_nucleic\_acid\_metabolic\_process | 270 | 1 | 0.609127 | -0.087909 | 563 | 374.738378 | 484.79 | 594.841622 | 0.861083 |
| GO:0051172\_negative\_regulation\_of\_nitrogen\_compound\_metabolic\_process | 271 | 1 | 0.606879 | -0.087280 | 564 | 375.364097 | 485.32 | 595.275903 | 0.860496 |
| GO:0010558\_negative\_regulation\_of\_macromolecule\_biosynthetic\_process | 274 | 1 | 0.600235 | -0.085422 | 565 | 376.501379 | 486.29 | 596.078621 | 0.860690 |
| GO:0048534\_hemopoietic\_or\_lymphoid\_organ\_development | 277 | 1 | 0.593734 | -0.083606 | 566 | 377.873064 | 487.54 | 597.206936 | 0.861378 |
| GO:0031327\_negative\_regulation\_of\_cellular\_biosynthetic\_process | 282 | 1 | 0.583207 | -0.080671 | 567 | 379.977733 | 489.34 | 598.702267 | 0.863034 |
| GO:0009890\_negative\_regulation\_of\_biosynthetic\_process | 284 | 1 | 0.579100 | -0.079528 | 568 | 381.003603 | 490.25 | 599.496397 | 0.863116 |
| GO:0051234\_establishment\_of\_localization | 729 | 3 | 0.676808 | -0.074031 | 569 | 383.156337 | 491.97 | 600.783663 | 0.864622 |
| GO:0002520\_immune\_system\_development | 295 | 1 | 0.557506 | -0.073539 | 570 | 384.479855 | 493.09 | 601.700145 | 0.865070 |
| GO:0048598\_embryonic\_morphogenesis | 299 | 1 | 0.550048 | -0.071481 | 571 | 385.039951 | 493.59 | 602.140049 | 0.864431 |
| GO:0006950\_response\_to\_stress | 549 | 2 | 0.599141 | -0.063574 | 572 | 388.978584 | 497.0 | 605.021416 | 0.868881 |
| GO:0030182\_neuron\_differentiation | 356 | 1 | 0.461978 | -0.047874 | 573 | 398.742168 | 505.27 | 611.797832 | 0.881798 |
| GO:0050877\_neurological\_system\_process | 390 | 1 | 0.421703 | -0.037768 | 574 | 404.713206 | 509.91 | 615.106794 | 0.888345 |
| GO:0032501\_multicellular\_organismal\_process | 2183 | 10 | 0.753387 | -0.033796 | 575 | 405.773060 | 510.87 | 615.966940 | 0.888470 |
| GO:0042592\_homeostatic\_process | 419 | 1 | 0.392516 | -0.030867 | 576 | 407.203851 | 511.86 | 616.516149 | 0.888646 |
| GO:0006810\_transport | 718 | 2 | 0.458118 | -0.023698 | 577 | 412.167927 | 515.72 | 619.272073 | 0.893795 |
| GO:0002376\_immune\_system\_process | 505 | 1 | 0.325672 | -0.016957 | 578 | 414.701519 | 517.57 | 620.438481 | 0.895450 |
| GO:0003008\_system\_process | 516 | 1 | 0.318729 | -0.015702 | 579 | 415.256719 | 518.0 | 620.743281 | 0.894646 |
| GO:0006954\_inflammatory\_response | 96 | 0 | 0.000000 | -0.000000 | 581 | 425.919706 | 525.56 | 625.200294 | 0.904578 |
| GO:0070661\_leukocyte\_proliferation | 96 | 0 | 0.000000 | -0.000000 | 581 | 425.919706 | 525.56 | 625.200294 | 0.904578 |
| GO:0002683\_negative\_regulation\_of\_immune\_system\_process | 56 | 0 | 0.000000 | -0.000000 | 588 | 434.356786 | 533.16 | 631.963214 | 0.906735 |
| GO:0002703\_regulation\_of\_leukocyte\_mediated\_immunity | 56 | 0 | 0.000000 | -0.000000 | 588 | 434.356786 | 533.16 | 631.963214 | 0.906735 |
| GO:0006790\_sulfur\_metabolic\_process | 56 | 0 | 0.000000 | -0.000000 | 588 | 434.356786 | 533.16 | 631.963214 | 0.906735 |
| GO:0009187\_cyclic\_nucleotide\_metabolic\_process | 56 | 0 | 0.000000 | -0.000000 | 588 | 434.356786 | 533.16 | 631.963214 | 0.906735 |
| GO:0042089\_cytokine\_biosynthetic\_process | 56 | 0 | 0.000000 | -0.000000 | 588 | 434.356786 | 533.16 | 631.963214 | 0.906735 |
| GO:0042107\_cytokine\_metabolic\_process | 56 | 0 | 0.000000 | -0.000000 | 588 | 434.356786 | 533.16 | 631.963214 | 0.906735 |
| GO:0046486\_glycerolipid\_metabolic\_process | 56 | 0 | 0.000000 | -0.000000 | 588 | 434.356786 | 533.16 | 631.963214 | 0.906735 |
| GO:0001934\_positive\_regulation\_of\_protein\_amino\_acid\_phosphorylation | 29 | 0 | 0.000000 | -0.000000 | 606 | 453.739923 | 551.77 | 649.800077 | 0.910512 |
| GO:0006417\_regulation\_of\_translation | 29 | 0 | 0.000000 | -0.000000 | 606 | 453.739923 | 551.77 | 649.800077 | 0.910512 |
| GO:0006641\_triglyceride\_metabolic\_process | 29 | 0 | 0.000000 | -0.000000 | 606 | 453.739923 | 551.77 | 649.800077 | 0.910512 |
| GO:0006909\_phagocytosis | 29 | 0 | 0.000000 | -0.000000 | 606 | 453.739923 | 551.77 | 649.800077 | 0.910512 |
| GO:0007190\_activation\_of\_adenylate\_cyclase\_activity | 29 | 0 | 0.000000 | -0.000000 | 606 | 453.739923 | 551.77 | 649.800077 | 0.910512 |
| GO:0010564\_regulation\_of\_cell\_cycle\_process | 29 | 0 | 0.000000 | -0.000000 | 606 | 453.739923 | 551.77 | 649.800077 | 0.910512 |
| GO:0016447\_somatic\_recombination\_of\_immunoglobulin\_gene\_segments | 29 | 0 | 0.000000 | -0.000000 | 606 | 453.739923 | 551.77 | 649.800077 | 0.910512 |
| GO:0042176\_regulation\_of\_protein\_catabolic\_process | 29 | 0 | 0.000000 | -0.000000 | 606 | 453.739923 | 551.77 | 649.800077 | 0.910512 |
| GO:0042490\_mechanoreceptor\_differentiation | 29 | 0 | 0.000000 | -0.000000 | 606 | 453.739923 | 551.77 | 649.800077 | 0.910512 |
| GO:0042770\_DNA\_damage\_response\_\_signal\_transduction | 29 | 0 | 0.000000 | -0.000000 | 606 | 453.739923 | 551.77 | 649.800077 | 0.910512 |
| GO:0043281\_regulation\_of\_caspase\_activity | 29 | 0 | 0.000000 | -0.000000 | 606 | 453.739923 | 551.77 | 649.800077 | 0.910512 |
| GO:0044270\_nitrogen\_compound\_catabolic\_process | 29 | 0 | 0.000000 | -0.000000 | 606 | 453.739923 | 551.77 | 649.800077 | 0.910512 |
| GO:0046634\_regulation\_of\_alpha-beta\_T\_cell\_activation | 29 | 0 | 0.000000 | -0.000000 | 606 | 453.739923 | 551.77 | 649.800077 | 0.910512 |
| GO:0048066\_pigmentation\_during\_development | 29 | 0 | 0.000000 | -0.000000 | 606 | 453.739923 | 551.77 | 649.800077 | 0.910512 |
| GO:0051301\_cell\_division | 29 | 0 | 0.000000 | -0.000000 | 606 | 453.739923 | 551.77 | 649.800077 | 0.910512 |
| GO:0052548\_regulation\_of\_endopeptidase\_activity | 29 | 0 | 0.000000 | -0.000000 | 606 | 453.739923 | 551.77 | 649.800077 | 0.910512 |
| GO:0060041\_retina\_development\_in\_camera-type\_eye | 29 | 0 | 0.000000 | -0.000000 | 606 | 453.739923 | 551.77 | 649.800077 | 0.910512 |
| GO:0070302\_regulation\_of\_stress-activated\_protein\_kinase\_signaling\_pathway | 29 | 0 | 0.000000 | -0.000000 | 606 | 453.739923 | 551.77 | 649.800077 | 0.910512 |
| GO:0032940\_secretion\_by\_cell | 149 | 0 | 0.000000 | -0.000000 | 607 | 454.224844 | 552.17 | 650.115156 | 0.909671 |
| GO:0000002\_mitochondrial\_genome\_maintenance | 9 | 0 | 0.000000 | -0.000000 | 724 | 570.130341 | 666.08 | 762.029659 | 0.920000 |
| GO:0000186\_activation\_of\_MAPKK\_activity | 9 | 0 | 0.000000 | -0.000000 | 724 | 570.130341 | 666.08 | 762.029659 | 0.920000 |
| GO:0001539\_ciliary\_or\_flagellar\_motility | 9 | 0 | 0.000000 | -0.000000 | 724 | 570.130341 | 666.08 | 762.029659 | 0.920000 |
| GO:0001542\_ovulation\_from\_ovarian\_follicle | 9 | 0 | 0.000000 | -0.000000 | 724 | 570.130341 | 666.08 | 762.029659 | 0.920000 |
| GO:0001667\_ameboidal\_cell\_migration | 9 | 0 | 0.000000 | -0.000000 | 724 | 570.130341 | 666.08 | 762.029659 | 0.920000 |
| GO:0001676\_long-chain\_fatty\_acid\_metabolic\_process | 9 | 0 | 0.000000 | -0.000000 | 724 | 570.130341 | 666.08 | 762.029659 | 0.920000 |
| GO:0001935\_endothelial\_cell\_proliferation | 9 | 0 | 0.000000 | -0.000000 | 724 | 570.130341 | 666.08 | 762.029659 | 0.920000 |
| GO:0002021\_response\_to\_dietary\_excess | 9 | 0 | 0.000000 | -0.000000 | 724 | 570.130341 | 666.08 | 762.029659 | 0.920000 |
| GO:0002028\_regulation\_of\_sodium\_ion\_transport | 9 | 0 | 0.000000 | -0.000000 | 724 | 570.130341 | 666.08 | 762.029659 | 0.920000 |
| GO:0002221\_pattern\_recognition\_receptor\_signaling\_pathway | 9 | 0 | 0.000000 | -0.000000 | 724 | 570.130341 | 666.08 | 762.029659 | 0.920000 |
| GO:0002292\_T\_cell\_differentiation\_during\_immune\_response | 9 | 0 | 0.000000 | -0.000000 | 724 | 570.130341 | 666.08 | 762.029659 | 0.920000 |
| GO:0002293\_alpha-beta\_T\_cell\_differentiation\_during\_immune\_response | 9 | 0 | 0.000000 | -0.000000 | 724 | 570.130341 | 666.08 | 762.029659 | 0.920000 |
| GO:0002294\_CD4-positive\_\_alpha-beta\_T\_cell\_differentiation\_during\_immune\_response | 9 | 0 | 0.000000 | -0.000000 | 724 | 570.130341 | 666.08 | 762.029659 | 0.920000 |
| GO:0002507\_tolerance\_induction | 9 | 0 | 0.000000 | -0.000000 | 724 | 570.130341 | 666.08 | 762.029659 | 0.920000 |
| GO:0002886\_regulation\_of\_myeloid\_leukocyte\_mediated\_immunity | 9 | 0 | 0.000000 | -0.000000 | 724 | 570.130341 | 666.08 | 762.029659 | 0.920000 |
| GO:0006007\_glucose\_catabolic\_process | 9 | 0 | 0.000000 | -0.000000 | 724 | 570.130341 | 666.08 | 762.029659 | 0.920000 |
| GO:0006182\_cGMP\_biosynthetic\_process | 9 | 0 | 0.000000 | -0.000000 | 724 | 570.130341 | 666.08 | 762.029659 | 0.920000 |
| GO:0006309\_DNA\_fragmentation\_involved\_in\_apoptosis | 9 | 0 | 0.000000 | -0.000000 | 724 | 570.130341 | 666.08 | 762.029659 | 0.920000 |
| GO:0006364\_rRNA\_processing | 9 | 0 | 0.000000 | -0.000000 | 724 | 570.130341 | 666.08 | 762.029659 | 0.920000 |
| GO:0006476\_protein\_amino\_acid\_deacetylation | 9 | 0 | 0.000000 | -0.000000 | 724 | 570.130341 | 666.08 | 762.029659 | 0.920000 |
| GO:0006595\_polyamine\_metabolic\_process | 9 | 0 | 0.000000 | -0.000000 | 724 | 570.130341 | 666.08 | 762.029659 | 0.920000 |
| GO:0006611\_protein\_export\_from\_nucleus | 9 | 0 | 0.000000 | -0.000000 | 724 | 570.130341 | 666.08 | 762.029659 | 0.920000 |
| GO:0006910\_phagocytosis\_\_recognition | 9 | 0 | 0.000000 | -0.000000 | 724 | 570.130341 | 666.08 | 762.029659 | 0.920000 |
| GO:0006911\_phagocytosis\_\_engulfment | 9 | 0 | 0.000000 | -0.000000 | 724 | 570.130341 | 666.08 | 762.029659 | 0.920000 |
| GO:0007128\_meiotic\_prophase\_I | 9 | 0 | 0.000000 | -0.000000 | 724 | 570.130341 | 666.08 | 762.029659 | 0.920000 |
| GO:0007193\_inhibition\_of\_adenylate\_cyclase\_activity\_by\_G-protein\_signaling | 9 | 0 | 0.000000 | -0.000000 | 724 | 570.130341 | 666.08 | 762.029659 | 0.920000 |
| GO:0007379\_segment\_specification | 9 | 0 | 0.000000 | -0.000000 | 724 | 570.130341 | 666.08 | 762.029659 | 0.920000 |
| GO:0007617\_mating\_behavior | 9 | 0 | 0.000000 | -0.000000 | 724 | 570.130341 | 666.08 | 762.029659 | 0.920000 |
| GO:0009451\_RNA\_modification | 9 | 0 | 0.000000 | -0.000000 | 724 | 570.130341 | 666.08 | 762.029659 | 0.920000 |
| GO:0010165\_response\_to\_X-ray | 9 | 0 | 0.000000 | -0.000000 | 724 | 570.130341 | 666.08 | 762.029659 | 0.920000 |
| GO:0010675\_regulation\_of\_cellular\_carbohydrate\_metabolic\_process | 9 | 0 | 0.000000 | -0.000000 | 724 | 570.130341 | 666.08 | 762.029659 | 0.920000 |
| GO:0014037\_Schwann\_cell\_differentiation | 9 | 0 | 0.000000 | -0.000000 | 724 | 570.130341 | 666.08 | 762.029659 | 0.920000 |
| GO:0014073\_response\_to\_tropane | 9 | 0 | 0.000000 | -0.000000 | 724 | 570.130341 | 666.08 | 762.029659 | 0.920000 |
| GO:0015695\_organic\_cation\_transport | 9 | 0 | 0.000000 | -0.000000 | 724 | 570.130341 | 666.08 | 762.029659 | 0.920000 |
| GO:0016072\_rRNA\_metabolic\_process | 9 | 0 | 0.000000 | -0.000000 | 724 | 570.130341 | 666.08 | 762.029659 | 0.920000 |
| GO:0016601\_Rac\_protein\_signal\_transduction | 9 | 0 | 0.000000 | -0.000000 | 724 | 570.130341 | 666.08 | 762.029659 | 0.920000 |
| GO:0017145\_stem\_cell\_division | 9 | 0 | 0.000000 | -0.000000 | 724 | 570.130341 | 666.08 | 762.029659 | 0.920000 |
| GO:0019320\_hexose\_catabolic\_process | 9 | 0 | 0.000000 | -0.000000 | 724 | 570.130341 | 666.08 | 762.029659 | 0.920000 |
| GO:0021544\_subpallium\_development | 9 | 0 | 0.000000 | -0.000000 | 724 | 570.130341 | 666.08 | 762.029659 | 0.920000 |
| GO:0021936\_regulation\_of\_granule\_cell\_precursor\_proliferation | 9 | 0 | 0.000000 | -0.000000 | 724 | 570.130341 | 666.08 | 762.029659 | 0.920000 |
| GO:0021940\_positive\_regulation\_of\_granule\_cell\_precursor\_proliferation | 9 | 0 | 0.000000 | -0.000000 | 724 | 570.130341 | 666.08 | 762.029659 | 0.920000 |
| GO:0030048\_actin\_filament-based\_movement | 9 | 0 | 0.000000 | -0.000000 | 724 | 570.130341 | 666.08 | 762.029659 | 0.920000 |
| GO:0030279\_negative\_regulation\_of\_ossification | 9 | 0 | 0.000000 | -0.000000 | 724 | 570.130341 | 666.08 | 762.029659 | 0.920000 |
| GO:0030325\_adrenal\_gland\_development | 9 | 0 | 0.000000 | -0.000000 | 724 | 570.130341 | 666.08 | 762.029659 | 0.920000 |
| GO:0030728\_ovulation | 9 | 0 | 0.000000 | -0.000000 | 724 | 570.130341 | 666.08 | 762.029659 | 0.920000 |
| GO:0031023\_microtubule\_organizing\_center\_organization | 9 | 0 | 0.000000 | -0.000000 | 724 | 570.130341 | 666.08 | 762.029659 | 0.920000 |
| GO:0032388\_positive\_regulation\_of\_intracellular\_transport | 9 | 0 | 0.000000 | -0.000000 | 724 | 570.130341 | 666.08 | 762.029659 | 0.920000 |
| GO:0032606\_type\_I\_interferon\_production | 9 | 0 | 0.000000 | -0.000000 | 724 | 570.130341 | 666.08 | 762.029659 | 0.920000 |
| GO:0032814\_regulation\_of\_natural\_killer\_cell\_activation | 9 | 0 | 0.000000 | -0.000000 | 724 | 570.130341 | 666.08 | 762.029659 | 0.920000 |
| GO:0032816\_positive\_regulation\_of\_natural\_killer\_cell\_activation | 9 | 0 | 0.000000 | -0.000000 | 724 | 570.130341 | 666.08 | 762.029659 | 0.920000 |
| GO:0032963\_collagen\_metabolic\_process | 9 | 0 | 0.000000 | -0.000000 | 724 | 570.130341 | 666.08 | 762.029659 | 0.920000 |
| GO:0033028\_myeloid\_cell\_apoptosis | 9 | 0 | 0.000000 | -0.000000 | 724 | 570.130341 | 666.08 | 762.029659 | 0.920000 |
| GO:0033143\_regulation\_of\_steroid\_hormone\_receptor\_signaling\_pathway | 9 | 0 | 0.000000 | -0.000000 | 724 | 570.130341 | 666.08 | 762.029659 | 0.920000 |
| GO:0033151\_V(D)J\_recombination | 9 | 0 | 0.000000 | -0.000000 | 724 | 570.130341 | 666.08 | 762.029659 | 0.920000 |
| GO:0033344\_cholesterol\_efflux | 9 | 0 | 0.000000 | -0.000000 | 724 | 570.130341 | 666.08 | 762.029659 | 0.920000 |
| GO:0034605\_cellular\_response\_to\_heat | 9 | 0 | 0.000000 | -0.000000 | 724 | 570.130341 | 666.08 | 762.029659 | 0.920000 |
| GO:0035088\_establishment\_or\_maintenance\_of\_apical\_basal\_cell\_polarity | 9 | 0 | 0.000000 | -0.000000 | 724 | 570.130341 | 666.08 | 762.029659 | 0.920000 |
| GO:0035162\_embryonic\_hemopoiesis | 9 | 0 | 0.000000 | -0.000000 | 724 | 570.130341 | 666.08 | 762.029659 | 0.920000 |
| GO:0040020\_regulation\_of\_meiosis | 9 | 0 | 0.000000 | -0.000000 | 724 | 570.130341 | 666.08 | 762.029659 | 0.920000 |
| GO:0042093\_T-helper\_cell\_differentiation | 9 | 0 | 0.000000 | -0.000000 | 724 | 570.130341 | 666.08 | 762.029659 | 0.920000 |
| GO:0042220\_response\_to\_cocaine | 9 | 0 | 0.000000 | -0.000000 | 724 | 570.130341 | 666.08 | 762.029659 | 0.920000 |
| GO:0042402\_biogenic\_amine\_catabolic\_process | 9 | 0 | 0.000000 | -0.000000 | 724 | 570.130341 | 666.08 | 762.029659 | 0.920000 |
| GO:0042509\_regulation\_of\_tyrosine\_phosphorylation\_of\_STAT\_protein | 9 | 0 | 0.000000 | -0.000000 | 724 | 570.130341 | 666.08 | 762.029659 | 0.920000 |
| GO:0042640\_anagen | 9 | 0 | 0.000000 | -0.000000 | 724 | 570.130341 | 666.08 | 762.029659 | 0.920000 |
| GO:0043242\_negative\_regulation\_of\_protein\_complex\_disassembly | 9 | 0 | 0.000000 | -0.000000 | 724 | 570.130341 | 666.08 | 762.029659 | 0.920000 |
| GO:0043299\_leukocyte\_degranulation | 9 | 0 | 0.000000 | -0.000000 | 724 | 570.130341 | 666.08 | 762.029659 | 0.920000 |
| GO:0043383\_negative\_T\_cell\_selection | 9 | 0 | 0.000000 | -0.000000 | 724 | 570.130341 | 666.08 | 762.029659 | 0.920000 |
| GO:0043409\_negative\_regulation\_of\_MAPKKK\_cascade | 9 | 0 | 0.000000 | -0.000000 | 724 | 570.130341 | 666.08 | 762.029659 | 0.920000 |
| GO:0043433\_negative\_regulation\_of\_transcription\_factor\_activity | 9 | 0 | 0.000000 | -0.000000 | 724 | 570.130341 | 666.08 | 762.029659 | 0.920000 |
| GO:0043603\_cellular\_amide\_metabolic\_process | 9 | 0 | 0.000000 | -0.000000 | 724 | 570.130341 | 666.08 | 762.029659 | 0.920000 |
| GO:0045060\_negative\_thymic\_T\_cell\_selection | 9 | 0 | 0.000000 | -0.000000 | 724 | 570.130341 | 666.08 | 762.029659 | 0.920000 |
| GO:0045109\_intermediate\_filament\_organization | 9 | 0 | 0.000000 | -0.000000 | 724 | 570.130341 | 666.08 | 762.029659 | 0.920000 |
| GO:0045136\_development\_of\_secondary\_sexual\_characteristics | 9 | 0 | 0.000000 | -0.000000 | 724 | 570.130341 | 666.08 | 762.029659 | 0.920000 |
| GO:0045185\_maintenance\_of\_protein\_location | 9 | 0 | 0.000000 | -0.000000 | 724 | 570.130341 | 666.08 | 762.029659 | 0.920000 |
| GO:0045214\_sarcomere\_organization | 9 | 0 | 0.000000 | -0.000000 | 724 | 570.130341 | 666.08 | 762.029659 | 0.920000 |
| GO:0045620\_negative\_regulation\_of\_lymphocyte\_differentiation | 9 | 0 | 0.000000 | -0.000000 | 724 | 570.130341 | 666.08 | 762.029659 | 0.920000 |
| GO:0045646\_regulation\_of\_erythrocyte\_differentiation | 9 | 0 | 0.000000 | -0.000000 | 724 | 570.130341 | 666.08 | 762.029659 | 0.920000 |
| GO:0045671\_negative\_regulation\_of\_osteoclast\_differentiation | 9 | 0 | 0.000000 | -0.000000 | 724 | 570.130341 | 666.08 | 762.029659 | 0.920000 |
| GO:0045766\_positive\_regulation\_of\_angiogenesis | 9 | 0 | 0.000000 | -0.000000 | 724 | 570.130341 | 666.08 | 762.029659 | 0.920000 |
| GO:0045830\_positive\_regulation\_of\_isotype\_switching | 9 | 0 | 0.000000 | -0.000000 | 724 | 570.130341 | 666.08 | 762.029659 | 0.920000 |
| GO:0045884\_regulation\_of\_survival\_gene\_product\_expression | 9 | 0 | 0.000000 | -0.000000 | 724 | 570.130341 | 666.08 | 762.029659 | 0.920000 |
| GO:0046006\_regulation\_of\_activated\_T\_cell\_proliferation | 9 | 0 | 0.000000 | -0.000000 | 724 | 570.130341 | 666.08 | 762.029659 | 0.920000 |
| GO:0046324\_regulation\_of\_glucose\_import | 9 | 0 | 0.000000 | -0.000000 | 724 | 570.130341 | 666.08 | 762.029659 | 0.920000 |
| GO:0046365\_monosaccharide\_catabolic\_process | 9 | 0 | 0.000000 | -0.000000 | 724 | 570.130341 | 666.08 | 762.029659 | 0.920000 |
| GO:0046636\_negative\_regulation\_of\_alpha-beta\_T\_cell\_activation | 9 | 0 | 0.000000 | -0.000000 | 724 | 570.130341 | 666.08 | 762.029659 | 0.920000 |
| GO:0046641\_positive\_regulation\_of\_alpha-beta\_T\_cell\_proliferation | 9 | 0 | 0.000000 | -0.000000 | 724 | 570.130341 | 666.08 | 762.029659 | 0.920000 |
| GO:0046888\_negative\_regulation\_of\_hormone\_secretion | 9 | 0 | 0.000000 | -0.000000 | 724 | 570.130341 | 666.08 | 762.029659 | 0.920000 |
| GO:0048070\_regulation\_of\_pigmentation\_during\_development | 9 | 0 | 0.000000 | -0.000000 | 724 | 570.130341 | 666.08 | 762.029659 | 0.920000 |
| GO:0048146\_positive\_regulation\_of\_fibroblast\_proliferation | 9 | 0 | 0.000000 | -0.000000 | 724 | 570.130341 | 666.08 | 762.029659 | 0.920000 |
| GO:0048284\_organelle\_fusion | 9 | 0 | 0.000000 | -0.000000 | 724 | 570.130341 | 666.08 | 762.029659 | 0.920000 |
| GO:0048488\_synaptic\_vesicle\_endocytosis | 9 | 0 | 0.000000 | -0.000000 | 724 | 570.130341 | 666.08 | 762.029659 | 0.920000 |
| GO:0048569\_post-embryonic\_organ\_development | 9 | 0 | 0.000000 | -0.000000 | 724 | 570.130341 | 666.08 | 762.029659 | 0.920000 |
| GO:0048708\_astrocyte\_differentiation | 9 | 0 | 0.000000 | -0.000000 | 724 | 570.130341 | 666.08 | 762.029659 | 0.920000 |
| GO:0050433\_regulation\_of\_catecholamine\_secretion | 9 | 0 | 0.000000 | -0.000000 | 724 | 570.130341 | 666.08 | 762.029659 | 0.920000 |
| GO:0050856\_regulation\_of\_T\_cell\_receptor\_signaling\_pathway | 9 | 0 | 0.000000 | -0.000000 | 724 | 570.130341 | 666.08 | 762.029659 | 0.920000 |
| GO:0050884\_neuromuscular\_process\_controlling\_posture | 9 | 0 | 0.000000 | -0.000000 | 724 | 570.130341 | 666.08 | 762.029659 | 0.920000 |
| GO:0050910\_detection\_of\_mechanical\_stimulus\_involved\_in\_sensory\_perception\_of\_sound | 9 | 0 | 0.000000 | -0.000000 | 724 | 570.130341 | 666.08 | 762.029659 | 0.920000 |
| GO:0050918\_positive\_chemotaxis | 9 | 0 | 0.000000 | -0.000000 | 724 | 570.130341 | 666.08 | 762.029659 | 0.920000 |
| GO:0051023\_regulation\_of\_immunoglobulin\_secretion | 9 | 0 | 0.000000 | -0.000000 | 724 | 570.130341 | 666.08 | 762.029659 | 0.920000 |
| GO:0051297\_centrosome\_organization | 9 | 0 | 0.000000 | -0.000000 | 724 | 570.130341 | 666.08 | 762.029659 | 0.920000 |
| GO:0051324\_prophase | 9 | 0 | 0.000000 | -0.000000 | 724 | 570.130341 | 666.08 | 762.029659 | 0.920000 |
| GO:0051607\_defense\_response\_to\_virus | 9 | 0 | 0.000000 | -0.000000 | 724 | 570.130341 | 666.08 | 762.029659 | 0.920000 |
| GO:0051647\_nucleus\_localization | 9 | 0 | 0.000000 | -0.000000 | 724 | 570.130341 | 666.08 | 762.029659 | 0.920000 |
| GO:0051896\_regulation\_of\_protein\_kinase\_B\_signaling\_cascade | 9 | 0 | 0.000000 | -0.000000 | 724 | 570.130341 | 666.08 | 762.029659 | 0.920000 |
| GO:0051932\_synaptic\_transmission\_\_GABAergic | 9 | 0 | 0.000000 | -0.000000 | 724 | 570.130341 | 666.08 | 762.029659 | 0.920000 |
| GO:0055012\_ventricular\_cardiac\_muscle\_cell\_differentiation | 9 | 0 | 0.000000 | -0.000000 | 724 | 570.130341 | 666.08 | 762.029659 | 0.920000 |
| GO:0055013\_cardiac\_muscle\_cell\_development | 9 | 0 | 0.000000 | -0.000000 | 724 | 570.130341 | 666.08 | 762.029659 | 0.920000 |
| GO:0060052\_neurofilament\_cytoskeleton\_organization | 9 | 0 | 0.000000 | -0.000000 | 724 | 570.130341 | 666.08 | 762.029659 | 0.920000 |
| GO:0060081\_membrane\_hyperpolarization | 9 | 0 | 0.000000 | -0.000000 | 724 | 570.130341 | 666.08 | 762.029659 | 0.920000 |
| GO:0060119\_inner\_ear\_receptor\_cell\_development | 9 | 0 | 0.000000 | -0.000000 | 724 | 570.130341 | 666.08 | 762.029659 | 0.920000 |
| GO:0060122\_inner\_ear\_receptor\_stereocilium\_organization | 9 | 0 | 0.000000 | -0.000000 | 724 | 570.130341 | 666.08 | 762.029659 | 0.920000 |
| GO:0060325\_face\_morphogenesis | 9 | 0 | 0.000000 | -0.000000 | 724 | 570.130341 | 666.08 | 762.029659 | 0.920000 |
| GO:0060513\_prostatic\_bud\_formation | 9 | 0 | 0.000000 | -0.000000 | 724 | 570.130341 | 666.08 | 762.029659 | 0.920000 |
| GO:0060602\_branch\_elongation\_of\_an\_epithelium | 9 | 0 | 0.000000 | -0.000000 | 724 | 570.130341 | 666.08 | 762.029659 | 0.920000 |
| GO:0060693\_regulation\_of\_branching\_involved\_in\_salivary\_gland\_morphogenesis | 9 | 0 | 0.000000 | -0.000000 | 724 | 570.130341 | 666.08 | 762.029659 | 0.920000 |
| GO:0070306\_lens\_fiber\_cell\_differentiation | 9 | 0 | 0.000000 | -0.000000 | 724 | 570.130341 | 666.08 | 762.029659 | 0.920000 |
| GO:0090048\_negative\_regulation\_of\_transcription\_regulator\_activity | 9 | 0 | 0.000000 | -0.000000 | 724 | 570.130341 | 666.08 | 762.029659 | 0.920000 |
| GO:0035239\_tube\_morphogenesis | 143 | 0 | 0.000000 | -0.000000 | 725 | 570.673731 | 666.49 | 762.306269 | 0.919297 |
| GO:0001508\_regulation\_of\_action\_potential | 43 | 0 | 0.000000 | -0.000000 | 739 | 586.013130 | 680.55 | 775.086870 | 0.920907 |
| GO:0001841\_neural\_tube\_formation | 43 | 0 | 0.000000 | -0.000000 | 739 | 586.013130 | 680.55 | 775.086870 | 0.920907 |
| GO:0001894\_tissue\_homeostasis | 43 | 0 | 0.000000 | -0.000000 | 739 | 586.013130 | 680.55 | 775.086870 | 0.920907 |
| GO:0002819\_regulation\_of\_adaptive\_immune\_response | 43 | 0 | 0.000000 | -0.000000 | 739 | 586.013130 | 680.55 | 775.086870 | 0.920907 |
| GO:0002822\_regulation\_of\_adaptive\_immune\_response\_based\_on\_somatic\_recombination\_of\_immune\_receptors\_built\_from\_immunoglobulin\_superfamily\_domains | 43 | 0 | 0.000000 | -0.000000 | 739 | 586.013130 | 680.55 | 775.086870 | 0.920907 |
| GO:0006766\_vitamin\_metabolic\_process | 43 | 0 | 0.000000 | -0.000000 | 739 | 586.013130 | 680.55 | 775.086870 | 0.920907 |
| GO:0009582\_detection\_of\_abiotic\_stimulus | 43 | 0 | 0.000000 | -0.000000 | 739 | 586.013130 | 680.55 | 775.086870 | 0.920907 |
| GO:0019637\_organophosphate\_metabolic\_process | 43 | 0 | 0.000000 | -0.000000 | 739 | 586.013130 | 680.55 | 775.086870 | 0.920907 |
| GO:0030814\_regulation\_of\_cAMP\_metabolic\_process | 43 | 0 | 0.000000 | -0.000000 | 739 | 586.013130 | 680.55 | 775.086870 | 0.920907 |
| GO:0031098\_stress-activated\_protein\_kinase\_signaling\_pathway | 43 | 0 | 0.000000 | -0.000000 | 739 | 586.013130 | 680.55 | 775.086870 | 0.920907 |
| GO:0032446\_protein\_modification\_by\_small\_protein\_conjugation | 43 | 0 | 0.000000 | -0.000000 | 739 | 586.013130 | 680.55 | 775.086870 | 0.920907 |
| GO:0046879\_hormone\_secretion | 43 | 0 | 0.000000 | -0.000000 | 739 | 586.013130 | 680.55 | 775.086870 | 0.920907 |
| GO:0048762\_mesenchymal\_cell\_differentiation | 43 | 0 | 0.000000 | -0.000000 | 739 | 586.013130 | 680.55 | 775.086870 | 0.920907 |
| GO:0051604\_protein\_maturation | 43 | 0 | 0.000000 | -0.000000 | 739 | 586.013130 | 680.55 | 775.086870 | 0.920907 |
| GO:0006812\_cation\_transport | 146 | 0 | 0.000000 | -0.000000 | 740 | 587.614296 | 681.82 | 776.025704 | 0.921378 |
| GO:0000086\_G2\_M\_transition\_of\_mitotic\_cell\_cycle | 4 | 0 |  |  |  |  |  |  |  |  |
| GO:0000305\_response\_to\_oxygen\_radical | 4 | 0 |  |  |  |  |  |  |  |  |
| GO:0001661\_conditioned\_taste\_aversion | 4 | 0 |  |  |  |  |  |  |  |  |
| GO:0001678\_cellular\_glucose\_homeostasis | 4 | 0 |  |  |  |  |  |  |  |  |
| GO:0001777\_T\_cell\_homeostatic\_proliferation | 4 | 0 |  |  |  |  |  |  |  |  |
| GO:0001794\_type\_IIa\_hypersensitivity | 4 | 0 |  |  |  |  |  |  |  |  |
| GO:0001796\_regulation\_of\_type\_IIa\_hypersensitivity | 4 | 0 |  |  |  |  |  |  |  |  |
| GO:0001798\_positive\_regulation\_of\_type\_IIa\_hypersensitivity | 4 | 0 |  |  |  |  |  |  |  |  |
| GO:0001810\_regulation\_of\_type\_I\_hypersensitivity | 4 | 0 |  |  |  |  |  |  |  |  |
| GO:0001820\_serotonin\_secretion | 4 | 0 |  |  |  |  |  |  |  |  |
| GO:0001835\_blastocyst\_hatching | 4 | 0 |  |  |  |  |  |  |  |  |
| GO:0001842\_neural\_fold\_formation | 4 | 0 |  |  |  |  |  |  |  |  |
| GO:0001881\_receptor\_recycling | 4 | 0 |  |  |  |  |  |  |  |  |
| GO:0001938\_positive\_regulation\_of\_endothelial\_cell\_proliferation | 4 | 0 |  |  |  |  |  |  |  |  |
| GO:0001978\_regulation\_of\_systemic\_arterial\_blood\_pressure\_by\_carotid\_sinus\_baroreceptor\_feedback | 4 | 0 |  |  |  |  |  |  |  |  |
| GO:0002035\_brain\_renin-angiotensin\_system | 4 | 0 |  |  |  |  |  |  |  |  |
| GO:0002051\_osteoblast\_fate\_commitment | 4 | 0 |  |  |  |  |  |  |  |  |
| GO:0002220\_innate\_immune\_response\_activating\_cell\_surface\_receptor\_signaling\_pathway | 4 | 0 |  |  |  |  |  |  |  |  |
| GO:0002249\_lymphocyte\_anergy | 4 | 0 |  |  |  |  |  |  |  |  |
| GO:0002312\_B\_cell\_activation\_during\_immune\_response | 4 | 0 |  |  |  |  |  |  |  |  |
| GO:0002313\_mature\_B\_cell\_differentiation\_during\_immune\_response | 4 | 0 |  |  |  |  |  |  |  |  |
| GO:0002318\_myeloid\_progenitor\_cell\_differentiation | 4 | 0 |  |  |  |  |  |  |  |  |
| GO:0002326\_B\_cell\_lineage\_commitment | 4 | 0 |  |  |  |  |  |  |  |  |
| GO:0002347\_response\_to\_tumor\_cell | 4 | 0 |  |  |  |  |  |  |  |  |
| GO:0002418\_immune\_response\_to\_tumor\_cell | 4 | 0 |  |  |  |  |  |  |  |  |
| GO:0002445\_type\_II\_hypersensitivity | 4 | 0 |  |  |  |  |  |  |  |  |
| GO:0002544\_chronic\_inflammatory\_response | 4 | 0 |  |  |  |  |  |  |  |  |
| GO:0002636\_positive\_regulation\_of\_germinal\_center\_formation | 4 | 0 |  |  |  |  |  |  |  |  |
| GO:0002667\_regulation\_of\_T\_cell\_anergy | 4 | 0 |  |  |  |  |  |  |  |  |
| GO:0002669\_positive\_regulation\_of\_T\_cell\_anergy | 4 | 0 |  |  |  |  |  |  |  |  |
| GO:0002687\_positive\_regulation\_of\_leukocyte\_migration | 4 | 0 |  |  |  |  |  |  |  |  |
| GO:0002702\_positive\_regulation\_of\_production\_of\_molecular\_mediator\_of\_immune\_response | 4 | 0 |  |  |  |  |  |  |  |  |
| GO:0002718\_regulation\_of\_cytokine\_production\_during\_immune\_response | 4 | 0 |  |  |  |  |  |  |  |  |
| GO:0002829\_negative\_regulation\_of\_T-helper\_2\_type\_immune\_response | 4 | 0 |  |  |  |  |  |  |  |  |
| GO:0002833\_positive\_regulation\_of\_response\_to\_biotic\_stimulus | 4 | 0 |  |  |  |  |  |  |  |  |
| GO:0002834\_regulation\_of\_response\_to\_tumor\_cell | 4 | 0 |  |  |  |  |  |  |  |  |
| GO:0002836\_positive\_regulation\_of\_response\_to\_tumor\_cell | 4 | 0 |  |  |  |  |  |  |  |  |
| GO:0002837\_regulation\_of\_immune\_response\_to\_tumor\_cell | 4 | 0 |  |  |  |  |  |  |  |  |
| GO:0002839\_positive\_regulation\_of\_immune\_response\_to\_tumor\_cell | 4 | 0 |  |  |  |  |  |  |  |  |
| GO:0002870\_T\_cell\_anergy | 4 | 0 |  |  |  |  |  |  |  |  |
| GO:0002888\_positive\_regulation\_of\_myeloid\_leukocyte\_mediated\_immunity | 4 | 0 |  |  |  |  |  |  |  |  |
| GO:0002892\_regulation\_of\_type\_II\_hypersensitivity | 4 | 0 |  |  |  |  |  |  |  |  |
| GO:0002894\_positive\_regulation\_of\_type\_II\_hypersensitivity | 4 | 0 |  |  |  |  |  |  |  |  |
| GO:0002911\_regulation\_of\_lymphocyte\_anergy | 4 | 0 |  |  |  |  |  |  |  |  |
| GO:0002913\_positive\_regulation\_of\_lymphocyte\_anergy | 4 | 0 |  |  |  |  |  |  |  |  |
| GO:0002923\_regulation\_of\_humoral\_immune\_response\_mediated\_by\_circulating\_immunoglobulin | 4 | 0 |  |  |  |  |  |  |  |  |
| GO:0003025\_regulation\_of\_systemic\_arterial\_blood\_pressure\_by\_baroreceptor\_feedback | 4 | 0 |  |  |  |  |  |  |  |  |
| GO:0003091\_renal\_water\_homeostasis | 4 | 0 |  |  |  |  |  |  |  |  |
| GO:0005978\_glycogen\_biosynthetic\_process | 4 | 0 |  |  |  |  |  |  |  |  |
| GO:0006012\_galactose\_metabolic\_process | 4 | 0 |  |  |  |  |  |  |  |  |
| GO:0006085\_acetyl-CoA\_biosynthetic\_process | 4 | 0 |  |  |  |  |  |  |  |  |
| GO:0006111\_regulation\_of\_gluconeogenesis | 4 | 0 |  |  |  |  |  |  |  |  |
| GO:0006144\_purine\_base\_metabolic\_process | 4 | 0 |  |  |  |  |  |  |  |  |
| GO:0006290\_pyrimidine\_dimer\_repair | 4 | 0 |  |  |  |  |  |  |  |  |
| GO:0006334\_nucleosome\_assembly | 4 | 0 |  |  |  |  |  |  |  |  |
| GO:0006534\_cysteine\_metabolic\_process | 4 | 0 |  |  |  |  |  |  |  |  |
| GO:0006547\_histidine\_metabolic\_process | 4 | 0 |  |  |  |  |  |  |  |  |
| GO:0006548\_histidine\_catabolic\_process | 4 | 0 |  |  |  |  |  |  |  |  |
| GO:0006555\_methionine\_metabolic\_process | 4 | 0 |  |  |  |  |  |  |  |  |
| GO:0006599\_phosphagen\_metabolic\_process | 4 | 0 |  |  |  |  |  |  |  |  |
| GO:0006623\_protein\_targeting\_to\_vacuole | 4 | 0 |  |  |  |  |  |  |  |  |
| GO:0006626\_protein\_targeting\_to\_mitochondrion | 4 | 0 |  |  |  |  |  |  |  |  |
| GO:0006684\_sphingomyelin\_metabolic\_process | 4 | 0 |  |  |  |  |  |  |  |  |
| GO:0006688\_glycosphingolipid\_biosynthetic\_process | 4 | 0 |  |  |  |  |  |  |  |  |
| GO:0006707\_cholesterol\_catabolic\_process | 4 | 0 |  |  |  |  |  |  |  |  |
| GO:0006739\_NADP\_metabolic\_process | 4 | 0 |  |  |  |  |  |  |  |  |
| GO:0006835\_dicarboxylic\_acid\_transport | 4 | 0 |  |  |  |  |  |  |  |  |
| GO:0006837\_serotonin\_transport | 4 | 0 |  |  |  |  |  |  |  |  |
| GO:0006888\_ER\_to\_Golgi\_vesicle-mediated\_transport | 4 | 0 |  |  |  |  |  |  |  |  |
| GO:0006906\_vesicle\_fusion | 4 | 0 |  |  |  |  |  |  |  |  |
| GO:0006927\_transformed\_cell\_apoptosis | 4 | 0 |  |  |  |  |  |  |  |  |
| GO:0006972\_hyperosmotic\_response | 4 | 0 |  |  |  |  |  |  |  |  |
| GO:0007028\_cytoplasm\_organization | 4 | 0 |  |  |  |  |  |  |  |  |
| GO:0007031\_peroxisome\_organization | 4 | 0 |  |  |  |  |  |  |  |  |
| GO:0007066\_female\_meiosis\_sister\_chromatid\_cohesion | 4 | 0 |  |  |  |  |  |  |  |  |
| GO:0007144\_female\_meiosis\_I | 4 | 0 |  |  |  |  |  |  |  |  |
| GO:0007184\_SMAD\_protein\_nuclear\_translocation | 4 | 0 |  |  |  |  |  |  |  |  |
| GO:0007216\_metabotropic\_glutamate\_receptor\_signaling\_pathway | 4 | 0 |  |  |  |  |  |  |  |  |
| GO:0007342\_fusion\_of\_sperm\_to\_egg\_plasma\_membrane | 4 | 0 |  |  |  |  |  |  |  |  |
| GO:0007386\_compartment\_specification | 4 | 0 |  |  |  |  |  |  |  |  |
| GO:0008053\_mitochondrial\_fusion | 4 | 0 |  |  |  |  |  |  |  |  |
| GO:0008207\_C21-steroid\_hormone\_metabolic\_process | 4 | 0 |  |  |  |  |  |  |  |  |
| GO:0008215\_spermine\_metabolic\_process | 4 | 0 |  |  |  |  |  |  |  |  |
| GO:0009065\_glutamine\_family\_amino\_acid\_catabolic\_process | 4 | 0 |  |  |  |  |  |  |  |  |
| GO:0009075\_histidine\_family\_amino\_acid\_metabolic\_process | 4 | 0 |  |  |  |  |  |  |  |  |
| GO:0009077\_histidine\_family\_amino\_acid\_catabolic\_process | 4 | 0 |  |  |  |  |  |  |  |  |
| GO:0009134\_nucleoside\_diphosphate\_catabolic\_process | 4 | 0 |  |  |  |  |  |  |  |  |
| GO:0009163\_nucleoside\_biosynthetic\_process | 4 | 0 |  |  |  |  |  |  |  |  |
| GO:0009225\_nucleotide-sugar\_metabolic\_process | 4 | 0 |  |  |  |  |  |  |  |  |
| GO:0009250\_glucan\_biosynthetic\_process | 4 | 0 |  |  |  |  |  |  |  |  |
| GO:0009404\_toxin\_metabolic\_process | 4 | 0 |  |  |  |  |  |  |  |  |
| GO:0009593\_detection\_of\_chemical\_stimulus | 4 | 0 |  |  |  |  |  |  |  |  |
| GO:0009595\_detection\_of\_biotic\_stimulus | 4 | 0 |  |  |  |  |  |  |  |  |
| GO:0009755\_hormone-mediated\_signaling | 4 | 0 |  |  |  |  |  |  |  |  |
| GO:0009912\_auditory\_receptor\_cell\_fate\_commitment | 4 | 0 |  |  |  |  |  |  |  |  |
| GO:0010224\_response\_to\_UV-B | 4 | 0 |  |  |  |  |  |  |  |  |
| GO:0010453\_regulation\_of\_cell\_fate\_commitment | 4 | 0 |  |  |  |  |  |  |  |  |
| GO:0010506\_regulation\_of\_autophagy | 4 | 0 |  |  |  |  |  |  |  |  |
| GO:0010631\_epithelial\_cell\_migration | 4 | 0 |  |  |  |  |  |  |  |  |
| GO:0010812\_negative\_regulation\_of\_cell-substrate\_adhesion | 4 | 0 |  |  |  |  |  |  |  |  |
| GO:0010829\_negative\_regulation\_of\_glucose\_transport | 4 | 0 |  |  |  |  |  |  |  |  |
| GO:0014002\_astrocyte\_development | 4 | 0 |  |  |  |  |  |  |  |  |
| GO:0014832\_urinary\_bladder\_smooth\_muscle\_contraction | 4 | 0 |  |  |  |  |  |  |  |  |
| GO:0014848\_urinary\_tract\_smooth\_muscle\_contraction | 4 | 0 |  |  |  |  |  |  |  |  |
| GO:0015701\_bicarbonate\_transport | 4 | 0 |  |  |  |  |  |  |  |  |
| GO:0015809\_arginine\_transport | 4 | 0 |  |  |  |  |  |  |  |  |
| GO:0015850\_organic\_alcohol\_transport | 4 | 0 |  |  |  |  |  |  |  |  |
| GO:0015858\_nucleoside\_transport | 4 | 0 |  |  |  |  |  |  |  |  |
| GO:0016068\_type\_I\_hypersensitivity | 4 | 0 |  |  |  |  |  |  |  |  |
| GO:0016127\_sterol\_catabolic\_process | 4 | 0 |  |  |  |  |  |  |  |  |
| GO:0016198\_axon\_choice\_point\_recognition | 4 | 0 |  |  |  |  |  |  |  |  |
| GO:0016338\_calcium-independent\_cell-cell\_adhesion | 4 | 0 |  |  |  |  |  |  |  |  |
| GO:0018198\_peptidyl-cysteine\_modification | 4 | 0 |  |  |  |  |  |  |  |  |
| GO:0018409\_peptide\_or\_protein\_amino-terminal\_blocking | 4 | 0 |  |  |  |  |  |  |  |  |
| GO:0019377\_glycolipid\_catabolic\_process | 4 | 0 |  |  |  |  |  |  |  |  |
| GO:0019432\_triglyceride\_biosynthetic\_process | 4 | 0 |  |  |  |  |  |  |  |  |
| GO:0019530\_taurine\_metabolic\_process | 4 | 0 |  |  |  |  |  |  |  |  |
| GO:0021535\_cell\_migration\_in\_hindbrain | 4 | 0 |  |  |  |  |  |  |  |  |
| GO:0021542\_dentate\_gyrus\_development | 4 | 0 |  |  |  |  |  |  |  |  |
| GO:0021561\_facial\_nerve\_development | 4 | 0 |  |  |  |  |  |  |  |  |
| GO:0021569\_rhombomere\_3\_development | 4 | 0 |  |  |  |  |  |  |  |  |
| GO:0021571\_rhombomere\_5\_development | 4 | 0 |  |  |  |  |  |  |  |  |
| GO:0021604\_cranial\_nerve\_structural\_organization | 4 | 0 |  |  |  |  |  |  |  |  |
| GO:0021610\_facial\_nerve\_morphogenesis | 4 | 0 |  |  |  |  |  |  |  |  |
| GO:0021612\_facial\_nerve\_structural\_organization | 4 | 0 |  |  |  |  |  |  |  |  |
| GO:0021631\_optic\_nerve\_morphogenesis | 4 | 0 |  |  |  |  |  |  |  |  |
| GO:0021681\_cerebellar\_granular\_layer\_development | 4 | 0 |  |  |  |  |  |  |  |  |
| GO:0021683\_cerebellar\_granular\_layer\_morphogenesis | 4 | 0 |  |  |  |  |  |  |  |  |
| GO:0021684\_cerebellar\_granular\_layer\_formation | 4 | 0 |  |  |  |  |  |  |  |  |
| GO:0021707\_cerebellar\_granule\_cell\_differentiation | 4 | 0 |  |  |  |  |  |  |  |  |
| GO:0021801\_cerebral\_cortex\_radial\_glia\_guided\_migration | 4 | 0 |  |  |  |  |  |  |  |  |
| GO:0021830\_interneuron\_migration\_from\_the\_subpallium\_to\_the\_cortex | 4 | 0 |  |  |  |  |  |  |  |  |
| GO:0021853\_cerebral\_cortex\_GABAergic\_interneuron\_migration | 4 | 0 |  |  |  |  |  |  |  |  |
| GO:0021894\_cerebral\_cortex\_GABAergic\_interneuron\_development | 4 | 0 |  |  |  |  |  |  |  |  |
| GO:0021910\_smoothened\_signaling\_pathway\_involved\_in\_ventral\_spinal\_cord\_patterning | 4 | 0 |  |  |  |  |  |  |  |  |
| GO:0021938\_smoothened\_signaling\_pathway\_involved\_in\_regulation\_of\_granule\_cell\_precursor\_cell\_proliferation | 4 | 0 |  |  |  |  |  |  |  |  |
| GO:0021978\_telencephalon\_regionalization | 4 | 0 |  |  |  |  |  |  |  |  |
| GO:0022011\_myelination\_in\_the\_peripheral\_nervous\_system | 4 | 0 |  |  |  |  |  |  |  |  |
| GO:0030146\_diuresis | 4 | 0 |  |  |  |  |  |  |  |  |
| GO:0030300\_regulation\_of\_intestinal\_cholesterol\_absorption | 4 | 0 |  |  |  |  |  |  |  |  |
| GO:0030800\_negative\_regulation\_of\_cyclic\_nucleotide\_metabolic\_process | 4 | 0 |  |  |  |  |  |  |  |  |
| GO:0030803\_negative\_regulation\_of\_cyclic\_nucleotide\_biosynthetic\_process | 4 | 0 |  |  |  |  |  |  |  |  |
| GO:0030809\_negative\_regulation\_of\_nucleotide\_biosynthetic\_process | 4 | 0 |  |  |  |  |  |  |  |  |
| GO:0030815\_negative\_regulation\_of\_cAMP\_metabolic\_process | 4 | 0 |  |  |  |  |  |  |  |  |
| GO:0030816\_positive\_regulation\_of\_cAMP\_metabolic\_process | 4 | 0 |  |  |  |  |  |  |  |  |
| GO:0030818\_negative\_regulation\_of\_cAMP\_biosynthetic\_process | 4 | 0 |  |  |  |  |  |  |  |  |
| GO:0030819\_positive\_regulation\_of\_cAMP\_biosynthetic\_process | 4 | 0 |  |  |  |  |  |  |  |  |
| GO:0030826\_regulation\_of\_cGMP\_biosynthetic\_process | 4 | 0 |  |  |  |  |  |  |  |  |
| GO:0030859\_polarized\_epithelial\_cell\_differentiation | 4 | 0 |  |  |  |  |  |  |  |  |
| GO:0030949\_positive\_regulation\_of\_vascular\_endothelial\_growth\_factor\_receptor\_signaling\_pathway | 4 | 0 |  |  |  |  |  |  |  |  |
| GO:0031113\_regulation\_of\_microtubule\_polymerization | 4 | 0 |  |  |  |  |  |  |  |  |
| GO:0031365\_N-terminal\_protein\_amino\_acid\_modification | 4 | 0 |  |  |  |  |  |  |  |  |
| GO:0031424\_keratinization | 4 | 0 |  |  |  |  |  |  |  |  |
| GO:0031557\_induction\_of\_programmed\_cell\_death\_in\_response\_to\_chemical\_stimulus | 4 | 0 |  |  |  |  |  |  |  |  |
| GO:0031558\_induction\_of\_apoptosis\_in\_response\_to\_chemical\_stimulus | 4 | 0 |  |  |  |  |  |  |  |  |
| GO:0031623\_receptor\_internalization | 4 | 0 |  |  |  |  |  |  |  |  |
| GO:0032088\_negative\_regulation\_of\_NF-kappaB\_transcription\_factor\_activity | 4 | 0 |  |  |  |  |  |  |  |  |
| GO:0032098\_regulation\_of\_appetite | 4 | 0 |  |  |  |  |  |  |  |  |
| GO:0032105\_negative\_regulation\_of\_response\_to\_extracellular\_stimulus | 4 | 0 |  |  |  |  |  |  |  |  |
| GO:0032108\_negative\_regulation\_of\_response\_to\_nutrient\_levels | 4 | 0 |  |  |  |  |  |  |  |  |
| GO:0032225\_regulation\_of\_synaptic\_transmission\_\_dopaminergic | 4 | 0 |  |  |  |  |  |  |  |  |
| GO:0032292\_ensheathment\_of\_axons\_in\_the\_peripheral\_nervous\_system | 4 | 0 |  |  |  |  |  |  |  |  |
| GO:0032321\_positive\_regulation\_of\_Rho\_GTPase\_activity | 4 | 0 |  |  |  |  |  |  |  |  |
| GO:0032371\_regulation\_of\_sterol\_transport | 4 | 0 |  |  |  |  |  |  |  |  |
| GO:0032374\_regulation\_of\_cholesterol\_transport | 4 | 0 |  |  |  |  |  |  |  |  |
| GO:0032401\_establishment\_of\_melanosome\_localization | 4 | 0 |  |  |  |  |  |  |  |  |
| GO:0032608\_interferon-beta\_production | 4 | 0 |  |  |  |  |  |  |  |  |
| GO:0032611\_interleukin-1\_beta\_production | 4 | 0 |  |  |  |  |  |  |  |  |
| GO:0032612\_interleukin-1\_production | 4 | 0 |  |  |  |  |  |  |  |  |
| GO:0032648\_regulation\_of\_interferon-beta\_production | 4 | 0 |  |  |  |  |  |  |  |  |
| GO:0032651\_regulation\_of\_interleukin-1\_beta\_production | 4 | 0 |  |  |  |  |  |  |  |  |
| GO:0032652\_regulation\_of\_interleukin-1\_production | 4 | 0 |  |  |  |  |  |  |  |  |
| GO:0032689\_negative\_regulation\_of\_interferon-gamma\_production | 4 | 0 |  |  |  |  |  |  |  |  |
| GO:0032713\_negative\_regulation\_of\_interleukin-4\_production | 4 | 0 |  |  |  |  |  |  |  |  |
| GO:0032715\_negative\_regulation\_of\_interleukin-6\_production | 4 | 0 |  |  |  |  |  |  |  |  |
| GO:0032733\_positive\_regulation\_of\_interleukin-10\_production | 4 | 0 |  |  |  |  |  |  |  |  |
| GO:0032835\_glomerulus\_development | 4 | 0 |  |  |  |  |  |  |  |  |
| GO:0032872\_regulation\_of\_stress-activated\_MAPK\_cascade | 4 | 0 |  |  |  |  |  |  |  |  |
| GO:0032922\_circadian\_regulation\_of\_gene\_expression | 4 | 0 |  |  |  |  |  |  |  |  |
| GO:0033026\_negative\_regulation\_of\_mast\_cell\_apoptosis | 4 | 0 |  |  |  |  |  |  |  |  |
| GO:0033079\_immature\_T\_cell\_proliferation | 4 | 0 |  |  |  |  |  |  |  |  |
| GO:0033083\_regulation\_of\_immature\_T\_cell\_proliferation | 4 | 0 |  |  |  |  |  |  |  |  |
| GO:0033089\_positive\_regulation\_of\_T\_cell\_differentiation\_in\_the\_thymus | 4 | 0 |  |  |  |  |  |  |  |  |
| GO:0033135\_regulation\_of\_peptidyl-serine\_phosphorylation | 4 | 0 |  |  |  |  |  |  |  |  |
| GO:0033299\_secretion\_of\_lysosomal\_enzymes | 4 | 0 |  |  |  |  |  |  |  |  |
| GO:0033327\_Leydig\_cell\_differentiation | 4 | 0 |  |  |  |  |  |  |  |  |
| GO:0033363\_secretory\_granule\_organization | 4 | 0 |  |  |  |  |  |  |  |  |
| GO:0033599\_regulation\_of\_mammary\_gland\_epithelial\_cell\_proliferation | 4 | 0 |  |  |  |  |  |  |  |  |
| GO:0033865\_nucleoside\_bisphosphate\_metabolic\_process | 4 | 0 |  |  |  |  |  |  |  |  |
| GO:0034204\_lipid\_translocation | 4 | 0 |  |  |  |  |  |  |  |  |
| GO:0034404\_nucleobase\_\_nucleoside\_and\_nucleotide\_biosynthetic\_process | 4 | 0 |  |  |  |  |  |  |  |  |
| GO:0034587\_piRNA\_metabolic\_process | 4 | 0 |  |  |  |  |  |  |  |  |
| GO:0034614\_cellular\_response\_to\_reactive\_oxygen\_species | 4 | 0 |  |  |  |  |  |  |  |  |
| GO:0034654\_nucleobase\_\_nucleoside\_\_nucleotide\_and\_nucleic\_acid\_biosynthetic\_process | 4 | 0 |  |  |  |  |  |  |  |  |
| GO:0035020\_regulation\_of\_Rac\_protein\_signal\_transduction | 4 | 0 |  |  |  |  |  |  |  |  |
| GO:0035082\_axoneme\_assembly | 4 | 0 |  |  |  |  |  |  |  |  |
| GO:0035188\_hatching | 4 | 0 |  |  |  |  |  |  |  |  |
| GO:0035235\_ionotropic\_glutamate\_receptor\_signaling\_pathway | 4 | 0 |  |  |  |  |  |  |  |  |
| GO:0042345\_regulation\_of\_NF-kappaB\_import\_into\_nucleus | 4 | 0 |  |  |  |  |  |  |  |  |
| GO:0042348\_NF-kappaB\_import\_into\_nucleus | 4 | 0 |  |  |  |  |  |  |  |  |
| GO:0042359\_vitamin\_D\_metabolic\_process | 4 | 0 |  |  |  |  |  |  |  |  |
| GO:0042428\_serotonin\_metabolic\_process | 4 | 0 |  |  |  |  |  |  |  |  |
| GO:0042451\_purine\_nucleoside\_biosynthetic\_process | 4 | 0 |  |  |  |  |  |  |  |  |
| GO:0042455\_ribonucleoside\_biosynthetic\_process | 4 | 0 |  |  |  |  |  |  |  |  |
| GO:0042473\_outer\_ear\_morphogenesis | 4 | 0 |  |  |  |  |  |  |  |  |
| GO:0042522\_regulation\_of\_tyrosine\_phosphorylation\_of\_Stat5\_protein | 4 | 0 |  |  |  |  |  |  |  |  |
| GO:0042535\_positive\_regulation\_of\_tumor\_necrosis\_factor\_biosynthetic\_process | 4 | 0 |  |  |  |  |  |  |  |  |
| GO:0042541\_hemoglobin\_biosynthetic\_process | 4 | 0 |  |  |  |  |  |  |  |  |
| GO:0042558\_pteridine\_and\_derivative\_metabolic\_process | 4 | 0 |  |  |  |  |  |  |  |  |
| GO:0042634\_regulation\_of\_hair\_cycle | 4 | 0 |  |  |  |  |  |  |  |  |
| GO:0042744\_hydrogen\_peroxide\_catabolic\_process | 4 | 0 |  |  |  |  |  |  |  |  |
| GO:0042773\_ATP\_synthesis\_coupled\_electron\_transport | 4 | 0 |  |  |  |  |  |  |  |  |
| GO:0042775\_mitochondrial\_ATP\_synthesis\_coupled\_electron\_transport | 4 | 0 |  |  |  |  |  |  |  |  |
| GO:0042832\_defense\_response\_to\_protozoan | 4 | 0 |  |  |  |  |  |  |  |  |
| GO:0042982\_amyloid\_precursor\_protein\_metabolic\_process | 4 | 0 |  |  |  |  |  |  |  |  |
| GO:0042992\_negative\_regulation\_of\_transcription\_factor\_import\_into\_nucleus | 4 | 0 |  |  |  |  |  |  |  |  |
| GO:0043043\_peptide\_biosynthetic\_process | 4 | 0 |  |  |  |  |  |  |  |  |
| GO:0043129\_surfactant\_homeostasis | 4 | 0 |  |  |  |  |  |  |  |  |
| GO:0043374\_CD8-positive\_\_alpha-beta\_T\_cell\_differentiation | 4 | 0 |  |  |  |  |  |  |  |  |
| GO:0043470\_regulation\_of\_carbohydrate\_catabolic\_process | 4 | 0 |  |  |  |  |  |  |  |  |
| GO:0043471\_regulation\_of\_cellular\_carbohydrate\_catabolic\_process | 4 | 0 |  |  |  |  |  |  |  |  |
| GO:0043484\_regulation\_of\_RNA\_splicing | 4 | 0 |  |  |  |  |  |  |  |  |
| GO:0043500\_muscle\_adaptation | 4 | 0 |  |  |  |  |  |  |  |  |
| GO:0043534\_blood\_vessel\_endothelial\_cell\_migration | 4 | 0 |  |  |  |  |  |  |  |  |
| GO:0043691\_reverse\_cholesterol\_transport | 4 | 0 |  |  |  |  |  |  |  |  |
| GO:0044243\_multicellular\_organismal\_catabolic\_process | 4 | 0 |  |  |  |  |  |  |  |  |
| GO:0044403\_symbiosis\_\_encompassing\_mutualism\_through\_parasitism | 4 | 0 |  |  |  |  |  |  |  |  |
| GO:0044419\_interspecies\_interaction\_between\_organisms | 4 | 0 |  |  |  |  |  |  |  |  |
| GO:0045066\_regulatory\_T\_cell\_differentiation | 4 | 0 |  |  |  |  |  |  |  |  |
| GO:0045078\_positive\_regulation\_of\_interferon-gamma\_biosynthetic\_process | 4 | 0 |  |  |  |  |  |  |  |  |
| GO:0045332\_phospholipid\_translocation | 4 | 0 |  |  |  |  |  |  |  |  |
| GO:0045346\_regulation\_of\_MHC\_class\_II\_biosynthetic\_process | 4 | 0 |  |  |  |  |  |  |  |  |
| GO:0045350\_interferon-beta\_biosynthetic\_process | 4 | 0 |  |  |  |  |  |  |  |  |
| GO:0045357\_regulation\_of\_interferon-beta\_biosynthetic\_process | 4 | 0 |  |  |  |  |  |  |  |  |
| GO:0045359\_positive\_regulation\_of\_interferon-beta\_biosynthetic\_process | 4 | 0 |  |  |  |  |  |  |  |  |
| GO:0045600\_positive\_regulation\_of\_fat\_cell\_differentiation | 4 | 0 |  |  |  |  |  |  |  |  |
| GO:0045616\_regulation\_of\_keratinocyte\_differentiation | 4 | 0 |  |  |  |  |  |  |  |  |
| GO:0045624\_positive\_regulation\_of\_T-helper\_cell\_differentiation | 4 | 0 |  |  |  |  |  |  |  |  |
| GO:0045628\_regulation\_of\_T-helper\_2\_cell\_differentiation | 4 | 0 |  |  |  |  |  |  |  |  |
| GO:0045634\_regulation\_of\_melanocyte\_differentiation | 4 | 0 |  |  |  |  |  |  |  |  |
| GO:0045647\_negative\_regulation\_of\_erythrocyte\_differentiation | 4 | 0 |  |  |  |  |  |  |  |  |
| GO:0045672\_positive\_regulation\_of\_osteoclast\_differentiation | 4 | 0 |  |  |  |  |  |  |  |  |
| GO:0045684\_positive\_regulation\_of\_epidermis\_development | 4 | 0 |  |  |  |  |  |  |  |  |
| GO:0045736\_negative\_regulation\_of\_cyclin-dependent\_protein\_kinase\_activity | 4 | 0 |  |  |  |  |  |  |  |  |
| GO:0045742\_positive\_regulation\_of\_epidermal\_growth\_factor\_receptor\_signaling\_pathway | 4 | 0 |  |  |  |  |  |  |  |  |
| GO:0045747\_positive\_regulation\_of\_Notch\_signaling\_pathway | 4 | 0 |  |  |  |  |  |  |  |  |
| GO:0045767\_regulation\_of\_anti-apoptosis | 4 | 0 |  |  |  |  |  |  |  |  |
| GO:0045779\_negative\_regulation\_of\_bone\_resorption | 4 | 0 |  |  |  |  |  |  |  |  |
| GO:0045923\_positive\_regulation\_of\_fatty\_acid\_metabolic\_process | 4 | 0 |  |  |  |  |  |  |  |  |
| GO:0045930\_negative\_regulation\_of\_mitotic\_cell\_cycle | 4 | 0 |  |  |  |  |  |  |  |  |
| GO:0045940\_positive\_regulation\_of\_steroid\_metabolic\_process | 4 | 0 |  |  |  |  |  |  |  |  |
| GO:0045980\_negative\_regulation\_of\_nucleotide\_metabolic\_process | 4 | 0 |  |  |  |  |  |  |  |  |
| GO:0046129\_purine\_ribonucleoside\_biosynthetic\_process | 4 | 0 |  |  |  |  |  |  |  |  |
| GO:0046173\_polyol\_biosynthetic\_process | 4 | 0 |  |  |  |  |  |  |  |  |
| GO:0046541\_saliva\_secretion | 4 | 0 |  |  |  |  |  |  |  |  |
| GO:0046548\_retinal\_rod\_cell\_development | 4 | 0 |  |  |  |  |  |  |  |  |
| GO:0046579\_positive\_regulation\_of\_Ras\_protein\_signal\_transduction | 4 | 0 |  |  |  |  |  |  |  |  |
| GO:0046639\_negative\_regulation\_of\_alpha-beta\_T\_cell\_differentiation | 4 | 0 |  |  |  |  |  |  |  |  |
| GO:0046642\_negative\_regulation\_of\_alpha-beta\_T\_cell\_proliferation | 4 | 0 |  |  |  |  |  |  |  |  |
| GO:0046668\_regulation\_of\_retinal\_cell\_programmed\_cell\_death | 4 | 0 |  |  |  |  |  |  |  |  |
| GO:0046686\_response\_to\_cadmium\_ion | 4 | 0 |  |  |  |  |  |  |  |  |
| GO:0046835\_carbohydrate\_phosphorylation | 4 | 0 |  |  |  |  |  |  |  |  |
| GO:0046902\_regulation\_of\_mitochondrial\_membrane\_permeability | 4 | 0 |  |  |  |  |  |  |  |  |
| GO:0047496\_vesicle\_transport\_along\_microtubule | 4 | 0 |  |  |  |  |  |  |  |  |
| GO:0048011\_nerve\_growth\_factor\_receptor\_signaling\_pathway | 4 | 0 |  |  |  |  |  |  |  |  |
| GO:0048024\_regulation\_of\_nuclear\_mRNA\_splicing\_\_via\_spliceosome | 4 | 0 |  |  |  |  |  |  |  |  |
| GO:0048240\_sperm\_capacitation | 4 | 0 |  |  |  |  |  |  |  |  |
| GO:0048341\_paraxial\_mesoderm\_formation | 4 | 0 |  |  |  |  |  |  |  |  |
| GO:0048484\_enteric\_nervous\_system\_development | 4 | 0 |  |  |  |  |  |  |  |  |
| GO:0048512\_circadian\_behavior | 4 | 0 |  |  |  |  |  |  |  |  |
| GO:0048558\_embryonic\_gut\_morphogenesis | 4 | 0 |  |  |  |  |  |  |  |  |
| GO:0048639\_positive\_regulation\_of\_developmental\_growth | 4 | 0 |  |  |  |  |  |  |  |  |
| GO:0048710\_regulation\_of\_astrocyte\_differentiation | 4 | 0 |  |  |  |  |  |  |  |  |
| GO:0048841\_regulation\_of\_axon\_extension\_involved\_in\_axon\_guidance | 4 | 0 |  |  |  |  |  |  |  |  |
| GO:0048843\_negative\_regulation\_of\_axon\_extension\_involved\_in\_axon\_guidance | 4 | 0 |  |  |  |  |  |  |  |  |
| GO:0048846\_axon\_extension\_involved\_in\_axon\_guidance | 4 | 0 |  |  |  |  |  |  |  |  |
| GO:0048875\_chemical\_homeostasis\_within\_a\_tissue | 4 | 0 |  |  |  |  |  |  |  |  |
| GO:0048935\_peripheral\_nervous\_system\_neuron\_development | 4 | 0 |  |  |  |  |  |  |  |  |
| GO:0050702\_interleukin-1\_beta\_secretion | 4 | 0 |  |  |  |  |  |  |  |  |
| GO:0050704\_regulation\_of\_interleukin-1\_secretion | 4 | 0 |  |  |  |  |  |  |  |  |
| GO:0050706\_regulation\_of\_interleukin-1\_beta\_secretion | 4 | 0 |  |  |  |  |  |  |  |  |
| GO:0050716\_positive\_regulation\_of\_interleukin-1\_secretion | 4 | 0 |  |  |  |  |  |  |  |  |
| GO:0050718\_positive\_regulation\_of\_interleukin-1\_beta\_secretion | 4 | 0 |  |  |  |  |  |  |  |  |
| GO:0050820\_positive\_regulation\_of\_coagulation | 4 | 0 |  |  |  |  |  |  |  |  |
| GO:0050891\_multicellular\_organismal\_water\_homeostasis | 4 | 0 |  |  |  |  |  |  |  |  |
| GO:0050919\_negative\_chemotaxis | 4 | 0 |  |  |  |  |  |  |  |  |
| GO:0050932\_regulation\_of\_pigment\_cell\_differentiation | 4 | 0 |  |  |  |  |  |  |  |  |
| GO:0050961\_detection\_of\_temperature\_stimulus\_involved\_in\_sensory\_perception | 4 | 0 |  |  |  |  |  |  |  |  |
| GO:0050965\_detection\_of\_temperature\_stimulus\_involved\_in\_sensory\_perception\_of\_pain | 4 | 0 |  |  |  |  |  |  |  |  |
| GO:0050994\_regulation\_of\_lipid\_catabolic\_process | 4 | 0 |  |  |  |  |  |  |  |  |
| GO:0051024\_positive\_regulation\_of\_immunoglobulin\_secretion | 4 | 0 |  |  |  |  |  |  |  |  |
| GO:0051055\_negative\_regulation\_of\_lipid\_biosynthetic\_process | 4 | 0 |  |  |  |  |  |  |  |  |
| GO:0051148\_negative\_regulation\_of\_muscle\_cell\_differentiation | 4 | 0 |  |  |  |  |  |  |  |  |
| GO:0051205\_protein\_insertion\_into\_membrane | 4 | 0 |  |  |  |  |  |  |  |  |
| GO:0051225\_spindle\_assembly | 4 | 0 |  |  |  |  |  |  |  |  |
| GO:0051341\_regulation\_of\_oxidoreductase\_activity | 4 | 0 |  |  |  |  |  |  |  |  |
| GO:0051452\_intracellular\_pH\_reduction | 4 | 0 |  |  |  |  |  |  |  |  |
| GO:0051567\_histone\_H3-K9\_methylation | 4 | 0 |  |  |  |  |  |  |  |  |
| GO:0051642\_centrosome\_localization | 4 | 0 |  |  |  |  |  |  |  |  |
| GO:0051797\_regulation\_of\_hair\_follicle\_development | 4 | 0 |  |  |  |  |  |  |  |  |
| GO:0051897\_positive\_regulation\_of\_protein\_kinase\_B\_signaling\_cascade | 4 | 0 |  |  |  |  |  |  |  |  |
| GO:0051904\_pigment\_granule\_transport | 4 | 0 |  |  |  |  |  |  |  |  |
| GO:0055009\_atrial\_cardiac\_muscle\_morphogenesis | 4 | 0 |  |  |  |  |  |  |  |  |
| GO:0060008\_Sertoli\_cell\_differentiation | 4 | 0 |  |  |  |  |  |  |  |  |
| GO:0060011\_Sertoli\_cell\_proliferation | 4 | 0 |  |  |  |  |  |  |  |  |
| GO:0060057\_apoptosis\_involved\_in\_mammary\_gland\_involution | 4 | 0 |  |  |  |  |  |  |  |  |
| GO:0060058\_positive\_regulation\_of\_apoptosis\_involved\_in\_mammary\_gland\_involution | 4 | 0 |  |  |  |  |  |  |  |  |
| GO:0060065\_uterus\_development | 4 | 0 |  |  |  |  |  |  |  |  |
| GO:0060087\_relaxation\_of\_vascular\_smooth\_muscle | 4 | 0 |  |  |  |  |  |  |  |  |
| GO:0060120\_inner\_ear\_receptor\_cell\_fate\_commitment | 4 | 0 |  |  |  |  |  |  |  |  |
| GO:0060157\_urinary\_bladder\_development | 4 | 0 |  |  |  |  |  |  |  |  |
| GO:0060158\_activation\_of\_phospholipase\_C\_activity\_by\_dopamine\_receptor\_signaling\_pathway | 4 | 0 |  |  |  |  |  |  |  |  |
| GO:0060164\_regulation\_of\_timing\_of\_neuron\_differentiation | 4 | 0 |  |  |  |  |  |  |  |  |
| GO:0060235\_lens\_induction\_in\_camera-type\_eye | 4 | 0 |  |  |  |  |  |  |  |  |
| GO:0060291\_long-term\_synaptic\_potentiation | 4 | 0 |  |  |  |  |  |  |  |  |
| GO:0060412\_ventricular\_septum\_morphogenesis | 4 | 0 |  |  |  |  |  |  |  |  |
| GO:0060459\_left\_lung\_development | 4 | 0 |  |  |  |  |  |  |  |  |
| GO:0060528\_secretory\_columnal\_luminar\_epithelial\_cell\_differentiation\_involved\_in\_prostate\_glandular\_acinus\_development | 4 | 0 |  |  |  |  |  |  |  |  |
| GO:0060561\_apoptosis\_involved\_in\_morphogenesis | 4 | 0 |  |  |  |  |  |  |  |  |
| GO:0060592\_mammary\_gland\_formation | 4 | 0 |  |  |  |  |  |  |  |  |
| GO:0060644\_mammary\_gland\_epithelial\_cell\_differentiation | 4 | 0 |  |  |  |  |  |  |  |  |
| GO:0060666\_dichotomous\_subdivision\_of\_terminal\_units\_involved\_in\_salivary\_gland\_branching | 4 | 0 |  |  |  |  |  |  |  |  |
| GO:0060737\_prostate\_gland\_morphogenetic\_growth | 4 | 0 |  |  |  |  |  |  |  |  |
| GO:0060743\_epithelial\_cell\_maturation\_involved\_in\_prostate\_gland\_development | 4 | 0 |  |  |  |  |  |  |  |  |
| GO:0060751\_mammary\_gland\_duct\_branch\_elongation | 4 | 0 |  |  |  |  |  |  |  |  |
| GO:0060900\_embryonic\_camera-type\_eye\_formation | 4 | 0 |  |  |  |  |  |  |  |  |
| GO:0070059\_apoptosis\_in\_response\_to\_endoplasmic\_reticulum\_stress | 4 | 0 |  |  |  |  |  |  |  |  |
| GO:0070254\_mucus\_secretion | 4 | 0 |  |  |  |  |  |  |  |  |
| GO:0070255\_regulation\_of\_mucus\_secretion | 4 | 0 |  |  |  |  |  |  |  |  |
| GO:0070301\_cellular\_response\_to\_hydrogen\_peroxide | 4 | 0 |  |  |  |  |  |  |  |  |
| GO:0070585\_protein\_localization\_in\_mitochondrion | 4 | 0 |  |  |  |  |  |  |  |  |
| GO:0000060\_protein\_import\_into\_nucleus\_\_translocation | 14 | 0 | 0.000000 | -0.000000 | 802 | 649.963876 | 742.72 | 835.476124 | 0.926085 |
| GO:0000077\_DNA\_damage\_checkpoint | 14 | 0 | 0.000000 | -0.000000 | 802 | 649.963876 | 742.72 | 835.476124 | 0.926085 |
| GO:0001502\_cartilage\_condensation | 14 | 0 | 0.000000 | -0.000000 | 802 | 649.963876 | 742.72 | 835.476124 | 0.926085 |
| GO:0001829\_trophectodermal\_cell\_differentiation | 14 | 0 | 0.000000 | -0.000000 | 802 | 649.963876 | 742.72 | 835.476124 | 0.926085 |
| GO:0002027\_regulation\_of\_heart\_rate | 14 | 0 | 0.000000 | -0.000000 | 802 | 649.963876 | 742.72 | 835.476124 | 0.926085 |
| GO:0002262\_myeloid\_cell\_homeostasis | 14 | 0 | 0.000000 | -0.000000 | 802 | 649.963876 | 742.72 | 835.476124 | 0.926085 |
| GO:0002698\_negative\_regulation\_of\_immune\_effector\_process | 14 | 0 | 0.000000 | -0.000000 | 802 | 649.963876 | 742.72 | 835.476124 | 0.926085 |
| GO:0006304\_DNA\_modification | 14 | 0 | 0.000000 | -0.000000 | 802 | 649.963876 | 742.72 | 835.476124 | 0.926085 |
| GO:0006305\_DNA\_alkylation | 14 | 0 | 0.000000 | -0.000000 | 802 | 649.963876 | 742.72 | 835.476124 | 0.926085 |
| GO:0006306\_DNA\_methylation | 14 | 0 | 0.000000 | -0.000000 | 802 | 649.963876 | 742.72 | 835.476124 | 0.926085 |
| GO:0006695\_cholesterol\_biosynthetic\_process | 14 | 0 | 0.000000 | -0.000000 | 802 | 649.963876 | 742.72 | 835.476124 | 0.926085 |
| GO:0006914\_autophagy | 14 | 0 | 0.000000 | -0.000000 | 802 | 649.963876 | 742.72 | 835.476124 | 0.926085 |
| GO:0006970\_response\_to\_osmotic\_stress | 14 | 0 | 0.000000 | -0.000000 | 802 | 649.963876 | 742.72 | 835.476124 | 0.926085 |
| GO:0007157\_heterophilic\_cell\_adhesion | 14 | 0 | 0.000000 | -0.000000 | 802 | 649.963876 | 742.72 | 835.476124 | 0.926085 |
| GO:0007530\_sex\_determination | 14 | 0 | 0.000000 | -0.000000 | 802 | 649.963876 | 742.72 | 835.476124 | 0.926085 |
| GO:0007589\_body\_fluid\_secretion | 14 | 0 | 0.000000 | -0.000000 | 802 | 649.963876 | 742.72 | 835.476124 | 0.926085 |
| GO:0008064\_regulation\_of\_actin\_polymerization\_or\_depolymerization | 14 | 0 | 0.000000 | -0.000000 | 802 | 649.963876 | 742.72 | 835.476124 | 0.926085 |
| GO:0008306\_associative\_learning | 14 | 0 | 0.000000 | -0.000000 | 802 | 649.963876 | 742.72 | 835.476124 | 0.926085 |
| GO:0008630\_DNA\_damage\_response\_\_signal\_transduction\_resulting\_in\_induction\_of\_apoptosis | 14 | 0 | 0.000000 | -0.000000 | 802 | 649.963876 | 742.72 | 835.476124 | 0.926085 |
| GO:0009108\_coenzyme\_biosynthetic\_process | 14 | 0 | 0.000000 | -0.000000 | 802 | 649.963876 | 742.72 | 835.476124 | 0.926085 |
| GO:0009267\_cellular\_response\_to\_starvation | 14 | 0 | 0.000000 | -0.000000 | 802 | 649.963876 | 742.72 | 835.476124 | 0.926085 |
| GO:0009895\_negative\_regulation\_of\_catabolic\_process | 14 | 0 | 0.000000 | -0.000000 | 802 | 649.963876 | 742.72 | 835.476124 | 0.926085 |
| GO:0010332\_response\_to\_gamma\_radiation | 14 | 0 | 0.000000 | -0.000000 | 802 | 649.963876 | 742.72 | 835.476124 | 0.926085 |
| GO:0014855\_striated\_muscle\_cell\_proliferation | 14 | 0 | 0.000000 | -0.000000 | 802 | 649.963876 | 742.72 | 835.476124 | 0.926085 |
| GO:0016573\_histone\_acetylation | 14 | 0 | 0.000000 | -0.000000 | 802 | 649.963876 | 742.72 | 835.476124 | 0.926085 |
| GO:0018130\_heterocycle\_biosynthetic\_process | 14 | 0 | 0.000000 | -0.000000 | 802 | 649.963876 | 742.72 | 835.476124 | 0.926085 |
| GO:0019217\_regulation\_of\_fatty\_acid\_metabolic\_process | 14 | 0 | 0.000000 | -0.000000 | 802 | 649.963876 | 742.72 | 835.476124 | 0.926085 |
| GO:0021782\_glial\_cell\_development | 14 | 0 | 0.000000 | -0.000000 | 802 | 649.963876 | 742.72 | 835.476124 | 0.926085 |
| GO:0021904\_dorsal\_ventral\_neural\_tube\_patterning | 14 | 0 | 0.000000 | -0.000000 | 802 | 649.963876 | 742.72 | 835.476124 | 0.926085 |
| GO:0030032\_lamellipodium\_assembly | 14 | 0 | 0.000000 | -0.000000 | 802 | 649.963876 | 742.72 | 835.476124 | 0.926085 |
| GO:0030148\_sphingolipid\_biosynthetic\_process | 14 | 0 | 0.000000 | -0.000000 | 802 | 649.963876 | 742.72 | 835.476124 | 0.926085 |
| GO:0030162\_regulation\_of\_proteolysis | 14 | 0 | 0.000000 | -0.000000 | 802 | 649.963876 | 742.72 | 835.476124 | 0.926085 |
| GO:0030832\_regulation\_of\_actin\_filament\_length | 14 | 0 | 0.000000 | -0.000000 | 802 | 649.963876 | 742.72 | 835.476124 | 0.926085 |
| GO:0031099\_regeneration | 14 | 0 | 0.000000 | -0.000000 | 802 | 649.963876 | 742.72 | 835.476124 | 0.926085 |
| GO:0031346\_positive\_regulation\_of\_cell\_projection\_organization | 14 | 0 | 0.000000 | -0.000000 | 802 | 649.963876 | 742.72 | 835.476124 | 0.926085 |
| GO:0031663\_lipopolysaccharide-mediated\_signaling\_pathway | 14 | 0 | 0.000000 | -0.000000 | 802 | 649.963876 | 742.72 | 835.476124 | 0.926085 |
| GO:0032271\_regulation\_of\_protein\_polymerization | 14 | 0 | 0.000000 | -0.000000 | 802 | 649.963876 | 742.72 | 835.476124 | 0.926085 |
| GO:0033044\_regulation\_of\_chromosome\_organization | 14 | 0 | 0.000000 | -0.000000 | 802 | 649.963876 | 742.72 | 835.476124 | 0.926085 |
| GO:0034104\_negative\_regulation\_of\_tissue\_remodeling | 14 | 0 | 0.000000 | -0.000000 | 802 | 649.963876 | 742.72 | 835.476124 | 0.926085 |
| GO:0034623\_cellular\_macromolecular\_complex\_disassembly | 14 | 0 | 0.000000 | -0.000000 | 802 | 649.963876 | 742.72 | 835.476124 | 0.926085 |
| GO:0035036\_sperm-egg\_recognition | 14 | 0 | 0.000000 | -0.000000 | 802 | 649.963876 | 742.72 | 835.476124 | 0.926085 |
| GO:0042310\_vasoconstriction | 14 | 0 | 0.000000 | -0.000000 | 802 | 649.963876 | 742.72 | 835.476124 | 0.926085 |
| GO:0042573\_retinoic\_acid\_metabolic\_process | 14 | 0 | 0.000000 | -0.000000 | 802 | 649.963876 | 742.72 | 835.476124 | 0.926085 |
| GO:0043123\_positive\_regulation\_of\_I-kappaB\_kinase\_NF-kappaB\_cascade | 14 | 0 | 0.000000 | -0.000000 | 802 | 649.963876 | 742.72 | 835.476124 | 0.926085 |
| GO:0043254\_regulation\_of\_protein\_complex\_assembly | 14 | 0 | 0.000000 | -0.000000 | 802 | 649.963876 | 742.72 | 835.476124 | 0.926085 |
| GO:0043491\_protein\_kinase\_B\_signaling\_cascade | 14 | 0 | 0.000000 | -0.000000 | 802 | 649.963876 | 742.72 | 835.476124 | 0.926085 |
| GO:0044236\_multicellular\_organismal\_metabolic\_process | 14 | 0 | 0.000000 | -0.000000 | 802 | 649.963876 | 742.72 | 835.476124 | 0.926085 |
| GO:0045061\_thymic\_T\_cell\_selection | 14 | 0 | 0.000000 | -0.000000 | 802 | 649.963876 | 742.72 | 835.476124 | 0.926085 |
| GO:0045453\_bone\_resorption | 14 | 0 | 0.000000 | -0.000000 | 802 | 649.963876 | 742.72 | 835.476124 | 0.926085 |
| GO:0045598\_regulation\_of\_fat\_cell\_differentiation | 14 | 0 | 0.000000 | -0.000000 | 802 | 649.963876 | 742.72 | 835.476124 | 0.926085 |
| GO:0045732\_positive\_regulation\_of\_protein\_catabolic\_process | 14 | 0 | 0.000000 | -0.000000 | 802 | 649.963876 | 742.72 | 835.476124 | 0.926085 |
| GO:0048048\_embryonic\_eye\_morphogenesis | 14 | 0 | 0.000000 | -0.000000 | 802 | 649.963876 | 742.72 | 835.476124 | 0.926085 |
| GO:0048545\_response\_to\_steroid\_hormone\_stimulus | 14 | 0 | 0.000000 | -0.000000 | 802 | 649.963876 | 742.72 | 835.476124 | 0.926085 |
| GO:0048844\_artery\_morphogenesis | 14 | 0 | 0.000000 | -0.000000 | 802 | 649.963876 | 742.72 | 835.476124 | 0.926085 |
| GO:0050810\_regulation\_of\_steroid\_biosynthetic\_process | 14 | 0 | 0.000000 | -0.000000 | 802 | 649.963876 | 742.72 | 835.476124 | 0.926085 |
| GO:0051017\_actin\_filament\_bundle\_formation | 14 | 0 | 0.000000 | -0.000000 | 802 | 649.963876 | 742.72 | 835.476124 | 0.926085 |
| GO:0051053\_negative\_regulation\_of\_DNA\_metabolic\_process | 14 | 0 | 0.000000 | -0.000000 | 802 | 649.963876 | 742.72 | 835.476124 | 0.926085 |
| GO:0051054\_positive\_regulation\_of\_DNA\_metabolic\_process | 14 | 0 | 0.000000 | -0.000000 | 802 | 649.963876 | 742.72 | 835.476124 | 0.926085 |
| GO:0051100\_negative\_regulation\_of\_binding | 14 | 0 | 0.000000 | -0.000000 | 802 | 649.963876 | 742.72 | 835.476124 | 0.926085 |
| GO:0051952\_regulation\_of\_amine\_transport | 14 | 0 | 0.000000 | -0.000000 | 802 | 649.963876 | 742.72 | 835.476124 | 0.926085 |
| GO:0060716\_labyrinthine\_layer\_blood\_vessel\_development | 14 | 0 | 0.000000 | -0.000000 | 802 | 649.963876 | 742.72 | 835.476124 | 0.926085 |
| GO:0060840\_artery\_development | 14 | 0 | 0.000000 | -0.000000 | 802 | 649.963876 | 742.72 | 835.476124 | 0.926085 |
| GO:0000079\_regulation\_of\_cyclin-dependent\_protein\_kinase\_activity | 7 | 0 | 0.000000 | -0.000000 | 971 | 826.838855 | 917.06 | 1007.281145 | 0.944449 |
| GO:0000188\_inactivation\_of\_MAPK\_activity | 7 | 0 | 0.000000 | -0.000000 | 971 | 826.838855 | 917.06 | 1007.281145 | 0.944449 |
| GO:0001504\_neurotransmitter\_uptake | 7 | 0 | 0.000000 | -0.000000 | 971 | 826.838855 | 917.06 | 1007.281145 | 0.944449 |
| GO:0001556\_oocyte\_maturation | 7 | 0 | 0.000000 | -0.000000 | 971 | 826.838855 | 917.06 | 1007.281145 | 0.944449 |
| GO:0001573\_ganglioside\_metabolic\_process | 7 | 0 | 0.000000 | -0.000000 | 971 | 826.838855 | 917.06 | 1007.281145 | 0.944449 |
| GO:0001736\_establishment\_of\_planar\_polarity | 7 | 0 | 0.000000 | -0.000000 | 971 | 826.838855 | 917.06 | 1007.281145 | 0.944449 |
| GO:0001839\_neural\_plate\_morphogenesis | 7 | 0 | 0.000000 | -0.000000 | 971 | 826.838855 | 917.06 | 1007.281145 | 0.944449 |
| GO:0001936\_regulation\_of\_endothelial\_cell\_proliferation | 7 | 0 | 0.000000 | -0.000000 | 971 | 826.838855 | 917.06 | 1007.281145 | 0.944449 |
| GO:0001967\_suckling\_behavior | 7 | 0 | 0.000000 | -0.000000 | 971 | 826.838855 | 917.06 | 1007.281145 | 0.944449 |
| GO:0002011\_morphogenesis\_of\_an\_epithelial\_sheet | 7 | 0 | 0.000000 | -0.000000 | 971 | 826.838855 | 917.06 | 1007.281145 | 0.944449 |
| GO:0002063\_chondrocyte\_development | 7 | 0 | 0.000000 | -0.000000 | 971 | 826.838855 | 917.06 | 1007.281145 | 0.944449 |
| GO:0002067\_glandular\_epithelial\_cell\_differentiation | 7 | 0 | 0.000000 | -0.000000 | 971 | 826.838855 | 917.06 | 1007.281145 | 0.944449 |
| GO:0002076\_osteoblast\_development | 7 | 0 | 0.000000 | -0.000000 | 971 | 826.838855 | 917.06 | 1007.281145 | 0.944449 |
| GO:0002087\_regulation\_of\_respiratory\_gaseous\_exchange\_by\_neurological\_system\_process | 7 | 0 | 0.000000 | -0.000000 | 971 | 826.838855 | 917.06 | 1007.281145 | 0.944449 |
| GO:0002093\_auditory\_receptor\_cell\_morphogenesis | 7 | 0 | 0.000000 | -0.000000 | 971 | 826.838855 | 917.06 | 1007.281145 | 0.944449 |
| GO:0002224\_toll-like\_receptor\_signaling\_pathway | 7 | 0 | 0.000000 | -0.000000 | 971 | 826.838855 | 917.06 | 1007.281145 | 0.944449 |
| GO:0002455\_humoral\_immune\_response\_mediated\_by\_circulating\_immunoglobulin | 7 | 0 | 0.000000 | -0.000000 | 971 | 826.838855 | 917.06 | 1007.281145 | 0.944449 |
| GO:0002643\_regulation\_of\_tolerance\_induction | 7 | 0 | 0.000000 | -0.000000 | 971 | 826.838855 | 917.06 | 1007.281145 | 0.944449 |
| GO:0002645\_positive\_regulation\_of\_tolerance\_induction | 7 | 0 | 0.000000 | -0.000000 | 971 | 826.838855 | 917.06 | 1007.281145 | 0.944449 |
| GO:0002714\_positive\_regulation\_of\_B\_cell\_mediated\_immunity | 7 | 0 | 0.000000 | -0.000000 | 971 | 826.838855 | 917.06 | 1007.281145 | 0.944449 |
| GO:0002792\_negative\_regulation\_of\_peptide\_secretion | 7 | 0 | 0.000000 | -0.000000 | 971 | 826.838855 | 917.06 | 1007.281145 | 0.944449 |
| GO:0002793\_positive\_regulation\_of\_peptide\_secretion | 7 | 0 | 0.000000 | -0.000000 | 971 | 826.838855 | 917.06 | 1007.281145 | 0.944449 |
| GO:0002828\_regulation\_of\_T-helper\_2\_type\_immune\_response | 7 | 0 | 0.000000 | -0.000000 | 971 | 826.838855 | 917.06 | 1007.281145 | 0.944449 |
| GO:0002863\_positive\_regulation\_of\_inflammatory\_response\_to\_antigenic\_stimulus | 7 | 0 | 0.000000 | -0.000000 | 971 | 826.838855 | 917.06 | 1007.281145 | 0.944449 |
| GO:0002891\_positive\_regulation\_of\_immunoglobulin\_mediated\_immune\_response | 7 | 0 | 0.000000 | -0.000000 | 971 | 826.838855 | 917.06 | 1007.281145 | 0.944449 |
| GO:0003084\_positive\_regulation\_of\_systemic\_arterial\_blood\_pressure | 7 | 0 | 0.000000 | -0.000000 | 971 | 826.838855 | 917.06 | 1007.281145 | 0.944449 |
| GO:0003085\_negative\_regulation\_of\_systemic\_arterial\_blood\_pressure | 7 | 0 | 0.000000 | -0.000000 | 971 | 826.838855 | 917.06 | 1007.281145 | 0.944449 |
| GO:0006041\_glucosamine\_metabolic\_process | 7 | 0 | 0.000000 | -0.000000 | 971 | 826.838855 | 917.06 | 1007.281145 | 0.944449 |
| GO:0006044\_N-acetylglucosamine\_metabolic\_process | 7 | 0 | 0.000000 | -0.000000 | 971 | 826.838855 | 917.06 | 1007.281145 | 0.944449 |
| GO:0006096\_glycolysis | 7 | 0 | 0.000000 | -0.000000 | 971 | 826.838855 | 917.06 | 1007.281145 | 0.944449 |
| GO:0006119\_oxidative\_phosphorylation | 7 | 0 | 0.000000 | -0.000000 | 971 | 826.838855 | 917.06 | 1007.281145 | 0.944449 |
| GO:0006275\_regulation\_of\_DNA\_replication | 7 | 0 | 0.000000 | -0.000000 | 971 | 826.838855 | 917.06 | 1007.281145 | 0.944449 |
| GO:0006298\_mismatch\_repair | 7 | 0 | 0.000000 | -0.000000 | 971 | 826.838855 | 917.06 | 1007.281145 | 0.944449 |
| GO:0006352\_transcription\_initiation | 7 | 0 | 0.000000 | -0.000000 | 971 | 826.838855 | 917.06 | 1007.281145 | 0.944449 |
| GO:0006401\_RNA\_catabolic\_process | 7 | 0 | 0.000000 | -0.000000 | 971 | 826.838855 | 917.06 | 1007.281145 | 0.944449 |
| GO:0006406\_mRNA\_export\_from\_nucleus | 7 | 0 | 0.000000 | -0.000000 | 971 | 826.838855 | 917.06 | 1007.281145 | 0.944449 |
| GO:0006505\_GPI\_anchor\_metabolic\_process | 7 | 0 | 0.000000 | -0.000000 | 971 | 826.838855 | 917.06 | 1007.281145 | 0.944449 |
| GO:0006516\_glycoprotein\_catabolic\_process | 7 | 0 | 0.000000 | -0.000000 | 971 | 826.838855 | 917.06 | 1007.281145 | 0.944449 |
| GO:0006612\_protein\_targeting\_to\_membrane | 7 | 0 | 0.000000 | -0.000000 | 971 | 826.838855 | 917.06 | 1007.281145 | 0.944449 |
| GO:0006769\_nicotinamide\_metabolic\_process | 7 | 0 | 0.000000 | -0.000000 | 971 | 826.838855 | 917.06 | 1007.281145 | 0.944449 |
| GO:0006783\_heme\_biosynthetic\_process | 7 | 0 | 0.000000 | -0.000000 | 971 | 826.838855 | 917.06 | 1007.281145 | 0.944449 |
| GO:0006818\_hydrogen\_transport | 7 | 0 | 0.000000 | -0.000000 | 971 | 826.838855 | 917.06 | 1007.281145 | 0.944449 |
| GO:0006878\_cellular\_copper\_ion\_homeostasis | 7 | 0 | 0.000000 | -0.000000 | 971 | 826.838855 | 917.06 | 1007.281145 | 0.944449 |
| GO:0006884\_cell\_volume\_homeostasis | 7 | 0 | 0.000000 | -0.000000 | 971 | 826.838855 | 917.06 | 1007.281145 | 0.944449 |
| GO:0007019\_microtubule\_depolymerization | 7 | 0 | 0.000000 | -0.000000 | 971 | 826.838855 | 917.06 | 1007.281145 | 0.944449 |
| GO:0007026\_negative\_regulation\_of\_microtubule\_depolymerization | 7 | 0 | 0.000000 | -0.000000 | 971 | 826.838855 | 917.06 | 1007.281145 | 0.944449 |
| GO:0007034\_vacuolar\_transport | 7 | 0 | 0.000000 | -0.000000 | 971 | 826.838855 | 917.06 | 1007.281145 | 0.944449 |
| GO:0007062\_sister\_chromatid\_cohesion | 7 | 0 | 0.000000 | -0.000000 | 971 | 826.838855 | 917.06 | 1007.281145 | 0.944449 |
| GO:0007130\_synaptonemal\_complex\_assembly | 7 | 0 | 0.000000 | -0.000000 | 971 | 826.838855 | 917.06 | 1007.281145 | 0.944449 |
| GO:0007164\_establishment\_of\_tissue\_polarity | 7 | 0 | 0.000000 | -0.000000 | 971 | 826.838855 | 917.06 | 1007.281145 | 0.944449 |
| GO:0007191\_activation\_of\_adenylate\_cyclase\_activity\_by\_dopamine\_receptor\_signaling\_pathway | 7 | 0 | 0.000000 | -0.000000 | 971 | 826.838855 | 917.06 | 1007.281145 | 0.944449 |
| GO:0007271\_synaptic\_transmission\_\_cholinergic | 7 | 0 | 0.000000 | -0.000000 | 971 | 826.838855 | 917.06 | 1007.281145 | 0.944449 |
| GO:0007413\_axonal\_fasciculation | 7 | 0 | 0.000000 | -0.000000 | 971 | 826.838855 | 917.06 | 1007.281145 | 0.944449 |
| GO:0007440\_foregut\_morphogenesis | 7 | 0 | 0.000000 | -0.000000 | 971 | 826.838855 | 917.06 | 1007.281145 | 0.944449 |
| GO:0007616\_long-term\_memory | 7 | 0 | 0.000000 | -0.000000 | 971 | 826.838855 | 917.06 | 1007.281145 | 0.944449 |
| GO:0008033\_tRNA\_processing | 7 | 0 | 0.000000 | -0.000000 | 971 | 826.838855 | 917.06 | 1007.281145 | 0.944449 |
| GO:0008299\_isoprenoid\_biosynthetic\_process | 7 | 0 | 0.000000 | -0.000000 | 971 | 826.838855 | 917.06 | 1007.281145 | 0.944449 |
| GO:0008340\_determination\_of\_adult\_lifespan | 7 | 0 | 0.000000 | -0.000000 | 971 | 826.838855 | 917.06 | 1007.281145 | 0.944449 |
| GO:0009150\_purine\_ribonucleotide\_metabolic\_process | 7 | 0 | 0.000000 | -0.000000 | 971 | 826.838855 | 917.06 | 1007.281145 | 0.944449 |
| GO:0009259\_ribonucleotide\_metabolic\_process | 7 | 0 | 0.000000 | -0.000000 | 971 | 826.838855 | 917.06 | 1007.281145 | 0.944449 |
| GO:0009311\_oligosaccharide\_metabolic\_process | 7 | 0 | 0.000000 | -0.000000 | 971 | 826.838855 | 917.06 | 1007.281145 | 0.944449 |
| GO:0009820\_alkaloid\_metabolic\_process | 7 | 0 | 0.000000 | -0.000000 | 971 | 826.838855 | 917.06 | 1007.281145 | 0.944449 |
| GO:0010469\_regulation\_of\_receptor\_activity | 7 | 0 | 0.000000 | -0.000000 | 971 | 826.838855 | 917.06 | 1007.281145 | 0.944449 |
| GO:0010948\_negative\_regulation\_of\_cell\_cycle\_process | 7 | 0 | 0.000000 | -0.000000 | 971 | 826.838855 | 917.06 | 1007.281145 | 0.944449 |
| GO:0014047\_glutamate\_secretion | 7 | 0 | 0.000000 | -0.000000 | 971 | 826.838855 | 917.06 | 1007.281145 | 0.944449 |
| GO:0014066\_regulation\_of\_phosphoinositide\_3-kinase\_cascade | 7 | 0 | 0.000000 | -0.000000 | 971 | 826.838855 | 917.06 | 1007.281145 | 0.944449 |
| GO:0014821\_phasic\_smooth\_muscle\_contraction | 7 | 0 | 0.000000 | -0.000000 | 971 | 826.838855 | 917.06 | 1007.281145 | 0.944449 |
| GO:0015697\_quaternary\_ammonium\_group\_transport | 7 | 0 | 0.000000 | -0.000000 | 971 | 826.838855 | 917.06 | 1007.281145 | 0.944449 |
| GO:0015813\_L-glutamate\_transport | 7 | 0 | 0.000000 | -0.000000 | 971 | 826.838855 | 917.06 | 1007.281145 | 0.944449 |
| GO:0015908\_fatty\_acid\_transport | 7 | 0 | 0.000000 | -0.000000 | 971 | 826.838855 | 917.06 | 1007.281145 | 0.944449 |
| GO:0015914\_phospholipid\_transport | 7 | 0 | 0.000000 | -0.000000 | 971 | 826.838855 | 917.06 | 1007.281145 | 0.944449 |
| GO:0015992\_proton\_transport | 7 | 0 | 0.000000 | -0.000000 | 971 | 826.838855 | 917.06 | 1007.281145 | 0.944449 |
| GO:0016339\_calcium-dependent\_cell-cell\_adhesion | 7 | 0 | 0.000000 | -0.000000 | 971 | 826.838855 | 917.06 | 1007.281145 | 0.944449 |
| GO:0016575\_histone\_deacetylation | 7 | 0 | 0.000000 | -0.000000 | 971 | 826.838855 | 917.06 | 1007.281145 | 0.944449 |
| GO:0019362\_pyridine\_nucleotide\_metabolic\_process | 7 | 0 | 0.000000 | -0.000000 | 971 | 826.838855 | 917.06 | 1007.281145 | 0.944449 |
| GO:0019800\_peptide\_cross-linking\_via\_chondroitin\_4-sulfate\_glycosaminoglycan | 7 | 0 | 0.000000 | -0.000000 | 971 | 826.838855 | 917.06 | 1007.281145 | 0.944449 |
| GO:0020027\_hemoglobin\_metabolic\_process | 7 | 0 | 0.000000 | -0.000000 | 971 | 826.838855 | 917.06 | 1007.281145 | 0.944449 |
| GO:0021516\_dorsal\_spinal\_cord\_development | 7 | 0 | 0.000000 | -0.000000 | 971 | 826.838855 | 917.06 | 1007.281145 | 0.944449 |
| GO:0021546\_rhombomere\_development | 7 | 0 | 0.000000 | -0.000000 | 971 | 826.838855 | 917.06 | 1007.281145 | 0.944449 |
| GO:0021756\_striatum\_development | 7 | 0 | 0.000000 | -0.000000 | 971 | 826.838855 | 917.06 | 1007.281145 | 0.944449 |
| GO:0021884\_forebrain\_neuron\_development | 7 | 0 | 0.000000 | -0.000000 | 971 | 826.838855 | 917.06 | 1007.281145 | 0.944449 |
| GO:0021984\_adenohypophysis\_development | 7 | 0 | 0.000000 | -0.000000 | 971 | 826.838855 | 917.06 | 1007.281145 | 0.944449 |
| GO:0022407\_regulation\_of\_cell-cell\_adhesion | 7 | 0 | 0.000000 | -0.000000 | 971 | 826.838855 | 917.06 | 1007.281145 | 0.944449 |
| GO:0022618\_ribonucleoprotein\_complex\_assembly | 7 | 0 | 0.000000 | -0.000000 | 971 | 826.838855 | 917.06 | 1007.281145 | 0.944449 |
| GO:0030104\_water\_homeostasis | 7 | 0 | 0.000000 | -0.000000 | 971 | 826.838855 | 917.06 | 1007.281145 | 0.944449 |
| GO:0030201\_heparan\_sulfate\_proteoglycan\_metabolic\_process | 7 | 0 | 0.000000 | -0.000000 | 971 | 826.838855 | 917.06 | 1007.281145 | 0.944449 |
| GO:0030432\_peristalsis | 7 | 0 | 0.000000 | -0.000000 | 971 | 826.838855 | 917.06 | 1007.281145 | 0.944449 |
| GO:0030517\_negative\_regulation\_of\_axon\_extension | 7 | 0 | 0.000000 | -0.000000 | 971 | 826.838855 | 917.06 | 1007.281145 | 0.944449 |
| GO:0030520\_estrogen\_receptor\_signaling\_pathway | 7 | 0 | 0.000000 | -0.000000 | 971 | 826.838855 | 917.06 | 1007.281145 | 0.944449 |
| GO:0030903\_notochord\_development | 7 | 0 | 0.000000 | -0.000000 | 971 | 826.838855 | 917.06 | 1007.281145 | 0.944449 |
| GO:0031017\_exocrine\_pancreas\_development | 7 | 0 | 0.000000 | -0.000000 | 971 | 826.838855 | 917.06 | 1007.281145 | 0.944449 |
| GO:0031114\_regulation\_of\_microtubule\_depolymerization | 7 | 0 | 0.000000 | -0.000000 | 971 | 826.838855 | 917.06 | 1007.281145 | 0.944449 |
| GO:0031124\_mRNA\_3'-end\_processing | 7 | 0 | 0.000000 | -0.000000 | 971 | 826.838855 | 917.06 | 1007.281145 | 0.944449 |
| GO:0031497\_chromatin\_assembly | 7 | 0 | 0.000000 | -0.000000 | 971 | 826.838855 | 917.06 | 1007.281145 | 0.944449 |
| GO:0032104\_regulation\_of\_response\_to\_extracellular\_stimulus | 7 | 0 | 0.000000 | -0.000000 | 971 | 826.838855 | 917.06 | 1007.281145 | 0.944449 |
| GO:0032107\_regulation\_of\_response\_to\_nutrient\_levels | 7 | 0 | 0.000000 | -0.000000 | 971 | 826.838855 | 917.06 | 1007.281145 | 0.944449 |
| GO:0032228\_regulation\_of\_synaptic\_transmission\_\_GABAergic | 7 | 0 | 0.000000 | -0.000000 | 971 | 826.838855 | 917.06 | 1007.281145 | 0.944449 |
| GO:0032319\_regulation\_of\_Rho\_GTPase\_activity | 7 | 0 | 0.000000 | -0.000000 | 971 | 826.838855 | 917.06 | 1007.281145 | 0.944449 |
| GO:0032387\_negative\_regulation\_of\_intracellular\_transport | 7 | 0 | 0.000000 | -0.000000 | 971 | 826.838855 | 917.06 | 1007.281145 | 0.944449 |
| GO:0032507\_maintenance\_of\_protein\_location\_in\_cell | 7 | 0 | 0.000000 | -0.000000 | 971 | 826.838855 | 917.06 | 1007.281145 | 0.944449 |
| GO:0033032\_regulation\_of\_myeloid\_cell\_apoptosis | 7 | 0 | 0.000000 | -0.000000 | 971 | 826.838855 | 917.06 | 1007.281145 | 0.944449 |
| GO:0033057\_reproductive\_behavior\_in\_a\_multicellular\_organism | 7 | 0 | 0.000000 | -0.000000 | 971 | 826.838855 | 917.06 | 1007.281145 | 0.944449 |
| GO:0034599\_cellular\_response\_to\_oxidative\_stress | 7 | 0 | 0.000000 | -0.000000 | 971 | 826.838855 | 917.06 | 1007.281145 | 0.944449 |
| GO:0042033\_chemokine\_biosynthetic\_process | 7 | 0 | 0.000000 | -0.000000 | 971 | 826.838855 | 917.06 | 1007.281145 | 0.944449 |
| GO:0042133\_neurotransmitter\_metabolic\_process | 7 | 0 | 0.000000 | -0.000000 | 971 | 826.838855 | 917.06 | 1007.281145 | 0.944449 |
| GO:0042168\_heme\_metabolic\_process | 7 | 0 | 0.000000 | -0.000000 | 971 | 826.838855 | 917.06 | 1007.281145 | 0.944449 |
| GO:0042415\_norepinephrine\_metabolic\_process | 7 | 0 | 0.000000 | -0.000000 | 971 | 826.838855 | 917.06 | 1007.281145 | 0.944449 |
| GO:0042438\_melanin\_biosynthetic\_process | 7 | 0 | 0.000000 | -0.000000 | 971 | 826.838855 | 917.06 | 1007.281145 | 0.944449 |
| GO:0042503\_tyrosine\_phosphorylation\_of\_Stat3\_protein | 7 | 0 | 0.000000 | -0.000000 | 971 | 826.838855 | 917.06 | 1007.281145 | 0.944449 |
| GO:0042572\_retinol\_metabolic\_process | 7 | 0 | 0.000000 | -0.000000 | 971 | 826.838855 | 917.06 | 1007.281145 | 0.944449 |
| GO:0043353\_enucleate\_erythrocyte\_differentiation | 7 | 0 | 0.000000 | -0.000000 | 971 | 826.838855 | 917.06 | 1007.281145 | 0.944449 |
| GO:0043372\_positive\_regulation\_of\_CD4-positive\_\_alpha\_beta\_T\_cell\_differentiation | 7 | 0 | 0.000000 | -0.000000 | 971 | 826.838855 | 917.06 | 1007.281145 | 0.944449 |
| GO:0043449\_cellular\_alkene\_metabolic\_process | 7 | 0 | 0.000000 | -0.000000 | 971 | 826.838855 | 917.06 | 1007.281145 | 0.944449 |
| GO:0043507\_positive\_regulation\_of\_JUN\_kinase\_activity | 7 | 0 | 0.000000 | -0.000000 | 971 | 826.838855 | 917.06 | 1007.281145 | 0.944449 |
| GO:0043567\_regulation\_of\_insulin-like\_growth\_factor\_receptor\_signaling\_pathway | 7 | 0 | 0.000000 | -0.000000 | 971 | 826.838855 | 917.06 | 1007.281145 | 0.944449 |
| GO:0043584\_nose\_development | 7 | 0 | 0.000000 | -0.000000 | 971 | 826.838855 | 917.06 | 1007.281145 | 0.944449 |
| GO:0044065\_regulation\_of\_respiratory\_system\_process | 7 | 0 | 0.000000 | -0.000000 | 971 | 826.838855 | 917.06 | 1007.281145 | 0.944449 |
| GO:0044275\_cellular\_carbohydrate\_catabolic\_process | 7 | 0 | 0.000000 | -0.000000 | 971 | 826.838855 | 917.06 | 1007.281145 | 0.944449 |
| GO:0045059\_positive\_thymic\_T\_cell\_selection | 7 | 0 | 0.000000 | -0.000000 | 971 | 826.838855 | 917.06 | 1007.281145 | 0.944449 |
| GO:0045073\_regulation\_of\_chemokine\_biosynthetic\_process | 7 | 0 | 0.000000 | -0.000000 | 971 | 826.838855 | 917.06 | 1007.281145 | 0.944449 |
| GO:0045581\_negative\_regulation\_of\_T\_cell\_differentiation | 7 | 0 | 0.000000 | -0.000000 | 971 | 826.838855 | 917.06 | 1007.281145 | 0.944449 |
| GO:0045599\_negative\_regulation\_of\_fat\_cell\_differentiation | 7 | 0 | 0.000000 | -0.000000 | 971 | 826.838855 | 917.06 | 1007.281145 | 0.944449 |
| GO:0045604\_regulation\_of\_epidermal\_cell\_differentiation | 7 | 0 | 0.000000 | -0.000000 | 971 | 826.838855 | 917.06 | 1007.281145 | 0.944449 |
| GO:0045668\_negative\_regulation\_of\_osteoblast\_differentiation | 7 | 0 | 0.000000 | -0.000000 | 971 | 826.838855 | 917.06 | 1007.281145 | 0.944449 |
| GO:0045823\_positive\_regulation\_of\_heart\_contraction | 7 | 0 | 0.000000 | -0.000000 | 971 | 826.838855 | 917.06 | 1007.281145 | 0.944449 |
| GO:0045840\_positive\_regulation\_of\_mitosis | 7 | 0 | 0.000000 | -0.000000 | 971 | 826.838855 | 917.06 | 1007.281145 | 0.944449 |
| GO:0045862\_positive\_regulation\_of\_proteolysis | 7 | 0 | 0.000000 | -0.000000 | 971 | 826.838855 | 917.06 | 1007.281145 | 0.944449 |
| GO:0045879\_negative\_regulation\_of\_smoothened\_signaling\_pathway | 7 | 0 | 0.000000 | -0.000000 | 971 | 826.838855 | 917.06 | 1007.281145 | 0.944449 |
| GO:0045880\_positive\_regulation\_of\_smoothened\_signaling\_pathway | 7 | 0 | 0.000000 | -0.000000 | 971 | 826.838855 | 917.06 | 1007.281145 | 0.944449 |
| GO:0045986\_negative\_regulation\_of\_smooth\_muscle\_contraction | 7 | 0 | 0.000000 | -0.000000 | 971 | 826.838855 | 917.06 | 1007.281145 | 0.944449 |
| GO:0046496\_nicotinamide\_nucleotide\_metabolic\_process | 7 | 0 | 0.000000 | -0.000000 | 971 | 826.838855 | 917.06 | 1007.281145 | 0.944449 |
| GO:0046504\_glycerol\_ether\_biosynthetic\_process | 7 | 0 | 0.000000 | -0.000000 | 971 | 826.838855 | 917.06 | 1007.281145 | 0.944449 |
| GO:0046513\_ceramide\_biosynthetic\_process | 7 | 0 | 0.000000 | -0.000000 | 971 | 826.838855 | 917.06 | 1007.281145 | 0.944449 |
| GO:0046520\_sphingoid\_biosynthetic\_process | 7 | 0 | 0.000000 | -0.000000 | 971 | 826.838855 | 917.06 | 1007.281145 | 0.944449 |
| GO:0046543\_development\_of\_secondary\_female\_sexual\_characteristics | 7 | 0 | 0.000000 | -0.000000 | 971 | 826.838855 | 917.06 | 1007.281145 | 0.944449 |
| GO:0046626\_regulation\_of\_insulin\_receptor\_signaling\_pathway | 7 | 0 | 0.000000 | -0.000000 | 971 | 826.838855 | 917.06 | 1007.281145 | 0.944449 |
| GO:0046676\_negative\_regulation\_of\_insulin\_secretion | 7 | 0 | 0.000000 | -0.000000 | 971 | 826.838855 | 917.06 | 1007.281145 | 0.944449 |
| GO:0046677\_response\_to\_antibiotic | 7 | 0 | 0.000000 | -0.000000 | 971 | 826.838855 | 917.06 | 1007.281145 | 0.944449 |
| GO:0046823\_negative\_regulation\_of\_nucleocytoplasmic\_transport | 7 | 0 | 0.000000 | -0.000000 | 971 | 826.838855 | 917.06 | 1007.281145 | 0.944449 |
| GO:0046824\_positive\_regulation\_of\_nucleocytoplasmic\_transport | 7 | 0 | 0.000000 | -0.000000 | 971 | 826.838855 | 917.06 | 1007.281145 | 0.944449 |
| GO:0046847\_filopodium\_assembly | 7 | 0 | 0.000000 | -0.000000 | 971 | 826.838855 | 917.06 | 1007.281145 | 0.944449 |
| GO:0048148\_behavioral\_response\_to\_cocaine | 7 | 0 | 0.000000 | -0.000000 | 971 | 826.838855 | 917.06 | 1007.281145 | 0.944449 |
| GO:0048304\_positive\_regulation\_of\_isotype\_switching\_to\_IgG\_isotypes | 7 | 0 | 0.000000 | -0.000000 | 971 | 826.838855 | 917.06 | 1007.281145 | 0.944449 |
| GO:0048486\_parasympathetic\_nervous\_system\_development | 7 | 0 | 0.000000 | -0.000000 | 971 | 826.838855 | 917.06 | 1007.281145 | 0.944449 |
| GO:0048537\_mucosal-associated\_lymphoid\_tissue\_development | 7 | 0 | 0.000000 | -0.000000 | 971 | 826.838855 | 917.06 | 1007.281145 | 0.944449 |
| GO:0048753\_pigment\_granule\_organization | 7 | 0 | 0.000000 | -0.000000 | 971 | 826.838855 | 917.06 | 1007.281145 | 0.944449 |
| GO:0048814\_regulation\_of\_dendrite\_morphogenesis | 7 | 0 | 0.000000 | -0.000000 | 971 | 826.838855 | 917.06 | 1007.281145 | 0.944449 |
| GO:0048857\_neural\_nucleus\_development | 7 | 0 | 0.000000 | -0.000000 | 971 | 826.838855 | 917.06 | 1007.281145 | 0.944449 |
| GO:0050755\_chemokine\_metabolic\_process | 7 | 0 | 0.000000 | -0.000000 | 971 | 826.838855 | 917.06 | 1007.281145 | 0.944449 |
| GO:0050773\_regulation\_of\_dendrite\_development | 7 | 0 | 0.000000 | -0.000000 | 971 | 826.838855 | 917.06 | 1007.281145 | 0.944449 |
| GO:0051028\_mRNA\_transport | 7 | 0 | 0.000000 | -0.000000 | 971 | 826.838855 | 917.06 | 1007.281145 | 0.944449 |
| GO:0051785\_positive\_regulation\_of\_nuclear\_division | 7 | 0 | 0.000000 | -0.000000 | 971 | 826.838855 | 917.06 | 1007.281145 | 0.944449 |
| GO:0051928\_positive\_regulation\_of\_calcium\_ion\_transport | 7 | 0 | 0.000000 | -0.000000 | 971 | 826.838855 | 917.06 | 1007.281145 | 0.944449 |
| GO:0055069\_zinc\_ion\_homeostasis | 7 | 0 | 0.000000 | -0.000000 | 971 | 826.838855 | 917.06 | 1007.281145 | 0.944449 |
| GO:0055070\_copper\_ion\_homeostasis | 7 | 0 | 0.000000 | -0.000000 | 971 | 826.838855 | 917.06 | 1007.281145 | 0.944449 |
| GO:0060037\_pharyngeal\_system\_development | 7 | 0 | 0.000000 | -0.000000 | 971 | 826.838855 | 917.06 | 1007.281145 | 0.944449 |
| GO:0060080\_regulation\_of\_inhibitory\_postsynaptic\_membrane\_potential | 7 | 0 | 0.000000 | -0.000000 | 971 | 826.838855 | 917.06 | 1007.281145 | 0.944449 |
| GO:0060088\_auditory\_receptor\_cell\_stereocilium\_organization | 7 | 0 | 0.000000 | -0.000000 | 971 | 826.838855 | 917.06 | 1007.281145 | 0.944449 |
| GO:0060117\_auditory\_receptor\_cell\_development | 7 | 0 | 0.000000 | -0.000000 | 971 | 826.838855 | 917.06 | 1007.281145 | 0.944449 |
| GO:0060441\_branching\_involved\_in\_lung\_morphogenesis | 7 | 0 | 0.000000 | -0.000000 | 971 | 826.838855 | 917.06 | 1007.281145 | 0.944449 |
| GO:0060526\_prostate\_glandular\_acinus\_morphogenesis | 7 | 0 | 0.000000 | -0.000000 | 971 | 826.838855 | 917.06 | 1007.281145 | 0.944449 |
| GO:0060527\_prostate\_epithelial\_cord\_arborization\_involved\_in\_prostate\_glandular\_acinus\_morphogenesis | 7 | 0 | 0.000000 | -0.000000 | 971 | 826.838855 | 917.06 | 1007.281145 | 0.944449 |
| GO:0060664\_epithelial\_cell\_proliferation\_involved\_in\_salivary\_gland\_morphogenesis | 7 | 0 | 0.000000 | -0.000000 | 971 | 826.838855 | 917.06 | 1007.281145 | 0.944449 |
| GO:0060687\_regulation\_of\_branching\_involved\_in\_prostate\_gland\_morphogenesis | 7 | 0 | 0.000000 | -0.000000 | 971 | 826.838855 | 917.06 | 1007.281145 | 0.944449 |
| GO:0060770\_negative\_regulation\_of\_epithelial\_cell\_proliferation\_involved\_in\_prostate\_gland\_development | 7 | 0 | 0.000000 | -0.000000 | 971 | 826.838855 | 917.06 | 1007.281145 | 0.944449 |
| GO:0060788\_ectodermal\_placode\_formation | 7 | 0 | 0.000000 | -0.000000 | 971 | 826.838855 | 917.06 | 1007.281145 | 0.944449 |
| GO:0060795\_cell\_fate\_commitment\_involved\_in\_the\_formation\_of\_primary\_germ\_layers | 7 | 0 | 0.000000 | -0.000000 | 971 | 826.838855 | 917.06 | 1007.281145 | 0.944449 |
| GO:0070228\_regulation\_of\_lymphocyte\_apoptosis | 7 | 0 | 0.000000 | -0.000000 | 971 | 826.838855 | 917.06 | 1007.281145 | 0.944449 |
| GO:0070646\_protein\_modification\_by\_small\_protein\_removal | 7 | 0 | 0.000000 | -0.000000 | 971 | 826.838855 | 917.06 | 1007.281145 | 0.944449 |
| GO:0002200\_somatic\_diversification\_of\_immune\_receptors | 34 | 0 | 0.000000 | -0.000000 | 984 | 842.725508 | 931.87 | 1021.014492 | 0.947022 |
| GO:0002237\_response\_to\_molecule\_of\_bacterial\_origin | 34 | 0 | 0.000000 | -0.000000 | 984 | 842.725508 | 931.87 | 1021.014492 | 0.947022 |
| GO:0002699\_positive\_regulation\_of\_immune\_effector\_process | 34 | 0 | 0.000000 | -0.000000 | 984 | 842.725508 | 931.87 | 1021.014492 | 0.947022 |
| GO:0007269\_neurotransmitter\_secretion | 34 | 0 | 0.000000 | -0.000000 | 984 | 842.725508 | 931.87 | 1021.014492 | 0.947022 |
| GO:0007338\_single\_fertilization | 34 | 0 | 0.000000 | -0.000000 | 984 | 842.725508 | 931.87 | 1021.014492 | 0.947022 |
| GO:0016054\_organic\_acid\_catabolic\_process | 34 | 0 | 0.000000 | -0.000000 | 984 | 842.725508 | 931.87 | 1021.014492 | 0.947022 |
| GO:0019882\_antigen\_processing\_and\_presentation | 34 | 0 | 0.000000 | -0.000000 | 984 | 842.725508 | 931.87 | 1021.014492 | 0.947022 |
| GO:0046395\_carboxylic\_acid\_catabolic\_process | 34 | 0 | 0.000000 | -0.000000 | 984 | 842.725508 | 931.87 | 1021.014492 | 0.947022 |
| GO:0050730\_regulation\_of\_peptidyl-tyrosine\_phosphorylation | 34 | 0 | 0.000000 | -0.000000 | 984 | 842.725508 | 931.87 | 1021.014492 | 0.947022 |
| GO:0051047\_positive\_regulation\_of\_secretion | 34 | 0 | 0.000000 | -0.000000 | 984 | 842.725508 | 931.87 | 1021.014492 | 0.947022 |
| GO:0051052\_regulation\_of\_DNA\_metabolic\_process | 34 | 0 | 0.000000 | -0.000000 | 984 | 842.725508 | 931.87 | 1021.014492 | 0.947022 |
| GO:0060443\_mammary\_gland\_morphogenesis | 34 | 0 | 0.000000 | -0.000000 | 984 | 842.725508 | 931.87 | 1021.014492 | 0.947022 |
| GO:0060711\_labyrinthine\_layer\_development | 34 | 0 | 0.000000 | -0.000000 | 984 | 842.725508 | 931.87 | 1021.014492 | 0.947022 |
| GO:0008610\_lipid\_biosynthetic\_process | 94 | 0 | 0.000000 | -0.000000 | 988 | 846.750114 | 935.22 | 1023.689886 | 0.946579 |
| GO:0032943\_mononuclear\_cell\_proliferation | 94 | 0 | 0.000000 | -0.000000 | 988 | 846.750114 | 935.22 | 1023.689886 | 0.946579 |
| GO:0034984\_cellular\_response\_to\_DNA\_damage\_stimulus | 94 | 0 | 0.000000 | -0.000000 | 988 | 846.750114 | 935.22 | 1023.689886 | 0.946579 |
| GO:0046651\_lymphocyte\_proliferation | 94 | 0 | 0.000000 | -0.000000 | 988 | 846.750114 | 935.22 | 1023.689886 | 0.946579 |
| GO:0000278\_mitotic\_cell\_cycle | 80 | 0 | 0.000000 | -0.000000 | 992 | 851.596776 | 939.26 | 1026.923224 | 0.946835 |
| GO:0002250\_adaptive\_immune\_response | 80 | 0 | 0.000000 | -0.000000 | 992 | 851.596776 | 939.26 | 1026.923224 | 0.946835 |
| GO:0002460\_adaptive\_immune\_response\_based\_on\_somatic\_recombination\_of\_immune\_receptors\_built\_from\_immunoglobulin\_superfamily\_domains | 80 | 0 | 0.000000 | -0.000000 | 992 | 851.596776 | 939.26 | 1026.923224 | 0.946835 |
| GO:0006631\_fatty\_acid\_metabolic\_process | 80 | 0 | 0.000000 | -0.000000 | 992 | 851.596776 | 939.26 | 1026.923224 | 0.946835 |
| GO:0015674\_di-\_\_tri-valent\_inorganic\_cation\_transport | 79 | 0 | 0.000000 | -0.000000 | 994 | 853.006681 | 940.5 | 1027.993319 | 0.946177 |
| GO:0051046\_regulation\_of\_secretion | 79 | 0 | 0.000000 | -0.000000 | 994 | 853.006681 | 940.5 | 1027.993319 | 0.946177 |
| GO:0009416\_response\_to\_light\_stimulus | 74 | 0 | 0.000000 | -0.000000 | 996 | 854.406382 | 941.68 | 1028.953618 | 0.945462 |
| GO:0048771\_tissue\_remodeling | 74 | 0 | 0.000000 | -0.000000 | 996 | 854.406382 | 941.68 | 1028.953618 | 0.945462 |
| GO:0000280\_nuclear\_division | 24 | 0 | 0.000000 | -0.000000 | 1022 | 880.519465 | 966.6 | 1052.680535 | 0.945793 |
| GO:0001541\_ovarian\_follicle\_development | 24 | 0 | 0.000000 | -0.000000 | 1022 | 880.519465 | 966.6 | 1052.680535 | 0.945793 |
| GO:0002381\_immunoglobulin\_production\_during\_immune\_response | 24 | 0 | 0.000000 | -0.000000 | 1022 | 880.519465 | 966.6 | 1052.680535 | 0.945793 |
| GO:0006650\_glycerophospholipid\_metabolic\_process | 24 | 0 | 0.000000 | -0.000000 | 1022 | 880.519465 | 966.6 | 1052.680535 | 0.945793 |
| GO:0006941\_striated\_muscle\_contraction | 24 | 0 | 0.000000 | -0.000000 | 1022 | 880.519465 | 966.6 | 1052.680535 | 0.945793 |
| GO:0006959\_humoral\_immune\_response | 24 | 0 | 0.000000 | -0.000000 | 1022 | 880.519465 | 966.6 | 1052.680535 | 0.945793 |
| GO:0007050\_cell\_cycle\_arrest | 24 | 0 | 0.000000 | -0.000000 | 1022 | 880.519465 | 966.6 | 1052.680535 | 0.945793 |
| GO:0007067\_mitosis | 24 | 0 | 0.000000 | -0.000000 | 1022 | 880.519465 | 966.6 | 1052.680535 | 0.945793 |
| GO:0007204\_elevation\_of\_cytosolic\_calcium\_ion\_concentration | 24 | 0 | 0.000000 | -0.000000 | 1022 | 880.519465 | 966.6 | 1052.680535 | 0.945793 |
| GO:0007259\_JAK-STAT\_cascade | 24 | 0 | 0.000000 | -0.000000 | 1022 | 880.519465 | 966.6 | 1052.680535 | 0.945793 |
| GO:0007266\_Rho\_protein\_signal\_transduction | 24 | 0 | 0.000000 | -0.000000 | 1022 | 880.519465 | 966.6 | 1052.680535 | 0.945793 |
| GO:0007632\_visual\_behavior | 24 | 0 | 0.000000 | -0.000000 | 1022 | 880.519465 | 966.6 | 1052.680535 | 0.945793 |
| GO:0008629\_induction\_of\_apoptosis\_by\_intracellular\_signals | 24 | 0 | 0.000000 | -0.000000 | 1022 | 880.519465 | 966.6 | 1052.680535 | 0.945793 |
| GO:0009612\_response\_to\_mechanical\_stimulus | 24 | 0 | 0.000000 | -0.000000 | 1022 | 880.519465 | 966.6 | 1052.680535 | 0.945793 |
| GO:0014070\_response\_to\_organic\_cyclic\_substance | 24 | 0 | 0.000000 | -0.000000 | 1022 | 880.519465 | 966.6 | 1052.680535 | 0.945793 |
| GO:0032386\_regulation\_of\_intracellular\_transport | 24 | 0 | 0.000000 | -0.000000 | 1022 | 880.519465 | 966.6 | 1052.680535 | 0.945793 |
| GO:0042158\_lipoprotein\_biosynthetic\_process | 24 | 0 | 0.000000 | -0.000000 | 1022 | 880.519465 | 966.6 | 1052.680535 | 0.945793 |
| GO:0042632\_cholesterol\_homeostasis | 24 | 0 | 0.000000 | -0.000000 | 1022 | 880.519465 | 966.6 | 1052.680535 | 0.945793 |
| GO:0043410\_positive\_regulation\_of\_MAPKKK\_cascade | 24 | 0 | 0.000000 | -0.000000 | 1022 | 880.519465 | 966.6 | 1052.680535 | 0.945793 |
| GO:0043588\_skin\_development | 24 | 0 | 0.000000 | -0.000000 | 1022 | 880.519465 | 966.6 | 1052.680535 | 0.945793 |
| GO:0048002\_antigen\_processing\_and\_presentation\_of\_peptide\_antigen | 24 | 0 | 0.000000 | -0.000000 | 1022 | 880.519465 | 966.6 | 1052.680535 | 0.945793 |
| GO:0048546\_digestive\_tract\_morphogenesis | 24 | 0 | 0.000000 | -0.000000 | 1022 | 880.519465 | 966.6 | 1052.680535 | 0.945793 |
| GO:0055092\_sterol\_homeostasis | 24 | 0 | 0.000000 | -0.000000 | 1022 | 880.519465 | 966.6 | 1052.680535 | 0.945793 |
| GO:0060078\_regulation\_of\_postsynaptic\_membrane\_potential | 24 | 0 | 0.000000 | -0.000000 | 1022 | 880.519465 | 966.6 | 1052.680535 | 0.945793 |
| GO:0060113\_inner\_ear\_receptor\_cell\_differentiation | 24 | 0 | 0.000000 | -0.000000 | 1022 | 880.519465 | 966.6 | 1052.680535 | 0.945793 |
| GO:0070667\_negative\_regulation\_of\_mast\_cell\_proliferation | 24 | 0 | 0.000000 | -0.000000 | 1022 | 880.519465 | 966.6 | 1052.680535 | 0.945793 |
| GO:0000302\_response\_to\_reactive\_oxygen\_species | 16 | 0 | 0.000000 | -0.000000 | 1066 | 925.453027 | 1010.24 | 1095.026973 | 0.947692 |
| GO:0001933\_negative\_regulation\_of\_protein\_amino\_acid\_phosphorylation | 16 | 0 | 0.000000 | -0.000000 | 1066 | 925.453027 | 1010.24 | 1095.026973 | 0.947692 |
| GO:0003044\_regulation\_of\_systemic\_arterial\_blood\_pressure\_mediated\_by\_a\_chemical\_signal | 16 | 0 | 0.000000 | -0.000000 | 1066 | 925.453027 | 1010.24 | 1095.026973 | 0.947692 |
| GO:0006664\_glycolipid\_metabolic\_process | 16 | 0 | 0.000000 | -0.000000 | 1066 | 925.453027 | 1010.24 | 1095.026973 | 0.947692 |
| GO:0006821\_chloride\_transport | 16 | 0 | 0.000000 | -0.000000 | 1066 | 925.453027 | 1010.24 | 1095.026973 | 0.947692 |
| GO:0007033\_vacuole\_organization | 16 | 0 | 0.000000 | -0.000000 | 1066 | 925.453027 | 1010.24 | 1095.026973 | 0.947692 |
| GO:0007156\_homophilic\_cell\_adhesion | 16 | 0 | 0.000000 | -0.000000 | 1066 | 925.453027 | 1010.24 | 1095.026973 | 0.947692 |
| GO:0007602\_phototransduction | 16 | 0 | 0.000000 | -0.000000 | 1066 | 925.453027 | 1010.24 | 1095.026973 | 0.947692 |
| GO:0008654\_phospholipid\_biosynthetic\_process | 16 | 0 | 0.000000 | -0.000000 | 1066 | 925.453027 | 1010.24 | 1095.026973 | 0.947692 |
| GO:0009988\_cell-cell\_recognition | 16 | 0 | 0.000000 | -0.000000 | 1066 | 925.453027 | 1010.24 | 1095.026973 | 0.947692 |
| GO:0010038\_response\_to\_metal\_ion | 16 | 0 | 0.000000 | -0.000000 | 1066 | 925.453027 | 1010.24 | 1095.026973 | 0.947692 |
| GO:0010243\_response\_to\_organic\_nitrogen | 16 | 0 | 0.000000 | -0.000000 | 1066 | 925.453027 | 1010.24 | 1095.026973 | 0.947692 |
| GO:0010876\_lipid\_localization | 16 | 0 | 0.000000 | -0.000000 | 1066 | 925.453027 | 1010.24 | 1095.026973 | 0.947692 |
| GO:0014075\_response\_to\_amine\_stimulus | 16 | 0 | 0.000000 | -0.000000 | 1066 | 925.453027 | 1010.24 | 1095.026973 | 0.947692 |
| GO:0016126\_sterol\_biosynthetic\_process | 16 | 0 | 0.000000 | -0.000000 | 1066 | 925.453027 | 1010.24 | 1095.026973 | 0.947692 |
| GO:0019722\_calcium-mediated\_signaling | 16 | 0 | 0.000000 | -0.000000 | 1066 | 925.453027 | 1010.24 | 1095.026973 | 0.947692 |
| GO:0019915\_lipid\_storage | 16 | 0 | 0.000000 | -0.000000 | 1066 | 925.453027 | 1010.24 | 1095.026973 | 0.947692 |
| GO:0021696\_cerebellar\_cortex\_morphogenesis | 16 | 0 | 0.000000 | -0.000000 | 1066 | 925.453027 | 1010.24 | 1095.026973 | 0.947692 |
| GO:0030890\_positive\_regulation\_of\_B\_cell\_proliferation | 16 | 0 | 0.000000 | -0.000000 | 1066 | 925.453027 | 1010.24 | 1095.026973 | 0.947692 |
| GO:0031345\_negative\_regulation\_of\_cell\_projection\_organization | 16 | 0 | 0.000000 | -0.000000 | 1066 | 925.453027 | 1010.24 | 1095.026973 | 0.947692 |
| GO:0031570\_DNA\_integrity\_checkpoint | 16 | 0 | 0.000000 | -0.000000 | 1066 | 925.453027 | 1010.24 | 1095.026973 | 0.947692 |
| GO:0031669\_cellular\_response\_to\_nutrient\_levels | 16 | 0 | 0.000000 | -0.000000 | 1066 | 925.453027 | 1010.24 | 1095.026973 | 0.947692 |
| GO:0032663\_regulation\_of\_interleukin-2\_production | 16 | 0 | 0.000000 | -0.000000 | 1066 | 925.453027 | 1010.24 | 1095.026973 | 0.947692 |
| GO:0032956\_regulation\_of\_actin\_cytoskeleton\_organization | 16 | 0 | 0.000000 | -0.000000 | 1066 | 925.453027 | 1010.24 | 1095.026973 | 0.947692 |
| GO:0042311\_vasodilation | 16 | 0 | 0.000000 | -0.000000 | 1066 | 925.453027 | 1010.24 | 1095.026973 | 0.947692 |
| GO:0042594\_response\_to\_starvation | 16 | 0 | 0.000000 | -0.000000 | 1066 | 925.453027 | 1010.24 | 1095.026973 | 0.947692 |
| GO:0042596\_fear\_response | 16 | 0 | 0.000000 | -0.000000 | 1066 | 925.453027 | 1010.24 | 1095.026973 | 0.947692 |
| GO:0043087\_regulation\_of\_GTPase\_activity | 16 | 0 | 0.000000 | -0.000000 | 1066 | 925.453027 | 1010.24 | 1095.026973 | 0.947692 |
| GO:0043122\_regulation\_of\_I-kappaB\_kinase\_NF-kappaB\_cascade | 16 | 0 | 0.000000 | -0.000000 | 1066 | 925.453027 | 1010.24 | 1095.026973 | 0.947692 |
| GO:0043367\_CD4-positive\_\_alpha\_beta\_T\_cell\_differentiation | 16 | 0 | 0.000000 | -0.000000 | 1066 | 925.453027 | 1010.24 | 1095.026973 | 0.947692 |
| GO:0045104\_intermediate\_filament\_cytoskeleton\_organization | 16 | 0 | 0.000000 | -0.000000 | 1066 | 925.453027 | 1010.24 | 1095.026973 | 0.947692 |
| GO:0046148\_pigment\_biosynthetic\_process | 16 | 0 | 0.000000 | -0.000000 | 1066 | 925.453027 | 1010.24 | 1095.026973 | 0.947692 |
| GO:0046467\_membrane\_lipid\_biosynthetic\_process | 16 | 0 | 0.000000 | -0.000000 | 1066 | 925.453027 | 1010.24 | 1095.026973 | 0.947692 |
| GO:0046633\_alpha-beta\_T\_cell\_proliferation | 16 | 0 | 0.000000 | -0.000000 | 1066 | 925.453027 | 1010.24 | 1095.026973 | 0.947692 |
| GO:0046700\_heterocycle\_catabolic\_process | 16 | 0 | 0.000000 | -0.000000 | 1066 | 925.453027 | 1010.24 | 1095.026973 | 0.947692 |
| GO:0048015\_phosphoinositide-mediated\_signaling | 16 | 0 | 0.000000 | -0.000000 | 1066 | 925.453027 | 1010.24 | 1095.026973 | 0.947692 |
| GO:0048286\_lung\_alveolus\_development | 16 | 0 | 0.000000 | -0.000000 | 1066 | 925.453027 | 1010.24 | 1095.026973 | 0.947692 |
| GO:0048483\_autonomic\_nervous\_system\_development | 16 | 0 | 0.000000 | -0.000000 | 1066 | 925.453027 | 1010.24 | 1095.026973 | 0.947692 |
| GO:0050974\_detection\_of\_mechanical\_stimulus\_involved\_in\_sensory\_perception | 16 | 0 | 0.000000 | -0.000000 | 1066 | 925.453027 | 1010.24 | 1095.026973 | 0.947692 |
| GO:0051048\_negative\_regulation\_of\_secretion | 16 | 0 | 0.000000 | -0.000000 | 1066 | 925.453027 | 1010.24 | 1095.026973 | 0.947692 |
| GO:0051937\_catecholamine\_transport | 16 | 0 | 0.000000 | -0.000000 | 1066 | 925.453027 | 1010.24 | 1095.026973 | 0.947692 |
| GO:0055007\_cardiac\_muscle\_cell\_differentiation | 16 | 0 | 0.000000 | -0.000000 | 1066 | 925.453027 | 1010.24 | 1095.026973 | 0.947692 |
| GO:0060193\_positive\_regulation\_of\_lipase\_activity | 16 | 0 | 0.000000 | -0.000000 | 1066 | 925.453027 | 1010.24 | 1095.026973 | 0.947692 |
| GO:0060713\_labyrinthine\_layer\_morphogenesis | 16 | 0 | 0.000000 | -0.000000 | 1066 | 925.453027 | 1010.24 | 1095.026973 | 0.947692 |
| GO:0001843\_neural\_tube\_closure | 33 | 0 | 0.000000 | -0.000000 | 1077 | 938.883427 | 1022.62 | 1106.356573 | 0.949508 |
| GO:0002562\_somatic\_diversification\_of\_immune\_receptors\_via\_germline\_recombination\_within\_a\_single\_locus | 33 | 0 | 0.000000 | -0.000000 | 1077 | 938.883427 | 1022.62 | 1106.356573 | 0.949508 |
| GO:0006643\_membrane\_lipid\_metabolic\_process | 33 | 0 | 0.000000 | -0.000000 | 1077 | 938.883427 | 1022.62 | 1106.356573 | 0.949508 |
| GO:0007188\_G-protein\_signaling\_\_coupled\_to\_cAMP\_nucleotide\_second\_messenger | 33 | 0 | 0.000000 | -0.000000 | 1077 | 938.883427 | 1022.62 | 1106.356573 | 0.949508 |
| GO:0007270\_nerve-nerve\_synaptic\_transmission | 33 | 0 | 0.000000 | -0.000000 | 1077 | 938.883427 | 1022.62 | 1106.356573 | 0.949508 |
| GO:0007565\_female\_pregnancy | 33 | 0 | 0.000000 | -0.000000 | 1077 | 938.883427 | 1022.62 | 1106.356573 | 0.949508 |
| GO:0008643\_carbohydrate\_transport | 33 | 0 | 0.000000 | -0.000000 | 1077 | 938.883427 | 1022.62 | 1106.356573 | 0.949508 |
| GO:0016444\_somatic\_cell\_DNA\_recombination | 33 | 0 | 0.000000 | -0.000000 | 1077 | 938.883427 | 1022.62 | 1106.356573 | 0.949508 |
| GO:0022037\_metencephalon\_development | 33 | 0 | 0.000000 | -0.000000 | 1077 | 938.883427 | 1022.62 | 1106.356573 | 0.949508 |
| GO:0042108\_positive\_regulation\_of\_cytokine\_biosynthetic\_process | 33 | 0 | 0.000000 | -0.000000 | 1077 | 938.883427 | 1022.62 | 1106.356573 | 0.949508 |
| GO:0060606\_tube\_closure | 33 | 0 | 0.000000 | -0.000000 | 1077 | 938.883427 | 1022.62 | 1106.356573 | 0.949508 |
| GO:0006974\_response\_to\_DNA\_damage\_stimulus | 113 | 0 | 0.000000 | -0.000000 | 1078 | 939.973693 | 1023.64 | 1107.306307 | 0.949573 |
| GO:0001910\_regulation\_of\_leukocyte\_mediated\_cytotoxicity | 27 | 0 | 0.000000 | -0.000000 | 1094 | 956.168054 | 1039.14 | 1122.111946 | 0.949854 |
| GO:0002761\_regulation\_of\_myeloid\_leukocyte\_differentiation | 27 | 0 | 0.000000 | -0.000000 | 1094 | 956.168054 | 1039.14 | 1122.111946 | 0.949854 |
| GO:0006479\_protein\_amino\_acid\_methylation | 27 | 0 | 0.000000 | -0.000000 | 1094 | 956.168054 | 1039.14 | 1122.111946 | 0.949854 |
| GO:0007422\_peripheral\_nervous\_system\_development | 27 | 0 | 0.000000 | -0.000000 | 1094 | 956.168054 | 1039.14 | 1122.111946 | 0.949854 |
| GO:0008213\_protein\_amino\_acid\_alkylation | 27 | 0 | 0.000000 | -0.000000 | 1094 | 956.168054 | 1039.14 | 1122.111946 | 0.949854 |
| GO:0008286\_insulin\_receptor\_signaling\_pathway | 27 | 0 | 0.000000 | -0.000000 | 1094 | 956.168054 | 1039.14 | 1122.111946 | 0.949854 |
| GO:0009411\_response\_to\_UV | 27 | 0 | 0.000000 | -0.000000 | 1094 | 956.168054 | 1039.14 | 1122.111946 | 0.949854 |
| GO:0010638\_positive\_regulation\_of\_organelle\_organization | 27 | 0 | 0.000000 | -0.000000 | 1094 | 956.168054 | 1039.14 | 1122.111946 | 0.949854 |
| GO:0016050\_vesicle\_organization | 27 | 0 | 0.000000 | -0.000000 | 1094 | 956.168054 | 1039.14 | 1122.111946 | 0.949854 |
| GO:0019884\_antigen\_processing\_and\_presentation\_of\_exogenous\_antigen | 27 | 0 | 0.000000 | -0.000000 | 1094 | 956.168054 | 1039.14 | 1122.111946 | 0.949854 |
| GO:0030100\_regulation\_of\_endocytosis | 27 | 0 | 0.000000 | -0.000000 | 1094 | 956.168054 | 1039.14 | 1122.111946 | 0.949854 |
| GO:0031016\_pancreas\_development | 27 | 0 | 0.000000 | -0.000000 | 1094 | 956.168054 | 1039.14 | 1122.111946 | 0.949854 |
| GO:0031341\_regulation\_of\_cell\_killing | 27 | 0 | 0.000000 | -0.000000 | 1094 | 956.168054 | 1039.14 | 1122.111946 | 0.949854 |
| GO:0032496\_response\_to\_lipopolysaccharide | 27 | 0 | 0.000000 | -0.000000 | 1094 | 956.168054 | 1039.14 | 1122.111946 | 0.949854 |
| GO:0051272\_positive\_regulation\_of\_cell\_motion | 27 | 0 | 0.000000 | -0.000000 | 1094 | 956.168054 | 1039.14 | 1122.111946 | 0.949854 |
| GO:0070482\_response\_to\_oxygen\_levels | 27 | 0 | 0.000000 | -0.000000 | 1094 | 956.168054 | 1039.14 | 1122.111946 | 0.949854 |
| GO:0010647\_positive\_regulation\_of\_cell\_communication | 110 | 0 | 0.000000 | -0.000000 | 1096 | 958.643720 | 1041.17 | 1123.696280 | 0.949973 |
| GO:0055080\_cation\_homeostasis | 110 | 0 | 0.000000 | -0.000000 | 1096 | 958.643720 | 1041.17 | 1123.696280 | 0.949973 |
| GO:0050776\_regulation\_of\_immune\_response | 130 | 0 | 0.000000 | -0.000000 | 1097 | 960.364969 | 1042.54 | 1124.715031 | 0.950356 |
| GO:0006936\_muscle\_contraction | 73 | 0 | 0.000000 | -0.000000 | 1100 | 964.088309 | 1045.76 | 1127.431691 | 0.950691 |
| GO:0048706\_embryonic\_skeletal\_system\_development | 73 | 0 | 0.000000 | -0.000000 | 1100 | 964.088309 | 1045.76 | 1127.431691 | 0.950691 |
| GO:0051336\_regulation\_of\_hydrolase\_activity | 73 | 0 | 0.000000 | -0.000000 | 1100 | 964.088309 | 1045.76 | 1127.431691 | 0.950691 |
| GO:0000012\_single\_strand\_break\_repair | 2 | 0 |  |  |  |  |  |  |  |  |
| GO:0000019\_regulation\_of\_mitotic\_recombination | 2 | 0 |  |  |  |  |  |  |  |  |
| GO:0000076\_DNA\_replication\_checkpoint | 2 | 0 |  |  |  |  |  |  |  |  |
| GO:0000080\_G1\_phase\_of\_mitotic\_cell\_cycle | 2 | 0 |  |  |  |  |  |  |  |  |
| GO:0000083\_regulation\_of\_transcription\_of\_G1\_S-phase\_of\_mitotic\_cell\_cycle | 2 | 0 |  |  |  |  |  |  |  |  |
| GO:0000085\_G2\_phase\_of\_mitotic\_cell\_cycle | 2 | 0 |  |  |  |  |  |  |  |  |
| GO:0000289\_nuclear-transcribed\_mRNA\_poly(A)\_tail\_shortening | 2 | 0 |  |  |  |  |  |  |  |  |
| GO:0000381\_regulation\_of\_alternative\_nuclear\_mRNA\_splicing\_\_via\_spliceosome | 2 | 0 |  |  |  |  |  |  |  |  |
| GO:0000712\_resolution\_of\_meiotic\_joint\_molecules\_as\_recombinants | 2 | 0 |  |  |  |  |  |  |  |  |
| GO:0000720\_pyrimidine\_dimer\_repair\_by\_nucleotide-excision\_repair | 2 | 0 |  |  |  |  |  |  |  |  |
| GO:0001302\_replicative\_cell\_aging | 2 | 0 |  |  |  |  |  |  |  |  |
| GO:0001306\_age-dependent\_response\_to\_oxidative\_stress | 2 | 0 |  |  |  |  |  |  |  |  |
| GO:0001514\_selenocysteine\_incorporation | 2 | 0 |  |  |  |  |  |  |  |  |
| GO:0001522\_pseudouridine\_synthesis | 2 | 0 |  |  |  |  |  |  |  |  |
| GO:0001543\_ovarian\_follicle\_rupture | 2 | 0 |  |  |  |  |  |  |  |  |
| GO:0001561\_fatty\_acid\_alpha-oxidation | 2 | 0 |  |  |  |  |  |  |  |  |
| GO:0001675\_acrosome\_assembly | 2 | 0 |  |  |  |  |  |  |  |  |
| GO:0001743\_optic\_placode\_formation | 2 | 0 |  |  |  |  |  |  |  |  |
| GO:0001767\_establishment\_of\_lymphocyte\_polarity | 2 | 0 |  |  |  |  |  |  |  |  |
| GO:0001768\_establishment\_of\_T\_cell\_polarity | 2 | 0 |  |  |  |  |  |  |  |  |
| GO:0001771\_formation\_of\_immunological\_synapse | 2 | 0 |  |  |  |  |  |  |  |  |
| GO:0001774\_microglial\_cell\_activation | 2 | 0 |  |  |  |  |  |  |  |  |
| GO:0001781\_neutrophil\_apoptosis | 2 | 0 |  |  |  |  |  |  |  |  |
| GO:0001787\_natural\_killer\_cell\_proliferation | 2 | 0 |  |  |  |  |  |  |  |  |
| GO:0001788\_antibody-dependent\_cellular\_cytotoxicity | 2 | 0 |  |  |  |  |  |  |  |  |
| GO:0001806\_type\_IV\_hypersensitivity | 2 | 0 |  |  |  |  |  |  |  |  |
| GO:0001807\_regulation\_of\_type\_IV\_hypersensitivity | 2 | 0 |  |  |  |  |  |  |  |  |
| GO:0001808\_negative\_regulation\_of\_type\_IV\_hypersensitivity | 2 | 0 |  |  |  |  |  |  |  |  |
| GO:0001823\_mesonephros\_development | 2 | 0 |  |  |  |  |  |  |  |  |
| GO:0001845\_phagolysosome\_formation | 2 | 0 |  |  |  |  |  |  |  |  |
| GO:0001866\_NK\_T\_cell\_proliferation | 2 | 0 |  |  |  |  |  |  |  |  |
| GO:0001879\_detection\_of\_yeast | 2 | 0 |  |  |  |  |  |  |  |  |
| GO:0001886\_endothelial\_cell\_morphogenesis | 2 | 0 |  |  |  |  |  |  |  |  |
| GO:0001919\_regulation\_of\_receptor\_recycling | 2 | 0 |  |  |  |  |  |  |  |  |
| GO:0001954\_positive\_regulation\_of\_cell-matrix\_adhesion | 2 | 0 |  |  |  |  |  |  |  |  |
| GO:0001977\_renal\_system\_process\_involved\_in\_regulation\_of\_blood\_volume | 2 | 0 |  |  |  |  |  |  |  |  |
| GO:0001982\_baroreceptor\_response\_to\_decreased\_systemic\_arterial\_blood\_pressure | 2 | 0 |  |  |  |  |  |  |  |  |
| GO:0001983\_baroreceptor\_response\_to\_increased\_systemic\_arterial\_blood\_pressure | 2 | 0 |  |  |  |  |  |  |  |  |
| GO:0001992\_regulation\_of\_systemic\_arterial\_blood\_pressure\_by\_vasopressin | 2 | 0 |  |  |  |  |  |  |  |  |
| GO:0001997\_positive\_regulation\_of\_the\_force\_of\_heart\_contraction\_by\_epinephrine-norepinephrine | 2 | 0 |  |  |  |  |  |  |  |  |
| GO:0001998\_angiotensin\_mediated\_vasoconstriction\_involved\_in\_regulation\_of\_systemic\_arterial\_blood\_pressure | 2 | 0 |  |  |  |  |  |  |  |  |
| GO:0001999\_renal\_response\_to\_blood\_flow\_during\_renin-angiotensin\_regulation\_of\_systemic\_arterial\_blood\_pressure | 2 | 0 |  |  |  |  |  |  |  |  |
| GO:0002018\_renin-angiotensin\_regulation\_of\_aldosterone\_production | 2 | 0 |  |  |  |  |  |  |  |  |
| GO:0002019\_regulation\_of\_renal\_output\_by\_angiotensin | 2 | 0 |  |  |  |  |  |  |  |  |
| GO:0002024\_diet\_induced\_thermogenesis | 2 | 0 |  |  |  |  |  |  |  |  |
| GO:0002025\_vasodilation\_by\_norepinephrine-epinephrine\_involved\_in\_regulation\_of\_systemic\_arterial\_blood\_pressure | 2 | 0 |  |  |  |  |  |  |  |  |
| GO:0002029\_desensitization\_of\_G-protein\_coupled\_receptor\_protein\_signaling\_pathway | 2 | 0 |  |  |  |  |  |  |  |  |
| GO:0002033\_vasodilation\_by\_angiotensin\_involved\_in\_regulation\_of\_systemic\_arterial\_blood\_pressure | 2 | 0 |  |  |  |  |  |  |  |  |
| GO:0002066\_columnar\_cuboidal\_epithelial\_cell\_development | 2 | 0 |  |  |  |  |  |  |  |  |
| GO:0002072\_optic\_cup\_morphogenesis\_involved\_in\_camera-type\_eye\_development | 2 | 0 |  |  |  |  |  |  |  |  |
| GO:0002074\_extraocular\_skeletal\_muscle\_development | 2 | 0 |  |  |  |  |  |  |  |  |
| GO:0002138\_retinoic\_acid\_biosynthetic\_process | 2 | 0 |  |  |  |  |  |  |  |  |
| GO:0002223\_stimulatory\_C-type\_lectin\_receptor\_signaling\_pathway | 2 | 0 |  |  |  |  |  |  |  |  |
| GO:0002246\_healing\_during\_inflammatory\_response | 2 | 0 |  |  |  |  |  |  |  |  |
| GO:0002251\_organ\_or\_tissue\_specific\_immune\_response | 2 | 0 |  |  |  |  |  |  |  |  |
| GO:0002266\_follicular\_dendritic\_cell\_activation | 2 | 0 |  |  |  |  |  |  |  |  |
| GO:0002268\_follicular\_dendritic\_cell\_differentiation | 2 | 0 |  |  |  |  |  |  |  |  |
| GO:0002327\_immature\_B\_cell\_differentiation | 2 | 0 |  |  |  |  |  |  |  |  |
| GO:0002329\_pre-B\_cell\_differentiation | 2 | 0 |  |  |  |  |  |  |  |  |
| GO:0002339\_B\_cell\_selection | 2 | 0 |  |  |  |  |  |  |  |  |
| GO:0002352\_B\_cell\_negative\_selection | 2 | 0 |  |  |  |  |  |  |  |  |
| GO:0002358\_B\_cell\_homeostatic\_proliferation | 2 | 0 |  |  |  |  |  |  |  |  |
| GO:0002385\_mucosal\_immune\_response | 2 | 0 |  |  |  |  |  |  |  |  |
| GO:0002514\_B\_cell\_tolerance\_induction | 2 | 0 |  |  |  |  |  |  |  |  |
| GO:0002523\_leukocyte\_migration\_during\_inflammatory\_response | 2 | 0 |  |  |  |  |  |  |  |  |
| GO:0002536\_respiratory\_burst\_during\_acute\_inflammatory\_response | 2 | 0 |  |  |  |  |  |  |  |  |
| GO:0002537\_production\_of\_nitric\_oxide\_during\_acute\_inflammatory\_response | 2 | 0 |  |  |  |  |  |  |  |  |
| GO:0002576\_platelet\_degranulation | 2 | 0 |  |  |  |  |  |  |  |  |
| GO:0002639\_positive\_regulation\_of\_immunoglobulin\_production | 2 | 0 |  |  |  |  |  |  |  |  |
| GO:0002661\_regulation\_of\_B\_cell\_tolerance\_induction | 2 | 0 |  |  |  |  |  |  |  |  |
| GO:0002663\_positive\_regulation\_of\_B\_cell\_tolerance\_induction | 2 | 0 |  |  |  |  |  |  |  |  |
| GO:0002676\_regulation\_of\_chronic\_inflammatory\_response | 2 | 0 |  |  |  |  |  |  |  |  |
| GO:0002679\_respiratory\_burst\_during\_defense\_response | 2 | 0 |  |  |  |  |  |  |  |  |
| GO:0002686\_negative\_regulation\_of\_leukocyte\_migration | 2 | 0 |  |  |  |  |  |  |  |  |
| GO:0002720\_positive\_regulation\_of\_cytokine\_production\_during\_immune\_response | 2 | 0 |  |  |  |  |  |  |  |  |
| GO:0002752\_cell\_surface\_pattern\_recognition\_receptor\_signaling\_pathway | 2 | 0 |  |  |  |  |  |  |  |  |
| GO:0002755\_MyD88-dependent\_toll-like\_receptor\_signaling\_pathway | 2 | 0 |  |  |  |  |  |  |  |  |
| GO:0002765\_immune\_response-inhibiting\_signal\_transduction | 2 | 0 |  |  |  |  |  |  |  |  |
| GO:0002921\_negative\_regulation\_of\_humoral\_immune\_response | 2 | 0 |  |  |  |  |  |  |  |  |
| GO:0002922\_positive\_regulation\_of\_humoral\_immune\_response | 2 | 0 |  |  |  |  |  |  |  |  |
| GO:0002924\_negative\_regulation\_of\_humoral\_immune\_response\_mediated\_by\_circulating\_immunoglobulin | 2 | 0 |  |  |  |  |  |  |  |  |
| GO:0002925\_positive\_regulation\_of\_humoral\_immune\_response\_mediated\_by\_circulating\_immunoglobulin | 2 | 0 |  |  |  |  |  |  |  |  |
| GO:0003057\_regulation\_of\_the\_force\_of\_heart\_contraction\_by\_chemical\_signal | 2 | 0 |  |  |  |  |  |  |  |  |
| GO:0003099\_positive\_regulation\_of\_the\_force\_of\_heart\_contraction\_by\_chemical\_signal | 2 | 0 |  |  |  |  |  |  |  |  |
| GO:0005981\_regulation\_of\_glycogen\_catabolic\_process | 2 | 0 |  |  |  |  |  |  |  |  |
| GO:0006021\_inositol\_biosynthetic\_process | 2 | 0 |  |  |  |  |  |  |  |  |
| GO:0006042\_glucosamine\_biosynthetic\_process | 2 | 0 |  |  |  |  |  |  |  |  |
| GO:0006045\_N-acetylglucosamine\_biosynthetic\_process | 2 | 0 |  |  |  |  |  |  |  |  |
| GO:0006048\_UDP-N-acetylglucosamine\_biosynthetic\_process | 2 | 0 |  |  |  |  |  |  |  |  |
| GO:0006054\_N-acetylneuraminate\_metabolic\_process | 2 | 0 |  |  |  |  |  |  |  |  |
| GO:0006059\_hexitol\_metabolic\_process | 2 | 0 |  |  |  |  |  |  |  |  |
| GO:0006063\_uronic\_acid\_metabolic\_process | 2 | 0 |  |  |  |  |  |  |  |  |
| GO:0006068\_ethanol\_catabolic\_process | 2 | 0 |  |  |  |  |  |  |  |  |
| GO:0006083\_acetate\_metabolic\_process | 2 | 0 |  |  |  |  |  |  |  |  |
| GO:0006089\_lactate\_metabolic\_process | 2 | 0 |  |  |  |  |  |  |  |  |
| GO:0006105\_succinate\_metabolic\_process | 2 | 0 |  |  |  |  |  |  |  |  |
| GO:0006106\_fumarate\_metabolic\_process | 2 | 0 |  |  |  |  |  |  |  |  |
| GO:0006110\_regulation\_of\_glycolysis | 2 | 0 |  |  |  |  |  |  |  |  |
| GO:0006113\_fermentation | 2 | 0 |  |  |  |  |  |  |  |  |
| GO:0006114\_glycerol\_biosynthetic\_process | 2 | 0 |  |  |  |  |  |  |  |  |
| GO:0006122\_mitochondrial\_electron\_transport\_\_ubiquinol\_to\_cytochrome\_c | 2 | 0 |  |  |  |  |  |  |  |  |
| GO:0006152\_purine\_nucleoside\_catabolic\_process | 2 | 0 |  |  |  |  |  |  |  |  |
| GO:0006168\_adenine\_salvage | 2 | 0 |  |  |  |  |  |  |  |  |
| GO:0006200\_ATP\_catabolic\_process | 2 | 0 |  |  |  |  |  |  |  |  |
| GO:0006206\_pyrimidine\_base\_metabolic\_process | 2 | 0 |  |  |  |  |  |  |  |  |
| GO:0006213\_pyrimidine\_nucleoside\_metabolic\_process | 2 | 0 |  |  |  |  |  |  |  |  |
| GO:0006265\_DNA\_topological\_change | 2 | 0 |  |  |  |  |  |  |  |  |
| GO:0006278\_RNA-dependent\_DNA\_replication | 2 | 0 |  |  |  |  |  |  |  |  |
| GO:0006312\_mitotic\_recombination | 2 | 0 |  |  |  |  |  |  |  |  |
| GO:0006398\_histone\_mRNA\_3'-end\_processing | 2 | 0 |  |  |  |  |  |  |  |  |
| GO:0006418\_tRNA\_aminoacylation\_for\_protein\_translation | 2 | 0 |  |  |  |  |  |  |  |  |
| GO:0006451\_translational\_readthrough | 2 | 0 |  |  |  |  |  |  |  |  |
| GO:0006477\_protein\_amino\_acid\_sulfation | 2 | 0 |  |  |  |  |  |  |  |  |
| GO:0006482\_protein\_amino\_acid\_demethylation | 2 | 0 |  |  |  |  |  |  |  |  |
| GO:0006499\_N-terminal\_protein\_myristoylation | 2 | 0 |  |  |  |  |  |  |  |  |
| GO:0006525\_arginine\_metabolic\_process | 2 | 0 |  |  |  |  |  |  |  |  |
| GO:0006527\_arginine\_catabolic\_process | 2 | 0 |  |  |  |  |  |  |  |  |
| GO:0006532\_aspartate\_biosynthetic\_process | 2 | 0 |  |  |  |  |  |  |  |  |
| GO:0006538\_glutamate\_catabolic\_process | 2 | 0 |  |  |  |  |  |  |  |  |
| GO:0006558\_L-phenylalanine\_metabolic\_process | 2 | 0 |  |  |  |  |  |  |  |  |
| GO:0006563\_L-serine\_metabolic\_process | 2 | 0 |  |  |  |  |  |  |  |  |
| GO:0006566\_threonine\_metabolic\_process | 2 | 0 |  |  |  |  |  |  |  |  |
| GO:0006568\_tryptophan\_metabolic\_process | 2 | 0 |  |  |  |  |  |  |  |  |
| GO:0006583\_melanin\_biosynthetic\_process\_from\_tyrosine | 2 | 0 |  |  |  |  |  |  |  |  |
| GO:0006600\_creatine\_metabolic\_process | 2 | 0 |  |  |  |  |  |  |  |  |
| GO:0006603\_phosphocreatine\_metabolic\_process | 2 | 0 |  |  |  |  |  |  |  |  |
| GO:0006610\_ribosomal\_protein\_import\_into\_nucleus | 2 | 0 |  |  |  |  |  |  |  |  |
| GO:0006642\_triglyceride\_mobilization | 2 | 0 |  |  |  |  |  |  |  |  |
| GO:0006649\_phospholipid\_transfer\_to\_membrane | 2 | 0 |  |  |  |  |  |  |  |  |
| GO:0006681\_galactosylceramide\_metabolic\_process | 2 | 0 |  |  |  |  |  |  |  |  |
| GO:0006686\_sphingomyelin\_biosynthetic\_process | 2 | 0 |  |  |  |  |  |  |  |  |
| GO:0006702\_androgen\_biosynthetic\_process | 2 | 0 |  |  |  |  |  |  |  |  |
| GO:0006750\_glutathione\_biosynthetic\_process | 2 | 0 |  |  |  |  |  |  |  |  |
| GO:0006760\_folic\_acid\_and\_derivative\_metabolic\_process | 2 | 0 |  |  |  |  |  |  |  |  |
| GO:0006808\_regulation\_of\_nitrogen\_utilization | 2 | 0 |  |  |  |  |  |  |  |  |
| GO:0006868\_glutamine\_transport | 2 | 0 |  |  |  |  |  |  |  |  |
| GO:0006907\_pinocytosis | 2 | 0 |  |  |  |  |  |  |  |  |
| GO:0006925\_inflammatory\_cell\_apoptosis | 2 | 0 |  |  |  |  |  |  |  |  |
| GO:0006977\_DNA\_damage\_response\_\_signal\_transduction\_by\_p53\_class\_mediator\_resulting\_in\_cell\_cycle\_arrest | 2 | 0 |  |  |  |  |  |  |  |  |
| GO:0006991\_response\_to\_sterol\_depletion | 2 | 0 |  |  |  |  |  |  |  |  |
| GO:0007004\_telomere\_maintenance\_via\_telomerase | 2 | 0 |  |  |  |  |  |  |  |  |
| GO:0007020\_microtubule\_nucleation | 2 | 0 |  |  |  |  |  |  |  |  |
| GO:0007030\_Golgi\_organization | 2 | 0 |  |  |  |  |  |  |  |  |
| GO:0007035\_vacuolar\_acidification | 2 | 0 |  |  |  |  |  |  |  |  |
| GO:0007042\_lysosomal\_lumen\_acidification | 2 | 0 |  |  |  |  |  |  |  |  |
| GO:0007060\_male\_meiosis\_chromosome\_segregation | 2 | 0 |  |  |  |  |  |  |  |  |
| GO:0007089\_traversing\_start\_control\_point\_of\_mitotic\_cell\_cycle | 2 | 0 |  |  |  |  |  |  |  |  |
| GO:0007094\_mitotic\_cell\_cycle\_spindle\_assembly\_checkpoint | 2 | 0 |  |  |  |  |  |  |  |  |
| GO:0007097\_nuclear\_migration | 2 | 0 |  |  |  |  |  |  |  |  |
| GO:0007100\_mitotic\_centrosome\_separation | 2 | 0 |  |  |  |  |  |  |  |  |
| GO:0007132\_meiotic\_metaphase\_I | 2 | 0 |  |  |  |  |  |  |  |  |
| GO:0007171\_activation\_of\_transmembrane\_receptor\_protein\_tyrosine\_kinase\_activity | 2 | 0 |  |  |  |  |  |  |  |  |
| GO:0007182\_common-partner\_SMAD\_protein\_phosphorylation | 2 | 0 |  |  |  |  |  |  |  |  |
| GO:0007185\_transmembrane\_receptor\_protein\_tyrosine\_phosphatase\_signaling\_pathway | 2 | 0 |  |  |  |  |  |  |  |  |
| GO:0007205\_activation\_of\_protein\_kinase\_C\_activity\_by\_G-protein\_coupled\_receptor\_protein\_signaling\_pathway | 2 | 0 |  |  |  |  |  |  |  |  |
| GO:0007210\_serotonin\_receptor\_signaling\_pathway | 2 | 0 |  |  |  |  |  |  |  |  |
| GO:0007220\_Notch\_receptor\_processing | 2 | 0 |  |  |  |  |  |  |  |  |
| GO:0007256\_activation\_of\_JNKK\_activity | 2 | 0 |  |  |  |  |  |  |  |  |
| GO:0007258\_JUN\_phosphorylation | 2 | 0 |  |  |  |  |  |  |  |  |
| GO:0007263\_nitric\_oxide\_mediated\_signal\_transduction | 2 | 0 |  |  |  |  |  |  |  |  |
| GO:0007289\_spermatid\_nucleus\_differentiation | 2 | 0 |  |  |  |  |  |  |  |  |
| GO:0007343\_egg\_activation | 2 | 0 |  |  |  |  |  |  |  |  |
| GO:0007351\_tripartite\_regional\_subdivision | 2 | 0 |  |  |  |  |  |  |  |  |
| GO:0007418\_ventral\_midline\_development | 2 | 0 |  |  |  |  |  |  |  |  |
| GO:0007494\_midgut\_development | 2 | 0 |  |  |  |  |  |  |  |  |
| GO:0007527\_adult\_somatic\_muscle\_development | 2 | 0 |  |  |  |  |  |  |  |  |
| GO:0007549\_dosage\_compensation | 2 | 0 |  |  |  |  |  |  |  |  |
| GO:0007571\_age-dependent\_general\_metabolic\_decline | 2 | 0 |  |  |  |  |  |  |  |  |
| GO:0007603\_phototransduction\_\_visible\_light | 2 | 0 |  |  |  |  |  |  |  |  |
| GO:0007619\_courtship\_behavior | 2 | 0 |  |  |  |  |  |  |  |  |
| GO:0008065\_establishment\_of\_blood-nerve\_barrier | 2 | 0 |  |  |  |  |  |  |  |  |
| GO:0008089\_anterograde\_axon\_cargo\_transport | 2 | 0 |  |  |  |  |  |  |  |  |
| GO:0008210\_estrogen\_metabolic\_process | 2 | 0 |  |  |  |  |  |  |  |  |
| GO:0008212\_mineralocorticoid\_metabolic\_process | 2 | 0 |  |  |  |  |  |  |  |  |
| GO:0008214\_protein\_amino\_acid\_dealkylation | 2 | 0 |  |  |  |  |  |  |  |  |
| GO:0008228\_opsonization | 2 | 0 |  |  |  |  |  |  |  |  |
| GO:0008272\_sulfate\_transport | 2 | 0 |  |  |  |  |  |  |  |  |
| GO:0008291\_acetylcholine\_metabolic\_process | 2 | 0 |  |  |  |  |  |  |  |  |
| GO:0008298\_intracellular\_mRNA\_localization | 2 | 0 |  |  |  |  |  |  |  |  |
| GO:0008334\_histone\_mRNA\_metabolic\_process | 2 | 0 |  |  |  |  |  |  |  |  |
| GO:0008356\_asymmetric\_cell\_division | 2 | 0 |  |  |  |  |  |  |  |  |
| GO:0008594\_photoreceptor\_cell\_morphogenesis | 2 | 0 |  |  |  |  |  |  |  |  |
| GO:0008595\_determination\_of\_anterior\_posterior\_axis\_\_embryo | 2 | 0 |  |  |  |  |  |  |  |  |
| GO:0008608\_attachment\_of\_spindle\_microtubules\_to\_kinetochore | 2 | 0 |  |  |  |  |  |  |  |  |
| GO:0008616\_queuosine\_biosynthetic\_process | 2 | 0 |  |  |  |  |  |  |  |  |
| GO:0008617\_guanosine\_metabolic\_process | 2 | 0 |  |  |  |  |  |  |  |  |
| GO:0008618\_7-methylguanosine\_metabolic\_process | 2 | 0 |  |  |  |  |  |  |  |  |
| GO:0008634\_negative\_regulation\_of\_survival\_gene\_product\_expression | 2 | 0 |  |  |  |  |  |  |  |  |
| GO:0009048\_dosage\_compensation\_\_by\_inactivation\_of\_X\_chromosome | 2 | 0 |  |  |  |  |  |  |  |  |
| GO:0009070\_serine\_family\_amino\_acid\_biosynthetic\_process | 2 | 0 |  |  |  |  |  |  |  |  |
| GO:0009071\_serine\_family\_amino\_acid\_catabolic\_process | 2 | 0 |  |  |  |  |  |  |  |  |
| GO:0009074\_aromatic\_amino\_acid\_family\_catabolic\_process | 2 | 0 |  |  |  |  |  |  |  |  |
| GO:0009083\_branched\_chain\_family\_amino\_acid\_catabolic\_process | 2 | 0 |  |  |  |  |  |  |  |  |
| GO:0009093\_cysteine\_catabolic\_process | 2 | 0 |  |  |  |  |  |  |  |  |
| GO:0009120\_deoxyribonucleoside\_metabolic\_process | 2 | 0 |  |  |  |  |  |  |  |  |
| GO:0009125\_nucleoside\_monophosphate\_catabolic\_process | 2 | 0 |  |  |  |  |  |  |  |  |
| GO:0009126\_purine\_nucleoside\_monophosphate\_metabolic\_process | 2 | 0 |  |  |  |  |  |  |  |  |
| GO:0009161\_ribonucleoside\_monophosphate\_metabolic\_process | 2 | 0 |  |  |  |  |  |  |  |  |
| GO:0009164\_nucleoside\_catabolic\_process | 2 | 0 |  |  |  |  |  |  |  |  |
| GO:0009167\_purine\_ribonucleoside\_monophosphate\_metabolic\_process | 2 | 0 |  |  |  |  |  |  |  |  |
| GO:0009203\_ribonucleoside\_triphosphate\_catabolic\_process | 2 | 0 |  |  |  |  |  |  |  |  |
| GO:0009207\_purine\_ribonucleoside\_triphosphate\_catabolic\_process | 2 | 0 |  |  |  |  |  |  |  |  |
| GO:0009219\_pyrimidine\_deoxyribonucleotide\_metabolic\_process | 2 | 0 |  |  |  |  |  |  |  |  |
| GO:0009268\_response\_to\_pH | 2 | 0 |  |  |  |  |  |  |  |  |
| GO:0009313\_oligosaccharide\_catabolic\_process | 2 | 0 |  |  |  |  |  |  |  |  |
| GO:0009395\_phospholipid\_catabolic\_process | 2 | 0 |  |  |  |  |  |  |  |  |
| GO:0009435\_NAD\_biosynthetic\_process | 2 | 0 |  |  |  |  |  |  |  |  |
| GO:0009608\_response\_to\_symbiont | 2 | 0 |  |  |  |  |  |  |  |  |
| GO:0009609\_response\_to\_symbiotic\_bacterium | 2 | 0 |  |  |  |  |  |  |  |  |
| GO:0009649\_entrainment\_of\_circadian\_clock | 2 | 0 |  |  |  |  |  |  |  |  |
| GO:0009996\_negative\_regulation\_of\_cell\_fate\_specification | 2 | 0 |  |  |  |  |  |  |  |  |
| GO:0010002\_cardioblast\_differentiation | 2 | 0 |  |  |  |  |  |  |  |  |
| GO:0010149\_senescence | 2 | 0 |  |  |  |  |  |  |  |  |
| GO:0010225\_response\_to\_UV-C | 2 | 0 |  |  |  |  |  |  |  |  |
| GO:0010389\_regulation\_of\_G2\_M\_transition\_of\_mitotic\_cell\_cycle | 2 | 0 |  |  |  |  |  |  |  |  |
| GO:0010458\_exit\_from\_mitosis | 2 | 0 |  |  |  |  |  |  |  |  |
| GO:0010459\_negative\_regulation\_of\_heart\_rate | 2 | 0 |  |  |  |  |  |  |  |  |
| GO:0010559\_regulation\_of\_glycoprotein\_biosynthetic\_process | 2 | 0 |  |  |  |  |  |  |  |  |
| GO:0010633\_negative\_regulation\_of\_epithelial\_cell\_migration | 2 | 0 |  |  |  |  |  |  |  |  |
| GO:0010677\_negative\_regulation\_of\_cellular\_carbohydrate\_metabolic\_process | 2 | 0 |  |  |  |  |  |  |  |  |
| GO:0010718\_positive\_regulation\_of\_epithelial\_to\_mesenchymal\_transition | 2 | 0 |  |  |  |  |  |  |  |  |
| GO:0010742\_foam\_cell\_differentiation | 2 | 0 |  |  |  |  |  |  |  |  |
| GO:0010743\_regulation\_of\_foam\_cell\_differentiation | 2 | 0 |  |  |  |  |  |  |  |  |
| GO:0010744\_positive\_regulation\_of\_foam\_cell\_differentiation | 2 | 0 |  |  |  |  |  |  |  |  |
| GO:0010765\_positive\_regulation\_of\_sodium\_ion\_transport | 2 | 0 |  |  |  |  |  |  |  |  |
| GO:0010766\_negative\_regulation\_of\_sodium\_ion\_transport | 2 | 0 |  |  |  |  |  |  |  |  |
| GO:0010770\_positive\_regulation\_of\_cell\_morphogenesis\_involved\_in\_differentiation | 2 | 0 |  |  |  |  |  |  |  |  |
| GO:0010771\_negative\_regulation\_of\_cell\_morphogenesis\_involved\_in\_differentiation | 2 | 0 |  |  |  |  |  |  |  |  |
| GO:0010824\_regulation\_of\_centrosome\_duplication | 2 | 0 |  |  |  |  |  |  |  |  |
| GO:0010833\_telomere\_maintenance\_via\_telomere\_lengthening | 2 | 0 |  |  |  |  |  |  |  |  |
| GO:0010862\_positive\_regulation\_of\_pathway-restricted\_SMAD\_protein\_phosphorylation | 2 | 0 |  |  |  |  |  |  |  |  |
| GO:0010872\_regulation\_of\_cholesterol\_esterification | 2 | 0 |  |  |  |  |  |  |  |  |
| GO:0010878\_cholesterol\_storage | 2 | 0 |  |  |  |  |  |  |  |  |
| GO:0010885\_regulation\_of\_cholesterol\_storage | 2 | 0 |  |  |  |  |  |  |  |  |
| GO:0010886\_positive\_regulation\_of\_cholesterol\_storage | 2 | 0 |  |  |  |  |  |  |  |  |
| GO:0010891\_negative\_regulation\_of\_sequestering\_of\_triglyceride | 2 | 0 |  |  |  |  |  |  |  |  |
| GO:0010896\_regulation\_of\_triglyceride\_catabolic\_process | 2 | 0 |  |  |  |  |  |  |  |  |
| GO:0010898\_positive\_regulation\_of\_triglyceride\_catabolic\_process | 2 | 0 |  |  |  |  |  |  |  |  |
| GO:0010907\_positive\_regulation\_of\_glucose\_metabolic\_process | 2 | 0 |  |  |  |  |  |  |  |  |
| GO:0014028\_notochord\_formation | 2 | 0 |  |  |  |  |  |  |  |  |
| GO:0014048\_regulation\_of\_glutamate\_secretion | 2 | 0 |  |  |  |  |  |  |  |  |
| GO:0014052\_regulation\_of\_gamma-aminobutyric\_acid\_secretion | 2 | 0 |  |  |  |  |  |  |  |  |
| GO:0014054\_positive\_regulation\_of\_gamma-aminobutyric\_acid\_secretion | 2 | 0 |  |  |  |  |  |  |  |  |
| GO:0014055\_acetylcholine\_secretion | 2 | 0 |  |  |  |  |  |  |  |  |
| GO:0014056\_regulation\_of\_acetylcholine\_secretion | 2 | 0 |  |  |  |  |  |  |  |  |
| GO:0014067\_negative\_regulation\_of\_phosphoinositide\_3-kinase\_cascade | 2 | 0 |  |  |  |  |  |  |  |  |
| GO:0014745\_negative\_regulation\_of\_muscle\_adaptation | 2 | 0 |  |  |  |  |  |  |  |  |
| GO:0014829\_vascular\_smooth\_muscle\_contraction | 2 | 0 |  |  |  |  |  |  |  |  |
| GO:0014850\_response\_to\_muscle\_activity | 2 | 0 |  |  |  |  |  |  |  |  |
| GO:0014866\_skeletal\_myofibril\_assembly | 2 | 0 |  |  |  |  |  |  |  |  |
| GO:0014888\_striated\_muscle\_adaptation | 2 | 0 |  |  |  |  |  |  |  |  |
| GO:0014916\_regulation\_of\_lung\_blood\_pressure | 2 | 0 |  |  |  |  |  |  |  |  |
| GO:0015671\_oxygen\_transport | 2 | 0 |  |  |  |  |  |  |  |  |
| GO:0015696\_ammonium\_transport | 2 | 0 |  |  |  |  |  |  |  |  |
| GO:0015732\_prostaglandin\_transport | 2 | 0 |  |  |  |  |  |  |  |  |
| GO:0015819\_lysine\_transport | 2 | 0 |  |  |  |  |  |  |  |  |
| GO:0015840\_urea\_transport | 2 | 0 |  |  |  |  |  |  |  |  |
| GO:0015860\_purine\_nucleoside\_transport | 2 | 0 |  |  |  |  |  |  |  |  |
| GO:0015870\_acetylcholine\_transport | 2 | 0 |  |  |  |  |  |  |  |  |
| GO:0015937\_coenzyme\_A\_biosynthetic\_process | 2 | 0 |  |  |  |  |  |  |  |  |
| GO:0016045\_detection\_of\_bacterium | 2 | 0 |  |  |  |  |  |  |  |  |
| GO:0016046\_detection\_of\_fungus | 2 | 0 |  |  |  |  |  |  |  |  |
| GO:0016080\_synaptic\_vesicle\_targeting | 2 | 0 |  |  |  |  |  |  |  |  |
| GO:0016199\_axon\_midline\_choice\_point\_recognition | 2 | 0 |  |  |  |  |  |  |  |  |
| GO:0016226\_iron-sulfur\_cluster\_assembly | 2 | 0 |  |  |  |  |  |  |  |  |
| GO:0016233\_telomere\_capping | 2 | 0 |  |  |  |  |  |  |  |  |
| GO:0016242\_negative\_regulation\_of\_macroautophagy | 2 | 0 |  |  |  |  |  |  |  |  |
| GO:0016441\_posttranscriptional\_gene\_silencing | 2 | 0 |  |  |  |  |  |  |  |  |
| GO:0016540\_protein\_autoprocessing | 2 | 0 |  |  |  |  |  |  |  |  |
| GO:0016558\_protein\_import\_into\_peroxisome\_matrix | 2 | 0 |  |  |  |  |  |  |  |  |
| GO:0016572\_histone\_phosphorylation | 2 | 0 |  |  |  |  |  |  |  |  |
| GO:0016577\_histone\_demethylation | 2 | 0 |  |  |  |  |  |  |  |  |
| GO:0016584\_nucleosome\_positioning | 2 | 0 |  |  |  |  |  |  |  |  |
| GO:0016926\_protein\_desumoylation | 2 | 0 |  |  |  |  |  |  |  |  |
| GO:0017014\_protein\_amino\_acid\_nitrosylation | 2 | 0 |  |  |  |  |  |  |  |  |
| GO:0017144\_drug\_metabolic\_process | 2 | 0 |  |  |  |  |  |  |  |  |
| GO:0018094\_protein\_polyglycylation | 2 | 0 |  |  |  |  |  |  |  |  |
| GO:0018119\_peptidyl-cysteine\_S-nitrosylation | 2 | 0 |  |  |  |  |  |  |  |  |
| GO:0018125\_peptidyl-cysteine\_methylation | 2 | 0 |  |  |  |  |  |  |  |  |
| GO:0018205\_peptidyl-lysine\_modification | 2 | 0 |  |  |  |  |  |  |  |  |
| GO:0018319\_protein\_amino\_acid\_myristoylation | 2 | 0 |  |  |  |  |  |  |  |  |
| GO:0018377\_protein\_myristoylation | 2 | 0 |  |  |  |  |  |  |  |  |
| GO:0018401\_peptidyl-proline\_hydroxylation\_to\_4-hydroxy-L-proline | 2 | 0 |  |  |  |  |  |  |  |  |
| GO:0018993\_somatic\_sex\_determination | 2 | 0 |  |  |  |  |  |  |  |  |
| GO:0019067\_viral\_assembly\_\_maturation\_\_egress\_\_and\_release | 2 | 0 |  |  |  |  |  |  |  |  |
| GO:0019322\_pentose\_biosynthetic\_process | 2 | 0 |  |  |  |  |  |  |  |  |
| GO:0019370\_leukotriene\_biosynthetic\_process | 2 | 0 |  |  |  |  |  |  |  |  |
| GO:0019374\_galactolipid\_metabolic\_process | 2 | 0 |  |  |  |  |  |  |  |  |
| GO:0019401\_alditol\_biosynthetic\_process | 2 | 0 |  |  |  |  |  |  |  |  |
| GO:0019448\_L-cysteine\_catabolic\_process | 2 | 0 |  |  |  |  |  |  |  |  |
| GO:0019452\_L-cysteine\_catabolic\_process\_to\_taurine | 2 | 0 |  |  |  |  |  |  |  |  |
| GO:0019471\_4-hydroxyproline\_metabolic\_process | 2 | 0 |  |  |  |  |  |  |  |  |
| GO:0019511\_peptidyl-proline\_hydroxylation | 2 | 0 |  |  |  |  |  |  |  |  |
| GO:0019550\_glutamate\_catabolic\_process\_to\_aspartate | 2 | 0 |  |  |  |  |  |  |  |  |
| GO:0019551\_glutamate\_catabolic\_process\_to\_2-oxoglutarate | 2 | 0 |  |  |  |  |  |  |  |  |
| GO:0019585\_glucuronate\_metabolic\_process | 2 | 0 |  |  |  |  |  |  |  |  |
| GO:0019730\_antimicrobial\_humoral\_response | 2 | 0 |  |  |  |  |  |  |  |  |
| GO:0019740\_nitrogen\_utilization | 2 | 0 |  |  |  |  |  |  |  |  |
| GO:0019853\_L-ascorbic\_acid\_biosynthetic\_process | 2 | 0 |  |  |  |  |  |  |  |  |
| GO:0021506\_anterior\_neuropore\_closure | 2 | 0 |  |  |  |  |  |  |  |  |
| GO:0021524\_visceral\_motor\_neuron\_differentiation | 2 | 0 |  |  |  |  |  |  |  |  |
| GO:0021526\_medial\_motor\_column\_neuron\_differentiation | 2 | 0 |  |  |  |  |  |  |  |  |
| GO:0021557\_oculomotor\_nerve\_development | 2 | 0 |  |  |  |  |  |  |  |  |
| GO:0021558\_trochlear\_nerve\_development | 2 | 0 |  |  |  |  |  |  |  |  |
| GO:0021562\_vestibulocochlear\_nerve\_development | 2 | 0 |  |  |  |  |  |  |  |  |
| GO:0021568\_rhombomere\_2\_development | 2 | 0 |  |  |  |  |  |  |  |  |
| GO:0021578\_hindbrain\_maturation | 2 | 0 |  |  |  |  |  |  |  |  |
| GO:0021593\_rhombomere\_morphogenesis | 2 | 0 |  |  |  |  |  |  |  |  |
| GO:0021626\_central\_nervous\_system\_maturation | 2 | 0 |  |  |  |  |  |  |  |  |
| GO:0021658\_rhombomere\_3\_morphogenesis | 2 | 0 |  |  |  |  |  |  |  |  |
| GO:0021754\_facial\_nucleus\_development | 2 | 0 |  |  |  |  |  |  |  |  |
| GO:0021775\_smoothened\_signaling\_pathway\_involved\_in\_ventral\_spinal\_cord\_interneuron\_specification | 2 | 0 |  |  |  |  |  |  |  |  |
| GO:0021776\_smoothened\_signaling\_pathway\_involved\_in\_spinal\_cord\_motor\_neuron\_cell\_fate\_specification | 2 | 0 |  |  |  |  |  |  |  |  |
| GO:0021796\_cerebral\_cortex\_regionalization | 2 | 0 |  |  |  |  |  |  |  |  |
| GO:0021831\_embryonic\_olfactory\_bulb\_interneuron\_precursor\_migration | 2 | 0 |  |  |  |  |  |  |  |  |
| GO:0021869\_forebrain\_ventricular\_zone\_progenitor\_cell\_division | 2 | 0 |  |  |  |  |  |  |  |  |
| GO:0021873\_forebrain\_neuroblast\_division | 2 | 0 |  |  |  |  |  |  |  |  |
| GO:0021882\_regulation\_of\_transcription\_from\_RNA\_polymerase\_II\_promoter\_involved\_in\_forebrain\_neuron\_fate\_commitment | 2 | 0 |  |  |  |  |  |  |  |  |
| GO:0021893\_cerebral\_cortex\_GABAergic\_interneuron\_fate\_commitment | 2 | 0 |  |  |  |  |  |  |  |  |
| GO:0021898\_commitment\_of\_multipotent\_stem\_cells\_to\_the\_neuronal\_lineage\_in\_the\_forebrain | 2 | 0 |  |  |  |  |  |  |  |  |
| GO:0021932\_hindbrain\_radial\_glia\_guided\_cell\_migration | 2 | 0 |  |  |  |  |  |  |  |  |
| GO:0021965\_spinal\_cord\_ventral\_commissure\_morphogenesis | 2 | 0 |  |  |  |  |  |  |  |  |
| GO:0021985\_neurohypophysis\_development | 2 | 0 |  |  |  |  |  |  |  |  |
| GO:0021990\_neural\_plate\_formation | 2 | 0 |  |  |  |  |  |  |  |  |
| GO:0021995\_neuropore\_closure | 2 | 0 |  |  |  |  |  |  |  |  |
| GO:0022028\_tangential\_migration\_from\_the\_subventricular\_zone\_to\_the\_olfactory\_bulb | 2 | 0 |  |  |  |  |  |  |  |  |
| GO:0022401\_adaptation\_of\_signaling\_pathway | 2 | 0 |  |  |  |  |  |  |  |  |
| GO:0022408\_negative\_regulation\_of\_cell-cell\_adhesion | 2 | 0 |  |  |  |  |  |  |  |  |
| GO:0022410\_circadian\_sleep\_wake\_cycle\_process | 2 | 0 |  |  |  |  |  |  |  |  |
| GO:0030046\_parallel\_actin\_filament\_bundle\_formation | 2 | 0 |  |  |  |  |  |  |  |  |
| GO:0030049\_muscle\_filament\_sliding | 2 | 0 |  |  |  |  |  |  |  |  |
| GO:0030050\_vesicle\_transport\_along\_actin\_filament | 2 | 0 |  |  |  |  |  |  |  |  |
| GO:0030071\_regulation\_of\_mitotic\_metaphase\_anaphase\_transition | 2 | 0 |  |  |  |  |  |  |  |  |
| GO:0030147\_natriuresis | 2 | 0 |  |  |  |  |  |  |  |  |
| GO:0030174\_regulation\_of\_DNA\_replication\_initiation | 2 | 0 |  |  |  |  |  |  |  |  |
| GO:0030202\_heparin\_metabolic\_process | 2 | 0 |  |  |  |  |  |  |  |  |
| GO:0030219\_megakaryocyte\_differentiation | 2 | 0 |  |  |  |  |  |  |  |  |
| GO:0030223\_neutrophil\_differentiation | 2 | 0 |  |  |  |  |  |  |  |  |
| GO:0030240\_muscle\_thin\_filament\_assembly | 2 | 0 |  |  |  |  |  |  |  |  |
| GO:0030259\_lipid\_glycosylation | 2 | 0 |  |  |  |  |  |  |  |  |
| GO:0030397\_membrane\_disassembly | 2 | 0 |  |  |  |  |  |  |  |  |
| GO:0030502\_negative\_regulation\_of\_bone\_mineralization | 2 | 0 |  |  |  |  |  |  |  |  |
| GO:0030644\_cellular\_chloride\_ion\_homeostasis | 2 | 0 |  |  |  |  |  |  |  |  |
| GO:0030825\_positive\_regulation\_of\_cGMP\_metabolic\_process | 2 | 0 |  |  |  |  |  |  |  |  |
| GO:0030828\_positive\_regulation\_of\_cGMP\_biosynthetic\_process | 2 | 0 |  |  |  |  |  |  |  |  |
| GO:0030835\_negative\_regulation\_of\_actin\_filament\_depolymerization | 2 | 0 |  |  |  |  |  |  |  |  |
| GO:0030837\_negative\_regulation\_of\_actin\_filament\_polymerization | 2 | 0 |  |  |  |  |  |  |  |  |
| GO:0030852\_regulation\_of\_granulocyte\_differentiation | 2 | 0 |  |  |  |  |  |  |  |  |
| GO:0030885\_regulation\_of\_myeloid\_dendritic\_cell\_activation | 2 | 0 |  |  |  |  |  |  |  |  |
| GO:0030910\_olfactory\_placode\_formation | 2 | 0 |  |  |  |  |  |  |  |  |
| GO:0030948\_negative\_regulation\_of\_vascular\_endothelial\_growth\_factor\_receptor\_signaling\_pathway | 2 | 0 |  |  |  |  |  |  |  |  |
| GO:0030953\_spindle\_astral\_microtubule\_organization | 2 | 0 |  |  |  |  |  |  |  |  |
| GO:0031050\_dsRNA\_fragmentation | 2 | 0 |  |  |  |  |  |  |  |  |
| GO:0031061\_negative\_regulation\_of\_histone\_methylation | 2 | 0 |  |  |  |  |  |  |  |  |
| GO:0031119\_tRNA\_pseudouridine\_synthesis | 2 | 0 |  |  |  |  |  |  |  |  |
| GO:0031163\_metallo-sulfur\_cluster\_assembly | 2 | 0 |  |  |  |  |  |  |  |  |
| GO:0031223\_auditory\_behavior | 2 | 0 |  |  |  |  |  |  |  |  |
| GO:0031296\_B\_cell\_costimulation | 2 | 0 |  |  |  |  |  |  |  |  |
| GO:0031338\_regulation\_of\_vesicle\_fusion | 2 | 0 |  |  |  |  |  |  |  |  |
| GO:0031573\_intra-S\_DNA\_damage\_checkpoint | 2 | 0 |  |  |  |  |  |  |  |  |
| GO:0031577\_spindle\_checkpoint | 2 | 0 |  |  |  |  |  |  |  |  |
| GO:0031629\_synaptic\_vesicle\_fusion\_to\_presynaptic\_membrane | 2 | 0 |  |  |  |  |  |  |  |  |
| GO:0031630\_regulation\_of\_synaptic\_vesicle\_fusion\_to\_presynaptic\_membrane | 2 | 0 |  |  |  |  |  |  |  |  |
| GO:0031664\_regulation\_of\_lipopolysaccharide-mediated\_signaling\_pathway | 2 | 0 |  |  |  |  |  |  |  |  |
| GO:0031670\_cellular\_response\_to\_nutrient | 2 | 0 |  |  |  |  |  |  |  |  |
| GO:0031848\_protection\_from\_non-homologous\_end\_joining\_at\_telomere | 2 | 0 |  |  |  |  |  |  |  |  |
| GO:0031946\_regulation\_of\_glucocorticoid\_biosynthetic\_process | 2 | 0 |  |  |  |  |  |  |  |  |
| GO:0031952\_regulation\_of\_protein\_amino\_acid\_autophosphorylation | 2 | 0 |  |  |  |  |  |  |  |  |
| GO:0031953\_negative\_regulation\_of\_protein\_amino\_acid\_autophosphorylation | 2 | 0 |  |  |  |  |  |  |  |  |
| GO:0031958\_corticosteroid\_receptor\_signaling\_pathway | 2 | 0 |  |  |  |  |  |  |  |  |
| GO:0031987\_locomotion\_involved\_in\_locomotory\_behavior | 2 | 0 |  |  |  |  |  |  |  |  |
| GO:0032096\_negative\_regulation\_of\_response\_to\_food | 2 | 0 |  |  |  |  |  |  |  |  |
| GO:0032099\_negative\_regulation\_of\_appetite | 2 | 0 |  |  |  |  |  |  |  |  |
| GO:0032106\_positive\_regulation\_of\_response\_to\_extracellular\_stimulus | 2 | 0 |  |  |  |  |  |  |  |  |
| GO:0032109\_positive\_regulation\_of\_response\_to\_nutrient\_levels | 2 | 0 |  |  |  |  |  |  |  |  |
| GO:0032226\_positive\_regulation\_of\_synaptic\_transmission\_\_dopaminergic | 2 | 0 |  |  |  |  |  |  |  |  |
| GO:0032230\_positive\_regulation\_of\_synaptic\_transmission\_\_GABAergic | 2 | 0 |  |  |  |  |  |  |  |  |
| GO:0032234\_regulation\_of\_calcium\_ion\_transport\_via\_store-operated\_calcium\_channel\_activity | 2 | 0 |  |  |  |  |  |  |  |  |
| GO:0032236\_positive\_regulation\_of\_calcium\_ion\_transport\_via\_store-operated\_calcium\_channel\_activity | 2 | 0 |  |  |  |  |  |  |  |  |
| GO:0032297\_negative\_regulation\_of\_DNA\_replication\_initiation | 2 | 0 |  |  |  |  |  |  |  |  |
| GO:0032309\_icosanoid\_secretion | 2 | 0 |  |  |  |  |  |  |  |  |
| GO:0032328\_alanine\_transport | 2 | 0 |  |  |  |  |  |  |  |  |
| GO:0032341\_aldosterone\_metabolic\_process | 2 | 0 |  |  |  |  |  |  |  |  |
| GO:0032351\_negative\_regulation\_of\_hormone\_metabolic\_process | 2 | 0 |  |  |  |  |  |  |  |  |
| GO:0032353\_negative\_regulation\_of\_hormone\_biosynthetic\_process | 2 | 0 |  |  |  |  |  |  |  |  |
| GO:0032435\_negative\_regulation\_of\_proteasomal\_ubiquitin-dependent\_protein\_catabolic\_process | 2 | 0 |  |  |  |  |  |  |  |  |
| GO:0032471\_reduction\_of\_endoplasmic\_reticulum\_calcium\_ion\_concentration | 2 | 0 |  |  |  |  |  |  |  |  |
| GO:0032481\_positive\_regulation\_of\_type\_I\_interferon\_production | 2 | 0 |  |  |  |  |  |  |  |  |
| GO:0032488\_Cdc42\_protein\_signal\_transduction | 2 | 0 |  |  |  |  |  |  |  |  |
| GO:0032489\_regulation\_of\_Cdc42\_protein\_signal\_transduction | 2 | 0 |  |  |  |  |  |  |  |  |
| GO:0032495\_response\_to\_muramyl\_dipeptide | 2 | 0 |  |  |  |  |  |  |  |  |
| GO:0032604\_granulocyte\_macrophage\_colony-stimulating\_factor\_production | 2 | 0 |  |  |  |  |  |  |  |  |
| GO:0032616\_interleukin-13\_production | 2 | 0 |  |  |  |  |  |  |  |  |
| GO:0032645\_regulation\_of\_granulocyte\_macrophage\_colony-stimulating\_factor\_production | 2 | 0 |  |  |  |  |  |  |  |  |
| GO:0032672\_regulation\_of\_interleukin-3\_production | 2 | 0 |  |  |  |  |  |  |  |  |
| GO:0032695\_negative\_regulation\_of\_interleukin-12\_production | 2 | 0 |  |  |  |  |  |  |  |  |
| GO:0032714\_negative\_regulation\_of\_interleukin-5\_production | 2 | 0 |  |  |  |  |  |  |  |  |
| GO:0032722\_positive\_regulation\_of\_chemokine\_production | 2 | 0 |  |  |  |  |  |  |  |  |
| GO:0032743\_positive\_regulation\_of\_interleukin-2\_production | 2 | 0 |  |  |  |  |  |  |  |  |
| GO:0032762\_mast\_cell\_cytokine\_production | 2 | 0 |  |  |  |  |  |  |  |  |
| GO:0032763\_regulation\_of\_mast\_cell\_cytokine\_production | 2 | 0 |  |  |  |  |  |  |  |  |
| GO:0032768\_regulation\_of\_monooxygenase\_activity | 2 | 0 |  |  |  |  |  |  |  |  |
| GO:0032788\_saturated\_monocarboxylic\_acid\_metabolic\_process | 2 | 0 |  |  |  |  |  |  |  |  |
| GO:0032789\_unsaturated\_monocarboxylic\_acid\_metabolic\_process | 2 | 0 |  |  |  |  |  |  |  |  |
| GO:0032796\_uropod\_organization | 2 | 0 |  |  |  |  |  |  |  |  |
| GO:0032800\_receptor\_biosynthetic\_process | 2 | 0 |  |  |  |  |  |  |  |  |
| GO:0032801\_receptor\_catabolic\_process | 2 | 0 |  |  |  |  |  |  |  |  |
| GO:0032829\_regulation\_of\_CD4-positive\_\_CD25-positive\_\_alpha-beta\_regulatory\_T\_cell\_differentiation | 2 | 0 |  |  |  |  |  |  |  |  |
| GO:0032831\_positive\_regulation\_of\_CD4-positive\_\_CD25-positive\_\_alpha-beta\_regulatory\_T\_cell\_differentiation | 2 | 0 |  |  |  |  |  |  |  |  |
| GO:0032892\_positive\_regulation\_of\_organic\_acid\_transport | 2 | 0 |  |  |  |  |  |  |  |  |
| GO:0032905\_transforming\_growth\_factor-beta1\_production | 2 | 0 |  |  |  |  |  |  |  |  |
| GO:0032908\_regulation\_of\_transforming\_growth\_factor-beta1\_production | 2 | 0 |  |  |  |  |  |  |  |  |
| GO:0032914\_positive\_regulation\_of\_transforming\_growth\_factor-beta1\_production | 2 | 0 |  |  |  |  |  |  |  |  |
| GO:0032933\_SREBP-mediated\_signaling\_pathway | 2 | 0 |  |  |  |  |  |  |  |  |
| GO:0032957\_inositol\_trisphosphate\_metabolic\_process | 2 | 0 |  |  |  |  |  |  |  |  |
| GO:0032958\_inositol\_phosphate\_biosynthetic\_process | 2 | 0 |  |  |  |  |  |  |  |  |
| GO:0032959\_inositol\_trisphosphate\_biosynthetic\_process | 2 | 0 |  |  |  |  |  |  |  |  |
| GO:0033092\_positive\_regulation\_of\_immature\_T\_cell\_proliferation\_in\_the\_thymus | 2 | 0 |  |  |  |  |  |  |  |  |
| GO:0033119\_negative\_regulation\_of\_RNA\_splicing | 2 | 0 |  |  |  |  |  |  |  |  |
| GO:0033136\_serine\_phosphorylation\_of\_STAT3\_protein | 2 | 0 |  |  |  |  |  |  |  |  |
| GO:0033145\_positive\_regulation\_of\_steroid\_hormone\_receptor\_signaling\_pathway | 2 | 0 |  |  |  |  |  |  |  |  |
| GO:0033147\_negative\_regulation\_of\_estrogen\_receptor\_signaling\_pathway | 2 | 0 |  |  |  |  |  |  |  |  |
| GO:0033148\_positive\_regulation\_of\_estrogen\_receptor\_signaling\_pathway | 2 | 0 |  |  |  |  |  |  |  |  |
| GO:0033194\_response\_to\_hydroperoxide | 2 | 0 |  |  |  |  |  |  |  |  |
| GO:0033275\_actin-myosin\_filament\_sliding | 2 | 0 |  |  |  |  |  |  |  |  |
| GO:0033280\_response\_to\_vitamin\_D | 2 | 0 |  |  |  |  |  |  |  |  |
| GO:0033364\_mast\_cell\_secretory\_granule\_organization | 2 | 0 |  |  |  |  |  |  |  |  |
| GO:0033504\_floor\_plate\_development | 2 | 0 |  |  |  |  |  |  |  |  |
| GO:0033603\_positive\_regulation\_of\_dopamine\_secretion | 2 | 0 |  |  |  |  |  |  |  |  |
| GO:0033605\_positive\_regulation\_of\_catecholamine\_secretion | 2 | 0 |  |  |  |  |  |  |  |  |
| GO:0033622\_integrin\_activation | 2 | 0 |  |  |  |  |  |  |  |  |
| GO:0033623\_regulation\_of\_integrin\_activation | 2 | 0 |  |  |  |  |  |  |  |  |
| GO:0033625\_positive\_regulation\_of\_integrin\_activation | 2 | 0 |  |  |  |  |  |  |  |  |
| GO:0033700\_phospholipid\_efflux | 2 | 0 |  |  |  |  |  |  |  |  |
| GO:0034142\_toll-like\_receptor\_4\_signaling\_pathway | 2 | 0 |  |  |  |  |  |  |  |  |
| GO:0034310\_monohydric\_alcohol\_catabolic\_process | 2 | 0 |  |  |  |  |  |  |  |  |
| GO:0034341\_response\_to\_interferon-gamma | 2 | 0 |  |  |  |  |  |  |  |  |
| GO:0034370\_triglyceride-rich\_lipoprotein\_particle\_remodeling | 2 | 0 |  |  |  |  |  |  |  |  |
| GO:0034374\_low-density\_lipoprotein\_particle\_remodeling | 2 | 0 |  |  |  |  |  |  |  |  |
| GO:0034377\_plasma\_lipoprotein\_particle\_assembly | 2 | 0 |  |  |  |  |  |  |  |  |
| GO:0034384\_high-density\_lipoprotein\_particle\_clearance | 2 | 0 |  |  |  |  |  |  |  |  |
| GO:0034433\_steroid\_esterification | 2 | 0 |  |  |  |  |  |  |  |  |
| GO:0034434\_sterol\_esterification | 2 | 0 |  |  |  |  |  |  |  |  |
| GO:0034435\_cholesterol\_esterification | 2 | 0 |  |  |  |  |  |  |  |  |
| GO:0034453\_microtubule\_anchoring | 2 | 0 |  |  |  |  |  |  |  |  |
| GO:0034644\_cellular\_response\_to\_UV | 2 | 0 |  |  |  |  |  |  |  |  |
| GO:0034755\_iron\_ion\_transmembrane\_transport | 2 | 0 |  |  |  |  |  |  |  |  |
| GO:0034764\_positive\_regulation\_of\_transmembrane\_transport | 2 | 0 |  |  |  |  |  |  |  |  |
| GO:0035021\_negative\_regulation\_of\_Rac\_protein\_signal\_transduction | 2 | 0 |  |  |  |  |  |  |  |  |
| GO:0035054\_embryonic\_heart\_tube\_anterior\_posterior\_pattern\_formation | 2 | 0 |  |  |  |  |  |  |  |  |
| GO:0035092\_sperm\_chromatin\_condensation | 2 | 0 |  |  |  |  |  |  |  |  |
| GO:0035110\_leg\_morphogenesis | 2 | 0 |  |  |  |  |  |  |  |  |
| GO:0035117\_embryonic\_arm\_morphogenesis | 2 | 0 |  |  |  |  |  |  |  |  |
| GO:0035120\_post-embryonic\_appendage\_morphogenesis | 2 | 0 |  |  |  |  |  |  |  |  |
| GO:0035127\_post-embryonic\_limb\_morphogenesis | 2 | 0 |  |  |  |  |  |  |  |  |
| GO:0035129\_post-embryonic\_hindlimb\_morphogenesis | 2 | 0 |  |  |  |  |  |  |  |  |
| GO:0035140\_arm\_morphogenesis | 2 | 0 |  |  |  |  |  |  |  |  |
| GO:0035194\_posttranscriptional\_gene\_silencing\_by\_RNA | 2 | 0 |  |  |  |  |  |  |  |  |
| GO:0035195\_gene\_silencing\_by\_miRNA | 2 | 0 |  |  |  |  |  |  |  |  |
| GO:0035196\_gene\_silencing\_by\_miRNA\_\_production\_of\_miRNAs | 2 | 0 |  |  |  |  |  |  |  |  |
| GO:0035315\_hair\_cell\_differentiation | 2 | 0 |  |  |  |  |  |  |  |  |
| GO:0040009\_regulation\_of\_growth\_rate | 2 | 0 |  |  |  |  |  |  |  |  |
| GO:0040037\_negative\_regulation\_of\_fibroblast\_growth\_factor\_receptor\_signaling\_pathway | 2 | 0 |  |  |  |  |  |  |  |  |
| GO:0042119\_neutrophil\_activation | 2 | 0 |  |  |  |  |  |  |  |  |
| GO:0042147\_retrograde\_transport\_\_endosome\_to\_Golgi | 2 | 0 |  |  |  |  |  |  |  |  |
| GO:0042223\_interleukin-3\_biosynthetic\_process | 2 | 0 |  |  |  |  |  |  |  |  |
| GO:0042249\_establishment\_of\_polarity\_of\_embryonic\_epithelium | 2 | 0 |  |  |  |  |  |  |  |  |
| GO:0042253\_granulocyte\_macrophage\_colony-stimulating\_factor\_biosynthetic\_process | 2 | 0 |  |  |  |  |  |  |  |  |
| GO:0042270\_protection\_from\_natural\_killer\_cell\_mediated\_cytotoxicity | 2 | 0 |  |  |  |  |  |  |  |  |
| GO:0042274\_ribosomal\_small\_subunit\_biogenesis | 2 | 0 |  |  |  |  |  |  |  |  |
| GO:0042312\_regulation\_of\_vasodilation | 2 | 0 |  |  |  |  |  |  |  |  |
| GO:0042346\_positive\_regulation\_of\_NF-kappaB\_import\_into\_nucleus | 2 | 0 |  |  |  |  |  |  |  |  |
| GO:0042396\_phosphagen\_biosynthetic\_process | 2 | 0 |  |  |  |  |  |  |  |  |
| GO:0042454\_ribonucleoside\_catabolic\_process | 2 | 0 |  |  |  |  |  |  |  |  |
| GO:0042482\_positive\_regulation\_of\_odontogenesis | 2 | 0 |  |  |  |  |  |  |  |  |
| GO:0042483\_negative\_regulation\_of\_odontogenesis | 2 | 0 |  |  |  |  |  |  |  |  |
| GO:0042488\_positive\_regulation\_of\_odontogenesis\_of\_dentine-containing\_tooth | 2 | 0 |  |  |  |  |  |  |  |  |
| GO:0042501\_serine\_phosphorylation\_of\_STAT\_protein | 2 | 0 |  |  |  |  |  |  |  |  |
| GO:0042517\_positive\_regulation\_of\_tyrosine\_phosphorylation\_of\_Stat3\_protein | 2 | 0 |  |  |  |  |  |  |  |  |
| GO:0042532\_negative\_regulation\_of\_tyrosine\_phosphorylation\_of\_STAT\_protein | 2 | 0 |  |  |  |  |  |  |  |  |
| GO:0042559\_pteridine\_and\_derivative\_biosynthetic\_process | 2 | 0 |  |  |  |  |  |  |  |  |
| GO:0042730\_fibrinolysis | 2 | 0 |  |  |  |  |  |  |  |  |
| GO:0042749\_regulation\_of\_circadian\_sleep\_wake\_cycle | 2 | 0 |  |  |  |  |  |  |  |  |
| GO:0042886\_amide\_transport | 2 | 0 |  |  |  |  |  |  |  |  |
| GO:0042921\_glucocorticoid\_receptor\_signaling\_pathway | 2 | 0 |  |  |  |  |  |  |  |  |
| GO:0042987\_amyloid\_precursor\_protein\_catabolic\_process | 2 | 0 |  |  |  |  |  |  |  |  |
| GO:0042993\_positive\_regulation\_of\_transcription\_factor\_import\_into\_nucleus | 2 | 0 |  |  |  |  |  |  |  |  |
| GO:0042994\_cytoplasmic\_sequestering\_of\_transcription\_factor | 2 | 0 |  |  |  |  |  |  |  |  |
| GO:0043032\_positive\_regulation\_of\_macrophage\_activation | 2 | 0 |  |  |  |  |  |  |  |  |
| GO:0043038\_amino\_acid\_activation | 2 | 0 |  |  |  |  |  |  |  |  |
| GO:0043039\_tRNA\_aminoacylation | 2 | 0 |  |  |  |  |  |  |  |  |
| GO:0043084\_penile\_erection | 2 | 0 |  |  |  |  |  |  |  |  |
| GO:0043088\_regulation\_of\_Cdc42\_GTPase\_activity | 2 | 0 |  |  |  |  |  |  |  |  |
| GO:0043089\_positive\_regulation\_of\_Cdc42\_GTPase\_activity | 2 | 0 |  |  |  |  |  |  |  |  |
| GO:0043096\_purine\_base\_salvage | 2 | 0 |  |  |  |  |  |  |  |  |
| GO:0043247\_telomere\_maintenance\_in\_response\_to\_DNA\_damage | 2 | 0 |  |  |  |  |  |  |  |  |
| GO:0043297\_apical\_junction\_assembly | 2 | 0 |  |  |  |  |  |  |  |  |
| GO:0043312\_neutrophil\_degranulation | 2 | 0 |  |  |  |  |  |  |  |  |
| GO:0043320\_natural\_killer\_cell\_degranulation | 2 | 0 |  |  |  |  |  |  |  |  |
| GO:0043366\_beta\_selection | 2 | 0 |  |  |  |  |  |  |  |  |
| GO:0043450\_alkene\_biosynthetic\_process | 2 | 0 |  |  |  |  |  |  |  |  |
| GO:0043476\_pigment\_accumulation | 2 | 0 |  |  |  |  |  |  |  |  |
| GO:0043490\_malate-aspartate\_shuttle | 2 | 0 |  |  |  |  |  |  |  |  |
| GO:0043502\_regulation\_of\_muscle\_adaptation | 2 | 0 |  |  |  |  |  |  |  |  |
| GO:0043516\_regulation\_of\_DNA\_damage\_response\_\_signal\_transduction\_by\_p53\_class\_mediator | 2 | 0 |  |  |  |  |  |  |  |  |
| GO:0043568\_positive\_regulation\_of\_insulin-like\_growth\_factor\_receptor\_signaling\_pathway | 2 | 0 |  |  |  |  |  |  |  |  |
| GO:0043589\_skin\_morphogenesis | 2 | 0 |  |  |  |  |  |  |  |  |
| GO:0043618\_regulation\_of\_transcription\_from\_RNA\_polymerase\_II\_promoter\_in\_response\_to\_stress | 2 | 0 |  |  |  |  |  |  |  |  |
| GO:0043619\_regulation\_of\_transcription\_from\_RNA\_polymerase\_II\_promoter\_in\_response\_to\_oxidative\_stress | 2 | 0 |  |  |  |  |  |  |  |  |
| GO:0043620\_regulation\_of\_transcription\_in\_response\_to\_stress | 2 | 0 |  |  |  |  |  |  |  |  |
| GO:0043647\_inositol\_phosphate\_metabolic\_process | 2 | 0 |  |  |  |  |  |  |  |  |
| GO:0043654\_recognition\_of\_apoptotic\_cell | 2 | 0 |  |  |  |  |  |  |  |  |
| GO:0043966\_histone\_H3\_acetylation | 2 | 0 |  |  |  |  |  |  |  |  |
| GO:0043967\_histone\_H4\_acetylation | 2 | 0 |  |  |  |  |  |  |  |  |
| GO:0044070\_regulation\_of\_anion\_transport | 2 | 0 |  |  |  |  |  |  |  |  |
| GO:0044246\_regulation\_of\_multicellular\_organismal\_metabolic\_process | 2 | 0 |  |  |  |  |  |  |  |  |
| GO:0044253\_positive\_regulation\_of\_multicellular\_organismal\_metabolic\_process | 2 | 0 |  |  |  |  |  |  |  |  |
| GO:0044268\_multicellular\_organismal\_protein\_metabolic\_process | 2 | 0 |  |  |  |  |  |  |  |  |
| GO:0045005\_maintenance\_of\_fidelity\_during\_DNA-dependent\_DNA\_replication | 2 | 0 |  |  |  |  |  |  |  |  |
| GO:0045010\_actin\_nucleation | 2 | 0 |  |  |  |  |  |  |  |  |
| GO:0045077\_negative\_regulation\_of\_interferon-gamma\_biosynthetic\_process | 2 | 0 |  |  |  |  |  |  |  |  |
| GO:0045079\_negative\_regulation\_of\_chemokine\_biosynthetic\_process | 2 | 0 |  |  |  |  |  |  |  |  |
| GO:0045116\_protein\_neddylation | 2 | 0 |  |  |  |  |  |  |  |  |
| GO:0045187\_regulation\_of\_circadian\_sleep\_wake\_cycle\_\_sleep | 2 | 0 |  |  |  |  |  |  |  |  |
| GO:0045212\_neurotransmitter\_receptor\_biosynthetic\_process | 2 | 0 |  |  |  |  |  |  |  |  |
| GO:0045399\_regulation\_of\_interleukin-3\_biosynthetic\_process | 2 | 0 |  |  |  |  |  |  |  |  |
| GO:0045401\_positive\_regulation\_of\_interleukin-3\_biosynthetic\_process | 2 | 0 |  |  |  |  |  |  |  |  |
| GO:0045409\_negative\_regulation\_of\_interleukin-6\_biosynthetic\_process | 2 | 0 |  |  |  |  |  |  |  |  |
| GO:0045423\_regulation\_of\_granulocyte\_macrophage\_colony-stimulating\_factor\_biosynthetic\_process | 2 | 0 |  |  |  |  |  |  |  |  |
| GO:0045425\_positive\_regulation\_of\_granulocyte\_macrophage\_colony-stimulating\_factor\_biosynthetic\_process | 2 | 0 |  |  |  |  |  |  |  |  |
| GO:0045475\_locomotor\_rhythm | 2 | 0 |  |  |  |  |  |  |  |  |
| GO:0045578\_negative\_regulation\_of\_B\_cell\_differentiation | 2 | 0 |  |  |  |  |  |  |  |  |
| GO:0045589\_regulation\_of\_regulatory\_T\_cell\_differentiation | 2 | 0 |  |  |  |  |  |  |  |  |
| GO:0045591\_positive\_regulation\_of\_regulatory\_T\_cell\_differentiation | 2 | 0 |  |  |  |  |  |  |  |  |
| GO:0045608\_negative\_regulation\_of\_auditory\_receptor\_cell\_differentiation | 2 | 0 |  |  |  |  |  |  |  |  |
| GO:0045627\_positive\_regulation\_of\_T-helper\_1\_cell\_differentiation | 2 | 0 |  |  |  |  |  |  |  |  |
| GO:0045629\_negative\_regulation\_of\_T-helper\_2\_cell\_differentiation | 2 | 0 |  |  |  |  |  |  |  |  |
| GO:0045630\_positive\_regulation\_of\_T-helper\_2\_cell\_differentiation | 2 | 0 |  |  |  |  |  |  |  |  |
| GO:0045632\_negative\_regulation\_of\_mechanoreceptor\_differentiation | 2 | 0 |  |  |  |  |  |  |  |  |
| GO:0045636\_positive\_regulation\_of\_melanocyte\_differentiation | 2 | 0 |  |  |  |  |  |  |  |  |
| GO:0045655\_regulation\_of\_monocyte\_differentiation | 2 | 0 |  |  |  |  |  |  |  |  |
| GO:0045658\_regulation\_of\_neutrophil\_differentiation | 2 | 0 |  |  |  |  |  |  |  |  |
| GO:0045662\_negative\_regulation\_of\_myoblast\_differentiation | 2 | 0 |  |  |  |  |  |  |  |  |
| GO:0045663\_positive\_regulation\_of\_myoblast\_differentiation | 2 | 0 |  |  |  |  |  |  |  |  |
| GO:0045683\_negative\_regulation\_of\_epidermis\_development | 2 | 0 |  |  |  |  |  |  |  |  |
| GO:0045737\_positive\_regulation\_of\_cyclin-dependent\_protein\_kinase\_activity | 2 | 0 |  |  |  |  |  |  |  |  |
| GO:0045739\_positive\_regulation\_of\_DNA\_repair | 2 | 0 |  |  |  |  |  |  |  |  |
| GO:0045741\_positive\_regulation\_of\_epidermal\_growth\_factor\_receptor\_activity | 2 | 0 |  |  |  |  |  |  |  |  |
| GO:0045743\_positive\_regulation\_of\_fibroblast\_growth\_factor\_receptor\_signaling\_pathway | 2 | 0 |  |  |  |  |  |  |  |  |
| GO:0045749\_negative\_regulation\_of\_S\_phase\_of\_mitotic\_cell\_cycle | 2 | 0 |  |  |  |  |  |  |  |  |
| GO:0045819\_positive\_regulation\_of\_glycogen\_catabolic\_process | 2 | 0 |  |  |  |  |  |  |  |  |
| GO:0045821\_positive\_regulation\_of\_glycolysis | 2 | 0 |  |  |  |  |  |  |  |  |
| GO:0045835\_negative\_regulation\_of\_meiosis | 2 | 0 |  |  |  |  |  |  |  |  |
| GO:0045836\_positive\_regulation\_of\_meiosis | 2 | 0 |  |  |  |  |  |  |  |  |
| GO:0045839\_negative\_regulation\_of\_mitosis | 2 | 0 |  |  |  |  |  |  |  |  |
| GO:0045841\_negative\_regulation\_of\_mitotic\_metaphase\_anaphase\_transition | 2 | 0 |  |  |  |  |  |  |  |  |
| GO:0045872\_positive\_regulation\_of\_rhodopsin\_gene\_expression | 2 | 0 |  |  |  |  |  |  |  |  |
| GO:0045912\_negative\_regulation\_of\_carbohydrate\_metabolic\_process | 2 | 0 |  |  |  |  |  |  |  |  |
| GO:0045948\_positive\_regulation\_of\_translational\_initiation | 2 | 0 |  |  |  |  |  |  |  |  |
| GO:0045950\_negative\_regulation\_of\_mitotic\_recombination | 2 | 0 |  |  |  |  |  |  |  |  |
| GO:0046033\_AMP\_metabolic\_process | 2 | 0 |  |  |  |  |  |  |  |  |
| GO:0046070\_dGTP\_metabolic\_process | 2 | 0 |  |  |  |  |  |  |  |  |
| GO:0046083\_adenine\_metabolic\_process | 2 | 0 |  |  |  |  |  |  |  |  |
| GO:0046085\_adenosine\_metabolic\_process | 2 | 0 |  |  |  |  |  |  |  |  |
| GO:0046100\_hypoxanthine\_metabolic\_process | 2 | 0 |  |  |  |  |  |  |  |  |
| GO:0046114\_guanosine\_biosynthetic\_process | 2 | 0 |  |  |  |  |  |  |  |  |
| GO:0046116\_queuosine\_metabolic\_process | 2 | 0 |  |  |  |  |  |  |  |  |
| GO:0046118\_7-methylguanosine\_biosynthetic\_process | 2 | 0 |  |  |  |  |  |  |  |  |
| GO:0046130\_purine\_ribonucleoside\_catabolic\_process | 2 | 0 |  |  |  |  |  |  |  |  |
| GO:0046146\_tetrahydrobiopterin\_metabolic\_process | 2 | 0 |  |  |  |  |  |  |  |  |
| GO:0046185\_aldehyde\_catabolic\_process | 2 | 0 |  |  |  |  |  |  |  |  |
| GO:0046208\_spermine\_catabolic\_process | 2 | 0 |  |  |  |  |  |  |  |  |
| GO:0046349\_amino\_sugar\_biosynthetic\_process | 2 | 0 |  |  |  |  |  |  |  |  |
| GO:0046439\_L-cysteine\_metabolic\_process | 2 | 0 |  |  |  |  |  |  |  |  |
| GO:0046500\_S-adenosylmethionine\_metabolic\_process | 2 | 0 |  |  |  |  |  |  |  |  |
| GO:0046501\_protoporphyrinogen\_IX\_metabolic\_process | 2 | 0 |  |  |  |  |  |  |  |  |
| GO:0046514\_ceramide\_catabolic\_process | 2 | 0 |  |  |  |  |  |  |  |  |
| GO:0046521\_sphingoid\_catabolic\_process | 2 | 0 |  |  |  |  |  |  |  |  |
| GO:0046532\_regulation\_of\_photoreceptor\_cell\_differentiation | 2 | 0 |  |  |  |  |  |  |  |  |
| GO:0046533\_negative\_regulation\_of\_photoreceptor\_cell\_differentiation | 2 | 0 |  |  |  |  |  |  |  |  |
| GO:0046544\_development\_of\_secondary\_male\_sexual\_characteristics | 2 | 0 |  |  |  |  |  |  |  |  |
| GO:0046619\_optic\_placode\_formation\_involved\_in\_camera-type\_eye | 2 | 0 |  |  |  |  |  |  |  |  |
| GO:0046950\_cellular\_ketone\_body\_metabolic\_process | 2 | 0 |  |  |  |  |  |  |  |  |
| GO:0046984\_regulation\_of\_hemoglobin\_biosynthetic\_process | 2 | 0 |  |  |  |  |  |  |  |  |
| GO:0047484\_regulation\_of\_response\_to\_osmotic\_stress | 2 | 0 |  |  |  |  |  |  |  |  |
| GO:0048025\_negative\_regulation\_of\_nuclear\_mRNA\_splicing\_\_via\_spliceosome | 2 | 0 |  |  |  |  |  |  |  |  |
| GO:0048134\_germ-line\_cyst\_formation | 2 | 0 |  |  |  |  |  |  |  |  |
| GO:0048136\_male\_germ-line\_cyst\_formation | 2 | 0 |  |  |  |  |  |  |  |  |
| GO:0048172\_regulation\_of\_short-term\_neuronal\_synaptic\_plasticity | 2 | 0 |  |  |  |  |  |  |  |  |
| GO:0048295\_positive\_regulation\_of\_isotype\_switching\_to\_IgE\_isotypes | 2 | 0 |  |  |  |  |  |  |  |  |
| GO:0048342\_paraxial\_mesodermal\_cell\_differentiation | 2 | 0 |  |  |  |  |  |  |  |  |
| GO:0048343\_paraxial\_mesodermal\_cell\_fate\_commitment | 2 | 0 |  |  |  |  |  |  |  |  |
| GO:0048552\_regulation\_of\_metalloenzyme\_activity | 2 | 0 |  |  |  |  |  |  |  |  |
| GO:0048554\_positive\_regulation\_of\_metalloenzyme\_activity | 2 | 0 |  |  |  |  |  |  |  |  |
| GO:0048619\_embryonic\_hindgut\_morphogenesis | 2 | 0 |  |  |  |  |  |  |  |  |
| GO:0048625\_myoblast\_cell\_fate\_commitment | 2 | 0 |  |  |  |  |  |  |  |  |
| GO:0048627\_myoblast\_development | 2 | 0 |  |  |  |  |  |  |  |  |
| GO:0048643\_positive\_regulation\_of\_skeletal\_muscle\_tissue\_development | 2 | 0 |  |  |  |  |  |  |  |  |
| GO:0048661\_positive\_regulation\_of\_smooth\_muscle\_cell\_proliferation | 2 | 0 |  |  |  |  |  |  |  |  |
| GO:0048670\_regulation\_of\_collateral\_sprouting | 2 | 0 |  |  |  |  |  |  |  |  |
| GO:0048671\_negative\_regulation\_of\_collateral\_sprouting | 2 | 0 |  |  |  |  |  |  |  |  |
| GO:0048677\_axon\_extension\_involved\_in\_regeneration | 2 | 0 |  |  |  |  |  |  |  |  |
| GO:0048679\_regulation\_of\_axon\_regeneration | 2 | 0 |  |  |  |  |  |  |  |  |
| GO:0048682\_sprouting\_of\_injured\_axon | 2 | 0 |  |  |  |  |  |  |  |  |
| GO:0048702\_embryonic\_neurocranium\_morphogenesis | 2 | 0 |  |  |  |  |  |  |  |  |
| GO:0048711\_positive\_regulation\_of\_astrocyte\_differentiation | 2 | 0 |  |  |  |  |  |  |  |  |
| GO:0048712\_negative\_regulation\_of\_astrocyte\_differentiation | 2 | 0 |  |  |  |  |  |  |  |  |
| GO:0048739\_cardiac\_muscle\_fiber\_development | 2 | 0 |  |  |  |  |  |  |  |  |
| GO:0048807\_female\_genitalia\_morphogenesis | 2 | 0 |  |  |  |  |  |  |  |  |
| GO:0048808\_male\_genitalia\_morphogenesis | 2 | 0 |  |  |  |  |  |  |  |  |
| GO:0048840\_otolith\_development | 2 | 0 |  |  |  |  |  |  |  |  |
| GO:0048850\_hypophysis\_morphogenesis | 2 | 0 |  |  |  |  |  |  |  |  |
| GO:0048867\_stem\_cell\_fate\_determination | 2 | 0 |  |  |  |  |  |  |  |  |
| GO:0050000\_chromosome\_localization | 2 | 0 |  |  |  |  |  |  |  |  |
| GO:0050686\_negative\_regulation\_of\_mRNA\_processing | 2 | 0 |  |  |  |  |  |  |  |  |
| GO:0050688\_regulation\_of\_defense\_response\_to\_virus | 2 | 0 |  |  |  |  |  |  |  |  |
| GO:0050746\_regulation\_of\_lipoprotein\_metabolic\_process | 2 | 0 |  |  |  |  |  |  |  |  |
| GO:0050779\_RNA\_destabilization | 2 | 0 |  |  |  |  |  |  |  |  |
| GO:0050792\_regulation\_of\_viral\_reproduction | 2 | 0 |  |  |  |  |  |  |  |  |
| GO:0050802\_circadian\_sleep\_wake\_cycle\_\_sleep | 2 | 0 |  |  |  |  |  |  |  |  |
| GO:0050847\_progesterone\_receptor\_signaling\_pathway | 2 | 0 |  |  |  |  |  |  |  |  |
| GO:0050855\_regulation\_of\_B\_cell\_receptor\_signaling\_pathway | 2 | 0 |  |  |  |  |  |  |  |  |
| GO:0050883\_musculoskeletal\_movement\_\_spinal\_reflex\_action | 2 | 0 |  |  |  |  |  |  |  |  |
| GO:0050901\_leukocyte\_tethering\_or\_rolling | 2 | 0 |  |  |  |  |  |  |  |  |
| GO:0050907\_detection\_of\_chemical\_stimulus\_involved\_in\_sensory\_perception | 2 | 0 |  |  |  |  |  |  |  |  |
| GO:0050917\_sensory\_perception\_of\_umami\_taste | 2 | 0 |  |  |  |  |  |  |  |  |
| GO:0050942\_positive\_regulation\_of\_pigment\_cell\_differentiation | 2 | 0 |  |  |  |  |  |  |  |  |
| GO:0050955\_thermoception | 2 | 0 |  |  |  |  |  |  |  |  |
| GO:0050968\_detection\_of\_chemical\_stimulus\_involved\_in\_sensory\_perception\_of\_pain | 2 | 0 |  |  |  |  |  |  |  |  |
| GO:0050973\_detection\_of\_mechanical\_stimulus\_involved\_in\_equilibrioception | 2 | 0 |  |  |  |  |  |  |  |  |
| GO:0050999\_regulation\_of\_nitric-oxide\_synthase\_activity | 2 | 0 |  |  |  |  |  |  |  |  |
| GO:0051004\_regulation\_of\_lipoprotein\_lipase\_activity | 2 | 0 |  |  |  |  |  |  |  |  |
| GO:0051014\_actin\_filament\_severing | 2 | 0 |  |  |  |  |  |  |  |  |
| GO:0051026\_chiasma\_formation | 2 | 0 |  |  |  |  |  |  |  |  |
| GO:0051081\_nuclear\_envelope\_disassembly | 2 | 0 |  |  |  |  |  |  |  |  |
| GO:0051132\_NK\_T\_cell\_activation | 2 | 0 |  |  |  |  |  |  |  |  |
| GO:0051133\_regulation\_of\_NK\_T\_cell\_activation | 2 | 0 |  |  |  |  |  |  |  |  |
| GO:0051135\_positive\_regulation\_of\_NK\_T\_cell\_activation | 2 | 0 |  |  |  |  |  |  |  |  |
| GO:0051150\_regulation\_of\_smooth\_muscle\_cell\_differentiation | 2 | 0 |  |  |  |  |  |  |  |  |
| GO:0051220\_cytoplasmic\_sequestering\_of\_protein | 2 | 0 |  |  |  |  |  |  |  |  |
| GO:0051279\_regulation\_of\_release\_of\_sequestered\_calcium\_ion\_into\_cytosol | 2 | 0 |  |  |  |  |  |  |  |  |
| GO:0051293\_establishment\_of\_spindle\_localization | 2 | 0 |  |  |  |  |  |  |  |  |
| GO:0051295\_establishment\_of\_meiotic\_spindle\_localization | 2 | 0 |  |  |  |  |  |  |  |  |
| GO:0051299\_centrosome\_separation | 2 | 0 |  |  |  |  |  |  |  |  |
| GO:0051303\_establishment\_of\_chromosome\_localization | 2 | 0 |  |  |  |  |  |  |  |  |
| GO:0051304\_chromosome\_separation | 2 | 0 |  |  |  |  |  |  |  |  |
| GO:0051307\_meiotic\_chromosome\_separation | 2 | 0 |  |  |  |  |  |  |  |  |
| GO:0051313\_attachment\_of\_spindle\_microtubules\_to\_chromosome | 2 | 0 |  |  |  |  |  |  |  |  |
| GO:0051318\_G1\_phase | 2 | 0 |  |  |  |  |  |  |  |  |
| GO:0051319\_G2\_phase | 2 | 0 |  |  |  |  |  |  |  |  |
| GO:0051353\_positive\_regulation\_of\_oxidoreductase\_activity | 2 | 0 |  |  |  |  |  |  |  |  |
| GO:0051451\_myoblast\_migration | 2 | 0 |  |  |  |  |  |  |  |  |
| GO:0051489\_regulation\_of\_filopodium\_assembly | 2 | 0 |  |  |  |  |  |  |  |  |
| GO:0051491\_positive\_regulation\_of\_filopodium\_assembly | 2 | 0 |  |  |  |  |  |  |  |  |
| GO:0051541\_elastin\_metabolic\_process | 2 | 0 |  |  |  |  |  |  |  |  |
| GO:0051546\_keratinocyte\_migration | 2 | 0 |  |  |  |  |  |  |  |  |
| GO:0051563\_smooth\_endoplasmic\_reticulum\_calcium\_ion\_homeostasis | 2 | 0 |  |  |  |  |  |  |  |  |
| GO:0051590\_positive\_regulation\_of\_neurotransmitter\_transport | 2 | 0 |  |  |  |  |  |  |  |  |
| GO:0051602\_response\_to\_electrical\_stimulus | 2 | 0 |  |  |  |  |  |  |  |  |
| GO:0051608\_histamine\_transport | 2 | 0 |  |  |  |  |  |  |  |  |
| GO:0051643\_ER\_localization | 2 | 0 |  |  |  |  |  |  |  |  |
| GO:0051653\_spindle\_localization | 2 | 0 |  |  |  |  |  |  |  |  |
| GO:0051657\_maintenance\_of\_organelle\_location | 2 | 0 |  |  |  |  |  |  |  |  |
| GO:0051702\_interaction\_with\_symbiont | 2 | 0 |  |  |  |  |  |  |  |  |
| GO:0051781\_positive\_regulation\_of\_cell\_division | 2 | 0 |  |  |  |  |  |  |  |  |
| GO:0051784\_negative\_regulation\_of\_nuclear\_division | 2 | 0 |  |  |  |  |  |  |  |  |
| GO:0051890\_regulation\_of\_cardioblast\_differentiation | 2 | 0 |  |  |  |  |  |  |  |  |
| GO:0051891\_positive\_regulation\_of\_cardioblast\_differentiation | 2 | 0 |  |  |  |  |  |  |  |  |
| GO:0051923\_sulfation | 2 | 0 |  |  |  |  |  |  |  |  |
| GO:0051938\_L-glutamate\_import | 2 | 0 |  |  |  |  |  |  |  |  |
| GO:0051957\_positive\_regulation\_of\_amino\_acid\_transport | 2 | 0 |  |  |  |  |  |  |  |  |
| GO:0051988\_regulation\_of\_attachment\_of\_spindle\_microtubules\_to\_kinetochore | 2 | 0 |  |  |  |  |  |  |  |  |
| GO:0055057\_neuroblast\_division | 2 | 0 |  |  |  |  |  |  |  |  |
| GO:0055064\_chloride\_ion\_homeostasis | 2 | 0 |  |  |  |  |  |  |  |  |
| GO:0055075\_potassium\_ion\_homeostasis | 2 | 0 |  |  |  |  |  |  |  |  |
| GO:0055090\_acylglycerol\_homeostasis | 2 | 0 |  |  |  |  |  |  |  |  |
| GO:0055091\_phospholipid\_homeostasis | 2 | 0 |  |  |  |  |  |  |  |  |
| GO:0060012\_synaptic\_transmission\_\_glycinergic | 2 | 0 |  |  |  |  |  |  |  |  |
| GO:0060023\_soft\_palate\_development | 2 | 0 |  |  |  |  |  |  |  |  |
| GO:0060032\_notochord\_regression | 2 | 0 |  |  |  |  |  |  |  |  |
| GO:0060039\_pericardium\_development | 2 | 0 |  |  |  |  |  |  |  |  |
| GO:0060044\_negative\_regulation\_of\_cardiac\_muscle\_cell\_proliferation | 2 | 0 |  |  |  |  |  |  |  |  |
| GO:0060060\_post-embryonic\_retina\_morphogenesis\_in\_camera-type\_eye | 2 | 0 |  |  |  |  |  |  |  |  |
| GO:0060083\_smooth\_muscle\_contraction\_involved\_in\_micturition | 2 | 0 |  |  |  |  |  |  |  |  |
| GO:0060124\_positive\_regulation\_of\_growth\_hormone\_secretion | 2 | 0 |  |  |  |  |  |  |  |  |
| GO:0060133\_somatotropin\_secreting\_cell\_development | 2 | 0 |  |  |  |  |  |  |  |  |
| GO:0060155\_platelet\_dense\_granule\_organization | 2 | 0 |  |  |  |  |  |  |  |  |
| GO:0060159\_regulation\_of\_dopamine\_receptor\_signaling\_pathway | 2 | 0 |  |  |  |  |  |  |  |  |
| GO:0060160\_negative\_regulation\_of\_dopamine\_receptor\_signaling\_pathway | 2 | 0 |  |  |  |  |  |  |  |  |
| GO:0060166\_olfactory\_pit\_development | 2 | 0 |  |  |  |  |  |  |  |  |
| GO:0060179\_male\_mating\_behavior | 2 | 0 |  |  |  |  |  |  |  |  |
| GO:0060180\_female\_mating\_behavior | 2 | 0 |  |  |  |  |  |  |  |  |
| GO:0060214\_endocardium\_formation | 2 | 0 |  |  |  |  |  |  |  |  |
| GO:0060218\_hemopoietic\_stem\_cell\_differentiation | 2 | 0 |  |  |  |  |  |  |  |  |
| GO:0060259\_regulation\_of\_feeding\_behavior | 2 | 0 |  |  |  |  |  |  |  |  |
| GO:0060260\_regulation\_of\_transcription\_initiation\_from\_RNA\_polymerase\_II\_promoter | 2 | 0 |  |  |  |  |  |  |  |  |
| GO:0060292\_long\_term\_synaptic\_depression | 2 | 0 |  |  |  |  |  |  |  |  |
| GO:0060318\_definitive\_erythrocyte\_differentiation | 2 | 0 |  |  |  |  |  |  |  |  |
| GO:0060346\_bone\_trabecula\_formation | 2 | 0 |  |  |  |  |  |  |  |  |
| GO:0060363\_cranial\_suture\_morphogenesis | 2 | 0 |  |  |  |  |  |  |  |  |
| GO:0060393\_regulation\_of\_pathway-restricted\_SMAD\_protein\_phosphorylation | 2 | 0 |  |  |  |  |  |  |  |  |
| GO:0060397\_JAK-STAT\_cascade\_involved\_in\_growth\_hormone\_signaling\_pathway | 2 | 0 |  |  |  |  |  |  |  |  |
| GO:0060426\_lung\_vasculature\_development | 2 | 0 |  |  |  |  |  |  |  |  |
| GO:0060430\_lung\_saccule\_development | 2 | 0 |  |  |  |  |  |  |  |  |
| GO:0060434\_bronchus\_morphogenesis | 2 | 0 |  |  |  |  |  |  |  |  |
| GO:0060439\_trachea\_morphogenesis | 2 | 0 |  |  |  |  |  |  |  |  |
| GO:0060458\_right\_lung\_development | 2 | 0 |  |  |  |  |  |  |  |  |
| GO:0060462\_lung\_lobe\_development | 2 | 0 |  |  |  |  |  |  |  |  |
| GO:0060463\_lung\_lobe\_morphogenesis | 2 | 0 |  |  |  |  |  |  |  |  |
| GO:0060479\_lung\_cell\_differentiation | 2 | 0 |  |  |  |  |  |  |  |  |
| GO:0060487\_lung\_epithelial\_cell\_differentiation | 2 | 0 |  |  |  |  |  |  |  |  |
| GO:0060516\_primary\_prostatic\_bud\_elongation | 2 | 0 |  |  |  |  |  |  |  |  |
| GO:0060529\_squamous\_basal\_epithelial\_stem\_cell\_differentiation\_involved\_in\_prostate\_gland\_acinus\_development | 2 | 0 |  |  |  |  |  |  |  |  |
| GO:0060534\_trachea\_cartilage\_development | 2 | 0 |  |  |  |  |  |  |  |  |
| GO:0060599\_lateral\_sprouting\_involved\_in\_mammary\_gland\_duct\_morphogenesis | 2 | 0 |  |  |  |  |  |  |  |  |
| GO:0060612\_adipose\_tissue\_development | 2 | 0 |  |  |  |  |  |  |  |  |
| GO:0060615\_mammary\_gland\_bud\_formation | 2 | 0 |  |  |  |  |  |  |  |  |
| GO:0060667\_branch\_elongation\_involved\_in\_salivary\_gland\_morphogenesis | 2 | 0 |  |  |  |  |  |  |  |  |
| GO:0060690\_epithelial\_cell\_differentiation\_involved\_in\_salivary\_gland\_development | 2 | 0 |  |  |  |  |  |  |  |  |
| GO:0060738\_epithelial-mesenchymal\_signaling\_involved\_in\_prostate\_gland\_development | 2 | 0 |  |  |  |  |  |  |  |  |
| GO:0060741\_prostate\_gland\_stromal\_morphogenesis | 2 | 0 |  |  |  |  |  |  |  |  |
| GO:0060763\_mammary\_duct\_terminal\_end\_bud\_growth | 2 | 0 |  |  |  |  |  |  |  |  |
| GO:0060765\_regulation\_of\_androgen\_receptor\_signaling\_pathway | 2 | 0 |  |  |  |  |  |  |  |  |
| GO:0060766\_negative\_regulation\_of\_androgen\_receptor\_signaling\_pathway | 2 | 0 |  |  |  |  |  |  |  |  |
| GO:0060769\_positive\_regulation\_of\_epithelial\_cell\_proliferation\_involved\_in\_prostate\_gland\_development | 2 | 0 |  |  |  |  |  |  |  |  |
| GO:0065005\_protein-lipid\_complex\_assembly | 2 | 0 |  |  |  |  |  |  |  |  |
| GO:0070076\_histone\_lysine\_demethylation | 2 | 0 |  |  |  |  |  |  |  |  |
| GO:0070168\_negative\_regulation\_of\_biomineral\_formation | 2 | 0 |  |  |  |  |  |  |  |  |
| GO:0070252\_actin-mediated\_cell\_contraction | 2 | 0 |  |  |  |  |  |  |  |  |
| GO:0070256\_negative\_regulation\_of\_mucus\_secretion | 2 | 0 |  |  |  |  |  |  |  |  |
| GO:0070257\_positive\_regulation\_of\_mucus\_secretion | 2 | 0 |  |  |  |  |  |  |  |  |
| GO:0070570\_regulation\_of\_neuron\_projection\_regeneration | 2 | 0 |  |  |  |  |  |  |  |  |
| GO:0070723\_response\_to\_cholesterol | 2 | 0 |  |  |  |  |  |  |  |  |
| GO:0090030\_regulation\_of\_steroid\_hormone\_biosynthetic\_process | 2 | 0 |  |  |  |  |  |  |  |  |
| GO:0018193\_peptidyl-amino\_acid\_modification | 97 | 0 | 0.000000 | -0.000000 | 1102 | 967.818445 | 1048.91 | 1130.001555 | 0.951824 |
| GO:0060341\_regulation\_of\_cellular\_localization | 97 | 0 | 0.000000 | -0.000000 | 1102 | 967.818445 | 1048.91 | 1130.001555 | 0.951824 |
| GO:0048705\_skeletal\_system\_morphogenesis | 111 | 0 | 0.000000 | -0.000000 | 1103 | 968.371340 | 1049.44 | 1130.508660 | 0.951442 |
| GO:0006576\_biogenic\_amine\_metabolic\_process | 53 | 0 | 0.000000 | -0.000000 | 1109 | 974.418573 | 1055.29 | 1136.161427 | 0.951569 |
| GO:0006935\_chemotaxis | 53 | 0 | 0.000000 | -0.000000 | 1109 | 974.418573 | 1055.29 | 1136.161427 | 0.951569 |
| GO:0042330\_taxis | 53 | 0 | 0.000000 | -0.000000 | 1109 | 974.418573 | 1055.29 | 1136.161427 | 0.951569 |
| GO:0046942\_carboxylic\_acid\_transport | 53 | 0 | 0.000000 | -0.000000 | 1109 | 974.418573 | 1055.29 | 1136.161427 | 0.951569 |
| GO:0050905\_neuromuscular\_process | 53 | 0 | 0.000000 | -0.000000 | 1109 | 974.418573 | 1055.29 | 1136.161427 | 0.951569 |
| GO:0055085\_transmembrane\_transport | 53 | 0 | 0.000000 | -0.000000 | 1109 | 974.418573 | 1055.29 | 1136.161427 | 0.951569 |
| GO:0007186\_G-protein\_coupled\_receptor\_protein\_signaling\_pathway | 144 | 0 | 0.000000 | -0.000000 | 1110 | 975.255756 | 1055.98 | 1136.704244 | 0.951333 |
| GO:0000910\_cytokinesis | 8 | 0 | 0.000000 | -0.000000 | 1232 | 1103.274183 | 1181.98 | 1260.685817 | 0.959399 |
| GO:0001783\_B\_cell\_apoptosis | 8 | 0 | 0.000000 | -0.000000 | 1232 | 1103.274183 | 1181.98 | 1260.685817 | 0.959399 |
| GO:0001833\_inner\_cell\_mass\_cell\_proliferation | 8 | 0 | 0.000000 | -0.000000 | 1232 | 1103.274183 | 1181.98 | 1260.685817 | 0.959399 |
| GO:0001840\_neural\_plate\_development | 8 | 0 | 0.000000 | -0.000000 | 1232 | 1103.274183 | 1181.98 | 1260.685817 | 0.959399 |
| GO:0001893\_maternal\_placenta\_development | 8 | 0 | 0.000000 | -0.000000 | 1232 | 1103.274183 | 1181.98 | 1260.685817 | 0.959399 |
| GO:0001911\_negative\_regulation\_of\_leukocyte\_mediated\_cytotoxicity | 8 | 0 | 0.000000 | -0.000000 | 1232 | 1103.274183 | 1181.98 | 1260.685817 | 0.959399 |
| GO:0001916\_positive\_regulation\_of\_T\_cell\_mediated\_cytotoxicity | 8 | 0 | 0.000000 | -0.000000 | 1232 | 1103.274183 | 1181.98 | 1260.685817 | 0.959399 |
| GO:0002065\_columnar\_cuboidal\_epithelial\_cell\_differentiation | 8 | 0 | 0.000000 | -0.000000 | 1232 | 1103.274183 | 1181.98 | 1260.685817 | 0.959399 |
| GO:0002320\_lymphoid\_progenitor\_cell\_differentiation | 8 | 0 | 0.000000 | -0.000000 | 1232 | 1103.274183 | 1181.98 | 1260.685817 | 0.959399 |
| GO:0002438\_acute\_inflammatory\_response\_to\_antigenic\_stimulus | 8 | 0 | 0.000000 | -0.000000 | 1232 | 1103.274183 | 1181.98 | 1260.685817 | 0.959399 |
| GO:0002524\_hypersensitivity | 8 | 0 | 0.000000 | -0.000000 | 1232 | 1103.274183 | 1181.98 | 1260.685817 | 0.959399 |
| GO:0002566\_somatic\_diversification\_of\_immune\_receptors\_via\_somatic\_mutation | 8 | 0 | 0.000000 | -0.000000 | 1232 | 1103.274183 | 1181.98 | 1260.685817 | 0.959399 |
| GO:0002864\_regulation\_of\_acute\_inflammatory\_response\_to\_antigenic\_stimulus | 8 | 0 | 0.000000 | -0.000000 | 1232 | 1103.274183 | 1181.98 | 1260.685817 | 0.959399 |
| GO:0002883\_regulation\_of\_hypersensitivity | 8 | 0 | 0.000000 | -0.000000 | 1232 | 1103.274183 | 1181.98 | 1260.685817 | 0.959399 |
| GO:0003081\_regulation\_of\_systemic\_arterial\_blood\_pressure\_by\_renin-angiotensin | 8 | 0 | 0.000000 | -0.000000 | 1232 | 1103.274183 | 1181.98 | 1260.685817 | 0.959399 |
| GO:0006195\_purine\_nucleotide\_catabolic\_process | 8 | 0 | 0.000000 | -0.000000 | 1232 | 1103.274183 | 1181.98 | 1260.685817 | 0.959399 |
| GO:0006284\_base-excision\_repair | 8 | 0 | 0.000000 | -0.000000 | 1232 | 1103.274183 | 1181.98 | 1260.685817 | 0.959399 |
| GO:0006349\_genetic\_imprinting | 8 | 0 | 0.000000 | -0.000000 | 1232 | 1103.274183 | 1181.98 | 1260.685817 | 0.959399 |
| GO:0006360\_transcription\_from\_RNA\_polymerase\_I\_promoter | 8 | 0 | 0.000000 | -0.000000 | 1232 | 1103.274183 | 1181.98 | 1260.685817 | 0.959399 |
| GO:0006399\_tRNA\_metabolic\_process | 8 | 0 | 0.000000 | -0.000000 | 1232 | 1103.274183 | 1181.98 | 1260.685817 | 0.959399 |
| GO:0006458\_'de\_novo'\_protein\_folding | 8 | 0 | 0.000000 | -0.000000 | 1232 | 1103.274183 | 1181.98 | 1260.685817 | 0.959399 |
| GO:0006493\_protein\_amino\_acid\_O-linked\_glycosylation | 8 | 0 | 0.000000 | -0.000000 | 1232 | 1103.274183 | 1181.98 | 1260.685817 | 0.959399 |
| GO:0006582\_melanin\_metabolic\_process | 8 | 0 | 0.000000 | -0.000000 | 1232 | 1103.274183 | 1181.98 | 1260.685817 | 0.959399 |
| GO:0006733\_oxidoreduction\_coenzyme\_metabolic\_process | 8 | 0 | 0.000000 | -0.000000 | 1232 | 1103.274183 | 1181.98 | 1260.685817 | 0.959399 |
| GO:0006829\_zinc\_ion\_transport | 8 | 0 | 0.000000 | -0.000000 | 1232 | 1103.274183 | 1181.98 | 1260.685817 | 0.959399 |
| GO:0007098\_centrosome\_cycle | 8 | 0 | 0.000000 | -0.000000 | 1232 | 1103.274183 | 1181.98 | 1260.685817 | 0.959399 |
| GO:0007131\_reciprocal\_meiotic\_recombination | 8 | 0 | 0.000000 | -0.000000 | 1232 | 1103.274183 | 1181.98 | 1260.685817 | 0.959399 |
| GO:0007141\_male\_meiosis\_I | 8 | 0 | 0.000000 | -0.000000 | 1232 | 1103.274183 | 1181.98 | 1260.685817 | 0.959399 |
| GO:0007625\_grooming\_behavior | 8 | 0 | 0.000000 | -0.000000 | 1232 | 1103.274183 | 1181.98 | 1260.685817 | 0.959399 |
| GO:0008593\_regulation\_of\_Notch\_signaling\_pathway | 8 | 0 | 0.000000 | -0.000000 | 1232 | 1103.274183 | 1181.98 | 1260.685817 | 0.959399 |
| GO:0009072\_aromatic\_amino\_acid\_family\_metabolic\_process | 8 | 0 | 0.000000 | -0.000000 | 1232 | 1103.274183 | 1181.98 | 1260.685817 | 0.959399 |
| GO:0009746\_response\_to\_hexose\_stimulus | 8 | 0 | 0.000000 | -0.000000 | 1232 | 1103.274183 | 1181.98 | 1260.685817 | 0.959399 |
| GO:0009749\_response\_to\_glucose\_stimulus | 8 | 0 | 0.000000 | -0.000000 | 1232 | 1103.274183 | 1181.98 | 1260.685817 | 0.959399 |
| GO:0014014\_negative\_regulation\_of\_gliogenesis | 8 | 0 | 0.000000 | -0.000000 | 1232 | 1103.274183 | 1181.98 | 1260.685817 | 0.959399 |
| GO:0014046\_dopamine\_secretion | 8 | 0 | 0.000000 | -0.000000 | 1232 | 1103.274183 | 1181.98 | 1260.685817 | 0.959399 |
| GO:0014059\_regulation\_of\_dopamine\_secretion | 8 | 0 | 0.000000 | -0.000000 | 1232 | 1103.274183 | 1181.98 | 1260.685817 | 0.959399 |
| GO:0014065\_phosphoinositide\_3-kinase\_cascade | 8 | 0 | 0.000000 | -0.000000 | 1232 | 1103.274183 | 1181.98 | 1260.685817 | 0.959399 |
| GO:0015800\_acidic\_amino\_acid\_transport | 8 | 0 | 0.000000 | -0.000000 | 1232 | 1103.274183 | 1181.98 | 1260.685817 | 0.959399 |
| GO:0015804\_neutral\_amino\_acid\_transport | 8 | 0 | 0.000000 | -0.000000 | 1232 | 1103.274183 | 1181.98 | 1260.685817 | 0.959399 |
| GO:0016236\_macroautophagy | 8 | 0 | 0.000000 | -0.000000 | 1232 | 1103.274183 | 1181.98 | 1260.685817 | 0.959399 |
| GO:0016446\_somatic\_hypermutation\_of\_immunoglobulin\_genes | 8 | 0 | 0.000000 | -0.000000 | 1232 | 1103.274183 | 1181.98 | 1260.685817 | 0.959399 |
| GO:0018107\_peptidyl-threonine\_phosphorylation | 8 | 0 | 0.000000 | -0.000000 | 1232 | 1103.274183 | 1181.98 | 1260.685817 | 0.959399 |
| GO:0018210\_peptidyl-threonine\_modification | 8 | 0 | 0.000000 | -0.000000 | 1232 | 1103.274183 | 1181.98 | 1260.685817 | 0.959399 |
| GO:0018345\_protein\_palmitoylation | 8 | 0 | 0.000000 | -0.000000 | 1232 | 1103.274183 | 1181.98 | 1260.685817 | 0.959399 |
| GO:0019229\_regulation\_of\_vasoconstriction | 8 | 0 | 0.000000 | -0.000000 | 1232 | 1103.274183 | 1181.98 | 1260.685817 | 0.959399 |
| GO:0019400\_alditol\_metabolic\_process | 8 | 0 | 0.000000 | -0.000000 | 1232 | 1103.274183 | 1181.98 | 1260.685817 | 0.959399 |
| GO:0021692\_cerebellar\_Purkinje\_cell\_layer\_morphogenesis | 8 | 0 | 0.000000 | -0.000000 | 1232 | 1103.274183 | 1181.98 | 1260.685817 | 0.959399 |
| GO:0021694\_cerebellar\_Purkinje\_cell\_layer\_formation | 8 | 0 | 0.000000 | -0.000000 | 1232 | 1103.274183 | 1181.98 | 1260.685817 | 0.959399 |
| GO:0021702\_cerebellar\_Purkinje\_cell\_differentiation | 8 | 0 | 0.000000 | -0.000000 | 1232 | 1103.274183 | 1181.98 | 1260.685817 | 0.959399 |
| GO:0021799\_cerebral\_cortex\_radially\_oriented\_cell\_migration | 8 | 0 | 0.000000 | -0.000000 | 1232 | 1103.274183 | 1181.98 | 1260.685817 | 0.959399 |
| GO:0022898\_regulation\_of\_transmembrane\_transporter\_activity | 8 | 0 | 0.000000 | -0.000000 | 1232 | 1103.274183 | 1181.98 | 1260.685817 | 0.959399 |
| GO:0030035\_microspike\_assembly | 8 | 0 | 0.000000 | -0.000000 | 1232 | 1103.274183 | 1181.98 | 1260.685817 | 0.959399 |
| GO:0030193\_regulation\_of\_blood\_coagulation | 8 | 0 | 0.000000 | -0.000000 | 1232 | 1103.274183 | 1181.98 | 1260.685817 | 0.959399 |
| GO:0030204\_chondroitin\_sulfate\_metabolic\_process | 8 | 0 | 0.000000 | -0.000000 | 1232 | 1103.274183 | 1181.98 | 1260.685817 | 0.959399 |
| GO:0030500\_regulation\_of\_bone\_mineralization | 8 | 0 | 0.000000 | -0.000000 | 1232 | 1103.274183 | 1181.98 | 1260.685817 | 0.959399 |
| GO:0030511\_positive\_regulation\_of\_transforming\_growth\_factor\_beta\_receptor\_signaling\_pathway | 8 | 0 | 0.000000 | -0.000000 | 1232 | 1103.274183 | 1181.98 | 1260.685817 | 0.959399 |
| GO:0031102\_neuron\_projection\_regeneration | 8 | 0 | 0.000000 | -0.000000 | 1232 | 1103.274183 | 1181.98 | 1260.685817 | 0.959399 |
| GO:0031103\_axon\_regeneration | 8 | 0 | 0.000000 | -0.000000 | 1232 | 1103.274183 | 1181.98 | 1260.685817 | 0.959399 |
| GO:0031111\_negative\_regulation\_of\_microtubule\_polymerization\_or\_depolymerization | 8 | 0 | 0.000000 | -0.000000 | 1232 | 1103.274183 | 1181.98 | 1260.685817 | 0.959399 |
| GO:0031123\_RNA\_3'-end\_processing | 8 | 0 | 0.000000 | -0.000000 | 1232 | 1103.274183 | 1181.98 | 1260.685817 | 0.959399 |
| GO:0031294\_lymphocyte\_costimulation | 8 | 0 | 0.000000 | -0.000000 | 1232 | 1103.274183 | 1181.98 | 1260.685817 | 0.959399 |
| GO:0031295\_T\_cell\_costimulation | 8 | 0 | 0.000000 | -0.000000 | 1232 | 1103.274183 | 1181.98 | 1260.685817 | 0.959399 |
| GO:0031334\_positive\_regulation\_of\_protein\_complex\_assembly | 8 | 0 | 0.000000 | -0.000000 | 1232 | 1103.274183 | 1181.98 | 1260.685817 | 0.959399 |
| GO:0031342\_negative\_regulation\_of\_cell\_killing | 8 | 0 | 0.000000 | -0.000000 | 1232 | 1103.274183 | 1181.98 | 1260.685817 | 0.959399 |
| GO:0031396\_regulation\_of\_protein\_ubiquitination | 8 | 0 | 0.000000 | -0.000000 | 1232 | 1103.274183 | 1181.98 | 1260.685817 | 0.959399 |
| GO:0032094\_response\_to\_food | 8 | 0 | 0.000000 | -0.000000 | 1232 | 1103.274183 | 1181.98 | 1260.685817 | 0.959399 |
| GO:0032273\_positive\_regulation\_of\_protein\_polymerization | 8 | 0 | 0.000000 | -0.000000 | 1232 | 1103.274183 | 1181.98 | 1260.685817 | 0.959399 |
| GO:0032409\_regulation\_of\_transporter\_activity | 8 | 0 | 0.000000 | -0.000000 | 1232 | 1103.274183 | 1181.98 | 1260.685817 | 0.959399 |
| GO:0032412\_regulation\_of\_ion\_transmembrane\_transporter\_activity | 8 | 0 | 0.000000 | -0.000000 | 1232 | 1103.274183 | 1181.98 | 1260.685817 | 0.959399 |
| GO:0032613\_interleukin-10\_production | 8 | 0 | 0.000000 | -0.000000 | 1232 | 1103.274183 | 1181.98 | 1260.685817 | 0.959399 |
| GO:0033198\_response\_to\_ATP | 8 | 0 | 0.000000 | -0.000000 | 1232 | 1103.274183 | 1181.98 | 1260.685817 | 0.959399 |
| GO:0034284\_response\_to\_monosaccharide\_stimulus | 8 | 0 | 0.000000 | -0.000000 | 1232 | 1103.274183 | 1181.98 | 1260.685817 | 0.959399 |
| GO:0034728\_nucleosome\_organization | 8 | 0 | 0.000000 | -0.000000 | 1232 | 1103.274183 | 1181.98 | 1260.685817 | 0.959399 |
| GO:0035023\_regulation\_of\_Rho\_protein\_signal\_transduction | 8 | 0 | 0.000000 | -0.000000 | 1232 | 1103.274183 | 1181.98 | 1260.685817 | 0.959399 |
| GO:0035112\_genitalia\_morphogenesis | 8 | 0 | 0.000000 | -0.000000 | 1232 | 1103.274183 | 1181.98 | 1260.685817 | 0.959399 |
| GO:0040017\_positive\_regulation\_of\_locomotion | 8 | 0 | 0.000000 | -0.000000 | 1232 | 1103.274183 | 1181.98 | 1260.685817 | 0.959399 |
| GO:0042074\_cell\_migration\_involved\_in\_gastrulation | 8 | 0 | 0.000000 | -0.000000 | 1232 | 1103.274183 | 1181.98 | 1260.685817 | 0.959399 |
| GO:0042090\_interleukin-12\_biosynthetic\_process | 8 | 0 | 0.000000 | -0.000000 | 1232 | 1103.274183 | 1181.98 | 1260.685817 | 0.959399 |
| GO:0042092\_T-helper\_2\_type\_immune\_response | 8 | 0 | 0.000000 | -0.000000 | 1232 | 1103.274183 | 1181.98 | 1260.685817 | 0.959399 |
| GO:0042095\_interferon-gamma\_biosynthetic\_process | 8 | 0 | 0.000000 | -0.000000 | 1232 | 1103.274183 | 1181.98 | 1260.685817 | 0.959399 |
| GO:0042104\_positive\_regulation\_of\_activated\_T\_cell\_proliferation | 8 | 0 | 0.000000 | -0.000000 | 1232 | 1103.274183 | 1181.98 | 1260.685817 | 0.959399 |
| GO:0042226\_interleukin-6\_biosynthetic\_process | 8 | 0 | 0.000000 | -0.000000 | 1232 | 1103.274183 | 1181.98 | 1260.685817 | 0.959399 |
| GO:0042304\_regulation\_of\_fatty\_acid\_biosynthetic\_process | 8 | 0 | 0.000000 | -0.000000 | 1232 | 1103.274183 | 1181.98 | 1260.685817 | 0.959399 |
| GO:0042423\_catecholamine\_biosynthetic\_process | 8 | 0 | 0.000000 | -0.000000 | 1232 | 1103.274183 | 1181.98 | 1260.685817 | 0.959399 |
| GO:0042771\_DNA\_damage\_response\_\_signal\_transduction\_by\_p53\_class\_mediator\_resulting\_in\_induction\_of\_apoptosis | 8 | 0 | 0.000000 | -0.000000 | 1232 | 1103.274183 | 1181.98 | 1260.685817 | 0.959399 |
| GO:0042990\_regulation\_of\_transcription\_factor\_import\_into\_nucleus | 8 | 0 | 0.000000 | -0.000000 | 1232 | 1103.274183 | 1181.98 | 1260.685817 | 0.959399 |
| GO:0042991\_transcription\_factor\_import\_into\_nucleus | 8 | 0 | 0.000000 | -0.000000 | 1232 | 1103.274183 | 1181.98 | 1260.685817 | 0.959399 |
| GO:0043011\_myeloid\_dendritic\_cell\_differentiation | 8 | 0 | 0.000000 | -0.000000 | 1232 | 1103.274183 | 1181.98 | 1260.685817 | 0.959399 |
| GO:0043368\_positive\_T\_cell\_selection | 8 | 0 | 0.000000 | -0.000000 | 1232 | 1103.274183 | 1181.98 | 1260.685817 | 0.959399 |
| GO:0043370\_regulation\_of\_CD4-positive\_\_alpha\_beta\_T\_cell\_differentiation | 8 | 0 | 0.000000 | -0.000000 | 1232 | 1103.274183 | 1181.98 | 1260.685817 | 0.959399 |
| GO:0043542\_endothelial\_cell\_migration | 8 | 0 | 0.000000 | -0.000000 | 1232 | 1103.274183 | 1181.98 | 1260.685817 | 0.959399 |
| GO:0043616\_keratinocyte\_proliferation | 8 | 0 | 0.000000 | -0.000000 | 1232 | 1103.274183 | 1181.98 | 1260.685817 | 0.959399 |
| GO:0045075\_regulation\_of\_interleukin-12\_biosynthetic\_process | 8 | 0 | 0.000000 | -0.000000 | 1232 | 1103.274183 | 1181.98 | 1260.685817 | 0.959399 |
| GO:0045086\_positive\_regulation\_of\_interleukin-2\_biosynthetic\_process | 8 | 0 | 0.000000 | -0.000000 | 1232 | 1103.274183 | 1181.98 | 1260.685817 | 0.959399 |
| GO:0045351\_type\_I\_interferon\_biosynthetic\_process | 8 | 0 | 0.000000 | -0.000000 | 1232 | 1103.274183 | 1181.98 | 1260.685817 | 0.959399 |
| GO:0045408\_regulation\_of\_interleukin-6\_biosynthetic\_process | 8 | 0 | 0.000000 | -0.000000 | 1232 | 1103.274183 | 1181.98 | 1260.685817 | 0.959399 |
| GO:0045494\_photoreceptor\_cell\_maintenance | 8 | 0 | 0.000000 | -0.000000 | 1232 | 1103.274183 | 1181.98 | 1260.685817 | 0.959399 |
| GO:0045686\_negative\_regulation\_of\_glial\_cell\_differentiation | 8 | 0 | 0.000000 | -0.000000 | 1232 | 1103.274183 | 1181.98 | 1260.685817 | 0.959399 |
| GO:0045910\_negative\_regulation\_of\_DNA\_recombination | 8 | 0 | 0.000000 | -0.000000 | 1232 | 1103.274183 | 1181.98 | 1260.685817 | 0.959399 |
| GO:0045921\_positive\_regulation\_of\_exocytosis | 8 | 0 | 0.000000 | -0.000000 | 1232 | 1103.274183 | 1181.98 | 1260.685817 | 0.959399 |
| GO:0045932\_negative\_regulation\_of\_muscle\_contraction | 8 | 0 | 0.000000 | -0.000000 | 1232 | 1103.274183 | 1181.98 | 1260.685817 | 0.959399 |
| GO:0046470\_phosphatidylcholine\_metabolic\_process | 8 | 0 | 0.000000 | -0.000000 | 1232 | 1103.274183 | 1181.98 | 1260.685817 | 0.959399 |
| GO:0048266\_behavioral\_response\_to\_pain | 8 | 0 | 0.000000 | -0.000000 | 1232 | 1103.274183 | 1181.98 | 1260.685817 | 0.959399 |
| GO:0048520\_positive\_regulation\_of\_behavior | 8 | 0 | 0.000000 | -0.000000 | 1232 | 1103.274183 | 1181.98 | 1260.685817 | 0.959399 |
| GO:0048557\_embryonic\_digestive\_tract\_morphogenesis | 8 | 0 | 0.000000 | -0.000000 | 1232 | 1103.274183 | 1181.98 | 1260.685817 | 0.959399 |
| GO:0050707\_regulation\_of\_cytokine\_secretion | 8 | 0 | 0.000000 | -0.000000 | 1232 | 1103.274183 | 1181.98 | 1260.685817 | 0.959399 |
| GO:0050909\_sensory\_perception\_of\_taste | 8 | 0 | 0.000000 | -0.000000 | 1232 | 1103.274183 | 1181.98 | 1260.685817 | 0.959399 |
| GO:0050920\_regulation\_of\_chemotaxis | 8 | 0 | 0.000000 | -0.000000 | 1232 | 1103.274183 | 1181.98 | 1260.685817 | 0.959399 |
| GO:0050921\_positive\_regulation\_of\_chemotaxis | 8 | 0 | 0.000000 | -0.000000 | 1232 | 1103.274183 | 1181.98 | 1260.685817 | 0.959399 |
| GO:0050926\_regulation\_of\_positive\_chemotaxis | 8 | 0 | 0.000000 | -0.000000 | 1232 | 1103.274183 | 1181.98 | 1260.685817 | 0.959399 |
| GO:0050927\_positive\_regulation\_of\_positive\_chemotaxis | 8 | 0 | 0.000000 | -0.000000 | 1232 | 1103.274183 | 1181.98 | 1260.685817 | 0.959399 |
| GO:0050930\_induction\_of\_positive\_chemotaxis | 8 | 0 | 0.000000 | -0.000000 | 1232 | 1103.274183 | 1181.98 | 1260.685817 | 0.959399 |
| GO:0051084\_'de\_novo'\_posttranslational\_protein\_folding | 8 | 0 | 0.000000 | -0.000000 | 1232 | 1103.274183 | 1181.98 | 1260.685817 | 0.959399 |
| GO:0051181\_cofactor\_transport | 8 | 0 | 0.000000 | -0.000000 | 1232 | 1103.274183 | 1181.98 | 1260.685817 | 0.959399 |
| GO:0060043\_regulation\_of\_cardiac\_muscle\_cell\_proliferation | 8 | 0 | 0.000000 | -0.000000 | 1232 | 1103.274183 | 1181.98 | 1260.685817 | 0.959399 |
| GO:0060347\_heart\_trabecula\_formation | 8 | 0 | 0.000000 | -0.000000 | 1232 | 1103.274183 | 1181.98 | 1260.685817 | 0.959399 |
| GO:0060670\_branching\_involved\_in\_embryonic\_placenta\_morphogenesis | 8 | 0 | 0.000000 | -0.000000 | 1232 | 1103.274183 | 1181.98 | 1260.685817 | 0.959399 |
| GO:0060712\_spongiotrophoblast\_layer\_development | 8 | 0 | 0.000000 | -0.000000 | 1232 | 1103.274183 | 1181.98 | 1260.685817 | 0.959399 |
| GO:0070167\_regulation\_of\_biomineral\_formation | 8 | 0 | 0.000000 | -0.000000 | 1232 | 1103.274183 | 1181.98 | 1260.685817 | 0.959399 |
| GO:0070193\_synaptonemal\_complex\_organization | 8 | 0 | 0.000000 | -0.000000 | 1232 | 1103.274183 | 1181.98 | 1260.685817 | 0.959399 |
| GO:0070231\_T\_cell\_apoptosis | 8 | 0 | 0.000000 | -0.000000 | 1232 | 1103.274183 | 1181.98 | 1260.685817 | 0.959399 |
| GO:0070584\_mitochondrion\_morphogenesis | 8 | 0 | 0.000000 | -0.000000 | 1232 | 1103.274183 | 1181.98 | 1260.685817 | 0.959399 |
| GO:0007281\_germ\_cell\_development | 75 | 0 | 0.000000 | -0.000000 | 1234 | 1106.311875 | 1184.66 | 1263.008125 | 0.960016 |
| GO:0051050\_positive\_regulation\_of\_transport | 75 | 0 | 0.000000 | -0.000000 | 1234 | 1106.311875 | 1184.66 | 1263.008125 | 0.960016 |
| GO:0003013\_circulatory\_system\_process | 103 | 0 | 0.000000 | -0.000000 | 1236 | 1108.658326 | 1186.8 | 1264.941674 | 0.960194 |
| GO:0008015\_blood\_circulation | 103 | 0 | 0.000000 | -0.000000 | 1236 | 1108.658326 | 1186.8 | 1264.941674 | 0.960194 |
| GO:0001819\_positive\_regulation\_of\_cytokine\_production | 36 | 0 | 0.000000 | -0.000000 | 1249 | 1124.307345 | 1201.29 | 1278.272655 | 0.961801 |
| GO:0001889\_liver\_development | 36 | 0 | 0.000000 | -0.000000 | 1249 | 1124.307345 | 1201.29 | 1278.272655 | 0.961801 |
| GO:0007187\_G-protein\_signaling\_\_coupled\_to\_cyclic\_nucleotide\_second\_messenger | 36 | 0 | 0.000000 | -0.000000 | 1249 | 1124.307345 | 1201.29 | 1278.272655 | 0.961801 |
| GO:0007631\_feeding\_behavior | 36 | 0 | 0.000000 | -0.000000 | 1249 | 1124.307345 | 1201.29 | 1278.272655 | 0.961801 |
| GO:0014020\_primary\_neural\_tube\_formation | 36 | 0 | 0.000000 | -0.000000 | 1249 | 1124.307345 | 1201.29 | 1278.272655 | 0.961801 |
| GO:0019228\_regulation\_of\_action\_potential\_in\_neuron | 36 | 0 | 0.000000 | -0.000000 | 1249 | 1124.307345 | 1201.29 | 1278.272655 | 0.961801 |
| GO:0022602\_ovulation\_cycle\_process | 36 | 0 | 0.000000 | -0.000000 | 1249 | 1124.307345 | 1201.29 | 1278.272655 | 0.961801 |
| GO:0030072\_peptide\_hormone\_secretion | 36 | 0 | 0.000000 | -0.000000 | 1249 | 1124.307345 | 1201.29 | 1278.272655 | 0.961801 |
| GO:0030278\_regulation\_of\_ossification | 36 | 0 | 0.000000 | -0.000000 | 1249 | 1124.307345 | 1201.29 | 1278.272655 | 0.961801 |
| GO:0042742\_defense\_response\_to\_bacterium | 36 | 0 | 0.000000 | -0.000000 | 1249 | 1124.307345 | 1201.29 | 1278.272655 | 0.961801 |
| GO:0050851\_antigen\_receptor-mediated\_signaling\_pathway | 36 | 0 | 0.000000 | -0.000000 | 1249 | 1124.307345 | 1201.29 | 1278.272655 | 0.961801 |
| GO:0050900\_leukocyte\_migration | 36 | 0 | 0.000000 | -0.000000 | 1249 | 1124.307345 | 1201.29 | 1278.272655 | 0.961801 |
| GO:0051223\_regulation\_of\_protein\_transport | 36 | 0 | 0.000000 | -0.000000 | 1249 | 1124.307345 | 1201.29 | 1278.272655 | 0.961801 |
| GO:0000165\_MAPKKK\_cascade | 114 | 0 | 0.000000 | -0.000000 | 1250 | 1125.564141 | 1202.35 | 1279.135859 | 0.961880 |
| GO:0051186\_cofactor\_metabolic\_process | 63 | 0 | 0.000000 | -0.000000 | 1253 | 1130.279090 | 1206.52 | 1282.760910 | 0.962905 |
| GO:0070662\_mast\_cell\_proliferation | 63 | 0 | 0.000000 | -0.000000 | 1253 | 1130.279090 | 1206.52 | 1282.760910 | 0.962905 |
| GO:0070666\_regulation\_of\_mast\_cell\_proliferation | 63 | 0 | 0.000000 | -0.000000 | 1253 | 1130.279090 | 1206.52 | 1282.760910 | 0.962905 |
| GO:0001932\_regulation\_of\_protein\_amino\_acid\_phosphorylation | 69 | 0 | 0.000000 | -0.000000 | 1257 | 1134.740240 | 1210.35 | 1285.959760 | 0.962888 |
| GO:0006816\_calcium\_ion\_transport | 69 | 0 | 0.000000 | -0.000000 | 1257 | 1134.740240 | 1210.35 | 1285.959760 | 0.962888 |
| GO:0032101\_regulation\_of\_response\_to\_external\_stimulus | 69 | 0 | 0.000000 | -0.000000 | 1257 | 1134.740240 | 1210.35 | 1285.959760 | 0.962888 |
| GO:0055065\_metal\_ion\_homeostasis | 69 | 0 | 0.000000 | -0.000000 | 1257 | 1134.740240 | 1210.35 | 1285.959760 | 0.962888 |
| GO:0001776\_leukocyte\_homeostasis | 41 | 0 | 0.000000 | -0.000000 | 1273 | 1151.536917 | 1225.91 | 1300.283083 | 0.963009 |
| GO:0002429\_immune\_response-activating\_cell\_surface\_receptor\_signaling\_pathway | 41 | 0 | 0.000000 | -0.000000 | 1273 | 1151.536917 | 1225.91 | 1300.283083 | 0.963009 |
| GO:0006836\_neurotransmitter\_transport | 41 | 0 | 0.000000 | -0.000000 | 1273 | 1151.536917 | 1225.91 | 1300.283083 | 0.963009 |
| GO:0006865\_amino\_acid\_transport | 41 | 0 | 0.000000 | -0.000000 | 1273 | 1151.536917 | 1225.91 | 1300.283083 | 0.963009 |
| GO:0006979\_response\_to\_oxidative\_stress | 41 | 0 | 0.000000 | -0.000000 | 1273 | 1151.536917 | 1225.91 | 1300.283083 | 0.963009 |
| GO:0007254\_JNK\_cascade | 41 | 0 | 0.000000 | -0.000000 | 1273 | 1151.536917 | 1225.91 | 1300.283083 | 0.963009 |
| GO:0008585\_female\_gonad\_development | 41 | 0 | 0.000000 | -0.000000 | 1273 | 1151.536917 | 1225.91 | 1300.283083 | 0.963009 |
| GO:0009894\_regulation\_of\_catabolic\_process | 41 | 0 | 0.000000 | -0.000000 | 1273 | 1151.536917 | 1225.91 | 1300.283083 | 0.963009 |
| GO:0015833\_peptide\_transport | 41 | 0 | 0.000000 | -0.000000 | 1273 | 1151.536917 | 1225.91 | 1300.283083 | 0.963009 |
| GO:0019216\_regulation\_of\_lipid\_metabolic\_process | 41 | 0 | 0.000000 | -0.000000 | 1273 | 1151.536917 | 1225.91 | 1300.283083 | 0.963009 |
| GO:0019748\_secondary\_metabolic\_process | 41 | 0 | 0.000000 | -0.000000 | 1273 | 1151.536917 | 1225.91 | 1300.283083 | 0.963009 |
| GO:0030817\_regulation\_of\_cAMP\_biosynthetic\_process | 41 | 0 | 0.000000 | -0.000000 | 1273 | 1151.536917 | 1225.91 | 1300.283083 | 0.963009 |
| GO:0031344\_regulation\_of\_cell\_projection\_organization | 41 | 0 | 0.000000 | -0.000000 | 1273 | 1151.536917 | 1225.91 | 1300.283083 | 0.963009 |
| GO:0032844\_regulation\_of\_homeostatic\_process | 41 | 0 | 0.000000 | -0.000000 | 1273 | 1151.536917 | 1225.91 | 1300.283083 | 0.963009 |
| GO:0033077\_T\_cell\_differentiation\_in\_the\_thymus | 41 | 0 | 0.000000 | -0.000000 | 1273 | 1151.536917 | 1225.91 | 1300.283083 | 0.963009 |
| GO:0050864\_regulation\_of\_B\_cell\_activation | 41 | 0 | 0.000000 | -0.000000 | 1273 | 1151.536917 | 1225.91 | 1300.283083 | 0.963009 |
| GO:0044057\_regulation\_of\_system\_process | 133 | 0 | 0.000000 | -0.000000 | 1274 | 1152.606288 | 1226.85 | 1301.093712 | 0.962991 |
| GO:0016568\_chromatin\_modification | 72 | 0 | 0.000000 | -0.000000 | 1279 | 1160.806361 | 1234.2 | 1307.593639 | 0.964973 |
| GO:0042098\_T\_cell\_proliferation | 72 | 0 | 0.000000 | -0.000000 | 1279 | 1160.806361 | 1234.2 | 1307.593639 | 0.964973 |
| GO:0044262\_cellular\_carbohydrate\_metabolic\_process | 72 | 0 | 0.000000 | -0.000000 | 1279 | 1160.806361 | 1234.2 | 1307.593639 | 0.964973 |
| GO:0048839\_inner\_ear\_development | 72 | 0 | 0.000000 | -0.000000 | 1279 | 1160.806361 | 1234.2 | 1307.593639 | 0.964973 |
| GO:0051347\_positive\_regulation\_of\_transferase\_activity | 72 | 0 | 0.000000 | -0.000000 | 1279 | 1160.806361 | 1234.2 | 1307.593639 | 0.964973 |
| GO:0001505\_regulation\_of\_neurotransmitter\_levels | 48 | 0 | 0.000000 | -0.000000 | 1283 | 1166.255295 | 1239.35 | 1312.444705 | 0.965978 |
| GO:0009101\_glycoprotein\_biosynthetic\_process | 48 | 0 | 0.000000 | -0.000000 | 1283 | 1166.255295 | 1239.35 | 1312.444705 | 0.965978 |
| GO:0034504\_protein\_localization\_in\_nucleus | 48 | 0 | 0.000000 | -0.000000 | 1283 | 1166.255295 | 1239.35 | 1312.444705 | 0.965978 |
| GO:0046849\_bone\_remodeling | 48 | 0 | 0.000000 | -0.000000 | 1283 | 1166.255295 | 1239.35 | 1312.444705 | 0.965978 |
| GO:0002253\_activation\_of\_immune\_response | 54 | 0 | 0.000000 | -0.000000 | 1286 | 1173.045328 | 1245.68 | 1318.314672 | 0.968647 |
| GO:0006412\_translation | 54 | 0 | 0.000000 | -0.000000 | 1286 | 1173.045328 | 1245.68 | 1318.314672 | 0.968647 |
| GO:0015849\_organic\_acid\_transport | 54 | 0 | 0.000000 | -0.000000 | 1286 | 1173.045328 | 1245.68 | 1318.314672 | 0.968647 |
| GO:0002440\_production\_of\_molecular\_mediator\_of\_immune\_response | 49 | 0 | 0.000000 | -0.000000 | 1295 | 1182.691950 | 1254.6 | 1326.508050 | 0.968803 |
| GO:0003015\_heart\_process | 49 | 0 | 0.000000 | -0.000000 | 1295 | 1182.691950 | 1254.6 | 1326.508050 | 0.968803 |
| GO:0006725\_cellular\_aromatic\_compound\_metabolic\_process | 49 | 0 | 0.000000 | -0.000000 | 1295 | 1182.691950 | 1254.6 | 1326.508050 | 0.968803 |
| GO:0007606\_sensory\_perception\_of\_chemical\_stimulus | 49 | 0 | 0.000000 | -0.000000 | 1295 | 1182.691950 | 1254.6 | 1326.508050 | 0.968803 |
| GO:0034101\_erythrocyte\_homeostasis | 49 | 0 | 0.000000 | -0.000000 | 1295 | 1182.691950 | 1254.6 | 1326.508050 | 0.968803 |
| GO:0042035\_regulation\_of\_cytokine\_biosynthetic\_process | 49 | 0 | 0.000000 | -0.000000 | 1295 | 1182.691950 | 1254.6 | 1326.508050 | 0.968803 |
| GO:0043473\_pigmentation | 49 | 0 | 0.000000 | -0.000000 | 1295 | 1182.691950 | 1254.6 | 1326.508050 | 0.968803 |
| GO:0046660\_female\_sex\_differentiation | 49 | 0 | 0.000000 | -0.000000 | 1295 | 1182.691950 | 1254.6 | 1326.508050 | 0.968803 |
| GO:0060047\_heart\_contraction | 49 | 0 | 0.000000 | -0.000000 | 1295 | 1182.691950 | 1254.6 | 1326.508050 | 0.968803 |
| GO:0001525\_angiogenesis | 100 | 0 | 0.000000 | -0.000000 | 1296 | 1183.274382 | 1255.14 | 1327.005618 | 0.968472 |
| GO:0007611\_learning\_or\_memory | 70 | 0 | 0.000000 | -0.000000 | 1299 | 1187.064193 | 1258.41 | 1329.755807 | 0.968753 |
| GO:0009617\_response\_to\_bacterium | 70 | 0 | 0.000000 | -0.000000 | 1299 | 1187.064193 | 1258.41 | 1329.755807 | 0.968753 |
| GO:0070838\_divalent\_metal\_ion\_transport | 70 | 0 | 0.000000 | -0.000000 | 1299 | 1187.064193 | 1258.41 | 1329.755807 | 0.968753 |
| GO:0003018\_vascular\_process\_in\_circulatory\_system | 31 | 0 | 0.000000 | -0.000000 | 1329 | 1215.221295 | 1285.05 | 1354.878705 | 0.966930 |
| GO:0006486\_protein\_amino\_acid\_glycosylation | 31 | 0 | 0.000000 | -0.000000 | 1329 | 1215.221295 | 1285.05 | 1354.878705 | 0.966930 |
| GO:0006639\_acylglycerol\_metabolic\_process | 31 | 0 | 0.000000 | -0.000000 | 1329 | 1215.221295 | 1285.05 | 1354.878705 | 0.966930 |
| GO:0006665\_sphingolipid\_metabolic\_process | 31 | 0 | 0.000000 | -0.000000 | 1329 | 1215.221295 | 1285.05 | 1354.878705 | 0.966930 |
| GO:0006694\_steroid\_biosynthetic\_process | 31 | 0 | 0.000000 | -0.000000 | 1329 | 1215.221295 | 1285.05 | 1354.878705 | 0.966930 |
| GO:0006939\_smooth\_muscle\_contraction | 31 | 0 | 0.000000 | -0.000000 | 1329 | 1215.221295 | 1285.05 | 1354.878705 | 0.966930 |
| GO:0008645\_hexose\_transport | 31 | 0 | 0.000000 | -0.000000 | 1329 | 1215.221295 | 1285.05 | 1354.878705 | 0.966930 |
| GO:0009306\_protein\_secretion | 31 | 0 | 0.000000 | -0.000000 | 1329 | 1215.221295 | 1285.05 | 1354.878705 | 0.966930 |
| GO:0010562\_positive\_regulation\_of\_phosphorus\_metabolic\_process | 31 | 0 | 0.000000 | -0.000000 | 1329 | 1215.221295 | 1285.05 | 1354.878705 | 0.966930 |
| GO:0015749\_monosaccharide\_transport | 31 | 0 | 0.000000 | -0.000000 | 1329 | 1215.221295 | 1285.05 | 1354.878705 | 0.966930 |
| GO:0015758\_glucose\_transport | 31 | 0 | 0.000000 | -0.000000 | 1329 | 1215.221295 | 1285.05 | 1354.878705 | 0.966930 |
| GO:0021954\_central\_nervous\_system\_neuron\_development | 31 | 0 | 0.000000 | -0.000000 | 1329 | 1215.221295 | 1285.05 | 1354.878705 | 0.966930 |
| GO:0033555\_multicellular\_organismal\_response\_to\_stress | 31 | 0 | 0.000000 | -0.000000 | 1329 | 1215.221295 | 1285.05 | 1354.878705 | 0.966930 |
| GO:0035150\_regulation\_of\_tube\_size | 31 | 0 | 0.000000 | -0.000000 | 1329 | 1215.221295 | 1285.05 | 1354.878705 | 0.966930 |
| GO:0042157\_lipoprotein\_metabolic\_process | 31 | 0 | 0.000000 | -0.000000 | 1329 | 1215.221295 | 1285.05 | 1354.878705 | 0.966930 |
| GO:0042327\_positive\_regulation\_of\_phosphorylation | 31 | 0 | 0.000000 | -0.000000 | 1329 | 1215.221295 | 1285.05 | 1354.878705 | 0.966930 |
| GO:0043269\_regulation\_of\_ion\_transport | 31 | 0 | 0.000000 | -0.000000 | 1329 | 1215.221295 | 1285.05 | 1354.878705 | 0.966930 |
| GO:0043413\_biopolymer\_glycosylation | 31 | 0 | 0.000000 | -0.000000 | 1329 | 1215.221295 | 1285.05 | 1354.878705 | 0.966930 |
| GO:0045088\_regulation\_of\_innate\_immune\_response | 31 | 0 | 0.000000 | -0.000000 | 1329 | 1215.221295 | 1285.05 | 1354.878705 | 0.966930 |
| GO:0045937\_positive\_regulation\_of\_phosphate\_metabolic\_process | 31 | 0 | 0.000000 | -0.000000 | 1329 | 1215.221295 | 1285.05 | 1354.878705 | 0.966930 |
| GO:0046632\_alpha-beta\_T\_cell\_differentiation | 31 | 0 | 0.000000 | -0.000000 | 1329 | 1215.221295 | 1285.05 | 1354.878705 | 0.966930 |
| GO:0048167\_regulation\_of\_synaptic\_plasticity | 31 | 0 | 0.000000 | -0.000000 | 1329 | 1215.221295 | 1285.05 | 1354.878705 | 0.966930 |
| GO:0048562\_embryonic\_organ\_morphogenesis | 31 | 0 | 0.000000 | -0.000000 | 1329 | 1215.221295 | 1285.05 | 1354.878705 | 0.966930 |
| GO:0050868\_negative\_regulation\_of\_T\_cell\_activation | 31 | 0 | 0.000000 | -0.000000 | 1329 | 1215.221295 | 1285.05 | 1354.878705 | 0.966930 |
| GO:0050880\_regulation\_of\_blood\_vessel\_size | 31 | 0 | 0.000000 | -0.000000 | 1329 | 1215.221295 | 1285.05 | 1354.878705 | 0.966930 |
| GO:0051640\_organelle\_localization | 31 | 0 | 0.000000 | -0.000000 | 1329 | 1215.221295 | 1285.05 | 1354.878705 | 0.966930 |
| GO:0051899\_membrane\_depolarization | 31 | 0 | 0.000000 | -0.000000 | 1329 | 1215.221295 | 1285.05 | 1354.878705 | 0.966930 |
| GO:0055088\_lipid\_homeostasis | 31 | 0 | 0.000000 | -0.000000 | 1329 | 1215.221295 | 1285.05 | 1354.878705 | 0.966930 |
| GO:0060512\_prostate\_gland\_morphogenesis | 31 | 0 | 0.000000 | -0.000000 | 1329 | 1215.221295 | 1285.05 | 1354.878705 | 0.966930 |
| GO:0070085\_glycosylation | 31 | 0 | 0.000000 | -0.000000 | 1329 | 1215.221295 | 1285.05 | 1354.878705 | 0.966930 |
| GO:0002764\_immune\_response-regulating\_signal\_transduction | 51 | 0 | 0.000000 | -0.000000 | 1336 | 1223.552601 | 1292.86 | 1362.167399 | 0.967710 |
| GO:0006520\_cellular\_amino\_acid\_metabolic\_process | 51 | 0 | 0.000000 | -0.000000 | 1336 | 1223.552601 | 1292.86 | 1362.167399 | 0.967710 |
| GO:0006887\_exocytosis | 51 | 0 | 0.000000 | -0.000000 | 1336 | 1223.552601 | 1292.86 | 1362.167399 | 0.967710 |
| GO:0007601\_visual\_perception | 51 | 0 | 0.000000 | -0.000000 | 1336 | 1223.552601 | 1292.86 | 1362.167399 | 0.967710 |
| GO:0016569\_covalent\_chromatin\_modification | 51 | 0 | 0.000000 | -0.000000 | 1336 | 1223.552601 | 1292.86 | 1362.167399 | 0.967710 |
| GO:0043408\_regulation\_of\_MAPKKK\_cascade | 51 | 0 | 0.000000 | -0.000000 | 1336 | 1223.552601 | 1292.86 | 1362.167399 | 0.967710 |
| GO:0044106\_cellular\_amine\_metabolic\_process | 51 | 0 | 0.000000 | -0.000000 | 1336 | 1223.552601 | 1292.86 | 1362.167399 | 0.967710 |
| GO:0000084\_S\_phase\_of\_mitotic\_cell\_cycle | 3 | 0 |  |  |  |  |  |  |  |  |
| GO:0000089\_mitotic\_metaphase | 3 | 0 |  |  |  |  |  |  |  |  |
| GO:0000098\_sulfur\_amino\_acid\_catabolic\_process | 3 | 0 |  |  |  |  |  |  |  |  |
| GO:0000103\_sulfate\_assimilation | 3 | 0 |  |  |  |  |  |  |  |  |
| GO:0000212\_meiotic\_spindle\_organization | 3 | 0 |  |  |  |  |  |  |  |  |
| GO:0000281\_cytokinesis\_after\_mitosis | 3 | 0 |  |  |  |  |  |  |  |  |
| GO:0000303\_response\_to\_superoxide | 3 | 0 |  |  |  |  |  |  |  |  |
| GO:0000380\_alternative\_nuclear\_mRNA\_splicing\_\_via\_spliceosome | 3 | 0 |  |  |  |  |  |  |  |  |
| GO:0001516\_prostaglandin\_biosynthetic\_process | 3 | 0 |  |  |  |  |  |  |  |  |
| GO:0001553\_luteinization | 3 | 0 |  |  |  |  |  |  |  |  |
| GO:0001574\_ganglioside\_biosynthetic\_process | 3 | 0 |  |  |  |  |  |  |  |  |
| GO:0001705\_ectoderm\_formation | 3 | 0 |  |  |  |  |  |  |  |  |
| GO:0001711\_endodermal\_cell\_fate\_commitment | 3 | 0 |  |  |  |  |  |  |  |  |
| GO:0001757\_somite\_specification | 3 | 0 |  |  |  |  |  |  |  |  |
| GO:0001778\_plasma\_membrane\_repair | 3 | 0 |  |  |  |  |  |  |  |  |
| GO:0001780\_neutrophil\_homeostasis | 3 | 0 |  |  |  |  |  |  |  |  |
| GO:0001802\_type\_III\_hypersensitivity | 3 | 0 |  |  |  |  |  |  |  |  |
| GO:0001803\_regulation\_of\_type\_III\_hypersensitivity | 3 | 0 |  |  |  |  |  |  |  |  |
| GO:0001805\_positive\_regulation\_of\_type\_III\_hypersensitivity | 3 | 0 |  |  |  |  |  |  |  |  |
| GO:0001812\_positive\_regulation\_of\_type\_I\_hypersensitivity | 3 | 0 |  |  |  |  |  |  |  |  |
| GO:0001831\_trophectodermal\_cellular\_morphogenesis | 3 | 0 |  |  |  |  |  |  |  |  |
| GO:0001844\_protein\_insertion\_into\_mitochondrial\_membrane\_during\_induction\_of\_apoptosis | 3 | 0 |  |  |  |  |  |  |  |  |
| GO:0001878\_response\_to\_yeast | 3 | 0 |  |  |  |  |  |  |  |  |
| GO:0001895\_retina\_homeostasis | 3 | 0 |  |  |  |  |  |  |  |  |
| GO:0001915\_negative\_regulation\_of\_T\_cell\_mediated\_cytotoxicity | 3 | 0 |  |  |  |  |  |  |  |  |
| GO:0001937\_negative\_regulation\_of\_endothelial\_cell\_proliferation | 3 | 0 |  |  |  |  |  |  |  |  |
| GO:0001953\_negative\_regulation\_of\_cell-matrix\_adhesion | 3 | 0 |  |  |  |  |  |  |  |  |
| GO:0001955\_blood\_vessel\_maturation | 3 | 0 |  |  |  |  |  |  |  |  |
| GO:0001960\_negative\_regulation\_of\_cytokine-mediated\_signaling\_pathway | 3 | 0 |  |  |  |  |  |  |  |  |
| GO:0001973\_adenosine\_receptor\_signaling\_pathway | 3 | 0 |  |  |  |  |  |  |  |  |
| GO:0001996\_positive\_regulation\_of\_heart\_rate\_by\_epinephrine-norepinephrine | 3 | 0 |  |  |  |  |  |  |  |  |
| GO:0002034\_regulation\_of\_blood\_vessel\_size\_by\_renin-angiotensin | 3 | 0 |  |  |  |  |  |  |  |  |
| GO:0002238\_response\_to\_molecule\_of\_fungal\_origin | 3 | 0 |  |  |  |  |  |  |  |  |
| GO:0002275\_myeloid\_cell\_activation\_during\_immune\_response | 3 | 0 |  |  |  |  |  |  |  |  |
| GO:0002281\_macrophage\_activation\_during\_immune\_response | 3 | 0 |  |  |  |  |  |  |  |  |
| GO:0002309\_T\_cell\_proliferation\_during\_immune\_response | 3 | 0 |  |  |  |  |  |  |  |  |
| GO:0002361\_CD4-positive\_\_CD25-positive\_\_alpha-beta\_regulatory\_T\_cell\_differentiation | 3 | 0 |  |  |  |  |  |  |  |  |
| GO:0002369\_T\_cell\_cytokine\_production | 3 | 0 |  |  |  |  |  |  |  |  |
| GO:0002428\_antigen\_processing\_and\_presentation\_of\_peptide\_antigen\_via\_MHC\_class\_Ib | 3 | 0 |  |  |  |  |  |  |  |  |
| GO:0002446\_neutrophil\_mediated\_immunity | 3 | 0 |  |  |  |  |  |  |  |  |
| GO:0002477\_antigen\_processing\_and\_presentation\_of\_exogenous\_peptide\_antigen\_via\_MHC\_class\_Ib | 3 | 0 |  |  |  |  |  |  |  |  |
| GO:0002481\_antigen\_processing\_and\_presentation\_of\_exogenous\_protein\_antigen\_via\_MHC\_class\_Ib\_\_TAP-dependent | 3 | 0 |  |  |  |  |  |  |  |  |
| GO:0002513\_tolerance\_induction\_to\_self\_antigen | 3 | 0 |  |  |  |  |  |  |  |  |
| GO:0002568\_somatic\_diversification\_of\_T\_cell\_receptor\_genes | 3 | 0 |  |  |  |  |  |  |  |  |
| GO:0002674\_negative\_regulation\_of\_acute\_inflammatory\_response | 3 | 0 |  |  |  |  |  |  |  |  |
| GO:0002681\_somatic\_recombination\_of\_T\_cell\_receptor\_gene\_segments | 3 | 0 |  |  |  |  |  |  |  |  |
| GO:0002713\_negative\_regulation\_of\_B\_cell\_mediated\_immunity | 3 | 0 |  |  |  |  |  |  |  |  |
| GO:0002827\_positive\_regulation\_of\_T-helper\_1\_type\_immune\_response | 3 | 0 |  |  |  |  |  |  |  |  |
| GO:0002865\_negative\_regulation\_of\_acute\_inflammatory\_response\_to\_antigenic\_stimulus | 3 | 0 |  |  |  |  |  |  |  |  |
| GO:0002884\_negative\_regulation\_of\_hypersensitivity | 3 | 0 |  |  |  |  |  |  |  |  |
| GO:0002890\_negative\_regulation\_of\_immunoglobulin\_mediated\_immune\_response | 3 | 0 |  |  |  |  |  |  |  |  |
| GO:0002904\_positive\_regulation\_of\_B\_cell\_apoptosis | 3 | 0 |  |  |  |  |  |  |  |  |
| GO:0003009\_skeletal\_muscle\_contraction | 3 | 0 |  |  |  |  |  |  |  |  |
| GO:0003072\_renal\_control\_of\_peripheral\_vascular\_resistance\_involved\_in\_regulation\_of\_systemic\_arterial\_blood\_pressure | 3 | 0 |  |  |  |  |  |  |  |  |
| GO:0006047\_UDP-N-acetylglucosamine\_metabolic\_process | 3 | 0 |  |  |  |  |  |  |  |  |
| GO:0006067\_ethanol\_metabolic\_process | 3 | 0 |  |  |  |  |  |  |  |  |
| GO:0006072\_glycerol-3-phosphate\_metabolic\_process | 3 | 0 |  |  |  |  |  |  |  |  |
| GO:0006103\_2-oxoglutarate\_metabolic\_process | 3 | 0 |  |  |  |  |  |  |  |  |
| GO:0006107\_oxaloacetate\_metabolic\_process | 3 | 0 |  |  |  |  |  |  |  |  |
| GO:0006220\_pyrimidine\_nucleotide\_metabolic\_process | 3 | 0 |  |  |  |  |  |  |  |  |
| GO:0006266\_DNA\_ligation | 3 | 0 |  |  |  |  |  |  |  |  |
| GO:0006282\_regulation\_of\_DNA\_repair | 3 | 0 |  |  |  |  |  |  |  |  |
| GO:0006287\_base-excision\_repair\_\_gap-filling | 3 | 0 |  |  |  |  |  |  |  |  |
| GO:0006301\_postreplication\_repair | 3 | 0 |  |  |  |  |  |  |  |  |
| GO:0006361\_transcription\_initiation\_from\_RNA\_polymerase\_I\_promoter | 3 | 0 |  |  |  |  |  |  |  |  |
| GO:0006367\_transcription\_initiation\_from\_RNA\_polymerase\_II\_promoter | 3 | 0 |  |  |  |  |  |  |  |  |
| GO:0006414\_translational\_elongation | 3 | 0 |  |  |  |  |  |  |  |  |
| GO:0006491\_N-glycan\_processing | 3 | 0 |  |  |  |  |  |  |  |  |
| GO:0006498\_N-terminal\_protein\_lipidation | 3 | 0 |  |  |  |  |  |  |  |  |
| GO:0006531\_aspartate\_metabolic\_process | 3 | 0 |  |  |  |  |  |  |  |  |
| GO:0006598\_polyamine\_catabolic\_process | 3 | 0 |  |  |  |  |  |  |  |  |
| GO:0006620\_posttranslational\_protein\_targeting\_to\_membrane | 3 | 0 |  |  |  |  |  |  |  |  |
| GO:0006625\_protein\_targeting\_to\_peroxisome | 3 | 0 |  |  |  |  |  |  |  |  |
| GO:0006651\_diacylglycerol\_biosynthetic\_process | 3 | 0 |  |  |  |  |  |  |  |  |
| GO:0006670\_sphingosine\_metabolic\_process | 3 | 0 |  |  |  |  |  |  |  |  |
| GO:0006677\_glycosylceramide\_metabolic\_process | 3 | 0 |  |  |  |  |  |  |  |  |
| GO:0006689\_ganglioside\_catabolic\_process | 3 | 0 |  |  |  |  |  |  |  |  |
| GO:0006699\_bile\_acid\_biosynthetic\_process | 3 | 0 |  |  |  |  |  |  |  |  |
| GO:0006791\_sulfur\_utilization | 3 | 0 |  |  |  |  |  |  |  |  |
| GO:0006817\_phosphate\_transport | 3 | 0 |  |  |  |  |  |  |  |  |
| GO:0006825\_copper\_ion\_transport | 3 | 0 |  |  |  |  |  |  |  |  |
| GO:0006828\_manganese\_ion\_transport | 3 | 0 |  |  |  |  |  |  |  |  |
| GO:0006857\_oligopeptide\_transport | 3 | 0 |  |  |  |  |  |  |  |  |
| GO:0006892\_post-Golgi\_vesicle-mediated\_transport | 3 | 0 |  |  |  |  |  |  |  |  |
| GO:0006904\_vesicle\_docking\_during\_exocytosis | 3 | 0 |  |  |  |  |  |  |  |  |
| GO:0006926\_virus-infected\_cell\_apoptosis | 3 | 0 |  |  |  |  |  |  |  |  |
| GO:0006953\_acute-phase\_response | 3 | 0 |  |  |  |  |  |  |  |  |
| GO:0007000\_nucleolus\_organization | 3 | 0 |  |  |  |  |  |  |  |  |
| GO:0007041\_lysosomal\_transport | 3 | 0 |  |  |  |  |  |  |  |  |
| GO:0007043\_cell-cell\_junction\_assembly | 3 | 0 |  |  |  |  |  |  |  |  |
| GO:0007090\_regulation\_of\_S\_phase\_of\_mitotic\_cell\_cycle | 3 | 0 |  |  |  |  |  |  |  |  |
| GO:0007195\_inhibition\_of\_adenylate\_cyclase\_activity\_by\_dopamine\_receptor\_signaling\_pathway | 3 | 0 |  |  |  |  |  |  |  |  |
| GO:0007199\_G-protein\_signaling\_\_coupled\_to\_cGMP\_nucleotide\_second\_messenger | 3 | 0 |  |  |  |  |  |  |  |  |
| GO:0007213\_muscarinic\_acetylcholine\_receptor\_signaling\_pathway | 3 | 0 |  |  |  |  |  |  |  |  |
| GO:0007250\_activation\_of\_NF-kappaB-inducing\_kinase\_activity | 3 | 0 |  |  |  |  |  |  |  |  |
| GO:0007252\_I-kappaB\_phosphorylation | 3 | 0 |  |  |  |  |  |  |  |  |
| GO:0007262\_STAT\_protein\_nuclear\_translocation | 3 | 0 |  |  |  |  |  |  |  |  |
| GO:0007288\_sperm\_axoneme\_assembly | 3 | 0 |  |  |  |  |  |  |  |  |
| GO:0007350\_blastoderm\_segmentation | 3 | 0 |  |  |  |  |  |  |  |  |
| GO:0007403\_glial\_cell\_fate\_determination | 3 | 0 |  |  |  |  |  |  |  |  |
| GO:0007412\_axon\_target\_recognition | 3 | 0 |  |  |  |  |  |  |  |  |
| GO:0007468\_regulation\_of\_rhodopsin\_gene\_expression | 3 | 0 |  |  |  |  |  |  |  |  |
| GO:0007525\_somatic\_muscle\_development | 3 | 0 |  |  |  |  |  |  |  |  |
| GO:0007635\_chemosensory\_behavior | 3 | 0 |  |  |  |  |  |  |  |  |
| GO:0008090\_retrograde\_axon\_cargo\_transport | 3 | 0 |  |  |  |  |  |  |  |  |
| GO:0008347\_glial\_cell\_migration | 3 | 0 |  |  |  |  |  |  |  |  |
| GO:0008635\_activation\_of\_caspase\_activity\_by\_cytochrome\_c | 3 | 0 |  |  |  |  |  |  |  |  |
| GO:0009060\_aerobic\_respiration | 3 | 0 |  |  |  |  |  |  |  |  |
| GO:0009081\_branched\_chain\_family\_amino\_acid\_metabolic\_process | 3 | 0 |  |  |  |  |  |  |  |  |
| GO:0009086\_methionine\_biosynthetic\_process | 3 | 0 |  |  |  |  |  |  |  |  |
| GO:0009135\_purine\_nucleoside\_diphosphate\_metabolic\_process | 3 | 0 |  |  |  |  |  |  |  |  |
| GO:0009137\_purine\_nucleoside\_diphosphate\_catabolic\_process | 3 | 0 |  |  |  |  |  |  |  |  |
| GO:0009155\_purine\_deoxyribonucleotide\_catabolic\_process | 3 | 0 |  |  |  |  |  |  |  |  |
| GO:0009179\_purine\_ribonucleoside\_diphosphate\_metabolic\_process | 3 | 0 |  |  |  |  |  |  |  |  |
| GO:0009181\_purine\_ribonucleoside\_diphosphate\_catabolic\_process | 3 | 0 |  |  |  |  |  |  |  |  |
| GO:0009185\_ribonucleoside\_diphosphate\_metabolic\_process | 3 | 0 |  |  |  |  |  |  |  |  |
| GO:0009191\_ribonucleoside\_diphosphate\_catabolic\_process | 3 | 0 |  |  |  |  |  |  |  |  |
| GO:0009199\_ribonucleoside\_triphosphate\_metabolic\_process | 3 | 0 |  |  |  |  |  |  |  |  |
| GO:0009204\_deoxyribonucleoside\_triphosphate\_catabolic\_process | 3 | 0 |  |  |  |  |  |  |  |  |
| GO:0009205\_purine\_ribonucleoside\_triphosphate\_metabolic\_process | 3 | 0 |  |  |  |  |  |  |  |  |
| GO:0009217\_purine\_deoxyribonucleoside\_triphosphate\_catabolic\_process | 3 | 0 |  |  |  |  |  |  |  |  |
| GO:0009448\_gamma-aminobutyric\_acid\_metabolic\_process | 3 | 0 |  |  |  |  |  |  |  |  |
| GO:0010043\_response\_to\_zinc\_ion | 3 | 0 |  |  |  |  |  |  |  |  |
| GO:0010159\_specification\_of\_organ\_position | 3 | 0 |  |  |  |  |  |  |  |  |
| GO:0010172\_embryonic\_body\_morphogenesis | 3 | 0 |  |  |  |  |  |  |  |  |
| GO:0010216\_maintenance\_of\_DNA\_methylation | 3 | 0 |  |  |  |  |  |  |  |  |
| GO:0010273\_detoxification\_of\_copper\_ion | 3 | 0 |  |  |  |  |  |  |  |  |
| GO:0010454\_negative\_regulation\_of\_cell\_fate\_commitment | 3 | 0 |  |  |  |  |  |  |  |  |
| GO:0010507\_negative\_regulation\_of\_autophagy | 3 | 0 |  |  |  |  |  |  |  |  |
| GO:0010524\_positive\_regulation\_of\_calcium\_ion\_transport\_into\_cytosol | 3 | 0 |  |  |  |  |  |  |  |  |
| GO:0010573\_vascular\_endothelial\_growth\_factor\_production | 3 | 0 |  |  |  |  |  |  |  |  |
| GO:0010574\_regulation\_of\_vascular\_endothelial\_growth\_factor\_production | 3 | 0 |  |  |  |  |  |  |  |  |
| GO:0010575\_positive\_regulation\_vascular\_endothelial\_growth\_factor\_production | 3 | 0 |  |  |  |  |  |  |  |  |
| GO:0010632\_regulation\_of\_epithelial\_cell\_migration | 3 | 0 |  |  |  |  |  |  |  |  |
| GO:0010717\_regulation\_of\_epithelial\_to\_mesenchymal\_transition | 3 | 0 |  |  |  |  |  |  |  |  |
| GO:0010884\_positive\_regulation\_of\_lipid\_storage | 3 | 0 |  |  |  |  |  |  |  |  |
| GO:0010888\_negative\_regulation\_of\_lipid\_storage | 3 | 0 |  |  |  |  |  |  |  |  |
| GO:0010889\_regulation\_of\_sequestering\_of\_triglyceride | 3 | 0 |  |  |  |  |  |  |  |  |
| GO:0010893\_positive\_regulation\_of\_steroid\_biosynthetic\_process | 3 | 0 |  |  |  |  |  |  |  |  |
| GO:0010894\_negative\_regulation\_of\_steroid\_biosynthetic\_process | 3 | 0 |  |  |  |  |  |  |  |  |
| GO:0010998\_regulation\_of\_translational\_initiation\_by\_eIF2\_alpha\_phosphorylation | 3 | 0 |  |  |  |  |  |  |  |  |
| GO:0010999\_regulation\_of\_eIF2\_alpha\_phosphorylation\_by\_heme | 3 | 0 |  |  |  |  |  |  |  |  |
| GO:0014074\_response\_to\_purine | 3 | 0 |  |  |  |  |  |  |  |  |
| GO:0014909\_smooth\_muscle\_cell\_migration | 3 | 0 |  |  |  |  |  |  |  |  |
| GO:0015669\_gas\_transport | 3 | 0 |  |  |  |  |  |  |  |  |
| GO:0015760\_glucose-6-phosphate\_transport | 3 | 0 |  |  |  |  |  |  |  |  |
| GO:0015816\_glycine\_transport | 3 | 0 |  |  |  |  |  |  |  |  |
| GO:0015838\_betaine\_transport | 3 | 0 |  |  |  |  |  |  |  |  |
| GO:0015871\_choline\_transport | 3 | 0 |  |  |  |  |  |  |  |  |
| GO:0015879\_carnitine\_transport | 3 | 0 |  |  |  |  |  |  |  |  |
| GO:0015893\_drug\_transport | 3 | 0 |  |  |  |  |  |  |  |  |
| GO:0015909\_long-chain\_fatty\_acid\_transport | 3 | 0 |  |  |  |  |  |  |  |  |
| GO:0015936\_coenzyme\_A\_metabolic\_process | 3 | 0 |  |  |  |  |  |  |  |  |
| GO:0015988\_energy\_coupled\_proton\_transport\_\_against\_electrochemical\_gradient | 3 | 0 |  |  |  |  |  |  |  |  |
| GO:0015991\_ATP\_hydrolysis\_coupled\_proton\_transport | 3 | 0 |  |  |  |  |  |  |  |  |
| GO:0016241\_regulation\_of\_macroautophagy | 3 | 0 |  |  |  |  |  |  |  |  |
| GO:0016322\_neuron\_remodeling | 3 | 0 |  |  |  |  |  |  |  |  |
| GO:0016556\_mRNA\_modification | 3 | 0 |  |  |  |  |  |  |  |  |
| GO:0016973\_poly(A)+\_mRNA\_export\_from\_nucleus | 3 | 0 |  |  |  |  |  |  |  |  |
| GO:0018196\_peptidyl-asparagine\_modification | 3 | 0 |  |  |  |  |  |  |  |  |
| GO:0018208\_peptidyl-proline\_modification | 3 | 0 |  |  |  |  |  |  |  |  |
| GO:0018279\_protein\_amino\_acid\_N-linked\_glycosylation\_via\_asparagine | 3 | 0 |  |  |  |  |  |  |  |  |
| GO:0018894\_dibenzo-p-dioxin\_metabolic\_process | 3 | 0 |  |  |  |  |  |  |  |  |
| GO:0019058\_viral\_infectious\_cycle | 3 | 0 |  |  |  |  |  |  |  |  |
| GO:0019230\_proprioception | 3 | 0 |  |  |  |  |  |  |  |  |
| GO:0019236\_response\_to\_pheromone | 3 | 0 |  |  |  |  |  |  |  |  |
| GO:0019359\_nicotinamide\_nucleotide\_biosynthetic\_process | 3 | 0 |  |  |  |  |  |  |  |  |
| GO:0019363\_pyridine\_nucleotide\_biosynthetic\_process | 3 | 0 |  |  |  |  |  |  |  |  |
| GO:0019438\_aromatic\_compound\_biosynthetic\_process | 3 | 0 |  |  |  |  |  |  |  |  |
| GO:0019439\_aromatic\_compound\_catabolic\_process | 3 | 0 |  |  |  |  |  |  |  |  |
| GO:0019605\_butyrate\_metabolic\_process | 3 | 0 |  |  |  |  |  |  |  |  |
| GO:0019614\_catechol\_catabolic\_process | 3 | 0 |  |  |  |  |  |  |  |  |
| GO:0019674\_NAD\_metabolic\_process | 3 | 0 |  |  |  |  |  |  |  |  |
| GO:0019852\_L-ascorbic\_acid\_metabolic\_process | 3 | 0 |  |  |  |  |  |  |  |  |
| GO:0019934\_cGMP-mediated\_signaling | 3 | 0 |  |  |  |  |  |  |  |  |
| GO:0019987\_negative\_regulation\_of\_anti-apoptosis | 3 | 0 |  |  |  |  |  |  |  |  |
| GO:0021527\_spinal\_cord\_association\_neuron\_differentiation | 3 | 0 |  |  |  |  |  |  |  |  |
| GO:0021529\_spinal\_cord\_oligodendrocyte\_cell\_differentiation | 3 | 0 |  |  |  |  |  |  |  |  |
| GO:0021530\_spinal\_cord\_oligodendrocyte\_cell\_fate\_specification | 3 | 0 |  |  |  |  |  |  |  |  |
| GO:0021555\_midbrain-hindbrain\_boundary\_morphogenesis | 3 | 0 |  |  |  |  |  |  |  |  |
| GO:0021563\_glossopharyngeal\_nerve\_development | 3 | 0 |  |  |  |  |  |  |  |  |
| GO:0021570\_rhombomere\_4\_development | 3 | 0 |  |  |  |  |  |  |  |  |
| GO:0021591\_ventricular\_system\_development | 3 | 0 |  |  |  |  |  |  |  |  |
| GO:0021615\_glossopharyngeal\_nerve\_morphogenesis | 3 | 0 |  |  |  |  |  |  |  |  |
| GO:0021794\_thalamus\_development | 3 | 0 |  |  |  |  |  |  |  |  |
| GO:0021800\_cerebral\_cortex\_tangential\_migration | 3 | 0 |  |  |  |  |  |  |  |  |
| GO:0021819\_layer\_formation\_in\_the\_cerebral\_cortex | 3 | 0 |  |  |  |  |  |  |  |  |
| GO:0021859\_pyramidal\_neuron\_differentiation | 3 | 0 |  |  |  |  |  |  |  |  |
| GO:0021860\_pyramidal\_neuron\_development | 3 | 0 |  |  |  |  |  |  |  |  |
| GO:0021889\_olfactory\_bulb\_interneuron\_differentiation | 3 | 0 |  |  |  |  |  |  |  |  |
| GO:0021891\_olfactory\_bulb\_interneuron\_development | 3 | 0 |  |  |  |  |  |  |  |  |
| GO:0021979\_hypothalamus\_cell\_differentiation | 3 | 0 |  |  |  |  |  |  |  |  |
| GO:0022010\_myelination\_in\_the\_central\_nervous\_system | 3 | 0 |  |  |  |  |  |  |  |  |
| GO:0022027\_interkinetic\_nuclear\_migration | 3 | 0 |  |  |  |  |  |  |  |  |
| GO:0022406\_membrane\_docking | 3 | 0 |  |  |  |  |  |  |  |  |
| GO:0030091\_protein\_repair | 3 | 0 |  |  |  |  |  |  |  |  |
| GO:0030195\_negative\_regulation\_of\_blood\_coagulation | 3 | 0 |  |  |  |  |  |  |  |  |
| GO:0030224\_monocyte\_differentiation | 3 | 0 |  |  |  |  |  |  |  |  |
| GO:0030307\_positive\_regulation\_of\_cell\_growth | 3 | 0 |  |  |  |  |  |  |  |  |
| GO:0030319\_cellular\_di-\_\_tri-valent\_inorganic\_anion\_homeostasis | 3 | 0 |  |  |  |  |  |  |  |  |
| GO:0030320\_cellular\_monovalent\_inorganic\_anion\_homeostasis | 3 | 0 |  |  |  |  |  |  |  |  |
| GO:0030321\_transepithelial\_chloride\_transport | 3 | 0 |  |  |  |  |  |  |  |  |
| GO:0030501\_positive\_regulation\_of\_bone\_mineralization | 3 | 0 |  |  |  |  |  |  |  |  |
| GO:0030513\_positive\_regulation\_of\_BMP\_signaling\_pathway | 3 | 0 |  |  |  |  |  |  |  |  |
| GO:0030538\_embryonic\_genitalia\_morphogenesis | 3 | 0 |  |  |  |  |  |  |  |  |
| GO:0030540\_female\_genitalia\_development | 3 | 0 |  |  |  |  |  |  |  |  |
| GO:0030574\_collagen\_catabolic\_process | 3 | 0 |  |  |  |  |  |  |  |  |
| GO:0030643\_cellular\_phosphate\_ion\_homeostasis | 3 | 0 |  |  |  |  |  |  |  |  |
| GO:0030718\_germ-line\_stem\_cell\_maintenance | 3 | 0 |  |  |  |  |  |  |  |  |
| GO:0030730\_sequestering\_of\_triglyceride | 3 | 0 |  |  |  |  |  |  |  |  |
| GO:0030836\_positive\_regulation\_of\_actin\_filament\_depolymerization | 3 | 0 |  |  |  |  |  |  |  |  |
| GO:0030916\_otic\_vesicle\_formation | 3 | 0 |  |  |  |  |  |  |  |  |
| GO:0031000\_response\_to\_caffeine | 3 | 0 |  |  |  |  |  |  |  |  |
| GO:0031063\_regulation\_of\_histone\_deacetylation | 3 | 0 |  |  |  |  |  |  |  |  |
| GO:0031065\_positive\_regulation\_of\_histone\_deacetylation | 3 | 0 |  |  |  |  |  |  |  |  |
| GO:0031112\_positive\_regulation\_of\_microtubule\_polymerization\_or\_depolymerization | 3 | 0 |  |  |  |  |  |  |  |  |
| GO:0031116\_positive\_regulation\_of\_microtubule\_polymerization | 3 | 0 |  |  |  |  |  |  |  |  |
| GO:0031133\_regulation\_of\_axon\_diameter | 3 | 0 |  |  |  |  |  |  |  |  |
| GO:0031282\_regulation\_of\_guanylate\_cyclase\_activity | 3 | 0 |  |  |  |  |  |  |  |  |
| GO:0031333\_negative\_regulation\_of\_protein\_complex\_assembly | 3 | 0 |  |  |  |  |  |  |  |  |
| GO:0031397\_negative\_regulation\_of\_protein\_ubiquitination | 3 | 0 |  |  |  |  |  |  |  |  |
| GO:0031398\_positive\_regulation\_of\_protein\_ubiquitination | 3 | 0 |  |  |  |  |  |  |  |  |
| GO:0031503\_protein\_complex\_localization | 3 | 0 |  |  |  |  |  |  |  |  |
| GO:0031571\_G1\_DNA\_damage\_checkpoint | 3 | 0 |  |  |  |  |  |  |  |  |
| GO:0031579\_membrane\_raft\_organization | 3 | 0 |  |  |  |  |  |  |  |  |
| GO:0031638\_zymogen\_activation | 3 | 0 |  |  |  |  |  |  |  |  |
| GO:0031641\_regulation\_of\_myelination | 3 | 0 |  |  |  |  |  |  |  |  |
| GO:0031642\_negative\_regulation\_of\_myelination | 3 | 0 |  |  |  |  |  |  |  |  |
| GO:0031649\_heat\_generation | 3 | 0 |  |  |  |  |  |  |  |  |
| GO:0031943\_regulation\_of\_glucocorticoid\_metabolic\_process | 3 | 0 |  |  |  |  |  |  |  |  |
| GO:0032020\_ISG15-protein\_conjugation | 3 | 0 |  |  |  |  |  |  |  |  |
| GO:0032060\_bleb\_formation | 3 | 0 |  |  |  |  |  |  |  |  |
| GO:0032095\_regulation\_of\_response\_to\_food | 3 | 0 |  |  |  |  |  |  |  |  |
| GO:0032272\_negative\_regulation\_of\_protein\_polymerization | 3 | 0 |  |  |  |  |  |  |  |  |
| GO:0032288\_myelin\_assembly | 3 | 0 |  |  |  |  |  |  |  |  |
| GO:0032291\_ensheathment\_of\_axons\_in\_the\_central\_nervous\_system | 3 | 0 |  |  |  |  |  |  |  |  |
| GO:0032355\_response\_to\_estradiol\_stimulus | 3 | 0 |  |  |  |  |  |  |  |  |
| GO:0032402\_melanosome\_transport | 3 | 0 |  |  |  |  |  |  |  |  |
| GO:0032411\_positive\_regulation\_of\_transporter\_activity | 3 | 0 |  |  |  |  |  |  |  |  |
| GO:0032414\_positive\_regulation\_of\_ion\_transmembrane\_transporter\_activity | 3 | 0 |  |  |  |  |  |  |  |  |
| GO:0032436\_positive\_regulation\_of\_proteasomal\_ubiquitin-dependent\_protein\_catabolic\_process | 3 | 0 |  |  |  |  |  |  |  |  |
| GO:0032536\_regulation\_of\_cell\_projection\_size | 3 | 0 |  |  |  |  |  |  |  |  |
| GO:0032632\_interleukin-3\_production | 3 | 0 |  |  |  |  |  |  |  |  |
| GO:0032634\_interleukin-5\_production | 3 | 0 |  |  |  |  |  |  |  |  |
| GO:0032674\_regulation\_of\_interleukin-5\_production | 3 | 0 |  |  |  |  |  |  |  |  |
| GO:0032703\_negative\_regulation\_of\_interleukin-2\_production | 3 | 0 |  |  |  |  |  |  |  |  |
| GO:0032753\_positive\_regulation\_of\_interleukin-4\_production | 3 | 0 |  |  |  |  |  |  |  |  |
| GO:0032823\_regulation\_of\_natural\_killer\_cell\_differentiation | 3 | 0 |  |  |  |  |  |  |  |  |
| GO:0032825\_positive\_regulation\_of\_natural\_killer\_cell\_differentiation | 3 | 0 |  |  |  |  |  |  |  |  |
| GO:0032856\_activation\_of\_Ras\_GTPase\_activity | 3 | 0 |  |  |  |  |  |  |  |  |
| GO:0032862\_activation\_of\_Rho\_GTPase\_activity | 3 | 0 |  |  |  |  |  |  |  |  |
| GO:0032874\_positive\_regulation\_of\_stress-activated\_MAPK\_cascade | 3 | 0 |  |  |  |  |  |  |  |  |
| GO:0032881\_regulation\_of\_polysaccharide\_metabolic\_process | 3 | 0 |  |  |  |  |  |  |  |  |
| GO:0032890\_regulation\_of\_organic\_acid\_transport | 3 | 0 |  |  |  |  |  |  |  |  |
| GO:0033058\_directional\_locomotion | 3 | 0 |  |  |  |  |  |  |  |  |
| GO:0033080\_immature\_T\_cell\_proliferation\_in\_the\_thymus | 3 | 0 |  |  |  |  |  |  |  |  |
| GO:0033084\_regulation\_of\_immature\_T\_cell\_proliferation\_in\_the\_thymus | 3 | 0 |  |  |  |  |  |  |  |  |
| GO:0033091\_positive\_regulation\_of\_immature\_T\_cell\_proliferation | 3 | 0 |  |  |  |  |  |  |  |  |
| GO:0033137\_negative\_regulation\_of\_peptidyl-serine\_phosphorylation | 3 | 0 |  |  |  |  |  |  |  |  |
| GO:0033153\_T\_cell\_receptor\_V(D)J\_recombination | 3 | 0 |  |  |  |  |  |  |  |  |
| GO:0033209\_tumor\_necrosis\_factor-mediated\_signaling\_pathway | 3 | 0 |  |  |  |  |  |  |  |  |
| GO:0033261\_regulation\_of\_S\_phase | 3 | 0 |  |  |  |  |  |  |  |  |
| GO:0033600\_negative\_regulation\_of\_mammary\_gland\_epithelial\_cell\_proliferation | 3 | 0 |  |  |  |  |  |  |  |  |
| GO:0033631\_cell-cell\_adhesion\_mediated\_by\_integrin | 3 | 0 |  |  |  |  |  |  |  |  |
| GO:0033993\_response\_to\_lipid | 3 | 0 |  |  |  |  |  |  |  |  |
| GO:0034220\_ion\_transmembrane\_transport | 3 | 0 |  |  |  |  |  |  |  |  |
| GO:0034308\_monohydric\_alcohol\_metabolic\_process | 3 | 0 |  |  |  |  |  |  |  |  |
| GO:0034313\_diol\_catabolic\_process | 3 | 0 |  |  |  |  |  |  |  |  |
| GO:0034331\_cell\_junction\_maintenance | 3 | 0 |  |  |  |  |  |  |  |  |
| GO:0034332\_adherens\_junction\_organization | 3 | 0 |  |  |  |  |  |  |  |  |
| GO:0034375\_high-density\_lipoprotein\_particle\_remodeling | 3 | 0 |  |  |  |  |  |  |  |  |
| GO:0034381\_lipoprotein\_particle\_clearance | 3 | 0 |  |  |  |  |  |  |  |  |
| GO:0034612\_response\_to\_tumor\_necrosis\_factor | 3 | 0 |  |  |  |  |  |  |  |  |
| GO:0034655\_nucleobase\_\_nucleoside\_\_nucleotide\_and\_nucleic\_acid\_catabolic\_process | 3 | 0 |  |  |  |  |  |  |  |  |
| GO:0034656\_nucleobase\_\_nucleoside\_and\_nucleotide\_catabolic\_process | 3 | 0 |  |  |  |  |  |  |  |  |
| GO:0035067\_negative\_regulation\_of\_histone\_acetylation | 3 | 0 |  |  |  |  |  |  |  |  |
| GO:0035084\_flagellar\_axoneme\_assembly | 3 | 0 |  |  |  |  |  |  |  |  |
| GO:0035166\_post-embryonic\_hemopoiesis | 3 | 0 |  |  |  |  |  |  |  |  |
| GO:0035283\_central\_nervous\_system\_segmentation | 3 | 0 |  |  |  |  |  |  |  |  |
| GO:0035284\_brain\_segmentation | 3 | 0 |  |  |  |  |  |  |  |  |
| GO:0042097\_interleukin-4\_biosynthetic\_process | 3 | 0 |  |  |  |  |  |  |  |  |
| GO:0042135\_neurotransmitter\_catabolic\_process | 3 | 0 |  |  |  |  |  |  |  |  |
| GO:0042271\_susceptibility\_to\_natural\_killer\_cell\_mediated\_cytotoxicity | 3 | 0 |  |  |  |  |  |  |  |  |
| GO:0042273\_ribosomal\_large\_subunit\_biogenesis | 3 | 0 |  |  |  |  |  |  |  |  |
| GO:0042375\_quinone\_cofactor\_metabolic\_process | 3 | 0 |  |  |  |  |  |  |  |  |
| GO:0042420\_dopamine\_catabolic\_process | 3 | 0 |  |  |  |  |  |  |  |  |
| GO:0042421\_norepinephrine\_biosynthetic\_process | 3 | 0 |  |  |  |  |  |  |  |  |
| GO:0042424\_catecholamine\_catabolic\_process | 3 | 0 |  |  |  |  |  |  |  |  |
| GO:0042447\_hormone\_catabolic\_process | 3 | 0 |  |  |  |  |  |  |  |  |
| GO:0042448\_progesterone\_metabolic\_process | 3 | 0 |  |  |  |  |  |  |  |  |
| GO:0042523\_positive\_regulation\_of\_tyrosine\_phosphorylation\_of\_Stat5\_protein | 3 | 0 |  |  |  |  |  |  |  |  |
| GO:0042659\_regulation\_of\_cell\_fate\_specification | 3 | 0 |  |  |  |  |  |  |  |  |
| GO:0042668\_auditory\_receptor\_cell\_fate\_determination | 3 | 0 |  |  |  |  |  |  |  |  |
| GO:0042670\_retinal\_cone\_cell\_differentiation | 3 | 0 |  |  |  |  |  |  |  |  |
| GO:0042693\_muscle\_cell\_fate\_commitment | 3 | 0 |  |  |  |  |  |  |  |  |
| GO:0042711\_maternal\_behavior | 3 | 0 |  |  |  |  |  |  |  |  |
| GO:0042745\_circadian\_sleep\_wake\_cycle | 3 | 0 |  |  |  |  |  |  |  |  |
| GO:0042759\_long-chain\_fatty\_acid\_biosynthetic\_process | 3 | 0 |  |  |  |  |  |  |  |  |
| GO:0042787\_protein\_ubiquitination\_during\_ubiquitin-dependent\_protein\_catabolic\_process | 3 | 0 |  |  |  |  |  |  |  |  |
| GO:0043045\_DNA\_methylation\_during\_embryonic\_development | 3 | 0 |  |  |  |  |  |  |  |  |
| GO:0043090\_amino\_acid\_import | 3 | 0 |  |  |  |  |  |  |  |  |
| GO:0043092\_L-amino\_acid\_import | 3 | 0 |  |  |  |  |  |  |  |  |
| GO:0043149\_stress\_fiber\_formation | 3 | 0 |  |  |  |  |  |  |  |  |
| GO:0043200\_response\_to\_amino\_acid\_stimulus | 3 | 0 |  |  |  |  |  |  |  |  |
| GO:0043243\_positive\_regulation\_of\_protein\_complex\_disassembly | 3 | 0 |  |  |  |  |  |  |  |  |
| GO:0043249\_erythrocyte\_maturation | 3 | 0 |  |  |  |  |  |  |  |  |
| GO:0043267\_negative\_regulation\_of\_potassium\_ion\_transport | 3 | 0 |  |  |  |  |  |  |  |  |
| GO:0043371\_negative\_regulation\_of\_CD4-positive\_\_alpha\_beta\_T\_cell\_differentiation | 3 | 0 |  |  |  |  |  |  |  |  |
| GO:0043462\_regulation\_of\_ATPase\_activity | 3 | 0 |  |  |  |  |  |  |  |  |
| GO:0043569\_negative\_regulation\_of\_insulin-like\_growth\_factor\_receptor\_signaling\_pathway | 3 | 0 |  |  |  |  |  |  |  |  |
| GO:0043574\_peroxisomal\_transport | 3 | 0 |  |  |  |  |  |  |  |  |
| GO:0043586\_tongue\_development | 3 | 0 |  |  |  |  |  |  |  |  |
| GO:0043900\_regulation\_of\_multi-organism\_process | 3 | 0 |  |  |  |  |  |  |  |  |
| GO:0043954\_cellular\_component\_maintenance | 3 | 0 |  |  |  |  |  |  |  |  |
| GO:0044030\_regulation\_of\_DNA\_methylation | 3 | 0 |  |  |  |  |  |  |  |  |
| GO:0044089\_positive\_regulation\_of\_cellular\_component\_biogenesis | 3 | 0 |  |  |  |  |  |  |  |  |
| GO:0044273\_sulfur\_compound\_catabolic\_process | 3 | 0 |  |  |  |  |  |  |  |  |
| GO:0045047\_protein\_targeting\_to\_ER | 3 | 0 |  |  |  |  |  |  |  |  |
| GO:0045085\_negative\_regulation\_of\_interleukin-2\_biosynthetic\_process | 3 | 0 |  |  |  |  |  |  |  |  |
| GO:0045110\_intermediate\_filament\_bundle\_assembly | 3 | 0 |  |  |  |  |  |  |  |  |
| GO:0045143\_homologous\_chromosome\_segregation | 3 | 0 |  |  |  |  |  |  |  |  |
| GO:0045198\_establishment\_of\_epithelial\_cell\_apical\_basal\_polarity | 3 | 0 |  |  |  |  |  |  |  |  |
| GO:0045217\_cell-cell\_junction\_maintenance | 3 | 0 |  |  |  |  |  |  |  |  |
| GO:0045348\_positive\_regulation\_of\_MHC\_class\_II\_biosynthetic\_process | 3 | 0 |  |  |  |  |  |  |  |  |
| GO:0045402\_regulation\_of\_interleukin-4\_biosynthetic\_process | 3 | 0 |  |  |  |  |  |  |  |  |
| GO:0045404\_positive\_regulation\_of\_interleukin-4\_biosynthetic\_process | 3 | 0 |  |  |  |  |  |  |  |  |
| GO:0045542\_positive\_regulation\_of\_cholesterol\_biosynthetic\_process | 3 | 0 |  |  |  |  |  |  |  |  |
| GO:0045607\_regulation\_of\_auditory\_receptor\_cell\_differentiation | 3 | 0 |  |  |  |  |  |  |  |  |
| GO:0045623\_negative\_regulation\_of\_T-helper\_cell\_differentiation | 3 | 0 |  |  |  |  |  |  |  |  |
| GO:0045625\_regulation\_of\_T-helper\_1\_cell\_differentiation | 3 | 0 |  |  |  |  |  |  |  |  |
| GO:0045631\_regulation\_of\_mechanoreceptor\_differentiation | 3 | 0 |  |  |  |  |  |  |  |  |
| GO:0045717\_negative\_regulation\_of\_fatty\_acid\_biosynthetic\_process | 3 | 0 |  |  |  |  |  |  |  |  |
| GO:0045723\_positive\_regulation\_of\_fatty\_acid\_biosynthetic\_process | 3 | 0 |  |  |  |  |  |  |  |  |
| GO:0045746\_negative\_regulation\_of\_Notch\_signaling\_pathway | 3 | 0 |  |  |  |  |  |  |  |  |
| GO:0045806\_negative\_regulation\_of\_endocytosis | 3 | 0 |  |  |  |  |  |  |  |  |
| GO:0045829\_negative\_regulation\_of\_isotype\_switching | 3 | 0 |  |  |  |  |  |  |  |  |
| GO:0045844\_positive\_regulation\_of\_striated\_muscle\_development | 3 | 0 |  |  |  |  |  |  |  |  |
| GO:0045907\_positive\_regulation\_of\_vasoconstriction | 3 | 0 |  |  |  |  |  |  |  |  |
| GO:0045922\_negative\_regulation\_of\_fatty\_acid\_metabolic\_process | 3 | 0 |  |  |  |  |  |  |  |  |
| GO:0045939\_negative\_regulation\_of\_steroid\_metabolic\_process | 3 | 0 |  |  |  |  |  |  |  |  |
| GO:0046013\_regulation\_of\_T\_cell\_homeostatic\_proliferation | 3 | 0 |  |  |  |  |  |  |  |  |
| GO:0046034\_ATP\_metabolic\_process | 3 | 0 |  |  |  |  |  |  |  |  |
| GO:0046325\_negative\_regulation\_of\_glucose\_import | 3 | 0 |  |  |  |  |  |  |  |  |
| GO:0046426\_negative\_regulation\_of\_JAK-STAT\_cascade | 3 | 0 |  |  |  |  |  |  |  |  |
| GO:0046457\_prostanoid\_biosynthetic\_process | 3 | 0 |  |  |  |  |  |  |  |  |
| GO:0046479\_glycosphingolipid\_catabolic\_process | 3 | 0 |  |  |  |  |  |  |  |  |
| GO:0046488\_phosphatidylinositol\_metabolic\_process | 3 | 0 |  |  |  |  |  |  |  |  |
| GO:0046549\_retinal\_cone\_cell\_development | 3 | 0 |  |  |  |  |  |  |  |  |
| GO:0046605\_regulation\_of\_centrosome\_cycle | 3 | 0 |  |  |  |  |  |  |  |  |
| GO:0046688\_response\_to\_copper\_ion | 3 | 0 |  |  |  |  |  |  |  |  |
| GO:0046717\_acid\_secretion | 3 | 0 |  |  |  |  |  |  |  |  |
| GO:0046825\_regulation\_of\_protein\_export\_from\_nucleus | 3 | 0 |  |  |  |  |  |  |  |  |
| GO:0046885\_regulation\_of\_hormone\_biosynthetic\_process | 3 | 0 |  |  |  |  |  |  |  |  |
| GO:0048003\_antigen\_processing\_and\_presentation\_of\_lipid\_antigen\_via\_MHC\_class\_Ib | 3 | 0 |  |  |  |  |  |  |  |  |
| GO:0048007\_antigen\_processing\_and\_presentation\_\_exogenous\_lipid\_antigen\_via\_MHC\_class\_Ib | 3 | 0 |  |  |  |  |  |  |  |  |
| GO:0048012\_hepatocyte\_growth\_factor\_receptor\_signaling\_pathway | 3 | 0 |  |  |  |  |  |  |  |  |
| GO:0048050\_post-embryonic\_eye\_morphogenesis | 3 | 0 |  |  |  |  |  |  |  |  |
| GO:0048087\_positive\_regulation\_of\_pigmentation\_during\_development | 3 | 0 |  |  |  |  |  |  |  |  |
| GO:0048246\_macrophage\_chemotaxis | 3 | 0 |  |  |  |  |  |  |  |  |
| GO:0048251\_elastic\_fiber\_assembly | 3 | 0 |  |  |  |  |  |  |  |  |
| GO:0048278\_vesicle\_docking | 3 | 0 |  |  |  |  |  |  |  |  |
| GO:0048294\_negative\_regulation\_of\_isotype\_switching\_to\_IgE\_isotypes | 3 | 0 |  |  |  |  |  |  |  |  |
| GO:0048318\_axial\_mesoderm\_development | 3 | 0 |  |  |  |  |  |  |  |  |
| GO:0048597\_post-embryonic\_camera-type\_eye\_morphogenesis | 3 | 0 |  |  |  |  |  |  |  |  |
| GO:0048636\_positive\_regulation\_of\_muscle\_development | 3 | 0 |  |  |  |  |  |  |  |  |
| GO:0048660\_regulation\_of\_smooth\_muscle\_cell\_proliferation | 3 | 0 |  |  |  |  |  |  |  |  |
| GO:0048668\_collateral\_sprouting | 3 | 0 |  |  |  |  |  |  |  |  |
| GO:0048676\_axon\_extension\_involved\_in\_development | 3 | 0 |  |  |  |  |  |  |  |  |
| GO:0048755\_branching\_morphogenesis\_of\_a\_nerve | 3 | 0 |  |  |  |  |  |  |  |  |
| GO:0048845\_venous\_blood\_vessel\_morphogenesis | 3 | 0 |  |  |  |  |  |  |  |  |
| GO:0048852\_diencephalon\_morphogenesis | 3 | 0 |  |  |  |  |  |  |  |  |
| GO:0048859\_formation\_of\_anatomical\_boundary | 3 | 0 |  |  |  |  |  |  |  |  |
| GO:0048865\_stem\_cell\_fate\_commitment | 3 | 0 |  |  |  |  |  |  |  |  |
| GO:0050435\_beta-amyloid\_metabolic\_process | 3 | 0 |  |  |  |  |  |  |  |  |
| GO:0050650\_chondroitin\_sulfate\_proteoglycan\_biosynthetic\_process | 3 | 0 |  |  |  |  |  |  |  |  |
| GO:0050703\_interleukin-1\_alpha\_secretion | 3 | 0 |  |  |  |  |  |  |  |  |
| GO:0050705\_regulation\_of\_interleukin-1\_alpha\_secretion | 3 | 0 |  |  |  |  |  |  |  |  |
| GO:0050709\_negative\_regulation\_of\_protein\_secretion | 3 | 0 |  |  |  |  |  |  |  |  |
| GO:0050710\_negative\_regulation\_of\_cytokine\_secretion | 3 | 0 |  |  |  |  |  |  |  |  |
| GO:0050717\_positive\_regulation\_of\_interleukin-1\_alpha\_secretion | 3 | 0 |  |  |  |  |  |  |  |  |
| GO:0050774\_negative\_regulation\_of\_dendrite\_morphogenesis | 3 | 0 |  |  |  |  |  |  |  |  |
| GO:0050857\_positive\_regulation\_of\_antigen\_receptor-mediated\_signaling\_pathway | 3 | 0 |  |  |  |  |  |  |  |  |
| GO:0050882\_voluntary\_musculoskeletal\_movement | 3 | 0 |  |  |  |  |  |  |  |  |
| GO:0050913\_sensory\_perception\_of\_bitter\_taste | 3 | 0 |  |  |  |  |  |  |  |  |
| GO:0050957\_equilibrioception | 3 | 0 |  |  |  |  |  |  |  |  |
| GO:0050996\_positive\_regulation\_of\_lipid\_catabolic\_process | 3 | 0 |  |  |  |  |  |  |  |  |
| GO:0051149\_positive\_regulation\_of\_muscle\_cell\_differentiation | 3 | 0 |  |  |  |  |  |  |  |  |
| GO:0051153\_regulation\_of\_striated\_muscle\_cell\_differentiation | 3 | 0 |  |  |  |  |  |  |  |  |
| GO:0051204\_protein\_insertion\_into\_mitochondrial\_membrane | 3 | 0 |  |  |  |  |  |  |  |  |
| GO:0051291\_protein\_heterooligomerization | 3 | 0 |  |  |  |  |  |  |  |  |
| GO:0051302\_regulation\_of\_cell\_division | 3 | 0 |  |  |  |  |  |  |  |  |
| GO:0051320\_S\_phase | 3 | 0 |  |  |  |  |  |  |  |  |
| GO:0051450\_myoblast\_proliferation | 3 | 0 |  |  |  |  |  |  |  |  |
| GO:0051583\_dopamine\_uptake | 3 | 0 |  |  |  |  |  |  |  |  |
| GO:0051798\_positive\_regulation\_of\_hair\_follicle\_development | 3 | 0 |  |  |  |  |  |  |  |  |
| GO:0051882\_mitochondrial\_depolarization | 3 | 0 |  |  |  |  |  |  |  |  |
| GO:0051900\_regulation\_of\_mitochondrial\_depolarization | 3 | 0 |  |  |  |  |  |  |  |  |
| GO:0051925\_regulation\_of\_calcium\_ion\_transport\_via\_voltage-gated\_calcium\_channel\_activity | 3 | 0 |  |  |  |  |  |  |  |  |
| GO:0051926\_negative\_regulation\_of\_calcium\_ion\_transport | 3 | 0 |  |  |  |  |  |  |  |  |
| GO:0051930\_regulation\_of\_sensory\_perception\_of\_pain | 3 | 0 |  |  |  |  |  |  |  |  |
| GO:0051931\_regulation\_of\_sensory\_perception | 3 | 0 |  |  |  |  |  |  |  |  |
| GO:0051934\_catecholamine\_uptake\_during\_transmission\_of\_nerve\_impulse | 3 | 0 |  |  |  |  |  |  |  |  |
| GO:0051955\_regulation\_of\_amino\_acid\_transport | 3 | 0 |  |  |  |  |  |  |  |  |
| GO:0051962\_positive\_regulation\_of\_nervous\_system\_development | 3 | 0 |  |  |  |  |  |  |  |  |
| GO:0051965\_positive\_regulation\_of\_synaptogenesis | 3 | 0 |  |  |  |  |  |  |  |  |
| GO:0051967\_negative\_regulation\_of\_synaptic\_transmission\_\_glutamatergic | 3 | 0 |  |  |  |  |  |  |  |  |
| GO:0051983\_regulation\_of\_chromosome\_segregation | 3 | 0 |  |  |  |  |  |  |  |  |
| GO:0055061\_di-\_\_tri-valent\_inorganic\_anion\_homeostasis | 3 | 0 |  |  |  |  |  |  |  |  |
| GO:0055062\_phosphate\_ion\_homeostasis | 3 | 0 |  |  |  |  |  |  |  |  |
| GO:0055083\_monovalent\_inorganic\_anion\_homeostasis | 3 | 0 |  |  |  |  |  |  |  |  |
| GO:0055117\_regulation\_of\_cardiac\_muscle\_contraction | 3 | 0 |  |  |  |  |  |  |  |  |
| GO:0060009\_Sertoli\_cell\_development | 3 | 0 |  |  |  |  |  |  |  |  |
| GO:0060024\_rhythmic\_synaptic\_transmission | 3 | 0 |  |  |  |  |  |  |  |  |
| GO:0060040\_retinal\_bipolar\_neuron\_differentiation | 3 | 0 |  |  |  |  |  |  |  |  |
| GO:0060055\_angiogenesis\_involved\_in\_wound\_healing | 3 | 0 |  |  |  |  |  |  |  |  |
| GO:0060084\_synaptic\_transmission\_involved\_in\_micturition | 3 | 0 |  |  |  |  |  |  |  |  |
| GO:0060123\_regulation\_of\_growth\_hormone\_secretion | 3 | 0 |  |  |  |  |  |  |  |  |
| GO:0060126\_somatotropin\_secreting\_cell\_differentiation | 3 | 0 |  |  |  |  |  |  |  |  |
| GO:0060192\_negative\_regulation\_of\_lipase\_activity | 3 | 0 |  |  |  |  |  |  |  |  |
| GO:0060219\_camera-type\_eye\_photoreceptor\_cell\_differentiation | 3 | 0 |  |  |  |  |  |  |  |  |
| GO:0060285\_ciliary\_cell\_motility | 3 | 0 |  |  |  |  |  |  |  |  |
| GO:0060294\_cilium\_movement\_involved\_in\_ciliary\_motility | 3 | 0 |  |  |  |  |  |  |  |  |
| GO:0060295\_regulation\_of\_cilium\_movement\_involved\_in\_ciliary\_motility | 3 | 0 |  |  |  |  |  |  |  |  |
| GO:0060296\_regulation\_of\_cilium\_beat\_frequency\_involved\_in\_ciliary\_motility | 3 | 0 |  |  |  |  |  |  |  |  |
| GO:0060314\_regulation\_of\_ryanodine-sensitive\_calcium-release\_channel\_activity | 3 | 0 |  |  |  |  |  |  |  |  |
| GO:0060396\_growth\_hormone\_receptor\_signaling\_pathway | 3 | 0 |  |  |  |  |  |  |  |  |
| GO:0060416\_response\_to\_growth\_hormone\_stimulus | 3 | 0 |  |  |  |  |  |  |  |  |
| GO:0060428\_lung\_epithelium\_development | 3 | 0 |  |  |  |  |  |  |  |  |
| GO:0060433\_bronchus\_development | 3 | 0 |  |  |  |  |  |  |  |  |
| GO:0060435\_bronchiole\_development | 3 | 0 |  |  |  |  |  |  |  |  |
| GO:0060460\_left\_lung\_morphogenesis | 3 | 0 |  |  |  |  |  |  |  |  |
| GO:0060491\_regulation\_of\_cell\_projection\_assembly | 3 | 0 |  |  |  |  |  |  |  |  |
| GO:0060523\_prostate\_epithelial\_cord\_elongation | 3 | 0 |  |  |  |  |  |  |  |  |
| GO:0060586\_multicellular\_organismal\_iron\_ion\_homeostasis | 3 | 0 |  |  |  |  |  |  |  |  |
| GO:0060596\_mammary\_placode\_formation | 3 | 0 |  |  |  |  |  |  |  |  |
| GO:0060632\_regulation\_of\_microtubule-based\_movement | 3 | 0 |  |  |  |  |  |  |  |  |
| GO:0060648\_mammary\_gland\_bud\_morphogenesis | 3 | 0 |  |  |  |  |  |  |  |  |
| GO:0060684\_epithelial-mesenchymal\_cell\_signaling | 3 | 0 |  |  |  |  |  |  |  |  |
| GO:0060686\_negative\_regulation\_of\_prostatic\_bud\_formation | 3 | 0 |  |  |  |  |  |  |  |  |
| GO:0060689\_cell\_differentiation\_involved\_in\_salivary\_gland\_development | 3 | 0 |  |  |  |  |  |  |  |  |
| GO:0060708\_spongiotrophoblast\_differentiation | 3 | 0 |  |  |  |  |  |  |  |  |
| GO:0060746\_parental\_behavior | 3 | 0 |  |  |  |  |  |  |  |  |
| GO:0060748\_tertiary\_branching\_involved\_in\_mammary\_gland\_duct\_morphogenesis | 3 | 0 |  |  |  |  |  |  |  |  |
| GO:0060750\_epithelial\_cell\_proliferation\_involved\_in\_mammary\_gland\_duct\_elongation | 3 | 0 |  |  |  |  |  |  |  |  |
| GO:0060841\_venous\_blood\_vessel\_development | 3 | 0 |  |  |  |  |  |  |  |  |
| GO:0070102\_interleukin-6-mediated\_signaling\_pathway | 3 | 0 |  |  |  |  |  |  |  |  |
| GO:0070169\_positive\_regulation\_of\_biomineral\_formation | 3 | 0 |  |  |  |  |  |  |  |  |
| GO:0070206\_protein\_trimerization | 3 | 0 |  |  |  |  |  |  |  |  |
| GO:0070207\_protein\_homotrimerization | 3 | 0 |  |  |  |  |  |  |  |  |
| GO:0070229\_negative\_regulation\_of\_lymphocyte\_apoptosis | 3 | 0 |  |  |  |  |  |  |  |  |
| GO:0070230\_positive\_regulation\_of\_lymphocyte\_apoptosis | 3 | 0 |  |  |  |  |  |  |  |  |
| GO:0070232\_regulation\_of\_T\_cell\_apoptosis | 3 | 0 |  |  |  |  |  |  |  |  |
| GO:0070233\_negative\_regulation\_of\_T\_cell\_apoptosis | 3 | 0 |  |  |  |  |  |  |  |  |
| GO:0070242\_thymocyte\_apoptosis | 3 | 0 |  |  |  |  |  |  |  |  |
| GO:0070243\_regulation\_of\_thymocyte\_apoptosis | 3 | 0 |  |  |  |  |  |  |  |  |
| GO:0070244\_negative\_regulation\_of\_thymocyte\_apoptosis | 3 | 0 |  |  |  |  |  |  |  |  |
| GO:0070307\_lens\_fiber\_cell\_development | 3 | 0 |  |  |  |  |  |  |  |  |
| GO:0070309\_lens\_fiber\_cell\_morphogenesis | 3 | 0 |  |  |  |  |  |  |  |  |
| GO:0070423\_nucleotide-binding\_oligomerization\_domain\_containing\_signaling\_pathway | 3 | 0 |  |  |  |  |  |  |  |  |
| GO:0070427\_nucleotide-binding\_oligomerization\_domain\_containing\_1\_signaling\_pathway | 3 | 0 |  |  |  |  |  |  |  |  |
| GO:0070431\_nucleotide-binding\_oligomerization\_domain\_containing\_2\_signaling\_pathway | 3 | 0 |  |  |  |  |  |  |  |  |
| GO:0070633\_transepithelial\_transport | 3 | 0 |  |  |  |  |  |  |  |  |
| GO:0070846\_Hsp90\_deacetylation | 3 | 0 |  |  |  |  |  |  |  |  |
| GO:0070873\_regulation\_of\_glycogen\_metabolic\_process | 3 | 0 |  |  |  |  |  |  |  |  |
| GO:0070875\_positive\_regulation\_of\_glycogen\_metabolic\_process | 3 | 0 |  |  |  |  |  |  |  |  |
| GO:0001503\_ossification | 88 | 0 | 0.000000 | -0.000000 | 1338 | 1225.684868 | 1294.65 | 1363.615132 | 0.967601 |
| GO:0048754\_branching\_morphogenesis\_of\_a\_tube | 88 | 0 | 0.000000 | -0.000000 | 1338 | 1225.684868 | 1294.65 | 1363.615132 | 0.967601 |
| GO:0001759\_induction\_of\_an\_organ | 15 | 0 | 0.000000 | -0.000000 | 1389 | 1278.161668 | 1345.81 | 1413.458332 | 0.968906 |
| GO:0001782\_B\_cell\_homeostasis | 15 | 0 | 0.000000 | -0.000000 | 1389 | 1278.161668 | 1345.81 | 1413.458332 | 0.968906 |
| GO:0001964\_startle\_response | 15 | 0 | 0.000000 | -0.000000 | 1389 | 1278.161668 | 1345.81 | 1413.458332 | 0.968906 |
| GO:0002286\_T\_cell\_activation\_during\_immune\_response | 15 | 0 | 0.000000 | -0.000000 | 1389 | 1278.161668 | 1345.81 | 1413.458332 | 0.968906 |
| GO:0002495\_antigen\_processing\_and\_presentation\_of\_peptide\_antigen\_via\_MHC\_class\_II | 15 | 0 | 0.000000 | -0.000000 | 1389 | 1278.161668 | 1345.81 | 1413.458332 | 0.968906 |
| GO:0002504\_antigen\_processing\_and\_presentation\_of\_peptide\_or\_polysaccharide\_antigen\_via\_MHC\_class\_II | 15 | 0 | 0.000000 | -0.000000 | 1389 | 1278.161668 | 1345.81 | 1413.458332 | 0.968906 |
| GO:0002709\_regulation\_of\_T\_cell\_mediated\_immunity | 15 | 0 | 0.000000 | -0.000000 | 1389 | 1278.161668 | 1345.81 | 1413.458332 | 0.968906 |
| GO:0006473\_protein\_amino\_acid\_acetylation | 15 | 0 | 0.000000 | -0.000000 | 1389 | 1278.161668 | 1345.81 | 1413.458332 | 0.968906 |
| GO:0006487\_protein\_amino\_acid\_N-linked\_glycosylation | 15 | 0 | 0.000000 | -0.000000 | 1389 | 1278.161668 | 1345.81 | 1413.458332 | 0.968906 |
| GO:0006749\_glutathione\_metabolic\_process | 15 | 0 | 0.000000 | -0.000000 | 1389 | 1278.161668 | 1345.81 | 1413.458332 | 0.968906 |
| GO:0006885\_regulation\_of\_pH | 15 | 0 | 0.000000 | -0.000000 | 1389 | 1278.161668 | 1345.81 | 1413.458332 | 0.968906 |
| GO:0007040\_lysosome\_organization | 15 | 0 | 0.000000 | -0.000000 | 1389 | 1278.161668 | 1345.81 | 1413.458332 | 0.968906 |
| GO:0007200\_activation\_of\_phospholipase\_C\_activity\_by\_G-protein\_coupled\_receptor\_protein\_signaling\_pathway\_coupled\_to\_IP3\_second\_messenger | 15 | 0 | 0.000000 | -0.000000 | 1389 | 1278.161668 | 1345.81 | 1413.458332 | 0.968906 |
| GO:0007202\_activation\_of\_phospholipase\_C\_activity | 15 | 0 | 0.000000 | -0.000000 | 1389 | 1278.161668 | 1345.81 | 1413.458332 | 0.968906 |
| GO:0007218\_neuropeptide\_signaling\_pathway | 15 | 0 | 0.000000 | -0.000000 | 1389 | 1278.161668 | 1345.81 | 1413.458332 | 0.968906 |
| GO:0007588\_excretion | 15 | 0 | 0.000000 | -0.000000 | 1389 | 1278.161668 | 1345.81 | 1413.458332 | 0.968906 |
| GO:0007618\_mating | 15 | 0 | 0.000000 | -0.000000 | 1389 | 1278.161668 | 1345.81 | 1413.458332 | 0.968906 |
| GO:0008543\_fibroblast\_growth\_factor\_receptor\_signaling\_pathway | 15 | 0 | 0.000000 | -0.000000 | 1389 | 1278.161668 | 1345.81 | 1413.458332 | 0.968906 |
| GO:0009062\_fatty\_acid\_catabolic\_process | 15 | 0 | 0.000000 | -0.000000 | 1389 | 1278.161668 | 1345.81 | 1413.458332 | 0.968906 |
| GO:0010092\_specification\_of\_organ\_identity | 15 | 0 | 0.000000 | -0.000000 | 1389 | 1278.161668 | 1345.81 | 1413.458332 | 0.968906 |
| GO:0010171\_body\_morphogenesis | 15 | 0 | 0.000000 | -0.000000 | 1389 | 1278.161668 | 1345.81 | 1413.458332 | 0.968906 |
| GO:0010518\_positive\_regulation\_of\_phospholipase\_activity | 15 | 0 | 0.000000 | -0.000000 | 1389 | 1278.161668 | 1345.81 | 1413.458332 | 0.968906 |
| GO:0010863\_positive\_regulation\_of\_phospholipase\_C\_activity | 15 | 0 | 0.000000 | -0.000000 | 1389 | 1278.161668 | 1345.81 | 1413.458332 | 0.968906 |
| GO:0015931\_nucleobase\_\_nucleoside\_\_nucleotide\_and\_nucleic\_acid\_transport | 15 | 0 | 0.000000 | -0.000000 | 1389 | 1278.161668 | 1345.81 | 1413.458332 | 0.968906 |
| GO:0019886\_antigen\_processing\_and\_presentation\_of\_exogenous\_peptide\_antigen\_via\_MHC\_class\_II | 15 | 0 | 0.000000 | -0.000000 | 1389 | 1278.161668 | 1345.81 | 1413.458332 | 0.968906 |
| GO:0021795\_cerebral\_cortex\_cell\_migration | 15 | 0 | 0.000000 | -0.000000 | 1389 | 1278.161668 | 1345.81 | 1413.458332 | 0.968906 |
| GO:0022600\_digestive\_system\_process | 15 | 0 | 0.000000 | -0.000000 | 1389 | 1278.161668 | 1345.81 | 1413.458332 | 0.968906 |
| GO:0030041\_actin\_filament\_polymerization | 15 | 0 | 0.000000 | -0.000000 | 1389 | 1278.161668 | 1345.81 | 1413.458332 | 0.968906 |
| GO:0031069\_hair\_follicle\_morphogenesis | 15 | 0 | 0.000000 | -0.000000 | 1389 | 1278.161668 | 1345.81 | 1413.458332 | 0.968906 |
| GO:0031076\_embryonic\_camera-type\_eye\_development | 15 | 0 | 0.000000 | -0.000000 | 1389 | 1278.161668 | 1345.81 | 1413.458332 | 0.968906 |
| GO:0031329\_regulation\_of\_cellular\_catabolic\_process | 15 | 0 | 0.000000 | -0.000000 | 1389 | 1278.161668 | 1345.81 | 1413.458332 | 0.968906 |
| GO:0035116\_embryonic\_hindlimb\_morphogenesis | 15 | 0 | 0.000000 | -0.000000 | 1389 | 1278.161668 | 1345.81 | 1413.458332 | 0.968906 |
| GO:0035249\_synaptic\_transmission\_\_glutamatergic | 15 | 0 | 0.000000 | -0.000000 | 1389 | 1278.161668 | 1345.81 | 1413.458332 | 0.968906 |
| GO:0042306\_regulation\_of\_protein\_import\_into\_nucleus | 15 | 0 | 0.000000 | -0.000000 | 1389 | 1278.161668 | 1345.81 | 1413.458332 | 0.968906 |
| GO:0045666\_positive\_regulation\_of\_neuron\_differentiation | 15 | 0 | 0.000000 | -0.000000 | 1389 | 1278.161668 | 1345.81 | 1413.458332 | 0.968906 |
| GO:0046164\_alcohol\_catabolic\_process | 15 | 0 | 0.000000 | -0.000000 | 1389 | 1278.161668 | 1345.81 | 1413.458332 | 0.968906 |
| GO:0046638\_positive\_regulation\_of\_alpha-beta\_T\_cell\_differentiation | 15 | 0 | 0.000000 | -0.000000 | 1389 | 1278.161668 | 1345.81 | 1413.458332 | 0.968906 |
| GO:0048008\_platelet-derived\_growth\_factor\_receptor\_signaling\_pathway | 15 | 0 | 0.000000 | -0.000000 | 1389 | 1278.161668 | 1345.81 | 1413.458332 | 0.968906 |
| GO:0048010\_vascular\_endothelial\_growth\_factor\_receptor\_signaling\_pathway | 15 | 0 | 0.000000 | -0.000000 | 1389 | 1278.161668 | 1345.81 | 1413.458332 | 0.968906 |
| GO:0048144\_fibroblast\_proliferation | 15 | 0 | 0.000000 | -0.000000 | 1389 | 1278.161668 | 1345.81 | 1413.458332 | 0.968906 |
| GO:0048145\_regulation\_of\_fibroblast\_proliferation | 15 | 0 | 0.000000 | -0.000000 | 1389 | 1278.161668 | 1345.81 | 1413.458332 | 0.968906 |
| GO:0048610\_reproductive\_cellular\_process | 15 | 0 | 0.000000 | -0.000000 | 1389 | 1278.161668 | 1345.81 | 1413.458332 | 0.968906 |
| GO:0050729\_positive\_regulation\_of\_inflammatory\_response | 15 | 0 | 0.000000 | -0.000000 | 1389 | 1278.161668 | 1345.81 | 1413.458332 | 0.968906 |
| GO:0050796\_regulation\_of\_insulin\_secretion | 15 | 0 | 0.000000 | -0.000000 | 1389 | 1278.161668 | 1345.81 | 1413.458332 | 0.968906 |
| GO:0050798\_activated\_T\_cell\_proliferation | 15 | 0 | 0.000000 | -0.000000 | 1389 | 1278.161668 | 1345.81 | 1413.458332 | 0.968906 |
| GO:0055010\_ventricular\_cardiac\_muscle\_morphogenesis | 15 | 0 | 0.000000 | -0.000000 | 1389 | 1278.161668 | 1345.81 | 1413.458332 | 0.968906 |
| GO:0060322\_head\_development | 15 | 0 | 0.000000 | -0.000000 | 1389 | 1278.161668 | 1345.81 | 1413.458332 | 0.968906 |
| GO:0060425\_lung\_morphogenesis | 15 | 0 | 0.000000 | -0.000000 | 1389 | 1278.161668 | 1345.81 | 1413.458332 | 0.968906 |
| GO:0060442\_branching\_involved\_in\_prostate\_gland\_morphogenesis | 15 | 0 | 0.000000 | -0.000000 | 1389 | 1278.161668 | 1345.81 | 1413.458332 | 0.968906 |
| GO:0070227\_lymphocyte\_apoptosis | 15 | 0 | 0.000000 | -0.000000 | 1389 | 1278.161668 | 1345.81 | 1413.458332 | 0.968906 |
| GO:0070507\_regulation\_of\_microtubule\_cytoskeleton\_organization | 15 | 0 | 0.000000 | -0.000000 | 1389 | 1278.161668 | 1345.81 | 1413.458332 | 0.968906 |
| GO:0048872\_homeostasis\_of\_number\_of\_cells | 105 | 0 | 0.000000 | -0.000000 | 1390 | 1278.939337 | 1346.36 | 1413.780663 | 0.968604 |
| GO:0001656\_metanephros\_development | 50 | 0 | 0.000000 | -0.000000 | 1398 | 1285.501787 | 1352.26 | 1419.018213 | 0.967282 |
| GO:0002573\_myeloid\_leukocyte\_differentiation | 50 | 0 | 0.000000 | -0.000000 | 1398 | 1285.501787 | 1352.26 | 1419.018213 | 0.967282 |
| GO:0007015\_actin\_filament\_organization | 50 | 0 | 0.000000 | -0.000000 | 1398 | 1285.501787 | 1352.26 | 1419.018213 | 0.967282 |
| GO:0009190\_cyclic\_nucleotide\_biosynthetic\_process | 50 | 0 | 0.000000 | -0.000000 | 1398 | 1285.501787 | 1352.26 | 1419.018213 | 0.967282 |
| GO:0017038\_protein\_import | 50 | 0 | 0.000000 | -0.000000 | 1398 | 1285.501787 | 1352.26 | 1419.018213 | 0.967282 |
| GO:0042129\_regulation\_of\_T\_cell\_proliferation | 50 | 0 | 0.000000 | -0.000000 | 1398 | 1285.501787 | 1352.26 | 1419.018213 | 0.967282 |
| GO:0051606\_detection\_of\_stimulus | 50 | 0 | 0.000000 | -0.000000 | 1398 | 1285.501787 | 1352.26 | 1419.018213 | 0.967282 |
| GO:0070647\_protein\_modification\_by\_small\_protein\_conjugation\_or\_removal | 50 | 0 | 0.000000 | -0.000000 | 1398 | 1285.501787 | 1352.26 | 1419.018213 | 0.967282 |
| GO:0001818\_negative\_regulation\_of\_cytokine\_production | 18 | 0 | 0.000000 | -0.000000 | 1444 | 1334.779768 | 1399.95 | 1465.120232 | 0.969494 |
| GO:0001825\_blastocyst\_formation | 18 | 0 | 0.000000 | -0.000000 | 1444 | 1334.779768 | 1399.95 | 1465.120232 | 0.969494 |
| GO:0001974\_blood\_vessel\_remodeling | 18 | 0 | 0.000000 | -0.000000 | 1444 | 1334.779768 | 1399.95 | 1465.120232 | 0.969494 |
| GO:0002064\_epithelial\_cell\_development | 18 | 0 | 0.000000 | -0.000000 | 1444 | 1334.779768 | 1399.95 | 1465.120232 | 0.969494 |
| GO:0002285\_lymphocyte\_activation\_during\_immune\_response | 18 | 0 | 0.000000 | -0.000000 | 1444 | 1334.779768 | 1399.95 | 1465.120232 | 0.969494 |
| GO:0002715\_regulation\_of\_natural\_killer\_cell\_mediated\_immunity | 18 | 0 | 0.000000 | -0.000000 | 1444 | 1334.779768 | 1399.95 | 1465.120232 | 0.969494 |
| GO:0003014\_renal\_system\_process | 18 | 0 | 0.000000 | -0.000000 | 1444 | 1334.779768 | 1399.95 | 1465.120232 | 0.969494 |
| GO:0006022\_aminoglycan\_metabolic\_process | 18 | 0 | 0.000000 | -0.000000 | 1444 | 1334.779768 | 1399.95 | 1465.120232 | 0.969494 |
| GO:0006940\_regulation\_of\_smooth\_muscle\_contraction | 18 | 0 | 0.000000 | -0.000000 | 1444 | 1334.779768 | 1399.95 | 1465.120232 | 0.969494 |
| GO:0007140\_male\_meiosis | 18 | 0 | 0.000000 | -0.000000 | 1444 | 1334.779768 | 1399.95 | 1465.120232 | 0.969494 |
| GO:0007608\_sensory\_perception\_of\_smell | 18 | 0 | 0.000000 | -0.000000 | 1444 | 1334.779768 | 1399.95 | 1465.120232 | 0.969494 |
| GO:0008589\_regulation\_of\_smoothened\_signaling\_pathway | 18 | 0 | 0.000000 | -0.000000 | 1444 | 1334.779768 | 1399.95 | 1465.120232 | 0.969494 |
| GO:0009063\_cellular\_amino\_acid\_catabolic\_process | 18 | 0 | 0.000000 | -0.000000 | 1444 | 1334.779768 | 1399.95 | 1465.120232 | 0.969494 |
| GO:0010553\_negative\_regulation\_of\_specific\_transcription\_from\_RNA\_polymerase\_II\_promoter | 18 | 0 | 0.000000 | -0.000000 | 1444 | 1334.779768 | 1399.95 | 1465.120232 | 0.969494 |
| GO:0015711\_organic\_anion\_transport | 18 | 0 | 0.000000 | -0.000000 | 1444 | 1334.779768 | 1399.95 | 1465.120232 | 0.969494 |
| GO:0016458\_gene\_silencing | 18 | 0 | 0.000000 | -0.000000 | 1444 | 1334.779768 | 1399.95 | 1465.120232 | 0.969494 |
| GO:0021885\_forebrain\_cell\_migration | 18 | 0 | 0.000000 | -0.000000 | 1444 | 1334.779768 | 1399.95 | 1465.120232 | 0.969494 |
| GO:0030203\_glycosaminoglycan\_metabolic\_process | 18 | 0 | 0.000000 | -0.000000 | 1444 | 1334.779768 | 1399.95 | 1465.120232 | 0.969494 |
| GO:0030282\_bone\_mineralization | 18 | 0 | 0.000000 | -0.000000 | 1444 | 1334.779768 | 1399.95 | 1465.120232 | 0.969494 |
| GO:0030318\_melanocyte\_differentiation | 18 | 0 | 0.000000 | -0.000000 | 1444 | 1334.779768 | 1399.95 | 1465.120232 | 0.969494 |
| GO:0030336\_negative\_regulation\_of\_cell\_migration | 18 | 0 | 0.000000 | -0.000000 | 1444 | 1334.779768 | 1399.95 | 1465.120232 | 0.969494 |
| GO:0030510\_regulation\_of\_BMP\_signaling\_pathway | 18 | 0 | 0.000000 | -0.000000 | 1444 | 1334.779768 | 1399.95 | 1465.120232 | 0.969494 |
| GO:0030901\_midbrain\_development | 18 | 0 | 0.000000 | -0.000000 | 1444 | 1334.779768 | 1399.95 | 1465.120232 | 0.969494 |
| GO:0032623\_interleukin-2\_production | 18 | 0 | 0.000000 | -0.000000 | 1444 | 1334.779768 | 1399.95 | 1465.120232 | 0.969494 |
| GO:0032984\_macromolecular\_complex\_disassembly | 18 | 0 | 0.000000 | -0.000000 | 1444 | 1334.779768 | 1399.95 | 1465.120232 | 0.969494 |
| GO:0033157\_regulation\_of\_intracellular\_protein\_transport | 18 | 0 | 0.000000 | -0.000000 | 1444 | 1334.779768 | 1399.95 | 1465.120232 | 0.969494 |
| GO:0035051\_cardiac\_cell\_differentiation | 18 | 0 | 0.000000 | -0.000000 | 1444 | 1334.779768 | 1399.95 | 1465.120232 | 0.969494 |
| GO:0042269\_regulation\_of\_natural\_killer\_cell\_mediated\_cytotoxicity | 18 | 0 | 0.000000 | -0.000000 | 1444 | 1334.779768 | 1399.95 | 1465.120232 | 0.969494 |
| GO:0043029\_T\_cell\_homeostasis | 18 | 0 | 0.000000 | -0.000000 | 1444 | 1334.779768 | 1399.95 | 1465.120232 | 0.969494 |
| GO:0044272\_sulfur\_compound\_biosynthetic\_process | 18 | 0 | 0.000000 | -0.000000 | 1444 | 1334.779768 | 1399.95 | 1465.120232 | 0.969494 |
| GO:0045058\_T\_cell\_selection | 18 | 0 | 0.000000 | -0.000000 | 1444 | 1334.779768 | 1399.95 | 1465.120232 | 0.969494 |
| GO:0045103\_intermediate\_filament-based\_process | 18 | 0 | 0.000000 | -0.000000 | 1444 | 1334.779768 | 1399.95 | 1465.120232 | 0.969494 |
| GO:0045638\_negative\_regulation\_of\_myeloid\_cell\_differentiation | 18 | 0 | 0.000000 | -0.000000 | 1444 | 1334.779768 | 1399.95 | 1465.120232 | 0.969494 |
| GO:0045807\_positive\_regulation\_of\_endocytosis | 18 | 0 | 0.000000 | -0.000000 | 1444 | 1334.779768 | 1399.95 | 1465.120232 | 0.969494 |
| GO:0048535\_lymph\_node\_development | 18 | 0 | 0.000000 | -0.000000 | 1444 | 1334.779768 | 1399.95 | 1465.120232 | 0.969494 |
| GO:0048730\_epidermis\_morphogenesis | 18 | 0 | 0.000000 | -0.000000 | 1444 | 1334.779768 | 1399.95 | 1465.120232 | 0.969494 |
| GO:0048813\_dendrite\_morphogenesis | 18 | 0 | 0.000000 | -0.000000 | 1444 | 1334.779768 | 1399.95 | 1465.120232 | 0.969494 |
| GO:0050731\_positive\_regulation\_of\_peptidyl-tyrosine\_phosphorylation | 18 | 0 | 0.000000 | -0.000000 | 1444 | 1334.779768 | 1399.95 | 1465.120232 | 0.969494 |
| GO:0050982\_detection\_of\_mechanical\_stimulus | 18 | 0 | 0.000000 | -0.000000 | 1444 | 1334.779768 | 1399.95 | 1465.120232 | 0.969494 |
| GO:0051168\_nuclear\_export | 18 | 0 | 0.000000 | -0.000000 | 1444 | 1334.779768 | 1399.95 | 1465.120232 | 0.969494 |
| GO:0051222\_positive\_regulation\_of\_protein\_transport | 18 | 0 | 0.000000 | -0.000000 | 1444 | 1334.779768 | 1399.95 | 1465.120232 | 0.969494 |
| GO:0051924\_regulation\_of\_calcium\_ion\_transport | 18 | 0 | 0.000000 | -0.000000 | 1444 | 1334.779768 | 1399.95 | 1465.120232 | 0.969494 |
| GO:0055008\_cardiac\_muscle\_tissue\_morphogenesis | 18 | 0 | 0.000000 | -0.000000 | 1444 | 1334.779768 | 1399.95 | 1465.120232 | 0.969494 |
| GO:0060415\_muscle\_tissue\_morphogenesis | 18 | 0 | 0.000000 | -0.000000 | 1444 | 1334.779768 | 1399.95 | 1465.120232 | 0.969494 |
| GO:0060571\_morphogenesis\_of\_an\_epithelial\_fold | 18 | 0 | 0.000000 | -0.000000 | 1444 | 1334.779768 | 1399.95 | 1465.120232 | 0.969494 |
| GO:0060674\_placenta\_blood\_vessel\_development | 18 | 0 | 0.000000 | -0.000000 | 1444 | 1334.779768 | 1399.95 | 1465.120232 | 0.969494 |
| GO:0002757\_immune\_response-activating\_signal\_transduction | 47 | 0 | 0.000000 | -0.000000 | 1455 | 1345.481274 | 1409.43 | 1473.378726 | 0.968680 |
| GO:0006140\_regulation\_of\_nucleotide\_metabolic\_process | 47 | 0 | 0.000000 | -0.000000 | 1455 | 1345.481274 | 1409.43 | 1473.378726 | 0.968680 |
| GO:0006396\_RNA\_processing | 47 | 0 | 0.000000 | -0.000000 | 1455 | 1345.481274 | 1409.43 | 1473.378726 | 0.968680 |
| GO:0016570\_histone\_modification | 47 | 0 | 0.000000 | -0.000000 | 1455 | 1345.481274 | 1409.43 | 1473.378726 | 0.968680 |
| GO:0030183\_B\_cell\_differentiation | 47 | 0 | 0.000000 | -0.000000 | 1455 | 1345.481274 | 1409.43 | 1473.378726 | 0.968680 |
| GO:0030799\_regulation\_of\_cyclic\_nucleotide\_metabolic\_process | 47 | 0 | 0.000000 | -0.000000 | 1455 | 1345.481274 | 1409.43 | 1473.378726 | 0.968680 |
| GO:0031667\_response\_to\_nutrient\_levels | 47 | 0 | 0.000000 | -0.000000 | 1455 | 1345.481274 | 1409.43 | 1473.378726 | 0.968680 |
| GO:0034754\_cellular\_hormone\_metabolic\_process | 47 | 0 | 0.000000 | -0.000000 | 1455 | 1345.481274 | 1409.43 | 1473.378726 | 0.968680 |
| GO:0045087\_innate\_immune\_response | 47 | 0 | 0.000000 | -0.000000 | 1455 | 1345.481274 | 1409.43 | 1473.378726 | 0.968680 |
| GO:0048871\_multicellular\_organismal\_homeostasis | 47 | 0 | 0.000000 | -0.000000 | 1455 | 1345.481274 | 1409.43 | 1473.378726 | 0.968680 |
| GO:0060627\_regulation\_of\_vesicle-mediated\_transport | 47 | 0 | 0.000000 | -0.000000 | 1455 | 1345.481274 | 1409.43 | 1473.378726 | 0.968680 |
| GO:0030902\_hindbrain\_development | 58 | 0 | 0.000000 | -0.000000 | 1458 | 1348.697879 | 1412.42 | 1476.142121 | 0.968738 |
| GO:0033043\_regulation\_of\_organelle\_organization | 58 | 0 | 0.000000 | -0.000000 | 1458 | 1348.697879 | 1412.42 | 1476.142121 | 0.968738 |
| GO:0050804\_regulation\_of\_synaptic\_transmission | 58 | 0 | 0.000000 | -0.000000 | 1458 | 1348.697879 | 1412.42 | 1476.142121 | 0.968738 |
| GO:0001817\_regulation\_of\_cytokine\_production | 99 | 0 | 0.000000 | -0.000000 | 1461 | 1351.235651 | 1414.64 | 1478.044349 | 0.968268 |
| GO:0060348\_bone\_development | 99 | 0 | 0.000000 | -0.000000 | 1461 | 1351.235651 | 1414.64 | 1478.044349 | 0.968268 |
| GO:0060562\_epithelial\_tube\_morphogenesis | 99 | 0 | 0.000000 | -0.000000 | 1461 | 1351.235651 | 1414.64 | 1478.044349 | 0.968268 |
| GO:0000209\_protein\_polyubiquitination | 10 | 0 | 0.000000 | -0.000000 | 1578 | 1469.792536 | 1531.68 | 1593.567464 | 0.970646 |
| GO:0000724\_double-strand\_break\_repair\_via\_homologous\_recombination | 10 | 0 | 0.000000 | -0.000000 | 1578 | 1469.792536 | 1531.68 | 1593.567464 | 0.970646 |
| GO:0000725\_recombinational\_repair | 10 | 0 | 0.000000 | -0.000000 | 1578 | 1469.792536 | 1531.68 | 1593.567464 | 0.970646 |
| GO:0001578\_microtubule\_bundle\_formation | 10 | 0 | 0.000000 | -0.000000 | 1578 | 1469.792536 | 1531.68 | 1593.567464 | 0.970646 |
| GO:0001659\_temperature\_homeostasis | 10 | 0 | 0.000000 | -0.000000 | 1578 | 1469.792536 | 1531.68 | 1593.567464 | 0.970646 |
| GO:0001773\_myeloid\_dendritic\_cell\_activation | 10 | 0 | 0.000000 | -0.000000 | 1578 | 1469.792536 | 1531.68 | 1593.567464 | 0.970646 |
| GO:0001832\_blastocyst\_growth | 10 | 0 | 0.000000 | -0.000000 | 1578 | 1469.792536 | 1531.68 | 1593.567464 | 0.970646 |
| GO:0001914\_regulation\_of\_T\_cell\_mediated\_cytotoxicity | 10 | 0 | 0.000000 | -0.000000 | 1578 | 1469.792536 | 1531.68 | 1593.567464 | 0.970646 |
| GO:0001990\_regulation\_of\_systemic\_arterial\_blood\_pressure\_by\_hormone | 10 | 0 | 0.000000 | -0.000000 | 1578 | 1469.792536 | 1531.68 | 1593.567464 | 0.970646 |
| GO:0002070\_epithelial\_cell\_maturation | 10 | 0 | 0.000000 | -0.000000 | 1578 | 1469.792536 | 1531.68 | 1593.567464 | 0.970646 |
| GO:0002673\_regulation\_of\_acute\_inflammatory\_response | 10 | 0 | 0.000000 | -0.000000 | 1578 | 1469.792536 | 1531.68 | 1593.567464 | 0.970646 |
| GO:0002711\_positive\_regulation\_of\_T\_cell\_mediated\_immunity | 10 | 0 | 0.000000 | -0.000000 | 1578 | 1469.792536 | 1531.68 | 1593.567464 | 0.970646 |
| GO:0002762\_negative\_regulation\_of\_myeloid\_leukocyte\_differentiation | 10 | 0 | 0.000000 | -0.000000 | 1578 | 1469.792536 | 1531.68 | 1593.567464 | 0.970646 |
| GO:0006040\_amino\_sugar\_metabolic\_process | 10 | 0 | 0.000000 | -0.000000 | 1578 | 1469.792536 | 1531.68 | 1593.567464 | 0.970646 |
| GO:0006081\_cellular\_aldehyde\_metabolic\_process | 10 | 0 | 0.000000 | -0.000000 | 1578 | 1469.792536 | 1531.68 | 1593.567464 | 0.970646 |
| GO:0006109\_regulation\_of\_carbohydrate\_metabolic\_process | 10 | 0 | 0.000000 | -0.000000 | 1578 | 1469.792536 | 1531.68 | 1593.567464 | 0.970646 |
| GO:0006289\_nucleotide-excision\_repair | 10 | 0 | 0.000000 | -0.000000 | 1578 | 1469.792536 | 1531.68 | 1593.567464 | 0.970646 |
| GO:0006342\_chromatin\_silencing | 10 | 0 | 0.000000 | -0.000000 | 1578 | 1469.792536 | 1531.68 | 1593.567464 | 0.970646 |
| GO:0006405\_RNA\_export\_from\_nucleus | 10 | 0 | 0.000000 | -0.000000 | 1578 | 1469.792536 | 1531.68 | 1593.567464 | 0.970646 |
| GO:0006801\_superoxide\_metabolic\_process | 10 | 0 | 0.000000 | -0.000000 | 1578 | 1469.792536 | 1531.68 | 1593.567464 | 0.970646 |
| GO:0006805\_xenobiotic\_metabolic\_process | 10 | 0 | 0.000000 | -0.000000 | 1578 | 1469.792536 | 1531.68 | 1593.567464 | 0.970646 |
| GO:0006826\_iron\_ion\_transport | 10 | 0 | 0.000000 | -0.000000 | 1578 | 1469.792536 | 1531.68 | 1593.567464 | 0.970646 |
| GO:0006921\_cell\_structure\_disassembly\_during\_apoptosis | 10 | 0 | 0.000000 | -0.000000 | 1578 | 1469.792536 | 1531.68 | 1593.567464 | 0.970646 |
| GO:0006968\_cellular\_defense\_response | 10 | 0 | 0.000000 | -0.000000 | 1578 | 1469.792536 | 1531.68 | 1593.567464 | 0.970646 |
| GO:0007006\_mitochondrial\_membrane\_organization | 10 | 0 | 0.000000 | -0.000000 | 1578 | 1469.792536 | 1531.68 | 1593.567464 | 0.970646 |
| GO:0007044\_cell-substrate\_junction\_assembly | 10 | 0 | 0.000000 | -0.000000 | 1578 | 1469.792536 | 1531.68 | 1593.567464 | 0.970646 |
| GO:0007093\_mitotic\_cell\_cycle\_checkpoint | 10 | 0 | 0.000000 | -0.000000 | 1578 | 1469.792536 | 1531.68 | 1593.567464 | 0.970646 |
| GO:0007194\_negative\_regulation\_of\_adenylate\_cyclase\_activity | 10 | 0 | 0.000000 | -0.000000 | 1578 | 1469.792536 | 1531.68 | 1593.567464 | 0.970646 |
| GO:0008088\_axon\_cargo\_transport | 10 | 0 | 0.000000 | -0.000000 | 1578 | 1469.792536 | 1531.68 | 1593.567464 | 0.970646 |
| GO:0008206\_bile\_acid\_metabolic\_process | 10 | 0 | 0.000000 | -0.000000 | 1578 | 1469.792536 | 1531.68 | 1593.567464 | 0.970646 |
| GO:0008211\_glucocorticoid\_metabolic\_process | 10 | 0 | 0.000000 | -0.000000 | 1578 | 1469.792536 | 1531.68 | 1593.567464 | 0.970646 |
| GO:0009066\_aspartate\_family\_amino\_acid\_metabolic\_process | 10 | 0 | 0.000000 | -0.000000 | 1578 | 1469.792536 | 1531.68 | 1593.567464 | 0.970646 |
| GO:0009110\_vitamin\_biosynthetic\_process | 10 | 0 | 0.000000 | -0.000000 | 1578 | 1469.792536 | 1531.68 | 1593.567464 | 0.970646 |
| GO:0009620\_response\_to\_fungus | 10 | 0 | 0.000000 | -0.000000 | 1578 | 1469.792536 | 1531.68 | 1593.567464 | 0.970646 |
| GO:0009743\_response\_to\_carbohydrate\_stimulus | 10 | 0 | 0.000000 | -0.000000 | 1578 | 1469.792536 | 1531.68 | 1593.567464 | 0.970646 |
| GO:0009948\_anterior\_posterior\_axis\_specification | 10 | 0 | 0.000000 | -0.000000 | 1578 | 1469.792536 | 1531.68 | 1593.567464 | 0.970646 |
| GO:0010827\_regulation\_of\_glucose\_transport | 10 | 0 | 0.000000 | -0.000000 | 1578 | 1469.792536 | 1531.68 | 1593.567464 | 0.970646 |
| GO:0015718\_monocarboxylic\_acid\_transport | 10 | 0 | 0.000000 | -0.000000 | 1578 | 1469.792536 | 1531.68 | 1593.567464 | 0.970646 |
| GO:0016197\_endosome\_transport | 10 | 0 | 0.000000 | -0.000000 | 1578 | 1469.792536 | 1531.68 | 1593.567464 | 0.970646 |
| GO:0016486\_peptide\_hormone\_processing | 10 | 0 | 0.000000 | -0.000000 | 1578 | 1469.792536 | 1531.68 | 1593.567464 | 0.970646 |
| GO:0017156\_calcium\_ion-dependent\_exocytosis | 10 | 0 | 0.000000 | -0.000000 | 1578 | 1469.792536 | 1531.68 | 1593.567464 | 0.970646 |
| GO:0018149\_peptide\_cross-linking | 10 | 0 | 0.000000 | -0.000000 | 1578 | 1469.792536 | 1531.68 | 1593.567464 | 0.970646 |
| GO:0021534\_cell\_proliferation\_in\_hindbrain | 10 | 0 | 0.000000 | -0.000000 | 1578 | 1469.792536 | 1531.68 | 1593.567464 | 0.970646 |
| GO:0021895\_cerebral\_cortex\_neuron\_differentiation | 10 | 0 | 0.000000 | -0.000000 | 1578 | 1469.792536 | 1531.68 | 1593.567464 | 0.970646 |
| GO:0021924\_cell\_proliferation\_in\_the\_external\_granule\_layer | 10 | 0 | 0.000000 | -0.000000 | 1578 | 1469.792536 | 1531.68 | 1593.567464 | 0.970646 |
| GO:0021930\_granule\_cell\_precursor\_proliferation | 10 | 0 | 0.000000 | -0.000000 | 1578 | 1469.792536 | 1531.68 | 1593.567464 | 0.970646 |
| GO:0021952\_central\_nervous\_system\_projection\_neuron\_axonogenesis | 10 | 0 | 0.000000 | -0.000000 | 1578 | 1469.792536 | 1531.68 | 1593.567464 | 0.970646 |
| GO:0030168\_platelet\_activation | 10 | 0 | 0.000000 | -0.000000 | 1578 | 1469.792536 | 1531.68 | 1593.567464 | 0.970646 |
| GO:0030833\_regulation\_of\_actin\_filament\_polymerization | 10 | 0 | 0.000000 | -0.000000 | 1578 | 1469.792536 | 1531.68 | 1593.567464 | 0.970646 |
| GO:0031018\_endocrine\_pancreas\_development | 10 | 0 | 0.000000 | -0.000000 | 1578 | 1469.792536 | 1531.68 | 1593.567464 | 0.970646 |
| GO:0031280\_negative\_regulation\_of\_cyclase\_activity | 10 | 0 | 0.000000 | -0.000000 | 1578 | 1469.792536 | 1531.68 | 1593.567464 | 0.970646 |
| GO:0031331\_positive\_regulation\_of\_cellular\_catabolic\_process | 10 | 0 | 0.000000 | -0.000000 | 1578 | 1469.792536 | 1531.68 | 1593.567464 | 0.970646 |
| GO:0031645\_negative\_regulation\_of\_neurological\_system\_process | 10 | 0 | 0.000000 | -0.000000 | 1578 | 1469.792536 | 1531.68 | 1593.567464 | 0.970646 |
| GO:0032318\_regulation\_of\_Ras\_GTPase\_activity | 10 | 0 | 0.000000 | -0.000000 | 1578 | 1469.792536 | 1531.68 | 1593.567464 | 0.970646 |
| GO:0032602\_chemokine\_production | 10 | 0 | 0.000000 | -0.000000 | 1578 | 1469.792536 | 1531.68 | 1593.567464 | 0.970646 |
| GO:0032633\_interleukin-4\_production | 10 | 0 | 0.000000 | -0.000000 | 1578 | 1469.792536 | 1531.68 | 1593.567464 | 0.970646 |
| GO:0032642\_regulation\_of\_chemokine\_production | 10 | 0 | 0.000000 | -0.000000 | 1578 | 1469.792536 | 1531.68 | 1593.567464 | 0.970646 |
| GO:0032673\_regulation\_of\_interleukin-4\_production | 10 | 0 | 0.000000 | -0.000000 | 1578 | 1469.792536 | 1531.68 | 1593.567464 | 0.970646 |
| GO:0032760\_positive\_regulation\_of\_tumor\_necrosis\_factor\_production | 10 | 0 | 0.000000 | -0.000000 | 1578 | 1469.792536 | 1531.68 | 1593.567464 | 0.970646 |
| GO:0033081\_regulation\_of\_T\_cell\_differentiation\_in\_the\_thymus | 10 | 0 | 0.000000 | -0.000000 | 1578 | 1469.792536 | 1531.68 | 1593.567464 | 0.970646 |
| GO:0034105\_positive\_regulation\_of\_tissue\_remodeling | 10 | 0 | 0.000000 | -0.000000 | 1578 | 1469.792536 | 1531.68 | 1593.567464 | 0.970646 |
| GO:0034637\_cellular\_carbohydrate\_biosynthetic\_process | 10 | 0 | 0.000000 | -0.000000 | 1578 | 1469.792536 | 1531.68 | 1593.567464 | 0.970646 |
| GO:0040015\_negative\_regulation\_of\_multicellular\_organism\_growth | 10 | 0 | 0.000000 | -0.000000 | 1578 | 1469.792536 | 1531.68 | 1593.567464 | 0.970646 |
| GO:0042088\_T-helper\_1\_type\_immune\_response | 10 | 0 | 0.000000 | -0.000000 | 1578 | 1469.792536 | 1531.68 | 1593.567464 | 0.970646 |
| GO:0042116\_macrophage\_activation | 10 | 0 | 0.000000 | -0.000000 | 1578 | 1469.792536 | 1531.68 | 1593.567464 | 0.970646 |
| GO:0042177\_negative\_regulation\_of\_protein\_catabolic\_process | 10 | 0 | 0.000000 | -0.000000 | 1578 | 1469.792536 | 1531.68 | 1593.567464 | 0.970646 |
| GO:0042755\_eating\_behavior | 10 | 0 | 0.000000 | -0.000000 | 1578 | 1469.792536 | 1531.68 | 1593.567464 | 0.970646 |
| GO:0043330\_response\_to\_exogenous\_dsRNA | 10 | 0 | 0.000000 | -0.000000 | 1578 | 1469.792536 | 1531.68 | 1593.567464 | 0.970646 |
| GO:0043488\_regulation\_of\_mRNA\_stability | 10 | 0 | 0.000000 | -0.000000 | 1578 | 1469.792536 | 1531.68 | 1593.567464 | 0.970646 |
| GO:0043506\_regulation\_of\_JUN\_kinase\_activity | 10 | 0 | 0.000000 | -0.000000 | 1578 | 1469.792536 | 1531.68 | 1593.567464 | 0.970646 |
| GO:0043525\_positive\_regulation\_of\_neuron\_apoptosis | 10 | 0 | 0.000000 | -0.000000 | 1578 | 1469.792536 | 1531.68 | 1593.567464 | 0.970646 |
| GO:0044259\_multicellular\_organismal\_macromolecule\_metabolic\_process | 10 | 0 | 0.000000 | -0.000000 | 1578 | 1469.792536 | 1531.68 | 1593.567464 | 0.970646 |
| GO:0045132\_meiotic\_chromosome\_segregation | 10 | 0 | 0.000000 | -0.000000 | 1578 | 1469.792536 | 1531.68 | 1593.567464 | 0.970646 |
| GO:0045446\_endothelial\_cell\_differentiation | 10 | 0 | 0.000000 | -0.000000 | 1578 | 1469.792536 | 1531.68 | 1593.567464 | 0.970646 |
| GO:0045576\_mast\_cell\_activation | 10 | 0 | 0.000000 | -0.000000 | 1578 | 1469.792536 | 1531.68 | 1593.567464 | 0.970646 |
| GO:0045669\_positive\_regulation\_of\_osteoblast\_differentiation | 10 | 0 | 0.000000 | -0.000000 | 1578 | 1469.792536 | 1531.68 | 1593.567464 | 0.970646 |
| GO:0045776\_negative\_regulation\_of\_blood\_pressure | 10 | 0 | 0.000000 | -0.000000 | 1578 | 1469.792536 | 1531.68 | 1593.567464 | 0.970646 |
| GO:0045777\_positive\_regulation\_of\_blood\_pressure | 10 | 0 | 0.000000 | -0.000000 | 1578 | 1469.792536 | 1531.68 | 1593.567464 | 0.970646 |
| GO:0045814\_negative\_regulation\_of\_gene\_expression\_\_epigenetic | 10 | 0 | 0.000000 | -0.000000 | 1578 | 1469.792536 | 1531.68 | 1593.567464 | 0.970646 |
| GO:0045911\_positive\_regulation\_of\_DNA\_recombination | 10 | 0 | 0.000000 | -0.000000 | 1578 | 1469.792536 | 1531.68 | 1593.567464 | 0.970646 |
| GO:0046887\_positive\_regulation\_of\_hormone\_secretion | 10 | 0 | 0.000000 | -0.000000 | 1578 | 1469.792536 | 1531.68 | 1593.567464 | 0.970646 |
| GO:0048291\_isotype\_switching\_to\_IgG\_isotypes | 10 | 0 | 0.000000 | -0.000000 | 1578 | 1469.792536 | 1531.68 | 1593.567464 | 0.970646 |
| GO:0048302\_regulation\_of\_isotype\_switching\_to\_IgG\_isotypes | 10 | 0 | 0.000000 | -0.000000 | 1578 | 1469.792536 | 1531.68 | 1593.567464 | 0.970646 |
| GO:0048339\_paraxial\_mesoderm\_development | 10 | 0 | 0.000000 | -0.000000 | 1578 | 1469.792536 | 1531.68 | 1593.567464 | 0.970646 |
| GO:0048384\_retinoic\_acid\_receptor\_signaling\_pathway | 10 | 0 | 0.000000 | -0.000000 | 1578 | 1469.792536 | 1531.68 | 1593.567464 | 0.970646 |
| GO:0048596\_embryonic\_camera-type\_eye\_morphogenesis | 10 | 0 | 0.000000 | -0.000000 | 1578 | 1469.792536 | 1531.68 | 1593.567464 | 0.970646 |
| GO:0048738\_cardiac\_muscle\_tissue\_development | 10 | 0 | 0.000000 | -0.000000 | 1578 | 1469.792536 | 1531.68 | 1593.567464 | 0.970646 |
| GO:0050654\_chondroitin\_sulfate\_proteoglycan\_metabolic\_process | 10 | 0 | 0.000000 | -0.000000 | 1578 | 1469.792536 | 1531.68 | 1593.567464 | 0.970646 |
| GO:0050657\_nucleic\_acid\_transport | 10 | 0 | 0.000000 | -0.000000 | 1578 | 1469.792536 | 1531.68 | 1593.567464 | 0.970646 |
| GO:0050658\_RNA\_transport | 10 | 0 | 0.000000 | -0.000000 | 1578 | 1469.792536 | 1531.68 | 1593.567464 | 0.970646 |
| GO:0050663\_cytokine\_secretion | 10 | 0 | 0.000000 | -0.000000 | 1578 | 1469.792536 | 1531.68 | 1593.567464 | 0.970646 |
| GO:0050714\_positive\_regulation\_of\_protein\_secretion | 10 | 0 | 0.000000 | -0.000000 | 1578 | 1469.792536 | 1531.68 | 1593.567464 | 0.970646 |
| GO:0050879\_multicellular\_organismal\_movement | 10 | 0 | 0.000000 | -0.000000 | 1578 | 1469.792536 | 1531.68 | 1593.567464 | 0.970646 |
| GO:0050881\_musculoskeletal\_movement | 10 | 0 | 0.000000 | -0.000000 | 1578 | 1469.792536 | 1531.68 | 1593.567464 | 0.970646 |
| GO:0050886\_endocrine\_process | 10 | 0 | 0.000000 | -0.000000 | 1578 | 1469.792536 | 1531.68 | 1593.567464 | 0.970646 |
| GO:0050892\_intestinal\_absorption | 10 | 0 | 0.000000 | -0.000000 | 1578 | 1469.792536 | 1531.68 | 1593.567464 | 0.970646 |
| GO:0051147\_regulation\_of\_muscle\_cell\_differentiation | 10 | 0 | 0.000000 | -0.000000 | 1578 | 1469.792536 | 1531.68 | 1593.567464 | 0.970646 |
| GO:0051208\_sequestering\_of\_calcium\_ion | 10 | 0 | 0.000000 | -0.000000 | 1578 | 1469.792536 | 1531.68 | 1593.567464 | 0.970646 |
| GO:0051209\_release\_of\_sequestered\_calcium\_ion\_into\_cytosol | 10 | 0 | 0.000000 | -0.000000 | 1578 | 1469.792536 | 1531.68 | 1593.567464 | 0.970646 |
| GO:0051224\_negative\_regulation\_of\_protein\_transport | 10 | 0 | 0.000000 | -0.000000 | 1578 | 1469.792536 | 1531.68 | 1593.567464 | 0.970646 |
| GO:0051236\_establishment\_of\_RNA\_localization | 10 | 0 | 0.000000 | -0.000000 | 1578 | 1469.792536 | 1531.68 | 1593.567464 | 0.970646 |
| GO:0051238\_sequestering\_of\_metal\_ion | 10 | 0 | 0.000000 | -0.000000 | 1578 | 1469.792536 | 1531.68 | 1593.567464 | 0.970646 |
| GO:0051262\_protein\_tetramerization | 10 | 0 | 0.000000 | -0.000000 | 1578 | 1469.792536 | 1531.68 | 1593.567464 | 0.970646 |
| GO:0051282\_regulation\_of\_sequestering\_of\_calcium\_ion | 10 | 0 | 0.000000 | -0.000000 | 1578 | 1469.792536 | 1531.68 | 1593.567464 | 0.970646 |
| GO:0051283\_negative\_regulation\_of\_sequestering\_of\_calcium\_ion | 10 | 0 | 0.000000 | -0.000000 | 1578 | 1469.792536 | 1531.68 | 1593.567464 | 0.970646 |
| GO:0051350\_negative\_regulation\_of\_lyase\_activity | 10 | 0 | 0.000000 | -0.000000 | 1578 | 1469.792536 | 1531.68 | 1593.567464 | 0.970646 |
| GO:0051445\_regulation\_of\_meiotic\_cell\_cycle | 10 | 0 | 0.000000 | -0.000000 | 1578 | 1469.792536 | 1531.68 | 1593.567464 | 0.970646 |
| GO:0051650\_establishment\_of\_vesicle\_localization | 10 | 0 | 0.000000 | -0.000000 | 1578 | 1469.792536 | 1531.68 | 1593.567464 | 0.970646 |
| GO:0051651\_maintenance\_of\_location\_in\_cell | 10 | 0 | 0.000000 | -0.000000 | 1578 | 1469.792536 | 1531.68 | 1593.567464 | 0.970646 |
| GO:0060135\_maternal\_process\_involved\_in\_female\_pregnancy | 10 | 0 | 0.000000 | -0.000000 | 1578 | 1469.792536 | 1531.68 | 1593.567464 | 0.970646 |
| GO:0060216\_definitive\_hemopoiesis | 10 | 0 | 0.000000 | -0.000000 | 1578 | 1469.792536 | 1531.68 | 1593.567464 | 0.970646 |
| GO:0060323\_head\_morphogenesis | 10 | 0 | 0.000000 | -0.000000 | 1578 | 1469.792536 | 1531.68 | 1593.567464 | 0.970646 |
| GO:0060343\_trabecula\_formation | 10 | 0 | 0.000000 | -0.000000 | 1578 | 1469.792536 | 1531.68 | 1593.567464 | 0.970646 |
| GO:0060601\_lateral\_sprouting\_from\_an\_epithelium | 10 | 0 | 0.000000 | -0.000000 | 1578 | 1469.792536 | 1531.68 | 1593.567464 | 0.970646 |
| GO:0060669\_embryonic\_placenta\_morphogenesis | 10 | 0 | 0.000000 | -0.000000 | 1578 | 1469.792536 | 1531.68 | 1593.567464 | 0.970646 |
| GO:0060706\_cell\_differentiation\_involved\_in\_embryonic\_placenta\_development | 10 | 0 | 0.000000 | -0.000000 | 1578 | 1469.792536 | 1531.68 | 1593.567464 | 0.970646 |
| GO:0060768\_regulation\_of\_epithelial\_cell\_proliferation\_involved\_in\_prostate\_gland\_development | 10 | 0 | 0.000000 | -0.000000 | 1578 | 1469.792536 | 1531.68 | 1593.567464 | 0.970646 |
| GO:0001824\_blastocyst\_development | 40 | 0 | 0.000000 | -0.000000 | 1590 | 1480.738842 | 1542.05 | 1603.361158 | 0.969843 |
| GO:0007346\_regulation\_of\_mitotic\_cell\_cycle | 40 | 0 | 0.000000 | -0.000000 | 1590 | 1480.738842 | 1542.05 | 1603.361158 | 0.969843 |
| GO:0007599\_hemostasis | 40 | 0 | 0.000000 | -0.000000 | 1590 | 1480.738842 | 1542.05 | 1603.361158 | 0.969843 |
| GO:0008203\_cholesterol\_metabolic\_process | 40 | 0 | 0.000000 | -0.000000 | 1590 | 1480.738842 | 1542.05 | 1603.361158 | 0.969843 |
| GO:0014031\_mesenchymal\_cell\_development | 40 | 0 | 0.000000 | -0.000000 | 1590 | 1480.738842 | 1542.05 | 1603.361158 | 0.969843 |
| GO:0016071\_mRNA\_metabolic\_process | 40 | 0 | 0.000000 | -0.000000 | 1590 | 1480.738842 | 1542.05 | 1603.361158 | 0.969843 |
| GO:0016358\_dendrite\_development | 40 | 0 | 0.000000 | -0.000000 | 1590 | 1480.738842 | 1542.05 | 1603.361158 | 0.969843 |
| GO:0016485\_protein\_processing | 40 | 0 | 0.000000 | -0.000000 | 1590 | 1480.738842 | 1542.05 | 1603.361158 | 0.969843 |
| GO:0017015\_regulation\_of\_transforming\_growth\_factor\_beta\_receptor\_signaling\_pathway | 40 | 0 | 0.000000 | -0.000000 | 1590 | 1480.738842 | 1542.05 | 1603.361158 | 0.969843 |
| GO:0019935\_cyclic-nucleotide-mediated\_signaling | 40 | 0 | 0.000000 | -0.000000 | 1590 | 1480.738842 | 1542.05 | 1603.361158 | 0.969843 |
| GO:0046850\_regulation\_of\_bone\_remodeling | 40 | 0 | 0.000000 | -0.000000 | 1590 | 1480.738842 | 1542.05 | 1603.361158 | 0.969843 |
| GO:0051129\_negative\_regulation\_of\_cellular\_component\_organization | 40 | 0 | 0.000000 | -0.000000 | 1590 | 1480.738842 | 1542.05 | 1603.361158 | 0.969843 |
| GO:0021700\_developmental\_maturation | 81 | 0 | 0.000000 | -0.000000 | 1591 | 1481.509112 | 1542.73 | 1603.950888 | 0.969661 |
| GO:0009791\_post-embryonic\_development | 67 | 0 | 0.000000 | -0.000000 | 1595 | 1485.451075 | 1546.33 | 1607.208925 | 0.969486 |
| GO:0031347\_regulation\_of\_defense\_response | 67 | 0 | 0.000000 | -0.000000 | 1595 | 1485.451075 | 1546.33 | 1607.208925 | 0.969486 |
| GO:0042445\_hormone\_metabolic\_process | 67 | 0 | 0.000000 | -0.000000 | 1595 | 1485.451075 | 1546.33 | 1607.208925 | 0.969486 |
| GO:0051247\_positive\_regulation\_of\_protein\_metabolic\_process | 67 | 0 | 0.000000 | -0.000000 | 1595 | 1485.451075 | 1546.33 | 1607.208925 | 0.969486 |
| GO:0048584\_positive\_regulation\_of\_response\_to\_stimulus | 115 | 0 | 0.000000 | -0.000000 | 1596 | 1486.755463 | 1547.29 | 1607.824537 | 0.969480 |
| GO:0001890\_placenta\_development | 77 | 0 | 0.000000 | -0.000000 | 1598 | 1488.008566 | 1548.47 | 1608.931434 | 0.969005 |
| GO:0051241\_negative\_regulation\_of\_multicellular\_organismal\_process | 77 | 0 | 0.000000 | -0.000000 | 1598 | 1488.008566 | 1548.47 | 1608.931434 | 0.969005 |
| GO:0006519\_cellular\_amino\_acid\_and\_derivative\_metabolic\_process | 118 | 0 | 0.000000 | -0.000000 | 1599 | 1489.665186 | 1549.88 | 1610.094814 | 0.969281 |
| GO:0030326\_embryonic\_limb\_morphogenesis | 78 | 0 | 0.000000 | -0.000000 | 1601 | 1494.593365 | 1554.52 | 1614.446635 | 0.970968 |
| GO:0035113\_embryonic\_appendage\_morphogenesis | 78 | 0 | 0.000000 | -0.000000 | 1601 | 1494.593365 | 1554.52 | 1614.446635 | 0.970968 |
| GO:0042471\_ear\_morphogenesis | 65 | 0 | 0.000000 | -0.000000 | 1603 | 1496.788026 | 1556.52 | 1616.251974 | 0.971004 |
| GO:0048511\_rhythmic\_process | 65 | 0 | 0.000000 | -0.000000 | 1603 | 1496.788026 | 1556.52 | 1616.251974 | 0.971004 |
| GO:0001912\_positive\_regulation\_of\_leukocyte\_mediated\_cytotoxicity | 20 | 0 | 0.000000 | -0.000000 | 1631 | 1524.877355 | 1583.92 | 1642.962645 | 0.971134 |
| GO:0005977\_glycogen\_metabolic\_process | 20 | 0 | 0.000000 | -0.000000 | 1631 | 1524.877355 | 1583.92 | 1642.962645 | 0.971134 |
| GO:0006073\_cellular\_glucan\_metabolic\_process | 20 | 0 | 0.000000 | -0.000000 | 1631 | 1524.877355 | 1583.92 | 1642.962645 | 0.971134 |
| GO:0006518\_peptide\_metabolic\_process | 20 | 0 | 0.000000 | -0.000000 | 1631 | 1524.877355 | 1583.92 | 1642.962645 | 0.971134 |
| GO:0006584\_catecholamine\_metabolic\_process | 20 | 0 | 0.000000 | -0.000000 | 1631 | 1524.877355 | 1583.92 | 1642.962645 | 0.971134 |
| GO:0007586\_digestion | 20 | 0 | 0.000000 | -0.000000 | 1631 | 1524.877355 | 1583.92 | 1642.962645 | 0.971134 |
| GO:0009615\_response\_to\_virus | 20 | 0 | 0.000000 | -0.000000 | 1631 | 1524.877355 | 1583.92 | 1642.962645 | 0.971134 |
| GO:0009712\_catechol\_metabolic\_process | 20 | 0 | 0.000000 | -0.000000 | 1631 | 1524.877355 | 1583.92 | 1642.962645 | 0.971134 |
| GO:0010927\_cellular\_component\_assembly\_involved\_in\_morphogenesis | 20 | 0 | 0.000000 | -0.000000 | 1631 | 1524.877355 | 1583.92 | 1642.962645 | 0.971134 |
| GO:0016571\_histone\_methylation | 20 | 0 | 0.000000 | -0.000000 | 1631 | 1524.877355 | 1583.92 | 1642.962645 | 0.971134 |
| GO:0018209\_peptidyl-serine\_modification | 20 | 0 | 0.000000 | -0.000000 | 1631 | 1524.877355 | 1583.92 | 1642.962645 | 0.971134 |
| GO:0018958\_phenol\_metabolic\_process | 20 | 0 | 0.000000 | -0.000000 | 1631 | 1524.877355 | 1583.92 | 1642.962645 | 0.971134 |
| GO:0021695\_cerebellar\_cortex\_development | 20 | 0 | 0.000000 | -0.000000 | 1631 | 1524.877355 | 1583.92 | 1642.962645 | 0.971134 |
| GO:0031128\_developmental\_induction | 20 | 0 | 0.000000 | -0.000000 | 1631 | 1524.877355 | 1583.92 | 1642.962645 | 0.971134 |
| GO:0031214\_biomineral\_formation | 20 | 0 | 0.000000 | -0.000000 | 1631 | 1524.877355 | 1583.92 | 1642.962645 | 0.971134 |
| GO:0031343\_positive\_regulation\_of\_cell\_killing | 20 | 0 | 0.000000 | -0.000000 | 1631 | 1524.877355 | 1583.92 | 1642.962645 | 0.971134 |
| GO:0032582\_negative\_regulation\_of\_gene-specific\_transcription | 20 | 0 | 0.000000 | -0.000000 | 1631 | 1524.877355 | 1583.92 | 1642.962645 | 0.971134 |
| GO:0032640\_tumor\_necrosis\_factor\_production | 20 | 0 | 0.000000 | -0.000000 | 1631 | 1524.877355 | 1583.92 | 1642.962645 | 0.971134 |
| GO:0032680\_regulation\_of\_tumor\_necrosis\_factor\_production | 20 | 0 | 0.000000 | -0.000000 | 1631 | 1524.877355 | 1583.92 | 1642.962645 | 0.971134 |
| GO:0034311\_diol\_metabolic\_process | 20 | 0 | 0.000000 | -0.000000 | 1631 | 1524.877355 | 1583.92 | 1642.962645 | 0.971134 |
| GO:0042326\_negative\_regulation\_of\_phosphorylation | 20 | 0 | 0.000000 | -0.000000 | 1631 | 1524.877355 | 1583.92 | 1642.962645 | 0.971134 |
| GO:0044042\_glucan\_metabolic\_process | 20 | 0 | 0.000000 | -0.000000 | 1631 | 1524.877355 | 1583.92 | 1642.962645 | 0.971134 |
| GO:0045017\_glycerolipid\_biosynthetic\_process | 20 | 0 | 0.000000 | -0.000000 | 1631 | 1524.877355 | 1583.92 | 1642.962645 | 0.971134 |
| GO:0045168\_cell-cell\_signaling\_involved\_in\_cell\_fate\_specification | 20 | 0 | 0.000000 | -0.000000 | 1631 | 1524.877355 | 1583.92 | 1642.962645 | 0.971134 |
| GO:0045639\_positive\_regulation\_of\_myeloid\_cell\_differentiation | 20 | 0 | 0.000000 | -0.000000 | 1631 | 1524.877355 | 1583.92 | 1642.962645 | 0.971134 |
| GO:0046822\_regulation\_of\_nucleocytoplasmic\_transport | 20 | 0 | 0.000000 | -0.000000 | 1631 | 1524.877355 | 1583.92 | 1642.962645 | 0.971134 |
| GO:0048806\_genitalia\_development | 20 | 0 | 0.000000 | -0.000000 | 1631 | 1524.877355 | 1583.92 | 1642.962645 | 0.971134 |
| GO:0060191\_regulation\_of\_lipase\_activity | 20 | 0 | 0.000000 | -0.000000 | 1631 | 1524.877355 | 1583.92 | 1642.962645 | 0.971134 |
| GO:0006897\_endocytosis | 86 | 0 | 0.000000 | -0.000000 | 1633 | 1528.803813 | 1587.39 | 1645.976187 | 0.972070 |
| GO:0010324\_membrane\_invagination | 86 | 0 | 0.000000 | -0.000000 | 1633 | 1528.803813 | 1587.39 | 1645.976187 | 0.972070 |
| GO:0000027\_ribosomal\_large\_subunit\_assembly | 1 | 0 |  |  |  |  |  |  |  |  |
| GO:0000042\_protein\_targeting\_to\_Golgi | 1 | 0 |  |  |  |  |  |  |  |  |
| GO:0000046\_autophagic\_vacuole\_fusion | 1 | 0 |  |  |  |  |  |  |  |  |
| GO:0000050\_urea\_cycle | 1 | 0 |  |  |  |  |  |  |  |  |
| GO:0000054\_ribosome\_export\_from\_nucleus | 1 | 0 |  |  |  |  |  |  |  |  |
| GO:0000055\_ribosomal\_large\_subunit\_export\_from\_nucleus | 1 | 0 |  |  |  |  |  |  |  |  |
| GO:0000056\_ribosomal\_small\_subunit\_export\_from\_nucleus | 1 | 0 |  |  |  |  |  |  |  |  |
| GO:0000072\_M\_phase\_specific\_microtubule\_process | 1 | 0 |  |  |  |  |  |  |  |  |
| GO:0000101\_sulfur\_amino\_acid\_transport | 1 | 0 |  |  |  |  |  |  |  |  |
| GO:0000147\_actin\_cortical\_patch\_assembly | 1 | 0 |  |  |  |  |  |  |  |  |
| GO:0000154\_rRNA\_modification | 1 | 0 |  |  |  |  |  |  |  |  |
| GO:0000183\_chromatin\_silencing\_at\_rDNA | 1 | 0 |  |  |  |  |  |  |  |  |
| GO:0000185\_activation\_of\_MAPKKK\_activity | 1 | 0 |  |  |  |  |  |  |  |  |
| GO:0000238\_zygotene | 1 | 0 |  |  |  |  |  |  |  |  |
| GO:0000255\_allantoin\_metabolic\_process | 1 | 0 |  |  |  |  |  |  |  |  |
| GO:0000266\_mitochondrial\_fission | 1 | 0 |  |  |  |  |  |  |  |  |
| GO:0000273\_lipoic\_acid\_metabolic\_process | 1 | 0 |  |  |  |  |  |  |  |  |
| GO:0000301\_retrograde\_transport\_\_vesicle\_recycling\_within\_Golgi | 1 | 0 |  |  |  |  |  |  |  |  |
| GO:0000394\_RNA\_splicing\_\_via\_endonucleolytic\_cleavage\_and\_ligation | 1 | 0 |  |  |  |  |  |  |  |  |
| GO:0000429\_regulation\_of\_transcription\_from\_RNA\_polymerase\_II\_promoter\_by\_carbon\_catabolites | 1 | 0 |  |  |  |  |  |  |  |  |
| GO:0000430\_regulation\_of\_transcription\_from\_RNA\_polymerase\_II\_promoter\_by\_glucose | 1 | 0 |  |  |  |  |  |  |  |  |
| GO:0000432\_positive\_regulation\_of\_transcription\_from\_RNA\_polymerase\_II\_promoter\_by\_glucose | 1 | 0 |  |  |  |  |  |  |  |  |
| GO:0000436\_positive\_regulation\_of\_transcription\_from\_RNA\_polymerase\_II\_promoter\_by\_carbon\_catabolites | 1 | 0 |  |  |  |  |  |  |  |  |
| GO:0000448\_cleavage\_in\_ITS2\_between\_5.8S\_rRNA\_and\_LSU-rRNA\_of\_tricistronic\_rRNA\_transcript\_(SSU-rRNA\_\_5.8S\_rRNA\_\_LSU-rRNA) | 1 | 0 |  |  |  |  |  |  |  |  |
| GO:0000460\_maturation\_of\_5.8S\_rRNA | 1 | 0 |  |  |  |  |  |  |  |  |
| GO:0000463\_maturation\_of\_LSU-rRNA\_from\_tricistronic\_rRNA\_transcript\_(SSU-rRNA\_\_5.8S\_rRNA\_\_LSU-rRNA) | 1 | 0 |  |  |  |  |  |  |  |  |
| GO:0000466\_maturation\_of\_5.8S\_rRNA\_from\_tricistronic\_rRNA\_transcript\_(SSU-rRNA\_\_5.8S\_rRNA\_\_LSU-rRNA) | 1 | 0 |  |  |  |  |  |  |  |  |
| GO:0000469\_cleavages\_during\_rRNA\_processing | 1 | 0 |  |  |  |  |  |  |  |  |
| GO:0000470\_maturation\_of\_LSU-rRNA | 1 | 0 |  |  |  |  |  |  |  |  |
| GO:0000478\_endonucleolytic\_cleavages\_during\_rRNA\_processing | 1 | 0 |  |  |  |  |  |  |  |  |
| GO:0000479\_endonucleolytic\_cleavage\_of\_tricistronic\_rRNA\_transcript\_(SSU-rRNA\_\_5.8S\_rRNA\_\_LSU-rRNA) | 1 | 0 |  |  |  |  |  |  |  |  |
| GO:0000705\_achiasmate\_meiosis\_I | 1 | 0 |  |  |  |  |  |  |  |  |
| GO:0000966\_RNA\_5'-end\_processing | 1 | 0 |  |  |  |  |  |  |  |  |
| GO:0001300\_chronological\_cell\_aging | 1 | 0 |  |  |  |  |  |  |  |  |
| GO:0001547\_antral\_ovarian\_follicle\_growth | 1 | 0 |  |  |  |  |  |  |  |  |
| GO:0001555\_oocyte\_growth | 1 | 0 |  |  |  |  |  |  |  |  |
| GO:0001560\_regulation\_of\_cell\_growth\_by\_extracellular\_stimulus | 1 | 0 |  |  |  |  |  |  |  |  |
| GO:0001660\_fever | 1 | 0 |  |  |  |  |  |  |  |  |
| GO:0001696\_gastric\_acid\_secretion | 1 | 0 |  |  |  |  |  |  |  |  |
| GO:0001712\_ectodermal\_cell\_fate\_commitment | 1 | 0 |  |  |  |  |  |  |  |  |
| GO:0001714\_endodermal\_cell\_fate\_specification | 1 | 0 |  |  |  |  |  |  |  |  |
| GO:0001762\_beta-alanine\_transport | 1 | 0 |  |  |  |  |  |  |  |  |
| GO:0001766\_membrane\_raft\_polarization | 1 | 0 |  |  |  |  |  |  |  |  |
| GO:0001811\_negative\_regulation\_of\_type\_I\_hypersensitivity | 1 | 0 |  |  |  |  |  |  |  |  |
| GO:0001821\_histamine\_secretion | 1 | 0 |  |  |  |  |  |  |  |  |
| GO:0001826\_inner\_cell\_mass\_cell\_differentiation | 1 | 0 |  |  |  |  |  |  |  |  |
| GO:0001830\_trophectodermal\_cell\_fate\_commitment | 1 | 0 |  |  |  |  |  |  |  |  |
| GO:0001834\_trophectodermal\_cell\_proliferation | 1 | 0 |  |  |  |  |  |  |  |  |
| GO:0001867\_complement\_activation\_\_lectin\_pathway | 1 | 0 |  |  |  |  |  |  |  |  |
| GO:0001887\_selenium\_metabolic\_process | 1 | 0 |  |  |  |  |  |  |  |  |
| GO:0001922\_B-1\_B\_cell\_homeostasis | 1 | 0 |  |  |  |  |  |  |  |  |
| GO:0001923\_B-1\_B\_cell\_differentiation | 1 | 0 |  |  |  |  |  |  |  |  |
| GO:0001941\_postsynaptic\_membrane\_organization | 1 | 0 |  |  |  |  |  |  |  |  |
| GO:0001946\_lymphangiogenesis | 1 | 0 |  |  |  |  |  |  |  |  |
| GO:0001956\_positive\_regulation\_of\_neurotransmitter\_secretion | 1 | 0 |  |  |  |  |  |  |  |  |
| GO:0001961\_positive\_regulation\_of\_cytokine-mediated\_signaling\_pathway | 1 | 0 |  |  |  |  |  |  |  |  |
| GO:0001979\_regulation\_of\_systemic\_arterial\_blood\_pressure\_by\_chemoreceptor\_signaling | 1 | 0 |  |  |  |  |  |  |  |  |
| GO:0001980\_regulation\_of\_systemic\_arterial\_blood\_pressure\_by\_ischemic\_conditions | 1 | 0 |  |  |  |  |  |  |  |  |
| GO:0001984\_vasodilation\_of\_artery\_during\_baroreceptor\_response\_to\_increased\_systemic\_arterial\_blood\_pressure | 1 | 0 |  |  |  |  |  |  |  |  |
| GO:0001985\_negative\_regulation\_of\_heart\_rate\_in\_baroreceptor\_response\_to\_increased\_systemic\_arterial\_blood\_pressure | 1 | 0 |  |  |  |  |  |  |  |  |
| GO:0001987\_vasoconstriction\_of\_artery\_involved\_in\_baroreceptor\_response\_to\_lowering\_of\_systemic\_arterial\_blood\_pressure | 1 | 0 |  |  |  |  |  |  |  |  |
| GO:0001988\_positive\_regulation\_of\_heart\_rate\_in\_baroreceptor\_response\_to\_decreased\_systemic\_arterial\_blood\_pressure | 1 | 0 |  |  |  |  |  |  |  |  |
| GO:0001994\_norepinephrine-epinephrine\_vasoconstriction\_involved\_in\_regulation\_of\_systemic\_arterial\_blood\_pressure | 1 | 0 |  |  |  |  |  |  |  |  |
| GO:0002001\_renin\_secretion\_into\_blood\_stream | 1 | 0 |  |  |  |  |  |  |  |  |
| GO:0002002\_regulation\_of\_angiotensin\_levels\_in\_blood | 1 | 0 |  |  |  |  |  |  |  |  |
| GO:0002003\_angiotensin\_maturation | 1 | 0 |  |  |  |  |  |  |  |  |
| GO:0002007\_detection\_of\_hypoxic\_conditions\_in\_blood\_by\_chemoreceptor\_signaling | 1 | 0 |  |  |  |  |  |  |  |  |
| GO:0002017\_regulation\_of\_blood\_volume\_by\_renal\_aldosterone | 1 | 0 |  |  |  |  |  |  |  |  |
| GO:0002023\_reduction\_of\_food\_intake\_in\_response\_to\_dietary\_excess | 1 | 0 |  |  |  |  |  |  |  |  |
| GO:0002031\_G-protein\_coupled\_receptor\_internalization | 1 | 0 |  |  |  |  |  |  |  |  |
| GO:0002036\_regulation\_of\_L-glutamate\_transport | 1 | 0 |  |  |  |  |  |  |  |  |
| GO:0002040\_sprouting\_angiogenesis | 1 | 0 |  |  |  |  |  |  |  |  |
| GO:0002041\_intussusceptive\_angiogenesis | 1 | 0 |  |  |  |  |  |  |  |  |
| GO:0002068\_glandular\_epithelial\_cell\_development | 1 | 0 |  |  |  |  |  |  |  |  |
| GO:0002069\_columnar\_cuboidal\_epithelial\_cell\_maturation | 1 | 0 |  |  |  |  |  |  |  |  |
| GO:0002071\_glandular\_epithelial\_cell\_maturation | 1 | 0 |  |  |  |  |  |  |  |  |
| GO:0002082\_regulation\_of\_oxidative\_phosphorylation | 1 | 0 |  |  |  |  |  |  |  |  |
| GO:0002084\_protein\_depalmitoylation | 1 | 0 |  |  |  |  |  |  |  |  |
| GO:0002085\_inhibition\_of\_neuroepithelial\_cell\_differentiation | 1 | 0 |  |  |  |  |  |  |  |  |
| GO:0002086\_diaphragm\_contraction | 1 | 0 |  |  |  |  |  |  |  |  |
| GO:0002118\_aggressive\_behavior | 1 | 0 |  |  |  |  |  |  |  |  |
| GO:0002121\_inter-male\_aggressive\_behavior | 1 | 0 |  |  |  |  |  |  |  |  |
| GO:0002124\_territorial\_aggressive\_behavior | 1 | 0 |  |  |  |  |  |  |  |  |
| GO:0002227\_innate\_immune\_response\_in\_mucosa | 1 | 0 |  |  |  |  |  |  |  |  |
| GO:0002232\_leukocyte\_chemotaxis\_during\_inflammatory\_response | 1 | 0 |  |  |  |  |  |  |  |  |
| GO:0002248\_connective\_tissue\_replacement\_during\_inflammatory\_response | 1 | 0 |  |  |  |  |  |  |  |  |
| GO:0002282\_microglial\_cell\_activation\_during\_immune\_response | 1 | 0 |  |  |  |  |  |  |  |  |
| GO:0002287\_alpha-beta\_T\_cell\_activation\_during\_immune\_response | 1 | 0 |  |  |  |  |  |  |  |  |
| GO:0002314\_germinal\_center\_B\_cell\_differentiation | 1 | 0 |  |  |  |  |  |  |  |  |
| GO:0002315\_marginal\_zone\_B\_cell\_differentiation | 1 | 0 |  |  |  |  |  |  |  |  |
| GO:0002316\_follicular\_B\_cell\_differentiation | 1 | 0 |  |  |  |  |  |  |  |  |
| GO:0002317\_plasma\_cell\_differentiation | 1 | 0 |  |  |  |  |  |  |  |  |
| GO:0002349\_histamine\_production\_during\_acute\_inflammatory\_response | 1 | 0 |  |  |  |  |  |  |  |  |
| GO:0002351\_serotonin\_production\_during\_acute\_inflammatory\_response | 1 | 0 |  |  |  |  |  |  |  |  |
| GO:0002355\_detection\_of\_tumor\_cell | 1 | 0 |  |  |  |  |  |  |  |  |
| GO:0002370\_natural\_killer\_cell\_cytokine\_production | 1 | 0 |  |  |  |  |  |  |  |  |
| GO:0002371\_dendritic\_cell\_cytokine\_production | 1 | 0 |  |  |  |  |  |  |  |  |
| GO:0002380\_immunoglobulin\_secretion\_during\_immune\_response | 1 | 0 |  |  |  |  |  |  |  |  |
| GO:0002396\_MHC\_protein\_complex\_assembly | 1 | 0 |  |  |  |  |  |  |  |  |
| GO:0002397\_MHC\_class\_I\_protein\_complex\_assembly | 1 | 0 |  |  |  |  |  |  |  |  |
| GO:0002420\_natural\_killer\_cell\_mediated\_cytotoxicity\_directed\_against\_tumor\_cell\_target | 1 | 0 |  |  |  |  |  |  |  |  |
| GO:0002423\_natural\_killer\_cell\_mediated\_immune\_response\_to\_tumor\_cell | 1 | 0 |  |  |  |  |  |  |  |  |
| GO:0002424\_T\_cell\_mediated\_immune\_response\_to\_tumor\_cell | 1 | 0 |  |  |  |  |  |  |  |  |
| GO:0002426\_immunoglobulin\_production\_in\_mucosal\_tissue | 1 | 0 |  |  |  |  |  |  |  |  |
| GO:0002431\_Fc\_receptor\_mediated\_stimulatory\_signaling\_pathway | 1 | 0 |  |  |  |  |  |  |  |  |
| GO:0002432\_granuloma\_formation | 1 | 0 |  |  |  |  |  |  |  |  |
| GO:0002441\_histamine\_secretion\_during\_acute\_inflammatory\_response | 1 | 0 |  |  |  |  |  |  |  |  |
| GO:0002442\_serotonin\_secretion\_during\_acute\_inflammatory\_response | 1 | 0 |  |  |  |  |  |  |  |  |
| GO:0002457\_T\_cell\_antigen\_processing\_and\_presentation | 1 | 0 |  |  |  |  |  |  |  |  |
| GO:0002458\_peripheral\_T\_cell\_tolerance\_induction | 1 | 0 |  |  |  |  |  |  |  |  |
| GO:0002461\_tolerance\_induction\_dependent\_upon\_immune\_response | 1 | 0 |  |  |  |  |  |  |  |  |
| GO:0002465\_peripheral\_tolerance\_induction | 1 | 0 |  |  |  |  |  |  |  |  |
| GO:0002468\_dendritic\_cell\_antigen\_processing\_and\_presentation | 1 | 0 |  |  |  |  |  |  |  |  |
| GO:0002476\_antigen\_processing\_and\_presentation\_of\_endogenous\_peptide\_antigen\_via\_MHC\_class\_Ib | 1 | 0 |  |  |  |  |  |  |  |  |
| GO:0002479\_antigen\_processing\_and\_presentation\_of\_exogenous\_peptide\_antigen\_via\_MHC\_class\_I\_\_TAP-dependent | 1 | 0 |  |  |  |  |  |  |  |  |
| GO:0002483\_antigen\_processing\_and\_presentation\_of\_endogenous\_peptide\_antigen | 1 | 0 |  |  |  |  |  |  |  |  |
| GO:0002501\_peptide\_antigen\_assembly\_with\_MHC\_protein\_complex | 1 | 0 |  |  |  |  |  |  |  |  |
| GO:0002502\_peptide\_antigen\_assembly\_with\_MHC\_class\_I\_protein\_complex | 1 | 0 |  |  |  |  |  |  |  |  |
| GO:0002508\_central\_tolerance\_induction | 1 | 0 |  |  |  |  |  |  |  |  |
| GO:0002510\_central\_B\_cell\_tolerance\_induction | 1 | 0 |  |  |  |  |  |  |  |  |
| GO:0002545\_chronic\_inflammatory\_response\_to\_non-antigenic\_stimulus | 1 | 0 |  |  |  |  |  |  |  |  |
| GO:0002553\_histamine\_secretion\_by\_mast\_cell | 1 | 0 |  |  |  |  |  |  |  |  |
| GO:0002554\_serotonin\_secretion\_by\_platelet | 1 | 0 |  |  |  |  |  |  |  |  |
| GO:0002572\_pro-T\_cell\_differentiation | 1 | 0 |  |  |  |  |  |  |  |  |
| GO:0002577\_regulation\_of\_antigen\_processing\_and\_presentation | 1 | 0 |  |  |  |  |  |  |  |  |
| GO:0002579\_positive\_regulation\_of\_antigen\_processing\_and\_presentation | 1 | 0 |  |  |  |  |  |  |  |  |
| GO:0002604\_regulation\_of\_dendritic\_cell\_antigen\_processing\_and\_presentation | 1 | 0 |  |  |  |  |  |  |  |  |
| GO:0002606\_positive\_regulation\_of\_dendritic\_cell\_antigen\_processing\_and\_presentation | 1 | 0 |  |  |  |  |  |  |  |  |
| GO:0002635\_negative\_regulation\_of\_germinal\_center\_formation | 1 | 0 |  |  |  |  |  |  |  |  |
| GO:0002646\_regulation\_of\_central\_tolerance\_induction | 1 | 0 |  |  |  |  |  |  |  |  |
| GO:0002648\_positive\_regulation\_of\_central\_tolerance\_induction | 1 | 0 |  |  |  |  |  |  |  |  |
| GO:0002649\_regulation\_of\_tolerance\_induction\_to\_self\_antigen | 1 | 0 |  |  |  |  |  |  |  |  |
| GO:0002651\_positive\_regulation\_of\_tolerance\_induction\_to\_self\_antigen | 1 | 0 |  |  |  |  |  |  |  |  |
| GO:0002652\_regulation\_of\_tolerance\_induction\_dependent\_upon\_immune\_response | 1 | 0 |  |  |  |  |  |  |  |  |
| GO:0002654\_positive\_regulation\_of\_tolerance\_induction\_dependent\_upon\_immune\_response | 1 | 0 |  |  |  |  |  |  |  |  |
| GO:0002658\_regulation\_of\_peripheral\_tolerance\_induction | 1 | 0 |  |  |  |  |  |  |  |  |
| GO:0002660\_positive\_regulation\_of\_peripheral\_tolerance\_induction | 1 | 0 |  |  |  |  |  |  |  |  |
| GO:0002677\_negative\_regulation\_of\_chronic\_inflammatory\_response | 1 | 0 |  |  |  |  |  |  |  |  |
| GO:0002678\_positive\_regulation\_of\_chronic\_inflammatory\_response | 1 | 0 |  |  |  |  |  |  |  |  |
| GO:0002701\_negative\_regulation\_of\_production\_of\_molecular\_mediator\_of\_immune\_response | 1 | 0 |  |  |  |  |  |  |  |  |
| GO:0002719\_negative\_regulation\_of\_cytokine\_production\_during\_immune\_response | 1 | 0 |  |  |  |  |  |  |  |  |
| GO:0002724\_regulation\_of\_T\_cell\_cytokine\_production | 1 | 0 |  |  |  |  |  |  |  |  |
| GO:0002727\_regulation\_of\_natural\_killer\_cell\_cytokine\_production | 1 | 0 |  |  |  |  |  |  |  |  |
| GO:0002729\_positive\_regulation\_of\_natural\_killer\_cell\_cytokine\_production | 1 | 0 |  |  |  |  |  |  |  |  |
| GO:0002730\_regulation\_of\_dendritic\_cell\_cytokine\_production | 1 | 0 |  |  |  |  |  |  |  |  |
| GO:0002756\_MyD88-independent\_toll-like\_receptor\_signaling\_pathway | 1 | 0 |  |  |  |  |  |  |  |  |
| GO:0002767\_immune\_response-inhibiting\_cell\_surface\_receptor\_signaling\_pathway | 1 | 0 |  |  |  |  |  |  |  |  |
| GO:0002769\_natural\_killer\_cell\_inhibitory\_signaling\_pathway | 1 | 0 |  |  |  |  |  |  |  |  |
| GO:0002840\_regulation\_of\_T\_cell\_mediated\_immune\_response\_to\_tumor\_cell | 1 | 0 |  |  |  |  |  |  |  |  |
| GO:0002842\_positive\_regulation\_of\_T\_cell\_mediated\_immune\_response\_to\_tumor\_cell | 1 | 0 |  |  |  |  |  |  |  |  |
| GO:0002849\_regulation\_of\_peripheral\_T\_cell\_tolerance\_induction | 1 | 0 |  |  |  |  |  |  |  |  |
| GO:0002851\_positive\_regulation\_of\_peripheral\_T\_cell\_tolerance\_induction | 1 | 0 |  |  |  |  |  |  |  |  |
| GO:0002855\_regulation\_of\_natural\_killer\_cell\_mediated\_immune\_response\_to\_tumor\_cell | 1 | 0 |  |  |  |  |  |  |  |  |
| GO:0002857\_positive\_regulation\_of\_natural\_killer\_cell\_mediated\_immune\_response\_to\_tumor\_cell | 1 | 0 |  |  |  |  |  |  |  |  |
| GO:0002858\_regulation\_of\_natural\_killer\_cell\_mediated\_cytotoxicity\_directed\_against\_tumor\_cell\_target | 1 | 0 |  |  |  |  |  |  |  |  |
| GO:0002860\_positive\_regulation\_of\_natural\_killer\_cell\_mediated\_cytotoxicity\_directed\_against\_tumor\_cell\_target | 1 | 0 |  |  |  |  |  |  |  |  |
| GO:0002880\_regulation\_of\_chronic\_inflammatory\_response\_to\_non-antigenic\_stimulus | 1 | 0 |  |  |  |  |  |  |  |  |
| GO:0002882\_positive\_regulation\_of\_chronic\_inflammatory\_response\_to\_non-antigenic\_stimulus | 1 | 0 |  |  |  |  |  |  |  |  |
| GO:0002895\_regulation\_of\_central\_B\_cell\_tolerance\_induction | 1 | 0 |  |  |  |  |  |  |  |  |
| GO:0002897\_positive\_regulation\_of\_central\_B\_cell\_tolerance\_induction | 1 | 0 |  |  |  |  |  |  |  |  |
| GO:0002901\_mature\_B\_cell\_apoptosis | 1 | 0 |  |  |  |  |  |  |  |  |
| GO:0002903\_negative\_regulation\_of\_B\_cell\_apoptosis | 1 | 0 |  |  |  |  |  |  |  |  |
| GO:0002905\_regulation\_of\_mature\_B\_cell\_apoptosis | 1 | 0 |  |  |  |  |  |  |  |  |
| GO:0002906\_negative\_regulation\_of\_mature\_B\_cell\_apoptosis | 1 | 0 |  |  |  |  |  |  |  |  |
| GO:0003011\_involuntary\_skeletal\_muscle\_contraction | 1 | 0 |  |  |  |  |  |  |  |  |
| GO:0003027\_regulation\_of\_systemic\_arterial\_blood\_pressure\_by\_carotid\_body\_chemoreceptor\_signaling | 1 | 0 |  |  |  |  |  |  |  |  |
| GO:0003029\_detection\_of\_hypoxic\_conditions\_in\_blood\_by\_carotid\_body\_chemoreceptor\_signaling | 1 | 0 |  |  |  |  |  |  |  |  |
| GO:0003032\_detection\_of\_oxygen | 1 | 0 |  |  |  |  |  |  |  |  |
| GO:0003056\_regulation\_of\_vascular\_smooth\_muscle\_contraction | 1 | 0 |  |  |  |  |  |  |  |  |
| GO:0003062\_regulation\_of\_heart\_rate\_by\_chemical\_signal | 1 | 0 |  |  |  |  |  |  |  |  |
| GO:0003065\_positive\_regulation\_of\_heart\_rate\_by\_epinephrine | 1 | 0 |  |  |  |  |  |  |  |  |
| GO:0003068\_regulation\_of\_systemic\_arterial\_blood\_pressure\_by\_acetylcholine | 1 | 0 |  |  |  |  |  |  |  |  |
| GO:0003069\_vasodilation\_by\_acetylcholine\_involved\_in\_regulation\_of\_systemic\_arterial\_blood\_pressure | 1 | 0 |  |  |  |  |  |  |  |  |
| GO:0003070\_regulation\_of\_systemic\_arterial\_blood\_pressure\_by\_neurotransmitter | 1 | 0 |  |  |  |  |  |  |  |  |
| GO:0003097\_renal\_water\_transport | 1 | 0 |  |  |  |  |  |  |  |  |
| GO:0005979\_regulation\_of\_glycogen\_biosynthetic\_process | 1 | 0 |  |  |  |  |  |  |  |  |
| GO:0005984\_disaccharide\_metabolic\_process | 1 | 0 |  |  |  |  |  |  |  |  |
| GO:0005988\_lactose\_metabolic\_process | 1 | 0 |  |  |  |  |  |  |  |  |
| GO:0005989\_lactose\_biosynthetic\_process | 1 | 0 |  |  |  |  |  |  |  |  |
| GO:0005997\_xylulose\_metabolic\_process | 1 | 0 |  |  |  |  |  |  |  |  |
| GO:0006000\_fructose\_metabolic\_process | 1 | 0 |  |  |  |  |  |  |  |  |
| GO:0006002\_fructose\_6-phosphate\_metabolic\_process | 1 | 0 |  |  |  |  |  |  |  |  |
| GO:0006004\_fucose\_metabolic\_process | 1 | 0 |  |  |  |  |  |  |  |  |
| GO:0006013\_mannose\_metabolic\_process | 1 | 0 |  |  |  |  |  |  |  |  |
| GO:0006060\_sorbitol\_metabolic\_process | 1 | 0 |  |  |  |  |  |  |  |  |
| GO:0006064\_glucuronate\_catabolic\_process | 1 | 0 |  |  |  |  |  |  |  |  |
| GO:0006086\_acetyl-CoA\_biosynthetic\_process\_from\_pyruvate | 1 | 0 |  |  |  |  |  |  |  |  |
| GO:0006098\_pentose-phosphate\_shunt | 1 | 0 |  |  |  |  |  |  |  |  |
| GO:0006101\_citrate\_metabolic\_process | 1 | 0 |  |  |  |  |  |  |  |  |
| GO:0006104\_succinyl-CoA\_metabolic\_process | 1 | 0 |  |  |  |  |  |  |  |  |
| GO:0006116\_NADH\_oxidation | 1 | 0 |  |  |  |  |  |  |  |  |
| GO:0006120\_mitochondrial\_electron\_transport\_\_NADH\_to\_ubiquinone | 1 | 0 |  |  |  |  |  |  |  |  |
| GO:0006154\_adenosine\_catabolic\_process | 1 | 0 |  |  |  |  |  |  |  |  |
| GO:0006157\_deoxyadenosine\_catabolic\_process | 1 | 0 |  |  |  |  |  |  |  |  |
| GO:0006167\_AMP\_biosynthetic\_process | 1 | 0 |  |  |  |  |  |  |  |  |
| GO:0006178\_guanine\_salvage | 1 | 0 |  |  |  |  |  |  |  |  |
| GO:0006196\_AMP\_catabolic\_process | 1 | 0 |  |  |  |  |  |  |  |  |
| GO:0006203\_dGTP\_catabolic\_process | 1 | 0 |  |  |  |  |  |  |  |  |
| GO:0006208\_pyrimidine\_base\_catabolic\_process | 1 | 0 |  |  |  |  |  |  |  |  |
| GO:0006221\_pyrimidine\_nucleotide\_biosynthetic\_process | 1 | 0 |  |  |  |  |  |  |  |  |
| GO:0006235\_dTTP\_biosynthetic\_process | 1 | 0 |  |  |  |  |  |  |  |  |
| GO:0006244\_pyrimidine\_nucleotide\_catabolic\_process | 1 | 0 |  |  |  |  |  |  |  |  |
| GO:0006269\_DNA\_replication\_\_synthesis\_of\_RNA\_primer | 1 | 0 |  |  |  |  |  |  |  |  |
| GO:0006283\_transcription-coupled\_nucleotide-excision\_repair | 1 | 0 |  |  |  |  |  |  |  |  |
| GO:0006296\_nucleotide-excision\_repair\_\_DNA\_incision\_\_5'-to\_lesion | 1 | 0 |  |  |  |  |  |  |  |  |
| GO:0006307\_DNA\_dealkylation | 1 | 0 |  |  |  |  |  |  |  |  |
| GO:0006337\_nucleosome\_disassembly | 1 | 0 |  |  |  |  |  |  |  |  |
| GO:0006344\_maintenance\_of\_chromatin\_silencing | 1 | 0 |  |  |  |  |  |  |  |  |
| GO:0006356\_regulation\_of\_transcription\_from\_RNA\_polymerase\_I\_promoter | 1 | 0 |  |  |  |  |  |  |  |  |
| GO:0006388\_tRNA\_splicing\_\_via\_endonucleolytic\_cleavage\_and\_ligation | 1 | 0 |  |  |  |  |  |  |  |  |
| GO:0006407\_rRNA\_export\_from\_nucleus | 1 | 0 |  |  |  |  |  |  |  |  |
| GO:0006419\_alanyl-tRNA\_aminoacylation | 1 | 0 |  |  |  |  |  |  |  |  |
| GO:0006434\_seryl-tRNA\_aminoacylation | 1 | 0 |  |  |  |  |  |  |  |  |
| GO:0006447\_regulation\_of\_translational\_initiation\_by\_iron | 1 | 0 |  |  |  |  |  |  |  |  |
| GO:0006463\_steroid\_hormone\_receptor\_complex\_assembly | 1 | 0 |  |  |  |  |  |  |  |  |
| GO:0006467\_protein\_thiol-disulfide\_exchange | 1 | 0 |  |  |  |  |  |  |  |  |
| GO:0006474\_N-terminal\_protein\_amino\_acid\_acetylation | 1 | 0 |  |  |  |  |  |  |  |  |
| GO:0006481\_C-terminal\_protein\_amino\_acid\_methylation | 1 | 0 |  |  |  |  |  |  |  |  |
| GO:0006488\_dolichol-linked\_oligosaccharide\_biosynthetic\_process | 1 | 0 |  |  |  |  |  |  |  |  |
| GO:0006494\_protein\_amino\_acid\_terminal\_glycosylation | 1 | 0 |  |  |  |  |  |  |  |  |
| GO:0006496\_protein\_amino\_acid\_terminal\_N-glycosylation | 1 | 0 |  |  |  |  |  |  |  |  |
| GO:0006500\_N-terminal\_protein\_palmitoylation | 1 | 0 |  |  |  |  |  |  |  |  |
| GO:0006507\_GPI\_anchor\_release | 1 | 0 |  |  |  |  |  |  |  |  |
| GO:0006537\_glutamate\_biosynthetic\_process | 1 | 0 |  |  |  |  |  |  |  |  |
| GO:0006544\_glycine\_metabolic\_process | 1 | 0 |  |  |  |  |  |  |  |  |
| GO:0006549\_isoleucine\_metabolic\_process | 1 | 0 |  |  |  |  |  |  |  |  |
| GO:0006553\_lysine\_metabolic\_process | 1 | 0 |  |  |  |  |  |  |  |  |
| GO:0006554\_lysine\_catabolic\_process | 1 | 0 |  |  |  |  |  |  |  |  |
| GO:0006556\_S-adenosylmethionine\_biosynthetic\_process | 1 | 0 |  |  |  |  |  |  |  |  |
| GO:0006559\_L-phenylalanine\_catabolic\_process | 1 | 0 |  |  |  |  |  |  |  |  |
| GO:0006569\_tryptophan\_catabolic\_process | 1 | 0 |  |  |  |  |  |  |  |  |
| GO:0006572\_tyrosine\_catabolic\_process | 1 | 0 |  |  |  |  |  |  |  |  |
| GO:0006573\_valine\_metabolic\_process | 1 | 0 |  |  |  |  |  |  |  |  |
| GO:0006581\_acetylcholine\_catabolic\_process | 1 | 0 |  |  |  |  |  |  |  |  |
| GO:0006585\_dopamine\_biosynthetic\_process\_from\_tyrosine | 1 | 0 |  |  |  |  |  |  |  |  |
| GO:0006590\_thyroid\_hormone\_generation | 1 | 0 |  |  |  |  |  |  |  |  |
| GO:0006591\_ornithine\_metabolic\_process | 1 | 0 |  |  |  |  |  |  |  |  |
| GO:0006596\_polyamine\_biosynthetic\_process | 1 | 0 |  |  |  |  |  |  |  |  |
| GO:0006597\_spermine\_biosynthetic\_process | 1 | 0 |  |  |  |  |  |  |  |  |
| GO:0006601\_creatine\_biosynthetic\_process | 1 | 0 |  |  |  |  |  |  |  |  |
| GO:0006613\_cotranslational\_protein\_targeting\_to\_membrane | 1 | 0 |  |  |  |  |  |  |  |  |
| GO:0006622\_protein\_targeting\_to\_lysosome | 1 | 0 |  |  |  |  |  |  |  |  |
| GO:0006627\_mitochondrial\_protein\_processing\_during\_import | 1 | 0 |  |  |  |  |  |  |  |  |
| GO:0006653\_lecithin\_metabolic\_process | 1 | 0 |  |  |  |  |  |  |  |  |
| GO:0006654\_phosphatidic\_acid\_biosynthetic\_process | 1 | 0 |  |  |  |  |  |  |  |  |
| GO:0006658\_phosphatidylserine\_metabolic\_process | 1 | 0 |  |  |  |  |  |  |  |  |
| GO:0006659\_phosphatidylserine\_biosynthetic\_process | 1 | 0 |  |  |  |  |  |  |  |  |
| GO:0006667\_sphinganine\_metabolic\_process | 1 | 0 |  |  |  |  |  |  |  |  |
| GO:0006668\_sphinganine-1-phosphate\_metabolic\_process | 1 | 0 |  |  |  |  |  |  |  |  |
| GO:0006678\_glucosylceramide\_metabolic\_process | 1 | 0 |  |  |  |  |  |  |  |  |
| GO:0006682\_galactosylceramide\_biosynthetic\_process | 1 | 0 |  |  |  |  |  |  |  |  |
| GO:0006685\_sphingomyelin\_catabolic\_process | 1 | 0 |  |  |  |  |  |  |  |  |
| GO:0006700\_C21-steroid\_hormone\_biosynthetic\_process | 1 | 0 |  |  |  |  |  |  |  |  |
| GO:0006705\_mineralocorticoid\_biosynthetic\_process | 1 | 0 |  |  |  |  |  |  |  |  |
| GO:0006709\_progesterone\_catabolic\_process | 1 | 0 |  |  |  |  |  |  |  |  |
| GO:0006729\_tetrahydrobiopterin\_biosynthetic\_process | 1 | 0 |  |  |  |  |  |  |  |  |
| GO:0006734\_NADH\_metabolic\_process | 1 | 0 |  |  |  |  |  |  |  |  |
| GO:0006740\_NADPH\_regeneration | 1 | 0 |  |  |  |  |  |  |  |  |
| GO:0006741\_NADP\_biosynthetic\_process | 1 | 0 |  |  |  |  |  |  |  |  |
| GO:0006743\_ubiquinone\_metabolic\_process | 1 | 0 |  |  |  |  |  |  |  |  |
| GO:0006744\_ubiquinone\_biosynthetic\_process | 1 | 0 |  |  |  |  |  |  |  |  |
| GO:0006772\_thiamin\_metabolic\_process | 1 | 0 |  |  |  |  |  |  |  |  |
| GO:0006784\_heme\_a\_biosynthetic\_process | 1 | 0 |  |  |  |  |  |  |  |  |
| GO:0006797\_polyphosphate\_metabolic\_process | 1 | 0 |  |  |  |  |  |  |  |  |
| GO:0006798\_polyphosphate\_catabolic\_process | 1 | 0 |  |  |  |  |  |  |  |  |
| GO:0006824\_cobalt\_ion\_transport | 1 | 0 |  |  |  |  |  |  |  |  |
| GO:0006842\_tricarboxylic\_acid\_transport | 1 | 0 |  |  |  |  |  |  |  |  |
| GO:0006844\_acyl\_carnitine\_transport | 1 | 0 |  |  |  |  |  |  |  |  |
| GO:0006855\_multidrug\_transport | 1 | 0 |  |  |  |  |  |  |  |  |
| GO:0006863\_purine\_transport | 1 | 0 |  |  |  |  |  |  |  |  |
| GO:0006890\_retrograde\_vesicle-mediated\_transport\_\_Golgi\_to\_ER | 1 | 0 |  |  |  |  |  |  |  |  |
| GO:0006891\_intra-Golgi\_vesicle-mediated\_transport | 1 | 0 |  |  |  |  |  |  |  |  |
| GO:0006893\_Golgi\_to\_plasma\_membrane\_transport | 1 | 0 |  |  |  |  |  |  |  |  |
| GO:0006895\_Golgi\_to\_endosome\_transport | 1 | 0 |  |  |  |  |  |  |  |  |
| GO:0006896\_Golgi\_to\_vacuole\_transport | 1 | 0 |  |  |  |  |  |  |  |  |
| GO:0006900\_membrane\_budding | 1 | 0 |  |  |  |  |  |  |  |  |
| GO:0006930\_substrate-bound\_cell\_migration\_\_cell\_extension | 1 | 0 |  |  |  |  |  |  |  |  |
| GO:0006931\_substrate-bound\_cell\_migration\_\_cell\_attachment\_to\_substrate | 1 | 0 |  |  |  |  |  |  |  |  |
| GO:0006933\_negative\_regulation\_of\_cell\_adhesion\_involved\_in\_substrate-bound\_cell\_migration | 1 | 0 |  |  |  |  |  |  |  |  |
| GO:0006957\_complement\_activation\_\_alternative\_pathway | 1 | 0 |  |  |  |  |  |  |  |  |
| GO:0006958\_complement\_activation\_\_classical\_pathway | 1 | 0 |  |  |  |  |  |  |  |  |
| GO:0006978\_DNA\_damage\_response\_\_signal\_transduction\_by\_p53\_class\_mediator\_resulting\_in\_transcription\_of\_p21\_class\_mediator | 1 | 0 |  |  |  |  |  |  |  |  |
| GO:0007016\_cytoskeletal\_anchoring\_at\_plasma\_membrane | 1 | 0 |  |  |  |  |  |  |  |  |
| GO:0007021\_tubulin\_complex\_assembly | 1 | 0 |  |  |  |  |  |  |  |  |
| GO:0007052\_mitotic\_spindle\_organization | 1 | 0 |  |  |  |  |  |  |  |  |
| GO:0007056\_spindle\_assembly\_involved\_in\_female\_meiosis | 1 | 0 |  |  |  |  |  |  |  |  |
| GO:0007057\_spindle\_assembly\_involved\_in\_female\_meiosis\_I | 1 | 0 |  |  |  |  |  |  |  |  |
| GO:0007063\_regulation\_of\_sister\_chromatid\_cohesion | 1 | 0 |  |  |  |  |  |  |  |  |
| GO:0007065\_male\_meiosis\_sister\_chromatid\_cohesion | 1 | 0 |  |  |  |  |  |  |  |  |
| GO:0007076\_mitotic\_chromosome\_condensation | 1 | 0 |  |  |  |  |  |  |  |  |
| GO:0007095\_mitotic\_cell\_cycle\_G2\_M\_transition\_DNA\_damage\_checkpoint | 1 | 0 |  |  |  |  |  |  |  |  |
| GO:0007096\_regulation\_of\_exit\_from\_mitosis | 1 | 0 |  |  |  |  |  |  |  |  |
| GO:0007158\_neuron\_adhesion | 1 | 0 |  |  |  |  |  |  |  |  |
| GO:0007168\_receptor\_guanylyl\_cyclase\_signaling\_pathway | 1 | 0 |  |  |  |  |  |  |  |  |
| GO:0007197\_inhibition\_of\_adenylate\_cyclase\_activity\_by\_muscarinic\_acetylcholine\_receptor\_signaling\_pathway | 1 | 0 |  |  |  |  |  |  |  |  |
| GO:0007207\_activation\_of\_phospholipase\_C\_activity\_by\_muscarinic\_acetylcholine\_receptor\_signaling\_pathway | 1 | 0 |  |  |  |  |  |  |  |  |
| GO:0007208\_activation\_of\_phospholipase\_C\_activity\_by\_serotonin\_receptor\_signaling\_pathway | 1 | 0 |  |  |  |  |  |  |  |  |
| GO:0007217\_tachykinin\_receptor\_signaling\_pathway | 1 | 0 |  |  |  |  |  |  |  |  |
| GO:0007221\_positive\_regulation\_of\_transcription\_of\_Notch\_receptor\_target | 1 | 0 |  |  |  |  |  |  |  |  |
| GO:0007223\_Wnt\_receptor\_signaling\_pathway\_\_calcium\_modulating\_pathway | 1 | 0 |  |  |  |  |  |  |  |  |
| GO:0007225\_patched\_ligand\_processing | 1 | 0 |  |  |  |  |  |  |  |  |
| GO:0007227\_signal\_transduction\_downstream\_of\_smoothened | 1 | 0 |  |  |  |  |  |  |  |  |
| GO:0007228\_positive\_regulation\_of\_hh\_target\_transcription\_factor\_activity | 1 | 0 |  |  |  |  |  |  |  |  |
| GO:0007231\_osmosensory\_signaling\_pathway | 1 | 0 |  |  |  |  |  |  |  |  |
| GO:0007284\_spermatogonial\_cell\_division | 1 | 0 |  |  |  |  |  |  |  |  |
| GO:0007290\_spermatid\_nucleus\_elongation | 1 | 0 |  |  |  |  |  |  |  |  |
| GO:0007296\_vitellogenesis | 1 | 0 |  |  |  |  |  |  |  |  |
| GO:0007321\_sperm\_displacement | 1 | 0 |  |  |  |  |  |  |  |  |
| GO:0007380\_specification\_of\_segmental\_identity\_\_head | 1 | 0 |  |  |  |  |  |  |  |  |
| GO:0007382\_specification\_of\_segmental\_identity\_\_maxillary\_segment | 1 | 0 |  |  |  |  |  |  |  |  |
| GO:0007400\_neuroblast\_fate\_determination | 1 | 0 |  |  |  |  |  |  |  |  |
| GO:0007402\_ganglion\_mother\_cell\_fate\_determination | 1 | 0 |  |  |  |  |  |  |  |  |
| GO:0007495\_visceral\_mesoderm-endoderm\_interaction\_involved\_in\_midgut\_development | 1 | 0 |  |  |  |  |  |  |  |  |
| GO:0007497\_posterior\_midgut\_development | 1 | 0 |  |  |  |  |  |  |  |  |
| GO:0007499\_ectoderm\_and\_mesoderm\_interaction | 1 | 0 |  |  |  |  |  |  |  |  |
| GO:0007500\_mesodermal\_cell\_fate\_determination | 1 | 0 |  |  |  |  |  |  |  |  |
| GO:0007509\_mesoderm\_migration | 1 | 0 |  |  |  |  |  |  |  |  |
| GO:0007518\_myoblast\_cell\_fate\_determination | 1 | 0 |  |  |  |  |  |  |  |  |
| GO:0007521\_muscle\_cell\_fate\_determination | 1 | 0 |  |  |  |  |  |  |  |  |
| GO:0007522\_visceral\_muscle\_development | 1 | 0 |  |  |  |  |  |  |  |  |
| GO:0007529\_establishment\_of\_synaptic\_specificity\_at\_neuromuscular\_junction | 1 | 0 |  |  |  |  |  |  |  |  |
| GO:0007538\_primary\_sex\_determination | 1 | 0 |  |  |  |  |  |  |  |  |
| GO:0007542\_primary\_sex\_determination\_\_germ-line | 1 | 0 |  |  |  |  |  |  |  |  |
| GO:0007567\_parturition | 1 | 0 |  |  |  |  |  |  |  |  |
| GO:0007614\_short-term\_memory | 1 | 0 |  |  |  |  |  |  |  |  |
| GO:0007621\_negative\_regulation\_of\_female\_receptivity | 1 | 0 |  |  |  |  |  |  |  |  |
| GO:0008049\_male\_courtship\_behavior | 1 | 0 |  |  |  |  |  |  |  |  |
| GO:0008050\_female\_courtship\_behavior | 1 | 0 |  |  |  |  |  |  |  |  |
| GO:0008052\_sensory\_organ\_boundary\_specification | 1 | 0 |  |  |  |  |  |  |  |  |
| GO:0008054\_cyclin\_catabolic\_process | 1 | 0 |  |  |  |  |  |  |  |  |
[truncated: 255,364 more chars]
