# Supplementary material for: RedundancyMiner: De-replication of redundant GO categories in microarray and proteomics analysis
Source: BMC Bioinformatics. 2011 Feb 10;12:52. doi: 10.1186/1471-2105-12-52 (PMC3223614; doi:10.1186/1471-2105-12-52)
Supplement: Additional file 8 — Retinal development HTGM download. compressed package of the results of running HTGM on the retinal development genes list. [file 1471-2105-12-52-S8.ZIP › SCENARIO_2_MODIFIED/total.txt.total.txt.dir/Exp1_BestClusterMap_LEIGS_KM_24.csv.join.24.txt.dir/Exp1_BestClusterMap_LEIGS_KM_24.csv.join.24.txt.change.gce.html]

Gene Category Report for Exp1\_BestClusterMap\_LEIGS\_KM\_24.csv.join.24.txt

# Gene Category Report for Exp1\_BestClusterMap\_LEIGS\_KM\_24.csv.join.24.txt

| HYPERLINKED GO CATEGORY | HYPERLINKED GENE NAME | TOTAL GENES | CHANGED GENES | ENRICHMENT | LOG10(p) | CUMULATIVE NUMBER OF CATEGORIES | CUMULATIVE RANDOMS MEAN | FALSE DISCOVERY RATE |
| --- | --- | --- | --- | --- | --- | --- | --- | --- |
| GO:0006605\_protein\_targeting | YWHAB | 86 | 4 | 7.932817 | -2.843546 | 1 | 1.14 | 1.140000 |
| GO:0006605\_protein\_targeting | YWHAQ | 86 | 4 | 7.932817 | -2.843546 | 1 | 1.14 | 1.140000 |
| GO:0006605\_protein\_targeting | NUTF2 | 86 | 4 | 7.932817 | -2.843546 | 1 | 1.14 | 1.140000 |
| GO:0006605\_protein\_targeting | PRDX1 | 86 | 4 | 7.932817 | -2.843546 | 1 | 1.14 | 1.140000 |
| GO:0006886\_intracellular\_protein\_transport | YWHAB | 122 | 4 | 5.591985 | -2.289606 | 2 | 3.94 | 1.970000 |
| GO:0006886\_intracellular\_protein\_transport | YWHAQ | 122 | 4 | 5.591985 | -2.289606 | 2 | 3.94 | 1.970000 |
| GO:0006886\_intracellular\_protein\_transport | NUTF2 | 122 | 4 | 5.591985 | -2.289606 | 2 | 3.94 | 1.970000 |
| GO:0006886\_intracellular\_protein\_transport | PRDX1 | 122 | 4 | 5.591985 | -2.289606 | 2 | 3.94 | 1.970000 |
| GO:0006283\_transcription-coupled\_nucleotide-excision\_repair | HMGN1 | 1 | 1 |  |  |  |  |  |  |
| GO:0006556\_S-adenosylmethionine\_biosynthetic\_process | MAT2A | 1 | 1 |  |  |  |  |  |  |
| GO:0032607\_interferon-alpha\_production | HSPD1 | 1 | 1 |  |  |  |  |  |  |
| GO:0032647\_regulation\_of\_interferon-alpha\_production | HSPD1 | 1 | 1 |  |  |  |  |  |  |
| GO:0032727\_positive\_regulation\_of\_interferon-alpha\_production | HSPD1 | 1 | 1 |  |  |  |  |  |  |
| GO:0034613\_cellular\_protein\_localization | YWHAB | 139 | 4 | 4.908074 | -2.089904 | 3 | 5.67 | 1.890000 |
| GO:0034613\_cellular\_protein\_localization | YWHAQ | 139 | 4 | 4.908074 | -2.089904 | 3 | 5.67 | 1.890000 |
| GO:0034613\_cellular\_protein\_localization | NUTF2 | 139 | 4 | 4.908074 | -2.089904 | 3 | 5.67 | 1.890000 |
| GO:0034613\_cellular\_protein\_localization | PRDX1 | 139 | 4 | 4.908074 | -2.089904 | 3 | 5.67 | 1.890000 |
| GO:0070727\_cellular\_macromolecule\_localization | YWHAB | 141 | 4 | 4.838455 | -2.068295 | 4 | 6.1 | 1.525000 |
| GO:0070727\_cellular\_macromolecule\_localization | YWHAQ | 141 | 4 | 4.838455 | -2.068295 | 4 | 6.1 | 1.525000 |
| GO:0070727\_cellular\_macromolecule\_localization | NUTF2 | 141 | 4 | 4.838455 | -2.068295 | 4 | 6.1 | 1.525000 |
| GO:0070727\_cellular\_macromolecule\_localization | PRDX1 | 141 | 4 | 4.838455 | -2.068295 | 4 | 6.1 | 1.525000 |
| GO:0009987\_cellular\_process | HMGN1 | 3868 | 27 | 1.190538 | -2.051399 | 5 | 6.14 | 1.228000 |
| GO:0009987\_cellular\_process | NDN | 3868 | 27 | 1.190538 | -2.051399 | 5 | 6.14 | 1.228000 |
| GO:0009987\_cellular\_process | HAX1 | 3868 | 27 | 1.190538 | -2.051399 | 5 | 6.14 | 1.228000 |
| GO:0009987\_cellular\_process | GJA1 | 3868 | 27 | 1.190538 | -2.051399 | 5 | 6.14 | 1.228000 |
| GO:0009987\_cellular\_process | CBX3 | 3868 | 27 | 1.190538 | -2.051399 | 5 | 6.14 | 1.228000 |
| GO:0009987\_cellular\_process | FKBP1A | 3868 | 27 | 1.190538 | -2.051399 | 5 | 6.14 | 1.228000 |
| GO:0009987\_cellular\_process | PRDX1 | 3868 | 27 | 1.190538 | -2.051399 | 5 | 6.14 | 1.228000 |
| GO:0009987\_cellular\_process | RPA1 | 3868 | 27 | 1.190538 | -2.051399 | 5 | 6.14 | 1.228000 |
| GO:0009987\_cellular\_process | PRPF19 | 3868 | 27 | 1.190538 | -2.051399 | 5 | 6.14 | 1.228000 |
| GO:0009987\_cellular\_process | OAZ1 | 3868 | 27 | 1.190538 | -2.051399 | 5 | 6.14 | 1.228000 |
| GO:0009987\_cellular\_process | TUBB5 | 3868 | 27 | 1.190538 | -2.051399 | 5 | 6.14 | 1.228000 |
| GO:0009987\_cellular\_process | HSPA5 | 3868 | 27 | 1.190538 | -2.051399 | 5 | 6.14 | 1.228000 |
| GO:0009987\_cellular\_process | HSPA8 | 3868 | 27 | 1.190538 | -2.051399 | 5 | 6.14 | 1.228000 |
| GO:0009987\_cellular\_process | IMPDH2 | 3868 | 27 | 1.190538 | -2.051399 | 5 | 6.14 | 1.228000 |
| GO:0009987\_cellular\_process | GDI2 | 3868 | 27 | 1.190538 | -2.051399 | 5 | 6.14 | 1.228000 |
| GO:0009987\_cellular\_process | MAT2A | 3868 | 27 | 1.190538 | -2.051399 | 5 | 6.14 | 1.228000 |
| GO:0009987\_cellular\_process | CYCS | 3868 | 27 | 1.190538 | -2.051399 | 5 | 6.14 | 1.228000 |
| GO:0009987\_cellular\_process | TRIM28 | 3868 | 27 | 1.190538 | -2.051399 | 5 | 6.14 | 1.228000 |
| GO:0009987\_cellular\_process | YWHAB | 3868 | 27 | 1.190538 | -2.051399 | 5 | 6.14 | 1.228000 |
| GO:0009987\_cellular\_process | RBBP7 | 3868 | 27 | 1.190538 | -2.051399 | 5 | 6.14 | 1.228000 |
| GO:0009987\_cellular\_process | DDX5 | 3868 | 27 | 1.190538 | -2.051399 | 5 | 6.14 | 1.228000 |
| GO:0009987\_cellular\_process | SFRS1 | 3868 | 27 | 1.190538 | -2.051399 | 5 | 6.14 | 1.228000 |
| GO:0009987\_cellular\_process | RPL29 | 3868 | 27 | 1.190538 | -2.051399 | 5 | 6.14 | 1.228000 |
| GO:0009987\_cellular\_process | CFL1 | 3868 | 27 | 1.190538 | -2.051399 | 5 | 6.14 | 1.228000 |
| GO:0009987\_cellular\_process | YWHAQ | 3868 | 27 | 1.190538 | -2.051399 | 5 | 6.14 | 1.228000 |
| GO:0009987\_cellular\_process | NUTF2 | 3868 | 27 | 1.190538 | -2.051399 | 5 | 6.14 | 1.228000 |
| GO:0009987\_cellular\_process | HSPD1 | 3868 | 27 | 1.190538 | -2.051399 | 5 | 6.14 | 1.228000 |
| GO:0006800\_oxygen\_and\_reactive\_oxygen\_species\_metabolic\_process | CYCS | 26 | 2 | 13.119658 | -2.005824 | 6 | 6.68 | 1.113333 |
| GO:0006800\_oxygen\_and\_reactive\_oxygen\_species\_metabolic\_process | PRDX1 | 26 | 2 | 13.119658 | -2.005824 | 6 | 6.68 | 1.113333 |
| GO:0000720\_pyrimidine\_dimer\_repair\_by\_nucleotide-excision\_repair | HMGN1 | 2 | 1 |  |  |  |  |  |  |
| GO:0010225\_response\_to\_UV-C | HMGN1 | 2 | 1 |  |  |  |  |  |  |
| GO:0032481\_positive\_regulation\_of\_type\_I\_interferon\_production | HSPD1 | 2 | 1 |  |  |  |  |  |  |
| GO:0046500\_S-adenosylmethionine\_metabolic\_process | MAT2A | 2 | 1 |  |  |  |  |  |  |
| GO:0048711\_positive\_regulation\_of\_astrocyte\_differentiation | PRPF19 | 2 | 1 |  |  |  |  |  |  |
| GO:0008347\_glial\_cell\_migration | NDN | 3 | 1 |  |  |  |  |  |  |
| GO:0008635\_activation\_of\_caspase\_activity\_by\_cytochrome\_c | CYCS | 3 | 1 |  |  |  |  |  |  |
| GO:0014074\_response\_to\_purine | FKBP1A | 3 | 1 |  |  |  |  |  |  |
| GO:0030836\_positive\_regulation\_of\_actin\_filament\_depolymerization | CFL1 | 3 | 1 |  |  |  |  |  |  |
| GO:0031000\_response\_to\_caffeine | FKBP1A | 3 | 1 |  |  |  |  |  |  |
| GO:0043200\_response\_to\_amino\_acid\_stimulus | CFL1 | 3 | 1 |  |  |  |  |  |  |
| GO:0043243\_positive\_regulation\_of\_protein\_complex\_disassembly | CFL1 | 3 | 1 |  |  |  |  |  |  |
| GO:0045844\_positive\_regulation\_of\_striated\_muscle\_development | GJA1 | 3 | 1 |  |  |  |  |  |  |
| GO:0048050\_post-embryonic\_eye\_morphogenesis | HMGN1 | 3 | 1 |  |  |  |  |  |  |
| GO:0048597\_post-embryonic\_camera-type\_eye\_morphogenesis | HMGN1 | 3 | 1 |  |  |  |  |  |  |
| GO:0048636\_positive\_regulation\_of\_muscle\_development | GJA1 | 3 | 1 |  |  |  |  |  |  |
| GO:0048676\_axon\_extension\_involved\_in\_development | NDN | 3 | 1 |  |  |  |  |  |  |
| GO:0060314\_regulation\_of\_ryanodine-sensitive\_calcium-release\_channel\_activity | FKBP1A | 3 | 1 |  |  |  |  |  |  |
| GO:0015031\_protein\_transport | YWHAB | 175 | 4 | 3.898413 | -1.748491 | 7 | 10.78 | 1.540000 |
| GO:0015031\_protein\_transport | YWHAQ | 175 | 4 | 3.898413 | -1.748491 | 7 | 10.78 | 1.540000 |
| GO:0015031\_protein\_transport | NUTF2 | 175 | 4 | 3.898413 | -1.748491 | 7 | 10.78 | 1.540000 |
| GO:0015031\_protein\_transport | PRDX1 | 175 | 4 | 3.898413 | -1.748491 | 7 | 10.78 | 1.540000 |
| GO:0045184\_establishment\_of\_protein\_localization | YWHAB | 180 | 4 | 3.790123 | -1.707823 | 8 | 11.82 | 1.477500 |
| GO:0045184\_establishment\_of\_protein\_localization | YWHAQ | 180 | 4 | 3.790123 | -1.707823 | 8 | 11.82 | 1.477500 |
| GO:0045184\_establishment\_of\_protein\_localization | NUTF2 | 180 | 4 | 3.790123 | -1.707823 | 8 | 11.82 | 1.477500 |
| GO:0045184\_establishment\_of\_protein\_localization | PRDX1 | 180 | 4 | 3.790123 | -1.707823 | 8 | 11.82 | 1.477500 |
| GO:0014031\_mesenchymal\_cell\_development | CFL1 | 40 | 2 | 8.527778 | -1.647465 | 9 | 13.25 | 1.472222 |
| GO:0014031\_mesenchymal\_cell\_development | TRIM28 | 40 | 2 | 8.527778 | -1.647465 | 9 | 13.25 | 1.472222 |
| GO:0001842\_neural\_fold\_formation | CFL1 | 4 | 1 |  |  |  |  |  |  |
| GO:0006290\_pyrimidine\_dimer\_repair | HMGN1 | 4 | 1 |  |  |  |  |  |  |
| GO:0010224\_response\_to\_UV-B | HMGN1 | 4 | 1 |  |  |  |  |  |  |
| GO:0032872\_regulation\_of\_stress-activated\_MAPK\_cascade | PRDX1 | 4 | 1 |  |  |  |  |  |  |
| GO:0042345\_regulation\_of\_NF-kappaB\_import\_into\_nucleus | PRDX1 | 4 | 1 |  |  |  |  |  |  |
| GO:0042348\_NF-kappaB\_import\_into\_nucleus | PRDX1 | 4 | 1 |  |  |  |  |  |  |
| GO:0048011\_nerve\_growth\_factor\_receptor\_signaling\_pathway | NDN | 4 | 1 |  |  |  |  |  |  |
| GO:0048710\_regulation\_of\_astrocyte\_differentiation | PRPF19 | 4 | 1 |  |  |  |  |  |  |
| GO:0051225\_spindle\_assembly | TUBB5 | 4 | 1 |  |  |  |  |  |  |
| GO:0046907\_intracellular\_transport | YWHAB | 194 | 4 | 3.516609 | -1.600968 | 10 | 14.32 | 1.432000 |
| GO:0046907\_intracellular\_transport | YWHAQ | 194 | 4 | 3.516609 | -1.600968 | 10 | 14.32 | 1.432000 |
| GO:0046907\_intracellular\_transport | NUTF2 | 194 | 4 | 3.516609 | -1.600968 | 10 | 14.32 | 1.432000 |
| GO:0046907\_intracellular\_transport | PRDX1 | 194 | 4 | 3.516609 | -1.600968 | 10 | 14.32 | 1.432000 |
| GO:0048762\_mesenchymal\_cell\_differentiation | CFL1 | 43 | 2 | 7.932817 | -1.588542 | 11 | 15.06 | 1.369091 |
| GO:0048762\_mesenchymal\_cell\_differentiation | TRIM28 | 43 | 2 | 7.932817 | -1.588542 | 11 | 15.06 | 1.369091 |
| GO:0033554\_cellular\_response\_to\_stress | HMGN1 | 196 | 4 | 3.480726 | -1.586486 | 12 | 15.07 | 1.255833 |
| GO:0033554\_cellular\_response\_to\_stress | RPA1 | 196 | 4 | 3.480726 | -1.586486 | 12 | 15.07 | 1.255833 |
| GO:0033554\_cellular\_response\_to\_stress | HSPA5 | 196 | 4 | 3.480726 | -1.586486 | 12 | 15.07 | 1.255833 |
| GO:0033554\_cellular\_response\_to\_stress | PRDX1 | 196 | 4 | 3.480726 | -1.586486 | 12 | 15.07 | 1.255833 |
| GO:0008152\_metabolic\_process | HMGN1 | 2133 | 18 | 1.439287 | -1.578090 | 13 | 15.22 | 1.170769 |
| GO:0008152\_metabolic\_process | NDN | 2133 | 18 | 1.439287 | -1.578090 | 13 | 15.22 | 1.170769 |
| GO:0008152\_metabolic\_process | MAT2A | 2133 | 18 | 1.439287 | -1.578090 | 13 | 15.22 | 1.170769 |
| GO:0008152\_metabolic\_process | CYCS | 2133 | 18 | 1.439287 | -1.578090 | 13 | 15.22 | 1.170769 |
| GO:0008152\_metabolic\_process | TRIM28 | 2133 | 18 | 1.439287 | -1.578090 | 13 | 15.22 | 1.170769 |
| GO:0008152\_metabolic\_process | CBX3 | 2133 | 18 | 1.439287 | -1.578090 | 13 | 15.22 | 1.170769 |
| GO:0008152\_metabolic\_process | FKBP1A | 2133 | 18 | 1.439287 | -1.578090 | 13 | 15.22 | 1.170769 |
| GO:0008152\_metabolic\_process | RBBP7 | 2133 | 18 | 1.439287 | -1.578090 | 13 | 15.22 | 1.170769 |
| GO:0008152\_metabolic\_process | DDX5 | 2133 | 18 | 1.439287 | -1.578090 | 13 | 15.22 | 1.170769 |
| GO:0008152\_metabolic\_process | SFRS1 | 2133 | 18 | 1.439287 | -1.578090 | 13 | 15.22 | 1.170769 |
| GO:0008152\_metabolic\_process | PRDX1 | 2133 | 18 | 1.439287 | -1.578090 | 13 | 15.22 | 1.170769 |
| GO:0008152\_metabolic\_process | RPL29 | 2133 | 18 | 1.439287 | -1.578090 | 13 | 15.22 | 1.170769 |
| GO:0008152\_metabolic\_process | RPA1 | 2133 | 18 | 1.439287 | -1.578090 | 13 | 15.22 | 1.170769 |
| GO:0008152\_metabolic\_process | PRPF19 | 2133 | 18 | 1.439287 | -1.578090 | 13 | 15.22 | 1.170769 |
| GO:0008152\_metabolic\_process | OAZ1 | 2133 | 18 | 1.439287 | -1.578090 | 13 | 15.22 | 1.170769 |
| GO:0008152\_metabolic\_process | CFL1 | 2133 | 18 | 1.439287 | -1.578090 | 13 | 15.22 | 1.170769 |
| GO:0008152\_metabolic\_process | HSPA8 | 2133 | 18 | 1.439287 | -1.578090 | 13 | 15.22 | 1.170769 |
| GO:0008152\_metabolic\_process | IMPDH2 | 2133 | 18 | 1.439287 | -1.578090 | 13 | 15.22 | 1.170769 |
| GO:0006606\_protein\_import\_into\_nucleus | NUTF2 | 44 | 2 | 7.752525 | -1.569894 | 16 | 16.01 | 1.000625 |
| GO:0006606\_protein\_import\_into\_nucleus | PRDX1 | 44 | 2 | 7.752525 | -1.569894 | 16 | 16.01 | 1.000625 |
| GO:0051170\_nuclear\_import | NUTF2 | 44 | 2 | 7.752525 | -1.569894 | 16 | 16.01 | 1.000625 |
| GO:0051170\_nuclear\_import | PRDX1 | 44 | 2 | 7.752525 | -1.569894 | 16 | 16.01 | 1.000625 |
| GO:0060485\_mesenchyme\_development | CFL1 | 44 | 2 | 7.752525 | -1.569894 | 16 | 16.01 | 1.000625 |
| GO:0060485\_mesenchyme\_development | TRIM28 | 44 | 2 | 7.752525 | -1.569894 | 16 | 16.01 | 1.000625 |
| GO:0044237\_cellular\_metabolic\_process | HMGN1 | 1974 | 17 | 1.468817 | -1.554181 | 17 | 16.16 | 0.950588 |
| GO:0044237\_cellular\_metabolic\_process | MAT2A | 1974 | 17 | 1.468817 | -1.554181 | 17 | 16.16 | 0.950588 |
| GO:0044237\_cellular\_metabolic\_process | NDN | 1974 | 17 | 1.468817 | -1.554181 | 17 | 16.16 | 0.950588 |
| GO:0044237\_cellular\_metabolic\_process | CYCS | 1974 | 17 | 1.468817 | -1.554181 | 17 | 16.16 | 0.950588 |
| GO:0044237\_cellular\_metabolic\_process | TRIM28 | 1974 | 17 | 1.468817 | -1.554181 | 17 | 16.16 | 0.950588 |
| GO:0044237\_cellular\_metabolic\_process | CBX3 | 1974 | 17 | 1.468817 | -1.554181 | 17 | 16.16 | 0.950588 |
| GO:0044237\_cellular\_metabolic\_process | FKBP1A | 1974 | 17 | 1.468817 | -1.554181 | 17 | 16.16 | 0.950588 |
| GO:0044237\_cellular\_metabolic\_process | RBBP7 | 1974 | 17 | 1.468817 | -1.554181 | 17 | 16.16 | 0.950588 |
| GO:0044237\_cellular\_metabolic\_process | DDX5 | 1974 | 17 | 1.468817 | -1.554181 | 17 | 16.16 | 0.950588 |
| GO:0044237\_cellular\_metabolic\_process | SFRS1 | 1974 | 17 | 1.468817 | -1.554181 | 17 | 16.16 | 0.950588 |
| GO:0044237\_cellular\_metabolic\_process | PRDX1 | 1974 | 17 | 1.468817 | -1.554181 | 17 | 16.16 | 0.950588 |
| GO:0044237\_cellular\_metabolic\_process | RPL29 | 1974 | 17 | 1.468817 | -1.554181 | 17 | 16.16 | 0.950588 |
| GO:0044237\_cellular\_metabolic\_process | RPA1 | 1974 | 17 | 1.468817 | -1.554181 | 17 | 16.16 | 0.950588 |
| GO:0044237\_cellular\_metabolic\_process | OAZ1 | 1974 | 17 | 1.468817 | -1.554181 | 17 | 16.16 | 0.950588 |
| GO:0044237\_cellular\_metabolic\_process | CFL1 | 1974 | 17 | 1.468817 | -1.554181 | 17 | 16.16 | 0.950588 |
| GO:0044237\_cellular\_metabolic\_process | HSPA8 | 1974 | 17 | 1.468817 | -1.554181 | 17 | 16.16 | 0.950588 |
| GO:0044237\_cellular\_metabolic\_process | IMPDH2 | 1974 | 17 | 1.468817 | -1.554181 | 17 | 16.16 | 0.950588 |
| GO:0000097\_sulfur\_amino\_acid\_biosynthetic\_process | MAT2A | 5 | 1 | 34.111111 | -1.537802 | 27 | 24.78 | 0.917778 |
| GO:0006983\_ER\_overload\_response | HSPA5 | 5 | 1 | 34.111111 | -1.537802 | 27 | 24.78 | 0.917778 |
| GO:0014015\_positive\_regulation\_of\_gliogenesis | PRPF19 | 5 | 1 | 34.111111 | -1.537802 | 27 | 24.78 | 0.917778 |
| GO:0019430\_removal\_of\_superoxide\_radicals | PRDX1 | 5 | 1 | 34.111111 | -1.537802 | 27 | 24.78 | 0.917778 |
| GO:0030042\_actin\_filament\_depolymerization | CFL1 | 5 | 1 | 34.111111 | -1.537802 | 27 | 24.78 | 0.917778 |
| GO:0030834\_regulation\_of\_actin\_filament\_depolymerization | CFL1 | 5 | 1 | 34.111111 | -1.537802 | 27 | 24.78 | 0.917778 |
| GO:0032479\_regulation\_of\_type\_I\_interferon\_production | HSPD1 | 5 | 1 | 34.111111 | -1.537802 | 27 | 24.78 | 0.917778 |
| GO:0043403\_skeletal\_muscle\_regeneration | GJA1 | 5 | 1 | 34.111111 | -1.537802 | 27 | 24.78 | 0.917778 |
| GO:0045687\_positive\_regulation\_of\_glial\_cell\_differentiation | PRPF19 | 5 | 1 | 34.111111 | -1.537802 | 27 | 24.78 | 0.917778 |
| GO:0051403\_stress-activated\_MAPK\_cascade | PRDX1 | 5 | 1 | 34.111111 | -1.537802 | 27 | 24.78 | 0.917778 |
| GO:0042063\_gliogenesis | PRPF19 | 46 | 2 | 7.415459 | -1.533955 | 28 | 25.11 | 0.896786 |
| GO:0042063\_gliogenesis | NDN | 46 | 2 | 7.415459 | -1.533955 | 28 | 25.11 | 0.896786 |
| GO:0034504\_protein\_localization\_in\_nucleus | NUTF2 | 48 | 2 | 7.106481 | -1.499694 | 29 | 26.05 | 0.898276 |
| GO:0034504\_protein\_localization\_in\_nucleus | PRDX1 | 48 | 2 | 7.106481 | -1.499694 | 29 | 26.05 | 0.898276 |
| GO:0003015\_heart\_process | GJA1 | 49 | 2 | 6.961451 | -1.483149 | 31 | 26.64 | 0.859355 |
| GO:0003015\_heart\_process | SFRS1 | 49 | 2 | 6.961451 | -1.483149 | 31 | 26.64 | 0.859355 |
| GO:0060047\_heart\_contraction | GJA1 | 49 | 2 | 6.961451 | -1.483149 | 31 | 26.64 | 0.859355 |
| GO:0060047\_heart\_contraction | SFRS1 | 49 | 2 | 6.961451 | -1.483149 | 31 | 26.64 | 0.859355 |
| GO:0008283\_cell\_proliferation | HMGN1 | 544 | 7 | 2.194649 | -1.478768 | 32 | 26.67 | 0.833438 |
| GO:0008283\_cell\_proliferation | RPA1 | 544 | 7 | 2.194649 | -1.478768 | 32 | 26.67 | 0.833438 |
| GO:0008283\_cell\_proliferation | PRPF19 | 544 | 7 | 2.194649 | -1.478768 | 32 | 26.67 | 0.833438 |
| GO:0008283\_cell\_proliferation | FKBP1A | 544 | 7 | 2.194649 | -1.478768 | 32 | 26.67 | 0.833438 |
| GO:0008283\_cell\_proliferation | PRDX1 | 544 | 7 | 2.194649 | -1.478768 | 32 | 26.67 | 0.833438 |
| GO:0008283\_cell\_proliferation | IMPDH2 | 544 | 7 | 2.194649 | -1.478768 | 32 | 26.67 | 0.833438 |
| GO:0008283\_cell\_proliferation | RPL29 | 544 | 7 | 2.194649 | -1.478768 | 32 | 26.67 | 0.833438 |
| GO:0050789\_regulation\_of\_biological\_process | HMGN1 | 2357 | 19 | 1.374864 | -1.467393 | 33 | 26.86 | 0.813939 |
| GO:0050789\_regulation\_of\_biological\_process | GDI2 | 2357 | 19 | 1.374864 | -1.467393 | 33 | 26.86 | 0.813939 |
| GO:0050789\_regulation\_of\_biological\_process | HAX1 | 2357 | 19 | 1.374864 | -1.467393 | 33 | 26.86 | 0.813939 |
| GO:0050789\_regulation\_of\_biological\_process | NDN | 2357 | 19 | 1.374864 | -1.467393 | 33 | 26.86 | 0.813939 |
| GO:0050789\_regulation\_of\_biological\_process | TRIM28 | 2357 | 19 | 1.374864 | -1.467393 | 33 | 26.86 | 0.813939 |
| GO:0050789\_regulation\_of\_biological\_process | CYCS | 2357 | 19 | 1.374864 | -1.467393 | 33 | 26.86 | 0.813939 |
| GO:0050789\_regulation\_of\_biological\_process | CBX3 | 2357 | 19 | 1.374864 | -1.467393 | 33 | 26.86 | 0.813939 |
| GO:0050789\_regulation\_of\_biological\_process | GJA1 | 2357 | 19 | 1.374864 | -1.467393 | 33 | 26.86 | 0.813939 |
| GO:0050789\_regulation\_of\_biological\_process | FKBP1A | 2357 | 19 | 1.374864 | -1.467393 | 33 | 26.86 | 0.813939 |
| GO:0050789\_regulation\_of\_biological\_process | RBBP7 | 2357 | 19 | 1.374864 | -1.467393 | 33 | 26.86 | 0.813939 |
| GO:0050789\_regulation\_of\_biological\_process | DDX5 | 2357 | 19 | 1.374864 | -1.467393 | 33 | 26.86 | 0.813939 |
| GO:0050789\_regulation\_of\_biological\_process | PRDX1 | 2357 | 19 | 1.374864 | -1.467393 | 33 | 26.86 | 0.813939 |
| GO:0050789\_regulation\_of\_biological\_process | RPA1 | 2357 | 19 | 1.374864 | -1.467393 | 33 | 26.86 | 0.813939 |
| GO:0050789\_regulation\_of\_biological\_process | PRPF19 | 2357 | 19 | 1.374864 | -1.467393 | 33 | 26.86 | 0.813939 |
| GO:0050789\_regulation\_of\_biological\_process | CFL1 | 2357 | 19 | 1.374864 | -1.467393 | 33 | 26.86 | 0.813939 |
| GO:0050789\_regulation\_of\_biological\_process | YWHAQ | 2357 | 19 | 1.374864 | -1.467393 | 33 | 26.86 | 0.813939 |
| GO:0050789\_regulation\_of\_biological\_process | HSPA5 | 2357 | 19 | 1.374864 | -1.467393 | 33 | 26.86 | 0.813939 |
| GO:0050789\_regulation\_of\_biological\_process | HSPD1 | 2357 | 19 | 1.374864 | -1.467393 | 33 | 26.86 | 0.813939 |
| GO:0050789\_regulation\_of\_biological\_process | HSPA8 | 2357 | 19 | 1.374864 | -1.467393 | 33 | 26.86 | 0.813939 |
| GO:0017038\_protein\_import | NUTF2 | 50 | 2 | 6.822222 | -1.466973 | 34 | 27.14 | 0.798235 |
| GO:0017038\_protein\_import | PRDX1 | 50 | 2 | 6.822222 | -1.466973 | 34 | 27.14 | 0.798235 |
| GO:0031077\_post-embryonic\_camera-type\_eye\_development | HMGN1 | 6 | 1 | 28.425926 | -1.459845 | 38 | 33.53 | 0.882368 |
| GO:0042246\_tissue\_regeneration | GJA1 | 6 | 1 | 28.425926 | -1.459845 | 38 | 33.53 | 0.882368 |
| GO:0048563\_post-embryonic\_organ\_morphogenesis | HMGN1 | 6 | 1 | 28.425926 | -1.459845 | 38 | 33.53 | 0.882368 |
| GO:0051085\_chaperone\_mediated\_protein\_folding\_requiring\_cofactor | HSPA8 | 6 | 1 | 28.425926 | -1.459845 | 38 | 33.53 | 0.882368 |
| GO:0050794\_regulation\_of\_cellular\_process | HMGN1 | 2190 | 18 | 1.401826 | -1.450485 | 39 | 33.85 | 0.867949 |
| GO:0050794\_regulation\_of\_cellular\_process | GDI2 | 2190 | 18 | 1.401826 | -1.450485 | 39 | 33.85 | 0.867949 |
| GO:0050794\_regulation\_of\_cellular\_process | NDN | 2190 | 18 | 1.401826 | -1.450485 | 39 | 33.85 | 0.867949 |
| GO:0050794\_regulation\_of\_cellular\_process | HAX1 | 2190 | 18 | 1.401826 | -1.450485 | 39 | 33.85 | 0.867949 |
| GO:0050794\_regulation\_of\_cellular\_process | CYCS | 2190 | 18 | 1.401826 | -1.450485 | 39 | 33.85 | 0.867949 |
| GO:0050794\_regulation\_of\_cellular\_process | TRIM28 | 2190 | 18 | 1.401826 | -1.450485 | 39 | 33.85 | 0.867949 |
| GO:0050794\_regulation\_of\_cellular\_process | CBX3 | 2190 | 18 | 1.401826 | -1.450485 | 39 | 33.85 | 0.867949 |
| GO:0050794\_regulation\_of\_cellular\_process | FKBP1A | 2190 | 18 | 1.401826 | -1.450485 | 39 | 33.85 | 0.867949 |
| GO:0050794\_regulation\_of\_cellular\_process | DDX5 | 2190 | 18 | 1.401826 | -1.450485 | 39 | 33.85 | 0.867949 |
| GO:0050794\_regulation\_of\_cellular\_process | RBBP7 | 2190 | 18 | 1.401826 | -1.450485 | 39 | 33.85 | 0.867949 |
| GO:0050794\_regulation\_of\_cellular\_process | PRDX1 | 2190 | 18 | 1.401826 | -1.450485 | 39 | 33.85 | 0.867949 |
| GO:0050794\_regulation\_of\_cellular\_process | PRPF19 | 2190 | 18 | 1.401826 | -1.450485 | 39 | 33.85 | 0.867949 |
| GO:0050794\_regulation\_of\_cellular\_process | RPA1 | 2190 | 18 | 1.401826 | -1.450485 | 39 | 33.85 | 0.867949 |
| GO:0050794\_regulation\_of\_cellular\_process | CFL1 | 2190 | 18 | 1.401826 | -1.450485 | 39 | 33.85 | 0.867949 |
| GO:0050794\_regulation\_of\_cellular\_process | YWHAQ | 2190 | 18 | 1.401826 | -1.450485 | 39 | 33.85 | 0.867949 |
| GO:0050794\_regulation\_of\_cellular\_process | HSPD1 | 2190 | 18 | 1.401826 | -1.450485 | 39 | 33.85 | 0.867949 |
| GO:0050794\_regulation\_of\_cellular\_process | HSPA5 | 2190 | 18 | 1.401826 | -1.450485 | 39 | 33.85 | 0.867949 |
| GO:0050794\_regulation\_of\_cellular\_process | HSPA8 | 2190 | 18 | 1.401826 | -1.450485 | 39 | 33.85 | 0.867949 |
| GO:0040007\_growth | PRPF19 | 217 | 4 | 3.143881 | -1.444822 | 40 | 34.03 | 0.850750 |
| GO:0040007\_growth | NDN | 217 | 4 | 3.143881 | -1.444822 | 40 | 34.03 | 0.850750 |
| GO:0040007\_growth | GJA1 | 217 | 4 | 3.143881 | -1.444822 | 40 | 34.03 | 0.850750 |
| GO:0040007\_growth | RPL29 | 217 | 4 | 3.143881 | -1.444822 | 40 | 34.03 | 0.850750 |
| GO:0001701\_in\_utero\_embryonic\_development | RPA1 | 221 | 4 | 3.086978 | -1.419812 | 41 | 34.79 | 0.848537 |
| GO:0001701\_in\_utero\_embryonic\_development | PRPF19 | 221 | 4 | 3.086978 | -1.419812 | 41 | 34.79 | 0.848537 |
| GO:0001701\_in\_utero\_embryonic\_development | GJA1 | 221 | 4 | 3.086978 | -1.419812 | 41 | 34.79 | 0.848537 |
| GO:0001701\_in\_utero\_embryonic\_development | SFRS1 | 221 | 4 | 3.086978 | -1.419812 | 41 | 34.79 | 0.848537 |
| GO:0051276\_chromosome\_organization | RPA1 | 129 | 3 | 3.966408 | -1.414719 | 42 | 34.85 | 0.829762 |
| GO:0051276\_chromosome\_organization | HMGN1 | 129 | 3 | 3.966408 | -1.414719 | 42 | 34.85 | 0.829762 |
| GO:0051276\_chromosome\_organization | RBBP7 | 129 | 3 | 3.966408 | -1.414719 | 42 | 34.85 | 0.829762 |
| GO:0001839\_neural\_plate\_morphogenesis | CFL1 | 7 | 1 | 24.365079 | -1.394121 | 44 | 42.83 | 0.973409 |
| GO:0007413\_axonal\_fasciculation | NDN | 7 | 1 | 24.365079 | -1.394121 | 44 | 42.83 | 0.973409 |
| GO:0001764\_neuron\_migration | NDN | 57 | 2 | 5.984405 | -1.362930 | 46 | 44.46 | 0.966522 |
| GO:0001764\_neuron\_migration | GJA1 | 57 | 2 | 5.984405 | -1.362930 | 46 | 44.46 | 0.966522 |
| GO:0033365\_protein\_localization\_in\_organelle | NUTF2 | 57 | 2 | 5.984405 | -1.362930 | 46 | 44.46 | 0.966522 |
| GO:0033365\_protein\_localization\_in\_organelle | PRDX1 | 57 | 2 | 5.984405 | -1.362930 | 46 | 44.46 | 0.966522 |
| GO:0044238\_primary\_metabolic\_process | HMGN1 | 1905 | 16 | 1.432488 | -1.338425 | 47 | 45.01 | 0.957660 |
| GO:0044238\_primary\_metabolic\_process | NDN | 1905 | 16 | 1.432488 | -1.338425 | 47 | 45.01 | 0.957660 |
| GO:0044238\_primary\_metabolic\_process | MAT2A | 1905 | 16 | 1.432488 | -1.338425 | 47 | 45.01 | 0.957660 |
| GO:0044238\_primary\_metabolic\_process | TRIM28 | 1905 | 16 | 1.432488 | -1.338425 | 47 | 45.01 | 0.957660 |
| GO:0044238\_primary\_metabolic\_process | CBX3 | 1905 | 16 | 1.432488 | -1.338425 | 47 | 45.01 | 0.957660 |
| GO:0044238\_primary\_metabolic\_process | FKBP1A | 1905 | 16 | 1.432488 | -1.338425 | 47 | 45.01 | 0.957660 |
| GO:0044238\_primary\_metabolic\_process | RBBP7 | 1905 | 16 | 1.432488 | -1.338425 | 47 | 45.01 | 0.957660 |
| GO:0044238\_primary\_metabolic\_process | DDX5 | 1905 | 16 | 1.432488 | -1.338425 | 47 | 45.01 | 0.957660 |
| GO:0044238\_primary\_metabolic\_process | SFRS1 | 1905 | 16 | 1.432488 | -1.338425 | 47 | 45.01 | 0.957660 |
| GO:0044238\_primary\_metabolic\_process | RPL29 | 1905 | 16 | 1.432488 | -1.338425 | 47 | 45.01 | 0.957660 |
| GO:0044238\_primary\_metabolic\_process | RPA1 | 1905 | 16 | 1.432488 | -1.338425 | 47 | 45.01 | 0.957660 |
| GO:0044238\_primary\_metabolic\_process | PRPF19 | 1905 | 16 | 1.432488 | -1.338425 | 47 | 45.01 | 0.957660 |
| GO:0044238\_primary\_metabolic\_process | OAZ1 | 1905 | 16 | 1.432488 | -1.338425 | 47 | 45.01 | 0.957660 |
| GO:0044238\_primary\_metabolic\_process | CFL1 | 1905 | 16 | 1.432488 | -1.338425 | 47 | 45.01 | 0.957660 |
| GO:0044238\_primary\_metabolic\_process | IMPDH2 | 1905 | 16 | 1.432488 | -1.338425 | 47 | 45.01 | 0.957660 |
| GO:0044238\_primary\_metabolic\_process | HSPA8 | 1905 | 16 | 1.432488 | -1.338425 | 47 | 45.01 | 0.957660 |
| GO:0000910\_cytokinesis | CFL1 | 8 | 1 | 21.319444 | -1.337351 | 58 | 51.32 | 0.884828 |
| GO:0001833\_inner\_cell\_mass\_cell\_proliferation | PRPF19 | 8 | 1 | 21.319444 | -1.337351 | 58 | 51.32 | 0.884828 |
| GO:0001840\_neural\_plate\_development | CFL1 | 8 | 1 | 21.319444 | -1.337351 | 58 | 51.32 | 0.884828 |
| GO:0006458\_'de\_novo'\_protein\_folding | HSPA8 | 8 | 1 | 21.319444 | -1.337351 | 58 | 51.32 | 0.884828 |
| GO:0022898\_regulation\_of\_transmembrane\_transporter\_activity | FKBP1A | 8 | 1 | 21.319444 | -1.337351 | 58 | 51.32 | 0.884828 |
| GO:0032409\_regulation\_of\_transporter\_activity | FKBP1A | 8 | 1 | 21.319444 | -1.337351 | 58 | 51.32 | 0.884828 |
| GO:0032412\_regulation\_of\_ion\_transmembrane\_transporter\_activity | FKBP1A | 8 | 1 | 21.319444 | -1.337351 | 58 | 51.32 | 0.884828 |
| GO:0042990\_regulation\_of\_transcription\_factor\_import\_into\_nucleus | PRDX1 | 8 | 1 | 21.319444 | -1.337351 | 58 | 51.32 | 0.884828 |
| GO:0042991\_transcription\_factor\_import\_into\_nucleus | PRDX1 | 8 | 1 | 21.319444 | -1.337351 | 58 | 51.32 | 0.884828 |
| GO:0051084\_'de\_novo'\_posttranslational\_protein\_folding | HSPA8 | 8 | 1 | 21.319444 | -1.337351 | 58 | 51.32 | 0.884828 |
| GO:0060347\_heart\_trabecula\_formation | FKBP1A | 8 | 1 | 21.319444 | -1.337351 | 58 | 51.32 | 0.884828 |
| GO:0006874\_cellular\_calcium\_ion\_homeostasis | NDN | 61 | 2 | 5.591985 | -1.309703 | 59 | 52.29 | 0.886271 |
| GO:0006874\_cellular\_calcium\_ion\_homeostasis | FKBP1A | 61 | 2 | 5.591985 | -1.309703 | 59 | 52.29 | 0.886271 |
| GO:0006139\_nucleobase\_\_nucleoside\_\_nucleotide\_and\_nucleic\_acid\_metabolic\_process | HMGN1 | 1002 | 10 | 1.702151 | -1.293675 | 60 | 53.12 | 0.885333 |
| GO:0006139\_nucleobase\_\_nucleoside\_\_nucleotide\_and\_nucleic\_acid\_metabolic\_process | RPA1 | 1002 | 10 | 1.702151 | -1.293675 | 60 | 53.12 | 0.885333 |
| GO:0006139\_nucleobase\_\_nucleoside\_\_nucleotide\_and\_nucleic\_acid\_metabolic\_process | MAT2A | 1002 | 10 | 1.702151 | -1.293675 | 60 | 53.12 | 0.885333 |
| GO:0006139\_nucleobase\_\_nucleoside\_\_nucleotide\_and\_nucleic\_acid\_metabolic\_process | NDN | 1002 | 10 | 1.702151 | -1.293675 | 60 | 53.12 | 0.885333 |
| GO:0006139\_nucleobase\_\_nucleoside\_\_nucleotide\_and\_nucleic\_acid\_metabolic\_process | TRIM28 | 1002 | 10 | 1.702151 | -1.293675 | 60 | 53.12 | 0.885333 |
| GO:0006139\_nucleobase\_\_nucleoside\_\_nucleotide\_and\_nucleic\_acid\_metabolic\_process | CBX3 | 1002 | 10 | 1.702151 | -1.293675 | 60 | 53.12 | 0.885333 |
| GO:0006139\_nucleobase\_\_nucleoside\_\_nucleotide\_and\_nucleic\_acid\_metabolic\_process | SFRS1 | 1002 | 10 | 1.702151 | -1.293675 | 60 | 53.12 | 0.885333 |
| GO:0006139\_nucleobase\_\_nucleoside\_\_nucleotide\_and\_nucleic\_acid\_metabolic\_process | DDX5 | 1002 | 10 | 1.702151 | -1.293675 | 60 | 53.12 | 0.885333 |
| GO:0006139\_nucleobase\_\_nucleoside\_\_nucleotide\_and\_nucleic\_acid\_metabolic\_process | RBBP7 | 1002 | 10 | 1.702151 | -1.293675 | 60 | 53.12 | 0.885333 |
| GO:0006139\_nucleobase\_\_nucleoside\_\_nucleotide\_and\_nucleic\_acid\_metabolic\_process | IMPDH2 | 1002 | 10 | 1.702151 | -1.293675 | 60 | 53.12 | 0.885333 |
| GO:0006807\_nitrogen\_compound\_metabolic\_process | HMGN1 | 1147 | 11 | 1.635668 | -1.292371 | 61 | 53.13 | 0.870984 |
| GO:0006807\_nitrogen\_compound\_metabolic\_process | RPA1 | 1147 | 11 | 1.635668 | -1.292371 | 61 | 53.13 | 0.870984 |
| GO:0006807\_nitrogen\_compound\_metabolic\_process | OAZ1 | 1147 | 11 | 1.635668 | -1.292371 | 61 | 53.13 | 0.870984 |
| GO:0006807\_nitrogen\_compound\_metabolic\_process | MAT2A | 1147 | 11 | 1.635668 | -1.292371 | 61 | 53.13 | 0.870984 |
| GO:0006807\_nitrogen\_compound\_metabolic\_process | NDN | 1147 | 11 | 1.635668 | -1.292371 | 61 | 53.13 | 0.870984 |
| GO:0006807\_nitrogen\_compound\_metabolic\_process | TRIM28 | 1147 | 11 | 1.635668 | -1.292371 | 61 | 53.13 | 0.870984 |
| GO:0006807\_nitrogen\_compound\_metabolic\_process | CBX3 | 1147 | 11 | 1.635668 | -1.292371 | 61 | 53.13 | 0.870984 |
| GO:0006807\_nitrogen\_compound\_metabolic\_process | SFRS1 | 1147 | 11 | 1.635668 | -1.292371 | 61 | 53.13 | 0.870984 |
| GO:0006807\_nitrogen\_compound\_metabolic\_process | DDX5 | 1147 | 11 | 1.635668 | -1.292371 | 61 | 53.13 | 0.870984 |
| GO:0006807\_nitrogen\_compound\_metabolic\_process | RBBP7 | 1147 | 11 | 1.635668 | -1.292371 | 61 | 53.13 | 0.870984 |
| GO:0006807\_nitrogen\_compound\_metabolic\_process | IMPDH2 | 1147 | 11 | 1.635668 | -1.292371 | 61 | 53.13 | 0.870984 |
| GO:0006595\_polyamine\_metabolic\_process | OAZ1 | 9 | 1 | 18.950617 | -1.287420 | 65 | 59.32 | 0.912615 |
| GO:0032606\_type\_I\_interferon\_production | HSPD1 | 9 | 1 | 18.950617 | -1.287420 | 65 | 59.32 | 0.912615 |
| GO:0048569\_post-embryonic\_organ\_development | HMGN1 | 9 | 1 | 18.950617 | -1.287420 | 65 | 59.32 | 0.912615 |
| GO:0048708\_astrocyte\_differentiation | PRPF19 | 9 | 1 | 18.950617 | -1.287420 | 65 | 59.32 | 0.912615 |
| GO:0006875\_cellular\_metal\_ion\_homeostasis | NDN | 64 | 2 | 5.329861 | -1.272302 | 67 | 59.99 | 0.895373 |
| GO:0006875\_cellular\_metal\_ion\_homeostasis | FKBP1A | 64 | 2 | 5.329861 | -1.272302 | 67 | 59.99 | 0.895373 |
| GO:0055074\_calcium\_ion\_homeostasis | NDN | 64 | 2 | 5.329861 | -1.272302 | 67 | 59.99 | 0.895373 |
| GO:0055074\_calcium\_ion\_homeostasis | FKBP1A | 64 | 2 | 5.329861 | -1.272302 | 67 | 59.99 | 0.895373 |
| GO:0008104\_protein\_localization | YWHAB | 251 | 4 | 2.718017 | -1.249266 | 68 | 60.62 | 0.891471 |
| GO:0008104\_protein\_localization | YWHAQ | 251 | 4 | 2.718017 | -1.249266 | 68 | 60.62 | 0.891471 |
| GO:0008104\_protein\_localization | NUTF2 | 251 | 4 | 2.718017 | -1.249266 | 68 | 60.62 | 0.891471 |
| GO:0008104\_protein\_localization | PRDX1 | 251 | 4 | 2.718017 | -1.249266 | 68 | 60.62 | 0.891471 |
| GO:0000724\_double-strand\_break\_repair\_via\_homologous\_recombination | RPA1 | 10 | 1 | 17.055556 | -1.242883 | 80 | 67.74 | 0.846750 |
| GO:0000725\_recombinational\_repair | RPA1 | 10 | 1 | 17.055556 | -1.242883 | 80 | 67.74 | 0.846750 |
| GO:0001832\_blastocyst\_growth | PRPF19 | 10 | 1 | 17.055556 | -1.242883 | 80 | 67.74 | 0.846750 |
| GO:0002070\_epithelial\_cell\_maturation | GJA1 | 10 | 1 | 17.055556 | -1.242883 | 80 | 67.74 | 0.846750 |
| GO:0006289\_nucleotide-excision\_repair | HMGN1 | 10 | 1 | 17.055556 | -1.242883 | 80 | 67.74 | 0.846750 |
| GO:0006801\_superoxide\_metabolic\_process | PRDX1 | 10 | 1 | 17.055556 | -1.242883 | 80 | 67.74 | 0.846750 |
| GO:0051208\_sequestering\_of\_calcium\_ion | FKBP1A | 10 | 1 | 17.055556 | -1.242883 | 80 | 67.74 | 0.846750 |
| GO:0051209\_release\_of\_sequestered\_calcium\_ion\_into\_cytosol | FKBP1A | 10 | 1 | 17.055556 | -1.242883 | 80 | 67.74 | 0.846750 |
| GO:0051238\_sequestering\_of\_metal\_ion | FKBP1A | 10 | 1 | 17.055556 | -1.242883 | 80 | 67.74 | 0.846750 |
| GO:0051282\_regulation\_of\_sequestering\_of\_calcium\_ion | FKBP1A | 10 | 1 | 17.055556 | -1.242883 | 80 | 67.74 | 0.846750 |
| GO:0051283\_negative\_regulation\_of\_sequestering\_of\_calcium\_ion | FKBP1A | 10 | 1 | 17.055556 | -1.242883 | 80 | 67.74 | 0.846750 |
| GO:0060343\_trabecula\_formation | FKBP1A | 10 | 1 | 17.055556 | -1.242883 | 80 | 67.74 | 0.846750 |
| GO:0043009\_chordate\_embryonic\_development | RPA1 | 365 | 5 | 2.336377 | -1.238932 | 81 | 67.88 | 0.838025 |
| GO:0043009\_chordate\_embryonic\_development | PRPF19 | 365 | 5 | 2.336377 | -1.238932 | 81 | 67.88 | 0.838025 |
| GO:0043009\_chordate\_embryonic\_development | CFL1 | 365 | 5 | 2.336377 | -1.238932 | 81 | 67.88 | 0.838025 |
| GO:0043009\_chordate\_embryonic\_development | GJA1 | 365 | 5 | 2.336377 | -1.238932 | 81 | 67.88 | 0.838025 |
| GO:0043009\_chordate\_embryonic\_development | SFRS1 | 365 | 5 | 2.336377 | -1.238932 | 81 | 67.88 | 0.838025 |
| GO:0003007\_heart\_morphogenesis | GJA1 | 67 | 2 | 5.091211 | -1.236837 | 82 | 68.28 | 0.832683 |
| GO:0003007\_heart\_morphogenesis | FKBP1A | 67 | 2 | 5.091211 | -1.236837 | 82 | 68.28 | 0.832683 |
| GO:0009792\_embryonic\_development\_ending\_in\_birth\_or\_egg\_hatching | RPA1 | 368 | 5 | 2.317331 | -1.226339 | 83 | 68.5 | 0.825301 |
| GO:0009792\_embryonic\_development\_ending\_in\_birth\_or\_egg\_hatching | PRPF19 | 368 | 5 | 2.317331 | -1.226339 | 83 | 68.5 | 0.825301 |
| GO:0009792\_embryonic\_development\_ending\_in\_birth\_or\_egg\_hatching | CFL1 | 368 | 5 | 2.317331 | -1.226339 | 83 | 68.5 | 0.825301 |
| GO:0009792\_embryonic\_development\_ending\_in\_birth\_or\_egg\_hatching | GJA1 | 368 | 5 | 2.317331 | -1.226339 | 83 | 68.5 | 0.825301 |
| GO:0009792\_embryonic\_development\_ending\_in\_birth\_or\_egg\_hatching | SFRS1 | 368 | 5 | 2.317331 | -1.226339 | 83 | 68.5 | 0.825301 |
| GO:0055065\_metal\_ion\_homeostasis | NDN | 69 | 2 | 4.943639 | -1.214184 | 84 | 69.07 | 0.822262 |
| GO:0055065\_metal\_ion\_homeostasis | FKBP1A | 69 | 2 | 4.943639 | -1.214184 | 84 | 69.07 | 0.822262 |
| GO:0000096\_sulfur\_amino\_acid\_metabolic\_process | MAT2A | 11 | 1 | 15.505051 | -1.202709 | 92 | 74.57 | 0.810543 |
| GO:0001101\_response\_to\_acid | CFL1 | 11 | 1 | 15.505051 | -1.202709 | 92 | 74.57 | 0.810543 |
| GO:0001837\_epithelial\_to\_mesenchymal\_transition | TRIM28 | 11 | 1 | 15.505051 | -1.202709 | 92 | 74.57 | 0.810543 |
| GO:0007051\_spindle\_organization | TUBB5 | 11 | 1 | 15.505051 | -1.202709 | 92 | 74.57 | 0.810543 |
| GO:0008652\_cellular\_amino\_acid\_biosynthetic\_process | MAT2A | 11 | 1 | 15.505051 | -1.202709 | 92 | 74.57 | 0.810543 |
| GO:0014013\_regulation\_of\_gliogenesis | PRPF19 | 11 | 1 | 15.505051 | -1.202709 | 92 | 74.57 | 0.810543 |
| GO:0034762\_regulation\_of\_transmembrane\_transport | FKBP1A | 11 | 1 | 15.505051 | -1.202709 | 92 | 74.57 | 0.810543 |
| GO:0045685\_regulation\_of\_glial\_cell\_differentiation | PRPF19 | 11 | 1 | 15.505051 | -1.202709 | 92 | 74.57 | 0.810543 |
| GO:0006281\_DNA\_repair | HMGN1 | 71 | 2 | 4.804382 | -1.192270 | 94 | 75.27 | 0.800745 |
| GO:0006281\_DNA\_repair | RPA1 | 71 | 2 | 4.804382 | -1.192270 | 94 | 75.27 | 0.800745 |
| GO:0006913\_nucleocytoplasmic\_transport | NUTF2 | 71 | 2 | 4.804382 | -1.192270 | 94 | 75.27 | 0.800745 |
| GO:0006913\_nucleocytoplasmic\_transport | PRDX1 | 71 | 2 | 4.804382 | -1.192270 | 94 | 75.27 | 0.800745 |
| GO:0007264\_small\_GTPase\_mediated\_signal\_transduction | GDI2 | 72 | 2 | 4.737654 | -1.181576 | 96 | 75.96 | 0.791250 |
| GO:0007264\_small\_GTPase\_mediated\_signal\_transduction | YWHAQ | 72 | 2 | 4.737654 | -1.181576 | 96 | 75.96 | 0.791250 |
| GO:0051169\_nuclear\_transport | NUTF2 | 72 | 2 | 4.737654 | -1.181576 | 96 | 75.96 | 0.791250 |
| GO:0051169\_nuclear\_transport | PRDX1 | 72 | 2 | 4.737654 | -1.181576 | 96 | 75.96 | 0.791250 |
| GO:0006936\_muscle\_contraction | FKBP1A | 73 | 2 | 4.672755 | -1.171053 | 97 | 76.52 | 0.788866 |
| GO:0006936\_muscle\_contraction | SFRS1 | 73 | 2 | 4.672755 | -1.171053 | 97 | 76.52 | 0.788866 |
| GO:0008038\_neuron\_recognition | NDN | 12 | 1 | 14.212963 | -1.166139 | 107 | 81.97 | 0.766075 |
| GO:0009886\_post-embryonic\_morphogenesis | HMGN1 | 12 | 1 | 14.212963 | -1.166139 | 107 | 81.97 | 0.766075 |
| GO:0030010\_establishment\_of\_cell\_polarity | CFL1 | 12 | 1 | 14.212963 | -1.166139 | 107 | 81.97 | 0.766075 |
| GO:0035050\_embryonic\_heart\_tube\_development | GJA1 | 12 | 1 | 14.212963 | -1.166139 | 107 | 81.97 | 0.766075 |
| GO:0042278\_purine\_nucleoside\_metabolic\_process | MAT2A | 12 | 1 | 14.212963 | -1.166139 | 107 | 81.97 | 0.766075 |
| GO:0042743\_hydrogen\_peroxide\_metabolic\_process | CYCS | 12 | 1 | 14.212963 | -1.166139 | 107 | 81.97 | 0.766075 |
| GO:0043624\_cellular\_protein\_complex\_disassembly | CFL1 | 12 | 1 | 14.212963 | -1.166139 | 107 | 81.97 | 0.766075 |
| GO:0046128\_purine\_ribonucleoside\_metabolic\_process | MAT2A | 12 | 1 | 14.212963 | -1.166139 | 107 | 81.97 | 0.766075 |
| GO:0051261\_protein\_depolymerization | CFL1 | 12 | 1 | 14.212963 | -1.166139 | 107 | 81.97 | 0.766075 |
| GO:0060048\_cardiac\_muscle\_contraction | SFRS1 | 12 | 1 | 14.212963 | -1.166139 | 107 | 81.97 | 0.766075 |
| GO:0048589\_developmental\_growth | PRPF19 | 75 | 2 | 4.548148 | -1.150495 | 108 | 82.96 | 0.768148 |
| GO:0048589\_developmental\_growth | GJA1 | 75 | 2 | 4.548148 | -1.150495 | 108 | 82.96 | 0.768148 |
| GO:0051716\_cellular\_response\_to\_stimulus | HMGN1 | 273 | 4 | 2.498982 | -1.140546 | 109 | 83.4 | 0.765138 |
| GO:0051716\_cellular\_response\_to\_stimulus | RPA1 | 273 | 4 | 2.498982 | -1.140546 | 109 | 83.4 | 0.765138 |
| GO:0051716\_cellular\_response\_to\_stimulus | HSPA5 | 273 | 4 | 2.498982 | -1.140546 | 109 | 83.4 | 0.765138 |
| GO:0051716\_cellular\_response\_to\_stimulus | PRDX1 | 273 | 4 | 2.498982 | -1.140546 | 109 | 83.4 | 0.765138 |
| GO:0003012\_muscle\_system\_process | FKBP1A | 76 | 2 | 4.488304 | -1.140452 | 110 | 83.58 | 0.759818 |
| GO:0003012\_muscle\_system\_process | SFRS1 | 76 | 2 | 4.488304 | -1.140452 | 110 | 83.58 | 0.759818 |
| GO:0033036\_macromolecule\_localization | YWHAB | 274 | 4 | 2.489862 | -1.135889 | 111 | 84.0 | 0.756757 |
| GO:0033036\_macromolecule\_localization | YWHAQ | 274 | 4 | 2.489862 | -1.135889 | 111 | 84.0 | 0.756757 |
| GO:0033036\_macromolecule\_localization | NUTF2 | 274 | 4 | 2.489862 | -1.135889 | 111 | 84.0 | 0.756757 |
| GO:0033036\_macromolecule\_localization | PRDX1 | 274 | 4 | 2.489862 | -1.135889 | 111 | 84.0 | 0.756757 |
| GO:0007512\_adult\_heart\_development | GJA1 | 13 | 1 | 13.119658 | -1.132594 | 119 | 89.26 | 0.750084 |
| GO:0009119\_ribonucleoside\_metabolic\_process | MAT2A | 13 | 1 | 13.119658 | -1.132594 | 119 | 89.26 | 0.750084 |
| GO:0032729\_positive\_regulation\_of\_interferon-gamma\_production | HSPD1 | 13 | 1 | 13.119658 | -1.132594 | 119 | 89.26 | 0.750084 |
| GO:0043241\_protein\_complex\_disassembly | CFL1 | 13 | 1 | 13.119658 | -1.132594 | 119 | 89.26 | 0.750084 |
| GO:0043244\_regulation\_of\_protein\_complex\_disassembly | CFL1 | 13 | 1 | 13.119658 | -1.132594 | 119 | 89.26 | 0.750084 |
| GO:0051495\_positive\_regulation\_of\_cytoskeleton\_organization | CFL1 | 13 | 1 | 13.119658 | -1.132594 | 119 | 89.26 | 0.750084 |
| GO:0060401\_cytosolic\_calcium\_ion\_transport | FKBP1A | 13 | 1 | 13.119658 | -1.132594 | 119 | 89.26 | 0.750084 |
| GO:0060402\_calcium\_ion\_transport\_into\_cytosol | FKBP1A | 13 | 1 | 13.119658 | -1.132594 | 119 | 89.26 | 0.750084 |
| GO:0008064\_regulation\_of\_actin\_polymerization\_or\_depolymerization | CFL1 | 14 | 1 | 12.182540 | -1.101625 | 123 | 95.9 | 0.779675 |
| GO:0030832\_regulation\_of\_actin\_filament\_length | CFL1 | 14 | 1 | 12.182540 | -1.101625 | 123 | 95.9 | 0.779675 |
| GO:0031099\_regeneration | GJA1 | 14 | 1 | 12.182540 | -1.101625 | 123 | 95.9 | 0.779675 |
| GO:0034623\_cellular\_macromolecular\_complex\_disassembly | CFL1 | 14 | 1 | 12.182540 | -1.101625 | 123 | 95.9 | 0.779675 |
| GO:0006325\_chromatin\_organization | HMGN1 | 83 | 2 | 4.109772 | -1.074194 | 125 | 97.35 | 0.778800 |
| GO:0006325\_chromatin\_organization | RBBP7 | 83 | 2 | 4.109772 | -1.074194 | 125 | 97.35 | 0.778800 |
| GO:0006575\_cellular\_amino\_acid\_derivative\_metabolic\_process | OAZ1 | 83 | 2 | 4.109772 | -1.074194 | 125 | 97.35 | 0.778800 |
| GO:0006575\_cellular\_amino\_acid\_derivative\_metabolic\_process | MAT2A | 83 | 2 | 4.109772 | -1.074194 | 125 | 97.35 | 0.778800 |
| GO:0034960\_cellular\_biopolymer\_metabolic\_process | HMGN1 | 1395 | 12 | 1.467145 | -1.073444 | 126 | 97.39 | 0.772937 |
| GO:0034960\_cellular\_biopolymer\_metabolic\_process | RPA1 | 1395 | 12 | 1.467145 | -1.073444 | 126 | 97.39 | 0.772937 |
| GO:0034960\_cellular\_biopolymer\_metabolic\_process | NDN | 1395 | 12 | 1.467145 | -1.073444 | 126 | 97.39 | 0.772937 |
| GO:0034960\_cellular\_biopolymer\_metabolic\_process | CFL1 | 1395 | 12 | 1.467145 | -1.073444 | 126 | 97.39 | 0.772937 |
| GO:0034960\_cellular\_biopolymer\_metabolic\_process | TRIM28 | 1395 | 12 | 1.467145 | -1.073444 | 126 | 97.39 | 0.772937 |
| GO:0034960\_cellular\_biopolymer\_metabolic\_process | CBX3 | 1395 | 12 | 1.467145 | -1.073444 | 126 | 97.39 | 0.772937 |
| GO:0034960\_cellular\_biopolymer\_metabolic\_process | FKBP1A | 1395 | 12 | 1.467145 | -1.073444 | 126 | 97.39 | 0.772937 |
| GO:0034960\_cellular\_biopolymer\_metabolic\_process | DDX5 | 1395 | 12 | 1.467145 | -1.073444 | 126 | 97.39 | 0.772937 |
| GO:0034960\_cellular\_biopolymer\_metabolic\_process | RBBP7 | 1395 | 12 | 1.467145 | -1.073444 | 126 | 97.39 | 0.772937 |
| GO:0034960\_cellular\_biopolymer\_metabolic\_process | SFRS1 | 1395 | 12 | 1.467145 | -1.073444 | 126 | 97.39 | 0.772937 |
| GO:0034960\_cellular\_biopolymer\_metabolic\_process | HSPA8 | 1395 | 12 | 1.467145 | -1.073444 | 126 | 97.39 | 0.772937 |
| GO:0034960\_cellular\_biopolymer\_metabolic\_process | RPL29 | 1395 | 12 | 1.467145 | -1.073444 | 126 | 97.39 | 0.772937 |
| GO:0009116\_nucleoside\_metabolic\_process | MAT2A | 15 | 1 | 11.370370 | -1.072877 | 129 | 101.83 | 0.789380 |
| GO:0042306\_regulation\_of\_protein\_import\_into\_nucleus | PRDX1 | 15 | 1 | 11.370370 | -1.072877 | 129 | 101.83 | 0.789380 |
| GO:0055010\_ventricular\_cardiac\_muscle\_morphogenesis | FKBP1A | 15 | 1 | 11.370370 | -1.072877 | 129 | 101.83 | 0.789380 |
| GO:0030005\_cellular\_di-\_\_tri-valent\_inorganic\_cation\_homeostasis | NDN | 84 | 2 | 4.060847 | -1.065264 | 130 | 102.34 | 0.787231 |
| GO:0030005\_cellular\_di-\_\_tri-valent\_inorganic\_cation\_homeostasis | FKBP1A | 84 | 2 | 4.060847 | -1.065264 | 130 | 102.34 | 0.787231 |
| GO:0000279\_M\_phase | RPA1 | 85 | 2 | 4.013072 | -1.056457 | 131 | 102.79 | 0.784656 |
| GO:0000279\_M\_phase | TUBB5 | 85 | 2 | 4.013072 | -1.056457 | 131 | 102.79 | 0.784656 |
| GO:0000302\_response\_to\_reactive\_oxygen\_species | PRDX1 | 16 | 1 | 10.659722 | -1.046063 | 137 | 107.54 | 0.784964 |
| GO:0001933\_negative\_regulation\_of\_protein\_amino\_acid\_phosphorylation | FKBP1A | 16 | 1 | 10.659722 | -1.046063 | 137 | 107.54 | 0.784964 |
| GO:0010243\_response\_to\_organic\_nitrogen | CFL1 | 16 | 1 | 10.659722 | -1.046063 | 137 | 107.54 | 0.784964 |
| GO:0014075\_response\_to\_amine\_stimulus | CFL1 | 16 | 1 | 10.659722 | -1.046063 | 137 | 107.54 | 0.784964 |
| GO:0032956\_regulation\_of\_actin\_cytoskeleton\_organization | CFL1 | 16 | 1 | 10.659722 | -1.046063 | 137 | 107.54 | 0.784964 |
| GO:0034976\_response\_to\_endoplasmic\_reticulum\_stress | HSPA5 | 16 | 1 | 10.659722 | -1.046063 | 137 | 107.54 | 0.784964 |
| GO:0006919\_activation\_of\_caspase\_activity | CYCS | 17 | 1 | 10.032680 | -1.020947 | 144 | 114.0 | 0.791667 |
| GO:0006984\_ER-nuclear\_signaling\_pathway | HSPA5 | 17 | 1 | 10.032680 | -1.020947 | 144 | 114.0 | 0.791667 |
| GO:0008380\_RNA\_splicing | SFRS1 | 17 | 1 | 10.032680 | -1.020947 | 144 | 114.0 | 0.791667 |
| GO:0032535\_regulation\_of\_cellular\_component\_size | CFL1 | 17 | 1 | 10.032680 | -1.020947 | 144 | 114.0 | 0.791667 |
| GO:0032970\_regulation\_of\_actin\_filament-based\_process | CFL1 | 17 | 1 | 10.032680 | -1.020947 | 144 | 114.0 | 0.791667 |
| GO:0042398\_cellular\_amino\_acid\_derivative\_biosynthetic\_process | MAT2A | 17 | 1 | 10.032680 | -1.020947 | 144 | 114.0 | 0.791667 |
| GO:0048873\_homeostasis\_of\_number\_of\_cells\_within\_a\_tissue | RPA1 | 17 | 1 | 10.032680 | -1.020947 | 144 | 114.0 | 0.791667 |
| GO:0030003\_cellular\_cation\_homeostasis | NDN | 90 | 2 | 3.790123 | -1.014186 | 145 | 114.52 | 0.789793 |
| GO:0030003\_cellular\_cation\_homeostasis | FKBP1A | 90 | 2 | 3.790123 | -1.014186 | 145 | 114.52 | 0.789793 |
| GO:0065007\_biological\_regulation | HMGN1 | 2593 | 19 | 1.249732 | -1.007167 | 146 | 114.64 | 0.785205 |
| GO:0065007\_biological\_regulation | GDI2 | 2593 | 19 | 1.249732 | -1.007167 | 146 | 114.64 | 0.785205 |
| GO:0065007\_biological\_regulation | HAX1 | 2593 | 19 | 1.249732 | -1.007167 | 146 | 114.64 | 0.785205 |
| GO:0065007\_biological\_regulation | NDN | 2593 | 19 | 1.249732 | -1.007167 | 146 | 114.64 | 0.785205 |
| GO:0065007\_biological\_regulation | TRIM28 | 2593 | 19 | 1.249732 | -1.007167 | 146 | 114.64 | 0.785205 |
| GO:0065007\_biological\_regulation | CYCS | 2593 | 19 | 1.249732 | -1.007167 | 146 | 114.64 | 0.785205 |
| GO:0065007\_biological\_regulation | CBX3 | 2593 | 19 | 1.249732 | -1.007167 | 146 | 114.64 | 0.785205 |
| GO:0065007\_biological\_regulation | GJA1 | 2593 | 19 | 1.249732 | -1.007167 | 146 | 114.64 | 0.785205 |
| GO:0065007\_biological\_regulation | FKBP1A | 2593 | 19 | 1.249732 | -1.007167 | 146 | 114.64 | 0.785205 |
| GO:0065007\_biological\_regulation | RBBP7 | 2593 | 19 | 1.249732 | -1.007167 | 146 | 114.64 | 0.785205 |
| GO:0065007\_biological\_regulation | DDX5 | 2593 | 19 | 1.249732 | -1.007167 | 146 | 114.64 | 0.785205 |
| GO:0065007\_biological\_regulation | PRDX1 | 2593 | 19 | 1.249732 | -1.007167 | 146 | 114.64 | 0.785205 |
| GO:0065007\_biological\_regulation | RPA1 | 2593 | 19 | 1.249732 | -1.007167 | 146 | 114.64 | 0.785205 |
| GO:0065007\_biological\_regulation | PRPF19 | 2593 | 19 | 1.249732 | -1.007167 | 146 | 114.64 | 0.785205 |
| GO:0065007\_biological\_regulation | CFL1 | 2593 | 19 | 1.249732 | -1.007167 | 146 | 114.64 | 0.785205 |
| GO:0065007\_biological\_regulation | YWHAQ | 2593 | 19 | 1.249732 | -1.007167 | 146 | 114.64 | 0.785205 |
| GO:0065007\_biological\_regulation | HSPA5 | 2593 | 19 | 1.249732 | -1.007167 | 146 | 114.64 | 0.785205 |
| GO:0065007\_biological\_regulation | HSPD1 | 2593 | 19 | 1.249732 | -1.007167 | 146 | 114.64 | 0.785205 |
| GO:0065007\_biological\_regulation | HSPA8 | 2593 | 19 | 1.249732 | -1.007167 | 146 | 114.64 | 0.785205 |
| GO:0002064\_epithelial\_cell\_development | GJA1 | 18 | 1 | 9.475309 | -0.997336 | 154 | 120.38 | 0.781688 |
| GO:0006457\_protein\_folding | HSPA8 | 18 | 1 | 9.475309 | -0.997336 | 154 | 120.38 | 0.781688 |
| GO:0032984\_macromolecular\_complex\_disassembly | CFL1 | 18 | 1 | 9.475309 | -0.997336 | 154 | 120.38 | 0.781688 |
| GO:0033157\_regulation\_of\_intracellular\_protein\_transport | PRDX1 | 18 | 1 | 9.475309 | -0.997336 | 154 | 120.38 | 0.781688 |
| GO:0044272\_sulfur\_compound\_biosynthetic\_process | MAT2A | 18 | 1 | 9.475309 | -0.997336 | 154 | 120.38 | 0.781688 |
| GO:0055008\_cardiac\_muscle\_tissue\_morphogenesis | FKBP1A | 18 | 1 | 9.475309 | -0.997336 | 154 | 120.38 | 0.781688 |
| GO:0060415\_muscle\_tissue\_morphogenesis | FKBP1A | 18 | 1 | 9.475309 | -0.997336 | 154 | 120.38 | 0.781688 |
| GO:0060571\_morphogenesis\_of\_an\_epithelial\_fold | CFL1 | 18 | 1 | 9.475309 | -0.997336 | 154 | 120.38 | 0.781688 |
| GO:0051094\_positive\_regulation\_of\_developmental\_process | PRPF19 | 308 | 4 | 2.215007 | -0.990368 | 155 | 120.53 | 0.777613 |
| GO:0051094\_positive\_regulation\_of\_developmental\_process | CYCS | 308 | 4 | 2.215007 | -0.990368 | 155 | 120.53 | 0.777613 |
| GO:0051094\_positive\_regulation\_of\_developmental\_process | GJA1 | 308 | 4 | 2.215007 | -0.990368 | 155 | 120.53 | 0.777613 |
| GO:0051094\_positive\_regulation\_of\_developmental\_process | PRDX1 | 308 | 4 | 2.215007 | -0.990368 | 155 | 120.53 | 0.777613 |
| GO:0055066\_di-\_\_tri-valent\_inorganic\_cation\_homeostasis | NDN | 93 | 2 | 3.667861 | -0.990140 | 156 | 120.85 | 0.774679 |
| GO:0055066\_di-\_\_tri-valent\_inorganic\_cation\_homeostasis | FKBP1A | 93 | 2 | 3.667861 | -0.990140 | 156 | 120.85 | 0.774679 |
| GO:0032943\_mononuclear\_cell\_proliferation | FKBP1A | 94 | 2 | 3.628842 | -0.982329 | 159 | 121.85 | 0.766352 |
| GO:0032943\_mononuclear\_cell\_proliferation | IMPDH2 | 94 | 2 | 3.628842 | -0.982329 | 159 | 121.85 | 0.766352 |
| GO:0034984\_cellular\_response\_to\_DNA\_damage\_stimulus | HMGN1 | 94 | 2 | 3.628842 | -0.982329 | 159 | 121.85 | 0.766352 |
| GO:0034984\_cellular\_response\_to\_DNA\_damage\_stimulus | RPA1 | 94 | 2 | 3.628842 | -0.982329 | 159 | 121.85 | 0.766352 |
| GO:0046651\_lymphocyte\_proliferation | FKBP1A | 94 | 2 | 3.628842 | -0.982329 | 159 | 121.85 | 0.766352 |
| GO:0046651\_lymphocyte\_proliferation | IMPDH2 | 94 | 2 | 3.628842 | -0.982329 | 159 | 121.85 | 0.766352 |
| GO:0006338\_chromatin\_remodeling | RBBP7 | 19 | 1 | 8.976608 | -0.975066 | 162 | 126.01 | 0.777840 |
| GO:0010952\_positive\_regulation\_of\_peptidase\_activity | CYCS | 19 | 1 | 8.976608 | -0.975066 | 162 | 126.01 | 0.777840 |
| GO:0043280\_positive\_regulation\_of\_caspase\_activity | CYCS | 19 | 1 | 8.976608 | -0.975066 | 162 | 126.01 | 0.777840 |
| GO:0044260\_cellular\_macromolecule\_metabolic\_process | HMGN1 | 1447 | 12 | 1.414421 | -0.971179 | 163 | 126.32 | 0.774969 |
| GO:0044260\_cellular\_macromolecule\_metabolic\_process | RPA1 | 1447 | 12 | 1.414421 | -0.971179 | 163 | 126.32 | 0.774969 |
| GO:0044260\_cellular\_macromolecule\_metabolic\_process | NDN | 1447 | 12 | 1.414421 | -0.971179 | 163 | 126.32 | 0.774969 |
| GO:0044260\_cellular\_macromolecule\_metabolic\_process | CFL1 | 1447 | 12 | 1.414421 | -0.971179 | 163 | 126.32 | 0.774969 |
| GO:0044260\_cellular\_macromolecule\_metabolic\_process | TRIM28 | 1447 | 12 | 1.414421 | -0.971179 | 163 | 126.32 | 0.774969 |
| GO:0044260\_cellular\_macromolecule\_metabolic\_process | CBX3 | 1447 | 12 | 1.414421 | -0.971179 | 163 | 126.32 | 0.774969 |
| GO:0044260\_cellular\_macromolecule\_metabolic\_process | FKBP1A | 1447 | 12 | 1.414421 | -0.971179 | 163 | 126.32 | 0.774969 |
| GO:0044260\_cellular\_macromolecule\_metabolic\_process | SFRS1 | 1447 | 12 | 1.414421 | -0.971179 | 163 | 126.32 | 0.774969 |
| GO:0044260\_cellular\_macromolecule\_metabolic\_process | DDX5 | 1447 | 12 | 1.414421 | -0.971179 | 163 | 126.32 | 0.774969 |
| GO:0044260\_cellular\_macromolecule\_metabolic\_process | RBBP7 | 1447 | 12 | 1.414421 | -0.971179 | 163 | 126.32 | 0.774969 |
| GO:0044260\_cellular\_macromolecule\_metabolic\_process | HSPA8 | 1447 | 12 | 1.414421 | -0.971179 | 163 | 126.32 | 0.774969 |
| GO:0044260\_cellular\_macromolecule\_metabolic\_process | RPL29 | 1447 | 12 | 1.414421 | -0.971179 | 163 | 126.32 | 0.774969 |
| GO:0070661\_leukocyte\_proliferation | FKBP1A | 96 | 2 | 3.553241 | -0.967002 | 164 | 127.04 | 0.774634 |
| GO:0070661\_leukocyte\_proliferation | IMPDH2 | 96 | 2 | 3.553241 | -0.967002 | 164 | 127.04 | 0.774634 |
| GO:0048518\_positive\_regulation\_of\_biological\_process | PRPF19 | 995 | 9 | 1.542714 | -0.963622 | 165 | 127.22 | 0.771030 |
| GO:0048518\_positive\_regulation\_of\_biological\_process | RPA1 | 995 | 9 | 1.542714 | -0.963622 | 165 | 127.22 | 0.771030 |
| GO:0048518\_positive\_regulation\_of\_biological\_process | CYCS | 995 | 9 | 1.542714 | -0.963622 | 165 | 127.22 | 0.771030 |
| GO:0048518\_positive\_regulation\_of\_biological\_process | CFL1 | 995 | 9 | 1.542714 | -0.963622 | 165 | 127.22 | 0.771030 |
| GO:0048518\_positive\_regulation\_of\_biological\_process | TRIM28 | 995 | 9 | 1.542714 | -0.963622 | 165 | 127.22 | 0.771030 |
| GO:0048518\_positive\_regulation\_of\_biological\_process | GJA1 | 995 | 9 | 1.542714 | -0.963622 | 165 | 127.22 | 0.771030 |
| GO:0048518\_positive\_regulation\_of\_biological\_process | HSPD1 | 995 | 9 | 1.542714 | -0.963622 | 165 | 127.22 | 0.771030 |
| GO:0048518\_positive\_regulation\_of\_biological\_process | DDX5 | 995 | 9 | 1.542714 | -0.963622 | 165 | 127.22 | 0.771030 |
| GO:0048518\_positive\_regulation\_of\_biological\_process | PRDX1 | 995 | 9 | 1.542714 | -0.963622 | 165 | 127.22 | 0.771030 |
| GO:0042326\_negative\_regulation\_of\_phosphorylation | FKBP1A | 20 | 1 | 8.527778 | -0.954000 | 167 | 130.93 | 0.784012 |
| GO:0046822\_regulation\_of\_nucleocytoplasmic\_transport | PRDX1 | 20 | 1 | 8.527778 | -0.954000 | 167 | 130.93 | 0.784012 |
| GO:0006996\_organelle\_organization | RPA1 | 449 | 5 | 1.899282 | -0.934736 | 168 | 132.82 | 0.790595 |
| GO:0006996\_organelle\_organization | HMGN1 | 449 | 5 | 1.899282 | -0.934736 | 168 | 132.82 | 0.790595 |
| GO:0006996\_organelle\_organization | CFL1 | 449 | 5 | 1.899282 | -0.934736 | 168 | 132.82 | 0.790595 |
| GO:0006996\_organelle\_organization | TUBB5 | 449 | 5 | 1.899282 | -0.934736 | 168 | 132.82 | 0.790595 |
| GO:0006996\_organelle\_organization | RBBP7 | 449 | 5 | 1.899282 | -0.934736 | 168 | 132.82 | 0.790595 |
| GO:0001755\_neural\_crest\_cell\_migration | CFL1 | 21 | 1 | 8.121693 | -0.934019 | 176 | 137.4 | 0.780682 |
| GO:0008154\_actin\_polymerization\_or\_depolymerization | CFL1 | 21 | 1 | 8.121693 | -0.934019 | 176 | 137.4 | 0.780682 |
| GO:0010563\_negative\_regulation\_of\_phosphorus\_metabolic\_process | FKBP1A | 21 | 1 | 8.121693 | -0.934019 | 176 | 137.4 | 0.780682 |
| GO:0016202\_regulation\_of\_striated\_muscle\_tissue\_development | GJA1 | 21 | 1 | 8.121693 | -0.934019 | 176 | 137.4 | 0.780682 |
| GO:0043279\_response\_to\_alkaloid | FKBP1A | 21 | 1 | 8.121693 | -0.934019 | 176 | 137.4 | 0.780682 |
| GO:0045936\_negative\_regulation\_of\_phosphate\_metabolic\_process | FKBP1A | 21 | 1 | 8.121693 | -0.934019 | 176 | 137.4 | 0.780682 |
| GO:0048634\_regulation\_of\_muscle\_development | GJA1 | 21 | 1 | 8.121693 | -0.934019 | 176 | 137.4 | 0.780682 |
| GO:0048675\_axon\_extension | NDN | 21 | 1 | 8.121693 | -0.934019 | 176 | 137.4 | 0.780682 |
| GO:0003013\_circulatory\_system\_process | GJA1 | 103 | 2 | 3.311758 | -0.916242 | 178 | 138.31 | 0.777022 |
| GO:0003013\_circulatory\_system\_process | SFRS1 | 103 | 2 | 3.311758 | -0.916242 | 178 | 138.31 | 0.777022 |
| GO:0008015\_blood\_circulation | GJA1 | 103 | 2 | 3.311758 | -0.916242 | 178 | 138.31 | 0.777022 |
| GO:0008015\_blood\_circulation | SFRS1 | 103 | 2 | 3.311758 | -0.916242 | 178 | 138.31 | 0.777022 |
| GO:0001558\_regulation\_of\_cell\_growth | NDN | 22 | 1 | 7.752525 | -0.915024 | 182 | 143.65 | 0.789286 |
| GO:0001947\_heart\_looping | GJA1 | 22 | 1 | 7.752525 | -0.915024 | 182 | 143.65 | 0.789286 |
| GO:0009309\_amine\_biosynthetic\_process | MAT2A | 22 | 1 | 7.752525 | -0.915024 | 182 | 143.65 | 0.789286 |
| GO:0032649\_regulation\_of\_interferon-gamma\_production | HSPD1 | 22 | 1 | 7.752525 | -0.915024 | 182 | 143.65 | 0.789286 |
| GO:0055086\_nucleobase\_\_nucleoside\_and\_nucleotide\_metabolic\_process | MAT2A | 104 | 2 | 3.279915 | -0.909333 | 183 | 143.96 | 0.786667 |
| GO:0055086\_nucleobase\_\_nucleoside\_and\_nucleotide\_metabolic\_process | IMPDH2 | 104 | 2 | 3.279915 | -0.909333 | 183 | 143.96 | 0.786667 |
| GO:0010605\_negative\_regulation\_of\_macromolecule\_metabolic\_process | TRIM28 | 331 | 4 | 2.061094 | -0.904315 | 184 | 144.48 | 0.785217 |
| GO:0010605\_negative\_regulation\_of\_macromolecule\_metabolic\_process | CBX3 | 331 | 4 | 2.061094 | -0.904315 | 184 | 144.48 | 0.785217 |
| GO:0010605\_negative\_regulation\_of\_macromolecule\_metabolic\_process | FKBP1A | 331 | 4 | 2.061094 | -0.904315 | 184 | 144.48 | 0.785217 |
| GO:0010605\_negative\_regulation\_of\_macromolecule\_metabolic\_process | RBBP7 | 331 | 4 | 2.061094 | -0.904315 | 184 | 144.48 | 0.785217 |
| GO:0048872\_homeostasis\_of\_number\_of\_cells | RPA1 | 105 | 2 | 3.248677 | -0.902504 | 185 | 144.6 | 0.781622 |
| GO:0048872\_homeostasis\_of\_number\_of\_cells | PRDX1 | 105 | 2 | 3.248677 | -0.902504 | 185 | 144.6 | 0.781622 |
| GO:0031324\_negative\_regulation\_of\_cellular\_metabolic\_process | TRIM28 | 332 | 4 | 2.054886 | -0.900773 | 186 | 144.94 | 0.779247 |
| GO:0031324\_negative\_regulation\_of\_cellular\_metabolic\_process | CBX3 | 332 | 4 | 2.054886 | -0.900773 | 186 | 144.94 | 0.779247 |
| GO:0031324\_negative\_regulation\_of\_cellular\_metabolic\_process | FKBP1A | 332 | 4 | 2.054886 | -0.900773 | 186 | 144.94 | 0.779247 |
| GO:0031324\_negative\_regulation\_of\_cellular\_metabolic\_process | RBBP7 | 332 | 4 | 2.054886 | -0.900773 | 186 | 144.94 | 0.779247 |
| GO:0002228\_natural\_killer\_cell\_mediated\_immunity | PRDX1 | 23 | 1 | 7.415459 | -0.896926 | 190 | 148.25 | 0.780263 |
| GO:0007163\_establishment\_or\_maintenance\_of\_cell\_polarity | CFL1 | 23 | 1 | 7.415459 | -0.896926 | 190 | 148.25 | 0.780263 |
| GO:0030512\_negative\_regulation\_of\_transforming\_growth\_factor\_beta\_receptor\_signaling\_pathway | HSPA5 | 23 | 1 | 7.415459 | -0.896926 | 190 | 148.25 | 0.780263 |
| GO:0042267\_natural\_killer\_cell\_mediated\_cytotoxicity | PRDX1 | 23 | 1 | 7.415459 | -0.896926 | 190 | 148.25 | 0.780263 |
| GO:0043283\_biopolymer\_metabolic\_process | HMGN1 | 1490 | 12 | 1.373602 | -0.892382 | 191 | 148.52 | 0.777592 |
| GO:0043283\_biopolymer\_metabolic\_process | RPA1 | 1490 | 12 | 1.373602 | -0.892382 | 191 | 148.52 | 0.777592 |
| GO:0043283\_biopolymer\_metabolic\_process | NDN | 1490 | 12 | 1.373602 | -0.892382 | 191 | 148.52 | 0.777592 |
| GO:0043283\_biopolymer\_metabolic\_process | CFL1 | 1490 | 12 | 1.373602 | -0.892382 | 191 | 148.52 | 0.777592 |
| GO:0043283\_biopolymer\_metabolic\_process | TRIM28 | 1490 | 12 | 1.373602 | -0.892382 | 191 | 148.52 | 0.777592 |
| GO:0043283\_biopolymer\_metabolic\_process | CBX3 | 1490 | 12 | 1.373602 | -0.892382 | 191 | 148.52 | 0.777592 |
| GO:0043283\_biopolymer\_metabolic\_process | FKBP1A | 1490 | 12 | 1.373602 | -0.892382 | 191 | 148.52 | 0.777592 |
| GO:0043283\_biopolymer\_metabolic\_process | DDX5 | 1490 | 12 | 1.373602 | -0.892382 | 191 | 148.52 | 0.777592 |
| GO:0043283\_biopolymer\_metabolic\_process | RBBP7 | 1490 | 12 | 1.373602 | -0.892382 | 191 | 148.52 | 0.777592 |
| GO:0043283\_biopolymer\_metabolic\_process | SFRS1 | 1490 | 12 | 1.373602 | -0.892382 | 191 | 148.52 | 0.777592 |
| GO:0043283\_biopolymer\_metabolic\_process | HSPA8 | 1490 | 12 | 1.373602 | -0.892382 | 191 | 148.52 | 0.777592 |
| GO:0043283\_biopolymer\_metabolic\_process | RPL29 | 1490 | 12 | 1.373602 | -0.892382 | 191 | 148.52 | 0.777592 |
| GO:0006941\_striated\_muscle\_contraction | SFRS1 | 24 | 1 | 7.106481 | -0.879649 | 195 | 152.63 | 0.782718 |
| GO:0007204\_elevation\_of\_cytosolic\_calcium\_ion\_concentration | FKBP1A | 24 | 1 | 7.106481 | -0.879649 | 195 | 152.63 | 0.782718 |
| GO:0014070\_response\_to\_organic\_cyclic\_substance | FKBP1A | 24 | 1 | 7.106481 | -0.879649 | 195 | 152.63 | 0.782718 |
| GO:0032386\_regulation\_of\_intracellular\_transport | PRDX1 | 24 | 1 | 7.106481 | -0.879649 | 195 | 152.63 | 0.782718 |
| GO:0045892\_negative\_regulation\_of\_transcription\_\_DNA-dependent | TRIM28 | 218 | 3 | 2.347095 | -0.875218 | 196 | 153.2 | 0.781633 |
| GO:0045892\_negative\_regulation\_of\_transcription\_\_DNA-dependent | CBX3 | 218 | 3 | 2.347095 | -0.875218 | 196 | 153.2 | 0.781633 |
| GO:0045892\_negative\_regulation\_of\_transcription\_\_DNA-dependent | RBBP7 | 218 | 3 | 2.347095 | -0.875218 | 196 | 153.2 | 0.781633 |
| GO:0055080\_cation\_homeostasis | NDN | 110 | 2 | 3.101010 | -0.869516 | 197 | 154.02 | 0.781827 |
| GO:0055080\_cation\_homeostasis | FKBP1A | 110 | 2 | 3.101010 | -0.869516 | 197 | 154.02 | 0.781827 |
| GO:0051253\_negative\_regulation\_of\_RNA\_metabolic\_process | TRIM28 | 220 | 3 | 2.325758 | -0.866570 | 198 | 154.14 | 0.778485 |
| GO:0051253\_negative\_regulation\_of\_RNA\_metabolic\_process | CBX3 | 220 | 3 | 2.325758 | -0.866570 | 198 | 154.14 | 0.778485 |
| GO:0051253\_negative\_regulation\_of\_RNA\_metabolic\_process | RBBP7 | 220 | 3 | 2.325758 | -0.866570 | 198 | 154.14 | 0.778485 |
| GO:0051649\_establishment\_of\_localization\_in\_cell | YWHAB | 342 | 4 | 1.994802 | -0.866202 | 199 | 154.29 | 0.775327 |
| GO:0051649\_establishment\_of\_localization\_in\_cell | YWHAQ | 342 | 4 | 1.994802 | -0.866202 | 199 | 154.29 | 0.775327 |
| GO:0051649\_establishment\_of\_localization\_in\_cell | NUTF2 | 342 | 4 | 1.994802 | -0.866202 | 199 | 154.29 | 0.775327 |
| GO:0051649\_establishment\_of\_localization\_in\_cell | PRDX1 | 342 | 4 | 1.994802 | -0.866202 | 199 | 154.29 | 0.775327 |
| GO:0048522\_positive\_regulation\_of\_cellular\_process | PRPF19 | 895 | 8 | 1.524519 | -0.863259 | 200 | 154.39 | 0.771950 |
| GO:0048522\_positive\_regulation\_of\_cellular\_process | RPA1 | 895 | 8 | 1.524519 | -0.863259 | 200 | 154.39 | 0.771950 |
| GO:0048522\_positive\_regulation\_of\_cellular\_process | TRIM28 | 895 | 8 | 1.524519 | -0.863259 | 200 | 154.39 | 0.771950 |
| GO:0048522\_positive\_regulation\_of\_cellular\_process | CYCS | 895 | 8 | 1.524519 | -0.863259 | 200 | 154.39 | 0.771950 |
| GO:0048522\_positive\_regulation\_of\_cellular\_process | CFL1 | 895 | 8 | 1.524519 | -0.863259 | 200 | 154.39 | 0.771950 |
| GO:0048522\_positive\_regulation\_of\_cellular\_process | HSPD1 | 895 | 8 | 1.524519 | -0.863259 | 200 | 154.39 | 0.771950 |
| GO:0048522\_positive\_regulation\_of\_cellular\_process | DDX5 | 895 | 8 | 1.524519 | -0.863259 | 200 | 154.39 | 0.771950 |
| GO:0048522\_positive\_regulation\_of\_cellular\_process | PRDX1 | 895 | 8 | 1.524519 | -0.863259 | 200 | 154.39 | 0.771950 |
| GO:0006302\_double-strand\_break\_repair | RPA1 | 25 | 1 | 6.822222 | -0.863126 | 202 | 157.61 | 0.780248 |
| GO:0031400\_negative\_regulation\_of\_protein\_modification\_process | FKBP1A | 25 | 1 | 6.822222 | -0.863126 | 202 | 157.61 | 0.780248 |
| GO:0006974\_response\_to\_DNA\_damage\_stimulus | HMGN1 | 113 | 2 | 3.018682 | -0.850597 | 203 | 158.33 | 0.779951 |
| GO:0006974\_response\_to\_DNA\_damage\_stimulus | RPA1 | 113 | 2 | 3.018682 | -0.850597 | 203 | 158.33 | 0.779951 |
| GO:0019233\_sensory\_perception\_of\_pain | NDN | 26 | 1 | 6.559829 | -0.847297 | 208 | 161.68 | 0.777308 |
| GO:0032609\_interferon-gamma\_production | HSPD1 | 26 | 1 | 6.559829 | -0.847297 | 208 | 161.68 | 0.777308 |
| GO:0045665\_negative\_regulation\_of\_neuron\_differentiation | PRPF19 | 26 | 1 | 6.559829 | -0.847297 | 208 | 161.68 | 0.777308 |
| GO:0051235\_maintenance\_of\_location | FKBP1A | 26 | 1 | 6.559829 | -0.847297 | 208 | 161.68 | 0.777308 |
| GO:0051480\_cytosolic\_calcium\_ion\_homeostasis | FKBP1A | 26 | 1 | 6.559829 | -0.847297 | 208 | 161.68 | 0.777308 |
| GO:0009892\_negative\_regulation\_of\_metabolic\_process | TRIM28 | 348 | 4 | 1.960409 | -0.846177 | 209 | 161.77 | 0.774019 |
| GO:0009892\_negative\_regulation\_of\_metabolic\_process | CBX3 | 348 | 4 | 1.960409 | -0.846177 | 209 | 161.77 | 0.774019 |
| GO:0009892\_negative\_regulation\_of\_metabolic\_process | FKBP1A | 348 | 4 | 1.960409 | -0.846177 | 209 | 161.77 | 0.774019 |
| GO:0009892\_negative\_regulation\_of\_metabolic\_process | RBBP7 | 348 | 4 | 1.960409 | -0.846177 | 209 | 161.77 | 0.774019 |
| GO:0046649\_lymphocyte\_activation | FKBP1A | 228 | 3 | 2.244152 | -0.833034 | 210 | 162.94 | 0.775905 |
| GO:0046649\_lymphocyte\_activation | HSPD1 | 228 | 3 | 2.244152 | -0.833034 | 210 | 162.94 | 0.775905 |
| GO:0046649\_lymphocyte\_activation | IMPDH2 | 228 | 3 | 2.244152 | -0.833034 | 210 | 162.94 | 0.775905 |
| GO:0009411\_response\_to\_UV | HMGN1 | 27 | 1 | 6.316872 | -0.832109 | 212 | 165.72 | 0.781698 |
| GO:0010638\_positive\_regulation\_of\_organelle\_organization | CFL1 | 27 | 1 | 6.316872 | -0.832109 | 212 | 165.72 | 0.781698 |
| GO:0006519\_cellular\_amino\_acid\_and\_derivative\_metabolic\_process | OAZ1 | 118 | 2 | 2.890772 | -0.820411 | 213 | 166.69 | 0.782582 |
| GO:0006519\_cellular\_amino\_acid\_and\_derivative\_metabolic\_process | MAT2A | 118 | 2 | 2.890772 | -0.820411 | 213 | 166.69 | 0.782582 |
| GO:0007585\_respiratory\_gaseous\_exchange | NDN | 28 | 1 | 6.091270 | -0.817517 | 215 | 169.28 | 0.787349 |
| GO:0043193\_positive\_regulation\_of\_gene-specific\_transcription | TRIM28 | 28 | 1 | 6.091270 | -0.817517 | 215 | 169.28 | 0.787349 |
| GO:0022403\_cell\_cycle\_phase | RPA1 | 119 | 2 | 2.866480 | -0.814565 | 216 | 169.49 | 0.784676 |
| GO:0022403\_cell\_cycle\_phase | TUBB5 | 119 | 2 | 2.866480 | -0.814565 | 216 | 169.49 | 0.784676 |
| GO:0016477\_cell\_migration | NDN | 234 | 3 | 2.186610 | -0.808937 | 217 | 169.91 | 0.782995 |
| GO:0016477\_cell\_migration | CFL1 | 234 | 3 | 2.186610 | -0.808937 | 217 | 169.91 | 0.782995 |
| GO:0016477\_cell\_migration | GJA1 | 234 | 3 | 2.186610 | -0.808937 | 217 | 169.91 | 0.782995 |
| GO:0043281\_regulation\_of\_caspase\_activity | CYCS | 29 | 1 | 5.881226 | -0.803479 | 222 | 173.23 | 0.780315 |
| GO:0050769\_positive\_regulation\_of\_neurogenesis | PRPF19 | 29 | 1 | 5.881226 | -0.803479 | 222 | 173.23 | 0.780315 |
| GO:0051301\_cell\_division | CFL1 | 29 | 1 | 5.881226 | -0.803479 | 222 | 173.23 | 0.780315 |
| GO:0052548\_regulation\_of\_endopeptidase\_activity | CYCS | 29 | 1 | 5.881226 | -0.803479 | 222 | 173.23 | 0.780315 |
| GO:0070302\_regulation\_of\_stress-activated\_protein\_kinase\_signaling\_pathway | PRDX1 | 29 | 1 | 5.881226 | -0.803479 | 222 | 173.23 | 0.780315 |
| GO:0006468\_protein\_amino\_acid\_phosphorylation | CFL1 | 237 | 3 | 2.158931 | -0.797212 | 223 | 175.12 | 0.785291 |
| GO:0006468\_protein\_amino\_acid\_phosphorylation | TRIM28 | 237 | 3 | 2.158931 | -0.797212 | 223 | 175.12 | 0.785291 |
| GO:0006468\_protein\_amino\_acid\_phosphorylation | FKBP1A | 237 | 3 | 2.158931 | -0.797212 | 223 | 175.12 | 0.785291 |
| GO:0007049\_cell\_cycle | RPA1 | 238 | 3 | 2.149860 | -0.793349 | 224 | 175.17 | 0.782009 |
| GO:0007049\_cell\_cycle | TUBB5 | 238 | 3 | 2.149860 | -0.793349 | 224 | 175.17 | 0.782009 |
| GO:0007049\_cell\_cycle | HSPA8 | 238 | 3 | 2.149860 | -0.793349 | 224 | 175.17 | 0.782009 |
| GO:0014032\_neural\_crest\_cell\_development | CFL1 | 30 | 1 | 5.685185 | -0.789956 | 228 | 179.21 | 0.786009 |
| GO:0014033\_neural\_crest\_cell\_differentiation | CFL1 | 30 | 1 | 5.685185 | -0.789956 | 228 | 179.21 | 0.786009 |
| GO:0022411\_cellular\_component\_disassembly | CFL1 | 30 | 1 | 5.685185 | -0.789956 | 228 | 179.21 | 0.786009 |
| GO:0052547\_regulation\_of\_peptidase\_activity | CYCS | 30 | 1 | 5.685185 | -0.789956 | 228 | 179.21 | 0.786009 |
| GO:0009308\_amine\_metabolic\_process | OAZ1 | 124 | 2 | 2.750896 | -0.786235 | 229 | 179.47 | 0.783712 |
| GO:0009308\_amine\_metabolic\_process | MAT2A | 124 | 2 | 2.750896 | -0.786235 | 229 | 179.47 | 0.783712 |
| GO:0051641\_cellular\_localization | YWHAB | 370 | 4 | 1.843844 | -0.777030 | 230 | 180.42 | 0.784435 |
| GO:0051641\_cellular\_localization | YWHAQ | 370 | 4 | 1.843844 | -0.777030 | 230 | 180.42 | 0.784435 |
| GO:0051641\_cellular\_localization | NUTF2 | 370 | 4 | 1.843844 | -0.777030 | 230 | 180.42 | 0.784435 |
| GO:0051641\_cellular\_localization | PRDX1 | 370 | 4 | 1.843844 | -0.777030 | 230 | 180.42 | 0.784435 |
| GO:0016049\_cell\_growth | NDN | 31 | 1 | 5.501792 | -0.776914 | 231 | 185.2 | 0.801732 |
| GO:0002376\_immune\_system\_process | RPA1 | 505 | 5 | 1.688669 | -0.776850 | 232 | 185.36 | 0.798966 |
| GO:0002376\_immune\_system\_process | FKBP1A | 505 | 5 | 1.688669 | -0.776850 | 232 | 185.36 | 0.798966 |
| GO:0002376\_immune\_system\_process | HSPD1 | 505 | 5 | 1.688669 | -0.776850 | 232 | 185.36 | 0.798966 |
| GO:0002376\_immune\_system\_process | PRDX1 | 505 | 5 | 1.688669 | -0.776850 | 232 | 185.36 | 0.798966 |
| GO:0002376\_immune\_system\_process | IMPDH2 | 505 | 5 | 1.688669 | -0.776850 | 232 | 185.36 | 0.798966 |
| GO:0051493\_regulation\_of\_cytoskeleton\_organization | CFL1 | 32 | 1 | 5.329861 | -0.764324 | 233 | 188.36 | 0.808412 |
| GO:0045321\_leukocyte\_activation | FKBP1A | 248 | 3 | 2.063172 | -0.755954 | 234 | 189.08 | 0.808034 |
| GO:0045321\_leukocyte\_activation | HSPD1 | 248 | 3 | 2.063172 | -0.755954 | 234 | 189.08 | 0.808034 |
| GO:0045321\_leukocyte\_activation | IMPDH2 | 248 | 3 | 2.063172 | -0.755954 | 234 | 189.08 | 0.808034 |
| GO:0043170\_macromolecule\_metabolic\_process | HMGN1 | 1576 | 12 | 1.298646 | -0.749326 | 235 | 192.46 | 0.818979 |
| GO:0043170\_macromolecule\_metabolic\_process | RPA1 | 1576 | 12 | 1.298646 | -0.749326 | 235 | 192.46 | 0.818979 |
| GO:0043170\_macromolecule\_metabolic\_process | NDN | 1576 | 12 | 1.298646 | -0.749326 | 235 | 192.46 | 0.818979 |
| GO:0043170\_macromolecule\_metabolic\_process | CFL1 | 1576 | 12 | 1.298646 | -0.749326 | 235 | 192.46 | 0.818979 |
| GO:0043170\_macromolecule\_metabolic\_process | TRIM28 | 1576 | 12 | 1.298646 | -0.749326 | 235 | 192.46 | 0.818979 |
| GO:0043170\_macromolecule\_metabolic\_process | CBX3 | 1576 | 12 | 1.298646 | -0.749326 | 235 | 192.46 | 0.818979 |
| GO:0043170\_macromolecule\_metabolic\_process | FKBP1A | 1576 | 12 | 1.298646 | -0.749326 | 235 | 192.46 | 0.818979 |
| GO:0043170\_macromolecule\_metabolic\_process | SFRS1 | 1576 | 12 | 1.298646 | -0.749326 | 235 | 192.46 | 0.818979 |
| GO:0043170\_macromolecule\_metabolic\_process | RBBP7 | 1576 | 12 | 1.298646 | -0.749326 | 235 | 192.46 | 0.818979 |
| GO:0043170\_macromolecule\_metabolic\_process | DDX5 | 1576 | 12 | 1.298646 | -0.749326 | 235 | 192.46 | 0.818979 |
| GO:0043170\_macromolecule\_metabolic\_process | HSPA8 | 1576 | 12 | 1.298646 | -0.749326 | 235 | 192.46 | 0.818979 |
| GO:0043170\_macromolecule\_metabolic\_process | RPL29 | 1576 | 12 | 1.298646 | -0.749326 | 235 | 192.46 | 0.818979 |
| GO:0010720\_positive\_regulation\_of\_cell\_development | PRPF19 | 34 | 1 | 5.016340 | -0.740389 | 236 | 196.3 | 0.831780 |
| GO:0016481\_negative\_regulation\_of\_transcription | TRIM28 | 253 | 3 | 2.022398 | -0.738055 | 237 | 196.91 | 0.830844 |
| GO:0016481\_negative\_regulation\_of\_transcription | CBX3 | 253 | 3 | 2.022398 | -0.738055 | 237 | 196.91 | 0.830844 |
| GO:0016481\_negative\_regulation\_of\_transcription | RBBP7 | 253 | 3 | 2.022398 | -0.738055 | 237 | 196.91 | 0.830844 |
| GO:0048729\_tissue\_morphogenesis | CFL1 | 255 | 3 | 2.006536 | -0.731038 | 238 | 197.7 | 0.830672 |
| GO:0048729\_tissue\_morphogenesis | GJA1 | 255 | 3 | 2.006536 | -0.731038 | 238 | 197.7 | 0.830672 |
| GO:0048729\_tissue\_morphogenesis | FKBP1A | 255 | 3 | 2.006536 | -0.731038 | 238 | 197.7 | 0.830672 |
| GO:0001906\_cell\_killing | PRDX1 | 35 | 1 | 4.873016 | -0.728995 | 240 | 200.31 | 0.834625 |
| GO:0001909\_leukocyte\_mediated\_cytotoxicity | PRDX1 | 35 | 1 | 4.873016 | -0.728995 | 240 | 200.31 | 0.834625 |
| GO:0048870\_cell\_motility | NDN | 257 | 3 | 1.990921 | -0.724101 | 241 | 201.19 | 0.834813 |
| GO:0048870\_cell\_motility | CFL1 | 257 | 3 | 1.990921 | -0.724101 | 241 | 201.19 | 0.834813 |
| GO:0048870\_cell\_motility | GJA1 | 257 | 3 | 1.990921 | -0.724101 | 241 | 201.19 | 0.834813 |
| GO:0001819\_positive\_regulation\_of\_cytokine\_production | HSPD1 | 36 | 1 | 4.737654 | -0.717955 | 244 | 204.66 | 0.838770 |
| GO:0014020\_primary\_neural\_tube\_formation | CFL1 | 36 | 1 | 4.737654 | -0.717955 | 244 | 204.66 | 0.838770 |
| GO:0051223\_regulation\_of\_protein\_transport | PRDX1 | 36 | 1 | 4.737654 | -0.717955 | 244 | 204.66 | 0.838770 |
| GO:0001775\_cell\_activation | FKBP1A | 262 | 3 | 1.952926 | -0.707096 | 246 | 207.94 | 0.845285 |
| GO:0001775\_cell\_activation | HSPD1 | 262 | 3 | 1.952926 | -0.707096 | 246 | 207.94 | 0.845285 |
| GO:0001775\_cell\_activation | IMPDH2 | 262 | 3 | 1.952926 | -0.707096 | 246 | 207.94 | 0.845285 |
| GO:0010629\_negative\_regulation\_of\_gene\_expression | TRIM28 | 262 | 3 | 1.952926 | -0.707096 | 246 | 207.94 | 0.845285 |
| GO:0010629\_negative\_regulation\_of\_gene\_expression | CBX3 | 262 | 3 | 1.952926 | -0.707096 | 246 | 207.94 | 0.845285 |
| GO:0010629\_negative\_regulation\_of\_gene\_expression | RBBP7 | 262 | 3 | 1.952926 | -0.707096 | 246 | 207.94 | 0.845285 |
| GO:0008016\_regulation\_of\_heart\_contraction | GJA1 | 38 | 1 | 4.488304 | -0.696859 | 250 | 212.3 | 0.849200 |
| GO:0016053\_organic\_acid\_biosynthetic\_process | MAT2A | 38 | 1 | 4.488304 | -0.696859 | 250 | 212.3 | 0.849200 |
| GO:0046394\_carboxylic\_acid\_biosynthetic\_process | MAT2A | 38 | 1 | 4.488304 | -0.696859 | 250 | 212.3 | 0.849200 |
| GO:0046777\_protein\_amino\_acid\_autophosphorylation | TRIM28 | 38 | 1 | 4.488304 | -0.696859 | 250 | 212.3 | 0.849200 |
| GO:0008037\_cell\_recognition | NDN | 39 | 1 | 4.373219 | -0.686769 | 252 | 215.49 | 0.855119 |
| GO:0070201\_regulation\_of\_establishment\_of\_protein\_localization | PRDX1 | 39 | 1 | 4.373219 | -0.686769 | 252 | 215.49 | 0.855119 |
| GO:0045934\_negative\_regulation\_of\_nucleobase\_\_nucleoside\_\_nucleotide\_and\_nucleic\_acid\_metabolic\_process | TRIM28 | 270 | 3 | 1.895062 | -0.680858 | 253 | 216.22 | 0.854625 |
| GO:0045934\_negative\_regulation\_of\_nucleobase\_\_nucleoside\_\_nucleotide\_and\_nucleic\_acid\_metabolic\_process | CBX3 | 270 | 3 | 1.895062 | -0.680858 | 253 | 216.22 | 0.854625 |
| GO:0045934\_negative\_regulation\_of\_nucleobase\_\_nucleoside\_\_nucleotide\_and\_nucleic\_acid\_metabolic\_process | RBBP7 | 270 | 3 | 1.895062 | -0.680858 | 253 | 216.22 | 0.854625 |
| GO:0051172\_negative\_regulation\_of\_nitrogen\_compound\_metabolic\_process | TRIM28 | 271 | 3 | 1.888069 | -0.677659 | 254 | 216.38 | 0.851890 |
| GO:0051172\_negative\_regulation\_of\_nitrogen\_compound\_metabolic\_process | CBX3 | 271 | 3 | 1.888069 | -0.677659 | 254 | 216.38 | 0.851890 |
| GO:0051172\_negative\_regulation\_of\_nitrogen\_compound\_metabolic\_process | RBBP7 | 271 | 3 | 1.888069 | -0.677659 | 254 | 216.38 | 0.851890 |
| GO:0001824\_blastocyst\_development | PRPF19 | 40 | 1 | 4.263889 | -0.676964 | 256 | 219.52 | 0.857500 |
| GO:0017015\_regulation\_of\_transforming\_growth\_factor\_beta\_receptor\_signaling\_pathway | HSPA5 | 40 | 1 | 4.263889 | -0.676964 | 256 | 219.52 | 0.857500 |
| GO:0006950\_response\_to\_stress | HMGN1 | 549 | 5 | 1.553329 | -0.671940 | 257 | 220.07 | 0.856304 |
| GO:0006950\_response\_to\_stress | RPA1 | 549 | 5 | 1.553329 | -0.671940 | 257 | 220.07 | 0.856304 |
| GO:0006950\_response\_to\_stress | GJA1 | 549 | 5 | 1.553329 | -0.671940 | 257 | 220.07 | 0.856304 |
| GO:0006950\_response\_to\_stress | HSPA5 | 549 | 5 | 1.553329 | -0.671940 | 257 | 220.07 | 0.856304 |
| GO:0006950\_response\_to\_stress | PRDX1 | 549 | 5 | 1.553329 | -0.671940 | 257 | 220.07 | 0.856304 |
| GO:0010558\_negative\_regulation\_of\_macromolecule\_biosynthetic\_process | TRIM28 | 274 | 3 | 1.867397 | -0.668167 | 258 | 220.65 | 0.855233 |
| GO:0010558\_negative\_regulation\_of\_macromolecule\_biosynthetic\_process | CBX3 | 274 | 3 | 1.867397 | -0.668167 | 258 | 220.65 | 0.855233 |
| GO:0010558\_negative\_regulation\_of\_macromolecule\_biosynthetic\_process | RBBP7 | 274 | 3 | 1.867397 | -0.668167 | 258 | 220.65 | 0.855233 |
| GO:0006979\_response\_to\_oxidative\_stress | PRDX1 | 41 | 1 | 4.159892 | -0.667430 | 260 | 224.77 | 0.864500 |
| GO:0032844\_regulation\_of\_homeostatic\_process | FKBP1A | 41 | 1 | 4.159892 | -0.667430 | 260 | 224.77 | 0.864500 |
| GO:0007242\_intracellular\_signaling\_cascade | GDI2 | 411 | 4 | 1.659908 | -0.663959 | 261 | 224.9 | 0.861686 |
| GO:0007242\_intracellular\_signaling\_cascade | YWHAQ | 411 | 4 | 1.659908 | -0.663959 | 261 | 224.9 | 0.861686 |
| GO:0007242\_intracellular\_signaling\_cascade | HSPA5 | 411 | 4 | 1.659908 | -0.663959 | 261 | 224.9 | 0.861686 |
| GO:0007242\_intracellular\_signaling\_cascade | PRDX1 | 411 | 4 | 1.659908 | -0.663959 | 261 | 224.9 | 0.861686 |
| GO:0008361\_regulation\_of\_cell\_size | NDN | 42 | 1 | 4.060847 | -0.658152 | 265 | 228.93 | 0.863887 |
| GO:0019221\_cytokine-mediated\_signaling\_pathway | FKBP1A | 42 | 1 | 4.060847 | -0.658152 | 265 | 228.93 | 0.863887 |
| GO:0051345\_positive\_regulation\_of\_hydrolase\_activity | CYCS | 42 | 1 | 4.060847 | -0.658152 | 265 | 228.93 | 0.863887 |
| GO:0080135\_regulation\_of\_cellular\_response\_to\_stress | PRDX1 | 42 | 1 | 4.060847 | -0.658152 | 265 | 228.93 | 0.863887 |
| GO:0032268\_regulation\_of\_cellular\_protein\_metabolic\_process | CFL1 | 152 | 2 | 2.244152 | -0.650852 | 266 | 229.59 | 0.863120 |
| GO:0032268\_regulation\_of\_cellular\_protein\_metabolic\_process | FKBP1A | 152 | 2 | 2.244152 | -0.650852 | 266 | 229.59 | 0.863120 |
| GO:0044267\_cellular\_protein\_metabolic\_process | CFL1 | 559 | 5 | 1.525542 | -0.650120 | 267 | 229.7 | 0.860300 |
| GO:0044267\_cellular\_protein\_metabolic\_process | TRIM28 | 559 | 5 | 1.525542 | -0.650120 | 267 | 229.7 | 0.860300 |
| GO:0044267\_cellular\_protein\_metabolic\_process | FKBP1A | 559 | 5 | 1.525542 | -0.650120 | 267 | 229.7 | 0.860300 |
| GO:0044267\_cellular\_protein\_metabolic\_process | HSPA8 | 559 | 5 | 1.525542 | -0.650120 | 267 | 229.7 | 0.860300 |
| GO:0044267\_cellular\_protein\_metabolic\_process | RPL29 | 559 | 5 | 1.525542 | -0.650120 | 267 | 229.7 | 0.860300 |
| GO:0001841\_neural\_tube\_formation | CFL1 | 43 | 1 | 3.966408 | -0.649120 | 271 | 233.29 | 0.860849 |
| GO:0001894\_tissue\_homeostasis | RPA1 | 43 | 1 | 3.966408 | -0.649120 | 271 | 233.29 | 0.860849 |
| GO:0010001\_glial\_cell\_differentiation | PRPF19 | 43 | 1 | 3.966408 | -0.649120 | 271 | 233.29 | 0.860849 |
| GO:0031098\_stress-activated\_protein\_kinase\_signaling\_pathway | PRDX1 | 43 | 1 | 3.966408 | -0.649120 | 271 | 233.29 | 0.860849 |
| GO:0042592\_homeostatic\_process | RPA1 | 419 | 4 | 1.628215 | -0.644007 | 272 | 233.72 | 0.859265 |
| GO:0042592\_homeostatic\_process | NDN | 419 | 4 | 1.628215 | -0.644007 | 272 | 233.72 | 0.859265 |
| GO:0042592\_homeostatic\_process | FKBP1A | 419 | 4 | 1.628215 | -0.644007 | 272 | 233.72 | 0.859265 |
| GO:0042592\_homeostatic\_process | PRDX1 | 419 | 4 | 1.628215 | -0.644007 | 272 | 233.72 | 0.859265 |
| GO:0031327\_negative\_regulation\_of\_cellular\_biosynthetic\_process | TRIM28 | 282 | 3 | 1.814421 | -0.643599 | 273 | 233.84 | 0.856557 |
| GO:0031327\_negative\_regulation\_of\_cellular\_biosynthetic\_process | CBX3 | 282 | 3 | 1.814421 | -0.643599 | 273 | 233.84 | 0.856557 |
| GO:0031327\_negative\_regulation\_of\_cellular\_biosynthetic\_process | RBBP7 | 282 | 3 | 1.814421 | -0.643599 | 273 | 233.84 | 0.856557 |
| GO:0000902\_cell\_morphogenesis | NDN | 283 | 3 | 1.808009 | -0.640602 | 274 | 234.14 | 0.854526 |
| GO:0000902\_cell\_morphogenesis | CFL1 | 283 | 3 | 1.808009 | -0.640602 | 274 | 234.14 | 0.854526 |
| GO:0000902\_cell\_morphogenesis | TRIM28 | 283 | 3 | 1.808009 | -0.640602 | 274 | 234.14 | 0.854526 |
| GO:0048593\_camera-type\_eye\_morphogenesis | HMGN1 | 44 | 1 | 3.876263 | -0.640322 | 275 | 238.47 | 0.867164 |
| GO:0022402\_cell\_cycle\_process | RPA1 | 155 | 2 | 2.200717 | -0.638304 | 276 | 238.77 | 0.865109 |
| GO:0022402\_cell\_cycle\_process | TUBB5 | 155 | 2 | 2.200717 | -0.638304 | 276 | 238.77 | 0.865109 |
| GO:0009890\_negative\_regulation\_of\_biosynthetic\_process | TRIM28 | 284 | 3 | 1.801643 | -0.637621 | 277 | 238.98 | 0.862744 |
| GO:0009890\_negative\_regulation\_of\_biosynthetic\_process | CBX3 | 284 | 3 | 1.801643 | -0.637621 | 277 | 238.98 | 0.862744 |
| GO:0009890\_negative\_regulation\_of\_biosynthetic\_process | RBBP7 | 284 | 3 | 1.801643 | -0.637621 | 277 | 238.98 | 0.862744 |
| GO:0009058\_biosynthetic\_process | PRPF19 | 1175 | 9 | 1.306383 | -0.633427 | 278 | 239.36 | 0.861007 |
| GO:0009058\_biosynthetic\_process | MAT2A | 1175 | 9 | 1.306383 | -0.633427 | 278 | 239.36 | 0.861007 |
| GO:0009058\_biosynthetic\_process | NDN | 1175 | 9 | 1.306383 | -0.633427 | 278 | 239.36 | 0.861007 |
| GO:0009058\_biosynthetic\_process | TRIM28 | 1175 | 9 | 1.306383 | -0.633427 | 278 | 239.36 | 0.861007 |
| GO:0009058\_biosynthetic\_process | CBX3 | 1175 | 9 | 1.306383 | -0.633427 | 278 | 239.36 | 0.861007 |
| GO:0009058\_biosynthetic\_process | RBBP7 | 1175 | 9 | 1.306383 | -0.633427 | 278 | 239.36 | 0.861007 |
| GO:0009058\_biosynthetic\_process | DDX5 | 1175 | 9 | 1.306383 | -0.633427 | 278 | 239.36 | 0.861007 |
| GO:0009058\_biosynthetic\_process | IMPDH2 | 1175 | 9 | 1.306383 | -0.633427 | 278 | 239.36 | 0.861007 |
| GO:0009058\_biosynthetic\_process | RPL29 | 1175 | 9 | 1.306383 | -0.633427 | 278 | 239.36 | 0.861007 |
| GO:0009790\_embryonic\_development | RPA1 | 567 | 5 | 1.504017 | -0.633162 | 279 | 239.54 | 0.858566 |
| GO:0009790\_embryonic\_development | PRPF19 | 567 | 5 | 1.504017 | -0.633162 | 279 | 239.54 | 0.858566 |
| GO:0009790\_embryonic\_development | CFL1 | 567 | 5 | 1.504017 | -0.633162 | 279 | 239.54 | 0.858566 |
| GO:0009790\_embryonic\_development | GJA1 | 567 | 5 | 1.504017 | -0.633162 | 279 | 239.54 | 0.858566 |
| GO:0009790\_embryonic\_development | SFRS1 | 567 | 5 | 1.504017 | -0.633162 | 279 | 239.54 | 0.858566 |
| GO:0001838\_embryonic\_epithelial\_tube\_formation | CFL1 | 45 | 1 | 3.790123 | -0.631748 | 280 | 240.87 | 0.860250 |
| GO:0006396\_RNA\_processing | SFRS1 | 47 | 1 | 3.628842 | -0.615230 | 283 | 247.14 | 0.873286 |
| GO:0045087\_innate\_immune\_response | PRDX1 | 47 | 1 | 3.628842 | -0.615230 | 283 | 247.14 | 0.873286 |
| GO:0048871\_multicellular\_organismal\_homeostasis | RPA1 | 47 | 1 | 3.628842 | -0.615230 | 283 | 247.14 | 0.873286 |
| GO:0032269\_negative\_regulation\_of\_cellular\_protein\_metabolic\_process | FKBP1A | 48 | 1 | 3.553241 | -0.607268 | 284 | 249.13 | 0.877218 |
| GO:0042110\_T\_cell\_activation | FKBP1A | 163 | 2 | 2.092706 | -0.606384 | 285 | 249.64 | 0.875930 |
| GO:0042110\_T\_cell\_activation | HSPD1 | 163 | 2 | 2.092706 | -0.606384 | 285 | 249.64 | 0.875930 |
| GO:0040011\_locomotion | NDN | 295 | 3 | 1.734463 | -0.605854 | 286 | 250.28 | 0.875105 |
| GO:0040011\_locomotion | CFL1 | 295 | 3 | 1.734463 | -0.605854 | 286 | 250.28 | 0.875105 |
| GO:0040011\_locomotion | GJA1 | 295 | 3 | 1.734463 | -0.605854 | 286 | 250.28 | 0.875105 |
| GO:0006725\_cellular\_aromatic\_compound\_metabolic\_process | MAT2A | 49 | 1 | 3.480726 | -0.599495 | 288 | 253.06 | 0.878681 |
| GO:0034101\_erythrocyte\_homeostasis | PRDX1 | 49 | 1 | 3.480726 | -0.599495 | 288 | 253.06 | 0.878681 |
| GO:0006259\_DNA\_metabolic\_process | HMGN1 | 165 | 2 | 2.067340 | -0.598735 | 289 | 253.57 | 0.877405 |
| GO:0006259\_DNA\_metabolic\_process | RPA1 | 165 | 2 | 2.067340 | -0.598735 | 289 | 253.57 | 0.877405 |
| GO:0043065\_positive\_regulation\_of\_apoptosis | CYCS | 166 | 2 | 2.054886 | -0.594958 | 290 | 254.0 | 0.875862 |
| GO:0043065\_positive\_regulation\_of\_apoptosis | PRDX1 | 166 | 2 | 2.054886 | -0.594958 | 290 | 254.0 | 0.875862 |
| GO:0007015\_actin\_filament\_organization | CFL1 | 50 | 1 | 3.411111 | -0.591901 | 291 | 256.14 | 0.880206 |
| GO:0010942\_positive\_regulation\_of\_cell\_death | CYCS | 167 | 2 | 2.042582 | -0.591213 | 294 | 256.84 | 0.873605 |
| GO:0010942\_positive\_regulation\_of\_cell\_death | PRDX1 | 167 | 2 | 2.042582 | -0.591213 | 294 | 256.84 | 0.873605 |
| GO:0043068\_positive\_regulation\_of\_programmed\_cell\_death | CYCS | 167 | 2 | 2.042582 | -0.591213 | 294 | 256.84 | 0.873605 |
| GO:0043068\_positive\_regulation\_of\_programmed\_cell\_death | PRDX1 | 167 | 2 | 2.042582 | -0.591213 | 294 | 256.84 | 0.873605 |
| GO:0051049\_regulation\_of\_transport | FKBP1A | 167 | 2 | 2.042582 | -0.591213 | 294 | 256.84 | 0.873605 |
| GO:0051049\_regulation\_of\_transport | PRDX1 | 167 | 2 | 2.042582 | -0.591213 | 294 | 256.84 | 0.873605 |
| GO:0006520\_cellular\_amino\_acid\_metabolic\_process | MAT2A | 51 | 1 | 3.344227 | -0.584480 | 299 | 259.94 | 0.869365 |
| GO:0032583\_regulation\_of\_gene-specific\_transcription | TRIM28 | 51 | 1 | 3.344227 | -0.584480 | 299 | 259.94 | 0.869365 |
| GO:0032880\_regulation\_of\_protein\_localization | PRDX1 | 51 | 1 | 3.344227 | -0.584480 | 299 | 259.94 | 0.869365 |
| GO:0043408\_regulation\_of\_MAPKKK\_cascade | PRDX1 | 51 | 1 | 3.344227 | -0.584480 | 299 | 259.94 | 0.869365 |
| GO:0044106\_cellular\_amine\_metabolic\_process | MAT2A | 51 | 1 | 3.344227 | -0.584480 | 299 | 259.94 | 0.869365 |
| GO:0051246\_regulation\_of\_protein\_metabolic\_process | CFL1 | 170 | 2 | 2.006536 | -0.580158 | 300 | 260.55 | 0.868500 |
| GO:0051246\_regulation\_of\_protein\_metabolic\_process | FKBP1A | 170 | 2 | 2.006536 | -0.580158 | 300 | 260.55 | 0.868500 |
| GO:0032989\_cellular\_component\_morphogenesis | NDN | 307 | 3 | 1.666667 | -0.573224 | 301 | 263.23 | 0.874518 |
| GO:0032989\_cellular\_component\_morphogenesis | CFL1 | 307 | 3 | 1.666667 | -0.573224 | 301 | 263.23 | 0.874518 |
| GO:0032989\_cellular\_component\_morphogenesis | TRIM28 | 307 | 3 | 1.666667 | -0.573224 | 301 | 263.23 | 0.874518 |
| GO:0051179\_localization | NDN | 1058 | 8 | 1.289645 | -0.572893 | 302 | 263.82 | 0.873576 |
| GO:0051179\_localization | CFL1 | 1058 | 8 | 1.289645 | -0.572893 | 302 | 263.82 | 0.873576 |
| GO:0051179\_localization | YWHAB | 1058 | 8 | 1.289645 | -0.572893 | 302 | 263.82 | 0.873576 |
| GO:0051179\_localization | YWHAQ | 1058 | 8 | 1.289645 | -0.572893 | 302 | 263.82 | 0.873576 |
| GO:0051179\_localization | GJA1 | 1058 | 8 | 1.289645 | -0.572893 | 302 | 263.82 | 0.873576 |
| GO:0051179\_localization | NUTF2 | 1058 | 8 | 1.289645 | -0.572893 | 302 | 263.82 | 0.873576 |
| GO:0051179\_localization | FKBP1A | 1058 | 8 | 1.289645 | -0.572893 | 302 | 263.82 | 0.873576 |
| GO:0051179\_localization | PRDX1 | 1058 | 8 | 1.289645 | -0.572893 | 302 | 263.82 | 0.873576 |
| GO:0006576\_biogenic\_amine\_metabolic\_process | OAZ1 | 53 | 1 | 3.218029 | -0.570129 | 305 | 266.02 | 0.872197 |
| GO:0051248\_negative\_regulation\_of\_protein\_metabolic\_process | FKBP1A | 53 | 1 | 3.218029 | -0.570129 | 305 | 266.02 | 0.872197 |
| GO:0055085\_transmembrane\_transport | FKBP1A | 53 | 1 | 3.218029 | -0.570129 | 305 | 266.02 | 0.872197 |
| GO:0010467\_gene\_expression | NDN | 905 | 7 | 1.319214 | -0.568027 | 306 | 266.8 | 0.871895 |
| GO:0010467\_gene\_expression | TRIM28 | 905 | 7 | 1.319214 | -0.568027 | 306 | 266.8 | 0.871895 |
| GO:0010467\_gene\_expression | CBX3 | 905 | 7 | 1.319214 | -0.568027 | 306 | 266.8 | 0.871895 |
| GO:0010467\_gene\_expression | SFRS1 | 905 | 7 | 1.319214 | -0.568027 | 306 | 266.8 | 0.871895 |
| GO:0010467\_gene\_expression | DDX5 | 905 | 7 | 1.319214 | -0.568027 | 306 | 266.8 | 0.871895 |
| GO:0010467\_gene\_expression | RBBP7 | 905 | 7 | 1.319214 | -0.568027 | 306 | 266.8 | 0.871895 |
| GO:0010467\_gene\_expression | RPL29 | 905 | 7 | 1.319214 | -0.568027 | 306 | 266.8 | 0.871895 |
| GO:0016310\_phosphorylation | CFL1 | 309 | 3 | 1.655879 | -0.567979 | 307 | 266.99 | 0.869674 |
| GO:0016310\_phosphorylation | TRIM28 | 309 | 3 | 1.655879 | -0.567979 | 307 | 266.99 | 0.869674 |
| GO:0016310\_phosphorylation | FKBP1A | 309 | 3 | 1.655879 | -0.567979 | 307 | 266.99 | 0.869674 |
| GO:0006164\_purine\_nucleotide\_biosynthetic\_process | IMPDH2 | 54 | 1 | 3.158436 | -0.563187 | 310 | 269.05 | 0.867903 |
| GO:0006412\_translation | RPL29 | 54 | 1 | 3.158436 | -0.563187 | 310 | 269.05 | 0.867903 |
| GO:0044271\_nitrogen\_compound\_biosynthetic\_process | MAT2A | 54 | 1 | 3.158436 | -0.563187 | 310 | 269.05 | 0.867903 |
| GO:0000122\_negative\_regulation\_of\_transcription\_from\_RNA\_polymerase\_II\_promoter | TRIM28 | 175 | 2 | 1.949206 | -0.562321 | 311 | 269.71 | 0.867235 |
| GO:0000122\_negative\_regulation\_of\_transcription\_from\_RNA\_polymerase\_II\_promoter | RBBP7 | 175 | 2 | 1.949206 | -0.562321 | 311 | 269.71 | 0.867235 |
| GO:0006873\_cellular\_ion\_homeostasis | NDN | 176 | 2 | 1.938131 | -0.558838 | 312 | 270.33 | 0.866442 |
| GO:0006873\_cellular\_ion\_homeostasis | FKBP1A | 176 | 2 | 1.938131 | -0.558838 | 312 | 270.33 | 0.866442 |
| GO:0006310\_DNA\_recombination | RPA1 | 55 | 1 | 3.101010 | -0.556394 | 315 | 272.07 | 0.863714 |
| GO:0007126\_meiosis | RPA1 | 55 | 1 | 3.101010 | -0.556394 | 315 | 272.07 | 0.863714 |
| GO:0051327\_M\_phase\_of\_meiotic\_cell\_cycle | RPA1 | 55 | 1 | 3.101010 | -0.556394 | 315 | 272.07 | 0.863714 |
| GO:0007165\_signal\_transduction | GDI2 | 915 | 7 | 1.304797 | -0.552472 | 316 | 272.55 | 0.862500 |
| GO:0007165\_signal\_transduction | HAX1 | 915 | 7 | 1.304797 | -0.552472 | 316 | 272.55 | 0.862500 |
| GO:0007165\_signal\_transduction | NDN | 915 | 7 | 1.304797 | -0.552472 | 316 | 272.55 | 0.862500 |
| GO:0007165\_signal\_transduction | YWHAQ | 915 | 7 | 1.304797 | -0.552472 | 316 | 272.55 | 0.862500 |
| GO:0007165\_signal\_transduction | FKBP1A | 915 | 7 | 1.304797 | -0.552472 | 316 | 272.55 | 0.862500 |
| GO:0007165\_signal\_transduction | HSPA5 | 915 | 7 | 1.304797 | -0.552472 | 316 | 272.55 | 0.862500 |
| GO:0007165\_signal\_transduction | PRDX1 | 915 | 7 | 1.304797 | -0.552472 | 316 | 272.55 | 0.862500 |
| GO:0006790\_sulfur\_metabolic\_process | MAT2A | 56 | 1 | 3.045635 | -0.549742 | 319 | 275.01 | 0.862100 |
| GO:0050678\_regulation\_of\_epithelial\_cell\_proliferation | HMGN1 | 56 | 1 | 3.045635 | -0.549742 | 319 | 275.01 | 0.862100 |
| GO:0051321\_meiotic\_cell\_cycle | RPA1 | 56 | 1 | 3.045635 | -0.549742 | 319 | 275.01 | 0.862100 |
| GO:0000226\_microtubule\_cytoskeleton\_organization | TUBB5 | 57 | 1 | 2.992203 | -0.543229 | 320 | 278.97 | 0.871781 |
| GO:0055082\_cellular\_chemical\_homeostasis | NDN | 181 | 2 | 1.884592 | -0.541834 | 321 | 279.39 | 0.870374 |
| GO:0055082\_cellular\_chemical\_homeostasis | FKBP1A | 181 | 2 | 1.884592 | -0.541834 | 321 | 279.39 | 0.870374 |
| GO:0033043\_regulation\_of\_organelle\_organization | CFL1 | 58 | 1 | 2.940613 | -0.536847 | 322 | 280.73 | 0.871832 |
| GO:0080090\_regulation\_of\_primary\_metabolic\_process | NDN | 926 | 7 | 1.289297 | -0.535786 | 323 | 280.81 | 0.869381 |
| GO:0080090\_regulation\_of\_primary\_metabolic\_process | CFL1 | 926 | 7 | 1.289297 | -0.535786 | 323 | 280.81 | 0.869381 |
| GO:0080090\_regulation\_of\_primary\_metabolic\_process | TRIM28 | 926 | 7 | 1.289297 | -0.535786 | 323 | 280.81 | 0.869381 |
| GO:0080090\_regulation\_of\_primary\_metabolic\_process | CBX3 | 926 | 7 | 1.289297 | -0.535786 | 323 | 280.81 | 0.869381 |
| GO:0080090\_regulation\_of\_primary\_metabolic\_process | FKBP1A | 926 | 7 | 1.289297 | -0.535786 | 323 | 280.81 | 0.869381 |
| GO:0080090\_regulation\_of\_primary\_metabolic\_process | RBBP7 | 926 | 7 | 1.289297 | -0.535786 | 323 | 280.81 | 0.869381 |
| GO:0080090\_regulation\_of\_primary\_metabolic\_process | DDX5 | 926 | 7 | 1.289297 | -0.535786 | 323 | 280.81 | 0.869381 |
| GO:0048469\_cell\_maturation | GJA1 | 59 | 1 | 2.890772 | -0.530594 | 325 | 282.61 | 0.869569 |
| GO:0050870\_positive\_regulation\_of\_T\_cell\_activation | HSPD1 | 59 | 1 | 2.890772 | -0.530594 | 325 | 282.61 | 0.869569 |
| GO:0048523\_negative\_regulation\_of\_cellular\_process | PRPF19 | 774 | 6 | 1.322136 | -0.530057 | 326 | 282.79 | 0.867454 |
| GO:0048523\_negative\_regulation\_of\_cellular\_process | TRIM28 | 774 | 6 | 1.322136 | -0.530057 | 326 | 282.79 | 0.867454 |
| GO:0048523\_negative\_regulation\_of\_cellular\_process | CBX3 | 774 | 6 | 1.322136 | -0.530057 | 326 | 282.79 | 0.867454 |
| GO:0048523\_negative\_regulation\_of\_cellular\_process | FKBP1A | 774 | 6 | 1.322136 | -0.530057 | 326 | 282.79 | 0.867454 |
| GO:0048523\_negative\_regulation\_of\_cellular\_process | HSPA5 | 774 | 6 | 1.322136 | -0.530057 | 326 | 282.79 | 0.867454 |
| GO:0048523\_negative\_regulation\_of\_cellular\_process | RBBP7 | 774 | 6 | 1.322136 | -0.530057 | 326 | 282.79 | 0.867454 |
| GO:0007010\_cytoskeleton\_organization | CFL1 | 185 | 2 | 1.843844 | -0.528701 | 327 | 283.31 | 0.866391 |
| GO:0007010\_cytoskeleton\_organization | TUBB5 | 185 | 2 | 1.843844 | -0.528701 | 327 | 283.31 | 0.866391 |
| GO:0060255\_regulation\_of\_macromolecule\_metabolic\_process | NDN | 936 | 7 | 1.275522 | -0.520995 | 328 | 284.92 | 0.868659 |
| GO:0060255\_regulation\_of\_macromolecule\_metabolic\_process | CFL1 | 936 | 7 | 1.275522 | -0.520995 | 328 | 284.92 | 0.868659 |
| GO:0060255\_regulation\_of\_macromolecule\_metabolic\_process | TRIM28 | 936 | 7 | 1.275522 | -0.520995 | 328 | 284.92 | 0.868659 |
| GO:0060255\_regulation\_of\_macromolecule\_metabolic\_process | CBX3 | 936 | 7 | 1.275522 | -0.520995 | 328 | 284.92 | 0.868659 |
| GO:0060255\_regulation\_of\_macromolecule\_metabolic\_process | FKBP1A | 936 | 7 | 1.275522 | -0.520995 | 328 | 284.92 | 0.868659 |
| GO:0060255\_regulation\_of\_macromolecule\_metabolic\_process | RBBP7 | 936 | 7 | 1.275522 | -0.520995 | 328 | 284.92 | 0.868659 |
| GO:0060255\_regulation\_of\_macromolecule\_metabolic\_process | DDX5 | 936 | 7 | 1.275522 | -0.520995 | 328 | 284.92 | 0.868659 |
| GO:0007154\_cell\_communication | GDI2 | 1096 | 8 | 1.244931 | -0.518607 | 329 | 285.23 | 0.866960 |
| GO:0007154\_cell\_communication | HAX1 | 1096 | 8 | 1.244931 | -0.518607 | 329 | 285.23 | 0.866960 |
| GO:0007154\_cell\_communication | NDN | 1096 | 8 | 1.244931 | -0.518607 | 329 | 285.23 | 0.866960 |
| GO:0007154\_cell\_communication | YWHAQ | 1096 | 8 | 1.244931 | -0.518607 | 329 | 285.23 | 0.866960 |
| GO:0007154\_cell\_communication | GJA1 | 1096 | 8 | 1.244931 | -0.518607 | 329 | 285.23 | 0.866960 |
| GO:0007154\_cell\_communication | FKBP1A | 1096 | 8 | 1.244931 | -0.518607 | 329 | 285.23 | 0.866960 |
| GO:0007154\_cell\_communication | HSPA5 | 1096 | 8 | 1.244931 | -0.518607 | 329 | 285.23 | 0.866960 |
| GO:0007154\_cell\_communication | PRDX1 | 1096 | 8 | 1.244931 | -0.518607 | 329 | 285.23 | 0.866960 |
| GO:0032270\_positive\_regulation\_of\_cellular\_protein\_metabolic\_process | CFL1 | 61 | 1 | 2.795993 | -0.518455 | 330 | 286.69 | 0.868758 |
| GO:0006928\_cell\_motion | NDN | 330 | 3 | 1.550505 | -0.516004 | 332 | 287.37 | 0.865572 |
| GO:0006928\_cell\_motion | CFL1 | 330 | 3 | 1.550505 | -0.516004 | 332 | 287.37 | 0.865572 |
| GO:0006928\_cell\_motion | GJA1 | 330 | 3 | 1.550505 | -0.516004 | 332 | 287.37 | 0.865572 |
| GO:0051674\_localization\_of\_cell | NDN | 330 | 3 | 1.550505 | -0.516004 | 332 | 287.37 | 0.865572 |
| GO:0051674\_localization\_of\_cell | CFL1 | 330 | 3 | 1.550505 | -0.516004 | 332 | 287.37 | 0.865572 |
| GO:0051674\_localization\_of\_cell | GJA1 | 330 | 3 | 1.550505 | -0.516004 | 332 | 287.37 | 0.865572 |
| GO:0030855\_epithelial\_cell\_differentiation | GJA1 | 62 | 1 | 2.750896 | -0.512562 | 333 | 290.37 | 0.871982 |
| GO:0009165\_nucleotide\_biosynthetic\_process | IMPDH2 | 63 | 1 | 2.707231 | -0.506780 | 334 | 292.18 | 0.874790 |
| GO:0042060\_wound\_healing | GJA1 | 64 | 1 | 2.664931 | -0.501106 | 335 | 293.73 | 0.876806 |
| GO:0007507\_heart\_development | GJA1 | 195 | 2 | 1.749288 | -0.497572 | 337 | 294.88 | 0.875015 |
| GO:0007507\_heart\_development | FKBP1A | 195 | 2 | 1.749288 | -0.497572 | 337 | 294.88 | 0.875015 |
| GO:0019725\_cellular\_homeostasis | NDN | 195 | 2 | 1.749288 | -0.497572 | 337 | 294.88 | 0.875015 |
| GO:0019725\_cellular\_homeostasis | FKBP1A | 195 | 2 | 1.749288 | -0.497572 | 337 | 294.88 | 0.875015 |
| GO:0006793\_phosphorus\_metabolic\_process | CFL1 | 340 | 3 | 1.504902 | -0.493101 | 339 | 296.68 | 0.875162 |
| GO:0006793\_phosphorus\_metabolic\_process | TRIM28 | 340 | 3 | 1.504902 | -0.493101 | 339 | 296.68 | 0.875162 |
| GO:0006793\_phosphorus\_metabolic\_process | FKBP1A | 340 | 3 | 1.504902 | -0.493101 | 339 | 296.68 | 0.875162 |
| GO:0006796\_phosphate\_metabolic\_process | CFL1 | 340 | 3 | 1.504902 | -0.493101 | 339 | 296.68 | 0.875162 |
| GO:0006796\_phosphate\_metabolic\_process | TRIM28 | 340 | 3 | 1.504902 | -0.493101 | 339 | 296.68 | 0.875162 |
| GO:0006796\_phosphate\_metabolic\_process | FKBP1A | 340 | 3 | 1.504902 | -0.493101 | 339 | 296.68 | 0.875162 |
| GO:0050801\_ion\_homeostasis | NDN | 197 | 2 | 1.731528 | -0.491622 | 340 | 297.01 | 0.873559 |
| GO:0050801\_ion\_homeostasis | FKBP1A | 197 | 2 | 1.731528 | -0.491622 | 340 | 297.01 | 0.873559 |
| GO:0007179\_transforming\_growth\_factor\_beta\_receptor\_signaling\_pathway | HSPA5 | 66 | 1 | 2.584175 | -0.490071 | 343 | 298.59 | 0.870525 |
| GO:0031589\_cell-substrate\_adhesion | RPL29 | 66 | 1 | 2.584175 | -0.490071 | 343 | 298.59 | 0.870525 |
| GO:0051130\_positive\_regulation\_of\_cellular\_component\_organization | CFL1 | 66 | 1 | 2.584175 | -0.490071 | 343 | 298.59 | 0.870525 |
| GO:0002009\_morphogenesis\_of\_an\_epithelium | CFL1 | 198 | 2 | 1.722783 | -0.488680 | 345 | 299.2 | 0.867246 |
| GO:0002009\_morphogenesis\_of\_an\_epithelium | GJA1 | 198 | 2 | 1.722783 | -0.488680 | 345 | 299.2 | 0.867246 |
| GO:0060429\_epithelium\_development | CFL1 | 198 | 2 | 1.722783 | -0.488680 | 345 | 299.2 | 0.867246 |
| GO:0060429\_epithelium\_development | GJA1 | 198 | 2 | 1.722783 | -0.488680 | 345 | 299.2 | 0.867246 |
| GO:0000904\_cell\_morphogenesis\_involved\_in\_differentiation | NDN | 199 | 2 | 1.714126 | -0.485760 | 346 | 299.51 | 0.865636 |
| GO:0000904\_cell\_morphogenesis\_involved\_in\_differentiation | TRIM28 | 199 | 2 | 1.714126 | -0.485760 | 346 | 299.51 | 0.865636 |
| GO:0009791\_post-embryonic\_development | HMGN1 | 67 | 1 | 2.545605 | -0.484703 | 348 | 301.01 | 0.864971 |
| GO:0051247\_positive\_regulation\_of\_protein\_metabolic\_process | CFL1 | 67 | 1 | 2.545605 | -0.484703 | 348 | 301.01 | 0.864971 |
| GO:0034961\_cellular\_biopolymer\_biosynthetic\_process | NDN | 804 | 6 | 1.272803 | -0.483915 | 349 | 301.14 | 0.862865 |
| GO:0034961\_cellular\_biopolymer\_biosynthetic\_process | TRIM28 | 804 | 6 | 1.272803 | -0.483915 | 349 | 301.14 | 0.862865 |
| GO:0034961\_cellular\_biopolymer\_biosynthetic\_process | CBX3 | 804 | 6 | 1.272803 | -0.483915 | 349 | 301.14 | 0.862865 |
| GO:0034961\_cellular\_biopolymer\_biosynthetic\_process | DDX5 | 804 | 6 | 1.272803 | -0.483915 | 349 | 301.14 | 0.862865 |
| GO:0034961\_cellular\_biopolymer\_biosynthetic\_process | RBBP7 | 804 | 6 | 1.272803 | -0.483915 | 349 | 301.14 | 0.862865 |
| GO:0034961\_cellular\_biopolymer\_biosynthetic\_process | RPL29 | 804 | 6 | 1.272803 | -0.483915 | 349 | 301.14 | 0.862865 |
| GO:0016043\_cellular\_component\_organization | RPA1 | 964 | 7 | 1.238474 | -0.481420 | 350 | 301.39 | 0.861114 |
| GO:0016043\_cellular\_component\_organization | HMGN1 | 964 | 7 | 1.238474 | -0.481420 | 350 | 301.39 | 0.861114 |
| GO:0016043\_cellular\_component\_organization | NDN | 964 | 7 | 1.238474 | -0.481420 | 350 | 301.39 | 0.861114 |
| GO:0016043\_cellular\_component\_organization | TRIM28 | 964 | 7 | 1.238474 | -0.481420 | 350 | 301.39 | 0.861114 |
| GO:0016043\_cellular\_component\_organization | CFL1 | 964 | 7 | 1.238474 | -0.481420 | 350 | 301.39 | 0.861114 |
| GO:0016043\_cellular\_component\_organization | TUBB5 | 964 | 7 | 1.238474 | -0.481420 | 350 | 301.39 | 0.861114 |
| GO:0016043\_cellular\_component\_organization | RBBP7 | 964 | 7 | 1.238474 | -0.481420 | 350 | 301.39 | 0.861114 |
| GO:0043284\_biopolymer\_biosynthetic\_process | NDN | 807 | 6 | 1.268071 | -0.479504 | 351 | 301.75 | 0.859687 |
| GO:0043284\_biopolymer\_biosynthetic\_process | TRIM28 | 807 | 6 | 1.268071 | -0.479504 | 351 | 301.75 | 0.859687 |
| GO:0043284\_biopolymer\_biosynthetic\_process | CBX3 | 807 | 6 | 1.268071 | -0.479504 | 351 | 301.75 | 0.859687 |
| GO:0043284\_biopolymer\_biosynthetic\_process | DDX5 | 807 | 6 | 1.268071 | -0.479504 | 351 | 301.75 | 0.859687 |
| GO:0043284\_biopolymer\_biosynthetic\_process | RBBP7 | 807 | 6 | 1.268071 | -0.479504 | 351 | 301.75 | 0.859687 |
| GO:0043284\_biopolymer\_biosynthetic\_process | RPL29 | 807 | 6 | 1.268071 | -0.479504 | 351 | 301.75 | 0.859687 |
| GO:0001932\_regulation\_of\_protein\_amino\_acid\_phosphorylation | FKBP1A | 69 | 1 | 2.471820 | -0.474251 | 353 | 304.7 | 0.863173 |
| GO:0006816\_calcium\_ion\_transport | FKBP1A | 69 | 1 | 2.471820 | -0.474251 | 353 | 304.7 | 0.863173 |
| GO:0048468\_cell\_development | PRPF19 | 654 | 5 | 1.303942 | -0.474023 | 354 | 304.88 | 0.861243 |
| GO:0048468\_cell\_development | NDN | 654 | 5 | 1.303942 | -0.474023 | 354 | 304.88 | 0.861243 |
| GO:0048468\_cell\_development | CFL1 | 654 | 5 | 1.303942 | -0.474023 | 354 | 304.88 | 0.861243 |
| GO:0048468\_cell\_development | TRIM28 | 654 | 5 | 1.303942 | -0.474023 | 354 | 304.88 | 0.861243 |
| GO:0048468\_cell\_development | GJA1 | 654 | 5 | 1.303942 | -0.474023 | 354 | 304.88 | 0.861243 |
| GO:0019538\_protein\_metabolic\_process | CFL1 | 655 | 5 | 1.301951 | -0.472435 | 355 | 305.05 | 0.859296 |
| GO:0019538\_protein\_metabolic\_process | TRIM28 | 655 | 5 | 1.301951 | -0.472435 | 355 | 305.05 | 0.859296 |
| GO:0019538\_protein\_metabolic\_process | FKBP1A | 655 | 5 | 1.301951 | -0.472435 | 355 | 305.05 | 0.859296 |
| GO:0019538\_protein\_metabolic\_process | HSPA8 | 655 | 5 | 1.301951 | -0.472435 | 355 | 305.05 | 0.859296 |
| GO:0019538\_protein\_metabolic\_process | RPL29 | 655 | 5 | 1.301951 | -0.472435 | 355 | 305.05 | 0.859296 |
| GO:0048592\_eye\_morphogenesis | HMGN1 | 70 | 1 | 2.436508 | -0.469162 | 357 | 306.57 | 0.858739 |
| GO:0070838\_divalent\_metal\_ion\_transport | FKBP1A | 70 | 1 | 2.436508 | -0.469162 | 357 | 306.57 | 0.858739 |
| GO:0016070\_RNA\_metabolic\_process | NDN | 658 | 5 | 1.296015 | -0.467699 | 358 | 307.33 | 0.858464 |
| GO:0016070\_RNA\_metabolic\_process | TRIM28 | 658 | 5 | 1.296015 | -0.467699 | 358 | 307.33 | 0.858464 |
| GO:0016070\_RNA\_metabolic\_process | CBX3 | 658 | 5 | 1.296015 | -0.467699 | 358 | 307.33 | 0.858464 |
| GO:0016070\_RNA\_metabolic\_process | RBBP7 | 658 | 5 | 1.296015 | -0.467699 | 358 | 307.33 | 0.858464 |
| GO:0016070\_RNA\_metabolic\_process | SFRS1 | 658 | 5 | 1.296015 | -0.467699 | 358 | 307.33 | 0.858464 |
| GO:0016331\_morphogenesis\_of\_embryonic\_epithelium | CFL1 | 71 | 1 | 2.402191 | -0.464161 | 359 | 309.08 | 0.860947 |
| GO:0016568\_chromatin\_modification | RBBP7 | 72 | 1 | 2.368827 | -0.459244 | 363 | 312.69 | 0.861405 |
| GO:0021915\_neural\_tube\_development | CFL1 | 72 | 1 | 2.368827 | -0.459244 | 363 | 312.69 | 0.861405 |
| GO:0042098\_T\_cell\_proliferation | FKBP1A | 72 | 1 | 2.368827 | -0.459244 | 363 | 312.69 | 0.861405 |
| GO:0050673\_epithelial\_cell\_proliferation | HMGN1 | 72 | 1 | 2.368827 | -0.459244 | 363 | 312.69 | 0.861405 |
| GO:0006163\_purine\_nucleotide\_metabolic\_process | IMPDH2 | 73 | 1 | 2.336377 | -0.454411 | 365 | 314.43 | 0.861452 |
| GO:0051336\_regulation\_of\_hydrolase\_activity | CYCS | 73 | 1 | 2.336377 | -0.454411 | 365 | 314.43 | 0.861452 |
| GO:0035295\_tube\_development | CFL1 | 212 | 2 | 1.609015 | -0.449662 | 366 | 315.22 | 0.861257 |
| GO:0035295\_tube\_development | GJA1 | 212 | 2 | 1.609015 | -0.449662 | 366 | 315.22 | 0.861257 |
| GO:0009416\_response\_to\_light\_stimulus | HMGN1 | 74 | 1 | 2.304805 | -0.449657 | 367 | 315.76 | 0.860381 |
| GO:0044249\_cellular\_biosynthetic\_process | NDN | 1150 | 8 | 1.186473 | -0.448808 | 368 | 315.88 | 0.858370 |
| GO:0044249\_cellular\_biosynthetic\_process | MAT2A | 1150 | 8 | 1.186473 | -0.448808 | 368 | 315.88 | 0.858370 |
| GO:0044249\_cellular\_biosynthetic\_process | TRIM28 | 1150 | 8 | 1.186473 | -0.448808 | 368 | 315.88 | 0.858370 |
| GO:0044249\_cellular\_biosynthetic\_process | CBX3 | 1150 | 8 | 1.186473 | -0.448808 | 368 | 315.88 | 0.858370 |
| GO:0044249\_cellular\_biosynthetic\_process | DDX5 | 1150 | 8 | 1.186473 | -0.448808 | 368 | 315.88 | 0.858370 |
| GO:0044249\_cellular\_biosynthetic\_process | RBBP7 | 1150 | 8 | 1.186473 | -0.448808 | 368 | 315.88 | 0.858370 |
| GO:0044249\_cellular\_biosynthetic\_process | IMPDH2 | 1150 | 8 | 1.186473 | -0.448808 | 368 | 315.88 | 0.858370 |
| GO:0044249\_cellular\_biosynthetic\_process | RPL29 | 1150 | 8 | 1.186473 | -0.448808 | 368 | 315.88 | 0.858370 |
| GO:0003008\_system\_process | NDN | 516 | 4 | 1.322136 | -0.445528 | 369 | 316.4 | 0.857453 |
| GO:0003008\_system\_process | GJA1 | 516 | 4 | 1.322136 | -0.445528 | 369 | 316.4 | 0.857453 |
| GO:0003008\_system\_process | FKBP1A | 516 | 4 | 1.322136 | -0.445528 | 369 | 316.4 | 0.857453 |
| GO:0003008\_system\_process | SFRS1 | 516 | 4 | 1.322136 | -0.445528 | 369 | 316.4 | 0.857453 |
| GO:0034621\_cellular\_macromolecular\_complex\_subunit\_organization | CFL1 | 76 | 1 | 2.244152 | -0.440384 | 370 | 319.58 | 0.863730 |
| GO:0045449\_regulation\_of\_transcription | NDN | 676 | 5 | 1.261506 | -0.440191 | 371 | 319.83 | 0.862075 |
| GO:0045449\_regulation\_of\_transcription | TRIM28 | 676 | 5 | 1.261506 | -0.440191 | 371 | 319.83 | 0.862075 |
| GO:0045449\_regulation\_of\_transcription | CBX3 | 676 | 5 | 1.261506 | -0.440191 | 371 | 319.83 | 0.862075 |
| GO:0045449\_regulation\_of\_transcription | DDX5 | 676 | 5 | 1.261506 | -0.440191 | 371 | 319.83 | 0.862075 |
| GO:0045449\_regulation\_of\_transcription | RBBP7 | 676 | 5 | 1.261506 | -0.440191 | 371 | 319.83 | 0.862075 |
| GO:0010033\_response\_to\_organic\_substance | CFL1 | 216 | 2 | 1.579218 | -0.439213 | 372 | 320.06 | 0.860376 |
| GO:0010033\_response\_to\_organic\_substance | FKBP1A | 216 | 2 | 1.579218 | -0.439213 | 372 | 320.06 | 0.860376 |
| GO:0051251\_positive\_regulation\_of\_lymphocyte\_activation | HSPD1 | 78 | 1 | 2.186610 | -0.431407 | 373 | 323.75 | 0.867962 |
| GO:0009888\_tissue\_development | TRIM28 | 525 | 4 | 1.299471 | -0.430536 | 374 | 324.1 | 0.866578 |
| GO:0009888\_tissue\_development | CFL1 | 525 | 4 | 1.299471 | -0.430536 | 374 | 324.1 | 0.866578 |
| GO:0009888\_tissue\_development | GJA1 | 525 | 4 | 1.299471 | -0.430536 | 374 | 324.1 | 0.866578 |
| GO:0009888\_tissue\_development | FKBP1A | 525 | 4 | 1.299471 | -0.430536 | 374 | 324.1 | 0.866578 |
| GO:0015674\_di-\_\_tri-valent\_inorganic\_cation\_transport | FKBP1A | 79 | 1 | 2.158931 | -0.427025 | 375 | 325.22 | 0.867253 |
| GO:0021700\_developmental\_maturation | GJA1 | 81 | 1 | 2.105624 | -0.418465 | 376 | 327.71 | 0.871569 |
| GO:0031323\_regulation\_of\_cellular\_metabolic\_process | NDN | 1015 | 7 | 1.176245 | -0.415865 | 377 | 327.86 | 0.869655 |
| GO:0031323\_regulation\_of\_cellular\_metabolic\_process | CFL1 | 1015 | 7 | 1.176245 | -0.415865 | 377 | 327.86 | 0.869655 |
| GO:0031323\_regulation\_of\_cellular\_metabolic\_process | TRIM28 | 1015 | 7 | 1.176245 | -0.415865 | 377 | 327.86 | 0.869655 |
| GO:0031323\_regulation\_of\_cellular\_metabolic\_process | CBX3 | 1015 | 7 | 1.176245 | -0.415865 | 377 | 327.86 | 0.869655 |
| GO:0031323\_regulation\_of\_cellular\_metabolic\_process | FKBP1A | 1015 | 7 | 1.176245 | -0.415865 | 377 | 327.86 | 0.869655 |
| GO:0031323\_regulation\_of\_cellular\_metabolic\_process | RBBP7 | 1015 | 7 | 1.176245 | -0.415865 | 377 | 327.86 | 0.869655 |
| GO:0031323\_regulation\_of\_cellular\_metabolic\_process | DDX5 | 1015 | 7 | 1.176245 | -0.415865 | 377 | 327.86 | 0.869655 |
| GO:0065008\_regulation\_of\_biological\_quality | RPA1 | 693 | 5 | 1.230560 | -0.415585 | 378 | 328.02 | 0.867778 |
| GO:0065008\_regulation\_of\_biological\_quality | NDN | 693 | 5 | 1.230560 | -0.415585 | 378 | 328.02 | 0.867778 |
| GO:0065008\_regulation\_of\_biological\_quality | CFL1 | 693 | 5 | 1.230560 | -0.415585 | 378 | 328.02 | 0.867778 |
| GO:0065008\_regulation\_of\_biological\_quality | FKBP1A | 693 | 5 | 1.230560 | -0.415585 | 378 | 328.02 | 0.867778 |
| GO:0065008\_regulation\_of\_biological\_quality | PRDX1 | 693 | 5 | 1.230560 | -0.415585 | 378 | 328.02 | 0.867778 |
| GO:0002696\_positive\_regulation\_of\_leukocyte\_activation | HSPD1 | 82 | 1 | 2.079946 | -0.414284 | 381 | 329.65 | 0.865223 |
| GO:0010627\_regulation\_of\_protein\_kinase\_cascade | PRDX1 | 82 | 1 | 2.079946 | -0.414284 | 381 | 329.65 | 0.865223 |
| GO:0045664\_regulation\_of\_neuron\_differentiation | PRPF19 | 82 | 1 | 2.079946 | -0.414284 | 381 | 329.65 | 0.865223 |
| GO:0007017\_microtubule-based\_process | TUBB5 | 83 | 1 | 2.054886 | -0.410166 | 383 | 331.66 | 0.865953 |
| GO:0050867\_positive\_regulation\_of\_cell\_activation | HSPD1 | 83 | 1 | 2.054886 | -0.410166 | 383 | 331.66 | 0.865953 |
| GO:0048519\_negative\_regulation\_of\_biological\_process | PRPF19 | 859 | 6 | 1.191308 | -0.408455 | 384 | 332.49 | 0.865859 |
| GO:0048519\_negative\_regulation\_of\_biological\_process | TRIM28 | 859 | 6 | 1.191308 | -0.408455 | 384 | 332.49 | 0.865859 |
| GO:0048519\_negative\_regulation\_of\_biological\_process | CBX3 | 859 | 6 | 1.191308 | -0.408455 | 384 | 332.49 | 0.865859 |
| GO:0048519\_negative\_regulation\_of\_biological\_process | FKBP1A | 859 | 6 | 1.191308 | -0.408455 | 384 | 332.49 | 0.865859 |
| GO:0048519\_negative\_regulation\_of\_biological\_process | HSPA5 | 859 | 6 | 1.191308 | -0.408455 | 384 | 332.49 | 0.865859 |
| GO:0048519\_negative\_regulation\_of\_biological\_process | RBBP7 | 859 | 6 | 1.191308 | -0.408455 | 384 | 332.49 | 0.865859 |
| GO:0007167\_enzyme\_linked\_receptor\_protein\_signaling\_pathway | NDN | 229 | 2 | 1.489568 | -0.407191 | 385 | 332.79 | 0.864390 |
| GO:0007167\_enzyme\_linked\_receptor\_protein\_signaling\_pathway | HSPA5 | 229 | 2 | 1.489568 | -0.407191 | 385 | 332.79 | 0.864390 |
| GO:0043687\_post-translational\_protein\_modification | CFL1 | 384 | 3 | 1.332465 | -0.404481 | 386 | 333.66 | 0.864404 |
| GO:0043687\_post-translational\_protein\_modification | TRIM28 | 384 | 3 | 1.332465 | -0.404481 | 386 | 333.66 | 0.864404 |
| GO:0043687\_post-translational\_protein\_modification | FKBP1A | 384 | 3 | 1.332465 | -0.404481 | 386 | 333.66 | 0.864404 |
| GO:0006350\_transcription | NDN | 701 | 5 | 1.216516 | -0.404446 | 387 | 333.9 | 0.862791 |
| GO:0006350\_transcription | TRIM28 | 701 | 5 | 1.216516 | -0.404446 | 387 | 333.9 | 0.862791 |
| GO:0006350\_transcription | CBX3 | 701 | 5 | 1.216516 | -0.404446 | 387 | 333.9 | 0.862791 |
| GO:0006350\_transcription | DDX5 | 701 | 5 | 1.216516 | -0.404446 | 387 | 333.9 | 0.862791 |
| GO:0006350\_transcription | RBBP7 | 701 | 5 | 1.216516 | -0.404446 | 387 | 333.9 | 0.862791 |
| GO:0002449\_lymphocyte\_mediated\_immunity | PRDX1 | 85 | 1 | 2.006536 | -0.402115 | 388 | 335.16 | 0.863814 |
| GO:0034641\_cellular\_nitrogen\_compound\_metabolic\_process | MAT2A | 86 | 1 | 1.983204 | -0.398179 | 389 | 337.94 | 0.868740 |
| GO:0007178\_transmembrane\_receptor\_protein\_serine\_threonine\_kinase\_signaling\_pathway | HSPA5 | 87 | 1 | 1.960409 | -0.394301 | 390 | 341.39 | 0.875359 |
| GO:0050863\_regulation\_of\_T\_cell\_activation | HSPD1 | 88 | 1 | 1.938131 | -0.390479 | 391 | 343.1 | 0.877494 |
| GO:0048699\_generation\_of\_neurons | PRPF19 | 396 | 3 | 1.292088 | -0.383330 | 392 | 344.18 | 0.878010 |
| GO:0048699\_generation\_of\_neurons | NDN | 396 | 3 | 1.292088 | -0.383330 | 392 | 344.18 | 0.878010 |
| GO:0048699\_generation\_of\_neurons | GJA1 | 396 | 3 | 1.292088 | -0.383330 | 392 | 344.18 | 0.878010 |
| GO:0035264\_multicellular\_organism\_growth | RPL29 | 90 | 1 | 1.895062 | -0.382999 | 393 | 345.48 | 0.879084 |
| GO:0006810\_transport | YWHAB | 718 | 5 | 1.187713 | -0.381673 | 394 | 345.64 | 0.877259 |
| GO:0006810\_transport | YWHAQ | 718 | 5 | 1.187713 | -0.381673 | 394 | 345.64 | 0.877259 |
| GO:0006810\_transport | NUTF2 | 718 | 5 | 1.187713 | -0.381673 | 394 | 345.64 | 0.877259 |
| GO:0006810\_transport | FKBP1A | 718 | 5 | 1.187713 | -0.381673 | 394 | 345.64 | 0.877259 |
| GO:0006810\_transport | PRDX1 | 718 | 5 | 1.187713 | -0.381673 | 394 | 345.64 | 0.877259 |
| GO:0002443\_leukocyte\_mediated\_immunity | PRDX1 | 91 | 1 | 1.874237 | -0.379338 | 396 | 346.72 | 0.875556 |
| GO:0031399\_regulation\_of\_protein\_modification\_process | FKBP1A | 91 | 1 | 1.874237 | -0.379338 | 396 | 346.72 | 0.875556 |
| GO:0009719\_response\_to\_endogenous\_stimulus | CFL1 | 92 | 1 | 1.853865 | -0.375729 | 397 | 347.88 | 0.876272 |
| GO:0006753\_nucleoside\_phosphate\_metabolic\_process | IMPDH2 | 94 | 1 | 1.814421 | -0.368660 | 400 | 351.51 | 0.878775 |
| GO:0008610\_lipid\_biosynthetic\_process | PRPF19 | 94 | 1 | 1.814421 | -0.368660 | 400 | 351.51 | 0.878775 |
| GO:0009117\_nucleotide\_metabolic\_process | IMPDH2 | 94 | 1 | 1.814421 | -0.368660 | 400 | 351.51 | 0.878775 |
| GO:0051234\_establishment\_of\_localization | YWHAB | 729 | 5 | 1.169791 | -0.367563 | 401 | 351.69 | 0.877032 |
| GO:0051234\_establishment\_of\_localization | YWHAQ | 729 | 5 | 1.169791 | -0.367563 | 401 | 351.69 | 0.877032 |
| GO:0051234\_establishment\_of\_localization | NUTF2 | 729 | 5 | 1.169791 | -0.367563 | 401 | 351.69 | 0.877032 |
| GO:0051234\_establishment\_of\_localization | FKBP1A | 729 | 5 | 1.169791 | -0.367563 | 401 | 351.69 | 0.877032 |
| GO:0051234\_establishment\_of\_localization | PRDX1 | 729 | 5 | 1.169791 | -0.367563 | 401 | 351.69 | 0.877032 |
| GO:0032879\_regulation\_of\_localization | FKBP1A | 248 | 2 | 1.375448 | -0.365166 | 402 | 353.13 | 0.878433 |
| GO:0032879\_regulation\_of\_localization | PRDX1 | 248 | 2 | 1.375448 | -0.365166 | 402 | 353.13 | 0.878433 |
| GO:0060249\_anatomical\_structure\_homeostasis | RPA1 | 96 | 1 | 1.776620 | -0.361784 | 403 | 355.33 | 0.881712 |
| GO:0042221\_response\_to\_chemical\_stimulus | CFL1 | 409 | 3 | 1.251019 | -0.361698 | 404 | 355.51 | 0.879975 |
| GO:0042221\_response\_to\_chemical\_stimulus | FKBP1A | 409 | 3 | 1.251019 | -0.361698 | 404 | 355.51 | 0.879975 |
| GO:0042221\_response\_to\_chemical\_stimulus | PRDX1 | 409 | 3 | 1.251019 | -0.361698 | 404 | 355.51 | 0.879975 |
| GO:0060341\_regulation\_of\_cellular\_localization | PRDX1 | 97 | 1 | 1.758305 | -0.358416 | 405 | 357.21 | 0.882000 |
| GO:0034645\_cellular\_macromolecule\_biosynthetic\_process | NDN | 901 | 6 | 1.135775 | -0.357955 | 406 | 357.4 | 0.880296 |
| GO:0034645\_cellular\_macromolecule\_biosynthetic\_process | TRIM28 | 901 | 6 | 1.135775 | -0.357955 | 406 | 357.4 | 0.880296 |
| GO:0034645\_cellular\_macromolecule\_biosynthetic\_process | CBX3 | 901 | 6 | 1.135775 | -0.357955 | 406 | 357.4 | 0.880296 |
| GO:0034645\_cellular\_macromolecule\_biosynthetic\_process | DDX5 | 901 | 6 | 1.135775 | -0.357955 | 406 | 357.4 | 0.880296 |
| GO:0034645\_cellular\_macromolecule\_biosynthetic\_process | RBBP7 | 901 | 6 | 1.135775 | -0.357955 | 406 | 357.4 | 0.880296 |
| GO:0034645\_cellular\_macromolecule\_biosynthetic\_process | RPL29 | 901 | 6 | 1.135775 | -0.357955 | 406 | 357.4 | 0.880296 |
| GO:0006355\_regulation\_of\_transcription\_\_DNA-dependent | NDN | 575 | 4 | 1.186473 | -0.355708 | 407 | 357.87 | 0.879287 |
| GO:0006355\_regulation\_of\_transcription\_\_DNA-dependent | TRIM28 | 575 | 4 | 1.186473 | -0.355708 | 407 | 357.87 | 0.879287 |
| GO:0006355\_regulation\_of\_transcription\_\_DNA-dependent | CBX3 | 575 | 4 | 1.186473 | -0.355708 | 407 | 357.87 | 0.879287 |
| GO:0006355\_regulation\_of\_transcription\_\_DNA-dependent | RBBP7 | 575 | 4 | 1.186473 | -0.355708 | 407 | 357.87 | 0.879287 |
| GO:0009314\_response\_to\_radiation | HMGN1 | 98 | 1 | 1.740363 | -0.355092 | 408 | 359.11 | 0.880172 |
| GO:0048878\_chemical\_homeostasis | NDN | 254 | 2 | 1.342957 | -0.352946 | 409 | 359.94 | 0.880049 |
| GO:0048878\_chemical\_homeostasis | FKBP1A | 254 | 2 | 1.342957 | -0.352946 | 409 | 359.94 | 0.880049 |
| GO:0001817\_regulation\_of\_cytokine\_production | HSPD1 | 99 | 1 | 1.722783 | -0.351813 | 411 | 361.53 | 0.879635 |
| GO:0060562\_epithelial\_tube\_morphogenesis | CFL1 | 99 | 1 | 1.722783 | -0.351813 | 411 | 361.53 | 0.879635 |
| GO:0009966\_regulation\_of\_signal\_transduction | HSPA5 | 256 | 2 | 1.332465 | -0.348976 | 412 | 361.96 | 0.878544 |
| GO:0009966\_regulation\_of\_signal\_transduction | PRDX1 | 256 | 2 | 1.332465 | -0.348976 | 412 | 361.96 | 0.878544 |
| GO:0010556\_regulation\_of\_macromolecule\_biosynthetic\_process | NDN | 745 | 5 | 1.144668 | -0.347877 | 413 | 362.5 | 0.877724 |
| GO:0010556\_regulation\_of\_macromolecule\_biosynthetic\_process | TRIM28 | 745 | 5 | 1.144668 | -0.347877 | 413 | 362.5 | 0.877724 |
| GO:0010556\_regulation\_of\_macromolecule\_biosynthetic\_process | CBX3 | 745 | 5 | 1.144668 | -0.347877 | 413 | 362.5 | 0.877724 |
| GO:0010556\_regulation\_of\_macromolecule\_biosynthetic\_process | RBBP7 | 745 | 5 | 1.144668 | -0.347877 | 413 | 362.5 | 0.877724 |
| GO:0010556\_regulation\_of\_macromolecule\_biosynthetic\_process | DDX5 | 745 | 5 | 1.144668 | -0.347877 | 413 | 362.5 | 0.877724 |
| GO:0009059\_macromolecule\_biosynthetic\_process | NDN | 910 | 6 | 1.124542 | -0.347866 | 414 | 362.74 | 0.876184 |
| GO:0009059\_macromolecule\_biosynthetic\_process | TRIM28 | 910 | 6 | 1.124542 | -0.347866 | 414 | 362.74 | 0.876184 |
| GO:0009059\_macromolecule\_biosynthetic\_process | CBX3 | 910 | 6 | 1.124542 | -0.347866 | 414 | 362.74 | 0.876184 |
| GO:0009059\_macromolecule\_biosynthetic\_process | RBBP7 | 910 | 6 | 1.124542 | -0.347866 | 414 | 362.74 | 0.876184 |
| GO:0009059\_macromolecule\_biosynthetic\_process | DDX5 | 910 | 6 | 1.124542 | -0.347866 | 414 | 362.74 | 0.876184 |
| GO:0009059\_macromolecule\_biosynthetic\_process | RPL29 | 910 | 6 | 1.124542 | -0.347866 | 414 | 362.74 | 0.876184 |
| GO:0030036\_actin\_cytoskeleton\_organization | CFL1 | 102 | 1 | 1.672113 | -0.342232 | 415 | 364.29 | 0.877807 |
| GO:0022008\_neurogenesis | PRPF19 | 423 | 3 | 1.209614 | -0.339790 | 416 | 364.47 | 0.876130 |
| GO:0022008\_neurogenesis | NDN | 423 | 3 | 1.209614 | -0.339790 | 416 | 364.47 | 0.876130 |
| GO:0022008\_neurogenesis | GJA1 | 423 | 3 | 1.209614 | -0.339790 | 416 | 364.47 | 0.876130 |
| GO:0009968\_negative\_regulation\_of\_signal\_transduction | HSPA5 | 103 | 1 | 1.655879 | -0.339121 | 417 | 365.85 | 0.877338 |
| GO:0050767\_regulation\_of\_neurogenesis | PRPF19 | 104 | 1 | 1.639957 | -0.336050 | 418 | 367.57 | 0.879354 |
| GO:0051252\_regulation\_of\_RNA\_metabolic\_process | NDN | 590 | 4 | 1.156309 | -0.335793 | 419 | 367.74 | 0.877661 |
| GO:0051252\_regulation\_of\_RNA\_metabolic\_process | TRIM28 | 590 | 4 | 1.156309 | -0.335793 | 419 | 367.74 | 0.877661 |
| GO:0051252\_regulation\_of\_RNA\_metabolic\_process | CBX3 | 590 | 4 | 1.156309 | -0.335793 | 419 | 367.74 | 0.877661 |
| GO:0051252\_regulation\_of\_RNA\_metabolic\_process | RBBP7 | 590 | 4 | 1.156309 | -0.335793 | 419 | 367.74 | 0.877661 |
| GO:0019222\_regulation\_of\_metabolic\_process | NDN | 1088 | 7 | 1.097324 | -0.335168 | 420 | 368.19 | 0.876643 |
| GO:0019222\_regulation\_of\_metabolic\_process | CFL1 | 1088 | 7 | 1.097324 | -0.335168 | 420 | 368.19 | 0.876643 |
| GO:0019222\_regulation\_of\_metabolic\_process | TRIM28 | 1088 | 7 | 1.097324 | -0.335168 | 420 | 368.19 | 0.876643 |
| GO:0019222\_regulation\_of\_metabolic\_process | CBX3 | 1088 | 7 | 1.097324 | -0.335168 | 420 | 368.19 | 0.876643 |
| GO:0019222\_regulation\_of\_metabolic\_process | FKBP1A | 1088 | 7 | 1.097324 | -0.335168 | 420 | 368.19 | 0.876643 |
| GO:0019222\_regulation\_of\_metabolic\_process | DDX5 | 1088 | 7 | 1.097324 | -0.335168 | 420 | 368.19 | 0.876643 |
| GO:0019222\_regulation\_of\_metabolic\_process | RBBP7 | 1088 | 7 | 1.097324 | -0.335168 | 420 | 368.19 | 0.876643 |
| GO:0019219\_regulation\_of\_nucleobase\_\_nucleoside\_\_nucleotide\_and\_nucleic\_acid\_metabolic\_process | NDN | 757 | 5 | 1.126523 | -0.333740 | 421 | 368.72 | 0.875819 |
| GO:0019219\_regulation\_of\_nucleobase\_\_nucleoside\_\_nucleotide\_and\_nucleic\_acid\_metabolic\_process | TRIM28 | 757 | 5 | 1.126523 | -0.333740 | 421 | 368.72 | 0.875819 |
| GO:0019219\_regulation\_of\_nucleobase\_\_nucleoside\_\_nucleotide\_and\_nucleic\_acid\_metabolic\_process | CBX3 | 757 | 5 | 1.126523 | -0.333740 | 421 | 368.72 | 0.875819 |
| GO:0019219\_regulation\_of\_nucleobase\_\_nucleoside\_\_nucleotide\_and\_nucleic\_acid\_metabolic\_process | RBBP7 | 757 | 5 | 1.126523 | -0.333740 | 421 | 368.72 | 0.875819 |
| GO:0019219\_regulation\_of\_nucleobase\_\_nucleoside\_\_nucleotide\_and\_nucleic\_acid\_metabolic\_process | DDX5 | 757 | 5 | 1.126523 | -0.333740 | 421 | 368.72 | 0.875819 |
| GO:0006351\_transcription\_\_DNA-dependent | NDN | 594 | 4 | 1.148522 | -0.330663 | 422 | 369.62 | 0.875877 |
| GO:0006351\_transcription\_\_DNA-dependent | TRIM28 | 594 | 4 | 1.148522 | -0.330663 | 422 | 369.62 | 0.875877 |
| GO:0006351\_transcription\_\_DNA-dependent | CBX3 | 594 | 4 | 1.148522 | -0.330663 | 422 | 369.62 | 0.875877 |
| GO:0006351\_transcription\_\_DNA-dependent | RBBP7 | 594 | 4 | 1.148522 | -0.330663 | 422 | 369.62 | 0.875877 |
| GO:0032774\_RNA\_biosynthetic\_process | NDN | 595 | 4 | 1.146592 | -0.329392 | 423 | 370.34 | 0.875508 |
| GO:0032774\_RNA\_biosynthetic\_process | TRIM28 | 595 | 4 | 1.146592 | -0.329392 | 423 | 370.34 | 0.875508 |
| GO:0032774\_RNA\_biosynthetic\_process | CBX3 | 595 | 4 | 1.146592 | -0.329392 | 423 | 370.34 | 0.875508 |
| GO:0032774\_RNA\_biosynthetic\_process | RBBP7 | 595 | 4 | 1.146592 | -0.329392 | 423 | 370.34 | 0.875508 |
| GO:0007166\_cell\_surface\_receptor\_linked\_signal\_transduction | HAX1 | 597 | 4 | 1.142751 | -0.326864 | 424 | 371.06 | 0.875142 |
| GO:0007166\_cell\_surface\_receptor\_linked\_signal\_transduction | NDN | 597 | 4 | 1.142751 | -0.326864 | 424 | 371.06 | 0.875142 |
| GO:0007166\_cell\_surface\_receptor\_linked\_signal\_transduction | FKBP1A | 597 | 4 | 1.142751 | -0.326864 | 424 | 371.06 | 0.875142 |
| GO:0007166\_cell\_surface\_receptor\_linked\_signal\_transduction | HSPA5 | 597 | 4 | 1.142751 | -0.326864 | 424 | 371.06 | 0.875142 |
| GO:0010604\_positive\_regulation\_of\_macromolecule\_metabolic\_process | CFL1 | 433 | 3 | 1.181678 | -0.324967 | 425 | 371.67 | 0.874518 |
| GO:0010604\_positive\_regulation\_of\_macromolecule\_metabolic\_process | TRIM28 | 433 | 3 | 1.181678 | -0.324967 | 425 | 371.67 | 0.874518 |
| GO:0010604\_positive\_regulation\_of\_macromolecule\_metabolic\_process | DDX5 | 433 | 3 | 1.181678 | -0.324967 | 425 | 371.67 | 0.874518 |
| GO:0051240\_positive\_regulation\_of\_multicellular\_organismal\_process | HSPD1 | 108 | 1 | 1.579218 | -0.324148 | 426 | 372.5 | 0.874413 |
| GO:0030029\_actin\_filament-based\_process | CFL1 | 109 | 1 | 1.564730 | -0.321265 | 427 | 373.23 | 0.874075 |
| GO:0010648\_negative\_regulation\_of\_cell\_communication | HSPA5 | 110 | 1 | 1.550505 | -0.318418 | 429 | 374.98 | 0.874079 |
| GO:0043010\_camera-type\_eye\_development | HMGN1 | 110 | 1 | 1.550505 | -0.318418 | 429 | 374.98 | 0.874079 |
| GO:0051171\_regulation\_of\_nitrogen\_compound\_metabolic\_process | NDN | 771 | 5 | 1.106067 | -0.317897 | 430 | 375.21 | 0.872581 |
| GO:0051171\_regulation\_of\_nitrogen\_compound\_metabolic\_process | TRIM28 | 771 | 5 | 1.106067 | -0.317897 | 430 | 375.21 | 0.872581 |
| GO:0051171\_regulation\_of\_nitrogen\_compound\_metabolic\_process | CBX3 | 771 | 5 | 1.106067 | -0.317897 | 430 | 375.21 | 0.872581 |
| GO:0051171\_regulation\_of\_nitrogen\_compound\_metabolic\_process | RBBP7 | 771 | 5 | 1.106067 | -0.317897 | 430 | 375.21 | 0.872581 |
| GO:0051171\_regulation\_of\_nitrogen\_compound\_metabolic\_process | DDX5 | 771 | 5 | 1.106067 | -0.317897 | 430 | 375.21 | 0.872581 |
| GO:0050896\_response\_to\_stimulus | HMGN1 | 1107 | 7 | 1.078490 | -0.316453 | 431 | 375.71 | 0.871717 |
| GO:0050896\_response\_to\_stimulus | RPA1 | 1107 | 7 | 1.078490 | -0.316453 | 431 | 375.71 | 0.871717 |
| GO:0050896\_response\_to\_stimulus | CFL1 | 1107 | 7 | 1.078490 | -0.316453 | 431 | 375.71 | 0.871717 |
| GO:0050896\_response\_to\_stimulus | GJA1 | 1107 | 7 | 1.078490 | -0.316453 | 431 | 375.71 | 0.871717 |
| GO:0050896\_response\_to\_stimulus | FKBP1A | 1107 | 7 | 1.078490 | -0.316453 | 431 | 375.71 | 0.871717 |
| GO:0050896\_response\_to\_stimulus | HSPA5 | 1107 | 7 | 1.078490 | -0.316453 | 431 | 375.71 | 0.871717 |
| GO:0050896\_response\_to\_stimulus | PRDX1 | 1107 | 7 | 1.078490 | -0.316453 | 431 | 375.71 | 0.871717 |
| GO:0006464\_protein\_modification\_process | CFL1 | 439 | 3 | 1.165528 | -0.316386 | 432 | 375.97 | 0.870301 |
| GO:0006464\_protein\_modification\_process | TRIM28 | 439 | 3 | 1.165528 | -0.316386 | 432 | 375.97 | 0.870301 |
| GO:0006464\_protein\_modification\_process | FKBP1A | 439 | 3 | 1.165528 | -0.316386 | 432 | 375.97 | 0.870301 |
| GO:0051249\_regulation\_of\_lymphocyte\_activation | HSPD1 | 112 | 1 | 1.522817 | -0.312827 | 433 | 377.64 | 0.872148 |
| GO:0031325\_positive\_regulation\_of\_cellular\_metabolic\_process | CFL1 | 442 | 3 | 1.157617 | -0.312182 | 434 | 378.06 | 0.871106 |
| GO:0031325\_positive\_regulation\_of\_cellular\_metabolic\_process | TRIM28 | 442 | 3 | 1.157617 | -0.312182 | 434 | 378.06 | 0.871106 |
| GO:0031325\_positive\_regulation\_of\_cellular\_metabolic\_process | DDX5 | 442 | 3 | 1.157617 | -0.312182 | 434 | 378.06 | 0.871106 |
| GO:0010468\_regulation\_of\_gene\_expression | NDN | 778 | 5 | 1.096115 | -0.310230 | 435 | 378.54 | 0.870207 |
| GO:0010468\_regulation\_of\_gene\_expression | TRIM28 | 778 | 5 | 1.096115 | -0.310230 | 435 | 378.54 | 0.870207 |
| GO:0010468\_regulation\_of\_gene\_expression | CBX3 | 778 | 5 | 1.096115 | -0.310230 | 435 | 378.54 | 0.870207 |
| GO:0010468\_regulation\_of\_gene\_expression | RBBP7 | 778 | 5 | 1.096115 | -0.310230 | 435 | 378.54 | 0.870207 |
| GO:0010468\_regulation\_of\_gene\_expression | DDX5 | 778 | 5 | 1.096115 | -0.310230 | 435 | 378.54 | 0.870207 |
| GO:0048646\_anatomical\_structure\_formation\_involved\_in\_morphogenesis | CFL1 | 277 | 2 | 1.231448 | -0.310190 | 436 | 379.14 | 0.869587 |
| GO:0048646\_anatomical\_structure\_formation\_involved\_in\_morphogenesis | FKBP1A | 277 | 2 | 1.231448 | -0.310190 | 436 | 379.14 | 0.869587 |
| GO:0040008\_regulation\_of\_growth | NDN | 113 | 1 | 1.509341 | -0.310082 | 437 | 379.87 | 0.869268 |
| GO:0000165\_MAPKKK\_cascade | PRDX1 | 114 | 1 | 1.496101 | -0.307370 | 439 | 381.14 | 0.868200 |
| GO:0009607\_response\_to\_biotic\_stimulus | HSPA5 | 114 | 1 | 1.496101 | -0.307370 | 439 | 381.14 | 0.868200 |
| GO:0065009\_regulation\_of\_molecular\_function | CYCS | 279 | 2 | 1.222620 | -0.306753 | 440 | 381.7 | 0.867500 |
| GO:0065009\_regulation\_of\_molecular\_function | FKBP1A | 279 | 2 | 1.222620 | -0.306753 | 440 | 381.7 | 0.867500 |
| GO:0010926\_anatomical\_structure\_formation | CFL1 | 447 | 3 | 1.144668 | -0.305297 | 441 | 382.0 | 0.866213 |
| GO:0010926\_anatomical\_structure\_formation | TUBB5 | 447 | 3 | 1.144668 | -0.305297 | 441 | 382.0 | 0.866213 |
| GO:0010926\_anatomical\_structure\_formation | FKBP1A | 447 | 3 | 1.144668 | -0.305297 | 441 | 382.0 | 0.866213 |
| GO:0046483\_heterocycle\_metabolic\_process | IMPDH2 | 116 | 1 | 1.470307 | -0.302043 | 443 | 384.1 | 0.867043 |
| GO:0080134\_regulation\_of\_response\_to\_stress | PRDX1 | 116 | 1 | 1.470307 | -0.302043 | 443 | 384.1 | 0.867043 |
| GO:0043933\_macromolecular\_complex\_subunit\_organization | CFL1 | 117 | 1 | 1.457740 | -0.299426 | 444 | 385.44 | 0.868108 |
| GO:0009653\_anatomical\_structure\_morphogenesis | HMGN1 | 958 | 6 | 1.068198 | -0.298078 | 445 | 385.88 | 0.867146 |
| GO:0009653\_anatomical\_structure\_morphogenesis | NDN | 958 | 6 | 1.068198 | -0.298078 | 445 | 385.88 | 0.867146 |
| GO:0009653\_anatomical\_structure\_morphogenesis | TRIM28 | 958 | 6 | 1.068198 | -0.298078 | 445 | 385.88 | 0.867146 |
| GO:0009653\_anatomical\_structure\_morphogenesis | CFL1 | 958 | 6 | 1.068198 | -0.298078 | 445 | 385.88 | 0.867146 |
| GO:0009653\_anatomical\_structure\_morphogenesis | GJA1 | 958 | 6 | 1.068198 | -0.298078 | 445 | 385.88 | 0.867146 |
| GO:0009653\_anatomical\_structure\_morphogenesis | FKBP1A | 958 | 6 | 1.068198 | -0.298078 | 445 | 385.88 | 0.867146 |
| GO:0007399\_nervous\_system\_development | PRPF19 | 621 | 4 | 1.098587 | -0.297908 | 446 | 386.14 | 0.865785 |
| GO:0007399\_nervous\_system\_development | NDN | 621 | 4 | 1.098587 | -0.297908 | 446 | 386.14 | 0.865785 |
| GO:0007399\_nervous\_system\_development | CFL1 | 621 | 4 | 1.098587 | -0.297908 | 446 | 386.14 | 0.865785 |
| GO:0007399\_nervous\_system\_development | GJA1 | 621 | 4 | 1.098587 | -0.297908 | 446 | 386.14 | 0.865785 |
| GO:0051960\_regulation\_of\_nervous\_system\_development | PRPF19 | 118 | 1 | 1.445386 | -0.296841 | 447 | 387.0 | 0.865772 |
| GO:0007417\_central\_nervous\_system\_development | PRPF19 | 287 | 2 | 1.188540 | -0.293421 | 448 | 388.24 | 0.866607 |
| GO:0007417\_central\_nervous\_system\_development | NDN | 287 | 2 | 1.188540 | -0.293421 | 448 | 388.24 | 0.866607 |
| GO:0014706\_striated\_muscle\_tissue\_development | GJA1 | 120 | 1 | 1.421296 | -0.291759 | 449 | 388.75 | 0.865813 |
| GO:0009893\_positive\_regulation\_of\_metabolic\_process | CFL1 | 458 | 3 | 1.117176 | -0.290679 | 451 | 389.3 | 0.863193 |
| GO:0009893\_positive\_regulation\_of\_metabolic\_process | TRIM28 | 458 | 3 | 1.117176 | -0.290679 | 451 | 389.3 | 0.863193 |
| GO:0009893\_positive\_regulation\_of\_metabolic\_process | DDX5 | 458 | 3 | 1.117176 | -0.290679 | 451 | 389.3 | 0.863193 |
| GO:0043412\_biopolymer\_modification | CFL1 | 458 | 3 | 1.117176 | -0.290679 | 451 | 389.3 | 0.863193 |
| GO:0043412\_biopolymer\_modification | TRIM28 | 458 | 3 | 1.117176 | -0.290679 | 451 | 389.3 | 0.863193 |
| GO:0043412\_biopolymer\_modification | FKBP1A | 458 | 3 | 1.117176 | -0.290679 | 451 | 389.3 | 0.863193 |
| GO:0002694\_regulation\_of\_leukocyte\_activation | HSPD1 | 121 | 1 | 1.409550 | -0.289262 | 455 | 390.77 | 0.858835 |
| GO:0006917\_induction\_of\_apoptosis | PRDX1 | 121 | 1 | 1.409550 | -0.289262 | 455 | 390.77 | 0.858835 |
| GO:0012502\_induction\_of\_programmed\_cell\_death | PRDX1 | 121 | 1 | 1.409550 | -0.289262 | 455 | 390.77 | 0.858835 |
| GO:0051726\_regulation\_of\_cell\_cycle | HSPA8 | 121 | 1 | 1.409550 | -0.289262 | 455 | 390.77 | 0.858835 |
| GO:0001816\_cytokine\_production | HSPD1 | 122 | 1 | 1.397996 | -0.286794 | 460 | 393.08 | 0.854522 |
| GO:0002252\_immune\_effector\_process | PRDX1 | 122 | 1 | 1.397996 | -0.286794 | 460 | 393.08 | 0.854522 |
| GO:0030001\_metal\_ion\_transport | FKBP1A | 122 | 1 | 1.397996 | -0.286794 | 460 | 393.08 | 0.854522 |
| GO:0050865\_regulation\_of\_cell\_activation | HSPD1 | 122 | 1 | 1.397996 | -0.286794 | 460 | 393.08 | 0.854522 |
| GO:0060284\_regulation\_of\_cell\_development | PRPF19 | 122 | 1 | 1.397996 | -0.286794 | 460 | 393.08 | 0.854522 |
| GO:0031326\_regulation\_of\_cellular\_biosynthetic\_process | NDN | 812 | 5 | 1.050219 | -0.275275 | 461 | 396.64 | 0.860390 |
| GO:0031326\_regulation\_of\_cellular\_biosynthetic\_process | TRIM28 | 812 | 5 | 1.050219 | -0.275275 | 461 | 396.64 | 0.860390 |
| GO:0031326\_regulation\_of\_cellular\_biosynthetic\_process | CBX3 | 812 | 5 | 1.050219 | -0.275275 | 461 | 396.64 | 0.860390 |
| GO:0031326\_regulation\_of\_cellular\_biosynthetic\_process | DDX5 | 812 | 5 | 1.050219 | -0.275275 | 461 | 396.64 | 0.860390 |
| GO:0031326\_regulation\_of\_cellular\_biosynthetic\_process | RBBP7 | 812 | 5 | 1.050219 | -0.275275 | 461 | 396.64 | 0.860390 |
| GO:0009887\_organ\_morphogenesis | HMGN1 | 642 | 4 | 1.062651 | -0.274549 | 462 | 397.14 | 0.859610 |
| GO:0009887\_organ\_morphogenesis | CFL1 | 642 | 4 | 1.062651 | -0.274549 | 462 | 397.14 | 0.859610 |
| GO:0009887\_organ\_morphogenesis | GJA1 | 642 | 4 | 1.062651 | -0.274549 | 462 | 397.14 | 0.859610 |
| GO:0009887\_organ\_morphogenesis | FKBP1A | 642 | 4 | 1.062651 | -0.274549 | 462 | 397.14 | 0.859610 |
| GO:0045597\_positive\_regulation\_of\_cell\_differentiation | PRPF19 | 128 | 1 | 1.332465 | -0.272559 | 464 | 398.18 | 0.858147 |
| GO:0060537\_muscle\_tissue\_development | GJA1 | 128 | 1 | 1.332465 | -0.272559 | 464 | 398.18 | 0.858147 |
| GO:0009889\_regulation\_of\_biosynthetic\_process | NDN | 815 | 5 | 1.046353 | -0.272364 | 465 | 398.46 | 0.856903 |
| GO:0009889\_regulation\_of\_biosynthetic\_process | TRIM28 | 815 | 5 | 1.046353 | -0.272364 | 465 | 398.46 | 0.856903 |
| GO:0009889\_regulation\_of\_biosynthetic\_process | CBX3 | 815 | 5 | 1.046353 | -0.272364 | 465 | 398.46 | 0.856903 |
| GO:0009889\_regulation\_of\_biosynthetic\_process | DDX5 | 815 | 5 | 1.046353 | -0.272364 | 465 | 398.46 | 0.856903 |
| GO:0009889\_regulation\_of\_biosynthetic\_process | RBBP7 | 815 | 5 | 1.046353 | -0.272364 | 465 | 398.46 | 0.856903 |
| GO:0044057\_regulation\_of\_system\_process | GJA1 | 133 | 1 | 1.282373 | -0.261402 | 466 | 402.12 | 0.862918 |
| GO:0001654\_eye\_development | HMGN1 | 136 | 1 | 1.254085 | -0.254993 | 467 | 404.99 | 0.867216 |
| GO:0007169\_transmembrane\_receptor\_protein\_tyrosine\_kinase\_signaling\_pathway | NDN | 139 | 1 | 1.227018 | -0.248786 | 468 | 406.47 | 0.868526 |
| GO:0035239\_tube\_morphogenesis | CFL1 | 143 | 1 | 1.192696 | -0.240809 | 469 | 408.38 | 0.870746 |
| GO:0045596\_negative\_regulation\_of\_cell\_differentiation | PRPF19 | 144 | 1 | 1.184414 | -0.238866 | 470 | 409.35 | 0.870957 |
| GO:0006812\_cation\_transport | FKBP1A | 146 | 1 | 1.168189 | -0.235039 | 471 | 410.8 | 0.872187 |
| GO:0010646\_regulation\_of\_cell\_communication | HSPA5 | 330 | 2 | 1.033670 | -0.231711 | 472 | 412.31 | 0.873538 |
| GO:0010646\_regulation\_of\_cell\_communication | PRDX1 | 330 | 2 | 1.033670 | -0.231711 | 472 | 412.31 | 0.873538 |
| GO:0002684\_positive\_regulation\_of\_immune\_system\_process | HSPD1 | 148 | 1 | 1.152402 | -0.231290 | 474 | 413.04 | 0.871392 |
| GO:0043085\_positive\_regulation\_of\_catalytic\_activity | CYCS | 148 | 1 | 1.152402 | -0.231290 | 474 | 413.04 | 0.871392 |
| GO:0007517\_muscle\_organ\_development | GJA1 | 153 | 1 | 1.114742 | -0.222241 | 475 | 415.82 | 0.875411 |
| GO:0045941\_positive\_regulation\_of\_transcription | TRIM28 | 338 | 2 | 1.009204 | -0.221838 | 476 | 416.0 | 0.873950 |
| GO:0045941\_positive\_regulation\_of\_transcription | DDX5 | 338 | 2 | 1.009204 | -0.221838 | 476 | 416.0 | 0.873950 |
| GO:0050793\_regulation\_of\_developmental\_process | PRPF19 | 703 | 4 | 0.970444 | -0.215911 | 477 | 419.16 | 0.878742 |
| GO:0050793\_regulation\_of\_developmental\_process | CYCS | 703 | 4 | 0.970444 | -0.215911 | 477 | 419.16 | 0.878742 |
| GO:0050793\_regulation\_of\_developmental\_process | GJA1 | 703 | 4 | 0.970444 | -0.215911 | 477 | 419.16 | 0.878742 |
| GO:0050793\_regulation\_of\_developmental\_process | PRDX1 | 703 | 4 | 0.970444 | -0.215911 | 477 | 419.16 | 0.878742 |
| GO:0007409\_axonogenesis | NDN | 158 | 1 | 1.079466 | -0.213629 | 479 | 420.92 | 0.878747 |
| GO:0048514\_blood\_vessel\_morphogenesis | GJA1 | 158 | 1 | 1.079466 | -0.213629 | 479 | 420.92 | 0.878747 |
| GO:0010628\_positive\_regulation\_of\_gene\_expression | TRIM28 | 346 | 2 | 0.985870 | -0.212405 | 480 | 421.3 | 0.877708 |
| GO:0010628\_positive\_regulation\_of\_gene\_expression | DDX5 | 346 | 2 | 0.985870 | -0.212405 | 480 | 421.3 | 0.877708 |
| GO:0051128\_regulation\_of\_cellular\_component\_organization | CFL1 | 160 | 1 | 1.065972 | -0.210301 | 481 | 421.79 | 0.876902 |
| GO:0009628\_response\_to\_abiotic\_stimulus | HMGN1 | 162 | 1 | 1.052812 | -0.207036 | 482 | 422.8 | 0.877178 |
| GO:0045935\_positive\_regulation\_of\_nucleobase\_\_nucleoside\_\_nucleotide\_and\_nucleic\_acid\_metabolic\_process | TRIM28 | 352 | 2 | 0.969066 | -0.205605 | 483 | 423.15 | 0.876087 |
| GO:0045935\_positive\_regulation\_of\_nucleobase\_\_nucleoside\_\_nucleotide\_and\_nucleic\_acid\_metabolic\_process | DDX5 | 352 | 2 | 0.969066 | -0.205605 | 483 | 423.15 | 0.876087 |
| GO:0042325\_regulation\_of\_phosphorylation | FKBP1A | 164 | 1 | 1.039973 | -0.203834 | 484 | 424.41 | 0.876880 |
| GO:0019220\_regulation\_of\_phosphate\_metabolic\_process | FKBP1A | 165 | 1 | 1.033670 | -0.202255 | 486 | 425.86 | 0.876255 |
| GO:0051174\_regulation\_of\_phosphorus\_metabolic\_process | FKBP1A | 165 | 1 | 1.033670 | -0.202255 | 486 | 425.86 | 0.876255 |
| GO:0030182\_neuron\_differentiation | PRPF19 | 356 | 2 | 0.958177 | -0.201197 | 487 | 426.19 | 0.875133 |
| GO:0030182\_neuron\_differentiation | NDN | 356 | 2 | 0.958177 | -0.201197 | 487 | 426.19 | 0.875133 |
| GO:0042981\_regulation\_of\_apoptosis | CYCS | 360 | 2 | 0.947531 | -0.196887 | 488 | 428.31 | 0.877684 |
| GO:0042981\_regulation\_of\_apoptosis | PRDX1 | 360 | 2 | 0.947531 | -0.196887 | 488 | 428.31 | 0.877684 |
| GO:0051173\_positive\_regulation\_of\_nitrogen\_compound\_metabolic\_process | TRIM28 | 361 | 2 | 0.944906 | -0.195824 | 489 | 428.73 | 0.876748 |
| GO:0051173\_positive\_regulation\_of\_nitrogen\_compound\_metabolic\_process | DDX5 | 361 | 2 | 0.944906 | -0.195824 | 489 | 428.73 | 0.876748 |
| GO:0048812\_neuron\_projection\_morphogenesis | NDN | 170 | 1 | 1.003268 | -0.194582 | 490 | 429.77 | 0.877082 |
| GO:0010941\_regulation\_of\_cell\_death | CYCS | 365 | 2 | 0.934551 | -0.191632 | 492 | 431.37 | 0.876768 |
| GO:0010941\_regulation\_of\_cell\_death | PRDX1 | 365 | 2 | 0.934551 | -0.191632 | 492 | 431.37 | 0.876768 |
| GO:0043067\_regulation\_of\_programmed\_cell\_death | CYCS | 365 | 2 | 0.934551 | -0.191632 | 492 | 431.37 | 0.876768 |
| GO:0043067\_regulation\_of\_programmed\_cell\_death | PRDX1 | 365 | 2 | 0.934551 | -0.191632 | 492 | 431.37 | 0.876768 |
| GO:0007600\_sensory\_perception | NDN | 172 | 1 | 0.991602 | -0.191612 | 494 | 432.14 | 0.874777 |
| GO:0009611\_response\_to\_wounding | GJA1 | 172 | 1 | 0.991602 | -0.191612 | 494 | 432.14 | 0.874777 |
| GO:0044093\_positive\_regulation\_of\_molecular\_function | CYCS | 173 | 1 | 0.985870 | -0.190148 | 496 | 433.31 | 0.873609 |
| GO:0048667\_cell\_morphogenesis\_involved\_in\_neuron\_differentiation | NDN | 173 | 1 | 0.985870 | -0.190148 | 496 | 433.31 | 0.873609 |
| GO:0048858\_cell\_projection\_morphogenesis | NDN | 176 | 1 | 0.969066 | -0.185834 | 497 | 437.07 | 0.879416 |
| GO:0010557\_positive\_regulation\_of\_macromolecule\_biosynthetic\_process | TRIM28 | 371 | 2 | 0.919437 | -0.185517 | 498 | 437.27 | 0.878052 |
| GO:0010557\_positive\_regulation\_of\_macromolecule\_biosynthetic\_process | DDX5 | 371 | 2 | 0.919437 | -0.185517 | 498 | 437.27 | 0.878052 |
| GO:0019752\_carboxylic\_acid\_metabolic\_process | MAT2A | 181 | 1 | 0.942296 | -0.178903 | 500 | 441.37 | 0.882740 |
| GO:0043436\_oxoacid\_metabolic\_process | MAT2A | 181 | 1 | 0.942296 | -0.178903 | 500 | 441.37 | 0.882740 |
| GO:0006082\_organic\_acid\_metabolic\_process | MAT2A | 182 | 1 | 0.937118 | -0.177554 | 501 | 442.3 | 0.882834 |
| GO:0042180\_cellular\_ketone\_metabolic\_process | MAT2A | 183 | 1 | 0.931998 | -0.176217 | 502 | 442.87 | 0.882211 |
| GO:0032990\_cell\_part\_morphogenesis | NDN | 184 | 1 | 0.926932 | -0.174892 | 503 | 443.82 | 0.882346 |
| GO:0006811\_ion\_transport | FKBP1A | 186 | 1 | 0.916965 | -0.172277 | 506 | 446.04 | 0.881502 |
| GO:0007155\_cell\_adhesion | RPL29 | 186 | 1 | 0.916965 | -0.172277 | 506 | 446.04 | 0.881502 |
| GO:0022610\_biological\_adhesion | RPL29 | 186 | 1 | 0.916965 | -0.172277 | 506 | 446.04 | 0.881502 |
| GO:0006952\_defense\_response | PRDX1 | 187 | 1 | 0.912062 | -0.170987 | 507 | 446.59 | 0.880848 |
| GO:0031328\_positive\_regulation\_of\_cellular\_biosynthetic\_process | TRIM28 | 387 | 2 | 0.881424 | -0.170165 | 508 | 446.81 | 0.879547 |
| GO:0031328\_positive\_regulation\_of\_cellular\_biosynthetic\_process | DDX5 | 387 | 2 | 0.881424 | -0.170165 | 508 | 446.81 | 0.879547 |
| GO:0009891\_positive\_regulation\_of\_biosynthetic\_process | TRIM28 | 388 | 2 | 0.879152 | -0.169249 | 509 | 447.4 | 0.878978 |
| GO:0009891\_positive\_regulation\_of\_biosynthetic\_process | DDX5 | 388 | 2 | 0.879152 | -0.169249 | 509 | 447.4 | 0.878978 |
| GO:0042127\_regulation\_of\_cell\_proliferation | HMGN1 | 393 | 2 | 0.867967 | -0.164746 | 510 | 448.34 | 0.879098 |
| GO:0042127\_regulation\_of\_cell\_proliferation | RPA1 | 393 | 2 | 0.867967 | -0.164746 | 510 | 448.34 | 0.879098 |
| GO:0051239\_regulation\_of\_multicellular\_organismal\_process | PRPF19 | 587 | 3 | 0.871664 | -0.162646 | 511 | 448.8 | 0.878278 |
| GO:0051239\_regulation\_of\_multicellular\_organismal\_process | GJA1 | 587 | 3 | 0.871664 | -0.162646 | 511 | 448.8 | 0.878278 |
| GO:0051239\_regulation\_of\_multicellular\_organismal\_process | HSPD1 | 587 | 3 | 0.871664 | -0.162646 | 511 | 448.8 | 0.878278 |
| GO:0031175\_neuron\_projection\_development | NDN | 197 | 1 | 0.865764 | -0.158692 | 512 | 452.97 | 0.884707 |
| GO:0001568\_blood\_vessel\_development | GJA1 | 203 | 1 | 0.840175 | -0.151809 | 513 | 455.93 | 0.888752 |
| GO:0022607\_cellular\_component\_assembly | TUBB5 | 204 | 1 | 0.836057 | -0.150696 | 514 | 456.57 | 0.888268 |
| GO:0006955\_immune\_response | PRDX1 | 205 | 1 | 0.831978 | -0.149592 | 516 | 457.33 | 0.886298 |
| GO:0007243\_protein\_kinase\_cascade | PRDX1 | 205 | 1 | 0.831978 | -0.149592 | 516 | 457.33 | 0.886298 |
| GO:0001944\_vasculature\_development | GJA1 | 208 | 1 | 0.819979 | -0.146336 | 518 | 458.26 | 0.884672 |
| GO:0008284\_positive\_regulation\_of\_cell\_proliferation | RPA1 | 208 | 1 | 0.819979 | -0.146336 | 518 | 458.26 | 0.884672 |
| GO:0032502\_developmental\_process | HMGN1 | 2060 | 11 | 0.910734 | -0.137818 | 519 | 460.83 | 0.887919 |
| GO:0032502\_developmental\_process | RPA1 | 2060 | 11 | 0.910734 | -0.137818 | 519 | 460.83 | 0.887919 |
| GO:0032502\_developmental\_process | PRPF19 | 2060 | 11 | 0.910734 | -0.137818 | 519 | 460.83 | 0.887919 |
| GO:0032502\_developmental\_process | NDN | 2060 | 11 | 0.910734 | -0.137818 | 519 | 460.83 | 0.887919 |
| GO:0032502\_developmental\_process | CYCS | 2060 | 11 | 0.910734 | -0.137818 | 519 | 460.83 | 0.887919 |
| GO:0032502\_developmental\_process | TRIM28 | 2060 | 11 | 0.910734 | -0.137818 | 519 | 460.83 | 0.887919 |
| GO:0032502\_developmental\_process | CFL1 | 2060 | 11 | 0.910734 | -0.137818 | 519 | 460.83 | 0.887919 |
| GO:0032502\_developmental\_process | GJA1 | 2060 | 11 | 0.910734 | -0.137818 | 519 | 460.83 | 0.887919 |
| GO:0032502\_developmental\_process | FKBP1A | 2060 | 11 | 0.910734 | -0.137818 | 519 | 460.83 | 0.887919 |
| GO:0032502\_developmental\_process | SFRS1 | 2060 | 11 | 0.910734 | -0.137818 | 519 | 460.83 | 0.887919 |
| GO:0032502\_developmental\_process | PRDX1 | 2060 | 11 | 0.910734 | -0.137818 | 519 | 460.83 | 0.887919 |
| GO:0006915\_apoptosis | CYCS | 427 | 2 | 0.798855 | -0.137160 | 520 | 461.06 | 0.886654 |
| GO:0006915\_apoptosis | PRDX1 | 427 | 2 | 0.798855 | -0.137160 | 520 | 461.06 | 0.886654 |
| GO:0048583\_regulation\_of\_response\_to\_stimulus | PRDX1 | 217 | 1 | 0.785970 | -0.137044 | 521 | 461.82 | 0.886411 |
| GO:0007423\_sensory\_organ\_development | HMGN1 | 219 | 1 | 0.778792 | -0.135072 | 522 | 463.18 | 0.887318 |
| GO:0012501\_programmed\_cell\_death | CYCS | 433 | 2 | 0.787785 | -0.132795 | 523 | 464.31 | 0.887782 |
| GO:0012501\_programmed\_cell\_death | PRDX1 | 433 | 2 | 0.787785 | -0.132795 | 523 | 464.31 | 0.887782 |
| GO:0006357\_regulation\_of\_transcription\_from\_RNA\_polymerase\_II\_promoter | TRIM28 | 435 | 2 | 0.784163 | -0.131371 | 524 | 464.53 | 0.886508 |
| GO:0006357\_regulation\_of\_transcription\_from\_RNA\_polymerase\_II\_promoter | RBBP7 | 435 | 2 | 0.784163 | -0.131371 | 524 | 464.53 | 0.886508 |
| GO:0002682\_regulation\_of\_immune\_system\_process | HSPD1 | 228 | 1 | 0.748051 | -0.126583 | 525 | 466.27 | 0.888133 |
| GO:0006366\_transcription\_from\_RNA\_polymerase\_II\_promoter | TRIM28 | 444 | 2 | 0.768268 | -0.125146 | 527 | 467.68 | 0.887438 |
| GO:0006366\_transcription\_from\_RNA\_polymerase\_II\_promoter | RBBP7 | 444 | 2 | 0.768268 | -0.125146 | 527 | 467.68 | 0.887438 |
| GO:0008219\_cell\_death | CYCS | 444 | 2 | 0.768268 | -0.125146 | 527 | 467.68 | 0.887438 |
| GO:0008219\_cell\_death | PRDX1 | 444 | 2 | 0.768268 | -0.125146 | 527 | 467.68 | 0.887438 |
| GO:0050790\_regulation\_of\_catalytic\_activity | CYCS | 233 | 1 | 0.731998 | -0.122127 | 529 | 469.02 | 0.886616 |
| GO:0050890\_cognition | NDN | 233 | 1 | 0.731998 | -0.122127 | 529 | 469.02 | 0.886616 |
| GO:0016265\_death | CYCS | 450 | 2 | 0.758025 | -0.121159 | 530 | 469.85 | 0.886509 |
| GO:0016265\_death | PRDX1 | 450 | 2 | 0.758025 | -0.121159 | 530 | 469.85 | 0.886509 |
| GO:0044085\_cellular\_component\_biogenesis | TUBB5 | 237 | 1 | 0.719644 | -0.118688 | 531 | 471.5 | 0.887947 |
| GO:0007275\_multicellular\_organismal\_development | HMGN1 | 1760 | 9 | 0.872159 | -0.117856 | 532 | 471.68 | 0.886617 |
| GO:0007275\_multicellular\_organismal\_development | RPA1 | 1760 | 9 | 0.872159 | -0.117856 | 532 | 471.68 | 0.886617 |
| GO:0007275\_multicellular\_organismal\_development | PRPF19 | 1760 | 9 | 0.872159 | -0.117856 | 532 | 471.68 | 0.886617 |
| GO:0007275\_multicellular\_organismal\_development | NDN | 1760 | 9 | 0.872159 | -0.117856 | 532 | 471.68 | 0.886617 |
| GO:0007275\_multicellular\_organismal\_development | TRIM28 | 1760 | 9 | 0.872159 | -0.117856 | 532 | 471.68 | 0.886617 |
| GO:0007275\_multicellular\_organismal\_development | CFL1 | 1760 | 9 | 0.872159 | -0.117856 | 532 | 471.68 | 0.886617 |
| GO:0007275\_multicellular\_organismal\_development | GJA1 | 1760 | 9 | 0.872159 | -0.117856 | 532 | 471.68 | 0.886617 |
| GO:0007275\_multicellular\_organismal\_development | FKBP1A | 1760 | 9 | 0.872159 | -0.117856 | 532 | 471.68 | 0.886617 |
| GO:0007275\_multicellular\_organismal\_development | SFRS1 | 1760 | 9 | 0.872159 | -0.117856 | 532 | 471.68 | 0.886617 |
| GO:0030154\_cell\_differentiation | PRPF19 | 1060 | 5 | 0.804507 | -0.108387 | 533 | 474.29 | 0.889850 |
| GO:0030154\_cell\_differentiation | NDN | 1060 | 5 | 0.804507 | -0.108387 | 533 | 474.29 | 0.889850 |
| GO:0030154\_cell\_differentiation | CFL1 | 1060 | 5 | 0.804507 | -0.108387 | 533 | 474.29 | 0.889850 |
| GO:0030154\_cell\_differentiation | TRIM28 | 1060 | 5 | 0.804507 | -0.108387 | 533 | 474.29 | 0.889850 |
| GO:0030154\_cell\_differentiation | GJA1 | 1060 | 5 | 0.804507 | -0.108387 | 533 | 474.29 | 0.889850 |
| GO:0048731\_system\_development | HMGN1 | 1609 | 8 | 0.848008 | -0.107637 | 534 | 475.01 | 0.889532 |
| GO:0048731\_system\_development | PRPF19 | 1609 | 8 | 0.848008 | -0.107637 | 534 | 475.01 | 0.889532 |
| GO:0048731\_system\_development | RPA1 | 1609 | 8 | 0.848008 | -0.107637 | 534 | 475.01 | 0.889532 |
| GO:0048731\_system\_development | NDN | 1609 | 8 | 0.848008 | -0.107637 | 534 | 475.01 | 0.889532 |
| GO:0048731\_system\_development | TRIM28 | 1609 | 8 | 0.848008 | -0.107637 | 534 | 475.01 | 0.889532 |
| GO:0048731\_system\_development | CFL1 | 1609 | 8 | 0.848008 | -0.107637 | 534 | 475.01 | 0.889532 |
| GO:0048731\_system\_development | GJA1 | 1609 | 8 | 0.848008 | -0.107637 | 534 | 475.01 | 0.889532 |
| GO:0048731\_system\_development | FKBP1A | 1609 | 8 | 0.848008 | -0.107637 | 534 | 475.01 | 0.889532 |
| GO:0007267\_cell-cell\_signaling | GJA1 | 252 | 1 | 0.676808 | -0.106713 | 535 | 475.64 | 0.889047 |
| GO:0030097\_hemopoiesis | RPA1 | 253 | 1 | 0.674133 | -0.105964 | 536 | 476.38 | 0.888769 |
| GO:0048666\_neuron\_development | NDN | 262 | 1 | 0.650975 | -0.099470 | 537 | 479.01 | 0.892011 |
| GO:0030030\_cell\_projection\_organization | NDN | 263 | 1 | 0.648500 | -0.098775 | 538 | 479.67 | 0.891580 |
| GO:0032501\_multicellular\_organismal\_process | HMGN1 | 2183 | 11 | 0.859419 | -0.090143 | 539 | 483.68 | 0.897365 |
| GO:0032501\_multicellular\_organismal\_process | RPA1 | 2183 | 11 | 0.859419 | -0.090143 | 539 | 483.68 | 0.897365 |
| GO:0032501\_multicellular\_organismal\_process | PRPF19 | 2183 | 11 | 0.859419 | -0.090143 | 539 | 483.68 | 0.897365 |
| GO:0032501\_multicellular\_organismal\_process | NDN | 2183 | 11 | 0.859419 | -0.090143 | 539 | 483.68 | 0.897365 |
| GO:0032501\_multicellular\_organismal\_process | TRIM28 | 2183 | 11 | 0.859419 | -0.090143 | 539 | 483.68 | 0.897365 |
| GO:0032501\_multicellular\_organismal\_process | CFL1 | 2183 | 11 | 0.859419 | -0.090143 | 539 | 483.68 | 0.897365 |
| GO:0032501\_multicellular\_organismal\_process | GJA1 | 2183 | 11 | 0.859419 | -0.090143 | 539 | 483.68 | 0.897365 |
| GO:0032501\_multicellular\_organismal\_process | FKBP1A | 2183 | 11 | 0.859419 | -0.090143 | 539 | 483.68 | 0.897365 |
| GO:0032501\_multicellular\_organismal\_process | HSPD1 | 2183 | 11 | 0.859419 | -0.090143 | 539 | 483.68 | 0.897365 |
| GO:0032501\_multicellular\_organismal\_process | SFRS1 | 2183 | 11 | 0.859419 | -0.090143 | 539 | 483.68 | 0.897365 |
| GO:0032501\_multicellular\_organismal\_process | RPL29 | 2183 | 11 | 0.859419 | -0.090143 | 539 | 483.68 | 0.897365 |
| GO:0048534\_hemopoietic\_or\_lymphoid\_organ\_development | RPA1 | 277 | 1 | 0.615724 | -0.089585 | 540 | 484.45 | 0.897130 |
| GO:0048869\_cellular\_developmental\_process | PRPF19 | 1113 | 5 | 0.766197 | -0.087360 | 541 | 485.8 | 0.897967 |
| GO:0048869\_cellular\_developmental\_process | NDN | 1113 | 5 | 0.766197 | -0.087360 | 541 | 485.8 | 0.897967 |
| GO:0048869\_cellular\_developmental\_process | CFL1 | 1113 | 5 | 0.766197 | -0.087360 | 541 | 485.8 | 0.897967 |
| GO:0048869\_cellular\_developmental\_process | TRIM28 | 1113 | 5 | 0.766197 | -0.087360 | 541 | 485.8 | 0.897967 |
| GO:0048869\_cellular\_developmental\_process | GJA1 | 1113 | 5 | 0.766197 | -0.087360 | 541 | 485.8 | 0.897967 |
| GO:0006629\_lipid\_metabolic\_process | PRPF19 | 285 | 1 | 0.598441 | -0.084750 | 542 | 487.59 | 0.899613 |
| GO:0048856\_anatomical\_structure\_development | RPA1 | 1688 | 8 | 0.808320 | -0.080340 | 543 | 488.63 | 0.899871 |
| GO:0048856\_anatomical\_structure\_development | HMGN1 | 1688 | 8 | 0.808320 | -0.080340 | 543 | 488.63 | 0.899871 |
| GO:0048856\_anatomical\_structure\_development | PRPF19 | 1688 | 8 | 0.808320 | -0.080340 | 543 | 488.63 | 0.899871 |
| GO:0048856\_anatomical\_structure\_development | NDN | 1688 | 8 | 0.808320 | -0.080340 | 543 | 488.63 | 0.899871 |
| GO:0048856\_anatomical\_structure\_development | TRIM28 | 1688 | 8 | 0.808320 | -0.080340 | 543 | 488.63 | 0.899871 |
| GO:0048856\_anatomical\_structure\_development | CFL1 | 1688 | 8 | 0.808320 | -0.080340 | 543 | 488.63 | 0.899871 |
| GO:0048856\_anatomical\_structure\_development | GJA1 | 1688 | 8 | 0.808320 | -0.080340 | 543 | 488.63 | 0.899871 |
| GO:0048856\_anatomical\_structure\_development | FKBP1A | 1688 | 8 | 0.808320 | -0.080340 | 543 | 488.63 | 0.899871 |
| GO:0002520\_immune\_system\_development | RPA1 | 295 | 1 | 0.578154 | -0.079094 | 545 | 489.57 | 0.898294 |
| GO:0045595\_regulation\_of\_cell\_differentiation | PRPF19 | 295 | 1 | 0.578154 | -0.079094 | 545 | 489.57 | 0.898294 |
| GO:0048598\_embryonic\_morphogenesis | CFL1 | 299 | 1 | 0.570420 | -0.076945 | 546 | 490.04 | 0.897509 |
| GO:0045893\_positive\_regulation\_of\_transcription\_\_DNA-dependent | TRIM28 | 306 | 1 | 0.557371 | -0.073331 | 548 | 491.43 | 0.896770 |
| GO:0051254\_positive\_regulation\_of\_RNA\_metabolic\_process | TRIM28 | 306 | 1 | 0.557371 | -0.073331 | 548 | 491.43 | 0.896770 |
| GO:0048513\_organ\_development | RPA1 | 1365 | 6 | 0.749695 | -0.067475 | 549 | 493.47 | 0.898852 |
| GO:0048513\_organ\_development | HMGN1 | 1365 | 6 | 0.749695 | -0.067475 | 549 | 493.47 | 0.898852 |
| GO:0048513\_organ\_development | TRIM28 | 1365 | 6 | 0.749695 | -0.067475 | 549 | 493.47 | 0.898852 |
| GO:0048513\_organ\_development | CFL1 | 1365 | 6 | 0.749695 | -0.067475 | 549 | 493.47 | 0.898852 |
| GO:0048513\_organ\_development | GJA1 | 1365 | 6 | 0.749695 | -0.067475 | 549 | 493.47 | 0.898852 |
| GO:0048513\_organ\_development | FKBP1A | 1365 | 6 | 0.749695 | -0.067475 | 549 | 493.47 | 0.898852 |
| GO:0051093\_negative\_regulation\_of\_developmental\_process | PRPF19 | 331 | 1 | 0.515274 | -0.061813 | 550 | 495.84 | 0.901527 |
| GO:0009605\_response\_to\_external\_stimulus | GJA1 | 339 | 1 | 0.503114 | -0.058539 | 551 | 497.59 | 0.903067 |
| GO:0050877\_neurological\_system\_process | NDN | 390 | 1 | 0.437322 | -0.041458 | 552 | 505.76 | 0.916232 |
| GO:0008150\_biological\_process | HMGN1 | 4605 | 27 | 1.000000 | 0.000000 | 1594 | 1585.91 | 0.994925 |
| GO:0008150\_biological\_process | NDN | 4605 | 27 | 1.000000 | 0.000000 | 1594 | 1585.91 | 0.994925 |
| GO:0008150\_biological\_process | HAX1 | 4605 | 27 | 1.000000 | 0.000000 | 1594 | 1585.91 | 0.994925 |
| GO:0008150\_biological\_process | CBX3 | 4605 | 27 | 1.000000 | 0.000000 | 1594 | 1585.91 | 0.994925 |
| GO:0008150\_biological\_process | GJA1 | 4605 | 27 | 1.000000 | 0.000000 | 1594 | 1585.91 | 0.994925 |
| GO:0008150\_biological\_process | FKBP1A | 4605 | 27 | 1.000000 | 0.000000 | 1594 | 1585.91 | 0.994925 |
| GO:0008150\_biological\_process | PRDX1 | 4605 | 27 | 1.000000 | 0.000000 | 1594 | 1585.91 | 0.994925 |
| GO:0008150\_biological\_process | PRPF19 | 4605 | 27 | 1.000000 | 0.000000 | 1594 | 1585.91 | 0.994925 |
| GO:0008150\_biological\_process | RPA1 | 4605 | 27 | 1.000000 | 0.000000 | 1594 | 1585.91 | 0.994925 |
| GO:0008150\_biological\_process | OAZ1 | 4605 | 27 | 1.000000 | 0.000000 | 1594 | 1585.91 | 0.994925 |
| GO:0008150\_biological\_process | TUBB5 | 4605 | 27 | 1.000000 | 0.000000 | 1594 | 1585.91 | 0.994925 |
| GO:0008150\_biological\_process | HSPA5 | 4605 | 27 | 1.000000 | 0.000000 | 1594 | 1585.91 | 0.994925 |
| GO:0008150\_biological\_process | IMPDH2 | 4605 | 27 | 1.000000 | 0.000000 | 1594 | 1585.91 | 0.994925 |
| GO:0008150\_biological\_process | HSPA8 | 4605 | 27 | 1.000000 | 0.000000 | 1594 | 1585.91 | 0.994925 |
| GO:0008150\_biological\_process | GDI2 | 4605 | 27 | 1.000000 | 0.000000 | 1594 | 1585.91 | 0.994925 |
| GO:0008150\_biological\_process | MAT2A | 4605 | 27 | 1.000000 | 0.000000 | 1594 | 1585.91 | 0.994925 |
| GO:0008150\_biological\_process | TRIM28 | 4605 | 27 | 1.000000 | 0.000000 | 1594 | 1585.91 | 0.994925 |
| GO:0008150\_biological\_process | CYCS | 4605 | 27 | 1.000000 | 0.000000 | 1594 | 1585.91 | 0.994925 |
| GO:0008150\_biological\_process | YWHAB | 4605 | 27 | 1.000000 | 0.000000 | 1594 | 1585.91 | 0.994925 |
| GO:0008150\_biological\_process | SFRS1 | 4605 | 27 | 1.000000 | 0.000000 | 1594 | 1585.91 | 0.994925 |
| GO:0008150\_biological\_process | DDX5 | 4605 | 27 | 1.000000 | 0.000000 | 1594 | 1585.91 | 0.994925 |
| GO:0008150\_biological\_process | RBBP7 | 4605 | 27 | 1.000000 | 0.000000 | 1594 | 1585.91 | 0.994925 |
| GO:0008150\_biological\_process | RPL29 | 4605 | 27 | 1.000000 | 0.000000 | 1594 | 1585.91 | 0.994925 |
| GO:0008150\_biological\_process | CFL1 | 4605 | 27 | 1.000000 | 0.000000 | 1594 | 1585.91 | 0.994925 |
| GO:0008150\_biological\_process | YWHAQ | 4605 | 27 | 1.000000 | 0.000000 | 1594 | 1585.91 | 0.994925 |
| GO:0008150\_biological\_process | NUTF2 | 4605 | 27 | 1.000000 | 0.000000 | 1594 | 1585.91 | 0.994925 |
| GO:0008150\_biological\_process | HSPD1 | 4605 | 27 | 1.000000 | 0.000000 | 1594 | 1585.91 | 0.994925 |
