# Supplementary material for: RedundancyMiner: De-replication of redundant GO categories in microarray and proteomics analysis
Source: BMC Bioinformatics. 2011 Feb 10;12:52. doi: 10.1186/1471-2105-12-52 (PMC3223614; doi:10.1186/1471-2105-12-52)
Supplement: Additional file 8 — Retinal development HTGM download. compressed package of the results of running HTGM on the retinal development genes list. [file 1471-2105-12-52-S8.ZIP › SCENARIO_2_MODIFIED/total.txt.total.txt.dir/Exp1_BestClusterMap_LEIGS_KM_24.csv.join.24.txt.dir/Exp1_BestClusterMap_LEIGS_KM_24.csv.join.24.txt.change.html]

Category Summary Report for Exp1\_BestClusterMap\_LEIGS\_KM\_24.csv.join.24.txt

# Category Summary Report for Exp1\_BestClusterMap\_LEIGS\_KM\_24.csv.join.24.txt

| HYPERLINKED GO CATEGORY | TOTAL GENES | CHANGED GENES | ENRICHMENT | LOG10(p) | CUMULATIVE NUMBER OF CATEGORIES | CUMULATIVE RANDOMS LOWER BOUND | CUMULATIVE RANDOMS MEAN | CUMULATIVE RANDOMS UPPER BOUND | FALSE DISCOVERY RATE |
| --- | --- | --- | --- | --- | --- | --- | --- | --- | --- |
| GO:0006605\_protein\_targeting | 86 | 4 | 7.932817 | -2.843546 | 1 | -0.963484 | 1.14 | 3.243484 | 1.140000 |
| GO:0006886\_intracellular\_protein\_transport | 122 | 4 | 5.591985 | -2.289606 | 2 | -1.379717 | 3.94 | 9.259717 | 1.970000 |
| GO:0006283\_transcription-coupled\_nucleotide-excision\_repair | 1 | 1 |  |  |  |  |  |  |  |  |
| GO:0006556\_S-adenosylmethionine\_biosynthetic\_process | 1 | 1 |  |  |  |  |  |  |  |  |
| GO:0032607\_interferon-alpha\_production | 1 | 1 |  |  |  |  |  |  |  |  |
| GO:0032647\_regulation\_of\_interferon-alpha\_production | 1 | 1 |  |  |  |  |  |  |  |  |
| GO:0032727\_positive\_regulation\_of\_interferon-alpha\_production | 1 | 1 |  |  |  |  |  |  |  |  |
| GO:0034613\_cellular\_protein\_localization | 139 | 4 | 4.908074 | -2.089904 | 3 | -1.573325 | 5.67 | 12.913325 | 1.890000 |
| GO:0070727\_cellular\_macromolecule\_localization | 141 | 4 | 4.838455 | -2.068295 | 4 | -1.613952 | 6.1 | 13.813952 | 1.525000 |
| GO:0009987\_cellular\_process | 3868 | 27 | 1.190538 | -2.051399 | 5 | -1.598165 | 6.14 | 13.878165 | 1.228000 |
| GO:0006800\_oxygen\_and\_reactive\_oxygen\_species\_metabolic\_process | 26 | 2 | 13.119658 | -2.005824 | 6 | -1.846702 | 6.68 | 15.206702 | 1.113333 |
| GO:0000720\_pyrimidine\_dimer\_repair\_by\_nucleotide-excision\_repair | 2 | 1 |  |  |  |  |  |  |  |  |
| GO:0010225\_response\_to\_UV-C | 2 | 1 |  |  |  |  |  |  |  |  |
| GO:0032481\_positive\_regulation\_of\_type\_I\_interferon\_production | 2 | 1 |  |  |  |  |  |  |  |  |
| GO:0046500\_S-adenosylmethionine\_metabolic\_process | 2 | 1 |  |  |  |  |  |  |  |  |
| GO:0048711\_positive\_regulation\_of\_astrocyte\_differentiation | 2 | 1 |  |  |  |  |  |  |  |  |
| GO:0008347\_glial\_cell\_migration | 3 | 1 |  |  |  |  |  |  |  |  |
| GO:0008635\_activation\_of\_caspase\_activity\_by\_cytochrome\_c | 3 | 1 |  |  |  |  |  |  |  |  |
| GO:0014074\_response\_to\_purine | 3 | 1 |  |  |  |  |  |  |  |  |
| GO:0030836\_positive\_regulation\_of\_actin\_filament\_depolymerization | 3 | 1 |  |  |  |  |  |  |  |  |
| GO:0031000\_response\_to\_caffeine | 3 | 1 |  |  |  |  |  |  |  |  |
| GO:0043200\_response\_to\_amino\_acid\_stimulus | 3 | 1 |  |  |  |  |  |  |  |  |
| GO:0043243\_positive\_regulation\_of\_protein\_complex\_disassembly | 3 | 1 |  |  |  |  |  |  |  |  |
| GO:0045844\_positive\_regulation\_of\_striated\_muscle\_development | 3 | 1 |  |  |  |  |  |  |  |  |
| GO:0048050\_post-embryonic\_eye\_morphogenesis | 3 | 1 |  |  |  |  |  |  |  |  |
| GO:0048597\_post-embryonic\_camera-type\_eye\_morphogenesis | 3 | 1 |  |  |  |  |  |  |  |  |
| GO:0048636\_positive\_regulation\_of\_muscle\_development | 3 | 1 |  |  |  |  |  |  |  |  |
| GO:0048676\_axon\_extension\_involved\_in\_development | 3 | 1 |  |  |  |  |  |  |  |  |
| GO:0060314\_regulation\_of\_ryanodine-sensitive\_calcium-release\_channel\_activity | 3 | 1 |  |  |  |  |  |  |  |  |
| GO:0015031\_protein\_transport | 175 | 4 | 3.898413 | -1.748491 | 7 | -1.478644 | 10.78 | 23.038644 | 1.540000 |
| GO:0045184\_establishment\_of\_protein\_localization | 180 | 4 | 3.790123 | -1.707823 | 8 | -1.462007 | 11.82 | 25.102007 | 1.477500 |
| GO:0014031\_mesenchymal\_cell\_development | 40 | 2 | 8.527778 | -1.647465 | 9 | -0.977854 | 13.25 | 27.477854 | 1.472222 |
| GO:0001842\_neural\_fold\_formation | 4 | 1 |  |  |  |  |  |  |  |  |
| GO:0006290\_pyrimidine\_dimer\_repair | 4 | 1 |  |  |  |  |  |  |  |  |
| GO:0010224\_response\_to\_UV-B | 4 | 1 |  |  |  |  |  |  |  |  |
| GO:0032872\_regulation\_of\_stress-activated\_MAPK\_cascade | 4 | 1 |  |  |  |  |  |  |  |  |
| GO:0042345\_regulation\_of\_NF-kappaB\_import\_into\_nucleus | 4 | 1 |  |  |  |  |  |  |  |  |
| GO:0042348\_NF-kappaB\_import\_into\_nucleus | 4 | 1 |  |  |  |  |  |  |  |  |
| GO:0048011\_nerve\_growth\_factor\_receptor\_signaling\_pathway | 4 | 1 |  |  |  |  |  |  |  |  |
| GO:0048710\_regulation\_of\_astrocyte\_differentiation | 4 | 1 |  |  |  |  |  |  |  |  |
| GO:0051225\_spindle\_assembly | 4 | 1 |  |  |  |  |  |  |  |  |
| GO:0046907\_intracellular\_transport | 194 | 4 | 3.516609 | -1.600968 | 10 | -0.660715 | 14.32 | 29.300715 | 1.432000 |
| GO:0048762\_mesenchymal\_cell\_differentiation | 43 | 2 | 7.932817 | -1.588542 | 11 | -0.444851 | 15.06 | 30.564851 | 1.369091 |
| GO:0033554\_cellular\_response\_to\_stress | 196 | 4 | 3.480726 | -1.586486 | 12 | -0.439694 | 15.07 | 30.579694 | 1.255833 |
| GO:0008152\_metabolic\_process | 2133 | 18 | 1.439287 | -1.578090 | 13 | -0.476348 | 15.22 | 30.916348 | 1.170769 |
| GO:0006606\_protein\_import\_into\_nucleus | 44 | 2 | 7.752525 | -1.569894 | 16 | -0.393064 | 16.01 | 32.413064 | 1.000625 |
| GO:0051170\_nuclear\_import | 44 | 2 | 7.752525 | -1.569894 | 16 | -0.393064 | 16.01 | 32.413064 | 1.000625 |
| GO:0060485\_mesenchyme\_development | 44 | 2 | 7.752525 | -1.569894 | 16 | -0.393064 | 16.01 | 32.413064 | 1.000625 |
| GO:0044237\_cellular\_metabolic\_process | 1974 | 17 | 1.468817 | -1.554181 | 17 | -0.344496 | 16.16 | 32.664496 | 0.950588 |
| GO:0000097\_sulfur\_amino\_acid\_biosynthetic\_process | 5 | 1 | 34.111111 | -1.537802 | 27 | 5.077885 | 24.78 | 44.482115 | 0.917778 |
| GO:0006983\_ER\_overload\_response | 5 | 1 | 34.111111 | -1.537802 | 27 | 5.077885 | 24.78 | 44.482115 | 0.917778 |
| GO:0014015\_positive\_regulation\_of\_gliogenesis | 5 | 1 | 34.111111 | -1.537802 | 27 | 5.077885 | 24.78 | 44.482115 | 0.917778 |
| GO:0019430\_removal\_of\_superoxide\_radicals | 5 | 1 | 34.111111 | -1.537802 | 27 | 5.077885 | 24.78 | 44.482115 | 0.917778 |
| GO:0030042\_actin\_filament\_depolymerization | 5 | 1 | 34.111111 | -1.537802 | 27 | 5.077885 | 24.78 | 44.482115 | 0.917778 |
| GO:0030834\_regulation\_of\_actin\_filament\_depolymerization | 5 | 1 | 34.111111 | -1.537802 | 27 | 5.077885 | 24.78 | 44.482115 | 0.917778 |
| GO:0032479\_regulation\_of\_type\_I\_interferon\_production | 5 | 1 | 34.111111 | -1.537802 | 27 | 5.077885 | 24.78 | 44.482115 | 0.917778 |
| GO:0043403\_skeletal\_muscle\_regeneration | 5 | 1 | 34.111111 | -1.537802 | 27 | 5.077885 | 24.78 | 44.482115 | 0.917778 |
| GO:0045687\_positive\_regulation\_of\_glial\_cell\_differentiation | 5 | 1 | 34.111111 | -1.537802 | 27 | 5.077885 | 24.78 | 44.482115 | 0.917778 |
| GO:0051403\_stress-activated\_MAPK\_cascade | 5 | 1 | 34.111111 | -1.537802 | 27 | 5.077885 | 24.78 | 44.482115 | 0.917778 |
| GO:0042063\_gliogenesis | 46 | 2 | 7.415459 | -1.533955 | 28 | 5.260111 | 25.11 | 44.959889 | 0.896786 |
| GO:0034504\_protein\_localization\_in\_nucleus | 48 | 2 | 7.106481 | -1.499694 | 29 | 5.693890 | 26.05 | 46.406110 | 0.898276 |
| GO:0003015\_heart\_process | 49 | 2 | 6.961451 | -1.483149 | 31 | 5.901357 | 26.64 | 47.378643 | 0.859355 |
| GO:0060047\_heart\_contraction | 49 | 2 | 6.961451 | -1.483149 | 31 | 5.901357 | 26.64 | 47.378643 | 0.859355 |
| GO:0008283\_cell\_proliferation | 544 | 7 | 2.194649 | -1.478768 | 32 | 5.849432 | 26.67 | 47.490568 | 0.833438 |
| GO:0050789\_regulation\_of\_biological\_process | 2357 | 19 | 1.374864 | -1.467393 | 33 | 5.842922 | 26.86 | 47.877078 | 0.813939 |
| GO:0017038\_protein\_import | 50 | 2 | 6.822222 | -1.466973 | 34 | 5.852635 | 27.14 | 48.427365 | 0.798235 |
| GO:0031077\_post-embryonic\_camera-type\_eye\_development | 6 | 1 | 28.425926 | -1.459845 | 38 | 10.307556 | 33.53 | 56.752444 | 0.882368 |
| GO:0042246\_tissue\_regeneration | 6 | 1 | 28.425926 | -1.459845 | 38 | 10.307556 | 33.53 | 56.752444 | 0.882368 |
| GO:0048563\_post-embryonic\_organ\_morphogenesis | 6 | 1 | 28.425926 | -1.459845 | 38 | 10.307556 | 33.53 | 56.752444 | 0.882368 |
| GO:0051085\_chaperone\_mediated\_protein\_folding\_requiring\_cofactor | 6 | 1 | 28.425926 | -1.459845 | 38 | 10.307556 | 33.53 | 56.752444 | 0.882368 |
| GO:0050794\_regulation\_of\_cellular\_process | 2190 | 18 | 1.401826 | -1.450485 | 39 | 10.390869 | 33.85 | 57.309131 | 0.867949 |
| GO:0040007\_growth | 217 | 4 | 3.143881 | -1.444822 | 40 | 10.421469 | 34.03 | 57.638531 | 0.850750 |
| GO:0001701\_in\_utero\_embryonic\_development | 221 | 4 | 3.086978 | -1.419812 | 41 | 10.779777 | 34.79 | 58.800223 | 0.848537 |
| GO:0051276\_chromosome\_organization | 129 | 3 | 3.966408 | -1.414719 | 42 | 10.822921 | 34.85 | 58.877079 | 0.829762 |
| GO:0001839\_neural\_plate\_morphogenesis | 7 | 1 | 24.365079 | -1.394121 | 44 | 16.505661 | 42.83 | 69.154339 | 0.973409 |
| GO:0007413\_axonal\_fasciculation | 7 | 1 | 24.365079 | -1.394121 | 44 | 16.505661 | 42.83 | 69.154339 | 0.973409 |
| GO:0001764\_neuron\_migration | 57 | 2 | 5.984405 | -1.362930 | 46 | 17.197557 | 44.46 | 71.722443 | 0.966522 |
| GO:0033365\_protein\_localization\_in\_organelle | 57 | 2 | 5.984405 | -1.362930 | 46 | 17.197557 | 44.46 | 71.722443 | 0.966522 |
| GO:0044238\_primary\_metabolic\_process | 1905 | 16 | 1.432488 | -1.338425 | 47 | 17.342605 | 45.01 | 72.677395 | 0.957660 |
| GO:0000910\_cytokinesis | 8 | 1 | 21.319444 | -1.337351 | 58 | 21.927195 | 51.32 | 80.712805 | 0.884828 |
| GO:0001833\_inner\_cell\_mass\_cell\_proliferation | 8 | 1 | 21.319444 | -1.337351 | 58 | 21.927195 | 51.32 | 80.712805 | 0.884828 |
| GO:0001840\_neural\_plate\_development | 8 | 1 | 21.319444 | -1.337351 | 58 | 21.927195 | 51.32 | 80.712805 | 0.884828 |
| GO:0006458\_'de\_novo'\_protein\_folding | 8 | 1 | 21.319444 | -1.337351 | 58 | 21.927195 | 51.32 | 80.712805 | 0.884828 |
| GO:0022898\_regulation\_of\_transmembrane\_transporter\_activity | 8 | 1 | 21.319444 | -1.337351 | 58 | 21.927195 | 51.32 | 80.712805 | 0.884828 |
| GO:0032409\_regulation\_of\_transporter\_activity | 8 | 1 | 21.319444 | -1.337351 | 58 | 21.927195 | 51.32 | 80.712805 | 0.884828 |
| GO:0032412\_regulation\_of\_ion\_transmembrane\_transporter\_activity | 8 | 1 | 21.319444 | -1.337351 | 58 | 21.927195 | 51.32 | 80.712805 | 0.884828 |
| GO:0042990\_regulation\_of\_transcription\_factor\_import\_into\_nucleus | 8 | 1 | 21.319444 | -1.337351 | 58 | 21.927195 | 51.32 | 80.712805 | 0.884828 |
| GO:0042991\_transcription\_factor\_import\_into\_nucleus | 8 | 1 | 21.319444 | -1.337351 | 58 | 21.927195 | 51.32 | 80.712805 | 0.884828 |
| GO:0051084\_'de\_novo'\_posttranslational\_protein\_folding | 8 | 1 | 21.319444 | -1.337351 | 58 | 21.927195 | 51.32 | 80.712805 | 0.884828 |
| GO:0060347\_heart\_trabecula\_formation | 8 | 1 | 21.319444 | -1.337351 | 58 | 21.927195 | 51.32 | 80.712805 | 0.884828 |
| GO:0006874\_cellular\_calcium\_ion\_homeostasis | 61 | 2 | 5.591985 | -1.309703 | 59 | 22.324938 | 52.29 | 82.255062 | 0.886271 |
| GO:0006139\_nucleobase\_\_nucleoside\_\_nucleotide\_and\_nucleic\_acid\_metabolic\_process | 1002 | 10 | 1.702151 | -1.293675 | 60 | 22.634938 | 53.12 | 83.605062 | 0.885333 |
| GO:0006807\_nitrogen\_compound\_metabolic\_process | 1147 | 11 | 1.635668 | -1.292371 | 61 | 22.639513 | 53.13 | 83.620487 | 0.870984 |
| GO:0006595\_polyamine\_metabolic\_process | 9 | 1 | 18.950617 | -1.287420 | 65 | 26.700715 | 59.32 | 91.939285 | 0.912615 |
| GO:0032606\_type\_I\_interferon\_production | 9 | 1 | 18.950617 | -1.287420 | 65 | 26.700715 | 59.32 | 91.939285 | 0.912615 |
| GO:0048569\_post-embryonic\_organ\_development | 9 | 1 | 18.950617 | -1.287420 | 65 | 26.700715 | 59.32 | 91.939285 | 0.912615 |
| GO:0048708\_astrocyte\_differentiation | 9 | 1 | 18.950617 | -1.287420 | 65 | 26.700715 | 59.32 | 91.939285 | 0.912615 |
| GO:0006875\_cellular\_metal\_ion\_homeostasis | 64 | 2 | 5.329861 | -1.272302 | 67 | 26.986941 | 59.99 | 92.993059 | 0.895373 |
| GO:0055074\_calcium\_ion\_homeostasis | 64 | 2 | 5.329861 | -1.272302 | 67 | 26.986941 | 59.99 | 92.993059 | 0.895373 |
| GO:0008104\_protein\_localization | 251 | 4 | 2.718017 | -1.249266 | 68 | 27.165741 | 60.62 | 94.074259 | 0.891471 |
| GO:0000724\_double-strand\_break\_repair\_via\_homologous\_recombination | 10 | 1 | 17.055556 | -1.242883 | 80 | 32.738811 | 67.74 | 102.741189 | 0.846750 |
| GO:0000725\_recombinational\_repair | 10 | 1 | 17.055556 | -1.242883 | 80 | 32.738811 | 67.74 | 102.741189 | 0.846750 |
| GO:0001832\_blastocyst\_growth | 10 | 1 | 17.055556 | -1.242883 | 80 | 32.738811 | 67.74 | 102.741189 | 0.846750 |
| GO:0002070\_epithelial\_cell\_maturation | 10 | 1 | 17.055556 | -1.242883 | 80 | 32.738811 | 67.74 | 102.741189 | 0.846750 |
| GO:0006289\_nucleotide-excision\_repair | 10 | 1 | 17.055556 | -1.242883 | 80 | 32.738811 | 67.74 | 102.741189 | 0.846750 |
| GO:0006801\_superoxide\_metabolic\_process | 10 | 1 | 17.055556 | -1.242883 | 80 | 32.738811 | 67.74 | 102.741189 | 0.846750 |
| GO:0051208\_sequestering\_of\_calcium\_ion | 10 | 1 | 17.055556 | -1.242883 | 80 | 32.738811 | 67.74 | 102.741189 | 0.846750 |
| GO:0051209\_release\_of\_sequestered\_calcium\_ion\_into\_cytosol | 10 | 1 | 17.055556 | -1.242883 | 80 | 32.738811 | 67.74 | 102.741189 | 0.846750 |
| GO:0051238\_sequestering\_of\_metal\_ion | 10 | 1 | 17.055556 | -1.242883 | 80 | 32.738811 | 67.74 | 102.741189 | 0.846750 |
| GO:0051282\_regulation\_of\_sequestering\_of\_calcium\_ion | 10 | 1 | 17.055556 | -1.242883 | 80 | 32.738811 | 67.74 | 102.741189 | 0.846750 |
| GO:0051283\_negative\_regulation\_of\_sequestering\_of\_calcium\_ion | 10 | 1 | 17.055556 | -1.242883 | 80 | 32.738811 | 67.74 | 102.741189 | 0.846750 |
| GO:0060343\_trabecula\_formation | 10 | 1 | 17.055556 | -1.242883 | 80 | 32.738811 | 67.74 | 102.741189 | 0.846750 |
| GO:0043009\_chordate\_embryonic\_development | 365 | 5 | 2.336377 | -1.238932 | 81 | 32.693260 | 67.88 | 103.066740 | 0.838025 |
| GO:0003007\_heart\_morphogenesis | 67 | 2 | 5.091211 | -1.236837 | 82 | 32.714701 | 68.28 | 103.845299 | 0.832683 |
| GO:0009792\_embryonic\_development\_ending\_in\_birth\_or\_egg\_hatching | 368 | 5 | 2.317331 | -1.226339 | 83 | 32.808986 | 68.5 | 104.191014 | 0.825301 |
| GO:0055065\_metal\_ion\_homeostasis | 69 | 2 | 4.943639 | -1.214184 | 84 | 33.023382 | 69.07 | 105.116618 | 0.822262 |
| GO:0000096\_sulfur\_amino\_acid\_metabolic\_process | 11 | 1 | 15.505051 | -1.202709 | 92 | 36.838826 | 74.57 | 112.301174 | 0.810543 |
| GO:0001101\_response\_to\_acid | 11 | 1 | 15.505051 | -1.202709 | 92 | 36.838826 | 74.57 | 112.301174 | 0.810543 |
| GO:0001837\_epithelial\_to\_mesenchymal\_transition | 11 | 1 | 15.505051 | -1.202709 | 92 | 36.838826 | 74.57 | 112.301174 | 0.810543 |
| GO:0007051\_spindle\_organization | 11 | 1 | 15.505051 | -1.202709 | 92 | 36.838826 | 74.57 | 112.301174 | 0.810543 |
| GO:0008652\_cellular\_amino\_acid\_biosynthetic\_process | 11 | 1 | 15.505051 | -1.202709 | 92 | 36.838826 | 74.57 | 112.301174 | 0.810543 |
| GO:0014013\_regulation\_of\_gliogenesis | 11 | 1 | 15.505051 | -1.202709 | 92 | 36.838826 | 74.57 | 112.301174 | 0.810543 |
| GO:0034762\_regulation\_of\_transmembrane\_transport | 11 | 1 | 15.505051 | -1.202709 | 92 | 36.838826 | 74.57 | 112.301174 | 0.810543 |
| GO:0045685\_regulation\_of\_glial\_cell\_differentiation | 11 | 1 | 15.505051 | -1.202709 | 92 | 36.838826 | 74.57 | 112.301174 | 0.810543 |
| GO:0006281\_DNA\_repair | 71 | 2 | 4.804382 | -1.192270 | 94 | 37.273494 | 75.27 | 113.266506 | 0.800745 |
| GO:0006913\_nucleocytoplasmic\_transport | 71 | 2 | 4.804382 | -1.192270 | 94 | 37.273494 | 75.27 | 113.266506 | 0.800745 |
| GO:0007264\_small\_GTPase\_mediated\_signal\_transduction | 72 | 2 | 4.737654 | -1.181576 | 96 | 37.598635 | 75.96 | 114.321365 | 0.791250 |
| GO:0051169\_nuclear\_transport | 72 | 2 | 4.737654 | -1.181576 | 96 | 37.598635 | 75.96 | 114.321365 | 0.791250 |
| GO:0006936\_muscle\_contraction | 73 | 2 | 4.672755 | -1.171053 | 97 | 37.838843 | 76.52 | 115.201157 | 0.788866 |
| GO:0008038\_neuron\_recognition | 12 | 1 | 14.212963 | -1.166139 | 107 | 41.449035 | 81.97 | 122.490965 | 0.766075 |
| GO:0009886\_post-embryonic\_morphogenesis | 12 | 1 | 14.212963 | -1.166139 | 107 | 41.449035 | 81.97 | 122.490965 | 0.766075 |
| GO:0030010\_establishment\_of\_cell\_polarity | 12 | 1 | 14.212963 | -1.166139 | 107 | 41.449035 | 81.97 | 122.490965 | 0.766075 |
| GO:0035050\_embryonic\_heart\_tube\_development | 12 | 1 | 14.212963 | -1.166139 | 107 | 41.449035 | 81.97 | 122.490965 | 0.766075 |
| GO:0042278\_purine\_nucleoside\_metabolic\_process | 12 | 1 | 14.212963 | -1.166139 | 107 | 41.449035 | 81.97 | 122.490965 | 0.766075 |
| GO:0042743\_hydrogen\_peroxide\_metabolic\_process | 12 | 1 | 14.212963 | -1.166139 | 107 | 41.449035 | 81.97 | 122.490965 | 0.766075 |
| GO:0043624\_cellular\_protein\_complex\_disassembly | 12 | 1 | 14.212963 | -1.166139 | 107 | 41.449035 | 81.97 | 122.490965 | 0.766075 |
| GO:0046128\_purine\_ribonucleoside\_metabolic\_process | 12 | 1 | 14.212963 | -1.166139 | 107 | 41.449035 | 81.97 | 122.490965 | 0.766075 |
| GO:0051261\_protein\_depolymerization | 12 | 1 | 14.212963 | -1.166139 | 107 | 41.449035 | 81.97 | 122.490965 | 0.766075 |
| GO:0060048\_cardiac\_muscle\_contraction | 12 | 1 | 14.212963 | -1.166139 | 107 | 41.449035 | 81.97 | 122.490965 | 0.766075 |
| GO:0048589\_developmental\_growth | 75 | 2 | 4.548148 | -1.150495 | 108 | 41.784858 | 82.96 | 124.135142 | 0.768148 |
| GO:0051716\_cellular\_response\_to\_stimulus | 273 | 4 | 2.498982 | -1.140546 | 109 | 41.914941 | 83.4 | 124.885059 | 0.765138 |
| GO:0003012\_muscle\_system\_process | 76 | 2 | 4.488304 | -1.140452 | 110 | 42.080291 | 83.58 | 125.079709 | 0.759818 |
| GO:0033036\_macromolecule\_localization | 274 | 4 | 2.489862 | -1.135889 | 111 | 42.222571 | 84.0 | 125.777429 | 0.756757 |
| GO:0007512\_adult\_heart\_development | 13 | 1 | 13.119658 | -1.132594 | 119 | 45.551225 | 89.26 | 132.968775 | 0.750084 |
| GO:0009119\_ribonucleoside\_metabolic\_process | 13 | 1 | 13.119658 | -1.132594 | 119 | 45.551225 | 89.26 | 132.968775 | 0.750084 |
| GO:0032729\_positive\_regulation\_of\_interferon-gamma\_production | 13 | 1 | 13.119658 | -1.132594 | 119 | 45.551225 | 89.26 | 132.968775 | 0.750084 |
| GO:0043241\_protein\_complex\_disassembly | 13 | 1 | 13.119658 | -1.132594 | 119 | 45.551225 | 89.26 | 132.968775 | 0.750084 |
| GO:0043244\_regulation\_of\_protein\_complex\_disassembly | 13 | 1 | 13.119658 | -1.132594 | 119 | 45.551225 | 89.26 | 132.968775 | 0.750084 |
| GO:0051495\_positive\_regulation\_of\_cytoskeleton\_organization | 13 | 1 | 13.119658 | -1.132594 | 119 | 45.551225 | 89.26 | 132.968775 | 0.750084 |
| GO:0060401\_cytosolic\_calcium\_ion\_transport | 13 | 1 | 13.119658 | -1.132594 | 119 | 45.551225 | 89.26 | 132.968775 | 0.750084 |
| GO:0060402\_calcium\_ion\_transport\_into\_cytosol | 13 | 1 | 13.119658 | -1.132594 | 119 | 45.551225 | 89.26 | 132.968775 | 0.750084 |
| GO:0008064\_regulation\_of\_actin\_polymerization\_or\_depolymerization | 14 | 1 | 12.182540 | -1.101625 | 123 | 49.740089 | 95.9 | 142.059911 | 0.779675 |
| GO:0030832\_regulation\_of\_actin\_filament\_length | 14 | 1 | 12.182540 | -1.101625 | 123 | 49.740089 | 95.9 | 142.059911 | 0.779675 |
| GO:0031099\_regeneration | 14 | 1 | 12.182540 | -1.101625 | 123 | 49.740089 | 95.9 | 142.059911 | 0.779675 |
| GO:0034623\_cellular\_macromolecular\_complex\_disassembly | 14 | 1 | 12.182540 | -1.101625 | 123 | 49.740089 | 95.9 | 142.059911 | 0.779675 |
| GO:0006325\_chromatin\_organization | 83 | 2 | 4.109772 | -1.074194 | 125 | 50.639334 | 97.35 | 144.060666 | 0.778800 |
| GO:0006575\_cellular\_amino\_acid\_derivative\_metabolic\_process | 83 | 2 | 4.109772 | -1.074194 | 125 | 50.639334 | 97.35 | 144.060666 | 0.778800 |
| GO:0034960\_cellular\_biopolymer\_metabolic\_process | 1395 | 12 | 1.467145 | -1.073444 | 126 | 50.658898 | 97.39 | 144.121102 | 0.772937 |
| GO:0009116\_nucleoside\_metabolic\_process | 15 | 1 | 11.370370 | -1.072877 | 129 | 53.490490 | 101.83 | 150.169510 | 0.789380 |
| GO:0042306\_regulation\_of\_protein\_import\_into\_nucleus | 15 | 1 | 11.370370 | -1.072877 | 129 | 53.490490 | 101.83 | 150.169510 | 0.789380 |
| GO:0055010\_ventricular\_cardiac\_muscle\_morphogenesis | 15 | 1 | 11.370370 | -1.072877 | 129 | 53.490490 | 101.83 | 150.169510 | 0.789380 |
| GO:0030005\_cellular\_di-\_\_tri-valent\_inorganic\_cation\_homeostasis | 84 | 2 | 4.060847 | -1.065264 | 130 | 53.793611 | 102.34 | 150.886389 | 0.787231 |
| GO:0000279\_M\_phase | 85 | 2 | 4.013072 | -1.056457 | 131 | 54.145278 | 102.79 | 151.434722 | 0.784656 |
| GO:0000302\_response\_to\_reactive\_oxygen\_species | 16 | 1 | 10.659722 | -1.046063 | 137 | 57.701185 | 107.54 | 157.378815 | 0.784964 |
| GO:0001933\_negative\_regulation\_of\_protein\_amino\_acid\_phosphorylation | 16 | 1 | 10.659722 | -1.046063 | 137 | 57.701185 | 107.54 | 157.378815 | 0.784964 |
| GO:0010243\_response\_to\_organic\_nitrogen | 16 | 1 | 10.659722 | -1.046063 | 137 | 57.701185 | 107.54 | 157.378815 | 0.784964 |
| GO:0014075\_response\_to\_amine\_stimulus | 16 | 1 | 10.659722 | -1.046063 | 137 | 57.701185 | 107.54 | 157.378815 | 0.784964 |
| GO:0032956\_regulation\_of\_actin\_cytoskeleton\_organization | 16 | 1 | 10.659722 | -1.046063 | 137 | 57.701185 | 107.54 | 157.378815 | 0.784964 |
| GO:0034976\_response\_to\_endoplasmic\_reticulum\_stress | 16 | 1 | 10.659722 | -1.046063 | 137 | 57.701185 | 107.54 | 157.378815 | 0.784964 |
| GO:0006919\_activation\_of\_caspase\_activity | 17 | 1 | 10.032680 | -1.020947 | 144 | 62.300011 | 114.0 | 165.699989 | 0.791667 |
| GO:0006984\_ER-nuclear\_signaling\_pathway | 17 | 1 | 10.032680 | -1.020947 | 144 | 62.300011 | 114.0 | 165.699989 | 0.791667 |
| GO:0008380\_RNA\_splicing | 17 | 1 | 10.032680 | -1.020947 | 144 | 62.300011 | 114.0 | 165.699989 | 0.791667 |
| GO:0032535\_regulation\_of\_cellular\_component\_size | 17 | 1 | 10.032680 | -1.020947 | 144 | 62.300011 | 114.0 | 165.699989 | 0.791667 |
| GO:0032970\_regulation\_of\_actin\_filament-based\_process | 17 | 1 | 10.032680 | -1.020947 | 144 | 62.300011 | 114.0 | 165.699989 | 0.791667 |
| GO:0042398\_cellular\_amino\_acid\_derivative\_biosynthetic\_process | 17 | 1 | 10.032680 | -1.020947 | 144 | 62.300011 | 114.0 | 165.699989 | 0.791667 |
| GO:0048873\_homeostasis\_of\_number\_of\_cells\_within\_a\_tissue | 17 | 1 | 10.032680 | -1.020947 | 144 | 62.300011 | 114.0 | 165.699989 | 0.791667 |
| GO:0030003\_cellular\_cation\_homeostasis | 90 | 2 | 3.790123 | -1.014186 | 145 | 62.616928 | 114.52 | 166.423072 | 0.789793 |
| GO:0065007\_biological\_regulation | 2593 | 19 | 1.249732 | -1.007167 | 146 | 62.660105 | 114.64 | 166.619895 | 0.785205 |
| GO:0002064\_epithelial\_cell\_development | 18 | 1 | 9.475309 | -0.997336 | 154 | 66.272031 | 120.38 | 174.487969 | 0.781688 |
| GO:0006457\_protein\_folding | 18 | 1 | 9.475309 | -0.997336 | 154 | 66.272031 | 120.38 | 174.487969 | 0.781688 |
| GO:0032984\_macromolecular\_complex\_disassembly | 18 | 1 | 9.475309 | -0.997336 | 154 | 66.272031 | 120.38 | 174.487969 | 0.781688 |
| GO:0033157\_regulation\_of\_intracellular\_protein\_transport | 18 | 1 | 9.475309 | -0.997336 | 154 | 66.272031 | 120.38 | 174.487969 | 0.781688 |
| GO:0044272\_sulfur\_compound\_biosynthetic\_process | 18 | 1 | 9.475309 | -0.997336 | 154 | 66.272031 | 120.38 | 174.487969 | 0.781688 |
| GO:0055008\_cardiac\_muscle\_tissue\_morphogenesis | 18 | 1 | 9.475309 | -0.997336 | 154 | 66.272031 | 120.38 | 174.487969 | 0.781688 |
| GO:0060415\_muscle\_tissue\_morphogenesis | 18 | 1 | 9.475309 | -0.997336 | 154 | 66.272031 | 120.38 | 174.487969 | 0.781688 |
| GO:0060571\_morphogenesis\_of\_an\_epithelial\_fold | 18 | 1 | 9.475309 | -0.997336 | 154 | 66.272031 | 120.38 | 174.487969 | 0.781688 |
| GO:0051094\_positive\_regulation\_of\_developmental\_process | 308 | 4 | 2.215007 | -0.990368 | 155 | 66.313551 | 120.53 | 174.746449 | 0.777613 |
| GO:0055066\_di-\_\_tri-valent\_inorganic\_cation\_homeostasis | 93 | 2 | 3.667861 | -0.990140 | 156 | 66.465403 | 120.85 | 175.234597 | 0.774679 |
| GO:0032943\_mononuclear\_cell\_proliferation | 94 | 2 | 3.628842 | -0.982329 | 159 | 67.068086 | 121.85 | 176.631914 | 0.766352 |
| GO:0034984\_cellular\_response\_to\_DNA\_damage\_stimulus | 94 | 2 | 3.628842 | -0.982329 | 159 | 67.068086 | 121.85 | 176.631914 | 0.766352 |
| GO:0046651\_lymphocyte\_proliferation | 94 | 2 | 3.628842 | -0.982329 | 159 | 67.068086 | 121.85 | 176.631914 | 0.766352 |
| GO:0006338\_chromatin\_remodeling | 19 | 1 | 8.976608 | -0.975066 | 162 | 70.313946 | 126.01 | 181.706054 | 0.777840 |
| GO:0010952\_positive\_regulation\_of\_peptidase\_activity | 19 | 1 | 8.976608 | -0.975066 | 162 | 70.313946 | 126.01 | 181.706054 | 0.777840 |
| GO:0043280\_positive\_regulation\_of\_caspase\_activity | 19 | 1 | 8.976608 | -0.975066 | 162 | 70.313946 | 126.01 | 181.706054 | 0.777840 |
| GO:0044260\_cellular\_macromolecule\_metabolic\_process | 1447 | 12 | 1.414421 | -0.971179 | 163 | 70.480425 | 126.32 | 182.159575 | 0.774969 |
| GO:0070661\_leukocyte\_proliferation | 96 | 2 | 3.553241 | -0.967002 | 164 | 70.808530 | 127.04 | 183.271470 | 0.774634 |
| GO:0048518\_positive\_regulation\_of\_biological\_process | 995 | 9 | 1.542714 | -0.963622 | 165 | 70.937958 | 127.22 | 183.502042 | 0.771030 |
| GO:0042326\_negative\_regulation\_of\_phosphorylation | 20 | 1 | 8.527778 | -0.954000 | 167 | 73.763531 | 130.93 | 188.096469 | 0.784012 |
| GO:0046822\_regulation\_of\_nucleocytoplasmic\_transport | 20 | 1 | 8.527778 | -0.954000 | 167 | 73.763531 | 130.93 | 188.096469 | 0.784012 |
| GO:0006996\_organelle\_organization | 449 | 5 | 1.899282 | -0.934736 | 168 | 74.879351 | 132.82 | 190.760649 | 0.790595 |
| GO:0001755\_neural\_crest\_cell\_migration | 21 | 1 | 8.121693 | -0.934019 | 176 | 77.858486 | 137.4 | 196.941514 | 0.780682 |
| GO:0008154\_actin\_polymerization\_or\_depolymerization | 21 | 1 | 8.121693 | -0.934019 | 176 | 77.858486 | 137.4 | 196.941514 | 0.780682 |
| GO:0010563\_negative\_regulation\_of\_phosphorus\_metabolic\_process | 21 | 1 | 8.121693 | -0.934019 | 176 | 77.858486 | 137.4 | 196.941514 | 0.780682 |
| GO:0016202\_regulation\_of\_striated\_muscle\_tissue\_development | 21 | 1 | 8.121693 | -0.934019 | 176 | 77.858486 | 137.4 | 196.941514 | 0.780682 |
| GO:0043279\_response\_to\_alkaloid | 21 | 1 | 8.121693 | -0.934019 | 176 | 77.858486 | 137.4 | 196.941514 | 0.780682 |
| GO:0045936\_negative\_regulation\_of\_phosphate\_metabolic\_process | 21 | 1 | 8.121693 | -0.934019 | 176 | 77.858486 | 137.4 | 196.941514 | 0.780682 |
| GO:0048634\_regulation\_of\_muscle\_development | 21 | 1 | 8.121693 | -0.934019 | 176 | 77.858486 | 137.4 | 196.941514 | 0.780682 |
| GO:0048675\_axon\_extension | 21 | 1 | 8.121693 | -0.934019 | 176 | 77.858486 | 137.4 | 196.941514 | 0.780682 |
| GO:0003013\_circulatory\_system\_process | 103 | 2 | 3.311758 | -0.916242 | 178 | 78.372205 | 138.31 | 198.247795 | 0.777022 |
| GO:0008015\_blood\_circulation | 103 | 2 | 3.311758 | -0.916242 | 178 | 78.372205 | 138.31 | 198.247795 | 0.777022 |
| GO:0001558\_regulation\_of\_cell\_growth | 22 | 1 | 7.752525 | -0.915024 | 182 | 82.175792 | 143.65 | 205.124208 | 0.789286 |
| GO:0001947\_heart\_looping | 22 | 1 | 7.752525 | -0.915024 | 182 | 82.175792 | 143.65 | 205.124208 | 0.789286 |
| GO:0009309\_amine\_biosynthetic\_process | 22 | 1 | 7.752525 | -0.915024 | 182 | 82.175792 | 143.65 | 205.124208 | 0.789286 |
| GO:0032649\_regulation\_of\_interferon-gamma\_production | 22 | 1 | 7.752525 | -0.915024 | 182 | 82.175792 | 143.65 | 205.124208 | 0.789286 |
| GO:0055086\_nucleobase\_\_nucleoside\_and\_nucleotide\_metabolic\_process | 104 | 2 | 3.279915 | -0.909333 | 183 | 82.417386 | 143.96 | 205.502614 | 0.786667 |
| GO:0010605\_negative\_regulation\_of\_macromolecule\_metabolic\_process | 331 | 4 | 2.061094 | -0.904315 | 184 | 82.669034 | 144.48 | 206.290966 | 0.785217 |
| GO:0048872\_homeostasis\_of\_number\_of\_cells | 105 | 2 | 3.248677 | -0.902504 | 185 | 82.757744 | 144.6 | 206.442256 | 0.781622 |
| GO:0031324\_negative\_regulation\_of\_cellular\_metabolic\_process | 332 | 4 | 2.054886 | -0.900773 | 186 | 82.931069 | 144.94 | 206.948931 | 0.779247 |
| GO:0002228\_natural\_killer\_cell\_mediated\_immunity | 23 | 1 | 7.415459 | -0.896926 | 190 | 85.258679 | 148.25 | 211.241321 | 0.780263 |
| GO:0007163\_establishment\_or\_maintenance\_of\_cell\_polarity | 23 | 1 | 7.415459 | -0.896926 | 190 | 85.258679 | 148.25 | 211.241321 | 0.780263 |
| GO:0030512\_negative\_regulation\_of\_transforming\_growth\_factor\_beta\_receptor\_signaling\_pathway | 23 | 1 | 7.415459 | -0.896926 | 190 | 85.258679 | 148.25 | 211.241321 | 0.780263 |
| GO:0042267\_natural\_killer\_cell\_mediated\_cytotoxicity | 23 | 1 | 7.415459 | -0.896926 | 190 | 85.258679 | 148.25 | 211.241321 | 0.780263 |
| GO:0043283\_biopolymer\_metabolic\_process | 1490 | 12 | 1.373602 | -0.892382 | 191 | 85.441809 | 148.52 | 211.598191 | 0.777592 |
| GO:0006941\_striated\_muscle\_contraction | 24 | 1 | 7.106481 | -0.879649 | 195 | 88.378340 | 152.63 | 216.881660 | 0.782718 |
| GO:0007204\_elevation\_of\_cytosolic\_calcium\_ion\_concentration | 24 | 1 | 7.106481 | -0.879649 | 195 | 88.378340 | 152.63 | 216.881660 | 0.782718 |
| GO:0014070\_response\_to\_organic\_cyclic\_substance | 24 | 1 | 7.106481 | -0.879649 | 195 | 88.378340 | 152.63 | 216.881660 | 0.782718 |
| GO:0032386\_regulation\_of\_intracellular\_transport | 24 | 1 | 7.106481 | -0.879649 | 195 | 88.378340 | 152.63 | 216.881660 | 0.782718 |
| GO:0045892\_negative\_regulation\_of\_transcription\_\_DNA-dependent | 218 | 3 | 2.347095 | -0.875218 | 196 | 88.814168 | 153.2 | 217.585832 | 0.781633 |
| GO:0055080\_cation\_homeostasis | 110 | 2 | 3.101010 | -0.869516 | 197 | 89.300094 | 154.02 | 218.739906 | 0.781827 |
| GO:0051253\_negative\_regulation\_of\_RNA\_metabolic\_process | 220 | 3 | 2.325758 | -0.866570 | 198 | 89.412753 | 154.14 | 218.867247 | 0.778485 |
| GO:0051649\_establishment\_of\_localization\_in\_cell | 342 | 4 | 1.994802 | -0.866202 | 199 | 89.551787 | 154.29 | 219.028213 | 0.775327 |
| GO:0048522\_positive\_regulation\_of\_cellular\_process | 895 | 8 | 1.524519 | -0.863259 | 200 | 89.605837 | 154.39 | 219.174163 | 0.771950 |
| GO:0006302\_double-strand\_break\_repair | 25 | 1 | 6.822222 | -0.863126 | 202 | 91.630987 | 157.61 | 223.589013 | 0.780248 |
| GO:0031400\_negative\_regulation\_of\_protein\_modification\_process | 25 | 1 | 6.822222 | -0.863126 | 202 | 91.630987 | 157.61 | 223.589013 | 0.780248 |
| GO:0006974\_response\_to\_DNA\_damage\_stimulus | 113 | 2 | 3.018682 | -0.850597 | 203 | 92.142624 | 158.33 | 224.517376 | 0.779951 |
| GO:0019233\_sensory\_perception\_of\_pain | 26 | 1 | 6.559829 | -0.847297 | 208 | 94.511453 | 161.68 | 228.848547 | 0.777308 |
| GO:0032609\_interferon-gamma\_production | 26 | 1 | 6.559829 | -0.847297 | 208 | 94.511453 | 161.68 | 228.848547 | 0.777308 |
| GO:0045665\_negative\_regulation\_of\_neuron\_differentiation | 26 | 1 | 6.559829 | -0.847297 | 208 | 94.511453 | 161.68 | 228.848547 | 0.777308 |
| GO:0051235\_maintenance\_of\_location | 26 | 1 | 6.559829 | -0.847297 | 208 | 94.511453 | 161.68 | 228.848547 | 0.777308 |
| GO:0051480\_cytosolic\_calcium\_ion\_homeostasis | 26 | 1 | 6.559829 | -0.847297 | 208 | 94.511453 | 161.68 | 228.848547 | 0.777308 |
| GO:0009892\_negative\_regulation\_of\_metabolic\_process | 348 | 4 | 1.960409 | -0.846177 | 209 | 94.500775 | 161.77 | 229.039225 | 0.774019 |
| GO:0046649\_lymphocyte\_activation | 228 | 3 | 2.244152 | -0.833034 | 210 | 95.257763 | 162.94 | 230.622237 | 0.775905 |
| GO:0009411\_response\_to\_UV | 27 | 1 | 6.316872 | -0.832109 | 212 | 97.311825 | 165.72 | 234.128175 | 0.781698 |
| GO:0010638\_positive\_regulation\_of\_organelle\_organization | 27 | 1 | 6.316872 | -0.832109 | 212 | 97.311825 | 165.72 | 234.128175 | 0.781698 |
| GO:0006519\_cellular\_amino\_acid\_and\_derivative\_metabolic\_process | 118 | 2 | 2.890772 | -0.820411 | 213 | 97.968972 | 166.69 | 235.411028 | 0.782582 |
| GO:0007585\_respiratory\_gaseous\_exchange | 28 | 1 | 6.091270 | -0.817517 | 215 | 99.942430 | 169.28 | 238.617570 | 0.787349 |
| GO:0043193\_positive\_regulation\_of\_gene-specific\_transcription | 28 | 1 | 6.091270 | -0.817517 | 215 | 99.942430 | 169.28 | 238.617570 | 0.787349 |
| GO:0022403\_cell\_cycle\_phase | 119 | 2 | 2.866480 | -0.814565 | 216 | 100.028217 | 169.49 | 238.951783 | 0.784676 |
| GO:0016477\_cell\_migration | 234 | 3 | 2.186610 | -0.808937 | 217 | 100.389842 | 169.91 | 239.430158 | 0.782995 |
| GO:0043281\_regulation\_of\_caspase\_activity | 29 | 1 | 5.881226 | -0.803479 | 222 | 102.704862 | 173.23 | 243.755138 | 0.780315 |
| GO:0050769\_positive\_regulation\_of\_neurogenesis | 29 | 1 | 5.881226 | -0.803479 | 222 | 102.704862 | 173.23 | 243.755138 | 0.780315 |
| GO:0051301\_cell\_division | 29 | 1 | 5.881226 | -0.803479 | 222 | 102.704862 | 173.23 | 243.755138 | 0.780315 |
| GO:0052548\_regulation\_of\_endopeptidase\_activity | 29 | 1 | 5.881226 | -0.803479 | 222 | 102.704862 | 173.23 | 243.755138 | 0.780315 |
| GO:0070302\_regulation\_of\_stress-activated\_protein\_kinase\_signaling\_pathway | 29 | 1 | 5.881226 | -0.803479 | 222 | 102.704862 | 173.23 | 243.755138 | 0.780315 |
| GO:0006468\_protein\_amino\_acid\_phosphorylation | 237 | 3 | 2.158931 | -0.797212 | 223 | 103.760435 | 175.12 | 246.479565 | 0.785291 |
| GO:0007049\_cell\_cycle | 238 | 3 | 2.149860 | -0.793349 | 224 | 103.783295 | 175.17 | 246.556705 | 0.782009 |
| GO:0014032\_neural\_crest\_cell\_development | 30 | 1 | 5.685185 | -0.789956 | 228 | 106.590013 | 179.21 | 251.829987 | 0.786009 |
| GO:0014033\_neural\_crest\_cell\_differentiation | 30 | 1 | 5.685185 | -0.789956 | 228 | 106.590013 | 179.21 | 251.829987 | 0.786009 |
| GO:0022411\_cellular\_component\_disassembly | 30 | 1 | 5.685185 | -0.789956 | 228 | 106.590013 | 179.21 | 251.829987 | 0.786009 |
| GO:0052547\_regulation\_of\_peptidase\_activity | 30 | 1 | 5.685185 | -0.789956 | 228 | 106.590013 | 179.21 | 251.829987 | 0.786009 |
| GO:0009308\_amine\_metabolic\_process | 124 | 2 | 2.750896 | -0.786235 | 229 | 106.710197 | 179.47 | 252.229803 | 0.783712 |
| GO:0051641\_cellular\_localization | 370 | 4 | 1.843844 | -0.777030 | 230 | 107.323526 | 180.42 | 253.516474 | 0.784435 |
| GO:0016049\_cell\_growth | 31 | 1 | 5.501792 | -0.776914 | 231 | 110.860076 | 185.2 | 259.539924 | 0.801732 |
| GO:0002376\_immune\_system\_process | 505 | 5 | 1.688669 | -0.776850 | 232 | 110.937168 | 185.36 | 259.782832 | 0.798966 |
| GO:0051493\_regulation\_of\_cytoskeleton\_organization | 32 | 1 | 5.329861 | -0.764324 | 233 | 113.325527 | 188.36 | 263.394473 | 0.808412 |
| GO:0045321\_leukocyte\_activation | 248 | 3 | 2.063172 | -0.755954 | 234 | 113.833244 | 189.08 | 264.326756 | 0.808034 |
| GO:0043170\_macromolecule\_metabolic\_process | 1576 | 12 | 1.298646 | -0.749326 | 235 | 116.132897 | 192.46 | 268.787103 | 0.818979 |
| GO:0010720\_positive\_regulation\_of\_cell\_development | 34 | 1 | 5.016340 | -0.740389 | 236 | 118.877442 | 196.3 | 273.722558 | 0.831780 |
| GO:0016481\_negative\_regulation\_of\_transcription | 253 | 3 | 2.022398 | -0.738055 | 237 | 119.247028 | 196.91 | 274.572972 | 0.830844 |
| GO:0048729\_tissue\_morphogenesis | 255 | 3 | 2.006536 | -0.731038 | 238 | 119.701878 | 197.7 | 275.698122 | 0.830672 |
| GO:0001906\_cell\_killing | 35 | 1 | 4.873016 | -0.728995 | 240 | 121.863892 | 200.31 | 278.756108 | 0.834625 |
| GO:0001909\_leukocyte\_mediated\_cytotoxicity | 35 | 1 | 4.873016 | -0.728995 | 240 | 121.863892 | 200.31 | 278.756108 | 0.834625 |
| GO:0048870\_cell\_motility | 257 | 3 | 1.990921 | -0.724101 | 241 | 122.576806 | 201.19 | 279.803194 | 0.834813 |
| GO:0001819\_positive\_regulation\_of\_cytokine\_production | 36 | 1 | 4.737654 | -0.717955 | 244 | 125.179589 | 204.66 | 284.140411 | 0.838770 |
| GO:0014020\_primary\_neural\_tube\_formation | 36 | 1 | 4.737654 | -0.717955 | 244 | 125.179589 | 204.66 | 284.140411 | 0.838770 |
| GO:0051223\_regulation\_of\_protein\_transport | 36 | 1 | 4.737654 | -0.717955 | 244 | 125.179589 | 204.66 | 284.140411 | 0.838770 |
| GO:0001775\_cell\_activation | 262 | 3 | 1.952926 | -0.707096 | 246 | 127.513885 | 207.94 | 288.366115 | 0.845285 |
| GO:0010629\_negative\_regulation\_of\_gene\_expression | 262 | 3 | 1.952926 | -0.707096 | 246 | 127.513885 | 207.94 | 288.366115 | 0.845285 |
| GO:0008016\_regulation\_of\_heart\_contraction | 38 | 1 | 4.488304 | -0.696859 | 250 | 130.702874 | 212.3 | 293.897126 | 0.849200 |
| GO:0016053\_organic\_acid\_biosynthetic\_process | 38 | 1 | 4.488304 | -0.696859 | 250 | 130.702874 | 212.3 | 293.897126 | 0.849200 |
| GO:0046394\_carboxylic\_acid\_biosynthetic\_process | 38 | 1 | 4.488304 | -0.696859 | 250 | 130.702874 | 212.3 | 293.897126 | 0.849200 |
| GO:0046777\_protein\_amino\_acid\_autophosphorylation | 38 | 1 | 4.488304 | -0.696859 | 250 | 130.702874 | 212.3 | 293.897126 | 0.849200 |
| GO:0008037\_cell\_recognition | 39 | 1 | 4.373219 | -0.686769 | 252 | 133.113862 | 215.49 | 297.866138 | 0.855119 |
| GO:0070201\_regulation\_of\_establishment\_of\_protein\_localization | 39 | 1 | 4.373219 | -0.686769 | 252 | 133.113862 | 215.49 | 297.866138 | 0.855119 |
| GO:0045934\_negative\_regulation\_of\_nucleobase\_\_nucleoside\_\_nucleotide\_and\_nucleic\_acid\_metabolic\_process | 270 | 3 | 1.895062 | -0.680858 | 253 | 133.597140 | 216.22 | 298.842860 | 0.854625 |
| GO:0051172\_negative\_regulation\_of\_nitrogen\_compound\_metabolic\_process | 271 | 3 | 1.888069 | -0.677659 | 254 | 133.705540 | 216.38 | 299.054460 | 0.851890 |
| GO:0001824\_blastocyst\_development | 40 | 1 | 4.263889 | -0.676964 | 256 | 136.133050 | 219.52 | 302.906950 | 0.857500 |
| GO:0017015\_regulation\_of\_transforming\_growth\_factor\_beta\_receptor\_signaling\_pathway | 40 | 1 | 4.263889 | -0.676964 | 256 | 136.133050 | 219.52 | 302.906950 | 0.857500 |
| GO:0006950\_response\_to\_stress | 549 | 5 | 1.553329 | -0.671940 | 257 | 136.502420 | 220.07 | 303.637580 | 0.856304 |
| GO:0010558\_negative\_regulation\_of\_macromolecule\_biosynthetic\_process | 274 | 3 | 1.867397 | -0.668167 | 258 | 136.991438 | 220.65 | 304.308562 | 0.855233 |
| GO:0006979\_response\_to\_oxidative\_stress | 41 | 1 | 4.159892 | -0.667430 | 260 | 140.260141 | 224.77 | 309.279859 | 0.864500 |
| GO:0032844\_regulation\_of\_homeostatic\_process | 41 | 1 | 4.159892 | -0.667430 | 260 | 140.260141 | 224.77 | 309.279859 | 0.864500 |
| GO:0007242\_intracellular\_signaling\_cascade | 411 | 4 | 1.659908 | -0.663959 | 261 | 140.348957 | 224.9 | 309.451043 | 0.861686 |
| GO:0008361\_regulation\_of\_cell\_size | 42 | 1 | 4.060847 | -0.658152 | 265 | 143.171677 | 228.93 | 314.688323 | 0.863887 |
| GO:0019221\_cytokine-mediated\_signaling\_pathway | 42 | 1 | 4.060847 | -0.658152 | 265 | 143.171677 | 228.93 | 314.688323 | 0.863887 |
| GO:0051345\_positive\_regulation\_of\_hydrolase\_activity | 42 | 1 | 4.060847 | -0.658152 | 265 | 143.171677 | 228.93 | 314.688323 | 0.863887 |
| GO:0080135\_regulation\_of\_cellular\_response\_to\_stress | 42 | 1 | 4.060847 | -0.658152 | 265 | 143.171677 | 228.93 | 314.688323 | 0.863887 |
| GO:0032268\_regulation\_of\_cellular\_protein\_metabolic\_process | 152 | 2 | 2.244152 | -0.650852 | 266 | 143.718462 | 229.59 | 315.461538 | 0.863120 |
| GO:0044267\_cellular\_protein\_metabolic\_process | 559 | 5 | 1.525542 | -0.650120 | 267 | 143.850060 | 229.7 | 315.549940 | 0.860300 |
| GO:0001841\_neural\_tube\_formation | 43 | 1 | 3.966408 | -0.649120 | 271 | 146.535930 | 233.29 | 320.044070 | 0.860849 |
| GO:0001894\_tissue\_homeostasis | 43 | 1 | 3.966408 | -0.649120 | 271 | 146.535930 | 233.29 | 320.044070 | 0.860849 |
| GO:0010001\_glial\_cell\_differentiation | 43 | 1 | 3.966408 | -0.649120 | 271 | 146.535930 | 233.29 | 320.044070 | 0.860849 |
| GO:0031098\_stress-activated\_protein\_kinase\_signaling\_pathway | 43 | 1 | 3.966408 | -0.649120 | 271 | 146.535930 | 233.29 | 320.044070 | 0.860849 |
| GO:0042592\_homeostatic\_process | 419 | 4 | 1.628215 | -0.644007 | 272 | 146.929520 | 233.72 | 320.510480 | 0.859265 |
| GO:0031327\_negative\_regulation\_of\_cellular\_biosynthetic\_process | 282 | 3 | 1.814421 | -0.643599 | 273 | 147.007092 | 233.84 | 320.672908 | 0.856557 |
| GO:0000902\_cell\_morphogenesis | 283 | 3 | 1.808009 | -0.640602 | 274 | 147.141913 | 234.14 | 321.138087 | 0.854526 |
| GO:0048593\_camera-type\_eye\_morphogenesis | 44 | 1 | 3.876263 | -0.640322 | 275 | 150.361544 | 238.47 | 326.578456 | 0.867164 |
| GO:0022402\_cell\_cycle\_process | 155 | 2 | 2.200717 | -0.638304 | 276 | 150.532275 | 238.77 | 327.007725 | 0.865109 |
| GO:0009890\_negative\_regulation\_of\_biosynthetic\_process | 284 | 3 | 1.801643 | -0.637621 | 277 | 150.672802 | 238.98 | 327.287198 | 0.862744 |
| GO:0009058\_biosynthetic\_process | 1175 | 9 | 1.306383 | -0.633427 | 278 | 150.876078 | 239.36 | 327.843922 | 0.861007 |
| GO:0009790\_embryonic\_development | 567 | 5 | 1.504017 | -0.633162 | 279 | 151.101079 | 239.54 | 327.978921 | 0.858566 |
| GO:0001838\_embryonic\_epithelial\_tube\_formation | 45 | 1 | 3.790123 | -0.631748 | 280 | 152.026629 | 240.87 | 329.713371 | 0.860250 |
| GO:0006396\_RNA\_processing | 47 | 1 | 3.628842 | -0.615230 | 283 | 156.722448 | 247.14 | 337.557552 | 0.873286 |
| GO:0045087\_innate\_immune\_response | 47 | 1 | 3.628842 | -0.615230 | 283 | 156.722448 | 247.14 | 337.557552 | 0.873286 |
| GO:0048871\_multicellular\_organismal\_homeostasis | 47 | 1 | 3.628842 | -0.615230 | 283 | 156.722448 | 247.14 | 337.557552 | 0.873286 |
| GO:0032269\_negative\_regulation\_of\_cellular\_protein\_metabolic\_process | 48 | 1 | 3.553241 | -0.607268 | 284 | 158.083929 | 249.13 | 340.176071 | 0.877218 |
| GO:0042110\_T\_cell\_activation | 163 | 2 | 2.092706 | -0.606384 | 285 | 158.526626 | 249.64 | 340.753374 | 0.875930 |
| GO:0040011\_locomotion | 295 | 3 | 1.734463 | -0.605854 | 286 | 159.000312 | 250.28 | 341.559688 | 0.875105 |
| GO:0006725\_cellular\_aromatic\_compound\_metabolic\_process | 49 | 1 | 3.480726 | -0.599495 | 288 | 161.044321 | 253.06 | 345.075679 | 0.878681 |
| GO:0034101\_erythrocyte\_homeostasis | 49 | 1 | 3.480726 | -0.599495 | 288 | 161.044321 | 253.06 | 345.075679 | 0.878681 |
| GO:0006259\_DNA\_metabolic\_process | 165 | 2 | 2.067340 | -0.598735 | 289 | 161.365325 | 253.57 | 345.774675 | 0.877405 |
| GO:0043065\_positive\_regulation\_of\_apoptosis | 166 | 2 | 2.054886 | -0.594958 | 290 | 161.785275 | 254.0 | 346.214725 | 0.875862 |
| GO:0007015\_actin\_filament\_organization | 50 | 1 | 3.411111 | -0.591901 | 291 | 163.449353 | 256.14 | 348.830647 | 0.880206 |
| GO:0010942\_positive\_regulation\_of\_cell\_death | 167 | 2 | 2.042582 | -0.591213 | 294 | 163.952024 | 256.84 | 349.727976 | 0.873605 |
| GO:0043068\_positive\_regulation\_of\_programmed\_cell\_death | 167 | 2 | 2.042582 | -0.591213 | 294 | 163.952024 | 256.84 | 349.727976 | 0.873605 |
| GO:0051049\_regulation\_of\_transport | 167 | 2 | 2.042582 | -0.591213 | 294 | 163.952024 | 256.84 | 349.727976 | 0.873605 |
| GO:0006520\_cellular\_amino\_acid\_metabolic\_process | 51 | 1 | 3.344227 | -0.584480 | 299 | 166.709990 | 259.94 | 353.170010 | 0.869365 |
| GO:0032583\_regulation\_of\_gene-specific\_transcription | 51 | 1 | 3.344227 | -0.584480 | 299 | 166.709990 | 259.94 | 353.170010 | 0.869365 |
| GO:0032880\_regulation\_of\_protein\_localization | 51 | 1 | 3.344227 | -0.584480 | 299 | 166.709990 | 259.94 | 353.170010 | 0.869365 |
| GO:0043408\_regulation\_of\_MAPKKK\_cascade | 51 | 1 | 3.344227 | -0.584480 | 299 | 166.709990 | 259.94 | 353.170010 | 0.869365 |
| GO:0044106\_cellular\_amine\_metabolic\_process | 51 | 1 | 3.344227 | -0.584480 | 299 | 166.709990 | 259.94 | 353.170010 | 0.869365 |
| GO:0051246\_regulation\_of\_protein\_metabolic\_process | 170 | 2 | 2.006536 | -0.580158 | 300 | 166.903947 | 260.55 | 354.196053 | 0.868500 |
| GO:0032989\_cellular\_component\_morphogenesis | 307 | 3 | 1.666667 | -0.573224 | 301 | 168.838717 | 263.23 | 357.621283 | 0.874518 |
| GO:0051179\_localization | 1058 | 8 | 1.289645 | -0.572893 | 302 | 169.323634 | 263.82 | 358.316366 | 0.873576 |
| GO:0006576\_biogenic\_amine\_metabolic\_process | 53 | 1 | 3.218029 | -0.570129 | 305 | 171.292962 | 266.02 | 360.747038 | 0.872197 |
| GO:0051248\_negative\_regulation\_of\_protein\_metabolic\_process | 53 | 1 | 3.218029 | -0.570129 | 305 | 171.292962 | 266.02 | 360.747038 | 0.872197 |
| GO:0055085\_transmembrane\_transport | 53 | 1 | 3.218029 | -0.570129 | 305 | 171.292962 | 266.02 | 360.747038 | 0.872197 |
| GO:0010467\_gene\_expression | 905 | 7 | 1.319214 | -0.568027 | 306 | 171.894837 | 266.8 | 361.705163 | 0.871895 |
| GO:0016310\_phosphorylation | 309 | 3 | 1.655879 | -0.567979 | 307 | 172.065629 | 266.99 | 361.914371 | 0.869674 |
| GO:0006164\_purine\_nucleotide\_biosynthetic\_process | 54 | 1 | 3.158436 | -0.563187 | 310 | 173.939073 | 269.05 | 364.160927 | 0.867903 |
| GO:0006412\_translation | 54 | 1 | 3.158436 | -0.563187 | 310 | 173.939073 | 269.05 | 364.160927 | 0.867903 |
| GO:0044271\_nitrogen\_compound\_biosynthetic\_process | 54 | 1 | 3.158436 | -0.563187 | 310 | 173.939073 | 269.05 | 364.160927 | 0.867903 |
| GO:0000122\_negative\_regulation\_of\_transcription\_from\_RNA\_polymerase\_II\_promoter | 175 | 2 | 1.949206 | -0.562321 | 311 | 174.494954 | 269.71 | 364.925046 | 0.867235 |
| GO:0006873\_cellular\_ion\_homeostasis | 176 | 2 | 1.938131 | -0.558838 | 312 | 174.976002 | 270.33 | 365.683998 | 0.866442 |
| GO:0006310\_DNA\_recombination | 55 | 1 | 3.101010 | -0.556394 | 315 | 176.378339 | 272.07 | 367.761661 | 0.863714 |
| GO:0007126\_meiosis | 55 | 1 | 3.101010 | -0.556394 | 315 | 176.378339 | 272.07 | 367.761661 | 0.863714 |
| GO:0051327\_M\_phase\_of\_meiotic\_cell\_cycle | 55 | 1 | 3.101010 | -0.556394 | 315 | 176.378339 | 272.07 | 367.761661 | 0.863714 |
| GO:0007165\_signal\_transduction | 915 | 7 | 1.304797 | -0.552472 | 316 | 176.745446 | 272.55 | 368.354554 | 0.862500 |
| GO:0006790\_sulfur\_metabolic\_process | 56 | 1 | 3.045635 | -0.549742 | 319 | 178.710017 | 275.01 | 371.309983 | 0.862100 |
| GO:0050678\_regulation\_of\_epithelial\_cell\_proliferation | 56 | 1 | 3.045635 | -0.549742 | 319 | 178.710017 | 275.01 | 371.309983 | 0.862100 |
| GO:0051321\_meiotic\_cell\_cycle | 56 | 1 | 3.045635 | -0.549742 | 319 | 178.710017 | 275.01 | 371.309983 | 0.862100 |
| GO:0000226\_microtubule\_cytoskeleton\_organization | 57 | 1 | 2.992203 | -0.543229 | 320 | 182.640446 | 278.97 | 375.299554 | 0.871781 |
| GO:0055082\_cellular\_chemical\_homeostasis | 181 | 2 | 1.884592 | -0.541834 | 321 | 183.051488 | 279.39 | 375.728512 | 0.870374 |
| GO:0033043\_regulation\_of\_organelle\_organization | 58 | 1 | 2.940613 | -0.536847 | 322 | 184.076307 | 280.73 | 377.383693 | 0.871832 |
| GO:0080090\_regulation\_of\_primary\_metabolic\_process | 926 | 7 | 1.289297 | -0.535786 | 323 | 184.122260 | 280.81 | 377.497740 | 0.869381 |
| GO:0048469\_cell\_maturation | 59 | 1 | 2.890772 | -0.530594 | 325 | 185.506505 | 282.61 | 379.713495 | 0.869569 |
| GO:0050870\_positive\_regulation\_of\_T\_cell\_activation | 59 | 1 | 2.890772 | -0.530594 | 325 | 185.506505 | 282.61 | 379.713495 | 0.869569 |
| GO:0048523\_negative\_regulation\_of\_cellular\_process | 774 | 6 | 1.322136 | -0.530057 | 326 | 185.598292 | 282.79 | 379.981708 | 0.867454 |
| GO:0007010\_cytoskeleton\_organization | 185 | 2 | 1.843844 | -0.528701 | 327 | 185.893712 | 283.31 | 380.726288 | 0.866391 |
| GO:0060255\_regulation\_of\_macromolecule\_metabolic\_process | 936 | 7 | 1.275522 | -0.520995 | 328 | 187.080234 | 284.92 | 382.759766 | 0.868659 |
| GO:0007154\_cell\_communication | 1096 | 8 | 1.244931 | -0.518607 | 329 | 187.355121 | 285.23 | 383.104879 | 0.866960 |
| GO:0032270\_positive\_regulation\_of\_cellular\_protein\_metabolic\_process | 61 | 1 | 2.795993 | -0.518455 | 330 | 188.522580 | 286.69 | 384.857420 | 0.868758 |
| GO:0006928\_cell\_motion | 330 | 3 | 1.550505 | -0.516004 | 332 | 189.065520 | 287.37 | 385.674480 | 0.865572 |
| GO:0051674\_localization\_of\_cell | 330 | 3 | 1.550505 | -0.516004 | 332 | 189.065520 | 287.37 | 385.674480 | 0.865572 |
| GO:0030855\_epithelial\_cell\_differentiation | 62 | 1 | 2.750896 | -0.512562 | 333 | 191.353049 | 290.37 | 389.386951 | 0.871982 |
| GO:0009165\_nucleotide\_biosynthetic\_process | 63 | 1 | 2.707231 | -0.506780 | 334 | 192.858730 | 292.18 | 391.501270 | 0.874790 |
| GO:0042060\_wound\_healing | 64 | 1 | 2.664931 | -0.501106 | 335 | 193.992177 | 293.73 | 393.467823 | 0.876806 |
| GO:0007507\_heart\_development | 195 | 2 | 1.749288 | -0.497572 | 337 | 194.843110 | 294.88 | 394.916890 | 0.875015 |
| GO:0019725\_cellular\_homeostasis | 195 | 2 | 1.749288 | -0.497572 | 337 | 194.843110 | 294.88 | 394.916890 | 0.875015 |
| GO:0006793\_phosphorus\_metabolic\_process | 340 | 3 | 1.504902 | -0.493101 | 339 | 196.423372 | 296.68 | 396.936628 | 0.875162 |
| GO:0006796\_phosphate\_metabolic\_process | 340 | 3 | 1.504902 | -0.493101 | 339 | 196.423372 | 296.68 | 396.936628 | 0.875162 |
| GO:0050801\_ion\_homeostasis | 197 | 2 | 1.731528 | -0.491622 | 340 | 196.661768 | 297.01 | 397.358232 | 0.873559 |
| GO:0007179\_transforming\_growth\_factor\_beta\_receptor\_signaling\_pathway | 66 | 1 | 2.584175 | -0.490071 | 343 | 198.109428 | 298.59 | 399.070572 | 0.870525 |
| GO:0031589\_cell-substrate\_adhesion | 66 | 1 | 2.584175 | -0.490071 | 343 | 198.109428 | 298.59 | 399.070572 | 0.870525 |
| GO:0051130\_positive\_regulation\_of\_cellular\_component\_organization | 66 | 1 | 2.584175 | -0.490071 | 343 | 198.109428 | 298.59 | 399.070572 | 0.870525 |
| GO:0002009\_morphogenesis\_of\_an\_epithelium | 198 | 2 | 1.722783 | -0.488680 | 345 | 198.654116 | 299.2 | 399.745884 | 0.867246 |
| GO:0060429\_epithelium\_development | 198 | 2 | 1.722783 | -0.488680 | 345 | 198.654116 | 299.2 | 399.745884 | 0.867246 |
| GO:0000904\_cell\_morphogenesis\_involved\_in\_differentiation | 199 | 2 | 1.714126 | -0.485760 | 346 | 198.952012 | 299.51 | 400.067988 | 0.865636 |
| GO:0009791\_post-embryonic\_development | 67 | 1 | 2.545605 | -0.484703 | 348 | 200.223648 | 301.01 | 401.796352 | 0.864971 |
| GO:0051247\_positive\_regulation\_of\_protein\_metabolic\_process | 67 | 1 | 2.545605 | -0.484703 | 348 | 200.223648 | 301.01 | 401.796352 | 0.864971 |
| GO:0034961\_cellular\_biopolymer\_biosynthetic\_process | 804 | 6 | 1.272803 | -0.483915 | 349 | 200.298588 | 301.14 | 401.981412 | 0.862865 |
| GO:0016043\_cellular\_component\_organization | 964 | 7 | 1.238474 | -0.481420 | 350 | 200.524564 | 301.39 | 402.255436 | 0.861114 |
| GO:0043284\_biopolymer\_biosynthetic\_process | 807 | 6 | 1.268071 | -0.479504 | 351 | 200.792025 | 301.75 | 402.707975 | 0.859687 |
| GO:0001932\_regulation\_of\_protein\_amino\_acid\_phosphorylation | 69 | 1 | 2.471820 | -0.474251 | 353 | 202.978703 | 304.7 | 406.421297 | 0.863173 |
| GO:0006816\_calcium\_ion\_transport | 69 | 1 | 2.471820 | -0.474251 | 353 | 202.978703 | 304.7 | 406.421297 | 0.863173 |
| GO:0048468\_cell\_development | 654 | 5 | 1.303942 | -0.474023 | 354 | 203.116531 | 304.88 | 406.643469 | 0.861243 |
| GO:0019538\_protein\_metabolic\_process | 655 | 5 | 1.301951 | -0.472435 | 355 | 203.289698 | 305.05 | 406.810302 | 0.859296 |
| GO:0048592\_eye\_morphogenesis | 70 | 1 | 2.436508 | -0.469162 | 357 | 204.413430 | 306.57 | 408.726570 | 0.858739 |
| GO:0070838\_divalent\_metal\_ion\_transport | 70 | 1 | 2.436508 | -0.469162 | 357 | 204.413430 | 306.57 | 408.726570 | 0.858739 |
| GO:0016070\_RNA\_metabolic\_process | 658 | 5 | 1.296015 | -0.467699 | 358 | 205.098726 | 307.33 | 409.561274 | 0.858464 |
| GO:0016331\_morphogenesis\_of\_embryonic\_epithelium | 71 | 1 | 2.402191 | -0.464161 | 359 | 206.479797 | 309.08 | 411.680203 | 0.860947 |
| GO:0016568\_chromatin\_modification | 72 | 1 | 2.368827 | -0.459244 | 363 | 209.514295 | 312.69 | 415.865705 | 0.861405 |
| GO:0021915\_neural\_tube\_development | 72 | 1 | 2.368827 | -0.459244 | 363 | 209.514295 | 312.69 | 415.865705 | 0.861405 |
| GO:0042098\_T\_cell\_proliferation | 72 | 1 | 2.368827 | -0.459244 | 363 | 209.514295 | 312.69 | 415.865705 | 0.861405 |
| GO:0050673\_epithelial\_cell\_proliferation | 72 | 1 | 2.368827 | -0.459244 | 363 | 209.514295 | 312.69 | 415.865705 | 0.861405 |
| GO:0006163\_purine\_nucleotide\_metabolic\_process | 73 | 1 | 2.336377 | -0.454411 | 365 | 210.974915 | 314.43 | 417.885085 | 0.861452 |
| GO:0051336\_regulation\_of\_hydrolase\_activity | 73 | 1 | 2.336377 | -0.454411 | 365 | 210.974915 | 314.43 | 417.885085 | 0.861452 |
| GO:0035295\_tube\_development | 212 | 2 | 1.609015 | -0.449662 | 366 | 211.656856 | 315.22 | 418.783144 | 0.861257 |
| GO:0009416\_response\_to\_light\_stimulus | 74 | 1 | 2.304805 | -0.449657 | 367 | 212.075638 | 315.76 | 419.444362 | 0.860381 |
| GO:0044249\_cellular\_biosynthetic\_process | 1150 | 8 | 1.186473 | -0.448808 | 368 | 212.171368 | 315.88 | 419.588632 | 0.858370 |
| GO:0003008\_system\_process | 516 | 4 | 1.322136 | -0.445528 | 369 | 212.725880 | 316.4 | 420.074120 | 0.857453 |
| GO:0034621\_cellular\_macromolecular\_complex\_subunit\_organization | 76 | 1 | 2.244152 | -0.440384 | 370 | 215.494450 | 319.58 | 423.665550 | 0.863730 |
| GO:0045449\_regulation\_of\_transcription | 676 | 5 | 1.261506 | -0.440191 | 371 | 215.756109 | 319.83 | 423.903891 | 0.862075 |
| GO:0010033\_response\_to\_organic\_substance | 216 | 2 | 1.579218 | -0.439213 | 372 | 215.937130 | 320.06 | 424.182870 | 0.860376 |
| GO:0051251\_positive\_regulation\_of\_lymphocyte\_activation | 78 | 1 | 2.186610 | -0.431407 | 373 | 219.096037 | 323.75 | 428.403963 | 0.867962 |
| GO:0009888\_tissue\_development | 525 | 4 | 1.299471 | -0.430536 | 374 | 219.342801 | 324.1 | 428.857199 | 0.866578 |
| GO:0015674\_di-\_\_tri-valent\_inorganic\_cation\_transport | 79 | 1 | 2.158931 | -0.427025 | 375 | 220.152482 | 325.22 | 430.287518 | 0.867253 |
| GO:0021700\_developmental\_maturation | 81 | 1 | 2.105624 | -0.418465 | 376 | 222.053008 | 327.71 | 433.366992 | 0.871569 |
| GO:0031323\_regulation\_of\_cellular\_metabolic\_process | 1015 | 7 | 1.176245 | -0.415865 | 377 | 222.163654 | 327.86 | 433.556346 | 0.869655 |
| GO:0065008\_regulation\_of\_biological\_quality | 693 | 5 | 1.230560 | -0.415585 | 378 | 222.307699 | 328.02 | 433.732301 | 0.867778 |
| GO:0002696\_positive\_regulation\_of\_leukocyte\_activation | 82 | 1 | 2.079946 | -0.414284 | 381 | 223.523111 | 329.65 | 435.776889 | 0.865223 |
| GO:0010627\_regulation\_of\_protein\_kinase\_cascade | 82 | 1 | 2.079946 | -0.414284 | 381 | 223.523111 | 329.65 | 435.776889 | 0.865223 |
| GO:0045664\_regulation\_of\_neuron\_differentiation | 82 | 1 | 2.079946 | -0.414284 | 381 | 223.523111 | 329.65 | 435.776889 | 0.865223 |
| GO:0007017\_microtubule-based\_process | 83 | 1 | 2.054886 | -0.410166 | 383 | 225.384275 | 331.66 | 437.935725 | 0.865953 |
| GO:0050867\_positive\_regulation\_of\_cell\_activation | 83 | 1 | 2.054886 | -0.410166 | 383 | 225.384275 | 331.66 | 437.935725 | 0.865953 |
| GO:0048519\_negative\_regulation\_of\_biological\_process | 859 | 6 | 1.191308 | -0.408455 | 384 | 226.031064 | 332.49 | 438.948936 | 0.865859 |
| GO:0007167\_enzyme\_linked\_receptor\_protein\_signaling\_pathway | 229 | 2 | 1.489568 | -0.407191 | 385 | 226.302338 | 332.79 | 439.277662 | 0.864390 |
| GO:0043687\_post-translational\_protein\_modification | 384 | 3 | 1.332465 | -0.404481 | 386 | 227.045977 | 333.66 | 440.274023 | 0.864404 |
| GO:0006350\_transcription | 701 | 5 | 1.216516 | -0.404446 | 387 | 227.280929 | 333.9 | 440.519071 | 0.862791 |
| GO:0002449\_lymphocyte\_mediated\_immunity | 85 | 1 | 2.006536 | -0.402115 | 388 | 228.241290 | 335.16 | 442.078710 | 0.863814 |
| GO:0034641\_cellular\_nitrogen\_compound\_metabolic\_process | 86 | 1 | 1.983204 | -0.398179 | 389 | 230.599908 | 337.94 | 445.280092 | 0.868740 |
| GO:0007178\_transmembrane\_receptor\_protein\_serine\_threonine\_kinase\_signaling\_pathway | 87 | 1 | 1.960409 | -0.394301 | 390 | 233.639456 | 341.39 | 449.140544 | 0.875359 |
| GO:0050863\_regulation\_of\_T\_cell\_activation | 88 | 1 | 1.938131 | -0.390479 | 391 | 234.994879 | 343.1 | 451.205121 | 0.877494 |
| GO:0048699\_generation\_of\_neurons | 396 | 3 | 1.292088 | -0.383330 | 392 | 236.022765 | 344.18 | 452.337235 | 0.878010 |
| GO:0035264\_multicellular\_organism\_growth | 90 | 1 | 1.895062 | -0.382999 | 393 | 237.231550 | 345.48 | 453.728450 | 0.879084 |
| GO:0006810\_transport | 718 | 5 | 1.187713 | -0.381673 | 394 | 237.351056 | 345.64 | 453.928944 | 0.877259 |
| GO:0002443\_leukocyte\_mediated\_immunity | 91 | 1 | 1.874237 | -0.379338 | 396 | 238.202154 | 346.72 | 455.237846 | 0.875556 |
| GO:0031399\_regulation\_of\_protein\_modification\_process | 91 | 1 | 1.874237 | -0.379338 | 396 | 238.202154 | 346.72 | 455.237846 | 0.875556 |
| GO:0009719\_response\_to\_endogenous\_stimulus | 92 | 1 | 1.853865 | -0.375729 | 397 | 239.106741 | 347.88 | 456.653259 | 0.876272 |
| GO:0006753\_nucleoside\_phosphate\_metabolic\_process | 94 | 1 | 1.814421 | -0.368660 | 400 | 242.383302 | 351.51 | 460.636698 | 0.878775 |
| GO:0008610\_lipid\_biosynthetic\_process | 94 | 1 | 1.814421 | -0.368660 | 400 | 242.383302 | 351.51 | 460.636698 | 0.878775 |
| GO:0009117\_nucleotide\_metabolic\_process | 94 | 1 | 1.814421 | -0.368660 | 400 | 242.383302 | 351.51 | 460.636698 | 0.878775 |
| GO:0051234\_establishment\_of\_localization | 729 | 5 | 1.169791 | -0.367563 | 401 | 242.498046 | 351.69 | 460.881954 | 0.877032 |
| GO:0032879\_regulation\_of\_localization | 248 | 2 | 1.375448 | -0.365166 | 402 | 243.555188 | 353.13 | 462.704812 | 0.878433 |
| GO:0060249\_anatomical\_structure\_homeostasis | 96 | 1 | 1.776620 | -0.361784 | 403 | 245.800146 | 355.33 | 464.859854 | 0.881712 |
| GO:0042221\_response\_to\_chemical\_stimulus | 409 | 3 | 1.251019 | -0.361698 | 404 | 246.035821 | 355.51 | 464.984179 | 0.879975 |
| GO:0060341\_regulation\_of\_cellular\_localization | 97 | 1 | 1.758305 | -0.358416 | 405 | 247.532668 | 357.21 | 466.887332 | 0.882000 |
| GO:0034645\_cellular\_macromolecule\_biosynthetic\_process | 901 | 6 | 1.135775 | -0.357955 | 406 | 247.731721 | 357.4 | 467.068279 | 0.880296 |
| GO:0006355\_regulation\_of\_transcription\_\_DNA-dependent | 575 | 4 | 1.186473 | -0.355708 | 407 | 248.118614 | 357.87 | 467.621386 | 0.879287 |
| GO:0009314\_response\_to\_radiation | 98 | 1 | 1.740363 | -0.355092 | 408 | 249.303384 | 359.11 | 468.916616 | 0.880172 |
| GO:0048878\_chemical\_homeostasis | 254 | 2 | 1.342957 | -0.352946 | 409 | 250.099636 | 359.94 | 469.780364 | 0.880049 |
| GO:0001817\_regulation\_of\_cytokine\_production | 99 | 1 | 1.722783 | -0.351813 | 411 | 251.531703 | 361.53 | 471.528297 | 0.879635 |
| GO:0060562\_epithelial\_tube\_morphogenesis | 99 | 1 | 1.722783 | -0.351813 | 411 | 251.531703 | 361.53 | 471.528297 | 0.879635 |
| GO:0009966\_regulation\_of\_signal\_transduction | 256 | 2 | 1.332465 | -0.348976 | 412 | 251.917132 | 361.96 | 472.002868 | 0.878544 |
| GO:0010556\_regulation\_of\_macromolecule\_biosynthetic\_process | 745 | 5 | 1.144668 | -0.347877 | 413 | 252.386606 | 362.5 | 472.613394 | 0.877724 |
| GO:0009059\_macromolecule\_biosynthetic\_process | 910 | 6 | 1.124542 | -0.347866 | 414 | 252.615863 | 362.74 | 472.864137 | 0.876184 |
| GO:0030036\_actin\_cytoskeleton\_organization | 102 | 1 | 1.672113 | -0.342232 | 415 | 254.133335 | 364.29 | 474.446665 | 0.877807 |
| GO:0022008\_neurogenesis | 423 | 3 | 1.209614 | -0.339790 | 416 | 254.350831 | 364.47 | 474.589169 | 0.876130 |
| GO:0009968\_negative\_regulation\_of\_signal\_transduction | 103 | 1 | 1.655879 | -0.339121 | 417 | 255.589942 | 365.85 | 476.110058 | 0.877338 |
| GO:0050767\_regulation\_of\_neurogenesis | 104 | 1 | 1.639957 | -0.336050 | 418 | 257.060779 | 367.57 | 478.079221 | 0.879354 |
| GO:0051252\_regulation\_of\_RNA\_metabolic\_process | 590 | 4 | 1.156309 | -0.335793 | 419 | 257.217218 | 367.74 | 478.262782 | 0.877661 |
| GO:0019222\_regulation\_of\_metabolic\_process | 1088 | 7 | 1.097324 | -0.335168 | 420 | 257.663282 | 368.19 | 478.716718 | 0.876643 |
| GO:0019219\_regulation\_of\_nucleobase\_\_nucleoside\_\_nucleotide\_and\_nucleic\_acid\_metabolic\_process | 757 | 5 | 1.126523 | -0.333740 | 421 | 258.140527 | 368.72 | 479.299473 | 0.875819 |
| GO:0006351\_transcription\_\_DNA-dependent | 594 | 4 | 1.148522 | -0.330663 | 422 | 258.952806 | 369.62 | 480.287194 | 0.875877 |
| GO:0032774\_RNA\_biosynthetic\_process | 595 | 4 | 1.146592 | -0.329392 | 423 | 259.587549 | 370.34 | 481.092451 | 0.875508 |
| GO:0007166\_cell\_surface\_receptor\_linked\_signal\_transduction | 597 | 4 | 1.142751 | -0.326864 | 424 | 260.192182 | 371.06 | 481.927818 | 0.875142 |
| GO:0010604\_positive\_regulation\_of\_macromolecule\_metabolic\_process | 433 | 3 | 1.181678 | -0.324967 | 425 | 260.777655 | 371.67 | 482.562345 | 0.874518 |
| GO:0051240\_positive\_regulation\_of\_multicellular\_organismal\_process | 108 | 1 | 1.579218 | -0.324148 | 426 | 261.555979 | 372.5 | 483.444021 | 0.874413 |
| GO:0030029\_actin\_filament-based\_process | 109 | 1 | 1.564730 | -0.321265 | 427 | 262.326196 | 373.23 | 484.133804 | 0.874075 |
| GO:0010648\_negative\_regulation\_of\_cell\_communication | 110 | 1 | 1.550505 | -0.318418 | 429 | 263.981958 | 374.98 | 485.978042 | 0.874079 |
| GO:0043010\_camera-type\_eye\_development | 110 | 1 | 1.550505 | -0.318418 | 429 | 263.981958 | 374.98 | 485.978042 | 0.874079 |
| GO:0051171\_regulation\_of\_nitrogen\_compound\_metabolic\_process | 771 | 5 | 1.106067 | -0.317897 | 430 | 264.184633 | 375.21 | 486.235367 | 0.872581 |
| GO:0050896\_response\_to\_stimulus | 1107 | 7 | 1.078490 | -0.316453 | 431 | 264.581692 | 375.71 | 486.838308 | 0.871717 |
| GO:0006464\_protein\_modification\_process | 439 | 3 | 1.165528 | -0.316386 | 432 | 264.743100 | 375.97 | 487.196900 | 0.870301 |
| GO:0051249\_regulation\_of\_lymphocyte\_activation | 112 | 1 | 1.522817 | -0.312827 | 433 | 266.181758 | 377.64 | 489.098242 | 0.872148 |
| GO:0031325\_positive\_regulation\_of\_cellular\_metabolic\_process | 442 | 3 | 1.157617 | -0.312182 | 434 | 266.546009 | 378.06 | 489.573991 | 0.871106 |
| GO:0010468\_regulation\_of\_gene\_expression | 778 | 5 | 1.096115 | -0.310230 | 435 | 267.073792 | 378.54 | 490.006208 | 0.870207 |
| GO:0048646\_anatomical\_structure\_formation\_involved\_in\_morphogenesis | 277 | 2 | 1.231448 | -0.310190 | 436 | 267.632876 | 379.14 | 490.647124 | 0.869587 |
| GO:0040008\_regulation\_of\_growth | 113 | 1 | 1.509341 | -0.310082 | 437 | 268.231455 | 379.87 | 491.508545 | 0.869268 |
| GO:0000165\_MAPKKK\_cascade | 114 | 1 | 1.496101 | -0.307370 | 439 | 269.345815 | 381.14 | 492.934185 | 0.868200 |
| GO:0009607\_response\_to\_biotic\_stimulus | 114 | 1 | 1.496101 | -0.307370 | 439 | 269.345815 | 381.14 | 492.934185 | 0.868200 |
| GO:0065009\_regulation\_of\_molecular\_function | 279 | 2 | 1.222620 | -0.306753 | 440 | 269.865842 | 381.7 | 493.534158 | 0.867500 |
| GO:0010926\_anatomical\_structure\_formation | 447 | 3 | 1.144668 | -0.305297 | 441 | 270.134280 | 382.0 | 493.865720 | 0.866213 |
| GO:0046483\_heterocycle\_metabolic\_process | 116 | 1 | 1.470307 | -0.302043 | 443 | 271.953136 | 384.1 | 496.246864 | 0.867043 |
| GO:0080134\_regulation\_of\_response\_to\_stress | 116 | 1 | 1.470307 | -0.302043 | 443 | 271.953136 | 384.1 | 496.246864 | 0.867043 |
| GO:0043933\_macromolecular\_complex\_subunit\_organization | 117 | 1 | 1.457740 | -0.299426 | 444 | 273.102007 | 385.44 | 497.777993 | 0.868108 |
| GO:0009653\_anatomical\_structure\_morphogenesis | 958 | 6 | 1.068198 | -0.298078 | 445 | 273.525288 | 385.88 | 498.234712 | 0.867146 |
| GO:0007399\_nervous\_system\_development | 621 | 4 | 1.098587 | -0.297908 | 446 | 273.802214 | 386.14 | 498.477786 | 0.865785 |
| GO:0051960\_regulation\_of\_nervous\_system\_development | 118 | 1 | 1.445386 | -0.296841 | 447 | 274.584016 | 387.0 | 499.415984 | 0.865772 |
| GO:0007417\_central\_nervous\_system\_development | 287 | 2 | 1.188540 | -0.293421 | 448 | 275.711833 | 388.24 | 500.768167 | 0.866607 |
| GO:0014706\_striated\_muscle\_tissue\_development | 120 | 1 | 1.421296 | -0.291759 | 449 | 276.080789 | 388.75 | 501.419211 | 0.865813 |
| GO:0009893\_positive\_regulation\_of\_metabolic\_process | 458 | 3 | 1.117176 | -0.290679 | 451 | 276.605858 | 389.3 | 501.994142 | 0.863193 |
| GO:0043412\_biopolymer\_modification | 458 | 3 | 1.117176 | -0.290679 | 451 | 276.605858 | 389.3 | 501.994142 | 0.863193 |
| GO:0002694\_regulation\_of\_leukocyte\_activation | 121 | 1 | 1.409550 | -0.289262 | 455 | 278.114464 | 390.77 | 503.425536 | 0.858835 |
| GO:0006917\_induction\_of\_apoptosis | 121 | 1 | 1.409550 | -0.289262 | 455 | 278.114464 | 390.77 | 503.425536 | 0.858835 |
| GO:0012502\_induction\_of\_programmed\_cell\_death | 121 | 1 | 1.409550 | -0.289262 | 455 | 278.114464 | 390.77 | 503.425536 | 0.858835 |
| GO:0051726\_regulation\_of\_cell\_cycle | 121 | 1 | 1.409550 | -0.289262 | 455 | 278.114464 | 390.77 | 503.425536 | 0.858835 |
| GO:0001816\_cytokine\_production | 122 | 1 | 1.397996 | -0.286794 | 460 | 280.182560 | 393.08 | 505.977440 | 0.854522 |
| GO:0002252\_immune\_effector\_process | 122 | 1 | 1.397996 | -0.286794 | 460 | 280.182560 | 393.08 | 505.977440 | 0.854522 |
| GO:0030001\_metal\_ion\_transport | 122 | 1 | 1.397996 | -0.286794 | 460 | 280.182560 | 393.08 | 505.977440 | 0.854522 |
| GO:0050865\_regulation\_of\_cell\_activation | 122 | 1 | 1.397996 | -0.286794 | 460 | 280.182560 | 393.08 | 505.977440 | 0.854522 |
| GO:0060284\_regulation\_of\_cell\_development | 122 | 1 | 1.397996 | -0.286794 | 460 | 280.182560 | 393.08 | 505.977440 | 0.854522 |
| GO:0031326\_regulation\_of\_cellular\_biosynthetic\_process | 812 | 5 | 1.050219 | -0.275275 | 461 | 283.497155 | 396.64 | 509.782845 | 0.860390 |
| GO:0009887\_organ\_morphogenesis | 642 | 4 | 1.062651 | -0.274549 | 462 | 284.017199 | 397.14 | 510.262801 | 0.859610 |
| GO:0045597\_positive\_regulation\_of\_cell\_differentiation | 128 | 1 | 1.332465 | -0.272559 | 464 | 284.928481 | 398.18 | 511.431519 | 0.858147 |
| GO:0060537\_muscle\_tissue\_development | 128 | 1 | 1.332465 | -0.272559 | 464 | 284.928481 | 398.18 | 511.431519 | 0.858147 |
| GO:0009889\_regulation\_of\_biosynthetic\_process | 815 | 5 | 1.046353 | -0.272364 | 465 | 285.190908 | 398.46 | 511.729092 | 0.856903 |
| GO:0044057\_regulation\_of\_system\_process | 133 | 1 | 1.282373 | -0.261402 | 466 | 288.878658 | 402.12 | 515.361342 | 0.862918 |
| GO:0001654\_eye\_development | 136 | 1 | 1.254085 | -0.254993 | 467 | 291.605208 | 404.99 | 518.374792 | 0.867216 |
| GO:0007169\_transmembrane\_receptor\_protein\_tyrosine\_kinase\_signaling\_pathway | 139 | 1 | 1.227018 | -0.248786 | 468 | 292.985034 | 406.47 | 519.954966 | 0.868526 |
| GO:0035239\_tube\_morphogenesis | 143 | 1 | 1.192696 | -0.240809 | 469 | 294.709132 | 408.38 | 522.050868 | 0.870746 |
| GO:0045596\_negative\_regulation\_of\_cell\_differentiation | 144 | 1 | 1.184414 | -0.238866 | 470 | 295.584214 | 409.35 | 523.115786 | 0.870957 |
| GO:0006812\_cation\_transport | 146 | 1 | 1.168189 | -0.235039 | 471 | 297.082647 | 410.8 | 524.517353 | 0.872187 |
| GO:0010646\_regulation\_of\_cell\_communication | 330 | 2 | 1.033670 | -0.231711 | 472 | 298.448690 | 412.31 | 526.171310 | 0.873538 |
| GO:0002684\_positive\_regulation\_of\_immune\_system\_process | 148 | 1 | 1.152402 | -0.231290 | 474 | 299.218332 | 413.04 | 526.861668 | 0.871392 |
| GO:0043085\_positive\_regulation\_of\_catalytic\_activity | 148 | 1 | 1.152402 | -0.231290 | 474 | 299.218332 | 413.04 | 526.861668 | 0.871392 |
| GO:0007517\_muscle\_organ\_development | 153 | 1 | 1.114742 | -0.222241 | 475 | 301.827764 | 415.82 | 529.812236 | 0.875411 |
| GO:0045941\_positive\_regulation\_of\_transcription | 338 | 2 | 1.009204 | -0.221838 | 476 | 302.026673 | 416.0 | 529.973327 | 0.873950 |
| GO:0050793\_regulation\_of\_developmental\_process | 703 | 4 | 0.970444 | -0.215911 | 477 | 305.138939 | 419.16 | 533.181061 | 0.878742 |
| GO:0007409\_axonogenesis | 158 | 1 | 1.079466 | -0.213629 | 479 | 306.830838 | 420.92 | 535.009162 | 0.878747 |
| GO:0048514\_blood\_vessel\_morphogenesis | 158 | 1 | 1.079466 | -0.213629 | 479 | 306.830838 | 420.92 | 535.009162 | 0.878747 |
| GO:0010628\_positive\_regulation\_of\_gene\_expression | 346 | 2 | 0.985870 | -0.212405 | 480 | 307.206338 | 421.3 | 535.393662 | 0.877708 |
| GO:0051128\_regulation\_of\_cellular\_component\_organization | 160 | 1 | 1.065972 | -0.210301 | 481 | 307.651833 | 421.79 | 535.928167 | 0.876902 |
| GO:0009628\_response\_to\_abiotic\_stimulus | 162 | 1 | 1.052812 | -0.207036 | 482 | 308.659824 | 422.8 | 536.940176 | 0.877178 |
| GO:0045935\_positive\_regulation\_of\_nucleobase\_\_nucleoside\_\_nucleotide\_and\_nucleic\_acid\_metabolic\_process | 352 | 2 | 0.969066 | -0.205605 | 483 | 309.019968 | 423.15 | 537.280032 | 0.876087 |
| GO:0042325\_regulation\_of\_phosphorylation | 164 | 1 | 1.039973 | -0.203834 | 484 | 310.222037 | 424.41 | 538.597963 | 0.876880 |
| GO:0019220\_regulation\_of\_phosphate\_metabolic\_process | 165 | 1 | 1.033670 | -0.202255 | 486 | 311.623667 | 425.86 | 540.096333 | 0.876255 |
| GO:0051174\_regulation\_of\_phosphorus\_metabolic\_process | 165 | 1 | 1.033670 | -0.202255 | 486 | 311.623667 | 425.86 | 540.096333 | 0.876255 |
| GO:0030182\_neuron\_differentiation | 356 | 2 | 0.958177 | -0.201197 | 487 | 311.962538 | 426.19 | 540.417462 | 0.875133 |
| GO:0042981\_regulation\_of\_apoptosis | 360 | 2 | 0.947531 | -0.196887 | 488 | 314.152064 | 428.31 | 542.467936 | 0.877684 |
| GO:0051173\_positive\_regulation\_of\_nitrogen\_compound\_metabolic\_process | 361 | 2 | 0.944906 | -0.195824 | 489 | 314.572758 | 428.73 | 542.887242 | 0.876748 |
| GO:0048812\_neuron\_projection\_morphogenesis | 170 | 1 | 1.003268 | -0.194582 | 490 | 315.530056 | 429.77 | 544.009944 | 0.877082 |
| GO:0010941\_regulation\_of\_cell\_death | 365 | 2 | 0.934551 | -0.191632 | 492 | 317.122559 | 431.37 | 545.617441 | 0.876768 |
| GO:0043067\_regulation\_of\_programmed\_cell\_death | 365 | 2 | 0.934551 | -0.191632 | 492 | 317.122559 | 431.37 | 545.617441 | 0.876768 |
| GO:0007600\_sensory\_perception | 172 | 1 | 0.991602 | -0.191612 | 494 | 317.957086 | 432.14 | 546.322914 | 0.874777 |
| GO:0009611\_response\_to\_wounding | 172 | 1 | 0.991602 | -0.191612 | 494 | 317.957086 | 432.14 | 546.322914 | 0.874777 |
| GO:0044093\_positive\_regulation\_of\_molecular\_function | 173 | 1 | 0.985870 | -0.190148 | 496 | 319.025339 | 433.31 | 547.594661 | 0.873609 |
| GO:0048667\_cell\_morphogenesis\_involved\_in\_neuron\_differentiation | 173 | 1 | 0.985870 | -0.190148 | 496 | 319.025339 | 433.31 | 547.594661 | 0.873609 |
| GO:0048858\_cell\_projection\_morphogenesis | 176 | 1 | 0.969066 | -0.185834 | 497 | 322.690669 | 437.07 | 551.449331 | 0.879416 |
| GO:0010557\_positive\_regulation\_of\_macromolecule\_biosynthetic\_process | 371 | 2 | 0.919437 | -0.185517 | 498 | 322.840731 | 437.27 | 551.699269 | 0.878052 |
| GO:0019752\_carboxylic\_acid\_metabolic\_process | 181 | 1 | 0.942296 | -0.178903 | 500 | 326.592336 | 441.37 | 556.147664 | 0.882740 |
| GO:0043436\_oxoacid\_metabolic\_process | 181 | 1 | 0.942296 | -0.178903 | 500 | 326.592336 | 441.37 | 556.147664 | 0.882740 |
| GO:0006082\_organic\_acid\_metabolic\_process | 182 | 1 | 0.937118 | -0.177554 | 501 | 327.386374 | 442.3 | 557.213626 | 0.882834 |
| GO:0042180\_cellular\_ketone\_metabolic\_process | 183 | 1 | 0.931998 | -0.176217 | 502 | 327.839073 | 442.87 | 557.900927 | 0.882211 |
| GO:0032990\_cell\_part\_morphogenesis | 184 | 1 | 0.926932 | -0.174892 | 503 | 328.755997 | 443.82 | 558.884003 | 0.882346 |
| GO:0006811\_ion\_transport | 186 | 1 | 0.916965 | -0.172277 | 506 | 331.039436 | 446.04 | 561.040564 | 0.881502 |
| GO:0007155\_cell\_adhesion | 186 | 1 | 0.916965 | -0.172277 | 506 | 331.039436 | 446.04 | 561.040564 | 0.881502 |
| GO:0022610\_biological\_adhesion | 186 | 1 | 0.916965 | -0.172277 | 506 | 331.039436 | 446.04 | 561.040564 | 0.881502 |
| GO:0006952\_defense\_response | 187 | 1 | 0.912062 | -0.170987 | 507 | 331.616125 | 446.59 | 561.563875 | 0.880848 |
| GO:0031328\_positive\_regulation\_of\_cellular\_biosynthetic\_process | 387 | 2 | 0.881424 | -0.170165 | 508 | 331.814023 | 446.81 | 561.805977 | 0.879547 |
| GO:0009891\_positive\_regulation\_of\_biosynthetic\_process | 388 | 2 | 0.879152 | -0.169249 | 509 | 332.338400 | 447.4 | 562.461600 | 0.878978 |
| GO:0042127\_regulation\_of\_cell\_proliferation | 393 | 2 | 0.867967 | -0.164746 | 510 | 333.270656 | 448.34 | 563.409344 | 0.879098 |
| GO:0051239\_regulation\_of\_multicellular\_organismal\_process | 587 | 3 | 0.871664 | -0.162646 | 511 | 333.858030 | 448.8 | 563.741970 | 0.878278 |
| GO:0031175\_neuron\_projection\_development | 197 | 1 | 0.865764 | -0.158692 | 512 | 338.299153 | 452.97 | 567.640847 | 0.884707 |
| GO:0001568\_blood\_vessel\_development | 203 | 1 | 0.840175 | -0.151809 | 513 | 341.506433 | 455.93 | 570.353567 | 0.888752 |
| GO:0022607\_cellular\_component\_assembly | 204 | 1 | 0.836057 | -0.150696 | 514 | 342.106715 | 456.57 | 571.033285 | 0.888268 |
| GO:0006955\_immune\_response | 205 | 1 | 0.831978 | -0.149592 | 516 | 342.976300 | 457.33 | 571.683700 | 0.886298 |
| GO:0007243\_protein\_kinase\_cascade | 205 | 1 | 0.831978 | -0.149592 | 516 | 342.976300 | 457.33 | 571.683700 | 0.886298 |
| GO:0001944\_vasculature\_development | 208 | 1 | 0.819979 | -0.146336 | 518 | 344.022730 | 458.26 | 572.497270 | 0.884672 |
| GO:0008284\_positive\_regulation\_of\_cell\_proliferation | 208 | 1 | 0.819979 | -0.146336 | 518 | 344.022730 | 458.26 | 572.497270 | 0.884672 |
| GO:0032502\_developmental\_process | 2060 | 11 | 0.910734 | -0.137818 | 519 | 346.619221 | 460.83 | 575.040779 | 0.887919 |
| GO:0006915\_apoptosis | 427 | 2 | 0.798855 | -0.137160 | 520 | 346.885508 | 461.06 | 575.234492 | 0.886654 |
| GO:0048583\_regulation\_of\_response\_to\_stimulus | 217 | 1 | 0.785970 | -0.137044 | 521 | 347.714398 | 461.82 | 575.925602 | 0.886411 |
| GO:0007423\_sensory\_organ\_development | 219 | 1 | 0.778792 | -0.135072 | 522 | 349.263640 | 463.18 | 577.096360 | 0.887318 |
| GO:0012501\_programmed\_cell\_death | 433 | 2 | 0.787785 | -0.132795 | 523 | 350.523322 | 464.31 | 578.096678 | 0.887782 |
| GO:0006357\_regulation\_of\_transcription\_from\_RNA\_polymerase\_II\_promoter | 435 | 2 | 0.784163 | -0.131371 | 524 | 350.684770 | 464.53 | 578.375230 | 0.886508 |
| GO:0002682\_regulation\_of\_immune\_system\_process | 228 | 1 | 0.748051 | -0.126583 | 525 | 352.793009 | 466.27 | 579.746991 | 0.888133 |
| GO:0006366\_transcription\_from\_RNA\_polymerase\_II\_promoter | 444 | 2 | 0.768268 | -0.125146 | 527 | 354.206123 | 467.68 | 581.153877 | 0.887438 |
| GO:0008219\_cell\_death | 444 | 2 | 0.768268 | -0.125146 | 527 | 354.206123 | 467.68 | 581.153877 | 0.887438 |
| GO:0050790\_regulation\_of\_catalytic\_activity | 233 | 1 | 0.731998 | -0.122127 | 529 | 355.626257 | 469.02 | 582.413743 | 0.886616 |
| GO:0050890\_cognition | 233 | 1 | 0.731998 | -0.122127 | 529 | 355.626257 | 469.02 | 582.413743 | 0.886616 |
| GO:0016265\_death | 450 | 2 | 0.758025 | -0.121159 | 530 | 356.507988 | 469.85 | 583.192012 | 0.886509 |
| GO:0044085\_cellular\_component\_biogenesis | 237 | 1 | 0.719644 | -0.118688 | 531 | 358.308600 | 471.5 | 584.691400 | 0.887947 |
| GO:0007275\_multicellular\_organismal\_development | 1760 | 9 | 0.872159 | -0.117856 | 532 | 358.538194 | 471.68 | 584.821806 | 0.886617 |
| GO:0030154\_cell\_differentiation | 1060 | 5 | 0.804507 | -0.108387 | 533 | 361.300209 | 474.29 | 587.279791 | 0.889850 |
| GO:0048731\_system\_development | 1609 | 8 | 0.848008 | -0.107637 | 534 | 362.187041 | 475.01 | 587.832959 | 0.889532 |
| GO:0007267\_cell-cell\_signaling | 252 | 1 | 0.676808 | -0.106713 | 535 | 362.954999 | 475.64 | 588.325001 | 0.889047 |
| GO:0030097\_hemopoiesis | 253 | 1 | 0.674133 | -0.105964 | 536 | 363.862367 | 476.38 | 588.897633 | 0.888769 |
| GO:0048666\_neuron\_development | 262 | 1 | 0.650975 | -0.099470 | 537 | 366.816040 | 479.01 | 591.203960 | 0.892011 |
| GO:0030030\_cell\_projection\_organization | 263 | 1 | 0.648500 | -0.098775 | 538 | 367.463205 | 479.67 | 591.876795 | 0.891580 |
| GO:0032501\_multicellular\_organismal\_process | 2183 | 11 | 0.859419 | -0.090143 | 539 | 372.119908 | 483.68 | 595.240092 | 0.897365 |
| GO:0048534\_hemopoietic\_or\_lymphoid\_organ\_development | 277 | 1 | 0.615724 | -0.089585 | 540 | 373.041445 | 484.45 | 595.858555 | 0.897130 |
| GO:0048869\_cellular\_developmental\_process | 1113 | 5 | 0.766197 | -0.087360 | 541 | 374.607656 | 485.8 | 596.992344 | 0.897967 |
| GO:0006629\_lipid\_metabolic\_process | 285 | 1 | 0.598441 | -0.084750 | 542 | 376.668325 | 487.59 | 598.511675 | 0.899613 |
| GO:0048856\_anatomical\_structure\_development | 1688 | 8 | 0.808320 | -0.080340 | 543 | 377.964460 | 488.63 | 599.295540 | 0.899871 |
| GO:0002520\_immune\_system\_development | 295 | 1 | 0.578154 | -0.079094 | 545 | 378.981194 | 489.57 | 600.158806 | 0.898294 |
| GO:0045595\_regulation\_of\_cell\_differentiation | 295 | 1 | 0.578154 | -0.079094 | 545 | 378.981194 | 489.57 | 600.158806 | 0.898294 |
| GO:0048598\_embryonic\_morphogenesis | 299 | 1 | 0.570420 | -0.076945 | 546 | 379.582648 | 490.04 | 600.497352 | 0.897509 |
| GO:0045893\_positive\_regulation\_of\_transcription\_\_DNA-dependent | 306 | 1 | 0.557371 | -0.073331 | 548 | 381.112071 | 491.43 | 601.747929 | 0.896770 |
| GO:0051254\_positive\_regulation\_of\_RNA\_metabolic\_process | 306 | 1 | 0.557371 | -0.073331 | 548 | 381.112071 | 491.43 | 601.747929 | 0.896770 |
| GO:0048513\_organ\_development | 1365 | 6 | 0.749695 | -0.067475 | 549 | 383.548039 | 493.47 | 603.391961 | 0.898852 |
| GO:0051093\_negative\_regulation\_of\_developmental\_process | 331 | 1 | 0.515274 | -0.061813 | 550 | 386.308424 | 495.84 | 605.371576 | 0.901527 |
| GO:0009605\_response\_to\_external\_stimulus | 339 | 1 | 0.503114 | -0.058539 | 551 | 388.445938 | 497.59 | 606.734062 | 0.903067 |
| GO:0050877\_neurological\_system\_process | 390 | 1 | 0.437322 | -0.041458 | 552 | 398.911848 | 505.76 | 612.608152 | 0.916232 |
| GO:0001708\_cell\_fate\_specification | 56 | 0 | 0.000000 | -0.000000 | 559 | 426.809343 | 527.01 | 627.210657 | 0.942773 |
| GO:0002683\_negative\_regulation\_of\_immune\_system\_process | 56 | 0 | 0.000000 | -0.000000 | 559 | 426.809343 | 527.01 | 627.210657 | 0.942773 |
| GO:0002703\_regulation\_of\_leukocyte\_mediated\_immunity | 56 | 0 | 0.000000 | -0.000000 | 559 | 426.809343 | 527.01 | 627.210657 | 0.942773 |
| GO:0009187\_cyclic\_nucleotide\_metabolic\_process | 56 | 0 | 0.000000 | -0.000000 | 559 | 426.809343 | 527.01 | 627.210657 | 0.942773 |
| GO:0042089\_cytokine\_biosynthetic\_process | 56 | 0 | 0.000000 | -0.000000 | 559 | 426.809343 | 527.01 | 627.210657 | 0.942773 |
| GO:0042107\_cytokine\_metabolic\_process | 56 | 0 | 0.000000 | -0.000000 | 559 | 426.809343 | 527.01 | 627.210657 | 0.942773 |
| GO:0046486\_glycerolipid\_metabolic\_process | 56 | 0 | 0.000000 | -0.000000 | 559 | 426.809343 | 527.01 | 627.210657 | 0.942773 |
| GO:0001783\_B\_cell\_apoptosis | 8 | 0 | 0.000000 | -0.000000 | 680 | 555.612893 | 653.73 | 751.847107 | 0.961368 |
| GO:0001893\_maternal\_placenta\_development | 8 | 0 | 0.000000 | -0.000000 | 680 | 555.612893 | 653.73 | 751.847107 | 0.961368 |
| GO:0001911\_negative\_regulation\_of\_leukocyte\_mediated\_cytotoxicity | 8 | 0 | 0.000000 | -0.000000 | 680 | 555.612893 | 653.73 | 751.847107 | 0.961368 |
| GO:0001916\_positive\_regulation\_of\_T\_cell\_mediated\_cytotoxicity | 8 | 0 | 0.000000 | -0.000000 | 680 | 555.612893 | 653.73 | 751.847107 | 0.961368 |
| GO:0002065\_columnar\_cuboidal\_epithelial\_cell\_differentiation | 8 | 0 | 0.000000 | -0.000000 | 680 | 555.612893 | 653.73 | 751.847107 | 0.961368 |
| GO:0002320\_lymphoid\_progenitor\_cell\_differentiation | 8 | 0 | 0.000000 | -0.000000 | 680 | 555.612893 | 653.73 | 751.847107 | 0.961368 |
| GO:0002438\_acute\_inflammatory\_response\_to\_antigenic\_stimulus | 8 | 0 | 0.000000 | -0.000000 | 680 | 555.612893 | 653.73 | 751.847107 | 0.961368 |
| GO:0002524\_hypersensitivity | 8 | 0 | 0.000000 | -0.000000 | 680 | 555.612893 | 653.73 | 751.847107 | 0.961368 |
| GO:0002566\_somatic\_diversification\_of\_immune\_receptors\_via\_somatic\_mutation | 8 | 0 | 0.000000 | -0.000000 | 680 | 555.612893 | 653.73 | 751.847107 | 0.961368 |
| GO:0002864\_regulation\_of\_acute\_inflammatory\_response\_to\_antigenic\_stimulus | 8 | 0 | 0.000000 | -0.000000 | 680 | 555.612893 | 653.73 | 751.847107 | 0.961368 |
| GO:0002883\_regulation\_of\_hypersensitivity | 8 | 0 | 0.000000 | -0.000000 | 680 | 555.612893 | 653.73 | 751.847107 | 0.961368 |
| GO:0003081\_regulation\_of\_systemic\_arterial\_blood\_pressure\_by\_renin-angiotensin | 8 | 0 | 0.000000 | -0.000000 | 680 | 555.612893 | 653.73 | 751.847107 | 0.961368 |
| GO:0006020\_inositol\_metabolic\_process | 8 | 0 | 0.000000 | -0.000000 | 680 | 555.612893 | 653.73 | 751.847107 | 0.961368 |
| GO:0006195\_purine\_nucleotide\_catabolic\_process | 8 | 0 | 0.000000 | -0.000000 | 680 | 555.612893 | 653.73 | 751.847107 | 0.961368 |
| GO:0006284\_base-excision\_repair | 8 | 0 | 0.000000 | -0.000000 | 680 | 555.612893 | 653.73 | 751.847107 | 0.961368 |
| GO:0006349\_genetic\_imprinting | 8 | 0 | 0.000000 | -0.000000 | 680 | 555.612893 | 653.73 | 751.847107 | 0.961368 |
| GO:0006360\_transcription\_from\_RNA\_polymerase\_I\_promoter | 8 | 0 | 0.000000 | -0.000000 | 680 | 555.612893 | 653.73 | 751.847107 | 0.961368 |
| GO:0006399\_tRNA\_metabolic\_process | 8 | 0 | 0.000000 | -0.000000 | 680 | 555.612893 | 653.73 | 751.847107 | 0.961368 |
| GO:0006493\_protein\_amino\_acid\_O-linked\_glycosylation | 8 | 0 | 0.000000 | -0.000000 | 680 | 555.612893 | 653.73 | 751.847107 | 0.961368 |
| GO:0006582\_melanin\_metabolic\_process | 8 | 0 | 0.000000 | -0.000000 | 680 | 555.612893 | 653.73 | 751.847107 | 0.961368 |
| GO:0006733\_oxidoreduction\_coenzyme\_metabolic\_process | 8 | 0 | 0.000000 | -0.000000 | 680 | 555.612893 | 653.73 | 751.847107 | 0.961368 |
| GO:0006829\_zinc\_ion\_transport | 8 | 0 | 0.000000 | -0.000000 | 680 | 555.612893 | 653.73 | 751.847107 | 0.961368 |
| GO:0007009\_plasma\_membrane\_organization | 8 | 0 | 0.000000 | -0.000000 | 680 | 555.612893 | 653.73 | 751.847107 | 0.961368 |
| GO:0007098\_centrosome\_cycle | 8 | 0 | 0.000000 | -0.000000 | 680 | 555.612893 | 653.73 | 751.847107 | 0.961368 |
| GO:0007131\_reciprocal\_meiotic\_recombination | 8 | 0 | 0.000000 | -0.000000 | 680 | 555.612893 | 653.73 | 751.847107 | 0.961368 |
| GO:0007141\_male\_meiosis\_I | 8 | 0 | 0.000000 | -0.000000 | 680 | 555.612893 | 653.73 | 751.847107 | 0.961368 |
| GO:0007625\_grooming\_behavior | 8 | 0 | 0.000000 | -0.000000 | 680 | 555.612893 | 653.73 | 751.847107 | 0.961368 |
| GO:0008105\_asymmetric\_protein\_localization | 8 | 0 | 0.000000 | -0.000000 | 680 | 555.612893 | 653.73 | 751.847107 | 0.961368 |
| GO:0008593\_regulation\_of\_Notch\_signaling\_pathway | 8 | 0 | 0.000000 | -0.000000 | 680 | 555.612893 | 653.73 | 751.847107 | 0.961368 |
| GO:0009072\_aromatic\_amino\_acid\_family\_metabolic\_process | 8 | 0 | 0.000000 | -0.000000 | 680 | 555.612893 | 653.73 | 751.847107 | 0.961368 |
| GO:0009144\_purine\_nucleoside\_triphosphate\_metabolic\_process | 8 | 0 | 0.000000 | -0.000000 | 680 | 555.612893 | 653.73 | 751.847107 | 0.961368 |
| GO:0009746\_response\_to\_hexose\_stimulus | 8 | 0 | 0.000000 | -0.000000 | 680 | 555.612893 | 653.73 | 751.847107 | 0.961368 |
| GO:0009749\_response\_to\_glucose\_stimulus | 8 | 0 | 0.000000 | -0.000000 | 680 | 555.612893 | 653.73 | 751.847107 | 0.961368 |
| GO:0014014\_negative\_regulation\_of\_gliogenesis | 8 | 0 | 0.000000 | -0.000000 | 680 | 555.612893 | 653.73 | 751.847107 | 0.961368 |
| GO:0014046\_dopamine\_secretion | 8 | 0 | 0.000000 | -0.000000 | 680 | 555.612893 | 653.73 | 751.847107 | 0.961368 |
| GO:0014059\_regulation\_of\_dopamine\_secretion | 8 | 0 | 0.000000 | -0.000000 | 680 | 555.612893 | 653.73 | 751.847107 | 0.961368 |
| GO:0014065\_phosphoinositide\_3-kinase\_cascade | 8 | 0 | 0.000000 | -0.000000 | 680 | 555.612893 | 653.73 | 751.847107 | 0.961368 |
| GO:0015800\_acidic\_amino\_acid\_transport | 8 | 0 | 0.000000 | -0.000000 | 680 | 555.612893 | 653.73 | 751.847107 | 0.961368 |
| GO:0015804\_neutral\_amino\_acid\_transport | 8 | 0 | 0.000000 | -0.000000 | 680 | 555.612893 | 653.73 | 751.847107 | 0.961368 |
| GO:0016236\_macroautophagy | 8 | 0 | 0.000000 | -0.000000 | 680 | 555.612893 | 653.73 | 751.847107 | 0.961368 |
| GO:0016446\_somatic\_hypermutation\_of\_immunoglobulin\_genes | 8 | 0 | 0.000000 | -0.000000 | 680 | 555.612893 | 653.73 | 751.847107 | 0.961368 |
| GO:0018107\_peptidyl-threonine\_phosphorylation | 8 | 0 | 0.000000 | -0.000000 | 680 | 555.612893 | 653.73 | 751.847107 | 0.961368 |
| GO:0018210\_peptidyl-threonine\_modification | 8 | 0 | 0.000000 | -0.000000 | 680 | 555.612893 | 653.73 | 751.847107 | 0.961368 |
| GO:0018345\_protein\_palmitoylation | 8 | 0 | 0.000000 | -0.000000 | 680 | 555.612893 | 653.73 | 751.847107 | 0.961368 |
| GO:0019229\_regulation\_of\_vasoconstriction | 8 | 0 | 0.000000 | -0.000000 | 680 | 555.612893 | 653.73 | 751.847107 | 0.961368 |
| GO:0019400\_alditol\_metabolic\_process | 8 | 0 | 0.000000 | -0.000000 | 680 | 555.612893 | 653.73 | 751.847107 | 0.961368 |
| GO:0021692\_cerebellar\_Purkinje\_cell\_layer\_morphogenesis | 8 | 0 | 0.000000 | -0.000000 | 680 | 555.612893 | 653.73 | 751.847107 | 0.961368 |
| GO:0021694\_cerebellar\_Purkinje\_cell\_layer\_formation | 8 | 0 | 0.000000 | -0.000000 | 680 | 555.612893 | 653.73 | 751.847107 | 0.961368 |
| GO:0021702\_cerebellar\_Purkinje\_cell\_differentiation | 8 | 0 | 0.000000 | -0.000000 | 680 | 555.612893 | 653.73 | 751.847107 | 0.961368 |
| GO:0021781\_glial\_cell\_fate\_commitment | 8 | 0 | 0.000000 | -0.000000 | 680 | 555.612893 | 653.73 | 751.847107 | 0.961368 |
| GO:0021799\_cerebral\_cortex\_radially\_oriented\_cell\_migration | 8 | 0 | 0.000000 | -0.000000 | 680 | 555.612893 | 653.73 | 751.847107 | 0.961368 |
| GO:0030035\_microspike\_assembly | 8 | 0 | 0.000000 | -0.000000 | 680 | 555.612893 | 653.73 | 751.847107 | 0.961368 |
| GO:0030193\_regulation\_of\_blood\_coagulation | 8 | 0 | 0.000000 | -0.000000 | 680 | 555.612893 | 653.73 | 751.847107 | 0.961368 |
| GO:0030204\_chondroitin\_sulfate\_metabolic\_process | 8 | 0 | 0.000000 | -0.000000 | 680 | 555.612893 | 653.73 | 751.847107 | 0.961368 |
| GO:0030500\_regulation\_of\_bone\_mineralization | 8 | 0 | 0.000000 | -0.000000 | 680 | 555.612893 | 653.73 | 751.847107 | 0.961368 |
| GO:0030511\_positive\_regulation\_of\_transforming\_growth\_factor\_beta\_receptor\_signaling\_pathway | 8 | 0 | 0.000000 | -0.000000 | 680 | 555.612893 | 653.73 | 751.847107 | 0.961368 |
| GO:0031102\_neuron\_projection\_regeneration | 8 | 0 | 0.000000 | -0.000000 | 680 | 555.612893 | 653.73 | 751.847107 | 0.961368 |
| GO:0031103\_axon\_regeneration | 8 | 0 | 0.000000 | -0.000000 | 680 | 555.612893 | 653.73 | 751.847107 | 0.961368 |
| GO:0031111\_negative\_regulation\_of\_microtubule\_polymerization\_or\_depolymerization | 8 | 0 | 0.000000 | -0.000000 | 680 | 555.612893 | 653.73 | 751.847107 | 0.961368 |
| GO:0031123\_RNA\_3'-end\_processing | 8 | 0 | 0.000000 | -0.000000 | 680 | 555.612893 | 653.73 | 751.847107 | 0.961368 |
| GO:0031294\_lymphocyte\_costimulation | 8 | 0 | 0.000000 | -0.000000 | 680 | 555.612893 | 653.73 | 751.847107 | 0.961368 |
| GO:0031295\_T\_cell\_costimulation | 8 | 0 | 0.000000 | -0.000000 | 680 | 555.612893 | 653.73 | 751.847107 | 0.961368 |
| GO:0031334\_positive\_regulation\_of\_protein\_complex\_assembly | 8 | 0 | 0.000000 | -0.000000 | 680 | 555.612893 | 653.73 | 751.847107 | 0.961368 |
| GO:0031342\_negative\_regulation\_of\_cell\_killing | 8 | 0 | 0.000000 | -0.000000 | 680 | 555.612893 | 653.73 | 751.847107 | 0.961368 |
| GO:0031396\_regulation\_of\_protein\_ubiquitination | 8 | 0 | 0.000000 | -0.000000 | 680 | 555.612893 | 653.73 | 751.847107 | 0.961368 |
| GO:0032094\_response\_to\_food | 8 | 0 | 0.000000 | -0.000000 | 680 | 555.612893 | 653.73 | 751.847107 | 0.961368 |
| GO:0032273\_positive\_regulation\_of\_protein\_polymerization | 8 | 0 | 0.000000 | -0.000000 | 680 | 555.612893 | 653.73 | 751.847107 | 0.961368 |
| GO:0032613\_interleukin-10\_production | 8 | 0 | 0.000000 | -0.000000 | 680 | 555.612893 | 653.73 | 751.847107 | 0.961368 |
| GO:0033198\_response\_to\_ATP | 8 | 0 | 0.000000 | -0.000000 | 680 | 555.612893 | 653.73 | 751.847107 | 0.961368 |
| GO:0034284\_response\_to\_monosaccharide\_stimulus | 8 | 0 | 0.000000 | -0.000000 | 680 | 555.612893 | 653.73 | 751.847107 | 0.961368 |
| GO:0034728\_nucleosome\_organization | 8 | 0 | 0.000000 | -0.000000 | 680 | 555.612893 | 653.73 | 751.847107 | 0.961368 |
| GO:0035023\_regulation\_of\_Rho\_protein\_signal\_transduction | 8 | 0 | 0.000000 | -0.000000 | 680 | 555.612893 | 653.73 | 751.847107 | 0.961368 |
| GO:0035112\_genitalia\_morphogenesis | 8 | 0 | 0.000000 | -0.000000 | 680 | 555.612893 | 653.73 | 751.847107 | 0.961368 |
| GO:0040017\_positive\_regulation\_of\_locomotion | 8 | 0 | 0.000000 | -0.000000 | 680 | 555.612893 | 653.73 | 751.847107 | 0.961368 |
| GO:0040034\_regulation\_of\_development\_\_heterochronic | 8 | 0 | 0.000000 | -0.000000 | 680 | 555.612893 | 653.73 | 751.847107 | 0.961368 |
| GO:0042074\_cell\_migration\_involved\_in\_gastrulation | 8 | 0 | 0.000000 | -0.000000 | 680 | 555.612893 | 653.73 | 751.847107 | 0.961368 |
| GO:0042090\_interleukin-12\_biosynthetic\_process | 8 | 0 | 0.000000 | -0.000000 | 680 | 555.612893 | 653.73 | 751.847107 | 0.961368 |
| GO:0042092\_T-helper\_2\_type\_immune\_response | 8 | 0 | 0.000000 | -0.000000 | 680 | 555.612893 | 653.73 | 751.847107 | 0.961368 |
| GO:0042095\_interferon-gamma\_biosynthetic\_process | 8 | 0 | 0.000000 | -0.000000 | 680 | 555.612893 | 653.73 | 751.847107 | 0.961368 |
| GO:0042104\_positive\_regulation\_of\_activated\_T\_cell\_proliferation | 8 | 0 | 0.000000 | -0.000000 | 680 | 555.612893 | 653.73 | 751.847107 | 0.961368 |
| GO:0042226\_interleukin-6\_biosynthetic\_process | 8 | 0 | 0.000000 | -0.000000 | 680 | 555.612893 | 653.73 | 751.847107 | 0.961368 |
| GO:0042304\_regulation\_of\_fatty\_acid\_biosynthetic\_process | 8 | 0 | 0.000000 | -0.000000 | 680 | 555.612893 | 653.73 | 751.847107 | 0.961368 |
| GO:0042423\_catecholamine\_biosynthetic\_process | 8 | 0 | 0.000000 | -0.000000 | 680 | 555.612893 | 653.73 | 751.847107 | 0.961368 |
| GO:0042771\_DNA\_damage\_response\_\_signal\_transduction\_by\_p53\_class\_mediator\_resulting\_in\_induction\_of\_apoptosis | 8 | 0 | 0.000000 | -0.000000 | 680 | 555.612893 | 653.73 | 751.847107 | 0.961368 |
| GO:0043011\_myeloid\_dendritic\_cell\_differentiation | 8 | 0 | 0.000000 | -0.000000 | 680 | 555.612893 | 653.73 | 751.847107 | 0.961368 |
| GO:0043368\_positive\_T\_cell\_selection | 8 | 0 | 0.000000 | -0.000000 | 680 | 555.612893 | 653.73 | 751.847107 | 0.961368 |
| GO:0043370\_regulation\_of\_CD4-positive\_\_alpha\_beta\_T\_cell\_differentiation | 8 | 0 | 0.000000 | -0.000000 | 680 | 555.612893 | 653.73 | 751.847107 | 0.961368 |
| GO:0043542\_endothelial\_cell\_migration | 8 | 0 | 0.000000 | -0.000000 | 680 | 555.612893 | 653.73 | 751.847107 | 0.961368 |
| GO:0043616\_keratinocyte\_proliferation | 8 | 0 | 0.000000 | -0.000000 | 680 | 555.612893 | 653.73 | 751.847107 | 0.961368 |
| GO:0045075\_regulation\_of\_interleukin-12\_biosynthetic\_process | 8 | 0 | 0.000000 | -0.000000 | 680 | 555.612893 | 653.73 | 751.847107 | 0.961368 |
| GO:0045086\_positive\_regulation\_of\_interleukin-2\_biosynthetic\_process | 8 | 0 | 0.000000 | -0.000000 | 680 | 555.612893 | 653.73 | 751.847107 | 0.961368 |
| GO:0045351\_type\_I\_interferon\_biosynthetic\_process | 8 | 0 | 0.000000 | -0.000000 | 680 | 555.612893 | 653.73 | 751.847107 | 0.961368 |
| GO:0045408\_regulation\_of\_interleukin-6\_biosynthetic\_process | 8 | 0 | 0.000000 | -0.000000 | 680 | 555.612893 | 653.73 | 751.847107 | 0.961368 |
| GO:0045429\_positive\_regulation\_of\_nitric\_oxide\_biosynthetic\_process | 8 | 0 | 0.000000 | -0.000000 | 680 | 555.612893 | 653.73 | 751.847107 | 0.961368 |
| GO:0045494\_photoreceptor\_cell\_maintenance | 8 | 0 | 0.000000 | -0.000000 | 680 | 555.612893 | 653.73 | 751.847107 | 0.961368 |
| GO:0045686\_negative\_regulation\_of\_glial\_cell\_differentiation | 8 | 0 | 0.000000 | -0.000000 | 680 | 555.612893 | 653.73 | 751.847107 | 0.961368 |
| GO:0045910\_negative\_regulation\_of\_DNA\_recombination | 8 | 0 | 0.000000 | -0.000000 | 680 | 555.612893 | 653.73 | 751.847107 | 0.961368 |
| GO:0045921\_positive\_regulation\_of\_exocytosis | 8 | 0 | 0.000000 | -0.000000 | 680 | 555.612893 | 653.73 | 751.847107 | 0.961368 |
| GO:0045932\_negative\_regulation\_of\_muscle\_contraction | 8 | 0 | 0.000000 | -0.000000 | 680 | 555.612893 | 653.73 | 751.847107 | 0.961368 |
| GO:0046470\_phosphatidylcholine\_metabolic\_process | 8 | 0 | 0.000000 | -0.000000 | 680 | 555.612893 | 653.73 | 751.847107 | 0.961368 |
| GO:0048266\_behavioral\_response\_to\_pain | 8 | 0 | 0.000000 | -0.000000 | 680 | 555.612893 | 653.73 | 751.847107 | 0.961368 |
| GO:0048505\_regulation\_of\_timing\_of\_cell\_differentiation | 8 | 0 | 0.000000 | -0.000000 | 680 | 555.612893 | 653.73 | 751.847107 | 0.961368 |
| GO:0048520\_positive\_regulation\_of\_behavior | 8 | 0 | 0.000000 | -0.000000 | 680 | 555.612893 | 653.73 | 751.847107 | 0.961368 |
| GO:0048557\_embryonic\_digestive\_tract\_morphogenesis | 8 | 0 | 0.000000 | -0.000000 | 680 | 555.612893 | 653.73 | 751.847107 | 0.961368 |
| GO:0048638\_regulation\_of\_developmental\_growth | 8 | 0 | 0.000000 | -0.000000 | 680 | 555.612893 | 653.73 | 751.847107 | 0.961368 |
| GO:0048742\_regulation\_of\_skeletal\_muscle\_fiber\_development | 8 | 0 | 0.000000 | -0.000000 | 680 | 555.612893 | 653.73 | 751.847107 | 0.961368 |
| GO:0050707\_regulation\_of\_cytokine\_secretion | 8 | 0 | 0.000000 | -0.000000 | 680 | 555.612893 | 653.73 | 751.847107 | 0.961368 |
| GO:0050909\_sensory\_perception\_of\_taste | 8 | 0 | 0.000000 | -0.000000 | 680 | 555.612893 | 653.73 | 751.847107 | 0.961368 |
| GO:0050920\_regulation\_of\_chemotaxis | 8 | 0 | 0.000000 | -0.000000 | 680 | 555.612893 | 653.73 | 751.847107 | 0.961368 |
| GO:0050921\_positive\_regulation\_of\_chemotaxis | 8 | 0 | 0.000000 | -0.000000 | 680 | 555.612893 | 653.73 | 751.847107 | 0.961368 |
| GO:0050926\_regulation\_of\_positive\_chemotaxis | 8 | 0 | 0.000000 | -0.000000 | 680 | 555.612893 | 653.73 | 751.847107 | 0.961368 |
| GO:0050927\_positive\_regulation\_of\_positive\_chemotaxis | 8 | 0 | 0.000000 | -0.000000 | 680 | 555.612893 | 653.73 | 751.847107 | 0.961368 |
| GO:0050930\_induction\_of\_positive\_chemotaxis | 8 | 0 | 0.000000 | -0.000000 | 680 | 555.612893 | 653.73 | 751.847107 | 0.961368 |
| GO:0051181\_cofactor\_transport | 8 | 0 | 0.000000 | -0.000000 | 680 | 555.612893 | 653.73 | 751.847107 | 0.961368 |
| GO:0060043\_regulation\_of\_cardiac\_muscle\_cell\_proliferation | 8 | 0 | 0.000000 | -0.000000 | 680 | 555.612893 | 653.73 | 751.847107 | 0.961368 |
| GO:0060670\_branching\_involved\_in\_embryonic\_placenta\_morphogenesis | 8 | 0 | 0.000000 | -0.000000 | 680 | 555.612893 | 653.73 | 751.847107 | 0.961368 |
| GO:0060712\_spongiotrophoblast\_layer\_development | 8 | 0 | 0.000000 | -0.000000 | 680 | 555.612893 | 653.73 | 751.847107 | 0.961368 |
| GO:0070167\_regulation\_of\_biomineral\_formation | 8 | 0 | 0.000000 | -0.000000 | 680 | 555.612893 | 653.73 | 751.847107 | 0.961368 |
| GO:0070193\_synaptonemal\_complex\_organization | 8 | 0 | 0.000000 | -0.000000 | 680 | 555.612893 | 653.73 | 751.847107 | 0.961368 |
| GO:0070231\_T\_cell\_apoptosis | 8 | 0 | 0.000000 | -0.000000 | 680 | 555.612893 | 653.73 | 751.847107 | 0.961368 |
| GO:0070584\_mitochondrion\_morphogenesis | 8 | 0 | 0.000000 | -0.000000 | 680 | 555.612893 | 653.73 | 751.847107 | 0.961368 |
| GO:0007626\_locomotory\_behavior | 163 | 0 | 0.000000 | -0.000000 | 681 | 556.644533 | 654.53 | 752.415467 | 0.961131 |
| GO:0002062\_chondrocyte\_differentiation | 28 | 0 | 0.000000 | -0.000000 | 697 | 572.714164 | 669.86 | 767.005836 | 0.961062 |
| GO:0002088\_lens\_development\_in\_camera-type\_eye | 28 | 0 | 0.000000 | -0.000000 | 697 | 572.714164 | 669.86 | 767.005836 | 0.961062 |
| GO:0002705\_positive\_regulation\_of\_leukocyte\_mediated\_immunity | 28 | 0 | 0.000000 | -0.000000 | 697 | 572.714164 | 669.86 | 767.005836 | 0.961062 |
| GO:0002708\_positive\_regulation\_of\_lymphocyte\_mediated\_immunity | 28 | 0 | 0.000000 | -0.000000 | 697 | 572.714164 | 669.86 | 767.005836 | 0.961062 |
| GO:0006470\_protein\_amino\_acid\_dephosphorylation | 28 | 0 | 0.000000 | -0.000000 | 697 | 572.714164 | 669.86 | 767.005836 | 0.961062 |
| GO:0006997\_nucleus\_organization | 28 | 0 | 0.000000 | -0.000000 | 697 | 572.714164 | 669.86 | 767.005836 | 0.961062 |
| GO:0007127\_meiosis\_I | 28 | 0 | 0.000000 | -0.000000 | 697 | 572.714164 | 669.86 | 767.005836 | 0.961062 |
| GO:0021549\_cerebellum\_development | 28 | 0 | 0.000000 | -0.000000 | 697 | 572.714164 | 669.86 | 767.005836 | 0.961062 |
| GO:0030073\_insulin\_secretion | 28 | 0 | 0.000000 | -0.000000 | 697 | 572.714164 | 669.86 | 767.005836 | 0.961062 |
| GO:0030111\_regulation\_of\_Wnt\_receptor\_signaling\_pathway | 28 | 0 | 0.000000 | -0.000000 | 697 | 572.714164 | 669.86 | 767.005836 | 0.961062 |
| GO:0042100\_B\_cell\_proliferation | 28 | 0 | 0.000000 | -0.000000 | 697 | 572.714164 | 669.86 | 767.005836 | 0.961062 |
| GO:0045926\_negative\_regulation\_of\_growth | 28 | 0 | 0.000000 | -0.000000 | 697 | 572.714164 | 669.86 | 767.005836 | 0.961062 |
| GO:0046328\_regulation\_of\_JNK\_cascade | 28 | 0 | 0.000000 | -0.000000 | 697 | 572.714164 | 669.86 | 767.005836 | 0.961062 |
| GO:0048863\_stem\_cell\_differentiation | 28 | 0 | 0.000000 | -0.000000 | 697 | 572.714164 | 669.86 | 767.005836 | 0.961062 |
| GO:0050871\_positive\_regulation\_of\_B\_cell\_activation | 28 | 0 | 0.000000 | -0.000000 | 697 | 572.714164 | 669.86 | 767.005836 | 0.961062 |
| GO:0051188\_cofactor\_biosynthetic\_process | 28 | 0 | 0.000000 | -0.000000 | 697 | 572.714164 | 669.86 | 767.005836 | 0.961062 |
| GO:0048771\_tissue\_remodeling | 74 | 0 | 0.000000 | -0.000000 | 698 | 574.212334 | 671.17 | 768.127666 | 0.961562 |
| GO:0006954\_inflammatory\_response | 96 | 0 | 0.000000 | -0.000000 | 701 | 578.011815 | 674.48 | 770.948185 | 0.962168 |
| GO:0048736\_appendage\_development | 96 | 0 | 0.000000 | -0.000000 | 701 | 578.011815 | 674.48 | 770.948185 | 0.962168 |
| GO:0060173\_limb\_development | 96 | 0 | 0.000000 | -0.000000 | 701 | 578.011815 | 674.48 | 770.948185 | 0.962168 |
| GO:0002440\_production\_of\_molecular\_mediator\_of\_immune\_response | 49 | 0 | 0.000000 | -0.000000 | 709 | 587.960459 | 683.52 | 779.079541 | 0.964062 |
| GO:0007606\_sensory\_perception\_of\_chemical\_stimulus | 49 | 0 | 0.000000 | -0.000000 | 709 | 587.960459 | 683.52 | 779.079541 | 0.964062 |
| GO:0021543\_pallium\_development | 49 | 0 | 0.000000 | -0.000000 | 709 | 587.960459 | 683.52 | 779.079541 | 0.964062 |
| GO:0042035\_regulation\_of\_cytokine\_biosynthetic\_process | 49 | 0 | 0.000000 | -0.000000 | 709 | 587.960459 | 683.52 | 779.079541 | 0.964062 |
| GO:0043473\_pigmentation | 49 | 0 | 0.000000 | -0.000000 | 709 | 587.960459 | 683.52 | 779.079541 | 0.964062 |
| GO:0046660\_female\_sex\_differentiation | 49 | 0 | 0.000000 | -0.000000 | 709 | 587.960459 | 683.52 | 779.079541 | 0.964062 |
| GO:0046661\_male\_sex\_differentiation | 49 | 0 | 0.000000 | -0.000000 | 709 | 587.960459 | 683.52 | 779.079541 | 0.964062 |
| GO:0048741\_skeletal\_muscle\_fiber\_development | 49 | 0 | 0.000000 | -0.000000 | 709 | 587.960459 | 683.52 | 779.079541 | 0.964062 |
| GO:0007281\_germ\_cell\_development | 75 | 0 | 0.000000 | -0.000000 | 712 | 590.879589 | 686.14 | 781.400411 | 0.963680 |
| GO:0044265\_cellular\_macromolecule\_catabolic\_process | 75 | 0 | 0.000000 | -0.000000 | 712 | 590.879589 | 686.14 | 781.400411 | 0.963680 |
| GO:0051050\_positive\_regulation\_of\_transport | 75 | 0 | 0.000000 | -0.000000 | 712 | 590.879589 | 686.14 | 781.400411 | 0.963680 |
| GO:0001843\_neural\_tube\_closure | 33 | 0 | 0.000000 | -0.000000 | 727 | 603.903019 | 698.18 | 792.456981 | 0.960358 |
| GO:0002562\_somatic\_diversification\_of\_immune\_receptors\_via\_germline\_recombination\_within\_a\_single\_locus | 33 | 0 | 0.000000 | -0.000000 | 727 | 603.903019 | 698.18 | 792.456981 | 0.960358 |
| GO:0006643\_membrane\_lipid\_metabolic\_process | 33 | 0 | 0.000000 | -0.000000 | 727 | 603.903019 | 698.18 | 792.456981 | 0.960358 |
| GO:0007188\_G-protein\_signaling\_\_coupled\_to\_cAMP\_nucleotide\_second\_messenger | 33 | 0 | 0.000000 | -0.000000 | 727 | 603.903019 | 698.18 | 792.456981 | 0.960358 |
| GO:0007270\_nerve-nerve\_synaptic\_transmission | 33 | 0 | 0.000000 | -0.000000 | 727 | 603.903019 | 698.18 | 792.456981 | 0.960358 |
| GO:0007431\_salivary\_gland\_development | 33 | 0 | 0.000000 | -0.000000 | 727 | 603.903019 | 698.18 | 792.456981 | 0.960358 |
| GO:0007565\_female\_pregnancy | 33 | 0 | 0.000000 | -0.000000 | 727 | 603.903019 | 698.18 | 792.456981 | 0.960358 |
| GO:0008584\_male\_gonad\_development | 33 | 0 | 0.000000 | -0.000000 | 727 | 603.903019 | 698.18 | 792.456981 | 0.960358 |
| GO:0008643\_carbohydrate\_transport | 33 | 0 | 0.000000 | -0.000000 | 727 | 603.903019 | 698.18 | 792.456981 | 0.960358 |
| GO:0016444\_somatic\_cell\_DNA\_recombination | 33 | 0 | 0.000000 | -0.000000 | 727 | 603.903019 | 698.18 | 792.456981 | 0.960358 |
| GO:0021536\_diencephalon\_development | 33 | 0 | 0.000000 | -0.000000 | 727 | 603.903019 | 698.18 | 792.456981 | 0.960358 |
| GO:0021987\_cerebral\_cortex\_development | 33 | 0 | 0.000000 | -0.000000 | 727 | 603.903019 | 698.18 | 792.456981 | 0.960358 |
| GO:0022037\_metencephalon\_development | 33 | 0 | 0.000000 | -0.000000 | 727 | 603.903019 | 698.18 | 792.456981 | 0.960358 |
| GO:0042108\_positive\_regulation\_of\_cytokine\_biosynthetic\_process | 33 | 0 | 0.000000 | -0.000000 | 727 | 603.903019 | 698.18 | 792.456981 | 0.960358 |
| GO:0060606\_tube\_closure | 33 | 0 | 0.000000 | -0.000000 | 727 | 603.903019 | 698.18 | 792.456981 | 0.960358 |
| GO:0007268\_synaptic\_transmission | 154 | 0 | 0.000000 | -0.000000 | 728 | 604.442965 | 698.58 | 792.717035 | 0.959588 |
| GO:0001759\_induction\_of\_an\_organ | 15 | 0 | 0.000000 | -0.000000 | 781 | 657.568692 | 749.93 | 842.291308 | 0.960218 |
| GO:0001782\_B\_cell\_homeostasis | 15 | 0 | 0.000000 | -0.000000 | 781 | 657.568692 | 749.93 | 842.291308 | 0.960218 |
| GO:0001964\_startle\_response | 15 | 0 | 0.000000 | -0.000000 | 781 | 657.568692 | 749.93 | 842.291308 | 0.960218 |
| GO:0002286\_T\_cell\_activation\_during\_immune\_response | 15 | 0 | 0.000000 | -0.000000 | 781 | 657.568692 | 749.93 | 842.291308 | 0.960218 |
| GO:0002495\_antigen\_processing\_and\_presentation\_of\_peptide\_antigen\_via\_MHC\_class\_II | 15 | 0 | 0.000000 | -0.000000 | 781 | 657.568692 | 749.93 | 842.291308 | 0.960218 |
| GO:0002504\_antigen\_processing\_and\_presentation\_of\_peptide\_or\_polysaccharide\_antigen\_via\_MHC\_class\_II | 15 | 0 | 0.000000 | -0.000000 | 781 | 657.568692 | 749.93 | 842.291308 | 0.960218 |
| GO:0002709\_regulation\_of\_T\_cell\_mediated\_immunity | 15 | 0 | 0.000000 | -0.000000 | 781 | 657.568692 | 749.93 | 842.291308 | 0.960218 |
| GO:0006473\_protein\_amino\_acid\_acetylation | 15 | 0 | 0.000000 | -0.000000 | 781 | 657.568692 | 749.93 | 842.291308 | 0.960218 |
| GO:0006487\_protein\_amino\_acid\_N-linked\_glycosylation | 15 | 0 | 0.000000 | -0.000000 | 781 | 657.568692 | 749.93 | 842.291308 | 0.960218 |
| GO:0006749\_glutathione\_metabolic\_process | 15 | 0 | 0.000000 | -0.000000 | 781 | 657.568692 | 749.93 | 842.291308 | 0.960218 |
| GO:0006885\_regulation\_of\_pH | 15 | 0 | 0.000000 | -0.000000 | 781 | 657.568692 | 749.93 | 842.291308 | 0.960218 |
| GO:0007040\_lysosome\_organization | 15 | 0 | 0.000000 | -0.000000 | 781 | 657.568692 | 749.93 | 842.291308 | 0.960218 |
| GO:0007173\_epidermal\_growth\_factor\_receptor\_signaling\_pathway | 15 | 0 | 0.000000 | -0.000000 | 781 | 657.568692 | 749.93 | 842.291308 | 0.960218 |
| GO:0007200\_activation\_of\_phospholipase\_C\_activity\_by\_G-protein\_coupled\_receptor\_protein\_signaling\_pathway\_coupled\_to\_IP3\_second\_messenger | 15 | 0 | 0.000000 | -0.000000 | 781 | 657.568692 | 749.93 | 842.291308 | 0.960218 |
| GO:0007202\_activation\_of\_phospholipase\_C\_activity | 15 | 0 | 0.000000 | -0.000000 | 781 | 657.568692 | 749.93 | 842.291308 | 0.960218 |
| GO:0007218\_neuropeptide\_signaling\_pathway | 15 | 0 | 0.000000 | -0.000000 | 781 | 657.568692 | 749.93 | 842.291308 | 0.960218 |
| GO:0007588\_excretion | 15 | 0 | 0.000000 | -0.000000 | 781 | 657.568692 | 749.93 | 842.291308 | 0.960218 |
| GO:0007618\_mating | 15 | 0 | 0.000000 | -0.000000 | 781 | 657.568692 | 749.93 | 842.291308 | 0.960218 |
| GO:0008543\_fibroblast\_growth\_factor\_receptor\_signaling\_pathway | 15 | 0 | 0.000000 | -0.000000 | 781 | 657.568692 | 749.93 | 842.291308 | 0.960218 |
| GO:0009062\_fatty\_acid\_catabolic\_process | 15 | 0 | 0.000000 | -0.000000 | 781 | 657.568692 | 749.93 | 842.291308 | 0.960218 |
| GO:0010092\_specification\_of\_organ\_identity | 15 | 0 | 0.000000 | -0.000000 | 781 | 657.568692 | 749.93 | 842.291308 | 0.960218 |
| GO:0010171\_body\_morphogenesis | 15 | 0 | 0.000000 | -0.000000 | 781 | 657.568692 | 749.93 | 842.291308 | 0.960218 |
| GO:0010518\_positive\_regulation\_of\_phospholipase\_activity | 15 | 0 | 0.000000 | -0.000000 | 781 | 657.568692 | 749.93 | 842.291308 | 0.960218 |
| GO:0010863\_positive\_regulation\_of\_phospholipase\_C\_activity | 15 | 0 | 0.000000 | -0.000000 | 781 | 657.568692 | 749.93 | 842.291308 | 0.960218 |
| GO:0015931\_nucleobase\_\_nucleoside\_\_nucleotide\_and\_nucleic\_acid\_transport | 15 | 0 | 0.000000 | -0.000000 | 781 | 657.568692 | 749.93 | 842.291308 | 0.960218 |
| GO:0019886\_antigen\_processing\_and\_presentation\_of\_exogenous\_peptide\_antigen\_via\_MHC\_class\_II | 15 | 0 | 0.000000 | -0.000000 | 781 | 657.568692 | 749.93 | 842.291308 | 0.960218 |
| GO:0021795\_cerebral\_cortex\_cell\_migration | 15 | 0 | 0.000000 | -0.000000 | 781 | 657.568692 | 749.93 | 842.291308 | 0.960218 |
| GO:0021872\_generation\_of\_neurons\_in\_the\_forebrain | 15 | 0 | 0.000000 | -0.000000 | 781 | 657.568692 | 749.93 | 842.291308 | 0.960218 |
| GO:0022600\_digestive\_system\_process | 15 | 0 | 0.000000 | -0.000000 | 781 | 657.568692 | 749.93 | 842.291308 | 0.960218 |
| GO:0030041\_actin\_filament\_polymerization | 15 | 0 | 0.000000 | -0.000000 | 781 | 657.568692 | 749.93 | 842.291308 | 0.960218 |
| GO:0031069\_hair\_follicle\_morphogenesis | 15 | 0 | 0.000000 | -0.000000 | 781 | 657.568692 | 749.93 | 842.291308 | 0.960218 |
| GO:0031076\_embryonic\_camera-type\_eye\_development | 15 | 0 | 0.000000 | -0.000000 | 781 | 657.568692 | 749.93 | 842.291308 | 0.960218 |
| GO:0031329\_regulation\_of\_cellular\_catabolic\_process | 15 | 0 | 0.000000 | -0.000000 | 781 | 657.568692 | 749.93 | 842.291308 | 0.960218 |
| GO:0035116\_embryonic\_hindlimb\_morphogenesis | 15 | 0 | 0.000000 | -0.000000 | 781 | 657.568692 | 749.93 | 842.291308 | 0.960218 |
| GO:0035249\_synaptic\_transmission\_\_glutamatergic | 15 | 0 | 0.000000 | -0.000000 | 781 | 657.568692 | 749.93 | 842.291308 | 0.960218 |
| GO:0045666\_positive\_regulation\_of\_neuron\_differentiation | 15 | 0 | 0.000000 | -0.000000 | 781 | 657.568692 | 749.93 | 842.291308 | 0.960218 |
| GO:0046164\_alcohol\_catabolic\_process | 15 | 0 | 0.000000 | -0.000000 | 781 | 657.568692 | 749.93 | 842.291308 | 0.960218 |
| GO:0046638\_positive\_regulation\_of\_alpha-beta\_T\_cell\_differentiation | 15 | 0 | 0.000000 | -0.000000 | 781 | 657.568692 | 749.93 | 842.291308 | 0.960218 |
| GO:0048008\_platelet-derived\_growth\_factor\_receptor\_signaling\_pathway | 15 | 0 | 0.000000 | -0.000000 | 781 | 657.568692 | 749.93 | 842.291308 | 0.960218 |
| GO:0048010\_vascular\_endothelial\_growth\_factor\_receptor\_signaling\_pathway | 15 | 0 | 0.000000 | -0.000000 | 781 | 657.568692 | 749.93 | 842.291308 | 0.960218 |
| GO:0048144\_fibroblast\_proliferation | 15 | 0 | 0.000000 | -0.000000 | 781 | 657.568692 | 749.93 | 842.291308 | 0.960218 |
| GO:0048145\_regulation\_of\_fibroblast\_proliferation | 15 | 0 | 0.000000 | -0.000000 | 781 | 657.568692 | 749.93 | 842.291308 | 0.960218 |
| GO:0048610\_reproductive\_cellular\_process | 15 | 0 | 0.000000 | -0.000000 | 781 | 657.568692 | 749.93 | 842.291308 | 0.960218 |
| GO:0048709\_oligodendrocyte\_differentiation | 15 | 0 | 0.000000 | -0.000000 | 781 | 657.568692 | 749.93 | 842.291308 | 0.960218 |
| GO:0050729\_positive\_regulation\_of\_inflammatory\_response | 15 | 0 | 0.000000 | -0.000000 | 781 | 657.568692 | 749.93 | 842.291308 | 0.960218 |
| GO:0050796\_regulation\_of\_insulin\_secretion | 15 | 0 | 0.000000 | -0.000000 | 781 | 657.568692 | 749.93 | 842.291308 | 0.960218 |
| GO:0050798\_activated\_T\_cell\_proliferation | 15 | 0 | 0.000000 | -0.000000 | 781 | 657.568692 | 749.93 | 842.291308 | 0.960218 |
| GO:0060322\_head\_development | 15 | 0 | 0.000000 | -0.000000 | 781 | 657.568692 | 749.93 | 842.291308 | 0.960218 |
| GO:0060425\_lung\_morphogenesis | 15 | 0 | 0.000000 | -0.000000 | 781 | 657.568692 | 749.93 | 842.291308 | 0.960218 |
| GO:0060442\_branching\_involved\_in\_prostate\_gland\_morphogenesis | 15 | 0 | 0.000000 | -0.000000 | 781 | 657.568692 | 749.93 | 842.291308 | 0.960218 |
| GO:0060749\_mammary\_gland\_alveolus\_development | 15 | 0 | 0.000000 | -0.000000 | 781 | 657.568692 | 749.93 | 842.291308 | 0.960218 |
| GO:0070227\_lymphocyte\_apoptosis | 15 | 0 | 0.000000 | -0.000000 | 781 | 657.568692 | 749.93 | 842.291308 | 0.960218 |
| GO:0070507\_regulation\_of\_microtubule\_cytoskeleton\_organization | 15 | 0 | 0.000000 | -0.000000 | 781 | 657.568692 | 749.93 | 842.291308 | 0.960218 |
| GO:0007186\_G-protein\_coupled\_receptor\_protein\_signaling\_pathway | 144 | 0 | 0.000000 | -0.000000 | 782 | 658.538625 | 750.73 | 842.921375 | 0.960013 |
| GO:0000209\_protein\_polyubiquitination | 10 | 0 | 0.000000 | -0.000000 | 894 | 777.442001 | 867.86 | 958.277999 | 0.970761 |
| GO:0001578\_microtubule\_bundle\_formation | 10 | 0 | 0.000000 | -0.000000 | 894 | 777.442001 | 867.86 | 958.277999 | 0.970761 |
| GO:0001659\_temperature\_homeostasis | 10 | 0 | 0.000000 | -0.000000 | 894 | 777.442001 | 867.86 | 958.277999 | 0.970761 |
| GO:0001773\_myeloid\_dendritic\_cell\_activation | 10 | 0 | 0.000000 | -0.000000 | 894 | 777.442001 | 867.86 | 958.277999 | 0.970761 |
| GO:0001914\_regulation\_of\_T\_cell\_mediated\_cytotoxicity | 10 | 0 | 0.000000 | -0.000000 | 894 | 777.442001 | 867.86 | 958.277999 | 0.970761 |
| GO:0001990\_regulation\_of\_systemic\_arterial\_blood\_pressure\_by\_hormone | 10 | 0 | 0.000000 | -0.000000 | 894 | 777.442001 | 867.86 | 958.277999 | 0.970761 |
| GO:0002673\_regulation\_of\_acute\_inflammatory\_response | 10 | 0 | 0.000000 | -0.000000 | 894 | 777.442001 | 867.86 | 958.277999 | 0.970761 |
| GO:0002711\_positive\_regulation\_of\_T\_cell\_mediated\_immunity | 10 | 0 | 0.000000 | -0.000000 | 894 | 777.442001 | 867.86 | 958.277999 | 0.970761 |
| GO:0002762\_negative\_regulation\_of\_myeloid\_leukocyte\_differentiation | 10 | 0 | 0.000000 | -0.000000 | 894 | 777.442001 | 867.86 | 958.277999 | 0.970761 |
| GO:0006040\_amino\_sugar\_metabolic\_process | 10 | 0 | 0.000000 | -0.000000 | 894 | 777.442001 | 867.86 | 958.277999 | 0.970761 |
| GO:0006081\_cellular\_aldehyde\_metabolic\_process | 10 | 0 | 0.000000 | -0.000000 | 894 | 777.442001 | 867.86 | 958.277999 | 0.970761 |
| GO:0006109\_regulation\_of\_carbohydrate\_metabolic\_process | 10 | 0 | 0.000000 | -0.000000 | 894 | 777.442001 | 867.86 | 958.277999 | 0.970761 |
| GO:0006342\_chromatin\_silencing | 10 | 0 | 0.000000 | -0.000000 | 894 | 777.442001 | 867.86 | 958.277999 | 0.970761 |
| GO:0006405\_RNA\_export\_from\_nucleus | 10 | 0 | 0.000000 | -0.000000 | 894 | 777.442001 | 867.86 | 958.277999 | 0.970761 |
| GO:0006805\_xenobiotic\_metabolic\_process | 10 | 0 | 0.000000 | -0.000000 | 894 | 777.442001 | 867.86 | 958.277999 | 0.970761 |
| GO:0006826\_iron\_ion\_transport | 10 | 0 | 0.000000 | -0.000000 | 894 | 777.442001 | 867.86 | 958.277999 | 0.970761 |
| GO:0006921\_cell\_structure\_disassembly\_during\_apoptosis | 10 | 0 | 0.000000 | -0.000000 | 894 | 777.442001 | 867.86 | 958.277999 | 0.970761 |
| GO:0006968\_cellular\_defense\_response | 10 | 0 | 0.000000 | -0.000000 | 894 | 777.442001 | 867.86 | 958.277999 | 0.970761 |
| GO:0007006\_mitochondrial\_membrane\_organization | 10 | 0 | 0.000000 | -0.000000 | 894 | 777.442001 | 867.86 | 958.277999 | 0.970761 |
| GO:0007044\_cell-substrate\_junction\_assembly | 10 | 0 | 0.000000 | -0.000000 | 894 | 777.442001 | 867.86 | 958.277999 | 0.970761 |
| GO:0007093\_mitotic\_cell\_cycle\_checkpoint | 10 | 0 | 0.000000 | -0.000000 | 894 | 777.442001 | 867.86 | 958.277999 | 0.970761 |
| GO:0007172\_signal\_complex\_assembly | 10 | 0 | 0.000000 | -0.000000 | 894 | 777.442001 | 867.86 | 958.277999 | 0.970761 |
| GO:0007194\_negative\_regulation\_of\_adenylate\_cyclase\_activity | 10 | 0 | 0.000000 | -0.000000 | 894 | 777.442001 | 867.86 | 958.277999 | 0.970761 |
| GO:0008088\_axon\_cargo\_transport | 10 | 0 | 0.000000 | -0.000000 | 894 | 777.442001 | 867.86 | 958.277999 | 0.970761 |
| GO:0008206\_bile\_acid\_metabolic\_process | 10 | 0 | 0.000000 | -0.000000 | 894 | 777.442001 | 867.86 | 958.277999 | 0.970761 |
| GO:0008211\_glucocorticoid\_metabolic\_process | 10 | 0 | 0.000000 | -0.000000 | 894 | 777.442001 | 867.86 | 958.277999 | 0.970761 |
| GO:0009066\_aspartate\_family\_amino\_acid\_metabolic\_process | 10 | 0 | 0.000000 | -0.000000 | 894 | 777.442001 | 867.86 | 958.277999 | 0.970761 |
| GO:0009110\_vitamin\_biosynthetic\_process | 10 | 0 | 0.000000 | -0.000000 | 894 | 777.442001 | 867.86 | 958.277999 | 0.970761 |
| GO:0009620\_response\_to\_fungus | 10 | 0 | 0.000000 | -0.000000 | 894 | 777.442001 | 867.86 | 958.277999 | 0.970761 |
| GO:0009743\_response\_to\_carbohydrate\_stimulus | 10 | 0 | 0.000000 | -0.000000 | 894 | 777.442001 | 867.86 | 958.277999 | 0.970761 |
| GO:0009948\_anterior\_posterior\_axis\_specification | 10 | 0 | 0.000000 | -0.000000 | 894 | 777.442001 | 867.86 | 958.277999 | 0.970761 |
| GO:0010827\_regulation\_of\_glucose\_transport | 10 | 0 | 0.000000 | -0.000000 | 894 | 777.442001 | 867.86 | 958.277999 | 0.970761 |
| GO:0015718\_monocarboxylic\_acid\_transport | 10 | 0 | 0.000000 | -0.000000 | 894 | 777.442001 | 867.86 | 958.277999 | 0.970761 |
| GO:0016197\_endosome\_transport | 10 | 0 | 0.000000 | -0.000000 | 894 | 777.442001 | 867.86 | 958.277999 | 0.970761 |
| GO:0016486\_peptide\_hormone\_processing | 10 | 0 | 0.000000 | -0.000000 | 894 | 777.442001 | 867.86 | 958.277999 | 0.970761 |
| GO:0017156\_calcium\_ion-dependent\_exocytosis | 10 | 0 | 0.000000 | -0.000000 | 894 | 777.442001 | 867.86 | 958.277999 | 0.970761 |
| GO:0018149\_peptide\_cross-linking | 10 | 0 | 0.000000 | -0.000000 | 894 | 777.442001 | 867.86 | 958.277999 | 0.970761 |
| GO:0019321\_pentose\_metabolic\_process | 10 | 0 | 0.000000 | -0.000000 | 894 | 777.442001 | 867.86 | 958.277999 | 0.970761 |
| GO:0021534\_cell\_proliferation\_in\_hindbrain | 10 | 0 | 0.000000 | -0.000000 | 894 | 777.442001 | 867.86 | 958.277999 | 0.970761 |
| GO:0021871\_forebrain\_regionalization | 10 | 0 | 0.000000 | -0.000000 | 894 | 777.442001 | 867.86 | 958.277999 | 0.970761 |
| GO:0021895\_cerebral\_cortex\_neuron\_differentiation | 10 | 0 | 0.000000 | -0.000000 | 894 | 777.442001 | 867.86 | 958.277999 | 0.970761 |
| GO:0021924\_cell\_proliferation\_in\_the\_external\_granule\_layer | 10 | 0 | 0.000000 | -0.000000 | 894 | 777.442001 | 867.86 | 958.277999 | 0.970761 |
| GO:0021930\_granule\_cell\_precursor\_proliferation | 10 | 0 | 0.000000 | -0.000000 | 894 | 777.442001 | 867.86 | 958.277999 | 0.970761 |
| GO:0021952\_central\_nervous\_system\_projection\_neuron\_axonogenesis | 10 | 0 | 0.000000 | -0.000000 | 894 | 777.442001 | 867.86 | 958.277999 | 0.970761 |
| GO:0022900\_electron\_transport\_chain | 10 | 0 | 0.000000 | -0.000000 | 894 | 777.442001 | 867.86 | 958.277999 | 0.970761 |
| GO:0022904\_respiratory\_electron\_transport\_chain | 10 | 0 | 0.000000 | -0.000000 | 894 | 777.442001 | 867.86 | 958.277999 | 0.970761 |
| GO:0030168\_platelet\_activation | 10 | 0 | 0.000000 | -0.000000 | 894 | 777.442001 | 867.86 | 958.277999 | 0.970761 |
| GO:0030833\_regulation\_of\_actin\_filament\_polymerization | 10 | 0 | 0.000000 | -0.000000 | 894 | 777.442001 | 867.86 | 958.277999 | 0.970761 |
| GO:0031018\_endocrine\_pancreas\_development | 10 | 0 | 0.000000 | -0.000000 | 894 | 777.442001 | 867.86 | 958.277999 | 0.970761 |
| GO:0031280\_negative\_regulation\_of\_cyclase\_activity | 10 | 0 | 0.000000 | -0.000000 | 894 | 777.442001 | 867.86 | 958.277999 | 0.970761 |
| GO:0031331\_positive\_regulation\_of\_cellular\_catabolic\_process | 10 | 0 | 0.000000 | -0.000000 | 894 | 777.442001 | 867.86 | 958.277999 | 0.970761 |
| GO:0031645\_negative\_regulation\_of\_neurological\_system\_process | 10 | 0 | 0.000000 | -0.000000 | 894 | 777.442001 | 867.86 | 958.277999 | 0.970761 |
| GO:0032318\_regulation\_of\_Ras\_GTPase\_activity | 10 | 0 | 0.000000 | -0.000000 | 894 | 777.442001 | 867.86 | 958.277999 | 0.970761 |
| GO:0032602\_chemokine\_production | 10 | 0 | 0.000000 | -0.000000 | 894 | 777.442001 | 867.86 | 958.277999 | 0.970761 |
| GO:0032633\_interleukin-4\_production | 10 | 0 | 0.000000 | -0.000000 | 894 | 777.442001 | 867.86 | 958.277999 | 0.970761 |
| GO:0032642\_regulation\_of\_chemokine\_production | 10 | 0 | 0.000000 | -0.000000 | 894 | 777.442001 | 867.86 | 958.277999 | 0.970761 |
| GO:0032673\_regulation\_of\_interleukin-4\_production | 10 | 0 | 0.000000 | -0.000000 | 894 | 777.442001 | 867.86 | 958.277999 | 0.970761 |
| GO:0032760\_positive\_regulation\_of\_tumor\_necrosis\_factor\_production | 10 | 0 | 0.000000 | -0.000000 | 894 | 777.442001 | 867.86 | 958.277999 | 0.970761 |
| GO:0033081\_regulation\_of\_T\_cell\_differentiation\_in\_the\_thymus | 10 | 0 | 0.000000 | -0.000000 | 894 | 777.442001 | 867.86 | 958.277999 | 0.970761 |
| GO:0034105\_positive\_regulation\_of\_tissue\_remodeling | 10 | 0 | 0.000000 | -0.000000 | 894 | 777.442001 | 867.86 | 958.277999 | 0.970761 |
| GO:0034637\_cellular\_carbohydrate\_biosynthetic\_process | 10 | 0 | 0.000000 | -0.000000 | 894 | 777.442001 | 867.86 | 958.277999 | 0.970761 |
| GO:0040015\_negative\_regulation\_of\_multicellular\_organism\_growth | 10 | 0 | 0.000000 | -0.000000 | 894 | 777.442001 | 867.86 | 958.277999 | 0.970761 |
| GO:0042088\_T-helper\_1\_type\_immune\_response | 10 | 0 | 0.000000 | -0.000000 | 894 | 777.442001 | 867.86 | 958.277999 | 0.970761 |
| GO:0042116\_macrophage\_activation | 10 | 0 | 0.000000 | -0.000000 | 894 | 777.442001 | 867.86 | 958.277999 | 0.970761 |
| GO:0042177\_negative\_regulation\_of\_protein\_catabolic\_process | 10 | 0 | 0.000000 | -0.000000 | 894 | 777.442001 | 867.86 | 958.277999 | 0.970761 |
| GO:0042755\_eating\_behavior | 10 | 0 | 0.000000 | -0.000000 | 894 | 777.442001 | 867.86 | 958.277999 | 0.970761 |
| GO:0043113\_receptor\_clustering | 10 | 0 | 0.000000 | -0.000000 | 894 | 777.442001 | 867.86 | 958.277999 | 0.970761 |
| GO:0043330\_response\_to\_exogenous\_dsRNA | 10 | 0 | 0.000000 | -0.000000 | 894 | 777.442001 | 867.86 | 958.277999 | 0.970761 |
| GO:0043488\_regulation\_of\_mRNA\_stability | 10 | 0 | 0.000000 | -0.000000 | 894 | 777.442001 | 867.86 | 958.277999 | 0.970761 |
| GO:0043506\_regulation\_of\_JUN\_kinase\_activity | 10 | 0 | 0.000000 | -0.000000 | 894 | 777.442001 | 867.86 | 958.277999 | 0.970761 |
| GO:0043525\_positive\_regulation\_of\_neuron\_apoptosis | 10 | 0 | 0.000000 | -0.000000 | 894 | 777.442001 | 867.86 | 958.277999 | 0.970761 |
| GO:0044259\_multicellular\_organismal\_macromolecule\_metabolic\_process | 10 | 0 | 0.000000 | -0.000000 | 894 | 777.442001 | 867.86 | 958.277999 | 0.970761 |
| GO:0045132\_meiotic\_chromosome\_segregation | 10 | 0 | 0.000000 | -0.000000 | 894 | 777.442001 | 867.86 | 958.277999 | 0.970761 |
| GO:0045446\_endothelial\_cell\_differentiation | 10 | 0 | 0.000000 | -0.000000 | 894 | 777.442001 | 867.86 | 958.277999 | 0.970761 |
| GO:0045576\_mast\_cell\_activation | 10 | 0 | 0.000000 | -0.000000 | 894 | 777.442001 | 867.86 | 958.277999 | 0.970761 |
| GO:0045669\_positive\_regulation\_of\_osteoblast\_differentiation | 10 | 0 | 0.000000 | -0.000000 | 894 | 777.442001 | 867.86 | 958.277999 | 0.970761 |
| GO:0045776\_negative\_regulation\_of\_blood\_pressure | 10 | 0 | 0.000000 | -0.000000 | 894 | 777.442001 | 867.86 | 958.277999 | 0.970761 |
| GO:0045777\_positive\_regulation\_of\_blood\_pressure | 10 | 0 | 0.000000 | -0.000000 | 894 | 777.442001 | 867.86 | 958.277999 | 0.970761 |
| GO:0045814\_negative\_regulation\_of\_gene\_expression\_\_epigenetic | 10 | 0 | 0.000000 | -0.000000 | 894 | 777.442001 | 867.86 | 958.277999 | 0.970761 |
| GO:0045911\_positive\_regulation\_of\_DNA\_recombination | 10 | 0 | 0.000000 | -0.000000 | 894 | 777.442001 | 867.86 | 958.277999 | 0.970761 |
| GO:0046887\_positive\_regulation\_of\_hormone\_secretion | 10 | 0 | 0.000000 | -0.000000 | 894 | 777.442001 | 867.86 | 958.277999 | 0.970761 |
| GO:0048291\_isotype\_switching\_to\_IgG\_isotypes | 10 | 0 | 0.000000 | -0.000000 | 894 | 777.442001 | 867.86 | 958.277999 | 0.970761 |
| GO:0048302\_regulation\_of\_isotype\_switching\_to\_IgG\_isotypes | 10 | 0 | 0.000000 | -0.000000 | 894 | 777.442001 | 867.86 | 958.277999 | 0.970761 |
| GO:0048339\_paraxial\_mesoderm\_development | 10 | 0 | 0.000000 | -0.000000 | 894 | 777.442001 | 867.86 | 958.277999 | 0.970761 |
| GO:0048384\_retinoic\_acid\_receptor\_signaling\_pathway | 10 | 0 | 0.000000 | -0.000000 | 894 | 777.442001 | 867.86 | 958.277999 | 0.970761 |
| GO:0048596\_embryonic\_camera-type\_eye\_morphogenesis | 10 | 0 | 0.000000 | -0.000000 | 894 | 777.442001 | 867.86 | 958.277999 | 0.970761 |
| GO:0048641\_regulation\_of\_skeletal\_muscle\_tissue\_development | 10 | 0 | 0.000000 | -0.000000 | 894 | 777.442001 | 867.86 | 958.277999 | 0.970761 |
| GO:0048738\_cardiac\_muscle\_tissue\_development | 10 | 0 | 0.000000 | -0.000000 | 894 | 777.442001 | 867.86 | 958.277999 | 0.970761 |
| GO:0050654\_chondroitin\_sulfate\_proteoglycan\_metabolic\_process | 10 | 0 | 0.000000 | -0.000000 | 894 | 777.442001 | 867.86 | 958.277999 | 0.970761 |
| GO:0050657\_nucleic\_acid\_transport | 10 | 0 | 0.000000 | -0.000000 | 894 | 777.442001 | 867.86 | 958.277999 | 0.970761 |
| GO:0050658\_RNA\_transport | 10 | 0 | 0.000000 | -0.000000 | 894 | 777.442001 | 867.86 | 958.277999 | 0.970761 |
| GO:0050663\_cytokine\_secretion | 10 | 0 | 0.000000 | -0.000000 | 894 | 777.442001 | 867.86 | 958.277999 | 0.970761 |
| GO:0050714\_positive\_regulation\_of\_protein\_secretion | 10 | 0 | 0.000000 | -0.000000 | 894 | 777.442001 | 867.86 | 958.277999 | 0.970761 |
| GO:0050879\_multicellular\_organismal\_movement | 10 | 0 | 0.000000 | -0.000000 | 894 | 777.442001 | 867.86 | 958.277999 | 0.970761 |
| GO:0050881\_musculoskeletal\_movement | 10 | 0 | 0.000000 | -0.000000 | 894 | 777.442001 | 867.86 | 958.277999 | 0.970761 |
| GO:0050886\_endocrine\_process | 10 | 0 | 0.000000 | -0.000000 | 894 | 777.442001 | 867.86 | 958.277999 | 0.970761 |
| GO:0050892\_intestinal\_absorption | 10 | 0 | 0.000000 | -0.000000 | 894 | 777.442001 | 867.86 | 958.277999 | 0.970761 |
| GO:0051147\_regulation\_of\_muscle\_cell\_differentiation | 10 | 0 | 0.000000 | -0.000000 | 894 | 777.442001 | 867.86 | 958.277999 | 0.970761 |
| GO:0051224\_negative\_regulation\_of\_protein\_transport | 10 | 0 | 0.000000 | -0.000000 | 894 | 777.442001 | 867.86 | 958.277999 | 0.970761 |
| GO:0051236\_establishment\_of\_RNA\_localization | 10 | 0 | 0.000000 | -0.000000 | 894 | 777.442001 | 867.86 | 958.277999 | 0.970761 |
| GO:0051262\_protein\_tetramerization | 10 | 0 | 0.000000 | -0.000000 | 894 | 777.442001 | 867.86 | 958.277999 | 0.970761 |
| GO:0051350\_negative\_regulation\_of\_lyase\_activity | 10 | 0 | 0.000000 | -0.000000 | 894 | 777.442001 | 867.86 | 958.277999 | 0.970761 |
| GO:0051445\_regulation\_of\_meiotic\_cell\_cycle | 10 | 0 | 0.000000 | -0.000000 | 894 | 777.442001 | 867.86 | 958.277999 | 0.970761 |
| GO:0051650\_establishment\_of\_vesicle\_localization | 10 | 0 | 0.000000 | -0.000000 | 894 | 777.442001 | 867.86 | 958.277999 | 0.970761 |
| GO:0051651\_maintenance\_of\_location\_in\_cell | 10 | 0 | 0.000000 | -0.000000 | 894 | 777.442001 | 867.86 | 958.277999 | 0.970761 |
| GO:0060135\_maternal\_process\_involved\_in\_female\_pregnancy | 10 | 0 | 0.000000 | -0.000000 | 894 | 777.442001 | 867.86 | 958.277999 | 0.970761 |
| GO:0060216\_definitive\_hemopoiesis | 10 | 0 | 0.000000 | -0.000000 | 894 | 777.442001 | 867.86 | 958.277999 | 0.970761 |
| GO:0060323\_head\_morphogenesis | 10 | 0 | 0.000000 | -0.000000 | 894 | 777.442001 | 867.86 | 958.277999 | 0.970761 |
| GO:0060601\_lateral\_sprouting\_from\_an\_epithelium | 10 | 0 | 0.000000 | -0.000000 | 894 | 777.442001 | 867.86 | 958.277999 | 0.970761 |
| GO:0060669\_embryonic\_placenta\_morphogenesis | 10 | 0 | 0.000000 | -0.000000 | 894 | 777.442001 | 867.86 | 958.277999 | 0.970761 |
| GO:0060706\_cell\_differentiation\_involved\_in\_embryonic\_placenta\_development | 10 | 0 | 0.000000 | -0.000000 | 894 | 777.442001 | 867.86 | 958.277999 | 0.970761 |
| GO:0060768\_regulation\_of\_epithelial\_cell\_proliferation\_involved\_in\_prostate\_gland\_development | 10 | 0 | 0.000000 | -0.000000 | 894 | 777.442001 | 867.86 | 958.277999 | 0.970761 |
| GO:0018193\_peptidyl-amino\_acid\_modification | 97 | 0 | 0.000000 | -0.000000 | 895 | 778.700390 | 868.9 | 959.099610 | 0.970838 |
| GO:0002200\_somatic\_diversification\_of\_immune\_receptors | 34 | 0 | 0.000000 | -0.000000 | 912 | 794.459263 | 883.7 | 972.940737 | 0.968969 |
| GO:0002237\_response\_to\_molecule\_of\_bacterial\_origin | 34 | 0 | 0.000000 | -0.000000 | 912 | 794.459263 | 883.7 | 972.940737 | 0.968969 |
| GO:0002699\_positive\_regulation\_of\_immune\_effector\_process | 34 | 0 | 0.000000 | -0.000000 | 912 | 794.459263 | 883.7 | 972.940737 | 0.968969 |
| GO:0007269\_neurotransmitter\_secretion | 34 | 0 | 0.000000 | -0.000000 | 912 | 794.459263 | 883.7 | 972.940737 | 0.968969 |
| GO:0007338\_single\_fertilization | 34 | 0 | 0.000000 | -0.000000 | 912 | 794.459263 | 883.7 | 972.940737 | 0.968969 |
| GO:0007568\_aging | 34 | 0 | 0.000000 | -0.000000 | 912 | 794.459263 | 883.7 | 972.940737 | 0.968969 |
| GO:0010721\_negative\_regulation\_of\_cell\_development | 34 | 0 | 0.000000 | -0.000000 | 912 | 794.459263 | 883.7 | 972.940737 | 0.968969 |
| GO:0016054\_organic\_acid\_catabolic\_process | 34 | 0 | 0.000000 | -0.000000 | 912 | 794.459263 | 883.7 | 972.940737 | 0.968969 |
| GO:0019882\_antigen\_processing\_and\_presentation | 34 | 0 | 0.000000 | -0.000000 | 912 | 794.459263 | 883.7 | 972.940737 | 0.968969 |
| GO:0030509\_BMP\_signaling\_pathway | 34 | 0 | 0.000000 | -0.000000 | 912 | 794.459263 | 883.7 | 972.940737 | 0.968969 |
| GO:0045927\_positive\_regulation\_of\_growth | 34 | 0 | 0.000000 | -0.000000 | 912 | 794.459263 | 883.7 | 972.940737 | 0.968969 |
| GO:0046395\_carboxylic\_acid\_catabolic\_process | 34 | 0 | 0.000000 | -0.000000 | 912 | 794.459263 | 883.7 | 972.940737 | 0.968969 |
| GO:0050730\_regulation\_of\_peptidyl-tyrosine\_phosphorylation | 34 | 0 | 0.000000 | -0.000000 | 912 | 794.459263 | 883.7 | 972.940737 | 0.968969 |
| GO:0051047\_positive\_regulation\_of\_secretion | 34 | 0 | 0.000000 | -0.000000 | 912 | 794.459263 | 883.7 | 972.940737 | 0.968969 |
| GO:0051052\_regulation\_of\_DNA\_metabolic\_process | 34 | 0 | 0.000000 | -0.000000 | 912 | 794.459263 | 883.7 | 972.940737 | 0.968969 |
| GO:0060443\_mammary\_gland\_morphogenesis | 34 | 0 | 0.000000 | -0.000000 | 912 | 794.459263 | 883.7 | 972.940737 | 0.968969 |
| GO:0060711\_labyrinthine\_layer\_development | 34 | 0 | 0.000000 | -0.000000 | 912 | 794.459263 | 883.7 | 972.940737 | 0.968969 |
| GO:0016044\_membrane\_organization | 140 | 0 | 0.000000 | -0.000000 | 913 | 797.191669 | 885.96 | 974.728331 | 0.970383 |
| GO:0000027\_ribosomal\_large\_subunit\_assembly | 1 | 0 |  |  |  |  |  |  |  |  |
| GO:0000042\_protein\_targeting\_to\_Golgi | 1 | 0 |  |  |  |  |  |  |  |  |
| GO:0000046\_autophagic\_vacuole\_fusion | 1 | 0 |  |  |  |  |  |  |  |  |
| GO:0000050\_urea\_cycle | 1 | 0 |  |  |  |  |  |  |  |  |
| GO:0000054\_ribosome\_export\_from\_nucleus | 1 | 0 |  |  |  |  |  |  |  |  |
| GO:0000055\_ribosomal\_large\_subunit\_export\_from\_nucleus | 1 | 0 |  |  |  |  |  |  |  |  |
| GO:0000056\_ribosomal\_small\_subunit\_export\_from\_nucleus | 1 | 0 |  |  |  |  |  |  |  |  |
| GO:0000072\_M\_phase\_specific\_microtubule\_process | 1 | 0 |  |  |  |  |  |  |  |  |
| GO:0000101\_sulfur\_amino\_acid\_transport | 1 | 0 |  |  |  |  |  |  |  |  |
| GO:0000147\_actin\_cortical\_patch\_assembly | 1 | 0 |  |  |  |  |  |  |  |  |
| GO:0000154\_rRNA\_modification | 1 | 0 |  |  |  |  |  |  |  |  |
| GO:0000183\_chromatin\_silencing\_at\_rDNA | 1 | 0 |  |  |  |  |  |  |  |  |
| GO:0000185\_activation\_of\_MAPKKK\_activity | 1 | 0 |  |  |  |  |  |  |  |  |
| GO:0000238\_zygotene | 1 | 0 |  |  |  |  |  |  |  |  |
| GO:0000255\_allantoin\_metabolic\_process | 1 | 0 |  |  |  |  |  |  |  |  |
| GO:0000266\_mitochondrial\_fission | 1 | 0 |  |  |  |  |  |  |  |  |
| GO:0000273\_lipoic\_acid\_metabolic\_process | 1 | 0 |  |  |  |  |  |  |  |  |
| GO:0000301\_retrograde\_transport\_\_vesicle\_recycling\_within\_Golgi | 1 | 0 |  |  |  |  |  |  |  |  |
| GO:0000394\_RNA\_splicing\_\_via\_endonucleolytic\_cleavage\_and\_ligation | 1 | 0 |  |  |  |  |  |  |  |  |
| GO:0000429\_regulation\_of\_transcription\_from\_RNA\_polymerase\_II\_promoter\_by\_carbon\_catabolites | 1 | 0 |  |  |  |  |  |  |  |  |
| GO:0000430\_regulation\_of\_transcription\_from\_RNA\_polymerase\_II\_promoter\_by\_glucose | 1 | 0 |  |  |  |  |  |  |  |  |
| GO:0000432\_positive\_regulation\_of\_transcription\_from\_RNA\_polymerase\_II\_promoter\_by\_glucose | 1 | 0 |  |  |  |  |  |  |  |  |
| GO:0000436\_positive\_regulation\_of\_transcription\_from\_RNA\_polymerase\_II\_promoter\_by\_carbon\_catabolites | 1 | 0 |  |  |  |  |  |  |  |  |
| GO:0000448\_cleavage\_in\_ITS2\_between\_5.8S\_rRNA\_and\_LSU-rRNA\_of\_tricistronic\_rRNA\_transcript\_(SSU-rRNA\_\_5.8S\_rRNA\_\_LSU-rRNA) | 1 | 0 |  |  |  |  |  |  |  |  |
| GO:0000460\_maturation\_of\_5.8S\_rRNA | 1 | 0 |  |  |  |  |  |  |  |  |
| GO:0000463\_maturation\_of\_LSU-rRNA\_from\_tricistronic\_rRNA\_transcript\_(SSU-rRNA\_\_5.8S\_rRNA\_\_LSU-rRNA) | 1 | 0 |  |  |  |  |  |  |  |  |
| GO:0000466\_maturation\_of\_5.8S\_rRNA\_from\_tricistronic\_rRNA\_transcript\_(SSU-rRNA\_\_5.8S\_rRNA\_\_LSU-rRNA) | 1 | 0 |  |  |  |  |  |  |  |  |
| GO:0000469\_cleavages\_during\_rRNA\_processing | 1 | 0 |  |  |  |  |  |  |  |  |
| GO:0000470\_maturation\_of\_LSU-rRNA | 1 | 0 |  |  |  |  |  |  |  |  |
| GO:0000478\_endonucleolytic\_cleavages\_during\_rRNA\_processing | 1 | 0 |  |  |  |  |  |  |  |  |
| GO:0000479\_endonucleolytic\_cleavage\_of\_tricistronic\_rRNA\_transcript\_(SSU-rRNA\_\_5.8S\_rRNA\_\_LSU-rRNA) | 1 | 0 |  |  |  |  |  |  |  |  |
| GO:0000705\_achiasmate\_meiosis\_I | 1 | 0 |  |  |  |  |  |  |  |  |
| GO:0000966\_RNA\_5'-end\_processing | 1 | 0 |  |  |  |  |  |  |  |  |
| GO:0001300\_chronological\_cell\_aging | 1 | 0 |  |  |  |  |  |  |  |  |
| GO:0001547\_antral\_ovarian\_follicle\_growth | 1 | 0 |  |  |  |  |  |  |  |  |
| GO:0001555\_oocyte\_growth | 1 | 0 |  |  |  |  |  |  |  |  |
| GO:0001560\_regulation\_of\_cell\_growth\_by\_extracellular\_stimulus | 1 | 0 |  |  |  |  |  |  |  |  |
| GO:0001660\_fever | 1 | 0 |  |  |  |  |  |  |  |  |
| GO:0001696\_gastric\_acid\_secretion | 1 | 0 |  |  |  |  |  |  |  |  |
| GO:0001712\_ectodermal\_cell\_fate\_commitment | 1 | 0 |  |  |  |  |  |  |  |  |
| GO:0001714\_endodermal\_cell\_fate\_specification | 1 | 0 |  |  |  |  |  |  |  |  |
| GO:0001762\_beta-alanine\_transport | 1 | 0 |  |  |  |  |  |  |  |  |
| GO:0001766\_membrane\_raft\_polarization | 1 | 0 |  |  |  |  |  |  |  |  |
| GO:0001811\_negative\_regulation\_of\_type\_I\_hypersensitivity | 1 | 0 |  |  |  |  |  |  |  |  |
| GO:0001821\_histamine\_secretion | 1 | 0 |  |  |  |  |  |  |  |  |
| GO:0001826\_inner\_cell\_mass\_cell\_differentiation | 1 | 0 |  |  |  |  |  |  |  |  |
| GO:0001830\_trophectodermal\_cell\_fate\_commitment | 1 | 0 |  |  |  |  |  |  |  |  |
| GO:0001834\_trophectodermal\_cell\_proliferation | 1 | 0 |  |  |  |  |  |  |  |  |
| GO:0001867\_complement\_activation\_\_lectin\_pathway | 1 | 0 |  |  |  |  |  |  |  |  |
| GO:0001880\_Mullerian\_duct\_regression | 1 | 0 |  |  |  |  |  |  |  |  |
| GO:0001887\_selenium\_metabolic\_process | 1 | 0 |  |  |  |  |  |  |  |  |
| GO:0001922\_B-1\_B\_cell\_homeostasis | 1 | 0 |  |  |  |  |  |  |  |  |
| GO:0001923\_B-1\_B\_cell\_differentiation | 1 | 0 |  |  |  |  |  |  |  |  |
| GO:0001941\_postsynaptic\_membrane\_organization | 1 | 0 |  |  |  |  |  |  |  |  |
| GO:0001946\_lymphangiogenesis | 1 | 0 |  |  |  |  |  |  |  |  |
| GO:0001956\_positive\_regulation\_of\_neurotransmitter\_secretion | 1 | 0 |  |  |  |  |  |  |  |  |
| GO:0001961\_positive\_regulation\_of\_cytokine-mediated\_signaling\_pathway | 1 | 0 |  |  |  |  |  |  |  |  |
| GO:0001979\_regulation\_of\_systemic\_arterial\_blood\_pressure\_by\_chemoreceptor\_signaling | 1 | 0 |  |  |  |  |  |  |  |  |
| GO:0001980\_regulation\_of\_systemic\_arterial\_blood\_pressure\_by\_ischemic\_conditions | 1 | 0 |  |  |  |  |  |  |  |  |
| GO:0001984\_vasodilation\_of\_artery\_during\_baroreceptor\_response\_to\_increased\_systemic\_arterial\_blood\_pressure | 1 | 0 |  |  |  |  |  |  |  |  |
| GO:0001985\_negative\_regulation\_of\_heart\_rate\_in\_baroreceptor\_response\_to\_increased\_systemic\_arterial\_blood\_pressure | 1 | 0 |  |  |  |  |  |  |  |  |
| GO:0001987\_vasoconstriction\_of\_artery\_involved\_in\_baroreceptor\_response\_to\_lowering\_of\_systemic\_arterial\_blood\_pressure | 1 | 0 |  |  |  |  |  |  |  |  |
| GO:0001988\_positive\_regulation\_of\_heart\_rate\_in\_baroreceptor\_response\_to\_decreased\_systemic\_arterial\_blood\_pressure | 1 | 0 |  |  |  |  |  |  |  |  |
| GO:0001994\_norepinephrine-epinephrine\_vasoconstriction\_involved\_in\_regulation\_of\_systemic\_arterial\_blood\_pressure | 1 | 0 |  |  |  |  |  |  |  |  |
| GO:0002001\_renin\_secretion\_into\_blood\_stream | 1 | 0 |  |  |  |  |  |  |  |  |
| GO:0002002\_regulation\_of\_angiotensin\_levels\_in\_blood | 1 | 0 |  |  |  |  |  |  |  |  |
| GO:0002003\_angiotensin\_maturation | 1 | 0 |  |  |  |  |  |  |  |  |
| GO:0002007\_detection\_of\_hypoxic\_conditions\_in\_blood\_by\_chemoreceptor\_signaling | 1 | 0 |  |  |  |  |  |  |  |  |
| GO:0002017\_regulation\_of\_blood\_volume\_by\_renal\_aldosterone | 1 | 0 |  |  |  |  |  |  |  |  |
| GO:0002023\_reduction\_of\_food\_intake\_in\_response\_to\_dietary\_excess | 1 | 0 |  |  |  |  |  |  |  |  |
| GO:0002031\_G-protein\_coupled\_receptor\_internalization | 1 | 0 |  |  |  |  |  |  |  |  |
| GO:0002036\_regulation\_of\_L-glutamate\_transport | 1 | 0 |  |  |  |  |  |  |  |  |
| GO:0002040\_sprouting\_angiogenesis | 1 | 0 |  |  |  |  |  |  |  |  |
| GO:0002041\_intussusceptive\_angiogenesis | 1 | 0 |  |  |  |  |  |  |  |  |
| GO:0002068\_glandular\_epithelial\_cell\_development | 1 | 0 |  |  |  |  |  |  |  |  |
| GO:0002069\_columnar\_cuboidal\_epithelial\_cell\_maturation | 1 | 0 |  |  |  |  |  |  |  |  |
| GO:0002071\_glandular\_epithelial\_cell\_maturation | 1 | 0 |  |  |  |  |  |  |  |  |
| GO:0002082\_regulation\_of\_oxidative\_phosphorylation | 1 | 0 |  |  |  |  |  |  |  |  |
| GO:0002084\_protein\_depalmitoylation | 1 | 0 |  |  |  |  |  |  |  |  |
| GO:0002085\_inhibition\_of\_neuroepithelial\_cell\_differentiation | 1 | 0 |  |  |  |  |  |  |  |  |
| GO:0002086\_diaphragm\_contraction | 1 | 0 |  |  |  |  |  |  |  |  |
| GO:0002118\_aggressive\_behavior | 1 | 0 |  |  |  |  |  |  |  |  |
| GO:0002121\_inter-male\_aggressive\_behavior | 1 | 0 |  |  |  |  |  |  |  |  |
| GO:0002124\_territorial\_aggressive\_behavior | 1 | 0 |  |  |  |  |  |  |  |  |
| GO:0002227\_innate\_immune\_response\_in\_mucosa | 1 | 0 |  |  |  |  |  |  |  |  |
| GO:0002232\_leukocyte\_chemotaxis\_during\_inflammatory\_response | 1 | 0 |  |  |  |  |  |  |  |  |
| GO:0002248\_connective\_tissue\_replacement\_during\_inflammatory\_response | 1 | 0 |  |  |  |  |  |  |  |  |
| GO:0002282\_microglial\_cell\_activation\_during\_immune\_response | 1 | 0 |  |  |  |  |  |  |  |  |
| GO:0002287\_alpha-beta\_T\_cell\_activation\_during\_immune\_response | 1 | 0 |  |  |  |  |  |  |  |  |
| GO:0002314\_germinal\_center\_B\_cell\_differentiation | 1 | 0 |  |  |  |  |  |  |  |  |
| GO:0002315\_marginal\_zone\_B\_cell\_differentiation | 1 | 0 |  |  |  |  |  |  |  |  |
| GO:0002316\_follicular\_B\_cell\_differentiation | 1 | 0 |  |  |  |  |  |  |  |  |
| GO:0002317\_plasma\_cell\_differentiation | 1 | 0 |  |  |  |  |  |  |  |  |
| GO:0002349\_histamine\_production\_during\_acute\_inflammatory\_response | 1 | 0 |  |  |  |  |  |  |  |  |
| GO:0002351\_serotonin\_production\_during\_acute\_inflammatory\_response | 1 | 0 |  |  |  |  |  |  |  |  |
| GO:0002355\_detection\_of\_tumor\_cell | 1 | 0 |  |  |  |  |  |  |  |  |
| GO:0002370\_natural\_killer\_cell\_cytokine\_production | 1 | 0 |  |  |  |  |  |  |  |  |
| GO:0002371\_dendritic\_cell\_cytokine\_production | 1 | 0 |  |  |  |  |  |  |  |  |
| GO:0002380\_immunoglobulin\_secretion\_during\_immune\_response | 1 | 0 |  |  |  |  |  |  |  |  |
| GO:0002396\_MHC\_protein\_complex\_assembly | 1 | 0 |  |  |  |  |  |  |  |  |
| GO:0002397\_MHC\_class\_I\_protein\_complex\_assembly | 1 | 0 |  |  |  |  |  |  |  |  |
| GO:0002420\_natural\_killer\_cell\_mediated\_cytotoxicity\_directed\_against\_tumor\_cell\_target | 1 | 0 |  |  |  |  |  |  |  |  |
| GO:0002423\_natural\_killer\_cell\_mediated\_immune\_response\_to\_tumor\_cell | 1 | 0 |  |  |  |  |  |  |  |  |
| GO:0002424\_T\_cell\_mediated\_immune\_response\_to\_tumor\_cell | 1 | 0 |  |  |  |  |  |  |  |  |
| GO:0002426\_immunoglobulin\_production\_in\_mucosal\_tissue | 1 | 0 |  |  |  |  |  |  |  |  |
| GO:0002431\_Fc\_receptor\_mediated\_stimulatory\_signaling\_pathway | 1 | 0 |  |  |  |  |  |  |  |  |
| GO:0002432\_granuloma\_formation | 1 | 0 |  |  |  |  |  |  |  |  |
| GO:0002441\_histamine\_secretion\_during\_acute\_inflammatory\_response | 1 | 0 |  |  |  |  |  |  |  |  |
| GO:0002442\_serotonin\_secretion\_during\_acute\_inflammatory\_response | 1 | 0 |  |  |  |  |  |  |  |  |
| GO:0002457\_T\_cell\_antigen\_processing\_and\_presentation | 1 | 0 |  |  |  |  |  |  |  |  |
| GO:0002458\_peripheral\_T\_cell\_tolerance\_induction | 1 | 0 |  |  |  |  |  |  |  |  |
| GO:0002461\_tolerance\_induction\_dependent\_upon\_immune\_response | 1 | 0 |  |  |  |  |  |  |  |  |
| GO:0002465\_peripheral\_tolerance\_induction | 1 | 0 |  |  |  |  |  |  |  |  |
| GO:0002468\_dendritic\_cell\_antigen\_processing\_and\_presentation | 1 | 0 |  |  |  |  |  |  |  |  |
| GO:0002476\_antigen\_processing\_and\_presentation\_of\_endogenous\_peptide\_antigen\_via\_MHC\_class\_Ib | 1 | 0 |  |  |  |  |  |  |  |  |
| GO:0002479\_antigen\_processing\_and\_presentation\_of\_exogenous\_peptide\_antigen\_via\_MHC\_class\_I\_\_TAP-dependent | 1 | 0 |  |  |  |  |  |  |  |  |
| GO:0002483\_antigen\_processing\_and\_presentation\_of\_endogenous\_peptide\_antigen | 1 | 0 |  |  |  |  |  |  |  |  |
| GO:0002501\_peptide\_antigen\_assembly\_with\_MHC\_protein\_complex | 1 | 0 |  |  |  |  |  |  |  |  |
| GO:0002502\_peptide\_antigen\_assembly\_with\_MHC\_class\_I\_protein\_complex | 1 | 0 |  |  |  |  |  |  |  |  |
| GO:0002508\_central\_tolerance\_induction | 1 | 0 |  |  |  |  |  |  |  |  |
| GO:0002510\_central\_B\_cell\_tolerance\_induction | 1 | 0 |  |  |  |  |  |  |  |  |
| GO:0002545\_chronic\_inflammatory\_response\_to\_non-antigenic\_stimulus | 1 | 0 |  |  |  |  |  |  |  |  |
| GO:0002553\_histamine\_secretion\_by\_mast\_cell | 1 | 0 |  |  |  |  |  |  |  |  |
| GO:0002554\_serotonin\_secretion\_by\_platelet | 1 | 0 |  |  |  |  |  |  |  |  |
| GO:0002572\_pro-T\_cell\_differentiation | 1 | 0 |  |  |  |  |  |  |  |  |
| GO:0002577\_regulation\_of\_antigen\_processing\_and\_presentation | 1 | 0 |  |  |  |  |  |  |  |  |
| GO:0002579\_positive\_regulation\_of\_antigen\_processing\_and\_presentation | 1 | 0 |  |  |  |  |  |  |  |  |
| GO:0002604\_regulation\_of\_dendritic\_cell\_antigen\_processing\_and\_presentation | 1 | 0 |  |  |  |  |  |  |  |  |
| GO:0002606\_positive\_regulation\_of\_dendritic\_cell\_antigen\_processing\_and\_presentation | 1 | 0 |  |  |  |  |  |  |  |  |
| GO:0002635\_negative\_regulation\_of\_germinal\_center\_formation | 1 | 0 |  |  |  |  |  |  |  |  |
| GO:0002646\_regulation\_of\_central\_tolerance\_induction | 1 | 0 |  |  |  |  |  |  |  |  |
| GO:0002648\_positive\_regulation\_of\_central\_tolerance\_induction | 1 | 0 |  |  |  |  |  |  |  |  |
| GO:0002649\_regulation\_of\_tolerance\_induction\_to\_self\_antigen | 1 | 0 |  |  |  |  |  |  |  |  |
| GO:0002651\_positive\_regulation\_of\_tolerance\_induction\_to\_self\_antigen | 1 | 0 |  |  |  |  |  |  |  |  |
| GO:0002652\_regulation\_of\_tolerance\_induction\_dependent\_upon\_immune\_response | 1 | 0 |  |  |  |  |  |  |  |  |
| GO:0002654\_positive\_regulation\_of\_tolerance\_induction\_dependent\_upon\_immune\_response | 1 | 0 |  |  |  |  |  |  |  |  |
| GO:0002658\_regulation\_of\_peripheral\_tolerance\_induction | 1 | 0 |  |  |  |  |  |  |  |  |
| GO:0002660\_positive\_regulation\_of\_peripheral\_tolerance\_induction | 1 | 0 |  |  |  |  |  |  |  |  |
| GO:0002677\_negative\_regulation\_of\_chronic\_inflammatory\_response | 1 | 0 |  |  |  |  |  |  |  |  |
| GO:0002678\_positive\_regulation\_of\_chronic\_inflammatory\_response | 1 | 0 |  |  |  |  |  |  |  |  |
| GO:0002701\_negative\_regulation\_of\_production\_of\_molecular\_mediator\_of\_immune\_response | 1 | 0 |  |  |  |  |  |  |  |  |
| GO:0002719\_negative\_regulation\_of\_cytokine\_production\_during\_immune\_response | 1 | 0 |  |  |  |  |  |  |  |  |
| GO:0002724\_regulation\_of\_T\_cell\_cytokine\_production | 1 | 0 |  |  |  |  |  |  |  |  |
| GO:0002727\_regulation\_of\_natural\_killer\_cell\_cytokine\_production | 1 | 0 |  |  |  |  |  |  |  |  |
| GO:0002729\_positive\_regulation\_of\_natural\_killer\_cell\_cytokine\_production | 1 | 0 |  |  |  |  |  |  |  |  |
| GO:0002730\_regulation\_of\_dendritic\_cell\_cytokine\_production | 1 | 0 |  |  |  |  |  |  |  |  |
| GO:0002756\_MyD88-independent\_toll-like\_receptor\_signaling\_pathway | 1 | 0 |  |  |  |  |  |  |  |  |
| GO:0002767\_immune\_response-inhibiting\_cell\_surface\_receptor\_signaling\_pathway | 1 | 0 |  |  |  |  |  |  |  |  |
| GO:0002769\_natural\_killer\_cell\_inhibitory\_signaling\_pathway | 1 | 0 |  |  |  |  |  |  |  |  |
| GO:0002840\_regulation\_of\_T\_cell\_mediated\_immune\_response\_to\_tumor\_cell | 1 | 0 |  |  |  |  |  |  |  |  |
| GO:0002842\_positive\_regulation\_of\_T\_cell\_mediated\_immune\_response\_to\_tumor\_cell | 1 | 0 |  |  |  |  |  |  |  |  |
| GO:0002849\_regulation\_of\_peripheral\_T\_cell\_tolerance\_induction | 1 | 0 |  |  |  |  |  |  |  |  |
| GO:0002851\_positive\_regulation\_of\_peripheral\_T\_cell\_tolerance\_induction | 1 | 0 |  |  |  |  |  |  |  |  |
| GO:0002855\_regulation\_of\_natural\_killer\_cell\_mediated\_immune\_response\_to\_tumor\_cell | 1 | 0 |  |  |  |  |  |  |  |  |
| GO:0002857\_positive\_regulation\_of\_natural\_killer\_cell\_mediated\_immune\_response\_to\_tumor\_cell | 1 | 0 |  |  |  |  |  |  |  |  |
| GO:0002858\_regulation\_of\_natural\_killer\_cell\_mediated\_cytotoxicity\_directed\_against\_tumor\_cell\_target | 1 | 0 |  |  |  |  |  |  |  |  |
| GO:0002860\_positive\_regulation\_of\_natural\_killer\_cell\_mediated\_cytotoxicity\_directed\_against\_tumor\_cell\_target | 1 | 0 |  |  |  |  |  |  |  |  |
| GO:0002880\_regulation\_of\_chronic\_inflammatory\_response\_to\_non-antigenic\_stimulus | 1 | 0 |  |  |  |  |  |  |  |  |
| GO:0002882\_positive\_regulation\_of\_chronic\_inflammatory\_response\_to\_non-antigenic\_stimulus | 1 | 0 |  |  |  |  |  |  |  |  |
| GO:0002895\_regulation\_of\_central\_B\_cell\_tolerance\_induction | 1 | 0 |  |  |  |  |  |  |  |  |
| GO:0002897\_positive\_regulation\_of\_central\_B\_cell\_tolerance\_induction | 1 | 0 |  |  |  |  |  |  |  |  |
| GO:0002901\_mature\_B\_cell\_apoptosis | 1 | 0 |  |  |  |  |  |  |  |  |
| GO:0002903\_negative\_regulation\_of\_B\_cell\_apoptosis | 1 | 0 |  |  |  |  |  |  |  |  |
| GO:0002905\_regulation\_of\_mature\_B\_cell\_apoptosis | 1 | 0 |  |  |  |  |  |  |  |  |
| GO:0002906\_negative\_regulation\_of\_mature\_B\_cell\_apoptosis | 1 | 0 |  |  |  |  |  |  |  |  |
| GO:0003011\_involuntary\_skeletal\_muscle\_contraction | 1 | 0 |  |  |  |  |  |  |  |  |
| GO:0003027\_regulation\_of\_systemic\_arterial\_blood\_pressure\_by\_carotid\_body\_chemoreceptor\_signaling | 1 | 0 |  |  |  |  |  |  |  |  |
| GO:0003029\_detection\_of\_hypoxic\_conditions\_in\_blood\_by\_carotid\_body\_chemoreceptor\_signaling | 1 | 0 |  |  |  |  |  |  |  |  |
| GO:0003032\_detection\_of\_oxygen | 1 | 0 |  |  |  |  |  |  |  |  |
| GO:0003056\_regulation\_of\_vascular\_smooth\_muscle\_contraction | 1 | 0 |  |  |  |  |  |  |  |  |
| GO:0003062\_regulation\_of\_heart\_rate\_by\_chemical\_signal | 1 | 0 |  |  |  |  |  |  |  |  |
| GO:0003065\_positive\_regulation\_of\_heart\_rate\_by\_epinephrine | 1 | 0 |  |  |  |  |  |  |  |  |
| GO:0003068\_regulation\_of\_systemic\_arterial\_blood\_pressure\_by\_acetylcholine | 1 | 0 |  |  |  |  |  |  |  |  |
| GO:0003069\_vasodilation\_by\_acetylcholine\_involved\_in\_regulation\_of\_systemic\_arterial\_blood\_pressure | 1 | 0 |  |  |  |  |  |  |  |  |
| GO:0003070\_regulation\_of\_systemic\_arterial\_blood\_pressure\_by\_neurotransmitter | 1 | 0 |  |  |  |  |  |  |  |  |
| GO:0003097\_renal\_water\_transport | 1 | 0 |  |  |  |  |  |  |  |  |
| GO:0005979\_regulation\_of\_glycogen\_biosynthetic\_process | 1 | 0 |  |  |  |  |  |  |  |  |
| GO:0005984\_disaccharide\_metabolic\_process | 1 | 0 |  |  |  |  |  |  |  |  |
| GO:0005988\_lactose\_metabolic\_process | 1 | 0 |  |  |  |  |  |  |  |  |
| GO:0005989\_lactose\_biosynthetic\_process | 1 | 0 |  |  |  |  |  |  |  |  |
| GO:0005997\_xylulose\_metabolic\_process | 1 | 0 |  |  |  |  |  |  |  |  |
| GO:0006000\_fructose\_metabolic\_process | 1 | 0 |  |  |  |  |  |  |  |  |
| GO:0006002\_fructose\_6-phosphate\_metabolic\_process | 1 | 0 |  |  |  |  |  |  |  |  |
| GO:0006004\_fucose\_metabolic\_process | 1 | 0 |  |  |  |  |  |  |  |  |
| GO:0006013\_mannose\_metabolic\_process | 1 | 0 |  |  |  |  |  |  |  |  |
| GO:0006060\_sorbitol\_metabolic\_process | 1 | 0 |  |  |  |  |  |  |  |  |
| GO:0006064\_glucuronate\_catabolic\_process | 1 | 0 |  |  |  |  |  |  |  |  |
| GO:0006086\_acetyl-CoA\_biosynthetic\_process\_from\_pyruvate | 1 | 0 |  |  |  |  |  |  |  |  |
| GO:0006098\_pentose-phosphate\_shunt | 1 | 0 |  |  |  |  |  |  |  |  |
| GO:0006101\_citrate\_metabolic\_process | 1 | 0 |  |  |  |  |  |  |  |  |
| GO:0006104\_succinyl-CoA\_metabolic\_process | 1 | 0 |  |  |  |  |  |  |  |  |
| GO:0006116\_NADH\_oxidation | 1 | 0 |  |  |  |  |  |  |  |  |
| GO:0006120\_mitochondrial\_electron\_transport\_\_NADH\_to\_ubiquinone | 1 | 0 |  |  |  |  |  |  |  |  |
| GO:0006154\_adenosine\_catabolic\_process | 1 | 0 |  |  |  |  |  |  |  |  |
| GO:0006157\_deoxyadenosine\_catabolic\_process | 1 | 0 |  |  |  |  |  |  |  |  |
| GO:0006167\_AMP\_biosynthetic\_process | 1 | 0 |  |  |  |  |  |  |  |  |
| GO:0006175\_dATP\_biosynthetic\_process | 1 | 0 |  |  |  |  |  |  |  |  |
| GO:0006178\_guanine\_salvage | 1 | 0 |  |  |  |  |  |  |  |  |
| GO:0006196\_AMP\_catabolic\_process | 1 | 0 |  |  |  |  |  |  |  |  |
| GO:0006203\_dGTP\_catabolic\_process | 1 | 0 |  |  |  |  |  |  |  |  |
| GO:0006208\_pyrimidine\_base\_catabolic\_process | 1 | 0 |  |  |  |  |  |  |  |  |
| GO:0006221\_pyrimidine\_nucleotide\_biosynthetic\_process | 1 | 0 |  |  |  |  |  |  |  |  |
| GO:0006235\_dTTP\_biosynthetic\_process | 1 | 0 |  |  |  |  |  |  |  |  |
| GO:0006244\_pyrimidine\_nucleotide\_catabolic\_process | 1 | 0 |  |  |  |  |  |  |  |  |
| GO:0006269\_DNA\_replication\_\_synthesis\_of\_RNA\_primer | 1 | 0 |  |  |  |  |  |  |  |  |
| GO:0006296\_nucleotide-excision\_repair\_\_DNA\_incision\_\_5'-to\_lesion | 1 | 0 |  |  |  |  |  |  |  |  |
| GO:0006307\_DNA\_dealkylation | 1 | 0 |  |  |  |  |  |  |  |  |
| GO:0006337\_nucleosome\_disassembly | 1 | 0 |  |  |  |  |  |  |  |  |
| GO:0006344\_maintenance\_of\_chromatin\_silencing | 1 | 0 |  |  |  |  |  |  |  |  |
| GO:0006356\_regulation\_of\_transcription\_from\_RNA\_polymerase\_I\_promoter | 1 | 0 |  |  |  |  |  |  |  |  |
| GO:0006388\_tRNA\_splicing\_\_via\_endonucleolytic\_cleavage\_and\_ligation | 1 | 0 |  |  |  |  |  |  |  |  |
| GO:0006407\_rRNA\_export\_from\_nucleus | 1 | 0 |  |  |  |  |  |  |  |  |
| GO:0006419\_alanyl-tRNA\_aminoacylation | 1 | 0 |  |  |  |  |  |  |  |  |
| GO:0006434\_seryl-tRNA\_aminoacylation | 1 | 0 |  |  |  |  |  |  |  |  |
| GO:0006447\_regulation\_of\_translational\_initiation\_by\_iron | 1 | 0 |  |  |  |  |  |  |  |  |
| GO:0006463\_steroid\_hormone\_receptor\_complex\_assembly | 1 | 0 |  |  |  |  |  |  |  |  |
| GO:0006467\_protein\_thiol-disulfide\_exchange | 1 | 0 |  |  |  |  |  |  |  |  |
| GO:0006474\_N-terminal\_protein\_amino\_acid\_acetylation | 1 | 0 |  |  |  |  |  |  |  |  |
| GO:0006481\_C-terminal\_protein\_amino\_acid\_methylation | 1 | 0 |  |  |  |  |  |  |  |  |
| GO:0006488\_dolichol-linked\_oligosaccharide\_biosynthetic\_process | 1 | 0 |  |  |  |  |  |  |  |  |
| GO:0006494\_protein\_amino\_acid\_terminal\_glycosylation | 1 | 0 |  |  |  |  |  |  |  |  |
| GO:0006496\_protein\_amino\_acid\_terminal\_N-glycosylation | 1 | 0 |  |  |  |  |  |  |  |  |
| GO:0006500\_N-terminal\_protein\_palmitoylation | 1 | 0 |  |  |  |  |  |  |  |  |
| GO:0006507\_GPI\_anchor\_release | 1 | 0 |  |  |  |  |  |  |  |  |
| GO:0006537\_glutamate\_biosynthetic\_process | 1 | 0 |  |  |  |  |  |  |  |  |
| GO:0006544\_glycine\_metabolic\_process | 1 | 0 |  |  |  |  |  |  |  |  |
| GO:0006549\_isoleucine\_metabolic\_process | 1 | 0 |  |  |  |  |  |  |  |  |
| GO:0006553\_lysine\_metabolic\_process | 1 | 0 |  |  |  |  |  |  |  |  |
| GO:0006554\_lysine\_catabolic\_process | 1 | 0 |  |  |  |  |  |  |  |  |
| GO:0006559\_L-phenylalanine\_catabolic\_process | 1 | 0 |  |  |  |  |  |  |  |  |
| GO:0006569\_tryptophan\_catabolic\_process | 1 | 0 |  |  |  |  |  |  |  |  |
| GO:0006572\_tyrosine\_catabolic\_process | 1 | 0 |  |  |  |  |  |  |  |  |
| GO:0006573\_valine\_metabolic\_process | 1 | 0 |  |  |  |  |  |  |  |  |
| GO:0006581\_acetylcholine\_catabolic\_process | 1 | 0 |  |  |  |  |  |  |  |  |
| GO:0006585\_dopamine\_biosynthetic\_process\_from\_tyrosine | 1 | 0 |  |  |  |  |  |  |  |  |
| GO:0006590\_thyroid\_hormone\_generation | 1 | 0 |  |  |  |  |  |  |  |  |
| GO:0006591\_ornithine\_metabolic\_process | 1 | 0 |  |  |  |  |  |  |  |  |
| GO:0006596\_polyamine\_biosynthetic\_process | 1 | 0 |  |  |  |  |  |  |  |  |
| GO:0006597\_spermine\_biosynthetic\_process | 1 | 0 |  |  |  |  |  |  |  |  |
| GO:0006601\_creatine\_biosynthetic\_process | 1 | 0 |  |  |  |  |  |  |  |  |
| GO:0006613\_cotranslational\_protein\_targeting\_to\_membrane | 1 | 0 |  |  |  |  |  |  |  |  |
| GO:0006622\_protein\_targeting\_to\_lysosome | 1 | 0 |  |  |  |  |  |  |  |  |
| GO:0006627\_mitochondrial\_protein\_processing\_during\_import | 1 | 0 |  |  |  |  |  |  |  |  |
| GO:0006653\_lecithin\_metabolic\_process | 1 | 0 |  |  |  |  |  |  |  |  |
| GO:0006654\_phosphatidic\_acid\_biosynthetic\_process | 1 | 0 |  |  |  |  |  |  |  |  |
| GO:0006658\_phosphatidylserine\_metabolic\_process | 1 | 0 |  |  |  |  |  |  |  |  |
| GO:0006659\_phosphatidylserine\_biosynthetic\_process | 1 | 0 |  |  |  |  |  |  |  |  |
| GO:0006667\_sphinganine\_metabolic\_process | 1 | 0 |  |  |  |  |  |  |  |  |
| GO:0006668\_sphinganine-1-phosphate\_metabolic\_process | 1 | 0 |  |  |  |  |  |  |  |  |
| GO:0006678\_glucosylceramide\_metabolic\_process | 1 | 0 |  |  |  |  |  |  |  |  |
| GO:0006682\_galactosylceramide\_biosynthetic\_process | 1 | 0 |  |  |  |  |  |  |  |  |
| GO:0006685\_sphingomyelin\_catabolic\_process | 1 | 0 |  |  |  |  |  |  |  |  |
| GO:0006700\_C21-steroid\_hormone\_biosynthetic\_process | 1 | 0 |  |  |  |  |  |  |  |  |
| GO:0006705\_mineralocorticoid\_biosynthetic\_process | 1 | 0 |  |  |  |  |  |  |  |  |
| GO:0006709\_progesterone\_catabolic\_process | 1 | 0 |  |  |  |  |  |  |  |  |
| GO:0006729\_tetrahydrobiopterin\_biosynthetic\_process | 1 | 0 |  |  |  |  |  |  |  |  |
| GO:0006734\_NADH\_metabolic\_process | 1 | 0 |  |  |  |  |  |  |  |  |
| GO:0006740\_NADPH\_regeneration | 1 | 0 |  |  |  |  |  |  |  |  |
| GO:0006741\_NADP\_biosynthetic\_process | 1 | 0 |  |  |  |  |  |  |  |  |
| GO:0006743\_ubiquinone\_metabolic\_process | 1 | 0 |  |  |  |  |  |  |  |  |
| GO:0006744\_ubiquinone\_biosynthetic\_process | 1 | 0 |  |  |  |  |  |  |  |  |
| GO:0006772\_thiamin\_metabolic\_process | 1 | 0 |  |  |  |  |  |  |  |  |
| GO:0006784\_heme\_a\_biosynthetic\_process | 1 | 0 |  |  |  |  |  |  |  |  |
| GO:0006797\_polyphosphate\_metabolic\_process | 1 | 0 |  |  |  |  |  |  |  |  |
| GO:0006798\_polyphosphate\_catabolic\_process | 1 | 0 |  |  |  |  |  |  |  |  |
| GO:0006824\_cobalt\_ion\_transport | 1 | 0 |  |  |  |  |  |  |  |  |
| GO:0006842\_tricarboxylic\_acid\_transport | 1 | 0 |  |  |  |  |  |  |  |  |
| GO:0006844\_acyl\_carnitine\_transport | 1 | 0 |  |  |  |  |  |  |  |  |
| GO:0006855\_multidrug\_transport | 1 | 0 |  |  |  |  |  |  |  |  |
| GO:0006863\_purine\_transport | 1 | 0 |  |  |  |  |  |  |  |  |
| GO:0006890\_retrograde\_vesicle-mediated\_transport\_\_Golgi\_to\_ER | 1 | 0 |  |  |  |  |  |  |  |  |
| GO:0006891\_intra-Golgi\_vesicle-mediated\_transport | 1 | 0 |  |  |  |  |  |  |  |  |
| GO:0006893\_Golgi\_to\_plasma\_membrane\_transport | 1 | 0 |  |  |  |  |  |  |  |  |
| GO:0006895\_Golgi\_to\_endosome\_transport | 1 | 0 |  |  |  |  |  |  |  |  |
| GO:0006896\_Golgi\_to\_vacuole\_transport | 1 | 0 |  |  |  |  |  |  |  |  |
| GO:0006900\_membrane\_budding | 1 | 0 |  |  |  |  |  |  |  |  |
| GO:0006930\_substrate-bound\_cell\_migration\_\_cell\_extension | 1 | 0 |  |  |  |  |  |  |  |  |
| GO:0006931\_substrate-bound\_cell\_migration\_\_cell\_attachment\_to\_substrate | 1 | 0 |  |  |  |  |  |  |  |  |
| GO:0006933\_negative\_regulation\_of\_cell\_adhesion\_involved\_in\_substrate-bound\_cell\_migration | 1 | 0 |  |  |  |  |  |  |  |  |
| GO:0006957\_complement\_activation\_\_alternative\_pathway | 1 | 0 |  |  |  |  |  |  |  |  |
| GO:0006958\_complement\_activation\_\_classical\_pathway | 1 | 0 |  |  |  |  |  |  |  |  |
| GO:0006978\_DNA\_damage\_response\_\_signal\_transduction\_by\_p53\_class\_mediator\_resulting\_in\_transcription\_of\_p21\_class\_mediator | 1 | 0 |  |  |  |  |  |  |  |  |
| GO:0007016\_cytoskeletal\_anchoring\_at\_plasma\_membrane | 1 | 0 |  |  |  |  |  |  |  |  |
| GO:0007021\_tubulin\_complex\_assembly | 1 | 0 |  |  |  |  |  |  |  |  |
| GO:0007052\_mitotic\_spindle\_organization | 1 | 0 |  |  |  |  |  |  |  |  |
| GO:0007056\_spindle\_assembly\_involved\_in\_female\_meiosis | 1 | 0 |  |  |  |  |  |  |  |  |
| GO:0007057\_spindle\_assembly\_involved\_in\_female\_meiosis\_I | 1 | 0 |  |  |  |  |  |  |  |  |
| GO:0007063\_regulation\_of\_sister\_chromatid\_cohesion | 1 | 0 |  |  |  |  |  |  |  |  |
| GO:0007065\_male\_meiosis\_sister\_chromatid\_cohesion | 1 | 0 |  |  |  |  |  |  |  |  |
| GO:0007076\_mitotic\_chromosome\_condensation | 1 | 0 |  |  |  |  |  |  |  |  |
| GO:0007095\_mitotic\_cell\_cycle\_G2\_M\_transition\_DNA\_damage\_checkpoint | 1 | 0 |  |  |  |  |  |  |  |  |
| GO:0007096\_regulation\_of\_exit\_from\_mitosis | 1 | 0 |  |  |  |  |  |  |  |  |
| GO:0007158\_neuron\_adhesion | 1 | 0 |  |  |  |  |  |  |  |  |
| GO:0007168\_receptor\_guanylyl\_cyclase\_signaling\_pathway | 1 | 0 |  |  |  |  |  |  |  |  |
| GO:0007197\_inhibition\_of\_adenylate\_cyclase\_activity\_by\_muscarinic\_acetylcholine\_receptor\_signaling\_pathway | 1 | 0 |  |  |  |  |  |  |  |  |
| GO:0007207\_activation\_of\_phospholipase\_C\_activity\_by\_muscarinic\_acetylcholine\_receptor\_signaling\_pathway | 1 | 0 |  |  |  |  |  |  |  |  |
| GO:0007208\_activation\_of\_phospholipase\_C\_activity\_by\_serotonin\_receptor\_signaling\_pathway | 1 | 0 |  |  |  |  |  |  |  |  |
| GO:0007217\_tachykinin\_receptor\_signaling\_pathway | 1 | 0 |  |  |  |  |  |  |  |  |
| GO:0007221\_positive\_regulation\_of\_transcription\_of\_Notch\_receptor\_target | 1 | 0 |  |  |  |  |  |  |  |  |
| GO:0007223\_Wnt\_receptor\_signaling\_pathway\_\_calcium\_modulating\_pathway | 1 | 0 |  |  |  |  |  |  |  |  |
| GO:0007225\_patched\_ligand\_processing | 1 | 0 |  |  |  |  |  |  |  |  |
| GO:0007227\_signal\_transduction\_downstream\_of\_smoothened | 1 | 0 |  |  |  |  |  |  |  |  |
| GO:0007228\_positive\_regulation\_of\_hh\_target\_transcription\_factor\_activity | 1 | 0 |  |  |  |  |  |  |  |  |
| GO:0007231\_osmosensory\_signaling\_pathway | 1 | 0 |  |  |  |  |  |  |  |  |
| GO:0007284\_spermatogonial\_cell\_division | 1 | 0 |  |  |  |  |  |  |  |  |
| GO:0007290\_spermatid\_nucleus\_elongation | 1 | 0 |  |  |  |  |  |  |  |  |
| GO:0007296\_vitellogenesis | 1 | 0 |  |  |  |  |  |  |  |  |
| GO:0007321\_sperm\_displacement | 1 | 0 |  |  |  |  |  |  |  |  |
| GO:0007380\_specification\_of\_segmental\_identity\_\_head | 1 | 0 |  |  |  |  |  |  |  |  |
| GO:0007382\_specification\_of\_segmental\_identity\_\_maxillary\_segment | 1 | 0 |  |  |  |  |  |  |  |  |
| GO:0007400\_neuroblast\_fate\_determination | 1 | 0 |  |  |  |  |  |  |  |  |
| GO:0007402\_ganglion\_mother\_cell\_fate\_determination | 1 | 0 |  |  |  |  |  |  |  |  |
| GO:0007495\_visceral\_mesoderm-endoderm\_interaction\_involved\_in\_midgut\_development | 1 | 0 |  |  |  |  |  |  |  |  |
| GO:0007497\_posterior\_midgut\_development | 1 | 0 |  |  |  |  |  |  |  |  |
| GO:0007499\_ectoderm\_and\_mesoderm\_interaction | 1 | 0 |  |  |  |  |  |  |  |  |
| GO:0007500\_mesodermal\_cell\_fate\_determination | 1 | 0 |  |  |  |  |  |  |  |  |
| GO:0007509\_mesoderm\_migration | 1 | 0 |  |  |  |  |  |  |  |  |
| GO:0007518\_myoblast\_cell\_fate\_determination | 1 | 0 |  |  |  |  |  |  |  |  |
| GO:0007521\_muscle\_cell\_fate\_determination | 1 | 0 |  |  |  |  |  |  |  |  |
| GO:0007522\_visceral\_muscle\_development | 1 | 0 |  |  |  |  |  |  |  |  |
| GO:0007529\_establishment\_of\_synaptic\_specificity\_at\_neuromuscular\_junction | 1 | 0 |  |  |  |  |  |  |  |  |
| GO:0007538\_primary\_sex\_determination | 1 | 0 |  |  |  |  |  |  |  |  |
| GO:0007542\_primary\_sex\_determination\_\_germ-line | 1 | 0 |  |  |  |  |  |  |  |  |
| GO:0007567\_parturition | 1 | 0 |  |  |  |  |  |  |  |  |
| GO:0007614\_short-term\_memory | 1 | 0 |  |  |  |  |  |  |  |  |
| GO:0007621\_negative\_regulation\_of\_female\_receptivity | 1 | 0 |  |  |  |  |  |  |  |  |
| GO:0008049\_male\_courtship\_behavior | 1 | 0 |  |  |  |  |  |  |  |  |
| GO:0008050\_female\_courtship\_behavior | 1 | 0 |  |  |  |  |  |  |  |  |
| GO:0008052\_sensory\_organ\_boundary\_specification | 1 | 0 |  |  |  |  |  |  |  |  |
| GO:0008054\_cyclin\_catabolic\_process | 1 | 0 |  |  |  |  |  |  |  |  |
| GO:0008057\_eye\_pigment\_granule\_organization | 1 | 0 |  |  |  |  |  |  |  |  |
| GO:0008078\_mesodermal\_cell\_migration | 1 | 0 |  |  |  |  |  |  |  |  |
| GO:0008208\_C21-steroid\_hormone\_catabolic\_process | 1 | 0 |  |  |  |  |  |  |  |  |
| GO:0008216\_spermidine\_metabolic\_process | 1 | 0 |  |  |  |  |  |  |  |  |
| GO:0008292\_acetylcholine\_biosynthetic\_process | 1 | 0 |  |  |  |  |  |  |  |  |
| GO:0008295\_spermidine\_biosynthetic\_process | 1 | 0 |  |  |  |  |  |  |  |  |
| GO:0008300\_isoprenoid\_catabolic\_process | 1 | 0 |  |  |  |  |  |  |  |  |
| GO:0008333\_endosome\_to\_lysosome\_transport | 1 | 0 |  |  |  |  |  |  |  |  |
| GO:0008355\_olfactory\_learning | 1 | 0 |  |  |  |  |  |  |  |  |
| GO:0008611\_ether\_lipid\_biosynthetic\_process | 1 | 0 |  |  |  |  |  |  |  |  |
| GO:0008626\_induction\_of\_apoptosis\_by\_granzyme | 1 | 0 |  |  |  |  |  |  |  |  |
| GO:0008633\_activation\_of\_pro-apoptotic\_gene\_products | 1 | 0 |  |  |  |  |  |  |  |  |
| GO:0008653\_lipopolysaccharide\_metabolic\_process | 1 | 0 |  |  |  |  |  |  |  |  |
| GO:0009068\_aspartate\_family\_amino\_acid\_catabolic\_process | 1 | 0 |  |  |  |  |  |  |  |  |
| GO:0009084\_glutamine\_family\_amino\_acid\_biosynthetic\_process | 1 | 0 |  |  |  |  |  |  |  |  |
| GO:0009088\_threonine\_biosynthetic\_process | 1 | 0 |  |  |  |  |  |  |  |  |
| GO:0009105\_lipoic\_acid\_biosynthetic\_process | 1 | 0 |  |  |  |  |  |  |  |  |
| GO:0009109\_coenzyme\_catabolic\_process | 1 | 0 |  |  |  |  |  |  |  |  |
| GO:0009111\_vitamin\_catabolic\_process | 1 | 0 |  |  |  |  |  |  |  |  |
| GO:0009113\_purine\_base\_biosynthetic\_process | 1 | 0 |  |  |  |  |  |  |  |  |
| GO:0009127\_purine\_nucleoside\_monophosphate\_biosynthetic\_process | 1 | 0 |  |  |  |  |  |  |  |  |
| GO:0009128\_purine\_nucleoside\_monophosphate\_catabolic\_process | 1 | 0 |  |  |  |  |  |  |  |  |
| GO:0009129\_pyrimidine\_nucleoside\_monophosphate\_metabolic\_process | 1 | 0 |  |  |  |  |  |  |  |  |
| GO:0009131\_pyrimidine\_nucleoside\_monophosphate\_catabolic\_process | 1 | 0 |  |  |  |  |  |  |  |  |
| GO:0009133\_nucleoside\_diphosphate\_biosynthetic\_process | 1 | 0 |  |  |  |  |  |  |  |  |
| GO:0009145\_purine\_nucleoside\_triphosphate\_biosynthetic\_process | 1 | 0 |  |  |  |  |  |  |  |  |
| GO:0009147\_pyrimidine\_nucleoside\_triphosphate\_metabolic\_process | 1 | 0 |  |  |  |  |  |  |  |  |
| GO:0009148\_pyrimidine\_nucleoside\_triphosphate\_biosynthetic\_process | 1 | 0 |  |  |  |  |  |  |  |  |
| GO:0009152\_purine\_ribonucleotide\_biosynthetic\_process | 1 | 0 |  |  |  |  |  |  |  |  |
| GO:0009153\_purine\_deoxyribonucleotide\_biosynthetic\_process | 1 | 0 |  |  |  |  |  |  |  |  |
| GO:0009156\_ribonucleoside\_monophosphate\_biosynthetic\_process | 1 | 0 |  |  |  |  |  |  |  |  |
| GO:0009158\_ribonucleoside\_monophosphate\_catabolic\_process | 1 | 0 |  |  |  |  |  |  |  |  |
| GO:0009159\_deoxyribonucleoside\_monophosphate\_catabolic\_process | 1 | 0 |  |  |  |  |  |  |  |  |
| GO:0009162\_deoxyribonucleoside\_monophosphate\_metabolic\_process | 1 | 0 |  |  |  |  |  |  |  |  |
| GO:0009168\_purine\_ribonucleoside\_monophosphate\_biosynthetic\_process | 1 | 0 |  |  |  |  |  |  |  |  |
| GO:0009169\_purine\_ribonucleoside\_monophosphate\_catabolic\_process | 1 | 0 |  |  |  |  |  |  |  |  |
| GO:0009176\_pyrimidine\_deoxyribonucleoside\_monophosphate\_metabolic\_process | 1 | 0 |  |  |  |  |  |  |  |  |
| GO:0009178\_pyrimidine\_deoxyribonucleoside\_monophosphate\_catabolic\_process | 1 | 0 |  |  |  |  |  |  |  |  |
| GO:0009211\_pyrimidine\_deoxyribonucleoside\_triphosphate\_metabolic\_process | 1 | 0 |  |  |  |  |  |  |  |  |
| GO:0009212\_pyrimidine\_deoxyribonucleoside\_triphosphate\_biosynthetic\_process | 1 | 0 |  |  |  |  |  |  |  |  |
| GO:0009216\_purine\_deoxyribonucleoside\_triphosphate\_biosynthetic\_process | 1 | 0 |  |  |  |  |  |  |  |  |
| GO:0009221\_pyrimidine\_deoxyribonucleotide\_biosynthetic\_process | 1 | 0 |  |  |  |  |  |  |  |  |
| GO:0009223\_pyrimidine\_deoxyribonucleotide\_catabolic\_process | 1 | 0 |  |  |  |  |  |  |  |  |
| GO:0009260\_ribonucleotide\_biosynthetic\_process | 1 | 0 |  |  |  |  |  |  |  |  |
| GO:0009405\_pathogenesis | 1 | 0 |  |  |  |  |  |  |  |  |
| GO:0009414\_response\_to\_water\_deprivation | 1 | 0 |  |  |  |  |  |  |  |  |
| GO:0009415\_response\_to\_water | 1 | 0 |  |  |  |  |  |  |  |  |
| GO:0009449\_gamma-aminobutyric\_acid\_biosynthetic\_process | 1 | 0 |  |  |  |  |  |  |  |  |
| GO:0009450\_gamma-aminobutyric\_acid\_catabolic\_process | 1 | 0 |  |  |  |  |  |  |  |  |
| GO:0009589\_detection\_of\_UV | 1 | 0 |  |  |  |  |  |  |  |  |
| GO:0009590\_detection\_of\_gravity | 1 | 0 |  |  |  |  |  |  |  |  |
| GO:0009624\_response\_to\_nematode | 1 | 0 |  |  |  |  |  |  |  |  |
| GO:0009629\_response\_to\_gravity | 1 | 0 |  |  |  |  |  |  |  |  |
| GO:0009648\_photoperiodism | 1 | 0 |  |  |  |  |  |  |  |  |
| GO:0009690\_cytokinin\_metabolic\_process | 1 | 0 |  |  |  |  |  |  |  |  |
| GO:0009691\_cytokinin\_biosynthetic\_process | 1 | 0 |  |  |  |  |  |  |  |  |
| GO:0009786\_regulation\_of\_asymmetric\_cell\_division | 1 | 0 |  |  |  |  |  |  |  |  |
| GO:0009794\_regulation\_of\_mitotic\_cell\_cycle\_\_embryonic | 1 | 0 |  |  |  |  |  |  |  |  |
| GO:0009956\_radial\_pattern\_formation | 1 | 0 |  |  |  |  |  |  |  |  |
| GO:0009957\_epidermal\_cell\_fate\_specification | 1 | 0 |  |  |  |  |  |  |  |  |
| GO:0009992\_cellular\_water\_homeostasis | 1 | 0 |  |  |  |  |  |  |  |  |
| GO:0010032\_meiotic\_chromosome\_condensation | 1 | 0 |  |  |  |  |  |  |  |  |
| GO:0010039\_response\_to\_iron\_ion | 1 | 0 |  |  |  |  |  |  |  |  |
| GO:0010042\_response\_to\_manganese\_ion | 1 | 0 |  |  |  |  |  |  |  |  |
| GO:0010045\_response\_to\_nickel\_ion | 1 | 0 |  |  |  |  |  |  |  |  |
| GO:0010046\_response\_to\_mycotoxin | 1 | 0 |  |  |  |  |  |  |  |  |
| GO:0010107\_potassium\_ion\_import | 1 | 0 |  |  |  |  |  |  |  |  |
| GO:0010155\_regulation\_of\_proton\_transport | 1 | 0 |  |  |  |  |  |  |  |  |
| GO:0010160\_formation\_of\_organ\_boundary | 1 | 0 |  |  |  |  |  |  |  |  |
| GO:0010260\_organ\_senescence | 1 | 0 |  |  |  |  |  |  |  |  |
| GO:0010310\_regulation\_of\_hydrogen\_peroxide\_metabolic\_process | 1 | 0 |  |  |  |  |  |  |  |  |
| GO:0010447\_response\_to\_acidity | 1 | 0 |  |  |  |  |  |  |  |  |
| GO:0010452\_histone\_H3-K36\_methylation | 1 | 0 |  |  |  |  |  |  |  |  |
| GO:0010455\_positive\_regulation\_of\_cell\_fate\_commitment | 1 | 0 |  |  |  |  |  |  |  |  |
| GO:0010470\_regulation\_of\_gastrulation | 1 | 0 |  |  |  |  |  |  |  |  |
| GO:0010508\_positive\_regulation\_of\_autophagy | 1 | 0 |  |  |  |  |  |  |  |  |
| GO:0010519\_negative\_regulation\_of\_phospholipase\_activity | 1 | 0 |  |  |  |  |  |  |  |  |
| GO:0010520\_regulation\_of\_reciprocal\_meiotic\_recombination | 1 | 0 |  |  |  |  |  |  |  |  |
| GO:0010523\_negative\_regulation\_of\_calcium\_ion\_transport\_into\_cytosol | 1 | 0 |  |  |  |  |  |  |  |  |
| GO:0010543\_regulation\_of\_platelet\_activation | 1 | 0 |  |  |  |  |  |  |  |  |
| GO:0010561\_negative\_regulation\_of\_glycoprotein\_biosynthetic\_process | 1 | 0 |  |  |  |  |  |  |  |  |
| GO:0010569\_regulation\_of\_double-strand\_break\_repair\_via\_homologous\_recombination | 1 | 0 |  |  |  |  |  |  |  |  |
| GO:0010572\_positive\_regulation\_of\_platelet\_activation | 1 | 0 |  |  |  |  |  |  |  |  |
| GO:0010594\_regulation\_of\_endothelial\_cell\_migration | 1 | 0 |  |  |  |  |  |  |  |  |
| GO:0010596\_negative\_regulation\_of\_endothelial\_cell\_migration | 1 | 0 |  |  |  |  |  |  |  |  |
| GO:0010611\_regulation\_of\_cardiac\_muscle\_hypertrophy | 1 | 0 |  |  |  |  |  |  |  |  |
| GO:0010612\_regulation\_of\_cardiac\_muscle\_adaptation | 1 | 0 |  |  |  |  |  |  |  |  |
| GO:0010614\_negative\_regulation\_of\_cardiac\_muscle\_hypertrophy | 1 | 0 |  |  |  |  |  |  |  |  |
| GO:0010616\_negative\_regulation\_of\_cardiac\_muscle\_adaptation | 1 | 0 |  |  |  |  |  |  |  |  |
| GO:0010634\_positive\_regulation\_of\_epithelial\_cell\_migration | 1 | 0 |  |  |  |  |  |  |  |  |
| GO:0010656\_negative\_regulation\_of\_muscle\_cell\_apoptosis | 1 | 0 |  |  |  |  |  |  |  |  |
| GO:0010657\_muscle\_cell\_apoptosis | 1 | 0 |  |  |  |  |  |  |  |  |
| GO:0010658\_striated\_muscle\_cell\_apoptosis | 1 | 0 |  |  |  |  |  |  |  |  |
| GO:0010659\_cardiac\_muscle\_cell\_apoptosis | 1 | 0 |  |  |  |  |  |  |  |  |
| GO:0010660\_regulation\_of\_muscle\_cell\_apoptosis | 1 | 0 |  |  |  |  |  |  |  |  |
| GO:0010662\_regulation\_of\_striated\_muscle\_cell\_apoptosis | 1 | 0 |  |  |  |  |  |  |  |  |
| GO:0010664\_negative\_regulation\_of\_striated\_muscle\_cell\_apoptosis | 1 | 0 |  |  |  |  |  |  |  |  |
| GO:0010665\_regulation\_of\_cardiac\_muscle\_cell\_apoptosis | 1 | 0 |  |  |  |  |  |  |  |  |
| GO:0010667\_negative\_regulation\_of\_cardiac\_muscle\_cell\_apoptosis | 1 | 0 |  |  |  |  |  |  |  |  |
| GO:0010668\_ectodermal\_cell\_differentiation | 1 | 0 |  |  |  |  |  |  |  |  |
| GO:0010671\_negative\_regulation\_of\_oxygen\_and\_reactive\_oxygen\_species\_metabolic\_process | 1 | 0 |  |  |  |  |  |  |  |  |
| GO:0010719\_negative\_regulation\_of\_epithelial\_to\_mesenchymal\_transition | 1 | 0 |  |  |  |  |  |  |  |  |
| GO:0010735\_positive\_regulation\_of\_transcription\_via\_serum\_response\_element\_binding | 1 | 0 |  |  |  |  |  |  |  |  |
| GO:0010825\_positive\_regulation\_of\_centrosome\_duplication | 1 | 0 |  |  |  |  |  |  |  |  |
| GO:0010845\_positive\_regulation\_of\_reciprocal\_meiotic\_recombination | 1 | 0 |  |  |  |  |  |  |  |  |
| GO:0010850\_chemoreceptor\_signaling\_pathway\_involved\_in\_regulation\_of\_blood\_pressure | 1 | 0 |  |  |  |  |  |  |  |  |
| GO:0010873\_positive\_regulation\_of\_cholesterol\_esterification | 1 | 0 |  |  |  |  |  |  |  |  |
| GO:0010880\_regulation\_of\_release\_of\_sequestered\_calcium\_ion\_into\_cytosol\_by\_sarcoplasmic\_reticulum | 1 | 0 |  |  |  |  |  |  |  |  |
| GO:0010881\_regulation\_of\_cardiac\_muscle\_contraction\_by\_regulation\_of\_the\_release\_of\_sequestered\_calcium\_ion | 1 | 0 |  |  |  |  |  |  |  |  |
| GO:0010882\_regulation\_of\_cardiac\_muscle\_contraction\_by\_calcium\_ion\_signaling | 1 | 0 |  |  |  |  |  |  |  |  |
| GO:0010890\_positive\_regulation\_of\_sequestering\_of\_triglyceride | 1 | 0 |  |  |  |  |  |  |  |  |
| GO:0010919\_regulation\_of\_inositol\_phosphate\_biosynthetic\_process | 1 | 0 |  |  |  |  |  |  |  |  |
| GO:0010931\_macrophage\_tolerance\_induction | 1 | 0 |  |  |  |  |  |  |  |  |
| GO:0010932\_regulation\_of\_macrophage\_tolerance\_induction | 1 | 0 |  |  |  |  |  |  |  |  |
| GO:0010933\_positive\_regulation\_of\_macrophage\_tolerance\_induction | 1 | 0 |  |  |  |  |  |  |  |  |
| GO:0010934\_macrophage\_cytokine\_production | 1 | 0 |  |  |  |  |  |  |  |  |
| GO:0010935\_regulation\_of\_macrophage\_cytokine\_production | 1 | 0 |  |  |  |  |  |  |  |  |
| GO:0010936\_negative\_regulation\_of\_macrophage\_cytokine\_production | 1 | 0 |  |  |  |  |  |  |  |  |
| GO:0010953\_regulation\_of\_protein\_maturation\_by\_peptide\_bond\_cleavage | 1 | 0 |  |  |  |  |  |  |  |  |
| GO:0010962\_regulation\_of\_glucan\_biosynthetic\_process | 1 | 0 |  |  |  |  |  |  |  |  |
| GO:0010966\_regulation\_of\_phosphate\_transport | 1 | 0 |  |  |  |  |  |  |  |  |
| GO:0014012\_axon\_regeneration\_in\_the\_peripheral\_nervous\_system | 1 | 0 |  |  |  |  |  |  |  |  |
| GO:0014016\_neuroblast\_differentiation | 1 | 0 |  |  |  |  |  |  |  |  |
| GO:0014017\_neuroblast\_fate\_commitment | 1 | 0 |  |  |  |  |  |  |  |  |
| GO:0014041\_regulation\_of\_neuron\_maturation | 1 | 0 |  |  |  |  |  |  |  |  |
| GO:0014042\_positive\_regulation\_of\_neuron\_maturation | 1 | 0 |  |  |  |  |  |  |  |  |
| GO:0014049\_positive\_regulation\_of\_glutamate\_secretion | 1 | 0 |  |  |  |  |  |  |  |  |
| GO:0014061\_regulation\_of\_norepinephrine\_secretion | 1 | 0 |  |  |  |  |  |  |  |  |
| GO:0014071\_response\_to\_cycloalkane | 1 | 0 |  |  |  |  |  |  |  |  |
| GO:0014707\_branchiomeric\_skeletal\_muscle\_development | 1 | 0 |  |  |  |  |  |  |  |  |
| GO:0014738\_regulation\_of\_muscle\_hyperplasia | 1 | 0 |  |  |  |  |  |  |  |  |
| GO:0014740\_negative\_regulation\_of\_muscle\_hyperplasia | 1 | 0 |  |  |  |  |  |  |  |  |
| GO:0014741\_negative\_regulation\_of\_muscle\_hypertrophy | 1 | 0 |  |  |  |  |  |  |  |  |
| GO:0014743\_regulation\_of\_muscle\_hypertrophy | 1 | 0 |  |  |  |  |  |  |  |  |
| GO:0014805\_smooth\_muscle\_adaptation | 1 | 0 |  |  |  |  |  |  |  |  |
| GO:0014806\_smooth\_muscle\_hyperplasia | 1 | 0 |  |  |  |  |  |  |  |  |
| GO:0014807\_regulation\_of\_somitogenesis | 1 | 0 |  |  |  |  |  |  |  |  |
| GO:0014808\_release\_of\_sequestered\_calcium\_ion\_into\_cytosol\_by\_sarcoplasmic\_reticulum | 1 | 0 |  |  |  |  |  |  |  |  |
| GO:0014813\_satellite\_cell\_commitment | 1 | 0 |  |  |  |  |  |  |  |  |
| GO:0014816\_satellite\_cell\_differentiation | 1 | 0 |  |  |  |  |  |  |  |  |
| GO:0014819\_regulation\_of\_skeletal\_muscle\_contraction | 1 | 0 |  |  |  |  |  |  |  |  |
| GO:0014852\_regulation\_of\_skeletal\_muscle\_contraction\_by\_neural\_stimulation\_via\_neuromuscular\_junction | 1 | 0 |  |  |  |  |  |  |  |  |
| GO:0014853\_regulation\_of\_excitatory\_postsynaptic\_membrane\_potential\_involved\_in\_skeletal\_muscle\_contraction | 1 | 0 |  |  |  |  |  |  |  |  |
| GO:0014856\_skeletal\_muscle\_cell\_proliferation | 1 | 0 |  |  |  |  |  |  |  |  |
| GO:0014857\_regulation\_of\_skeletal\_muscle\_cell\_proliferation | 1 | 0 |  |  |  |  |  |  |  |  |
| GO:0014858\_positive\_regulation\_of\_skeletal\_muscle\_cell\_proliferation | 1 | 0 |  |  |  |  |  |  |  |  |
| GO:0014887\_cardiac\_muscle\_adaptation | 1 | 0 |  |  |  |  |  |  |  |  |
| GO:0014889\_muscle\_atrophy | 1 | 0 |  |  |  |  |  |  |  |  |
| GO:0014896\_muscle\_hypertrophy | 1 | 0 |  |  |  |  |  |  |  |  |
| GO:0014897\_striated\_muscle\_hypertrophy | 1 | 0 |  |  |  |  |  |  |  |  |
| GO:0014898\_cardiac\_muscle\_hypertrophy | 1 | 0 |  |  |  |  |  |  |  |  |
| GO:0014900\_muscle\_hyperplasia | 1 | 0 |  |  |  |  |  |  |  |  |
| GO:0014910\_regulation\_of\_smooth\_muscle\_cell\_migration | 1 | 0 |  |  |  |  |  |  |  |  |
| GO:0014911\_positive\_regulation\_of\_smooth\_muscle\_cell\_migration | 1 | 0 |  |  |  |  |  |  |  |  |
| GO:0015014\_heparan\_sulfate\_proteoglycan\_biosynthetic\_process\_\_polysaccharide\_chain\_biosynthetic\_process | 1 | 0 |  |  |  |  |  |  |  |  |
| GO:0015074\_DNA\_integration | 1 | 0 |  |  |  |  |  |  |  |  |
| GO:0015670\_carbon\_dioxide\_transport | 1 | 0 |  |  |  |  |  |  |  |  |
| GO:0015677\_copper\_ion\_import | 1 | 0 |  |  |  |  |  |  |  |  |
| GO:0015680\_intracellular\_copper\_ion\_transport | 1 | 0 |  |  |  |  |  |  |  |  |
| GO:0015684\_ferrous\_iron\_transport | 1 | 0 |  |  |  |  |  |  |  |  |
| GO:0015707\_nitrite\_transport | 1 | 0 |  |  |  |  |  |  |  |  |
| GO:0015724\_formate\_transport | 1 | 0 |  |  |  |  |  |  |  |  |
| GO:0015734\_taurine\_transport | 1 | 0 |  |  |  |  |  |  |  |  |
| GO:0015740\_C4-dicarboxylate\_transport | 1 | 0 |  |  |  |  |  |  |  |  |
| GO:0015744\_succinate\_transport | 1 | 0 |  |  |  |  |  |  |  |  |
| GO:0015746\_citrate\_transport | 1 | 0 |  |  |  |  |  |  |  |  |
| GO:0015747\_urate\_transport | 1 | 0 |  |  |  |  |  |  |  |  |
| GO:0015791\_polyol\_transport | 1 | 0 |  |  |  |  |  |  |  |  |
| GO:0015798\_myo-inositol\_transport | 1 | 0 |  |  |  |  |  |  |  |  |
| GO:0015808\_L-alanine\_transport | 1 | 0 |  |  |  |  |  |  |  |  |
| GO:0015810\_aspartate\_transport | 1 | 0 |  |  |  |  |  |  |  |  |
| GO:0015811\_L-cystine\_transport | 1 | 0 |  |  |  |  |  |  |  |  |
| GO:0015817\_histidine\_transport | 1 | 0 |  |  |  |  |  |  |  |  |
| GO:0015822\_ornithine\_transport | 1 | 0 |  |  |  |  |  |  |  |  |
| GO:0015824\_proline\_transport | 1 | 0 |  |  |  |  |  |  |  |  |
| GO:0015851\_nucleobase\_transport | 1 | 0 |  |  |  |  |  |  |  |  |
| GO:0015864\_pyrimidine\_nucleoside\_transport | 1 | 0 |  |  |  |  |  |  |  |  |
| GO:0015874\_norepinephrine\_transport | 1 | 0 |  |  |  |  |  |  |  |  |
| GO:0015881\_creatine\_transport | 1 | 0 |  |  |  |  |  |  |  |  |
| GO:0015884\_folic\_acid\_transport | 1 | 0 |  |  |  |  |  |  |  |  |
| GO:0015886\_heme\_transport | 1 | 0 |  |  |  |  |  |  |  |  |
| GO:0015888\_thiamin\_transport | 1 | 0 |  |  |  |  |  |  |  |  |
| GO:0015938\_coenzyme\_A\_catabolic\_process | 1 | 0 |  |  |  |  |  |  |  |  |
| GO:0015939\_pantothenate\_metabolic\_process | 1 | 0 |  |  |  |  |  |  |  |  |
| GO:0016073\_snRNA\_metabolic\_process | 1 | 0 |  |  |  |  |  |  |  |  |
| GO:0016074\_snoRNA\_metabolic\_process | 1 | 0 |  |  |  |  |  |  |  |  |
| GO:0016082\_synaptic\_vesicle\_priming | 1 | 0 |  |  |  |  |  |  |  |  |
| GO:0016090\_prenol\_metabolic\_process | 1 | 0 |  |  |  |  |  |  |  |  |
| GO:0016093\_polyprenol\_metabolic\_process | 1 | 0 |  |  |  |  |  |  |  |  |
| GO:0016180\_snRNA\_processing | 1 | 0 |  |  |  |  |  |  |  |  |
| GO:0016239\_positive\_regulation\_of\_macroautophagy | 1 | 0 |  |  |  |  |  |  |  |  |
| GO:0016246\_RNA\_interference | 1 | 0 |  |  |  |  |  |  |  |  |
| GO:0016255\_attachment\_of\_GPI\_anchor\_to\_protein | 1 | 0 |  |  |  |  |  |  |  |  |
| GO:0016333\_morphogenesis\_of\_follicular\_epithelium | 1 | 0 |  |  |  |  |  |  |  |  |
| GO:0016340\_calcium-dependent\_cell-matrix\_adhesion | 1 | 0 |  |  |  |  |  |  |  |  |
| GO:0016344\_meiotic\_chromosome\_movement\_towards\_spindle\_pole | 1 | 0 |  |  |  |  |  |  |  |  |
| GO:0016482\_cytoplasmic\_transport | 1 | 0 |  |  |  |  |  |  |  |  |
| GO:0016553\_base\_conversion\_or\_substitution\_editing | 1 | 0 |  |  |  |  |  |  |  |  |
| GO:0016554\_cytidine\_to\_uridine\_editing | 1 | 0 |  |  |  |  |  |  |  |  |
| GO:0016560\_protein\_import\_into\_peroxisome\_matrix\_\_docking | 1 | 0 |  |  |  |  |  |  |  |  |
| GO:0016578\_histone\_deubiquitination | 1 | 0 |  |  |  |  |  |  |  |  |
| GO:0016598\_protein\_arginylation | 1 | 0 |  |  |  |  |  |  |  |  |
| GO:0017004\_cytochrome\_complex\_assembly | 1 | 0 |  |  |  |  |  |  |  |  |
| GO:0018022\_peptidyl-lysine\_methylation | 1 | 0 |  |  |  |  |  |  |  |  |
| GO:0018023\_peptidyl-lysine\_trimethylation | 1 | 0 |  |  |  |  |  |  |  |  |
| GO:0018120\_peptidyl-arginine\_ADP-ribosylation | 1 | 0 |  |  |  |  |  |  |  |  |
| GO:0018126\_protein\_amino\_acid\_hydroxylation | 1 | 0 |  |  |  |  |  |  |  |  |
| GO:0018146\_keratan\_sulfate\_biosynthetic\_process | 1 | 0 |  |  |  |  |  |  |  |  |
| GO:0018158\_protein\_amino\_acid\_oxidation | 1 | 0 |  |  |  |  |  |  |  |  |
| GO:0018195\_peptidyl-arginine\_modification | 1 | 0 |  |  |  |  |  |  |  |  |
| GO:0018197\_peptidyl-aspartic\_acid\_modification | 1 | 0 |  |  |  |  |  |  |  |  |
| GO:0018282\_metal\_incorporation\_into\_metallo-sulfur\_cluster | 1 | 0 |  |  |  |  |  |  |  |  |
| GO:0018283\_iron\_incorporation\_into\_metallo-sulfur\_cluster | 1 | 0 |  |  |  |  |  |  |  |  |
| GO:0018318\_protein\_amino\_acid\_palmitoylation | 1 | 0 |  |  |  |  |  |  |  |  |
| GO:0018342\_protein\_prenylation | 1 | 0 |  |  |  |  |  |  |  |  |
| GO:0018344\_protein\_geranylgeranylation | 1 | 0 |  |  |  |  |  |  |  |  |
| GO:0018410\_peptide\_or\_protein\_carboxyl-terminal\_blocking | 1 | 0 |  |  |  |  |  |  |  |  |
| GO:0018916\_nitrobenzene\_metabolic\_process | 1 | 0 |  |  |  |  |  |  |  |  |
| GO:0018931\_naphthalene\_metabolic\_process | 1 | 0 |  |  |  |  |  |  |  |  |
| GO:0018992\_germ-line\_sex\_determination | 1 | 0 |  |  |  |  |  |  |  |  |
| GO:0019042\_latent\_virus\_infection | 1 | 0 |  |  |  |  |  |  |  |  |
| GO:0019046\_reactivation\_of\_latent\_virus | 1 | 0 |  |  |  |  |  |  |  |  |
| GO:0019047\_provirus\_integration | 1 | 0 |  |  |  |  |  |  |  |  |
| GO:0019076\_release\_of\_virus\_from\_host | 1 | 0 |  |  |  |  |  |  |  |  |
| GO:0019079\_viral\_genome\_replication | 1 | 0 |  |  |  |  |  |  |  |  |
| GO:0019100\_male\_germ-line\_sex\_determination | 1 | 0 |  |  |  |  |  |  |  |  |
| GO:0019101\_female\_somatic\_sex\_determination | 1 | 0 |  |  |  |  |  |  |  |  |
| GO:0019102\_male\_somatic\_sex\_determination | 1 | 0 |  |  |  |  |  |  |  |  |
| GO:0019255\_glucose\_1-phosphate\_metabolic\_process | 1 | 0 |  |  |  |  |  |  |  |  |
| GO:0019276\_UDP-N-acetylgalactosamine\_metabolic\_process | 1 | 0 |  |  |  |  |  |  |  |  |
| GO:0019344\_cysteine\_biosynthetic\_process | 1 | 0 |  |  |  |  |  |  |  |  |
| GO:0019348\_dolichol\_metabolic\_process | 1 | 0 |  |  |  |  |  |  |  |  |
| GO:0019375\_galactolipid\_biosynthetic\_process | 1 | 0 |  |  |  |  |  |  |  |  |
| GO:0019402\_galactitol\_metabolic\_process | 1 | 0 |  |  |  |  |  |  |  |  |
| GO:0019441\_tryptophan\_catabolic\_process\_to\_kynurenine | 1 | 0 |  |  |  |  |  |  |  |  |
| GO:0019477\_L-lysine\_catabolic\_process | 1 | 0 |  |  |  |  |  |  |  |  |
| GO:0019510\_S-adenosylhomocysteine\_catabolic\_process | 1 | 0 |  |  |  |  |  |  |  |  |
| GO:0019532\_oxalate\_transport | 1 | 0 |  |  |  |  |  |  |  |  |
| GO:0019626\_short-chain\_fatty\_acid\_catabolic\_process | 1 | 0 |  |  |  |  |  |  |  |  |
| GO:0019627\_urea\_metabolic\_process | 1 | 0 |  |  |  |  |  |  |  |  |
| GO:0019676\_ammonia\_assimilation\_cycle | 1 | 0 |  |  |  |  |  |  |  |  |
| GO:0019682\_glyceraldehyde-3-phosphate\_metabolic\_process | 1 | 0 |  |  |  |  |  |  |  |  |
| GO:0019695\_choline\_metabolic\_process | 1 | 0 |  |  |  |  |  |  |  |  |
| GO:0019731\_antibacterial\_humoral\_response | 1 | 0 |  |  |  |  |  |  |  |  |
| GO:0019794\_nonprotein\_amino\_acid\_metabolic\_process | 1 | 0 |  |  |  |  |  |  |  |  |
| GO:0019858\_cytosine\_metabolic\_process | 1 | 0 |  |  |  |  |  |  |  |  |
| GO:0019883\_antigen\_processing\_and\_presentation\_of\_endogenous\_antigen | 1 | 0 |  |  |  |  |  |  |  |  |
| GO:0019889\_pteridine\_metabolic\_process | 1 | 0 |  |  |  |  |  |  |  |  |
| GO:0019896\_axon\_transport\_of\_mitochondrion | 1 | 0 |  |  |  |  |  |  |  |  |
| GO:0021508\_floor\_plate\_formation | 1 | 0 |  |  |  |  |  |  |  |  |
| GO:0021528\_commissural\_neuron\_differentiation\_in\_the\_spinal\_cord | 1 | 0 |  |  |  |  |  |  |  |  |
| GO:0021572\_rhombomere\_6\_development | 1 | 0 |  |  |  |  |  |  |  |  |
| GO:0021577\_hindbrain\_structural\_organization | 1 | 0 |  |  |  |  |  |  |  |  |
| GO:0021586\_pons\_maturation | 1 | 0 |  |  |  |  |  |  |  |  |
| GO:0021589\_cerebellum\_structural\_organization | 1 | 0 |  |  |  |  |  |  |  |  |
| GO:0021590\_cerebellum\_maturation | 1 | 0 |  |  |  |  |  |  |  |  |
| GO:0021592\_fourth\_ventricle\_development | 1 | 0 |  |  |  |  |  |  |  |  |
| GO:0021594\_rhombomere\_formation | 1 | 0 |  |  |  |  |  |  |  |  |
| GO:0021660\_rhombomere\_3\_formation | 1 | 0 |  |  |  |  |  |  |  |  |
| GO:0021664\_rhombomere\_5\_morphogenesis | 1 | 0 |  |  |  |  |  |  |  |  |
| GO:0021666\_rhombomere\_5\_formation | 1 | 0 |  |  |  |  |  |  |  |  |
| GO:0021670\_lateral\_ventricle\_development | 1 | 0 |  |  |  |  |  |  |  |  |
| GO:0021678\_third\_ventricle\_development | 1 | 0 |  |  |  |  |  |  |  |  |
| GO:0021679\_cerebellar\_molecular\_layer\_development | 1 | 0 |  |  |  |  |  |  |  |  |
| GO:0021703\_locus\_ceruleus\_development | 1 | 0 |  |  |  |  |  |  |  |  |
| GO:0021732\_midbrain-hindbrain\_boundary\_maturation | 1 | 0 |  |  |  |  |  |  |  |  |
| GO:0021747\_cochlear\_nucleus\_development | 1 | 0 |  |  |  |  |  |  |  |  |
| GO:0021750\_vestibular\_nucleus\_development | 1 | 0 |  |  |  |  |  |  |  |  |
| GO:0021759\_globus\_pallidus\_development | 1 | 0 |  |  |  |  |  |  |  |  |
| GO:0021768\_nucleus\_accumbens\_development | 1 | 0 |  |  |  |  |  |  |  |  |
| GO:0021771\_lateral\_geniculate\_nucleus\_development | 1 | 0 |  |  |  |  |  |  |  |  |
| GO:0021812\_neuronal-glial\_interaction\_involved\_in\_cerebral\_cortex\_radial\_glia\_guided\_migration | 1 | 0 |  |  |  |  |  |  |  |  |
| GO:0021813\_cell-cell\_adhesion\_involved\_in\_neuronal-glial\_interactions\_involved\_in\_cerebral\_cortex\_radial\_glia\_guided\_migration | 1 | 0 |  |  |  |  |  |  |  |  |
| GO:0021870\_Cajal-Retzius\_cell\_differentiation | 1 | 0 |  |  |  |  |  |  |  |  |
| GO:0021874\_Wnt\_receptor\_signaling\_pathway\_in\_forebrain\_neuroblast\_division | 1 | 0 |  |  |  |  |  |  |  |  |
| GO:0021896\_forebrain\_astrocyte\_differentiation | 1 | 0 |  |  |  |  |  |  |  |  |
| GO:0021897\_forebrain\_astrocyte\_development | 1 | 0 |  |  |  |  |  |  |  |  |
| GO:0021902\_commitment\_of\_a\_neuronal\_cell\_to\_a\_specific\_type\_of\_neuron\_in\_the\_forebrain | 1 | 0 |  |  |  |  |  |  |  |  |
| GO:0021905\_forebrain-midbrain\_boundary\_formation | 1 | 0 |  |  |  |  |  |  |  |  |
| GO:0021914\_negative\_regulation\_of\_smoothened\_signaling\_pathway\_involved\_in\_ventral\_spinal\_cord\_patterning | 1 | 0 |  |  |  |  |  |  |  |  |
| GO:0021917\_somatic\_motor\_neuron\_fate\_commitment | 1 | 0 |  |  |  |  |  |  |  |  |
| GO:0021918\_regulation\_of\_transcription\_from\_RNA\_polymerase\_II\_promoter\_involved\_in\_somatic\_motor\_neuron\_fate\_commitment | 1 | 0 |  |  |  |  |  |  |  |  |
| GO:0021933\_radial\_glia\_guided\_migration\_of\_granule\_cell | 1 | 0 |  |  |  |  |  |  |  |  |
| GO:0021934\_hindbrain\_tangential\_cell\_migration | 1 | 0 |  |  |  |  |  |  |  |  |
| GO:0021935\_granule\_cell\_precursor\_tangential\_migration | 1 | 0 |  |  |  |  |  |  |  |  |
| GO:0021942\_radial\_glia\_guided\_migration\_of\_Purkinje\_cell | 1 | 0 |  |  |  |  |  |  |  |  |
| GO:0021960\_anterior\_commissure\_morphogenesis | 1 | 0 |  |  |  |  |  |  |  |  |
| GO:0021997\_neural\_plate\_axis\_specification | 1 | 0 |  |  |  |  |  |  |  |  |
| GO:0021999\_neural\_plate\_anterior\_posterior\_pattern\_formation | 1 | 0 |  |  |  |  |  |  |  |  |
| GO:0022004\_midbrain-hindbrain\_boundary\_maturation\_during\_brain\_development | 1 | 0 |  |  |  |  |  |  |  |  |
| GO:0022038\_corpus\_callosum\_development | 1 | 0 |  |  |  |  |  |  |  |  |
| GO:0022605\_oogenesis\_stage | 1 | 0 |  |  |  |  |  |  |  |  |
| GO:0030011\_maintenance\_of\_cell\_polarity | 1 | 0 |  |  |  |  |  |  |  |  |
| GO:0030069\_lysogeny | 1 | 0 |  |  |  |  |  |  |  |  |
| GO:0030070\_insulin\_processing | 1 | 0 |  |  |  |  |  |  |  |  |
| GO:0030092\_regulation\_of\_flagellum\_assembly | 1 | 0 |  |  |  |  |  |  |  |  |
| GO:0030103\_vasopressin\_secretion | 1 | 0 |  |  |  |  |  |  |  |  |
| GO:0030194\_positive\_regulation\_of\_blood\_coagulation | 1 | 0 |  |  |  |  |  |  |  |  |
| GO:0030206\_chondroitin\_sulfate\_biosynthetic\_process | 1 | 0 |  |  |  |  |  |  |  |  |
| GO:0030210\_heparin\_biosynthetic\_process | 1 | 0 |  |  |  |  |  |  |  |  |
| GO:0030220\_platelet\_formation | 1 | 0 |  |  |  |  |  |  |  |  |
| GO:0030222\_eosinophil\_differentiation | 1 | 0 |  |  |  |  |  |  |  |  |
| GO:0030237\_female\_sex\_determination | 1 | 0 |  |  |  |  |  |  |  |  |
| GO:0030264\_nuclear\_fragmentation\_during\_apoptosis | 1 | 0 |  |  |  |  |  |  |  |  |
| GO:0030322\_stabilization\_of\_membrane\_potential | 1 | 0 |  |  |  |  |  |  |  |  |
| GO:0030327\_prenylated\_protein\_catabolic\_process | 1 | 0 |  |  |  |  |  |  |  |  |
| GO:0030328\_prenylcysteine\_catabolic\_process | 1 | 0 |  |  |  |  |  |  |  |  |
| GO:0030329\_prenylcysteine\_metabolic\_process | 1 | 0 |  |  |  |  |  |  |  |  |
| GO:0030382\_sperm\_mitochondrion\_organization | 1 | 0 |  |  |  |  |  |  |  |  |
| GO:0030389\_fructosamine\_metabolic\_process | 1 | 0 |  |  |  |  |  |  |  |  |
| GO:0030422\_RNA\_interference\_\_production\_of\_siRNA | 1 | 0 |  |  |  |  |  |  |  |  |
| GO:0030449\_regulation\_of\_complement\_activation | 1 | 0 |  |  |  |  |  |  |  |  |
| GO:0030497\_fatty\_acid\_elongation | 1 | 0 |  |  |  |  |  |  |  |  |
| GO:0030575\_nuclear\_body\_organization | 1 | 0 |  |  |  |  |  |  |  |  |
| GO:0030578\_PML\_body\_organization | 1 | 0 |  |  |  |  |  |  |  |  |
| GO:0030853\_negative\_regulation\_of\_granulocyte\_differentiation | 1 | 0 |  |  |  |  |  |  |  |  |
| GO:0030854\_positive\_regulation\_of\_granulocyte\_differentiation | 1 | 0 |  |  |  |  |  |  |  |  |
| GO:0030886\_negative\_regulation\_of\_myeloid\_dendritic\_cell\_activation | 1 | 0 |  |  |  |  |  |  |  |  |
| GO:0030913\_paranodal\_junction\_assembly | 1 | 0 |  |  |  |  |  |  |  |  |
| GO:0031033\_myosin\_filament\_assembly\_or\_disassembly | 1 | 0 |  |  |  |  |  |  |  |  |
| GO:0031034\_myosin\_filament\_assembly | 1 | 0 |  |  |  |  |  |  |  |  |
| GO:0031055\_chromatin\_remodeling\_at\_centromere | 1 | 0 |  |  |  |  |  |  |  |  |
| GO:0031062\_positive\_regulation\_of\_histone\_methylation | 1 | 0 |  |  |  |  |  |  |  |  |
| GO:0031115\_negative\_regulation\_of\_microtubule\_polymerization | 1 | 0 |  |  |  |  |  |  |  |  |
| GO:0031129\_inductive\_cell-cell\_signaling | 1 | 0 |  |  |  |  |  |  |  |  |
| GO:0031284\_positive\_regulation\_of\_guanylate\_cyclase\_activity | 1 | 0 |  |  |  |  |  |  |  |  |
| GO:0031498\_chromatin\_disassembly | 1 | 0 |  |  |  |  |  |  |  |  |
| GO:0031507\_heterochromatin\_formation | 1 | 0 |  |  |  |  |  |  |  |  |
| GO:0031508\_centromeric\_heterochromatin\_formation | 1 | 0 |  |  |  |  |  |  |  |  |
| GO:0031529\_ruffle\_organization | 1 | 0 |  |  |  |  |  |  |  |  |
| GO:0031536\_positive\_regulation\_of\_exit\_from\_mitosis | 1 | 0 |  |  |  |  |  |  |  |  |
| GO:0031572\_G2\_M\_transition\_DNA\_damage\_checkpoint | 1 | 0 |  |  |  |  |  |  |  |  |
| GO:0031576\_G2\_M\_transition\_checkpoint | 1 | 0 |  |  |  |  |  |  |  |  |
| GO:0031580\_membrane\_raft\_distribution | 1 | 0 |  |  |  |  |  |  |  |  |
| GO:0031583\_activation\_of\_phospholipase\_D\_activity\_by\_G-protein\_coupled\_receptor\_protein\_signaling\_pathway | 1 | 0 |  |  |  |  |  |  |  |  |
| GO:0031584\_activation\_of\_phospholipase\_D\_activity | 1 | 0 |  |  |  |  |  |  |  |  |
| GO:0031585\_regulation\_of\_inositol-1\_4\_5-triphosphate\_receptor\_activity | 1 | 0 |  |  |  |  |  |  |  |  |
| GO:0031639\_plasminogen\_activation | 1 | 0 |  |  |  |  |  |  |  |  |
| GO:0031648\_protein\_destabilization | 1 | 0 |  |  |  |  |  |  |  |  |
| GO:0031665\_negative\_regulation\_of\_lipopolysaccharide-mediated\_signaling\_pathway | 1 | 0 |  |  |  |  |  |  |  |  |
| GO:0031914\_negative\_regulation\_of\_synaptic\_plasticity | 1 | 0 |  |  |  |  |  |  |  |  |
| GO:0031944\_negative\_regulation\_of\_glucocorticoid\_metabolic\_process | 1 | 0 |  |  |  |  |  |  |  |  |
| GO:0031947\_negative\_regulation\_of\_glucocorticoid\_biosynthetic\_process | 1 | 0 |  |  |  |  |  |  |  |  |
| GO:0032025\_response\_to\_cobalt\_ion | 1 | 0 |  |  |  |  |  |  |  |  |
| GO:0032026\_response\_to\_magnesium\_ion | 1 | 0 |  |  |  |  |  |  |  |  |
| GO:0032048\_cardiolipin\_metabolic\_process | 1 | 0 |  |  |  |  |  |  |  |  |
| GO:0032066\_nucleolus\_to\_nucleoplasm\_transport | 1 | 0 |  |  |  |  |  |  |  |  |
| GO:0032091\_negative\_regulation\_of\_protein\_binding | 1 | 0 |  |  |  |  |  |  |  |  |
| GO:0032092\_positive\_regulation\_of\_protein\_binding | 1 | 0 |  |  |  |  |  |  |  |  |
| GO:0032097\_positive\_regulation\_of\_response\_to\_food | 1 | 0 |  |  |  |  |  |  |  |  |
| GO:0032100\_positive\_regulation\_of\_appetite | 1 | 0 |  |  |  |  |  |  |  |  |
| GO:0032204\_regulation\_of\_telomere\_maintenance | 1 | 0 |  |  |  |  |  |  |  |  |
| GO:0032206\_positive\_regulation\_of\_telomere\_maintenance | 1 | 0 |  |  |  |  |  |  |  |  |
| GO:0032222\_regulation\_of\_synaptic\_transmission\_\_cholinergic | 1 | 0 |  |  |  |  |  |  |  |  |
| GO:0032224\_positive\_regulation\_of\_synaptic\_transmission\_\_cholinergic | 1 | 0 |  |  |  |  |  |  |  |  |
| GO:0032229\_negative\_regulation\_of\_synaptic\_transmission\_\_GABAergic | 1 | 0 |  |  |  |  |  |  |  |  |
| GO:0032237\_activation\_of\_store-operated\_calcium\_channel\_activity | 1 | 0 |  |  |  |  |  |  |  |  |
| GO:0032239\_regulation\_of\_nucleobase\_\_nucleoside\_\_nucleotide\_and\_nucleic\_acid\_transport | 1 | 0 |  |  |  |  |  |  |  |  |
| GO:0032252\_secretory\_granule\_localization | 1 | 0 |  |  |  |  |  |  |  |  |
| GO:0032274\_gonadotropin\_secretion | 1 | 0 |  |  |  |  |  |  |  |  |
| GO:0032275\_luteinizing\_hormone\_secretion | 1 | 0 |  |  |  |  |  |  |  |  |
| GO:0032287\_myelin\_maintenance\_in\_the\_peripheral\_nervous\_system | 1 | 0 |  |  |  |  |  |  |  |  |
| GO:0032289\_myelin\_formation\_in\_the\_central\_nervous\_system | 1 | 0 |  |  |  |  |  |  |  |  |
| GO:0032303\_regulation\_of\_icosanoid\_secretion | 1 | 0 |  |  |  |  |  |  |  |  |
| GO:0032305\_positive\_regulation\_of\_icosanoid\_secretion | 1 | 0 |  |  |  |  |  |  |  |  |
| GO:0032306\_regulation\_of\_prostaglandin\_secretion | 1 | 0 |  |  |  |  |  |  |  |  |
| GO:0032308\_positive\_regulation\_of\_prostaglandin\_secretion | 1 | 0 |  |  |  |  |  |  |  |  |
| GO:0032310\_prostaglandin\_secretion | 1 | 0 |  |  |  |  |  |  |  |  |
| GO:0032313\_regulation\_of\_Rab\_GTPase\_activity | 1 | 0 |  |  |  |  |  |  |  |  |
| GO:0032314\_regulation\_of\_Rac\_GTPase\_activity | 1 | 0 |  |  |  |  |  |  |  |  |
| GO:0032317\_regulation\_of\_Rap\_GTPase\_activity | 1 | 0 |  |  |  |  |  |  |  |  |
| GO:0032324\_molybdopterin\_cofactor\_biosynthetic\_process | 1 | 0 |  |  |  |  |  |  |  |  |
| GO:0032329\_serine\_transport | 1 | 0 |  |  |  |  |  |  |  |  |
| GO:0032342\_aldosterone\_biosynthetic\_process | 1 | 0 |  |  |  |  |  |  |  |  |
| GO:0032344\_regulation\_of\_aldosterone\_metabolic\_process | 1 | 0 |  |  |  |  |  |  |  |  |
| GO:0032365\_intracellular\_lipid\_transport | 1 | 0 |  |  |  |  |  |  |  |  |
| GO:0032366\_intracellular\_sterol\_transport | 1 | 0 |  |  |  |  |  |  |  |  |
| GO:0032367\_intracellular\_cholesterol\_transport | 1 | 0 |  |  |  |  |  |  |  |  |
| GO:0032370\_positive\_regulation\_of\_lipid\_transport | 1 | 0 |  |  |  |  |  |  |  |  |
| GO:0032410\_negative\_regulation\_of\_transporter\_activity | 1 | 0 |  |  |  |  |  |  |  |  |
| GO:0032413\_negative\_regulation\_of\_ion\_transmembrane\_transporter\_activity | 1 | 0 |  |  |  |  |  |  |  |  |
| GO:0032429\_regulation\_of\_phospholipase\_A2\_activity | 1 | 0 |  |  |  |  |  |  |  |  |
| GO:0032474\_otolith\_morphogenesis | 1 | 0 |  |  |  |  |  |  |  |  |
| GO:0032482\_Rab\_protein\_signal\_transduction | 1 | 0 |  |  |  |  |  |  |  |  |
| GO:0032483\_regulation\_of\_Rab\_protein\_signal\_transduction | 1 | 0 |  |  |  |  |  |  |  |  |
| GO:0032486\_Rap\_protein\_signal\_transduction | 1 | 0 |  |  |  |  |  |  |  |  |
| GO:0032487\_regulation\_of\_Rap\_protein\_signal\_transduction | 1 | 0 |  |  |  |  |  |  |  |  |
| GO:0032594\_protein\_transport\_within\_lipid\_bilayer | 1 | 0 |  |  |  |  |  |  |  |  |
| GO:0032599\_protein\_transport\_out\_of\_membrane\_raft | 1 | 0 |  |  |  |  |  |  |  |  |
| GO:0032600\_chemokine\_receptor\_transport\_out\_of\_membrane\_raft | 1 | 0 |  |  |  |  |  |  |  |  |
| GO:0032621\_interleukin-18\_production | 1 | 0 |  |  |  |  |  |  |  |  |
| GO:0032656\_regulation\_of\_interleukin-13\_production | 1 | 0 |  |  |  |  |  |  |  |  |
| GO:0032682\_negative\_regulation\_of\_chemokine\_production | 1 | 0 |  |  |  |  |  |  |  |  |
| GO:0032691\_negative\_regulation\_of\_interleukin-1\_beta\_production | 1 | 0 |  |  |  |  |  |  |  |  |
| GO:0032692\_negative\_regulation\_of\_interleukin-1\_production | 1 | 0 |  |  |  |  |  |  |  |  |
| GO:0032693\_negative\_regulation\_of\_interleukin-10\_production | 1 | 0 |  |  |  |  |  |  |  |  |
| GO:0032696\_negative\_regulation\_of\_interleukin-13\_production | 1 | 0 |  |  |  |  |  |  |  |  |
| GO:0032731\_positive\_regulation\_of\_interleukin-1\_beta\_production | 1 | 0 |  |  |  |  |  |  |  |  |
| GO:0032732\_positive\_regulation\_of\_interleukin-1\_production | 1 | 0 |  |  |  |  |  |  |  |  |
| GO:0032735\_positive\_regulation\_of\_interleukin-12\_production | 1 | 0 |  |  |  |  |  |  |  |  |
| GO:0032764\_negative\_regulation\_of\_mast\_cell\_cytokine\_production | 1 | 0 |  |  |  |  |  |  |  |  |
| GO:0032765\_positive\_regulation\_of\_mast\_cell\_cytokine\_production | 1 | 0 |  |  |  |  |  |  |  |  |
| GO:0032769\_negative\_regulation\_of\_monooxygenase\_activity | 1 | 0 |  |  |  |  |  |  |  |  |
| GO:0032781\_positive\_regulation\_of\_ATPase\_activity | 1 | 0 |  |  |  |  |  |  |  |  |
| GO:0032790\_ribosome\_disassembly | 1 | 0 |  |  |  |  |  |  |  |  |
| GO:0032799\_low-density\_lipoprotein\_receptor\_metabolic\_process | 1 | 0 |  |  |  |  |  |  |  |  |
| GO:0032802\_low-density\_lipoprotein\_receptor\_catabolic\_process | 1 | 0 |  |  |  |  |  |  |  |  |
| GO:0032803\_regulation\_of\_low-density\_lipoprotein\_receptor\_catabolic\_process | 1 | 0 |  |  |  |  |  |  |  |  |
| GO:0032817\_regulation\_of\_natural\_killer\_cell\_proliferation | 1 | 0 |  |  |  |  |  |  |  |  |
| GO:0032819\_positive\_regulation\_of\_natural\_killer\_cell\_proliferation | 1 | 0 |  |  |  |  |  |  |  |  |
| GO:0032836\_glomerular\_basement\_membrane\_development | 1 | 0 |  |  |  |  |  |  |  |  |
| GO:0032855\_positive\_regulation\_of\_Rac\_GTPase\_activity | 1 | 0 |  |  |  |  |  |  |  |  |
| GO:0032863\_activation\_of\_Rac\_GTPase\_activity | 1 | 0 |  |  |  |  |  |  |  |  |
| GO:0032864\_activation\_of\_Cdc42\_GTPase\_activity | 1 | 0 |  |  |  |  |  |  |  |  |
| GO:0032885\_regulation\_of\_polysaccharide\_biosynthetic\_process | 1 | 0 |  |  |  |  |  |  |  |  |
| GO:0032907\_transforming\_growth\_factor-beta3\_production | 1 | 0 |  |  |  |  |  |  |  |  |
| GO:0032910\_regulation\_of\_transforming\_growth\_factor-beta3\_production | 1 | 0 |  |  |  |  |  |  |  |  |
| GO:0032913\_negative\_regulation\_of\_transforming\_growth\_factor-beta3\_production | 1 | 0 |  |  |  |  |  |  |  |  |
| GO:0032924\_activin\_receptor\_signaling\_pathway | 1 | 0 |  |  |  |  |  |  |  |  |
| GO:0032925\_regulation\_of\_activin\_receptor\_signaling\_pathway | 1 | 0 |  |  |  |  |  |  |  |  |
| GO:0032960\_regulation\_of\_inositol\_trisphosphate\_biosynthetic\_process | 1 | 0 |  |  |  |  |  |  |  |  |
| GO:0032962\_positive\_regulation\_of\_inositol\_trisphosphate\_biosynthetic\_process | 1 | 0 |  |  |  |  |  |  |  |  |
| GO:0032964\_collagen\_biosynthetic\_process | 1 | 0 |  |  |  |  |  |  |  |  |
| GO:0032971\_regulation\_of\_muscle\_filament\_sliding | 1 | 0 |  |  |  |  |  |  |  |  |
| GO:0032972\_regulation\_of\_muscle\_filament\_sliding\_speed | 1 | 0 |  |  |  |  |  |  |  |  |
| GO:0032986\_protein-DNA\_complex\_disassembly | 1 | 0 |  |  |  |  |  |  |  |  |
| GO:0032988\_ribonucleoprotein\_complex\_disassembly | 1 | 0 |  |  |  |  |  |  |  |  |
| GO:0033037\_polysaccharide\_localization | 1 | 0 |  |  |  |  |  |  |  |  |
| GO:0033078\_extrathymic\_T\_cell\_differentiation | 1 | 0 |  |  |  |  |  |  |  |  |
| GO:0033085\_negative\_regulation\_of\_T\_cell\_differentiation\_in\_the\_thymus | 1 | 0 |  |  |  |  |  |  |  |  |
| GO:0033087\_negative\_regulation\_of\_immature\_T\_cell\_proliferation | 1 | 0 |  |  |  |  |  |  |  |  |
| GO:0033088\_negative\_regulation\_of\_immature\_T\_cell\_proliferation\_in\_the\_thymus | 1 | 0 |  |  |  |  |  |  |  |  |
| GO:0033108\_mitochondrial\_respiratory\_chain\_complex\_assembly | 1 | 0 |  |  |  |  |  |  |  |  |
| GO:0033127\_regulation\_of\_histone\_phosphorylation | 1 | 0 |  |  |  |  |  |  |  |  |
| GO:0033128\_negative\_regulation\_of\_histone\_phosphorylation | 1 | 0 |  |  |  |  |  |  |  |  |
| GO:0033138\_positive\_regulation\_of\_peptidyl-serine\_phosphorylation | 1 | 0 |  |  |  |  |  |  |  |  |
| GO:0033158\_regulation\_of\_protein\_import\_into\_nucleus\_\_translocation | 1 | 0 |  |  |  |  |  |  |  |  |
| GO:0033160\_positive\_regulation\_of\_protein\_import\_into\_nucleus\_\_translocation | 1 | 0 |  |  |  |  |  |  |  |  |
| GO:0033169\_histone\_H3-K9\_demethylation | 1 | 0 |  |  |  |  |  |  |  |  |
| GO:0033206\_cytokinesis\_after\_meiosis | 1 | 0 |  |  |  |  |  |  |  |  |
| GO:0033240\_positive\_regulation\_of\_cellular\_amine\_metabolic\_process | 1 | 0 |  |  |  |  |  |  |  |  |
| GO:0033313\_meiotic\_cell\_cycle\_checkpoint | 1 | 0 |  |  |  |  |  |  |  |  |
| GO:0033315\_meiotic\_cell\_cycle\_DNA\_replication\_checkpoint | 1 | 0 |  |  |  |  |  |  |  |  |
| GO:0033326\_cerebrospinal\_fluid\_secretion | 1 | 0 |  |  |  |  |  |  |  |  |
| GO:0033366\_protein\_localization\_in\_secretory\_granule | 1 | 0 |  |  |  |  |  |  |  |  |
| GO:0033367\_protein\_localization\_in\_mast\_cell\_secretory\_granule | 1 | 0 |  |  |  |  |  |  |  |  |
| GO:0033368\_protease\_localization\_in\_mast\_cell\_secretory\_granule | 1 | 0 |  |  |  |  |  |  |  |  |
| GO:0033370\_maintenance\_of\_protein\_location\_in\_mast\_cell\_secretory\_granule | 1 | 0 |  |  |  |  |  |  |  |  |
| GO:0033371\_T\_cell\_secretory\_granule\_organization | 1 | 0 |  |  |  |  |  |  |  |  |
| GO:0033373\_maintenance\_of\_protease\_location\_in\_mast\_cell\_secretory\_granule | 1 | 0 |  |  |  |  |  |  |  |  |
| GO:0033374\_protein\_localization\_in\_T\_cell\_secretory\_granule | 1 | 0 |  |  |  |  |  |  |  |  |
| GO:0033375\_protease\_localization\_in\_T\_cell\_secretory\_granule | 1 | 0 |  |  |  |  |  |  |  |  |
| GO:0033377\_maintenance\_of\_protein\_location\_in\_T\_cell\_secretory\_granule | 1 | 0 |  |  |  |  |  |  |  |  |
| GO:0033379\_maintenance\_of\_protease\_location\_in\_T\_cell\_secretory\_granule | 1 | 0 |  |  |  |  |  |  |  |  |
| GO:0033380\_granzyme\_B\_localization\_in\_T\_cell\_secretory\_granule | 1 | 0 |  |  |  |  |  |  |  |  |
| GO:0033382\_maintenance\_of\_granzyme\_B\_location\_in\_T\_cell\_secretory\_granule | 1 | 0 |  |  |  |  |  |  |  |  |
| GO:0033483\_gas\_homeostasis | 1 | 0 |  |  |  |  |  |  |  |  |
| GO:0033484\_nitric\_oxide\_homeostasis | 1 | 0 |  |  |  |  |  |  |  |  |
| GO:0033505\_floor\_plate\_morphogenesis | 1 | 0 |  |  |  |  |  |  |  |  |
| GO:0033522\_histone\_H2A\_ubiquitination | 1 | 0 |  |  |  |  |  |  |  |  |
| GO:0033523\_histone\_H2B\_ubiquitination | 1 | 0 |  |  |  |  |  |  |  |  |
| GO:0033574\_response\_to\_testosterone\_stimulus | 1 | 0 |  |  |  |  |  |  |  |  |
| GO:0033606\_chemokine\_receptor\_transport\_within\_lipid\_bilayer | 1 | 0 |  |  |  |  |  |  |  |  |
| GO:0033628\_regulation\_of\_cell\_adhesion\_mediated\_by\_integrin | 1 | 0 |  |  |  |  |  |  |  |  |
| GO:0033630\_positive\_regulation\_of\_cell\_adhesion\_mediated\_by\_integrin | 1 | 0 |  |  |  |  |  |  |  |  |
| GO:0033632\_regulation\_of\_cell-cell\_adhesion\_mediated\_by\_integrin | 1 | 0 |  |  |  |  |  |  |  |  |
| GO:0033634\_positive\_regulation\_of\_cell-cell\_adhesion\_mediated\_by\_integrin | 1 | 0 |  |  |  |  |  |  |  |  |
| GO:0033683\_nucleotide-excision\_repair\_\_DNA\_incision | 1 | 0 |  |  |  |  |  |  |  |  |
| GO:0033687\_osteoblast\_proliferation | 1 | 0 |  |  |  |  |  |  |  |  |
| GO:0033688\_regulation\_of\_osteoblast\_proliferation | 1 | 0 |  |  |  |  |  |  |  |  |
| GO:0033689\_negative\_regulation\_of\_osteoblast\_proliferation | 1 | 0 |  |  |  |  |  |  |  |  |
| GO:0033750\_ribosome\_localization | 1 | 0 |  |  |  |  |  |  |  |  |
| GO:0033753\_establishment\_of\_ribosome\_localization | 1 | 0 |  |  |  |  |  |  |  |  |
| GO:0033866\_nucleoside\_bisphosphate\_biosynthetic\_process | 1 | 0 |  |  |  |  |  |  |  |  |
| GO:0033875\_ribonucleoside\_bisphosphate\_metabolic\_process | 1 | 0 |  |  |  |  |  |  |  |  |
| GO:0034030\_ribonucleoside\_bisphosphate\_biosynthetic\_process | 1 | 0 |  |  |  |  |  |  |  |  |
| GO:0034032\_purine\_nucleoside\_bisphosphate\_metabolic\_process | 1 | 0 |  |  |  |  |  |  |  |  |
| GO:0034033\_purine\_nucleoside\_bisphosphate\_biosynthetic\_process | 1 | 0 |  |  |  |  |  |  |  |  |
| GO:0034035\_purine\_ribonucleoside\_bisphosphate\_metabolic\_process | 1 | 0 |  |  |  |  |  |  |  |  |
| GO:0034036\_purine\_ribonucleoside\_bisphosphate\_biosynthetic\_process | 1 | 0 |  |  |  |  |  |  |  |  |
| GO:0034067\_protein\_localization\_in\_Golgi\_apparatus | 1 | 0 |  |  |  |  |  |  |  |  |
| GO:0034102\_erythrocyte\_clearance | 1 | 0 |  |  |  |  |  |  |  |  |
| GO:0034106\_regulation\_of\_erythrocyte\_clearance | 1 | 0 |  |  |  |  |  |  |  |  |
| GO:0034107\_negative\_regulation\_of\_erythrocyte\_clearance | 1 | 0 |  |  |  |  |  |  |  |  |
| GO:0034110\_regulation\_of\_homotypic\_cell-cell\_adhesion | 1 | 0 |  |  |  |  |  |  |  |  |
| GO:0034111\_negative\_regulation\_of\_homotypic\_cell-cell\_adhesion | 1 | 0 |  |  |  |  |  |  |  |  |
| GO:0034113\_heterotypic\_cell-cell\_adhesion | 1 | 0 |  |  |  |  |  |  |  |  |
| GO:0034117\_erythrocyte\_aggregation | 1 | 0 |  |  |  |  |  |  |  |  |
| GO:0034118\_regulation\_of\_erythrocyte\_aggregation | 1 | 0 |  |  |  |  |  |  |  |  |
| GO:0034119\_negative\_regulation\_of\_erythrocyte\_aggregation | 1 | 0 |  |  |  |  |  |  |  |  |
| GO:0034121\_regulation\_of\_toll-like\_receptor\_signaling\_pathway | 1 | 0 |  |  |  |  |  |  |  |  |
| GO:0034122\_negative\_regulation\_of\_toll-like\_receptor\_signaling\_pathway | 1 | 0 |  |  |  |  |  |  |  |  |
| GO:0034230\_enkephalin\_processing | 1 | 0 |  |  |  |  |  |  |  |  |
| GO:0034372\_very-low-density\_lipoprotein\_particle\_remodeling | 1 | 0 |  |  |  |  |  |  |  |  |
| GO:0034379\_very-low-density\_lipoprotein\_particle\_assembly | 1 | 0 |  |  |  |  |  |  |  |  |
| GO:0034380\_high-density\_lipoprotein\_particle\_assembly | 1 | 0 |  |  |  |  |  |  |  |  |
| GO:0034394\_protein\_localization\_at\_cell\_surface | 1 | 0 |  |  |  |  |  |  |  |  |
| GO:0034405\_response\_to\_fluid\_shear\_stress | 1 | 0 |  |  |  |  |  |  |  |  |
| GO:0034472\_snRNA\_3'-end\_processing | 1 | 0 |  |  |  |  |  |  |  |  |
| GO:0034474\_U2\_snRNA\_3'-end\_processing | 1 | 0 |  |  |  |  |  |  |  |  |
| GO:0034502\_protein\_localization\_to\_chromosome | 1 | 0 |  |  |  |  |  |  |  |  |
| GO:0034505\_tooth\_mineralization | 1 | 0 |  |  |  |  |  |  |  |  |
| GO:0034508\_centromere\_complex\_assembly | 1 | 0 |  |  |  |  |  |  |  |  |
| GO:0034633\_retinol\_transport | 1 | 0 |  |  |  |  |  |  |  |  |
| GO:0034643\_mitochondrion\_localization\_\_microtubule-mediated | 1 | 0 |  |  |  |  |  |  |  |  |
| GO:0034969\_histone\_arginine\_methylation | 1 | 0 |  |  |  |  |  |  |  |  |
| GO:0034982\_mitochondrial\_protein\_processing | 1 | 0 |  |  |  |  |  |  |  |  |
| GO:0035022\_positive\_regulation\_of\_Rac\_protein\_signal\_transduction | 1 | 0 |  |  |  |  |  |  |  |  |
| GO:0035024\_negative\_regulation\_of\_Rho\_protein\_signal\_transduction | 1 | 0 |  |  |  |  |  |  |  |  |
| GO:0035026\_leading\_edge\_cell\_differentiation | 1 | 0 |  |  |  |  |  |  |  |  |
| GO:0035037\_sperm\_entry | 1 | 0 |  |  |  |  |  |  |  |  |
| GO:0035039\_male\_pronucleus\_formation | 1 | 0 |  |  |  |  |  |  |  |  |
| GO:0035066\_positive\_regulation\_of\_histone\_acetylation | 1 | 0 |  |  |  |  |  |  |  |  |
| GO:0035083\_cilium\_axoneme\_assembly | 1 | 0 |  |  |  |  |  |  |  |  |
| GO:0035090\_maintenance\_of\_apical\_basal\_cell\_polarity | 1 | 0 |  |  |  |  |  |  |  |  |
| GO:0035106\_operant\_conditioning | 1 | 0 |  |  |  |  |  |  |  |  |
| GO:0035172\_hemocyte\_proliferation | 1 | 0 |  |  |  |  |  |  |  |  |
| GO:0035227\_regulation\_of\_glutamate-cysteine\_ligase\_activity | 1 | 0 |  |  |  |  |  |  |  |  |
| GO:0035229\_positive\_regulation\_of\_glutamate-cysteine\_ligase\_activity | 1 | 0 |  |  |  |  |  |  |  |  |
| GO:0035260\_internal\_genitalia\_morphogenesis | 1 | 0 |  |  |  |  |  |  |  |  |
| GO:0035262\_gonad\_morphogenesis | 1 | 0 |  |  |  |  |  |  |  |  |
| GO:0035287\_head\_segmentation | 1 | 0 |  |  |  |  |  |  |  |  |
| GO:0035289\_posterior\_head\_segmentation | 1 | 0 |  |  |  |  |  |  |  |  |
| GO:0035303\_regulation\_of\_dephosphorylation | 1 | 0 |  |  |  |  |  |  |  |  |
| GO:0035304\_regulation\_of\_protein\_amino\_acid\_dephosphorylation | 1 | 0 |  |  |  |  |  |  |  |  |
| GO:0035305\_negative\_regulation\_of\_dephosphorylation | 1 | 0 |  |  |  |  |  |  |  |  |
| GO:0035308\_negative\_regulation\_of\_protein\_amino\_acid\_dephosphorylation | 1 | 0 |  |  |  |  |  |  |  |  |
| GO:0035313\_wound\_healing\_\_spreading\_of\_epidermal\_cells | 1 | 0 |  |  |  |  |  |  |  |  |
| GO:0040013\_negative\_regulation\_of\_locomotion | 1 | 0 |  |  |  |  |  |  |  |  |
| GO:0040019\_positive\_regulation\_of\_embryonic\_development | 1 | 0 |  |  |  |  |  |  |  |  |
| GO:0040032\_post-embryonic\_body\_morphogenesis | 1 | 0 |  |  |  |  |  |  |  |  |
| GO:0040038\_polar\_body\_extrusion\_after\_meiotic\_divisions | 1 | 0 |  |  |  |  |  |  |  |  |
| GO:0042026\_protein\_refolding | 1 | 0 |  |  |  |  |  |  |  |  |
| GO:0042048\_olfactory\_behavior | 1 | 0 |  |  |  |  |  |  |  |  |
| GO:0042059\_negative\_regulation\_of\_epidermal\_growth\_factor\_receptor\_signaling\_pathway | 1 | 0 |  |  |  |  |  |  |  |  |
| GO:0042073\_intraflagellar\_transport | 1 | 0 |  |  |  |  |  |  |  |  |
| GO:0042078\_germ-line\_stem\_cell\_division | 1 | 0 |  |  |  |  |  |  |  |  |
| GO:0042091\_interleukin-10\_biosynthetic\_process | 1 | 0 |  |  |  |  |  |  |  |  |
| GO:0042103\_positive\_regulation\_of\_T\_cell\_homeostatic\_proliferation | 1 | 0 |  |  |  |  |  |  |  |  |
| GO:0042136\_neurotransmitter\_biosynthetic\_process | 1 | 0 |  |  |  |  |  |  |  |  |
| GO:0042137\_sequestering\_of\_neurotransmitter | 1 | 0 |  |  |  |  |  |  |  |  |
| GO:0042138\_meiotic\_DNA\_double-strand\_break\_formation | 1 | 0 |  |  |  |  |  |  |  |  |
| GO:0042178\_xenobiotic\_catabolic\_process | 1 | 0 |  |  |  |  |  |  |  |  |
| GO:0042225\_interleukin-5\_biosynthetic\_process | 1 | 0 |  |  |  |  |  |  |  |  |
| GO:0042231\_interleukin-13\_biosynthetic\_process | 1 | 0 |  |  |  |  |  |  |  |  |
| GO:0042255\_ribosome\_assembly | 1 | 0 |  |  |  |  |  |  |  |  |
| GO:0042257\_ribosomal\_subunit\_assembly | 1 | 0 |  |  |  |  |  |  |  |  |
| GO:0042264\_peptidyl-aspartic\_acid\_hydroxylation | 1 | 0 |  |  |  |  |  |  |  |  |
| GO:0042276\_error-prone\_postreplication\_DNA\_repair | 1 | 0 |  |  |  |  |  |  |  |  |
| GO:0042297\_vocal\_learning | 1 | 0 |  |  |  |  |  |  |  |  |
| GO:0042309\_homoiothermy | 1 | 0 |  |  |  |  |  |  |  |  |
| GO:0042320\_regulation\_of\_circadian\_sleep\_wake\_cycle\_\_REM\_sleep | 1 | 0 |  |  |  |  |  |  |  |  |
| GO:0042339\_keratan\_sulfate\_metabolic\_process | 1 | 0 |  |  |  |  |  |  |  |  |
| GO:0042347\_negative\_regulation\_of\_NF-kappaB\_import\_into\_nucleus | 1 | 0 |  |  |  |  |  |  |  |  |
| GO:0042360\_vitamin\_E\_metabolic\_process | 1 | 0 |  |  |  |  |  |  |  |  |
| GO:0042363\_fat-soluble\_vitamin\_catabolic\_process | 1 | 0 |  |  |  |  |  |  |  |  |
| GO:0042369\_vitamin\_D\_catabolic\_process | 1 | 0 |  |  |  |  |  |  |  |  |
| GO:0042373\_vitamin\_K\_metabolic\_process | 1 | 0 |  |  |  |  |  |  |  |  |
| GO:0042404\_thyroid\_hormone\_catabolic\_process | 1 | 0 |  |  |  |  |  |  |  |  |
| GO:0042414\_epinephrine\_metabolic\_process | 1 | 0 |  |  |  |  |  |  |  |  |
| GO:0042436\_indole\_derivative\_catabolic\_process | 1 | 0 |  |  |  |  |  |  |  |  |
| GO:0042489\_negative\_regulation\_of\_odontogenesis\_of\_dentine-containing\_tooth | 1 | 0 |  |  |  |  |  |  |  |  |
| GO:0042508\_tyrosine\_phosphorylation\_of\_Stat1\_protein | 1 | 0 |  |  |  |  |  |  |  |  |
| GO:0042518\_negative\_regulation\_of\_tyrosine\_phosphorylation\_of\_Stat3\_protein | 1 | 0 |  |  |  |  |  |  |  |  |
| GO:0042524\_negative\_regulation\_of\_tyrosine\_phosphorylation\_of\_Stat5\_protein | 1 | 0 |  |  |  |  |  |  |  |  |
| GO:0042536\_negative\_regulation\_of\_tumor\_necrosis\_factor\_biosynthetic\_process | 1 | 0 |  |  |  |  |  |  |  |  |
| GO:0042538\_hyperosmotic\_salinity\_response | 1 | 0 |  |  |  |  |  |  |  |  |
| GO:0042628\_mating\_plug\_formation | 1 | 0 |  |  |  |  |  |  |  |  |
| GO:0042631\_cellular\_response\_to\_water\_deprivation | 1 | 0 |  |  |  |  |  |  |  |  |
| GO:0042637\_catagen | 1 | 0 |  |  |  |  |  |  |  |  |
| GO:0042660\_positive\_regulation\_of\_cell\_fate\_specification | 1 | 0 |  |  |  |  |  |  |  |  |
| GO:0042663\_regulation\_of\_endodermal\_cell\_fate\_specification | 1 | 0 |  |  |  |  |  |  |  |  |
| GO:0042664\_negative\_regulation\_of\_endodermal\_cell\_fate\_specification | 1 | 0 |  |  |  |  |  |  |  |  |
| GO:0042667\_auditory\_receptor\_cell\_fate\_specification | 1 | 0 |  |  |  |  |  |  |  |  |
| GO:0042694\_muscle\_cell\_fate\_specification | 1 | 0 |  |  |  |  |  |  |  |  |
| GO:0042706\_eye\_photoreceptor\_cell\_fate\_commitment | 1 | 0 |  |  |  |  |  |  |  |  |
| GO:0042713\_sperm\_ejaculation | 1 | 0 |  |  |  |  |  |  |  |  |
| GO:0042723\_thiamin\_and\_derivative\_metabolic\_process | 1 | 0 |  |  |  |  |  |  |  |  |
| GO:0042737\_drug\_catabolic\_process | 1 | 0 |  |  |  |  |  |  |  |  |
| GO:0042738\_exogenous\_drug\_catabolic\_process | 1 | 0 |  |  |  |  |  |  |  |  |
| GO:0042747\_circadian\_sleep\_wake\_cycle\_\_REM\_sleep | 1 | 0 |  |  |  |  |  |  |  |  |
| GO:0042748\_circadian\_sleep\_wake\_cycle\_\_non-REM\_sleep | 1 | 0 |  |  |  |  |  |  |  |  |
| GO:0042772\_DNA\_damage\_response\_\_signal\_transduction\_resulting\_in\_transcription | 1 | 0 |  |  |  |  |  |  |  |  |
| GO:0042790\_transcription\_of\_nuclear\_rRNA\_large\_RNA\_polymerase\_I\_transcript | 1 | 0 |  |  |  |  |  |  |  |  |
| GO:0042839\_D-glucuronate\_metabolic\_process | 1 | 0 |  |  |  |  |  |  |  |  |
| GO:0042840\_D-glucuronate\_catabolic\_process | 1 | 0 |  |  |  |  |  |  |  |  |
| GO:0042891\_antibiotic\_transport | 1 | 0 |  |  |  |  |  |  |  |  |
| GO:0042892\_chloramphenicol\_transport | 1 | 0 |  |  |  |  |  |  |  |  |
| GO:0042940\_D-amino\_acid\_transport | 1 | 0 |  |  |  |  |  |  |  |  |
| GO:0042941\_D-alanine\_transport | 1 | 0 |  |  |  |  |  |  |  |  |
| GO:0042942\_D-serine\_transport | 1 | 0 |  |  |  |  |  |  |  |  |
| GO:0042983\_amyloid\_precursor\_protein\_biosynthetic\_process | 1 | 0 |  |  |  |  |  |  |  |  |
| GO:0042984\_regulation\_of\_amyloid\_precursor\_protein\_biosynthetic\_process | 1 | 0 |  |  |  |  |  |  |  |  |
| GO:0042985\_negative\_regulation\_of\_amyloid\_precursor\_protein\_biosynthetic\_process | 1 | 0 |  |  |  |  |  |  |  |  |
| GO:0042989\_sequestering\_of\_actin\_monomers | 1 | 0 |  |  |  |  |  |  |  |  |
| GO:0043044\_ATP-dependent\_chromatin\_remodeling | 1 | 0 |  |  |  |  |  |  |  |  |
| GO:0043056\_forward\_locomotion | 1 | 0 |  |  |  |  |  |  |  |  |
| GO:0043060\_meiotic\_metaphase\_I\_plate\_congression | 1 | 0 |  |  |  |  |  |  |  |  |
| GO:0043091\_L-arginine\_import | 1 | 0 |  |  |  |  |  |  |  |  |
| GO:0043124\_negative\_regulation\_of\_I-kappaB\_kinase\_NF-kappaB\_cascade | 1 | 0 |  |  |  |  |  |  |  |  |
| GO:0043132\_NAD\_transport | 1 | 0 |  |  |  |  |  |  |  |  |
| GO:0043153\_entrainment\_of\_circadian\_clock\_by\_photoperiod | 1 | 0 |  |  |  |  |  |  |  |  |
| GO:0043171\_peptide\_catabolic\_process | 1 | 0 |  |  |  |  |  |  |  |  |
| GO:0043179\_rhythmic\_excitation | 1 | 0 |  |  |  |  |  |  |  |  |
| GO:0043206\_fibril\_organization | 1 | 0 |  |  |  |  |  |  |  |  |
| GO:0043217\_myelin\_maintenance | 1 | 0 |  |  |  |  |  |  |  |  |
| GO:0043313\_regulation\_of\_neutrophil\_degranulation | 1 | 0 |  |  |  |  |  |  |  |  |
| GO:0043316\_cytotoxic\_T\_cell\_degranulation | 1 | 0 |  |  |  |  |  |  |  |  |
| GO:0043369\_CD4-positive\_or\_CD8-positive\_\_alpha-beta\_T\_cell\_lineage\_commitment | 1 | 0 |  |  |  |  |  |  |  |  |
| GO:0043375\_CD8-positive\_\_alpha-beta\_T\_cell\_lineage\_commitment | 1 | 0 |  |  |  |  |  |  |  |  |
| GO:0043379\_memory\_T\_cell\_differentiation | 1 | 0 |  |  |  |  |  |  |  |  |
| GO:0043380\_regulation\_of\_memory\_T\_cell\_differentiation | 1 | 0 |  |  |  |  |  |  |  |  |
| GO:0043400\_cortisol\_secretion | 1 | 0 |  |  |  |  |  |  |  |  |
| GO:0043415\_positive\_regulation\_of\_skeletal\_muscle\_regeneration | 1 | 0 |  |  |  |  |  |  |  |  |
| GO:0043416\_regulation\_of\_skeletal\_muscle\_regeneration | 1 | 0 |  |  |  |  |  |  |  |  |
| GO:0043437\_butanoic\_acid\_metabolic\_process | 1 | 0 |  |  |  |  |  |  |  |  |
| GO:0043438\_acetoacetic\_acid\_metabolic\_process | 1 | 0 |  |  |  |  |  |  |  |  |
| GO:0043480\_pigment\_accumulation\_in\_tissues | 1 | 0 |  |  |  |  |  |  |  |  |
| GO:0043482\_cellular\_pigment\_accumulation | 1 | 0 |  |  |  |  |  |  |  |  |
| GO:0043486\_histone\_exchange | 1 | 0 |  |  |  |  |  |  |  |  |
| GO:0043496\_regulation\_of\_protein\_homodimerization\_activity | 1 | 0 |  |  |  |  |  |  |  |  |
| GO:0043501\_skeletal\_muscle\_adaptation | 1 | 0 |  |  |  |  |  |  |  |  |
| GO:0043508\_negative\_regulation\_of\_JUN\_kinase\_activity | 1 | 0 |  |  |  |  |  |  |  |  |
| GO:0043517\_positive\_regulation\_of\_DNA\_damage\_response\_\_signal\_transduction\_by\_p53\_class\_mediator | 1 | 0 |  |  |  |  |  |  |  |  |
| GO:0043535\_regulation\_of\_blood\_vessel\_endothelial\_cell\_migration | 1 | 0 |  |  |  |  |  |  |  |  |
| GO:0043537\_negative\_regulation\_of\_blood\_vessel\_endothelial\_cell\_migration | 1 | 0 |  |  |  |  |  |  |  |  |
| GO:0043545\_molybdopterin\_cofactor\_metabolic\_process | 1 | 0 |  |  |  |  |  |  |  |  |
| GO:0043587\_tongue\_morphogenesis | 1 | 0 |  |  |  |  |  |  |  |  |
| GO:0043604\_amide\_biosynthetic\_process | 1 | 0 |  |  |  |  |  |  |  |  |
| GO:0043628\_ncRNA\_3'-end\_processing | 1 | 0 |  |  |  |  |  |  |  |  |
| GO:0044254\_multicellular\_organismal\_protein\_catabolic\_process | 1 | 0 |  |  |  |  |  |  |  |  |
| GO:0044256\_protein\_digestion | 1 | 0 |  |  |  |  |  |  |  |  |
| GO:0044266\_multicellular\_organismal\_macromolecule\_catabolic\_process | 1 | 0 |  |  |  |  |  |  |  |  |
| GO:0045004\_DNA\_replication\_proofreading | 1 | 0 |  |  |  |  |  |  |  |  |
| GO:0045019\_negative\_regulation\_of\_nitric\_oxide\_biosynthetic\_process | 1 | 0 |  |  |  |  |  |  |  |  |
| GO:0045020\_error-prone\_DNA\_repair | 1 | 0 |  |  |  |  |  |  |  |  |
| GO:0045022\_early\_endosome\_to\_late\_endosome\_transport | 1 | 0 |  |  |  |  |  |  |  |  |
| GO:0045062\_extrathymic\_T\_cell\_selection | 1 | 0 |  |  |  |  |  |  |  |  |
| GO:0045069\_regulation\_of\_viral\_genome\_replication | 1 | 0 |  |  |  |  |  |  |  |  |
| GO:0045074\_regulation\_of\_interleukin-10\_biosynthetic\_process | 1 | 0 |  |  |  |  |  |  |  |  |
| GO:0045082\_positive\_regulation\_of\_interleukin-10\_biosynthetic\_process | 1 | 0 |  |  |  |  |  |  |  |  |
| GO:0045083\_negative\_regulation\_of\_interleukin-12\_biosynthetic\_process | 1 | 0 |  |  |  |  |  |  |  |  |
| GO:0045112\_integrin\_biosynthetic\_process | 1 | 0 |  |  |  |  |  |  |  |  |
| GO:0045113\_regulation\_of\_integrin\_biosynthetic\_process | 1 | 0 |  |  |  |  |  |  |  |  |
| GO:0045188\_regulation\_of\_circadian\_sleep\_wake\_cycle\_\_non-REM\_sleep | 1 | 0 |  |  |  |  |  |  |  |  |
| GO:0045210\_FasL\_biosynthetic\_process | 1 | 0 |  |  |  |  |  |  |  |  |
| GO:0045297\_post-mating\_behavior | 1 | 0 |  |  |  |  |  |  |  |  |
| GO:0045299\_otolith\_mineralization | 1 | 0 |  |  |  |  |  |  |  |  |
| GO:0045329\_carnitine\_biosynthetic\_process | 1 | 0 |  |  |  |  |  |  |  |  |
| GO:0045341\_MHC\_class\_I\_biosynthetic\_process | 1 | 0 |  |  |  |  |  |  |  |  |
| GO:0045343\_regulation\_of\_MHC\_class\_I\_biosynthetic\_process | 1 | 0 |  |  |  |  |  |  |  |  |
| GO:0045347\_negative\_regulation\_of\_MHC\_class\_II\_biosynthetic\_process | 1 | 0 |  |  |  |  |  |  |  |  |
| GO:0045405\_regulation\_of\_interleukin-5\_biosynthetic\_process | 1 | 0 |  |  |  |  |  |  |  |  |
| GO:0045407\_positive\_regulation\_of\_interleukin-5\_biosynthetic\_process | 1 | 0 |  |  |  |  |  |  |  |  |
| GO:0045426\_quinone\_cofactor\_biosynthetic\_process | 1 | 0 |  |  |  |  |  |  |  |  |
| GO:0045448\_mitotic\_cell\_cycle\_\_embryonic | 1 | 0 |  |  |  |  |  |  |  |  |
| GO:0045454\_cell\_redox\_homeostasis | 1 | 0 |  |  |  |  |  |  |  |  |
| GO:0045583\_regulation\_of\_cytotoxic\_T\_cell\_differentiation | 1 | 0 |  |  |  |  |  |  |  |  |
| GO:0045585\_positive\_regulation\_of\_cytotoxic\_T\_cell\_differentiation | 1 | 0 |  |  |  |  |  |  |  |  |
| GO:0045601\_regulation\_of\_endothelial\_cell\_differentiation | 1 | 0 |  |  |  |  |  |  |  |  |
| GO:0045602\_negative\_regulation\_of\_endothelial\_cell\_differentiation | 1 | 0 |  |  |  |  |  |  |  |  |
| GO:0045605\_negative\_regulation\_of\_epidermal\_cell\_differentiation | 1 | 0 |  |  |  |  |  |  |  |  |
| GO:0045606\_positive\_regulation\_of\_epidermal\_cell\_differentiation | 1 | 0 |  |  |  |  |  |  |  |  |
| GO:0045609\_positive\_regulation\_of\_auditory\_receptor\_cell\_differentiation | 1 | 0 |  |  |  |  |  |  |  |  |
| GO:0045617\_negative\_regulation\_of\_keratinocyte\_differentiation | 1 | 0 |  |  |  |  |  |  |  |  |
| GO:0045618\_positive\_regulation\_of\_keratinocyte\_differentiation | 1 | 0 |  |  |  |  |  |  |  |  |
| GO:0045626\_negative\_regulation\_of\_T-helper\_1\_cell\_differentiation | 1 | 0 |  |  |  |  |  |  |  |  |
| GO:0045633\_positive\_regulation\_of\_mechanoreceptor\_differentiation | 1 | 0 |  |  |  |  |  |  |  |  |
| GO:0045650\_negative\_regulation\_of\_macrophage\_differentiation | 1 | 0 |  |  |  |  |  |  |  |  |
| GO:0045656\_negative\_regulation\_of\_monocyte\_differentiation | 1 | 0 |  |  |  |  |  |  |  |  |
| GO:0045657\_positive\_regulation\_of\_monocyte\_differentiation | 1 | 0 |  |  |  |  |  |  |  |  |
| GO:0045659\_negative\_regulation\_of\_neutrophil\_differentiation | 1 | 0 |  |  |  |  |  |  |  |  |
| GO:0045660\_positive\_regulation\_of\_neutrophil\_differentiation | 1 | 0 |  |  |  |  |  |  |  |  |
| GO:0045721\_negative\_regulation\_of\_gluconeogenesis | 1 | 0 |  |  |  |  |  |  |  |  |
| GO:0045724\_positive\_regulation\_of\_flagellum\_assembly | 1 | 0 |  |  |  |  |  |  |  |  |
| GO:0045725\_positive\_regulation\_of\_glycogen\_biosynthetic\_process | 1 | 0 |  |  |  |  |  |  |  |  |
| GO:0045740\_positive\_regulation\_of\_DNA\_replication | 1 | 0 |  |  |  |  |  |  |  |  |
| GO:0045759\_negative\_regulation\_of\_action\_potential | 1 | 0 |  |  |  |  |  |  |  |  |
| GO:0045768\_positive\_regulation\_of\_anti-apoptosis | 1 | 0 |  |  |  |  |  |  |  |  |
| GO:0045769\_negative\_regulation\_of\_asymmetric\_cell\_division | 1 | 0 |  |  |  |  |  |  |  |  |
| GO:0045794\_negative\_regulation\_of\_cell\_volume | 1 | 0 |  |  |  |  |  |  |  |  |
| GO:0045815\_positive\_regulation\_of\_gene\_expression\_\_epigenetic | 1 | 0 |  |  |  |  |  |  |  |  |
| GO:0045818\_negative\_regulation\_of\_glycogen\_catabolic\_process | 1 | 0 |  |  |  |  |  |  |  |  |
| GO:0045842\_positive\_regulation\_of\_mitotic\_metaphase\_anaphase\_transition | 1 | 0 |  |  |  |  |  |  |  |  |
| GO:0045875\_negative\_regulation\_of\_sister\_chromatid\_cohesion | 1 | 0 |  |  |  |  |  |  |  |  |
| GO:0045898\_regulation\_of\_transcriptional\_preinitiation\_complex\_assembly | 1 | 0 |  |  |  |  |  |  |  |  |
| GO:0045899\_positive\_regulation\_of\_transcriptional\_preinitiation\_complex\_assembly | 1 | 0 |  |  |  |  |  |  |  |  |
| GO:0045906\_negative\_regulation\_of\_vasoconstriction | 1 | 0 |  |  |  |  |  |  |  |  |
| GO:0045908\_negative\_regulation\_of\_vasodilation | 1 | 0 |  |  |  |  |  |  |  |  |
| GO:0045909\_positive\_regulation\_of\_vasodilation | 1 | 0 |  |  |  |  |  |  |  |  |
| GO:0045915\_positive\_regulation\_of\_catecholamine\_metabolic\_process | 1 | 0 |  |  |  |  |  |  |  |  |
| GO:0045920\_negative\_regulation\_of\_exocytosis | 1 | 0 |  |  |  |  |  |  |  |  |
| GO:0045924\_regulation\_of\_female\_receptivity | 1 | 0 |  |  |  |  |  |  |  |  |
| GO:0045947\_negative\_regulation\_of\_translational\_initiation | 1 | 0 |  |  |  |  |  |  |  |  |
| GO:0045955\_negative\_regulation\_of\_calcium\_ion-dependent\_exocytosis | 1 | 0 |  |  |  |  |  |  |  |  |
| GO:0045956\_positive\_regulation\_of\_calcium\_ion-dependent\_exocytosis | 1 | 0 |  |  |  |  |  |  |  |  |
| GO:0045964\_positive\_regulation\_of\_dopamine\_metabolic\_process | 1 | 0 |  |  |  |  |  |  |  |  |
| GO:0045988\_negative\_regulation\_of\_striated\_muscle\_contraction | 1 | 0 |  |  |  |  |  |  |  |  |
| GO:0045989\_positive\_regulation\_of\_striated\_muscle\_contraction | 1 | 0 |  |  |  |  |  |  |  |  |
| GO:0045990\_regulation\_of\_transcription\_by\_carbon\_catabolites | 1 | 0 |  |  |  |  |  |  |  |  |
| GO:0045991\_positive\_regulation\_of\_transcription\_by\_carbon\_catabolites | 1 | 0 |  |  |  |  |  |  |  |  |
| GO:0045994\_positive\_regulation\_of\_translational\_initiation\_by\_iron | 1 | 0 |  |  |  |  |  |  |  |  |
| GO:0046007\_negative\_regulation\_of\_activated\_T\_cell\_proliferation | 1 | 0 |  |  |  |  |  |  |  |  |
| GO:0046014\_negative\_regulation\_of\_T\_cell\_homeostatic\_proliferation | 1 | 0 |  |  |  |  |  |  |  |  |
| GO:0046015\_regulation\_of\_transcription\_by\_glucose | 1 | 0 |  |  |  |  |  |  |  |  |
| GO:0046016\_positive\_regulation\_of\_transcription\_by\_glucose | 1 | 0 |  |  |  |  |  |  |  |  |
| GO:0046031\_ADP\_metabolic\_process | 1 | 0 |  |  |  |  |  |  |  |  |
| GO:0046032\_ADP\_catabolic\_process | 1 | 0 |  |  |  |  |  |  |  |  |
| GO:0046061\_dATP\_catabolic\_process | 1 | 0 |  |  |  |  |  |  |  |  |
| GO:0046075\_dTTP\_metabolic\_process | 1 | 0 |  |  |  |  |  |  |  |  |
| GO:0046078\_dUMP\_metabolic\_process | 1 | 0 |  |  |  |  |  |  |  |  |
| GO:0046079\_dUMP\_catabolic\_process | 1 | 0 |  |  |  |  |  |  |  |  |
| GO:0046086\_adenosine\_biosynthetic\_process | 1 | 0 |  |  |  |  |  |  |  |  |
| GO:0046090\_deoxyadenosine\_metabolic\_process | 1 | 0 |  |  |  |  |  |  |  |  |
| GO:0046098\_guanine\_metabolic\_process | 1 | 0 |  |  |  |  |  |  |  |  |
| GO:0046101\_hypoxanthine\_biosynthetic\_process | 1 | 0 |  |  |  |  |  |  |  |  |
| GO:0046102\_inosine\_metabolic\_process | 1 | 0 |  |  |  |  |  |  |  |  |
| GO:0046103\_inosine\_biosynthetic\_process | 1 | 0 |  |  |  |  |  |  |  |  |
| GO:0046108\_uridine\_metabolic\_process | 1 | 0 |  |  |  |  |  |  |  |  |
| GO:0046110\_xanthine\_metabolic\_process | 1 | 0 |  |  |  |  |  |  |  |  |
| GO:0046111\_xanthine\_biosynthetic\_process | 1 | 0 |  |  |  |  |  |  |  |  |
| GO:0046112\_nucleobase\_biosynthetic\_process | 1 | 0 |  |  |  |  |  |  |  |  |
| GO:0046113\_nucleobase\_catabolic\_process | 1 | 0 |  |  |  |  |  |  |  |  |
| GO:0046121\_deoxyribonucleoside\_catabolic\_process | 1 | 0 |  |  |  |  |  |  |  |  |
| GO:0046122\_purine\_deoxyribonucleoside\_metabolic\_process | 1 | 0 |  |  |  |  |  |  |  |  |
| GO:0046124\_purine\_deoxyribonucleoside\_catabolic\_process | 1 | 0 |  |  |  |  |  |  |  |  |
| GO:0046125\_pyrimidine\_deoxyribonucleoside\_metabolic\_process | 1 | 0 |  |  |  |  |  |  |  |  |
| GO:0046131\_pyrimidine\_ribonucleoside\_metabolic\_process | 1 | 0 |  |  |  |  |  |  |  |  |
| GO:0046160\_heme\_a\_metabolic\_process | 1 | 0 |  |  |  |  |  |  |  |  |
| GO:0046218\_indolalkylamine\_catabolic\_process | 1 | 0 |  |  |  |  |  |  |  |  |
| GO:0046292\_formaldehyde\_metabolic\_process | 1 | 0 |  |  |  |  |  |  |  |  |
| GO:0046294\_formaldehyde\_catabolic\_process | 1 | 0 |  |  |  |  |  |  |  |  |
| GO:0046314\_phosphocreatine\_biosynthetic\_process | 1 | 0 |  |  |  |  |  |  |  |  |
| GO:0046327\_glycerol\_biosynthetic\_process\_from\_pyruvate | 1 | 0 |  |  |  |  |  |  |  |  |
| GO:0046329\_negative\_regulation\_of\_JNK\_cascade | 1 | 0 |  |  |  |  |  |  |  |  |
| GO:0046340\_diacylglycerol\_catabolic\_process | 1 | 0 |  |  |  |  |  |  |  |  |
| GO:0046351\_disaccharide\_biosynthetic\_process | 1 | 0 |  |  |  |  |  |  |  |  |
| GO:0046356\_acetyl-CoA\_catabolic\_process | 1 | 0 |  |  |  |  |  |  |  |  |
| GO:0046358\_butyrate\_biosynthetic\_process | 1 | 0 |  |  |  |  |  |  |  |  |
| GO:0046359\_butyrate\_catabolic\_process | 1 | 0 |  |  |  |  |  |  |  |  |
| GO:0046381\_CMP-N-acetylneuraminate\_metabolic\_process | 1 | 0 |  |  |  |  |  |  |  |  |
| GO:0046415\_urate\_metabolic\_process | 1 | 0 |  |  |  |  |  |  |  |  |
| GO:0046416\_D-amino\_acid\_metabolic\_process | 1 | 0 |  |  |  |  |  |  |  |  |
| GO:0046434\_organophosphate\_catabolic\_process | 1 | 0 |  |  |  |  |  |  |  |  |
| GO:0046437\_D-amino\_acid\_biosynthetic\_process | 1 | 0 |  |  |  |  |  |  |  |  |
| GO:0046440\_L-lysine\_metabolic\_process | 1 | 0 |  |  |  |  |  |  |  |  |
| GO:0046449\_creatinine\_metabolic\_process | 1 | 0 |  |  |  |  |  |  |  |  |
| GO:0046471\_phosphatidylglycerol\_metabolic\_process | 1 | 0 |  |  |  |  |  |  |  |  |
| GO:0046473\_phosphatidic\_acid\_metabolic\_process | 1 | 0 |  |  |  |  |  |  |  |  |
| GO:0046476\_glycosylceramide\_biosynthetic\_process | 1 | 0 |  |  |  |  |  |  |  |  |
| GO:0046477\_glycosylceramide\_catabolic\_process | 1 | 0 |  |  |  |  |  |  |  |  |
| GO:0046485\_ether\_lipid\_metabolic\_process | 1 | 0 |  |  |  |  |  |  |  |  |
| GO:0046487\_glyoxylate\_metabolic\_process | 1 | 0 |  |  |  |  |  |  |  |  |
| GO:0046498\_S-adenosylhomocysteine\_metabolic\_process | 1 | 0 |  |  |  |  |  |  |  |  |
| GO:0046552\_photoreceptor\_cell\_fate\_commitment | 1 | 0 |  |  |  |  |  |  |  |  |
| GO:0046586\_regulation\_of\_calcium-dependent\_cell-cell\_adhesion | 1 | 0 |  |  |  |  |  |  |  |  |
| GO:0046587\_positive\_regulation\_of\_calcium-dependent\_cell-cell\_adhesion | 1 | 0 |  |  |  |  |  |  |  |  |
| GO:0046602\_regulation\_of\_mitotic\_centrosome\_separation | 1 | 0 |  |  |  |  |  |  |  |  |
| GO:0046604\_positive\_regulation\_of\_mitotic\_centrosome\_separation | 1 | 0 |  |  |  |  |  |  |  |  |
| GO:0046607\_positive\_regulation\_of\_centrosome\_cycle | 1 | 0 |  |  |  |  |  |  |  |  |
| GO:0046655\_folic\_acid\_metabolic\_process | 1 | 0 |  |  |  |  |  |  |  |  |
| GO:0046671\_negative\_regulation\_of\_retinal\_cell\_programmed\_cell\_death | 1 | 0 |  |  |  |  |  |  |  |  |
| GO:0046685\_response\_to\_arsenic | 1 | 0 |  |  |  |  |  |  |  |  |
| GO:0046692\_sperm\_competition | 1 | 0 |  |  |  |  |  |  |  |  |
| GO:0046707\_IDP\_metabolic\_process | 1 | 0 |  |  |  |  |  |  |  |  |
| GO:0046709\_IDP\_catabolic\_process | 1 | 0 |  |  |  |  |  |  |  |  |
| GO:0046724\_oxalic\_acid\_secretion | 1 | 0 |  |  |  |  |  |  |  |  |
| GO:0046753\_non-lytic\_viral\_release | 1 | 0 |  |  |  |  |  |  |  |  |
| GO:0046755\_non-lytic\_virus\_budding | 1 | 0 |  |  |  |  |  |  |  |  |
| GO:0046826\_negative\_regulation\_of\_protein\_export\_from\_nucleus | 1 | 0 |  |  |  |  |  |  |  |  |
| GO:0046827\_positive\_regulation\_of\_protein\_export\_from\_nucleus | 1 | 0 |  |  |  |  |  |  |  |  |
| GO:0046831\_regulation\_of\_RNA\_export\_from\_nucleus | 1 | 0 |  |  |  |  |  |  |  |  |
| GO:0046834\_lipid\_phosphorylation | 1 | 0 |  |  |  |  |  |  |  |  |
| GO:0046853\_inositol\_and\_derivative\_phosphorylation | 1 | 0 |  |  |  |  |  |  |  |  |
| GO:0046864\_isoprenoid\_transport | 1 | 0 |  |  |  |  |  |  |  |  |
| GO:0046865\_terpenoid\_transport | 1 | 0 |  |  |  |  |  |  |  |  |
| GO:0046877\_regulation\_of\_saliva\_secretion | 1 | 0 |  |  |  |  |  |  |  |  |
| GO:0046878\_positive\_regulation\_of\_saliva\_secretion | 1 | 0 |  |  |  |  |  |  |  |  |
| GO:0046884\_follicle-stimulating\_hormone\_secretion | 1 | 0 |  |  |  |  |  |  |  |  |
| GO:0046898\_response\_to\_cycloheximide | 1 | 0 |  |  |  |  |  |  |  |  |
| GO:0046929\_negative\_regulation\_of\_neurotransmitter\_secretion | 1 | 0 |  |  |  |  |  |  |  |  |
| GO:0046931\_pore\_complex\_biogenesis | 1 | 0 |  |  |  |  |  |  |  |  |
| GO:0046949\_acyl-CoA\_biosynthetic\_process | 1 | 0 |  |  |  |  |  |  |  |  |
| GO:0046958\_nonassociative\_learning | 1 | 0 |  |  |  |  |  |  |  |  |
| GO:0046960\_sensitization | 1 | 0 |  |  |  |  |  |  |  |  |
| GO:0046986\_negative\_regulation\_of\_hemoglobin\_biosynthetic\_process | 1 | 0 |  |  |  |  |  |  |  |  |
| GO:0047497\_mitochondrion\_transport\_along\_microtubule | 1 | 0 |  |  |  |  |  |  |  |  |
| GO:0048047\_mating\_behavior\_\_sex\_discrimination | 1 | 0 |  |  |  |  |  |  |  |  |
| GO:0048133\_male\_germ-line\_stem\_cell\_division | 1 | 0 |  |  |  |  |  |  |  |  |
| GO:0048137\_spermatocyte\_division | 1 | 0 |  |  |  |  |  |  |  |  |
| GO:0048143\_astrocyte\_activation | 1 | 0 |  |  |  |  |  |  |  |  |
| GO:0048170\_positive\_regulation\_of\_long-term\_neuronal\_synaptic\_plasticity | 1 | 0 |  |  |  |  |  |  |  |  |
| GO:0048199\_vesicle\_targeting\_\_to\_\_from\_or\_within\_Golgi | 1 | 0 |  |  |  |  |  |  |  |  |
| GO:0048241\_epinephrine\_transport | 1 | 0 |  |  |  |  |  |  |  |  |
| GO:0048242\_epinephrine\_secretion | 1 | 0 |  |  |  |  |  |  |  |  |
| GO:0048243\_norepinephrine\_secretion | 1 | 0 |  |  |  |  |  |  |  |  |
| GO:0048247\_lymphocyte\_chemotaxis | 1 | 0 |  |  |  |  |  |  |  |  |
| GO:0048250\_mitochondrial\_iron\_ion\_transport | 1 | 0 |  |  |  |  |  |  |  |  |
| GO:0048259\_regulation\_of\_receptor-mediated\_endocytosis | 1 | 0 |  |  |  |  |  |  |  |  |
| GO:0048260\_positive\_regulation\_of\_receptor-mediated\_endocytosis | 1 | 0 |  |  |  |  |  |  |  |  |
| GO:0048290\_isotype\_switching\_to\_IgA\_isotypes | 1 | 0 |  |  |  |  |  |  |  |  |
| GO:0048296\_regulation\_of\_isotype\_switching\_to\_IgA\_isotypes | 1 | 0 |  |  |  |  |  |  |  |  |
| GO:0048298\_positive\_regulation\_of\_isotype\_switching\_to\_IgA\_isotypes | 1 | 0 |  |  |  |  |  |  |  |  |
| GO:0048319\_axial\_mesoderm\_morphogenesis | 1 | 0 |  |  |  |  |  |  |  |  |
| GO:0048320\_axial\_mesoderm\_formation | 1 | 0 |  |  |  |  |  |  |  |  |
| GO:0048385\_regulation\_of\_retinoic\_acid\_receptor\_signaling\_pathway | 1 | 0 |  |  |  |  |  |  |  |  |
| GO:0048387\_negative\_regulation\_of\_retinoic\_acid\_receptor\_signaling\_pathway | 1 | 0 |  |  |  |  |  |  |  |  |
| GO:0048388\_endosomal\_lumen\_acidification | 1 | 0 |  |  |  |  |  |  |  |  |
| GO:0048389\_intermediate\_mesoderm\_development | 1 | 0 |  |  |  |  |  |  |  |  |
| GO:0048478\_replication\_fork\_protection | 1 | 0 |  |  |  |  |  |  |  |  |
| GO:0048496\_maintenance\_of\_organ\_identity | 1 | 0 |  |  |  |  |  |  |  |  |
| GO:0048525\_negative\_regulation\_of\_viral\_reproduction | 1 | 0 |  |  |  |  |  |  |  |  |
| GO:0048539\_bone\_marrow\_development | 1 | 0 |  |  |  |  |  |  |  |  |
| GO:0048548\_regulation\_of\_pinocytosis | 1 | 0 |  |  |  |  |  |  |  |  |
| GO:0048549\_positive\_regulation\_of\_pinocytosis | 1 | 0 |  |  |  |  |  |  |  |  |
| GO:0048553\_negative\_regulation\_of\_metalloenzyme\_activity | 1 | 0 |  |  |  |  |  |  |  |  |
| GO:0048588\_developmental\_cell\_growth | 1 | 0 |  |  |  |  |  |  |  |  |
| GO:0048601\_oocyte\_morphogenesis | 1 | 0 |  |  |  |  |  |  |  |  |
| GO:0048621\_post-embryonic\_gut\_morphogenesis | 1 | 0 |  |  |  |  |  |  |  |  |
| GO:0048640\_negative\_regulation\_of\_developmental\_growth | 1 | 0 |  |  |  |  |  |  |  |  |
| GO:0048642\_negative\_regulation\_of\_skeletal\_muscle\_tissue\_development | 1 | 0 |  |  |  |  |  |  |  |  |
| GO:0048669\_collateral\_sprouting\_in\_the\_absence\_of\_injury | 1 | 0 |  |  |  |  |  |  |  |  |
| GO:0048680\_positive\_regulation\_of\_axon\_regeneration | 1 | 0 |  |  |  |  |  |  |  |  |
| GO:0048681\_negative\_regulation\_of\_axon\_regeneration | 1 | 0 |  |  |  |  |  |  |  |  |
| GO:0048686\_regulation\_of\_sprouting\_of\_injured\_axon | 1 | 0 |  |  |  |  |  |  |  |  |
| GO:0048687\_positive\_regulation\_of\_sprouting\_of\_injured\_axon | 1 | 0 |  |  |  |  |  |  |  |  |
| GO:0048690\_regulation\_of\_axon\_extension\_involved\_in\_regeneration | 1 | 0 |  |  |  |  |  |  |  |  |
| GO:0048691\_positive\_regulation\_of\_axon\_extension\_involved\_in\_regeneration | 1 | 0 |  |  |  |  |  |  |  |  |
| GO:0048714\_positive\_regulation\_of\_oligodendrocyte\_differentiation | 1 | 0 |  |  |  |  |  |  |  |  |
| GO:0048733\_sebaceous\_gland\_development | 1 | 0 |  |  |  |  |  |  |  |  |
| GO:0048743\_positive\_regulation\_of\_skeletal\_muscle\_fiber\_development | 1 | 0 |  |  |  |  |  |  |  |  |
| GO:0048752\_semicircular\_canal\_morphogenesis | 1 | 0 |  |  |  |  |  |  |  |  |
| GO:0048773\_erythrophore\_differentiation | 1 | 0 |  |  |  |  |  |  |  |  |
| GO:0048790\_maintenance\_of\_presynaptic\_active\_zone\_structure | 1 | 0 |  |  |  |  |  |  |  |  |
| GO:0048791\_calcium\_ion-dependent\_exocytosis\_of\_neurotransmitter | 1 | 0 |  |  |  |  |  |  |  |  |
| GO:0048822\_enucleate\_erythrocyte\_development | 1 | 0 |  |  |  |  |  |  |  |  |
| GO:0048866\_stem\_cell\_fate\_specification | 1 | 0 |  |  |  |  |  |  |  |  |
| GO:0048936\_peripheral\_nervous\_system\_neuron\_axonogenesis | 1 | 0 |  |  |  |  |  |  |  |  |
| GO:0050427\_3'-phosphoadenosine\_5'-phosphosulfate\_metabolic\_process | 1 | 0 |  |  |  |  |  |  |  |  |
| GO:0050428\_3'-phosphoadenosine\_5'-phosphosulfate\_biosynthetic\_process | 1 | 0 |  |  |  |  |  |  |  |  |
| GO:0050482\_arachidonic\_acid\_secretion | 1 | 0 |  |  |  |  |  |  |  |  |
| GO:0050667\_homocysteine\_metabolic\_process | 1 | 0 |  |  |  |  |  |  |  |  |
| GO:0050674\_urothelial\_cell\_proliferation | 1 | 0 |  |  |  |  |  |  |  |  |
| GO:0050675\_regulation\_of\_urothelial\_cell\_proliferation | 1 | 0 |  |  |  |  |  |  |  |  |
| GO:0050677\_positive\_regulation\_of\_urothelial\_cell\_proliferation | 1 | 0 |  |  |  |  |  |  |  |  |
| GO:0050691\_regulation\_of\_defense\_response\_to\_virus\_by\_host | 1 | 0 |  |  |  |  |  |  |  |  |
| GO:0050748\_negative\_regulation\_of\_lipoprotein\_metabolic\_process | 1 | 0 |  |  |  |  |  |  |  |  |
| GO:0050757\_thymidylate\_synthase\_biosynthetic\_process | 1 | 0 |  |  |  |  |  |  |  |  |
| GO:0050758\_regulation\_of\_thymidylate\_synthase\_biosynthetic\_process | 1 | 0 |  |  |  |  |  |  |  |  |
| GO:0050760\_negative\_regulation\_of\_thymidylate\_synthase\_biosynthetic\_process | 1 | 0 |  |  |  |  |  |  |  |  |
| GO:0050812\_regulation\_of\_acyl-CoA\_biosynthetic\_process | 1 | 0 |  |  |  |  |  |  |  |  |
| GO:0050832\_defense\_response\_to\_fungus | 1 | 0 |  |  |  |  |  |  |  |  |
| GO:0050861\_positive\_regulation\_of\_B\_cell\_receptor\_signaling\_pathway | 1 | 0 |  |  |  |  |  |  |  |  |
| GO:0050862\_positive\_regulation\_of\_T\_cell\_receptor\_signaling\_pathway | 1 | 0 |  |  |  |  |  |  |  |  |
| GO:0050916\_sensory\_perception\_of\_sweet\_taste | 1 | 0 |  |  |  |  |  |  |  |  |
| GO:0050975\_sensory\_perception\_of\_touch | 1 | 0 |  |  |  |  |  |  |  |  |
| GO:0050995\_negative\_regulation\_of\_lipid\_catabolic\_process | 1 | 0 |  |  |  |  |  |  |  |  |
| GO:0051001\_negative\_regulation\_of\_nitric-oxide\_synthase\_activity | 1 | 0 |  |  |  |  |  |  |  |  |
| GO:0051005\_negative\_regulation\_of\_lipoprotein\_lipase\_activity | 1 | 0 |  |  |  |  |  |  |  |  |
| GO:0051006\_positive\_regulation\_of\_lipoprotein\_lipase\_activity | 1 | 0 |  |  |  |  |  |  |  |  |
| GO:0051016\_barbed-end\_actin\_filament\_capping | 1 | 0 |  |  |  |  |  |  |  |  |
| GO:0051029\_rRNA\_transport | 1 | 0 |  |  |  |  |  |  |  |  |
| GO:0051043\_regulation\_of\_membrane\_protein\_ectodomain\_proteolysis | 1 | 0 |  |  |  |  |  |  |  |  |
| GO:0051044\_positive\_regulation\_of\_membrane\_protein\_ectodomain\_proteolysis | 1 | 0 |  |  |  |  |  |  |  |  |
| GO:0051088\_PMA-inducible\_membrane\_protein\_ectodomain\_proteolysis | 1 | 0 |  |  |  |  |  |  |  |  |
| GO:0051102\_DNA\_ligation\_during\_DNA\_recombination | 1 | 0 |  |  |  |  |  |  |  |  |
| GO:0051103\_DNA\_ligation\_during\_DNA\_repair | 1 | 0 |  |  |  |  |  |  |  |  |
| GO:0051123\_transcriptional\_preinitiation\_complex\_assembly | 1 | 0 |  |  |  |  |  |  |  |  |
| GO:0051125\_regulation\_of\_actin\_nucleation | 1 | 0 |  |  |  |  |  |  |  |  |
| GO:0051127\_positive\_regulation\_of\_actin\_nucleation | 1 | 0 |  |  |  |  |  |  |  |  |
| GO:0051151\_negative\_regulation\_of\_smooth\_muscle\_cell\_differentiation | 1 | 0 |  |  |  |  |  |  |  |  |
| GO:0051154\_negative\_regulation\_of\_striated\_muscle\_cell\_differentiation | 1 | 0 |  |  |  |  |  |  |  |  |
| GO:0051155\_positive\_regulation\_of\_striated\_muscle\_cell\_differentiation | 1 | 0 |  |  |  |  |  |  |  |  |
| GO:0051156\_glucose\_6-phosphate\_metabolic\_process | 1 | 0 |  |  |  |  |  |  |  |  |
| GO:0051187\_cofactor\_catabolic\_process | 1 | 0 |  |  |  |  |  |  |  |  |
| GO:0051189\_prosthetic\_group\_metabolic\_process | 1 | 0 |  |  |  |  |  |  |  |  |
| GO:0051193\_regulation\_of\_cofactor\_metabolic\_process | 1 | 0 |  |  |  |  |  |  |  |  |
| GO:0051196\_regulation\_of\_coenzyme\_metabolic\_process | 1 | 0 |  |  |  |  |  |  |  |  |
| GO:0051255\_spindle\_midzone\_assembly | 1 | 0 |  |  |  |  |  |  |  |  |
| GO:0051257\_spindle\_midzone\_assembly\_involved\_in\_meiosis | 1 | 0 |  |  |  |  |  |  |  |  |
| GO:0051281\_positive\_regulation\_of\_release\_of\_sequestered\_calcium\_ion\_into\_cytosol | 1 | 0 |  |  |  |  |  |  |  |  |
| GO:0051290\_protein\_heterotetramerization | 1 | 0 |  |  |  |  |  |  |  |  |
| GO:0051305\_chromosome\_movement\_towards\_spindle\_pole | 1 | 0 |  |  |  |  |  |  |  |  |
| GO:0051310\_metaphase\_plate\_congression | 1 | 0 |  |  |  |  |  |  |  |  |
| GO:0051311\_meiotic\_metaphase\_plate\_congression | 1 | 0 |  |  |  |  |  |  |  |  |
| GO:0051340\_regulation\_of\_ligase\_activity | 1 | 0 |  |  |  |  |  |  |  |  |
| GO:0051351\_positive\_regulation\_of\_ligase\_activity | 1 | 0 |  |  |  |  |  |  |  |  |
| GO:0051354\_negative\_regulation\_of\_oxidoreductase\_activity | 1 | 0 |  |  |  |  |  |  |  |  |
| GO:0051355\_proprioception\_during\_equilibrioception | 1 | 0 |  |  |  |  |  |  |  |  |
| GO:0051383\_kinetochore\_organization | 1 | 0 |  |  |  |  |  |  |  |  |
| GO:0051386\_regulation\_of\_nerve\_growth\_factor\_receptor\_signaling\_pathway | 1 | 0 |  |  |  |  |  |  |  |  |
| GO:0051409\_response\_to\_nitrosative\_stress | 1 | 0 |  |  |  |  |  |  |  |  |
| GO:0051457\_maintenance\_of\_protein\_location\_in\_nucleus | 1 | 0 |  |  |  |  |  |  |  |  |
| GO:0051462\_regulation\_of\_cortisol\_secretion | 1 | 0 |  |  |  |  |  |  |  |  |
| GO:0051463\_negative\_regulation\_of\_cortisol\_secretion | 1 | 0 |  |  |  |  |  |  |  |  |
| GO:0051481\_reduction\_of\_cytosolic\_calcium\_ion\_concentration | 1 | 0 |  |  |  |  |  |  |  |  |
| GO:0051482\_elevation\_of\_cytosolic\_calcium\_ion\_concentration\_during\_G-protein\_signaling\_\_coupled\_to\_IP3\_second\_messenger\_(phospholipase\_C\_activating) | 1 | 0 |  |  |  |  |  |  |  |  |
| GO:0051542\_elastin\_biosynthetic\_process | 1 | 0 |  |  |  |  |  |  |  |  |
| GO:0051568\_histone\_H3-K4\_methylation | 1 | 0 |  |  |  |  |  |  |  |  |
| GO:0051569\_regulation\_of\_histone\_H3-K4\_methylation | 1 | 0 |  |  |  |  |  |  |  |  |
| GO:0051570\_regulation\_of\_histone\_H3-K9\_methylation | 1 | 0 |  |  |  |  |  |  |  |  |
| GO:0051573\_negative\_regulation\_of\_histone\_H3-K9\_methylation | 1 | 0 |  |  |  |  |  |  |  |  |
| GO:0051580\_regulation\_of\_neurotransmitter\_uptake | 1 | 0 |  |  |  |  |  |  |  |  |
| GO:0051582\_positive\_regulation\_of\_neurotransmitter\_uptake | 1 | 0 |  |  |  |  |  |  |  |  |
| GO:0051584\_regulation\_of\_dopamine\_uptake | 1 | 0 |  |  |  |  |  |  |  |  |
| GO:0051586\_positive\_regulation\_of\_dopamine\_uptake | 1 | 0 |  |  |  |  |  |  |  |  |
| GO:0051589\_negative\_regulation\_of\_neurotransmitter\_transport | 1 | 0 |  |  |  |  |  |  |  |  |
| GO:0051593\_response\_to\_folic\_acid | 1 | 0 |  |  |  |  |  |  |  |  |
| GO:0051615\_histamine\_uptake | 1 | 0 |  |  |  |  |  |  |  |  |
| GO:0051646\_mitochondrion\_localization | 1 | 0 |  |  |  |  |  |  |  |  |
| GO:0051654\_establishment\_of\_mitochondrion\_localization | 1 | 0 |  |  |  |  |  |  |  |  |
| GO:0051661\_maintenance\_of\_centrosome\_location | 1 | 0 |  |  |  |  |  |  |  |  |
| GO:0051665\_membrane\_raft\_localization | 1 | 0 |  |  |  |  |  |  |  |  |
| GO:0051685\_maintenance\_of\_ER\_location | 1 | 0 |  |  |  |  |  |  |  |  |
| GO:0051693\_actin\_filament\_capping | 1 | 0 |  |  |  |  |  |  |  |  |
| GO:0051701\_interaction\_with\_host | 1 | 0 |  |  |  |  |  |  |  |  |
| GO:0051754\_meiotic\_sister\_chromatid\_cohesion\_\_centromeric | 1 | 0 |  |  |  |  |  |  |  |  |
| GO:0051782\_negative\_regulation\_of\_cell\_division | 1 | 0 |  |  |  |  |  |  |  |  |
| GO:0051790\_short-chain\_fatty\_acid\_biosynthetic\_process | 1 | 0 |  |  |  |  |  |  |  |  |
| GO:0051799\_negative\_regulation\_of\_hair\_follicle\_development | 1 | 0 |  |  |  |  |  |  |  |  |
| GO:0051823\_regulation\_of\_synapse\_structural\_plasticity | 1 | 0 |  |  |  |  |  |  |  |  |
| GO:0051865\_protein\_autoubiquitination | 1 | 0 |  |  |  |  |  |  |  |  |
| GO:0051901\_positive\_regulation\_of\_mitochondrial\_depolarization | 1 | 0 |  |  |  |  |  |  |  |  |
| GO:0051917\_regulation\_of\_fibrinolysis | 1 | 0 |  |  |  |  |  |  |  |  |
| GO:0051918\_negative\_regulation\_of\_fibrinolysis | 1 | 0 |  |  |  |  |  |  |  |  |
| GO:0051929\_positive\_regulation\_of\_calcium\_ion\_transport\_via\_voltage-gated\_calcium\_channel\_activity | 1 | 0 |  |  |  |  |  |  |  |  |
| GO:0051933\_amino\_acid\_uptake\_during\_transmission\_of\_nerve\_impulse | 1 | 0 |  |  |  |  |  |  |  |  |
| GO:0051935\_glutamate\_uptake\_during\_transmission\_of\_nerve\_impulse | 1 | 0 |  |  |  |  |  |  |  |  |
| GO:0051940\_regulation\_of\_catecholamine\_uptake\_during\_transmission\_of\_nerve\_impulse | 1 | 0 |  |  |  |  |  |  |  |  |
| GO:0051944\_positive\_regulation\_of\_catecholamine\_uptake\_during\_transmission\_of\_nerve\_impulse | 1 | 0 |  |  |  |  |  |  |  |  |
| GO:0051961\_negative\_regulation\_of\_nervous\_system\_development | 1 | 0 |  |  |  |  |  |  |  |  |
| GO:0051964\_negative\_regulation\_of\_synaptogenesis | 1 | 0 |  |  |  |  |  |  |  |  |
| GO:0051968\_positive\_regulation\_of\_synaptic\_transmission\_\_glutamatergic | 1 | 0 |  |  |  |  |  |  |  |  |
| GO:0051984\_positive\_regulation\_of\_chromosome\_segregation | 1 | 0 |  |  |  |  |  |  |  |  |
| GO:0051987\_positive\_regulation\_of\_attachment\_of\_spindle\_microtubules\_to\_kinetochore | 1 | 0 |  |  |  |  |  |  |  |  |
| GO:0052173\_response\_to\_defenses\_of\_other\_organism\_during\_symbiotic\_interaction | 1 | 0 |  |  |  |  |  |  |  |  |
| GO:0052200\_response\_to\_host\_defenses | 1 | 0 |  |  |  |  |  |  |  |  |
| GO:0052551\_response\_to\_defense-related\_nitric\_oxide\_production\_by\_other\_organism\_during\_symbiotic\_interaction | 1 | 0 |  |  |  |  |  |  |  |  |
| GO:0052564\_response\_to\_immune\_response\_of\_other\_organism\_during\_symbiotic\_interaction | 1 | 0 |  |  |  |  |  |  |  |  |
| GO:0052565\_response\_to\_defense-related\_host\_nitric\_oxide\_production | 1 | 0 |  |  |  |  |  |  |  |  |
| GO:0052572\_response\_to\_host\_immune\_response | 1 | 0 |  |  |  |  |  |  |  |  |
| GO:0055005\_ventricular\_cardiac\_myofibril\_development | 1 | 0 |  |  |  |  |  |  |  |  |
| GO:0055011\_atrial\_cardiac\_muscle\_cell\_differentiation | 1 | 0 |  |  |  |  |  |  |  |  |
| GO:0055014\_atrial\_cardiac\_muscle\_cell\_development | 1 | 0 |  |  |  |  |  |  |  |  |
| GO:0055078\_sodium\_ion\_homeostasis | 1 | 0 |  |  |  |  |  |  |  |  |
| GO:0055089\_fatty\_acid\_homeostasis | 1 | 0 |  |  |  |  |  |  |  |  |
| GO:0055093\_response\_to\_hyperoxia | 1 | 0 |  |  |  |  |  |  |  |  |
| GO:0060003\_copper\_ion\_export | 1 | 0 |  |  |  |  |  |  |  |  |
| GO:0060005\_vestibular\_reflex | 1 | 0 |  |  |  |  |  |  |  |  |
| GO:0060014\_granulosa\_cell\_differentiation | 1 | 0 |  |  |  |  |  |  |  |  |
| GO:0060018\_astrocyte\_fate\_commitment | 1 | 0 |  |  |  |  |  |  |  |  |
| GO:0060020\_Bergmann\_glial\_cell\_differentiation | 1 | 0 |  |  |  |  |  |  |  |  |
| GO:0060022\_hard\_palate\_development | 1 | 0 |  |  |  |  |  |  |  |  |
| GO:0060034\_notochord\_cell\_differentiation | 1 | 0 |  |  |  |  |  |  |  |  |
| GO:0060035\_notochord\_cell\_development | 1 | 0 |  |  |  |  |  |  |  |  |
| GO:0060046\_regulation\_of\_acrosome\_reaction | 1 | 0 |  |  |  |  |  |  |  |  |
| GO:0060054\_positive\_regulation\_of\_epithelial\_cell\_proliferation\_involved\_in\_wound\_healing | 1 | 0 |  |  |  |  |  |  |  |  |
| GO:0060059\_embryonic\_retina\_morphogenesis\_in\_camera-type\_eye | 1 | 0 |  |  |  |  |  |  |  |  |
| GO:0060061\_Spemann\_organizer\_formation | 1 | 0 |  |  |  |  |  |  |  |  |
| GO:0060064\_Spemann\_organizer\_formation\_at\_the\_anterior\_end\_of\_the\_primitive\_streak | 1 | 0 |  |  |  |  |  |  |  |  |
| GO:0060071\_Wnt\_receptor\_signaling\_pathway\_\_planar\_cell\_polarity\_pathway | 1 | 0 |  |  |  |  |  |  |  |  |
| GO:0060075\_regulation\_of\_resting\_membrane\_potential | 1 | 0 |  |  |  |  |  |  |  |  |
| GO:0060082\_eye\_blink\_reflex | 1 | 0 |  |  |  |  |  |  |  |  |
| GO:0060112\_generation\_of\_ovulation\_cycle\_rhythm | 1 | 0 |  |  |  |  |  |  |  |  |
| GO:0060125\_negative\_regulation\_of\_growth\_hormone\_secretion | 1 | 0 |  |  |  |  |  |  |  |  |
| GO:0060151\_peroxisome\_localization | 1 | 0 |  |  |  |  |  |  |  |  |
| GO:0060152\_microtubule-based\_peroxisome\_localization | 1 | 0 |  |  |  |  |  |  |  |  |
| GO:0060161\_positive\_regulation\_of\_dopamine\_receptor\_signaling\_pathway | 1 | 0 |  |  |  |  |  |  |  |  |
| GO:0060163\_subpallium\_neuron\_fate\_commitment | 1 | 0 |  |  |  |  |  |  |  |  |
| GO:0060165\_regulation\_of\_timing\_of\_subpallium\_neuron\_differentiation | 1 | 0 |  |  |  |  |  |  |  |  |
| GO:0060174\_limb\_bud\_formation | 1 | 0 |  |  |  |  |  |  |  |  |
| GO:0060177\_regulation\_of\_angiotensin\_metabolic\_process | 1 | 0 |  |  |  |  |  |  |  |  |
| GO:0060197\_cloacal\_septation | 1 | 0 |  |  |  |  |  |  |  |  |
| GO:0060215\_primitive\_hemopoiesis | 1 | 0 |  |  |  |  |  |  |  |  |
| GO:0060231\_mesenchymal\_to\_epithelial\_transition | 1 | 0 |  |  |  |  |  |  |  |  |
| GO:0060254\_regulation\_of\_N-terminal\_protein\_palmitoylation | 1 | 0 |  |  |  |  |  |  |  |  |
| GO:0060261\_positive\_regulation\_of\_transcription\_initiation\_from\_RNA\_polymerase\_II\_promoter | 1 | 0 |  |  |  |  |  |  |  |  |
| GO:0060262\_negative\_regulation\_of\_N-terminal\_protein\_palmitoylation | 1 | 0 |  |  |  |  |  |  |  |  |
| GO:0060263\_regulation\_of\_respiratory\_burst | 1 | 0 |  |  |  |  |  |  |  |  |
| GO:0060264\_regulation\_of\_respiratory\_burst\_during\_acute\_inflammatory\_response | 1 | 0 |  |  |  |  |  |  |  |  |
| GO:0060265\_positive\_regulation\_of\_respiratory\_burst\_during\_acute\_inflammatory\_response | 1 | 0 |  |  |  |  |  |  |  |  |
| GO:0060267\_positive\_regulation\_of\_respiratory\_burst | 1 | 0 |  |  |  |  |  |  |  |  |
| GO:0060272\_embryonic\_skeletal\_joint\_morphogenesis | 1 | 0 |  |  |  |  |  |  |  |  |
| GO:0060297\_regulation\_of\_sarcomere\_organization | 1 | 0 |  |  |  |  |  |  |  |  |
| GO:0060298\_positive\_regulation\_of\_sarcomere\_organization | 1 | 0 |  |  |  |  |  |  |  |  |
| GO:0060315\_negative\_regulation\_of\_ryanodine-sensitive\_calcium-release\_channel\_activity | 1 | 0 |  |  |  |  |  |  |  |  |
| GO:0060319\_primitive\_erythrocyte\_differentiation | 1 | 0 |  |  |  |  |  |  |  |  |
| GO:0060371\_regulation\_of\_atrial\_cardiomyocyte\_membrane\_depolarization | 1 | 0 |  |  |  |  |  |  |  |  |
| GO:0060374\_mast\_cell\_differentiation | 1 | 0 |  |  |  |  |  |  |  |  |
| GO:0060375\_regulation\_of\_mast\_cell\_differentiation | 1 | 0 |  |  |  |  |  |  |  |  |
| GO:0060376\_positive\_regulation\_of\_mast\_cell\_differentiation | 1 | 0 |  |  |  |  |  |  |  |  |
| GO:0060390\_regulation\_of\_SMAD\_protein\_nuclear\_translocation | 1 | 0 |  |  |  |  |  |  |  |  |
| GO:0060391\_positive\_regulation\_of\_SMAD\_protein\_nuclear\_translocation | 1 | 0 |  |  |  |  |  |  |  |  |
| GO:0060398\_regulation\_of\_growth\_hormone\_receptor\_signaling\_pathway | 1 | 0 |  |  |  |  |  |  |  |  |
| GO:0060399\_positive\_regulation\_of\_growth\_hormone\_receptor\_signaling\_pathway | 1 | 0 |  |  |  |  |  |  |  |  |
| GO:0060405\_regulation\_of\_penile\_erection | 1 | 0 |  |  |  |  |  |  |  |  |
| GO:0060407\_negative\_regulation\_of\_penile\_erection | 1 | 0 |  |  |  |  |  |  |  |  |
| GO:0060413\_atrial\_septum\_morphogenesis | 1 | 0 |  |  |  |  |  |  |  |  |
| GO:0060414\_aorta\_smooth\_muscle\_tissue\_morphogenesis | 1 | 0 |  |  |  |  |  |  |  |  |
| GO:0060419\_heart\_growth | 1 | 0 |  |  |  |  |  |  |  |  |
| GO:0060420\_regulation\_of\_heart\_growth | 1 | 0 |  |  |  |  |  |  |  |  |
| GO:0060421\_positive\_regulation\_of\_heart\_growth | 1 | 0 |  |  |  |  |  |  |  |  |
| GO:0060431\_primary\_lung\_bud\_formation | 1 | 0 |  |  |  |  |  |  |  |  |
| GO:0060436\_bronchiole\_morphogenesis | 1 | 0 |  |  |  |  |  |  |  |  |
| GO:0060440\_trachea\_formation | 1 | 0 |  |  |  |  |  |  |  |  |
| GO:0060449\_bud\_elongation\_involved\_in\_lung\_branching | 1 | 0 |  |  |  |  |  |  |  |  |
| GO:0060456\_positive\_regulation\_of\_digestive\_system\_process | 1 | 0 |  |  |  |  |  |  |  |  |
| GO:0060461\_right\_lung\_morphogenesis | 1 | 0 |  |  |  |  |  |  |  |  |
| GO:0060481\_lobar\_bronchus\_epithelium\_development | 1 | 0 |  |  |  |  |  |  |  |  |
| GO:0060482\_lobar\_bronchus\_development | 1 | 0 |  |  |  |  |  |  |  |  |
| GO:0060484\_lung-associated\_mesenchyme\_development | 1 | 0 |  |  |  |  |  |  |  |  |
| GO:0060486\_Clara\_cell\_differentiation | 1 | 0 |  |  |  |  |  |  |  |  |
| GO:0060510\_Type\_II\_pneumocyte\_differentiation | 1 | 0 |  |  |  |  |  |  |  |  |
| GO:0060514\_prostate\_induction | 1 | 0 |  |  |  |  |  |  |  |  |
| GO:0060515\_prostate\_field\_specification | 1 | 0 |  |  |  |  |  |  |  |  |
| GO:0060517\_epithelial\_cell\_proliferation\_involved\_in\_prostatic\_bud\_elongation | 1 | 0 |  |  |  |  |  |  |  |  |
| GO:0060520\_activation\_of\_prostate\_induction\_by\_androgen\_receptor\_signaling\_pathway | 1 | 0 |  |  |  |  |  |  |  |  |
| GO:0060535\_trachea\_cartilage\_morphogenesis | 1 | 0 |  |  |  |  |  |  |  |  |
| GO:0060536\_cartilage\_morphogenesis | 1 | 0 |  |  |  |  |  |  |  |  |
| GO:0060563\_neuroepithelial\_cell\_differentiation | 1 | 0 |  |  |  |  |  |  |  |  |
| GO:0060577\_pulmonary\_vein\_morphogenesis | 1 | 0 |  |  |  |  |  |  |  |  |
| GO:0060578\_superior\_vena\_cava\_morphogenesis | 1 | 0 |  |  |  |  |  |  |  |  |
| GO:0060584\_regulation\_of\_prostaglandin-endoperoxide\_synthase\_activity | 1 | 0 |  |  |  |  |  |  |  |  |
| GO:0060585\_positive\_regulation\_of\_prostaglandin-endoperoxidase\_synthase\_activity | 1 | 0 |  |  |  |  |  |  |  |  |
| GO:0060598\_dichotomous\_subdivision\_of\_terminal\_units\_involved\_in\_mammary\_gland\_duct\_morphogenesis | 1 | 0 |  |  |  |  |  |  |  |  |
| GO:0060611\_mammary\_gland\_fat\_development | 1 | 0 |  |  |  |  |  |  |  |  |
| GO:0060618\_nipple\_development | 1 | 0 |  |  |  |  |  |  |  |  |
| GO:0060631\_regulation\_of\_meiosis\_I | 1 | 0 |  |  |  |  |  |  |  |  |
| GO:0060649\_mammary\_gland\_bud\_elongation | 1 | 0 |  |  |  |  |  |  |  |  |
| GO:0060658\_nipple\_morphogenesis | 1 | 0 |  |  |  |  |  |  |  |  |
| GO:0060659\_nipple\_sheath\_formation | 1 | 0 |  |  |  |  |  |  |  |  |
| GO:0060668\_regulation\_of\_branching\_involved\_in\_salivary\_gland\_morphogenesis\_by\_extracellular\_matrix-epithelial\_cell\_signaling | 1 | 0 |  |  |  |  |  |  |  |  |
| GO:0060683\_regulation\_of\_branching\_involved\_in\_salivary\_gland\_morphogenesis\_by\_epithelial-mesenchymal\_signaling | 1 | 0 |  |  |  |  |  |  |  |  |
| GO:0060691\_epithelial\_cell\_maturation\_involved\_in\_salivary\_gland\_development | 1 | 0 |  |  |  |  |  |  |  |  |
| GO:0060709\_glycogen\_cell\_development\_involved\_in\_embryonic\_placenta\_development | 1 | 0 |  |  |  |  |  |  |  |  |
| GO:0060732\_positive\_regulation\_of\_inositol\_phosphate\_biosynthetic\_process | 1 | 0 |  |  |  |  |  |  |  |  |
| GO:0060739\_mesenchymal-epithelial\_cell\_signaling\_involved\_in\_prostate\_gland\_development | 1 | 0 |  |  |  |  |  |  |  |  |
| GO:0060781\_mesenchymal\_cell\_proliferation\_involved\_in\_prostate\_gland\_development | 1 | 0 |  |  |  |  |  |  |  |  |
| GO:0060782\_regulation\_of\_mesenchymal\_cell\_proliferation\_involved\_in\_prostate\_gland\_development | 1 | 0 |  |  |  |  |  |  |  |  |
| GO:0060783\_mesenchymal\_smoothened\_signaling\_pathway\_involved\_in\_prostate\_gland\_development | 1 | 0 |  |  |  |  |  |  |  |  |
| GO:0060872\_semicircular\_canal\_development | 1 | 0 |  |  |  |  |  |  |  |  |
| GO:0060896\_neural\_plate\_pattern\_specification | 1 | 0 |  |  |  |  |  |  |  |  |
| GO:0070091\_glucagon\_secretion | 1 | 0 |  |  |  |  |  |  |  |  |
| GO:0070162\_adiponectin\_secretion | 1 | 0 |  |  |  |  |  |  |  |  |
| GO:0070163\_regulation\_of\_adiponectin\_secretion | 1 | 0 |  |  |  |  |  |  |  |  |
| GO:0070164\_negative\_regulation\_of\_adiponectin\_secretion | 1 | 0 |  |  |  |  |  |  |  |  |
| GO:0070178\_D-serine\_metabolic\_process | 1 | 0 |  |  |  |  |  |  |  |  |
| GO:0070179\_D-serine\_biosynthetic\_process | 1 | 0 |  |  |  |  |  |  |  |  |
| GO:0070296\_sarcoplasmic\_reticulum\_calcium\_ion\_transport | 1 | 0 |  |  |  |  |  |  |  |  |
| GO:0070303\_negative\_regulation\_of\_stress-activated\_protein\_kinase\_signaling\_pathway | 1 | 0 |  |  |  |  |  |  |  |  |
| GO:0070328\_triglyceride\_homeostasis | 1 | 0 |  |  |  |  |  |  |  |  |
| GO:0070365\_hepatocyte\_differentiation | 1 | 0 |  |  |  |  |  |  |  |  |
| GO:0070384\_Harderian\_gland\_development | 1 | 0 |  |  |  |  |  |  |  |  |
| GO:0070391\_response\_to\_lipoteichoic\_acid | 1 | 0 |  |  |  |  |  |  |  |  |
| GO:0070424\_regulation\_of\_nucleotide-binding\_oligomerization\_domain\_containing\_signaling\_pathway | 1 | 0 |  |  |  |  |  |  |  |  |
| GO:0070426\_positive\_regulation\_of\_nucleotide-binding\_oligomerization\_domain\_containing\_signaling\_pathway | 1 | 0 |  |  |  |  |  |  |  |  |
| GO:0070428\_regulation\_of\_nucleotide-binding\_oligomerization\_domain\_containing\_1\_signaling\_pathway | 1 | 0 |  |  |  |  |  |  |  |  |
| GO:0070430\_positive\_regulation\_of\_nucleotide-binding\_oligomerization\_domain\_containing\_1\_signaling\_pathway | 1 | 0 |  |  |  |  |  |  |  |  |
| GO:0070432\_regulation\_of\_nucleotide-binding\_oligomerization\_domain\_containing\_2\_signaling\_pathway | 1 | 0 |  |  |  |  |  |  |  |  |
| GO:0070434\_positive\_regulation\_of\_nucleotide-binding\_oligomerization\_domain\_containing\_2\_signaling\_pathway | 1 | 0 |  |  |  |  |  |  |  |  |
| GO:0070493\_thrombin\_receptor\_signaling\_pathway | 1 | 0 |  |  |  |  |  |  |  |  |
| GO:0070508\_cholesterol\_import | 1 | 0 |  |  |  |  |  |  |  |  |
| GO:0070527\_platelet\_aggregation | 1 | 0 |  |  |  |  |  |  |  |  |
| GO:0070528\_protein\_kinase\_C\_signaling\_cascade | 1 | 0 |  |  |  |  |  |  |  |  |
| GO:0070555\_response\_to\_interleukin-1 | 1 | 0 |  |  |  |  |  |  |  |  |
| GO:0070560\_protein\_secretion\_by\_platelet | 1 | 0 |  |  |  |  |  |  |  |  |
| GO:0070561\_vitamin\_D\_receptor\_signaling\_pathway | 1 | 0 |  |  |  |  |  |  |  |  |
| GO:0070562\_regulation\_of\_vitamin\_D\_receptor\_signaling\_pathway | 1 | 0 |  |  |  |  |  |  |  |  |
| GO:0070571\_negative\_regulation\_of\_neuron\_projection\_regeneration | 1 | 0 |  |  |  |  |  |  |  |  |
| GO:0070572\_positive\_regulation\_of\_neuron\_projection\_regeneration | 1 | 0 |  |  |  |  |  |  |  |  |
| GO:0070613\_regulation\_of\_protein\_processing | 1 | 0 |  |  |  |  |  |  |  |  |
| GO:0070627\_ferrous\_iron\_import | 1 | 0 |  |  |  |  |  |  |  |  |
| GO:0070669\_response\_to\_interleukin-2 | 1 | 0 |  |  |  |  |  |  |  |  |
| GO:0070670\_response\_to\_interleukin-4 | 1 | 0 |  |  |  |  |  |  |  |  |
| GO:0070671\_response\_to\_interleukin-12 | 1 | 0 |  |  |  |  |  |  |  |  |
| GO:0070672\_response\_to\_interleukin-15 | 1 | 0 |  |  |  |  |  |  |  |  |
| GO:0070673\_response\_to\_interleukin-18 | 1 | 0 |  |  |  |  |  |  |  |  |
| GO:0070828\_heterochromatin\_organization | 1 | 0 |  |  |  |  |  |  |  |  |
| GO:0070874\_negative\_regulation\_of\_glycogen\_metabolic\_process | 1 | 0 |  |  |  |  |  |  |  |  |
| GO:0075136\_response\_to\_host | 1 | 0 |  |  |  |  |  |  |  |  |
| GO:0080010\_regulation\_of\_oxygen\_and\_reactive\_oxygen\_species\_metabolic\_process | 1 | 0 |  |  |  |  |  |  |  |  |
| GO:0090032\_negative\_regulation\_of\_steroid\_hormone\_biosynthetic\_process | 1 | 0 |  |  |  |  |  |  |  |  |
| GO:0000084\_S\_phase\_of\_mitotic\_cell\_cycle | 3 | 0 |  |  |  |  |  |  |  |  |
| GO:0000089\_mitotic\_metaphase | 3 | 0 |  |  |  |  |  |  |  |  |
| GO:0000098\_sulfur\_amino\_acid\_catabolic\_process | 3 | 0 |  |  |  |  |  |  |  |  |
| GO:0000103\_sulfate\_assimilation | 3 | 0 |  |  |  |  |  |  |  |  |
| GO:0000212\_meiotic\_spindle\_organization | 3 | 0 |  |  |  |  |  |  |  |  |
| GO:0000281\_cytokinesis\_after\_mitosis | 3 | 0 |  |  |  |  |  |  |  |  |
| GO:0000303\_response\_to\_superoxide | 3 | 0 |  |  |  |  |  |  |  |  |
| GO:0000320\_re-entry\_into\_mitotic\_cell\_cycle | 3 | 0 |  |  |  |  |  |  |  |  |
| GO:0000380\_alternative\_nuclear\_mRNA\_splicing\_\_via\_spliceosome | 3 | 0 |  |  |  |  |  |  |  |  |
| GO:0001516\_prostaglandin\_biosynthetic\_process | 3 | 0 |  |  |  |  |  |  |  |  |
| GO:0001553\_luteinization | 3 | 0 |  |  |  |  |  |  |  |  |
| GO:0001574\_ganglioside\_biosynthetic\_process | 3 | 0 |  |  |  |  |  |  |  |  |
| GO:0001705\_ectoderm\_formation | 3 | 0 |  |  |  |  |  |  |  |  |
| GO:0001711\_endodermal\_cell\_fate\_commitment | 3 | 0 |  |  |  |  |  |  |  |  |
| GO:0001757\_somite\_specification | 3 | 0 |  |  |  |  |  |  |  |  |
| GO:0001778\_plasma\_membrane\_repair | 3 | 0 |  |  |  |  |  |  |  |  |
| GO:0001780\_neutrophil\_homeostasis | 3 | 0 |  |  |  |  |  |  |  |  |
| GO:0001802\_type\_III\_hypersensitivity | 3 | 0 |  |  |  |  |  |  |  |  |
| GO:0001803\_regulation\_of\_type\_III\_hypersensitivity | 3 | 0 |  |  |  |  |  |  |  |  |
| GO:0001805\_positive\_regulation\_of\_type\_III\_hypersensitivity | 3 | 0 |  |  |  |  |  |  |  |  |
| GO:0001812\_positive\_regulation\_of\_type\_I\_hypersensitivity | 3 | 0 |  |  |  |  |  |  |  |  |
| GO:0001831\_trophectodermal\_cellular\_morphogenesis | 3 | 0 |  |  |  |  |  |  |  |  |
| GO:0001844\_protein\_insertion\_into\_mitochondrial\_membrane\_during\_induction\_of\_apoptosis | 3 | 0 |  |  |  |  |  |  |  |  |
| GO:0001878\_response\_to\_yeast | 3 | 0 |  |  |  |  |  |  |  |  |
| GO:0001895\_retina\_homeostasis | 3 | 0 |  |  |  |  |  |  |  |  |
| GO:0001915\_negative\_regulation\_of\_T\_cell\_mediated\_cytotoxicity | 3 | 0 |  |  |  |  |  |  |  |  |
| GO:0001937\_negative\_regulation\_of\_endothelial\_cell\_proliferation | 3 | 0 |  |  |  |  |  |  |  |  |
| GO:0001953\_negative\_regulation\_of\_cell-matrix\_adhesion | 3 | 0 |  |  |  |  |  |  |  |  |
| GO:0001955\_blood\_vessel\_maturation | 3 | 0 |  |  |  |  |  |  |  |  |
| GO:0001960\_negative\_regulation\_of\_cytokine-mediated\_signaling\_pathway | 3 | 0 |  |  |  |  |  |  |  |  |
| GO:0001973\_adenosine\_receptor\_signaling\_pathway | 3 | 0 |  |  |  |  |  |  |  |  |
| GO:0001996\_positive\_regulation\_of\_heart\_rate\_by\_epinephrine-norepinephrine | 3 | 0 |  |  |  |  |  |  |  |  |
| GO:0002034\_regulation\_of\_blood\_vessel\_size\_by\_renin-angiotensin | 3 | 0 |  |  |  |  |  |  |  |  |
| GO:0002238\_response\_to\_molecule\_of\_fungal\_origin | 3 | 0 |  |  |  |  |  |  |  |  |
| GO:0002275\_myeloid\_cell\_activation\_during\_immune\_response | 3 | 0 |  |  |  |  |  |  |  |  |
| GO:0002281\_macrophage\_activation\_during\_immune\_response | 3 | 0 |  |  |  |  |  |  |  |  |
| GO:0002309\_T\_cell\_proliferation\_during\_immune\_response | 3 | 0 |  |  |  |  |  |  |  |  |
| GO:0002361\_CD4-positive\_\_CD25-positive\_\_alpha-beta\_regulatory\_T\_cell\_differentiation | 3 | 0 |  |  |  |  |  |  |  |  |
| GO:0002369\_T\_cell\_cytokine\_production | 3 | 0 |  |  |  |  |  |  |  |  |
| GO:0002428\_antigen\_processing\_and\_presentation\_of\_peptide\_antigen\_via\_MHC\_class\_Ib | 3 | 0 |  |  |  |  |  |  |  |  |
| GO:0002446\_neutrophil\_mediated\_immunity | 3 | 0 |  |  |  |  |  |  |  |  |
| GO:0002477\_antigen\_processing\_and\_presentation\_of\_exogenous\_peptide\_antigen\_via\_MHC\_class\_Ib | 3 | 0 |  |  |  |  |  |  |  |  |
| GO:0002481\_antigen\_processing\_and\_presentation\_of\_exogenous\_protein\_antigen\_via\_MHC\_class\_Ib\_\_TAP-dependent | 3 | 0 |  |  |  |  |  |  |  |  |
| GO:0002513\_tolerance\_induction\_to\_self\_antigen | 3 | 0 |  |  |  |  |  |  |  |  |
| GO:0002568\_somatic\_diversification\_of\_T\_cell\_receptor\_genes | 3 | 0 |  |  |  |  |  |  |  |  |
| GO:0002674\_negative\_regulation\_of\_acute\_inflammatory\_response | 3 | 0 |  |  |  |  |  |  |  |  |
| GO:0002681\_somatic\_recombination\_of\_T\_cell\_receptor\_gene\_segments | 3 | 0 |  |  |  |  |  |  |  |  |
| GO:0002713\_negative\_regulation\_of\_B\_cell\_mediated\_immunity | 3 | 0 |  |  |  |  |  |  |  |  |
| GO:0002827\_positive\_regulation\_of\_T-helper\_1\_type\_immune\_response | 3 | 0 |  |  |  |  |  |  |  |  |
| GO:0002865\_negative\_regulation\_of\_acute\_inflammatory\_response\_to\_antigenic\_stimulus | 3 | 0 |  |  |  |  |  |  |  |  |
| GO:0002884\_negative\_regulation\_of\_hypersensitivity | 3 | 0 |  |  |  |  |  |  |  |  |
| GO:0002890\_negative\_regulation\_of\_immunoglobulin\_mediated\_immune\_response | 3 | 0 |  |  |  |  |  |  |  |  |
| GO:0002904\_positive\_regulation\_of\_B\_cell\_apoptosis | 3 | 0 |  |  |  |  |  |  |  |  |
| GO:0003009\_skeletal\_muscle\_contraction | 3 | 0 |  |  |  |  |  |  |  |  |
| GO:0003072\_renal\_control\_of\_peripheral\_vascular\_resistance\_involved\_in\_regulation\_of\_systemic\_arterial\_blood\_pressure | 3 | 0 |  |  |  |  |  |  |  |  |
| GO:0006047\_UDP-N-acetylglucosamine\_metabolic\_process | 3 | 0 |  |  |  |  |  |  |  |  |
| GO:0006067\_ethanol\_metabolic\_process | 3 | 0 |  |  |  |  |  |  |  |  |
| GO:0006072\_glycerol-3-phosphate\_metabolic\_process | 3 | 0 |  |  |  |  |  |  |  |  |
| GO:0006103\_2-oxoglutarate\_metabolic\_process | 3 | 0 |  |  |  |  |  |  |  |  |
| GO:0006107\_oxaloacetate\_metabolic\_process | 3 | 0 |  |  |  |  |  |  |  |  |
| GO:0006166\_purine\_ribonucleoside\_salvage | 3 | 0 |  |  |  |  |  |  |  |  |
| GO:0006220\_pyrimidine\_nucleotide\_metabolic\_process | 3 | 0 |  |  |  |  |  |  |  |  |
| GO:0006266\_DNA\_ligation | 3 | 0 |  |  |  |  |  |  |  |  |
| GO:0006282\_regulation\_of\_DNA\_repair | 3 | 0 |  |  |  |  |  |  |  |  |
| GO:0006287\_base-excision\_repair\_\_gap-filling | 3 | 0 |  |  |  |  |  |  |  |  |
| GO:0006301\_postreplication\_repair | 3 | 0 |  |  |  |  |  |  |  |  |
| GO:0006361\_transcription\_initiation\_from\_RNA\_polymerase\_I\_promoter | 3 | 0 |  |  |  |  |  |  |  |  |
| GO:0006367\_transcription\_initiation\_from\_RNA\_polymerase\_II\_promoter | 3 | 0 |  |  |  |  |  |  |  |  |
| GO:0006414\_translational\_elongation | 3 | 0 |  |  |  |  |  |  |  |  |
| GO:0006491\_N-glycan\_processing | 3 | 0 |  |  |  |  |  |  |  |  |
| GO:0006498\_N-terminal\_protein\_lipidation | 3 | 0 |  |  |  |  |  |  |  |  |
| GO:0006531\_aspartate\_metabolic\_process | 3 | 0 |  |  |  |  |  |  |  |  |
| GO:0006598\_polyamine\_catabolic\_process | 3 | 0 |  |  |  |  |  |  |  |  |
| GO:0006620\_posttranslational\_protein\_targeting\_to\_membrane | 3 | 0 |  |  |  |  |  |  |  |  |
| GO:0006625\_protein\_targeting\_to\_peroxisome | 3 | 0 |  |  |  |  |  |  |  |  |
| GO:0006651\_diacylglycerol\_biosynthetic\_process | 3 | 0 |  |  |  |  |  |  |  |  |
| GO:0006670\_sphingosine\_metabolic\_process | 3 | 0 |  |  |  |  |  |  |  |  |
| GO:0006677\_glycosylceramide\_metabolic\_process | 3 | 0 |  |  |  |  |  |  |  |  |
| GO:0006689\_ganglioside\_catabolic\_process | 3 | 0 |  |  |  |  |  |  |  |  |
| GO:0006699\_bile\_acid\_biosynthetic\_process | 3 | 0 |  |  |  |  |  |  |  |  |
| GO:0006791\_sulfur\_utilization | 3 | 0 |  |  |  |  |  |  |  |  |
| GO:0006817\_phosphate\_transport | 3 | 0 |  |  |  |  |  |  |  |  |
| GO:0006825\_copper\_ion\_transport | 3 | 0 |  |  |  |  |  |  |  |  |
| GO:0006828\_manganese\_ion\_transport | 3 | 0 |  |  |  |  |  |  |  |  |
| GO:0006857\_oligopeptide\_transport | 3 | 0 |  |  |  |  |  |  |  |  |
| GO:0006892\_post-Golgi\_vesicle-mediated\_transport | 3 | 0 |  |  |  |  |  |  |  |  |
| GO:0006904\_vesicle\_docking\_during\_exocytosis | 3 | 0 |  |  |  |  |  |  |  |  |
| GO:0006926\_virus-infected\_cell\_apoptosis | 3 | 0 |  |  |  |  |  |  |  |  |
| GO:0006953\_acute-phase\_response | 3 | 0 |  |  |  |  |  |  |  |  |
| GO:0007000\_nucleolus\_organization | 3 | 0 |  |  |  |  |  |  |  |  |
| GO:0007041\_lysosomal\_transport | 3 | 0 |  |  |  |  |  |  |  |  |
| GO:0007043\_cell-cell\_junction\_assembly | 3 | 0 |  |  |  |  |  |  |  |  |
| GO:0007090\_regulation\_of\_S\_phase\_of\_mitotic\_cell\_cycle | 3 | 0 |  |  |  |  |  |  |  |  |
| GO:0007195\_inhibition\_of\_adenylate\_cyclase\_activity\_by\_dopamine\_receptor\_signaling\_pathway | 3 | 0 |  |  |  |  |  |  |  |  |
| GO:0007199\_G-protein\_signaling\_\_coupled\_to\_cGMP\_nucleotide\_second\_messenger | 3 | 0 |  |  |  |  |  |  |  |  |
| GO:0007213\_muscarinic\_acetylcholine\_receptor\_signaling\_pathway | 3 | 0 |  |  |  |  |  |  |  |  |
| GO:0007250\_activation\_of\_NF-kappaB-inducing\_kinase\_activity | 3 | 0 |  |  |  |  |  |  |  |  |
| GO:0007252\_I-kappaB\_phosphorylation | 3 | 0 |  |  |  |  |  |  |  |  |
| GO:0007262\_STAT\_protein\_nuclear\_translocation | 3 | 0 |  |  |  |  |  |  |  |  |
| GO:0007288\_sperm\_axoneme\_assembly | 3 | 0 |  |  |  |  |  |  |  |  |
| GO:0007350\_blastoderm\_segmentation | 3 | 0 |  |  |  |  |  |  |  |  |
| GO:0007403\_glial\_cell\_fate\_determination | 3 | 0 |  |  |  |  |  |  |  |  |
| GO:0007412\_axon\_target\_recognition | 3 | 0 |  |  |  |  |  |  |  |  |
| GO:0007468\_regulation\_of\_rhodopsin\_gene\_expression | 3 | 0 |  |  |  |  |  |  |  |  |
| GO:0007525\_somatic\_muscle\_development | 3 | 0 |  |  |  |  |  |  |  |  |
| GO:0007635\_chemosensory\_behavior | 3 | 0 |  |  |  |  |  |  |  |  |
| GO:0008090\_retrograde\_axon\_cargo\_transport | 3 | 0 |  |  |  |  |  |  |  |  |
| GO:0009060\_aerobic\_respiration | 3 | 0 |  |  |  |  |  |  |  |  |
| GO:0009081\_branched\_chain\_family\_amino\_acid\_metabolic\_process | 3 | 0 |  |  |  |  |  |  |  |  |
| GO:0009086\_methionine\_biosynthetic\_process | 3 | 0 |  |  |  |  |  |  |  |  |
| GO:0009135\_purine\_nucleoside\_diphosphate\_metabolic\_process | 3 | 0 |  |  |  |  |  |  |  |  |
| GO:0009137\_purine\_nucleoside\_diphosphate\_catabolic\_process | 3 | 0 |  |  |  |  |  |  |  |  |
| GO:0009155\_purine\_deoxyribonucleotide\_catabolic\_process | 3 | 0 |  |  |  |  |  |  |  |  |
| GO:0009179\_purine\_ribonucleoside\_diphosphate\_metabolic\_process | 3 | 0 |  |  |  |  |  |  |  |  |
| GO:0009181\_purine\_ribonucleoside\_diphosphate\_catabolic\_process | 3 | 0 |  |  |  |  |  |  |  |  |
| GO:0009185\_ribonucleoside\_diphosphate\_metabolic\_process | 3 | 0 |  |  |  |  |  |  |  |  |
| GO:0009191\_ribonucleoside\_diphosphate\_catabolic\_process | 3 | 0 |  |  |  |  |  |  |  |  |
| GO:0009199\_ribonucleoside\_triphosphate\_metabolic\_process | 3 | 0 |  |  |  |  |  |  |  |  |
| GO:0009204\_deoxyribonucleoside\_triphosphate\_catabolic\_process | 3 | 0 |  |  |  |  |  |  |  |  |
| GO:0009205\_purine\_ribonucleoside\_triphosphate\_metabolic\_process | 3 | 0 |  |  |  |  |  |  |  |  |
| GO:0009217\_purine\_deoxyribonucleoside\_triphosphate\_catabolic\_process | 3 | 0 |  |  |  |  |  |  |  |  |
| GO:0009448\_gamma-aminobutyric\_acid\_metabolic\_process | 3 | 0 |  |  |  |  |  |  |  |  |
| GO:0010043\_response\_to\_zinc\_ion | 3 | 0 |  |  |  |  |  |  |  |  |
| GO:0010159\_specification\_of\_organ\_position | 3 | 0 |  |  |  |  |  |  |  |  |
| GO:0010172\_embryonic\_body\_morphogenesis | 3 | 0 |  |  |  |  |  |  |  |  |
| GO:0010216\_maintenance\_of\_DNA\_methylation | 3 | 0 |  |  |  |  |  |  |  |  |
| GO:0010273\_detoxification\_of\_copper\_ion | 3 | 0 |  |  |  |  |  |  |  |  |
| GO:0010454\_negative\_regulation\_of\_cell\_fate\_commitment | 3 | 0 |  |  |  |  |  |  |  |  |
| GO:0010507\_negative\_regulation\_of\_autophagy | 3 | 0 |  |  |  |  |  |  |  |  |
| GO:0010524\_positive\_regulation\_of\_calcium\_ion\_transport\_into\_cytosol | 3 | 0 |  |  |  |  |  |  |  |  |
| GO:0010573\_vascular\_endothelial\_growth\_factor\_production | 3 | 0 |  |  |  |  |  |  |  |  |
| GO:0010574\_regulation\_of\_vascular\_endothelial\_growth\_factor\_production | 3 | 0 |  |  |  |  |  |  |  |  |
| GO:0010575\_positive\_regulation\_vascular\_endothelial\_growth\_factor\_production | 3 | 0 |  |  |  |  |  |  |  |  |
| GO:0010632\_regulation\_of\_epithelial\_cell\_migration | 3 | 0 |  |  |  |  |  |  |  |  |
| GO:0010717\_regulation\_of\_epithelial\_to\_mesenchymal\_transition | 3 | 0 |  |  |  |  |  |  |  |  |
| GO:0010884\_positive\_regulation\_of\_lipid\_storage | 3 | 0 |  |  |  |  |  |  |  |  |
| GO:0010888\_negative\_regulation\_of\_lipid\_storage | 3 | 0 |  |  |  |  |  |  |  |  |
| GO:0010889\_regulation\_of\_sequestering\_of\_triglyceride | 3 | 0 |  |  |  |  |  |  |  |  |
| GO:0010893\_positive\_regulation\_of\_steroid\_biosynthetic\_process | 3 | 0 |  |  |  |  |  |  |  |  |
| GO:0010894\_negative\_regulation\_of\_steroid\_biosynthetic\_process | 3 | 0 |  |  |  |  |  |  |  |  |
| GO:0010998\_regulation\_of\_translational\_initiation\_by\_eIF2\_alpha\_phosphorylation | 3 | 0 |  |  |  |  |  |  |  |  |
| GO:0010999\_regulation\_of\_eIF2\_alpha\_phosphorylation\_by\_heme | 3 | 0 |  |  |  |  |  |  |  |  |
| GO:0014909\_smooth\_muscle\_cell\_migration | 3 | 0 |  |  |  |  |  |  |  |  |
| GO:0015669\_gas\_transport | 3 | 0 |  |  |  |  |  |  |  |  |
| GO:0015760\_glucose-6-phosphate\_transport | 3 | 0 |  |  |  |  |  |  |  |  |
| GO:0015816\_glycine\_transport | 3 | 0 |  |  |  |  |  |  |  |  |
| GO:0015838\_betaine\_transport | 3 | 0 |  |  |  |  |  |  |  |  |
| GO:0015871\_choline\_transport | 3 | 0 |  |  |  |  |  |  |  |  |
| GO:0015879\_carnitine\_transport | 3 | 0 |  |  |  |  |  |  |  |  |
| GO:0015893\_drug\_transport | 3 | 0 |  |  |  |  |  |  |  |  |
| GO:0015909\_long-chain\_fatty\_acid\_transport | 3 | 0 |  |  |  |  |  |  |  |  |
| GO:0015936\_coenzyme\_A\_metabolic\_process | 3 | 0 |  |  |  |  |  |  |  |  |
| GO:0015988\_energy\_coupled\_proton\_transport\_\_against\_electrochemical\_gradient | 3 | 0 |  |  |  |  |  |  |  |  |
| GO:0015991\_ATP\_hydrolysis\_coupled\_proton\_transport | 3 | 0 |  |  |  |  |  |  |  |  |
| GO:0016241\_regulation\_of\_macroautophagy | 3 | 0 |  |  |  |  |  |  |  |  |
| GO:0016322\_neuron\_remodeling | 3 | 0 |  |  |  |  |  |  |  |  |
| GO:0016556\_mRNA\_modification | 3 | 0 |  |  |  |  |  |  |  |  |
| GO:0016973\_poly(A)+\_mRNA\_export\_from\_nucleus | 3 | 0 |  |  |  |  |  |  |  |  |
| GO:0018196\_peptidyl-asparagine\_modification | 3 | 0 |  |  |  |  |  |  |  |  |
| GO:0018208\_peptidyl-proline\_modification | 3 | 0 |  |  |  |  |  |  |  |  |
| GO:0018279\_protein\_amino\_acid\_N-linked\_glycosylation\_via\_asparagine | 3 | 0 |  |  |  |  |  |  |  |  |
| GO:0018894\_dibenzo-p-dioxin\_metabolic\_process | 3 | 0 |  |  |  |  |  |  |  |  |
| GO:0019058\_viral\_infectious\_cycle | 3 | 0 |  |  |  |  |  |  |  |  |
| GO:0019230\_proprioception | 3 | 0 |  |  |  |  |  |  |  |  |
| GO:0019236\_response\_to\_pheromone | 3 | 0 |  |  |  |  |  |  |  |  |
| GO:0019359\_nicotinamide\_nucleotide\_biosynthetic\_process | 3 | 0 |  |  |  |  |  |  |  |  |
| GO:0019363\_pyridine\_nucleotide\_biosynthetic\_process | 3 | 0 |  |  |  |  |  |  |  |  |
| GO:0019438\_aromatic\_compound\_biosynthetic\_process | 3 | 0 |  |  |  |  |  |  |  |  |
| GO:0019439\_aromatic\_compound\_catabolic\_process | 3 | 0 |  |  |  |  |  |  |  |  |
| GO:0019605\_butyrate\_metabolic\_process | 3 | 0 |  |  |  |  |  |  |  |  |
| GO:0019614\_catechol\_catabolic\_process | 3 | 0 |  |  |  |  |  |  |  |  |
| GO:0019674\_NAD\_metabolic\_process | 3 | 0 |  |  |  |  |  |  |  |  |
| GO:0019852\_L-ascorbic\_acid\_metabolic\_process | 3 | 0 |  |  |  |  |  |  |  |  |
| GO:0019934\_cGMP-mediated\_signaling | 3 | 0 |  |  |  |  |  |  |  |  |
| GO:0019987\_negative\_regulation\_of\_anti-apoptosis | 3 | 0 |  |  |  |  |  |  |  |  |
| GO:0021527\_spinal\_cord\_association\_neuron\_differentiation | 3 | 0 |  |  |  |  |  |  |  |  |
| GO:0021529\_spinal\_cord\_oligodendrocyte\_cell\_differentiation | 3 | 0 |  |  |  |  |  |  |  |  |
| GO:0021530\_spinal\_cord\_oligodendrocyte\_cell\_fate\_specification | 3 | 0 |  |  |  |  |  |  |  |  |
| GO:0021555\_midbrain-hindbrain\_boundary\_morphogenesis | 3 | 0 |  |  |  |  |  |  |  |  |
| GO:0021563\_glossopharyngeal\_nerve\_development | 3 | 0 |  |  |  |  |  |  |  |  |
| GO:0021570\_rhombomere\_4\_development | 3 | 0 |  |  |  |  |  |  |  |  |
| GO:0021591\_ventricular\_system\_development | 3 | 0 |  |  |  |  |  |  |  |  |
| GO:0021615\_glossopharyngeal\_nerve\_morphogenesis | 3 | 0 |  |  |  |  |  |  |  |  |
| GO:0021794\_thalamus\_development | 3 | 0 |  |  |  |  |  |  |  |  |
| GO:0021797\_forebrain\_anterior\_posterior\_pattern\_formation | 3 | 0 |  |  |  |  |  |  |  |  |
| GO:0021798\_forebrain\_dorsal\_ventral\_pattern\_formation | 3 | 0 |  |  |  |  |  |  |  |  |
| GO:0021800\_cerebral\_cortex\_tangential\_migration | 3 | 0 |  |  |  |  |  |  |  |  |
| GO:0021819\_layer\_formation\_in\_the\_cerebral\_cortex | 3 | 0 |  |  |  |  |  |  |  |  |
| GO:0021859\_pyramidal\_neuron\_differentiation | 3 | 0 |  |  |  |  |  |  |  |  |
| GO:0021860\_pyramidal\_neuron\_development | 3 | 0 |  |  |  |  |  |  |  |  |
| GO:0021889\_olfactory\_bulb\_interneuron\_differentiation | 3 | 0 |  |  |  |  |  |  |  |  |
| GO:0021891\_olfactory\_bulb\_interneuron\_development | 3 | 0 |  |  |  |  |  |  |  |  |
| GO:0021912\_regulation\_of\_transcription\_from\_RNA\_polymerase\_II\_promoter\_involved\_in\_spinal\_cord\_motor\_neuron\_fate\_specification | 3 | 0 |  |  |  |  |  |  |  |  |
| GO:0021979\_hypothalamus\_cell\_differentiation | 3 | 0 |  |  |  |  |  |  |  |  |
| GO:0022010\_myelination\_in\_the\_central\_nervous\_system | 3 | 0 |  |  |  |  |  |  |  |  |
| GO:0022027\_interkinetic\_nuclear\_migration | 3 | 0 |  |  |  |  |  |  |  |  |
| GO:0022406\_membrane\_docking | 3 | 0 |  |  |  |  |  |  |  |  |
| GO:0030033\_microvillus\_assembly | 3 | 0 |  |  |  |  |  |  |  |  |
| GO:0030091\_protein\_repair | 3 | 0 |  |  |  |  |  |  |  |  |
| GO:0030195\_negative\_regulation\_of\_blood\_coagulation | 3 | 0 |  |  |  |  |  |  |  |  |
| GO:0030224\_monocyte\_differentiation | 3 | 0 |  |  |  |  |  |  |  |  |
| GO:0030307\_positive\_regulation\_of\_cell\_growth | 3 | 0 |  |  |  |  |  |  |  |  |
| GO:0030319\_cellular\_di-\_\_tri-valent\_inorganic\_anion\_homeostasis | 3 | 0 |  |  |  |  |  |  |  |  |
| GO:0030320\_cellular\_monovalent\_inorganic\_anion\_homeostasis | 3 | 0 |  |  |  |  |  |  |  |  |
| GO:0030321\_transepithelial\_chloride\_transport | 3 | 0 |  |  |  |  |  |  |  |  |
| GO:0030501\_positive\_regulation\_of\_bone\_mineralization | 3 | 0 |  |  |  |  |  |  |  |  |
| GO:0030513\_positive\_regulation\_of\_BMP\_signaling\_pathway | 3 | 0 |  |  |  |  |  |  |  |  |
| GO:0030538\_embryonic\_genitalia\_morphogenesis | 3 | 0 |  |  |  |  |  |  |  |  |
| GO:0030540\_female\_genitalia\_development | 3 | 0 |  |  |  |  |  |  |  |  |
| GO:0030574\_collagen\_catabolic\_process | 3 | 0 |  |  |  |  |  |  |  |  |
| GO:0030643\_cellular\_phosphate\_ion\_homeostasis | 3 | 0 |  |  |  |  |  |  |  |  |
| GO:0030718\_germ-line\_stem\_cell\_maintenance | 3 | 0 |  |  |  |  |  |  |  |  |
| GO:0030730\_sequestering\_of\_triglyceride | 3 | 0 |  |  |  |  |  |  |  |  |
| GO:0030857\_negative\_regulation\_of\_epithelial\_cell\_differentiation | 3 | 0 |  |  |  |  |  |  |  |  |
| GO:0030916\_otic\_vesicle\_formation | 3 | 0 |  |  |  |  |  |  |  |  |
| GO:0031063\_regulation\_of\_histone\_deacetylation | 3 | 0 |  |  |  |  |  |  |  |  |
| GO:0031065\_positive\_regulation\_of\_histone\_deacetylation | 3 | 0 |  |  |  |  |  |  |  |  |
| GO:0031112\_positive\_regulation\_of\_microtubule\_polymerization\_or\_depolymerization | 3 | 0 |  |  |  |  |  |  |  |  |
| GO:0031116\_positive\_regulation\_of\_microtubule\_polymerization | 3 | 0 |  |  |  |  |  |  |  |  |
| GO:0031133\_regulation\_of\_axon\_diameter | 3 | 0 |  |  |  |  |  |  |  |  |
| GO:0031282\_regulation\_of\_guanylate\_cyclase\_activity | 3 | 0 |  |  |  |  |  |  |  |  |
| GO:0031333\_negative\_regulation\_of\_protein\_complex\_assembly | 3 | 0 |  |  |  |  |  |  |  |  |
| GO:0031397\_negative\_regulation\_of\_protein\_ubiquitination | 3 | 0 |  |  |  |  |  |  |  |  |
| GO:0031398\_positive\_regulation\_of\_protein\_ubiquitination | 3 | 0 |  |  |  |  |  |  |  |  |
| GO:0031503\_protein\_complex\_localization | 3 | 0 |  |  |  |  |  |  |  |  |
| GO:0031571\_G1\_DNA\_damage\_checkpoint | 3 | 0 |  |  |  |  |  |  |  |  |
| GO:0031579\_membrane\_raft\_organization | 3 | 0 |  |  |  |  |  |  |  |  |
| GO:0031638\_zymogen\_activation | 3 | 0 |  |  |  |  |  |  |  |  |
| GO:0031641\_regulation\_of\_myelination | 3 | 0 |  |  |  |  |  |  |  |  |
| GO:0031642\_negative\_regulation\_of\_myelination | 3 | 0 |  |  |  |  |  |  |  |  |
| GO:0031649\_heat\_generation | 3 | 0 |  |  |  |  |  |  |  |  |
| GO:0031943\_regulation\_of\_glucocorticoid\_metabolic\_process | 3 | 0 |  |  |  |  |  |  |  |  |
| GO:0032020\_ISG15-protein\_conjugation | 3 | 0 |  |  |  |  |  |  |  |  |
| GO:0032060\_bleb\_formation | 3 | 0 |  |  |  |  |  |  |  |  |
| GO:0032095\_regulation\_of\_response\_to\_food | 3 | 0 |  |  |  |  |  |  |  |  |
| GO:0032272\_negative\_regulation\_of\_protein\_polymerization | 3 | 0 |  |  |  |  |  |  |  |  |
| GO:0032288\_myelin\_assembly | 3 | 0 |  |  |  |  |  |  |  |  |
| GO:0032291\_ensheathment\_of\_axons\_in\_the\_central\_nervous\_system | 3 | 0 |  |  |  |  |  |  |  |  |
| GO:0032355\_response\_to\_estradiol\_stimulus | 3 | 0 |  |  |  |  |  |  |  |  |
| GO:0032402\_melanosome\_transport | 3 | 0 |  |  |  |  |  |  |  |  |
| GO:0032411\_positive\_regulation\_of\_transporter\_activity | 3 | 0 |  |  |  |  |  |  |  |  |
| GO:0032414\_positive\_regulation\_of\_ion\_transmembrane\_transporter\_activity | 3 | 0 |  |  |  |  |  |  |  |  |
| GO:0032436\_positive\_regulation\_of\_proteasomal\_ubiquitin-dependent\_protein\_catabolic\_process | 3 | 0 |  |  |  |  |  |  |  |  |
| GO:0032528\_microvillus\_organization | 3 | 0 |  |  |  |  |  |  |  |  |
| GO:0032536\_regulation\_of\_cell\_projection\_size | 3 | 0 |  |  |  |  |  |  |  |  |
| GO:0032632\_interleukin-3\_production | 3 | 0 |  |  |  |  |  |  |  |  |
| GO:0032634\_interleukin-5\_production | 3 | 0 |  |  |  |  |  |  |  |  |
| GO:0032674\_regulation\_of\_interleukin-5\_production | 3 | 0 |  |  |  |  |  |  |  |  |
| GO:0032703\_negative\_regulation\_of\_interleukin-2\_production | 3 | 0 |  |  |  |  |  |  |  |  |
| GO:0032753\_positive\_regulation\_of\_interleukin-4\_production | 3 | 0 |  |  |  |  |  |  |  |  |
| GO:0032823\_regulation\_of\_natural\_killer\_cell\_differentiation | 3 | 0 |  |  |  |  |  |  |  |  |
| GO:0032825\_positive\_regulation\_of\_natural\_killer\_cell\_differentiation | 3 | 0 |  |  |  |  |  |  |  |  |
| GO:0032856\_activation\_of\_Ras\_GTPase\_activity | 3 | 0 |  |  |  |  |  |  |  |  |
| GO:0032862\_activation\_of\_Rho\_GTPase\_activity | 3 | 0 |  |  |  |  |  |  |  |  |
| GO:0032874\_positive\_regulation\_of\_stress-activated\_MAPK\_cascade | 3 | 0 |  |  |  |  |  |  |  |  |
| GO:0032881\_regulation\_of\_polysaccharide\_metabolic\_process | 3 | 0 |  |  |  |  |  |  |  |  |
| GO:0032890\_regulation\_of\_organic\_acid\_transport | 3 | 0 |  |  |  |  |  |  |  |  |
| GO:0033058\_directional\_locomotion | 3 | 0 |  |  |  |  |  |  |  |  |
| GO:0033080\_immature\_T\_cell\_proliferation\_in\_the\_thymus | 3 | 0 |  |  |  |  |  |  |  |  |
| GO:0033084\_regulation\_of\_immature\_T\_cell\_proliferation\_in\_the\_thymus | 3 | 0 |  |  |  |  |  |  |  |  |
| GO:0033091\_positive\_regulation\_of\_immature\_T\_cell\_proliferation | 3 | 0 |  |  |  |  |  |  |  |  |
| GO:0033137\_negative\_regulation\_of\_peptidyl-serine\_phosphorylation | 3 | 0 |  |  |  |  |  |  |  |  |
| GO:0033153\_T\_cell\_receptor\_V(D)J\_recombination | 3 | 0 |  |  |  |  |  |  |  |  |
| GO:0033209\_tumor\_necrosis\_factor-mediated\_signaling\_pathway | 3 | 0 |  |  |  |  |  |  |  |  |
| GO:0033261\_regulation\_of\_S\_phase | 3 | 0 |  |  |  |  |  |  |  |  |
| GO:0033600\_negative\_regulation\_of\_mammary\_gland\_epithelial\_cell\_proliferation | 3 | 0 |  |  |  |  |  |  |  |  |
| GO:0033631\_cell-cell\_adhesion\_mediated\_by\_integrin | 3 | 0 |  |  |  |  |  |  |  |  |
| GO:0033993\_response\_to\_lipid | 3 | 0 |  |  |  |  |  |  |  |  |
| GO:0034220\_ion\_transmembrane\_transport | 3 | 0 |  |  |  |  |  |  |  |  |
| GO:0034308\_monohydric\_alcohol\_metabolic\_process | 3 | 0 |  |  |  |  |  |  |  |  |
| GO:0034313\_diol\_catabolic\_process | 3 | 0 |  |  |  |  |  |  |  |  |
| GO:0034331\_cell\_junction\_maintenance | 3 | 0 |  |  |  |  |  |  |  |  |
| GO:0034332\_adherens\_junction\_organization | 3 | 0 |  |  |  |  |  |  |  |  |
| GO:0034375\_high-density\_lipoprotein\_particle\_remodeling | 3 | 0 |  |  |  |  |  |  |  |  |
| GO:0034381\_lipoprotein\_particle\_clearance | 3 | 0 |  |  |  |  |  |  |  |  |
| GO:0034612\_response\_to\_tumor\_necrosis\_factor | 3 | 0 |  |  |  |  |  |  |  |  |
| GO:0034655\_nucleobase\_\_nucleoside\_\_nucleotide\_and\_nucleic\_acid\_catabolic\_process | 3 | 0 |  |  |  |  |  |  |  |  |
| GO:0034656\_nucleobase\_\_nucleoside\_and\_nucleotide\_catabolic\_process | 3 | 0 |  |  |  |  |  |  |  |  |
| GO:0035067\_negative\_regulation\_of\_histone\_acetylation | 3 | 0 |  |  |  |  |  |  |  |  |
| GO:0035084\_flagellar\_axoneme\_assembly | 3 | 0 |  |  |  |  |  |  |  |  |
| GO:0035166\_post-embryonic\_hemopoiesis | 3 | 0 |  |  |  |  |  |  |  |  |
| GO:0035283\_central\_nervous\_system\_segmentation | 3 | 0 |  |  |  |  |  |  |  |  |
| GO:0035284\_brain\_segmentation | 3 | 0 |  |  |  |  |  |  |  |  |
| GO:0042097\_interleukin-4\_biosynthetic\_process | 3 | 0 |  |  |  |  |  |  |  |  |
| GO:0042135\_neurotransmitter\_catabolic\_process | 3 | 0 |  |  |  |  |  |  |  |  |
| GO:0042271\_susceptibility\_to\_natural\_killer\_cell\_mediated\_cytotoxicity | 3 | 0 |  |  |  |  |  |  |  |  |
| GO:0042273\_ribosomal\_large\_subunit\_biogenesis | 3 | 0 |  |  |  |  |  |  |  |  |
| GO:0042375\_quinone\_cofactor\_metabolic\_process | 3 | 0 |  |  |  |  |  |  |  |  |
| GO:0042420\_dopamine\_catabolic\_process | 3 | 0 |  |  |  |  |  |  |  |  |
| GO:0042421\_norepinephrine\_biosynthetic\_process | 3 | 0 |  |  |  |  |  |  |  |  |
| GO:0042424\_catecholamine\_catabolic\_process | 3 | 0 |  |  |  |  |  |  |  |  |
| GO:0042447\_hormone\_catabolic\_process | 3 | 0 |  |  |  |  |  |  |  |  |
| GO:0042448\_progesterone\_metabolic\_process | 3 | 0 |  |  |  |  |  |  |  |  |
| GO:0042523\_positive\_regulation\_of\_tyrosine\_phosphorylation\_of\_Stat5\_protein | 3 | 0 |  |  |  |  |  |  |  |  |
| GO:0042659\_regulation\_of\_cell\_fate\_specification | 3 | 0 |  |  |  |  |  |  |  |  |
| GO:0042668\_auditory\_receptor\_cell\_fate\_determination | 3 | 0 |  |  |  |  |  |  |  |  |
| GO:0042670\_retinal\_cone\_cell\_differentiation | 3 | 0 |  |  |  |  |  |  |  |  |
| GO:0042693\_muscle\_cell\_fate\_commitment | 3 | 0 |  |  |  |  |  |  |  |  |
| GO:0042711\_maternal\_behavior | 3 | 0 |  |  |  |  |  |  |  |  |
| GO:0042745\_circadian\_sleep\_wake\_cycle | 3 | 0 |  |  |  |  |  |  |  |  |
| GO:0042759\_long-chain\_fatty\_acid\_biosynthetic\_process | 3 | 0 |  |  |  |  |  |  |  |  |
| GO:0042787\_protein\_ubiquitination\_during\_ubiquitin-dependent\_protein\_catabolic\_process | 3 | 0 |  |  |  |  |  |  |  |  |
| GO:0043045\_DNA\_methylation\_during\_embryonic\_development | 3 | 0 |  |  |  |  |  |  |  |  |
| GO:0043090\_amino\_acid\_import | 3 | 0 |  |  |  |  |  |  |  |  |
| GO:0043092\_L-amino\_acid\_import | 3 | 0 |  |  |  |  |  |  |  |  |
| GO:0043094\_cellular\_metabolic\_compound\_salvage | 3 | 0 |  |  |  |  |  |  |  |  |
| GO:0043101\_purine\_salvage | 3 | 0 |  |  |  |  |  |  |  |  |
| GO:0043149\_stress\_fiber\_formation | 3 | 0 |  |  |  |  |  |  |  |  |
| GO:0043174\_nucleoside\_salvage | 3 | 0 |  |  |  |  |  |  |  |  |
| GO:0043249\_erythrocyte\_maturation | 3 | 0 |  |  |  |  |  |  |  |  |
| GO:0043267\_negative\_regulation\_of\_potassium\_ion\_transport | 3 | 0 |  |  |  |  |  |  |  |  |
| GO:0043371\_negative\_regulation\_of\_CD4-positive\_\_alpha\_beta\_T\_cell\_differentiation | 3 | 0 |  |  |  |  |  |  |  |  |
| GO:0043462\_regulation\_of\_ATPase\_activity | 3 | 0 |  |  |  |  |  |  |  |  |
| GO:0043569\_negative\_regulation\_of\_insulin-like\_growth\_factor\_receptor\_signaling\_pathway | 3 | 0 |  |  |  |  |  |  |  |  |
| GO:0043574\_peroxisomal\_transport | 3 | 0 |  |  |  |  |  |  |  |  |
| GO:0043586\_tongue\_development | 3 | 0 |  |  |  |  |  |  |  |  |
| GO:0043900\_regulation\_of\_multi-organism\_process | 3 | 0 |  |  |  |  |  |  |  |  |
| GO:0043954\_cellular\_component\_maintenance | 3 | 0 |  |  |  |  |  |  |  |  |
| GO:0044030\_regulation\_of\_DNA\_methylation | 3 | 0 |  |  |  |  |  |  |  |  |
| GO:0044089\_positive\_regulation\_of\_cellular\_component\_biogenesis | 3 | 0 |  |  |  |  |  |  |  |  |
| GO:0044273\_sulfur\_compound\_catabolic\_process | 3 | 0 |  |  |  |  |  |  |  |  |
| GO:0045047\_protein\_targeting\_to\_ER | 3 | 0 |  |  |  |  |  |  |  |  |
| GO:0045085\_negative\_regulation\_of\_interleukin-2\_biosynthetic\_process | 3 | 0 |  |  |  |  |  |  |  |  |
| GO:0045110\_intermediate\_filament\_bundle\_assembly | 3 | 0 |  |  |  |  |  |  |  |  |
| GO:0045143\_homologous\_chromosome\_segregation | 3 | 0 |  |  |  |  |  |  |  |  |
| GO:0045198\_establishment\_of\_epithelial\_cell\_apical\_basal\_polarity | 3 | 0 |  |  |  |  |  |  |  |  |
| GO:0045217\_cell-cell\_junction\_maintenance | 3 | 0 |  |  |  |  |  |  |  |  |
| GO:0045348\_positive\_regulation\_of\_MHC\_class\_II\_biosynthetic\_process | 3 | 0 |  |  |  |  |  |  |  |  |
| GO:0045402\_regulation\_of\_interleukin-4\_biosynthetic\_process | 3 | 0 |  |  |  |  |  |  |  |  |
| GO:0045404\_positive\_regulation\_of\_interleukin-4\_biosynthetic\_process | 3 | 0 |  |  |  |  |  |  |  |  |
| GO:0045542\_positive\_regulation\_of\_cholesterol\_biosynthetic\_process | 3 | 0 |  |  |  |  |  |  |  |  |
| GO:0045607\_regulation\_of\_auditory\_receptor\_cell\_differentiation | 3 | 0 |  |  |  |  |  |  |  |  |
| GO:0045623\_negative\_regulation\_of\_T-helper\_cell\_differentiation | 3 | 0 |  |  |  |  |  |  |  |  |
| GO:0045625\_regulation\_of\_T-helper\_1\_cell\_differentiation | 3 | 0 |  |  |  |  |  |  |  |  |
| GO:0045631\_regulation\_of\_mechanoreceptor\_differentiation | 3 | 0 |  |  |  |  |  |  |  |  |
| GO:0045717\_negative\_regulation\_of\_fatty\_acid\_biosynthetic\_process | 3 | 0 |  |  |  |  |  |  |  |  |
| GO:0045723\_positive\_regulation\_of\_fatty\_acid\_biosynthetic\_process | 3 | 0 |  |  |  |  |  |  |  |  |
| GO:0045746\_negative\_regulation\_of\_Notch\_signaling\_pathway | 3 | 0 |  |  |  |  |  |  |  |  |
| GO:0045806\_negative\_regulation\_of\_endocytosis | 3 | 0 |  |  |  |  |  |  |  |  |
| GO:0045829\_negative\_regulation\_of\_isotype\_switching | 3 | 0 |  |  |  |  |  |  |  |  |
| GO:0045907\_positive\_regulation\_of\_vasoconstriction | 3 | 0 |  |  |  |  |  |  |  |  |
| GO:0045922\_negative\_regulation\_of\_fatty\_acid\_metabolic\_process | 3 | 0 |  |  |  |  |  |  |  |  |
| GO:0045939\_negative\_regulation\_of\_steroid\_metabolic\_process | 3 | 0 |  |  |  |  |  |  |  |  |
| GO:0046013\_regulation\_of\_T\_cell\_homeostatic\_proliferation | 3 | 0 |  |  |  |  |  |  |  |  |
| GO:0046034\_ATP\_metabolic\_process | 3 | 0 |  |  |  |  |  |  |  |  |
| GO:0046325\_negative\_regulation\_of\_glucose\_import | 3 | 0 |  |  |  |  |  |  |  |  |
| GO:0046426\_negative\_regulation\_of\_JAK-STAT\_cascade | 3 | 0 |  |  |  |  |  |  |  |  |
| GO:0046457\_prostanoid\_biosynthetic\_process | 3 | 0 |  |  |  |  |  |  |  |  |
| GO:0046479\_glycosphingolipid\_catabolic\_process | 3 | 0 |  |  |  |  |  |  |  |  |
| GO:0046488\_phosphatidylinositol\_metabolic\_process | 3 | 0 |  |  |  |  |  |  |  |  |
| GO:0046549\_retinal\_cone\_cell\_development | 3 | 0 |  |  |  |  |  |  |  |  |
| GO:0046605\_regulation\_of\_centrosome\_cycle | 3 | 0 |  |  |  |  |  |  |  |  |
| GO:0046688\_response\_to\_copper\_ion | 3 | 0 |  |  |  |  |  |  |  |  |
| GO:0046717\_acid\_secretion | 3 | 0 |  |  |  |  |  |  |  |  |
| GO:0046825\_regulation\_of\_protein\_export\_from\_nucleus | 3 | 0 |  |  |  |  |  |  |  |  |
| GO:0046885\_regulation\_of\_hormone\_biosynthetic\_process | 3 | 0 |  |  |  |  |  |  |  |  |
| GO:0048003\_antigen\_processing\_and\_presentation\_of\_lipid\_antigen\_via\_MHC\_class\_Ib | 3 | 0 |  |  |  |  |  |  |  |  |
| GO:0048007\_antigen\_processing\_and\_presentation\_\_exogenous\_lipid\_antigen\_via\_MHC\_class\_Ib | 3 | 0 |  |  |  |  |  |  |  |  |
| GO:0048012\_hepatocyte\_growth\_factor\_receptor\_signaling\_pathway | 3 | 0 |  |  |  |  |  |  |  |  |
| GO:0048087\_positive\_regulation\_of\_pigmentation\_during\_development | 3 | 0 |  |  |  |  |  |  |  |  |
| GO:0048246\_macrophage\_chemotaxis | 3 | 0 |  |  |  |  |  |  |  |  |
| GO:0048251\_elastic\_fiber\_assembly | 3 | 0 |  |  |  |  |  |  |  |  |
| GO:0048278\_vesicle\_docking | 3 | 0 |  |  |  |  |  |  |  |  |
| GO:0048294\_negative\_regulation\_of\_isotype\_switching\_to\_IgE\_isotypes | 3 | 0 |  |  |  |  |  |  |  |  |
| GO:0048318\_axial\_mesoderm\_development | 3 | 0 |  |  |  |  |  |  |  |  |
| GO:0048660\_regulation\_of\_smooth\_muscle\_cell\_proliferation | 3 | 0 |  |  |  |  |  |  |  |  |
| GO:0048668\_collateral\_sprouting | 3 | 0 |  |  |  |  |  |  |  |  |
| GO:0048755\_branching\_morphogenesis\_of\_a\_nerve | 3 | 0 |  |  |  |  |  |  |  |  |
| GO:0048845\_venous\_blood\_vessel\_morphogenesis | 3 | 0 |  |  |  |  |  |  |  |  |
| GO:0048852\_diencephalon\_morphogenesis | 3 | 0 |  |  |  |  |  |  |  |  |
| GO:0048859\_formation\_of\_anatomical\_boundary | 3 | 0 |  |  |  |  |  |  |  |  |
| GO:0048865\_stem\_cell\_fate\_commitment | 3 | 0 |  |  |  |  |  |  |  |  |
| GO:0050435\_beta-amyloid\_metabolic\_process | 3 | 0 |  |  |  |  |  |  |  |  |
| GO:0050650\_chondroitin\_sulfate\_proteoglycan\_biosynthetic\_process | 3 | 0 |  |  |  |  |  |  |  |  |
| GO:0050703\_interleukin-1\_alpha\_secretion | 3 | 0 |  |  |  |  |  |  |  |  |
| GO:0050705\_regulation\_of\_interleukin-1\_alpha\_secretion | 3 | 0 |  |  |  |  |  |  |  |  |
| GO:0050709\_negative\_regulation\_of\_protein\_secretion | 3 | 0 |  |  |  |  |  |  |  |  |
| GO:0050710\_negative\_regulation\_of\_cytokine\_secretion | 3 | 0 |  |  |  |  |  |  |  |  |
| GO:0050717\_positive\_regulation\_of\_interleukin-1\_alpha\_secretion | 3 | 0 |  |  |  |  |  |  |  |  |
| GO:0050774\_negative\_regulation\_of\_dendrite\_morphogenesis | 3 | 0 |  |  |  |  |  |  |  |  |
| GO:0050857\_positive\_regulation\_of\_antigen\_receptor-mediated\_signaling\_pathway | 3 | 0 |  |  |  |  |  |  |  |  |
| GO:0050882\_voluntary\_musculoskeletal\_movement | 3 | 0 |  |  |  |  |  |  |  |  |
| GO:0050913\_sensory\_perception\_of\_bitter\_taste | 3 | 0 |  |  |  |  |  |  |  |  |
| GO:0050957\_equilibrioception | 3 | 0 |  |  |  |  |  |  |  |  |
| GO:0050996\_positive\_regulation\_of\_lipid\_catabolic\_process | 3 | 0 |  |  |  |  |  |  |  |  |
| GO:0051149\_positive\_regulation\_of\_muscle\_cell\_differentiation | 3 | 0 |  |  |  |  |  |  |  |  |
| GO:0051153\_regulation\_of\_striated\_muscle\_cell\_differentiation | 3 | 0 |  |  |  |  |  |  |  |  |
| GO:0051204\_protein\_insertion\_into\_mitochondrial\_membrane | 3 | 0 |  |  |  |  |  |  |  |  |
| GO:0051291\_protein\_heterooligomerization | 3 | 0 |  |  |  |  |  |  |  |  |
| GO:0051302\_regulation\_of\_cell\_division | 3 | 0 |  |  |  |  |  |  |  |  |
| GO:0051320\_S\_phase | 3 | 0 |  |  |  |  |  |  |  |  |
| GO:0051450\_myoblast\_proliferation | 3 | 0 |  |  |  |  |  |  |  |  |
| GO:0051583\_dopamine\_uptake | 3 | 0 |  |  |  |  |  |  |  |  |
| GO:0051798\_positive\_regulation\_of\_hair\_follicle\_development | 3 | 0 |  |  |  |  |  |  |  |  |
| GO:0051882\_mitochondrial\_depolarization | 3 | 0 |  |  |  |  |  |  |  |  |
| GO:0051900\_regulation\_of\_mitochondrial\_depolarization | 3 | 0 |  |  |  |  |  |  |  |  |
| GO:0051925\_regulation\_of\_calcium\_ion\_transport\_via\_voltage-gated\_calcium\_channel\_activity | 3 | 0 |  |  |  |  |  |  |  |  |
| GO:0051926\_negative\_regulation\_of\_calcium\_ion\_transport | 3 | 0 |  |  |  |  |  |  |  |  |
| GO:0051930\_regulation\_of\_sensory\_perception\_of\_pain | 3 | 0 |  |  |  |  |  |  |  |  |
| GO:0051931\_regulation\_of\_sensory\_perception | 3 | 0 |  |  |  |  |  |  |  |  |
| GO:0051934\_catecholamine\_uptake\_during\_transmission\_of\_nerve\_impulse | 3 | 0 |  |  |  |  |  |  |  |  |
| GO:0051955\_regulation\_of\_amino\_acid\_transport | 3 | 0 |  |  |  |  |  |  |  |  |
| GO:0051962\_positive\_regulation\_of\_nervous\_system\_development | 3 | 0 |  |  |  |  |  |  |  |  |
| GO:0051965\_positive\_regulation\_of\_synaptogenesis | 3 | 0 |  |  |  |  |  |  |  |  |
| GO:0051967\_negative\_regulation\_of\_synaptic\_transmission\_\_glutamatergic | 3 | 0 |  |  |  |  |  |  |  |  |
| GO:0051983\_regulation\_of\_chromosome\_segregation | 3 | 0 |  |  |  |  |  |  |  |  |
| GO:0055061\_di-\_\_tri-valent\_inorganic\_anion\_homeostasis | 3 | 0 |  |  |  |  |  |  |  |  |
| GO:0055062\_phosphate\_ion\_homeostasis | 3 | 0 |  |  |  |  |  |  |  |  |
| GO:0055083\_monovalent\_inorganic\_anion\_homeostasis | 3 | 0 |  |  |  |  |  |  |  |  |
| GO:0055117\_regulation\_of\_cardiac\_muscle\_contraction | 3 | 0 |  |  |  |  |  |  |  |  |
| GO:0060009\_Sertoli\_cell\_development | 3 | 0 |  |  |  |  |  |  |  |  |
| GO:0060024\_rhythmic\_synaptic\_transmission | 3 | 0 |  |  |  |  |  |  |  |  |
| GO:0060033\_anatomical\_structure\_regression | 3 | 0 |  |  |  |  |  |  |  |  |
| GO:0060040\_retinal\_bipolar\_neuron\_differentiation | 3 | 0 |  |  |  |  |  |  |  |  |
| GO:0060055\_angiogenesis\_involved\_in\_wound\_healing | 3 | 0 |  |  |  |  |  |  |  |  |
| GO:0060084\_synaptic\_transmission\_involved\_in\_micturition | 3 | 0 |  |  |  |  |  |  |  |  |
| GO:0060123\_regulation\_of\_growth\_hormone\_secretion | 3 | 0 |  |  |  |  |  |  |  |  |
| GO:0060126\_somatotropin\_secreting\_cell\_differentiation | 3 | 0 |  |  |  |  |  |  |  |  |
| GO:0060192\_negative\_regulation\_of\_lipase\_activity | 3 | 0 |  |  |  |  |  |  |  |  |
| GO:0060219\_camera-type\_eye\_photoreceptor\_cell\_differentiation | 3 | 0 |  |  |  |  |  |  |  |  |
| GO:0060285\_ciliary\_cell\_motility | 3 | 0 |  |  |  |  |  |  |  |  |
| GO:0060294\_cilium\_movement\_involved\_in\_ciliary\_motility | 3 | 0 |  |  |  |  |  |  |  |  |
| GO:0060295\_regulation\_of\_cilium\_movement\_involved\_in\_ciliary\_motility | 3 | 0 |  |  |  |  |  |  |  |  |
| GO:0060296\_regulation\_of\_cilium\_beat\_frequency\_involved\_in\_ciliary\_motility | 3 | 0 |  |  |  |  |  |  |  |  |
| GO:0060396\_growth\_hormone\_receptor\_signaling\_pathway | 3 | 0 |  |  |  |  |  |  |  |  |
| GO:0060416\_response\_to\_growth\_hormone\_stimulus | 3 | 0 |  |  |  |  |  |  |  |  |
| GO:0060428\_lung\_epithelium\_development | 3 | 0 |  |  |  |  |  |  |  |  |
| GO:0060433\_bronchus\_development | 3 | 0 |  |  |  |  |  |  |  |  |
| GO:0060435\_bronchiole\_development | 3 | 0 |  |  |  |  |  |  |  |  |
| GO:0060460\_left\_lung\_morphogenesis | 3 | 0 |  |  |  |  |  |  |  |  |
| GO:0060491\_regulation\_of\_cell\_projection\_assembly | 3 | 0 |  |  |  |  |  |  |  |  |
| GO:0060523\_prostate\_epithelial\_cord\_elongation | 3 | 0 |  |  |  |  |  |  |  |  |
| GO:0060586\_multicellular\_organismal\_iron\_ion\_homeostasis | 3 | 0 |  |  |  |  |  |  |  |  |
| GO:0060596\_mammary\_placode\_formation | 3 | 0 |  |  |  |  |  |  |  |  |
| GO:0060632\_regulation\_of\_microtubule-based\_movement | 3 | 0 |  |  |  |  |  |  |  |  |
| GO:0060648\_mammary\_gland\_bud\_morphogenesis | 3 | 0 |  |  |  |  |  |  |  |  |
| GO:0060684\_epithelial-mesenchymal\_cell\_signaling | 3 | 0 |  |  |  |  |  |  |  |  |
| GO:0060686\_negative\_regulation\_of\_prostatic\_bud\_formation | 3 | 0 |  |  |  |  |  |  |  |  |
| GO:0060689\_cell\_differentiation\_involved\_in\_salivary\_gland\_development | 3 | 0 |  |  |  |  |  |  |  |  |
| GO:0060708\_spongiotrophoblast\_differentiation | 3 | 0 |  |  |  |  |  |  |  |  |
| GO:0060746\_parental\_behavior | 3 | 0 |  |  |  |  |  |  |  |  |
| GO:0060748\_tertiary\_branching\_involved\_in\_mammary\_gland\_duct\_morphogenesis | 3 | 0 |  |  |  |  |  |  |  |  |
| GO:0060750\_epithelial\_cell\_proliferation\_involved\_in\_mammary\_gland\_duct\_elongation | 3 | 0 |  |  |  |  |  |  |  |  |
| GO:0060841\_venous\_blood\_vessel\_development | 3 | 0 |  |  |  |  |  |  |  |  |
| GO:0070102\_interleukin-6-mediated\_signaling\_pathway | 3 | 0 |  |  |  |  |  |  |  |  |
| GO:0070169\_positive\_regulation\_of\_biomineral\_formation | 3 | 0 |  |  |  |  |  |  |  |  |
| GO:0070206\_protein\_trimerization | 3 | 0 |  |  |  |  |  |  |  |  |
| GO:0070207\_protein\_homotrimerization | 3 | 0 |  |  |  |  |  |  |  |  |
| GO:0070229\_negative\_regulation\_of\_lymphocyte\_apoptosis | 3 | 0 |  |  |  |  |  |  |  |  |
| GO:0070230\_positive\_regulation\_of\_lymphocyte\_apoptosis | 3 | 0 |  |  |  |  |  |  |  |  |
| GO:0070232\_regulation\_of\_T\_cell\_apoptosis | 3 | 0 |  |  |  |  |  |  |  |  |
| GO:0070233\_negative\_regulation\_of\_T\_cell\_apoptosis | 3 | 0 |  |  |  |  |  |  |  |  |
| GO:0070242\_thymocyte\_apoptosis | 3 | 0 |  |  |  |  |  |  |  |  |
| GO:0070243\_regulation\_of\_thymocyte\_apoptosis | 3 | 0 |  |  |  |  |  |  |  |  |
| GO:0070244\_negative\_regulation\_of\_thymocyte\_apoptosis | 3 | 0 |  |  |  |  |  |  |  |  |
| GO:0070307\_lens\_fiber\_cell\_development | 3 | 0 |  |  |  |  |  |  |  |  |
| GO:0070309\_lens\_fiber\_cell\_morphogenesis | 3 | 0 |  |  |  |  |  |  |  |  |
| GO:0070423\_nucleotide-binding\_oligomerization\_domain\_containing\_signaling\_pathway | 3 | 0 |  |  |  |  |  |  |  |  |
| GO:0070427\_nucleotide-binding\_oligomerization\_domain\_containing\_1\_signaling\_pathway | 3 | 0 |  |  |  |  |  |  |  |  |
| GO:0070431\_nucleotide-binding\_oligomerization\_domain\_containing\_2\_signaling\_pathway | 3 | 0 |  |  |  |  |  |  |  |  |
| GO:0070633\_transepithelial\_transport | 3 | 0 |  |  |  |  |  |  |  |  |
| GO:0070846\_Hsp90\_deacetylation | 3 | 0 |  |  |  |  |  |  |  |  |
| GO:0070873\_regulation\_of\_glycogen\_metabolic\_process | 3 | 0 |  |  |  |  |  |  |  |  |
| GO:0070875\_positive\_regulation\_of\_glycogen\_metabolic\_process | 3 | 0 |  |  |  |  |  |  |  |  |
| GO:0032787\_monocarboxylic\_acid\_metabolic\_process | 130 | 0 | 0.000000 | -0.000000 | 916 | 798.806517 | 887.28 | 975.753483 | 0.968646 |
| GO:0045165\_cell\_fate\_commitment | 130 | 0 | 0.000000 | -0.000000 | 916 | 798.806517 | 887.28 | 975.753483 | 0.968646 |
| GO:0050776\_regulation\_of\_immune\_response | 130 | 0 | 0.000000 | -0.000000 | 916 | 798.806517 | 887.28 | 975.753483 | 0.968646 |
| GO:0043549\_regulation\_of\_kinase\_activity | 112 | 0 | 0.000000 | -0.000000 | 917 | 801.261995 | 889.26 | 977.258005 | 0.969749 |
| GO:0007346\_regulation\_of\_mitotic\_cell\_cycle | 40 | 0 | 0.000000 | -0.000000 | 927 | 812.202818 | 899.5 | 986.797182 | 0.970334 |
| GO:0007599\_hemostasis | 40 | 0 | 0.000000 | -0.000000 | 927 | 812.202818 | 899.5 | 986.797182 | 0.970334 |
| GO:0008203\_cholesterol\_metabolic\_process | 40 | 0 | 0.000000 | -0.000000 | 927 | 812.202818 | 899.5 | 986.797182 | 0.970334 |
| GO:0016071\_mRNA\_metabolic\_process | 40 | 0 | 0.000000 | -0.000000 | 927 | 812.202818 | 899.5 | 986.797182 | 0.970334 |
| GO:0016358\_dendrite\_development | 40 | 0 | 0.000000 | -0.000000 | 927 | 812.202818 | 899.5 | 986.797182 | 0.970334 |
| GO:0016485\_protein\_processing | 40 | 0 | 0.000000 | -0.000000 | 927 | 812.202818 | 899.5 | 986.797182 | 0.970334 |
| GO:0019935\_cyclic-nucleotide-mediated\_signaling | 40 | 0 | 0.000000 | -0.000000 | 927 | 812.202818 | 899.5 | 986.797182 | 0.970334 |
| GO:0035272\_exocrine\_system\_development | 40 | 0 | 0.000000 | -0.000000 | 927 | 812.202818 | 899.5 | 986.797182 | 0.970334 |
| GO:0046850\_regulation\_of\_bone\_remodeling | 40 | 0 | 0.000000 | -0.000000 | 927 | 812.202818 | 899.5 | 986.797182 | 0.970334 |
| GO:0051129\_negative\_regulation\_of\_cellular\_component\_organization | 40 | 0 | 0.000000 | -0.000000 | 927 | 812.202818 | 899.5 | 986.797182 | 0.970334 |
| GO:0007283\_spermatogenesis | 134 | 0 | 0.000000 | -0.000000 | 929 | 813.375032 | 900.56 | 987.744968 | 0.969386 |
| GO:0048232\_male\_gamete\_generation | 134 | 0 | 0.000000 | -0.000000 | 929 | 813.375032 | 900.56 | 987.744968 | 0.969386 |
| GO:0048584\_positive\_regulation\_of\_response\_to\_stimulus | 115 | 0 | 0.000000 | -0.000000 | 931 | 815.779998 | 902.52 | 989.260002 | 0.969409 |
| GO:0051338\_regulation\_of\_transferase\_activity | 115 | 0 | 0.000000 | -0.000000 | 931 | 815.779998 | 902.52 | 989.260002 | 0.969409 |
| GO:0002697\_regulation\_of\_immune\_effector\_process | 68 | 0 | 0.000000 | -0.000000 | 935 | 818.876053 | 905.18 | 991.483947 | 0.968107 |
| GO:0019932\_second-messenger-mediated\_signaling | 68 | 0 | 0.000000 | -0.000000 | 935 | 818.876053 | 905.18 | 991.483947 | 0.968107 |
| GO:0034962\_cellular\_biopolymer\_catabolic\_process | 68 | 0 | 0.000000 | -0.000000 | 935 | 818.876053 | 905.18 | 991.483947 | 0.968107 |
| GO:0042692\_muscle\_cell\_differentiation | 68 | 0 | 0.000000 | -0.000000 | 935 | 818.876053 | 905.18 | 991.483947 | 0.968107 |
| GO:0051046\_regulation\_of\_secretion | 79 | 0 | 0.000000 | -0.000000 | 936 | 820.361507 | 906.42 | 992.478493 | 0.968397 |
| GO:0000086\_G2\_M\_transition\_of\_mitotic\_cell\_cycle | 4 | 0 |  |  |  |  |  |  |  |  |
| GO:0000305\_response\_to\_oxygen\_radical | 4 | 0 |  |  |  |  |  |  |  |  |
| GO:0001661\_conditioned\_taste\_aversion | 4 | 0 |  |  |  |  |  |  |  |  |
| GO:0001678\_cellular\_glucose\_homeostasis | 4 | 0 |  |  |  |  |  |  |  |  |
| GO:0001777\_T\_cell\_homeostatic\_proliferation | 4 | 0 |  |  |  |  |  |  |  |  |
| GO:0001794\_type\_IIa\_hypersensitivity | 4 | 0 |  |  |  |  |  |  |  |  |
| GO:0001796\_regulation\_of\_type\_IIa\_hypersensitivity | 4 | 0 |  |  |  |  |  |  |  |  |
| GO:0001798\_positive\_regulation\_of\_type\_IIa\_hypersensitivity | 4 | 0 |  |  |  |  |  |  |  |  |
| GO:0001810\_regulation\_of\_type\_I\_hypersensitivity | 4 | 0 |  |  |  |  |  |  |  |  |
| GO:0001820\_serotonin\_secretion | 4 | 0 |  |  |  |  |  |  |  |  |
| GO:0001835\_blastocyst\_hatching | 4 | 0 |  |  |  |  |  |  |  |  |
| GO:0001881\_receptor\_recycling | 4 | 0 |  |  |  |  |  |  |  |  |
| GO:0001938\_positive\_regulation\_of\_endothelial\_cell\_proliferation | 4 | 0 |  |  |  |  |  |  |  |  |
| GO:0001978\_regulation\_of\_systemic\_arterial\_blood\_pressure\_by\_carotid\_sinus\_baroreceptor\_feedback | 4 | 0 |  |  |  |  |  |  |  |  |
| GO:0002035\_brain\_renin-angiotensin\_system | 4 | 0 |  |  |  |  |  |  |  |  |
| GO:0002051\_osteoblast\_fate\_commitment | 4 | 0 |  |  |  |  |  |  |  |  |
| GO:0002220\_innate\_immune\_response\_activating\_cell\_surface\_receptor\_signaling\_pathway | 4 | 0 |  |  |  |  |  |  |  |  |
| GO:0002249\_lymphocyte\_anergy | 4 | 0 |  |  |  |  |  |  |  |  |
| GO:0002312\_B\_cell\_activation\_during\_immune\_response | 4 | 0 |  |  |  |  |  |  |  |  |
| GO:0002313\_mature\_B\_cell\_differentiation\_during\_immune\_response | 4 | 0 |  |  |  |  |  |  |  |  |
| GO:0002318\_myeloid\_progenitor\_cell\_differentiation | 4 | 0 |  |  |  |  |  |  |  |  |
| GO:0002326\_B\_cell\_lineage\_commitment | 4 | 0 |  |  |  |  |  |  |  |  |
| GO:0002347\_response\_to\_tumor\_cell | 4 | 0 |  |  |  |  |  |  |  |  |
| GO:0002418\_immune\_response\_to\_tumor\_cell | 4 | 0 |  |  |  |  |  |  |  |  |
| GO:0002445\_type\_II\_hypersensitivity | 4 | 0 |  |  |  |  |  |  |  |  |
| GO:0002544\_chronic\_inflammatory\_response | 4 | 0 |  |  |  |  |  |  |  |  |
| GO:0002636\_positive\_regulation\_of\_germinal\_center\_formation | 4 | 0 |  |  |  |  |  |  |  |  |
| GO:0002667\_regulation\_of\_T\_cell\_anergy | 4 | 0 |  |  |  |  |  |  |  |  |
| GO:0002669\_positive\_regulation\_of\_T\_cell\_anergy | 4 | 0 |  |  |  |  |  |  |  |  |
| GO:0002687\_positive\_regulation\_of\_leukocyte\_migration | 4 | 0 |  |  |  |  |  |  |  |  |
| GO:0002702\_positive\_regulation\_of\_production\_of\_molecular\_mediator\_of\_immune\_response | 4 | 0 |  |  |  |  |  |  |  |  |
| GO:0002718\_regulation\_of\_cytokine\_production\_during\_immune\_response | 4 | 0 |  |  |  |  |  |  |  |  |
| GO:0002829\_negative\_regulation\_of\_T-helper\_2\_type\_immune\_response | 4 | 0 |  |  |  |  |  |  |  |  |
| GO:0002833\_positive\_regulation\_of\_response\_to\_biotic\_stimulus | 4 | 0 |  |  |  |  |  |  |  |  |
| GO:0002834\_regulation\_of\_response\_to\_tumor\_cell | 4 | 0 |  |  |  |  |  |  |  |  |
| GO:0002836\_positive\_regulation\_of\_response\_to\_tumor\_cell | 4 | 0 |  |  |  |  |  |  |  |  |
| GO:0002837\_regulation\_of\_immune\_response\_to\_tumor\_cell | 4 | 0 |  |  |  |  |  |  |  |  |
| GO:0002839\_positive\_regulation\_of\_immune\_response\_to\_tumor\_cell | 4 | 0 |  |  |  |  |  |  |  |  |
| GO:0002870\_T\_cell\_anergy | 4 | 0 |  |  |  |  |  |  |  |  |
| GO:0002888\_positive\_regulation\_of\_myeloid\_leukocyte\_mediated\_immunity | 4 | 0 |  |  |  |  |  |  |  |  |
| GO:0002892\_regulation\_of\_type\_II\_hypersensitivity | 4 | 0 |  |  |  |  |  |  |  |  |
| GO:0002894\_positive\_regulation\_of\_type\_II\_hypersensitivity | 4 | 0 |  |  |  |  |  |  |  |  |
| GO:0002911\_regulation\_of\_lymphocyte\_anergy | 4 | 0 |  |  |  |  |  |  |  |  |
| GO:0002913\_positive\_regulation\_of\_lymphocyte\_anergy | 4 | 0 |  |  |  |  |  |  |  |  |
| GO:0002923\_regulation\_of\_humoral\_immune\_response\_mediated\_by\_circulating\_immunoglobulin | 4 | 0 |  |  |  |  |  |  |  |  |
| GO:0003025\_regulation\_of\_systemic\_arterial\_blood\_pressure\_by\_baroreceptor\_feedback | 4 | 0 |  |  |  |  |  |  |  |  |
| GO:0003091\_renal\_water\_homeostasis | 4 | 0 |  |  |  |  |  |  |  |  |
| GO:0005978\_glycogen\_biosynthetic\_process | 4 | 0 |  |  |  |  |  |  |  |  |
| GO:0006012\_galactose\_metabolic\_process | 4 | 0 |  |  |  |  |  |  |  |  |
| GO:0006085\_acetyl-CoA\_biosynthetic\_process | 4 | 0 |  |  |  |  |  |  |  |  |
| GO:0006111\_regulation\_of\_gluconeogenesis | 4 | 0 |  |  |  |  |  |  |  |  |
| GO:0006144\_purine\_base\_metabolic\_process | 4 | 0 |  |  |  |  |  |  |  |  |
| GO:0006334\_nucleosome\_assembly | 4 | 0 |  |  |  |  |  |  |  |  |
| GO:0006534\_cysteine\_metabolic\_process | 4 | 0 |  |  |  |  |  |  |  |  |
| GO:0006547\_histidine\_metabolic\_process | 4 | 0 |  |  |  |  |  |  |  |  |
| GO:0006548\_histidine\_catabolic\_process | 4 | 0 |  |  |  |  |  |  |  |  |
| GO:0006555\_methionine\_metabolic\_process | 4 | 0 |  |  |  |  |  |  |  |  |
| GO:0006599\_phosphagen\_metabolic\_process | 4 | 0 |  |  |  |  |  |  |  |  |
| GO:0006623\_protein\_targeting\_to\_vacuole | 4 | 0 |  |  |  |  |  |  |  |  |
| GO:0006626\_protein\_targeting\_to\_mitochondrion | 4 | 0 |  |  |  |  |  |  |  |  |
| GO:0006684\_sphingomyelin\_metabolic\_process | 4 | 0 |  |  |  |  |  |  |  |  |
| GO:0006688\_glycosphingolipid\_biosynthetic\_process | 4 | 0 |  |  |  |  |  |  |  |  |
| GO:0006707\_cholesterol\_catabolic\_process | 4 | 0 |  |  |  |  |  |  |  |  |
| GO:0006739\_NADP\_metabolic\_process | 4 | 0 |  |  |  |  |  |  |  |  |
| GO:0006835\_dicarboxylic\_acid\_transport | 4 | 0 |  |  |  |  |  |  |  |  |
| GO:0006837\_serotonin\_transport | 4 | 0 |  |  |  |  |  |  |  |  |
| GO:0006888\_ER\_to\_Golgi\_vesicle-mediated\_transport | 4 | 0 |  |  |  |  |  |  |  |  |
| GO:0006906\_vesicle\_fusion | 4 | 0 |  |  |  |  |  |  |  |  |
| GO:0006927\_transformed\_cell\_apoptosis | 4 | 0 |  |  |  |  |  |  |  |  |
| GO:0006972\_hyperosmotic\_response | 4 | 0 |  |  |  |  |  |  |  |  |
| GO:0007028\_cytoplasm\_organization | 4 | 0 |  |  |  |  |  |  |  |  |
| GO:0007031\_peroxisome\_organization | 4 | 0 |  |  |  |  |  |  |  |  |
| GO:0007066\_female\_meiosis\_sister\_chromatid\_cohesion | 4 | 0 |  |  |  |  |  |  |  |  |
| GO:0007144\_female\_meiosis\_I | 4 | 0 |  |  |  |  |  |  |  |  |
| GO:0007184\_SMAD\_protein\_nuclear\_translocation | 4 | 0 |  |  |  |  |  |  |  |  |
| GO:0007216\_metabotropic\_glutamate\_receptor\_signaling\_pathway | 4 | 0 |  |  |  |  |  |  |  |  |
| GO:0007342\_fusion\_of\_sperm\_to\_egg\_plasma\_membrane | 4 | 0 |  |  |  |  |  |  |  |  |
| GO:0007386\_compartment\_specification | 4 | 0 |  |  |  |  |  |  |  |  |
| GO:0008053\_mitochondrial\_fusion | 4 | 0 |  |  |  |  |  |  |  |  |
| GO:0008207\_C21-steroid\_hormone\_metabolic\_process | 4 | 0 |  |  |  |  |  |  |  |  |
| GO:0008215\_spermine\_metabolic\_process | 4 | 0 |  |  |  |  |  |  |  |  |
| GO:0009065\_glutamine\_family\_amino\_acid\_catabolic\_process | 4 | 0 |  |  |  |  |  |  |  |  |
| GO:0009075\_histidine\_family\_amino\_acid\_metabolic\_process | 4 | 0 |  |  |  |  |  |  |  |  |
| GO:0009077\_histidine\_family\_amino\_acid\_catabolic\_process | 4 | 0 |  |  |  |  |  |  |  |  |
| GO:0009134\_nucleoside\_diphosphate\_catabolic\_process | 4 | 0 |  |  |  |  |  |  |  |  |
| GO:0009163\_nucleoside\_biosynthetic\_process | 4 | 0 |  |  |  |  |  |  |  |  |
| GO:0009225\_nucleotide-sugar\_metabolic\_process | 4 | 0 |  |  |  |  |  |  |  |  |
| GO:0009250\_glucan\_biosynthetic\_process | 4 | 0 |  |  |  |  |  |  |  |  |
| GO:0009404\_toxin\_metabolic\_process | 4 | 0 |  |  |  |  |  |  |  |  |
| GO:0009593\_detection\_of\_chemical\_stimulus | 4 | 0 |  |  |  |  |  |  |  |  |
| GO:0009595\_detection\_of\_biotic\_stimulus | 4 | 0 |  |  |  |  |  |  |  |  |
| GO:0009755\_hormone-mediated\_signaling | 4 | 0 |  |  |  |  |  |  |  |  |
| GO:0009912\_auditory\_receptor\_cell\_fate\_commitment | 4 | 0 |  |  |  |  |  |  |  |  |
| GO:0010453\_regulation\_of\_cell\_fate\_commitment | 4 | 0 |  |  |  |  |  |  |  |  |
| GO:0010506\_regulation\_of\_autophagy | 4 | 0 |  |  |  |  |  |  |  |  |
| GO:0010631\_epithelial\_cell\_migration | 4 | 0 |  |  |  |  |  |  |  |  |
| GO:0010812\_negative\_regulation\_of\_cell-substrate\_adhesion | 4 | 0 |  |  |  |  |  |  |  |  |
| GO:0010829\_negative\_regulation\_of\_glucose\_transport | 4 | 0 |  |  |  |  |  |  |  |  |
| GO:0014002\_astrocyte\_development | 4 | 0 |  |  |  |  |  |  |  |  |
| GO:0014832\_urinary\_bladder\_smooth\_muscle\_contraction | 4 | 0 |  |  |  |  |  |  |  |  |
| GO:0014848\_urinary\_tract\_smooth\_muscle\_contraction | 4 | 0 |  |  |  |  |  |  |  |  |
| GO:0015701\_bicarbonate\_transport | 4 | 0 |  |  |  |  |  |  |  |  |
| GO:0015809\_arginine\_transport | 4 | 0 |  |  |  |  |  |  |  |  |
| GO:0015850\_organic\_alcohol\_transport | 4 | 0 |  |  |  |  |  |  |  |  |
| GO:0015858\_nucleoside\_transport | 4 | 0 |  |  |  |  |  |  |  |  |
| GO:0016068\_type\_I\_hypersensitivity | 4 | 0 |  |  |  |  |  |  |  |  |
| GO:0016127\_sterol\_catabolic\_process | 4 | 0 |  |  |  |  |  |  |  |  |
| GO:0016198\_axon\_choice\_point\_recognition | 4 | 0 |  |  |  |  |  |  |  |  |
| GO:0016338\_calcium-independent\_cell-cell\_adhesion | 4 | 0 |  |  |  |  |  |  |  |  |
| GO:0018198\_peptidyl-cysteine\_modification | 4 | 0 |  |  |  |  |  |  |  |  |
| GO:0018409\_peptide\_or\_protein\_amino-terminal\_blocking | 4 | 0 |  |  |  |  |  |  |  |  |
| GO:0019377\_glycolipid\_catabolic\_process | 4 | 0 |  |  |  |  |  |  |  |  |
| GO:0019432\_triglyceride\_biosynthetic\_process | 4 | 0 |  |  |  |  |  |  |  |  |
| GO:0019530\_taurine\_metabolic\_process | 4 | 0 |  |  |  |  |  |  |  |  |
| GO:0021523\_somatic\_motor\_neuron\_differentiation | 4 | 0 |  |  |  |  |  |  |  |  |
| GO:0021535\_cell\_migration\_in\_hindbrain | 4 | 0 |  |  |  |  |  |  |  |  |
| GO:0021542\_dentate\_gyrus\_development | 4 | 0 |  |  |  |  |  |  |  |  |
| GO:0021561\_facial\_nerve\_development | 4 | 0 |  |  |  |  |  |  |  |  |
| GO:0021569\_rhombomere\_3\_development | 4 | 0 |  |  |  |  |  |  |  |  |
| GO:0021571\_rhombomere\_5\_development | 4 | 0 |  |  |  |  |  |  |  |  |
| GO:0021604\_cranial\_nerve\_structural\_organization | 4 | 0 |  |  |  |  |  |  |  |  |
| GO:0021610\_facial\_nerve\_morphogenesis | 4 | 0 |  |  |  |  |  |  |  |  |
| GO:0021612\_facial\_nerve\_structural\_organization | 4 | 0 |  |  |  |  |  |  |  |  |
| GO:0021631\_optic\_nerve\_morphogenesis | 4 | 0 |  |  |  |  |  |  |  |  |
| GO:0021681\_cerebellar\_granular\_layer\_development | 4 | 0 |  |  |  |  |  |  |  |  |
| GO:0021683\_cerebellar\_granular\_layer\_morphogenesis | 4 | 0 |  |  |  |  |  |  |  |  |
| GO:0021684\_cerebellar\_granular\_layer\_formation | 4 | 0 |  |  |  |  |  |  |  |  |
| GO:0021707\_cerebellar\_granule\_cell\_differentiation | 4 | 0 |  |  |  |  |  |  |  |  |
| GO:0021778\_oligodendrocyte\_cell\_fate\_specification | 4 | 0 |  |  |  |  |  |  |  |  |
| GO:0021779\_oligodendrocyte\_cell\_fate\_commitment | 4 | 0 |  |  |  |  |  |  |  |  |
| GO:0021780\_glial\_cell\_fate\_specification | 4 | 0 |  |  |  |  |  |  |  |  |
| GO:0021801\_cerebral\_cortex\_radial\_glia\_guided\_migration | 4 | 0 |  |  |  |  |  |  |  |  |
| GO:0021830\_interneuron\_migration\_from\_the\_subpallium\_to\_the\_cortex | 4 | 0 |  |  |  |  |  |  |  |  |
| GO:0021853\_cerebral\_cortex\_GABAergic\_interneuron\_migration | 4 | 0 |  |  |  |  |  |  |  |  |
| GO:0021877\_forebrain\_neuron\_fate\_commitment | 4 | 0 |  |  |  |  |  |  |  |  |
| GO:0021894\_cerebral\_cortex\_GABAergic\_interneuron\_development | 4 | 0 |  |  |  |  |  |  |  |  |
| GO:0021910\_smoothened\_signaling\_pathway\_involved\_in\_ventral\_spinal\_cord\_patterning | 4 | 0 |  |  |  |  |  |  |  |  |
| GO:0021913\_regulation\_of\_transcription\_from\_RNA\_polymerase\_II\_promoter\_involved\_in\_ventral\_spinal\_cord\_interneuron\_specification | 4 | 0 |  |  |  |  |  |  |  |  |
| GO:0021938\_smoothened\_signaling\_pathway\_involved\_in\_regulation\_of\_granule\_cell\_precursor\_cell\_proliferation | 4 | 0 |  |  |  |  |  |  |  |  |
| GO:0021978\_telencephalon\_regionalization | 4 | 0 |  |  |  |  |  |  |  |  |
| GO:0022011\_myelination\_in\_the\_peripheral\_nervous\_system | 4 | 0 |  |  |  |  |  |  |  |  |
| GO:0030146\_diuresis | 4 | 0 |  |  |  |  |  |  |  |  |
| GO:0030300\_regulation\_of\_intestinal\_cholesterol\_absorption | 4 | 0 |  |  |  |  |  |  |  |  |
| GO:0030800\_negative\_regulation\_of\_cyclic\_nucleotide\_metabolic\_process | 4 | 0 |  |  |  |  |  |  |  |  |
| GO:0030803\_negative\_regulation\_of\_cyclic\_nucleotide\_biosynthetic\_process | 4 | 0 |  |  |  |  |  |  |  |  |
| GO:0030809\_negative\_regulation\_of\_nucleotide\_biosynthetic\_process | 4 | 0 |  |  |  |  |  |  |  |  |
| GO:0030815\_negative\_regulation\_of\_cAMP\_metabolic\_process | 4 | 0 |  |  |  |  |  |  |  |  |
| GO:0030816\_positive\_regulation\_of\_cAMP\_metabolic\_process | 4 | 0 |  |  |  |  |  |  |  |  |
| GO:0030818\_negative\_regulation\_of\_cAMP\_biosynthetic\_process | 4 | 0 |  |  |  |  |  |  |  |  |
| GO:0030819\_positive\_regulation\_of\_cAMP\_biosynthetic\_process | 4 | 0 |  |  |  |  |  |  |  |  |
| GO:0030826\_regulation\_of\_cGMP\_biosynthetic\_process | 4 | 0 |  |  |  |  |  |  |  |  |
| GO:0030858\_positive\_regulation\_of\_epithelial\_cell\_differentiation | 4 | 0 |  |  |  |  |  |  |  |  |
| GO:0030859\_polarized\_epithelial\_cell\_differentiation | 4 | 0 |  |  |  |  |  |  |  |  |
| GO:0030949\_positive\_regulation\_of\_vascular\_endothelial\_growth\_factor\_receptor\_signaling\_pathway | 4 | 0 |  |  |  |  |  |  |  |  |
| GO:0031113\_regulation\_of\_microtubule\_polymerization | 4 | 0 |  |  |  |  |  |  |  |  |
| GO:0031365\_N-terminal\_protein\_amino\_acid\_modification | 4 | 0 |  |  |  |  |  |  |  |  |
| GO:0031424\_keratinization | 4 | 0 |  |  |  |  |  |  |  |  |
| GO:0031557\_induction\_of\_programmed\_cell\_death\_in\_response\_to\_chemical\_stimulus | 4 | 0 |  |  |  |  |  |  |  |  |
| GO:0031558\_induction\_of\_apoptosis\_in\_response\_to\_chemical\_stimulus | 4 | 0 |  |  |  |  |  |  |  |  |
| GO:0031623\_receptor\_internalization | 4 | 0 |  |  |  |  |  |  |  |  |
| GO:0032088\_negative\_regulation\_of\_NF-kappaB\_transcription\_factor\_activity | 4 | 0 |  |  |  |  |  |  |  |  |
| GO:0032098\_regulation\_of\_appetite | 4 | 0 |  |  |  |  |  |  |  |  |
| GO:0032105\_negative\_regulation\_of\_response\_to\_extracellular\_stimulus | 4 | 0 |  |  |  |  |  |  |  |  |
| GO:0032108\_negative\_regulation\_of\_response\_to\_nutrient\_levels | 4 | 0 |  |  |  |  |  |  |  |  |
| GO:0032225\_regulation\_of\_synaptic\_transmission\_\_dopaminergic | 4 | 0 |  |  |  |  |  |  |  |  |
| GO:0032292\_ensheathment\_of\_axons\_in\_the\_peripheral\_nervous\_system | 4 | 0 |  |  |  |  |  |  |  |  |
| GO:0032321\_positive\_regulation\_of\_Rho\_GTPase\_activity | 4 | 0 |  |  |  |  |  |  |  |  |
| GO:0032371\_regulation\_of\_sterol\_transport | 4 | 0 |  |  |  |  |  |  |  |  |
| GO:0032374\_regulation\_of\_cholesterol\_transport | 4 | 0 |  |  |  |  |  |  |  |  |
| GO:0032401\_establishment\_of\_melanosome\_localization | 4 | 0 |  |  |  |  |  |  |  |  |
| GO:0032608\_interferon-beta\_production | 4 | 0 |  |  |  |  |  |  |  |  |
| GO:0032611\_interleukin-1\_beta\_production | 4 | 0 |  |  |  |  |  |  |  |  |
| GO:0032612\_interleukin-1\_production | 4 | 0 |  |  |  |  |  |  |  |  |
| GO:0032648\_regulation\_of\_interferon-beta\_production | 4 | 0 |  |  |  |  |  |  |  |  |
| GO:0032651\_regulation\_of\_interleukin-1\_beta\_production | 4 | 0 |  |  |  |  |  |  |  |  |
| GO:0032652\_regulation\_of\_interleukin-1\_production | 4 | 0 |  |  |  |  |  |  |  |  |
| GO:0032689\_negative\_regulation\_of\_interferon-gamma\_production | 4 | 0 |  |  |  |  |  |  |  |  |
| GO:0032713\_negative\_regulation\_of\_interleukin-4\_production | 4 | 0 |  |  |  |  |  |  |  |  |
| GO:0032715\_negative\_regulation\_of\_interleukin-6\_production | 4 | 0 |  |  |  |  |  |  |  |  |
| GO:0032733\_positive\_regulation\_of\_interleukin-10\_production | 4 | 0 |  |  |  |  |  |  |  |  |
| GO:0032808\_lacrimal\_gland\_development | 4 | 0 |  |  |  |  |  |  |  |  |
| GO:0032835\_glomerulus\_development | 4 | 0 |  |  |  |  |  |  |  |  |
| GO:0032922\_circadian\_regulation\_of\_gene\_expression | 4 | 0 |  |  |  |  |  |  |  |  |
| GO:0033026\_negative\_regulation\_of\_mast\_cell\_apoptosis | 4 | 0 |  |  |  |  |  |  |  |  |
| GO:0033079\_immature\_T\_cell\_proliferation | 4 | 0 |  |  |  |  |  |  |  |  |
| GO:0033083\_regulation\_of\_immature\_T\_cell\_proliferation | 4 | 0 |  |  |  |  |  |  |  |  |
| GO:0033089\_positive\_regulation\_of\_T\_cell\_differentiation\_in\_the\_thymus | 4 | 0 |  |  |  |  |  |  |  |  |
| GO:0033135\_regulation\_of\_peptidyl-serine\_phosphorylation | 4 | 0 |  |  |  |  |  |  |  |  |
| GO:0033299\_secretion\_of\_lysosomal\_enzymes | 4 | 0 |  |  |  |  |  |  |  |  |
| GO:0033327\_Leydig\_cell\_differentiation | 4 | 0 |  |  |  |  |  |  |  |  |
| GO:0033363\_secretory\_granule\_organization | 4 | 0 |  |  |  |  |  |  |  |  |
| GO:0033599\_regulation\_of\_mammary\_gland\_epithelial\_cell\_proliferation | 4 | 0 |  |  |  |  |  |  |  |  |
| GO:0033865\_nucleoside\_bisphosphate\_metabolic\_process | 4 | 0 |  |  |  |  |  |  |  |  |
| GO:0034204\_lipid\_translocation | 4 | 0 |  |  |  |  |  |  |  |  |
| GO:0034404\_nucleobase\_\_nucleoside\_and\_nucleotide\_biosynthetic\_process | 4 | 0 |  |  |  |  |  |  |  |  |
| GO:0034587\_piRNA\_metabolic\_process | 4 | 0 |  |  |  |  |  |  |  |  |
| GO:0034614\_cellular\_response\_to\_reactive\_oxygen\_species | 4 | 0 |  |  |  |  |  |  |  |  |
| GO:0034654\_nucleobase\_\_nucleoside\_\_nucleotide\_and\_nucleic\_acid\_biosynthetic\_process | 4 | 0 |  |  |  |  |  |  |  |  |
| GO:0035020\_regulation\_of\_Rac\_protein\_signal\_transduction | 4 | 0 |  |  |  |  |  |  |  |  |
| GO:0035082\_axoneme\_assembly | 4 | 0 |  |  |  |  |  |  |  |  |
| GO:0035188\_hatching | 4 | 0 |  |  |  |  |  |  |  |  |
| GO:0035235\_ionotropic\_glutamate\_receptor\_signaling\_pathway | 4 | 0 |  |  |  |  |  |  |  |  |
| GO:0042359\_vitamin\_D\_metabolic\_process | 4 | 0 |  |  |  |  |  |  |  |  |
| GO:0042428\_serotonin\_metabolic\_process | 4 | 0 |  |  |  |  |  |  |  |  |
| GO:0042451\_purine\_nucleoside\_biosynthetic\_process | 4 | 0 |  |  |  |  |  |  |  |  |
| GO:0042455\_ribonucleoside\_biosynthetic\_process | 4 | 0 |  |  |  |  |  |  |  |  |
| GO:0042473\_outer\_ear\_morphogenesis | 4 | 0 |  |  |  |  |  |  |  |  |
| GO:0042522\_regulation\_of\_tyrosine\_phosphorylation\_of\_Stat5\_protein | 4 | 0 |  |  |  |  |  |  |  |  |
| GO:0042535\_positive\_regulation\_of\_tumor\_necrosis\_factor\_biosynthetic\_process | 4 | 0 |  |  |  |  |  |  |  |  |
| GO:0042541\_hemoglobin\_biosynthetic\_process | 4 | 0 |  |  |  |  |  |  |  |  |
| GO:0042558\_pteridine\_and\_derivative\_metabolic\_process | 4 | 0 |  |  |  |  |  |  |  |  |
| GO:0042634\_regulation\_of\_hair\_cycle | 4 | 0 |  |  |  |  |  |  |  |  |
| GO:0042744\_hydrogen\_peroxide\_catabolic\_process | 4 | 0 |  |  |  |  |  |  |  |  |
| GO:0042773\_ATP\_synthesis\_coupled\_electron\_transport | 4 | 0 |  |  |  |  |  |  |  |  |
| GO:0042775\_mitochondrial\_ATP\_synthesis\_coupled\_electron\_transport | 4 | 0 |  |  |  |  |  |  |  |  |
| GO:0042832\_defense\_response\_to\_protozoan | 4 | 0 |  |  |  |  |  |  |  |  |
| GO:0042982\_amyloid\_precursor\_protein\_metabolic\_process | 4 | 0 |  |  |  |  |  |  |  |  |
| GO:0042992\_negative\_regulation\_of\_transcription\_factor\_import\_into\_nucleus | 4 | 0 |  |  |  |  |  |  |  |  |
| GO:0043043\_peptide\_biosynthetic\_process | 4 | 0 |  |  |  |  |  |  |  |  |
| GO:0043129\_surfactant\_homeostasis | 4 | 0 |  |  |  |  |  |  |  |  |
| GO:0043374\_CD8-positive\_\_alpha-beta\_T\_cell\_differentiation | 4 | 0 |  |  |  |  |  |  |  |  |
| GO:0043470\_regulation\_of\_carbohydrate\_catabolic\_process | 4 | 0 |  |  |  |  |  |  |  |  |
| GO:0043471\_regulation\_of\_cellular\_carbohydrate\_catabolic\_process | 4 | 0 |  |  |  |  |  |  |  |  |
| GO:0043484\_regulation\_of\_RNA\_splicing | 4 | 0 |  |  |  |  |  |  |  |  |
| GO:0043500\_muscle\_adaptation | 4 | 0 |  |  |  |  |  |  |  |  |
| GO:0043534\_blood\_vessel\_endothelial\_cell\_migration | 4 | 0 |  |  |  |  |  |  |  |  |
| GO:0043691\_reverse\_cholesterol\_transport | 4 | 0 |  |  |  |  |  |  |  |  |
| GO:0044243\_multicellular\_organismal\_catabolic\_process | 4 | 0 |  |  |  |  |  |  |  |  |
| GO:0044403\_symbiosis\_\_encompassing\_mutualism\_through\_parasitism | 4 | 0 |  |  |  |  |  |  |  |  |
| GO:0044419\_interspecies\_interaction\_between\_organisms | 4 | 0 |  |  |  |  |  |  |  |  |
| GO:0045066\_regulatory\_T\_cell\_differentiation | 4 | 0 |  |  |  |  |  |  |  |  |
| GO:0045078\_positive\_regulation\_of\_interferon-gamma\_biosynthetic\_process | 4 | 0 |  |  |  |  |  |  |  |  |
| GO:0045332\_phospholipid\_translocation | 4 | 0 |  |  |  |  |  |  |  |  |
| GO:0045346\_regulation\_of\_MHC\_class\_II\_biosynthetic\_process | 4 | 0 |  |  |  |  |  |  |  |  |
| GO:0045350\_interferon-beta\_biosynthetic\_process | 4 | 0 |  |  |  |  |  |  |  |  |
| GO:0045357\_regulation\_of\_interferon-beta\_biosynthetic\_process | 4 | 0 |  |  |  |  |  |  |  |  |
| GO:0045359\_positive\_regulation\_of\_interferon-beta\_biosynthetic\_process | 4 | 0 |  |  |  |  |  |  |  |  |
| GO:0045600\_positive\_regulation\_of\_fat\_cell\_differentiation | 4 | 0 |  |  |  |  |  |  |  |  |
| GO:0045616\_regulation\_of\_keratinocyte\_differentiation | 4 | 0 |  |  |  |  |  |  |  |  |
| GO:0045624\_positive\_regulation\_of\_T-helper\_cell\_differentiation | 4 | 0 |  |  |  |  |  |  |  |  |
| GO:0045628\_regulation\_of\_T-helper\_2\_cell\_differentiation | 4 | 0 |  |  |  |  |  |  |  |  |
| GO:0045634\_regulation\_of\_melanocyte\_differentiation | 4 | 0 |  |  |  |  |  |  |  |  |
| GO:0045647\_negative\_regulation\_of\_erythrocyte\_differentiation | 4 | 0 |  |  |  |  |  |  |  |  |
| GO:0045672\_positive\_regulation\_of\_osteoclast\_differentiation | 4 | 0 |  |  |  |  |  |  |  |  |
| GO:0045684\_positive\_regulation\_of\_epidermis\_development | 4 | 0 |  |  |  |  |  |  |  |  |
| GO:0045736\_negative\_regulation\_of\_cyclin-dependent\_protein\_kinase\_activity | 4 | 0 |  |  |  |  |  |  |  |  |
| GO:0045742\_positive\_regulation\_of\_epidermal\_growth\_factor\_receptor\_signaling\_pathway | 4 | 0 |  |  |  |  |  |  |  |  |
| GO:0045747\_positive\_regulation\_of\_Notch\_signaling\_pathway | 4 | 0 |  |  |  |  |  |  |  |  |
| GO:0045767\_regulation\_of\_anti-apoptosis | 4 | 0 |  |  |  |  |  |  |  |  |
| GO:0045779\_negative\_regulation\_of\_bone\_resorption | 4 | 0 |  |  |  |  |  |  |  |  |
| GO:0045923\_positive\_regulation\_of\_fatty\_acid\_metabolic\_process | 4 | 0 |  |  |  |  |  |  |  |  |
| GO:0045930\_negative\_regulation\_of\_mitotic\_cell\_cycle | 4 | 0 |  |  |  |  |  |  |  |  |
| GO:0045940\_positive\_regulation\_of\_steroid\_metabolic\_process | 4 | 0 |  |  |  |  |  |  |  |  |
| GO:0045980\_negative\_regulation\_of\_nucleotide\_metabolic\_process | 4 | 0 |  |  |  |  |  |  |  |  |
| GO:0046129\_purine\_ribonucleoside\_biosynthetic\_process | 4 | 0 |  |  |  |  |  |  |  |  |
| GO:0046173\_polyol\_biosynthetic\_process | 4 | 0 |  |  |  |  |  |  |  |  |
| GO:0046541\_saliva\_secretion | 4 | 0 |  |  |  |  |  |  |  |  |
| GO:0046548\_retinal\_rod\_cell\_development | 4 | 0 |  |  |  |  |  |  |  |  |
| GO:0046579\_positive\_regulation\_of\_Ras\_protein\_signal\_transduction | 4 | 0 |  |  |  |  |  |  |  |  |
| GO:0046639\_negative\_regulation\_of\_alpha-beta\_T\_cell\_differentiation | 4 | 0 |  |  |  |  |  |  |  |  |
| GO:0046642\_negative\_regulation\_of\_alpha-beta\_T\_cell\_proliferation | 4 | 0 |  |  |  |  |  |  |  |  |
| GO:0046668\_regulation\_of\_retinal\_cell\_programmed\_cell\_death | 4 | 0 |  |  |  |  |  |  |  |  |
| GO:0046686\_response\_to\_cadmium\_ion | 4 | 0 |  |  |  |  |  |  |  |  |
| GO:0046835\_carbohydrate\_phosphorylation | 4 | 0 |  |  |  |  |  |  |  |  |
| GO:0046902\_regulation\_of\_mitochondrial\_membrane\_permeability | 4 | 0 |  |  |  |  |  |  |  |  |
| GO:0047496\_vesicle\_transport\_along\_microtubule | 4 | 0 |  |  |  |  |  |  |  |  |
| GO:0048024\_regulation\_of\_nuclear\_mRNA\_splicing\_\_via\_spliceosome | 4 | 0 |  |  |  |  |  |  |  |  |
| GO:0048240\_sperm\_capacitation | 4 | 0 |  |  |  |  |  |  |  |  |
| GO:0048341\_paraxial\_mesoderm\_formation | 4 | 0 |  |  |  |  |  |  |  |  |
| GO:0048484\_enteric\_nervous\_system\_development | 4 | 0 |  |  |  |  |  |  |  |  |
| GO:0048512\_circadian\_behavior | 4 | 0 |  |  |  |  |  |  |  |  |
| GO:0048558\_embryonic\_gut\_morphogenesis | 4 | 0 |  |  |  |  |  |  |  |  |
| GO:0048639\_positive\_regulation\_of\_developmental\_growth | 4 | 0 |  |  |  |  |  |  |  |  |
| GO:0048841\_regulation\_of\_axon\_extension\_involved\_in\_axon\_guidance | 4 | 0 |  |  |  |  |  |  |  |  |
| GO:0048843\_negative\_regulation\_of\_axon\_extension\_involved\_in\_axon\_guidance | 4 | 0 |  |  |  |  |  |  |  |  |
| GO:0048846\_axon\_extension\_involved\_in\_axon\_guidance | 4 | 0 |  |  |  |  |  |  |  |  |
| GO:0048875\_chemical\_homeostasis\_within\_a\_tissue | 4 | 0 |  |  |  |  |  |  |  |  |
| GO:0048935\_peripheral\_nervous\_system\_neuron\_development | 4 | 0 |  |  |  |  |  |  |  |  |
| GO:0050702\_interleukin-1\_beta\_secretion | 4 | 0 |  |  |  |  |  |  |  |  |
| GO:0050704\_regulation\_of\_interleukin-1\_secretion | 4 | 0 |  |  |  |  |  |  |  |  |
| GO:0050706\_regulation\_of\_interleukin-1\_beta\_secretion | 4 | 0 |  |  |  |  |  |  |  |  |
| GO:0050716\_positive\_regulation\_of\_interleukin-1\_secretion | 4 | 0 |  |  |  |  |  |  |  |  |
| GO:0050718\_positive\_regulation\_of\_interleukin-1\_beta\_secretion | 4 | 0 |  |  |  |  |  |  |  |  |
| GO:0050820\_positive\_regulation\_of\_coagulation | 4 | 0 |  |  |  |  |  |  |  |  |
| GO:0050891\_multicellular\_organismal\_water\_homeostasis | 4 | 0 |  |  |  |  |  |  |  |  |
| GO:0050919\_negative\_chemotaxis | 4 | 0 |  |  |  |  |  |  |  |  |
| GO:0050932\_regulation\_of\_pigment\_cell\_differentiation | 4 | 0 |  |  |  |  |  |  |  |  |
| GO:0050961\_detection\_of\_temperature\_stimulus\_involved\_in\_sensory\_perception | 4 | 0 |  |  |  |  |  |  |  |  |
| GO:0050965\_detection\_of\_temperature\_stimulus\_involved\_in\_sensory\_perception\_of\_pain | 4 | 0 |  |  |  |  |  |  |  |  |
| GO:0050994\_regulation\_of\_lipid\_catabolic\_process | 4 | 0 |  |  |  |  |  |  |  |  |
| GO:0051024\_positive\_regulation\_of\_immunoglobulin\_secretion | 4 | 0 |  |  |  |  |  |  |  |  |
| GO:0051055\_negative\_regulation\_of\_lipid\_biosynthetic\_process | 4 | 0 |  |  |  |  |  |  |  |  |
| GO:0051124\_synaptic\_growth\_at\_neuromuscular\_junction | 4 | 0 |  |  |  |  |  |  |  |  |
| GO:0051148\_negative\_regulation\_of\_muscle\_cell\_differentiation | 4 | 0 |  |  |  |  |  |  |  |  |
| GO:0051205\_protein\_insertion\_into\_membrane | 4 | 0 |  |  |  |  |  |  |  |  |
| GO:0051341\_regulation\_of\_oxidoreductase\_activity | 4 | 0 |  |  |  |  |  |  |  |  |
| GO:0051452\_intracellular\_pH\_reduction | 4 | 0 |  |  |  |  |  |  |  |  |
| GO:0051567\_histone\_H3-K9\_methylation | 4 | 0 |  |  |  |  |  |  |  |  |
| GO:0051642\_centrosome\_localization | 4 | 0 |  |  |  |  |  |  |  |  |
| GO:0051797\_regulation\_of\_hair\_follicle\_development | 4 | 0 |  |  |  |  |  |  |  |  |
| GO:0051897\_positive\_regulation\_of\_protein\_kinase\_B\_signaling\_cascade | 4 | 0 |  |  |  |  |  |  |  |  |
| GO:0051904\_pigment\_granule\_transport | 4 | 0 |  |  |  |  |  |  |  |  |
| GO:0055009\_atrial\_cardiac\_muscle\_morphogenesis | 4 | 0 |  |  |  |  |  |  |  |  |
| GO:0060008\_Sertoli\_cell\_differentiation | 4 | 0 |  |  |  |  |  |  |  |  |
| GO:0060011\_Sertoli\_cell\_proliferation | 4 | 0 |  |  |  |  |  |  |  |  |
| GO:0060057\_apoptosis\_involved\_in\_mammary\_gland\_involution | 4 | 0 |  |  |  |  |  |  |  |  |
| GO:0060058\_positive\_regulation\_of\_apoptosis\_involved\_in\_mammary\_gland\_involution | 4 | 0 |  |  |  |  |  |  |  |  |
| GO:0060065\_uterus\_development | 4 | 0 |  |  |  |  |  |  |  |  |
| GO:0060087\_relaxation\_of\_vascular\_smooth\_muscle | 4 | 0 |  |  |  |  |  |  |  |  |
| GO:0060120\_inner\_ear\_receptor\_cell\_fate\_commitment | 4 | 0 |  |  |  |  |  |  |  |  |
| GO:0060157\_urinary\_bladder\_development | 4 | 0 |  |  |  |  |  |  |  |  |
| GO:0060158\_activation\_of\_phospholipase\_C\_activity\_by\_dopamine\_receptor\_signaling\_pathway | 4 | 0 |  |  |  |  |  |  |  |  |
| GO:0060164\_regulation\_of\_timing\_of\_neuron\_differentiation | 4 | 0 |  |  |  |  |  |  |  |  |
| GO:0060235\_lens\_induction\_in\_camera-type\_eye | 4 | 0 |  |  |  |  |  |  |  |  |
| GO:0060291\_long-term\_synaptic\_potentiation | 4 | 0 |  |  |  |  |  |  |  |  |
| GO:0060412\_ventricular\_septum\_morphogenesis | 4 | 0 |  |  |  |  |  |  |  |  |
| GO:0060459\_left\_lung\_development | 4 | 0 |  |  |  |  |  |  |  |  |
| GO:0060528\_secretory\_columnal\_luminar\_epithelial\_cell\_differentiation\_involved\_in\_prostate\_glandular\_acinus\_development | 4 | 0 |  |  |  |  |  |  |  |  |
| GO:0060561\_apoptosis\_involved\_in\_morphogenesis | 4 | 0 |  |  |  |  |  |  |  |  |
| GO:0060592\_mammary\_gland\_formation | 4 | 0 |  |  |  |  |  |  |  |  |
| GO:0060644\_mammary\_gland\_epithelial\_cell\_differentiation | 4 | 0 |  |  |  |  |  |  |  |  |
| GO:0060666\_dichotomous\_subdivision\_of\_terminal\_units\_involved\_in\_salivary\_gland\_branching | 4 | 0 |  |  |  |  |  |  |  |  |
| GO:0060737\_prostate\_gland\_morphogenetic\_growth | 4 | 0 |  |  |  |  |  |  |  |  |
| GO:0060743\_epithelial\_cell\_maturation\_involved\_in\_prostate\_gland\_development | 4 | 0 |  |  |  |  |  |  |  |  |
| GO:0060751\_mammary\_gland\_duct\_branch\_elongation | 4 | 0 |  |  |  |  |  |  |  |  |
| GO:0060900\_embryonic\_camera-type\_eye\_formation | 4 | 0 |  |  |  |  |  |  |  |  |
| GO:0070059\_apoptosis\_in\_response\_to\_endoplasmic\_reticulum\_stress | 4 | 0 |  |  |  |  |  |  |  |  |
| GO:0070254\_mucus\_secretion | 4 | 0 |  |  |  |  |  |  |  |  |
| GO:0070255\_regulation\_of\_mucus\_secretion | 4 | 0 |  |  |  |  |  |  |  |  |
| GO:0070301\_cellular\_response\_to\_hydrogen\_peroxide | 4 | 0 |  |  |  |  |  |  |  |  |
| GO:0070585\_protein\_localization\_in\_mitochondrion | 4 | 0 |  |  |  |  |  |  |  |  |
| GO:0010647\_positive\_regulation\_of\_cell\_communication | 110 | 0 | 0.000000 | -0.000000 | 937 | 822.814305 | 908.47 | 994.125695 | 0.969552 |
| GO:0048706\_embryonic\_skeletal\_system\_development | 73 | 0 | 0.000000 | -0.000000 | 939 | 826.495485 | 911.68 | 996.864515 | 0.970905 |
| GO:0051270\_regulation\_of\_cell\_motion | 73 | 0 | 0.000000 | -0.000000 | 939 | 826.495485 | 911.68 | 996.864515 | 0.970905 |
| GO:0006935\_chemotaxis | 53 | 0 | 0.000000 | -0.000000 | 944 | 836.380186 | 920.65 | 1004.919814 | 0.975265 |
| GO:0030031\_cell\_projection\_assembly | 53 | 0 | 0.000000 | -0.000000 | 944 | 836.380186 | 920.65 | 1004.919814 | 0.975265 |
| GO:0042330\_taxis | 53 | 0 | 0.000000 | -0.000000 | 944 | 836.380186 | 920.65 | 1004.919814 | 0.975265 |
| GO:0046942\_carboxylic\_acid\_transport | 53 | 0 | 0.000000 | -0.000000 | 944 | 836.380186 | 920.65 | 1004.919814 | 0.975265 |
| GO:0050905\_neuromuscular\_process | 53 | 0 | 0.000000 | -0.000000 | 944 | 836.380186 | 920.65 | 1004.919814 | 0.975265 |
| GO:0000278\_mitotic\_cell\_cycle | 80 | 0 | 0.000000 | -0.000000 | 949 | 840.099890 | 923.75 | 1007.400110 | 0.973393 |
| GO:0002250\_adaptive\_immune\_response | 80 | 0 | 0.000000 | -0.000000 | 949 | 840.099890 | 923.75 | 1007.400110 | 0.973393 |
| GO:0002460\_adaptive\_immune\_response\_based\_on\_somatic\_recombination\_of\_immune\_receptors\_built\_from\_immunoglobulin\_superfamily\_domains | 80 | 0 | 0.000000 | -0.000000 | 949 | 840.099890 | 923.75 | 1007.400110 | 0.973393 |
| GO:0006631\_fatty\_acid\_metabolic\_process | 80 | 0 | 0.000000 | -0.000000 | 949 | 840.099890 | 923.75 | 1007.400110 | 0.973393 |
| GO:0044092\_negative\_regulation\_of\_molecular\_function | 80 | 0 | 0.000000 | -0.000000 | 949 | 840.099890 | 923.75 | 1007.400110 | 0.973393 |
| GO:0001656\_metanephros\_development | 50 | 0 | 0.000000 | -0.000000 | 955 | 846.842519 | 929.74 | 1012.637481 | 0.973550 |
| GO:0002573\_myeloid\_leukocyte\_differentiation | 50 | 0 | 0.000000 | -0.000000 | 955 | 846.842519 | 929.74 | 1012.637481 | 0.973550 |
| GO:0009190\_cyclic\_nucleotide\_biosynthetic\_process | 50 | 0 | 0.000000 | -0.000000 | 955 | 846.842519 | 929.74 | 1012.637481 | 0.973550 |
| GO:0042129\_regulation\_of\_T\_cell\_proliferation | 50 | 0 | 0.000000 | -0.000000 | 955 | 846.842519 | 929.74 | 1012.637481 | 0.973550 |
| GO:0051606\_detection\_of\_stimulus | 50 | 0 | 0.000000 | -0.000000 | 955 | 846.842519 | 929.74 | 1012.637481 | 0.973550 |
| GO:0070647\_protein\_modification\_by\_small\_protein\_conjugation\_or\_removal | 50 | 0 | 0.000000 | -0.000000 | 955 | 846.842519 | 929.74 | 1012.637481 | 0.973550 |
| GO:0002764\_immune\_response-regulating\_signal\_transduction | 51 | 0 | 0.000000 | -0.000000 | 960 | 854.872717 | 937.36 | 1019.847283 | 0.976417 |
| GO:0006887\_exocytosis | 51 | 0 | 0.000000 | -0.000000 | 960 | 854.872717 | 937.36 | 1019.847283 | 0.976417 |
| GO:0007601\_visual\_perception | 51 | 0 | 0.000000 | -0.000000 | 960 | 854.872717 | 937.36 | 1019.847283 | 0.976417 |
| GO:0016569\_covalent\_chromatin\_modification | 51 | 0 | 0.000000 | -0.000000 | 960 | 854.872717 | 937.36 | 1019.847283 | 0.976417 |
| GO:0048747\_muscle\_fiber\_development | 51 | 0 | 0.000000 | -0.000000 | 960 | 854.872717 | 937.36 | 1019.847283 | 0.976417 |
| GO:0001818\_negative\_regulation\_of\_cytokine\_production | 18 | 0 | 0.000000 | -0.000000 | 1005 | 904.456742 | 985.06 | 1065.663258 | 0.980159 |
| GO:0001825\_blastocyst\_formation | 18 | 0 | 0.000000 | -0.000000 | 1005 | 904.456742 | 985.06 | 1065.663258 | 0.980159 |
| GO:0001974\_blood\_vessel\_remodeling | 18 | 0 | 0.000000 | -0.000000 | 1005 | 904.456742 | 985.06 | 1065.663258 | 0.980159 |
| GO:0002285\_lymphocyte\_activation\_during\_immune\_response | 18 | 0 | 0.000000 | -0.000000 | 1005 | 904.456742 | 985.06 | 1065.663258 | 0.980159 |
| GO:0002715\_regulation\_of\_natural\_killer\_cell\_mediated\_immunity | 18 | 0 | 0.000000 | -0.000000 | 1005 | 904.456742 | 985.06 | 1065.663258 | 0.980159 |
| GO:0003014\_renal\_system\_process | 18 | 0 | 0.000000 | -0.000000 | 1005 | 904.456742 | 985.06 | 1065.663258 | 0.980159 |
| GO:0006022\_aminoglycan\_metabolic\_process | 18 | 0 | 0.000000 | -0.000000 | 1005 | 904.456742 | 985.06 | 1065.663258 | 0.980159 |
| GO:0006940\_regulation\_of\_smooth\_muscle\_contraction | 18 | 0 | 0.000000 | -0.000000 | 1005 | 904.456742 | 985.06 | 1065.663258 | 0.980159 |
| GO:0007140\_male\_meiosis | 18 | 0 | 0.000000 | -0.000000 | 1005 | 904.456742 | 985.06 | 1065.663258 | 0.980159 |
| GO:0007608\_sensory\_perception\_of\_smell | 18 | 0 | 0.000000 | -0.000000 | 1005 | 904.456742 | 985.06 | 1065.663258 | 0.980159 |
| GO:0008589\_regulation\_of\_smoothened\_signaling\_pathway | 18 | 0 | 0.000000 | -0.000000 | 1005 | 904.456742 | 985.06 | 1065.663258 | 0.980159 |
| GO:0009063\_cellular\_amino\_acid\_catabolic\_process | 18 | 0 | 0.000000 | -0.000000 | 1005 | 904.456742 | 985.06 | 1065.663258 | 0.980159 |
| GO:0010498\_proteasomal\_protein\_catabolic\_process | 18 | 0 | 0.000000 | -0.000000 | 1005 | 904.456742 | 985.06 | 1065.663258 | 0.980159 |
| GO:0010553\_negative\_regulation\_of\_specific\_transcription\_from\_RNA\_polymerase\_II\_promoter | 18 | 0 | 0.000000 | -0.000000 | 1005 | 904.456742 | 985.06 | 1065.663258 | 0.980159 |
| GO:0015711\_organic\_anion\_transport | 18 | 0 | 0.000000 | -0.000000 | 1005 | 904.456742 | 985.06 | 1065.663258 | 0.980159 |
| GO:0016458\_gene\_silencing | 18 | 0 | 0.000000 | -0.000000 | 1005 | 904.456742 | 985.06 | 1065.663258 | 0.980159 |
| GO:0021517\_ventral\_spinal\_cord\_development | 18 | 0 | 0.000000 | -0.000000 | 1005 | 904.456742 | 985.06 | 1065.663258 | 0.980159 |
| GO:0021885\_forebrain\_cell\_migration | 18 | 0 | 0.000000 | -0.000000 | 1005 | 904.456742 | 985.06 | 1065.663258 | 0.980159 |
| GO:0030178\_negative\_regulation\_of\_Wnt\_receptor\_signaling\_pathway | 18 | 0 | 0.000000 | -0.000000 | 1005 | 904.456742 | 985.06 | 1065.663258 | 0.980159 |
| GO:0030203\_glycosaminoglycan\_metabolic\_process | 18 | 0 | 0.000000 | -0.000000 | 1005 | 904.456742 | 985.06 | 1065.663258 | 0.980159 |
| GO:0030282\_bone\_mineralization | 18 | 0 | 0.000000 | -0.000000 | 1005 | 904.456742 | 985.06 | 1065.663258 | 0.980159 |
| GO:0030318\_melanocyte\_differentiation | 18 | 0 | 0.000000 | -0.000000 | 1005 | 904.456742 | 985.06 | 1065.663258 | 0.980159 |
| GO:0030336\_negative\_regulation\_of\_cell\_migration | 18 | 0 | 0.000000 | -0.000000 | 1005 | 904.456742 | 985.06 | 1065.663258 | 0.980159 |
| GO:0030510\_regulation\_of\_BMP\_signaling\_pathway | 18 | 0 | 0.000000 | -0.000000 | 1005 | 904.456742 | 985.06 | 1065.663258 | 0.980159 |
| GO:0030901\_midbrain\_development | 18 | 0 | 0.000000 | -0.000000 | 1005 | 904.456742 | 985.06 | 1065.663258 | 0.980159 |
| GO:0032623\_interleukin-2\_production | 18 | 0 | 0.000000 | -0.000000 | 1005 | 904.456742 | 985.06 | 1065.663258 | 0.980159 |
| GO:0035051\_cardiac\_cell\_differentiation | 18 | 0 | 0.000000 | -0.000000 | 1005 | 904.456742 | 985.06 | 1065.663258 | 0.980159 |
| GO:0042269\_regulation\_of\_natural\_killer\_cell\_mediated\_cytotoxicity | 18 | 0 | 0.000000 | -0.000000 | 1005 | 904.456742 | 985.06 | 1065.663258 | 0.980159 |
| GO:0043029\_T\_cell\_homeostasis | 18 | 0 | 0.000000 | -0.000000 | 1005 | 904.456742 | 985.06 | 1065.663258 | 0.980159 |
| GO:0043161\_proteasomal\_ubiquitin-dependent\_protein\_catabolic\_process | 18 | 0 | 0.000000 | -0.000000 | 1005 | 904.456742 | 985.06 | 1065.663258 | 0.980159 |
| GO:0045058\_T\_cell\_selection | 18 | 0 | 0.000000 | -0.000000 | 1005 | 904.456742 | 985.06 | 1065.663258 | 0.980159 |
| GO:0045103\_intermediate\_filament-based\_process | 18 | 0 | 0.000000 | -0.000000 | 1005 | 904.456742 | 985.06 | 1065.663258 | 0.980159 |
| GO:0045638\_negative\_regulation\_of\_myeloid\_cell\_differentiation | 18 | 0 | 0.000000 | -0.000000 | 1005 | 904.456742 | 985.06 | 1065.663258 | 0.980159 |
| GO:0045807\_positive\_regulation\_of\_endocytosis | 18 | 0 | 0.000000 | -0.000000 | 1005 | 904.456742 | 985.06 | 1065.663258 | 0.980159 |
| GO:0046578\_regulation\_of\_Ras\_protein\_signal\_transduction | 18 | 0 | 0.000000 | -0.000000 | 1005 | 904.456742 | 985.06 | 1065.663258 | 0.980159 |
| GO:0046620\_regulation\_of\_organ\_growth | 18 | 0 | 0.000000 | -0.000000 | 1005 | 904.456742 | 985.06 | 1065.663258 | 0.980159 |
| GO:0048535\_lymph\_node\_development | 18 | 0 | 0.000000 | -0.000000 | 1005 | 904.456742 | 985.06 | 1065.663258 | 0.980159 |
| GO:0048730\_epidermis\_morphogenesis | 18 | 0 | 0.000000 | -0.000000 | 1005 | 904.456742 | 985.06 | 1065.663258 | 0.980159 |
| GO:0048813\_dendrite\_morphogenesis | 18 | 0 | 0.000000 | -0.000000 | 1005 | 904.456742 | 985.06 | 1065.663258 | 0.980159 |
| GO:0050731\_positive\_regulation\_of\_peptidyl-tyrosine\_phosphorylation | 18 | 0 | 0.000000 | -0.000000 | 1005 | 904.456742 | 985.06 | 1065.663258 | 0.980159 |
| GO:0050982\_detection\_of\_mechanical\_stimulus | 18 | 0 | 0.000000 | -0.000000 | 1005 | 904.456742 | 985.06 | 1065.663258 | 0.980159 |
| GO:0051168\_nuclear\_export | 18 | 0 | 0.000000 | -0.000000 | 1005 | 904.456742 | 985.06 | 1065.663258 | 0.980159 |
| GO:0051222\_positive\_regulation\_of\_protein\_transport | 18 | 0 | 0.000000 | -0.000000 | 1005 | 904.456742 | 985.06 | 1065.663258 | 0.980159 |
| GO:0051924\_regulation\_of\_calcium\_ion\_transport | 18 | 0 | 0.000000 | -0.000000 | 1005 | 904.456742 | 985.06 | 1065.663258 | 0.980159 |
| GO:0060674\_placenta\_blood\_vessel\_development | 18 | 0 | 0.000000 | -0.000000 | 1005 | 904.456742 | 985.06 | 1065.663258 | 0.980159 |
| GO:0001570\_vasculogenesis | 38 | 0 | 0.000000 | -0.000000 | 1021 | 921.975387 | 1001.22 | 1080.464613 | 0.980627 |
| GO:0001649\_osteoblast\_differentiation | 38 | 0 | 0.000000 | -0.000000 | 1021 | 921.975387 | 1001.22 | 1080.464613 | 0.980627 |
| GO:0001657\_ureteric\_bud\_development | 38 | 0 | 0.000000 | -0.000000 | 1021 | 921.975387 | 1001.22 | 1080.464613 | 0.980627 |
| GO:0002695\_negative\_regulation\_of\_leukocyte\_activation | 38 | 0 | 0.000000 | -0.000000 | 1021 | 921.975387 | 1001.22 | 1080.464613 | 0.980627 |
| GO:0006820\_anion\_transport | 38 | 0 | 0.000000 | -0.000000 | 1021 | 921.975387 | 1001.22 | 1080.464613 | 0.980627 |
| GO:0007596\_blood\_coagulation | 38 | 0 | 0.000000 | -0.000000 | 1021 | 921.975387 | 1001.22 | 1080.464613 | 0.980627 |
| GO:0010975\_regulation\_of\_neuron\_projection\_development | 38 | 0 | 0.000000 | -0.000000 | 1021 | 921.975387 | 1001.22 | 1080.464613 | 0.980627 |
| GO:0016042\_lipid\_catabolic\_process | 38 | 0 | 0.000000 | -0.000000 | 1021 | 921.975387 | 1001.22 | 1080.464613 | 0.980627 |
| GO:0031401\_positive\_regulation\_of\_protein\_modification\_process | 38 | 0 | 0.000000 | -0.000000 | 1021 | 921.975387 | 1001.22 | 1080.464613 | 0.980627 |
| GO:0032259\_methylation | 38 | 0 | 0.000000 | -0.000000 | 1021 | 921.975387 | 1001.22 | 1080.464613 | 0.980627 |
| GO:0042493\_response\_to\_drug | 38 | 0 | 0.000000 | -0.000000 | 1021 | 921.975387 | 1001.22 | 1080.464613 | 0.980627 |
| GO:0043414\_biopolymer\_methylation | 38 | 0 | 0.000000 | -0.000000 | 1021 | 921.975387 | 1001.22 | 1080.464613 | 0.980627 |
| GO:0045580\_regulation\_of\_T\_cell\_differentiation | 38 | 0 | 0.000000 | -0.000000 | 1021 | 921.975387 | 1001.22 | 1080.464613 | 0.980627 |
| GO:0050727\_regulation\_of\_inflammatory\_response | 38 | 0 | 0.000000 | -0.000000 | 1021 | 921.975387 | 1001.22 | 1080.464613 | 0.980627 |
| GO:0050866\_negative\_regulation\_of\_cell\_activation | 38 | 0 | 0.000000 | -0.000000 | 1021 | 921.975387 | 1001.22 | 1080.464613 | 0.980627 |
| GO:0051348\_negative\_regulation\_of\_transferase\_activity | 38 | 0 | 0.000000 | -0.000000 | 1021 | 921.975387 | 1001.22 | 1080.464613 | 0.980627 |
| GO:0005975\_carbohydrate\_metabolic\_process | 146 | 0 | 0.000000 | -0.000000 | 1023 | 923.464320 | 1002.44 | 1081.415680 | 0.979902 |
| GO:0030900\_forebrain\_development | 146 | 0 | 0.000000 | -0.000000 | 1023 | 923.464320 | 1002.44 | 1081.415680 | 0.979902 |
| GO:0032940\_secretion\_by\_cell | 149 | 0 | 0.000000 | -0.000000 | 1024 | 923.972602 | 1002.85 | 1081.727398 | 0.979346 |
| GO:0000060\_protein\_import\_into\_nucleus\_\_translocation | 14 | 0 | 0.000000 | -0.000000 | 1085 | 985.693067 | 1063.16 | 1140.626933 | 0.979871 |
| GO:0000077\_DNA\_damage\_checkpoint | 14 | 0 | 0.000000 | -0.000000 | 1085 | 985.693067 | 1063.16 | 1140.626933 | 0.979871 |
| GO:0001502\_cartilage\_condensation | 14 | 0 | 0.000000 | -0.000000 | 1085 | 985.693067 | 1063.16 | 1140.626933 | 0.979871 |
| GO:0001829\_trophectodermal\_cell\_differentiation | 14 | 0 | 0.000000 | -0.000000 | 1085 | 985.693067 | 1063.16 | 1140.626933 | 0.979871 |
| GO:0002027\_regulation\_of\_heart\_rate | 14 | 0 | 0.000000 | -0.000000 | 1085 | 985.693067 | 1063.16 | 1140.626933 | 0.979871 |
| GO:0002262\_myeloid\_cell\_homeostasis | 14 | 0 | 0.000000 | -0.000000 | 1085 | 985.693067 | 1063.16 | 1140.626933 | 0.979871 |
| GO:0002698\_negative\_regulation\_of\_immune\_effector\_process | 14 | 0 | 0.000000 | -0.000000 | 1085 | 985.693067 | 1063.16 | 1140.626933 | 0.979871 |
| GO:0006304\_DNA\_modification | 14 | 0 | 0.000000 | -0.000000 | 1085 | 985.693067 | 1063.16 | 1140.626933 | 0.979871 |
| GO:0006305\_DNA\_alkylation | 14 | 0 | 0.000000 | -0.000000 | 1085 | 985.693067 | 1063.16 | 1140.626933 | 0.979871 |
| GO:0006306\_DNA\_methylation | 14 | 0 | 0.000000 | -0.000000 | 1085 | 985.693067 | 1063.16 | 1140.626933 | 0.979871 |
| GO:0006695\_cholesterol\_biosynthetic\_process | 14 | 0 | 0.000000 | -0.000000 | 1085 | 985.693067 | 1063.16 | 1140.626933 | 0.979871 |
| GO:0006809\_nitric\_oxide\_biosynthetic\_process | 14 | 0 | 0.000000 | -0.000000 | 1085 | 985.693067 | 1063.16 | 1140.626933 | 0.979871 |
| GO:0006914\_autophagy | 14 | 0 | 0.000000 | -0.000000 | 1085 | 985.693067 | 1063.16 | 1140.626933 | 0.979871 |
| GO:0006970\_response\_to\_osmotic\_stress | 14 | 0 | 0.000000 | -0.000000 | 1085 | 985.693067 | 1063.16 | 1140.626933 | 0.979871 |
| GO:0007157\_heterophilic\_cell\_adhesion | 14 | 0 | 0.000000 | -0.000000 | 1085 | 985.693067 | 1063.16 | 1140.626933 | 0.979871 |
| GO:0007530\_sex\_determination | 14 | 0 | 0.000000 | -0.000000 | 1085 | 985.693067 | 1063.16 | 1140.626933 | 0.979871 |
| GO:0007589\_body\_fluid\_secretion | 14 | 0 | 0.000000 | -0.000000 | 1085 | 985.693067 | 1063.16 | 1140.626933 | 0.979871 |
| GO:0008306\_associative\_learning | 14 | 0 | 0.000000 | -0.000000 | 1085 | 985.693067 | 1063.16 | 1140.626933 | 0.979871 |
| GO:0008630\_DNA\_damage\_response\_\_signal\_transduction\_resulting\_in\_induction\_of\_apoptosis | 14 | 0 | 0.000000 | -0.000000 | 1085 | 985.693067 | 1063.16 | 1140.626933 | 0.979871 |
| GO:0009108\_coenzyme\_biosynthetic\_process | 14 | 0 | 0.000000 | -0.000000 | 1085 | 985.693067 | 1063.16 | 1140.626933 | 0.979871 |
| GO:0009267\_cellular\_response\_to\_starvation | 14 | 0 | 0.000000 | -0.000000 | 1085 | 985.693067 | 1063.16 | 1140.626933 | 0.979871 |
| GO:0009895\_negative\_regulation\_of\_catabolic\_process | 14 | 0 | 0.000000 | -0.000000 | 1085 | 985.693067 | 1063.16 | 1140.626933 | 0.979871 |
| GO:0010332\_response\_to\_gamma\_radiation | 14 | 0 | 0.000000 | -0.000000 | 1085 | 985.693067 | 1063.16 | 1140.626933 | 0.979871 |
| GO:0014855\_striated\_muscle\_cell\_proliferation | 14 | 0 | 0.000000 | -0.000000 | 1085 | 985.693067 | 1063.16 | 1140.626933 | 0.979871 |
| GO:0016573\_histone\_acetylation | 14 | 0 | 0.000000 | -0.000000 | 1085 | 985.693067 | 1063.16 | 1140.626933 | 0.979871 |
| GO:0018130\_heterocycle\_biosynthetic\_process | 14 | 0 | 0.000000 | -0.000000 | 1085 | 985.693067 | 1063.16 | 1140.626933 | 0.979871 |
| GO:0019217\_regulation\_of\_fatty\_acid\_metabolic\_process | 14 | 0 | 0.000000 | -0.000000 | 1085 | 985.693067 | 1063.16 | 1140.626933 | 0.979871 |
| GO:0021782\_glial\_cell\_development | 14 | 0 | 0.000000 | -0.000000 | 1085 | 985.693067 | 1063.16 | 1140.626933 | 0.979871 |
| GO:0021904\_dorsal\_ventral\_neural\_tube\_patterning | 14 | 0 | 0.000000 | -0.000000 | 1085 | 985.693067 | 1063.16 | 1140.626933 | 0.979871 |
| GO:0030032\_lamellipodium\_assembly | 14 | 0 | 0.000000 | -0.000000 | 1085 | 985.693067 | 1063.16 | 1140.626933 | 0.979871 |
| GO:0030148\_sphingolipid\_biosynthetic\_process | 14 | 0 | 0.000000 | -0.000000 | 1085 | 985.693067 | 1063.16 | 1140.626933 | 0.979871 |
| GO:0030162\_regulation\_of\_proteolysis | 14 | 0 | 0.000000 | -0.000000 | 1085 | 985.693067 | 1063.16 | 1140.626933 | 0.979871 |
| GO:0031346\_positive\_regulation\_of\_cell\_projection\_organization | 14 | 0 | 0.000000 | -0.000000 | 1085 | 985.693067 | 1063.16 | 1140.626933 | 0.979871 |
| GO:0031663\_lipopolysaccharide-mediated\_signaling\_pathway | 14 | 0 | 0.000000 | -0.000000 | 1085 | 985.693067 | 1063.16 | 1140.626933 | 0.979871 |
| GO:0032271\_regulation\_of\_protein\_polymerization | 14 | 0 | 0.000000 | -0.000000 | 1085 | 985.693067 | 1063.16 | 1140.626933 | 0.979871 |
| GO:0033044\_regulation\_of\_chromosome\_organization | 14 | 0 | 0.000000 | -0.000000 | 1085 | 985.693067 | 1063.16 | 1140.626933 | 0.979871 |
| GO:0034104\_negative\_regulation\_of\_tissue\_remodeling | 14 | 0 | 0.000000 | -0.000000 | 1085 | 985.693067 | 1063.16 | 1140.626933 | 0.979871 |
| GO:0035036\_sperm-egg\_recognition | 14 | 0 | 0.000000 | -0.000000 | 1085 | 985.693067 | 1063.16 | 1140.626933 | 0.979871 |
| GO:0042310\_vasoconstriction | 14 | 0 | 0.000000 | -0.000000 | 1085 | 985.693067 | 1063.16 | 1140.626933 | 0.979871 |
| GO:0042573\_retinoic\_acid\_metabolic\_process | 14 | 0 | 0.000000 | -0.000000 | 1085 | 985.693067 | 1063.16 | 1140.626933 | 0.979871 |
| GO:0043123\_positive\_regulation\_of\_I-kappaB\_kinase\_NF-kappaB\_cascade | 14 | 0 | 0.000000 | -0.000000 | 1085 | 985.693067 | 1063.16 | 1140.626933 | 0.979871 |
| GO:0043254\_regulation\_of\_protein\_complex\_assembly | 14 | 0 | 0.000000 | -0.000000 | 1085 | 985.693067 | 1063.16 | 1140.626933 | 0.979871 |
| GO:0043491\_protein\_kinase\_B\_signaling\_cascade | 14 | 0 | 0.000000 | -0.000000 | 1085 | 985.693067 | 1063.16 | 1140.626933 | 0.979871 |
| GO:0044236\_multicellular\_organismal\_metabolic\_process | 14 | 0 | 0.000000 | -0.000000 | 1085 | 985.693067 | 1063.16 | 1140.626933 | 0.979871 |
| GO:0045061\_thymic\_T\_cell\_selection | 14 | 0 | 0.000000 | -0.000000 | 1085 | 985.693067 | 1063.16 | 1140.626933 | 0.979871 |
| GO:0045453\_bone\_resorption | 14 | 0 | 0.000000 | -0.000000 | 1085 | 985.693067 | 1063.16 | 1140.626933 | 0.979871 |
| GO:0045598\_regulation\_of\_fat\_cell\_differentiation | 14 | 0 | 0.000000 | -0.000000 | 1085 | 985.693067 | 1063.16 | 1140.626933 | 0.979871 |
| GO:0045732\_positive\_regulation\_of\_protein\_catabolic\_process | 14 | 0 | 0.000000 | -0.000000 | 1085 | 985.693067 | 1063.16 | 1140.626933 | 0.979871 |
| GO:0046209\_nitric\_oxide\_metabolic\_process | 14 | 0 | 0.000000 | -0.000000 | 1085 | 985.693067 | 1063.16 | 1140.626933 | 0.979871 |
| GO:0048048\_embryonic\_eye\_morphogenesis | 14 | 0 | 0.000000 | -0.000000 | 1085 | 985.693067 | 1063.16 | 1140.626933 | 0.979871 |
| GO:0048545\_response\_to\_steroid\_hormone\_stimulus | 14 | 0 | 0.000000 | -0.000000 | 1085 | 985.693067 | 1063.16 | 1140.626933 | 0.979871 |
| GO:0048665\_neuron\_fate\_specification | 14 | 0 | 0.000000 | -0.000000 | 1085 | 985.693067 | 1063.16 | 1140.626933 | 0.979871 |
| GO:0048844\_artery\_morphogenesis | 14 | 0 | 0.000000 | -0.000000 | 1085 | 985.693067 | 1063.16 | 1140.626933 | 0.979871 |
| GO:0050810\_regulation\_of\_steroid\_biosynthetic\_process | 14 | 0 | 0.000000 | -0.000000 | 1085 | 985.693067 | 1063.16 | 1140.626933 | 0.979871 |
| GO:0051017\_actin\_filament\_bundle\_formation | 14 | 0 | 0.000000 | -0.000000 | 1085 | 985.693067 | 1063.16 | 1140.626933 | 0.979871 |
| GO:0051053\_negative\_regulation\_of\_DNA\_metabolic\_process | 14 | 0 | 0.000000 | -0.000000 | 1085 | 985.693067 | 1063.16 | 1140.626933 | 0.979871 |
| GO:0051054\_positive\_regulation\_of\_DNA\_metabolic\_process | 14 | 0 | 0.000000 | -0.000000 | 1085 | 985.693067 | 1063.16 | 1140.626933 | 0.979871 |
| GO:0051100\_negative\_regulation\_of\_binding | 14 | 0 | 0.000000 | -0.000000 | 1085 | 985.693067 | 1063.16 | 1140.626933 | 0.979871 |
| GO:0051952\_regulation\_of\_amine\_transport | 14 | 0 | 0.000000 | -0.000000 | 1085 | 985.693067 | 1063.16 | 1140.626933 | 0.979871 |
| GO:0060716\_labyrinthine\_layer\_blood\_vessel\_development | 14 | 0 | 0.000000 | -0.000000 | 1085 | 985.693067 | 1063.16 | 1140.626933 | 0.979871 |
| GO:0060840\_artery\_development | 14 | 0 | 0.000000 | -0.000000 | 1085 | 985.693067 | 1063.16 | 1140.626933 | 0.979871 |
| GO:0001508\_regulation\_of\_action\_potential | 43 | 0 | 0.000000 | -0.000000 | 1098 | 1000.714786 | 1076.97 | 1153.225214 | 0.980847 |
| GO:0002819\_regulation\_of\_adaptive\_immune\_response | 43 | 0 | 0.000000 | -0.000000 | 1098 | 1000.714786 | 1076.97 | 1153.225214 | 0.980847 |
| GO:0002822\_regulation\_of\_adaptive\_immune\_response\_based\_on\_somatic\_recombination\_of\_immune\_receptors\_built\_from\_immunoglobulin\_superfamily\_domains | 43 | 0 | 0.000000 | -0.000000 | 1098 | 1000.714786 | 1076.97 | 1153.225214 | 0.980847 |
| GO:0006766\_vitamin\_metabolic\_process | 43 | 0 | 0.000000 | -0.000000 | 1098 | 1000.714786 | 1076.97 | 1153.225214 | 0.980847 |
| GO:0007224\_smoothened\_signaling\_pathway | 43 | 0 | 0.000000 | -0.000000 | 1098 | 1000.714786 | 1076.97 | 1153.225214 | 0.980847 |
| GO:0009582\_detection\_of\_abiotic\_stimulus | 43 | 0 | 0.000000 | -0.000000 | 1098 | 1000.714786 | 1076.97 | 1153.225214 | 0.980847 |
| GO:0019637\_organophosphate\_metabolic\_process | 43 | 0 | 0.000000 | -0.000000 | 1098 | 1000.714786 | 1076.97 | 1153.225214 | 0.980847 |
| GO:0030814\_regulation\_of\_cAMP\_metabolic\_process | 43 | 0 | 0.000000 | -0.000000 | 1098 | 1000.714786 | 1076.97 | 1153.225214 | 0.980847 |
| GO:0032446\_protein\_modification\_by\_small\_protein\_conjugation | 43 | 0 | 0.000000 | -0.000000 | 1098 | 1000.714786 | 1076.97 | 1153.225214 | 0.980847 |
| GO:0032868\_response\_to\_insulin\_stimulus | 43 | 0 | 0.000000 | -0.000000 | 1098 | 1000.714786 | 1076.97 | 1153.225214 | 0.980847 |
| GO:0046879\_hormone\_secretion | 43 | 0 | 0.000000 | -0.000000 | 1098 | 1000.714786 | 1076.97 | 1153.225214 | 0.980847 |
| GO:0051604\_protein\_maturation | 43 | 0 | 0.000000 | -0.000000 | 1098 | 1000.714786 | 1076.97 | 1153.225214 | 0.980847 |
| GO:0051789\_response\_to\_protein\_stimulus | 43 | 0 | 0.000000 | -0.000000 | 1098 | 1000.714786 | 1076.97 | 1153.225214 | 0.980847 |
| GO:0002757\_immune\_response-activating\_signal\_transduction | 47 | 0 | 0.000000 | -0.000000 | 1107 | 1010.653599 | 1086.04 | 1161.426401 | 0.981066 |
| GO:0006140\_regulation\_of\_nucleotide\_metabolic\_process | 47 | 0 | 0.000000 | -0.000000 | 1107 | 1010.653599 | 1086.04 | 1161.426401 | 0.981066 |
| GO:0016570\_histone\_modification | 47 | 0 | 0.000000 | -0.000000 | 1107 | 1010.653599 | 1086.04 | 1161.426401 | 0.981066 |
| GO:0030183\_B\_cell\_differentiation | 47 | 0 | 0.000000 | -0.000000 | 1107 | 1010.653599 | 1086.04 | 1161.426401 | 0.981066 |
| GO:0030799\_regulation\_of\_cyclic\_nucleotide\_metabolic\_process | 47 | 0 | 0.000000 | -0.000000 | 1107 | 1010.653599 | 1086.04 | 1161.426401 | 0.981066 |
| GO:0031667\_response\_to\_nutrient\_levels | 47 | 0 | 0.000000 | -0.000000 | 1107 | 1010.653599 | 1086.04 | 1161.426401 | 0.981066 |
| GO:0034754\_cellular\_hormone\_metabolic\_process | 47 | 0 | 0.000000 | -0.000000 | 1107 | 1010.653599 | 1086.04 | 1161.426401 | 0.981066 |
| GO:0045619\_regulation\_of\_lymphocyte\_differentiation | 47 | 0 | 0.000000 | -0.000000 | 1107 | 1010.653599 | 1086.04 | 1161.426401 | 0.981066 |
| GO:0060627\_regulation\_of\_vesicle-mediated\_transport | 47 | 0 | 0.000000 | -0.000000 | 1107 | 1010.653599 | 1086.04 | 1161.426401 | 0.981066 |
| GO:0051704\_multi-organism\_process | 157 | 0 | 0.000000 | -0.000000 | 1108 | 1011.253881 | 1086.53 | 1161.806119 | 0.980623 |
| GO:0001776\_leukocyte\_homeostasis | 41 | 0 | 0.000000 | -0.000000 | 1126 | 1028.056277 | 1102.38 | 1176.703723 | 0.979023 |
| GO:0002429\_immune\_response-activating\_cell\_surface\_receptor\_signaling\_pathway | 41 | 0 | 0.000000 | -0.000000 | 1126 | 1028.056277 | 1102.38 | 1176.703723 | 0.979023 |
| GO:0006260\_DNA\_replication | 41 | 0 | 0.000000 | -0.000000 | 1126 | 1028.056277 | 1102.38 | 1176.703723 | 0.979023 |
| GO:0006836\_neurotransmitter\_transport | 41 | 0 | 0.000000 | -0.000000 | 1126 | 1028.056277 | 1102.38 | 1176.703723 | 0.979023 |
| GO:0006865\_amino\_acid\_transport | 41 | 0 | 0.000000 | -0.000000 | 1126 | 1028.056277 | 1102.38 | 1176.703723 | 0.979023 |
| GO:0007254\_JNK\_cascade | 41 | 0 | 0.000000 | -0.000000 | 1126 | 1028.056277 | 1102.38 | 1176.703723 | 0.979023 |
| GO:0008585\_female\_gonad\_development | 41 | 0 | 0.000000 | -0.000000 | 1126 | 1028.056277 | 1102.38 | 1176.703723 | 0.979023 |
| GO:0009894\_regulation\_of\_catabolic\_process | 41 | 0 | 0.000000 | -0.000000 | 1126 | 1028.056277 | 1102.38 | 1176.703723 | 0.979023 |
| GO:0010551\_regulation\_of\_specific\_transcription\_from\_RNA\_polymerase\_II\_promoter | 41 | 0 | 0.000000 | -0.000000 | 1126 | 1028.056277 | 1102.38 | 1176.703723 | 0.979023 |
| GO:0015833\_peptide\_transport | 41 | 0 | 0.000000 | -0.000000 | 1126 | 1028.056277 | 1102.38 | 1176.703723 | 0.979023 |
| GO:0015980\_energy\_derivation\_by\_oxidation\_of\_organic\_compounds | 41 | 0 | 0.000000 | -0.000000 | 1126 | 1028.056277 | 1102.38 | 1176.703723 | 0.979023 |
| GO:0019216\_regulation\_of\_lipid\_metabolic\_process | 41 | 0 | 0.000000 | -0.000000 | 1126 | 1028.056277 | 1102.38 | 1176.703723 | 0.979023 |
| GO:0019748\_secondary\_metabolic\_process | 41 | 0 | 0.000000 | -0.000000 | 1126 | 1028.056277 | 1102.38 | 1176.703723 | 0.979023 |
| GO:0030817\_regulation\_of\_cAMP\_biosynthetic\_process | 41 | 0 | 0.000000 | -0.000000 | 1126 | 1028.056277 | 1102.38 | 1176.703723 | 0.979023 |
| GO:0031344\_regulation\_of\_cell\_projection\_organization | 41 | 0 | 0.000000 | -0.000000 | 1126 | 1028.056277 | 1102.38 | 1176.703723 | 0.979023 |
| GO:0032569\_specific\_transcription\_from\_RNA\_polymerase\_II\_promoter | 41 | 0 | 0.000000 | -0.000000 | 1126 | 1028.056277 | 1102.38 | 1176.703723 | 0.979023 |
| GO:0033077\_T\_cell\_differentiation\_in\_the\_thymus | 41 | 0 | 0.000000 | -0.000000 | 1126 | 1028.056277 | 1102.38 | 1176.703723 | 0.979023 |
| GO:0050864\_regulation\_of\_B\_cell\_activation | 41 | 0 | 0.000000 | -0.000000 | 1126 | 1028.056277 | 1102.38 | 1176.703723 | 0.979023 |
| GO:0030163\_protein\_catabolic\_process | 101 | 0 | 0.000000 | -0.000000 | 1127 | 1028.763278 | 1103.01 | 1177.256722 | 0.978713 |
| GO:0000045\_autophagic\_vacuole\_formation | 5 | 0 | 0.000000 | -0.000000 | 1405 | 1311.645954 | 1382.75 | 1453.854046 | 0.984164 |
| GO:0000070\_mitotic\_sister\_chromatid\_segregation | 5 | 0 | 0.000000 | -0.000000 | 1405 | 1311.645954 | 1382.75 | 1453.854046 | 0.984164 |
| GO:0000239\_pachytene | 5 | 0 | 0.000000 | -0.000000 | 1405 | 1311.645954 | 1382.75 | 1453.854046 | 0.984164 |
| GO:0000272\_polysaccharide\_catabolic\_process | 5 | 0 | 0.000000 | -0.000000 | 1405 | 1311.645954 | 1382.75 | 1453.854046 | 0.984164 |
| GO:0000288\_nuclear-transcribed\_mRNA\_catabolic\_process\_\_deadenylation-dependent\_decay | 5 | 0 | 0.000000 | -0.000000 | 1405 | 1311.645954 | 1382.75 | 1453.854046 | 0.984164 |
| GO:0000578\_embryonic\_axis\_specification | 5 | 0 | 0.000000 | -0.000000 | 1405 | 1311.645954 | 1382.75 | 1453.854046 | 0.984164 |
| GO:0000726\_non-recombinational\_repair | 5 | 0 | 0.000000 | -0.000000 | 1405 | 1311.645954 | 1382.75 | 1453.854046 | 0.984164 |
| GO:0000819\_sister\_chromatid\_segregation | 5 | 0 | 0.000000 | -0.000000 | 1405 | 1311.645954 | 1382.75 | 1453.854046 | 0.984164 |
| GO:0000956\_nuclear-transcribed\_mRNA\_catabolic\_process | 5 | 0 | 0.000000 | -0.000000 | 1405 | 1311.645954 | 1382.75 | 1453.854046 | 0.984164 |
| GO:0001562\_response\_to\_protozoan | 5 | 0 | 0.000000 | -0.000000 | 1405 | 1311.645954 | 1382.75 | 1453.854046 | 0.984164 |
| GO:0001706\_endoderm\_formation | 5 | 0 | 0.000000 | -0.000000 | 1405 | 1311.645954 | 1382.75 | 1453.854046 | 0.984164 |
| GO:0001865\_NK\_T\_cell\_differentiation | 5 | 0 | 0.000000 | -0.000000 | 1405 | 1311.645954 | 1382.75 | 1453.854046 | 0.984164 |
| GO:0001945\_lymph\_vessel\_development | 5 | 0 | 0.000000 | -0.000000 | 1405 | 1311.645954 | 1382.75 | 1453.854046 | 0.984164 |
| GO:0001957\_intramembranous\_ossification | 5 | 0 | 0.000000 | -0.000000 | 1405 | 1311.645954 | 1382.75 | 1453.854046 | 0.984164 |
| GO:0001959\_regulation\_of\_cytokine-mediated\_signaling\_pathway | 5 | 0 | 0.000000 | -0.000000 | 1405 | 1311.645954 | 1382.75 | 1453.854046 | 0.984164 |
| GO:0001991\_regulation\_of\_systemic\_arterial\_blood\_pressure\_by\_circulatory\_renin-angiotensin | 5 | 0 | 0.000000 | -0.000000 | 1405 | 1311.645954 | 1382.75 | 1453.854046 | 0.984164 |
| GO:0001993\_regulation\_of\_systemic\_arterial\_blood\_pressure\_by\_norepinephrine-epinephrine | 5 | 0 | 0.000000 | -0.000000 | 1405 | 1311.645954 | 1382.75 | 1453.854046 | 0.984164 |
| GO:0002328\_pro-B\_cell\_differentiation | 5 | 0 | 0.000000 | -0.000000 | 1405 | 1311.645954 | 1382.75 | 1453.854046 | 0.984164 |
| GO:0002448\_mast\_cell\_mediated\_immunity | 5 | 0 | 0.000000 | -0.000000 | 1405 | 1311.645954 | 1382.75 | 1453.854046 | 0.984164 |
| GO:0002517\_T\_cell\_tolerance\_induction | 5 | 0 | 0.000000 | -0.000000 | 1405 | 1311.645954 | 1382.75 | 1453.854046 | 0.984164 |
| GO:0002634\_regulation\_of\_germinal\_center\_formation | 5 | 0 | 0.000000 | -0.000000 | 1405 | 1311.645954 | 1382.75 | 1453.854046 | 0.984164 |
| GO:0002664\_regulation\_of\_T\_cell\_tolerance\_induction | 5 | 0 | 0.000000 | -0.000000 | 1405 | 1311.645954 | 1382.75 | 1453.854046 | 0.984164 |
| GO:0002666\_positive\_regulation\_of\_T\_cell\_tolerance\_induction | 5 | 0 | 0.000000 | -0.000000 | 1405 | 1311.645954 | 1382.75 | 1453.854046 | 0.984164 |
| GO:0002710\_negative\_regulation\_of\_T\_cell\_mediated\_immunity | 5 | 0 | 0.000000 | -0.000000 | 1405 | 1311.645954 | 1382.75 | 1453.854046 | 0.984164 |
| GO:0002716\_negative\_regulation\_of\_natural\_killer\_cell\_mediated\_immunity | 5 | 0 | 0.000000 | -0.000000 | 1405 | 1311.645954 | 1382.75 | 1453.854046 | 0.984164 |
| GO:0002825\_regulation\_of\_T-helper\_1\_type\_immune\_response | 5 | 0 | 0.000000 | -0.000000 | 1405 | 1311.645954 | 1382.75 | 1453.854046 | 0.984164 |
| GO:0002862\_negative\_regulation\_of\_inflammatory\_response\_to\_antigenic\_stimulus | 5 | 0 | 0.000000 | -0.000000 | 1405 | 1311.645954 | 1382.75 | 1453.854046 | 0.984164 |
| GO:0002866\_positive\_regulation\_of\_acute\_inflammatory\_response\_to\_antigenic\_stimulus | 5 | 0 | 0.000000 | -0.000000 | 1405 | 1311.645954 | 1382.75 | 1453.854046 | 0.984164 |
| GO:0002885\_positive\_regulation\_of\_hypersensitivity | 5 | 0 | 0.000000 | -0.000000 | 1405 | 1311.645954 | 1382.75 | 1453.854046 | 0.984164 |
| GO:0002902\_regulation\_of\_B\_cell\_apoptosis | 5 | 0 | 0.000000 | -0.000000 | 1405 | 1311.645954 | 1382.75 | 1453.854046 | 0.984164 |
| GO:0003071\_renal\_system\_process\_involved\_in\_regulation\_of\_systemic\_arterial\_blood\_pressure | 5 | 0 | 0.000000 | -0.000000 | 1405 | 1311.645954 | 1382.75 | 1453.854046 | 0.984164 |
| GO:0005980\_glycogen\_catabolic\_process | 5 | 0 | 0.000000 | -0.000000 | 1405 | 1311.645954 | 1382.75 | 1453.854046 | 0.984164 |
| GO:0006023\_aminoglycan\_biosynthetic\_process | 5 | 0 | 0.000000 | -0.000000 | 1405 | 1311.645954 | 1382.75 | 1453.854046 | 0.984164 |
| GO:0006024\_glycosaminoglycan\_biosynthetic\_process | 5 | 0 | 0.000000 | -0.000000 | 1405 | 1311.645954 | 1382.75 | 1453.854046 | 0.984164 |
| GO:0006268\_DNA\_unwinding\_during\_replication | 5 | 0 | 0.000000 | -0.000000 | 1405 | 1311.645954 | 1382.75 | 1453.854046 | 0.984164 |
| GO:0006270\_DNA\_replication\_initiation | 5 | 0 | 0.000000 | -0.000000 | 1405 | 1311.645954 | 1382.75 | 1453.854046 | 0.984164 |
| GO:0006303\_double-strand\_break\_repair\_via\_nonhomologous\_end\_joining | 5 | 0 | 0.000000 | -0.000000 | 1405 | 1311.645954 | 1382.75 | 1453.854046 | 0.984164 |
| GO:0006346\_methylation-dependent\_chromatin\_silencing | 5 | 0 | 0.000000 | -0.000000 | 1405 | 1311.645954 | 1382.75 | 1453.854046 | 0.984164 |
| GO:0006376\_mRNA\_splice\_site\_selection | 5 | 0 | 0.000000 | -0.000000 | 1405 | 1311.645954 | 1382.75 | 1453.854046 | 0.984164 |
| GO:0006378\_mRNA\_polyadenylation | 5 | 0 | 0.000000 | -0.000000 | 1405 | 1311.645954 | 1382.75 | 1453.854046 | 0.984164 |
| GO:0006400\_tRNA\_modification | 5 | 0 | 0.000000 | -0.000000 | 1405 | 1311.645954 | 1382.75 | 1453.854046 | 0.984164 |
| GO:0006506\_GPI\_anchor\_biosynthetic\_process | 5 | 0 | 0.000000 | -0.000000 | 1405 | 1311.645954 | 1382.75 | 1453.854046 | 0.984164 |
| GO:0006509\_membrane\_protein\_ectodomain\_proteolysis | 5 | 0 | 0.000000 | -0.000000 | 1405 | 1311.645954 | 1382.75 | 1453.854046 | 0.984164 |
| GO:0006515\_misfolded\_or\_incompletely\_synthesized\_protein\_catabolic\_process | 5 | 0 | 0.000000 | -0.000000 | 1405 | 1311.645954 | 1382.75 | 1453.854046 | 0.984164 |
| GO:0006541\_glutamine\_metabolic\_process | 5 | 0 | 0.000000 | -0.000000 | 1405 | 1311.645954 | 1382.75 | 1453.854046 | 0.984164 |
| GO:0006570\_tyrosine\_metabolic\_process | 5 | 0 | 0.000000 | -0.000000 | 1405 | 1311.645954 | 1382.75 | 1453.854046 | 0.984164 |
| GO:0006577\_betaine\_metabolic\_process | 5 | 0 | 0.000000 | -0.000000 | 1405 | 1311.645954 | 1382.75 | 1453.854046 | 0.984164 |
| GO:0006586\_indolalkylamine\_metabolic\_process | 5 | 0 | 0.000000 | -0.000000 | 1405 | 1311.645954 | 1382.75 | 1453.854046 | 0.984164 |
| GO:0006636\_unsaturated\_fatty\_acid\_biosynthetic\_process | 5 | 0 | 0.000000 | -0.000000 | 1405 | 1311.645954 | 1382.75 | 1453.854046 | 0.984164 |
| GO:0006691\_leukotriene\_metabolic\_process | 5 | 0 | 0.000000 | -0.000000 | 1405 | 1311.645954 | 1382.75 | 1453.854046 | 0.984164 |
| GO:0006704\_glucocorticoid\_biosynthetic\_process | 5 | 0 | 0.000000 | -0.000000 | 1405 | 1311.645954 | 1382.75 | 1453.854046 | 0.984164 |
| GO:0006903\_vesicle\_targeting | 5 | 0 | 0.000000 | -0.000000 | 1405 | 1311.645954 | 1382.75 | 1453.854046 | 0.984164 |
| GO:0006924\_activation-induced\_cell\_death\_of\_T\_cells | 5 | 0 | 0.000000 | -0.000000 | 1405 | 1311.645954 | 1382.75 | 1453.854046 | 0.984164 |
| GO:0006929\_substrate-bound\_cell\_migration | 5 | 0 | 0.000000 | -0.000000 | 1405 | 1311.645954 | 1382.75 | 1453.854046 | 0.984164 |
| GO:0007029\_endoplasmic\_reticulum\_organization | 5 | 0 | 0.000000 | -0.000000 | 1405 | 1311.645954 | 1382.75 | 1453.854046 | 0.984164 |
| GO:0007091\_mitotic\_metaphase\_anaphase\_transition | 5 | 0 | 0.000000 | -0.000000 | 1405 | 1311.645954 | 1382.75 | 1453.854046 | 0.984164 |
| GO:0007320\_insemination | 5 | 0 | 0.000000 | -0.000000 | 1405 | 1311.645954 | 1382.75 | 1453.854046 | 0.984164 |
| GO:0007622\_rhythmic\_behavior | 5 | 0 | 0.000000 | -0.000000 | 1405 | 1311.645954 | 1382.75 | 1453.854046 | 0.984164 |
| GO:0007638\_mechanosensory\_behavior | 5 | 0 | 0.000000 | -0.000000 | 1405 | 1311.645954 | 1382.75 | 1453.854046 | 0.984164 |
| GO:0008343\_adult\_feeding\_behavior | 5 | 0 | 0.000000 | -0.000000 | 1405 | 1311.645954 | 1382.75 | 1453.854046 | 0.984164 |
| GO:0008631\_induction\_of\_apoptosis\_by\_oxidative\_stress | 5 | 0 | 0.000000 | -0.000000 | 1405 | 1311.645954 | 1382.75 | 1453.854046 | 0.984164 |
| GO:0009132\_nucleoside\_diphosphate\_metabolic\_process | 5 | 0 | 0.000000 | -0.000000 | 1405 | 1311.645954 | 1382.75 | 1453.854046 | 0.984164 |
| GO:0009146\_purine\_nucleoside\_triphosphate\_catabolic\_process | 5 | 0 | 0.000000 | -0.000000 | 1405 | 1311.645954 | 1382.75 | 1453.854046 | 0.984164 |
| GO:0009151\_purine\_deoxyribonucleotide\_metabolic\_process | 5 | 0 | 0.000000 | -0.000000 | 1405 | 1311.645954 | 1382.75 | 1453.854046 | 0.984164 |
| GO:0009154\_purine\_ribonucleotide\_catabolic\_process | 5 | 0 | 0.000000 | -0.000000 | 1405 | 1311.645954 | 1382.75 | 1453.854046 | 0.984164 |
| GO:0009215\_purine\_deoxyribonucleoside\_triphosphate\_metabolic\_process | 5 | 0 | 0.000000 | -0.000000 | 1405 | 1311.645954 | 1382.75 | 1453.854046 | 0.984164 |
| GO:0009251\_glucan\_catabolic\_process | 5 | 0 | 0.000000 | -0.000000 | 1405 | 1311.645954 | 1382.75 | 1453.854046 | 0.984164 |
| GO:0009261\_ribonucleotide\_catabolic\_process | 5 | 0 | 0.000000 | -0.000000 | 1405 | 1311.645954 | 1382.75 | 1453.854046 | 0.984164 |
| GO:0009263\_deoxyribonucleotide\_biosynthetic\_process | 5 | 0 | 0.000000 | -0.000000 | 1405 | 1311.645954 | 1382.75 | 1453.854046 | 0.984164 |
| GO:0009264\_deoxyribonucleotide\_catabolic\_process | 5 | 0 | 0.000000 | -0.000000 | 1405 | 1311.645954 | 1382.75 | 1453.854046 | 0.984164 |
| GO:0009296\_flagellum\_assembly | 5 | 0 | 0.000000 | -0.000000 | 1405 | 1311.645954 | 1382.75 | 1453.854046 | 0.984164 |
| GO:0009303\_rRNA\_transcription | 5 | 0 | 0.000000 | -0.000000 | 1405 | 1311.645954 | 1382.75 | 1453.854046 | 0.984164 |
| GO:0009312\_oligosaccharide\_biosynthetic\_process | 5 | 0 | 0.000000 | -0.000000 | 1405 | 1311.645954 | 1382.75 | 1453.854046 | 0.984164 |
| GO:0009437\_carnitine\_metabolic\_process | 5 | 0 | 0.000000 | -0.000000 | 1405 | 1311.645954 | 1382.75 | 1453.854046 | 0.984164 |
| GO:0009950\_dorsal\_ventral\_axis\_specification | 5 | 0 | 0.000000 | -0.000000 | 1405 | 1311.645954 | 1382.75 | 1453.854046 | 0.984164 |
| GO:0010460\_positive\_regulation\_of\_heart\_rate | 5 | 0 | 0.000000 | -0.000000 | 1405 | 1311.645954 | 1382.75 | 1453.854046 | 0.984164 |
| GO:0010522\_regulation\_of\_calcium\_ion\_transport\_into\_cytosol | 5 | 0 | 0.000000 | -0.000000 | 1405 | 1311.645954 | 1382.75 | 1453.854046 | 0.984164 |
| GO:0010676\_positive\_regulation\_of\_cellular\_carbohydrate\_metabolic\_process | 5 | 0 | 0.000000 | -0.000000 | 1405 | 1311.645954 | 1382.75 | 1453.854046 | 0.984164 |
| GO:0010761\_fibroblast\_migration | 5 | 0 | 0.000000 | -0.000000 | 1405 | 1311.645954 | 1382.75 | 1453.854046 | 0.984164 |
| GO:0010828\_positive\_regulation\_of\_glucose\_transport | 5 | 0 | 0.000000 | -0.000000 | 1405 | 1311.645954 | 1382.75 | 1453.854046 | 0.984164 |
| GO:0014044\_Schwann\_cell\_development | 5 | 0 | 0.000000 | -0.000000 | 1405 | 1311.645954 | 1382.75 | 1453.854046 | 0.984164 |
| GO:0014068\_positive\_regulation\_of\_phosphoinositide\_3-kinase\_cascade | 5 | 0 | 0.000000 | -0.000000 | 1405 | 1311.645954 | 1382.75 | 1453.854046 | 0.984164 |
| GO:0015802\_basic\_amino\_acid\_transport | 5 | 0 | 0.000000 | -0.000000 | 1405 | 1311.645954 | 1382.75 | 1453.854046 | 0.984164 |
| GO:0016048\_detection\_of\_temperature\_stimulus | 5 | 0 | 0.000000 | -0.000000 | 1405 | 1311.645954 | 1382.75 | 1453.854046 | 0.984164 |
| GO:0016188\_synaptic\_vesicle\_maturation | 5 | 0 | 0.000000 | -0.000000 | 1405 | 1311.645954 | 1382.75 | 1453.854046 | 0.984164 |
| GO:0016579\_protein\_deubiquitination | 5 | 0 | 0.000000 | -0.000000 | 1405 | 1311.645954 | 1382.75 | 1453.854046 | 0.984164 |
| GO:0017148\_negative\_regulation\_of\_translation | 5 | 0 | 0.000000 | -0.000000 | 1405 | 1311.645954 | 1382.75 | 1453.854046 | 0.984164 |
| GO:0017158\_regulation\_of\_calcium\_ion-dependent\_exocytosis | 5 | 0 | 0.000000 | -0.000000 | 1405 | 1311.645954 | 1382.75 | 1453.854046 | 0.984164 |
| GO:0019227\_neuronal\_action\_potential\_propagation | 5 | 0 | 0.000000 | -0.000000 | 1405 | 1311.645954 | 1382.75 | 1453.854046 | 0.984164 |
| GO:0021554\_optic\_nerve\_development | 5 | 0 | 0.000000 | -0.000000 | 1405 | 1311.645954 | 1382.75 | 1453.854046 | 0.984164 |
| GO:0021772\_olfactory\_bulb\_development | 5 | 0 | 0.000000 | -0.000000 | 1405 | 1311.645954 | 1382.75 | 1453.854046 | 0.984164 |
| GO:0021826\_substrate-independent\_telencephalic\_tangential\_migration | 5 | 0 | 0.000000 | -0.000000 | 1405 | 1311.645954 | 1382.75 | 1453.854046 | 0.984164 |
| GO:0021843\_substrate-independent\_telencephalic\_tangential\_interneuron\_migration | 5 | 0 | 0.000000 | -0.000000 | 1405 | 1311.645954 | 1382.75 | 1453.854046 | 0.984164 |
| GO:0021854\_hypothalamus\_development | 5 | 0 | 0.000000 | -0.000000 | 1405 | 1311.645954 | 1382.75 | 1453.854046 | 0.984164 |
| GO:0021988\_olfactory\_lobe\_development | 5 | 0 | 0.000000 | -0.000000 | 1405 | 1311.645954 | 1382.75 | 1453.854046 | 0.984164 |
| GO:0022415\_viral\_reproductive\_process | 5 | 0 | 0.000000 | -0.000000 | 1405 | 1311.645954 | 1382.75 | 1453.854046 | 0.984164 |
| GO:0030004\_cellular\_monovalent\_inorganic\_cation\_homeostasis | 5 | 0 | 0.000000 | -0.000000 | 1405 | 1311.645954 | 1382.75 | 1453.854046 | 0.984164 |
| GO:0030177\_positive\_regulation\_of\_Wnt\_receptor\_signaling\_pathway | 5 | 0 | 0.000000 | -0.000000 | 1405 | 1311.645954 | 1382.75 | 1453.854046 | 0.984164 |
| GO:0030261\_chromosome\_condensation | 5 | 0 | 0.000000 | -0.000000 | 1405 | 1311.645954 | 1382.75 | 1453.854046 | 0.984164 |
| GO:0030299\_intestinal\_cholesterol\_absorption | 5 | 0 | 0.000000 | -0.000000 | 1405 | 1311.645954 | 1382.75 | 1453.854046 | 0.984164 |
| GO:0030431\_sleep | 5 | 0 | 0.000000 | -0.000000 | 1405 | 1311.645954 | 1382.75 | 1453.854046 | 0.984164 |
| GO:0030433\_ER-associated\_protein\_catabolic\_process | 5 | 0 | 0.000000 | -0.000000 | 1405 | 1311.645954 | 1382.75 | 1453.854046 | 0.984164 |
| GO:0030641\_regulation\_of\_cellular\_pH | 5 | 0 | 0.000000 | -0.000000 | 1405 | 1311.645954 | 1382.75 | 1453.854046 | 0.984164 |
| GO:0030801\_positive\_regulation\_of\_cyclic\_nucleotide\_metabolic\_process | 5 | 0 | 0.000000 | -0.000000 | 1405 | 1311.645954 | 1382.75 | 1453.854046 | 0.984164 |
| GO:0030804\_positive\_regulation\_of\_cyclic\_nucleotide\_biosynthetic\_process | 5 | 0 | 0.000000 | -0.000000 | 1405 | 1311.645954 | 1382.75 | 1453.854046 | 0.984164 |
| GO:0030810\_positive\_regulation\_of\_nucleotide\_biosynthetic\_process | 5 | 0 | 0.000000 | -0.000000 | 1405 | 1311.645954 | 1382.75 | 1453.854046 | 0.984164 |
| GO:0030823\_regulation\_of\_cGMP\_metabolic\_process | 5 | 0 | 0.000000 | -0.000000 | 1405 | 1311.645954 | 1382.75 | 1453.854046 | 0.984164 |
| GO:0030838\_positive\_regulation\_of\_actin\_filament\_polymerization | 5 | 0 | 0.000000 | -0.000000 | 1405 | 1311.645954 | 1382.75 | 1453.854046 | 0.984164 |
| GO:0030851\_granulocyte\_differentiation | 5 | 0 | 0.000000 | -0.000000 | 1405 | 1311.645954 | 1382.75 | 1453.854046 | 0.984164 |
| GO:0030866\_cortical\_actin\_cytoskeleton\_organization | 5 | 0 | 0.000000 | -0.000000 | 1405 | 1311.645954 | 1382.75 | 1453.854046 | 0.984164 |
| GO:0030889\_negative\_regulation\_of\_B\_cell\_proliferation | 5 | 0 | 0.000000 | -0.000000 | 1405 | 1311.645954 | 1382.75 | 1453.854046 | 0.984164 |
| GO:0030917\_midbrain-hindbrain\_boundary\_development | 5 | 0 | 0.000000 | -0.000000 | 1405 | 1311.645954 | 1382.75 | 1453.854046 | 0.984164 |
| GO:0031047\_gene\_silencing\_by\_RNA | 5 | 0 | 0.000000 | -0.000000 | 1405 | 1311.645954 | 1382.75 | 1453.854046 | 0.984164 |
| GO:0031057\_negative\_regulation\_of\_histone\_modification | 5 | 0 | 0.000000 | -0.000000 | 1405 | 1311.645954 | 1382.75 | 1453.854046 | 0.984164 |
| GO:0031058\_positive\_regulation\_of\_histone\_modification | 5 | 0 | 0.000000 | -0.000000 | 1405 | 1311.645954 | 1382.75 | 1453.854046 | 0.984164 |
| GO:0031060\_regulation\_of\_histone\_methylation | 5 | 0 | 0.000000 | -0.000000 | 1405 | 1311.645954 | 1382.75 | 1453.854046 | 0.984164 |
| GO:0031122\_cytoplasmic\_microtubule\_organization | 5 | 0 | 0.000000 | -0.000000 | 1405 | 1311.645954 | 1382.75 | 1453.854046 | 0.984164 |
| GO:0031532\_actin\_cytoskeleton\_reorganization | 5 | 0 | 0.000000 | -0.000000 | 1405 | 1311.645954 | 1382.75 | 1453.854046 | 0.984164 |
| GO:0032024\_positive\_regulation\_of\_insulin\_secretion | 5 | 0 | 0.000000 | -0.000000 | 1405 | 1311.645954 | 1382.75 | 1453.854046 | 0.984164 |
| GO:0032320\_positive\_regulation\_of\_Ras\_GTPase\_activity | 5 | 0 | 0.000000 | -0.000000 | 1405 | 1311.645954 | 1382.75 | 1453.854046 | 0.984164 |
| GO:0032332\_positive\_regulation\_of\_chondrocyte\_differentiation | 5 | 0 | 0.000000 | -0.000000 | 1405 | 1311.645954 | 1382.75 | 1453.854046 | 0.984164 |
| GO:0032350\_regulation\_of\_hormone\_metabolic\_process | 5 | 0 | 0.000000 | -0.000000 | 1405 | 1311.645954 | 1382.75 | 1453.854046 | 0.984164 |
| GO:0032368\_regulation\_of\_lipid\_transport | 5 | 0 | 0.000000 | -0.000000 | 1405 | 1311.645954 | 1382.75 | 1453.854046 | 0.984164 |
| GO:0032400\_melanosome\_localization | 5 | 0 | 0.000000 | -0.000000 | 1405 | 1311.645954 | 1382.75 | 1453.854046 | 0.984164 |
| GO:0032434\_regulation\_of\_proteasomal\_ubiquitin-dependent\_protein\_catabolic\_process | 5 | 0 | 0.000000 | -0.000000 | 1405 | 1311.645954 | 1382.75 | 1453.854046 | 0.984164 |
| GO:0032494\_response\_to\_peptidoglycan | 5 | 0 | 0.000000 | -0.000000 | 1405 | 1311.645954 | 1382.75 | 1453.854046 | 0.984164 |
| GO:0032508\_DNA\_duplex\_unwinding | 5 | 0 | 0.000000 | -0.000000 | 1405 | 1311.645954 | 1382.75 | 1453.854046 | 0.984164 |
| GO:0032720\_negative\_regulation\_of\_tumor\_necrosis\_factor\_production | 5 | 0 | 0.000000 | -0.000000 | 1405 | 1311.645954 | 1382.75 | 1453.854046 | 0.984164 |
| GO:0032845\_negative\_regulation\_of\_homeostatic\_process | 5 | 0 | 0.000000 | -0.000000 | 1405 | 1311.645954 | 1382.75 | 1453.854046 | 0.984164 |
| GO:0032846\_positive\_regulation\_of\_homeostatic\_process | 5 | 0 | 0.000000 | -0.000000 | 1405 | 1311.645954 | 1382.75 | 1453.854046 | 0.984164 |
| GO:0033003\_regulation\_of\_mast\_cell\_activation | 5 | 0 | 0.000000 | -0.000000 | 1405 | 1311.645954 | 1382.75 | 1453.854046 | 0.984164 |
| GO:0033005\_positive\_regulation\_of\_mast\_cell\_activation | 5 | 0 | 0.000000 | -0.000000 | 1405 | 1311.645954 | 1382.75 | 1453.854046 | 0.984164 |
| GO:0033023\_mast\_cell\_homeostasis | 5 | 0 | 0.000000 | -0.000000 | 1405 | 1311.645954 | 1382.75 | 1453.854046 | 0.984164 |
| GO:0033024\_mast\_cell\_apoptosis | 5 | 0 | 0.000000 | -0.000000 | 1405 | 1311.645954 | 1382.75 | 1453.854046 | 0.984164 |
| GO:0033025\_regulation\_of\_mast\_cell\_apoptosis | 5 | 0 | 0.000000 | -0.000000 | 1405 | 1311.645954 | 1382.75 | 1453.854046 | 0.984164 |
| GO:0033033\_negative\_regulation\_of\_myeloid\_cell\_apoptosis | 5 | 0 | 0.000000 | -0.000000 | 1405 | 1311.645954 | 1382.75 | 1453.854046 | 0.984164 |
| GO:0033144\_negative\_regulation\_of\_steroid\_hormone\_receptor\_signaling\_pathway | 5 | 0 | 0.000000 | -0.000000 | 1405 | 1311.645954 | 1382.75 | 1453.854046 | 0.984164 |
| GO:0033146\_regulation\_of\_estrogen\_receptor\_signaling\_pathway | 5 | 0 | 0.000000 | -0.000000 | 1405 | 1311.645954 | 1382.75 | 1453.854046 | 0.984164 |
| GO:0033152\_immunoglobulin\_V(D)J\_recombination | 5 | 0 | 0.000000 | -0.000000 | 1405 | 1311.645954 | 1382.75 | 1453.854046 | 0.984164 |
| GO:0033205\_cytokinesis\_during\_cell\_cycle | 5 | 0 | 0.000000 | -0.000000 | 1405 | 1311.645954 | 1382.75 | 1453.854046 | 0.984164 |
| GO:0033619\_membrane\_protein\_proteolysis | 5 | 0 | 0.000000 | -0.000000 | 1405 | 1311.645954 | 1382.75 | 1453.854046 | 0.984164 |
| GO:0033627\_cell\_adhesion\_mediated\_by\_integrin | 5 | 0 | 0.000000 | -0.000000 | 1405 | 1311.645954 | 1382.75 | 1453.854046 | 0.984164 |
| GO:0033692\_cellular\_polysaccharide\_biosynthetic\_process | 5 | 0 | 0.000000 | -0.000000 | 1405 | 1311.645954 | 1382.75 | 1453.854046 | 0.984164 |
| GO:0034109\_homotypic\_cell-cell\_adhesion | 5 | 0 | 0.000000 | -0.000000 | 1405 | 1311.645954 | 1382.75 | 1453.854046 | 0.984164 |
| GO:0034367\_macromolecular\_complex\_remodeling | 5 | 0 | 0.000000 | -0.000000 | 1405 | 1311.645954 | 1382.75 | 1453.854046 | 0.984164 |
| GO:0034368\_protein-lipid\_complex\_remodeling | 5 | 0 | 0.000000 | -0.000000 | 1405 | 1311.645954 | 1382.75 | 1453.854046 | 0.984164 |
| GO:0034369\_plasma\_lipoprotein\_particle\_remodeling | 5 | 0 | 0.000000 | -0.000000 | 1405 | 1311.645954 | 1382.75 | 1453.854046 | 0.984164 |
| GO:0035058\_sensory\_cilium\_assembly | 5 | 0 | 0.000000 | -0.000000 | 1405 | 1311.645954 | 1382.75 | 1453.854046 | 0.984164 |
| GO:0035065\_regulation\_of\_histone\_acetylation | 5 | 0 | 0.000000 | -0.000000 | 1405 | 1311.645954 | 1382.75 | 1453.854046 | 0.984164 |
| GO:0035089\_establishment\_of\_apical\_basal\_cell\_polarity | 5 | 0 | 0.000000 | -0.000000 | 1405 | 1311.645954 | 1382.75 | 1453.854046 | 0.984164 |
| GO:0035095\_behavioral\_response\_to\_nicotine | 5 | 0 | 0.000000 | -0.000000 | 1405 | 1311.645954 | 1382.75 | 1453.854046 | 0.984164 |
| GO:0035234\_germ\_cell\_programmed\_cell\_death | 5 | 0 | 0.000000 | -0.000000 | 1405 | 1311.645954 | 1382.75 | 1453.854046 | 0.984164 |
| GO:0035238\_vitamin\_A\_biosynthetic\_process | 5 | 0 | 0.000000 | -0.000000 | 1405 | 1311.645954 | 1382.75 | 1453.854046 | 0.984164 |
| GO:0042159\_lipoprotein\_catabolic\_process | 5 | 0 | 0.000000 | -0.000000 | 1405 | 1311.645954 | 1382.75 | 1453.854046 | 0.984164 |
| GO:0042362\_fat-soluble\_vitamin\_biosynthetic\_process | 5 | 0 | 0.000000 | -0.000000 | 1405 | 1311.645954 | 1382.75 | 1453.854046 | 0.984164 |
| GO:0042364\_water-soluble\_vitamin\_biosynthetic\_process | 5 | 0 | 0.000000 | -0.000000 | 1405 | 1311.645954 | 1382.75 | 1453.854046 | 0.984164 |
| GO:0042416\_dopamine\_biosynthetic\_process | 5 | 0 | 0.000000 | -0.000000 | 1405 | 1311.645954 | 1382.75 | 1453.854046 | 0.984164 |
| GO:0042430\_indole\_and\_derivative\_metabolic\_process | 5 | 0 | 0.000000 | -0.000000 | 1405 | 1311.645954 | 1382.75 | 1453.854046 | 0.984164 |
| GO:0042434\_indole\_derivative\_metabolic\_process | 5 | 0 | 0.000000 | -0.000000 | 1405 | 1311.645954 | 1382.75 | 1453.854046 | 0.984164 |
| GO:0042487\_regulation\_of\_odontogenesis\_of\_dentine-containing\_tooth | 5 | 0 | 0.000000 | -0.000000 | 1405 | 1311.645954 | 1382.75 | 1453.854046 | 0.984164 |
| GO:0042506\_tyrosine\_phosphorylation\_of\_Stat5\_protein | 5 | 0 | 0.000000 | -0.000000 | 1405 | 1311.645954 | 1382.75 | 1453.854046 | 0.984164 |
| GO:0042516\_regulation\_of\_tyrosine\_phosphorylation\_of\_Stat3\_protein | 5 | 0 | 0.000000 | -0.000000 | 1405 | 1311.645954 | 1382.75 | 1453.854046 | 0.984164 |
| GO:0042531\_positive\_regulation\_of\_tyrosine\_phosphorylation\_of\_STAT\_protein | 5 | 0 | 0.000000 | -0.000000 | 1405 | 1311.645954 | 1382.75 | 1453.854046 | 0.984164 |
| GO:0042533\_tumor\_necrosis\_factor\_biosynthetic\_process | 5 | 0 | 0.000000 | -0.000000 | 1405 | 1311.645954 | 1382.75 | 1453.854046 | 0.984164 |
| GO:0042534\_regulation\_of\_tumor\_necrosis\_factor\_biosynthetic\_process | 5 | 0 | 0.000000 | -0.000000 | 1405 | 1311.645954 | 1382.75 | 1453.854046 | 0.984164 |
| GO:0042537\_benzene\_and\_derivative\_metabolic\_process | 5 | 0 | 0.000000 | -0.000000 | 1405 | 1311.645954 | 1382.75 | 1453.854046 | 0.984164 |
| GO:0042554\_superoxide\_anion\_generation | 5 | 0 | 0.000000 | -0.000000 | 1405 | 1311.645954 | 1382.75 | 1453.854046 | 0.984164 |
| GO:0042574\_retinal\_metabolic\_process | 5 | 0 | 0.000000 | -0.000000 | 1405 | 1311.645954 | 1382.75 | 1453.854046 | 0.984164 |
| GO:0042590\_antigen\_processing\_and\_presentation\_of\_exogenous\_peptide\_antigen\_via\_MHC\_class\_I | 5 | 0 | 0.000000 | -0.000000 | 1405 | 1311.645954 | 1382.75 | 1453.854046 | 0.984164 |
| GO:0042695\_thelarche | 5 | 0 | 0.000000 | -0.000000 | 1405 | 1311.645954 | 1382.75 | 1453.854046 | 0.984164 |
| GO:0042752\_regulation\_of\_circadian\_rhythm | 5 | 0 | 0.000000 | -0.000000 | 1405 | 1311.645954 | 1382.75 | 1453.854046 | 0.984164 |
| GO:0042756\_drinking\_behavior | 5 | 0 | 0.000000 | -0.000000 | 1405 | 1311.645954 | 1382.75 | 1453.854046 | 0.984164 |
| GO:0042904\_9-cis-retinoic\_acid\_biosynthetic\_process | 5 | 0 | 0.000000 | -0.000000 | 1405 | 1311.645954 | 1382.75 | 1453.854046 | 0.984164 |
| GO:0042905\_9-cis-retinoic\_acid\_metabolic\_process | 5 | 0 | 0.000000 | -0.000000 | 1405 | 1311.645954 | 1382.75 | 1453.854046 | 0.984164 |
| GO:0043030\_regulation\_of\_macrophage\_activation | 5 | 0 | 0.000000 | -0.000000 | 1405 | 1311.645954 | 1382.75 | 1453.854046 | 0.984164 |
| GO:0043046\_DNA\_methylation\_during\_gametogenesis | 5 | 0 | 0.000000 | -0.000000 | 1405 | 1311.645954 | 1382.75 | 1453.854046 | 0.984164 |
| GO:0043266\_regulation\_of\_potassium\_ion\_transport | 5 | 0 | 0.000000 | -0.000000 | 1405 | 1311.645954 | 1382.75 | 1453.854046 | 0.984164 |
| GO:0043277\_apoptotic\_cell\_clearance | 5 | 0 | 0.000000 | -0.000000 | 1405 | 1311.645954 | 1382.75 | 1453.854046 | 0.984164 |
| GO:0043288\_apocarotenoid\_metabolic\_process | 5 | 0 | 0.000000 | -0.000000 | 1405 | 1311.645954 | 1382.75 | 1453.854046 | 0.984164 |
| GO:0043302\_positive\_regulation\_of\_leukocyte\_degranulation | 5 | 0 | 0.000000 | -0.000000 | 1405 | 1311.645954 | 1382.75 | 1453.854046 | 0.984164 |
| GO:0043303\_mast\_cell\_degranulation | 5 | 0 | 0.000000 | -0.000000 | 1405 | 1311.645954 | 1382.75 | 1453.854046 | 0.984164 |
| GO:0043304\_regulation\_of\_mast\_cell\_degranulation | 5 | 0 | 0.000000 | -0.000000 | 1405 | 1311.645954 | 1382.75 | 1453.854046 | 0.984164 |
| GO:0043306\_positive\_regulation\_of\_mast\_cell\_degranulation | 5 | 0 | 0.000000 | -0.000000 | 1405 | 1311.645954 | 1382.75 | 1453.854046 | 0.984164 |
| GO:0043393\_regulation\_of\_protein\_binding | 5 | 0 | 0.000000 | -0.000000 | 1405 | 1311.645954 | 1382.75 | 1453.854046 | 0.984164 |
| GO:0043489\_RNA\_stabilization | 5 | 0 | 0.000000 | -0.000000 | 1405 | 1311.645954 | 1382.75 | 1453.854046 | 0.984164 |
| GO:0043526\_neuroprotection | 5 | 0 | 0.000000 | -0.000000 | 1405 | 1311.645954 | 1382.75 | 1453.854046 | 0.984164 |
| GO:0043550\_regulation\_of\_lipid\_kinase\_activity | 5 | 0 | 0.000000 | -0.000000 | 1405 | 1311.645954 | 1382.75 | 1453.854046 | 0.984164 |
| GO:0043551\_regulation\_of\_phosphoinositide\_3-kinase\_activity | 5 | 0 | 0.000000 | -0.000000 | 1405 | 1311.645954 | 1382.75 | 1453.854046 | 0.984164 |
| GO:0043552\_positive\_regulation\_of\_phosphoinositide\_3-kinase\_activity | 5 | 0 | 0.000000 | -0.000000 | 1405 | 1311.645954 | 1382.75 | 1453.854046 | 0.984164 |
| GO:0043555\_regulation\_of\_translation\_in\_response\_to\_stress | 5 | 0 | 0.000000 | -0.000000 | 1405 | 1311.645954 | 1382.75 | 1453.854046 | 0.984164 |
| GO:0043558\_regulation\_of\_translational\_initiation\_in\_response\_to\_stress | 5 | 0 | 0.000000 | -0.000000 | 1405 | 1311.645954 | 1382.75 | 1453.854046 | 0.984164 |
| GO:0043631\_RNA\_polyadenylation | 5 | 0 | 0.000000 | -0.000000 | 1405 | 1311.645954 | 1382.75 | 1453.854046 | 0.984164 |
| GO:0043648\_dicarboxylic\_acid\_metabolic\_process | 5 | 0 | 0.000000 | -0.000000 | 1405 | 1311.645954 | 1382.75 | 1453.854046 | 0.984164 |
| GO:0044058\_regulation\_of\_digestive\_system\_process | 5 | 0 | 0.000000 | -0.000000 | 1405 | 1311.645954 | 1382.75 | 1453.854046 | 0.984164 |
| GO:0044241\_lipid\_digestion | 5 | 0 | 0.000000 | -0.000000 | 1405 | 1311.645954 | 1382.75 | 1453.854046 | 0.984164 |
| GO:0044247\_cellular\_polysaccharide\_catabolic\_process | 5 | 0 | 0.000000 | -0.000000 | 1405 | 1311.645954 | 1382.75 | 1453.854046 | 0.984164 |
| GO:0045063\_T-helper\_1\_cell\_differentiation | 5 | 0 | 0.000000 | -0.000000 | 1405 | 1311.645954 | 1382.75 | 1453.854046 | 0.984164 |
| GO:0045064\_T-helper\_2\_cell\_differentiation | 5 | 0 | 0.000000 | -0.000000 | 1405 | 1311.645954 | 1382.75 | 1453.854046 | 0.984164 |
| GO:0045080\_positive\_regulation\_of\_chemokine\_biosynthetic\_process | 5 | 0 | 0.000000 | -0.000000 | 1405 | 1311.645954 | 1382.75 | 1453.854046 | 0.984164 |
| GO:0045123\_cellular\_extravasation | 5 | 0 | 0.000000 | -0.000000 | 1405 | 1311.645954 | 1382.75 | 1453.854046 | 0.984164 |
| GO:0045197\_establishment\_or\_maintenance\_of\_epithelial\_cell\_apical\_basal\_polarity | 5 | 0 | 0.000000 | -0.000000 | 1405 | 1311.645954 | 1382.75 | 1453.854046 | 0.984164 |
| GO:0045213\_neurotransmitter\_receptor\_metabolic\_process | 5 | 0 | 0.000000 | -0.000000 | 1405 | 1311.645954 | 1382.75 | 1453.854046 | 0.984164 |
| GO:0045342\_MHC\_class\_II\_biosynthetic\_process | 5 | 0 | 0.000000 | -0.000000 | 1405 | 1311.645954 | 1382.75 | 1453.854046 | 0.984164 |
| GO:0045410\_positive\_regulation\_of\_interleukin-6\_biosynthetic\_process | 5 | 0 | 0.000000 | -0.000000 | 1405 | 1311.645954 | 1382.75 | 1453.854046 | 0.984164 |
| GO:0045586\_regulation\_of\_gamma-delta\_T\_cell\_differentiation | 5 | 0 | 0.000000 | -0.000000 | 1405 | 1311.645954 | 1382.75 | 1453.854046 | 0.984164 |
| GO:0045588\_positive\_regulation\_of\_gamma-delta\_T\_cell\_differentiation | 5 | 0 | 0.000000 | -0.000000 | 1405 | 1311.645954 | 1382.75 | 1453.854046 | 0.984164 |
| GO:0045622\_regulation\_of\_T-helper\_cell\_differentiation | 5 | 0 | 0.000000 | -0.000000 | 1405 | 1311.645954 | 1382.75 | 1453.854046 | 0.984164 |
| GO:0045648\_positive\_regulation\_of\_erythrocyte\_differentiation | 5 | 0 | 0.000000 | -0.000000 | 1405 | 1311.645954 | 1382.75 | 1453.854046 | 0.984164 |
| GO:0045651\_positive\_regulation\_of\_macrophage\_differentiation | 5 | 0 | 0.000000 | -0.000000 | 1405 | 1311.645954 | 1382.75 | 1453.854046 | 0.984164 |
| GO:0045661\_regulation\_of\_myoblast\_differentiation | 5 | 0 | 0.000000 | -0.000000 | 1405 | 1311.645954 | 1382.75 | 1453.854046 | 0.984164 |
| GO:0045730\_respiratory\_burst | 5 | 0 | 0.000000 | -0.000000 | 1405 | 1311.645954 | 1382.75 | 1453.854046 | 0.984164 |
| GO:0045744\_negative\_regulation\_of\_G-protein\_coupled\_receptor\_protein\_signaling\_pathway | 5 | 0 | 0.000000 | -0.000000 | 1405 | 1311.645954 | 1382.75 | 1453.854046 | 0.984164 |
| GO:0045745\_positive\_regulation\_of\_G-protein\_coupled\_receptor\_protein\_signaling\_pathway | 5 | 0 | 0.000000 | -0.000000 | 1405 | 1311.645954 | 1382.75 | 1453.854046 | 0.984164 |
| GO:0045773\_positive\_regulation\_of\_axon\_extension | 5 | 0 | 0.000000 | -0.000000 | 1405 | 1311.645954 | 1382.75 | 1453.854046 | 0.984164 |
| GO:0045793\_positive\_regulation\_of\_cell\_size | 5 | 0 | 0.000000 | -0.000000 | 1405 | 1311.645954 | 1382.75 | 1453.854046 | 0.984164 |
| GO:0045851\_pH\_reduction | 5 | 0 | 0.000000 | -0.000000 | 1405 | 1311.645954 | 1382.75 | 1453.854046 | 0.984164 |
| GO:0045885\_positive\_regulation\_of\_survival\_gene\_product\_expression | 5 | 0 | 0.000000 | -0.000000 | 1405 | 1311.645954 | 1382.75 | 1453.854046 | 0.984164 |
| GO:0045953\_negative\_regulation\_of\_natural\_killer\_cell\_mediated\_cytotoxicity | 5 | 0 | 0.000000 | -0.000000 | 1405 | 1311.645954 | 1382.75 | 1453.854046 | 0.984164 |
| GO:0045981\_positive\_regulation\_of\_nucleotide\_metabolic\_process | 5 | 0 | 0.000000 | -0.000000 | 1405 | 1311.645954 | 1382.75 | 1453.854046 | 0.984164 |
| GO:0045987\_positive\_regulation\_of\_smooth\_muscle\_contraction | 5 | 0 | 0.000000 | -0.000000 | 1405 | 1311.645954 | 1382.75 | 1453.854046 | 0.984164 |
| GO:0046326\_positive\_regulation\_of\_glucose\_import | 5 | 0 | 0.000000 | -0.000000 | 1405 | 1311.645954 | 1382.75 | 1453.854046 | 0.984164 |
| GO:0046339\_diacylglycerol\_metabolic\_process | 5 | 0 | 0.000000 | -0.000000 | 1405 | 1311.645954 | 1382.75 | 1453.854046 | 0.984164 |
| GO:0046456\_icosanoid\_biosynthetic\_process | 5 | 0 | 0.000000 | -0.000000 | 1405 | 1311.645954 | 1382.75 | 1453.854046 | 0.984164 |
| GO:0046459\_short-chain\_fatty\_acid\_metabolic\_process | 5 | 0 | 0.000000 | -0.000000 | 1405 | 1311.645954 | 1382.75 | 1453.854046 | 0.984164 |
| GO:0046489\_phosphoinositide\_biosynthetic\_process | 5 | 0 | 0.000000 | -0.000000 | 1405 | 1311.645954 | 1382.75 | 1453.854046 | 0.984164 |
| GO:0046621\_negative\_regulation\_of\_organ\_growth | 5 | 0 | 0.000000 | -0.000000 | 1405 | 1311.645954 | 1382.75 | 1453.854046 | 0.984164 |
| GO:0046643\_regulation\_of\_gamma-delta\_T\_cell\_activation | 5 | 0 | 0.000000 | -0.000000 | 1405 | 1311.645954 | 1382.75 | 1453.854046 | 0.984164 |
| GO:0046645\_positive\_regulation\_of\_gamma-delta\_T\_cell\_activation | 5 | 0 | 0.000000 | -0.000000 | 1405 | 1311.645954 | 1382.75 | 1453.854046 | 0.984164 |
| GO:0046697\_decidualization | 5 | 0 | 0.000000 | -0.000000 | 1405 | 1311.645954 | 1382.75 | 1453.854046 | 0.984164 |
| GO:0046785\_microtubule\_polymerization | 5 | 0 | 0.000000 | -0.000000 | 1405 | 1311.645954 | 1382.75 | 1453.854046 | 0.984164 |
| GO:0048013\_ephrin\_receptor\_signaling\_pathway | 5 | 0 | 0.000000 | -0.000000 | 1405 | 1311.645954 | 1382.75 | 1453.854046 | 0.984164 |
| GO:0048149\_behavioral\_response\_to\_ethanol | 5 | 0 | 0.000000 | -0.000000 | 1405 | 1311.645954 | 1382.75 | 1453.854046 | 0.984164 |
| GO:0048255\_mRNA\_stabilization | 5 | 0 | 0.000000 | -0.000000 | 1405 | 1311.645954 | 1382.75 | 1453.854046 | 0.984164 |
| GO:0048289\_isotype\_switching\_to\_IgE\_isotypes | 5 | 0 | 0.000000 | -0.000000 | 1405 | 1311.645954 | 1382.75 | 1453.854046 | 0.984164 |
| GO:0048293\_regulation\_of\_isotype\_switching\_to\_IgE\_isotypes | 5 | 0 | 0.000000 | -0.000000 | 1405 | 1311.645954 | 1382.75 | 1453.854046 | 0.984164 |
| GO:0048485\_sympathetic\_nervous\_system\_development | 5 | 0 | 0.000000 | -0.000000 | 1405 | 1311.645954 | 1382.75 | 1453.854046 | 0.984164 |
| GO:0048532\_anatomical\_structure\_arrangement | 5 | 0 | 0.000000 | -0.000000 | 1405 | 1311.645954 | 1382.75 | 1453.854046 | 0.984164 |
| GO:0048570\_notochord\_morphogenesis | 5 | 0 | 0.000000 | -0.000000 | 1405 | 1311.645954 | 1382.75 | 1453.854046 | 0.984164 |
| GO:0048659\_smooth\_muscle\_cell\_proliferation | 5 | 0 | 0.000000 | -0.000000 | 1405 | 1311.645954 | 1382.75 | 1453.854046 | 0.984164 |
| GO:0048664\_neuron\_fate\_determination | 5 | 0 | 0.000000 | -0.000000 | 1405 | 1311.645954 | 1382.75 | 1453.854046 | 0.984164 |
| GO:0048715\_negative\_regulation\_of\_oligodendrocyte\_differentiation | 5 | 0 | 0.000000 | -0.000000 | 1405 | 1311.645954 | 1382.75 | 1453.854046 | 0.984164 |
| GO:0048934\_peripheral\_nervous\_system\_neuron\_differentiation | 5 | 0 | 0.000000 | -0.000000 | 1405 | 1311.645954 | 1382.75 | 1453.854046 | 0.984164 |
| GO:0050665\_hydrogen\_peroxide\_biosynthetic\_process | 5 | 0 | 0.000000 | -0.000000 | 1405 | 1311.645954 | 1382.75 | 1453.854046 | 0.984164 |
| GO:0050701\_interleukin-1\_secretion | 5 | 0 | 0.000000 | -0.000000 | 1405 | 1311.645954 | 1382.75 | 1453.854046 | 0.984164 |
| GO:0050715\_positive\_regulation\_of\_cytokine\_secretion | 5 | 0 | 0.000000 | -0.000000 | 1405 | 1311.645954 | 1382.75 | 1453.854046 | 0.984164 |
| GO:0050819\_negative\_regulation\_of\_coagulation | 5 | 0 | 0.000000 | -0.000000 | 1405 | 1311.645954 | 1382.75 | 1453.854046 | 0.984164 |
| GO:0050858\_negative\_regulation\_of\_antigen\_receptor-mediated\_signaling\_pathway | 5 | 0 | 0.000000 | -0.000000 | 1405 | 1311.645954 | 1382.75 | 1453.854046 | 0.984164 |
| GO:0050860\_negative\_regulation\_of\_T\_cell\_receptor\_signaling\_pathway | 5 | 0 | 0.000000 | -0.000000 | 1405 | 1311.645954 | 1382.75 | 1453.854046 | 0.984164 |
| GO:0051057\_positive\_regulation\_of\_small\_GTPase\_mediated\_signal\_transduction | 5 | 0 | 0.000000 | -0.000000 | 1405 | 1311.645954 | 1382.75 | 1453.854046 | 0.984164 |
| GO:0051136\_regulation\_of\_NK\_T\_cell\_differentiation | 5 | 0 | 0.000000 | -0.000000 | 1405 | 1311.645954 | 1382.75 | 1453.854046 | 0.984164 |
| GO:0051138\_positive\_regulation\_of\_NK\_T\_cell\_differentiation | 5 | 0 | 0.000000 | -0.000000 | 1405 | 1311.645954 | 1382.75 | 1453.854046 | 0.984164 |
| GO:0051177\_meiotic\_sister\_chromatid\_cohesion | 5 | 0 | 0.000000 | -0.000000 | 1405 | 1311.645954 | 1382.75 | 1453.854046 | 0.984164 |
| GO:0051289\_protein\_homotetramerization | 5 | 0 | 0.000000 | -0.000000 | 1405 | 1311.645954 | 1382.75 | 1453.854046 | 0.984164 |
| GO:0051298\_centrosome\_duplication | 5 | 0 | 0.000000 | -0.000000 | 1405 | 1311.645954 | 1382.75 | 1453.854046 | 0.984164 |
| GO:0051323\_metaphase | 5 | 0 | 0.000000 | -0.000000 | 1405 | 1311.645954 | 1382.75 | 1453.854046 | 0.984164 |
| GO:0051453\_regulation\_of\_intracellular\_pH | 5 | 0 | 0.000000 | -0.000000 | 1405 | 1311.645954 | 1382.75 | 1453.854046 | 0.984164 |
| GO:0051668\_localization\_within\_membrane | 5 | 0 | 0.000000 | -0.000000 | 1405 | 1311.645954 | 1382.75 | 1453.854046 | 0.984164 |
| GO:0051898\_negative\_regulation\_of\_protein\_kinase\_B\_signaling\_cascade | 5 | 0 | 0.000000 | -0.000000 | 1405 | 1311.645954 | 1382.75 | 1453.854046 | 0.984164 |
| GO:0051905\_establishment\_of\_pigment\_granule\_localization | 5 | 0 | 0.000000 | -0.000000 | 1405 | 1311.645954 | 1382.75 | 1453.854046 | 0.984164 |
| GO:0051954\_positive\_regulation\_of\_amine\_transport | 5 | 0 | 0.000000 | -0.000000 | 1405 | 1311.645954 | 1382.75 | 1453.854046 | 0.984164 |
| GO:0051966\_regulation\_of\_synaptic\_transmission\_\_glutamatergic | 5 | 0 | 0.000000 | -0.000000 | 1405 | 1311.645954 | 1382.75 | 1453.854046 | 0.984164 |
| GO:0055003\_cardiac\_myofibril\_assembly | 5 | 0 | 0.000000 | -0.000000 | 1405 | 1311.645954 | 1382.75 | 1453.854046 | 0.984164 |
| GO:0055015\_ventricular\_cardiac\_muscle\_cell\_development | 5 | 0 | 0.000000 | -0.000000 | 1405 | 1311.645954 | 1382.75 | 1453.854046 | 0.984164 |
| GO:0060045\_positive\_regulation\_of\_cardiac\_muscle\_cell\_proliferation | 5 | 0 | 0.000000 | -0.000000 | 1405 | 1311.645954 | 1382.75 | 1453.854046 | 0.984164 |
| GO:0060073\_micturition | 5 | 0 | 0.000000 | -0.000000 | 1405 | 1311.645954 | 1382.75 | 1453.854046 | 0.984164 |
| GO:0060351\_cartilage\_development\_involved\_in\_endochondral\_bone\_morphogenesis | 5 | 0 | 0.000000 | -0.000000 | 1405 | 1311.645954 | 1382.75 | 1453.854046 | 0.984164 |
| GO:0060438\_trachea\_development | 5 | 0 | 0.000000 | -0.000000 | 1405 | 1311.645954 | 1382.75 | 1453.854046 | 0.984164 |
| GO:0060600\_dichotomous\_subdivision\_of\_an\_epithelial\_terminal\_unit | 5 | 0 | 0.000000 | -0.000000 | 1405 | 1311.645954 | 1382.75 | 1453.854046 | 0.984164 |
| GO:0060605\_tube\_lumen\_cavitation | 5 | 0 | 0.000000 | -0.000000 | 1405 | 1311.645954 | 1382.75 | 1453.854046 | 0.984164 |
| GO:0060662\_salivary\_gland\_cavitation | 5 | 0 | 0.000000 | -0.000000 | 1405 | 1311.645954 | 1382.75 | 1453.854046 | 0.984164 |
| GO:0060665\_regulation\_of\_branching\_involved\_in\_salivary\_gland\_morphogenesis\_by\_mesenchymal-epithelial\_signaling | 5 | 0 | 0.000000 | -0.000000 | 1405 | 1311.645954 | 1382.75 | 1453.854046 | 0.984164 |
| GO:0060707\_trophoblast\_giant\_cell\_differentiation | 5 | 0 | 0.000000 | -0.000000 | 1405 | 1311.645954 | 1382.75 | 1453.854046 | 0.984164 |
| GO:0060744\_mammary\_gland\_branching\_involved\_in\_thelarche | 5 | 0 | 0.000000 | -0.000000 | 1405 | 1311.645954 | 1382.75 | 1453.854046 | 0.984164 |
| GO:0060745\_mammary\_gland\_branching\_involved\_in\_pregnancy | 5 | 0 | 0.000000 | -0.000000 | 1405 | 1311.645954 | 1382.75 | 1453.854046 | 0.984164 |
| GO:0060762\_regulation\_of\_branching\_involved\_in\_mammary\_gland\_duct\_morphogenesis | 5 | 0 | 0.000000 | -0.000000 | 1405 | 1311.645954 | 1382.75 | 1453.854046 | 0.984164 |
| GO:0070371\_ERK1\_and\_ERK2\_cascade | 5 | 0 | 0.000000 | -0.000000 | 1405 | 1311.645954 | 1382.75 | 1453.854046 | 0.984164 |
| GO:0070372\_regulation\_of\_ERK1\_and\_ERK2\_cascade | 5 | 0 | 0.000000 | -0.000000 | 1405 | 1311.645954 | 1382.75 | 1453.854046 | 0.984164 |
| GO:0070374\_positive\_regulation\_of\_ERK1\_and\_ERK2\_cascade | 5 | 0 | 0.000000 | -0.000000 | 1405 | 1311.645954 | 1382.75 | 1453.854046 | 0.984164 |
| GO:0000002\_mitochondrial\_genome\_maintenance | 9 | 0 | 0.000000 | -0.000000 | 1521 | 1428.786514 | 1497.45 | 1566.113486 | 0.984517 |
| GO:0000186\_activation\_of\_MAPKK\_activity | 9 | 0 | 0.000000 | -0.000000 | 1521 | 1428.786514 | 1497.45 | 1566.113486 | 0.984517 |
[truncated: 255,585 more chars]
