# Supplementary figures and images for: RedundancyMiner: De-replication of redundant GO categories in microarray and proteomics analysis
Source: BMC Bioinformatics. 2011 Feb 10;12:52. doi: 10.1186/1471-2105-12-52 (PMC3223614; doi:10.1186/1471-2105-12-52)

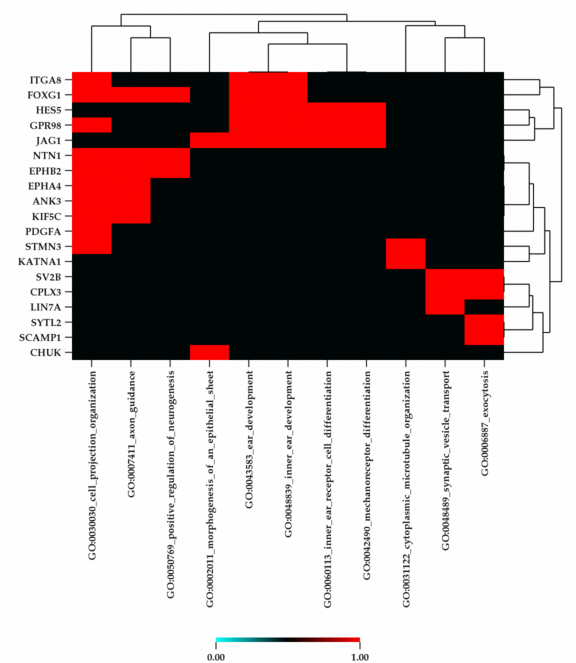

Supplement: Additional file 8 — Retinal development HTGM download. compressed package of the results of running HTGM on the retinal development genes list. [file 1471-2105-12-52-S8.ZIP › SCENARIO_2_MODIFIED/total.txt.total.txt.dir/Exp1_BestClusterMap_LEIGS_KM_24.csv.join.21.txt.dir/Exp1_BestClusterMap_LEIGS_KM_24.csv.join.21.txt.change.gce.CIM.dir/cgi_user_matrix.gif]

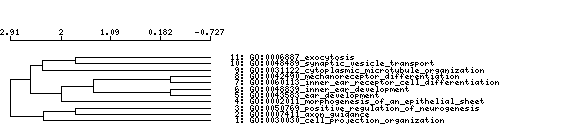

Supplement: Additional file 8 — Retinal development HTGM download. compressed package of the results of running HTGM on the retinal development genes list. [file 1471-2105-12-52-S8.ZIP › SCENARIO_2_MODIFIED/total.txt.total.txt.dir/Exp1_BestClusterMap_LEIGS_KM_24.csv.join.21.txt.dir/Exp1_BestClusterMap_LEIGS_KM_24.csv.join.21.txt.change.gce.CIM.dir/xplclust.gif]

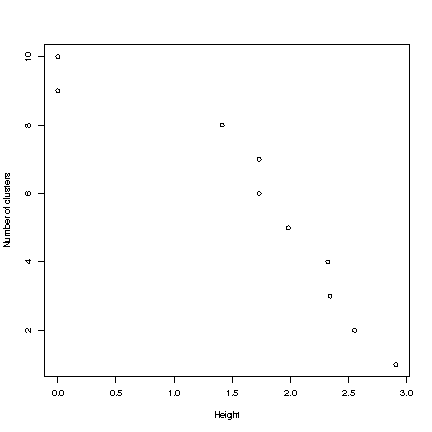

Supplement: Additional file 8 — Retinal development HTGM download. compressed package of the results of running HTGM on the retinal development genes list. [file 1471-2105-12-52-S8.ZIP › SCENARIO_2_MODIFIED/total.txt.total.txt.dir/Exp1_BestClusterMap_LEIGS_KM_24.csv.join.21.txt.dir/Exp1_BestClusterMap_LEIGS_KM_24.csv.join.21.txt.change.gce.CIM.dir/xplot.png]

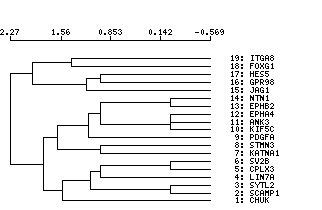

Supplement: Additional file 8 — Retinal development HTGM download. compressed package of the results of running HTGM on the retinal development genes list. [file 1471-2105-12-52-S8.ZIP › SCENARIO_2_MODIFIED/total.txt.total.txt.dir/Exp1_BestClusterMap_LEIGS_KM_24.csv.join.21.txt.dir/Exp1_BestClusterMap_LEIGS_KM_24.csv.join.21.txt.change.gce.CIM.dir/yplclust.gif]

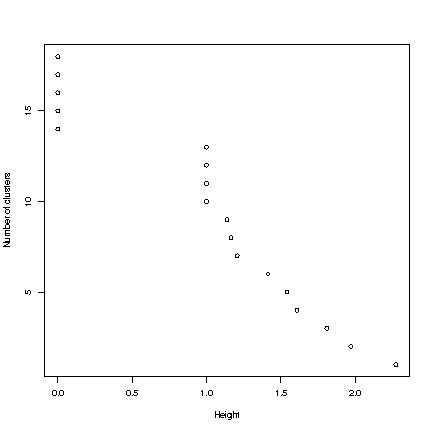

Supplement: Additional file 8 — Retinal development HTGM download. compressed package of the results of running HTGM on the retinal development genes list. [file 1471-2105-12-52-S8.ZIP › SCENARIO_2_MODIFIED/total.txt.total.txt.dir/Exp1_BestClusterMap_LEIGS_KM_24.csv.join.21.txt.dir/Exp1_BestClusterMap_LEIGS_KM_24.csv.join.21.txt.change.gce.CIM.dir/yplot.png]

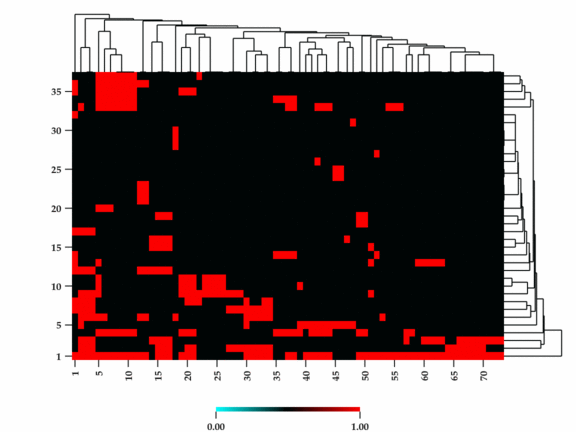

Supplement: Additional file 8 — Retinal development HTGM download. compressed package of the results of running HTGM on the retinal development genes list. [file 1471-2105-12-52-S8.ZIP › SCENARIO_2_MODIFIED/total.txt.total.txt.dir/Exp1_BestClusterMap_LEIGS_KM_24.csv.join.22.txt.dir/Exp1_BestClusterMap_LEIGS_KM_24.csv.join.22.txt.change.gce.CIM.dir/cgi_user_matrix.gif]

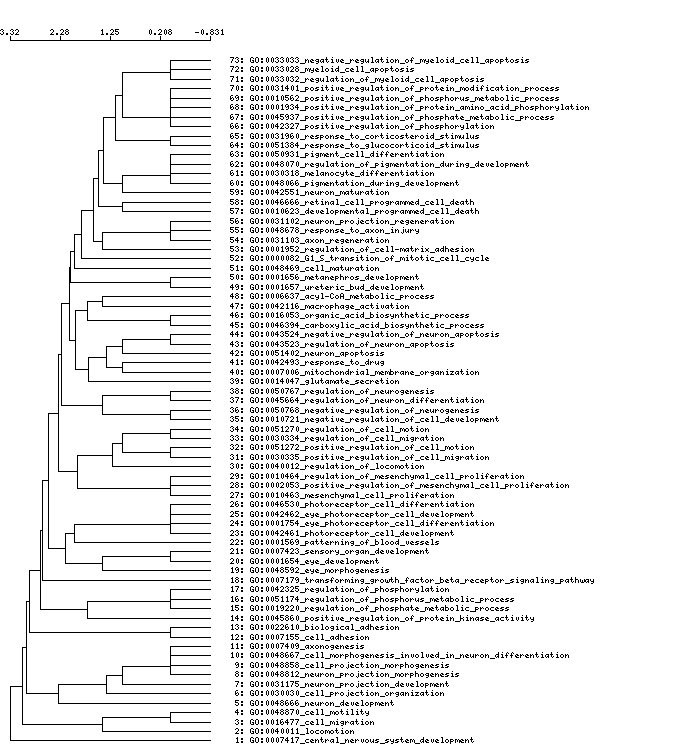

Supplement: Additional file 8 — Retinal development HTGM download. compressed package of the results of running HTGM on the retinal development genes list. [file 1471-2105-12-52-S8.ZIP › SCENARIO_2_MODIFIED/total.txt.total.txt.dir/Exp1_BestClusterMap_LEIGS_KM_24.csv.join.22.txt.dir/Exp1_BestClusterMap_LEIGS_KM_24.csv.join.22.txt.change.gce.CIM.dir/xplclust.gif]

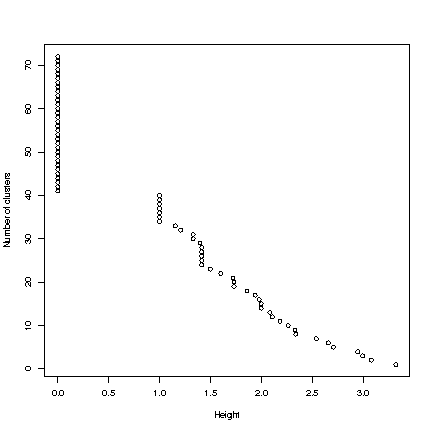

Supplement: Additional file 8 — Retinal development HTGM download. compressed package of the results of running HTGM on the retinal development genes list. [file 1471-2105-12-52-S8.ZIP › SCENARIO_2_MODIFIED/total.txt.total.txt.dir/Exp1_BestClusterMap_LEIGS_KM_24.csv.join.22.txt.dir/Exp1_BestClusterMap_LEIGS_KM_24.csv.join.22.txt.change.gce.CIM.dir/xplot.png]

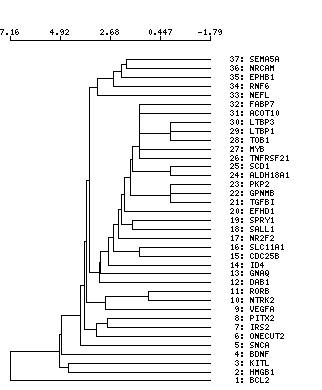

Supplement: Additional file 8 — Retinal development HTGM download. compressed package of the results of running HTGM on the retinal development genes list. [file 1471-2105-12-52-S8.ZIP › SCENARIO_2_MODIFIED/total.txt.total.txt.dir/Exp1_BestClusterMap_LEIGS_KM_24.csv.join.22.txt.dir/Exp1_BestClusterMap_LEIGS_KM_24.csv.join.22.txt.change.gce.CIM.dir/yplclust.gif]

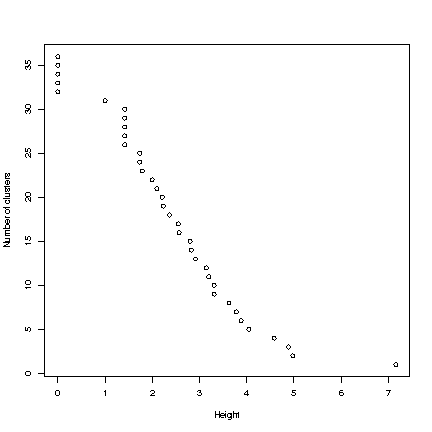

Supplement: Additional file 8 — Retinal development HTGM download. compressed package of the results of running HTGM on the retinal development genes list. [file 1471-2105-12-52-S8.ZIP › SCENARIO_2_MODIFIED/total.txt.total.txt.dir/Exp1_BestClusterMap_LEIGS_KM_24.csv.join.22.txt.dir/Exp1_BestClusterMap_LEIGS_KM_24.csv.join.22.txt.change.gce.CIM.dir/yplot.png]

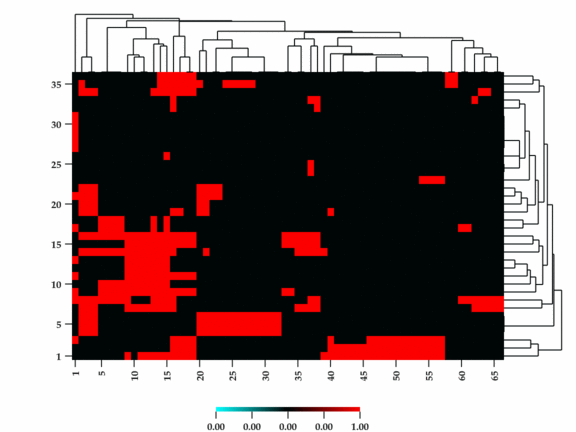

Supplement: Additional file 10 — Kinetochore genes HTGM download. compressed package of the results of running HTGM on the kinetochore genes list. [file 1471-2105-12-52-S10.ZIP › work405493610/total.txt405493610.dir/kinetochore.txt.dir/kinetochore.txt.change.gce.CIM.dir/cgi_user_matrix.gif]

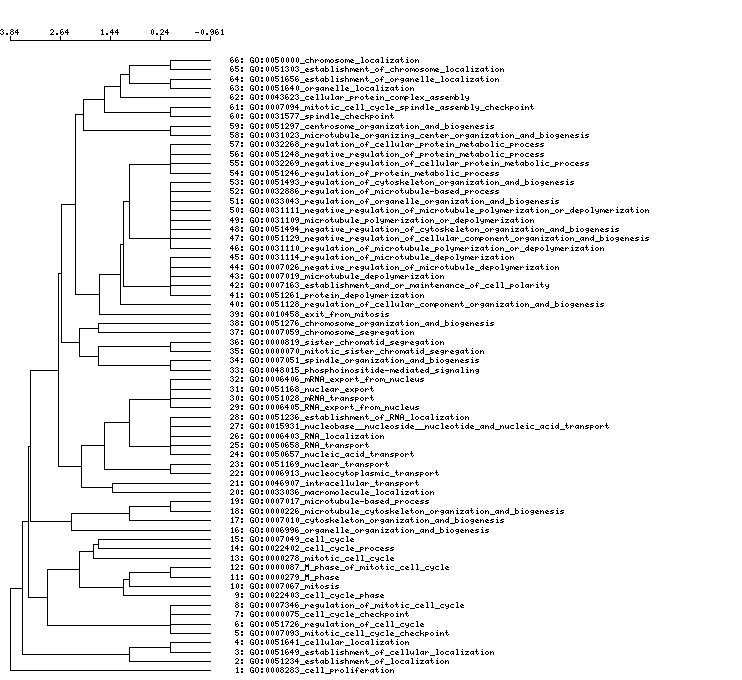

Supplement: Additional file 10 — Kinetochore genes HTGM download. compressed package of the results of running HTGM on the kinetochore genes list. [file 1471-2105-12-52-S10.ZIP › work405493610/total.txt405493610.dir/kinetochore.txt.dir/kinetochore.txt.change.gce.CIM.dir/xplclust.gif]

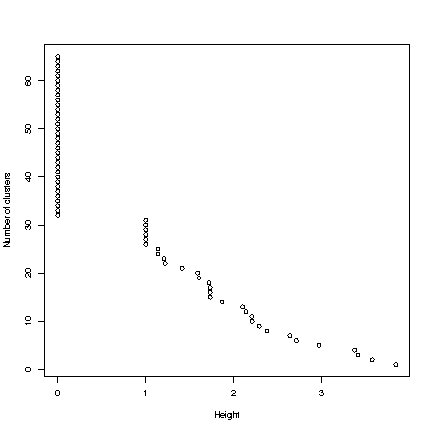

Supplement: Additional file 10 — Kinetochore genes HTGM download. compressed package of the results of running HTGM on the kinetochore genes list. [file 1471-2105-12-52-S10.ZIP › work405493610/total.txt405493610.dir/kinetochore.txt.dir/kinetochore.txt.change.gce.CIM.dir/xplot.png]

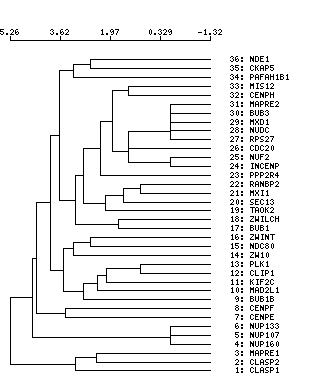

Supplement: Additional file 10 — Kinetochore genes HTGM download. compressed package of the results of running HTGM on the kinetochore genes list. [file 1471-2105-12-52-S10.ZIP › work405493610/total.txt405493610.dir/kinetochore.txt.dir/kinetochore.txt.change.gce.CIM.dir/yplclust.gif]

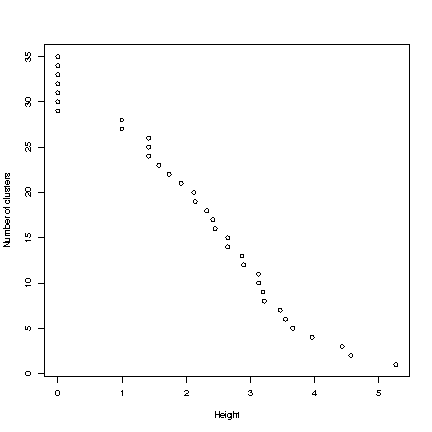

Supplement: Additional file 10 — Kinetochore genes HTGM download. compressed package of the results of running HTGM on the kinetochore genes list. [file 1471-2105-12-52-S10.ZIP › work405493610/total.txt405493610.dir/kinetochore.txt.dir/kinetochore.txt.change.gce.CIM.dir/yplot.png]

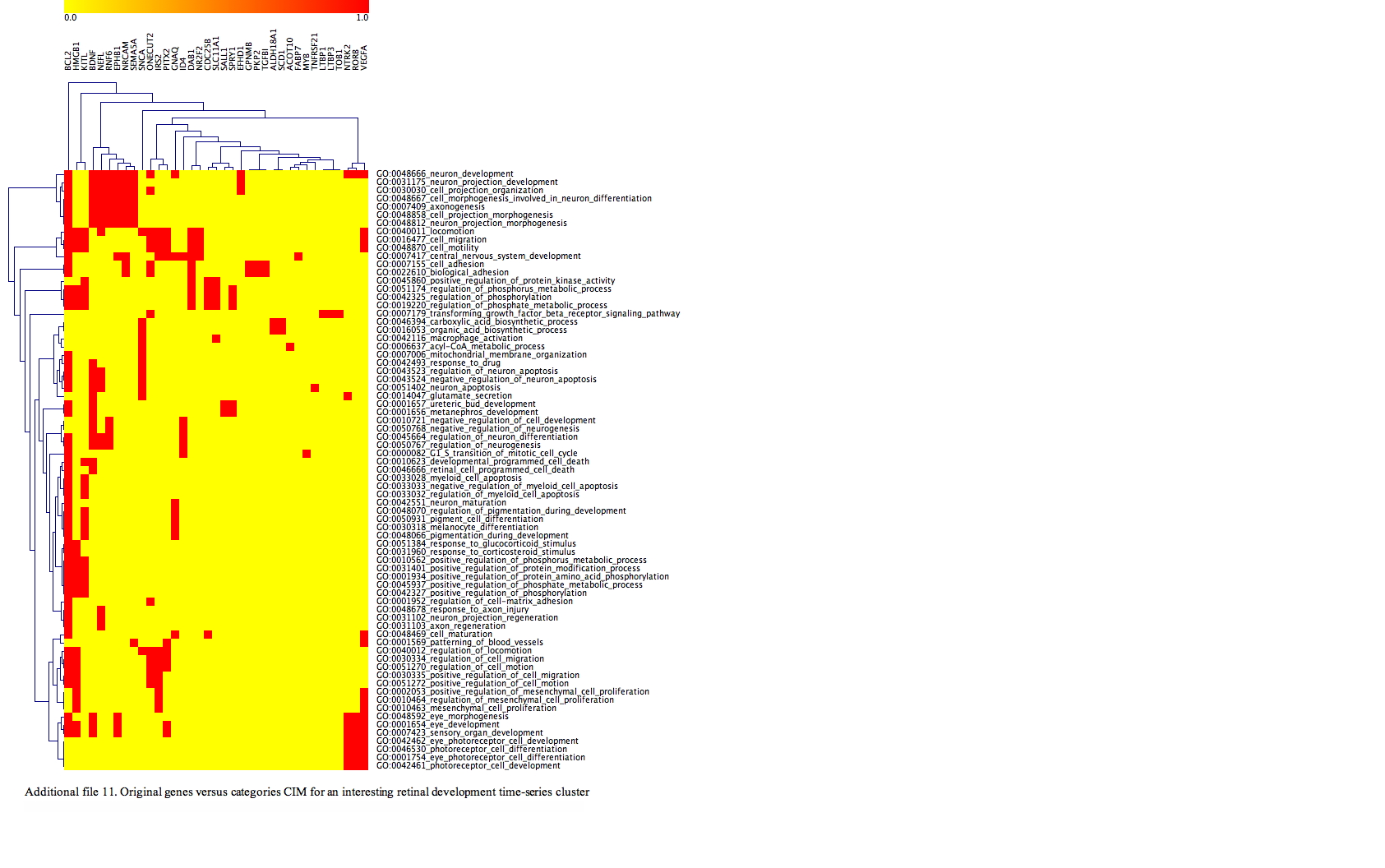

Supplement: Additional file 11 — Original genes versus categories CIM for an interesting retinal development time-series cluster. .png image of original genes versus categories CIM for an interesting retinal development time-series cluster. [file 1471-2105-12-52-S11.PNG]

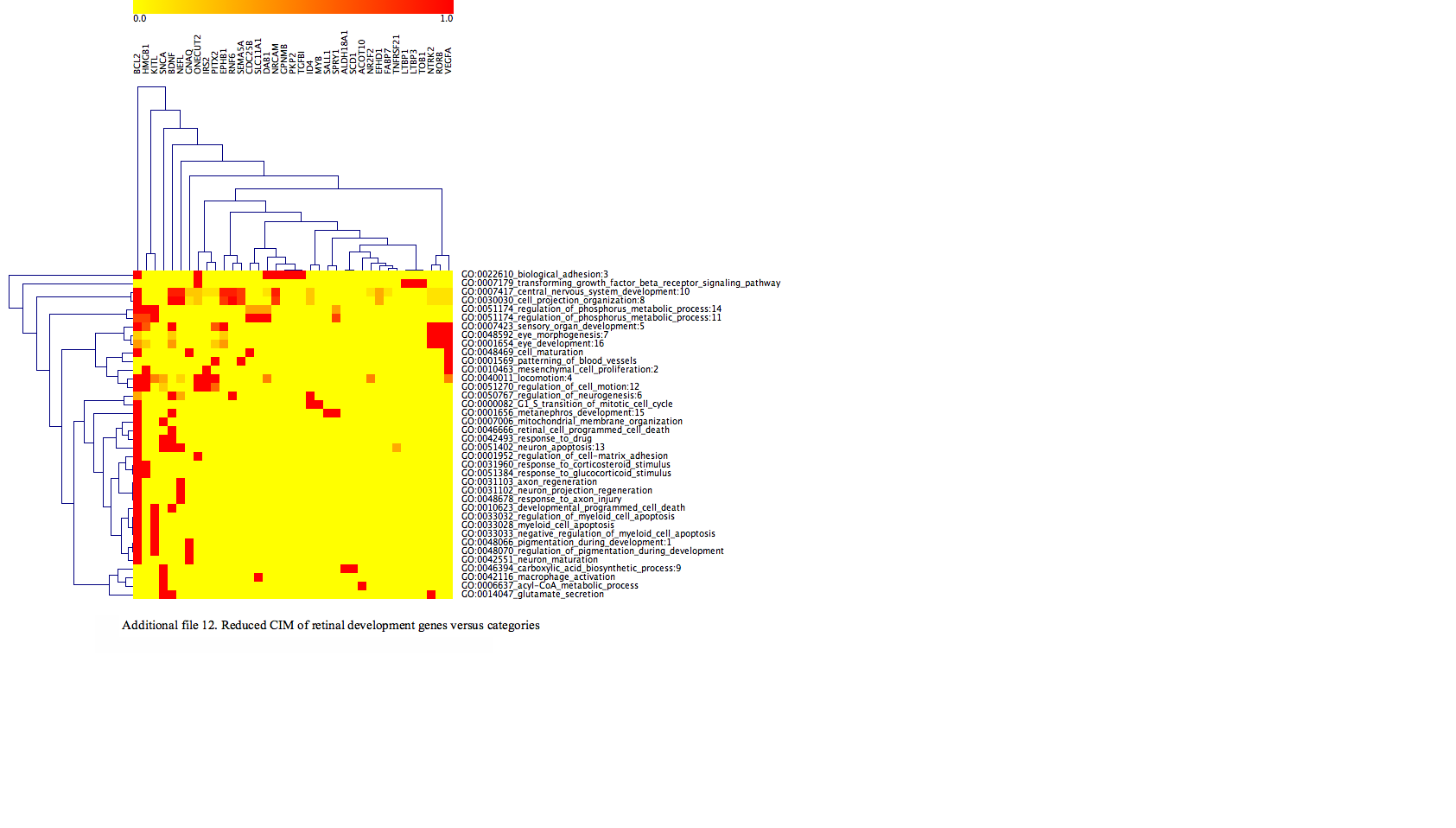

Supplement: Additional file 12 — Reduced CIM of retinal development genes versus categories. .png image of reduced CIM of retinal development genes versus categories. [file 1471-2105-12-52-S12.PNG]

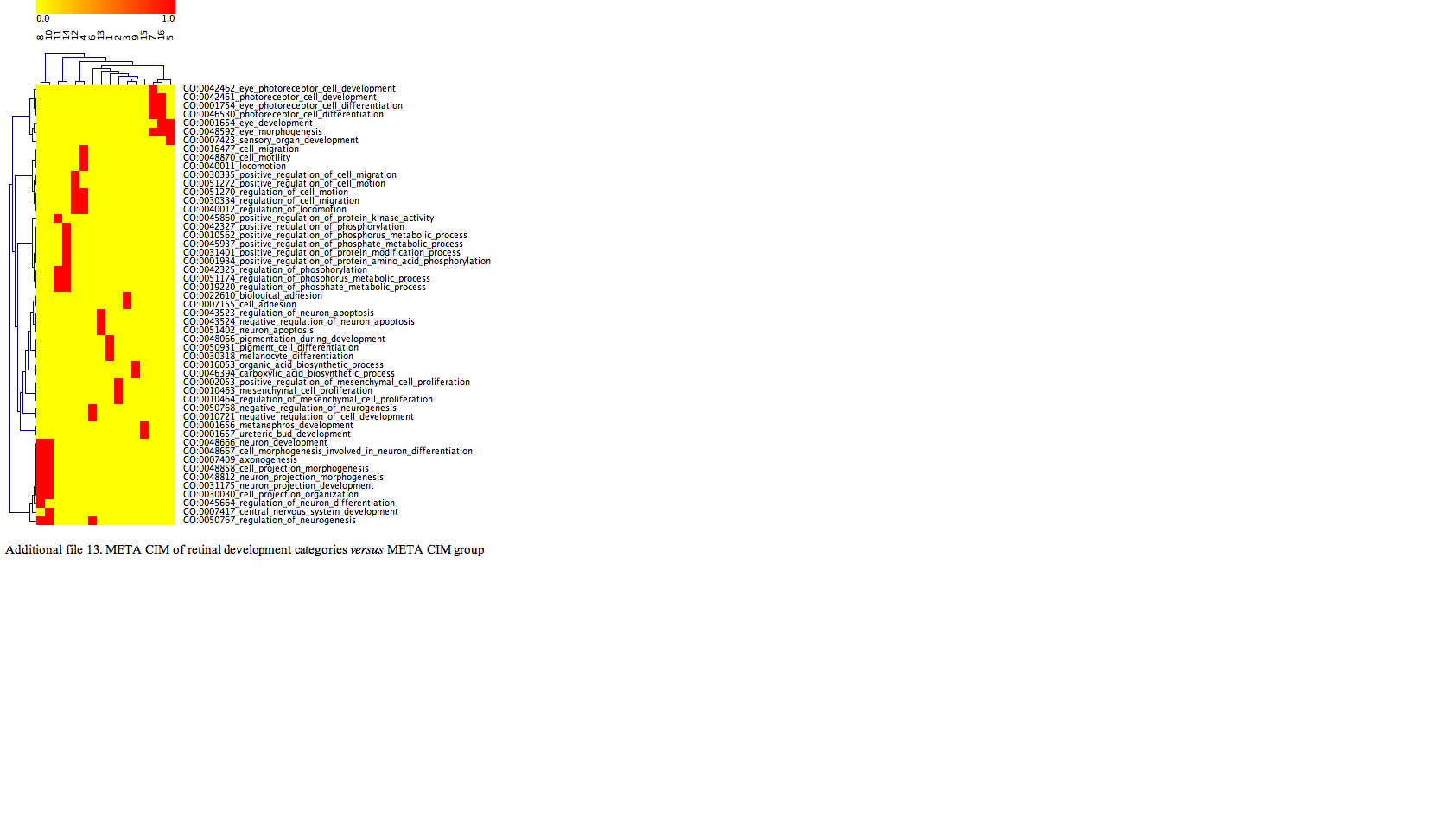

Supplement: Additional file 13 — META CIM of retinal development categories versus META CIM group. .png image of META CIM of retinal development categories versus META CIM group. [file 1471-2105-12-52-S13.PNG]

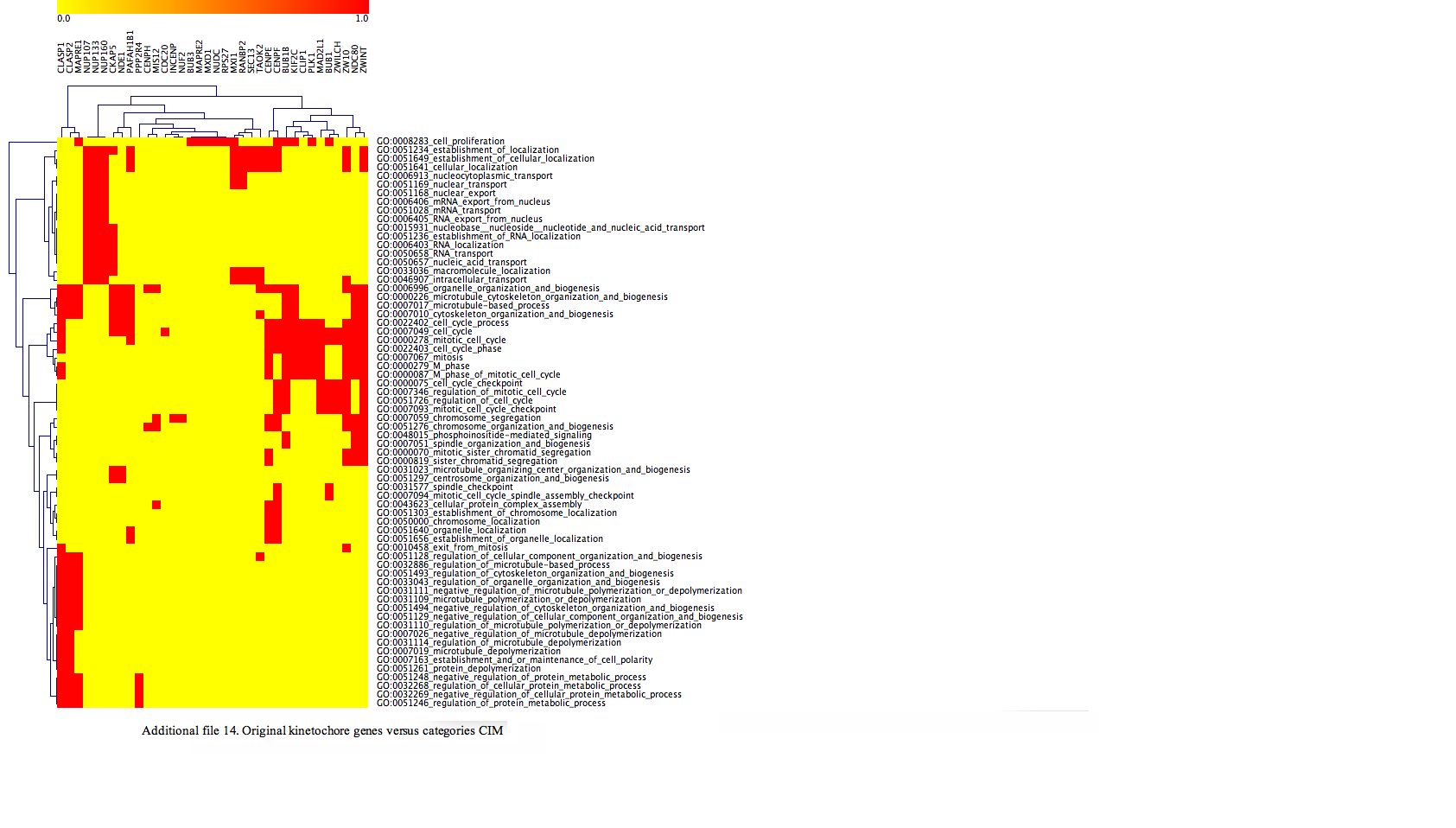

Supplement: Additional file 14 — Original kinetochore genes versus categories CIM. .png image of original kinetochore genes versus categories CIM. [file 1471-2105-12-52-S14.PNG]

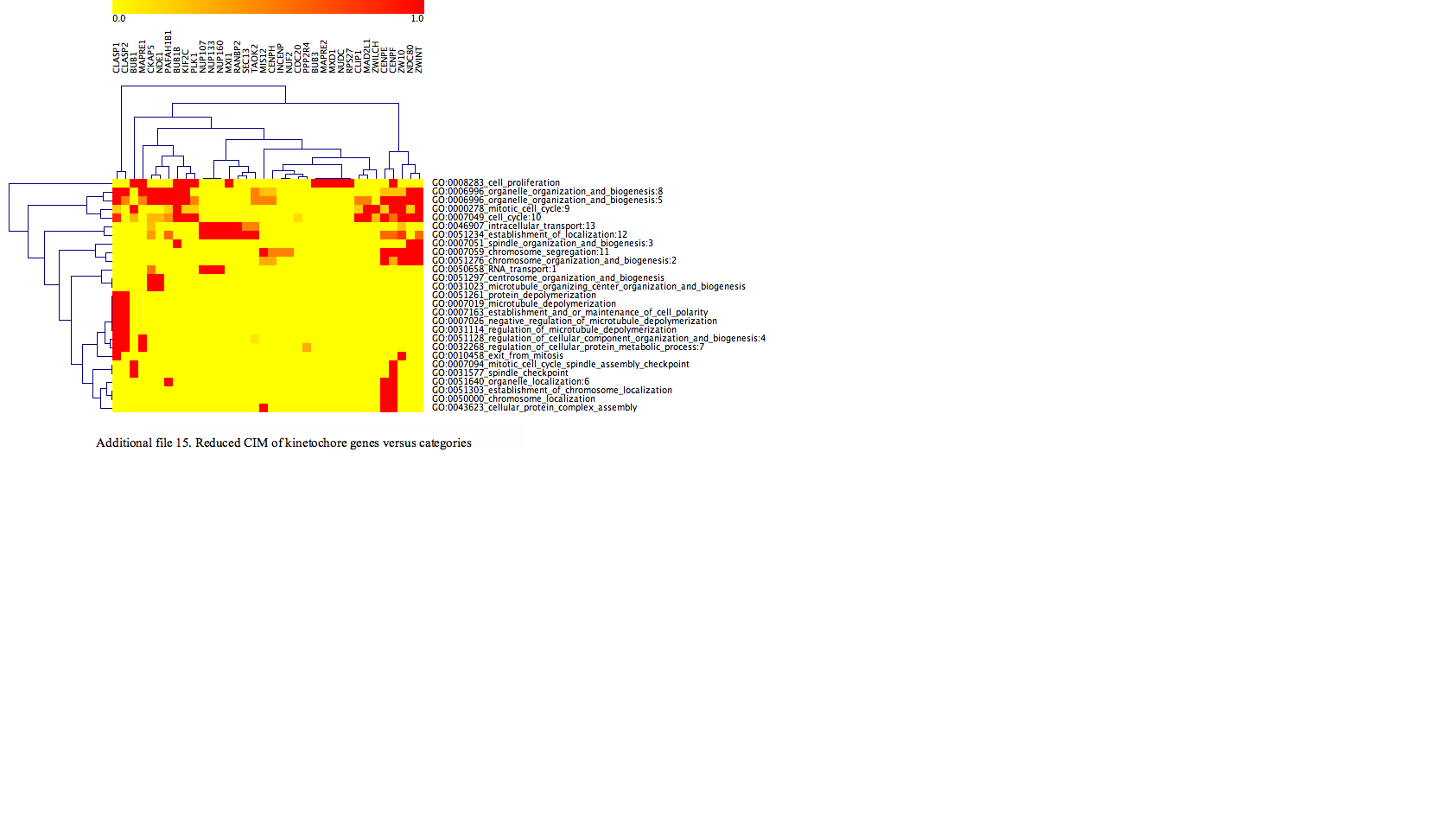

Supplement: Additional file 15 — Reduced CIM of kinetochore genes versus categories. .png image of reduced CIM of kinetochore genes versus categories. [file 1471-2105-12-52-S15.PNG]

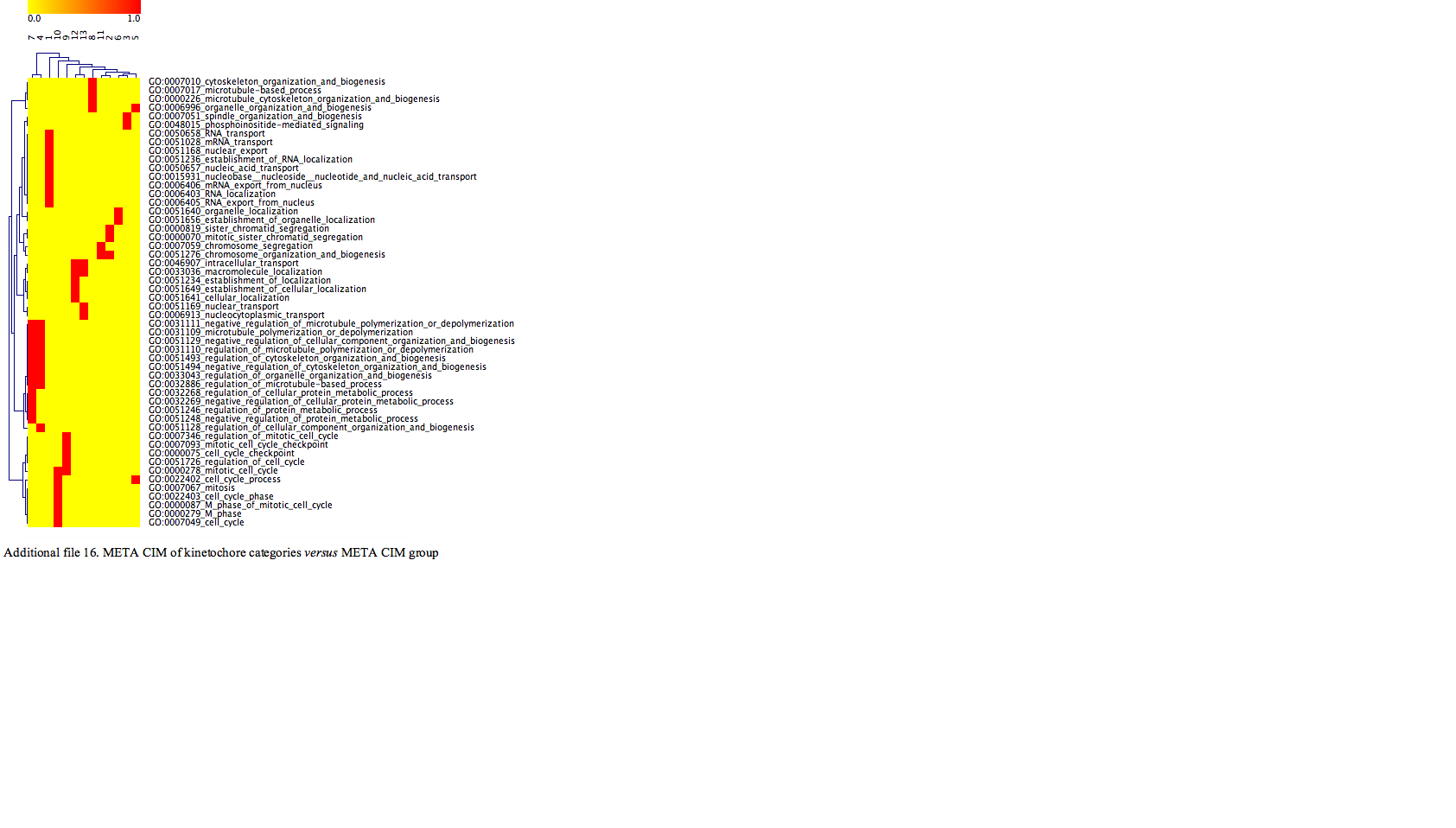

Supplement: Additional file 16 — META CIM of kinetochore categories versus META CIM group. .png image of META CIM of kinetochore categories versus META CIM group. [file 1471-2105-12-52-S16.PNG]

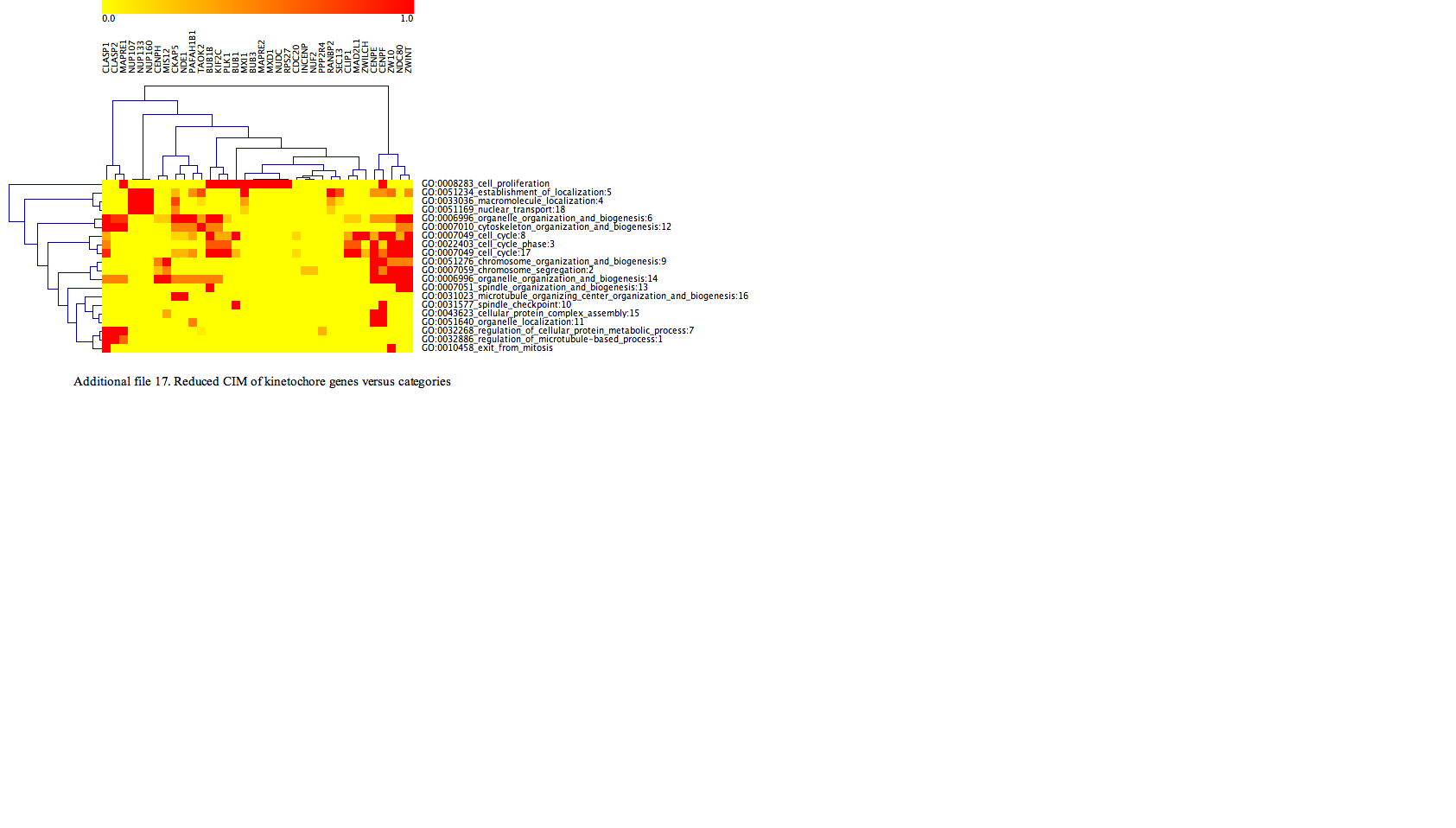

Supplement: Additional file 17 — Reduced CIM of kinetochore genes versus categories. .png image of reduced CIM of kinetochore genes versus categories. [file 1471-2105-12-52-S17.PNG]

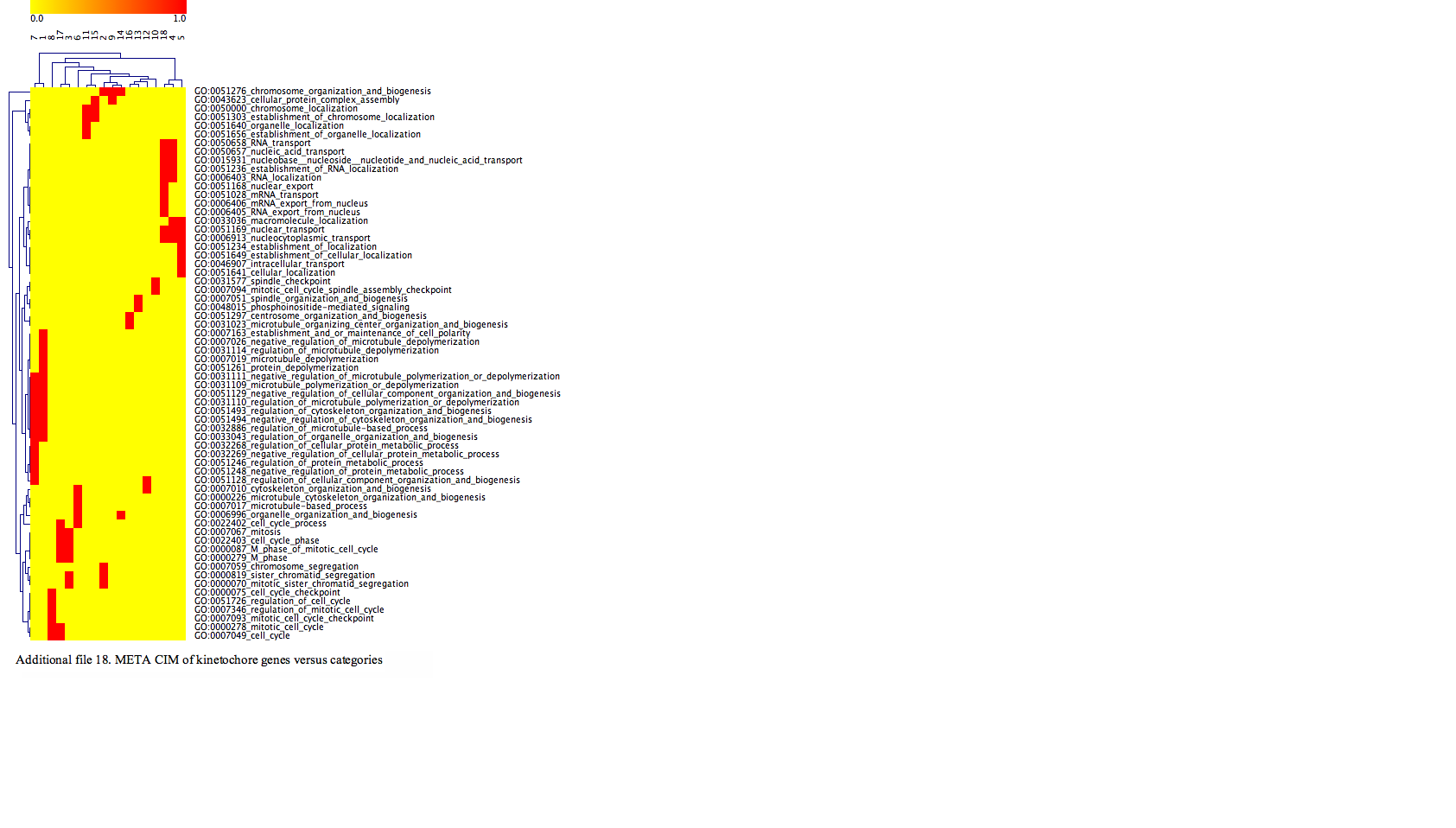

Supplement: Additional file 18 — META CIM of kinetochore genes versus categories. .png image of META CIM of kinetochore genes versus categories. [file 1471-2105-12-52-S18.PNG]
