# Supplementary material for: RedundancyMiner: De-replication of redundant GO categories in microarray and proteomics analysis
Source: BMC Bioinformatics. 2011 Feb 10;12:52. doi: 10.1186/1471-2105-12-52 (PMC3223614; doi:10.1186/1471-2105-12-52)
Supplement: Additional file 9 — Kinetochore genes. table listing kinetochore genes. [file 1471-2105-12-52-S9.DOC]

Additional file 9. Kinetochore genes

| | APITD1 | CENPT | CLASP1 | NDC80 | RANBP2 | | --- | --- | --- | --- | --- | | CENPA | INCENP | CLASP2 | NDE1 | RANGAP1 | | CENPB | ITGB3BP | CLIP1 | NDEL1 | RCC2 | | CENPC1 | MIS12 | COX7A2L | NSL1 | RPS27 | | CENPE | MLF1IP | DSN1 | NUDC | SEC13 | | CENPF | AHCTF1 | fam33a | NUF2 | SEH1L | | CENPH | BUB1 | IDUA | NUP107 | SGOL1 | | CENPI | BUB1B | KIF18A | NUP133 | SPC24 | | CENPK | BUB3 | KIF2A | NUP160 | SPC25 | | CENPL | c18orf24 | KIF2C | NUP37 | TAOK2 | | CENPM | C21orf45 | MAD2L1 | NUP43 | XPO1 | | CENPN | CDC20 | MAPRE1 | NUP85 | ZW10 | | CENPO | CDCA8 | MAPRE2 | PAFAH1B1 | ZWILCH | | CENPP | cep192 | MXD1 | PLK1 | ZWINT | | CENPQ | CKAP5 | MXI1 | PPP2R4 |  | |
| --- | --- | --- | --- | --- | --- | --- | --- | --- | --- | --- | --- | --- | --- | --- | --- | --- | --- | --- | --- | --- | --- | --- | --- | --- | --- | --- | --- | --- | --- | --- | --- | --- | --- | --- | --- | --- | --- | --- | --- | --- | --- | --- | --- | --- | --- | --- | --- | --- | --- | --- | --- | --- | --- | --- | --- | --- | --- | --- | --- | --- | --- | --- | --- | --- | --- | --- | --- | --- | --- | --- | --- | --- | --- | --- | --- |
