# Supplementary material for: RedundancyMiner: De-replication of redundant GO categories in microarray and proteomics analysis
Source: BMC Bioinformatics. 2011 Feb 10;12:52. doi: 10.1186/1471-2105-12-52 (PMC3223614; doi:10.1186/1471-2105-12-52)
Supplement: Additional file 10 — Kinetochore genes HTGM download. compressed package of the results of running HTGM on the kinetochore genes list. [file 1471-2105-12-52-S10.ZIP › work405493610/index.html]

HTGM Job Summary

# HTGM Job Summary

| Input and Parameters | | |
| --- | --- | --- |
| Type | File | Description |
| Total File | total.txt | User-submitted list of total genes in the experiment or analysis |
| Changed File | kinetochore.txt | User-submitted list of changed genes in the experiment or analysis |
| Summary of options | userinputparam.txt | A summary of the parameters selected by the user for this job |
| Database version | GODBVersion.txt | The version of the GO database used to process this job |
| --- | | ||| Results | | |
| --- | --- | --- |
| Type | File | Description |
| Archive Results (Zip) | HighThruputResult405493610.zip | An archive of all of the result files for this job |
| Browsable Results (HTML) | Browse Results | A browsable collection of pages to navigate the result files for this job |
