# Supplementary material for: RedundancyMiner: De-replication of redundant GO categories in microarray and proteomics analysis
Source: BMC Bioinformatics. 2011 Feb 10;12:52. doi: 10.1186/1471-2105-12-52 (PMC3223614; doi:10.1186/1471-2105-12-52)
Supplement: Additional file 10 — Kinetochore genes HTGM download. compressed package of the results of running HTGM on the kinetochore genes list. [file 1471-2105-12-52-S10.ZIP › work405493610/total.txt405493610.dir/index.html]

Results - total.txt

# Results - total.txt

| Integrative Results | | |
| --- | --- | --- |
| Type | File | Description |
| Integrative Category Report | total.txt.report | List of the number of significantly changed categories in each changed genes file |
| Total Versus Total Report | total.txt.total.tvt | GO category mapping of all genes in total file |
| Clustered Image Map(CIM) data: Significant categories versus genes in those categories | | || Integrative CIM Data | total.txt.change.series.CIM | Matrix of significant categories for changed genes against those genes |
| --- | | ||| Results for Each Changed File | | |
| --- | --- | --- |
| kinetochore.txt.dir/ | | |  |
| --- | | ||| Navigation | | |
| --- | --- | --- |
| Up to Parent(HTGM Job Summary) | | |
