# Supplementary material for: RedundancyMiner: De-replication of redundant GO categories in microarray and proteomics analysis
Source: BMC Bioinformatics. 2011 Feb 10;12:52. doi: 10.1186/1471-2105-12-52 (PMC3223614; doi:10.1186/1471-2105-12-52)
Supplement: Additional file 10 — Kinetochore genes HTGM download. compressed package of the results of running HTGM on the kinetochore genes list. [file 1471-2105-12-52-S10.ZIP › work405493610/total.txt405493610.dir/kinetochore.txt.dir/kinetochore.txt.change.gce.CIM.dir/cgi_user_x.html]

**X-axis Names**   
Cluster is based on euclidean distance  
Cluster method is: average  
plclust  
height plot  

|  |
| --- |
| 1.GO:0008283\_cell\_proliferation |
| 2.GO:0051234\_establishment\_of\_localization |
| 3.GO:0051649\_establishment\_of\_cellular\_localization |
| 4.GO:0051641\_cellular\_localization |
| 5.GO:0007093\_mitotic\_cell\_cycle\_checkpoint |
| 6.GO:0051726\_regulation\_of\_cell\_cycle |
| 7.GO:0000075\_cell\_cycle\_checkpoint |
| 8.GO:0007346\_regulation\_of\_mitotic\_cell\_cycle |
| 9.GO:0022403\_cell\_cycle\_phase |
| 10.GO:0007067\_mitosis |
| 11.GO:0000279\_M\_phase |
| 12.GO:0000087\_M\_phase\_of\_mitotic\_cell\_cycle |
| 13.GO:0000278\_mitotic\_cell\_cycle |
| 14.GO:0022402\_cell\_cycle\_process |
| 15.GO:0007049\_cell\_cycle |
| 16.GO:0006996\_organelle\_organization\_and\_biogenesis |
| 17.GO:0007010\_cytoskeleton\_organization\_and\_biogenesis |
| 18.GO:0000226\_microtubule\_cytoskeleton\_organization\_and\_biogenesis |
| 19.GO:0007017\_microtubule-based\_process |
| 20.GO:0033036\_macromolecule\_localization |
| 21.GO:0046907\_intracellular\_transport |
| 22.GO:0006913\_nucleocytoplasmic\_transport |
| 23.GO:0051169\_nuclear\_transport |
| 24.GO:0050657\_nucleic\_acid\_transport |
| 25.GO:0050658\_RNA\_transport |
| 26.GO:0006403\_RNA\_localization |
| 27.GO:0015931\_nucleobase\_\_nucleoside\_\_nucleotide\_and\_nucleic\_acid\_transport |
| 28.GO:0051236\_establishment\_of\_RNA\_localization |
| 29.GO:0006405\_RNA\_export\_from\_nucleus |
| 30.GO:0051028\_mRNA\_transport |
| 31.GO:0051168\_nuclear\_export |
| 32.GO:0006406\_mRNA\_export\_from\_nucleus |
| 33.GO:0048015\_phosphoinositide-mediated\_signaling |
| 34.GO:0007051\_spindle\_organization\_and\_biogenesis |
| 35.GO:0000070\_mitotic\_sister\_chromatid\_segregation |
| 36.GO:0000819\_sister\_chromatid\_segregation |
| 37.GO:0007059\_chromosome\_segregation |
| 38.GO:0051276\_chromosome\_organization\_and\_biogenesis |
| 39.GO:0010458\_exit\_from\_mitosis |
| 40.GO:0051128\_regulation\_of\_cellular\_component\_organization\_and\_biogenesis |
| 41.GO:0051261\_protein\_depolymerization |
| 42.GO:0007163\_establishment\_and\_or\_maintenance\_of\_cell\_polarity |
| 43.GO:0007019\_microtubule\_depolymerization |
| 44.GO:0007026\_negative\_regulation\_of\_microtubule\_depolymerization |
| 45.GO:0031114\_regulation\_of\_microtubule\_depolymerization |
| 46.GO:0031110\_regulation\_of\_microtubule\_polymerization\_or\_depolymerization |
| 47.GO:0051129\_negative\_regulation\_of\_cellular\_component\_organization\_and\_biogenesis |
| 48.GO:0051494\_negative\_regulation\_of\_cytoskeleton\_organization\_and\_biogenesis |
| 49.GO:0031109\_microtubule\_polymerization\_or\_depolymerization |
| 50.GO:0031111\_negative\_regulation\_of\_microtubule\_polymerization\_or\_depolymerization |
| 51.GO:0033043\_regulation\_of\_organelle\_organization\_and\_biogenesis |
| 52.GO:0032886\_regulation\_of\_microtubule-based\_process |
| 53.GO:0051493\_regulation\_of\_cytoskeleton\_organization\_and\_biogenesis |
| 54.GO:0051246\_regulation\_of\_protein\_metabolic\_process |
| 55.GO:0032269\_negative\_regulation\_of\_cellular\_protein\_metabolic\_process |
| 56.GO:0051248\_negative\_regulation\_of\_protein\_metabolic\_process |
| 57.GO:0032268\_regulation\_of\_cellular\_protein\_metabolic\_process |
| 58.GO:0031023\_microtubule\_organizing\_center\_organization\_and\_biogenesis |
| 59.GO:0051297\_centrosome\_organization\_and\_biogenesis |
| 60.GO:0031577\_spindle\_checkpoint |
| 61.GO:0007094\_mitotic\_cell\_cycle\_spindle\_assembly\_checkpoint |
| 62.GO:0043623\_cellular\_protein\_complex\_assembly |
| 63.GO:0051640\_organelle\_localization |
| 64.GO:0051656\_establishment\_of\_organelle\_localization |
| 65.GO:0051303\_establishment\_of\_chromosome\_localization |
| 66.GO:0050000\_chromosome\_localization |
