# Supplementary material for: RedundancyMiner: De-replication of redundant GO categories in microarray and proteomics analysis
Source: BMC Bioinformatics. 2011 Feb 10;12:52. doi: 10.1186/1471-2105-12-52 (PMC3223614; doi:10.1186/1471-2105-12-52)
Supplement: Additional file 10 — Kinetochore genes HTGM download. compressed package of the results of running HTGM on the kinetochore genes list. [file 1471-2105-12-52-S10.ZIP › work405493610/total.txt405493610.dir/kinetochore.txt.dir/kinetochore.txt.change.gce.CIM.dir/cgi_user_y.html]

**Y-axis Names**   
Cluster is based on euclidean distance  
Cluster method is: average  
plclust  
height plot  

|  |
| --- |
| 1.CLASP1 |
| 2.CLASP2 |
| 3.MAPRE1 |
| 4.NUP160 |
| 5.NUP107 |
| 6.NUP133 |
| 7.CENPE |
| 8.CENPF |
| 9.BUB1B |
| 10.MAD2L1 |
| 11.KIF2C |
| 12.CLIP1 |
| 13.PLK1 |
| 14.ZW10 |
| 15.NDC80 |
| 16.ZWINT |
| 17.BUB1 |
| 18.ZWILCH |
| 19.TAOK2 |
| 20.SEC13 |
| 21.MXI1 |
| 22.RANBP2 |
| 23.PPP2R4 |
| 24.INCENP |
| 25.NUF2 |
| 26.CDC20 |
| 27.RPS27 |
| 28.NUDC |
| 29.MXD1 |
| 30.BUB3 |
| 31.MAPRE2 |
| 32.CENPH |
| 33.MIS12 |
| 34.PAFAH1B1 |
| 35.CKAP5 |
| 36.NDE1 |
