# Supplementary material for: RedundancyMiner: De-replication of redundant GO categories in microarray and proteomics analysis
Source: BMC Bioinformatics. 2011 Feb 10;12:52. doi: 10.1186/1471-2105-12-52 (PMC3223614; doi:10.1186/1471-2105-12-52)
Supplement: Additional file 10 — Kinetochore genes HTGM download. compressed package of the results of running HTGM on the kinetochore genes list. [file 1471-2105-12-52-S10.ZIP › work405493610/total.txt405493610.dir/kinetochore.txt.dir/kinetochore.txt.change.gce.html]

Gene Category Report for kinetochore.txt

# Gene Category Report for kinetochore.txt

| HYPERLINKED GO CATEGORY | HYPERLINKED GENE NAME | TOTAL GENES | CHANGED GENES | ENRICHMENT | LOG10(p) | CUMULATIVE NUMBER OF CATEGORIES | CUMULATIVE RANDOMS MEAN | FALSE DISCOVERY RATE |
| --- | --- | --- | --- | --- | --- | --- | --- | --- |
| GO:0000226\_microtubule\_cytoskeleton\_organization\_and\_biogenesis | BUB1B | 58 | 10 | 25.735913 | -11.634038 | 1 | 0.0 | 0.000000 |
| GO:0000226\_microtubule\_cytoskeleton\_organization\_and\_biogenesis | ZWINT | 58 | 10 | 25.735913 | -11.634038 | 1 | 0.0 | 0.000000 |
| GO:0000226\_microtubule\_cytoskeleton\_organization\_and\_biogenesis | MAPRE1 | 58 | 10 | 25.735913 | -11.634038 | 1 | 0.0 | 0.000000 |
| GO:0000226\_microtubule\_cytoskeleton\_organization\_and\_biogenesis | PAFAH1B1 | 58 | 10 | 25.735913 | -11.634038 | 1 | 0.0 | 0.000000 |
| GO:0000226\_microtubule\_cytoskeleton\_organization\_and\_biogenesis | CKAP5 | 58 | 10 | 25.735913 | -11.634038 | 1 | 0.0 | 0.000000 |
| GO:0000226\_microtubule\_cytoskeleton\_organization\_and\_biogenesis | CLASP2 | 58 | 10 | 25.735913 | -11.634038 | 1 | 0.0 | 0.000000 |
| GO:0000226\_microtubule\_cytoskeleton\_organization\_and\_biogenesis | KIF2C | 58 | 10 | 25.735913 | -11.634038 | 1 | 0.0 | 0.000000 |
| GO:0000226\_microtubule\_cytoskeleton\_organization\_and\_biogenesis | NDC80 | 58 | 10 | 25.735913 | -11.634038 | 1 | 0.0 | 0.000000 |
| GO:0000226\_microtubule\_cytoskeleton\_organization\_and\_biogenesis | CLASP1 | 58 | 10 | 25.735913 | -11.634038 | 1 | 0.0 | 0.000000 |
| GO:0000226\_microtubule\_cytoskeleton\_organization\_and\_biogenesis | NDE1 | 58 | 10 | 25.735913 | -11.634038 | 1 | 0.0 | 0.000000 |
| GO:0000278\_mitotic\_cell\_cycle | ZW10 | 183 | 14 | 11.419432 | -11.315199 | 2 | 0.0 | 0.000000 |
| GO:0000278\_mitotic\_cell\_cycle | CENPF | 183 | 14 | 11.419432 | -11.315199 | 2 | 0.0 | 0.000000 |
| GO:0000278\_mitotic\_cell\_cycle | CENPE | 183 | 14 | 11.419432 | -11.315199 | 2 | 0.0 | 0.000000 |
| GO:0000278\_mitotic\_cell\_cycle | MAD2L1 | 183 | 14 | 11.419432 | -11.315199 | 2 | 0.0 | 0.000000 |
| GO:0000278\_mitotic\_cell\_cycle | CLIP1 | 183 | 14 | 11.419432 | -11.315199 | 2 | 0.0 | 0.000000 |
| GO:0000278\_mitotic\_cell\_cycle | BUB1 | 183 | 14 | 11.419432 | -11.315199 | 2 | 0.0 | 0.000000 |
| GO:0000278\_mitotic\_cell\_cycle | NDC80 | 183 | 14 | 11.419432 | -11.315199 | 2 | 0.0 | 0.000000 |
| GO:0000278\_mitotic\_cell\_cycle | BUB1B | 183 | 14 | 11.419432 | -11.315199 | 2 | 0.0 | 0.000000 |
| GO:0000278\_mitotic\_cell\_cycle | ZWINT | 183 | 14 | 11.419432 | -11.315199 | 2 | 0.0 | 0.000000 |
| GO:0000278\_mitotic\_cell\_cycle | ZWILCH | 183 | 14 | 11.419432 | -11.315199 | 2 | 0.0 | 0.000000 |
| GO:0000278\_mitotic\_cell\_cycle | PAFAH1B1 | 183 | 14 | 11.419432 | -11.315199 | 2 | 0.0 | 0.000000 |
| GO:0000278\_mitotic\_cell\_cycle | KIF2C | 183 | 14 | 11.419432 | -11.315199 | 2 | 0.0 | 0.000000 |
| GO:0000278\_mitotic\_cell\_cycle | CLASP1 | 183 | 14 | 11.419432 | -11.315199 | 2 | 0.0 | 0.000000 |
| GO:0000278\_mitotic\_cell\_cycle | PLK1 | 183 | 14 | 11.419432 | -11.315199 | 2 | 0.0 | 0.000000 |
| GO:0007049\_cell\_cycle | CDC20 | 349 | 17 | 7.270948 | -10.678022 | 3 | 0.0 | 0.000000 |
| GO:0007049\_cell\_cycle | ZW10 | 349 | 17 | 7.270948 | -10.678022 | 3 | 0.0 | 0.000000 |
| GO:0007049\_cell\_cycle | CENPF | 349 | 17 | 7.270948 | -10.678022 | 3 | 0.0 | 0.000000 |
| GO:0007049\_cell\_cycle | CENPE | 349 | 17 | 7.270948 | -10.678022 | 3 | 0.0 | 0.000000 |
| GO:0007049\_cell\_cycle | MAD2L1 | 349 | 17 | 7.270948 | -10.678022 | 3 | 0.0 | 0.000000 |
| GO:0007049\_cell\_cycle | CLIP1 | 349 | 17 | 7.270948 | -10.678022 | 3 | 0.0 | 0.000000 |
| GO:0007049\_cell\_cycle | BUB1 | 349 | 17 | 7.270948 | -10.678022 | 3 | 0.0 | 0.000000 |
| GO:0007049\_cell\_cycle | CKAP5 | 349 | 17 | 7.270948 | -10.678022 | 3 | 0.0 | 0.000000 |
| GO:0007049\_cell\_cycle | NDC80 | 349 | 17 | 7.270948 | -10.678022 | 3 | 0.0 | 0.000000 |
| GO:0007049\_cell\_cycle | NDE1 | 349 | 17 | 7.270948 | -10.678022 | 3 | 0.0 | 0.000000 |
| GO:0007049\_cell\_cycle | BUB1B | 349 | 17 | 7.270948 | -10.678022 | 3 | 0.0 | 0.000000 |
| GO:0007049\_cell\_cycle | ZWINT | 349 | 17 | 7.270948 | -10.678022 | 3 | 0.0 | 0.000000 |
| GO:0007049\_cell\_cycle | ZWILCH | 349 | 17 | 7.270948 | -10.678022 | 3 | 0.0 | 0.000000 |
| GO:0007049\_cell\_cycle | PAFAH1B1 | 349 | 17 | 7.270948 | -10.678022 | 3 | 0.0 | 0.000000 |
| GO:0007049\_cell\_cycle | KIF2C | 349 | 17 | 7.270948 | -10.678022 | 3 | 0.0 | 0.000000 |
| GO:0007049\_cell\_cycle | CLASP1 | 349 | 17 | 7.270948 | -10.678022 | 3 | 0.0 | 0.000000 |
| GO:0007049\_cell\_cycle | PLK1 | 349 | 17 | 7.270948 | -10.678022 | 3 | 0.0 | 0.000000 |
| GO:0007059\_chromosome\_segregation | INCENP | 38 | 8 | 31.424904 | -10.079699 | 4 | 0.0 | 0.000000 |
| GO:0007059\_chromosome\_segregation | MIS12 | 38 | 8 | 31.424904 | -10.079699 | 4 | 0.0 | 0.000000 |
| GO:0007059\_chromosome\_segregation | ZWINT | 38 | 8 | 31.424904 | -10.079699 | 4 | 0.0 | 0.000000 |
| GO:0007059\_chromosome\_segregation | NUF2 | 38 | 8 | 31.424904 | -10.079699 | 4 | 0.0 | 0.000000 |
| GO:0007059\_chromosome\_segregation | CENPF | 38 | 8 | 31.424904 | -10.079699 | 4 | 0.0 | 0.000000 |
| GO:0007059\_chromosome\_segregation | ZW10 | 38 | 8 | 31.424904 | -10.079699 | 4 | 0.0 | 0.000000 |
| GO:0007059\_chromosome\_segregation | CENPE | 38 | 8 | 31.424904 | -10.079699 | 4 | 0.0 | 0.000000 |
| GO:0007059\_chromosome\_segregation | NDC80 | 38 | 8 | 31.424904 | -10.079699 | 4 | 0.0 | 0.000000 |
| GO:0000087\_M\_phase\_of\_mitotic\_cell\_cycle | BUB1B | 91 | 10 | 16.403109 | -9.610265 | 6 | 0.0 | 0.000000 |
| GO:0000087\_M\_phase\_of\_mitotic\_cell\_cycle | ZWINT | 91 | 10 | 16.403109 | -9.610265 | 6 | 0.0 | 0.000000 |
| GO:0000087\_M\_phase\_of\_mitotic\_cell\_cycle | ZW10 | 91 | 10 | 16.403109 | -9.610265 | 6 | 0.0 | 0.000000 |
| GO:0000087\_M\_phase\_of\_mitotic\_cell\_cycle | CENPE | 91 | 10 | 16.403109 | -9.610265 | 6 | 0.0 | 0.000000 |
| GO:0000087\_M\_phase\_of\_mitotic\_cell\_cycle | CLIP1 | 91 | 10 | 16.403109 | -9.610265 | 6 | 0.0 | 0.000000 |
| GO:0000087\_M\_phase\_of\_mitotic\_cell\_cycle | MAD2L1 | 91 | 10 | 16.403109 | -9.610265 | 6 | 0.0 | 0.000000 |
| GO:0000087\_M\_phase\_of\_mitotic\_cell\_cycle | CLASP1 | 91 | 10 | 16.403109 | -9.610265 | 6 | 0.0 | 0.000000 |
| GO:0000087\_M\_phase\_of\_mitotic\_cell\_cycle | KIF2C | 91 | 10 | 16.403109 | -9.610265 | 6 | 0.0 | 0.000000 |
| GO:0000087\_M\_phase\_of\_mitotic\_cell\_cycle | NDC80 | 91 | 10 | 16.403109 | -9.610265 | 6 | 0.0 | 0.000000 |
| GO:0000087\_M\_phase\_of\_mitotic\_cell\_cycle | PLK1 | 91 | 10 | 16.403109 | -9.610265 | 6 | 0.0 | 0.000000 |
| GO:0007017\_microtubule-based\_process | BUB1B | 91 | 10 | 16.403109 | -9.610265 | 6 | 0.0 | 0.000000 |
| GO:0007017\_microtubule-based\_process | ZWINT | 91 | 10 | 16.403109 | -9.610265 | 6 | 0.0 | 0.000000 |
| GO:0007017\_microtubule-based\_process | MAPRE1 | 91 | 10 | 16.403109 | -9.610265 | 6 | 0.0 | 0.000000 |
| GO:0007017\_microtubule-based\_process | PAFAH1B1 | 91 | 10 | 16.403109 | -9.610265 | 6 | 0.0 | 0.000000 |
| GO:0007017\_microtubule-based\_process | CKAP5 | 91 | 10 | 16.403109 | -9.610265 | 6 | 0.0 | 0.000000 |
| GO:0007017\_microtubule-based\_process | CLASP2 | 91 | 10 | 16.403109 | -9.610265 | 6 | 0.0 | 0.000000 |
| GO:0007017\_microtubule-based\_process | KIF2C | 91 | 10 | 16.403109 | -9.610265 | 6 | 0.0 | 0.000000 |
| GO:0007017\_microtubule-based\_process | NDC80 | 91 | 10 | 16.403109 | -9.610265 | 6 | 0.0 | 0.000000 |
| GO:0007017\_microtubule-based\_process | CLASP1 | 91 | 10 | 16.403109 | -9.610265 | 6 | 0.0 | 0.000000 |
| GO:0007017\_microtubule-based\_process | NDE1 | 91 | 10 | 16.403109 | -9.610265 | 6 | 0.0 | 0.000000 |
| GO:0022402\_cell\_cycle\_process | ZW10 | 245 | 14 | 8.529617 | -9.598482 | 7 | 0.0 | 0.000000 |
| GO:0022402\_cell\_cycle\_process | CENPF | 245 | 14 | 8.529617 | -9.598482 | 7 | 0.0 | 0.000000 |
| GO:0022402\_cell\_cycle\_process | CENPE | 245 | 14 | 8.529617 | -9.598482 | 7 | 0.0 | 0.000000 |
| GO:0022402\_cell\_cycle\_process | MAD2L1 | 245 | 14 | 8.529617 | -9.598482 | 7 | 0.0 | 0.000000 |
| GO:0022402\_cell\_cycle\_process | CLIP1 | 245 | 14 | 8.529617 | -9.598482 | 7 | 0.0 | 0.000000 |
| GO:0022402\_cell\_cycle\_process | CKAP5 | 245 | 14 | 8.529617 | -9.598482 | 7 | 0.0 | 0.000000 |
| GO:0022402\_cell\_cycle\_process | NDC80 | 245 | 14 | 8.529617 | -9.598482 | 7 | 0.0 | 0.000000 |
| GO:0022402\_cell\_cycle\_process | NDE1 | 245 | 14 | 8.529617 | -9.598482 | 7 | 0.0 | 0.000000 |
| GO:0022402\_cell\_cycle\_process | BUB1B | 245 | 14 | 8.529617 | -9.598482 | 7 | 0.0 | 0.000000 |
| GO:0022402\_cell\_cycle\_process | ZWINT | 245 | 14 | 8.529617 | -9.598482 | 7 | 0.0 | 0.000000 |
| GO:0022402\_cell\_cycle\_process | PAFAH1B1 | 245 | 14 | 8.529617 | -9.598482 | 7 | 0.0 | 0.000000 |
| GO:0022402\_cell\_cycle\_process | CLASP1 | 245 | 14 | 8.529617 | -9.598482 | 7 | 0.0 | 0.000000 |
| GO:0022402\_cell\_cycle\_process | KIF2C | 245 | 14 | 8.529617 | -9.598482 | 7 | 0.0 | 0.000000 |
| GO:0022402\_cell\_cycle\_process | PLK1 | 245 | 14 | 8.529617 | -9.598482 | 7 | 0.0 | 0.000000 |
| GO:0007093\_mitotic\_cell\_cycle\_checkpoint | BUB1B | 28 | 7 | 37.317073 | -9.422662 | 8 | 0.0 | 0.000000 |
| GO:0007093\_mitotic\_cell\_cycle\_checkpoint | ZWINT | 28 | 7 | 37.317073 | -9.422662 | 8 | 0.0 | 0.000000 |
| GO:0007093\_mitotic\_cell\_cycle\_checkpoint | ZWILCH | 28 | 7 | 37.317073 | -9.422662 | 8 | 0.0 | 0.000000 |
| GO:0007093\_mitotic\_cell\_cycle\_checkpoint | ZW10 | 28 | 7 | 37.317073 | -9.422662 | 8 | 0.0 | 0.000000 |
| GO:0007093\_mitotic\_cell\_cycle\_checkpoint | CENPF | 28 | 7 | 37.317073 | -9.422662 | 8 | 0.0 | 0.000000 |
| GO:0007093\_mitotic\_cell\_cycle\_checkpoint | MAD2L1 | 28 | 7 | 37.317073 | -9.422662 | 8 | 0.0 | 0.000000 |
| GO:0007093\_mitotic\_cell\_cycle\_checkpoint | BUB1 | 28 | 7 | 37.317073 | -9.422662 | 8 | 0.0 | 0.000000 |
| GO:0051383\_kinetochore\_organization\_and\_biogenesis | MIS12 | 4 | 4 |  |  |  |  |  |  |
| GO:0051383\_kinetochore\_organization\_and\_biogenesis | CENPF | 4 | 4 |  |  |  |  |  |  |
| GO:0051383\_kinetochore\_organization\_and\_biogenesis | CENPE | 4 | 4 |  |  |  |  |  |  |
| GO:0051383\_kinetochore\_organization\_and\_biogenesis | CENPH | 4 | 4 |  |  |  |  |  |  |
| GO:0007067\_mitosis | BUB1B | 88 | 9 | 15.266075 | -8.379757 | 9 | 0.0 | 0.000000 |
| GO:0007067\_mitosis | ZWINT | 88 | 9 | 15.266075 | -8.379757 | 9 | 0.0 | 0.000000 |
| GO:0007067\_mitosis | ZW10 | 88 | 9 | 15.266075 | -8.379757 | 9 | 0.0 | 0.000000 |
| GO:0007067\_mitosis | CENPE | 88 | 9 | 15.266075 | -8.379757 | 9 | 0.0 | 0.000000 |
| GO:0007067\_mitosis | CLIP1 | 88 | 9 | 15.266075 | -8.379757 | 9 | 0.0 | 0.000000 |
| GO:0007067\_mitosis | MAD2L1 | 88 | 9 | 15.266075 | -8.379757 | 9 | 0.0 | 0.000000 |
| GO:0007067\_mitosis | KIF2C | 88 | 9 | 15.266075 | -8.379757 | 9 | 0.0 | 0.000000 |
| GO:0007067\_mitosis | NDC80 | 88 | 9 | 15.266075 | -8.379757 | 9 | 0.0 | 0.000000 |
| GO:0007067\_mitosis | PLK1 | 88 | 9 | 15.266075 | -8.379757 | 9 | 0.0 | 0.000000 |
| GO:0000279\_M\_phase | BUB1B | 125 | 10 | 11.941463 | -8.238396 | 10 | 0.0 | 0.000000 |
| GO:0000279\_M\_phase | ZWINT | 125 | 10 | 11.941463 | -8.238396 | 10 | 0.0 | 0.000000 |
| GO:0000279\_M\_phase | ZW10 | 125 | 10 | 11.941463 | -8.238396 | 10 | 0.0 | 0.000000 |
| GO:0000279\_M\_phase | CENPE | 125 | 10 | 11.941463 | -8.238396 | 10 | 0.0 | 0.000000 |
| GO:0000279\_M\_phase | CLIP1 | 125 | 10 | 11.941463 | -8.238396 | 10 | 0.0 | 0.000000 |
| GO:0000279\_M\_phase | MAD2L1 | 125 | 10 | 11.941463 | -8.238396 | 10 | 0.0 | 0.000000 |
| GO:0000279\_M\_phase | CLASP1 | 125 | 10 | 11.941463 | -8.238396 | 10 | 0.0 | 0.000000 |
| GO:0000279\_M\_phase | KIF2C | 125 | 10 | 11.941463 | -8.238396 | 10 | 0.0 | 0.000000 |
| GO:0000279\_M\_phase | NDC80 | 125 | 10 | 11.941463 | -8.238396 | 10 | 0.0 | 0.000000 |
| GO:0000279\_M\_phase | PLK1 | 125 | 10 | 11.941463 | -8.238396 | 10 | 0.0 | 0.000000 |
| GO:0022403\_cell\_cycle\_phase | BUB1B | 187 | 11 | 8.780488 | -7.639150 | 11 | 0.0 | 0.000000 |
| GO:0022403\_cell\_cycle\_phase | ZWINT | 187 | 11 | 8.780488 | -7.639150 | 11 | 0.0 | 0.000000 |
| GO:0022403\_cell\_cycle\_phase | CENPF | 187 | 11 | 8.780488 | -7.639150 | 11 | 0.0 | 0.000000 |
| GO:0022403\_cell\_cycle\_phase | ZW10 | 187 | 11 | 8.780488 | -7.639150 | 11 | 0.0 | 0.000000 |
| GO:0022403\_cell\_cycle\_phase | CENPE | 187 | 11 | 8.780488 | -7.639150 | 11 | 0.0 | 0.000000 |
| GO:0022403\_cell\_cycle\_phase | CLIP1 | 187 | 11 | 8.780488 | -7.639150 | 11 | 0.0 | 0.000000 |
| GO:0022403\_cell\_cycle\_phase | MAD2L1 | 187 | 11 | 8.780488 | -7.639150 | 11 | 0.0 | 0.000000 |
| GO:0022403\_cell\_cycle\_phase | CLASP1 | 187 | 11 | 8.780488 | -7.639150 | 11 | 0.0 | 0.000000 |
| GO:0022403\_cell\_cycle\_phase | KIF2C | 187 | 11 | 8.780488 | -7.639150 | 11 | 0.0 | 0.000000 |
| GO:0022403\_cell\_cycle\_phase | NDC80 | 187 | 11 | 8.780488 | -7.639150 | 11 | 0.0 | 0.000000 |
| GO:0022403\_cell\_cycle\_phase | PLK1 | 187 | 11 | 8.780488 | -7.639150 | 11 | 0.0 | 0.000000 |
| GO:0007346\_regulation\_of\_mitotic\_cell\_cycle | BUB1B | 50 | 7 | 20.897561 | -7.543240 | 12 | 0.0 | 0.000000 |
| GO:0007346\_regulation\_of\_mitotic\_cell\_cycle | ZWINT | 50 | 7 | 20.897561 | -7.543240 | 12 | 0.0 | 0.000000 |
| GO:0007346\_regulation\_of\_mitotic\_cell\_cycle | ZWILCH | 50 | 7 | 20.897561 | -7.543240 | 12 | 0.0 | 0.000000 |
| GO:0007346\_regulation\_of\_mitotic\_cell\_cycle | ZW10 | 50 | 7 | 20.897561 | -7.543240 | 12 | 0.0 | 0.000000 |
| GO:0007346\_regulation\_of\_mitotic\_cell\_cycle | CENPF | 50 | 7 | 20.897561 | -7.543240 | 12 | 0.0 | 0.000000 |
| GO:0007346\_regulation\_of\_mitotic\_cell\_cycle | MAD2L1 | 50 | 7 | 20.897561 | -7.543240 | 12 | 0.0 | 0.000000 |
| GO:0007346\_regulation\_of\_mitotic\_cell\_cycle | BUB1 | 50 | 7 | 20.897561 | -7.543240 | 12 | 0.0 | 0.000000 |
| GO:0000075\_cell\_cycle\_checkpoint | BUB1B | 52 | 7 | 20.093809 | -7.420582 | 13 | 0.0 | 0.000000 |
| GO:0000075\_cell\_cycle\_checkpoint | ZWINT | 52 | 7 | 20.093809 | -7.420582 | 13 | 0.0 | 0.000000 |
| GO:0000075\_cell\_cycle\_checkpoint | ZWILCH | 52 | 7 | 20.093809 | -7.420582 | 13 | 0.0 | 0.000000 |
| GO:0000075\_cell\_cycle\_checkpoint | ZW10 | 52 | 7 | 20.093809 | -7.420582 | 13 | 0.0 | 0.000000 |
| GO:0000075\_cell\_cycle\_checkpoint | CENPF | 52 | 7 | 20.093809 | -7.420582 | 13 | 0.0 | 0.000000 |
| GO:0000075\_cell\_cycle\_checkpoint | MAD2L1 | 52 | 7 | 20.093809 | -7.420582 | 13 | 0.0 | 0.000000 |
| GO:0000075\_cell\_cycle\_checkpoint | BUB1 | 52 | 7 | 20.093809 | -7.420582 | 13 | 0.0 | 0.000000 |
| GO:0051382\_kinetochore\_assembly | MIS12 | 3 | 3 |  |  |  |  |  |  |
| GO:0051382\_kinetochore\_assembly | CENPF | 3 | 3 |  |  |  |  |  |  |
| GO:0051382\_kinetochore\_assembly | CENPE | 3 | 3 |  |  |  |  |  |  |
| GO:0006996\_organelle\_organization\_and\_biogenesis | MIS12 | 571 | 16 | 4.182649 | -6.521159 | 14 | 0.0 | 0.000000 |
| GO:0006996\_organelle\_organization\_and\_biogenesis | TAOK2 | 571 | 16 | 4.182649 | -6.521159 | 14 | 0.0 | 0.000000 |
| GO:0006996\_organelle\_organization\_and\_biogenesis | CENPF | 571 | 16 | 4.182649 | -6.521159 | 14 | 0.0 | 0.000000 |
| GO:0006996\_organelle\_organization\_and\_biogenesis | ZW10 | 571 | 16 | 4.182649 | -6.521159 | 14 | 0.0 | 0.000000 |
| GO:0006996\_organelle\_organization\_and\_biogenesis | CENPE | 571 | 16 | 4.182649 | -6.521159 | 14 | 0.0 | 0.000000 |
| GO:0006996\_organelle\_organization\_and\_biogenesis | CKAP5 | 571 | 16 | 4.182649 | -6.521159 | 14 | 0.0 | 0.000000 |
| GO:0006996\_organelle\_organization\_and\_biogenesis | NDC80 | 571 | 16 | 4.182649 | -6.521159 | 14 | 0.0 | 0.000000 |
| GO:0006996\_organelle\_organization\_and\_biogenesis | NDE1 | 571 | 16 | 4.182649 | -6.521159 | 14 | 0.0 | 0.000000 |
| GO:0006996\_organelle\_organization\_and\_biogenesis | BUB1B | 571 | 16 | 4.182649 | -6.521159 | 14 | 0.0 | 0.000000 |
| GO:0006996\_organelle\_organization\_and\_biogenesis | ZWINT | 571 | 16 | 4.182649 | -6.521159 | 14 | 0.0 | 0.000000 |
| GO:0006996\_organelle\_organization\_and\_biogenesis | MAPRE1 | 571 | 16 | 4.182649 | -6.521159 | 14 | 0.0 | 0.000000 |
| GO:0006996\_organelle\_organization\_and\_biogenesis | CENPH | 571 | 16 | 4.182649 | -6.521159 | 14 | 0.0 | 0.000000 |
| GO:0006996\_organelle\_organization\_and\_biogenesis | PAFAH1B1 | 571 | 16 | 4.182649 | -6.521159 | 14 | 0.0 | 0.000000 |
| GO:0006996\_organelle\_organization\_and\_biogenesis | CLASP1 | 571 | 16 | 4.182649 | -6.521159 | 14 | 0.0 | 0.000000 |
| GO:0006996\_organelle\_organization\_and\_biogenesis | KIF2C | 571 | 16 | 4.182649 | -6.521159 | 14 | 0.0 | 0.000000 |
| GO:0006996\_organelle\_organization\_and\_biogenesis | CLASP2 | 571 | 16 | 4.182649 | -6.521159 | 14 | 0.0 | 0.000000 |
| GO:0007010\_cytoskeleton\_organization\_and\_biogenesis | BUB1B | 253 | 11 | 6.489926 | -6.293175 | 15 | 0.0 | 0.000000 |
| GO:0007010\_cytoskeleton\_organization\_and\_biogenesis | ZWINT | 253 | 11 | 6.489926 | -6.293175 | 15 | 0.0 | 0.000000 |
| GO:0007010\_cytoskeleton\_organization\_and\_biogenesis | TAOK2 | 253 | 11 | 6.489926 | -6.293175 | 15 | 0.0 | 0.000000 |
| GO:0007010\_cytoskeleton\_organization\_and\_biogenesis | MAPRE1 | 253 | 11 | 6.489926 | -6.293175 | 15 | 0.0 | 0.000000 |
| GO:0007010\_cytoskeleton\_organization\_and\_biogenesis | PAFAH1B1 | 253 | 11 | 6.489926 | -6.293175 | 15 | 0.0 | 0.000000 |
| GO:0007010\_cytoskeleton\_organization\_and\_biogenesis | CKAP5 | 253 | 11 | 6.489926 | -6.293175 | 15 | 0.0 | 0.000000 |
| GO:0007010\_cytoskeleton\_organization\_and\_biogenesis | CLASP2 | 253 | 11 | 6.489926 | -6.293175 | 15 | 0.0 | 0.000000 |
| GO:0007010\_cytoskeleton\_organization\_and\_biogenesis | KIF2C | 253 | 11 | 6.489926 | -6.293175 | 15 | 0.0 | 0.000000 |
| GO:0007010\_cytoskeleton\_organization\_and\_biogenesis | NDC80 | 253 | 11 | 6.489926 | -6.293175 | 15 | 0.0 | 0.000000 |
| GO:0007010\_cytoskeleton\_organization\_and\_biogenesis | CLASP1 | 253 | 11 | 6.489926 | -6.293175 | 15 | 0.0 | 0.000000 |
| GO:0007010\_cytoskeleton\_organization\_and\_biogenesis | NDE1 | 253 | 11 | 6.489926 | -6.293175 | 15 | 0.0 | 0.000000 |
| GO:0016043\_cellular\_component\_organization\_and\_biogenesis | MXI1 | 1240 | 22 | 2.648308 | -5.673135 | 16 | 0.0 | 0.000000 |
| GO:0016043\_cellular\_component\_organization\_and\_biogenesis | MIS12 | 1240 | 22 | 2.648308 | -5.673135 | 16 | 0.0 | 0.000000 |
| GO:0016043\_cellular\_component\_organization\_and\_biogenesis | TAOK2 | 1240 | 22 | 2.648308 | -5.673135 | 16 | 0.0 | 0.000000 |
| GO:0016043\_cellular\_component\_organization\_and\_biogenesis | CENPF | 1240 | 22 | 2.648308 | -5.673135 | 16 | 0.0 | 0.000000 |
| GO:0016043\_cellular\_component\_organization\_and\_biogenesis | ZW10 | 1240 | 22 | 2.648308 | -5.673135 | 16 | 0.0 | 0.000000 |
| GO:0016043\_cellular\_component\_organization\_and\_biogenesis | NUP160 | 1240 | 22 | 2.648308 | -5.673135 | 16 | 0.0 | 0.000000 |
| GO:0016043\_cellular\_component\_organization\_and\_biogenesis | CENPE | 1240 | 22 | 2.648308 | -5.673135 | 16 | 0.0 | 0.000000 |
| GO:0016043\_cellular\_component\_organization\_and\_biogenesis | CKAP5 | 1240 | 22 | 2.648308 | -5.673135 | 16 | 0.0 | 0.000000 |
| GO:0016043\_cellular\_component\_organization\_and\_biogenesis | SEC13 | 1240 | 22 | 2.648308 | -5.673135 | 16 | 0.0 | 0.000000 |
| GO:0016043\_cellular\_component\_organization\_and\_biogenesis | NDC80 | 1240 | 22 | 2.648308 | -5.673135 | 16 | 0.0 | 0.000000 |
| GO:0016043\_cellular\_component\_organization\_and\_biogenesis | NDE1 | 1240 | 22 | 2.648308 | -5.673135 | 16 | 0.0 | 0.000000 |
| GO:0016043\_cellular\_component\_organization\_and\_biogenesis | BUB1B | 1240 | 22 | 2.648308 | -5.673135 | 16 | 0.0 | 0.000000 |
| GO:0016043\_cellular\_component\_organization\_and\_biogenesis | ZWINT | 1240 | 22 | 2.648308 | -5.673135 | 16 | 0.0 | 0.000000 |
| GO:0016043\_cellular\_component\_organization\_and\_biogenesis | MAPRE1 | 1240 | 22 | 2.648308 | -5.673135 | 16 | 0.0 | 0.000000 |
| GO:0016043\_cellular\_component\_organization\_and\_biogenesis | NUP107 | 1240 | 22 | 2.648308 | -5.673135 | 16 | 0.0 | 0.000000 |
| GO:0016043\_cellular\_component\_organization\_and\_biogenesis | RANBP2 | 1240 | 22 | 2.648308 | -5.673135 | 16 | 0.0 | 0.000000 |
| GO:0016043\_cellular\_component\_organization\_and\_biogenesis | NUP133 | 1240 | 22 | 2.648308 | -5.673135 | 16 | 0.0 | 0.000000 |
| GO:0016043\_cellular\_component\_organization\_and\_biogenesis | CENPH | 1240 | 22 | 2.648308 | -5.673135 | 16 | 0.0 | 0.000000 |
| GO:0016043\_cellular\_component\_organization\_and\_biogenesis | PAFAH1B1 | 1240 | 22 | 2.648308 | -5.673135 | 16 | 0.0 | 0.000000 |
| GO:0016043\_cellular\_component\_organization\_and\_biogenesis | CLASP1 | 1240 | 22 | 2.648308 | -5.673135 | 16 | 0.0 | 0.000000 |
| GO:0016043\_cellular\_component\_organization\_and\_biogenesis | KIF2C | 1240 | 22 | 2.648308 | -5.673135 | 16 | 0.0 | 0.000000 |
| GO:0016043\_cellular\_component\_organization\_and\_biogenesis | CLASP2 | 1240 | 22 | 2.648308 | -5.673135 | 16 | 0.0 | 0.000000 |
| GO:0031111\_negative\_regulation\_of\_microtubule\_polymerization\_or\_depolymerization | MAPRE1 | 6 | 3 | 74.634146 | -5.259186 | 17 | 0.0 | 0.000000 |
| GO:0031111\_negative\_regulation\_of\_microtubule\_polymerization\_or\_depolymerization | CLASP1 | 6 | 3 | 74.634146 | -5.259186 | 17 | 0.0 | 0.000000 |
| GO:0031111\_negative\_regulation\_of\_microtubule\_polymerization\_or\_depolymerization | CLASP2 | 6 | 3 | 74.634146 | -5.259186 | 17 | 0.0 | 0.000000 |
| GO:0051649\_establishment\_of\_cellular\_localization | MXI1 | 410 | 12 | 4.368828 | -5.034276 | 18 | 0.0 | 0.000000 |
| GO:0051649\_establishment\_of\_cellular\_localization | ZWINT | 410 | 12 | 4.368828 | -5.034276 | 18 | 0.0 | 0.000000 |
| GO:0051649\_establishment\_of\_cellular\_localization | TAOK2 | 410 | 12 | 4.368828 | -5.034276 | 18 | 0.0 | 0.000000 |
| GO:0051649\_establishment\_of\_cellular\_localization | CENPF | 410 | 12 | 4.368828 | -5.034276 | 18 | 0.0 | 0.000000 |
| GO:0051649\_establishment\_of\_cellular\_localization | NUP107 | 410 | 12 | 4.368828 | -5.034276 | 18 | 0.0 | 0.000000 |
| GO:0051649\_establishment\_of\_cellular\_localization | RANBP2 | 410 | 12 | 4.368828 | -5.034276 | 18 | 0.0 | 0.000000 |
| GO:0051649\_establishment\_of\_cellular\_localization | ZW10 | 410 | 12 | 4.368828 | -5.034276 | 18 | 0.0 | 0.000000 |
| GO:0051649\_establishment\_of\_cellular\_localization | NUP160 | 410 | 12 | 4.368828 | -5.034276 | 18 | 0.0 | 0.000000 |
| GO:0051649\_establishment\_of\_cellular\_localization | CENPE | 410 | 12 | 4.368828 | -5.034276 | 18 | 0.0 | 0.000000 |
| GO:0051649\_establishment\_of\_cellular\_localization | NUP133 | 410 | 12 | 4.368828 | -5.034276 | 18 | 0.0 | 0.000000 |
| GO:0051649\_establishment\_of\_cellular\_localization | PAFAH1B1 | 410 | 12 | 4.368828 | -5.034276 | 18 | 0.0 | 0.000000 |
| GO:0051649\_establishment\_of\_cellular\_localization | SEC13 | 410 | 12 | 4.368828 | -5.034276 | 18 | 0.0 | 0.000000 |
| GO:0000070\_mitotic\_sister\_chromatid\_segregation | ZWINT | 21 | 4 | 28.432056 | -5.019623 | 19 | 0.0 | 0.000000 |
| GO:0000070\_mitotic\_sister\_chromatid\_segregation | ZW10 | 21 | 4 | 28.432056 | -5.019623 | 19 | 0.0 | 0.000000 |
| GO:0000070\_mitotic\_sister\_chromatid\_segregation | CENPE | 21 | 4 | 28.432056 | -5.019623 | 19 | 0.0 | 0.000000 |
| GO:0000070\_mitotic\_sister\_chromatid\_segregation | NDC80 | 21 | 4 | 28.432056 | -5.019623 | 19 | 0.0 | 0.000000 |
| GO:0000819\_sister\_chromatid\_segregation | ZWINT | 22 | 4 | 27.139690 | -4.934577 | 20 | 0.0 | 0.000000 |
| GO:0000819\_sister\_chromatid\_segregation | ZW10 | 22 | 4 | 27.139690 | -4.934577 | 20 | 0.0 | 0.000000 |
| GO:0000819\_sister\_chromatid\_segregation | CENPE | 22 | 4 | 27.139690 | -4.934577 | 20 | 0.0 | 0.000000 |
| GO:0000819\_sister\_chromatid\_segregation | NDC80 | 22 | 4 | 27.139690 | -4.934577 | 20 | 0.0 | 0.000000 |
| GO:0051641\_cellular\_localization | MXI1 | 429 | 12 | 4.175337 | -4.833159 | 21 | 0.0 | 0.000000 |
| GO:0051641\_cellular\_localization | ZWINT | 429 | 12 | 4.175337 | -4.833159 | 21 | 0.0 | 0.000000 |
| GO:0051641\_cellular\_localization | TAOK2 | 429 | 12 | 4.175337 | -4.833159 | 21 | 0.0 | 0.000000 |
| GO:0051641\_cellular\_localization | NUP107 | 429 | 12 | 4.175337 | -4.833159 | 21 | 0.0 | 0.000000 |
| GO:0051641\_cellular\_localization | RANBP2 | 429 | 12 | 4.175337 | -4.833159 | 21 | 0.0 | 0.000000 |
| GO:0051641\_cellular\_localization | ZW10 | 429 | 12 | 4.175337 | -4.833159 | 21 | 0.0 | 0.000000 |
| GO:0051641\_cellular\_localization | CENPF | 429 | 12 | 4.175337 | -4.833159 | 21 | 0.0 | 0.000000 |
| GO:0051641\_cellular\_localization | NUP160 | 429 | 12 | 4.175337 | -4.833159 | 21 | 0.0 | 0.000000 |
| GO:0051641\_cellular\_localization | NUP133 | 429 | 12 | 4.175337 | -4.833159 | 21 | 0.0 | 0.000000 |
| GO:0051641\_cellular\_localization | CENPE | 429 | 12 | 4.175337 | -4.833159 | 21 | 0.0 | 0.000000 |
| GO:0051641\_cellular\_localization | PAFAH1B1 | 429 | 12 | 4.175337 | -4.833159 | 21 | 0.0 | 0.000000 |
| GO:0051641\_cellular\_localization | SEC13 | 429 | 12 | 4.175337 | -4.833159 | 21 | 0.0 | 0.000000 |
| GO:0031110\_regulation\_of\_microtubule\_polymerization\_or\_depolymerization | MAPRE1 | 9 | 3 | 49.756098 | -4.642017 | 22 | 0.0 | 0.000000 |
| GO:0031110\_regulation\_of\_microtubule\_polymerization\_or\_depolymerization | CLASP1 | 9 | 3 | 49.756098 | -4.642017 | 22 | 0.0 | 0.000000 |
| GO:0031110\_regulation\_of\_microtubule\_polymerization\_or\_depolymerization | CLASP2 | 9 | 3 | 49.756098 | -4.642017 | 22 | 0.0 | 0.000000 |
| GO:0050657\_nucleic\_acid\_transport | NUP107 | 26 | 4 | 22.964353 | -4.632567 | 25 | 0.0 | 0.000000 |
| GO:0050657\_nucleic\_acid\_transport | NUP160 | 26 | 4 | 22.964353 | -4.632567 | 25 | 0.0 | 0.000000 |
| GO:0050657\_nucleic\_acid\_transport | NUP133 | 26 | 4 | 22.964353 | -4.632567 | 25 | 0.0 | 0.000000 |
| GO:0050657\_nucleic\_acid\_transport | CKAP5 | 26 | 4 | 22.964353 | -4.632567 | 25 | 0.0 | 0.000000 |
| GO:0050658\_RNA\_transport | NUP107 | 26 | 4 | 22.964353 | -4.632567 | 25 | 0.0 | 0.000000 |
| GO:0050658\_RNA\_transport | NUP160 | 26 | 4 | 22.964353 | -4.632567 | 25 | 0.0 | 0.000000 |
| GO:0050658\_RNA\_transport | NUP133 | 26 | 4 | 22.964353 | -4.632567 | 25 | 0.0 | 0.000000 |
| GO:0050658\_RNA\_transport | CKAP5 | 26 | 4 | 22.964353 | -4.632567 | 25 | 0.0 | 0.000000 |
| GO:0051236\_establishment\_of\_RNA\_localization | NUP107 | 26 | 4 | 22.964353 | -4.632567 | 25 | 0.0 | 0.000000 |
| GO:0051236\_establishment\_of\_RNA\_localization | NUP160 | 26 | 4 | 22.964353 | -4.632567 | 25 | 0.0 | 0.000000 |
| GO:0051236\_establishment\_of\_RNA\_localization | NUP133 | 26 | 4 | 22.964353 | -4.632567 | 25 | 0.0 | 0.000000 |
| GO:0051236\_establishment\_of\_RNA\_localization | CKAP5 | 26 | 4 | 22.964353 | -4.632567 | 25 | 0.0 | 0.000000 |
| GO:0006403\_RNA\_localization | NUP107 | 27 | 4 | 22.113821 | -4.565035 | 26 | 0.0 | 0.000000 |
| GO:0006403\_RNA\_localization | NUP160 | 27 | 4 | 22.113821 | -4.565035 | 26 | 0.0 | 0.000000 |
| GO:0006403\_RNA\_localization | NUP133 | 27 | 4 | 22.113821 | -4.565035 | 26 | 0.0 | 0.000000 |
| GO:0006403\_RNA\_localization | CKAP5 | 27 | 4 | 22.113821 | -4.565035 | 26 | 0.0 | 0.000000 |
| GO:0008283\_cell\_proliferation | BUB1B | 475 | 12 | 3.770988 | -4.388387 | 27 | 0.0 | 0.000000 |
| GO:0008283\_cell\_proliferation | MXI1 | 475 | 12 | 3.770988 | -4.388387 | 27 | 0.0 | 0.000000 |
| GO:0008283\_cell\_proliferation | MAPRE1 | 475 | 12 | 3.770988 | -4.388387 | 27 | 0.0 | 0.000000 |
| GO:0008283\_cell\_proliferation | CENPF | 475 | 12 | 3.770988 | -4.388387 | 27 | 0.0 | 0.000000 |
| GO:0008283\_cell\_proliferation | BUB3 | 475 | 12 | 3.770988 | -4.388387 | 27 | 0.0 | 0.000000 |
| GO:0008283\_cell\_proliferation | MXD1 | 475 | 12 | 3.770988 | -4.388387 | 27 | 0.0 | 0.000000 |
| GO:0008283\_cell\_proliferation | NUDC | 475 | 12 | 3.770988 | -4.388387 | 27 | 0.0 | 0.000000 |
| GO:0008283\_cell\_proliferation | BUB1 | 475 | 12 | 3.770988 | -4.388387 | 27 | 0.0 | 0.000000 |
| GO:0008283\_cell\_proliferation | MAPRE2 | 475 | 12 | 3.770988 | -4.388387 | 27 | 0.0 | 0.000000 |
| GO:0008283\_cell\_proliferation | KIF2C | 475 | 12 | 3.770988 | -4.388387 | 27 | 0.0 | 0.000000 |
| GO:0008283\_cell\_proliferation | RPS27 | 475 | 12 | 3.770988 | -4.388387 | 27 | 0.0 | 0.000000 |
| GO:0008283\_cell\_proliferation | PLK1 | 475 | 12 | 3.770988 | -4.388387 | 27 | 0.0 | 0.000000 |
| GO:0030951\_establishment\_and\_or\_maintenance\_of\_microtubule\_cytoskeleton\_polarity | CKAP5 | 2 | 2 |  |  |  |  |  |  |
| GO:0030951\_establishment\_and\_or\_maintenance\_of\_microtubule\_cytoskeleton\_polarity | KIF2C | 2 | 2 |  |  |  |  |  |  |
| GO:0030952\_establishment\_and\_or\_maintenance\_of\_cytoskeleton\_polarity | CKAP5 | 2 | 2 |  |  |  |  |  |  |
| GO:0030952\_establishment\_and\_or\_maintenance\_of\_cytoskeleton\_polarity | KIF2C | 2 | 2 |  |  |  |  |  |  |
| GO:0031109\_microtubule\_polymerization\_or\_depolymerization | MAPRE1 | 11 | 3 | 40.709534 | -4.352864 | 29 | 0.0 | 0.000000 |
| GO:0031109\_microtubule\_polymerization\_or\_depolymerization | CLASP1 | 11 | 3 | 40.709534 | -4.352864 | 29 | 0.0 | 0.000000 |
| GO:0031109\_microtubule\_polymerization\_or\_depolymerization | CLASP2 | 11 | 3 | 40.709534 | -4.352864 | 29 | 0.0 | 0.000000 |
| GO:0032886\_regulation\_of\_microtubule-based\_process | MAPRE1 | 11 | 3 | 40.709534 | -4.352864 | 29 | 0.0 | 0.000000 |
| GO:0032886\_regulation\_of\_microtubule-based\_process | CLASP1 | 11 | 3 | 40.709534 | -4.352864 | 29 | 0.0 | 0.000000 |
| GO:0032886\_regulation\_of\_microtubule-based\_process | CLASP2 | 11 | 3 | 40.709534 | -4.352864 | 29 | 0.0 | 0.000000 |
| GO:0051726\_regulation\_of\_cell\_cycle | BUB1B | 156 | 7 | 6.697936 | -4.178528 | 30 | 0.03 | 0.001000 |
| GO:0051726\_regulation\_of\_cell\_cycle | ZWINT | 156 | 7 | 6.697936 | -4.178528 | 30 | 0.03 | 0.001000 |
| GO:0051726\_regulation\_of\_cell\_cycle | ZWILCH | 156 | 7 | 6.697936 | -4.178528 | 30 | 0.03 | 0.001000 |
| GO:0051726\_regulation\_of\_cell\_cycle | ZW10 | 156 | 7 | 6.697936 | -4.178528 | 30 | 0.03 | 0.001000 |
| GO:0051726\_regulation\_of\_cell\_cycle | CENPF | 156 | 7 | 6.697936 | -4.178528 | 30 | 0.03 | 0.001000 |
| GO:0051726\_regulation\_of\_cell\_cycle | MAD2L1 | 156 | 7 | 6.697936 | -4.178528 | 30 | 0.03 | 0.001000 |
| GO:0051726\_regulation\_of\_cell\_cycle | BUB1 | 156 | 7 | 6.697936 | -4.178528 | 30 | 0.03 | 0.001000 |
| GO:0015931\_nucleobase\_\_nucleoside\_\_nucleotide\_and\_nucleic\_acid\_transport | NUP107 | 34 | 4 | 17.560976 | -4.157740 | 31 | 0.03 | 0.000968 |
| GO:0015931\_nucleobase\_\_nucleoside\_\_nucleotide\_and\_nucleic\_acid\_transport | NUP160 | 34 | 4 | 17.560976 | -4.157740 | 31 | 0.03 | 0.000968 |
| GO:0015931\_nucleobase\_\_nucleoside\_\_nucleotide\_and\_nucleic\_acid\_transport | NUP133 | 34 | 4 | 17.560976 | -4.157740 | 31 | 0.03 | 0.000968 |
| GO:0015931\_nucleobase\_\_nucleoside\_\_nucleotide\_and\_nucleic\_acid\_transport | CKAP5 | 34 | 4 | 17.560976 | -4.157740 | 31 | 0.03 | 0.000968 |
| GO:0051494\_negative\_regulation\_of\_cytoskeleton\_organization\_and\_biogenesis | MAPRE1 | 13 | 3 | 34.446529 | -4.118032 | 32 | 0.04 | 0.001250 |
| GO:0051494\_negative\_regulation\_of\_cytoskeleton\_organization\_and\_biogenesis | CLASP1 | 13 | 3 | 34.446529 | -4.118032 | 32 | 0.04 | 0.001250 |
| GO:0051494\_negative\_regulation\_of\_cytoskeleton\_organization\_and\_biogenesis | CLASP2 | 13 | 3 | 34.446529 | -4.118032 | 32 | 0.04 | 0.001250 |
| GO:0032269\_negative\_regulation\_of\_cellular\_protein\_metabolic\_process | MAPRE1 | 37 | 4 | 16.137113 | -4.010501 | 33 | 0.05 | 0.001515 |
| GO:0032269\_negative\_regulation\_of\_cellular\_protein\_metabolic\_process | PPP2R4 | 37 | 4 | 16.137113 | -4.010501 | 33 | 0.05 | 0.001515 |
| GO:0032269\_negative\_regulation\_of\_cellular\_protein\_metabolic\_process | CLASP1 | 37 | 4 | 16.137113 | -4.010501 | 33 | 0.05 | 0.001515 |
| GO:0032269\_negative\_regulation\_of\_cellular\_protein\_metabolic\_process | CLASP2 | 37 | 4 | 16.137113 | -4.010501 | 33 | 0.05 | 0.001515 |
| GO:0051248\_negative\_regulation\_of\_protein\_metabolic\_process | MAPRE1 | 40 | 4 | 14.926829 | -3.875747 | 34 | 0.05 | 0.001471 |
| GO:0051248\_negative\_regulation\_of\_protein\_metabolic\_process | PPP2R4 | 40 | 4 | 14.926829 | -3.875747 | 34 | 0.05 | 0.001471 |
| GO:0051248\_negative\_regulation\_of\_protein\_metabolic\_process | CLASP1 | 40 | 4 | 14.926829 | -3.875747 | 34 | 0.05 | 0.001471 |
| GO:0051248\_negative\_regulation\_of\_protein\_metabolic\_process | CLASP2 | 40 | 4 | 14.926829 | -3.875747 | 34 | 0.05 | 0.001471 |
| GO:0051276\_chromosome\_organization\_and\_biogenesis | MIS12 | 175 | 7 | 5.970732 | -3.863256 | 35 | 0.05 | 0.001429 |
| GO:0051276\_chromosome\_organization\_and\_biogenesis | ZWINT | 175 | 7 | 5.970732 | -3.863256 | 35 | 0.05 | 0.001429 |
| GO:0051276\_chromosome\_organization\_and\_biogenesis | CENPF | 175 | 7 | 5.970732 | -3.863256 | 35 | 0.05 | 0.001429 |
| GO:0051276\_chromosome\_organization\_and\_biogenesis | ZW10 | 175 | 7 | 5.970732 | -3.863256 | 35 | 0.05 | 0.001429 |
| GO:0051276\_chromosome\_organization\_and\_biogenesis | CENPE | 175 | 7 | 5.970732 | -3.863256 | 35 | 0.05 | 0.001429 |
| GO:0051276\_chromosome\_organization\_and\_biogenesis | CENPH | 175 | 7 | 5.970732 | -3.863256 | 35 | 0.05 | 0.001429 |
| GO:0051276\_chromosome\_organization\_and\_biogenesis | NDC80 | 175 | 7 | 5.970732 | -3.863256 | 35 | 0.05 | 0.001429 |
| GO:0006406\_mRNA\_export\_from\_nucleus | NUP107 | 19 | 3 | 23.568678 | -3.600215 | 38 | 0.08 | 0.002105 |
| GO:0006406\_mRNA\_export\_from\_nucleus | NUP160 | 19 | 3 | 23.568678 | -3.600215 | 38 | 0.08 | 0.002105 |
| GO:0006406\_mRNA\_export\_from\_nucleus | NUP133 | 19 | 3 | 23.568678 | -3.600215 | 38 | 0.08 | 0.002105 |
| GO:0007051\_spindle\_organization\_and\_biogenesis | BUB1B | 19 | 3 | 23.568678 | -3.600215 | 38 | 0.08 | 0.002105 |
| GO:0007051\_spindle\_organization\_and\_biogenesis | ZWINT | 19 | 3 | 23.568678 | -3.600215 | 38 | 0.08 | 0.002105 |
| GO:0007051\_spindle\_organization\_and\_biogenesis | NDC80 | 19 | 3 | 23.568678 | -3.600215 | 38 | 0.08 | 0.002105 |
| GO:0051028\_mRNA\_transport | NUP107 | 19 | 3 | 23.568678 | -3.600215 | 38 | 0.08 | 0.002105 |
| GO:0051028\_mRNA\_transport | NUP160 | 19 | 3 | 23.568678 | -3.600215 | 38 | 0.08 | 0.002105 |
| GO:0051028\_mRNA\_transport | NUP133 | 19 | 3 | 23.568678 | -3.600215 | 38 | 0.08 | 0.002105 |
| GO:0051310\_metaphase\_plate\_congression | CENPF | 4 | 2 |  |  |  |  |  |  |
| GO:0051310\_metaphase\_plate\_congression | CENPE | 4 | 2 |  |  |  |  |  |  |
| GO:0051656\_establishment\_of\_organelle\_localization | CENPF | 20 | 3 | 22.390244 | -3.531656 | 39 | 0.1 | 0.002564 |
| GO:0051656\_establishment\_of\_organelle\_localization | CENPE | 20 | 3 | 22.390244 | -3.531656 | 39 | 0.1 | 0.002564 |
| GO:0051656\_establishment\_of\_organelle\_localization | PAFAH1B1 | 20 | 3 | 22.390244 | -3.531656 | 39 | 0.1 | 0.002564 |
| GO:0033036\_macromolecule\_localization | MXI1 | 267 | 8 | 4.472458 | -3.492545 | 40 | 0.12 | 0.003000 |
| GO:0033036\_macromolecule\_localization | TAOK2 | 267 | 8 | 4.472458 | -3.492545 | 40 | 0.12 | 0.003000 |
| GO:0033036\_macromolecule\_localization | RANBP2 | 267 | 8 | 4.472458 | -3.492545 | 40 | 0.12 | 0.003000 |
| GO:0033036\_macromolecule\_localization | NUP107 | 267 | 8 | 4.472458 | -3.492545 | 40 | 0.12 | 0.003000 |
| GO:0033036\_macromolecule\_localization | NUP160 | 267 | 8 | 4.472458 | -3.492545 | 40 | 0.12 | 0.003000 |
| GO:0033036\_macromolecule\_localization | NUP133 | 267 | 8 | 4.472458 | -3.492545 | 40 | 0.12 | 0.003000 |
| GO:0033036\_macromolecule\_localization | CKAP5 | 267 | 8 | 4.472458 | -3.492545 | 40 | 0.12 | 0.003000 |
| GO:0033036\_macromolecule\_localization | SEC13 | 267 | 8 | 4.472458 | -3.492545 | 40 | 0.12 | 0.003000 |
| GO:0006913\_nucleocytoplasmic\_transport | MXI1 | 92 | 5 | 8.112407 | -3.473158 | 41 | 0.12 | 0.002927 |
| GO:0006913\_nucleocytoplasmic\_transport | RANBP2 | 92 | 5 | 8.112407 | -3.473158 | 41 | 0.12 | 0.002927 |
| GO:0006913\_nucleocytoplasmic\_transport | NUP107 | 92 | 5 | 8.112407 | -3.473158 | 41 | 0.12 | 0.002927 |
| GO:0006913\_nucleocytoplasmic\_transport | NUP160 | 92 | 5 | 8.112407 | -3.473158 | 41 | 0.12 | 0.002927 |
| GO:0006913\_nucleocytoplasmic\_transport | NUP133 | 92 | 5 | 8.112407 | -3.473158 | 41 | 0.12 | 0.002927 |
| GO:0051169\_nuclear\_transport | MXI1 | 93 | 5 | 8.025177 | -3.451291 | 42 | 0.12 | 0.002857 |
| GO:0051169\_nuclear\_transport | RANBP2 | 93 | 5 | 8.025177 | -3.451291 | 42 | 0.12 | 0.002857 |
| GO:0051169\_nuclear\_transport | NUP107 | 93 | 5 | 8.025177 | -3.451291 | 42 | 0.12 | 0.002857 |
| GO:0051169\_nuclear\_transport | NUP160 | 93 | 5 | 8.025177 | -3.451291 | 42 | 0.12 | 0.002857 |
| GO:0051169\_nuclear\_transport | NUP133 | 93 | 5 | 8.025177 | -3.451291 | 42 | 0.12 | 0.002857 |
| GO:0007026\_negative\_regulation\_of\_microtubule\_depolymerization | CLASP1 | 5 | 2 | 59.707317 | -3.364134 | 44 | 0.23 | 0.005227 |
| GO:0007026\_negative\_regulation\_of\_microtubule\_depolymerization | CLASP2 | 5 | 2 | 59.707317 | -3.364134 | 44 | 0.23 | 0.005227 |
| GO:0031114\_regulation\_of\_microtubule\_depolymerization | CLASP1 | 5 | 2 | 59.707317 | -3.364134 | 44 | 0.23 | 0.005227 |
| GO:0031114\_regulation\_of\_microtubule\_depolymerization | CLASP2 | 5 | 2 | 59.707317 | -3.364134 | 44 | 0.23 | 0.005227 |
| GO:0006405\_RNA\_export\_from\_nucleus | NUP107 | 24 | 3 | 18.658537 | -3.290435 | 45 | 0.28 | 0.006222 |
| GO:0006405\_RNA\_export\_from\_nucleus | NUP160 | 24 | 3 | 18.658537 | -3.290435 | 45 | 0.28 | 0.006222 |
| GO:0006405\_RNA\_export\_from\_nucleus | NUP133 | 24 | 3 | 18.658537 | -3.290435 | 45 | 0.28 | 0.006222 |
| GO:0007019\_microtubule\_depolymerization | CLASP1 | 6 | 2 | 49.756098 | -3.189891 | 49 | 0.39 | 0.007959 |
| GO:0007019\_microtubule\_depolymerization | CLASP2 | 6 | 2 | 49.756098 | -3.189891 | 49 | 0.39 | 0.007959 |
| GO:0007094\_mitotic\_cell\_cycle\_spindle\_assembly\_checkpoint | CENPF | 6 | 2 | 49.756098 | -3.189891 | 49 | 0.39 | 0.007959 |
| GO:0007094\_mitotic\_cell\_cycle\_spindle\_assembly\_checkpoint | BUB1 | 6 | 2 | 49.756098 | -3.189891 | 49 | 0.39 | 0.007959 |
| GO:0050000\_chromosome\_localization | CENPF | 6 | 2 | 49.756098 | -3.189891 | 49 | 0.39 | 0.007959 |
| GO:0050000\_chromosome\_localization | CENPE | 6 | 2 | 49.756098 | -3.189891 | 49 | 0.39 | 0.007959 |
| GO:0051303\_establishment\_of\_chromosome\_localization | CENPF | 6 | 2 | 49.756098 | -3.189891 | 49 | 0.39 | 0.007959 |
| GO:0051303\_establishment\_of\_chromosome\_localization | CENPE | 6 | 2 | 49.756098 | -3.189891 | 49 | 0.39 | 0.007959 |
| GO:0051640\_organelle\_localization | CENPF | 27 | 3 | 16.585366 | -3.136579 | 50 | 0.43 | 0.008600 |
| GO:0051640\_organelle\_localization | CENPE | 27 | 3 | 16.585366 | -3.136579 | 50 | 0.43 | 0.008600 |
| GO:0051640\_organelle\_localization | PAFAH1B1 | 27 | 3 | 16.585366 | -3.136579 | 50 | 0.43 | 0.008600 |
| GO:0031577\_spindle\_checkpoint | CENPF | 7 | 2 | 42.648084 | -3.045609 | 51 | 0.54 | 0.010588 |
| GO:0031577\_spindle\_checkpoint | BUB1 | 7 | 2 | 42.648084 | -3.045609 | 51 | 0.54 | 0.010588 |
| GO:0051129\_negative\_regulation\_of\_cellular\_component\_organization\_and\_biogenesis | MAPRE1 | 33 | 3 | 13.569845 | -2.877939 | 52 | 0.72 | 0.013846 |
| GO:0051129\_negative\_regulation\_of\_cellular\_component\_organization\_and\_biogenesis | CLASP1 | 33 | 3 | 13.569845 | -2.877939 | 52 | 0.72 | 0.013846 |
| GO:0051129\_negative\_regulation\_of\_cellular\_component\_organization\_and\_biogenesis | CLASP2 | 33 | 3 | 13.569845 | -2.877939 | 52 | 0.72 | 0.013846 |
| GO:0046907\_intracellular\_transport | MXI1 | 342 | 8 | 3.491656 | -2.782559 | 53 | 0.84 | 0.015849 |
| GO:0046907\_intracellular\_transport | TAOK2 | 342 | 8 | 3.491656 | -2.782559 | 53 | 0.84 | 0.015849 |
| GO:0046907\_intracellular\_transport | NUP107 | 342 | 8 | 3.491656 | -2.782559 | 53 | 0.84 | 0.015849 |
| GO:0046907\_intracellular\_transport | ZW10 | 342 | 8 | 3.491656 | -2.782559 | 53 | 0.84 | 0.015849 |
| GO:0046907\_intracellular\_transport | RANBP2 | 342 | 8 | 3.491656 | -2.782559 | 53 | 0.84 | 0.015849 |
| GO:0046907\_intracellular\_transport | NUP160 | 342 | 8 | 3.491656 | -2.782559 | 53 | 0.84 | 0.015849 |
| GO:0046907\_intracellular\_transport | NUP133 | 342 | 8 | 3.491656 | -2.782559 | 53 | 0.84 | 0.015849 |
| GO:0046907\_intracellular\_transport | SEC13 | 342 | 8 | 3.491656 | -2.782559 | 53 | 0.84 | 0.015849 |
| GO:0051168\_nuclear\_export | NUP107 | 36 | 3 | 12.439024 | -2.767164 | 54 | 0.86 | 0.015926 |
| GO:0051168\_nuclear\_export | NUP160 | 36 | 3 | 12.439024 | -2.767164 | 54 | 0.86 | 0.015926 |
| GO:0051168\_nuclear\_export | NUP133 | 36 | 3 | 12.439024 | -2.767164 | 54 | 0.86 | 0.015926 |
| GO:0051493\_regulation\_of\_cytoskeleton\_organization\_and\_biogenesis | MAPRE1 | 37 | 3 | 12.102835 | -2.732457 | 55 | 0.87 | 0.015818 |
| GO:0051493\_regulation\_of\_cytoskeleton\_organization\_and\_biogenesis | CLASP1 | 37 | 3 | 12.102835 | -2.732457 | 55 | 0.87 | 0.015818 |
| GO:0051493\_regulation\_of\_cytoskeleton\_organization\_and\_biogenesis | CLASP2 | 37 | 3 | 12.102835 | -2.732457 | 55 | 0.87 | 0.015818 |
| GO:0010458\_exit\_from\_mitosis | ZW10 | 10 | 2 | 29.853659 | -2.720151 | 57 | 1.08 | 0.018947 |
| GO:0010458\_exit\_from\_mitosis | CLASP1 | 10 | 2 | 29.853659 | -2.720151 | 57 | 1.08 | 0.018947 |
| GO:0051261\_protein\_depolymerization | CLASP1 | 10 | 2 | 29.853659 | -2.720151 | 57 | 1.08 | 0.018947 |
| GO:0051261\_protein\_depolymerization | CLASP2 | 10 | 2 | 29.853659 | -2.720151 | 57 | 1.08 | 0.018947 |
| GO:0043623\_cellular\_protein\_complex\_assembly | MIS12 | 40 | 3 | 11.195122 | -2.634165 | 58 | 1.29 | 0.022241 |
| GO:0043623\_cellular\_protein\_complex\_assembly | CENPF | 40 | 3 | 11.195122 | -2.634165 | 58 | 1.29 | 0.022241 |
| GO:0043623\_cellular\_protein\_complex\_assembly | CENPE | 40 | 3 | 11.195122 | -2.634165 | 58 | 1.29 | 0.022241 |
| GO:0051297\_centrosome\_organization\_and\_biogenesis | CKAP5 | 13 | 2 | 22.964353 | -2.486797 | 59 | 1.73 | 0.029322 |
| GO:0051297\_centrosome\_organization\_and\_biogenesis | NDE1 | 13 | 2 | 22.964353 | -2.486797 | 59 | 1.73 | 0.029322 |
| GO:0031023\_microtubule\_organizing\_center\_organization\_and\_biogenesis | CKAP5 | 14 | 2 | 21.324042 | -2.421691 | 60 | 1.92 | 0.032000 |
| GO:0031023\_microtubule\_organizing\_center\_organization\_and\_biogenesis | NDE1 | 14 | 2 | 21.324042 | -2.421691 | 60 | 1.92 | 0.032000 |
| GO:0033043\_regulation\_of\_organelle\_organization\_and\_biogenesis | MAPRE1 | 49 | 3 | 9.138875 | -2.381648 | 61 | 1.99 | 0.032623 |
| GO:0033043\_regulation\_of\_organelle\_organization\_and\_biogenesis | CLASP1 | 49 | 3 | 9.138875 | -2.381648 | 61 | 1.99 | 0.032623 |
| GO:0033043\_regulation\_of\_organelle\_organization\_and\_biogenesis | CLASP2 | 49 | 3 | 9.138875 | -2.381648 | 61 | 1.99 | 0.032623 |
| GO:0051234\_establishment\_of\_localization | MXI1 | 902 | 13 | 2.151317 | -2.341718 | 62 | 2.26 | 0.036452 |
| GO:0051234\_establishment\_of\_localization | TAOK2 | 902 | 13 | 2.151317 | -2.341718 | 62 | 2.26 | 0.036452 |
| GO:0051234\_establishment\_of\_localization | ZW10 | 902 | 13 | 2.151317 | -2.341718 | 62 | 2.26 | 0.036452 |
| GO:0051234\_establishment\_of\_localization | CENPF | 902 | 13 | 2.151317 | -2.341718 | 62 | 2.26 | 0.036452 |
| GO:0051234\_establishment\_of\_localization | NUP160 | 902 | 13 | 2.151317 | -2.341718 | 62 | 2.26 | 0.036452 |
| GO:0051234\_establishment\_of\_localization | CENPE | 902 | 13 | 2.151317 | -2.341718 | 62 | 2.26 | 0.036452 |
| GO:0051234\_establishment\_of\_localization | CKAP5 | 902 | 13 | 2.151317 | -2.341718 | 62 | 2.26 | 0.036452 |
| GO:0051234\_establishment\_of\_localization | SEC13 | 902 | 13 | 2.151317 | -2.341718 | 62 | 2.26 | 0.036452 |
| GO:0051234\_establishment\_of\_localization | ZWINT | 902 | 13 | 2.151317 | -2.341718 | 62 | 2.26 | 0.036452 |
| GO:0051234\_establishment\_of\_localization | RANBP2 | 902 | 13 | 2.151317 | -2.341718 | 62 | 2.26 | 0.036452 |
| GO:0051234\_establishment\_of\_localization | NUP107 | 902 | 13 | 2.151317 | -2.341718 | 62 | 2.26 | 0.036452 |
| GO:0051234\_establishment\_of\_localization | NUP133 | 902 | 13 | 2.151317 | -2.341718 | 62 | 2.26 | 0.036452 |
| GO:0051234\_establishment\_of\_localization | PAFAH1B1 | 902 | 13 | 2.151317 | -2.341718 | 62 | 2.26 | 0.036452 |
| GO:0048015\_phosphoinositide-mediated\_signaling | BUB1B | 52 | 3 | 8.611632 | -2.308668 | 63 | 2.37 | 0.037619 |
| GO:0048015\_phosphoinositide-mediated\_signaling | ZWINT | 52 | 3 | 8.611632 | -2.308668 | 63 | 2.37 | 0.037619 |
| GO:0048015\_phosphoinositide-mediated\_signaling | NDC80 | 52 | 3 | 8.611632 | -2.308668 | 63 | 2.37 | 0.037619 |
| GO:0000089\_mitotic\_metaphase | CENPE | 1 | 1 |  |  |  |  |  |  |
| GO:0000132\_establishment\_of\_mitotic\_spindle\_orientation | PAFAH1B1 | 1 | 1 |  |  |  |  |  |  |
| GO:0005984\_disaccharide\_metabolic\_process | IDUA | 1 | 1 |  |  |  |  |  |  |
| GO:0031115\_negative\_regulation\_of\_microtubule\_polymerization | MAPRE1 | 1 | 1 |  |  |  |  |  |  |
| GO:0032515\_negative\_regulation\_of\_phosphoprotein\_phosphatase\_activity | PPP2R4 | 1 | 1 |  |  |  |  |  |  |
| GO:0032516\_positive\_regulation\_of\_phosphoprotein\_phosphatase\_activity | PPP2R4 | 1 | 1 |  |  |  |  |  |  |
| GO:0035305\_negative\_regulation\_of\_dephosphorylation | PPP2R4 | 1 | 1 |  |  |  |  |  |  |
| GO:0035308\_negative\_regulation\_of\_protein\_amino\_acid\_dephosphorylation | PPP2R4 | 1 | 1 |  |  |  |  |  |  |
| GO:0051294\_establishment\_of\_spindle\_orientation | PAFAH1B1 | 1 | 1 |  |  |  |  |  |  |
| GO:0051323\_metaphase | CENPE | 1 | 1 |  |  |  |  |  |  |
| GO:0007163\_establishment\_and\_or\_maintenance\_of\_cell\_polarity | CLASP1 | 21 | 2 | 14.216028 | -2.071379 | 64 | 3.99 | 0.062344 |
| GO:0007163\_establishment\_and\_or\_maintenance\_of\_cell\_polarity | CLASP2 | 21 | 2 | 14.216028 | -2.071379 | 64 | 3.99 | 0.062344 |
| GO:0009987\_cellular\_process | MIS12 | 5174 | 40 | 1.153988 | -2.068409 | 65 | 4.0 | 0.061538 |
| GO:0009987\_cellular\_process | TAOK2 | 5174 | 40 | 1.153988 | -2.068409 | 65 | 4.0 | 0.061538 |
| GO:0009987\_cellular\_process | ZW10 | 5174 | 40 | 1.153988 | -2.068409 | 65 | 4.0 | 0.061538 |
| GO:0009987\_cellular\_process | ITGB3BP | 5174 | 40 | 1.153988 | -2.068409 | 65 | 4.0 | 0.061538 |
| GO:0009987\_cellular\_process | CENPE | 5174 | 40 | 1.153988 | -2.068409 | 65 | 4.0 | 0.061538 |
| GO:0009987\_cellular\_process | CLIP1 | 5174 | 40 | 1.153988 | -2.068409 | 65 | 4.0 | 0.061538 |
| GO:0009987\_cellular\_process | MAD2L1 | 5174 | 40 | 1.153988 | -2.068409 | 65 | 4.0 | 0.061538 |
| GO:0009987\_cellular\_process | CKAP5 | 5174 | 40 | 1.153988 | -2.068409 | 65 | 4.0 | 0.061538 |
| GO:0009987\_cellular\_process | NDC80 | 5174 | 40 | 1.153988 | -2.068409 | 65 | 4.0 | 0.061538 |
| GO:0009987\_cellular\_process | NUP85 | 5174 | 40 | 1.153988 | -2.068409 | 65 | 4.0 | 0.061538 |
| GO:0009987\_cellular\_process | INCENP | 5174 | 40 | 1.153988 | -2.068409 | 65 | 4.0 | 0.061538 |
| GO:0009987\_cellular\_process | MAPRE1 | 5174 | 40 | 1.153988 | -2.068409 | 65 | 4.0 | 0.061538 |
| GO:0009987\_cellular\_process | ZWILCH | 5174 | 40 | 1.153988 | -2.068409 | 65 | 4.0 | 0.061538 |
| GO:0009987\_cellular\_process | RANBP2 | 5174 | 40 | 1.153988 | -2.068409 | 65 | 4.0 | 0.061538 |
| GO:0009987\_cellular\_process | MXD1 | 5174 | 40 | 1.153988 | -2.068409 | 65 | 4.0 | 0.061538 |
| GO:0009987\_cellular\_process | CENPH | 5174 | 40 | 1.153988 | -2.068409 | 65 | 4.0 | 0.061538 |
| GO:0009987\_cellular\_process | PAFAH1B1 | 5174 | 40 | 1.153988 | -2.068409 | 65 | 4.0 | 0.061538 |
| GO:0009987\_cellular\_process | IDUA | 5174 | 40 | 1.153988 | -2.068409 | 65 | 4.0 | 0.061538 |
| GO:0009987\_cellular\_process | CLASP1 | 5174 | 40 | 1.153988 | -2.068409 | 65 | 4.0 | 0.061538 |
| GO:0009987\_cellular\_process | KIF2C | 5174 | 40 | 1.153988 | -2.068409 | 65 | 4.0 | 0.061538 |
| GO:0009987\_cellular\_process | MXI1 | 5174 | 40 | 1.153988 | -2.068409 | 65 | 4.0 | 0.061538 |
| GO:0009987\_cellular\_process | CDC20 | 5174 | 40 | 1.153988 | -2.068409 | 65 | 4.0 | 0.061538 |
| GO:0009987\_cellular\_process | NUF2 | 5174 | 40 | 1.153988 | -2.068409 | 65 | 4.0 | 0.061538 |
| GO:0009987\_cellular\_process | BUB3 | 5174 | 40 | 1.153988 | -2.068409 | 65 | 4.0 | 0.061538 |
| GO:0009987\_cellular\_process | CENPF | 5174 | 40 | 1.153988 | -2.068409 | 65 | 4.0 | 0.061538 |
| GO:0009987\_cellular\_process | NUP160 | 5174 | 40 | 1.153988 | -2.068409 | 65 | 4.0 | 0.061538 |
| GO:0009987\_cellular\_process | NUDC | 5174 | 40 | 1.153988 | -2.068409 | 65 | 4.0 | 0.061538 |
| GO:0009987\_cellular\_process | BUB1 | 5174 | 40 | 1.153988 | -2.068409 | 65 | 4.0 | 0.061538 |
| GO:0009987\_cellular\_process | MAPRE2 | 5174 | 40 | 1.153988 | -2.068409 | 65 | 4.0 | 0.061538 |
| GO:0009987\_cellular\_process | RANGAP1 | 5174 | 40 | 1.153988 | -2.068409 | 65 | 4.0 | 0.061538 |
| GO:0009987\_cellular\_process | SEC13 | 5174 | 40 | 1.153988 | -2.068409 | 65 | 4.0 | 0.061538 |
| GO:0009987\_cellular\_process | RPS27 | 5174 | 40 | 1.153988 | -2.068409 | 65 | 4.0 | 0.061538 |
| GO:0009987\_cellular\_process | NDE1 | 5174 | 40 | 1.153988 | -2.068409 | 65 | 4.0 | 0.061538 |
| GO:0009987\_cellular\_process | BUB1B | 5174 | 40 | 1.153988 | -2.068409 | 65 | 4.0 | 0.061538 |
| GO:0009987\_cellular\_process | ZWINT | 5174 | 40 | 1.153988 | -2.068409 | 65 | 4.0 | 0.061538 |
| GO:0009987\_cellular\_process | NUP107 | 5174 | 40 | 1.153988 | -2.068409 | 65 | 4.0 | 0.061538 |
| GO:0009987\_cellular\_process | NUP133 | 5174 | 40 | 1.153988 | -2.068409 | 65 | 4.0 | 0.061538 |
| GO:0009987\_cellular\_process | PPP2R4 | 5174 | 40 | 1.153988 | -2.068409 | 65 | 4.0 | 0.061538 |
| GO:0009987\_cellular\_process | CLASP2 | 5174 | 40 | 1.153988 | -2.068409 | 65 | 4.0 | 0.061538 |
| GO:0009987\_cellular\_process | PLK1 | 5174 | 40 | 1.153988 | -2.068409 | 65 | 4.0 | 0.061538 |
| GO:0051128\_regulation\_of\_cellular\_component\_organization\_and\_biogenesis | MAPRE1 | 124 | 4 | 4.815106 | -2.040267 | 66 | 4.06 | 0.061515 |
| GO:0051128\_regulation\_of\_cellular\_component\_organization\_and\_biogenesis | TAOK2 | 124 | 4 | 4.815106 | -2.040267 | 66 | 4.06 | 0.061515 |
| GO:0051128\_regulation\_of\_cellular\_component\_organization\_and\_biogenesis | CLASP1 | 124 | 4 | 4.815106 | -2.040267 | 66 | 4.06 | 0.061515 |
| GO:0051128\_regulation\_of\_cellular\_component\_organization\_and\_biogenesis | CLASP2 | 124 | 4 | 4.815106 | -2.040267 | 66 | 4.06 | 0.061515 |
| GO:0032268\_regulation\_of\_cellular\_protein\_metabolic\_process | MAPRE1 | 134 | 4 | 4.455770 | -1.924778 | 67 | 5.11 | 0.076269 |
| GO:0032268\_regulation\_of\_cellular\_protein\_metabolic\_process | PPP2R4 | 134 | 4 | 4.455770 | -1.924778 | 67 | 5.11 | 0.076269 |
| GO:0032268\_regulation\_of\_cellular\_protein\_metabolic\_process | CLASP1 | 134 | 4 | 4.455770 | -1.924778 | 67 | 5.11 | 0.076269 |
| GO:0032268\_regulation\_of\_cellular\_protein\_metabolic\_process | CLASP2 | 134 | 4 | 4.455770 | -1.924778 | 67 | 5.11 | 0.076269 |
| GO:0007079\_mitotic\_chromosome\_movement\_towards\_spindle\_pole | CENPE | 2 | 1 |  |  |  |  |  |  |
| GO:0032272\_negative\_regulation\_of\_protein\_polymerization | MAPRE1 | 2 | 1 |  |  |  |  |  |  |
| GO:0035304\_regulation\_of\_protein\_amino\_acid\_dephosphorylation | PPP2R4 | 2 | 1 |  |  |  |  |  |  |
| GO:0035306\_positive\_regulation\_of\_dephosphorylation | PPP2R4 | 2 | 1 |  |  |  |  |  |  |
| GO:0035307\_positive\_regulation\_of\_protein\_amino\_acid\_dephosphorylation | PPP2R4 | 2 | 1 |  |  |  |  |  |  |
| GO:0051300\_spindle\_pole\_body\_organization\_and\_biogenesis | CKAP5 | 2 | 1 |  |  |  |  |  |  |
| GO:0051305\_chromosome\_movement\_towards\_spindle\_pole | CENPE | 2 | 1 |  |  |  |  |  |  |
| GO:0051246\_regulation\_of\_protein\_metabolic\_process | MAPRE1 | 146 | 4 | 4.089542 | -1.799138 | 68 | 6.6 | 0.097059 |
| GO:0051246\_regulation\_of\_protein\_metabolic\_process | PPP2R4 | 146 | 4 | 4.089542 | -1.799138 | 68 | 6.6 | 0.097059 |
| GO:0051246\_regulation\_of\_protein\_metabolic\_process | CLASP1 | 146 | 4 | 4.089542 | -1.799138 | 68 | 6.6 | 0.097059 |
| GO:0051246\_regulation\_of\_protein\_metabolic\_process | CLASP2 | 146 | 4 | 4.089542 | -1.799138 | 68 | 6.6 | 0.097059 |
| GO:0000186\_activation\_of\_MAPKK\_activity | TAOK2 | 3 | 1 |  |  |  |  |  |  |
| GO:0007080\_mitotic\_metaphase\_plate\_congression | CENPE | 3 | 1 |  |  |  |  |  |  |
| GO:0040001\_establishment\_of\_mitotic\_spindle\_localization | PAFAH1B1 | 3 | 1 |  |  |  |  |  |  |
| GO:0043666\_regulation\_of\_phosphoprotein\_phosphatase\_activity | PPP2R4 | 3 | 1 |  |  |  |  |  |  |
| GO:0051004\_regulation\_of\_lipoprotein\_lipase\_activity | PPP2R4 | 3 | 1 |  |  |  |  |  |  |
| GO:0051293\_establishment\_of\_spindle\_localization | PAFAH1B1 | 3 | 1 |  |  |  |  |  |  |
| GO:0051653\_spindle\_localization | PAFAH1B1 | 3 | 1 |  |  |  |  |  |  |
| GO:0051179\_localization | MXI1 | 1090 | 13 | 1.780264 | -1.660437 | 69 | 8.76 | 0.126957 |
| GO:0051179\_localization | TAOK2 | 1090 | 13 | 1.780264 | -1.660437 | 69 | 8.76 | 0.126957 |
| GO:0051179\_localization | ZW10 | 1090 | 13 | 1.780264 | -1.660437 | 69 | 8.76 | 0.126957 |
| GO:0051179\_localization | CENPF | 1090 | 13 | 1.780264 | -1.660437 | 69 | 8.76 | 0.126957 |
| GO:0051179\_localization | NUP160 | 1090 | 13 | 1.780264 | -1.660437 | 69 | 8.76 | 0.126957 |
| GO:0051179\_localization | CENPE | 1090 | 13 | 1.780264 | -1.660437 | 69 | 8.76 | 0.126957 |
| GO:0051179\_localization | CKAP5 | 1090 | 13 | 1.780264 | -1.660437 | 69 | 8.76 | 0.126957 |
| GO:0051179\_localization | SEC13 | 1090 | 13 | 1.780264 | -1.660437 | 69 | 8.76 | 0.126957 |
| GO:0051179\_localization | ZWINT | 1090 | 13 | 1.780264 | -1.660437 | 69 | 8.76 | 0.126957 |
| GO:0051179\_localization | NUP107 | 1090 | 13 | 1.780264 | -1.660437 | 69 | 8.76 | 0.126957 |
| GO:0051179\_localization | RANBP2 | 1090 | 13 | 1.780264 | -1.660437 | 69 | 8.76 | 0.126957 |
| GO:0051179\_localization | NUP133 | 1090 | 13 | 1.780264 | -1.660437 | 69 | 8.76 | 0.126957 |
| GO:0051179\_localization | PAFAH1B1 | 1090 | 13 | 1.780264 | -1.660437 | 69 | 8.76 | 0.126957 |
| GO:0000085\_G2\_phase\_of\_mitotic\_cell\_cycle | CENPF | 4 | 1 |  |  |  |  |  |  |
| GO:0001578\_microtubule\_bundle\_formation | CLASP1 | 4 | 1 |  |  |  |  |  |  |
| GO:0031113\_regulation\_of\_microtubule\_polymerization | MAPRE1 | 4 | 1 |  |  |  |  |  |  |
| GO:0051319\_G2\_phase | CENPF | 4 | 1 |  |  |  |  |  |  |
| GO:0006461\_protein\_complex\_assembly | MIS12 | 174 | 4 | 3.431455 | -1.549580 | 70 | 10.67 | 0.152429 |
| GO:0006461\_protein\_complex\_assembly | ZW10 | 174 | 4 | 3.431455 | -1.549580 | 70 | 10.67 | 0.152429 |
| GO:0006461\_protein\_complex\_assembly | CENPF | 174 | 4 | 3.431455 | -1.549580 | 70 | 10.67 | 0.152429 |
| GO:0006461\_protein\_complex\_assembly | CENPE | 174 | 4 | 3.431455 | -1.549580 | 70 | 10.67 | 0.152429 |
| GO:0006886\_intracellular\_protein\_transport | MXI1 | 176 | 4 | 3.392461 | -1.533699 | 71 | 11.12 | 0.156620 |
| GO:0006886\_intracellular\_protein\_transport | TAOK2 | 176 | 4 | 3.392461 | -1.533699 | 71 | 11.12 | 0.156620 |
| GO:0006886\_intracellular\_protein\_transport | RANBP2 | 176 | 4 | 3.392461 | -1.533699 | 71 | 11.12 | 0.156620 |
| GO:0006886\_intracellular\_protein\_transport | SEC13 | 176 | 4 | 3.392461 | -1.533699 | 71 | 11.12 | 0.156620 |
| GO:0035303\_regulation\_of\_dephosphorylation | PPP2R4 | 5 | 1 | 29.853659 | -1.480676 | 73 | 18.31 | 0.250822 |
| GO:0046785\_microtubule\_polymerization | MAPRE1 | 5 | 1 | 29.853659 | -1.480676 | 73 | 18.31 | 0.250822 |
| GO:0031324\_negative\_regulation\_of\_cellular\_metabolic\_process | MAPRE1 | 273 | 5 | 2.733852 | -1.464328 | 74 | 18.56 | 0.250811 |
| GO:0031324\_negative\_regulation\_of\_cellular\_metabolic\_process | CENPF | 273 | 5 | 2.733852 | -1.464328 | 74 | 18.56 | 0.250811 |
| GO:0031324\_negative\_regulation\_of\_cellular\_metabolic\_process | CLASP1 | 273 | 5 | 2.733852 | -1.464328 | 74 | 18.56 | 0.250811 |
| GO:0031324\_negative\_regulation\_of\_cellular\_metabolic\_process | CLASP2 | 273 | 5 | 2.733852 | -1.464328 | 74 | 18.56 | 0.250811 |
| GO:0031324\_negative\_regulation\_of\_cellular\_metabolic\_process | PPP2R4 | 273 | 5 | 2.733852 | -1.464328 | 74 | 18.56 | 0.250811 |
| GO:0009892\_negative\_regulation\_of\_metabolic\_process | MAPRE1 | 277 | 5 | 2.694374 | -1.440979 | 75 | 19.12 | 0.254933 |
| GO:0009892\_negative\_regulation\_of\_metabolic\_process | CENPF | 277 | 5 | 2.694374 | -1.440979 | 75 | 19.12 | 0.254933 |
| GO:0009892\_negative\_regulation\_of\_metabolic\_process | CLASP1 | 277 | 5 | 2.694374 | -1.440979 | 75 | 19.12 | 0.254933 |
| GO:0009892\_negative\_regulation\_of\_metabolic\_process | CLASP2 | 277 | 5 | 2.694374 | -1.440979 | 75 | 19.12 | 0.254933 |
| GO:0009892\_negative\_regulation\_of\_metabolic\_process | PPP2R4 | 277 | 5 | 2.694374 | -1.440979 | 75 | 19.12 | 0.254933 |
| GO:0046330\_positive\_regulation\_of\_JNK\_cascade | TAOK2 | 6 | 1 | 24.878049 | -1.402911 | 78 | 24.8 | 0.317949 |
| GO:0051298\_centrosome\_duplication | NDE1 | 6 | 1 | 24.878049 | -1.402911 | 78 | 24.8 | 0.317949 |
| GO:0060191\_regulation\_of\_lipase\_activity | PPP2R4 | 6 | 1 | 24.878049 | -1.402911 | 78 | 24.8 | 0.317949 |
| GO:0019932\_second-messenger-mediated\_signaling | BUB1B | 114 | 3 | 3.928113 | -1.395730 | 79 | 24.89 | 0.315063 |
| GO:0019932\_second-messenger-mediated\_signaling | ZWINT | 114 | 3 | 3.928113 | -1.395730 | 79 | 24.89 | 0.315063 |
| GO:0019932\_second-messenger-mediated\_signaling | NDC80 | 114 | 3 | 3.928113 | -1.395730 | 79 | 24.89 | 0.315063 |
| GO:0015031\_protein\_transport | MXI1 | 195 | 4 | 3.061914 | -1.393475 | 80 | 24.94 | 0.311750 |
| GO:0015031\_protein\_transport | TAOK2 | 195 | 4 | 3.061914 | -1.393475 | 80 | 24.94 | 0.311750 |
| GO:0015031\_protein\_transport | RANBP2 | 195 | 4 | 3.061914 | -1.393475 | 80 | 24.94 | 0.311750 |
| GO:0015031\_protein\_transport | SEC13 | 195 | 4 | 3.061914 | -1.393475 | 80 | 24.94 | 0.311750 |
| GO:0000902\_cell\_morphogenesis | TAOK2 | 116 | 3 | 3.860387 | -1.376832 | 82 | 25.26 | 0.308049 |
| GO:0000902\_cell\_morphogenesis | CLASP1 | 116 | 3 | 3.860387 | -1.376832 | 82 | 25.26 | 0.308049 |
| GO:0000902\_cell\_morphogenesis | CLASP2 | 116 | 3 | 3.860387 | -1.376832 | 82 | 25.26 | 0.308049 |
| GO:0032989\_cellular\_structure\_morphogenesis | TAOK2 | 116 | 3 | 3.860387 | -1.376832 | 82 | 25.26 | 0.308049 |
| GO:0032989\_cellular\_structure\_morphogenesis | CLASP1 | 116 | 3 | 3.860387 | -1.376832 | 82 | 25.26 | 0.308049 |
| GO:0032989\_cellular\_structure\_morphogenesis | CLASP2 | 116 | 3 | 3.860387 | -1.376832 | 82 | 25.26 | 0.308049 |
| GO:0006606\_protein\_import\_into\_nucleus | MXI1 | 50 | 2 | 5.970732 | -1.358349 | 83 | 25.87 | 0.311687 |
| GO:0006606\_protein\_import\_into\_nucleus | RANBP2 | 50 | 2 | 5.970732 | -1.358349 | 83 | 25.87 | 0.311687 |
| GO:0051170\_nuclear\_import | MXI1 | 51 | 2 | 5.853659 | -1.342786 | 84 | 26.22 | 0.312143 |
| GO:0051170\_nuclear\_import | RANBP2 | 51 | 2 | 5.853659 | -1.342786 | 84 | 26.22 | 0.312143 |
| GO:0006605\_protein\_targeting | MXI1 | 126 | 3 | 3.554007 | -1.287968 | 85 | 31.95 | 0.375882 |
| GO:0006605\_protein\_targeting | TAOK2 | 126 | 3 | 3.554007 | -1.287968 | 85 | 31.95 | 0.375882 |
| GO:0006605\_protein\_targeting | RANBP2 | 126 | 3 | 3.554007 | -1.287968 | 85 | 31.95 | 0.375882 |
| GO:0045184\_establishment\_of\_protein\_localization | MXI1 | 216 | 4 | 2.764228 | -1.257829 | 86 | 37.06 | 0.430930 |
| GO:0045184\_establishment\_of\_protein\_localization | TAOK2 | 216 | 4 | 2.764228 | -1.257829 | 86 | 37.06 | 0.430930 |
| GO:0045184\_establishment\_of\_protein\_localization | RANBP2 | 216 | 4 | 2.764228 | -1.257829 | 86 | 37.06 | 0.430930 |
| GO:0045184\_establishment\_of\_protein\_localization | SEC13 | 216 | 4 | 2.764228 | -1.257829 | 86 | 37.06 | 0.430930 |
| GO:0007096\_regulation\_of\_exit\_from\_mitosis | ZW10 | 9 | 1 | 16.585366 | -1.231060 | 90 | 41.34 | 0.459333 |
| GO:0016202\_regulation\_of\_striated\_muscle\_development | CENPF | 9 | 1 | 16.585366 | -1.231060 | 90 | 41.34 | 0.459333 |
| GO:0042994\_cytoplasmic\_sequestering\_of\_transcription\_factor | MXI1 | 9 | 1 | 16.585366 | -1.231060 | 90 | 41.34 | 0.459333 |
| GO:0048634\_regulation\_of\_muscle\_development | CENPF | 9 | 1 | 16.585366 | -1.231060 | 90 | 41.34 | 0.459333 |
| GO:0007098\_centrosome\_cycle | NDE1 | 10 | 1 | 14.926829 | -1.186713 | 94 | 48.21 | 0.512872 |
| GO:0042308\_negative\_regulation\_of\_protein\_import\_into\_nucleus | MXI1 | 10 | 1 | 14.926829 | -1.186713 | 94 | 48.21 | 0.512872 |
| GO:0042992\_negative\_regulation\_of\_transcription\_factor\_import\_into\_nucleus | MXI1 | 10 | 1 | 14.926829 | -1.186713 | 94 | 48.21 | 0.512872 |
| GO:0051220\_cytoplasmic\_sequestering\_of\_protein | MXI1 | 10 | 1 | 14.926829 | -1.186713 | 94 | 48.21 | 0.512872 |
| GO:0046823\_negative\_regulation\_of\_nucleocytoplasmic\_transport | MXI1 | 11 | 1 | 13.569845 | -1.146730 | 96 | 52.8 | 0.550000 |
| GO:0048041\_focal\_adhesion\_formation | TAOK2 | 11 | 1 | 13.569845 | -1.146730 | 96 | 52.8 | 0.550000 |
| GO:0017038\_protein\_import | MXI1 | 67 | 2 | 4.455770 | -1.132578 | 97 | 53.64 | 0.552990 |
| GO:0017038\_protein\_import | RANBP2 | 67 | 2 | 4.455770 | -1.132578 | 97 | 53.64 | 0.552990 |
| GO:0008104\_protein\_localization | MXI1 | 242 | 4 | 2.467245 | -1.112552 | 98 | 54.01 | 0.551122 |
| GO:0008104\_protein\_localization | TAOK2 | 242 | 4 | 2.467245 | -1.112552 | 98 | 54.01 | 0.551122 |
| GO:0008104\_protein\_localization | RANBP2 | 242 | 4 | 2.467245 | -1.112552 | 98 | 54.01 | 0.551122 |
| GO:0008104\_protein\_localization | SEC13 | 242 | 4 | 2.467245 | -1.112552 | 98 | 54.01 | 0.551122 |
| GO:0031400\_negative\_regulation\_of\_protein\_modification\_process | PPP2R4 | 12 | 1 | 12.439024 | -1.110349 | 102 | 58.99 | 0.578333 |
| GO:0032271\_regulation\_of\_protein\_polymerization | MAPRE1 | 12 | 1 | 12.439024 | -1.110349 | 102 | 58.99 | 0.578333 |
| GO:0046328\_regulation\_of\_JNK\_cascade | TAOK2 | 12 | 1 | 12.439024 | -1.110349 | 102 | 58.99 | 0.578333 |
| GO:0051224\_negative\_regulation\_of\_protein\_transport | MXI1 | 12 | 1 | 12.439024 | -1.110349 | 102 | 58.99 | 0.578333 |
| GO:0006612\_protein\_targeting\_to\_membrane | TAOK2 | 13 | 1 | 11.482176 | -1.076993 | 106 | 64.35 | 0.607075 |
| GO:0032387\_negative\_regulation\_of\_intracellular\_transport | MXI1 | 13 | 1 | 11.482176 | -1.076993 | 106 | 64.35 | 0.607075 |
| GO:0043410\_positive\_regulation\_of\_MAPKKK\_cascade | TAOK2 | 13 | 1 | 11.482176 | -1.076993 | 106 | 64.35 | 0.607075 |
| GO:0051346\_negative\_regulation\_of\_hydrolase\_activity | PPP2R4 | 13 | 1 | 11.482176 | -1.076993 | 106 | 64.35 | 0.607075 |
| GO:0010563\_negative\_regulation\_of\_phosphorus\_metabolic\_process | PPP2R4 | 15 | 1 | 9.951220 | -1.017654 | 108 | 71.71 | 0.663981 |
| GO:0045936\_negative\_regulation\_of\_phosphate\_metabolic\_process | PPP2R4 | 15 | 1 | 9.951220 | -1.017654 | 108 | 71.71 | 0.663981 |
| GO:0008360\_regulation\_of\_cell\_shape | TAOK2 | 16 | 1 | 9.329268 | -0.991027 | 109 | 77.03 | 0.706697 |
| GO:0006810\_transport | MXI1 | 844 | 9 | 1.591724 | -0.989819 | 110 | 77.06 | 0.700545 |
| GO:0006810\_transport | TAOK2 | 844 | 9 | 1.591724 | -0.989819 | 110 | 77.06 | 0.700545 |
| GO:0006810\_transport | ZW10 | 844 | 9 | 1.591724 | -0.989819 | 110 | 77.06 | 0.700545 |
| GO:0006810\_transport | NUP107 | 844 | 9 | 1.591724 | -0.989819 | 110 | 77.06 | 0.700545 |
| GO:0006810\_transport | RANBP2 | 844 | 9 | 1.591724 | -0.989819 | 110 | 77.06 | 0.700545 |
| GO:0006810\_transport | NUP160 | 844 | 9 | 1.591724 | -0.989819 | 110 | 77.06 | 0.700545 |
| GO:0006810\_transport | NUP133 | 844 | 9 | 1.591724 | -0.989819 | 110 | 77.06 | 0.700545 |
| GO:0006810\_transport | CKAP5 | 844 | 9 | 1.591724 | -0.989819 | 110 | 77.06 | 0.700545 |
| GO:0006810\_transport | SEC13 | 844 | 9 | 1.591724 | -0.989819 | 110 | 77.06 | 0.700545 |
| GO:0022604\_regulation\_of\_cell\_morphogenesis | TAOK2 | 17 | 1 | 8.780488 | -0.966099 | 111 | 80.73 | 0.727297 |
| GO:0042990\_regulation\_of\_transcription\_factor\_import\_into\_nucleus | MXI1 | 18 | 1 | 8.292683 | -0.942675 | 113 | 84.89 | 0.751239 |
| GO:0042991\_transcription\_factor\_import\_into\_nucleus | MXI1 | 18 | 1 | 8.292683 | -0.942675 | 113 | 84.89 | 0.751239 |
| GO:0032507\_maintenance\_of\_cellular\_protein\_localization | MXI1 | 19 | 1 | 7.856226 | -0.920592 | 116 | 89.31 | 0.769914 |
| GO:0042306\_regulation\_of\_protein\_import\_into\_nucleus | MXI1 | 19 | 1 | 7.856226 | -0.920592 | 116 | 89.31 | 0.769914 |
| GO:0051258\_protein\_polymerization | MAPRE1 | 19 | 1 | 7.856226 | -0.920592 | 116 | 89.31 | 0.769914 |
| GO:0051651\_maintenance\_of\_cellular\_localization | MXI1 | 20 | 1 | 7.463415 | -0.899712 | 117 | 94.17 | 0.804872 |
| GO:0065003\_macromolecular\_complex\_assembly | MIS12 | 293 | 4 | 2.037792 | -0.882701 | 118 | 95.26 | 0.807288 |
| GO:0065003\_macromolecular\_complex\_assembly | ZW10 | 293 | 4 | 2.037792 | -0.882701 | 118 | 95.26 | 0.807288 |
| GO:0065003\_macromolecular\_complex\_assembly | CENPF | 293 | 4 | 2.037792 | -0.882701 | 118 | 95.26 | 0.807288 |
| GO:0065003\_macromolecular\_complex\_assembly | CENPE | 293 | 4 | 2.037792 | -0.882701 | 118 | 95.26 | 0.807288 |
| GO:0045185\_maintenance\_of\_protein\_localization | MXI1 | 21 | 1 | 7.108014 | -0.879918 | 119 | 98.3 | 0.826050 |
| GO:0032880\_regulation\_of\_protein\_localization | MXI1 | 22 | 1 | 6.784922 | -0.861108 | 122 | 101.54 | 0.832295 |
| GO:0033157\_regulation\_of\_intracellular\_protein\_transport | MXI1 | 22 | 1 | 6.784922 | -0.861108 | 122 | 101.54 | 0.832295 |
| GO:0043408\_regulation\_of\_MAPKKK\_cascade | TAOK2 | 22 | 1 | 6.784922 | -0.861108 | 122 | 101.54 | 0.832295 |
| GO:0006888\_ER\_to\_Golgi\_vesicle-mediated\_transport | ZW10 | 23 | 1 | 6.489926 | -0.843195 | 125 | 105.52 | 0.844160 |
| GO:0042493\_response\_to\_drug | CENPF | 23 | 1 | 6.489926 | -0.843195 | 125 | 105.52 | 0.844160 |
| GO:0051223\_regulation\_of\_protein\_transport | MXI1 | 23 | 1 | 6.489926 | -0.843195 | 125 | 105.52 | 0.844160 |
| GO:0051051\_negative\_regulation\_of\_transport | MXI1 | 24 | 1 | 6.219512 | -0.826102 | 126 | 108.54 | 0.861429 |
| GO:0022607\_cellular\_component\_assembly | MIS12 | 310 | 4 | 1.926042 | -0.818773 | 127 | 109.26 | 0.860315 |
| GO:0022607\_cellular\_component\_assembly | ZW10 | 310 | 4 | 1.926042 | -0.818773 | 127 | 109.26 | 0.860315 |
| GO:0022607\_cellular\_component\_assembly | CENPF | 310 | 4 | 1.926042 | -0.818773 | 127 | 109.26 | 0.860315 |
| GO:0022607\_cellular\_component\_assembly | CENPE | 310 | 4 | 1.926042 | -0.818773 | 127 | 109.26 | 0.860315 |
| GO:0000910\_cytokinesis | INCENP | 26 | 1 | 5.741088 | -0.794118 | 129 | 114.93 | 0.890930 |
| GO:0046822\_regulation\_of\_nucleocytoplasmic\_transport | MXI1 | 26 | 1 | 5.741088 | -0.794118 | 129 | 114.93 | 0.890930 |
| GO:0019221\_cytokine\_and\_chemokine\_mediated\_signaling\_pathway | NUP85 | 28 | 1 | 5.331010 | -0.764705 | 131 | 120.3 | 0.918321 |
| GO:0022603\_regulation\_of\_anatomical\_structure\_morphogenesis | TAOK2 | 28 | 1 | 5.331010 | -0.764705 | 131 | 120.3 | 0.918321 |
| GO:0007088\_regulation\_of\_mitosis | ZW10 | 31 | 1 | 4.815106 | -0.724648 | 137 | 129.83 | 0.947664 |
| GO:0010562\_positive\_regulation\_of\_phosphorus\_metabolic\_process | PPP2R4 | 31 | 1 | 4.815106 | -0.724648 | 137 | 129.83 | 0.947664 |
| GO:0032386\_regulation\_of\_intracellular\_transport | MXI1 | 31 | 1 | 4.815106 | -0.724648 | 137 | 129.83 | 0.947664 |
| GO:0045937\_positive\_regulation\_of\_phosphate\_metabolic\_process | PPP2R4 | 31 | 1 | 4.815106 | -0.724648 | 137 | 129.83 | 0.947664 |
| GO:0051235\_maintenance\_of\_localization | MXI1 | 31 | 1 | 4.815106 | -0.724648 | 137 | 129.83 | 0.947664 |
| GO:0051301\_cell\_division | INCENP | 31 | 1 | 4.815106 | -0.724648 | 137 | 129.83 | 0.947664 |
| GO:0007548\_sex\_differentiation | CENPI | 33 | 1 | 4.523282 | -0.700253 | 138 | 136.38 | 0.988261 |
| GO:0003006\_reproductive\_developmental\_process | CENPI | 34 | 1 | 4.390244 | -0.688665 | 140 | 139.38 | 0.995571 |
| GO:0032147\_activation\_of\_protein\_kinase\_activity | TAOK2 | 34 | 1 | 4.390244 | -0.688665 | 140 | 139.38 | 0.995571 |
| GO:0014706\_striated\_muscle\_development | CENPF | 35 | 1 | 4.264808 | -0.677451 | 142 | 140.9 | 0.992254 |
| GO:0031401\_positive\_regulation\_of\_protein\_modification\_process | PPP2R4 | 35 | 1 | 4.264808 | -0.677451 | 142 | 140.9 | 0.992254 |
| GO:0007126\_meiosis | ZW10 | 36 | 1 | 4.146341 | -0.666590 | 144 | 144.82 | 1.005694 |
| GO:0051327\_M\_phase\_of\_meiotic\_cell\_cycle | ZW10 | 36 | 1 | 4.146341 | -0.666590 | 144 | 144.82 | 1.005694 |
| GO:0051321\_meiotic\_cell\_cycle | ZW10 | 37 | 1 | 4.034278 | -0.656063 | 145 | 147.59 | 1.017862 |
| GO:0048523\_negative\_regulation\_of\_cellular\_process | MXI1 | 621 | 6 | 1.442206 | -0.634093 | 146 | 152.36 | 1.043562 |
| GO:0048523\_negative\_regulation\_of\_cellular\_process | MAPRE1 | 621 | 6 | 1.442206 | -0.634093 | 146 | 152.36 | 1.043562 |
| GO:0048523\_negative\_regulation\_of\_cellular\_process | CENPF | 621 | 6 | 1.442206 | -0.634093 | 146 | 152.36 | 1.043562 |
| GO:0048523\_negative\_regulation\_of\_cellular\_process | CLASP1 | 621 | 6 | 1.442206 | -0.634093 | 146 | 152.36 | 1.043562 |
| GO:0048523\_negative\_regulation\_of\_cellular\_process | CLASP2 | 621 | 6 | 1.442206 | -0.634093 | 146 | 152.36 | 1.043562 |
| GO:0048523\_negative\_regulation\_of\_cellular\_process | PPP2R4 | 621 | 6 | 1.442206 | -0.634093 | 146 | 152.36 | 1.043562 |
| GO:0019538\_protein\_metabolic\_process | CDC20 | 1292 | 11 | 1.270860 | -0.631127 | 147 | 152.7 | 1.038776 |
| GO:0019538\_protein\_metabolic\_process | MIS12 | 1292 | 11 | 1.270860 | -0.631127 | 147 | 152.7 | 1.038776 |
| GO:0019538\_protein\_metabolic\_process | MAPRE1 | 1292 | 11 | 1.270860 | -0.631127 | 147 | 152.7 | 1.038776 |
| GO:0019538\_protein\_metabolic\_process | TAOK2 | 1292 | 11 | 1.270860 | -0.631127 | 147 | 152.7 | 1.038776 |
| GO:0019538\_protein\_metabolic\_process | ZW10 | 1292 | 11 | 1.270860 | -0.631127 | 147 | 152.7 | 1.038776 |
| GO:0019538\_protein\_metabolic\_process | CENPF | 1292 | 11 | 1.270860 | -0.631127 | 147 | 152.7 | 1.038776 |
| GO:0019538\_protein\_metabolic\_process | CENPE | 1292 | 11 | 1.270860 | -0.631127 | 147 | 152.7 | 1.038776 |
| GO:0019538\_protein\_metabolic\_process | CLASP1 | 1292 | 11 | 1.270860 | -0.631127 | 147 | 152.7 | 1.038776 |
| GO:0019538\_protein\_metabolic\_process | CLASP2 | 1292 | 11 | 1.270860 | -0.631127 | 147 | 152.7 | 1.038776 |
| GO:0019538\_protein\_metabolic\_process | PPP2R4 | 1292 | 11 | 1.270860 | -0.631127 | 147 | 152.7 | 1.038776 |
| GO:0019538\_protein\_metabolic\_process | RPS27 | 1292 | 11 | 1.270860 | -0.631127 | 147 | 152.7 | 1.038776 |
| GO:0007160\_cell-matrix\_adhesion | TAOK2 | 40 | 1 | 3.731707 | -0.626314 | 148 | 154.76 | 1.045676 |
| GO:0031589\_cell-substrate\_adhesion | TAOK2 | 41 | 1 | 3.640690 | -0.616957 | 149 | 157.15 | 1.054698 |
| GO:0010564\_regulation\_of\_cell\_cycle\_process | ZW10 | 43 | 1 | 3.471356 | -0.599001 | 150 | 160.4 | 1.069333 |
| GO:0044267\_cellular\_protein\_metabolic\_process | CDC20 | 1179 | 10 | 1.266058 | -0.594094 | 151 | 160.71 | 1.064305 |
| GO:0044267\_cellular\_protein\_metabolic\_process | MIS12 | 1179 | 10 | 1.266058 | -0.594094 | 151 | 160.71 | 1.064305 |
| GO:0044267\_cellular\_protein\_metabolic\_process | MAPRE1 | 1179 | 10 | 1.266058 | -0.594094 | 151 | 160.71 | 1.064305 |
| GO:0044267\_cellular\_protein\_metabolic\_process | TAOK2 | 1179 | 10 | 1.266058 | -0.594094 | 151 | 160.71 | 1.064305 |
| GO:0044267\_cellular\_protein\_metabolic\_process | CENPF | 1179 | 10 | 1.266058 | -0.594094 | 151 | 160.71 | 1.064305 |
| GO:0044267\_cellular\_protein\_metabolic\_process | CENPE | 1179 | 10 | 1.266058 | -0.594094 | 151 | 160.71 | 1.064305 |
| GO:0044267\_cellular\_protein\_metabolic\_process | CLASP1 | 1179 | 10 | 1.266058 | -0.594094 | 151 | 160.71 | 1.064305 |
| GO:0044267\_cellular\_protein\_metabolic\_process | CLASP2 | 1179 | 10 | 1.266058 | -0.594094 | 151 | 160.71 | 1.064305 |
| GO:0044267\_cellular\_protein\_metabolic\_process | PPP2R4 | 1179 | 10 | 1.266058 | -0.594094 | 151 | 160.71 | 1.064305 |
| GO:0044267\_cellular\_protein\_metabolic\_process | RPS27 | 1179 | 10 | 1.266058 | -0.594094 | 151 | 160.71 | 1.064305 |
| GO:0007254\_JNK\_cascade | TAOK2 | 48 | 1 | 3.109756 | -0.558026 | 152 | 168.21 | 1.106645 |
| GO:0048519\_negative\_regulation\_of\_biological\_process | MXI1 | 664 | 6 | 1.348810 | -0.549539 | 153 | 170.75 | 1.116013 |
| GO:0048519\_negative\_regulation\_of\_biological\_process | MAPRE1 | 664 | 6 | 1.348810 | -0.549539 | 153 | 170.75 | 1.116013 |
| GO:0048519\_negative\_regulation\_of\_biological\_process | CENPF | 664 | 6 | 1.348810 | -0.549539 | 153 | 170.75 | 1.116013 |
| GO:0048519\_negative\_regulation\_of\_biological\_process | CLASP1 | 664 | 6 | 1.348810 | -0.549539 | 153 | 170.75 | 1.116013 |
| GO:0048519\_negative\_regulation\_of\_biological\_process | CLASP2 | 664 | 6 | 1.348810 | -0.549539 | 153 | 170.75 | 1.116013 |
| GO:0048519\_negative\_regulation\_of\_biological\_process | PPP2R4 | 664 | 6 | 1.348810 | -0.549539 | 153 | 170.75 | 1.116013 |
| GO:0031098\_stress-activated\_protein\_kinase\_signaling\_pathway | TAOK2 | 50 | 1 | 2.985366 | -0.543006 | 155 | 172.76 | 1.114581 |
| GO:0031399\_regulation\_of\_protein\_modification\_process | PPP2R4 | 50 | 1 | 2.985366 | -0.543006 | 155 | 172.76 | 1.114581 |
| GO:0044260\_cellular\_macromolecule\_metabolic\_process | CDC20 | 1219 | 10 | 1.224514 | -0.536345 | 156 | 173.37 | 1.111346 |
| GO:0044260\_cellular\_macromolecule\_metabolic\_process | MIS12 | 1219 | 10 | 1.224514 | -0.536345 | 156 | 173.37 | 1.111346 |
| GO:0044260\_cellular\_macromolecule\_metabolic\_process | MAPRE1 | 1219 | 10 | 1.224514 | -0.536345 | 156 | 173.37 | 1.111346 |
| GO:0044260\_cellular\_macromolecule\_metabolic\_process | TAOK2 | 1219 | 10 | 1.224514 | -0.536345 | 156 | 173.37 | 1.111346 |
| GO:0044260\_cellular\_macromolecule\_metabolic\_process | CENPF | 1219 | 10 | 1.224514 | -0.536345 | 156 | 173.37 | 1.111346 |
| GO:0044260\_cellular\_macromolecule\_metabolic\_process | CENPE | 1219 | 10 | 1.224514 | -0.536345 | 156 | 173.37 | 1.111346 |
| GO:0044260\_cellular\_macromolecule\_metabolic\_process | CLASP1 | 1219 | 10 | 1.224514 | -0.536345 | 156 | 173.37 | 1.111346 |
| GO:0044260\_cellular\_macromolecule\_metabolic\_process | CLASP2 | 1219 | 10 | 1.224514 | -0.536345 | 156 | 173.37 | 1.111346 |
| GO:0044260\_cellular\_macromolecule\_metabolic\_process | PPP2R4 | 1219 | 10 | 1.224514 | -0.536345 | 156 | 173.37 | 1.111346 |
| GO:0044260\_cellular\_macromolecule\_metabolic\_process | RPS27 | 1219 | 10 | 1.224514 | -0.536345 | 156 | 173.37 | 1.111346 |
| GO:0043085\_positive\_regulation\_of\_catalytic\_activity | TAOK2 | 162 | 2 | 1.842818 | -0.528625 | 157 | 175.96 | 1.120764 |
| GO:0043085\_positive\_regulation\_of\_catalytic\_activity | PPP2R4 | 162 | 2 | 1.842818 | -0.528625 | 157 | 175.96 | 1.120764 |
| GO:0050794\_regulation\_of\_cellular\_process | MXI1 | 1656 | 13 | 1.171792 | -0.518349 | 158 | 178.37 | 1.128924 |
| GO:0050794\_regulation\_of\_cellular\_process | TAOK2 | 1656 | 13 | 1.171792 | -0.518349 | 158 | 178.37 | 1.128924 |
| GO:0050794\_regulation\_of\_cellular\_process | CENPF | 1656 | 13 | 1.171792 | -0.518349 | 158 | 178.37 | 1.128924 |
| GO:0050794\_regulation\_of\_cellular\_process | ZW10 | 1656 | 13 | 1.171792 | -0.518349 | 158 | 178.37 | 1.128924 |
| GO:0050794\_regulation\_of\_cellular\_process | BUB1 | 1656 | 13 | 1.171792 | -0.518349 | 158 | 178.37 | 1.128924 |
| GO:0050794\_regulation\_of\_cellular\_process | MAD2L1 | 1656 | 13 | 1.171792 | -0.518349 | 158 | 178.37 | 1.128924 |
| GO:0050794\_regulation\_of\_cellular\_process | BUB1B | 1656 | 13 | 1.171792 | -0.518349 | 158 | 178.37 | 1.128924 |
| GO:0050794\_regulation\_of\_cellular\_process | ZWINT | 1656 | 13 | 1.171792 | -0.518349 | 158 | 178.37 | 1.128924 |
| GO:0050794\_regulation\_of\_cellular\_process | MAPRE1 | 1656 | 13 | 1.171792 | -0.518349 | 158 | 178.37 | 1.128924 |
| GO:0050794\_regulation\_of\_cellular\_process | ZWILCH | 1656 | 13 | 1.171792 | -0.518349 | 158 | 178.37 | 1.128924 |
| GO:0050794\_regulation\_of\_cellular\_process | PPP2R4 | 1656 | 13 | 1.171792 | -0.518349 | 158 | 178.37 | 1.128924 |
| GO:0050794\_regulation\_of\_cellular\_process | CLASP2 | 1656 | 13 | 1.171792 | -0.518349 | 158 | 178.37 | 1.128924 |
| GO:0050794\_regulation\_of\_cellular\_process | CLASP1 | 1656 | 13 | 1.171792 | -0.518349 | 158 | 178.37 | 1.128924 |
| GO:0032270\_positive\_regulation\_of\_cellular\_protein\_metabolic\_process | PPP2R4 | 56 | 1 | 2.665505 | -0.501881 | 159 | 182.89 | 1.150252 |
| GO:0019220\_regulation\_of\_phosphate\_metabolic\_process | PPP2R4 | 57 | 1 | 2.618742 | -0.495538 | 161 | 184.52 | 1.146087 |
| GO:0051174\_regulation\_of\_phosphorus\_metabolic\_process | PPP2R4 | 57 | 1 | 2.618742 | -0.495538 | 161 | 184.52 | 1.146087 |
| GO:0051247\_positive\_regulation\_of\_protein\_metabolic\_process | PPP2R4 | 59 | 1 | 2.529971 | -0.483245 | 162 | 188.48 | 1.163457 |
| GO:0051345\_positive\_regulation\_of\_hydrolase\_activity | PPP2R4 | 61 | 1 | 2.447021 | -0.471445 | 163 | 192.92 | 1.183558 |
| GO:0006470\_protein\_amino\_acid\_dephosphorylation | PPP2R4 | 63 | 1 | 2.369338 | -0.460106 | 166 | 195.53 | 1.177892 |
| GO:0043086\_negative\_regulation\_of\_catalytic\_activity | PPP2R4 | 63 | 1 | 2.369338 | -0.460106 | 166 | 195.53 | 1.177892 |
| GO:0048193\_Golgi\_vesicle\_transport | ZW10 | 63 | 1 | 2.369338 | -0.460106 | 166 | 195.53 | 1.177892 |
| GO:0006511\_ubiquitin-dependent\_protein\_catabolic\_process | CDC20 | 67 | 1 | 2.227885 | -0.438698 | 171 | 202.06 | 1.181637 |
| GO:0019941\_modification-dependent\_protein\_catabolic\_process | CDC20 | 67 | 1 | 2.227885 | -0.438698 | 171 | 202.06 | 1.181637 |
| GO:0043632\_modification-dependent\_macromolecule\_catabolic\_process | CDC20 | 67 | 1 | 2.227885 | -0.438698 | 171 | 202.06 | 1.181637 |
| GO:0044257\_cellular\_protein\_catabolic\_process | CDC20 | 67 | 1 | 2.227885 | -0.438698 | 171 | 202.06 | 1.181637 |
| GO:0051603\_proteolysis\_involved\_in\_cellular\_protein\_catabolic\_process | CDC20 | 67 | 1 | 2.227885 | -0.438698 | 171 | 202.06 | 1.181637 |
| GO:0006928\_cell\_motility | TAOK2 | 191 | 2 | 1.563019 | -0.434399 | 173 | 202.9 | 1.172832 |
| GO:0006928\_cell\_motility | PAFAH1B1 | 191 | 2 | 1.563019 | -0.434399 | 173 | 202.9 | 1.172832 |
| GO:0051674\_localization\_of\_cell | TAOK2 | 191 | 2 | 1.563019 | -0.434399 | 173 | 202.9 | 1.172832 |
| GO:0051674\_localization\_of\_cell | PAFAH1B1 | 191 | 2 | 1.563019 | -0.434399 | 173 | 202.9 | 1.172832 |
| GO:0051329\_interphase\_of\_mitotic\_cell\_cycle | CENPF | 70 | 1 | 2.132404 | -0.423655 | 174 | 206.63 | 1.187529 |
| GO:0016311\_dephosphorylation | PPP2R4 | 71 | 1 | 2.102370 | -0.418818 | 175 | 208.56 | 1.191771 |
| GO:0051049\_regulation\_of\_transport | MXI1 | 73 | 1 | 2.044771 | -0.409397 | 176 | 211.22 | 1.200114 |
| GO:0051325\_interphase | CENPF | 75 | 1 | 1.990244 | -0.400296 | 177 | 212.67 | 1.201525 |
| GO:0050789\_regulation\_of\_biological\_process | MXI1 | 1765 | 13 | 1.099427 | -0.399138 | 178 | 212.81 | 1.195562 |
| GO:0050789\_regulation\_of\_biological\_process | TAOK2 | 1765 | 13 | 1.099427 | -0.399138 | 178 | 212.81 | 1.195562 |
| GO:0050789\_regulation\_of\_biological\_process | CENPF | 1765 | 13 | 1.099427 | -0.399138 | 178 | 212.81 | 1.195562 |
| GO:0050789\_regulation\_of\_biological\_process | ZW10 | 1765 | 13 | 1.099427 | -0.399138 | 178 | 212.81 | 1.195562 |
| GO:0050789\_regulation\_of\_biological\_process | BUB1 | 1765 | 13 | 1.099427 | -0.399138 | 178 | 212.81 | 1.195562 |
| GO:0050789\_regulation\_of\_biological\_process | MAD2L1 | 1765 | 13 | 1.099427 | -0.399138 | 178 | 212.81 | 1.195562 |
| GO:0050789\_regulation\_of\_biological\_process | BUB1B | 1765 | 13 | 1.099427 | -0.399138 | 178 | 212.81 | 1.195562 |
| GO:0050789\_regulation\_of\_biological\_process | ZWINT | 1765 | 13 | 1.099427 | -0.399138 | 178 | 212.81 | 1.195562 |
| GO:0050789\_regulation\_of\_biological\_process | MAPRE1 | 1765 | 13 | 1.099427 | -0.399138 | 178 | 212.81 | 1.195562 |
| GO:0050789\_regulation\_of\_biological\_process | ZWILCH | 1765 | 13 | 1.099427 | -0.399138 | 178 | 212.81 | 1.195562 |
| GO:0050789\_regulation\_of\_biological\_process | PPP2R4 | 1765 | 13 | 1.099427 | -0.399138 | 178 | 212.81 | 1.195562 |
| GO:0050789\_regulation\_of\_biological\_process | CLASP2 | 1765 | 13 | 1.099427 | -0.399138 | 178 | 212.81 | 1.195562 |
| GO:0050789\_regulation\_of\_biological\_process | CLASP1 | 1765 | 13 | 1.099427 | -0.399138 | 178 | 212.81 | 1.195562 |
| GO:0001558\_regulation\_of\_cell\_growth | TAOK2 | 80 | 1 | 1.865854 | -0.378836 | 179 | 217.04 | 1.212514 |
| GO:0009653\_anatomical\_structure\_morphogenesis | TAOK2 | 360 | 3 | 1.243902 | -0.359604 | 180 | 224.14 | 1.245222 |
| GO:0009653\_anatomical\_structure\_morphogenesis | CLASP1 | 360 | 3 | 1.243902 | -0.359604 | 180 | 224.14 | 1.245222 |
| GO:0009653\_anatomical\_structure\_morphogenesis | CLASP2 | 360 | 3 | 1.243902 | -0.359604 | 180 | 224.14 | 1.245222 |
| GO:0030163\_protein\_catabolic\_process | CDC20 | 85 | 1 | 1.756098 | -0.359040 | 181 | 225.38 | 1.245193 |
| GO:0051336\_regulation\_of\_hydrolase\_activity | PPP2R4 | 86 | 1 | 1.735678 | -0.355263 | 182 | 226.44 | 1.244176 |
| GO:0033674\_positive\_regulation\_of\_kinase\_activity | TAOK2 | 88 | 1 | 1.696231 | -0.347879 | 184 | 229.42 | 1.246848 |
| GO:0045860\_positive\_regulation\_of\_protein\_kinase\_activity | TAOK2 | 88 | 1 | 1.696231 | -0.347879 | 184 | 229.42 | 1.246848 |
| GO:0007517\_muscle\_development | CENPF | 89 | 1 | 1.677172 | -0.344270 | 185 | 230.25 | 1.244595 |
| GO:0040008\_regulation\_of\_growth | TAOK2 | 91 | 1 | 1.640311 | -0.337210 | 187 | 233.26 | 1.247380 |
| GO:0051347\_positive\_regulation\_of\_transferase\_activity | TAOK2 | 91 | 1 | 1.640311 | -0.337210 | 187 | 233.26 | 1.247380 |
| GO:0032879\_regulation\_of\_localization | MXI1 | 93 | 1 | 1.605035 | -0.330354 | 188 | 235.65 | 1.253457 |
| GO:0045045\_secretory\_pathway | ZW10 | 94 | 1 | 1.587961 | -0.327000 | 189 | 236.15 | 1.249471 |
| GO:0016049\_cell\_growth | TAOK2 | 98 | 1 | 1.523146 | -0.314050 | 190 | 239.83 | 1.262263 |
| GO:0008361\_regulation\_of\_cell\_size | TAOK2 | 99 | 1 | 1.507761 | -0.310925 | 191 | 240.79 | 1.260681 |
| GO:0016477\_cell\_migration | TAOK2 | 103 | 1 | 1.449207 | -0.298843 | 192 | 244.77 | 1.274844 |
| GO:0030036\_actin\_cytoskeleton\_organization\_and\_biogenesis | TAOK2 | 110 | 1 | 1.356984 | -0.279192 | 193 | 250.17 | 1.296218 |
| GO:0000165\_MAPKKK\_cascade | TAOK2 | 111 | 1 | 1.344759 | -0.276528 | 194 | 250.76 | 1.292577 |
| GO:0050790\_regulation\_of\_catalytic\_activity | TAOK2 | 269 | 2 | 1.109801 | -0.264455 | 195 | 254.6 | 1.305641 |
| GO:0050790\_regulation\_of\_catalytic\_activity | PPP2R4 | 269 | 2 | 1.109801 | -0.264455 | 195 | 254.6 | 1.305641 |
| GO:0040007\_growth | TAOK2 | 119 | 1 | 1.254355 | -0.256383 | 196 | 258.15 | 1.317092 |
| GO:0030029\_actin\_filament-based\_process | TAOK2 | 120 | 1 | 1.243902 | -0.254002 | 197 | 259.13 | 1.315381 |
| GO:0009967\_positive\_regulation\_of\_signal\_transduction | TAOK2 | 121 | 1 | 1.233622 | -0.251650 | 198 | 260.72 | 1.316768 |
| GO:0032940\_secretion\_by\_cell | ZW10 | 125 | 1 | 1.194146 | -0.242519 | 199 | 264.23 | 1.327789 |
| GO:0044262\_cellular\_carbohydrate\_metabolic\_process | IDUA | 129 | 1 | 1.157119 | -0.233809 | 200 | 266.54 | 1.332700 |
| GO:0044265\_cellular\_macromolecule\_catabolic\_process | CDC20 | 130 | 1 | 1.148218 | -0.231695 | 201 | 267.56 | 1.331144 |
| GO:0043285\_biopolymer\_catabolic\_process | CDC20 | 135 | 1 | 1.105691 | -0.221478 | 202 | 272.08 | 1.346931 |
| GO:0008285\_negative\_regulation\_of\_cell\_proliferation | MXI1 | 137 | 1 | 1.089550 | -0.217551 | 203 | 273.46 | 1.347094 |
| GO:0022414\_reproductive\_process | CENPI | 142 | 1 | 1.051185 | -0.208107 | 204 | 277.1 | 1.358333 |
| GO:0007165\_signal\_transduction | BUB1B | 1564 | 10 | 0.954401 | -0.202548 | 205 | 278.73 | 1.359659 |
| GO:0007165\_signal\_transduction | ZWINT | 1564 | 10 | 0.954401 | -0.202548 | 205 | 278.73 | 1.359659 |
| GO:0007165\_signal\_transduction | TAOK2 | 1564 | 10 | 0.954401 | -0.202548 | 205 | 278.73 | 1.359659 |
| GO:0007165\_signal\_transduction | ITGB3BP | 1564 | 10 | 0.954401 | -0.202548 | 205 | 278.73 | 1.359659 |
| GO:0007165\_signal\_transduction | PAFAH1B1 | 1564 | 10 | 0.954401 | -0.202548 | 205 | 278.73 | 1.359659 |
| GO:0007165\_signal\_transduction | RANGAP1 | 1564 | 10 | 0.954401 | -0.202548 | 205 | 278.73 | 1.359659 |
| GO:0007165\_signal\_transduction | MAPRE2 | 1564 | 10 | 0.954401 | -0.202548 | 205 | 278.73 | 1.359659 |
| GO:0007165\_signal\_transduction | NDC80 | 1564 | 10 | 0.954401 | -0.202548 | 205 | 278.73 | 1.359659 |
| GO:0007165\_signal\_transduction | NUP85 | 1564 | 10 | 0.954401 | -0.202548 | 205 | 278.73 | 1.359659 |
| GO:0007165\_signal\_transduction | RPS27 | 1564 | 10 | 0.954401 | -0.202548 | 205 | 278.73 | 1.359659 |
| GO:0065007\_biological\_regulation | MXI1 | 2021 | 13 | 0.960162 | -0.201413 | 206 | 278.86 | 1.353689 |
| GO:0065007\_biological\_regulation | TAOK2 | 2021 | 13 | 0.960162 | -0.201413 | 206 | 278.86 | 1.353689 |
| GO:0065007\_biological\_regulation | CENPF | 2021 | 13 | 0.960162 | -0.201413 | 206 | 278.86 | 1.353689 |
| GO:0065007\_biological\_regulation | ZW10 | 2021 | 13 | 0.960162 | -0.201413 | 206 | 278.86 | 1.353689 |
| GO:0065007\_biological\_regulation | BUB1 | 2021 | 13 | 0.960162 | -0.201413 | 206 | 278.86 | 1.353689 |
| GO:0065007\_biological\_regulation | MAD2L1 | 2021 | 13 | 0.960162 | -0.201413 | 206 | 278.86 | 1.353689 |
| GO:0065007\_biological\_regulation | BUB1B | 2021 | 13 | 0.960162 | -0.201413 | 206 | 278.86 | 1.353689 |
| GO:0065007\_biological\_regulation | ZWINT | 2021 | 13 | 0.960162 | -0.201413 | 206 | 278.86 | 1.353689 |
| GO:0065007\_biological\_regulation | MAPRE1 | 2021 | 13 | 0.960162 | -0.201413 | 206 | 278.86 | 1.353689 |
| GO:0065007\_biological\_regulation | ZWILCH | 2021 | 13 | 0.960162 | -0.201413 | 206 | 278.86 | 1.353689 |
| GO:0065007\_biological\_regulation | PPP2R4 | 2021 | 13 | 0.960162 | -0.201413 | 206 | 278.86 | 1.353689 |
| GO:0065007\_biological\_regulation | CLASP2 | 2021 | 13 | 0.960162 | -0.201413 | 206 | 278.86 | 1.353689 |
| GO:0065007\_biological\_regulation | CLASP1 | 2021 | 13 | 0.960162 | -0.201413 | 206 | 278.86 | 1.353689 |
| GO:0065009\_regulation\_of\_molecular\_function | TAOK2 | 314 | 2 | 0.950753 | -0.200870 | 207 | 279.7 | 1.351208 |
| GO:0065009\_regulation\_of\_molecular\_function | PPP2R4 | 314 | 2 | 0.950753 | -0.200870 | 207 | 279.7 | 1.351208 |
| GO:0007155\_cell\_adhesion | TAOK2 | 327 | 2 | 0.912956 | -0.185677 | 209 | 282.58 | 1.352057 |
| GO:0007155\_cell\_adhesion | ITGB3BP | 327 | 2 | 0.912956 | -0.185677 | 209 | 282.58 | 1.352057 |
| GO:0022610\_biological\_adhesion | TAOK2 | 327 | 2 | 0.912956 | -0.185677 | 209 | 282.58 | 1.352057 |
| GO:0022610\_biological\_adhesion | ITGB3BP | 327 | 2 | 0.912956 | -0.185677 | 209 | 282.58 | 1.352057 |
| GO:0007242\_intracellular\_signaling\_cascade | BUB1B | 665 | 4 | 0.897854 | -0.177013 | 210 | 284.38 | 1.354190 |
| GO:0007242\_intracellular\_signaling\_cascade | ZWINT | 665 | 4 | 0.897854 | -0.177013 | 210 | 284.38 | 1.354190 |
| GO:0007242\_intracellular\_signaling\_cascade | TAOK2 | 665 | 4 | 0.897854 | -0.177013 | 210 | 284.38 | 1.354190 |
| GO:0007242\_intracellular\_signaling\_cascade | NDC80 | 665 | 4 | 0.897854 | -0.177013 | 210 | 284.38 | 1.354190 |
| GO:0045859\_regulation\_of\_protein\_kinase\_activity | TAOK2 | 161 | 1 | 0.927132 | -0.176513 | 211 | 284.74 | 1.349479 |
| GO:0043549\_regulation\_of\_kinase\_activity | TAOK2 | 163 | 1 | 0.915756 | -0.173537 | 212 | 285.6 | 1.347170 |
| GO:0006412\_translation | RPS27 | 165 | 1 | 0.904656 | -0.170620 | 213 | 286.8 | 1.346479 |
| GO:0051338\_regulation\_of\_transferase\_activity | TAOK2 | 167 | 1 | 0.893822 | -0.167761 | 214 | 287.75 | 1.344626 |
| GO:0009057\_macromolecule\_catabolic\_process | CDC20 | 170 | 1 | 0.878049 | -0.163579 | 215 | 289.57 | 1.346837 |
| GO:0046903\_secretion | ZW10 | 178 | 1 | 0.838586 | -0.153012 | 216 | 294.99 | 1.365694 |
| GO:0005975\_carbohydrate\_metabolic\_process | IDUA | 186 | 1 | 0.802518 | -0.143233 | 217 | 297.84 | 1.372535 |
| GO:0031323\_regulation\_of\_cellular\_metabolic\_process | MAPRE1 | 883 | 5 | 0.845234 | -0.139725 | 218 | 299.26 | 1.372752 |
| GO:0031323\_regulation\_of\_cellular\_metabolic\_process | CENPF | 883 | 5 | 0.845234 | -0.139725 | 218 | 299.26 | 1.372752 |
| GO:0031323\_regulation\_of\_cellular\_metabolic\_process | CLASP1 | 883 | 5 | 0.845234 | -0.139725 | 218 | 299.26 | 1.372752 |
| GO:0031323\_regulation\_of\_cellular\_metabolic\_process | CLASP2 | 883 | 5 | 0.845234 | -0.139725 | 218 | 299.26 | 1.372752 |
| GO:0031323\_regulation\_of\_cellular\_metabolic\_process | PPP2R4 | 883 | 5 | 0.845234 | -0.139725 | 218 | 299.26 | 1.372752 |
| GO:0019222\_regulation\_of\_metabolic\_process | MAPRE1 | 898 | 5 | 0.831115 | -0.131218 | 219 | 301.57 | 1.377032 |
| GO:0019222\_regulation\_of\_metabolic\_process | CENPF | 898 | 5 | 0.831115 | -0.131218 | 219 | 301.57 | 1.377032 |
| GO:0019222\_regulation\_of\_metabolic\_process | CLASP1 | 898 | 5 | 0.831115 | -0.131218 | 219 | 301.57 | 1.377032 |
| GO:0019222\_regulation\_of\_metabolic\_process | CLASP2 | 898 | 5 | 0.831115 | -0.131218 | 219 | 301.57 | 1.377032 |
| GO:0019222\_regulation\_of\_metabolic\_process | PPP2R4 | 898 | 5 | 0.831115 | -0.131218 | 219 | 301.57 | 1.377032 |
| GO:0016481\_negative\_regulation\_of\_transcription | CENPF | 202 | 1 | 0.738952 | -0.125752 | 220 | 303.1 | 1.377727 |
| GO:0006508\_proteolysis | CDC20 | 204 | 1 | 0.731707 | -0.123742 | 221 | 303.72 | 1.374299 |
| GO:0048856\_anatomical\_structure\_development | TAOK2 | 925 | 5 | 0.806856 | -0.117055 | 222 | 304.96 | 1.373694 |
| GO:0048856\_anatomical\_structure\_development | CENPF | 925 | 5 | 0.806856 | -0.117055 | 222 | 304.96 | 1.373694 |
| GO:0048856\_anatomical\_structure\_development | PAFAH1B1 | 925 | 5 | 0.806856 | -0.117055 | 222 | 304.96 | 1.373694 |
| GO:0048856\_anatomical\_structure\_development | CLASP1 | 925 | 5 | 0.806856 | -0.117055 | 222 | 304.96 | 1.373694 |
| GO:0048856\_anatomical\_structure\_development | CLASP2 | 925 | 5 | 0.806856 | -0.117055 | 222 | 304.96 | 1.373694 |
| GO:0007154\_cell\_communication | BUB1B | 1733 | 10 | 0.861329 | -0.116520 | 223 | 305.11 | 1.368206 |
| GO:0007154\_cell\_communication | ZWINT | 1733 | 10 | 0.861329 | -0.116520 | 223 | 305.11 | 1.368206 |
| GO:0007154\_cell\_communication | TAOK2 | 1733 | 10 | 0.861329 | -0.116520 | 223 | 305.11 | 1.368206 |
| GO:0007154\_cell\_communication | ITGB3BP | 1733 | 10 | 0.861329 | -0.116520 | 223 | 305.11 | 1.368206 |
| GO:0007154\_cell\_communication | PAFAH1B1 | 1733 | 10 | 0.861329 | -0.116520 | 223 | 305.11 | 1.368206 |
| GO:0007154\_cell\_communication | RANGAP1 | 1733 | 10 | 0.861329 | -0.116520 | 223 | 305.11 | 1.368206 |
| GO:0007154\_cell\_communication | MAPRE2 | 1733 | 10 | 0.861329 | -0.116520 | 223 | 305.11 | 1.368206 |
| GO:0007154\_cell\_communication | NDC80 | 1733 | 10 | 0.861329 | -0.116520 | 223 | 305.11 | 1.368206 |
| GO:0007154\_cell\_communication | NUP85 | 1733 | 10 | 0.861329 | -0.116520 | 223 | 305.11 | 1.368206 |
| GO:0007154\_cell\_communication | RPS27 | 1733 | 10 | 0.861329 | -0.116520 | 223 | 305.11 | 1.368206 |
| GO:0045934\_negative\_regulation\_of\_nucleobase\_\_nucleoside\_\_nucleotide\_and\_nucleic\_acid\_metabolic\_process | CENPF | 223 | 1 | 0.669365 | -0.106349 | 224 | 307.46 | 1.372589 |
| GO:0016192\_vesicle-mediated\_transport | ZW10 | 227 | 1 | 0.657570 | -0.103044 | 225 | 308.44 | 1.370844 |
| GO:0009966\_regulation\_of\_signal\_transduction | TAOK2 | 232 | 1 | 0.643398 | -0.099071 | 226 | 309.85 | 1.371018 |
| GO:0000003\_reproduction | CENPI | 237 | 1 | 0.629824 | -0.095266 | 228 | 310.83 | 1.363289 |
| GO:0031325\_positive\_regulation\_of\_cellular\_metabolic\_process | PPP2R4 | 237 | 1 | 0.629824 | -0.095266 | 228 | 310.83 | 1.363289 |
| GO:0044248\_cellular\_catabolic\_process | CDC20 | 239 | 1 | 0.624554 | -0.093788 | 229 | 311.72 | 1.361223 |
| GO:0009893\_positive\_regulation\_of\_metabolic\_process | PPP2R4 | 244 | 1 | 0.611755 | -0.090204 | 230 | 313.13 | 1.361435 |
| GO:0050793\_regulation\_of\_developmental\_process | TAOK2 | 455 | 2 | 0.656124 | -0.086087 | 231 | 314.31 | 1.360649 |
| GO:0050793\_regulation\_of\_developmental\_process | CENPF | 455 | 2 | 0.656124 | -0.086087 | 231 | 314.31 | 1.360649 |
| GO:0032502\_developmental\_process | TAOK2 | 1506 | 8 | 0.792926 | -0.082952 | 232 | 314.85 | 1.357112 |
| GO:0032502\_developmental\_process | MXD1 | 1506 | 8 | 0.792926 | -0.082952 | 232 | 314.85 | 1.357112 |
| GO:0032502\_developmental\_process | CENPF | 1506 | 8 | 0.792926 | -0.082952 | 232 | 314.85 | 1.357112 |
| GO:0032502\_developmental\_process | NUDC | 1506 | 8 | 0.792926 | -0.082952 | 232 | 314.85 | 1.357112 |
| GO:0032502\_developmental\_process | PAFAH1B1 | 1506 | 8 | 0.792926 | -0.082952 | 232 | 314.85 | 1.357112 |
| GO:0032502\_developmental\_process | CENPI | 1506 | 8 | 0.792926 | -0.082952 | 232 | 314.85 | 1.357112 |
| GO:0032502\_developmental\_process | CLASP1 | 1506 | 8 | 0.792926 | -0.082952 | 232 | 314.85 | 1.357112 |
| GO:0032502\_developmental\_process | CLASP2 | 1506 | 8 | 0.792926 | -0.082952 | 232 | 314.85 | 1.357112 |
| GO:0009056\_catabolic\_process | CDC20 | 257 | 1 | 0.580810 | -0.081559 | 233 | 315.2 | 1.352790 |
| GO:0006793\_phosphorus\_metabolic\_process | TAOK2 | 466 | 2 | 0.640636 | -0.080572 | 235 | 315.78 | 1.343745 |
| GO:0006793\_phosphorus\_metabolic\_process | PPP2R4 | 466 | 2 | 0.640636 | -0.080572 | 235 | 315.78 | 1.343745 |
| GO:0006796\_phosphate\_metabolic\_process | TAOK2 | 466 | 2 | 0.640636 | -0.080572 | 235 | 315.78 | 1.343745 |
| GO:0006796\_phosphate\_metabolic\_process | PPP2R4 | 466 | 2 | 0.640636 | -0.080572 | 235 | 315.78 | 1.343745 |
| GO:0042127\_regulation\_of\_cell\_proliferation | MXI1 | 279 | 1 | 0.535012 | -0.068889 | 236 | 320.57 | 1.358347 |
| GO:0007243\_protein\_kinase\_cascade | TAOK2 | 294 | 1 | 0.507715 | -0.061459 | 237 | 322.39 | 1.360295 |
| GO:0042221\_response\_to\_chemical\_stimulus | CENPF | 298 | 1 | 0.500900 | -0.059623 | 238 | 323.26 | 1.358235 |
| GO:0009059\_macromolecule\_biosynthetic\_process | RPS27 | 302 | 1 | 0.494266 | -0.057845 | 239 | 324.0 | 1.355649 |
| GO:0043687\_post-translational\_protein\_modification | TAOK2 | 529 | 2 | 0.564341 | -0.055027 | 240 | 324.87 | 1.353625 |
| GO:0043687\_post-translational\_protein\_modification | PPP2R4 | 529 | 2 | 0.564341 | -0.055027 | 240 | 324.87 | 1.353625 |
| GO:0006468\_protein\_amino\_acid\_phosphorylation | TAOK2 | 316 | 1 | 0.472368 | -0.052045 | 241 | 325.7 | 1.351452 |
| GO:0044249\_cellular\_biosynthetic\_process | RPS27 | 325 | 1 | 0.459287 | -0.048639 | 242 | 327.02 | 1.351322 |
| GO:0007275\_multicellular\_organismal\_development | MXD1 | 975 | 4 | 0.612383 | -0.040622 | 243 | 329.71 | 1.356831 |
| GO:0007275\_multicellular\_organismal\_development | CENPF | 975 | 4 | 0.612383 | -0.040622 | 243 | 329.71 | 1.356831 |
| GO:0007275\_multicellular\_organismal\_development | NUDC | 975 | 4 | 0.612383 | -0.040622 | 243 | 329.71 | 1.356831 |
| GO:0007275\_multicellular\_organismal\_development | PAFAH1B1 | 975 | 4 | 0.612383 | -0.040622 | 243 | 329.71 | 1.356831 |
| GO:0007399\_nervous\_system\_development | PAFAH1B1 | 350 | 1 | 0.426481 | -0.040332 | 244 | 329.99 | 1.352418 |
| GO:0016310\_phosphorylation | TAOK2 | 385 | 1 | 0.387710 | -0.031072 | 245 | 331.42 | 1.352735 |
| GO:0048522\_positive\_regulation\_of\_cellular\_process | TAOK2 | 634 | 2 | 0.470878 | -0.028803 | 246 | 331.8 | 1.348780 |
| GO:0048522\_positive\_regulation\_of\_cellular\_process | PPP2R4 | 634 | 2 | 0.470878 | -0.028803 | 246 | 331.8 | 1.348780 |
| GO:0065008\_regulation\_of\_biological\_quality | TAOK2 | 428 | 1 | 0.348758 | -0.022578 | 247 | 333.23 | 1.349109 |
| GO:0006915\_apoptosis | TAOK2 | 430 | 1 | 0.347136 | -0.022245 | 248 | 333.55 | 1.344960 |
| GO:0048518\_positive\_regulation\_of\_biological\_process | TAOK2 | 678 | 2 | 0.440319 | -0.021840 | 249 | 333.8 | 1.340562 |
| GO:0048518\_positive\_regulation\_of\_biological\_process | PPP2R4 | 678 | 2 | 0.440319 | -0.021840 | 249 | 333.8 | 1.340562 |
| GO:0012501\_programmed\_cell\_death | TAOK2 | 435 | 1 | 0.343146 | -0.021435 | 250 | 333.98 | 1.335920 |
| GO:0006464\_protein\_modification\_process | TAOK2 | 686 | 2 | 0.435185 | -0.020760 | 251 | 334.46 | 1.332510 |
| GO:0006464\_protein\_modification\_process | PPP2R4 | 686 | 2 | 0.435185 | -0.020760 | 251 | 334.46 | 1.332510 |
| GO:0008219\_cell\_death | TAOK2 | 444 | 1 | 0.336190 | -0.020051 | 253 | 334.89 | 1.323676 |
| GO:0016265\_death | TAOK2 | 444 | 1 | 0.336190 | -0.020051 | 253 | 334.89 | 1.323676 |
| GO:0043412\_biopolymer\_modification | TAOK2 | 708 | 2 | 0.421662 | -0.018047 | 254 | 335.69 | 1.321614 |
| GO:0043412\_biopolymer\_modification | PPP2R4 | 708 | 2 | 0.421662 | -0.018047 | 254 | 335.69 | 1.321614 |
| GO:0043170\_macromolecule\_metabolic\_process | CDC20 | 2397 | 11 | 0.685003 | -0.015183 | 255 | 336.37 | 1.319098 |
| GO:0043170\_macromolecule\_metabolic\_process | MIS12 | 2397 | 11 | 0.685003 | -0.015183 | 255 | 336.37 | 1.319098 |
| GO:0043170\_macromolecule\_metabolic\_process | MAPRE1 | 2397 | 11 | 0.685003 | -0.015183 | 255 | 336.37 | 1.319098 |
| GO:0043170\_macromolecule\_metabolic\_process | TAOK2 | 2397 | 11 | 0.685003 | -0.015183 | 255 | 336.37 | 1.319098 |
| GO:0043170\_macromolecule\_metabolic\_process | ZW10 | 2397 | 11 | 0.685003 | -0.015183 | 255 | 336.37 | 1.319098 |
| GO:0043170\_macromolecule\_metabolic\_process | CENPF | 2397 | 11 | 0.685003 | -0.015183 | 255 | 336.37 | 1.319098 |
| GO:0043170\_macromolecule\_metabolic\_process | CENPE | 2397 | 11 | 0.685003 | -0.015183 | 255 | 336.37 | 1.319098 |
| GO:0043170\_macromolecule\_metabolic\_process | CLASP1 | 2397 | 11 | 0.685003 | -0.015183 | 255 | 336.37 | 1.319098 |
| GO:0043170\_macromolecule\_metabolic\_process | CLASP2 | 2397 | 11 | 0.685003 | -0.015183 | 255 | 336.37 | 1.319098 |
| GO:0043170\_macromolecule\_metabolic\_process | PPP2R4 | 2397 | 11 | 0.685003 | -0.015183 | 255 | 336.37 | 1.319098 |
| GO:0043170\_macromolecule\_metabolic\_process | RPS27 | 2397 | 11 | 0.685003 | -0.015183 | 255 | 336.37 | 1.319098 |
| GO:0009058\_biosynthetic\_process | RPS27 | 506 | 1 | 0.294997 | -0.012653 | 256 | 336.8 | 1.315625 |
| GO:0048513\_organ\_development | CENPF | 520 | 1 | 0.287054 | -0.011401 | 257 | 337.01 | 1.311323 |
| GO:0048731\_system\_development | CENPF | 780 | 2 | 0.382739 | -0.011337 | 258 | 337.06 | 1.306434 |
| GO:0048731\_system\_development | PAFAH1B1 | 780 | 2 | 0.382739 | -0.011337 | 258 | 337.06 | 1.306434 |
| GO:0006950\_response\_to\_stress | TAOK2 | 524 | 1 | 0.284863 | -0.011066 | 259 | 337.18 | 1.301853 |
| GO:0007166\_cell\_surface\_receptor\_linked\_signal\_transduction | NUP85 | 540 | 1 | 0.276423 | -0.009823 | 260 | 337.44 | 1.297846 |
| GO:0048468\_cell\_development | TAOK2 | 575 | 1 | 0.259597 | -0.007562 | 261 | 337.86 | 1.294483 |
| GO:0045449\_regulation\_of\_transcription | CENPF | 681 | 1 | 0.219190 | -0.003401 | 262 | 338.73 | 1.292863 |
| GO:0032501\_multicellular\_organismal\_process | MXD1 | 1413 | 4 | 0.422557 | -0.003400 | 263 | 338.73 | 1.287947 |
| GO:0032501\_multicellular\_organismal\_process | CENPF | 1413 | 4 | 0.422557 | -0.003400 | 263 | 338.73 | 1.287947 |
| GO:0032501\_multicellular\_organismal\_process | NUDC | 1413 | 4 | 0.422557 | -0.003400 | 263 | 338.73 | 1.287947 |
| GO:0032501\_multicellular\_organismal\_process | PAFAH1B1 | 1413 | 4 | 0.422557 | -0.003400 | 263 | 338.73 | 1.287947 |
| GO:0030154\_cell\_differentiation | TAOK2 | 704 | 1 | 0.212029 | -0.002855 | 265 | 338.99 | 1.279208 |
| GO:0048869\_cellular\_developmental\_process | TAOK2 | 704 | 1 | 0.212029 | -0.002855 | 265 | 338.99 | 1.279208 |
| GO:0044238\_primary\_metabolic\_process | CDC20 | 2900 | 12 | 0.617662 | -0.002477 | 266 | 339.21 | 1.275226 |
| GO:0044238\_primary\_metabolic\_process | MIS12 | 2900 | 12 | 0.617662 | -0.002477 | 266 | 339.21 | 1.275226 |
| GO:0044238\_primary\_metabolic\_process | MAPRE1 | 2900 | 12 | 0.617662 | -0.002477 | 266 | 339.21 | 1.275226 |
| GO:0044238\_primary\_metabolic\_process | TAOK2 | 2900 | 12 | 0.617662 | -0.002477 | 266 | 339.21 | 1.275226 |
| GO:0044238\_primary\_metabolic\_process | ZW10 | 2900 | 12 | 0.617662 | -0.002477 | 266 | 339.21 | 1.275226 |
| GO:0044238\_primary\_metabolic\_process | CENPF | 2900 | 12 | 0.617662 | -0.002477 | 266 | 339.21 | 1.275226 |
| GO:0044238\_primary\_metabolic\_process | CENPE | 2900 | 12 | 0.617662 | -0.002477 | 266 | 339.21 | 1.275226 |
| GO:0044238\_primary\_metabolic\_process | IDUA | 2900 | 12 | 0.617662 | -0.002477 | 266 | 339.21 | 1.275226 |
| GO:0044238\_primary\_metabolic\_process | CLASP1 | 2900 | 12 | 0.617662 | -0.002477 | 266 | 339.21 | 1.275226 |
| GO:0044238\_primary\_metabolic\_process | CLASP2 | 2900 | 12 | 0.617662 | -0.002477 | 266 | 339.21 | 1.275226 |
| GO:0044238\_primary\_metabolic\_process | PPP2R4 | 2900 | 12 | 0.617662 | -0.002477 | 266 | 339.21 | 1.275226 |
| GO:0044238\_primary\_metabolic\_process | RPS27 | 2900 | 12 | 0.617662 | -0.002477 | 266 | 339.21 | 1.275226 |
| GO:0019219\_regulation\_of\_nucleobase\_\_nucleoside\_\_nucleotide\_and\_nucleic\_acid\_metabolic\_process | CENPF | 728 | 1 | 0.205039 | -0.002377 | 267 | 339.25 | 1.270599 |
| GO:0050896\_response\_to\_stimulus | TAOK2 | 1013 | 2 | 0.294705 | -0.002339 | 268 | 339.31 | 1.266082 |
| GO:0050896\_response\_to\_stimulus | CENPF | 1013 | 2 | 0.294705 | -0.002339 | 268 | 339.31 | 1.266082 |
| GO:0010468\_regulation\_of\_gene\_expression | CENPF | 750 | 1 | 0.199024 | -0.002008 | 269 | 339.36 | 1.261561 |
| GO:0006350\_transcription | CENPF | 845 | 1 | 0.176649 | -0.000962 | 270 | 339.45 | 1.257222 |
| GO:0044237\_cellular\_metabolic\_process | CDC20 | 2921 | 11 | 0.562120 | -0.000791 | 271 | 339.48 | 1.252694 |
| GO:0044237\_cellular\_metabolic\_process | MIS12 | 2921 | 11 | 0.562120 | -0.000791 | 271 | 339.48 | 1.252694 |
| GO:0044237\_cellular\_metabolic\_process | MAPRE1 | 2921 | 11 | 0.562120 | -0.000791 | 271 | 339.48 | 1.252694 |
| GO:0044237\_cellular\_metabolic\_process | TAOK2 | 2921 | 11 | 0.562120 | -0.000791 | 271 | 339.48 | 1.252694 |
| GO:0044237\_cellular\_metabolic\_process | CENPF | 2921 | 11 | 0.562120 | -0.000791 | 271 | 339.48 | 1.252694 |
| GO:0044237\_cellular\_metabolic\_process | CENPE | 2921 | 11 | 0.562120 | -0.000791 | 271 | 339.48 | 1.252694 |
| GO:0044237\_cellular\_metabolic\_process | IDUA | 2921 | 11 | 0.562120 | -0.000791 | 271 | 339.48 | 1.252694 |
| GO:0044237\_cellular\_metabolic\_process | CLASP1 | 2921 | 11 | 0.562120 | -0.000791 | 271 | 339.48 | 1.252694 |
| GO:0044237\_cellular\_metabolic\_process | CLASP2 | 2921 | 11 | 0.562120 | -0.000791 | 271 | 339.48 | 1.252694 |
| GO:0044237\_cellular\_metabolic\_process | PPP2R4 | 2921 | 11 | 0.562120 | -0.000791 | 271 | 339.48 | 1.252694 |
| GO:0044237\_cellular\_metabolic\_process | RPS27 | 2921 | 11 | 0.562120 | -0.000791 | 271 | 339.48 | 1.252694 |
| GO:0010467\_gene\_expression | CENPF | 1201 | 2 | 0.248573 | -0.000601 | 272 | 339.53 | 1.248272 |
| GO:0010467\_gene\_expression | RPS27 | 1201 | 2 | 0.248573 | -0.000601 | 272 | 339.53 | 1.248272 |
| GO:0008152\_metabolic\_process | CDC20 | 3126 | 12 | 0.573007 | -0.000586 | 273 | 339.55 | 1.243773 |
| GO:0008152\_metabolic\_process | MIS12 | 3126 | 12 | 0.573007 | -0.000586 | 273 | 339.55 | 1.243773 |
| GO:0008152\_metabolic\_process | TAOK2 | 3126 | 12 | 0.573007 | -0.000586 | 273 | 339.55 | 1.243773 |
| GO:0008152\_metabolic\_process | MAPRE1 | 3126 | 12 | 0.573007 | -0.000586 | 273 | 339.55 | 1.243773 |
| GO:0008152\_metabolic\_process | ZW10 | 3126 | 12 | 0.573007 | -0.000586 | 273 | 339.55 | 1.243773 |
| GO:0008152\_metabolic\_process | CENPF | 3126 | 12 | 0.573007 | -0.000586 | 273 | 339.55 | 1.243773 |
| GO:0008152\_metabolic\_process | CENPE | 3126 | 12 | 0.573007 | -0.000586 | 273 | 339.55 | 1.243773 |
| GO:0008152\_metabolic\_process | IDUA | 3126 | 12 | 0.573007 | -0.000586 | 273 | 339.55 | 1.243773 |
| GO:0008152\_metabolic\_process | CLASP1 | 3126 | 12 | 0.573007 | -0.000586 | 273 | 339.55 | 1.243773 |
| GO:0008152\_metabolic\_process | CLASP2 | 3126 | 12 | 0.573007 | -0.000586 | 273 | 339.55 | 1.243773 |
| GO:0008152\_metabolic\_process | PPP2R4 | 3126 | 12 | 0.573007 | -0.000586 | 273 | 339.55 | 1.243773 |
| GO:0008152\_metabolic\_process | RPS27 | 3126 | 12 | 0.573007 | -0.000586 | 273 | 339.55 | 1.243773 |
| GO:0043283\_biopolymer\_metabolic\_process | CDC20 | 1839 | 3 | 0.243505 | -0.000031 | 274 | 339.64 | 1.239562 |
| GO:0043283\_biopolymer\_metabolic\_process | TAOK2 | 1839 | 3 | 0.243505 | -0.000031 | 274 | 339.64 | 1.239562 |
| GO:0043283\_biopolymer\_metabolic\_process | PPP2R4 | 1839 | 3 | 0.243505 | -0.000031 | 274 | 339.64 | 1.239562 |
| GO:0006139\_nucleobase\_\_nucleoside\_\_nucleotide\_and\_nucleic\_acid\_metabolic\_process | CENPF | 1365 | 1 | 0.109354 | -0.000013 | 275 | 339.64 | 1.235055 |
| GO:0008150\_biological\_process | MIS12 | 6120 | 41 | 1.000000 | 0.000000 | 545 | 583.65 | 1.070917 |
| GO:0008150\_biological\_process | TAOK2 | 6120 | 41 | 1.000000 | 0.000000 | 545 | 583.65 | 1.070917 |
| GO:0008150\_biological\_process | ZW10 | 6120 | 41 | 1.000000 | 0.000000 | 545 | 583.65 | 1.070917 |
| GO:0008150\_biological\_process | ITGB3BP | 6120 | 41 | 1.000000 | 0.000000 | 545 | 583.65 | 1.070917 |
| GO:0008150\_biological\_process | CENPE | 6120 | 41 | 1.000000 | 0.000000 | 545 | 583.65 | 1.070917 |
| GO:0008150\_biological\_process | MAD2L1 | 6120 | 41 | 1.000000 | 0.000000 | 545 | 583.65 | 1.070917 |
| GO:0008150\_biological\_process | CLIP1 | 6120 | 41 | 1.000000 | 0.000000 | 545 | 583.65 | 1.070917 |
| GO:0008150\_biological\_process | CKAP5 | 6120 | 41 | 1.000000 | 0.000000 | 545 | 583.65 | 1.070917 |
| GO:0008150\_biological\_process | NUP85 | 6120 | 41 | 1.000000 | 0.000000 | 545 | 583.65 | 1.070917 |
| GO:0008150\_biological\_process | NDC80 | 6120 | 41 | 1.000000 | 0.000000 | 545 | 583.65 | 1.070917 |
| GO:0008150\_biological\_process | INCENP | 6120 | 41 | 1.000000 | 0.000000 | 545 | 583.65 | 1.070917 |
| GO:0008150\_biological\_process | MAPRE1 | 6120 | 41 | 1.000000 | 0.000000 | 545 | 583.65 | 1.070917 |
| GO:0008150\_biological\_process | ZWILCH | 6120 | 41 | 1.000000 | 0.000000 | 545 | 583.65 | 1.070917 |
| GO:0008150\_biological\_process | RANBP2 | 6120 | 41 | 1.000000 | 0.000000 | 545 | 583.65 | 1.070917 |
| GO:0008150\_biological\_process | MXD1 | 6120 | 41 | 1.000000 | 0.000000 | 545 | 583.65 | 1.070917 |
| GO:0008150\_biological\_process | CENPH | 6120 | 41 | 1.000000 | 0.000000 | 545 | 583.65 | 1.070917 |
| GO:0008150\_biological\_process | CENPI | 6120 | 41 | 1.000000 | 0.000000 | 545 | 583.65 | 1.070917 |
| GO:0008150\_biological\_process | PAFAH1B1 | 6120 | 41 | 1.000000 | 0.000000 | 545 | 583.65 | 1.070917 |
| GO:0008150\_biological\_process | IDUA | 6120 | 41 | 1.000000 | 0.000000 | 545 | 583.65 | 1.070917 |
| GO:0008150\_biological\_process | CLASP1 | 6120 | 41 | 1.000000 | 0.000000 | 545 | 583.65 | 1.070917 |
| GO:0008150\_biological\_process | KIF2C | 6120 | 41 | 1.000000 | 0.000000 | 545 | 583.65 | 1.070917 |
| GO:0008150\_biological\_process | MXI1 | 6120 | 41 | 1.000000 | 0.000000 | 545 | 583.65 | 1.070917 |
| GO:0008150\_biological\_process | CDC20 | 6120 | 41 | 1.000000 | 0.000000 | 545 | 583.65 | 1.070917 |
| GO:0008150\_biological\_process | NUF2 | 6120 | 41 | 1.000000 | 0.000000 | 545 | 583.65 | 1.070917 |
| GO:0008150\_biological\_process | BUB3 | 6120 | 41 | 1.000000 | 0.000000 | 545 | 583.65 | 1.070917 |
| GO:0008150\_biological\_process | CENPF | 6120 | 41 | 1.000000 | 0.000000 | 545 | 583.65 | 1.070917 |
| GO:0008150\_biological\_process | NUP160 | 6120 | 41 | 1.000000 | 0.000000 | 545 | 583.65 | 1.070917 |
| GO:0008150\_biological\_process | NUDC | 6120 | 41 | 1.000000 | 0.000000 | 545 | 583.65 | 1.070917 |
| GO:0008150\_biological\_process | BUB1 | 6120 | 41 | 1.000000 | 0.000000 | 545 | 583.65 | 1.070917 |
| GO:0008150\_biological\_process | RANGAP1 | 6120 | 41 | 1.000000 | 0.000000 | 545 | 583.65 | 1.070917 |
| GO:0008150\_biological\_process | MAPRE2 | 6120 | 41 | 1.000000 | 0.000000 | 545 | 583.65 | 1.070917 |
| GO:0008150\_biological\_process | SEC13 | 6120 | 41 | 1.000000 | 0.000000 | 545 | 583.65 | 1.070917 |
| GO:0008150\_biological\_process | RPS27 | 6120 | 41 | 1.000000 | 0.000000 | 545 | 583.65 | 1.070917 |
| GO:0008150\_biological\_process | NDE1 | 6120 | 41 | 1.000000 | 0.000000 | 545 | 583.65 | 1.070917 |
| GO:0008150\_biological\_process | BUB1B | 6120 | 41 | 1.000000 | 0.000000 | 545 | 583.65 | 1.070917 |
| GO:0008150\_biological\_process | ZWINT | 6120 | 41 | 1.000000 | 0.000000 | 545 | 583.65 | 1.070917 |
| GO:0008150\_biological\_process | NUP107 | 6120 | 41 | 1.000000 | 0.000000 | 545 | 583.65 | 1.070917 |
| GO:0008150\_biological\_process | NUP133 | 6120 | 41 | 1.000000 | 0.000000 | 545 | 583.65 | 1.070917 |
| GO:0008150\_biological\_process | CLASP2 | 6120 | 41 | 1.000000 | 0.000000 | 545 | 583.65 | 1.070917 |
| GO:0008150\_biological\_process | PPP2R4 | 6120 | 41 | 1.000000 | 0.000000 | 545 | 583.65 | 1.070917 |
| GO:0008150\_biological\_process | PLK1 | 6120 | 41 | 1.000000 | 0.000000 | 545 | 583.65 | 1.070917 |
