# Supplementary material for: RedundancyMiner: De-replication of redundant GO categories in microarray and proteomics analysis
Source: BMC Bioinformatics. 2011 Feb 10;12:52. doi: 10.1186/1471-2105-12-52 (PMC3223614; doi:10.1186/1471-2105-12-52)
Supplement: Additional file 10 — Kinetochore genes HTGM download. compressed package of the results of running HTGM on the kinetochore genes list. [file 1471-2105-12-52-S10.ZIP › work405493610/total.txt405493610.dir/kinetochore.txt.dir/kinetochore.txt.change.html]

Category Summary Report for kinetochore.txt

# Category Summary Report for kinetochore.txt

| HYPERLINKED GO CATEGORY | TOTAL GENES | CHANGED GENES | ENRICHMENT | LOG10(p) | CUMULATIVE NUMBER OF CATEGORIES | CUMULATIVE RANDOMS LOWER BOUND | CUMULATIVE RANDOMS MEAN | CUMULATIVE RANDOMS UPPER BOUND | FALSE DISCOVERY RATE |
| --- | --- | --- | --- | --- | --- | --- | --- | --- | --- |
| GO:0000226\_microtubule\_cytoskeleton\_organization\_and\_biogenesis | 58 | 10 | 25.735913 | -11.634038 | 1 | 0.000000 | 0.0 | 0.000000 | 0.000000 |
| GO:0000278\_mitotic\_cell\_cycle | 183 | 14 | 11.419432 | -11.315199 | 2 | 0.000000 | 0.0 | 0.000000 | 0.000000 |
| GO:0007049\_cell\_cycle | 349 | 17 | 7.270948 | -10.678022 | 3 | 0.000000 | 0.0 | 0.000000 | 0.000000 |
| GO:0007059\_chromosome\_segregation | 38 | 8 | 31.424904 | -10.079699 | 4 | 0.000000 | 0.0 | 0.000000 | 0.000000 |
| GO:0000087\_M\_phase\_of\_mitotic\_cell\_cycle | 91 | 10 | 16.403109 | -9.610265 | 6 | 0.000000 | 0.0 | 0.000000 | 0.000000 |
| GO:0007017\_microtubule-based\_process | 91 | 10 | 16.403109 | -9.610265 | 6 | 0.000000 | 0.0 | 0.000000 | 0.000000 |
| GO:0022402\_cell\_cycle\_process | 245 | 14 | 8.529617 | -9.598482 | 7 | 0.000000 | 0.0 | 0.000000 | 0.000000 |
| GO:0007093\_mitotic\_cell\_cycle\_checkpoint | 28 | 7 | 37.317073 | -9.422662 | 8 | 0.000000 | 0.0 | 0.000000 | 0.000000 |
| GO:0051383\_kinetochore\_organization\_and\_biogenesis | 4 | 4 |  |  |  |  |  |  |  |  |
| GO:0007067\_mitosis | 88 | 9 | 15.266075 | -8.379757 | 9 | 0.000000 | 0.0 | 0.000000 | 0.000000 |
| GO:0000279\_M\_phase | 125 | 10 | 11.941463 | -8.238396 | 10 | 0.000000 | 0.0 | 0.000000 | 0.000000 |
| GO:0022403\_cell\_cycle\_phase | 187 | 11 | 8.780488 | -7.639150 | 11 | 0.000000 | 0.0 | 0.000000 | 0.000000 |
| GO:0007346\_regulation\_of\_mitotic\_cell\_cycle | 50 | 7 | 20.897561 | -7.543240 | 12 | 0.000000 | 0.0 | 0.000000 | 0.000000 |
| GO:0000075\_cell\_cycle\_checkpoint | 52 | 7 | 20.093809 | -7.420582 | 13 | 0.000000 | 0.0 | 0.000000 | 0.000000 |
| GO:0051382\_kinetochore\_assembly | 3 | 3 |  |  |  |  |  |  |  |  |
| GO:0006996\_organelle\_organization\_and\_biogenesis | 571 | 16 | 4.182649 | -6.521159 | 14 | 0.000000 | 0.0 | 0.000000 | 0.000000 |
| GO:0007010\_cytoskeleton\_organization\_and\_biogenesis | 253 | 11 | 6.489926 | -6.293175 | 15 | 0.000000 | 0.0 | 0.000000 | 0.000000 |
| GO:0016043\_cellular\_component\_organization\_and\_biogenesis | 1240 | 22 | 2.648308 | -5.673135 | 16 | 0.000000 | 0.0 | 0.000000 | 0.000000 |
| GO:0031111\_negative\_regulation\_of\_microtubule\_polymerization\_or\_depolymerization | 6 | 3 | 74.634146 | -5.259186 | 17 | 0.000000 | 0.0 | 0.000000 | 0.000000 |
| GO:0051649\_establishment\_of\_cellular\_localization | 410 | 12 | 4.368828 | -5.034276 | 18 | 0.000000 | 0.0 | 0.000000 | 0.000000 |
| GO:0000070\_mitotic\_sister\_chromatid\_segregation | 21 | 4 | 28.432056 | -5.019623 | 19 | 0.000000 | 0.0 | 0.000000 | 0.000000 |
| GO:0000819\_sister\_chromatid\_segregation | 22 | 4 | 27.139690 | -4.934577 | 20 | 0.000000 | 0.0 | 0.000000 | 0.000000 |
| GO:0051641\_cellular\_localization | 429 | 12 | 4.175337 | -4.833159 | 21 | 0.000000 | 0.0 | 0.000000 | 0.000000 |
| GO:0031110\_regulation\_of\_microtubule\_polymerization\_or\_depolymerization | 9 | 3 | 49.756098 | -4.642017 | 22 | 0.000000 | 0.0 | 0.000000 | 0.000000 |
| GO:0050657\_nucleic\_acid\_transport | 26 | 4 | 22.964353 | -4.632567 | 25 | 0.000000 | 0.0 | 0.000000 | 0.000000 |
| GO:0050658\_RNA\_transport | 26 | 4 | 22.964353 | -4.632567 | 25 | 0.000000 | 0.0 | 0.000000 | 0.000000 |
| GO:0051236\_establishment\_of\_RNA\_localization | 26 | 4 | 22.964353 | -4.632567 | 25 | 0.000000 | 0.0 | 0.000000 | 0.000000 |
| GO:0006403\_RNA\_localization | 27 | 4 | 22.113821 | -4.565035 | 26 | 0.000000 | 0.0 | 0.000000 | 0.000000 |
| GO:0008283\_cell\_proliferation | 475 | 12 | 3.770988 | -4.388387 | 27 | 0.000000 | 0.0 | 0.000000 | 0.000000 |
| GO:0030951\_establishment\_and\_or\_maintenance\_of\_microtubule\_cytoskeleton\_polarity | 2 | 2 |  |  |  |  |  |  |  |  |
| GO:0030952\_establishment\_and\_or\_maintenance\_of\_cytoskeleton\_polarity | 2 | 2 |  |  |  |  |  |  |  |  |
| GO:0031109\_microtubule\_polymerization\_or\_depolymerization | 11 | 3 | 40.709534 | -4.352864 | 29 | 0.000000 | 0.0 | 0.000000 | 0.000000 |
| GO:0032886\_regulation\_of\_microtubule-based\_process | 11 | 3 | 40.709534 | -4.352864 | 29 | 0.000000 | 0.0 | 0.000000 | 0.000000 |
| GO:0051726\_regulation\_of\_cell\_cycle | 156 | 7 | 6.697936 | -4.178528 | 30 | -0.141447 | 0.03 | 0.201447 | 0.001000 |
| GO:0015931\_nucleobase\_\_nucleoside\_\_nucleotide\_and\_nucleic\_acid\_transport | 34 | 4 | 17.560976 | -4.157740 | 31 | -0.141447 | 0.03 | 0.201447 | 0.000968 |
| GO:0051494\_negative\_regulation\_of\_cytoskeleton\_organization\_and\_biogenesis | 13 | 3 | 34.446529 | -4.118032 | 32 | -0.156946 | 0.04 | 0.236946 | 0.001250 |
| GO:0032269\_negative\_regulation\_of\_cellular\_protein\_metabolic\_process | 37 | 4 | 16.137113 | -4.010501 | 33 | -0.169043 | 0.05 | 0.269043 | 0.001515 |
| GO:0051248\_negative\_regulation\_of\_protein\_metabolic\_process | 40 | 4 | 14.926829 | -3.875747 | 34 | -0.169043 | 0.05 | 0.269043 | 0.001471 |
| GO:0051276\_chromosome\_organization\_and\_biogenesis | 175 | 7 | 5.970732 | -3.863256 | 35 | -0.169043 | 0.05 | 0.269043 | 0.001429 |
| GO:0006406\_mRNA\_export\_from\_nucleus | 19 | 3 | 23.568678 | -3.600215 | 38 | -0.362217 | 0.08 | 0.522217 | 0.002105 |
| GO:0007051\_spindle\_organization\_and\_biogenesis | 19 | 3 | 23.568678 | -3.600215 | 38 | -0.362217 | 0.08 | 0.522217 | 0.002105 |
| GO:0051028\_mRNA\_transport | 19 | 3 | 23.568678 | -3.600215 | 38 | -0.362217 | 0.08 | 0.522217 | 0.002105 |
| GO:0051310\_metaphase\_plate\_congression | 4 | 2 |  |  |  |  |  |  |  |  |
| GO:0051656\_establishment\_of\_organelle\_localization | 20 | 3 | 22.390244 | -3.531656 | 39 | -0.527646 | 0.1 | 0.727646 | 0.002564 |
| GO:0033036\_macromolecule\_localization | 267 | 8 | 4.472458 | -3.492545 | 40 | -0.699953 | 0.12 | 0.939953 | 0.003000 |
| GO:0006913\_nucleocytoplasmic\_transport | 92 | 5 | 8.112407 | -3.473158 | 41 | -0.699953 | 0.12 | 0.939953 | 0.002927 |
| GO:0051169\_nuclear\_transport | 93 | 5 | 8.025177 | -3.451291 | 42 | -0.699953 | 0.12 | 0.939953 | 0.002857 |
| GO:0007026\_negative\_regulation\_of\_microtubule\_depolymerization | 5 | 2 | 59.707317 | -3.364134 | 44 | -0.851105 | 0.23 | 1.311105 | 0.005227 |
| GO:0031114\_regulation\_of\_microtubule\_depolymerization | 5 | 2 | 59.707317 | -3.364134 | 44 | -0.851105 | 0.23 | 1.311105 | 0.005227 |
| GO:0006405\_RNA\_export\_from\_nucleus | 24 | 3 | 18.658537 | -3.290435 | 45 | -1.007704 | 0.28 | 1.567704 | 0.006222 |
| GO:0007019\_microtubule\_depolymerization | 6 | 2 | 49.756098 | -3.189891 | 49 | -0.965088 | 0.39 | 1.745088 | 0.007959 |
| GO:0007094\_mitotic\_cell\_cycle\_spindle\_assembly\_checkpoint | 6 | 2 | 49.756098 | -3.189891 | 49 | -0.965088 | 0.39 | 1.745088 | 0.007959 |
| GO:0050000\_chromosome\_localization | 6 | 2 | 49.756098 | -3.189891 | 49 | -0.965088 | 0.39 | 1.745088 | 0.007959 |
| GO:0051303\_establishment\_of\_chromosome\_localization | 6 | 2 | 49.756098 | -3.189891 | 49 | -0.965088 | 0.39 | 1.745088 | 0.007959 |
| GO:0051640\_organelle\_localization | 27 | 3 | 16.585366 | -3.136579 | 50 | -0.964470 | 0.43 | 1.824470 | 0.008600 |
| GO:0031577\_spindle\_checkpoint | 7 | 2 | 42.648084 | -3.045609 | 51 | -1.058105 | 0.54 | 2.138105 | 0.010588 |
| GO:0051129\_negative\_regulation\_of\_cellular\_component\_organization\_and\_biogenesis | 33 | 3 | 13.569845 | -2.877939 | 52 | -1.187349 | 0.72 | 2.627349 | 0.013846 |
| GO:0046907\_intracellular\_transport | 342 | 8 | 3.491656 | -2.782559 | 53 | -1.193656 | 0.84 | 2.873656 | 0.015849 |
| GO:0051168\_nuclear\_export | 36 | 3 | 12.439024 | -2.767164 | 54 | -1.262606 | 0.86 | 2.982606 | 0.015926 |
| GO:0051493\_regulation\_of\_cytoskeleton\_organization\_and\_biogenesis | 37 | 3 | 12.102835 | -2.732457 | 55 | -1.260372 | 0.87 | 3.000372 | 0.015818 |
| GO:0010458\_exit\_from\_mitosis | 10 | 2 | 29.853659 | -2.720151 | 57 | -1.284211 | 1.08 | 3.444211 | 0.018947 |
| GO:0051261\_protein\_depolymerization | 10 | 2 | 29.853659 | -2.720151 | 57 | -1.284211 | 1.08 | 3.444211 | 0.018947 |
| GO:0043623\_cellular\_protein\_complex\_assembly | 40 | 3 | 11.195122 | -2.634165 | 58 | -1.627364 | 1.29 | 4.207364 | 0.022241 |
| GO:0051297\_centrosome\_organization\_and\_biogenesis | 13 | 2 | 22.964353 | -2.486797 | 59 | -1.611974 | 1.73 | 5.071974 | 0.029322 |
| GO:0031023\_microtubule\_organizing\_center\_organization\_and\_biogenesis | 14 | 2 | 21.324042 | -2.421691 | 60 | -1.598149 | 1.92 | 5.438149 | 0.032000 |
| GO:0033043\_regulation\_of\_organelle\_organization\_and\_biogenesis | 49 | 3 | 9.138875 | -2.381648 | 61 | -1.587412 | 1.99 | 5.567412 | 0.032623 |
| GO:0051234\_establishment\_of\_localization | 902 | 13 | 2.151317 | -2.341718 | 62 | -1.693313 | 2.26 | 6.213313 | 0.036452 |
| GO:0048015\_phosphoinositide-mediated\_signaling | 52 | 3 | 8.611632 | -2.308668 | 63 | -1.666838 | 2.37 | 6.406838 | 0.037619 |
| GO:0000089\_mitotic\_metaphase | 1 | 1 |  |  |  |  |  |  |  |  |
| GO:0000132\_establishment\_of\_mitotic\_spindle\_orientation | 1 | 1 |  |  |  |  |  |  |  |  |
| GO:0005984\_disaccharide\_metabolic\_process | 1 | 1 |  |  |  |  |  |  |  |  |
| GO:0031115\_negative\_regulation\_of\_microtubule\_polymerization | 1 | 1 |  |  |  |  |  |  |  |  |
| GO:0032515\_negative\_regulation\_of\_phosphoprotein\_phosphatase\_activity | 1 | 1 |  |  |  |  |  |  |  |  |
| GO:0032516\_positive\_regulation\_of\_phosphoprotein\_phosphatase\_activity | 1 | 1 |  |  |  |  |  |  |  |  |
| GO:0035305\_negative\_regulation\_of\_dephosphorylation | 1 | 1 |  |  |  |  |  |  |  |  |
| GO:0035308\_negative\_regulation\_of\_protein\_amino\_acid\_dephosphorylation | 1 | 1 |  |  |  |  |  |  |  |  |
| GO:0051294\_establishment\_of\_spindle\_orientation | 1 | 1 |  |  |  |  |  |  |  |  |
| GO:0051323\_metaphase | 1 | 1 |  |  |  |  |  |  |  |  |
| GO:0007163\_establishment\_and\_or\_maintenance\_of\_cell\_polarity | 21 | 2 | 14.216028 | -2.071379 | 64 | -1.321499 | 3.99 | 9.301499 | 0.062344 |
| GO:0009987\_cellular\_process | 5174 | 40 | 1.153988 | -2.068409 | 65 | -1.321958 | 4.0 | 9.321958 | 0.061538 |
| GO:0051128\_regulation\_of\_cellular\_component\_organization\_and\_biogenesis | 124 | 4 | 4.815106 | -2.040267 | 66 | -1.297559 | 4.06 | 9.417559 | 0.061515 |
| GO:0032268\_regulation\_of\_cellular\_protein\_metabolic\_process | 134 | 4 | 4.455770 | -1.924778 | 67 | -0.928421 | 5.11 | 11.148421 | 0.076269 |
| GO:0007079\_mitotic\_chromosome\_movement\_towards\_spindle\_pole | 2 | 1 |  |  |  |  |  |  |  |  |
| GO:0032272\_negative\_regulation\_of\_protein\_polymerization | 2 | 1 |  |  |  |  |  |  |  |  |
| GO:0035304\_regulation\_of\_protein\_amino\_acid\_dephosphorylation | 2 | 1 |  |  |  |  |  |  |  |  |
| GO:0035306\_positive\_regulation\_of\_dephosphorylation | 2 | 1 |  |  |  |  |  |  |  |  |
| GO:0035307\_positive\_regulation\_of\_protein\_amino\_acid\_dephosphorylation | 2 | 1 |  |  |  |  |  |  |  |  |
| GO:0051300\_spindle\_pole\_body\_organization\_and\_biogenesis | 2 | 1 |  |  |  |  |  |  |  |  |
| GO:0051305\_chromosome\_movement\_towards\_spindle\_pole | 2 | 1 |  |  |  |  |  |  |  |  |
| GO:0051246\_regulation\_of\_protein\_metabolic\_process | 146 | 4 | 4.089542 | -1.799138 | 68 | -0.361655 | 6.6 | 13.561655 | 0.097059 |
| GO:0000186\_activation\_of\_MAPKK\_activity | 3 | 1 |  |  |  |  |  |  |  |  |
| GO:0007080\_mitotic\_metaphase\_plate\_congression | 3 | 1 |  |  |  |  |  |  |  |  |
| GO:0040001\_establishment\_of\_mitotic\_spindle\_localization | 3 | 1 |  |  |  |  |  |  |  |  |
| GO:0043666\_regulation\_of\_phosphoprotein\_phosphatase\_activity | 3 | 1 |  |  |  |  |  |  |  |  |
| GO:0051004\_regulation\_of\_lipoprotein\_lipase\_activity | 3 | 1 |  |  |  |  |  |  |  |  |
| GO:0051293\_establishment\_of\_spindle\_localization | 3 | 1 |  |  |  |  |  |  |  |  |
| GO:0051653\_spindle\_localization | 3 | 1 |  |  |  |  |  |  |  |  |
| GO:0051179\_localization | 1090 | 13 | 1.780264 | -1.660437 | 69 | 0.340302 | 8.76 | 17.179698 | 0.126957 |
| GO:0000085\_G2\_phase\_of\_mitotic\_cell\_cycle | 4 | 1 |  |  |  |  |  |  |  |  |
| GO:0001578\_microtubule\_bundle\_formation | 4 | 1 |  |  |  |  |  |  |  |  |
| GO:0031113\_regulation\_of\_microtubule\_polymerization | 4 | 1 |  |  |  |  |  |  |  |  |
| GO:0051319\_G2\_phase | 4 | 1 |  |  |  |  |  |  |  |  |
| GO:0006461\_protein\_complex\_assembly | 174 | 4 | 3.431455 | -1.549580 | 70 | 1.138495 | 10.67 | 20.201505 | 0.152429 |
| GO:0006886\_intracellular\_protein\_transport | 176 | 4 | 3.392461 | -1.533699 | 71 | 1.381728 | 11.12 | 20.858272 | 0.156620 |
| GO:0035303\_regulation\_of\_dephosphorylation | 5 | 1 | 29.853659 | -1.480676 | 73 | 6.561203 | 18.31 | 30.058797 | 0.250822 |
| GO:0046785\_microtubule\_polymerization | 5 | 1 | 29.853659 | -1.480676 | 73 | 6.561203 | 18.31 | 30.058797 | 0.250822 |
| GO:0031324\_negative\_regulation\_of\_cellular\_metabolic\_process | 273 | 5 | 2.733852 | -1.464328 | 74 | 6.655226 | 18.56 | 30.464774 | 0.250811 |
| GO:0009892\_negative\_regulation\_of\_metabolic\_process | 277 | 5 | 2.694374 | -1.440979 | 75 | 7.050103 | 19.12 | 31.189897 | 0.254933 |
| GO:0046330\_positive\_regulation\_of\_JNK\_cascade | 6 | 1 | 24.878049 | -1.402911 | 78 | 10.655008 | 24.8 | 38.944992 | 0.317949 |
| GO:0051298\_centrosome\_duplication | 6 | 1 | 24.878049 | -1.402911 | 78 | 10.655008 | 24.8 | 38.944992 | 0.317949 |
| GO:0060191\_regulation\_of\_lipase\_activity | 6 | 1 | 24.878049 | -1.402911 | 78 | 10.655008 | 24.8 | 38.944992 | 0.317949 |
| GO:0019932\_second-messenger-mediated\_signaling | 114 | 3 | 3.928113 | -1.395730 | 79 | 10.680958 | 24.89 | 39.099042 | 0.315063 |
| GO:0015031\_protein\_transport | 195 | 4 | 3.061914 | -1.393475 | 80 | 10.703314 | 24.94 | 39.176686 | 0.311750 |
| GO:0000902\_cell\_morphogenesis | 116 | 3 | 3.860387 | -1.376832 | 82 | 10.965394 | 25.26 | 39.554606 | 0.308049 |
| GO:0032989\_cellular\_structure\_morphogenesis | 116 | 3 | 3.860387 | -1.376832 | 82 | 10.965394 | 25.26 | 39.554606 | 0.308049 |
| GO:0006606\_protein\_import\_into\_nucleus | 50 | 2 | 5.970732 | -1.358349 | 83 | 11.362840 | 25.87 | 40.377160 | 0.311687 |
| GO:0051170\_nuclear\_import | 51 | 2 | 5.853659 | -1.342786 | 84 | 11.603294 | 26.22 | 40.836706 | 0.312143 |
| GO:0006605\_protein\_targeting | 126 | 3 | 3.554007 | -1.287968 | 85 | 15.238318 | 31.95 | 48.661682 | 0.375882 |
| GO:0045184\_establishment\_of\_protein\_localization | 216 | 4 | 2.764228 | -1.257829 | 86 | 18.718779 | 37.06 | 55.401221 | 0.430930 |
| GO:0007096\_regulation\_of\_exit\_from\_mitosis | 9 | 1 | 16.585366 | -1.231060 | 90 | 21.516425 | 41.34 | 61.163575 | 0.459333 |
| GO:0016202\_regulation\_of\_striated\_muscle\_development | 9 | 1 | 16.585366 | -1.231060 | 90 | 21.516425 | 41.34 | 61.163575 | 0.459333 |
| GO:0042994\_cytoplasmic\_sequestering\_of\_transcription\_factor | 9 | 1 | 16.585366 | -1.231060 | 90 | 21.516425 | 41.34 | 61.163575 | 0.459333 |
| GO:0048634\_regulation\_of\_muscle\_development | 9 | 1 | 16.585366 | -1.231060 | 90 | 21.516425 | 41.34 | 61.163575 | 0.459333 |
| GO:0007098\_centrosome\_cycle | 10 | 1 | 14.926829 | -1.186713 | 94 | 26.546693 | 48.21 | 69.873307 | 0.512872 |
| GO:0042308\_negative\_regulation\_of\_protein\_import\_into\_nucleus | 10 | 1 | 14.926829 | -1.186713 | 94 | 26.546693 | 48.21 | 69.873307 | 0.512872 |
| GO:0042992\_negative\_regulation\_of\_transcription\_factor\_import\_into\_nucleus | 10 | 1 | 14.926829 | -1.186713 | 94 | 26.546693 | 48.21 | 69.873307 | 0.512872 |
| GO:0051220\_cytoplasmic\_sequestering\_of\_protein | 10 | 1 | 14.926829 | -1.186713 | 94 | 26.546693 | 48.21 | 69.873307 | 0.512872 |
| GO:0046823\_negative\_regulation\_of\_nucleocytoplasmic\_transport | 11 | 1 | 13.569845 | -1.146730 | 96 | 29.754152 | 52.8 | 75.845848 | 0.550000 |
| GO:0048041\_focal\_adhesion\_formation | 11 | 1 | 13.569845 | -1.146730 | 96 | 29.754152 | 52.8 | 75.845848 | 0.550000 |
| GO:0017038\_protein\_import | 67 | 2 | 4.455770 | -1.132578 | 97 | 30.380150 | 53.64 | 76.899850 | 0.552990 |
| GO:0008104\_protein\_localization | 242 | 4 | 2.467245 | -1.112552 | 98 | 30.612903 | 54.01 | 77.407097 | 0.551122 |
| GO:0031400\_negative\_regulation\_of\_protein\_modification\_process | 12 | 1 | 12.439024 | -1.110349 | 102 | 34.332141 | 58.99 | 83.647859 | 0.578333 |
| GO:0032271\_regulation\_of\_protein\_polymerization | 12 | 1 | 12.439024 | -1.110349 | 102 | 34.332141 | 58.99 | 83.647859 | 0.578333 |
| GO:0046328\_regulation\_of\_JNK\_cascade | 12 | 1 | 12.439024 | -1.110349 | 102 | 34.332141 | 58.99 | 83.647859 | 0.578333 |
| GO:0051224\_negative\_regulation\_of\_protein\_transport | 12 | 1 | 12.439024 | -1.110349 | 102 | 34.332141 | 58.99 | 83.647859 | 0.578333 |
| GO:0006612\_protein\_targeting\_to\_membrane | 13 | 1 | 11.482176 | -1.076993 | 106 | 38.478376 | 64.35 | 90.221624 | 0.607075 |
| GO:0032387\_negative\_regulation\_of\_intracellular\_transport | 13 | 1 | 11.482176 | -1.076993 | 106 | 38.478376 | 64.35 | 90.221624 | 0.607075 |
| GO:0043410\_positive\_regulation\_of\_MAPKKK\_cascade | 13 | 1 | 11.482176 | -1.076993 | 106 | 38.478376 | 64.35 | 90.221624 | 0.607075 |
| GO:0051346\_negative\_regulation\_of\_hydrolase\_activity | 13 | 1 | 11.482176 | -1.076993 | 106 | 38.478376 | 64.35 | 90.221624 | 0.607075 |
| GO:0010563\_negative\_regulation\_of\_phosphorus\_metabolic\_process | 15 | 1 | 9.951220 | -1.017654 | 108 | 43.397425 | 71.71 | 100.022575 | 0.663981 |
| GO:0045936\_negative\_regulation\_of\_phosphate\_metabolic\_process | 15 | 1 | 9.951220 | -1.017654 | 108 | 43.397425 | 71.71 | 100.022575 | 0.663981 |
| GO:0008360\_regulation\_of\_cell\_shape | 16 | 1 | 9.329268 | -0.991027 | 109 | 47.203751 | 77.03 | 106.856249 | 0.706697 |
| GO:0006810\_transport | 844 | 9 | 1.591724 | -0.989819 | 110 | 47.225162 | 77.06 | 106.894838 | 0.700545 |
| GO:0022604\_regulation\_of\_cell\_morphogenesis | 17 | 1 | 8.780488 | -0.966099 | 111 | 49.911910 | 80.73 | 111.548090 | 0.727297 |
| GO:0042990\_regulation\_of\_transcription\_factor\_import\_into\_nucleus | 18 | 1 | 8.292683 | -0.942675 | 113 | 53.102862 | 84.89 | 116.677138 | 0.751239 |
| GO:0042991\_transcription\_factor\_import\_into\_nucleus | 18 | 1 | 8.292683 | -0.942675 | 113 | 53.102862 | 84.89 | 116.677138 | 0.751239 |
| GO:0032507\_maintenance\_of\_cellular\_protein\_localization | 19 | 1 | 7.856226 | -0.920592 | 116 | 56.364776 | 89.31 | 122.255224 | 0.769914 |
| GO:0042306\_regulation\_of\_protein\_import\_into\_nucleus | 19 | 1 | 7.856226 | -0.920592 | 116 | 56.364776 | 89.31 | 122.255224 | 0.769914 |
| GO:0051258\_protein\_polymerization | 19 | 1 | 7.856226 | -0.920592 | 116 | 56.364776 | 89.31 | 122.255224 | 0.769914 |
| GO:0051651\_maintenance\_of\_cellular\_localization | 20 | 1 | 7.463415 | -0.899712 | 117 | 59.814739 | 94.17 | 128.525261 | 0.804872 |
| GO:0065003\_macromolecular\_complex\_assembly | 293 | 4 | 2.037792 | -0.882701 | 118 | 60.619969 | 95.26 | 129.900031 | 0.807288 |
| GO:0045185\_maintenance\_of\_protein\_localization | 21 | 1 | 7.108014 | -0.879918 | 119 | 62.535196 | 98.3 | 134.064804 | 0.826050 |
| GO:0032880\_regulation\_of\_protein\_localization | 22 | 1 | 6.784922 | -0.861108 | 122 | 64.724484 | 101.54 | 138.355516 | 0.832295 |
| GO:0033157\_regulation\_of\_intracellular\_protein\_transport | 22 | 1 | 6.784922 | -0.861108 | 122 | 64.724484 | 101.54 | 138.355516 | 0.832295 |
| GO:0043408\_regulation\_of\_MAPKKK\_cascade | 22 | 1 | 6.784922 | -0.861108 | 122 | 64.724484 | 101.54 | 138.355516 | 0.832295 |
| GO:0006888\_ER\_to\_Golgi\_vesicle-mediated\_transport | 23 | 1 | 6.489926 | -0.843195 | 125 | 67.548059 | 105.52 | 143.491941 | 0.844160 |
| GO:0042493\_response\_to\_drug | 23 | 1 | 6.489926 | -0.843195 | 125 | 67.548059 | 105.52 | 143.491941 | 0.844160 |
| GO:0051223\_regulation\_of\_protein\_transport | 23 | 1 | 6.489926 | -0.843195 | 125 | 67.548059 | 105.52 | 143.491941 | 0.844160 |
| GO:0051051\_negative\_regulation\_of\_transport | 24 | 1 | 6.219512 | -0.826102 | 126 | 69.651810 | 108.54 | 147.428190 | 0.861429 |
| GO:0022607\_cellular\_component\_assembly | 310 | 4 | 1.926042 | -0.818773 | 127 | 70.215188 | 109.26 | 148.304812 | 0.860315 |
| GO:0000910\_cytokinesis | 26 | 1 | 5.741088 | -0.794118 | 129 | 74.663651 | 114.93 | 155.196349 | 0.890930 |
| GO:0046822\_regulation\_of\_nucleocytoplasmic\_transport | 26 | 1 | 5.741088 | -0.794118 | 129 | 74.663651 | 114.93 | 155.196349 | 0.890930 |
| GO:0019221\_cytokine\_and\_chemokine\_mediated\_signaling\_pathway | 28 | 1 | 5.331010 | -0.764705 | 131 | 78.788775 | 120.3 | 161.811225 | 0.918321 |
| GO:0022603\_regulation\_of\_anatomical\_structure\_morphogenesis | 28 | 1 | 5.331010 | -0.764705 | 131 | 78.788775 | 120.3 | 161.811225 | 0.918321 |
| GO:0007088\_regulation\_of\_mitosis | 31 | 1 | 4.815106 | -0.724648 | 137 | 85.970939 | 129.83 | 173.689061 | 0.947664 |
| GO:0010562\_positive\_regulation\_of\_phosphorus\_metabolic\_process | 31 | 1 | 4.815106 | -0.724648 | 137 | 85.970939 | 129.83 | 173.689061 | 0.947664 |
| GO:0032386\_regulation\_of\_intracellular\_transport | 31 | 1 | 4.815106 | -0.724648 | 137 | 85.970939 | 129.83 | 173.689061 | 0.947664 |
| GO:0045937\_positive\_regulation\_of\_phosphate\_metabolic\_process | 31 | 1 | 4.815106 | -0.724648 | 137 | 85.970939 | 129.83 | 173.689061 | 0.947664 |
| GO:0051235\_maintenance\_of\_localization | 31 | 1 | 4.815106 | -0.724648 | 137 | 85.970939 | 129.83 | 173.689061 | 0.947664 |
| GO:0051301\_cell\_division | 31 | 1 | 4.815106 | -0.724648 | 137 | 85.970939 | 129.83 | 173.689061 | 0.947664 |
| GO:0007548\_sex\_differentiation | 33 | 1 | 4.523282 | -0.700253 | 138 | 90.975693 | 136.38 | 181.784307 | 0.988261 |
| GO:0003006\_reproductive\_developmental\_process | 34 | 1 | 4.390244 | -0.688665 | 140 | 93.392566 | 139.38 | 185.367434 | 0.995571 |
| GO:0032147\_activation\_of\_protein\_kinase\_activity | 34 | 1 | 4.390244 | -0.688665 | 140 | 93.392566 | 139.38 | 185.367434 | 0.995571 |
| GO:0014706\_striated\_muscle\_development | 35 | 1 | 4.264808 | -0.677451 | 142 | 94.614653 | 140.9 | 187.185347 | 0.992254 |
| GO:0031401\_positive\_regulation\_of\_protein\_modification\_process | 35 | 1 | 4.264808 | -0.677451 | 142 | 94.614653 | 140.9 | 187.185347 | 0.992254 |
| GO:0007126\_meiosis | 36 | 1 | 4.146341 | -0.666590 | 144 | 97.773627 | 144.82 | 191.866373 | 1.005694 |
| GO:0051327\_M\_phase\_of\_meiotic\_cell\_cycle | 36 | 1 | 4.146341 | -0.666590 | 144 | 97.773627 | 144.82 | 191.866373 | 1.005694 |
| GO:0051321\_meiotic\_cell\_cycle | 37 | 1 | 4.034278 | -0.656063 | 145 | 100.037351 | 147.59 | 195.142649 | 1.017862 |
| GO:0048523\_negative\_regulation\_of\_cellular\_process | 621 | 6 | 1.442206 | -0.634093 | 146 | 103.872676 | 152.36 | 200.847324 | 1.043562 |
| GO:0019538\_protein\_metabolic\_process | 1292 | 11 | 1.270860 | -0.631127 | 147 | 104.199349 | 152.7 | 201.200651 | 1.038776 |
| GO:0007160\_cell-matrix\_adhesion | 40 | 1 | 3.731707 | -0.626314 | 148 | 106.173491 | 154.76 | 203.346509 | 1.045676 |
| GO:0031589\_cell-substrate\_adhesion | 41 | 1 | 3.640690 | -0.616957 | 149 | 108.096664 | 157.15 | 206.203336 | 1.054698 |
| GO:0010564\_regulation\_of\_cell\_cycle\_process | 43 | 1 | 3.471356 | -0.599001 | 150 | 110.518727 | 160.4 | 210.281273 | 1.069333 |
| GO:0044267\_cellular\_protein\_metabolic\_process | 1179 | 10 | 1.266058 | -0.594094 | 151 | 110.741668 | 160.71 | 210.678332 | 1.064305 |
| GO:0007254\_JNK\_cascade | 48 | 1 | 3.109756 | -0.558026 | 152 | 117.020849 | 168.21 | 219.399151 | 1.106645 |
| GO:0048519\_negative\_regulation\_of\_biological\_process | 664 | 6 | 1.348810 | -0.549539 | 153 | 119.005996 | 170.75 | 222.494004 | 1.116013 |
| GO:0031098\_stress-activated\_protein\_kinase\_signaling\_pathway | 50 | 1 | 2.985366 | -0.543006 | 155 | 120.495884 | 172.76 | 225.024116 | 1.114581 |
| GO:0031399\_regulation\_of\_protein\_modification\_process | 50 | 1 | 2.985366 | -0.543006 | 155 | 120.495884 | 172.76 | 225.024116 | 1.114581 |
| GO:0044260\_cellular\_macromolecule\_metabolic\_process | 1219 | 10 | 1.224514 | -0.536345 | 156 | 120.919416 | 173.37 | 225.820584 | 1.111346 |
| GO:0043085\_positive\_regulation\_of\_catalytic\_activity | 162 | 2 | 1.842818 | -0.528625 | 157 | 123.195434 | 175.96 | 228.724566 | 1.120764 |
| GO:0050794\_regulation\_of\_cellular\_process | 1656 | 13 | 1.171792 | -0.518349 | 158 | 125.118613 | 178.37 | 231.621387 | 1.128924 |
| GO:0032270\_positive\_regulation\_of\_cellular\_protein\_metabolic\_process | 56 | 1 | 2.665505 | -0.501881 | 159 | 128.813568 | 182.89 | 236.966432 | 1.150252 |
| GO:0019220\_regulation\_of\_phosphate\_metabolic\_process | 57 | 1 | 2.618742 | -0.495538 | 161 | 130.083032 | 184.52 | 238.956968 | 1.146087 |
| GO:0051174\_regulation\_of\_phosphorus\_metabolic\_process | 57 | 1 | 2.618742 | -0.495538 | 161 | 130.083032 | 184.52 | 238.956968 | 1.146087 |
| GO:0051247\_positive\_regulation\_of\_protein\_metabolic\_process | 59 | 1 | 2.529971 | -0.483245 | 162 | 133.544320 | 188.48 | 243.415680 | 1.163457 |
| GO:0051345\_positive\_regulation\_of\_hydrolase\_activity | 61 | 1 | 2.447021 | -0.471445 | 163 | 137.442335 | 192.92 | 248.397665 | 1.183558 |
| GO:0006470\_protein\_amino\_acid\_dephosphorylation | 63 | 1 | 2.369338 | -0.460106 | 166 | 140.004511 | 195.53 | 251.055489 | 1.177892 |
| GO:0043086\_negative\_regulation\_of\_catalytic\_activity | 63 | 1 | 2.369338 | -0.460106 | 166 | 140.004511 | 195.53 | 251.055489 | 1.177892 |
| GO:0048193\_Golgi\_vesicle\_transport | 63 | 1 | 2.369338 | -0.460106 | 166 | 140.004511 | 195.53 | 251.055489 | 1.177892 |
| GO:0006511\_ubiquitin-dependent\_protein\_catabolic\_process | 67 | 1 | 2.227885 | -0.438698 | 171 | 145.388731 | 202.06 | 258.731269 | 1.181637 |
| GO:0019941\_modification-dependent\_protein\_catabolic\_process | 67 | 1 | 2.227885 | -0.438698 | 171 | 145.388731 | 202.06 | 258.731269 | 1.181637 |
| GO:0043632\_modification-dependent\_macromolecule\_catabolic\_process | 67 | 1 | 2.227885 | -0.438698 | 171 | 145.388731 | 202.06 | 258.731269 | 1.181637 |
| GO:0044257\_cellular\_protein\_catabolic\_process | 67 | 1 | 2.227885 | -0.438698 | 171 | 145.388731 | 202.06 | 258.731269 | 1.181637 |
| GO:0051603\_proteolysis\_involved\_in\_cellular\_protein\_catabolic\_process | 67 | 1 | 2.227885 | -0.438698 | 171 | 145.388731 | 202.06 | 258.731269 | 1.181637 |
| GO:0006928\_cell\_motility | 191 | 2 | 1.563019 | -0.434399 | 173 | 146.175876 | 202.9 | 259.624124 | 1.172832 |
| GO:0051674\_localization\_of\_cell | 191 | 2 | 1.563019 | -0.434399 | 173 | 146.175876 | 202.9 | 259.624124 | 1.172832 |
| GO:0051329\_interphase\_of\_mitotic\_cell\_cycle | 70 | 1 | 2.132404 | -0.423655 | 174 | 149.386474 | 206.63 | 263.873526 | 1.187529 |
| GO:0016311\_dephosphorylation | 71 | 1 | 2.102370 | -0.418818 | 175 | 151.029802 | 208.56 | 266.090198 | 1.191771 |
| GO:0051049\_regulation\_of\_transport | 73 | 1 | 2.044771 | -0.409397 | 176 | 153.363232 | 211.22 | 269.076768 | 1.200114 |
| GO:0051325\_interphase | 75 | 1 | 1.990244 | -0.400296 | 177 | 154.564722 | 212.67 | 270.775278 | 1.201525 |
| GO:0050789\_regulation\_of\_biological\_process | 1765 | 13 | 1.099427 | -0.399138 | 178 | 154.635811 | 212.81 | 270.984189 | 1.195562 |
| GO:0001558\_regulation\_of\_cell\_growth | 80 | 1 | 1.865854 | -0.378836 | 179 | 158.420887 | 217.04 | 275.659113 | 1.212514 |
| GO:0009653\_anatomical\_structure\_morphogenesis | 360 | 3 | 1.243902 | -0.359604 | 180 | 164.952911 | 224.14 | 283.327089 | 1.245222 |
| GO:0030163\_protein\_catabolic\_process | 85 | 1 | 1.756098 | -0.359040 | 181 | 165.949066 | 225.38 | 284.810934 | 1.245193 |
| GO:0051336\_regulation\_of\_hydrolase\_activity | 86 | 1 | 1.735678 | -0.355263 | 182 | 166.980937 | 226.44 | 285.899063 | 1.244176 |
| GO:0033674\_positive\_regulation\_of\_kinase\_activity | 88 | 1 | 1.696231 | -0.347879 | 184 | 169.475045 | 229.42 | 289.364955 | 1.246848 |
| GO:0045860\_positive\_regulation\_of\_protein\_kinase\_activity | 88 | 1 | 1.696231 | -0.347879 | 184 | 169.475045 | 229.42 | 289.364955 | 1.246848 |
| GO:0007517\_muscle\_development | 89 | 1 | 1.677172 | -0.344270 | 185 | 170.217959 | 230.25 | 290.282041 | 1.244595 |
| GO:0040008\_regulation\_of\_growth | 91 | 1 | 1.640311 | -0.337210 | 187 | 172.979742 | 233.26 | 293.540258 | 1.247380 |
| GO:0051347\_positive\_regulation\_of\_transferase\_activity | 91 | 1 | 1.640311 | -0.337210 | 187 | 172.979742 | 233.26 | 293.540258 | 1.247380 |
| GO:0032879\_regulation\_of\_localization | 93 | 1 | 1.605035 | -0.330354 | 188 | 174.797243 | 235.65 | 296.502757 | 1.253457 |
| GO:0045045\_secretory\_pathway | 94 | 1 | 1.587961 | -0.327000 | 189 | 175.260570 | 236.15 | 297.039430 | 1.249471 |
| GO:0016049\_cell\_growth | 98 | 1 | 1.523146 | -0.314050 | 190 | 178.262913 | 239.83 | 301.397087 | 1.262263 |
| GO:0008361\_regulation\_of\_cell\_size | 99 | 1 | 1.507761 | -0.310925 | 191 | 179.283608 | 240.79 | 302.296392 | 1.260681 |
| GO:0016477\_cell\_migration | 103 | 1 | 1.449207 | -0.298843 | 192 | 182.857900 | 244.77 | 306.682100 | 1.274844 |
| GO:0030036\_actin\_cytoskeleton\_organization\_and\_biogenesis | 110 | 1 | 1.356984 | -0.279192 | 193 | 188.198834 | 250.17 | 312.141166 | 1.296218 |
| GO:0000165\_MAPKKK\_cascade | 111 | 1 | 1.344759 | -0.276528 | 194 | 188.700869 | 250.76 | 312.819131 | 1.292577 |
| GO:0050790\_regulation\_of\_catalytic\_activity | 269 | 2 | 1.109801 | -0.264455 | 195 | 192.099414 | 254.6 | 317.100586 | 1.305641 |
| GO:0040007\_growth | 119 | 1 | 1.254355 | -0.256383 | 196 | 195.689447 | 258.15 | 320.610553 | 1.317092 |
| GO:0030029\_actin\_filament-based\_process | 120 | 1 | 1.243902 | -0.254002 | 197 | 196.728457 | 259.13 | 321.531543 | 1.315381 |
| GO:0009967\_positive\_regulation\_of\_signal\_transduction | 121 | 1 | 1.233622 | -0.251650 | 198 | 198.115731 | 260.72 | 323.324269 | 1.316768 |
| GO:0032940\_secretion\_by\_cell | 125 | 1 | 1.194146 | -0.242519 | 199 | 201.682748 | 264.23 | 326.777252 | 1.327789 |
| GO:0044262\_cellular\_carbohydrate\_metabolic\_process | 129 | 1 | 1.157119 | -0.233809 | 200 | 203.833626 | 266.54 | 329.246374 | 1.332700 |
| GO:0044265\_cellular\_macromolecule\_catabolic\_process | 130 | 1 | 1.148218 | -0.231695 | 201 | 204.848005 | 267.56 | 330.271995 | 1.331144 |
| GO:0043285\_biopolymer\_catabolic\_process | 135 | 1 | 1.105691 | -0.221478 | 202 | 209.120549 | 272.08 | 335.039451 | 1.346931 |
| GO:0008285\_negative\_regulation\_of\_cell\_proliferation | 137 | 1 | 1.089550 | -0.217551 | 203 | 210.658176 | 273.46 | 336.261824 | 1.347094 |
| GO:0022414\_reproductive\_process | 142 | 1 | 1.051185 | -0.208107 | 204 | 214.252984 | 277.1 | 339.947016 | 1.358333 |
| GO:0007165\_signal\_transduction | 1564 | 10 | 0.954401 | -0.202548 | 205 | 215.853360 | 278.73 | 341.606640 | 1.359659 |
| GO:0065007\_biological\_regulation | 2021 | 13 | 0.960162 | -0.201413 | 206 | 215.946075 | 278.86 | 341.773925 | 1.353689 |
| GO:0065009\_regulation\_of\_molecular\_function | 314 | 2 | 0.950753 | -0.200870 | 207 | 216.770265 | 279.7 | 342.629735 | 1.351208 |
| GO:0007155\_cell\_adhesion | 327 | 2 | 0.912956 | -0.185677 | 209 | 219.794783 | 282.58 | 345.365217 | 1.352057 |
| GO:0022610\_biological\_adhesion | 327 | 2 | 0.912956 | -0.185677 | 209 | 219.794783 | 282.58 | 345.365217 | 1.352057 |
| GO:0007242\_intracellular\_signaling\_cascade | 665 | 4 | 0.897854 | -0.177013 | 210 | 221.568629 | 284.38 | 347.191371 | 1.354190 |
| GO:0045859\_regulation\_of\_protein\_kinase\_activity | 161 | 1 | 0.927132 | -0.176513 | 211 | 221.943130 | 284.74 | 347.536870 | 1.349479 |
| GO:0043549\_regulation\_of\_kinase\_activity | 163 | 1 | 0.915756 | -0.173537 | 212 | 222.815778 | 285.6 | 348.384222 | 1.347170 |
| GO:0006412\_translation | 165 | 1 | 0.904656 | -0.170620 | 213 | 223.928157 | 286.8 | 349.671843 | 1.346479 |
| GO:0051338\_regulation\_of\_transferase\_activity | 167 | 1 | 0.893822 | -0.167761 | 214 | 224.952365 | 287.75 | 350.547635 | 1.344626 |
| GO:0009057\_macromolecule\_catabolic\_process | 170 | 1 | 0.878049 | -0.163579 | 215 | 227.011703 | 289.57 | 352.128297 | 1.346837 |
| GO:0046903\_secretion | 178 | 1 | 0.838586 | -0.153012 | 216 | 232.270729 | 294.99 | 357.709271 | 1.365694 |
| GO:0005975\_carbohydrate\_metabolic\_process | 186 | 1 | 0.802518 | -0.143233 | 217 | 235.224011 | 297.84 | 360.455989 | 1.372535 |
| GO:0031323\_regulation\_of\_cellular\_metabolic\_process | 883 | 5 | 0.845234 | -0.139725 | 218 | 236.574539 | 299.26 | 361.945461 | 1.372752 |
| GO:0019222\_regulation\_of\_metabolic\_process | 898 | 5 | 0.831115 | -0.131218 | 219 | 238.951022 | 301.57 | 364.188978 | 1.377032 |
| GO:0016481\_negative\_regulation\_of\_transcription | 202 | 1 | 0.738952 | -0.125752 | 220 | 240.486789 | 303.1 | 365.713211 | 1.377727 |
| GO:0006508\_proteolysis | 204 | 1 | 0.731707 | -0.123742 | 221 | 241.046712 | 303.72 | 366.393288 | 1.374299 |
| GO:0048856\_anatomical\_structure\_development | 925 | 5 | 0.806856 | -0.117055 | 222 | 242.276585 | 304.96 | 367.643415 | 1.373694 |
| GO:0007154\_cell\_communication | 1733 | 10 | 0.861329 | -0.116520 | 223 | 242.485111 | 305.11 | 367.734889 | 1.368206 |
| GO:0045934\_negative\_regulation\_of\_nucleobase\_\_nucleoside\_\_nucleotide\_and\_nucleic\_acid\_metabolic\_process | 223 | 1 | 0.669365 | -0.106349 | 224 | 244.834059 | 307.46 | 370.085941 | 1.372589 |
| GO:0016192\_vesicle-mediated\_transport | 227 | 1 | 0.657570 | -0.103044 | 225 | 245.943403 | 308.44 | 370.936597 | 1.370844 |
| GO:0009966\_regulation\_of\_signal\_transduction | 232 | 1 | 0.643398 | -0.099071 | 226 | 247.345798 | 309.85 | 372.354202 | 1.371018 |
| GO:0000003\_reproduction | 237 | 1 | 0.629824 | -0.095266 | 228 | 248.403469 | 310.83 | 373.256531 | 1.363289 |
| GO:0031325\_positive\_regulation\_of\_cellular\_metabolic\_process | 237 | 1 | 0.629824 | -0.095266 | 228 | 248.403469 | 310.83 | 373.256531 | 1.363289 |
| GO:0044248\_cellular\_catabolic\_process | 239 | 1 | 0.624554 | -0.093788 | 229 | 249.468136 | 311.72 | 373.971864 | 1.361223 |
| GO:0009893\_positive\_regulation\_of\_metabolic\_process | 244 | 1 | 0.611755 | -0.090204 | 230 | 251.033032 | 313.13 | 375.226968 | 1.361435 |
| GO:0050793\_regulation\_of\_developmental\_process | 455 | 2 | 0.656124 | -0.086087 | 231 | 252.385691 | 314.31 | 376.234309 | 1.360649 |
| GO:0032502\_developmental\_process | 1506 | 8 | 0.792926 | -0.082952 | 232 | 252.890519 | 314.85 | 376.809481 | 1.357112 |
| GO:0009056\_catabolic\_process | 257 | 1 | 0.580810 | -0.081559 | 233 | 253.467930 | 315.2 | 376.932070 | 1.352790 |
| GO:0006793\_phosphorus\_metabolic\_process | 466 | 2 | 0.640636 | -0.080572 | 235 | 254.135109 | 315.78 | 377.424891 | 1.343745 |
| GO:0006796\_phosphate\_metabolic\_process | 466 | 2 | 0.640636 | -0.080572 | 235 | 254.135109 | 315.78 | 377.424891 | 1.343745 |
| GO:0042127\_regulation\_of\_cell\_proliferation | 279 | 1 | 0.535012 | -0.068889 | 236 | 259.695190 | 320.57 | 381.444810 | 1.358347 |
| GO:0007243\_protein\_kinase\_cascade | 294 | 1 | 0.507715 | -0.061459 | 237 | 261.785321 | 322.39 | 382.994679 | 1.360295 |
| GO:0042221\_response\_to\_chemical\_stimulus | 298 | 1 | 0.500900 | -0.059623 | 238 | 262.830290 | 323.26 | 383.689710 | 1.358235 |
| GO:0009059\_macromolecule\_biosynthetic\_process | 302 | 1 | 0.494266 | -0.057845 | 239 | 263.661895 | 324.0 | 384.338105 | 1.355649 |
| GO:0043687\_post-translational\_protein\_modification | 529 | 2 | 0.564341 | -0.055027 | 240 | 264.942830 | 324.87 | 384.797170 | 1.353625 |
| GO:0006468\_protein\_amino\_acid\_phosphorylation | 316 | 1 | 0.472368 | -0.052045 | 241 | 266.023685 | 325.7 | 385.376315 | 1.351452 |
| GO:0044249\_cellular\_biosynthetic\_process | 325 | 1 | 0.459287 | -0.048639 | 242 | 267.500547 | 327.02 | 386.539453 | 1.351322 |
| GO:0007275\_multicellular\_organismal\_development | 975 | 4 | 0.612383 | -0.040622 | 243 | 270.597490 | 329.71 | 388.822510 | 1.356831 |
| GO:0007399\_nervous\_system\_development | 350 | 1 | 0.426481 | -0.040332 | 244 | 270.859346 | 329.99 | 389.120654 | 1.352418 |
| GO:0016310\_phosphorylation | 385 | 1 | 0.387710 | -0.031072 | 245 | 272.742456 | 331.42 | 390.097544 | 1.352735 |
| GO:0048522\_positive\_regulation\_of\_cellular\_process | 634 | 2 | 0.470878 | -0.028803 | 246 | 273.282973 | 331.8 | 390.317027 | 1.348780 |
| GO:0065008\_regulation\_of\_biological\_quality | 428 | 1 | 0.348758 | -0.022578 | 247 | 275.180238 | 333.23 | 391.279762 | 1.349109 |
| GO:0006915\_apoptosis | 430 | 1 | 0.347136 | -0.022245 | 248 | 275.577109 | 333.55 | 391.522891 | 1.344960 |
| GO:0048518\_positive\_regulation\_of\_biological\_process | 678 | 2 | 0.440319 | -0.021840 | 249 | 275.916278 | 333.8 | 391.683722 | 1.340562 |
| GO:0012501\_programmed\_cell\_death | 435 | 1 | 0.343146 | -0.021435 | 250 | 276.114258 | 333.98 | 391.845742 | 1.335920 |
| GO:0006464\_protein\_modification\_process | 686 | 2 | 0.435185 | -0.020760 | 251 | 276.802281 | 334.46 | 392.117719 | 1.332510 |
| GO:0008219\_cell\_death | 444 | 1 | 0.336190 | -0.020051 | 253 | 277.278467 | 334.89 | 392.501533 | 1.323676 |
| GO:0016265\_death | 444 | 1 | 0.336190 | -0.020051 | 253 | 277.278467 | 334.89 | 392.501533 | 1.323676 |
| GO:0043412\_biopolymer\_modification | 708 | 2 | 0.421662 | -0.018047 | 254 | 278.529461 | 335.69 | 392.850539 | 1.321614 |
| GO:0043170\_macromolecule\_metabolic\_process | 2397 | 11 | 0.685003 | -0.015183 | 255 | 279.327635 | 336.37 | 393.412365 | 1.319098 |
| GO:0009058\_biosynthetic\_process | 506 | 1 | 0.294997 | -0.012653 | 256 | 279.932264 | 336.8 | 393.667736 | 1.315625 |
| GO:0048513\_organ\_development | 520 | 1 | 0.287054 | -0.011401 | 257 | 280.250273 | 337.01 | 393.769727 | 1.311323 |
| GO:0048731\_system\_development | 780 | 2 | 0.382739 | -0.011337 | 258 | 280.340627 | 337.06 | 393.779373 | 1.306434 |
| GO:0006950\_response\_to\_stress | 524 | 1 | 0.284863 | -0.011066 | 259 | 280.508988 | 337.18 | 393.851012 | 1.301853 |
| GO:0007166\_cell\_surface\_receptor\_linked\_signal\_transduction | 540 | 1 | 0.276423 | -0.009823 | 260 | 280.793422 | 337.44 | 394.086578 | 1.297846 |
| GO:0048468\_cell\_development | 575 | 1 | 0.259597 | -0.007562 | 261 | 281.392253 | 337.86 | 394.327747 | 1.294483 |
| GO:0045449\_regulation\_of\_transcription | 681 | 1 | 0.219190 | -0.003401 | 262 | 282.842874 | 338.73 | 394.617126 | 1.292863 |
| GO:0032501\_multicellular\_organismal\_process | 1413 | 4 | 0.422557 | -0.003400 | 263 | 282.842874 | 338.73 | 394.617126 | 1.287947 |
| GO:0030154\_cell\_differentiation | 704 | 1 | 0.212029 | -0.002855 | 265 | 283.330230 | 338.99 | 394.649770 | 1.279208 |
| GO:0048869\_cellular\_developmental\_process | 704 | 1 | 0.212029 | -0.002855 | 265 | 283.330230 | 338.99 | 394.649770 | 1.279208 |
| GO:0044238\_primary\_metabolic\_process | 2900 | 12 | 0.617662 | -0.002477 | 266 | 283.706011 | 339.21 | 394.713989 | 1.275226 |
| GO:0019219\_regulation\_of\_nucleobase\_\_nucleoside\_\_nucleotide\_and\_nucleic\_acid\_metabolic\_process | 728 | 1 | 0.205039 | -0.002377 | 267 | 283.739445 | 339.25 | 394.760555 | 1.270599 |
| GO:0050896\_response\_to\_stimulus | 1013 | 2 | 0.294705 | -0.002339 | 268 | 283.826324 | 339.31 | 394.793676 | 1.266082 |
| GO:0010468\_regulation\_of\_gene\_expression | 750 | 1 | 0.199024 | -0.002008 | 269 | 283.918973 | 339.36 | 394.801027 | 1.261561 |
| GO:0006350\_transcription | 845 | 1 | 0.176649 | -0.000962 | 270 | 284.081925 | 339.45 | 394.818075 | 1.257222 |
| GO:0044237\_cellular\_metabolic\_process | 2921 | 11 | 0.562120 | -0.000791 | 271 | 284.134350 | 339.48 | 394.825650 | 1.252694 |
| GO:0010467\_gene\_expression | 1201 | 2 | 0.248573 | -0.000601 | 272 | 284.254482 | 339.53 | 394.805518 | 1.248272 |
| GO:0008152\_metabolic\_process | 3126 | 12 | 0.573007 | -0.000586 | 273 | 284.303925 | 339.55 | 394.796075 | 1.243773 |
| GO:0043283\_biopolymer\_metabolic\_process | 1839 | 3 | 0.243505 | -0.000031 | 274 | 284.501695 | 339.64 | 394.778305 | 1.239562 |
| GO:0006139\_nucleobase\_\_nucleoside\_\_nucleotide\_and\_nucleic\_acid\_metabolic\_process | 1365 | 1 | 0.109354 | -0.000013 | 275 | 284.501695 | 339.64 | 394.778305 | 1.235055 |
| GO:0006281\_DNA\_repair | 138 | 0 | 0.000000 | -0.000000 | 276 | 285.350819 | 340.51 | 395.669181 | 1.233732 |
| GO:0006006\_glucose\_metabolic\_process | 32 | 0 | 0.000000 | -0.000000 | 282 | 290.569901 | 345.32 | 400.070099 | 1.224539 |
| GO:0006413\_translational\_initiation | 32 | 0 | 0.000000 | -0.000000 | 282 | 290.569901 | 345.32 | 400.070099 | 1.224539 |
| GO:0007259\_JAK-STAT\_cascade | 32 | 0 | 0.000000 | -0.000000 | 282 | 290.569901 | 345.32 | 400.070099 | 1.224539 |
| GO:0022411\_cellular\_component\_disassembly | 32 | 0 | 0.000000 | -0.000000 | 282 | 290.569901 | 345.32 | 400.070099 | 1.224539 |
| GO:0042089\_cytokine\_biosynthetic\_process | 32 | 0 | 0.000000 | -0.000000 | 282 | 290.569901 | 345.32 | 400.070099 | 1.224539 |
| GO:0048771\_tissue\_remodeling | 32 | 0 | 0.000000 | -0.000000 | 282 | 290.569901 | 345.32 | 400.070099 | 1.224539 |
| GO:0000079\_regulation\_of\_cyclin-dependent\_protein\_kinase\_activity | 41 | 0 | 0.000000 | -0.000000 | 291 | 298.592331 | 353.01 | 407.427669 | 1.213093 |
| GO:0002252\_immune\_effector\_process | 41 | 0 | 0.000000 | -0.000000 | 291 | 298.592331 | 353.01 | 407.427669 | 1.213093 |
| GO:0006650\_glycerophospholipid\_metabolic\_process | 41 | 0 | 0.000000 | -0.000000 | 291 | 298.592331 | 353.01 | 407.427669 | 1.213093 |
| GO:0008654\_phospholipid\_biosynthetic\_process | 41 | 0 | 0.000000 | -0.000000 | 291 | 298.592331 | 353.01 | 407.427669 | 1.213093 |
| GO:0009416\_response\_to\_light\_stimulus | 41 | 0 | 0.000000 | -0.000000 | 291 | 298.592331 | 353.01 | 407.427669 | 1.213093 |
| GO:0009725\_response\_to\_hormone\_stimulus | 41 | 0 | 0.000000 | -0.000000 | 291 | 298.592331 | 353.01 | 407.427669 | 1.213093 |
| GO:0015849\_organic\_acid\_transport | 41 | 0 | 0.000000 | -0.000000 | 291 | 298.592331 | 353.01 | 407.427669 | 1.213093 |
| GO:0030099\_myeloid\_cell\_differentiation | 41 | 0 | 0.000000 | -0.000000 | 291 | 298.592331 | 353.01 | 407.427669 | 1.213093 |
| GO:0046942\_carboxylic\_acid\_transport | 41 | 0 | 0.000000 | -0.000000 | 291 | 298.592331 | 353.01 | 407.427669 | 1.213093 |
| GO:0006665\_sphingolipid\_metabolic\_process | 37 | 0 | 0.000000 | -0.000000 | 297 | 306.092458 | 360.18 | 414.267542 | 1.212727 |
| GO:0006986\_response\_to\_unfolded\_protein | 37 | 0 | 0.000000 | -0.000000 | 297 | 306.092458 | 360.18 | 414.267542 | 1.212727 |
| GO:0015837\_amine\_transport | 37 | 0 | 0.000000 | -0.000000 | 297 | 306.092458 | 360.18 | 414.267542 | 1.212727 |
| GO:0045786\_negative\_regulation\_of\_cell\_cycle | 37 | 0 | 0.000000 | -0.000000 | 297 | 306.092458 | 360.18 | 414.267542 | 1.212727 |
| GO:0051046\_regulation\_of\_secretion | 37 | 0 | 0.000000 | -0.000000 | 297 | 306.092458 | 360.18 | 414.267542 | 1.212727 |
| GO:0051789\_response\_to\_protein\_stimulus | 37 | 0 | 0.000000 | -0.000000 | 297 | 306.092458 | 360.18 | 414.267542 | 1.212727 |
| GO:0048878\_chemical\_homeostasis | 143 | 0 | 0.000000 | -0.000000 | 298 | 306.658302 | 360.61 | 414.561698 | 1.210101 |
| GO:0016567\_protein\_ubiquitination | 51 | 0 | 0.000000 | -0.000000 | 300 | 308.982666 | 362.78 | 416.577334 | 1.209267 |
| GO:0045926\_negative\_regulation\_of\_growth | 51 | 0 | 0.000000 | -0.000000 | 300 | 308.982666 | 362.78 | 416.577334 | 1.209267 |
| GO:0000038\_very-long-chain\_fatty\_acid\_metabolic\_process | 5 | 0 | 0.000000 | -0.000000 | 488 | 496.600854 | 548.4 | 600.199146 | 1.123770 |
| GO:0000389\_nuclear\_mRNA\_3'-splice\_site\_recognition | 5 | 0 | 0.000000 | -0.000000 | 488 | 496.600854 | 548.4 | 600.199146 | 1.123770 |
| GO:0000578\_embryonic\_axis\_specification | 5 | 0 | 0.000000 | -0.000000 | 488 | 496.600854 | 548.4 | 600.199146 | 1.123770 |
| GO:0001523\_retinoid\_metabolic\_process | 5 | 0 | 0.000000 | -0.000000 | 488 | 496.600854 | 548.4 | 600.199146 | 1.123770 |
| GO:0001654\_eye\_development | 5 | 0 | 0.000000 | -0.000000 | 488 | 496.600854 | 548.4 | 600.199146 | 1.123770 |
| GO:0001704\_formation\_of\_primary\_germ\_layer | 5 | 0 | 0.000000 | -0.000000 | 488 | 496.600854 | 548.4 | 600.199146 | 1.123770 |
| GO:0001707\_mesoderm\_formation | 5 | 0 | 0.000000 | -0.000000 | 488 | 496.600854 | 548.4 | 600.199146 | 1.123770 |
| GO:0001763\_morphogenesis\_of\_a\_branching\_structure | 5 | 0 | 0.000000 | -0.000000 | 488 | 496.600854 | 548.4 | 600.199146 | 1.123770 |
| GO:0001764\_neuron\_migration | 5 | 0 | 0.000000 | -0.000000 | 488 | 496.600854 | 548.4 | 600.199146 | 1.123770 |
| GO:0001840\_neural\_plate\_development | 5 | 0 | 0.000000 | -0.000000 | 488 | 496.600854 | 548.4 | 600.199146 | 1.123770 |
| GO:0001937\_negative\_regulation\_of\_endothelial\_cell\_proliferation | 5 | 0 | 0.000000 | -0.000000 | 488 | 496.600854 | 548.4 | 600.199146 | 1.123770 |
| GO:0001953\_negative\_regulation\_of\_cell-matrix\_adhesion | 5 | 0 | 0.000000 | -0.000000 | 488 | 496.600854 | 548.4 | 600.199146 | 1.123770 |
| GO:0002237\_response\_to\_molecule\_of\_bacterial\_origin | 5 | 0 | 0.000000 | -0.000000 | 488 | 496.600854 | 548.4 | 600.199146 | 1.123770 |
| GO:0002700\_regulation\_of\_production\_of\_molecular\_mediator\_of\_immune\_response | 5 | 0 | 0.000000 | -0.000000 | 488 | 496.600854 | 548.4 | 600.199146 | 1.123770 |
| GO:0002702\_positive\_regulation\_of\_production\_of\_molecular\_mediator\_of\_immune\_response | 5 | 0 | 0.000000 | -0.000000 | 488 | 496.600854 | 548.4 | 600.199146 | 1.123770 |
| GO:0002711\_positive\_regulation\_of\_T\_cell\_mediated\_immunity | 5 | 0 | 0.000000 | -0.000000 | 488 | 496.600854 | 548.4 | 600.199146 | 1.123770 |
| GO:0002718\_regulation\_of\_cytokine\_production\_during\_immune\_response | 5 | 0 | 0.000000 | -0.000000 | 488 | 496.600854 | 548.4 | 600.199146 | 1.123770 |
| GO:0002720\_positive\_regulation\_of\_cytokine\_production\_during\_immune\_response | 5 | 0 | 0.000000 | -0.000000 | 488 | 496.600854 | 548.4 | 600.199146 | 1.123770 |
| GO:0002791\_regulation\_of\_peptide\_secretion | 5 | 0 | 0.000000 | -0.000000 | 488 | 496.600854 | 548.4 | 600.199146 | 1.123770 |
| GO:0002821\_positive\_regulation\_of\_adaptive\_immune\_response | 5 | 0 | 0.000000 | -0.000000 | 488 | 496.600854 | 548.4 | 600.199146 | 1.123770 |
| GO:0002824\_positive\_regulation\_of\_adaptive\_immune\_response\_based\_on\_somatic\_recombination\_of\_immune\_receptors\_built\_from\_immunoglobulin\_superfamily\_domains | 5 | 0 | 0.000000 | -0.000000 | 488 | 496.600854 | 548.4 | 600.199146 | 1.123770 |
| GO:0006020\_inositol\_metabolic\_process | 5 | 0 | 0.000000 | -0.000000 | 488 | 496.600854 | 548.4 | 600.199146 | 1.123770 |
| GO:0006094\_gluconeogenesis | 5 | 0 | 0.000000 | -0.000000 | 488 | 496.600854 | 548.4 | 600.199146 | 1.123770 |
| GO:0006110\_regulation\_of\_glycolysis | 5 | 0 | 0.000000 | -0.000000 | 488 | 496.600854 | 548.4 | 600.199146 | 1.123770 |
| GO:0006140\_regulation\_of\_nucleotide\_metabolic\_process | 5 | 0 | 0.000000 | -0.000000 | 488 | 496.600854 | 548.4 | 600.199146 | 1.123770 |
| GO:0006144\_purine\_base\_metabolic\_process | 5 | 0 | 0.000000 | -0.000000 | 488 | 496.600854 | 548.4 | 600.199146 | 1.123770 |
| GO:0006268\_DNA\_unwinding\_during\_replication | 5 | 0 | 0.000000 | -0.000000 | 488 | 496.600854 | 548.4 | 600.199146 | 1.123770 |
| GO:0006390\_transcription\_from\_mitochondrial\_promoter | 5 | 0 | 0.000000 | -0.000000 | 488 | 496.600854 | 548.4 | 600.199146 | 1.123770 |
| GO:0006400\_tRNA\_modification | 5 | 0 | 0.000000 | -0.000000 | 488 | 496.600854 | 548.4 | 600.199146 | 1.123770 |
| GO:0006536\_glutamate\_metabolic\_process | 5 | 0 | 0.000000 | -0.000000 | 488 | 496.600854 | 548.4 | 600.199146 | 1.123770 |
| GO:0006570\_tyrosine\_metabolic\_process | 5 | 0 | 0.000000 | -0.000000 | 488 | 496.600854 | 548.4 | 600.199146 | 1.123770 |
| GO:0006691\_leukotriene\_metabolic\_process | 5 | 0 | 0.000000 | -0.000000 | 488 | 496.600854 | 548.4 | 600.199146 | 1.123770 |
| GO:0006721\_terpenoid\_metabolic\_process | 5 | 0 | 0.000000 | -0.000000 | 488 | 496.600854 | 548.4 | 600.199146 | 1.123770 |
| GO:0006826\_iron\_ion\_transport | 5 | 0 | 0.000000 | -0.000000 | 488 | 496.600854 | 548.4 | 600.199146 | 1.123770 |
| GO:0006893\_Golgi\_to\_plasma\_membrane\_transport | 5 | 0 | 0.000000 | -0.000000 | 488 | 496.600854 | 548.4 | 600.199146 | 1.123770 |
| GO:0006929\_substrate-bound\_cell\_migration | 5 | 0 | 0.000000 | -0.000000 | 488 | 496.600854 | 548.4 | 600.199146 | 1.123770 |
| GO:0006970\_response\_to\_osmotic\_stress | 5 | 0 | 0.000000 | -0.000000 | 488 | 496.600854 | 548.4 | 600.199146 | 1.123770 |
| GO:0006977\_DNA\_damage\_response\_\_signal\_transduction\_by\_p53\_class\_mediator\_resulting\_in\_cell\_cycle\_arrest | 5 | 0 | 0.000000 | -0.000000 | 488 | 496.600854 | 548.4 | 600.199146 | 1.123770 |
| GO:0007009\_plasma\_membrane\_organization\_and\_biogenesis | 5 | 0 | 0.000000 | -0.000000 | 488 | 496.600854 | 548.4 | 600.199146 | 1.123770 |
| GO:0007016\_cytoskeletal\_anchoring | 5 | 0 | 0.000000 | -0.000000 | 488 | 496.600854 | 548.4 | 600.199146 | 1.123770 |
| GO:0007025\_beta-tubulin\_folding | 5 | 0 | 0.000000 | -0.000000 | 488 | 496.600854 | 548.4 | 600.199146 | 1.123770 |
| GO:0007062\_sister\_chromatid\_cohesion | 5 | 0 | 0.000000 | -0.000000 | 488 | 496.600854 | 548.4 | 600.199146 | 1.123770 |
| GO:0007091\_mitotic\_metaphase\_anaphase\_transition | 5 | 0 | 0.000000 | -0.000000 | 488 | 496.600854 | 548.4 | 600.199146 | 1.123770 |
| GO:0007220\_Notch\_receptor\_processing | 5 | 0 | 0.000000 | -0.000000 | 488 | 496.600854 | 548.4 | 600.199146 | 1.123770 |
| GO:0007271\_synaptic\_transmission\_\_cholinergic | 5 | 0 | 0.000000 | -0.000000 | 488 | 496.600854 | 548.4 | 600.199146 | 1.123770 |
| GO:0007342\_fusion\_of\_sperm\_to\_egg\_plasma\_membrane | 5 | 0 | 0.000000 | -0.000000 | 488 | 496.600854 | 548.4 | 600.199146 | 1.123770 |
| GO:0007350\_blastoderm\_segmentation | 5 | 0 | 0.000000 | -0.000000 | 488 | 496.600854 | 548.4 | 600.199146 | 1.123770 |
| GO:0007351\_tripartite\_regional\_subdivision | 5 | 0 | 0.000000 | -0.000000 | 488 | 496.600854 | 548.4 | 600.199146 | 1.123770 |
| GO:0007567\_parturition | 5 | 0 | 0.000000 | -0.000000 | 488 | 496.600854 | 548.4 | 600.199146 | 1.123770 |
| GO:0008089\_anterograde\_axon\_cargo\_transport | 5 | 0 | 0.000000 | -0.000000 | 488 | 496.600854 | 548.4 | 600.199146 | 1.123770 |
| GO:0008105\_asymmetric\_protein\_localization | 5 | 0 | 0.000000 | -0.000000 | 488 | 496.600854 | 548.4 | 600.199146 | 1.123770 |
| GO:0008272\_sulfate\_transport | 5 | 0 | 0.000000 | -0.000000 | 488 | 496.600854 | 548.4 | 600.199146 | 1.123770 |
| GO:0008333\_endosome\_to\_lysosome\_transport | 5 | 0 | 0.000000 | -0.000000 | 488 | 496.600854 | 548.4 | 600.199146 | 1.123770 |
| GO:0008595\_determination\_of\_anterior\_posterior\_axis\_\_embryo | 5 | 0 | 0.000000 | -0.000000 | 488 | 496.600854 | 548.4 | 600.199146 | 1.123770 |
| GO:0009074\_aromatic\_amino\_acid\_family\_catabolic\_process | 5 | 0 | 0.000000 | -0.000000 | 488 | 496.600854 | 548.4 | 600.199146 | 1.123770 |
| GO:0009083\_branched\_chain\_family\_amino\_acid\_catabolic\_process | 5 | 0 | 0.000000 | -0.000000 | 488 | 496.600854 | 548.4 | 600.199146 | 1.123770 |
| GO:0009110\_vitamin\_biosynthetic\_process | 5 | 0 | 0.000000 | -0.000000 | 488 | 496.600854 | 548.4 | 600.199146 | 1.123770 |
| GO:0009119\_ribonucleoside\_metabolic\_process | 5 | 0 | 0.000000 | -0.000000 | 488 | 496.600854 | 548.4 | 600.199146 | 1.123770 |
| GO:0009156\_ribonucleoside\_monophosphate\_biosynthetic\_process | 5 | 0 | 0.000000 | -0.000000 | 488 | 496.600854 | 548.4 | 600.199146 | 1.123770 |
| GO:0009299\_mRNA\_transcription | 5 | 0 | 0.000000 | -0.000000 | 488 | 496.600854 | 548.4 | 600.199146 | 1.123770 |
| GO:0009312\_oligosaccharide\_biosynthetic\_process | 5 | 0 | 0.000000 | -0.000000 | 488 | 496.600854 | 548.4 | 600.199146 | 1.123770 |
| GO:0009395\_phospholipid\_catabolic\_process | 5 | 0 | 0.000000 | -0.000000 | 488 | 496.600854 | 548.4 | 600.199146 | 1.123770 |
| GO:0009405\_pathogenesis | 5 | 0 | 0.000000 | -0.000000 | 488 | 496.600854 | 548.4 | 600.199146 | 1.123770 |
| GO:0009650\_UV\_protection | 5 | 0 | 0.000000 | -0.000000 | 488 | 496.600854 | 548.4 | 600.199146 | 1.123770 |
| GO:0009948\_anterior\_posterior\_axis\_specification | 5 | 0 | 0.000000 | -0.000000 | 488 | 496.600854 | 548.4 | 600.199146 | 1.123770 |
| GO:0009994\_oocyte\_differentiation | 5 | 0 | 0.000000 | -0.000000 | 488 | 496.600854 | 548.4 | 600.199146 | 1.123770 |
| GO:0010165\_response\_to\_X-ray | 5 | 0 | 0.000000 | -0.000000 | 488 | 496.600854 | 548.4 | 600.199146 | 1.123770 |
| GO:0010257\_NADH\_dehydrogenase\_complex\_assembly | 5 | 0 | 0.000000 | -0.000000 | 488 | 496.600854 | 548.4 | 600.199146 | 1.123770 |
| GO:0010551\_regulation\_of\_specific\_transcription\_from\_RNA\_polymerase\_II\_promoter | 5 | 0 | 0.000000 | -0.000000 | 488 | 496.600854 | 548.4 | 600.199146 | 1.123770 |
| GO:0010552\_positive\_regulation\_of\_specific\_transcription\_from\_RNA\_polymerase\_II\_promoter | 5 | 0 | 0.000000 | -0.000000 | 488 | 496.600854 | 548.4 | 600.199146 | 1.123770 |
| GO:0014003\_oligodendrocyte\_development | 5 | 0 | 0.000000 | -0.000000 | 488 | 496.600854 | 548.4 | 600.199146 | 1.123770 |
| GO:0014070\_response\_to\_organic\_cyclic\_substance | 5 | 0 | 0.000000 | -0.000000 | 488 | 496.600854 | 548.4 | 600.199146 | 1.123770 |
| GO:0015671\_oxygen\_transport | 5 | 0 | 0.000000 | -0.000000 | 488 | 496.600854 | 548.4 | 600.199146 | 1.123770 |
| GO:0015780\_nucleotide-sugar\_transport | 5 | 0 | 0.000000 | -0.000000 | 488 | 496.600854 | 548.4 | 600.199146 | 1.123770 |
| GO:0015781\_pyrimidine\_nucleotide-sugar\_transport | 5 | 0 | 0.000000 | -0.000000 | 488 | 496.600854 | 548.4 | 600.199146 | 1.123770 |
| GO:0015908\_fatty\_acid\_transport | 5 | 0 | 0.000000 | -0.000000 | 488 | 496.600854 | 548.4 | 600.199146 | 1.123770 |
| GO:0016101\_diterpenoid\_metabolic\_process | 5 | 0 | 0.000000 | -0.000000 | 488 | 496.600854 | 548.4 | 600.199146 | 1.123770 |
| GO:0016255\_attachment\_of\_GPI\_anchor\_to\_protein | 5 | 0 | 0.000000 | -0.000000 | 488 | 496.600854 | 548.4 | 600.199146 | 1.123770 |
| GO:0016331\_morphogenesis\_of\_embryonic\_epithelium | 5 | 0 | 0.000000 | -0.000000 | 488 | 496.600854 | 548.4 | 600.199146 | 1.123770 |
| GO:0016571\_histone\_methylation | 5 | 0 | 0.000000 | -0.000000 | 488 | 496.600854 | 548.4 | 600.199146 | 1.123770 |
| GO:0017156\_calcium\_ion-dependent\_exocytosis | 5 | 0 | 0.000000 | -0.000000 | 488 | 496.600854 | 548.4 | 600.199146 | 1.123770 |
| GO:0018065\_protein-cofactor\_linkage | 5 | 0 | 0.000000 | -0.000000 | 488 | 496.600854 | 548.4 | 600.199146 | 1.123770 |
| GO:0018196\_peptidyl-asparagine\_modification | 5 | 0 | 0.000000 | -0.000000 | 488 | 496.600854 | 548.4 | 600.199146 | 1.123770 |
| GO:0018279\_protein\_amino\_acid\_N-linked\_glycosylation\_via\_asparagine | 5 | 0 | 0.000000 | -0.000000 | 488 | 496.600854 | 548.4 | 600.199146 | 1.123770 |
| GO:0019218\_regulation\_of\_steroid\_metabolic\_process | 5 | 0 | 0.000000 | -0.000000 | 488 | 496.600854 | 548.4 | 600.199146 | 1.123770 |
| GO:0022407\_regulation\_of\_cell-cell\_adhesion | 5 | 0 | 0.000000 | -0.000000 | 488 | 496.600854 | 548.4 | 600.199146 | 1.123770 |
| GO:0022600\_digestive\_system\_process | 5 | 0 | 0.000000 | -0.000000 | 488 | 496.600854 | 548.4 | 600.199146 | 1.123770 |
| GO:0030032\_lamellipodium\_biogenesis | 5 | 0 | 0.000000 | -0.000000 | 488 | 496.600854 | 548.4 | 600.199146 | 1.123770 |
| GO:0030307\_positive\_regulation\_of\_cell\_growth | 5 | 0 | 0.000000 | -0.000000 | 488 | 496.600854 | 548.4 | 600.199146 | 1.123770 |
| GO:0030317\_sperm\_motility | 5 | 0 | 0.000000 | -0.000000 | 488 | 496.600854 | 548.4 | 600.199146 | 1.123770 |
| GO:0030326\_embryonic\_limb\_morphogenesis | 5 | 0 | 0.000000 | -0.000000 | 488 | 496.600854 | 548.4 | 600.199146 | 1.123770 |
| GO:0030511\_positive\_regulation\_of\_transforming\_growth\_factor\_beta\_receptor\_signaling\_pathway | 5 | 0 | 0.000000 | -0.000000 | 488 | 496.600854 | 548.4 | 600.199146 | 1.123770 |
| GO:0030838\_positive\_regulation\_of\_actin\_filament\_polymerization | 5 | 0 | 0.000000 | -0.000000 | 488 | 496.600854 | 548.4 | 600.199146 | 1.123770 |
| GO:0031268\_pseudopodium\_organization\_and\_biogenesis | 5 | 0 | 0.000000 | -0.000000 | 488 | 496.600854 | 548.4 | 600.199146 | 1.123770 |
| GO:0031269\_pseudopodium\_formation | 5 | 0 | 0.000000 | -0.000000 | 488 | 496.600854 | 548.4 | 600.199146 | 1.123770 |
| GO:0031272\_regulation\_of\_pseudopodium\_formation | 5 | 0 | 0.000000 | -0.000000 | 488 | 496.600854 | 548.4 | 600.199146 | 1.123770 |
| GO:0031274\_positive\_regulation\_of\_pseudopodium\_formation | 5 | 0 | 0.000000 | -0.000000 | 488 | 496.600854 | 548.4 | 600.199146 | 1.123770 |
| GO:0031529\_ruffle\_organization\_and\_biogenesis | 5 | 0 | 0.000000 | -0.000000 | 488 | 496.600854 | 548.4 | 600.199146 | 1.123770 |
| GO:0031572\_G2\_M\_transition\_DNA\_damage\_checkpoint | 5 | 0 | 0.000000 | -0.000000 | 488 | 496.600854 | 548.4 | 600.199146 | 1.123770 |
| GO:0031576\_G2\_M\_transition\_checkpoint | 5 | 0 | 0.000000 | -0.000000 | 488 | 496.600854 | 548.4 | 600.199146 | 1.123770 |
| GO:0032204\_regulation\_of\_telomere\_maintenance | 5 | 0 | 0.000000 | -0.000000 | 488 | 496.600854 | 548.4 | 600.199146 | 1.123770 |
| GO:0032233\_positive\_regulation\_of\_actin\_filament\_bundle\_formation | 5 | 0 | 0.000000 | -0.000000 | 488 | 496.600854 | 548.4 | 600.199146 | 1.123770 |
| GO:0032365\_intracellular\_lipid\_transport | 5 | 0 | 0.000000 | -0.000000 | 488 | 496.600854 | 548.4 | 600.199146 | 1.123770 |
| GO:0032366\_intracellular\_sterol\_transport | 5 | 0 | 0.000000 | -0.000000 | 488 | 496.600854 | 548.4 | 600.199146 | 1.123770 |
| GO:0032367\_intracellular\_cholesterol\_transport | 5 | 0 | 0.000000 | -0.000000 | 488 | 496.600854 | 548.4 | 600.199146 | 1.123770 |
| GO:0032569\_specific\_transcription\_from\_RNA\_polymerase\_II\_promoter | 5 | 0 | 0.000000 | -0.000000 | 488 | 496.600854 | 548.4 | 600.199146 | 1.123770 |
| GO:0032594\_protein\_transport\_within\_lipid\_bilayer | 5 | 0 | 0.000000 | -0.000000 | 488 | 496.600854 | 548.4 | 600.199146 | 1.123770 |
| GO:0032602\_chemokine\_production | 5 | 0 | 0.000000 | -0.000000 | 488 | 496.600854 | 548.4 | 600.199146 | 1.123770 |
| GO:0032924\_activin\_receptor\_signaling\_pathway | 5 | 0 | 0.000000 | -0.000000 | 488 | 496.600854 | 548.4 | 600.199146 | 1.123770 |
| GO:0032925\_regulation\_of\_activin\_receptor\_signaling\_pathway | 5 | 0 | 0.000000 | -0.000000 | 488 | 496.600854 | 548.4 | 600.199146 | 1.123770 |
| GO:0032963\_collagen\_metabolic\_process | 5 | 0 | 0.000000 | -0.000000 | 488 | 496.600854 | 548.4 | 600.199146 | 1.123770 |
| GO:0032981\_mitochondrial\_respiratory\_chain\_complex\_I\_assembly | 5 | 0 | 0.000000 | -0.000000 | 488 | 496.600854 | 548.4 | 600.199146 | 1.123770 |
| GO:0033108\_mitochondrial\_respiratory\_chain\_complex\_assembly | 5 | 0 | 0.000000 | -0.000000 | 488 | 496.600854 | 548.4 | 600.199146 | 1.123770 |
| GO:0033135\_regulation\_of\_peptidyl-serine\_phosphorylation | 5 | 0 | 0.000000 | -0.000000 | 488 | 496.600854 | 548.4 | 600.199146 | 1.123770 |
| GO:0033138\_positive\_regulation\_of\_peptidyl-serine\_phosphorylation | 5 | 0 | 0.000000 | -0.000000 | 488 | 496.600854 | 548.4 | 600.199146 | 1.123770 |
| GO:0035113\_embryonic\_appendage\_morphogenesis | 5 | 0 | 0.000000 | -0.000000 | 488 | 496.600854 | 548.4 | 600.199146 | 1.123770 |
| GO:0042033\_chemokine\_biosynthetic\_process | 5 | 0 | 0.000000 | -0.000000 | 488 | 496.600854 | 548.4 | 600.199146 | 1.123770 |
| GO:0042094\_interleukin-2\_biosynthetic\_process | 5 | 0 | 0.000000 | -0.000000 | 488 | 496.600854 | 548.4 | 600.199146 | 1.123770 |
| GO:0042219\_amino\_acid\_derivative\_catabolic\_process | 5 | 0 | 0.000000 | -0.000000 | 488 | 496.600854 | 548.4 | 600.199146 | 1.123770 |
| GO:0042226\_interleukin-6\_biosynthetic\_process | 5 | 0 | 0.000000 | -0.000000 | 488 | 496.600854 | 548.4 | 600.199146 | 1.123770 |
| GO:0042311\_vasodilation | 5 | 0 | 0.000000 | -0.000000 | 488 | 496.600854 | 548.4 | 600.199146 | 1.123770 |
| GO:0042347\_negative\_regulation\_of\_NF-kappaB\_import\_into\_nucleus | 5 | 0 | 0.000000 | -0.000000 | 488 | 496.600854 | 548.4 | 600.199146 | 1.123770 |
| GO:0042533\_tumor\_necrosis\_factor\_biosynthetic\_process | 5 | 0 | 0.000000 | -0.000000 | 488 | 496.600854 | 548.4 | 600.199146 | 1.123770 |
| GO:0042534\_regulation\_of\_tumor\_necrosis\_factor\_biosynthetic\_process | 5 | 0 | 0.000000 | -0.000000 | 488 | 496.600854 | 548.4 | 600.199146 | 1.123770 |
| GO:0042535\_positive\_regulation\_of\_tumor\_necrosis\_factor\_biosynthetic\_process | 5 | 0 | 0.000000 | -0.000000 | 488 | 496.600854 | 548.4 | 600.199146 | 1.123770 |
| GO:0042769\_DNA\_damage\_response\_\_detection\_of\_DNA\_damage | 5 | 0 | 0.000000 | -0.000000 | 488 | 496.600854 | 548.4 | 600.199146 | 1.123770 |
| GO:0042771\_DNA\_damage\_response\_\_signal\_transduction\_by\_p53\_class\_mediator\_resulting\_in\_induction\_of\_apoptosis | 5 | 0 | 0.000000 | -0.000000 | 488 | 496.600854 | 548.4 | 600.199146 | 1.123770 |
| GO:0043244\_regulation\_of\_protein\_complex\_disassembly | 5 | 0 | 0.000000 | -0.000000 | 488 | 496.600854 | 548.4 | 600.199146 | 1.123770 |
| GO:0043449\_alkene\_metabolic\_process | 5 | 0 | 0.000000 | -0.000000 | 488 | 496.600854 | 548.4 | 600.199146 | 1.123770 |
| GO:0043470\_regulation\_of\_carbohydrate\_catabolic\_process | 5 | 0 | 0.000000 | -0.000000 | 488 | 496.600854 | 548.4 | 600.199146 | 1.123770 |
| GO:0043471\_regulation\_of\_cellular\_carbohydrate\_catabolic\_process | 5 | 0 | 0.000000 | -0.000000 | 488 | 496.600854 | 548.4 | 600.199146 | 1.123770 |
| GO:0043525\_positive\_regulation\_of\_neuron\_apoptosis | 5 | 0 | 0.000000 | -0.000000 | 488 | 496.600854 | 548.4 | 600.199146 | 1.123770 |
| GO:0045071\_negative\_regulation\_of\_viral\_genome\_replication | 5 | 0 | 0.000000 | -0.000000 | 488 | 496.600854 | 548.4 | 600.199146 | 1.123770 |
| GO:0045073\_regulation\_of\_chemokine\_biosynthetic\_process | 5 | 0 | 0.000000 | -0.000000 | 488 | 496.600854 | 548.4 | 600.199146 | 1.123770 |
| GO:0045076\_regulation\_of\_interleukin-2\_biosynthetic\_process | 5 | 0 | 0.000000 | -0.000000 | 488 | 496.600854 | 548.4 | 600.199146 | 1.123770 |
| GO:0045078\_positive\_regulation\_of\_interferon-gamma\_biosynthetic\_process | 5 | 0 | 0.000000 | -0.000000 | 488 | 496.600854 | 548.4 | 600.199146 | 1.123770 |
| GO:0045088\_regulation\_of\_innate\_immune\_response | 5 | 0 | 0.000000 | -0.000000 | 488 | 496.600854 | 548.4 | 600.199146 | 1.123770 |
| GO:0045161\_neuronal\_ion\_channel\_clustering | 5 | 0 | 0.000000 | -0.000000 | 488 | 496.600854 | 548.4 | 600.199146 | 1.123770 |
| GO:0045414\_regulation\_of\_interleukin-8\_biosynthetic\_process | 5 | 0 | 0.000000 | -0.000000 | 488 | 496.600854 | 548.4 | 600.199146 | 1.123770 |
| GO:0045428\_regulation\_of\_nitric\_oxide\_biosynthetic\_process | 5 | 0 | 0.000000 | -0.000000 | 488 | 496.600854 | 548.4 | 600.199146 | 1.123770 |
| GO:0045446\_endothelial\_cell\_differentiation | 5 | 0 | 0.000000 | -0.000000 | 488 | 496.600854 | 548.4 | 600.199146 | 1.123770 |
| GO:0045453\_bone\_resorption | 5 | 0 | 0.000000 | -0.000000 | 488 | 496.600854 | 548.4 | 600.199146 | 1.123770 |
| GO:0045671\_negative\_regulation\_of\_osteoclast\_differentiation | 5 | 0 | 0.000000 | -0.000000 | 488 | 496.600854 | 548.4 | 600.199146 | 1.123770 |
| GO:0045739\_positive\_regulation\_of\_DNA\_repair | 5 | 0 | 0.000000 | -0.000000 | 488 | 496.600854 | 548.4 | 600.199146 | 1.123770 |
| GO:0045749\_negative\_regulation\_of\_S\_phase\_of\_mitotic\_cell\_cycle | 5 | 0 | 0.000000 | -0.000000 | 488 | 496.600854 | 548.4 | 600.199146 | 1.123770 |
| GO:0045815\_positive\_regulation\_of\_gene\_expression\_\_epigenetic | 5 | 0 | 0.000000 | -0.000000 | 488 | 496.600854 | 548.4 | 600.199146 | 1.123770 |
| GO:0045834\_positive\_regulation\_of\_lipid\_metabolic\_process | 5 | 0 | 0.000000 | -0.000000 | 488 | 496.600854 | 548.4 | 600.199146 | 1.123770 |
| GO:0045843\_negative\_regulation\_of\_striated\_muscle\_development | 5 | 0 | 0.000000 | -0.000000 | 488 | 496.600854 | 548.4 | 600.199146 | 1.123770 |
| GO:0045862\_positive\_regulation\_of\_proteolysis | 5 | 0 | 0.000000 | -0.000000 | 488 | 496.600854 | 548.4 | 600.199146 | 1.123770 |
| GO:0045913\_positive\_regulation\_of\_carbohydrate\_metabolic\_process | 5 | 0 | 0.000000 | -0.000000 | 488 | 496.600854 | 548.4 | 600.199146 | 1.123770 |
| GO:0045947\_negative\_regulation\_of\_translational\_initiation | 5 | 0 | 0.000000 | -0.000000 | 488 | 496.600854 | 548.4 | 600.199146 | 1.123770 |
| GO:0046426\_negative\_regulation\_of\_JAK-STAT\_cascade | 5 | 0 | 0.000000 | -0.000000 | 488 | 496.600854 | 548.4 | 600.199146 | 1.123770 |
| GO:0046460\_neutral\_lipid\_biosynthetic\_process | 5 | 0 | 0.000000 | -0.000000 | 488 | 496.600854 | 548.4 | 600.199146 | 1.123770 |
| GO:0046463\_acylglycerol\_biosynthetic\_process | 5 | 0 | 0.000000 | -0.000000 | 488 | 496.600854 | 548.4 | 600.199146 | 1.123770 |
| GO:0046513\_ceramide\_biosynthetic\_process | 5 | 0 | 0.000000 | -0.000000 | 488 | 496.600854 | 548.4 | 600.199146 | 1.123770 |
| GO:0046887\_positive\_regulation\_of\_hormone\_secretion | 5 | 0 | 0.000000 | -0.000000 | 488 | 496.600854 | 548.4 | 600.199146 | 1.123770 |
| GO:0046902\_regulation\_of\_mitochondrial\_membrane\_permeability | 5 | 0 | 0.000000 | -0.000000 | 488 | 496.600854 | 548.4 | 600.199146 | 1.123770 |
| GO:0047497\_mitochondrion\_transport\_along\_microtubule | 5 | 0 | 0.000000 | -0.000000 | 488 | 496.600854 | 548.4 | 600.199146 | 1.123770 |
| GO:0048009\_insulin-like\_growth\_factor\_receptor\_signaling\_pathway | 5 | 0 | 0.000000 | -0.000000 | 488 | 496.600854 | 548.4 | 600.199146 | 1.123770 |
| GO:0048069\_eye\_pigmentation | 5 | 0 | 0.000000 | -0.000000 | 488 | 496.600854 | 548.4 | 600.199146 | 1.123770 |
| GO:0048259\_regulation\_of\_receptor-mediated\_endocytosis | 5 | 0 | 0.000000 | -0.000000 | 488 | 496.600854 | 548.4 | 600.199146 | 1.123770 |
| GO:0048525\_negative\_regulation\_of\_viral\_reproduction | 5 | 0 | 0.000000 | -0.000000 | 488 | 496.600854 | 548.4 | 600.199146 | 1.123770 |
| GO:0048627\_myoblast\_development | 5 | 0 | 0.000000 | -0.000000 | 488 | 496.600854 | 548.4 | 600.199146 | 1.123770 |
| GO:0048628\_myoblast\_maturation | 5 | 0 | 0.000000 | -0.000000 | 488 | 496.600854 | 548.4 | 600.199146 | 1.123770 |
| GO:0048635\_negative\_regulation\_of\_muscle\_development | 5 | 0 | 0.000000 | -0.000000 | 488 | 496.600854 | 548.4 | 600.199146 | 1.123770 |
| GO:0048661\_positive\_regulation\_of\_smooth\_muscle\_cell\_proliferation | 5 | 0 | 0.000000 | -0.000000 | 488 | 496.600854 | 548.4 | 600.199146 | 1.123770 |
| GO:0048675\_axon\_extension | 5 | 0 | 0.000000 | -0.000000 | 488 | 496.600854 | 548.4 | 600.199146 | 1.123770 |
| GO:0048709\_oligodendrocyte\_differentiation | 5 | 0 | 0.000000 | -0.000000 | 488 | 496.600854 | 548.4 | 600.199146 | 1.123770 |
| GO:0050684\_regulation\_of\_mRNA\_processing | 5 | 0 | 0.000000 | -0.000000 | 488 | 496.600854 | 548.4 | 600.199146 | 1.123770 |
| GO:0050688\_regulation\_of\_defense\_response\_to\_virus | 5 | 0 | 0.000000 | -0.000000 | 488 | 496.600854 | 548.4 | 600.199146 | 1.123770 |
| GO:0050691\_regulation\_of\_defense\_response\_to\_virus\_by\_host | 5 | 0 | 0.000000 | -0.000000 | 488 | 496.600854 | 548.4 | 600.199146 | 1.123770 |
| GO:0050706\_regulation\_of\_interleukin-1\_beta\_secretion | 5 | 0 | 0.000000 | -0.000000 | 488 | 496.600854 | 548.4 | 600.199146 | 1.123770 |
| GO:0050709\_negative\_regulation\_of\_protein\_secretion | 5 | 0 | 0.000000 | -0.000000 | 488 | 496.600854 | 548.4 | 600.199146 | 1.123770 |
| GO:0050718\_positive\_regulation\_of\_interleukin-1\_beta\_secretion | 5 | 0 | 0.000000 | -0.000000 | 488 | 496.600854 | 548.4 | 600.199146 | 1.123770 |
| GO:0050755\_chemokine\_metabolic\_process | 5 | 0 | 0.000000 | -0.000000 | 488 | 496.600854 | 548.4 | 600.199146 | 1.123770 |
| GO:0050772\_positive\_regulation\_of\_axonogenesis | 5 | 0 | 0.000000 | -0.000000 | 488 | 496.600854 | 548.4 | 600.199146 | 1.123770 |
| GO:0050777\_negative\_regulation\_of\_immune\_response | 5 | 0 | 0.000000 | -0.000000 | 488 | 496.600854 | 548.4 | 600.199146 | 1.123770 |
| GO:0050796\_regulation\_of\_insulin\_secretion | 5 | 0 | 0.000000 | -0.000000 | 488 | 496.600854 | 548.4 | 600.199146 | 1.123770 |
| GO:0050853\_B\_cell\_receptor\_signaling\_pathway | 5 | 0 | 0.000000 | -0.000000 | 488 | 496.600854 | 548.4 | 600.199146 | 1.123770 |
| GO:0050854\_regulation\_of\_antigen\_receptor-mediated\_signaling\_pathway | 5 | 0 | 0.000000 | -0.000000 | 488 | 496.600854 | 548.4 | 600.199146 | 1.123770 |
| GO:0050930\_induction\_of\_positive\_chemotaxis | 5 | 0 | 0.000000 | -0.000000 | 488 | 496.600854 | 548.4 | 600.199146 | 1.123770 |
| GO:0051173\_positive\_regulation\_of\_nitrogen\_compound\_metabolic\_process | 5 | 0 | 0.000000 | -0.000000 | 488 | 496.600854 | 548.4 | 600.199146 | 1.123770 |
| GO:0051208\_sequestering\_of\_calcium\_ion | 5 | 0 | 0.000000 | -0.000000 | 488 | 496.600854 | 548.4 | 600.199146 | 1.123770 |
| GO:0051216\_cartilage\_development | 5 | 0 | 0.000000 | -0.000000 | 488 | 496.600854 | 548.4 | 600.199146 | 1.123770 |
| GO:0051291\_protein\_heterooligomerization | 5 | 0 | 0.000000 | -0.000000 | 488 | 496.600854 | 548.4 | 600.199146 | 1.123770 |
| GO:0051340\_regulation\_of\_ligase\_activity | 5 | 0 | 0.000000 | -0.000000 | 488 | 496.600854 | 548.4 | 600.199146 | 1.123770 |
| GO:0051592\_response\_to\_calcium\_ion | 5 | 0 | 0.000000 | -0.000000 | 488 | 496.600854 | 548.4 | 600.199146 | 1.123770 |
| GO:0051924\_regulation\_of\_calcium\_ion\_transport | 5 | 0 | 0.000000 | -0.000000 | 488 | 496.600854 | 548.4 | 600.199146 | 1.123770 |
| GO:0006512\_ubiquitin\_cycle | 61 | 0 | 0.000000 | -0.000000 | 491 | 499.388894 | 551.04 | 602.691106 | 1.122281 |
| GO:0019318\_hexose\_metabolic\_process | 61 | 0 | 0.000000 | -0.000000 | 491 | 499.388894 | 551.04 | 602.691106 | 1.122281 |
| GO:0051707\_response\_to\_other\_organism | 61 | 0 | 0.000000 | -0.000000 | 491 | 499.388894 | 551.04 | 602.691106 | 1.122281 |
| GO:0006120\_mitochondrial\_electron\_transport\_\_NADH\_to\_ubiquinone | 34 | 0 | 0.000000 | -0.000000 | 500 | 509.457992 | 560.62 | 611.782008 | 1.121240 |
| GO:0007204\_elevation\_of\_cytosolic\_calcium\_ion\_concentration | 34 | 0 | 0.000000 | -0.000000 | 500 | 509.457992 | 560.62 | 611.782008 | 1.121240 |
| GO:0007586\_digestion | 34 | 0 | 0.000000 | -0.000000 | 500 | 509.457992 | 560.62 | 611.782008 | 1.121240 |
| GO:0007605\_sensory\_perception\_of\_sound | 34 | 0 | 0.000000 | -0.000000 | 500 | 509.457992 | 560.62 | 611.782008 | 1.121240 |
| GO:0016197\_endosome\_transport | 34 | 0 | 0.000000 | -0.000000 | 500 | 509.457992 | 560.62 | 611.782008 | 1.121240 |
| GO:0042773\_ATP\_synthesis\_coupled\_electron\_transport | 34 | 0 | 0.000000 | -0.000000 | 500 | 509.457992 | 560.62 | 611.782008 | 1.121240 |
| GO:0042775\_organelle\_ATP\_synthesis\_coupled\_electron\_transport | 34 | 0 | 0.000000 | -0.000000 | 500 | 509.457992 | 560.62 | 611.782008 | 1.121240 |
| GO:0050954\_sensory\_perception\_of\_mechanical\_stimulus | 34 | 0 | 0.000000 | -0.000000 | 500 | 509.457992 | 560.62 | 611.782008 | 1.121240 |
| GO:0051480\_cytosolic\_calcium\_ion\_homeostasis | 34 | 0 | 0.000000 | -0.000000 | 500 | 509.457992 | 560.62 | 611.782008 | 1.121240 |
| GO:0007610\_behavior | 115 | 0 | 0.000000 | -0.000000 | 502 | 510.573019 | 561.57 | 612.566981 | 1.118665 |
| GO:0016337\_cell-cell\_adhesion | 115 | 0 | 0.000000 | -0.000000 | 502 | 510.573019 | 561.57 | 612.566981 | 1.118665 |
| GO:0001501\_skeletal\_development | 90 | 0 | 0.000000 | -0.000000 | 503 | 511.109439 | 562.15 | 613.190561 | 1.117594 |
| GO:0006807\_nitrogen\_compound\_metabolic\_process | 144 | 0 | 0.000000 | -0.000000 | 504 | 511.494574 | 562.47 | 613.445426 | 1.116012 |
| GO:0048666\_neuron\_development | 65 | 0 | 0.000000 | -0.000000 | 505 | 512.239613 | 563.12 | 614.000387 | 1.115089 |
| GO:0002376\_immune\_system\_process | 359 | 0 | 0.000000 | 0.000000 | 545 | 537.776354 | 583.65 | 629.523646 | 1.070917 |
| GO:0003008\_system\_process | 424 | 0 | 0.000000 | 0.000000 | 545 | 537.776354 | 583.65 | 629.523646 | 1.070917 |
| GO:0006082\_organic\_acid\_metabolic\_process | 179 | 0 | 0.000000 | 0.000000 | 545 | 537.776354 | 583.65 | 629.523646 | 1.070917 |
| GO:0006259\_DNA\_metabolic\_process | 267 | 0 | 0.000000 | 0.000000 | 545 | 537.776354 | 583.65 | 629.523646 | 1.070917 |
| GO:0006351\_transcription\_\_DNA-dependent | 707 | 0 | 0.000000 | 0.000000 | 545 | 537.776354 | 583.65 | 629.523646 | 1.070917 |
| GO:0006355\_regulation\_of\_transcription\_\_DNA-dependent | 557 | 0 | 0.000000 | 0.000000 | 545 | 537.776354 | 583.65 | 629.523646 | 1.070917 |
| GO:0006357\_regulation\_of\_transcription\_from\_RNA\_polymerase\_II\_promoter | 260 | 0 | 0.000000 | 0.000000 | 545 | 537.776354 | 583.65 | 629.523646 | 1.070917 |
| GO:0006366\_transcription\_from\_RNA\_polymerase\_II\_promoter | 405 | 0 | 0.000000 | 0.000000 | 545 | 537.776354 | 583.65 | 629.523646 | 1.070917 |
| GO:0006396\_RNA\_processing | 188 | 0 | 0.000000 | 0.000000 | 545 | 537.776354 | 583.65 | 629.523646 | 1.070917 |
| GO:0006629\_lipid\_metabolic\_process | 323 | 0 | 0.000000 | 0.000000 | 545 | 537.776354 | 583.65 | 629.523646 | 1.070917 |
| GO:0006811\_ion\_transport | 174 | 0 | 0.000000 | 0.000000 | 545 | 537.776354 | 583.65 | 629.523646 | 1.070917 |
| GO:0006917\_induction\_of\_apoptosis | 150 | 0 | 0.000000 | 0.000000 | 545 | 537.776354 | 583.65 | 629.523646 | 1.070917 |
| GO:0006952\_defense\_response | 239 | 0 | 0.000000 | 0.000000 | 545 | 537.776354 | 583.65 | 629.523646 | 1.070917 |
| GO:0006955\_immune\_response | 243 | 0 | 0.000000 | 0.000000 | 545 | 537.776354 | 583.65 | 629.523646 | 1.070917 |
| GO:0006974\_response\_to\_DNA\_damage\_stimulus | 173 | 0 | 0.000000 | 0.000000 | 545 | 537.776354 | 583.65 | 629.523646 | 1.070917 |
| GO:0007167\_enzyme\_linked\_receptor\_protein\_signaling\_pathway | 165 | 0 | 0.000000 | 0.000000 | 545 | 537.776354 | 583.65 | 629.523646 | 1.070917 |
| GO:0007186\_G-protein\_coupled\_receptor\_protein\_signaling\_pathway | 221 | 0 | 0.000000 | 0.000000 | 545 | 537.776354 | 583.65 | 629.523646 | 1.070917 |
| GO:0007267\_cell-cell\_signaling | 307 | 0 | 0.000000 | 0.000000 | 545 | 537.776354 | 583.65 | 629.523646 | 1.070917 |
| GO:0008150\_biological\_process | 6120 | 41 | 1.000000 | 0.000000 | 545 | 537.776354 | 583.65 | 629.523646 | 1.070917 |
| GO:0009605\_response\_to\_external\_stimulus | 279 | 0 | 0.000000 | 0.000000 | 545 | 537.776354 | 583.65 | 629.523646 | 1.070917 |
| GO:0009611\_response\_to\_wounding | 188 | 0 | 0.000000 | 0.000000 | 545 | 537.776354 | 583.65 | 629.523646 | 1.070917 |
| GO:0009719\_response\_to\_endogenous\_stimulus | 213 | 0 | 0.000000 | 0.000000 | 545 | 537.776354 | 583.65 | 629.523646 | 1.070917 |
| GO:0012502\_induction\_of\_programmed\_cell\_death | 151 | 0 | 0.000000 | 0.000000 | 545 | 537.776354 | 583.65 | 629.523646 | 1.070917 |
| GO:0016070\_RNA\_metabolic\_process | 937 | 0 | 0.000000 | 0.000000 | 545 | 537.776354 | 583.65 | 629.523646 | 1.070917 |
| GO:0019752\_carboxylic\_acid\_metabolic\_process | 177 | 0 | 0.000000 | 0.000000 | 545 | 537.776354 | 583.65 | 629.523646 | 1.070917 |
| GO:0032774\_RNA\_biosynthetic\_process | 709 | 0 | 0.000000 | 0.000000 | 545 | 537.776354 | 583.65 | 629.523646 | 1.070917 |
| GO:0042592\_homeostatic\_process | 195 | 0 | 0.000000 | 0.000000 | 545 | 537.776354 | 583.65 | 629.523646 | 1.070917 |
| GO:0042981\_regulation\_of\_apoptosis | 343 | 0 | 0.000000 | 0.000000 | 545 | 537.776354 | 583.65 | 629.523646 | 1.070917 |
| GO:0043065\_positive\_regulation\_of\_apoptosis | 170 | 0 | 0.000000 | 0.000000 | 545 | 537.776354 | 583.65 | 629.523646 | 1.070917 |
| GO:0043066\_negative\_regulation\_of\_apoptosis | 154 | 0 | 0.000000 | 0.000000 | 545 | 537.776354 | 583.65 | 629.523646 | 1.070917 |
| GO:0043067\_regulation\_of\_programmed\_cell\_death | 348 | 0 | 0.000000 | 0.000000 | 545 | 537.776354 | 583.65 | 629.523646 | 1.070917 |
| GO:0043068\_positive\_regulation\_of\_programmed\_cell\_death | 173 | 0 | 0.000000 | 0.000000 | 545 | 537.776354 | 583.65 | 629.523646 | 1.070917 |
| GO:0043069\_negative\_regulation\_of\_programmed\_cell\_death | 156 | 0 | 0.000000 | 0.000000 | 545 | 537.776354 | 583.65 | 629.523646 | 1.070917 |
| GO:0044255\_cellular\_lipid\_metabolic\_process | 249 | 0 | 0.000000 | 0.000000 | 545 | 537.776354 | 583.65 | 629.523646 | 1.070917 |
| GO:0045935\_positive\_regulation\_of\_nucleobase\_\_nucleoside\_\_nucleotide\_and\_nucleic\_acid\_metabolic\_process | 169 | 0 | 0.000000 | 0.000000 | 545 | 537.776354 | 583.65 | 629.523646 | 1.070917 |
| GO:0045941\_positive\_regulation\_of\_transcription | 158 | 0 | 0.000000 | 0.000000 | 545 | 537.776354 | 583.65 | 629.523646 | 1.070917 |
| GO:0050877\_neurological\_system\_process | 270 | 0 | 0.000000 | 0.000000 | 545 | 537.776354 | 583.65 | 629.523646 | 1.070917 |
| GO:0051093\_negative\_regulation\_of\_developmental\_process | 199 | 0 | 0.000000 | 0.000000 | 545 | 537.776354 | 583.65 | 629.523646 | 1.070917 |
| GO:0051094\_positive\_regulation\_of\_developmental\_process | 216 | 0 | 0.000000 | 0.000000 | 545 | 537.776354 | 583.65 | 629.523646 | 1.070917 |
| GO:0051252\_regulation\_of\_RNA\_metabolic\_process | 568 | 0 | 0.000000 | 0.000000 | 545 | 537.776354 | 583.65 | 629.523646 | 1.070917 |
| GO:0000076\_DNA\_replication\_checkpoint | 6 | 0 | 0.000000 | 0.000000 | 676 | 673.192876 | 717.5 | 761.807124 | 1.061391 |
| GO:0000737\_DNA\_catabolic\_process\_\_endonucleolytic | 6 | 0 | 0.000000 | 0.000000 | 676 | 673.192876 | 717.5 | 761.807124 | 1.061391 |
| GO:0001516\_prostaglandin\_biosynthetic\_process | 6 | 0 | 0.000000 | 0.000000 | 676 | 673.192876 | 717.5 | 761.807124 | 1.061391 |
| GO:0001776\_leukocyte\_homeostasis | 6 | 0 | 0.000000 | 0.000000 | 676 | 673.192876 | 717.5 | 761.807124 | 1.061391 |
| GO:0001938\_positive\_regulation\_of\_endothelial\_cell\_proliferation | 6 | 0 | 0.000000 | 0.000000 | 676 | 673.192876 | 717.5 | 761.807124 | 1.061391 |
| GO:0002562\_somatic\_diversification\_of\_immune\_receptors\_via\_germline\_recombination\_within\_a\_single\_locus | 6 | 0 | 0.000000 | 0.000000 | 676 | 673.192876 | 717.5 | 761.807124 | 1.061391 |
| GO:0002705\_positive\_regulation\_of\_leukocyte\_mediated\_immunity | 6 | 0 | 0.000000 | 0.000000 | 676 | 673.192876 | 717.5 | 761.807124 | 1.061391 |
| GO:0002708\_positive\_regulation\_of\_lymphocyte\_mediated\_immunity | 6 | 0 | 0.000000 | 0.000000 | 676 | 673.192876 | 717.5 | 761.807124 | 1.061391 |
| GO:0002709\_regulation\_of\_T\_cell\_mediated\_immunity | 6 | 0 | 0.000000 | 0.000000 | 676 | 673.192876 | 717.5 | 761.807124 | 1.061391 |
| GO:0002831\_regulation\_of\_response\_to\_biotic\_stimulus | 6 | 0 | 0.000000 | 0.000000 | 676 | 673.192876 | 717.5 | 761.807124 | 1.061391 |
| GO:0005513\_detection\_of\_calcium\_ion | 6 | 0 | 0.000000 | 0.000000 | 676 | 673.192876 | 717.5 | 761.807124 | 1.061391 |
| GO:0006090\_pyruvate\_metabolic\_process | 6 | 0 | 0.000000 | 0.000000 | 676 | 673.192876 | 717.5 | 761.807124 | 1.061391 |
| GO:0006283\_transcription-coupled\_nucleotide-excision\_repair | 6 | 0 | 0.000000 | 0.000000 | 676 | 673.192876 | 717.5 | 761.807124 | 1.061391 |
| GO:0006303\_double-strand\_break\_repair\_via\_nonhomologous\_end\_joining | 6 | 0 | 0.000000 | 0.000000 | 676 | 673.192876 | 717.5 | 761.807124 | 1.061391 |
| GO:0006474\_N-terminal\_protein\_amino\_acid\_acetylation | 6 | 0 | 0.000000 | 0.000000 | 676 | 673.192876 | 717.5 | 761.807124 | 1.061391 |
| GO:0006477\_protein\_amino\_acid\_sulfation | 6 | 0 | 0.000000 | 0.000000 | 676 | 673.192876 | 717.5 | 761.807124 | 1.061391 |
| GO:0006491\_N-glycan\_processing | 6 | 0 | 0.000000 | 0.000000 | 676 | 673.192876 | 717.5 | 761.807124 | 1.061391 |
| GO:0006544\_glycine\_metabolic\_process | 6 | 0 | 0.000000 | 0.000000 | 676 | 673.192876 | 717.5 | 761.807124 | 1.061391 |
| GO:0006595\_polyamine\_metabolic\_process | 6 | 0 | 0.000000 | 0.000000 | 676 | 673.192876 | 717.5 | 761.807124 | 1.061391 |
| GO:0006637\_acyl-CoA\_metabolic\_process | 6 | 0 | 0.000000 | 0.000000 | 676 | 673.192876 | 717.5 | 761.807124 | 1.061391 |
| GO:0006739\_NADP\_metabolic\_process | 6 | 0 | 0.000000 | 0.000000 | 676 | 673.192876 | 717.5 | 761.807124 | 1.061391 |
| GO:0006769\_nicotinamide\_metabolic\_process | 6 | 0 | 0.000000 | 0.000000 | 676 | 673.192876 | 717.5 | 761.807124 | 1.061391 |
| GO:0006878\_cellular\_copper\_ion\_homeostasis | 6 | 0 | 0.000000 | 0.000000 | 676 | 673.192876 | 717.5 | 761.807124 | 1.061391 |
| GO:0006942\_regulation\_of\_striated\_muscle\_contraction | 6 | 0 | 0.000000 | 0.000000 | 676 | 673.192876 | 717.5 | 761.807124 | 1.061391 |
| GO:0006953\_acute-phase\_response | 6 | 0 | 0.000000 | 0.000000 | 676 | 673.192876 | 717.5 | 761.807124 | 1.061391 |
| GO:0007020\_microtubule\_nucleation | 6 | 0 | 0.000000 | 0.000000 | 676 | 673.192876 | 717.5 | 761.807124 | 1.061391 |
| GO:0007159\_leukocyte\_adhesion | 6 | 0 | 0.000000 | 0.000000 | 676 | 673.192876 | 717.5 | 761.807124 | 1.061391 |
| GO:0007185\_transmembrane\_receptor\_protein\_tyrosine\_phosphatase\_signaling\_pathway | 6 | 0 | 0.000000 | 0.000000 | 676 | 673.192876 | 717.5 | 761.807124 | 1.061391 |
| GO:0007194\_negative\_regulation\_of\_adenylate\_cyclase\_activity | 6 | 0 | 0.000000 | 0.000000 | 676 | 673.192876 | 717.5 | 761.807124 | 1.061391 |
| GO:0007516\_hemocyte\_development | 6 | 0 | 0.000000 | 0.000000 | 676 | 673.192876 | 717.5 | 761.807124 | 1.061391 |
| GO:0007589\_body\_fluid\_secretion | 6 | 0 | 0.000000 | 0.000000 | 676 | 673.192876 | 717.5 | 761.807124 | 1.061391 |
| GO:0007631\_feeding\_behavior | 6 | 0 | 0.000000 | 0.000000 | 676 | 673.192876 | 717.5 | 761.807124 | 1.061391 |
| GO:0008088\_axon\_cargo\_transport | 6 | 0 | 0.000000 | 0.000000 | 676 | 673.192876 | 717.5 | 761.807124 | 1.061391 |
| GO:0008209\_androgen\_metabolic\_process | 6 | 0 | 0.000000 | 0.000000 | 676 | 673.192876 | 717.5 | 761.807124 | 1.061391 |
| GO:0008535\_respiratory\_chain\_complex\_IV\_assembly | 6 | 0 | 0.000000 | 0.000000 | 676 | 673.192876 | 717.5 | 761.807124 | 1.061391 |
| GO:0009068\_aspartate\_family\_amino\_acid\_catabolic\_process | 6 | 0 | 0.000000 | 0.000000 | 676 | 673.192876 | 717.5 | 761.807124 | 1.061391 |
| GO:0009124\_nucleoside\_monophosphate\_biosynthetic\_process | 6 | 0 | 0.000000 | 0.000000 | 676 | 673.192876 | 717.5 | 761.807124 | 1.061391 |
| GO:0009142\_nucleoside\_triphosphate\_biosynthetic\_process | 6 | 0 | 0.000000 | 0.000000 | 676 | 673.192876 | 717.5 | 761.807124 | 1.061391 |
| GO:0009152\_purine\_ribonucleotide\_biosynthetic\_process | 6 | 0 | 0.000000 | 0.000000 | 676 | 673.192876 | 717.5 | 761.807124 | 1.061391 |
| GO:0009161\_ribonucleoside\_monophosphate\_metabolic\_process | 6 | 0 | 0.000000 | 0.000000 | 676 | 673.192876 | 717.5 | 761.807124 | 1.061391 |
| GO:0009303\_rRNA\_transcription | 6 | 0 | 0.000000 | 0.000000 | 676 | 673.192876 | 717.5 | 761.807124 | 1.061391 |
| GO:0009595\_detection\_of\_biotic\_stimulus | 6 | 0 | 0.000000 | 0.000000 | 676 | 673.192876 | 717.5 | 761.807124 | 1.061391 |
| GO:0009746\_response\_to\_hexose\_stimulus | 6 | 0 | 0.000000 | 0.000000 | 676 | 673.192876 | 717.5 | 761.807124 | 1.061391 |
| GO:0009749\_response\_to\_glucose\_stimulus | 6 | 0 | 0.000000 | 0.000000 | 676 | 673.192876 | 717.5 | 761.807124 | 1.061391 |
| GO:0009755\_hormone-mediated\_signaling | 6 | 0 | 0.000000 | 0.000000 | 676 | 673.192876 | 717.5 | 761.807124 | 1.061391 |
| GO:0009896\_positive\_regulation\_of\_catabolic\_process | 6 | 0 | 0.000000 | 0.000000 | 676 | 673.192876 | 717.5 | 761.807124 | 1.061391 |
| GO:0009913\_epidermal\_cell\_differentiation | 6 | 0 | 0.000000 | 0.000000 | 676 | 673.192876 | 717.5 | 761.807124 | 1.061391 |
| GO:0010001\_glial\_cell\_differentiation | 6 | 0 | 0.000000 | 0.000000 | 676 | 673.192876 | 717.5 | 761.807124 | 1.061391 |
| GO:0015669\_gas\_transport | 6 | 0 | 0.000000 | 0.000000 | 676 | 673.192876 | 717.5 | 761.807124 | 1.061391 |
| GO:0015695\_organic\_cation\_transport | 6 | 0 | 0.000000 | 0.000000 | 676 | 673.192876 | 717.5 | 761.807124 | 1.061391 |
| GO:0015884\_folic\_acid\_transport | 6 | 0 | 0.000000 | 0.000000 | 676 | 673.192876 | 717.5 | 761.807124 | 1.061391 |
| GO:0016064\_immunoglobulin\_mediated\_immune\_response | 6 | 0 | 0.000000 | 0.000000 | 676 | 673.192876 | 717.5 | 761.807124 | 1.061391 |
| GO:0016444\_somatic\_cell\_DNA\_recombination | 6 | 0 | 0.000000 | 0.000000 | 676 | 673.192876 | 717.5 | 761.807124 | 1.061391 |
| GO:0016445\_somatic\_diversification\_of\_immunoglobulins | 6 | 0 | 0.000000 | 0.000000 | 676 | 673.192876 | 717.5 | 761.807124 | 1.061391 |
| GO:0016601\_Rac\_protein\_signal\_transduction | 6 | 0 | 0.000000 | 0.000000 | 676 | 673.192876 | 717.5 | 761.807124 | 1.061391 |
| GO:0018210\_peptidyl-threonine\_modification | 6 | 0 | 0.000000 | 0.000000 | 676 | 673.192876 | 717.5 | 761.807124 | 1.061391 |
| GO:0019362\_pyridine\_nucleotide\_metabolic\_process | 6 | 0 | 0.000000 | 0.000000 | 676 | 673.192876 | 717.5 | 761.807124 | 1.061391 |
| GO:0021782\_glial\_cell\_development | 6 | 0 | 0.000000 | 0.000000 | 676 | 673.192876 | 717.5 | 761.807124 | 1.061391 |
| GO:0030193\_regulation\_of\_blood\_coagulation | 6 | 0 | 0.000000 | 0.000000 | 676 | 673.192876 | 717.5 | 761.807124 | 1.061391 |
| GO:0030199\_collagen\_fibril\_organization | 6 | 0 | 0.000000 | 0.000000 | 676 | 673.192876 | 717.5 | 761.807124 | 1.061391 |
| GO:0030258\_lipid\_modification | 6 | 0 | 0.000000 | 0.000000 | 676 | 673.192876 | 717.5 | 761.807124 | 1.061391 |
| GO:0030282\_bone\_mineralization | 6 | 0 | 0.000000 | 0.000000 | 676 | 673.192876 | 717.5 | 761.807124 | 1.061391 |
| GO:0030316\_osteoclast\_differentiation | 6 | 0 | 0.000000 | 0.000000 | 676 | 673.192876 | 717.5 | 761.807124 | 1.061391 |
| GO:0030503\_regulation\_of\_cell\_redox\_homeostasis | 6 | 0 | 0.000000 | 0.000000 | 676 | 673.192876 | 717.5 | 761.807124 | 1.061391 |
| GO:0030509\_BMP\_signaling\_pathway | 6 | 0 | 0.000000 | 0.000000 | 676 | 673.192876 | 717.5 | 761.807124 | 1.061391 |
| GO:0030879\_mammary\_gland\_development | 6 | 0 | 0.000000 | 0.000000 | 676 | 673.192876 | 717.5 | 761.807124 | 1.061391 |
| GO:0031280\_negative\_regulation\_of\_cyclase\_activity | 6 | 0 | 0.000000 | 0.000000 | 676 | 673.192876 | 717.5 | 761.807124 | 1.061391 |
| GO:0031344\_regulation\_of\_cell\_projection\_organization\_and\_biogenesis | 6 | 0 | 0.000000 | 0.000000 | 676 | 673.192876 | 717.5 | 761.807124 | 1.061391 |
| GO:0031346\_positive\_regulation\_of\_cell\_projection\_organization\_and\_biogenesis | 6 | 0 | 0.000000 | 0.000000 | 676 | 673.192876 | 717.5 | 761.807124 | 1.061391 |
| GO:0031349\_positive\_regulation\_of\_defense\_response | 6 | 0 | 0.000000 | 0.000000 | 676 | 673.192876 | 717.5 | 761.807124 | 1.061391 |
| GO:0031398\_positive\_regulation\_of\_protein\_ubiquitination | 6 | 0 | 0.000000 | 0.000000 | 676 | 673.192876 | 717.5 | 761.807124 | 1.061391 |
| GO:0031571\_G1\_DNA\_damage\_checkpoint | 6 | 0 | 0.000000 | 0.000000 | 676 | 673.192876 | 717.5 | 761.807124 | 1.061391 |
| GO:0031960\_response\_to\_corticosteroid\_stimulus | 6 | 0 | 0.000000 | 0.000000 | 676 | 673.192876 | 717.5 | 761.807124 | 1.061391 |
| GO:0032297\_negative\_regulation\_of\_DNA\_replication\_initiation | 6 | 0 | 0.000000 | 0.000000 | 676 | 673.192876 | 717.5 | 761.807124 | 1.061391 |
| GO:0032640\_tumor\_necrosis\_factor\_production | 6 | 0 | 0.000000 | 0.000000 | 676 | 673.192876 | 717.5 | 761.807124 | 1.061391 |
| GO:0032868\_response\_to\_insulin\_stimulus | 6 | 0 | 0.000000 | 0.000000 | 676 | 673.192876 | 717.5 | 761.807124 | 1.061391 |
| GO:0033500\_carbohydrate\_homeostasis | 6 | 0 | 0.000000 | 0.000000 | 676 | 673.192876 | 717.5 | 761.807124 | 1.061391 |
| GO:0033683\_nucleotide-excision\_repair\_\_DNA\_incision | 6 | 0 | 0.000000 | 0.000000 | 676 | 673.192876 | 717.5 | 761.807124 | 1.061391 |
| GO:0034284\_response\_to\_monosaccharide\_stimulus | 6 | 0 | 0.000000 | 0.000000 | 676 | 673.192876 | 717.5 | 761.807124 | 1.061391 |
| GO:0035107\_appendage\_morphogenesis | 6 | 0 | 0.000000 | 0.000000 | 676 | 673.192876 | 717.5 | 761.807124 | 1.061391 |
| GO:0035108\_limb\_morphogenesis | 6 | 0 | 0.000000 | 0.000000 | 676 | 673.192876 | 717.5 | 761.807124 | 1.061391 |
| GO:0035270\_endocrine\_system\_development | 6 | 0 | 0.000000 | 0.000000 | 676 | 673.192876 | 717.5 | 761.807124 | 1.061391 |
| GO:0035282\_segmentation | 6 | 0 | 0.000000 | 0.000000 | 676 | 673.192876 | 717.5 | 761.807124 | 1.061391 |
| GO:0042095\_interferon-gamma\_biosynthetic\_process | 6 | 0 | 0.000000 | 0.000000 | 676 | 673.192876 | 717.5 | 761.807124 | 1.061391 |
| GO:0042228\_interleukin-8\_biosynthetic\_process | 6 | 0 | 0.000000 | 0.000000 | 676 | 673.192876 | 717.5 | 761.807124 | 1.061391 |
| GO:0042255\_ribosome\_assembly | 6 | 0 | 0.000000 | 0.000000 | 676 | 673.192876 | 717.5 | 761.807124 | 1.061391 |
| GO:0042401\_biogenic\_amine\_biosynthetic\_process | 6 | 0 | 0.000000 | 0.000000 | 676 | 673.192876 | 717.5 | 761.807124 | 1.061391 |
| GO:0042593\_glucose\_homeostasis | 6 | 0 | 0.000000 | 0.000000 | 676 | 673.192876 | 717.5 | 761.807124 | 1.061391 |
| GO:0043462\_regulation\_of\_ATPase\_activity | 6 | 0 | 0.000000 | 0.000000 | 676 | 673.192876 | 717.5 | 761.807124 | 1.061391 |
| GO:0043648\_dicarboxylic\_acid\_metabolic\_process | 6 | 0 | 0.000000 | 0.000000 | 676 | 673.192876 | 717.5 | 761.807124 | 1.061391 |
| GO:0043681\_protein\_import\_into\_mitochondrion | 6 | 0 | 0.000000 | 0.000000 | 676 | 673.192876 | 717.5 | 761.807124 | 1.061391 |
| GO:0045017\_glycerolipid\_biosynthetic\_process | 6 | 0 | 0.000000 | 0.000000 | 676 | 673.192876 | 717.5 | 761.807124 | 1.061391 |
| GO:0045058\_T\_cell\_selection | 6 | 0 | 0.000000 | 0.000000 | 676 | 673.192876 | 717.5 | 761.807124 | 1.061391 |
| GO:0045072\_regulation\_of\_interferon-gamma\_biosynthetic\_process | 6 | 0 | 0.000000 | 0.000000 | 676 | 673.192876 | 717.5 | 761.807124 | 1.061391 |
| GO:0045090\_retroviral\_genome\_replication | 6 | 0 | 0.000000 | 0.000000 | 676 | 673.192876 | 717.5 | 761.807124 | 1.061391 |
| GO:0045103\_intermediate\_filament-based\_process | 6 | 0 | 0.000000 | 0.000000 | 676 | 673.192876 | 717.5 | 761.807124 | 1.061391 |
| GO:0045104\_intermediate\_filament\_cytoskeleton\_organization\_and\_biogenesis | 6 | 0 | 0.000000 | 0.000000 | 676 | 673.192876 | 717.5 | 761.807124 | 1.061391 |
| GO:0045454\_cell\_redox\_homeostasis | 6 | 0 | 0.000000 | 0.000000 | 676 | 673.192876 | 717.5 | 761.807124 | 1.061391 |
| GO:0045661\_regulation\_of\_myoblast\_differentiation | 6 | 0 | 0.000000 | 0.000000 | 676 | 673.192876 | 717.5 | 761.807124 | 1.061391 |
| GO:0045670\_regulation\_of\_osteoclast\_differentiation | 6 | 0 | 0.000000 | 0.000000 | 676 | 673.192876 | 717.5 | 761.807124 | 1.061391 |
| GO:0045742\_positive\_regulation\_of\_epidermal\_growth\_factor\_receptor\_signaling\_pathway | 6 | 0 | 0.000000 | 0.000000 | 676 | 673.192876 | 717.5 | 761.807124 | 1.061391 |
| GO:0045793\_positive\_regulation\_of\_cell\_size | 6 | 0 | 0.000000 | 0.000000 | 676 | 673.192876 | 717.5 | 761.807124 | 1.061391 |
| GO:0045807\_positive\_regulation\_of\_endocytosis | 6 | 0 | 0.000000 | 0.000000 | 676 | 673.192876 | 717.5 | 761.807124 | 1.061391 |
| GO:0045927\_positive\_regulation\_of\_growth | 6 | 0 | 0.000000 | 0.000000 | 676 | 673.192876 | 717.5 | 761.807124 | 1.061391 |
| GO:0046034\_ATP\_metabolic\_process | 6 | 0 | 0.000000 | 0.000000 | 676 | 673.192876 | 717.5 | 761.807124 | 1.061391 |
| GO:0046457\_prostanoid\_biosynthetic\_process | 6 | 0 | 0.000000 | 0.000000 | 676 | 673.192876 | 717.5 | 761.807124 | 1.061391 |
| GO:0046504\_glycerol\_ether\_biosynthetic\_process | 6 | 0 | 0.000000 | 0.000000 | 676 | 673.192876 | 717.5 | 761.807124 | 1.061391 |
| GO:0046520\_sphingoid\_biosynthetic\_process | 6 | 0 | 0.000000 | 0.000000 | 676 | 673.192876 | 717.5 | 761.807124 | 1.061391 |
| GO:0046847\_filopodium\_formation | 6 | 0 | 0.000000 | 0.000000 | 676 | 673.192876 | 717.5 | 761.807124 | 1.061391 |
| GO:0046890\_regulation\_of\_lipid\_biosynthetic\_process | 6 | 0 | 0.000000 | 0.000000 | 676 | 673.192876 | 717.5 | 761.807124 | 1.061391 |
| GO:0048332\_mesoderm\_morphogenesis | 6 | 0 | 0.000000 | 0.000000 | 676 | 673.192876 | 717.5 | 761.807124 | 1.061391 |
| GO:0048477\_oogenesis | 6 | 0 | 0.000000 | 0.000000 | 676 | 673.192876 | 717.5 | 761.807124 | 1.061391 |
| GO:0048524\_positive\_regulation\_of\_viral\_reproduction | 6 | 0 | 0.000000 | 0.000000 | 676 | 673.192876 | 717.5 | 761.807124 | 1.061391 |
| GO:0048736\_appendage\_development | 6 | 0 | 0.000000 | 0.000000 | 676 | 673.192876 | 717.5 | 761.807124 | 1.061391 |
| GO:0050702\_interleukin-1\_beta\_secretion | 6 | 0 | 0.000000 | 0.000000 | 676 | 673.192876 | 717.5 | 761.807124 | 1.061391 |
| GO:0050704\_regulation\_of\_interleukin-1\_secretion | 6 | 0 | 0.000000 | 0.000000 | 676 | 673.192876 | 717.5 | 761.807124 | 1.061391 |
| GO:0050716\_positive\_regulation\_of\_interleukin-1\_secretion | 6 | 0 | 0.000000 | 0.000000 | 676 | 673.192876 | 717.5 | 761.807124 | 1.061391 |
| GO:0050871\_positive\_regulation\_of\_B\_cell\_activation | 6 | 0 | 0.000000 | 0.000000 | 676 | 673.192876 | 717.5 | 761.807124 | 1.061391 |
| GO:0050909\_sensory\_perception\_of\_taste | 6 | 0 | 0.000000 | 0.000000 | 676 | 673.192876 | 717.5 | 761.807124 | 1.061391 |
| GO:0050918\_positive\_chemotaxis | 6 | 0 | 0.000000 | 0.000000 | 676 | 673.192876 | 717.5 | 761.807124 | 1.061391 |
| GO:0050926\_regulation\_of\_positive\_chemotaxis | 6 | 0 | 0.000000 | 0.000000 | 676 | 673.192876 | 717.5 | 761.807124 | 1.061391 |
| GO:0050927\_positive\_regulation\_of\_positive\_chemotaxis | 6 | 0 | 0.000000 | 0.000000 | 676 | 673.192876 | 717.5 | 761.807124 | 1.061391 |
| GO:0051171\_regulation\_of\_nitrogen\_compound\_metabolic\_process | 6 | 0 | 0.000000 | 0.000000 | 676 | 673.192876 | 717.5 | 761.807124 | 1.061391 |
| GO:0051289\_protein\_homotetramerization | 6 | 0 | 0.000000 | 0.000000 | 676 | 673.192876 | 717.5 | 761.807124 | 1.061391 |
| GO:0051350\_negative\_regulation\_of\_lyase\_activity | 6 | 0 | 0.000000 | 0.000000 | 676 | 673.192876 | 717.5 | 761.807124 | 1.061391 |
| GO:0051384\_response\_to\_glucocorticoid\_stimulus | 6 | 0 | 0.000000 | 0.000000 | 676 | 673.192876 | 717.5 | 761.807124 | 1.061391 |
| GO:0051457\_maintenance\_of\_protein\_localization\_in\_nucleus | 6 | 0 | 0.000000 | 0.000000 | 676 | 673.192876 | 717.5 | 761.807124 | 1.061391 |
| GO:0051923\_sulfation | 6 | 0 | 0.000000 | 0.000000 | 676 | 673.192876 | 717.5 | 761.807124 | 1.061391 |
| GO:0055010\_ventricular\_cardiac\_muscle\_morphogenesis | 6 | 0 | 0.000000 | 0.000000 | 676 | 673.192876 | 717.5 | 761.807124 | 1.061391 |
| GO:0055070\_copper\_ion\_homeostasis | 6 | 0 | 0.000000 | 0.000000 | 676 | 673.192876 | 717.5 | 761.807124 | 1.061391 |
| GO:0060173\_limb\_development | 6 | 0 | 0.000000 | 0.000000 | 676 | 673.192876 | 717.5 | 761.807124 | 1.061391 |
| GO:0002694\_regulation\_of\_leukocyte\_activation | 36 | 0 | 0.000000 | 0.000000 | 688 | 685.561313 | 729.12 | 772.678687 | 1.059767 |
| GO:0007200\_G-protein\_signaling\_\_coupled\_to\_IP3\_second\_messenger\_(phospholipase\_C\_activating) | 36 | 0 | 0.000000 | 0.000000 | 688 | 685.561313 | 729.12 | 772.678687 | 1.059767 |
| GO:0016042\_lipid\_catabolic\_process | 36 | 0 | 0.000000 | 0.000000 | 688 | 685.561313 | 729.12 | 772.678687 | 1.059767 |
| GO:0016052\_carbohydrate\_catabolic\_process | 36 | 0 | 0.000000 | 0.000000 | 688 | 685.561313 | 729.12 | 772.678687 | 1.059767 |
| GO:0022900\_electron\_transport\_chain | 36 | 0 | 0.000000 | 0.000000 | 688 | 685.561313 | 729.12 | 772.678687 | 1.059767 |
| GO:0022904\_respiratory\_electron\_transport\_chain | 36 | 0 | 0.000000 | 0.000000 | 688 | 685.561313 | 729.12 | 772.678687 | 1.059767 |
| GO:0030518\_steroid\_hormone\_receptor\_signaling\_pathway | 36 | 0 | 0.000000 | 0.000000 | 688 | 685.561313 | 729.12 | 772.678687 | 1.059767 |
| GO:0044242\_cellular\_lipid\_catabolic\_process | 36 | 0 | 0.000000 | 0.000000 | 688 | 685.561313 | 729.12 | 772.678687 | 1.059767 |
| GO:0044275\_cellular\_carbohydrate\_catabolic\_process | 36 | 0 | 0.000000 | 0.000000 | 688 | 685.561313 | 729.12 | 772.678687 | 1.059767 |
| GO:0050865\_regulation\_of\_cell\_activation | 36 | 0 | 0.000000 | 0.000000 | 688 | 685.561313 | 729.12 | 772.678687 | 1.059767 |
| GO:0051130\_positive\_regulation\_of\_cellular\_component\_organization\_and\_biogenesis | 36 | 0 | 0.000000 | 0.000000 | 688 | 685.561313 | 729.12 | 772.678687 | 1.059767 |
| GO:0055114\_oxidation\_reduction | 36 | 0 | 0.000000 | 0.000000 | 688 | 685.561313 | 729.12 | 772.678687 | 1.059767 |
| GO:0001944\_vasculature\_development | 56 | 0 | 0.000000 | 0.000000 | 695 | 692.809125 | 735.54 | 778.270875 | 1.058331 |
| GO:0006753\_nucleoside\_phosphate\_metabolic\_process | 56 | 0 | 0.000000 | 0.000000 | 695 | 692.809125 | 735.54 | 778.270875 | 1.058331 |
| GO:0007050\_cell\_cycle\_arrest | 56 | 0 | 0.000000 | 0.000000 | 695 | 692.809125 | 735.54 | 778.270875 | 1.058331 |
| GO:0009117\_nucleotide\_metabolic\_process | 56 | 0 | 0.000000 | 0.000000 | 695 | 692.809125 | 735.54 | 778.270875 | 1.058331 |
| GO:0031175\_neurite\_development | 56 | 0 | 0.000000 | 0.000000 | 695 | 692.809125 | 735.54 | 778.270875 | 1.058331 |
| GO:0051098\_regulation\_of\_binding | 56 | 0 | 0.000000 | 0.000000 | 695 | 692.809125 | 735.54 | 778.270875 | 1.058331 |
| GO:0065004\_protein-DNA\_complex\_assembly | 56 | 0 | 0.000000 | 0.000000 | 695 | 692.809125 | 735.54 | 778.270875 | 1.058331 |
| GO:0006519\_amino\_acid\_and\_derivative\_metabolic\_process | 101 | 0 | 0.000000 | 0.000000 | 697 | 693.908565 | 736.5 | 779.091435 | 1.056671 |
| GO:0008380\_RNA\_splicing | 101 | 0 | 0.000000 | 0.000000 | 697 | 693.908565 | 736.5 | 779.091435 | 1.056671 |
| GO:0009887\_organ\_morphogenesis | 139 | 0 | 0.000000 | 0.000000 | 698 | 694.392564 | 736.85 | 779.307436 | 1.055659 |
| GO:0009310\_amine\_catabolic\_process | 28 | 0 | 0.000000 | 0.000000 | 704 | 702.299898 | 744.24 | 786.180102 | 1.057159 |
| GO:0009890\_negative\_regulation\_of\_biosynthetic\_process | 28 | 0 | 0.000000 | 0.000000 | 704 | 702.299898 | 744.24 | 786.180102 | 1.057159 |
| GO:0010033\_response\_to\_organic\_substance | 28 | 0 | 0.000000 | 0.000000 | 704 | 702.299898 | 744.24 | 786.180102 | 1.057159 |
| GO:0016569\_covalent\_chromatin\_modification | 28 | 0 | 0.000000 | 0.000000 | 704 | 702.299898 | 744.24 | 786.180102 | 1.057159 |
| GO:0030384\_phosphoinositide\_metabolic\_process | 28 | 0 | 0.000000 | 0.000000 | 704 | 702.299898 | 744.24 | 786.180102 | 1.057159 |
| GO:0046474\_glycerophospholipid\_biosynthetic\_process | 28 | 0 | 0.000000 | 0.000000 | 704 | 702.299898 | 744.24 | 786.180102 | 1.057159 |
| GO:0007596\_blood\_coagulation | 44 | 0 | 0.000000 | 0.000000 | 708 | 705.486127 | 747.12 | 788.753873 | 1.055254 |
| GO:0009968\_negative\_regulation\_of\_signal\_transduction | 44 | 0 | 0.000000 | 0.000000 | 708 | 705.486127 | 747.12 | 788.753873 | 1.055254 |
| GO:0052547\_regulation\_of\_peptidase\_activity | 44 | 0 | 0.000000 | 0.000000 | 708 | 705.486127 | 747.12 | 788.753873 | 1.055254 |
| GO:0052548\_regulation\_of\_endopeptidase\_activity | 44 | 0 | 0.000000 | 0.000000 | 708 | 705.486127 | 747.12 | 788.753873 | 1.055254 |
| GO:0001816\_cytokine\_production | 60 | 0 | 0.000000 | 0.000000 | 711 | 707.596052 | 748.95 | 790.303948 | 1.053376 |
| GO:0007005\_mitochondrion\_organization\_and\_biogenesis | 60 | 0 | 0.000000 | 0.000000 | 711 | 707.596052 | 748.95 | 790.303948 | 1.053376 |
| GO:0051186\_cofactor\_metabolic\_process | 60 | 0 | 0.000000 | 0.000000 | 711 | 707.596052 | 748.95 | 790.303948 | 1.053376 |
| GO:0007276\_gamete\_generation | 104 | 0 | 0.000000 | 0.000000 | 713 | 708.679453 | 750.03 | 791.380547 | 1.051935 |
| GO:0007417\_central\_nervous\_system\_development | 104 | 0 | 0.000000 | 0.000000 | 713 | 708.679453 | 750.03 | 791.380547 | 1.051935 |
| GO:0002009\_morphogenesis\_of\_an\_epithelium | 18 | 0 | 0.000000 | 0.000000 | 734 | 729.625234 | 770.26 | 810.894766 | 1.049401 |
| GO:0002573\_myeloid\_leukocyte\_differentiation | 18 | 0 | 0.000000 | 0.000000 | 734 | 729.625234 | 770.26 | 810.894766 | 1.049401 |
| GO:0005976\_polysaccharide\_metabolic\_process | 18 | 0 | 0.000000 | 0.000000 | 734 | 729.625234 | 770.26 | 810.894766 | 1.049401 |
| GO:0006040\_amino\_sugar\_metabolic\_process | 18 | 0 | 0.000000 | 0.000000 | 734 | 729.625234 | 770.26 | 810.894766 | 1.049401 |
| GO:0006364\_rRNA\_processing | 18 | 0 | 0.000000 | 0.000000 | 734 | 729.625234 | 770.26 | 810.894766 | 1.049401 |
| GO:0006473\_protein\_amino\_acid\_acetylation | 18 | 0 | 0.000000 | 0.000000 | 734 | 729.625234 | 770.26 | 810.894766 | 1.049401 |
| GO:0006493\_protein\_amino\_acid\_O-linked\_glycosylation | 18 | 0 | 0.000000 | 0.000000 | 734 | 729.625234 | 770.26 | 810.894766 | 1.049401 |
| GO:0006633\_fatty\_acid\_biosynthetic\_process | 18 | 0 | 0.000000 | 0.000000 | 734 | 729.625234 | 770.26 | 810.894766 | 1.049401 |
| GO:0006800\_oxygen\_and\_reactive\_oxygen\_species\_metabolic\_process | 18 | 0 | 0.000000 | 0.000000 | 734 | 729.625234 | 770.26 | 810.894766 | 1.049401 |
| GO:0007131\_meiotic\_recombination | 18 | 0 | 0.000000 | 0.000000 | 734 | 729.625234 | 770.26 | 810.894766 | 1.049401 |
| GO:0008645\_hexose\_transport | 18 | 0 | 0.000000 | 0.000000 | 734 | 729.625234 | 770.26 | 810.894766 | 1.049401 |
| GO:0015749\_monosaccharide\_transport | 18 | 0 | 0.000000 | 0.000000 | 734 | 729.625234 | 770.26 | 810.894766 | 1.049401 |
| GO:0015758\_glucose\_transport | 18 | 0 | 0.000000 | 0.000000 | 734 | 729.625234 | 770.26 | 810.894766 | 1.049401 |
| GO:0030262\_apoptotic\_nuclear\_changes | 18 | 0 | 0.000000 | 0.000000 | 734 | 729.625234 | 770.26 | 810.894766 | 1.049401 |
| GO:0031347\_regulation\_of\_defense\_response | 18 | 0 | 0.000000 | 0.000000 | 734 | 729.625234 | 770.26 | 810.894766 | 1.049401 |
| GO:0031667\_response\_to\_nutrient\_levels | 18 | 0 | 0.000000 | 0.000000 | 734 | 729.625234 | 770.26 | 810.894766 | 1.049401 |
| GO:0043284\_biopolymer\_biosynthetic\_process | 18 | 0 | 0.000000 | 0.000000 | 734 | 729.625234 | 770.26 | 810.894766 | 1.049401 |
| GO:0045087\_innate\_immune\_response | 18 | 0 | 0.000000 | 0.000000 | 734 | 729.625234 | 770.26 | 810.894766 | 1.049401 |
| GO:0046148\_pigment\_biosynthetic\_process | 18 | 0 | 0.000000 | 0.000000 | 734 | 729.625234 | 770.26 | 810.894766 | 1.049401 |
| GO:0046519\_sphingoid\_metabolic\_process | 18 | 0 | 0.000000 | 0.000000 | 734 | 729.625234 | 770.26 | 810.894766 | 1.049401 |
| GO:0051047\_positive\_regulation\_of\_secretion | 18 | 0 | 0.000000 | 0.000000 | 734 | 729.625234 | 770.26 | 810.894766 | 1.049401 |
| GO:0006874\_cellular\_calcium\_ion\_homeostasis | 55 | 0 | 0.000000 | 0.000000 | 738 | 732.789387 | 773.18 | 813.570613 | 1.047669 |
| GO:0046467\_membrane\_lipid\_biosynthetic\_process | 55 | 0 | 0.000000 | 0.000000 | 738 | 732.789387 | 773.18 | 813.570613 | 1.047669 |
| GO:0050878\_regulation\_of\_body\_fluid\_levels | 55 | 0 | 0.000000 | 0.000000 | 738 | 732.789387 | 773.18 | 813.570613 | 1.047669 |
| GO:0055074\_calcium\_ion\_homeostasis | 55 | 0 | 0.000000 | 0.000000 | 738 | 732.789387 | 773.18 | 813.570613 | 1.047669 |
| GO:0006643\_membrane\_lipid\_metabolic\_process | 103 | 0 | 0.000000 | 0.000000 | 739 | 733.982465 | 774.24 | 814.497535 | 1.047686 |
| GO:0000738\_DNA\_catabolic\_process\_\_exonucleolytic | 4 | 0 |  |  |  |  |  |  |  |  |
| GO:0001502\_cartilage\_condensation | 4 | 0 |  |  |  |  |  |  |  |  |
| GO:0001539\_ciliary\_or\_flagellar\_motility | 4 | 0 |  |  |  |  |  |  |  |  |
| GO:0001708\_cell\_fate\_specification | 4 | 0 |  |  |  |  |  |  |  |  |
| GO:0001818\_negative\_regulation\_of\_cytokine\_production | 4 | 0 |  |  |  |  |  |  |  |  |
| GO:0001838\_embryonic\_epithelial\_tube\_formation | 4 | 0 |  |  |  |  |  |  |  |  |
| GO:0001839\_neural\_plate\_morphogenesis | 4 | 0 |  |  |  |  |  |  |  |  |
| GO:0001841\_neural\_tube\_formation | 4 | 0 |  |  |  |  |  |  |  |  |
| GO:0001843\_neural\_tube\_closure | 4 | 0 |  |  |  |  |  |  |  |  |
| GO:0001910\_regulation\_of\_leukocyte\_mediated\_cytotoxicity | 4 | 0 |  |  |  |  |  |  |  |  |
| GO:0001942\_hair\_follicle\_development | 4 | 0 |  |  |  |  |  |  |  |  |
| GO:0002204\_somatic\_recombination\_of\_immunoglobulin\_genes\_during\_immune\_response | 4 | 0 |  |  |  |  |  |  |  |  |
| GO:0002208\_somatic\_diversification\_of\_immunoglobulins\_during\_immune\_response | 4 | 0 |  |  |  |  |  |  |  |  |
| GO:0002260\_lymphocyte\_homeostasis | 4 | 0 |  |  |  |  |  |  |  |  |
| GO:0002369\_T\_cell\_cytokine\_production | 4 | 0 |  |  |  |  |  |  |  |  |
| GO:0002381\_immunoglobulin\_production\_during\_immune\_response | 4 | 0 |  |  |  |  |  |  |  |  |
| GO:0002541\_activation\_of\_plasma\_proteins\_during\_acute\_inflammatory\_response | 4 | 0 |  |  |  |  |  |  |  |  |
| GO:0002724\_regulation\_of\_T\_cell\_cytokine\_production | 4 | 0 |  |  |  |  |  |  |  |  |
| GO:0002726\_positive\_regulation\_of\_T\_cell\_cytokine\_production | 4 | 0 |  |  |  |  |  |  |  |  |
| GO:0002763\_positive\_regulation\_of\_myeloid\_leukocyte\_differentiation | 4 | 0 |  |  |  |  |  |  |  |  |
| GO:0005978\_glycogen\_biosynthetic\_process | 4 | 0 |  |  |  |  |  |  |  |  |
| GO:0006012\_galactose\_metabolic\_process | 4 | 0 |  |  |  |  |  |  |  |  |
| GO:0006026\_aminoglycan\_catabolic\_process | 4 | 0 |  |  |  |  |  |  |  |  |
| GO:0006027\_glycosaminoglycan\_catabolic\_process | 4 | 0 |  |  |  |  |  |  |  |  |
| GO:0006098\_pentose-phosphate\_shunt | 4 | 0 |  |  |  |  |  |  |  |  |
| GO:0006206\_pyrimidine\_base\_metabolic\_process | 4 | 0 |  |  |  |  |  |  |  |  |
| GO:0006213\_pyrimidine\_nucleoside\_metabolic\_process | 4 | 0 |  |  |  |  |  |  |  |  |
| GO:0006337\_nucleosome\_disassembly | 4 | 0 |  |  |  |  |  |  |  |  |
| GO:0006353\_transcription\_termination | 4 | 0 |  |  |  |  |  |  |  |  |
| GO:0006356\_regulation\_of\_transcription\_from\_RNA\_polymerase\_I\_promoter | 4 | 0 |  |  |  |  |  |  |  |  |
| GO:0006358\_regulation\_of\_transcription\_from\_RNA\_polymerase\_II\_promoter\_\_global | 4 | 0 |  |  |  |  |  |  |  |  |
| GO:0006415\_translational\_termination | 4 | 0 |  |  |  |  |  |  |  |  |
| GO:0006449\_regulation\_of\_translational\_termination | 4 | 0 |  |  |  |  |  |  |  |  |
| GO:0006531\_aspartate\_metabolic\_process | 4 | 0 |  |  |  |  |  |  |  |  |
| GO:0006533\_aspartate\_catabolic\_process | 4 | 0 |  |  |  |  |  |  |  |  |
| GO:0006636\_unsaturated\_fatty\_acid\_biosynthetic\_process | 4 | 0 |  |  |  |  |  |  |  |  |
| GO:0006658\_phosphatidylserine\_metabolic\_process | 4 | 0 |  |  |  |  |  |  |  |  |
| GO:0006726\_eye\_pigment\_biosynthetic\_process | 4 | 0 |  |  |  |  |  |  |  |  |
| GO:0006740\_NADPH\_regeneration | 4 | 0 |  |  |  |  |  |  |  |  |
| GO:0006743\_ubiquinone\_metabolic\_process | 4 | 0 |  |  |  |  |  |  |  |  |
| GO:0006744\_ubiquinone\_biosynthetic\_process | 4 | 0 |  |  |  |  |  |  |  |  |
| GO:0006884\_cell\_volume\_homeostasis | 4 | 0 |  |  |  |  |  |  |  |  |
| GO:0006906\_vesicle\_fusion | 4 | 0 |  |  |  |  |  |  |  |  |
| GO:0006911\_phagocytosis\_\_engulfment | 4 | 0 |  |  |  |  |  |  |  |  |
| GO:0006956\_complement\_activation | 4 | 0 |  |  |  |  |  |  |  |  |
| GO:0007032\_endosome\_organization\_and\_biogenesis | 4 | 0 |  |  |  |  |  |  |  |  |
| GO:0007042\_lysosomal\_lumen\_acidification | 4 | 0 |  |  |  |  |  |  |  |  |
| GO:0007089\_traversing\_start\_control\_point\_of\_mitotic\_cell\_cycle | 4 | 0 |  |  |  |  |  |  |  |  |
| GO:0007158\_neuron\_adhesion | 4 | 0 |  |  |  |  |  |  |  |  |
| GO:0007164\_establishment\_of\_tissue\_polarity | 4 | 0 |  |  |  |  |  |  |  |  |
| GO:0007181\_transforming\_growth\_factor\_beta\_receptor\_complex\_assembly | 4 | 0 |  |  |  |  |  |  |  |  |
| GO:0007183\_SMAD\_protein\_complex\_assembly | 4 | 0 |  |  |  |  |  |  |  |  |
| GO:0007213\_acetylcholine\_receptor\_signaling\_\_muscarinic\_pathway | 4 | 0 |  |  |  |  |  |  |  |  |
| GO:0007224\_smoothened\_signaling\_pathway | 4 | 0 |  |  |  |  |  |  |  |  |
| GO:0007253\_cytoplasmic\_sequestering\_of\_NF-kappaB | 4 | 0 |  |  |  |  |  |  |  |  |
| GO:0007368\_determination\_of\_left\_right\_symmetry | 4 | 0 |  |  |  |  |  |  |  |  |
| GO:0007528\_neuromuscular\_junction\_development | 4 | 0 |  |  |  |  |  |  |  |  |
| GO:0007595\_lactation | 4 | 0 |  |  |  |  |  |  |  |  |
| GO:0007613\_memory | 4 | 0 |  |  |  |  |  |  |  |  |
| GO:0008038\_neuron\_recognition | 4 | 0 |  |  |  |  |  |  |  |  |
| GO:0008299\_isoprenoid\_biosynthetic\_process | 4 | 0 |  |  |  |  |  |  |  |  |
| GO:0008634\_negative\_regulation\_of\_survival\_gene\_product\_expression | 4 | 0 |  |  |  |  |  |  |  |  |
| GO:0009071\_serine\_family\_amino\_acid\_catabolic\_process | 4 | 0 |  |  |  |  |  |  |  |  |
| GO:0009201\_ribonucleoside\_triphosphate\_biosynthetic\_process | 4 | 0 |  |  |  |  |  |  |  |  |
| GO:0009218\_pyrimidine\_ribonucleotide\_metabolic\_process | 4 | 0 |  |  |  |  |  |  |  |  |
| GO:0009249\_protein\_lipoylation | 4 | 0 |  |  |  |  |  |  |  |  |
| GO:0009250\_glucan\_biosynthetic\_process | 4 | 0 |  |  |  |  |  |  |  |  |
| GO:0009262\_deoxyribonucleotide\_metabolic\_process | 4 | 0 |  |  |  |  |  |  |  |  |
| GO:0009268\_response\_to\_pH | 4 | 0 |  |  |  |  |  |  |  |  |
| GO:0009799\_determination\_of\_symmetry | 4 | 0 |  |  |  |  |  |  |  |  |
| GO:0009855\_determination\_of\_bilateral\_symmetry | 4 | 0 |  |  |  |  |  |  |  |  |
| GO:0009895\_negative\_regulation\_of\_catabolic\_process | 4 | 0 |  |  |  |  |  |  |  |  |
| GO:0009953\_dorsal\_ventral\_pattern\_formation | 4 | 0 |  |  |  |  |  |  |  |  |
| GO:0014020\_primary\_neural\_tube\_formation | 4 | 0 |  |  |  |  |  |  |  |  |
| GO:0014902\_myotube\_differentiation | 4 | 0 |  |  |  |  |  |  |  |  |
| GO:0015701\_bicarbonate\_transport | 4 | 0 |  |  |  |  |  |  |  |  |
| GO:0015807\_L-amino\_acid\_transport | 4 | 0 |  |  |  |  |  |  |  |  |
| GO:0015844\_monoamine\_transport | 4 | 0 |  |  |  |  |  |  |  |  |
| GO:0015858\_nucleoside\_transport | 4 | 0 |  |  |  |  |  |  |  |  |
| GO:0016358\_dendrite\_development | 4 | 0 |  |  |  |  |  |  |  |  |
| GO:0016447\_somatic\_recombination\_of\_immunoglobulin\_gene\_segments | 4 | 0 |  |  |  |  |  |  |  |  |
| GO:0016486\_peptide\_hormone\_processing | 4 | 0 |  |  |  |  |  |  |  |  |
| GO:0016579\_protein\_deubiquitination | 4 | 0 |  |  |  |  |  |  |  |  |
| GO:0017157\_regulation\_of\_exocytosis | 4 | 0 |  |  |  |  |  |  |  |  |
| GO:0018958\_phenol\_metabolic\_process | 4 | 0 |  |  |  |  |  |  |  |  |
| GO:0019217\_regulation\_of\_fatty\_acid\_metabolic\_process | 4 | 0 |  |  |  |  |  |  |  |  |
| GO:0019369\_arachidonic\_acid\_metabolic\_process | 4 | 0 |  |  |  |  |  |  |  |  |
| GO:0019438\_aromatic\_compound\_biosynthetic\_process | 4 | 0 |  |  |  |  |  |  |  |  |
| GO:0019883\_antigen\_processing\_and\_presentation\_of\_endogenous\_antigen | 4 | 0 |  |  |  |  |  |  |  |  |
| GO:0019987\_negative\_regulation\_of\_anti-apoptosis | 4 | 0 |  |  |  |  |  |  |  |  |
| GO:0021915\_neural\_tube\_development | 4 | 0 |  |  |  |  |  |  |  |  |
| GO:0022404\_molting\_cycle\_process | 4 | 0 |  |  |  |  |  |  |  |  |
| GO:0022405\_hair\_cycle\_process | 4 | 0 |  |  |  |  |  |  |  |  |
| GO:0030042\_actin\_filament\_depolymerization | 4 | 0 |  |  |  |  |  |  |  |  |
| GO:0030049\_muscle\_filament\_sliding | 4 | 0 |  |  |  |  |  |  |  |  |
| GO:0030071\_regulation\_of\_mitotic\_metaphase\_anaphase\_transition | 4 | 0 |  |  |  |  |  |  |  |  |
| GO:0030091\_protein\_repair | 4 | 0 |  |  |  |  |  |  |  |  |
| GO:0030104\_water\_homeostasis | 4 | 0 |  |  |  |  |  |  |  |  |
| GO:0030224\_monocyte\_differentiation | 4 | 0 |  |  |  |  |  |  |  |  |
| GO:0030323\_respiratory\_tube\_development | 4 | 0 |  |  |  |  |  |  |  |  |
| GO:0030500\_regulation\_of\_bone\_mineralization | 4 | 0 |  |  |  |  |  |  |  |  |
| GO:0030516\_regulation\_of\_axon\_extension | 4 | 0 |  |  |  |  |  |  |  |  |
| GO:0030799\_regulation\_of\_cyclic\_nucleotide\_metabolic\_process | 4 | 0 |  |  |  |  |  |  |  |  |
| GO:0030802\_regulation\_of\_cyclic\_nucleotide\_biosynthetic\_process | 4 | 0 |  |  |  |  |  |  |  |  |
| GO:0030808\_regulation\_of\_nucleotide\_biosynthetic\_process | 4 | 0 |  |  |  |  |  |  |  |  |
| GO:0030834\_regulation\_of\_actin\_filament\_depolymerization | 4 | 0 |  |  |  |  |  |  |  |  |
| GO:0030835\_negative\_regulation\_of\_actin\_filament\_depolymerization | 4 | 0 |  |  |  |  |  |  |  |  |
| GO:0030865\_cortical\_cytoskeleton\_organization\_and\_biogenesis | 4 | 0 |  |  |  |  |  |  |  |  |
| GO:0030888\_regulation\_of\_B\_cell\_proliferation | 4 | 0 |  |  |  |  |  |  |  |  |
| GO:0030890\_positive\_regulation\_of\_B\_cell\_proliferation | 4 | 0 |  |  |  |  |  |  |  |  |
| GO:0030900\_forebrain\_development | 4 | 0 |  |  |  |  |  |  |  |  |
| GO:0031056\_regulation\_of\_histone\_modification | 4 | 0 |  |  |  |  |  |  |  |  |
| GO:0031099\_regeneration | 4 | 0 |  |  |  |  |  |  |  |  |
| GO:0031112\_positive\_regulation\_of\_microtubule\_polymerization\_or\_depolymerization | 4 | 0 |  |  |  |  |  |  |  |  |
| GO:0031331\_positive\_regulation\_of\_cellular\_catabolic\_process | 4 | 0 |  |  |  |  |  |  |  |  |
| GO:0031341\_regulation\_of\_cell\_killing | 4 | 0 |  |  |  |  |  |  |  |  |
| GO:0031348\_negative\_regulation\_of\_defense\_response | 4 | 0 |  |  |  |  |  |  |  |  |
| GO:0031498\_chromatin\_disassembly | 4 | 0 |  |  |  |  |  |  |  |  |
| GO:0032088\_inhibition\_of\_NF-kappaB\_transcription\_factor | 4 | 0 |  |  |  |  |  |  |  |  |
| GO:0032102\_negative\_regulation\_of\_response\_to\_external\_stimulus | 4 | 0 |  |  |  |  |  |  |  |  |
| GO:0032205\_negative\_regulation\_of\_telomere\_maintenance | 4 | 0 |  |  |  |  |  |  |  |  |
| GO:0032274\_gonadotropin\_secretion | 4 | 0 |  |  |  |  |  |  |  |  |
| GO:0032364\_oxygen\_homeostasis | 4 | 0 |  |  |  |  |  |  |  |  |
| GO:0032615\_interleukin-12\_production | 4 | 0 |  |  |  |  |  |  |  |  |
| GO:0032663\_regulation\_of\_interleukin-2\_production | 4 | 0 |  |  |  |  |  |  |  |  |
| GO:0032743\_positive\_regulation\_of\_interleukin-2\_production | 4 | 0 |  |  |  |  |  |  |  |  |
| GO:0032768\_regulation\_of\_monooxygenase\_activity | 4 | 0 |  |  |  |  |  |  |  |  |
| GO:0032856\_activation\_of\_Ras\_GTPase\_activity | 4 | 0 |  |  |  |  |  |  |  |  |
| GO:0032862\_activation\_of\_Rho\_GTPase\_activity | 4 | 0 |  |  |  |  |  |  |  |  |
| GO:0032869\_cellular\_response\_to\_insulin\_stimulus | 4 | 0 |  |  |  |  |  |  |  |  |
| GO:0032870\_cellular\_response\_to\_hormone\_stimulus | 4 | 0 |  |  |  |  |  |  |  |  |
| GO:0032906\_transforming\_growth\_factor-beta2\_production | 4 | 0 |  |  |  |  |  |  |  |  |
| GO:0032909\_regulation\_of\_transforming\_growth\_factor-beta2\_production | 4 | 0 |  |  |  |  |  |  |  |  |
| GO:0033077\_T\_cell\_differentiation\_in\_the\_thymus | 4 | 0 |  |  |  |  |  |  |  |  |
| GO:0033275\_actin-myosin\_filament\_sliding | 4 | 0 |  |  |  |  |  |  |  |  |
| GO:0033483\_gas\_homeostasis | 4 | 0 |  |  |  |  |  |  |  |  |
| GO:0033559\_unsaturated\_fatty\_acid\_metabolic\_process | 4 | 0 |  |  |  |  |  |  |  |  |
| GO:0035088\_establishment\_and\_or\_maintenance\_of\_apical\_basal\_cell\_polarity | 4 | 0 |  |  |  |  |  |  |  |  |
| GO:0042088\_T-helper\_1\_type\_immune\_response | 4 | 0 |  |  |  |  |  |  |  |  |
| GO:0042246\_tissue\_regeneration | 4 | 0 |  |  |  |  |  |  |  |  |
| GO:0042278\_purine\_nucleoside\_metabolic\_process | 4 | 0 |  |  |  |  |  |  |  |  |
| GO:0042303\_molting\_cycle | 4 | 0 |  |  |  |  |  |  |  |  |
| GO:0042310\_vasoconstriction | 4 | 0 |  |  |  |  |  |  |  |  |
| GO:0042312\_regulation\_of\_vasodilation | 4 | 0 |  |  |  |  |  |  |  |  |
| GO:0042346\_positive\_regulation\_of\_NF-kappaB\_import\_into\_nucleus | 4 | 0 |  |  |  |  |  |  |  |  |
| GO:0042375\_quinone\_cofactor\_metabolic\_process | 4 | 0 |  |  |  |  |  |  |  |  |
| GO:0042441\_eye\_pigment\_metabolic\_process | 4 | 0 |  |  |  |  |  |  |  |  |
| GO:0042447\_hormone\_catabolic\_process | 4 | 0 |  |  |  |  |  |  |  |  |
| GO:0042461\_photoreceptor\_cell\_development | 4 | 0 |  |  |  |  |  |  |  |  |
| GO:0042475\_odontogenesis\_of\_dentine-containing\_teeth | 4 | 0 |  |  |  |  |  |  |  |  |
| GO:0042518\_negative\_regulation\_of\_tyrosine\_phosphorylation\_of\_Stat3\_protein | 4 | 0 |  |  |  |  |  |  |  |  |
| GO:0042532\_negative\_regulation\_of\_tyrosine\_phosphorylation\_of\_STAT\_protein | 4 | 0 |  |  |  |  |  |  |  |  |
| GO:0042542\_response\_to\_hydrogen\_peroxide | 4 | 0 |  |  |  |  |  |  |  |  |
| GO:0042559\_pteridine\_and\_derivative\_biosynthetic\_process | 4 | 0 |  |  |  |  |  |  |  |  |
| GO:0042572\_retinol\_metabolic\_process | 4 | 0 |  |  |  |  |  |  |  |  |
| GO:0042633\_hair\_cycle | 4 | 0 |  |  |  |  |  |  |  |  |
| GO:0043094\_metabolic\_compound\_salvage | 4 | 0 |  |  |  |  |  |  |  |  |
| GO:0043114\_regulation\_of\_vascular\_permeability | 4 | 0 |  |  |  |  |  |  |  |  |
| GO:0043124\_negative\_regulation\_of\_I-kappaB\_kinase\_NF-kappaB\_cascade | 4 | 0 |  |  |  |  |  |  |  |  |
| GO:0043254\_regulation\_of\_protein\_complex\_assembly | 4 | 0 |  |  |  |  |  |  |  |  |
| GO:0043255\_regulation\_of\_carbohydrate\_biosynthetic\_process | 4 | 0 |  |  |  |  |  |  |  |  |
| GO:0043279\_response\_to\_alkaloid | 4 | 0 |  |  |  |  |  |  |  |  |
| GO:0043409\_negative\_regulation\_of\_MAPKKK\_cascade | 4 | 0 |  |  |  |  |  |  |  |  |
| GO:0043487\_regulation\_of\_RNA\_stability | 4 | 0 |  |  |  |  |  |  |  |  |
| GO:0043488\_regulation\_of\_mRNA\_stability | 4 | 0 |  |  |  |  |  |  |  |  |
| GO:0043497\_regulation\_of\_protein\_heterodimerization\_activity | 4 | 0 |  |  |  |  |  |  |  |  |
| GO:0043524\_negative\_regulation\_of\_neuron\_apoptosis | 4 | 0 |  |  |  |  |  |  |  |  |
| GO:0043534\_blood\_vessel\_endothelial\_cell\_migration | 4 | 0 |  |  |  |  |  |  |  |  |
| GO:0043550\_regulation\_of\_lipid\_kinase\_activity | 4 | 0 |  |  |  |  |  |  |  |  |
| GO:0043583\_ear\_development | 4 | 0 |  |  |  |  |  |  |  |  |
| GO:0045070\_positive\_regulation\_of\_viral\_genome\_replication | 4 | 0 |  |  |  |  |  |  |  |  |
| GO:0045086\_positive\_regulation\_of\_interleukin-2\_biosynthetic\_process | 4 | 0 |  |  |  |  |  |  |  |  |
| GO:0045089\_positive\_regulation\_of\_innate\_immune\_response | 4 | 0 |  |  |  |  |  |  |  |  |
| GO:0045091\_regulation\_of\_retroviral\_genome\_replication | 4 | 0 |  |  |  |  |  |  |  |  |
| GO:0045176\_apical\_protein\_localization | 4 | 0 |  |  |  |  |  |  |  |  |
| GO:0045190\_isotype\_switching | 4 | 0 |  |  |  |  |  |  |  |  |
| GO:0045191\_regulation\_of\_isotype\_switching | 4 | 0 |  |  |  |  |  |  |  |  |
| GO:0045197\_establishment\_and\_or\_maintenance\_of\_epithelial\_cell\_polarity | 4 | 0 |  |  |  |  |  |  |  |  |
| GO:0045408\_regulation\_of\_interleukin-6\_biosynthetic\_process | 4 | 0 |  |  |  |  |  |  |  |  |
| GO:0045426\_quinone\_cofactor\_biosynthetic\_process | 4 | 0 |  |  |  |  |  |  |  |  |
| GO:0045429\_positive\_regulation\_of\_nitric\_oxide\_biosynthetic\_process | 4 | 0 |  |  |  |  |  |  |  |  |
| GO:0045494\_photoreceptor\_cell\_maintenance | 4 | 0 |  |  |  |  |  |  |  |  |
| GO:0045582\_positive\_regulation\_of\_T\_cell\_differentiation | 4 | 0 |  |  |  |  |  |  |  |  |
| GO:0045621\_positive\_regulation\_of\_lymphocyte\_differentiation | 4 | 0 |  |  |  |  |  |  |  |  |
| GO:0045648\_positive\_regulation\_of\_erythrocyte\_differentiation | 4 | 0 |  |  |  |  |  |  |  |  |
| GO:0045667\_regulation\_of\_osteoblast\_differentiation | 4 | 0 |  |  |  |  |  |  |  |  |
| GO:0045682\_regulation\_of\_epidermis\_development | 4 | 0 |  |  |  |  |  |  |  |  |
| GO:0045741\_positive\_regulation\_of\_epidermal\_growth\_factor\_receptor\_activity | 4 | 0 |  |  |  |  |  |  |  |  |
| GO:0045833\_negative\_regulation\_of\_lipid\_metabolic\_process | 4 | 0 |  |  |  |  |  |  |  |  |
| GO:0045931\_positive\_regulation\_of\_mitotic\_cell\_cycle | 4 | 0 |  |  |  |  |  |  |  |  |
| GO:0045948\_positive\_regulation\_of\_translational\_initiation | 4 | 0 |  |  |  |  |  |  |  |  |
| GO:0046112\_nucleobase\_biosynthetic\_process | 4 | 0 |  |  |  |  |  |  |  |  |
| GO:0046128\_purine\_ribonucleoside\_metabolic\_process | 4 | 0 |  |  |  |  |  |  |  |  |
| GO:0046323\_glucose\_import | 4 | 0 |  |  |  |  |  |  |  |  |
| GO:0046329\_negative\_regulation\_of\_JNK\_cascade | 4 | 0 |  |  |  |  |  |  |  |  |
| GO:0046459\_short-chain\_fatty\_acid\_metabolic\_process | 4 | 0 |  |  |  |  |  |  |  |  |
| GO:0046488\_phosphatidylinositol\_metabolic\_process | 4 | 0 |  |  |  |  |  |  |  |  |
| GO:0046530\_photoreceptor\_cell\_differentiation | 4 | 0 |  |  |  |  |  |  |  |  |
| GO:0046631\_alpha-beta\_T\_cell\_activation | 4 | 0 |  |  |  |  |  |  |  |  |
| GO:0046884\_follicle-stimulating\_hormone\_secretion | 4 | 0 |  |  |  |  |  |  |  |  |
| GO:0048002\_antigen\_processing\_and\_presentation\_of\_peptide\_antigen | 4 | 0 |  |  |  |  |  |  |  |  |
| GO:0048168\_regulation\_of\_neuronal\_synaptic\_plasticity | 4 | 0 |  |  |  |  |  |  |  |  |
| GO:0048247\_lymphocyte\_chemotaxis | 4 | 0 |  |  |  |  |  |  |  |  |
| GO:0048488\_synaptic\_vesicle\_endocytosis | 4 | 0 |  |  |  |  |  |  |  |  |
| GO:0048599\_oocyte\_development | 4 | 0 |  |  |  |  |  |  |  |  |
| GO:0048678\_response\_to\_axon\_injury | 4 | 0 |  |  |  |  |  |  |  |  |
| GO:0048839\_inner\_ear\_development | 4 | 0 |  |  |  |  |  |  |  |  |
| GO:0050732\_negative\_regulation\_of\_peptidyl-tyrosine\_phosphorylation | 4 | 0 |  |  |  |  |  |  |  |  |
| GO:0050768\_negative\_regulation\_of\_neurogenesis | 4 | 0 |  |  |  |  |  |  |  |  |
| GO:0050803\_regulation\_of\_synapse\_structure\_and\_activity | 4 | 0 |  |  |  |  |  |  |  |  |
| GO:0050810\_regulation\_of\_steroid\_biosynthetic\_process | 4 | 0 |  |  |  |  |  |  |  |  |
| GO:0050819\_negative\_regulation\_of\_coagulation | 4 | 0 |  |  |  |  |  |  |  |  |
| GO:0050857\_positive\_regulation\_of\_antigen\_receptor-mediated\_signaling\_pathway | 4 | 0 |  |  |  |  |  |  |  |  |
| GO:0050868\_negative\_regulation\_of\_T\_cell\_activation | 4 | 0 |  |  |  |  |  |  |  |  |
| GO:0050905\_neuromuscular\_process | 4 | 0 |  |  |  |  |  |  |  |  |
| GO:0050999\_regulation\_of\_nitric-oxide\_synthase\_activity | 4 | 0 |  |  |  |  |  |  |  |  |
| GO:0051014\_actin\_filament\_severing | 4 | 0 |  |  |  |  |  |  |  |  |
| GO:0051055\_negative\_regulation\_of\_lipid\_biosynthetic\_process | 4 | 0 |  |  |  |  |  |  |  |  |
| GO:0051103\_DNA\_ligation\_during\_DNA\_repair | 4 | 0 |  |  |  |  |  |  |  |  |
| GO:0051148\_negative\_regulation\_of\_muscle\_cell\_differentiation | 4 | 0 |  |  |  |  |  |  |  |  |
| GO:0051205\_protein\_insertion\_into\_membrane | 4 | 0 |  |  |  |  |  |  |  |  |
| GO:0051353\_positive\_regulation\_of\_oxidoreductase\_activity | 4 | 0 |  |  |  |  |  |  |  |  |
| GO:0051496\_positive\_regulation\_of\_stress\_fiber\_formation | 4 | 0 |  |  |  |  |  |  |  |  |
| GO:0060048\_cardiac\_muscle\_contraction | 4 | 0 |  |  |  |  |  |  |  |  |
| GO:0060193\_positive\_regulation\_of\_lipase\_activity | 4 | 0 |  |  |  |  |  |  |  |  |
| GO:0006954\_inflammatory\_response | 121 | 0 | 0.000000 | 0.000000 | 741 | 735.590484 | 775.68 | 815.769516 | 1.046802 |
| GO:0019953\_sexual\_reproduction | 121 | 0 | 0.000000 | 0.000000 | 741 | 735.590484 | 775.68 | 815.769516 | 1.046802 |
| GO:0050801\_ion\_homeostasis | 118 | 0 | 0.000000 | 0.000000 | 742 | 736.231007 | 776.19 | 816.148993 | 1.046078 |
| GO:0055082\_cellular\_chemical\_homeostasis | 113 | 0 | 0.000000 | 0.000000 | 743 | 736.945362 | 776.72 | 816.494638 | 1.045384 |
| GO:0006325\_establishment\_and\_or\_maintenance\_of\_chromatin\_architecture | 119 | 0 | 0.000000 | 0.000000 | 744 | 737.974057 | 777.61 | 817.245943 | 1.045175 |
| GO:0000904\_cellular\_morphogenesis\_during\_differentiation | 52 | 0 | 0.000000 | 0.000000 | 747 | 741.755497 | 781.1 | 820.444503 | 1.045649 |
| GO:0016485\_protein\_processing | 52 | 0 | 0.000000 | 0.000000 | 747 | 741.755497 | 781.1 | 820.444503 | 1.045649 |
| GO:0042325\_regulation\_of\_phosphorylation | 52 | 0 | 0.000000 | 0.000000 | 747 | 741.755497 | 781.1 | 820.444503 | 1.045649 |
| GO:0006921\_cell\_structure\_disassembly\_during\_apoptosis | 17 | 0 | 0.000000 | 0.000000 | 769 | 763.043889 | 801.65 | 840.256111 | 1.042458 |
| GO:0006941\_striated\_muscle\_contraction | 17 | 0 | 0.000000 | 0.000000 | 769 | 763.043889 | 801.65 | 840.256111 | 1.042458 |
| GO:0009062\_fatty\_acid\_catabolic\_process | 17 | 0 | 0.000000 | 0.000000 | 769 | 763.043889 | 801.65 | 840.256111 | 1.042458 |
| GO:0009259\_ribonucleotide\_metabolic\_process | 17 | 0 | 0.000000 | 0.000000 | 769 | 763.043889 | 801.65 | 840.256111 | 1.042458 |
| GO:0016054\_organic\_acid\_catabolic\_process | 17 | 0 | 0.000000 | 0.000000 | 769 | 763.043889 | 801.65 | 840.256111 | 1.042458 |
| GO:0019395\_fatty\_acid\_oxidation | 17 | 0 | 0.000000 | 0.000000 | 769 | 763.043889 | 801.65 | 840.256111 | 1.042458 |
| GO:0019722\_calcium-mediated\_signaling | 17 | 0 | 0.000000 | 0.000000 | 769 | 763.043889 | 801.65 | 840.256111 | 1.042458 |
| GO:0021700\_developmental\_maturation | 17 | 0 | 0.000000 | 0.000000 | 769 | 763.043889 | 801.65 | 840.256111 | 1.042458 |
| GO:0030166\_proteoglycan\_biosynthetic\_process | 17 | 0 | 0.000000 | 0.000000 | 769 | 763.043889 | 801.65 | 840.256111 | 1.042458 |
| GO:0030217\_T\_cell\_differentiation | 17 | 0 | 0.000000 | 0.000000 | 769 | 763.043889 | 801.65 | 840.256111 | 1.042458 |
| GO:0031279\_regulation\_of\_cyclase\_activity | 17 | 0 | 0.000000 | 0.000000 | 769 | 763.043889 | 801.65 | 840.256111 | 1.042458 |
| GO:0032535\_regulation\_of\_cellular\_component\_size | 17 | 0 | 0.000000 | 0.000000 | 769 | 763.043889 | 801.65 | 840.256111 | 1.042458 |
| GO:0043507\_positive\_regulation\_of\_JNK\_activity | 17 | 0 | 0.000000 | 0.000000 | 769 | 763.043889 | 801.65 | 840.256111 | 1.042458 |
| GO:0045761\_regulation\_of\_adenylate\_cyclase\_activity | 17 | 0 | 0.000000 | 0.000000 | 769 | 763.043889 | 801.65 | 840.256111 | 1.042458 |
| GO:0046395\_carboxylic\_acid\_catabolic\_process | 17 | 0 | 0.000000 | 0.000000 | 769 | 763.043889 | 801.65 | 840.256111 | 1.042458 |
| GO:0050708\_regulation\_of\_protein\_secretion | 17 | 0 | 0.000000 | 0.000000 | 769 | 763.043889 | 801.65 | 840.256111 | 1.042458 |
| GO:0050870\_positive\_regulation\_of\_T\_cell\_activation | 17 | 0 | 0.000000 | 0.000000 | 769 | 763.043889 | 801.65 | 840.256111 | 1.042458 |
| GO:0051053\_negative\_regulation\_of\_DNA\_metabolic\_process | 17 | 0 | 0.000000 | 0.000000 | 769 | 763.043889 | 801.65 | 840.256111 | 1.042458 |
| GO:0051092\_activation\_of\_NF-kappaB\_transcription\_factor | 17 | 0 | 0.000000 | 0.000000 | 769 | 763.043889 | 801.65 | 840.256111 | 1.042458 |
| GO:0051339\_regulation\_of\_lyase\_activity | 17 | 0 | 0.000000 | 0.000000 | 769 | 763.043889 | 801.65 | 840.256111 | 1.042458 |
| GO:0051402\_neuron\_apoptosis | 17 | 0 | 0.000000 | 0.000000 | 769 | 763.043889 | 801.65 | 840.256111 | 1.042458 |
| GO:0051701\_interaction\_with\_host | 17 | 0 | 0.000000 | 0.000000 | 769 | 763.043889 | 801.65 | 840.256111 | 1.042458 |
| GO:0006790\_sulfur\_metabolic\_process | 43 | 0 | 0.000000 | 0.000000 | 772 | 766.277618 | 804.55 | 842.822382 | 1.042163 |
| GO:0009891\_positive\_regulation\_of\_biosynthetic\_process | 43 | 0 | 0.000000 | 0.000000 | 772 | 766.277618 | 804.55 | 842.822382 | 1.042163 |
| GO:0043281\_regulation\_of\_caspase\_activity | 43 | 0 | 0.000000 | 0.000000 | 772 | 766.277618 | 804.55 | 842.822382 | 1.042163 |
| GO:0000187\_activation\_of\_MAPK\_activity | 39 | 0 | 0.000000 | 0.000000 | 776 | 769.464018 | 807.65 | 845.835982 | 1.040786 |
| GO:0007179\_transforming\_growth\_factor\_beta\_receptor\_signaling\_pathway | 39 | 0 | 0.000000 | 0.000000 | 776 | 769.464018 | 807.65 | 845.835982 | 1.040786 |
| GO:0007266\_Rho\_protein\_signal\_transduction | 39 | 0 | 0.000000 | 0.000000 | 776 | 769.464018 | 807.65 | 845.835982 | 1.040786 |
| GO:0051348\_negative\_regulation\_of\_transferase\_activity | 39 | 0 | 0.000000 | 0.000000 | 776 | 769.464018 | 807.65 | 845.835982 | 1.040786 |
| GO:0006916\_anti-apoptosis | 123 | 0 | 0.000000 | 0.000000 | 778 | 771.129172 | 809.11 | 847.090828 | 1.039987 |
| GO:0007268\_synaptic\_transmission | 123 | 0 | 0.000000 | 0.000000 | 778 | 771.129172 | 809.11 | 847.090828 | 1.039987 |
| GO:0006813\_potassium\_ion\_transport | 40 | 0 | 0.000000 | 0.000000 | 785 | 779.209645 | 816.96 | 854.710355 | 1.040713 |
| GO:0007420\_brain\_development | 40 | 0 | 0.000000 | 0.000000 | 785 | 779.209645 | 816.96 | 854.710355 | 1.040713 |
| GO:0009615\_response\_to\_virus | 40 | 0 | 0.000000 | 0.000000 | 785 | 779.209645 | 816.96 | 854.710355 | 1.040713 |
| GO:0022415\_viral\_reproductive\_process | 40 | 0 | 0.000000 | 0.000000 | 785 | 779.209645 | 816.96 | 854.710355 | 1.040713 |
| GO:0030155\_regulation\_of\_cell\_adhesion | 40 | 0 | 0.000000 | 0.000000 | 785 | 779.209645 | 816.96 | 854.710355 | 1.040713 |
| GO:0031497\_chromatin\_assembly | 40 | 0 | 0.000000 | 0.000000 | 785 | 779.209645 | 816.96 | 854.710355 | 1.040713 |
| GO:0048584\_positive\_regulation\_of\_response\_to\_stimulus | 40 | 0 | 0.000000 | 0.000000 | 785 | 779.209645 | 816.96 | 854.710355 | 1.040713 |
| GO:0006119\_oxidative\_phosphorylation | 38 | 0 | 0.000000 | 0.000000 | 790 | 783.982788 | 821.56 | 859.137212 | 1.039949 |
| GO:0006469\_negative\_regulation\_of\_protein\_kinase\_activity | 38 | 0 | 0.000000 | 0.000000 | 790 | 783.982788 | 821.56 | 859.137212 | 1.039949 |
| GO:0030522\_intracellular\_receptor-mediated\_signaling\_pathway | 38 | 0 | 0.000000 | 0.000000 | 790 | 783.982788 | 821.56 | 859.137212 | 1.039949 |
| GO:0033673\_negative\_regulation\_of\_kinase\_activity | 38 | 0 | 0.000000 | 0.000000 | 790 | 783.982788 | 821.56 | 859.137212 | 1.039949 |
| GO:0051090\_regulation\_of\_transcription\_factor\_activity | 38 | 0 | 0.000000 | 0.000000 | 790 | 783.982788 | 821.56 | 859.137212 | 1.039949 |
| GO:0000002\_mitochondrial\_genome\_maintenance | 2 | 0 |  |  |  |  |  |  |  |  |
| GO:0000012\_single\_strand\_break\_repair | 2 | 0 |  |  |  |  |  |  |  |  |
| GO:0000019\_regulation\_of\_mitotic\_recombination | 2 | 0 |  |  |  |  |  |  |  |  |
| GO:0000022\_mitotic\_spindle\_elongation | 2 | 0 |  |  |  |  |  |  |  |  |
| GO:0000050\_urea\_cycle | 2 | 0 |  |  |  |  |  |  |  |  |
| GO:0000051\_urea\_cycle\_intermediate\_metabolic\_process | 2 | 0 |  |  |  |  |  |  |  |  |
| GO:0000059\_protein\_import\_into\_nucleus\_\_docking | 2 | 0 |  |  |  |  |  |  |  |  |
| GO:0000066\_mitochondrial\_ornithine\_transport | 2 | 0 |  |  |  |  |  |  |  |  |
| GO:0000266\_mitochondrial\_fission | 2 | 0 |  |  |  |  |  |  |  |  |
| GO:0000303\_response\_to\_superoxide | 2 | 0 |  |  |  |  |  |  |  |  |
| GO:0000729\_DNA\_double-strand\_break\_processing | 2 | 0 |  |  |  |  |  |  |  |  |
| GO:0000921\_septin\_ring\_assembly | 2 | 0 |  |  |  |  |  |  |  |  |
| GO:0001510\_RNA\_methylation | 2 | 0 |  |  |  |  |  |  |  |  |
| GO:0001514\_selenocysteine\_incorporation | 2 | 0 |  |  |  |  |  |  |  |  |
| GO:0001522\_pseudouridine\_synthesis | 2 | 0 |  |  |  |  |  |  |  |  |
| GO:0001547\_antral\_ovarian\_follicle\_growth | 2 | 0 |  |  |  |  |  |  |  |  |
| GO:0001550\_ovarian\_cumulus\_expansion | 2 | 0 |  |  |  |  |  |  |  |  |
| GO:0001556\_oocyte\_maturation | 2 | 0 |  |  |  |  |  |  |  |  |
| GO:0001569\_patterning\_of\_blood\_vessels | 2 | 0 |  |  |  |  |  |  |  |  |
| GO:0001672\_regulation\_of\_chromatin\_assembly\_or\_disassembly | 2 | 0 |  |  |  |  |  |  |  |  |
| GO:0001676\_long-chain\_fatty\_acid\_metabolic\_process | 2 | 0 |  |  |  |  |  |  |  |  |
| GO:0001736\_establishment\_of\_planar\_polarity | 2 | 0 |  |  |  |  |  |  |  |  |
| GO:0001738\_morphogenesis\_of\_a\_polarized\_epithelium | 2 | 0 |  |  |  |  |  |  |  |  |
| GO:0001766\_membrane\_raft\_polarization | 2 | 0 |  |  |  |  |  |  |  |  |
| GO:0001773\_myeloid\_dendritic\_cell\_activation | 2 | 0 |  |  |  |  |  |  |  |  |
| GO:0001783\_B\_cell\_apoptosis | 2 | 0 |  |  |  |  |  |  |  |  |
| GO:0001824\_blastocyst\_development | 2 | 0 |  |  |  |  |  |  |  |  |
| GO:0001880\_Mullerian\_duct\_regression | 2 | 0 |  |  |  |  |  |  |  |  |
| GO:0001890\_placenta\_development | 2 | 0 |  |  |  |  |  |  |  |  |
| GO:0001897\_cytolysis\_by\_symbiont\_of\_host\_cells | 2 | 0 |  |  |  |  |  |  |  |  |
| GO:0001907\_killing\_by\_symbiont\_of\_host\_cells | 2 | 0 |  |  |  |  |  |  |  |  |
| GO:0001912\_positive\_regulation\_of\_leukocyte\_mediated\_cytotoxicity | 2 | 0 |  |  |  |  |  |  |  |  |
| GO:0001914\_regulation\_of\_T\_cell\_mediated\_cytotoxicity | 2 | 0 |  |  |  |  |  |  |  |  |
| GO:0001945\_lymph\_vessel\_development | 2 | 0 |  |  |  |  |  |  |  |  |
| GO:0001946\_lymphangiogenesis | 2 | 0 |  |  |  |  |  |  |  |  |
| GO:0001960\_negative\_regulation\_of\_cytokine\_and\_chemokine\_mediated\_signaling\_pathway | 2 | 0 |  |  |  |  |  |  |  |  |
| GO:0001963\_synaptic\_transmission\_\_dopaminergic | 2 | 0 |  |  |  |  |  |  |  |  |
| GO:0001990\_regulation\_of\_systemic\_arterial\_blood\_pressure\_by\_hormone | 2 | 0 |  |  |  |  |  |  |  |  |
| GO:0001991\_regulation\_of\_systemic\_arterial\_blood\_pressure\_by\_circulatory\_renin-angiotensin | 2 | 0 |  |  |  |  |  |  |  |  |
| GO:0002002\_regulation\_of\_angiotensin\_levels\_in\_blood | 2 | 0 |  |  |  |  |  |  |  |  |
| GO:0002003\_angiotensin\_maturation | 2 | 0 |  |  |  |  |  |  |  |  |
| GO:0002262\_myeloid\_cell\_homeostasis | 2 | 0 |  |  |  |  |  |  |  |  |
| GO:0002285\_lymphocyte\_activation\_during\_immune\_response | 2 | 0 |  |  |  |  |  |  |  |  |
| GO:0002286\_T\_cell\_activation\_during\_immune\_response | 2 | 0 |  |  |  |  |  |  |  |  |
| GO:0002292\_T\_cell\_differentiation\_during\_immune\_response | 2 | 0 |  |  |  |  |  |  |  |  |
| GO:0002293\_alpha-beta\_T\_cell\_differentiation\_during\_immune\_response | 2 | 0 |  |  |  |  |  |  |  |  |
| GO:0002294\_CD4-positive\_\_alpha-beta\_T\_cell\_differentiation\_during\_immune\_response | 2 | 0 |  |  |  |  |  |  |  |  |
| GO:0002374\_cytokine\_secretion\_during\_immune\_response | 2 | 0 |  |  |  |  |  |  |  |  |
| GO:0002378\_immunoglobulin\_biosynthetic\_process | 2 | 0 |  |  |  |  |  |  |  |  |
| GO:0002474\_antigen\_processing\_and\_presentation\_of\_peptide\_antigen\_via\_MHC\_class\_I | 2 | 0 |  |  |  |  |  |  |  |  |
| GO:0002483\_antigen\_processing\_and\_presentation\_of\_endogenous\_peptide\_antigen | 2 | 0 |  |  |  |  |  |  |  |  |
| GO:0002698\_negative\_regulation\_of\_immune\_effector\_process | 2 | 0 |  |  |  |  |  |  |  |  |
| GO:0002717\_positive\_regulation\_of\_natural\_killer\_cell\_mediated\_immunity | 2 | 0 |  |  |  |  |  |  |  |  |
| GO:0002739\_regulation\_of\_cytokine\_secretion\_during\_immune\_response | 2 | 0 |  |  |  |  |  |  |  |  |
| GO:0002740\_negative\_regulation\_of\_cytokine\_secretion\_during\_immune\_response | 2 | 0 |  |  |  |  |  |  |  |  |
| GO:0002793\_positive\_regulation\_of\_peptide\_secretion | 2 | 0 |  |  |  |  |  |  |  |  |
| GO:0002902\_regulation\_of\_B\_cell\_apoptosis | 2 | 0 |  |  |  |  |  |  |  |  |
| GO:0002903\_negative\_regulation\_of\_B\_cell\_apoptosis | 2 | 0 |  |  |  |  |  |  |  |  |
| GO:0003007\_heart\_morphogenesis | 2 | 0 |  |  |  |  |  |  |  |  |
| GO:0003014\_renal\_system\_process | 2 | 0 |  |  |  |  |  |  |  |  |
| GO:0003044\_regulation\_of\_systemic\_arterial\_blood\_pressure\_mediated\_by\_a\_chemical\_signal | 2 | 0 |  |  |  |  |  |  |  |  |
| GO:0003081\_regulation\_of\_systemic\_arterial\_blood\_pressure\_by\_renin-angiotensin | 2 | 0 |  |  |  |  |  |  |  |  |
| GO:0003091\_renal\_water\_homeostasis | 2 | 0 |  |  |  |  |  |  |  |  |
| GO:0005980\_glycogen\_catabolic\_process | 2 | 0 |  |  |  |  |  |  |  |  |
| GO:0006003\_fructose\_2\_6-bisphosphate\_metabolic\_process | 2 | 0 |  |  |  |  |  |  |  |  |
| GO:0006005\_L-fucose\_biosynthetic\_process | 2 | 0 |  |  |  |  |  |  |  |  |
| GO:0006011\_UDP-glucose\_metabolic\_process | 2 | 0 |  |  |  |  |  |  |  |  |
| GO:0006047\_UDP-N-acetylglucosamine\_metabolic\_process | 2 | 0 |  |  |  |  |  |  |  |  |
| GO:0006054\_N-acetylneuraminate\_metabolic\_process | 2 | 0 |  |  |  |  |  |  |  |  |
| GO:0006071\_glycerol\_metabolic\_process | 2 | 0 |  |  |  |  |  |  |  |  |
| GO:0006085\_acetyl-CoA\_biosynthetic\_process | 2 | 0 |  |  |  |  |  |  |  |  |
| GO:0006101\_citrate\_metabolic\_process | 2 | 0 |  |  |  |  |  |  |  |  |
| GO:0006108\_malate\_metabolic\_process | 2 | 0 |  |  |  |  |  |  |  |  |
| GO:0006111\_regulation\_of\_gluconeogenesis | 2 | 0 |  |  |  |  |  |  |  |  |
| GO:0006182\_cGMP\_biosynthetic\_process | 2 | 0 |  |  |  |  |  |  |  |  |
| GO:0006269\_DNA\_replication\_\_synthesis\_of\_RNA\_primer | 2 | 0 |  |  |  |  |  |  |  |  |
| GO:0006271\_DNA\_strand\_elongation\_during\_DNA\_replication | 2 | 0 |  |  |  |  |  |  |  |  |
| GO:0006288\_base-excision\_repair\_\_DNA\_ligation | 2 | 0 |  |  |  |  |  |  |  |  |
| GO:0006307\_DNA\_dealkylation | 2 | 0 |  |  |  |  |  |  |  |  |
| GO:0006345\_loss\_of\_chromatin\_silencing | 2 | 0 |  |  |  |  |  |  |  |  |
| GO:0006349\_genetic\_imprinting | 2 | 0 |  |  |  |  |  |  |  |  |
| GO:0006370\_mRNA\_capping | 2 | 0 |  |  |  |  |  |  |  |  |
| GO:0006398\_histone\_mRNA\_3'-end\_processing | 2 | 0 |  |  |  |  |  |  |  |  |
| GO:0006410\_transcription\_\_RNA-dependent | 2 | 0 |  |  |  |  |  |  |  |  |
| GO:0006422\_aspartyl-tRNA\_aminoacylation | 2 | 0 |  |  |  |  |  |  |  |  |
| GO:0006451\_translational\_readthrough | 2 | 0 |  |  |  |  |  |  |  |  |
| GO:0006458\_'de\_novo'\_protein\_folding | 2 | 0 |  |  |  |  |  |  |  |  |
| GO:0006465\_signal\_peptide\_processing | 2 | 0 |  |  |  |  |  |  |  |  |
| GO:0006475\_internal\_protein\_amino\_acid\_acetylation | 2 | 0 |  |  |  |  |  |  |  |  |
| GO:0006478\_peptidyl-tyrosine\_sulfation | 2 | 0 |  |  |  |  |  |  |  |  |
| GO:0006481\_C-terminal\_protein\_amino\_acid\_methylation | 2 | 0 |  |  |  |  |  |  |  |  |
| GO:0006488\_dolichol-linked\_oligosaccharide\_biosynthetic\_process | 2 | 0 |  |  |  |  |  |  |  |  |
| GO:0006517\_protein\_deglycosylation | 2 | 0 |  |  |  |  |  |  |  |  |
| GO:0006525\_arginine\_metabolic\_process | 2 | 0 |  |  |  |  |  |  |  |  |
| GO:0006527\_arginine\_catabolic\_process | 2 | 0 |  |  |  |  |  |  |  |  |
| GO:0006538\_glutamate\_catabolic\_process | 2 | 0 |  |  |  |  |  |  |  |  |
| GO:0006551\_leucine\_metabolic\_process | 2 | 0 |  |  |  |  |  |  |  |  |
| GO:0006552\_leucine\_catabolic\_process | 2 | 0 |  |  |  |  |  |  |  |  |
| GO:0006558\_L-phenylalanine\_metabolic\_process | 2 | 0 |  |  |  |  |  |  |  |  |
| GO:0006559\_L-phenylalanine\_catabolic\_process | 2 | 0 |  |  |  |  |  |  |  |  |
| GO:0006561\_proline\_biosynthetic\_process | 2 | 0 |  |  |  |  |  |  |  |  |
| GO:0006573\_valine\_metabolic\_process | 2 | 0 |  |  |  |  |  |  |  |  |
| GO:0006582\_melanin\_metabolic\_process | 2 | 0 |  |  |  |  |  |  |  |  |
| GO:0006583\_melanin\_biosynthetic\_process\_from\_tyrosine | 2 | 0 |  |  |  |  |  |  |  |  |
| GO:0006586\_indolalkylamine\_metabolic\_process | 2 | 0 |  |  |  |  |  |  |  |  |
| GO:0006599\_phosphagen\_metabolic\_process | 2 | 0 |  |  |  |  |  |  |  |  |
| GO:0006600\_creatine\_metabolic\_process | 2 | 0 |  |  |  |  |  |  |  |  |
| GO:0006601\_creatine\_biosynthetic\_process | 2 | 0 |  |  |  |  |  |  |  |  |
| GO:0006651\_diacylglycerol\_biosynthetic\_process | 2 | 0 |  |  |  |  |  |  |  |  |
| GO:0006654\_phosphatidic\_acid\_biosynthetic\_process | 2 | 0 |  |  |  |  |  |  |  |  |
| GO:0006663\_platelet\_activating\_factor\_biosynthetic\_process | 2 | 0 |  |  |  |  |  |  |  |  |
| GO:0006679\_glucosylceramide\_biosynthetic\_process | 2 | 0 |  |  |  |  |  |  |  |  |
| GO:0006685\_sphingomyelin\_catabolic\_process | 2 | 0 |  |  |  |  |  |  |  |  |
| GO:0006702\_androgen\_biosynthetic\_process | 2 | 0 |  |  |  |  |  |  |  |  |
| GO:0006710\_androgen\_catabolic\_process | 2 | 0 |  |  |  |  |  |  |  |  |
| GO:0006729\_tetrahydrobiopterin\_biosynthetic\_process | 2 | 0 |  |  |  |  |  |  |  |  |
| GO:0006754\_ATP\_biosynthetic\_process | 2 | 0 |  |  |  |  |  |  |  |  |
| GO:0006760\_folic\_acid\_and\_derivative\_metabolic\_process | 2 | 0 |  |  |  |  |  |  |  |  |
| GO:0006772\_thiamin\_metabolic\_process | 2 | 0 |  |  |  |  |  |  |  |  |
| GO:0006777\_Mo-molybdopterin\_cofactor\_biosynthetic\_process | 2 | 0 |  |  |  |  |  |  |  |  |
| GO:0006780\_uroporphyrinogen\_III\_biosynthetic\_process | 2 | 0 |  |  |  |  |  |  |  |  |
| GO:0006784\_heme\_a\_biosynthetic\_process | 2 | 0 |  |  |  |  |  |  |  |  |
| GO:0006829\_zinc\_ion\_transport | 2 | 0 |  |  |  |  |  |  |  |  |
| GO:0006863\_purine\_transport | 2 | 0 |  |  |  |  |  |  |  |  |
| GO:0006880\_intracellular\_sequestering\_of\_iron\_ion | 2 | 0 |  |  |  |  |  |  |  |  |
| GO:0006882\_cellular\_zinc\_ion\_homeostasis | 2 | 0 |  |  |  |  |  |  |  |  |
| GO:0006883\_cellular\_sodium\_ion\_homeostasis | 2 | 0 |  |  |  |  |  |  |  |  |
| GO:0006900\_membrane\_budding | 2 | 0 |  |  |  |  |  |  |  |  |
| GO:0006901\_vesicle\_coating | 2 | 0 |  |  |  |  |  |  |  |  |
| GO:0006922\_cleavage\_of\_lamin | 2 | 0 |  |  |  |  |  |  |  |  |
| GO:0006923\_cleavage\_of\_cytoskeletal\_proteins\_during\_apoptosis | 2 | 0 |  |  |  |  |  |  |  |  |
| GO:0006924\_activated\_T\_cell\_apoptosis | 2 | 0 |  |  |  |  |  |  |  |  |
| GO:0006927\_transformed\_cell\_apoptosis | 2 | 0 |  |  |  |  |  |  |  |  |
| GO:0006972\_hyperosmotic\_response | 2 | 0 |  |  |  |  |  |  |  |  |
| GO:0006975\_DNA\_damage\_induced\_protein\_phosphorylation | 2 | 0 |  |  |  |  |  |  |  |  |
| GO:0006978\_DNA\_damage\_response\_\_signal\_transduction\_by\_p53\_class\_mediator\_resulting\_in\_transcription\_of\_p21\_class\_mediator | 2 | 0 |  |  |  |  |  |  |  |  |
| GO:0006998\_nuclear\_membrane\_organization\_and\_biogenesis | 2 | 0 |  |  |  |  |  |  |  |  |
| GO:0006999\_nuclear\_pore\_organization\_and\_biogenesis | 2 | 0 |  |  |  |  |  |  |  |  |
| GO:0007007\_inner\_mitochondrial\_membrane\_organization\_and\_biogenesis | 2 | 0 |  |  |  |  |  |  |  |  |
| GO:0007023\_post-chaperonin\_tubulin\_folding\_pathway | 2 | 0 |  |  |  |  |  |  |  |  |
| GO:0007028\_cytoplasm\_organization\_and\_biogenesis | 2 | 0 |  |  |  |  |  |  |  |  |
| GO:0007044\_cell-substrate\_junction\_assembly | 2 | 0 |  |  |  |  |  |  |  |  |
| GO:0007095\_mitotic\_cell\_cycle\_G2\_M\_transition\_DNA\_damage\_checkpoint | 2 | 0 |  |  |  |  |  |  |  |  |
| GO:0007099\_centriole\_replication | 2 | 0 |  |  |  |  |  |  |  |  |
| GO:0007128\_meiotic\_prophase\_I | 2 | 0 |  |  |  |  |  |  |  |  |
| GO:0007168\_receptor\_guanylyl\_cyclase\_signaling\_pathway | 2 | 0 |  |  |  |  |  |  |  |  |
| GO:0007175\_negative\_regulation\_of\_epidermal\_growth\_factor\_receptor\_activity | 2 | 0 |  |  |  |  |  |  |  |  |
| GO:0007191\_dopamine\_receptor\_\_adenylate\_cyclase\_activating\_pathway | 2 | 0 |  |  |  |  |  |  |  |  |
| GO:0007207\_muscarinic\_acetylcholine\_receptor\_\_phospholipase\_C\_activating\_pathway | 2 | 0 |  |  |  |  |  |  |  |  |
| GO:0007231\_osmosensory\_signaling\_pathway | 2 | 0 |  |  |  |  |  |  |  |  |
| GO:0007308\_oocyte\_construction | 2 | 0 |  |  |  |  |  |  |  |  |
| GO:0007309\_oocyte\_axis\_determination | 2 | 0 |  |  |  |  |  |  |  |  |
| GO:0007340\_acrosome\_reaction | 2 | 0 |  |  |  |  |  |  |  |  |
| GO:0007386\_compartment\_specification | 2 | 0 |  |  |  |  |  |  |  |  |
| GO:0007405\_neuroblast\_proliferation | 2 | 0 |  |  |  |  |  |  |  |  |
| GO:0007418\_ventral\_midline\_development | 2 | 0 |  |  |  |  |  |  |  |  |
| GO:0007439\_ectodermal\_gut\_development | 2 | 0 |  |  |  |  |  |  |  |  |
| GO:0007442\_hindgut\_morphogenesis | 2 | 0 |  |  |  |  |  |  |  |  |
| GO:0007492\_endoderm\_development | 2 | 0 |  |  |  |  |  |  |  |  |
| GO:0007500\_mesodermal\_cell\_fate\_determination | 2 | 0 |  |  |  |  |  |  |  |  |
| GO:0007549\_dosage\_compensation | 2 | 0 |  |  |  |  |  |  |  |  |
| GO:0007603\_phototransduction\_\_visible\_light | 2 | 0 |  |  |  |  |  |  |  |  |
| GO:0007612\_learning | 2 | 0 |  |  |  |  |  |  |  |  |
| GO:0007635\_chemosensory\_behavior | 2 | 0 |  |  |  |  |  |  |  |  |
| GO:0008053\_mitochondrial\_fusion | 2 | 0 |  |  |  |  |  |  |  |  |
| GO:0008054\_cyclin\_catabolic\_process | 2 | 0 |  |  |  |  |  |  |  |  |
| GO:0008216\_spermidine\_metabolic\_process | 2 | 0 |  |  |  |  |  |  |  |  |
| GO:0008347\_glial\_cell\_migration | 2 | 0 |  |  |  |  |  |  |  |  |
| GO:0008582\_regulation\_of\_synaptic\_growth\_at\_neuromuscular\_junction | 2 | 0 |  |  |  |  |  |  |  |  |
| GO:0008588\_release\_of\_cytoplasmic\_sequestered\_NF-kappaB | 2 | 0 |  |  |  |  |  |  |  |  |
| GO:0008617\_guanosine\_metabolic\_process | 2 | 0 |  |  |  |  |  |  |  |  |
| GO:0008653\_lipopolysaccharide\_metabolic\_process | 2 | 0 |  |  |  |  |  |  |  |  |
| GO:0009084\_glutamine\_family\_amino\_acid\_biosynthetic\_process | 2 | 0 |  |  |  |  |  |  |  |  |
| GO:0009103\_lipopolysaccharide\_biosynthetic\_process | 2 | 0 |  |  |  |  |  |  |  |  |
| GO:0009127\_purine\_nucleoside\_monophosphate\_biosynthetic\_process | 2 | 0 |  |  |  |  |  |  |  |  |
| GO:0009134\_nucleoside\_diphosphate\_catabolic\_process | 2 | 0 |  |  |  |  |  |  |  |  |
| GO:0009143\_nucleoside\_triphosphate\_catabolic\_process | 2 | 0 |  |  |  |  |  |  |  |  |
| GO:0009168\_purine\_ribonucleoside\_monophosphate\_biosynthetic\_process | 2 | 0 |  |  |  |  |  |  |  |  |
| GO:0009191\_ribonucleoside\_diphosphate\_catabolic\_process | 2 | 0 |  |  |  |  |  |  |  |  |
| GO:0009219\_pyrimidine\_deoxyribonucleotide\_metabolic\_process | 2 | 0 |  |  |  |  |  |  |  |  |
| GO:0009223\_pyrimidine\_deoxyribonucleotide\_catabolic\_process | 2 | 0 |  |  |  |  |  |  |  |  |
| GO:0009263\_deoxyribonucleotide\_biosynthetic\_process | 2 | 0 |  |  |  |  |  |  |  |  |
| GO:0009264\_deoxyribonucleotide\_catabolic\_process | 2 | 0 |  |  |  |  |  |  |  |  |
| GO:0009386\_translational\_attenuation | 2 | 0 |  |  |  |  |  |  |  |  |
| GO:0009439\_cyanate\_metabolic\_process | 2 | 0 |  |  |  |  |  |  |  |  |
| GO:0009440\_cyanate\_catabolic\_process | 2 | 0 |  |  |  |  |  |  |  |  |
| GO:0009448\_gamma-aminobutyric\_acid\_metabolic\_process | 2 | 0 |  |  |  |  |  |  |  |  |
| GO:0009450\_gamma-aminobutyric\_acid\_catabolic\_process | 2 | 0 |  |  |  |  |  |  |  |  |
| GO:0009452\_RNA\_capping | 2 | 0 |  |  |  |  |  |  |  |  |
| GO:0009620\_response\_to\_fungus | 2 | 0 |  |  |  |  |  |  |  |  |
| GO:0009649\_entrainment\_of\_circadian\_clock | 2 | 0 |  |  |  |  |  |  |  |  |
| GO:0009726\_detection\_of\_endogenous\_stimulus | 2 | 0 |  |  |  |  |  |  |  |  |
| GO:0010155\_regulation\_of\_proton\_transport | 2 | 0 |  |  |  |  |  |  |  |  |
| GO:0010225\_response\_to\_UV-C | 2 | 0 |  |  |  |  |  |  |  |  |
| GO:0010332\_response\_to\_gamma\_radiation | 2 | 0 |  |  |  |  |  |  |  |  |
| GO:0015014\_heparan\_sulfate\_proteoglycan\_biosynthetic\_process\_\_polysaccharide\_chain\_biosynthetic\_process | 2 | 0 |  |  |  |  |  |  |  |  |
| GO:0015696\_ammonium\_transport | 2 | 0 |  |  |  |  |  |  |  |  |
| GO:0015788\_UDP-N-acetylglucosamine\_transport | 2 | 0 |  |  |  |  |  |  |  |  |
| GO:0015791\_polyol\_transport | 2 | 0 |  |  |  |  |  |  |  |  |
| GO:0015801\_aromatic\_amino\_acid\_transport | 2 | 0 |  |  |  |  |  |  |  |  |
| GO:0015811\_L-cystine\_transport | 2 | 0 |  |  |  |  |  |  |  |  |
| GO:0015822\_ornithine\_transport | 2 | 0 |  |  |  |  |  |  |  |  |
| GO:0015860\_purine\_nucleoside\_transport | 2 | 0 |  |  |  |  |  |  |  |  |
| GO:0015871\_choline\_transport | 2 | 0 |  |  |  |  |  |  |  |  |
| GO:0015893\_drug\_transport | 2 | 0 |  |  |  |  |  |  |  |  |
| GO:0016075\_rRNA\_catabolic\_process | 2 | 0 |  |  |  |  |  |  |  |  |
| GO:0016080\_synaptic\_vesicle\_targeting | 2 | 0 |  |  |  |  |  |  |  |  |
| GO:0016226\_iron-sulfur\_cluster\_assembly | 2 | 0 |  |  |  |  |  |  |  |  |
| GO:0016244\_non-apoptotic\_programmed\_cell\_death | 2 | 0 |  |  |  |  |  |  |  |  |
| GO:0016246\_RNA\_interference | 2 | 0 |  |  |  |  |  |  |  |  |
| GO:0016322\_neuron\_remodeling | 2 | 0 |  |  |  |  |  |  |  |  |
| GO:0016557\_peroxisome\_membrane\_biogenesis | 2 | 0 |  |  |  |  |  |  |  |  |
| GO:0016561\_protein\_import\_into\_peroxisome\_matrix\_\_translocation | 2 | 0 |  |  |  |  |  |  |  |  |
| GO:0016572\_histone\_phosphorylation | 2 | 0 |  |  |  |  |  |  |  |  |
| GO:0016574\_histone\_ubiquitination | 2 | 0 |  |  |  |  |  |  |  |  |
| GO:0016577\_histone\_demethylation | 2 | 0 |  |  |  |  |  |  |  |  |
| GO:0016584\_nucleosome\_positioning | 2 | 0 |  |  |  |  |  |  |  |  |
| GO:0016925\_protein\_sumoylation | 2 | 0 |  |  |  |  |  |  |  |  |
| GO:0017144\_drug\_metabolic\_process | 2 | 0 |  |  |  |  |  |  |  |  |
| GO:0018076\_N-terminal\_peptidyl-lysine\_acetylation | 2 | 0 |  |  |  |  |  |  |  |  |
| GO:0018195\_peptidyl-arginine\_modification | 2 | 0 |  |  |  |  |  |  |  |  |
| GO:0018216\_peptidyl-arginine\_methylation | 2 | 0 |  |  |  |  |  |  |  |  |
| GO:0018343\_protein\_farnesylation | 2 | 0 |  |  |  |  |  |  |  |  |
| GO:0018344\_protein\_geranylgeranylation | 2 | 0 |  |  |  |  |  |  |  |  |
| GO:0018347\_protein\_amino\_acid\_farnesylation | 2 | 0 |  |  |  |  |  |  |  |  |
| GO:0018348\_protein\_amino\_acid\_geranylgeranylation | 2 | 0 |  |  |  |  |  |  |  |  |
| GO:0018394\_peptidyl-lysine\_acetylation | 2 | 0 |  |  |  |  |  |  |  |  |
| GO:0018410\_peptide\_or\_protein\_carboxyl-terminal\_blocking | 2 | 0 |  |  |  |  |  |  |  |  |
| GO:0019042\_latent\_virus\_infection | 2 | 0 |  |  |  |  |  |  |  |  |
| GO:0019046\_reactivation\_of\_latent\_virus | 2 | 0 |  |  |  |  |  |  |  |  |
| GO:0019049\_evasion\_of\_host\_defenses\_by\_virus | 2 | 0 |  |  |  |  |  |  |  |  |
| GO:0019276\_UDP-N-acetylgalactosamine\_metabolic\_process | 2 | 0 |  |  |  |  |  |  |  |  |
| GO:0019370\_leukotriene\_biosynthetic\_process | 2 | 0 |  |  |  |  |  |  |  |  |
| GO:0019509\_methionine\_salvage | 2 | 0 |  |  |  |  |  |  |  |  |
| GO:0019605\_butyrate\_metabolic\_process | 2 | 0 |  |  |  |  |  |  |  |  |
| GO:0019626\_short-chain\_fatty\_acid\_catabolic\_process | 2 | 0 |  |  |  |  |  |  |  |  |
| GO:0019627\_urea\_metabolic\_process | 2 | 0 |  |  |  |  |  |  |  |  |
| GO:0019720\_Mo-molybdopterin\_cofactor\_metabolic\_process | 2 | 0 |  |  |  |  |  |  |  |  |
| GO:0019751\_polyol\_metabolic\_process | 2 | 0 |  |  |  |  |  |  |  |  |
| GO:0019754\_one-carbon\_compound\_catabolic\_process | 2 | 0 |  |  |  |  |  |  |  |  |
| GO:0019836\_hemolysis\_by\_symbiont\_of\_host\_red\_blood\_cells | 2 | 0 |  |  |  |  |  |  |  |  |
| GO:0019885\_antigen\_processing\_and\_presentation\_of\_endogenous\_peptide\_antigen\_via\_MHC\_class\_I | 2 | 0 |  |  |  |  |  |  |  |  |
| GO:0019896\_axon\_transport\_of\_mitochondrion | 2 | 0 |  |  |  |  |  |  |  |  |
| GO:0021517\_ventral\_spinal\_cord\_development | 2 | 0 |  |  |  |  |  |  |  |  |
| GO:0021536\_diencephalon\_development | 2 | 0 |  |  |  |  |  |  |  |  |
| GO:0021983\_pituitary\_gland\_development | 2 | 0 |  |  |  |  |  |  |  |  |
| GO:0022409\_positive\_regulation\_of\_cell-cell\_adhesion | 2 | 0 |  |  |  |  |  |  |  |  |
| GO:0022605\_oogenesis\_stage | 2 | 0 |  |  |  |  |  |  |  |  |
| GO:0022616\_DNA\_strand\_elongation | 2 | 0 |  |  |  |  |  |  |  |  |
| GO:0030146\_diuresis | 2 | 0 |  |  |  |  |  |  |  |  |
| GO:0030147\_natriuresis | 2 | 0 |  |  |  |  |  |  |  |  |
| GO:0030150\_protein\_import\_into\_mitochondrial\_matrix | 2 | 0 |  |  |  |  |  |  |  |  |
| GO:0030185\_nitric\_oxide\_transport | 2 | 0 |  |  |  |  |  |  |  |  |
| GO:0030205\_dermatan\_sulfate\_metabolic\_process | 2 | 0 |  |  |  |  |  |  |  |  |
| GO:0030208\_dermatan\_sulfate\_biosynthetic\_process | 2 | 0 |  |  |  |  |  |  |  |  |
| GO:0030239\_myofibril\_assembly | 2 | 0 |  |  |  |  |  |  |  |  |
| GO:0030252\_growth\_hormone\_secretion | 2 | 0 |  |  |  |  |  |  |  |  |
| GO:0030279\_negative\_regulation\_of\_ossification | 2 | 0 |  |  |  |  |  |  |  |  |
| GO:0030309\_poly-N-acetyllactosamine\_metabolic\_process | 2 | 0 |  |  |  |  |  |  |  |  |
| GO:0030311\_poly-N-acetyllactosamine\_biosynthetic\_process | 2 | 0 |  |  |  |  |  |  |  |  |
| GO:0030318\_melanocyte\_differentiation | 2 | 0 |  |  |  |  |  |  |  |  |
| GO:0030325\_adrenal\_gland\_development | 2 | 0 |  |  |  |  |  |  |  |  |
| GO:0030490\_maturation\_of\_SSU-rRNA | 2 | 0 |  |  |  |  |  |  |  |  |
| GO:0030501\_positive\_regulation\_of\_bone\_mineralization | 2 | 0 |  |  |  |  |  |  |  |  |
| GO:0030505\_inorganic\_diphosphate\_transport | 2 | 0 |  |  |  |  |  |  |  |  |
| GO:0030510\_regulation\_of\_BMP\_signaling\_pathway | 2 | 0 |  |  |  |  |  |  |  |  |
| GO:0030514\_negative\_regulation\_of\_BMP\_signaling\_pathway | 2 | 0 |  |  |  |  |  |  |  |  |
| GO:0030539\_male\_genitalia\_development | 2 | 0 |  |  |  |  |  |  |  |  |
| GO:0030801\_positive\_regulation\_of\_cyclic\_nucleotide\_metabolic\_process | 2 | 0 |  |  |  |  |  |  |  |  |
| GO:0030804\_positive\_regulation\_of\_cyclic\_nucleotide\_biosynthetic\_process | 2 | 0 |  |  |  |  |  |  |  |  |
| GO:0030810\_positive\_regulation\_of\_nucleotide\_biosynthetic\_process | 2 | 0 |  |  |  |  |  |  |  |  |
| GO:0030814\_regulation\_of\_cAMP\_metabolic\_process | 2 | 0 |  |  |  |  |  |  |  |  |
| GO:0030817\_regulation\_of\_cAMP\_biosynthetic\_process | 2 | 0 |  |  |  |  |  |  |  |  |
| GO:0030823\_regulation\_of\_cGMP\_metabolic\_process | 2 | 0 |  |  |  |  |  |  |  |  |
| GO:0030826\_regulation\_of\_cGMP\_biosynthetic\_process | 2 | 0 |  |  |  |  |  |  |  |  |
| GO:0030850\_prostate\_gland\_development | 2 | 0 |  |  |  |  |  |  |  |  |
| GO:0030851\_granulocyte\_differentiation | 2 | 0 |  |  |  |  |  |  |  |  |
| GO:0030947\_regulation\_of\_vascular\_endothelial\_growth\_factor\_receptor\_signaling\_pathway | 2 | 0 |  |  |  |  |  |  |  |  |
| GO:0031069\_hair\_follicle\_morphogenesis | 2 | 0 |  |  |  |  |  |  |  |  |
| GO:0031106\_septin\_ring\_organization | 2 | 0 |  |  |  |  |  |  |  |  |
| GO:0031163\_metallo-sulfur\_cluster\_assembly | 2 | 0 |  |  |  |  |  |  |  |  |
| GO:0031334\_positive\_regulation\_of\_protein\_complex\_assembly | 2 | 0 |  |  |  |  |  |  |  |  |
| GO:0031343\_positive\_regulation\_of\_cell\_killing | 2 | 0 |  |  |  |  |  |  |  |  |
| GO:0031440\_regulation\_of\_mRNA\_3'-end\_processing | 2 | 0 |  |  |  |  |  |  |  |  |
| GO:0031503\_protein\_complex\_localization | 2 | 0 |  |  |  |  |  |  |  |  |
| GO:0031536\_positive\_regulation\_of\_exit\_from\_mitosis | 2 | 0 |  |  |  |  |  |  |  |  |
| GO:0031580\_membrane\_raft\_distribution | 2 | 0 |  |  |  |  |  |  |  |  |
| GO:0031638\_zymogen\_activation | 2 | 0 |  |  |  |  |  |  |  |  |
| GO:0031640\_killing\_of\_cells\_of\_another\_organism | 2 | 0 |  |  |  |  |  |  |  |  |
| GO:0031670\_cellular\_response\_to\_nutrient | 2 | 0 |  |  |  |  |  |  |  |  |
| GO:0032024\_positive\_regulation\_of\_insulin\_secretion | 2 | 0 |  |  |  |  |  |  |  |  |
| GO:0032055\_negative\_regulation\_of\_translation\_in\_response\_to\_stress | 2 | 0 |  |  |  |  |  |  |  |  |
| GO:0032185\_septin\_cytoskeleton\_organization\_and\_biogenesis | 2 | 0 |  |  |  |  |  |  |  |  |
| GO:0032234\_regulation\_of\_calcium\_ion\_transport\_via\_store-operated\_calcium\_channel | 2 | 0 |  |  |  |  |  |  |  |  |
| GO:0032278\_positive\_regulation\_of\_gonadotropin\_secretion | 2 | 0 |  |  |  |  |  |  |  |  |
| GO:0032324\_molybdopterin\_cofactor\_biosynthetic\_process | 2 | 0 |  |  |  |  |  |  |  |  |
| GO:0032368\_regulation\_of\_lipid\_transport | 2 | 0 |  |  |  |  |  |  |  |  |
| GO:0032371\_regulation\_of\_sterol\_transport | 2 | 0 |  |  |  |  |  |  |  |  |
| GO:0032374\_regulation\_of\_cholesterol\_transport | 2 | 0 |  |  |  |  |  |  |  |  |
| GO:0032415\_regulation\_of\_sodium:hydrogen\_antiporter\_activity | 2 | 0 |  |  |  |  |  |  |  |  |
| GO:0032608\_interferon-beta\_production | 2 | 0 |  |  |  |  |  |  |  |  |
| GO:0032612\_interleukin-1\_production | 2 | 0 |  |  |  |  |  |  |  |  |
| GO:0032641\_lymphotoxin\_A\_production | 2 | 0 |  |  |  |  |  |  |  |  |
| GO:0032677\_regulation\_of\_interleukin-8\_production | 2 | 0 |  |  |  |  |  |  |  |  |
| GO:0032715\_negative\_regulation\_of\_interleukin-6\_production | 2 | 0 |  |  |  |  |  |  |  |  |
| GO:0032757\_positive\_regulation\_of\_interleukin-8\_production | 2 | 0 |  |  |  |  |  |  |  |  |
| GO:0032769\_negative\_regulation\_of\_monooxygenase\_activity | 2 | 0 |  |  |  |  |  |  |  |  |
| GO:0032780\_negative\_regulation\_of\_ATPase\_activity | 2 | 0 |  |  |  |  |  |  |  |  |
| GO:0032799\_low-density\_lipoprotein\_receptor\_metabolic\_process | 2 | 0 |  |  |  |  |  |  |  |  |
| GO:0032801\_receptor\_catabolic\_process | 2 | 0 |  |  |  |  |  |  |  |  |
| GO:0032855\_positive\_regulation\_of\_Rac\_GTPase\_activity | 2 | 0 |  |  |  |  |  |  |  |  |
| GO:0032933\_SREBP-mediated\_signaling\_pathway | 2 | 0 |  |  |  |  |  |  |  |  |
| GO:0033143\_regulation\_of\_steroid\_hormone\_receptor\_signaling\_pathway | 2 | 0 |  |  |  |  |  |  |  |  |
| GO:0033151\_V(D)J\_recombination | 2 | 0 |  |  |  |  |  |  |  |  |
| GO:0033363\_secretory\_granule\_organization\_and\_biogenesis | 2 | 0 |  |  |  |  |  |  |  |  |
| GO:0033598\_mammary\_gland\_epithelial\_cell\_proliferation | 2 | 0 |  |  |  |  |  |  |  |  |
| GO:0033599\_regulation\_of\_mammary\_gland\_epithelial\_cell\_proliferation | 2 | 0 |  |  |  |  |  |  |  |  |
| GO:0033700\_phospholipid\_efflux | 2 | 0 |  |  |  |  |  |  |  |  |
| GO:0034097\_response\_to\_cytokine\_stimulus | 2 | 0 |  |  |  |  |  |  |  |  |
| GO:0034105\_positive\_regulation\_of\_tissue\_remodeling | 2 | 0 |  |  |  |  |  |  |  |  |
| GO:0034331\_cell\_junction\_maintenance | 2 | 0 |  |  |  |  |  |  |  |  |
| GO:0035065\_regulation\_of\_histone\_acetylation | 2 | 0 |  |  |  |  |  |  |  |  |
| GO:0035104\_positive\_regulation\_of\_sterol\_regulatory\_element\_binding\_protein\_target\_gene\_transcription | 2 | 0 |  |  |  |  |  |  |  |  |
| GO:0035264\_multicellular\_organism\_growth | 2 | 0 |  |  |  |  |  |  |  |  |
| GO:0035272\_exocrine\_system\_development | 2 | 0 |  |  |  |  |  |  |  |  |
| GO:0040014\_regulation\_of\_multicellular\_organism\_growth | 2 | 0 |  |  |  |  |  |  |  |  |
| GO:0042059\_negative\_regulation\_of\_epidermal\_growth\_factor\_receptor\_signaling\_pathway | 2 | 0 |  |  |  |  |  |  |  |  |
| GO:0042062\_long-term\_strengthening\_of\_neuromuscular\_junction | 2 | 0 |  |  |  |  |  |  |  |  |
| GO:0042093\_T-helper\_cell\_differentiation | 2 | 0 |  |  |  |  |  |  |  |  |
| GO:0042104\_positive\_regulation\_of\_activated\_T\_cell\_proliferation | 2 | 0 |  |  |  |  |  |  |  |  |
| GO:0042109\_lymphotoxin\_A\_biosynthetic\_process | 2 | 0 |  |  |  |  |  |  |  |  |
| GO:0042119\_neutrophil\_activation | 2 | 0 |  |  |  |  |  |  |  |  |
| GO:0042130\_negative\_regulation\_of\_T\_cell\_proliferation | 2 | 0 |  |  |  |  |  |  |  |  |
| GO:0042135\_neurotransmitter\_catabolic\_process | 2 | 0 |  |  |  |  |  |  |  |  |
| GO:0042136\_neurotransmitter\_biosynthetic\_process | 2 | 0 |  |  |  |  |  |  |  |  |
| GO:0042167\_heme\_catabolic\_process | 2 | 0 |  |  |  |  |  |  |  |  |
| GO:0042274\_ribosomal\_small\_subunit\_biogenesis\_and\_assembly | 2 | 0 |  |  |  |  |  |  |  |  |
| GO:0042304\_regulation\_of\_fatty\_acid\_biosynthetic\_process | 2 | 0 |  |  |  |  |  |  |  |  |
| GO:0042339\_keratan\_sulfate\_metabolic\_process | 2 | 0 |  |  |  |  |  |  |  |  |
| GO:0042350\_GDP-L-fucose\_biosynthetic\_process | 2 | 0 |  |  |  |  |  |  |  |  |
| GO:0042351\_'de\_novo'\_GDP-L-fucose\_biosynthetic\_process | 2 | 0 |  |  |  |  |  |  |  |  |
| GO:0042353\_fucose\_biosynthetic\_process | 2 | 0 |  |  |  |  |  |  |  |  |
| GO:0042384\_cilium\_biogenesis | 2 | 0 |  |  |  |  |  |  |  |  |
| GO:0042396\_phosphagen\_biosynthetic\_process | 2 | 0 |  |  |  |  |  |  |  |  |
| GO:0042403\_thyroid\_hormone\_metabolic\_process | 2 | 0 |  |  |  |  |  |  |  |  |
| GO:0042430\_indole\_and\_derivative\_metabolic\_process | 2 | 0 |  |  |  |  |  |  |  |  |
| GO:0042434\_indole\_derivative\_metabolic\_process | 2 | 0 |  |  |  |  |  |  |  |  |
| GO:0042438\_melanin\_biosynthetic\_process | 2 | 0 |  |  |  |  |  |  |  |  |
| GO:0042439\_ethanolamine\_and\_derivative\_metabolic\_process | 2 | 0 |  |  |  |  |  |  |  |  |
| GO:0042448\_progesterone\_metabolic\_process | 2 | 0 |  |  |  |  |  |  |  |  |
| GO:0042454\_ribonucleoside\_catabolic\_process | 2 | 0 |  |  |  |  |  |  |  |  |
| GO:0042490\_mechanoreceptor\_differentiation | 2 | 0 |  |  |  |  |  |  |  |  |
| GO:0042491\_auditory\_receptor\_cell\_differentiation | 2 | 0 |  |  |  |  |  |  |  |  |
| GO:0042506\_tyrosine\_phosphorylation\_of\_Stat5\_protein | 2 | 0 |  |  |  |  |  |  |  |  |
| GO:0042522\_regulation\_of\_tyrosine\_phosphorylation\_of\_Stat5\_protein | 2 | 0 |  |  |  |  |  |  |  |  |
| GO:0042723\_thiamin\_and\_derivative\_metabolic\_process | 2 | 0 |  |  |  |  |  |  |  |  |
| GO:0042733\_embryonic\_digit\_morphogenesis | 2 | 0 |  |  |  |  |  |  |  |  |
| GO:0042752\_regulation\_of\_circadian\_rhythm | 2 | 0 |  |  |  |  |  |  |  |  |
| GO:0042772\_DNA\_damage\_response\_\_signal\_transduction\_resulting\_in\_transcription | 2 | 0 |  |  |  |  |  |  |  |  |
| GO:0043000\_Golgi\_to\_plasma\_membrane\_CFTR\_protein\_transport | 2 | 0 |  |  |  |  |  |  |  |  |
| GO:0043006\_calcium-dependent\_phospholipase\_A2\_activation | 2 | 0 |  |  |  |  |  |  |  |  |
| GO:0043010\_camera-type\_eye\_development | 2 | 0 |  |  |  |  |  |  |  |  |
| GO:0043070\_regulation\_of\_non-apoptotic\_programmed\_cell\_death | 2 | 0 |  |  |  |  |  |  |  |  |
| GO:0043072\_negative\_regulation\_of\_non-apoptotic\_programmed\_cell\_death | 2 | 0 |  |  |  |  |  |  |  |  |
| GO:0043102\_amino\_acid\_salvage | 2 | 0 |  |  |  |  |  |  |  |  |
| GO:0043113\_receptor\_clustering | 2 | 0 |  |  |  |  |  |  |  |  |
| GO:0043162\_ubiquitin-dependent\_protein\_catabolic\_process\_via\_the\_multivesicular\_body\_pathway | 2 | 0 |  |  |  |  |  |  |  |  |
| GO:0043163\_cell\_envelope\_organization\_and\_biogenesis | 2 | 0 |  |  |  |  |  |  |  |  |
| GO:0043171\_peptide\_catabolic\_process | 2 | 0 |  |  |  |  |  |  |  |  |
| GO:0043206\_fibril\_organization\_and\_biogenesis | 2 | 0 |  |  |  |  |  |  |  |  |
| GO:0043270\_positive\_regulation\_of\_ion\_transport | 2 | 0 |  |  |  |  |  |  |  |  |
| GO:0043271\_negative\_regulation\_of\_ion\_transport | 2 | 0 |  |  |  |  |  |  |  |  |
| GO:0043297\_apical\_junction\_assembly | 2 | 0 |  |  |  |  |  |  |  |  |
| GO:0043331\_response\_to\_dsRNA | 2 | 0 |  |  |  |  |  |  |  |  |
| GO:0043367\_CD4-positive\_\_alpha\_beta\_T\_cell\_differentiation | 2 | 0 |  |  |  |  |  |  |  |  |
| GO:0043368\_positive\_T\_cell\_selection | 2 | 0 |  |  |  |  |  |  |  |  |
| GO:0043370\_regulation\_of\_CD4-positive\_\_alpha\_beta\_T\_cell\_differentiation | 2 | 0 |  |  |  |  |  |  |  |  |
| GO:0043450\_alkene\_biosynthetic\_process | 2 | 0 |  |  |  |  |  |  |  |  |
| GO:0043484\_regulation\_of\_RNA\_splicing | 2 | 0 |  |  |  |  |  |  |  |  |
| GO:0043489\_RNA\_stabilization | 2 | 0 |  |  |  |  |  |  |  |  |
| GO:0043490\_malate-aspartate\_shuttle | 2 | 0 |  |  |  |  |  |  |  |  |
| GO:0043508\_negative\_regulation\_of\_JNK\_activity | 2 | 0 |  |  |  |  |  |  |  |  |
| GO:0043526\_neuroprotection | 2 | 0 |  |  |  |  |  |  |  |  |
| GO:0043536\_positive\_regulation\_of\_blood\_vessel\_endothelial\_cell\_migration | 2 | 0 |  |  |  |  |  |  |  |  |
| GO:0043555\_regulation\_of\_translation\_in\_response\_to\_stress | 2 | 0 |  |  |  |  |  |  |  |  |
| GO:0043570\_maintenance\_of\_DNA\_repeat\_elements | 2 | 0 |  |  |  |  |  |  |  |  |
| GO:0043603\_amide\_metabolic\_process | 2 | 0 |  |  |  |  |  |  |  |  |
| GO:0044003\_modification\_by\_symbiont\_of\_host\_morphology\_or\_physiology | 2 | 0 |  |  |  |  |  |  |  |  |
| GO:0044004\_disruption\_by\_symbiont\_of\_host\_cells | 2 | 0 |  |  |  |  |  |  |  |  |
| GO:0044413\_avoidance\_of\_host\_defenses | 2 | 0 |  |  |  |  |  |  |  |  |
| GO:0044415\_evasion\_or\_tolerance\_of\_host\_defenses | 2 | 0 |  |  |  |  |  |  |  |  |
| GO:0045002\_double-strand\_break\_repair\_via\_single-strand\_annealing | 2 | 0 |  |  |  |  |  |  |  |  |
| GO:0045003\_double-strand\_break\_repair\_via\_synthesis-dependent\_strand\_annealing | 2 | 0 |  |  |  |  |  |  |  |  |
| GO:0045010\_actin\_nucleation | 2 | 0 |  |  |  |  |  |  |  |  |
| GO:0045046\_protein\_import\_into\_peroxisome\_membrane | 2 | 0 |  |  |  |  |  |  |  |  |
| GO:0045059\_positive\_thymic\_T\_cell\_selection | 2 | 0 |  |  |  |  |  |  |  |  |
| GO:0045080\_positive\_regulation\_of\_chemokine\_biosynthetic\_process | 2 | 0 |  |  |  |  |  |  |  |  |
| GO:0045084\_positive\_regulation\_of\_interleukin-12\_biosynthetic\_process | 2 | 0 |  |  |  |  |  |  |  |  |
| GO:0045124\_regulation\_of\_bone\_resorption | 2 | 0 |  |  |  |  |  |  |  |  |
| GO:0045162\_clustering\_of\_voltage-gated\_sodium\_channels | 2 | 0 |  |  |  |  |  |  |  |  |
| GO:0045217\_cell-cell\_junction\_maintenance | 2 | 0 |  |  |  |  |  |  |  |  |
| GO:0045229\_external\_encapsulating\_structure\_organization\_and\_biogenesis | 2 | 0 |  |  |  |  |  |  |  |  |
| GO:0045348\_positive\_regulation\_of\_MHC\_class\_II\_biosynthetic\_process | 2 | 0 |  |  |  |  |  |  |  |  |
| GO:0045350\_interferon-beta\_biosynthetic\_process | 2 | 0 |  |  |  |  |  |  |  |  |
| GO:0045356\_positive\_regulation\_of\_interferon-alpha\_biosynthetic\_process | 2 | 0 |  |  |  |  |  |  |  |  |
| GO:0045357\_regulation\_of\_interferon-beta\_biosynthetic\_process | 2 | 0 |  |  |  |  |  |  |  |  |
| GO:0045359\_positive\_regulation\_of\_interferon-beta\_biosynthetic\_process | 2 | 0 |  |  |  |  |  |  |  |  |
| GO:0045415\_negative\_regulation\_of\_interleukin-8\_biosynthetic\_process | 2 | 0 |  |  |  |  |  |  |  |  |
| GO:0045471\_response\_to\_ethanol | 2 | 0 |  |  |  |  |  |  |  |  |
| GO:0045541\_negative\_regulation\_of\_cholesterol\_biosynthetic\_process | 2 | 0 |  |  |  |  |  |  |  |  |
| GO:0045600\_positive\_regulation\_of\_fat\_cell\_differentiation | 2 | 0 |  |  |  |  |  |  |  |  |
| GO:0045601\_regulation\_of\_endothelial\_cell\_differentiation | 2 | 0 |  |  |  |  |  |  |  |  |
| GO:0045616\_regulation\_of\_keratinocyte\_differentiation | 2 | 0 |  |  |  |  |  |  |  |  |
| GO:0045618\_positive\_regulation\_of\_keratinocyte\_differentiation | 2 | 0 |  |  |  |  |  |  |  |  |
| GO:0045620\_negative\_regulation\_of\_lymphocyte\_differentiation | 2 | 0 |  |  |  |  |  |  |  |  |
| GO:0045622\_regulation\_of\_T-helper\_cell\_differentiation | 2 | 0 |  |  |  |  |  |  |  |  |
| GO:0045647\_negative\_regulation\_of\_erythrocyte\_differentiation | 2 | 0 |  |  |  |  |  |  |  |  |
| GO:0045651\_positive\_regulation\_of\_macrophage\_differentiation | 2 | 0 |  |  |  |  |  |  |  |  |
| GO:0045652\_regulation\_of\_megakaryocyte\_differentiation | 2 | 0 |  |  |  |  |  |  |  |  |
| GO:0045725\_positive\_regulation\_of\_glycogen\_biosynthetic\_process | 2 | 0 |  |  |  |  |  |  |  |  |
| GO:0045778\_positive\_regulation\_of\_ossification | 2 | 0 |  |  |  |  |  |  |  |  |
| GO:0045821\_positive\_regulation\_of\_glycolysis | 2 | 0 |  |  |  |  |  |  |  |  |
| GO:0045842\_positive\_regulation\_of\_mitotic\_metaphase\_anaphase\_transition | 2 | 0 |  |  |  |  |  |  |  |  |
| GO:0045869\_negative\_regulation\_of\_retroviral\_genome\_replication | 2 | 0 |  |  |  |  |  |  |  |  |
| GO:0045885\_positive\_regulation\_of\_survival\_gene\_product\_expression | 2 | 0 |  |  |  |  |  |  |  |  |
| GO:0045898\_regulation\_of\_transcriptional\_preinitiation\_complex\_assembly | 2 | 0 |  |  |  |  |  |  |  |  |
| GO:0045909\_positive\_regulation\_of\_vasodilation | 2 | 0 |  |  |  |  |  |  |  |  |
| GO:0045912\_negative\_regulation\_of\_carbohydrate\_metabolic\_process | 2 | 0 |  |  |  |  |  |  |  |  |
| GO:0045933\_positive\_regulation\_of\_muscle\_contraction | 2 | 0 |  |  |  |  |  |  |  |  |
| GO:0045939\_negative\_regulation\_of\_steroid\_metabolic\_process | 2 | 0 |  |  |  |  |  |  |  |  |
| GO:0045940\_positive\_regulation\_of\_steroid\_metabolic\_process | 2 | 0 |  |  |  |  |  |  |  |  |
| GO:0045954\_positive\_regulation\_of\_natural\_killer\_cell\_mediated\_cytotoxicity | 2 | 0 |  |  |  |  |  |  |  |  |
| GO:0045980\_negative\_regulation\_of\_nucleotide\_metabolic\_process | 2 | 0 |  |  |  |  |  |  |  |  |
| GO:0045981\_positive\_regulation\_of\_nucleotide\_metabolic\_process | 2 | 0 |  |  |  |  |  |  |  |  |
| GO:0045987\_positive\_regulation\_of\_smooth\_muscle\_contraction | 2 | 0 |  |  |  |  |  |  |  |  |
| GO:0046006\_regulation\_of\_activated\_T\_cell\_proliferation | 2 | 0 |  |  |  |  |  |  |  |  |
| GO:0046033\_AMP\_metabolic\_process | 2 | 0 |  |  |  |  |  |  |  |  |
| GO:0046068\_cGMP\_metabolic\_process | 2 | 0 |  |  |  |  |  |  |  |  |
| GO:0046135\_pyrimidine\_nucleoside\_catabolic\_process | 2 | 0 |  |  |  |  |  |  |  |  |
| GO:0046146\_tetrahydrobiopterin\_metabolic\_process | 2 | 0 |  |  |  |  |  |  |  |  |
| GO:0046149\_pigment\_catabolic\_process | 2 | 0 |  |  |  |  |  |  |  |  |
| GO:0046160\_heme\_a\_metabolic\_process | 2 | 0 |  |  |  |  |  |  |  |  |
| GO:0046320\_regulation\_of\_fatty\_acid\_oxidation | 2 | 0 |  |  |  |  |  |  |  |  |
| GO:0046321\_positive\_regulation\_of\_fatty\_acid\_oxidation | 2 | 0 |  |  |  |  |  |  |  |  |
| GO:0046325\_negative\_regulation\_of\_glucose\_import | 2 | 0 |  |  |  |  |  |  |  |  |
| GO:0046349\_amino\_sugar\_biosynthetic\_process | 2 | 0 |  |  |  |  |  |  |  |  |
| GO:0046359\_butyrate\_catabolic\_process | 2 | 0 |  |  |  |  |  |  |  |  |
| GO:0046368\_GDP-L-fucose\_metabolic\_process | 2 | 0 |  |  |  |  |  |  |  |  |
| GO:0046469\_platelet\_activating\_factor\_metabolic\_process | 2 | 0 |  |  |  |  |  |  |  |  |
| GO:0046473\_phosphatidic\_acid\_metabolic\_process | 2 | 0 |  |  |  |  |  |  |  |  |
| GO:0046475\_glycerophospholipid\_catabolic\_process | 2 | 0 |  |  |  |  |  |  |  |  |
| GO:0046476\_glycosylceramide\_biosynthetic\_process | 2 | 0 |  |  |  |  |  |  |  |  |
| GO:0046479\_glycosphingolipid\_catabolic\_process | 2 | 0 |  |  |  |  |  |  |  |  |
| GO:0046502\_uroporphyrinogen\_III\_metabolic\_process | 2 | 0 |  |  |  |  |  |  |  |  |
| GO:0046580\_negative\_regulation\_of\_Ras\_protein\_signal\_transduction | 2 | 0 |  |  |  |  |  |  |  |  |
| GO:0046627\_negative\_regulation\_of\_insulin\_receptor\_signaling\_pathway | 2 | 0 |  |  |  |  |  |  |  |  |
| GO:0046632\_alpha-beta\_T\_cell\_differentiation | 2 | 0 |  |  |  |  |  |  |  |  |
| GO:0046633\_alpha-beta\_T\_cell\_proliferation | 2 | 0 |  |  |  |  |  |  |  |  |
| GO:0046635\_positive\_regulation\_of\_alpha-beta\_T\_cell\_activation | 2 | 0 |  |  |  |  |  |  |  |  |
| GO:0046637\_regulation\_of\_alpha-beta\_T\_cell\_differentiation | 2 | 0 |  |  |  |  |  |  |  |  |
| GO:0046719\_regulation\_of\_viral\_protein\_levels\_in\_host\_cell | 2 | 0 |  |  |  |  |  |  |  |  |
| GO:0046836\_glycolipid\_transport | 2 | 0 |  |  |  |  |  |  |  |  |
| GO:0046839\_phospholipid\_dephosphorylation | 2 | 0 |  |  |  |  |  |  |  |  |
| GO:0046852\_positive\_regulation\_of\_bone\_remodeling | 2 | 0 |  |  |  |  |  |  |  |  |
| GO:0046881\_positive\_regulation\_of\_follicle-stimulating\_hormone\_secretion | 2 | 0 |  |  |  |  |  |  |  |  |
| GO:0046885\_regulation\_of\_hormone\_biosynthetic\_process | 2 | 0 |  |  |  |  |  |  |  |  |
| GO:0046928\_regulation\_of\_neurotransmitter\_secretion | 2 | 0 |  |  |  |  |  |  |  |  |
| GO:0046967\_cytosol\_to\_ER\_transport | 2 | 0 |  |  |  |  |  |  |  |  |
| GO:0047496\_vesicle\_transport\_along\_microtubule | 2 | 0 |  |  |  |  |  |  |  |  |
| GO:0048016\_inositol\_phosphate-mediated\_signaling | 2 | 0 |  |  |  |  |  |  |  |  |
| GO:0048096\_chromatin-mediated\_maintenance\_of\_transcription | 2 | 0 |  |  |  |  |  |  |  |  |
| GO:0048102\_autophagic\_cell\_death | 2 | 0 |  |  |  |  |  |  |  |  |
| GO:0048147\_negative\_regulation\_of\_fibroblast\_proliferation | 2 | 0 |  |  |  |  |  |  |  |  |
| GO:0048165\_fused\_antrum\_stage\_\_oogenesis | 2 | 0 |  |  |  |  |  |  |  |  |
| GO:0048255\_mRNA\_stabilization | 2 | 0 |  |  |  |  |  |  |  |  |
| GO:0048261\_negative\_regulation\_of\_receptor-mediated\_endocytosis | 2 | 0 |  |  |  |  |  |  |  |  |
| GO:0048268\_clathrin\_cage\_assembly | 2 | 0 |  |  |  |  |  |  |  |  |
| GO:0048284\_organelle\_fusion | 2 | 0 |  |  |  |  |  |  |  |  |
| GO:0048285\_organelle\_fission | 2 | 0 |  |  |  |  |  |  |  |  |
| GO:0048290\_isotype\_switching\_to\_IgA\_isotypes | 2 | 0 |  |  |  |  |  |  |  |  |
| GO:0048296\_regulation\_of\_isotype\_switching\_to\_IgA\_isotypes | 2 | 0 |  |  |  |  |  |  |  |  |
| GO:0048298\_positive\_regulation\_of\_isotype\_switching\_to\_IgA\_isotypes | 2 | 0 |  |  |  |  |  |  |  |  |
| GO:0048538\_thymus\_development | 2 | 0 |  |  |  |  |  |  |  |  |
| GO:0048546\_digestive\_tract\_morphogenesis | 2 | 0 |  |  |  |  |  |  |  |  |
| GO:0048547\_gut\_morphogenesis | 2 | 0 |  |  |  |  |  |  |  |  |
| GO:0048565\_gut\_development | 2 | 0 |  |  |  |  |  |  |  |  |
| GO:0048566\_embryonic\_gut\_development | 2 | 0 |  |  |  |  |  |  |  |  |
| GO:0048567\_ectodermal\_gut\_morphogenesis | 2 | 0 |  |  |  |  |  |  |  |  |
| GO:0048662\_negative\_regulation\_of\_smooth\_muscle\_cell\_proliferation | 2 | 0 |  |  |  |  |  |  |  |  |
| GO:0048663\_neuron\_fate\_commitment | 2 | 0 |  |  |  |  |  |  |  |  |
| GO:0048704\_embryonic\_skeletal\_morphogenesis | 2 | 0 |  |  |  |  |  |  |  |  |
| GO:0048705\_skeletal\_morphogenesis | 2 | 0 |  |  |  |  |  |  |  |  |
| GO:0048706\_embryonic\_skeletal\_development | 2 | 0 |  |  |  |  |  |  |  |  |
| GO:0048820\_hair\_follicle\_maturation | 2 | 0 |  |  |  |  |  |  |  |  |
| GO:0050434\_positive\_regulation\_of\_viral\_transcription | 2 | 0 |  |  |  |  |  |  |  |  |
| GO:0050651\_dermatan\_sulfate\_proteoglycan\_biosynthetic\_process | 2 | 0 |  |  |  |  |  |  |  |  |
| GO:0050665\_hydrogen\_peroxide\_biosynthetic\_process | 2 | 0 |  |  |  |  |  |  |  |  |
| GO:0050686\_negative\_regulation\_of\_mRNA\_processing | 2 | 0 |  |  |  |  |  |  |  |  |
| GO:0050729\_positive\_regulation\_of\_inflammatory\_response | 2 | 0 |  |  |  |  |  |  |  |  |
| GO:0050764\_regulation\_of\_phagocytosis | 2 | 0 |  |  |  |  |  |  |  |  |
| GO:0050766\_positive\_regulation\_of\_phagocytosis | 2 | 0 |  |  |  |  |  |  |  |  |
| GO:0050773\_regulation\_of\_dendrite\_development | 2 | 0 |  |  |  |  |  |  |  |  |
| GO:0050850\_positive\_regulation\_of\_calcium-mediated\_signaling | 2 | 0 |  |  |  |  |  |  |  |  |
| GO:0050862\_positive\_regulation\_of\_T\_cell\_receptor\_signaling\_pathway | 2 | 0 |  |  |  |  |  |  |  |  |
| GO:0050869\_negative\_regulation\_of\_B\_cell\_activation | 2 | 0 |  |  |  |  |  |  |  |  |
| GO:0050885\_neuromuscular\_process\_controlling\_balance | 2 | 0 |  |  |  |  |  |  |  |  |
| GO:0050886\_endocrine\_process | 2 | 0 |  |  |  |  |  |  |  |  |
| GO:0050891\_multicellular\_organismal\_water\_homeostasis | 2 | 0 |  |  |  |  |  |  |  |  |
| GO:0050931\_pigment\_cell\_differentiation | 2 | 0 |  |  |  |  |  |  |  |  |
| GO:0050957\_equilibrioception | 2 | 0 |  |  |  |  |  |  |  |  |
| GO:0051001\_negative\_regulation\_of\_nitric-oxide\_synthase\_activity | 2 | 0 |  |  |  |  |  |  |  |  |
| GO:0051043\_regulation\_of\_membrane\_protein\_ectodomain\_proteolysis | 2 | 0 |  |  |  |  |  |  |  |  |
| GO:0051058\_negative\_regulation\_of\_small\_GTPase\_mediated\_signal\_transduction | 2 | 0 |  |  |  |  |  |  |  |  |
| GO:0051084\_'de\_novo'\_posttranslational\_protein\_folding | 2 | 0 |  |  |  |  |  |  |  |  |
| GO:0051123\_transcriptional\_preinitiation\_complex\_assembly | 2 | 0 |  |  |  |  |  |  |  |  |
| GO:0051124\_synaptic\_growth\_at\_neuromuscular\_junction | 2 | 0 |  |  |  |  |  |  |  |  |
| GO:0051131\_chaperone-mediated\_protein\_complex\_assembly | 2 | 0 |  |  |  |  |  |  |  |  |
| GO:0051231\_spindle\_elongation | 2 | 0 |  |  |  |  |  |  |  |  |
| GO:0051665\_membrane\_raft\_localization | 2 | 0 |  |  |  |  |  |  |  |  |
| GO:0051715\_cytolysis\_of\_cells\_of\_another\_organism | 2 | 0 |  |  |  |  |  |  |  |  |
| GO:0051797\_regulation\_of\_hair\_follicle\_development | 2 | 0 |  |  |  |  |  |  |  |  |
| GO:0051798\_positive\_regulation\_of\_hair\_follicle\_development | 2 | 0 |  |  |  |  |  |  |  |  |
| GO:0051801\_cytolysis\_of\_cells\_in\_other\_organism\_during\_symbiotic\_interaction | 2 | 0 |  |  |  |  |  |  |  |  |
| GO:0051817\_modification\_of\_morphology\_or\_physiology\_of\_other\_organism\_during\_symbiotic\_interaction | 2 | 0 |  |  |  |  |  |  |  |  |
| GO:0051818\_disruption\_of\_cells\_of\_other\_organism\_during\_symbiotic\_interaction | 2 | 0 |  |  |  |  |  |  |  |  |
| GO:0051832\_avoidance\_of\_defenses\_of\_other\_organism\_during\_symbiotic\_interaction | 2 | 0 |  |  |  |  |  |  |  |  |
| GO:0051834\_evasion\_or\_tolerance\_of\_defenses\_of\_other\_organism\_during\_symbiotic\_interaction | 2 | 0 |  |  |  |  |  |  |  |  |
| GO:0051883\_killing\_of\_cells\_in\_other\_organism\_during\_symbiotic\_interaction | 2 | 0 |  |  |  |  |  |  |  |  |
| GO:0051895\_negative\_regulation\_of\_focal\_adhesion\_formation | 2 | 0 |  |  |  |  |  |  |  |  |
| GO:0051900\_regulation\_of\_mitochondrial\_depolarization | 2 | 0 |  |  |  |  |  |  |  |  |
| GO:0051902\_negative\_regulation\_of\_mitochondrial\_depolarization | 2 | 0 |  |  |  |  |  |  |  |  |
| GO:0051928\_positive\_regulation\_of\_calcium\_ion\_transport | 2 | 0 |  |  |  |  |  |  |  |  |
| GO:0051963\_regulation\_of\_synaptogenesis | 2 | 0 |  |  |  |  |  |  |  |  |
| GO:0051972\_regulation\_of\_telomerase\_activity | 2 | 0 |  |  |  |  |  |  |  |  |
| GO:0051974\_negative\_regulation\_of\_telomerase\_activity | 2 | 0 |  |  |  |  |  |  |  |  |
| GO:0052025\_modification\_by\_symbiont\_of\_host\_cell\_membrane | 2 | 0 |  |  |  |  |  |  |  |  |
| GO:0052043\_modification\_by\_symbiont\_of\_host\_cellular\_component | 2 | 0 |  |  |  |  |  |  |  |  |
| GO:0052111\_modification\_by\_symbiont\_of\_host\_structure | 2 | 0 |  |  |  |  |  |  |  |  |
| GO:0052173\_response\_to\_defenses\_of\_other\_organism\_during\_symbiotic\_interaction | 2 | 0 |  |  |  |  |  |  |  |  |
| GO:0052185\_modification\_of\_structure\_of\_other\_organism\_during\_symbiotic\_interaction | 2 | 0 |  |  |  |  |  |  |  |  |
| GO:0052188\_modification\_of\_cellular\_component\_in\_other\_organism\_during\_symbiotic\_interaction | 2 | 0 |  |  |  |  |  |  |  |  |
| GO:0052200\_response\_to\_host\_defenses | 2 | 0 |  |  |  |  |  |  |  |  |
| GO:0052331\_hemolysis\_by\_organism\_of\_red\_blood\_cells\_in\_other\_organism\_during\_symbiotic\_interaction | 2 | 0 |  |  |  |  |  |  |  |  |
| GO:0052332\_modification\_by\_organism\_of\_cell\_membrane\_in\_other\_organism\_during\_symbiotic\_interaction | 2 | 0 |  |  |  |  |  |  |  |  |
| GO:0055001\_muscle\_cell\_development | 2 | 0 |  |  |  |  |  |  |  |  |
| GO:0055002\_striated\_muscle\_cell\_development | 2 | 0 |  |  |  |  |  |  |  |  |
| GO:0055069\_zinc\_ion\_homeostasis | 2 | 0 |  |  |  |  |  |  |  |  |
| GO:0055078\_sodium\_ion\_homeostasis | 2 | 0 |  |  |  |  |  |  |  |  |
| GO:0055091\_phospholipid\_homeostasis | 2 | 0 |  |  |  |  |  |  |  |  |
| GO:0060021\_palate\_development | 2 | 0 |  |  |  |  |  |  |  |  |
| GO:0060023\_soft\_palate\_development | 2 | 0 |  |  |  |  |  |  |  |  |
| GO:0060033\_anatomical\_structure\_regression | 2 | 0 |  |  |  |  |  |  |  |  |
| GO:0060052\_neurofilament\_cytoskeleton\_organization\_and\_biogenesis | 2 | 0 |  |  |  |  |  |  |  |  |
| GO:0060113\_inner\_ear\_receptor\_cell\_differentiation | 2 | 0 |  |  |  |  |  |  |  |  |
| GO:0060158\_dopamine\_receptor\_\_phospholipase\_C\_activating\_pathway | 2 | 0 |  |  |  |  |  |  |  |  |
| GO:0060177\_regulation\_of\_angiotensin\_metabolic\_process | 2 | 0 |  |  |  |  |  |  |  |  |
| GO:0065002\_intracellular\_protein\_transport\_across\_a\_membrane | 2 | 0 |  |  |  |  |  |  |  |  |
| GO:0008632\_apoptotic\_program | 66 | 0 | 0.000000 | 0.000000 | 793 | 786.134349 | 823.51 | 860.885651 | 1.038474 |
| GO:0022618\_protein-RNA\_complex\_assembly | 66 | 0 | 0.000000 | 0.000000 | 793 | 786.134349 | 823.51 | 860.885651 | 1.038474 |
| GO:0031326\_regulation\_of\_cellular\_biosynthetic\_process | 66 | 0 | 0.000000 | 0.000000 | 793 | 786.134349 | 823.51 | 860.885651 | 1.038474 |
| GO:0003013\_circulatory\_system\_process | 80 | 0 | 0.000000 | 0.000000 | 796 | 789.228473 | 826.25 | 863.271527 | 1.038003 |
| GO:0008015\_blood\_circulation | 80 | 0 | 0.000000 | 0.000000 | 796 | 789.228473 | 826.25 | 863.271527 | 1.038003 |
| GO:0030182\_neuron\_differentiation | 80 | 0 | 0.000000 | 0.000000 | 796 | 789.228473 | 826.25 | 863.271527 | 1.038003 |
| GO:0030003\_cellular\_cation\_homeostasis | 87 | 0 | 0.000000 | 0.000000 | 799 | 791.908789 | 828.45 | 864.991211 | 1.036859 |
| GO:0045321\_leukocyte\_activation | 87 | 0 | 0.000000 | 0.000000 | 799 | 791.908789 | 828.45 | 864.991211 | 1.036859 |
| GO:0048699\_generation\_of\_neurons | 87 | 0 | 0.000000 | 0.000000 | 799 | 791.908789 | 828.45 | 864.991211 | 1.036859 |
| GO:0001525\_angiogenesis | 48 | 0 | 0.000000 | 0.000000 | 803 | 795.721327 | 832.08 | 868.438673 | 1.036214 |
| GO:0007187\_G-protein\_signaling\_\_coupled\_to\_cyclic\_nucleotide\_second\_messenger | 48 | 0 | 0.000000 | 0.000000 | 803 | 795.721327 | 832.08 | 868.438673 | 1.036214 |
| GO:0016051\_carbohydrate\_biosynthetic\_process | 48 | 0 | 0.000000 | 0.000000 | 803 | 795.721327 | 832.08 | 868.438673 | 1.036214 |
| GO:0043406\_positive\_regulation\_of\_MAP\_kinase\_activity | 48 | 0 | 0.000000 | 0.000000 | 803 | 795.721327 | 832.08 | 868.438673 | 1.036214 |
| GO:0006091\_generation\_of\_precursor\_metabolites\_and\_energy | 100 | 0 | 0.000000 | 0.000000 | 805 | 796.908729 | 833.1 | 869.291271 | 1.034907 |
| GO:0009889\_regulation\_of\_biosynthetic\_process | 100 | 0 | 0.000000 | 0.000000 | 805 | 796.908729 | 833.1 | 869.291271 | 1.034907 |
| GO:0000184\_mRNA\_catabolic\_process\_\_nonsense-mediated\_decay | 8 | 0 | 0.000000 | 0.000000 | 900 | 888.673786 | 923.69 | 958.706214 | 1.026322 |
| GO:0000726\_non-recombinational\_repair | 8 | 0 | 0.000000 | 0.000000 | 900 | 888.673786 | 923.69 | 958.706214 | 1.026322 |
| GO:0001570\_vasculogenesis | 8 | 0 | 0.000000 | 0.000000 | 900 | 888.673786 | 923.69 | 958.706214 | 1.026322 |
| GO:0001701\_in\_utero\_embryonic\_development | 8 | 0 | 0.000000 | 0.000000 | 900 | 888.673786 | 923.69 | 958.706214 | 1.026322 |
| GO:0001822\_kidney\_development | 8 | 0 | 0.000000 | 0.000000 | 900 | 888.673786 | 923.69 | 958.706214 | 1.026322 |
| GO:0001894\_tissue\_homeostasis | 8 | 0 | 0.000000 | 0.000000 | 900 | 888.673786 | 923.69 | 958.706214 | 1.026322 |
| GO:0001909\_leukocyte\_mediated\_cytotoxicity | 8 | 0 | 0.000000 | 0.000000 | 900 | 888.673786 | 923.69 | 958.706214 | 1.026322 |
| GO:0001952\_regulation\_of\_cell-matrix\_adhesion | 8 | 0 | 0.000000 | 0.000000 | 900 | 888.673786 | 923.69 | 958.706214 | 1.026322 |
| GO:0002200\_somatic\_diversification\_of\_immune\_receptors | 8 | 0 | 0.000000 | 0.000000 | 900 | 888.673786 | 923.69 | 958.706214 | 1.026322 |
| GO:0002367\_cytokine\_production\_during\_immune\_response | 8 | 0 | 0.000000 | 0.000000 | 900 | 888.673786 | 923.69 | 958.706214 | 1.026322 |
| GO:0002377\_immunoglobulin\_production | 8 | 0 | 0.000000 | 0.000000 | 900 | 888.673786 | 923.69 | 958.706214 | 1.026322 |
| GO:0002456\_T\_cell\_mediated\_immunity | 8 | 0 | 0.000000 | 0.000000 | 900 | 888.673786 | 923.69 | 958.706214 | 1.026322 |
| GO:0002695\_negative\_regulation\_of\_leukocyte\_activation | 8 | 0 | 0.000000 | 0.000000 | 900 | 888.673786 | 923.69 | 958.706214 | 1.026322 |
| GO:0002703\_regulation\_of\_leukocyte\_mediated\_immunity | 8 | 0 | 0.000000 | 0.000000 | 900 | 888.673786 | 923.69 | 958.706214 | 1.026322 |
| GO:0002706\_regulation\_of\_lymphocyte\_mediated\_immunity | 8 | 0 | 0.000000 | 0.000000 | 900 | 888.673786 | 923.69 | 958.706214 | 1.026322 |
| GO:0006081\_aldehyde\_metabolic\_process | 8 | 0 | 0.000000 | 0.000000 | 900 | 888.673786 | 923.69 | 958.706214 | 1.026322 |
| GO:0006164\_purine\_nucleotide\_biosynthetic\_process | 8 | 0 | 0.000000 | 0.000000 | 900 | 888.673786 | 923.69 | 958.706214 | 1.026322 |
| GO:0006171\_cAMP\_biosynthetic\_process | 8 | 0 | 0.000000 | 0.000000 | 900 | 888.673786 | 923.69 | 958.706214 | 1.026322 |
| GO:0006220\_pyrimidine\_nucleotide\_metabolic\_process | 8 | 0 | 0.000000 | 0.000000 | 900 | 888.673786 | 923.69 | 958.706214 | 1.026322 |
| GO:0006282\_regulation\_of\_DNA\_repair | 8 | 0 | 0.000000 | 0.000000 | 900 | 888.673786 | 923.69 | 958.706214 | 1.026322 |
| GO:0006360\_transcription\_from\_RNA\_polymerase\_I\_promoter | 8 | 0 | 0.000000 | 0.000000 | 900 | 888.673786 | 923.69 | 958.706214 | 1.026322 |
| GO:0006378\_mRNA\_polyadenylation | 8 | 0 | 0.000000 | 0.000000 | 900 | 888.673786 | 923.69 | 958.706214 | 1.026322 |
| GO:0006379\_mRNA\_cleavage | 8 | 0 | 0.000000 | 0.000000 | 900 | 888.673786 | 923.69 | 958.706214 | 1.026322 |
| GO:0006414\_translational\_elongation | 8 | 0 | 0.000000 | 0.000000 | 900 | 888.673786 | 923.69 | 958.706214 | 1.026322 |
| GO:0006476\_protein\_amino\_acid\_deacetylation | 8 | 0 | 0.000000 | 0.000000 | 900 | 888.673786 | 923.69 | 958.706214 | 1.026322 |
| GO:0006684\_sphingomyelin\_metabolic\_process | 8 | 0 | 0.000000 | 0.000000 | 900 | 888.673786 | 923.69 | 958.706214 | 1.026322 |
| GO:0006749\_glutathione\_metabolic\_process | 8 | 0 | 0.000000 | 0.000000 | 900 | 888.673786 | 923.69 | 958.706214 | 1.026322 |
| GO:0006904\_vesicle\_docking\_during\_exocytosis | 8 | 0 | 0.000000 | 0.000000 | 900 | 888.673786 | 923.69 | 958.706214 | 1.026322 |
| GO:0007030\_Golgi\_organization\_and\_biogenesis | 8 | 0 | 0.000000 | 0.000000 | 900 | 888.673786 | 923.69 | 958.706214 | 1.026322 |
| GO:0007176\_regulation\_of\_epidermal\_growth\_factor\_receptor\_activity | 8 | 0 | 0.000000 | 0.000000 | 900 | 888.673786 | 923.69 | 958.706214 | 1.026322 |
| GO:0007286\_spermatid\_development | 8 | 0 | 0.000000 | 0.000000 | 900 | 888.673786 | 923.69 | 958.706214 | 1.026322 |
| GO:0009081\_branched\_chain\_family\_amino\_acid\_metabolic\_process | 8 | 0 | 0.000000 | 0.000000 | 900 | 888.673786 | 923.69 | 958.706214 | 1.026322 |
| GO:0009199\_ribonucleoside\_triphosphate\_metabolic\_process | 8 | 0 | 0.000000 | 0.000000 | 900 | 888.673786 | 923.69 | 958.706214 | 1.026322 |
| GO:0009743\_response\_to\_carbohydrate\_stimulus | 8 | 0 | 0.000000 | 0.000000 | 900 | 888.673786 | 923.69 | 958.706214 | 1.026322 |
| GO:0009798\_axis\_specification | 8 | 0 | 0.000000 | 0.000000 | 900 | 888.673786 | 923.69 | 958.706214 | 1.026322 |
| GO:0010466\_negative\_regulation\_of\_peptidase\_activity | 8 | 0 | 0.000000 | 0.000000 | 900 | 888.673786 | 923.69 | 958.706214 | 1.026322 |
| GO:0010469\_regulation\_of\_receptor\_activity | 8 | 0 | 0.000000 | 0.000000 | 900 | 888.673786 | 923.69 | 958.706214 | 1.026322 |
| GO:0015800\_acidic\_amino\_acid\_transport | 8 | 0 | 0.000000 | 0.000000 | 900 | 888.673786 | 923.69 | 958.706214 | 1.026322 |
| GO:0015813\_L-glutamate\_transport | 8 | 0 | 0.000000 | 0.000000 | 900 | 888.673786 | 923.69 | 958.706214 | 1.026322 |
| GO:0016236\_macroautophagy | 8 | 0 | 0.000000 | 0.000000 | 900 | 888.673786 | 923.69 | 958.706214 | 1.026322 |
| GO:0016573\_histone\_acetylation | 8 | 0 | 0.000000 | 0.000000 | 900 | 888.673786 | 923.69 | 958.706214 | 1.026322 |
| GO:0016575\_histone\_deacetylation | 8 | 0 | 0.000000 | 0.000000 | 900 | 888.673786 | 923.69 | 958.706214 | 1.026322 |
| GO:0018149\_peptide\_cross-linking | 8 | 0 | 0.000000 | 0.000000 | 900 | 888.673786 | 923.69 | 958.706214 | 1.026322 |
| GO:0019048\_virus-host\_interaction | 8 | 0 | 0.000000 | 0.000000 | 900 | 888.673786 | 923.69 | 958.706214 | 1.026322 |
| GO:0019059\_initiation\_of\_viral\_infection | 8 | 0 | 0.000000 | 0.000000 | 900 | 888.673786 | 923.69 | 958.706214 | 1.026322 |
| GO:0019319\_hexose\_biosynthetic\_process | 8 | 0 | 0.000000 | 0.000000 | 900 | 888.673786 | 923.69 | 958.706214 | 1.026322 |
| GO:0030149\_sphingolipid\_catabolic\_process | 8 | 0 | 0.000000 | 0.000000 | 900 | 888.673786 | 923.69 | 958.706214 | 1.026322 |
| GO:0030174\_regulation\_of\_DNA\_replication\_initiation | 8 | 0 | 0.000000 | 0.000000 | 900 | 888.673786 | 923.69 | 958.706214 | 1.026322 |
| GO:0030260\_entry\_into\_host\_cell | 8 | 0 | 0.000000 | 0.000000 | 900 | 888.673786 | 923.69 | 958.706214 | 1.026322 |
| GO:0030833\_regulation\_of\_actin\_filament\_polymerization | 8 | 0 | 0.000000 | 0.000000 | 900 | 888.673786 | 923.69 | 958.706214 | 1.026322 |
| GO:0031329\_regulation\_of\_cellular\_catabolic\_process | 8 | 0 | 0.000000 | 0.000000 | 900 | 888.673786 | 923.69 | 958.706214 | 1.026322 |
| GO:0032273\_positive\_regulation\_of\_protein\_polymerization | 8 | 0 | 0.000000 | 0.000000 | 900 | 888.673786 | 923.69 | 958.706214 | 1.026322 |
| GO:0032320\_positive\_regulation\_of\_Ras\_GTPase\_activity | 8 | 0 | 0.000000 | 0.000000 | 900 | 888.673786 | 923.69 | 958.706214 | 1.026322 |
| GO:0032321\_positive\_regulation\_of\_Rho\_GTPase\_activity | 8 | 0 | 0.000000 | 0.000000 | 900 | 888.673786 | 923.69 | 958.706214 | 1.026322 |
| GO:0032489\_regulation\_of\_Cdc42\_protein\_signal\_transduction | 8 | 0 | 0.000000 | 0.000000 | 900 | 888.673786 | 923.69 | 958.706214 | 1.026322 |
| GO:0032635\_interleukin-6\_production | 8 | 0 | 0.000000 | 0.000000 | 900 | 888.673786 | 923.69 | 958.706214 | 1.026322 |
| GO:0032637\_interleukin-8\_production | 8 | 0 | 0.000000 | 0.000000 | 900 | 888.673786 | 923.69 | 958.706214 | 1.026322 |
| GO:0042036\_negative\_regulation\_of\_cytokine\_biosynthetic\_process | 8 | 0 | 0.000000 | 0.000000 | 900 | 888.673786 | 923.69 | 958.706214 | 1.026322 |
| GO:0042116\_macrophage\_activation | 8 | 0 | 0.000000 | 0.000000 | 900 | 888.673786 | 923.69 | 958.706214 | 1.026322 |
| GO:0042133\_neurotransmitter\_metabolic\_process | 8 | 0 | 0.000000 | 0.000000 | 900 | 888.673786 | 923.69 | 958.706214 | 1.026322 |
| GO:0042147\_retrograde\_transport\_\_endosome\_to\_Golgi | 8 | 0 | 0.000000 | 0.000000 | 900 | 888.673786 | 923.69 | 958.706214 | 1.026322 |
| GO:0042476\_odontogenesis | 8 | 0 | 0.000000 | 0.000000 | 900 | 888.673786 | 923.69 | 958.706214 | 1.026322 |
| GO:0042551\_neuron\_maturation | 8 | 0 | 0.000000 | 0.000000 | 900 | 888.673786 | 923.69 | 958.706214 | 1.026322 |
| GO:0042594\_response\_to\_starvation | 8 | 0 | 0.000000 | 0.000000 | 900 | 888.673786 | 923.69 | 958.706214 | 1.026322 |
| GO:0043088\_regulation\_of\_Cdc42\_GTPase\_activity | 8 | 0 | 0.000000 | 0.000000 | 900 | 888.673786 | 923.69 | 958.706214 | 1.026322 |
| GO:0043112\_receptor\_metabolic\_process | 8 | 0 | 0.000000 | 0.000000 | 900 | 888.673786 | 923.69 | 958.706214 | 1.026322 |
| GO:0043149\_stress\_fiber\_formation | 8 | 0 | 0.000000 | 0.000000 | 900 | 888.673786 | 923.69 | 958.706214 | 1.026322 |
| GO:0043154\_negative\_regulation\_of\_caspase\_activity | 8 | 0 | 0.000000 | 0.000000 | 900 | 888.673786 | 923.69 | 958.706214 | 1.026322 |
| GO:0043269\_regulation\_of\_ion\_transport | 8 | 0 | 0.000000 | 0.000000 | 900 | 888.673786 | 923.69 | 958.706214 | 1.026322 |
| GO:0043393\_regulation\_of\_protein\_binding | 8 | 0 | 0.000000 | 0.000000 | 900 | 888.673786 | 923.69 | 958.706214 | 1.026322 |
| GO:0043473\_pigmentation | 8 | 0 | 0.000000 | 0.000000 | 900 | 888.673786 | 923.69 | 958.706214 | 1.026322 |
| GO:0043631\_RNA\_polyadenylation | 8 | 0 | 0.000000 | 0.000000 | 900 | 888.673786 | 923.69 | 958.706214 | 1.026322 |
| GO:0044409\_entry\_into\_host | 8 | 0 | 0.000000 | 0.000000 | 900 | 888.673786 | 923.69 | 958.706214 | 1.026322 |
| GO:0045026\_plasma\_membrane\_fusion | 8 | 0 | 0.000000 | 0.000000 | 900 | 888.673786 | 923.69 | 958.706214 | 1.026322 |
| GO:0045580\_regulation\_of\_T\_cell\_differentiation | 8 | 0 | 0.000000 | 0.000000 | 900 | 888.673786 | 923.69 | 958.706214 | 1.026322 |
| GO:0045646\_regulation\_of\_erythrocyte\_differentiation | 8 | 0 | 0.000000 | 0.000000 | 900 | 888.673786 | 923.69 | 958.706214 | 1.026322 |
| GO:0046165\_alcohol\_biosynthetic\_process | 8 | 0 | 0.000000 | 0.000000 | 900 | 888.673786 | 923.69 | 958.706214 | 1.026322 |
| GO:0046364\_monosaccharide\_biosynthetic\_process | 8 | 0 | 0.000000 | 0.000000 | 900 | 888.673786 | 923.69 | 958.706214 | 1.026322 |
| GO:0046718\_entry\_of\_virus\_into\_host\_cell | 8 | 0 | 0.000000 | 0.000000 | 900 | 888.673786 | 923.69 | 958.706214 | 1.026322 |
| GO:0048146\_positive\_regulation\_of\_fibroblast\_proliferation | 8 | 0 | 0.000000 | 0.000000 | 900 | 888.673786 | 923.69 | 958.706214 | 1.026322 |
| GO:0048167\_regulation\_of\_synaptic\_plasticity | 8 | 0 | 0.000000 | 0.000000 | 900 | 888.673786 | 923.69 | 958.706214 | 1.026322 |
| GO:0048659\_smooth\_muscle\_cell\_proliferation | 8 | 0 | 0.000000 | 0.000000 | 900 | 888.673786 | 923.69 | 958.706214 | 1.026322 |
| GO:0048730\_epidermis\_morphogenesis | 8 | 0 | 0.000000 | 0.000000 | 900 | 888.673786 | 923.69 | 958.706214 | 1.026322 |
| GO:0048871\_multicellular\_organismal\_homeostasis | 8 | 0 | 0.000000 | 0.000000 | 900 | 888.673786 | 923.69 | 958.706214 | 1.026322 |
| GO:0050654\_chondroitin\_sulfate\_proteoglycan\_metabolic\_process | 8 | 0 | 0.000000 | 0.000000 | 900 | 888.673786 | 923.69 | 958.706214 | 1.026322 |
| GO:0050727\_regulation\_of\_inflammatory\_response | 8 | 0 | 0.000000 | 0.000000 | 900 | 888.673786 | 923.69 | 958.706214 | 1.026322 |
| GO:0050866\_negative\_regulation\_of\_cell\_activation | 8 | 0 | 0.000000 | 0.000000 | 900 | 888.673786 | 923.69 | 958.706214 | 1.026322 |
| GO:0050920\_regulation\_of\_chemotaxis | 8 | 0 | 0.000000 | 0.000000 | 900 | 888.673786 | 923.69 | 958.706214 | 1.026322 |
| GO:0051222\_positive\_regulation\_of\_protein\_transport | 8 | 0 | 0.000000 | 0.000000 | 900 | 888.673786 | 923.69 | 958.706214 | 1.026322 |
| GO:0051605\_protein\_maturation\_via\_proteolysis | 8 | 0 | 0.000000 | 0.000000 | 900 | 888.673786 | 923.69 | 958.706214 | 1.026322 |
| GO:0051668\_localization\_within\_membrane | 8 | 0 | 0.000000 | 0.000000 | 900 | 888.673786 | 923.69 | 958.706214 | 1.026322 |
| GO:0051806\_entry\_into\_cell\_of\_other\_organism\_during\_symbiotic\_interaction | 8 | 0 | 0.000000 | 0.000000 | 900 | 888.673786 | 923.69 | 958.706214 | 1.026322 |
| GO:0051828\_entry\_into\_other\_organism\_during\_symbiotic\_interaction | 8 | 0 | 0.000000 | 0.000000 | 900 | 888.673786 | 923.69 | 958.706214 | 1.026322 |
| GO:0052126\_movement\_in\_host\_environment | 8 | 0 | 0.000000 | 0.000000 | 900 | 888.673786 | 923.69 | 958.706214 | 1.026322 |
| GO:0052192\_movement\_in\_environment\_of\_other\_organism\_during\_symbiotic\_interaction | 8 | 0 | 0.000000 | 0.000000 | 900 | 888.673786 | 923.69 | 958.706214 | 1.026322 |
| GO:0016071\_mRNA\_metabolic\_process | 105 | 0 | 0.000000 | 0.000000 | 901 | 889.170039 | 924.16 | 959.149961 | 1.025705 |
| GO:0006260\_DNA\_replication | 122 | 0 | 0.000000 | 0.000000 | 902 | 889.595969 | 924.58 | 959.564031 | 1.025033 |
| GO:0002697\_regulation\_of\_immune\_effector\_process | 14 | 0 | 0.000000 | 0.000000 | 928 | 914.757139 | 948.99 | 983.222861 | 1.022619 |
| GO:0006284\_base-excision\_repair | 14 | 0 | 0.000000 | 0.000000 | 928 | 914.757139 | 948.99 | 983.222861 | 1.022619 |
| GO:0007219\_Notch\_signaling\_pathway | 14 | 0 | 0.000000 | 0.000000 | 928 | 914.757139 | 948.99 | 983.222861 | 1.022619 |
| GO:0008064\_regulation\_of\_actin\_polymerization\_and\_or\_depolymerization | 14 | 0 | 0.000000 | 0.000000 | 928 | 914.757139 | 948.99 | 983.222861 | 1.022619 |
| GO:0009581\_detection\_of\_external\_stimulus | 14 | 0 | 0.000000 | 0.000000 | 928 | 914.757139 | 948.99 | 983.222861 | 1.022619 |
| GO:0009792\_embryonic\_development\_ending\_in\_birth\_or\_egg\_hatching | 14 | 0 | 0.000000 | 0.000000 | 928 | 914.757139 | 948.99 | 983.222861 | 1.022619 |
| GO:0030041\_actin\_filament\_polymerization | 14 | 0 | 0.000000 | 0.000000 | 928 | 914.757139 | 948.99 | 983.222861 | 1.022619 |
| GO:0030111\_regulation\_of\_Wnt\_receptor\_signaling\_pathway | 14 | 0 | 0.000000 | 0.000000 | 928 | 914.757139 | 948.99 | 983.222861 | 1.022619 |
| GO:0030595\_leukocyte\_chemotaxis | 14 | 0 | 0.000000 | 0.000000 | 928 | 914.757139 | 948.99 | 983.222861 | 1.022619 |
| GO:0031327\_negative\_regulation\_of\_cellular\_biosynthetic\_process | 14 | 0 | 0.000000 | 0.000000 | 928 | 914.757139 | 948.99 | 983.222861 | 1.022619 |
| GO:0032504\_multicellular\_organism\_reproduction | 14 | 0 | 0.000000 | 0.000000 | 928 | 914.757139 | 948.99 | 983.222861 | 1.022619 |
| GO:0042326\_negative\_regulation\_of\_phosphorylation | 14 | 0 | 0.000000 | 0.000000 | 928 | 914.757139 | 948.99 | 983.222861 | 1.022619 |
| GO:0043009\_chordate\_embryonic\_development | 14 | 0 | 0.000000 | 0.000000 | 928 | 914.757139 | 948.99 | 983.222861 | 1.022619 |
| GO:0043193\_positive\_regulation\_of\_gene-specific\_transcription | 14 | 0 | 0.000000 | 0.000000 | 928 | 914.757139 | 948.99 | 983.222861 | 1.022619 |
| GO:0043241\_protein\_complex\_disassembly | 14 | 0 | 0.000000 | 0.000000 | 928 | 914.757139 | 948.99 | 983.222861 | 1.022619 |
| GO:0045216\_cell-cell\_junction\_assembly\_and\_maintenance | 14 | 0 | 0.000000 | 0.000000 | 928 | 914.757139 | 948.99 | 983.222861 | 1.022619 |
| GO:0048545\_response\_to\_steroid\_hormone\_stimulus | 14 | 0 | 0.000000 | 0.000000 | 928 | 914.757139 | 948.99 | 983.222861 | 1.022619 |
| GO:0048609\_reproductive\_process\_in\_a\_multicellular\_organism | 14 | 0 | 0.000000 | 0.000000 | 928 | 914.757139 | 948.99 | 983.222861 | 1.022619 |
| GO:0048729\_tissue\_morphogenesis | 14 | 0 | 0.000000 | 0.000000 | 928 | 914.757139 | 948.99 | 983.222861 | 1.022619 |
| GO:0050731\_positive\_regulation\_of\_peptidyl-tyrosine\_phosphorylation | 14 | 0 | 0.000000 | 0.000000 | 928 | 914.757139 | 948.99 | 983.222861 | 1.022619 |
| GO:0050767\_regulation\_of\_neurogenesis | 14 | 0 | 0.000000 | 0.000000 | 928 | 914.757139 | 948.99 | 983.222861 | 1.022619 |
| GO:0051017\_actin\_filament\_bundle\_formation | 14 | 0 | 0.000000 | 0.000000 | 928 | 914.757139 | 948.99 | 983.222861 | 1.022619 |
| GO:0051262\_protein\_tetramerization | 14 | 0 | 0.000000 | 0.000000 | 928 | 914.757139 | 948.99 | 983.222861 | 1.022619 |
| GO:0051272\_positive\_regulation\_of\_cell\_motility | 14 | 0 | 0.000000 | 0.000000 | 928 | 914.757139 | 948.99 | 983.222861 | 1.022619 |
| GO:0051318\_G1\_phase | 14 | 0 | 0.000000 | 0.000000 | 928 | 914.757139 | 948.99 | 983.222861 | 1.022619 |
| GO:0051495\_positive\_regulation\_of\_cytoskeleton\_organization\_and\_biogenesis | 14 | 0 | 0.000000 | 0.000000 | 928 | 914.757139 | 948.99 | 983.222861 | 1.022619 |
| GO:0001932\_regulation\_of\_protein\_amino\_acid\_phosphorylation | 33 | 0 | 0.000000 | 0.000000 | 941 | 927.806355 | 960.97 | 994.133645 | 1.021222 |
| GO:0006334\_nucleosome\_assembly | 33 | 0 | 0.000000 | 0.000000 | 941 | 927.806355 | 960.97 | 994.133645 | 1.021222 |
| GO:0006338\_chromatin\_remodeling | 33 | 0 | 0.000000 | 0.000000 | 941 | 927.806355 | 960.97 | 994.133645 | 1.021222 |
| GO:0006839\_mitochondrial\_transport | 33 | 0 | 0.000000 | 0.000000 | 941 | 927.806355 | 960.97 | 994.133645 | 1.021222 |
| GO:0010557\_positive\_regulation\_of\_macromolecule\_biosynthetic\_process | 33 | 0 | 0.000000 | 0.000000 | 941 | 927.806355 | 960.97 | 994.133645 | 1.021222 |
| GO:0015674\_di-\_\_tri-valent\_inorganic\_cation\_transport | 33 | 0 | 0.000000 | 0.000000 | 941 | 927.806355 | 960.97 | 994.133645 | 1.021222 |
| GO:0016540\_protein\_autoprocessing | 33 | 0 | 0.000000 | 0.000000 | 941 | 927.806355 | 960.97 | 994.133645 | 1.021222 |
| GO:0019058\_viral\_infectious\_cycle | 33 | 0 | 0.000000 | 0.000000 | 941 | 927.806355 | 960.97 | 994.133645 | 1.021222 |
| GO:0040029\_regulation\_of\_gene\_expression\_\_epigenetic | 33 | 0 | 0.000000 | 0.000000 | 941 | 927.806355 | 960.97 | 994.133645 | 1.021222 |
| GO:0042107\_cytokine\_metabolic\_process | 33 | 0 | 0.000000 | 0.000000 | 941 | 927.806355 | 960.97 | 994.133645 | 1.021222 |
| GO:0042770\_DNA\_damage\_response\_\_signal\_transduction | 33 | 0 | 0.000000 | 0.000000 | 941 | 927.806355 | 960.97 | 994.133645 | 1.021222 |
| GO:0050776\_regulation\_of\_immune\_response | 33 | 0 | 0.000000 | 0.000000 | 941 | 927.806355 | 960.97 | 994.133645 | 1.021222 |
| GO:0051249\_regulation\_of\_lymphocyte\_activation | 33 | 0 | 0.000000 | 0.000000 | 941 | 927.806355 | 960.97 | 994.133645 | 1.021222 |
| GO:0008284\_positive\_regulation\_of\_cell\_proliferation | 135 | 0 | 0.000000 | 0.000000 | 942 | 928.885892 | 961.83 | 994.774108 | 1.021051 |
| GO:0009888\_tissue\_development | 127 | 0 | 0.000000 | 0.000000 | 943 | 929.430200 | 962.34 | 995.249800 | 1.020509 |
| GO:0005996\_monosaccharide\_metabolic\_process | 62 | 0 | 0.000000 | 0.000000 | 945 | 930.804543 | 963.7 | 996.595457 | 1.019788 |
| GO:0008544\_epidermis\_development | 62 | 0 | 0.000000 | 0.000000 | 945 | 930.804543 | 963.7 | 996.595457 | 1.019788 |
| GO:0000209\_protein\_polyubiquitination | 11 | 0 | 0.000000 | 0.000000 | 999 | 986.062440 | 1017.8 | 1049.537560 | 1.018819 |
| GO:0002790\_peptide\_secretion | 11 | 0 | 0.000000 | 0.000000 | 999 | 986.062440 | 1017.8 | 1049.537560 | 1.018819 |
| GO:0006096\_glycolysis | 11 | 0 | 0.000000 | 0.000000 | 999 | 986.062440 | 1017.8 | 1049.537560 | 1.018819 |
| GO:0006278\_RNA-dependent\_DNA\_replication | 11 | 0 | 0.000000 | 0.000000 | 999 | 986.062440 | 1017.8 | 1049.537560 | 1.018819 |
| GO:0006304\_DNA\_modification | 11 | 0 | 0.000000 | 0.000000 | 999 | 986.062440 | 1017.8 | 1049.537560 | 1.018819 |
| GO:0006471\_protein\_amino\_acid\_ADP-ribosylation | 11 | 0 | 0.000000 | 0.000000 | 999 | 986.062440 | 1017.8 | 1049.537560 | 1.018819 |
| GO:0006479\_protein\_amino\_acid\_methylation | 11 | 0 | 0.000000 | 0.000000 | 999 | 986.062440 | 1017.8 | 1049.537560 | 1.018819 |
| GO:0006509\_membrane\_protein\_ectodomain\_proteolysis | 11 | 0 | 0.000000 | 0.000000 | 999 | 986.062440 | 1017.8 | 1049.537560 | 1.018819 |
| GO:0006662\_glycerol\_ether\_metabolic\_process | 11 | 0 | 0.000000 | 0.000000 | 999 | 986.062440 | 1017.8 | 1049.537560 | 1.018819 |
| GO:0006695\_cholesterol\_biosynthetic\_process | 11 | 0 | 0.000000 | 0.000000 | 999 | 986.062440 | 1017.8 | 1049.537560 | 1.018819 |
| GO:0006809\_nitric\_oxide\_biosynthetic\_process | 11 | 0 | 0.000000 | 0.000000 | 999 | 986.062440 | 1017.8 | 1049.537560 | 1.018819 |
| GO:0007004\_telomere\_maintenance\_via\_telomerase | 11 | 0 | 0.000000 | 0.000000 | 999 | 986.062440 | 1017.8 | 1049.537560 | 1.018819 |
| GO:0007006\_mitochondrial\_membrane\_organization\_and\_biogenesis | 11 | 0 | 0.000000 | 0.000000 | 999 | 986.062440 | 1017.8 | 1049.537560 | 1.018819 |
| GO:0007040\_lysosome\_organization\_and\_biogenesis | 11 | 0 | 0.000000 | 0.000000 | 999 | 986.062440 | 1017.8 | 1049.537560 | 1.018819 |
| GO:0007043\_cell-cell\_junction\_assembly | 11 | 0 | 0.000000 | 0.000000 | 999 | 986.062440 | 1017.8 | 1049.537560 | 1.018819 |
| GO:0007052\_mitotic\_spindle\_organization\_and\_biogenesis | 11 | 0 | 0.000000 | 0.000000 | 999 | 986.062440 | 1017.8 | 1049.537560 | 1.018819 |
| GO:0007422\_peripheral\_nervous\_system\_development | 11 | 0 | 0.000000 | 0.000000 | 999 | 986.062440 | 1017.8 | 1049.537560 | 1.018819 |
| GO:0008213\_protein\_amino\_acid\_alkylation | 11 | 0 | 0.000000 | 0.000000 | 999 | 986.062440 | 1017.8 | 1049.537560 | 1.018819 |
| GO:0008585\_female\_gonad\_development | 11 | 0 | 0.000000 | 0.000000 | 999 | 986.062440 | 1017.8 | 1049.537560 | 1.018819 |
| GO:0009069\_serine\_family\_amino\_acid\_metabolic\_process | 11 | 0 | 0.000000 | 0.000000 | 999 | 986.062440 | 1017.8 | 1049.537560 | 1.018819 |
| GO:0009150\_purine\_ribonucleotide\_metabolic\_process | 11 | 0 | 0.000000 | 0.000000 | 999 | 986.062440 | 1017.8 | 1049.537560 | 1.018819 |
| GO:0009311\_oligosaccharide\_metabolic\_process | 11 | 0 | 0.000000 | 0.000000 | 999 | 986.062440 | 1017.8 | 1049.537560 | 1.018819 |
| GO:0009410\_response\_to\_xenobiotic\_stimulus | 11 | 0 | 0.000000 | 0.000000 | 999 | 986.062440 | 1017.8 | 1049.537560 | 1.018819 |
| GO:0009593\_detection\_of\_chemical\_stimulus | 11 | 0 | 0.000000 | 0.000000 | 999 | 986.062440 | 1017.8 | 1049.537560 | 1.018819 |
| GO:0030004\_cellular\_monovalent\_inorganic\_cation\_homeostasis | 11 | 0 | 0.000000 | 0.000000 | 999 | 986.062440 | 1017.8 | 1049.537560 | 1.018819 |
| GO:0030072\_peptide\_hormone\_secretion | 11 | 0 | 0.000000 | 0.000000 | 999 | 986.062440 | 1017.8 | 1049.537560 | 1.018819 |
| GO:0030335\_positive\_regulation\_of\_cell\_migration | 11 | 0 | 0.000000 | 0.000000 | 999 | 986.062440 | 1017.8 | 1049.537560 | 1.018819 |
| GO:0030336\_negative\_regulation\_of\_cell\_migration | 11 | 0 | 0.000000 | 0.000000 | 999 | 986.062440 | 1017.8 | 1049.537560 | 1.018819 |
| GO:0031281\_positive\_regulation\_of\_cyclase\_activity | 11 | 0 | 0.000000 | 0.000000 | 999 | 986.062440 | 1017.8 | 1049.537560 | 1.018819 |
| GO:0031396\_regulation\_of\_protein\_ubiquitination | 11 | 0 | 0.000000 | 0.000000 | 999 | 986.062440 | 1017.8 | 1049.537560 | 1.018819 |
| GO:0031644\_regulation\_of\_neurological\_system\_process | 11 | 0 | 0.000000 | 0.000000 | 999 | 986.062440 | 1017.8 | 1049.537560 | 1.018819 |
| GO:0032844\_regulation\_of\_homeostatic\_process | 11 | 0 | 0.000000 | 0.000000 | 999 | 986.062440 | 1017.8 | 1049.537560 | 1.018819 |
| GO:0033261\_regulation\_of\_S\_phase | 11 | 0 | 0.000000 | 0.000000 | 999 | 986.062440 | 1017.8 | 1049.537560 | 1.018819 |
| GO:0033554\_cellular\_response\_to\_stress | 11 | 0 | 0.000000 | 0.000000 | 999 | 986.062440 | 1017.8 | 1049.537560 | 1.018819 |
| GO:0033619\_membrane\_protein\_proteolysis | 11 | 0 | 0.000000 | 0.000000 | 999 | 986.062440 | 1017.8 | 1049.537560 | 1.018819 |
| GO:0035150\_regulation\_of\_tube\_size | 11 | 0 | 0.000000 | 0.000000 | 999 | 986.062440 | 1017.8 | 1049.537560 | 1.018819 |
| GO:0042058\_regulation\_of\_epidermal\_growth\_factor\_receptor\_signaling\_pathway | 11 | 0 | 0.000000 | 0.000000 | 999 | 986.062440 | 1017.8 | 1049.537560 | 1.018819 |
| GO:0042354\_L-fucose\_metabolic\_process | 11 | 0 | 0.000000 | 0.000000 | 999 | 986.062440 | 1017.8 | 1049.537560 | 1.018819 |
| GO:0042509\_regulation\_of\_tyrosine\_phosphorylation\_of\_STAT\_protein | 11 | 0 | 0.000000 | 0.000000 | 999 | 986.062440 | 1017.8 | 1049.537560 | 1.018819 |
| GO:0043434\_response\_to\_peptide\_hormone\_stimulus | 11 | 0 | 0.000000 | 0.000000 | 999 | 986.062440 | 1017.8 | 1049.537560 | 1.018819 |
| GO:0043523\_regulation\_of\_neuron\_apoptosis | 11 | 0 | 0.000000 | 0.000000 | 999 | 986.062440 | 1017.8 | 1049.537560 | 1.018819 |
| GO:0043542\_endothelial\_cell\_migration | 11 | 0 | 0.000000 | 0.000000 | 999 | 986.062440 | 1017.8 | 1049.537560 | 1.018819 |
| GO:0045762\_positive\_regulation\_of\_adenylate\_cyclase\_activity | 11 | 0 | 0.000000 | 0.000000 | 999 | 986.062440 | 1017.8 | 1049.537560 | 1.018819 |
| GO:0046209\_nitric\_oxide\_metabolic\_process | 11 | 0 | 0.000000 | 0.000000 | 999 | 986.062440 | 1017.8 | 1049.537560 | 1.018819 |
| GO:0046466\_membrane\_lipid\_catabolic\_process | 11 | 0 | 0.000000 | 0.000000 | 999 | 986.062440 | 1017.8 | 1049.537560 | 1.018819 |
| GO:0046486\_glycerolipid\_metabolic\_process | 11 | 0 | 0.000000 | 0.000000 | 999 | 986.062440 | 1017.8 | 1049.537560 | 1.018819 |
| GO:0046546\_development\_of\_primary\_male\_sexual\_characteristics | 11 | 0 | 0.000000 | 0.000000 | 999 | 986.062440 | 1017.8 | 1049.537560 | 1.018819 |
| GO:0048589\_developmental\_growth | 11 | 0 | 0.000000 | 0.000000 | 999 | 986.062440 | 1017.8 | 1049.537560 | 1.018819 |
| GO:0050707\_regulation\_of\_cytokine\_secretion | 11 | 0 | 0.000000 | 0.000000 | 999 | 986.062440 | 1017.8 | 1049.537560 | 1.018819 |
| GO:0050852\_T\_cell\_receptor\_signaling\_pathway | 11 | 0 | 0.000000 | 0.000000 | 999 | 986.062440 | 1017.8 | 1049.537560 | 1.018819 |
| GO:0050880\_regulation\_of\_blood\_vessel\_size | 11 | 0 | 0.000000 | 0.000000 | 999 | 986.062440 | 1017.8 | 1049.537560 | 1.018819 |
| GO:0051181\_cofactor\_transport | 11 | 0 | 0.000000 | 0.000000 | 999 | 986.062440 | 1017.8 | 1049.537560 | 1.018819 |
| GO:0051349\_positive\_regulation\_of\_lyase\_activity | 11 | 0 | 0.000000 | 0.000000 | 999 | 986.062440 | 1017.8 | 1049.537560 | 1.018819 |
| GO:0051648\_vesicle\_localization | 11 | 0 | 0.000000 | 0.000000 | 999 | 986.062440 | 1017.8 | 1049.537560 | 1.018819 |
| GO:0006323\_DNA\_packaging | 58 | 0 | 0.000000 | 0.000000 | 1003 | 989.609581 | 1021.1 | 1052.590419 | 1.018046 |
| GO:0009314\_response\_to\_radiation | 58 | 0 | 0.000000 | 0.000000 | 1003 | 989.609581 | 1021.1 | 1052.590419 | 1.018046 |
| GO:0042060\_wound\_healing | 58 | 0 | 0.000000 | 0.000000 | 1003 | 989.609581 | 1021.1 | 1052.590419 | 1.018046 |
| GO:0048646\_anatomical\_structure\_formation | 58 | 0 | 0.000000 | 0.000000 | 1003 | 989.609581 | 1021.1 | 1052.590419 | 1.018046 |
| GO:0045944\_positive\_regulation\_of\_transcription\_from\_RNA\_polymerase\_II\_promoter | 64 | 0 | 0.000000 | 0.000000 | 1004 | 990.285434 | 1021.67 | 1053.054566 | 1.017600 |
| GO:0000377\_RNA\_splicing\_\_via\_transesterification\_reactions\_with\_bulged\_adenosine\_as\_nucleophile | 45 | 0 | 0.000000 | 0.000000 | 1010 | 995.196507 | 1026.49 | 1057.783493 | 1.016327 |
| GO:0000398\_nuclear\_mRNA\_splicing\_\_via\_spliceosome | 45 | 0 | 0.000000 | 0.000000 | 1010 | 995.196507 | 1026.49 | 1057.783493 | 1.016327 |
| GO:0006979\_response\_to\_oxidative\_stress | 45 | 0 | 0.000000 | 0.000000 | 1010 | 995.196507 | 1026.49 | 1057.783493 | 1.016327 |
| GO:0016032\_viral\_reproduction | 45 | 0 | 0.000000 | 0.000000 | 1010 | 995.196507 | 1026.49 | 1057.783493 | 1.016327 |
| GO:0050817\_coagulation | 45 | 0 | 0.000000 | 0.000000 | 1010 | 995.196507 | 1026.49 | 1057.783493 | 1.016327 |
| GO:0051052\_regulation\_of\_DNA\_metabolic\_process | 45 | 0 | 0.000000 | 0.000000 | 1010 | 995.196507 | 1026.49 | 1057.783493 | 1.016327 |
| GO:0002253\_activation\_of\_immune\_response | 19 | 0 | 0.000000 | 0.000000 | 1033 | 1021.919237 | 1052.27 | 1082.620763 | 1.018654 |
| GO:0006023\_aminoglycan\_biosynthetic\_process | 19 | 0 | 0.000000 | 0.000000 | 1033 | 1021.919237 | 1052.27 | 1082.620763 | 1.018654 |
| GO:0006024\_glycosaminoglycan\_biosynthetic\_process | 19 | 0 | 0.000000 | 0.000000 | 1033 | 1021.919237 | 1052.27 | 1082.620763 | 1.018654 |
| GO:0006505\_GPI\_anchor\_metabolic\_process | 19 | 0 | 0.000000 | 0.000000 | 1033 | 1021.919237 | 1052.27 | 1082.620763 | 1.018654 |
| GO:0006506\_GPI\_anchor\_biosynthetic\_process | 19 | 0 | 0.000000 | 0.000000 | 1033 | 1021.919237 | 1052.27 | 1082.620763 | 1.018654 |
| GO:0006664\_glycolipid\_metabolic\_process | 19 | 0 | 0.000000 | 0.000000 | 1033 | 1021.919237 | 1052.27 | 1082.620763 | 1.018654 |
| GO:0006690\_icosanoid\_metabolic\_process | 19 | 0 | 0.000000 | 0.000000 | 1033 | 1021.919237 | 1052.27 | 1082.620763 | 1.018654 |
| GO:0006814\_sodium\_ion\_transport | 19 | 0 | 0.000000 | 0.000000 | 1033 | 1021.919237 | 1052.27 | 1082.620763 | 1.018654 |
| GO:0006892\_post-Golgi\_vesicle-mediated\_transport | 19 | 0 | 0.000000 | 0.000000 | 1033 | 1021.919237 | 1052.27 | 1082.620763 | 1.018654 |
| GO:0007338\_single\_fertilization | 19 | 0 | 0.000000 | 0.000000 | 1033 | 1021.919237 | 1052.27 | 1082.620763 | 1.018654 |
| GO:0009617\_response\_to\_bacterium | 19 | 0 | 0.000000 | 0.000000 | 1033 | 1021.919237 | 1052.27 | 1082.620763 | 1.018654 |
| GO:0018209\_peptidyl-serine\_modification | 19 | 0 | 0.000000 | 0.000000 | 1033 | 1021.919237 | 1052.27 | 1082.620763 | 1.018654 |
| GO:0030198\_extracellular\_matrix\_organization\_and\_biogenesis | 19 | 0 | 0.000000 | 0.000000 | 1033 | 1021.919237 | 1052.27 | 1082.620763 | 1.018654 |
| GO:0035239\_tube\_morphogenesis | 19 | 0 | 0.000000 | 0.000000 | 1033 | 1021.919237 | 1052.27 | 1082.620763 | 1.018654 |
| GO:0043087\_regulation\_of\_GTPase\_activity | 19 | 0 | 0.000000 | 0.000000 | 1033 | 1021.919237 | 1052.27 | 1082.620763 | 1.018654 |
| GO:0043392\_negative\_regulation\_of\_DNA\_binding | 19 | 0 | 0.000000 | 0.000000 | 1033 | 1021.919237 | 1052.27 | 1082.620763 | 1.018654 |
| GO:0043407\_negative\_regulation\_of\_MAP\_kinase\_activity | 19 | 0 | 0.000000 | 0.000000 | 1033 | 1021.919237 | 1052.27 | 1082.620763 | 1.018654 |
| GO:0043506\_regulation\_of\_JNK\_activity | 19 | 0 | 0.000000 | 0.000000 | 1033 | 1021.919237 | 1052.27 | 1082.620763 | 1.018654 |
| GO:0043543\_protein\_amino\_acid\_acylation | 19 | 0 | 0.000000 | 0.000000 | 1033 | 1021.919237 | 1052.27 | 1082.620763 | 1.018654 |
| GO:0046879\_hormone\_secretion | 19 | 0 | 0.000000 | 0.000000 | 1033 | 1021.919237 | 1052.27 | 1082.620763 | 1.018654 |
| GO:0050673\_epithelial\_cell\_proliferation | 19 | 0 | 0.000000 | 0.000000 | 1033 | 1021.919237 | 1052.27 | 1082.620763 | 1.018654 |
| GO:0050678\_regulation\_of\_epithelial\_cell\_proliferation | 19 | 0 | 0.000000 | 0.000000 | 1033 | 1021.919237 | 1052.27 | 1082.620763 | 1.018654 |
| GO:0050900\_leukocyte\_migration | 19 | 0 | 0.000000 | 0.000000 | 1033 | 1021.919237 | 1052.27 | 1082.620763 | 1.018654 |
| GO:0048534\_hemopoietic\_or\_lymphoid\_organ\_development | 91 | 0 | 0.000000 | 0.000000 | 1036 | 1026.327589 | 1056.05 | 1085.772411 | 1.019353 |
| GO:0051239\_regulation\_of\_multicellular\_organismal\_process | 91 | 0 | 0.000000 | 0.000000 | 1036 | 1026.327589 | 1056.05 | 1085.772411 | 1.019353 |
| GO:0055080\_cation\_homeostasis | 91 | 0 | 0.000000 | 0.000000 | 1036 | 1026.327589 | 1056.05 | 1085.772411 | 1.019353 |
| GO:0009790\_embryonic\_development | 57 | 0 | 0.000000 | 0.000000 | 1037 | 1028.475075 | 1057.92 | 1087.364925 | 1.020174 |
| GO:0006875\_cellular\_metal\_ion\_homeostasis | 59 | 0 | 0.000000 | 0.000000 | 1039 | 1030.868931 | 1060.01 | 1089.151069 | 1.020221 |
| GO:0055065\_metal\_ion\_homeostasis | 59 | 0 | 0.000000 | 0.000000 | 1039 | 1030.868931 | 1060.01 | 1089.151069 | 1.020221 |
| GO:0001817\_regulation\_of\_cytokine\_production | 23 | 0 | 0.000000 | 0.000000 | 1060 | 1052.672051 | 1080.75 | 1108.827949 | 1.019575 |
| GO:0001934\_positive\_regulation\_of\_protein\_amino\_acid\_phosphorylation | 23 | 0 | 0.000000 | 0.000000 | 1060 | 1052.672051 | 1080.75 | 1108.827949 | 1.019575 |
| GO:0003015\_heart\_process | 23 | 0 | 0.000000 | 0.000000 | 1060 | 1052.672051 | 1080.75 | 1108.827949 | 1.019575 |
| GO:0006308\_DNA\_catabolic\_process | 23 | 0 | 0.000000 | 0.000000 | 1060 | 1052.672051 | 1080.75 | 1108.827949 | 1.019575 |
| GO:0006352\_transcription\_initiation | 23 | 0 | 0.000000 | 0.000000 | 1060 | 1052.672051 | 1080.75 | 1108.827949 | 1.019575 |
| GO:0006575\_amino\_acid\_derivative\_metabolic\_process | 23 | 0 | 0.000000 | 0.000000 | 1060 | 1052.672051 | 1080.75 | 1108.827949 | 1.019575 |
| GO:0008277\_regulation\_of\_G-protein\_coupled\_receptor\_protein\_signaling\_pathway | 23 | 0 | 0.000000 | 0.000000 | 1060 | 1052.672051 | 1080.75 | 1108.827949 | 1.019575 |
| GO:0008643\_carbohydrate\_transport | 23 | 0 | 0.000000 | 0.000000 | 1060 | 1052.672051 | 1080.75 | 1108.827949 | 1.019575 |
| GO:0009991\_response\_to\_extracellular\_stimulus | 23 | 0 | 0.000000 | 0.000000 | 1060 | 1052.672051 | 1080.75 | 1108.827949 | 1.019575 |
| GO:0019079\_viral\_genome\_replication | 23 | 0 | 0.000000 | 0.000000 | 1060 | 1052.672051 | 1080.75 | 1108.827949 | 1.019575 |
| GO:0030031\_cell\_projection\_biogenesis | 23 | 0 | 0.000000 | 0.000000 | 1060 | 1052.672051 | 1080.75 | 1108.827949 | 1.019575 |
| GO:0031570\_DNA\_integrity\_checkpoint | 23 | 0 | 0.000000 | 0.000000 | 1060 | 1052.672051 | 1080.75 | 1108.827949 | 1.019575 |
| GO:0032259\_methylation | 23 | 0 | 0.000000 | 0.000000 | 1060 | 1052.672051 | 1080.75 | 1108.827949 | 1.019575 |
| GO:0032956\_regulation\_of\_actin\_cytoskeleton\_organization\_and\_biogenesis | 23 | 0 | 0.000000 | 0.000000 | 1060 | 1052.672051 | 1080.75 | 1108.827949 | 1.019575 |
| GO:0042113\_B\_cell\_activation | 23 | 0 | 0.000000 | 0.000000 | 1060 | 1052.672051 | 1080.75 | 1108.827949 | 1.019575 |
| GO:0043388\_positive\_regulation\_of\_DNA\_binding | 23 | 0 | 0.000000 | 0.000000 | 1060 | 1052.672051 | 1080.75 | 1108.827949 | 1.019575 |
| GO:0043414\_biopolymer\_methylation | 23 | 0 | 0.000000 | 0.000000 | 1060 | 1052.672051 | 1080.75 | 1108.827949 | 1.019575 |
| GO:0045137\_development\_of\_primary\_sexual\_characteristics | 23 | 0 | 0.000000 | 0.000000 | 1060 | 1052.672051 | 1080.75 | 1108.827949 | 1.019575 |
| GO:0045787\_positive\_regulation\_of\_cell\_cycle | 23 | 0 | 0.000000 | 0.000000 | 1060 | 1052.672051 | 1080.75 | 1108.827949 | 1.019575 |
| GO:0051716\_cellular\_response\_to\_stimulus | 23 | 0 | 0.000000 | 0.000000 | 1060 | 1052.672051 | 1080.75 | 1108.827949 | 1.019575 |
| GO:0060047\_heart\_contraction | 23 | 0 | 0.000000 | 0.000000 | 1060 | 1052.672051 | 1080.75 | 1108.827949 | 1.019575 |
| GO:0006968\_cellular\_defense\_response | 50 | 0 | 0.000000 | 0.000000 | 1061 | 1056.706083 | 1084.3 | 1111.893917 | 1.021960 |
| GO:0006066\_alcohol\_metabolic\_process | 106 | 0 | 0.000000 | 0.000000 | 1062 | 1057.228827 | 1084.81 | 1112.391173 | 1.021478 |
| GO:0002682\_regulation\_of\_immune\_system\_process | 68 | 0 | 0.000000 | 0.000000 | 1065 | 1059.513157 | 1086.79 | 1114.066843 | 1.020460 |
| GO:0008202\_steroid\_metabolic\_process | 68 | 0 | 0.000000 | 0.000000 | 1065 | 1059.513157 | 1086.79 | 1114.066843 | 1.020460 |
| GO:0018193\_peptidyl-amino\_acid\_modification | 68 | 0 | 0.000000 | 0.000000 | 1065 | 1059.513157 | 1086.79 | 1114.066843 | 1.020460 |
| GO:0006732\_coenzyme\_metabolic\_process | 42 | 0 | 0.000000 | 0.000000 | 1069 | 1062.762465 | 1089.72 | 1116.677535 | 1.019383 |
| GO:0007565\_female\_pregnancy | 42 | 0 | 0.000000 | 0.000000 | 1069 | 1062.762465 | 1089.72 | 1116.677535 | 1.019383 |
| GO:0043062\_extracellular\_structure\_organization\_and\_biogenesis | 42 | 0 | 0.000000 | 0.000000 | 1069 | 1062.762465 | 1089.72 | 1116.677535 | 1.019383 |
| GO:0051259\_protein\_oligomerization | 42 | 0 | 0.000000 | 0.000000 | 1069 | 1062.762465 | 1089.72 | 1116.677535 | 1.019383 |
| GO:0009607\_response\_to\_biotic\_stimulus | 102 | 0 | 0.000000 | 0.000000 | 1070 | 1063.267377 | 1090.21 | 1117.152623 | 1.018888 |
| GO:0006873\_cellular\_ion\_homeostasis | 112 | 0 | 0.000000 | 0.000000 | 1072 | 1064.476795 | 1091.27 | 1118.063205 | 1.017976 |
| GO:0008610\_lipid\_biosynthetic\_process | 112 | 0 | 0.000000 | 0.000000 | 1072 | 1064.476795 | 1091.27 | 1118.063205 | 1.017976 |
| GO:0006644\_phospholipid\_metabolic\_process | 70 | 0 | 0.000000 | 0.000000 | 1076 | 1067.793295 | 1094.44 | 1121.086705 | 1.017138 |
| GO:0007398\_ectoderm\_development | 70 | 0 | 0.000000 | 0.000000 | 1076 | 1067.793295 | 1094.44 | 1121.086705 | 1.017138 |
| GO:0016568\_chromatin\_modification | 70 | 0 | 0.000000 | 0.000000 | 1076 | 1067.793295 | 1094.44 | 1121.086705 | 1.017138 |
| GO:0043405\_regulation\_of\_MAP\_kinase\_activity | 70 | 0 | 0.000000 | 0.000000 | 1076 | 1067.793295 | 1094.44 | 1121.086705 | 1.017138 |
| GO:0000045\_autophagic\_vacuole\_formation | 7 | 0 | 0.000000 | 0.000000 | 1180 | 1169.694130 | 1194.65 | 1219.605870 | 1.012415 |
| GO:0000096\_sulfur\_amino\_acid\_metabolic\_process | 7 | 0 | 0.000000 | 0.000000 | 1180 | 1169.694130 | 1194.65 | 1219.605870 | 1.012415 |
| GO:0000302\_response\_to\_reactive\_oxygen\_species | 7 | 0 | 0.000000 | 0.000000 | 1180 | 1169.694130 | 1194.65 | 1219.605870 | 1.012415 |
| GO:0000387\_spliceosomal\_snRNP\_biogenesis | 7 | 0 | 0.000000 | 0.000000 | 1180 | 1169.694130 | 1194.65 | 1219.605870 | 1.012415 |
| GO:0000724\_double-strand\_break\_repair\_via\_homologous\_recombination | 7 | 0 | 0.000000 | 0.000000 | 1180 | 1169.694130 | 1194.65 | 1219.605870 | 1.012415 |
| GO:0000725\_recombinational\_repair | 7 | 0 | 0.000000 | 0.000000 | 1180 | 1169.694130 | 1194.65 | 1219.605870 | 1.012415 |
| GO:0000731\_DNA\_synthesis\_during\_DNA\_repair | 7 | 0 | 0.000000 | 0.000000 | 1180 | 1169.694130 | 1194.65 | 1219.605870 | 1.012415 |
| GO:0001933\_negative\_regulation\_of\_protein\_amino\_acid\_phosphorylation | 7 | 0 | 0.000000 | 0.000000 | 1180 | 1169.694130 | 1194.65 | 1219.605870 | 1.012415 |
| GO:0002228\_natural\_killer\_cell\_mediated\_immunity | 7 | 0 | 0.000000 | 0.000000 | 1180 | 1169.694130 | 1194.65 | 1219.605870 | 1.012415 |
| GO:0002699\_positive\_regulation\_of\_immune\_effector\_process | 7 | 0 | 0.000000 | 0.000000 | 1180 | 1169.694130 | 1194.65 | 1219.605870 | 1.012415 |
| GO:0002762\_negative\_regulation\_of\_myeloid\_leukocyte\_differentiation | 7 | 0 | 0.000000 | 0.000000 | 1180 | 1169.694130 | 1194.65 | 1219.605870 | 1.012415 |
| GO:0002819\_regulation\_of\_adaptive\_immune\_response | 7 | 0 | 0.000000 | 0.000000 | 1180 | 1169.694130 | 1194.65 | 1219.605870 | 1.012415 |
| GO:0002822\_regulation\_of\_adaptive\_immune\_response\_based\_on\_somatic\_recombination\_of\_immune\_receptors\_built\_from\_immunoglobulin\_superfamily\_domains | 7 | 0 | 0.000000 | 0.000000 | 1180 | 1169.694130 | 1194.65 | 1219.605870 | 1.012415 |
| GO:0006099\_tricarboxylic\_acid\_cycle | 7 | 0 | 0.000000 | 0.000000 | 1180 | 1169.694130 | 1194.65 | 1219.605870 | 1.012415 |
| GO:0006266\_DNA\_ligation | 7 | 0 | 0.000000 | 0.000000 | 1180 | 1169.694130 | 1194.65 | 1219.605870 | 1.012415 |
| GO:0006301\_postreplication\_repair | 7 | 0 | 0.000000 | 0.000000 | 1180 | 1169.694130 | 1194.65 | 1219.605870 | 1.012415 |
| GO:0006312\_mitotic\_recombination | 7 | 0 | 0.000000 | 0.000000 | 1180 | 1169.694130 | 1194.65 | 1219.605870 | 1.012415 |
| GO:0006342\_chromatin\_silencing | 7 | 0 | 0.000000 | 0.000000 | 1180 | 1169.694130 | 1194.65 | 1219.605870 | 1.012415 |
| GO:0006359\_regulation\_of\_transcription\_from\_RNA\_polymerase\_III\_promoter | 7 | 0 | 0.000000 | 0.000000 | 1180 | 1169.694130 | 1194.65 | 1219.605870 | 1.012415 |
| GO:0006368\_RNA\_elongation\_from\_RNA\_polymerase\_II\_promoter | 7 | 0 | 0.000000 | 0.000000 | 1180 | 1169.694130 | 1194.65 | 1219.605870 | 1.012415 |
| GO:0006518\_peptide\_metabolic\_process | 7 | 0 | 0.000000 | 0.000000 | 1180 | 1169.694130 | 1194.65 | 1219.605870 | 1.012415 |
| GO:0006641\_triacylglycerol\_metabolic\_process | 7 | 0 | 0.000000 | 0.000000 | 1180 | 1169.694130 | 1194.65 | 1219.605870 | 1.012415 |
| GO:0006688\_glycosphingolipid\_biosynthetic\_process | 7 | 0 | 0.000000 | 0.000000 | 1180 | 1169.694130 | 1194.65 | 1219.605870 | 1.012415 |
| GO:0006706\_steroid\_catabolic\_process | 7 | 0 | 0.000000 | 0.000000 | 1180 | 1169.694130 | 1194.65 | 1219.605870 | 1.012415 |
| GO:0006775\_fat-soluble\_vitamin\_metabolic\_process | 7 | 0 | 0.000000 | 0.000000 | 1180 | 1169.694130 | 1194.65 | 1219.605870 | 1.012415 |
| GO:0006776\_vitamin\_A\_metabolic\_process | 7 | 0 | 0.000000 | 0.000000 | 1180 | 1169.694130 | 1194.65 | 1219.605870 | 1.012415 |
| GO:0006801\_superoxide\_metabolic\_process | 7 | 0 | 0.000000 | 0.000000 | 1180 | 1169.694130 | 1194.65 | 1219.605870 | 1.012415 |
| GO:0006825\_copper\_ion\_transport | 7 | 0 | 0.000000 | 0.000000 | 1180 | 1169.694130 | 1194.65 | 1219.605870 | 1.012415 |
| GO:0006833\_water\_transport | 7 | 0 | 0.000000 | 0.000000 | 1180 | 1169.694130 | 1194.65 | 1219.605870 | 1.012415 |
| GO:0006895\_Golgi\_to\_endosome\_transport | 7 | 0 | 0.000000 | 0.000000 | 1180 | 1169.694130 | 1194.65 | 1219.605870 | 1.012415 |
| GO:0006940\_regulation\_of\_smooth\_muscle\_contraction | 7 | 0 | 0.000000 | 0.000000 | 1180 | 1169.694130 | 1194.65 | 1219.605870 | 1.012415 |
| GO:0007021\_tubulin\_folding | 7 | 0 | 0.000000 | 0.000000 | 1180 | 1169.694130 | 1194.65 | 1219.605870 | 1.012415 |
| GO:0007090\_regulation\_of\_S\_phase\_of\_mitotic\_cell\_cycle | 7 | 0 | 0.000000 | 0.000000 | 1180 | 1169.694130 | 1194.65 | 1219.605870 | 1.012415 |
| GO:0007157\_heterophilic\_cell\_adhesion | 7 | 0 | 0.000000 | 0.000000 | 1180 | 1169.694130 | 1194.65 | 1219.605870 | 1.012415 |
| GO:0007214\_gamma-aminobutyric\_acid\_signaling\_pathway | 7 | 0 | 0.000000 | 0.000000 | 1180 | 1169.694130 | 1194.65 | 1219.605870 | 1.012415 |
| GO:0007263\_nitric\_oxide\_mediated\_signal\_transduction | 7 | 0 | 0.000000 | 0.000000 | 1180 | 1169.694130 | 1194.65 | 1219.605870 | 1.012415 |
| GO:0007369\_gastrulation | 7 | 0 | 0.000000 | 0.000000 | 1180 | 1169.694130 | 1194.65 | 1219.605870 | 1.012415 |
| GO:0007566\_embryo\_implantation | 7 | 0 | 0.000000 | 0.000000 | 1180 | 1169.694130 | 1194.65 | 1219.605870 | 1.012415 |
| GO:0008635\_caspase\_activation\_via\_cytochrome\_c | 7 | 0 | 0.000000 | 0.000000 | 1180 | 1169.694130 | 1194.65 | 1219.605870 | 1.012415 |
| GO:0009065\_glutamine\_family\_amino\_acid\_catabolic\_process | 7 | 0 | 0.000000 | 0.000000 | 1180 | 1169.694130 | 1194.65 | 1219.605870 | 1.012415 |
| GO:0009072\_aromatic\_amino\_acid\_family\_metabolic\_process | 7 | 0 | 0.000000 | 0.000000 | 1180 | 1169.694130 | 1194.65 | 1219.605870 | 1.012415 |
| GO:0009109\_coenzyme\_catabolic\_process | 7 | 0 | 0.000000 | 0.000000 | 1180 | 1169.694130 | 1194.65 | 1219.605870 | 1.012415 |
| GO:0009123\_nucleoside\_monophosphate\_metabolic\_process | 7 | 0 | 0.000000 | 0.000000 | 1180 | 1169.694130 | 1194.65 | 1219.605870 | 1.012415 |
| GO:0009144\_purine\_nucleoside\_triphosphate\_metabolic\_process | 7 | 0 | 0.000000 | 0.000000 | 1180 | 1169.694130 | 1194.65 | 1219.605870 | 1.012415 |
| GO:0009166\_nucleotide\_catabolic\_process | 7 | 0 | 0.000000 | 0.000000 | 1180 | 1169.694130 | 1194.65 | 1219.605870 | 1.012415 |
| GO:0009205\_purine\_ribonucleoside\_triphosphate\_metabolic\_process | 7 | 0 | 0.000000 | 0.000000 | 1180 | 1169.694130 | 1194.65 | 1219.605870 | 1.012415 |
| GO:0009267\_cellular\_response\_to\_starvation | 7 | 0 | 0.000000 | 0.000000 | 1180 | 1169.694130 | 1194.65 | 1219.605870 | 1.012415 |
| GO:0015012\_heparan\_sulfate\_proteoglycan\_biosynthetic\_process | 7 | 0 | 0.000000 | 0.000000 | 1180 | 1169.694130 | 1194.65 | 1219.605870 | 1.012415 |
| GO:0016079\_synaptic\_vesicle\_exocytosis | 7 | 0 | 0.000000 | 0.000000 | 1180 | 1169.694130 | 1194.65 | 1219.605870 | 1.012415 |
| GO:0018409\_peptide\_or\_protein\_amino-terminal\_blocking | 7 | 0 | 0.000000 | 0.000000 | 1180 | 1169.694130 | 1194.65 | 1219.605870 | 1.012415 |
| GO:0019439\_aromatic\_compound\_catabolic\_process | 7 | 0 | 0.000000 | 0.000000 | 1180 | 1169.694130 | 1194.65 | 1219.605870 | 1.012415 |
| GO:0019724\_B\_cell\_mediated\_immunity | 7 | 0 | 0.000000 | 0.000000 | 1180 | 1169.694130 | 1194.65 | 1219.605870 | 1.012415 |
| GO:0019882\_antigen\_processing\_and\_presentation | 7 | 0 | 0.000000 | 0.000000 | 1180 | 1169.694130 | 1194.65 | 1219.605870 | 1.012415 |
| GO:0030035\_microspike\_biogenesis | 7 | 0 | 0.000000 | 0.000000 | 1180 | 1169.694130 | 1194.65 | 1219.605870 | 1.012415 |
| GO:0030178\_negative\_regulation\_of\_Wnt\_receptor\_signaling\_pathway | 7 | 0 | 0.000000 | 0.000000 | 1180 | 1169.694130 | 1194.65 | 1219.605870 | 1.012415 |
| GO:0030204\_chondroitin\_sulfate\_metabolic\_process | 7 | 0 | 0.000000 | 0.000000 | 1180 | 1169.694130 | 1194.65 | 1219.605870 | 1.012415 |
| GO:0030206\_chondroitin\_sulfate\_biosynthetic\_process | 7 | 0 | 0.000000 | 0.000000 | 1180 | 1169.694130 | 1194.65 | 1219.605870 | 1.012415 |
| GO:0030225\_macrophage\_differentiation | 7 | 0 | 0.000000 | 0.000000 | 1180 | 1169.694130 | 1194.65 | 1219.605870 | 1.012415 |
| GO:0030278\_regulation\_of\_ossification | 7 | 0 | 0.000000 | 0.000000 | 1180 | 1169.694130 | 1194.65 | 1219.605870 | 1.012415 |
| GO:0030520\_estrogen\_receptor\_signaling\_pathway | 7 | 0 | 0.000000 | 0.000000 | 1180 | 1169.694130 | 1194.65 | 1219.605870 | 1.012415 |
| GO:0030593\_neutrophil\_chemotaxis | 7 | 0 | 0.000000 | 0.000000 | 1180 | 1169.694130 | 1194.65 | 1219.605870 | 1.012415 |
| GO:0031507\_heterochromatin\_formation | 7 | 0 | 0.000000 | 0.000000 | 1180 | 1169.694130 | 1194.65 | 1219.605870 | 1.012415 |
| GO:0031532\_actin\_cytoskeleton\_reorganization | 7 | 0 | 0.000000 | 0.000000 | 1180 | 1169.694130 | 1194.65 | 1219.605870 | 1.012415 |
| GO:0032231\_regulation\_of\_actin\_filament\_bundle\_formation | 7 | 0 | 0.000000 | 0.000000 | 1180 | 1169.694130 | 1194.65 | 1219.605870 | 1.012415 |
| GO:0032392\_DNA\_geometric\_change | 7 | 0 | 0.000000 | 0.000000 | 1180 | 1169.694130 | 1194.65 | 1219.605870 | 1.012415 |
| GO:0032508\_DNA\_duplex\_unwinding | 7 | 0 | 0.000000 | 0.000000 | 1180 | 1169.694130 | 1194.65 | 1219.605870 | 1.012415 |
| GO:0032609\_interferon-gamma\_production | 7 | 0 | 0.000000 | 0.000000 | 1180 | 1169.694130 | 1194.65 | 1219.605870 | 1.012415 |
| GO:0042044\_fluid\_transport | 7 | 0 | 0.000000 | 0.000000 | 1180 | 1169.694130 | 1194.65 | 1219.605870 | 1.012415 |
| GO:0042100\_B\_cell\_proliferation | 7 | 0 | 0.000000 | 0.000000 | 1180 | 1169.694130 | 1194.65 | 1219.605870 | 1.012415 |
| GO:0042267\_natural\_killer\_cell\_mediated\_cytotoxicity | 7 | 0 | 0.000000 | 0.000000 | 1180 | 1169.694130 | 1194.65 | 1219.605870 | 1.012415 |
| GO:0042307\_positive\_regulation\_of\_protein\_import\_into\_nucleus | 7 | 0 | 0.000000 | 0.000000 | 1180 | 1169.694130 | 1194.65 | 1219.605870 | 1.012415 |
| GO:0042386\_hemocyte\_differentiation | 7 | 0 | 0.000000 | 0.000000 | 1180 | 1169.694130 | 1194.65 | 1219.605870 | 1.012415 |
| GO:0042503\_tyrosine\_phosphorylation\_of\_Stat3\_protein | 7 | 0 | 0.000000 | 0.000000 | 1180 | 1169.694130 | 1194.65 | 1219.605870 | 1.012415 |
| GO:0042516\_regulation\_of\_tyrosine\_phosphorylation\_of\_Stat3\_protein | 7 | 0 | 0.000000 | 0.000000 | 1180 | 1169.694130 | 1194.65 | 1219.605870 | 1.012415 |
| GO:0042531\_positive\_regulation\_of\_tyrosine\_phosphorylation\_of\_STAT\_protein | 7 | 0 | 0.000000 | 0.000000 | 1180 | 1169.694130 | 1194.65 | 1219.605870 | 1.012415 |
| GO:0042558\_pteridine\_and\_derivative\_metabolic\_process | 7 | 0 | 0.000000 | 0.000000 | 1180 | 1169.694130 | 1194.65 | 1219.605870 | 1.012415 |
| GO:0042987\_amyloid\_precursor\_protein\_catabolic\_process | 7 | 0 | 0.000000 | 0.000000 | 1180 | 1169.694130 | 1194.65 | 1219.605870 | 1.012415 |
| GO:0042993\_positive\_regulation\_of\_transcription\_factor\_import\_into\_nucleus | 7 | 0 | 0.000000 | 0.000000 | 1180 | 1169.694130 | 1194.65 | 1219.605870 | 1.012415 |
| GO:0043627\_response\_to\_estrogen\_stimulus | 7 | 0 | 0.000000 | 0.000000 | 1180 | 1169.694130 | 1194.65 | 1219.605870 | 1.012415 |
| GO:0045444\_fat\_cell\_differentiation | 7 | 0 | 0.000000 | 0.000000 | 1180 | 1169.694130 | 1194.65 | 1219.605870 | 1.012415 |
| GO:0045736\_negative\_regulation\_of\_cyclin-dependent\_protein\_kinase\_activity | 7 | 0 | 0.000000 | 0.000000 | 1180 | 1169.694130 | 1194.65 | 1219.605870 | 1.012415 |
| GO:0045768\_positive\_regulation\_of\_anti-apoptosis | 7 | 0 | 0.000000 | 0.000000 | 1180 | 1169.694130 | 1194.65 | 1219.605870 | 1.012415 |
| GO:0045851\_pH\_reduction | 7 | 0 | 0.000000 | 0.000000 | 1180 | 1169.694130 | 1194.65 | 1219.605870 | 1.012415 |
| GO:0045884\_regulation\_of\_survival\_gene\_product\_expression | 7 | 0 | 0.000000 | 0.000000 | 1180 | 1169.694130 | 1194.65 | 1219.605870 | 1.012415 |
| GO:0046356\_acetyl-CoA\_catabolic\_process | 7 | 0 | 0.000000 | 0.000000 | 1180 | 1169.694130 | 1194.65 | 1219.605870 | 1.012415 |
| GO:0046427\_positive\_regulation\_of\_JAK-STAT\_cascade | 7 | 0 | 0.000000 | 0.000000 | 1180 | 1169.694130 | 1194.65 | 1219.605870 | 1.012415 |
| GO:0046888\_negative\_regulation\_of\_hormone\_secretion | 7 | 0 | 0.000000 | 0.000000 | 1180 | 1169.694130 | 1194.65 | 1219.605870 | 1.012415 |
| GO:0048066\_pigmentation\_during\_development | 7 | 0 | 0.000000 | 0.000000 | 1180 | 1169.694130 | 1194.65 | 1219.605870 | 1.012415 |
| GO:0048520\_positive\_regulation\_of\_behavior | 7 | 0 | 0.000000 | 0.000000 | 1180 | 1169.694130 | 1194.65 | 1219.605870 | 1.012415 |
| GO:0048644\_muscle\_morphogenesis | 7 | 0 | 0.000000 | 0.000000 | 1180 | 1169.694130 | 1194.65 | 1219.605870 | 1.012415 |
| GO:0048660\_regulation\_of\_smooth\_muscle\_cell\_proliferation | 7 | 0 | 0.000000 | 0.000000 | 1180 | 1169.694130 | 1194.65 | 1219.605870 | 1.012415 |
| GO:0050650\_chondroitin\_sulfate\_proteoglycan\_biosynthetic\_process | 7 | 0 | 0.000000 | 0.000000 | 1180 | 1169.694130 | 1194.65 | 1219.605870 | 1.012415 |
| GO:0050701\_interleukin-1\_secretion | 7 | 0 | 0.000000 | 0.000000 | 1180 | 1169.694130 | 1194.65 | 1219.605870 | 1.012415 |
| GO:0050769\_positive\_regulation\_of\_neurogenesis | 7 | 0 | 0.000000 | 0.000000 | 1180 | 1169.694130 | 1194.65 | 1219.605870 | 1.012415 |
| GO:0050818\_regulation\_of\_coagulation | 7 | 0 | 0.000000 | 0.000000 | 1180 | 1169.694130 | 1194.65 | 1219.605870 | 1.012415 |
| GO:0050921\_positive\_regulation\_of\_chemotaxis | 7 | 0 | 0.000000 | 0.000000 | 1180 | 1169.694130 | 1194.65 | 1219.605870 | 1.012415 |
| GO:0051147\_regulation\_of\_muscle\_cell\_differentiation | 7 | 0 | 0.000000 | 0.000000 | 1180 | 1169.694130 | 1194.65 | 1219.605870 | 1.012415 |
| GO:0051238\_sequestering\_of\_metal\_ion | 7 | 0 | 0.000000 | 0.000000 | 1180 | 1169.694130 | 1194.65 | 1219.605870 | 1.012415 |
| GO:0051250\_negative\_regulation\_of\_lymphocyte\_activation | 7 | 0 | 0.000000 | 0.000000 | 1180 | 1169.694130 | 1194.65 | 1219.605870 | 1.012415 |
| GO:0051452\_intracellular\_pH\_reduction | 7 | 0 | 0.000000 | 0.000000 | 1180 | 1169.694130 | 1194.65 | 1219.605870 | 1.012415 |
| GO:0051453\_regulation\_of\_intracellular\_pH | 7 | 0 | 0.000000 | 0.000000 | 1180 | 1169.694130 | 1194.65 | 1219.605870 | 1.012415 |
| GO:0051492\_regulation\_of\_stress\_fiber\_formation | 7 | 0 | 0.000000 | 0.000000 | 1180 | 1169.694130 | 1194.65 | 1219.605870 | 1.012415 |
| GO:0051881\_regulation\_of\_mitochondrial\_membrane\_potential | 7 | 0 | 0.000000 | 0.000000 | 1180 | 1169.694130 | 1194.65 | 1219.605870 | 1.012415 |
| GO:0055008\_cardiac\_muscle\_morphogensis | 7 | 0 | 0.000000 | 0.000000 | 1180 | 1169.694130 | 1194.65 | 1219.605870 | 1.012415 |
| GO:0009308\_amine\_metabolic\_process | 130 | 0 | 0.000000 | 0.000000 | 1182 | 1171.251023 | 1195.87 | 1220.488977 | 1.011734 |
| GO:0051704\_multi-organism\_process | 130 | 0 | 0.000000 | 0.000000 | 1182 | 1171.251023 | 1195.87 | 1220.488977 | 1.011734 |
| GO:0003001\_generation\_of\_a\_signal\_involved\_in\_cell-cell\_signaling | 31 | 0 | 0.000000 | 0.000000 | 1190 | 1183.629857 | 1207.23 | 1230.830143 | 1.014479 |
| GO:0006887\_exocytosis | 31 | 0 | 0.000000 | 0.000000 | 1190 | 1183.629857 | 1207.23 | 1230.830143 | 1.014479 |
| GO:0006919\_caspase\_activation | 31 | 0 | 0.000000 | 0.000000 | 1190 | 1183.629857 | 1207.23 | 1230.830143 | 1.014479 |
| GO:0018212\_peptidyl-tyrosine\_modification | 31 | 0 | 0.000000 | 0.000000 | 1190 | 1183.629857 | 1207.23 | 1230.830143 | 1.014479 |
| GO:0019933\_cAMP-mediated\_signaling | 31 | 0 | 0.000000 | 0.000000 | 1190 | 1183.629857 | 1207.23 | 1230.830143 | 1.014479 |
| GO:0030098\_lymphocyte\_differentiation | 31 | 0 | 0.000000 | 0.000000 | 1190 | 1183.629857 | 1207.23 | 1230.830143 | 1.014479 |
| GO:0046777\_protein\_amino\_acid\_autophosphorylation | 31 | 0 | 0.000000 | 0.000000 | 1190 | 1183.629857 | 1207.23 | 1230.830143 | 1.014479 |
| GO:0046849\_bone\_remodeling | 31 | 0 | 0.000000 | 0.000000 | 1190 | 1183.629857 | 1207.23 | 1230.830143 | 1.014479 |
| GO:0007249\_I-kappaB\_kinase\_NF-kappaB\_cascade | 111 | 0 | 0.000000 | 0.000000 | 1191 | 1184.861106 | 1208.33 | 1231.798894 | 1.014551 |
| GO:0001568\_blood\_vessel\_development | 53 | 0 | 0.000000 | 0.000000 | 1196 | 1188.458546 | 1211.66 | 1234.861454 | 1.013094 |
| GO:0006310\_DNA\_recombination | 53 | 0 | 0.000000 | 0.000000 | 1196 | 1188.458546 | 1211.66 | 1234.861454 | 1.013094 |
| GO:0019935\_cyclic-nucleotide-mediated\_signaling | 53 | 0 | 0.000000 | 0.000000 | 1196 | 1188.458546 | 1211.66 | 1234.861454 | 1.013094 |
| GO:0032446\_protein\_modification\_by\_small\_protein\_conjugation | 53 | 0 | 0.000000 | 0.000000 | 1196 | 1188.458546 | 1211.66 | 1234.861454 | 1.013094 |
| GO:0048514\_blood\_vessel\_morphogenesis | 53 | 0 | 0.000000 | 0.000000 | 1196 | 1188.458546 | 1211.66 | 1234.861454 | 1.013094 |
| GO:0000090\_mitotic\_anaphase | 3 | 0 |  |  |  |  |  |  |  |  |
| GO:0000097\_sulfur\_amino\_acid\_biosynthetic\_process | 3 | 0 |  |  |  |  |  |  |  |  |
| GO:0000101\_sulfur\_amino\_acid\_transport | 3 | 0 |  |  |  |  |  |  |  |  |
| GO:0000185\_activation\_of\_MAPKKK\_activity | 3 | 0 |  |  |  |  |  |  |  |  |
| GO:0000244\_assembly\_of\_spliceosomal\_tri-snRNP | 3 | 0 |  |  |  |  |  |  |  |  |
| GO:0000272\_polysaccharide\_catabolic\_process | 3 | 0 |  |  |  |  |  |  |  |  |
| GO:0000305\_response\_to\_oxygen\_radical | 3 | 0 |  |  |  |  |  |  |  |  |
| GO:0000768\_syncytium\_formation\_by\_plasma\_membrane\_fusion | 3 | 0 |  |  |  |  |  |  |  |  |
| GO:0001504\_neurotransmitter\_uptake | 3 | 0 |  |  |  |  |  |  |  |  |
| GO:0001573\_ganglioside\_metabolic\_process | 3 | 0 |  |  |  |  |  |  |  |  |
| GO:0001580\_detection\_of\_chemical\_stimulus\_involved\_in\_sensory\_perception\_of\_bitter\_taste | 3 | 0 |  |  |  |  |  |  |  |  |
| GO:0001656\_metanephros\_development | 3 | 0 |  |  |  |  |  |  |  |  |
| GO:0001657\_ureteric\_bud\_development | 3 | 0 |  |  |  |  |  |  |  |  |
| GO:0001710\_mesodermal\_cell\_fate\_commitment | 3 | 0 |  |  |  |  |  |  |  |  |
| GO:0001754\_eye\_photoreceptor\_cell\_differentiation | 3 | 0 |  |  |  |  |  |  |  |  |
| GO:0001755\_neural\_crest\_cell\_migration | 3 | 0 |  |  |  |  |  |  |  |  |
| GO:0001889\_liver\_development | 3 | 0 |  |  |  |  |  |  |  |  |
| GO:0001913\_T\_cell\_mediated\_cytotoxicity | 3 | 0 |  |  |  |  |  |  |  |  |
| GO:0001954\_positive\_regulation\_of\_cell-matrix\_adhesion | 3 | 0 |  |  |  |  |  |  |  |  |
| GO:0001959\_regulation\_of\_cytokine\_and\_chemokine\_mediated\_signaling\_pathway | 3 | 0 |  |  |  |  |  |  |  |  |
| GO:0002026\_regulation\_of\_the\_force\_of\_heart\_contraction | 3 | 0 |  |  |  |  |  |  |  |  |
| GO:0002076\_osteoblast\_development | 3 | 0 |  |  |  |  |  |  |  |  |
| GO:0002230\_positive\_regulation\_of\_defense\_response\_to\_virus\_by\_host | 3 | 0 |  |  |  |  |  |  |  |  |
| GO:0002263\_cell\_activation\_during\_immune\_response | 3 | 0 |  |  |  |  |  |  |  |  |
| GO:0002366\_leukocyte\_activation\_during\_immune\_response | 3 | 0 |  |  |  |  |  |  |  |  |
| GO:0002715\_regulation\_of\_natural\_killer\_cell\_mediated\_immunity | 3 | 0 |  |  |  |  |  |  |  |  |
| GO:0003009\_skeletal\_muscle\_contraction | 3 | 0 |  |  |  |  |  |  |  |  |
| GO:0003073\_regulation\_of\_systemic\_arterial\_blood\_pressure | 3 | 0 |  |  |  |  |  |  |  |  |
| GO:0005979\_regulation\_of\_glycogen\_biosynthetic\_process | 3 | 0 |  |  |  |  |  |  |  |  |
| GO:0006002\_fructose\_6-phosphate\_metabolic\_process | 3 | 0 |  |  |  |  |  |  |  |  |
| GO:0006102\_isocitrate\_metabolic\_process | 3 | 0 |  |  |  |  |  |  |  |  |
| GO:0006104\_succinyl-CoA\_metabolic\_process | 3 | 0 |  |  |  |  |  |  |  |  |
| GO:0006221\_pyrimidine\_nucleotide\_biosynthetic\_process | 3 | 0 |  |  |  |  |  |  |  |  |
| GO:0006244\_pyrimidine\_nucleotide\_catabolic\_process | 3 | 0 |  |  |  |  |  |  |  |  |
| GO:0006295\_nucleotide-excision\_repair\_\_DNA\_incision\_\_3'-to\_lesion | 3 | 0 |  |  |  |  |  |  |  |  |
| GO:0006296\_nucleotide-excision\_repair\_\_DNA\_incision\_\_5'-to\_lesion | 3 | 0 |  |  |  |  |  |  |  |  |
| GO:0006384\_transcription\_initiation\_from\_RNA\_polymerase\_III\_promoter | 3 | 0 |  |  |  |  |  |  |  |  |
| GO:0006467\_protein\_thiol-disulfide\_exchange | 3 | 0 |  |  |  |  |  |  |  |  |
| GO:0006482\_protein\_amino\_acid\_demethylation | 3 | 0 |  |  |  |  |  |  |  |  |
| GO:0006546\_glycine\_catabolic\_process | 3 | 0 |  |  |  |  |  |  |  |  |
| GO:0006555\_methionine\_metabolic\_process | 3 | 0 |  |  |  |  |  |  |  |  |
| GO:0006560\_proline\_metabolic\_process | 3 | 0 |  |  |  |  |  |  |  |  |
| GO:0006563\_L-serine\_metabolic\_process | 3 | 0 |  |  |  |  |  |  |  |  |
| GO:0006572\_tyrosine\_catabolic\_process | 3 | 0 |  |  |  |  |  |  |  |  |
| GO:0006584\_catecholamine\_metabolic\_process | 3 | 0 |  |  |  |  |  |  |  |  |
| GO:0006596\_polyamine\_biosynthetic\_process | 3 | 0 |  |  |  |  |  |  |  |  |
| GO:0006613\_cotranslational\_protein\_targeting\_to\_membrane | 3 | 0 |  |  |  |  |  |  |  |  |
| GO:0006621\_protein\_retention\_in\_ER | 3 | 0 |  |  |  |  |  |  |  |  |
| GO:0006622\_protein\_targeting\_to\_lysosome | 3 | 0 |  |  |  |  |  |  |  |  |
| GO:0006623\_protein\_targeting\_to\_vacuole | 3 | 0 |  |  |  |  |  |  |  |  |
| GO:0006656\_phosphatidylcholine\_biosynthetic\_process | 3 | 0 |  |  |  |  |  |  |  |  |
| GO:0006661\_phosphatidylinositol\_biosynthetic\_process | 3 | 0 |  |  |  |  |  |  |  |  |
| GO:0006677\_glycosylceramide\_metabolic\_process | 3 | 0 |  |  |  |  |  |  |  |  |
| GO:0006678\_glucosylceramide\_metabolic\_process | 3 | 0 |  |  |  |  |  |  |  |  |
| GO:0006686\_sphingomyelin\_biosynthetic\_process | 3 | 0 |  |  |  |  |  |  |  |  |
| GO:0006699\_bile\_acid\_biosynthetic\_process | 3 | 0 |  |  |  |  |  |  |  |  |
| GO:0006750\_glutathione\_biosynthetic\_process | 3 | 0 |  |  |  |  |  |  |  |  |
| GO:0006752\_group\_transfer\_coenzyme\_metabolic\_process | 3 | 0 |  |  |  |  |  |  |  |  |
| GO:0006787\_porphyrin\_catabolic\_process | 3 | 0 |  |  |  |  |  |  |  |  |
| GO:0006817\_phosphate\_transport | 3 | 0 |  |  |  |  |  |  |  |  |
| GO:0006835\_dicarboxylic\_acid\_transport | 3 | 0 |  |  |  |  |  |  |  |  |
| GO:0006930\_substrate-bound\_cell\_migration\_\_cell\_extension | 3 | 0 |  |  |  |  |  |  |  |  |
| GO:0006949\_syncytium\_formation | 3 | 0 |  |  |  |  |  |  |  |  |
| GO:0006980\_redox\_signal\_response | 3 | 0 |  |  |  |  |  |  |  |  |
| GO:0006983\_ER\_overload\_response | 3 | 0 |  |  |  |  |  |  |  |  |
| GO:0007029\_endoplasmic\_reticulum\_organization\_and\_biogenesis | 3 | 0 |  |  |  |  |  |  |  |  |
| GO:0007129\_synapsis | 3 | 0 |  |  |  |  |  |  |  |  |
| GO:0007130\_synaptonemal\_complex\_assembly | 3 | 0 |  |  |  |  |  |  |  |  |
| GO:0007140\_male\_meiosis | 3 | 0 |  |  |  |  |  |  |  |  |
| GO:0007171\_activation\_of\_transmembrane\_receptor\_protein\_tyrosine\_kinase\_activity | 3 | 0 |  |  |  |  |  |  |  |  |
| GO:0007193\_G-protein\_signaling\_\_adenylate\_cyclase\_inhibiting\_pathway | 3 | 0 |  |  |  |  |  |  |  |  |
| GO:0007212\_dopamine\_receptor\_signaling\_pathway | 3 | 0 |  |  |  |  |  |  |  |  |
| GO:0007216\_metabotropic\_glutamate\_receptor\_signaling\_pathway | 3 | 0 |  |  |  |  |  |  |  |  |
| GO:0007252\_I-kappaB\_phosphorylation | 3 | 0 |  |  |  |  |  |  |  |  |
| GO:0007270\_nerve-nerve\_synaptic\_transmission | 3 | 0 |  |  |  |  |  |  |  |  |
| GO:0007274\_neuromuscular\_synaptic\_transmission | 3 | 0 |  |  |  |  |  |  |  |  |
| GO:0007413\_axonal\_fasciculation | 3 | 0 |  |  |  |  |  |  |  |  |
| GO:0007520\_myoblast\_fusion | 3 | 0 |  |  |  |  |  |  |  |  |
| GO:0007530\_sex\_determination | 3 | 0 |  |  |  |  |  |  |  |  |
| GO:0007632\_visual\_behavior | 3 | 0 |  |  |  |  |  |  |  |  |
| GO:0008207\_C21-steroid\_hormone\_metabolic\_process | 3 | 0 |  |  |  |  |  |  |  |  |
| GO:0008210\_estrogen\_metabolic\_process | 3 | 0 |  |  |  |  |  |  |  |  |
| GO:0008211\_glucocorticoid\_metabolic\_process | 3 | 0 |  |  |  |  |  |  |  |  |
| GO:0008214\_protein\_amino\_acid\_dealkylation | 3 | 0 |  |  |  |  |  |  |  |  |
| GO:0008334\_histone\_mRNA\_metabolic\_process | 3 | 0 |  |  |  |  |  |  |  |  |
| GO:0008354\_germ\_cell\_migration | 3 | 0 |  |  |  |  |  |  |  |  |
| GO:0008589\_regulation\_of\_smoothened\_signaling\_pathway | 3 | 0 |  |  |  |  |  |  |  |  |
| GO:0008631\_induction\_of\_apoptosis\_by\_oxidative\_stress | 3 | 0 |  |  |  |  |  |  |  |  |
| GO:0009070\_serine\_family\_amino\_acid\_biosynthetic\_process | 3 | 0 |  |  |  |  |  |  |  |  |
| GO:0009113\_purine\_base\_biosynthetic\_process | 3 | 0 |  |  |  |  |  |  |  |  |
| GO:0009126\_purine\_nucleoside\_monophosphate\_metabolic\_process | 3 | 0 |  |  |  |  |  |  |  |  |
| GO:0009132\_nucleoside\_diphosphate\_metabolic\_process | 3 | 0 |  |  |  |  |  |  |  |  |
| GO:0009145\_purine\_nucleoside\_triphosphate\_biosynthetic\_process | 3 | 0 |  |  |  |  |  |  |  |  |
| GO:0009164\_nucleoside\_catabolic\_process | 3 | 0 |  |  |  |  |  |  |  |  |
| GO:0009167\_purine\_ribonucleoside\_monophosphate\_metabolic\_process | 3 | 0 |  |  |  |  |  |  |  |  |
| GO:0009185\_ribonucleoside\_diphosphate\_metabolic\_process | 3 | 0 |  |  |  |  |  |  |  |  |
| GO:0009206\_purine\_ribonucleoside\_triphosphate\_biosynthetic\_process | 3 | 0 |  |  |  |  |  |  |  |  |
| GO:0009220\_pyrimidine\_ribonucleotide\_biosynthetic\_process | 3 | 0 |  |  |  |  |  |  |  |  |
| GO:0009226\_nucleotide-sugar\_biosynthetic\_process | 3 | 0 |  |  |  |  |  |  |  |  |
| GO:0009251\_glucan\_catabolic\_process | 3 | 0 |  |  |  |  |  |  |  |  |
| GO:0009261\_ribonucleotide\_catabolic\_process | 3 | 0 |  |  |  |  |  |  |  |  |
| GO:0009301\_snRNA\_transcription | 3 | 0 |  |  |  |  |  |  |  |  |
| GO:0009409\_response\_to\_cold | 3 | 0 |  |  |  |  |  |  |  |  |
| GO:0010043\_response\_to\_zinc\_ion | 3 | 0 |  |  |  |  |  |  |  |  |
| GO:0010224\_response\_to\_UV-B | 3 | 0 |  |  |  |  |  |  |  |  |
| GO:0010517\_regulation\_of\_phospholipase\_activity | 3 | 0 |  |  |  |  |  |  |  |  |
| GO:0010518\_positive\_regulation\_of\_phospholipase\_activity | 3 | 0 |  |  |  |  |  |  |  |  |
| GO:0014032\_neural\_crest\_cell\_development | 3 | 0 |  |  |  |  |  |  |  |  |
| GO:0014033\_neural\_crest\_cell\_differentiation | 3 | 0 |  |  |  |  |  |  |  |  |
| GO:0014065\_phosphoinositide\_3-kinase\_cascade | 3 | 0 |  |  |  |  |  |  |  |  |
| GO:0015015\_heparan\_sulfate\_proteoglycan\_biosynthetic\_process\_\_enzymatic\_modification | 3 | 0 |  |  |  |  |  |  |  |  |
| GO:0015711\_organic\_anion\_transport | 3 | 0 |  |  |  |  |  |  |  |  |
| GO:0015721\_bile\_acid\_and\_bile\_salt\_transport | 3 | 0 |  |  |  |  |  |  |  |  |
| GO:0015802\_basic\_amino\_acid\_transport | 3 | 0 |  |  |  |  |  |  |  |  |
| GO:0015810\_aspartate\_transport | 3 | 0 |  |  |  |  |  |  |  |  |
| GO:0015851\_nucleobase\_transport | 3 | 0 |  |  |  |  |  |  |  |  |
| GO:0015879\_carnitine\_transport | 3 | 0 |  |  |  |  |  |  |  |  |
| GO:0015889\_cobalamin\_transport | 3 | 0 |  |  |  |  |  |  |  |  |
| GO:0015914\_phospholipid\_transport | 3 | 0 |  |  |  |  |  |  |  |  |
| GO:0015917\_aminophospholipid\_transport | 3 | 0 |  |  |  |  |  |  |  |  |
| GO:0016045\_detection\_of\_bacterium | 3 | 0 |  |  |  |  |  |  |  |  |
| GO:0016081\_synaptic\_vesicle\_docking\_during\_exocytosis | 3 | 0 |  |  |  |  |  |  |  |  |
| GO:0016441\_posttranscriptional\_gene\_silencing | 3 | 0 |  |  |  |  |  |  |  |  |
| GO:0016553\_base\_conversion\_or\_substitution\_editing | 3 | 0 |  |  |  |  |  |  |  |  |
| GO:0017121\_phospholipid\_scrambling | 3 | 0 |  |  |  |  |  |  |  |  |
| GO:0018103\_protein\_amino\_acid\_C-linked\_glycosylation | 3 | 0 |  |  |  |  |  |  |  |  |
| GO:0018107\_peptidyl-threonine\_phosphorylation | 3 | 0 |  |  |  |  |  |  |  |  |
| GO:0018205\_peptidyl-lysine\_modification | 3 | 0 |  |  |  |  |  |  |  |  |
| GO:0018206\_peptidyl-methionine\_modification | 3 | 0 |  |  |  |  |  |  |  |  |
| GO:0018211\_peptidyl-tryptophan\_modification | 3 | 0 |  |  |  |  |  |  |  |  |
| GO:0018242\_protein\_amino\_acid\_O-linked\_glycosylation\_via\_serine | 3 | 0 |  |  |  |  |  |  |  |  |
| GO:0018243\_protein\_amino\_acid\_O-linked\_glycosylation\_via\_threonine | 3 | 0 |  |  |  |  |  |  |  |  |
| GO:0018317\_protein\_amino\_acid\_C-linked\_glycosylation\_via\_tryptophan | 3 | 0 |  |  |  |  |  |  |  |  |
| GO:0018342\_protein\_prenylation | 3 | 0 |  |  |  |  |  |  |  |  |
| GO:0018346\_protein\_amino\_acid\_prenylation | 3 | 0 |  |  |  |  |  |  |  |  |
| GO:0018406\_protein\_amino\_acid\_C-linked\_glycosylation\_via\_2'-alpha-mannosyl-L-tryptophan | 3 | 0 |  |  |  |  |  |  |  |  |
| GO:0019080\_viral\_genome\_expression | 3 | 0 |  |  |  |  |  |  |  |  |
| GO:0019083\_viral\_transcription | 3 | 0 |  |  |  |  |  |  |  |  |
| GO:0019229\_regulation\_of\_vasoconstriction | 3 | 0 |  |  |  |  |  |  |  |  |
| GO:0019321\_pentose\_metabolic\_process | 3 | 0 |  |  |  |  |  |  |  |  |
| GO:0019377\_glycolipid\_catabolic\_process | 3 | 0 |  |  |  |  |  |  |  |  |
| GO:0019432\_triacylglycerol\_biosynthetic\_process | 3 | 0 |  |  |  |  |  |  |  |  |
| GO:0019934\_cGMP-mediated\_signaling | 3 | 0 |  |  |  |  |  |  |  |  |
| GO:0020027\_hemoglobin\_metabolic\_process | 3 | 0 |  |  |  |  |  |  |  |  |
| GO:0021510\_spinal\_cord\_development | 3 | 0 |  |  |  |  |  |  |  |  |
| GO:0021515\_cell\_differentiation\_in\_spinal\_cord | 3 | 0 |  |  |  |  |  |  |  |  |
| GO:0022408\_negative\_regulation\_of\_cell-cell\_adhesion | 3 | 0 |  |  |  |  |  |  |  |  |
| GO:0022898\_regulation\_of\_transmembrane\_transporter\_activity | 3 | 0 |  |  |  |  |  |  |  |  |
| GO:0030010\_establishment\_of\_cell\_polarity | 3 | 0 |  |  |  |  |  |  |  |  |
| GO:0030195\_negative\_regulation\_of\_blood\_coagulation | 3 | 0 |  |  |  |  |  |  |  |  |
| GO:0030219\_megakaryocyte\_differentiation | 3 | 0 |  |  |  |  |  |  |  |  |
| GO:0030263\_apoptotic\_chromosome\_condensation | 3 | 0 |  |  |  |  |  |  |  |  |
| GO:0030299\_cholesterol\_absorption | 3 | 0 |  |  |  |  |  |  |  |  |
| GO:0030324\_lung\_development | 3 | 0 |  |  |  |  |  |  |  |  |
| GO:0030512\_negative\_regulation\_of\_transforming\_growth\_factor\_beta\_receptor\_signaling\_pathway | 3 | 0 |  |  |  |  |  |  |  |  |
| GO:0030574\_collagen\_catabolic\_process | 3 | 0 |  |  |  |  |  |  |  |  |
| GO:0030866\_cortical\_actin\_cytoskeleton\_organization\_and\_biogenesis | 3 | 0 |  |  |  |  |  |  |  |  |
| GO:0030902\_hindbrain\_development | 3 | 0 |  |  |  |  |  |  |  |  |
| GO:0030970\_retrograde\_protein\_transport\_\_ER\_to\_cytosol | 3 | 0 |  |  |  |  |  |  |  |  |
| GO:0031032\_actomyosin\_structure\_organization\_and\_biogenesis | 3 | 0 |  |  |  |  |  |  |  |  |
| GO:0031047\_gene\_silencing\_by\_RNA | 3 | 0 |  |  |  |  |  |  |  |  |
| GO:0031058\_positive\_regulation\_of\_histone\_modification | 3 | 0 |  |  |  |  |  |  |  |  |
| GO:0031102\_neurite\_regeneration | 3 | 0 |  |  |  |  |  |  |  |  |
| GO:0031103\_axon\_regeneration | 3 | 0 |  |  |  |  |  |  |  |  |
| GO:0031116\_positive\_regulation\_of\_microtubule\_polymerization | 3 | 0 |  |  |  |  |  |  |  |  |
| GO:0031397\_negative\_regulation\_of\_protein\_ubiquitination | 3 | 0 |  |  |  |  |  |  |  |  |
| GO:0031579\_membrane\_raft\_organization\_and\_biogenesis | 3 | 0 |  |  |  |  |  |  |  |  |
| GO:0031646\_positive\_regulation\_of\_neurological\_system\_process | 3 | 0 |  |  |  |  |  |  |  |  |
| GO:0031958\_corticosteroid\_receptor\_signaling\_pathway | 3 | 0 |  |  |  |  |  |  |  |  |
| GO:0032020\_ISG15-protein\_conjugation | 3 | 0 |  |  |  |  |  |  |  |  |
| GO:0032239\_regulation\_of\_nucleobase\_\_nucleoside\_\_nucleotide\_and\_nucleic\_acid\_transport | 3 | 0 |  |  |  |  |  |  |  |  |
| GO:0032276\_regulation\_of\_gonadotropin\_secretion | 3 | 0 |  |  |  |  |  |  |  |  |
| GO:0032277\_negative\_regulation\_of\_gonadotropin\_secretion | 3 | 0 |  |  |  |  |  |  |  |  |
| GO:0032350\_regulation\_of\_hormone\_metabolic\_process | 3 | 0 |  |  |  |  |  |  |  |  |
| GO:0032409\_regulation\_of\_transporter\_activity | 3 | 0 |  |  |  |  |  |  |  |  |
| GO:0032412\_regulation\_of\_ion\_transmembrane\_transporter\_activity | 3 | 0 |  |  |  |  |  |  |  |  |
| GO:0032429\_regulation\_of\_phospholipase\_A2\_activity | 3 | 0 |  |  |  |  |  |  |  |  |
| GO:0032430\_positive\_regulation\_of\_phospholipase\_A2\_activity | 3 | 0 |  |  |  |  |  |  |  |  |
| GO:0032431\_activation\_of\_phospholipase\_A2 | 3 | 0 |  |  |  |  |  |  |  |  |
| GO:0032582\_negative\_regulation\_of\_gene-specific\_transcription | 3 | 0 |  |  |  |  |  |  |  |  |
| GO:0032606\_type\_I\_interferon\_production | 3 | 0 |  |  |  |  |  |  |  |  |
| GO:0032607\_interferon-alpha\_production | 3 | 0 |  |  |  |  |  |  |  |  |
| GO:0032675\_regulation\_of\_interleukin-6\_production | 3 | 0 |  |  |  |  |  |  |  |  |
| GO:0032781\_positive\_regulation\_of\_ATPase\_activity | 3 | 0 |  |  |  |  |  |  |  |  |
| GO:0032800\_receptor\_biosynthetic\_process | 3 | 0 |  |  |  |  |  |  |  |  |
| GO:0032845\_negative\_regulation\_of\_homeostatic\_process | 3 | 0 |  |  |  |  |  |  |  |  |
| GO:0032881\_regulation\_of\_polysaccharide\_metabolic\_process | 3 | 0 |  |  |  |  |  |  |  |  |
| GO:0032885\_regulation\_of\_polysaccharide\_biosynthetic\_process | 3 | 0 |  |  |  |  |  |  |  |  |
| GO:0032927\_positive\_regulation\_of\_activin\_receptor\_signaling\_pathway | 3 | 0 |  |  |  |  |  |  |  |  |
| GO:0032945\_negative\_regulation\_of\_mononuclear\_cell\_proliferation | 3 | 0 |  |  |  |  |  |  |  |  |
| GO:0033015\_tetrapyrrole\_catabolic\_process | 3 | 0 |  |  |  |  |  |  |  |  |
| GO:0033205\_cytokinesis\_during\_cell\_cycle | 3 | 0 |  |  |  |  |  |  |  |  |
| GO:0033209\_tumor\_necrosis\_factor-mediated\_signaling\_pathway | 3 | 0 |  |  |  |  |  |  |  |  |
| GO:0033344\_cholesterol\_efflux | 3 | 0 |  |  |  |  |  |  |  |  |
| GO:0033365\_protein\_localization\_in\_organelle | 3 | 0 |  |  |  |  |  |  |  |  |
| GO:0034104\_negative\_regulation\_of\_tissue\_remodeling | 3 | 0 |  |  |  |  |  |  |  |  |
| GO:0035051\_cardiac\_cell\_differentiation | 3 | 0 |  |  |  |  |  |  |  |  |
| GO:0035094\_response\_to\_nicotine | 3 | 0 |  |  |  |  |  |  |  |  |
| GO:0035194\_posttranscriptional\_gene\_silencing\_by\_RNA | 3 | 0 |  |  |  |  |  |  |  |  |
| GO:0035268\_protein\_amino\_acid\_mannosylation | 3 | 0 |  |  |  |  |  |  |  |  |
| GO:0035269\_protein\_amino\_acid\_O-linked\_mannosylation | 3 | 0 |  |  |  |  |  |  |  |  |
| GO:0035315\_hair\_cell\_differentiation | 3 | 0 |  |  |  |  |  |  |  |  |
| GO:0042090\_interleukin-12\_biosynthetic\_process | 3 | 0 |  |  |  |  |  |  |  |  |
| GO:0042092\_T-helper\_2\_type\_immune\_response | 3 | 0 |  |  |  |  |  |  |  |  |
| GO:0042149\_cellular\_response\_to\_glucose\_starvation | 3 | 0 |  |  |  |  |  |  |  |  |
| GO:0042177\_negative\_regulation\_of\_protein\_catabolic\_process | 3 | 0 |  |  |  |  |  |  |  |  |
| GO:0042269\_regulation\_of\_natural\_killer\_cell\_mediated\_cytotoxicity | 3 | 0 |  |  |  |  |  |  |  |  |
| GO:0042364\_water-soluble\_vitamin\_biosynthetic\_process | 3 | 0 |  |  |  |  |  |  |  |  |
| GO:0042402\_biogenic\_amine\_catabolic\_process | 3 | 0 |  |  |  |  |  |  |  |  |
| GO:0042417\_dopamine\_metabolic\_process | 3 | 0 |  |  |  |  |  |  |  |  |
| GO:0042462\_eye\_photoreceptor\_cell\_development | 3 | 0 |  |  |  |  |  |  |  |  |
| GO:0042471\_ear\_morphogenesis | 3 | 0 |  |  |  |  |  |  |  |  |
| GO:0042472\_inner\_ear\_morphogenesis | 3 | 0 |  |  |  |  |  |  |  |  |
| GO:0042517\_positive\_regulation\_of\_tyrosine\_phosphorylation\_of\_Stat3\_protein | 3 | 0 |  |  |  |  |  |  |  |  |
| GO:0042541\_hemoglobin\_biosynthetic\_process | 3 | 0 |  |  |  |  |  |  |  |  |
| GO:0042554\_superoxide\_release | 3 | 0 |  |  |  |  |  |  |  |  |
| GO:0042743\_hydrogen\_peroxide\_metabolic\_process | 3 | 0 |  |  |  |  |  |  |  |  |
| GO:0042787\_protein\_ubiquitination\_during\_ubiquitin-dependent\_protein\_catabolic\_process | 3 | 0 |  |  |  |  |  |  |  |  |
| GO:0042921\_glucocorticoid\_receptor\_signaling\_pathway | 3 | 0 |  |  |  |  |  |  |  |  |
| GO:0043001\_Golgi\_to\_plasma\_membrane\_protein\_transport | 3 | 0 |  |  |  |  |  |  |  |  |
| GO:0043029\_T\_cell\_homeostasis | 3 | 0 |  |  |  |  |  |  |  |  |
| GO:0043030\_regulation\_of\_macrophage\_activation | 3 | 0 |  |  |  |  |  |  |  |  |
| GO:0043089\_positive\_regulation\_of\_Cdc42\_GTPase\_activity | 3 | 0 |  |  |  |  |  |  |  |  |
| GO:0043467\_regulation\_of\_generation\_of\_precursor\_metabolites\_and\_energy | 3 | 0 |  |  |  |  |  |  |  |  |
| GO:0043496\_regulation\_of\_protein\_homodimerization\_activity | 3 | 0 |  |  |  |  |  |  |  |  |
| GO:0043535\_regulation\_of\_blood\_vessel\_endothelial\_cell\_migration | 3 | 0 |  |  |  |  |  |  |  |  |
| GO:0043545\_molybdopterin\_cofactor\_metabolic\_process | 3 | 0 |  |  |  |  |  |  |  |  |
| GO:0043588\_skin\_development | 3 | 0 |  |  |  |  |  |  |  |  |
| GO:0043616\_keratinocyte\_proliferation | 3 | 0 |  |  |  |  |  |  |  |  |
| GO:0043691\_reverse\_cholesterol\_transport | 3 | 0 |  |  |  |  |  |  |  |  |
| GO:0044236\_multicellular\_organismal\_metabolic\_process | 3 | 0 |  |  |  |  |  |  |  |  |
| GO:0044241\_lipid\_digestion | 3 | 0 |  |  |  |  |  |  |  |  |
| GO:0044243\_multicellular\_organismal\_catabolic\_process | 3 | 0 |  |  |  |  |  |  |  |  |
| GO:0044247\_cellular\_polysaccharide\_catabolic\_process | 3 | 0 |  |  |  |  |  |  |  |  |
| GO:0044254\_multicellular\_organismal\_protein\_catabolic\_process | 3 | 0 |  |  |  |  |  |  |  |  |
| GO:0044256\_protein\_digestion | 3 | 0 |  |  |  |  |  |  |  |  |
| GO:0044259\_multicellular\_organismal\_macromolecule\_metabolic\_process | 3 | 0 |  |  |  |  |  |  |  |  |
| GO:0044266\_multicellular\_organismal\_macromolecule\_catabolic\_process | 3 | 0 |  |  |  |  |  |  |  |  |
| GO:0044268\_multicellular\_organismal\_protein\_metabolic\_process | 3 | 0 |  |  |  |  |  |  |  |  |
| GO:0045022\_early\_endosome\_to\_late\_endosome\_transport | 3 | 0 |  |  |  |  |  |  |  |  |
| GO:0045055\_regulated\_secretory\_pathway | 3 | 0 |  |  |  |  |  |  |  |  |
| GO:0045061\_thymic\_T\_cell\_selection | 3 | 0 |  |  |  |  |  |  |  |  |
| GO:0045075\_regulation\_of\_interleukin-12\_biosynthetic\_process | 3 | 0 |  |  |  |  |  |  |  |  |
| GO:0045079\_negative\_regulation\_of\_chemokine\_biosynthetic\_process | 3 | 0 |  |  |  |  |  |  |  |  |
| GO:0045109\_intermediate\_filament\_organization | 3 | 0 |  |  |  |  |  |  |  |  |
| GO:0045123\_cellular\_extravasation | 3 | 0 |  |  |  |  |  |  |  |  |
| GO:0045342\_MHC\_class\_II\_biosynthetic\_process | 3 | 0 |  |  |  |  |  |  |  |  |
| GO:0045346\_regulation\_of\_MHC\_class\_II\_biosynthetic\_process | 3 | 0 |  |  |  |  |  |  |  |  |
| GO:0045349\_interferon-alpha\_biosynthetic\_process | 3 | 0 |  |  |  |  |  |  |  |  |
| GO:0045351\_type\_I\_interferon\_biosynthetic\_process | 3 | 0 |  |  |  |  |  |  |  |  |
| GO:0045354\_regulation\_of\_interferon-alpha\_biosynthetic\_process | 3 | 0 |  |  |  |  |  |  |  |  |
| GO:0045410\_positive\_regulation\_of\_interleukin-6\_biosynthetic\_process | 3 | 0 |  |  |  |  |  |  |  |  |
| GO:0045416\_positive\_regulation\_of\_interleukin-8\_biosynthetic\_process | 3 | 0 |  |  |  |  |  |  |  |  |
| GO:0045540\_regulation\_of\_cholesterol\_biosynthetic\_process | 3 | 0 |  |  |  |  |  |  |  |  |
| GO:0045598\_regulation\_of\_fat\_cell\_differentiation | 3 | 0 |  |  |  |  |  |  |  |  |
| GO:0045649\_regulation\_of\_macrophage\_differentiation | 3 | 0 |  |  |  |  |  |  |  |  |
| GO:0045662\_negative\_regulation\_of\_myoblast\_differentiation | 3 | 0 |  |  |  |  |  |  |  |  |
| GO:0045663\_positive\_regulation\_of\_myoblast\_differentiation | 3 | 0 |  |  |  |  |  |  |  |  |
| GO:0045668\_negative\_regulation\_of\_osteoblast\_differentiation | 3 | 0 |  |  |  |  |  |  |  |  |
| GO:0045684\_positive\_regulation\_of\_epidermis\_development | 3 | 0 |  |  |  |  |  |  |  |  |
| GO:0045730\_respiratory\_burst | 3 | 0 |  |  |  |  |  |  |  |  |
| GO:0045732\_positive\_regulation\_of\_protein\_catabolic\_process | 3 | 0 |  |  |  |  |  |  |  |  |
| GO:0045737\_positive\_regulation\_of\_cyclin-dependent\_protein\_kinase\_activity | 3 | 0 |  |  |  |  |  |  |  |  |
| GO:0045740\_positive\_regulation\_of\_DNA\_replication | 3 | 0 |  |  |  |  |  |  |  |  |
| GO:0045806\_negative\_regulation\_of\_endocytosis | 3 | 0 |  |  |  |  |  |  |  |  |
| GO:0045817\_positive\_regulation\_of\_transcription\_from\_RNA\_polymerase\_II\_promoter\_\_global | 3 | 0 |  |  |  |  |  |  |  |  |
| GO:0045830\_positive\_regulation\_of\_isotype\_switching | 3 | 0 |  |  |  |  |  |  |  |  |
| GO:0045861\_negative\_regulation\_of\_proteolysis | 3 | 0 |  |  |  |  |  |  |  |  |
| GO:0045911\_positive\_regulation\_of\_DNA\_recombination | 3 | 0 |  |  |  |  |  |  |  |  |
| GO:0045923\_positive\_regulation\_of\_fatty\_acid\_metabolic\_process | 3 | 0 |  |  |  |  |  |  |  |  |
| GO:0045932\_negative\_regulation\_of\_muscle\_contraction | 3 | 0 |  |  |  |  |  |  |  |  |
| GO:0046324\_regulation\_of\_glucose\_import | 3 | 0 |  |  |  |  |  |  |  |  |
| GO:0046339\_diacylglycerol\_metabolic\_process | 3 | 0 |  |  |  |  |  |  |  |  |
| GO:0046470\_phosphatidylcholine\_metabolic\_process | 3 | 0 |  |  |  |  |  |  |  |  |
| GO:0046579\_positive\_regulation\_of\_Ras\_protein\_signal\_transduction | 3 | 0 |  |  |  |  |  |  |  |  |
| GO:0046626\_regulation\_of\_insulin\_receptor\_signaling\_pathway | 3 | 0 |  |  |  |  |  |  |  |  |
| GO:0046634\_regulation\_of\_alpha-beta\_T\_cell\_activation | 3 | 0 |  |  |  |  |  |  |  |  |
| GO:0046716\_muscle\_maintenance | 3 | 0 |  |  |  |  |  |  |  |  |
| GO:0046782\_regulation\_of\_viral\_transcription | 3 | 0 |  |  |  |  |  |  |  |  |
| GO:0046825\_regulation\_of\_protein\_export\_from\_nucleus | 3 | 0 |  |  |  |  |  |  |  |  |
| GO:0046831\_regulation\_of\_RNA\_export\_from\_nucleus | 3 | 0 |  |  |  |  |  |  |  |  |
| GO:0046834\_lipid\_phosphorylation | 3 | 0 |  |  |  |  |  |  |  |  |
| GO:0046851\_negative\_regulation\_of\_bone\_remodeling | 3 | 0 |  |  |  |  |  |  |  |  |
| GO:0046854\_phosphoinositide\_phosphorylation | 3 | 0 |  |  |  |  |  |  |  |  |
| GO:0046880\_regulation\_of\_follicle-stimulating\_hormone\_secretion | 3 | 0 |  |  |  |  |  |  |  |  |
| GO:0046882\_negative\_regulation\_of\_follicle-stimulating\_hormone\_secretion | 3 | 0 |  |  |  |  |  |  |  |  |
| GO:0046889\_positive\_regulation\_of\_lipid\_biosynthetic\_process | 3 | 0 |  |  |  |  |  |  |  |  |
| GO:0048010\_vascular\_endothelial\_growth\_factor\_receptor\_signaling\_pathway | 3 | 0 |  |  |  |  |  |  |  |  |
| GO:0048011\_nerve\_growth\_factor\_receptor\_signaling\_pathway | 3 | 0 |  |  |  |  |  |  |  |  |
| GO:0048246\_macrophage\_chemotaxis | 3 | 0 |  |  |  |  |  |  |  |  |
| GO:0048260\_positive\_regulation\_of\_receptor-mediated\_endocytosis | 3 | 0 |  |  |  |  |  |  |  |  |
| GO:0048333\_mesodermal\_cell\_differentiation | 3 | 0 |  |  |  |  |  |  |  |  |
| GO:0048496\_maintenance\_of\_organ\_identity | 3 | 0 |  |  |  |  |  |  |  |  |
| GO:0048568\_embryonic\_organ\_development | 3 | 0 |  |  |  |  |  |  |  |  |
| GO:0048592\_eye\_morphogenesis | 3 | 0 |  |  |  |  |  |  |  |  |
| GO:0048638\_regulation\_of\_developmental\_growth | 3 | 0 |  |  |  |  |  |  |  |  |
| GO:0048754\_branching\_morphogenesis\_of\_a\_tube | 3 | 0 |  |  |  |  |  |  |  |  |
| GO:0048806\_genitalia\_development | 3 | 0 |  |  |  |  |  |  |  |  |
| GO:0050655\_dermatan\_sulfate\_proteoglycan\_metabolic\_process | 3 | 0 |  |  |  |  |  |  |  |  |
| GO:0050672\_negative\_regulation\_of\_lymphocyte\_proliferation | 3 | 0 |  |  |  |  |  |  |  |  |
| GO:0050710\_negative\_regulation\_of\_cytokine\_secretion | 3 | 0 |  |  |  |  |  |  |  |  |
| GO:0050728\_negative\_regulation\_of\_inflammatory\_response | 3 | 0 |  |  |  |  |  |  |  |  |
| GO:0050771\_negative\_regulation\_of\_axonogenesis | 3 | 0 |  |  |  |  |  |  |  |  |
| GO:0050798\_activated\_T\_cell\_proliferation | 3 | 0 |  |  |  |  |  |  |  |  |
| GO:0050806\_positive\_regulation\_of\_synaptic\_transmission | 3 | 0 |  |  |  |  |  |  |  |  |
| GO:0050807\_regulation\_of\_synapse\_organization\_and\_biogenesis | 3 | 0 |  |  |  |  |  |  |  |  |
| GO:0050848\_regulation\_of\_calcium-mediated\_signaling | 3 | 0 |  |  |  |  |  |  |  |  |
| GO:0050856\_regulation\_of\_T\_cell\_receptor\_signaling\_pathway | 3 | 0 |  |  |  |  |  |  |  |  |
| GO:0050879\_multicellular\_organismal\_movement | 3 | 0 |  |  |  |  |  |  |  |  |
| GO:0050881\_musculoskeletal\_movement | 3 | 0 |  |  |  |  |  |  |  |  |
| GO:0050890\_cognition | 3 | 0 |  |  |  |  |  |  |  |  |
| GO:0050892\_intestinal\_absorption | 3 | 0 |  |  |  |  |  |  |  |  |
| GO:0050907\_detection\_of\_chemical\_stimulus\_involved\_in\_sensory\_perception | 3 | 0 |  |  |  |  |  |  |  |  |
| GO:0050912\_detection\_of\_chemical\_stimulus\_involved\_in\_sensory\_perception\_of\_taste | 3 | 0 |  |  |  |  |  |  |  |  |
| GO:0050913\_sensory\_perception\_of\_bitter\_taste | 3 | 0 |  |  |  |  |  |  |  |  |
| GO:0051016\_barbed-end\_actin\_filament\_capping | 3 | 0 |  |  |  |  |  |  |  |  |
| GO:0051057\_positive\_regulation\_of\_small\_GTPase\_mediated\_signal\_transduction | 3 | 0 |  |  |  |  |  |  |  |  |
| GO:0051146\_striated\_muscle\_cell\_differentiation | 3 | 0 |  |  |  |  |  |  |  |  |
| GO:0051149\_positive\_regulation\_of\_muscle\_cell\_differentiation | 3 | 0 |  |  |  |  |  |  |  |  |
| GO:0051209\_release\_of\_sequestered\_calcium\_ion\_into\_cytosol | 3 | 0 |  |  |  |  |  |  |  |  |
| GO:0051282\_regulation\_of\_sequestering\_of\_calcium\_ion | 3 | 0 |  |  |  |  |  |  |  |  |
| GO:0051283\_negative\_regulation\_of\_sequestering\_of\_calcium\_ion | 3 | 0 |  |  |  |  |  |  |  |  |
| GO:0051322\_anaphase | 3 | 0 |  |  |  |  |  |  |  |  |
| GO:0051324\_prophase | 3 | 0 |  |  |  |  |  |  |  |  |
| GO:0051351\_positive\_regulation\_of\_ligase\_activity | 3 | 0 |  |  |  |  |  |  |  |  |
| GO:0051354\_negative\_regulation\_of\_oxidoreductase\_activity | 3 | 0 |  |  |  |  |  |  |  |  |
| GO:0051403\_stress-activated\_MAPK\_cascade | 3 | 0 |  |  |  |  |  |  |  |  |
| GO:0051438\_regulation\_of\_ubiquitin-protein\_ligase\_activity | 3 | 0 |  |  |  |  |  |  |  |  |
| GO:0051591\_response\_to\_cAMP | 3 | 0 |  |  |  |  |  |  |  |  |
| GO:0051646\_mitochondrion\_localization | 3 | 0 |  |  |  |  |  |  |  |  |
| GO:0051693\_actin\_filament\_capping | 3 | 0 |  |  |  |  |  |  |  |  |
| GO:0051775\_response\_to\_redox\_state | 3 | 0 |  |  |  |  |  |  |  |  |
| GO:0051865\_protein\_autoubiquitination | 3 | 0 |  |  |  |  |  |  |  |  |
| GO:0051882\_mitochondrial\_depolarization | 3 | 0 |  |  |  |  |  |  |  |  |
| GO:0051893\_regulation\_of\_focal\_adhesion\_formation | 3 | 0 |  |  |  |  |  |  |  |  |
| GO:0051899\_membrane\_depolarization | 3 | 0 |  |  |  |  |  |  |  |  |
| GO:0051971\_positive\_regulation\_of\_transmission\_of\_nerve\_impulse | 3 | 0 |  |  |  |  |  |  |  |  |
| GO:0048583\_regulation\_of\_response\_to\_stimulus | 63 | 0 | 0.000000 | 0.000000 | 1197 | 1191.398172 | 1214.39 | 1237.381828 | 1.014528 |
| GO:0006520\_amino\_acid\_metabolic\_process | 79 | 0 | 0.000000 | 0.000000 | 1201 | 1194.191904 | 1216.87 | 1239.548096 | 1.013214 |
| GO:0007601\_visual\_perception | 79 | 0 | 0.000000 | 0.000000 | 1201 | 1194.191904 | 1216.87 | 1239.548096 | 1.013214 |
| GO:0015672\_monovalent\_inorganic\_cation\_transport | 79 | 0 | 0.000000 | 0.000000 | 1201 | 1194.191904 | 1216.87 | 1239.548096 | 1.013214 |
| GO:0050953\_sensory\_perception\_of\_light\_stimulus | 79 | 0 | 0.000000 | 0.000000 | 1201 | 1194.191904 | 1216.87 | 1239.548096 | 1.013214 |
| GO:0000080\_G1\_phase\_of\_mitotic\_cell\_cycle | 13 | 0 | 0.000000 | 0.000000 | 1240 | 1236.410915 | 1258.05 | 1279.689085 | 1.014556 |
| GO:0003018\_vascular\_process\_in\_circulatory\_system | 13 | 0 | 0.000000 | 0.000000 | 1240 | 1236.410915 | 1258.05 | 1279.689085 | 1.014556 |
| GO:0006635\_fatty\_acid\_beta-oxidation | 13 | 0 | 0.000000 | 0.000000 | 1240 | 1236.410915 | 1258.05 | 1279.689085 | 1.014556 |
| GO:0006687\_glycosphingolipid\_metabolic\_process | 13 | 0 | 0.000000 | 0.000000 | 1240 | 1236.410915 | 1258.05 | 1279.689085 | 1.014556 |
| GO:0006767\_water-soluble\_vitamin\_metabolic\_process | 13 | 0 | 0.000000 | 0.000000 | 1240 | 1236.410915 | 1258.05 | 1279.689085 | 1.014556 |
| GO:0006885\_regulation\_of\_pH | 13 | 0 | 0.000000 | 0.000000 | 1240 | 1236.410915 | 1258.05 | 1279.689085 | 1.014556 |
| GO:0007033\_vacuole\_organization\_and\_biogenesis | 13 | 0 | 0.000000 | 0.000000 | 1240 | 1236.410915 | 1258.05 | 1279.689085 | 1.014556 |
| GO:0007034\_vacuolar\_transport | 13 | 0 | 0.000000 | 0.000000 | 1240 | 1236.410915 | 1258.05 | 1279.689085 | 1.014556 |
| GO:0007189\_G-protein\_signaling\_\_adenylate\_cyclase\_activating\_pathway | 13 | 0 | 0.000000 | 0.000000 | 1240 | 1236.410915 | 1258.05 | 1279.689085 | 1.014556 |
| GO:0007260\_tyrosine\_phosphorylation\_of\_STAT\_protein | 13 | 0 | 0.000000 | 0.000000 | 1240 | 1236.410915 | 1258.05 | 1279.689085 | 1.014556 |
| GO:0007269\_neurotransmitter\_secretion | 13 | 0 | 0.000000 | 0.000000 | 1240 | 1236.410915 | 1258.05 | 1279.689085 | 1.014556 |
| GO:0007272\_ensheathment\_of\_neurons | 13 | 0 | 0.000000 | 0.000000 | 1240 | 1236.410915 | 1258.05 | 1279.689085 | 1.014556 |
| GO:0007623\_circadian\_rhythm | 13 | 0 | 0.000000 | 0.000000 | 1240 | 1236.410915 | 1258.05 | 1279.689085 | 1.014556 |
| GO:0008033\_tRNA\_processing | 13 | 0 | 0.000000 | 0.000000 | 1240 | 1236.410915 | 1258.05 | 1279.689085 | 1.014556 |
| GO:0008037\_cell\_recognition | 13 | 0 | 0.000000 | 0.000000 | 1240 | 1236.410915 | 1258.05 | 1279.689085 | 1.014556 |
| GO:0008156\_negative\_regulation\_of\_DNA\_replication | 13 | 0 | 0.000000 | 0.000000 | 1240 | 1236.410915 | 1258.05 | 1279.689085 | 1.014556 |
| GO:0008366\_axon\_ensheathment | 13 | 0 | 0.000000 | 0.000000 | 1240 | 1236.410915 | 1258.05 | 1279.689085 | 1.014556 |
| GO:0008630\_DNA\_damage\_response\_\_signal\_transduction\_resulting\_in\_induction\_of\_apoptosis | 13 | 0 | 0.000000 | 0.000000 | 1240 | 1236.410915 | 1258.05 | 1279.689085 | 1.014556 |
| GO:0009108\_coenzyme\_biosynthetic\_process | 13 | 0 | 0.000000 | 0.000000 | 1240 | 1236.410915 | 1258.05 | 1279.689085 | 1.014556 |
| GO:0015918\_sterol\_transport | 13 | 0 | 0.000000 | 0.000000 | 1240 | 1236.410915 | 1258.05 | 1279.689085 | 1.014556 |
| GO:0019216\_regulation\_of\_lipid\_metabolic\_process | 13 | 0 | 0.000000 | 0.000000 | 1240 | 1236.410915 | 1258.05 | 1279.689085 | 1.014556 |
| GO:0030162\_regulation\_of\_proteolysis | 13 | 0 | 0.000000 | 0.000000 | 1240 | 1236.410915 | 1258.05 | 1279.689085 | 1.014556 |
| GO:0030301\_cholesterol\_transport | 13 | 0 | 0.000000 | 0.000000 | 1240 | 1236.410915 | 1258.05 | 1279.689085 | 1.014556 |
| GO:0030330\_DNA\_damage\_response\_\_signal\_transduction\_by\_p53\_class\_mediator | 13 | 0 | 0.000000 | 0.000000 | 1240 | 1236.410915 | 1258.05 | 1279.689085 | 1.014556 |
| GO:0031123\_RNA\_3'-end\_processing | 13 | 0 | 0.000000 | 0.000000 | 1240 | 1236.410915 | 1258.05 | 1279.689085 | 1.014556 |
| GO:0032318\_regulation\_of\_Ras\_GTPase\_activity | 13 | 0 | 0.000000 | 0.000000 | 1240 | 1236.410915 | 1258.05 | 1279.689085 | 1.014556 |
| GO:0032319\_regulation\_of\_Rho\_GTPase\_activity | 13 | 0 | 0.000000 | 0.000000 | 1240 | 1236.410915 | 1258.05 | 1279.689085 | 1.014556 |
| GO:0034329\_cell\_junction\_assembly | 13 | 0 | 0.000000 | 0.000000 | 1240 | 1236.410915 | 1258.05 | 1279.689085 | 1.014556 |
| GO:0043574\_peroxisomal\_transport | 13 | 0 | 0.000000 | 0.000000 | 1240 | 1236.410915 | 1258.05 | 1279.689085 | 1.014556 |
| GO:0043624\_cellular\_protein\_complex\_disassembly | 13 | 0 | 0.000000 | 0.000000 | 1240 | 1236.410915 | 1258.05 | 1279.689085 | 1.014556 |
| GO:0045840\_positive\_regulation\_of\_mitosis | 13 | 0 | 0.000000 | 0.000000 | 1240 | 1236.410915 | 1258.05 | 1279.689085 | 1.014556 |
| GO:0046425\_regulation\_of\_JAK-STAT\_cascade | 13 | 0 | 0.000000 | 0.000000 | 1240 | 1236.410915 | 1258.05 | 1279.689085 | 1.014556 |
| GO:0046661\_male\_sex\_differentiation | 13 | 0 | 0.000000 | 0.000000 | 1240 | 1236.410915 | 1258.05 | 1279.689085 | 1.014556 |
| GO:0046883\_regulation\_of\_hormone\_secretion | 13 | 0 | 0.000000 | 0.000000 | 1240 | 1236.410915 | 1258.05 | 1279.689085 | 1.014556 |
| GO:0050663\_cytokine\_secretion | 13 | 0 | 0.000000 | 0.000000 | 1240 | 1236.410915 | 1258.05 | 1279.689085 | 1.014556 |
| GO:0050906\_detection\_of\_stimulus\_involved\_in\_sensory\_perception | 13 | 0 | 0.000000 | 0.000000 | 1240 | 1236.410915 | 1258.05 | 1279.689085 | 1.014556 |
| GO:0051180\_vitamin\_transport | 13 | 0 | 0.000000 | 0.000000 | 1240 | 1236.410915 | 1258.05 | 1279.689085 | 1.014556 |
| GO:0051271\_negative\_regulation\_of\_cell\_motility | 13 | 0 | 0.000000 | 0.000000 | 1240 | 1236.410915 | 1258.05 | 1279.689085 | 1.014556 |
| GO:0051607\_defense\_response\_to\_virus | 13 | 0 | 0.000000 | 0.000000 | 1240 | 1236.410915 | 1258.05 | 1279.689085 | 1.014556 |
| GO:0002521\_leukocyte\_differentiation | 47 | 0 | 0.000000 | 0.000000 | 1241 | 1237.347903 | 1258.79 | 1280.232097 | 1.014335 |
| GO:0006631\_fatty\_acid\_metabolic\_process | 67 | 0 | 0.000000 | 0.000000 | 1242 | 1242.210130 | 1262.9 | 1283.589870 | 1.016828 |
| GO:0000018\_regulation\_of\_DNA\_recombination | 10 | 0 | 0.000000 | 0.000000 | 1322 | 1324.273001 | 1343.36 | 1362.446999 | 1.016157 |
| GO:0000060\_protein\_import\_into\_nucleus\_\_translocation | 10 | 0 | 0.000000 | 0.000000 | 1322 | 1324.273001 | 1343.36 | 1362.446999 | 1.016157 |
| GO:0000271\_polysaccharide\_biosynthetic\_process | 10 | 0 | 0.000000 | 0.000000 | 1322 | 1324.273001 | 1343.36 | 1362.446999 | 1.016157 |
| GO:0001541\_ovarian\_follicle\_development | 10 | 0 | 0.000000 | 0.000000 | 1322 | 1324.273001 | 1343.36 | 1362.446999 | 1.016157 |
| GO:0001709\_cell\_fate\_determination | 10 | 0 | 0.000000 | 0.000000 | 1322 | 1324.273001 | 1343.36 | 1362.446999 | 1.016157 |
| GO:0001906\_cell\_killing | 10 | 0 | 0.000000 | 0.000000 | 1322 | 1324.273001 | 1343.36 | 1362.446999 | 1.016157 |
| GO:0001936\_regulation\_of\_endothelial\_cell\_proliferation | 10 | 0 | 0.000000 | 0.000000 | 1322 | 1324.273001 | 1343.36 | 1362.446999 | 1.016157 |
| GO:0002526\_acute\_inflammatory\_response | 10 | 0 | 0.000000 | 0.000000 | 1322 | 1324.273001 | 1343.36 | 1362.446999 | 1.016157 |
| GO:0002761\_regulation\_of\_myeloid\_leukocyte\_differentiation | 10 | 0 | 0.000000 | 0.000000 | 1322 | 1324.273001 | 1343.36 | 1362.446999 | 1.016157 |
| GO:0006044\_N-acetylglucosamine\_metabolic\_process | 10 | 0 | 0.000000 | 0.000000 | 1322 | 1324.273001 | 1343.36 | 1362.446999 | 1.016157 |
| GO:0006073\_glucan\_metabolic\_process | 10 | 0 | 0.000000 | 0.000000 | 1322 | 1324.273001 | 1343.36 | 1362.446999 | 1.016157 |
| GO:0006305\_DNA\_alkylation | 10 | 0 | 0.000000 | 0.000000 | 1322 | 1324.273001 | 1343.36 | 1362.446999 | 1.016157 |
| GO:0006306\_DNA\_methylation | 10 | 0 | 0.000000 | 0.000000 | 1322 | 1324.273001 | 1343.36 | 1362.446999 | 1.016157 |
| GO:0006638\_neutral\_lipid\_metabolic\_process | 10 | 0 | 0.000000 | 0.000000 | 1322 | 1324.273001 | 1343.36 | 1362.446999 | 1.016157 |
| GO:0006639\_acylglycerol\_metabolic\_process | 10 | 0 | 0.000000 | 0.000000 | 1322 | 1324.273001 | 1343.36 | 1362.446999 | 1.016157 |
| GO:0006720\_isoprenoid\_metabolic\_process | 10 | 0 | 0.000000 | 0.000000 | 1322 | 1324.273001 | 1343.36 | 1362.446999 | 1.016157 |
| GO:0006733\_oxidoreduction\_coenzyme\_metabolic\_process | 10 | 0 | 0.000000 | 0.000000 | 1322 | 1324.273001 | 1343.36 | 1362.446999 | 1.016157 |
| GO:0006783\_heme\_biosynthetic\_process | 10 | 0 | 0.000000 | 0.000000 | 1322 | 1324.273001 | 1343.36 | 1362.446999 | 1.016157 |
| GO:0006805\_xenobiotic\_metabolic\_process | 10 | 0 | 0.000000 | 0.000000 | 1322 | 1324.273001 | 1343.36 | 1362.446999 | 1.016157 |
| GO:0006821\_chloride\_transport | 10 | 0 | 0.000000 | 0.000000 | 1322 | 1324.273001 | 1343.36 | 1362.446999 | 1.016157 |
| GO:0006836\_neurotransmitter\_transport | 10 | 0 | 0.000000 | 0.000000 | 1322 | 1324.273001 | 1343.36 | 1362.446999 | 1.016157 |
| GO:0006890\_retrograde\_vesicle-mediated\_transport\_\_Golgi\_to\_ER | 10 | 0 | 0.000000 | 0.000000 | 1322 | 1324.273001 | 1343.36 | 1362.446999 | 1.016157 |
| GO:0006903\_vesicle\_targeting | 10 | 0 | 0.000000 | 0.000000 | 1322 | 1324.273001 | 1343.36 | 1362.446999 | 1.016157 |
| GO:0006914\_autophagy | 10 | 0 | 0.000000 | 0.000000 | 1322 | 1324.273001 | 1343.36 | 1362.446999 | 1.016157 |
| GO:0007041\_lysosomal\_transport | 10 | 0 | 0.000000 | 0.000000 | 1322 | 1324.273001 | 1343.36 | 1362.446999 | 1.016157 |
| GO:0007076\_mitotic\_chromosome\_condensation | 10 | 0 | 0.000000 | 0.000000 | 1322 | 1324.273001 | 1343.36 | 1362.446999 | 1.016157 |
| GO:0007190\_activation\_of\_adenylate\_cyclase\_activity | 10 | 0 | 0.000000 | 0.000000 | 1322 | 1324.273001 | 1343.36 | 1362.446999 | 1.016157 |
| GO:0007202\_activation\_of\_phospholipase\_C\_activity | 10 | 0 | 0.000000 | 0.000000 | 1322 | 1324.273001 | 1343.36 | 1362.446999 | 1.016157 |
| GO:0007205\_activation\_of\_protein\_kinase\_C\_activity | 10 | 0 | 0.000000 | 0.000000 | 1322 | 1324.273001 | 1343.36 | 1362.446999 | 1.016157 |
| GO:0007215\_glutamate\_signaling\_pathway | 10 | 0 | 0.000000 | 0.000000 | 1322 | 1324.273001 | 1343.36 | 1362.446999 | 1.016157 |
| GO:0007218\_neuropeptide\_signaling\_pathway | 10 | 0 | 0.000000 | 0.000000 | 1322 | 1324.273001 | 1343.36 | 1362.446999 | 1.016157 |
| GO:0007281\_germ\_cell\_development | 10 | 0 | 0.000000 | 0.000000 | 1322 | 1324.273001 | 1343.36 | 1362.446999 | 1.016157 |
| GO:0007423\_sensory\_organ\_development | 10 | 0 | 0.000000 | 0.000000 | 1322 | 1324.273001 | 1343.36 | 1362.446999 | 1.016157 |
| GO:0007569\_cell\_aging | 10 | 0 | 0.000000 | 0.000000 | 1322 | 1324.273001 | 1343.36 | 1362.446999 | 1.016157 |
| GO:0007584\_response\_to\_nutrient | 10 | 0 | 0.000000 | 0.000000 | 1322 | 1324.273001 | 1343.36 | 1362.446999 | 1.016157 |
| GO:0007602\_phototransduction | 10 | 0 | 0.000000 | 0.000000 | 1322 | 1324.273001 | 1343.36 | 1362.446999 | 1.016157 |
| GO:0008625\_induction\_of\_apoptosis\_via\_death\_domain\_receptors | 10 | 0 | 0.000000 | 0.000000 | 1322 | 1324.273001 | 1343.36 | 1362.446999 | 1.016157 |
| GO:0009066\_aspartate\_family\_amino\_acid\_metabolic\_process | 10 | 0 | 0.000000 | 0.000000 | 1322 | 1324.273001 | 1343.36 | 1362.446999 | 1.016157 |
| GO:0009190\_cyclic\_nucleotide\_biosynthetic\_process | 10 | 0 | 0.000000 | 0.000000 | 1322 | 1324.273001 | 1343.36 | 1362.446999 | 1.016157 |
| GO:0009247\_glycolipid\_biosynthetic\_process | 10 | 0 | 0.000000 | 0.000000 | 1322 | 1324.273001 | 1343.36 | 1362.446999 | 1.016157 |
| GO:0009260\_ribonucleotide\_biosynthetic\_process | 10 | 0 | 0.000000 | 0.000000 | 1322 | 1324.273001 | 1343.36 | 1362.446999 | 1.016157 |
| GO:0009583\_detection\_of\_light\_stimulus | 10 | 0 | 0.000000 | 0.000000 | 1322 | 1324.273001 | 1343.36 | 1362.446999 | 1.016157 |
| GO:0009584\_detection\_of\_visible\_light | 10 | 0 | 0.000000 | 0.000000 | 1322 | 1324.273001 | 1343.36 | 1362.446999 | 1.016157 |
| GO:0009880\_embryonic\_pattern\_specification | 10 | 0 | 0.000000 | 0.000000 | 1322 | 1324.273001 | 1343.36 | 1362.446999 | 1.016157 |
| GO:0009952\_anterior\_posterior\_pattern\_formation | 10 | 0 | 0.000000 | 0.000000 | 1322 | 1324.273001 | 1343.36 | 1362.446999 | 1.016157 |
| GO:0015718\_monocarboxylic\_acid\_transport | 10 | 0 | 0.000000 | 0.000000 | 1322 | 1324.273001 | 1343.36 | 1362.446999 | 1.016157 |
| GO:0016050\_vesicle\_organization\_and\_biogenesis | 10 | 0 | 0.000000 | 0.000000 | 1322 | 1324.273001 | 1343.36 | 1362.446999 | 1.016157 |
| GO:0016073\_snRNA\_metabolic\_process | 10 | 0 | 0.000000 | 0.000000 | 1322 | 1324.273001 | 1343.36 | 1362.446999 | 1.016157 |
| GO:0016180\_snRNA\_processing | 10 | 0 | 0.000000 | 0.000000 | 1322 | 1324.273001 | 1343.36 | 1362.446999 | 1.016157 |
| GO:0016558\_protein\_import\_into\_peroxisome\_matrix | 10 | 0 | 0.000000 | 0.000000 | 1322 | 1324.273001 | 1343.36 | 1362.446999 | 1.016157 |
| GO:0022602\_ovulation\_cycle\_process | 10 | 0 | 0.000000 | 0.000000 | 1322 | 1324.273001 | 1343.36 | 1362.446999 | 1.016157 |
| GO:0030048\_actin\_filament-based\_movement | 10 | 0 | 0.000000 | 0.000000 | 1322 | 1324.273001 | 1343.36 | 1362.446999 | 1.016157 |
| GO:0030101\_natural\_killer\_cell\_activation | 10 | 0 | 0.000000 | 0.000000 | 1322 | 1324.273001 | 1343.36 | 1362.446999 | 1.016157 |
| GO:0030168\_platelet\_activation | 10 | 0 | 0.000000 | 0.000000 | 1322 | 1324.273001 | 1343.36 | 1362.446999 | 1.016157 |
| GO:0031365\_N-terminal\_protein\_amino\_acid\_modification | 10 | 0 | 0.000000 | 0.000000 | 1322 | 1324.273001 | 1343.36 | 1362.446999 | 1.016157 |
| GO:0031575\_G1\_S\_transition\_checkpoint | 10 | 0 | 0.000000 | 0.000000 | 1322 | 1324.273001 | 1343.36 | 1362.446999 | 1.016157 |
| GO:0032388\_positive\_regulation\_of\_intracellular\_transport | 10 | 0 | 0.000000 | 0.000000 | 1322 | 1324.273001 | 1343.36 | 1362.446999 | 1.016157 |
| GO:0032488\_Cdc42\_protein\_signal\_transduction | 10 | 0 | 0.000000 | 0.000000 | 1322 | 1324.273001 | 1343.36 | 1362.446999 | 1.016157 |
| GO:0033692\_cellular\_polysaccharide\_biosynthetic\_process | 10 | 0 | 0.000000 | 0.000000 | 1322 | 1324.273001 | 1343.36 | 1362.446999 | 1.016157 |
| GO:0042063\_gliogenesis | 10 | 0 | 0.000000 | 0.000000 | 1322 | 1324.273001 | 1343.36 | 1362.446999 | 1.016157 |
| GO:0042102\_positive\_regulation\_of\_T\_cell\_proliferation | 10 | 0 | 0.000000 | 0.000000 | 1322 | 1324.273001 | 1343.36 | 1362.446999 | 1.016157 |
| GO:0042345\_regulation\_of\_NF-kappaB\_import\_into\_nucleus | 10 | 0 | 0.000000 | 0.000000 | 1322 | 1324.273001 | 1343.36 | 1362.446999 | 1.016157 |
| GO:0042348\_NF-kappaB\_import\_into\_nucleus | 10 | 0 | 0.000000 | 0.000000 | 1322 | 1324.273001 | 1343.36 | 1362.446999 | 1.016157 |
| GO:0042552\_myelination | 10 | 0 | 0.000000 | 0.000000 | 1322 | 1324.273001 | 1343.36 | 1362.446999 | 1.016157 |
| GO:0042698\_ovulation\_cycle | 10 | 0 | 0.000000 | 0.000000 | 1322 | 1324.273001 | 1343.36 | 1362.446999 | 1.016157 |
| GO:0042982\_amyloid\_precursor\_protein\_metabolic\_process | 10 | 0 | 0.000000 | 0.000000 | 1322 | 1324.273001 | 1343.36 | 1362.446999 | 1.016157 |
| GO:0045638\_negative\_regulation\_of\_myeloid\_cell\_differentiation | 10 | 0 | 0.000000 | 0.000000 | 1322 | 1324.273001 | 1343.36 | 1362.446999 | 1.016157 |
| GO:0045814\_negative\_regulation\_of\_gene\_expression\_\_epigenetic | 10 | 0 | 0.000000 | 0.000000 | 1322 | 1324.273001 | 1343.36 | 1362.446999 | 1.016157 |
| GO:0048144\_fibroblast\_proliferation | 10 | 0 | 0.000000 | 0.000000 | 1322 | 1324.273001 | 1343.36 | 1362.446999 | 1.016157 |
| GO:0048145\_regulation\_of\_fibroblast\_proliferation | 10 | 0 | 0.000000 | 0.000000 | 1322 | 1324.273001 | 1343.36 | 1362.446999 | 1.016157 |
| GO:0048585\_negative\_regulation\_of\_response\_to\_stimulus | 10 | 0 | 0.000000 | 0.000000 | 1322 | 1324.273001 | 1343.36 | 1362.446999 | 1.016157 |
| GO:0050715\_positive\_regulation\_of\_cytokine\_secretion | 10 | 0 | 0.000000 | 0.000000 | 1322 | 1324.273001 | 1343.36 | 1362.446999 | 1.016157 |
| GO:0050770\_regulation\_of\_axonogenesis | 10 | 0 | 0.000000 | 0.000000 | 1322 | 1324.273001 | 1343.36 | 1362.446999 | 1.016157 |
| GO:0050804\_regulation\_of\_synaptic\_transmission | 10 | 0 | 0.000000 | 0.000000 | 1322 | 1324.273001 | 1343.36 | 1362.446999 | 1.016157 |
| GO:0050908\_detection\_of\_light\_stimulus\_involved\_in\_visual\_perception | 10 | 0 | 0.000000 | 0.000000 | 1322 | 1324.273001 | 1343.36 | 1362.446999 | 1.016157 |
| GO:0050962\_detection\_of\_light\_stimulus\_involved\_in\_sensory\_perception | 10 | 0 | 0.000000 | 0.000000 | 1322 | 1324.273001 | 1343.36 | 1362.446999 | 1.016157 |
| GO:0051187\_cofactor\_catabolic\_process | 10 | 0 | 0.000000 | 0.000000 | 1322 | 1324.273001 | 1343.36 | 1362.446999 | 1.016157 |
| GO:0051604\_protein\_maturation | 10 | 0 | 0.000000 | 0.000000 | 1322 | 1324.273001 | 1343.36 | 1362.446999 | 1.016157 |
| GO:0051650\_establishment\_of\_vesicle\_localization | 10 | 0 | 0.000000 | 0.000000 | 1322 | 1324.273001 | 1343.36 | 1362.446999 | 1.016157 |
| GO:0051969\_regulation\_of\_transmission\_of\_nerve\_impulse | 10 | 0 | 0.000000 | 0.000000 | 1322 | 1324.273001 | 1343.36 | 1362.446999 | 1.016157 |
| GO:0007169\_transmembrane\_receptor\_protein\_tyrosine\_kinase\_signaling\_pathway | 109 | 0 | 0.000000 | 0.000000 | 1323 | 1324.805699 | 1343.87 | 1362.934301 | 1.015775 |
| GO:0000122\_negative\_regulation\_of\_transcription\_from\_RNA\_polymerase\_II\_promoter | 83 | 0 | 0.000000 | 0.000000 | 1329 | 1328.606238 | 1347.58 | 1366.553762 | 1.013980 |
| GO:0006897\_endocytosis | 83 | 0 | 0.000000 | 0.000000 | 1329 | 1328.606238 | 1347.58 | 1366.553762 | 1.013980 |
| GO:0007283\_spermatogenesis | 83 | 0 | 0.000000 | 0.000000 | 1329 | 1328.606238 | 1347.58 | 1366.553762 | 1.013980 |
| GO:0010324\_membrane\_invagination | 83 | 0 | 0.000000 | 0.000000 | 1329 | 1328.606238 | 1347.58 | 1366.553762 | 1.013980 |
| GO:0043122\_regulation\_of\_I-kappaB\_kinase\_NF-kappaB\_cascade | 83 | 0 | 0.000000 | 0.000000 | 1329 | 1328.606238 | 1347.58 | 1366.553762 | 1.013980 |
| GO:0048232\_male\_gamete\_generation | 83 | 0 | 0.000000 | 0.000000 | 1329 | 1328.606238 | 1347.58 | 1366.553762 | 1.013980 |
| GO:0001503\_ossification | 27 | 0 | 0.000000 | 0.000000 | 1342 | 1341.750976 | 1360.08 | 1378.409024 | 1.013472 |
| GO:0001666\_response\_to\_hypoxia | 27 | 0 | 0.000000 | 0.000000 | 1342 | 1341.750976 | 1360.08 | 1378.409024 | 1.013472 |
| GO:0006401\_RNA\_catabolic\_process | 27 | 0 | 0.000000 | 0.000000 | 1342 | 1341.750976 | 1360.08 | 1378.409024 | 1.013472 |
| GO:0006694\_steroid\_biosynthetic\_process | 27 | 0 | 0.000000 | 0.000000 | 1342 | 1341.750976 | 1360.08 | 1378.409024 | 1.013472 |
| GO:0008624\_induction\_of\_apoptosis\_by\_extracellular\_signals | 27 | 0 | 0.000000 | 0.000000 | 1342 | 1341.750976 | 1360.08 | 1378.409024 | 1.013472 |
| GO:0009165\_nucleotide\_biosynthetic\_process | 27 | 0 | 0.000000 | 0.000000 | 1342 | 1341.750976 | 1360.08 | 1378.409024 | 1.013472 |
| GO:0009306\_protein\_secretion | 27 | 0 | 0.000000 | 0.000000 | 1342 | 1341.750976 | 1360.08 | 1378.409024 | 1.013472 |
| GO:0016570\_histone\_modification | 27 | 0 | 0.000000 | 0.000000 | 1342 | 1341.750976 | 1360.08 | 1378.409024 | 1.013472 |
| GO:0031214\_biomineral\_formation | 27 | 0 | 0.000000 | 0.000000 | 1342 | 1341.750976 | 1360.08 | 1378.409024 | 1.013472 |
| GO:0042157\_lipoprotein\_metabolic\_process | 27 | 0 | 0.000000 | 0.000000 | 1342 | 1341.750976 | 1360.08 | 1378.409024 | 1.013472 |
| GO:0044271\_nitrogen\_compound\_biosynthetic\_process | 27 | 0 | 0.000000 | 0.000000 | 1342 | 1341.750976 | 1360.08 | 1378.409024 | 1.013472 |
| GO:0046164\_alcohol\_catabolic\_process | 27 | 0 | 0.000000 | 0.000000 | 1342 | 1341.750976 | 1360.08 | 1378.409024 | 1.013472 |
| GO:0046365\_monosaccharide\_catabolic\_process | 27 | 0 | 0.000000 | 0.000000 | 1342 | 1341.750976 | 1360.08 | 1378.409024 | 1.013472 |
| GO:0000245\_spliceosome\_assembly | 25 | 0 | 0.000000 | 0.000000 | 1352 | 1350.553476 | 1368.63 | 1386.706524 | 1.012300 |
| GO:0006446\_regulation\_of\_translational\_initiation | 25 | 0 | 0.000000 | 0.000000 | 1352 | 1350.553476 | 1368.63 | 1386.706524 | 1.012300 |
| GO:0006997\_nuclear\_organization\_and\_biogenesis | 25 | 0 | 0.000000 | 0.000000 | 1352 | 1350.553476 | 1368.63 | 1386.706524 | 1.012300 |
| GO:0007015\_actin\_filament\_organization | 25 | 0 | 0.000000 | 0.000000 | 1352 | 1350.553476 | 1368.63 | 1386.706524 | 1.012300 |
| GO:0007588\_excretion | 25 | 0 | 0.000000 | 0.000000 | 1352 | 1350.553476 | 1368.63 | 1386.706524 | 1.012300 |
| GO:0030521\_androgen\_receptor\_signaling\_pathway | 25 | 0 | 0.000000 | 0.000000 | 1352 | 1350.553476 | 1368.63 | 1386.706524 | 1.012300 |
| GO:0045765\_regulation\_of\_angiogenesis | 25 | 0 | 0.000000 | 0.000000 | 1352 | 1350.553476 | 1368.63 | 1386.706524 | 1.012300 |
| GO:0051056\_regulation\_of\_small\_GTPase\_mediated\_signal\_transduction | 25 | 0 | 0.000000 | 0.000000 | 1352 | 1350.553476 | 1368.63 | 1386.706524 | 1.012300 |
| GO:0051099\_positive\_regulation\_of\_binding | 25 | 0 | 0.000000 | 0.000000 | 1352 | 1350.553476 | 1368.63 | 1386.706524 | 1.012300 |
| GO:0051188\_cofactor\_biosynthetic\_process | 25 | 0 | 0.000000 | 0.000000 | 1352 | 1350.553476 | 1368.63 | 1386.706524 | 1.012300 |
| GO:0006725\_aromatic\_compound\_metabolic\_process | 30 | 0 | 0.000000 | 0.000000 | 1362 | 1358.707606 | 1376.55 | 1394.392394 | 1.010683 |
| GO:0006730\_one-carbon\_compound\_metabolic\_process | 30 | 0 | 0.000000 | 0.000000 | 1362 | 1358.707606 | 1376.55 | 1394.392394 | 1.010683 |
| GO:0007389\_pattern\_specification\_process | 30 | 0 | 0.000000 | 0.000000 | 1362 | 1358.707606 | 1376.55 | 1394.392394 | 1.010683 |
| GO:0007507\_heart\_development | 30 | 0 | 0.000000 | 0.000000 | 1362 | 1358.707606 | 1376.55 | 1394.392394 | 1.010683 |
| GO:0016125\_sterol\_metabolic\_process | 30 | 0 | 0.000000 | 0.000000 | 1362 | 1358.707606 | 1376.55 | 1394.392394 | 1.010683 |
| GO:0030705\_cytoskeleton-dependent\_intracellular\_transport | 30 | 0 | 0.000000 | 0.000000 | 1362 | 1358.707606 | 1376.55 | 1394.392394 | 1.010683 |
| GO:0042035\_regulation\_of\_cytokine\_biosynthetic\_process | 30 | 0 | 0.000000 | 0.000000 | 1362 | 1358.707606 | 1376.55 | 1394.392394 | 1.010683 |
| GO:0044270\_nitrogen\_compound\_catabolic\_process | 30 | 0 | 0.000000 | 0.000000 | 1362 | 1358.707606 | 1376.55 | 1394.392394 | 1.010683 |
| GO:0046483\_heterocycle\_metabolic\_process | 30 | 0 | 0.000000 | 0.000000 | 1362 | 1358.707606 | 1376.55 | 1394.392394 | 1.010683 |
| GO:0051606\_detection\_of\_stimulus | 30 | 0 | 0.000000 | 0.000000 | 1362 | 1358.707606 | 1376.55 | 1394.392394 | 1.010683 |
| GO:0000723\_telomere\_maintenance | 21 | 0 | 0.000000 | 0.000000 | 1386 | 1383.691911 | 1400.39 | 1417.088089 | 1.010382 |
| GO:0002250\_adaptive\_immune\_response | 21 | 0 | 0.000000 | 0.000000 | 1386 | 1383.691911 | 1400.39 | 1417.088089 | 1.010382 |
| GO:0006275\_regulation\_of\_DNA\_replication | 21 | 0 | 0.000000 | 0.000000 | 1386 | 1383.691911 | 1400.39 | 1417.088089 | 1.010382 |
| GO:0006937\_regulation\_of\_muscle\_contraction | 21 | 0 | 0.000000 | 0.000000 | 1386 | 1383.691911 | 1400.39 | 1417.088089 | 1.010382 |
| GO:0007127\_meiosis\_I | 21 | 0 | 0.000000 | 0.000000 | 1386 | 1383.691911 | 1400.39 | 1417.088089 | 1.010382 |
| GO:0007156\_homophilic\_cell\_adhesion | 21 | 0 | 0.000000 | 0.000000 | 1386 | 1383.691911 | 1400.39 | 1417.088089 | 1.010382 |
| GO:0007162\_negative\_regulation\_of\_cell\_adhesion | 21 | 0 | 0.000000 | 0.000000 | 1386 | 1383.691911 | 1400.39 | 1417.088089 | 1.010382 |
| GO:0007229\_integrin-mediated\_signaling\_pathway | 21 | 0 | 0.000000 | 0.000000 | 1386 | 1383.691911 | 1400.39 | 1417.088089 | 1.010382 |
| GO:0008406\_gonad\_development | 21 | 0 | 0.000000 | 0.000000 | 1386 | 1383.691911 | 1400.39 | 1417.088089 | 1.010382 |
| GO:0010558\_negative\_regulation\_of\_macromolecule\_biosynthetic\_process | 21 | 0 | 0.000000 | 0.000000 | 1386 | 1383.691911 | 1400.39 | 1417.088089 | 1.010382 |
| GO:0015698\_inorganic\_anion\_transport | 21 | 0 | 0.000000 | 0.000000 | 1386 | 1383.691911 | 1400.39 | 1417.088089 | 1.010382 |
| GO:0015992\_proton\_transport | 21 | 0 | 0.000000 | 0.000000 | 1386 | 1383.691911 | 1400.39 | 1417.088089 | 1.010382 |
| GO:0016072\_rRNA\_metabolic\_process | 21 | 0 | 0.000000 | 0.000000 | 1386 | 1383.691911 | 1400.39 | 1417.088089 | 1.010382 |
| GO:0031647\_regulation\_of\_protein\_stability | 21 | 0 | 0.000000 | 0.000000 | 1386 | 1383.691911 | 1400.39 | 1417.088089 | 1.010382 |
| GO:0032200\_telomere\_organization\_and\_biogenesis | 21 | 0 | 0.000000 | 0.000000 | 1386 | 1383.691911 | 1400.39 | 1417.088089 | 1.010382 |
| GO:0032943\_mononuclear\_cell\_proliferation | 21 | 0 | 0.000000 | 0.000000 | 1386 | 1383.691911 | 1400.39 | 1417.088089 | 1.010382 |
| GO:0042108\_positive\_regulation\_of\_cytokine\_biosynthetic\_process | 21 | 0 | 0.000000 | 0.000000 | 1386 | 1383.691911 | 1400.39 | 1417.088089 | 1.010382 |
| GO:0044272\_sulfur\_compound\_biosynthetic\_process | 21 | 0 | 0.000000 | 0.000000 | 1386 | 1383.691911 | 1400.39 | 1417.088089 | 1.010382 |
| GO:0046578\_regulation\_of\_Ras\_protein\_signal\_transduction | 21 | 0 | 0.000000 | 0.000000 | 1386 | 1383.691911 | 1400.39 | 1417.088089 | 1.010382 |
| GO:0046651\_lymphocyte\_proliferation | 21 | 0 | 0.000000 | 0.000000 | 1386 | 1383.691911 | 1400.39 | 1417.088089 | 1.010382 |
| GO:0048608\_reproductive\_structure\_development | 21 | 0 | 0.000000 | 0.000000 | 1386 | 1383.691911 | 1400.39 | 1417.088089 | 1.010382 |
| GO:0048872\_homeostasis\_of\_number\_of\_cells | 21 | 0 | 0.000000 | 0.000000 | 1386 | 1383.691911 | 1400.39 | 1417.088089 | 1.010382 |
| GO:0051091\_positive\_regulation\_of\_transcription\_factor\_activity | 21 | 0 | 0.000000 | 0.000000 | 1386 | 1383.691911 | 1400.39 | 1417.088089 | 1.010382 |
| GO:0051251\_positive\_regulation\_of\_lymphocyte\_activation | 21 | 0 | 0.000000 | 0.000000 | 1386 | 1383.691911 | 1400.39 | 1417.088089 | 1.010382 |
| GO:0006029\_proteoglycan\_metabolic\_process | 24 | 0 | 0.000000 | 0.000000 | 1398 | 1396.122707 | 1412.22 | 1428.317293 | 1.010172 |
| GO:0006497\_protein\_amino\_acid\_lipidation | 24 | 0 | 0.000000 | 0.000000 | 1398 | 1396.122707 | 1412.22 | 1428.317293 | 1.010172 |
| GO:0008154\_actin\_polymerization\_and\_or\_depolymerization | 24 | 0 | 0.000000 | 0.000000 | 1398 | 1396.122707 | 1412.22 | 1428.317293 | 1.010172 |
| GO:0009411\_response\_to\_UV | 24 | 0 | 0.000000 | 0.000000 | 1398 | 1396.122707 | 1412.22 | 1428.317293 | 1.010172 |
| GO:0016053\_organic\_acid\_biosynthetic\_process | 24 | 0 | 0.000000 | 0.000000 | 1398 | 1396.122707 | 1412.22 | 1428.317293 | 1.010172 |
| GO:0032970\_regulation\_of\_actin\_filament-based\_process | 24 | 0 | 0.000000 | 0.000000 | 1398 | 1396.122707 | 1412.22 | 1428.317293 | 1.010172 |
| GO:0042158\_lipoprotein\_biosynthetic\_process | 24 | 0 | 0.000000 | 0.000000 | 1398 | 1396.122707 | 1412.22 | 1428.317293 | 1.010172 |
| GO:0042254\_ribosome\_biogenesis\_and\_assembly | 24 | 0 | 0.000000 | 0.000000 | 1398 | 1396.122707 | 1412.22 | 1428.317293 | 1.010172 |
| GO:0042391\_regulation\_of\_membrane\_potential | 24 | 0 | 0.000000 | 0.000000 | 1398 | 1396.122707 | 1412.22 | 1428.317293 | 1.010172 |
| GO:0046394\_carboxylic\_acid\_biosynthetic\_process | 24 | 0 | 0.000000 | 0.000000 | 1398 | 1396.122707 | 1412.22 | 1428.317293 | 1.010172 |
| GO:0048511\_rhythmic\_process | 24 | 0 | 0.000000 | 0.000000 | 1398 | 1396.122707 | 1412.22 | 1428.317293 | 1.010172 |
| GO:0050808\_synapse\_organization\_and\_biogenesis | 24 | 0 | 0.000000 | 0.000000 | 1398 | 1396.122707 | 1412.22 | 1428.317293 | 1.010172 |
| GO:0030097\_hemopoiesis | 89 | 0 | 0.000000 | 0.000000 | 1399 | 1397.447478 | 1413.32 | 1429.192522 | 1.010236 |
| GO:0006022\_aminoglycan\_metabolic\_process | 26 | 0 | 0.000000 | 0.000000 | 1416 | 1416.701677 | 1431.75 | 1446.798323 | 1.011123 |
| GO:0006302\_double-strand\_break\_repair | 26 | 0 | 0.000000 | 0.000000 | 1416 | 1416.701677 | 1431.75 | 1446.798323 | 1.011123 |
| GO:0006816\_calcium\_ion\_transport | 26 | 0 | 0.000000 | 0.000000 | 1416 | 1416.701677 | 1431.75 | 1446.798323 | 1.011123 |
| GO:0006865\_amino\_acid\_transport | 26 | 0 | 0.000000 | 0.000000 | 1416 | 1416.701677 | 1431.75 | 1446.798323 | 1.011123 |
| GO:0006959\_humoral\_immune\_response | 26 | 0 | 0.000000 | 0.000000 | 1416 | 1416.701677 | 1431.75 | 1446.798323 | 1.011123 |
| GO:0007173\_epidermal\_growth\_factor\_receptor\_signaling\_pathway | 26 | 0 | 0.000000 | 0.000000 | 1416 | 1416.701677 | 1431.75 | 1446.798323 | 1.011123 |
| GO:0007519\_skeletal\_muscle\_development | 26 | 0 | 0.000000 | 0.000000 | 1416 | 1416.701677 | 1431.75 | 1446.798323 | 1.011123 |
| GO:0008629\_induction\_of\_apoptosis\_by\_intracellular\_signals | 26 | 0 | 0.000000 | 0.000000 | 1416 | 1416.701677 | 1431.75 | 1446.798323 | 1.011123 |
| GO:0009063\_amino\_acid\_catabolic\_process | 26 | 0 | 0.000000 | 0.000000 | 1416 | 1416.701677 | 1431.75 | 1446.798323 | 1.011123 |
| GO:0019320\_hexose\_catabolic\_process | 26 | 0 | 0.000000 | 0.000000 | 1416 | 1416.701677 | 1431.75 | 1446.798323 | 1.011123 |
| GO:0019748\_secondary\_metabolic\_process | 26 | 0 | 0.000000 | 0.000000 | 1416 | 1416.701677 | 1431.75 | 1446.798323 | 1.011123 |
| GO:0030203\_glycosaminoglycan\_metabolic\_process | 26 | 0 | 0.000000 | 0.000000 | 1416 | 1416.701677 | 1431.75 | 1446.798323 | 1.011123 |
| GO:0045596\_negative\_regulation\_of\_cell\_differentiation | 26 | 0 | 0.000000 | 0.000000 | 1416 | 1416.701677 | 1431.75 | 1446.798323 | 1.011123 |
| GO:0045597\_positive\_regulation\_of\_cell\_differentiation | 26 | 0 | 0.000000 | 0.000000 | 1416 | 1416.701677 | 1431.75 | 1446.798323 | 1.011123 |
| GO:0050778\_positive\_regulation\_of\_immune\_response | 26 | 0 | 0.000000 | 0.000000 | 1416 | 1416.701677 | 1431.75 | 1446.798323 | 1.011123 |
| GO:0050863\_regulation\_of\_T\_cell\_activation | 26 | 0 | 0.000000 | 0.000000 | 1416 | 1416.701677 | 1431.75 | 1446.798323 | 1.011123 |
| GO:0051240\_positive\_regulation\_of\_multicellular\_organismal\_process | 26 | 0 | 0.000000 | 0.000000 | 1416 | 1416.701677 | 1431.75 | 1446.798323 | 1.011123 |
| GO:0006261\_DNA-dependent\_DNA\_replication | 49 | 0 | 0.000000 | 0.000000 | 1423 | 1422.851579 | 1437.4 | 1451.948421 | 1.010119 |
| GO:0006333\_chromatin\_assembly\_or\_disassembly | 49 | 0 | 0.000000 | 0.000000 | 1423 | 1422.851579 | 1437.4 | 1451.948421 | 1.010119 |
| GO:0006417\_regulation\_of\_translation | 49 | 0 | 0.000000 | 0.000000 | 1423 | 1422.851579 | 1437.4 | 1451.948421 | 1.010119 |
| GO:0007178\_transmembrane\_receptor\_protein\_serine\_threonine\_kinase\_signaling\_pathway | 49 | 0 | 0.000000 | 0.000000 | 1423 | 1422.851579 | 1437.4 | 1451.948421 | 1.010119 |
| GO:0007599\_hemostasis | 49 | 0 | 0.000000 | 0.000000 | 1423 | 1422.851579 | 1437.4 | 1451.948421 | 1.010119 |
| GO:0048667\_neuron\_morphogenesis\_during\_differentiation | 49 | 0 | 0.000000 | 0.000000 | 1423 | 1422.851579 | 1437.4 | 1451.948421 | 1.010119 |
| GO:0048812\_neurite\_morphogenesis | 49 | 0 | 0.000000 | 0.000000 | 1423 | 1422.851579 | 1437.4 | 1451.948421 | 1.010119 |
| GO:0000082\_G1\_S\_transition\_of\_mitotic\_cell\_cycle | 29 | 0 | 0.000000 | 0.000000 | 1435 | 1432.915049 | 1447.15 | 1461.384951 | 1.008467 |
| GO:0006487\_protein\_amino\_acid\_N-linked\_glycosylation | 29 | 0 | 0.000000 | 0.000000 | 1435 | 1432.915049 | 1447.15 | 1461.384951 | 1.008467 |
| GO:0006820\_anion\_transport | 29 | 0 | 0.000000 | 0.000000 | 1435 | 1432.915049 | 1447.15 | 1461.384951 | 1.008467 |
| GO:0006869\_lipid\_transport | 29 | 0 | 0.000000 | 0.000000 | 1435 | 1432.915049 | 1447.15 | 1461.384951 | 1.008467 |
| GO:0006898\_receptor-mediated\_endocytosis | 29 | 0 | 0.000000 | 0.000000 | 1435 | 1432.915049 | 1447.15 | 1461.384951 | 1.008467 |
| GO:0006944\_membrane\_fusion | 29 | 0 | 0.000000 | 0.000000 | 1435 | 1432.915049 | 1447.15 | 1461.384951 | 1.008467 |
| GO:0007188\_G-protein\_signaling\_\_coupled\_to\_cAMP\_nucleotide\_second\_messenger | 29 | 0 | 0.000000 | 0.000000 | 1435 | 1432.915049 | 1447.15 | 1461.384951 | 1.008467 |
| GO:0008203\_cholesterol\_metabolic\_process | 29 | 0 | 0.000000 | 0.000000 | 1435 | 1432.915049 | 1447.15 | 1461.384951 | 1.008467 |
| GO:0018108\_peptidyl-tyrosine\_phosphorylation | 29 | 0 | 0.000000 | 0.000000 | 1435 | 1432.915049 | 1447.15 | 1461.384951 | 1.008467 |
| GO:0030334\_regulation\_of\_cell\_migration | 29 | 0 | 0.000000 | 0.000000 | 1435 | 1432.915049 | 1447.15 | 1461.384951 | 1.008467 |
| GO:0042327\_positive\_regulation\_of\_phosphorylation | 29 | 0 | 0.000000 | 0.000000 | 1435 | 1432.915049 | 1447.15 | 1461.384951 | 1.008467 |
| GO:0042445\_hormone\_metabolic\_process | 29 | 0 | 0.000000 | 0.000000 | 1435 | 1432.915049 | 1447.15 | 1461.384951 | 1.008467 |
| GO:0006812\_cation\_transport | 133 | 0 | 0.000000 | 0.000000 | 1437 | 1434.276695 | 1448.22 | 1462.163305 | 1.007808 |
| GO:0019725\_cellular\_homeostasis | 133 | 0 | 0.000000 | 0.000000 | 1437 | 1434.276695 | 1448.22 | 1462.163305 | 1.007808 |
| GO:0000084\_S\_phase\_of\_mitotic\_cell\_cycle | 12 | 0 | 0.000000 | 0.000000 | 1497 | 1495.325022 | 1507.76 | 1520.194978 | 1.007188 |
| GO:0001935\_endothelial\_cell\_proliferation | 12 | 0 | 0.000000 | 0.000000 | 1497 | 1495.325022 | 1507.76 | 1520.194978 | 1.007188 |
| GO:0002274\_myeloid\_leukocyte\_activation | 12 | 0 | 0.000000 | 0.000000 | 1497 | 1495.325022 | 1507.76 | 1520.194978 | 1.007188 |
| GO:0006004\_fucose\_metabolic\_process | 12 | 0 | 0.000000 | 0.000000 | 1497 | 1495.325022 | 1507.76 | 1520.194978 | 1.007188 |
| GO:0006041\_glucosamine\_metabolic\_process | 12 | 0 | 0.000000 | 0.000000 | 1497 | 1495.325022 | 1507.76 | 1520.194978 | 1.007188 |
| GO:0006112\_energy\_reserve\_metabolic\_process | 12 | 0 | 0.000000 | 0.000000 | 1497 | 1495.325022 | 1507.76 | 1520.194978 | 1.007188 |
| GO:0006309\_DNA\_fragmentation\_during\_apoptosis | 12 | 0 | 0.000000 | 0.000000 | 1497 | 1495.325022 | 1507.76 | 1520.194978 | 1.007188 |
| GO:0006376\_mRNA\_splice\_site\_selection | 12 | 0 | 0.000000 | 0.000000 | 1497 | 1495.325022 | 1507.76 | 1520.194978 | 1.007188 |
| GO:0006402\_mRNA\_catabolic\_process | 12 | 0 | 0.000000 | 0.000000 | 1497 | 1495.325022 | 1507.76 | 1520.194978 | 1.007188 |
| GO:0006516\_glycoprotein\_catabolic\_process | 12 | 0 | 0.000000 | 0.000000 | 1497 | 1495.325022 | 1507.76 | 1520.194978 | 1.007188 |
| GO:0006607\_NLS-bearing\_substrate\_import\_into\_nucleus | 12 | 0 | 0.000000 | 0.000000 | 1497 | 1495.325022 | 1507.76 | 1520.194978 | 1.007188 |
| GO:0006611\_protein\_export\_from\_nucleus | 12 | 0 | 0.000000 | 0.000000 | 1497 | 1495.325022 | 1507.76 | 1520.194978 | 1.007188 |
| GO:0006625\_protein\_targeting\_to\_peroxisome | 12 | 0 | 0.000000 | 0.000000 | 1497 | 1495.325022 | 1507.76 | 1520.194978 | 1.007188 |
| GO:0006692\_prostanoid\_metabolic\_process | 12 | 0 | 0.000000 | 0.000000 | 1497 | 1495.325022 | 1507.76 | 1520.194978 | 1.007188 |
| GO:0006693\_prostaglandin\_metabolic\_process | 12 | 0 | 0.000000 | 0.000000 | 1497 | 1495.325022 | 1507.76 | 1520.194978 | 1.007188 |
| GO:0006779\_porphyrin\_biosynthetic\_process | 12 | 0 | 0.000000 | 0.000000 | 1497 | 1495.325022 | 1507.76 | 1520.194978 | 1.007188 |
| GO:0006879\_cellular\_iron\_ion\_homeostasis | 12 | 0 | 0.000000 | 0.000000 | 1497 | 1495.325022 | 1507.76 | 1520.194978 | 1.007188 |
| GO:0006909\_phagocytosis | 12 | 0 | 0.000000 | 0.000000 | 1497 | 1495.325022 | 1507.76 | 1520.194978 | 1.007188 |
| GO:0006939\_smooth\_muscle\_contraction | 12 | 0 | 0.000000 | 0.000000 | 1497 | 1495.325022 | 1507.76 | 1520.194978 | 1.007188 |
| GO:0007250\_activation\_of\_NF-kappaB-inducing\_kinase\_activity | 12 | 0 | 0.000000 | 0.000000 | 1497 | 1495.325022 | 1507.76 | 1520.194978 | 1.007188 |
| GO:0007292\_female\_gamete\_generation | 12 | 0 | 0.000000 | 0.000000 | 1497 | 1495.325022 | 1507.76 | 1520.194978 | 1.007188 |
| GO:0007585\_respiratory\_gaseous\_exchange | 12 | 0 | 0.000000 | 0.000000 | 1497 | 1495.325022 | 1507.76 | 1520.194978 | 1.007188 |
| GO:0007608\_sensory\_perception\_of\_smell | 12 | 0 | 0.000000 | 0.000000 | 1497 | 1495.325022 | 1507.76 | 1520.194978 | 1.007188 |
| GO:0007611\_learning\_and\_or\_memory | 12 | 0 | 0.000000 | 0.000000 | 1497 | 1495.325022 | 1507.76 | 1520.194978 | 1.007188 |
| GO:0008637\_apoptotic\_mitochondrial\_changes | 12 | 0 | 0.000000 | 0.000000 | 1497 | 1495.325022 | 1507.76 | 1520.194978 | 1.007188 |
| GO:0009064\_glutamine\_family\_amino\_acid\_metabolic\_process | 12 | 0 | 0.000000 | 0.000000 | 1497 | 1495.325022 | 1507.76 | 1520.194978 | 1.007188 |
| GO:0009116\_nucleoside\_metabolic\_process | 12 | 0 | 0.000000 | 0.000000 | 1497 | 1495.325022 | 1507.76 | 1520.194978 | 1.007188 |
| GO:0009141\_nucleoside\_triphosphate\_metabolic\_process | 12 | 0 | 0.000000 | 0.000000 | 1497 | 1495.325022 | 1507.76 | 1520.194978 | 1.007188 |
| GO:0009451\_RNA\_modification | 12 | 0 | 0.000000 | 0.000000 | 1497 | 1495.325022 | 1507.76 | 1520.194978 | 1.007188 |
| GO:0009582\_detection\_of\_abiotic\_stimulus | 12 | 0 | 0.000000 | 0.000000 | 1497 | 1495.325022 | 1507.76 | 1520.194978 | 1.007188 |
| GO:0010212\_response\_to\_ionizing\_radiation | 12 | 0 | 0.000000 | 0.000000 | 1497 | 1495.325022 | 1507.76 | 1520.194978 | 1.007188 |
| GO:0014031\_mesenchymal\_cell\_development | 12 | 0 | 0.000000 | 0.000000 | 1497 | 1495.325022 | 1507.76 | 1520.194978 | 1.007188 |
| GO:0015833\_peptide\_transport | 12 | 0 | 0.000000 | 0.000000 | 1497 | 1495.325022 | 1507.76 | 1520.194978 | 1.007188 |
| GO:0016126\_sterol\_biosynthetic\_process | 12 | 0 | 0.000000 | 0.000000 | 1497 | 1495.325022 | 1507.76 | 1520.194978 | 1.007188 |
| GO:0016458\_gene\_silencing | 12 | 0 | 0.000000 | 0.000000 | 1497 | 1495.325022 | 1507.76 | 1520.194978 | 1.007188 |
| GO:0016525\_negative\_regulation\_of\_angiogenesis | 12 | 0 | 0.000000 | 0.000000 | 1497 | 1495.325022 | 1507.76 | 1520.194978 | 1.007188 |
| GO:0030855\_epithelial\_cell\_differentiation | 12 | 0 | 0.000000 | 0.000000 | 1497 | 1495.325022 | 1507.76 | 1520.194978 | 1.007188 |
| GO:0031124\_mRNA\_3'-end\_processing | 12 | 0 | 0.000000 | 0.000000 | 1497 | 1495.325022 | 1507.76 | 1520.194978 | 1.007188 |
| GO:0031668\_cellular\_response\_to\_extracellular\_stimulus | 12 | 0 | 0.000000 | 0.000000 | 1497 | 1495.325022 | 1507.76 | 1520.194978 | 1.007188 |
| GO:0032946\_positive\_regulation\_of\_mononuclear\_cell\_proliferation | 12 | 0 | 0.000000 | 0.000000 | 1497 | 1495.325022 | 1507.76 | 1520.194978 | 1.007188 |
| GO:0033014\_tetrapyrrole\_biosynthetic\_process | 12 | 0 | 0.000000 | 0.000000 | 1497 | 1495.325022 | 1507.76 | 1520.194978 | 1.007188 |
| GO:0033044\_regulation\_of\_chromosome\_organization\_and\_biogenesis | 12 | 0 | 0.000000 | 0.000000 | 1497 | 1495.325022 | 1507.76 | 1520.194978 | 1.007188 |
| GO:0042129\_regulation\_of\_T\_cell\_proliferation | 12 | 0 | 0.000000 | 0.000000 | 1497 | 1495.325022 | 1507.76 | 1520.194978 | 1.007188 |
| GO:0042168\_heme\_metabolic\_process | 12 | 0 | 0.000000 | 0.000000 | 1497 | 1495.325022 | 1507.76 | 1520.194978 | 1.007188 |
| GO:0042632\_cholesterol\_homeostasis | 12 | 0 | 0.000000 | 0.000000 | 1497 | 1495.325022 | 1507.76 | 1520.194978 | 1.007188 |
| GO:0045069\_regulation\_of\_viral\_genome\_replication | 12 | 0 | 0.000000 | 0.000000 | 1497 | 1495.325022 | 1507.76 | 1520.194978 | 1.007188 |
| GO:0045767\_regulation\_of\_anti-apoptosis | 12 | 0 | 0.000000 | 0.000000 | 1497 | 1495.325022 | 1507.76 | 1520.194978 | 1.007188 |
| GO:0045785\_positive\_regulation\_of\_cell\_adhesion | 12 | 0 | 0.000000 | 0.000000 | 1497 | 1495.325022 | 1507.76 | 1520.194978 | 1.007188 |
| GO:0045930\_negative\_regulation\_of\_mitotic\_cell\_cycle | 12 | 0 | 0.000000 | 0.000000 | 1497 | 1495.325022 | 1507.76 | 1520.194978 | 1.007188 |
| GO:0046545\_development\_of\_primary\_female\_sexual\_characteristics | 12 | 0 | 0.000000 | 0.000000 | 1497 | 1495.325022 | 1507.76 | 1520.194978 | 1.007188 |
| GO:0046660\_female\_sex\_differentiation | 12 | 0 | 0.000000 | 0.000000 | 1497 | 1495.325022 | 1507.76 | 1520.194978 | 1.007188 |
| GO:0048489\_synaptic\_vesicle\_transport | 12 | 0 | 0.000000 | 0.000000 | 1497 | 1495.325022 | 1507.76 | 1520.194978 | 1.007188 |
| GO:0048732\_gland\_development | 12 | 0 | 0.000000 | 0.000000 | 1497 | 1495.325022 | 1507.76 | 1520.194978 | 1.007188 |
| GO:0048762\_mesenchymal\_cell\_differentiation | 12 | 0 | 0.000000 | 0.000000 | 1497 | 1495.325022 | 1507.76 | 1520.194978 | 1.007188 |
| GO:0050671\_positive\_regulation\_of\_lymphocyte\_proliferation | 12 | 0 | 0.000000 | 0.000000 | 1497 | 1495.325022 | 1507.76 | 1520.194978 | 1.007188 |
| GO:0050714\_positive\_regulation\_of\_protein\_secretion | 12 | 0 | 0.000000 | 0.000000 | 1497 | 1495.325022 | 1507.76 | 1520.194978 | 1.007188 |
| GO:0051054\_positive\_regulation\_of\_DNA\_metabolic\_process | 12 | 0 | 0.000000 | 0.000000 | 1497 | 1495.325022 | 1507.76 | 1520.194978 | 1.007188 |
| GO:0055072\_iron\_ion\_homeostasis | 12 | 0 | 0.000000 | 0.000000 | 1497 | 1495.325022 | 1507.76 | 1520.194978 | 1.007188 |
| GO:0055088\_lipid\_homeostasis | 12 | 0 | 0.000000 | 0.000000 | 1497 | 1495.325022 | 1507.76 | 1520.194978 | 1.007188 |
| GO:0055092\_sterol\_homeostasis | 12 | 0 | 0.000000 | 0.000000 | 1497 | 1495.325022 | 1507.76 | 1520.194978 | 1.007188 |
| GO:0001775\_cell\_activation | 96 | 0 | 0.000000 | 0.000000 | 1500 | 1497.392256 | 1509.41 | 1521.427744 | 1.006273 |
| GO:0002520\_immune\_system\_development | 96 | 0 | 0.000000 | 0.000000 | 1500 | 1497.392256 | 1509.41 | 1521.427744 | 1.006273 |
| GO:0022008\_neurogenesis | 96 | 0 | 0.000000 | 0.000000 | 1500 | 1497.392256 | 1509.41 | 1521.427744 | 1.006273 |
| GO:0006457\_protein\_folding | 69 | 0 | 0.000000 | 0.000000 | 1501 | 1498.010645 | 1509.99 | 1521.969355 | 1.005989 |
| GO:0006935\_chemotaxis | 81 | 0 | 0.000000 | 0.000000 | 1506 | 1501.191849 | 1512.9 | 1524.608151 | 1.004582 |
| GO:0030030\_cell\_projection\_organization\_and\_biogenesis | 81 | 0 | 0.000000 | 0.000000 | 1506 | 1501.191849 | 1512.9 | 1524.608151 | 1.004582 |
| GO:0032990\_cell\_part\_morphogenesis | 81 | 0 | 0.000000 | 0.000000 | 1506 | 1501.191849 | 1512.9 | 1524.608151 | 1.004582 |
| GO:0042330\_taxis | 81 | 0 | 0.000000 | 0.000000 | 1506 | 1501.191849 | 1512.9 | 1524.608151 | 1.004582 |
| GO:0048858\_cell\_projection\_morphogenesis | 81 | 0 | 0.000000 | 0.000000 | 1506 | 1501.191849 | 1512.9 | 1524.608151 | 1.004582 |
| GO:0000028\_ribosomal\_small\_subunit\_assembly\_and\_maintenance | 1 | 0 |  |  |  |  |  |  |  |  |
| GO:0000042\_protein\_targeting\_to\_Golgi | 1 | 0 |  |  |  |  |  |  |  |  |
| GO:0000072\_M\_phase\_specific\_microtubule\_process | 1 | 0 |  |  |  |  |  |  |  |  |
| GO:0000083\_G1\_S-specific\_transcription\_in\_mitotic\_cell\_cycle | 1 | 0 |  |  |  |  |  |  |  |  |
| GO:0000114\_G1-specific\_transcription\_in\_mitotic\_cell\_cycle | 1 | 0 |  |  |  |  |  |  |  |  |
| GO:0000115\_S-phase-specific\_transcription\_in\_mitotic\_cell\_cycle | 1 | 0 |  |  |  |  |  |  |  |  |
| GO:0000154\_rRNA\_modification | 1 | 0 |  |  |  |  |  |  |  |  |
| GO:0000161\_MAPKKK\_cascade\_during\_osmolarity\_sensing | 1 | 0 |  |  |  |  |  |  |  |  |
| GO:0000173\_inactivation\_of\_MAPK\_activity\_during\_osmolarity\_sensing | 1 | 0 |  |  |  |  |  |  |  |  |
| GO:0000183\_chromatin\_silencing\_at\_rDNA | 1 | 0 |  |  |  |  |  |  |  |  |
| GO:0000212\_meiotic\_spindle\_organization\_and\_biogenesis | 1 | 0 |  |  |  |  |  |  |  |  |
| GO:0000281\_cytokinesis\_after\_mitosis | 1 | 0 |  |  |  |  |  |  |  |  |
| GO:0000301\_retrograde\_transport\_\_vesicle\_recycling\_within\_Golgi | 1 | 0 |  |  |  |  |  |  |  |  |
| GO:0000320\_re-entry\_into\_mitotic\_cell\_cycle | 1 | 0 |  |  |  |  |  |  |  |  |
| GO:0000351\_assembly\_of\_spliceosomal\_tri-snRNP\_U4\_U6.U5 | 1 | 0 |  |  |  |  |  |  |  |  |
| GO:0000380\_alternative\_nuclear\_mRNA\_splicing\_\_via\_spliceosome | 1 | 0 |  |  |  |  |  |  |  |  |
| GO:0000717\_nucleotide-excision\_repair\_\_DNA\_duplex\_unwinding | 1 | 0 |  |  |  |  |  |  |  |  |
| GO:0000718\_nucleotide-excision\_repair\_\_DNA\_damage\_removal | 1 | 0 |  |  |  |  |  |  |  |  |
| GO:0000722\_telomere\_maintenance\_via\_recombination | 1 | 0 |  |  |  |  |  |  |  |  |
| GO:0000746\_conjugation | 1 | 0 |  |  |  |  |  |  |  |  |
| GO:0000747\_conjugation\_with\_cellular\_fusion | 1 | 0 |  |  |  |  |  |  |  |  |
| GO:0000912\_formation\_of\_actomyosin\_apparatus\_involved\_in\_cytokinesis | 1 | 0 |  |  |  |  |  |  |  |  |
| GO:0000915\_cytokinesis\_\_contractile\_ring\_formation | 1 | 0 |  |  |  |  |  |  |  |  |
| GO:0000920\_cell\_separation\_during\_cytokinesis | 1 | 0 |  |  |  |  |  |  |  |  |
| GO:0001301\_progressive\_alteration\_of\_chromatin\_during\_cell\_aging | 1 | 0 |  |  |  |  |  |  |  |  |
| GO:0001302\_replicative\_cell\_aging | 1 | 0 |  |  |  |  |  |  |  |  |
| GO:0001304\_progressive\_alteration\_of\_chromatin\_during\_replicative\_cell\_aging | 1 | 0 |  |  |  |  |  |  |  |  |
| GO:0001309\_age-dependent\_telomere\_shortening | 1 | 0 |  |  |  |  |  |  |  |  |
| GO:0001315\_age-dependent\_response\_to\_reactive\_oxygen\_species | 1 | 0 |  |  |  |  |  |  |  |  |
| GO:0001507\_acetylcholine\_catabolic\_process\_in\_synaptic\_cleft | 1 | 0 |  |  |  |  |  |  |  |  |
| GO:0001560\_regulation\_of\_cell\_growth\_by\_extracellular\_stimulus | 1 | 0 |  |  |  |  |  |  |  |  |
| GO:0001561\_fatty\_acid\_alpha-oxidation | 1 | 0 |  |  |  |  |  |  |  |  |
| GO:0001562\_response\_to\_protozoan | 1 | 0 |  |  |  |  |  |  |  |  |
| GO:0001574\_ganglioside\_biosynthetic\_process | 1 | 0 |  |  |  |  |  |  |  |  |
| GO:0001575\_globoside\_metabolic\_process | 1 | 0 |  |  |  |  |  |  |  |  |
| GO:0001658\_ureteric\_bud\_branching | 1 | 0 |  |  |  |  |  |  |  |  |
| GO:0001659\_temperature\_homeostasis | 1 | 0 |  |  |  |  |  |  |  |  |
| GO:0001675\_acrosome\_formation | 1 | 0 |  |  |  |  |  |  |  |  |
| GO:0001678\_cellular\_glucose\_homeostasis | 1 | 0 |  |  |  |  |  |  |  |  |
| GO:0001702\_gastrulation\_with\_mouth\_forming\_second | 1 | 0 |  |  |  |  |  |  |  |  |
| GO:0001774\_microglial\_cell\_activation | 1 | 0 |  |  |  |  |  |  |  |  |
| GO:0001780\_neutrophil\_homeostasis | 1 | 0 |  |  |  |  |  |  |  |  |
| GO:0001781\_neutrophil\_apoptosis | 1 | 0 |  |  |  |  |  |  |  |  |
| GO:0001782\_B\_cell\_homeostasis | 1 | 0 |  |  |  |  |  |  |  |  |
| GO:0001787\_natural\_killer\_cell\_proliferation | 1 | 0 |  |  |  |  |  |  |  |  |
| GO:0001825\_blastocyst\_formation | 1 | 0 |  |  |  |  |  |  |  |  |
| GO:0001829\_trophectodermal\_cell\_differentiation | 1 | 0 |  |  |  |  |  |  |  |  |
| GO:0001866\_NK\_T\_cell\_proliferation | 1 | 0 |  |  |  |  |  |  |  |  |
| GO:0001867\_complement\_activation\_\_lectin\_pathway | 1 | 0 |  |  |  |  |  |  |  |  |
| GO:0001881\_receptor\_recycling | 1 | 0 |  |  |  |  |  |  |  |  |
| GO:0001885\_endothelial\_cell\_development | 1 | 0 |  |  |  |  |  |  |  |  |
| GO:0001895\_retina\_homeostasis | 1 | 0 |  |  |  |  |  |  |  |  |
| GO:0001911\_negative\_regulation\_of\_leukocyte\_mediated\_cytotoxicity | 1 | 0 |  |  |  |  |  |  |  |  |
| GO:0001915\_negative\_regulation\_of\_T\_cell\_mediated\_cytotoxicity | 1 | 0 |  |  |  |  |  |  |  |  |
| GO:0001916\_positive\_regulation\_of\_T\_cell\_mediated\_cytotoxicity | 1 | 0 |  |  |  |  |  |  |  |  |
| GO:0001919\_regulation\_of\_receptor\_recycling | 1 | 0 |  |  |  |  |  |  |  |  |
| GO:0001920\_negative\_regulation\_of\_receptor\_recycling | 1 | 0 |  |  |  |  |  |  |  |  |
| GO:0001947\_heart\_looping | 1 | 0 |  |  |  |  |  |  |  |  |
| GO:0001958\_endochondral\_ossification | 1 | 0 |  |  |  |  |  |  |  |  |
| GO:0001964\_startle\_response | 1 | 0 |  |  |  |  |  |  |  |  |
| GO:0001976\_regulation\_of\_systemic\_arterial\_blood\_pressure\_by\_neurological\_process | 1 | 0 |  |  |  |  |  |  |  |  |
| GO:0002029\_desensitization\_of\_G-protein\_coupled\_receptor\_protein\_signaling\_pathway | 1 | 0 |  |  |  |  |  |  |  |  |
| GO:0002032\_desensitization\_of\_G-protein\_coupled\_receptor\_protein\_signaling\_pathway\_by\_arrestin | 1 | 0 |  |  |  |  |  |  |  |  |
| GO:0002040\_sprouting\_angiogenesis | 1 | 0 |  |  |  |  |  |  |  |  |
| GO:0002074\_extraocular\_skeletal\_muscle\_development | 1 | 0 |  |  |  |  |  |  |  |  |
| GO:0002084\_protein\_depalmitoylation | 1 | 0 |  |  |  |  |  |  |  |  |
| GO:0002086\_diaphragm\_contraction | 1 | 0 |  |  |  |  |  |  |  |  |
| GO:0002093\_auditory\_receptor\_cell\_morphogenesis | 1 | 0 |  |  |  |  |  |  |  |  |
| GO:0002244\_hemopoietic\_progenitor\_cell\_differentiation | 1 | 0 |  |  |  |  |  |  |  |  |
| GO:0002275\_myeloid\_cell\_activation\_during\_immune\_response | 1 | 0 |  |  |  |  |  |  |  |  |
| GO:0002281\_macrophage\_activation\_during\_immune\_response | 1 | 0 |  |  |  |  |  |  |  |  |
| GO:0002318\_myeloid\_progenitor\_cell\_differentiation | 1 | 0 |  |  |  |  |  |  |  |  |
| GO:0002326\_B\_cell\_lineage\_commitment | 1 | 0 |  |  |  |  |  |  |  |  |
| GO:0002347\_response\_to\_tumor\_cell | 1 | 0 |  |  |  |  |  |  |  |  |
| GO:0002418\_immune\_response\_to\_tumor\_cell | 1 | 0 |  |  |  |  |  |  |  |  |
| GO:0002420\_natural\_killer\_cell\_mediated\_cytotoxicity\_directed\_against\_tumor\_cell\_target | 1 | 0 |  |  |  |  |  |  |  |  |
| GO:0002423\_natural\_killer\_cell\_mediated\_immune\_response\_to\_tumor\_cell | 1 | 0 |  |  |  |  |  |  |  |  |
| GO:0002437\_inflammatory\_response\_to\_antigenic\_stimulus | 1 | 0 |  |  |  |  |  |  |  |  |
| GO:0002439\_chronic\_inflammatory\_response\_to\_antigenic\_stimulus | 1 | 0 |  |  |  |  |  |  |  |  |
| GO:0002455\_humoral\_immune\_response\_mediated\_by\_circulating\_immunoglobulin | 1 | 0 |  |  |  |  |  |  |  |  |
| GO:0002467\_germinal\_center\_formation | 1 | 0 |  |  |  |  |  |  |  |  |
| GO:0002475\_antigen\_processing\_and\_presentation\_via\_MHC\_class\_Ib | 1 | 0 |  |  |  |  |  |  |  |  |
| GO:0002478\_antigen\_processing\_and\_presentation\_of\_exogenous\_peptide\_antigen | 1 | 0 |  |  |  |  |  |  |  |  |
| GO:0002495\_antigen\_processing\_and\_presentation\_of\_peptide\_antigen\_via\_MHC\_class\_II | 1 | 0 |  |  |  |  |  |  |  |  |
| GO:0002504\_antigen\_processing\_and\_presentation\_of\_peptide\_or\_polysaccharide\_antigen\_via\_MHC\_class\_II | 1 | 0 |  |  |  |  |  |  |  |  |
| GO:0002544\_chronic\_inflammatory\_response | 1 | 0 |  |  |  |  |  |  |  |  |
| GO:0002566\_somatic\_diversification\_of\_immune\_receptors\_via\_somatic\_mutation | 1 | 0 |  |  |  |  |  |  |  |  |
| GO:0002634\_regulation\_of\_germinal\_center\_formation | 1 | 0 |  |  |  |  |  |  |  |  |
| GO:0002685\_regulation\_of\_leukocyte\_migration | 1 | 0 |  |  |  |  |  |  |  |  |
| GO:0002687\_positive\_regulation\_of\_leukocyte\_migration | 1 | 0 |  |  |  |  |  |  |  |  |
| GO:0002704\_negative\_regulation\_of\_leukocyte\_mediated\_immunity | 1 | 0 |  |  |  |  |  |  |  |  |
| GO:0002707\_negative\_regulation\_of\_lymphocyte\_mediated\_immunity | 1 | 0 |  |  |  |  |  |  |  |  |
| GO:0002710\_negative\_regulation\_of\_T\_cell\_mediated\_immunity | 1 | 0 |  |  |  |  |  |  |  |  |
| GO:0002792\_negative\_regulation\_of\_peptide\_secretion | 1 | 0 |  |  |  |  |  |  |  |  |
| GO:0002820\_negative\_regulation\_of\_adaptive\_immune\_response | 1 | 0 |  |  |  |  |  |  |  |  |
| GO:0002823\_negative\_regulation\_of\_adaptive\_immune\_response\_based\_on\_somatic\_recombination\_of\_immune\_receptors\_built\_from\_immunoglobulin\_superfamily\_domains | 1 | 0 |  |  |  |  |  |  |  |  |
| GO:0002832\_negative\_regulation\_of\_response\_to\_biotic\_stimulus | 1 | 0 |  |  |  |  |  |  |  |  |
| GO:0002833\_positive\_regulation\_of\_response\_to\_biotic\_stimulus | 1 | 0 |  |  |  |  |  |  |  |  |
| GO:0002834\_regulation\_of\_response\_to\_tumor\_cell | 1 | 0 |  |  |  |  |  |  |  |  |
| GO:0002836\_positive\_regulation\_of\_response\_to\_tumor\_cell | 1 | 0 |  |  |  |  |  |  |  |  |
| GO:0002837\_regulation\_of\_immune\_response\_to\_tumor\_cell | 1 | 0 |  |  |  |  |  |  |  |  |
| GO:0002839\_positive\_regulation\_of\_immune\_response\_to\_tumor\_cell | 1 | 0 |  |  |  |  |  |  |  |  |
| GO:0002855\_regulation\_of\_natural\_killer\_cell\_mediated\_immune\_response\_to\_tumor\_cell | 1 | 0 |  |  |  |  |  |  |  |  |
| GO:0002857\_positive\_regulation\_of\_natural\_killer\_cell\_mediated\_immune\_response\_to\_tumor\_cell | 1 | 0 |  |  |  |  |  |  |  |  |
| GO:0002858\_regulation\_of\_natural\_killer\_cell\_mediated\_cytotoxicity\_directed\_against\_tumor\_cell\_target | 1 | 0 |  |  |  |  |  |  |  |  |
| GO:0002860\_positive\_regulation\_of\_natural\_killer\_cell\_mediated\_cytotoxicity\_directed\_against\_tumor\_cell\_target | 1 | 0 |  |  |  |  |  |  |  |  |
| GO:0002901\_mature\_B\_cell\_apoptosis | 1 | 0 |  |  |  |  |  |  |  |  |
| GO:0002905\_regulation\_of\_mature\_B\_cell\_apoptosis | 1 | 0 |  |  |  |  |  |  |  |  |
| GO:0002906\_negative\_regulation\_of\_mature\_B\_cell\_apoptosis | 1 | 0 |  |  |  |  |  |  |  |  |
| GO:0003011\_involuntary\_skeletal\_muscle\_contraction | 1 | 0 |  |  |  |  |  |  |  |  |
| GO:0003016\_respiratory\_system\_process | 1 | 0 |  |  |  |  |  |  |  |  |
| GO:0003057\_regulation\_of\_the\_force\_of\_heart\_contraction\_by\_chemical\_signal | 1 | 0 |  |  |  |  |  |  |  |  |
| GO:0003108\_negative\_regulation\_of\_the\_force\_of\_heart\_contraction\_by\_chemical\_signal | 1 | 0 |  |  |  |  |  |  |  |  |
| GO:0005982\_starch\_metabolic\_process | 1 | 0 |  |  |  |  |  |  |  |  |
| GO:0005983\_starch\_catabolic\_process | 1 | 0 |  |  |  |  |  |  |  |  |
| GO:0006010\_glucose\_6-phosphate\_utilization | 1 | 0 |  |  |  |  |  |  |  |  |
| GO:0006013\_mannose\_metabolic\_process | 1 | 0 |  |  |  |  |  |  |  |  |
| GO:0006014\_D-ribose\_metabolic\_process | 1 | 0 |  |  |  |  |  |  |  |  |
| GO:0006042\_glucosamine\_biosynthetic\_process | 1 | 0 |  |  |  |  |  |  |  |  |
| GO:0006043\_glucosamine\_catabolic\_process | 1 | 0 |  |  |  |  |  |  |  |  |
| GO:0006045\_N-acetylglucosamine\_biosynthetic\_process | 1 | 0 |  |  |  |  |  |  |  |  |
| GO:0006048\_UDP-N-acetylglucosamine\_biosynthetic\_process | 1 | 0 |  |  |  |  |  |  |  |  |
| GO:0006050\_mannosamine\_metabolic\_process | 1 | 0 |  |  |  |  |  |  |  |  |
| GO:0006051\_N-acetylmannosamine\_metabolic\_process | 1 | 0 |  |  |  |  |  |  |  |  |
| GO:0006063\_uronic\_acid\_metabolic\_process | 1 | 0 |  |  |  |  |  |  |  |  |
| GO:0006065\_UDP-glucuronate\_biosynthetic\_process | 1 | 0 |  |  |  |  |  |  |  |  |
| GO:0006067\_ethanol\_metabolic\_process | 1 | 0 |  |  |  |  |  |  |  |  |
| GO:0006069\_ethanol\_oxidation | 1 | 0 |  |  |  |  |  |  |  |  |
| GO:0006105\_succinate\_metabolic\_process | 1 | 0 |  |  |  |  |  |  |  |  |
| GO:0006106\_fumarate\_metabolic\_process | 1 | 0 |  |  |  |  |  |  |  |  |
| GO:0006152\_purine\_nucleoside\_catabolic\_process | 1 | 0 |  |  |  |  |  |  |  |  |
| GO:0006166\_purine\_ribonucleoside\_salvage | 1 | 0 |  |  |  |  |  |  |  |  |
| GO:0006167\_AMP\_biosynthetic\_process | 1 | 0 |  |  |  |  |  |  |  |  |
| GO:0006172\_ADP\_biosynthetic\_process | 1 | 0 |  |  |  |  |  |  |  |  |
| GO:0006173\_dADP\_biosynthetic\_process | 1 | 0 |  |  |  |  |  |  |  |  |
| GO:0006183\_GTP\_biosynthetic\_process | 1 | 0 |  |  |  |  |  |  |  |  |
| GO:0006188\_IMP\_biosynthetic\_process | 1 | 0 |  |  |  |  |  |  |  |  |
| GO:0006189\_'de\_novo'\_IMP\_biosynthetic\_process | 1 | 0 |  |  |  |  |  |  |  |  |
| GO:0006195\_purine\_nucleotide\_catabolic\_process | 1 | 0 |  |  |  |  |  |  |  |  |
| GO:0006196\_AMP\_catabolic\_process | 1 | 0 |  |  |  |  |  |  |  |  |
| GO:0006207\_'de\_novo'\_pyrimidine\_base\_biosynthetic\_process | 1 | 0 |  |  |  |  |  |  |  |  |
| GO:0006208\_pyrimidine\_base\_catabolic\_process | 1 | 0 |  |  |  |  |  |  |  |  |
| GO:0006212\_uracil\_catabolic\_process | 1 | 0 |  |  |  |  |  |  |  |  |
| GO:0006214\_thymidine\_catabolic\_process | 1 | 0 |  |  |  |  |  |  |  |  |
| GO:0006216\_cytidine\_catabolic\_process | 1 | 0 |  |  |  |  |  |  |  |  |
| GO:0006222\_UMP\_biosynthetic\_process | 1 | 0 |  |  |  |  |  |  |  |  |
| GO:0006241\_CTP\_biosynthetic\_process | 1 | 0 |  |  |  |  |  |  |  |  |
| GO:0006256\_UDP\_catabolic\_process | 1 | 0 |  |  |  |  |  |  |  |  |
| GO:0006265\_DNA\_topological\_change | 1 | 0 |  |  |  |  |  |  |  |  |
| GO:0006272\_leading\_strand\_elongation | 1 | 0 |  |  |  |  |  |  |  |  |
| GO:0006273\_lagging\_strand\_elongation | 1 | 0 |  |  |  |  |  |  |  |  |
| GO:0006285\_base-excision\_repair\_\_AP\_site\_formation | 1 | 0 |  |  |  |  |  |  |  |  |
| GO:0006287\_base-excision\_repair\_\_gap-filling | 1 | 0 |  |  |  |  |  |  |  |  |
| GO:0006297\_nucleotide-excision\_repair\_\_DNA\_gap\_filling | 1 | 0 |  |  |  |  |  |  |  |  |
| GO:0006313\_transposition\_\_DNA-mediated | 1 | 0 |  |  |  |  |  |  |  |  |
| GO:0006335\_DNA\_replication-dependent\_nucleosome\_assembly | 1 | 0 |  |  |  |  |  |  |  |  |
| GO:0006346\_methylation-dependent\_chromatin\_silencing | 1 | 0 |  |  |  |  |  |  |  |  |
| GO:0006348\_chromatin\_silencing\_at\_telomere | 1 | 0 |  |  |  |  |  |  |  |  |
| GO:0006361\_transcription\_initiation\_from\_RNA\_polymerase\_I\_promoter | 1 | 0 |  |  |  |  |  |  |  |  |
| GO:0006369\_termination\_of\_RNA\_polymerase\_II\_transcription | 1 | 0 |  |  |  |  |  |  |  |  |
| GO:0006374\_nuclear\_mRNA\_splicing\_via\_U2-type\_spliceosome | 1 | 0 |  |  |  |  |  |  |  |  |
| GO:0006393\_termination\_of\_mitochondrial\_transcription | 1 | 0 |  |  |  |  |  |  |  |  |
| GO:0006407\_rRNA\_export\_from\_nucleus | 1 | 0 |  |  |  |  |  |  |  |  |
| GO:0006408\_snRNA\_export\_from\_nucleus | 1 | 0 |  |  |  |  |  |  |  |  |
| GO:0006419\_alanyl-tRNA\_aminoacylation | 1 | 0 |  |  |  |  |  |  |  |  |
| GO:0006420\_arginyl-tRNA\_aminoacylation | 1 | 0 |  |  |  |  |  |  |  |  |
| GO:0006431\_methionyl-tRNA\_aminoacylation | 1 | 0 |  |  |  |  |  |  |  |  |
| GO:0006434\_seryl-tRNA\_aminoacylation | 1 | 0 |  |  |  |  |  |  |  |  |
| GO:0006436\_tryptophanyl-tRNA\_aminoacylation | 1 | 0 |  |  |  |  |  |  |  |  |
| GO:0006437\_tyrosyl-tRNA\_aminoacylation | 1 | 0 |  |  |  |  |  |  |  |  |
| GO:0006447\_regulation\_of\_translational\_initiation\_by\_iron | 1 | 0 |  |  |  |  |  |  |  |  |
| GO:0006494\_protein\_amino\_acid\_terminal\_glycosylation | 1 | 0 |  |  |  |  |  |  |  |  |
| GO:0006496\_protein\_amino\_acid\_terminal\_N-glycosylation | 1 | 0 |  |  |  |  |  |  |  |  |
| GO:0006498\_N-terminal\_protein\_lipidation | 1 | 0 |  |  |  |  |  |  |  |  |
| GO:0006499\_N-terminal\_protein\_myristoylation | 1 | 0 |  |  |  |  |  |  |  |  |
| GO:0006510\_ATP-dependent\_proteolysis | 1 | 0 |  |  |  |  |  |  |  |  |
| GO:0006528\_asparagine\_metabolic\_process | 1 | 0 |  |  |  |  |  |  |  |  |
| GO:0006530\_asparagine\_catabolic\_process | 1 | 0 |  |  |  |  |  |  |  |  |
| GO:0006534\_cysteine\_metabolic\_process | 1 | 0 |  |  |  |  |  |  |  |  |
| GO:0006540\_glutamate\_decarboxylation\_to\_succinate | 1 | 0 |  |  |  |  |  |  |  |  |
| GO:0006541\_glutamine\_metabolic\_process | 1 | 0 |  |  |  |  |  |  |  |  |
| GO:0006543\_glutamine\_catabolic\_process | 1 | 0 |  |  |  |  |  |  |  |  |
| GO:0006545\_glycine\_biosynthetic\_process | 1 | 0 |  |  |  |  |  |  |  |  |
| GO:0006553\_lysine\_metabolic\_process | 1 | 0 |  |  |  |  |  |  |  |  |
| GO:0006554\_lysine\_catabolic\_process | 1 | 0 |  |  |  |  |  |  |  |  |
| GO:0006556\_S-adenosylmethionine\_biosynthetic\_process | 1 | 0 |  |  |  |  |  |  |  |  |
| GO:0006562\_proline\_catabolic\_process | 1 | 0 |  |  |  |  |  |  |  |  |
| GO:0006564\_L-serine\_biosynthetic\_process | 1 | 0 |  |  |  |  |  |  |  |  |
| GO:0006565\_L-serine\_catabolic\_process | 1 | 0 |  |  |  |  |  |  |  |  |
| GO:0006568\_tryptophan\_metabolic\_process | 1 | 0 |  |  |  |  |  |  |  |  |
| GO:0006569\_tryptophan\_catabolic\_process | 1 | 0 |  |  |  |  |  |  |  |  |
| GO:0006580\_ethanolamine\_metabolic\_process | 1 | 0 |  |  |  |  |  |  |  |  |
| GO:0006581\_acetylcholine\_catabolic\_process | 1 | 0 |  |  |  |  |  |  |  |  |
| GO:0006590\_thyroid\_hormone\_generation | 1 | 0 |  |  |  |  |  |  |  |  |
| GO:0006597\_spermine\_biosynthetic\_process | 1 | 0 |  |  |  |  |  |  |  |  |
| GO:0006598\_polyamine\_catabolic\_process | 1 | 0 |  |  |  |  |  |  |  |  |
| GO:0006608\_snRNP\_protein\_import\_into\_nucleus | 1 | 0 |  |  |  |  |  |  |  |  |
| GO:0006610\_ribosomal\_protein\_import\_into\_nucleus | 1 | 0 |  |  |  |  |  |  |  |  |
| GO:0006627\_mitochondrial\_protein\_processing\_during\_import | 1 | 0 |  |  |  |  |  |  |  |  |
| GO:0006646\_phosphatidylethanolamine\_biosynthetic\_process | 1 | 0 |  |  |  |  |  |  |  |  |
| GO:0006655\_phosphatidylglycerol\_biosynthetic\_process | 1 | 0 |  |  |  |  |  |  |  |  |
| GO:0006657\_CDP-choline\_pathway | 1 | 0 |  |  |  |  |  |  |  |  |
| GO:0006667\_sphinganine\_metabolic\_process | 1 | 0 |  |  |  |  |  |  |  |  |
| GO:0006668\_sphinganine-1-phosphate\_metabolic\_process | 1 | 0 |  |  |  |  |  |  |  |  |
| GO:0006669\_sphinganine-1-phosphate\_biosynthetic\_process | 1 | 0 |  |  |  |  |  |  |  |  |
| GO:0006670\_sphingosine\_metabolic\_process | 1 | 0 |  |  |  |  |  |  |  |  |
| GO:0006681\_galactosylceramide\_metabolic\_process | 1 | 0 |  |  |  |  |  |  |  |  |
| GO:0006689\_ganglioside\_catabolic\_process | 1 | 0 |  |  |  |  |  |  |  |  |
| GO:0006700\_C21-steroid\_hormone\_biosynthetic\_process | 1 | 0 |  |  |  |  |  |  |  |  |
| GO:0006701\_progesterone\_biosynthetic\_process | 1 | 0 |  |  |  |  |  |  |  |  |
| GO:0006703\_estrogen\_biosynthetic\_process | 1 | 0 |  |  |  |  |  |  |  |  |
| GO:0006704\_glucocorticoid\_biosynthetic\_process | 1 | 0 |  |  |  |  |  |  |  |  |
| GO:0006705\_mineralocorticoid\_biosynthetic\_process | 1 | 0 |  |  |  |  |  |  |  |  |
| GO:0006707\_cholesterol\_catabolic\_process | 1 | 0 |  |  |  |  |  |  |  |  |
| GO:0006711\_estrogen\_catabolic\_process | 1 | 0 |  |  |  |  |  |  |  |  |
| GO:0006713\_glucocorticoid\_catabolic\_process | 1 | 0 |  |  |  |  |  |  |  |  |
| GO:0006741\_NADP\_biosynthetic\_process | 1 | 0 |  |  |  |  |  |  |  |  |
| GO:0006746\_FADH2\_metabolic\_process | 1 | 0 |  |  |  |  |  |  |  |  |
| GO:0006768\_biotin\_metabolic\_process | 1 | 0 |  |  |  |  |  |  |  |  |
| GO:0006771\_riboflavin\_metabolic\_process | 1 | 0 |  |  |  |  |  |  |  |  |
| GO:0006781\_succinyl-CoA\_pathway | 1 | 0 |  |  |  |  |  |  |  |  |
| GO:0006789\_bilirubin\_conjugation | 1 | 0 |  |  |  |  |  |  |  |  |
| GO:0006797\_polyphosphate\_metabolic\_process | 1 | 0 |  |  |  |  |  |  |  |  |
| GO:0006824\_cobalt\_ion\_transport | 1 | 0 |  |  |  |  |  |  |  |  |
| GO:0006828\_manganese\_ion\_transport | 1 | 0 |  |  |  |  |  |  |  |  |
| GO:0006842\_tricarboxylic\_acid\_transport | 1 | 0 |  |  |  |  |  |  |  |  |
| GO:0006843\_mitochondrial\_citrate\_transport | 1 | 0 |  |  |  |  |  |  |  |  |
| GO:0006848\_pyruvate\_transport | 1 | 0 |  |  |  |  |  |  |  |  |
| GO:0006862\_nucleotide\_transport | 1 | 0 |  |  |  |  |  |  |  |  |
| GO:0006867\_asparagine\_transport | 1 | 0 |  |  |  |  |  |  |  |  |
| GO:0006868\_glutamine\_transport | 1 | 0 |  |  |  |  |  |  |  |  |
| GO:0006907\_pinocytosis | 1 | 0 |  |  |  |  |  |  |  |  |
| GO:0006910\_phagocytosis\_\_recognition | 1 | 0 |  |  |  |  |  |  |  |  |
| GO:0006926\_virus-infected\_cell\_apoptosis | 1 | 0 |  |  |  |  |  |  |  |  |
| GO:0006931\_substrate-bound\_cell\_migration\_\_cell\_attachment\_to\_substrate | 1 | 0 |  |  |  |  |  |  |  |  |
| GO:0006948\_induction\_by\_virus\_of\_cell-cell\_fusion\_in\_host | 1 | 0 |  |  |  |  |  |  |  |  |
| GO:0006957\_complement\_activation\_\_alternative\_pathway | 1 | 0 |  |  |  |  |  |  |  |  |
| GO:0006958\_complement\_activation\_\_classical\_pathway | 1 | 0 |  |  |  |  |  |  |  |  |
| GO:0006982\_response\_to\_lipid\_hydroperoxide | 1 | 0 |  |  |  |  |  |  |  |  |
| GO:0006987\_activation\_of\_signaling\_protein\_activity\_involved\_in\_unfolded\_protein\_response | 1 | 0 |  |  |  |  |  |  |  |  |
| GO:0006990\_positive\_regulation\_of\_gene-specific\_transcription\_involved\_in\_unfolded\_protein\_response | 1 | 0 |  |  |  |  |  |  |  |  |
| GO:0006991\_response\_to\_sterol\_depletion | 1 | 0 |  |  |  |  |  |  |  |  |
| GO:0006994\_positive\_regulation\_of\_sterol\_regulatory\_element\_binding\_protein\_target\_gene\_transcription\_involved\_in\_sterol\_depletion\_response | 1 | 0 |  |  |  |  |  |  |  |  |
| GO:0007022\_chaperonin-mediated\_tubulin\_folding | 1 | 0 |  |  |  |  |  |  |  |  |
| GO:0007035\_vacuolar\_acidification | 1 | 0 |  |  |  |  |  |  |  |  |
| GO:0007063\_regulation\_of\_sister\_chromatid\_cohesion | 1 | 0 |  |  |  |  |  |  |  |  |
| GO:0007064\_mitotic\_sister\_chromatid\_cohesion | 1 | 0 |  |  |  |  |  |  |  |  |
| GO:0007068\_negative\_regulation\_of\_transcription\_\_mitotic | 1 | 0 |  |  |  |  |  |  |  |  |
| GO:0007097\_nuclear\_migration | 1 | 0 |  |  |  |  |  |  |  |  |
| GO:0007108\_cytokinesis\_\_initiation\_of\_separation | 1 | 0 |  |  |  |  |  |  |  |  |
| GO:0007135\_meiosis\_II | 1 | 0 |  |  |  |  |  |  |  |  |
| GO:0007136\_meiotic\_prophase\_II | 1 | 0 |  |  |  |  |  |  |  |  |
| GO:0007141\_male\_meiosis\_I | 1 | 0 |  |  |  |  |  |  |  |  |
| GO:0007143\_female\_meiosis | 1 | 0 |  |  |  |  |  |  |  |  |
| GO:0007161\_calcium-independent\_cell-matrix\_adhesion | 1 | 0 |  |  |  |  |  |  |  |  |
| GO:0007184\_SMAD\_protein\_nuclear\_translocation | 1 | 0 |  |  |  |  |  |  |  |  |
| GO:0007195\_dopamine\_receptor\_\_adenylate\_cyclase\_inhibiting\_pathway | 1 | 0 |  |  |  |  |  |  |  |  |
| GO:0007199\_G-protein\_signaling\_\_coupled\_to\_cGMP\_nucleotide\_second\_messenger | 1 | 0 |  |  |  |  |  |  |  |  |
| GO:0007206\_metabotropic\_glutamate\_receptor\_\_phospholipase\_C\_activating\_pathway | 1 | 0 |  |  |  |  |  |  |  |  |
| GO:0007210\_serotonin\_receptor\_signaling\_pathway | 1 | 0 |  |  |  |  |  |  |  |  |
| GO:0007217\_tachykinin\_signaling\_pathway | 1 | 0 |  |  |  |  |  |  |  |  |
| GO:0007258\_JUN\_phosphorylation | 1 | 0 |  |  |  |  |  |  |  |  |
| GO:0007262\_STAT\_protein\_nuclear\_translocation | 1 | 0 |  |  |  |  |  |  |  |  |
| GO:0007339\_binding\_of\_sperm\_to\_zona\_pellucida | 1 | 0 |  |  |  |  |  |  |  |  |
| GO:0007379\_segment\_specification | 1 | 0 |  |  |  |  |  |  |  |  |
| GO:0007387\_anterior\_compartment\_specification | 1 | 0 |  |  |  |  |  |  |  |  |
| GO:0007388\_posterior\_compartment\_specification | 1 | 0 |  |  |  |  |  |  |  |  |
| GO:0007431\_salivary\_gland\_development | 1 | 0 |  |  |  |  |  |  |  |  |
| GO:0007435\_salivary\_gland\_morphogenesis | 1 | 0 |  |  |  |  |  |  |  |  |
| GO:0007443\_Malpighian\_tubule\_morphogenesis | 1 | 0 |  |  |  |  |  |  |  |  |
| GO:0007444\_imaginal\_disc\_development | 1 | 0 |  |  |  |  |  |  |  |  |
| GO:0007447\_imaginal\_disc\_pattern\_formation | 1 | 0 |  |  |  |  |  |  |  |  |
| GO:0007468\_regulation\_of\_rhodopsin\_gene\_expression | 1 | 0 |  |  |  |  |  |  |  |  |
| GO:0007499\_ectoderm\_and\_mesoderm\_interaction | 1 | 0 |  |  |  |  |  |  |  |  |
| GO:0007501\_mesodermal\_cell\_fate\_specification | 1 | 0 |  |  |  |  |  |  |  |  |
| GO:0007509\_mesoderm\_migration | 1 | 0 |  |  |  |  |  |  |  |  |
| GO:0007518\_myoblast\_cell\_fate\_determination | 1 | 0 |  |  |  |  |  |  |  |  |
| GO:0007525\_somatic\_muscle\_development | 1 | 0 |  |  |  |  |  |  |  |  |
| GO:0007616\_long-term\_memory | 1 | 0 |  |  |  |  |  |  |  |  |
| GO:0007617\_mating\_behavior | 1 | 0 |  |  |  |  |  |  |  |  |
| GO:0007618\_mating | 1 | 0 |  |  |  |  |  |  |  |  |
| GO:0007622\_rhythmic\_behavior | 1 | 0 |  |  |  |  |  |  |  |  |
| GO:0007624\_ultradian\_rhythm | 1 | 0 |  |  |  |  |  |  |  |  |
| GO:0008045\_motor\_axon\_guidance | 1 | 0 |  |  |  |  |  |  |  |  |
| GO:0008065\_establishment\_of\_blood-nerve\_barrier | 1 | 0 |  |  |  |  |  |  |  |  |
| GO:0008090\_retrograde\_axon\_cargo\_transport | 1 | 0 |  |  |  |  |  |  |  |  |
| GO:0008212\_mineralocorticoid\_metabolic\_process | 1 | 0 |  |  |  |  |  |  |  |  |
| GO:0008215\_spermine\_metabolic\_process | 1 | 0 |  |  |  |  |  |  |  |  |
| GO:0008291\_acetylcholine\_metabolic\_process | 1 | 0 |  |  |  |  |  |  |  |  |
| GO:0008295\_spermidine\_biosynthetic\_process | 1 | 0 |  |  |  |  |  |  |  |  |
| GO:0008298\_intracellular\_mRNA\_localization | 1 | 0 |  |  |  |  |  |  |  |  |
| GO:0008356\_asymmetric\_cell\_division | 1 | 0 |  |  |  |  |  |  |  |  |
| GO:0008593\_regulation\_of\_Notch\_signaling\_pathway | 1 | 0 |  |  |  |  |  |  |  |  |
| GO:0008611\_ether\_lipid\_biosynthetic\_process | 1 | 0 |  |  |  |  |  |  |  |  |
| GO:0008614\_pyridoxine\_metabolic\_process | 1 | 0 |  |  |  |  |  |  |  |  |
| GO:0008615\_pyridoxine\_biosynthetic\_process | 1 | 0 |  |  |  |  |  |  |  |  |
| GO:0008616\_queuosine\_biosynthetic\_process | 1 | 0 |  |  |  |  |  |  |  |  |
| GO:0008618\_7-methylguanosine\_metabolic\_process | 1 | 0 |  |  |  |  |  |  |  |  |
| GO:0008627\_induction\_of\_apoptosis\_by\_ionic\_changes | 1 | 0 |  |  |  |  |  |  |  |  |
| GO:0008628\_induction\_of\_apoptosis\_by\_hormones | 1 | 0 |  |  |  |  |  |  |  |  |
| GO:0008633\_activation\_of\_pro-apoptotic\_gene\_products | 1 | 0 |  |  |  |  |  |  |  |  |
| GO:0008655\_pyrimidine\_salvage | 1 | 0 |  |  |  |  |  |  |  |  |
| GO:0009051\_pentose-phosphate\_shunt\_\_oxidative\_branch | 1 | 0 |  |  |  |  |  |  |  |  |
| GO:0009052\_pentose-phosphate\_shunt\_\_non-oxidative\_branch | 1 | 0 |  |  |  |  |  |  |  |  |
| GO:0009067\_aspartate\_family\_amino\_acid\_biosynthetic\_process | 1 | 0 |  |  |  |  |  |  |  |  |
| GO:0009082\_branched\_chain\_family\_amino\_acid\_biosynthetic\_process | 1 | 0 |  |  |  |  |  |  |  |  |
| GO:0009120\_deoxyribonucleoside\_metabolic\_process | 1 | 0 |  |  |  |  |  |  |  |  |
| GO:0009125\_nucleoside\_monophosphate\_catabolic\_process | 1 | 0 |  |  |  |  |  |  |  |  |
| GO:0009128\_purine\_nucleoside\_monophosphate\_catabolic\_process | 1 | 0 |  |  |  |  |  |  |  |  |
| GO:0009129\_pyrimidine\_nucleoside\_monophosphate\_metabolic\_process | 1 | 0 |  |  |  |  |  |  |  |  |
| GO:0009130\_pyrimidine\_nucleoside\_monophosphate\_biosynthetic\_process | 1 | 0 |  |  |  |  |  |  |  |  |
| GO:0009133\_nucleoside\_diphosphate\_biosynthetic\_process | 1 | 0 |  |  |  |  |  |  |  |  |
| GO:0009135\_purine\_nucleoside\_diphosphate\_metabolic\_process | 1 | 0 |  |  |  |  |  |  |  |  |
| GO:0009136\_purine\_nucleoside\_diphosphate\_biosynthetic\_process | 1 | 0 |  |  |  |  |  |  |  |  |
| GO:0009138\_pyrimidine\_nucleoside\_diphosphate\_metabolic\_process | 1 | 0 |  |  |  |  |  |  |  |  |
| GO:0009140\_pyrimidine\_nucleoside\_diphosphate\_catabolic\_process | 1 | 0 |  |  |  |  |  |  |  |  |
| GO:0009147\_pyrimidine\_nucleoside\_triphosphate\_metabolic\_process | 1 | 0 |  |  |  |  |  |  |  |  |
| GO:0009151\_purine\_deoxyribonucleotide\_metabolic\_process | 1 | 0 |  |  |  |  |  |  |  |  |
| GO:0009153\_purine\_deoxyribonucleotide\_biosynthetic\_process | 1 | 0 |  |  |  |  |  |  |  |  |
| GO:0009154\_purine\_ribonucleotide\_catabolic\_process | 1 | 0 |  |  |  |  |  |  |  |  |
| GO:0009157\_deoxyribonucleoside\_monophosphate\_biosynthetic\_process | 1 | 0 |  |  |  |  |  |  |  |  |
| GO:0009158\_ribonucleoside\_monophosphate\_catabolic\_process | 1 | 0 |  |  |  |  |  |  |  |  |
| GO:0009162\_deoxyribonucleoside\_monophosphate\_metabolic\_process | 1 | 0 |  |  |  |  |  |  |  |  |
| GO:0009163\_nucleoside\_biosynthetic\_process | 1 | 0 |  |  |  |  |  |  |  |  |
| GO:0009169\_purine\_ribonucleoside\_monophosphate\_catabolic\_process | 1 | 0 |  |  |  |  |  |  |  |  |
| GO:0009173\_pyrimidine\_ribonucleoside\_monophosphate\_metabolic\_process | 1 | 0 |  |  |  |  |  |  |  |  |
| GO:0009174\_pyrimidine\_ribonucleoside\_monophosphate\_biosynthetic\_process | 1 | 0 |  |  |  |  |  |  |  |  |
| GO:0009179\_purine\_ribonucleoside\_diphosphate\_metabolic\_process | 1 | 0 |  |  |  |  |  |  |  |  |
| GO:0009180\_purine\_ribonucleoside\_diphosphate\_biosynthetic\_process | 1 | 0 |  |  |  |  |  |  |  |  |
| GO:0009182\_purine\_deoxyribonucleoside\_diphosphate\_metabolic\_process | 1 | 0 |  |  |  |  |  |  |  |  |
| GO:0009183\_purine\_deoxyribonucleoside\_diphosphate\_biosynthetic\_process | 1 | 0 |  |  |  |  |  |  |  |  |
| GO:0009186\_deoxyribonucleoside\_diphosphate\_metabolic\_process | 1 | 0 |  |  |  |  |  |  |  |  |
| GO:0009188\_ribonucleoside\_diphosphate\_biosynthetic\_process | 1 | 0 |  |  |  |  |  |  |  |  |
| GO:0009189\_deoxyribonucleoside\_diphosphate\_biosynthetic\_process | 1 | 0 |  |  |  |  |  |  |  |  |
| GO:0009193\_pyrimidine\_ribonucleoside\_diphosphate\_metabolic\_process | 1 | 0 |  |  |  |  |  |  |  |  |
| GO:0009195\_pyrimidine\_ribonucleoside\_diphosphate\_catabolic\_process | 1 | 0 |  |  |  |  |  |  |  |  |
| GO:0009208\_pyrimidine\_ribonucleoside\_triphosphate\_metabolic\_process | 1 | 0 |  |  |  |  |  |  |  |  |
| GO:0009209\_pyrimidine\_ribonucleoside\_triphosphate\_biosynthetic\_process | 1 | 0 |  |  |  |  |  |  |  |  |
| GO:0009222\_pyrimidine\_ribonucleotide\_catabolic\_process | 1 | 0 |  |  |  |  |  |  |  |  |
| GO:0009231\_riboflavin\_biosynthetic\_process | 1 | 0 |  |  |  |  |  |  |  |  |
| GO:0009292\_genetic\_transfer | 1 | 0 |  |  |  |  |  |  |  |  |
| GO:0009294\_DNA\_mediated\_transformation | 1 | 0 |  |  |  |  |  |  |  |  |
| GO:0009298\_GDP-mannose\_biosynthetic\_process | 1 | 0 |  |  |  |  |  |  |  |  |
| GO:0009304\_tRNA\_transcription | 1 | 0 |  |  |  |  |  |  |  |  |
| GO:0009399\_nitrogen\_fixation | 1 | 0 |  |  |  |  |  |  |  |  |
| GO:0009404\_toxin\_metabolic\_process | 1 | 0 |  |  |  |  |  |  |  |  |
| GO:0009441\_glycolate\_metabolic\_process | 1 | 0 |  |  |  |  |  |  |  |  |
| GO:0009597\_detection\_of\_virus | 1 | 0 |  |  |  |  |  |  |  |  |
| GO:0009612\_response\_to\_mechanical\_stimulus | 1 | 0 |  |  |  |  |  |  |  |  |
| GO:0009642\_response\_to\_light\_intensity | 1 | 0 |  |  |  |  |  |  |  |  |
| GO:0009648\_photoperiodism | 1 | 0 |  |  |  |  |  |  |  |  |
| GO:0009720\_detection\_of\_hormone\_stimulus | 1 | 0 |  |  |  |  |  |  |  |  |
| GO:0009730\_detection\_of\_carbohydrate\_stimulus | 1 | 0 |  |  |  |  |  |  |  |  |
| GO:0009732\_detection\_of\_hexose\_stimulus | 1 | 0 |  |  |  |  |  |  |  |  |
| GO:0009756\_carbohydrate\_mediated\_signaling | 1 | 0 |  |  |  |  |  |  |  |  |
| GO:0009791\_post-embryonic\_development | 1 | 0 |  |  |  |  |  |  |  |  |
| GO:0009826\_unidimensional\_cell\_growth | 1 | 0 |  |  |  |  |  |  |  |  |
| GO:0009912\_auditory\_receptor\_cell\_fate\_commitment | 1 | 0 |  |  |  |  |  |  |  |  |
| GO:0009935\_nutrient\_import | 1 | 0 |  |  |  |  |  |  |  |  |
| GO:0009950\_dorsal\_ventral\_axis\_specification | 1 | 0 |  |  |  |  |  |  |  |  |
| GO:0009954\_proximal\_distal\_pattern\_formation | 1 | 0 |  |  |  |  |  |  |  |  |
| GO:0009972\_cytidine\_deamination | 1 | 0 |  |  |  |  |  |  |  |  |
| GO:0009988\_cell-cell\_recognition | 1 | 0 |  |  |  |  |  |  |  |  |
| GO:0010002\_cardioblast\_differentiation | 1 | 0 |  |  |  |  |  |  |  |  |
| GO:0010039\_response\_to\_iron\_ion | 1 | 0 |  |  |  |  |  |  |  |  |
| GO:0010042\_response\_to\_manganese\_ion | 1 | 0 |  |  |  |  |  |  |  |  |
| GO:0010149\_senescence | 1 | 0 |  |  |  |  |  |  |  |  |
| GO:0010216\_maintenance\_of\_DNA\_methylation | 1 | 0 |  |  |  |  |  |  |  |  |
| GO:0010248\_establishment\_and\_or\_maintenance\_of\_transmembrane\_electrochemical\_gradient | 1 | 0 |  |  |  |  |  |  |  |  |
| GO:0010269\_response\_to\_selenium\_ion | 1 | 0 |  |  |  |  |  |  |  |  |
| GO:0010389\_regulation\_of\_G2\_M\_transition\_of\_mitotic\_cell\_cycle | 1 | 0 |  |  |  |  |  |  |  |  |
| GO:0010457\_centriole-centriole\_cohesion | 1 | 0 |  |  |  |  |  |  |  |  |
| GO:0010509\_polyamine\_homeostasis | 1 | 0 |  |  |  |  |  |  |  |  |
| GO:0010553\_negative\_regulation\_of\_specific\_transcription\_from\_RNA\_polymerase\_II\_promoter | 1 | 0 |  |  |  |  |  |  |  |  |
| GO:0010559\_regulation\_of\_glycoprotein\_biosynthetic\_process | 1 | 0 |  |  |  |  |  |  |  |  |
| GO:0010561\_negative\_regulation\_of\_glycoprotein\_biosynthetic\_process | 1 | 0 |  |  |  |  |  |  |  |  |
| GO:0014002\_astrocyte\_development | 1 | 0 |  |  |  |  |  |  |  |  |
| GO:0014009\_glial\_cell\_proliferation | 1 | 0 |  |  |  |  |  |  |  |  |
| GO:0014010\_Schwann\_cell\_proliferation | 1 | 0 |  |  |  |  |  |  |  |  |
| GO:0014037\_Schwann\_cell\_differentiation | 1 | 0 |  |  |  |  |  |  |  |  |
| GO:0014044\_Schwann\_cell\_development | 1 | 0 |  |  |  |  |  |  |  |  |
| GO:0014066\_regulation\_of\_phosphoinositide\_3-kinase\_cascade | 1 | 0 |  |  |  |  |  |  |  |  |
| GO:0014068\_positive\_regulation\_of\_phosphoinositide\_3-kinase\_cascade | 1 | 0 |  |  |  |  |  |  |  |  |
| GO:0014073\_response\_to\_tropane | 1 | 0 |  |  |  |  |  |  |  |  |
| GO:0014855\_striated\_muscle\_cell\_proliferation | 1 | 0 |  |  |  |  |  |  |  |  |
| GO:0015074\_DNA\_integration | 1 | 0 |  |  |  |  |  |  |  |  |
| GO:0015677\_copper\_ion\_import | 1 | 0 |  |  |  |  |  |  |  |  |
| GO:0015680\_intracellular\_copper\_ion\_transport | 1 | 0 |  |  |  |  |  |  |  |  |
| GO:0015697\_quaternary\_ammonium\_group\_transport | 1 | 0 |  |  |  |  |  |  |  |  |
| GO:0015728\_mevalonate\_transport | 1 | 0 |  |  |  |  |  |  |  |  |
| GO:0015746\_citrate\_transport | 1 | 0 |  |  |  |  |  |  |  |  |
| GO:0015755\_fructose\_transport | 1 | 0 |  |  |  |  |  |  |  |  |
| GO:0015760\_glucose-6-phosphate\_transport | 1 | 0 |  |  |  |  |  |  |  |  |
| GO:0015782\_CMP-sialic\_acid\_transport | 1 | 0 |  |  |  |  |  |  |  |  |
| GO:0015785\_UDP-galactose\_transport | 1 | 0 |  |  |  |  |  |  |  |  |
| GO:0015789\_UDP-N-acetylgalactosamine\_transport | 1 | 0 |  |  |  |  |  |  |  |  |
| GO:0015790\_UDP-xylose\_transport | 1 | 0 |  |  |  |  |  |  |  |  |
| GO:0015793\_glycerol\_transport | 1 | 0 |  |  |  |  |  |  |  |  |
| GO:0015805\_S-adenosylmethionine\_transport | 1 | 0 |  |  |  |  |  |  |  |  |
| GO:0015808\_L-alanine\_transport | 1 | 0 |  |  |  |  |  |  |  |  |
| GO:0015809\_arginine\_transport | 1 | 0 |  |  |  |  |  |  |  |  |
| GO:0015817\_histidine\_transport | 1 | 0 |  |  |  |  |  |  |  |  |
| GO:0015824\_proline\_transport | 1 | 0 |  |  |  |  |  |  |  |  |
| GO:0015825\_L-serine\_transport | 1 | 0 |  |  |  |  |  |  |  |  |
| GO:0015827\_tryptophan\_transport | 1 | 0 |  |  |  |  |  |  |  |  |
| GO:0015840\_urea\_transport | 1 | 0 |  |  |  |  |  |  |  |  |
| GO:0015853\_adenine\_transport | 1 | 0 |  |  |  |  |  |  |  |  |
| GO:0015855\_pyrimidine\_transport | 1 | 0 |  |  |  |  |  |  |  |  |
| GO:0015870\_acetylcholine\_transport | 1 | 0 |  |  |  |  |  |  |  |  |
| GO:0015872\_dopamine\_transport | 1 | 0 |  |  |  |  |  |  |  |  |
| GO:0015886\_heme\_transport | 1 | 0 |  |  |  |  |  |  |  |  |
| GO:0015888\_thiamin\_transport | 1 | 0 |  |  |  |  |  |  |  |  |
| GO:0015909\_long-chain\_fatty\_acid\_transport | 1 | 0 |  |  |  |  |  |  |  |  |
| GO:0015910\_peroxisomal\_long-chain\_fatty\_acid\_import | 1 | 0 |  |  |  |  |  |  |  |  |
| GO:0015919\_peroxisomal\_membrane\_transport | 1 | 0 |  |  |  |  |  |  |  |  |
| GO:0015936\_coenzyme\_A\_metabolic\_process | 1 | 0 |  |  |  |  |  |  |  |  |
| GO:0015937\_coenzyme\_A\_biosynthetic\_process | 1 | 0 |  |  |  |  |  |  |  |  |
| GO:0015942\_formate\_metabolic\_process | 1 | 0 |  |  |  |  |  |  |  |  |
| GO:0015956\_bis(5'-nucleosidyl)\_oligophosphate\_metabolic\_process | 1 | 0 |  |  |  |  |  |  |  |  |
| GO:0015958\_bis(5'-nucleosidyl)\_oligophosphate\_catabolic\_process | 1 | 0 |  |  |  |  |  |  |  |  |
| GO:0015959\_diadenosine\_polyphosphate\_metabolic\_process | 1 | 0 |  |  |  |  |  |  |  |  |
| GO:0015961\_diadenosine\_polyphosphate\_catabolic\_process | 1 | 0 |  |  |  |  |  |  |  |  |
| GO:0015988\_energy\_coupled\_proton\_transport\_\_against\_electrochemical\_gradient | 1 | 0 |  |  |  |  |  |  |  |  |
| GO:0015991\_ATP\_hydrolysis\_coupled\_proton\_transport | 1 | 0 |  |  |  |  |  |  |  |  |
| GO:0015993\_molecular\_hydrogen\_transport | 1 | 0 |  |  |  |  |  |  |  |  |
| GO:0016046\_detection\_of\_fungus | 1 | 0 |  |  |  |  |  |  |  |  |
| GO:0016078\_tRNA\_catabolic\_process | 1 | 0 |  |  |  |  |  |  |  |  |
| GO:0016090\_prenol\_metabolic\_process | 1 | 0 |  |  |  |  |  |  |  |  |
| GO:0016091\_prenol\_biosynthetic\_process | 1 | 0 |  |  |  |  |  |  |  |  |
| GO:0016093\_polyprenol\_metabolic\_process | 1 | 0 |  |  |  |  |  |  |  |  |
| GO:0016094\_polyprenol\_biosynthetic\_process | 1 | 0 |  |  |  |  |  |  |  |  |
| GO:0016127\_sterol\_catabolic\_process | 1 | 0 |  |  |  |  |  |  |  |  |
| GO:0016137\_glycoside\_metabolic\_process | 1 | 0 |  |  |  |  |  |  |  |  |
| GO:0016139\_glycoside\_catabolic\_process | 1 | 0 |  |  |  |  |  |  |  |  |
| GO:0016140\_O-glycoside\_metabolic\_process | 1 | 0 |  |  |  |  |  |  |  |  |
| GO:0016142\_O-glycoside\_catabolic\_process | 1 | 0 |  |  |  |  |  |  |  |  |
| GO:0016188\_synaptic\_vesicle\_maturation | 1 | 0 |  |  |  |  |  |  |  |  |
| GO:0016189\_synaptic\_vesicle\_to\_endosome\_fusion | 1 | 0 |  |  |  |  |  |  |  |  |
| GO:0016259\_selenocysteine\_metabolic\_process | 1 | 0 |  |  |  |  |  |  |  |  |
| GO:0016260\_selenocysteine\_biosynthetic\_process | 1 | 0 |  |  |  |  |  |  |  |  |
| GO:0016264\_gap\_junction\_assembly | 1 | 0 |  |  |  |  |  |  |  |  |
| GO:0016266\_O-glycan\_processing | 1 | 0 |  |  |  |  |  |  |  |  |
| GO:0016320\_endoplasmic\_reticulum\_membrane\_fusion | 1 | 0 |  |  |  |  |  |  |  |  |
| GO:0016446\_somatic\_hypermutation\_of\_immunoglobulin\_genes | 1 | 0 |  |  |  |  |  |  |  |  |
| GO:0016482\_cytoplasmic\_transport | 1 | 0 |  |  |  |  |  |  |  |  |
| GO:0016559\_peroxisome\_fission | 1 | 0 |  |  |  |  |  |  |  |  |
| GO:0016560\_protein\_import\_into\_peroxisome\_matrix\_\_docking | 1 | 0 |  |  |  |  |  |  |  |  |
| GO:0016598\_protein\_arginylation | 1 | 0 |  |  |  |  |  |  |  |  |
| GO:0017055\_negative\_regulation\_of\_transcriptional\_preinitiation\_complex\_assembly | 1 | 0 |  |  |  |  |  |  |  |  |
| GO:0017145\_stem\_cell\_division | 1 | 0 |  |  |  |  |  |  |  |  |
| GO:0017158\_regulation\_of\_calcium\_ion-dependent\_exocytosis | 1 | 0 |  |  |  |  |  |  |  |  |
| GO:0017182\_peptidyl-diphthamide\_metabolic\_process | 1 | 0 |  |  |  |  |  |  |  |  |
| GO:0017183\_peptidyl-diphthamide\_biosynthetic\_process\_from\_peptidyl-histidine | 1 | 0 |  |  |  |  |  |  |  |  |
| GO:0018146\_keratan\_sulfate\_biosynthetic\_process | 1 | 0 |  |  |  |  |  |  |  |  |
| GO:0018153\_isopeptide\_cross-linking\_via\_N6-(L-isoglutamyl)-L-lysine | 1 | 0 |  |  |  |  |  |  |  |  |
| GO:0018199\_peptidyl-glutamine\_modification | 1 | 0 |  |  |  |  |  |  |  |  |
| GO:0018202\_peptidyl-histidine\_modification | 1 | 0 |  |  |  |  |  |  |  |  |
| GO:0018208\_peptidyl-proline\_modification | 1 | 0 |  |  |  |  |  |  |  |  |
| GO:0018262\_isopeptide\_cross-linking | 1 | 0 |  |  |  |  |  |  |  |  |
| GO:0018277\_protein\_amino\_acid\_deamination | 1 | 0 |  |  |  |  |  |  |  |  |
| GO:0018282\_metal\_incorporation\_into\_metallo-sulfur\_cluster | 1 | 0 |  |  |  |  |  |  |  |  |
| GO:0018283\_iron\_incorporation\_into\_metallo-sulfur\_cluster | 1 | 0 |  |  |  |  |  |  |  |  |
| GO:0018319\_protein\_amino\_acid\_myristoylation | 1 | 0 |  |  |  |  |  |  |  |  |
| GO:0018352\_protein-pyridoxal-5-phosphate\_linkage | 1 | 0 |  |  |  |  |  |  |  |  |
| GO:0018377\_protein\_myristoylation | 1 | 0 |  |  |  |  |  |  |  |  |
| GO:0018401\_peptidyl-proline\_hydroxylation\_to\_4-hydroxy-L-proline | 1 | 0 |  |  |  |  |  |  |  |  |
| GO:0018872\_arsonoacetate\_metabolic\_process | 1 | 0 |  |  |  |  |  |  |  |  |
| GO:0019060\_intracellular\_transport\_of\_viral\_proteins\_in\_host\_cell | 1 | 0 |  |  |  |  |  |  |  |  |
| GO:0019062\_virion\_attachment\_to\_host\_cell\_surface\_receptor | 1 | 0 |  |  |  |  |  |  |  |  |
| GO:0019063\_virion\_penetration\_into\_host\_cell | 1 | 0 |  |  |  |  |  |  |  |  |
| GO:0019098\_reproductive\_behavior | 1 | 0 |  |  |  |  |  |  |  |  |
| GO:0019233\_sensory\_perception\_of\_pain | 1 | 0 |  |  |  |  |  |  |  |  |
| GO:0019303\_D-ribose\_catabolic\_process | 1 | 0 |  |  |  |  |  |  |  |  |
| GO:0019307\_mannose\_biosynthetic\_process | 1 | 0 |  |  |  |  |  |  |  |  |
| GO:0019310\_inositol\_catabolic\_process | 1 | 0 |  |  |  |  |  |  |  |  |
| GO:0019323\_pentose\_catabolic\_process | 1 | 0 |  |  |  |  |  |  |  |  |
| GO:0019348\_dolichol\_metabolic\_process | 1 | 0 |  |  |  |  |  |  |  |  |
| GO:0019363\_pyridine\_nucleotide\_biosynthetic\_process | 1 | 0 |  |  |  |  |  |  |  |  |
| GO:0019371\_cyclooxygenase\_pathway | 1 | 0 |  |  |  |  |  |  |  |  |
| GO:0019372\_lipoxygenase\_pathway | 1 | 0 |  |  |  |  |  |  |  |  |
| GO:0019374\_galactolipid\_metabolic\_process | 1 | 0 |  |  |  |  |  |  |  |  |
| GO:0019408\_dolichol\_biosynthetic\_process | 1 | 0 |  |  |  |  |  |  |  |  |
| GO:0019430\_removal\_of\_superoxide\_radicals | 1 | 0 |  |  |  |  |  |  |  |  |
| GO:0019433\_triacylglycerol\_catabolic\_process | 1 | 0 |  |  |  |  |  |  |  |  |
| GO:0019471\_4-hydroxyproline\_metabolic\_process | 1 | 0 |  |  |  |  |  |  |  |  |
| GO:0019511\_peptidyl-proline\_hydroxylation | 1 | 0 |  |  |  |  |  |  |  |  |
| GO:0019530\_taurine\_metabolic\_process | 1 | 0 |  |  |  |  |  |  |  |  |
| GO:0019532\_oxalate\_transport | 1 | 0 |  |  |  |  |  |  |  |  |
| GO:0019585\_glucuronate\_metabolic\_process | 1 | 0 |  |  |  |  |  |  |  |  |
| GO:0019673\_GDP-mannose\_metabolic\_process | 1 | 0 |  |  |  |  |  |  |  |  |
| GO:0019730\_antimicrobial\_humoral\_response | 1 | 0 |  |  |  |  |  |  |  |  |
| GO:0019731\_antibacterial\_humoral\_response | 1 | 0 |  |  |  |  |  |  |  |  |
| GO:0019747\_regulation\_of\_isoprenoid\_metabolic\_process | 1 | 0 |  |  |  |  |  |  |  |  |
| GO:0019827\_stem\_cell\_maintenance | 1 | 0 |  |  |  |  |  |  |  |  |
| GO:0019835\_cytolysis | 1 | 0 |  |  |  |  |  |  |  |  |
| GO:0019852\_L-ascorbic\_acid\_metabolic\_process | 1 | 0 |  |  |  |  |  |  |  |  |
| GO:0019856\_pyrimidine\_base\_biosynthetic\_process | 1 | 0 |  |  |  |  |  |  |  |  |
| GO:0019858\_cytosine\_metabolic\_process | 1 | 0 |  |  |  |  |  |  |  |  |
| GO:0019859\_thymine\_metabolic\_process | 1 | 0 |  |  |  |  |  |  |  |  |
| GO:0019860\_uracil\_metabolic\_process | 1 | 0 |  |  |  |  |  |  |  |  |
| GO:0019884\_antigen\_processing\_and\_presentation\_of\_exogenous\_antigen | 1 | 0 |  |  |  |  |  |  |  |  |
| GO:0019886\_antigen\_processing\_and\_presentation\_of\_exogenous\_peptide\_antigen\_via\_MHC\_class\_II | 1 | 0 |  |  |  |  |  |  |  |  |
| GO:0019915\_sequestering\_of\_lipid | 1 | 0 |  |  |  |  |  |  |  |  |
| GO:0021508\_floor\_plate\_formation | 1 | 0 |  |  |  |  |  |  |  |  |
| GO:0021511\_spinal\_cord\_patterning | 1 | 0 |  |  |  |  |  |  |  |  |
| GO:0021513\_spinal\_cord\_dorsal\_ventral\_patterning | 1 | 0 |  |  |  |  |  |  |  |  |
| GO:0021514\_ventral\_spinal\_cord\_interneuron\_differentiation | 1 | 0 |  |  |  |  |  |  |  |  |
| GO:0021516\_dorsal\_spinal\_cord\_development | 1 | 0 |  |  |  |  |  |  |  |  |
| GO:0021521\_ventral\_spinal\_cord\_interneuron\_specification | 1 | 0 |  |  |  |  |  |  |  |  |
| GO:0021522\_spinal\_cord\_motor\_neuron\_differentiation | 1 | 0 |  |  |  |  |  |  |  |  |
| GO:0021527\_spinal\_cord\_association\_neuron\_differentiation | 1 | 0 |  |  |  |  |  |  |  |  |
| GO:0021537\_telencephalon\_development | 1 | 0 |  |  |  |  |  |  |  |  |
| GO:0021544\_subpallium\_development | 1 | 0 |  |  |  |  |  |  |  |  |
| GO:0021549\_cerebellum\_development | 1 | 0 |  |  |  |  |  |  |  |  |
| GO:0021575\_hindbrain\_morphogenesis | 1 | 0 |  |  |  |  |  |  |  |  |
| GO:0021587\_cerebellum\_morphogenesis | 1 | 0 |  |  |  |  |  |  |  |  |
| GO:0021695\_cerebellar\_cortex\_development | 1 | 0 |  |  |  |  |  |  |  |  |
| GO:0021696\_cerebellar\_cortex\_morphogenesis | 1 | 0 |  |  |  |  |  |  |  |  |
| GO:0021756\_striatum\_development | 1 | 0 |  |  |  |  |  |  |  |  |
| GO:0021757\_caudate\_nucleus\_development | 1 | 0 |  |  |  |  |  |  |  |  |
| GO:0021758\_putamen\_development | 1 | 0 |  |  |  |  |  |  |  |  |
| GO:0021761\_limbic\_system\_development | 1 | 0 |  |  |  |  |  |  |  |  |
| GO:0021775\_smoothened\_signaling\_pathway\_involved\_in\_ventral\_spinal\_cord\_interneuron\_specification | 1 | 0 |  |  |  |  |  |  |  |  |
| GO:0021854\_hypothalamus\_development | 1 | 0 |  |  |  |  |  |  |  |  |
| GO:0021910\_smoothened\_signaling\_pathway\_in\_ventral\_spinal\_cord\_patterning | 1 | 0 |  |  |  |  |  |  |  |  |
| GO:0021952\_central\_nervous\_system\_projection\_neuron\_axonogenesis | 1 | 0 |  |  |  |  |  |  |  |  |
| GO:0021953\_central\_nervous\_system\_neuron\_differentiation | 1 | 0 |  |  |  |  |  |  |  |  |
| GO:0021954\_central\_nervous\_system\_neuron\_development | 1 | 0 |  |  |  |  |  |  |  |  |
| GO:0021955\_central\_nervous\_system\_neuron\_axonogenesis | 1 | 0 |  |  |  |  |  |  |  |  |
| GO:0021965\_spinal\_cord\_ventral\_commissure\_morphogenesis | 1 | 0 |  |  |  |  |  |  |  |  |
| GO:0021990\_neural\_plate\_formation | 1 | 0 |  |  |  |  |  |  |  |  |
| GO:0022011\_myelination\_in\_the\_peripheral\_nervous\_system | 1 | 0 |  |  |  |  |  |  |  |  |
| GO:0022037\_metencephalon\_development | 1 | 0 |  |  |  |  |  |  |  |  |
| GO:0022401\_adaptation\_of\_signaling\_pathway | 1 | 0 |  |  |  |  |  |  |  |  |
| GO:0022410\_circadian\_sleep\_wake\_cycle\_process | 1 | 0 |  |  |  |  |  |  |  |  |
| GO:0022417\_protein\_maturation\_via\_protein\_folding | 1 | 0 |  |  |  |  |  |  |  |  |
| GO:0022601\_menstrual\_cycle\_phase | 1 | 0 |  |  |  |  |  |  |  |  |
| GO:0022612\_gland\_morphogenesis | 1 | 0 |  |  |  |  |  |  |  |  |
| GO:0022617\_extracellular\_matrix\_disassembly | 1 | 0 |  |  |  |  |  |  |  |  |
| GO:0030002\_cellular\_anion\_homeostasis | 1 | 0 |  |  |  |  |  |  |  |  |
| GO:0030011\_maintenance\_of\_cell\_polarity | 1 | 0 |  |  |  |  |  |  |  |  |
| GO:0030026\_cellular\_manganese\_ion\_homeostasis | 1 | 0 |  |  |  |  |  |  |  |  |
| GO:0030033\_microvillus\_biogenesis | 1 | 0 |  |  |  |  |  |  |  |  |
| GO:0030037\_actin\_filament\_reorganization\_during\_cell\_cycle | 1 | 0 |  |  |  |  |  |  |  |  |
| GO:0030047\_actin\_modification | 1 | 0 |  |  |  |  |  |  |  |  |
| GO:0030070\_insulin\_processing | 1 | 0 |  |  |  |  |  |  |  |  |
| GO:0030157\_pancreatic\_juice\_secretion | 1 | 0 |  |  |  |  |  |  |  |  |
| GO:0030177\_positive\_regulation\_of\_Wnt\_receptor\_signaling\_pathway | 1 | 0 |  |  |  |  |  |  |  |  |
| GO:0030186\_melatonin\_metabolic\_process | 1 | 0 |  |  |  |  |  |  |  |  |
| GO:0030187\_melatonin\_biosynthetic\_process | 1 | 0 |  |  |  |  |  |  |  |  |
| GO:0030202\_heparin\_metabolic\_process | 1 | 0 |  |  |  |  |  |  |  |  |
| GO:0030210\_heparin\_biosynthetic\_process | 1 | 0 |  |  |  |  |  |  |  |  |
| GO:0030220\_platelet\_formation | 1 | 0 |  |  |  |  |  |  |  |  |
| GO:0030259\_lipid\_glycosylation | 1 | 0 |  |  |  |  |  |  |  |  |
| GO:0030264\_nuclear\_fragmentation\_during\_apoptosis | 1 | 0 |  |  |  |  |  |  |  |  |
| GO:0030277\_maintenance\_of\_gastrointestinal\_epithelium | 1 | 0 |  |  |  |  |  |  |  |  |
| GO:0030302\_deoxynucleotide\_transport | 1 | 0 |  |  |  |  |  |  |  |  |
| GO:0030319\_cellular\_di-\_\_tri-valent\_inorganic\_anion\_homeostasis | 1 | 0 |  |  |  |  |  |  |  |  |
| GO:0030321\_transepithelial\_chloride\_transport | 1 | 0 |  |  |  |  |  |  |  |  |
| GO:0030389\_fructosamine\_metabolic\_process | 1 | 0 |  |  |  |  |  |  |  |  |
| GO:0030393\_fructoselysine\_metabolic\_process | 1 | 0 |  |  |  |  |  |  |  |  |
| GO:0030423\_RNA\_interference\_\_targeting\_of\_mRNA\_for\_destruction | 1 | 0 |  |  |  |  |  |  |  |  |
| GO:0030431\_sleep | 1 | 0 |  |  |  |  |  |  |  |  |
| GO:0030488\_tRNA\_methylation | 1 | 0 |  |  |  |  |  |  |  |  |
| GO:0030502\_negative\_regulation\_of\_bone\_mineralization | 1 | 0 |  |  |  |  |  |  |  |  |
| GO:0030517\_negative\_regulation\_of\_axon\_extension | 1 | 0 |  |  |  |  |  |  |  |  |
| GO:0030534\_adult\_behavior | 1 | 0 |  |  |  |  |  |  |  |  |
| GO:0030540\_female\_genitalia\_development | 1 | 0 |  |  |  |  |  |  |  |  |
| GO:0030575\_nuclear\_body\_organization\_and\_biogenesis | 1 | 0 |  |  |  |  |  |  |  |  |
| GO:0030578\_PML\_body\_organization\_and\_biogenesis | 1 | 0 |  |  |  |  |  |  |  |  |
| GO:0030579\_ubiquitin-dependent\_SMAD\_protein\_catabolic\_process | 1 | 0 |  |  |  |  |  |  |  |  |
| GO:0030581\_intracellular\_protein\_transport\_in\_host | 1 | 0 |  |  |  |  |  |  |  |  |
| GO:0030643\_cellular\_phosphate\_ion\_homeostasis | 1 | 0 |  |  |  |  |  |  |  |  |
| GO:0030728\_ovulation | 1 | 0 |  |  |  |  |  |  |  |  |
| GO:0030730\_sequestering\_of\_triacylglycerol | 1 | 0 |  |  |  |  |  |  |  |  |
| GO:0030800\_negative\_regulation\_of\_cyclic\_nucleotide\_metabolic\_process | 1 | 0 |  |  |  |  |  |  |  |  |
| GO:0030803\_negative\_regulation\_of\_cyclic\_nucleotide\_biosynthetic\_process | 1 | 0 |  |  |  |  |  |  |  |  |
| GO:0030809\_negative\_regulation\_of\_nucleotide\_biosynthetic\_process | 1 | 0 |  |  |  |  |  |  |  |  |
| GO:0030816\_positive\_regulation\_of\_cAMP\_metabolic\_process | 1 | 0 |  |  |  |  |  |  |  |  |
| GO:0030819\_positive\_regulation\_of\_cAMP\_biosynthetic\_process | 1 | 0 |  |  |  |  |  |  |  |  |
| GO:0030824\_negative\_regulation\_of\_cGMP\_metabolic\_process | 1 | 0 |  |  |  |  |  |  |  |  |
| GO:0030825\_positive\_regulation\_of\_cGMP\_metabolic\_process | 1 | 0 |  |  |  |  |  |  |  |  |
| GO:0030827\_negative\_regulation\_of\_cGMP\_biosynthetic\_process | 1 | 0 |  |  |  |  |  |  |  |  |
| GO:0030828\_positive\_regulation\_of\_cGMP\_biosynthetic\_process | 1 | 0 |  |  |  |  |  |  |  |  |
| GO:0030837\_negative\_regulation\_of\_actin\_filament\_polymerization | 1 | 0 |  |  |  |  |  |  |  |  |
| GO:0030852\_regulation\_of\_granulocyte\_differentiation | 1 | 0 |  |  |  |  |  |  |  |  |
| GO:0030853\_negative\_regulation\_of\_granulocyte\_differentiation | 1 | 0 |  |  |  |  |  |  |  |  |
| GO:0030854\_positive\_regulation\_of\_granulocyte\_differentiation | 1 | 0 |  |  |  |  |  |  |  |  |
| GO:0030856\_regulation\_of\_epithelial\_cell\_differentiation | 1 | 0 |  |  |  |  |  |  |  |  |
| GO:0030878\_thyroid\_gland\_development | 1 | 0 |  |  |  |  |  |  |  |  |
| GO:0030885\_regulation\_of\_myeloid\_dendritic\_cell\_activation | 1 | 0 |  |  |  |  |  |  |  |  |
| GO:0030887\_positive\_regulation\_of\_myeloid\_dendritic\_cell\_activation | 1 | 0 |  |  |  |  |  |  |  |  |
| GO:0030901\_midbrain\_development | 1 | 0 |  |  |  |  |  |  |  |  |
| GO:0030903\_notochord\_development | 1 | 0 |  |  |  |  |  |  |  |  |
| GO:0030913\_paranodal\_junction\_assembly | 1 | 0 |  |  |  |  |  |  |  |  |
| GO:0030916\_otic\_vesicle\_formation | 1 | 0 |  |  |  |  |  |  |  |  |
| GO:0030948\_negative\_regulation\_of\_vascular\_endothelial\_growth\_factor\_receptor\_signaling\_pathway | 1 | 0 |  |  |  |  |  |  |  |  |
| GO:0030949\_positive\_regulation\_of\_vascular\_endothelial\_growth\_factor\_receptor\_signaling\_pathway | 1 | 0 |  |  |  |  |  |  |  |  |
| GO:0030967\_ER-nuclear\_sterol\_response\_pathway | 1 | 0 |  |  |  |  |  |  |  |  |
| GO:0030997\_regulation\_of\_centriole-centriole\_cohesion | 1 | 0 |  |  |  |  |  |  |  |  |
| GO:0031016\_pancreas\_development | 1 | 0 |  |  |  |  |  |  |  |  |
| GO:0031018\_endocrine\_pancreas\_development | 1 | 0 |  |  |  |  |  |  |  |  |
| GO:0031050\_dsRNA\_fragmentation | 1 | 0 |  |  |  |  |  |  |  |  |
| GO:0031053\_primary\_microRNA\_processing | 1 | 0 |  |  |  |  |  |  |  |  |
| GO:0031055\_chromatin\_remodeling\_at\_centromere | 1 | 0 |  |  |  |  |  |  |  |  |
| GO:0031057\_negative\_regulation\_of\_histone\_modification | 1 | 0 |  |  |  |  |  |  |  |  |
| GO:0031063\_regulation\_of\_histone\_deacetylation | 1 | 0 |  |  |  |  |  |  |  |  |
| GO:0031065\_positive\_regulation\_of\_histone\_deacetylation | 1 | 0 |  |  |  |  |  |  |  |  |
| GO:0031081\_nuclear\_pore\_distribution | 1 | 0 |  |  |  |  |  |  |  |  |
| GO:0031117\_positive\_regulation\_of\_microtubule\_depolymerization | 1 | 0 |  |  |  |  |  |  |  |  |
| GO:0031118\_rRNA\_pseudouridine\_synthesis | 1 | 0 |  |  |  |  |  |  |  |  |
| GO:0031122\_cytoplasmic\_microtubule\_organization\_and\_biogenesis | 1 | 0 |  |  |  |  |  |  |  |  |
| GO:0031293\_membrane\_protein\_intracellular\_domain\_proteolysis | 1 | 0 |  |  |  |  |  |  |  |  |
| GO:0031294\_lymphocyte\_costimulation | 1 | 0 |  |  |  |  |  |  |  |  |
| GO:0031295\_T\_cell\_costimulation | 1 | 0 |  |  |  |  |  |  |  |  |
| GO:0031330\_negative\_regulation\_of\_cellular\_catabolic\_process | 1 | 0 |  |  |  |  |  |  |  |  |
| GO:0031333\_negative\_regulation\_of\_protein\_complex\_assembly | 1 | 0 |  |  |  |  |  |  |  |  |
| GO:0031342\_negative\_regulation\_of\_cell\_killing | 1 | 0 |  |  |  |  |  |  |  |  |
| GO:0031441\_negative\_regulation\_of\_mRNA\_3'-end\_processing | 1 | 0 |  |  |  |  |  |  |  |  |
| GO:0031442\_positive\_regulation\_of\_mRNA\_3'-end\_processing | 1 | 0 |  |  |  |  |  |  |  |  |
| GO:0031508\_centric\_heterochromatin\_formation | 1 | 0 |  |  |  |  |  |  |  |  |
| GO:0031509\_telomeric\_heterochromatin\_formation | 1 | 0 |  |  |  |  |  |  |  |  |
| GO:0031557\_induction\_of\_programmed\_cell\_death\_in\_response\_to\_chemical\_stimulus | 1 | 0 |  |  |  |  |  |  |  |  |
| GO:0031573\_intra-S\_DNA\_damage\_checkpoint | 1 | 0 |  |  |  |  |  |  |  |  |
| GO:0031581\_hemidesmosome\_assembly | 1 | 0 |  |  |  |  |  |  |  |  |
| GO:0031641\_regulation\_of\_myelination | 1 | 0 |  |  |  |  |  |  |  |  |
| GO:0031642\_negative\_regulation\_of\_myelination | 1 | 0 |  |  |  |  |  |  |  |  |
| GO:0031645\_negative\_regulation\_of\_neurological\_system\_process | 1 | 0 |  |  |  |  |  |  |  |  |
| GO:0031648\_protein\_destabilization | 1 | 0 |  |  |  |  |  |  |  |  |
| GO:0031663\_lipopolysaccharide-mediated\_signaling\_pathway | 1 | 0 |  |  |  |  |  |  |  |  |
| GO:0031664\_regulation\_of\_lipopolysaccharide-mediated\_signaling\_pathway | 1 | 0 |  |  |  |  |  |  |  |  |
| GO:0031665\_negative\_regulation\_of\_lipopolysaccharide-mediated\_signaling\_pathway | 1 | 0 |  |  |  |  |  |  |  |  |
| GO:0031929\_TOR\_signaling\_pathway | 1 | 0 |  |  |  |  |  |  |  |  |
| GO:0031943\_regulation\_of\_glucocorticoid\_metabolic\_process | 1 | 0 |  |  |  |  |  |  |  |  |
| GO:0031952\_regulation\_of\_protein\_amino\_acid\_autophosphorylation | 1 | 0 |  |  |  |  |  |  |  |  |
| GO:0031953\_negative\_regulation\_of\_protein\_amino\_acid\_autophosphorylation | 1 | 0 |  |  |  |  |  |  |  |  |
| GO:0031998\_regulation\_of\_fatty\_acid\_beta-oxidation | 1 | 0 |  |  |  |  |  |  |  |  |
| GO:0032000\_positive\_regulation\_of\_fatty\_acid\_beta-oxidation | 1 | 0 |  |  |  |  |  |  |  |  |
| GO:0032006\_regulation\_of\_TOR\_signaling\_pathway | 1 | 0 |  |  |  |  |  |  |  |  |
| GO:0032007\_negative\_regulation\_of\_TOR\_signaling\_pathway | 1 | 0 |  |  |  |  |  |  |  |  |
| GO:0032023\_trypsinogen\_activation | 1 | 0 |  |  |  |  |  |  |  |  |
| GO:0032025\_response\_to\_cobalt\_ion | 1 | 0 |  |  |  |  |  |  |  |  |
| GO:0032026\_response\_to\_magnesium\_ion | 1 | 0 |  |  |  |  |  |  |  |  |
| GO:0032048\_cardiolipin\_metabolic\_process | 1 | 0 |  |  |  |  |  |  |  |  |
| GO:0032049\_cardiolipin\_biosynthetic\_process | 1 | 0 |  |  |  |  |  |  |  |  |
| GO:0032057\_negative\_regulation\_of\_translational\_initiation\_in\_response\_to\_stress | 1 | 0 |  |  |  |  |  |  |  |  |
| GO:0032066\_nucleolus\_to\_nucleoplasm\_transport | 1 | 0 |  |  |  |  |  |  |  |  |
| GO:0032069\_regulation\_of\_nuclease\_activity | 1 | 0 |  |  |  |  |  |  |  |  |
| GO:0032075\_positive\_regulation\_of\_nuclease\_activity | 1 | 0 |  |  |  |  |  |  |  |  |
| GO:0032091\_negative\_regulation\_of\_protein\_binding | 1 | 0 |  |  |  |  |  |  |  |  |
| GO:0032092\_positive\_regulation\_of\_protein\_binding | 1 | 0 |  |  |  |  |  |  |  |  |
| GO:0032148\_activation\_of\_protein\_kinase\_B\_activity | 1 | 0 |  |  |  |  |  |  |  |  |
| GO:0032196\_transposition | 1 | 0 |  |  |  |  |  |  |  |  |
| GO:0032232\_negative\_regulation\_of\_actin\_filament\_bundle\_formation | 1 | 0 |  |  |  |  |  |  |  |  |
| GO:0032235\_negative\_regulation\_of\_calcium\_ion\_transport\_via\_store-operated\_calcium\_channel | 1 | 0 |  |  |  |  |  |  |  |  |
| GO:0032236\_positive\_regulation\_of\_calcium\_ion\_transport\_via\_store-operated\_calcium\_channel | 1 | 0 |  |  |  |  |  |  |  |  |
| GO:0032237\_activation\_of\_store-operated\_calcium\_channel\_activity | 1 | 0 |  |  |  |  |  |  |  |  |
| GO:0032241\_positive\_regulation\_of\_nucleobase\_\_nucleoside\_\_nucleotide\_and\_nucleic\_acid\_transport | 1 | 0 |  |  |  |  |  |  |  |  |
| GO:0032275\_luteinizing\_hormone\_secretion | 1 | 0 |  |  |  |  |  |  |  |  |
| GO:0032287\_myelin\_maintenance\_in\_the\_peripheral\_nervous\_system | 1 | 0 |  |  |  |  |  |  |  |  |
| GO:0032288\_myelin\_formation | 1 | 0 |  |  |  |  |  |  |  |  |
| GO:0032292\_ensheathment\_of\_axons\_in\_the\_peripheral\_nervous\_system | 1 | 0 |  |  |  |  |  |  |  |  |
| GO:0032328\_alanine\_transport | 1 | 0 |  |  |  |  |  |  |  |  |
| GO:0032329\_serine\_transport | 1 | 0 |  |  |  |  |  |  |  |  |
| GO:0032341\_aldosterone\_metabolic\_process | 1 | 0 |  |  |  |  |  |  |  |  |
| GO:0032342\_aldosterone\_biosynthetic\_process | 1 | 0 |  |  |  |  |  |  |  |  |
| GO:0032344\_regulation\_of\_aldosterone\_metabolic\_process | 1 | 0 |  |  |  |  |  |  |  |  |
| GO:0032346\_positive\_regulation\_of\_aldosterone\_metabolic\_process | 1 | 0 |  |  |  |  |  |  |  |  |
| GO:0032347\_regulation\_of\_aldosterone\_biosynthetic\_process | 1 | 0 |  |  |  |  |  |  |  |  |
| GO:0032349\_positive\_regulation\_of\_aldosterone\_biosynthetic\_process | 1 | 0 |  |  |  |  |  |  |  |  |
| GO:0032352\_positive\_regulation\_of\_hormone\_metabolic\_process | 1 | 0 |  |  |  |  |  |  |  |  |
| GO:0032377\_regulation\_of\_intracellular\_lipid\_transport | 1 | 0 |  |  |  |  |  |  |  |  |
| GO:0032380\_regulation\_of\_intracellular\_sterol\_transport | 1 | 0 |  |  |  |  |  |  |  |  |
| GO:0032383\_regulation\_of\_intracellular\_cholesterol\_transport | 1 | 0 |  |  |  |  |  |  |  |  |
| GO:0032411\_positive\_regulation\_of\_transporter\_activity | 1 | 0 |  |  |  |  |  |  |  |  |
| GO:0032414\_positive\_regulation\_of\_ion\_transmembrane\_transporter\_activity | 1 | 0 |  |  |  |  |  |  |  |  |
| GO:0032417\_positive\_regulation\_of\_sodium:hydrogen\_antiporter\_activity | 1 | 0 |  |  |  |  |  |  |  |  |
| GO:0032423\_regulation\_of\_mismatch\_repair | 1 | 0 |  |  |  |  |  |  |  |  |
| GO:0032425\_positive\_regulation\_of\_mismatch\_repair | 1 | 0 |  |  |  |  |  |  |  |  |
| GO:0032434\_regulation\_of\_proteasomal\_ubiquitin-dependent\_protein\_catabolic\_process | 1 | 0 |  |  |  |  |  |  |  |  |
| GO:0032436\_positive\_regulation\_of\_proteasomal\_ubiquitin-dependent\_protein\_catabolic\_process | 1 | 0 |  |  |  |  |  |  |  |  |
| GO:0032468\_Golgi\_calcium\_ion\_homeostasis | 1 | 0 |  |  |  |  |  |  |  |  |
| GO:0032469\_endoplasmic\_reticulum\_calcium\_ion\_homeostasis | 1 | 0 |  |  |  |  |  |  |  |  |
| GO:0032472\_Golgi\_calcium\_ion\_transport | 1 | 0 |  |  |  |  |  |  |  |  |
| GO:0032490\_detection\_of\_molecule\_of\_bacterial\_origin | 1 | 0 |  |  |  |  |  |  |  |  |
| GO:0032494\_response\_to\_peptidoglycan | 1 | 0 |  |  |  |  |  |  |  |  |
| GO:0032495\_response\_to\_muramyl\_dipeptide | 1 | 0 |  |  |  |  |  |  |  |  |
| GO:0032496\_response\_to\_lipopolysaccharide | 1 | 0 |  |  |  |  |  |  |  |  |
| GO:0032498\_detection\_of\_muramyl\_dipeptide | 1 | 0 |  |  |  |  |  |  |  |  |
| GO:0032499\_detection\_of\_peptidoglycan | 1 | 0 |  |  |  |  |  |  |  |  |
| GO:0032506\_cytokinetic\_process | 1 | 0 |  |  |  |  |  |  |  |  |
| GO:0032509\_endosome\_transport\_via\_multivesicular\_body\_sorting\_pathway | 1 | 0 |  |  |  |  |  |  |  |  |
| GO:0032510\_endosome\_to\_lysosome\_transport\_via\_multivesicular\_body\_sorting\_pathway | 1 | 0 |  |  |  |  |  |  |  |  |
| GO:0032526\_response\_to\_retinoic\_acid | 1 | 0 |  |  |  |  |  |  |  |  |
| GO:0032528\_microvillus\_organization\_and\_biogenesis | 1 | 0 |  |  |  |  |  |  |  |  |
| GO:0032581\_ER-dependent\_peroxisome\_biogenesis | 1 | 0 |  |  |  |  |  |  |  |  |
| GO:0032595\_B\_cell\_receptor\_transport\_within\_lipid\_bilayer | 1 | 0 |  |  |  |  |  |  |  |  |
| GO:0032596\_protein\_transport\_into\_membrane\_raft | 1 | 0 |  |  |  |  |  |  |  |  |
| GO:0032597\_B\_cell\_receptor\_transport\_into\_membrane\_raft | 1 | 0 |  |  |  |  |  |  |  |  |
| GO:0032599\_protein\_transport\_out\_of\_membrane\_raft | 1 | 0 |  |  |  |  |  |  |  |  |
| GO:0032600\_chemokine\_receptor\_transport\_out\_of\_membrane\_raft | 1 | 0 |  |  |  |  |  |  |  |  |
| GO:0032601\_connective\_tissue\_growth\_factor\_production | 1 | 0 |  |  |  |  |  |  |  |  |
| GO:0032603\_fractalkine\_production | 1 | 0 |  |  |  |  |  |  |  |  |
| GO:0032604\_granulocyte\_macrophage\_colony-stimulating\_factor\_production | 1 | 0 |  |  |  |  |  |  |  |  |
| GO:0032605\_hepatocyte\_growth\_factor\_production | 1 | 0 |  |  |  |  |  |  |  |  |
| GO:0032610\_interleukin-1\_alpha\_production | 1 | 0 |  |  |  |  |  |  |  |  |
| GO:0032611\_interleukin-1\_beta\_production | 1 | 0 |  |  |  |  |  |  |  |  |
| GO:0032613\_interleukin-10\_production | 1 | 0 |  |  |  |  |  |  |  |  |
| GO:0032616\_interleukin-13\_production | 1 | 0 |  |  |  |  |  |  |  |  |
| GO:0032620\_interleukin-17\_production | 1 | 0 |  |  |  |  |  |  |  |  |
| GO:0032633\_interleukin-4\_production | 1 | 0 |  |  |  |  |  |  |  |  |
| GO:0032634\_interleukin-5\_production | 1 | 0 |  |  |  |  |  |  |  |  |
| GO:0032639\_TRAIL\_production | 1 | 0 |  |  |  |  |  |  |  |  |
| GO:0032642\_regulation\_of\_chemokine\_production | 1 | 0 |  |  |  |  |  |  |  |  |
| GO:0032649\_regulation\_of\_interferon-gamma\_production | 1 | 0 |  |  |  |  |  |  |  |  |
| GO:0032650\_regulation\_of\_interleukin-1\_alpha\_production | 1 | 0 |  |  |  |  |  |  |  |  |
| GO:0032652\_regulation\_of\_interleukin-1\_production | 1 | 0 |  |  |  |  |  |  |  |  |
| GO:0032655\_regulation\_of\_interleukin-12\_production | 1 | 0 |  |  |  |  |  |  |  |  |
| GO:0032660\_regulation\_of\_interleukin-17\_production | 1 | 0 |  |  |  |  |  |  |  |  |
| GO:0032680\_regulation\_of\_tumor\_necrosis\_factor\_production | 1 | 0 |  |  |  |  |  |  |  |  |
| GO:0032720\_negative\_regulation\_of\_tumor\_necrosis\_factor\_production | 1 | 0 |  |  |  |  |  |  |  |  |
| GO:0032722\_positive\_regulation\_of\_chemokine\_production | 1 | 0 |  |  |  |  |  |  |  |  |
| GO:0032729\_positive\_regulation\_of\_interferon-gamma\_production | 1 | 0 |  |  |  |  |  |  |  |  |
| GO:0032730\_positive\_regulation\_of\_interleukin-1\_alpha\_production | 1 | 0 |  |  |  |  |  |  |  |  |
| GO:0032732\_positive\_regulation\_of\_interleukin-1\_production | 1 | 0 |  |  |  |  |  |  |  |  |
| GO:0032735\_positive\_regulation\_of\_interleukin-12\_production | 1 | 0 |  |  |  |  |  |  |  |  |
| GO:0032740\_positive\_regulation\_of\_interleukin-17\_production | 1 | 0 |  |  |  |  |  |  |  |  |
| GO:0032762\_mast\_cell\_cytokine\_production | 1 | 0 |  |  |  |  |  |  |  |  |
| GO:0032763\_regulation\_of\_mast\_cell\_cytokine\_production | 1 | 0 |  |  |  |  |  |  |  |  |
| GO:0032765\_positive\_regulation\_of\_mast\_cell\_cytokine\_production | 1 | 0 |  |  |  |  |  |  |  |  |
| GO:0032770\_positive\_regulation\_of\_monooxygenase\_activity | 1 | 0 |  |  |  |  |  |  |  |  |
| GO:0032784\_regulation\_of\_RNA\_elongation | 1 | 0 |  |  |  |  |  |  |  |  |
| GO:0032786\_positive\_regulation\_of\_RNA\_elongation | 1 | 0 |  |  |  |  |  |  |  |  |
| GO:0032788\_saturated\_monocarboxylic\_acid\_metabolic\_process | 1 | 0 |  |  |  |  |  |  |  |  |
| GO:0032789\_unsaturated\_monocarboxylic\_acid\_metabolic\_process | 1 | 0 |  |  |  |  |  |  |  |  |
| GO:0032790\_ribosome\_disassembly | 1 | 0 |  |  |  |  |  |  |  |  |
| GO:0032802\_low-density\_lipoprotein\_receptor\_catabolic\_process | 1 | 0 |  |  |  |  |  |  |  |  |
| GO:0032803\_regulation\_of\_low-density\_lipoprotein\_receptor\_catabolic\_process | 1 | 0 |  |  |  |  |  |  |  |  |
| GO:0032804\_negative\_regulation\_of\_low-density\_lipoprotein\_receptor\_catabolic\_process | 1 | 0 |  |  |  |  |  |  |  |  |
| GO:0032814\_regulation\_of\_natural\_killer\_cell\_activation | 1 | 0 |  |  |  |  |  |  |  |  |
| GO:0032816\_positive\_regulation\_of\_natural\_killer\_cell\_activation | 1 | 0 |  |  |  |  |  |  |  |  |
| GO:0032835\_glomerulus\_development | 1 | 0 |  |  |  |  |  |  |  |  |
| GO:0032846\_positive\_regulation\_of\_homeostatic\_process | 1 | 0 |  |  |  |  |  |  |  |  |
| GO:0032847\_regulation\_of\_cellular\_pH\_reduction | 1 | 0 |  |  |  |  |  |  |  |  |
| GO:0032848\_negative\_regulation\_of\_cellular\_pH\_reduction | 1 | 0 |  |  |  |  |  |  |  |  |
| GO:0032863\_activation\_of\_Rac\_GTPase\_activity | 1 | 0 |  |  |  |  |  |  |  |  |
| GO:0032872\_regulation\_of\_stress-activated\_MAPK\_cascade | 1 | 0 |  |  |  |  |  |  |  |  |
| GO:0032874\_positive\_regulation\_of\_stress-activated\_MAPK\_cascade | 1 | 0 |  |  |  |  |  |  |  |  |
| GO:0032905\_transforming\_growth\_factor-beta1\_production | 1 | 0 |  |  |  |  |  |  |  |  |
| GO:0032907\_transforming\_growth\_factor-beta3\_production | 1 | 0 |  |  |  |  |  |  |  |  |
| GO:0032908\_regulation\_of\_transforming\_growth\_factor-beta1\_production | 1 | 0 |  |  |  |  |  |  |  |  |
| GO:0032910\_regulation\_of\_transforming\_growth\_factor-beta3\_production | 1 | 0 |  |  |  |  |  |  |  |  |
| GO:0032913\_negative\_regulation\_of\_transforming\_growth\_factor-beta3\_production | 1 | 0 |  |  |  |  |  |  |  |  |
| GO:0032914\_positive\_regulation\_of\_transforming\_growth\_factor-beta1\_production | 1 | 0 |  |  |  |  |  |  |  |  |
| GO:0032926\_negative\_regulation\_of\_activin\_receptor\_signaling\_pathway | 1 | 0 |  |  |  |  |  |  |  |  |
| GO:0032938\_negative\_regulation\_of\_translation\_in\_response\_to\_oxidative\_stress | 1 | 0 |  |  |  |  |  |  |  |  |
| GO:0032964\_collagen\_biosynthetic\_process | 1 | 0 |  |  |  |  |  |  |  |  |
| GO:0032971\_regulation\_of\_muscle\_filament\_sliding | 1 | 0 |  |  |  |  |  |  |  |  |
| GO:0032988\_protein-RNA\_complex\_disassembly | 1 | 0 |  |  |  |  |  |  |  |  |
| GO:0033028\_myeloid\_cell\_apoptosis | 1 | 0 |  |  |  |  |  |  |  |  |
| GO:0033059\_cellular\_pigmentation | 1 | 0 |  |  |  |  |  |  |  |  |
| GO:0033081\_regulation\_of\_T\_cell\_differentiation\_in\_the\_thymus | 1 | 0 |  |  |  |  |  |  |  |  |
| GO:0033119\_negative\_regulation\_of\_RNA\_splicing | 1 | 0 |  |  |  |  |  |  |  |  |
| GO:0033144\_negative\_regulation\_of\_steroid\_hormone\_receptor\_signaling\_pathway | 1 | 0 |  |  |  |  |  |  |  |  |
| GO:0033169\_histone\_H3-K9\_demethylation | 1 | 0 |  |  |  |  |  |  |  |  |
| GO:0033182\_regulation\_of\_histone\_ubiquitination | 1 | 0 |  |  |  |  |  |  |  |  |
| GO:0033189\_response\_to\_vitamin\_A | 1 | 0 |  |  |  |  |  |  |  |  |
| GO:0033194\_response\_to\_hydroperoxide | 1 | 0 |  |  |  |  |  |  |  |  |
| GO:0033238\_regulation\_of\_amine\_metabolic\_process | 1 | 0 |  |  |  |  |  |  |  |  |
| GO:0033240\_positive\_regulation\_of\_amine\_metabolic\_process | 1 | 0 |  |  |  |  |  |  |  |  |
| GO:0033260\_DNA\_replication\_during\_S\_phase | 1 | 0 |  |  |  |  |  |  |  |  |
| GO:0033262\_regulation\_of\_DNA\_replication\_during\_S\_phase | 1 | 0 |  |  |  |  |  |  |  |  |
| GO:0033273\_response\_to\_vitamin | 1 | 0 |  |  |  |  |  |  |  |  |
| GO:0033280\_response\_to\_vitamin\_D | 1 | 0 |  |  |  |  |  |  |  |  |
| GO:0033341\_regulation\_of\_collagen\_binding | 1 | 0 |  |  |  |  |  |  |  |  |
| GO:0033342\_negative\_regulation\_of\_collagen\_binding | 1 | 0 |  |  |  |  |  |  |  |  |
| GO:0033345\_asparagine\_catabolic\_process\_via\_L-aspartate | 1 | 0 |  |  |  |  |  |  |  |  |
| GO:0033364\_mast\_cell\_secretory\_granule\_organization\_and\_biogenesis | 1 | 0 |  |  |  |  |  |  |  |  |
| GO:0033366\_protein\_localization\_in\_secretory\_granule | 1 | 0 |  |  |  |  |  |  |  |  |
| GO:0033367\_protein\_localization\_in\_mast\_cell\_secretory\_granule | 1 | 0 |  |  |  |  |  |  |  |  |
| GO:0033368\_protease\_localization\_in\_mast\_cell\_secretory\_granule | 1 | 0 |  |  |  |  |  |  |  |  |
| GO:0033370\_maintenance\_of\_protein\_localization\_in\_mast\_cell\_secretory\_granule | 1 | 0 |  |  |  |  |  |  |  |  |
| GO:0033371\_T\_cell\_secretory\_granule\_organization\_and\_biogenesis | 1 | 0 |  |  |  |  |  |  |  |  |
| GO:0033373\_maintenance\_of\_protease\_localization\_in\_mast\_cell\_secretory\_granule | 1 | 0 |  |  |  |  |  |  |  |  |
| GO:0033374\_protein\_localization\_in\_T\_cell\_secretory\_granule | 1 | 0 |  |  |  |  |  |  |  |  |
| GO:0033375\_protease\_localization\_in\_T\_cell\_secretory\_granule | 1 | 0 |  |  |  |  |  |  |  |  |
| GO:0033377\_maintenance\_of\_protein\_localization\_in\_T\_cell\_secretory\_granule | 1 | 0 |  |  |  |  |  |  |  |  |
| GO:0033379\_maintenance\_of\_protease\_localization\_in\_T\_cell\_secretory\_granule | 1 | 0 |  |  |  |  |  |  |  |  |
| GO:0033380\_granzyme\_B\_localization\_in\_T\_cell\_secretory\_granule | 1 | 0 |  |  |  |  |  |  |  |  |
| GO:0033382\_maintenance\_of\_granzyme\_B\_localization\_in\_T\_cell\_secretory\_granule | 1 | 0 |  |  |  |  |  |  |  |  |
| GO:0033577\_protein\_amino\_acid\_glycosylation\_in\_endoplasmic\_reticulum | 1 | 0 |  |  |  |  |  |  |  |  |
| GO:0033595\_response\_to\_genistein | 1 | 0 |  |  |  |  |  |  |  |  |
| GO:0033600\_negative\_regulation\_of\_mammary\_gland\_epithelial\_cell\_proliferation | 1 | 0 |  |  |  |  |  |  |  |  |
| GO:0033606\_chemokine\_receptor\_transport\_within\_lipid\_bilayer | 1 | 0 |  |  |  |  |  |  |  |  |
| GO:0033693\_neurofilament\_bundle\_assembly | 1 | 0 |  |  |  |  |  |  |  |  |
| GO:0033860\_regulation\_of\_NAD(P)H\_oxidase\_activity | 1 | 0 |  |  |  |  |  |  |  |  |
| GO:0033861\_negative\_regulation\_of\_NAD(P)H\_oxidase\_activity | 1 | 0 |  |  |  |  |  |  |  |  |
| GO:0033865\_nucleoside\_bisphosphate\_metabolic\_process | 1 | 0 |  |  |  |  |  |  |  |  |
| GO:0033875\_ribonucleoside\_bisphosphate\_metabolic\_process | 1 | 0 |  |  |  |  |  |  |  |  |
| GO:0033962\_cytoplasmic\_mRNA\_processing\_body\_assembly | 1 | 0 |  |  |  |  |  |  |  |  |
| GO:0033993\_response\_to\_lipid | 1 | 0 |  |  |  |  |  |  |  |  |
| GO:0034032\_purine\_nucleoside\_bisphosphate\_metabolic\_process | 1 | 0 |  |  |  |  |  |  |  |  |
| GO:0034035\_purine\_ribonucleoside\_bisphosphate\_metabolic\_process | 1 | 0 |  |  |  |  |  |  |  |  |
| GO:0034063\_stress\_granule\_assembly | 1 | 0 |  |  |  |  |  |  |  |  |
| GO:0034213\_quinolinate\_catabolic\_process | 1 | 0 |  |  |  |  |  |  |  |  |
| GO:0034220\_transmembrane\_ion\_transport | 1 | 0 |  |  |  |  |  |  |  |  |
| GO:0034287\_detection\_of\_monosaccharide\_stimulus | 1 | 0 |  |  |  |  |  |  |  |  |
| GO:0034308\_monohydric\_alcohol\_metabolic\_process | 1 | 0 |  |  |  |  |  |  |  |  |
| GO:0035020\_regulation\_of\_Rac\_protein\_signal\_transduction | 1 | 0 |  |  |  |  |  |  |  |  |
| GO:0035024\_negative\_regulation\_of\_Rho\_protein\_signal\_transduction | 1 | 0 |  |  |  |  |  |  |  |  |
| GO:0035025\_positive\_regulation\_of\_Rho\_protein\_signal\_transduction | 1 | 0 |  |  |  |  |  |  |  |  |
| GO:0035036\_sperm-egg\_recognition | 1 | 0 |  |  |  |  |  |  |  |  |
| GO:0035050\_embryonic\_heart\_tube\_development | 1 | 0 |  |  |  |  |  |  |  |  |
| GO:0035058\_sensory\_cilium\_biogenesis | 1 | 0 |  |  |  |  |  |  |  |  |
| GO:0035066\_positive\_regulation\_of\_histone\_acetylation | 1 | 0 |  |  |  |  |  |  |  |  |
| GO:0035067\_negative\_regulation\_of\_histone\_acetylation | 1 | 0 |  |  |  |  |  |  |  |  |
| GO:0035081\_induction\_of\_programmed\_cell\_death\_by\_hormones | 1 | 0 |  |  |  |  |  |  |  |  |
| GO:0035090\_maintenance\_of\_apical\_basal\_cell\_polarity | 1 | 0 |  |  |  |  |  |  |  |  |
| GO:0035115\_embryonic\_forelimb\_morphogenesis | 1 | 0 |  |  |  |  |  |  |  |  |
| GO:0035117\_embryonic\_arm\_morphogenesis | 1 | 0 |  |  |  |  |  |  |  |  |
| GO:0035136\_forelimb\_morphogenesis | 1 | 0 |  |  |  |  |  |  |  |  |
| GO:0035140\_arm\_morphogenesis | 1 | 0 |  |  |  |  |  |  |  |  |
| GO:0035162\_embryonic\_hemopoiesis | 1 | 0 |  |  |  |  |  |  |  |  |
| GO:0035195\_gene\_silencing\_by\_miRNA | 1 | 0 |  |  |  |  |  |  |  |  |
| GO:0035196\_gene\_silencing\_by\_miRNA\_\_production\_of\_miRNAs | 1 | 0 |  |  |  |  |  |  |  |  |
| GO:0035235\_ionotropic\_glutamate\_receptor\_signaling\_pathway | 1 | 0 |  |  |  |  |  |  |  |  |
| GO:0035238\_vitamin\_A\_biosynthetic\_process | 1 | 0 |  |  |  |  |  |  |  |  |
| GO:0035246\_peptidyl-arginine\_N-methylation | 1 | 0 |  |  |  |  |  |  |  |  |
| GO:0035265\_organ\_growth | 1 | 0 |  |  |  |  |  |  |  |  |
| GO:0035313\_wound\_healing\_\_spreading\_of\_epidermal\_cells | 1 | 0 |  |  |  |  |  |  |  |  |
| GO:0040009\_regulation\_of\_growth\_rate | 1 | 0 |  |  |  |  |  |  |  |  |
| GO:0040015\_negative\_regulation\_of\_multicellular\_organism\_growth | 1 | 0 |  |  |  |  |  |  |  |  |
| GO:0040020\_regulation\_of\_meiosis | 1 | 0 |  |  |  |  |  |  |  |  |
| GO:0040023\_establishment\_of\_nucleus\_localization | 1 | 0 |  |  |  |  |  |  |  |  |
| GO:0042026\_protein\_refolding | 1 | 0 |  |  |  |  |  |  |  |  |
| GO:0042053\_regulation\_of\_dopamine\_metabolic\_process | 1 | 0 |  |  |  |  |  |  |  |  |
| GO:0042069\_regulation\_of\_catecholamine\_metabolic\_process | 1 | 0 |  |  |  |  |  |  |  |  |
| GO:0042074\_cell\_migration\_involved\_in\_gastrulation | 1 | 0 |  |  |  |  |  |  |  |  |
| GO:0042091\_interleukin-10\_biosynthetic\_process | 1 | 0 |  |  |  |  |  |  |  |  |
| GO:0042097\_interleukin-4\_biosynthetic\_process | 1 | 0 |  |  |  |  |  |  |  |  |
| GO:0042117\_monocyte\_activation | 1 | 0 |  |  |  |  |  |  |  |  |
| GO:0042159\_lipoprotein\_catabolic\_process | 1 | 0 |  |  |  |  |  |  |  |  |
| GO:0042220\_response\_to\_cocaine | 1 | 0 |  |  |  |  |  |  |  |  |
| GO:0042222\_interleukin-1\_biosynthetic\_process | 1 | 0 |  |  |  |  |  |  |  |  |
| GO:0042225\_interleukin-5\_biosynthetic\_process | 1 | 0 |  |  |  |  |  |  |  |  |
| GO:0042231\_interleukin-13\_biosynthetic\_process | 1 | 0 |  |  |  |  |  |  |  |  |
| GO:0042253\_granulocyte\_macrophage\_colony-stimulating\_factor\_biosynthetic\_process | 1 | 0 |  |  |  |  |  |  |  |  |
| GO:0042257\_ribosomal\_subunit\_assembly | 1 | 0 |  |  |  |  |  |  |  |  |
| GO:0042262\_DNA\_protection | 1 | 0 |  |  |  |  |  |  |  |  |
| GO:0042268\_regulation\_of\_cytolysis | 1 | 0 |  |  |  |  |  |  |  |  |
| GO:0042271\_susceptibility\_to\_natural\_killer\_cell\_mediated\_cytotoxicity | 1 | 0 |  |  |  |  |  |  |  |  |
| GO:0042276\_error-prone\_postreplication\_DNA\_repair | 1 | 0 |  |  |  |  |  |  |  |  |
| GO:0042313\_protein\_kinase\_C\_deactivation | 1 | 0 |  |  |  |  |  |  |  |  |
| GO:0042335\_cuticle\_development | 1 | 0 |  |  |  |  |  |  |  |  |
| GO:0042362\_fat-soluble\_vitamin\_biosynthetic\_process | 1 | 0 |  |  |  |  |  |  |  |  |
| GO:0042407\_cristae\_formation | 1 | 0 |  |  |  |  |  |  |  |  |
| GO:0042412\_taurine\_biosynthetic\_process | 1 | 0 |  |  |  |  |  |  |  |  |
| GO:0042416\_dopamine\_biosynthetic\_process | 1 | 0 |  |  |  |  |  |  |  |  |
| GO:0042423\_catecholamine\_biosynthetic\_process | 1 | 0 |  |  |  |  |  |  |  |  |
| GO:0042435\_indole\_derivative\_biosynthetic\_process | 1 | 0 |  |  |  |  |  |  |  |  |
| GO:0042436\_indole\_derivative\_catabolic\_process | 1 | 0 |  |  |  |  |  |  |  |  |
| GO:0042451\_purine\_nucleoside\_biosynthetic\_process | 1 | 0 |  |  |  |  |  |  |  |  |
| GO:0042455\_ribonucleoside\_biosynthetic\_process | 1 | 0 |  |  |  |  |  |  |  |  |
| GO:0042504\_tyrosine\_phosphorylation\_of\_Stat4\_protein | 1 | 0 |  |  |  |  |  |  |  |  |
| GO:0042519\_regulation\_of\_tyrosine\_phosphorylation\_of\_Stat4\_protein | 1 | 0 |  |  |  |  |  |  |  |  |
| GO:0042520\_positive\_regulation\_of\_tyrosine\_phosphorylation\_of\_Stat4\_protein | 1 | 0 |  |  |  |  |  |  |  |  |
| GO:0042523\_positive\_regulation\_of\_tyrosine\_phosphorylation\_of\_Stat5\_protein | 1 | 0 |  |  |  |  |  |  |  |  |
| GO:0042524\_negative\_regulation\_of\_tyrosine\_phosphorylation\_of\_Stat5\_protein | 1 | 0 |  |  |  |  |  |  |  |  |
| GO:0042573\_retinoic\_acid\_metabolic\_process | 1 | 0 |  |  |  |  |  |  |  |  |
| GO:0042637\_catagen | 1 | 0 |  |  |  |  |  |  |  |  |
| GO:0042640\_anagen | 1 | 0 |  |  |  |  |  |  |  |  |
| GO:0042670\_retinal\_cone\_cell\_differentiation | 1 | 0 |  |  |  |  |  |  |  |  |
| GO:0042700\_luteinizing\_hormone\_signaling\_pathway | 1 | 0 |  |  |  |  |  |  |  |  |
| GO:0042703\_menstruation | 1 | 0 |  |  |  |  |  |  |  |  |
| GO:0042726\_riboflavin\_and\_derivative\_metabolic\_process | 1 | 0 |  |  |  |  |  |  |  |  |
| GO:0042727\_riboflavin\_and\_derivative\_biosynthetic\_process | 1 | 0 |  |  |  |  |  |  |  |  |
| GO:0042730\_fibrinolysis | 1 | 0 |  |  |  |  |  |  |  |  |
| GO:0042744\_hydrogen\_peroxide\_catabolic\_process | 1 | 0 |  |  |  |  |  |  |  |  |
| GO:0042745\_circadian\_sleep\_wake\_cycle | 1 | 0 |  |  |  |  |  |  |  |  |
| GO:0042749\_regulation\_of\_circadian\_sleep\_wake\_cycle | 1 | 0 |  |  |  |  |  |  |  |  |
| GO:0042780\_tRNA\_3'-end\_processing | 1 | 0 |  |  |  |  |  |  |  |  |
| GO:0042789\_mRNA\_transcription\_from\_RNA\_polymerase\_II\_promoter | 1 | 0 |  |  |  |  |  |  |  |  |
| GO:0042795\_snRNA\_transcription\_from\_RNA\_polymerase\_II\_promoter | 1 | 0 |  |  |  |  |  |  |  |  |
| GO:0042796\_snRNA\_transcription\_from\_RNA\_polymerase\_III\_promoter | 1 | 0 |  |  |  |  |  |  |  |  |
| GO:0042816\_vitamin\_B6\_metabolic\_process | 1 | 0 |  |  |  |  |  |  |  |  |
| GO:0042819\_vitamin\_B6\_biosynthetic\_process | 1 | 0 |  |  |  |  |  |  |  |  |
| GO:0042832\_defense\_response\_to\_protozoan | 1 | 0 |  |  |  |  |  |  |  |  |
| GO:0042886\_amide\_transport | 1 | 0 |  |  |  |  |  |  |  |  |
| GO:0042904\_9-cis-retinoic\_acid\_biosynthetic\_process | 1 | 0 |  |  |  |  |  |  |  |  |
| GO:0042905\_9-cis-retinoic\_acid\_metabolic\_process | 1 | 0 |  |  |  |  |  |  |  |  |
| GO:0042953\_lipoprotein\_transport | 1 | 0 |  |  |  |  |  |  |  |  |
| GO:0042976\_activation\_of\_JAK\_protein | 1 | 0 |  |  |  |  |  |  |  |  |
| GO:0042977\_activation\_of\_JAK2\_protein | 1 | 0 |  |  |  |  |  |  |  |  |
| GO:0042983\_amyloid\_precursor\_protein\_biosynthetic\_process | 1 | 0 |  |  |  |  |  |  |  |  |
| GO:0042984\_regulation\_of\_amyloid\_precursor\_protein\_biosynthetic\_process | 1 | 0 |  |  |  |  |  |  |  |  |
| GO:0042985\_negative\_regulation\_of\_amyloid\_precursor\_protein\_biosynthetic\_process | 1 | 0 |  |  |  |  |  |  |  |  |
| GO:0042989\_sequestering\_of\_actin\_monomers | 1 | 0 |  |  |  |  |  |  |  |  |
| GO:0042996\_regulation\_of\_Golgi\_to\_plasma\_membrane\_protein\_transport | 1 | 0 |  |  |  |  |  |  |  |  |
| GO:0042997\_negative\_regulation\_of\_Golgi\_to\_plasma\_membrane\_protein\_transport | 1 | 0 |  |  |  |  |  |  |  |  |
| GO:0042999\_regulation\_of\_Golgi\_to\_plasma\_membrane\_CFTR\_protein\_transport | 1 | 0 |  |  |  |  |  |  |  |  |
| GO:0043002\_negative\_regulation\_of\_Golgi\_to\_plasma\_membrane\_CFTR\_protein\_transport | 1 | 0 |  |  |  |  |  |  |  |  |
| GO:0043004\_cytoplasmic\_sequestering\_of\_CFTR\_protein | 1 | 0 |  |  |  |  |  |  |  |  |
| GO:0043011\_myeloid\_dendritic\_cell\_differentiation | 1 | 0 |  |  |  |  |  |  |  |  |
| GO:0043012\_regulation\_of\_fusion\_of\_sperm\_to\_egg\_plasma\_membrane | 1 | 0 |  |  |  |  |  |  |  |  |
| GO:0043031\_negative\_regulation\_of\_macrophage\_activation | 1 | 0 |  |  |  |  |  |  |  |  |
| GO:0043043\_peptide\_biosynthetic\_process | 1 | 0 |  |  |  |  |  |  |  |  |
| GO:0043044\_ATP-dependent\_chromatin\_remodeling | 1 | 0 |  |  |  |  |  |  |  |  |
| GO:0043045\_DNA\_methylation\_during\_embryonic\_development | 1 | 0 |  |  |  |  |  |  |  |  |
| GO:0043046\_DNA\_methylation\_during\_gametogenesis | 1 | 0 |  |  |  |  |  |  |  |  |
| GO:0043101\_purine\_salvage | 1 | 0 |  |  |  |  |  |  |  |  |
| GO:0043116\_negative\_regulation\_of\_vascular\_permeability | 1 | 0 |  |  |  |  |  |  |  |  |
| GO:0043126\_regulation\_of\_1-phosphatidylinositol\_4-kinase\_activity | 1 | 0 |  |  |  |  |  |  |  |  |
| GO:0043128\_positive\_regulation\_of\_1-phosphatidylinositol\_4-kinase\_activity | 1 | 0 |  |  |  |  |  |  |  |  |
| GO:0043152\_induction\_of\_bacterial\_agglutination | 1 | 0 |  |  |  |  |  |  |  |  |
| GO:0043174\_nucleoside\_salvage | 1 | 0 |  |  |  |  |  |  |  |  |
| GO:0043181\_vacuolar\_sequestering | 1 | 0 |  |  |  |  |  |  |  |  |
| GO:0043217\_myelin\_maintenance | 1 | 0 |  |  |  |  |  |  |  |  |
| GO:0043248\_proteasome\_assembly | 1 | 0 |  |  |  |  |  |  |  |  |
| GO:0043353\_enucleate\_erythrocyte\_differentiation | 1 | 0 |  |  |  |  |  |  |  |  |
| GO:0043371\_negative\_regulation\_of\_CD4-positive\_\_alpha\_beta\_T\_cell\_differentiation | 1 | 0 |  |  |  |  |  |  |  |  |
| GO:0043372\_positive\_regulation\_of\_CD4-positive\_\_alpha\_beta\_T\_cell\_differentiation | 1 | 0 |  |  |  |  |  |  |  |  |
| GO:0043437\_butanoic\_acid\_metabolic\_process | 1 | 0 |  |  |  |  |  |  |  |  |
| GO:0043461\_proton-transporting\_ATP\_synthase\_complex\_assembly | 1 | 0 |  |  |  |  |  |  |  |  |
| GO:0043476\_pigment\_accumulation | 1 | 0 |  |  |  |  |  |  |  |  |
| GO:0043482\_cellular\_pigment\_accumulation | 1 | 0 |  |  |  |  |  |  |  |  |
| GO:0043486\_histone\_exchange | 1 | 0 |  |  |  |  |  |  |  |  |
| GO:0043491\_protein\_kinase\_B\_signaling\_cascade | 1 | 0 |  |  |  |  |  |  |  |  |
| GO:0043516\_regulation\_of\_DNA\_damage\_response\_\_signal\_transduction\_by\_p53\_class\_mediator | 1 | 0 |  |  |  |  |  |  |  |  |
| GO:0043517\_positive\_regulation\_of\_DNA\_damage\_response\_\_signal\_transduction\_by\_p53\_class\_mediator | 1 | 0 |  |  |  |  |  |  |  |  |
| GO:0043537\_negative\_regulation\_of\_blood\_vessel\_endothelial\_cell\_migration | 1 | 0 |  |  |  |  |  |  |  |  |
| GO:0043556\_regulation\_of\_translation\_in\_response\_to\_oxidative\_stress | 1 | 0 |  |  |  |  |  |  |  |  |
| GO:0043558\_regulation\_of\_translational\_initiation\_in\_response\_to\_stress | 1 | 0 |  |  |  |  |  |  |  |  |
| GO:0043567\_regulation\_of\_insulin-like\_growth\_factor\_receptor\_signaling\_pathway | 1 | 0 |  |  |  |  |  |  |  |  |
| GO:0043569\_negative\_regulation\_of\_insulin-like\_growth\_factor\_receptor\_signaling\_pathway | 1 | 0 |  |  |  |  |  |  |  |  |
| GO:0043586\_tongue\_development | 1 | 0 |  |  |  |  |  |  |  |  |
| GO:0043587\_tongue\_morphogenesis | 1 | 0 |  |  |  |  |  |  |  |  |
| GO:0043653\_mitochondrial\_fragmentation\_during\_apoptosis | 1 | 0 |  |  |  |  |  |  |  |  |
| GO:0043696\_dedifferentiation | 1 | 0 |  |  |  |  |  |  |  |  |
| GO:0043697\_cell\_dedifferentiation | 1 | 0 |  |  |  |  |  |  |  |  |
| GO:0044269\_glycerol\_ether\_catabolic\_process | 1 | 0 |  |  |  |  |  |  |  |  |
| GO:0045013\_negative\_regulation\_of\_transcription\_by\_carbon\_catabolites | 1 | 0 |  |  |  |  |  |  |  |  |
| GO:0045014\_negative\_regulation\_of\_transcription\_by\_glucose | 1 | 0 |  |  |  |  |  |  |  |  |
| GO:0045019\_negative\_regulation\_of\_nitric\_oxide\_biosynthetic\_process | 1 | 0 |  |  |  |  |  |  |  |  |
| GO:0045020\_error-prone\_DNA\_repair | 1 | 0 |  |  |  |  |  |  |  |  |
| GO:0045023\_G0\_to\_G1\_transition | 1 | 0 |  |  |  |  |  |  |  |  |
| GO:0045063\_T-helper\_1\_cell\_differentiation | 1 | 0 |  |  |  |  |  |  |  |  |
| GO:0045064\_T-helper\_2\_cell\_differentiation | 1 | 0 |  |  |  |  |  |  |  |  |
| GO:0045065\_cytotoxic\_T\_cell\_differentiation | 1 | 0 |  |  |  |  |  |  |  |  |
| GO:0045074\_regulation\_of\_interleukin-10\_biosynthetic\_process | 1 | 0 |  |  |  |  |  |  |  |  |
| GO:0045077\_negative\_regulation\_of\_interferon-gamma\_biosynthetic\_process | 1 | 0 |  |  |  |  |  |  |  |  |
| GO:0045082\_positive\_regulation\_of\_interleukin-10\_biosynthetic\_process | 1 | 0 |  |  |  |  |  |  |  |  |
| GO:0045110\_intermediate\_filament\_bundle\_assembly | 1 | 0 |  |  |  |  |  |  |  |  |
| GO:0045163\_clustering\_of\_voltage-gated\_potassium\_channels | 1 | 0 |  |  |  |  |  |  |  |  |
| GO:0045175\_basal\_protein\_localization | 1 | 0 |  |  |  |  |  |  |  |  |
| GO:0045187\_regulation\_of\_circadian\_sleep\_wake\_cycle\_\_sleep | 1 | 0 |  |  |  |  |  |  |  |  |
| GO:0045189\_connective\_tissue\_growth\_factor\_biosynthetic\_process | 1 | 0 |  |  |  |  |  |  |  |  |
| GO:0045196\_establishment\_and\_or\_maintenance\_of\_neuroblast\_polarity | 1 | 0 |  |  |  |  |  |  |  |  |
| GO:0045199\_maintenance\_of\_epithelial\_cell\_polarity | 1 | 0 |  |  |  |  |  |  |  |  |
| GO:0045200\_establishment\_of\_neuroblast\_polarity | 1 | 0 |  |  |  |  |  |  |  |  |
| GO:0045204\_MAPK\_export\_from\_nucleus | 1 | 0 |  |  |  |  |  |  |  |  |
| GO:0045208\_MAPK\_phosphatase\_export\_from\_nucleus | 1 | 0 |  |  |  |  |  |  |  |  |
| GO:0045209\_MAPK\_phosphatase\_export\_from\_nucleus\_\_leptomycin\_B\_sensitive | 1 | 0 |  |  |  |  |  |  |  |  |
| GO:0045214\_sarcomere\_organization | 1 | 0 |  |  |  |  |  |  |  |  |
| GO:0045341\_MHC\_class\_I\_biosynthetic\_process | 1 | 0 |  |  |  |  |  |  |  |  |
| GO:0045343\_regulation\_of\_MHC\_class\_I\_biosynthetic\_process | 1 | 0 |  |  |  |  |  |  |  |  |
| GO:0045345\_positive\_regulation\_of\_MHC\_class\_I\_biosynthetic\_process | 1 | 0 |  |  |  |  |  |  |  |  |
| GO:0045347\_negative\_regulation\_of\_MHC\_class\_II\_biosynthetic\_process | 1 | 0 |  |  |  |  |  |  |  |  |
| GO:0045355\_negative\_regulation\_of\_interferon-alpha\_biosynthetic\_process | 1 | 0 |  |  |  |  |  |  |  |  |
| GO:0045360\_regulation\_of\_interleukin-1\_biosynthetic\_process | 1 | 0 |  |  |  |  |  |  |  |  |
| GO:0045362\_positive\_regulation\_of\_interleukin-1\_biosynthetic\_process | 1 | 0 |  |  |  |  |  |  |  |  |
| GO:0045366\_regulation\_of\_interleukin-13\_biosynthetic\_process | 1 | 0 |  |  |  |  |  |  |  |  |
| GO:0045368\_positive\_regulation\_of\_interleukin-13\_biosynthetic\_process | 1 | 0 |  |  |  |  |  |  |  |  |
| GO:0045402\_regulation\_of\_interleukin-4\_biosynthetic\_process | 1 | 0 |  |  |  |  |  |  |  |  |
| GO:0045404\_positive\_regulation\_of\_interleukin-4\_biosynthetic\_process | 1 | 0 |  |  |  |  |  |  |  |  |
| GO:0045405\_regulation\_of\_interleukin-5\_biosynthetic\_process | 1 | 0 |  |  |  |  |  |  |  |  |
| GO:0045407\_positive\_regulation\_of\_interleukin-5\_biosynthetic\_process | 1 | 0 |  |  |  |  |  |  |  |  |
| GO:0045423\_regulation\_of\_granulocyte\_macrophage\_colony-stimulating\_factor\_biosynthetic\_process | 1 | 0 |  |  |  |  |  |  |  |  |
| GO:0045542\_positive\_regulation\_of\_cholesterol\_biosynthetic\_process | 1 | 0 |  |  |  |  |  |  |  |  |
| GO:0045553\_TRAIL\_biosynthetic\_process | 1 | 0 |  |  |  |  |  |  |  |  |
| GO:0045554\_regulation\_of\_TRAIL\_biosynthetic\_process | 1 | 0 |  |  |  |  |  |  |  |  |
| GO:0045556\_positive\_regulation\_of\_TRAIL\_biosynthetic\_process | 1 | 0 |  |  |  |  |  |  |  |  |
| GO:0045577\_regulation\_of\_B\_cell\_differentiation | 1 | 0 |  |  |  |  |  |  |  |  |
| GO:0045578\_negative\_regulation\_of\_B\_cell\_differentiation | 1 | 0 |  |  |  |  |  |  |  |  |
| GO:0045581\_negative\_regulation\_of\_T\_cell\_differentiation | 1 | 0 |  |  |  |  |  |  |  |  |
| GO:0045583\_regulation\_of\_cytotoxic\_T\_cell\_differentiation | 1 | 0 |  |  |  |  |  |  |  |  |
| GO:0045585\_positive\_regulation\_of\_cytotoxic\_T\_cell\_differentiation | 1 | 0 |  |  |  |  |  |  |  |  |
| GO:0045599\_negative\_regulation\_of\_fat\_cell\_differentiation | 1 | 0 |  |  |  |  |  |  |  |  |
| GO:0045602\_negative\_regulation\_of\_endothelial\_cell\_differentiation | 1 | 0 |  |  |  |  |  |  |  |  |
| GO:0045603\_positive\_regulation\_of\_endothelial\_cell\_differentiation | 1 | 0 |  |  |  |  |  |  |  |  |
| GO:0045604\_regulation\_of\_epidermal\_cell\_differentiation | 1 | 0 |  |  |  |  |  |  |  |  |
| GO:0045606\_positive\_regulation\_of\_epidermal\_cell\_differentiation | 1 | 0 |  |  |  |  |  |  |  |  |
| GO:0045610\_regulation\_of\_hemocyte\_differentiation | 1 | 0 |  |  |  |  |  |  |  |  |
| GO:0045623\_negative\_regulation\_of\_T-helper\_cell\_differentiation | 1 | 0 |  |  |  |  |  |  |  |  |
| GO:0045624\_positive\_regulation\_of\_T-helper\_cell\_differentiation | 1 | 0 |  |  |  |  |  |  |  |  |
| GO:0045625\_regulation\_of\_T-helper\_1\_cell\_differentiation | 1 | 0 |  |  |  |  |  |  |  |  |
| GO:0045627\_positive\_regulation\_of\_T-helper\_1\_cell\_differentiation | 1 | 0 |  |  |  |  |  |  |  |  |
| GO:0045628\_regulation\_of\_T-helper\_2\_cell\_differentiation | 1 | 0 |  |  |  |  |  |  |  |  |
| GO:0045629\_negative\_regulation\_of\_T-helper\_2\_cell\_differentiation | 1 | 0 |  |  |  |  |  |  |  |  |
| GO:0045650\_negative\_regulation\_of\_macrophage\_differentiation | 1 | 0 |  |  |  |  |  |  |  |  |
| GO:0045653\_negative\_regulation\_of\_megakaryocyte\_differentiation | 1 | 0 |  |  |  |  |  |  |  |  |
| GO:0045654\_positive\_regulation\_of\_megakaryocyte\_differentiation | 1 | 0 |  |  |  |  |  |  |  |  |
| GO:0045655\_regulation\_of\_monocyte\_differentiation | 1 | 0 |  |  |  |  |  |  |  |  |
| GO:0045657\_positive\_regulation\_of\_monocyte\_differentiation | 1 | 0 |  |  |  |  |  |  |  |  |
| GO:0045665\_negative\_regulation\_of\_neuron\_differentiation | 1 | 0 |  |  |  |  |  |  |  |  |
| GO:0045666\_positive\_regulation\_of\_neuron\_differentiation | 1 | 0 |  |  |  |  |  |  |  |  |
| GO:0045669\_positive\_regulation\_of\_osteoblast\_differentiation | 1 | 0 |  |  |  |  |  |  |  |  |
| GO:0045713\_low-density\_lipoprotein\_receptor\_biosynthetic\_process | 1 | 0 |  |  |  |  |  |  |  |  |
| GO:0045714\_regulation\_of\_low-density\_lipoprotein\_receptor\_biosynthetic\_process | 1 | 0 |  |  |  |  |  |  |  |  |
| GO:0045716\_positive\_regulation\_of\_low-density\_lipoprotein\_receptor\_biosynthetic\_process | 1 | 0 |  |  |  |  |  |  |  |  |
| GO:0045717\_negative\_regulation\_of\_fatty\_acid\_biosynthetic\_process | 1 | 0 |  |  |  |  |  |  |  |  |
| GO:0045719\_negative\_regulation\_of\_glycogen\_biosynthetic\_process | 1 | 0 |  |  |  |  |  |  |  |  |
| GO:0045721\_negative\_regulation\_of\_gluconeogenesis | 1 | 0 |  |  |  |  |  |  |  |  |
| GO:0045722\_positive\_regulation\_of\_gluconeogenesis | 1 | 0 |  |  |  |  |  |  |  |  |
| GO:0045723\_positive\_regulation\_of\_fatty\_acid\_biosynthetic\_process | 1 | 0 |  |  |  |  |  |  |  |  |
| GO:0045744\_negative\_regulation\_of\_G-protein\_coupled\_receptor\_protein\_signaling\_pathway | 1 | 0 |  |  |  |  |  |  |  |  |
| GO:0045773\_positive\_regulation\_of\_axon\_extension | 1 | 0 |  |  |  |  |  |  |  |  |
| GO:0045776\_negative\_regulation\_of\_blood\_pressure | 1 | 0 |  |  |  |  |  |  |  |  |
| GO:0045779\_negative\_regulation\_of\_bone\_resorption | 1 | 0 |  |  |  |  |  |  |  |  |
| GO:0045822\_negative\_regulation\_of\_heart\_contraction | 1 | 0 |  |  |  |  |  |  |  |  |
| GO:0045823\_positive\_regulation\_of\_heart\_contraction | 1 | 0 |  |  |  |  |  |  |  |  |
| GO:0045836\_positive\_regulation\_of\_meiosis | 1 | 0 |  |  |  |  |  |  |  |  |
| GO:0045837\_negative\_regulation\_of\_membrane\_potential | 1 | 0 |  |  |  |  |  |  |  |  |
| GO:0045839\_negative\_regulation\_of\_mitosis | 1 | 0 |  |  |  |  |  |  |  |  |
| GO:0045844\_positive\_regulation\_of\_striated\_muscle\_development | 1 | 0 |  |  |  |  |  |  |  |  |
| GO:0045870\_positive\_regulation\_of\_retroviral\_genome\_replication | 1 | 0 |  |  |  |  |  |  |  |  |
| GO:0045875\_negative\_regulation\_of\_sister\_chromatid\_cohesion | 1 | 0 |  |  |  |  |  |  |  |  |
| GO:0045896\_regulation\_of\_transcription\_\_mitotic | 1 | 0 |  |  |  |  |  |  |  |  |
| GO:0045906\_negative\_regulation\_of\_vasoconstriction | 1 | 0 |  |  |  |  |  |  |  |  |
| GO:0045915\_positive\_regulation\_of\_catecholamine\_metabolic\_process | 1 | 0 |  |  |  |  |  |  |  |  |
| GO:0045919\_positive\_regulation\_of\_cytolysis | 1 | 0 |  |  |  |  |  |  |  |  |
| GO:0045922\_negative\_regulation\_of\_fatty\_acid\_metabolic\_process | 1 | 0 |  |  |  |  |  |  |  |  |
| GO:0045964\_positive\_regulation\_of\_dopamine\_metabolic\_process | 1 | 0 |  |  |  |  |  |  |  |  |
| GO:0045986\_negative\_regulation\_of\_smooth\_muscle\_contraction | 1 | 0 |  |  |  |  |  |  |  |  |
[truncated: 55,684 more chars]
