# Supplementary material for: RedundancyMiner: De-replication of redundant GO categories in microarray and proteomics analysis
Source: BMC Bioinformatics. 2011 Feb 10;12:52. doi: 10.1186/1471-2105-12-52 (PMC3223614; doi:10.1186/1471-2105-12-52)
Supplement: Additional file 19 — Information for developers. table of the relationship of RM and HTGM files. [file 1471-2105-12-52-S19.DOC]

Additional File 19. Information for developers^1^

|  |  | **inputs to RedundancyMiner from HTGM** | | | | **RedundancyMiner**  **parameters** | | **obsoleted** | | | | | | **outputs of one perl module used as input to another** | | |
| --- | --- | --- | --- | --- | --- | --- | --- | --- | --- | --- | --- | --- | --- | --- | --- | --- |
| **module** | **function** | **gominerdir** | **type** | **gcefile**^3^ | **cimfile** | **pflag** | **mode**^4^ | **npair**^5^ | **ncimfile** | **randn** | **s2ns** | **fdr[1,2]flag** | **fdr[1,2]** | **simfile** | **cluster** | **pth**^3^ |
| prepareData.pl | Calculate the similarity scores between GO categories | NA | NA | .tvt  .gce  .CIM | .CIM | NA | NA | NA | NA | 0 | 0 | NA | NA | NA | NA | NA |
| prepareData_dir.pl^2^ |  | workXXXX | Change  Under  Over | NA | NA | NA | NA | NA | NA | 0 | 0 | NA | NA | NA | NA | NA |
| obtainStatistics.pl | Provide number of pairs for similarity thresholds | NA | NA | NA | NA | 1 | NA | NA | NA | NA | NA | 0 | NA | output of  prepareData[-dir].pl | NA | NA |
| multiCluster.pl | Iteratively merge pairs and generate new nodes for a given threshold | NA | NA | NA | NA | NA | NA | NA | NA | NA | NA | NA | 0 | output of  prepareData[-dir].pl | NA | log_10_(p) threshold, selected from output of obtainStatistics.pl |
| multiCluster_dir.pl^2^ |  | NA | NA | NA | NA | NA | NA | between 0 and 1 (default =0.05) | NA | NA | NA | NA | NA | output of  prepareData[-dir].pl | NA | NA |
| collapseCIM.pl | Regenerate  collapsed CIMs according to the new nodes | NA | NA | .tvt  .gce  .CIM | .CIM | NA | average=1  min=2  max=3 | NA | prefix for name of  collapsed CIM | NA | NA | NA | NA | NA | .clusterout  output of multiCluster[-dir].pl | NA |
| collapseCIM_dir.pl^2^ |  | NA | NA | NA | NA | NA | NA | NA | NA | NA | NA | NA | NA | NA | .clusterout  output of multiCluster[-dir].pl | NA |

^1^The best way for a developer to understand how to incorporate the perl modules into a program is to first run the java version in a terminal window (java -jar RedundancyMiner.jar) for the desired scenario(s). The proper form of the module calls will be shown in the terminal window during execution.

^2^The modules whose names contain “_dir” are the versions that are used for the “default” mode.

^3^Please see Additional file 4 for a description of the types of HTGM gene-category association files used by RedundancyMiner.

^4^The *mode* parameter specifies how the pixel values are computed for a cluster. For the individual gene *versus* category CIMs, the average is used (*e.g,* if 3 categories are combined, and a particular gene maps to 2 of them, the pixel value is 0.67). For the integrative experiment *versus* category CIMs, the mode should be set to 3 (the pseudocolor of the pixel is taken as the max pseudocolor over the combined categories. Because of the inverse mapping of the true FDR and the pseudocolor, the max pseudocolor corresponds to the min FDR).

^5^The p value threshold is set by the parameters *npair* and *pth* for default and custom modes, respectively.
