# Supplementary material for: RedundancyMiner: De-replication of redundant GO categories in microarray and proteomics analysis
Source: BMC Bioinformatics. 2011 Feb 10;12:52. doi: 10.1186/1471-2105-12-52 (PMC3223614; doi:10.1186/1471-2105-12-52)
Supplement: Additional file 3 — RedundancyMiner program package download. the code for running RedundancyMiner on your own computer. [file 1471-2105-12-52-S3.GZ › redundancyminer_pkg/Glitches.doc]

Glitches

- Not showing box for entering threshold in default mode

When performing step 3 of the custom mode, the java GUI does not instantly reveal the box into which the log_10_(p) threshold is to be entered. The solution is to click the mouse once or twice in the area, and the box will appear.

- Program freezing when using log_10_(p) threshold that is too permissive and leads to too much collapsing

The best approach is to start with a stringent threshold and successively request more permissive ones. You will notice that the response time gradually increases. At some point, the system freezes, because the computation requires more resource than is available. If this happens, you will need to kill and restart RedundancyMiner. Note that it is safest to save (ie, make a copy and rename) the temp directory containing the current computations to avoid losing all of your work. This can be done through the RedundancyMiner GUI or directly through the OS.
