# Supplementary material for: RedundancyMiner: De-replication of redundant GO categories in microarray and proteomics analysis
Source: BMC Bioinformatics. 2011 Feb 10;12:52. doi: 10.1186/1471-2105-12-52 (PMC3223614; doi:10.1186/1471-2105-12-52)
Supplement: Additional file 8 — Retinal development HTGM download. compressed package of the results of running HTGM on the retinal development genes list. [file 1471-2105-12-52-S8.ZIP › SCENARIO_2_MODIFIED/total.txt.total.txt.dir/Exp1_BestClusterMap_LEIGS_KM_24.csv.join.3.txt.dir/Exp1_BestClusterMap_LEIGS_KM_24.csv.join.3.txt.change.gce.html]

Gene Category Report for Exp1\_BestClusterMap\_LEIGS\_KM\_24.csv.join.3.txt

# Gene Category Report for Exp1\_BestClusterMap\_LEIGS\_KM\_24.csv.join.3.txt

| HYPERLINKED GO CATEGORY | HYPERLINKED GENE NAME | TOTAL GENES | CHANGED GENES | ENRICHMENT | LOG10(p) | CUMULATIVE NUMBER OF CATEGORIES | CUMULATIVE RANDOMS MEAN | FALSE DISCOVERY RATE |  |
| --- | --- | --- | --- | --- | --- | --- | --- | --- | --- |
| GO:0045595\_regulation\_of\_cell\_differentiation | NOTCH1 | 295 | 9 | 4.132104 | -3.693312 | 1 | 0.29 | 0.290000 |  |
| GO:0045595\_regulation\_of\_cell\_differentiation | BAX | 295 | 9 | 4.132104 | -3.693312 | 1 | 0.29 | 0.290000 |  |
| GO:0045595\_regulation\_of\_cell\_differentiation | VWC2 | 295 | 9 | 4.132104 | -3.693312 | 1 | 0.29 | 0.290000 |  |
| GO:0045595\_regulation\_of\_cell\_differentiation | RB1 | 295 | 9 | 4.132104 | -3.693312 | 1 | 0.29 | 0.290000 |  |
| GO:0045595\_regulation\_of\_cell\_differentiation | BAD | 295 | 9 | 4.132104 | -3.693312 | 1 | 0.29 | 0.290000 |  |
| GO:0045595\_regulation\_of\_cell\_differentiation | SEMA3A | 295 | 9 | 4.132104 | -3.693312 | 1 | 0.29 | 0.290000 |  |
| GO:0045595\_regulation\_of\_cell\_differentiation | VAX1 | 295 | 9 | 4.132104 | -3.693312 | 1 | 0.29 | 0.290000 |  |
| GO:0045595\_regulation\_of\_cell\_differentiation | RBPJ | 295 | 9 | 4.132104 | -3.693312 | 1 | 0.29 | 0.290000 |  |
| GO:0045595\_regulation\_of\_cell\_differentiation | CDH4 | 295 | 9 | 4.132104 | -3.693312 | 1 | 0.29 | 0.290000 |  |
| GO:0060284\_regulation\_of\_cell\_development | NOTCH1 | 122 | 6 | 6.661041 | -3.650057 | 2 | 0.29 | 0.145000 |  |
| GO:0060284\_regulation\_of\_cell\_development | BAX | 122 | 6 | 6.661041 | -3.650057 | 2 | 0.29 | 0.145000 |  |
| GO:0060284\_regulation\_of\_cell\_development | VWC2 | 122 | 6 | 6.661041 | -3.650057 | 2 | 0.29 | 0.145000 |  |
| GO:0060284\_regulation\_of\_cell\_development | SEMA3A | 122 | 6 | 6.661041 | -3.650057 | 2 | 0.29 | 0.145000 |  |
| GO:0060284\_regulation\_of\_cell\_development | VAX1 | 122 | 6 | 6.661041 | -3.650057 | 2 | 0.29 | 0.145000 |  |
| GO:0060284\_regulation\_of\_cell\_development | CDH4 | 122 | 6 | 6.661041 | -3.650057 | 2 | 0.29 | 0.145000 |  |
| GO:0051881\_regulation\_of\_mitochondrial\_membrane\_potential | GCLC | 6 | 2 | 45.147059 | -3.108346 | 3 | 1.22 | 0.406667 |  |
| GO:0051881\_regulation\_of\_mitochondrial\_membrane\_potential | BAX | 6 | 2 | 45.147059 | -3.108346 | 3 | 1.22 | 0.406667 |  |
| GO:0050767\_regulation\_of\_neurogenesis | NOTCH1 | 104 | 5 | 6.511595 | -3.054747 | 4 | 1.28 | 0.320000 |  |
| GO:0050767\_regulation\_of\_neurogenesis | VWC2 | 104 | 5 | 6.511595 | -3.054747 | 4 | 1.28 | 0.320000 |  |
| GO:0050767\_regulation\_of\_neurogenesis | VAX1 | 104 | 5 | 6.511595 | -3.054747 | 4 | 1.28 | 0.320000 |  |
| GO:0050767\_regulation\_of\_neurogenesis | SEMA3A | 104 | 5 | 6.511595 | -3.054747 | 4 | 1.28 | 0.320000 |  |
| GO:0050767\_regulation\_of\_neurogenesis | CDH4 | 104 | 5 | 6.511595 | -3.054747 | 4 | 1.28 | 0.320000 |  |
| GO:0007413\_axonal\_fasciculation | FEZF2 | 7 | 2 | 38.697479 | -2.964232 | 5 | 1.53 | 0.306000 |  |
| GO:0007413\_axonal\_fasciculation | SEMA3A | 7 | 2 | 38.697479 | -2.964232 | 5 | 1.53 | 0.306000 |  |
| GO:0032989\_cellular\_component\_morphogenesis | FEZF2 | 307 | 8 | 3.529412 | -2.853899 | 6 | 1.73 | 0.288333 |  |
| GO:0032989\_cellular\_component\_morphogenesis | NOTCH1 | 307 | 8 | 3.529412 | -2.853899 | 6 | 1.73 | 0.288333 |  |
| GO:0032989\_cellular\_component\_morphogenesis | PDPN | 307 | 8 | 3.529412 | -2.853899 | 6 | 1.73 | 0.288333 |  |
| GO:0032989\_cellular\_component\_morphogenesis | BAX | 307 | 8 | 3.529412 | -2.853899 | 6 | 1.73 | 0.288333 |  |
| GO:0032989\_cellular\_component\_morphogenesis | VAX1 | 307 | 8 | 3.529412 | -2.853899 | 6 | 1.73 | 0.288333 |  |
| GO:0032989\_cellular\_component\_morphogenesis | SEMA3A | 307 | 8 | 3.529412 | -2.853899 | 6 | 1.73 | 0.288333 |  |
| GO:0032989\_cellular\_component\_morphogenesis | RBPJ | 307 | 8 | 3.529412 | -2.853899 | 6 | 1.73 | 0.288333 |  |
| GO:0032989\_cellular\_component\_morphogenesis | CDH4 | 307 | 8 | 3.529412 | -2.853899 | 6 | 1.73 | 0.288333 |  |
| GO:0051960\_regulation\_of\_nervous\_system\_development | NOTCH1 | 118 | 5 | 5.739033 | -2.807512 | 7 | 2.17 | 0.310000 |  |
| GO:0051960\_regulation\_of\_nervous\_system\_development | VWC2 | 118 | 5 | 5.739033 | -2.807512 | 7 | 2.17 | 0.310000 |  |
| GO:0051960\_regulation\_of\_nervous\_system\_development | VAX1 | 118 | 5 | 5.739033 | -2.807512 | 7 | 2.17 | 0.310000 |  |
| GO:0051960\_regulation\_of\_nervous\_system\_development | SEMA3A | 118 | 5 | 5.739033 | -2.807512 | 7 | 2.17 | 0.310000 |  |
| GO:0051960\_regulation\_of\_nervous\_system\_development | CDH4 | 118 | 5 | 5.739033 | -2.807512 | 7 | 2.17 | 0.310000 |  |
| GO:0032990\_cell\_part\_morphogenesis | FEZF2 | 184 | 6 | 4.416560 | -2.703161 | 8 | 2.62 | 0.327500 |  |
| GO:0032990\_cell\_part\_morphogenesis | NOTCH1 | 184 | 6 | 4.416560 | -2.703161 | 8 | 2.62 | 0.327500 |  |
| GO:0032990\_cell\_part\_morphogenesis | BAX | 184 | 6 | 4.416560 | -2.703161 | 8 | 2.62 | 0.327500 |  |
| GO:0032990\_cell\_part\_morphogenesis | VAX1 | 184 | 6 | 4.416560 | -2.703161 | 8 | 2.62 | 0.327500 |  |
| GO:0032990\_cell\_part\_morphogenesis | SEMA3A | 184 | 6 | 4.416560 | -2.703161 | 8 | 2.62 | 0.327500 |  |
| GO:0032990\_cell\_part\_morphogenesis | CDH4 | 184 | 6 | 4.416560 | -2.703161 | 8 | 2.62 | 0.327500 |  |
| GO:0045597\_positive\_regulation\_of\_cell\_differentiation | NOTCH1 | 128 | 5 | 5.290671 | -2.650855 | 9 | 2.82 | 0.313333 |  |
| GO:0045597\_positive\_regulation\_of\_cell\_differentiation | VWC2 | 128 | 5 | 5.290671 | -2.650855 | 9 | 2.82 | 0.313333 |  |
| GO:0045597\_positive\_regulation\_of\_cell\_differentiation | RB1 | 128 | 5 | 5.290671 | -2.650855 | 9 | 2.82 | 0.313333 |  |
| GO:0045597\_positive\_regulation\_of\_cell\_differentiation | BAD | 128 | 5 | 5.290671 | -2.650855 | 9 | 2.82 | 0.313333 |  |
| GO:0045597\_positive\_regulation\_of\_cell\_differentiation | CDH4 | 128 | 5 | 5.290671 | -2.650855 | 9 | 2.82 | 0.313333 |  |
| GO:0006921\_cell\_structure\_disassembly\_during\_apoptosis | BAX | 10 | 2 | 27.088235 | -2.639276 | 10 | 3.11 | 0.311000 |  |
| GO:0006921\_cell\_structure\_disassembly\_during\_apoptosis | ENDOG | 10 | 2 | 27.088235 | -2.639276 | 10 | 3.11 | 0.311000 |  |
| GO:0001837\_epithelial\_to\_mesenchymal\_transition | NOTCH1 | 11 | 2 | 24.625668 | -2.554136 | 11 | 3.66 | 0.332727 |  |
| GO:0001837\_epithelial\_to\_mesenchymal\_transition | RBPJ | 11 | 2 | 24.625668 | -2.554136 | 11 | 3.66 | 0.332727 |  |
| GO:0007411\_axon\_guidance | FEZF2 | 82 | 4 | 6.606887 | -2.539881 | 13 | 3.82 | 0.293846 |  |
| GO:0007411\_axon\_guidance | VAX1 | 82 | 4 | 6.606887 | -2.539881 | 13 | 3.82 | 0.293846 |  |
| GO:0007411\_axon\_guidance | SEMA3A | 82 | 4 | 6.606887 | -2.539881 | 13 | 3.82 | 0.293846 |  |
| GO:0007411\_axon\_guidance | CDH4 | 82 | 4 | 6.606887 | -2.539881 | 13 | 3.82 | 0.293846 |  |
| GO:0045664\_regulation\_of\_neuron\_differentiation | NOTCH1 | 82 | 4 | 6.606887 | -2.539881 | 13 | 3.82 | 0.293846 |  |
| GO:0045664\_regulation\_of\_neuron\_differentiation | VWC2 | 82 | 4 | 6.606887 | -2.539881 | 13 | 3.82 | 0.293846 |  |
| GO:0045664\_regulation\_of\_neuron\_differentiation | SEMA3A | 82 | 4 | 6.606887 | -2.539881 | 13 | 3.82 | 0.293846 |  |
| GO:0045664\_regulation\_of\_neuron\_differentiation | CDH4 | 82 | 4 | 6.606887 | -2.539881 | 13 | 3.82 | 0.293846 |  |
| GO:0000904\_cell\_morphogenesis\_involved\_in\_differentiation | FEZF2 | 199 | 6 | 4.083654 | -2.530727 | 14 | 3.83 | 0.273571 |  |
| GO:0000904\_cell\_morphogenesis\_involved\_in\_differentiation | NOTCH1 | 199 | 6 | 4.083654 | -2.530727 | 14 | 3.83 | 0.273571 |  |
| GO:0000904\_cell\_morphogenesis\_involved\_in\_differentiation | VAX1 | 199 | 6 | 4.083654 | -2.530727 | 14 | 3.83 | 0.273571 |  |
| GO:0000904\_cell\_morphogenesis\_involved\_in\_differentiation | SEMA3A | 199 | 6 | 4.083654 | -2.530727 | 14 | 3.83 | 0.273571 |  |
| GO:0000904\_cell\_morphogenesis\_involved\_in\_differentiation | RBPJ | 199 | 6 | 4.083654 | -2.530727 | 14 | 3.83 | 0.273571 |  |
| GO:0000904\_cell\_morphogenesis\_involved\_in\_differentiation | CDH4 | 199 | 6 | 4.083654 | -2.530727 | 14 | 3.83 | 0.273571 |  |
| GO:0008038\_neuron\_recognition | FEZF2 | 12 | 2 | 22.573529 | -2.476965 | 17 | 4.28 | 0.251765 |  |
| GO:0008038\_neuron\_recognition | SEMA3A | 12 | 2 | 22.573529 | -2.476965 | 17 | 4.28 | 0.251765 |  |
| GO:0030262\_apoptotic\_nuclear\_changes | BAX | 12 | 2 | 22.573529 | -2.476965 | 17 | 4.28 | 0.251765 |  |
| GO:0030262\_apoptotic\_nuclear\_changes | ENDOG | 12 | 2 | 22.573529 | -2.476965 | 17 | 4.28 | 0.251765 |  |
| GO:0048820\_hair\_follicle\_maturation | NOTCH1 | 12 | 2 | 22.573529 | -2.476965 | 17 | 4.28 | 0.251765 |  |
| GO:0048820\_hair\_follicle\_maturation | RBPJ | 12 | 2 | 22.573529 | -2.476965 | 17 | 4.28 | 0.251765 |  |
| GO:0030516\_regulation\_of\_axon\_extension | SEMA3A | 13 | 2 | 20.837104 | -2.406423 | 18 | 4.91 | 0.272778 |  |
| GO:0030516\_regulation\_of\_axon\_extension | CDH4 | 13 | 2 | 20.837104 | -2.406423 | 18 | 4.91 | 0.272778 |  |
| GO:0000902\_cell\_morphogenesis | FEZF2 | 283 | 7 | 3.350135 | -2.405991 | 19 | 4.91 | 0.258421 |  |
| GO:0000902\_cell\_morphogenesis | NOTCH1 | 283 | 7 | 3.350135 | -2.405991 | 19 | 4.91 | 0.258421 |  |
| GO:0000902\_cell\_morphogenesis | PDPN | 283 | 7 | 3.350135 | -2.405991 | 19 | 4.91 | 0.258421 |  |
| GO:0000902\_cell\_morphogenesis | VAX1 | 283 | 7 | 3.350135 | -2.405991 | 19 | 4.91 | 0.258421 |  |
| GO:0000902\_cell\_morphogenesis | SEMA3A | 283 | 7 | 3.350135 | -2.405991 | 19 | 4.91 | 0.258421 |  |
| GO:0000902\_cell\_morphogenesis | RBPJ | 283 | 7 | 3.350135 | -2.405991 | 19 | 4.91 | 0.258421 |  |
| GO:0000902\_cell\_morphogenesis | CDH4 | 283 | 7 | 3.350135 | -2.405991 | 19 | 4.91 | 0.258421 |  |
| GO:0030183\_B\_cell\_differentiation | BAX | 47 | 3 | 8.645181 | -2.320770 | 20 | 5.8 | 0.290000 |  |
| GO:0030183\_B\_cell\_differentiation | BAD | 47 | 3 | 8.645181 | -2.320770 | 20 | 5.8 | 0.290000 |  |
| GO:0030183\_B\_cell\_differentiation | RBPJ | 47 | 3 | 8.645181 | -2.320770 | 20 | 5.8 | 0.290000 |  |
| GO:0007409\_axonogenesis | FEZF2 | 158 | 5 | 4.286113 | -2.255831 | 21 | 6.36 | 0.302857 |  |
| GO:0007409\_axonogenesis | NOTCH1 | 158 | 5 | 4.286113 | -2.255831 | 21 | 6.36 | 0.302857 |  |
| GO:0007409\_axonogenesis | VAX1 | 158 | 5 | 4.286113 | -2.255831 | 21 | 6.36 | 0.302857 |  |
| GO:0007409\_axonogenesis | SEMA3A | 158 | 5 | 4.286113 | -2.255831 | 21 | 6.36 | 0.302857 |  |
| GO:0007409\_axonogenesis | CDH4 | 158 | 5 | 4.286113 | -2.255831 | 21 | 6.36 | 0.302857 |  |
| GO:0051094\_positive\_regulation\_of\_developmental\_process | NOTCH1 | 308 | 7 | 3.078209 | -2.203456 | 22 | 6.93 | 0.315000 |  |
| GO:0051094\_positive\_regulation\_of\_developmental\_process | ENDOG | 308 | 7 | 3.078209 | -2.203456 | 22 | 6.93 | 0.315000 |  |
| GO:0051094\_positive\_regulation\_of\_developmental\_process | BAX | 308 | 7 | 3.078209 | -2.203456 | 22 | 6.93 | 0.315000 |  |
| GO:0051094\_positive\_regulation\_of\_developmental\_process | VWC2 | 308 | 7 | 3.078209 | -2.203456 | 22 | 6.93 | 0.315000 |  |
| GO:0051094\_positive\_regulation\_of\_developmental\_process | RB1 | 308 | 7 | 3.078209 | -2.203456 | 22 | 6.93 | 0.315000 |  |
| GO:0051094\_positive\_regulation\_of\_developmental\_process | BAD | 308 | 7 | 3.078209 | -2.203456 | 22 | 6.93 | 0.315000 |  |
| GO:0051094\_positive\_regulation\_of\_developmental\_process | CDH4 | 308 | 7 | 3.078209 | -2.203456 | 22 | 6.93 | 0.315000 |  |
| GO:0048699\_generation\_of\_neurons | FEZF2 | 396 | 8 | 2.736185 | -2.163354 | 23 | 7.43 | 0.323043 |  |
| GO:0048699\_generation\_of\_neurons | NOTCH1 | 396 | 8 | 2.736185 | -2.163354 | 23 | 7.43 | 0.323043 |  |
| GO:0048699\_generation\_of\_neurons | BAX | 396 | 8 | 2.736185 | -2.163354 | 23 | 7.43 | 0.323043 |  |
| GO:0048699\_generation\_of\_neurons | VWC2 | 396 | 8 | 2.736185 | -2.163354 | 23 | 7.43 | 0.323043 |  |
| GO:0048699\_generation\_of\_neurons | SEMA3A | 396 | 8 | 2.736185 | -2.163354 | 23 | 7.43 | 0.323043 |  |
| GO:0048699\_generation\_of\_neurons | VAX1 | 396 | 8 | 2.736185 | -2.163354 | 23 | 7.43 | 0.323043 |  |
| GO:0048699\_generation\_of\_neurons | RBPJ | 396 | 8 | 2.736185 | -2.163354 | 23 | 7.43 | 0.323043 |  |
| GO:0048699\_generation\_of\_neurons | CDH4 | 396 | 8 | 2.736185 | -2.163354 | 23 | 7.43 | 0.323043 |  |
| GO:0001946\_lymphangiogenesis | PDPN | 1 | 1 |  |  |  |  |  |  |
| GO:0002040\_sprouting\_angiogenesis | NOTCH1 | 1 | 1 |  |  |  |  |  |  |
| GO:0006060\_sorbitol\_metabolic\_process | SORD | 1 | 1 |  |  |  |  |  |  |
| GO:0007223\_Wnt\_receptor\_signaling\_pathway\_\_calcium\_modulating\_pathway | ROR2 | 1 | 1 |  |  |  |  |  |  |
| GO:0009957\_epidermal\_cell\_fate\_specification | RBPJ | 1 | 1 |  |  |  |  |  |  |
| GO:0014807\_regulation\_of\_somitogenesis | NOTCH1 | 1 | 1 |  |  |  |  |  |  |
| GO:0015884\_folic\_acid\_transport | PDPN | 1 | 1 |  |  |  |  |  |  |
| GO:0030264\_nuclear\_fragmentation\_during\_apoptosis | BAX | 1 | 1 |  |  |  |  |  |  |
| GO:0045210\_FasL\_biosynthetic\_process | PHLDA1 | 1 | 1 |  |  |  |  |  |  |
| GO:0045606\_positive\_regulation\_of\_epidermal\_cell\_differentiation | NOTCH1 | 1 | 1 |  |  |  |  |  |  |
| GO:0045618\_positive\_regulation\_of\_keratinocyte\_differentiation | NOTCH1 | 1 | 1 |  |  |  |  |  |  |
| GO:0046685\_response\_to\_arsenic | GCLC | 1 | 1 |  |  |  |  |  |  |
| GO:0048733\_sebaceous\_gland\_development | RBPJ | 1 | 1 |  |  |  |  |  |  |
| GO:0051281\_positive\_regulation\_of\_release\_of\_sequestered\_calcium\_ion\_into\_cytosol | BAX | 1 | 1 |  |  |  |  |  |  |
| GO:0048812\_neuron\_projection\_morphogenesis | FEZF2 | 170 | 5 | 3.983564 | -2.122409 | 24 | 7.94 | 0.330833 |  |
| GO:0048812\_neuron\_projection\_morphogenesis | NOTCH1 | 170 | 5 | 3.983564 | -2.122409 | 24 | 7.94 | 0.330833 |  |
| GO:0048812\_neuron\_projection\_morphogenesis | VAX1 | 170 | 5 | 3.983564 | -2.122409 | 24 | 7.94 | 0.330833 |  |
| GO:0048812\_neuron\_projection\_morphogenesis | SEMA3A | 170 | 5 | 3.983564 | -2.122409 | 24 | 7.94 | 0.330833 |  |
| GO:0048812\_neuron\_projection\_morphogenesis | CDH4 | 170 | 5 | 3.983564 | -2.122409 | 24 | 7.94 | 0.330833 |  |
| GO:0048667\_cell\_morphogenesis\_involved\_in\_neuron\_differentiation | FEZF2 | 173 | 5 | 3.914485 | -2.090851 | 25 | 8.09 | 0.323600 |  |
| GO:0048667\_cell\_morphogenesis\_involved\_in\_neuron\_differentiation | NOTCH1 | 173 | 5 | 3.914485 | -2.090851 | 25 | 8.09 | 0.323600 |  |
| GO:0048667\_cell\_morphogenesis\_involved\_in\_neuron\_differentiation | VAX1 | 173 | 5 | 3.914485 | -2.090851 | 25 | 8.09 | 0.323600 |  |
| GO:0048667\_cell\_morphogenesis\_involved\_in\_neuron\_differentiation | SEMA3A | 173 | 5 | 3.914485 | -2.090851 | 25 | 8.09 | 0.323600 |  |
| GO:0048667\_cell\_morphogenesis\_involved\_in\_neuron\_differentiation | CDH4 | 173 | 5 | 3.914485 | -2.090851 | 25 | 8.09 | 0.323600 |  |
| GO:0048858\_cell\_projection\_morphogenesis | FEZF2 | 176 | 5 | 3.847761 | -2.059960 | 26 | 8.76 | 0.336923 |  |
| GO:0048858\_cell\_projection\_morphogenesis | NOTCH1 | 176 | 5 | 3.847761 | -2.059960 | 26 | 8.76 | 0.336923 |  |
| GO:0048858\_cell\_projection\_morphogenesis | VAX1 | 176 | 5 | 3.847761 | -2.059960 | 26 | 8.76 | 0.336923 |  |
| GO:0048858\_cell\_projection\_morphogenesis | SEMA3A | 176 | 5 | 3.847761 | -2.059960 | 26 | 8.76 | 0.336923 |  |
| GO:0048858\_cell\_projection\_morphogenesis | CDH4 | 176 | 5 | 3.847761 | -2.059960 | 26 | 8.76 | 0.336923 |  |
| GO:0006928\_cell\_motion | FEZF2 | 330 | 7 | 2.872995 | -2.042371 | 28 | 8.87 | 0.316786 |  |
| GO:0006928\_cell\_motion | PDPN | 330 | 7 | 2.872995 | -2.042371 | 28 | 8.87 | 0.316786 |  |
| GO:0006928\_cell\_motion | RRAS2 | 330 | 7 | 2.872995 | -2.042371 | 28 | 8.87 | 0.316786 |  |
| GO:0006928\_cell\_motion | BAX | 330 | 7 | 2.872995 | -2.042371 | 28 | 8.87 | 0.316786 |  |
| GO:0006928\_cell\_motion | VAX1 | 330 | 7 | 2.872995 | -2.042371 | 28 | 8.87 | 0.316786 |  |
| GO:0006928\_cell\_motion | SEMA3A | 330 | 7 | 2.872995 | -2.042371 | 28 | 8.87 | 0.316786 |  |
| GO:0006928\_cell\_motion | CDH4 | 330 | 7 | 2.872995 | -2.042371 | 28 | 8.87 | 0.316786 |  |
| GO:0051674\_localization\_of\_cell | FEZF2 | 330 | 7 | 2.872995 | -2.042371 | 28 | 8.87 | 0.316786 |  |
| GO:0051674\_localization\_of\_cell | PDPN | 330 | 7 | 2.872995 | -2.042371 | 28 | 8.87 | 0.316786 |  |
| GO:0051674\_localization\_of\_cell | RRAS2 | 330 | 7 | 2.872995 | -2.042371 | 28 | 8.87 | 0.316786 |  |
| GO:0051674\_localization\_of\_cell | BAX | 330 | 7 | 2.872995 | -2.042371 | 28 | 8.87 | 0.316786 |  |
| GO:0051674\_localization\_of\_cell | VAX1 | 330 | 7 | 2.872995 | -2.042371 | 28 | 8.87 | 0.316786 |  |
| GO:0051674\_localization\_of\_cell | SEMA3A | 330 | 7 | 2.872995 | -2.042371 | 28 | 8.87 | 0.316786 |  |
| GO:0051674\_localization\_of\_cell | CDH4 | 330 | 7 | 2.872995 | -2.042371 | 28 | 8.87 | 0.316786 |  |
| GO:0050793\_regulation\_of\_developmental\_process | NOTCH1 | 703 | 11 | 2.119279 | -2.016103 | 29 | 9.42 | 0.324828 |  |
| GO:0050793\_regulation\_of\_developmental\_process | GCLC | 703 | 11 | 2.119279 | -2.016103 | 29 | 9.42 | 0.324828 |  |
| GO:0050793\_regulation\_of\_developmental\_process | ENDOG | 703 | 11 | 2.119279 | -2.016103 | 29 | 9.42 | 0.324828 |  |
| GO:0050793\_regulation\_of\_developmental\_process | BAX | 703 | 11 | 2.119279 | -2.016103 | 29 | 9.42 | 0.324828 |  |
| GO:0050793\_regulation\_of\_developmental\_process | VWC2 | 703 | 11 | 2.119279 | -2.016103 | 29 | 9.42 | 0.324828 |  |
| GO:0050793\_regulation\_of\_developmental\_process | RB1 | 703 | 11 | 2.119279 | -2.016103 | 29 | 9.42 | 0.324828 |  |
| GO:0050793\_regulation\_of\_developmental\_process | VAX1 | 703 | 11 | 2.119279 | -2.016103 | 29 | 9.42 | 0.324828 |  |
| GO:0050793\_regulation\_of\_developmental\_process | SEMA3A | 703 | 11 | 2.119279 | -2.016103 | 29 | 9.42 | 0.324828 |  |
| GO:0050793\_regulation\_of\_developmental\_process | BAD | 703 | 11 | 2.119279 | -2.016103 | 29 | 9.42 | 0.324828 |  |
| GO:0050793\_regulation\_of\_developmental\_process | RBPJ | 703 | 11 | 2.119279 | -2.016103 | 29 | 9.42 | 0.324828 |  |
| GO:0050793\_regulation\_of\_developmental\_process | CDH4 | 703 | 11 | 2.119279 | -2.016103 | 29 | 9.42 | 0.324828 |  |
| GO:0022008\_neurogenesis | FEZF2 | 423 | 8 | 2.561535 | -1.994127 | 30 | 9.57 | 0.319000 |  |
| GO:0022008\_neurogenesis | NOTCH1 | 423 | 8 | 2.561535 | -1.994127 | 30 | 9.57 | 0.319000 |  |
| GO:0022008\_neurogenesis | BAX | 423 | 8 | 2.561535 | -1.994127 | 30 | 9.57 | 0.319000 |  |
| GO:0022008\_neurogenesis | VWC2 | 423 | 8 | 2.561535 | -1.994127 | 30 | 9.57 | 0.319000 |  |
| GO:0022008\_neurogenesis | SEMA3A | 423 | 8 | 2.561535 | -1.994127 | 30 | 9.57 | 0.319000 |  |
| GO:0022008\_neurogenesis | VAX1 | 423 | 8 | 2.561535 | -1.994127 | 30 | 9.57 | 0.319000 |  |
| GO:0022008\_neurogenesis | RBPJ | 423 | 8 | 2.561535 | -1.994127 | 30 | 9.57 | 0.319000 |  |
| GO:0022008\_neurogenesis | CDH4 | 423 | 8 | 2.561535 | -1.994127 | 30 | 9.57 | 0.319000 |  |
| GO:0030216\_keratinocyte\_differentiation | NOTCH1 | 21 | 2 | 12.899160 | -1.992338 | 32 | 10.1 | 0.315625 |  |
| GO:0030216\_keratinocyte\_differentiation | RBPJ | 21 | 2 | 12.899160 | -1.992338 | 32 | 10.1 | 0.315625 |  |
| GO:0048675\_axon\_extension | SEMA3A | 21 | 2 | 12.899160 | -1.992338 | 32 | 10.1 | 0.315625 |  |
| GO:0048675\_axon\_extension | CDH4 | 21 | 2 | 12.899160 | -1.992338 | 32 | 10.1 | 0.315625 |  |
| GO:0008624\_induction\_of\_apoptosis\_by\_extracellular\_signals | BAX | 22 | 2 | 12.312834 | -1.952947 | 33 | 10.97 | 0.332424 |  |
| GO:0008624\_induction\_of\_apoptosis\_by\_extracellular\_signals | BAD | 22 | 2 | 12.312834 | -1.952947 | 33 | 10.97 | 0.332424 |  |
| GO:0030182\_neuron\_differentiation | FEZF2 | 356 | 7 | 2.663169 | -1.869739 | 34 | 12.44 | 0.365882 |  |
| GO:0030182\_neuron\_differentiation | NOTCH1 | 356 | 7 | 2.663169 | -1.869739 | 34 | 12.44 | 0.365882 |  |
| GO:0030182\_neuron\_differentiation | VWC2 | 356 | 7 | 2.663169 | -1.869739 | 34 | 12.44 | 0.365882 |  |
| GO:0030182\_neuron\_differentiation | VAX1 | 356 | 7 | 2.663169 | -1.869739 | 34 | 12.44 | 0.365882 |  |
| GO:0030182\_neuron\_differentiation | SEMA3A | 356 | 7 | 2.663169 | -1.869739 | 34 | 12.44 | 0.365882 |  |
| GO:0030182\_neuron\_differentiation | RBPJ | 356 | 7 | 2.663169 | -1.869739 | 34 | 12.44 | 0.365882 |  |
| GO:0030182\_neuron\_differentiation | CDH4 | 356 | 7 | 2.663169 | -1.869739 | 34 | 12.44 | 0.365882 |  |
| GO:0031175\_neuron\_projection\_development | FEZF2 | 197 | 5 | 3.437593 | -1.860655 | 35 | 12.52 | 0.357714 |  |
| GO:0031175\_neuron\_projection\_development | NOTCH1 | 197 | 5 | 3.437593 | -1.860655 | 35 | 12.52 | 0.357714 |  |
| GO:0031175\_neuron\_projection\_development | VAX1 | 197 | 5 | 3.437593 | -1.860655 | 35 | 12.52 | 0.357714 |  |
| GO:0031175\_neuron\_projection\_development | SEMA3A | 197 | 5 | 3.437593 | -1.860655 | 35 | 12.52 | 0.357714 |  |
| GO:0031175\_neuron\_projection\_development | CDH4 | 197 | 5 | 3.437593 | -1.860655 | 35 | 12.52 | 0.357714 |  |
| GO:0031400\_negative\_regulation\_of\_protein\_modification\_process | GCLC | 25 | 2 | 10.835294 | -1.845435 | 36 | 13.05 | 0.362500 |  |
| GO:0031400\_negative\_regulation\_of\_protein\_modification\_process | BAX | 25 | 2 | 10.835294 | -1.845435 | 36 | 13.05 | 0.362500 |  |
| GO:0002339\_B\_cell\_selection | BAX | 2 | 1 |  |  |  |  |  |  |
| GO:0002352\_B\_cell\_negative\_selection | BAX | 2 | 1 |  |  |  |  |  |  |
| GO:0002358\_B\_cell\_homeostatic\_proliferation | BAX | 2 | 1 |  |  |  |  |  |  |
| GO:0006059\_hexitol\_metabolic\_process | SORD | 2 | 1 |  |  |  |  |  |  |
| GO:0006122\_mitochondrial\_electron\_transport\_\_ubiquinol\_to\_cytochrome\_c | UQCRC1 | 2 | 1 |  |  |  |  |  |  |
| GO:0006750\_glutathione\_biosynthetic\_process | GCLC | 2 | 1 |  |  |  |  |  |  |
| GO:0006808\_regulation\_of\_nitrogen\_utilization | BAX | 2 | 1 |  |  |  |  |  |  |
| GO:0007527\_adult\_somatic\_muscle\_development | IFRD1 | 2 | 1 |  |  |  |  |  |  |
| GO:0010633\_negative\_regulation\_of\_epithelial\_cell\_migration | SEMA3A | 2 | 1 |  |  |  |  |  |  |
| GO:0015671\_oxygen\_transport | HBB-B1 | 2 | 1 |  |  |  |  |  |  |
| GO:0019740\_nitrogen\_utilization | BAX | 2 | 1 |  |  |  |  |  |  |
| GO:0032471\_reduction\_of\_endoplasmic\_reticulum\_calcium\_ion\_concentration | BAX | 2 | 1 |  |  |  |  |  |  |
| GO:0034644\_cellular\_response\_to\_UV | BAX | 2 | 1 |  |  |  |  |  |  |
| GO:0046532\_regulation\_of\_photoreceptor\_cell\_differentiation | NOTCH1 | 2 | 1 |  |  |  |  |  |  |
| GO:0046533\_negative\_regulation\_of\_photoreceptor\_cell\_differentiation | NOTCH1 | 2 | 1 |  |  |  |  |  |  |
| GO:0051279\_regulation\_of\_release\_of\_sequestered\_calcium\_ion\_into\_cytosol | BAX | 2 | 1 |  |  |  |  |  |  |
| GO:0051270\_regulation\_of\_cell\_motion | PDPN | 73 | 3 | 5.566076 | -1.793524 | 37 | 14.2 | 0.383784 |  |
| GO:0051270\_regulation\_of\_cell\_motion | RRAS2 | 73 | 3 | 5.566076 | -1.793524 | 37 | 14.2 | 0.383784 |  |
| GO:0051270\_regulation\_of\_cell\_motion | SEMA3A | 73 | 3 | 5.566076 | -1.793524 | 37 | 14.2 | 0.383784 |  |
| GO:0009913\_epidermal\_cell\_differentiation | NOTCH1 | 27 | 2 | 10.032680 | -1.781243 | 39 | 14.63 | 0.375128 |  |
| GO:0009913\_epidermal\_cell\_differentiation | RBPJ | 27 | 2 | 10.032680 | -1.781243 | 39 | 14.63 | 0.375128 |  |
| GO:0051272\_positive\_regulation\_of\_cell\_motion | PDPN | 27 | 2 | 10.032680 | -1.781243 | 39 | 14.63 | 0.375128 |  |
| GO:0051272\_positive\_regulation\_of\_cell\_motion | RRAS2 | 27 | 2 | 10.032680 | -1.781243 | 39 | 14.63 | 0.375128 |  |
| GO:0006997\_nucleus\_organization | BAX | 28 | 2 | 9.674370 | -1.751054 | 40 | 15.27 | 0.381750 |  |
| GO:0006997\_nucleus\_organization | ENDOG | 28 | 2 | 9.674370 | -1.751054 | 40 | 15.27 | 0.381750 |  |
| GO:0043281\_regulation\_of\_caspase\_activity | BAX | 29 | 2 | 9.340771 | -1.722015 | 43 | 15.85 | 0.368605 |  |
| GO:0043281\_regulation\_of\_caspase\_activity | BAD | 29 | 2 | 9.340771 | -1.722015 | 43 | 15.85 | 0.368605 |  |
| GO:0051301\_cell\_division | NOTCH1 | 29 | 2 | 9.340771 | -1.722015 | 43 | 15.85 | 0.368605 |  |
| GO:0051301\_cell\_division | RB1 | 29 | 2 | 9.340771 | -1.722015 | 43 | 15.85 | 0.368605 |  |
| GO:0052548\_regulation\_of\_endopeptidase\_activity | BAX | 29 | 2 | 9.340771 | -1.722015 | 43 | 15.85 | 0.368605 |  |
| GO:0052548\_regulation\_of\_endopeptidase\_activity | BAD | 29 | 2 | 9.340771 | -1.722015 | 43 | 15.85 | 0.368605 |  |
| GO:0022411\_cellular\_component\_disassembly | BAX | 30 | 2 | 9.029412 | -1.694046 | 45 | 16.55 | 0.367778 |  |
| GO:0022411\_cellular\_component\_disassembly | ENDOG | 30 | 2 | 9.029412 | -1.694046 | 45 | 16.55 | 0.367778 |  |
| GO:0052547\_regulation\_of\_peptidase\_activity | BAX | 30 | 2 | 9.029412 | -1.694046 | 45 | 16.55 | 0.367778 |  |
| GO:0052547\_regulation\_of\_peptidase\_activity | BAD | 30 | 2 | 9.029412 | -1.694046 | 45 | 16.55 | 0.367778 |  |
| GO:0045596\_negative\_regulation\_of\_cell\_differentiation | NOTCH1 | 144 | 4 | 3.762255 | -1.687592 | 46 | 16.7 | 0.363043 |  |
| GO:0045596\_negative\_regulation\_of\_cell\_differentiation | SEMA3A | 144 | 4 | 3.762255 | -1.687592 | 46 | 16.7 | 0.363043 |  |
| GO:0045596\_negative\_regulation\_of\_cell\_differentiation | VAX1 | 144 | 4 | 3.762255 | -1.687592 | 46 | 16.7 | 0.363043 |  |
| GO:0045596\_negative\_regulation\_of\_cell\_differentiation | RBPJ | 144 | 4 | 3.762255 | -1.687592 | 46 | 16.7 | 0.363043 |  |
| GO:0048869\_cellular\_developmental\_process | ALDH6A1 | 1113 | 14 | 1.703663 | -1.681411 | 47 | 16.76 | 0.356596 |  |
| GO:0048869\_cellular\_developmental\_process | PDPN | 1113 | 14 | 1.703663 | -1.681411 | 47 | 16.76 | 0.356596 |  |
| GO:0048869\_cellular\_developmental\_process | RB1 | 1113 | 14 | 1.703663 | -1.681411 | 47 | 16.76 | 0.356596 |  |
| GO:0048869\_cellular\_developmental\_process | BAD | 1113 | 14 | 1.703663 | -1.681411 | 47 | 16.76 | 0.356596 |  |
| GO:0048869\_cellular\_developmental\_process | VAX1 | 1113 | 14 | 1.703663 | -1.681411 | 47 | 16.76 | 0.356596 |  |
| GO:0048869\_cellular\_developmental\_process | CDH4 | 1113 | 14 | 1.703663 | -1.681411 | 47 | 16.76 | 0.356596 |  |
| GO:0048869\_cellular\_developmental\_process | FEZF2 | 1113 | 14 | 1.703663 | -1.681411 | 47 | 16.76 | 0.356596 |  |
| GO:0048869\_cellular\_developmental\_process | NOTCH1 | 1113 | 14 | 1.703663 | -1.681411 | 47 | 16.76 | 0.356596 |  |
| GO:0048869\_cellular\_developmental\_process | BAX | 1113 | 14 | 1.703663 | -1.681411 | 47 | 16.76 | 0.356596 |  |
| GO:0048869\_cellular\_developmental\_process | VWC2 | 1113 | 14 | 1.703663 | -1.681411 | 47 | 16.76 | 0.356596 |  |
| GO:0048869\_cellular\_developmental\_process | ROR2 | 1113 | 14 | 1.703663 | -1.681411 | 47 | 16.76 | 0.356596 |  |
| GO:0048869\_cellular\_developmental\_process | SEMA3A | 1113 | 14 | 1.703663 | -1.681411 | 47 | 16.76 | 0.356596 |  |
| GO:0048869\_cellular\_developmental\_process | RBPJ | 1113 | 14 | 1.703663 | -1.681411 | 47 | 16.76 | 0.356596 |  |
| GO:0048869\_cellular\_developmental\_process | IFRD1 | 1113 | 14 | 1.703663 | -1.681411 | 47 | 16.76 | 0.356596 |  |
| GO:0030900\_forebrain\_development | FEZF2 | 146 | 4 | 3.710717 | -1.667878 | 48 | 16.95 | 0.353125 |  |
| GO:0030900\_forebrain\_development | NOTCH1 | 146 | 4 | 3.710717 | -1.667878 | 48 | 16.95 | 0.353125 |  |
| GO:0030900\_forebrain\_development | BAX | 146 | 4 | 3.710717 | -1.667878 | 48 | 16.95 | 0.353125 |  |
| GO:0030900\_forebrain\_development | RBPJ | 146 | 4 | 3.710717 | -1.667878 | 48 | 16.95 | 0.353125 |  |
| GO:0048522\_positive\_regulation\_of\_cellular\_process | NOTCH1 | 895 | 12 | 1.815971 | -1.661009 | 49 | 17.76 | 0.362449 |  |
| GO:0048522\_positive\_regulation\_of\_cellular\_process | GCLC | 895 | 12 | 1.815971 | -1.661009 | 49 | 17.76 | 0.362449 |  |
| GO:0048522\_positive\_regulation\_of\_cellular\_process | PDPN | 895 | 12 | 1.815971 | -1.661009 | 49 | 17.76 | 0.362449 |  |
| GO:0048522\_positive\_regulation\_of\_cellular\_process | RRAS2 | 895 | 12 | 1.815971 | -1.661009 | 49 | 17.76 | 0.362449 |  |
| GO:0048522\_positive\_regulation\_of\_cellular\_process | ENDOG | 895 | 12 | 1.815971 | -1.661009 | 49 | 17.76 | 0.362449 |  |
| GO:0048522\_positive\_regulation\_of\_cellular\_process | BAX | 895 | 12 | 1.815971 | -1.661009 | 49 | 17.76 | 0.362449 |  |
| GO:0048522\_positive\_regulation\_of\_cellular\_process | VWC2 | 895 | 12 | 1.815971 | -1.661009 | 49 | 17.76 | 0.362449 |  |
| GO:0048522\_positive\_regulation\_of\_cellular\_process | NFYB | 895 | 12 | 1.815971 | -1.661009 | 49 | 17.76 | 0.362449 |  |
| GO:0048522\_positive\_regulation\_of\_cellular\_process | RB1 | 895 | 12 | 1.815971 | -1.661009 | 49 | 17.76 | 0.362449 |  |
| GO:0048522\_positive\_regulation\_of\_cellular\_process | BAD | 895 | 12 | 1.815971 | -1.661009 | 49 | 17.76 | 0.362449 |  |
| GO:0048522\_positive\_regulation\_of\_cellular\_process | RBPJ | 895 | 12 | 1.815971 | -1.661009 | 49 | 17.76 | 0.362449 |  |
| GO:0048522\_positive\_regulation\_of\_cellular\_process | CDH4 | 895 | 12 | 1.815971 | -1.661009 | 49 | 17.76 | 0.362449 |  |
| GO:0022603\_regulation\_of\_anatomical\_structure\_morphogenesis | NOTCH1 | 147 | 4 | 3.685474 | -1.658147 | 50 | 17.84 | 0.356800 |  |
| GO:0022603\_regulation\_of\_anatomical\_structure\_morphogenesis | BAX | 147 | 4 | 3.685474 | -1.658147 | 50 | 17.84 | 0.356800 |  |
| GO:0022603\_regulation\_of\_anatomical\_structure\_morphogenesis | SEMA3A | 147 | 4 | 3.685474 | -1.658147 | 50 | 17.84 | 0.356800 |  |
| GO:0022603\_regulation\_of\_anatomical\_structure\_morphogenesis | CDH4 | 147 | 4 | 3.685474 | -1.658147 | 50 | 17.84 | 0.356800 |  |
| GO:0001844\_protein\_insertion\_into\_mitochondrial\_membrane\_during\_induction\_of\_apoptosis | BAX | 3 | 1 |  |  |  |  |  |  |
| GO:0002904\_positive\_regulation\_of\_B\_cell\_apoptosis | BAX | 3 | 1 |  |  |  |  |  |  |
| GO:0007525\_somatic\_muscle\_development | IFRD1 | 3 | 1 |  |  |  |  |  |  |
| GO:0008635\_activation\_of\_caspase\_activity\_by\_cytochrome\_c | BAX | 3 | 1 |  |  |  |  |  |  |
| GO:0010524\_positive\_regulation\_of\_calcium\_ion\_transport\_into\_cytosol | BAX | 3 | 1 |  |  |  |  |  |  |
| GO:0010632\_regulation\_of\_epithelial\_cell\_migration | SEMA3A | 3 | 1 |  |  |  |  |  |  |
| GO:0010998\_regulation\_of\_translational\_initiation\_by\_eIF2\_alpha\_phosphorylation | HBB-B1 | 3 | 1 |  |  |  |  |  |  |
| GO:0010999\_regulation\_of\_eIF2\_alpha\_phosphorylation\_by\_heme | HBB-B1 | 3 | 1 |  |  |  |  |  |  |
| GO:0015669\_gas\_transport | HBB-B1 | 3 | 1 |  |  |  |  |  |  |
| GO:0019852\_L-ascorbic\_acid\_metabolic\_process | GCLC | 3 | 1 |  |  |  |  |  |  |
| GO:0019987\_negative\_regulation\_of\_anti-apoptosis | BAX | 3 | 1 |  |  |  |  |  |  |
| GO:0030538\_embryonic\_genitalia\_morphogenesis | ROR2 | 3 | 1 |  |  |  |  |  |  |
| GO:0031397\_negative\_regulation\_of\_protein\_ubiquitination | GCLC | 3 | 1 |  |  |  |  |  |  |
| GO:0032436\_positive\_regulation\_of\_proteasomal\_ubiquitin-dependent\_protein\_catabolic\_process | GCLC | 3 | 1 |  |  |  |  |  |  |
| GO:0033137\_negative\_regulation\_of\_peptidyl-serine\_phosphorylation | BAX | 3 | 1 |  |  |  |  |  |  |
| GO:0033209\_tumor\_necrosis\_factor-mediated\_signaling\_pathway | KRT18 | 3 | 1 |  |  |  |  |  |  |
| GO:0034612\_response\_to\_tumor\_necrosis\_factor | ENDOG | 3 | 1 |  |  |  |  |  |  |
| GO:0048050\_post-embryonic\_eye\_morphogenesis | BAX | 3 | 1 |  |  |  |  |  |  |
| GO:0048087\_positive\_regulation\_of\_pigmentation\_during\_development | BAX | 3 | 1 |  |  |  |  |  |  |
| GO:0048597\_post-embryonic\_camera-type\_eye\_morphogenesis | BAX | 3 | 1 |  |  |  |  |  |  |
| GO:0051204\_protein\_insertion\_into\_mitochondrial\_membrane | BAX | 3 | 1 |  |  |  |  |  |  |
| GO:0051882\_mitochondrial\_depolarization | GCLC | 3 | 1 |  |  |  |  |  |  |
| GO:0051900\_regulation\_of\_mitochondrial\_depolarization | GCLC | 3 | 1 |  |  |  |  |  |  |
| GO:0070230\_positive\_regulation\_of\_lymphocyte\_apoptosis | BAX | 3 | 1 |  |  |  |  |  |  |
| GO:0050768\_negative\_regulation\_of\_neurogenesis | SEMA3A | 32 | 2 | 8.465074 | -1.641038 | 52 | 18.35 | 0.352885 |  |
| GO:0050768\_negative\_regulation\_of\_neurogenesis | VAX1 | 32 | 2 | 8.465074 | -1.641038 | 52 | 18.35 | 0.352885 |  |
| GO:0050770\_regulation\_of\_axonogenesis | SEMA3A | 32 | 2 | 8.465074 | -1.641038 | 52 | 18.35 | 0.352885 |  |
| GO:0050770\_regulation\_of\_axonogenesis | CDH4 | 32 | 2 | 8.465074 | -1.641038 | 52 | 18.35 | 0.352885 |  |
| GO:0021536\_diencephalon\_development | BAX | 33 | 2 | 8.208556 | -1.615877 | 53 | 18.97 | 0.357925 |  |
| GO:0021536\_diencephalon\_development | RBPJ | 33 | 2 | 8.208556 | -1.615877 | 53 | 18.97 | 0.357925 |  |
| GO:0016337\_cell-cell\_adhesion | PDPN | 87 | 3 | 4.670385 | -1.592351 | 55 | 19.5 | 0.354545 |  |
| GO:0016337\_cell-cell\_adhesion | ROR2 | 87 | 3 | 4.670385 | -1.592351 | 55 | 19.5 | 0.354545 |  |
| GO:0016337\_cell-cell\_adhesion | CDH4 | 87 | 3 | 4.670385 | -1.592351 | 55 | 19.5 | 0.354545 |  |
| GO:0022612\_gland\_morphogenesis | NOTCH1 | 87 | 3 | 4.670385 | -1.592351 | 55 | 19.5 | 0.354545 |  |
| GO:0022612\_gland\_morphogenesis | BAX | 87 | 3 | 4.670385 | -1.592351 | 55 | 19.5 | 0.354545 |  |
| GO:0022612\_gland\_morphogenesis | SEMA3A | 87 | 3 | 4.670385 | -1.592351 | 55 | 19.5 | 0.354545 |  |
| GO:0010721\_negative\_regulation\_of\_cell\_development | SEMA3A | 34 | 2 | 7.967128 | -1.591539 | 56 | 19.98 | 0.356786 |  |
| GO:0010721\_negative\_regulation\_of\_cell\_development | VAX1 | 34 | 2 | 7.967128 | -1.591539 | 56 | 19.98 | 0.356786 |  |
| GO:0007420\_brain\_development | FEZF2 | 231 | 5 | 2.931627 | -1.589524 | 57 | 20.03 | 0.351404 |  |
| GO:0007420\_brain\_development | NOTCH1 | 231 | 5 | 2.931627 | -1.589524 | 57 | 20.03 | 0.351404 |  |
| GO:0007420\_brain\_development | BAX | 231 | 5 | 2.931627 | -1.589524 | 57 | 20.03 | 0.351404 |  |
| GO:0007420\_brain\_development | VAX1 | 231 | 5 | 2.931627 | -1.589524 | 57 | 20.03 | 0.351404 |  |
| GO:0007420\_brain\_development | RBPJ | 231 | 5 | 2.931627 | -1.589524 | 57 | 20.03 | 0.351404 |  |
| GO:0008285\_negative\_regulation\_of\_cell\_proliferation | BAX | 155 | 4 | 3.495256 | -1.583149 | 58 | 20.12 | 0.346897 |  |
| GO:0008285\_negative\_regulation\_of\_cell\_proliferation | RB1 | 155 | 4 | 3.495256 | -1.583149 | 58 | 20.12 | 0.346897 |  |
| GO:0008285\_negative\_regulation\_of\_cell\_proliferation | VAX1 | 155 | 4 | 3.495256 | -1.583149 | 58 | 20.12 | 0.346897 |  |
| GO:0008285\_negative\_regulation\_of\_cell\_proliferation | RBPJ | 155 | 4 | 3.495256 | -1.583149 | 58 | 20.12 | 0.346897 |  |
| GO:0001756\_somitogenesis | NOTCH1 | 35 | 2 | 7.739496 | -1.567974 | 59 | 20.83 | 0.353051 |  |
| GO:0001756\_somitogenesis | ROR2 | 35 | 2 | 7.739496 | -1.567974 | 59 | 20.83 | 0.353051 |  |
| GO:0042113\_B\_cell\_activation | BAX | 90 | 3 | 4.514706 | -1.554164 | 60 | 21.12 | 0.352000 |  |
| GO:0042113\_B\_cell\_activation | BAD | 90 | 3 | 4.514706 | -1.554164 | 60 | 21.12 | 0.352000 |  |
| GO:0042113\_B\_cell\_activation | RBPJ | 90 | 3 | 4.514706 | -1.554164 | 60 | 21.12 | 0.352000 |  |
| GO:0008544\_epidermis\_development | FRAS1 | 91 | 3 | 4.465094 | -1.541768 | 62 | 21.64 | 0.349032 |  |
| GO:0008544\_epidermis\_development | NOTCH1 | 91 | 3 | 4.465094 | -1.541768 | 62 | 21.64 | 0.349032 |  |
| GO:0008544\_epidermis\_development | RBPJ | 91 | 3 | 4.465094 | -1.541768 | 62 | 21.64 | 0.349032 |  |
| GO:0031399\_regulation\_of\_protein\_modification\_process | GCLC | 91 | 3 | 4.465094 | -1.541768 | 62 | 21.64 | 0.349032 |  |
| GO:0031399\_regulation\_of\_protein\_modification\_process | BAX | 91 | 3 | 4.465094 | -1.541768 | 62 | 21.64 | 0.349032 |  |
| GO:0031399\_regulation\_of\_protein\_modification\_process | HBB-B1 | 91 | 3 | 4.465094 | -1.541768 | 62 | 21.64 | 0.349032 |  |
| GO:0001777\_T\_cell\_homeostatic\_proliferation | BAX | 4 | 1 |  |  |  |  |  |  |
| GO:0007386\_compartment\_specification | NOTCH1 | 4 | 1 |  |  |  |  |  |  |
| GO:0008053\_mitochondrial\_fusion | BAX | 4 | 1 |  |  |  |  |  |  |
| GO:0010631\_epithelial\_cell\_migration | SEMA3A | 4 | 1 |  |  |  |  |  |  |
| GO:0031557\_induction\_of\_programmed\_cell\_death\_in\_response\_to\_chemical\_stimulus | BAX | 4 | 1 |  |  |  |  |  |  |
| GO:0031558\_induction\_of\_apoptosis\_in\_response\_to\_chemical\_stimulus | BAX | 4 | 1 |  |  |  |  |  |  |
| GO:0033135\_regulation\_of\_peptidyl-serine\_phosphorylation | BAX | 4 | 1 |  |  |  |  |  |  |
| GO:0033599\_regulation\_of\_mammary\_gland\_epithelial\_cell\_proliferation | BAX | 4 | 1 |  |  |  |  |  |  |
| GO:0042773\_ATP\_synthesis\_coupled\_electron\_transport | UQCRC1 | 4 | 1 |  |  |  |  |  |  |
| GO:0042775\_mitochondrial\_ATP\_synthesis\_coupled\_electron\_transport | UQCRC1 | 4 | 1 |  |  |  |  |  |  |
| GO:0043043\_peptide\_biosynthetic\_process | GCLC | 4 | 1 |  |  |  |  |  |  |
| GO:0045616\_regulation\_of\_keratinocyte\_differentiation | NOTCH1 | 4 | 1 |  |  |  |  |  |  |
| GO:0045684\_positive\_regulation\_of\_epidermis\_development | NOTCH1 | 4 | 1 |  |  |  |  |  |  |
| GO:0045767\_regulation\_of\_anti-apoptosis | BAX | 4 | 1 |  |  |  |  |  |  |
| GO:0046902\_regulation\_of\_mitochondrial\_membrane\_permeability | BAX | 4 | 1 |  |  |  |  |  |  |
| GO:0048841\_regulation\_of\_axon\_extension\_involved\_in\_axon\_guidance | SEMA3A | 4 | 1 |  |  |  |  |  |  |
| GO:0048843\_negative\_regulation\_of\_axon\_extension\_involved\_in\_axon\_guidance | SEMA3A | 4 | 1 |  |  |  |  |  |  |
| GO:0048846\_axon\_extension\_involved\_in\_axon\_guidance | SEMA3A | 4 | 1 |  |  |  |  |  |  |
| GO:0050919\_negative\_chemotaxis | SEMA3A | 4 | 1 |  |  |  |  |  |  |
| GO:0051205\_protein\_insertion\_into\_membrane | BAX | 4 | 1 |  |  |  |  |  |  |
| GO:0060011\_Sertoli\_cell\_proliferation | BAX | 4 | 1 |  |  |  |  |  |  |
| GO:0060057\_apoptosis\_involved\_in\_mammary\_gland\_involution | BAX | 4 | 1 |  |  |  |  |  |  |
| GO:0060058\_positive\_regulation\_of\_apoptosis\_involved\_in\_mammary\_gland\_involution | BAX | 4 | 1 |  |  |  |  |  |  |
| GO:0060528\_secretory\_columnal\_luminar\_epithelial\_cell\_differentiation\_involved\_in\_prostate\_glandular\_acinus\_development | NOTCH1 | 4 | 1 |  |  |  |  |  |  |
| GO:0060561\_apoptosis\_involved\_in\_morphogenesis | BAX | 4 | 1 |  |  |  |  |  |  |
| GO:0060666\_dichotomous\_subdivision\_of\_terminal\_units\_involved\_in\_salivary\_gland\_branching | SEMA3A | 4 | 1 |  |  |  |  |  |  |
| GO:0070059\_apoptosis\_in\_response\_to\_endoplasmic\_reticulum\_stress | BAX | 4 | 1 |  |  |  |  |  |  |
| GO:0002521\_leukocyte\_differentiation | BAX | 161 | 4 | 3.364998 | -1.530019 | 63 | 21.75 | 0.345238 |  |
| GO:0002521\_leukocyte\_differentiation | BAD | 161 | 4 | 3.364998 | -1.530019 | 63 | 21.75 | 0.345238 |  |
| GO:0002521\_leukocyte\_differentiation | RB1 | 161 | 4 | 3.364998 | -1.530019 | 63 | 21.75 | 0.345238 |  |
| GO:0002521\_leukocyte\_differentiation | RBPJ | 161 | 4 | 3.364998 | -1.530019 | 63 | 21.75 | 0.345238 |  |
| GO:0010975\_regulation\_of\_neuron\_projection\_development | SEMA3A | 38 | 2 | 7.128483 | -1.501498 | 64 | 23.3 | 0.364062 |  |
| GO:0010975\_regulation\_of\_neuron\_projection\_development | CDH4 | 38 | 2 | 7.128483 | -1.501498 | 64 | 23.3 | 0.364062 |  |
| GO:0051093\_negative\_regulation\_of\_developmental\_process | NOTCH1 | 331 | 6 | 2.455127 | -1.496012 | 65 | 23.34 | 0.359077 |  |
| GO:0051093\_negative\_regulation\_of\_developmental\_process | GCLC | 331 | 6 | 2.455127 | -1.496012 | 65 | 23.34 | 0.359077 |  |
| GO:0051093\_negative\_regulation\_of\_developmental\_process | BAX | 331 | 6 | 2.455127 | -1.496012 | 65 | 23.34 | 0.359077 |  |
| GO:0051093\_negative\_regulation\_of\_developmental\_process | VAX1 | 331 | 6 | 2.455127 | -1.496012 | 65 | 23.34 | 0.359077 |  |
| GO:0051093\_negative\_regulation\_of\_developmental\_process | SEMA3A | 331 | 6 | 2.455127 | -1.496012 | 65 | 23.34 | 0.359077 |  |
| GO:0051093\_negative\_regulation\_of\_developmental\_process | RBPJ | 331 | 6 | 2.455127 | -1.496012 | 65 | 23.34 | 0.359077 |  |
| GO:0043065\_positive\_regulation\_of\_apoptosis | NOTCH1 | 166 | 4 | 3.263643 | -1.487624 | 66 | 23.64 | 0.358182 |  |
| GO:0043065\_positive\_regulation\_of\_apoptosis | ENDOG | 166 | 4 | 3.263643 | -1.487624 | 66 | 23.64 | 0.358182 |  |
| GO:0043065\_positive\_regulation\_of\_apoptosis | BAX | 166 | 4 | 3.263643 | -1.487624 | 66 | 23.64 | 0.358182 |  |
| GO:0043065\_positive\_regulation\_of\_apoptosis | BAD | 166 | 4 | 3.263643 | -1.487624 | 66 | 23.64 | 0.358182 |  |
| GO:0030154\_cell\_differentiation | ALDH6A1 | 1060 | 13 | 1.661071 | -1.483095 | 67 | 23.7 | 0.353731 |  |
| GO:0030154\_cell\_differentiation | RB1 | 1060 | 13 | 1.661071 | -1.483095 | 67 | 23.7 | 0.353731 |  |
| GO:0030154\_cell\_differentiation | BAD | 1060 | 13 | 1.661071 | -1.483095 | 67 | 23.7 | 0.353731 |  |
| GO:0030154\_cell\_differentiation | VAX1 | 1060 | 13 | 1.661071 | -1.483095 | 67 | 23.7 | 0.353731 |  |
| GO:0030154\_cell\_differentiation | CDH4 | 1060 | 13 | 1.661071 | -1.483095 | 67 | 23.7 | 0.353731 |  |
| GO:0030154\_cell\_differentiation | FEZF2 | 1060 | 13 | 1.661071 | -1.483095 | 67 | 23.7 | 0.353731 |  |
| GO:0030154\_cell\_differentiation | NOTCH1 | 1060 | 13 | 1.661071 | -1.483095 | 67 | 23.7 | 0.353731 |  |
| GO:0030154\_cell\_differentiation | BAX | 1060 | 13 | 1.661071 | -1.483095 | 67 | 23.7 | 0.353731 |  |
| GO:0030154\_cell\_differentiation | VWC2 | 1060 | 13 | 1.661071 | -1.483095 | 67 | 23.7 | 0.353731 |  |
| GO:0030154\_cell\_differentiation | ROR2 | 1060 | 13 | 1.661071 | -1.483095 | 67 | 23.7 | 0.353731 |  |
| GO:0030154\_cell\_differentiation | SEMA3A | 1060 | 13 | 1.661071 | -1.483095 | 67 | 23.7 | 0.353731 |  |
| GO:0030154\_cell\_differentiation | RBPJ | 1060 | 13 | 1.661071 | -1.483095 | 67 | 23.7 | 0.353731 |  |
| GO:0030154\_cell\_differentiation | IFRD1 | 1060 | 13 | 1.661071 | -1.483095 | 67 | 23.7 | 0.353731 |  |
| GO:0008037\_cell\_recognition | FEZF2 | 39 | 2 | 6.945701 | -1.480621 | 68 | 24.27 | 0.356912 |  |
| GO:0008037\_cell\_recognition | SEMA3A | 39 | 2 | 6.945701 | -1.480621 | 68 | 24.27 | 0.356912 |  |
| GO:0010942\_positive\_regulation\_of\_cell\_death | NOTCH1 | 167 | 4 | 3.244100 | -1.479340 | 70 | 24.49 | 0.349857 |  |
| GO:0010942\_positive\_regulation\_of\_cell\_death | ENDOG | 167 | 4 | 3.244100 | -1.479340 | 70 | 24.49 | 0.349857 |  |
| GO:0010942\_positive\_regulation\_of\_cell\_death | BAX | 167 | 4 | 3.244100 | -1.479340 | 70 | 24.49 | 0.349857 |  |
| GO:0010942\_positive\_regulation\_of\_cell\_death | BAD | 167 | 4 | 3.244100 | -1.479340 | 70 | 24.49 | 0.349857 |  |
| GO:0043068\_positive\_regulation\_of\_programmed\_cell\_death | NOTCH1 | 167 | 4 | 3.244100 | -1.479340 | 70 | 24.49 | 0.349857 |  |
| GO:0043068\_positive\_regulation\_of\_programmed\_cell\_death | ENDOG | 167 | 4 | 3.244100 | -1.479340 | 70 | 24.49 | 0.349857 |  |
| GO:0043068\_positive\_regulation\_of\_programmed\_cell\_death | BAX | 167 | 4 | 3.244100 | -1.479340 | 70 | 24.49 | 0.349857 |  |
| GO:0043068\_positive\_regulation\_of\_programmed\_cell\_death | BAD | 167 | 4 | 3.244100 | -1.479340 | 70 | 24.49 | 0.349857 |  |
| GO:0014031\_mesenchymal\_cell\_development | NOTCH1 | 40 | 2 | 6.772059 | -1.460330 | 71 | 24.96 | 0.351549 |  |
| GO:0014031\_mesenchymal\_cell\_development | RBPJ | 40 | 2 | 6.772059 | -1.460330 | 71 | 24.96 | 0.351549 |  |
| GO:0007398\_ectoderm\_development | FRAS1 | 99 | 3 | 4.104278 | -1.448094 | 72 | 25.32 | 0.351667 |  |
| GO:0007398\_ectoderm\_development | NOTCH1 | 99 | 3 | 4.104278 | -1.448094 | 72 | 25.32 | 0.351667 |  |
| GO:0007398\_ectoderm\_development | RBPJ | 99 | 3 | 4.104278 | -1.448094 | 72 | 25.32 | 0.351667 |  |
| GO:0030097\_hemopoiesis | BAX | 253 | 5 | 2.676703 | -1.440680 | 73 | 25.51 | 0.349452 |  |
| GO:0030097\_hemopoiesis | HBB-B1 | 253 | 5 | 2.676703 | -1.440680 | 73 | 25.51 | 0.349452 |  |
| GO:0030097\_hemopoiesis | BAD | 253 | 5 | 2.676703 | -1.440680 | 73 | 25.51 | 0.349452 |  |
| GO:0030097\_hemopoiesis | RB1 | 253 | 5 | 2.676703 | -1.440680 | 73 | 25.51 | 0.349452 |  |
| GO:0030097\_hemopoiesis | RBPJ | 253 | 5 | 2.676703 | -1.440680 | 73 | 25.51 | 0.349452 |  |
| GO:0031344\_regulation\_of\_cell\_projection\_organization | SEMA3A | 41 | 2 | 6.606887 | -1.440594 | 75 | 26.28 | 0.350400 |  |
| GO:0031344\_regulation\_of\_cell\_projection\_organization | CDH4 | 41 | 2 | 6.606887 | -1.440594 | 75 | 26.28 | 0.350400 |  |
| GO:0032844\_regulation\_of\_homeostatic\_process | GCLC | 41 | 2 | 6.606887 | -1.440594 | 75 | 26.28 | 0.350400 |  |
| GO:0032844\_regulation\_of\_homeostatic\_process | BAX | 41 | 2 | 6.606887 | -1.440594 | 75 | 26.28 | 0.350400 |  |
| GO:0001945\_lymph\_vessel\_development | PDPN | 5 | 1 | 27.088235 | -1.439008 | 89 | 36.99 | 0.415618 |  |
| GO:0002902\_regulation\_of\_B\_cell\_apoptosis | BAX | 5 | 1 | 27.088235 | -1.439008 | 89 | 36.99 | 0.415618 |  |
| GO:0009303\_rRNA\_transcription | POLR1E | 5 | 1 | 27.088235 | -1.439008 | 89 | 36.99 | 0.415618 |  |
| GO:0010522\_regulation\_of\_calcium\_ion\_transport\_into\_cytosol | BAX | 5 | 1 | 27.088235 | -1.439008 | 89 | 36.99 | 0.415618 |  |
| GO:0021854\_hypothalamus\_development | BAX | 5 | 1 | 27.088235 | -1.439008 | 89 | 36.99 | 0.415618 |  |
| GO:0032434\_regulation\_of\_proteasomal\_ubiquitin-dependent\_protein\_catabolic\_process | GCLC | 5 | 1 | 27.088235 | -1.439008 | 89 | 36.99 | 0.415618 |  |
| GO:0032846\_positive\_regulation\_of\_homeostatic\_process | BAX | 5 | 1 | 27.088235 | -1.439008 | 89 | 36.99 | 0.415618 |  |
| GO:0035234\_germ\_cell\_programmed\_cell\_death | BAX | 5 | 1 | 27.088235 | -1.439008 | 89 | 36.99 | 0.415618 |  |
| GO:0043555\_regulation\_of\_translation\_in\_response\_to\_stress | HBB-B1 | 5 | 1 | 27.088235 | -1.439008 | 89 | 36.99 | 0.415618 |  |
| GO:0043558\_regulation\_of\_translational\_initiation\_in\_response\_to\_stress | HBB-B1 | 5 | 1 | 27.088235 | -1.439008 | 89 | 36.99 | 0.415618 |  |
| GO:0045651\_positive\_regulation\_of\_macrophage\_differentiation | RB1 | 5 | 1 | 27.088235 | -1.439008 | 89 | 36.99 | 0.415618 |  |
| GO:0045773\_positive\_regulation\_of\_axon\_extension | CDH4 | 5 | 1 | 27.088235 | -1.439008 | 89 | 36.99 | 0.415618 |  |
| GO:0051668\_localization\_within\_membrane | BAX | 5 | 1 | 27.088235 | -1.439008 | 89 | 36.99 | 0.415618 |  |
| GO:0060600\_dichotomous\_subdivision\_of\_an\_epithelial\_terminal\_unit | SEMA3A | 5 | 1 | 27.088235 | -1.439008 | 89 | 36.99 | 0.415618 |  |
| GO:0010769\_regulation\_of\_cell\_morphogenesis\_involved\_in\_differentiation | SEMA3A | 42 | 2 | 6.449580 | -1.421388 | 91 | 38.12 | 0.418901 |  |
| GO:0010769\_regulation\_of\_cell\_morphogenesis\_involved\_in\_differentiation | CDH4 | 42 | 2 | 6.449580 | -1.421388 | 91 | 38.12 | 0.418901 |  |
| GO:0019221\_cytokine-mediated\_signaling\_pathway | KRT18 | 42 | 2 | 6.449580 | -1.421388 | 91 | 38.12 | 0.418901 |  |
| GO:0019221\_cytokine-mediated\_signaling\_pathway | BAD | 42 | 2 | 6.449580 | -1.421388 | 91 | 38.12 | 0.418901 |  |
| GO:0048762\_mesenchymal\_cell\_differentiation | NOTCH1 | 43 | 2 | 6.299590 | -1.402686 | 92 | 39.32 | 0.427391 |  |
| GO:0048762\_mesenchymal\_cell\_differentiation | RBPJ | 43 | 2 | 6.299590 | -1.402686 | 92 | 39.32 | 0.427391 |  |
| GO:0048666\_neuron\_development | FEZF2 | 262 | 5 | 2.584755 | -1.384767 | 93 | 39.62 | 0.426022 |  |
| GO:0048666\_neuron\_development | NOTCH1 | 262 | 5 | 2.584755 | -1.384767 | 93 | 39.62 | 0.426022 |  |
| GO:0048666\_neuron\_development | VAX1 | 262 | 5 | 2.584755 | -1.384767 | 93 | 39.62 | 0.426022 |  |
| GO:0048666\_neuron\_development | SEMA3A | 262 | 5 | 2.584755 | -1.384767 | 93 | 39.62 | 0.426022 |  |
| GO:0048666\_neuron\_development | CDH4 | 262 | 5 | 2.584755 | -1.384767 | 93 | 39.62 | 0.426022 |  |
| GO:0048732\_gland\_development | NOTCH1 | 179 | 4 | 3.026618 | -1.384654 | 94 | 39.74 | 0.422766 |  |
| GO:0048732\_gland\_development | BAX | 179 | 4 | 3.026618 | -1.384654 | 94 | 39.74 | 0.422766 |  |
| GO:0048732\_gland\_development | SEMA3A | 179 | 4 | 3.026618 | -1.384654 | 94 | 39.74 | 0.422766 |  |
| GO:0048732\_gland\_development | RBPJ | 179 | 4 | 3.026618 | -1.384654 | 94 | 39.74 | 0.422766 |  |
| GO:0001942\_hair\_follicle\_development | NOTCH1 | 44 | 2 | 6.156417 | -1.384463 | 101 | 40.42 | 0.400198 |  |
| GO:0001942\_hair\_follicle\_development | RBPJ | 44 | 2 | 6.156417 | -1.384463 | 101 | 40.42 | 0.400198 |  |
| GO:0022404\_molting\_cycle\_process | NOTCH1 | 44 | 2 | 6.156417 | -1.384463 | 101 | 40.42 | 0.400198 |  |
| GO:0022404\_molting\_cycle\_process | RBPJ | 44 | 2 | 6.156417 | -1.384463 | 101 | 40.42 | 0.400198 |  |
| GO:0022405\_hair\_cycle\_process | NOTCH1 | 44 | 2 | 6.156417 | -1.384463 | 101 | 40.42 | 0.400198 |  |
| GO:0022405\_hair\_cycle\_process | RBPJ | 44 | 2 | 6.156417 | -1.384463 | 101 | 40.42 | 0.400198 |  |
| GO:0035282\_segmentation | NOTCH1 | 44 | 2 | 6.156417 | -1.384463 | 101 | 40.42 | 0.400198 |  |
| GO:0035282\_segmentation | ROR2 | 44 | 2 | 6.156417 | -1.384463 | 101 | 40.42 | 0.400198 |  |
| GO:0042303\_molting\_cycle | NOTCH1 | 44 | 2 | 6.156417 | -1.384463 | 101 | 40.42 | 0.400198 |  |
| GO:0042303\_molting\_cycle | RBPJ | 44 | 2 | 6.156417 | -1.384463 | 101 | 40.42 | 0.400198 |  |
| GO:0042633\_hair\_cycle | NOTCH1 | 44 | 2 | 6.156417 | -1.384463 | 101 | 40.42 | 0.400198 |  |
| GO:0042633\_hair\_cycle | RBPJ | 44 | 2 | 6.156417 | -1.384463 | 101 | 40.42 | 0.400198 |  |
| GO:0060485\_mesenchyme\_development | NOTCH1 | 44 | 2 | 6.156417 | -1.384463 | 101 | 40.42 | 0.400198 |  |
| GO:0060485\_mesenchyme\_development | RBPJ | 44 | 2 | 6.156417 | -1.384463 | 101 | 40.42 | 0.400198 |  |
| GO:0030030\_cell\_projection\_organization | FEZF2 | 263 | 5 | 2.574927 | -1.378718 | 102 | 40.55 | 0.397549 |  |
| GO:0030030\_cell\_projection\_organization | NOTCH1 | 263 | 5 | 2.574927 | -1.378718 | 102 | 40.55 | 0.397549 |  |
| GO:0030030\_cell\_projection\_organization | VAX1 | 263 | 5 | 2.574927 | -1.378718 | 102 | 40.55 | 0.397549 |  |
| GO:0030030\_cell\_projection\_organization | SEMA3A | 263 | 5 | 2.574927 | -1.378718 | 102 | 40.55 | 0.397549 |  |
| GO:0030030\_cell\_projection\_organization | CDH4 | 263 | 5 | 2.574927 | -1.378718 | 102 | 40.55 | 0.397549 |  |
| GO:0006692\_prostanoid\_metabolic\_process | PDPN | 6 | 1 | 22.573529 | -1.361379 | 117 | 49.29 | 0.421282 |  |
| GO:0006693\_prostaglandin\_metabolic\_process | PDPN | 6 | 1 | 22.573529 | -1.361379 | 117 | 49.29 | 0.421282 |  |
| GO:0007406\_negative\_regulation\_of\_neuroblast\_proliferation | VAX1 | 6 | 1 | 22.573529 | -1.361379 | 117 | 49.29 | 0.421282 |  |
| GO:0009651\_response\_to\_salt\_stress | BAX | 6 | 1 | 22.573529 | -1.361379 | 117 | 49.29 | 0.421282 |  |
| GO:0031077\_post-embryonic\_camera-type\_eye\_development | BAX | 6 | 1 | 22.573529 | -1.361379 | 117 | 49.29 | 0.421282 |  |
| GO:0032469\_endoplasmic\_reticulum\_calcium\_ion\_homeostasis | BAX | 6 | 1 | 22.573529 | -1.361379 | 117 | 49.29 | 0.421282 |  |
| GO:0045579\_positive\_regulation\_of\_B\_cell\_differentiation | BAD | 6 | 1 | 22.573529 | -1.361379 | 117 | 49.29 | 0.421282 |  |
| GO:0045649\_regulation\_of\_macrophage\_differentiation | RB1 | 6 | 1 | 22.573529 | -1.361379 | 117 | 49.29 | 0.421282 |  |
| GO:0046666\_retinal\_cell\_programmed\_cell\_death | BAX | 6 | 1 | 22.573529 | -1.361379 | 117 | 49.29 | 0.421282 |  |
| GO:0048103\_somatic\_stem\_cell\_division | NOTCH1 | 6 | 1 | 22.573529 | -1.361379 | 117 | 49.29 | 0.421282 |  |
| GO:0048147\_negative\_regulation\_of\_fibroblast\_proliferation | BAX | 6 | 1 | 22.573529 | -1.361379 | 117 | 49.29 | 0.421282 |  |
| GO:0048563\_post-embryonic\_organ\_morphogenesis | BAX | 6 | 1 | 22.573529 | -1.361379 | 117 | 49.29 | 0.421282 |  |
| GO:0051180\_vitamin\_transport | PDPN | 6 | 1 | 22.573529 | -1.361379 | 117 | 49.29 | 0.421282 |  |
| GO:0060056\_mammary\_gland\_involution | BAX | 6 | 1 | 22.573529 | -1.361379 | 117 | 49.29 | 0.421282 |  |
| GO:0060068\_vagina\_development | BAX | 6 | 1 | 22.573529 | -1.361379 | 117 | 49.29 | 0.421282 |  |
| GO:0007155\_cell\_adhesion | PDPN | 186 | 4 | 2.912713 | -1.333145 | 119 | 50.43 | 0.423782 |  |
| GO:0007155\_cell\_adhesion | VWC2 | 186 | 4 | 2.912713 | -1.333145 | 119 | 50.43 | 0.423782 |  |
| GO:0007155\_cell\_adhesion | ROR2 | 186 | 4 | 2.912713 | -1.333145 | 119 | 50.43 | 0.423782 |  |
| GO:0007155\_cell\_adhesion | CDH4 | 186 | 4 | 2.912713 | -1.333145 | 119 | 50.43 | 0.423782 |  |
| GO:0022610\_biological\_adhesion | PDPN | 186 | 4 | 2.912713 | -1.333145 | 119 | 50.43 | 0.423782 |  |
| GO:0022610\_biological\_adhesion | VWC2 | 186 | 4 | 2.912713 | -1.333145 | 119 | 50.43 | 0.423782 |  |
| GO:0022610\_biological\_adhesion | ROR2 | 186 | 4 | 2.912713 | -1.333145 | 119 | 50.43 | 0.423782 |  |
| GO:0022610\_biological\_adhesion | CDH4 | 186 | 4 | 2.912713 | -1.333145 | 119 | 50.43 | 0.423782 |  |
| GO:0048518\_positive\_regulation\_of\_biological\_process | NOTCH1 | 995 | 12 | 1.633461 | -1.331405 | 120 | 51.13 | 0.426083 |  |
| GO:0048518\_positive\_regulation\_of\_biological\_process | GCLC | 995 | 12 | 1.633461 | -1.331405 | 120 | 51.13 | 0.426083 |  |
| GO:0048518\_positive\_regulation\_of\_biological\_process | PDPN | 995 | 12 | 1.633461 | -1.331405 | 120 | 51.13 | 0.426083 |  |
| GO:0048518\_positive\_regulation\_of\_biological\_process | RRAS2 | 995 | 12 | 1.633461 | -1.331405 | 120 | 51.13 | 0.426083 |  |
| GO:0048518\_positive\_regulation\_of\_biological\_process | ENDOG | 995 | 12 | 1.633461 | -1.331405 | 120 | 51.13 | 0.426083 |  |
| GO:0048518\_positive\_regulation\_of\_biological\_process | BAX | 995 | 12 | 1.633461 | -1.331405 | 120 | 51.13 | 0.426083 |  |
| GO:0048518\_positive\_regulation\_of\_biological\_process | VWC2 | 995 | 12 | 1.633461 | -1.331405 | 120 | 51.13 | 0.426083 |  |
| GO:0048518\_positive\_regulation\_of\_biological\_process | NFYB | 995 | 12 | 1.633461 | -1.331405 | 120 | 51.13 | 0.426083 |  |
| GO:0048518\_positive\_regulation\_of\_biological\_process | RB1 | 995 | 12 | 1.633461 | -1.331405 | 120 | 51.13 | 0.426083 |  |
| GO:0048518\_positive\_regulation\_of\_biological\_process | BAD | 995 | 12 | 1.633461 | -1.331405 | 120 | 51.13 | 0.426083 |  |
| GO:0048518\_positive\_regulation\_of\_biological\_process | RBPJ | 995 | 12 | 1.633461 | -1.331405 | 120 | 51.13 | 0.426083 |  |
| GO:0048518\_positive\_regulation\_of\_biological\_process | CDH4 | 995 | 12 | 1.633461 | -1.331405 | 120 | 51.13 | 0.426083 |  |
| GO:0032269\_negative\_regulation\_of\_cellular\_protein\_metabolic\_process | GCLC | 48 | 2 | 5.643382 | -1.315961 | 121 | 51.75 | 0.427686 |  |
| GO:0032269\_negative\_regulation\_of\_cellular\_protein\_metabolic\_process | BAX | 48 | 2 | 5.643382 | -1.315961 | 121 | 51.75 | 0.427686 |  |
| GO:0048534\_hemopoietic\_or\_lymphoid\_organ\_development | BAX | 277 | 5 | 2.444787 | -1.297260 | 122 | 52.78 | 0.432623 |  |
| GO:0048534\_hemopoietic\_or\_lymphoid\_organ\_development | HBB-B1 | 277 | 5 | 2.444787 | -1.297260 | 122 | 52.78 | 0.432623 |  |
| GO:0048534\_hemopoietic\_or\_lymphoid\_organ\_development | BAD | 277 | 5 | 2.444787 | -1.297260 | 122 | 52.78 | 0.432623 |  |
| GO:0048534\_hemopoietic\_or\_lymphoid\_organ\_development | RB1 | 277 | 5 | 2.444787 | -1.297260 | 122 | 52.78 | 0.432623 |  |
| GO:0048534\_hemopoietic\_or\_lymphoid\_organ\_development | RBPJ | 277 | 5 | 2.444787 | -1.297260 | 122 | 52.78 | 0.432623 |  |
| GO:0002067\_glandular\_epithelial\_cell\_differentiation | NOTCH1 | 7 | 1 | 19.348739 | -1.295983 | 134 | 61.9 | 0.461940 |  |
| GO:0006119\_oxidative\_phosphorylation | UQCRC1 | 7 | 1 | 19.348739 | -1.295983 | 134 | 61.9 | 0.461940 |  |
| GO:0006406\_mRNA\_export\_from\_nucleus | NUP133 | 7 | 1 | 19.348739 | -1.295983 | 134 | 61.9 | 0.461940 |  |
| GO:0007440\_foregut\_morphogenesis | NOTCH1 | 7 | 1 | 19.348739 | -1.295983 | 134 | 61.9 | 0.461940 |  |
| GO:0030517\_negative\_regulation\_of\_axon\_extension | SEMA3A | 7 | 1 | 19.348739 | -1.295983 | 134 | 61.9 | 0.461940 |  |
| GO:0043353\_enucleate\_erythrocyte\_differentiation | RB1 | 7 | 1 | 19.348739 | -1.295983 | 134 | 61.9 | 0.461940 |  |
| GO:0045604\_regulation\_of\_epidermal\_cell\_differentiation | NOTCH1 | 7 | 1 | 19.348739 | -1.295983 | 134 | 61.9 | 0.461940 |  |
| GO:0045862\_positive\_regulation\_of\_proteolysis | GCLC | 7 | 1 | 19.348739 | -1.295983 | 134 | 61.9 | 0.461940 |  |
| GO:0046677\_response\_to\_antibiotic | ENDOG | 7 | 1 | 19.348739 | -1.295983 | 134 | 61.9 | 0.461940 |  |
| GO:0051028\_mRNA\_transport | NUP133 | 7 | 1 | 19.348739 | -1.295983 | 134 | 61.9 | 0.461940 |  |
| GO:0051928\_positive\_regulation\_of\_calcium\_ion\_transport | BAX | 7 | 1 | 19.348739 | -1.295983 | 134 | 61.9 | 0.461940 |  |
| GO:0070228\_regulation\_of\_lymphocyte\_apoptosis | BAX | 7 | 1 | 19.348739 | -1.295983 | 134 | 61.9 | 0.461940 |  |
| GO:0046907\_intracellular\_transport | SEC23A | 194 | 4 | 2.792602 | -1.277316 | 135 | 62.78 | 0.465037 |  |
| GO:0046907\_intracellular\_transport | NUP133 | 194 | 4 | 2.792602 | -1.277316 | 135 | 62.78 | 0.465037 |  |
| GO:0046907\_intracellular\_transport | BAX | 194 | 4 | 2.792602 | -1.277316 | 135 | 62.78 | 0.465037 |  |
| GO:0046907\_intracellular\_transport | SPIRE1 | 194 | 4 | 2.792602 | -1.277316 | 135 | 62.78 | 0.465037 |  |
| GO:0007417\_central\_nervous\_system\_development | FEZF2 | 287 | 5 | 2.359602 | -1.242546 | 136 | 64.7 | 0.475735 |  |
| GO:0007417\_central\_nervous\_system\_development | NOTCH1 | 287 | 5 | 2.359602 | -1.242546 | 136 | 64.7 | 0.475735 |  |
| GO:0007417\_central\_nervous\_system\_development | BAX | 287 | 5 | 2.359602 | -1.242546 | 136 | 64.7 | 0.475735 |  |
| GO:0007417\_central\_nervous\_system\_development | VAX1 | 287 | 5 | 2.359602 | -1.242546 | 136 | 64.7 | 0.475735 |  |
| GO:0007417\_central\_nervous\_system\_development | RBPJ | 287 | 5 | 2.359602 | -1.242546 | 136 | 64.7 | 0.475735 |  |
| GO:0001783\_B\_cell\_apoptosis | BAX | 8 | 1 | 16.930147 | -1.239541 | 145 | 72.98 | 0.503310 |  |
| GO:0002065\_columnar\_cuboidal\_epithelial\_cell\_differentiation | NOTCH1 | 8 | 1 | 16.930147 | -1.239541 | 145 | 72.98 | 0.503310 |  |
| GO:0019400\_alditol\_metabolic\_process | SORD | 8 | 1 | 16.930147 | -1.239541 | 145 | 72.98 | 0.503310 |  |
| GO:0031396\_regulation\_of\_protein\_ubiquitination | GCLC | 8 | 1 | 16.930147 | -1.239541 | 145 | 72.98 | 0.503310 |  |
| GO:0035112\_genitalia\_morphogenesis | ROR2 | 8 | 1 | 16.930147 | -1.239541 | 145 | 72.98 | 0.503310 |  |
| GO:0040034\_regulation\_of\_development\_\_heterochronic | RBPJ | 8 | 1 | 16.930147 | -1.239541 | 145 | 72.98 | 0.503310 |  |
| GO:0048505\_regulation\_of\_timing\_of\_cell\_differentiation | RBPJ | 8 | 1 | 16.930147 | -1.239541 | 145 | 72.98 | 0.503310 |  |
| GO:0051181\_cofactor\_transport | PDPN | 8 | 1 | 16.930147 | -1.239541 | 145 | 72.98 | 0.503310 |  |
| GO:0070584\_mitochondrion\_morphogenesis | BAX | 8 | 1 | 16.930147 | -1.239541 | 145 | 72.98 | 0.503310 |  |
| GO:0051248\_negative\_regulation\_of\_protein\_metabolic\_process | GCLC | 53 | 2 | 5.110988 | -1.238894 | 146 | 73.28 | 0.501918 |  |
| GO:0051248\_negative\_regulation\_of\_protein\_metabolic\_process | BAX | 53 | 2 | 5.110988 | -1.238894 | 146 | 73.28 | 0.501918 |  |
| GO:0051239\_regulation\_of\_multicellular\_organismal\_process | NOTCH1 | 587 | 8 | 1.845876 | -1.226748 | 147 | 73.73 | 0.501565 |  |
| GO:0051239\_regulation\_of\_multicellular\_organismal\_process | BAX | 587 | 8 | 1.845876 | -1.226748 | 147 | 73.73 | 0.501565 |  |
| GO:0051239\_regulation\_of\_multicellular\_organismal\_process | VWC2 | 587 | 8 | 1.845876 | -1.226748 | 147 | 73.73 | 0.501565 |  |
| GO:0051239\_regulation\_of\_multicellular\_organismal\_process | VAX1 | 587 | 8 | 1.845876 | -1.226748 | 147 | 73.73 | 0.501565 |  |
| GO:0051239\_regulation\_of\_multicellular\_organismal\_process | RB1 | 587 | 8 | 1.845876 | -1.226748 | 147 | 73.73 | 0.501565 |  |
| GO:0051239\_regulation\_of\_multicellular\_organismal\_process | BAD | 587 | 8 | 1.845876 | -1.226748 | 147 | 73.73 | 0.501565 |  |
| GO:0051239\_regulation\_of\_multicellular\_organismal\_process | SEMA3A | 587 | 8 | 1.845876 | -1.226748 | 147 | 73.73 | 0.501565 |  |
| GO:0051239\_regulation\_of\_multicellular\_organismal\_process | CDH4 | 587 | 8 | 1.845876 | -1.226748 | 147 | 73.73 | 0.501565 |  |
| GO:0030098\_lymphocyte\_differentiation | BAX | 124 | 3 | 3.276803 | -1.205845 | 148 | 75.44 | 0.509730 |  |
| GO:0030098\_lymphocyte\_differentiation | BAD | 124 | 3 | 3.276803 | -1.205845 | 148 | 75.44 | 0.509730 |  |
| GO:0030098\_lymphocyte\_differentiation | RBPJ | 124 | 3 | 3.276803 | -1.205845 | 148 | 75.44 | 0.509730 |  |
| GO:0006351\_transcription\_\_DNA-dependent | PKNOX2 | 594 | 8 | 1.824124 | -1.201597 | 149 | 75.67 | 0.507852 |  |
| GO:0006351\_transcription\_\_DNA-dependent | NOTCH1 | 594 | 8 | 1.824124 | -1.201597 | 149 | 75.67 | 0.507852 |  |
| GO:0006351\_transcription\_\_DNA-dependent | POLR1E | 594 | 8 | 1.824124 | -1.201597 | 149 | 75.67 | 0.507852 |  |
| GO:0006351\_transcription\_\_DNA-dependent | NR1D2 | 594 | 8 | 1.824124 | -1.201597 | 149 | 75.67 | 0.507852 |  |
| GO:0006351\_transcription\_\_DNA-dependent | NFYB | 594 | 8 | 1.824124 | -1.201597 | 149 | 75.67 | 0.507852 |  |
| GO:0006351\_transcription\_\_DNA-dependent | RB1 | 594 | 8 | 1.824124 | -1.201597 | 149 | 75.67 | 0.507852 |  |
| GO:0006351\_transcription\_\_DNA-dependent | VAX1 | 594 | 8 | 1.824124 | -1.201597 | 149 | 75.67 | 0.507852 |  |
| GO:0006351\_transcription\_\_DNA-dependent | RBPJ | 594 | 8 | 1.824124 | -1.201597 | 149 | 75.67 | 0.507852 |  |
| GO:0002520\_immune\_system\_development | BAX | 295 | 5 | 2.295613 | -1.200700 | 150 | 75.83 | 0.505533 |  |
| GO:0002520\_immune\_system\_development | HBB-B1 | 295 | 5 | 2.295613 | -1.200700 | 150 | 75.83 | 0.505533 |  |
| GO:0002520\_immune\_system\_development | BAD | 295 | 5 | 2.295613 | -1.200700 | 150 | 75.83 | 0.505533 |  |
| GO:0002520\_immune\_system\_development | RB1 | 295 | 5 | 2.295613 | -1.200700 | 150 | 75.83 | 0.505533 |  |
| GO:0002520\_immune\_system\_development | RBPJ | 295 | 5 | 2.295613 | -1.200700 | 150 | 75.83 | 0.505533 |  |
| GO:0032774\_RNA\_biosynthetic\_process | PKNOX2 | 595 | 8 | 1.821058 | -1.198044 | 151 | 75.96 | 0.503046 |  |
| GO:0032774\_RNA\_biosynthetic\_process | NOTCH1 | 595 | 8 | 1.821058 | -1.198044 | 151 | 75.96 | 0.503046 |  |
| GO:0032774\_RNA\_biosynthetic\_process | POLR1E | 595 | 8 | 1.821058 | -1.198044 | 151 | 75.96 | 0.503046 |  |
| GO:0032774\_RNA\_biosynthetic\_process | NR1D2 | 595 | 8 | 1.821058 | -1.198044 | 151 | 75.96 | 0.503046 |  |
| GO:0032774\_RNA\_biosynthetic\_process | NFYB | 595 | 8 | 1.821058 | -1.198044 | 151 | 75.96 | 0.503046 |  |
| GO:0032774\_RNA\_biosynthetic\_process | RB1 | 595 | 8 | 1.821058 | -1.198044 | 151 | 75.96 | 0.503046 |  |
| GO:0032774\_RNA\_biosynthetic\_process | VAX1 | 595 | 8 | 1.821058 | -1.198044 | 151 | 75.96 | 0.503046 |  |
| GO:0032774\_RNA\_biosynthetic\_process | RBPJ | 595 | 8 | 1.821058 | -1.198044 | 151 | 75.96 | 0.503046 |  |
| GO:0001708\_cell\_fate\_specification | NOTCH1 | 56 | 2 | 4.837185 | -1.196535 | 153 | 76.68 | 0.501176 |  |
| GO:0001708\_cell\_fate\_specification | RBPJ | 56 | 2 | 4.837185 | -1.196535 | 153 | 76.68 | 0.501176 |  |
| GO:0050678\_regulation\_of\_epithelial\_cell\_proliferation | NOTCH1 | 56 | 2 | 4.837185 | -1.196535 | 153 | 76.68 | 0.501176 |  |
| GO:0050678\_regulation\_of\_epithelial\_cell\_proliferation | BAX | 56 | 2 | 4.837185 | -1.196535 | 153 | 76.68 | 0.501176 |  |
| GO:0006007\_glucose\_catabolic\_process | BAD | 9 | 1 | 15.049020 | -1.189936 | 163 | 84.54 | 0.518650 |  |
| GO:0006309\_DNA\_fragmentation\_involved\_in\_apoptosis | ENDOG | 9 | 1 | 15.049020 | -1.189936 | 163 | 84.54 | 0.518650 |  |
| GO:0017145\_stem\_cell\_division | NOTCH1 | 9 | 1 | 15.049020 | -1.189936 | 163 | 84.54 | 0.518650 |  |
| GO:0019320\_hexose\_catabolic\_process | BAD | 9 | 1 | 15.049020 | -1.189936 | 163 | 84.54 | 0.518650 |  |
| GO:0042640\_anagen | NOTCH1 | 9 | 1 | 15.049020 | -1.189936 | 163 | 84.54 | 0.518650 |  |
| GO:0045136\_development\_of\_secondary\_sexual\_characteristics | BAX | 9 | 1 | 15.049020 | -1.189936 | 163 | 84.54 | 0.518650 |  |
| GO:0046365\_monosaccharide\_catabolic\_process | BAD | 9 | 1 | 15.049020 | -1.189936 | 163 | 84.54 | 0.518650 |  |
| GO:0048070\_regulation\_of\_pigmentation\_during\_development | BAX | 9 | 1 | 15.049020 | -1.189936 | 163 | 84.54 | 0.518650 |  |
| GO:0048284\_organelle\_fusion | BAX | 9 | 1 | 15.049020 | -1.189936 | 163 | 84.54 | 0.518650 |  |
| GO:0048569\_post-embryonic\_organ\_development | BAX | 9 | 1 | 15.049020 | -1.189936 | 163 | 84.54 | 0.518650 |  |
| GO:0001944\_vasculature\_development | NOTCH1 | 208 | 4 | 2.604638 | -1.186656 | 164 | 84.7 | 0.516463 |  |
| GO:0001944\_vasculature\_development | PDPN | 208 | 4 | 2.604638 | -1.186656 | 164 | 84.7 | 0.516463 |  |
| GO:0001944\_vasculature\_development | BAX | 208 | 4 | 2.604638 | -1.186656 | 164 | 84.7 | 0.516463 |  |
| GO:0001944\_vasculature\_development | RBPJ | 208 | 4 | 2.604638 | -1.186656 | 164 | 84.7 | 0.516463 |  |
| GO:0001764\_neuron\_migration | BAX | 57 | 2 | 4.752322 | -1.182992 | 165 | 85.47 | 0.518000 |  |
| GO:0001764\_neuron\_migration | VAX1 | 57 | 2 | 4.752322 | -1.182992 | 165 | 85.47 | 0.518000 |  |
| GO:0050794\_regulation\_of\_cellular\_process | GCLC | 2190 | 21 | 1.298751 | -1.169817 | 166 | 85.77 | 0.516687 |  |
| GO:0050794\_regulation\_of\_cellular\_process | PDPN | 2190 | 21 | 1.298751 | -1.169817 | 166 | 85.77 | 0.516687 |  |
| GO:0050794\_regulation\_of\_cellular\_process | NFYB | 2190 | 21 | 1.298751 | -1.169817 | 166 | 85.77 | 0.516687 |  |
| GO:0050794\_regulation\_of\_cellular\_process | VAX1 | 2190 | 21 | 1.298751 | -1.169817 | 166 | 85.77 | 0.516687 |  |
| GO:0050794\_regulation\_of\_cellular\_process | RB1 | 2190 | 21 | 1.298751 | -1.169817 | 166 | 85.77 | 0.516687 |  |
| GO:0050794\_regulation\_of\_cellular\_process | BAD | 2190 | 21 | 1.298751 | -1.169817 | 166 | 85.77 | 0.516687 |  |
| GO:0050794\_regulation\_of\_cellular\_process | RGS16 | 2190 | 21 | 1.298751 | -1.169817 | 166 | 85.77 | 0.516687 |  |
| GO:0050794\_regulation\_of\_cellular\_process | CDH4 | 2190 | 21 | 1.298751 | -1.169817 | 166 | 85.77 | 0.516687 |  |
| GO:0050794\_regulation\_of\_cellular\_process | PKNOX2 | 2190 | 21 | 1.298751 | -1.169817 | 166 | 85.77 | 0.516687 |  |
| GO:0050794\_regulation\_of\_cellular\_process | NOTCH1 | 2190 | 21 | 1.298751 | -1.169817 | 166 | 85.77 | 0.516687 |  |
| GO:0050794\_regulation\_of\_cellular\_process | KRT18 | 2190 | 21 | 1.298751 | -1.169817 | 166 | 85.77 | 0.516687 |  |
| GO:0050794\_regulation\_of\_cellular\_process | NR1D2 | 2190 | 21 | 1.298751 | -1.169817 | 166 | 85.77 | 0.516687 |  |
| GO:0050794\_regulation\_of\_cellular\_process | BAX | 2190 | 21 | 1.298751 | -1.169817 | 166 | 85.77 | 0.516687 |  |
| GO:0050794\_regulation\_of\_cellular\_process | RRAS2 | 2190 | 21 | 1.298751 | -1.169817 | 166 | 85.77 | 0.516687 |  |
| GO:0050794\_regulation\_of\_cellular\_process | SOS1 | 2190 | 21 | 1.298751 | -1.169817 | 166 | 85.77 | 0.516687 |  |
| GO:0050794\_regulation\_of\_cellular\_process | ENDOG | 2190 | 21 | 1.298751 | -1.169817 | 166 | 85.77 | 0.516687 |  |
| GO:0050794\_regulation\_of\_cellular\_process | ROR2 | 2190 | 21 | 1.298751 | -1.169817 | 166 | 85.77 | 0.516687 |  |
| GO:0050794\_regulation\_of\_cellular\_process | VWC2 | 2190 | 21 | 1.298751 | -1.169817 | 166 | 85.77 | 0.516687 |  |
| GO:0050794\_regulation\_of\_cellular\_process | HBB-B1 | 2190 | 21 | 1.298751 | -1.169817 | 166 | 85.77 | 0.516687 |  |
| GO:0050794\_regulation\_of\_cellular\_process | SEMA3A | 2190 | 21 | 1.298751 | -1.169817 | 166 | 85.77 | 0.516687 |  |
| GO:0050794\_regulation\_of\_cellular\_process | RBPJ | 2190 | 21 | 1.298751 | -1.169817 | 166 | 85.77 | 0.516687 |  |
| GO:0030334\_regulation\_of\_cell\_migration | RRAS2 | 59 | 2 | 4.591226 | -1.156710 | 167 | 86.69 | 0.519102 |  |
| GO:0030334\_regulation\_of\_cell\_migration | SEMA3A | 59 | 2 | 4.591226 | -1.156710 | 167 | 86.69 | 0.519102 |  |
| GO:0051179\_localization | FRAS1 | 1058 | 12 | 1.536195 | -1.153593 | 168 | 86.95 | 0.517560 |  |
| GO:0051179\_localization | SEC23A | 1058 | 12 | 1.536195 | -1.153593 | 168 | 86.95 | 0.517560 |  |
| GO:0051179\_localization | FEZF2 | 1058 | 12 | 1.536195 | -1.153593 | 168 | 86.95 | 0.517560 |  |
| GO:0051179\_localization | NUP133 | 1058 | 12 | 1.536195 | -1.153593 | 168 | 86.95 | 0.517560 |  |
| GO:0051179\_localization | PDPN | 1058 | 12 | 1.536195 | -1.153593 | 168 | 86.95 | 0.517560 |  |
| GO:0051179\_localization | RRAS2 | 1058 | 12 | 1.536195 | -1.153593 | 168 | 86.95 | 0.517560 |  |
| GO:0051179\_localization | BAX | 1058 | 12 | 1.536195 | -1.153593 | 168 | 86.95 | 0.517560 |  |
| GO:0051179\_localization | HBB-B1 | 1058 | 12 | 1.536195 | -1.153593 | 168 | 86.95 | 0.517560 |  |
| GO:0051179\_localization | SPIRE1 | 1058 | 12 | 1.536195 | -1.153593 | 168 | 86.95 | 0.517560 |  |
| GO:0051179\_localization | VAX1 | 1058 | 12 | 1.536195 | -1.153593 | 168 | 86.95 | 0.517560 |  |
| GO:0051179\_localization | SEMA3A | 1058 | 12 | 1.536195 | -1.153593 | 168 | 86.95 | 0.517560 |  |
| GO:0051179\_localization | CDH4 | 1058 | 12 | 1.536195 | -1.153593 | 168 | 86.95 | 0.517560 |  |
| GO:0006405\_RNA\_export\_from\_nucleus | NUP133 | 10 | 1 | 13.544118 | -1.145724 | 185 | 95.67 | 0.517135 |  |
| GO:0007006\_mitochondrial\_membrane\_organization | BAX | 10 | 1 | 13.544118 | -1.145724 | 185 | 95.67 | 0.517135 |  |
| GO:0022900\_electron\_transport\_chain | UQCRC1 | 10 | 1 | 13.544118 | -1.145724 | 185 | 95.67 | 0.517135 |  |
| GO:0022904\_respiratory\_electron\_transport\_chain | UQCRC1 | 10 | 1 | 13.544118 | -1.145724 | 185 | 95.67 | 0.517135 |  |
| GO:0031331\_positive\_regulation\_of\_cellular\_catabolic\_process | GCLC | 10 | 1 | 13.544118 | -1.145724 | 185 | 95.67 | 0.517135 |  |
| GO:0034105\_positive\_regulation\_of\_tissue\_remodeling | BAX | 10 | 1 | 13.544118 | -1.145724 | 185 | 95.67 | 0.517135 |  |
| GO:0043525\_positive\_regulation\_of\_neuron\_apoptosis | BAX | 10 | 1 | 13.544118 | -1.145724 | 185 | 95.67 | 0.517135 |  |
| GO:0048339\_paraxial\_mesoderm\_development | NUP133 | 10 | 1 | 13.544118 | -1.145724 | 185 | 95.67 | 0.517135 |  |
| GO:0050657\_nucleic\_acid\_transport | NUP133 | 10 | 1 | 13.544118 | -1.145724 | 185 | 95.67 | 0.517135 |  |
| GO:0050658\_RNA\_transport | NUP133 | 10 | 1 | 13.544118 | -1.145724 | 185 | 95.67 | 0.517135 |  |
| GO:0051208\_sequestering\_of\_calcium\_ion | BAX | 10 | 1 | 13.544118 | -1.145724 | 185 | 95.67 | 0.517135 |  |
| GO:0051209\_release\_of\_sequestered\_calcium\_ion\_into\_cytosol | BAX | 10 | 1 | 13.544118 | -1.145724 | 185 | 95.67 | 0.517135 |  |
| GO:0051236\_establishment\_of\_RNA\_localization | NUP133 | 10 | 1 | 13.544118 | -1.145724 | 185 | 95.67 | 0.517135 |  |
| GO:0051238\_sequestering\_of\_metal\_ion | BAX | 10 | 1 | 13.544118 | -1.145724 | 185 | 95.67 | 0.517135 |  |
| GO:0051282\_regulation\_of\_sequestering\_of\_calcium\_ion | BAX | 10 | 1 | 13.544118 | -1.145724 | 185 | 95.67 | 0.517135 |  |
| GO:0051283\_negative\_regulation\_of\_sequestering\_of\_calcium\_ion | BAX | 10 | 1 | 13.544118 | -1.145724 | 185 | 95.67 | 0.517135 |  |
| GO:0060768\_regulation\_of\_epithelial\_cell\_proliferation\_involved\_in\_prostate\_gland\_development | NOTCH1 | 10 | 1 | 13.544118 | -1.145724 | 185 | 95.67 | 0.517135 |  |
| GO:0022604\_regulation\_of\_cell\_morphogenesis | SEMA3A | 62 | 2 | 4.369070 | -1.119164 | 186 | 97.48 | 0.524086 |  |
| GO:0022604\_regulation\_of\_cell\_morphogenesis | CDH4 | 62 | 2 | 4.369070 | -1.119164 | 186 | 97.48 | 0.524086 |  |
| GO:0007399\_nervous\_system\_development | FEZF2 | 621 | 8 | 1.744814 | -1.109119 | 187 | 97.87 | 0.523369 |  |
| GO:0007399\_nervous\_system\_development | NOTCH1 | 621 | 8 | 1.744814 | -1.109119 | 187 | 97.87 | 0.523369 |  |
| GO:0007399\_nervous\_system\_development | BAX | 621 | 8 | 1.744814 | -1.109119 | 187 | 97.87 | 0.523369 |  |
| GO:0007399\_nervous\_system\_development | VWC2 | 621 | 8 | 1.744814 | -1.109119 | 187 | 97.87 | 0.523369 |  |
| GO:0007399\_nervous\_system\_development | SEMA3A | 621 | 8 | 1.744814 | -1.109119 | 187 | 97.87 | 0.523369 |  |
| GO:0007399\_nervous\_system\_development | VAX1 | 621 | 8 | 1.744814 | -1.109119 | 187 | 97.87 | 0.523369 |  |
| GO:0007399\_nervous\_system\_development | RBPJ | 621 | 8 | 1.744814 | -1.109119 | 187 | 97.87 | 0.523369 |  |
| GO:0007399\_nervous\_system\_development | CDH4 | 621 | 8 | 1.744814 | -1.109119 | 187 | 97.87 | 0.523369 |  |
| GO:0000737\_DNA\_catabolic\_process\_\_endonucleolytic | ENDOG | 11 | 1 | 12.312834 | -1.105876 | 197 | 104.89 | 0.532437 |  |
| GO:0001101\_response\_to\_acid | BAX | 11 | 1 | 12.312834 | -1.105876 | 197 | 104.89 | 0.532437 |  |
| GO:0006690\_icosanoid\_metabolic\_process | PDPN | 11 | 1 | 12.312834 | -1.105876 | 197 | 104.89 | 0.532437 |  |
| GO:0008354\_germ\_cell\_migration | BAX | 11 | 1 | 12.312834 | -1.105876 | 197 | 104.89 | 0.532437 |  |
| GO:0033559\_unsaturated\_fatty\_acid\_metabolic\_process | PDPN | 11 | 1 | 12.312834 | -1.105876 | 197 | 104.89 | 0.532437 |  |
| GO:0043270\_positive\_regulation\_of\_ion\_transport | BAX | 11 | 1 | 12.312834 | -1.105876 | 197 | 104.89 | 0.532437 |  |
| GO:0048193\_Golgi\_vesicle\_transport | SPIRE1 | 11 | 1 | 12.312834 | -1.105876 | 197 | 104.89 | 0.532437 |  |
| GO:0048678\_response\_to\_axon\_injury | BAX | 11 | 1 | 12.312834 | -1.105876 | 197 | 104.89 | 0.532437 |  |
| GO:0050772\_positive\_regulation\_of\_axonogenesis | CDH4 | 11 | 1 | 12.312834 | -1.105876 | 197 | 104.89 | 0.532437 |  |
| GO:0060767\_epithelial\_cell\_proliferation\_involved\_in\_prostate\_gland\_development | NOTCH1 | 11 | 1 | 12.312834 | -1.105876 | 197 | 104.89 | 0.532437 |  |
| GO:0003006\_reproductive\_developmental\_process | NOTCH1 | 141 | 3 | 2.881727 | -1.073567 | 198 | 106.22 | 0.536465 |  |
| GO:0003006\_reproductive\_developmental\_process | BAX | 141 | 3 | 2.881727 | -1.073567 | 198 | 106.22 | 0.536465 |  |
| GO:0003006\_reproductive\_developmental\_process | ROR2 | 141 | 3 | 2.881727 | -1.073567 | 198 | 106.22 | 0.536465 |  |
| GO:0009888\_tissue\_development | FRAS1 | 525 | 7 | 1.805882 | -1.072154 | 199 | 106.66 | 0.535980 |  |
| GO:0009888\_tissue\_development | NUP133 | 525 | 7 | 1.805882 | -1.072154 | 199 | 106.66 | 0.535980 |  |
| GO:0009888\_tissue\_development | NOTCH1 | 525 | 7 | 1.805882 | -1.072154 | 199 | 106.66 | 0.535980 |  |
| GO:0009888\_tissue\_development | ROR2 | 525 | 7 | 1.805882 | -1.072154 | 199 | 106.66 | 0.535980 |  |
| GO:0009888\_tissue\_development | RB1 | 525 | 7 | 1.805882 | -1.072154 | 199 | 106.66 | 0.535980 |  |
| GO:0009888\_tissue\_development | SEMA3A | 525 | 7 | 1.805882 | -1.072154 | 199 | 106.66 | 0.535980 |  |
| GO:0009888\_tissue\_development | RBPJ | 525 | 7 | 1.805882 | -1.072154 | 199 | 106.66 | 0.535980 |  |
| GO:0002763\_positive\_regulation\_of\_myeloid\_leukocyte\_differentiation | RB1 | 12 | 1 | 11.286765 | -1.069630 | 212 | 113.72 | 0.536415 |  |
| GO:0006308\_DNA\_catabolic\_process | ENDOG | 12 | 1 | 11.286765 | -1.069630 | 212 | 113.72 | 0.536415 |  |
| GO:0006403\_RNA\_localization | NUP133 | 12 | 1 | 11.286765 | -1.069630 | 212 | 113.72 | 0.536415 |  |
| GO:0006413\_translational\_initiation | HBB-B1 | 12 | 1 | 11.286765 | -1.069630 | 212 | 113.72 | 0.536415 |  |
| GO:0006446\_regulation\_of\_translational\_initiation | HBB-B1 | 12 | 1 | 11.286765 | -1.069630 | 212 | 113.72 | 0.536415 |  |
| GO:0006839\_mitochondrial\_transport | BAX | 12 | 1 | 11.286765 | -1.069630 | 212 | 113.72 | 0.536415 |  |
| GO:0009886\_post-embryonic\_morphogenesis | BAX | 12 | 1 | 11.286765 | -1.069630 | 212 | 113.72 | 0.536415 |  |
| GO:0030225\_macrophage\_differentiation | RB1 | 12 | 1 | 11.286765 | -1.069630 | 212 | 113.72 | 0.536415 |  |
| GO:0030514\_negative\_regulation\_of\_BMP\_signaling\_pathway | VWC2 | 12 | 1 | 11.286765 | -1.069630 | 212 | 113.72 | 0.536415 |  |
| GO:0033598\_mammary\_gland\_epithelial\_cell\_proliferation | BAX | 12 | 1 | 11.286765 | -1.069630 | 212 | 113.72 | 0.536415 |  |
| GO:0045995\_regulation\_of\_embryonic\_development | NOTCH1 | 12 | 1 | 11.286765 | -1.069630 | 212 | 113.72 | 0.536415 |  |
| GO:0055114\_oxidation\_reduction | UQCRC1 | 12 | 1 | 11.286765 | -1.069630 | 212 | 113.72 | 0.536415 |  |
| GO:0060525\_prostate\_glandular\_acinus\_development | NOTCH1 | 12 | 1 | 11.286765 | -1.069630 | 212 | 113.72 | 0.536415 |  |
| GO:0034962\_cellular\_biopolymer\_catabolic\_process | GCLC | 68 | 2 | 3.983564 | -1.050067 | 214 | 115.15 | 0.538084 |  |
| GO:0034962\_cellular\_biopolymer\_catabolic\_process | ENDOG | 68 | 2 | 3.983564 | -1.050067 | 214 | 115.15 | 0.538084 |  |
| GO:0042692\_muscle\_cell\_differentiation | RB1 | 68 | 2 | 3.983564 | -1.050067 | 214 | 115.15 | 0.538084 |  |
| GO:0042692\_muscle\_cell\_differentiation | IFRD1 | 68 | 2 | 3.983564 | -1.050067 | 214 | 115.15 | 0.538084 |  |
| GO:0006915\_apoptosis | NOTCH1 | 427 | 6 | 1.903155 | -1.049672 | 215 | 115.21 | 0.535860 |  |
| GO:0006915\_apoptosis | KRT18 | 427 | 6 | 1.903155 | -1.049672 | 215 | 115.21 | 0.535860 |  |
| GO:0006915\_apoptosis | GCLC | 427 | 6 | 1.903155 | -1.049672 | 215 | 115.21 | 0.535860 |  |
| GO:0006915\_apoptosis | ENDOG | 427 | 6 | 1.903155 | -1.049672 | 215 | 115.21 | 0.535860 |  |
| GO:0006915\_apoptosis | BAX | 427 | 6 | 1.903155 | -1.049672 | 215 | 115.21 | 0.535860 |  |
| GO:0006915\_apoptosis | BAD | 427 | 6 | 1.903155 | -1.049672 | 215 | 115.21 | 0.535860 |  |
| GO:0001932\_regulation\_of\_protein\_amino\_acid\_phosphorylation | BAX | 69 | 2 | 3.925831 | -1.039250 | 216 | 115.77 | 0.535972 |  |
| GO:0001932\_regulation\_of\_protein\_amino\_acid\_phosphorylation | HBB-B1 | 69 | 2 | 3.925831 | -1.039250 | 216 | 115.77 | 0.535972 |  |
| GO:0016477\_cell\_migration | RRAS2 | 234 | 4 | 2.315234 | -1.038612 | 217 | 115.86 | 0.533917 |  |
| GO:0016477\_cell\_migration | BAX | 234 | 4 | 2.315234 | -1.038612 | 217 | 115.86 | 0.533917 |  |
| GO:0016477\_cell\_migration | SEMA3A | 234 | 4 | 2.315234 | -1.038612 | 217 | 115.86 | 0.533917 |  |
| GO:0016477\_cell\_migration | VAX1 | 234 | 4 | 2.315234 | -1.038612 | 217 | 115.86 | 0.533917 |  |
| GO:0001836\_release\_of\_cytochrome\_c\_from\_mitochondria | BAX | 13 | 1 | 10.418552 | -1.036409 | 232 | 122.58 | 0.528362 |  |
| GO:0006687\_glycosphingolipid\_metabolic\_process | BAX | 13 | 1 | 10.418552 | -1.036409 | 232 | 122.58 | 0.528362 |  |
| GO:0006833\_water\_transport | PDPN | 13 | 1 | 10.418552 | -1.036409 | 232 | 122.58 | 0.528362 |  |
| GO:0007439\_ectodermal\_gut\_development | NOTCH1 | 13 | 1 | 10.418552 | -1.036409 | 232 | 122.58 | 0.528362 |  |
| GO:0009410\_response\_to\_xenobiotic\_stimulus | GCLC | 13 | 1 | 10.418552 | -1.036409 | 232 | 122.58 | 0.528362 |  |
| GO:0010623\_developmental\_programmed\_cell\_death | BAX | 13 | 1 | 10.418552 | -1.036409 | 232 | 122.58 | 0.528362 |  |
| GO:0018105\_peptidyl-serine\_phosphorylation | BAX | 13 | 1 | 10.418552 | -1.036409 | 232 | 122.58 | 0.528362 |  |
| GO:0042044\_fluid\_transport | PDPN | 13 | 1 | 10.418552 | -1.036409 | 232 | 122.58 | 0.528362 |  |
| GO:0045577\_regulation\_of\_B\_cell\_differentiation | BAD | 13 | 1 | 10.418552 | -1.036409 | 232 | 122.58 | 0.528362 |  |
| GO:0045682\_regulation\_of\_epidermis\_development | NOTCH1 | 13 | 1 | 10.418552 | -1.036409 | 232 | 122.58 | 0.528362 |  |
| GO:0048567\_ectodermal\_gut\_morphogenesis | NOTCH1 | 13 | 1 | 10.418552 | -1.036409 | 232 | 122.58 | 0.528362 |  |
| GO:0050771\_negative\_regulation\_of\_axonogenesis | SEMA3A | 13 | 1 | 10.418552 | -1.036409 | 232 | 122.58 | 0.528362 |  |
| GO:0060401\_cytosolic\_calcium\_ion\_transport | BAX | 13 | 1 | 10.418552 | -1.036409 | 232 | 122.58 | 0.528362 |  |
| GO:0060402\_calcium\_ion\_transport\_into\_cytosol | BAX | 13 | 1 | 10.418552 | -1.036409 | 232 | 122.58 | 0.528362 |  |
| GO:0060742\_epithelial\_cell\_differentiation\_involved\_in\_prostate\_gland\_development | NOTCH1 | 13 | 1 | 10.418552 | -1.036409 | 232 | 122.58 | 0.528362 |  |
| GO:0010605\_negative\_regulation\_of\_macromolecule\_metabolic\_process | GCLC | 331 | 5 | 2.045939 | -1.031157 | 233 | 122.92 | 0.527554 |  |
| GO:0010605\_negative\_regulation\_of\_macromolecule\_metabolic\_process | BAX | 331 | 5 | 2.045939 | -1.031157 | 233 | 122.92 | 0.527554 |  |
| GO:0010605\_negative\_regulation\_of\_macromolecule\_metabolic\_process | RB1 | 331 | 5 | 2.045939 | -1.031157 | 233 | 122.92 | 0.527554 |  |
| GO:0010605\_negative\_regulation\_of\_macromolecule\_metabolic\_process | VAX1 | 331 | 5 | 2.045939 | -1.031157 | 233 | 122.92 | 0.527554 |  |
| GO:0010605\_negative\_regulation\_of\_macromolecule\_metabolic\_process | RBPJ | 331 | 5 | 2.045939 | -1.031157 | 233 | 122.92 | 0.527554 |  |
| GO:0012501\_programmed\_cell\_death | NOTCH1 | 433 | 6 | 1.876783 | -1.026950 | 234 | 123.66 | 0.528462 |  |
| GO:0012501\_programmed\_cell\_death | KRT18 | 433 | 6 | 1.876783 | -1.026950 | 234 | 123.66 | 0.528462 |  |
| GO:0012501\_programmed\_cell\_death | GCLC | 433 | 6 | 1.876783 | -1.026950 | 234 | 123.66 | 0.528462 |  |
| GO:0012501\_programmed\_cell\_death | ENDOG | 433 | 6 | 1.876783 | -1.026950 | 234 | 123.66 | 0.528462 |  |
| GO:0012501\_programmed\_cell\_death | BAX | 433 | 6 | 1.876783 | -1.026950 | 234 | 123.66 | 0.528462 |  |
| GO:0012501\_programmed\_cell\_death | BAD | 433 | 6 | 1.876783 | -1.026950 | 234 | 123.66 | 0.528462 |  |
| GO:0031324\_negative\_regulation\_of\_cellular\_metabolic\_process | GCLC | 332 | 5 | 2.039777 | -1.026844 | 235 | 123.77 | 0.526681 |  |
| GO:0031324\_negative\_regulation\_of\_cellular\_metabolic\_process | BAX | 332 | 5 | 2.039777 | -1.026844 | 235 | 123.77 | 0.526681 |  |
| GO:0031324\_negative\_regulation\_of\_cellular\_metabolic\_process | RB1 | 332 | 5 | 2.039777 | -1.026844 | 235 | 123.77 | 0.526681 |  |
| GO:0031324\_negative\_regulation\_of\_cellular\_metabolic\_process | VAX1 | 332 | 5 | 2.039777 | -1.026844 | 235 | 123.77 | 0.526681 |  |
| GO:0031324\_negative\_regulation\_of\_cellular\_metabolic\_process | RBPJ | 332 | 5 | 2.039777 | -1.026844 | 235 | 123.77 | 0.526681 |  |
| GO:0048731\_system\_development | FRAS1 | 1609 | 16 | 1.346836 | -1.012261 | 236 | 124.73 | 0.528517 |  |
| GO:0048731\_system\_development | NUP133 | 1609 | 16 | 1.346836 | -1.012261 | 236 | 124.73 | 0.528517 |  |
| GO:0048731\_system\_development | PDPN | 1609 | 16 | 1.346836 | -1.012261 | 236 | 124.73 | 0.528517 |  |
| GO:0048731\_system\_development | RB1 | 1609 | 16 | 1.346836 | -1.012261 | 236 | 124.73 | 0.528517 |  |
| GO:0048731\_system\_development | BAD | 1609 | 16 | 1.346836 | -1.012261 | 236 | 124.73 | 0.528517 |  |
| GO:0048731\_system\_development | VAX1 | 1609 | 16 | 1.346836 | -1.012261 | 236 | 124.73 | 0.528517 |  |
| GO:0048731\_system\_development | CDH4 | 1609 | 16 | 1.346836 | -1.012261 | 236 | 124.73 | 0.528517 |  |
| GO:0048731\_system\_development | FEZF2 | 1609 | 16 | 1.346836 | -1.012261 | 236 | 124.73 | 0.528517 |  |
| GO:0048731\_system\_development | NOTCH1 | 1609 | 16 | 1.346836 | -1.012261 | 236 | 124.73 | 0.528517 |  |
| GO:0048731\_system\_development | BAX | 1609 | 16 | 1.346836 | -1.012261 | 236 | 124.73 | 0.528517 |  |
| GO:0048731\_system\_development | ROR2 | 1609 | 16 | 1.346836 | -1.012261 | 236 | 124.73 | 0.528517 |  |
| GO:0048731\_system\_development | VWC2 | 1609 | 16 | 1.346836 | -1.012261 | 236 | 124.73 | 0.528517 |  |
| GO:0048731\_system\_development | HBB-B1 | 1609 | 16 | 1.346836 | -1.012261 | 236 | 124.73 | 0.528517 |  |
| GO:0048731\_system\_development | SEMA3A | 1609 | 16 | 1.346836 | -1.012261 | 236 | 124.73 | 0.528517 |  |
| GO:0048731\_system\_development | RBPJ | 1609 | 16 | 1.346836 | -1.012261 | 236 | 124.73 | 0.528517 |  |
| GO:0048731\_system\_development | IFRD1 | 1609 | 16 | 1.346836 | -1.012261 | 236 | 124.73 | 0.528517 |  |
| GO:0007264\_small\_GTPase\_mediated\_signal\_transduction | SOS1 | 72 | 2 | 3.762255 | -1.007883 | 239 | 125.76 | 0.526192 |  |
| GO:0007264\_small\_GTPase\_mediated\_signal\_transduction | RRAS2 | 72 | 2 | 3.762255 | -1.007883 | 239 | 125.76 | 0.526192 |  |
| GO:0040012\_regulation\_of\_locomotion | RRAS2 | 72 | 2 | 3.762255 | -1.007883 | 239 | 125.76 | 0.526192 |  |
| GO:0040012\_regulation\_of\_locomotion | SEMA3A | 72 | 2 | 3.762255 | -1.007883 | 239 | 125.76 | 0.526192 |  |
| GO:0050673\_epithelial\_cell\_proliferation | NOTCH1 | 72 | 2 | 3.762255 | -1.007883 | 239 | 125.76 | 0.526192 |  |
| GO:0050673\_epithelial\_cell\_proliferation | BAX | 72 | 2 | 3.762255 | -1.007883 | 239 | 125.76 | 0.526192 |  |
| GO:0008283\_cell\_proliferation | NOTCH1 | 544 | 7 | 1.742809 | -1.007644 | 240 | 125.79 | 0.524125 |  |
| GO:0008283\_cell\_proliferation | PDPN | 544 | 7 | 1.742809 | -1.007644 | 240 | 125.79 | 0.524125 |  |
| GO:0008283\_cell\_proliferation | BAX | 544 | 7 | 1.742809 | -1.007644 | 240 | 125.79 | 0.524125 |  |
| GO:0008283\_cell\_proliferation | BAD | 544 | 7 | 1.742809 | -1.007644 | 240 | 125.79 | 0.524125 |  |
| GO:0008283\_cell\_proliferation | RB1 | 544 | 7 | 1.742809 | -1.007644 | 240 | 125.79 | 0.524125 |  |
| GO:0008283\_cell\_proliferation | VAX1 | 544 | 7 | 1.742809 | -1.007644 | 240 | 125.79 | 0.524125 |  |
| GO:0008283\_cell\_proliferation | RBPJ | 544 | 7 | 1.742809 | -1.007644 | 240 | 125.79 | 0.524125 |  |
| GO:0001502\_cartilage\_condensation | ROR2 | 14 | 1 | 9.674370 | -1.005764 | 250 | 131.93 | 0.527720 |  |
| GO:0002027\_regulation\_of\_heart\_rate | SEMA3A | 14 | 1 | 9.674370 | -1.005764 | 250 | 131.93 | 0.527720 |  |
| GO:0002262\_myeloid\_cell\_homeostasis | BAX | 14 | 1 | 9.674370 | -1.005764 | 250 | 131.93 | 0.527720 |  |
| GO:0006970\_response\_to\_osmotic\_stress | BAX | 14 | 1 | 9.674370 | -1.005764 | 250 | 131.93 | 0.527720 |  |
| GO:0007157\_heterophilic\_cell\_adhesion | CDH4 | 14 | 1 | 9.674370 | -1.005764 | 250 | 131.93 | 0.527720 |  |
| GO:0009108\_coenzyme\_biosynthetic\_process | GCLC | 14 | 1 | 9.674370 | -1.005764 | 250 | 131.93 | 0.527720 |  |
| GO:0010332\_response\_to\_gamma\_radiation | BAX | 14 | 1 | 9.674370 | -1.005764 | 250 | 131.93 | 0.527720 |  |
| GO:0030162\_regulation\_of\_proteolysis | GCLC | 14 | 1 | 9.674370 | -1.005764 | 250 | 131.93 | 0.527720 |  |
| GO:0031346\_positive\_regulation\_of\_cell\_projection\_organization | CDH4 | 14 | 1 | 9.674370 | -1.005764 | 250 | 131.93 | 0.527720 |  |
| GO:0045732\_positive\_regulation\_of\_protein\_catabolic\_process | GCLC | 14 | 1 | 9.674370 | -1.005764 | 250 | 131.93 | 0.527720 |  |
| GO:0048468\_cell\_development | FEZF2 | 654 | 8 | 1.656773 | -1.005188 | 251 | 132.02 | 0.525976 |  |
| GO:0048468\_cell\_development | NOTCH1 | 654 | 8 | 1.656773 | -1.005188 | 251 | 132.02 | 0.525976 |  |
| GO:0048468\_cell\_development | BAX | 654 | 8 | 1.656773 | -1.005188 | 251 | 132.02 | 0.525976 |  |
| GO:0048468\_cell\_development | VWC2 | 654 | 8 | 1.656773 | -1.005188 | 251 | 132.02 | 0.525976 |  |
| GO:0048468\_cell\_development | VAX1 | 654 | 8 | 1.656773 | -1.005188 | 251 | 132.02 | 0.525976 |  |
| GO:0048468\_cell\_development | SEMA3A | 654 | 8 | 1.656773 | -1.005188 | 251 | 132.02 | 0.525976 |  |
| GO:0048468\_cell\_development | RBPJ | 654 | 8 | 1.656773 | -1.005188 | 251 | 132.02 | 0.525976 |  |
| GO:0048468\_cell\_development | CDH4 | 654 | 8 | 1.656773 | -1.005188 | 251 | 132.02 | 0.525976 |  |
| GO:0032268\_regulation\_of\_cellular\_protein\_metabolic\_process | GCLC | 152 | 3 | 2.673181 | -0.998482 | 252 | 132.33 | 0.525119 |  |
| GO:0032268\_regulation\_of\_cellular\_protein\_metabolic\_process | BAX | 152 | 3 | 2.673181 | -0.998482 | 252 | 132.33 | 0.525119 |  |
| GO:0032268\_regulation\_of\_cellular\_protein\_metabolic\_process | HBB-B1 | 152 | 3 | 2.673181 | -0.998482 | 252 | 132.33 | 0.525119 |  |
| GO:0051336\_regulation\_of\_hydrolase\_activity | BAX | 73 | 2 | 3.710717 | -0.997773 | 253 | 132.73 | 0.524625 |  |
| GO:0051336\_regulation\_of\_hydrolase\_activity | BAD | 73 | 2 | 3.710717 | -0.997773 | 253 | 132.73 | 0.524625 |  |
| GO:0048513\_organ\_development | FRAS1 | 1365 | 14 | 1.389140 | -0.996609 | 254 | 132.83 | 0.522953 |  |
| GO:0048513\_organ\_development | NUP133 | 1365 | 14 | 1.389140 | -0.996609 | 254 | 132.83 | 0.522953 |  |
| GO:0048513\_organ\_development | PDPN | 1365 | 14 | 1.389140 | -0.996609 | 254 | 132.83 | 0.522953 |  |
| GO:0048513\_organ\_development | RB1 | 1365 | 14 | 1.389140 | -0.996609 | 254 | 132.83 | 0.522953 |  |
| GO:0048513\_organ\_development | VAX1 | 1365 | 14 | 1.389140 | -0.996609 | 254 | 132.83 | 0.522953 |  |
| GO:0048513\_organ\_development | BAD | 1365 | 14 | 1.389140 | -0.996609 | 254 | 132.83 | 0.522953 |  |
| GO:0048513\_organ\_development | FEZF2 | 1365 | 14 | 1.389140 | -0.996609 | 254 | 132.83 | 0.522953 |  |
| GO:0048513\_organ\_development | NOTCH1 | 1365 | 14 | 1.389140 | -0.996609 | 254 | 132.83 | 0.522953 |  |
| GO:0048513\_organ\_development | BAX | 1365 | 14 | 1.389140 | -0.996609 | 254 | 132.83 | 0.522953 |  |
| GO:0048513\_organ\_development | ROR2 | 1365 | 14 | 1.389140 | -0.996609 | 254 | 132.83 | 0.522953 |  |
| GO:0048513\_organ\_development | HBB-B1 | 1365 | 14 | 1.389140 | -0.996609 | 254 | 132.83 | 0.522953 |  |
| GO:0048513\_organ\_development | SEMA3A | 1365 | 14 | 1.389140 | -0.996609 | 254 | 132.83 | 0.522953 |  |
| GO:0048513\_organ\_development | RBPJ | 1365 | 14 | 1.389140 | -0.996609 | 254 | 132.83 | 0.522953 |  |
| GO:0048513\_organ\_development | IFRD1 | 1365 | 14 | 1.389140 | -0.996609 | 254 | 132.83 | 0.522953 |  |
| GO:0016070\_RNA\_metabolic\_process | PKNOX2 | 658 | 8 | 1.646701 | -0.993225 | 255 | 133.14 | 0.522118 |  |
| GO:0016070\_RNA\_metabolic\_process | NOTCH1 | 658 | 8 | 1.646701 | -0.993225 | 255 | 133.14 | 0.522118 |  |
| GO:0016070\_RNA\_metabolic\_process | POLR1E | 658 | 8 | 1.646701 | -0.993225 | 255 | 133.14 | 0.522118 |  |
| GO:0016070\_RNA\_metabolic\_process | NR1D2 | 658 | 8 | 1.646701 | -0.993225 | 255 | 133.14 | 0.522118 |  |
| GO:0016070\_RNA\_metabolic\_process | NFYB | 658 | 8 | 1.646701 | -0.993225 | 255 | 133.14 | 0.522118 |  |
| GO:0016070\_RNA\_metabolic\_process | RB1 | 658 | 8 | 1.646701 | -0.993225 | 255 | 133.14 | 0.522118 |  |
| GO:0016070\_RNA\_metabolic\_process | VAX1 | 658 | 8 | 1.646701 | -0.993225 | 255 | 133.14 | 0.522118 |  |
| GO:0016070\_RNA\_metabolic\_process | RBPJ | 658 | 8 | 1.646701 | -0.993225 | 255 | 133.14 | 0.522118 |  |
| GO:0008219\_cell\_death | NOTCH1 | 444 | 6 | 1.830286 | -0.986593 | 256 | 134.0 | 0.523438 |  |
| GO:0008219\_cell\_death | KRT18 | 444 | 6 | 1.830286 | -0.986593 | 256 | 134.0 | 0.523438 |  |
| GO:0008219\_cell\_death | GCLC | 444 | 6 | 1.830286 | -0.986593 | 256 | 134.0 | 0.523438 |  |
| GO:0008219\_cell\_death | ENDOG | 444 | 6 | 1.830286 | -0.986593 | 256 | 134.0 | 0.523438 |  |
| GO:0008219\_cell\_death | BAX | 444 | 6 | 1.830286 | -0.986593 | 256 | 134.0 | 0.523438 |  |
| GO:0008219\_cell\_death | BAD | 444 | 6 | 1.830286 | -0.986593 | 256 | 134.0 | 0.523438 |  |
| GO:0044265\_cellular\_macromolecule\_catabolic\_process | GCLC | 75 | 2 | 3.611765 | -0.978039 | 257 | 134.88 | 0.524825 |  |
| GO:0044265\_cellular\_macromolecule\_catabolic\_process | ENDOG | 75 | 2 | 3.611765 | -0.978039 | 257 | 134.88 | 0.524825 |  |
| GO:0001782\_B\_cell\_homeostasis | BAX | 15 | 1 | 9.029412 | -0.977338 | 268 | 140.49 | 0.524216 |  |
| GO:0006749\_glutathione\_metabolic\_process | GCLC | 15 | 1 | 9.029412 | -0.977338 | 268 | 140.49 | 0.524216 |  |
| GO:0015931\_nucleobase\_\_nucleoside\_\_nucleotide\_and\_nucleic\_acid\_transport | NUP133 | 15 | 1 | 9.029412 | -0.977338 | 268 | 140.49 | 0.524216 |  |
| GO:0031069\_hair\_follicle\_morphogenesis | NOTCH1 | 15 | 1 | 9.029412 | -0.977338 | 268 | 140.49 | 0.524216 |  |
| GO:0031329\_regulation\_of\_cellular\_catabolic\_process | GCLC | 15 | 1 | 9.029412 | -0.977338 | 268 | 140.49 | 0.524216 |  |
| GO:0035116\_embryonic\_hindlimb\_morphogenesis | NOTCH1 | 15 | 1 | 9.029412 | -0.977338 | 268 | 140.49 | 0.524216 |  |
| GO:0045666\_positive\_regulation\_of\_neuron\_differentiation | VWC2 | 15 | 1 | 9.029412 | -0.977338 | 268 | 140.49 | 0.524216 |  |
| GO:0046164\_alcohol\_catabolic\_process | BAD | 15 | 1 | 9.029412 | -0.977338 | 268 | 140.49 | 0.524216 |  |
| GO:0048144\_fibroblast\_proliferation | BAX | 15 | 1 | 9.029412 | -0.977338 | 268 | 140.49 | 0.524216 |  |
| GO:0048145\_regulation\_of\_fibroblast\_proliferation | BAX | 15 | 1 | 9.029412 | -0.977338 | 268 | 140.49 | 0.524216 |  |
| GO:0070227\_lymphocyte\_apoptosis | BAX | 15 | 1 | 9.029412 | -0.977338 | 268 | 140.49 | 0.524216 |  |
| GO:0032879\_regulation\_of\_localization | PDPN | 248 | 4 | 2.184535 | -0.968161 | 269 | 141.57 | 0.526283 |  |
| GO:0032879\_regulation\_of\_localization | RRAS2 | 248 | 4 | 2.184535 | -0.968161 | 269 | 141.57 | 0.526283 |  |
| GO:0032879\_regulation\_of\_localization | BAX | 248 | 4 | 2.184535 | -0.968161 | 269 | 141.57 | 0.526283 |  |
| GO:0032879\_regulation\_of\_localization | SEMA3A | 248 | 4 | 2.184535 | -0.968161 | 269 | 141.57 | 0.526283 |  |
| GO:0007275\_multicellular\_organismal\_development | FRAS1 | 1760 | 17 | 1.308239 | -0.965950 | 270 | 141.7 | 0.524815 |  |
| GO:0007275\_multicellular\_organismal\_development | NUP133 | 1760 | 17 | 1.308239 | -0.965950 | 270 | 141.7 | 0.524815 |  |
| GO:0007275\_multicellular\_organismal\_development | PDPN | 1760 | 17 | 1.308239 | -0.965950 | 270 | 141.7 | 0.524815 |  |
| GO:0007275\_multicellular\_organismal\_development | RB1 | 1760 | 17 | 1.308239 | -0.965950 | 270 | 141.7 | 0.524815 |  |
| GO:0007275\_multicellular\_organismal\_development | BAD | 1760 | 17 | 1.308239 | -0.965950 | 270 | 141.7 | 0.524815 |  |
| GO:0007275\_multicellular\_organismal\_development | VAX1 | 1760 | 17 | 1.308239 | -0.965950 | 270 | 141.7 | 0.524815 |  |
| GO:0007275\_multicellular\_organismal\_development | CDH4 | 1760 | 17 | 1.308239 | -0.965950 | 270 | 141.7 | 0.524815 |  |
| GO:0007275\_multicellular\_organismal\_development | FEZF2 | 1760 | 17 | 1.308239 | -0.965950 | 270 | 141.7 | 0.524815 |  |
| GO:0007275\_multicellular\_organismal\_development | NOTCH1 | 1760 | 17 | 1.308239 | -0.965950 | 270 | 141.7 | 0.524815 |  |
| GO:0007275\_multicellular\_organismal\_development | ENDOG | 1760 | 17 | 1.308239 | -0.965950 | 270 | 141.7 | 0.524815 |  |
| GO:0007275\_multicellular\_organismal\_development | BAX | 1760 | 17 | 1.308239 | -0.965950 | 270 | 141.7 | 0.524815 |  |
| GO:0007275\_multicellular\_organismal\_development | VWC2 | 1760 | 17 | 1.308239 | -0.965950 | 270 | 141.7 | 0.524815 |  |
| GO:0007275\_multicellular\_organismal\_development | ROR2 | 1760 | 17 | 1.308239 | -0.965950 | 270 | 141.7 | 0.524815 |  |
| GO:0007275\_multicellular\_organismal\_development | HBB-B1 | 1760 | 17 | 1.308239 | -0.965950 | 270 | 141.7 | 0.524815 |  |
| GO:0007275\_multicellular\_organismal\_development | SEMA3A | 1760 | 17 | 1.308239 | -0.965950 | 270 | 141.7 | 0.524815 |  |
| GO:0007275\_multicellular\_organismal\_development | RBPJ | 1760 | 17 | 1.308239 | -0.965950 | 270 | 141.7 | 0.524815 |  |
| GO:0007275\_multicellular\_organismal\_development | IFRD1 | 1760 | 17 | 1.308239 | -0.965950 | 270 | 141.7 | 0.524815 |  |
| GO:0016265\_death | NOTCH1 | 450 | 6 | 1.805882 | -0.965264 | 271 | 141.75 | 0.523063 |  |
| GO:0016265\_death | KRT18 | 450 | 6 | 1.805882 | -0.965264 | 271 | 141.75 | 0.523063 |  |
| GO:0016265\_death | GCLC | 450 | 6 | 1.805882 | -0.965264 | 271 | 141.75 | 0.523063 |  |
| GO:0016265\_death | ENDOG | 450 | 6 | 1.805882 | -0.965264 | 271 | 141.75 | 0.523063 |  |
| GO:0016265\_death | BAX | 450 | 6 | 1.805882 | -0.965264 | 271 | 141.75 | 0.523063 |  |
| GO:0016265\_death | BAD | 450 | 6 | 1.805882 | -0.965264 | 271 | 141.75 | 0.523063 |  |
| GO:0048514\_blood\_vessel\_morphogenesis | NOTCH1 | 158 | 3 | 2.571668 | -0.960482 | 272 | 142.18 | 0.522721 |  |
| GO:0048514\_blood\_vessel\_morphogenesis | BAX | 158 | 3 | 2.571668 | -0.960482 | 272 | 142.18 | 0.522721 |  |
| GO:0048514\_blood\_vessel\_morphogenesis | RBPJ | 158 | 3 | 2.571668 | -0.960482 | 272 | 142.18 | 0.522721 |  |
| GO:0009892\_negative\_regulation\_of\_metabolic\_process | GCLC | 348 | 5 | 1.945994 | -0.960452 | 273 | 142.29 | 0.521209 |  |
| GO:0009892\_negative\_regulation\_of\_metabolic\_process | BAX | 348 | 5 | 1.945994 | -0.960452 | 273 | 142.29 | 0.521209 |  |
| GO:0009892\_negative\_regulation\_of\_metabolic\_process | RB1 | 348 | 5 | 1.945994 | -0.960452 | 273 | 142.29 | 0.521209 |  |
| GO:0009892\_negative\_regulation\_of\_metabolic\_process | VAX1 | 348 | 5 | 1.945994 | -0.960452 | 273 | 142.29 | 0.521209 |  |
| GO:0009892\_negative\_regulation\_of\_metabolic\_process | RBPJ | 348 | 5 | 1.945994 | -0.960452 | 273 | 142.29 | 0.521209 |  |
| GO:0034645\_cellular\_macromolecule\_biosynthetic\_process | PKNOX2 | 901 | 10 | 1.503232 | -0.953522 | 274 | 142.7 | 0.520803 |  |
| GO:0034645\_cellular\_macromolecule\_biosynthetic\_process | NOTCH1 | 901 | 10 | 1.503232 | -0.953522 | 274 | 142.7 | 0.520803 |  |
| GO:0034645\_cellular\_macromolecule\_biosynthetic\_process | POLR1E | 901 | 10 | 1.503232 | -0.953522 | 274 | 142.7 | 0.520803 |  |
| GO:0034645\_cellular\_macromolecule\_biosynthetic\_process | NR1D2 | 901 | 10 | 1.503232 | -0.953522 | 274 | 142.7 | 0.520803 |  |
| GO:0034645\_cellular\_macromolecule\_biosynthetic\_process | HBB-B1 | 901 | 10 | 1.503232 | -0.953522 | 274 | 142.7 | 0.520803 |  |
| GO:0034645\_cellular\_macromolecule\_biosynthetic\_process | NFYB | 901 | 10 | 1.503232 | -0.953522 | 274 | 142.7 | 0.520803 |  |
| GO:0034645\_cellular\_macromolecule\_biosynthetic\_process | RB1 | 901 | 10 | 1.503232 | -0.953522 | 274 | 142.7 | 0.520803 |  |
| GO:0034645\_cellular\_macromolecule\_biosynthetic\_process | VAX1 | 901 | 10 | 1.503232 | -0.953522 | 274 | 142.7 | 0.520803 |  |
| GO:0034645\_cellular\_macromolecule\_biosynthetic\_process | RBPJ | 901 | 10 | 1.503232 | -0.953522 | 274 | 142.7 | 0.520803 |  |
| GO:0034645\_cellular\_macromolecule\_biosynthetic\_process | PHLDA1 | 901 | 10 | 1.503232 | -0.953522 | 274 | 142.7 | 0.520803 |  |
| GO:0001933\_negative\_regulation\_of\_protein\_amino\_acid\_phosphorylation | BAX | 16 | 1 | 8.465074 | -0.950846 | 280 | 147.39 | 0.526393 |  |
| GO:0006664\_glycolipid\_metabolic\_process | BAX | 16 | 1 | 8.465074 | -0.950846 | 280 | 147.39 | 0.526393 |  |
| GO:0007156\_homophilic\_cell\_adhesion | CDH4 | 16 | 1 | 8.465074 | -0.950846 | 280 | 147.39 | 0.526393 |  |
| GO:0019751\_polyol\_metabolic\_process | SORD | 16 | 1 | 8.465074 | -0.950846 | 280 | 147.39 | 0.526393 |  |
| GO:0031345\_negative\_regulation\_of\_cell\_projection\_organization | SEMA3A | 16 | 1 | 8.465074 | -0.950846 | 280 | 147.39 | 0.526393 |  |
| GO:0048286\_lung\_alveolus\_development | PDPN | 16 | 1 | 8.465074 | -0.950846 | 280 | 147.39 | 0.526393 |  |
| GO:0009059\_macromolecule\_biosynthetic\_process | PKNOX2 | 910 | 10 | 1.488365 | -0.931171 | 281 | 150.51 | 0.535623 |  |
| GO:0009059\_macromolecule\_biosynthetic\_process | NOTCH1 | 910 | 10 | 1.488365 | -0.931171 | 281 | 150.51 | 0.535623 |  |
| GO:0009059\_macromolecule\_biosynthetic\_process | POLR1E | 910 | 10 | 1.488365 | -0.931171 | 281 | 150.51 | 0.535623 |  |
| GO:0009059\_macromolecule\_biosynthetic\_process | NR1D2 | 910 | 10 | 1.488365 | -0.931171 | 281 | 150.51 | 0.535623 |  |
| GO:0009059\_macromolecule\_biosynthetic\_process | NFYB | 910 | 10 | 1.488365 | -0.931171 | 281 | 150.51 | 0.535623 |  |
| GO:0009059\_macromolecule\_biosynthetic\_process | HBB-B1 | 910 | 10 | 1.488365 | -0.931171 | 281 | 150.51 | 0.535623 |  |
| GO:0009059\_macromolecule\_biosynthetic\_process | VAX1 | 910 | 10 | 1.488365 | -0.931171 | 281 | 150.51 | 0.535623 |  |
| GO:0009059\_macromolecule\_biosynthetic\_process | RB1 | 910 | 10 | 1.488365 | -0.931171 | 281 | 150.51 | 0.535623 |  |
| GO:0009059\_macromolecule\_biosynthetic\_process | RBPJ | 910 | 10 | 1.488365 | -0.931171 | 281 | 150.51 | 0.535623 |  |
| GO:0009059\_macromolecule\_biosynthetic\_process | PHLDA1 | 910 | 10 | 1.488365 | -0.931171 | 281 | 150.51 | 0.535623 |  |
| GO:0006767\_water-soluble\_vitamin\_metabolic\_process | GCLC | 17 | 1 | 7.967128 | -0.926051 | 290 | 156.11 | 0.538310 |  |
| GO:0006919\_activation\_of\_caspase\_activity | BAX | 17 | 1 | 7.967128 | -0.926051 | 290 | 156.11 | 0.538310 |  |
| GO:0010035\_response\_to\_inorganic\_substance | GCLC | 17 | 1 | 7.967128 | -0.926051 | 290 | 156.11 | 0.538310 |  |
| GO:0016052\_carbohydrate\_catabolic\_process | BAD | 17 | 1 | 7.967128 | -0.926051 | 290 | 156.11 | 0.538310 |  |
| GO:0042398\_cellular\_amino\_acid\_derivative\_biosynthetic\_process | GCLC | 17 | 1 | 7.967128 | -0.926051 | 290 | 156.11 | 0.538310 |  |
| GO:0045333\_cellular\_respiration | UQCRC1 | 17 | 1 | 7.967128 | -0.926051 | 290 | 156.11 | 0.538310 |  |
| GO:0045786\_negative\_regulation\_of\_cell\_cycle | RB1 | 17 | 1 | 7.967128 | -0.926051 | 290 | 156.11 | 0.538310 |  |
| GO:0048265\_response\_to\_pain | TAC1 | 17 | 1 | 7.967128 | -0.926051 | 290 | 156.11 | 0.538310 |  |
| GO:0048873\_homeostasis\_of\_number\_of\_cells\_within\_a\_tissue | BAX | 17 | 1 | 7.967128 | -0.926051 | 290 | 156.11 | 0.538310 |  |
| GO:0048870\_cell\_motility | RRAS2 | 257 | 4 | 2.108034 | -0.925838 | 291 | 156.23 | 0.536873 |  |
| GO:0048870\_cell\_motility | BAX | 257 | 4 | 2.108034 | -0.925838 | 291 | 156.23 | 0.536873 |  |
| GO:0048870\_cell\_motility | SEMA3A | 257 | 4 | 2.108034 | -0.925838 | 291 | 156.23 | 0.536873 |  |
| GO:0048870\_cell\_motility | VAX1 | 257 | 4 | 2.108034 | -0.925838 | 291 | 156.23 | 0.536873 |  |
| GO:0021700\_developmental\_maturation | NOTCH1 | 81 | 2 | 3.344227 | -0.922446 | 292 | 156.49 | 0.535925 |  |
| GO:0021700\_developmental\_maturation | RBPJ | 81 | 2 | 3.344227 | -0.922446 | 292 | 156.49 | 0.535925 |  |
| GO:0042981\_regulation\_of\_apoptosis | NOTCH1 | 360 | 5 | 1.881127 | -0.913702 | 293 | 157.03 | 0.535939 |  |
| GO:0042981\_regulation\_of\_apoptosis | GCLC | 360 | 5 | 1.881127 | -0.913702 | 293 | 157.03 | 0.535939 |  |
| GO:0042981\_regulation\_of\_apoptosis | ENDOG | 360 | 5 | 1.881127 | -0.913702 | 293 | 157.03 | 0.535939 |  |
| GO:0042981\_regulation\_of\_apoptosis | BAX | 360 | 5 | 1.881127 | -0.913702 | 293 | 157.03 | 0.535939 |  |
| GO:0042981\_regulation\_of\_apoptosis | BAD | 360 | 5 | 1.881127 | -0.913702 | 293 | 157.03 | 0.535939 |  |
| GO:0006355\_regulation\_of\_transcription\_\_DNA-dependent | PKNOX2 | 575 | 7 | 1.648849 | -0.910358 | 294 | 157.68 | 0.536327 |  |
| GO:0006355\_regulation\_of\_transcription\_\_DNA-dependent | NOTCH1 | 575 | 7 | 1.648849 | -0.910358 | 294 | 157.68 | 0.536327 |  |
| GO:0006355\_regulation\_of\_transcription\_\_DNA-dependent | NR1D2 | 575 | 7 | 1.648849 | -0.910358 | 294 | 157.68 | 0.536327 |  |
| GO:0006355\_regulation\_of\_transcription\_\_DNA-dependent | NFYB | 575 | 7 | 1.648849 | -0.910358 | 294 | 157.68 | 0.536327 |  |
| GO:0006355\_regulation\_of\_transcription\_\_DNA-dependent | RB1 | 575 | 7 | 1.648849 | -0.910358 | 294 | 157.68 | 0.536327 |  |
| GO:0006355\_regulation\_of\_transcription\_\_DNA-dependent | VAX1 | 575 | 7 | 1.648849 | -0.910358 | 294 | 157.68 | 0.536327 |  |
| GO:0006355\_regulation\_of\_transcription\_\_DNA-dependent | RBPJ | 575 | 7 | 1.648849 | -0.910358 | 294 | 157.68 | 0.536327 |  |
| GO:0034961\_cellular\_biopolymer\_biosynthetic\_process | PKNOX2 | 804 | 9 | 1.516133 | -0.904254 | 295 | 158.79 | 0.538271 |  |
| GO:0034961\_cellular\_biopolymer\_biosynthetic\_process | NOTCH1 | 804 | 9 | 1.516133 | -0.904254 | 295 | 158.79 | 0.538271 |  |
| GO:0034961\_cellular\_biopolymer\_biosynthetic\_process | POLR1E | 804 | 9 | 1.516133 | -0.904254 | 295 | 158.79 | 0.538271 |  |
| GO:0034961\_cellular\_biopolymer\_biosynthetic\_process | NR1D2 | 804 | 9 | 1.516133 | -0.904254 | 295 | 158.79 | 0.538271 |  |
| GO:0034961\_cellular\_biopolymer\_biosynthetic\_process | HBB-B1 | 804 | 9 | 1.516133 | -0.904254 | 295 | 158.79 | 0.538271 |  |
| GO:0034961\_cellular\_biopolymer\_biosynthetic\_process | NFYB | 804 | 9 | 1.516133 | -0.904254 | 295 | 158.79 | 0.538271 |  |
| GO:0034961\_cellular\_biopolymer\_biosynthetic\_process | RB1 | 804 | 9 | 1.516133 | -0.904254 | 295 | 158.79 | 0.538271 |  |
| GO:0034961\_cellular\_biopolymer\_biosynthetic\_process | VAX1 | 804 | 9 | 1.516133 | -0.904254 | 295 | 158.79 | 0.538271 |  |
| GO:0034961\_cellular\_biopolymer\_biosynthetic\_process | RBPJ | 804 | 9 | 1.516133 | -0.904254 | 295 | 158.79 | 0.538271 |  |
| GO:0001974\_blood\_vessel\_remodeling | BAX | 18 | 1 | 7.524510 | -0.902760 | 305 | 165.57 | 0.542852 |  |
| GO:0010498\_proteasomal\_protein\_catabolic\_process | GCLC | 18 | 1 | 7.524510 | -0.902760 | 305 | 165.57 | 0.542852 |  |
| GO:0030336\_negative\_regulation\_of\_cell\_migration | SEMA3A | 18 | 1 | 7.524510 | -0.902760 | 305 | 165.57 | 0.542852 |  |
| GO:0030510\_regulation\_of\_BMP\_signaling\_pathway | VWC2 | 18 | 1 | 7.524510 | -0.902760 | 305 | 165.57 | 0.542852 |  |
| GO:0043029\_T\_cell\_homeostasis | BAX | 18 | 1 | 7.524510 | -0.902760 | 305 | 165.57 | 0.542852 |  |
| GO:0043161\_proteasomal\_ubiquitin-dependent\_protein\_catabolic\_process | GCLC | 18 | 1 | 7.524510 | -0.902760 | 305 | 165.57 | 0.542852 |  |
| GO:0044272\_sulfur\_compound\_biosynthetic\_process | GCLC | 18 | 1 | 7.524510 | -0.902760 | 305 | 165.57 | 0.542852 |  |
| GO:0048730\_epidermis\_morphogenesis | NOTCH1 | 18 | 1 | 7.524510 | -0.902760 | 305 | 165.57 | 0.542852 |  |
| GO:0051168\_nuclear\_export | NUP133 | 18 | 1 | 7.524510 | -0.902760 | 305 | 165.57 | 0.542852 |  |
| GO:0051924\_regulation\_of\_calcium\_ion\_transport | BAX | 18 | 1 | 7.524510 | -0.902760 | 305 | 165.57 | 0.542852 |  |
| GO:0043284\_biopolymer\_biosynthetic\_process | PKNOX2 | 807 | 9 | 1.510496 | -0.896648 | 306 | 165.83 | 0.541928 |  |
| GO:0043284\_biopolymer\_biosynthetic\_process | NOTCH1 | 807 | 9 | 1.510496 | -0.896648 | 306 | 165.83 | 0.541928 |  |
| GO:0043284\_biopolymer\_biosynthetic\_process | POLR1E | 807 | 9 | 1.510496 | -0.896648 | 306 | 165.83 | 0.541928 |  |
| GO:0043284\_biopolymer\_biosynthetic\_process | NR1D2 | 807 | 9 | 1.510496 | -0.896648 | 306 | 165.83 | 0.541928 |  |
| GO:0043284\_biopolymer\_biosynthetic\_process | HBB-B1 | 807 | 9 | 1.510496 | -0.896648 | 306 | 165.83 | 0.541928 |  |
| GO:0043284\_biopolymer\_biosynthetic\_process | NFYB | 807 | 9 | 1.510496 | -0.896648 | 306 | 165.83 | 0.541928 |  |
| GO:0043284\_biopolymer\_biosynthetic\_process | RB1 | 807 | 9 | 1.510496 | -0.896648 | 306 | 165.83 | 0.541928 |  |
| GO:0043284\_biopolymer\_biosynthetic\_process | VAX1 | 807 | 9 | 1.510496 | -0.896648 | 306 | 165.83 | 0.541928 |  |
| GO:0043284\_biopolymer\_biosynthetic\_process | RBPJ | 807 | 9 | 1.510496 | -0.896648 | 306 | 165.83 | 0.541928 |  |
| GO:0010941\_regulation\_of\_cell\_death | NOTCH1 | 365 | 5 | 1.855359 | -0.894944 | 308 | 166.19 | 0.539578 |  |
| GO:0010941\_regulation\_of\_cell\_death | GCLC | 365 | 5 | 1.855359 | -0.894944 | 308 | 166.19 | 0.539578 |  |
| GO:0010941\_regulation\_of\_cell\_death | ENDOG | 365 | 5 | 1.855359 | -0.894944 | 308 | 166.19 | 0.539578 |  |
| GO:0010941\_regulation\_of\_cell\_death | BAX | 365 | 5 | 1.855359 | -0.894944 | 308 | 166.19 | 0.539578 |  |
| GO:0010941\_regulation\_of\_cell\_death | BAD | 365 | 5 | 1.855359 | -0.894944 | 308 | 166.19 | 0.539578 |  |
| GO:0043067\_regulation\_of\_programmed\_cell\_death | NOTCH1 | 365 | 5 | 1.855359 | -0.894944 | 308 | 166.19 | 0.539578 |  |
| GO:0043067\_regulation\_of\_programmed\_cell\_death | GCLC | 365 | 5 | 1.855359 | -0.894944 | 308 | 166.19 | 0.539578 |  |
| GO:0043067\_regulation\_of\_programmed\_cell\_death | ENDOG | 365 | 5 | 1.855359 | -0.894944 | 308 | 166.19 | 0.539578 |  |
| GO:0043067\_regulation\_of\_programmed\_cell\_death | BAX | 365 | 5 | 1.855359 | -0.894944 | 308 | 166.19 | 0.539578 |  |
| GO:0043067\_regulation\_of\_programmed\_cell\_death | BAD | 365 | 5 | 1.855359 | -0.894944 | 308 | 166.19 | 0.539578 |  |
| GO:0032502\_developmental\_process | FRAS1 | 2060 | 19 | 1.249215 | -0.894401 | 309 | 166.23 | 0.537961 |  |
| GO:0032502\_developmental\_process | ALDH6A1 | 2060 | 19 | 1.249215 | -0.894401 | 309 | 166.23 | 0.537961 |  |
| GO:0032502\_developmental\_process | NUP133 | 2060 | 19 | 1.249215 | -0.894401 | 309 | 166.23 | 0.537961 |  |
| GO:0032502\_developmental\_process | GCLC | 2060 | 19 | 1.249215 | -0.894401 | 309 | 166.23 | 0.537961 |  |
| GO:0032502\_developmental\_process | PDPN | 2060 | 19 | 1.249215 | -0.894401 | 309 | 166.23 | 0.537961 |  |
| GO:0032502\_developmental\_process | RB1 | 2060 | 19 | 1.249215 | -0.894401 | 309 | 166.23 | 0.537961 |  |
| GO:0032502\_developmental\_process | BAD | 2060 | 19 | 1.249215 | -0.894401 | 309 | 166.23 | 0.537961 |  |
| GO:0032502\_developmental\_process | VAX1 | 2060 | 19 | 1.249215 | -0.894401 | 309 | 166.23 | 0.537961 |  |
| GO:0032502\_developmental\_process | CDH4 | 2060 | 19 | 1.249215 | -0.894401 | 309 | 166.23 | 0.537961 |  |
| GO:0032502\_developmental\_process | FEZF2 | 2060 | 19 | 1.249215 | -0.894401 | 309 | 166.23 | 0.537961 |  |
| GO:0032502\_developmental\_process | NOTCH1 | 2060 | 19 | 1.249215 | -0.894401 | 309 | 166.23 | 0.537961 |  |
| GO:0032502\_developmental\_process | BAX | 2060 | 19 | 1.249215 | -0.894401 | 309 | 166.23 | 0.537961 |  |
| GO:0032502\_developmental\_process | ENDOG | 2060 | 19 | 1.249215 | -0.894401 | 309 | 166.23 | 0.537961 |  |
| GO:0032502\_developmental\_process | VWC2 | 2060 | 19 | 1.249215 | -0.894401 | 309 | 166.23 | 0.537961 |  |
| GO:0032502\_developmental\_process | ROR2 | 2060 | 19 | 1.249215 | -0.894401 | 309 | 166.23 | 0.537961 |  |
| GO:0032502\_developmental\_process | HBB-B1 | 2060 | 19 | 1.249215 | -0.894401 | 309 | 166.23 | 0.537961 |  |
| GO:0032502\_developmental\_process | SEMA3A | 2060 | 19 | 1.249215 | -0.894401 | 309 | 166.23 | 0.537961 |  |
| GO:0032502\_developmental\_process | RBPJ | 2060 | 19 | 1.249215 | -0.894401 | 309 | 166.23 | 0.537961 |  |
| GO:0032502\_developmental\_process | IFRD1 | 2060 | 19 | 1.249215 | -0.894401 | 309 | 166.23 | 0.537961 |  |
| GO:0080090\_regulation\_of\_primary\_metabolic\_process | PKNOX2 | 926 | 10 | 1.462648 | -0.892530 | 310 | 166.55 | 0.537258 |  |
| GO:0080090\_regulation\_of\_primary\_metabolic\_process | NOTCH1 | 926 | 10 | 1.462648 | -0.892530 | 310 | 166.55 | 0.537258 |  |
| GO:0080090\_regulation\_of\_primary\_metabolic\_process | GCLC | 926 | 10 | 1.462648 | -0.892530 | 310 | 166.55 | 0.537258 |  |
| GO:0080090\_regulation\_of\_primary\_metabolic\_process | NR1D2 | 926 | 10 | 1.462648 | -0.892530 | 310 | 166.55 | 0.537258 |  |
| GO:0080090\_regulation\_of\_primary\_metabolic\_process | BAX | 926 | 10 | 1.462648 | -0.892530 | 310 | 166.55 | 0.537258 |  |
| GO:0080090\_regulation\_of\_primary\_metabolic\_process | HBB-B1 | 926 | 10 | 1.462648 | -0.892530 | 310 | 166.55 | 0.537258 |  |
| GO:0080090\_regulation\_of\_primary\_metabolic\_process | NFYB | 926 | 10 | 1.462648 | -0.892530 | 310 | 166.55 | 0.537258 |  |
| GO:0080090\_regulation\_of\_primary\_metabolic\_process | VAX1 | 926 | 10 | 1.462648 | -0.892530 | 310 | 166.55 | 0.537258 |  |
| GO:0080090\_regulation\_of\_primary\_metabolic\_process | RB1 | 926 | 10 | 1.462648 | -0.892530 | 310 | 166.55 | 0.537258 |  |
| GO:0080090\_regulation\_of\_primary\_metabolic\_process | RBPJ | 926 | 10 | 1.462648 | -0.892530 | 310 | 166.55 | 0.537258 |  |
| GO:0051246\_regulation\_of\_protein\_metabolic\_process | GCLC | 170 | 3 | 2.390138 | -0.889994 | 311 | 166.73 | 0.536109 |  |
| GO:0051246\_regulation\_of\_protein\_metabolic\_process | BAX | 170 | 3 | 2.390138 | -0.889994 | 311 | 166.73 | 0.536109 |  |
| GO:0051246\_regulation\_of\_protein\_metabolic\_process | HBB-B1 | 170 | 3 | 2.390138 | -0.889994 | 311 | 166.73 | 0.536109 |  |
| GO:0010952\_positive\_regulation\_of\_peptidase\_activity | BAX | 19 | 1 | 7.128483 | -0.880810 | 314 | 171.69 | 0.546783 |  |
| GO:0043280\_positive\_regulation\_of\_caspase\_activity | BAX | 19 | 1 | 7.128483 | -0.880810 | 314 | 171.69 | 0.546783 |  |
| GO:0048547\_gut\_morphogenesis | NOTCH1 | 19 | 1 | 7.128483 | -0.880810 | 314 | 171.69 | 0.546783 |  |
| GO:0006350\_transcription | PKNOX2 | 701 | 8 | 1.545691 | -0.872606 | 315 | 173.39 | 0.550444 |  |
| GO:0006350\_transcription | NOTCH1 | 701 | 8 | 1.545691 | -0.872606 | 315 | 173.39 | 0.550444 |  |
| GO:0006350\_transcription | POLR1E | 701 | 8 | 1.545691 | -0.872606 | 315 | 173.39 | 0.550444 |  |
| GO:0006350\_transcription | NR1D2 | 701 | 8 | 1.545691 | -0.872606 | 315 | 173.39 | 0.550444 |  |
| GO:0006350\_transcription | NFYB | 701 | 8 | 1.545691 | -0.872606 | 315 | 173.39 | 0.550444 |  |
| GO:0006350\_transcription | RB1 | 701 | 8 | 1.545691 | -0.872606 | 315 | 173.39 | 0.550444 |  |
| GO:0006350\_transcription | VAX1 | 701 | 8 | 1.545691 | -0.872606 | 315 | 173.39 | 0.550444 |  |
| GO:0006350\_transcription | RBPJ | 701 | 8 | 1.545691 | -0.872606 | 315 | 173.39 | 0.550444 |  |
| GO:0060255\_regulation\_of\_macromolecule\_metabolic\_process | PKNOX2 | 936 | 10 | 1.447021 | -0.869073 | 316 | 174.33 | 0.551677 |  |
| GO:0060255\_regulation\_of\_macromolecule\_metabolic\_process | NOTCH1 | 936 | 10 | 1.447021 | -0.869073 | 316 | 174.33 | 0.551677 |  |
| GO:0060255\_regulation\_of\_macromolecule\_metabolic\_process | GCLC | 936 | 10 | 1.447021 | -0.869073 | 316 | 174.33 | 0.551677 |  |
| GO:0060255\_regulation\_of\_macromolecule\_metabolic\_process | NR1D2 | 936 | 10 | 1.447021 | -0.869073 | 316 | 174.33 | 0.551677 |  |
| GO:0060255\_regulation\_of\_macromolecule\_metabolic\_process | BAX | 936 | 10 | 1.447021 | -0.869073 | 316 | 174.33 | 0.551677 |  |
| GO:0060255\_regulation\_of\_macromolecule\_metabolic\_process | NFYB | 936 | 10 | 1.447021 | -0.869073 | 316 | 174.33 | 0.551677 |  |
| GO:0060255\_regulation\_of\_macromolecule\_metabolic\_process | HBB-B1 | 936 | 10 | 1.447021 | -0.869073 | 316 | 174.33 | 0.551677 |  |
| GO:0060255\_regulation\_of\_macromolecule\_metabolic\_process | VAX1 | 936 | 10 | 1.447021 | -0.869073 | 316 | 174.33 | 0.551677 |  |
| GO:0060255\_regulation\_of\_macromolecule\_metabolic\_process | RB1 | 936 | 10 | 1.447021 | -0.869073 | 316 | 174.33 | 0.551677 |  |
| GO:0060255\_regulation\_of\_macromolecule\_metabolic\_process | RBPJ | 936 | 10 | 1.447021 | -0.869073 | 316 | 174.33 | 0.551677 |  |
| GO:0051252\_regulation\_of\_RNA\_metabolic\_process | PKNOX2 | 590 | 7 | 1.606929 | -0.866559 | 317 | 174.72 | 0.551167 |  |
| GO:0051252\_regulation\_of\_RNA\_metabolic\_process | NOTCH1 | 590 | 7 | 1.606929 | -0.866559 | 317 | 174.72 | 0.551167 |  |
| GO:0051252\_regulation\_of\_RNA\_metabolic\_process | NR1D2 | 590 | 7 | 1.606929 | -0.866559 | 317 | 174.72 | 0.551167 |  |
| GO:0051252\_regulation\_of\_RNA\_metabolic\_process | NFYB | 590 | 7 | 1.606929 | -0.866559 | 317 | 174.72 | 0.551167 |  |
| GO:0051252\_regulation\_of\_RNA\_metabolic\_process | RB1 | 590 | 7 | 1.606929 | -0.866559 | 317 | 174.72 | 0.551167 |  |
| GO:0051252\_regulation\_of\_RNA\_metabolic\_process | VAX1 | 590 | 7 | 1.606929 | -0.866559 | 317 | 174.72 | 0.551167 |  |
| GO:0051252\_regulation\_of\_RNA\_metabolic\_process | RBPJ | 590 | 7 | 1.606929 | -0.866559 | 317 | 174.72 | 0.551167 |  |
| GO:0006518\_peptide\_metabolic\_process | GCLC | 20 | 1 | 6.772059 | -0.860063 | 322 | 179.79 | 0.558354 |  |
| GO:0018209\_peptidyl-serine\_modification | BAX | 20 | 1 | 6.772059 | -0.860063 | 322 | 179.79 | 0.558354 |  |
| GO:0042326\_negative\_regulation\_of\_phosphorylation | BAX | 20 | 1 | 6.772059 | -0.860063 | 322 | 179.79 | 0.558354 |  |
| GO:0045639\_positive\_regulation\_of\_myeloid\_cell\_differentiation | RB1 | 20 | 1 | 6.772059 | -0.860063 | 322 | 179.79 | 0.558354 |  |
| GO:0048806\_genitalia\_development | ROR2 | 20 | 1 | 6.772059 | -0.860063 | 322 | 179.79 | 0.558354 |  |
| GO:0043066\_negative\_regulation\_of\_apoptosis | NOTCH1 | 176 | 3 | 2.308656 | -0.857244 | 323 | 180.11 | 0.557616 |  |
| GO:0043066\_negative\_regulation\_of\_apoptosis | GCLC | 176 | 3 | 2.308656 | -0.857244 | 323 | 180.11 | 0.557616 |  |
| GO:0043066\_negative\_regulation\_of\_apoptosis | BAX | 176 | 3 | 2.308656 | -0.857244 | 323 | 180.11 | 0.557616 |  |
| GO:0048856\_anatomical\_structure\_development | FRAS1 | 1688 | 16 | 1.283803 | -0.855745 | 324 | 180.3 | 0.556481 |  |
| GO:0048856\_anatomical\_structure\_development | NUP133 | 1688 | 16 | 1.283803 | -0.855745 | 324 | 180.3 | 0.556481 |  |
| GO:0048856\_anatomical\_structure\_development | PDPN | 1688 | 16 | 1.283803 | -0.855745 | 324 | 180.3 | 0.556481 |  |
| GO:0048856\_anatomical\_structure\_development | BAD | 1688 | 16 | 1.283803 | -0.855745 | 324 | 180.3 | 0.556481 |  |
| GO:0048856\_anatomical\_structure\_development | VAX1 | 1688 | 16 | 1.283803 | -0.855745 | 324 | 180.3 | 0.556481 |  |
| GO:0048856\_anatomical\_structure\_development | RB1 | 1688 | 16 | 1.283803 | -0.855745 | 324 | 180.3 | 0.556481 |  |
| GO:0048856\_anatomical\_structure\_development | CDH4 | 1688 | 16 | 1.283803 | -0.855745 | 324 | 180.3 | 0.556481 |  |
| GO:0048856\_anatomical\_structure\_development | FEZF2 | 1688 | 16 | 1.283803 | -0.855745 | 324 | 180.3 | 0.556481 |  |
| GO:0048856\_anatomical\_structure\_development | NOTCH1 | 1688 | 16 | 1.283803 | -0.855745 | 324 | 180.3 | 0.556481 |  |
| GO:0048856\_anatomical\_structure\_development | BAX | 1688 | 16 | 1.283803 | -0.855745 | 324 | 180.3 | 0.556481 |  |
| GO:0048856\_anatomical\_structure\_development | VWC2 | 1688 | 16 | 1.283803 | -0.855745 | 324 | 180.3 | 0.556481 |  |
| GO:0048856\_anatomical\_structure\_development | ROR2 | 1688 | 16 | 1.283803 | -0.855745 | 324 | 180.3 | 0.556481 |  |
| GO:0048856\_anatomical\_structure\_development | HBB-B1 | 1688 | 16 | 1.283803 | -0.855745 | 324 | 180.3 | 0.556481 |  |
| GO:0048856\_anatomical\_structure\_development | SEMA3A | 1688 | 16 | 1.283803 | -0.855745 | 324 | 180.3 | 0.556481 |  |
| GO:0048856\_anatomical\_structure\_development | RBPJ | 1688 | 16 | 1.283803 | -0.855745 | 324 | 180.3 | 0.556481 |  |
| GO:0048856\_anatomical\_structure\_development | IFRD1 | 1688 | 16 | 1.283803 | -0.855745 | 324 | 180.3 | 0.556481 |  |
| GO:0033036\_macromolecule\_localization | FRAS1 | 274 | 4 | 1.977243 | -0.851583 | 325 | 180.85 | 0.556462 |  |
| GO:0033036\_macromolecule\_localization | SEC23A | 274 | 4 | 1.977243 | -0.851583 | 325 | 180.85 | 0.556462 |  |
| GO:0033036\_macromolecule\_localization | NUP133 | 274 | 4 | 1.977243 | -0.851583 | 325 | 180.85 | 0.556462 |  |
| GO:0033036\_macromolecule\_localization | BAX | 274 | 4 | 1.977243 | -0.851583 | 325 | 180.85 | 0.556462 |  |
| GO:0030324\_lung\_development | NOTCH1 | 90 | 2 | 3.009804 | -0.847855 | 326 | 181.26 | 0.556012 |  |
| GO:0030324\_lung\_development | PDPN | 90 | 2 | 3.009804 | -0.847855 | 326 | 181.26 | 0.556012 |  |
| GO:0050789\_regulation\_of\_biological\_process | GCLC | 2357 | 21 | 1.206731 | -0.844814 | 327 | 181.36 | 0.554618 |  |
| GO:0050789\_regulation\_of\_biological\_process | PDPN | 2357 | 21 | 1.206731 | -0.844814 | 327 | 181.36 | 0.554618 |  |
| GO:0050789\_regulation\_of\_biological\_process | NFYB | 2357 | 21 | 1.206731 | -0.844814 | 327 | 181.36 | 0.554618 |  |
| GO:0050789\_regulation\_of\_biological\_process | BAD | 2357 | 21 | 1.206731 | -0.844814 | 327 | 181.36 | 0.554618 |  |
| GO:0050789\_regulation\_of\_biological\_process | RB1 | 2357 | 21 | 1.206731 | -0.844814 | 327 | 181.36 | 0.554618 |  |
| GO:0050789\_regulation\_of\_biological\_process | VAX1 | 2357 | 21 | 1.206731 | -0.844814 | 327 | 181.36 | 0.554618 |  |
| GO:0050789\_regulation\_of\_biological\_process | RGS16 | 2357 | 21 | 1.206731 | -0.844814 | 327 | 181.36 | 0.554618 |  |
| GO:0050789\_regulation\_of\_biological\_process | CDH4 | 2357 | 21 | 1.206731 | -0.844814 | 327 | 181.36 | 0.554618 |  |
| GO:0050789\_regulation\_of\_biological\_process | PKNOX2 | 2357 | 21 | 1.206731 | -0.844814 | 327 | 181.36 | 0.554618 |  |
| GO:0050789\_regulation\_of\_biological\_process | NOTCH1 | 2357 | 21 | 1.206731 | -0.844814 | 327 | 181.36 | 0.554618 |  |
| GO:0050789\_regulation\_of\_biological\_process | KRT18 | 2357 | 21 | 1.206731 | -0.844814 | 327 | 181.36 | 0.554618 |  |
| GO:0050789\_regulation\_of\_biological\_process | NR1D2 | 2357 | 21 | 1.206731 | -0.844814 | 327 | 181.36 | 0.554618 |  |
| GO:0050789\_regulation\_of\_biological\_process | SOS1 | 2357 | 21 | 1.206731 | -0.844814 | 327 | 181.36 | 0.554618 |  |
| GO:0050789\_regulation\_of\_biological\_process | ENDOG | 2357 | 21 | 1.206731 | -0.844814 | 327 | 181.36 | 0.554618 |  |
| GO:0050789\_regulation\_of\_biological\_process | BAX | 2357 | 21 | 1.206731 | -0.844814 | 327 | 181.36 | 0.554618 |  |
| GO:0050789\_regulation\_of\_biological\_process | RRAS2 | 2357 | 21 | 1.206731 | -0.844814 | 327 | 181.36 | 0.554618 |  |
| GO:0050789\_regulation\_of\_biological\_process | ROR2 | 2357 | 21 | 1.206731 | -0.844814 | 327 | 181.36 | 0.554618 |  |
| GO:0050789\_regulation\_of\_biological\_process | VWC2 | 2357 | 21 | 1.206731 | -0.844814 | 327 | 181.36 | 0.554618 |  |
| GO:0050789\_regulation\_of\_biological\_process | HBB-B1 | 2357 | 21 | 1.206731 | -0.844814 | 327 | 181.36 | 0.554618 |  |
| GO:0050789\_regulation\_of\_biological\_process | SEMA3A | 2357 | 21 | 1.206731 | -0.844814 | 327 | 181.36 | 0.554618 |  |
| GO:0050789\_regulation\_of\_biological\_process | RBPJ | 2357 | 21 | 1.206731 | -0.844814 | 327 | 181.36 | 0.554618 |  |
| GO:0043069\_negative\_regulation\_of\_programmed\_cell\_death | NOTCH1 | 179 | 3 | 2.269964 | -0.841442 | 329 | 181.74 | 0.552401 |  |
| GO:0043069\_negative\_regulation\_of\_programmed\_cell\_death | GCLC | 179 | 3 | 2.269964 | -0.841442 | 329 | 181.74 | 0.552401 |  |
| GO:0043069\_negative\_regulation\_of\_programmed\_cell\_death | BAX | 179 | 3 | 2.269964 | -0.841442 | 329 | 181.74 | 0.552401 |  |
| GO:0060548\_negative\_regulation\_of\_cell\_death | NOTCH1 | 179 | 3 | 2.269964 | -0.841442 | 329 | 181.74 | 0.552401 |  |
| GO:0060548\_negative\_regulation\_of\_cell\_death | GCLC | 179 | 3 | 2.269964 | -0.841442 | 329 | 181.74 | 0.552401 |  |
| GO:0060548\_negative\_regulation\_of\_cell\_death | BAX | 179 | 3 | 2.269964 | -0.841442 | 329 | 181.74 | 0.552401 |  |
| GO:0006944\_membrane\_fusion | BAX | 21 | 1 | 6.449580 | -0.840402 | 334 | 187.22 | 0.560539 |  |
| GO:0008637\_apoptotic\_mitochondrial\_changes | BAX | 21 | 1 | 6.449580 | -0.840402 | 334 | 187.22 | 0.560539 |  |
| GO:0010563\_negative\_regulation\_of\_phosphorus\_metabolic\_process | BAX | 21 | 1 | 6.449580 | -0.840402 | 334 | 187.22 | 0.560539 |  |
| GO:0045936\_negative\_regulation\_of\_phosphate\_metabolic\_process | BAX | 21 | 1 | 6.449580 | -0.840402 | 334 | 187.22 | 0.560539 |  |
| GO:0051271\_negative\_regulation\_of\_cell\_motion | SEMA3A | 21 | 1 | 6.449580 | -0.840402 | 334 | 187.22 | 0.560539 |  |
| GO:0030323\_respiratory\_tube\_development | NOTCH1 | 92 | 2 | 2.944373 | -0.832528 | 335 | 188.36 | 0.562269 |  |
| GO:0030323\_respiratory\_tube\_development | PDPN | 92 | 2 | 2.944373 | -0.832528 | 335 | 188.36 | 0.562269 |  |
| GO:0035107\_appendage\_morphogenesis | NOTCH1 | 93 | 2 | 2.912713 | -0.825020 | 337 | 190.0 | 0.563798 |  |
| GO:0035107\_appendage\_morphogenesis | BAX | 93 | 2 | 2.912713 | -0.825020 | 337 | 190.0 | 0.563798 |  |
| GO:0035108\_limb\_morphogenesis | NOTCH1 | 93 | 2 | 2.912713 | -0.825020 | 337 | 190.0 | 0.563798 |  |
| GO:0035108\_limb\_morphogenesis | BAX | 93 | 2 | 2.912713 | -0.825020 | 337 | 190.0 | 0.563798 |  |
| GO:0009896\_positive\_regulation\_of\_catabolic\_process | GCLC | 22 | 1 | 6.156417 | -0.821725 | 341 | 196.68 | 0.576774 |  |
| GO:0030335\_positive\_regulation\_of\_cell\_migration | RRAS2 | 22 | 1 | 6.156417 | -0.821725 | 341 | 196.68 | 0.576774 |  |
| GO:0034097\_response\_to\_cytokine\_stimulus | ENDOG | 22 | 1 | 6.156417 | -0.821725 | 341 | 196.68 | 0.576774 |  |
| GO:0051260\_protein\_homooligomerization | BAX | 22 | 1 | 6.156417 | -0.821725 | 341 | 196.68 | 0.576774 |  |
| GO:0009653\_anatomical\_structure\_morphogenesis | FRAS1 | 958 | 10 | 1.413791 | -0.819283 | 342 | 196.94 | 0.575848 |  |
| GO:0009653\_anatomical\_structure\_morphogenesis | FEZF2 | 958 | 10 | 1.413791 | -0.819283 | 342 | 196.94 | 0.575848 |  |
| GO:0009653\_anatomical\_structure\_morphogenesis | NOTCH1 | 958 | 10 | 1.413791 | -0.819283 | 342 | 196.94 | 0.575848 |  |
| GO:0009653\_anatomical\_structure\_morphogenesis | PDPN | 958 | 10 | 1.413791 | -0.819283 | 342 | 196.94 | 0.575848 |  |
| GO:0009653\_anatomical\_structure\_morphogenesis | BAX | 958 | 10 | 1.413791 | -0.819283 | 342 | 196.94 | 0.575848 |  |
| GO:0009653\_anatomical\_structure\_morphogenesis | ROR2 | 958 | 10 | 1.413791 | -0.819283 | 342 | 196.94 | 0.575848 |  |
| GO:0009653\_anatomical\_structure\_morphogenesis | VAX1 | 958 | 10 | 1.413791 | -0.819283 | 342 | 196.94 | 0.575848 |  |
| GO:0009653\_anatomical\_structure\_morphogenesis | SEMA3A | 958 | 10 | 1.413791 | -0.819283 | 342 | 196.94 | 0.575848 |  |
| GO:0009653\_anatomical\_structure\_morphogenesis | RBPJ | 958 | 10 | 1.413791 | -0.819283 | 342 | 196.94 | 0.575848 |  |
| GO:0009653\_anatomical\_structure\_morphogenesis | CDH4 | 958 | 10 | 1.413791 | -0.819283 | 342 | 196.94 | 0.575848 |  |
| GO:0042391\_regulation\_of\_membrane\_potential | GCLC | 95 | 2 | 2.851393 | -0.810304 | 343 | 198.78 | 0.579534 |  |
| GO:0042391\_regulation\_of\_membrane\_potential | BAX | 95 | 2 | 2.851393 | -0.810304 | 343 | 198.78 | 0.579534 |  |
| GO:0000082\_G1\_S\_transition\_of\_mitotic\_cell\_cycle | RB1 | 23 | 1 | 5.888747 | -0.803944 | 346 | 203.33 | 0.587659 |  |
| GO:0030512\_negative\_regulation\_of\_transforming\_growth\_factor\_beta\_receptor\_signaling\_pathway | VWC2 | 23 | 1 | 5.888747 | -0.803944 | 346 | 203.33 | 0.587659 |  |
| GO:0060445\_branching\_involved\_in\_salivary\_gland\_morphogenesis | SEMA3A | 23 | 1 | 5.888747 | -0.803944 | 346 | 203.33 | 0.587659 |  |
| GO:0048736\_appendage\_development | NOTCH1 | 96 | 2 | 2.821691 | -0.803091 | 348 | 203.96 | 0.586092 |  |
| GO:0048736\_appendage\_development | BAX | 96 | 2 | 2.821691 | -0.803091 | 348 | 203.96 | 0.586092 |  |
| GO:0060173\_limb\_development | NOTCH1 | 96 | 2 | 2.821691 | -0.803091 | 348 | 203.96 | 0.586092 |  |
| GO:0060173\_limb\_development | BAX | 96 | 2 | 2.821691 | -0.803091 | 348 | 203.96 | 0.586092 |  |
| GO:0042127\_regulation\_of\_cell\_proliferation | NOTCH1 | 393 | 5 | 1.723170 | -0.797114 | 349 | 204.42 | 0.585731 |  |
| GO:0042127\_regulation\_of\_cell\_proliferation | BAX | 393 | 5 | 1.723170 | -0.797114 | 349 | 204.42 | 0.585731 |  |
| GO:0042127\_regulation\_of\_cell\_proliferation | RB1 | 393 | 5 | 1.723170 | -0.797114 | 349 | 204.42 | 0.585731 |  |
| GO:0042127\_regulation\_of\_cell\_proliferation | VAX1 | 393 | 5 | 1.723170 | -0.797114 | 349 | 204.42 | 0.585731 |  |
| GO:0042127\_regulation\_of\_cell\_proliferation | RBPJ | 393 | 5 | 1.723170 | -0.797114 | 349 | 204.42 | 0.585731 |  |
| GO:0007548\_sex\_differentiation | BAX | 98 | 2 | 2.764106 | -0.788946 | 351 | 205.59 | 0.585726 |  |
| GO:0007548\_sex\_differentiation | ROR2 | 98 | 2 | 2.764106 | -0.788946 | 351 | 205.59 | 0.585726 |  |
| GO:0060541\_respiratory\_system\_development | NOTCH1 | 98 | 2 | 2.764106 | -0.788946 | 351 | 205.59 | 0.585726 |  |
| GO:0060541\_respiratory\_system\_development | PDPN | 98 | 2 | 2.764106 | -0.788946 | 351 | 205.59 | 0.585726 |  |
| GO:0001541\_ovarian\_follicle\_development | BAX | 24 | 1 | 5.643382 | -0.786983 | 357 | 210.18 | 0.588739 |  |
| GO:0007050\_cell\_cycle\_arrest | RB1 | 24 | 1 | 5.643382 | -0.786983 | 357 | 210.18 | 0.588739 |  |
| GO:0007204\_elevation\_of\_cytosolic\_calcium\_ion\_concentration | BAX | 24 | 1 | 5.643382 | -0.786983 | 357 | 210.18 | 0.588739 |  |
| GO:0008629\_induction\_of\_apoptosis\_by\_intracellular\_signals | BAX | 24 | 1 | 5.643382 | -0.786983 | 357 | 210.18 | 0.588739 |  |
| GO:0043588\_skin\_development | FRAS1 | 24 | 1 | 5.643382 | -0.786983 | 357 | 210.18 | 0.588739 |  |
| GO:0048546\_digestive\_tract\_morphogenesis | NOTCH1 | 24 | 1 | 5.643382 | -0.786983 | 357 | 210.18 | 0.588739 |  |
| GO:0001525\_angiogenesis | NOTCH1 | 100 | 2 | 2.708824 | -0.775163 | 358 | 211.04 | 0.589497 |  |
| GO:0001525\_angiogenesis | RBPJ | 100 | 2 | 2.708824 | -0.775163 | 358 | 211.04 | 0.589497 |  |
| GO:0007492\_endoderm\_development | NOTCH1 | 25 | 1 | 5.417647 | -0.770776 | 363 | 215.34 | 0.593223 |  |
| GO:0010811\_positive\_regulation\_of\_cell-substrate\_adhesion | VWC2 | 25 | 1 | 5.417647 | -0.770776 | 363 | 215.34 | 0.593223 |  |
| GO:0021983\_pituitary\_gland\_development | RBPJ | 25 | 1 | 5.417647 | -0.770776 | 363 | 215.34 | 0.593223 |  |
| GO:0035137\_hindlimb\_morphogenesis | NOTCH1 | 25 | 1 | 5.417647 | -0.770776 | 363 | 215.34 | 0.593223 |  |
| GO:0055123\_digestive\_system\_development | NOTCH1 | 25 | 1 | 5.417647 | -0.770776 | 363 | 215.34 | 0.593223 |  |
| GO:0040011\_locomotion | BAX | 295 | 4 | 1.836491 | -0.768972 | 364 | 215.72 | 0.592637 |  |
| GO:0040011\_locomotion | RRAS2 | 295 | 4 | 1.836491 | -0.768972 | 364 | 215.72 | 0.592637 |  |
| GO:0040011\_locomotion | VAX1 | 295 | 4 | 1.836491 | -0.768972 | 364 | 215.72 | 0.592637 |  |
| GO:0040011\_locomotion | SEMA3A | 295 | 4 | 1.836491 | -0.768972 | 364 | 215.72 | 0.592637 |  |
| GO:0010556\_regulation\_of\_macromolecule\_biosynthetic\_process | PKNOX2 | 745 | 8 | 1.454402 | -0.763016 | 365 | 216.59 | 0.593397 |  |
| GO:0010556\_regulation\_of\_macromolecule\_biosynthetic\_process | NOTCH1 | 745 | 8 | 1.454402 | -0.763016 | 365 | 216.59 | 0.593397 |  |
| GO:0010556\_regulation\_of\_macromolecule\_biosynthetic\_process | NR1D2 | 745 | 8 | 1.454402 | -0.763016 | 365 | 216.59 | 0.593397 |  |
| GO:0010556\_regulation\_of\_macromolecule\_biosynthetic\_process | NFYB | 745 | 8 | 1.454402 | -0.763016 | 365 | 216.59 | 0.593397 |  |
| GO:0010556\_regulation\_of\_macromolecule\_biosynthetic\_process | HBB-B1 | 745 | 8 | 1.454402 | -0.763016 | 365 | 216.59 | 0.593397 |  |
| GO:0010556\_regulation\_of\_macromolecule\_biosynthetic\_process | VAX1 | 745 | 8 | 1.454402 | -0.763016 | 365 | 216.59 | 0.593397 |  |
| GO:0010556\_regulation\_of\_macromolecule\_biosynthetic\_process | RB1 | 745 | 8 | 1.454402 | -0.763016 | 365 | 216.59 | 0.593397 |  |
| GO:0010556\_regulation\_of\_macromolecule\_biosynthetic\_process | RBPJ | 745 | 8 | 1.454402 | -0.763016 | 365 | 216.59 | 0.593397 |  |
| GO:0007405\_neuroblast\_proliferation | VAX1 | 26 | 1 | 5.209276 | -0.755262 | 375 | 221.05 | 0.589467 |  |
| GO:0009636\_response\_to\_toxin | BAX | 26 | 1 | 5.209276 | -0.755262 | 375 | 221.05 | 0.589467 |  |
| GO:0010212\_response\_to\_ionizing\_radiation | BAX | 26 | 1 | 5.209276 | -0.755262 | 375 | 221.05 | 0.589467 |  |
| GO:0010959\_regulation\_of\_metal\_ion\_transport | BAX | 26 | 1 | 5.209276 | -0.755262 | 375 | 221.05 | 0.589467 |  |
| GO:0019233\_sensory\_perception\_of\_pain | TAC1 | 26 | 1 | 5.209276 | -0.755262 | 375 | 221.05 | 0.589467 |  |
| GO:0045665\_negative\_regulation\_of\_neuron\_differentiation | NOTCH1 | 26 | 1 | 5.209276 | -0.755262 | 375 | 221.05 | 0.589467 |  |
| GO:0046530\_photoreceptor\_cell\_differentiation | NOTCH1 | 26 | 1 | 5.209276 | -0.755262 | 375 | 221.05 | 0.589467 |  |
| GO:0050873\_brown\_fat\_cell\_differentiation | ALDH6A1 | 26 | 1 | 5.209276 | -0.755262 | 375 | 221.05 | 0.589467 |  |
| GO:0051235\_maintenance\_of\_location | BAX | 26 | 1 | 5.209276 | -0.755262 | 375 | 221.05 | 0.589467 |  |
| GO:0051480\_cytosolic\_calcium\_ion\_homeostasis | BAX | 26 | 1 | 5.209276 | -0.755262 | 375 | 221.05 | 0.589467 |  |
| GO:0002009\_morphogenesis\_of\_an\_epithelium | FRAS1 | 198 | 3 | 2.052139 | -0.749348 | 377 | 222.17 | 0.589310 |  |
| GO:0002009\_morphogenesis\_of\_an\_epithelium | NOTCH1 | 198 | 3 | 2.052139 | -0.749348 | 377 | 222.17 | 0.589310 |  |
| GO:0002009\_morphogenesis\_of\_an\_epithelium | SEMA3A | 198 | 3 | 2.052139 | -0.749348 | 377 | 222.17 | 0.589310 |  |
| GO:0060429\_epithelium\_development | FRAS1 | 198 | 3 | 2.052139 | -0.749348 | 377 | 222.17 | 0.589310 |  |
| GO:0060429\_epithelium\_development | NOTCH1 | 198 | 3 | 2.052139 | -0.749348 | 377 | 222.17 | 0.589310 |  |
| GO:0060429\_epithelium\_development | SEMA3A | 198 | 3 | 2.052139 | -0.749348 | 377 | 222.17 | 0.589310 |  |
| GO:0048872\_homeostasis\_of\_number\_of\_cells | BAX | 105 | 2 | 2.579832 | -0.742192 | 378 | 222.96 | 0.589841 |  |
| GO:0048872\_homeostasis\_of\_number\_of\_cells | RB1 | 105 | 2 | 2.579832 | -0.742192 | 378 | 222.96 | 0.589841 |  |
| GO:0002761\_regulation\_of\_myeloid\_leukocyte\_differentiation | RB1 | 27 | 1 | 5.016340 | -0.740389 | 381 | 226.35 | 0.594094 |  |
| GO:0009411\_response\_to\_UV | BAX | 27 | 1 | 5.016340 | -0.740389 | 381 | 226.35 | 0.594094 |  |
| GO:0045582\_positive\_regulation\_of\_T\_cell\_differentiation | BAD | 27 | 1 | 5.016340 | -0.740389 | 381 | 226.35 | 0.594094 |  |
| GO:0009987\_cellular\_process | SORD | 3868 | 31 | 1.085490 | -0.737305 | 382 | 226.48 | 0.592880 |  |
| GO:0009987\_cellular\_process | GCLC | 3868 | 31 | 1.085490 | -0.737305 | 382 | 226.48 | 0.592880 |  |
| GO:0009987\_cellular\_process | UQCRC1 | 3868 | 31 | 1.085490 | -0.737305 | 382 | 226.48 | 0.592880 |  |
| GO:0009987\_cellular\_process | NFYB | 3868 | 31 | 1.085490 | -0.737305 | 382 | 226.48 | 0.592880 |  |
| GO:0009987\_cellular\_process | CDH4 | 3868 | 31 | 1.085490 | -0.737305 | 382 | 226.48 | 0.592880 |  |
| GO:0009987\_cellular\_process | NR1D2 | 3868 | 31 | 1.085490 | -0.737305 | 382 | 226.48 | 0.592880 |  |
| GO:0009987\_cellular\_process | SOS1 | 3868 | 31 | 1.085490 | -0.737305 | 382 | 226.48 | 0.592880 |  |
| GO:0009987\_cellular\_process | VWC2 | 3868 | 31 | 1.085490 | -0.737305 | 382 | 226.48 | 0.592880 |  |
| GO:0009987\_cellular\_process | HBB-B1 | 3868 | 31 | 1.085490 | -0.737305 | 382 | 226.48 | 0.592880 |  |
| GO:0009987\_cellular\_process | SEMA3A | 3868 | 31 | 1.085490 | -0.737305 | 382 | 226.48 | 0.592880 |  |
| GO:0009987\_cellular\_process | IFRD1 | 3868 | 31 | 1.085490 | -0.737305 | 382 | 226.48 | 0.592880 |  |
| GO:0009987\_cellular\_process | PHLDA1 | 3868 | 31 | 1.085490 | -0.737305 | 382 | 226.48 | 0.592880 |  |
| GO:0009987\_cellular\_process | SEC23A | 3868 | 31 | 1.085490 | -0.737305 | 382 | 226.48 | 0.592880 |  |
| GO:0009987\_cellular\_process | NUP133 | 3868 | 31 | 1.085490 | -0.737305 | 382 | 226.48 | 0.592880 |  |
| GO:0009987\_cellular\_process | ALDH6A1 | 3868 | 31 | 1.085490 | -0.737305 | 382 | 226.48 | 0.592880 |  |
| GO:0009987\_cellular\_process | POLR1E | 3868 | 31 | 1.085490 | -0.737305 | 382 | 226.48 | 0.592880 |  |
| GO:0009987\_cellular\_process | PDPN | 3868 | 31 | 1.085490 | -0.737305 | 382 | 226.48 | 0.592880 |  |
| GO:0009987\_cellular\_process | SPIRE1 | 3868 | 31 | 1.085490 | -0.737305 | 382 | 226.48 | 0.592880 |  |
| GO:0009987\_cellular\_process | VAX1 | 3868 | 31 | 1.085490 | -0.737305 | 382 | 226.48 | 0.592880 |  |
| GO:0009987\_cellular\_process | BAD | 3868 | 31 | 1.085490 | -0.737305 | 382 | 226.48 | 0.592880 |  |
| GO:0009987\_cellular\_process | RB1 | 3868 | 31 | 1.085490 | -0.737305 | 382 | 226.48 | 0.592880 |  |
| GO:0009987\_cellular\_process | RGS16 | 3868 | 31 | 1.085490 | -0.737305 | 382 | 226.48 | 0.592880 |  |
| GO:0009987\_cellular\_process | PKNOX2 | 3868 | 31 | 1.085490 | -0.737305 | 382 | 226.48 | 0.592880 |  |
| GO:0009987\_cellular\_process | FEZF2 | 3868 | 31 | 1.085490 | -0.737305 | 382 | 226.48 | 0.592880 |  |
| GO:0009987\_cellular\_process | NOTCH1 | 3868 | 31 | 1.085490 | -0.737305 | 382 | 226.48 | 0.592880 |  |
| GO:0009987\_cellular\_process | KRT18 | 3868 | 31 | 1.085490 | -0.737305 | 382 | 226.48 | 0.592880 |  |
| GO:0009987\_cellular\_process | RRAS2 | 3868 | 31 | 1.085490 | -0.737305 | 382 | 226.48 | 0.592880 |  |
| GO:0009987\_cellular\_process | BAX | 3868 | 31 | 1.085490 | -0.737305 | 382 | 226.48 | 0.592880 |  |
| GO:0009987\_cellular\_process | ENDOG | 3868 | 31 | 1.085490 | -0.737305 | 382 | 226.48 | 0.592880 |  |
| GO:0009987\_cellular\_process | ROR2 | 3868 | 31 | 1.085490 | -0.737305 | 382 | 226.48 | 0.592880 |  |
| GO:0009987\_cellular\_process | RBPJ | 3868 | 31 | 1.085490 | -0.737305 | 382 | 226.48 | 0.592880 |  |
| GO:0001568\_blood\_vessel\_development | NOTCH1 | 203 | 3 | 2.001594 | -0.727192 | 383 | 227.5 | 0.593995 |  |
| GO:0001568\_blood\_vessel\_development | BAX | 203 | 3 | 2.001594 | -0.727192 | 383 | 227.5 | 0.593995 |  |
| GO:0001568\_blood\_vessel\_development | RBPJ | 203 | 3 | 2.001594 | -0.727192 | 383 | 227.5 | 0.593995 |  |
| GO:0042100\_B\_cell\_proliferation | BAX | 28 | 1 | 4.837185 | -0.726111 | 386 | 230.54 | 0.597254 |  |
| GO:0050871\_positive\_regulation\_of\_B\_cell\_activation | BAD | 28 | 1 | 4.837185 | -0.726111 | 386 | 230.54 | 0.597254 |  |
| GO:0051188\_cofactor\_biosynthetic\_process | GCLC | 28 | 1 | 4.837185 | -0.726111 | 386 | 230.54 | 0.597254 |  |
| GO:0006417\_regulation\_of\_translation | HBB-B1 | 29 | 1 | 4.670385 | -0.712385 | 393 | 235.65 | 0.599618 |  |
| GO:0021761\_limbic\_system\_development | BAX | 29 | 1 | 4.670385 | -0.712385 | 393 | 235.65 | 0.599618 |  |
| GO:0042176\_regulation\_of\_protein\_catabolic\_process | GCLC | 29 | 1 | 4.670385 | -0.712385 | 393 | 235.65 | 0.599618 |  |
| GO:0045621\_positive\_regulation\_of\_lymphocyte\_differentiation | BAD | 29 | 1 | 4.670385 | -0.712385 | 393 | 235.65 | 0.599618 |  |
| GO:0048066\_pigmentation\_during\_development | BAX | 29 | 1 | 4.670385 | -0.712385 | 393 | 235.65 | 0.599618 |  |
| GO:0050769\_positive\_regulation\_of\_neurogenesis | CDH4 | 29 | 1 | 4.670385 | -0.712385 | 393 | 235.65 | 0.599618 |  |
| GO:0060041\_retina\_development\_in\_camera-type\_eye | BAX | 29 | 1 | 4.670385 | -0.712385 | 393 | 235.65 | 0.599618 |  |
| GO:0043010\_camera-type\_eye\_development | BAX | 110 | 2 | 2.462567 | -0.711187 | 394 | 236.16 | 0.599391 |  |
| GO:0043010\_camera-type\_eye\_development | VAX1 | 110 | 2 | 2.462567 | -0.711187 | 394 | 236.16 | 0.599391 |  |
| GO:0051171\_regulation\_of\_nitrogen\_compound\_metabolic\_process | PKNOX2 | 771 | 8 | 1.405356 | -0.704159 | 395 | 236.87 | 0.599671 |  |
| GO:0051171\_regulation\_of\_nitrogen\_compound\_metabolic\_process | NOTCH1 | 771 | 8 | 1.405356 | -0.704159 | 395 | 236.87 | 0.599671 |  |
| GO:0051171\_regulation\_of\_nitrogen\_compound\_metabolic\_process | NR1D2 | 771 | 8 | 1.405356 | -0.704159 | 395 | 236.87 | 0.599671 |  |
| GO:0051171\_regulation\_of\_nitrogen\_compound\_metabolic\_process | BAX | 771 | 8 | 1.405356 | -0.704159 | 395 | 236.87 | 0.599671 |  |
| GO:0051171\_regulation\_of\_nitrogen\_compound\_metabolic\_process | NFYB | 771 | 8 | 1.405356 | -0.704159 | 395 | 236.87 | 0.599671 |  |
| GO:0051171\_regulation\_of\_nitrogen\_compound\_metabolic\_process | VAX1 | 771 | 8 | 1.405356 | -0.704159 | 395 | 236.87 | 0.599671 |  |
| GO:0051171\_regulation\_of\_nitrogen\_compound\_metabolic\_process | RB1 | 771 | 8 | 1.405356 | -0.704159 | 395 | 236.87 | 0.599671 |  |
| GO:0051171\_regulation\_of\_nitrogen\_compound\_metabolic\_process | RBPJ | 771 | 8 | 1.405356 | -0.704159 | 395 | 236.87 | 0.599671 |  |
| GO:0031323\_regulation\_of\_cellular\_metabolic\_process | PKNOX2 | 1015 | 10 | 1.334396 | -0.701189 | 396 | 237.01 | 0.598510 |  |
| GO:0031323\_regulation\_of\_cellular\_metabolic\_process | NOTCH1 | 1015 | 10 | 1.334396 | -0.701189 | 396 | 237.01 | 0.598510 |  |
| GO:0031323\_regulation\_of\_cellular\_metabolic\_process | GCLC | 1015 | 10 | 1.334396 | -0.701189 | 396 | 237.01 | 0.598510 |  |
| GO:0031323\_regulation\_of\_cellular\_metabolic\_process | NR1D2 | 1015 | 10 | 1.334396 | -0.701189 | 396 | 237.01 | 0.598510 |  |
| GO:0031323\_regulation\_of\_cellular\_metabolic\_process | BAX | 1015 | 10 | 1.334396 | -0.701189 | 396 | 237.01 | 0.598510 |  |
| GO:0031323\_regulation\_of\_cellular\_metabolic\_process | HBB-B1 | 1015 | 10 | 1.334396 | -0.701189 | 396 | 237.01 | 0.598510 |  |
| GO:0031323\_regulation\_of\_cellular\_metabolic\_process | NFYB | 1015 | 10 | 1.334396 | -0.701189 | 396 | 237.01 | 0.598510 |  |
| GO:0031323\_regulation\_of\_cellular\_metabolic\_process | VAX1 | 1015 | 10 | 1.334396 | -0.701189 | 396 | 237.01 | 0.598510 |  |
| GO:0031323\_regulation\_of\_cellular\_metabolic\_process | RB1 | 1015 | 10 | 1.334396 | -0.701189 | 396 | 237.01 | 0.598510 |  |
| GO:0031323\_regulation\_of\_cellular\_metabolic\_process | RBPJ | 1015 | 10 | 1.334396 | -0.701189 | 396 | 237.01 | 0.598510 |  |
| GO:0002260\_lymphocyte\_homeostasis | BAX | 30 | 1 | 4.514706 | -0.699175 | 405 | 241.95 | 0.597407 |  |
| GO:0007219\_Notch\_signaling\_pathway | RBPJ | 30 | 1 | 4.514706 | -0.699175 | 405 | 241.95 | 0.597407 |  |
| GO:0007435\_salivary\_gland\_morphogenesis | SEMA3A | 30 | 1 | 4.514706 | -0.699175 | 405 | 241.95 | 0.597407 |  |
| GO:0033500\_carbohydrate\_homeostasis | BAD | 30 | 1 | 4.514706 | -0.699175 | 405 | 241.95 | 0.597407 |  |
| GO:0042593\_glucose\_homeostasis | BAD | 30 | 1 | 4.514706 | -0.699175 | 405 | 241.95 | 0.597407 |  |
| GO:0048565\_gut\_development | NOTCH1 | 30 | 1 | 4.514706 | -0.699175 | 405 | 241.95 | 0.597407 |  |
| GO:0051146\_striated\_muscle\_cell\_differentiation | RB1 | 30 | 1 | 4.514706 | -0.699175 | 405 | 241.95 | 0.597407 |  |
| GO:0060021\_palate\_development | VAX1 | 30 | 1 | 4.514706 | -0.699175 | 405 | 241.95 | 0.597407 |  |
| GO:0060740\_prostate\_gland\_epithelium\_morphogenesis | NOTCH1 | 30 | 1 | 4.514706 | -0.699175 | 405 | 241.95 | 0.597407 |  |
| GO:0048523\_negative\_regulation\_of\_cellular\_process | NOTCH1 | 774 | 8 | 1.399909 | -0.697631 | 406 | 242.17 | 0.596478 |  |
| GO:0048523\_negative\_regulation\_of\_cellular\_process | GCLC | 774 | 8 | 1.399909 | -0.697631 | 406 | 242.17 | 0.596478 |  |
| GO:0048523\_negative\_regulation\_of\_cellular\_process | BAX | 774 | 8 | 1.399909 | -0.697631 | 406 | 242.17 | 0.596478 |  |
| GO:0048523\_negative\_regulation\_of\_cellular\_process | VWC2 | 774 | 8 | 1.399909 | -0.697631 | 406 | 242.17 | 0.596478 |  |
| GO:0048523\_negative\_regulation\_of\_cellular\_process | SEMA3A | 774 | 8 | 1.399909 | -0.697631 | 406 | 242.17 | 0.596478 |  |
| GO:0048523\_negative\_regulation\_of\_cellular\_process | RB1 | 774 | 8 | 1.399909 | -0.697631 | 406 | 242.17 | 0.596478 |  |
| GO:0048523\_negative\_regulation\_of\_cellular\_process | VAX1 | 774 | 8 | 1.399909 | -0.697631 | 406 | 242.17 | 0.596478 |  |
| GO:0048523\_negative\_regulation\_of\_cellular\_process | RBPJ | 774 | 8 | 1.399909 | -0.697631 | 406 | 242.17 | 0.596478 |  |
| GO:0010468\_regulation\_of\_gene\_expression | PKNOX2 | 778 | 8 | 1.392711 | -0.689010 | 407 | 243.16 | 0.597445 |  |
| GO:0010468\_regulation\_of\_gene\_expression | NOTCH1 | 778 | 8 | 1.392711 | -0.689010 | 407 | 243.16 | 0.597445 |  |
| GO:0010468\_regulation\_of\_gene\_expression | NR1D2 | 778 | 8 | 1.392711 | -0.689010 | 407 | 243.16 | 0.597445 |  |
| GO:0010468\_regulation\_of\_gene\_expression | NFYB | 778 | 8 | 1.392711 | -0.689010 | 407 | 243.16 | 0.597445 |  |
| GO:0010468\_regulation\_of\_gene\_expression | HBB-B1 | 778 | 8 | 1.392711 | -0.689010 | 407 | 243.16 | 0.597445 |  |
| GO:0010468\_regulation\_of\_gene\_expression | VAX1 | 778 | 8 | 1.392711 | -0.689010 | 407 | 243.16 | 0.597445 |  |
| GO:0010468\_regulation\_of\_gene\_expression | RB1 | 778 | 8 | 1.392711 | -0.689010 | 407 | 243.16 | 0.597445 |  |
| GO:0010468\_regulation\_of\_gene\_expression | RBPJ | 778 | 8 | 1.392711 | -0.689010 | 407 | 243.16 | 0.597445 |  |
| GO:0006665\_sphingolipid\_metabolic\_process | BAX | 31 | 1 | 4.369070 | -0.686445 | 413 | 249.29 | 0.603608 |  |
| GO:0033555\_multicellular\_organismal\_response\_to\_stress | TAC1 | 31 | 1 | 4.369070 | -0.686445 | 413 | 249.29 | 0.603608 |  |
| GO:0043269\_regulation\_of\_ion\_transport | BAX | 31 | 1 | 4.369070 | -0.686445 | 413 | 249.29 | 0.603608 |  |
| GO:0048562\_embryonic\_organ\_morphogenesis | ROR2 | 31 | 1 | 4.369070 | -0.686445 | 413 | 249.29 | 0.603608 |  |
| GO:0051899\_membrane\_depolarization | GCLC | 31 | 1 | 4.369070 | -0.686445 | 413 | 249.29 | 0.603608 |  |
| GO:0060512\_prostate\_gland\_morphogenesis | NOTCH1 | 31 | 1 | 4.369070 | -0.686445 | 413 | 249.29 | 0.603608 |  |
| GO:0065007\_biological\_regulation | GCLC | 2593 | 22 | 1.149135 | -0.682397 | 414 | 249.81 | 0.603406 |  |
| GO:0065007\_biological\_regulation | PDPN | 2593 | 22 | 1.149135 | -0.682397 | 414 | 249.81 | 0.603406 |  |
| GO:0065007\_biological\_regulation | CRHBP | 2593 | 22 | 1.149135 | -0.682397 | 414 | 249.81 | 0.603406 |  |
| GO:0065007\_biological\_regulation | NFYB | 2593 | 22 | 1.149135 | -0.682397 | 414 | 249.81 | 0.603406 |  |
| GO:0065007\_biological\_regulation | BAD | 2593 | 22 | 1.149135 | -0.682397 | 414 | 249.81 | 0.603406 |  |
| GO:0065007\_biological\_regulation | RB1 | 2593 | 22 | 1.149135 | -0.682397 | 414 | 249.81 | 0.603406 |  |
| GO:0065007\_biological\_regulation | VAX1 | 2593 | 22 | 1.149135 | -0.682397 | 414 | 249.81 | 0.603406 |  |
| GO:0065007\_biological\_regulation | RGS16 | 2593 | 22 | 1.149135 | -0.682397 | 414 | 249.81 | 0.603406 |  |
| GO:0065007\_biological\_regulation | CDH4 | 2593 | 22 | 1.149135 | -0.682397 | 414 | 249.81 | 0.603406 |  |
| GO:0065007\_biological\_regulation | PKNOX2 | 2593 | 22 | 1.149135 | -0.682397 | 414 | 249.81 | 0.603406 |  |
| GO:0065007\_biological\_regulation | NOTCH1 | 2593 | 22 | 1.149135 | -0.682397 | 414 | 249.81 | 0.603406 |  |
| GO:0065007\_biological\_regulation | KRT18 | 2593 | 22 | 1.149135 | -0.682397 | 414 | 249.81 | 0.603406 |  |
| GO:0065007\_biological\_regulation | NR1D2 | 2593 | 22 | 1.149135 | -0.682397 | 414 | 249.81 | 0.603406 |  |
| GO:0065007\_biological\_regulation | SOS1 | 2593 | 22 | 1.149135 | -0.682397 | 414 | 249.81 | 0.603406 |  |
| GO:0065007\_biological\_regulation | ENDOG | 2593 | 22 | 1.149135 | -0.682397 | 414 | 249.81 | 0.603406 |  |
| GO:0065007\_biological\_regulation | BAX | 2593 | 22 | 1.149135 | -0.682397 | 414 | 249.81 | 0.603406 |  |
| GO:0065007\_biological\_regulation | RRAS2 | 2593 | 22 | 1.149135 | -0.682397 | 414 | 249.81 | 0.603406 |  |
| GO:0065007\_biological\_regulation | ROR2 | 2593 | 22 | 1.149135 | -0.682397 | 414 | 249.81 | 0.603406 |  |
| GO:0065007\_biological\_regulation | VWC2 | 2593 | 22 | 1.149135 | -0.682397 | 414 | 249.81 | 0.603406 |  |
| GO:0065007\_biological\_regulation | HBB-B1 | 2593 | 22 | 1.149135 | -0.682397 | 414 | 249.81 | 0.603406 |  |
| GO:0065007\_biological\_regulation | SEMA3A | 2593 | 22 | 1.149135 | -0.682397 | 414 | 249.81 | 0.603406 |  |
| GO:0065007\_biological\_regulation | RBPJ | 2593 | 22 | 1.149135 | -0.682397 | 414 | 249.81 | 0.603406 |  |
| GO:0044249\_cellular\_biosynthetic\_process | PKNOX2 | 1150 | 11 | 1.295524 | -0.680482 | 415 | 250.2 | 0.602892 |  |
| GO:0044249\_cellular\_biosynthetic\_process | NOTCH1 | 1150 | 11 | 1.295524 | -0.680482 | 415 | 250.2 | 0.602892 |  |
| GO:0044249\_cellular\_biosynthetic\_process | GCLC | 1150 | 11 | 1.295524 | -0.680482 | 415 | 250.2 | 0.602892 |  |
| GO:0044249\_cellular\_biosynthetic\_process | POLR1E | 1150 | 11 | 1.295524 | -0.680482 | 415 | 250.2 | 0.602892 |  |
| GO:0044249\_cellular\_biosynthetic\_process | NR1D2 | 1150 | 11 | 1.295524 | -0.680482 | 415 | 250.2 | 0.602892 |  |
| GO:0044249\_cellular\_biosynthetic\_process | NFYB | 1150 | 11 | 1.295524 | -0.680482 | 415 | 250.2 | 0.602892 |  |
| GO:0044249\_cellular\_biosynthetic\_process | HBB-B1 | 1150 | 11 | 1.295524 | -0.680482 | 415 | 250.2 | 0.602892 |  |
| GO:0044249\_cellular\_biosynthetic\_process | RB1 | 1150 | 11 | 1.295524 | -0.680482 | 415 | 250.2 | 0.602892 |  |
| GO:0044249\_cellular\_biosynthetic\_process | VAX1 | 1150 | 11 | 1.295524 | -0.680482 | 415 | 250.2 | 0.602892 |  |
| GO:0044249\_cellular\_biosynthetic\_process | RBPJ | 1150 | 11 | 1.295524 | -0.680482 | 415 | 250.2 | 0.602892 |  |
| GO:0044249\_cellular\_biosynthetic\_process | PHLDA1 | 1150 | 11 | 1.295524 | -0.680482 | 415 | 250.2 | 0.602892 |  |
| GO:0010467\_gene\_expression | PKNOX2 | 905 | 9 | 1.346929 | -0.676656 | 416 | 250.36 | 0.601827 |  |
| GO:0010467\_gene\_expression | NOTCH1 | 905 | 9 | 1.346929 | -0.676656 | 416 | 250.36 | 0.601827 |  |
| GO:0010467\_gene\_expression | POLR1E | 905 | 9 | 1.346929 | -0.676656 | 416 | 250.36 | 0.601827 |  |
| GO:0010467\_gene\_expression | NR1D2 | 905 | 9 | 1.346929 | -0.676656 | 416 | 250.36 | 0.601827 |  |
| GO:0010467\_gene\_expression | HBB-B1 | 905 | 9 | 1.346929 | -0.676656 | 416 | 250.36 | 0.601827 |  |
| GO:0010467\_gene\_expression | NFYB | 905 | 9 | 1.346929 | -0.676656 | 416 | 250.36 | 0.601827 |  |
| GO:0010467\_gene\_expression | RB1 | 905 | 9 | 1.346929 | -0.676656 | 416 | 250.36 | 0.601827 |  |
| GO:0010467\_gene\_expression | VAX1 | 905 | 9 | 1.346929 | -0.676656 | 416 | 250.36 | 0.601827 |  |
| GO:0010467\_gene\_expression | RBPJ | 905 | 9 | 1.346929 | -0.676656 | 416 | 250.36 | 0.601827 |  |
| GO:0048608\_reproductive\_structure\_development | NOTCH1 | 116 | 2 | 2.335193 | -0.676325 | 417 | 250.85 | 0.601559 |  |
| GO:0048608\_reproductive\_structure\_development | BAX | 116 | 2 | 2.335193 | -0.676325 | 417 | 250.85 | 0.601559 |  |
| GO:0051259\_protein\_oligomerization | BAX | 32 | 1 | 4.232537 | -0.674166 | 418 | 253.93 | 0.607488 |  |
| GO:0006357\_regulation\_of\_transcription\_from\_RNA\_polymerase\_II\_promoter | PKNOX2 | 435 | 5 | 1.556795 | -0.670494 | 419 | 254.58 | 0.607589 |  |
| GO:0006357\_regulation\_of\_transcription\_from\_RNA\_polymerase\_II\_promoter | NOTCH1 | 435 | 5 | 1.556795 | -0.670494 | 419 | 254.58 | 0.607589 |  |
| GO:0006357\_regulation\_of\_transcription\_from\_RNA\_polymerase\_II\_promoter | RB1 | 435 | 5 | 1.556795 | -0.670494 | 419 | 254.58 | 0.607589 |  |
| GO:0006357\_regulation\_of\_transcription\_from\_RNA\_polymerase\_II\_promoter | VAX1 | 435 | 5 | 1.556795 | -0.670494 | 419 | 254.58 | 0.607589 |  |
| GO:0006357\_regulation\_of\_transcription\_from\_RNA\_polymerase\_II\_promoter | RBPJ | 435 | 5 | 1.556795 | -0.670494 | 419 | 254.58 | 0.607589 |  |
| GO:0045892\_negative\_regulation\_of\_transcription\_\_DNA-dependent | RB1 | 218 | 3 | 1.863869 | -0.665266 | 420 | 255.25 | 0.607738 |  |
| GO:0045892\_negative\_regulation\_of\_transcription\_\_DNA-dependent | VAX1 | 218 | 3 | 1.863869 | -0.665266 | 420 | 255.25 | 0.607738 |  |
| GO:0045892\_negative\_regulation\_of\_transcription\_\_DNA-dependent | RBPJ | 218 | 3 | 1.863869 | -0.665266 | 420 | 255.25 | 0.607738 |  |
| GO:0006643\_membrane\_lipid\_metabolic\_process | BAX | 33 | 1 | 4.104278 | -0.662310 | 424 | 258.4 | 0.609434 |  |
| GO:0007431\_salivary\_gland\_development | SEMA3A | 33 | 1 | 4.104278 | -0.662310 | 424 | 258.4 | 0.609434 |  |
| GO:0008584\_male\_gonad\_development | BAX | 33 | 1 | 4.104278 | -0.662310 | 424 | 258.4 | 0.609434 |  |
| GO:0021987\_cerebral\_cortex\_development | BAX | 33 | 1 | 4.104278 | -0.662310 | 424 | 258.4 | 0.609434 |  |
| GO:0051253\_negative\_regulation\_of\_RNA\_metabolic\_process | RB1 | 220 | 3 | 1.846925 | -0.657488 | 425 | 259.02 | 0.609459 |  |
| GO:0051253\_negative\_regulation\_of\_RNA\_metabolic\_process | VAX1 | 220 | 3 | 1.846925 | -0.657488 | 425 | 259.02 | 0.609459 |  |
| GO:0051253\_negative\_regulation\_of\_RNA\_metabolic\_process | RBPJ | 220 | 3 | 1.846925 | -0.657488 | 425 | 259.02 | 0.609459 |  |
| GO:0007165\_signal\_transduction | KRT18 | 915 | 9 | 1.332208 | -0.657051 | 426 | 259.09 | 0.608192 |  |
| GO:0007165\_signal\_transduction | PDPN | 915 | 9 | 1.332208 | -0.657051 | 426 | 259.09 | 0.608192 |  |
| GO:0007165\_signal\_transduction | SOS1 | 915 | 9 | 1.332208 | -0.657051 | 426 | 259.09 | 0.608192 |  |
| GO:0007165\_signal\_transduction | RRAS2 | 915 | 9 | 1.332208 | -0.657051 | 426 | 259.09 | 0.608192 |  |
| GO:0007165\_signal\_transduction | VWC2 | 915 | 9 | 1.332208 | -0.657051 | 426 | 259.09 | 0.608192 |  |
| GO:0007165\_signal\_transduction | ROR2 | 915 | 9 | 1.332208 | -0.657051 | 426 | 259.09 | 0.608192 |  |
| GO:0007165\_signal\_transduction | BAD | 915 | 9 | 1.332208 | -0.657051 | 426 | 259.09 | 0.608192 |  |
| GO:0007165\_signal\_transduction | RBPJ | 915 | 9 | 1.332208 | -0.657051 | 426 | 259.09 | 0.608192 |  |
| GO:0007165\_signal\_transduction | RGS16 | 915 | 9 | 1.332208 | -0.657051 | 426 | 259.09 | 0.608192 |  |
| GO:0045449\_regulation\_of\_transcription | PKNOX2 | 676 | 7 | 1.402497 | -0.650925 | 427 | 259.67 | 0.608126 |  |
| GO:0045449\_regulation\_of\_transcription | NOTCH1 | 676 | 7 | 1.402497 | -0.650925 | 427 | 259.67 | 0.608126 |  |
| GO:0045449\_regulation\_of\_transcription | NR1D2 | 676 | 7 | 1.402497 | -0.650925 | 427 | 259.67 | 0.608126 |  |
| GO:0045449\_regulation\_of\_transcription | NFYB | 676 | 7 | 1.402497 | -0.650925 | 427 | 259.67 | 0.608126 |  |
| GO:0045449\_regulation\_of\_transcription | RB1 | 676 | 7 | 1.402497 | -0.650925 | 427 | 259.67 | 0.608126 |  |
| GO:0045449\_regulation\_of\_transcription | VAX1 | 676 | 7 | 1.402497 | -0.650925 | 427 | 259.67 | 0.608126 |  |
| GO:0045449\_regulation\_of\_transcription | RBPJ | 676 | 7 | 1.402497 | -0.650925 | 427 | 259.67 | 0.608126 |  |
| GO:0010720\_positive\_regulation\_of\_cell\_development | CDH4 | 34 | 1 | 3.983564 | -0.650851 | 430 | 263.49 | 0.612767 |  |
| GO:0030509\_BMP\_signaling\_pathway | VWC2 | 34 | 1 | 3.983564 | -0.650851 | 430 | 263.49 | 0.612767 |  |
| GO:0060443\_mammary\_gland\_morphogenesis | BAX | 34 | 1 | 3.983564 | -0.650851 | 430 | 263.49 | 0.612767 |  |
| GO:0006917\_induction\_of\_apoptosis | BAX | 121 | 2 | 2.238697 | -0.649047 | 433 | 264.7 | 0.611316 |  |
| GO:0006917\_induction\_of\_apoptosis | BAD | 121 | 2 | 2.238697 | -0.649047 | 433 | 264.7 | 0.611316 |  |
| GO:0012502\_induction\_of\_programmed\_cell\_death | BAX | 121 | 2 | 2.238697 | -0.649047 | 433 | 264.7 | 0.611316 |  |
| GO:0012502\_induction\_of\_programmed\_cell\_death | BAD | 121 | 2 | 2.238697 | -0.649047 | 433 | 264.7 | 0.611316 |  |
| GO:0051726\_regulation\_of\_cell\_cycle | BAX | 121 | 2 | 2.238697 | -0.649047 | 433 | 264.7 | 0.611316 |  |
| GO:0051726\_regulation\_of\_cell\_cycle | RB1 | 121 | 2 | 2.238697 | -0.649047 | 433 | 264.7 | 0.611316 |  |
| GO:0006366\_transcription\_from\_RNA\_polymerase\_II\_promoter | PKNOX2 | 444 | 5 | 1.525238 | -0.646105 | 434 | 265.45 | 0.611636 |  |
| GO:0006366\_transcription\_from\_RNA\_polymerase\_II\_promoter | NOTCH1 | 444 | 5 | 1.525238 | -0.646105 | 434 | 265.45 | 0.611636 |  |
| GO:0006366\_transcription\_from\_RNA\_polymerase\_II\_promoter | RB1 | 444 | 5 | 1.525238 | -0.646105 | 434 | 265.45 | 0.611636 |  |
| GO:0006366\_transcription\_from\_RNA\_polymerase\_II\_promoter | VAX1 | 444 | 5 | 1.525238 | -0.646105 | 434 | 265.45 | 0.611636 |  |
| GO:0006366\_transcription\_from\_RNA\_polymerase\_II\_promoter | RBPJ | 444 | 5 | 1.525238 | -0.646105 | 434 | 265.45 | 0.611636 |  |
| GO:0010810\_regulation\_of\_cell-substrate\_adhesion | VWC2 | 35 | 1 | 3.869748 | -0.639766 | 438 | 269.67 | 0.615685 |  |
| GO:0016567\_protein\_ubiquitination | GCLC | 35 | 1 | 3.869748 | -0.639766 | 438 | 269.67 | 0.615685 |  |
| GO:0051325\_interphase | RB1 | 35 | 1 | 3.869748 | -0.639766 | 438 | 269.67 | 0.615685 |  |
| GO:0051329\_interphase\_of\_mitotic\_cell\_cycle | RB1 | 35 | 1 | 3.869748 | -0.639766 | 438 | 269.67 | 0.615685 |  |
| GO:0009058\_biosynthetic\_process | PKNOX2 | 1175 | 11 | 1.267960 | -0.636338 | 439 | 269.92 | 0.614852 |  |
| GO:0009058\_biosynthetic\_process | NOTCH1 | 1175 | 11 | 1.267960 | -0.636338 | 439 | 269.92 | 0.614852 |  |
| GO:0009058\_biosynthetic\_process | GCLC | 1175 | 11 | 1.267960 | -0.636338 | 439 | 269.92 | 0.614852 |  |
| GO:0009058\_biosynthetic\_process | POLR1E | 1175 | 11 | 1.267960 | -0.636338 | 439 | 269.92 | 0.614852 |  |
| GO:0009058\_biosynthetic\_process | NR1D2 | 1175 | 11 | 1.267960 | -0.636338 | 439 | 269.92 | 0.614852 |  |
| GO:0009058\_biosynthetic\_process | NFYB | 1175 | 11 | 1.267960 | -0.636338 | 439 | 269.92 | 0.614852 |  |
| GO:0009058\_biosynthetic\_process | HBB-B1 | 1175 | 11 | 1.267960 | -0.636338 | 439 | 269.92 | 0.614852 |  |
| GO:0009058\_biosynthetic\_process | VAX1 | 1175 | 11 | 1.267960 | -0.636338 | 439 | 269.92 | 0.614852 |  |
| GO:0009058\_biosynthetic\_process | RB1 | 1175 | 11 | 1.267960 | -0.636338 | 439 | 269.92 | 0.614852 |  |
| GO:0009058\_biosynthetic\_process | RBPJ | 1175 | 11 | 1.267960 | -0.636338 | 439 | 269.92 | 0.614852 |  |
| GO:0009058\_biosynthetic\_process | PHLDA1 | 1175 | 11 | 1.267960 | -0.636338 | 439 | 269.92 | 0.614852 |  |
| GO:0001889\_liver\_development | NOTCH1 | 36 | 1 | 3.762255 | -0.629035 | 443 | 275.0 | 0.620767 |  |
| GO:0007368\_determination\_of\_left\_right\_symmetry | NOTCH1 | 36 | 1 | 3.762255 | -0.629035 | 443 | 275.0 | 0.620767 |  |
| GO:0022602\_ovulation\_cycle\_process | BAX | 36 | 1 | 3.762255 | -0.629035 | 443 | 275.0 | 0.620767 |  |
| GO:0042742\_defense\_response\_to\_bacterium | RBPJ | 36 | 1 | 3.762255 | -0.629035 | 443 | 275.0 | 0.620767 |  |
| GO:0001763\_morphogenesis\_of\_a\_branching\_structure | NOTCH1 | 125 | 2 | 2.167059 | -0.628289 | 444 | 275.36 | 0.620180 |  |
| GO:0001763\_morphogenesis\_of\_a\_branching\_structure | SEMA3A | 125 | 2 | 2.167059 | -0.628289 | 444 | 275.36 | 0.620180 |  |
| GO:0046649\_lymphocyte\_activation | BAX | 228 | 3 | 1.782121 | -0.627416 | 445 | 275.98 | 0.620180 |  |
| GO:0046649\_lymphocyte\_activation | BAD | 228 | 3 | 1.782121 | -0.627416 | 445 | 275.98 | 0.620180 |  |
| GO:0046649\_lymphocyte\_activation | RBPJ | 228 | 3 | 1.782121 | -0.627416 | 445 | 275.98 | 0.620180 |  |
| GO:0031326\_regulation\_of\_cellular\_biosynthetic\_process | PKNOX2 | 812 | 8 | 1.334396 | -0.619390 | 446 | 277.12 | 0.621345 |  |
| GO:0031326\_regulation\_of\_cellular\_biosynthetic\_process | NOTCH1 | 812 | 8 | 1.334396 | -0.619390 | 446 | 277.12 | 0.621345 |  |
| GO:0031326\_regulation\_of\_cellular\_biosynthetic\_process | NR1D2 | 812 | 8 | 1.334396 | -0.619390 | 446 | 277.12 | 0.621345 |  |
| GO:0031326\_regulation\_of\_cellular\_biosynthetic\_process | NFYB | 812 | 8 | 1.334396 | -0.619390 | 446 | 277.12 | 0.621345 |  |
| GO:0031326\_regulation\_of\_cellular\_biosynthetic\_process | HBB-B1 | 812 | 8 | 1.334396 | -0.619390 | 446 | 277.12 | 0.621345 |  |
| GO:0031326\_regulation\_of\_cellular\_biosynthetic\_process | RB1 | 812 | 8 | 1.334396 | -0.619390 | 446 | 277.12 | 0.621345 |  |
| GO:0031326\_regulation\_of\_cellular\_biosynthetic\_process | VAX1 | 812 | 8 | 1.334396 | -0.619390 | 446 | 277.12 | 0.621345 |  |
| GO:0031326\_regulation\_of\_cellular\_biosynthetic\_process | RBPJ | 812 | 8 | 1.334396 | -0.619390 | 446 | 277.12 | 0.621345 |  |
| GO:0009799\_determination\_of\_symmetry | NOTCH1 | 37 | 1 | 3.660572 | -0.618636 | 450 | 279.72 | 0.621600 |  |
| GO:0009855\_determination\_of\_bilateral\_symmetry | NOTCH1 | 37 | 1 | 3.660572 | -0.618636 | 450 | 279.72 | 0.621600 |  |
| GO:0042698\_ovulation\_cycle | BAX | 37 | 1 | 3.660572 | -0.618636 | 450 | 279.72 | 0.621600 |  |
| GO:0045785\_positive\_regulation\_of\_cell\_adhesion | VWC2 | 37 | 1 | 3.660572 | -0.618636 | 450 | 279.72 | 0.621600 |  |
| GO:0051649\_establishment\_of\_localization\_in\_cell | SEC23A | 342 | 4 | 1.584107 | -0.614020 | 451 | 280.42 | 0.621774 |  |
| GO:0051649\_establishment\_of\_localization\_in\_cell | NUP133 | 342 | 4 | 1.584107 | -0.614020 | 451 | 280.42 | 0.621774 |  |
| GO:0051649\_establishment\_of\_localization\_in\_cell | BAX | 342 | 4 | 1.584107 | -0.614020 | 451 | 280.42 | 0.621774 |  |
| GO:0051649\_establishment\_of\_localization\_in\_cell | SPIRE1 | 342 | 4 | 1.584107 | -0.614020 | 451 | 280.42 | 0.621774 |  |
| GO:0009889\_regulation\_of\_biosynthetic\_process | PKNOX2 | 815 | 8 | 1.329484 | -0.613550 | 452 | 280.61 | 0.620819 |  |
| GO:0009889\_regulation\_of\_biosynthetic\_process | NOTCH1 | 815 | 8 | 1.329484 | -0.613550 | 452 | 280.61 | 0.620819 |  |
| GO:0009889\_regulation\_of\_biosynthetic\_process | NR1D2 | 815 | 8 | 1.329484 | -0.613550 | 452 | 280.61 | 0.620819 |  |
| GO:0009889\_regulation\_of\_biosynthetic\_process | HBB-B1 | 815 | 8 | 1.329484 | -0.613550 | 452 | 280.61 | 0.620819 |  |
| GO:0009889\_regulation\_of\_biosynthetic\_process | NFYB | 815 | 8 | 1.329484 | -0.613550 | 452 | 280.61 | 0.620819 |  |
| GO:0009889\_regulation\_of\_biosynthetic\_process | RB1 | 815 | 8 | 1.329484 | -0.613550 | 452 | 280.61 | 0.620819 |  |
| GO:0009889\_regulation\_of\_biosynthetic\_process | VAX1 | 815 | 8 | 1.329484 | -0.613550 | 452 | 280.61 | 0.620819 |  |
| GO:0009889\_regulation\_of\_biosynthetic\_process | RBPJ | 815 | 8 | 1.329484 | -0.613550 | 452 | 280.61 | 0.620819 |  |
| GO:0001655\_urogenital\_system\_development | NOTCH1 | 128 | 2 | 2.116268 | -0.613301 | 453 | 281.13 | 0.620596 |  |
| GO:0001655\_urogenital\_system\_development | BAX | 128 | 2 | 2.116268 | -0.613301 | 453 | 281.13 | 0.620596 |  |
| GO:0008016\_regulation\_of\_heart\_contraction | SEMA3A | 38 | 1 | 3.564241 | -0.608554 | 455 | 286.05 | 0.628681 |  |
| GO:0045580\_regulation\_of\_T\_cell\_differentiation | BAD | 38 | 1 | 3.564241 | -0.608554 | 455 | 286.05 | 0.628681 |  |
| GO:0043285\_biopolymer\_catabolic\_process | GCLC | 129 | 2 | 2.099863 | -0.608411 | 456 | 286.35 | 0.627961 |  |
| GO:0043285\_biopolymer\_catabolic\_process | ENDOG | 129 | 2 | 2.099863 | -0.608411 | 456 | 286.35 | 0.627961 |  |
| GO:0044260\_cellular\_macromolecule\_metabolic\_process | GCLC | 1447 | 13 | 1.216818 | -0.608050 | 457 | 286.64 | 0.627221 |  |
| GO:0044260\_cellular\_macromolecule\_metabolic\_process | POLR1E | 1447 | 13 | 1.216818 | -0.608050 | 457 | 286.64 | 0.627221 |  |
| GO:0044260\_cellular\_macromolecule\_metabolic\_process | NFYB | 1447 | 13 | 1.216818 | -0.608050 | 457 | 286.64 | 0.627221 |  |
| GO:0044260\_cellular\_macromolecule\_metabolic\_process | VAX1 | 1447 | 13 | 1.216818 | -0.608050 | 457 | 286.64 | 0.627221 |  |
| GO:0044260\_cellular\_macromolecule\_metabolic\_process | RB1 | 1447 | 13 | 1.216818 | -0.608050 | 457 | 286.64 | 0.627221 |  |
| GO:0044260\_cellular\_macromolecule\_metabolic\_process | PKNOX2 | 1447 | 13 | 1.216818 | -0.608050 | 457 | 286.64 | 0.627221 |  |
| GO:0044260\_cellular\_macromolecule\_metabolic\_process | NOTCH1 | 1447 | 13 | 1.216818 | -0.608050 | 457 | 286.64 | 0.627221 |  |
| GO:0044260\_cellular\_macromolecule\_metabolic\_process | NR1D2 | 1447 | 13 | 1.216818 | -0.608050 | 457 | 286.64 | 0.627221 |  |
| GO:0044260\_cellular\_macromolecule\_metabolic\_process | ENDOG | 1447 | 13 | 1.216818 | -0.608050 | 457 | 286.64 | 0.627221 |  |
| GO:0044260\_cellular\_macromolecule\_metabolic\_process | BAX | 1447 | 13 | 1.216818 | -0.608050 | 457 | 286.64 | 0.627221 |  |
| GO:0044260\_cellular\_macromolecule\_metabolic\_process | HBB-B1 | 1447 | 13 | 1.216818 | -0.608050 | 457 | 286.64 | 0.627221 |  |
| GO:0044260\_cellular\_macromolecule\_metabolic\_process | RBPJ | 1447 | 13 | 1.216818 | -0.608050 | 457 | 286.64 | 0.627221 |  |
| GO:0044260\_cellular\_macromolecule\_metabolic\_process | PHLDA1 | 1447 | 13 | 1.216818 | -0.608050 | 457 | 286.64 | 0.627221 |  |
| GO:0045165\_cell\_fate\_commitment | NOTCH1 | 130 | 2 | 2.083710 | -0.603573 | 458 | 287.32 | 0.627336 |  |
| GO:0045165\_cell\_fate\_commitment | RBPJ | 130 | 2 | 2.083710 | -0.603573 | 458 | 287.32 | 0.627336 |  |
| GO:0006511\_ubiquitin-dependent\_protein\_catabolic\_process | GCLC | 39 | 1 | 3.472851 | -0.598770 | 462 | 291.0 | 0.629870 |  |
| GO:0042475\_odontogenesis\_of\_dentine-containing\_tooth | BAX | 39 | 1 | 3.472851 | -0.598770 | 462 | 291.0 | 0.629870 |  |
| GO:0043524\_negative\_regulation\_of\_neuron\_apoptosis | BAX | 39 | 1 | 3.472851 | -0.598770 | 462 | 291.0 | 0.629870 |  |
| GO:0048663\_neuron\_fate\_commitment | NOTCH1 | 39 | 1 | 3.472851 | -0.598770 | 462 | 291.0 | 0.629870 |  |
| GO:0007049\_cell\_cycle | PDPN | 238 | 3 | 1.707242 | -0.592026 | 463 | 291.9 | 0.630454 |  |
| GO:0007049\_cell\_cycle | BAX | 238 | 3 | 1.707242 | -0.592026 | 463 | 291.9 | 0.630454 |  |
| GO:0007049\_cell\_cycle | RB1 | 238 | 3 | 1.707242 | -0.592026 | 463 | 291.9 | 0.630454 |  |
| GO:0009952\_anterior\_posterior\_pattern\_formation | NOTCH1 | 133 | 2 | 2.036709 | -0.589358 | 464 | 292.31 | 0.629978 |  |
| GO:0009952\_anterior\_posterior\_pattern\_formation | ROR2 | 133 | 2 | 2.036709 | -0.589358 | 464 | 292.31 | 0.629978 |  |
| GO:0016358\_dendrite\_development | FEZF2 | 40 | 1 | 3.386029 | -0.589271 | 468 | 295.28 | 0.630940 |  |
| GO:0017015\_regulation\_of\_transforming\_growth\_factor\_beta\_receptor\_signaling\_pathway | VWC2 | 40 | 1 | 3.386029 | -0.589271 | 468 | 295.28 | 0.630940 |  |
| GO:0035272\_exocrine\_system\_development | SEMA3A | 40 | 1 | 3.386029 | -0.589271 | 468 | 295.28 | 0.630940 |  |
| GO:0051129\_negative\_regulation\_of\_cellular\_component\_organization | SEMA3A | 40 | 1 | 3.386029 | -0.589271 | 468 | 295.28 | 0.630940 |  |
| GO:0001776\_leukocyte\_homeostasis | BAX | 41 | 1 | 3.303443 | -0.580041 | 475 | 300.84 | 0.633347 |  |
| GO:0006865\_amino\_acid\_transport | PDPN | 41 | 1 | 3.303443 | -0.580041 | 475 | 300.84 | 0.633347 |  |
| GO:0007254\_JNK\_cascade | ROR2 | 41 | 1 | 3.303443 | -0.580041 | 475 | 300.84 | 0.633347 |  |
| GO:0008585\_female\_gonad\_development | BAX | 41 | 1 | 3.303443 | -0.580041 | 475 | 300.84 | 0.633347 |  |
| GO:0009894\_regulation\_of\_catabolic\_process | GCLC | 41 | 1 | 3.303443 | -0.580041 | 475 | 300.84 | 0.633347 |  |
| GO:0015980\_energy\_derivation\_by\_oxidation\_of\_organic\_compounds | UQCRC1 | 41 | 1 | 3.303443 | -0.580041 | 475 | 300.84 | 0.633347 |  |
| GO:0050864\_regulation\_of\_B\_cell\_activation | BAD | 41 | 1 | 3.303443 | -0.580041 | 475 | 300.84 | 0.633347 |  |
| GO:0001654\_eye\_development | BAX | 136 | 2 | 1.991782 | -0.575580 | 476 | 301.3 | 0.632983 |  |
| GO:0001654\_eye\_development | VAX1 | 136 | 2 | 1.991782 | -0.575580 | 476 | 301.3 | 0.632983 |  |
| GO:0009056\_catabolic\_process | GCLC | 243 | 3 | 1.672113 | -0.575187 | 477 | 301.52 | 0.632117 |  |
| GO:0009056\_catabolic\_process | ENDOG | 243 | 3 | 1.672113 | -0.575187 | 477 | 301.52 | 0.632117 |  |
| GO:0009056\_catabolic\_process | BAD | 243 | 3 | 1.672113 | -0.575187 | 477 | 301.52 | 0.632117 |  |
| GO:0009057\_macromolecule\_catabolic\_process | GCLC | 137 | 2 | 1.977243 | -0.571080 | 478 | 301.82 | 0.631423 |  |
| GO:0009057\_macromolecule\_catabolic\_process | ENDOG | 137 | 2 | 1.977243 | -0.571080 | 478 | 301.82 | 0.631423 |  |
| GO:0006006\_glucose\_metabolic\_process | BAD | 42 | 1 | 3.224790 | -0.571068 | 487 | 306.08 | 0.628501 |  |
| GO:0019941\_modification-dependent\_protein\_catabolic\_process | GCLC | 42 | 1 | 3.224790 | -0.571068 | 487 | 306.08 | 0.628501 |  |
| GO:0042476\_odontogenesis | BAX | 42 | 1 | 3.224790 | -0.571068 | 487 | 306.08 | 0.628501 |  |
| GO:0043632\_modification-dependent\_macromolecule\_catabolic\_process | GCLC | 42 | 1 | 3.224790 | -0.571068 | 487 | 306.08 | 0.628501 |  |
| GO:0045637\_regulation\_of\_myeloid\_cell\_differentiation | RB1 | 42 | 1 | 3.224790 | -0.571068 | 487 | 306.08 | 0.628501 |  |
| GO:0048515\_spermatid\_differentiation | BAX | 42 | 1 | 3.224790 | -0.571068 | 487 | 306.08 | 0.628501 |  |
| GO:0051345\_positive\_regulation\_of\_hydrolase\_activity | BAX | 42 | 1 | 3.224790 | -0.571068 | 487 | 306.08 | 0.628501 |  |
| GO:0051603\_proteolysis\_involved\_in\_cellular\_protein\_catabolic\_process | GCLC | 42 | 1 | 3.224790 | -0.571068 | 487 | 306.08 | 0.628501 |  |
| GO:0080135\_regulation\_of\_cellular\_response\_to\_stress | HBB-B1 | 42 | 1 | 3.224790 | -0.571068 | 487 | 306.08 | 0.628501 |  |
| GO:0019222\_regulation\_of\_metabolic\_process | PKNOX2 | 1088 | 10 | 1.244864 | -0.570675 | 488 | 306.24 | 0.627541 |  |
| GO:0019222\_regulation\_of\_metabolic\_process | NOTCH1 | 1088 | 10 | 1.244864 | -0.570675 | 488 | 306.24 | 0.627541 |  |
| GO:0019222\_regulation\_of\_metabolic\_process | GCLC | 1088 | 10 | 1.244864 | -0.570675 | 488 | 306.24 | 0.627541 |  |
| GO:0019222\_regulation\_of\_metabolic\_process | NR1D2 | 1088 | 10 | 1.244864 | -0.570675 | 488 | 306.24 | 0.627541 |  |
| GO:0019222\_regulation\_of\_metabolic\_process | BAX | 1088 | 10 | 1.244864 | -0.570675 | 488 | 306.24 | 0.627541 |  |
| GO:0019222\_regulation\_of\_metabolic\_process | HBB-B1 | 1088 | 10 | 1.244864 | -0.570675 | 488 | 306.24 | 0.627541 |  |
| GO:0019222\_regulation\_of\_metabolic\_process | NFYB | 1088 | 10 | 1.244864 | -0.570675 | 488 | 306.24 | 0.627541 |  |
| GO:0019222\_regulation\_of\_metabolic\_process | RB1 | 1088 | 10 | 1.244864 | -0.570675 | 488 | 306.24 | 0.627541 |  |
| GO:0019222\_regulation\_of\_metabolic\_process | VAX1 | 1088 | 10 | 1.244864 | -0.570675 | 488 | 306.24 | 0.627541 |  |
| GO:0019222\_regulation\_of\_metabolic\_process | RBPJ | 1088 | 10 | 1.244864 | -0.570675 | 488 | 306.24 | 0.627541 |  |
| GO:0016043\_cellular\_component\_organization | FEZF2 | 964 | 9 | 1.264492 | -0.567713 | 489 | 306.51 | 0.626810 |  |
| GO:0016043\_cellular\_component\_organization | NOTCH1 | 964 | 9 | 1.264492 | -0.567713 | 489 | 306.51 | 0.626810 |  |
| GO:0016043\_cellular\_component\_organization | PDPN | 964 | 9 | 1.264492 | -0.567713 | 489 | 306.51 | 0.626810 |  |
| GO:0016043\_cellular\_component\_organization | BAX | 964 | 9 | 1.264492 | -0.567713 | 489 | 306.51 | 0.626810 |  |
| GO:0016043\_cellular\_component\_organization | ENDOG | 964 | 9 | 1.264492 | -0.567713 | 489 | 306.51 | 0.626810 |  |
| GO:0016043\_cellular\_component\_organization | VAX1 | 964 | 9 | 1.264492 | -0.567713 | 489 | 306.51 | 0.626810 |  |
| GO:0016043\_cellular\_component\_organization | SEMA3A | 964 | 9 | 1.264492 | -0.567713 | 489 | 306.51 | 0.626810 |  |
| GO:0016043\_cellular\_component\_organization | RBPJ | 964 | 9 | 1.264492 | -0.567713 | 489 | 306.51 | 0.626810 |  |
| GO:0016043\_cellular\_component\_organization | CDH4 | 964 | 9 | 1.264492 | -0.567713 | 489 | 306.51 | 0.626810 |  |
| GO:0008152\_metabolic\_process | GCLC | 2133 | 18 | 1.142964 | -0.565197 | 490 | 306.85 | 0.626224 |  |
| GO:0008152\_metabolic\_process | SORD | 2133 | 18 | 1.142964 | -0.565197 | 490 | 306.85 | 0.626224 |  |
| GO:0008152\_metabolic\_process | UQCRC1 | 2133 | 18 | 1.142964 | -0.565197 | 490 | 306.85 | 0.626224 |  |
| GO:0008152\_metabolic\_process | POLR1E | 2133 | 18 | 1.142964 | -0.565197 | 490 | 306.85 | 0.626224 |  |
| GO:0008152\_metabolic\_process | PDPN | 2133 | 18 | 1.142964 | -0.565197 | 490 | 306.85 | 0.626224 |  |
| GO:0008152\_metabolic\_process | CRHBP | 2133 | 18 | 1.142964 | -0.565197 | 490 | 306.85 | 0.626224 |  |
| GO:0008152\_metabolic\_process | NFYB | 2133 | 18 | 1.142964 | -0.565197 | 490 | 306.85 | 0.626224 |  |
| GO:0008152\_metabolic\_process | BAD | 2133 | 18 | 1.142964 | -0.565197 | 490 | 306.85 | 0.626224 |  |
| GO:0008152\_metabolic\_process | VAX1 | 2133 | 18 | 1.142964 | -0.565197 | 490 | 306.85 | 0.626224 |  |
| GO:0008152\_metabolic\_process | RB1 | 2133 | 18 | 1.142964 | -0.565197 | 490 | 306.85 | 0.626224 |  |
| GO:0008152\_metabolic\_process | PKNOX2 | 2133 | 18 | 1.142964 | -0.565197 | 490 | 306.85 | 0.626224 |  |
| GO:0008152\_metabolic\_process | NOTCH1 | 2133 | 18 | 1.142964 | -0.565197 | 490 | 306.85 | 0.626224 |  |
| GO:0008152\_metabolic\_process | NR1D2 | 2133 | 18 | 1.142964 | -0.565197 | 490 | 306.85 | 0.626224 |  |
| GO:0008152\_metabolic\_process | ENDOG | 2133 | 18 | 1.142964 | -0.565197 | 490 | 306.85 | 0.626224 |  |
| GO:0008152\_metabolic\_process | BAX | 2133 | 18 | 1.142964 | -0.565197 | 490 | 306.85 | 0.626224 |  |
| GO:0008152\_metabolic\_process | HBB-B1 | 2133 | 18 | 1.142964 | -0.565197 | 490 | 306.85 | 0.626224 |  |
| GO:0008152\_metabolic\_process | RBPJ | 2133 | 18 | 1.142964 | -0.565197 | 490 | 306.85 | 0.626224 |  |
| GO:0008152\_metabolic\_process | PHLDA1 | 2133 | 18 | 1.142964 | -0.565197 | 490 | 306.85 | 0.626224 |  |
| GO:0006810\_transport | FRAS1 | 718 | 7 | 1.320457 | -0.564422 | 491 | 307.01 | 0.625275 |  |
| GO:0006810\_transport | SEC23A | 718 | 7 | 1.320457 | -0.564422 | 491 | 307.01 | 0.625275 |  |
| GO:0006810\_transport | NUP133 | 718 | 7 | 1.320457 | -0.564422 | 491 | 307.01 | 0.625275 |  |
| GO:0006810\_transport | PDPN | 718 | 7 | 1.320457 | -0.564422 | 491 | 307.01 | 0.625275 |  |
| GO:0006810\_transport | BAX | 718 | 7 | 1.320457 | -0.564422 | 491 | 307.01 | 0.625275 |  |
| GO:0006810\_transport | HBB-B1 | 718 | 7 | 1.320457 | -0.564422 | 491 | 307.01 | 0.625275 |  |
| GO:0006810\_transport | SPIRE1 | 718 | 7 | 1.320457 | -0.564422 | 491 | 307.01 | 0.625275 |  |
| GO:0007166\_cell\_surface\_receptor\_linked\_signal\_transduction | KRT18 | 597 | 6 | 1.361218 | -0.563139 | 492 | 307.34 | 0.624675 |  |
| GO:0007166\_cell\_surface\_receptor\_linked\_signal\_transduction | VWC2 | 597 | 6 | 1.361218 | -0.563139 | 492 | 307.34 | 0.624675 |  |
| GO:0007166\_cell\_surface\_receptor\_linked\_signal\_transduction | ROR2 | 597 | 6 | 1.361218 | -0.563139 | 492 | 307.34 | 0.624675 |  |
| GO:0007166\_cell\_surface\_receptor\_linked\_signal\_transduction | BAD | 597 | 6 | 1.361218 | -0.563139 | 492 | 307.34 | 0.624675 |  |
| GO:0007166\_cell\_surface\_receptor\_linked\_signal\_transduction | RBPJ | 597 | 6 | 1.361218 | -0.563139 | 492 | 307.34 | 0.624675 |  |
| GO:0007166\_cell\_surface\_receptor\_linked\_signal\_transduction | RGS16 | 597 | 6 | 1.361218 | -0.563139 | 492 | 307.34 | 0.624675 |  |
| GO:0001894\_tissue\_homeostasis | BAX | 43 | 1 | 3.149795 | -0.562340 | 497 | 311.28 | 0.626318 |  |
| GO:0006766\_vitamin\_metabolic\_process | GCLC | 43 | 1 | 3.149795 | -0.562340 | 497 | 311.28 | 0.626318 |  |
| GO:0010001\_glial\_cell\_differentiation | NOTCH1 | 43 | 1 | 3.149795 | -0.562340 | 497 | 311.28 | 0.626318 |  |
| GO:0031098\_stress-activated\_protein\_kinase\_signaling\_pathway | ROR2 | 43 | 1 | 3.149795 | -0.562340 | 497 | 311.28 | 0.626318 |  |
| GO:0032446\_protein\_modification\_by\_small\_protein\_conjugation | GCLC | 43 | 1 | 3.149795 | -0.562340 | 497 | 311.28 | 0.626318 |  |
| GO:0034613\_cellular\_protein\_localization | SEC23A | 139 | 2 | 1.948794 | -0.562217 | 498 | 311.63 | 0.625763 |  |
| GO:0034613\_cellular\_protein\_localization | BAX | 139 | 2 | 1.948794 | -0.562217 | 498 | 311.63 | 0.625763 |  |
| GO:0045321\_leukocyte\_activation | BAX | 248 | 3 | 1.638401 | -0.558886 | 499 | 312.16 | 0.625571 |  |
| GO:0045321\_leukocyte\_activation | BAD | 248 | 3 | 1.638401 | -0.558886 | 499 | 312.16 | 0.625571 |  |
| GO:0045321\_leukocyte\_activation | RBPJ | 248 | 3 | 1.638401 | -0.558886 | 499 | 312.16 | 0.625571 |  |
| GO:0044257\_cellular\_protein\_catabolic\_process | GCLC | 44 | 1 | 3.078209 | -0.553845 | 502 | 317.62 | 0.632709 |  |
| GO:0046545\_development\_of\_primary\_female\_sexual\_characteristics | BAX | 44 | 1 | 3.078209 | -0.553845 | 502 | 317.62 | 0.632709 |  |
| GO:0048593\_camera-type\_eye\_morphogenesis | BAX | 44 | 1 | 3.078209 | -0.553845 | 502 | 317.62 | 0.632709 |  |
| GO:0070727\_cellular\_macromolecule\_localization | SEC23A | 141 | 2 | 1.921151 | -0.553530 | 503 | 317.97 | 0.632147 |  |
| GO:0070727\_cellular\_macromolecule\_localization | BAX | 141 | 2 | 1.921151 | -0.553530 | 503 | 317.97 | 0.632147 |  |
| GO:0008104\_protein\_localization | FRAS1 | 251 | 3 | 1.618819 | -0.549355 | 504 | 318.88 | 0.632698 |  |
| GO:0008104\_protein\_localization | SEC23A | 251 | 3 | 1.618819 | -0.549355 | 504 | 318.88 | 0.632698 |  |
| GO:0008104\_protein\_localization | BAX | 251 | 3 | 1.618819 | -0.549355 | 504 | 318.88 | 0.632698 |  |
| GO:0034103\_regulation\_of\_tissue\_remodeling | BAX | 45 | 1 | 3.009804 | -0.545573 | 506 | 320.3 | 0.633004 |  |
| GO:0046546\_development\_of\_primary\_male\_sexual\_characteristics | BAX | 45 | 1 | 3.009804 | -0.545573 | 506 | 320.3 | 0.633004 |  |
| GO:0043283\_biopolymer\_metabolic\_process | GCLC | 1490 | 13 | 1.181702 | -0.543893 | 507 | 320.7 | 0.632544 |  |
| GO:0043283\_biopolymer\_metabolic\_process | POLR1E | 1490 | 13 | 1.181702 | -0.543893 | 507 | 320.7 | 0.632544 |  |
| GO:0043283\_biopolymer\_metabolic\_process | NFYB | 1490 | 13 | 1.181702 | -0.543893 | 507 | 320.7 | 0.632544 |  |
| GO:0043283\_biopolymer\_metabolic\_process | VAX1 | 1490 | 13 | 1.181702 | -0.543893 | 507 | 320.7 | 0.632544 |  |
| GO:0043283\_biopolymer\_metabolic\_process | RB1 | 1490 | 13 | 1.181702 | -0.543893 | 507 | 320.7 | 0.632544 |  |
| GO:0043283\_biopolymer\_metabolic\_process | PKNOX2 | 1490 | 13 | 1.181702 | -0.543893 | 507 | 320.7 | 0.632544 |  |
| GO:0043283\_biopolymer\_metabolic\_process | NOTCH1 | 1490 | 13 | 1.181702 | -0.543893 | 507 | 320.7 | 0.632544 |  |
| GO:0043283\_biopolymer\_metabolic\_process | NR1D2 | 1490 | 13 | 1.181702 | -0.543893 | 507 | 320.7 | 0.632544 |  |
| GO:0043283\_biopolymer\_metabolic\_process | BAX | 1490 | 13 | 1.181702 | -0.543893 | 507 | 320.7 | 0.632544 |  |
| GO:0043283\_biopolymer\_metabolic\_process | ENDOG | 1490 | 13 | 1.181702 | -0.543893 | 507 | 320.7 | 0.632544 |  |
| GO:0043283\_biopolymer\_metabolic\_process | HBB-B1 | 1490 | 13 | 1.181702 | -0.543893 | 507 | 320.7 | 0.632544 |  |
| GO:0043283\_biopolymer\_metabolic\_process | RBPJ | 1490 | 13 | 1.181702 | -0.543893 | 507 | 320.7 | 0.632544 |  |
| GO:0043283\_biopolymer\_metabolic\_process | PHLDA1 | 1490 | 13 | 1.181702 | -0.543893 | 507 | 320.7 | 0.632544 |  |
| GO:0051234\_establishment\_of\_localization | FRAS1 | 729 | 7 | 1.300533 | -0.543526 | 508 | 320.86 | 0.631614 |  |
| GO:0051234\_establishment\_of\_localization | SEC23A | 729 | 7 | 1.300533 | -0.543526 | 508 | 320.86 | 0.631614 |  |
| GO:0051234\_establishment\_of\_localization | NUP133 | 729 | 7 | 1.300533 | -0.543526 | 508 | 320.86 | 0.631614 |  |
| GO:0051234\_establishment\_of\_localization | PDPN | 729 | 7 | 1.300533 | -0.543526 | 508 | 320.86 | 0.631614 |  |
| GO:0051234\_establishment\_of\_localization | BAX | 729 | 7 | 1.300533 | -0.543526 | 508 | 320.86 | 0.631614 |  |
| GO:0051234\_establishment\_of\_localization | HBB-B1 | 729 | 7 | 1.300533 | -0.543526 | 508 | 320.86 | 0.631614 |  |
| GO:0051234\_establishment\_of\_localization | SPIRE1 | 729 | 7 | 1.300533 | -0.543526 | 508 | 320.86 | 0.631614 |  |
| GO:0016481\_negative\_regulation\_of\_transcription | RB1 | 253 | 3 | 1.606022 | -0.543102 | 509 | 321.21 | 0.631061 |  |
| GO:0016481\_negative\_regulation\_of\_transcription | VAX1 | 253 | 3 | 1.606022 | -0.543102 | 509 | 321.21 | 0.631061 |  |
| GO:0016481\_negative\_regulation\_of\_transcription | RBPJ | 253 | 3 | 1.606022 | -0.543102 | 509 | 321.21 | 0.631061 |  |
| GO:0048878\_chemical\_homeostasis | GCLC | 254 | 3 | 1.599699 | -0.540005 | 510 | 322.11 | 0.631588 |  |
| GO:0048878\_chemical\_homeostasis | BAX | 254 | 3 | 1.599699 | -0.540005 | 510 | 322.11 | 0.631588 |  |
| GO:0048878\_chemical\_homeostasis | BAD | 254 | 3 | 1.599699 | -0.540005 | 510 | 322.11 | 0.631588 |  |
| GO:0051641\_cellular\_localization | SEC23A | 370 | 4 | 1.464229 | -0.537668 | 511 | 322.3 | 0.630724 |  |
| GO:0051641\_cellular\_localization | NUP133 | 370 | 4 | 1.464229 | -0.537668 | 511 | 322.3 | 0.630724 |  |
| GO:0051641\_cellular\_localization | BAX | 370 | 4 | 1.464229 | -0.537668 | 511 | 322.3 | 0.630724 |  |
| GO:0051641\_cellular\_localization | SPIRE1 | 370 | 4 | 1.464229 | -0.537668 | 511 | 322.3 | 0.630724 |  |
| GO:0006732\_coenzyme\_metabolic\_process | GCLC | 46 | 1 | 2.944373 | -0.537513 | 515 | 324.8 | 0.630680 |  |
| GO:0030218\_erythrocyte\_differentiation | RB1 | 46 | 1 | 2.944373 | -0.537513 | 515 | 324.8 | 0.630680 |  |
| GO:0030850\_prostate\_gland\_development | NOTCH1 | 46 | 1 | 2.944373 | -0.537513 | 515 | 324.8 | 0.630680 |  |
| GO:0042063\_gliogenesis | NOTCH1 | 46 | 1 | 2.944373 | -0.537513 | 515 | 324.8 | 0.630680 |  |
| GO:0048729\_tissue\_morphogenesis | FRAS1 | 255 | 3 | 1.593426 | -0.536927 | 516 | 325.08 | 0.630000 |  |
| GO:0048729\_tissue\_morphogenesis | NOTCH1 | 255 | 3 | 1.593426 | -0.536927 | 516 | 325.08 | 0.630000 |  |
| GO:0048729\_tissue\_morphogenesis | SEMA3A | 255 | 3 | 1.593426 | -0.536927 | 516 | 325.08 | 0.630000 |  |
| GO:0048519\_negative\_regulation\_of\_biological\_process | NOTCH1 | 859 | 8 | 1.261385 | -0.533170 | 517 | 325.66 | 0.629903 |  |
| GO:0048519\_negative\_regulation\_of\_biological\_process | GCLC | 859 | 8 | 1.261385 | -0.533170 | 517 | 325.66 | 0.629903 |  |
| GO:0048519\_negative\_regulation\_of\_biological\_process | BAX | 859 | 8 | 1.261385 | -0.533170 | 517 | 325.66 | 0.629903 |  |
| GO:0048519\_negative\_regulation\_of\_biological\_process | VWC2 | 859 | 8 | 1.261385 | -0.533170 | 517 | 325.66 | 0.629903 |  |
| GO:0048519\_negative\_regulation\_of\_biological\_process | SEMA3A | 859 | 8 | 1.261385 | -0.533170 | 517 | 325.66 | 0.629903 |  |
| GO:0048519\_negative\_regulation\_of\_biological\_process | RB1 | 859 | 8 | 1.261385 | -0.533170 | 517 | 325.66 | 0.629903 |  |
| GO:0048519\_negative\_regulation\_of\_biological\_process | VAX1 | 859 | 8 | 1.261385 | -0.533170 | 517 | 325.66 | 0.629903 |  |
| GO:0048519\_negative\_regulation\_of\_biological\_process | RBPJ | 859 | 8 | 1.261385 | -0.533170 | 517 | 325.66 | 0.629903 |  |
| GO:0005975\_carbohydrate\_metabolic\_process | SORD | 146 | 2 | 1.855359 | -0.532555 | 518 | 326.26 | 0.629846 |  |
| GO:0005975\_carbohydrate\_metabolic\_process | BAD | 146 | 2 | 1.855359 | -0.532555 | 518 | 326.26 | 0.629846 |  |
| GO:0045619\_regulation\_of\_lymphocyte\_differentiation | BAD | 47 | 1 | 2.881727 | -0.529657 | 520 | 329.86 | 0.634346 |  |
| GO:0048871\_multicellular\_organismal\_homeostasis | BAX | 47 | 1 | 2.881727 | -0.529657 | 520 | 329.86 | 0.634346 |  |
| GO:0007498\_mesoderm\_development | NUP133 | 48 | 1 | 2.821691 | -0.521996 | 523 | 332.43 | 0.635621 |  |
| GO:0009101\_glycoprotein\_biosynthetic\_process | PHLDA1 | 48 | 1 | 2.821691 | -0.521996 | 523 | 332.43 | 0.635621 |  |
| GO:0019318\_hexose\_metabolic\_process | BAD | 48 | 1 | 2.821691 | -0.521996 | 523 | 332.43 | 0.635621 |  |
| GO:0001775\_cell\_activation | BAX | 262 | 3 | 1.550853 | -0.515922 | 525 | 333.36 | 0.634971 |  |
| GO:0001775\_cell\_activation | BAD | 262 | 3 | 1.550853 | -0.515922 | 525 | 333.36 | 0.634971 |  |
| GO:0001775\_cell\_activation | RBPJ | 262 | 3 | 1.550853 | -0.515922 | 525 | 333.36 | 0.634971 |  |
| GO:0010629\_negative\_regulation\_of\_gene\_expression | RB1 | 262 | 3 | 1.550853 | -0.515922 | 525 | 333.36 | 0.634971 |  |
| GO:0010629\_negative\_regulation\_of\_gene\_expression | VAX1 | 262 | 3 | 1.550853 | -0.515922 | 525 | 333.36 | 0.634971 |  |
| GO:0010629\_negative\_regulation\_of\_gene\_expression | RBPJ | 262 | 3 | 1.550853 | -0.515922 | 525 | 333.36 | 0.634971 |  |
| GO:0003015\_heart\_process | SEMA3A | 49 | 1 | 2.764106 | -0.514522 | 532 | 337.1 | 0.633647 |  |
| GO:0021543\_pallium\_development | BAX | 49 | 1 | 2.764106 | -0.514522 | 532 | 337.1 | 0.633647 |  |
| GO:0034101\_erythrocyte\_homeostasis | RB1 | 49 | 1 | 2.764106 | -0.514522 | 532 | 337.1 | 0.633647 |  |
| GO:0043473\_pigmentation | BAX | 49 | 1 | 2.764106 | -0.514522 | 532 | 337.1 | 0.633647 |  |
| GO:0046660\_female\_sex\_differentiation | BAX | 49 | 1 | 2.764106 | -0.514522 | 532 | 337.1 | 0.633647 |  |
| GO:0046661\_male\_sex\_differentiation | BAX | 49 | 1 | 2.764106 | -0.514522 | 532 | 337.1 | 0.633647 |  |
| GO:0060047\_heart\_contraction | SEMA3A | 49 | 1 | 2.764106 | -0.514522 | 532 | 337.1 | 0.633647 |  |
| GO:0002573\_myeloid\_leukocyte\_differentiation | RB1 | 50 | 1 | 2.708824 | -0.507228 | 534 | 339.8 | 0.636330 |  |
| GO:0070647\_protein\_modification\_by\_small\_protein\_conjugation\_or\_removal | GCLC | 50 | 1 | 2.708824 | -0.507228 | 534 | 339.8 | 0.636330 |  |
| GO:0006139\_nucleobase\_\_nucleoside\_\_nucleotide\_and\_nucleic\_acid\_metabolic\_process | PKNOX2 | 1002 | 9 | 1.216538 | -0.505590 | 535 | 339.98 | 0.635477 |  |
| GO:0006139\_nucleobase\_\_nucleoside\_\_nucleotide\_and\_nucleic\_acid\_metabolic\_process | NOTCH1 | 1002 | 9 | 1.216538 | -0.505590 | 535 | 339.98 | 0.635477 |  |
| GO:0006139\_nucleobase\_\_nucleoside\_\_nucleotide\_and\_nucleic\_acid\_metabolic\_process | POLR1E | 1002 | 9 | 1.216538 | -0.505590 | 535 | 339.98 | 0.635477 |  |
| GO:0006139\_nucleobase\_\_nucleoside\_\_nucleotide\_and\_nucleic\_acid\_metabolic\_process | NR1D2 | 1002 | 9 | 1.216538 | -0.505590 | 535 | 339.98 | 0.635477 |  |
| GO:0006139\_nucleobase\_\_nucleoside\_\_nucleotide\_and\_nucleic\_acid\_metabolic\_process | ENDOG | 1002 | 9 | 1.216538 | -0.505590 | 535 | 339.98 | 0.635477 |  |
| GO:0006139\_nucleobase\_\_nucleoside\_\_nucleotide\_and\_nucleic\_acid\_metabolic\_process | NFYB | 1002 | 9 | 1.216538 | -0.505590 | 535 | 339.98 | 0.635477 |  |
| GO:0006139\_nucleobase\_\_nucleoside\_\_nucleotide\_and\_nucleic\_acid\_metabolic\_process | RB1 | 1002 | 9 | 1.216538 | -0.505590 | 535 | 339.98 | 0.635477 |  |
| GO:0006139\_nucleobase\_\_nucleoside\_\_nucleotide\_and\_nucleic\_acid\_metabolic\_process | VAX1 | 1002 | 9 | 1.216538 | -0.505590 | 535 | 339.98 | 0.635477 |  |
| GO:0006139\_nucleobase\_\_nucleoside\_\_nucleotide\_and\_nucleic\_acid\_metabolic\_process | RBPJ | 1002 | 9 | 1.216538 | -0.505590 | 535 | 339.98 | 0.635477 |  |
| GO:0007517\_muscle\_organ\_development | RB1 | 153 | 2 | 1.770473 | -0.504851 | 536 | 340.18 | 0.634664 |  |
| GO:0007517\_muscle\_organ\_development | IFRD1 | 153 | 2 | 1.770473 | -0.504851 | 536 | 340.18 | 0.634664 |  |
| GO:0002376\_immune\_system\_process | BAX | 505 | 5 | 1.341002 | -0.502192 | 537 | 340.55 | 0.634171 |  |
| GO:0002376\_immune\_system\_process | HBB-B1 | 505 | 5 | 1.341002 | -0.502192 | 537 | 340.55 | 0.634171 |  |
| GO:0002376\_immune\_system\_process | BAD | 505 | 5 | 1.341002 | -0.502192 | 537 | 340.55 | 0.634171 |  |
| GO:0002376\_immune\_system\_process | RB1 | 505 | 5 | 1.341002 | -0.502192 | 537 | 340.55 | 0.634171 |  |
| GO:0002376\_immune\_system\_process | RBPJ | 505 | 5 | 1.341002 | -0.502192 | 537 | 340.55 | 0.634171 |  |
| GO:0032501\_multicellular\_organismal\_process | FRAS1 | 2183 | 18 | 1.116785 | -0.499817 | 538 | 344.1 | 0.639591 |  |
| GO:0032501\_multicellular\_organismal\_process | NUP133 | 2183 | 18 | 1.116785 | -0.499817 | 538 | 344.1 | 0.639591 |  |
| GO:0032501\_multicellular\_organismal\_process | PDPN | 2183 | 18 | 1.116785 | -0.499817 | 538 | 344.1 | 0.639591 |  |
| GO:0032501\_multicellular\_organismal\_process | TAC1 | 2183 | 18 | 1.116785 | -0.499817 | 538 | 344.1 | 0.639591 |  |
| GO:0032501\_multicellular\_organismal\_process | RB1 | 2183 | 18 | 1.116785 | -0.499817 | 538 | 344.1 | 0.639591 |  |
| GO:0032501\_multicellular\_organismal\_process | BAD | 2183 | 18 | 1.116785 | -0.499817 | 538 | 344.1 | 0.639591 |  |
| GO:0032501\_multicellular\_organismal\_process | VAX1 | 2183 | 18 | 1.116785 | -0.499817 | 538 | 344.1 | 0.639591 |  |
| GO:0032501\_multicellular\_organismal\_process | CDH4 | 2183 | 18 | 1.116785 | -0.499817 | 538 | 344.1 | 0.639591 |  |
| GO:0032501\_multicellular\_organismal\_process | FEZF2 | 2183 | 18 | 1.116785 | -0.499817 | 538 | 344.1 | 0.639591 |  |
| GO:0032501\_multicellular\_organismal\_process | NOTCH1 | 2183 | 18 | 1.116785 | -0.499817 | 538 | 344.1 | 0.639591 |  |
| GO:0032501\_multicellular\_organismal\_process | ENDOG | 2183 | 18 | 1.116785 | -0.499817 | 538 | 344.1 | 0.639591 |  |
| GO:0032501\_multicellular\_organismal\_process | BAX | 2183 | 18 | 1.116785 | -0.499817 | 538 | 344.1 | 0.639591 |  |
| GO:0032501\_multicellular\_organismal\_process | VWC2 | 2183 | 18 | 1.116785 | -0.499817 | 538 | 344.1 | 0.639591 |  |
| GO:0032501\_multicellular\_organismal\_process | ROR2 | 2183 | 18 | 1.116785 | -0.499817 | 538 | 344.1 | 0.639591 |  |
| GO:0032501\_multicellular\_organismal\_process | HBB-B1 | 2183 | 18 | 1.116785 | -0.499817 | 538 | 344.1 | 0.639591 |  |
| GO:0032501\_multicellular\_organismal\_process | SEMA3A | 2183 | 18 | 1.116785 | -0.499817 | 538 | 344.1 | 0.639591 |  |
| GO:0032501\_multicellular\_organismal\_process | RBPJ | 2183 | 18 | 1.116785 | -0.499817 | 538 | 344.1 | 0.639591 |  |
| GO:0032501\_multicellular\_organismal\_process | IFRD1 | 2183 | 18 | 1.116785 | -0.499817 | 538 | 344.1 | 0.639591 |  |
| GO:0034960\_cellular\_biopolymer\_metabolic\_process | PKNOX2 | 1395 | 12 | 1.165085 | -0.495140 | 539 | 345.04 | 0.640148 |  |
| GO:0034960\_cellular\_biopolymer\_metabolic\_process | NOTCH1 | 1395 | 12 | 1.165085 | -0.495140 | 539 | 345.04 | 0.640148 |  |
| GO:0034960\_cellular\_biopolymer\_metabolic\_process | GCLC | 1395 | 12 | 1.165085 | -0.495140 | 539 | 345.04 | 0.640148 |  |
| GO:0034960\_cellular\_biopolymer\_metabolic\_process | POLR1E | 1395 | 12 | 1.165085 | -0.495140 | 539 | 345.04 | 0.640148 |  |
| GO:0034960\_cellular\_biopolymer\_metabolic\_process | NR1D2 | 1395 | 12 | 1.165085 | -0.495140 | 539 | 345.04 | 0.640148 |  |
| GO:0034960\_cellular\_biopolymer\_metabolic\_process | BAX | 1395 | 12 | 1.165085 | -0.495140 | 539 | 345.04 | 0.640148 |  |
| GO:0034960\_cellular\_biopolymer\_metabolic\_process | ENDOG | 1395 | 12 | 1.165085 | -0.495140 | 539 | 345.04 | 0.640148 |  |
| GO:0034960\_cellular\_biopolymer\_metabolic\_process | HBB-B1 | 1395 | 12 | 1.165085 | -0.495140 | 539 | 345.04 | 0.640148 |  |
| GO:0034960\_cellular\_biopolymer\_metabolic\_process | NFYB | 1395 | 12 | 1.165085 | -0.495140 | 539 | 345.04 | 0.640148 |  |
| GO:0034960\_cellular\_biopolymer\_metabolic\_process | RB1 | 1395 | 12 | 1.165085 | -0.495140 | 539 | 345.04 | 0.640148 |  |
| GO:0034960\_cellular\_biopolymer\_metabolic\_process | VAX1 | 1395 | 12 | 1.165085 | -0.495140 | 539 | 345.04 | 0.640148 |  |
| GO:0034960\_cellular\_biopolymer\_metabolic\_process | RBPJ | 1395 | 12 | 1.165085 | -0.495140 | 539 | 345.04 | 0.640148 |  |
| GO:0019219\_regulation\_of\_nucleobase\_\_nucleoside\_\_nucleotide\_and\_nucleic\_acid\_metabolic\_process | PKNOX2 | 757 | 7 | 1.252428 | -0.493373 | 540 | 345.36 | 0.639556 |  |
| GO:0019219\_regulation\_of\_nucleobase\_\_nucleoside\_\_nucleotide\_and\_nucleic\_acid\_metabolic\_process | NOTCH1 | 757 | 7 | 1.252428 | -0.493373 | 540 | 345.36 | 0.639556 |  |
| GO:0019219\_regulation\_of\_nucleobase\_\_nucleoside\_\_nucleotide\_and\_nucleic\_acid\_metabolic\_process | NR1D2 | 757 | 7 | 1.252428 | -0.493373 | 540 | 345.36 | 0.639556 |  |
| GO:0019219\_regulation\_of\_nucleobase\_\_nucleoside\_\_nucleotide\_and\_nucleic\_acid\_metabolic\_process | NFYB | 757 | 7 | 1.252428 | -0.493373 | 540 | 345.36 | 0.639556 |  |
| GO:0019219\_regulation\_of\_nucleobase\_\_nucleoside\_\_nucleotide\_and\_nucleic\_acid\_metabolic\_process | VAX1 | 757 | 7 | 1.252428 | -0.493373 | 540 | 345.36 | 0.639556 |  |
| GO:0019219\_regulation\_of\_nucleobase\_\_nucleoside\_\_nucleotide\_and\_nucleic\_acid\_metabolic\_process | RB1 | 757 | 7 | 1.252428 | -0.493373 | 540 | 345.36 | 0.639556 |  |
| GO:0019219\_regulation\_of\_nucleobase\_\_nucleoside\_\_nucleotide\_and\_nucleic\_acid\_metabolic\_process | RBPJ | 757 | 7 | 1.252428 | -0.493373 | 540 | 345.36 | 0.639556 |  |
| GO:0010608\_posttranscriptional\_regulation\_of\_gene\_expression | HBB-B1 | 52 | 1 | 2.604638 | -0.493147 | 541 | 346.71 | 0.640869 |  |
| GO:0045934\_negative\_regulation\_of\_nucleobase\_\_nucleoside\_\_nucleotide\_and\_nucleic\_acid\_metabolic\_process | RB1 | 270 | 3 | 1.504902 | -0.493015 | 542 | 346.92 | 0.640074 |  |
| GO:0045934\_negative\_regulation\_of\_nucleobase\_\_nucleoside\_\_nucleotide\_and\_nucleic\_acid\_metabolic\_process | VAX1 | 270 | 3 | 1.504902 | -0.493015 | 542 | 346.92 | 0.640074 |  |
| GO:0045934\_negative\_regulation\_of\_nucleobase\_\_nucleoside\_\_nucleotide\_and\_nucleic\_acid\_metabolic\_process | RBPJ | 270 | 3 | 1.504902 | -0.493015 | 542 | 346.92 | 0.640074 |  |
| GO:0051172\_negative\_regulation\_of\_nitrogen\_compound\_metabolic\_process | RB1 | 271 | 3 | 1.499349 | -0.490231 | 543 | 347.51 | 0.639982 |  |
| GO:0051172\_negative\_regulation\_of\_nitrogen\_compound\_metabolic\_process | VAX1 | 271 | 3 | 1.499349 | -0.490231 | 543 | 347.51 | 0.639982 |  |
| GO:0051172\_negative\_regulation\_of\_nitrogen\_compound\_metabolic\_process | RBPJ | 271 | 3 | 1.499349 | -0.490231 | 543 | 347.51 | 0.639982 |  |
| GO:0006935\_chemotaxis | SEMA3A | 53 | 1 | 2.555494 | -0.486349 | 547 | 350.57 | 0.640896 |  |
| GO:0042330\_taxis | SEMA3A | 53 | 1 | 2.555494 | -0.486349 | 547 | 350.57 | 0.640896 |  |
| GO:0046942\_carboxylic\_acid\_transport | PDPN | 53 | 1 | 2.555494 | -0.486349 | 547 | 350.57 | 0.640896 |  |
| GO:0055085\_transmembrane\_transport | PDPN | 53 | 1 | 2.555494 | -0.486349 | 547 | 350.57 | 0.640896 |  |
| GO:0006066\_alcohol\_metabolic\_process | SORD | 158 | 2 | 1.714445 | -0.486160 | 548 | 351.18 | 0.640839 |  |
| GO:0006066\_alcohol\_metabolic\_process | BAD | 158 | 2 | 1.714445 | -0.486160 | 548 | 351.18 | 0.640839 |  |
| GO:0051716\_cellular\_response\_to\_stimulus | BAX | 273 | 3 | 1.488365 | -0.484713 | 549 | 351.46 | 0.640182 |  |
| GO:0051716\_cellular\_response\_to\_stimulus | ROR2 | 273 | 3 | 1.488365 | -0.484713 | 549 | 351.46 | 0.640182 |  |
| GO:0051716\_cellular\_response\_to\_stimulus | HBB-B1 | 273 | 3 | 1.488365 | -0.484713 | 549 | 351.46 | 0.640182 |  |
| GO:0010558\_negative\_regulation\_of\_macromolecule\_biosynthetic\_process | RB1 | 274 | 3 | 1.482933 | -0.481980 | 550 | 351.99 | 0.639982 |  |
| GO:0010558\_negative\_regulation\_of\_macromolecule\_biosynthetic\_process | VAX1 | 274 | 3 | 1.482933 | -0.481980 | 550 | 351.99 | 0.639982 |  |
| GO:0010558\_negative\_regulation\_of\_macromolecule\_biosynthetic\_process | RBPJ | 274 | 3 | 1.482933 | -0.481980 | 550 | 351.99 | 0.639982 |  |
| GO:0006807\_nitrogen\_compound\_metabolic\_process | PKNOX2 | 1147 | 10 | 1.180830 | -0.480194 | 551 | 352.14 | 0.639093 |  |
| GO:0006807\_nitrogen\_compound\_metabolic\_process | NOTCH1 | 1147 | 10 | 1.180830 | -0.480194 | 551 | 352.14 | 0.639093 |  |
| GO:0006807\_nitrogen\_compound\_metabolic\_process | POLR1E | 1147 | 10 | 1.180830 | -0.480194 | 551 | 352.14 | 0.639093 |  |
| GO:0006807\_nitrogen\_compound\_metabolic\_process | NR1D2 | 1147 | 10 | 1.180830 | -0.480194 | 551 | 352.14 | 0.639093 |  |
| GO:0006807\_nitrogen\_compound\_metabolic\_process | BAX | 1147 | 10 | 1.180830 | -0.480194 | 551 | 352.14 | 0.639093 |  |
| GO:0006807\_nitrogen\_compound\_metabolic\_process | ENDOG | 1147 | 10 | 1.180830 | -0.480194 | 551 | 352.14 | 0.639093 |  |
| GO:0006807\_nitrogen\_compound\_metabolic\_process | NFYB | 1147 | 10 | 1.180830 | -0.480194 | 551 | 352.14 | 0.639093 |  |
| GO:0006807\_nitrogen\_compound\_metabolic\_process | RB1 | 1147 | 10 | 1.180830 | -0.480194 | 551 | 352.14 | 0.639093 |  |
| GO:0006807\_nitrogen\_compound\_metabolic\_process | VAX1 | 1147 | 10 | 1.180830 | -0.480194 | 551 | 352.14 | 0.639093 |  |
| GO:0006807\_nitrogen\_compound\_metabolic\_process | RBPJ | 1147 | 10 | 1.180830 | -0.480194 | 551 | 352.14 | 0.639093 |  |
| GO:0006091\_generation\_of\_precursor\_metabolites\_and\_energy | UQCRC1 | 54 | 1 | 2.508170 | -0.479703 | 556 | 354.58 | 0.637734 |  |
| GO:0006412\_translation | HBB-B1 | 54 | 1 | 2.508170 | -0.479703 | 556 | 354.58 | 0.637734 |  |
| GO:0007265\_Ras\_protein\_signal\_transduction | RRAS2 | 54 | 1 | 2.508170 | -0.479703 | 556 | 354.58 | 0.637734 |  |
| GO:0009566\_fertilization | BAX | 54 | 1 | 2.508170 | -0.479703 | 556 | 354.58 | 0.637734 |  |
| GO:0015849\_organic\_acid\_transport | PDPN | 54 | 1 | 2.508170 | -0.479703 | 556 | 354.58 | 0.637734 |  |
| GO:0051128\_regulation\_of\_cellular\_component\_organization | SEMA3A | 160 | 2 | 1.693015 | -0.478924 | 557 | 354.81 | 0.637002 |  |
| GO:0051128\_regulation\_of\_cellular\_component\_organization | CDH4 | 160 | 2 | 1.693015 | -0.478924 | 557 | 354.81 | 0.637002 |  |
| GO:0009887\_organ\_morphogenesis | FRAS1 | 642 | 6 | 1.265805 | -0.475761 | 558 | 355.11 | 0.636398 |  |
| GO:0009887\_organ\_morphogenesis | NOTCH1 | 642 | 6 | 1.265805 | -0.475761 | 558 | 355.11 | 0.636398 |  |
| GO:0009887\_organ\_morphogenesis | BAX | 642 | 6 | 1.265805 | -0.475761 | 558 | 355.11 | 0.636398 |  |
| GO:0009887\_organ\_morphogenesis | ROR2 | 642 | 6 | 1.265805 | -0.475761 | 558 | 355.11 | 0.636398 |  |
| GO:0009887\_organ\_morphogenesis | SEMA3A | 642 | 6 | 1.265805 | -0.475761 | 558 | 355.11 | 0.636398 |  |
| GO:0009887\_organ\_morphogenesis | RBPJ | 642 | 6 | 1.265805 | -0.475761 | 558 | 355.11 | 0.636398 |  |
| GO:0048568\_embryonic\_organ\_development | ROR2 | 55 | 1 | 2.462567 | -0.473205 | 559 | 357.8 | 0.640072 |  |
| GO:0007626\_locomotory\_behavior | FEZF2 | 163 | 2 | 1.661855 | -0.468317 | 561 | 358.91 | 0.639768 |  |
| GO:0007626\_locomotory\_behavior | SEMA3A | 163 | 2 | 1.661855 | -0.468317 | 561 | 358.91 | 0.639768 |  |
| GO:0042110\_T\_cell\_activation | BAX | 163 | 2 | 1.661855 | -0.468317 | 561 | 358.91 | 0.639768 |  |
| GO:0042110\_T\_cell\_activation | BAD | 163 | 2 | 1.661855 | -0.468317 | 561 | 358.91 | 0.639768 |  |
| GO:0006790\_sulfur\_metabolic\_process | GCLC | 56 | 1 | 2.418592 | -0.466848 | 562 | 361.62 | 0.643452 |  |
| GO:0042325\_regulation\_of\_phosphorylation | BAX | 164 | 2 | 1.651722 | -0.464845 | 563 | 362.19 | 0.643321 |  |
| GO:0042325\_regulation\_of\_phosphorylation | HBB-B1 | 164 | 2 | 1.651722 | -0.464845 | 563 | 362.19 | 0.643321 |  |
| GO:0019220\_regulation\_of\_phosphate\_metabolic\_process | BAX | 165 | 2 | 1.641711 | -0.461405 | 565 | 362.98 | 0.642442 |  |
| GO:0019220\_regulation\_of\_phosphate\_metabolic\_process | HBB-B1 | 165 | 2 | 1.641711 | -0.461405 | 565 | 362.98 | 0.642442 |  |
| GO:0051174\_regulation\_of\_phosphorus\_metabolic\_process | BAX | 165 | 2 | 1.641711 | -0.461405 | 565 | 362.98 | 0.642442 |  |
| GO:0051174\_regulation\_of\_phosphorus\_metabolic\_process | HBB-B1 | 165 | 2 | 1.641711 | -0.461405 | 565 | 362.98 | 0.642442 |  |
| GO:0031327\_negative\_regulation\_of\_cellular\_biosynthetic\_process | RB1 | 282 | 3 | 1.440864 | -0.460704 | 566 | 363.38 | 0.642014 |  |
| GO:0031327\_negative\_regulation\_of\_cellular\_biosynthetic\_process | VAX1 | 282 | 3 | 1.440864 | -0.460704 | 566 | 363.38 | 0.642014 |  |
| GO:0031327\_negative\_regulation\_of\_cellular\_biosynthetic\_process | RBPJ | 282 | 3 | 1.440864 | -0.460704 | 566 | 363.38 | 0.642014 |  |
| GO:0043523\_regulation\_of\_neuron\_apoptosis | BAX | 57 | 1 | 2.376161 | -0.460629 | 568 | 367.06 | 0.646232 |  |
| GO:0045444\_fat\_cell\_differentiation | ALDH6A1 | 57 | 1 | 2.376161 | -0.460629 | 568 | 367.06 | 0.646232 |  |
| GO:0009890\_negative\_regulation\_of\_biosynthetic\_process | RB1 | 284 | 3 | 1.430717 | -0.455544 | 569 | 368.02 | 0.646784 |  |
| GO:0009890\_negative\_regulation\_of\_biosynthetic\_process | VAX1 | 284 | 3 | 1.430717 | -0.455544 | 569 | 368.02 | 0.646784 |  |
| GO:0009890\_negative\_regulation\_of\_biosynthetic\_process | RBPJ | 284 | 3 | 1.430717 | -0.455544 | 569 | 368.02 | 0.646784 |  |
| GO:0016055\_Wnt\_receptor\_signaling\_pathway | ROR2 | 59 | 1 | 2.295613 | -0.448581 | 572 | 371.99 | 0.650332 |  |
| GO:0035270\_endocrine\_system\_development | RBPJ | 59 | 1 | 2.295613 | -0.448581 | 572 | 371.99 | 0.650332 |  |
| GO:0050870\_positive\_regulation\_of\_T\_cell\_activation | BAD | 59 | 1 | 2.295613 | -0.448581 | 572 | 371.99 | 0.650332 |  |
| GO:0042221\_response\_to\_chemical\_stimulus | GCLC | 409 | 4 | 1.324608 | -0.447107 | 573 | 372.65 | 0.650349 |  |
| GO:0042221\_response\_to\_chemical\_stimulus | ENDOG | 409 | 4 | 1.324608 | -0.447107 | 573 | 372.65 | 0.650349 |  |
| GO:0042221\_response\_to\_chemical\_stimulus | BAX | 409 | 4 | 1.324608 | -0.447107 | 573 | 372.65 | 0.650349 |  |
| GO:0042221\_response\_to\_chemical\_stimulus | SEMA3A | 409 | 4 | 1.324608 | -0.447107 | 573 | 372.65 | 0.650349 |  |
| GO:0009611\_response\_to\_wounding | BAX | 172 | 2 | 1.574897 | -0.438169 | 574 | 374.23 | 0.651969 |  |
| GO:0009611\_response\_to\_wounding | TAC1 | 172 | 2 | 1.574897 | -0.438169 | 574 | 374.23 | 0.651969 |  |
| GO:0006874\_cellular\_calcium\_ion\_homeostasis | BAX | 61 | 1 | 2.220347 | -0.437026 | 577 | 375.7 | 0.651127 |  |
| GO:0007005\_mitochondrion\_organization | BAX | 61 | 1 | 2.220347 | -0.437026 | 577 | 375.7 | 0.651127 |  |
| GO:0032270\_positive\_regulation\_of\_cellular\_protein\_metabolic\_process | GCLC | 61 | 1 | 2.220347 | -0.437026 | 577 | 375.7 | 0.651127 |  |
| GO:0044248\_cellular\_catabolic\_process | GCLC | 173 | 2 | 1.565794 | -0.434965 | 578 | 376.34 | 0.651107 |  |
| GO:0044248\_cellular\_catabolic\_process | ENDOG | 173 | 2 | 1.565794 | -0.434965 | 578 | 376.34 | 0.651107 |  |
| GO:0006916\_anti-apoptosis | BAX | 62 | 1 | 2.184535 | -0.431423 | 582 | 379.1 | 0.651375 |  |
| GO:0021537\_telencephalon\_development | BAX | 62 | 1 | 2.184535 | -0.431423 | 582 | 379.1 | 0.651375 |  |
| GO:0030155\_regulation\_of\_cell\_adhesion | VWC2 | 62 | 1 | 2.184535 | -0.431423 | 582 | 379.1 | 0.651375 |  |
| GO:0030855\_epithelial\_cell\_differentiation | NOTCH1 | 62 | 1 | 2.184535 | -0.431423 | 582 | 379.1 | 0.651375 |  |
| GO:0043170\_macromolecule\_metabolic\_process | GCLC | 1576 | 13 | 1.117218 | -0.430919 | 583 | 379.34 | 0.650669 |  |
| GO:0043170\_macromolecule\_metabolic\_process | POLR1E | 1576 | 13 | 1.117218 | -0.430919 | 583 | 379.34 | 0.650669 |  |
| GO:0043170\_macromolecule\_metabolic\_process | NFYB | 1576 | 13 | 1.117218 | -0.430919 | 583 | 379.34 | 0.650669 |  |
| GO:0043170\_macromolecule\_metabolic\_process | RB1 | 1576 | 13 | 1.117218 | -0.430919 | 583 | 379.34 | 0.650669 |  |
| GO:0043170\_macromolecule\_metabolic\_process | VAX1 | 1576 | 13 | 1.117218 | -0.430919 | 583 | 379.34 | 0.650669 |  |
| GO:0043170\_macromolecule\_metabolic\_process | PKNOX2 | 1576 | 13 | 1.117218 | -0.430919 | 583 | 379.34 | 0.650669 |  |
| GO:0043170\_macromolecule\_metabolic\_process | NOTCH1 | 1576 | 13 | 1.117218 | -0.430919 | 583 | 379.34 | 0.650669 |  |
| GO:0043170\_macromolecule\_metabolic\_process | NR1D2 | 1576 | 13 | 1.117218 | -0.430919 | 583 | 379.34 | 0.650669 |  |
| GO:0043170\_macromolecule\_metabolic\_process | ENDOG | 1576 | 13 | 1.117218 | -0.430919 | 583 | 379.34 | 0.650669 |  |
| GO:0043170\_macromolecule\_metabolic\_process | BAX | 1576 | 13 | 1.117218 | -0.430919 | 583 | 379.34 | 0.650669 |  |
| GO:0043170\_macromolecule\_metabolic\_process | HBB-B1 | 1576 | 13 | 1.117218 | -0.430919 | 583 | 379.34 | 0.650669 |  |
| GO:0043170\_macromolecule\_metabolic\_process | RBPJ | 1576 | 13 | 1.117218 | -0.430919 | 583 | 379.34 | 0.650669 |  |
| GO:0043170\_macromolecule\_metabolic\_process | PHLDA1 | 1576 | 13 | 1.117218 | -0.430919 | 583 | 379.34 | 0.650669 |  |
| GO:0044237\_cellular\_metabolic\_process | SORD | 1974 | 16 | 1.097801 | -0.430338 | 584 | 379.45 | 0.649743 |  |
| GO:0044237\_cellular\_metabolic\_process | GCLC | 1974 | 16 | 1.097801 | -0.430338 | 584 | 379.45 | 0.649743 |  |
| GO:0044237\_cellular\_metabolic\_process | UQCRC1 | 1974 | 16 | 1.097801 | -0.430338 | 584 | 379.45 | 0.649743 |  |
| GO:0044237\_cellular\_metabolic\_process | POLR1E | 1974 | 16 | 1.097801 | -0.430338 | 584 | 379.45 | 0.649743 |  |
| GO:0044237\_cellular\_metabolic\_process | PDPN | 1974 | 16 | 1.097801 | -0.430338 | 584 | 379.45 | 0.649743 |  |
| GO:0044237\_cellular\_metabolic\_process | NFYB | 1974 | 16 | 1.097801 | -0.430338 | 584 | 379.45 | 0.649743 |  |
| GO:0044237\_cellular\_metabolic\_process | VAX1 | 1974 | 16 | 1.097801 | -0.430338 | 584 | 379.45 | 0.649743 |  |
| GO:0044237\_cellular\_metabolic\_process | RB1 | 1974 | 16 | 1.097801 | -0.430338 | 584 | 379.45 | 0.649743 |  |
| GO:0044237\_cellular\_metabolic\_process | PKNOX2 | 1974 | 16 | 1.097801 | -0.430338 | 584 | 379.45 | 0.649743 |  |
| GO:0044237\_cellular\_metabolic\_process | NOTCH1 | 1974 | 16 | 1.097801 | -0.430338 | 584 | 379.45 | 0.649743 |  |
| GO:0044237\_cellular\_metabolic\_process | NR1D2 | 1974 | 16 | 1.097801 | -0.430338 | 584 | 379.45 | 0.649743 |  |
| GO:0044237\_cellular\_metabolic\_process | ENDOG | 1974 | 16 | 1.097801 | -0.430338 | 584 | 379.45 | 0.649743 |  |
| GO:0044237\_cellular\_metabolic\_process | BAX | 1974 | 16 | 1.097801 | -0.430338 | 584 | 379.45 | 0.649743 |  |
| GO:0044237\_cellular\_metabolic\_process | HBB-B1 | 1974 | 16 | 1.097801 | -0.430338 | 584 | 379.45 | 0.649743 |  |
| GO:0044237\_cellular\_metabolic\_process | RBPJ | 1974 | 16 | 1.097801 | -0.430338 | 584 | 379.45 | 0.649743 |  |
| GO:0044237\_cellular\_metabolic\_process | PHLDA1 | 1974 | 16 | 1.097801 | -0.430338 | 584 | 379.45 | 0.649743 |  |
| GO:0000122\_negative\_regulation\_of\_transcription\_from\_RNA\_polymerase\_II\_promoter | RB1 | 175 | 2 | 1.547899 | -0.428643 | 586 | 380.18 | 0.648771 |  |
| GO:0000122\_negative\_regulation\_of\_transcription\_from\_RNA\_polymerase\_II\_promoter | VAX1 | 175 | 2 | 1.547899 | -0.428643 | 586 | 380.18 | 0.648771 |  |
| GO:0015031\_protein\_transport | FRAS1 | 175 | 2 | 1.547899 | -0.428643 | 586 | 380.18 | 0.648771 |  |
| GO:0015031\_protein\_transport | SEC23A | 175 | 2 | 1.547899 | -0.428643 | 586 | 380.18 | 0.648771 |  |
| GO:0042592\_homeostatic\_process | GCLC | 419 | 4 | 1.292995 | -0.426448 | 587 | 381.34 | 0.649642 |  |
| GO:0042592\_homeostatic\_process | BAX | 419 | 4 | 1.292995 | -0.426448 | 587 | 381.34 | 0.649642 |  |
| GO:0042592\_homeostatic\_process | BAD | 419 | 4 | 1.292995 | -0.426448 | 587 | 381.34 | 0.649642 |  |
| GO:0042592\_homeostatic\_process | RB1 | 419 | 4 | 1.292995 | -0.426448 | 587 | 381.34 | 0.649642 |  |
| GO:0051186\_cofactor\_metabolic\_process | GCLC | 63 | 1 | 2.149860 | -0.425931 | 589 | 383.14 | 0.650492 |  |
| GO:0051216\_cartilage\_development | ROR2 | 63 | 1 | 2.149860 | -0.425931 | 589 | 383.14 | 0.650492 |  |
| GO:0006873\_cellular\_ion\_homeostasis | GCLC | 176 | 2 | 1.539104 | -0.425523 | 590 | 383.87 | 0.650627 |  |
| GO:0006873\_cellular\_ion\_homeostasis | BAX | 176 | 2 | 1.539104 | -0.425523 | 590 | 383.87 | 0.650627 |  |
| GO:0006875\_cellular\_metal\_ion\_homeostasis | BAX | 64 | 1 | 2.116268 | -0.420547 | 592 | 385.8 | 0.651689 |  |
| GO:0055074\_calcium\_ion\_homeostasis | BAX | 64 | 1 | 2.116268 | -0.420547 | 592 | 385.8 | 0.651689 |  |
| GO:0006950\_response\_to\_stress | BAX | 549 | 5 | 1.233526 | -0.417969 | 593 | 386.67 | 0.652057 |  |
| GO:0006950\_response\_to\_stress | ROR2 | 549 | 5 | 1.233526 | -0.417969 | 593 | 386.67 | 0.652057 |  |
| GO:0006950\_response\_to\_stress | TAC1 | 549 | 5 | 1.233526 | -0.417969 | 593 | 386.67 | 0.652057 |  |
| GO:0006950\_response\_to\_stress | HBB-B1 | 549 | 5 | 1.233526 | -0.417969 | 593 | 386.67 | 0.652057 |  |
| GO:0006950\_response\_to\_stress | RBPJ | 549 | 5 | 1.233526 | -0.417969 | 593 | 386.67 | 0.652057 |  |
| GO:0048511\_rhythmic\_process | BAX | 65 | 1 | 2.083710 | -0.415267 | 594 | 388.37 | 0.653822 |  |
| GO:0045184\_establishment\_of\_protein\_localization | FRAS1 | 180 | 2 | 1.504902 | -0.413310 | 595 | 388.93 | 0.653664 |  |
| GO:0045184\_establishment\_of\_protein\_localization | SEC23A | 180 | 2 | 1.504902 | -0.413310 | 595 | 388.93 | 0.653664 |  |
| GO:0019752\_carboxylic\_acid\_metabolic\_process | GCLC | 181 | 2 | 1.496588 | -0.410322 | 598 | 389.86 | 0.651940 |  |
| GO:0019752\_carboxylic\_acid\_metabolic\_process | PDPN | 181 | 2 | 1.496588 | -0.410322 | 598 | 389.86 | 0.651940 |  |
| GO:0043436\_oxoacid\_metabolic\_process | GCLC | 181 | 2 | 1.496588 | -0.410322 | 598 | 389.86 | 0.651940 |  |
| GO:0043436\_oxoacid\_metabolic\_process | PDPN | 181 | 2 | 1.496588 | -0.410322 | 598 | 389.86 | 0.651940 |  |
| GO:0055082\_cellular\_chemical\_homeostasis | GCLC | 181 | 2 | 1.496588 | -0.410322 | 598 | 389.86 | 0.651940 |  |
| GO:0055082\_cellular\_chemical\_homeostasis | BAX | 181 | 2 | 1.496588 | -0.410322 | 598 | 389.86 | 0.651940 |  |
| GO:0007179\_transforming\_growth\_factor\_beta\_receptor\_signaling\_pathway | VWC2 | 66 | 1 | 2.052139 | -0.410089 | 603 | 391.91 | 0.649934 |  |
| GO:0015837\_amine\_transport | PDPN | 66 | 1 | 2.052139 | -0.410089 | 603 | 391.91 | 0.649934 |  |
| GO:0031589\_cell-substrate\_adhesion | VWC2 | 66 | 1 | 2.052139 | -0.410089 | 603 | 391.91 | 0.649934 |  |
| GO:0051130\_positive\_regulation\_of\_cellular\_component\_organization | CDH4 | 66 | 1 | 2.052139 | -0.410089 | 603 | 391.91 | 0.649934 |  |
| GO:0051402\_neuron\_apoptosis | BAX | 66 | 1 | 2.052139 | -0.410089 | 603 | 391.91 | 0.649934 |  |
| GO:0006082\_organic\_acid\_metabolic\_process | GCLC | 182 | 2 | 1.488365 | -0.407360 | 604 | 392.15 | 0.649255 |  |
| GO:0006082\_organic\_acid\_metabolic\_process | PDPN | 182 | 2 | 1.488365 | -0.407360 | 604 | 392.15 | 0.649255 |  |
| GO:0009791\_post-embryonic\_development | BAX | 67 | 1 | 2.021510 | -0.405008 | 607 | 393.85 | 0.648847 |  |
| GO:0042445\_hormone\_metabolic\_process | CRHBP | 67 | 1 | 2.021510 | -0.405008 | 607 | 393.85 | 0.648847 |  |
| GO:0051247\_positive\_regulation\_of\_protein\_metabolic\_process | GCLC | 67 | 1 | 2.021510 | -0.405008 | 607 | 393.85 | 0.648847 |  |
| GO:0042180\_cellular\_ketone\_metabolic\_process | GCLC | 183 | 2 | 1.480231 | -0.404423 | 608 | 394.09 | 0.648174 |  |
| GO:0042180\_cellular\_ketone\_metabolic\_process | PDPN | 183 | 2 | 1.480231 | -0.404423 | 608 | 394.09 | 0.648174 |  |
| GO:0045893\_positive\_regulation\_of\_transcription\_\_DNA-dependent | NOTCH1 | 306 | 3 | 1.327855 | -0.402684 | 610 | 394.81 | 0.647230 |  |
| GO:0045893\_positive\_regulation\_of\_transcription\_\_DNA-dependent | NFYB | 306 | 3 | 1.327855 | -0.402684 | 610 | 394.81 | 0.647230 |  |
| GO:0045893\_positive\_regulation\_of\_transcription\_\_DNA-dependent | RB1 | 306 | 3 | 1.327855 | -0.402684 | 610 | 394.81 | 0.647230 |  |
| GO:0051254\_positive\_regulation\_of\_RNA\_metabolic\_process | NOTCH1 | 306 | 3 | 1.327855 | -0.402684 | 610 | 394.81 | 0.647230 |  |
| GO:0051254\_positive\_regulation\_of\_RNA\_metabolic\_process | NFYB | 306 | 3 | 1.327855 | -0.402684 | 610 | 394.81 | 0.647230 |  |
| GO:0051254\_positive\_regulation\_of\_RNA\_metabolic\_process | RB1 | 306 | 3 | 1.327855 | -0.402684 | 610 | 394.81 | 0.647230 |  |
| GO:0010604\_positive\_regulation\_of\_macromolecule\_metabolic\_process | NOTCH1 | 433 | 4 | 1.251189 | -0.399092 | 611 | 397.58 | 0.650704 |  |
| GO:0010604\_positive\_regulation\_of\_macromolecule\_metabolic\_process | GCLC | 433 | 4 | 1.251189 | -0.399092 | 611 | 397.58 | 0.650704 |  |
| GO:0010604\_positive\_regulation\_of\_macromolecule\_metabolic\_process | NFYB | 433 | 4 | 1.251189 | -0.399092 | 611 | 397.58 | 0.650704 |  |
| GO:0010604\_positive\_regulation\_of\_macromolecule\_metabolic\_process | RB1 | 433 | 4 | 1.251189 | -0.399092 | 611 | 397.58 | 0.650704 |  |
| GO:0016310\_phosphorylation | UQCRC1 | 309 | 3 | 1.314963 | -0.395993 | 612 | 398.22 | 0.650686 |  |
| GO:0016310\_phosphorylation | BAX | 309 | 3 | 1.314963 | -0.395993 | 612 | 398.22 | 0.650686 |  |
| GO:0016310\_phosphorylation | HBB-B1 | 309 | 3 | 1.314963 | -0.395993 | 612 | 398.22 | 0.650686 |  |
| GO:0005996\_monosaccharide\_metabolic\_process | BAD | 69 | 1 | 1.962916 | -0.395129 | 615 | 400.61 | 0.651398 |  |
| GO:0006816\_calcium\_ion\_transport | BAX | 69 | 1 | 1.962916 | -0.395129 | 615 | 400.61 | 0.651398 |  |
| GO:0055065\_metal\_ion\_homeostasis | BAX | 69 | 1 | 1.962916 | -0.395129 | 615 | 400.61 | 0.651398 |  |
| GO:0006952\_defense\_response | TAC1 | 187 | 2 | 1.448569 | -0.392920 | 616 | 400.85 | 0.650731 |  |
| GO:0006952\_defense\_response | RBPJ | 187 | 2 | 1.448569 | -0.392920 | 616 | 400.85 | 0.650731 |  |
| GO:0065008\_regulation\_of\_biological\_quality | GCLC | 693 | 6 | 1.172651 | -0.391663 | 617 | 401.25 | 0.650324 |  |
| GO:0065008\_regulation\_of\_biological\_quality | BAX | 693 | 6 | 1.172651 | -0.391663 | 617 | 401.25 | 0.650324 |  |
| GO:0065008\_regulation\_of\_biological\_quality | CRHBP | 693 | 6 | 1.172651 | -0.391663 | 617 | 401.25 | 0.650324 |  |
| GO:0065008\_regulation\_of\_biological\_quality | BAD | 693 | 6 | 1.172651 | -0.391663 | 617 | 401.25 | 0.650324 |  |
| GO:0065008\_regulation\_of\_biological\_quality | RB1 | 693 | 6 | 1.172651 | -0.391663 | 617 | 401.25 | 0.650324 |  |
| GO:0065008\_regulation\_of\_biological\_quality | SEMA3A | 693 | 6 | 1.172651 | -0.391663 | 617 | 401.25 | 0.650324 |  |
| GO:0008406\_gonad\_development | BAX | 70 | 1 | 1.934874 | -0.390325 | 621 | 402.75 | 0.648551 |  |
| GO:0009617\_response\_to\_bacterium | RBPJ | 70 | 1 | 1.934874 | -0.390325 | 621 | 402.75 | 0.648551 |  |
| GO:0048592\_eye\_morphogenesis | BAX | 70 | 1 | 1.934874 | -0.390325 | 621 | 402.75 | 0.648551 |  |
| GO:0070838\_divalent\_metal\_ion\_transport | BAX | 70 | 1 | 1.934874 | -0.390325 | 621 | 402.75 | 0.648551 |  |
| GO:0006913\_nucleocytoplasmic\_transport | NUP133 | 71 | 1 | 1.907622 | -0.385609 | 623 | 405.17 | 0.650353 |  |
| GO:0009100\_glycoprotein\_metabolic\_process | PHLDA1 | 71 | 1 | 1.907622 | -0.385609 | 623 | 405.17 | 0.650353 |  |
| GO:0031325\_positive\_regulation\_of\_cellular\_metabolic\_process | NOTCH1 | 442 | 4 | 1.225712 | -0.382418 | 624 | 405.66 | 0.650096 |  |
| GO:0031325\_positive\_regulation\_of\_cellular\_metabolic\_process | GCLC | 442 | 4 | 1.225712 | -0.382418 | 624 | 405.66 | 0.650096 |  |
| GO:0031325\_positive\_regulation\_of\_cellular\_metabolic\_process | NFYB | 442 | 4 | 1.225712 | -0.382418 | 624 | 405.66 | 0.650096 |  |
| GO:0031325\_positive\_regulation\_of\_cellular\_metabolic\_process | RB1 | 442 | 4 | 1.225712 | -0.382418 | 624 | 405.66 | 0.650096 |  |
| GO:0021915\_neural\_tube\_development | NOTCH1 | 72 | 1 | 1.881127 | -0.380976 | 629 | 409.05 | 0.650318 |  |
| GO:0030879\_mammary\_gland\_development | BAX | 72 | 1 | 1.881127 | -0.380976 | 629 | 409.05 | 0.650318 |  |
| GO:0042098\_T\_cell\_proliferation | BAX | 72 | 1 | 1.881127 | -0.380976 | 629 | 409.05 | 0.650318 |  |
| GO:0044262\_cellular\_carbohydrate\_metabolic\_process | SORD | 72 | 1 | 1.881127 | -0.380976 | 629 | 409.05 | 0.650318 |  |
| GO:0051169\_nuclear\_transport | NUP133 | 72 | 1 | 1.881127 | -0.380976 | 629 | 409.05 | 0.650318 |  |
| GO:0007154\_cell\_communication | KRT18 | 1096 | 9 | 1.112199 | -0.375474 | 630 | 412.26 | 0.654381 |  |
| GO:0007154\_cell\_communication | PDPN | 1096 | 9 | 1.112199 | -0.375474 | 630 | 412.26 | 0.654381 |  |
| GO:0007154\_cell\_communication | SOS1 | 1096 | 9 | 1.112199 | -0.375474 | 630 | 412.26 | 0.654381 |  |
| GO:0007154\_cell\_communication | RRAS2 | 1096 | 9 | 1.112199 | -0.375474 | 630 | 412.26 | 0.654381 |  |
| GO:0007154\_cell\_communication | VWC2 | 1096 | 9 | 1.112199 | -0.375474 | 630 | 412.26 | 0.654381 |  |
| GO:0007154\_cell\_communication | ROR2 | 1096 | 9 | 1.112199 | -0.375474 | 630 | 412.26 | 0.654381 |  |
| GO:0007154\_cell\_communication | BAD | 1096 | 9 | 1.112199 | -0.375474 | 630 | 412.26 | 0.654381 |  |
| GO:0007154\_cell\_communication | RBPJ | 1096 | 9 | 1.112199 | -0.375474 | 630 | 412.26 | 0.654381 |  |
| GO:0007154\_cell\_communication | RGS16 | 1096 | 9 | 1.112199 | -0.375474 | 630 | 412.26 | 0.654381 |  |
| GO:0009416\_response\_to\_light\_stimulus | BAX | 74 | 1 | 1.830286 | -0.371955 | 632 | 413.76 | 0.654684 |  |
| GO:0048771\_tissue\_remodeling | BAX | 74 | 1 | 1.830286 | -0.371955 | 632 | 413.76 | 0.654684 |  |
| GO:0003002\_regionalization | NOTCH1 | 195 | 2 | 1.389140 | -0.371041 | 635 | 414.64 | 0.652976 |  |
| GO:0003002\_regionalization | ROR2 | 195 | 2 | 1.389140 | -0.371041 | 635 | 414.64 | 0.652976 |  |
| GO:0007507\_heart\_development | NOTCH1 | 195 | 2 | 1.389140 | -0.371041 | 635 | 414.64 | 0.652976 |  |
| GO:0007507\_heart\_development | RBPJ | 195 | 2 | 1.389140 | -0.371041 | 635 | 414.64 | 0.652976 |  |
| GO:0019725\_cellular\_homeostasis | GCLC | 195 | 2 | 1.389140 | -0.371041 | 635 | 414.64 | 0.652976 |  |
| GO:0019725\_cellular\_homeostasis | BAX | 195 | 2 | 1.389140 | -0.371041 | 635 | 414.64 | 0.652976 |  |
| GO:0033554\_cellular\_response\_to\_stress | ROR2 | 196 | 2 | 1.382053 | -0.368406 | 636 | 415.19 | 0.652814 |  |
| GO:0033554\_cellular\_response\_to\_stress | HBB-B1 | 196 | 2 | 1.382053 | -0.368406 | 636 | 415.19 | 0.652814 |  |
| GO:0007281\_germ\_cell\_development | BAX | 75 | 1 | 1.805882 | -0.367563 | 639 | 416.56 | 0.651894 |  |
| GO:0048589\_developmental\_growth | NOTCH1 | 75 | 1 | 1.805882 | -0.367563 | 639 | 416.56 | 0.651894 |  |
| GO:0051050\_positive\_regulation\_of\_transport | BAX | 75 | 1 | 1.805882 | -0.367563 | 639 | 416.56 | 0.651894 |  |
| GO:0050801\_ion\_homeostasis | GCLC | 197 | 2 | 1.375037 | -0.365794 | 640 | 417.07 | 0.651672 |  |
| GO:0050801\_ion\_homeostasis | BAX | 197 | 2 | 1.375037 | -0.365794 | 640 | 417.07 | 0.651672 |  |
| GO:0006508\_proteolysis | GCLC | 76 | 1 | 1.782121 | -0.363245 | 641 | 418.37 | 0.652683 |  |
| GO:0050896\_response\_to\_stimulus | FEZF2 | 1107 | 9 | 1.101148 | -0.362226 | 642 | 418.89 | 0.652477 |  |
| GO:0050896\_response\_to\_stimulus | GCLC | 1107 | 9 | 1.101148 | -0.362226 | 642 | 418.89 | 0.652477 |  |
| GO:0050896\_response\_to\_stimulus | ENDOG | 1107 | 9 | 1.101148 | -0.362226 | 642 | 418.89 | 0.652477 |  |
| GO:0050896\_response\_to\_stimulus | BAX | 1107 | 9 | 1.101148 | -0.362226 | 642 | 418.89 | 0.652477 |  |
| GO:0050896\_response\_to\_stimulus | ROR2 | 1107 | 9 | 1.101148 | -0.362226 | 642 | 418.89 | 0.652477 |  |
| GO:0050896\_response\_to\_stimulus | TAC1 | 1107 | 9 | 1.101148 | -0.362226 | 642 | 418.89 | 0.652477 |  |
| GO:0050896\_response\_to\_stimulus | HBB-B1 | 1107 | 9 | 1.101148 | -0.362226 | 642 | 418.89 | 0.652477 |  |
| GO:0050896\_response\_to\_stimulus | SEMA3A | 1107 | 9 | 1.101148 | -0.362226 | 642 | 418.89 | 0.652477 |  |
| GO:0050896\_response\_to\_stimulus | RBPJ | 1107 | 9 | 1.101148 | -0.362226 | 642 | 418.89 | 0.652477 |  |
| GO:0044238\_primary\_metabolic\_process | SORD | 1905 | 15 | 1.066466 | -0.360629 | 643 | 419.31 | 0.652115 |  |
| GO:0044238\_primary\_metabolic\_process | GCLC | 1905 | 15 | 1.066466 | -0.360629 | 643 | 419.31 | 0.652115 |  |
| GO:0044238\_primary\_metabolic\_process | POLR1E | 1905 | 15 | 1.066466 | -0.360629 | 643 | 419.31 | 0.652115 |  |
| GO:0044238\_primary\_metabolic\_process | PDPN | 1905 | 15 | 1.066466 | -0.360629 | 643 | 419.31 | 0.652115 |  |
| GO:0044238\_primary\_metabolic\_process | NFYB | 1905 | 15 | 1.066466 | -0.360629 | 643 | 419.31 | 0.652115 |  |
| GO:0044238\_primary\_metabolic\_process | BAD | 1905 | 15 | 1.066466 | -0.360629 | 643 | 419.31 | 0.652115 |  |
| GO:0044238\_primary\_metabolic\_process | VAX1 | 1905 | 15 | 1.066466 | -0.360629 | 643 | 419.31 | 0.652115 |  |
| GO:0044238\_primary\_metabolic\_process | RB1 | 1905 | 15 | 1.066466 | -0.360629 | 643 | 419.31 | 0.652115 |  |
| GO:0044238\_primary\_metabolic\_process | PKNOX2 | 1905 | 15 | 1.066466 | -0.360629 | 643 | 419.31 | 0.652115 |  |
| GO:0044238\_primary\_metabolic\_process | NOTCH1 | 1905 | 15 | 1.066466 | -0.360629 | 643 | 419.31 | 0.652115 |  |
| GO:0044238\_primary\_metabolic\_process | NR1D2 | 1905 | 15 | 1.066466 | -0.360629 | 643 | 419.31 | 0.652115 |  |
| GO:0044238\_primary\_metabolic\_process | BAX | 1905 | 15 | 1.066466 | -0.360629 | 643 | 419.31 | 0.652115 |  |
| GO:0044238\_primary\_metabolic\_process | ENDOG | 1905 | 15 | 1.066466 | -0.360629 | 643 | 419.31 | 0.652115 |  |
| GO:0044238\_primary\_metabolic\_process | HBB-B1 | 1905 | 15 | 1.066466 | -0.360629 | 643 | 419.31 | 0.652115 |  |
| GO:0044238\_primary\_metabolic\_process | RBPJ | 1905 | 15 | 1.066466 | -0.360629 | 643 | 419.31 | 0.652115 |  |
| GO:0051241\_negative\_regulation\_of\_multicellular\_organismal\_process | NOTCH1 | 77 | 1 | 1.758976 | -0.359001 | 644 | 420.08 | 0.652298 |  |
| GO:0006461\_protein\_complex\_assembly | BAX | 78 | 1 | 1.736425 | -0.354829 | 649 | 422.64 | 0.651217 |  |
| GO:0030326\_embryonic\_limb\_morphogenesis | NOTCH1 | 78 | 1 | 1.736425 | -0.354829 | 649 | 422.64 | 0.651217 |  |
| GO:0035113\_embryonic\_appendage\_morphogenesis | NOTCH1 | 78 | 1 | 1.736425 | -0.354829 | 649 | 422.64 | 0.651217 |  |
| GO:0051251\_positive\_regulation\_of\_lymphocyte\_activation | BAD | 78 | 1 | 1.736425 | -0.354829 | 649 | 422.64 | 0.651217 |  |
| GO:0070271\_protein\_complex\_biogenesis | BAX | 78 | 1 | 1.736425 | -0.354829 | 649 | 422.64 | 0.651217 |  |
| GO:0009893\_positive\_regulation\_of\_metabolic\_process | NOTCH1 | 458 | 4 | 1.182892 | -0.354427 | 650 | 423.14 | 0.650985 |  |
| GO:0009893\_positive\_regulation\_of\_metabolic\_process | GCLC | 458 | 4 | 1.182892 | -0.354427 | 650 | 423.14 | 0.650985 |  |
| GO:0009893\_positive\_regulation\_of\_metabolic\_process | NFYB | 458 | 4 | 1.182892 | -0.354427 | 650 | 423.14 | 0.650985 |  |
| GO:0009893\_positive\_regulation\_of\_metabolic\_process | RB1 | 458 | 4 | 1.182892 | -0.354427 | 650 | 423.14 | 0.650985 |  |
| GO:0015674\_di-\_\_tri-valent\_inorganic\_cation\_transport | BAX | 79 | 1 | 1.714445 | -0.350726 | 651 | 424.71 | 0.652396 |  |
| GO:0000278\_mitotic\_cell\_cycle | RB1 | 80 | 1 | 1.693015 | -0.346692 | 653 | 427.97 | 0.655391 |  |
| GO:0006631\_fatty\_acid\_metabolic\_process | PDPN | 80 | 1 | 1.693015 | -0.346692 | 653 | 427.97 | 0.655391 |  |
| GO:0002696\_positive\_regulation\_of\_leukocyte\_activation | BAD | 82 | 1 | 1.651722 | -0.338819 | 654 | 431.9 | 0.660398 |  |
| GO:0008284\_positive\_regulation\_of\_cell\_proliferation | NOTCH1 | 208 | 2 | 1.302319 | -0.338410 | 655 | 432.56 | 0.660397 |  |
| GO:0008284\_positive\_regulation\_of\_cell\_proliferation | RBPJ | 208 | 2 | 1.302319 | -0.338410 | 655 | 432.56 | 0.660397 |  |
| GO:0045941\_positive\_regulation\_of\_transcription | NOTCH1 | 338 | 3 | 1.202141 | -0.336967 | 656 | 432.89 | 0.659893 |  |
| GO:0045941\_positive\_regulation\_of\_transcription | NFYB | 338 | 3 | 1.202141 | -0.336967 | 656 | 432.89 | 0.659893 |  |
| GO:0045941\_positive\_regulation\_of\_transcription | RB1 | 338 | 3 | 1.202141 | -0.336967 | 656 | 432.89 | 0.659893 |  |
| GO:0009605\_response\_to\_external\_stimulus | BAX | 339 | 3 | 1.198594 | -0.335101 | 657 | 433.23 | 0.659406 |  |
| GO:0009605\_response\_to\_external\_stimulus | TAC1 | 339 | 3 | 1.198594 | -0.335101 | 657 | 433.23 | 0.659406 |  |
| GO:0009605\_response\_to\_external\_stimulus | SEMA3A | 339 | 3 | 1.198594 | -0.335101 | 657 | 433.23 | 0.659406 |  |
| GO:0006575\_cellular\_amino\_acid\_derivative\_metabolic\_process | GCLC | 83 | 1 | 1.631821 | -0.334978 | 659 | 435.3 | 0.660546 |  |
| GO:0050867\_positive\_regulation\_of\_cell\_activation | BAD | 83 | 1 | 1.631821 | -0.334978 | 659 | 435.3 | 0.660546 |  |
| GO:0006793\_phosphorus\_metabolic\_process | UQCRC1 | 340 | 3 | 1.195069 | -0.333246 | 661 | 435.7 | 0.659153 |  |
| GO:0006793\_phosphorus\_metabolic\_process | BAX | 340 | 3 | 1.195069 | -0.333246 | 661 | 435.7 | 0.659153 |  |
| GO:0006793\_phosphorus\_metabolic\_process | HBB-B1 | 340 | 3 | 1.195069 | -0.333246 | 661 | 435.7 | 0.659153 |  |
| GO:0006796\_phosphate\_metabolic\_process | UQCRC1 | 340 | 3 | 1.195069 | -0.333246 | 661 | 435.7 | 0.659153 |  |
| GO:0006796\_phosphate\_metabolic\_process | BAX | 340 | 3 | 1.195069 | -0.333246 | 661 | 435.7 | 0.659153 |  |
| GO:0006796\_phosphate\_metabolic\_process | HBB-B1 | 340 | 3 | 1.195069 | -0.333246 | 661 | 435.7 | 0.659153 |  |
| GO:0030005\_cellular\_di-\_\_tri-valent\_inorganic\_cation\_homeostasis | BAX | 84 | 1 | 1.612395 | -0.331198 | 663 | 436.32 | 0.658100 |  |
| GO:0045137\_development\_of\_primary\_sexual\_characteristics | BAX | 84 | 1 | 1.612395 | -0.331198 | 663 | 436.32 | 0.658100 |  |
| GO:0035295\_tube\_development | NOTCH1 | 212 | 2 | 1.277747 | -0.329036 | 664 | 436.9 | 0.657982 |  |
| GO:0035295\_tube\_development | PDPN | 212 | 2 | 1.277747 | -0.329036 | 664 | 436.9 | 0.657982 |  |
| GO:0032504\_multicellular\_organism\_reproduction | BAX | 86 | 1 | 1.574897 | -0.323816 | 666 | 439.87 | 0.660465 |  |
| GO:0048609\_reproductive\_process\_in\_a\_multicellular\_organism | BAX | 86 | 1 | 1.574897 | -0.323816 | 666 | 439.87 | 0.660465 |  |
| GO:0010628\_positive\_regulation\_of\_gene\_expression | NOTCH1 | 346 | 3 | 1.174345 | -0.322331 | 667 | 440.19 | 0.659955 |  |
| GO:0010628\_positive\_regulation\_of\_gene\_expression | NFYB | 346 | 3 | 1.174345 | -0.322331 | 667 | 440.19 | 0.659955 |  |
| GO:0010628\_positive\_regulation\_of\_gene\_expression | RB1 | 346 | 3 | 1.174345 | -0.322331 | 667 | 440.19 | 0.659955 |  |
| GO:0001822\_kidney\_development | BAX | 87 | 1 | 1.556795 | -0.320212 | 669 | 442.63 | 0.661629 |  |
| GO:0007178\_transmembrane\_receptor\_protein\_serine\_threonine\_kinase\_signaling\_pathway | VWC2 | 87 | 1 | 1.556795 | -0.320212 | 669 | 442.63 | 0.661629 |  |
| GO:0048754\_branching\_morphogenesis\_of\_a\_tube | NOTCH1 | 88 | 1 | 1.539104 | -0.316663 | 671 | 445.07 | 0.663294 |  |
| GO:0050863\_regulation\_of\_T\_cell\_activation | BAD | 88 | 1 | 1.539104 | -0.316663 | 671 | 445.07 | 0.663294 |  |
| GO:0007423\_sensory\_organ\_development | BAX | 219 | 2 | 1.236906 | -0.313326 | 672 | 445.94 | 0.663601 |  |
| GO:0007423\_sensory\_organ\_development | VAX1 | 219 | 2 | 1.236906 | -0.313326 | 672 | 445.94 | 0.663601 |  |
| GO:0045935\_positive\_regulation\_of\_nucleobase\_\_nucleoside\_\_nucleotide\_and\_nucleic\_acid\_metabolic\_process | NOTCH1 | 352 | 3 | 1.154328 | -0.311778 | 673 | 446.42 | 0.663328 |  |
| GO:0045935\_positive\_regulation\_of\_nucleobase\_\_nucleoside\_\_nucleotide\_and\_nucleic\_acid\_metabolic\_process | NFYB | 352 | 3 | 1.154328 | -0.311778 | 673 | 446.42 | 0.663328 |  |
| GO:0045935\_positive\_regulation\_of\_nucleobase\_\_nucleoside\_\_nucleotide\_and\_nucleic\_acid\_metabolic\_process | RB1 | 352 | 3 | 1.154328 | -0.311778 | 673 | 446.42 | 0.663328 |  |
| GO:0030003\_cellular\_cation\_homeostasis | BAX | 90 | 1 | 1.504902 | -0.309727 | 674 | 448.17 | 0.664941 |  |
| GO:0001701\_in\_utero\_embryonic\_development | NOTCH1 | 221 | 2 | 1.225712 | -0.308993 | 675 | 448.41 | 0.664311 |  |
| GO:0001701\_in\_utero\_embryonic\_development | ENDOG | 221 | 2 | 1.225712 | -0.308993 | 675 | 448.41 | 0.664311 |  |
| GO:0030217\_T\_cell\_differentiation | BAD | 92 | 1 | 1.472187 | -0.302999 | 676 | 451.13 | 0.667352 |  |
| GO:0055066\_di-\_\_tri-valent\_inorganic\_cation\_homeostasis | BAX | 93 | 1 | 1.456357 | -0.299710 | 678 | 452.72 | 0.667729 |  |
| GO:0065003\_macromolecular\_complex\_assembly | BAX | 93 | 1 | 1.456357 | -0.299710 | 678 | 452.72 | 0.667729 |  |
| GO:0051173\_positive\_regulation\_of\_nitrogen\_compound\_metabolic\_process | NOTCH1 | 361 | 3 | 1.125550 | -0.296598 | 679 | 453.25 | 0.667526 |  |
| GO:0051173\_positive\_regulation\_of\_nitrogen\_compound\_metabolic\_process | NFYB | 361 | 3 | 1.125550 | -0.296598 | 679 | 453.25 | 0.667526 |  |
| GO:0051173\_positive\_regulation\_of\_nitrogen\_compound\_metabolic\_process | RB1 | 361 | 3 | 1.125550 | -0.296598 | 679 | 453.25 | 0.667526 |  |
| GO:0032943\_mononuclear\_cell\_proliferation | BAX | 94 | 1 | 1.440864 | -0.296469 | 681 | 455.49 | 0.668855 |  |
| GO:0046651\_lymphocyte\_proliferation | BAX | 94 | 1 | 1.440864 | -0.296469 | 681 | 455.49 | 0.668855 |  |
| GO:0002682\_regulation\_of\_immune\_system\_process | RB1 | 228 | 2 | 1.188080 | -0.294342 | 682 | 456.44 | 0.669267 |  |
| GO:0002682\_regulation\_of\_immune\_system\_process | BAD | 228 | 2 | 1.188080 | -0.294342 | 682 | 456.44 | 0.669267 |  |
| GO:0051707\_response\_to\_other\_organism | RBPJ | 95 | 1 | 1.425697 | -0.293276 | 683 | 457.31 | 0.669561 |  |
| GO:0006954\_inflammatory\_response | TAC1 | 96 | 1 | 1.410846 | -0.290129 | 686 | 459.5 | 0.669825 |  |
| GO:0060249\_anatomical\_structure\_homeostasis | BAX | 96 | 1 | 1.410846 | -0.290129 | 686 | 459.5 | 0.669825 |  |
| GO:0070661\_leukocyte\_proliferation | BAX | 96 | 1 | 1.410846 | -0.290129 | 686 | 459.5 | 0.669825 |  |
| GO:0043009\_chordate\_embryonic\_development | NOTCH1 | 365 | 3 | 1.113215 | -0.290092 | 687 | 460.15 | 0.669796 |  |
| GO:0043009\_chordate\_embryonic\_development | ENDOG | 365 | 3 | 1.113215 | -0.290092 | 687 | 460.15 | 0.669796 |  |
| GO:0043009\_chordate\_embryonic\_development | ROR2 | 365 | 3 | 1.113215 | -0.290092 | 687 | 460.15 | 0.669796 |  |
| GO:0018193\_peptidyl-amino\_acid\_modification | BAX | 97 | 1 | 1.396301 | -0.287028 | 688 | 461.84 | 0.671279 |  |
| GO:0009792\_embryonic\_development\_ending\_in\_birth\_or\_egg\_hatching | NOTCH1 | 368 | 3 | 1.104140 | -0.285307 | 689 | 462.19 | 0.670813 |  |
| GO:0009792\_embryonic\_development\_ending\_in\_birth\_or\_egg\_hatching | ENDOG | 368 | 3 | 1.104140 | -0.285307 | 689 | 462.19 | 0.670813 |  |
| GO:0009792\_embryonic\_development\_ending\_in\_birth\_or\_egg\_hatching | ROR2 | 368 | 3 | 1.104140 | -0.285307 | 689 | 462.19 | 0.670813 |  |
| GO:0050790\_regulation\_of\_catalytic\_activity | BAX | 233 | 2 | 1.162585 | -0.284347 | 690 | 462.98 | 0.670986 |  |
| GO:0050790\_regulation\_of\_catalytic\_activity | BAD | 233 | 2 | 1.162585 | -0.284347 | 690 | 462.98 | 0.670986 |  |
| GO:0009314\_response\_to\_radiation | BAX | 98 | 1 | 1.382053 | -0.283971 | 691 | 464.41 | 0.672084 |  |
| GO:0010557\_positive\_regulation\_of\_macromolecule\_biosynthetic\_process | NOTCH1 | 371 | 3 | 1.095212 | -0.280600 | 692 | 467.04 | 0.674913 |  |
| GO:0010557\_positive\_regulation\_of\_macromolecule\_biosynthetic\_process | NFYB | 371 | 3 | 1.095212 | -0.280600 | 692 | 467.04 | 0.674913 |  |
| GO:0010557\_positive\_regulation\_of\_macromolecule\_biosynthetic\_process | RB1 | 371 | 3 | 1.095212 | -0.280600 | 692 | 467.04 | 0.674913 |  |
| GO:0006468\_protein\_amino\_acid\_phosphorylation | BAX | 237 | 2 | 1.142964 | -0.276618 | 693 | 468.67 | 0.676291 |  |
| GO:0006468\_protein\_amino\_acid\_phosphorylation | HBB-B1 | 237 | 2 | 1.142964 | -0.276618 | 693 | 468.67 | 0.676291 |  |
| GO:0030163\_protein\_catabolic\_process | GCLC | 101 | 1 | 1.341002 | -0.275058 | 694 | 469.07 | 0.675893 |  |
| GO:0022414\_reproductive\_process | NOTCH1 | 376 | 3 | 1.080648 | -0.272926 | 695 | 469.94 | 0.676173 |  |
| GO:0022414\_reproductive\_process | BAX | 376 | 3 | 1.080648 | -0.272926 | 695 | 469.94 | 0.676173 |  |
| GO:0022414\_reproductive\_process | ROR2 | 376 | 3 | 1.080648 | -0.272926 | 695 | 469.94 | 0.676173 |  |
| GO:0003013\_circulatory\_system\_process | SEMA3A | 103 | 1 | 1.314963 | -0.269321 | 698 | 471.8 | 0.675931 |  |
| GO:0008015\_blood\_circulation | SEMA3A | 103 | 1 | 1.314963 | -0.269321 | 698 | 471.8 | 0.675931 |  |
| GO:0009968\_negative\_regulation\_of\_signal\_transduction | VWC2 | 103 | 1 | 1.314963 | -0.269321 | 698 | 471.8 | 0.675931 |  |
| GO:0000003\_reproduction | NOTCH1 | 379 | 3 | 1.072094 | -0.268423 | 699 | 472.38 | 0.675794 |  |
| GO:0000003\_reproduction | BAX | 379 | 3 | 1.072094 | -0.268423 | 699 | 472.38 | 0.675794 |  |
| GO:0000003\_reproduction | ROR2 | 379 | 3 | 1.072094 | -0.268423 | 699 | 472.38 | 0.675794 |  |
| GO:0043687\_post-translational\_protein\_modification | GCLC | 384 | 3 | 1.058134 | -0.261080 | 700 | 475.0 | 0.678571 |  |
| GO:0043687\_post-translational\_protein\_modification | BAX | 384 | 3 | 1.058134 | -0.261080 | 700 | 475.0 | 0.678571 |  |
| GO:0043687\_post-translational\_protein\_modification | HBB-B1 | 384 | 3 | 1.058134 | -0.261080 | 700 | 475.0 | 0.678571 |  |
| GO:0010817\_regulation\_of\_hormone\_levels | CRHBP | 106 | 1 | 1.277747 | -0.261009 | 701 | 475.3 | 0.678031 |  |
| GO:0031328\_positive\_regulation\_of\_cellular\_biosynthetic\_process | NOTCH1 | 387 | 3 | 1.049932 | -0.256770 | 702 | 476.22 | 0.678376 |  |
| GO:0031328\_positive\_regulation\_of\_cellular\_biosynthetic\_process | NFYB | 387 | 3 | 1.049932 | -0.256770 | 702 | 476.22 | 0.678376 |  |
| GO:0031328\_positive\_regulation\_of\_cellular\_biosynthetic\_process | RB1 | 387 | 3 | 1.049932 | -0.256770 | 702 | 476.22 | 0.678376 |  |
| GO:0030099\_myeloid\_cell\_differentiation | RB1 | 108 | 1 | 1.254085 | -0.255654 | 704 | 477.48 | 0.678239 |  |
| GO:0051240\_positive\_regulation\_of\_multicellular\_organismal\_process | BAX | 108 | 1 | 1.254085 | -0.255654 | 704 | 477.48 | 0.678239 |  |
| GO:0009891\_positive\_regulation\_of\_biosynthetic\_process | NOTCH1 | 388 | 3 | 1.047226 | -0.255349 | 705 | 477.79 | 0.677716 |  |
| GO:0009891\_positive\_regulation\_of\_biosynthetic\_process | NFYB | 388 | 3 | 1.047226 | -0.255349 | 705 | 477.79 | 0.677716 |  |
| GO:0009891\_positive\_regulation\_of\_biosynthetic\_process | RB1 | 388 | 3 | 1.047226 | -0.255349 | 705 | 477.79 | 0.677716 |  |
| GO:0007389\_pattern\_specification\_process | NOTCH1 | 250 | 2 | 1.083529 | -0.253038 | 706 | 478.05 | 0.677125 |  |
| GO:0007389\_pattern\_specification\_process | ROR2 | 250 | 2 | 1.083529 | -0.253038 | 706 | 478.05 | 0.677125 |  |
| GO:0010648\_negative\_regulation\_of\_cell\_communication | VWC2 | 110 | 1 | 1.231283 | -0.250441 | 708 | 480.64 | 0.678870 |  |
| GO:0055080\_cation\_homeostasis | BAX | 110 | 1 | 1.231283 | -0.250441 | 708 | 480.64 | 0.678870 |  |
| GO:0048705\_skeletal\_system\_morphogenesis | ROR2 | 111 | 1 | 1.220191 | -0.247886 | 709 | 482.48 | 0.680508 |  |
| GO:0051249\_regulation\_of\_lymphocyte\_activation | BAD | 112 | 1 | 1.209296 | -0.245364 | 710 | 483.55 | 0.681056 |  |
| GO:0006974\_response\_to\_DNA\_damage\_stimulus | BAX | 113 | 1 | 1.198594 | -0.242875 | 711 | 485.28 | 0.682532 |  |
| GO:0000165\_MAPKKK\_cascade | ROR2 | 114 | 1 | 1.188080 | -0.240419 | 713 | 486.77 | 0.682707 |  |
| GO:0009607\_response\_to\_biotic\_stimulus | RBPJ | 114 | 1 | 1.188080 | -0.240419 | 713 | 486.77 | 0.682707 |  |
| GO:0080134\_regulation\_of\_response\_to\_stress | HBB-B1 | 116 | 1 | 1.167596 | -0.235601 | 714 | 489.11 | 0.685028 |  |
| GO:0043933\_macromolecular\_complex\_subunit\_organization | BAX | 117 | 1 | 1.157617 | -0.233238 | 715 | 489.71 | 0.684909 |  |
| GO:0006519\_cellular\_amino\_acid\_and\_derivative\_metabolic\_process | GCLC | 118 | 1 | 1.147807 | -0.230906 | 716 | 491.52 | 0.686480 |  |
| GO:0044255\_cellular\_lipid\_metabolic\_process | PDPN | 264 | 2 | 1.026070 | -0.230040 | 717 | 492.03 | 0.686234 |  |
| GO:0044255\_cellular\_lipid\_metabolic\_process | BAX | 264 | 2 | 1.026070 | -0.230040 | 717 | 492.03 | 0.686234 |  |
| GO:0022403\_cell\_cycle\_phase | RB1 | 119 | 1 | 1.138161 | -0.228602 | 718 | 492.75 | 0.686281 |  |
| GO:0014706\_striated\_muscle\_tissue\_development | RB1 | 120 | 1 | 1.128676 | -0.226328 | 719 | 493.39 | 0.686217 |  |
| GO:0007242\_intracellular\_signaling\_cascade | SOS1 | 411 | 3 | 0.988622 | -0.224717 | 720 | 493.86 | 0.685917 |  |
| GO:0007242\_intracellular\_signaling\_cascade | RRAS2 | 411 | 3 | 0.988622 | -0.224717 | 720 | 493.86 | 0.685917 |  |
| GO:0007242\_intracellular\_signaling\_cascade | ROR2 | 411 | 3 | 0.988622 | -0.224717 | 720 | 493.86 | 0.685917 |  |
| GO:0002694\_regulation\_of\_leukocyte\_activation | BAD | 121 | 1 | 1.119349 | -0.224082 | 721 | 495.48 | 0.687212 |  |
| GO:0045944\_positive\_regulation\_of\_transcription\_from\_RNA\_polymerase\_II\_promoter | NOTCH1 | 269 | 2 | 1.006998 | -0.222375 | 722 | 495.79 | 0.686690 |  |
| GO:0045944\_positive\_regulation\_of\_transcription\_from\_RNA\_polymerase\_II\_promoter | RB1 | 269 | 2 | 1.006998 | -0.222375 | 722 | 495.79 | 0.686690 |  |
| GO:0006886\_intracellular\_protein\_transport | SEC23A | 122 | 1 | 1.110174 | -0.221864 | 725 | 498.04 | 0.686952 |  |
| GO:0030001\_metal\_ion\_transport | BAX | 122 | 1 | 1.110174 | -0.221864 | 725 | 498.04 | 0.686952 |  |
| GO:0050865\_regulation\_of\_cell\_activation | BAD | 122 | 1 | 1.110174 | -0.221864 | 725 | 498.04 | 0.686952 |  |
| GO:0048646\_anatomical\_structure\_formation\_involved\_in\_morphogenesis | NOTCH1 | 277 | 2 | 0.977915 | -0.210665 | 726 | 502.6 | 0.692287 |  |
| GO:0048646\_anatomical\_structure\_formation\_involved\_in\_morphogenesis | RBPJ | 277 | 2 | 0.977915 | -0.210665 | 726 | 502.6 | 0.692287 |  |
| GO:0060537\_muscle\_tissue\_development | RB1 | 128 | 1 | 1.058134 | -0.209117 | 727 | 504.21 | 0.693549 |  |
| GO:0007610\_behavior | FEZF2 | 279 | 2 | 0.970904 | -0.207840 | 729 | 504.84 | 0.692510 |  |
| GO:0007610\_behavior | SEMA3A | 279 | 2 | 0.970904 | -0.207840 | 729 | 504.84 | 0.692510 |  |
| GO:0065009\_regulation\_of\_molecular\_function | BAX | 279 | 2 | 0.970904 | -0.207840 | 729 | 504.84 | 0.692510 |  |
| GO:0065009\_regulation\_of\_molecular\_function | BAD | 279 | 2 | 0.970904 | -0.207840 | 729 | 504.84 | 0.692510 |  |
| GO:0032787\_monocarboxylic\_acid\_metabolic\_process | PDPN | 130 | 1 | 1.041855 | -0.205071 | 730 | 507.3 | 0.694932 |  |
| GO:0006629\_lipid\_metabolic\_process | PDPN | 285 | 2 | 0.950464 | -0.199600 | 731 | 509.31 | 0.696731 |  |
| GO:0006629\_lipid\_metabolic\_process | BAX | 285 | 2 | 0.950464 | -0.199600 | 731 | 509.31 | 0.696731 |  |
| GO:0044057\_regulation\_of\_system\_process | SEMA3A | 133 | 1 | 1.018355 | -0.199181 | 732 | 510.15 | 0.696926 |  |
| GO:0007283\_spermatogenesis | BAX | 134 | 1 | 1.010755 | -0.197264 | 734 | 511.65 | 0.697071 |  |
| GO:0048232\_male\_gamete\_generation | BAX | 134 | 1 | 1.010755 | -0.197264 | 734 | 511.65 | 0.697071 |  |
| GO:0006464\_protein\_modification\_process | GCLC | 439 | 3 | 0.925566 | -0.192229 | 735 | 513.07 | 0.698054 |  |
| GO:0006464\_protein\_modification\_process | BAX | 439 | 3 | 0.925566 | -0.192229 | 735 | 513.07 | 0.698054 |  |
| GO:0006464\_protein\_modification\_process | HBB-B1 | 439 | 3 | 0.925566 | -0.192229 | 735 | 513.07 | 0.698054 |  |
| GO:0016044\_membrane\_organization | BAX | 140 | 1 | 0.967437 | -0.186221 | 736 | 517.12 | 0.702609 |  |
| GO:0010926\_anatomical\_structure\_formation | NOTCH1 | 447 | 3 | 0.909001 | -0.183812 | 737 | 518.14 | 0.703039 |  |
| GO:0010926\_anatomical\_structure\_formation | BAX | 447 | 3 | 0.909001 | -0.183812 | 737 | 518.14 | 0.703039 |  |
| GO:0010926\_anatomical\_structure\_formation | RBPJ | 447 | 3 | 0.909001 | -0.183812 | 737 | 518.14 | 0.703039 |  |
| GO:0048598\_embryonic\_morphogenesis | NOTCH1 | 299 | 2 | 0.905961 | -0.181668 | 738 | 519.09 | 0.703374 |  |
| GO:0048598\_embryonic\_morphogenesis | ROR2 | 299 | 2 | 0.905961 | -0.181668 | 738 | 519.09 | 0.703374 |  |
| GO:0035239\_tube\_morphogenesis | NOTCH1 | 143 | 1 | 0.947141 | -0.180980 | 739 | 519.38 | 0.702815 |  |
| GO:0007186\_G-protein\_coupled\_receptor\_protein\_signaling\_pathway | RGS16 | 144 | 1 | 0.940564 | -0.179272 | 740 | 520.46 | 0.703324 |  |
| GO:0006812\_cation\_transport | BAX | 146 | 1 | 0.927679 | -0.175915 | 741 | 522.11 | 0.704602 |  |
| GO:0043412\_biopolymer\_modification | GCLC | 458 | 3 | 0.887169 | -0.172813 | 742 | 523.68 | 0.705768 |  |
| GO:0043412\_biopolymer\_modification | BAX | 458 | 3 | 0.887169 | -0.172813 | 742 | 523.68 | 0.705768 |  |
| GO:0043412\_biopolymer\_modification | HBB-B1 | 458 | 3 | 0.887169 | -0.172813 | 742 | 523.68 | 0.705768 |  |
| GO:0002684\_positive\_regulation\_of\_immune\_system\_process | BAD | 148 | 1 | 0.915143 | -0.172632 | 744 | 524.38 | 0.704812 |  |
| GO:0043085\_positive\_regulation\_of\_catalytic\_activity | BAX | 148 | 1 | 0.915143 | -0.172632 | 744 | 524.38 | 0.704812 |  |
| GO:0022402\_cell\_cycle\_process | RB1 | 155 | 1 | 0.873814 | -0.161700 | 745 | 528.51 | 0.709409 |  |
| GO:0051704\_multi-organism\_process | RBPJ | 157 | 1 | 0.862683 | -0.158727 | 746 | 529.12 | 0.709276 |  |
| GO:0009628\_response\_to\_abiotic\_stimulus | BAX | 162 | 1 | 0.836057 | -0.151571 | 747 | 533.0 | 0.713521 |  |
| GO:0006259\_DNA\_metabolic\_process | ENDOG | 165 | 1 | 0.820856 | -0.147457 | 748 | 536.12 | 0.716738 |  |
| GO:0051049\_regulation\_of\_transport | BAX | 167 | 1 | 0.811025 | -0.144786 | 749 | 538.17 | 0.718518 |  |
| GO:0007600\_sensory\_perception | TAC1 | 172 | 1 | 0.787449 | -0.138349 | 750 | 541.42 | 0.721893 |  |
| GO:0044093\_positive\_regulation\_of\_molecular\_function | BAX | 173 | 1 | 0.782897 | -0.137101 | 751 | 543.05 | 0.723103 |  |
| GO:0016192\_vesicle-mediated\_transport | SPIRE1 | 184 | 1 | 0.736093 | -0.124187 | 752 | 552.52 | 0.734734 |  |
| GO:0006811\_ion\_transport | BAX | 186 | 1 | 0.728178 | -0.121990 | 753 | 554.3 | 0.736122 |  |
| GO:0007276\_gamete\_generation | BAX | 188 | 1 | 0.720432 | -0.119836 | 754 | 555.73 | 0.737042 |  |
| GO:0022607\_cellular\_component\_assembly | BAX | 204 | 1 | 0.663927 | -0.104051 | 755 | 563.78 | 0.746728 |  |
| GO:0007243\_protein\_kinase\_cascade | ROR2 | 205 | 1 | 0.660689 | -0.103144 | 756 | 564.66 | 0.746905 |  |
| GO:0044267\_cellular\_protein\_metabolic\_process | GCLC | 559 | 3 | 0.726876 | -0.097107 | 757 | 566.96 | 0.748956 |  |
| GO:0044267\_cellular\_protein\_metabolic\_process | BAX | 559 | 3 | 0.726876 | -0.097107 | 757 | 566.96 | 0.748956 |  |
| GO:0044267\_cellular\_protein\_metabolic\_process | HBB-B1 | 559 | 3 | 0.726876 | -0.097107 | 757 | 566.96 | 0.748956 |  |
| GO:0010033\_response\_to\_organic\_substance | ENDOG | 216 | 1 | 0.627042 | -0.093714 | 758 | 568.17 | 0.749565 |  |
| GO:0040007\_growth | NOTCH1 | 217 | 1 | 0.624153 | -0.092904 | 760 | 568.78 | 0.748395 |  |
| GO:0048583\_regulation\_of\_response\_to\_stimulus | HBB-B1 | 217 | 1 | 0.624153 | -0.092904 | 760 | 568.78 | 0.748395 |  |
| GO:0009790\_embryonic\_development | NOTCH1 | 567 | 3 | 0.716620 | -0.092688 | 761 | 568.88 | 0.747543 |  |
| GO:0009790\_embryonic\_development | ENDOG | 567 | 3 | 0.716620 | -0.092688 | 761 | 568.88 | 0.747543 |  |
| GO:0009790\_embryonic\_development | ROR2 | 567 | 3 | 0.716620 | -0.092688 | 761 | 568.88 | 0.747543 |  |
| GO:0019953\_sexual\_reproduction | BAX | 228 | 1 | 0.594040 | -0.084482 | 762 | 572.55 | 0.751378 |  |
| GO:0007167\_enzyme\_linked\_receptor\_protein\_signaling\_pathway | VWC2 | 229 | 1 | 0.591446 | -0.083759 | 763 | 573.15 | 0.751180 |  |
| GO:0050890\_cognition | TAC1 | 233 | 1 | 0.581293 | -0.080929 | 764 | 575.0 | 0.752618 |  |
| GO:0001501\_skeletal\_system\_development | ROR2 | 236 | 1 | 0.573903 | -0.078874 | 765 | 576.15 | 0.753137 |  |
| GO:0044085\_cellular\_component\_biogenesis | BAX | 237 | 1 | 0.571482 | -0.078202 | 766 | 577.29 | 0.753642 |  |
| GO:0009966\_regulation\_of\_signal\_transduction | VWC2 | 256 | 1 | 0.529067 | -0.066515 | 767 | 583.47 | 0.760717 |  |
| GO:0006996\_organelle\_organization | BAX | 449 | 2 | 0.603301 | -0.066425 | 768 | 583.65 | 0.759961 |  |
| GO:0006996\_organelle\_organization | ENDOG | 449 | 2 | 0.603301 | -0.066425 | 768 | 583.65 | 0.759961 |  |
| GO:0019538\_protein\_metabolic\_process | GCLC | 655 | 3 | 0.620341 | -0.054929 | 769 | 589.31 | 0.766333 |  |
| GO:0019538\_protein\_metabolic\_process | BAX | 655 | 3 | 0.620341 | -0.054929 | 769 | 589.31 | 0.766333 |  |
| GO:0019538\_protein\_metabolic\_process | HBB-B1 | 655 | 3 | 0.620341 | -0.054929 | 769 | 589.31 | 0.766333 |  |
| GO:0003008\_system\_process | TAC1 | 516 | 2 | 0.524966 | -0.042019 | 770 | 594.49 | 0.772065 |  |
| GO:0003008\_system\_process | SEMA3A | 516 | 2 | 0.524966 | -0.042019 | 770 | 594.49 | 0.772065 |  |
| GO:0010646\_regulation\_of\_cell\_communication | VWC2 | 330 | 1 | 0.410428 | -0.035765 | 771 | 595.98 | 0.772996 |  |
| GO:0050877\_neurological\_system\_process | TAC1 | 390 | 1 | 0.347285 | -0.021727 | 772 | 602.84 | 0.780881 |  |
| GO:0008150\_biological\_process | FRAS1 | 4605 | 34 | 1.000000 | 0.000000 | 2669 | 2669.43 | 1.000161 |  |
| GO:0008150\_biological\_process | UQCRC1 | 4605 | 34 | 1.000000 | 0.000000 | 2669 | 2669.43 | 1.000161 |  |
| GO:0008150\_biological\_process | SORD | 4605 | 34 | 1.000000 | 0.000000 | 2669 | 2669.43 | 1.000161 |  |
| GO:0008150\_biological\_process | GCLC | 4605 | 34 | 1.000000 | 0.000000 | 2669 | 2669.43 | 1.000161 |  |
| GO:0008150\_biological\_process | CRHBP | 4605 | 34 | 1.000000 | 0.000000 | 2669 | 2669.43 | 1.000161 |  |
| GO:0008150\_biological\_process | TAC1 | 4605 | 34 | 1.000000 | 0.000000 | 2669 | 2669.43 | 1.000161 |  |
| GO:0008150\_biological\_process | NFYB | 4605 | 34 | 1.000000 | 0.000000 | 2669 | 2669.43 | 1.000161 |  |
| GO:0008150\_biological\_process | CDH4 | 4605 | 34 | 1.000000 | 0.000000 | 2669 | 2669.43 | 1.000161 |  |
| GO:0008150\_biological\_process | NR1D2 | 4605 | 34 | 1.000000 | 0.000000 | 2669 | 2669.43 | 1.000161 |  |
| GO:0008150\_biological\_process | SOS1 | 4605 | 34 | 1.000000 | 0.000000 | 2669 | 2669.43 | 1.000161 |  |
| GO:0008150\_biological\_process | VWC2 | 4605 | 34 | 1.000000 | 0.000000 | 2669 | 2669.43 | 1.000161 |  |
| GO:0008150\_biological\_process | HBB-B1 | 4605 | 34 | 1.000000 | 0.000000 | 2669 | 2669.43 | 1.000161 |  |
| GO:0008150\_biological\_process | SEMA3A | 4605 | 34 | 1.000000 | 0.000000 | 2669 | 2669.43 | 1.000161 |  |
| GO:0008150\_biological\_process | IFRD1 | 4605 | 34 | 1.000000 | 0.000000 | 2669 | 2669.43 | 1.000161 |  |
| GO:0008150\_biological\_process | PHLDA1 | 4605 | 34 | 1.000000 | 0.000000 | 2669 | 2669.43 | 1.000161 |  |
| GO:0008150\_biological\_process | SEC23A | 4605 | 34 | 1.000000 | 0.000000 | 2669 | 2669.43 | 1.000161 |  |
| GO:0008150\_biological\_process | NUP133 | 4605 | 34 | 1.000000 | 0.000000 | 2669 | 2669.43 | 1.000161 |  |
| GO:0008150\_biological\_process | ALDH6A1 | 4605 | 34 | 1.000000 | 0.000000 | 2669 | 2669.43 | 1.000161 |  |
| GO:0008150\_biological\_process | POLR1E | 4605 | 34 | 1.000000 | 0.000000 | 2669 | 2669.43 | 1.000161 |  |
| GO:0008150\_biological\_process | PDPN | 4605 | 34 | 1.000000 | 0.000000 | 2669 | 2669.43 | 1.000161 |  |
| GO:0008150\_biological\_process | SPIRE1 | 4605 | 34 | 1.000000 | 0.000000 | 2669 | 2669.43 | 1.000161 |  |
| GO:0008150\_biological\_process | RB1 | 4605 | 34 | 1.000000 | 0.000000 | 2669 | 2669.43 | 1.000161 |  |
| GO:0008150\_biological\_process | VAX1 | 4605 | 34 | 1.000000 | 0.000000 | 2669 | 2669.43 | 1.000161 |  |
| GO:0008150\_biological\_process | BAD | 4605 | 34 | 1.000000 | 0.000000 | 2669 | 2669.43 | 1.000161 |  |
| GO:0008150\_biological\_process | RGS16 | 4605 | 34 | 1.000000 | 0.000000 | 2669 | 2669.43 | 1.000161 |  |
| GO:0008150\_biological\_process | PKNOX2 | 4605 | 34 | 1.000000 | 0.000000 | 2669 | 2669.43 | 1.000161 |  |
| GO:0008150\_biological\_process | FEZF2 | 4605 | 34 | 1.000000 | 0.000000 | 2669 | 2669.43 | 1.000161 |  |
| GO:0008150\_biological\_process | NOTCH1 | 4605 | 34 | 1.000000 | 0.000000 | 2669 | 2669.43 | 1.000161 |  |
| GO:0008150\_biological\_process | KRT18 | 4605 | 34 | 1.000000 | 0.000000 | 2669 | 2669.43 | 1.000161 |  |
| GO:0008150\_biological\_process | ENDOG | 4605 | 34 | 1.000000 | 0.000000 | 2669 | 2669.43 | 1.000161 |  |
| GO:0008150\_biological\_process | BAX | 4605 | 34 | 1.000000 | 0.000000 | 2669 | 2669.43 | 1.000161 |  |
| GO:0008150\_biological\_process | RRAS2 | 4605 | 34 | 1.000000 | 0.000000 | 2669 | 2669.43 | 1.000161 |  |
| GO:0008150\_biological\_process | ROR2 | 4605 | 34 | 1.000000 | 0.000000 | 2669 | 2669.43 | 1.000161 |  |
| GO:0008150\_biological\_process | RBPJ | 4605 | 34 | 1.000000 | 0.000000 | 2669 | 2669.43 | 1.000161 |  |
