# Supplementary material for: RedundancyMiner: De-replication of redundant GO categories in microarray and proteomics analysis
Source: BMC Bioinformatics. 2011 Feb 10;12:52. doi: 10.1186/1471-2105-12-52 (PMC3223614; doi:10.1186/1471-2105-12-52)
Supplement: Additional file 8 — Retinal development HTGM download. compressed package of the results of running HTGM on the retinal development genes list. [file 1471-2105-12-52-S8.ZIP › SCENARIO_2_MODIFIED/total.txt.total.txt.dir/Exp1_BestClusterMap_LEIGS_KM_24.csv.join.4.txt.dir/Exp1_BestClusterMap_LEIGS_KM_24.csv.join.4.txt.change.gce.html]

Gene Category Report for Exp1\_BestClusterMap\_LEIGS\_KM\_24.csv.join.4.txt

# Gene Category Report for Exp1\_BestClusterMap\_LEIGS\_KM\_24.csv.join.4.txt

| HYPERLINKED GO CATEGORY | HYPERLINKED GENE NAME | TOTAL GENES | CHANGED GENES | ENRICHMENT | LOG10(p) | CUMULATIVE NUMBER OF CATEGORIES | CUMULATIVE RANDOMS MEAN | FALSE DISCOVERY RATE |  |
| --- | --- | --- | --- | --- | --- | --- | --- | --- | --- |
| GO:0006333\_chromatin\_assembly\_or\_disassembly | SMARCA5 | 11 | 2 | 31.010101 | -2.753827 | 1 | 1.56 | 1.560000 |  |
| GO:0006333\_chromatin\_assembly\_or\_disassembly | SMARCA2 | 11 | 2 | 31.010101 | -2.753827 | 1 | 1.56 | 1.560000 |  |
| GO:0034643\_mitochondrion\_localization\_\_microtubule-mediated | KIF1B | 1 | 1 |  |  |  |  |  |  |
| GO:0047497\_mitochondrion\_transport\_along\_microtubule | KIF1B | 1 | 1 |  |  |  |  |  |  |
| GO:0051646\_mitochondrion\_localization | KIF1B | 1 | 1 |  |  |  |  |  |  |
| GO:0051654\_establishment\_of\_mitochondrion\_localization | KIF1B | 1 | 1 |  |  |  |  |  |  |
| GO:0051656\_establishment\_of\_organelle\_localization | CDC42 | 21 | 2 | 16.243386 | -2.187661 | 2 | 5.05 | 2.525000 |  |
| GO:0051656\_establishment\_of\_organelle\_localization | KIF1B | 21 | 2 | 16.243386 | -2.187661 | 2 | 5.05 | 2.525000 |  |
| GO:0009954\_proximal\_distal\_pattern\_formation | PBX1 | 23 | 2 | 14.830918 | -2.109892 | 3 | 5.88 | 1.960000 |  |
| GO:0009954\_proximal\_distal\_pattern\_formation | PBX2 | 23 | 2 | 14.830918 | -2.109892 | 3 | 5.88 | 1.960000 |  |
| GO:0007097\_nuclear\_migration | CDC42 | 2 | 1 |  |  |  |  |  |  |
| GO:0008089\_anterograde\_axon\_cargo\_transport | KIF1B | 2 | 1 |  |  |  |  |  |  |
| GO:0032743\_positive\_regulation\_of\_interleukin-2\_production | CD83 | 2 | 1 |  |  |  |  |  |  |
| GO:0006325\_chromatin\_organization | SMARCA5 | 83 | 3 | 6.164659 | -1.917532 | 4 | 8.08 | 2.020000 |  |
| GO:0006325\_chromatin\_organization | SMARCA2 | 83 | 3 | 6.164659 | -1.917532 | 4 | 8.08 | 2.020000 |  |
| GO:0006325\_chromatin\_organization | MYST4 | 83 | 3 | 6.164659 | -1.917532 | 4 | 8.08 | 2.020000 |  |
| GO:0051640\_organelle\_localization | CDC42 | 31 | 2 | 11.003584 | -1.858070 | 5 | 9.46 | 1.892000 |  |
| GO:0051640\_organelle\_localization | KIF1B | 31 | 2 | 11.003584 | -1.858070 | 5 | 9.46 | 1.892000 |  |
| GO:0033631\_cell-cell\_adhesion\_mediated\_by\_integrin | L1CAM | 3 | 1 |  |  |  |  |  |  |
| GO:0034332\_adherens\_junction\_organization | CDC42 | 3 | 1 |  |  |  |  |  |  |
| GO:0006511\_ubiquitin-dependent\_protein\_catabolic\_process | RNF11 | 39 | 2 | 8.746439 | -1.668184 | 6 | 14.26 | 2.376667 |  |
| GO:0006511\_ubiquitin-dependent\_protein\_catabolic\_process | EDEM1 | 39 | 2 | 8.746439 | -1.668184 | 6 | 14.26 | 2.376667 |  |
| GO:0000086\_G2\_M\_transition\_of\_mitotic\_cell\_cycle | CHEK1 | 4 | 1 |  |  |  |  |  |  |
| GO:0006334\_nucleosome\_assembly | SMARCA2 | 4 | 1 |  |  |  |  |  |  |
| GO:0032713\_negative\_regulation\_of\_interleukin-4\_production | CD83 | 4 | 1 |  |  |  |  |  |  |
| GO:0032733\_positive\_regulation\_of\_interleukin-10\_production | CD83 | 4 | 1 |  |  |  |  |  |  |
| GO:0019941\_modification-dependent\_protein\_catabolic\_process | RNF11 | 42 | 2 | 8.121693 | -1.607670 | 9 | 16.28 | 1.808889 |  |
| GO:0019941\_modification-dependent\_protein\_catabolic\_process | EDEM1 | 42 | 2 | 8.121693 | -1.607670 | 9 | 16.28 | 1.808889 |  |
| GO:0043632\_modification-dependent\_macromolecule\_catabolic\_process | RNF11 | 42 | 2 | 8.121693 | -1.607670 | 9 | 16.28 | 1.808889 |  |
| GO:0043632\_modification-dependent\_macromolecule\_catabolic\_process | EDEM1 | 42 | 2 | 8.121693 | -1.607670 | 9 | 16.28 | 1.808889 |  |
| GO:0051603\_proteolysis\_involved\_in\_cellular\_protein\_catabolic\_process | RNF11 | 42 | 2 | 8.121693 | -1.607670 | 9 | 16.28 | 1.808889 |  |
| GO:0051603\_proteolysis\_involved\_in\_cellular\_protein\_catabolic\_process | EDEM1 | 42 | 2 | 8.121693 | -1.607670 | 9 | 16.28 | 1.808889 |  |
| GO:0044257\_cellular\_protein\_catabolic\_process | RNF11 | 44 | 2 | 7.752525 | -1.569894 | 10 | 18.41 | 1.841000 |  |
| GO:0044257\_cellular\_protein\_catabolic\_process | EDEM1 | 44 | 2 | 7.752525 | -1.569894 | 10 | 18.41 | 1.841000 |  |
| GO:0006515\_misfolded\_or\_incompletely\_synthesized\_protein\_catabolic\_process | EDEM1 | 5 | 1 | 34.111111 | -1.537802 | 18 | 27.51 | 1.528333 |  |
| GO:0030433\_ER-associated\_protein\_catabolic\_process | EDEM1 | 5 | 1 | 34.111111 | -1.537802 | 18 | 27.51 | 1.528333 |  |
| GO:0033627\_cell\_adhesion\_mediated\_by\_integrin | L1CAM | 5 | 1 | 34.111111 | -1.537802 | 18 | 27.51 | 1.528333 |  |
| GO:0034109\_homotypic\_cell-cell\_adhesion | L1CAM | 5 | 1 | 34.111111 | -1.537802 | 18 | 27.51 | 1.528333 |  |
| GO:0043550\_regulation\_of\_lipid\_kinase\_activity | CDC42 | 5 | 1 | 34.111111 | -1.537802 | 18 | 27.51 | 1.528333 |  |
| GO:0043551\_regulation\_of\_phosphoinositide\_3-kinase\_activity | CDC42 | 5 | 1 | 34.111111 | -1.537802 | 18 | 27.51 | 1.528333 |  |
| GO:0043552\_positive\_regulation\_of\_phosphoinositide\_3-kinase\_activity | CDC42 | 5 | 1 | 34.111111 | -1.537802 | 18 | 27.51 | 1.528333 |  |
| GO:0048664\_neuron\_fate\_determination | CDC42 | 5 | 1 | 34.111111 | -1.537802 | 18 | 27.51 | 1.528333 |  |
| GO:0022403\_cell\_cycle\_phase | CDC42 | 119 | 3 | 4.299720 | -1.503919 | 19 | 28.56 | 1.503158 |  |
| GO:0022403\_cell\_cycle\_phase | CHEK1 | 119 | 3 | 4.299720 | -1.503919 | 19 | 28.56 | 1.503158 |  |
| GO:0022403\_cell\_cycle\_phase | FANCA | 119 | 3 | 4.299720 | -1.503919 | 19 | 28.56 | 1.503158 |  |
| GO:0022409\_positive\_regulation\_of\_cell-cell\_adhesion | L1CAM | 6 | 1 | 28.425926 | -1.459845 | 24 | 36.48 | 1.520000 |  |
| GO:0032653\_regulation\_of\_interleukin-10\_production | CD83 | 6 | 1 | 28.425926 | -1.459845 | 24 | 36.48 | 1.520000 |  |
| GO:0040023\_establishment\_of\_nucleus\_localization | CDC42 | 6 | 1 | 28.425926 | -1.459845 | 24 | 36.48 | 1.520000 |  |
| GO:0060271\_cilium\_morphogenesis | KIF3A | 6 | 1 | 28.425926 | -1.459845 | 24 | 36.48 | 1.520000 |  |
| GO:0065004\_protein-DNA\_complex\_assembly | SMARCA2 | 6 | 1 | 28.425926 | -1.459845 | 24 | 36.48 | 1.520000 |  |
| GO:0030031\_cell\_projection\_assembly | CDC42 | 53 | 2 | 6.436059 | -1.420515 | 25 | 38.11 | 1.524400 |  |
| GO:0030031\_cell\_projection\_assembly | KIF3A | 53 | 2 | 6.436059 | -1.420515 | 25 | 38.11 | 1.524400 |  |
| GO:0043285\_biopolymer\_catabolic\_process | NGLY1 | 129 | 3 | 3.966408 | -1.414719 | 27 | 38.26 | 1.417037 |  |
| GO:0043285\_biopolymer\_catabolic\_process | RNF11 | 129 | 3 | 3.966408 | -1.414719 | 27 | 38.26 | 1.417037 |  |
| GO:0043285\_biopolymer\_catabolic\_process | EDEM1 | 129 | 3 | 3.966408 | -1.414719 | 27 | 38.26 | 1.417037 |  |
| GO:0051276\_chromosome\_organization | SMARCA5 | 129 | 3 | 3.966408 | -1.414719 | 27 | 38.26 | 1.417037 |  |
| GO:0051276\_chromosome\_organization | SMARCA2 | 129 | 3 | 3.966408 | -1.414719 | 27 | 38.26 | 1.417037 |  |
| GO:0051276\_chromosome\_organization | MYST4 | 129 | 3 | 3.966408 | -1.414719 | 27 | 38.26 | 1.417037 |  |
| GO:0000188\_inactivation\_of\_MAPK\_activity | DUSP9 | 7 | 1 | 24.365079 | -1.394121 | 34 | 45.68 | 1.343529 |  |
| GO:0006516\_glycoprotein\_catabolic\_process | NGLY1 | 7 | 1 | 24.365079 | -1.394121 | 34 | 45.68 | 1.343529 |  |
| GO:0015908\_fatty\_acid\_transport | CROT | 7 | 1 | 24.365079 | -1.394121 | 34 | 45.68 | 1.343529 |  |
| GO:0022407\_regulation\_of\_cell-cell\_adhesion | L1CAM | 7 | 1 | 24.365079 | -1.394121 | 34 | 45.68 | 1.343529 |  |
| GO:0031497\_chromatin\_assembly | SMARCA2 | 7 | 1 | 24.365079 | -1.394121 | 34 | 45.68 | 1.343529 |  |
| GO:0043372\_positive\_regulation\_of\_CD4-positive\_\_alpha\_beta\_T\_cell\_differentiation | CD83 | 7 | 1 | 24.365079 | -1.394121 | 34 | 45.68 | 1.343529 |  |
| GO:0046847\_filopodium\_assembly | CDC42 | 7 | 1 | 24.365079 | -1.394121 | 34 | 45.68 | 1.343529 |  |
| GO:0009057\_macromolecule\_catabolic\_process | NGLY1 | 137 | 3 | 3.734793 | -1.349143 | 35 | 47.96 | 1.370286 |  |
| GO:0009057\_macromolecule\_catabolic\_process | RNF11 | 137 | 3 | 3.734793 | -1.349143 | 35 | 47.96 | 1.370286 |  |
| GO:0009057\_macromolecule\_catabolic\_process | EDEM1 | 137 | 3 | 3.734793 | -1.349143 | 35 | 47.96 | 1.370286 |  |
| GO:0006493\_protein\_amino\_acid\_O-linked\_glycosylation | OGT | 8 | 1 | 21.319444 | -1.337351 | 40 | 54.24 | 1.356000 |  |
| GO:0030035\_microspike\_assembly | CDC42 | 8 | 1 | 21.319444 | -1.337351 | 40 | 54.24 | 1.356000 |  |
| GO:0032613\_interleukin-10\_production | CD83 | 8 | 1 | 21.319444 | -1.337351 | 40 | 54.24 | 1.356000 |  |
| GO:0034728\_nucleosome\_organization | SMARCA2 | 8 | 1 | 21.319444 | -1.337351 | 40 | 54.24 | 1.356000 |  |
| GO:0043370\_regulation\_of\_CD4-positive\_\_alpha\_beta\_T\_cell\_differentiation | CD83 | 8 | 1 | 21.319444 | -1.337351 | 40 | 54.24 | 1.356000 |  |
| GO:0009056\_catabolic\_process | NGLY1 | 243 | 4 | 2.807499 | -1.292021 | 41 | 56.38 | 1.375122 |  |
| GO:0009056\_catabolic\_process | GOPC | 243 | 4 | 2.807499 | -1.292021 | 41 | 56.38 | 1.375122 |  |
| GO:0009056\_catabolic\_process | RNF11 | 243 | 4 | 2.807499 | -1.292021 | 41 | 56.38 | 1.375122 |  |
| GO:0009056\_catabolic\_process | EDEM1 | 243 | 4 | 2.807499 | -1.292021 | 41 | 56.38 | 1.375122 |  |
| GO:0030325\_adrenal\_gland\_development | PBX1 | 9 | 1 | 18.950617 | -1.287420 | 45 | 62.8 | 1.395556 |  |
| GO:0035088\_establishment\_or\_maintenance\_of\_apical\_basal\_cell\_polarity | CDC42 | 9 | 1 | 18.950617 | -1.287420 | 45 | 62.8 | 1.395556 |  |
| GO:0035162\_embryonic\_hemopoiesis | PBX1 | 9 | 1 | 18.950617 | -1.287420 | 45 | 62.8 | 1.395556 |  |
| GO:0051647\_nucleus\_localization | CDC42 | 9 | 1 | 18.950617 | -1.287420 | 45 | 62.8 | 1.395556 |  |
| GO:0007389\_pattern\_specification\_process | KIF3A | 250 | 4 | 2.728889 | -1.254511 | 46 | 64.4 | 1.400000 |  |
| GO:0007389\_pattern\_specification\_process | SOSTDC1 | 250 | 4 | 2.728889 | -1.254511 | 46 | 64.4 | 1.400000 |  |
| GO:0007389\_pattern\_specification\_process | PBX1 | 250 | 4 | 2.728889 | -1.254511 | 46 | 64.4 | 1.400000 |  |
| GO:0007389\_pattern\_specification\_process | PBX2 | 250 | 4 | 2.728889 | -1.254511 | 46 | 64.4 | 1.400000 |  |
| GO:0008088\_axon\_cargo\_transport | KIF1B | 10 | 1 | 17.055556 | -1.242883 | 51 | 72.13 | 1.414314 |  |
| GO:0015718\_monocarboxylic\_acid\_transport | CROT | 10 | 1 | 17.055556 | -1.242883 | 51 | 72.13 | 1.414314 |  |
| GO:0016197\_endosome\_transport | CDC42 | 10 | 1 | 17.055556 | -1.242883 | 51 | 72.13 | 1.414314 |  |
| GO:0032633\_interleukin-4\_production | CD83 | 10 | 1 | 17.055556 | -1.242883 | 51 | 72.13 | 1.414314 |  |
| GO:0032673\_regulation\_of\_interleukin-4\_production | CD83 | 10 | 1 | 17.055556 | -1.242883 | 51 | 72.13 | 1.414314 |  |
| GO:0019932\_second-messenger-mediated\_signaling | L1CAM | 68 | 2 | 5.016340 | -1.225415 | 53 | 72.95 | 1.376415 |  |
| GO:0019932\_second-messenger-mediated\_signaling | CACNA1D | 68 | 2 | 5.016340 | -1.225415 | 53 | 72.95 | 1.376415 |  |
| GO:0034962\_cellular\_biopolymer\_catabolic\_process | RNF11 | 68 | 2 | 5.016340 | -1.225415 | 53 | 72.95 | 1.376415 |  |
| GO:0034962\_cellular\_biopolymer\_catabolic\_process | EDEM1 | 68 | 2 | 5.016340 | -1.225415 | 53 | 72.95 | 1.376415 |  |
| GO:0022402\_cell\_cycle\_process | CDC42 | 155 | 3 | 3.301075 | -1.217314 | 54 | 73.27 | 1.356852 |  |
| GO:0022402\_cell\_cycle\_process | CHEK1 | 155 | 3 | 3.301075 | -1.217314 | 54 | 73.27 | 1.356852 |  |
| GO:0022402\_cell\_cycle\_process | FANCA | 155 | 3 | 3.301075 | -1.217314 | 54 | 73.27 | 1.356852 |  |
| GO:0007088\_regulation\_of\_mitosis | CDC42 | 11 | 1 | 15.505051 | -1.202709 | 58 | 79.8 | 1.375862 |  |
| GO:0007159\_leukocyte\_adhesion | L1CAM | 11 | 1 | 15.505051 | -1.202709 | 58 | 79.8 | 1.375862 |  |
| GO:0045216\_cell-cell\_junction\_organization | CDC42 | 11 | 1 | 15.505051 | -1.202709 | 58 | 79.8 | 1.375862 |  |
| GO:0051783\_regulation\_of\_nuclear\_division | CDC42 | 11 | 1 | 15.505051 | -1.202709 | 58 | 79.8 | 1.375862 |  |
| GO:0009100\_glycoprotein\_metabolic\_process | NGLY1 | 71 | 2 | 4.804382 | -1.192270 | 59 | 80.6 | 1.366102 |  |
| GO:0009100\_glycoprotein\_metabolic\_process | OGT | 71 | 2 | 4.804382 | -1.192270 | 59 | 80.6 | 1.366102 |  |
| GO:0016568\_chromatin\_modification | SMARCA5 | 72 | 2 | 4.737654 | -1.181576 | 60 | 81.51 | 1.358500 |  |
| GO:0016568\_chromatin\_modification | MYST4 | 72 | 2 | 4.737654 | -1.181576 | 60 | 81.51 | 1.358500 |  |
| GO:0030514\_negative\_regulation\_of\_BMP\_signaling\_pathway | SOSTDC1 | 12 | 1 | 14.212963 | -1.166139 | 63 | 87.81 | 1.393810 |  |
| GO:0050848\_regulation\_of\_calcium-mediated\_signaling | L1CAM | 12 | 1 | 14.212963 | -1.166139 | 63 | 87.81 | 1.393810 |  |
| GO:0050850\_positive\_regulation\_of\_calcium-mediated\_signaling | L1CAM | 12 | 1 | 14.212963 | -1.166139 | 63 | 87.81 | 1.393810 |  |
| GO:0044265\_cellular\_macromolecule\_catabolic\_process | RNF11 | 75 | 2 | 4.548148 | -1.150495 | 64 | 89.07 | 1.391719 |  |
| GO:0044265\_cellular\_macromolecule\_catabolic\_process | EDEM1 | 75 | 2 | 4.548148 | -1.150495 | 64 | 89.07 | 1.391719 |  |
| GO:0006508\_proteolysis | RNF11 | 76 | 2 | 4.488304 | -1.140452 | 65 | 89.84 | 1.382154 |  |
| GO:0006508\_proteolysis | EDEM1 | 76 | 2 | 4.488304 | -1.140452 | 65 | 89.84 | 1.382154 |  |
| GO:0007274\_neuromuscular\_synaptic\_transmission | KIF1B | 13 | 1 | 13.119658 | -1.132594 | 67 | 95.82 | 1.430149 |  |
| GO:0010970\_microtubule-based\_transport | KIF1B | 13 | 1 | 13.119658 | -1.132594 | 67 | 95.82 | 1.430149 |  |
| GO:0030326\_embryonic\_limb\_morphogenesis | PBX1 | 78 | 2 | 4.373219 | -1.120819 | 69 | 96.76 | 1.402319 |  |
| GO:0030326\_embryonic\_limb\_morphogenesis | PBX2 | 78 | 2 | 4.373219 | -1.120819 | 69 | 96.76 | 1.402319 |  |
| GO:0035113\_embryonic\_appendage\_morphogenesis | PBX1 | 78 | 2 | 4.373219 | -1.120819 | 69 | 96.76 | 1.402319 |  |
| GO:0035113\_embryonic\_appendage\_morphogenesis | PBX2 | 78 | 2 | 4.373219 | -1.120819 | 69 | 96.76 | 1.402319 |  |
| GO:0044248\_cellular\_catabolic\_process | GOPC | 173 | 3 | 2.957611 | -1.103324 | 70 | 97.81 | 1.397286 |  |
| GO:0044248\_cellular\_catabolic\_process | RNF11 | 173 | 3 | 2.957611 | -1.103324 | 70 | 97.81 | 1.397286 |  |
| GO:0044248\_cellular\_catabolic\_process | EDEM1 | 173 | 3 | 2.957611 | -1.103324 | 70 | 97.81 | 1.397286 |  |
| GO:0000278\_mitotic\_cell\_cycle | CDC42 | 80 | 2 | 4.263889 | -1.101761 | 71 | 98.2 | 1.383099 |  |
| GO:0000278\_mitotic\_cell\_cycle | CHEK1 | 80 | 2 | 4.263889 | -1.101761 | 71 | 98.2 | 1.383099 |  |
| GO:0000077\_DNA\_damage\_checkpoint | CHEK1 | 14 | 1 | 12.182540 | -1.101625 | 76 | 103.31 | 1.359342 |  |
| GO:0006914\_autophagy | GOPC | 14 | 1 | 12.182540 | -1.101625 | 76 | 103.31 | 1.359342 |  |
| GO:0007157\_heterophilic\_cell\_adhesion | L1CAM | 14 | 1 | 12.182540 | -1.101625 | 76 | 103.31 | 1.359342 |  |
| GO:0016573\_histone\_acetylation | MYST4 | 14 | 1 | 12.182540 | -1.101625 | 76 | 103.31 | 1.359342 |  |
| GO:0021904\_dorsal\_ventral\_neural\_tube\_patterning | KIF3A | 14 | 1 | 12.182540 | -1.101625 | 76 | 103.31 | 1.359342 |  |
| GO:0000902\_cell\_morphogenesis | EPB4.1L2 | 283 | 4 | 2.410679 | -1.095002 | 77 | 103.52 | 1.344416 |  |
| GO:0000902\_cell\_morphogenesis | CDC42 | 283 | 4 | 2.410679 | -1.095002 | 77 | 103.52 | 1.344416 |  |
| GO:0000902\_cell\_morphogenesis | KIF3A | 283 | 4 | 2.410679 | -1.095002 | 77 | 103.52 | 1.344416 |  |
| GO:0000902\_cell\_morphogenesis | L1CAM | 283 | 4 | 2.410679 | -1.095002 | 77 | 103.52 | 1.344416 |  |
| GO:0006473\_protein\_amino\_acid\_acetylation | MYST4 | 15 | 1 | 11.370370 | -1.072877 | 79 | 109.85 | 1.390506 |  |
| GO:0046638\_positive\_regulation\_of\_alpha-beta\_T\_cell\_differentiation | CD83 | 15 | 1 | 11.370370 | -1.072877 | 79 | 109.85 | 1.390506 |  |
| GO:0007242\_intracellular\_signaling\_cascade | CDC42 | 411 | 5 | 2.074885 | -1.060805 | 80 | 110.61 | 1.382625 |  |
| GO:0007242\_intracellular\_signaling\_cascade | CHEK1 | 411 | 5 | 2.074885 | -1.060805 | 80 | 110.61 | 1.382625 |  |
| GO:0007242\_intracellular\_signaling\_cascade | L1CAM | 411 | 5 | 2.074885 | -1.060805 | 80 | 110.61 | 1.382625 |  |
| GO:0007242\_intracellular\_signaling\_cascade | DUSP9 | 411 | 5 | 2.074885 | -1.060805 | 80 | 110.61 | 1.382625 |  |
| GO:0007242\_intracellular\_signaling\_cascade | CACNA1D | 411 | 5 | 2.074885 | -1.060805 | 80 | 110.61 | 1.382625 |  |
| GO:0000279\_M\_phase | CDC42 | 85 | 2 | 4.013072 | -1.056457 | 81 | 111.03 | 1.370741 |  |
| GO:0000279\_M\_phase | FANCA | 85 | 2 | 4.013072 | -1.056457 | 81 | 111.03 | 1.370741 |  |
| GO:0007156\_homophilic\_cell\_adhesion | L1CAM | 16 | 1 | 10.659722 | -1.046063 | 86 | 115.91 | 1.347791 |  |
| GO:0019722\_calcium-mediated\_signaling | L1CAM | 16 | 1 | 10.659722 | -1.046063 | 86 | 115.91 | 1.347791 |  |
| GO:0031570\_DNA\_integrity\_checkpoint | CHEK1 | 16 | 1 | 10.659722 | -1.046063 | 86 | 115.91 | 1.347791 |  |
| GO:0032663\_regulation\_of\_interleukin-2\_production | CD83 | 16 | 1 | 10.659722 | -1.046063 | 86 | 115.91 | 1.347791 |  |
| GO:0043367\_CD4-positive\_\_alpha\_beta\_T\_cell\_differentiation | CD83 | 16 | 1 | 10.659722 | -1.046063 | 86 | 115.91 | 1.347791 |  |
| GO:0001822\_kidney\_development | KIF3A | 87 | 2 | 3.920817 | -1.039205 | 88 | 116.99 | 1.329432 |  |
| GO:0001822\_kidney\_development | PBX1 | 87 | 2 | 3.920817 | -1.039205 | 88 | 116.99 | 1.329432 |  |
| GO:0016337\_cell-cell\_adhesion | CDC42 | 87 | 2 | 3.920817 | -1.039205 | 88 | 116.99 | 1.329432 |  |
| GO:0016337\_cell-cell\_adhesion | L1CAM | 87 | 2 | 3.920817 | -1.039205 | 88 | 116.99 | 1.329432 |  |
| GO:0006323\_DNA\_packaging | SMARCA2 | 17 | 1 | 10.032680 | -1.020947 | 92 | 122.69 | 1.333587 |  |
| GO:0042384\_cilium\_assembly | KIF3A | 17 | 1 | 10.032680 | -1.020947 | 92 | 122.69 | 1.333587 |  |
| GO:0043407\_negative\_regulation\_of\_MAP\_kinase\_activity | DUSP9 | 17 | 1 | 10.032680 | -1.020947 | 92 | 122.69 | 1.333587 |  |
| GO:0046637\_regulation\_of\_alpha-beta\_T\_cell\_differentiation | CD83 | 17 | 1 | 10.032680 | -1.020947 | 92 | 122.69 | 1.333587 |  |
| GO:0001818\_negative\_regulation\_of\_cytokine\_production | CD83 | 18 | 1 | 9.475309 | -0.997336 | 98 | 129.2 | 1.318367 |  |
| GO:0007140\_male\_meiosis | FANCA | 18 | 1 | 9.475309 | -0.997336 | 98 | 129.2 | 1.318367 |  |
| GO:0010498\_proteasomal\_protein\_catabolic\_process | EDEM1 | 18 | 1 | 9.475309 | -0.997336 | 98 | 129.2 | 1.318367 |  |
| GO:0030510\_regulation\_of\_BMP\_signaling\_pathway | SOSTDC1 | 18 | 1 | 9.475309 | -0.997336 | 98 | 129.2 | 1.318367 |  |
| GO:0032623\_interleukin-2\_production | CD83 | 18 | 1 | 9.475309 | -0.997336 | 98 | 129.2 | 1.318367 |  |
| GO:0043161\_proteasomal\_ubiquitin-dependent\_protein\_catabolic\_process | EDEM1 | 18 | 1 | 9.475309 | -0.997336 | 98 | 129.2 | 1.318367 |  |
| GO:0032989\_cellular\_component\_morphogenesis | EPB4.1L2 | 307 | 4 | 2.222222 | -0.994319 | 99 | 129.27 | 1.305758 |  |
| GO:0032989\_cellular\_component\_morphogenesis | CDC42 | 307 | 4 | 2.222222 | -0.994319 | 99 | 129.27 | 1.305758 |  |
| GO:0032989\_cellular\_component\_morphogenesis | KIF3A | 307 | 4 | 2.222222 | -0.994319 | 99 | 129.27 | 1.305758 |  |
| GO:0032989\_cellular\_component\_morphogenesis | L1CAM | 307 | 4 | 2.222222 | -0.994319 | 99 | 129.27 | 1.305758 |  |
| GO:0035107\_appendage\_morphogenesis | PBX1 | 93 | 2 | 3.667861 | -0.990140 | 101 | 129.72 | 1.284356 |  |
| GO:0035107\_appendage\_morphogenesis | PBX2 | 93 | 2 | 3.667861 | -0.990140 | 101 | 129.72 | 1.284356 |  |
| GO:0035108\_limb\_morphogenesis | PBX1 | 93 | 2 | 3.667861 | -0.990140 | 101 | 129.72 | 1.284356 |  |
| GO:0035108\_limb\_morphogenesis | PBX2 | 93 | 2 | 3.667861 | -0.990140 | 101 | 129.72 | 1.284356 |  |
| GO:0003002\_regionalization | KIF3A | 195 | 3 | 2.623932 | -0.983092 | 102 | 130.23 | 1.276765 |  |
| GO:0003002\_regionalization | PBX1 | 195 | 3 | 2.623932 | -0.983092 | 102 | 130.23 | 1.276765 |  |
| GO:0003002\_regionalization | PBX2 | 195 | 3 | 2.623932 | -0.983092 | 102 | 130.23 | 1.276765 |  |
| GO:0006338\_chromatin\_remodeling | SMARCA5 | 19 | 1 | 8.976608 | -0.975066 | 104 | 134.9 | 1.297115 |  |
| GO:0048536\_spleen\_development | PBX1 | 19 | 1 | 8.976608 | -0.975066 | 104 | 134.9 | 1.297115 |  |
| GO:0048736\_appendage\_development | PBX1 | 96 | 2 | 3.553241 | -0.967002 | 106 | 136.09 | 1.283868 |  |
| GO:0048736\_appendage\_development | PBX2 | 96 | 2 | 3.553241 | -0.967002 | 106 | 136.09 | 1.283868 |  |
| GO:0060173\_limb\_development | PBX1 | 96 | 2 | 3.553241 | -0.967002 | 106 | 136.09 | 1.283868 |  |
| GO:0060173\_limb\_development | PBX2 | 96 | 2 | 3.553241 | -0.967002 | 106 | 136.09 | 1.283868 |  |
| GO:0008360\_regulation\_of\_cell\_shape | EPB4.1L2 | 20 | 1 | 8.527778 | -0.954000 | 107 | 139.92 | 1.307664 |  |
| GO:0022607\_cellular\_component\_assembly | CDC42 | 204 | 3 | 2.508170 | -0.938937 | 108 | 141.6 | 1.311111 |  |
| GO:0022607\_cellular\_component\_assembly | KIF3A | 204 | 3 | 2.508170 | -0.938937 | 108 | 141.6 | 1.311111 |  |
| GO:0022607\_cellular\_component\_assembly | SMARCA2 | 204 | 3 | 2.508170 | -0.938937 | 108 | 141.6 | 1.311111 |  |
| GO:0006996\_organelle\_organization | EPB4.1L2 | 449 | 5 | 1.899282 | -0.934736 | 109 | 141.86 | 1.301468 |  |
| GO:0006996\_organelle\_organization | CDC42 | 449 | 5 | 1.899282 | -0.934736 | 109 | 141.86 | 1.301468 |  |
| GO:0006996\_organelle\_organization | SMARCA5 | 449 | 5 | 1.899282 | -0.934736 | 109 | 141.86 | 1.301468 |  |
| GO:0006996\_organelle\_organization | SMARCA2 | 449 | 5 | 1.899282 | -0.934736 | 109 | 141.86 | 1.301468 |  |
| GO:0006996\_organelle\_organization | MYST4 | 449 | 5 | 1.899282 | -0.934736 | 109 | 141.86 | 1.301468 |  |
| GO:0000075\_cell\_cycle\_checkpoint | CHEK1 | 21 | 1 | 8.121693 | -0.934019 | 114 | 146.68 | 1.286667 |  |
| GO:0001709\_cell\_fate\_determination | CDC42 | 21 | 1 | 8.121693 | -0.934019 | 114 | 146.68 | 1.286667 |  |
| GO:0021532\_neural\_tube\_patterning | KIF3A | 21 | 1 | 8.121693 | -0.934019 | 114 | 146.68 | 1.286667 |  |
| GO:0034330\_cell\_junction\_organization | CDC42 | 21 | 1 | 8.121693 | -0.934019 | 114 | 146.68 | 1.286667 |  |
| GO:0048538\_thymus\_development | PBX1 | 21 | 1 | 8.121693 | -0.934019 | 114 | 146.68 | 1.286667 |  |
| GO:0030163\_protein\_catabolic\_process | RNF11 | 101 | 2 | 3.377338 | -0.930309 | 115 | 146.82 | 1.276696 |  |
| GO:0030163\_protein\_catabolic\_process | EDEM1 | 101 | 2 | 3.377338 | -0.930309 | 115 | 146.82 | 1.276696 |  |
| GO:0030705\_cytoskeleton-dependent\_intracellular\_transport | KIF1B | 22 | 1 | 7.752525 | -0.915024 | 117 | 153.47 | 1.311709 |  |
| GO:0046635\_positive\_regulation\_of\_alpha-beta\_T\_cell\_activation | CD83 | 22 | 1 | 7.752525 | -0.915024 | 117 | 153.47 | 1.311709 |  |
| GO:0007018\_microtubule-based\_movement | KIF1B | 23 | 1 | 7.415459 | -0.896926 | 120 | 157.88 | 1.315667 |  |
| GO:0007163\_establishment\_or\_maintenance\_of\_cell\_polarity | CDC42 | 23 | 1 | 7.415459 | -0.896926 | 120 | 157.88 | 1.315667 |  |
| GO:0030512\_negative\_regulation\_of\_transforming\_growth\_factor\_beta\_receptor\_signaling\_pathway | SOSTDC1 | 23 | 1 | 7.415459 | -0.896926 | 120 | 157.88 | 1.315667 |  |
| GO:0000280\_nuclear\_division | CDC42 | 24 | 1 | 7.106481 | -0.879649 | 124 | 162.37 | 1.309435 |  |
| GO:0007067\_mitosis | CDC42 | 24 | 1 | 7.106481 | -0.879649 | 124 | 162.37 | 1.309435 |  |
| GO:0007266\_Rho\_protein\_signal\_transduction | CDC42 | 24 | 1 | 7.106481 | -0.879649 | 124 | 162.37 | 1.309435 |  |
| GO:0014070\_response\_to\_organic\_cyclic\_substance | CD83 | 24 | 1 | 7.106481 | -0.879649 | 124 | 162.37 | 1.309435 |  |
| GO:0000087\_M\_phase\_of\_mitotic\_cell\_cycle | CDC42 | 25 | 1 | 6.822222 | -0.863126 | 127 | 167.61 | 1.319764 |  |
| GO:0043543\_protein\_amino\_acid\_acylation | MYST4 | 25 | 1 | 6.822222 | -0.863126 | 127 | 167.61 | 1.319764 |  |
| GO:0048285\_organelle\_fission | CDC42 | 25 | 1 | 6.822222 | -0.863126 | 127 | 167.61 | 1.319764 |  |
| GO:0043549\_regulation\_of\_kinase\_activity | CDC42 | 112 | 2 | 3.045635 | -0.856833 | 128 | 168.02 | 1.312656 |  |
| GO:0043549\_regulation\_of\_kinase\_activity | DUSP9 | 112 | 2 | 3.045635 | -0.856833 | 128 | 168.02 | 1.312656 |  |
| GO:0001658\_branching\_involved\_in\_ureteric\_bud\_morphogenesis | PBX1 | 26 | 1 | 6.559829 | -0.847297 | 131 | 171.71 | 1.310763 |  |
| GO:0045665\_negative\_regulation\_of\_neuron\_differentiation | PBX1 | 26 | 1 | 6.559829 | -0.847297 | 131 | 171.71 | 1.310763 |  |
| GO:0060675\_ureteric\_bud\_morphogenesis | PBX1 | 26 | 1 | 6.559829 | -0.847297 | 131 | 171.71 | 1.310763 |  |
| GO:0051338\_regulation\_of\_transferase\_activity | CDC42 | 115 | 2 | 2.966184 | -0.838326 | 132 | 172.33 | 1.305530 |  |
| GO:0051338\_regulation\_of\_transferase\_activity | DUSP9 | 115 | 2 | 2.966184 | -0.838326 | 132 | 172.33 | 1.305530 |  |
| GO:0045582\_positive\_regulation\_of\_T\_cell\_differentiation | CD83 | 27 | 1 | 6.316872 | -0.832109 | 133 | 175.96 | 1.323008 |  |
| GO:0050794\_regulation\_of\_cellular\_process | COPS2 | 2190 | 16 | 1.246068 | -0.818102 | 134 | 177.24 | 1.322687 |  |
| GO:0050794\_regulation\_of\_cellular\_process | KIF3A | 2190 | 16 | 1.246068 | -0.818102 | 134 | 177.24 | 1.322687 |  |
| GO:0050794\_regulation\_of\_cellular\_process | CHEK1 | 2190 | 16 | 1.246068 | -0.818102 | 134 | 177.24 | 1.322687 |  |
| GO:0050794\_regulation\_of\_cellular\_process | L1CAM | 2190 | 16 | 1.246068 | -0.818102 | 134 | 177.24 | 1.322687 |  |
| GO:0050794\_regulation\_of\_cellular\_process | EPB4.1L2 | 2190 | 16 | 1.246068 | -0.818102 | 134 | 177.24 | 1.322687 |  |
| GO:0050794\_regulation\_of\_cellular\_process | CD83 | 2190 | 16 | 1.246068 | -0.818102 | 134 | 177.24 | 1.322687 |  |
| GO:0050794\_regulation\_of\_cellular\_process | CDC42 | 2190 | 16 | 1.246068 | -0.818102 | 134 | 177.24 | 1.322687 |  |
| GO:0050794\_regulation\_of\_cellular\_process | SOSTDC1 | 2190 | 16 | 1.246068 | -0.818102 | 134 | 177.24 | 1.322687 |  |
| GO:0050794\_regulation\_of\_cellular\_process | SMARCA5 | 2190 | 16 | 1.246068 | -0.818102 | 134 | 177.24 | 1.322687 |  |
| GO:0050794\_regulation\_of\_cellular\_process | PBX1 | 2190 | 16 | 1.246068 | -0.818102 | 134 | 177.24 | 1.322687 |  |
| GO:0050794\_regulation\_of\_cellular\_process | DUSP9 | 2190 | 16 | 1.246068 | -0.818102 | 134 | 177.24 | 1.322687 |  |
| GO:0050794\_regulation\_of\_cellular\_process | LCOR | 2190 | 16 | 1.246068 | -0.818102 | 134 | 177.24 | 1.322687 |  |
| GO:0050794\_regulation\_of\_cellular\_process | FANCA | 2190 | 16 | 1.246068 | -0.818102 | 134 | 177.24 | 1.322687 |  |
| GO:0050794\_regulation\_of\_cellular\_process | SMARCA2 | 2190 | 16 | 1.246068 | -0.818102 | 134 | 177.24 | 1.322687 |  |
| GO:0050794\_regulation\_of\_cellular\_process | CACNA1D | 2190 | 16 | 1.246068 | -0.818102 | 134 | 177.24 | 1.322687 |  |
| GO:0050794\_regulation\_of\_cellular\_process | PBX2 | 2190 | 16 | 1.246068 | -0.818102 | 134 | 177.24 | 1.322687 |  |
| GO:0010564\_regulation\_of\_cell\_cycle\_process | CDC42 | 29 | 1 | 5.881226 | -0.803479 | 138 | 183.84 | 1.332174 |  |
| GO:0042770\_DNA\_damage\_response\_\_signal\_transduction | CHEK1 | 29 | 1 | 5.881226 | -0.803479 | 138 | 183.84 | 1.332174 |  |
| GO:0045621\_positive\_regulation\_of\_lymphocyte\_differentiation | CD83 | 29 | 1 | 5.881226 | -0.803479 | 138 | 183.84 | 1.332174 |  |
| GO:0046634\_regulation\_of\_alpha-beta\_T\_cell\_activation | CD83 | 29 | 1 | 5.881226 | -0.803479 | 138 | 183.84 | 1.332174 |  |
| GO:0051726\_regulation\_of\_cell\_cycle | CDC42 | 121 | 2 | 2.819100 | -0.803056 | 139 | 184.28 | 1.325755 |  |
| GO:0051726\_regulation\_of\_cell\_cycle | CHEK1 | 121 | 2 | 2.819100 | -0.803056 | 139 | 184.28 | 1.325755 |  |
| GO:0044085\_cellular\_component\_biogenesis | CDC42 | 237 | 3 | 2.158931 | -0.797212 | 140 | 185.7 | 1.326429 |  |
| GO:0044085\_cellular\_component\_biogenesis | KIF3A | 237 | 3 | 2.158931 | -0.797212 | 140 | 185.7 | 1.326429 |  |
| GO:0044085\_cellular\_component\_biogenesis | SMARCA2 | 237 | 3 | 2.158931 | -0.797212 | 140 | 185.7 | 1.326429 |  |
| GO:0007049\_cell\_cycle | CDC42 | 238 | 3 | 2.149860 | -0.793349 | 141 | 185.83 | 1.317943 |  |
| GO:0007049\_cell\_cycle | CHEK1 | 238 | 3 | 2.149860 | -0.793349 | 141 | 185.83 | 1.317943 |  |
| GO:0007049\_cell\_cycle | FANCA | 238 | 3 | 2.149860 | -0.793349 | 141 | 185.83 | 1.317943 |  |
| GO:0006486\_protein\_amino\_acid\_glycosylation | OGT | 31 | 1 | 5.501792 | -0.776914 | 145 | 196.33 | 1.354000 |  |
| GO:0043413\_biopolymer\_glycosylation | OGT | 31 | 1 | 5.501792 | -0.776914 | 145 | 196.33 | 1.354000 |  |
| GO:0046632\_alpha-beta\_T\_cell\_differentiation | CD83 | 31 | 1 | 5.501792 | -0.776914 | 145 | 196.33 | 1.354000 |  |
| GO:0070085\_glycosylation | OGT | 31 | 1 | 5.501792 | -0.776914 | 145 | 196.33 | 1.354000 |  |
| GO:0001655\_urogenital\_system\_development | KIF3A | 128 | 2 | 2.664931 | -0.764592 | 146 | 197.16 | 1.350411 |  |
| GO:0001655\_urogenital\_system\_development | PBX1 | 128 | 2 | 2.664931 | -0.764592 | 146 | 197.16 | 1.350411 |  |
| GO:0007188\_G-protein\_signaling\_\_coupled\_to\_cAMP\_nucleotide\_second\_messenger | CACNA1D | 33 | 1 | 5.168350 | -0.752158 | 149 | 204.33 | 1.371342 |  |
| GO:0007270\_nerve-nerve\_synaptic\_transmission | KIF1B | 33 | 1 | 5.168350 | -0.752158 | 149 | 204.33 | 1.371342 |  |
| GO:0008584\_male\_gonad\_development | FANCA | 33 | 1 | 5.168350 | -0.752158 | 149 | 204.33 | 1.371342 |  |
| GO:0030509\_BMP\_signaling\_pathway | SOSTDC1 | 34 | 1 | 5.016340 | -0.740389 | 150 | 208.52 | 1.390133 |  |
| GO:0009653\_anatomical\_structure\_morphogenesis | EPB4.1L2 | 958 | 8 | 1.424264 | -0.738901 | 151 | 208.62 | 1.381589 |  |
| GO:0009653\_anatomical\_structure\_morphogenesis | CDC42 | 958 | 8 | 1.424264 | -0.738901 | 151 | 208.62 | 1.381589 |  |
| GO:0009653\_anatomical\_structure\_morphogenesis | KIF3A | 958 | 8 | 1.424264 | -0.738901 | 151 | 208.62 | 1.381589 |  |
| GO:0009653\_anatomical\_structure\_morphogenesis | FREM2 | 958 | 8 | 1.424264 | -0.738901 | 151 | 208.62 | 1.381589 |  |
| GO:0009653\_anatomical\_structure\_morphogenesis | SOSTDC1 | 958 | 8 | 1.424264 | -0.738901 | 151 | 208.62 | 1.381589 |  |
| GO:0009653\_anatomical\_structure\_morphogenesis | L1CAM | 958 | 8 | 1.424264 | -0.738901 | 151 | 208.62 | 1.381589 |  |
| GO:0009653\_anatomical\_structure\_morphogenesis | PBX1 | 958 | 8 | 1.424264 | -0.738901 | 151 | 208.62 | 1.381589 |  |
| GO:0009653\_anatomical\_structure\_morphogenesis | PBX2 | 958 | 8 | 1.424264 | -0.738901 | 151 | 208.62 | 1.381589 |  |
| GO:0009952\_anterior\_posterior\_pattern\_formation | KIF3A | 133 | 2 | 2.564745 | -0.738723 | 152 | 208.95 | 1.374671 |  |
| GO:0009952\_anterior\_posterior\_pattern\_formation | PBX1 | 133 | 2 | 2.564745 | -0.738723 | 152 | 208.95 | 1.374671 |  |
| GO:0006869\_lipid\_transport | CROT | 35 | 1 | 4.873016 | -0.728995 | 155 | 212.32 | 1.369806 |  |
| GO:0051325\_interphase | CHEK1 | 35 | 1 | 4.873016 | -0.728995 | 155 | 212.32 | 1.369806 |  |
| GO:0051329\_interphase\_of\_mitotic\_cell\_cycle | CHEK1 | 35 | 1 | 4.873016 | -0.728995 | 155 | 212.32 | 1.369806 |  |
| GO:0001819\_positive\_regulation\_of\_cytokine\_production | CD83 | 36 | 1 | 4.737654 | -0.717955 | 161 | 216.94 | 1.347453 |  |
| GO:0006469\_negative\_regulation\_of\_protein\_kinase\_activity | DUSP9 | 36 | 1 | 4.737654 | -0.717955 | 161 | 216.94 | 1.347453 |  |
| GO:0007187\_G-protein\_signaling\_\_coupled\_to\_cyclic\_nucleotide\_second\_messenger | CACNA1D | 36 | 1 | 4.737654 | -0.717955 | 161 | 216.94 | 1.347453 |  |
| GO:0007368\_determination\_of\_left\_right\_symmetry | KIF3A | 36 | 1 | 4.737654 | -0.717955 | 161 | 216.94 | 1.347453 |  |
| GO:0030278\_regulation\_of\_ossification | PBX1 | 36 | 1 | 4.737654 | -0.717955 | 161 | 216.94 | 1.347453 |  |
| GO:0033673\_negative\_regulation\_of\_kinase\_activity | DUSP9 | 36 | 1 | 4.737654 | -0.717955 | 161 | 216.94 | 1.347453 |  |
| GO:0009799\_determination\_of\_symmetry | KIF3A | 37 | 1 | 4.609610 | -0.707249 | 165 | 219.74 | 1.331758 |  |
| GO:0009855\_determination\_of\_bilateral\_symmetry | KIF3A | 37 | 1 | 4.609610 | -0.707249 | 165 | 219.74 | 1.331758 |  |
| GO:0019933\_cAMP-mediated\_signaling | CACNA1D | 37 | 1 | 4.609610 | -0.707249 | 165 | 219.74 | 1.331758 |  |
| GO:0045785\_positive\_regulation\_of\_cell\_adhesion | L1CAM | 37 | 1 | 4.609610 | -0.707249 | 165 | 219.74 | 1.331758 |  |
| GO:0030030\_cell\_projection\_organization | CDC42 | 263 | 3 | 1.945501 | -0.703752 | 166 | 220.48 | 1.328193 |  |
| GO:0030030\_cell\_projection\_organization | KIF3A | 263 | 3 | 1.945501 | -0.703752 | 166 | 220.48 | 1.328193 |  |
| GO:0030030\_cell\_projection\_organization | L1CAM | 263 | 3 | 1.945501 | -0.703752 | 166 | 220.48 | 1.328193 |  |
| GO:0044260\_cellular\_macromolecule\_metabolic\_process | NGLY1 | 1447 | 11 | 1.296552 | -0.701423 | 167 | 220.63 | 1.321138 |  |
| GO:0044260\_cellular\_macromolecule\_metabolic\_process | COPS2 | 1447 | 11 | 1.296552 | -0.701423 | 167 | 220.63 | 1.321138 |  |
| GO:0044260\_cellular\_macromolecule\_metabolic\_process | SMARCA5 | 1447 | 11 | 1.296552 | -0.701423 | 167 | 220.63 | 1.321138 |  |
| GO:0044260\_cellular\_macromolecule\_metabolic\_process | RNF11 | 1447 | 11 | 1.296552 | -0.701423 | 167 | 220.63 | 1.321138 |  |
| GO:0044260\_cellular\_macromolecule\_metabolic\_process | CHEK1 | 1447 | 11 | 1.296552 | -0.701423 | 167 | 220.63 | 1.321138 |  |
| GO:0044260\_cellular\_macromolecule\_metabolic\_process | PBX1 | 1447 | 11 | 1.296552 | -0.701423 | 167 | 220.63 | 1.321138 |  |
| GO:0044260\_cellular\_macromolecule\_metabolic\_process | OGT | 1447 | 11 | 1.296552 | -0.701423 | 167 | 220.63 | 1.321138 |  |
| GO:0044260\_cellular\_macromolecule\_metabolic\_process | LCOR | 1447 | 11 | 1.296552 | -0.701423 | 167 | 220.63 | 1.321138 |  |
| GO:0044260\_cellular\_macromolecule\_metabolic\_process | EDEM1 | 1447 | 11 | 1.296552 | -0.701423 | 167 | 220.63 | 1.321138 |  |
| GO:0044260\_cellular\_macromolecule\_metabolic\_process | PBX2 | 1447 | 11 | 1.296552 | -0.701423 | 167 | 220.63 | 1.321138 |  |
| GO:0044260\_cellular\_macromolecule\_metabolic\_process | MYST4 | 1447 | 11 | 1.296552 | -0.701423 | 167 | 220.63 | 1.321138 |  |
| GO:0001657\_ureteric\_bud\_development | PBX1 | 38 | 1 | 4.488304 | -0.696859 | 170 | 224.74 | 1.322000 |  |
| GO:0045580\_regulation\_of\_T\_cell\_differentiation | CD83 | 38 | 1 | 4.488304 | -0.696859 | 170 | 224.74 | 1.322000 |  |
| GO:0051348\_negative\_regulation\_of\_transferase\_activity | DUSP9 | 38 | 1 | 4.488304 | -0.696859 | 170 | 224.74 | 1.322000 |  |
| GO:0042475\_odontogenesis\_of\_dentine-containing\_tooth | SOSTDC1 | 39 | 1 | 4.373219 | -0.686769 | 172 | 227.81 | 1.324477 |  |
| GO:0048663\_neuron\_fate\_commitment | CDC42 | 39 | 1 | 4.373219 | -0.686769 | 172 | 227.81 | 1.324477 |  |
| GO:0007346\_regulation\_of\_mitotic\_cell\_cycle | CDC42 | 40 | 1 | 4.263889 | -0.676964 | 176 | 231.75 | 1.316761 |  |
| GO:0017015\_regulation\_of\_transforming\_growth\_factor\_beta\_receptor\_signaling\_pathway | SOSTDC1 | 40 | 1 | 4.263889 | -0.676964 | 176 | 231.75 | 1.316761 |  |
| GO:0019935\_cyclic-nucleotide-mediated\_signaling | CACNA1D | 40 | 1 | 4.263889 | -0.676964 | 176 | 231.75 | 1.316761 |  |
| GO:0046850\_regulation\_of\_bone\_remodeling | PBX1 | 40 | 1 | 4.263889 | -0.676964 | 176 | 231.75 | 1.316761 |  |
| GO:0008585\_female\_gonad\_development | FANCA | 41 | 1 | 4.159892 | -0.667430 | 178 | 237.6 | 1.334831 |  |
| GO:0019216\_regulation\_of\_lipid\_metabolic\_process | CDC42 | 41 | 1 | 4.159892 | -0.667430 | 178 | 237.6 | 1.334831 |  |
| GO:0042476\_odontogenesis | SOSTDC1 | 42 | 1 | 4.060847 | -0.658152 | 179 | 242.27 | 1.353464 |  |
| GO:0044267\_cellular\_protein\_metabolic\_process | RNF11 | 559 | 5 | 1.525542 | -0.650120 | 180 | 243.09 | 1.350500 |  |
| GO:0044267\_cellular\_protein\_metabolic\_process | CHEK1 | 559 | 5 | 1.525542 | -0.650120 | 180 | 243.09 | 1.350500 |  |
| GO:0044267\_cellular\_protein\_metabolic\_process | OGT | 559 | 5 | 1.525542 | -0.650120 | 180 | 243.09 | 1.350500 |  |
| GO:0044267\_cellular\_protein\_metabolic\_process | EDEM1 | 559 | 5 | 1.525542 | -0.650120 | 180 | 243.09 | 1.350500 |  |
| GO:0044267\_cellular\_protein\_metabolic\_process | MYST4 | 559 | 5 | 1.525542 | -0.650120 | 180 | 243.09 | 1.350500 |  |
| GO:0007224\_smoothened\_signaling\_pathway | KIF3A | 43 | 1 | 3.966408 | -0.649120 | 181 | 246.78 | 1.363425 |  |
| GO:0044237\_cellular\_metabolic\_process | COPS2 | 1974 | 14 | 1.209614 | -0.647144 | 182 | 246.87 | 1.356429 |  |
| GO:0044237\_cellular\_metabolic\_process | CHEK1 | 1974 | 14 | 1.209614 | -0.647144 | 182 | 246.87 | 1.356429 |  |
| GO:0044237\_cellular\_metabolic\_process | EDEM1 | 1974 | 14 | 1.209614 | -0.647144 | 182 | 246.87 | 1.356429 |  |
| GO:0044237\_cellular\_metabolic\_process | NGLY1 | 1974 | 14 | 1.209614 | -0.647144 | 182 | 246.87 | 1.356429 |  |
| GO:0044237\_cellular\_metabolic\_process | CDC42 | 1974 | 14 | 1.209614 | -0.647144 | 182 | 246.87 | 1.356429 |  |
| GO:0044237\_cellular\_metabolic\_process | GOPC | 1974 | 14 | 1.209614 | -0.647144 | 182 | 246.87 | 1.356429 |  |
| GO:0044237\_cellular\_metabolic\_process | SMARCA5 | 1974 | 14 | 1.209614 | -0.647144 | 182 | 246.87 | 1.356429 |  |
| GO:0044237\_cellular\_metabolic\_process | RNF11 | 1974 | 14 | 1.209614 | -0.647144 | 182 | 246.87 | 1.356429 |  |
| GO:0044237\_cellular\_metabolic\_process | PBX1 | 1974 | 14 | 1.209614 | -0.647144 | 182 | 246.87 | 1.356429 |  |
| GO:0044237\_cellular\_metabolic\_process | OGT | 1974 | 14 | 1.209614 | -0.647144 | 182 | 246.87 | 1.356429 |  |
| GO:0044237\_cellular\_metabolic\_process | LCOR | 1974 | 14 | 1.209614 | -0.647144 | 182 | 246.87 | 1.356429 |  |
| GO:0044237\_cellular\_metabolic\_process | DUSP9 | 1974 | 14 | 1.209614 | -0.647144 | 182 | 246.87 | 1.356429 |  |
| GO:0044237\_cellular\_metabolic\_process | PBX2 | 1974 | 14 | 1.209614 | -0.647144 | 182 | 246.87 | 1.356429 |  |
| GO:0044237\_cellular\_metabolic\_process | MYST4 | 1974 | 14 | 1.209614 | -0.647144 | 182 | 246.87 | 1.356429 |  |
| GO:0046545\_development\_of\_primary\_female\_sexual\_characteristics | FANCA | 44 | 1 | 3.876263 | -0.640322 | 183 | 251.87 | 1.376339 |  |
| GO:0043283\_biopolymer\_metabolic\_process | NGLY1 | 1490 | 11 | 1.259135 | -0.637990 | 184 | 252.45 | 1.372011 |  |
| GO:0043283\_biopolymer\_metabolic\_process | COPS2 | 1490 | 11 | 1.259135 | -0.637990 | 184 | 252.45 | 1.372011 |  |
| GO:0043283\_biopolymer\_metabolic\_process | SMARCA5 | 1490 | 11 | 1.259135 | -0.637990 | 184 | 252.45 | 1.372011 |  |
| GO:0043283\_biopolymer\_metabolic\_process | RNF11 | 1490 | 11 | 1.259135 | -0.637990 | 184 | 252.45 | 1.372011 |  |
| GO:0043283\_biopolymer\_metabolic\_process | PBX1 | 1490 | 11 | 1.259135 | -0.637990 | 184 | 252.45 | 1.372011 |  |
| GO:0043283\_biopolymer\_metabolic\_process | CHEK1 | 1490 | 11 | 1.259135 | -0.637990 | 184 | 252.45 | 1.372011 |  |
| GO:0043283\_biopolymer\_metabolic\_process | OGT | 1490 | 11 | 1.259135 | -0.637990 | 184 | 252.45 | 1.372011 |  |
| GO:0043283\_biopolymer\_metabolic\_process | LCOR | 1490 | 11 | 1.259135 | -0.637990 | 184 | 252.45 | 1.372011 |  |
| GO:0043283\_biopolymer\_metabolic\_process | PBX2 | 1490 | 11 | 1.259135 | -0.637990 | 184 | 252.45 | 1.372011 |  |
| GO:0043283\_biopolymer\_metabolic\_process | EDEM1 | 1490 | 11 | 1.259135 | -0.637990 | 184 | 252.45 | 1.372011 |  |
| GO:0043283\_biopolymer\_metabolic\_process | MYST4 | 1490 | 11 | 1.259135 | -0.637990 | 184 | 252.45 | 1.372011 |  |
| GO:0009790\_embryonic\_development | KIF3A | 567 | 5 | 1.504017 | -0.633162 | 185 | 253.29 | 1.369135 |  |
| GO:0009790\_embryonic\_development | SMARCA5 | 567 | 5 | 1.504017 | -0.633162 | 185 | 253.29 | 1.369135 |  |
| GO:0009790\_embryonic\_development | PBX1 | 567 | 5 | 1.504017 | -0.633162 | 185 | 253.29 | 1.369135 |  |
| GO:0009790\_embryonic\_development | PBX2 | 567 | 5 | 1.504017 | -0.633162 | 185 | 253.29 | 1.369135 |  |
| GO:0009790\_embryonic\_development | YBX1 | 567 | 5 | 1.504017 | -0.633162 | 185 | 253.29 | 1.369135 |  |
| GO:0034103\_regulation\_of\_tissue\_remodeling | PBX1 | 45 | 1 | 3.790123 | -0.631748 | 187 | 254.53 | 1.361123 |  |
| GO:0046546\_development\_of\_primary\_male\_sexual\_characteristics | FANCA | 45 | 1 | 3.790123 | -0.631748 | 187 | 254.53 | 1.361123 |  |
| GO:0046631\_alpha-beta\_T\_cell\_activation | CD83 | 46 | 1 | 3.707729 | -0.623387 | 188 | 257.69 | 1.370691 |  |
| GO:0051128\_regulation\_of\_cellular\_component\_organization | EPB4.1L2 | 160 | 2 | 2.131944 | -0.618100 | 189 | 257.92 | 1.364656 |  |
| GO:0051128\_regulation\_of\_cellular\_component\_organization | CDC42 | 160 | 2 | 2.131944 | -0.618100 | 189 | 257.92 | 1.364656 |  |
| GO:0016570\_histone\_modification | MYST4 | 47 | 1 | 3.628842 | -0.615230 | 191 | 260.67 | 1.364764 |  |
| GO:0045619\_regulation\_of\_lymphocyte\_differentiation | CD83 | 47 | 1 | 3.628842 | -0.615230 | 191 | 260.67 | 1.364764 |  |
| GO:0009101\_glycoprotein\_biosynthetic\_process | OGT | 48 | 1 | 3.553241 | -0.607268 | 193 | 262.84 | 1.361865 |  |
| GO:0046849\_bone\_remodeling | PBX1 | 48 | 1 | 3.553241 | -0.607268 | 193 | 262.84 | 1.361865 |  |
| GO:0042325\_regulation\_of\_phosphorylation | CDC42 | 164 | 2 | 2.079946 | -0.602544 | 194 | 264.23 | 1.362010 |  |
| GO:0042325\_regulation\_of\_phosphorylation | DUSP9 | 164 | 2 | 2.079946 | -0.602544 | 194 | 264.23 | 1.362010 |  |
| GO:0046660\_female\_sex\_differentiation | FANCA | 49 | 1 | 3.480726 | -0.599495 | 196 | 266.98 | 1.362143 |  |
| GO:0046661\_male\_sex\_differentiation | FANCA | 49 | 1 | 3.480726 | -0.599495 | 196 | 266.98 | 1.362143 |  |
| GO:0019220\_regulation\_of\_phosphate\_metabolic\_process | CDC42 | 165 | 2 | 2.067340 | -0.598735 | 198 | 267.47 | 1.350859 |  |
| GO:0019220\_regulation\_of\_phosphate\_metabolic\_process | DUSP9 | 165 | 2 | 2.067340 | -0.598735 | 198 | 267.47 | 1.350859 |  |
| GO:0051174\_regulation\_of\_phosphorus\_metabolic\_process | CDC42 | 165 | 2 | 2.067340 | -0.598735 | 198 | 267.47 | 1.350859 |  |
| GO:0051174\_regulation\_of\_phosphorus\_metabolic\_process | DUSP9 | 165 | 2 | 2.067340 | -0.598735 | 198 | 267.47 | 1.350859 |  |
| GO:0001656\_metanephros\_development | PBX1 | 50 | 1 | 3.411111 | -0.591901 | 199 | 269.96 | 1.356583 |  |
| GO:0050789\_regulation\_of\_biological\_process | COPS2 | 2357 | 16 | 1.157781 | -0.586869 | 200 | 270.99 | 1.354950 |  |
| GO:0050789\_regulation\_of\_biological\_process | KIF3A | 2357 | 16 | 1.157781 | -0.586869 | 200 | 270.99 | 1.354950 |  |
| GO:0050789\_regulation\_of\_biological\_process | L1CAM | 2357 | 16 | 1.157781 | -0.586869 | 200 | 270.99 | 1.354950 |  |
| GO:0050789\_regulation\_of\_biological\_process | CHEK1 | 2357 | 16 | 1.157781 | -0.586869 | 200 | 270.99 | 1.354950 |  |
| GO:0050789\_regulation\_of\_biological\_process | EPB4.1L2 | 2357 | 16 | 1.157781 | -0.586869 | 200 | 270.99 | 1.354950 |  |
| GO:0050789\_regulation\_of\_biological\_process | CD83 | 2357 | 16 | 1.157781 | -0.586869 | 200 | 270.99 | 1.354950 |  |
| GO:0050789\_regulation\_of\_biological\_process | CDC42 | 2357 | 16 | 1.157781 | -0.586869 | 200 | 270.99 | 1.354950 |  |
| GO:0050789\_regulation\_of\_biological\_process | SOSTDC1 | 2357 | 16 | 1.157781 | -0.586869 | 200 | 270.99 | 1.354950 |  |
| GO:0050789\_regulation\_of\_biological\_process | SMARCA5 | 2357 | 16 | 1.157781 | -0.586869 | 200 | 270.99 | 1.354950 |  |
| GO:0050789\_regulation\_of\_biological\_process | PBX1 | 2357 | 16 | 1.157781 | -0.586869 | 200 | 270.99 | 1.354950 |  |
| GO:0050789\_regulation\_of\_biological\_process | LCOR | 2357 | 16 | 1.157781 | -0.586869 | 200 | 270.99 | 1.354950 |  |
| GO:0050789\_regulation\_of\_biological\_process | DUSP9 | 2357 | 16 | 1.157781 | -0.586869 | 200 | 270.99 | 1.354950 |  |
| GO:0050789\_regulation\_of\_biological\_process | FANCA | 2357 | 16 | 1.157781 | -0.586869 | 200 | 270.99 | 1.354950 |  |
| GO:0050789\_regulation\_of\_biological\_process | CACNA1D | 2357 | 16 | 1.157781 | -0.586869 | 200 | 270.99 | 1.354950 |  |
| GO:0050789\_regulation\_of\_biological\_process | PBX2 | 2357 | 16 | 1.157781 | -0.586869 | 200 | 270.99 | 1.354950 |  |
| GO:0050789\_regulation\_of\_biological\_process | SMARCA2 | 2357 | 16 | 1.157781 | -0.586869 | 200 | 270.99 | 1.354950 |  |
| GO:0016569\_covalent\_chromatin\_modification | MYST4 | 51 | 1 | 3.344227 | -0.584480 | 201 | 273.89 | 1.362637 |  |
| GO:0046942\_carboxylic\_acid\_transport | CROT | 53 | 1 | 3.218029 | -0.570129 | 202 | 280.2 | 1.387129 |  |
| GO:0016310\_phosphorylation | CDC42 | 309 | 3 | 1.655879 | -0.567979 | 203 | 281.18 | 1.385123 |  |
| GO:0016310\_phosphorylation | CHEK1 | 309 | 3 | 1.655879 | -0.567979 | 203 | 281.18 | 1.385123 |  |
| GO:0016310\_phosphorylation | DUSP9 | 309 | 3 | 1.655879 | -0.567979 | 203 | 281.18 | 1.385123 |  |
| GO:0007265\_Ras\_protein\_signal\_transduction | CDC42 | 54 | 1 | 3.158436 | -0.563187 | 206 | 283.5 | 1.376214 |  |
| GO:0015849\_organic\_acid\_transport | CROT | 54 | 1 | 3.158436 | -0.563187 | 206 | 283.5 | 1.376214 |  |
| GO:0043405\_regulation\_of\_MAP\_kinase\_activity | DUSP9 | 54 | 1 | 3.158436 | -0.563187 | 206 | 283.5 | 1.376214 |  |
| GO:0048858\_cell\_projection\_morphogenesis | KIF3A | 176 | 2 | 1.938131 | -0.558838 | 207 | 284.8 | 1.375845 |  |
| GO:0048858\_cell\_projection\_morphogenesis | L1CAM | 176 | 2 | 1.938131 | -0.558838 | 207 | 284.8 | 1.375845 |  |
| GO:0007126\_meiosis | FANCA | 55 | 1 | 3.101010 | -0.556394 | 211 | 286.69 | 1.358720 |  |
| GO:0007605\_sensory\_perception\_of\_sound | CACNA1D | 55 | 1 | 3.101010 | -0.556394 | 211 | 286.69 | 1.358720 |  |
| GO:0048568\_embryonic\_organ\_development | PBX1 | 55 | 1 | 3.101010 | -0.556394 | 211 | 286.69 | 1.358720 |  |
| GO:0051327\_M\_phase\_of\_meiotic\_cell\_cycle | FANCA | 55 | 1 | 3.101010 | -0.556394 | 211 | 286.69 | 1.358720 |  |
| GO:0007165\_signal\_transduction | CDC42 | 915 | 7 | 1.304797 | -0.552472 | 212 | 287.08 | 1.354151 |  |
| GO:0007165\_signal\_transduction | KIF3A | 915 | 7 | 1.304797 | -0.552472 | 212 | 287.08 | 1.354151 |  |
| GO:0007165\_signal\_transduction | SOSTDC1 | 915 | 7 | 1.304797 | -0.552472 | 212 | 287.08 | 1.354151 |  |
| GO:0007165\_signal\_transduction | CHEK1 | 915 | 7 | 1.304797 | -0.552472 | 212 | 287.08 | 1.354151 |  |
| GO:0007165\_signal\_transduction | L1CAM | 915 | 7 | 1.304797 | -0.552472 | 212 | 287.08 | 1.354151 |  |
| GO:0007165\_signal\_transduction | DUSP9 | 915 | 7 | 1.304797 | -0.552472 | 212 | 287.08 | 1.354151 |  |
| GO:0007165\_signal\_transduction | CACNA1D | 915 | 7 | 1.304797 | -0.552472 | 212 | 287.08 | 1.354151 |  |
| GO:0051321\_meiotic\_cell\_cycle | FANCA | 56 | 1 | 3.045635 | -0.549742 | 213 | 289.86 | 1.360845 |  |
| GO:0034960\_cellular\_biopolymer\_metabolic\_process | COPS2 | 1395 | 10 | 1.222620 | -0.547625 | 214 | 290.62 | 1.358037 |  |
| GO:0034960\_cellular\_biopolymer\_metabolic\_process | SMARCA5 | 1395 | 10 | 1.222620 | -0.547625 | 214 | 290.62 | 1.358037 |  |
| GO:0034960\_cellular\_biopolymer\_metabolic\_process | RNF11 | 1395 | 10 | 1.222620 | -0.547625 | 214 | 290.62 | 1.358037 |  |
| GO:0034960\_cellular\_biopolymer\_metabolic\_process | PBX1 | 1395 | 10 | 1.222620 | -0.547625 | 214 | 290.62 | 1.358037 |  |
| GO:0034960\_cellular\_biopolymer\_metabolic\_process | CHEK1 | 1395 | 10 | 1.222620 | -0.547625 | 214 | 290.62 | 1.358037 |  |
| GO:0034960\_cellular\_biopolymer\_metabolic\_process | OGT | 1395 | 10 | 1.222620 | -0.547625 | 214 | 290.62 | 1.358037 |  |
| GO:0034960\_cellular\_biopolymer\_metabolic\_process | LCOR | 1395 | 10 | 1.222620 | -0.547625 | 214 | 290.62 | 1.358037 |  |
| GO:0034960\_cellular\_biopolymer\_metabolic\_process | EDEM1 | 1395 | 10 | 1.222620 | -0.547625 | 214 | 290.62 | 1.358037 |  |
| GO:0034960\_cellular\_biopolymer\_metabolic\_process | PBX2 | 1395 | 10 | 1.222620 | -0.547625 | 214 | 290.62 | 1.358037 |  |
| GO:0034960\_cellular\_biopolymer\_metabolic\_process | MYST4 | 1395 | 10 | 1.222620 | -0.547625 | 214 | 290.62 | 1.358037 |  |
| GO:0009953\_dorsal\_ventral\_pattern\_formation | KIF3A | 57 | 1 | 2.992203 | -0.543229 | 216 | 293.47 | 1.358657 |  |
| GO:0045444\_fat\_cell\_differentiation | NOC3L | 57 | 1 | 2.992203 | -0.543229 | 216 | 293.47 | 1.358657 |  |
| GO:0033043\_regulation\_of\_organelle\_organization | CDC42 | 58 | 1 | 2.940613 | -0.536847 | 218 | 295.67 | 1.356284 |  |
| GO:0034622\_cellular\_macromolecular\_complex\_assembly | SMARCA2 | 58 | 1 | 2.940613 | -0.536847 | 218 | 295.67 | 1.356284 |  |
| GO:0032990\_cell\_part\_morphogenesis | KIF3A | 184 | 2 | 1.853865 | -0.531946 | 219 | 296.74 | 1.354977 |  |
| GO:0032990\_cell\_part\_morphogenesis | L1CAM | 184 | 2 | 1.853865 | -0.531946 | 219 | 296.74 | 1.354977 |  |
| GO:0035270\_endocrine\_system\_development | PBX1 | 59 | 1 | 2.890772 | -0.530594 | 221 | 297.89 | 1.347919 |  |
| GO:0050870\_positive\_regulation\_of\_T\_cell\_activation | CD83 | 59 | 1 | 2.890772 | -0.530594 | 221 | 297.89 | 1.347919 |  |
| GO:0007155\_cell\_adhesion | CDC42 | 186 | 2 | 1.833931 | -0.525480 | 223 | 299.08 | 1.341166 |  |
| GO:0007155\_cell\_adhesion | L1CAM | 186 | 2 | 1.833931 | -0.525480 | 223 | 299.08 | 1.341166 |  |
| GO:0022610\_biological\_adhesion | CDC42 | 186 | 2 | 1.833931 | -0.525480 | 223 | 299.08 | 1.341166 |  |
| GO:0022610\_biological\_adhesion | L1CAM | 186 | 2 | 1.833931 | -0.525480 | 223 | 299.08 | 1.341166 |  |
| GO:0043170\_macromolecule\_metabolic\_process | NGLY1 | 1576 | 11 | 1.190426 | -0.524230 | 224 | 299.59 | 1.337455 |  |
| GO:0043170\_macromolecule\_metabolic\_process | COPS2 | 1576 | 11 | 1.190426 | -0.524230 | 224 | 299.59 | 1.337455 |  |
| GO:0043170\_macromolecule\_metabolic\_process | SMARCA5 | 1576 | 11 | 1.190426 | -0.524230 | 224 | 299.59 | 1.337455 |  |
| GO:0043170\_macromolecule\_metabolic\_process | RNF11 | 1576 | 11 | 1.190426 | -0.524230 | 224 | 299.59 | 1.337455 |  |
| GO:0043170\_macromolecule\_metabolic\_process | CHEK1 | 1576 | 11 | 1.190426 | -0.524230 | 224 | 299.59 | 1.337455 |  |
| GO:0043170\_macromolecule\_metabolic\_process | PBX1 | 1576 | 11 | 1.190426 | -0.524230 | 224 | 299.59 | 1.337455 |  |
| GO:0043170\_macromolecule\_metabolic\_process | OGT | 1576 | 11 | 1.190426 | -0.524230 | 224 | 299.59 | 1.337455 |  |
| GO:0043170\_macromolecule\_metabolic\_process | LCOR | 1576 | 11 | 1.190426 | -0.524230 | 224 | 299.59 | 1.337455 |  |
| GO:0043170\_macromolecule\_metabolic\_process | PBX2 | 1576 | 11 | 1.190426 | -0.524230 | 224 | 299.59 | 1.337455 |  |
| GO:0043170\_macromolecule\_metabolic\_process | EDEM1 | 1576 | 11 | 1.190426 | -0.524230 | 224 | 299.59 | 1.337455 |  |
| GO:0043170\_macromolecule\_metabolic\_process | MYST4 | 1576 | 11 | 1.190426 | -0.524230 | 224 | 299.59 | 1.337455 |  |
| GO:0007154\_cell\_communication | CDC42 | 1096 | 8 | 1.244931 | -0.518607 | 225 | 300.45 | 1.335333 |  |
| GO:0007154\_cell\_communication | KIF1B | 1096 | 8 | 1.244931 | -0.518607 | 225 | 300.45 | 1.335333 |  |
| GO:0007154\_cell\_communication | KIF3A | 1096 | 8 | 1.244931 | -0.518607 | 225 | 300.45 | 1.335333 |  |
| GO:0007154\_cell\_communication | SOSTDC1 | 1096 | 8 | 1.244931 | -0.518607 | 225 | 300.45 | 1.335333 |  |
| GO:0007154\_cell\_communication | CHEK1 | 1096 | 8 | 1.244931 | -0.518607 | 225 | 300.45 | 1.335333 |  |
| GO:0007154\_cell\_communication | L1CAM | 1096 | 8 | 1.244931 | -0.518607 | 225 | 300.45 | 1.335333 |  |
| GO:0007154\_cell\_communication | DUSP9 | 1096 | 8 | 1.244931 | -0.518607 | 225 | 300.45 | 1.335333 |  |
| GO:0007154\_cell\_communication | CACNA1D | 1096 | 8 | 1.244931 | -0.518607 | 225 | 300.45 | 1.335333 |  |
| GO:0022604\_regulation\_of\_cell\_morphogenesis | EPB4.1L2 | 62 | 1 | 2.750896 | -0.512562 | 228 | 305.16 | 1.338421 |  |
| GO:0030155\_regulation\_of\_cell\_adhesion | L1CAM | 62 | 1 | 2.750896 | -0.512562 | 228 | 305.16 | 1.338421 |  |
| GO:0050954\_sensory\_perception\_of\_mechanical\_stimulus | CACNA1D | 62 | 1 | 2.750896 | -0.512562 | 228 | 305.16 | 1.338421 |  |
| GO:0046907\_intracellular\_transport | CDC42 | 194 | 2 | 1.758305 | -0.500581 | 229 | 308.93 | 1.349039 |  |
| GO:0046907\_intracellular\_transport | KIF1B | 194 | 2 | 1.758305 | -0.500581 | 229 | 308.93 | 1.349039 |  |
| GO:0043086\_negative\_regulation\_of\_catalytic\_activity | DUSP9 | 65 | 1 | 2.623932 | -0.495538 | 230 | 310.71 | 1.350913 |  |
| GO:0006793\_phosphorus\_metabolic\_process | CDC42 | 340 | 3 | 1.504902 | -0.493101 | 232 | 311.65 | 1.343319 |  |
| GO:0006793\_phosphorus\_metabolic\_process | CHEK1 | 340 | 3 | 1.504902 | -0.493101 | 232 | 311.65 | 1.343319 |  |
| GO:0006793\_phosphorus\_metabolic\_process | DUSP9 | 340 | 3 | 1.504902 | -0.493101 | 232 | 311.65 | 1.343319 |  |
| GO:0006796\_phosphate\_metabolic\_process | CDC42 | 340 | 3 | 1.504902 | -0.493101 | 232 | 311.65 | 1.343319 |  |
| GO:0006796\_phosphate\_metabolic\_process | CHEK1 | 340 | 3 | 1.504902 | -0.493101 | 232 | 311.65 | 1.343319 |  |
| GO:0006796\_phosphate\_metabolic\_process | DUSP9 | 340 | 3 | 1.504902 | -0.493101 | 232 | 311.65 | 1.343319 |  |
| GO:0007179\_transforming\_growth\_factor\_beta\_receptor\_signaling\_pathway | SOSTDC1 | 66 | 1 | 2.584175 | -0.490071 | 233 | 313.68 | 1.346266 |  |
| GO:0002009\_morphogenesis\_of\_an\_epithelium | FREM2 | 198 | 2 | 1.722783 | -0.488680 | 235 | 314.15 | 1.336809 |  |
| GO:0002009\_morphogenesis\_of\_an\_epithelium | PBX1 | 198 | 2 | 1.722783 | -0.488680 | 235 | 314.15 | 1.336809 |  |
| GO:0060429\_epithelium\_development | FREM2 | 198 | 2 | 1.722783 | -0.488680 | 235 | 314.15 | 1.336809 |  |
| GO:0060429\_epithelium\_development | PBX1 | 198 | 2 | 1.722783 | -0.488680 | 235 | 314.15 | 1.336809 |  |
| GO:0016043\_cellular\_component\_organization | EPB4.1L2 | 964 | 7 | 1.238474 | -0.481420 | 236 | 316.49 | 1.341059 |  |
| GO:0016043\_cellular\_component\_organization | CDC42 | 964 | 7 | 1.238474 | -0.481420 | 236 | 316.49 | 1.341059 |  |
| GO:0016043\_cellular\_component\_organization | KIF3A | 964 | 7 | 1.238474 | -0.481420 | 236 | 316.49 | 1.341059 |  |
| GO:0016043\_cellular\_component\_organization | SMARCA5 | 964 | 7 | 1.238474 | -0.481420 | 236 | 316.49 | 1.341059 |  |
| GO:0016043\_cellular\_component\_organization | L1CAM | 964 | 7 | 1.238474 | -0.481420 | 236 | 316.49 | 1.341059 |  |
| GO:0016043\_cellular\_component\_organization | SMARCA2 | 964 | 7 | 1.238474 | -0.481420 | 236 | 316.49 | 1.341059 |  |
| GO:0016043\_cellular\_component\_organization | MYST4 | 964 | 7 | 1.238474 | -0.481420 | 236 | 316.49 | 1.341059 |  |
| GO:0019538\_protein\_metabolic\_process | RNF11 | 655 | 5 | 1.301951 | -0.472435 | 237 | 320.09 | 1.350591 |  |
| GO:0019538\_protein\_metabolic\_process | CHEK1 | 655 | 5 | 1.301951 | -0.472435 | 237 | 320.09 | 1.350591 |  |
| GO:0019538\_protein\_metabolic\_process | OGT | 655 | 5 | 1.301951 | -0.472435 | 237 | 320.09 | 1.350591 |  |
| GO:0019538\_protein\_metabolic\_process | EDEM1 | 655 | 5 | 1.301951 | -0.472435 | 237 | 320.09 | 1.350591 |  |
| GO:0019538\_protein\_metabolic\_process | MYST4 | 655 | 5 | 1.301951 | -0.472435 | 237 | 320.09 | 1.350591 |  |
| GO:0008406\_gonad\_development | FANCA | 70 | 1 | 2.436508 | -0.469162 | 238 | 321.82 | 1.352185 |  |
| GO:0006281\_DNA\_repair | CHEK1 | 71 | 1 | 2.402191 | -0.464161 | 240 | 324.39 | 1.351625 |  |
| GO:0033674\_positive\_regulation\_of\_kinase\_activity | CDC42 | 71 | 1 | 2.402191 | -0.464161 | 240 | 324.39 | 1.351625 |  |
| GO:0007264\_small\_GTPase\_mediated\_signal\_transduction | CDC42 | 72 | 1 | 2.368827 | -0.459244 | 244 | 328.1 | 1.344672 |  |
| GO:0021915\_neural\_tube\_development | KIF3A | 72 | 1 | 2.368827 | -0.459244 | 244 | 328.1 | 1.344672 |  |
| GO:0044262\_cellular\_carbohydrate\_metabolic\_process | OGT | 72 | 1 | 2.368827 | -0.459244 | 244 | 328.1 | 1.344672 |  |
| GO:0051347\_positive\_regulation\_of\_transferase\_activity | CDC42 | 72 | 1 | 2.368827 | -0.459244 | 244 | 328.1 | 1.344672 |  |
| GO:0030182\_neuron\_differentiation | CDC42 | 356 | 3 | 1.437266 | -0.458704 | 245 | 328.29 | 1.339959 |  |
| GO:0030182\_neuron\_differentiation | L1CAM | 356 | 3 | 1.437266 | -0.458704 | 245 | 328.29 | 1.339959 |  |
| GO:0030182\_neuron\_differentiation | PBX1 | 356 | 3 | 1.437266 | -0.458704 | 245 | 328.29 | 1.339959 |  |
| GO:0008152\_metabolic\_process | COPS2 | 2133 | 14 | 1.119446 | -0.457027 | 246 | 328.38 | 1.334878 |  |
| GO:0008152\_metabolic\_process | CHEK1 | 2133 | 14 | 1.119446 | -0.457027 | 246 | 328.38 | 1.334878 |  |
| GO:0008152\_metabolic\_process | EDEM1 | 2133 | 14 | 1.119446 | -0.457027 | 246 | 328.38 | 1.334878 |  |
| GO:0008152\_metabolic\_process | NGLY1 | 2133 | 14 | 1.119446 | -0.457027 | 246 | 328.38 | 1.334878 |  |
| GO:0008152\_metabolic\_process | CDC42 | 2133 | 14 | 1.119446 | -0.457027 | 246 | 328.38 | 1.334878 |  |
| GO:0008152\_metabolic\_process | GOPC | 2133 | 14 | 1.119446 | -0.457027 | 246 | 328.38 | 1.334878 |  |
| GO:0008152\_metabolic\_process | SMARCA5 | 2133 | 14 | 1.119446 | -0.457027 | 246 | 328.38 | 1.334878 |  |
| GO:0008152\_metabolic\_process | RNF11 | 2133 | 14 | 1.119446 | -0.457027 | 246 | 328.38 | 1.334878 |  |
| GO:0008152\_metabolic\_process | PBX1 | 2133 | 14 | 1.119446 | -0.457027 | 246 | 328.38 | 1.334878 |  |
| GO:0008152\_metabolic\_process | OGT | 2133 | 14 | 1.119446 | -0.457027 | 246 | 328.38 | 1.334878 |  |
| GO:0008152\_metabolic\_process | LCOR | 2133 | 14 | 1.119446 | -0.457027 | 246 | 328.38 | 1.334878 |  |
| GO:0008152\_metabolic\_process | DUSP9 | 2133 | 14 | 1.119446 | -0.457027 | 246 | 328.38 | 1.334878 |  |
| GO:0008152\_metabolic\_process | PBX2 | 2133 | 14 | 1.119446 | -0.457027 | 246 | 328.38 | 1.334878 |  |
| GO:0008152\_metabolic\_process | MYST4 | 2133 | 14 | 1.119446 | -0.457027 | 246 | 328.38 | 1.334878 |  |
| GO:0048706\_embryonic\_skeletal\_system\_development | PBX1 | 73 | 1 | 2.336377 | -0.454411 | 247 | 329.92 | 1.335709 |  |
| GO:0009987\_cellular\_process | COPS2 | 3868 | 24 | 1.058256 | -0.453470 | 248 | 330.17 | 1.331331 |  |
| GO:0009987\_cellular\_process | KIF3A | 3868 | 24 | 1.058256 | -0.453470 | 248 | 330.17 | 1.331331 |  |
| GO:0009987\_cellular\_process | NOC3L | 3868 | 24 | 1.058256 | -0.453470 | 248 | 330.17 | 1.331331 |  |
| GO:0009987\_cellular\_process | L1CAM | 3868 | 24 | 1.058256 | -0.453470 | 248 | 330.17 | 1.331331 |  |
| GO:0009987\_cellular\_process | CHEK1 | 3868 | 24 | 1.058256 | -0.453470 | 248 | 330.17 | 1.331331 |  |
| GO:0009987\_cellular\_process | EDEM1 | 3868 | 24 | 1.058256 | -0.453470 | 248 | 330.17 | 1.331331 |  |
| GO:0009987\_cellular\_process | NGLY1 | 3868 | 24 | 1.058256 | -0.453470 | 248 | 330.17 | 1.331331 |  |
| GO:0009987\_cellular\_process | EPB4.1L2 | 3868 | 24 | 1.058256 | -0.453470 | 248 | 330.17 | 1.331331 |  |
| GO:0009987\_cellular\_process | CD83 | 3868 | 24 | 1.058256 | -0.453470 | 248 | 330.17 | 1.331331 |  |
| GO:0009987\_cellular\_process | CDC42 | 3868 | 24 | 1.058256 | -0.453470 | 248 | 330.17 | 1.331331 |  |
| GO:0009987\_cellular\_process | KIF1B | 3868 | 24 | 1.058256 | -0.453470 | 248 | 330.17 | 1.331331 |  |
| GO:0009987\_cellular\_process | SOSTDC1 | 3868 | 24 | 1.058256 | -0.453470 | 248 | 330.17 | 1.331331 |  |
| GO:0009987\_cellular\_process | GOPC | 3868 | 24 | 1.058256 | -0.453470 | 248 | 330.17 | 1.331331 |  |
| GO:0009987\_cellular\_process | SMARCA5 | 3868 | 24 | 1.058256 | -0.453470 | 248 | 330.17 | 1.331331 |  |
| GO:0009987\_cellular\_process | RNF11 | 3868 | 24 | 1.058256 | -0.453470 | 248 | 330.17 | 1.331331 |  |
| GO:0009987\_cellular\_process | PBX1 | 3868 | 24 | 1.058256 | -0.453470 | 248 | 330.17 | 1.331331 |  |
| GO:0009987\_cellular\_process | OGT | 3868 | 24 | 1.058256 | -0.453470 | 248 | 330.17 | 1.331331 |  |
| GO:0009987\_cellular\_process | LCOR | 3868 | 24 | 1.058256 | -0.453470 | 248 | 330.17 | 1.331331 |  |
| GO:0009987\_cellular\_process | DUSP9 | 3868 | 24 | 1.058256 | -0.453470 | 248 | 330.17 | 1.331331 |  |
| GO:0009987\_cellular\_process | PBX2 | 3868 | 24 | 1.058256 | -0.453470 | 248 | 330.17 | 1.331331 |  |
| GO:0009987\_cellular\_process | SMARCA2 | 3868 | 24 | 1.058256 | -0.453470 | 248 | 330.17 | 1.331331 |  |
| GO:0009987\_cellular\_process | FANCA | 3868 | 24 | 1.058256 | -0.453470 | 248 | 330.17 | 1.331331 |  |
| GO:0009987\_cellular\_process | CACNA1D | 3868 | 24 | 1.058256 | -0.453470 | 248 | 330.17 | 1.331331 |  |
| GO:0009987\_cellular\_process | MYST4 | 3868 | 24 | 1.058256 | -0.453470 | 248 | 330.17 | 1.331331 |  |
| GO:0048771\_tissue\_remodeling | PBX1 | 74 | 1 | 2.304805 | -0.449657 | 249 | 331.32 | 1.330602 |  |
| GO:0043009\_chordate\_embryonic\_development | KIF3A | 365 | 3 | 1.401826 | -0.440486 | 250 | 333.98 | 1.335920 |  |
| GO:0043009\_chordate\_embryonic\_development | PBX1 | 365 | 3 | 1.401826 | -0.440486 | 250 | 333.98 | 1.335920 |  |
| GO:0043009\_chordate\_embryonic\_development | YBX1 | 365 | 3 | 1.401826 | -0.440486 | 250 | 333.98 | 1.335920 |  |
| GO:0034621\_cellular\_macromolecular\_complex\_subunit\_organization | SMARCA2 | 76 | 1 | 2.244152 | -0.440384 | 251 | 335.2 | 1.335458 |  |
| GO:0045449\_regulation\_of\_transcription | COPS2 | 676 | 5 | 1.261506 | -0.440191 | 252 | 335.36 | 1.330794 |  |
| GO:0045449\_regulation\_of\_transcription | SMARCA5 | 676 | 5 | 1.261506 | -0.440191 | 252 | 335.36 | 1.330794 |  |
| GO:0045449\_regulation\_of\_transcription | PBX1 | 676 | 5 | 1.261506 | -0.440191 | 252 | 335.36 | 1.330794 |  |
| GO:0045449\_regulation\_of\_transcription | LCOR | 676 | 5 | 1.261506 | -0.440191 | 252 | 335.36 | 1.330794 |  |
| GO:0045449\_regulation\_of\_transcription | PBX2 | 676 | 5 | 1.261506 | -0.440191 | 252 | 335.36 | 1.330794 |  |
| GO:0051241\_negative\_regulation\_of\_multicellular\_organismal\_process | CD83 | 77 | 1 | 2.215007 | -0.435859 | 253 | 336.66 | 1.330672 |  |
| GO:0009792\_embryonic\_development\_ending\_in\_birth\_or\_egg\_hatching | KIF3A | 368 | 3 | 1.390399 | -0.434585 | 254 | 336.9 | 1.326378 |  |
| GO:0009792\_embryonic\_development\_ending\_in\_birth\_or\_egg\_hatching | PBX1 | 368 | 3 | 1.390399 | -0.434585 | 254 | 336.9 | 1.326378 |  |
| GO:0009792\_embryonic\_development\_ending\_in\_birth\_or\_egg\_hatching | YBX1 | 368 | 3 | 1.390399 | -0.434585 | 254 | 336.9 | 1.326378 |  |
| GO:0051251\_positive\_regulation\_of\_lymphocyte\_activation | CD83 | 78 | 1 | 2.186610 | -0.431407 | 255 | 339.7 | 1.332157 |  |
| GO:0001701\_in\_utero\_embryonic\_development | KIF3A | 221 | 2 | 1.543489 | -0.426556 | 256 | 341.51 | 1.334023 |  |
| GO:0001701\_in\_utero\_embryonic\_development | YBX1 | 221 | 2 | 1.543489 | -0.426556 | 256 | 341.51 | 1.334023 |  |
| GO:0044092\_negative\_regulation\_of\_molecular\_function | DUSP9 | 80 | 1 | 2.131944 | -0.422712 | 257 | 342.89 | 1.334202 |  |
| GO:0031323\_regulation\_of\_cellular\_metabolic\_process | CDC42 | 1015 | 7 | 1.176245 | -0.415865 | 258 | 343.69 | 1.332132 |  |
| GO:0031323\_regulation\_of\_cellular\_metabolic\_process | COPS2 | 1015 | 7 | 1.176245 | -0.415865 | 258 | 343.69 | 1.332132 |  |
| GO:0031323\_regulation\_of\_cellular\_metabolic\_process | SMARCA5 | 1015 | 7 | 1.176245 | -0.415865 | 258 | 343.69 | 1.332132 |  |
| GO:0031323\_regulation\_of\_cellular\_metabolic\_process | PBX1 | 1015 | 7 | 1.176245 | -0.415865 | 258 | 343.69 | 1.332132 |  |
| GO:0031323\_regulation\_of\_cellular\_metabolic\_process | DUSP9 | 1015 | 7 | 1.176245 | -0.415865 | 258 | 343.69 | 1.332132 |  |
| GO:0031323\_regulation\_of\_cellular\_metabolic\_process | LCOR | 1015 | 7 | 1.176245 | -0.415865 | 258 | 343.69 | 1.332132 |  |
| GO:0031323\_regulation\_of\_cellular\_metabolic\_process | PBX2 | 1015 | 7 | 1.176245 | -0.415865 | 258 | 343.69 | 1.332132 |  |
| GO:0002696\_positive\_regulation\_of\_leukocyte\_activation | CD83 | 82 | 1 | 2.079946 | -0.414284 | 261 | 345.26 | 1.322835 |  |
| GO:0007411\_axon\_guidance | L1CAM | 82 | 1 | 2.079946 | -0.414284 | 261 | 345.26 | 1.322835 |  |
| GO:0045664\_regulation\_of\_neuron\_differentiation | PBX1 | 82 | 1 | 2.079946 | -0.414284 | 261 | 345.26 | 1.322835 |  |
| GO:0007017\_microtubule-based\_process | KIF1B | 83 | 1 | 2.054886 | -0.410166 | 263 | 347.27 | 1.320418 |  |
| GO:0050867\_positive\_regulation\_of\_cell\_activation | CD83 | 83 | 1 | 2.054886 | -0.410166 | 263 | 347.27 | 1.320418 |  |
| GO:0048519\_negative\_regulation\_of\_biological\_process | CD83 | 859 | 6 | 1.191308 | -0.408455 | 264 | 348.22 | 1.319015 |  |
| GO:0048519\_negative\_regulation\_of\_biological\_process | COPS2 | 859 | 6 | 1.191308 | -0.408455 | 264 | 348.22 | 1.319015 |  |
| GO:0048519\_negative\_regulation\_of\_biological\_process | SOSTDC1 | 859 | 6 | 1.191308 | -0.408455 | 264 | 348.22 | 1.319015 |  |
| GO:0048519\_negative\_regulation\_of\_biological\_process | PBX1 | 859 | 6 | 1.191308 | -0.408455 | 264 | 348.22 | 1.319015 |  |
| GO:0048519\_negative\_regulation\_of\_biological\_process | LCOR | 859 | 6 | 1.191308 | -0.408455 | 264 | 348.22 | 1.319015 |  |
| GO:0048519\_negative\_regulation\_of\_biological\_process | SMARCA2 | 859 | 6 | 1.191308 | -0.408455 | 264 | 348.22 | 1.319015 |  |
| GO:0045137\_development\_of\_primary\_sexual\_characteristics | FANCA | 84 | 1 | 2.030423 | -0.406111 | 265 | 349.39 | 1.318453 |  |
| GO:0006350\_transcription | COPS2 | 701 | 5 | 1.216516 | -0.404446 | 266 | 349.78 | 1.314962 |  |
| GO:0006350\_transcription | SMARCA5 | 701 | 5 | 1.216516 | -0.404446 | 266 | 349.78 | 1.314962 |  |
| GO:0006350\_transcription | PBX1 | 701 | 5 | 1.216516 | -0.404446 | 266 | 349.78 | 1.314962 |  |
| GO:0006350\_transcription | LCOR | 701 | 5 | 1.216516 | -0.404446 | 266 | 349.78 | 1.314962 |  |
| GO:0006350\_transcription | PBX2 | 701 | 5 | 1.216516 | -0.404446 | 266 | 349.78 | 1.314962 |  |
| GO:0008283\_cell\_proliferation | COPS2 | 544 | 4 | 1.254085 | -0.400476 | 267 | 351.42 | 1.316180 |  |
| GO:0008283\_cell\_proliferation | PBX1 | 544 | 4 | 1.254085 | -0.400476 | 267 | 351.42 | 1.316180 |  |
| GO:0008283\_cell\_proliferation | FANCA | 544 | 4 | 1.254085 | -0.400476 | 267 | 351.42 | 1.316180 |  |
| GO:0008283\_cell\_proliferation | SMARCA2 | 544 | 4 | 1.254085 | -0.400476 | 267 | 351.42 | 1.316180 |  |
| GO:0050790\_regulation\_of\_catalytic\_activity | CDC42 | 233 | 2 | 1.463996 | -0.397897 | 268 | 354.52 | 1.322836 |  |
| GO:0050790\_regulation\_of\_catalytic\_activity | DUSP9 | 233 | 2 | 1.463996 | -0.397897 | 268 | 354.52 | 1.322836 |  |
| GO:0007178\_transmembrane\_receptor\_protein\_serine\_threonine\_kinase\_signaling\_pathway | SOSTDC1 | 87 | 1 | 1.960409 | -0.394301 | 269 | 357.31 | 1.328290 |  |
| GO:0001503\_ossification | PBX1 | 88 | 1 | 1.938131 | -0.390479 | 272 | 359.18 | 1.320515 |  |
| GO:0048754\_branching\_morphogenesis\_of\_a\_tube | PBX1 | 88 | 1 | 1.938131 | -0.390479 | 272 | 359.18 | 1.320515 |  |
| GO:0050863\_regulation\_of\_T\_cell\_activation | CD83 | 88 | 1 | 1.938131 | -0.390479 | 272 | 359.18 | 1.320515 |  |
| GO:0042127\_regulation\_of\_cell\_proliferation | PBX1 | 393 | 3 | 1.301951 | -0.388508 | 273 | 359.94 | 1.318462 |  |
| GO:0042127\_regulation\_of\_cell\_proliferation | FANCA | 393 | 3 | 1.301951 | -0.388508 | 273 | 359.94 | 1.318462 |  |
| GO:0042127\_regulation\_of\_cell\_proliferation | SMARCA2 | 393 | 3 | 1.301951 | -0.388508 | 273 | 359.94 | 1.318462 |  |
| GO:0048699\_generation\_of\_neurons | CDC42 | 396 | 3 | 1.292088 | -0.383330 | 274 | 360.47 | 1.315584 |  |
| GO:0048699\_generation\_of\_neurons | L1CAM | 396 | 3 | 1.292088 | -0.383330 | 274 | 360.47 | 1.315584 |  |
| GO:0048699\_generation\_of\_neurons | PBX1 | 396 | 3 | 1.292088 | -0.383330 | 274 | 360.47 | 1.315584 |  |
| GO:0030217\_T\_cell\_differentiation | CD83 | 92 | 1 | 1.853865 | -0.375729 | 275 | 363.92 | 1.323345 |  |
| GO:0065003\_macromolecular\_complex\_assembly | SMARCA2 | 93 | 1 | 1.833931 | -0.372170 | 276 | 365.54 | 1.324420 |  |
| GO:0034984\_cellular\_response\_to\_DNA\_damage\_stimulus | CHEK1 | 94 | 1 | 1.814421 | -0.368660 | 277 | 367.49 | 1.326679 |  |
| GO:0032502\_developmental\_process | KIF3A | 2060 | 13 | 1.076321 | -0.363967 | 278 | 369.79 | 1.330180 |  |
| GO:0032502\_developmental\_process | NOC3L | 2060 | 13 | 1.076321 | -0.363967 | 278 | 369.79 | 1.330180 |  |
| GO:0032502\_developmental\_process | L1CAM | 2060 | 13 | 1.076321 | -0.363967 | 278 | 369.79 | 1.330180 |  |
| GO:0032502\_developmental\_process | YBX1 | 2060 | 13 | 1.076321 | -0.363967 | 278 | 369.79 | 1.330180 |  |
| GO:0032502\_developmental\_process | EPB4.1L2 | 2060 | 13 | 1.076321 | -0.363967 | 278 | 369.79 | 1.330180 |  |
| GO:0032502\_developmental\_process | CD83 | 2060 | 13 | 1.076321 | -0.363967 | 278 | 369.79 | 1.330180 |  |
| GO:0032502\_developmental\_process | CDC42 | 2060 | 13 | 1.076321 | -0.363967 | 278 | 369.79 | 1.330180 |  |
| GO:0032502\_developmental\_process | FREM2 | 2060 | 13 | 1.076321 | -0.363967 | 278 | 369.79 | 1.330180 |  |
| GO:0032502\_developmental\_process | SOSTDC1 | 2060 | 13 | 1.076321 | -0.363967 | 278 | 369.79 | 1.330180 |  |
| GO:0032502\_developmental\_process | SMARCA5 | 2060 | 13 | 1.076321 | -0.363967 | 278 | 369.79 | 1.330180 |  |
| GO:0032502\_developmental\_process | PBX1 | 2060 | 13 | 1.076321 | -0.363967 | 278 | 369.79 | 1.330180 |  |
| GO:0032502\_developmental\_process | FANCA | 2060 | 13 | 1.076321 | -0.363967 | 278 | 369.79 | 1.330180 |  |
| GO:0032502\_developmental\_process | PBX2 | 2060 | 13 | 1.076321 | -0.363967 | 278 | 369.79 | 1.330180 |  |
| GO:0034645\_cellular\_macromolecule\_biosynthetic\_process | COPS2 | 901 | 6 | 1.135775 | -0.357955 | 279 | 373.2 | 1.337634 |  |
| GO:0034645\_cellular\_macromolecule\_biosynthetic\_process | SMARCA5 | 901 | 6 | 1.135775 | -0.357955 | 279 | 373.2 | 1.337634 |  |
| GO:0034645\_cellular\_macromolecule\_biosynthetic\_process | PBX1 | 901 | 6 | 1.135775 | -0.357955 | 279 | 373.2 | 1.337634 |  |
| GO:0034645\_cellular\_macromolecule\_biosynthetic\_process | OGT | 901 | 6 | 1.135775 | -0.357955 | 279 | 373.2 | 1.337634 |  |
| GO:0034645\_cellular\_macromolecule\_biosynthetic\_process | LCOR | 901 | 6 | 1.135775 | -0.357955 | 279 | 373.2 | 1.337634 |  |
| GO:0034645\_cellular\_macromolecule\_biosynthetic\_process | PBX2 | 901 | 6 | 1.135775 | -0.357955 | 279 | 373.2 | 1.337634 |  |
| GO:0006355\_regulation\_of\_transcription\_\_DNA-dependent | SMARCA5 | 575 | 4 | 1.186473 | -0.355708 | 280 | 373.63 | 1.334393 |  |
| GO:0006355\_regulation\_of\_transcription\_\_DNA-dependent | PBX1 | 575 | 4 | 1.186473 | -0.355708 | 280 | 373.63 | 1.334393 |  |
| GO:0006355\_regulation\_of\_transcription\_\_DNA-dependent | LCOR | 575 | 4 | 1.186473 | -0.355708 | 280 | 373.63 | 1.334393 |  |
| GO:0006355\_regulation\_of\_transcription\_\_DNA-dependent | PBX2 | 575 | 4 | 1.186473 | -0.355708 | 280 | 373.63 | 1.334393 |  |
| GO:0007548\_sex\_differentiation | FANCA | 98 | 1 | 1.740363 | -0.355092 | 282 | 374.92 | 1.329504 |  |
| GO:0009967\_positive\_regulation\_of\_signal\_transduction | L1CAM | 98 | 1 | 1.740363 | -0.355092 | 282 | 374.92 | 1.329504 |  |
| GO:0016481\_negative\_regulation\_of\_transcription | COPS2 | 253 | 2 | 1.348265 | -0.354950 | 284 | 375.35 | 1.321655 |  |
| GO:0016481\_negative\_regulation\_of\_transcription | LCOR | 253 | 2 | 1.348265 | -0.354950 | 284 | 375.35 | 1.321655 |  |
| GO:0030097\_hemopoiesis | CD83 | 253 | 2 | 1.348265 | -0.354950 | 284 | 375.35 | 1.321655 |  |
| GO:0030097\_hemopoiesis | PBX1 | 253 | 2 | 1.348265 | -0.354950 | 284 | 375.35 | 1.321655 |  |
| GO:0001817\_regulation\_of\_cytokine\_production | CD83 | 99 | 1 | 1.722783 | -0.351813 | 287 | 377.32 | 1.314704 |  |
| GO:0060348\_bone\_development | PBX1 | 99 | 1 | 1.722783 | -0.351813 | 287 | 377.32 | 1.314704 |  |
| GO:0060562\_epithelial\_tube\_morphogenesis | PBX1 | 99 | 1 | 1.722783 | -0.351813 | 287 | 377.32 | 1.314704 |  |
| GO:0048729\_tissue\_morphogenesis | FREM2 | 255 | 2 | 1.337691 | -0.350955 | 288 | 377.61 | 1.311146 |  |
| GO:0048729\_tissue\_morphogenesis | PBX1 | 255 | 2 | 1.337691 | -0.350955 | 288 | 377.61 | 1.311146 |  |
| GO:0009966\_regulation\_of\_signal\_transduction | SOSTDC1 | 256 | 2 | 1.332465 | -0.348976 | 289 | 377.83 | 1.307370 |  |
| GO:0009966\_regulation\_of\_signal\_transduction | L1CAM | 256 | 2 | 1.332465 | -0.348976 | 289 | 377.83 | 1.307370 |  |
| GO:0010556\_regulation\_of\_macromolecule\_biosynthetic\_process | COPS2 | 745 | 5 | 1.144668 | -0.347877 | 290 | 378.26 | 1.304345 |  |
| GO:0010556\_regulation\_of\_macromolecule\_biosynthetic\_process | SMARCA5 | 745 | 5 | 1.144668 | -0.347877 | 290 | 378.26 | 1.304345 |  |
| GO:0010556\_regulation\_of\_macromolecule\_biosynthetic\_process | PBX1 | 745 | 5 | 1.144668 | -0.347877 | 290 | 378.26 | 1.304345 |  |
| GO:0010556\_regulation\_of\_macromolecule\_biosynthetic\_process | LCOR | 745 | 5 | 1.144668 | -0.347877 | 290 | 378.26 | 1.304345 |  |
| GO:0010556\_regulation\_of\_macromolecule\_biosynthetic\_process | PBX2 | 745 | 5 | 1.144668 | -0.347877 | 290 | 378.26 | 1.304345 |  |
| GO:0009059\_macromolecule\_biosynthetic\_process | COPS2 | 910 | 6 | 1.124542 | -0.347866 | 291 | 378.43 | 1.300447 |  |
| GO:0009059\_macromolecule\_biosynthetic\_process | SMARCA5 | 910 | 6 | 1.124542 | -0.347866 | 291 | 378.43 | 1.300447 |  |
| GO:0009059\_macromolecule\_biosynthetic\_process | PBX1 | 910 | 6 | 1.124542 | -0.347866 | 291 | 378.43 | 1.300447 |  |
| GO:0009059\_macromolecule\_biosynthetic\_process | OGT | 910 | 6 | 1.124542 | -0.347866 | 291 | 378.43 | 1.300447 |  |
| GO:0009059\_macromolecule\_biosynthetic\_process | LCOR | 910 | 6 | 1.124542 | -0.347866 | 291 | 378.43 | 1.300447 |  |
| GO:0009059\_macromolecule\_biosynthetic\_process | PBX2 | 910 | 6 | 1.124542 | -0.347866 | 291 | 378.43 | 1.300447 |  |
| GO:0030036\_actin\_cytoskeleton\_organization | EPB4.1L2 | 102 | 1 | 1.672113 | -0.342232 | 292 | 380.08 | 1.301644 |  |
| GO:0022008\_neurogenesis | CDC42 | 423 | 3 | 1.209614 | -0.339790 | 293 | 380.29 | 1.297918 |  |
| GO:0022008\_neurogenesis | L1CAM | 423 | 3 | 1.209614 | -0.339790 | 293 | 380.29 | 1.297918 |  |
| GO:0022008\_neurogenesis | PBX1 | 423 | 3 | 1.209614 | -0.339790 | 293 | 380.29 | 1.297918 |  |
| GO:0065007\_biological\_regulation | COPS2 | 2593 | 16 | 1.052406 | -0.339704 | 294 | 380.39 | 1.293844 |  |
| GO:0065007\_biological\_regulation | KIF3A | 2593 | 16 | 1.052406 | -0.339704 | 294 | 380.39 | 1.293844 |  |
| GO:0065007\_biological\_regulation | L1CAM | 2593 | 16 | 1.052406 | -0.339704 | 294 | 380.39 | 1.293844 |  |
| GO:0065007\_biological\_regulation | CHEK1 | 2593 | 16 | 1.052406 | -0.339704 | 294 | 380.39 | 1.293844 |  |
| GO:0065007\_biological\_regulation | EPB4.1L2 | 2593 | 16 | 1.052406 | -0.339704 | 294 | 380.39 | 1.293844 |  |
| GO:0065007\_biological\_regulation | CD83 | 2593 | 16 | 1.052406 | -0.339704 | 294 | 380.39 | 1.293844 |  |
| GO:0065007\_biological\_regulation | CDC42 | 2593 | 16 | 1.052406 | -0.339704 | 294 | 380.39 | 1.293844 |  |
| GO:0065007\_biological\_regulation | SOSTDC1 | 2593 | 16 | 1.052406 | -0.339704 | 294 | 380.39 | 1.293844 |  |
| GO:0065007\_biological\_regulation | SMARCA5 | 2593 | 16 | 1.052406 | -0.339704 | 294 | 380.39 | 1.293844 |  |
| GO:0065007\_biological\_regulation | PBX1 | 2593 | 16 | 1.052406 | -0.339704 | 294 | 380.39 | 1.293844 |  |
| GO:0065007\_biological\_regulation | LCOR | 2593 | 16 | 1.052406 | -0.339704 | 294 | 380.39 | 1.293844 |  |
| GO:0065007\_biological\_regulation | DUSP9 | 2593 | 16 | 1.052406 | -0.339704 | 294 | 380.39 | 1.293844 |  |
| GO:0065007\_biological\_regulation | FANCA | 2593 | 16 | 1.052406 | -0.339704 | 294 | 380.39 | 1.293844 |  |
| GO:0065007\_biological\_regulation | CACNA1D | 2593 | 16 | 1.052406 | -0.339704 | 294 | 380.39 | 1.293844 |  |
| GO:0065007\_biological\_regulation | PBX2 | 2593 | 16 | 1.052406 | -0.339704 | 294 | 380.39 | 1.293844 |  |
| GO:0065007\_biological\_regulation | SMARCA2 | 2593 | 16 | 1.052406 | -0.339704 | 294 | 380.39 | 1.293844 |  |
| GO:0009968\_negative\_regulation\_of\_signal\_transduction | SOSTDC1 | 103 | 1 | 1.655879 | -0.339121 | 295 | 381.68 | 1.293831 |  |
| GO:0010629\_negative\_regulation\_of\_gene\_expression | COPS2 | 262 | 2 | 1.301951 | -0.337367 | 296 | 382.38 | 1.291824 |  |
| GO:0010629\_negative\_regulation\_of\_gene\_expression | LCOR | 262 | 2 | 1.301951 | -0.337367 | 296 | 382.38 | 1.291824 |  |
| GO:0050767\_regulation\_of\_neurogenesis | PBX1 | 104 | 1 | 1.639957 | -0.336050 | 297 | 383.11 | 1.289933 |  |
| GO:0051252\_regulation\_of\_RNA\_metabolic\_process | SMARCA5 | 590 | 4 | 1.156309 | -0.335793 | 298 | 383.24 | 1.286040 |  |
| GO:0051252\_regulation\_of\_RNA\_metabolic\_process | PBX1 | 590 | 4 | 1.156309 | -0.335793 | 298 | 383.24 | 1.286040 |  |
| GO:0051252\_regulation\_of\_RNA\_metabolic\_process | LCOR | 590 | 4 | 1.156309 | -0.335793 | 298 | 383.24 | 1.286040 |  |
| GO:0051252\_regulation\_of\_RNA\_metabolic\_process | PBX2 | 590 | 4 | 1.156309 | -0.335793 | 298 | 383.24 | 1.286040 |  |
| GO:0019222\_regulation\_of\_metabolic\_process | CDC42 | 1088 | 7 | 1.097324 | -0.335168 | 299 | 383.69 | 1.283244 |  |
| GO:0019222\_regulation\_of\_metabolic\_process | COPS2 | 1088 | 7 | 1.097324 | -0.335168 | 299 | 383.69 | 1.283244 |  |
| GO:0019222\_regulation\_of\_metabolic\_process | SMARCA5 | 1088 | 7 | 1.097324 | -0.335168 | 299 | 383.69 | 1.283244 |  |
| GO:0019222\_regulation\_of\_metabolic\_process | PBX1 | 1088 | 7 | 1.097324 | -0.335168 | 299 | 383.69 | 1.283244 |  |
| GO:0019222\_regulation\_of\_metabolic\_process | DUSP9 | 1088 | 7 | 1.097324 | -0.335168 | 299 | 383.69 | 1.283244 |  |
| GO:0019222\_regulation\_of\_metabolic\_process | LCOR | 1088 | 7 | 1.097324 | -0.335168 | 299 | 383.69 | 1.283244 |  |
| GO:0019222\_regulation\_of\_metabolic\_process | PBX2 | 1088 | 7 | 1.097324 | -0.335168 | 299 | 383.69 | 1.283244 |  |
| GO:0019219\_regulation\_of\_nucleobase\_\_nucleoside\_\_nucleotide\_and\_nucleic\_acid\_metabolic\_process | COPS2 | 757 | 5 | 1.126523 | -0.333740 | 300 | 384.06 | 1.280200 |  |
| GO:0019219\_regulation\_of\_nucleobase\_\_nucleoside\_\_nucleotide\_and\_nucleic\_acid\_metabolic\_process | SMARCA5 | 757 | 5 | 1.126523 | -0.333740 | 300 | 384.06 | 1.280200 |  |
| GO:0019219\_regulation\_of\_nucleobase\_\_nucleoside\_\_nucleotide\_and\_nucleic\_acid\_metabolic\_process | PBX1 | 757 | 5 | 1.126523 | -0.333740 | 300 | 384.06 | 1.280200 |  |
| GO:0019219\_regulation\_of\_nucleobase\_\_nucleoside\_\_nucleotide\_and\_nucleic\_acid\_metabolic\_process | LCOR | 757 | 5 | 1.126523 | -0.333740 | 300 | 384.06 | 1.280200 |  |
| GO:0019219\_regulation\_of\_nucleobase\_\_nucleoside\_\_nucleotide\_and\_nucleic\_acid\_metabolic\_process | PBX2 | 757 | 5 | 1.126523 | -0.333740 | 300 | 384.06 | 1.280200 |  |
| GO:0007275\_multicellular\_organismal\_development | CDC42 | 1760 | 11 | 1.065972 | -0.332294 | 301 | 384.84 | 1.278538 |  |
| GO:0007275\_multicellular\_organismal\_development | CD83 | 1760 | 11 | 1.065972 | -0.332294 | 301 | 384.84 | 1.278538 |  |
| GO:0007275\_multicellular\_organismal\_development | KIF3A | 1760 | 11 | 1.065972 | -0.332294 | 301 | 384.84 | 1.278538 |  |
| GO:0007275\_multicellular\_organismal\_development | FREM2 | 1760 | 11 | 1.065972 | -0.332294 | 301 | 384.84 | 1.278538 |  |
| GO:0007275\_multicellular\_organismal\_development | SOSTDC1 | 1760 | 11 | 1.065972 | -0.332294 | 301 | 384.84 | 1.278538 |  |
| GO:0007275\_multicellular\_organismal\_development | SMARCA5 | 1760 | 11 | 1.065972 | -0.332294 | 301 | 384.84 | 1.278538 |  |
| GO:0007275\_multicellular\_organismal\_development | L1CAM | 1760 | 11 | 1.065972 | -0.332294 | 301 | 384.84 | 1.278538 |  |
| GO:0007275\_multicellular\_organismal\_development | PBX1 | 1760 | 11 | 1.065972 | -0.332294 | 301 | 384.84 | 1.278538 |  |
| GO:0007275\_multicellular\_organismal\_development | FANCA | 1760 | 11 | 1.065972 | -0.332294 | 301 | 384.84 | 1.278538 |  |
| GO:0007275\_multicellular\_organismal\_development | PBX2 | 1760 | 11 | 1.065972 | -0.332294 | 301 | 384.84 | 1.278538 |  |
| GO:0007275\_multicellular\_organismal\_development | YBX1 | 1760 | 11 | 1.065972 | -0.332294 | 301 | 384.84 | 1.278538 |  |
| GO:0006351\_transcription\_\_DNA-dependent | SMARCA5 | 594 | 4 | 1.148522 | -0.330663 | 302 | 384.99 | 1.274801 |  |
| GO:0006351\_transcription\_\_DNA-dependent | PBX1 | 594 | 4 | 1.148522 | -0.330663 | 302 | 384.99 | 1.274801 |  |
| GO:0006351\_transcription\_\_DNA-dependent | LCOR | 594 | 4 | 1.148522 | -0.330663 | 302 | 384.99 | 1.274801 |  |
| GO:0006351\_transcription\_\_DNA-dependent | PBX2 | 594 | 4 | 1.148522 | -0.330663 | 302 | 384.99 | 1.274801 |  |
| GO:0080090\_regulation\_of\_primary\_metabolic\_process | COPS2 | 926 | 6 | 1.105112 | -0.330534 | 303 | 385.16 | 1.271155 |  |
| GO:0080090\_regulation\_of\_primary\_metabolic\_process | CDC42 | 926 | 6 | 1.105112 | -0.330534 | 303 | 385.16 | 1.271155 |  |
| GO:0080090\_regulation\_of\_primary\_metabolic\_process | SMARCA5 | 926 | 6 | 1.105112 | -0.330534 | 303 | 385.16 | 1.271155 |  |
| GO:0080090\_regulation\_of\_primary\_metabolic\_process | PBX1 | 926 | 6 | 1.105112 | -0.330534 | 303 | 385.16 | 1.271155 |  |
| GO:0080090\_regulation\_of\_primary\_metabolic\_process | LCOR | 926 | 6 | 1.105112 | -0.330534 | 303 | 385.16 | 1.271155 |  |
| GO:0080090\_regulation\_of\_primary\_metabolic\_process | PBX2 | 926 | 6 | 1.105112 | -0.330534 | 303 | 385.16 | 1.271155 |  |
| GO:0032774\_RNA\_biosynthetic\_process | SMARCA5 | 595 | 4 | 1.146592 | -0.329392 | 304 | 385.64 | 1.268553 |  |
| GO:0032774\_RNA\_biosynthetic\_process | PBX1 | 595 | 4 | 1.146592 | -0.329392 | 304 | 385.64 | 1.268553 |  |
| GO:0032774\_RNA\_biosynthetic\_process | LCOR | 595 | 4 | 1.146592 | -0.329392 | 304 | 385.64 | 1.268553 |  |
| GO:0032774\_RNA\_biosynthetic\_process | PBX2 | 595 | 4 | 1.146592 | -0.329392 | 304 | 385.64 | 1.268553 |  |
| GO:0045859\_regulation\_of\_protein\_kinase\_activity | DUSP9 | 107 | 1 | 1.593977 | -0.327068 | 305 | 386.11 | 1.265934 |  |
| GO:0007166\_cell\_surface\_receptor\_linked\_signal\_transduction | KIF3A | 597 | 4 | 1.142751 | -0.326864 | 306 | 386.35 | 1.262582 |  |
| GO:0007166\_cell\_surface\_receptor\_linked\_signal\_transduction | SOSTDC1 | 597 | 4 | 1.142751 | -0.326864 | 306 | 386.35 | 1.262582 |  |
| GO:0007166\_cell\_surface\_receptor\_linked\_signal\_transduction | L1CAM | 597 | 4 | 1.142751 | -0.326864 | 306 | 386.35 | 1.262582 |  |
| GO:0007166\_cell\_surface\_receptor\_linked\_signal\_transduction | CACNA1D | 597 | 4 | 1.142751 | -0.326864 | 306 | 386.35 | 1.262582 |  |
| GO:0045944\_positive\_regulation\_of\_transcription\_from\_RNA\_polymerase\_II\_promoter | PBX1 | 269 | 2 | 1.268071 | -0.324368 | 307 | 387.06 | 1.260782 |  |
| GO:0045944\_positive\_regulation\_of\_transcription\_from\_RNA\_polymerase\_II\_promoter | PBX2 | 269 | 2 | 1.268071 | -0.324368 | 307 | 387.06 | 1.260782 |  |
| GO:0051240\_positive\_regulation\_of\_multicellular\_organismal\_process | CD83 | 108 | 1 | 1.579218 | -0.324148 | 308 | 387.74 | 1.258896 |  |
| GO:0045934\_negative\_regulation\_of\_nucleobase\_\_nucleoside\_\_nucleotide\_and\_nucleic\_acid\_metabolic\_process | COPS2 | 270 | 2 | 1.263374 | -0.322557 | 309 | 387.91 | 1.255372 |  |
| GO:0045934\_negative\_regulation\_of\_nucleobase\_\_nucleoside\_\_nucleotide\_and\_nucleic\_acid\_metabolic\_process | LCOR | 270 | 2 | 1.263374 | -0.322557 | 309 | 387.91 | 1.255372 |  |
| GO:0006357\_regulation\_of\_transcription\_from\_RNA\_polymerase\_II\_promoter | PBX1 | 435 | 3 | 1.176245 | -0.322081 | 310 | 388.06 | 1.251806 |  |
| GO:0006357\_regulation\_of\_transcription\_from\_RNA\_polymerase\_II\_promoter | LCOR | 435 | 3 | 1.176245 | -0.322081 | 310 | 388.06 | 1.251806 |  |
| GO:0006357\_regulation\_of\_transcription\_from\_RNA\_polymerase\_II\_promoter | PBX2 | 435 | 3 | 1.176245 | -0.322081 | 310 | 388.06 | 1.251806 |  |
| GO:0030029\_actin\_filament-based\_process | EPB4.1L2 | 109 | 1 | 1.564730 | -0.321265 | 311 | 388.49 | 1.249164 |  |
| GO:0051172\_negative\_regulation\_of\_nitrogen\_compound\_metabolic\_process | COPS2 | 271 | 2 | 1.258713 | -0.320757 | 312 | 388.66 | 1.245705 |  |
| GO:0051172\_negative\_regulation\_of\_nitrogen\_compound\_metabolic\_process | LCOR | 271 | 2 | 1.258713 | -0.320757 | 312 | 388.66 | 1.245705 |  |
| GO:0010647\_positive\_regulation\_of\_cell\_communication | L1CAM | 110 | 1 | 1.550505 | -0.318418 | 314 | 390.05 | 1.242197 |  |
| GO:0010648\_negative\_regulation\_of\_cell\_communication | SOSTDC1 | 110 | 1 | 1.550505 | -0.318418 | 314 | 390.05 | 1.242197 |  |
| GO:0051171\_regulation\_of\_nitrogen\_compound\_metabolic\_process | COPS2 | 771 | 5 | 1.106067 | -0.317897 | 315 | 390.2 | 1.238730 |  |
| GO:0051171\_regulation\_of\_nitrogen\_compound\_metabolic\_process | SMARCA5 | 771 | 5 | 1.106067 | -0.317897 | 315 | 390.2 | 1.238730 |  |
| GO:0051171\_regulation\_of\_nitrogen\_compound\_metabolic\_process | PBX1 | 771 | 5 | 1.106067 | -0.317897 | 315 | 390.2 | 1.238730 |  |
| GO:0051171\_regulation\_of\_nitrogen\_compound\_metabolic\_process | LCOR | 771 | 5 | 1.106067 | -0.317897 | 315 | 390.2 | 1.238730 |  |
| GO:0051171\_regulation\_of\_nitrogen\_compound\_metabolic\_process | PBX2 | 771 | 5 | 1.106067 | -0.317897 | 315 | 390.2 | 1.238730 |  |
| GO:0006464\_protein\_modification\_process | CHEK1 | 439 | 3 | 1.165528 | -0.316386 | 316 | 391.17 | 1.237880 |  |
| GO:0006464\_protein\_modification\_process | OGT | 439 | 3 | 1.165528 | -0.316386 | 316 | 391.17 | 1.237880 |  |
| GO:0006464\_protein\_modification\_process | MYST4 | 439 | 3 | 1.165528 | -0.316386 | 316 | 391.17 | 1.237880 |  |
| GO:0010558\_negative\_regulation\_of\_macromolecule\_biosynthetic\_process | COPS2 | 274 | 2 | 1.244931 | -0.315424 | 317 | 392.0 | 1.236593 |  |
| GO:0010558\_negative\_regulation\_of\_macromolecule\_biosynthetic\_process | LCOR | 274 | 2 | 1.244931 | -0.315424 | 317 | 392.0 | 1.236593 |  |
| GO:0048523\_negative\_regulation\_of\_cellular\_process | COPS2 | 774 | 5 | 1.101780 | -0.314591 | 318 | 392.26 | 1.233522 |  |
| GO:0048523\_negative\_regulation\_of\_cellular\_process | SOSTDC1 | 774 | 5 | 1.101780 | -0.314591 | 318 | 392.26 | 1.233522 |  |
| GO:0048523\_negative\_regulation\_of\_cellular\_process | PBX1 | 774 | 5 | 1.101780 | -0.314591 | 318 | 392.26 | 1.233522 |  |
| GO:0048523\_negative\_regulation\_of\_cellular\_process | LCOR | 774 | 5 | 1.101780 | -0.314591 | 318 | 392.26 | 1.233522 |  |
| GO:0048523\_negative\_regulation\_of\_cellular\_process | SMARCA2 | 774 | 5 | 1.101780 | -0.314591 | 318 | 392.26 | 1.233522 |  |
| GO:0051249\_regulation\_of\_lymphocyte\_activation | CD83 | 112 | 1 | 1.522817 | -0.312827 | 319 | 392.94 | 1.231787 |  |
| GO:0048869\_cellular\_developmental\_process | EPB4.1L2 | 1113 | 7 | 1.072676 | -0.310726 | 320 | 393.49 | 1.229656 |  |
| GO:0048869\_cellular\_developmental\_process | CD83 | 1113 | 7 | 1.072676 | -0.310726 | 320 | 393.49 | 1.229656 |  |
| GO:0048869\_cellular\_developmental\_process | CDC42 | 1113 | 7 | 1.072676 | -0.310726 | 320 | 393.49 | 1.229656 |  |
| GO:0048869\_cellular\_developmental\_process | KIF3A | 1113 | 7 | 1.072676 | -0.310726 | 320 | 393.49 | 1.229656 |  |
| GO:0048869\_cellular\_developmental\_process | NOC3L | 1113 | 7 | 1.072676 | -0.310726 | 320 | 393.49 | 1.229656 |  |
| GO:0048869\_cellular\_developmental\_process | L1CAM | 1113 | 7 | 1.072676 | -0.310726 | 320 | 393.49 | 1.229656 |  |
| GO:0048869\_cellular\_developmental\_process | PBX1 | 1113 | 7 | 1.072676 | -0.310726 | 320 | 393.49 | 1.229656 |  |
| GO:0010468\_regulation\_of\_gene\_expression | COPS2 | 778 | 5 | 1.096115 | -0.310230 | 321 | 393.65 | 1.226324 |  |
| GO:0010468\_regulation\_of\_gene\_expression | SMARCA5 | 778 | 5 | 1.096115 | -0.310230 | 321 | 393.65 | 1.226324 |  |
| GO:0010468\_regulation\_of\_gene\_expression | PBX1 | 778 | 5 | 1.096115 | -0.310230 | 321 | 393.65 | 1.226324 |  |
| GO:0010468\_regulation\_of\_gene\_expression | LCOR | 778 | 5 | 1.096115 | -0.310230 | 321 | 393.65 | 1.226324 |  |
| GO:0010468\_regulation\_of\_gene\_expression | PBX2 | 778 | 5 | 1.096115 | -0.310230 | 321 | 393.65 | 1.226324 |  |
| GO:0048534\_hemopoietic\_or\_lymphoid\_organ\_development | CD83 | 277 | 2 | 1.231448 | -0.310190 | 322 | 394.15 | 1.224068 |  |
| GO:0048534\_hemopoietic\_or\_lymphoid\_organ\_development | PBX1 | 277 | 2 | 1.231448 | -0.310190 | 322 | 394.15 | 1.224068 |  |
| GO:0006974\_response\_to\_DNA\_damage\_stimulus | CHEK1 | 113 | 1 | 1.509341 | -0.310082 | 323 | 394.81 | 1.222322 |  |
| GO:0006366\_transcription\_from\_RNA\_polymerase\_II\_promoter | PBX1 | 444 | 3 | 1.152402 | -0.309410 | 324 | 395.19 | 1.219722 |  |
| GO:0006366\_transcription\_from\_RNA\_polymerase\_II\_promoter | LCOR | 444 | 3 | 1.152402 | -0.309410 | 324 | 395.19 | 1.219722 |  |
| GO:0006366\_transcription\_from\_RNA\_polymerase\_II\_promoter | PBX2 | 444 | 3 | 1.152402 | -0.309410 | 324 | 395.19 | 1.219722 |  |
| GO:0000165\_MAPKKK\_cascade | DUSP9 | 114 | 1 | 1.496101 | -0.307370 | 325 | 395.94 | 1.218277 |  |
| GO:0065009\_regulation\_of\_molecular\_function | CDC42 | 279 | 2 | 1.222620 | -0.306753 | 326 | 396.47 | 1.216166 |  |
| GO:0065009\_regulation\_of\_molecular\_function | DUSP9 | 279 | 2 | 1.222620 | -0.306753 | 326 | 396.47 | 1.216166 |  |
| GO:0010926\_anatomical\_structure\_formation | CDC42 | 447 | 3 | 1.144668 | -0.305297 | 327 | 396.72 | 1.213211 |  |
| GO:0010926\_anatomical\_structure\_formation | KIF3A | 447 | 3 | 1.144668 | -0.305297 | 327 | 396.72 | 1.213211 |  |
| GO:0010926\_anatomical\_structure\_formation | SMARCA2 | 447 | 3 | 1.144668 | -0.305297 | 327 | 396.72 | 1.213211 |  |
| GO:0048608\_reproductive\_structure\_development | FANCA | 116 | 1 | 1.470307 | -0.302043 | 328 | 398.79 | 1.215823 |  |
| GO:0031327\_negative\_regulation\_of\_cellular\_biosynthetic\_process | COPS2 | 282 | 2 | 1.209614 | -0.301677 | 329 | 399.12 | 1.213131 |  |
| GO:0031327\_negative\_regulation\_of\_cellular\_biosynthetic\_process | LCOR | 282 | 2 | 1.209614 | -0.301677 | 329 | 399.12 | 1.213131 |  |
| GO:0043933\_macromolecular\_complex\_subunit\_organization | SMARCA2 | 117 | 1 | 1.457740 | -0.299426 | 330 | 400.12 | 1.212485 |  |
| GO:0009890\_negative\_regulation\_of\_biosynthetic\_process | COPS2 | 284 | 2 | 1.201095 | -0.298344 | 331 | 400.29 | 1.209335 |  |
| GO:0009890\_negative\_regulation\_of\_biosynthetic\_process | LCOR | 284 | 2 | 1.201095 | -0.298344 | 331 | 400.29 | 1.209335 |  |
| GO:0007399\_nervous\_system\_development | CDC42 | 621 | 4 | 1.098587 | -0.297908 | 332 | 400.59 | 1.206596 |  |
| GO:0007399\_nervous\_system\_development | KIF3A | 621 | 4 | 1.098587 | -0.297908 | 332 | 400.59 | 1.206596 |  |
| GO:0007399\_nervous\_system\_development | L1CAM | 621 | 4 | 1.098587 | -0.297908 | 332 | 400.59 | 1.206596 |  |
| GO:0007399\_nervous\_system\_development | PBX1 | 621 | 4 | 1.098587 | -0.297908 | 332 | 400.59 | 1.206596 |  |
| GO:0051960\_regulation\_of\_nervous\_system\_development | PBX1 | 118 | 1 | 1.445386 | -0.296841 | 333 | 401.25 | 1.204955 |  |
| GO:0043412\_biopolymer\_modification | CHEK1 | 458 | 3 | 1.117176 | -0.290679 | 334 | 403.33 | 1.207575 |  |
| GO:0043412\_biopolymer\_modification | OGT | 458 | 3 | 1.117176 | -0.290679 | 334 | 403.33 | 1.207575 |  |
| GO:0043412\_biopolymer\_modification | MYST4 | 458 | 3 | 1.117176 | -0.290679 | 334 | 403.33 | 1.207575 |  |
| GO:0002694\_regulation\_of\_leukocyte\_activation | CD83 | 121 | 1 | 1.409550 | -0.289262 | 335 | 404.89 | 1.208627 |  |
| GO:0001816\_cytokine\_production | CD83 | 122 | 1 | 1.397996 | -0.286794 | 338 | 407.11 | 1.204467 |  |
| GO:0050865\_regulation\_of\_cell\_activation | CD83 | 122 | 1 | 1.397996 | -0.286794 | 338 | 407.11 | 1.204467 |  |
| GO:0060284\_regulation\_of\_cell\_development | PBX1 | 122 | 1 | 1.397996 | -0.286794 | 338 | 407.11 | 1.204467 |  |
| GO:0034961\_cellular\_biopolymer\_biosynthetic\_process | COPS2 | 804 | 5 | 1.060669 | -0.283170 | 339 | 407.25 | 1.201327 |  |
| GO:0034961\_cellular\_biopolymer\_biosynthetic\_process | SMARCA5 | 804 | 5 | 1.060669 | -0.283170 | 339 | 407.25 | 1.201327 |  |
| GO:0034961\_cellular\_biopolymer\_biosynthetic\_process | PBX1 | 804 | 5 | 1.060669 | -0.283170 | 339 | 407.25 | 1.201327 |  |
| GO:0034961\_cellular\_biopolymer\_biosynthetic\_process | LCOR | 804 | 5 | 1.060669 | -0.283170 | 339 | 407.25 | 1.201327 |  |
| GO:0034961\_cellular\_biopolymer\_biosynthetic\_process | PBX2 | 804 | 5 | 1.060669 | -0.283170 | 339 | 407.25 | 1.201327 |  |
| GO:0030098\_lymphocyte\_differentiation | CD83 | 124 | 1 | 1.375448 | -0.281941 | 340 | 407.85 | 1.199559 |  |
| GO:0002520\_immune\_system\_development | CD83 | 295 | 2 | 1.156309 | -0.280719 | 342 | 408.73 | 1.195117 |  |
| GO:0002520\_immune\_system\_development | PBX1 | 295 | 2 | 1.156309 | -0.280719 | 342 | 408.73 | 1.195117 |  |
| GO:0045595\_regulation\_of\_cell\_differentiation | CD83 | 295 | 2 | 1.156309 | -0.280719 | 342 | 408.73 | 1.195117 |  |
| GO:0045595\_regulation\_of\_cell\_differentiation | PBX1 | 295 | 2 | 1.156309 | -0.280719 | 342 | 408.73 | 1.195117 |  |
| GO:0043284\_biopolymer\_biosynthetic\_process | COPS2 | 807 | 5 | 1.056726 | -0.280186 | 343 | 408.87 | 1.192041 |  |
| GO:0043284\_biopolymer\_biosynthetic\_process | SMARCA5 | 807 | 5 | 1.056726 | -0.280186 | 343 | 408.87 | 1.192041 |  |
| GO:0043284\_biopolymer\_biosynthetic\_process | PBX1 | 807 | 5 | 1.056726 | -0.280186 | 343 | 408.87 | 1.192041 |  |
| GO:0043284\_biopolymer\_biosynthetic\_process | LCOR | 807 | 5 | 1.056726 | -0.280186 | 343 | 408.87 | 1.192041 |  |
| GO:0043284\_biopolymer\_biosynthetic\_process | PBX2 | 807 | 5 | 1.056726 | -0.280186 | 343 | 408.87 | 1.192041 |  |
| GO:0001763\_morphogenesis\_of\_a\_branching\_structure | PBX1 | 125 | 1 | 1.364444 | -0.279556 | 344 | 409.82 | 1.191337 |  |
| GO:0031326\_regulation\_of\_cellular\_biosynthetic\_process | COPS2 | 812 | 5 | 1.050219 | -0.275275 | 345 | 410.35 | 1.189420 |  |
| GO:0031326\_regulation\_of\_cellular\_biosynthetic\_process | SMARCA5 | 812 | 5 | 1.050219 | -0.275275 | 345 | 410.35 | 1.189420 |  |
| GO:0031326\_regulation\_of\_cellular\_biosynthetic\_process | PBX1 | 812 | 5 | 1.050219 | -0.275275 | 345 | 410.35 | 1.189420 |  |
| GO:0031326\_regulation\_of\_cellular\_biosynthetic\_process | LCOR | 812 | 5 | 1.050219 | -0.275275 | 345 | 410.35 | 1.189420 |  |
| GO:0031326\_regulation\_of\_cellular\_biosynthetic\_process | PBX2 | 812 | 5 | 1.050219 | -0.275275 | 345 | 410.35 | 1.189420 |  |
| GO:0048598\_embryonic\_morphogenesis | PBX1 | 299 | 2 | 1.140840 | -0.274594 | 346 | 410.66 | 1.186879 |  |
| GO:0048598\_embryonic\_morphogenesis | PBX2 | 299 | 2 | 1.140840 | -0.274594 | 346 | 410.66 | 1.186879 |  |
| GO:0045597\_positive\_regulation\_of\_cell\_differentiation | CD83 | 128 | 1 | 1.332465 | -0.272559 | 347 | 412.07 | 1.187522 |  |
| GO:0009889\_regulation\_of\_biosynthetic\_process | COPS2 | 815 | 5 | 1.046353 | -0.272364 | 348 | 412.19 | 1.184454 |  |
| GO:0009889\_regulation\_of\_biosynthetic\_process | SMARCA5 | 815 | 5 | 1.046353 | -0.272364 | 348 | 412.19 | 1.184454 |  |
| GO:0009889\_regulation\_of\_biosynthetic\_process | PBX1 | 815 | 5 | 1.046353 | -0.272364 | 348 | 412.19 | 1.184454 |  |
| GO:0009889\_regulation\_of\_biosynthetic\_process | LCOR | 815 | 5 | 1.046353 | -0.272364 | 348 | 412.19 | 1.184454 |  |
| GO:0009889\_regulation\_of\_biosynthetic\_process | PBX2 | 815 | 5 | 1.046353 | -0.272364 | 348 | 412.19 | 1.184454 |  |
| GO:0045165\_cell\_fate\_commitment | CDC42 | 130 | 1 | 1.311966 | -0.268023 | 349 | 414.11 | 1.186562 |  |
| GO:0045893\_positive\_regulation\_of\_transcription\_\_DNA-dependent | PBX1 | 306 | 2 | 1.114742 | -0.264218 | 351 | 414.77 | 1.181681 |  |
| GO:0045893\_positive\_regulation\_of\_transcription\_\_DNA-dependent | PBX2 | 306 | 2 | 1.114742 | -0.264218 | 351 | 414.77 | 1.181681 |  |
| GO:0051254\_positive\_regulation\_of\_RNA\_metabolic\_process | PBX1 | 306 | 2 | 1.114742 | -0.264218 | 351 | 414.77 | 1.181681 |  |
| GO:0051254\_positive\_regulation\_of\_RNA\_metabolic\_process | PBX2 | 306 | 2 | 1.114742 | -0.264218 | 351 | 414.77 | 1.181681 |  |
| GO:0032501\_multicellular\_organismal\_process | KIF3A | 2183 | 13 | 1.015677 | -0.263922 | 352 | 415.09 | 1.179233 |  |
| GO:0032501\_multicellular\_organismal\_process | L1CAM | 2183 | 13 | 1.015677 | -0.263922 | 352 | 415.09 | 1.179233 |  |
| GO:0032501\_multicellular\_organismal\_process | YBX1 | 2183 | 13 | 1.015677 | -0.263922 | 352 | 415.09 | 1.179233 |  |
| GO:0032501\_multicellular\_organismal\_process | CD83 | 2183 | 13 | 1.015677 | -0.263922 | 352 | 415.09 | 1.179233 |  |
| GO:0032501\_multicellular\_organismal\_process | CDC42 | 2183 | 13 | 1.015677 | -0.263922 | 352 | 415.09 | 1.179233 |  |
| GO:0032501\_multicellular\_organismal\_process | KIF1B | 2183 | 13 | 1.015677 | -0.263922 | 352 | 415.09 | 1.179233 |  |
| GO:0032501\_multicellular\_organismal\_process | FREM2 | 2183 | 13 | 1.015677 | -0.263922 | 352 | 415.09 | 1.179233 |  |
| GO:0032501\_multicellular\_organismal\_process | SOSTDC1 | 2183 | 13 | 1.015677 | -0.263922 | 352 | 415.09 | 1.179233 |  |
| GO:0032501\_multicellular\_organismal\_process | SMARCA5 | 2183 | 13 | 1.015677 | -0.263922 | 352 | 415.09 | 1.179233 |  |
| GO:0032501\_multicellular\_organismal\_process | PBX1 | 2183 | 13 | 1.015677 | -0.263922 | 352 | 415.09 | 1.179233 |  |
| GO:0032501\_multicellular\_organismal\_process | CACNA1D | 2183 | 13 | 1.015677 | -0.263922 | 352 | 415.09 | 1.179233 |  |
| GO:0032501\_multicellular\_organismal\_process | PBX2 | 2183 | 13 | 1.015677 | -0.263922 | 352 | 415.09 | 1.179233 |  |
| GO:0032501\_multicellular\_organismal\_process | FANCA | 2183 | 13 | 1.015677 | -0.263922 | 352 | 415.09 | 1.179233 |  |
| GO:0006139\_nucleobase\_\_nucleoside\_\_nucleotide\_and\_nucleic\_acid\_metabolic\_process | COPS2 | 1002 | 6 | 1.021291 | -0.257911 | 353 | 418.34 | 1.185099 |  |
| GO:0006139\_nucleobase\_\_nucleoside\_\_nucleotide\_and\_nucleic\_acid\_metabolic\_process | SMARCA5 | 1002 | 6 | 1.021291 | -0.257911 | 353 | 418.34 | 1.185099 |  |
| GO:0006139\_nucleobase\_\_nucleoside\_\_nucleotide\_and\_nucleic\_acid\_metabolic\_process | PBX1 | 1002 | 6 | 1.021291 | -0.257911 | 353 | 418.34 | 1.185099 |  |
| GO:0006139\_nucleobase\_\_nucleoside\_\_nucleotide\_and\_nucleic\_acid\_metabolic\_process | CHEK1 | 1002 | 6 | 1.021291 | -0.257911 | 353 | 418.34 | 1.185099 |  |
| GO:0006139\_nucleobase\_\_nucleoside\_\_nucleotide\_and\_nucleic\_acid\_metabolic\_process | LCOR | 1002 | 6 | 1.021291 | -0.257911 | 353 | 418.34 | 1.185099 |  |
| GO:0006139\_nucleobase\_\_nucleoside\_\_nucleotide\_and\_nucleic\_acid\_metabolic\_process | PBX2 | 1002 | 6 | 1.021291 | -0.257911 | 353 | 418.34 | 1.185099 |  |
| GO:0016070\_RNA\_metabolic\_process | SMARCA5 | 658 | 4 | 1.036812 | -0.257900 | 354 | 418.46 | 1.182090 |  |
| GO:0016070\_RNA\_metabolic\_process | PBX1 | 658 | 4 | 1.036812 | -0.257900 | 354 | 418.46 | 1.182090 |  |
| GO:0016070\_RNA\_metabolic\_process | LCOR | 658 | 4 | 1.036812 | -0.257900 | 354 | 418.46 | 1.182090 |  |
| GO:0016070\_RNA\_metabolic\_process | PBX2 | 658 | 4 | 1.036812 | -0.257900 | 354 | 418.46 | 1.182090 |  |
| GO:0048856\_anatomical\_structure\_development | EPB4.1L2 | 1688 | 10 | 1.010400 | -0.254869 | 355 | 419.13 | 1.180648 |  |
| GO:0048856\_anatomical\_structure\_development | CD83 | 1688 | 10 | 1.010400 | -0.254869 | 355 | 419.13 | 1.180648 |  |
| GO:0048856\_anatomical\_structure\_development | CDC42 | 1688 | 10 | 1.010400 | -0.254869 | 355 | 419.13 | 1.180648 |  |
| GO:0048856\_anatomical\_structure\_development | KIF3A | 1688 | 10 | 1.010400 | -0.254869 | 355 | 419.13 | 1.180648 |  |
| GO:0048856\_anatomical\_structure\_development | FREM2 | 1688 | 10 | 1.010400 | -0.254869 | 355 | 419.13 | 1.180648 |  |
| GO:0048856\_anatomical\_structure\_development | SOSTDC1 | 1688 | 10 | 1.010400 | -0.254869 | 355 | 419.13 | 1.180648 |  |
| GO:0048856\_anatomical\_structure\_development | L1CAM | 1688 | 10 | 1.010400 | -0.254869 | 355 | 419.13 | 1.180648 |  |
| GO:0048856\_anatomical\_structure\_development | PBX1 | 1688 | 10 | 1.010400 | -0.254869 | 355 | 419.13 | 1.180648 |  |
| GO:0048856\_anatomical\_structure\_development | FANCA | 1688 | 10 | 1.010400 | -0.254869 | 355 | 419.13 | 1.180648 |  |
| GO:0048856\_anatomical\_structure\_development | PBX2 | 1688 | 10 | 1.010400 | -0.254869 | 355 | 419.13 | 1.180648 |  |
| GO:0034613\_cellular\_protein\_localization | CDC42 | 139 | 1 | 1.227018 | -0.248786 | 356 | 420.48 | 1.181124 |  |
| GO:0003006\_reproductive\_developmental\_process | FANCA | 141 | 1 | 1.209614 | -0.244756 | 358 | 421.83 | 1.178296 |  |
| GO:0070727\_cellular\_macromolecule\_localization | CDC42 | 141 | 1 | 1.209614 | -0.244756 | 358 | 421.83 | 1.178296 |  |
| GO:0035239\_tube\_morphogenesis | PBX1 | 143 | 1 | 1.192696 | -0.240809 | 359 | 422.48 | 1.176825 |  |
| GO:0007186\_G-protein\_coupled\_receptor\_protein\_signaling\_pathway | CACNA1D | 144 | 1 | 1.184414 | -0.238866 | 361 | 423.4 | 1.172853 |  |
| GO:0045596\_negative\_regulation\_of\_cell\_differentiation | PBX1 | 144 | 1 | 1.184414 | -0.238866 | 361 | 423.4 | 1.172853 |  |
| GO:0005975\_carbohydrate\_metabolic\_process | OGT | 146 | 1 | 1.168189 | -0.235039 | 362 | 424.72 | 1.173260 |  |
| GO:0022603\_regulation\_of\_anatomical\_structure\_morphogenesis | EPB4.1L2 | 147 | 1 | 1.160242 | -0.233155 | 363 | 425.08 | 1.171019 |  |
| GO:0010646\_regulation\_of\_cell\_communication | SOSTDC1 | 330 | 2 | 1.033670 | -0.231711 | 364 | 426.25 | 1.171016 |  |
| GO:0010646\_regulation\_of\_cell\_communication | L1CAM | 330 | 2 | 1.033670 | -0.231711 | 364 | 426.25 | 1.171016 |  |
| GO:0002684\_positive\_regulation\_of\_immune\_system\_process | CD83 | 148 | 1 | 1.152402 | -0.231290 | 366 | 427.05 | 1.166803 |  |
| GO:0043085\_positive\_regulation\_of\_catalytic\_activity | CDC42 | 148 | 1 | 1.152402 | -0.231290 | 366 | 427.05 | 1.166803 |  |
| GO:0010605\_negative\_regulation\_of\_macromolecule\_metabolic\_process | COPS2 | 331 | 2 | 1.030547 | -0.230452 | 367 | 427.62 | 1.165177 |  |
| GO:0010605\_negative\_regulation\_of\_macromolecule\_metabolic\_process | LCOR | 331 | 2 | 1.030547 | -0.230452 | 367 | 427.62 | 1.165177 |  |
| GO:0031324\_negative\_regulation\_of\_cellular\_metabolic\_process | COPS2 | 332 | 2 | 1.027443 | -0.229200 | 368 | 428.3 | 1.163859 |  |
| GO:0031324\_negative\_regulation\_of\_cellular\_metabolic\_process | LCOR | 332 | 2 | 1.027443 | -0.229200 | 368 | 428.3 | 1.163859 |  |
| GO:0044238\_primary\_metabolic\_process | CDC42 | 1905 | 11 | 0.984835 | -0.222520 | 369 | 429.32 | 1.163469 |  |
| GO:0044238\_primary\_metabolic\_process | COPS2 | 1905 | 11 | 0.984835 | -0.222520 | 369 | 429.32 | 1.163469 |  |
| GO:0044238\_primary\_metabolic\_process | SMARCA5 | 1905 | 11 | 0.984835 | -0.222520 | 369 | 429.32 | 1.163469 |  |
| GO:0044238\_primary\_metabolic\_process | RNF11 | 1905 | 11 | 0.984835 | -0.222520 | 369 | 429.32 | 1.163469 |  |
| GO:0044238\_primary\_metabolic\_process | PBX1 | 1905 | 11 | 0.984835 | -0.222520 | 369 | 429.32 | 1.163469 |  |
| GO:0044238\_primary\_metabolic\_process | CHEK1 | 1905 | 11 | 0.984835 | -0.222520 | 369 | 429.32 | 1.163469 |  |
| GO:0044238\_primary\_metabolic\_process | OGT | 1905 | 11 | 0.984835 | -0.222520 | 369 | 429.32 | 1.163469 |  |
| GO:0044238\_primary\_metabolic\_process | LCOR | 1905 | 11 | 0.984835 | -0.222520 | 369 | 429.32 | 1.163469 |  |
| GO:0044238\_primary\_metabolic\_process | EDEM1 | 1905 | 11 | 0.984835 | -0.222520 | 369 | 429.32 | 1.163469 |  |
| GO:0044238\_primary\_metabolic\_process | PBX2 | 1905 | 11 | 0.984835 | -0.222520 | 369 | 429.32 | 1.163469 |  |
| GO:0044238\_primary\_metabolic\_process | MYST4 | 1905 | 11 | 0.984835 | -0.222520 | 369 | 429.32 | 1.163469 |  |
| GO:0045941\_positive\_regulation\_of\_transcription | PBX1 | 338 | 2 | 1.009204 | -0.221838 | 370 | 430.03 | 1.162243 |  |
| GO:0045941\_positive\_regulation\_of\_transcription | PBX2 | 338 | 2 | 1.009204 | -0.221838 | 370 | 430.03 | 1.162243 |  |
| GO:0007268\_synaptic\_transmission | KIF1B | 154 | 1 | 1.107504 | -0.220484 | 371 | 430.69 | 1.160889 |  |
| GO:0008285\_negative\_regulation\_of\_cell\_proliferation | SMARCA2 | 155 | 1 | 1.100358 | -0.218745 | 372 | 432.13 | 1.161640 |  |
| GO:0051649\_establishment\_of\_localization\_in\_cell | CDC42 | 342 | 2 | 0.997401 | -0.217068 | 373 | 432.53 | 1.159598 |  |
| GO:0051649\_establishment\_of\_localization\_in\_cell | KIF1B | 342 | 2 | 0.997401 | -0.217068 | 373 | 432.53 | 1.159598 |  |
| GO:0007409\_axonogenesis | L1CAM | 158 | 1 | 1.079466 | -0.213629 | 374 | 434.84 | 1.162674 |  |
| GO:0010628\_positive\_regulation\_of\_gene\_expression | PBX1 | 346 | 2 | 0.985870 | -0.212405 | 375 | 435.31 | 1.160827 |  |
| GO:0010628\_positive\_regulation\_of\_gene\_expression | PBX2 | 346 | 2 | 0.985870 | -0.212405 | 375 | 435.31 | 1.160827 |  |
| GO:0009892\_negative\_regulation\_of\_metabolic\_process | COPS2 | 348 | 2 | 0.980204 | -0.210113 | 376 | 436.06 | 1.159734 |  |
| GO:0009892\_negative\_regulation\_of\_metabolic\_process | LCOR | 348 | 2 | 0.980204 | -0.210113 | 376 | 436.06 | 1.159734 |  |
| GO:0002521\_leukocyte\_differentiation | CD83 | 161 | 1 | 1.059351 | -0.208661 | 377 | 436.43 | 1.157639 |  |
| GO:0045935\_positive\_regulation\_of\_nucleobase\_\_nucleoside\_\_nucleotide\_and\_nucleic\_acid\_metabolic\_process | PBX1 | 352 | 2 | 0.969066 | -0.205605 | 378 | 437.31 | 1.156905 |  |
| GO:0045935\_positive\_regulation\_of\_nucleobase\_\_nucleoside\_\_nucleotide\_and\_nucleic\_acid\_metabolic\_process | PBX2 | 352 | 2 | 0.969066 | -0.205605 | 378 | 437.31 | 1.156905 |  |
| GO:0042110\_T\_cell\_activation | CD83 | 163 | 1 | 1.046353 | -0.205427 | 379 | 438.08 | 1.155884 |  |
| GO:0006259\_DNA\_metabolic\_process | CHEK1 | 165 | 1 | 1.033670 | -0.202255 | 380 | 439.9 | 1.157632 |  |
| GO:0010467\_gene\_expression | COPS2 | 905 | 5 | 0.942296 | -0.196692 | 381 | 442.64 | 1.161785 |  |
| GO:0010467\_gene\_expression | SMARCA5 | 905 | 5 | 0.942296 | -0.196692 | 381 | 442.64 | 1.161785 |  |
| GO:0010467\_gene\_expression | PBX1 | 905 | 5 | 0.942296 | -0.196692 | 381 | 442.64 | 1.161785 |  |
| GO:0010467\_gene\_expression | LCOR | 905 | 5 | 0.942296 | -0.196692 | 381 | 442.64 | 1.161785 |  |
| GO:0010467\_gene\_expression | PBX2 | 905 | 5 | 0.942296 | -0.196692 | 381 | 442.64 | 1.161785 |  |
| GO:0051173\_positive\_regulation\_of\_nitrogen\_compound\_metabolic\_process | PBX1 | 361 | 2 | 0.944906 | -0.195824 | 382 | 442.94 | 1.159529 |  |
| GO:0051173\_positive\_regulation\_of\_nitrogen\_compound\_metabolic\_process | PBX2 | 361 | 2 | 0.944906 | -0.195824 | 382 | 442.94 | 1.159529 |  |
| GO:0048812\_neuron\_projection\_morphogenesis | L1CAM | 170 | 1 | 1.003268 | -0.194582 | 383 | 443.9 | 1.159008 |  |
| GO:0007600\_sensory\_perception | CACNA1D | 172 | 1 | 0.991602 | -0.191612 | 384 | 445.96 | 1.161354 |  |
| GO:0044093\_positive\_regulation\_of\_molecular\_function | CDC42 | 173 | 1 | 0.985870 | -0.190148 | 386 | 447.2 | 1.158549 |  |
| GO:0048667\_cell\_morphogenesis\_involved\_in\_neuron\_differentiation | L1CAM | 173 | 1 | 0.985870 | -0.190148 | 386 | 447.2 | 1.158549 |  |
| GO:0000122\_negative\_regulation\_of\_transcription\_from\_RNA\_polymerase\_II\_promoter | LCOR | 175 | 1 | 0.974603 | -0.187259 | 387 | 449.01 | 1.160233 |  |
| GO:0051641\_cellular\_localization | CDC42 | 370 | 2 | 0.921922 | -0.186522 | 388 | 449.66 | 1.158918 |  |
| GO:0051641\_cellular\_localization | KIF1B | 370 | 2 | 0.921922 | -0.186522 | 388 | 449.66 | 1.158918 |  |
| GO:0010557\_positive\_regulation\_of\_macromolecule\_biosynthetic\_process | PBX1 | 371 | 2 | 0.919437 | -0.185517 | 389 | 451.08 | 1.159589 |  |
| GO:0010557\_positive\_regulation\_of\_macromolecule\_biosynthetic\_process | PBX2 | 371 | 2 | 0.919437 | -0.185517 | 389 | 451.08 | 1.159589 |  |
| GO:0048732\_gland\_development | PBX1 | 179 | 1 | 0.952824 | -0.181638 | 390 | 452.87 | 1.161205 |  |
| GO:0060255\_regulation\_of\_macromolecule\_metabolic\_process | COPS2 | 936 | 5 | 0.911087 | -0.175261 | 391 | 456.78 | 1.168235 |  |
| GO:0060255\_regulation\_of\_macromolecule\_metabolic\_process | SMARCA5 | 936 | 5 | 0.911087 | -0.175261 | 391 | 456.78 | 1.168235 |  |
| GO:0060255\_regulation\_of\_macromolecule\_metabolic\_process | PBX1 | 936 | 5 | 0.911087 | -0.175261 | 391 | 456.78 | 1.168235 |  |
| GO:0060255\_regulation\_of\_macromolecule\_metabolic\_process | LCOR | 936 | 5 | 0.911087 | -0.175261 | 391 | 456.78 | 1.168235 |  |
| GO:0060255\_regulation\_of\_macromolecule\_metabolic\_process | PBX2 | 936 | 5 | 0.911087 | -0.175261 | 391 | 456.78 | 1.168235 |  |
| GO:0016192\_vesicle-mediated\_transport | CDC42 | 184 | 1 | 0.926932 | -0.174892 | 392 | 457.56 | 1.167245 |  |
| GO:0007010\_cytoskeleton\_organization | EPB4.1L2 | 185 | 1 | 0.921922 | -0.173579 | 393 | 458.2 | 1.165903 |  |
| GO:0043687\_post-translational\_protein\_modification | CHEK1 | 384 | 2 | 0.888310 | -0.172941 | 394 | 458.46 | 1.163604 |  |
| GO:0043687\_post-translational\_protein\_modification | MYST4 | 384 | 2 | 0.888310 | -0.172941 | 394 | 458.46 | 1.163604 |  |
| GO:0031328\_positive\_regulation\_of\_cellular\_biosynthetic\_process | PBX1 | 387 | 2 | 0.881424 | -0.170165 | 395 | 460.14 | 1.164911 |  |
| GO:0031328\_positive\_regulation\_of\_cellular\_biosynthetic\_process | PBX2 | 387 | 2 | 0.881424 | -0.170165 | 395 | 460.14 | 1.164911 |  |
| GO:0009891\_positive\_regulation\_of\_biosynthetic\_process | PBX1 | 388 | 2 | 0.879152 | -0.169249 | 396 | 460.79 | 1.163611 |  |
| GO:0009891\_positive\_regulation\_of\_biosynthetic\_process | PBX2 | 388 | 2 | 0.879152 | -0.169249 | 396 | 460.79 | 1.163611 |  |
| GO:0019226\_transmission\_of\_nerve\_impulse | KIF1B | 189 | 1 | 0.902410 | -0.168441 | 397 | 461.14 | 1.161562 |  |
| GO:0050877\_neurological\_system\_process | KIF1B | 390 | 2 | 0.874644 | -0.167433 | 398 | 461.35 | 1.159171 |  |
| GO:0050877\_neurological\_system\_process | CACNA1D | 390 | 2 | 0.874644 | -0.167433 | 398 | 461.35 | 1.159171 |  |
| GO:0007507\_heart\_development | KIF3A | 195 | 1 | 0.874644 | -0.161066 | 399 | 464.39 | 1.163885 |  |
| GO:0033554\_cellular\_response\_to\_stress | CHEK1 | 196 | 1 | 0.870181 | -0.159874 | 400 | 465.06 | 1.162650 |  |
| GO:0031175\_neuron\_projection\_development | L1CAM | 197 | 1 | 0.865764 | -0.158692 | 401 | 465.99 | 1.162070 |  |
| GO:0000904\_cell\_morphogenesis\_involved\_in\_differentiation | L1CAM | 199 | 1 | 0.857063 | -0.156358 | 402 | 468.04 | 1.164279 |  |
| GO:0006807\_nitrogen\_compound\_metabolic\_process | COPS2 | 1147 | 6 | 0.892183 | -0.156207 | 403 | 468.24 | 1.161886 |  |
| GO:0006807\_nitrogen\_compound\_metabolic\_process | SMARCA5 | 1147 | 6 | 0.892183 | -0.156207 | 403 | 468.24 | 1.161886 |  |
| GO:0006807\_nitrogen\_compound\_metabolic\_process | PBX1 | 1147 | 6 | 0.892183 | -0.156207 | 403 | 468.24 | 1.161886 |  |
| GO:0006807\_nitrogen\_compound\_metabolic\_process | CHEK1 | 1147 | 6 | 0.892183 | -0.156207 | 403 | 468.24 | 1.161886 |  |
| GO:0006807\_nitrogen\_compound\_metabolic\_process | LCOR | 1147 | 6 | 0.892183 | -0.156207 | 403 | 468.24 | 1.161886 |  |
| GO:0006807\_nitrogen\_compound\_metabolic\_process | PBX2 | 1147 | 6 | 0.892183 | -0.156207 | 403 | 468.24 | 1.161886 |  |
| GO:0044249\_cellular\_biosynthetic\_process | COPS2 | 1150 | 6 | 0.889855 | -0.154528 | 404 | 468.45 | 1.159530 |  |
| GO:0044249\_cellular\_biosynthetic\_process | SMARCA5 | 1150 | 6 | 0.889855 | -0.154528 | 404 | 468.45 | 1.159530 |  |
| GO:0044249\_cellular\_biosynthetic\_process | PBX1 | 1150 | 6 | 0.889855 | -0.154528 | 404 | 468.45 | 1.159530 |  |
| GO:0044249\_cellular\_biosynthetic\_process | OGT | 1150 | 6 | 0.889855 | -0.154528 | 404 | 468.45 | 1.159530 |  |
| GO:0044249\_cellular\_biosynthetic\_process | LCOR | 1150 | 6 | 0.889855 | -0.154528 | 404 | 468.45 | 1.159530 |  |
| GO:0044249\_cellular\_biosynthetic\_process | PBX2 | 1150 | 6 | 0.889855 | -0.154528 | 404 | 468.45 | 1.159530 |  |
| GO:0007243\_protein\_kinase\_cascade | DUSP9 | 205 | 1 | 0.831978 | -0.149592 | 405 | 470.44 | 1.161580 |  |
| GO:0008284\_positive\_regulation\_of\_cell\_proliferation | PBX1 | 208 | 1 | 0.819979 | -0.146336 | 406 | 471.44 | 1.161182 |  |
| GO:0035295\_tube\_development | PBX1 | 212 | 1 | 0.804507 | -0.142121 | 407 | 472.29 | 1.160418 |  |
| GO:0009058\_biosynthetic\_process | COPS2 | 1175 | 6 | 0.870922 | -0.141110 | 408 | 472.56 | 1.158235 |  |
| GO:0009058\_biosynthetic\_process | SMARCA5 | 1175 | 6 | 0.870922 | -0.141110 | 408 | 472.56 | 1.158235 |  |
| GO:0009058\_biosynthetic\_process | PBX1 | 1175 | 6 | 0.870922 | -0.141110 | 408 | 472.56 | 1.158235 |  |
| GO:0009058\_biosynthetic\_process | OGT | 1175 | 6 | 0.870922 | -0.141110 | 408 | 472.56 | 1.158235 |  |
| GO:0009058\_biosynthetic\_process | LCOR | 1175 | 6 | 0.870922 | -0.141110 | 408 | 472.56 | 1.158235 |  |
| GO:0009058\_biosynthetic\_process | PBX2 | 1175 | 6 | 0.870922 | -0.141110 | 408 | 472.56 | 1.158235 |  |
| GO:0010033\_response\_to\_organic\_substance | CD83 | 216 | 1 | 0.789609 | -0.138043 | 409 | 473.79 | 1.158411 |  |
| GO:0045892\_negative\_regulation\_of\_transcription\_\_DNA-dependent | LCOR | 218 | 1 | 0.782365 | -0.136054 | 410 | 475.57 | 1.159927 |  |
| GO:0051253\_negative\_regulation\_of\_RNA\_metabolic\_process | LCOR | 220 | 1 | 0.775253 | -0.134098 | 411 | 476.36 | 1.159027 |  |
| GO:0010604\_positive\_regulation\_of\_macromolecule\_metabolic\_process | PBX1 | 433 | 2 | 0.787785 | -0.132795 | 412 | 477.28 | 1.158447 |  |
| GO:0010604\_positive\_regulation\_of\_macromolecule\_metabolic\_process | PBX2 | 433 | 2 | 0.787785 | -0.132795 | 412 | 477.28 | 1.158447 |  |
| GO:0002682\_regulation\_of\_immune\_system\_process | CD83 | 228 | 1 | 0.748051 | -0.126583 | 414 | 479.24 | 1.157585 |  |
| GO:0046649\_lymphocyte\_activation | CD83 | 228 | 1 | 0.748051 | -0.126583 | 414 | 479.24 | 1.157585 |  |
| GO:0031325\_positive\_regulation\_of\_cellular\_metabolic\_process | PBX1 | 442 | 2 | 0.771745 | -0.126504 | 415 | 479.51 | 1.155446 |  |
| GO:0031325\_positive\_regulation\_of\_cellular\_metabolic\_process | PBX2 | 442 | 2 | 0.771745 | -0.126504 | 415 | 479.51 | 1.155446 |  |
| GO:0009887\_organ\_morphogenesis | FREM2 | 642 | 3 | 0.796989 | -0.126286 | 416 | 479.87 | 1.153534 |  |
| GO:0009887\_organ\_morphogenesis | SOSTDC1 | 642 | 3 | 0.796989 | -0.126286 | 416 | 479.87 | 1.153534 |  |
| GO:0009887\_organ\_morphogenesis | PBX1 | 642 | 3 | 0.796989 | -0.126286 | 416 | 479.87 | 1.153534 |  |
| GO:0007167\_enzyme\_linked\_receptor\_protein\_signaling\_pathway | SOSTDC1 | 229 | 1 | 0.744784 | -0.125677 | 417 | 480.14 | 1.151415 |  |
| GO:0050890\_cognition | CACNA1D | 233 | 1 | 0.731998 | -0.122127 | 418 | 482.05 | 1.153230 |  |
| GO:0001501\_skeletal\_system\_development | PBX1 | 236 | 1 | 0.722693 | -0.119537 | 419 | 483.36 | 1.153604 |  |
| GO:0048468\_cell\_development | CDC42 | 654 | 3 | 0.782365 | -0.119432 | 420 | 483.62 | 1.151476 |  |
| GO:0048468\_cell\_development | PBX1 | 654 | 3 | 0.782365 | -0.119432 | 420 | 483.62 | 1.151476 |  |
| GO:0048468\_cell\_development | L1CAM | 654 | 3 | 0.782365 | -0.119432 | 420 | 483.62 | 1.151476 |  |
| GO:0006468\_protein\_amino\_acid\_phosphorylation | CHEK1 | 237 | 1 | 0.719644 | -0.118688 | 421 | 484.45 | 1.150713 |  |
| GO:0009893\_positive\_regulation\_of\_metabolic\_process | PBX1 | 458 | 2 | 0.744784 | -0.116038 | 422 | 485.49 | 1.150450 |  |
| GO:0009893\_positive\_regulation\_of\_metabolic\_process | PBX2 | 458 | 2 | 0.744784 | -0.116038 | 422 | 485.49 | 1.150450 |  |
| GO:0045321\_leukocyte\_activation | CD83 | 248 | 1 | 0.687724 | -0.109771 | 423 | 486.66 | 1.150496 |  |
| GO:0030154\_cell\_differentiation | CDC42 | 1060 | 5 | 0.804507 | -0.108387 | 424 | 486.99 | 1.148561 |  |
| GO:0030154\_cell\_differentiation | CD83 | 1060 | 5 | 0.804507 | -0.108387 | 424 | 486.99 | 1.148561 |  |
| GO:0030154\_cell\_differentiation | NOC3L | 1060 | 5 | 0.804507 | -0.108387 | 424 | 486.99 | 1.148561 |  |
| GO:0030154\_cell\_differentiation | L1CAM | 1060 | 5 | 0.804507 | -0.108387 | 424 | 486.99 | 1.148561 |  |
| GO:0030154\_cell\_differentiation | PBX1 | 1060 | 5 | 0.804507 | -0.108387 | 424 | 486.99 | 1.148561 |  |
| GO:0048731\_system\_development | CDC42 | 1609 | 8 | 0.848008 | -0.107637 | 425 | 487.73 | 1.147600 |  |
| GO:0048731\_system\_development | CD83 | 1609 | 8 | 0.848008 | -0.107637 | 425 | 487.73 | 1.147600 |  |
| GO:0048731\_system\_development | KIF3A | 1609 | 8 | 0.848008 | -0.107637 | 425 | 487.73 | 1.147600 |  |
| GO:0048731\_system\_development | FREM2 | 1609 | 8 | 0.848008 | -0.107637 | 425 | 487.73 | 1.147600 |  |
| GO:0048731\_system\_development | SOSTDC1 | 1609 | 8 | 0.848008 | -0.107637 | 425 | 487.73 | 1.147600 |  |
| GO:0048731\_system\_development | L1CAM | 1609 | 8 | 0.848008 | -0.107637 | 425 | 487.73 | 1.147600 |  |
| GO:0048731\_system\_development | PBX1 | 1609 | 8 | 0.848008 | -0.107637 | 425 | 487.73 | 1.147600 |  |
| GO:0048731\_system\_development | FANCA | 1609 | 8 | 0.848008 | -0.107637 | 425 | 487.73 | 1.147600 |  |
| GO:0008104\_protein\_localization | CDC42 | 251 | 1 | 0.679504 | -0.107469 | 426 | 488.1 | 1.145775 |  |
| GO:0007267\_cell-cell\_signaling | KIF1B | 252 | 1 | 0.676808 | -0.106713 | 427 | 488.44 | 1.143888 |  |
| GO:0001775\_cell\_activation | CD83 | 262 | 1 | 0.650975 | -0.099470 | 429 | 491.72 | 1.146200 |  |
| GO:0048666\_neuron\_development | L1CAM | 262 | 1 | 0.650975 | -0.099470 | 429 | 491.72 | 1.146200 |  |
| GO:0048522\_positive\_regulation\_of\_cellular\_process | CD83 | 895 | 4 | 0.762259 | -0.097418 | 430 | 492.68 | 1.145767 |  |
| GO:0048522\_positive\_regulation\_of\_cellular\_process | L1CAM | 895 | 4 | 0.762259 | -0.097418 | 430 | 492.68 | 1.145767 |  |
| GO:0048522\_positive\_regulation\_of\_cellular\_process | PBX1 | 895 | 4 | 0.762259 | -0.097418 | 430 | 492.68 | 1.145767 |  |
| GO:0048522\_positive\_regulation\_of\_cellular\_process | PBX2 | 895 | 4 | 0.762259 | -0.097418 | 430 | 492.68 | 1.145767 |  |
| GO:0050793\_regulation\_of\_developmental\_process | EPB4.1L2 | 703 | 3 | 0.727833 | -0.094857 | 431 | 493.4 | 1.144780 |  |
| GO:0050793\_regulation\_of\_developmental\_process | CD83 | 703 | 3 | 0.727833 | -0.094857 | 431 | 493.4 | 1.144780 |  |
| GO:0050793\_regulation\_of\_developmental\_process | PBX1 | 703 | 3 | 0.727833 | -0.094857 | 431 | 493.4 | 1.144780 |  |
| GO:0051716\_cellular\_response\_to\_stimulus | CHEK1 | 273 | 1 | 0.624746 | -0.092113 | 432 | 494.99 | 1.145810 |  |
| GO:0033036\_macromolecule\_localization | CDC42 | 274 | 1 | 0.622466 | -0.091474 | 433 | 495.6 | 1.144573 |  |
| GO:0002376\_immune\_system\_process | CD83 | 505 | 2 | 0.675468 | -0.089956 | 434 | 496.21 | 1.143341 |  |
| GO:0002376\_immune\_system\_process | PBX1 | 505 | 2 | 0.675468 | -0.089956 | 434 | 496.21 | 1.143341 |  |
| GO:0006810\_transport | CDC42 | 718 | 3 | 0.712628 | -0.088322 | 435 | 498.12 | 1.145103 |  |
| GO:0006810\_transport | KIF1B | 718 | 3 | 0.712628 | -0.088322 | 435 | 498.12 | 1.145103 |  |
| GO:0006810\_transport | CROT | 718 | 3 | 0.712628 | -0.088322 | 435 | 498.12 | 1.145103 |  |
| GO:0006629\_lipid\_metabolic\_process | CDC42 | 285 | 1 | 0.598441 | -0.084750 | 436 | 499.79 | 1.146307 |  |
| GO:0003008\_system\_process | KIF1B | 516 | 2 | 0.661068 | -0.084729 | 437 | 499.98 | 1.144119 |  |
| GO:0003008\_system\_process | CACNA1D | 516 | 2 | 0.661068 | -0.084729 | 437 | 499.98 | 1.144119 |  |
| GO:0051234\_establishment\_of\_localization | CDC42 | 729 | 3 | 0.701875 | -0.083794 | 438 | 500.09 | 1.141758 |  |
| GO:0051234\_establishment\_of\_localization | KIF1B | 729 | 3 | 0.701875 | -0.083794 | 438 | 500.09 | 1.141758 |  |
| GO:0051234\_establishment\_of\_localization | CROT | 729 | 3 | 0.701875 | -0.083794 | 438 | 500.09 | 1.141758 |  |
| GO:0009888\_tissue\_development | FREM2 | 525 | 2 | 0.649735 | -0.080672 | 439 | 500.7 | 1.140547 |  |
| GO:0009888\_tissue\_development | PBX1 | 525 | 2 | 0.649735 | -0.080672 | 439 | 500.7 | 1.140547 |  |
| GO:0051094\_positive\_regulation\_of\_developmental\_process | CD83 | 308 | 1 | 0.553752 | -0.072332 | 440 | 504.12 | 1.145727 |  |
| GO:0048513\_organ\_development | CD83 | 1365 | 6 | 0.749695 | -0.067475 | 441 | 505.13 | 1.145420 |  |
| GO:0048513\_organ\_development | KIF3A | 1365 | 6 | 0.749695 | -0.067475 | 441 | 505.13 | 1.145420 |  |
| GO:0048513\_organ\_development | FREM2 | 1365 | 6 | 0.749695 | -0.067475 | 441 | 505.13 | 1.145420 |  |
| GO:0048513\_organ\_development | SOSTDC1 | 1365 | 6 | 0.749695 | -0.067475 | 441 | 505.13 | 1.145420 |  |
| GO:0048513\_organ\_development | PBX1 | 1365 | 6 | 0.749695 | -0.067475 | 441 | 505.13 | 1.145420 |  |
| GO:0048513\_organ\_development | FANCA | 1365 | 6 | 0.749695 | -0.067475 | 441 | 505.13 | 1.145420 |  |
| GO:0048518\_positive\_regulation\_of\_biological\_process | CD83 | 995 | 4 | 0.685650 | -0.062457 | 442 | 506.04 | 1.144887 |  |
| GO:0048518\_positive\_regulation\_of\_biological\_process | L1CAM | 995 | 4 | 0.685650 | -0.062457 | 442 | 506.04 | 1.144887 |  |
| GO:0048518\_positive\_regulation\_of\_biological\_process | PBX1 | 995 | 4 | 0.685650 | -0.062457 | 442 | 506.04 | 1.144887 |  |
| GO:0048518\_positive\_regulation\_of\_biological\_process | PBX2 | 995 | 4 | 0.685650 | -0.062457 | 442 | 506.04 | 1.144887 |  |
| GO:0006928\_cell\_motion | L1CAM | 330 | 1 | 0.516835 | -0.062236 | 444 | 506.89 | 1.141644 |  |
| GO:0051674\_localization\_of\_cell | L1CAM | 330 | 1 | 0.516835 | -0.062236 | 444 | 506.89 | 1.141644 |  |
| GO:0051093\_negative\_regulation\_of\_developmental\_process | PBX1 | 331 | 1 | 0.515274 | -0.061813 | 445 | 507.46 | 1.140360 |  |
| GO:0051239\_regulation\_of\_multicellular\_organismal\_process | CD83 | 587 | 2 | 0.581109 | -0.057378 | 446 | 510.02 | 1.143543 |  |
| GO:0051239\_regulation\_of\_multicellular\_organismal\_process | PBX1 | 587 | 2 | 0.581109 | -0.057378 | 446 | 510.02 | 1.143543 |  |
| GO:0051179\_localization | CDC42 | 1058 | 4 | 0.644823 | -0.046622 | 447 | 515.32 | 1.152841 |  |
| GO:0051179\_localization | KIF1B | 1058 | 4 | 0.644823 | -0.046622 | 447 | 515.32 | 1.152841 |  |
| GO:0051179\_localization | L1CAM | 1058 | 4 | 0.644823 | -0.046622 | 447 | 515.32 | 1.152841 |  |
| GO:0051179\_localization | CROT | 1058 | 4 | 0.644823 | -0.046622 | 447 | 515.32 | 1.152841 |  |
| GO:0022414\_reproductive\_process | FANCA | 376 | 1 | 0.453605 | -0.045565 | 448 | 515.8 | 1.151339 |  |
| GO:0000003\_reproduction | FANCA | 379 | 1 | 0.450015 | -0.044651 | 449 | 516.03 | 1.149287 |  |
| GO:0042221\_response\_to\_chemical\_stimulus | CD83 | 409 | 1 | 0.417006 | -0.036476 | 450 | 518.83 | 1.152956 |  |
| GO:0006950\_response\_to\_stress | CHEK1 | 549 | 1 | 0.310666 | -0.014184 | 451 | 526.22 | 1.166785 |  |
| GO:0065008\_regulation\_of\_biological\_quality | EPB4.1L2 | 693 | 1 | 0.246112 | -0.005273 | 452 | 528.88 | 1.170088 |  |
| GO:0050896\_response\_to\_stimulus | CD83 | 1107 | 2 | 0.308140 | -0.002438 | 453 | 529.64 | 1.169183 |  |
| GO:0050896\_response\_to\_stimulus | CHEK1 | 1107 | 2 | 0.308140 | -0.002438 | 453 | 529.64 | 1.169183 |  |
| GO:0008150\_biological\_process | COPS2 | 4605 | 27 | 1.000000 | 0.000000 | 1569 | 1590.71 | 1.013837 |  |
| GO:0008150\_biological\_process | NOC3L | 4605 | 27 | 1.000000 | 0.000000 | 1569 | 1590.71 | 1.013837 |  |
| GO:0008150\_biological\_process | CHEK1 | 4605 | 27 | 1.000000 | 0.000000 | 1569 | 1590.71 | 1.013837 |  |
| GO:0008150\_biological\_process | L1CAM | 4605 | 27 | 1.000000 | 0.000000 | 1569 | 1590.71 | 1.013837 |  |
| GO:0008150\_biological\_process | EDEM1 | 4605 | 27 | 1.000000 | 0.000000 | 1569 | 1590.71 | 1.013837 |  |
| GO:0008150\_biological\_process | YBX1 | 4605 | 27 | 1.000000 | 0.000000 | 1569 | 1590.71 | 1.013837 |  |
| GO:0008150\_biological\_process | NGLY1 | 4605 | 27 | 1.000000 | 0.000000 | 1569 | 1590.71 | 1.013837 |  |
| GO:0008150\_biological\_process | CDC42 | 4605 | 27 | 1.000000 | 0.000000 | 1569 | 1590.71 | 1.013837 |  |
| GO:0008150\_biological\_process | SOSTDC1 | 4605 | 27 | 1.000000 | 0.000000 | 1569 | 1590.71 | 1.013837 |  |
| GO:0008150\_biological\_process | GOPC | 4605 | 27 | 1.000000 | 0.000000 | 1569 | 1590.71 | 1.013837 |  |
| GO:0008150\_biological\_process | RNF11 | 4605 | 27 | 1.000000 | 0.000000 | 1569 | 1590.71 | 1.013837 |  |
| GO:0008150\_biological\_process | OGT | 4605 | 27 | 1.000000 | 0.000000 | 1569 | 1590.71 | 1.013837 |  |
| GO:0008150\_biological\_process | FANCA | 4605 | 27 | 1.000000 | 0.000000 | 1569 | 1590.71 | 1.013837 |  |
| GO:0008150\_biological\_process | MYST4 | 4605 | 27 | 1.000000 | 0.000000 | 1569 | 1590.71 | 1.013837 |  |
| GO:0008150\_biological\_process | KIF3A | 4605 | 27 | 1.000000 | 0.000000 | 1569 | 1590.71 | 1.013837 |  |
| GO:0008150\_biological\_process | EPB4.1L2 | 4605 | 27 | 1.000000 | 0.000000 | 1569 | 1590.71 | 1.013837 |  |
| GO:0008150\_biological\_process | CD83 | 4605 | 27 | 1.000000 | 0.000000 | 1569 | 1590.71 | 1.013837 |  |
| GO:0008150\_biological\_process | KIF1B | 4605 | 27 | 1.000000 | 0.000000 | 1569 | 1590.71 | 1.013837 |  |
| GO:0008150\_biological\_process | FREM2 | 4605 | 27 | 1.000000 | 0.000000 | 1569 | 1590.71 | 1.013837 |  |
| GO:0008150\_biological\_process | SMARCA5 | 4605 | 27 | 1.000000 | 0.000000 | 1569 | 1590.71 | 1.013837 |  |
| GO:0008150\_biological\_process | PBX1 | 4605 | 27 | 1.000000 | 0.000000 | 1569 | 1590.71 | 1.013837 |  |
| GO:0008150\_biological\_process | DUSP9 | 4605 | 27 | 1.000000 | 0.000000 | 1569 | 1590.71 | 1.013837 |  |
| GO:0008150\_biological\_process | LCOR | 4605 | 27 | 1.000000 | 0.000000 | 1569 | 1590.71 | 1.013837 |  |
| GO:0008150\_biological\_process | PBX2 | 4605 | 27 | 1.000000 | 0.000000 | 1569 | 1590.71 | 1.013837 |  |
| GO:0008150\_biological\_process | SMARCA2 | 4605 | 27 | 1.000000 | 0.000000 | 1569 | 1590.71 | 1.013837 |  |
| GO:0008150\_biological\_process | CACNA1D | 4605 | 27 | 1.000000 | 0.000000 | 1569 | 1590.71 | 1.013837 |  |
| GO:0008150\_biological\_process | CROT | 4605 | 27 | 1.000000 | 0.000000 | 1569 | 1590.71 | 1.013837 |  |
