# Supplementary material for: RedundancyMiner: De-replication of redundant GO categories in microarray and proteomics analysis
Source: BMC Bioinformatics. 2011 Feb 10;12:52. doi: 10.1186/1471-2105-12-52 (PMC3223614; doi:10.1186/1471-2105-12-52)
Supplement: Additional file 8 — Retinal development HTGM download. compressed package of the results of running HTGM on the retinal development genes list. [file 1471-2105-12-52-S8.ZIP › SCENARIO_2_MODIFIED/total.txt.total.txt.dir/Exp1_BestClusterMap_LEIGS_KM_24.csv.join.5.txt.dir/Exp1_BestClusterMap_LEIGS_KM_24.csv.join.5.txt.change.gce.html]

Gene Category Report for Exp1\_BestClusterMap\_LEIGS\_KM\_24.csv.join.5.txt

# Gene Category Report for Exp1\_BestClusterMap\_LEIGS\_KM\_24.csv.join.5.txt

| HYPERLINKED GO CATEGORY | HYPERLINKED GENE NAME | TOTAL GENES | CHANGED GENES | ENRICHMENT | LOG10(p) | CUMULATIVE NUMBER OF CATEGORIES | CUMULATIVE RANDOMS MEAN | FALSE DISCOVERY RATE |  |
| --- | --- | --- | --- | --- | --- | --- | --- | --- | --- |
| GO:0008156\_negative\_regulation\_of\_DNA\_replication | GMNN | 6 | 2 | 76.750000 | -3.575024 | 1 | 0.15 | 0.150000 |  |
| GO:0008156\_negative\_regulation\_of\_DNA\_replication | CDT1 | 6 | 2 | 76.750000 | -3.575024 | 1 | 0.15 | 0.150000 |  |
| GO:0006275\_regulation\_of\_DNA\_replication | GMNN | 7 | 2 | 65.785714 | -3.430029 | 2 | 0.21 | 0.105000 |  |
| GO:0006275\_regulation\_of\_DNA\_replication | CDT1 | 7 | 2 | 65.785714 | -3.430029 | 2 | 0.21 | 0.105000 |  |
| GO:0051053\_negative\_regulation\_of\_DNA\_metabolic\_process | GMNN | 14 | 2 | 32.892857 | -2.801132 | 3 | 0.92 | 0.306667 |  |
| GO:0051053\_negative\_regulation\_of\_DNA\_metabolic\_process | CDT1 | 14 | 2 | 32.892857 | -2.801132 | 3 | 0.92 | 0.306667 |  |
| GO:0051168\_nuclear\_export | NPM1 | 18 | 2 | 25.583333 | -2.580006 | 4 | 1.52 | 0.380000 |  |
| GO:0051168\_nuclear\_export | HSPA9 | 18 | 2 | 25.583333 | -2.580006 | 4 | 1.52 | 0.380000 |  |
| GO:0000054\_ribosome\_export\_from\_nucleus | NPM1 | 1 | 1 |  |  |  |  |  |  |
| GO:0000055\_ribosomal\_large\_subunit\_export\_from\_nucleus | NPM1 | 1 | 1 |  |  |  |  |  |  |
| GO:0000056\_ribosomal\_small\_subunit\_export\_from\_nucleus | NPM1 | 1 | 1 |  |  |  |  |  |  |
| GO:0006407\_rRNA\_export\_from\_nucleus | NPM1 | 1 | 1 |  |  |  |  |  |  |
| GO:0010825\_positive\_regulation\_of\_centrosome\_duplication | NPM1 | 1 | 1 |  |  |  |  |  |  |
| GO:0016560\_protein\_import\_into\_peroxisome\_matrix\_\_docking | PEX13 | 1 | 1 |  |  |  |  |  |  |
| GO:0033750\_ribosome\_localization | NPM1 | 1 | 1 |  |  |  |  |  |  |
| GO:0033753\_establishment\_of\_ribosome\_localization | NPM1 | 1 | 1 |  |  |  |  |  |  |
| GO:0051029\_rRNA\_transport | NPM1 | 1 | 1 |  |  |  |  |  |  |
| GO:0060151\_peroxisome\_localization | PEX13 | 1 | 1 |  |  |  |  |  |  |
| GO:0060152\_microtubule-based\_peroxisome\_localization | PEX13 | 1 | 1 |  |  |  |  |  |  |
| GO:0045934\_negative\_regulation\_of\_nucleobase\_\_nucleoside\_\_nucleotide\_and\_nucleic\_acid\_metabolic\_process | SIN3B | 270 | 5 | 4.263889 | -2.301564 | 5 | 2.72 | 0.544000 |  |
| GO:0045934\_negative\_regulation\_of\_nucleobase\_\_nucleoside\_\_nucleotide\_and\_nucleic\_acid\_metabolic\_process | GMNN | 270 | 5 | 4.263889 | -2.301564 | 5 | 2.72 | 0.544000 |  |
| GO:0045934\_negative\_regulation\_of\_nucleobase\_\_nucleoside\_\_nucleotide\_and\_nucleic\_acid\_metabolic\_process | NPM1 | 270 | 5 | 4.263889 | -2.301564 | 5 | 2.72 | 0.544000 |  |
| GO:0045934\_negative\_regulation\_of\_nucleobase\_\_nucleoside\_\_nucleotide\_and\_nucleic\_acid\_metabolic\_process | MXD3 | 270 | 5 | 4.263889 | -2.301564 | 5 | 2.72 | 0.544000 |  |
| GO:0045934\_negative\_regulation\_of\_nucleobase\_\_nucleoside\_\_nucleotide\_and\_nucleic\_acid\_metabolic\_process | CDT1 | 270 | 5 | 4.263889 | -2.301564 | 5 | 2.72 | 0.544000 |  |
| GO:0051172\_negative\_regulation\_of\_nitrogen\_compound\_metabolic\_process | SIN3B | 271 | 5 | 4.248155 | -2.294690 | 6 | 2.78 | 0.463333 |  |
| GO:0051172\_negative\_regulation\_of\_nitrogen\_compound\_metabolic\_process | GMNN | 271 | 5 | 4.248155 | -2.294690 | 6 | 2.78 | 0.463333 |  |
| GO:0051172\_negative\_regulation\_of\_nitrogen\_compound\_metabolic\_process | NPM1 | 271 | 5 | 4.248155 | -2.294690 | 6 | 2.78 | 0.463333 |  |
| GO:0051172\_negative\_regulation\_of\_nitrogen\_compound\_metabolic\_process | MXD3 | 271 | 5 | 4.248155 | -2.294690 | 6 | 2.78 | 0.463333 |  |
| GO:0051172\_negative\_regulation\_of\_nitrogen\_compound\_metabolic\_process | CDT1 | 271 | 5 | 4.248155 | -2.294690 | 6 | 2.78 | 0.463333 |  |
| GO:0010564\_regulation\_of\_cell\_cycle\_process | NPM1 | 29 | 2 | 15.879310 | -2.168598 | 7 | 3.57 | 0.510000 |  |
| GO:0010564\_regulation\_of\_cell\_cycle\_process | CDT1 | 29 | 2 | 15.879310 | -2.168598 | 7 | 3.57 | 0.510000 |  |
| GO:0051640\_organelle\_localization | NPM1 | 31 | 2 | 14.854839 | -2.111928 | 8 | 4.11 | 0.513750 |  |
| GO:0051640\_organelle\_localization | PEX13 | 31 | 2 | 14.854839 | -2.111928 | 8 | 4.11 | 0.513750 |  |
| GO:0000076\_DNA\_replication\_checkpoint | CDT1 | 2 | 1 |  |  |  |  |  |  |
| GO:0001561\_fatty\_acid\_alpha-oxidation | PEX13 | 2 | 1 |  |  |  |  |  |  |
| GO:0010824\_regulation\_of\_centrosome\_duplication | NPM1 | 2 | 1 |  |  |  |  |  |  |
| GO:0016558\_protein\_import\_into\_peroxisome\_matrix | PEX13 | 2 | 1 |  |  |  |  |  |  |
| GO:0030174\_regulation\_of\_DNA\_replication\_initiation | CDT1 | 2 | 1 |  |  |  |  |  |  |
| GO:0032297\_negative\_regulation\_of\_DNA\_replication\_initiation | CDT1 | 2 | 1 |  |  |  |  |  |  |
| GO:0033119\_negative\_regulation\_of\_RNA\_splicing | NPM1 | 2 | 1 |  |  |  |  |  |  |
| GO:0042274\_ribosomal\_small\_subunit\_biogenesis | NPM1 | 2 | 1 |  |  |  |  |  |  |
| GO:0043516\_regulation\_of\_DNA\_damage\_response\_\_signal\_transduction\_by\_p53\_class\_mediator | NPM1 | 2 | 1 |  |  |  |  |  |  |
| GO:0048025\_negative\_regulation\_of\_nuclear\_mRNA\_splicing\_\_via\_spliceosome | NPM1 | 2 | 1 |  |  |  |  |  |  |
| GO:0050686\_negative\_regulation\_of\_mRNA\_processing | NPM1 | 2 | 1 |  |  |  |  |  |  |
| GO:0051052\_regulation\_of\_DNA\_metabolic\_process | GMNN | 34 | 2 | 13.544118 | -2.033802 | 9 | 4.86 | 0.540000 |  |
| GO:0051052\_regulation\_of\_DNA\_metabolic\_process | CDT1 | 34 | 2 | 13.544118 | -2.033802 | 9 | 4.86 | 0.540000 |  |
| GO:0010605\_negative\_regulation\_of\_macromolecule\_metabolic\_process | SIN3B | 331 | 5 | 3.478097 | -1.930450 | 10 | 5.66 | 0.566000 |  |
| GO:0010605\_negative\_regulation\_of\_macromolecule\_metabolic\_process | GMNN | 331 | 5 | 3.478097 | -1.930450 | 10 | 5.66 | 0.566000 |  |
| GO:0010605\_negative\_regulation\_of\_macromolecule\_metabolic\_process | NPM1 | 331 | 5 | 3.478097 | -1.930450 | 10 | 5.66 | 0.566000 |  |
| GO:0010605\_negative\_regulation\_of\_macromolecule\_metabolic\_process | MXD3 | 331 | 5 | 3.478097 | -1.930450 | 10 | 5.66 | 0.566000 |  |
| GO:0010605\_negative\_regulation\_of\_macromolecule\_metabolic\_process | CDT1 | 331 | 5 | 3.478097 | -1.930450 | 10 | 5.66 | 0.566000 |  |
| GO:0031324\_negative\_regulation\_of\_cellular\_metabolic\_process | SIN3B | 332 | 5 | 3.467620 | -1.925079 | 11 | 5.69 | 0.517273 |  |
| GO:0031324\_negative\_regulation\_of\_cellular\_metabolic\_process | GMNN | 332 | 5 | 3.467620 | -1.925079 | 11 | 5.69 | 0.517273 |  |
| GO:0031324\_negative\_regulation\_of\_cellular\_metabolic\_process | NPM1 | 332 | 5 | 3.467620 | -1.925079 | 11 | 5.69 | 0.517273 |  |
| GO:0031324\_negative\_regulation\_of\_cellular\_metabolic\_process | MXD3 | 332 | 5 | 3.467620 | -1.925079 | 11 | 5.69 | 0.517273 |  |
| GO:0031324\_negative\_regulation\_of\_cellular\_metabolic\_process | CDT1 | 332 | 5 | 3.467620 | -1.925079 | 11 | 5.69 | 0.517273 |  |
| GO:0000084\_S\_phase\_of\_mitotic\_cell\_cycle | CDT1 | 3 | 1 |  |  |  |  |  |  |
| GO:0006625\_protein\_targeting\_to\_peroxisome | PEX13 | 3 | 1 |  |  |  |  |  |  |
| GO:0007090\_regulation\_of\_S\_phase\_of\_mitotic\_cell\_cycle | CDT1 | 3 | 1 |  |  |  |  |  |  |
| GO:0018196\_peptidyl-asparagine\_modification | STT3B | 3 | 1 |  |  |  |  |  |  |
| GO:0018279\_protein\_amino\_acid\_N-linked\_glycosylation\_via\_asparagine | STT3B | 3 | 1 |  |  |  |  |  |  |
| GO:0033261\_regulation\_of\_S\_phase | CDT1 | 3 | 1 |  |  |  |  |  |  |
| GO:0042273\_ribosomal\_large\_subunit\_biogenesis | NPM1 | 3 | 1 |  |  |  |  |  |  |
| GO:0043574\_peroxisomal\_transport | PEX13 | 3 | 1 |  |  |  |  |  |  |
| GO:0046605\_regulation\_of\_centrosome\_cycle | NPM1 | 3 | 1 |  |  |  |  |  |  |
| GO:0051320\_S\_phase | CDT1 | 3 | 1 |  |  |  |  |  |  |
| GO:0006260\_DNA\_replication | GMNN | 41 | 2 | 11.231707 | -1.876839 | 12 | 6.59 | 0.549167 |  |
| GO:0006260\_DNA\_replication | CDT1 | 41 | 2 | 11.231707 | -1.876839 | 12 | 6.59 | 0.549167 |  |
| GO:0009892\_negative\_regulation\_of\_metabolic\_process | SIN3B | 348 | 5 | 3.308190 | -1.841794 | 13 | 7.01 | 0.539231 |  |
| GO:0009892\_negative\_regulation\_of\_metabolic\_process | GMNN | 348 | 5 | 3.308190 | -1.841794 | 13 | 7.01 | 0.539231 |  |
| GO:0009892\_negative\_regulation\_of\_metabolic\_process | NPM1 | 348 | 5 | 3.308190 | -1.841794 | 13 | 7.01 | 0.539231 |  |
| GO:0009892\_negative\_regulation\_of\_metabolic\_process | MXD3 | 348 | 5 | 3.308190 | -1.841794 | 13 | 7.01 | 0.539231 |  |
| GO:0009892\_negative\_regulation\_of\_metabolic\_process | CDT1 | 348 | 5 | 3.308190 | -1.841794 | 13 | 7.01 | 0.539231 |  |
| GO:0051726\_regulation\_of\_cell\_cycle | GMNN | 121 | 3 | 5.708678 | -1.836999 | 14 | 7.26 | 0.518571 |  |
| GO:0051726\_regulation\_of\_cell\_cycle | NPM1 | 121 | 3 | 5.708678 | -1.836999 | 14 | 7.26 | 0.518571 |  |
| GO:0051726\_regulation\_of\_cell\_cycle | CDT1 | 121 | 3 | 5.708678 | -1.836999 | 14 | 7.26 | 0.518571 |  |
| GO:0043623\_cellular\_protein\_complex\_assembly | PEX13 | 45 | 2 | 10.233333 | -1.799521 | 15 | 7.9 | 0.526667 |  |
| GO:0043623\_cellular\_protein\_complex\_assembly | DVL1 | 45 | 2 | 10.233333 | -1.799521 | 15 | 7.9 | 0.526667 |  |
| GO:0007031\_peroxisome\_organization | PEX13 | 4 | 1 |  |  |  |  |  |  |
| GO:0043484\_regulation\_of\_RNA\_splicing | NPM1 | 4 | 1 |  |  |  |  |  |  |
| GO:0048024\_regulation\_of\_nuclear\_mRNA\_splicing\_\_via\_spliceosome | NPM1 | 4 | 1 |  |  |  |  |  |  |
| GO:0008104\_protein\_localization | NPM1 | 251 | 4 | 3.669323 | -1.679652 | 16 | 9.59 | 0.599375 |  |
| GO:0008104\_protein\_localization | ARF6 | 251 | 4 | 3.669323 | -1.679652 | 16 | 9.59 | 0.599375 |  |
| GO:0008104\_protein\_localization | PEX13 | 251 | 4 | 3.669323 | -1.679652 | 16 | 9.59 | 0.599375 |  |
| GO:0008104\_protein\_localization | HSPA9 | 251 | 4 | 3.669323 | -1.679652 | 16 | 9.59 | 0.599375 |  |
| GO:0006270\_DNA\_replication\_initiation | CDT1 | 5 | 1 | 46.050000 | -1.666815 | 19 | 15.74 | 0.828421 |  |
| GO:0008631\_induction\_of\_apoptosis\_by\_oxidative\_stress | RNF7 | 5 | 1 | 46.050000 | -1.666815 | 19 | 15.74 | 0.828421 |  |
| GO:0051298\_centrosome\_duplication | NPM1 | 5 | 1 | 46.050000 | -1.666815 | 19 | 15.74 | 0.828421 |  |
| GO:0034622\_cellular\_macromolecular\_complex\_assembly | PEX13 | 58 | 2 | 7.939655 | -1.591496 | 20 | 17.58 | 0.879000 |  |
| GO:0034622\_cellular\_macromolecular\_complex\_assembly | DVL1 | 58 | 2 | 7.939655 | -1.591496 | 20 | 17.58 | 0.879000 |  |
| GO:0050684\_regulation\_of\_mRNA\_processing | NPM1 | 6 | 1 | 38.375000 | -1.588529 | 21 | 21.93 | 1.044286 |  |
| GO:0010558\_negative\_regulation\_of\_macromolecule\_biosynthetic\_process | SIN3B | 274 | 4 | 3.361314 | -1.554639 | 23 | 22.43 | 0.975217 |  |
| GO:0010558\_negative\_regulation\_of\_macromolecule\_biosynthetic\_process | GMNN | 274 | 4 | 3.361314 | -1.554639 | 23 | 22.43 | 0.975217 |  |
| GO:0010558\_negative\_regulation\_of\_macromolecule\_biosynthetic\_process | MXD3 | 274 | 4 | 3.361314 | -1.554639 | 23 | 22.43 | 0.975217 |  |
| GO:0010558\_negative\_regulation\_of\_macromolecule\_biosynthetic\_process | CDT1 | 274 | 4 | 3.361314 | -1.554639 | 23 | 22.43 | 0.975217 |  |
| GO:0033036\_macromolecule\_localization | NPM1 | 274 | 4 | 3.361314 | -1.554639 | 23 | 22.43 | 0.975217 |  |
| GO:0033036\_macromolecule\_localization | ARF6 | 274 | 4 | 3.361314 | -1.554639 | 23 | 22.43 | 0.975217 |  |
| GO:0033036\_macromolecule\_localization | PEX13 | 274 | 4 | 3.361314 | -1.554639 | 23 | 22.43 | 0.975217 |  |
| GO:0033036\_macromolecule\_localization | HSPA9 | 274 | 4 | 3.361314 | -1.554639 | 23 | 22.43 | 0.975217 |  |
| GO:0001967\_suckling\_behavior | PEX13 | 7 | 1 | 32.892857 | -1.522477 | 25 | 28.68 | 1.147200 |  |
| GO:0006884\_cell\_volume\_homeostasis | NPM1 | 7 | 1 | 32.892857 | -1.522477 | 25 | 28.68 | 1.147200 |  |
| GO:0009987\_cellular\_process | ODC1 | 3868 | 20 | 1.190538 | -1.518288 | 26 | 28.75 | 1.105769 |  |
| GO:0009987\_cellular\_process | SLC38A2 | 3868 | 20 | 1.190538 | -1.518288 | 26 | 28.75 | 1.105769 |  |
| GO:0009987\_cellular\_process | SLA2 | 3868 | 20 | 1.190538 | -1.518288 | 26 | 28.75 | 1.105769 |  |
| GO:0009987\_cellular\_process | GMNN | 3868 | 20 | 1.190538 | -1.518288 | 26 | 28.75 | 1.105769 |  |
| GO:0009987\_cellular\_process | EIF5A | 3868 | 20 | 1.190538 | -1.518288 | 26 | 28.75 | 1.105769 |  |
| GO:0009987\_cellular\_process | ARF6 | 3868 | 20 | 1.190538 | -1.518288 | 26 | 28.75 | 1.105769 |  |
| GO:0009987\_cellular\_process | FZD5 | 3868 | 20 | 1.190538 | -1.518288 | 26 | 28.75 | 1.105769 |  |
| GO:0009987\_cellular\_process | VDAC3 | 3868 | 20 | 1.190538 | -1.518288 | 26 | 28.75 | 1.105769 |  |
| GO:0009987\_cellular\_process | MXD3 | 3868 | 20 | 1.190538 | -1.518288 | 26 | 28.75 | 1.105769 |  |
| GO:0009987\_cellular\_process | H19 | 3868 | 20 | 1.190538 | -1.518288 | 26 | 28.75 | 1.105769 |  |
| GO:0009987\_cellular\_process | DVL1 | 3868 | 20 | 1.190538 | -1.518288 | 26 | 28.75 | 1.105769 |  |
| GO:0009987\_cellular\_process | CDT1 | 3868 | 20 | 1.190538 | -1.518288 | 26 | 28.75 | 1.105769 |  |
| GO:0009987\_cellular\_process | STT3B | 3868 | 20 | 1.190538 | -1.518288 | 26 | 28.75 | 1.105769 |  |
| GO:0009987\_cellular\_process | SIN3B | 3868 | 20 | 1.190538 | -1.518288 | 26 | 28.75 | 1.105769 |  |
| GO:0009987\_cellular\_process | D0H4S114 | 3868 | 20 | 1.190538 | -1.518288 | 26 | 28.75 | 1.105769 |  |
| GO:0009987\_cellular\_process | RNF7 | 3868 | 20 | 1.190538 | -1.518288 | 26 | 28.75 | 1.105769 |  |
| GO:0009987\_cellular\_process | SMARCC1 | 3868 | 20 | 1.190538 | -1.518288 | 26 | 28.75 | 1.105769 |  |
| GO:0009987\_cellular\_process | NPM1 | 3868 | 20 | 1.190538 | -1.518288 | 26 | 28.75 | 1.105769 |  |
| GO:0009987\_cellular\_process | PEX13 | 3868 | 20 | 1.190538 | -1.518288 | 26 | 28.75 | 1.105769 |  |
| GO:0009987\_cellular\_process | HSPA9 | 3868 | 20 | 1.190538 | -1.518288 | 26 | 28.75 | 1.105769 |  |
| GO:0031327\_negative\_regulation\_of\_cellular\_biosynthetic\_process | SIN3B | 282 | 4 | 3.265957 | -1.514177 | 27 | 28.82 | 1.067407 |  |
| GO:0031327\_negative\_regulation\_of\_cellular\_biosynthetic\_process | GMNN | 282 | 4 | 3.265957 | -1.514177 | 27 | 28.82 | 1.067407 |  |
| GO:0031327\_negative\_regulation\_of\_cellular\_biosynthetic\_process | MXD3 | 282 | 4 | 3.265957 | -1.514177 | 27 | 28.82 | 1.067407 |  |
| GO:0031327\_negative\_regulation\_of\_cellular\_biosynthetic\_process | CDT1 | 282 | 4 | 3.265957 | -1.514177 | 27 | 28.82 | 1.067407 |  |
| GO:0009890\_negative\_regulation\_of\_biosynthetic\_process | SIN3B | 284 | 4 | 3.242958 | -1.504286 | 28 | 29.05 | 1.037500 |  |
| GO:0009890\_negative\_regulation\_of\_biosynthetic\_process | GMNN | 284 | 4 | 3.242958 | -1.504286 | 28 | 29.05 | 1.037500 |  |
| GO:0009890\_negative\_regulation\_of\_biosynthetic\_process | MXD3 | 284 | 4 | 3.242958 | -1.504286 | 28 | 29.05 | 1.037500 |  |
| GO:0009890\_negative\_regulation\_of\_biosynthetic\_process | CDT1 | 284 | 4 | 3.242958 | -1.504286 | 28 | 29.05 | 1.037500 |  |
| GO:0007098\_centrosome\_cycle | NPM1 | 8 | 1 | 28.781250 | -1.465379 | 29 | 33.97 | 1.171379 |  |
| GO:0006913\_nucleocytoplasmic\_transport | NPM1 | 71 | 2 | 6.485915 | -1.429020 | 30 | 34.98 | 1.166000 |  |
| GO:0006913\_nucleocytoplasmic\_transport | HSPA9 | 71 | 2 | 6.485915 | -1.429020 | 30 | 34.98 | 1.166000 |  |
| GO:0051169\_nuclear\_transport | NPM1 | 72 | 2 | 6.395833 | -1.417906 | 31 | 35.39 | 1.141613 |  |
| GO:0051169\_nuclear\_transport | HSPA9 | 72 | 2 | 6.395833 | -1.417906 | 31 | 35.39 | 1.141613 |  |
| GO:0015031\_protein\_transport | ARF6 | 175 | 3 | 3.947143 | -1.417681 | 32 | 35.53 | 1.110312 |  |
| GO:0015031\_protein\_transport | PEX13 | 175 | 3 | 3.947143 | -1.417681 | 32 | 35.53 | 1.110312 |  |
| GO:0015031\_protein\_transport | HSPA9 | 175 | 3 | 3.947143 | -1.417681 | 32 | 35.53 | 1.110312 |  |
| GO:0048523\_negative\_regulation\_of\_cellular\_process | SIN3B | 774 | 7 | 2.082364 | -1.416225 | 33 | 35.62 | 1.079394 |  |
| GO:0048523\_negative\_regulation\_of\_cellular\_process | RNF7 | 774 | 7 | 2.082364 | -1.416225 | 33 | 35.62 | 1.079394 |  |
| GO:0048523\_negative\_regulation\_of\_cellular\_process | GMNN | 774 | 7 | 2.082364 | -1.416225 | 33 | 35.62 | 1.079394 |  |
| GO:0048523\_negative\_regulation\_of\_cellular\_process | NPM1 | 774 | 7 | 2.082364 | -1.416225 | 33 | 35.62 | 1.079394 |  |
| GO:0048523\_negative\_regulation\_of\_cellular\_process | MXD3 | 774 | 7 | 2.082364 | -1.416225 | 33 | 35.62 | 1.079394 |  |
| GO:0048523\_negative\_regulation\_of\_cellular\_process | CDT1 | 774 | 7 | 2.082364 | -1.416225 | 33 | 35.62 | 1.079394 |  |
| GO:0048523\_negative\_regulation\_of\_cellular\_process | H19 | 774 | 7 | 2.082364 | -1.416225 | 33 | 35.62 | 1.079394 |  |
| GO:0006611\_protein\_export\_from\_nucleus | HSPA9 | 9 | 1 | 25.583333 | -1.415120 | 36 | 40.29 | 1.119167 |  |
| GO:0031023\_microtubule\_organizing\_center\_organization | NPM1 | 9 | 1 | 25.583333 | -1.415120 | 36 | 40.29 | 1.119167 |  |
| GO:0051297\_centrosome\_organization | NPM1 | 9 | 1 | 25.583333 | -1.415120 | 36 | 40.29 | 1.119167 |  |
| GO:0045184\_establishment\_of\_protein\_localization | ARF6 | 180 | 3 | 3.837500 | -1.386760 | 37 | 40.99 | 1.107838 |  |
| GO:0045184\_establishment\_of\_protein\_localization | PEX13 | 180 | 3 | 3.837500 | -1.386760 | 37 | 40.99 | 1.107838 |  |
| GO:0045184\_establishment\_of\_protein\_localization | HSPA9 | 180 | 3 | 3.837500 | -1.386760 | 37 | 40.99 | 1.107838 |  |
| GO:0034621\_cellular\_macromolecular\_complex\_subunit\_organization | PEX13 | 76 | 2 | 6.059211 | -1.375099 | 38 | 41.43 | 1.090263 |  |
| GO:0034621\_cellular\_macromolecular\_complex\_subunit\_organization | DVL1 | 76 | 2 | 6.059211 | -1.375099 | 38 | 41.43 | 1.090263 |  |
| GO:0006405\_RNA\_export\_from\_nucleus | NPM1 | 10 | 1 | 23.025000 | -1.370256 | 44 | 46.34 | 1.053182 |  |
| GO:0007172\_signal\_complex\_assembly | DVL1 | 10 | 1 | 23.025000 | -1.370256 | 44 | 46.34 | 1.053182 |  |
| GO:0043113\_receptor\_clustering | DVL1 | 10 | 1 | 23.025000 | -1.370256 | 44 | 46.34 | 1.053182 |  |
| GO:0050657\_nucleic\_acid\_transport | NPM1 | 10 | 1 | 23.025000 | -1.370256 | 44 | 46.34 | 1.053182 |  |
| GO:0050658\_RNA\_transport | NPM1 | 10 | 1 | 23.025000 | -1.370256 | 44 | 46.34 | 1.053182 |  |
| GO:0051236\_establishment\_of\_RNA\_localization | NPM1 | 10 | 1 | 23.025000 | -1.370256 | 44 | 46.34 | 1.053182 |  |
| GO:0006461\_protein\_complex\_assembly | PEX13 | 78 | 2 | 5.903846 | -1.354626 | 46 | 46.97 | 1.021087 |  |
| GO:0006461\_protein\_complex\_assembly | DVL1 | 78 | 2 | 5.903846 | -1.354626 | 46 | 46.97 | 1.021087 |  |
| GO:0070271\_protein\_complex\_biogenesis | PEX13 | 78 | 2 | 5.903846 | -1.354626 | 46 | 46.97 | 1.021087 |  |
| GO:0070271\_protein\_complex\_biogenesis | DVL1 | 78 | 2 | 5.903846 | -1.354626 | 46 | 46.97 | 1.021087 |  |
| GO:0007017\_microtubule-based\_process | NPM1 | 83 | 2 | 5.548193 | -1.305908 | 47 | 51.81 | 1.102340 |  |
| GO:0007017\_microtubule-based\_process | PEX13 | 83 | 2 | 5.548193 | -1.305908 | 47 | 51.81 | 1.102340 |  |
| GO:0046907\_intracellular\_transport | NPM1 | 194 | 3 | 3.560567 | -1.305413 | 48 | 51.86 | 1.080417 |  |
| GO:0046907\_intracellular\_transport | PEX13 | 194 | 3 | 3.560567 | -1.305413 | 48 | 51.86 | 1.080417 |  |
| GO:0046907\_intracellular\_transport | HSPA9 | 194 | 3 | 3.560567 | -1.305413 | 48 | 51.86 | 1.080417 |  |
| GO:0000375\_RNA\_splicing\_\_via\_transesterification\_reactions | NPM1 | 12 | 1 | 19.187500 | -1.292859 | 55 | 56.4 | 1.025455 |  |
| GO:0000377\_RNA\_splicing\_\_via\_transesterification\_reactions\_with\_bulged\_adenosine\_as\_nucleophile | NPM1 | 12 | 1 | 19.187500 | -1.292859 | 55 | 56.4 | 1.025455 |  |
| GO:0000398\_nuclear\_mRNA\_splicing\_\_via\_spliceosome | NPM1 | 12 | 1 | 19.187500 | -1.292859 | 55 | 56.4 | 1.025455 |  |
| GO:0001662\_behavioral\_fear\_response | VDAC3 | 12 | 1 | 19.187500 | -1.292859 | 55 | 56.4 | 1.025455 |  |
| GO:0002209\_behavioral\_defense\_response | VDAC3 | 12 | 1 | 19.187500 | -1.292859 | 55 | 56.4 | 1.025455 |  |
| GO:0006403\_RNA\_localization | NPM1 | 12 | 1 | 19.187500 | -1.292859 | 55 | 56.4 | 1.025455 |  |
| GO:0030330\_DNA\_damage\_response\_\_signal\_transduction\_by\_p53\_class\_mediator | NPM1 | 12 | 1 | 19.187500 | -1.292859 | 55 | 56.4 | 1.025455 |  |
| GO:0006605\_protein\_targeting | PEX13 | 86 | 2 | 5.354651 | -1.278234 | 56 | 56.92 | 1.016429 |  |
| GO:0006605\_protein\_targeting | HSPA9 | 86 | 2 | 5.354651 | -1.278234 | 56 | 56.92 | 1.016429 |  |
| GO:0051649\_establishment\_of\_localization\_in\_cell | NPM1 | 342 | 4 | 2.692982 | -1.251022 | 57 | 61.48 | 1.078596 |  |
| GO:0051649\_establishment\_of\_localization\_in\_cell | PEX13 | 342 | 4 | 2.692982 | -1.251022 | 57 | 61.48 | 1.078596 |  |
| GO:0051649\_establishment\_of\_localization\_in\_cell | HSPA9 | 342 | 4 | 2.692982 | -1.251022 | 57 | 61.48 | 1.078596 |  |
| GO:0051649\_establishment\_of\_localization\_in\_cell | DVL1 | 342 | 4 | 2.692982 | -1.251022 | 57 | 61.48 | 1.078596 |  |
| GO:0065003\_macromolecular\_complex\_assembly | PEX13 | 93 | 2 | 4.951613 | -1.217692 | 58 | 66.05 | 1.138793 |  |
| GO:0065003\_macromolecular\_complex\_assembly | DVL1 | 93 | 2 | 4.951613 | -1.217692 | 58 | 66.05 | 1.138793 |  |
| GO:0048519\_negative\_regulation\_of\_biological\_process | SIN3B | 859 | 7 | 1.876310 | -1.201161 | 59 | 66.59 | 1.128644 |  |
| GO:0048519\_negative\_regulation\_of\_biological\_process | RNF7 | 859 | 7 | 1.876310 | -1.201161 | 59 | 66.59 | 1.128644 |  |
| GO:0048519\_negative\_regulation\_of\_biological\_process | GMNN | 859 | 7 | 1.876310 | -1.201161 | 59 | 66.59 | 1.128644 |  |
| GO:0048519\_negative\_regulation\_of\_biological\_process | NPM1 | 859 | 7 | 1.876310 | -1.201161 | 59 | 66.59 | 1.128644 |  |
| GO:0048519\_negative\_regulation\_of\_biological\_process | MXD3 | 859 | 7 | 1.876310 | -1.201161 | 59 | 66.59 | 1.128644 |  |
| GO:0048519\_negative\_regulation\_of\_biological\_process | H19 | 859 | 7 | 1.876310 | -1.201161 | 59 | 66.59 | 1.128644 |  |
| GO:0048519\_negative\_regulation\_of\_biological\_process | CDT1 | 859 | 7 | 1.876310 | -1.201161 | 59 | 66.59 | 1.128644 |  |
| GO:0006487\_protein\_amino\_acid\_N-linked\_glycosylation | STT3B | 15 | 1 | 15.350000 | -1.198623 | 64 | 69.9 | 1.092188 |  |
| GO:0009062\_fatty\_acid\_catabolic\_process | PEX13 | 15 | 1 | 15.350000 | -1.198623 | 64 | 69.9 | 1.092188 |  |
| GO:0015931\_nucleobase\_\_nucleoside\_\_nucleotide\_and\_nucleic\_acid\_transport | NPM1 | 15 | 1 | 15.350000 | -1.198623 | 64 | 69.9 | 1.092188 |  |
| GO:0021795\_cerebral\_cortex\_cell\_migration | PEX13 | 15 | 1 | 15.350000 | -1.198623 | 64 | 69.9 | 1.092188 |  |
| GO:0070507\_regulation\_of\_microtubule\_cytoskeleton\_organization | NPM1 | 15 | 1 | 15.350000 | -1.198623 | 64 | 69.9 | 1.092188 |  |
| GO:0031570\_DNA\_integrity\_checkpoint | CDT1 | 16 | 1 | 14.390625 | -1.171484 | 66 | 74.02 | 1.121515 |  |
| GO:0042596\_fear\_response | VDAC3 | 16 | 1 | 14.390625 | -1.171484 | 66 | 74.02 | 1.121515 |  |
| GO:0051641\_cellular\_localization | NPM1 | 370 | 4 | 2.489189 | -1.148104 | 67 | 75.06 | 1.120299 |  |
| GO:0051641\_cellular\_localization | PEX13 | 370 | 4 | 2.489189 | -1.148104 | 67 | 75.06 | 1.120299 |  |
| GO:0051641\_cellular\_localization | HSPA9 | 370 | 4 | 2.489189 | -1.148104 | 67 | 75.06 | 1.120299 |  |
| GO:0051641\_cellular\_localization | DVL1 | 370 | 4 | 2.489189 | -1.148104 | 67 | 75.06 | 1.120299 |  |
| GO:0006261\_DNA-dependent\_DNA\_replication | CDT1 | 17 | 1 | 13.544118 | -1.146045 | 76 | 78.59 | 1.034079 |  |
| GO:0006323\_DNA\_packaging | SMARCC1 | 17 | 1 | 13.544118 | -1.146045 | 76 | 78.59 | 1.034079 |  |
| GO:0006919\_activation\_of\_caspase\_activity | RNF7 | 17 | 1 | 13.544118 | -1.146045 | 76 | 78.59 | 1.034079 |  |
| GO:0008380\_RNA\_splicing | NPM1 | 17 | 1 | 13.544118 | -1.146045 | 76 | 78.59 | 1.034079 |  |
| GO:0019395\_fatty\_acid\_oxidation | PEX13 | 17 | 1 | 13.544118 | -1.146045 | 76 | 78.59 | 1.034079 |  |
| GO:0022029\_telencephalon\_cell\_migration | PEX13 | 17 | 1 | 13.544118 | -1.146045 | 76 | 78.59 | 1.034079 |  |
| GO:0034440\_lipid\_oxidation | PEX13 | 17 | 1 | 13.544118 | -1.146045 | 76 | 78.59 | 1.034079 |  |
| GO:0042254\_ribosome\_biogenesis | NPM1 | 17 | 1 | 13.544118 | -1.146045 | 76 | 78.59 | 1.034079 |  |
| GO:0045786\_negative\_regulation\_of\_cell\_cycle | GMNN | 17 | 1 | 13.544118 | -1.146045 | 76 | 78.59 | 1.034079 |  |
| GO:0021885\_forebrain\_cell\_migration | PEX13 | 18 | 1 | 12.791667 | -1.122110 | 78 | 82.89 | 1.062692 |  |
| GO:0048813\_dendrite\_morphogenesis | DVL1 | 18 | 1 | 12.791667 | -1.122110 | 78 | 82.89 | 1.062692 |  |
| GO:0006810\_transport | SLC38A2 | 718 | 6 | 1.924095 | -1.107110 | 79 | 83.57 | 1.057848 |  |
| GO:0006810\_transport | NPM1 | 718 | 6 | 1.924095 | -1.107110 | 79 | 83.57 | 1.057848 |  |
| GO:0006810\_transport | ARF6 | 718 | 6 | 1.924095 | -1.107110 | 79 | 83.57 | 1.057848 |  |
| GO:0006810\_transport | PEX13 | 718 | 6 | 1.924095 | -1.107110 | 79 | 83.57 | 1.057848 |  |
| GO:0006810\_transport | HSPA9 | 718 | 6 | 1.924095 | -1.107110 | 79 | 83.57 | 1.057848 |  |
| GO:0006810\_transport | DVL1 | 718 | 6 | 1.924095 | -1.107110 | 79 | 83.57 | 1.057848 |  |
| GO:0010952\_positive\_regulation\_of\_peptidase\_activity | RNF7 | 19 | 1 | 12.118421 | -1.099518 | 81 | 86.2 | 1.064198 |  |
| GO:0043280\_positive\_regulation\_of\_caspase\_activity | RNF7 | 19 | 1 | 12.118421 | -1.099518 | 81 | 86.2 | 1.064198 |  |
| GO:0044085\_cellular\_component\_biogenesis | NPM1 | 237 | 3 | 2.914557 | -1.094759 | 82 | 86.65 | 1.056707 |  |
| GO:0044085\_cellular\_component\_biogenesis | PEX13 | 237 | 3 | 2.914557 | -1.094759 | 82 | 86.65 | 1.056707 |  |
| GO:0044085\_cellular\_component\_biogenesis | DVL1 | 237 | 3 | 2.914557 | -1.094759 | 82 | 86.65 | 1.056707 |  |
| GO:0007049\_cell\_cycle | GMNN | 238 | 3 | 2.902311 | -1.090444 | 83 | 86.78 | 1.045542 |  |
| GO:0007049\_cell\_cycle | NPM1 | 238 | 3 | 2.902311 | -1.090444 | 83 | 86.78 | 1.045542 |  |
| GO:0007049\_cell\_cycle | CDT1 | 238 | 3 | 2.902311 | -1.090444 | 83 | 86.78 | 1.045542 |  |
| GO:0051234\_establishment\_of\_localization | SLC38A2 | 729 | 6 | 1.895062 | -1.080945 | 84 | 87.3 | 1.039286 |  |
| GO:0051234\_establishment\_of\_localization | NPM1 | 729 | 6 | 1.895062 | -1.080945 | 84 | 87.3 | 1.039286 |  |
| GO:0051234\_establishment\_of\_localization | ARF6 | 729 | 6 | 1.895062 | -1.080945 | 84 | 87.3 | 1.039286 |  |
| GO:0051234\_establishment\_of\_localization | PEX13 | 729 | 6 | 1.895062 | -1.080945 | 84 | 87.3 | 1.039286 |  |
| GO:0051234\_establishment\_of\_localization | HSPA9 | 729 | 6 | 1.895062 | -1.080945 | 84 | 87.3 | 1.039286 |  |
| GO:0051234\_establishment\_of\_localization | DVL1 | 729 | 6 | 1.895062 | -1.080945 | 84 | 87.3 | 1.039286 |  |
| GO:0007528\_neuromuscular\_junction\_development | DVL1 | 20 | 1 | 11.512500 | -1.078130 | 85 | 89.84 | 1.056941 |  |
| GO:0000075\_cell\_cycle\_checkpoint | CDT1 | 21 | 1 | 10.964286 | -1.057828 | 88 | 93.94 | 1.067500 |  |
| GO:0008637\_apoptotic\_mitochondrial\_changes | RNF7 | 21 | 1 | 10.964286 | -1.057828 | 88 | 93.94 | 1.067500 |  |
| GO:0051656\_establishment\_of\_organelle\_localization | NPM1 | 21 | 1 | 10.964286 | -1.057828 | 88 | 93.94 | 1.067500 |  |
| GO:0043933\_macromolecular\_complex\_subunit\_organization | PEX13 | 117 | 2 | 3.935897 | -1.044016 | 89 | 94.74 | 1.064494 |  |
| GO:0043933\_macromolecular\_complex\_subunit\_organization | DVL1 | 117 | 2 | 3.935897 | -1.044016 | 89 | 94.74 | 1.064494 |  |
| GO:0030258\_lipid\_modification | PEX13 | 22 | 1 | 10.465909 | -1.038512 | 92 | 98.48 | 1.070435 |  |
| GO:0032886\_regulation\_of\_microtubule-based\_process | NPM1 | 22 | 1 | 10.465909 | -1.038512 | 92 | 98.48 | 1.070435 |  |
| GO:0043112\_receptor\_metabolic\_process | DVL1 | 22 | 1 | 10.465909 | -1.038512 | 92 | 98.48 | 1.070435 |  |
| GO:0006397\_mRNA\_processing | NPM1 | 23 | 1 | 10.010870 | -1.020094 | 95 | 101.52 | 1.068632 |  |
| GO:0022613\_ribonucleoprotein\_complex\_biogenesis | NPM1 | 23 | 1 | 10.010870 | -1.020094 | 95 | 101.52 | 1.068632 |  |
| GO:0051705\_behavioral\_interaction\_between\_organisms | PEX13 | 23 | 1 | 10.010870 | -1.020094 | 95 | 101.52 | 1.068632 |  |
| GO:0006886\_intracellular\_protein\_transport | PEX13 | 122 | 2 | 3.774590 | -1.013060 | 96 | 102.41 | 1.066771 |  |
| GO:0006886\_intracellular\_protein\_transport | HSPA9 | 122 | 2 | 3.774590 | -1.013060 | 96 | 102.41 | 1.066771 |  |
| GO:0008629\_induction\_of\_apoptosis\_by\_intracellular\_signals | RNF7 | 24 | 1 | 9.593750 | -1.002496 | 97 | 105.0 | 1.082474 |  |
| GO:0010638\_positive\_regulation\_of\_organelle\_organization | NPM1 | 27 | 1 | 8.527778 | -0.954000 | 98 | 113.68 | 1.160000 |  |
| GO:0042770\_DNA\_damage\_response\_\_signal\_transduction | NPM1 | 29 | 1 | 7.939655 | -0.924733 | 101 | 119.89 | 1.187030 |  |
| GO:0043281\_regulation\_of\_caspase\_activity | RNF7 | 29 | 1 | 7.939655 | -0.924733 | 101 | 119.89 | 1.187030 |  |
| GO:0052548\_regulation\_of\_endopeptidase\_activity | RNF7 | 29 | 1 | 7.939655 | -0.924733 | 101 | 119.89 | 1.187030 |  |
| GO:0034613\_cellular\_protein\_localization | PEX13 | 139 | 2 | 3.312950 | -0.918120 | 102 | 120.51 | 1.181471 |  |
| GO:0034613\_cellular\_protein\_localization | HSPA9 | 139 | 2 | 3.312950 | -0.918120 | 102 | 120.51 | 1.181471 |  |
| GO:0052547\_regulation\_of\_peptidase\_activity | RNF7 | 30 | 1 | 7.675000 | -0.910893 | 103 | 123.84 | 1.202330 |  |
| GO:0070727\_cellular\_macromolecule\_localization | PEX13 | 141 | 2 | 3.265957 | -0.907874 | 104 | 124.11 | 1.193365 |  |
| GO:0070727\_cellular\_macromolecule\_localization | HSPA9 | 141 | 2 | 3.265957 | -0.907874 | 104 | 124.11 | 1.193365 |  |
| GO:0006486\_protein\_amino\_acid\_glycosylation | STT3B | 31 | 1 | 7.427419 | -0.897536 | 109 | 127.95 | 1.173853 |  |
| GO:0016049\_cell\_growth | NPM1 | 31 | 1 | 7.427419 | -0.897536 | 109 | 127.95 | 1.173853 |  |
| GO:0033555\_multicellular\_organismal\_response\_to\_stress | VDAC3 | 31 | 1 | 7.427419 | -0.897536 | 109 | 127.95 | 1.173853 |  |
| GO:0043413\_biopolymer\_glycosylation | STT3B | 31 | 1 | 7.427419 | -0.897536 | 109 | 127.95 | 1.173853 |  |
| GO:0070085\_glycosylation | STT3B | 31 | 1 | 7.427419 | -0.897536 | 109 | 127.95 | 1.173853 |  |
| GO:0051493\_regulation\_of\_cytoskeleton\_organization | NPM1 | 32 | 1 | 7.195312 | -0.884629 | 110 | 129.88 | 1.180727 |  |
| GO:0043085\_positive\_regulation\_of\_catalytic\_activity | RNF7 | 148 | 2 | 3.111486 | -0.873363 | 111 | 131.06 | 1.180721 |  |
| GO:0043085\_positive\_regulation\_of\_catalytic\_activity | NPM1 | 148 | 2 | 3.111486 | -0.873363 | 111 | 131.06 | 1.180721 |  |
| GO:0007270\_nerve-nerve\_synaptic\_transmission | VDAC3 | 33 | 1 | 6.977273 | -0.872147 | 113 | 132.78 | 1.175044 |  |
| GO:0021987\_cerebral\_cortex\_development | PEX13 | 33 | 1 | 6.977273 | -0.872147 | 113 | 132.78 | 1.175044 |  |
| GO:0007269\_neurotransmitter\_secretion | DVL1 | 34 | 1 | 6.772059 | -0.860063 | 116 | 135.39 | 1.167155 |  |
| GO:0016054\_organic\_acid\_catabolic\_process | PEX13 | 34 | 1 | 6.772059 | -0.860063 | 116 | 135.39 | 1.167155 |  |
| GO:0046395\_carboxylic\_acid\_catabolic\_process | PEX13 | 34 | 1 | 6.772059 | -0.860063 | 116 | 135.39 | 1.167155 |  |
| GO:0044242\_cellular\_lipid\_catabolic\_process | PEX13 | 35 | 1 | 6.578571 | -0.848355 | 119 | 137.44 | 1.154958 |  |
| GO:0051325\_interphase | CDT1 | 35 | 1 | 6.578571 | -0.848355 | 119 | 137.44 | 1.154958 |  |
| GO:0051329\_interphase\_of\_mitotic\_cell\_cycle | CDT1 | 35 | 1 | 6.578571 | -0.848355 | 119 | 137.44 | 1.154958 |  |
| GO:0007268\_synaptic\_transmission | VDAC3 | 154 | 2 | 2.990260 | -0.845338 | 120 | 137.55 | 1.146250 |  |
| GO:0007268\_synaptic\_transmission | DVL1 | 154 | 2 | 2.990260 | -0.845338 | 120 | 137.55 | 1.146250 |  |
| GO:0022402\_cell\_cycle\_process | NPM1 | 155 | 2 | 2.970968 | -0.840799 | 121 | 138.11 | 1.141405 |  |
| GO:0022402\_cell\_cycle\_process | CDT1 | 155 | 2 | 2.970968 | -0.840799 | 121 | 138.11 | 1.141405 |  |
| GO:0001889\_liver\_development | ARF6 | 36 | 1 | 6.395833 | -0.837001 | 123 | 140.49 | 1.142195 |  |
| GO:0007631\_feeding\_behavior | PEX13 | 36 | 1 | 6.395833 | -0.837001 | 123 | 140.49 | 1.142195 |  |
| GO:0016042\_lipid\_catabolic\_process | PEX13 | 38 | 1 | 6.059211 | -0.815279 | 124 | 145.64 | 1.174516 |  |
| GO:0006259\_DNA\_metabolic\_process | GMNN | 165 | 2 | 2.790909 | -0.797314 | 125 | 149.85 | 1.198800 |  |
| GO:0006259\_DNA\_metabolic\_process | CDT1 | 165 | 2 | 2.790909 | -0.797314 | 125 | 149.85 | 1.198800 |  |
| GO:0007346\_regulation\_of\_mitotic\_cell\_cycle | CDT1 | 40 | 1 | 5.756250 | -0.794759 | 129 | 151.66 | 1.175659 |  |
| GO:0016071\_mRNA\_metabolic\_process | NPM1 | 40 | 1 | 5.756250 | -0.794759 | 129 | 151.66 | 1.175659 |  |
| GO:0016358\_dendrite\_development | DVL1 | 40 | 1 | 5.756250 | -0.794759 | 129 | 151.66 | 1.175659 |  |
| GO:0017015\_regulation\_of\_transforming\_growth\_factor\_beta\_receptor\_signaling\_pathway | D0H4S114 | 40 | 1 | 5.756250 | -0.794759 | 129 | 151.66 | 1.175659 |  |
| GO:0006836\_neurotransmitter\_transport | DVL1 | 41 | 1 | 5.615854 | -0.784913 | 131 | 155.17 | 1.184504 |  |
| GO:0006865\_amino\_acid\_transport | SLC38A2 | 41 | 1 | 5.615854 | -0.784913 | 131 | 155.17 | 1.184504 |  |
| GO:0008361\_regulation\_of\_cell\_size | NPM1 | 42 | 1 | 5.482143 | -0.775325 | 134 | 158.45 | 1.182463 |  |
| GO:0051345\_positive\_regulation\_of\_hydrolase\_activity | RNF7 | 42 | 1 | 5.482143 | -0.775325 | 134 | 158.45 | 1.182463 |  |
| GO:0080135\_regulation\_of\_cellular\_response\_to\_stress | NPM1 | 42 | 1 | 5.482143 | -0.775325 | 134 | 158.45 | 1.182463 |  |
| GO:0044093\_positive\_regulation\_of\_molecular\_function | RNF7 | 173 | 2 | 2.661850 | -0.764842 | 135 | 162.69 | 1.205111 |  |
| GO:0044093\_positive\_regulation\_of\_molecular\_function | NPM1 | 173 | 2 | 2.661850 | -0.764842 | 135 | 162.69 | 1.205111 |  |
| GO:0050808\_synapse\_organization | DVL1 | 44 | 1 | 5.232955 | -0.756875 | 136 | 165.98 | 1.220441 |  |
| GO:0007612\_learning | VDAC3 | 46 | 1 | 5.005435 | -0.739320 | 137 | 170.24 | 1.242628 |  |
| GO:0006396\_RNA\_processing | NPM1 | 47 | 1 | 4.898936 | -0.730855 | 138 | 173.15 | 1.254710 |  |
| GO:0001505\_regulation\_of\_neurotransmitter\_levels | DVL1 | 48 | 1 | 4.796875 | -0.722586 | 140 | 175.28 | 1.252000 |  |
| GO:0009101\_glycoprotein\_biosynthetic\_process | STT3B | 48 | 1 | 4.796875 | -0.722586 | 140 | 175.28 | 1.252000 |  |
| GO:0021543\_pallium\_development | PEX13 | 49 | 1 | 4.698980 | -0.714505 | 142 | 178.05 | 1.253873 |  |
| GO:0048741\_skeletal\_muscle\_fiber\_development | DVL1 | 49 | 1 | 4.698980 | -0.714505 | 142 | 178.05 | 1.253873 |  |
| GO:0017038\_protein\_import | PEX13 | 50 | 1 | 4.605000 | -0.706604 | 143 | 179.76 | 1.257063 |  |
| GO:0019226\_transmission\_of\_nerve\_impulse | VDAC3 | 189 | 2 | 2.436508 | -0.705295 | 144 | 179.91 | 1.249375 |  |
| GO:0019226\_transmission\_of\_nerve\_impulse | DVL1 | 189 | 2 | 2.436508 | -0.705295 | 144 | 179.91 | 1.249375 |  |
| GO:0048747\_muscle\_fiber\_development | DVL1 | 51 | 1 | 4.514706 | -0.698876 | 145 | 182.1 | 1.255862 |  |
| GO:0046942\_carboxylic\_acid\_transport | SLC38A2 | 53 | 1 | 4.344340 | -0.683915 | 147 | 185.33 | 1.260748 |  |
| GO:0055085\_transmembrane\_transport | SLC38A2 | 53 | 1 | 4.344340 | -0.683915 | 147 | 185.33 | 1.260748 |  |
| GO:0015849\_organic\_acid\_transport | SLC38A2 | 54 | 1 | 4.263889 | -0.676668 | 148 | 188.14 | 1.271216 |  |
| GO:0000226\_microtubule\_cytoskeleton\_organization | NPM1 | 57 | 1 | 4.039474 | -0.655797 | 150 | 195.13 | 1.300867 |  |
| GO:0001764\_neuron\_migration | PEX13 | 57 | 1 | 4.039474 | -0.655797 | 150 | 195.13 | 1.300867 |  |
| GO:0022607\_cellular\_component\_assembly | PEX13 | 204 | 2 | 2.257353 | -0.655132 | 151 | 195.31 | 1.293444 |  |
| GO:0022607\_cellular\_component\_assembly | DVL1 | 204 | 2 | 2.257353 | -0.655132 | 151 | 195.31 | 1.293444 |  |
| GO:0019219\_regulation\_of\_nucleobase\_\_nucleoside\_\_nucleotide\_and\_nucleic\_acid\_metabolic\_process | SIN3B | 757 | 5 | 1.520806 | -0.653462 | 152 | 195.44 | 1.285789 |  |
| GO:0019219\_regulation\_of\_nucleobase\_\_nucleoside\_\_nucleotide\_and\_nucleic\_acid\_metabolic\_process | GMNN | 757 | 5 | 1.520806 | -0.653462 | 152 | 195.44 | 1.285789 |  |
| GO:0019219\_regulation\_of\_nucleobase\_\_nucleoside\_\_nucleotide\_and\_nucleic\_acid\_metabolic\_process | NPM1 | 757 | 5 | 1.520806 | -0.653462 | 152 | 195.44 | 1.285789 |  |
| GO:0019219\_regulation\_of\_nucleobase\_\_nucleoside\_\_nucleotide\_and\_nucleic\_acid\_metabolic\_process | MXD3 | 757 | 5 | 1.520806 | -0.653462 | 152 | 195.44 | 1.285789 |  |
| GO:0019219\_regulation\_of\_nucleobase\_\_nucleoside\_\_nucleotide\_and\_nucleic\_acid\_metabolic\_process | CDT1 | 757 | 5 | 1.520806 | -0.653462 | 152 | 195.44 | 1.285789 |  |
| GO:0033043\_regulation\_of\_organelle\_organization | NPM1 | 58 | 1 | 3.969828 | -0.649113 | 153 | 196.98 | 1.287451 |  |
| GO:0008284\_positive\_regulation\_of\_cell\_proliferation | ODC1 | 208 | 2 | 2.213942 | -0.642572 | 154 | 197.57 | 1.282922 |  |
| GO:0008284\_positive\_regulation\_of\_cell\_proliferation | NPM1 | 208 | 2 | 2.213942 | -0.642572 | 154 | 197.57 | 1.282922 |  |
| GO:0016055\_Wnt\_receptor\_signaling\_pathway | DVL1 | 59 | 1 | 3.902542 | -0.642558 | 156 | 198.55 | 1.272756 |  |
| GO:0048469\_cell\_maturation | FZD5 | 59 | 1 | 3.902542 | -0.642558 | 156 | 198.55 | 1.272756 |  |
| GO:0051171\_regulation\_of\_nitrogen\_compound\_metabolic\_process | SIN3B | 771 | 5 | 1.493191 | -0.631044 | 157 | 199.48 | 1.270573 |  |
| GO:0051171\_regulation\_of\_nitrogen\_compound\_metabolic\_process | GMNN | 771 | 5 | 1.493191 | -0.631044 | 157 | 199.48 | 1.270573 |  |
| GO:0051171\_regulation\_of\_nitrogen\_compound\_metabolic\_process | NPM1 | 771 | 5 | 1.493191 | -0.631044 | 157 | 199.48 | 1.270573 |  |
| GO:0051171\_regulation\_of\_nitrogen\_compound\_metabolic\_process | MXD3 | 771 | 5 | 1.493191 | -0.631044 | 157 | 199.48 | 1.270573 |  |
| GO:0051171\_regulation\_of\_nitrogen\_compound\_metabolic\_process | CDT1 | 771 | 5 | 1.493191 | -0.631044 | 157 | 199.48 | 1.270573 |  |
| GO:0007005\_mitochondrion\_organization | RNF7 | 61 | 1 | 3.774590 | -0.629816 | 158 | 200.74 | 1.270506 |  |
| GO:0006916\_anti-apoptosis | RNF7 | 62 | 1 | 3.713710 | -0.623621 | 160 | 203.47 | 1.271688 |  |
| GO:0021537\_telencephalon\_development | PEX13 | 62 | 1 | 3.713710 | -0.623621 | 160 | 203.47 | 1.271688 |  |
| GO:0042127\_regulation\_of\_cell\_proliferation | ODC1 | 393 | 3 | 1.757634 | -0.618675 | 161 | 203.67 | 1.265031 |  |
| GO:0042127\_regulation\_of\_cell\_proliferation | NPM1 | 393 | 3 | 1.757634 | -0.618675 | 161 | 203.67 | 1.265031 |  |
| GO:0042127\_regulation\_of\_cell\_proliferation | H19 | 393 | 3 | 1.757634 | -0.618675 | 161 | 203.67 | 1.265031 |  |
| GO:0051253\_negative\_regulation\_of\_RNA\_metabolic\_process | SIN3B | 220 | 2 | 2.093182 | -0.606756 | 162 | 207.79 | 1.282654 |  |
| GO:0051253\_negative\_regulation\_of\_RNA\_metabolic\_process | NPM1 | 220 | 2 | 2.093182 | -0.606756 | 162 | 207.79 | 1.282654 |  |
| GO:0007179\_transforming\_growth\_factor\_beta\_receptor\_signaling\_pathway | D0H4S114 | 66 | 1 | 3.488636 | -0.599932 | 166 | 210.26 | 1.266627 |  |
| GO:0015837\_amine\_transport | SLC38A2 | 66 | 1 | 3.488636 | -0.599932 | 166 | 210.26 | 1.266627 |  |
| GO:0045860\_positive\_regulation\_of\_protein\_kinase\_activity | NPM1 | 66 | 1 | 3.488636 | -0.599932 | 166 | 210.26 | 1.266627 |  |
| GO:0051130\_positive\_regulation\_of\_cellular\_component\_organization | NPM1 | 66 | 1 | 3.488636 | -0.599932 | 166 | 210.26 | 1.266627 |  |
| GO:0007167\_enzyme\_linked\_receptor\_protein\_signaling\_pathway | D0H4S114 | 229 | 2 | 2.010917 | -0.581589 | 167 | 214.6 | 1.285030 |  |
| GO:0007167\_enzyme\_linked\_receptor\_protein\_signaling\_pathway | DVL1 | 229 | 2 | 2.010917 | -0.581589 | 167 | 214.6 | 1.285030 |  |
| GO:0007611\_learning\_or\_memory | VDAC3 | 70 | 1 | 3.289286 | -0.577833 | 168 | 216.21 | 1.286964 |  |
| GO:0009100\_glycoprotein\_metabolic\_process | STT3B | 71 | 1 | 3.242958 | -0.572535 | 170 | 218.08 | 1.282824 |  |
| GO:0033674\_positive\_regulation\_of\_kinase\_activity | NPM1 | 71 | 1 | 3.242958 | -0.572535 | 170 | 218.08 | 1.282824 |  |
| GO:0050790\_regulation\_of\_catalytic\_activity | RNF7 | 233 | 2 | 1.976395 | -0.570835 | 171 | 218.55 | 1.278070 |  |
| GO:0050790\_regulation\_of\_catalytic\_activity | NPM1 | 233 | 2 | 1.976395 | -0.570835 | 171 | 218.55 | 1.278070 |  |
| GO:0031326\_regulation\_of\_cellular\_biosynthetic\_process | SIN3B | 812 | 5 | 1.417796 | -0.569460 | 172 | 218.66 | 1.271279 |  |
| GO:0031326\_regulation\_of\_cellular\_biosynthetic\_process | GMNN | 812 | 5 | 1.417796 | -0.569460 | 172 | 218.66 | 1.271279 |  |
| GO:0031326\_regulation\_of\_cellular\_biosynthetic\_process | NPM1 | 812 | 5 | 1.417796 | -0.569460 | 172 | 218.66 | 1.271279 |  |
| GO:0031326\_regulation\_of\_cellular\_biosynthetic\_process | MXD3 | 812 | 5 | 1.417796 | -0.569460 | 172 | 218.66 | 1.271279 |  |
| GO:0031326\_regulation\_of\_cellular\_biosynthetic\_process | CDT1 | 812 | 5 | 1.417796 | -0.569460 | 172 | 218.66 | 1.271279 |  |
| GO:0044262\_cellular\_carbohydrate\_metabolic\_process | STT3B | 72 | 1 | 3.197917 | -0.567323 | 174 | 221.47 | 1.272816 |  |
| GO:0051347\_positive\_regulation\_of\_transferase\_activity | NPM1 | 72 | 1 | 3.197917 | -0.567323 | 174 | 221.47 | 1.272816 |  |
| GO:0009889\_regulation\_of\_biosynthetic\_process | SIN3B | 815 | 5 | 1.412577 | -0.565180 | 175 | 221.77 | 1.267257 |  |
| GO:0009889\_regulation\_of\_biosynthetic\_process | GMNN | 815 | 5 | 1.412577 | -0.565180 | 175 | 221.77 | 1.267257 |  |
| GO:0009889\_regulation\_of\_biosynthetic\_process | NPM1 | 815 | 5 | 1.412577 | -0.565180 | 175 | 221.77 | 1.267257 |  |
| GO:0009889\_regulation\_of\_biosynthetic\_process | MXD3 | 815 | 5 | 1.412577 | -0.565180 | 175 | 221.77 | 1.267257 |  |
| GO:0009889\_regulation\_of\_biosynthetic\_process | CDT1 | 815 | 5 | 1.412577 | -0.565180 | 175 | 221.77 | 1.267257 |  |
| GO:0051336\_regulation\_of\_hydrolase\_activity | RNF7 | 73 | 1 | 3.154110 | -0.562194 | 176 | 223.13 | 1.267784 |  |
| GO:0006915\_apoptosis | RNF7 | 427 | 3 | 1.617681 | -0.550236 | 177 | 225.56 | 1.274350 |  |
| GO:0006915\_apoptosis | EIF5A | 427 | 3 | 1.617681 | -0.550236 | 177 | 225.56 | 1.274350 |  |
| GO:0006915\_apoptosis | ARF6 | 427 | 3 | 1.617681 | -0.550236 | 177 | 225.56 | 1.274350 |  |
| GO:0012501\_programmed\_cell\_death | RNF7 | 433 | 3 | 1.595266 | -0.539043 | 178 | 228.17 | 1.281854 |  |
| GO:0012501\_programmed\_cell\_death | EIF5A | 433 | 3 | 1.595266 | -0.539043 | 178 | 228.17 | 1.281854 |  |
| GO:0012501\_programmed\_cell\_death | ARF6 | 433 | 3 | 1.595266 | -0.539043 | 178 | 228.17 | 1.281854 |  |
| GO:0007519\_skeletal\_muscle\_tissue\_development | DVL1 | 78 | 1 | 2.951923 | -0.537723 | 180 | 229.67 | 1.275944 |  |
| GO:0060538\_skeletal\_muscle\_organ\_development | DVL1 | 78 | 1 | 2.951923 | -0.537723 | 180 | 229.67 | 1.275944 |  |
| GO:0000278\_mitotic\_cell\_cycle | CDT1 | 80 | 1 | 2.878125 | -0.528443 | 182 | 231.98 | 1.274615 |  |
| GO:0006631\_fatty\_acid\_metabolic\_process | PEX13 | 80 | 1 | 2.878125 | -0.528443 | 182 | 231.98 | 1.274615 |  |
| GO:0021700\_developmental\_maturation | FZD5 | 81 | 1 | 2.842593 | -0.523906 | 183 | 232.97 | 1.273060 |  |
| GO:0007267\_cell-cell\_signaling | VDAC3 | 252 | 2 | 1.827381 | -0.523061 | 184 | 233.16 | 1.267174 |  |
| GO:0007267\_cell-cell\_signaling | DVL1 | 252 | 2 | 1.827381 | -0.523061 | 184 | 233.16 | 1.267174 |  |
| GO:0016481\_negative\_regulation\_of\_transcription | SIN3B | 253 | 2 | 1.820158 | -0.520688 | 185 | 234.04 | 1.265081 |  |
| GO:0016481\_negative\_regulation\_of\_transcription | MXD3 | 253 | 2 | 1.820158 | -0.520688 | 185 | 234.04 | 1.265081 |  |
| GO:0051179\_localization | SLC38A2 | 1058 | 6 | 1.305766 | -0.519558 | 186 | 234.19 | 1.259086 |  |
| GO:0051179\_localization | NPM1 | 1058 | 6 | 1.305766 | -0.519558 | 186 | 234.19 | 1.259086 |  |
| GO:0051179\_localization | ARF6 | 1058 | 6 | 1.305766 | -0.519558 | 186 | 234.19 | 1.259086 |  |
| GO:0051179\_localization | PEX13 | 1058 | 6 | 1.305766 | -0.519558 | 186 | 234.19 | 1.259086 |  |
| GO:0051179\_localization | DVL1 | 1058 | 6 | 1.305766 | -0.519558 | 186 | 234.19 | 1.259086 |  |
| GO:0051179\_localization | HSPA9 | 1058 | 6 | 1.305766 | -0.519558 | 186 | 234.19 | 1.259086 |  |
| GO:0008219\_cell\_death | RNF7 | 444 | 3 | 1.555743 | -0.519155 | 187 | 235.81 | 1.261016 |  |
| GO:0008219\_cell\_death | EIF5A | 444 | 3 | 1.555743 | -0.519155 | 187 | 235.81 | 1.261016 |  |
| GO:0008219\_cell\_death | ARF6 | 444 | 3 | 1.555743 | -0.519155 | 187 | 235.81 | 1.261016 |  |
| GO:0009966\_regulation\_of\_signal\_transduction | D0H4S114 | 256 | 2 | 1.798828 | -0.513649 | 188 | 238.57 | 1.268989 |  |
| GO:0009966\_regulation\_of\_signal\_transduction | NPM1 | 256 | 2 | 1.798828 | -0.513649 | 188 | 238.57 | 1.268989 |  |
| GO:0006996\_organelle\_organization | RNF7 | 449 | 3 | 1.538419 | -0.510377 | 189 | 239.61 | 1.267778 |  |
| GO:0006996\_organelle\_organization | NPM1 | 449 | 3 | 1.538419 | -0.510377 | 189 | 239.61 | 1.267778 |  |
| GO:0006996\_organelle\_organization | PEX13 | 449 | 3 | 1.538419 | -0.510377 | 189 | 239.61 | 1.267778 |  |
| GO:0016265\_death | RNF7 | 450 | 3 | 1.535000 | -0.508641 | 190 | 239.75 | 1.261842 |  |
| GO:0016265\_death | EIF5A | 450 | 3 | 1.535000 | -0.508641 | 190 | 239.75 | 1.261842 |  |
| GO:0016265\_death | ARF6 | 450 | 3 | 1.535000 | -0.508641 | 190 | 239.75 | 1.261842 |  |
| GO:0010629\_negative\_regulation\_of\_gene\_expression | SIN3B | 262 | 2 | 1.757634 | -0.499918 | 191 | 243.22 | 1.273403 |  |
| GO:0010629\_negative\_regulation\_of\_gene\_expression | MXD3 | 262 | 2 | 1.757634 | -0.499918 | 191 | 243.22 | 1.273403 |  |
| GO:0001822\_kidney\_development | ODC1 | 87 | 1 | 2.646552 | -0.498005 | 194 | 245.03 | 1.263041 |  |
| GO:0003001\_generation\_of\_a\_signal\_involved\_in\_cell-cell\_signaling | DVL1 | 87 | 1 | 2.646552 | -0.498005 | 194 | 245.03 | 1.263041 |  |
| GO:0007178\_transmembrane\_receptor\_protein\_serine\_threonine\_kinase\_signaling\_pathway | D0H4S114 | 87 | 1 | 2.646552 | -0.498005 | 194 | 245.03 | 1.263041 |  |
| GO:0034984\_cellular\_response\_to\_DNA\_damage\_stimulus | NPM1 | 94 | 1 | 2.449468 | -0.470362 | 195 | 254.41 | 1.304667 |  |
| GO:0007610\_behavior | PEX13 | 279 | 2 | 1.650538 | -0.463374 | 197 | 256.12 | 1.300102 |  |
| GO:0007610\_behavior | VDAC3 | 279 | 2 | 1.650538 | -0.463374 | 197 | 256.12 | 1.300102 |  |
| GO:0065009\_regulation\_of\_molecular\_function | RNF7 | 279 | 2 | 1.650538 | -0.463374 | 197 | 256.12 | 1.300102 |  |
| GO:0065009\_regulation\_of\_molecular\_function | NPM1 | 279 | 2 | 1.650538 | -0.463374 | 197 | 256.12 | 1.300102 |  |
| GO:0018193\_peptidyl-amino\_acid\_modification | STT3B | 97 | 1 | 2.373711 | -0.459267 | 198 | 258.16 | 1.303838 |  |
| GO:0034645\_cellular\_macromolecule\_biosynthetic\_process | STT3B | 901 | 5 | 1.277747 | -0.454293 | 199 | 260.15 | 1.307286 |  |
| GO:0034645\_cellular\_macromolecule\_biosynthetic\_process | SIN3B | 901 | 5 | 1.277747 | -0.454293 | 199 | 260.15 | 1.307286 |  |
| GO:0034645\_cellular\_macromolecule\_biosynthetic\_process | GMNN | 901 | 5 | 1.277747 | -0.454293 | 199 | 260.15 | 1.307286 |  |
| GO:0034645\_cellular\_macromolecule\_biosynthetic\_process | MXD3 | 901 | 5 | 1.277747 | -0.454293 | 199 | 260.15 | 1.307286 |  |
| GO:0034645\_cellular\_macromolecule\_biosynthetic\_process | CDT1 | 901 | 5 | 1.277747 | -0.454293 | 199 | 260.15 | 1.307286 |  |
| GO:0009059\_macromolecule\_biosynthetic\_process | STT3B | 910 | 5 | 1.265110 | -0.443905 | 200 | 263.29 | 1.316450 |  |
| GO:0009059\_macromolecule\_biosynthetic\_process | SIN3B | 910 | 5 | 1.265110 | -0.443905 | 200 | 263.29 | 1.316450 |  |
| GO:0009059\_macromolecule\_biosynthetic\_process | GMNN | 910 | 5 | 1.265110 | -0.443905 | 200 | 263.29 | 1.316450 |  |
| GO:0009059\_macromolecule\_biosynthetic\_process | MXD3 | 910 | 5 | 1.265110 | -0.443905 | 200 | 263.29 | 1.316450 |  |
| GO:0009059\_macromolecule\_biosynthetic\_process | CDT1 | 910 | 5 | 1.265110 | -0.443905 | 200 | 263.29 | 1.316450 |  |
| GO:0080090\_regulation\_of\_primary\_metabolic\_process | SIN3B | 926 | 5 | 1.243251 | -0.425960 | 201 | 267.18 | 1.329254 |  |
| GO:0080090\_regulation\_of\_primary\_metabolic\_process | GMNN | 926 | 5 | 1.243251 | -0.425960 | 201 | 267.18 | 1.329254 |  |
| GO:0080090\_regulation\_of\_primary\_metabolic\_process | NPM1 | 926 | 5 | 1.243251 | -0.425960 | 201 | 267.18 | 1.329254 |  |
| GO:0080090\_regulation\_of\_primary\_metabolic\_process | MXD3 | 926 | 5 | 1.243251 | -0.425960 | 201 | 267.18 | 1.329254 |  |
| GO:0080090\_regulation\_of\_primary\_metabolic\_process | CDT1 | 926 | 5 | 1.243251 | -0.425960 | 201 | 267.18 | 1.329254 |  |
| GO:0045859\_regulation\_of\_protein\_kinase\_activity | NPM1 | 107 | 1 | 2.151869 | -0.425118 | 202 | 267.64 | 1.324950 |  |
| GO:0044249\_cellular\_biosynthetic\_process | STT3B | 1150 | 6 | 1.201304 | -0.418373 | 203 | 269.65 | 1.328325 |  |
| GO:0044249\_cellular\_biosynthetic\_process | SIN3B | 1150 | 6 | 1.201304 | -0.418373 | 203 | 269.65 | 1.328325 |  |
| GO:0044249\_cellular\_biosynthetic\_process | GMNN | 1150 | 6 | 1.201304 | -0.418373 | 203 | 269.65 | 1.328325 |  |
| GO:0044249\_cellular\_biosynthetic\_process | NPM1 | 1150 | 6 | 1.201304 | -0.418373 | 203 | 269.65 | 1.328325 |  |
| GO:0044249\_cellular\_biosynthetic\_process | MXD3 | 1150 | 6 | 1.201304 | -0.418373 | 203 | 269.65 | 1.328325 |  |
| GO:0044249\_cellular\_biosynthetic\_process | CDT1 | 1150 | 6 | 1.201304 | -0.418373 | 203 | 269.65 | 1.328325 |  |
| GO:0060255\_regulation\_of\_macromolecule\_metabolic\_process | SIN3B | 936 | 5 | 1.229968 | -0.415074 | 204 | 271.05 | 1.328676 |  |
| GO:0060255\_regulation\_of\_macromolecule\_metabolic\_process | GMNN | 936 | 5 | 1.229968 | -0.415074 | 204 | 271.05 | 1.328676 |  |
| GO:0060255\_regulation\_of\_macromolecule\_metabolic\_process | NPM1 | 936 | 5 | 1.229968 | -0.415074 | 204 | 271.05 | 1.328676 |  |
| GO:0060255\_regulation\_of\_macromolecule\_metabolic\_process | MXD3 | 936 | 5 | 1.229968 | -0.415074 | 204 | 271.05 | 1.328676 |  |
| GO:0060255\_regulation\_of\_macromolecule\_metabolic\_process | CDT1 | 936 | 5 | 1.229968 | -0.415074 | 204 | 271.05 | 1.328676 |  |
| GO:0043549\_regulation\_of\_kinase\_activity | NPM1 | 112 | 1 | 2.055804 | -0.409496 | 205 | 272.99 | 1.331659 |  |
| GO:0006974\_response\_to\_DNA\_damage\_stimulus | NPM1 | 113 | 1 | 2.037611 | -0.406476 | 206 | 274.09 | 1.330534 |  |
| GO:0051338\_regulation\_of\_transferase\_activity | NPM1 | 115 | 1 | 2.002174 | -0.400537 | 207 | 276.19 | 1.334251 |  |
| GO:0080134\_regulation\_of\_response\_to\_stress | NPM1 | 116 | 1 | 1.984914 | -0.397616 | 208 | 277.24 | 1.332885 |  |
| GO:0009058\_biosynthetic\_process | STT3B | 1175 | 6 | 1.175745 | -0.393950 | 209 | 278.09 | 1.330574 |  |
| GO:0009058\_biosynthetic\_process | SIN3B | 1175 | 6 | 1.175745 | -0.393950 | 209 | 278.09 | 1.330574 |  |
| GO:0009058\_biosynthetic\_process | GMNN | 1175 | 6 | 1.175745 | -0.393950 | 209 | 278.09 | 1.330574 |  |
| GO:0009058\_biosynthetic\_process | NPM1 | 1175 | 6 | 1.175745 | -0.393950 | 209 | 278.09 | 1.330574 |  |
| GO:0009058\_biosynthetic\_process | MXD3 | 1175 | 6 | 1.175745 | -0.393950 | 209 | 278.09 | 1.330574 |  |
| GO:0009058\_biosynthetic\_process | CDT1 | 1175 | 6 | 1.175745 | -0.393950 | 209 | 278.09 | 1.330574 |  |
| GO:0022403\_cell\_cycle\_phase | CDT1 | 119 | 1 | 1.934874 | -0.389042 | 210 | 279.09 | 1.329000 |  |
| GO:0010556\_regulation\_of\_macromolecule\_biosynthetic\_process | SIN3B | 745 | 4 | 1.236242 | -0.387507 | 211 | 279.28 | 1.323602 |  |
| GO:0010556\_regulation\_of\_macromolecule\_biosynthetic\_process | GMNN | 745 | 4 | 1.236242 | -0.387507 | 211 | 279.28 | 1.323602 |  |
| GO:0010556\_regulation\_of\_macromolecule\_biosynthetic\_process | MXD3 | 745 | 4 | 1.236242 | -0.387507 | 211 | 279.28 | 1.323602 |  |
| GO:0010556\_regulation\_of\_macromolecule\_biosynthetic\_process | CDT1 | 745 | 4 | 1.236242 | -0.387507 | 211 | 279.28 | 1.323602 |  |
| GO:0014706\_striated\_muscle\_tissue\_development | DVL1 | 120 | 1 | 1.918750 | -0.386244 | 212 | 279.54 | 1.318585 |  |
| GO:0016043\_cellular\_component\_organization | RNF7 | 964 | 5 | 1.194243 | -0.385887 | 213 | 279.72 | 1.313239 |  |
| GO:0016043\_cellular\_component\_organization | SMARCC1 | 964 | 5 | 1.194243 | -0.385887 | 213 | 279.72 | 1.313239 |  |
| GO:0016043\_cellular\_component\_organization | NPM1 | 964 | 5 | 1.194243 | -0.385887 | 213 | 279.72 | 1.313239 |  |
| GO:0016043\_cellular\_component\_organization | PEX13 | 964 | 5 | 1.194243 | -0.385887 | 213 | 279.72 | 1.313239 |  |
| GO:0016043\_cellular\_component\_organization | DVL1 | 964 | 5 | 1.194243 | -0.385887 | 213 | 279.72 | 1.313239 |  |
| GO:0006917\_induction\_of\_apoptosis | RNF7 | 121 | 1 | 1.902893 | -0.383477 | 215 | 280.95 | 1.306744 |  |
| GO:0012502\_induction\_of\_programmed\_cell\_death | RNF7 | 121 | 1 | 1.902893 | -0.383477 | 215 | 280.95 | 1.306744 |  |
| GO:0043062\_extracellular\_structure\_organization | DVL1 | 125 | 1 | 1.842000 | -0.372694 | 216 | 284.24 | 1.315926 |  |
| GO:0010646\_regulation\_of\_cell\_communication | D0H4S114 | 330 | 2 | 1.395455 | -0.371263 | 217 | 284.98 | 1.313272 |  |
| GO:0010646\_regulation\_of\_cell\_communication | NPM1 | 330 | 2 | 1.395455 | -0.371263 | 217 | 284.98 | 1.313272 |  |
| GO:0008283\_cell\_proliferation | ODC1 | 544 | 3 | 1.269761 | -0.370055 | 218 | 285.19 | 1.308211 |  |
| GO:0008283\_cell\_proliferation | NPM1 | 544 | 3 | 1.269761 | -0.370055 | 218 | 285.19 | 1.308211 |  |
| GO:0008283\_cell\_proliferation | H19 | 544 | 3 | 1.269761 | -0.370055 | 218 | 285.19 | 1.308211 |  |
| GO:0001655\_urogenital\_system\_development | ODC1 | 128 | 1 | 1.798828 | -0.364894 | 220 | 286.9 | 1.304091 |  |
| GO:0060537\_muscle\_tissue\_development | DVL1 | 128 | 1 | 1.798828 | -0.364894 | 220 | 286.9 | 1.304091 |  |
| GO:0032787\_monocarboxylic\_acid\_metabolic\_process | PEX13 | 130 | 1 | 1.771154 | -0.359825 | 221 | 288.88 | 1.307149 |  |
| GO:0006139\_nucleobase\_\_nucleoside\_\_nucleotide\_and\_nucleic\_acid\_metabolic\_process | SIN3B | 1002 | 5 | 1.148952 | -0.349160 | 222 | 293.14 | 1.320450 |  |
| GO:0006139\_nucleobase\_\_nucleoside\_\_nucleotide\_and\_nucleic\_acid\_metabolic\_process | GMNN | 1002 | 5 | 1.148952 | -0.349160 | 222 | 293.14 | 1.320450 |  |
| GO:0006139\_nucleobase\_\_nucleoside\_\_nucleotide\_and\_nucleic\_acid\_metabolic\_process | NPM1 | 1002 | 5 | 1.148952 | -0.349160 | 222 | 293.14 | 1.320450 |  |
| GO:0006139\_nucleobase\_\_nucleoside\_\_nucleotide\_and\_nucleic\_acid\_metabolic\_process | MXD3 | 1002 | 5 | 1.148952 | -0.349160 | 222 | 293.14 | 1.320450 |  |
| GO:0006139\_nucleobase\_\_nucleoside\_\_nucleotide\_and\_nucleic\_acid\_metabolic\_process | CDT1 | 1002 | 5 | 1.148952 | -0.349160 | 222 | 293.14 | 1.320450 |  |
| GO:0007169\_transmembrane\_receptor\_protein\_tyrosine\_kinase\_signaling\_pathway | DVL1 | 139 | 1 | 1.656475 | -0.338217 | 223 | 295.85 | 1.326682 |  |
| GO:0031323\_regulation\_of\_cellular\_metabolic\_process | SIN3B | 1015 | 5 | 1.134236 | -0.337315 | 224 | 296.39 | 1.323170 |  |
| GO:0031323\_regulation\_of\_cellular\_metabolic\_process | GMNN | 1015 | 5 | 1.134236 | -0.337315 | 224 | 296.39 | 1.323170 |  |
| GO:0031323\_regulation\_of\_cellular\_metabolic\_process | NPM1 | 1015 | 5 | 1.134236 | -0.337315 | 224 | 296.39 | 1.323170 |  |
| GO:0031323\_regulation\_of\_cellular\_metabolic\_process | MXD3 | 1015 | 5 | 1.134236 | -0.337315 | 224 | 296.39 | 1.323170 |  |
| GO:0031323\_regulation\_of\_cellular\_metabolic\_process | CDT1 | 1015 | 5 | 1.134236 | -0.337315 | 224 | 296.39 | 1.323170 |  |
| GO:0034961\_cellular\_biopolymer\_biosynthetic\_process | SIN3B | 804 | 4 | 1.145522 | -0.326195 | 225 | 299.78 | 1.332356 |  |
| GO:0034961\_cellular\_biopolymer\_biosynthetic\_process | GMNN | 804 | 4 | 1.145522 | -0.326195 | 225 | 299.78 | 1.332356 |  |
| GO:0034961\_cellular\_biopolymer\_biosynthetic\_process | MXD3 | 804 | 4 | 1.145522 | -0.326195 | 225 | 299.78 | 1.332356 |  |
| GO:0034961\_cellular\_biopolymer\_biosynthetic\_process | CDT1 | 804 | 4 | 1.145522 | -0.326195 | 225 | 299.78 | 1.332356 |  |
| GO:0043284\_biopolymer\_biosynthetic\_process | SIN3B | 807 | 4 | 1.141264 | -0.323331 | 226 | 300.33 | 1.328894 |  |
| GO:0043284\_biopolymer\_biosynthetic\_process | GMNN | 807 | 4 | 1.141264 | -0.323331 | 226 | 300.33 | 1.328894 |  |
| GO:0043284\_biopolymer\_biosynthetic\_process | MXD3 | 807 | 4 | 1.141264 | -0.323331 | 226 | 300.33 | 1.328894 |  |
| GO:0043284\_biopolymer\_biosynthetic\_process | CDT1 | 807 | 4 | 1.141264 | -0.323331 | 226 | 300.33 | 1.328894 |  |
| GO:0005975\_carbohydrate\_metabolic\_process | STT3B | 146 | 1 | 1.577055 | -0.322654 | 228 | 301.32 | 1.321579 |  |
| GO:0030900\_forebrain\_development | PEX13 | 146 | 1 | 1.577055 | -0.322654 | 228 | 301.32 | 1.321579 |  |
| GO:0032940\_secretion\_by\_cell | DVL1 | 149 | 1 | 1.545302 | -0.316288 | 229 | 304.31 | 1.328865 |  |
| GO:0007517\_muscle\_organ\_development | DVL1 | 153 | 1 | 1.504902 | -0.308065 | 230 | 307.02 | 1.334870 |  |
| GO:0008285\_negative\_regulation\_of\_cell\_proliferation | H19 | 155 | 1 | 1.485484 | -0.304063 | 231 | 308.38 | 1.334978 |  |
| GO:0050794\_regulation\_of\_cellular\_process | ODC1 | 2190 | 10 | 1.051370 | -0.300548 | 232 | 308.98 | 1.331810 |  |
| GO:0050794\_regulation\_of\_cellular\_process | SIN3B | 2190 | 10 | 1.051370 | -0.300548 | 232 | 308.98 | 1.331810 |  |
| GO:0050794\_regulation\_of\_cellular\_process | D0H4S114 | 2190 | 10 | 1.051370 | -0.300548 | 232 | 308.98 | 1.331810 |  |
| GO:0050794\_regulation\_of\_cellular\_process | RNF7 | 2190 | 10 | 1.051370 | -0.300548 | 232 | 308.98 | 1.331810 |  |
| GO:0050794\_regulation\_of\_cellular\_process | GMNN | 2190 | 10 | 1.051370 | -0.300548 | 232 | 308.98 | 1.331810 |  |
| GO:0050794\_regulation\_of\_cellular\_process | NPM1 | 2190 | 10 | 1.051370 | -0.300548 | 232 | 308.98 | 1.331810 |  |
| GO:0050794\_regulation\_of\_cellular\_process | MXD3 | 2190 | 10 | 1.051370 | -0.300548 | 232 | 308.98 | 1.331810 |  |
| GO:0050794\_regulation\_of\_cellular\_process | H19 | 2190 | 10 | 1.051370 | -0.300548 | 232 | 308.98 | 1.331810 |  |
| GO:0050794\_regulation\_of\_cellular\_process | DVL1 | 2190 | 10 | 1.051370 | -0.300548 | 232 | 308.98 | 1.331810 |  |
| GO:0050794\_regulation\_of\_cellular\_process | CDT1 | 2190 | 10 | 1.051370 | -0.300548 | 232 | 308.98 | 1.331810 |  |
| GO:0051704\_multi-organism\_process | PEX13 | 157 | 1 | 1.466561 | -0.300130 | 233 | 309.4 | 1.327897 |  |
| GO:0051128\_regulation\_of\_cellular\_component\_organization | NPM1 | 160 | 1 | 1.439062 | -0.294360 | 234 | 311.28 | 1.330256 |  |
| GO:0007626\_locomotory\_behavior | PEX13 | 163 | 1 | 1.412577 | -0.288737 | 236 | 313.56 | 1.328644 |  |
| GO:0042110\_T\_cell\_activation | SLA2 | 163 | 1 | 1.412577 | -0.288737 | 236 | 313.56 | 1.328644 |  |
| GO:0050877\_neurological\_system\_process | VDAC3 | 390 | 2 | 1.180769 | -0.288339 | 237 | 313.88 | 1.324388 |  |
| GO:0050877\_neurological\_system\_process | DVL1 | 390 | 2 | 1.180769 | -0.288339 | 237 | 313.88 | 1.324388 |  |
| GO:0042325\_regulation\_of\_phosphorylation | NPM1 | 164 | 1 | 1.403963 | -0.286895 | 238 | 314.32 | 1.320672 |  |
| GO:0019220\_regulation\_of\_phosphate\_metabolic\_process | NPM1 | 165 | 1 | 1.395455 | -0.285068 | 240 | 315.82 | 1.315917 |  |
| GO:0051174\_regulation\_of\_phosphorus\_metabolic\_process | NPM1 | 165 | 1 | 1.395455 | -0.285068 | 240 | 315.82 | 1.315917 |  |
| GO:0043065\_positive\_regulation\_of\_apoptosis | RNF7 | 166 | 1 | 1.387048 | -0.283257 | 241 | 316.63 | 1.313817 |  |
| GO:0010942\_positive\_regulation\_of\_cell\_death | RNF7 | 167 | 1 | 1.378743 | -0.281461 | 243 | 317.68 | 1.307325 |  |
| GO:0043068\_positive\_regulation\_of\_programmed\_cell\_death | RNF7 | 167 | 1 | 1.378743 | -0.281461 | 243 | 317.68 | 1.307325 |  |
| GO:0048699\_generation\_of\_neurons | PEX13 | 396 | 2 | 1.162879 | -0.281231 | 244 | 317.94 | 1.303033 |  |
| GO:0048699\_generation\_of\_neurons | DVL1 | 396 | 2 | 1.162879 | -0.281231 | 244 | 317.94 | 1.303033 |  |
| GO:0019222\_regulation\_of\_metabolic\_process | SIN3B | 1088 | 5 | 1.058134 | -0.277024 | 245 | 318.27 | 1.299061 |  |
| GO:0019222\_regulation\_of\_metabolic\_process | GMNN | 1088 | 5 | 1.058134 | -0.277024 | 245 | 318.27 | 1.299061 |  |
| GO:0019222\_regulation\_of\_metabolic\_process | NPM1 | 1088 | 5 | 1.058134 | -0.277024 | 245 | 318.27 | 1.299061 |  |
| GO:0019222\_regulation\_of\_metabolic\_process | MXD3 | 1088 | 5 | 1.058134 | -0.277024 | 245 | 318.27 | 1.299061 |  |
| GO:0019222\_regulation\_of\_metabolic\_process | CDT1 | 1088 | 5 | 1.058134 | -0.277024 | 245 | 318.27 | 1.299061 |  |
| GO:0048812\_neuron\_projection\_morphogenesis | DVL1 | 170 | 1 | 1.354412 | -0.276162 | 246 | 318.99 | 1.296707 |  |
| GO:0044248\_cellular\_catabolic\_process | PEX13 | 173 | 1 | 1.330925 | -0.270994 | 248 | 321.11 | 1.294798 |  |
| GO:0048667\_cell\_morphogenesis\_involved\_in\_neuron\_differentiation | DVL1 | 173 | 1 | 1.330925 | -0.270994 | 248 | 321.11 | 1.294798 |  |
| GO:0046903\_secretion | DVL1 | 175 | 1 | 1.315714 | -0.267618 | 249 | 322.1 | 1.293574 |  |
| GO:0043066\_negative\_regulation\_of\_apoptosis | RNF7 | 176 | 1 | 1.308239 | -0.265951 | 251 | 323.51 | 1.288884 |  |
| GO:0048858\_cell\_projection\_morphogenesis | DVL1 | 176 | 1 | 1.308239 | -0.265951 | 251 | 323.51 | 1.288884 |  |
| GO:0043069\_negative\_regulation\_of\_programmed\_cell\_death | RNF7 | 179 | 1 | 1.286313 | -0.261030 | 253 | 325.42 | 1.286245 |  |
| GO:0060548\_negative\_regulation\_of\_cell\_death | RNF7 | 179 | 1 | 1.286313 | -0.261030 | 253 | 325.42 | 1.286245 |  |
| GO:0019752\_carboxylic\_acid\_metabolic\_process | PEX13 | 181 | 1 | 1.272099 | -0.257815 | 255 | 326.99 | 1.282314 |  |
| GO:0043436\_oxoacid\_metabolic\_process | PEX13 | 181 | 1 | 1.272099 | -0.257815 | 255 | 326.99 | 1.282314 |  |
| GO:0006082\_organic\_acid\_metabolic\_process | PEX13 | 182 | 1 | 1.265110 | -0.256226 | 256 | 327.38 | 1.278828 |  |
| GO:0043170\_macromolecule\_metabolic\_process | STT3B | 1576 | 7 | 1.022684 | -0.255979 | 257 | 327.55 | 1.274514 |  |
| GO:0043170\_macromolecule\_metabolic\_process | SIN3B | 1576 | 7 | 1.022684 | -0.255979 | 257 | 327.55 | 1.274514 |  |
| GO:0043170\_macromolecule\_metabolic\_process | GMNN | 1576 | 7 | 1.022684 | -0.255979 | 257 | 327.55 | 1.274514 |  |
| GO:0043170\_macromolecule\_metabolic\_process | NPM1 | 1576 | 7 | 1.022684 | -0.255979 | 257 | 327.55 | 1.274514 |  |
| GO:0043170\_macromolecule\_metabolic\_process | MXD3 | 1576 | 7 | 1.022684 | -0.255979 | 257 | 327.55 | 1.274514 |  |
| GO:0043170\_macromolecule\_metabolic\_process | DVL1 | 1576 | 7 | 1.022684 | -0.255979 | 257 | 327.55 | 1.274514 |  |
| GO:0043170\_macromolecule\_metabolic\_process | CDT1 | 1576 | 7 | 1.022684 | -0.255979 | 257 | 327.55 | 1.274514 |  |
| GO:0048468\_cell\_development | PEX13 | 654 | 3 | 1.056193 | -0.255133 | 258 | 328.19 | 1.272054 |  |
| GO:0048468\_cell\_development | FZD5 | 654 | 3 | 1.056193 | -0.255133 | 258 | 328.19 | 1.272054 |  |
| GO:0048468\_cell\_development | DVL1 | 654 | 3 | 1.056193 | -0.255133 | 258 | 328.19 | 1.272054 |  |
| GO:0042180\_cellular\_ketone\_metabolic\_process | PEX13 | 183 | 1 | 1.258197 | -0.254650 | 259 | 328.58 | 1.268649 |  |
| GO:0032990\_cell\_part\_morphogenesis | DVL1 | 184 | 1 | 1.251359 | -0.253087 | 260 | 329.74 | 1.268231 |  |
| GO:0007010\_cytoskeleton\_organization | NPM1 | 185 | 1 | 1.244595 | -0.251535 | 261 | 330.48 | 1.266207 |  |
| GO:0022008\_neurogenesis | PEX13 | 423 | 2 | 1.088652 | -0.251477 | 262 | 330.78 | 1.262519 |  |
| GO:0022008\_neurogenesis | DVL1 | 423 | 2 | 1.088652 | -0.251477 | 262 | 330.78 | 1.262519 |  |
| GO:0006952\_defense\_response | VDAC3 | 187 | 1 | 1.231283 | -0.248469 | 263 | 332.58 | 1.264563 |  |
| GO:0048513\_organ\_development | ODC1 | 1365 | 6 | 1.012088 | -0.244062 | 264 | 334.0 | 1.265152 |  |
| GO:0048513\_organ\_development | GMNN | 1365 | 6 | 1.012088 | -0.244062 | 264 | 334.0 | 1.265152 |  |
| GO:0048513\_organ\_development | SMARCC1 | 1365 | 6 | 1.012088 | -0.244062 | 264 | 334.0 | 1.265152 |  |
| GO:0048513\_organ\_development | PEX13 | 1365 | 6 | 1.012088 | -0.244062 | 264 | 334.0 | 1.265152 |  |
| GO:0048513\_organ\_development | ARF6 | 1365 | 6 | 1.012088 | -0.244062 | 264 | 334.0 | 1.265152 |  |
| GO:0048513\_organ\_development | DVL1 | 1365 | 6 | 1.012088 | -0.244062 | 264 | 334.0 | 1.265152 |  |
| GO:0019725\_cellular\_homeostasis | NPM1 | 195 | 1 | 1.180769 | -0.236670 | 265 | 337.49 | 1.273547 |  |
| GO:0006807\_nitrogen\_compound\_metabolic\_process | SIN3B | 1147 | 5 | 1.003705 | -0.235270 | 266 | 338.09 | 1.271015 |  |
| GO:0006807\_nitrogen\_compound\_metabolic\_process | GMNN | 1147 | 5 | 1.003705 | -0.235270 | 266 | 338.09 | 1.271015 |  |
| GO:0006807\_nitrogen\_compound\_metabolic\_process | NPM1 | 1147 | 5 | 1.003705 | -0.235270 | 266 | 338.09 | 1.271015 |  |
| GO:0006807\_nitrogen\_compound\_metabolic\_process | MXD3 | 1147 | 5 | 1.003705 | -0.235270 | 266 | 338.09 | 1.271015 |  |
| GO:0006807\_nitrogen\_compound\_metabolic\_process | CDT1 | 1147 | 5 | 1.003705 | -0.235270 | 266 | 338.09 | 1.271015 |  |
| GO:0033554\_cellular\_response\_to\_stress | NPM1 | 196 | 1 | 1.174745 | -0.235245 | 267 | 338.38 | 1.267341 |  |
| GO:0031175\_neuron\_projection\_development | DVL1 | 197 | 1 | 1.168782 | -0.233831 | 268 | 339.35 | 1.266231 |  |
| GO:0000904\_cell\_morphogenesis\_involved\_in\_differentiation | DVL1 | 199 | 1 | 1.157035 | -0.231035 | 269 | 341.17 | 1.268290 |  |
| GO:0010926\_anatomical\_structure\_formation | PEX13 | 447 | 2 | 1.030201 | -0.227805 | 270 | 342.1 | 1.267037 |  |
| GO:0010926\_anatomical\_structure\_formation | DVL1 | 447 | 2 | 1.030201 | -0.227805 | 270 | 342.1 | 1.267037 |  |
| GO:0034960\_cellular\_biopolymer\_metabolic\_process | STT3B | 1395 | 6 | 0.990323 | -0.225394 | 271 | 343.08 | 1.265978 |  |
| GO:0034960\_cellular\_biopolymer\_metabolic\_process | SIN3B | 1395 | 6 | 0.990323 | -0.225394 | 271 | 343.08 | 1.265978 |  |
| GO:0034960\_cellular\_biopolymer\_metabolic\_process | GMNN | 1395 | 6 | 0.990323 | -0.225394 | 271 | 343.08 | 1.265978 |  |
| GO:0034960\_cellular\_biopolymer\_metabolic\_process | NPM1 | 1395 | 6 | 0.990323 | -0.225394 | 271 | 343.08 | 1.265978 |  |
| GO:0034960\_cellular\_biopolymer\_metabolic\_process | MXD3 | 1395 | 6 | 0.990323 | -0.225394 | 271 | 343.08 | 1.265978 |  |
| GO:0034960\_cellular\_biopolymer\_metabolic\_process | CDT1 | 1395 | 6 | 0.990323 | -0.225394 | 271 | 343.08 | 1.265978 |  |
| GO:0040007\_growth | NPM1 | 217 | 1 | 1.061060 | -0.207650 | 273 | 348.65 | 1.277106 |  |
| GO:0048583\_regulation\_of\_response\_to\_stimulus | NPM1 | 217 | 1 | 1.061060 | -0.207650 | 273 | 348.65 | 1.277106 |  |
| GO:0045892\_negative\_regulation\_of\_transcription\_\_DNA-dependent | SIN3B | 218 | 1 | 1.056193 | -0.206438 | 274 | 349.03 | 1.273832 |  |
| GO:0050789\_regulation\_of\_biological\_process | ODC1 | 2357 | 10 | 0.976877 | -0.200996 | 275 | 350.91 | 1.276036 |  |
| GO:0050789\_regulation\_of\_biological\_process | D0H4S114 | 2357 | 10 | 0.976877 | -0.200996 | 275 | 350.91 | 1.276036 |  |
| GO:0050789\_regulation\_of\_biological\_process | SIN3B | 2357 | 10 | 0.976877 | -0.200996 | 275 | 350.91 | 1.276036 |  |
| GO:0050789\_regulation\_of\_biological\_process | RNF7 | 2357 | 10 | 0.976877 | -0.200996 | 275 | 350.91 | 1.276036 |  |
| GO:0050789\_regulation\_of\_biological\_process | GMNN | 2357 | 10 | 0.976877 | -0.200996 | 275 | 350.91 | 1.276036 |  |
| GO:0050789\_regulation\_of\_biological\_process | NPM1 | 2357 | 10 | 0.976877 | -0.200996 | 275 | 350.91 | 1.276036 |  |
| GO:0050789\_regulation\_of\_biological\_process | MXD3 | 2357 | 10 | 0.976877 | -0.200996 | 275 | 350.91 | 1.276036 |  |
| GO:0050789\_regulation\_of\_biological\_process | DVL1 | 2357 | 10 | 0.976877 | -0.200996 | 275 | 350.91 | 1.276036 |  |
| GO:0050789\_regulation\_of\_biological\_process | H19 | 2357 | 10 | 0.976877 | -0.200996 | 275 | 350.91 | 1.276036 |  |
| GO:0050789\_regulation\_of\_biological\_process | CDT1 | 2357 | 10 | 0.976877 | -0.200996 | 275 | 350.91 | 1.276036 |  |
| GO:0044260\_cellular\_macromolecule\_metabolic\_process | STT3B | 1447 | 6 | 0.954734 | -0.195786 | 276 | 351.58 | 1.273841 |  |
| GO:0044260\_cellular\_macromolecule\_metabolic\_process | SIN3B | 1447 | 6 | 0.954734 | -0.195786 | 276 | 351.58 | 1.273841 |  |
| GO:0044260\_cellular\_macromolecule\_metabolic\_process | GMNN | 1447 | 6 | 0.954734 | -0.195786 | 276 | 351.58 | 1.273841 |  |
| GO:0044260\_cellular\_macromolecule\_metabolic\_process | NPM1 | 1447 | 6 | 0.954734 | -0.195786 | 276 | 351.58 | 1.273841 |  |
| GO:0044260\_cellular\_macromolecule\_metabolic\_process | MXD3 | 1447 | 6 | 0.954734 | -0.195786 | 276 | 351.58 | 1.273841 |  |
| GO:0044260\_cellular\_macromolecule\_metabolic\_process | CDT1 | 1447 | 6 | 0.954734 | -0.195786 | 276 | 351.58 | 1.273841 |  |
| GO:0046649\_lymphocyte\_activation | SLA2 | 228 | 1 | 1.009868 | -0.194779 | 277 | 352.99 | 1.274332 |  |
| GO:0007420\_brain\_development | PEX13 | 231 | 1 | 0.996753 | -0.191437 | 278 | 353.89 | 1.272986 |  |
| GO:0050890\_cognition | VDAC3 | 233 | 1 | 0.988197 | -0.189247 | 279 | 354.61 | 1.271004 |  |
| GO:0016477\_cell\_migration | PEX13 | 234 | 1 | 0.983974 | -0.188164 | 280 | 355.0 | 1.267857 |  |
| GO:0009056\_catabolic\_process | PEX13 | 243 | 1 | 0.947531 | -0.178735 | 281 | 358.25 | 1.274911 |  |
| GO:0043283\_biopolymer\_metabolic\_process | STT3B | 1490 | 6 | 0.927181 | -0.173753 | 282 | 358.58 | 1.271560 |  |
| GO:0043283\_biopolymer\_metabolic\_process | SIN3B | 1490 | 6 | 0.927181 | -0.173753 | 282 | 358.58 | 1.271560 |  |
| GO:0043283\_biopolymer\_metabolic\_process | GMNN | 1490 | 6 | 0.927181 | -0.173753 | 282 | 358.58 | 1.271560 |  |
| GO:0043283\_biopolymer\_metabolic\_process | NPM1 | 1490 | 6 | 0.927181 | -0.173753 | 282 | 358.58 | 1.271560 |  |
| GO:0043283\_biopolymer\_metabolic\_process | MXD3 | 1490 | 6 | 0.927181 | -0.173753 | 282 | 358.58 | 1.271560 |  |
| GO:0043283\_biopolymer\_metabolic\_process | CDT1 | 1490 | 6 | 0.927181 | -0.173753 | 282 | 358.58 | 1.271560 |  |
| GO:0045321\_leukocyte\_activation | SLA2 | 248 | 1 | 0.928427 | -0.173739 | 283 | 359.38 | 1.269894 |  |
| GO:0003008\_system\_process | VDAC3 | 516 | 2 | 0.892442 | -0.171694 | 284 | 360.31 | 1.268697 |  |
| GO:0003008\_system\_process | DVL1 | 516 | 2 | 0.892442 | -0.171694 | 284 | 360.31 | 1.268697 |  |
| GO:0010468\_regulation\_of\_gene\_expression | SIN3B | 778 | 3 | 0.887853 | -0.166494 | 285 | 363.34 | 1.274877 |  |
| GO:0010468\_regulation\_of\_gene\_expression | NPM1 | 778 | 3 | 0.887853 | -0.166494 | 285 | 363.34 | 1.274877 |  |
| GO:0010468\_regulation\_of\_gene\_expression | MXD3 | 778 | 3 | 0.887853 | -0.166494 | 285 | 363.34 | 1.274877 |  |
| GO:0048870\_cell\_motility | PEX13 | 257 | 1 | 0.895914 | -0.165151 | 286 | 364.63 | 1.274930 |  |
| GO:0001775\_cell\_activation | SLA2 | 262 | 1 | 0.878817 | -0.160594 | 288 | 365.81 | 1.270174 |  |
| GO:0048666\_neuron\_development | DVL1 | 262 | 1 | 0.878817 | -0.160594 | 288 | 365.81 | 1.270174 |  |
| GO:0030030\_cell\_projection\_organization | DVL1 | 263 | 1 | 0.875475 | -0.159700 | 289 | 366.14 | 1.266920 |  |
| GO:0044255\_cellular\_lipid\_metabolic\_process | PEX13 | 264 | 1 | 0.872159 | -0.158812 | 290 | 366.54 | 1.263931 |  |
| GO:0051716\_cellular\_response\_to\_stimulus | NPM1 | 273 | 1 | 0.843407 | -0.151069 | 291 | 368.75 | 1.267182 |  |
| GO:0006950\_response\_to\_stress | NPM1 | 549 | 2 | 0.838798 | -0.149998 | 292 | 369.96 | 1.266986 |  |
| GO:0006950\_response\_to\_stress | VDAC3 | 549 | 2 | 0.838798 | -0.149998 | 292 | 369.96 | 1.266986 |  |
| GO:0000902\_cell\_morphogenesis | DVL1 | 283 | 1 | 0.813604 | -0.142966 | 293 | 373.04 | 1.273174 |  |
| GO:0006629\_lipid\_metabolic\_process | PEX13 | 285 | 1 | 0.807895 | -0.141406 | 294 | 373.82 | 1.271497 |  |
| GO:0007417\_central\_nervous\_system\_development | PEX13 | 287 | 1 | 0.802265 | -0.139864 | 295 | 374.2 | 1.268475 |  |
| GO:0040011\_locomotion | PEX13 | 295 | 1 | 0.780508 | -0.133885 | 296 | 376.29 | 1.271250 |  |
| GO:0007154\_cell\_communication | D0H4S114 | 1096 | 4 | 0.840328 | -0.132564 | 297 | 376.45 | 1.267508 |  |
| GO:0007154\_cell\_communication | NPM1 | 1096 | 4 | 0.840328 | -0.132564 | 297 | 376.45 | 1.267508 |  |
| GO:0007154\_cell\_communication | VDAC3 | 1096 | 4 | 0.840328 | -0.132564 | 297 | 376.45 | 1.267508 |  |
| GO:0007154\_cell\_communication | DVL1 | 1096 | 4 | 0.840328 | -0.132564 | 297 | 376.45 | 1.267508 |  |
| GO:0032502\_developmental\_process | ODC1 | 2060 | 8 | 0.894175 | -0.130338 | 298 | 377.04 | 1.265235 |  |
| GO:0032502\_developmental\_process | RNF7 | 2060 | 8 | 0.894175 | -0.130338 | 298 | 377.04 | 1.265235 |  |
| GO:0032502\_developmental\_process | GMNN | 2060 | 8 | 0.894175 | -0.130338 | 298 | 377.04 | 1.265235 |  |
| GO:0032502\_developmental\_process | SMARCC1 | 2060 | 8 | 0.894175 | -0.130338 | 298 | 377.04 | 1.265235 |  |
| GO:0032502\_developmental\_process | ARF6 | 2060 | 8 | 0.894175 | -0.130338 | 298 | 377.04 | 1.265235 |  |
| GO:0032502\_developmental\_process | PEX13 | 2060 | 8 | 0.894175 | -0.130338 | 298 | 377.04 | 1.265235 |  |
| GO:0032502\_developmental\_process | FZD5 | 2060 | 8 | 0.894175 | -0.130338 | 298 | 377.04 | 1.265235 |  |
| GO:0032502\_developmental\_process | DVL1 | 2060 | 8 | 0.894175 | -0.130338 | 298 | 377.04 | 1.265235 |  |
| GO:0051252\_regulation\_of\_RNA\_metabolic\_process | SIN3B | 590 | 2 | 0.780508 | -0.126774 | 299 | 377.87 | 1.263779 |  |
| GO:0051252\_regulation\_of\_RNA\_metabolic\_process | NPM1 | 590 | 2 | 0.780508 | -0.126774 | 299 | 377.87 | 1.263779 |  |
| GO:0032989\_cellular\_component\_morphogenesis | DVL1 | 307 | 1 | 0.750000 | -0.125444 | 300 | 378.95 | 1.263167 |  |
| GO:0051094\_positive\_regulation\_of\_developmental\_process | RNF7 | 308 | 1 | 0.747565 | -0.124768 | 301 | 379.72 | 1.261528 |  |
| GO:0016310\_phosphorylation | NPM1 | 309 | 1 | 0.745146 | -0.124095 | 302 | 380.76 | 1.260795 |  |
| GO:0007166\_cell\_surface\_receptor\_linked\_signal\_transduction | D0H4S114 | 597 | 2 | 0.771357 | -0.123176 | 303 | 381.31 | 1.258449 |  |
| GO:0007166\_cell\_surface\_receptor\_linked\_signal\_transduction | DVL1 | 597 | 2 | 0.771357 | -0.123176 | 303 | 381.31 | 1.258449 |  |
| GO:0048731\_system\_development | ODC1 | 1609 | 6 | 0.858608 | -0.122990 | 304 | 381.49 | 1.254901 |  |
| GO:0048731\_system\_development | GMNN | 1609 | 6 | 0.858608 | -0.122990 | 304 | 381.49 | 1.254901 |  |
| GO:0048731\_system\_development | SMARCC1 | 1609 | 6 | 0.858608 | -0.122990 | 304 | 381.49 | 1.254901 |  |
| GO:0048731\_system\_development | ARF6 | 1609 | 6 | 0.858608 | -0.122990 | 304 | 381.49 | 1.254901 |  |
| GO:0048731\_system\_development | PEX13 | 1609 | 6 | 0.858608 | -0.122990 | 304 | 381.49 | 1.254901 |  |
| GO:0048731\_system\_development | DVL1 | 1609 | 6 | 0.858608 | -0.122990 | 304 | 381.49 | 1.254901 |  |
| GO:0007399\_nervous\_system\_development | PEX13 | 621 | 2 | 0.741546 | -0.111577 | 305 | 382.07 | 1.252689 |  |
| GO:0007399\_nervous\_system\_development | DVL1 | 621 | 2 | 0.741546 | -0.111577 | 305 | 382.07 | 1.252689 |  |
| GO:0006928\_cell\_motion | PEX13 | 330 | 1 | 0.697727 | -0.110861 | 307 | 383.4 | 1.248860 |  |
| GO:0051674\_localization\_of\_cell | PEX13 | 330 | 1 | 0.697727 | -0.110861 | 307 | 383.4 | 1.248860 |  |
| GO:0051093\_negative\_regulation\_of\_developmental\_process | RNF7 | 331 | 1 | 0.695619 | -0.110271 | 308 | 384.13 | 1.247175 |  |
| GO:0048522\_positive\_regulation\_of\_cellular\_process | ODC1 | 895 | 3 | 0.771788 | -0.109819 | 309 | 384.36 | 1.243883 |  |
| GO:0048522\_positive\_regulation\_of\_cellular\_process | RNF7 | 895 | 3 | 0.771788 | -0.109819 | 309 | 384.36 | 1.243883 |  |
| GO:0048522\_positive\_regulation\_of\_cellular\_process | NPM1 | 895 | 3 | 0.771788 | -0.109819 | 309 | 384.36 | 1.243883 |  |
| GO:0010467\_gene\_expression | SIN3B | 905 | 3 | 0.763260 | -0.105902 | 310 | 385.59 | 1.243839 |  |
| GO:0010467\_gene\_expression | NPM1 | 905 | 3 | 0.763260 | -0.105902 | 310 | 385.59 | 1.243839 |  |
| GO:0010467\_gene\_expression | MXD3 | 905 | 3 | 0.763260 | -0.105902 | 310 | 385.59 | 1.243839 |  |
| GO:0008152\_metabolic\_process | STT3B | 2133 | 8 | 0.863572 | -0.105148 | 311 | 386.03 | 1.241254 |  |
| GO:0008152\_metabolic\_process | SIN3B | 2133 | 8 | 0.863572 | -0.105148 | 311 | 386.03 | 1.241254 |  |
| GO:0008152\_metabolic\_process | GMNN | 2133 | 8 | 0.863572 | -0.105148 | 311 | 386.03 | 1.241254 |  |
| GO:0008152\_metabolic\_process | NPM1 | 2133 | 8 | 0.863572 | -0.105148 | 311 | 386.03 | 1.241254 |  |
| GO:0008152\_metabolic\_process | PEX13 | 2133 | 8 | 0.863572 | -0.105148 | 311 | 386.03 | 1.241254 |  |
| GO:0008152\_metabolic\_process | MXD3 | 2133 | 8 | 0.863572 | -0.105148 | 311 | 386.03 | 1.241254 |  |
| GO:0008152\_metabolic\_process | DVL1 | 2133 | 8 | 0.863572 | -0.105148 | 311 | 386.03 | 1.241254 |  |
| GO:0008152\_metabolic\_process | CDT1 | 2133 | 8 | 0.863572 | -0.105148 | 311 | 386.03 | 1.241254 |  |
| GO:0006793\_phosphorus\_metabolic\_process | NPM1 | 340 | 1 | 0.677206 | -0.105108 | 313 | 386.75 | 1.235623 |  |
| GO:0006796\_phosphate\_metabolic\_process | NPM1 | 340 | 1 | 0.677206 | -0.105108 | 313 | 386.75 | 1.235623 |  |
| GO:0065007\_biological\_regulation | ODC1 | 2593 | 10 | 0.887968 | -0.103687 | 314 | 387.35 | 1.233599 |  |
| GO:0065007\_biological\_regulation | D0H4S114 | 2593 | 10 | 0.887968 | -0.103687 | 314 | 387.35 | 1.233599 |  |
| GO:0065007\_biological\_regulation | SIN3B | 2593 | 10 | 0.887968 | -0.103687 | 314 | 387.35 | 1.233599 |  |
| GO:0065007\_biological\_regulation | RNF7 | 2593 | 10 | 0.887968 | -0.103687 | 314 | 387.35 | 1.233599 |  |
| GO:0065007\_biological\_regulation | GMNN | 2593 | 10 | 0.887968 | -0.103687 | 314 | 387.35 | 1.233599 |  |
| GO:0065007\_biological\_regulation | NPM1 | 2593 | 10 | 0.887968 | -0.103687 | 314 | 387.35 | 1.233599 |  |
| GO:0065007\_biological\_regulation | MXD3 | 2593 | 10 | 0.887968 | -0.103687 | 314 | 387.35 | 1.233599 |  |
| GO:0065007\_biological\_regulation | DVL1 | 2593 | 10 | 0.887968 | -0.103687 | 314 | 387.35 | 1.233599 |  |
| GO:0065007\_biological\_regulation | H19 | 2593 | 10 | 0.887968 | -0.103687 | 314 | 387.35 | 1.233599 |  |
| GO:0065007\_biological\_regulation | CDT1 | 2593 | 10 | 0.887968 | -0.103687 | 314 | 387.35 | 1.233599 |  |
| GO:0044238\_primary\_metabolic\_process | STT3B | 1905 | 7 | 0.846063 | -0.103256 | 315 | 387.5 | 1.230159 |  |
| GO:0044238\_primary\_metabolic\_process | SIN3B | 1905 | 7 | 0.846063 | -0.103256 | 315 | 387.5 | 1.230159 |  |
| GO:0044238\_primary\_metabolic\_process | GMNN | 1905 | 7 | 0.846063 | -0.103256 | 315 | 387.5 | 1.230159 |  |
| GO:0044238\_primary\_metabolic\_process | NPM1 | 1905 | 7 | 0.846063 | -0.103256 | 315 | 387.5 | 1.230159 |  |
| GO:0044238\_primary\_metabolic\_process | PEX13 | 1905 | 7 | 0.846063 | -0.103256 | 315 | 387.5 | 1.230159 |  |
| GO:0044238\_primary\_metabolic\_process | MXD3 | 1905 | 7 | 0.846063 | -0.103256 | 315 | 387.5 | 1.230159 |  |
| GO:0044238\_primary\_metabolic\_process | CDT1 | 1905 | 7 | 0.846063 | -0.103256 | 315 | 387.5 | 1.230159 |  |
| GO:0009887\_organ\_morphogenesis | GMNN | 642 | 2 | 0.717290 | -0.102298 | 316 | 387.79 | 1.227184 |  |
| GO:0009887\_organ\_morphogenesis | SMARCC1 | 642 | 2 | 0.717290 | -0.102298 | 316 | 387.79 | 1.227184 |  |
| GO:0007165\_signal\_transduction | D0H4S114 | 915 | 3 | 0.754918 | -0.102111 | 317 | 388.0 | 1.223975 |  |
| GO:0007165\_signal\_transduction | NPM1 | 915 | 3 | 0.754918 | -0.102111 | 317 | 388.0 | 1.223975 |  |
| GO:0007165\_signal\_transduction | DVL1 | 915 | 3 | 0.754918 | -0.102111 | 317 | 388.0 | 1.223975 |  |
| GO:0030182\_neuron\_differentiation | DVL1 | 356 | 1 | 0.646770 | -0.096562 | 318 | 390.28 | 1.227296 |  |
| GO:0048856\_anatomical\_structure\_development | ODC1 | 1688 | 6 | 0.818424 | -0.096473 | 319 | 390.44 | 1.223950 |  |
| GO:0048856\_anatomical\_structure\_development | GMNN | 1688 | 6 | 0.818424 | -0.096473 | 319 | 390.44 | 1.223950 |  |
| GO:0048856\_anatomical\_structure\_development | SMARCC1 | 1688 | 6 | 0.818424 | -0.096473 | 319 | 390.44 | 1.223950 |  |
| GO:0048856\_anatomical\_structure\_development | ARF6 | 1688 | 6 | 0.818424 | -0.096473 | 319 | 390.44 | 1.223950 |  |
| GO:0048856\_anatomical\_structure\_development | PEX13 | 1688 | 6 | 0.818424 | -0.096473 | 319 | 390.44 | 1.223950 |  |
| GO:0048856\_anatomical\_structure\_development | DVL1 | 1688 | 6 | 0.818424 | -0.096473 | 319 | 390.44 | 1.223950 |  |
| GO:0016070\_RNA\_metabolic\_process | SIN3B | 658 | 2 | 0.699848 | -0.095729 | 320 | 390.7 | 1.220938 |  |
| GO:0016070\_RNA\_metabolic\_process | NPM1 | 658 | 2 | 0.699848 | -0.095729 | 320 | 390.7 | 1.220938 |  |
| GO:0042981\_regulation\_of\_apoptosis | RNF7 | 360 | 1 | 0.639583 | -0.094545 | 321 | 391.34 | 1.219128 |  |
| GO:0010941\_regulation\_of\_cell\_death | RNF7 | 365 | 1 | 0.630822 | -0.092085 | 323 | 392.48 | 1.215108 |  |
| GO:0043067\_regulation\_of\_programmed\_cell\_death | RNF7 | 365 | 1 | 0.630822 | -0.092085 | 323 | 392.48 | 1.215108 |  |
| GO:0045449\_regulation\_of\_transcription | SIN3B | 676 | 2 | 0.681213 | -0.088821 | 324 | 393.73 | 1.215216 |  |
| GO:0045449\_regulation\_of\_transcription | MXD3 | 676 | 2 | 0.681213 | -0.088821 | 324 | 393.73 | 1.215216 |  |
| GO:0009653\_anatomical\_structure\_morphogenesis | GMNN | 958 | 3 | 0.721033 | -0.087163 | 325 | 394.03 | 1.212400 |  |
| GO:0009653\_anatomical\_structure\_morphogenesis | SMARCC1 | 958 | 3 | 0.721033 | -0.087163 | 325 | 394.03 | 1.212400 |  |
| GO:0009653\_anatomical\_structure\_morphogenesis | DVL1 | 958 | 3 | 0.721033 | -0.087163 | 325 | 394.03 | 1.212400 |  |
| GO:0044237\_cellular\_metabolic\_process | STT3B | 1974 | 7 | 0.816489 | -0.083252 | 326 | 395.45 | 1.213037 |  |
| GO:0044237\_cellular\_metabolic\_process | SIN3B | 1974 | 7 | 0.816489 | -0.083252 | 326 | 395.45 | 1.213037 |  |
| GO:0044237\_cellular\_metabolic\_process | GMNN | 1974 | 7 | 0.816489 | -0.083252 | 326 | 395.45 | 1.213037 |  |
| GO:0044237\_cellular\_metabolic\_process | NPM1 | 1974 | 7 | 0.816489 | -0.083252 | 326 | 395.45 | 1.213037 |  |
| GO:0044237\_cellular\_metabolic\_process | PEX13 | 1974 | 7 | 0.816489 | -0.083252 | 326 | 395.45 | 1.213037 |  |
| GO:0044237\_cellular\_metabolic\_process | MXD3 | 1974 | 7 | 0.816489 | -0.083252 | 326 | 395.45 | 1.213037 |  |
| GO:0044237\_cellular\_metabolic\_process | CDT1 | 1974 | 7 | 0.816489 | -0.083252 | 326 | 395.45 | 1.213037 |  |
| GO:0065008\_regulation\_of\_biological\_quality | NPM1 | 693 | 2 | 0.664502 | -0.082734 | 327 | 395.66 | 1.209969 |  |
| GO:0065008\_regulation\_of\_biological\_quality | DVL1 | 693 | 2 | 0.664502 | -0.082734 | 327 | 395.66 | 1.209969 |  |
| GO:0031328\_positive\_regulation\_of\_cellular\_biosynthetic\_process | NPM1 | 387 | 1 | 0.594961 | -0.082045 | 328 | 396.09 | 1.207591 |  |
| GO:0009891\_positive\_regulation\_of\_biosynthetic\_process | NPM1 | 388 | 1 | 0.593428 | -0.081617 | 329 | 396.37 | 1.204772 |  |
| GO:0006350\_transcription | SIN3B | 701 | 2 | 0.656919 | -0.080008 | 330 | 396.88 | 1.202667 |  |
| GO:0006350\_transcription | MXD3 | 701 | 2 | 0.656919 | -0.080008 | 330 | 396.88 | 1.202667 |  |
| GO:0007275\_multicellular\_organismal\_development | ODC1 | 1760 | 6 | 0.784943 | -0.076527 | 331 | 397.97 | 1.202326 |  |
| GO:0007275\_multicellular\_organismal\_development | GMNN | 1760 | 6 | 0.784943 | -0.076527 | 331 | 397.97 | 1.202326 |  |
| GO:0007275\_multicellular\_organismal\_development | SMARCC1 | 1760 | 6 | 0.784943 | -0.076527 | 331 | 397.97 | 1.202326 |  |
| GO:0007275\_multicellular\_organismal\_development | ARF6 | 1760 | 6 | 0.784943 | -0.076527 | 331 | 397.97 | 1.202326 |  |
| GO:0007275\_multicellular\_organismal\_development | PEX13 | 1760 | 6 | 0.784943 | -0.076527 | 331 | 397.97 | 1.202326 |  |
| GO:0007275\_multicellular\_organismal\_development | DVL1 | 1760 | 6 | 0.784943 | -0.076527 | 331 | 397.97 | 1.202326 |  |
| GO:0048518\_positive\_regulation\_of\_biological\_process | ODC1 | 995 | 3 | 0.694221 | -0.075905 | 332 | 398.14 | 1.199217 |  |
| GO:0048518\_positive\_regulation\_of\_biological\_process | RNF7 | 995 | 3 | 0.694221 | -0.075905 | 332 | 398.14 | 1.199217 |  |
| GO:0048518\_positive\_regulation\_of\_biological\_process | NPM1 | 995 | 3 | 0.694221 | -0.075905 | 332 | 398.14 | 1.199217 |  |
| GO:0007242\_intracellular\_signaling\_cascade | NPM1 | 411 | 1 | 0.560219 | -0.072392 | 333 | 399.19 | 1.198769 |  |
| GO:0042592\_homeostatic\_process | NPM1 | 419 | 1 | 0.549523 | -0.069444 | 334 | 399.95 | 1.197455 |  |
| GO:0006464\_protein\_modification\_process | STT3B | 439 | 1 | 0.524487 | -0.062605 | 335 | 402.14 | 1.200418 |  |
| GO:0031325\_positive\_regulation\_of\_cellular\_metabolic\_process | NPM1 | 442 | 1 | 0.520928 | -0.061641 | 336 | 402.53 | 1.198006 |  |
| GO:0030154\_cell\_differentiation | PEX13 | 1060 | 3 | 0.651651 | -0.059233 | 337 | 404.08 | 1.199050 |  |
| GO:0030154\_cell\_differentiation | FZD5 | 1060 | 3 | 0.651651 | -0.059233 | 337 | 404.08 | 1.199050 |  |
| GO:0030154\_cell\_differentiation | DVL1 | 1060 | 3 | 0.651651 | -0.059233 | 337 | 404.08 | 1.199050 |  |
| GO:0009893\_positive\_regulation\_of\_metabolic\_process | NPM1 | 458 | 1 | 0.502729 | -0.056748 | 339 | 405.25 | 1.195428 |  |
| GO:0043412\_biopolymer\_modification | STT3B | 458 | 1 | 0.502729 | -0.056748 | 339 | 405.25 | 1.195428 |  |
| GO:0050896\_response\_to\_stimulus | NPM1 | 1107 | 3 | 0.623984 | -0.049299 | 340 | 406.62 | 1.195941 |  |
| GO:0050896\_response\_to\_stimulus | PEX13 | 1107 | 3 | 0.623984 | -0.049299 | 340 | 406.62 | 1.195941 |  |
| GO:0050896\_response\_to\_stimulus | VDAC3 | 1107 | 3 | 0.623984 | -0.049299 | 340 | 406.62 | 1.195941 |  |
| GO:0048869\_cellular\_developmental\_process | PEX13 | 1113 | 3 | 0.620620 | -0.048144 | 341 | 407.15 | 1.193988 |  |
| GO:0048869\_cellular\_developmental\_process | FZD5 | 1113 | 3 | 0.620620 | -0.048144 | 341 | 407.15 | 1.193988 |  |
| GO:0048869\_cellular\_developmental\_process | DVL1 | 1113 | 3 | 0.620620 | -0.048144 | 341 | 407.15 | 1.193988 |  |
| GO:0002376\_immune\_system\_process | SLA2 | 505 | 1 | 0.455941 | -0.044538 | 342 | 407.58 | 1.191754 |  |
| GO:0032501\_multicellular\_organismal\_process | ODC1 | 2183 | 7 | 0.738319 | -0.040612 | 343 | 408.33 | 1.190466 |  |
| GO:0032501\_multicellular\_organismal\_process | GMNN | 2183 | 7 | 0.738319 | -0.040612 | 343 | 408.33 | 1.190466 |  |
| GO:0032501\_multicellular\_organismal\_process | SMARCC1 | 2183 | 7 | 0.738319 | -0.040612 | 343 | 408.33 | 1.190466 |  |
| GO:0032501\_multicellular\_organismal\_process | ARF6 | 2183 | 7 | 0.738319 | -0.040612 | 343 | 408.33 | 1.190466 |  |
| GO:0032501\_multicellular\_organismal\_process | PEX13 | 2183 | 7 | 0.738319 | -0.040612 | 343 | 408.33 | 1.190466 |  |
| GO:0032501\_multicellular\_organismal\_process | VDAC3 | 2183 | 7 | 0.738319 | -0.040612 | 343 | 408.33 | 1.190466 |  |
| GO:0032501\_multicellular\_organismal\_process | DVL1 | 2183 | 7 | 0.738319 | -0.040612 | 343 | 408.33 | 1.190466 |  |
| GO:0009888\_tissue\_development | DVL1 | 525 | 1 | 0.438571 | -0.040181 | 344 | 408.52 | 1.187558 |  |
| GO:0044267\_cellular\_protein\_metabolic\_process | STT3B | 559 | 1 | 0.411896 | -0.033727 | 345 | 410.0 | 1.188406 |  |
| GO:0006355\_regulation\_of\_transcription\_\_DNA-dependent | SIN3B | 575 | 1 | 0.400435 | -0.031057 | 346 | 410.97 | 1.187775 |  |
| GO:0006351\_transcription\_\_DNA-dependent | SIN3B | 594 | 1 | 0.387626 | -0.028157 | 347 | 411.75 | 1.186599 |  |
| GO:0032774\_RNA\_biosynthetic\_process | SIN3B | 595 | 1 | 0.386975 | -0.028012 | 348 | 411.89 | 1.183592 |  |
| GO:0019538\_protein\_metabolic\_process | STT3B | 655 | 1 | 0.351527 | -0.020529 | 349 | 413.39 | 1.184499 |  |
| GO:0050793\_regulation\_of\_developmental\_process | RNF7 | 703 | 1 | 0.327525 | -0.015983 | 350 | 414.82 | 1.185200 |  |
| GO:0008150\_biological\_process | ODC1 | 4605 | 20 | 1.000000 | 0.000000 | 2059 | 2090.14 | 1.015124 |  |
| GO:0008150\_biological\_process | SLC38A2 | 4605 | 20 | 1.000000 | 0.000000 | 2059 | 2090.14 | 1.015124 |  |
| GO:0008150\_biological\_process | GMNN | 4605 | 20 | 1.000000 | 0.000000 | 2059 | 2090.14 | 1.015124 |  |
| GO:0008150\_biological\_process | SLA2 | 4605 | 20 | 1.000000 | 0.000000 | 2059 | 2090.14 | 1.015124 |  |
| GO:0008150\_biological\_process | EIF5A | 4605 | 20 | 1.000000 | 0.000000 | 2059 | 2090.14 | 1.015124 |  |
| GO:0008150\_biological\_process | ARF6 | 4605 | 20 | 1.000000 | 0.000000 | 2059 | 2090.14 | 1.015124 |  |
| GO:0008150\_biological\_process | FZD5 | 4605 | 20 | 1.000000 | 0.000000 | 2059 | 2090.14 | 1.015124 |  |
| GO:0008150\_biological\_process | VDAC3 | 4605 | 20 | 1.000000 | 0.000000 | 2059 | 2090.14 | 1.015124 |  |
| GO:0008150\_biological\_process | MXD3 | 4605 | 20 | 1.000000 | 0.000000 | 2059 | 2090.14 | 1.015124 |  |
| GO:0008150\_biological\_process | H19 | 4605 | 20 | 1.000000 | 0.000000 | 2059 | 2090.14 | 1.015124 |  |
| GO:0008150\_biological\_process | CDT1 | 4605 | 20 | 1.000000 | 0.000000 | 2059 | 2090.14 | 1.015124 |  |
| GO:0008150\_biological\_process | DVL1 | 4605 | 20 | 1.000000 | 0.000000 | 2059 | 2090.14 | 1.015124 |  |
| GO:0008150\_biological\_process | STT3B | 4605 | 20 | 1.000000 | 0.000000 | 2059 | 2090.14 | 1.015124 |  |
| GO:0008150\_biological\_process | SIN3B | 4605 | 20 | 1.000000 | 0.000000 | 2059 | 2090.14 | 1.015124 |  |
| GO:0008150\_biological\_process | D0H4S114 | 4605 | 20 | 1.000000 | 0.000000 | 2059 | 2090.14 | 1.015124 |  |
| GO:0008150\_biological\_process | RNF7 | 4605 | 20 | 1.000000 | 0.000000 | 2059 | 2090.14 | 1.015124 |  |
| GO:0008150\_biological\_process | SMARCC1 | 4605 | 20 | 1.000000 | 0.000000 | 2059 | 2090.14 | 1.015124 |  |
| GO:0008150\_biological\_process | NPM1 | 4605 | 20 | 1.000000 | 0.000000 | 2059 | 2090.14 | 1.015124 |  |
| GO:0008150\_biological\_process | PEX13 | 4605 | 20 | 1.000000 | 0.000000 | 2059 | 2090.14 | 1.015124 |  |
| GO:0008150\_biological\_process | HSPA9 | 4605 | 20 | 1.000000 | 0.000000 | 2059 | 2090.14 | 1.015124 |  |
