# Supplementary material for: RedundancyMiner: De-replication of redundant GO categories in microarray and proteomics analysis
Source: BMC Bioinformatics. 2011 Feb 10;12:52. doi: 10.1186/1471-2105-12-52 (PMC3223614; doi:10.1186/1471-2105-12-52)
Supplement: Additional file 8 — Retinal development HTGM download. compressed package of the results of running HTGM on the retinal development genes list. [file 1471-2105-12-52-S8.ZIP › SCENARIO_2_MODIFIED/total.txt.total.txt.dir/Exp1_BestClusterMap_LEIGS_KM_24.csv.join.5.txt.dir/Exp1_BestClusterMap_LEIGS_KM_24.csv.join.5.txt.change.html]

Category Summary Report for Exp1\_BestClusterMap\_LEIGS\_KM\_24.csv.join.5.txt

# Category Summary Report for Exp1\_BestClusterMap\_LEIGS\_KM\_24.csv.join.5.txt

| HYPERLINKED GO CATEGORY | TOTAL GENES | CHANGED GENES | ENRICHMENT | LOG10(p) | CUMULATIVE NUMBER OF CATEGORIES | CUMULATIVE RANDOMS LOWER BOUND | CUMULATIVE RANDOMS MEAN | CUMULATIVE RANDOMS UPPER BOUND | FALSE DISCOVERY RATE |  |
| --- | --- | --- | --- | --- | --- | --- | --- | --- | --- | --- |
| GO:0008156\_negative\_regulation\_of\_DNA\_replication | 6 | 2 | 76.750000 | -3.575024 | 1 | -0.369810 | 0.15 | 0.669810 | 0.150000 |  |
| GO:0006275\_regulation\_of\_DNA\_replication | 7 | 2 | 65.785714 | -3.430029 | 2 | -0.445898 | 0.21 | 0.865898 | 0.105000 |  |
| GO:0051053\_negative\_regulation\_of\_DNA\_metabolic\_process | 14 | 2 | 32.892857 | -2.801132 | 3 | -1.047822 | 0.92 | 2.887822 | 0.306667 |  |
| GO:0051168\_nuclear\_export | 18 | 2 | 25.583333 | -2.580006 | 4 | -0.919076 | 1.52 | 3.959076 | 0.380000 |  |
| GO:0000054\_ribosome\_export\_from\_nucleus | 1 | 1 |  |  |  |  |  |  |  |  |
| GO:0000055\_ribosomal\_large\_subunit\_export\_from\_nucleus | 1 | 1 |  |  |  |  |  |  |  |  |
| GO:0000056\_ribosomal\_small\_subunit\_export\_from\_nucleus | 1 | 1 |  |  |  |  |  |  |  |  |
| GO:0006407\_rRNA\_export\_from\_nucleus | 1 | 1 |  |  |  |  |  |  |  |  |
| GO:0010825\_positive\_regulation\_of\_centrosome\_duplication | 1 | 1 |  |  |  |  |  |  |  |  |
| GO:0016560\_protein\_import\_into\_peroxisome\_matrix\_\_docking | 1 | 1 |  |  |  |  |  |  |  |  |
| GO:0033750\_ribosome\_localization | 1 | 1 |  |  |  |  |  |  |  |  |
| GO:0033753\_establishment\_of\_ribosome\_localization | 1 | 1 |  |  |  |  |  |  |  |  |
| GO:0051029\_rRNA\_transport | 1 | 1 |  |  |  |  |  |  |  |  |
| GO:0060151\_peroxisome\_localization | 1 | 1 |  |  |  |  |  |  |  |  |
| GO:0060152\_microtubule-based\_peroxisome\_localization | 1 | 1 |  |  |  |  |  |  |  |  |
| GO:0045934\_negative\_regulation\_of\_nucleobase\_\_nucleoside\_\_nucleotide\_and\_nucleic\_acid\_metabolic\_process | 270 | 5 | 4.263889 | -2.301564 | 5 | -0.655730 | 2.72 | 6.095730 | 0.544000 |  |
| GO:0051172\_negative\_regulation\_of\_nitrogen\_compound\_metabolic\_process | 271 | 5 | 4.248155 | -2.294690 | 6 | -0.635887 | 2.78 | 6.195887 | 0.463333 |  |
| GO:0010564\_regulation\_of\_cell\_cycle\_process | 29 | 2 | 15.879310 | -2.168598 | 7 | -0.333521 | 3.57 | 7.473521 | 0.510000 |  |
| GO:0051640\_organelle\_localization | 31 | 2 | 14.854839 | -2.111928 | 8 | -0.198542 | 4.11 | 8.418542 | 0.513750 |  |
| GO:0000076\_DNA\_replication\_checkpoint | 2 | 1 |  |  |  |  |  |  |  |  |
| GO:0001561\_fatty\_acid\_alpha-oxidation | 2 | 1 |  |  |  |  |  |  |  |  |
| GO:0010824\_regulation\_of\_centrosome\_duplication | 2 | 1 |  |  |  |  |  |  |  |  |
| GO:0016558\_protein\_import\_into\_peroxisome\_matrix | 2 | 1 |  |  |  |  |  |  |  |  |
| GO:0030174\_regulation\_of\_DNA\_replication\_initiation | 2 | 1 |  |  |  |  |  |  |  |  |
| GO:0032297\_negative\_regulation\_of\_DNA\_replication\_initiation | 2 | 1 |  |  |  |  |  |  |  |  |
| GO:0033119\_negative\_regulation\_of\_RNA\_splicing | 2 | 1 |  |  |  |  |  |  |  |  |
| GO:0042274\_ribosomal\_small\_subunit\_biogenesis | 2 | 1 |  |  |  |  |  |  |  |  |
| GO:0043516\_regulation\_of\_DNA\_damage\_response\_\_signal\_transduction\_by\_p53\_class\_mediator | 2 | 1 |  |  |  |  |  |  |  |  |
| GO:0048025\_negative\_regulation\_of\_nuclear\_mRNA\_splicing\_\_via\_spliceosome | 2 | 1 |  |  |  |  |  |  |  |  |
| GO:0050686\_negative\_regulation\_of\_mRNA\_processing | 2 | 1 |  |  |  |  |  |  |  |  |
| GO:0051052\_regulation\_of\_DNA\_metabolic\_process | 34 | 2 | 13.544118 | -2.033802 | 9 | 0.090526 | 4.86 | 9.629474 | 0.540000 |  |
| GO:0010605\_negative\_regulation\_of\_macromolecule\_metabolic\_process | 331 | 5 | 3.478097 | -1.930450 | 10 | 0.511263 | 5.66 | 10.808737 | 0.566000 |  |
| GO:0031324\_negative\_regulation\_of\_cellular\_metabolic\_process | 332 | 5 | 3.467620 | -1.925079 | 11 | 0.532491 | 5.69 | 10.847509 | 0.517273 |  |
| GO:0000084\_S\_phase\_of\_mitotic\_cell\_cycle | 3 | 1 |  |  |  |  |  |  |  |  |
| GO:0006625\_protein\_targeting\_to\_peroxisome | 3 | 1 |  |  |  |  |  |  |  |  |
| GO:0007090\_regulation\_of\_S\_phase\_of\_mitotic\_cell\_cycle | 3 | 1 |  |  |  |  |  |  |  |  |
| GO:0018196\_peptidyl-asparagine\_modification | 3 | 1 |  |  |  |  |  |  |  |  |
| GO:0018279\_protein\_amino\_acid\_N-linked\_glycosylation\_via\_asparagine | 3 | 1 |  |  |  |  |  |  |  |  |
| GO:0033261\_regulation\_of\_S\_phase | 3 | 1 |  |  |  |  |  |  |  |  |
| GO:0042273\_ribosomal\_large\_subunit\_biogenesis | 3 | 1 |  |  |  |  |  |  |  |  |
| GO:0043574\_peroxisomal\_transport | 3 | 1 |  |  |  |  |  |  |  |  |
| GO:0046605\_regulation\_of\_centrosome\_cycle | 3 | 1 |  |  |  |  |  |  |  |  |
| GO:0051320\_S\_phase | 3 | 1 |  |  |  |  |  |  |  |  |
| GO:0006260\_DNA\_replication | 41 | 2 | 11.231707 | -1.876839 | 12 | 1.096027 | 6.59 | 12.083973 | 0.549167 |  |
| GO:0009892\_negative\_regulation\_of\_metabolic\_process | 348 | 5 | 3.308190 | -1.841794 | 13 | 1.290116 | 7.01 | 12.729884 | 0.539231 |  |
| GO:0051726\_regulation\_of\_cell\_cycle | 121 | 3 | 5.708678 | -1.836999 | 14 | 1.440111 | 7.26 | 13.079889 | 0.518571 |  |
| GO:0043623\_cellular\_protein\_complex\_assembly | 45 | 2 | 10.233333 | -1.799521 | 15 | 1.511089 | 7.9 | 14.288911 | 0.526667 |  |
| GO:0007031\_peroxisome\_organization | 4 | 1 |  |  |  |  |  |  |  |  |
| GO:0043484\_regulation\_of\_RNA\_splicing | 4 | 1 |  |  |  |  |  |  |  |  |
| GO:0048024\_regulation\_of\_nuclear\_mRNA\_splicing\_\_via\_spliceosome | 4 | 1 |  |  |  |  |  |  |  |  |
| GO:0008104\_protein\_localization | 251 | 4 | 3.669323 | -1.679652 | 16 | 1.877888 | 9.59 | 17.302112 | 0.599375 |  |
| GO:0006270\_DNA\_replication\_initiation | 5 | 1 | 46.050000 | -1.666815 | 19 | 5.563160 | 15.74 | 25.916840 | 0.828421 |  |
| GO:0008631\_induction\_of\_apoptosis\_by\_oxidative\_stress | 5 | 1 | 46.050000 | -1.666815 | 19 | 5.563160 | 15.74 | 25.916840 | 0.828421 |  |
| GO:0051298\_centrosome\_duplication | 5 | 1 | 46.050000 | -1.666815 | 19 | 5.563160 | 15.74 | 25.916840 | 0.828421 |  |
| GO:0034622\_cellular\_macromolecular\_complex\_assembly | 58 | 2 | 7.939655 | -1.591496 | 20 | 6.350367 | 17.58 | 28.809633 | 0.879000 |  |
| GO:0050684\_regulation\_of\_mRNA\_processing | 6 | 1 | 38.375000 | -1.588529 | 21 | 9.207432 | 21.93 | 34.652568 | 1.044286 |  |
| GO:0010558\_negative\_regulation\_of\_macromolecule\_biosynthetic\_process | 274 | 4 | 3.361314 | -1.554639 | 23 | 9.445740 | 22.43 | 35.414260 | 0.975217 |  |
| GO:0033036\_macromolecule\_localization | 274 | 4 | 3.361314 | -1.554639 | 23 | 9.445740 | 22.43 | 35.414260 | 0.975217 |  |
| GO:0001967\_suckling\_behavior | 7 | 1 | 32.892857 | -1.522477 | 25 | 13.315815 | 28.68 | 44.044185 | 1.147200 |  |
| GO:0006884\_cell\_volume\_homeostasis | 7 | 1 | 32.892857 | -1.522477 | 25 | 13.315815 | 28.68 | 44.044185 | 1.147200 |  |
| GO:0009987\_cellular\_process | 3868 | 20 | 1.190538 | -1.518288 | 26 | 13.346095 | 28.75 | 44.153905 | 1.105769 |  |
| GO:0031327\_negative\_regulation\_of\_cellular\_biosynthetic\_process | 282 | 4 | 3.265957 | -1.514177 | 27 | 13.374835 | 28.82 | 44.265165 | 1.067407 |  |
| GO:0009890\_negative\_regulation\_of\_biosynthetic\_process | 284 | 4 | 3.242958 | -1.504286 | 28 | 13.478480 | 29.05 | 44.621520 | 1.037500 |  |
| GO:0007098\_centrosome\_cycle | 8 | 1 | 28.781250 | -1.465379 | 29 | 16.360054 | 33.97 | 51.579946 | 1.171379 |  |
| GO:0006913\_nucleocytoplasmic\_transport | 71 | 2 | 6.485915 | -1.429020 | 30 | 17.051985 | 34.98 | 52.908015 | 1.166000 |  |
| GO:0051169\_nuclear\_transport | 72 | 2 | 6.395833 | -1.417906 | 31 | 17.317813 | 35.39 | 53.462187 | 1.141613 |  |
| GO:0015031\_protein\_transport | 175 | 3 | 3.947143 | -1.417681 | 32 | 17.418418 | 35.53 | 53.641582 | 1.110312 |  |
| GO:0048523\_negative\_regulation\_of\_cellular\_process | 774 | 7 | 2.082364 | -1.416225 | 33 | 17.460894 | 35.62 | 53.779106 | 1.079394 |  |
| GO:0006611\_protein\_export\_from\_nucleus | 9 | 1 | 25.583333 | -1.415120 | 36 | 20.207697 | 40.29 | 60.372303 | 1.119167 |  |
| GO:0031023\_microtubule\_organizing\_center\_organization | 9 | 1 | 25.583333 | -1.415120 | 36 | 20.207697 | 40.29 | 60.372303 | 1.119167 |  |
| GO:0051297\_centrosome\_organization | 9 | 1 | 25.583333 | -1.415120 | 36 | 20.207697 | 40.29 | 60.372303 | 1.119167 |  |
| GO:0045184\_establishment\_of\_protein\_localization | 180 | 3 | 3.837500 | -1.386760 | 37 | 20.779744 | 40.99 | 61.200256 | 1.107838 |  |
| GO:0034621\_cellular\_macromolecular\_complex\_subunit\_organization | 76 | 2 | 6.059211 | -1.375099 | 38 | 21.055102 | 41.43 | 61.804898 | 1.090263 |  |
| GO:0006405\_RNA\_export\_from\_nucleus | 10 | 1 | 23.025000 | -1.370256 | 44 | 24.333013 | 46.34 | 68.346987 | 1.053182 |  |
| GO:0007172\_signal\_complex\_assembly | 10 | 1 | 23.025000 | -1.370256 | 44 | 24.333013 | 46.34 | 68.346987 | 1.053182 |  |
| GO:0043113\_receptor\_clustering | 10 | 1 | 23.025000 | -1.370256 | 44 | 24.333013 | 46.34 | 68.346987 | 1.053182 |  |
| GO:0050657\_nucleic\_acid\_transport | 10 | 1 | 23.025000 | -1.370256 | 44 | 24.333013 | 46.34 | 68.346987 | 1.053182 |  |
| GO:0050658\_RNA\_transport | 10 | 1 | 23.025000 | -1.370256 | 44 | 24.333013 | 46.34 | 68.346987 | 1.053182 |  |
| GO:0051236\_establishment\_of\_RNA\_localization | 10 | 1 | 23.025000 | -1.370256 | 44 | 24.333013 | 46.34 | 68.346987 | 1.053182 |  |
| GO:0006461\_protein\_complex\_assembly | 78 | 2 | 5.903846 | -1.354626 | 46 | 24.765507 | 46.97 | 69.174493 | 1.021087 |  |
| GO:0070271\_protein\_complex\_biogenesis | 78 | 2 | 5.903846 | -1.354626 | 46 | 24.765507 | 46.97 | 69.174493 | 1.021087 |  |
| GO:0007017\_microtubule-based\_process | 83 | 2 | 5.548193 | -1.305908 | 47 | 27.453351 | 51.81 | 76.166649 | 1.102340 |  |
| GO:0046907\_intracellular\_transport | 194 | 3 | 3.560567 | -1.305413 | 48 | 27.474618 | 51.86 | 76.245382 | 1.080417 |  |
| GO:0000375\_RNA\_splicing\_\_via\_transesterification\_reactions | 12 | 1 | 19.187500 | -1.292859 | 55 | 30.436934 | 56.4 | 82.363066 | 1.025455 |  |
| GO:0000377\_RNA\_splicing\_\_via\_transesterification\_reactions\_with\_bulged\_adenosine\_as\_nucleophile | 12 | 1 | 19.187500 | -1.292859 | 55 | 30.436934 | 56.4 | 82.363066 | 1.025455 |  |
| GO:0000398\_nuclear\_mRNA\_splicing\_\_via\_spliceosome | 12 | 1 | 19.187500 | -1.292859 | 55 | 30.436934 | 56.4 | 82.363066 | 1.025455 |  |
| GO:0001662\_behavioral\_fear\_response | 12 | 1 | 19.187500 | -1.292859 | 55 | 30.436934 | 56.4 | 82.363066 | 1.025455 |  |
| GO:0002209\_behavioral\_defense\_response | 12 | 1 | 19.187500 | -1.292859 | 55 | 30.436934 | 56.4 | 82.363066 | 1.025455 |  |
| GO:0006403\_RNA\_localization | 12 | 1 | 19.187500 | -1.292859 | 55 | 30.436934 | 56.4 | 82.363066 | 1.025455 |  |
| GO:0030330\_DNA\_damage\_response\_\_signal\_transduction\_by\_p53\_class\_mediator | 12 | 1 | 19.187500 | -1.292859 | 55 | 30.436934 | 56.4 | 82.363066 | 1.025455 |  |
| GO:0006605\_protein\_targeting | 86 | 2 | 5.354651 | -1.278234 | 56 | 30.804220 | 56.92 | 83.035780 | 1.016429 |  |
| GO:0051649\_establishment\_of\_localization\_in\_cell | 342 | 4 | 2.692982 | -1.251022 | 57 | 34.308461 | 61.48 | 88.651539 | 1.078596 |  |
| GO:0065003\_macromolecular\_complex\_assembly | 93 | 2 | 4.951613 | -1.217692 | 58 | 37.581206 | 66.05 | 94.518794 | 1.138793 |  |
| GO:0048519\_negative\_regulation\_of\_biological\_process | 859 | 7 | 1.876310 | -1.201161 | 59 | 37.777441 | 66.59 | 95.402559 | 1.128644 |  |
| GO:0006487\_protein\_amino\_acid\_N-linked\_glycosylation | 15 | 1 | 15.350000 | -1.198623 | 64 | 39.928802 | 69.9 | 99.871198 | 1.092188 |  |
| GO:0009062\_fatty\_acid\_catabolic\_process | 15 | 1 | 15.350000 | -1.198623 | 64 | 39.928802 | 69.9 | 99.871198 | 1.092188 |  |
| GO:0015931\_nucleobase\_\_nucleoside\_\_nucleotide\_and\_nucleic\_acid\_transport | 15 | 1 | 15.350000 | -1.198623 | 64 | 39.928802 | 69.9 | 99.871198 | 1.092188 |  |
| GO:0021795\_cerebral\_cortex\_cell\_migration | 15 | 1 | 15.350000 | -1.198623 | 64 | 39.928802 | 69.9 | 99.871198 | 1.092188 |  |
| GO:0070507\_regulation\_of\_microtubule\_cytoskeleton\_organization | 15 | 1 | 15.350000 | -1.198623 | 64 | 39.928802 | 69.9 | 99.871198 | 1.092188 |  |
| GO:0031570\_DNA\_integrity\_checkpoint | 16 | 1 | 14.390625 | -1.171484 | 66 | 42.667659 | 74.02 | 105.372341 | 1.121515 |  |
| GO:0042596\_fear\_response | 16 | 1 | 14.390625 | -1.171484 | 66 | 42.667659 | 74.02 | 105.372341 | 1.121515 |  |
| GO:0051641\_cellular\_localization | 370 | 4 | 2.489189 | -1.148104 | 67 | 43.228747 | 75.06 | 106.891253 | 1.120299 |  |
| GO:0006261\_DNA-dependent\_DNA\_replication | 17 | 1 | 13.544118 | -1.146045 | 76 | 46.023954 | 78.59 | 111.156046 | 1.034079 |  |
| GO:0006323\_DNA\_packaging | 17 | 1 | 13.544118 | -1.146045 | 76 | 46.023954 | 78.59 | 111.156046 | 1.034079 |  |
| GO:0006919\_activation\_of\_caspase\_activity | 17 | 1 | 13.544118 | -1.146045 | 76 | 46.023954 | 78.59 | 111.156046 | 1.034079 |  |
| GO:0008380\_RNA\_splicing | 17 | 1 | 13.544118 | -1.146045 | 76 | 46.023954 | 78.59 | 111.156046 | 1.034079 |  |
| GO:0019395\_fatty\_acid\_oxidation | 17 | 1 | 13.544118 | -1.146045 | 76 | 46.023954 | 78.59 | 111.156046 | 1.034079 |  |
| GO:0022029\_telencephalon\_cell\_migration | 17 | 1 | 13.544118 | -1.146045 | 76 | 46.023954 | 78.59 | 111.156046 | 1.034079 |  |
| GO:0034440\_lipid\_oxidation | 17 | 1 | 13.544118 | -1.146045 | 76 | 46.023954 | 78.59 | 111.156046 | 1.034079 |  |
| GO:0042254\_ribosome\_biogenesis | 17 | 1 | 13.544118 | -1.146045 | 76 | 46.023954 | 78.59 | 111.156046 | 1.034079 |  |
| GO:0045786\_negative\_regulation\_of\_cell\_cycle | 17 | 1 | 13.544118 | -1.146045 | 76 | 46.023954 | 78.59 | 111.156046 | 1.034079 |  |
| GO:0021885\_forebrain\_cell\_migration | 18 | 1 | 12.791667 | -1.122110 | 78 | 49.122556 | 82.89 | 116.657444 | 1.062692 |  |
| GO:0048813\_dendrite\_morphogenesis | 18 | 1 | 12.791667 | -1.122110 | 78 | 49.122556 | 82.89 | 116.657444 | 1.062692 |  |
| GO:0006810\_transport | 718 | 6 | 1.924095 | -1.107110 | 79 | 49.504628 | 83.57 | 117.635372 | 1.057848 |  |
| GO:0010952\_positive\_regulation\_of\_peptidase\_activity | 19 | 1 | 12.118421 | -1.099518 | 81 | 51.746108 | 86.2 | 120.653892 | 1.064198 |  |
| GO:0043280\_positive\_regulation\_of\_caspase\_activity | 19 | 1 | 12.118421 | -1.099518 | 81 | 51.746108 | 86.2 | 120.653892 | 1.064198 |  |
| GO:0044085\_cellular\_component\_biogenesis | 237 | 3 | 2.914557 | -1.094759 | 82 | 52.078927 | 86.65 | 121.221073 | 1.056707 |  |
| GO:0007049\_cell\_cycle | 238 | 3 | 2.902311 | -1.090444 | 83 | 52.182581 | 86.78 | 121.377419 | 1.045542 |  |
| GO:0051234\_establishment\_of\_localization | 729 | 6 | 1.895062 | -1.080945 | 84 | 52.466340 | 87.3 | 122.133660 | 1.039286 |  |
| GO:0007528\_neuromuscular\_junction\_development | 20 | 1 | 11.512500 | -1.078130 | 85 | 54.400845 | 89.84 | 125.279155 | 1.056941 |  |
| GO:0000075\_cell\_cycle\_checkpoint | 21 | 1 | 10.964286 | -1.057828 | 88 | 56.975420 | 93.94 | 130.904580 | 1.067500 |  |
| GO:0008637\_apoptotic\_mitochondrial\_changes | 21 | 1 | 10.964286 | -1.057828 | 88 | 56.975420 | 93.94 | 130.904580 | 1.067500 |  |
| GO:0051656\_establishment\_of\_organelle\_localization | 21 | 1 | 10.964286 | -1.057828 | 88 | 56.975420 | 93.94 | 130.904580 | 1.067500 |  |
| GO:0043933\_macromolecular\_complex\_subunit\_organization | 117 | 2 | 3.935897 | -1.044016 | 89 | 57.389728 | 94.74 | 132.090272 | 1.064494 |  |
| GO:0030258\_lipid\_modification | 22 | 1 | 10.465909 | -1.038512 | 92 | 60.115064 | 98.48 | 136.844936 | 1.070435 |  |
| GO:0032886\_regulation\_of\_microtubule-based\_process | 22 | 1 | 10.465909 | -1.038512 | 92 | 60.115064 | 98.48 | 136.844936 | 1.070435 |  |
| GO:0043112\_receptor\_metabolic\_process | 22 | 1 | 10.465909 | -1.038512 | 92 | 60.115064 | 98.48 | 136.844936 | 1.070435 |  |
| GO:0006397\_mRNA\_processing | 23 | 1 | 10.010870 | -1.020094 | 95 | 61.971671 | 101.52 | 141.068329 | 1.068632 |  |
| GO:0022613\_ribonucleoprotein\_complex\_biogenesis | 23 | 1 | 10.010870 | -1.020094 | 95 | 61.971671 | 101.52 | 141.068329 | 1.068632 |  |
| GO:0051705\_behavioral\_interaction\_between\_organisms | 23 | 1 | 10.010870 | -1.020094 | 95 | 61.971671 | 101.52 | 141.068329 | 1.068632 |  |
| GO:0006886\_intracellular\_protein\_transport | 122 | 2 | 3.774590 | -1.013060 | 96 | 62.472145 | 102.41 | 142.347855 | 1.066771 |  |
| GO:0008629\_induction\_of\_apoptosis\_by\_intracellular\_signals | 24 | 1 | 9.593750 | -1.002496 | 97 | 64.198930 | 105.0 | 145.801070 | 1.082474 |  |
| GO:0010638\_positive\_regulation\_of\_organelle\_organization | 27 | 1 | 8.527778 | -0.954000 | 98 | 70.141586 | 113.68 | 157.218414 | 1.160000 |  |
| GO:0042770\_DNA\_damage\_response\_\_signal\_transduction | 29 | 1 | 7.939655 | -0.924733 | 101 | 74.542655 | 119.89 | 165.237345 | 1.187030 |  |
| GO:0043281\_regulation\_of\_caspase\_activity | 29 | 1 | 7.939655 | -0.924733 | 101 | 74.542655 | 119.89 | 165.237345 | 1.187030 |  |
| GO:0052548\_regulation\_of\_endopeptidase\_activity | 29 | 1 | 7.939655 | -0.924733 | 101 | 74.542655 | 119.89 | 165.237345 | 1.187030 |  |
| GO:0034613\_cellular\_protein\_localization | 139 | 2 | 3.312950 | -0.918120 | 102 | 74.985782 | 120.51 | 166.034218 | 1.181471 |  |
| GO:0052547\_regulation\_of\_peptidase\_activity | 30 | 1 | 7.675000 | -0.910893 | 103 | 77.126664 | 123.84 | 170.553336 | 1.202330 |  |
| GO:0070727\_cellular\_macromolecule\_localization | 141 | 2 | 3.265957 | -0.907874 | 104 | 77.368740 | 124.11 | 170.851260 | 1.193365 |  |
| GO:0006486\_protein\_amino\_acid\_glycosylation | 31 | 1 | 7.427419 | -0.897536 | 109 | 79.931196 | 127.95 | 175.968804 | 1.173853 |  |
| GO:0016049\_cell\_growth | 31 | 1 | 7.427419 | -0.897536 | 109 | 79.931196 | 127.95 | 175.968804 | 1.173853 |  |
| GO:0033555\_multicellular\_organismal\_response\_to\_stress | 31 | 1 | 7.427419 | -0.897536 | 109 | 79.931196 | 127.95 | 175.968804 | 1.173853 |  |
| GO:0043413\_biopolymer\_glycosylation | 31 | 1 | 7.427419 | -0.897536 | 109 | 79.931196 | 127.95 | 175.968804 | 1.173853 |  |
| GO:0070085\_glycosylation | 31 | 1 | 7.427419 | -0.897536 | 109 | 79.931196 | 127.95 | 175.968804 | 1.173853 |  |
| GO:0051493\_regulation\_of\_cytoskeleton\_organization | 32 | 1 | 7.195312 | -0.884629 | 110 | 81.310924 | 129.88 | 178.449076 | 1.180727 |  |
| GO:0043085\_positive\_regulation\_of\_catalytic\_activity | 148 | 2 | 3.111486 | -0.873363 | 111 | 81.824671 | 131.06 | 180.295329 | 1.180721 |  |
| GO:0007270\_nerve-nerve\_synaptic\_transmission | 33 | 1 | 6.977273 | -0.872147 | 113 | 83.031221 | 132.78 | 182.528779 | 1.175044 |  |
| GO:0021987\_cerebral\_cortex\_development | 33 | 1 | 6.977273 | -0.872147 | 113 | 83.031221 | 132.78 | 182.528779 | 1.175044 |  |
| GO:0007269\_neurotransmitter\_secretion | 34 | 1 | 6.772059 | -0.860063 | 116 | 84.937523 | 135.39 | 185.842477 | 1.167155 |  |
| GO:0016054\_organic\_acid\_catabolic\_process | 34 | 1 | 6.772059 | -0.860063 | 116 | 84.937523 | 135.39 | 185.842477 | 1.167155 |  |
| GO:0046395\_carboxylic\_acid\_catabolic\_process | 34 | 1 | 6.772059 | -0.860063 | 116 | 84.937523 | 135.39 | 185.842477 | 1.167155 |  |
| GO:0044242\_cellular\_lipid\_catabolic\_process | 35 | 1 | 6.578571 | -0.848355 | 119 | 86.636493 | 137.44 | 188.243507 | 1.154958 |  |
| GO:0051325\_interphase | 35 | 1 | 6.578571 | -0.848355 | 119 | 86.636493 | 137.44 | 188.243507 | 1.154958 |  |
| GO:0051329\_interphase\_of\_mitotic\_cell\_cycle | 35 | 1 | 6.578571 | -0.848355 | 119 | 86.636493 | 137.44 | 188.243507 | 1.154958 |  |
| GO:0007268\_synaptic\_transmission | 154 | 2 | 2.990260 | -0.845338 | 120 | 86.694615 | 137.55 | 188.405385 | 1.146250 |  |
| GO:0022402\_cell\_cycle\_process | 155 | 2 | 2.970968 | -0.840799 | 121 | 87.070919 | 138.11 | 189.149081 | 1.141405 |  |
| GO:0001889\_liver\_development | 36 | 1 | 6.395833 | -0.837001 | 123 | 88.807306 | 140.49 | 192.172694 | 1.142195 |  |
| GO:0007631\_feeding\_behavior | 36 | 1 | 6.395833 | -0.837001 | 123 | 88.807306 | 140.49 | 192.172694 | 1.142195 |  |
| GO:0016042\_lipid\_catabolic\_process | 38 | 1 | 6.059211 | -0.815279 | 124 | 92.366450 | 145.64 | 198.913550 | 1.174516 |  |
| GO:0006259\_DNA\_metabolic\_process | 165 | 2 | 2.790909 | -0.797314 | 125 | 95.341475 | 149.85 | 204.358525 | 1.198800 |  |
| GO:0007346\_regulation\_of\_mitotic\_cell\_cycle | 40 | 1 | 5.756250 | -0.794759 | 129 | 96.779925 | 151.66 | 206.540075 | 1.175659 |  |
| GO:0016071\_mRNA\_metabolic\_process | 40 | 1 | 5.756250 | -0.794759 | 129 | 96.779925 | 151.66 | 206.540075 | 1.175659 |  |
| GO:0016358\_dendrite\_development | 40 | 1 | 5.756250 | -0.794759 | 129 | 96.779925 | 151.66 | 206.540075 | 1.175659 |  |
| GO:0017015\_regulation\_of\_transforming\_growth\_factor\_beta\_receptor\_signaling\_pathway | 40 | 1 | 5.756250 | -0.794759 | 129 | 96.779925 | 151.66 | 206.540075 | 1.175659 |  |
| GO:0006836\_neurotransmitter\_transport | 41 | 1 | 5.615854 | -0.784913 | 131 | 99.172155 | 155.17 | 211.167845 | 1.184504 |  |
| GO:0006865\_amino\_acid\_transport | 41 | 1 | 5.615854 | -0.784913 | 131 | 99.172155 | 155.17 | 211.167845 | 1.184504 |  |
| GO:0008361\_regulation\_of\_cell\_size | 42 | 1 | 5.482143 | -0.775325 | 134 | 101.585750 | 158.45 | 215.314250 | 1.182463 |  |
| GO:0051345\_positive\_regulation\_of\_hydrolase\_activity | 42 | 1 | 5.482143 | -0.775325 | 134 | 101.585750 | 158.45 | 215.314250 | 1.182463 |  |
| GO:0080135\_regulation\_of\_cellular\_response\_to\_stress | 42 | 1 | 5.482143 | -0.775325 | 134 | 101.585750 | 158.45 | 215.314250 | 1.182463 |  |
| GO:0044093\_positive\_regulation\_of\_molecular\_function | 173 | 2 | 2.661850 | -0.764842 | 135 | 104.654362 | 162.69 | 220.725638 | 1.205111 |  |
| GO:0050808\_synapse\_organization | 44 | 1 | 5.232955 | -0.756875 | 136 | 106.316638 | 165.98 | 225.643362 | 1.220441 |  |
| GO:0007612\_learning | 46 | 1 | 5.005435 | -0.739320 | 137 | 109.029134 | 170.24 | 231.450866 | 1.242628 |  |
| GO:0006396\_RNA\_processing | 47 | 1 | 4.898936 | -0.730855 | 138 | 110.824628 | 173.15 | 235.475372 | 1.254710 |  |
| GO:0001505\_regulation\_of\_neurotransmitter\_levels | 48 | 1 | 4.796875 | -0.722586 | 140 | 112.451216 | 175.28 | 238.108784 | 1.252000 |  |
| GO:0009101\_glycoprotein\_biosynthetic\_process | 48 | 1 | 4.796875 | -0.722586 | 140 | 112.451216 | 175.28 | 238.108784 | 1.252000 |  |
| GO:0021543\_pallium\_development | 49 | 1 | 4.698980 | -0.714505 | 142 | 114.462730 | 178.05 | 241.637270 | 1.253873 |  |
| GO:0048741\_skeletal\_muscle\_fiber\_development | 49 | 1 | 4.698980 | -0.714505 | 142 | 114.462730 | 178.05 | 241.637270 | 1.253873 |  |
| GO:0017038\_protein\_import | 50 | 1 | 4.605000 | -0.706604 | 143 | 115.601402 | 179.76 | 243.918598 | 1.257063 |  |
| GO:0019226\_transmission\_of\_nerve\_impulse | 189 | 2 | 2.436508 | -0.705295 | 144 | 115.692394 | 179.91 | 244.127606 | 1.249375 |  |
| GO:0048747\_muscle\_fiber\_development | 51 | 1 | 4.514706 | -0.698876 | 145 | 117.131243 | 182.1 | 247.068757 | 1.255862 |  |
| GO:0046942\_carboxylic\_acid\_transport | 53 | 1 | 4.344340 | -0.683915 | 147 | 119.219432 | 185.33 | 251.440568 | 1.260748 |  |
| GO:0055085\_transmembrane\_transport | 53 | 1 | 4.344340 | -0.683915 | 147 | 119.219432 | 185.33 | 251.440568 | 1.260748 |  |
| GO:0015849\_organic\_acid\_transport | 54 | 1 | 4.263889 | -0.676668 | 148 | 121.268645 | 188.14 | 255.011355 | 1.271216 |  |
| GO:0000226\_microtubule\_cytoskeleton\_organization | 57 | 1 | 4.039474 | -0.655797 | 150 | 126.337433 | 195.13 | 263.922567 | 1.300867 |  |
| GO:0001764\_neuron\_migration | 57 | 1 | 4.039474 | -0.655797 | 150 | 126.337433 | 195.13 | 263.922567 | 1.300867 |  |
| GO:0022607\_cellular\_component\_assembly | 204 | 2 | 2.257353 | -0.655132 | 151 | 126.511701 | 195.31 | 264.108299 | 1.293444 |  |
| GO:0019219\_regulation\_of\_nucleobase\_\_nucleoside\_\_nucleotide\_and\_nucleic\_acid\_metabolic\_process | 757 | 5 | 1.520806 | -0.653462 | 152 | 126.573958 | 195.44 | 264.306042 | 1.285789 |  |
| GO:0033043\_regulation\_of\_organelle\_organization | 58 | 1 | 3.969828 | -0.649113 | 153 | 127.637638 | 196.98 | 266.322362 | 1.287451 |  |
| GO:0008284\_positive\_regulation\_of\_cell\_proliferation | 208 | 2 | 2.213942 | -0.642572 | 154 | 128.056357 | 197.57 | 267.083643 | 1.282922 |  |
| GO:0016055\_Wnt\_receptor\_signaling\_pathway | 59 | 1 | 3.902542 | -0.642558 | 156 | 128.800756 | 198.55 | 268.299244 | 1.272756 |  |
| GO:0048469\_cell\_maturation | 59 | 1 | 3.902542 | -0.642558 | 156 | 128.800756 | 198.55 | 268.299244 | 1.272756 |  |
| GO:0051171\_regulation\_of\_nitrogen\_compound\_metabolic\_process | 771 | 5 | 1.493191 | -0.631044 | 157 | 129.210033 | 199.48 | 269.749967 | 1.270573 |  |
| GO:0007005\_mitochondrion\_organization | 61 | 1 | 3.774590 | -0.629816 | 158 | 130.134592 | 200.74 | 271.345408 | 1.270506 |  |
| GO:0006916\_anti-apoptosis | 62 | 1 | 3.713710 | -0.623621 | 160 | 131.965751 | 203.47 | 274.974249 | 1.271688 |  |
| GO:0021537\_telencephalon\_development | 62 | 1 | 3.713710 | -0.623621 | 160 | 131.965751 | 203.47 | 274.974249 | 1.271688 |  |
| GO:0042127\_regulation\_of\_cell\_proliferation | 393 | 3 | 1.757634 | -0.618675 | 161 | 132.036808 | 203.67 | 275.303192 | 1.265031 |  |
| GO:0051253\_negative\_regulation\_of\_RNA\_metabolic\_process | 220 | 2 | 2.093182 | -0.606756 | 162 | 134.766924 | 207.79 | 280.813076 | 1.282654 |  |
| GO:0007179\_transforming\_growth\_factor\_beta\_receptor\_signaling\_pathway | 66 | 1 | 3.488636 | -0.599932 | 166 | 136.786357 | 210.26 | 283.733643 | 1.266627 |  |
| GO:0015837\_amine\_transport | 66 | 1 | 3.488636 | -0.599932 | 166 | 136.786357 | 210.26 | 283.733643 | 1.266627 |  |
| GO:0045860\_positive\_regulation\_of\_protein\_kinase\_activity | 66 | 1 | 3.488636 | -0.599932 | 166 | 136.786357 | 210.26 | 283.733643 | 1.266627 |  |
| GO:0051130\_positive\_regulation\_of\_cellular\_component\_organization | 66 | 1 | 3.488636 | -0.599932 | 166 | 136.786357 | 210.26 | 283.733643 | 1.266627 |  |
| GO:0007167\_enzyme\_linked\_receptor\_protein\_signaling\_pathway | 229 | 2 | 2.010917 | -0.581589 | 167 | 139.723806 | 214.6 | 289.476194 | 1.285030 |  |
| GO:0007611\_learning\_or\_memory | 70 | 1 | 3.289286 | -0.577833 | 168 | 140.960476 | 216.21 | 291.459524 | 1.286964 |  |
| GO:0009100\_glycoprotein\_metabolic\_process | 71 | 1 | 3.242958 | -0.572535 | 170 | 142.483559 | 218.08 | 293.676441 | 1.282824 |  |
| GO:0033674\_positive\_regulation\_of\_kinase\_activity | 71 | 1 | 3.242958 | -0.572535 | 170 | 142.483559 | 218.08 | 293.676441 | 1.282824 |  |
| GO:0050790\_regulation\_of\_catalytic\_activity | 233 | 2 | 1.976395 | -0.570835 | 171 | 142.797296 | 218.55 | 294.302704 | 1.278070 |  |
| GO:0031326\_regulation\_of\_cellular\_biosynthetic\_process | 812 | 5 | 1.417796 | -0.569460 | 172 | 142.844139 | 218.66 | 294.475861 | 1.271279 |  |
| GO:0044262\_cellular\_carbohydrate\_metabolic\_process | 72 | 1 | 3.197917 | -0.567323 | 174 | 145.123044 | 221.47 | 297.816956 | 1.272816 |  |
| GO:0051347\_positive\_regulation\_of\_transferase\_activity | 72 | 1 | 3.197917 | -0.567323 | 174 | 145.123044 | 221.47 | 297.816956 | 1.272816 |  |
| GO:0009889\_regulation\_of\_biosynthetic\_process | 815 | 5 | 1.412577 | -0.565180 | 175 | 145.306784 | 221.77 | 298.233216 | 1.267257 |  |
| GO:0051336\_regulation\_of\_hydrolase\_activity | 73 | 1 | 3.154110 | -0.562194 | 176 | 146.246689 | 223.13 | 300.013311 | 1.267784 |  |
| GO:0006915\_apoptosis | 427 | 3 | 1.617681 | -0.550236 | 177 | 148.027170 | 225.56 | 303.092830 | 1.274350 |  |
| GO:0012501\_programmed\_cell\_death | 433 | 3 | 1.595266 | -0.539043 | 178 | 149.995622 | 228.17 | 306.344378 | 1.281854 |  |
| GO:0007519\_skeletal\_muscle\_tissue\_development | 78 | 1 | 2.951923 | -0.537723 | 180 | 151.170170 | 229.67 | 308.169830 | 1.275944 |  |
| GO:0060538\_skeletal\_muscle\_organ\_development | 78 | 1 | 2.951923 | -0.537723 | 180 | 151.170170 | 229.67 | 308.169830 | 1.275944 |  |
| GO:0000278\_mitotic\_cell\_cycle | 80 | 1 | 2.878125 | -0.528443 | 182 | 152.610008 | 231.98 | 311.349992 | 1.274615 |  |
| GO:0006631\_fatty\_acid\_metabolic\_process | 80 | 1 | 2.878125 | -0.528443 | 182 | 152.610008 | 231.98 | 311.349992 | 1.274615 |  |
| GO:0021700\_developmental\_maturation | 81 | 1 | 2.842593 | -0.523906 | 183 | 153.378054 | 232.97 | 312.561946 | 1.273060 |  |
| GO:0007267\_cell-cell\_signaling | 252 | 2 | 1.827381 | -0.523061 | 184 | 153.494827 | 233.16 | 312.825173 | 1.267174 |  |
| GO:0016481\_negative\_regulation\_of\_transcription | 253 | 2 | 1.820158 | -0.520688 | 185 | 154.081182 | 234.04 | 313.998818 | 1.265081 |  |
| GO:0051179\_localization | 1058 | 6 | 1.305766 | -0.519558 | 186 | 154.164285 | 234.19 | 314.215715 | 1.259086 |  |
| GO:0008219\_cell\_death | 444 | 3 | 1.555743 | -0.519155 | 187 | 155.475259 | 235.81 | 316.144741 | 1.261016 |  |
| GO:0009966\_regulation\_of\_signal\_transduction | 256 | 2 | 1.798828 | -0.513649 | 188 | 157.464973 | 238.57 | 319.675027 | 1.268989 |  |
| GO:0006996\_organelle\_organization | 449 | 3 | 1.538419 | -0.510377 | 189 | 158.465050 | 239.61 | 320.754950 | 1.267778 |  |
| GO:0016265\_death | 450 | 3 | 1.535000 | -0.508641 | 190 | 158.579227 | 239.75 | 320.920773 | 1.261842 |  |
| GO:0010629\_negative\_regulation\_of\_gene\_expression | 262 | 2 | 1.757634 | -0.499918 | 191 | 161.400961 | 243.22 | 325.039039 | 1.273403 |  |
| GO:0001822\_kidney\_development | 87 | 1 | 2.646552 | -0.498005 | 194 | 162.822274 | 245.03 | 327.237726 | 1.263041 |  |
| GO:0003001\_generation\_of\_a\_signal\_involved\_in\_cell-cell\_signaling | 87 | 1 | 2.646552 | -0.498005 | 194 | 162.822274 | 245.03 | 327.237726 | 1.263041 |  |
| GO:0007178\_transmembrane\_receptor\_protein\_serine\_threonine\_kinase\_signaling\_pathway | 87 | 1 | 2.646552 | -0.498005 | 194 | 162.822274 | 245.03 | 327.237726 | 1.263041 |  |
| GO:0034984\_cellular\_response\_to\_DNA\_damage\_stimulus | 94 | 1 | 2.449468 | -0.470362 | 195 | 169.672843 | 254.41 | 339.147157 | 1.304667 |  |
| GO:0007610\_behavior | 279 | 2 | 1.650538 | -0.463374 | 197 | 171.099351 | 256.12 | 341.140649 | 1.300102 |  |
| GO:0065009\_regulation\_of\_molecular\_function | 279 | 2 | 1.650538 | -0.463374 | 197 | 171.099351 | 256.12 | 341.140649 | 1.300102 |  |
| GO:0018193\_peptidyl-amino\_acid\_modification | 97 | 1 | 2.373711 | -0.459267 | 198 | 172.921923 | 258.16 | 343.398077 | 1.303838 |  |
| GO:0034645\_cellular\_macromolecule\_biosynthetic\_process | 901 | 5 | 1.277747 | -0.454293 | 199 | 174.479929 | 260.15 | 345.820071 | 1.307286 |  |
| GO:0009059\_macromolecule\_biosynthetic\_process | 910 | 5 | 1.265110 | -0.443905 | 200 | 177.162388 | 263.29 | 349.417612 | 1.316450 |  |
| GO:0080090\_regulation\_of\_primary\_metabolic\_process | 926 | 5 | 1.243251 | -0.425960 | 201 | 180.272482 | 267.18 | 354.087518 | 1.329254 |  |
| GO:0045859\_regulation\_of\_protein\_kinase\_activity | 107 | 1 | 2.151869 | -0.425118 | 202 | 180.642900 | 267.64 | 354.637100 | 1.324950 |  |
| GO:0044249\_cellular\_biosynthetic\_process | 1150 | 6 | 1.201304 | -0.418373 | 203 | 182.164893 | 269.65 | 357.135107 | 1.328325 |  |
| GO:0060255\_regulation\_of\_macromolecule\_metabolic\_process | 936 | 5 | 1.229968 | -0.415074 | 204 | 183.346946 | 271.05 | 358.753054 | 1.328676 |  |
| GO:0043549\_regulation\_of\_kinase\_activity | 112 | 1 | 2.055804 | -0.409496 | 205 | 184.817248 | 272.99 | 361.162752 | 1.331659 |  |
| GO:0006974\_response\_to\_DNA\_damage\_stimulus | 113 | 1 | 2.037611 | -0.406476 | 206 | 185.794457 | 274.09 | 362.385543 | 1.330534 |  |
| GO:0051338\_regulation\_of\_transferase\_activity | 115 | 1 | 2.002174 | -0.400537 | 207 | 187.507876 | 276.19 | 364.872124 | 1.334251 |  |
| GO:0080134\_regulation\_of\_response\_to\_stress | 116 | 1 | 1.984914 | -0.397616 | 208 | 188.304418 | 277.24 | 366.175582 | 1.332885 |  |
| GO:0009058\_biosynthetic\_process | 1175 | 6 | 1.175745 | -0.393950 | 209 | 188.969256 | 278.09 | 367.210744 | 1.330574 |  |
| GO:0022403\_cell\_cycle\_phase | 119 | 1 | 1.934874 | -0.389042 | 210 | 189.827014 | 279.09 | 368.352986 | 1.329000 |  |
| GO:0010556\_regulation\_of\_macromolecule\_biosynthetic\_process | 745 | 4 | 1.236242 | -0.387507 | 211 | 189.970066 | 279.28 | 368.589934 | 1.323602 |  |
| GO:0014706\_striated\_muscle\_tissue\_development | 120 | 1 | 1.918750 | -0.386244 | 212 | 190.187399 | 279.54 | 368.892601 | 1.318585 |  |
| GO:0016043\_cellular\_component\_organization | 964 | 5 | 1.194243 | -0.385887 | 213 | 190.295456 | 279.72 | 369.144544 | 1.313239 |  |
| GO:0006917\_induction\_of\_apoptosis | 121 | 1 | 1.902893 | -0.383477 | 215 | 191.144894 | 280.95 | 370.755106 | 1.306744 |  |
| GO:0012502\_induction\_of\_programmed\_cell\_death | 121 | 1 | 1.902893 | -0.383477 | 215 | 191.144894 | 280.95 | 370.755106 | 1.306744 |  |
| GO:0043062\_extracellular\_structure\_organization | 125 | 1 | 1.842000 | -0.372694 | 216 | 193.531663 | 284.24 | 374.948337 | 1.315926 |  |
| GO:0010646\_regulation\_of\_cell\_communication | 330 | 2 | 1.395455 | -0.371263 | 217 | 194.201439 | 284.98 | 375.758561 | 1.313272 |  |
| GO:0008283\_cell\_proliferation | 544 | 3 | 1.269761 | -0.370055 | 218 | 194.313717 | 285.19 | 376.066283 | 1.308211 |  |
| GO:0001655\_urogenital\_system\_development | 128 | 1 | 1.798828 | -0.364894 | 220 | 195.887569 | 286.9 | 377.912431 | 1.304091 |  |
| GO:0060537\_muscle\_tissue\_development | 128 | 1 | 1.798828 | -0.364894 | 220 | 195.887569 | 286.9 | 377.912431 | 1.304091 |  |
| GO:0032787\_monocarboxylic\_acid\_metabolic\_process | 130 | 1 | 1.771154 | -0.359825 | 221 | 197.731406 | 288.88 | 380.028594 | 1.307149 |  |
| GO:0006139\_nucleobase\_\_nucleoside\_\_nucleotide\_and\_nucleic\_acid\_metabolic\_process | 1002 | 5 | 1.148952 | -0.349160 | 222 | 201.200844 | 293.14 | 385.079156 | 1.320450 |  |
| GO:0007169\_transmembrane\_receptor\_protein\_tyrosine\_kinase\_signaling\_pathway | 139 | 1 | 1.656475 | -0.338217 | 223 | 203.657911 | 295.85 | 388.042089 | 1.326682 |  |
| GO:0031323\_regulation\_of\_cellular\_metabolic\_process | 1015 | 5 | 1.134236 | -0.337315 | 224 | 204.070299 | 296.39 | 388.709701 | 1.323170 |  |
| GO:0034961\_cellular\_biopolymer\_biosynthetic\_process | 804 | 4 | 1.145522 | -0.326195 | 225 | 207.247114 | 299.78 | 392.312886 | 1.332356 |  |
| GO:0043284\_biopolymer\_biosynthetic\_process | 807 | 4 | 1.141264 | -0.323331 | 226 | 207.683931 | 300.33 | 392.976069 | 1.328894 |  |
| GO:0005975\_carbohydrate\_metabolic\_process | 146 | 1 | 1.577055 | -0.322654 | 228 | 208.552355 | 301.32 | 394.087645 | 1.321579 |  |
| GO:0030900\_forebrain\_development | 146 | 1 | 1.577055 | -0.322654 | 228 | 208.552355 | 301.32 | 394.087645 | 1.321579 |  |
| GO:0032940\_secretion\_by\_cell | 149 | 1 | 1.545302 | -0.316288 | 229 | 211.059854 | 304.31 | 397.560146 | 1.328865 |  |
| GO:0007517\_muscle\_organ\_development | 153 | 1 | 1.504902 | -0.308065 | 230 | 213.406030 | 307.02 | 400.633970 | 1.334870 |  |
| GO:0008285\_negative\_regulation\_of\_cell\_proliferation | 155 | 1 | 1.485484 | -0.304063 | 231 | 214.662201 | 308.38 | 402.097799 | 1.334978 |  |
| GO:0050794\_regulation\_of\_cellular\_process | 2190 | 10 | 1.051370 | -0.300548 | 232 | 215.151661 | 308.98 | 402.808339 | 1.331810 |  |
| GO:0051704\_multi-organism\_process | 157 | 1 | 1.466561 | -0.300130 | 233 | 215.531728 | 309.4 | 403.268272 | 1.327897 |  |
| GO:0051128\_regulation\_of\_cellular\_component\_organization | 160 | 1 | 1.439062 | -0.294360 | 234 | 217.088697 | 311.28 | 405.471303 | 1.330256 |  |
| GO:0007626\_locomotory\_behavior | 163 | 1 | 1.412577 | -0.288737 | 236 | 218.928410 | 313.56 | 408.191590 | 1.328644 |  |
| GO:0042110\_T\_cell\_activation | 163 | 1 | 1.412577 | -0.288737 | 236 | 218.928410 | 313.56 | 408.191590 | 1.328644 |  |
| GO:0050877\_neurological\_system\_process | 390 | 2 | 1.180769 | -0.288339 | 237 | 219.226429 | 313.88 | 408.533571 | 1.324388 |  |
| GO:0042325\_regulation\_of\_phosphorylation | 164 | 1 | 1.403963 | -0.286895 | 238 | 219.606516 | 314.32 | 409.033484 | 1.320672 |  |
| GO:0019220\_regulation\_of\_phosphate\_metabolic\_process | 165 | 1 | 1.395455 | -0.285068 | 240 | 220.913413 | 315.82 | 410.726587 | 1.315917 |  |
| GO:0051174\_regulation\_of\_phosphorus\_metabolic\_process | 165 | 1 | 1.395455 | -0.285068 | 240 | 220.913413 | 315.82 | 410.726587 | 1.315917 |  |
| GO:0043065\_positive\_regulation\_of\_apoptosis | 166 | 1 | 1.387048 | -0.283257 | 241 | 221.650719 | 316.63 | 411.609281 | 1.313817 |  |
| GO:0010942\_positive\_regulation\_of\_cell\_death | 167 | 1 | 1.378743 | -0.281461 | 243 | 222.555533 | 317.68 | 412.804467 | 1.307325 |  |
| GO:0043068\_positive\_regulation\_of\_programmed\_cell\_death | 167 | 1 | 1.378743 | -0.281461 | 243 | 222.555533 | 317.68 | 412.804467 | 1.307325 |  |
| GO:0048699\_generation\_of\_neurons | 396 | 2 | 1.162879 | -0.281231 | 244 | 222.787298 | 317.94 | 413.092702 | 1.303033 |  |
| GO:0019222\_regulation\_of\_metabolic\_process | 1088 | 5 | 1.058134 | -0.277024 | 245 | 223.092881 | 318.27 | 413.447119 | 1.299061 |  |
| GO:0048812\_neuron\_projection\_morphogenesis | 170 | 1 | 1.354412 | -0.276162 | 246 | 223.702186 | 318.99 | 414.277814 | 1.296707 |  |
| GO:0044248\_cellular\_catabolic\_process | 173 | 1 | 1.330925 | -0.270994 | 248 | 225.496627 | 321.11 | 416.723373 | 1.294798 |  |
| GO:0048667\_cell\_morphogenesis\_involved\_in\_neuron\_differentiation | 173 | 1 | 1.330925 | -0.270994 | 248 | 225.496627 | 321.11 | 416.723373 | 1.294798 |  |
| GO:0046903\_secretion | 175 | 1 | 1.315714 | -0.267618 | 249 | 226.499347 | 322.1 | 417.700653 | 1.293574 |  |
| GO:0043066\_negative\_regulation\_of\_apoptosis | 176 | 1 | 1.308239 | -0.265951 | 251 | 227.963671 | 323.51 | 419.056329 | 1.288884 |  |
| GO:0048858\_cell\_projection\_morphogenesis | 176 | 1 | 1.308239 | -0.265951 | 251 | 227.963671 | 323.51 | 419.056329 | 1.288884 |  |
| GO:0043069\_negative\_regulation\_of\_programmed\_cell\_death | 179 | 1 | 1.286313 | -0.261030 | 253 | 229.886392 | 325.42 | 420.953608 | 1.286245 |  |
| GO:0060548\_negative\_regulation\_of\_cell\_death | 179 | 1 | 1.286313 | -0.261030 | 253 | 229.886392 | 325.42 | 420.953608 | 1.286245 |  |
| GO:0019752\_carboxylic\_acid\_metabolic\_process | 181 | 1 | 1.272099 | -0.257815 | 255 | 231.491045 | 326.99 | 422.488955 | 1.282314 |  |
| GO:0043436\_oxoacid\_metabolic\_process | 181 | 1 | 1.272099 | -0.257815 | 255 | 231.491045 | 326.99 | 422.488955 | 1.282314 |  |
| GO:0006082\_organic\_acid\_metabolic\_process | 182 | 1 | 1.265110 | -0.256226 | 256 | 231.822224 | 327.38 | 422.937776 | 1.278828 |  |
| GO:0043170\_macromolecule\_metabolic\_process | 1576 | 7 | 1.022684 | -0.255979 | 257 | 231.891583 | 327.55 | 423.208417 | 1.274514 |  |
| GO:0048468\_cell\_development | 654 | 3 | 1.056193 | -0.255133 | 258 | 232.409137 | 328.19 | 423.970863 | 1.272054 |  |
| GO:0042180\_cellular\_ketone\_metabolic\_process | 183 | 1 | 1.258197 | -0.254650 | 259 | 232.738045 | 328.58 | 424.421955 | 1.268649 |  |
| GO:0032990\_cell\_part\_morphogenesis | 184 | 1 | 1.251359 | -0.253087 | 260 | 233.821409 | 329.74 | 425.658591 | 1.268231 |  |
| GO:0007010\_cytoskeleton\_organization | 185 | 1 | 1.244595 | -0.251535 | 261 | 234.613144 | 330.48 | 426.346856 | 1.266207 |  |
| GO:0022008\_neurogenesis | 423 | 2 | 1.088652 | -0.251477 | 262 | 234.873524 | 330.78 | 426.686476 | 1.262519 |  |
| GO:0006952\_defense\_response | 187 | 1 | 1.231283 | -0.248469 | 263 | 236.670933 | 332.58 | 428.489067 | 1.264563 |  |
| GO:0048513\_organ\_development | 1365 | 6 | 1.012088 | -0.244062 | 264 | 237.879180 | 334.0 | 430.120820 | 1.265152 |  |
| GO:0019725\_cellular\_homeostasis | 195 | 1 | 1.180769 | -0.236670 | 265 | 241.043909 | 337.49 | 433.936091 | 1.273547 |  |
| GO:0006807\_nitrogen\_compound\_metabolic\_process | 1147 | 5 | 1.003705 | -0.235270 | 266 | 241.686377 | 338.09 | 434.493623 | 1.271015 |  |
| GO:0033554\_cellular\_response\_to\_stress | 196 | 1 | 1.174745 | -0.235245 | 267 | 241.863002 | 338.38 | 434.896998 | 1.267341 |  |
| GO:0031175\_neuron\_projection\_development | 197 | 1 | 1.168782 | -0.233831 | 268 | 242.905284 | 339.35 | 435.794716 | 1.266231 |  |
| GO:0000904\_cell\_morphogenesis\_involved\_in\_differentiation | 199 | 1 | 1.157035 | -0.231035 | 269 | 244.540325 | 341.17 | 437.799675 | 1.268290 |  |
| GO:0010926\_anatomical\_structure\_formation | 447 | 2 | 1.030201 | -0.227805 | 270 | 245.337717 | 342.1 | 438.862283 | 1.267037 |  |
| GO:0034960\_cellular\_biopolymer\_metabolic\_process | 1395 | 6 | 0.990323 | -0.225394 | 271 | 246.415143 | 343.08 | 439.744857 | 1.265978 |  |
| GO:0040007\_growth | 217 | 1 | 1.061060 | -0.207650 | 273 | 251.735128 | 348.65 | 445.564872 | 1.277106 |  |
| GO:0048583\_regulation\_of\_response\_to\_stimulus | 217 | 1 | 1.061060 | -0.207650 | 273 | 251.735128 | 348.65 | 445.564872 | 1.277106 |  |
| GO:0045892\_negative\_regulation\_of\_transcription\_\_DNA-dependent | 218 | 1 | 1.056193 | -0.206438 | 274 | 252.131797 | 349.03 | 445.928203 | 1.273832 |  |
| GO:0050789\_regulation\_of\_biological\_process | 2357 | 10 | 0.976877 | -0.200996 | 275 | 254.281889 | 350.91 | 447.538111 | 1.276036 |  |
| GO:0044260\_cellular\_macromolecule\_metabolic\_process | 1447 | 6 | 0.954734 | -0.195786 | 276 | 254.966098 | 351.58 | 448.193902 | 1.273841 |  |
| GO:0046649\_lymphocyte\_activation | 228 | 1 | 1.009868 | -0.194779 | 277 | 256.123280 | 352.99 | 449.856720 | 1.274332 |  |
| GO:0007420\_brain\_development | 231 | 1 | 0.996753 | -0.191437 | 278 | 256.950375 | 353.89 | 450.829625 | 1.272986 |  |
| GO:0050890\_cognition | 233 | 1 | 0.988197 | -0.189247 | 279 | 257.475927 | 354.61 | 451.744073 | 1.271004 |  |
| GO:0016477\_cell\_migration | 234 | 1 | 0.983974 | -0.188164 | 280 | 257.837739 | 355.0 | 452.162261 | 1.267857 |  |
| GO:0009056\_catabolic\_process | 243 | 1 | 0.947531 | -0.178735 | 281 | 261.064935 | 358.25 | 455.435065 | 1.274911 |  |
| GO:0043283\_biopolymer\_metabolic\_process | 1490 | 6 | 0.927181 | -0.173753 | 282 | 261.448705 | 358.58 | 455.711295 | 1.271560 |  |
| GO:0045321\_leukocyte\_activation | 248 | 1 | 0.928427 | -0.173739 | 283 | 262.215890 | 359.38 | 456.544110 | 1.269894 |  |
| GO:0003008\_system\_process | 516 | 2 | 0.892442 | -0.171694 | 284 | 263.124590 | 360.31 | 457.495410 | 1.268697 |  |
| GO:0010468\_regulation\_of\_gene\_expression | 778 | 3 | 0.887853 | -0.166494 | 285 | 266.495000 | 363.34 | 460.185000 | 1.274877 |  |
| GO:0048870\_cell\_motility | 257 | 1 | 0.895914 | -0.165151 | 286 | 267.827415 | 364.63 | 461.432585 | 1.274930 |  |
| GO:0001775\_cell\_activation | 262 | 1 | 0.878817 | -0.160594 | 288 | 269.018994 | 365.81 | 462.601006 | 1.270174 |  |
| GO:0048666\_neuron\_development | 262 | 1 | 0.878817 | -0.160594 | 288 | 269.018994 | 365.81 | 462.601006 | 1.270174 |  |
| GO:0030030\_cell\_projection\_organization | 263 | 1 | 0.875475 | -0.159700 | 289 | 269.378915 | 366.14 | 462.901085 | 1.266920 |  |
| GO:0044255\_cellular\_lipid\_metabolic\_process | 264 | 1 | 0.872159 | -0.158812 | 290 | 269.763008 | 366.54 | 463.316992 | 1.263931 |  |
| GO:0051716\_cellular\_response\_to\_stimulus | 273 | 1 | 0.843407 | -0.151069 | 291 | 271.966228 | 368.75 | 465.533772 | 1.267182 |  |
| GO:0006950\_response\_to\_stress | 549 | 2 | 0.838798 | -0.149998 | 292 | 273.253956 | 369.96 | 466.666044 | 1.266986 |  |
| GO:0000902\_cell\_morphogenesis | 283 | 1 | 0.813604 | -0.142966 | 293 | 276.749308 | 373.04 | 469.330692 | 1.273174 |  |
| GO:0006629\_lipid\_metabolic\_process | 285 | 1 | 0.807895 | -0.141406 | 294 | 277.513945 | 373.82 | 470.126055 | 1.271497 |  |
| GO:0007417\_central\_nervous\_system\_development | 287 | 1 | 0.802265 | -0.139864 | 295 | 277.855500 | 374.2 | 470.544500 | 1.268475 |  |
| GO:0040011\_locomotion | 295 | 1 | 0.780508 | -0.133885 | 296 | 280.226228 | 376.29 | 472.353772 | 1.271250 |  |
| GO:0007154\_cell\_communication | 1096 | 4 | 0.840328 | -0.132564 | 297 | 280.461430 | 376.45 | 472.438570 | 1.267508 |  |
| GO:0032502\_developmental\_process | 2060 | 8 | 0.894175 | -0.130338 | 298 | 281.010446 | 377.04 | 473.069554 | 1.265235 |  |
| GO:0051252\_regulation\_of\_RNA\_metabolic\_process | 590 | 2 | 0.780508 | -0.126774 | 299 | 281.778329 | 377.87 | 473.961671 | 1.263779 |  |
| GO:0032989\_cellular\_component\_morphogenesis | 307 | 1 | 0.750000 | -0.125444 | 300 | 282.928498 | 378.95 | 474.971502 | 1.263167 |  |
| GO:0051094\_positive\_regulation\_of\_developmental\_process | 308 | 1 | 0.747565 | -0.124768 | 301 | 283.717887 | 379.72 | 475.722113 | 1.261528 |  |
| GO:0016310\_phosphorylation | 309 | 1 | 0.745146 | -0.124095 | 302 | 284.803558 | 380.76 | 476.716442 | 1.260795 |  |
| GO:0007166\_cell\_surface\_receptor\_linked\_signal\_transduction | 597 | 2 | 0.771357 | -0.123176 | 303 | 285.420576 | 381.31 | 477.199424 | 1.258449 |  |
| GO:0048731\_system\_development | 1609 | 6 | 0.858608 | -0.122990 | 304 | 285.616715 | 381.49 | 477.363285 | 1.254901 |  |
| GO:0007399\_nervous\_system\_development | 621 | 2 | 0.741546 | -0.111577 | 305 | 286.241423 | 382.07 | 477.898577 | 1.252689 |  |
| GO:0006928\_cell\_motion | 330 | 1 | 0.697727 | -0.110861 | 307 | 287.720611 | 383.4 | 479.079389 | 1.248860 |  |
| GO:0051674\_localization\_of\_cell | 330 | 1 | 0.697727 | -0.110861 | 307 | 287.720611 | 383.4 | 479.079389 | 1.248860 |  |
| GO:0051093\_negative\_regulation\_of\_developmental\_process | 331 | 1 | 0.695619 | -0.110271 | 308 | 288.507145 | 384.13 | 479.752855 | 1.247175 |  |
| GO:0048522\_positive\_regulation\_of\_cellular\_process | 895 | 3 | 0.771788 | -0.109819 | 309 | 288.791259 | 384.36 | 479.928741 | 1.243883 |  |
| GO:0010467\_gene\_expression | 905 | 3 | 0.763260 | -0.105902 | 310 | 290.317070 | 385.59 | 480.862930 | 1.243839 |  |
| GO:0008152\_metabolic\_process | 2133 | 8 | 0.863572 | -0.105148 | 311 | 290.773042 | 386.03 | 481.286958 | 1.241254 |  |
| GO:0006793\_phosphorus\_metabolic\_process | 340 | 1 | 0.677206 | -0.105108 | 313 | 291.538022 | 386.75 | 481.961978 | 1.235623 |  |
| GO:0006796\_phosphate\_metabolic\_process | 340 | 1 | 0.677206 | -0.105108 | 313 | 291.538022 | 386.75 | 481.961978 | 1.235623 |  |
| GO:0065007\_biological\_regulation | 2593 | 10 | 0.887968 | -0.103687 | 314 | 292.412447 | 387.35 | 482.287553 | 1.233599 |  |
| GO:0044238\_primary\_metabolic\_process | 1905 | 7 | 0.846063 | -0.103256 | 315 | 292.605002 | 387.5 | 482.394998 | 1.230159 |  |
| GO:0009887\_organ\_morphogenesis | 642 | 2 | 0.717290 | -0.102298 | 316 | 292.947835 | 387.79 | 482.632165 | 1.227184 |  |
| GO:0007165\_signal\_transduction | 915 | 3 | 0.754918 | -0.102111 | 317 | 293.191101 | 388.0 | 482.808899 | 1.223975 |  |
| GO:0030182\_neuron\_differentiation | 356 | 1 | 0.646770 | -0.096562 | 318 | 295.884627 | 390.28 | 484.675373 | 1.227296 |  |
| GO:0048856\_anatomical\_structure\_development | 1688 | 6 | 0.818424 | -0.096473 | 319 | 296.116216 | 390.44 | 484.763784 | 1.223950 |  |
| GO:0016070\_RNA\_metabolic\_process | 658 | 2 | 0.699848 | -0.095729 | 320 | 296.378446 | 390.7 | 485.021554 | 1.220938 |  |
| GO:0042981\_regulation\_of\_apoptosis | 360 | 1 | 0.639583 | -0.094545 | 321 | 297.244066 | 391.34 | 485.435934 | 1.219128 |  |
| GO:0010941\_regulation\_of\_cell\_death | 365 | 1 | 0.630822 | -0.092085 | 323 | 298.660220 | 392.48 | 486.299780 | 1.215108 |  |
| GO:0043067\_regulation\_of\_programmed\_cell\_death | 365 | 1 | 0.630822 | -0.092085 | 323 | 298.660220 | 392.48 | 486.299780 | 1.215108 |  |
| GO:0045449\_regulation\_of\_transcription | 676 | 2 | 0.681213 | -0.088821 | 324 | 300.198516 | 393.73 | 487.261484 | 1.215216 |  |
| GO:0009653\_anatomical\_structure\_morphogenesis | 958 | 3 | 0.721033 | -0.087163 | 325 | 300.527507 | 394.03 | 487.532493 | 1.212400 |  |
| GO:0044237\_cellular\_metabolic\_process | 1974 | 7 | 0.816489 | -0.083252 | 326 | 301.927964 | 395.45 | 488.972036 | 1.213037 |  |
| GO:0065008\_regulation\_of\_biological\_quality | 693 | 2 | 0.664502 | -0.082734 | 327 | 302.264650 | 395.66 | 489.055350 | 1.209969 |  |
| GO:0031328\_positive\_regulation\_of\_cellular\_biosynthetic\_process | 387 | 1 | 0.594961 | -0.082045 | 328 | 302.782851 | 396.09 | 489.397149 | 1.207591 |  |
| GO:0009891\_positive\_regulation\_of\_biosynthetic\_process | 388 | 1 | 0.593428 | -0.081617 | 329 | 303.159837 | 396.37 | 489.580163 | 1.204772 |  |
| GO:0006350\_transcription | 701 | 2 | 0.656919 | -0.080008 | 330 | 303.711284 | 396.88 | 490.048716 | 1.202667 |  |
| GO:0007275\_multicellular\_organismal\_development | 1760 | 6 | 0.784943 | -0.076527 | 331 | 304.962076 | 397.97 | 490.977924 | 1.202326 |  |
| GO:0048518\_positive\_regulation\_of\_biological\_process | 995 | 3 | 0.694221 | -0.075905 | 332 | 305.207308 | 398.14 | 491.072692 | 1.199217 |  |
| GO:0007242\_intracellular\_signaling\_cascade | 411 | 1 | 0.560219 | -0.072392 | 333 | 306.395158 | 399.19 | 491.984842 | 1.198769 |  |
| GO:0042592\_homeostatic\_process | 419 | 1 | 0.549523 | -0.069444 | 334 | 307.422158 | 399.95 | 492.477842 | 1.197455 |  |
| GO:0006464\_protein\_modification\_process | 439 | 1 | 0.524487 | -0.062605 | 335 | 310.248428 | 402.14 | 494.031572 | 1.200418 |  |
| GO:0031325\_positive\_regulation\_of\_cellular\_metabolic\_process | 442 | 1 | 0.520928 | -0.061641 | 336 | 310.749580 | 402.53 | 494.310420 | 1.198006 |  |
| GO:0030154\_cell\_differentiation | 1060 | 3 | 0.651651 | -0.059233 | 337 | 312.714635 | 404.08 | 495.445365 | 1.199050 |  |
| GO:0009893\_positive\_regulation\_of\_metabolic\_process | 458 | 1 | 0.502729 | -0.056748 | 339 | 314.184326 | 405.25 | 496.315674 | 1.195428 |  |
| GO:0043412\_biopolymer\_modification | 458 | 1 | 0.502729 | -0.056748 | 339 | 314.184326 | 405.25 | 496.315674 | 1.195428 |  |
| GO:0050896\_response\_to\_stimulus | 1107 | 3 | 0.623984 | -0.049299 | 340 | 316.096965 | 406.62 | 497.143035 | 1.195941 |  |
| GO:0048869\_cellular\_developmental\_process | 1113 | 3 | 0.620620 | -0.048144 | 341 | 316.771517 | 407.15 | 497.528483 | 1.193988 |  |
| GO:0002376\_immune\_system\_process | 505 | 1 | 0.455941 | -0.044538 | 342 | 317.346724 | 407.58 | 497.813276 | 1.191754 |  |
| GO:0032501\_multicellular\_organismal\_process | 2183 | 7 | 0.738319 | -0.040612 | 343 | 318.305530 | 408.33 | 498.354470 | 1.190466 |  |
| GO:0009888\_tissue\_development | 525 | 1 | 0.438571 | -0.040181 | 344 | 318.554745 | 408.52 | 498.485255 | 1.187558 |  |
| GO:0044267\_cellular\_protein\_metabolic\_process | 559 | 1 | 0.411896 | -0.033727 | 345 | 320.472283 | 410.0 | 499.527717 | 1.188406 |  |
| GO:0006355\_regulation\_of\_transcription\_\_DNA-dependent | 575 | 1 | 0.400435 | -0.031057 | 346 | 321.731419 | 410.97 | 500.208581 | 1.187775 |  |
| GO:0006351\_transcription\_\_DNA-dependent | 594 | 1 | 0.387626 | -0.028157 | 347 | 322.915302 | 411.75 | 500.584698 | 1.186599 |  |
| GO:0032774\_RNA\_biosynthetic\_process | 595 | 1 | 0.386975 | -0.028012 | 348 | 323.110407 | 411.89 | 500.669593 | 1.183592 |  |
| GO:0019538\_protein\_metabolic\_process | 655 | 1 | 0.351527 | -0.020529 | 349 | 325.164749 | 413.39 | 501.615251 | 1.184499 |  |
| GO:0050793\_regulation\_of\_developmental\_process | 703 | 1 | 0.327525 | -0.015983 | 350 | 327.300838 | 414.82 | 502.339162 | 1.185200 |  |
| GO:0002764\_immune\_response-regulating\_signal\_transduction | 51 | 0 | 0.000000 | -0.000000 | 359 | 342.362018 | 426.64 | 510.917982 | 1.188412 |  |
| GO:0006520\_cellular\_amino\_acid\_metabolic\_process | 51 | 0 | 0.000000 | -0.000000 | 359 | 342.362018 | 426.64 | 510.917982 | 1.188412 |  |
| GO:0006887\_exocytosis | 51 | 0 | 0.000000 | -0.000000 | 359 | 342.362018 | 426.64 | 510.917982 | 1.188412 |  |
| GO:0007601\_visual\_perception | 51 | 0 | 0.000000 | -0.000000 | 359 | 342.362018 | 426.64 | 510.917982 | 1.188412 |  |
| GO:0016569\_covalent\_chromatin\_modification | 51 | 0 | 0.000000 | -0.000000 | 359 | 342.362018 | 426.64 | 510.917982 | 1.188412 |  |
| GO:0032583\_regulation\_of\_gene-specific\_transcription | 51 | 0 | 0.000000 | -0.000000 | 359 | 342.362018 | 426.64 | 510.917982 | 1.188412 |  |
| GO:0032880\_regulation\_of\_protein\_localization | 51 | 0 | 0.000000 | -0.000000 | 359 | 342.362018 | 426.64 | 510.917982 | 1.188412 |  |
| GO:0043408\_regulation\_of\_MAPKKK\_cascade | 51 | 0 | 0.000000 | -0.000000 | 359 | 342.362018 | 426.64 | 510.917982 | 1.188412 |  |
| GO:0044106\_cellular\_amine\_metabolic\_process | 51 | 0 | 0.000000 | -0.000000 | 359 | 342.362018 | 426.64 | 510.917982 | 1.188412 |  |
| GO:0001708\_cell\_fate\_specification | 56 | 0 | 0.000000 | -0.000000 | 369 | 351.345524 | 434.83 | 518.314476 | 1.178401 |  |
| GO:0002683\_negative\_regulation\_of\_immune\_system\_process | 56 | 0 | 0.000000 | -0.000000 | 369 | 351.345524 | 434.83 | 518.314476 | 1.178401 |  |
| GO:0002703\_regulation\_of\_leukocyte\_mediated\_immunity | 56 | 0 | 0.000000 | -0.000000 | 369 | 351.345524 | 434.83 | 518.314476 | 1.178401 |  |
| GO:0006790\_sulfur\_metabolic\_process | 56 | 0 | 0.000000 | -0.000000 | 369 | 351.345524 | 434.83 | 518.314476 | 1.178401 |  |
| GO:0009187\_cyclic\_nucleotide\_metabolic\_process | 56 | 0 | 0.000000 | -0.000000 | 369 | 351.345524 | 434.83 | 518.314476 | 1.178401 |  |
| GO:0042089\_cytokine\_biosynthetic\_process | 56 | 0 | 0.000000 | -0.000000 | 369 | 351.345524 | 434.83 | 518.314476 | 1.178401 |  |
| GO:0042107\_cytokine\_metabolic\_process | 56 | 0 | 0.000000 | -0.000000 | 369 | 351.345524 | 434.83 | 518.314476 | 1.178401 |  |
| GO:0046486\_glycerolipid\_metabolic\_process | 56 | 0 | 0.000000 | -0.000000 | 369 | 351.345524 | 434.83 | 518.314476 | 1.178401 |  |
| GO:0050678\_regulation\_of\_epithelial\_cell\_proliferation | 56 | 0 | 0.000000 | -0.000000 | 369 | 351.345524 | 434.83 | 518.314476 | 1.178401 |  |
| GO:0051321\_meiotic\_cell\_cycle | 56 | 0 | 0.000000 | -0.000000 | 369 | 351.345524 | 434.83 | 518.314476 | 1.178401 |  |
| GO:0030029\_actin\_filament-based\_process | 109 | 0 | 0.000000 | -0.000000 | 370 | 351.915713 | 435.35 | 518.784287 | 1.176622 |  |
| GO:0001776\_leukocyte\_homeostasis | 41 | 0 | 0.000000 | -0.000000 | 387 | 369.658466 | 452.12 | 534.581534 | 1.168269 |  |
| GO:0002429\_immune\_response-activating\_cell\_surface\_receptor\_signaling\_pathway | 41 | 0 | 0.000000 | -0.000000 | 387 | 369.658466 | 452.12 | 534.581534 | 1.168269 |  |
| GO:0006979\_response\_to\_oxidative\_stress | 41 | 0 | 0.000000 | -0.000000 | 387 | 369.658466 | 452.12 | 534.581534 | 1.168269 |  |
| GO:0007254\_JNK\_cascade | 41 | 0 | 0.000000 | -0.000000 | 387 | 369.658466 | 452.12 | 534.581534 | 1.168269 |  |
| GO:0008585\_female\_gonad\_development | 41 | 0 | 0.000000 | -0.000000 | 387 | 369.658466 | 452.12 | 534.581534 | 1.168269 |  |
| GO:0009894\_regulation\_of\_catabolic\_process | 41 | 0 | 0.000000 | -0.000000 | 387 | 369.658466 | 452.12 | 534.581534 | 1.168269 |  |
| GO:0010551\_regulation\_of\_specific\_transcription\_from\_RNA\_polymerase\_II\_promoter | 41 | 0 | 0.000000 | -0.000000 | 387 | 369.658466 | 452.12 | 534.581534 | 1.168269 |  |
| GO:0015833\_peptide\_transport | 41 | 0 | 0.000000 | -0.000000 | 387 | 369.658466 | 452.12 | 534.581534 | 1.168269 |  |
| GO:0015980\_energy\_derivation\_by\_oxidation\_of\_organic\_compounds | 41 | 0 | 0.000000 | -0.000000 | 387 | 369.658466 | 452.12 | 534.581534 | 1.168269 |  |
| GO:0019216\_regulation\_of\_lipid\_metabolic\_process | 41 | 0 | 0.000000 | -0.000000 | 387 | 369.658466 | 452.12 | 534.581534 | 1.168269 |  |
| GO:0019748\_secondary\_metabolic\_process | 41 | 0 | 0.000000 | -0.000000 | 387 | 369.658466 | 452.12 | 534.581534 | 1.168269 |  |
| GO:0030817\_regulation\_of\_cAMP\_biosynthetic\_process | 41 | 0 | 0.000000 | -0.000000 | 387 | 369.658466 | 452.12 | 534.581534 | 1.168269 |  |
| GO:0031344\_regulation\_of\_cell\_projection\_organization | 41 | 0 | 0.000000 | -0.000000 | 387 | 369.658466 | 452.12 | 534.581534 | 1.168269 |  |
| GO:0032569\_specific\_transcription\_from\_RNA\_polymerase\_II\_promoter | 41 | 0 | 0.000000 | -0.000000 | 387 | 369.658466 | 452.12 | 534.581534 | 1.168269 |  |
| GO:0032844\_regulation\_of\_homeostatic\_process | 41 | 0 | 0.000000 | -0.000000 | 387 | 369.658466 | 452.12 | 534.581534 | 1.168269 |  |
| GO:0033077\_T\_cell\_differentiation\_in\_the\_thymus | 41 | 0 | 0.000000 | -0.000000 | 387 | 369.658466 | 452.12 | 534.581534 | 1.168269 |  |
| GO:0050864\_regulation\_of\_B\_cell\_activation | 41 | 0 | 0.000000 | -0.000000 | 387 | 369.658466 | 452.12 | 534.581534 | 1.168269 |  |
| GO:0000280\_nuclear\_division | 24 | 0 | 0.000000 | -0.000000 | 415 | 396.954734 | 478.42 | 559.885266 | 1.152819 |  |
| GO:0001541\_ovarian\_follicle\_development | 24 | 0 | 0.000000 | -0.000000 | 415 | 396.954734 | 478.42 | 559.885266 | 1.152819 |  |
| GO:0002381\_immunoglobulin\_production\_during\_immune\_response | 24 | 0 | 0.000000 | -0.000000 | 415 | 396.954734 | 478.42 | 559.885266 | 1.152819 |  |
| GO:0006650\_glycerophospholipid\_metabolic\_process | 24 | 0 | 0.000000 | -0.000000 | 415 | 396.954734 | 478.42 | 559.885266 | 1.152819 |  |
| GO:0006941\_striated\_muscle\_contraction | 24 | 0 | 0.000000 | -0.000000 | 415 | 396.954734 | 478.42 | 559.885266 | 1.152819 |  |
| GO:0006959\_humoral\_immune\_response | 24 | 0 | 0.000000 | -0.000000 | 415 | 396.954734 | 478.42 | 559.885266 | 1.152819 |  |
| GO:0007050\_cell\_cycle\_arrest | 24 | 0 | 0.000000 | -0.000000 | 415 | 396.954734 | 478.42 | 559.885266 | 1.152819 |  |
| GO:0007067\_mitosis | 24 | 0 | 0.000000 | -0.000000 | 415 | 396.954734 | 478.42 | 559.885266 | 1.152819 |  |
| GO:0007204\_elevation\_of\_cytosolic\_calcium\_ion\_concentration | 24 | 0 | 0.000000 | -0.000000 | 415 | 396.954734 | 478.42 | 559.885266 | 1.152819 |  |
| GO:0007259\_JAK-STAT\_cascade | 24 | 0 | 0.000000 | -0.000000 | 415 | 396.954734 | 478.42 | 559.885266 | 1.152819 |  |
| GO:0007266\_Rho\_protein\_signal\_transduction | 24 | 0 | 0.000000 | -0.000000 | 415 | 396.954734 | 478.42 | 559.885266 | 1.152819 |  |
| GO:0007632\_visual\_behavior | 24 | 0 | 0.000000 | -0.000000 | 415 | 396.954734 | 478.42 | 559.885266 | 1.152819 |  |
| GO:0009612\_response\_to\_mechanical\_stimulus | 24 | 0 | 0.000000 | -0.000000 | 415 | 396.954734 | 478.42 | 559.885266 | 1.152819 |  |
| GO:0014070\_response\_to\_organic\_cyclic\_substance | 24 | 0 | 0.000000 | -0.000000 | 415 | 396.954734 | 478.42 | 559.885266 | 1.152819 |  |
| GO:0021515\_cell\_differentiation\_in\_spinal\_cord | 24 | 0 | 0.000000 | -0.000000 | 415 | 396.954734 | 478.42 | 559.885266 | 1.152819 |  |
| GO:0032386\_regulation\_of\_intracellular\_transport | 24 | 0 | 0.000000 | -0.000000 | 415 | 396.954734 | 478.42 | 559.885266 | 1.152819 |  |
| GO:0042158\_lipoprotein\_biosynthetic\_process | 24 | 0 | 0.000000 | -0.000000 | 415 | 396.954734 | 478.42 | 559.885266 | 1.152819 |  |
| GO:0042632\_cholesterol\_homeostasis | 24 | 0 | 0.000000 | -0.000000 | 415 | 396.954734 | 478.42 | 559.885266 | 1.152819 |  |
| GO:0043410\_positive\_regulation\_of\_MAPKKK\_cascade | 24 | 0 | 0.000000 | -0.000000 | 415 | 396.954734 | 478.42 | 559.885266 | 1.152819 |  |
| GO:0043588\_skin\_development | 24 | 0 | 0.000000 | -0.000000 | 415 | 396.954734 | 478.42 | 559.885266 | 1.152819 |  |
| GO:0048002\_antigen\_processing\_and\_presentation\_of\_peptide\_antigen | 24 | 0 | 0.000000 | -0.000000 | 415 | 396.954734 | 478.42 | 559.885266 | 1.152819 |  |
| GO:0048546\_digestive\_tract\_morphogenesis | 24 | 0 | 0.000000 | -0.000000 | 415 | 396.954734 | 478.42 | 559.885266 | 1.152819 |  |
| GO:0050679\_positive\_regulation\_of\_epithelial\_cell\_proliferation | 24 | 0 | 0.000000 | -0.000000 | 415 | 396.954734 | 478.42 | 559.885266 | 1.152819 |  |
| GO:0051099\_positive\_regulation\_of\_binding | 24 | 0 | 0.000000 | -0.000000 | 415 | 396.954734 | 478.42 | 559.885266 | 1.152819 |  |
| GO:0055092\_sterol\_homeostasis | 24 | 0 | 0.000000 | -0.000000 | 415 | 396.954734 | 478.42 | 559.885266 | 1.152819 |  |
| GO:0060078\_regulation\_of\_postsynaptic\_membrane\_potential | 24 | 0 | 0.000000 | -0.000000 | 415 | 396.954734 | 478.42 | 559.885266 | 1.152819 |  |
| GO:0060113\_inner\_ear\_receptor\_cell\_differentiation | 24 | 0 | 0.000000 | -0.000000 | 415 | 396.954734 | 478.42 | 559.885266 | 1.152819 |  |
| GO:0070667\_negative\_regulation\_of\_mast\_cell\_proliferation | 24 | 0 | 0.000000 | -0.000000 | 415 | 396.954734 | 478.42 | 559.885266 | 1.152819 |  |
| GO:0001759\_induction\_of\_an\_organ | 15 | 0 | 0.000000 | -0.000000 | 466 | 450.789520 | 531.06 | 611.330480 | 1.139614 |  |
| GO:0001782\_B\_cell\_homeostasis | 15 | 0 | 0.000000 | -0.000000 | 466 | 450.789520 | 531.06 | 611.330480 | 1.139614 |  |
| GO:0001964\_startle\_response | 15 | 0 | 0.000000 | -0.000000 | 466 | 450.789520 | 531.06 | 611.330480 | 1.139614 |  |
| GO:0002286\_T\_cell\_activation\_during\_immune\_response | 15 | 0 | 0.000000 | -0.000000 | 466 | 450.789520 | 531.06 | 611.330480 | 1.139614 |  |
| GO:0002495\_antigen\_processing\_and\_presentation\_of\_peptide\_antigen\_via\_MHC\_class\_II | 15 | 0 | 0.000000 | -0.000000 | 466 | 450.789520 | 531.06 | 611.330480 | 1.139614 |  |
| GO:0002504\_antigen\_processing\_and\_presentation\_of\_peptide\_or\_polysaccharide\_antigen\_via\_MHC\_class\_II | 15 | 0 | 0.000000 | -0.000000 | 466 | 450.789520 | 531.06 | 611.330480 | 1.139614 |  |
| GO:0002709\_regulation\_of\_T\_cell\_mediated\_immunity | 15 | 0 | 0.000000 | -0.000000 | 466 | 450.789520 | 531.06 | 611.330480 | 1.139614 |  |
| GO:0006473\_protein\_amino\_acid\_acetylation | 15 | 0 | 0.000000 | -0.000000 | 466 | 450.789520 | 531.06 | 611.330480 | 1.139614 |  |
| GO:0006749\_glutathione\_metabolic\_process | 15 | 0 | 0.000000 | -0.000000 | 466 | 450.789520 | 531.06 | 611.330480 | 1.139614 |  |
| GO:0006885\_regulation\_of\_pH | 15 | 0 | 0.000000 | -0.000000 | 466 | 450.789520 | 531.06 | 611.330480 | 1.139614 |  |
| GO:0007040\_lysosome\_organization | 15 | 0 | 0.000000 | -0.000000 | 466 | 450.789520 | 531.06 | 611.330480 | 1.139614 |  |
| GO:0007173\_epidermal\_growth\_factor\_receptor\_signaling\_pathway | 15 | 0 | 0.000000 | -0.000000 | 466 | 450.789520 | 531.06 | 611.330480 | 1.139614 |  |
| GO:0007200\_activation\_of\_phospholipase\_C\_activity\_by\_G-protein\_coupled\_receptor\_protein\_signaling\_pathway\_coupled\_to\_IP3\_second\_messenger | 15 | 0 | 0.000000 | -0.000000 | 466 | 450.789520 | 531.06 | 611.330480 | 1.139614 |  |
| GO:0007202\_activation\_of\_phospholipase\_C\_activity | 15 | 0 | 0.000000 | -0.000000 | 466 | 450.789520 | 531.06 | 611.330480 | 1.139614 |  |
| GO:0007218\_neuropeptide\_signaling\_pathway | 15 | 0 | 0.000000 | -0.000000 | 466 | 450.789520 | 531.06 | 611.330480 | 1.139614 |  |
| GO:0007588\_excretion | 15 | 0 | 0.000000 | -0.000000 | 466 | 450.789520 | 531.06 | 611.330480 | 1.139614 |  |
| GO:0007618\_mating | 15 | 0 | 0.000000 | -0.000000 | 466 | 450.789520 | 531.06 | 611.330480 | 1.139614 |  |
| GO:0008543\_fibroblast\_growth\_factor\_receptor\_signaling\_pathway | 15 | 0 | 0.000000 | -0.000000 | 466 | 450.789520 | 531.06 | 611.330480 | 1.139614 |  |
| GO:0009116\_nucleoside\_metabolic\_process | 15 | 0 | 0.000000 | -0.000000 | 466 | 450.789520 | 531.06 | 611.330480 | 1.139614 |  |
| GO:0010092\_specification\_of\_organ\_identity | 15 | 0 | 0.000000 | -0.000000 | 466 | 450.789520 | 531.06 | 611.330480 | 1.139614 |  |
| GO:0010171\_body\_morphogenesis | 15 | 0 | 0.000000 | -0.000000 | 466 | 450.789520 | 531.06 | 611.330480 | 1.139614 |  |
| GO:0010518\_positive\_regulation\_of\_phospholipase\_activity | 15 | 0 | 0.000000 | -0.000000 | 466 | 450.789520 | 531.06 | 611.330480 | 1.139614 |  |
| GO:0010863\_positive\_regulation\_of\_phospholipase\_C\_activity | 15 | 0 | 0.000000 | -0.000000 | 466 | 450.789520 | 531.06 | 611.330480 | 1.139614 |  |
| GO:0019886\_antigen\_processing\_and\_presentation\_of\_exogenous\_peptide\_antigen\_via\_MHC\_class\_II | 15 | 0 | 0.000000 | -0.000000 | 466 | 450.789520 | 531.06 | 611.330480 | 1.139614 |  |
| GO:0021872\_generation\_of\_neurons\_in\_the\_forebrain | 15 | 0 | 0.000000 | -0.000000 | 466 | 450.789520 | 531.06 | 611.330480 | 1.139614 |  |
| GO:0022600\_digestive\_system\_process | 15 | 0 | 0.000000 | -0.000000 | 466 | 450.789520 | 531.06 | 611.330480 | 1.139614 |  |
| GO:0030041\_actin\_filament\_polymerization | 15 | 0 | 0.000000 | -0.000000 | 466 | 450.789520 | 531.06 | 611.330480 | 1.139614 |  |
| GO:0031069\_hair\_follicle\_morphogenesis | 15 | 0 | 0.000000 | -0.000000 | 466 | 450.789520 | 531.06 | 611.330480 | 1.139614 |  |
| GO:0031076\_embryonic\_camera-type\_eye\_development | 15 | 0 | 0.000000 | -0.000000 | 466 | 450.789520 | 531.06 | 611.330480 | 1.139614 |  |
| GO:0031329\_regulation\_of\_cellular\_catabolic\_process | 15 | 0 | 0.000000 | -0.000000 | 466 | 450.789520 | 531.06 | 611.330480 | 1.139614 |  |
| GO:0035116\_embryonic\_hindlimb\_morphogenesis | 15 | 0 | 0.000000 | -0.000000 | 466 | 450.789520 | 531.06 | 611.330480 | 1.139614 |  |
| GO:0035249\_synaptic\_transmission\_\_glutamatergic | 15 | 0 | 0.000000 | -0.000000 | 466 | 450.789520 | 531.06 | 611.330480 | 1.139614 |  |
| GO:0042306\_regulation\_of\_protein\_import\_into\_nucleus | 15 | 0 | 0.000000 | -0.000000 | 466 | 450.789520 | 531.06 | 611.330480 | 1.139614 |  |
| GO:0045666\_positive\_regulation\_of\_neuron\_differentiation | 15 | 0 | 0.000000 | -0.000000 | 466 | 450.789520 | 531.06 | 611.330480 | 1.139614 |  |
| GO:0046164\_alcohol\_catabolic\_process | 15 | 0 | 0.000000 | -0.000000 | 466 | 450.789520 | 531.06 | 611.330480 | 1.139614 |  |
| GO:0046638\_positive\_regulation\_of\_alpha-beta\_T\_cell\_differentiation | 15 | 0 | 0.000000 | -0.000000 | 466 | 450.789520 | 531.06 | 611.330480 | 1.139614 |  |
| GO:0048008\_platelet-derived\_growth\_factor\_receptor\_signaling\_pathway | 15 | 0 | 0.000000 | -0.000000 | 466 | 450.789520 | 531.06 | 611.330480 | 1.139614 |  |
| GO:0048010\_vascular\_endothelial\_growth\_factor\_receptor\_signaling\_pathway | 15 | 0 | 0.000000 | -0.000000 | 466 | 450.789520 | 531.06 | 611.330480 | 1.139614 |  |
| GO:0048144\_fibroblast\_proliferation | 15 | 0 | 0.000000 | -0.000000 | 466 | 450.789520 | 531.06 | 611.330480 | 1.139614 |  |
| GO:0048145\_regulation\_of\_fibroblast\_proliferation | 15 | 0 | 0.000000 | -0.000000 | 466 | 450.789520 | 531.06 | 611.330480 | 1.139614 |  |
| GO:0048610\_reproductive\_cellular\_process | 15 | 0 | 0.000000 | -0.000000 | 466 | 450.789520 | 531.06 | 611.330480 | 1.139614 |  |
| GO:0048709\_oligodendrocyte\_differentiation | 15 | 0 | 0.000000 | -0.000000 | 466 | 450.789520 | 531.06 | 611.330480 | 1.139614 |  |
| GO:0050729\_positive\_regulation\_of\_inflammatory\_response | 15 | 0 | 0.000000 | -0.000000 | 466 | 450.789520 | 531.06 | 611.330480 | 1.139614 |  |
| GO:0050796\_regulation\_of\_insulin\_secretion | 15 | 0 | 0.000000 | -0.000000 | 466 | 450.789520 | 531.06 | 611.330480 | 1.139614 |  |
| GO:0050798\_activated\_T\_cell\_proliferation | 15 | 0 | 0.000000 | -0.000000 | 466 | 450.789520 | 531.06 | 611.330480 | 1.139614 |  |
| GO:0055010\_ventricular\_cardiac\_muscle\_morphogenesis | 15 | 0 | 0.000000 | -0.000000 | 466 | 450.789520 | 531.06 | 611.330480 | 1.139614 |  |
| GO:0060322\_head\_development | 15 | 0 | 0.000000 | -0.000000 | 466 | 450.789520 | 531.06 | 611.330480 | 1.139614 |  |
| GO:0060425\_lung\_morphogenesis | 15 | 0 | 0.000000 | -0.000000 | 466 | 450.789520 | 531.06 | 611.330480 | 1.139614 |  |
| GO:0060442\_branching\_involved\_in\_prostate\_gland\_morphogenesis | 15 | 0 | 0.000000 | -0.000000 | 466 | 450.789520 | 531.06 | 611.330480 | 1.139614 |  |
| GO:0060749\_mammary\_gland\_alveolus\_development | 15 | 0 | 0.000000 | -0.000000 | 466 | 450.789520 | 531.06 | 611.330480 | 1.139614 |  |
| GO:0070227\_lymphocyte\_apoptosis | 15 | 0 | 0.000000 | -0.000000 | 466 | 450.789520 | 531.06 | 611.330480 | 1.139614 |  |
| GO:0006954\_inflammatory\_response | 96 | 0 | 0.000000 | -0.000000 | 471 | 455.221276 | 535.04 | 614.858724 | 1.135966 |  |
| GO:0048736\_appendage\_development | 96 | 0 | 0.000000 | -0.000000 | 471 | 455.221276 | 535.04 | 614.858724 | 1.135966 |  |
| GO:0060173\_limb\_development | 96 | 0 | 0.000000 | -0.000000 | 471 | 455.221276 | 535.04 | 614.858724 | 1.135966 |  |
| GO:0060249\_anatomical\_structure\_homeostasis | 96 | 0 | 0.000000 | -0.000000 | 471 | 455.221276 | 535.04 | 614.858724 | 1.135966 |  |
| GO:0070661\_leukocyte\_proliferation | 96 | 0 | 0.000000 | -0.000000 | 471 | 455.221276 | 535.04 | 614.858724 | 1.135966 |  |
| GO:0000079\_regulation\_of\_cyclin-dependent\_protein\_kinase\_activity | 7 | 0 | 0.000000 | -0.000000 | 650 | 633.874439 | 711.65 | 789.425561 | 1.094846 |  |
| GO:0000188\_inactivation\_of\_MAPK\_activity | 7 | 0 | 0.000000 | -0.000000 | 650 | 633.874439 | 711.65 | 789.425561 | 1.094846 |  |
| GO:0001504\_neurotransmitter\_uptake | 7 | 0 | 0.000000 | -0.000000 | 650 | 633.874439 | 711.65 | 789.425561 | 1.094846 |  |
| GO:0001556\_oocyte\_maturation | 7 | 0 | 0.000000 | -0.000000 | 650 | 633.874439 | 711.65 | 789.425561 | 1.094846 |  |
| GO:0001573\_ganglioside\_metabolic\_process | 7 | 0 | 0.000000 | -0.000000 | 650 | 633.874439 | 711.65 | 789.425561 | 1.094846 |  |
| GO:0001736\_establishment\_of\_planar\_polarity | 7 | 0 | 0.000000 | -0.000000 | 650 | 633.874439 | 711.65 | 789.425561 | 1.094846 |  |
| GO:0001839\_neural\_plate\_morphogenesis | 7 | 0 | 0.000000 | -0.000000 | 650 | 633.874439 | 711.65 | 789.425561 | 1.094846 |  |
| GO:0001936\_regulation\_of\_endothelial\_cell\_proliferation | 7 | 0 | 0.000000 | -0.000000 | 650 | 633.874439 | 711.65 | 789.425561 | 1.094846 |  |
| GO:0002011\_morphogenesis\_of\_an\_epithelial\_sheet | 7 | 0 | 0.000000 | -0.000000 | 650 | 633.874439 | 711.65 | 789.425561 | 1.094846 |  |
| GO:0002052\_positive\_regulation\_of\_neuroblast\_proliferation | 7 | 0 | 0.000000 | -0.000000 | 650 | 633.874439 | 711.65 | 789.425561 | 1.094846 |  |
| GO:0002063\_chondrocyte\_development | 7 | 0 | 0.000000 | -0.000000 | 650 | 633.874439 | 711.65 | 789.425561 | 1.094846 |  |
| GO:0002067\_glandular\_epithelial\_cell\_differentiation | 7 | 0 | 0.000000 | -0.000000 | 650 | 633.874439 | 711.65 | 789.425561 | 1.094846 |  |
| GO:0002076\_osteoblast\_development | 7 | 0 | 0.000000 | -0.000000 | 650 | 633.874439 | 711.65 | 789.425561 | 1.094846 |  |
| GO:0002087\_regulation\_of\_respiratory\_gaseous\_exchange\_by\_neurological\_system\_process | 7 | 0 | 0.000000 | -0.000000 | 650 | 633.874439 | 711.65 | 789.425561 | 1.094846 |  |
| GO:0002093\_auditory\_receptor\_cell\_morphogenesis | 7 | 0 | 0.000000 | -0.000000 | 650 | 633.874439 | 711.65 | 789.425561 | 1.094846 |  |
| GO:0002224\_toll-like\_receptor\_signaling\_pathway | 7 | 0 | 0.000000 | -0.000000 | 650 | 633.874439 | 711.65 | 789.425561 | 1.094846 |  |
| GO:0002455\_humoral\_immune\_response\_mediated\_by\_circulating\_immunoglobulin | 7 | 0 | 0.000000 | -0.000000 | 650 | 633.874439 | 711.65 | 789.425561 | 1.094846 |  |
| GO:0002643\_regulation\_of\_tolerance\_induction | 7 | 0 | 0.000000 | -0.000000 | 650 | 633.874439 | 711.65 | 789.425561 | 1.094846 |  |
| GO:0002645\_positive\_regulation\_of\_tolerance\_induction | 7 | 0 | 0.000000 | -0.000000 | 650 | 633.874439 | 711.65 | 789.425561 | 1.094846 |  |
| GO:0002714\_positive\_regulation\_of\_B\_cell\_mediated\_immunity | 7 | 0 | 0.000000 | -0.000000 | 650 | 633.874439 | 711.65 | 789.425561 | 1.094846 |  |
| GO:0002792\_negative\_regulation\_of\_peptide\_secretion | 7 | 0 | 0.000000 | -0.000000 | 650 | 633.874439 | 711.65 | 789.425561 | 1.094846 |  |
| GO:0002793\_positive\_regulation\_of\_peptide\_secretion | 7 | 0 | 0.000000 | -0.000000 | 650 | 633.874439 | 711.65 | 789.425561 | 1.094846 |  |
| GO:0002828\_regulation\_of\_T-helper\_2\_type\_immune\_response | 7 | 0 | 0.000000 | -0.000000 | 650 | 633.874439 | 711.65 | 789.425561 | 1.094846 |  |
| GO:0002863\_positive\_regulation\_of\_inflammatory\_response\_to\_antigenic\_stimulus | 7 | 0 | 0.000000 | -0.000000 | 650 | 633.874439 | 711.65 | 789.425561 | 1.094846 |  |
| GO:0002891\_positive\_regulation\_of\_immunoglobulin\_mediated\_immune\_response | 7 | 0 | 0.000000 | -0.000000 | 650 | 633.874439 | 711.65 | 789.425561 | 1.094846 |  |
| GO:0003084\_positive\_regulation\_of\_systemic\_arterial\_blood\_pressure | 7 | 0 | 0.000000 | -0.000000 | 650 | 633.874439 | 711.65 | 789.425561 | 1.094846 |  |
| GO:0003085\_negative\_regulation\_of\_systemic\_arterial\_blood\_pressure | 7 | 0 | 0.000000 | -0.000000 | 650 | 633.874439 | 711.65 | 789.425561 | 1.094846 |  |
| GO:0006014\_D-ribose\_metabolic\_process | 7 | 0 | 0.000000 | -0.000000 | 650 | 633.874439 | 711.65 | 789.425561 | 1.094846 |  |
| GO:0006041\_glucosamine\_metabolic\_process | 7 | 0 | 0.000000 | -0.000000 | 650 | 633.874439 | 711.65 | 789.425561 | 1.094846 |  |
| GO:0006044\_N-acetylglucosamine\_metabolic\_process | 7 | 0 | 0.000000 | -0.000000 | 650 | 633.874439 | 711.65 | 789.425561 | 1.094846 |  |
| GO:0006096\_glycolysis | 7 | 0 | 0.000000 | -0.000000 | 650 | 633.874439 | 711.65 | 789.425561 | 1.094846 |  |
| GO:0006119\_oxidative\_phosphorylation | 7 | 0 | 0.000000 | -0.000000 | 650 | 633.874439 | 711.65 | 789.425561 | 1.094846 |  |
| GO:0006298\_mismatch\_repair | 7 | 0 | 0.000000 | -0.000000 | 650 | 633.874439 | 711.65 | 789.425561 | 1.094846 |  |
| GO:0006352\_transcription\_initiation | 7 | 0 | 0.000000 | -0.000000 | 650 | 633.874439 | 711.65 | 789.425561 | 1.094846 |  |
| GO:0006401\_RNA\_catabolic\_process | 7 | 0 | 0.000000 | -0.000000 | 650 | 633.874439 | 711.65 | 789.425561 | 1.094846 |  |
| GO:0006406\_mRNA\_export\_from\_nucleus | 7 | 0 | 0.000000 | -0.000000 | 650 | 633.874439 | 711.65 | 789.425561 | 1.094846 |  |
| GO:0006505\_GPI\_anchor\_metabolic\_process | 7 | 0 | 0.000000 | -0.000000 | 650 | 633.874439 | 711.65 | 789.425561 | 1.094846 |  |
| GO:0006516\_glycoprotein\_catabolic\_process | 7 | 0 | 0.000000 | -0.000000 | 650 | 633.874439 | 711.65 | 789.425561 | 1.094846 |  |
| GO:0006612\_protein\_targeting\_to\_membrane | 7 | 0 | 0.000000 | -0.000000 | 650 | 633.874439 | 711.65 | 789.425561 | 1.094846 |  |
| GO:0006769\_nicotinamide\_metabolic\_process | 7 | 0 | 0.000000 | -0.000000 | 650 | 633.874439 | 711.65 | 789.425561 | 1.094846 |  |
| GO:0006783\_heme\_biosynthetic\_process | 7 | 0 | 0.000000 | -0.000000 | 650 | 633.874439 | 711.65 | 789.425561 | 1.094846 |  |
| GO:0006818\_hydrogen\_transport | 7 | 0 | 0.000000 | -0.000000 | 650 | 633.874439 | 711.65 | 789.425561 | 1.094846 |  |
| GO:0006878\_cellular\_copper\_ion\_homeostasis | 7 | 0 | 0.000000 | -0.000000 | 650 | 633.874439 | 711.65 | 789.425561 | 1.094846 |  |
| GO:0006949\_syncytium\_formation | 7 | 0 | 0.000000 | -0.000000 | 650 | 633.874439 | 711.65 | 789.425561 | 1.094846 |  |
| GO:0007019\_microtubule\_depolymerization | 7 | 0 | 0.000000 | -0.000000 | 650 | 633.874439 | 711.65 | 789.425561 | 1.094846 |  |
| GO:0007026\_negative\_regulation\_of\_microtubule\_depolymerization | 7 | 0 | 0.000000 | -0.000000 | 650 | 633.874439 | 711.65 | 789.425561 | 1.094846 |  |
| GO:0007034\_vacuolar\_transport | 7 | 0 | 0.000000 | -0.000000 | 650 | 633.874439 | 711.65 | 789.425561 | 1.094846 |  |
| GO:0007062\_sister\_chromatid\_cohesion | 7 | 0 | 0.000000 | -0.000000 | 650 | 633.874439 | 711.65 | 789.425561 | 1.094846 |  |
| GO:0007130\_synaptonemal\_complex\_assembly | 7 | 0 | 0.000000 | -0.000000 | 650 | 633.874439 | 711.65 | 789.425561 | 1.094846 |  |
| GO:0007164\_establishment\_of\_tissue\_polarity | 7 | 0 | 0.000000 | -0.000000 | 650 | 633.874439 | 711.65 | 789.425561 | 1.094846 |  |
| GO:0007191\_activation\_of\_adenylate\_cyclase\_activity\_by\_dopamine\_receptor\_signaling\_pathway | 7 | 0 | 0.000000 | -0.000000 | 650 | 633.874439 | 711.65 | 789.425561 | 1.094846 |  |
| GO:0007271\_synaptic\_transmission\_\_cholinergic | 7 | 0 | 0.000000 | -0.000000 | 650 | 633.874439 | 711.65 | 789.425561 | 1.094846 |  |
| GO:0007413\_axonal\_fasciculation | 7 | 0 | 0.000000 | -0.000000 | 650 | 633.874439 | 711.65 | 789.425561 | 1.094846 |  |
| GO:0007440\_foregut\_morphogenesis | 7 | 0 | 0.000000 | -0.000000 | 650 | 633.874439 | 711.65 | 789.425561 | 1.094846 |  |
| GO:0007616\_long-term\_memory | 7 | 0 | 0.000000 | -0.000000 | 650 | 633.874439 | 711.65 | 789.425561 | 1.094846 |  |
| GO:0008033\_tRNA\_processing | 7 | 0 | 0.000000 | -0.000000 | 650 | 633.874439 | 711.65 | 789.425561 | 1.094846 |  |
| GO:0008299\_isoprenoid\_biosynthetic\_process | 7 | 0 | 0.000000 | -0.000000 | 650 | 633.874439 | 711.65 | 789.425561 | 1.094846 |  |
| GO:0008340\_determination\_of\_adult\_lifespan | 7 | 0 | 0.000000 | -0.000000 | 650 | 633.874439 | 711.65 | 789.425561 | 1.094846 |  |
| GO:0009150\_purine\_ribonucleotide\_metabolic\_process | 7 | 0 | 0.000000 | -0.000000 | 650 | 633.874439 | 711.65 | 789.425561 | 1.094846 |  |
| GO:0009200\_deoxyribonucleoside\_triphosphate\_metabolic\_process | 7 | 0 | 0.000000 | -0.000000 | 650 | 633.874439 | 711.65 | 789.425561 | 1.094846 |  |
| GO:0009259\_ribonucleotide\_metabolic\_process | 7 | 0 | 0.000000 | -0.000000 | 650 | 633.874439 | 711.65 | 789.425561 | 1.094846 |  |
| GO:0009311\_oligosaccharide\_metabolic\_process | 7 | 0 | 0.000000 | -0.000000 | 650 | 633.874439 | 711.65 | 789.425561 | 1.094846 |  |
| GO:0009394\_2'-deoxyribonucleotide\_metabolic\_process | 7 | 0 | 0.000000 | -0.000000 | 650 | 633.874439 | 711.65 | 789.425561 | 1.094846 |  |
| GO:0009820\_alkaloid\_metabolic\_process | 7 | 0 | 0.000000 | -0.000000 | 650 | 633.874439 | 711.65 | 789.425561 | 1.094846 |  |
| GO:0010469\_regulation\_of\_receptor\_activity | 7 | 0 | 0.000000 | -0.000000 | 650 | 633.874439 | 711.65 | 789.425561 | 1.094846 |  |
| GO:0010948\_negative\_regulation\_of\_cell\_cycle\_process | 7 | 0 | 0.000000 | -0.000000 | 650 | 633.874439 | 711.65 | 789.425561 | 1.094846 |  |
| GO:0014047\_glutamate\_secretion | 7 | 0 | 0.000000 | -0.000000 | 650 | 633.874439 | 711.65 | 789.425561 | 1.094846 |  |
| GO:0014066\_regulation\_of\_phosphoinositide\_3-kinase\_cascade | 7 | 0 | 0.000000 | -0.000000 | 650 | 633.874439 | 711.65 | 789.425561 | 1.094846 |  |
| GO:0014821\_phasic\_smooth\_muscle\_contraction | 7 | 0 | 0.000000 | -0.000000 | 650 | 633.874439 | 711.65 | 789.425561 | 1.094846 |  |
| GO:0015697\_quaternary\_ammonium\_group\_transport | 7 | 0 | 0.000000 | -0.000000 | 650 | 633.874439 | 711.65 | 789.425561 | 1.094846 |  |
| GO:0015813\_L-glutamate\_transport | 7 | 0 | 0.000000 | -0.000000 | 650 | 633.874439 | 711.65 | 789.425561 | 1.094846 |  |
| GO:0015908\_fatty\_acid\_transport | 7 | 0 | 0.000000 | -0.000000 | 650 | 633.874439 | 711.65 | 789.425561 | 1.094846 |  |
| GO:0015914\_phospholipid\_transport | 7 | 0 | 0.000000 | -0.000000 | 650 | 633.874439 | 711.65 | 789.425561 | 1.094846 |  |
| GO:0015992\_proton\_transport | 7 | 0 | 0.000000 | -0.000000 | 650 | 633.874439 | 711.65 | 789.425561 | 1.094846 |  |
| GO:0016339\_calcium-dependent\_cell-cell\_adhesion | 7 | 0 | 0.000000 | -0.000000 | 650 | 633.874439 | 711.65 | 789.425561 | 1.094846 |  |
| GO:0016575\_histone\_deacetylation | 7 | 0 | 0.000000 | -0.000000 | 650 | 633.874439 | 711.65 | 789.425561 | 1.094846 |  |
| GO:0019362\_pyridine\_nucleotide\_metabolic\_process | 7 | 0 | 0.000000 | -0.000000 | 650 | 633.874439 | 711.65 | 789.425561 | 1.094846 |  |
| GO:0019692\_deoxyribose\_phosphate\_metabolic\_process | 7 | 0 | 0.000000 | -0.000000 | 650 | 633.874439 | 711.65 | 789.425561 | 1.094846 |  |
| GO:0019800\_peptide\_cross-linking\_via\_chondroitin\_4-sulfate\_glycosaminoglycan | 7 | 0 | 0.000000 | -0.000000 | 650 | 633.874439 | 711.65 | 789.425561 | 1.094846 |  |
| GO:0020027\_hemoglobin\_metabolic\_process | 7 | 0 | 0.000000 | -0.000000 | 650 | 633.874439 | 711.65 | 789.425561 | 1.094846 |  |
| GO:0021514\_ventral\_spinal\_cord\_interneuron\_differentiation | 7 | 0 | 0.000000 | -0.000000 | 650 | 633.874439 | 711.65 | 789.425561 | 1.094846 |  |
| GO:0021516\_dorsal\_spinal\_cord\_development | 7 | 0 | 0.000000 | -0.000000 | 650 | 633.874439 | 711.65 | 789.425561 | 1.094846 |  |
| GO:0021520\_spinal\_cord\_motor\_neuron\_cell\_fate\_specification | 7 | 0 | 0.000000 | -0.000000 | 650 | 633.874439 | 711.65 | 789.425561 | 1.094846 |  |
| GO:0021521\_ventral\_spinal\_cord\_interneuron\_specification | 7 | 0 | 0.000000 | -0.000000 | 650 | 633.874439 | 711.65 | 789.425561 | 1.094846 |  |
| GO:0021546\_rhombomere\_development | 7 | 0 | 0.000000 | -0.000000 | 650 | 633.874439 | 711.65 | 789.425561 | 1.094846 |  |
| GO:0021756\_striatum\_development | 7 | 0 | 0.000000 | -0.000000 | 650 | 633.874439 | 711.65 | 789.425561 | 1.094846 |  |
| GO:0021884\_forebrain\_neuron\_development | 7 | 0 | 0.000000 | -0.000000 | 650 | 633.874439 | 711.65 | 789.425561 | 1.094846 |  |
| GO:0021903\_rostrocaudal\_neural\_tube\_patterning | 7 | 0 | 0.000000 | -0.000000 | 650 | 633.874439 | 711.65 | 789.425561 | 1.094846 |  |
| GO:0021984\_adenohypophysis\_development | 7 | 0 | 0.000000 | -0.000000 | 650 | 633.874439 | 711.65 | 789.425561 | 1.094846 |  |
| GO:0022407\_regulation\_of\_cell-cell\_adhesion | 7 | 0 | 0.000000 | -0.000000 | 650 | 633.874439 | 711.65 | 789.425561 | 1.094846 |  |
| GO:0022618\_ribonucleoprotein\_complex\_assembly | 7 | 0 | 0.000000 | -0.000000 | 650 | 633.874439 | 711.65 | 789.425561 | 1.094846 |  |
| GO:0030104\_water\_homeostasis | 7 | 0 | 0.000000 | -0.000000 | 650 | 633.874439 | 711.65 | 789.425561 | 1.094846 |  |
| GO:0030201\_heparan\_sulfate\_proteoglycan\_metabolic\_process | 7 | 0 | 0.000000 | -0.000000 | 650 | 633.874439 | 711.65 | 789.425561 | 1.094846 |  |
| GO:0030432\_peristalsis | 7 | 0 | 0.000000 | -0.000000 | 650 | 633.874439 | 711.65 | 789.425561 | 1.094846 |  |
| GO:0030517\_negative\_regulation\_of\_axon\_extension | 7 | 0 | 0.000000 | -0.000000 | 650 | 633.874439 | 711.65 | 789.425561 | 1.094846 |  |
| GO:0030520\_estrogen\_receptor\_signaling\_pathway | 7 | 0 | 0.000000 | -0.000000 | 650 | 633.874439 | 711.65 | 789.425561 | 1.094846 |  |
| GO:0030521\_androgen\_receptor\_signaling\_pathway | 7 | 0 | 0.000000 | -0.000000 | 650 | 633.874439 | 711.65 | 789.425561 | 1.094846 |  |
| GO:0030903\_notochord\_development | 7 | 0 | 0.000000 | -0.000000 | 650 | 633.874439 | 711.65 | 789.425561 | 1.094846 |  |
| GO:0031017\_exocrine\_pancreas\_development | 7 | 0 | 0.000000 | -0.000000 | 650 | 633.874439 | 711.65 | 789.425561 | 1.094846 |  |
| GO:0031114\_regulation\_of\_microtubule\_depolymerization | 7 | 0 | 0.000000 | -0.000000 | 650 | 633.874439 | 711.65 | 789.425561 | 1.094846 |  |
| GO:0031124\_mRNA\_3'-end\_processing | 7 | 0 | 0.000000 | -0.000000 | 650 | 633.874439 | 711.65 | 789.425561 | 1.094846 |  |
| GO:0031497\_chromatin\_assembly | 7 | 0 | 0.000000 | -0.000000 | 650 | 633.874439 | 711.65 | 789.425561 | 1.094846 |  |
| GO:0032104\_regulation\_of\_response\_to\_extracellular\_stimulus | 7 | 0 | 0.000000 | -0.000000 | 650 | 633.874439 | 711.65 | 789.425561 | 1.094846 |  |
| GO:0032107\_regulation\_of\_response\_to\_nutrient\_levels | 7 | 0 | 0.000000 | -0.000000 | 650 | 633.874439 | 711.65 | 789.425561 | 1.094846 |  |
| GO:0032228\_regulation\_of\_synaptic\_transmission\_\_GABAergic | 7 | 0 | 0.000000 | -0.000000 | 650 | 633.874439 | 711.65 | 789.425561 | 1.094846 |  |
| GO:0032319\_regulation\_of\_Rho\_GTPase\_activity | 7 | 0 | 0.000000 | -0.000000 | 650 | 633.874439 | 711.65 | 789.425561 | 1.094846 |  |
| GO:0032387\_negative\_regulation\_of\_intracellular\_transport | 7 | 0 | 0.000000 | -0.000000 | 650 | 633.874439 | 711.65 | 789.425561 | 1.094846 |  |
| GO:0032507\_maintenance\_of\_protein\_location\_in\_cell | 7 | 0 | 0.000000 | -0.000000 | 650 | 633.874439 | 711.65 | 789.425561 | 1.094846 |  |
| GO:0033032\_regulation\_of\_myeloid\_cell\_apoptosis | 7 | 0 | 0.000000 | -0.000000 | 650 | 633.874439 | 711.65 | 789.425561 | 1.094846 |  |
| GO:0033057\_reproductive\_behavior\_in\_a\_multicellular\_organism | 7 | 0 | 0.000000 | -0.000000 | 650 | 633.874439 | 711.65 | 789.425561 | 1.094846 |  |
| GO:0034599\_cellular\_response\_to\_oxidative\_stress | 7 | 0 | 0.000000 | -0.000000 | 650 | 633.874439 | 711.65 | 789.425561 | 1.094846 |  |
| GO:0042033\_chemokine\_biosynthetic\_process | 7 | 0 | 0.000000 | -0.000000 | 650 | 633.874439 | 711.65 | 789.425561 | 1.094846 |  |
| GO:0042133\_neurotransmitter\_metabolic\_process | 7 | 0 | 0.000000 | -0.000000 | 650 | 633.874439 | 711.65 | 789.425561 | 1.094846 |  |
| GO:0042168\_heme\_metabolic\_process | 7 | 0 | 0.000000 | -0.000000 | 650 | 633.874439 | 711.65 | 789.425561 | 1.094846 |  |
| GO:0042415\_norepinephrine\_metabolic\_process | 7 | 0 | 0.000000 | -0.000000 | 650 | 633.874439 | 711.65 | 789.425561 | 1.094846 |  |
| GO:0042438\_melanin\_biosynthetic\_process | 7 | 0 | 0.000000 | -0.000000 | 650 | 633.874439 | 711.65 | 789.425561 | 1.094846 |  |
| GO:0042503\_tyrosine\_phosphorylation\_of\_Stat3\_protein | 7 | 0 | 0.000000 | -0.000000 | 650 | 633.874439 | 711.65 | 789.425561 | 1.094846 |  |
| GO:0042572\_retinol\_metabolic\_process | 7 | 0 | 0.000000 | -0.000000 | 650 | 633.874439 | 711.65 | 789.425561 | 1.094846 |  |
| GO:0043353\_enucleate\_erythrocyte\_differentiation | 7 | 0 | 0.000000 | -0.000000 | 650 | 633.874439 | 711.65 | 789.425561 | 1.094846 |  |
| GO:0043372\_positive\_regulation\_of\_CD4-positive\_\_alpha\_beta\_T\_cell\_differentiation | 7 | 0 | 0.000000 | -0.000000 | 650 | 633.874439 | 711.65 | 789.425561 | 1.094846 |  |
| GO:0043449\_cellular\_alkene\_metabolic\_process | 7 | 0 | 0.000000 | -0.000000 | 650 | 633.874439 | 711.65 | 789.425561 | 1.094846 |  |
| GO:0043507\_positive\_regulation\_of\_JUN\_kinase\_activity | 7 | 0 | 0.000000 | -0.000000 | 650 | 633.874439 | 711.65 | 789.425561 | 1.094846 |  |
| GO:0043567\_regulation\_of\_insulin-like\_growth\_factor\_receptor\_signaling\_pathway | 7 | 0 | 0.000000 | -0.000000 | 650 | 633.874439 | 711.65 | 789.425561 | 1.094846 |  |
| GO:0043584\_nose\_development | 7 | 0 | 0.000000 | -0.000000 | 650 | 633.874439 | 711.65 | 789.425561 | 1.094846 |  |
| GO:0044065\_regulation\_of\_respiratory\_system\_process | 7 | 0 | 0.000000 | -0.000000 | 650 | 633.874439 | 711.65 | 789.425561 | 1.094846 |  |
| GO:0044275\_cellular\_carbohydrate\_catabolic\_process | 7 | 0 | 0.000000 | -0.000000 | 650 | 633.874439 | 711.65 | 789.425561 | 1.094846 |  |
| GO:0045059\_positive\_thymic\_T\_cell\_selection | 7 | 0 | 0.000000 | -0.000000 | 650 | 633.874439 | 711.65 | 789.425561 | 1.094846 |  |
| GO:0045073\_regulation\_of\_chemokine\_biosynthetic\_process | 7 | 0 | 0.000000 | -0.000000 | 650 | 633.874439 | 711.65 | 789.425561 | 1.094846 |  |
| GO:0045581\_negative\_regulation\_of\_T\_cell\_differentiation | 7 | 0 | 0.000000 | -0.000000 | 650 | 633.874439 | 711.65 | 789.425561 | 1.094846 |  |
| GO:0045599\_negative\_regulation\_of\_fat\_cell\_differentiation | 7 | 0 | 0.000000 | -0.000000 | 650 | 633.874439 | 711.65 | 789.425561 | 1.094846 |  |
| GO:0045604\_regulation\_of\_epidermal\_cell\_differentiation | 7 | 0 | 0.000000 | -0.000000 | 650 | 633.874439 | 711.65 | 789.425561 | 1.094846 |  |
| GO:0045668\_negative\_regulation\_of\_osteoblast\_differentiation | 7 | 0 | 0.000000 | -0.000000 | 650 | 633.874439 | 711.65 | 789.425561 | 1.094846 |  |
| GO:0045823\_positive\_regulation\_of\_heart\_contraction | 7 | 0 | 0.000000 | -0.000000 | 650 | 633.874439 | 711.65 | 789.425561 | 1.094846 |  |
| GO:0045840\_positive\_regulation\_of\_mitosis | 7 | 0 | 0.000000 | -0.000000 | 650 | 633.874439 | 711.65 | 789.425561 | 1.094846 |  |
| GO:0045862\_positive\_regulation\_of\_proteolysis | 7 | 0 | 0.000000 | -0.000000 | 650 | 633.874439 | 711.65 | 789.425561 | 1.094846 |  |
| GO:0045879\_negative\_regulation\_of\_smoothened\_signaling\_pathway | 7 | 0 | 0.000000 | -0.000000 | 650 | 633.874439 | 711.65 | 789.425561 | 1.094846 |  |
| GO:0045880\_positive\_regulation\_of\_smoothened\_signaling\_pathway | 7 | 0 | 0.000000 | -0.000000 | 650 | 633.874439 | 711.65 | 789.425561 | 1.094846 |  |
| GO:0045986\_negative\_regulation\_of\_smooth\_muscle\_contraction | 7 | 0 | 0.000000 | -0.000000 | 650 | 633.874439 | 711.65 | 789.425561 | 1.094846 |  |
| GO:0046496\_nicotinamide\_nucleotide\_metabolic\_process | 7 | 0 | 0.000000 | -0.000000 | 650 | 633.874439 | 711.65 | 789.425561 | 1.094846 |  |
| GO:0046504\_glycerol\_ether\_biosynthetic\_process | 7 | 0 | 0.000000 | -0.000000 | 650 | 633.874439 | 711.65 | 789.425561 | 1.094846 |  |
| GO:0046513\_ceramide\_biosynthetic\_process | 7 | 0 | 0.000000 | -0.000000 | 650 | 633.874439 | 711.65 | 789.425561 | 1.094846 |  |
| GO:0046520\_sphingoid\_biosynthetic\_process | 7 | 0 | 0.000000 | -0.000000 | 650 | 633.874439 | 711.65 | 789.425561 | 1.094846 |  |
| GO:0046543\_development\_of\_secondary\_female\_sexual\_characteristics | 7 | 0 | 0.000000 | -0.000000 | 650 | 633.874439 | 711.65 | 789.425561 | 1.094846 |  |
| GO:0046622\_positive\_regulation\_of\_organ\_growth | 7 | 0 | 0.000000 | -0.000000 | 650 | 633.874439 | 711.65 | 789.425561 | 1.094846 |  |
| GO:0046626\_regulation\_of\_insulin\_receptor\_signaling\_pathway | 7 | 0 | 0.000000 | -0.000000 | 650 | 633.874439 | 711.65 | 789.425561 | 1.094846 |  |
| GO:0046676\_negative\_regulation\_of\_insulin\_secretion | 7 | 0 | 0.000000 | -0.000000 | 650 | 633.874439 | 711.65 | 789.425561 | 1.094846 |  |
| GO:0046677\_response\_to\_antibiotic | 7 | 0 | 0.000000 | -0.000000 | 650 | 633.874439 | 711.65 | 789.425561 | 1.094846 |  |
| GO:0046823\_negative\_regulation\_of\_nucleocytoplasmic\_transport | 7 | 0 | 0.000000 | -0.000000 | 650 | 633.874439 | 711.65 | 789.425561 | 1.094846 |  |
| GO:0046824\_positive\_regulation\_of\_nucleocytoplasmic\_transport | 7 | 0 | 0.000000 | -0.000000 | 650 | 633.874439 | 711.65 | 789.425561 | 1.094846 |  |
| GO:0046847\_filopodium\_assembly | 7 | 0 | 0.000000 | -0.000000 | 650 | 633.874439 | 711.65 | 789.425561 | 1.094846 |  |
| GO:0048148\_behavioral\_response\_to\_cocaine | 7 | 0 | 0.000000 | -0.000000 | 650 | 633.874439 | 711.65 | 789.425561 | 1.094846 |  |
| GO:0048304\_positive\_regulation\_of\_isotype\_switching\_to\_IgG\_isotypes | 7 | 0 | 0.000000 | -0.000000 | 650 | 633.874439 | 711.65 | 789.425561 | 1.094846 |  |
| GO:0048486\_parasympathetic\_nervous\_system\_development | 7 | 0 | 0.000000 | -0.000000 | 650 | 633.874439 | 711.65 | 789.425561 | 1.094846 |  |
| GO:0048537\_mucosal-associated\_lymphoid\_tissue\_development | 7 | 0 | 0.000000 | -0.000000 | 650 | 633.874439 | 711.65 | 789.425561 | 1.094846 |  |
| GO:0048753\_pigment\_granule\_organization | 7 | 0 | 0.000000 | -0.000000 | 650 | 633.874439 | 711.65 | 789.425561 | 1.094846 |  |
| GO:0048814\_regulation\_of\_dendrite\_morphogenesis | 7 | 0 | 0.000000 | -0.000000 | 650 | 633.874439 | 711.65 | 789.425561 | 1.094846 |  |
| GO:0048857\_neural\_nucleus\_development | 7 | 0 | 0.000000 | -0.000000 | 650 | 633.874439 | 711.65 | 789.425561 | 1.094846 |  |
| GO:0050755\_chemokine\_metabolic\_process | 7 | 0 | 0.000000 | -0.000000 | 650 | 633.874439 | 711.65 | 789.425561 | 1.094846 |  |
| GO:0050773\_regulation\_of\_dendrite\_development | 7 | 0 | 0.000000 | -0.000000 | 650 | 633.874439 | 711.65 | 789.425561 | 1.094846 |  |
| GO:0051028\_mRNA\_transport | 7 | 0 | 0.000000 | -0.000000 | 650 | 633.874439 | 711.65 | 789.425561 | 1.094846 |  |
| GO:0051785\_positive\_regulation\_of\_nuclear\_division | 7 | 0 | 0.000000 | -0.000000 | 650 | 633.874439 | 711.65 | 789.425561 | 1.094846 |  |
| GO:0051928\_positive\_regulation\_of\_calcium\_ion\_transport | 7 | 0 | 0.000000 | -0.000000 | 650 | 633.874439 | 711.65 | 789.425561 | 1.094846 |  |
| GO:0055069\_zinc\_ion\_homeostasis | 7 | 0 | 0.000000 | -0.000000 | 650 | 633.874439 | 711.65 | 789.425561 | 1.094846 |  |
| GO:0055070\_copper\_ion\_homeostasis | 7 | 0 | 0.000000 | -0.000000 | 650 | 633.874439 | 711.65 | 789.425561 | 1.094846 |  |
| GO:0060037\_pharyngeal\_system\_development | 7 | 0 | 0.000000 | -0.000000 | 650 | 633.874439 | 711.65 | 789.425561 | 1.094846 |  |
| GO:0060080\_regulation\_of\_inhibitory\_postsynaptic\_membrane\_potential | 7 | 0 | 0.000000 | -0.000000 | 650 | 633.874439 | 711.65 | 789.425561 | 1.094846 |  |
| GO:0060088\_auditory\_receptor\_cell\_stereocilium\_organization | 7 | 0 | 0.000000 | -0.000000 | 650 | 633.874439 | 711.65 | 789.425561 | 1.094846 |  |
| GO:0060117\_auditory\_receptor\_cell\_development | 7 | 0 | 0.000000 | -0.000000 | 650 | 633.874439 | 711.65 | 789.425561 | 1.094846 |  |
| GO:0060441\_branching\_involved\_in\_lung\_morphogenesis | 7 | 0 | 0.000000 | -0.000000 | 650 | 633.874439 | 711.65 | 789.425561 | 1.094846 |  |
| GO:0060526\_prostate\_glandular\_acinus\_morphogenesis | 7 | 0 | 0.000000 | -0.000000 | 650 | 633.874439 | 711.65 | 789.425561 | 1.094846 |  |
| GO:0060527\_prostate\_epithelial\_cord\_arborization\_involved\_in\_prostate\_glandular\_acinus\_morphogenesis | 7 | 0 | 0.000000 | -0.000000 | 650 | 633.874439 | 711.65 | 789.425561 | 1.094846 |  |
| GO:0060579\_ventral\_spinal\_cord\_interneuron\_fate\_commitment | 7 | 0 | 0.000000 | -0.000000 | 650 | 633.874439 | 711.65 | 789.425561 | 1.094846 |  |
| GO:0060664\_epithelial\_cell\_proliferation\_involved\_in\_salivary\_gland\_morphogenesis | 7 | 0 | 0.000000 | -0.000000 | 650 | 633.874439 | 711.65 | 789.425561 | 1.094846 |  |
| GO:0060687\_regulation\_of\_branching\_involved\_in\_prostate\_gland\_morphogenesis | 7 | 0 | 0.000000 | -0.000000 | 650 | 633.874439 | 711.65 | 789.425561 | 1.094846 |  |
| GO:0060770\_negative\_regulation\_of\_epithelial\_cell\_proliferation\_involved\_in\_prostate\_gland\_development | 7 | 0 | 0.000000 | -0.000000 | 650 | 633.874439 | 711.65 | 789.425561 | 1.094846 |  |
| GO:0060788\_ectodermal\_placode\_formation | 7 | 0 | 0.000000 | -0.000000 | 650 | 633.874439 | 711.65 | 789.425561 | 1.094846 |  |
| GO:0060795\_cell\_fate\_commitment\_involved\_in\_the\_formation\_of\_primary\_germ\_layers | 7 | 0 | 0.000000 | -0.000000 | 650 | 633.874439 | 711.65 | 789.425561 | 1.094846 |  |
| GO:0070228\_regulation\_of\_lymphocyte\_apoptosis | 7 | 0 | 0.000000 | -0.000000 | 650 | 633.874439 | 711.65 | 789.425561 | 1.094846 |  |
| GO:0070646\_protein\_modification\_by\_small\_protein\_removal | 7 | 0 | 0.000000 | -0.000000 | 650 | 633.874439 | 711.65 | 789.425561 | 1.094846 |  |
| GO:0000027\_ribosomal\_large\_subunit\_assembly | 1 | 0 |  |  |  |  |  |  |  |  |
| GO:0000042\_protein\_targeting\_to\_Golgi | 1 | 0 |  |  |  |  |  |  |  |  |
| GO:0000046\_autophagic\_vacuole\_fusion | 1 | 0 |  |  |  |  |  |  |  |  |
| GO:0000050\_urea\_cycle | 1 | 0 |  |  |  |  |  |  |  |  |
| GO:0000072\_M\_phase\_specific\_microtubule\_process | 1 | 0 |  |  |  |  |  |  |  |  |
| GO:0000101\_sulfur\_amino\_acid\_transport | 1 | 0 |  |  |  |  |  |  |  |  |
| GO:0000147\_actin\_cortical\_patch\_assembly | 1 | 0 |  |  |  |  |  |  |  |  |
| GO:0000154\_rRNA\_modification | 1 | 0 |  |  |  |  |  |  |  |  |
| GO:0000183\_chromatin\_silencing\_at\_rDNA | 1 | 0 |  |  |  |  |  |  |  |  |
| GO:0000185\_activation\_of\_MAPKKK\_activity | 1 | 0 |  |  |  |  |  |  |  |  |
| GO:0000238\_zygotene | 1 | 0 |  |  |  |  |  |  |  |  |
| GO:0000255\_allantoin\_metabolic\_process | 1 | 0 |  |  |  |  |  |  |  |  |
| GO:0000266\_mitochondrial\_fission | 1 | 0 |  |  |  |  |  |  |  |  |
| GO:0000273\_lipoic\_acid\_metabolic\_process | 1 | 0 |  |  |  |  |  |  |  |  |
| GO:0000301\_retrograde\_transport\_\_vesicle\_recycling\_within\_Golgi | 1 | 0 |  |  |  |  |  |  |  |  |
| GO:0000394\_RNA\_splicing\_\_via\_endonucleolytic\_cleavage\_and\_ligation | 1 | 0 |  |  |  |  |  |  |  |  |
| GO:0000429\_regulation\_of\_transcription\_from\_RNA\_polymerase\_II\_promoter\_by\_carbon\_catabolites | 1 | 0 |  |  |  |  |  |  |  |  |
| GO:0000430\_regulation\_of\_transcription\_from\_RNA\_polymerase\_II\_promoter\_by\_glucose | 1 | 0 |  |  |  |  |  |  |  |  |
| GO:0000432\_positive\_regulation\_of\_transcription\_from\_RNA\_polymerase\_II\_promoter\_by\_glucose | 1 | 0 |  |  |  |  |  |  |  |  |
| GO:0000436\_positive\_regulation\_of\_transcription\_from\_RNA\_polymerase\_II\_promoter\_by\_carbon\_catabolites | 1 | 0 |  |  |  |  |  |  |  |  |
| GO:0000448\_cleavage\_in\_ITS2\_between\_5.8S\_rRNA\_and\_LSU-rRNA\_of\_tricistronic\_rRNA\_transcript\_(SSU-rRNA\_\_5.8S\_rRNA\_\_LSU-rRNA) | 1 | 0 |  |  |  |  |  |  |  |  |
| GO:0000460\_maturation\_of\_5.8S\_rRNA | 1 | 0 |  |  |  |  |  |  |  |  |
| GO:0000463\_maturation\_of\_LSU-rRNA\_from\_tricistronic\_rRNA\_transcript\_(SSU-rRNA\_\_5.8S\_rRNA\_\_LSU-rRNA) | 1 | 0 |  |  |  |  |  |  |  |  |
| GO:0000466\_maturation\_of\_5.8S\_rRNA\_from\_tricistronic\_rRNA\_transcript\_(SSU-rRNA\_\_5.8S\_rRNA\_\_LSU-rRNA) | 1 | 0 |  |  |  |  |  |  |  |  |
| GO:0000469\_cleavages\_during\_rRNA\_processing | 1 | 0 |  |  |  |  |  |  |  |  |
| GO:0000470\_maturation\_of\_LSU-rRNA | 1 | 0 |  |  |  |  |  |  |  |  |
| GO:0000478\_endonucleolytic\_cleavages\_during\_rRNA\_processing | 1 | 0 |  |  |  |  |  |  |  |  |
| GO:0000479\_endonucleolytic\_cleavage\_of\_tricistronic\_rRNA\_transcript\_(SSU-rRNA\_\_5.8S\_rRNA\_\_LSU-rRNA) | 1 | 0 |  |  |  |  |  |  |  |  |
| GO:0000705\_achiasmate\_meiosis\_I | 1 | 0 |  |  |  |  |  |  |  |  |
| GO:0000966\_RNA\_5'-end\_processing | 1 | 0 |  |  |  |  |  |  |  |  |
| GO:0001300\_chronological\_cell\_aging | 1 | 0 |  |  |  |  |  |  |  |  |
| GO:0001547\_antral\_ovarian\_follicle\_growth | 1 | 0 |  |  |  |  |  |  |  |  |
| GO:0001555\_oocyte\_growth | 1 | 0 |  |  |  |  |  |  |  |  |
| GO:0001560\_regulation\_of\_cell\_growth\_by\_extracellular\_stimulus | 1 | 0 |  |  |  |  |  |  |  |  |
| GO:0001660\_fever | 1 | 0 |  |  |  |  |  |  |  |  |
| GO:0001696\_gastric\_acid\_secretion | 1 | 0 |  |  |  |  |  |  |  |  |
| GO:0001712\_ectodermal\_cell\_fate\_commitment | 1 | 0 |  |  |  |  |  |  |  |  |
| GO:0001714\_endodermal\_cell\_fate\_specification | 1 | 0 |  |  |  |  |  |  |  |  |
| GO:0001762\_beta-alanine\_transport | 1 | 0 |  |  |  |  |  |  |  |  |
| GO:0001766\_membrane\_raft\_polarization | 1 | 0 |  |  |  |  |  |  |  |  |
| GO:0001811\_negative\_regulation\_of\_type\_I\_hypersensitivity | 1 | 0 |  |  |  |  |  |  |  |  |
| GO:0001821\_histamine\_secretion | 1 | 0 |  |  |  |  |  |  |  |  |
| GO:0001826\_inner\_cell\_mass\_cell\_differentiation | 1 | 0 |  |  |  |  |  |  |  |  |
| GO:0001830\_trophectodermal\_cell\_fate\_commitment | 1 | 0 |  |  |  |  |  |  |  |  |
| GO:0001834\_trophectodermal\_cell\_proliferation | 1 | 0 |  |  |  |  |  |  |  |  |
| GO:0001867\_complement\_activation\_\_lectin\_pathway | 1 | 0 |  |  |  |  |  |  |  |  |
| GO:0001880\_Mullerian\_duct\_regression | 1 | 0 |  |  |  |  |  |  |  |  |
| GO:0001887\_selenium\_metabolic\_process | 1 | 0 |  |  |  |  |  |  |  |  |
| GO:0001922\_B-1\_B\_cell\_homeostasis | 1 | 0 |  |  |  |  |  |  |  |  |
| GO:0001923\_B-1\_B\_cell\_differentiation | 1 | 0 |  |  |  |  |  |  |  |  |
| GO:0001941\_postsynaptic\_membrane\_organization | 1 | 0 |  |  |  |  |  |  |  |  |
| GO:0001946\_lymphangiogenesis | 1 | 0 |  |  |  |  |  |  |  |  |
| GO:0001956\_positive\_regulation\_of\_neurotransmitter\_secretion | 1 | 0 |  |  |  |  |  |  |  |  |
| GO:0001961\_positive\_regulation\_of\_cytokine-mediated\_signaling\_pathway | 1 | 0 |  |  |  |  |  |  |  |  |
| GO:0001979\_regulation\_of\_systemic\_arterial\_blood\_pressure\_by\_chemoreceptor\_signaling | 1 | 0 |  |  |  |  |  |  |  |  |
| GO:0001980\_regulation\_of\_systemic\_arterial\_blood\_pressure\_by\_ischemic\_conditions | 1 | 0 |  |  |  |  |  |  |  |  |
| GO:0001984\_vasodilation\_of\_artery\_during\_baroreceptor\_response\_to\_increased\_systemic\_arterial\_blood\_pressure | 1 | 0 |  |  |  |  |  |  |  |  |
| GO:0001985\_negative\_regulation\_of\_heart\_rate\_in\_baroreceptor\_response\_to\_increased\_systemic\_arterial\_blood\_pressure | 1 | 0 |  |  |  |  |  |  |  |  |
| GO:0001987\_vasoconstriction\_of\_artery\_involved\_in\_baroreceptor\_response\_to\_lowering\_of\_systemic\_arterial\_blood\_pressure | 1 | 0 |  |  |  |  |  |  |  |  |
| GO:0001988\_positive\_regulation\_of\_heart\_rate\_in\_baroreceptor\_response\_to\_decreased\_systemic\_arterial\_blood\_pressure | 1 | 0 |  |  |  |  |  |  |  |  |
| GO:0001994\_norepinephrine-epinephrine\_vasoconstriction\_involved\_in\_regulation\_of\_systemic\_arterial\_blood\_pressure | 1 | 0 |  |  |  |  |  |  |  |  |
| GO:0002001\_renin\_secretion\_into\_blood\_stream | 1 | 0 |  |  |  |  |  |  |  |  |
| GO:0002002\_regulation\_of\_angiotensin\_levels\_in\_blood | 1 | 0 |  |  |  |  |  |  |  |  |
| GO:0002003\_angiotensin\_maturation | 1 | 0 |  |  |  |  |  |  |  |  |
| GO:0002007\_detection\_of\_hypoxic\_conditions\_in\_blood\_by\_chemoreceptor\_signaling | 1 | 0 |  |  |  |  |  |  |  |  |
| GO:0002017\_regulation\_of\_blood\_volume\_by\_renal\_aldosterone | 1 | 0 |  |  |  |  |  |  |  |  |
| GO:0002023\_reduction\_of\_food\_intake\_in\_response\_to\_dietary\_excess | 1 | 0 |  |  |  |  |  |  |  |  |
| GO:0002031\_G-protein\_coupled\_receptor\_internalization | 1 | 0 |  |  |  |  |  |  |  |  |
| GO:0002036\_regulation\_of\_L-glutamate\_transport | 1 | 0 |  |  |  |  |  |  |  |  |
| GO:0002040\_sprouting\_angiogenesis | 1 | 0 |  |  |  |  |  |  |  |  |
| GO:0002041\_intussusceptive\_angiogenesis | 1 | 0 |  |  |  |  |  |  |  |  |
| GO:0002068\_glandular\_epithelial\_cell\_development | 1 | 0 |  |  |  |  |  |  |  |  |
| GO:0002069\_columnar\_cuboidal\_epithelial\_cell\_maturation | 1 | 0 |  |  |  |  |  |  |  |  |
| GO:0002071\_glandular\_epithelial\_cell\_maturation | 1 | 0 |  |  |  |  |  |  |  |  |
| GO:0002082\_regulation\_of\_oxidative\_phosphorylation | 1 | 0 |  |  |  |  |  |  |  |  |
| GO:0002084\_protein\_depalmitoylation | 1 | 0 |  |  |  |  |  |  |  |  |
| GO:0002085\_inhibition\_of\_neuroepithelial\_cell\_differentiation | 1 | 0 |  |  |  |  |  |  |  |  |
| GO:0002086\_diaphragm\_contraction | 1 | 0 |  |  |  |  |  |  |  |  |
| GO:0002118\_aggressive\_behavior | 1 | 0 |  |  |  |  |  |  |  |  |
| GO:0002121\_inter-male\_aggressive\_behavior | 1 | 0 |  |  |  |  |  |  |  |  |
| GO:0002124\_territorial\_aggressive\_behavior | 1 | 0 |  |  |  |  |  |  |  |  |
| GO:0002227\_innate\_immune\_response\_in\_mucosa | 1 | 0 |  |  |  |  |  |  |  |  |
| GO:0002232\_leukocyte\_chemotaxis\_during\_inflammatory\_response | 1 | 0 |  |  |  |  |  |  |  |  |
| GO:0002248\_connective\_tissue\_replacement\_during\_inflammatory\_response | 1 | 0 |  |  |  |  |  |  |  |  |
| GO:0002282\_microglial\_cell\_activation\_during\_immune\_response | 1 | 0 |  |  |  |  |  |  |  |  |
| GO:0002287\_alpha-beta\_T\_cell\_activation\_during\_immune\_response | 1 | 0 |  |  |  |  |  |  |  |  |
| GO:0002314\_germinal\_center\_B\_cell\_differentiation | 1 | 0 |  |  |  |  |  |  |  |  |
| GO:0002315\_marginal\_zone\_B\_cell\_differentiation | 1 | 0 |  |  |  |  |  |  |  |  |
| GO:0002316\_follicular\_B\_cell\_differentiation | 1 | 0 |  |  |  |  |  |  |  |  |
| GO:0002317\_plasma\_cell\_differentiation | 1 | 0 |  |  |  |  |  |  |  |  |
| GO:0002349\_histamine\_production\_during\_acute\_inflammatory\_response | 1 | 0 |  |  |  |  |  |  |  |  |
| GO:0002351\_serotonin\_production\_during\_acute\_inflammatory\_response | 1 | 0 |  |  |  |  |  |  |  |  |
| GO:0002355\_detection\_of\_tumor\_cell | 1 | 0 |  |  |  |  |  |  |  |  |
| GO:0002370\_natural\_killer\_cell\_cytokine\_production | 1 | 0 |  |  |  |  |  |  |  |  |
| GO:0002371\_dendritic\_cell\_cytokine\_production | 1 | 0 |  |  |  |  |  |  |  |  |
| GO:0002380\_immunoglobulin\_secretion\_during\_immune\_response | 1 | 0 |  |  |  |  |  |  |  |  |
| GO:0002396\_MHC\_protein\_complex\_assembly | 1 | 0 |  |  |  |  |  |  |  |  |
| GO:0002397\_MHC\_class\_I\_protein\_complex\_assembly | 1 | 0 |  |  |  |  |  |  |  |  |
| GO:0002420\_natural\_killer\_cell\_mediated\_cytotoxicity\_directed\_against\_tumor\_cell\_target | 1 | 0 |  |  |  |  |  |  |  |  |
| GO:0002423\_natural\_killer\_cell\_mediated\_immune\_response\_to\_tumor\_cell | 1 | 0 |  |  |  |  |  |  |  |  |
| GO:0002424\_T\_cell\_mediated\_immune\_response\_to\_tumor\_cell | 1 | 0 |  |  |  |  |  |  |  |  |
| GO:0002426\_immunoglobulin\_production\_in\_mucosal\_tissue | 1 | 0 |  |  |  |  |  |  |  |  |
| GO:0002431\_Fc\_receptor\_mediated\_stimulatory\_signaling\_pathway | 1 | 0 |  |  |  |  |  |  |  |  |
| GO:0002432\_granuloma\_formation | 1 | 0 |  |  |  |  |  |  |  |  |
| GO:0002441\_histamine\_secretion\_during\_acute\_inflammatory\_response | 1 | 0 |  |  |  |  |  |  |  |  |
| GO:0002442\_serotonin\_secretion\_during\_acute\_inflammatory\_response | 1 | 0 |  |  |  |  |  |  |  |  |
| GO:0002457\_T\_cell\_antigen\_processing\_and\_presentation | 1 | 0 |  |  |  |  |  |  |  |  |
| GO:0002458\_peripheral\_T\_cell\_tolerance\_induction | 1 | 0 |  |  |  |  |  |  |  |  |
| GO:0002461\_tolerance\_induction\_dependent\_upon\_immune\_response | 1 | 0 |  |  |  |  |  |  |  |  |
| GO:0002465\_peripheral\_tolerance\_induction | 1 | 0 |  |  |  |  |  |  |  |  |
| GO:0002468\_dendritic\_cell\_antigen\_processing\_and\_presentation | 1 | 0 |  |  |  |  |  |  |  |  |
| GO:0002476\_antigen\_processing\_and\_presentation\_of\_endogenous\_peptide\_antigen\_via\_MHC\_class\_Ib | 1 | 0 |  |  |  |  |  |  |  |  |
| GO:0002479\_antigen\_processing\_and\_presentation\_of\_exogenous\_peptide\_antigen\_via\_MHC\_class\_I\_\_TAP-dependent | 1 | 0 |  |  |  |  |  |  |  |  |
| GO:0002483\_antigen\_processing\_and\_presentation\_of\_endogenous\_peptide\_antigen | 1 | 0 |  |  |  |  |  |  |  |  |
| GO:0002501\_peptide\_antigen\_assembly\_with\_MHC\_protein\_complex | 1 | 0 |  |  |  |  |  |  |  |  |
| GO:0002502\_peptide\_antigen\_assembly\_with\_MHC\_class\_I\_protein\_complex | 1 | 0 |  |  |  |  |  |  |  |  |
| GO:0002508\_central\_tolerance\_induction | 1 | 0 |  |  |  |  |  |  |  |  |
| GO:0002510\_central\_B\_cell\_tolerance\_induction | 1 | 0 |  |  |  |  |  |  |  |  |
| GO:0002545\_chronic\_inflammatory\_response\_to\_non-antigenic\_stimulus | 1 | 0 |  |  |  |  |  |  |  |  |
| GO:0002553\_histamine\_secretion\_by\_mast\_cell | 1 | 0 |  |  |  |  |  |  |  |  |
| GO:0002554\_serotonin\_secretion\_by\_platelet | 1 | 0 |  |  |  |  |  |  |  |  |
| GO:0002572\_pro-T\_cell\_differentiation | 1 | 0 |  |  |  |  |  |  |  |  |
| GO:0002577\_regulation\_of\_antigen\_processing\_and\_presentation | 1 | 0 |  |  |  |  |  |  |  |  |
| GO:0002579\_positive\_regulation\_of\_antigen\_processing\_and\_presentation | 1 | 0 |  |  |  |  |  |  |  |  |
| GO:0002604\_regulation\_of\_dendritic\_cell\_antigen\_processing\_and\_presentation | 1 | 0 |  |  |  |  |  |  |  |  |
| GO:0002606\_positive\_regulation\_of\_dendritic\_cell\_antigen\_processing\_and\_presentation | 1 | 0 |  |  |  |  |  |  |  |  |
| GO:0002635\_negative\_regulation\_of\_germinal\_center\_formation | 1 | 0 |  |  |  |  |  |  |  |  |
| GO:0002646\_regulation\_of\_central\_tolerance\_induction | 1 | 0 |  |  |  |  |  |  |  |  |
| GO:0002648\_positive\_regulation\_of\_central\_tolerance\_induction | 1 | 0 |  |  |  |  |  |  |  |  |
| GO:0002649\_regulation\_of\_tolerance\_induction\_to\_self\_antigen | 1 | 0 |  |  |  |  |  |  |  |  |
| GO:0002651\_positive\_regulation\_of\_tolerance\_induction\_to\_self\_antigen | 1 | 0 |  |  |  |  |  |  |  |  |
| GO:0002652\_regulation\_of\_tolerance\_induction\_dependent\_upon\_immune\_response | 1 | 0 |  |  |  |  |  |  |  |  |
| GO:0002654\_positive\_regulation\_of\_tolerance\_induction\_dependent\_upon\_immune\_response | 1 | 0 |  |  |  |  |  |  |  |  |
| GO:0002658\_regulation\_of\_peripheral\_tolerance\_induction | 1 | 0 |  |  |  |  |  |  |  |  |
| GO:0002660\_positive\_regulation\_of\_peripheral\_tolerance\_induction | 1 | 0 |  |  |  |  |  |  |  |  |
| GO:0002677\_negative\_regulation\_of\_chronic\_inflammatory\_response | 1 | 0 |  |  |  |  |  |  |  |  |
| GO:0002678\_positive\_regulation\_of\_chronic\_inflammatory\_response | 1 | 0 |  |  |  |  |  |  |  |  |
| GO:0002701\_negative\_regulation\_of\_production\_of\_molecular\_mediator\_of\_immune\_response | 1 | 0 |  |  |  |  |  |  |  |  |
| GO:0002719\_negative\_regulation\_of\_cytokine\_production\_during\_immune\_response | 1 | 0 |  |  |  |  |  |  |  |  |
| GO:0002724\_regulation\_of\_T\_cell\_cytokine\_production | 1 | 0 |  |  |  |  |  |  |  |  |
| GO:0002727\_regulation\_of\_natural\_killer\_cell\_cytokine\_production | 1 | 0 |  |  |  |  |  |  |  |  |
| GO:0002729\_positive\_regulation\_of\_natural\_killer\_cell\_cytokine\_production | 1 | 0 |  |  |  |  |  |  |  |  |
| GO:0002730\_regulation\_of\_dendritic\_cell\_cytokine\_production | 1 | 0 |  |  |  |  |  |  |  |  |
| GO:0002756\_MyD88-independent\_toll-like\_receptor\_signaling\_pathway | 1 | 0 |  |  |  |  |  |  |  |  |
| GO:0002767\_immune\_response-inhibiting\_cell\_surface\_receptor\_signaling\_pathway | 1 | 0 |  |  |  |  |  |  |  |  |
| GO:0002769\_natural\_killer\_cell\_inhibitory\_signaling\_pathway | 1 | 0 |  |  |  |  |  |  |  |  |
| GO:0002840\_regulation\_of\_T\_cell\_mediated\_immune\_response\_to\_tumor\_cell | 1 | 0 |  |  |  |  |  |  |  |  |
| GO:0002842\_positive\_regulation\_of\_T\_cell\_mediated\_immune\_response\_to\_tumor\_cell | 1 | 0 |  |  |  |  |  |  |  |  |
| GO:0002849\_regulation\_of\_peripheral\_T\_cell\_tolerance\_induction | 1 | 0 |  |  |  |  |  |  |  |  |
| GO:0002851\_positive\_regulation\_of\_peripheral\_T\_cell\_tolerance\_induction | 1 | 0 |  |  |  |  |  |  |  |  |
| GO:0002855\_regulation\_of\_natural\_killer\_cell\_mediated\_immune\_response\_to\_tumor\_cell | 1 | 0 |  |  |  |  |  |  |  |  |
| GO:0002857\_positive\_regulation\_of\_natural\_killer\_cell\_mediated\_immune\_response\_to\_tumor\_cell | 1 | 0 |  |  |  |  |  |  |  |  |
| GO:0002858\_regulation\_of\_natural\_killer\_cell\_mediated\_cytotoxicity\_directed\_against\_tumor\_cell\_target | 1 | 0 |  |  |  |  |  |  |  |  |
| GO:0002860\_positive\_regulation\_of\_natural\_killer\_cell\_mediated\_cytotoxicity\_directed\_against\_tumor\_cell\_target | 1 | 0 |  |  |  |  |  |  |  |  |
| GO:0002880\_regulation\_of\_chronic\_inflammatory\_response\_to\_non-antigenic\_stimulus | 1 | 0 |  |  |  |  |  |  |  |  |
| GO:0002882\_positive\_regulation\_of\_chronic\_inflammatory\_response\_to\_non-antigenic\_stimulus | 1 | 0 |  |  |  |  |  |  |  |  |
| GO:0002895\_regulation\_of\_central\_B\_cell\_tolerance\_induction | 1 | 0 |  |  |  |  |  |  |  |  |
| GO:0002897\_positive\_regulation\_of\_central\_B\_cell\_tolerance\_induction | 1 | 0 |  |  |  |  |  |  |  |  |
| GO:0002901\_mature\_B\_cell\_apoptosis | 1 | 0 |  |  |  |  |  |  |  |  |
| GO:0002903\_negative\_regulation\_of\_B\_cell\_apoptosis | 1 | 0 |  |  |  |  |  |  |  |  |
| GO:0002905\_regulation\_of\_mature\_B\_cell\_apoptosis | 1 | 0 |  |  |  |  |  |  |  |  |
| GO:0002906\_negative\_regulation\_of\_mature\_B\_cell\_apoptosis | 1 | 0 |  |  |  |  |  |  |  |  |
| GO:0003011\_involuntary\_skeletal\_muscle\_contraction | 1 | 0 |  |  |  |  |  |  |  |  |
| GO:0003027\_regulation\_of\_systemic\_arterial\_blood\_pressure\_by\_carotid\_body\_chemoreceptor\_signaling | 1 | 0 |  |  |  |  |  |  |  |  |
| GO:0003029\_detection\_of\_hypoxic\_conditions\_in\_blood\_by\_carotid\_body\_chemoreceptor\_signaling | 1 | 0 |  |  |  |  |  |  |  |  |
| GO:0003032\_detection\_of\_oxygen | 1 | 0 |  |  |  |  |  |  |  |  |
| GO:0003056\_regulation\_of\_vascular\_smooth\_muscle\_contraction | 1 | 0 |  |  |  |  |  |  |  |  |
| GO:0003062\_regulation\_of\_heart\_rate\_by\_chemical\_signal | 1 | 0 |  |  |  |  |  |  |  |  |
| GO:0003065\_positive\_regulation\_of\_heart\_rate\_by\_epinephrine | 1 | 0 |  |  |  |  |  |  |  |  |
| GO:0003068\_regulation\_of\_systemic\_arterial\_blood\_pressure\_by\_acetylcholine | 1 | 0 |  |  |  |  |  |  |  |  |
| GO:0003069\_vasodilation\_by\_acetylcholine\_involved\_in\_regulation\_of\_systemic\_arterial\_blood\_pressure | 1 | 0 |  |  |  |  |  |  |  |  |
| GO:0003070\_regulation\_of\_systemic\_arterial\_blood\_pressure\_by\_neurotransmitter | 1 | 0 |  |  |  |  |  |  |  |  |
| GO:0003097\_renal\_water\_transport | 1 | 0 |  |  |  |  |  |  |  |  |
| GO:0005979\_regulation\_of\_glycogen\_biosynthetic\_process | 1 | 0 |  |  |  |  |  |  |  |  |
| GO:0005984\_disaccharide\_metabolic\_process | 1 | 0 |  |  |  |  |  |  |  |  |
| GO:0005988\_lactose\_metabolic\_process | 1 | 0 |  |  |  |  |  |  |  |  |
| GO:0005989\_lactose\_biosynthetic\_process | 1 | 0 |  |  |  |  |  |  |  |  |
| GO:0005997\_xylulose\_metabolic\_process | 1 | 0 |  |  |  |  |  |  |  |  |
| GO:0006000\_fructose\_metabolic\_process | 1 | 0 |  |  |  |  |  |  |  |  |
| GO:0006002\_fructose\_6-phosphate\_metabolic\_process | 1 | 0 |  |  |  |  |  |  |  |  |
| GO:0006004\_fucose\_metabolic\_process | 1 | 0 |  |  |  |  |  |  |  |  |
| GO:0006013\_mannose\_metabolic\_process | 1 | 0 |  |  |  |  |  |  |  |  |
| GO:0006060\_sorbitol\_metabolic\_process | 1 | 0 |  |  |  |  |  |  |  |  |
| GO:0006064\_glucuronate\_catabolic\_process | 1 | 0 |  |  |  |  |  |  |  |  |
| GO:0006086\_acetyl-CoA\_biosynthetic\_process\_from\_pyruvate | 1 | 0 |  |  |  |  |  |  |  |  |
| GO:0006098\_pentose-phosphate\_shunt | 1 | 0 |  |  |  |  |  |  |  |  |
| GO:0006101\_citrate\_metabolic\_process | 1 | 0 |  |  |  |  |  |  |  |  |
| GO:0006104\_succinyl-CoA\_metabolic\_process | 1 | 0 |  |  |  |  |  |  |  |  |
| GO:0006116\_NADH\_oxidation | 1 | 0 |  |  |  |  |  |  |  |  |
| GO:0006120\_mitochondrial\_electron\_transport\_\_NADH\_to\_ubiquinone | 1 | 0 |  |  |  |  |  |  |  |  |
| GO:0006154\_adenosine\_catabolic\_process | 1 | 0 |  |  |  |  |  |  |  |  |
| GO:0006157\_deoxyadenosine\_catabolic\_process | 1 | 0 |  |  |  |  |  |  |  |  |
| GO:0006167\_AMP\_biosynthetic\_process | 1 | 0 |  |  |  |  |  |  |  |  |
| GO:0006175\_dATP\_biosynthetic\_process | 1 | 0 |  |  |  |  |  |  |  |  |
| GO:0006178\_guanine\_salvage | 1 | 0 |  |  |  |  |  |  |  |  |
| GO:0006196\_AMP\_catabolic\_process | 1 | 0 |  |  |  |  |  |  |  |  |
| GO:0006203\_dGTP\_catabolic\_process | 1 | 0 |  |  |  |  |  |  |  |  |
| GO:0006208\_pyrimidine\_base\_catabolic\_process | 1 | 0 |  |  |  |  |  |  |  |  |
| GO:0006221\_pyrimidine\_nucleotide\_biosynthetic\_process | 1 | 0 |  |  |  |  |  |  |  |  |
| GO:0006235\_dTTP\_biosynthetic\_process | 1 | 0 |  |  |  |  |  |  |  |  |
| GO:0006244\_pyrimidine\_nucleotide\_catabolic\_process | 1 | 0 |  |  |  |  |  |  |  |  |
| GO:0006269\_DNA\_replication\_\_synthesis\_of\_RNA\_primer | 1 | 0 |  |  |  |  |  |  |  |  |
| GO:0006283\_transcription-coupled\_nucleotide-excision\_repair | 1 | 0 |  |  |  |  |  |  |  |  |
| GO:0006296\_nucleotide-excision\_repair\_\_DNA\_incision\_\_5'-to\_lesion | 1 | 0 |  |  |  |  |  |  |  |  |
| GO:0006307\_DNA\_dealkylation | 1 | 0 |  |  |  |  |  |  |  |  |
| GO:0006337\_nucleosome\_disassembly | 1 | 0 |  |  |  |  |  |  |  |  |
| GO:0006344\_maintenance\_of\_chromatin\_silencing | 1 | 0 |  |  |  |  |  |  |  |  |
| GO:0006356\_regulation\_of\_transcription\_from\_RNA\_polymerase\_I\_promoter | 1 | 0 |  |  |  |  |  |  |  |  |
| GO:0006388\_tRNA\_splicing\_\_via\_endonucleolytic\_cleavage\_and\_ligation | 1 | 0 |  |  |  |  |  |  |  |  |
| GO:0006419\_alanyl-tRNA\_aminoacylation | 1 | 0 |  |  |  |  |  |  |  |  |
| GO:0006434\_seryl-tRNA\_aminoacylation | 1 | 0 |  |  |  |  |  |  |  |  |
| GO:0006447\_regulation\_of\_translational\_initiation\_by\_iron | 1 | 0 |  |  |  |  |  |  |  |  |
| GO:0006463\_steroid\_hormone\_receptor\_complex\_assembly | 1 | 0 |  |  |  |  |  |  |  |  |
| GO:0006467\_protein\_thiol-disulfide\_exchange | 1 | 0 |  |  |  |  |  |  |  |  |
| GO:0006474\_N-terminal\_protein\_amino\_acid\_acetylation | 1 | 0 |  |  |  |  |  |  |  |  |
| GO:0006481\_C-terminal\_protein\_amino\_acid\_methylation | 1 | 0 |  |  |  |  |  |  |  |  |
| GO:0006488\_dolichol-linked\_oligosaccharide\_biosynthetic\_process | 1 | 0 |  |  |  |  |  |  |  |  |
| GO:0006494\_protein\_amino\_acid\_terminal\_glycosylation | 1 | 0 |  |  |  |  |  |  |  |  |
| GO:0006496\_protein\_amino\_acid\_terminal\_N-glycosylation | 1 | 0 |  |  |  |  |  |  |  |  |
| GO:0006500\_N-terminal\_protein\_palmitoylation | 1 | 0 |  |  |  |  |  |  |  |  |
| GO:0006507\_GPI\_anchor\_release | 1 | 0 |  |  |  |  |  |  |  |  |
| GO:0006537\_glutamate\_biosynthetic\_process | 1 | 0 |  |  |  |  |  |  |  |  |
| GO:0006544\_glycine\_metabolic\_process | 1 | 0 |  |  |  |  |  |  |  |  |
| GO:0006549\_isoleucine\_metabolic\_process | 1 | 0 |  |  |  |  |  |  |  |  |
| GO:0006553\_lysine\_metabolic\_process | 1 | 0 |  |  |  |  |  |  |  |  |
| GO:0006554\_lysine\_catabolic\_process | 1 | 0 |  |  |  |  |  |  |  |  |
| GO:0006556\_S-adenosylmethionine\_biosynthetic\_process | 1 | 0 |  |  |  |  |  |  |  |  |
| GO:0006559\_L-phenylalanine\_catabolic\_process | 1 | 0 |  |  |  |  |  |  |  |  |
| GO:0006569\_tryptophan\_catabolic\_process | 1 | 0 |  |  |  |  |  |  |  |  |
| GO:0006572\_tyrosine\_catabolic\_process | 1 | 0 |  |  |  |  |  |  |  |  |
| GO:0006573\_valine\_metabolic\_process | 1 | 0 |  |  |  |  |  |  |  |  |
| GO:0006581\_acetylcholine\_catabolic\_process | 1 | 0 |  |  |  |  |  |  |  |  |
| GO:0006585\_dopamine\_biosynthetic\_process\_from\_tyrosine | 1 | 0 |  |  |  |  |  |  |  |  |
| GO:0006590\_thyroid\_hormone\_generation | 1 | 0 |  |  |  |  |  |  |  |  |
| GO:0006591\_ornithine\_metabolic\_process | 1 | 0 |  |  |  |  |  |  |  |  |
| GO:0006596\_polyamine\_biosynthetic\_process | 1 | 0 |  |  |  |  |  |  |  |  |
| GO:0006597\_spermine\_biosynthetic\_process | 1 | 0 |  |  |  |  |  |  |  |  |
| GO:0006601\_creatine\_biosynthetic\_process | 1 | 0 |  |  |  |  |  |  |  |  |
| GO:0006613\_cotranslational\_protein\_targeting\_to\_membrane | 1 | 0 |  |  |  |  |  |  |  |  |
| GO:0006622\_protein\_targeting\_to\_lysosome | 1 | 0 |  |  |  |  |  |  |  |  |
| GO:0006627\_mitochondrial\_protein\_processing\_during\_import | 1 | 0 |  |  |  |  |  |  |  |  |
| GO:0006653\_lecithin\_metabolic\_process | 1 | 0 |  |  |  |  |  |  |  |  |
| GO:0006654\_phosphatidic\_acid\_biosynthetic\_process | 1 | 0 |  |  |  |  |  |  |  |  |
| GO:0006658\_phosphatidylserine\_metabolic\_process | 1 | 0 |  |  |  |  |  |  |  |  |
| GO:0006659\_phosphatidylserine\_biosynthetic\_process | 1 | 0 |  |  |  |  |  |  |  |  |
| GO:0006667\_sphinganine\_metabolic\_process | 1 | 0 |  |  |  |  |  |  |  |  |
| GO:0006668\_sphinganine-1-phosphate\_metabolic\_process | 1 | 0 |  |  |  |  |  |  |  |  |
| GO:0006678\_glucosylceramide\_metabolic\_process | 1 | 0 |  |  |  |  |  |  |  |  |
| GO:0006682\_galactosylceramide\_biosynthetic\_process | 1 | 0 |  |  |  |  |  |  |  |  |
| GO:0006685\_sphingomyelin\_catabolic\_process | 1 | 0 |  |  |  |  |  |  |  |  |
| GO:0006700\_C21-steroid\_hormone\_biosynthetic\_process | 1 | 0 |  |  |  |  |  |  |  |  |
| GO:0006705\_mineralocorticoid\_biosynthetic\_process | 1 | 0 |  |  |  |  |  |  |  |  |
| GO:0006709\_progesterone\_catabolic\_process | 1 | 0 |  |  |  |  |  |  |  |  |
| GO:0006729\_tetrahydrobiopterin\_biosynthetic\_process | 1 | 0 |  |  |  |  |  |  |  |  |
| GO:0006734\_NADH\_metabolic\_process | 1 | 0 |  |  |  |  |  |  |  |  |
| GO:0006740\_NADPH\_regeneration | 1 | 0 |  |  |  |  |  |  |  |  |
| GO:0006741\_NADP\_biosynthetic\_process | 1 | 0 |  |  |  |  |  |  |  |  |
| GO:0006743\_ubiquinone\_metabolic\_process | 1 | 0 |  |  |  |  |  |  |  |  |
| GO:0006744\_ubiquinone\_biosynthetic\_process | 1 | 0 |  |  |  |  |  |  |  |  |
| GO:0006772\_thiamin\_metabolic\_process | 1 | 0 |  |  |  |  |  |  |  |  |
| GO:0006784\_heme\_a\_biosynthetic\_process | 1 | 0 |  |  |  |  |  |  |  |  |
| GO:0006797\_polyphosphate\_metabolic\_process | 1 | 0 |  |  |  |  |  |  |  |  |
| GO:0006798\_polyphosphate\_catabolic\_process | 1 | 0 |  |  |  |  |  |  |  |  |
| GO:0006824\_cobalt\_ion\_transport | 1 | 0 |  |  |  |  |  |  |  |  |
| GO:0006842\_tricarboxylic\_acid\_transport | 1 | 0 |  |  |  |  |  |  |  |  |
| GO:0006844\_acyl\_carnitine\_transport | 1 | 0 |  |  |  |  |  |  |  |  |
| GO:0006855\_multidrug\_transport | 1 | 0 |  |  |  |  |  |  |  |  |
| GO:0006863\_purine\_transport | 1 | 0 |  |  |  |  |  |  |  |  |
| GO:0006890\_retrograde\_vesicle-mediated\_transport\_\_Golgi\_to\_ER | 1 | 0 |  |  |  |  |  |  |  |  |
| GO:0006891\_intra-Golgi\_vesicle-mediated\_transport | 1 | 0 |  |  |  |  |  |  |  |  |
| GO:0006893\_Golgi\_to\_plasma\_membrane\_transport | 1 | 0 |  |  |  |  |  |  |  |  |
| GO:0006895\_Golgi\_to\_endosome\_transport | 1 | 0 |  |  |  |  |  |  |  |  |
| GO:0006896\_Golgi\_to\_vacuole\_transport | 1 | 0 |  |  |  |  |  |  |  |  |
| GO:0006900\_membrane\_budding | 1 | 0 |  |  |  |  |  |  |  |  |
| GO:0006930\_substrate-bound\_cell\_migration\_\_cell\_extension | 1 | 0 |  |  |  |  |  |  |  |  |
| GO:0006931\_substrate-bound\_cell\_migration\_\_cell\_attachment\_to\_substrate | 1 | 0 |  |  |  |  |  |  |  |  |
| GO:0006933\_negative\_regulation\_of\_cell\_adhesion\_involved\_in\_substrate-bound\_cell\_migration | 1 | 0 |  |  |  |  |  |  |  |  |
| GO:0006957\_complement\_activation\_\_alternative\_pathway | 1 | 0 |  |  |  |  |  |  |  |  |
| GO:0006958\_complement\_activation\_\_classical\_pathway | 1 | 0 |  |  |  |  |  |  |  |  |
| GO:0006978\_DNA\_damage\_response\_\_signal\_transduction\_by\_p53\_class\_mediator\_resulting\_in\_transcription\_of\_p21\_class\_mediator | 1 | 0 |  |  |  |  |  |  |  |  |
| GO:0007016\_cytoskeletal\_anchoring\_at\_plasma\_membrane | 1 | 0 |  |  |  |  |  |  |  |  |
| GO:0007021\_tubulin\_complex\_assembly | 1 | 0 |  |  |  |  |  |  |  |  |
| GO:0007052\_mitotic\_spindle\_organization | 1 | 0 |  |  |  |  |  |  |  |  |
| GO:0007056\_spindle\_assembly\_involved\_in\_female\_meiosis | 1 | 0 |  |  |  |  |  |  |  |  |
| GO:0007057\_spindle\_assembly\_involved\_in\_female\_meiosis\_I | 1 | 0 |  |  |  |  |  |  |  |  |
| GO:0007063\_regulation\_of\_sister\_chromatid\_cohesion | 1 | 0 |  |  |  |  |  |  |  |  |
| GO:0007065\_male\_meiosis\_sister\_chromatid\_cohesion | 1 | 0 |  |  |  |  |  |  |  |  |
| GO:0007076\_mitotic\_chromosome\_condensation | 1 | 0 |  |  |  |  |  |  |  |  |
| GO:0007095\_mitotic\_cell\_cycle\_G2\_M\_transition\_DNA\_damage\_checkpoint | 1 | 0 |  |  |  |  |  |  |  |  |
| GO:0007096\_regulation\_of\_exit\_from\_mitosis | 1 | 0 |  |  |  |  |  |  |  |  |
| GO:0007158\_neuron\_adhesion | 1 | 0 |  |  |  |  |  |  |  |  |
| GO:0007168\_receptor\_guanylyl\_cyclase\_signaling\_pathway | 1 | 0 |  |  |  |  |  |  |  |  |
| GO:0007197\_inhibition\_of\_adenylate\_cyclase\_activity\_by\_muscarinic\_acetylcholine\_receptor\_signaling\_pathway | 1 | 0 |  |  |  |  |  |  |  |  |
| GO:0007207\_activation\_of\_phospholipase\_C\_activity\_by\_muscarinic\_acetylcholine\_receptor\_signaling\_pathway | 1 | 0 |  |  |  |  |  |  |  |  |
| GO:0007208\_activation\_of\_phospholipase\_C\_activity\_by\_serotonin\_receptor\_signaling\_pathway | 1 | 0 |  |  |  |  |  |  |  |  |
| GO:0007217\_tachykinin\_receptor\_signaling\_pathway | 1 | 0 |  |  |  |  |  |  |  |  |
| GO:0007221\_positive\_regulation\_of\_transcription\_of\_Notch\_receptor\_target | 1 | 0 |  |  |  |  |  |  |  |  |
| GO:0007223\_Wnt\_receptor\_signaling\_pathway\_\_calcium\_modulating\_pathway | 1 | 0 |  |  |  |  |  |  |  |  |
| GO:0007225\_patched\_ligand\_processing | 1 | 0 |  |  |  |  |  |  |  |  |
| GO:0007227\_signal\_transduction\_downstream\_of\_smoothened | 1 | 0 |  |  |  |  |  |  |  |  |
| GO:0007228\_positive\_regulation\_of\_hh\_target\_transcription\_factor\_activity | 1 | 0 |  |  |  |  |  |  |  |  |
| GO:0007231\_osmosensory\_signaling\_pathway | 1 | 0 |  |  |  |  |  |  |  |  |
| GO:0007284\_spermatogonial\_cell\_division | 1 | 0 |  |  |  |  |  |  |  |  |
| GO:0007290\_spermatid\_nucleus\_elongation | 1 | 0 |  |  |  |  |  |  |  |  |
| GO:0007296\_vitellogenesis | 1 | 0 |  |  |  |  |  |  |  |  |
| GO:0007321\_sperm\_displacement | 1 | 0 |  |  |  |  |  |  |  |  |
| GO:0007380\_specification\_of\_segmental\_identity\_\_head | 1 | 0 |  |  |  |  |  |  |  |  |
| GO:0007382\_specification\_of\_segmental\_identity\_\_maxillary\_segment | 1 | 0 |  |  |  |  |  |  |  |  |
| GO:0007400\_neuroblast\_fate\_determination | 1 | 0 |  |  |  |  |  |  |  |  |
| GO:0007402\_ganglion\_mother\_cell\_fate\_determination | 1 | 0 |  |  |  |  |  |  |  |  |
| GO:0007495\_visceral\_mesoderm-endoderm\_interaction\_involved\_in\_midgut\_development | 1 | 0 |  |  |  |  |  |  |  |  |
| GO:0007497\_posterior\_midgut\_development | 1 | 0 |  |  |  |  |  |  |  |  |
| GO:0007499\_ectoderm\_and\_mesoderm\_interaction | 1 | 0 |  |  |  |  |  |  |  |  |
| GO:0007500\_mesodermal\_cell\_fate\_determination | 1 | 0 |  |  |  |  |  |  |  |  |
| GO:0007509\_mesoderm\_migration | 1 | 0 |  |  |  |  |  |  |  |  |
| GO:0007518\_myoblast\_cell\_fate\_determination | 1 | 0 |  |  |  |  |  |  |  |  |
| GO:0007521\_muscle\_cell\_fate\_determination | 1 | 0 |  |  |  |  |  |  |  |  |
| GO:0007522\_visceral\_muscle\_development | 1 | 0 |  |  |  |  |  |  |  |  |
| GO:0007529\_establishment\_of\_synaptic\_specificity\_at\_neuromuscular\_junction | 1 | 0 |  |  |  |  |  |  |  |  |
| GO:0007538\_primary\_sex\_determination | 1 | 0 |  |  |  |  |  |  |  |  |
| GO:0007542\_primary\_sex\_determination\_\_germ-line | 1 | 0 |  |  |  |  |  |  |  |  |
| GO:0007567\_parturition | 1 | 0 |  |  |  |  |  |  |  |  |
| GO:0007614\_short-term\_memory | 1 | 0 |  |  |  |  |  |  |  |  |
| GO:0007621\_negative\_regulation\_of\_female\_receptivity | 1 | 0 |  |  |  |  |  |  |  |  |
| GO:0008049\_male\_courtship\_behavior | 1 | 0 |  |  |  |  |  |  |  |  |
| GO:0008050\_female\_courtship\_behavior | 1 | 0 |  |  |  |  |  |  |  |  |
| GO:0008052\_sensory\_organ\_boundary\_specification | 1 | 0 |  |  |  |  |  |  |  |  |
| GO:0008054\_cyclin\_catabolic\_process | 1 | 0 |  |  |  |  |  |  |  |  |
| GO:0008057\_eye\_pigment\_granule\_organization | 1 | 0 |  |  |  |  |  |  |  |  |
| GO:0008078\_mesodermal\_cell\_migration | 1 | 0 |  |  |  |  |  |  |  |  |
| GO:0008208\_C21-steroid\_hormone\_catabolic\_process | 1 | 0 |  |  |  |  |  |  |  |  |
| GO:0008216\_spermidine\_metabolic\_process | 1 | 0 |  |  |  |  |  |  |  |  |
| GO:0008292\_acetylcholine\_biosynthetic\_process | 1 | 0 |  |  |  |  |  |  |  |  |
| GO:0008295\_spermidine\_biosynthetic\_process | 1 | 0 |  |  |  |  |  |  |  |  |
| GO:0008300\_isoprenoid\_catabolic\_process | 1 | 0 |  |  |  |  |  |  |  |  |
| GO:0008333\_endosome\_to\_lysosome\_transport | 1 | 0 |  |  |  |  |  |  |  |  |
| GO:0008355\_olfactory\_learning | 1 | 0 |  |  |  |  |  |  |  |  |
| GO:0008611\_ether\_lipid\_biosynthetic\_process | 1 | 0 |  |  |  |  |  |  |  |  |
| GO:0008626\_induction\_of\_apoptosis\_by\_granzyme | 1 | 0 |  |  |  |  |  |  |  |  |
| GO:0008633\_activation\_of\_pro-apoptotic\_gene\_products | 1 | 0 |  |  |  |  |  |  |  |  |
| GO:0008653\_lipopolysaccharide\_metabolic\_process | 1 | 0 |  |  |  |  |  |  |  |  |
| GO:0009068\_aspartate\_family\_amino\_acid\_catabolic\_process | 1 | 0 |  |  |  |  |  |  |  |  |
| GO:0009084\_glutamine\_family\_amino\_acid\_biosynthetic\_process | 1 | 0 |  |  |  |  |  |  |  |  |
| GO:0009088\_threonine\_biosynthetic\_process | 1 | 0 |  |  |  |  |  |  |  |  |
| GO:0009105\_lipoic\_acid\_biosynthetic\_process | 1 | 0 |  |  |  |  |  |  |  |  |
| GO:0009109\_coenzyme\_catabolic\_process | 1 | 0 |  |  |  |  |  |  |  |  |
| GO:0009111\_vitamin\_catabolic\_process | 1 | 0 |  |  |  |  |  |  |  |  |
| GO:0009113\_purine\_base\_biosynthetic\_process | 1 | 0 |  |  |  |  |  |  |  |  |
| GO:0009127\_purine\_nucleoside\_monophosphate\_biosynthetic\_process | 1 | 0 |  |  |  |  |  |  |  |  |
| GO:0009128\_purine\_nucleoside\_monophosphate\_catabolic\_process | 1 | 0 |  |  |  |  |  |  |  |  |
| GO:0009129\_pyrimidine\_nucleoside\_monophosphate\_metabolic\_process | 1 | 0 |  |  |  |  |  |  |  |  |
| GO:0009131\_pyrimidine\_nucleoside\_monophosphate\_catabolic\_process | 1 | 0 |  |  |  |  |  |  |  |  |
| GO:0009133\_nucleoside\_diphosphate\_biosynthetic\_process | 1 | 0 |  |  |  |  |  |  |  |  |
| GO:0009145\_purine\_nucleoside\_triphosphate\_biosynthetic\_process | 1 | 0 |  |  |  |  |  |  |  |  |
| GO:0009147\_pyrimidine\_nucleoside\_triphosphate\_metabolic\_process | 1 | 0 |  |  |  |  |  |  |  |  |
| GO:0009148\_pyrimidine\_nucleoside\_triphosphate\_biosynthetic\_process | 1 | 0 |  |  |  |  |  |  |  |  |
| GO:0009152\_purine\_ribonucleotide\_biosynthetic\_process | 1 | 0 |  |  |  |  |  |  |  |  |
| GO:0009153\_purine\_deoxyribonucleotide\_biosynthetic\_process | 1 | 0 |  |  |  |  |  |  |  |  |
| GO:0009156\_ribonucleoside\_monophosphate\_biosynthetic\_process | 1 | 0 |  |  |  |  |  |  |  |  |
| GO:0009158\_ribonucleoside\_monophosphate\_catabolic\_process | 1 | 0 |  |  |  |  |  |  |  |  |
| GO:0009159\_deoxyribonucleoside\_monophosphate\_catabolic\_process | 1 | 0 |  |  |  |  |  |  |  |  |
| GO:0009162\_deoxyribonucleoside\_monophosphate\_metabolic\_process | 1 | 0 |  |  |  |  |  |  |  |  |
| GO:0009168\_purine\_ribonucleoside\_monophosphate\_biosynthetic\_process | 1 | 0 |  |  |  |  |  |  |  |  |
| GO:0009169\_purine\_ribonucleoside\_monophosphate\_catabolic\_process | 1 | 0 |  |  |  |  |  |  |  |  |
| GO:0009176\_pyrimidine\_deoxyribonucleoside\_monophosphate\_metabolic\_process | 1 | 0 |  |  |  |  |  |  |  |  |
| GO:0009178\_pyrimidine\_deoxyribonucleoside\_monophosphate\_catabolic\_process | 1 | 0 |  |  |  |  |  |  |  |  |
| GO:0009211\_pyrimidine\_deoxyribonucleoside\_triphosphate\_metabolic\_process | 1 | 0 |  |  |  |  |  |  |  |  |
| GO:0009212\_pyrimidine\_deoxyribonucleoside\_triphosphate\_biosynthetic\_process | 1 | 0 |  |  |  |  |  |  |  |  |
| GO:0009216\_purine\_deoxyribonucleoside\_triphosphate\_biosynthetic\_process | 1 | 0 |  |  |  |  |  |  |  |  |
| GO:0009221\_pyrimidine\_deoxyribonucleotide\_biosynthetic\_process | 1 | 0 |  |  |  |  |  |  |  |  |
| GO:0009223\_pyrimidine\_deoxyribonucleotide\_catabolic\_process | 1 | 0 |  |  |  |  |  |  |  |  |
| GO:0009260\_ribonucleotide\_biosynthetic\_process | 1 | 0 |  |  |  |  |  |  |  |  |
| GO:0009405\_pathogenesis | 1 | 0 |  |  |  |  |  |  |  |  |
| GO:0009414\_response\_to\_water\_deprivation | 1 | 0 |  |  |  |  |  |  |  |  |
| GO:0009415\_response\_to\_water | 1 | 0 |  |  |  |  |  |  |  |  |
| GO:0009449\_gamma-aminobutyric\_acid\_biosynthetic\_process | 1 | 0 |  |  |  |  |  |  |  |  |
| GO:0009450\_gamma-aminobutyric\_acid\_catabolic\_process | 1 | 0 |  |  |  |  |  |  |  |  |
| GO:0009589\_detection\_of\_UV | 1 | 0 |  |  |  |  |  |  |  |  |
| GO:0009590\_detection\_of\_gravity | 1 | 0 |  |  |  |  |  |  |  |  |
| GO:0009624\_response\_to\_nematode | 1 | 0 |  |  |  |  |  |  |  |  |
| GO:0009629\_response\_to\_gravity | 1 | 0 |  |  |  |  |  |  |  |  |
| GO:0009648\_photoperiodism | 1 | 0 |  |  |  |  |  |  |  |  |
| GO:0009690\_cytokinin\_metabolic\_process | 1 | 0 |  |  |  |  |  |  |  |  |
| GO:0009691\_cytokinin\_biosynthetic\_process | 1 | 0 |  |  |  |  |  |  |  |  |
| GO:0009786\_regulation\_of\_asymmetric\_cell\_division | 1 | 0 |  |  |  |  |  |  |  |  |
| GO:0009794\_regulation\_of\_mitotic\_cell\_cycle\_\_embryonic | 1 | 0 |  |  |  |  |  |  |  |  |
| GO:0009956\_radial\_pattern\_formation | 1 | 0 |  |  |  |  |  |  |  |  |
| GO:0009957\_epidermal\_cell\_fate\_specification | 1 | 0 |  |  |  |  |  |  |  |  |
| GO:0009992\_cellular\_water\_homeostasis | 1 | 0 |  |  |  |  |  |  |  |  |
| GO:0010032\_meiotic\_chromosome\_condensation | 1 | 0 |  |  |  |  |  |  |  |  |
| GO:0010039\_response\_to\_iron\_ion | 1 | 0 |  |  |  |  |  |  |  |  |
| GO:0010042\_response\_to\_manganese\_ion | 1 | 0 |  |  |  |  |  |  |  |  |
| GO:0010045\_response\_to\_nickel\_ion | 1 | 0 |  |  |  |  |  |  |  |  |
| GO:0010046\_response\_to\_mycotoxin | 1 | 0 |  |  |  |  |  |  |  |  |
| GO:0010107\_potassium\_ion\_import | 1 | 0 |  |  |  |  |  |  |  |  |
| GO:0010155\_regulation\_of\_proton\_transport | 1 | 0 |  |  |  |  |  |  |  |  |
| GO:0010160\_formation\_of\_organ\_boundary | 1 | 0 |  |  |  |  |  |  |  |  |
| GO:0010260\_organ\_senescence | 1 | 0 |  |  |  |  |  |  |  |  |
| GO:0010310\_regulation\_of\_hydrogen\_peroxide\_metabolic\_process | 1 | 0 |  |  |  |  |  |  |  |  |
| GO:0010447\_response\_to\_acidity | 1 | 0 |  |  |  |  |  |  |  |  |
| GO:0010452\_histone\_H3-K36\_methylation | 1 | 0 |  |  |  |  |  |  |  |  |
| GO:0010455\_positive\_regulation\_of\_cell\_fate\_commitment | 1 | 0 |  |  |  |  |  |  |  |  |
| GO:0010470\_regulation\_of\_gastrulation | 1 | 0 |  |  |  |  |  |  |  |  |
| GO:0010508\_positive\_regulation\_of\_autophagy | 1 | 0 |  |  |  |  |  |  |  |  |
| GO:0010519\_negative\_regulation\_of\_phospholipase\_activity | 1 | 0 |  |  |  |  |  |  |  |  |
| GO:0010520\_regulation\_of\_reciprocal\_meiotic\_recombination | 1 | 0 |  |  |  |  |  |  |  |  |
| GO:0010523\_negative\_regulation\_of\_calcium\_ion\_transport\_into\_cytosol | 1 | 0 |  |  |  |  |  |  |  |  |
| GO:0010543\_regulation\_of\_platelet\_activation | 1 | 0 |  |  |  |  |  |  |  |  |
| GO:0010561\_negative\_regulation\_of\_glycoprotein\_biosynthetic\_process | 1 | 0 |  |  |  |  |  |  |  |  |
| GO:0010569\_regulation\_of\_double-strand\_break\_repair\_via\_homologous\_recombination | 1 | 0 |  |  |  |  |  |  |  |  |
| GO:0010572\_positive\_regulation\_of\_platelet\_activation | 1 | 0 |  |  |  |  |  |  |  |  |
| GO:0010594\_regulation\_of\_endothelial\_cell\_migration | 1 | 0 |  |  |  |  |  |  |  |  |
| GO:0010596\_negative\_regulation\_of\_endothelial\_cell\_migration | 1 | 0 |  |  |  |  |  |  |  |  |
| GO:0010611\_regulation\_of\_cardiac\_muscle\_hypertrophy | 1 | 0 |  |  |  |  |  |  |  |  |
| GO:0010612\_regulation\_of\_cardiac\_muscle\_adaptation | 1 | 0 |  |  |  |  |  |  |  |  |
| GO:0010614\_negative\_regulation\_of\_cardiac\_muscle\_hypertrophy | 1 | 0 |  |  |  |  |  |  |  |  |
| GO:0010616\_negative\_regulation\_of\_cardiac\_muscle\_adaptation | 1 | 0 |  |  |  |  |  |  |  |  |
| GO:0010634\_positive\_regulation\_of\_epithelial\_cell\_migration | 1 | 0 |  |  |  |  |  |  |  |  |
| GO:0010656\_negative\_regulation\_of\_muscle\_cell\_apoptosis | 1 | 0 |  |  |  |  |  |  |  |  |
| GO:0010657\_muscle\_cell\_apoptosis | 1 | 0 |  |  |  |  |  |  |  |  |
| GO:0010658\_striated\_muscle\_cell\_apoptosis | 1 | 0 |  |  |  |  |  |  |  |  |
| GO:0010659\_cardiac\_muscle\_cell\_apoptosis | 1 | 0 |  |  |  |  |  |  |  |  |
| GO:0010660\_regulation\_of\_muscle\_cell\_apoptosis | 1 | 0 |  |  |  |  |  |  |  |  |
| GO:0010662\_regulation\_of\_striated\_muscle\_cell\_apoptosis | 1 | 0 |  |  |  |  |  |  |  |  |
| GO:0010664\_negative\_regulation\_of\_striated\_muscle\_cell\_apoptosis | 1 | 0 |  |  |  |  |  |  |  |  |
| GO:0010665\_regulation\_of\_cardiac\_muscle\_cell\_apoptosis | 1 | 0 |  |  |  |  |  |  |  |  |
| GO:0010667\_negative\_regulation\_of\_cardiac\_muscle\_cell\_apoptosis | 1 | 0 |  |  |  |  |  |  |  |  |
| GO:0010668\_ectodermal\_cell\_differentiation | 1 | 0 |  |  |  |  |  |  |  |  |
| GO:0010671\_negative\_regulation\_of\_oxygen\_and\_reactive\_oxygen\_species\_metabolic\_process | 1 | 0 |  |  |  |  |  |  |  |  |
| GO:0010719\_negative\_regulation\_of\_epithelial\_to\_mesenchymal\_transition | 1 | 0 |  |  |  |  |  |  |  |  |
| GO:0010735\_positive\_regulation\_of\_transcription\_via\_serum\_response\_element\_binding | 1 | 0 |  |  |  |  |  |  |  |  |
| GO:0010845\_positive\_regulation\_of\_reciprocal\_meiotic\_recombination | 1 | 0 |  |  |  |  |  |  |  |  |
| GO:0010850\_chemoreceptor\_signaling\_pathway\_involved\_in\_regulation\_of\_blood\_pressure | 1 | 0 |  |  |  |  |  |  |  |  |
| GO:0010873\_positive\_regulation\_of\_cholesterol\_esterification | 1 | 0 |  |  |  |  |  |  |  |  |
| GO:0010880\_regulation\_of\_release\_of\_sequestered\_calcium\_ion\_into\_cytosol\_by\_sarcoplasmic\_reticulum | 1 | 0 |  |  |  |  |  |  |  |  |
| GO:0010881\_regulation\_of\_cardiac\_muscle\_contraction\_by\_regulation\_of\_the\_release\_of\_sequestered\_calcium\_ion | 1 | 0 |  |  |  |  |  |  |  |  |
| GO:0010882\_regulation\_of\_cardiac\_muscle\_contraction\_by\_calcium\_ion\_signaling | 1 | 0 |  |  |  |  |  |  |  |  |
| GO:0010890\_positive\_regulation\_of\_sequestering\_of\_triglyceride | 1 | 0 |  |  |  |  |  |  |  |  |
| GO:0010919\_regulation\_of\_inositol\_phosphate\_biosynthetic\_process | 1 | 0 |  |  |  |  |  |  |  |  |
| GO:0010931\_macrophage\_tolerance\_induction | 1 | 0 |  |  |  |  |  |  |  |  |
| GO:0010932\_regulation\_of\_macrophage\_tolerance\_induction | 1 | 0 |  |  |  |  |  |  |  |  |
| GO:0010933\_positive\_regulation\_of\_macrophage\_tolerance\_induction | 1 | 0 |  |  |  |  |  |  |  |  |
| GO:0010934\_macrophage\_cytokine\_production | 1 | 0 |  |  |  |  |  |  |  |  |
| GO:0010935\_regulation\_of\_macrophage\_cytokine\_production | 1 | 0 |  |  |  |  |  |  |  |  |
| GO:0010936\_negative\_regulation\_of\_macrophage\_cytokine\_production | 1 | 0 |  |  |  |  |  |  |  |  |
| GO:0010953\_regulation\_of\_protein\_maturation\_by\_peptide\_bond\_cleavage | 1 | 0 |  |  |  |  |  |  |  |  |
| GO:0010962\_regulation\_of\_glucan\_biosynthetic\_process | 1 | 0 |  |  |  |  |  |  |  |  |
| GO:0010966\_regulation\_of\_phosphate\_transport | 1 | 0 |  |  |  |  |  |  |  |  |
| GO:0014012\_axon\_regeneration\_in\_the\_peripheral\_nervous\_system | 1 | 0 |  |  |  |  |  |  |  |  |
| GO:0014016\_neuroblast\_differentiation | 1 | 0 |  |  |  |  |  |  |  |  |
| GO:0014017\_neuroblast\_fate\_commitment | 1 | 0 |  |  |  |  |  |  |  |  |
| GO:0014041\_regulation\_of\_neuron\_maturation | 1 | 0 |  |  |  |  |  |  |  |  |
| GO:0014042\_positive\_regulation\_of\_neuron\_maturation | 1 | 0 |  |  |  |  |  |  |  |  |
| GO:0014049\_positive\_regulation\_of\_glutamate\_secretion | 1 | 0 |  |  |  |  |  |  |  |  |
| GO:0014061\_regulation\_of\_norepinephrine\_secretion | 1 | 0 |  |  |  |  |  |  |  |  |
| GO:0014071\_response\_to\_cycloalkane | 1 | 0 |  |  |  |  |  |  |  |  |
| GO:0014707\_branchiomeric\_skeletal\_muscle\_development | 1 | 0 |  |  |  |  |  |  |  |  |
| GO:0014738\_regulation\_of\_muscle\_hyperplasia | 1 | 0 |  |  |  |  |  |  |  |  |
| GO:0014740\_negative\_regulation\_of\_muscle\_hyperplasia | 1 | 0 |  |  |  |  |  |  |  |  |
| GO:0014741\_negative\_regulation\_of\_muscle\_hypertrophy | 1 | 0 |  |  |  |  |  |  |  |  |
| GO:0014743\_regulation\_of\_muscle\_hypertrophy | 1 | 0 |  |  |  |  |  |  |  |  |
| GO:0014805\_smooth\_muscle\_adaptation | 1 | 0 |  |  |  |  |  |  |  |  |
| GO:0014806\_smooth\_muscle\_hyperplasia | 1 | 0 |  |  |  |  |  |  |  |  |
| GO:0014807\_regulation\_of\_somitogenesis | 1 | 0 |  |  |  |  |  |  |  |  |
| GO:0014808\_release\_of\_sequestered\_calcium\_ion\_into\_cytosol\_by\_sarcoplasmic\_reticulum | 1 | 0 |  |  |  |  |  |  |  |  |
| GO:0014813\_satellite\_cell\_commitment | 1 | 0 |  |  |  |  |  |  |  |  |
| GO:0014816\_satellite\_cell\_differentiation | 1 | 0 |  |  |  |  |  |  |  |  |
| GO:0014819\_regulation\_of\_skeletal\_muscle\_contraction | 1 | 0 |  |  |  |  |  |  |  |  |
| GO:0014852\_regulation\_of\_skeletal\_muscle\_contraction\_by\_neural\_stimulation\_via\_neuromuscular\_junction | 1 | 0 |  |  |  |  |  |  |  |  |
| GO:0014853\_regulation\_of\_excitatory\_postsynaptic\_membrane\_potential\_involved\_in\_skeletal\_muscle\_contraction | 1 | 0 |  |  |  |  |  |  |  |  |
| GO:0014856\_skeletal\_muscle\_cell\_proliferation | 1 | 0 |  |  |  |  |  |  |  |  |
| GO:0014857\_regulation\_of\_skeletal\_muscle\_cell\_proliferation | 1 | 0 |  |  |  |  |  |  |  |  |
| GO:0014858\_positive\_regulation\_of\_skeletal\_muscle\_cell\_proliferation | 1 | 0 |  |  |  |  |  |  |  |  |
| GO:0014887\_cardiac\_muscle\_adaptation | 1 | 0 |  |  |  |  |  |  |  |  |
| GO:0014889\_muscle\_atrophy | 1 | 0 |  |  |  |  |  |  |  |  |
| GO:0014896\_muscle\_hypertrophy | 1 | 0 |  |  |  |  |  |  |  |  |
| GO:0014897\_striated\_muscle\_hypertrophy | 1 | 0 |  |  |  |  |  |  |  |  |
| GO:0014898\_cardiac\_muscle\_hypertrophy | 1 | 0 |  |  |  |  |  |  |  |  |
| GO:0014900\_muscle\_hyperplasia | 1 | 0 |  |  |  |  |  |  |  |  |
| GO:0014910\_regulation\_of\_smooth\_muscle\_cell\_migration | 1 | 0 |  |  |  |  |  |  |  |  |
| GO:0014911\_positive\_regulation\_of\_smooth\_muscle\_cell\_migration | 1 | 0 |  |  |  |  |  |  |  |  |
| GO:0015014\_heparan\_sulfate\_proteoglycan\_biosynthetic\_process\_\_polysaccharide\_chain\_biosynthetic\_process | 1 | 0 |  |  |  |  |  |  |  |  |
| GO:0015074\_DNA\_integration | 1 | 0 |  |  |  |  |  |  |  |  |
| GO:0015670\_carbon\_dioxide\_transport | 1 | 0 |  |  |  |  |  |  |  |  |
| GO:0015677\_copper\_ion\_import | 1 | 0 |  |  |  |  |  |  |  |  |
| GO:0015680\_intracellular\_copper\_ion\_transport | 1 | 0 |  |  |  |  |  |  |  |  |
| GO:0015684\_ferrous\_iron\_transport | 1 | 0 |  |  |  |  |  |  |  |  |
| GO:0015707\_nitrite\_transport | 1 | 0 |  |  |  |  |  |  |  |  |
| GO:0015724\_formate\_transport | 1 | 0 |  |  |  |  |  |  |  |  |
| GO:0015734\_taurine\_transport | 1 | 0 |  |  |  |  |  |  |  |  |
| GO:0015740\_C4-dicarboxylate\_transport | 1 | 0 |  |  |  |  |  |  |  |  |
| GO:0015744\_succinate\_transport | 1 | 0 |  |  |  |  |  |  |  |  |
| GO:0015746\_citrate\_transport | 1 | 0 |  |  |  |  |  |  |  |  |
| GO:0015747\_urate\_transport | 1 | 0 |  |  |  |  |  |  |  |  |
| GO:0015791\_polyol\_transport | 1 | 0 |  |  |  |  |  |  |  |  |
| GO:0015798\_myo-inositol\_transport | 1 | 0 |  |  |  |  |  |  |  |  |
| GO:0015808\_L-alanine\_transport | 1 | 0 |  |  |  |  |  |  |  |  |
| GO:0015810\_aspartate\_transport | 1 | 0 |  |  |  |  |  |  |  |  |
| GO:0015811\_L-cystine\_transport | 1 | 0 |  |  |  |  |  |  |  |  |
| GO:0015817\_histidine\_transport | 1 | 0 |  |  |  |  |  |  |  |  |
| GO:0015822\_ornithine\_transport | 1 | 0 |  |  |  |  |  |  |  |  |
| GO:0015824\_proline\_transport | 1 | 0 |  |  |  |  |  |  |  |  |
| GO:0015851\_nucleobase\_transport | 1 | 0 |  |  |  |  |  |  |  |  |
| GO:0015864\_pyrimidine\_nucleoside\_transport | 1 | 0 |  |  |  |  |  |  |  |  |
| GO:0015874\_norepinephrine\_transport | 1 | 0 |  |  |  |  |  |  |  |  |
| GO:0015881\_creatine\_transport | 1 | 0 |  |  |  |  |  |  |  |  |
| GO:0015884\_folic\_acid\_transport | 1 | 0 |  |  |  |  |  |  |  |  |
| GO:0015886\_heme\_transport | 1 | 0 |  |  |  |  |  |  |  |  |
| GO:0015888\_thiamin\_transport | 1 | 0 |  |  |  |  |  |  |  |  |
| GO:0015938\_coenzyme\_A\_catabolic\_process | 1 | 0 |  |  |  |  |  |  |  |  |
| GO:0015939\_pantothenate\_metabolic\_process | 1 | 0 |  |  |  |  |  |  |  |  |
| GO:0016073\_snRNA\_metabolic\_process | 1 | 0 |  |  |  |  |  |  |  |  |
| GO:0016074\_snoRNA\_metabolic\_process | 1 | 0 |  |  |  |  |  |  |  |  |
| GO:0016082\_synaptic\_vesicle\_priming | 1 | 0 |  |  |  |  |  |  |  |  |
| GO:0016090\_prenol\_metabolic\_process | 1 | 0 |  |  |  |  |  |  |  |  |
| GO:0016093\_polyprenol\_metabolic\_process | 1 | 0 |  |  |  |  |  |  |  |  |
| GO:0016180\_snRNA\_processing | 1 | 0 |  |  |  |  |  |  |  |  |
| GO:0016239\_positive\_regulation\_of\_macroautophagy | 1 | 0 |  |  |  |  |  |  |  |  |
| GO:0016246\_RNA\_interference | 1 | 0 |  |  |  |  |  |  |  |  |
| GO:0016255\_attachment\_of\_GPI\_anchor\_to\_protein | 1 | 0 |  |  |  |  |  |  |  |  |
| GO:0016333\_morphogenesis\_of\_follicular\_epithelium | 1 | 0 |  |  |  |  |  |  |  |  |
| GO:0016340\_calcium-dependent\_cell-matrix\_adhesion | 1 | 0 |  |  |  |  |  |  |  |  |
| GO:0016344\_meiotic\_chromosome\_movement\_towards\_spindle\_pole | 1 | 0 |  |  |  |  |  |  |  |  |
| GO:0016482\_cytoplasmic\_transport | 1 | 0 |  |  |  |  |  |  |  |  |
| GO:0016553\_base\_conversion\_or\_substitution\_editing | 1 | 0 |  |  |  |  |  |  |  |  |
| GO:0016554\_cytidine\_to\_uridine\_editing | 1 | 0 |  |  |  |  |  |  |  |  |
| GO:0016578\_histone\_deubiquitination | 1 | 0 |  |  |  |  |  |  |  |  |
| GO:0016598\_protein\_arginylation | 1 | 0 |  |  |  |  |  |  |  |  |
| GO:0017004\_cytochrome\_complex\_assembly | 1 | 0 |  |  |  |  |  |  |  |  |
| GO:0018022\_peptidyl-lysine\_methylation | 1 | 0 |  |  |  |  |  |  |  |  |
| GO:0018023\_peptidyl-lysine\_trimethylation | 1 | 0 |  |  |  |  |  |  |  |  |
| GO:0018120\_peptidyl-arginine\_ADP-ribosylation | 1 | 0 |  |  |  |  |  |  |  |  |
| GO:0018126\_protein\_amino\_acid\_hydroxylation | 1 | 0 |  |  |  |  |  |  |  |  |
| GO:0018146\_keratan\_sulfate\_biosynthetic\_process | 1 | 0 |  |  |  |  |  |  |  |  |
| GO:0018158\_protein\_amino\_acid\_oxidation | 1 | 0 |  |  |  |  |  |  |  |  |
| GO:0018195\_peptidyl-arginine\_modification | 1 | 0 |  |  |  |  |  |  |  |  |
| GO:0018197\_peptidyl-aspartic\_acid\_modification | 1 | 0 |  |  |  |  |  |  |  |  |
| GO:0018282\_metal\_incorporation\_into\_metallo-sulfur\_cluster | 1 | 0 |  |  |  |  |  |  |  |  |
| GO:0018283\_iron\_incorporation\_into\_metallo-sulfur\_cluster | 1 | 0 |  |  |  |  |  |  |  |  |
| GO:0018318\_protein\_amino\_acid\_palmitoylation | 1 | 0 |  |  |  |  |  |  |  |  |
| GO:0018342\_protein\_prenylation | 1 | 0 |  |  |  |  |  |  |  |  |
| GO:0018344\_protein\_geranylgeranylation | 1 | 0 |  |  |  |  |  |  |  |  |
| GO:0018410\_peptide\_or\_protein\_carboxyl-terminal\_blocking | 1 | 0 |  |  |  |  |  |  |  |  |
| GO:0018916\_nitrobenzene\_metabolic\_process | 1 | 0 |  |  |  |  |  |  |  |  |
| GO:0018931\_naphthalene\_metabolic\_process | 1 | 0 |  |  |  |  |  |  |  |  |
| GO:0018992\_germ-line\_sex\_determination | 1 | 0 |  |  |  |  |  |  |  |  |
| GO:0019042\_latent\_virus\_infection | 1 | 0 |  |  |  |  |  |  |  |  |
| GO:0019046\_reactivation\_of\_latent\_virus | 1 | 0 |  |  |  |  |  |  |  |  |
| GO:0019047\_provirus\_integration | 1 | 0 |  |  |  |  |  |  |  |  |
| GO:0019076\_release\_of\_virus\_from\_host | 1 | 0 |  |  |  |  |  |  |  |  |
| GO:0019079\_viral\_genome\_replication | 1 | 0 |  |  |  |  |  |  |  |  |
| GO:0019100\_male\_germ-line\_sex\_determination | 1 | 0 |  |  |  |  |  |  |  |  |
| GO:0019101\_female\_somatic\_sex\_determination | 1 | 0 |  |  |  |  |  |  |  |  |
| GO:0019102\_male\_somatic\_sex\_determination | 1 | 0 |  |  |  |  |  |  |  |  |
| GO:0019255\_glucose\_1-phosphate\_metabolic\_process | 1 | 0 |  |  |  |  |  |  |  |  |
| GO:0019276\_UDP-N-acetylgalactosamine\_metabolic\_process | 1 | 0 |  |  |  |  |  |  |  |  |
| GO:0019344\_cysteine\_biosynthetic\_process | 1 | 0 |  |  |  |  |  |  |  |  |
| GO:0019348\_dolichol\_metabolic\_process | 1 | 0 |  |  |  |  |  |  |  |  |
| GO:0019375\_galactolipid\_biosynthetic\_process | 1 | 0 |  |  |  |  |  |  |  |  |
| GO:0019402\_galactitol\_metabolic\_process | 1 | 0 |  |  |  |  |  |  |  |  |
| GO:0019441\_tryptophan\_catabolic\_process\_to\_kynurenine | 1 | 0 |  |  |  |  |  |  |  |  |
| GO:0019477\_L-lysine\_catabolic\_process | 1 | 0 |  |  |  |  |  |  |  |  |
| GO:0019510\_S-adenosylhomocysteine\_catabolic\_process | 1 | 0 |  |  |  |  |  |  |  |  |
| GO:0019532\_oxalate\_transport | 1 | 0 |  |  |  |  |  |  |  |  |
| GO:0019626\_short-chain\_fatty\_acid\_catabolic\_process | 1 | 0 |  |  |  |  |  |  |  |  |
| GO:0019627\_urea\_metabolic\_process | 1 | 0 |  |  |  |  |  |  |  |  |
| GO:0019676\_ammonia\_assimilation\_cycle | 1 | 0 |  |  |  |  |  |  |  |  |
| GO:0019682\_glyceraldehyde-3-phosphate\_metabolic\_process | 1 | 0 |  |  |  |  |  |  |  |  |
| GO:0019695\_choline\_metabolic\_process | 1 | 0 |  |  |  |  |  |  |  |  |
| GO:0019731\_antibacterial\_humoral\_response | 1 | 0 |  |  |  |  |  |  |  |  |
| GO:0019794\_nonprotein\_amino\_acid\_metabolic\_process | 1 | 0 |  |  |  |  |  |  |  |  |
| GO:0019858\_cytosine\_metabolic\_process | 1 | 0 |  |  |  |  |  |  |  |  |
| GO:0019883\_antigen\_processing\_and\_presentation\_of\_endogenous\_antigen | 1 | 0 |  |  |  |  |  |  |  |  |
| GO:0019889\_pteridine\_metabolic\_process | 1 | 0 |  |  |  |  |  |  |  |  |
| GO:0019896\_axon\_transport\_of\_mitochondrion | 1 | 0 |  |  |  |  |  |  |  |  |
| GO:0021508\_floor\_plate\_formation | 1 | 0 |  |  |  |  |  |  |  |  |
| GO:0021528\_commissural\_neuron\_differentiation\_in\_the\_spinal\_cord | 1 | 0 |  |  |  |  |  |  |  |  |
| GO:0021572\_rhombomere\_6\_development | 1 | 0 |  |  |  |  |  |  |  |  |
| GO:0021577\_hindbrain\_structural\_organization | 1 | 0 |  |  |  |  |  |  |  |  |
| GO:0021586\_pons\_maturation | 1 | 0 |  |  |  |  |  |  |  |  |
| GO:0021589\_cerebellum\_structural\_organization | 1 | 0 |  |  |  |  |  |  |  |  |
| GO:0021590\_cerebellum\_maturation | 1 | 0 |  |  |  |  |  |  |  |  |
| GO:0021592\_fourth\_ventricle\_development | 1 | 0 |  |  |  |  |  |  |  |  |
| GO:0021594\_rhombomere\_formation | 1 | 0 |  |  |  |  |  |  |  |  |
| GO:0021660\_rhombomere\_3\_formation | 1 | 0 |  |  |  |  |  |  |  |  |
| GO:0021664\_rhombomere\_5\_morphogenesis | 1 | 0 |  |  |  |  |  |  |  |  |
| GO:0021666\_rhombomere\_5\_formation | 1 | 0 |  |  |  |  |  |  |  |  |
| GO:0021670\_lateral\_ventricle\_development | 1 | 0 |  |  |  |  |  |  |  |  |
| GO:0021678\_third\_ventricle\_development | 1 | 0 |  |  |  |  |  |  |  |  |
| GO:0021679\_cerebellar\_molecular\_layer\_development | 1 | 0 |  |  |  |  |  |  |  |  |
| GO:0021703\_locus\_ceruleus\_development | 1 | 0 |  |  |  |  |  |  |  |  |
| GO:0021732\_midbrain-hindbrain\_boundary\_maturation | 1 | 0 |  |  |  |  |  |  |  |  |
| GO:0021747\_cochlear\_nucleus\_development | 1 | 0 |  |  |  |  |  |  |  |  |
| GO:0021750\_vestibular\_nucleus\_development | 1 | 0 |  |  |  |  |  |  |  |  |
| GO:0021759\_globus\_pallidus\_development | 1 | 0 |  |  |  |  |  |  |  |  |
| GO:0021768\_nucleus\_accumbens\_development | 1 | 0 |  |  |  |  |  |  |  |  |
| GO:0021771\_lateral\_geniculate\_nucleus\_development | 1 | 0 |  |  |  |  |  |  |  |  |
| GO:0021812\_neuronal-glial\_interaction\_involved\_in\_cerebral\_cortex\_radial\_glia\_guided\_migration | 1 | 0 |  |  |  |  |  |  |  |  |
| GO:0021813\_cell-cell\_adhesion\_involved\_in\_neuronal-glial\_interactions\_involved\_in\_cerebral\_cortex\_radial\_glia\_guided\_migration | 1 | 0 |  |  |  |  |  |  |  |  |
| GO:0021870\_Cajal-Retzius\_cell\_differentiation | 1 | 0 |  |  |  |  |  |  |  |  |
| GO:0021874\_Wnt\_receptor\_signaling\_pathway\_in\_forebrain\_neuroblast\_division | 1 | 0 |  |  |  |  |  |  |  |  |
| GO:0021896\_forebrain\_astrocyte\_differentiation | 1 | 0 |  |  |  |  |  |  |  |  |
| GO:0021897\_forebrain\_astrocyte\_development | 1 | 0 |  |  |  |  |  |  |  |  |
| GO:0021902\_commitment\_of\_a\_neuronal\_cell\_to\_a\_specific\_type\_of\_neuron\_in\_the\_forebrain | 1 | 0 |  |  |  |  |  |  |  |  |
| GO:0021905\_forebrain-midbrain\_boundary\_formation | 1 | 0 |  |  |  |  |  |  |  |  |
| GO:0021914\_negative\_regulation\_of\_smoothened\_signaling\_pathway\_involved\_in\_ventral\_spinal\_cord\_patterning | 1 | 0 |  |  |  |  |  |  |  |  |
| GO:0021917\_somatic\_motor\_neuron\_fate\_commitment | 1 | 0 |  |  |  |  |  |  |  |  |
| GO:0021918\_regulation\_of\_transcription\_from\_RNA\_polymerase\_II\_promoter\_involved\_in\_somatic\_motor\_neuron\_fate\_commitment | 1 | 0 |  |  |  |  |  |  |  |  |
| GO:0021933\_radial\_glia\_guided\_migration\_of\_granule\_cell | 1 | 0 |  |  |  |  |  |  |  |  |
| GO:0021934\_hindbrain\_tangential\_cell\_migration | 1 | 0 |  |  |  |  |  |  |  |  |
| GO:0021935\_granule\_cell\_precursor\_tangential\_migration | 1 | 0 |  |  |  |  |  |  |  |  |
| GO:0021942\_radial\_glia\_guided\_migration\_of\_Purkinje\_cell | 1 | 0 |  |  |  |  |  |  |  |  |
| GO:0021960\_anterior\_commissure\_morphogenesis | 1 | 0 |  |  |  |  |  |  |  |  |
| GO:0021997\_neural\_plate\_axis\_specification | 1 | 0 |  |  |  |  |  |  |  |  |
| GO:0021999\_neural\_plate\_anterior\_posterior\_pattern\_formation | 1 | 0 |  |  |  |  |  |  |  |  |
| GO:0022004\_midbrain-hindbrain\_boundary\_maturation\_during\_brain\_development | 1 | 0 |  |  |  |  |  |  |  |  |
| GO:0022038\_corpus\_callosum\_development | 1 | 0 |  |  |  |  |  |  |  |  |
| GO:0022605\_oogenesis\_stage | 1 | 0 |  |  |  |  |  |  |  |  |
| GO:0030011\_maintenance\_of\_cell\_polarity | 1 | 0 |  |  |  |  |  |  |  |  |
| GO:0030069\_lysogeny | 1 | 0 |  |  |  |  |  |  |  |  |
| GO:0030070\_insulin\_processing | 1 | 0 |  |  |  |  |  |  |  |  |
| GO:0030092\_regulation\_of\_flagellum\_assembly | 1 | 0 |  |  |  |  |  |  |  |  |
| GO:0030103\_vasopressin\_secretion | 1 | 0 |  |  |  |  |  |  |  |  |
| GO:0030194\_positive\_regulation\_of\_blood\_coagulation | 1 | 0 |  |  |  |  |  |  |  |  |
| GO:0030206\_chondroitin\_sulfate\_biosynthetic\_process | 1 | 0 |  |  |  |  |  |  |  |  |
| GO:0030210\_heparin\_biosynthetic\_process | 1 | 0 |  |  |  |  |  |  |  |  |
| GO:0030220\_platelet\_formation | 1 | 0 |  |  |  |  |  |  |  |  |
| GO:0030222\_eosinophil\_differentiation | 1 | 0 |  |  |  |  |  |  |  |  |
| GO:0030237\_female\_sex\_determination | 1 | 0 |  |  |  |  |  |  |  |  |
| GO:0030264\_nuclear\_fragmentation\_during\_apoptosis | 1 | 0 |  |  |  |  |  |  |  |  |
| GO:0030322\_stabilization\_of\_membrane\_potential | 1 | 0 |  |  |  |  |  |  |  |  |
| GO:0030327\_prenylated\_protein\_catabolic\_process | 1 | 0 |  |  |  |  |  |  |  |  |
| GO:0030328\_prenylcysteine\_catabolic\_process | 1 | 0 |  |  |  |  |  |  |  |  |
| GO:0030329\_prenylcysteine\_metabolic\_process | 1 | 0 |  |  |  |  |  |  |  |  |
| GO:0030382\_sperm\_mitochondrion\_organization | 1 | 0 |  |  |  |  |  |  |  |  |
| GO:0030389\_fructosamine\_metabolic\_process | 1 | 0 |  |  |  |  |  |  |  |  |
| GO:0030422\_RNA\_interference\_\_production\_of\_siRNA | 1 | 0 |  |  |  |  |  |  |  |  |
| GO:0030449\_regulation\_of\_complement\_activation | 1 | 0 |  |  |  |  |  |  |  |  |
| GO:0030497\_fatty\_acid\_elongation | 1 | 0 |  |  |  |  |  |  |  |  |
| GO:0030575\_nuclear\_body\_organization | 1 | 0 |  |  |  |  |  |  |  |  |
| GO:0030578\_PML\_body\_organization | 1 | 0 |  |  |  |  |  |  |  |  |
| GO:0030853\_negative\_regulation\_of\_granulocyte\_differentiation | 1 | 0 |  |  |  |  |  |  |  |  |
| GO:0030854\_positive\_regulation\_of\_granulocyte\_differentiation | 1 | 0 |  |  |  |  |  |  |  |  |
| GO:0030886\_negative\_regulation\_of\_myeloid\_dendritic\_cell\_activation | 1 | 0 |  |  |  |  |  |  |  |  |
| GO:0030913\_paranodal\_junction\_assembly | 1 | 0 |  |  |  |  |  |  |  |  |
| GO:0031033\_myosin\_filament\_assembly\_or\_disassembly | 1 | 0 |  |  |  |  |  |  |  |  |
| GO:0031034\_myosin\_filament\_assembly | 1 | 0 |  |  |  |  |  |  |  |  |
| GO:0031055\_chromatin\_remodeling\_at\_centromere | 1 | 0 |  |  |  |  |  |  |  |  |
| GO:0031062\_positive\_regulation\_of\_histone\_methylation | 1 | 0 |  |  |  |  |  |  |  |  |
| GO:0031115\_negative\_regulation\_of\_microtubule\_polymerization | 1 | 0 |  |  |  |  |  |  |  |  |
| GO:0031129\_inductive\_cell-cell\_signaling | 1 | 0 |  |  |  |  |  |  |  |  |
| GO:0031284\_positive\_regulation\_of\_guanylate\_cyclase\_activity | 1 | 0 |  |  |  |  |  |  |  |  |
| GO:0031498\_chromatin\_disassembly | 1 | 0 |  |  |  |  |  |  |  |  |
| GO:0031507\_heterochromatin\_formation | 1 | 0 |  |  |  |  |  |  |  |  |
| GO:0031508\_centromeric\_heterochromatin\_formation | 1 | 0 |  |  |  |  |  |  |  |  |
| GO:0031529\_ruffle\_organization | 1 | 0 |  |  |  |  |  |  |  |  |
| GO:0031536\_positive\_regulation\_of\_exit\_from\_mitosis | 1 | 0 |  |  |  |  |  |  |  |  |
| GO:0031572\_G2\_M\_transition\_DNA\_damage\_checkpoint | 1 | 0 |  |  |  |  |  |  |  |  |
| GO:0031576\_G2\_M\_transition\_checkpoint | 1 | 0 |  |  |  |  |  |  |  |  |
| GO:0031580\_membrane\_raft\_distribution | 1 | 0 |  |  |  |  |  |  |  |  |
| GO:0031583\_activation\_of\_phospholipase\_D\_activity\_by\_G-protein\_coupled\_receptor\_protein\_signaling\_pathway | 1 | 0 |  |  |  |  |  |  |  |  |
| GO:0031584\_activation\_of\_phospholipase\_D\_activity | 1 | 0 |  |  |  |  |  |  |  |  |
| GO:0031585\_regulation\_of\_inositol-1\_4\_5-triphosphate\_receptor\_activity | 1 | 0 |  |  |  |  |  |  |  |  |
| GO:0031639\_plasminogen\_activation | 1 | 0 |  |  |  |  |  |  |  |  |
| GO:0031648\_protein\_destabilization | 1 | 0 |  |  |  |  |  |  |  |  |
| GO:0031665\_negative\_regulation\_of\_lipopolysaccharide-mediated\_signaling\_pathway | 1 | 0 |  |  |  |  |  |  |  |  |
| GO:0031914\_negative\_regulation\_of\_synaptic\_plasticity | 1 | 0 |  |  |  |  |  |  |  |  |
| GO:0031944\_negative\_regulation\_of\_glucocorticoid\_metabolic\_process | 1 | 0 |  |  |  |  |  |  |  |  |
| GO:0031947\_negative\_regulation\_of\_glucocorticoid\_biosynthetic\_process | 1 | 0 |  |  |  |  |  |  |  |  |
| GO:0032025\_response\_to\_cobalt\_ion | 1 | 0 |  |  |  |  |  |  |  |  |
| GO:0032026\_response\_to\_magnesium\_ion | 1 | 0 |  |  |  |  |  |  |  |  |
| GO:0032048\_cardiolipin\_metabolic\_process | 1 | 0 |  |  |  |  |  |  |  |  |
| GO:0032066\_nucleolus\_to\_nucleoplasm\_transport | 1 | 0 |  |  |  |  |  |  |  |  |
| GO:0032091\_negative\_regulation\_of\_protein\_binding | 1 | 0 |  |  |  |  |  |  |  |  |
| GO:0032092\_positive\_regulation\_of\_protein\_binding | 1 | 0 |  |  |  |  |  |  |  |  |
| GO:0032097\_positive\_regulation\_of\_response\_to\_food | 1 | 0 |  |  |  |  |  |  |  |  |
| GO:0032100\_positive\_regulation\_of\_appetite | 1 | 0 |  |  |  |  |  |  |  |  |
| GO:0032204\_regulation\_of\_telomere\_maintenance | 1 | 0 |  |  |  |  |  |  |  |  |
| GO:0032206\_positive\_regulation\_of\_telomere\_maintenance | 1 | 0 |  |  |  |  |  |  |  |  |
| GO:0032222\_regulation\_of\_synaptic\_transmission\_\_cholinergic | 1 | 0 |  |  |  |  |  |  |  |  |
| GO:0032224\_positive\_regulation\_of\_synaptic\_transmission\_\_cholinergic | 1 | 0 |  |  |  |  |  |  |  |  |
| GO:0032229\_negative\_regulation\_of\_synaptic\_transmission\_\_GABAergic | 1 | 0 |  |  |  |  |  |  |  |  |
| GO:0032237\_activation\_of\_store-operated\_calcium\_channel\_activity | 1 | 0 |  |  |  |  |  |  |  |  |
| GO:0032239\_regulation\_of\_nucleobase\_\_nucleoside\_\_nucleotide\_and\_nucleic\_acid\_transport | 1 | 0 |  |  |  |  |  |  |  |  |
| GO:0032252\_secretory\_granule\_localization | 1 | 0 |  |  |  |  |  |  |  |  |
| GO:0032274\_gonadotropin\_secretion | 1 | 0 |  |  |  |  |  |  |  |  |
| GO:0032275\_luteinizing\_hormone\_secretion | 1 | 0 |  |  |  |  |  |  |  |  |
| GO:0032287\_myelin\_maintenance\_in\_the\_peripheral\_nervous\_system | 1 | 0 |  |  |  |  |  |  |  |  |
| GO:0032289\_myelin\_formation\_in\_the\_central\_nervous\_system | 1 | 0 |  |  |  |  |  |  |  |  |
| GO:0032303\_regulation\_of\_icosanoid\_secretion | 1 | 0 |  |  |  |  |  |  |  |  |
| GO:0032305\_positive\_regulation\_of\_icosanoid\_secretion | 1 | 0 |  |  |  |  |  |  |  |  |
| GO:0032306\_regulation\_of\_prostaglandin\_secretion | 1 | 0 |  |  |  |  |  |  |  |  |
| GO:0032308\_positive\_regulation\_of\_prostaglandin\_secretion | 1 | 0 |  |  |  |  |  |  |  |  |
| GO:0032310\_prostaglandin\_secretion | 1 | 0 |  |  |  |  |  |  |  |  |
| GO:0032313\_regulation\_of\_Rab\_GTPase\_activity | 1 | 0 |  |  |  |  |  |  |  |  |
| GO:0032314\_regulation\_of\_Rac\_GTPase\_activity | 1 | 0 |  |  |  |  |  |  |  |  |
| GO:0032317\_regulation\_of\_Rap\_GTPase\_activity | 1 | 0 |  |  |  |  |  |  |  |  |
| GO:0032324\_molybdopterin\_cofactor\_biosynthetic\_process | 1 | 0 |  |  |  |  |  |  |  |  |
| GO:0032329\_serine\_transport | 1 | 0 |  |  |  |  |  |  |  |  |
| GO:0032342\_aldosterone\_biosynthetic\_process | 1 | 0 |  |  |  |  |  |  |  |  |
| GO:0032344\_regulation\_of\_aldosterone\_metabolic\_process | 1 | 0 |  |  |  |  |  |  |  |  |
| GO:0032365\_intracellular\_lipid\_transport | 1 | 0 |  |  |  |  |  |  |  |  |
| GO:0032366\_intracellular\_sterol\_transport | 1 | 0 |  |  |  |  |  |  |  |  |
| GO:0032367\_intracellular\_cholesterol\_transport | 1 | 0 |  |  |  |  |  |  |  |  |
| GO:0032370\_positive\_regulation\_of\_lipid\_transport | 1 | 0 |  |  |  |  |  |  |  |  |
| GO:0032410\_negative\_regulation\_of\_transporter\_activity | 1 | 0 |  |  |  |  |  |  |  |  |
| GO:0032413\_negative\_regulation\_of\_ion\_transmembrane\_transporter\_activity | 1 | 0 |  |  |  |  |  |  |  |  |
| GO:0032429\_regulation\_of\_phospholipase\_A2\_activity | 1 | 0 |  |  |  |  |  |  |  |  |
| GO:0032474\_otolith\_morphogenesis | 1 | 0 |  |  |  |  |  |  |  |  |
| GO:0032482\_Rab\_protein\_signal\_transduction | 1 | 0 |  |  |  |  |  |  |  |  |
| GO:0032483\_regulation\_of\_Rab\_protein\_signal\_transduction | 1 | 0 |  |  |  |  |  |  |  |  |
| GO:0032486\_Rap\_protein\_signal\_transduction | 1 | 0 |  |  |  |  |  |  |  |  |
| GO:0032487\_regulation\_of\_Rap\_protein\_signal\_transduction | 1 | 0 |  |  |  |  |  |  |  |  |
| GO:0032594\_protein\_transport\_within\_lipid\_bilayer | 1 | 0 |  |  |  |  |  |  |  |  |
| GO:0032599\_protein\_transport\_out\_of\_membrane\_raft | 1 | 0 |  |  |  |  |  |  |  |  |
| GO:0032600\_chemokine\_receptor\_transport\_out\_of\_membrane\_raft | 1 | 0 |  |  |  |  |  |  |  |  |
| GO:0032607\_interferon-alpha\_production | 1 | 0 |  |  |  |  |  |  |  |  |
| GO:0032621\_interleukin-18\_production | 1 | 0 |  |  |  |  |  |  |  |  |
| GO:0032647\_regulation\_of\_interferon-alpha\_production | 1 | 0 |  |  |  |  |  |  |  |  |
| GO:0032656\_regulation\_of\_interleukin-13\_production | 1 | 0 |  |  |  |  |  |  |  |  |
| GO:0032682\_negative\_regulation\_of\_chemokine\_production | 1 | 0 |  |  |  |  |  |  |  |  |
| GO:0032691\_negative\_regulation\_of\_interleukin-1\_beta\_production | 1 | 0 |  |  |  |  |  |  |  |  |
| GO:0032692\_negative\_regulation\_of\_interleukin-1\_production | 1 | 0 |  |  |  |  |  |  |  |  |
| GO:0032693\_negative\_regulation\_of\_interleukin-10\_production | 1 | 0 |  |  |  |  |  |  |  |  |
| GO:0032696\_negative\_regulation\_of\_interleukin-13\_production | 1 | 0 |  |  |  |  |  |  |  |  |
| GO:0032727\_positive\_regulation\_of\_interferon-alpha\_production | 1 | 0 |  |  |  |  |  |  |  |  |
| GO:0032731\_positive\_regulation\_of\_interleukin-1\_beta\_production | 1 | 0 |  |  |  |  |  |  |  |  |
| GO:0032732\_positive\_regulation\_of\_interleukin-1\_production | 1 | 0 |  |  |  |  |  |  |  |  |
| GO:0032735\_positive\_regulation\_of\_interleukin-12\_production | 1 | 0 |  |  |  |  |  |  |  |  |
| GO:0032764\_negative\_regulation\_of\_mast\_cell\_cytokine\_production | 1 | 0 |  |  |  |  |  |  |  |  |
| GO:0032765\_positive\_regulation\_of\_mast\_cell\_cytokine\_production | 1 | 0 |  |  |  |  |  |  |  |  |
| GO:0032769\_negative\_regulation\_of\_monooxygenase\_activity | 1 | 0 |  |  |  |  |  |  |  |  |
| GO:0032781\_positive\_regulation\_of\_ATPase\_activity | 1 | 0 |  |  |  |  |  |  |  |  |
| GO:0032790\_ribosome\_disassembly | 1 | 0 |  |  |  |  |  |  |  |  |
| GO:0032799\_low-density\_lipoprotein\_receptor\_metabolic\_process | 1 | 0 |  |  |  |  |  |  |  |  |
| GO:0032802\_low-density\_lipoprotein\_receptor\_catabolic\_process | 1 | 0 |  |  |  |  |  |  |  |  |
| GO:0032803\_regulation\_of\_low-density\_lipoprotein\_receptor\_catabolic\_process | 1 | 0 |  |  |  |  |  |  |  |  |
| GO:0032817\_regulation\_of\_natural\_killer\_cell\_proliferation | 1 | 0 |  |  |  |  |  |  |  |  |
| GO:0032819\_positive\_regulation\_of\_natural\_killer\_cell\_proliferation | 1 | 0 |  |  |  |  |  |  |  |  |
| GO:0032836\_glomerular\_basement\_membrane\_development | 1 | 0 |  |  |  |  |  |  |  |  |
| GO:0032855\_positive\_regulation\_of\_Rac\_GTPase\_activity | 1 | 0 |  |  |  |  |  |  |  |  |
| GO:0032863\_activation\_of\_Rac\_GTPase\_activity | 1 | 0 |  |  |  |  |  |  |  |  |
| GO:0032864\_activation\_of\_Cdc42\_GTPase\_activity | 1 | 0 |  |  |  |  |  |  |  |  |
| GO:0032885\_regulation\_of\_polysaccharide\_biosynthetic\_process | 1 | 0 |  |  |  |  |  |  |  |  |
| GO:0032907\_transforming\_growth\_factor-beta3\_production | 1 | 0 |  |  |  |  |  |  |  |  |
| GO:0032910\_regulation\_of\_transforming\_growth\_factor-beta3\_production | 1 | 0 |  |  |  |  |  |  |  |  |
| GO:0032913\_negative\_regulation\_of\_transforming\_growth\_factor-beta3\_production | 1 | 0 |  |  |  |  |  |  |  |  |
| GO:0032924\_activin\_receptor\_signaling\_pathway | 1 | 0 |  |  |  |  |  |  |  |  |
| GO:0032925\_regulation\_of\_activin\_receptor\_signaling\_pathway | 1 | 0 |  |  |  |  |  |  |  |  |
| GO:0032960\_regulation\_of\_inositol\_trisphosphate\_biosynthetic\_process | 1 | 0 |  |  |  |  |  |  |  |  |
| GO:0032962\_positive\_regulation\_of\_inositol\_trisphosphate\_biosynthetic\_process | 1 | 0 |  |  |  |  |  |  |  |  |
| GO:0032964\_collagen\_biosynthetic\_process | 1 | 0 |  |  |  |  |  |  |  |  |
| GO:0032971\_regulation\_of\_muscle\_filament\_sliding | 1 | 0 |  |  |  |  |  |  |  |  |
| GO:0032972\_regulation\_of\_muscle\_filament\_sliding\_speed | 1 | 0 |  |  |  |  |  |  |  |  |
| GO:0032986\_protein-DNA\_complex\_disassembly | 1 | 0 |  |  |  |  |  |  |  |  |
| GO:0032988\_ribonucleoprotein\_complex\_disassembly | 1 | 0 |  |  |  |  |  |  |  |  |
| GO:0033037\_polysaccharide\_localization | 1 | 0 |  |  |  |  |  |  |  |  |
| GO:0033078\_extrathymic\_T\_cell\_differentiation | 1 | 0 |  |  |  |  |  |  |  |  |
| GO:0033085\_negative\_regulation\_of\_T\_cell\_differentiation\_in\_the\_thymus | 1 | 0 |  |  |  |  |  |  |  |  |
| GO:0033087\_negative\_regulation\_of\_immature\_T\_cell\_proliferation | 1 | 0 |  |  |  |  |  |  |  |  |
| GO:0033088\_negative\_regulation\_of\_immature\_T\_cell\_proliferation\_in\_the\_thymus | 1 | 0 |  |  |  |  |  |  |  |  |
| GO:0033108\_mitochondrial\_respiratory\_chain\_complex\_assembly | 1 | 0 |  |  |  |  |  |  |  |  |
| GO:0033127\_regulation\_of\_histone\_phosphorylation | 1 | 0 |  |  |  |  |  |  |  |  |
| GO:0033128\_negative\_regulation\_of\_histone\_phosphorylation | 1 | 0 |  |  |  |  |  |  |  |  |
| GO:0033138\_positive\_regulation\_of\_peptidyl-serine\_phosphorylation | 1 | 0 |  |  |  |  |  |  |  |  |
| GO:0033158\_regulation\_of\_protein\_import\_into\_nucleus\_\_translocation | 1 | 0 |  |  |  |  |  |  |  |  |
| GO:0033160\_positive\_regulation\_of\_protein\_import\_into\_nucleus\_\_translocation | 1 | 0 |  |  |  |  |  |  |  |  |
| GO:0033169\_histone\_H3-K9\_demethylation | 1 | 0 |  |  |  |  |  |  |  |  |
| GO:0033206\_cytokinesis\_after\_meiosis | 1 | 0 |  |  |  |  |  |  |  |  |
| GO:0033240\_positive\_regulation\_of\_cellular\_amine\_metabolic\_process | 1 | 0 |  |  |  |  |  |  |  |  |
| GO:0033313\_meiotic\_cell\_cycle\_checkpoint | 1 | 0 |  |  |  |  |  |  |  |  |
| GO:0033315\_meiotic\_cell\_cycle\_DNA\_replication\_checkpoint | 1 | 0 |  |  |  |  |  |  |  |  |
| GO:0033326\_cerebrospinal\_fluid\_secretion | 1 | 0 |  |  |  |  |  |  |  |  |
| GO:0033366\_protein\_localization\_in\_secretory\_granule | 1 | 0 |  |  |  |  |  |  |  |  |
| GO:0033367\_protein\_localization\_in\_mast\_cell\_secretory\_granule | 1 | 0 |  |  |  |  |  |  |  |  |
| GO:0033368\_protease\_localization\_in\_mast\_cell\_secretory\_granule | 1 | 0 |  |  |  |  |  |  |  |  |
| GO:0033370\_maintenance\_of\_protein\_location\_in\_mast\_cell\_secretory\_granule | 1 | 0 |  |  |  |  |  |  |  |  |
| GO:0033371\_T\_cell\_secretory\_granule\_organization | 1 | 0 |  |  |  |  |  |  |  |  |
| GO:0033373\_maintenance\_of\_protease\_location\_in\_mast\_cell\_secretory\_granule | 1 | 0 |  |  |  |  |  |  |  |  |
| GO:0033374\_protein\_localization\_in\_T\_cell\_secretory\_granule | 1 | 0 |  |  |  |  |  |  |  |  |
| GO:0033375\_protease\_localization\_in\_T\_cell\_secretory\_granule | 1 | 0 |  |  |  |  |  |  |  |  |
| GO:0033377\_maintenance\_of\_protein\_location\_in\_T\_cell\_secretory\_granule | 1 | 0 |  |  |  |  |  |  |  |  |
| GO:0033379\_maintenance\_of\_protease\_location\_in\_T\_cell\_secretory\_granule | 1 | 0 |  |  |  |  |  |  |  |  |
| GO:0033380\_granzyme\_B\_localization\_in\_T\_cell\_secretory\_granule | 1 | 0 |  |  |  |  |  |  |  |  |
| GO:0033382\_maintenance\_of\_granzyme\_B\_location\_in\_T\_cell\_secretory\_granule | 1 | 0 |  |  |  |  |  |  |  |  |
| GO:0033483\_gas\_homeostasis | 1 | 0 |  |  |  |  |  |  |  |  |
| GO:0033484\_nitric\_oxide\_homeostasis | 1 | 0 |  |  |  |  |  |  |  |  |
| GO:0033505\_floor\_plate\_morphogenesis | 1 | 0 |  |  |  |  |  |  |  |  |
| GO:0033522\_histone\_H2A\_ubiquitination | 1 | 0 |  |  |  |  |  |  |  |  |
| GO:0033523\_histone\_H2B\_ubiquitination | 1 | 0 |  |  |  |  |  |  |  |  |
| GO:0033574\_response\_to\_testosterone\_stimulus | 1 | 0 |  |  |  |  |  |  |  |  |
| GO:0033606\_chemokine\_receptor\_transport\_within\_lipid\_bilayer | 1 | 0 |  |  |  |  |  |  |  |  |
| GO:0033628\_regulation\_of\_cell\_adhesion\_mediated\_by\_integrin | 1 | 0 |  |  |  |  |  |  |  |  |
| GO:0033630\_positive\_regulation\_of\_cell\_adhesion\_mediated\_by\_integrin | 1 | 0 |  |  |  |  |  |  |  |  |
| GO:0033632\_regulation\_of\_cell-cell\_adhesion\_mediated\_by\_integrin | 1 | 0 |  |  |  |  |  |  |  |  |
| GO:0033634\_positive\_regulation\_of\_cell-cell\_adhesion\_mediated\_by\_integrin | 1 | 0 |  |  |  |  |  |  |  |  |
| GO:0033683\_nucleotide-excision\_repair\_\_DNA\_incision | 1 | 0 |  |  |  |  |  |  |  |  |
| GO:0033687\_osteoblast\_proliferation | 1 | 0 |  |  |  |  |  |  |  |  |
| GO:0033688\_regulation\_of\_osteoblast\_proliferation | 1 | 0 |  |  |  |  |  |  |  |  |
| GO:0033689\_negative\_regulation\_of\_osteoblast\_proliferation | 1 | 0 |  |  |  |  |  |  |  |  |
| GO:0033866\_nucleoside\_bisphosphate\_biosynthetic\_process | 1 | 0 |  |  |  |  |  |  |  |  |
| GO:0033875\_ribonucleoside\_bisphosphate\_metabolic\_process | 1 | 0 |  |  |  |  |  |  |  |  |
| GO:0034030\_ribonucleoside\_bisphosphate\_biosynthetic\_process | 1 | 0 |  |  |  |  |  |  |  |  |
| GO:0034032\_purine\_nucleoside\_bisphosphate\_metabolic\_process | 1 | 0 |  |  |  |  |  |  |  |  |
| GO:0034033\_purine\_nucleoside\_bisphosphate\_biosynthetic\_process | 1 | 0 |  |  |  |  |  |  |  |  |
| GO:0034035\_purine\_ribonucleoside\_bisphosphate\_metabolic\_process | 1 | 0 |  |  |  |  |  |  |  |  |
| GO:0034036\_purine\_ribonucleoside\_bisphosphate\_biosynthetic\_process | 1 | 0 |  |  |  |  |  |  |  |  |
| GO:0034067\_protein\_localization\_in\_Golgi\_apparatus | 1 | 0 |  |  |  |  |  |  |  |  |
| GO:0034102\_erythrocyte\_clearance | 1 | 0 |  |  |  |  |  |  |  |  |
| GO:0034106\_regulation\_of\_erythrocyte\_clearance | 1 | 0 |  |  |  |  |  |  |  |  |
| GO:0034107\_negative\_regulation\_of\_erythrocyte\_clearance | 1 | 0 |  |  |  |  |  |  |  |  |
| GO:0034110\_regulation\_of\_homotypic\_cell-cell\_adhesion | 1 | 0 |  |  |  |  |  |  |  |  |
| GO:0034111\_negative\_regulation\_of\_homotypic\_cell-cell\_adhesion | 1 | 0 |  |  |  |  |  |  |  |  |
| GO:0034113\_heterotypic\_cell-cell\_adhesion | 1 | 0 |  |  |  |  |  |  |  |  |
| GO:0034117\_erythrocyte\_aggregation | 1 | 0 |  |  |  |  |  |  |  |  |
| GO:0034118\_regulation\_of\_erythrocyte\_aggregation | 1 | 0 |  |  |  |  |  |  |  |  |
| GO:0034119\_negative\_regulation\_of\_erythrocyte\_aggregation | 1 | 0 |  |  |  |  |  |  |  |  |
| GO:0034121\_regulation\_of\_toll-like\_receptor\_signaling\_pathway | 1 | 0 |  |  |  |  |  |  |  |  |
| GO:0034122\_negative\_regulation\_of\_toll-like\_receptor\_signaling\_pathway | 1 | 0 |  |  |  |  |  |  |  |  |
| GO:0034230\_enkephalin\_processing | 1 | 0 |  |  |  |  |  |  |  |  |
| GO:0034372\_very-low-density\_lipoprotein\_particle\_remodeling | 1 | 0 |  |  |  |  |  |  |  |  |
| GO:0034379\_very-low-density\_lipoprotein\_particle\_assembly | 1 | 0 |  |  |  |  |  |  |  |  |
| GO:0034380\_high-density\_lipoprotein\_particle\_assembly | 1 | 0 |  |  |  |  |  |  |  |  |
| GO:0034394\_protein\_localization\_at\_cell\_surface | 1 | 0 |  |  |  |  |  |  |  |  |
| GO:0034405\_response\_to\_fluid\_shear\_stress | 1 | 0 |  |  |  |  |  |  |  |  |
| GO:0034472\_snRNA\_3'-end\_processing | 1 | 0 |  |  |  |  |  |  |  |  |
| GO:0034474\_U2\_snRNA\_3'-end\_processing | 1 | 0 |  |  |  |  |  |  |  |  |
| GO:0034502\_protein\_localization\_to\_chromosome | 1 | 0 |  |  |  |  |  |  |  |  |
| GO:0034505\_tooth\_mineralization | 1 | 0 |  |  |  |  |  |  |  |  |
| GO:0034508\_centromere\_complex\_assembly | 1 | 0 |  |  |  |  |  |  |  |  |
| GO:0034633\_retinol\_transport | 1 | 0 |  |  |  |  |  |  |  |  |
| GO:0034643\_mitochondrion\_localization\_\_microtubule-mediated | 1 | 0 |  |  |  |  |  |  |  |  |
| GO:0034969\_histone\_arginine\_methylation | 1 | 0 |  |  |  |  |  |  |  |  |
| GO:0034982\_mitochondrial\_protein\_processing | 1 | 0 |  |  |  |  |  |  |  |  |
| GO:0035022\_positive\_regulation\_of\_Rac\_protein\_signal\_transduction | 1 | 0 |  |  |  |  |  |  |  |  |
| GO:0035024\_negative\_regulation\_of\_Rho\_protein\_signal\_transduction | 1 | 0 |  |  |  |  |  |  |  |  |
| GO:0035026\_leading\_edge\_cell\_differentiation | 1 | 0 |  |  |  |  |  |  |  |  |
| GO:0035037\_sperm\_entry | 1 | 0 |  |  |  |  |  |  |  |  |
| GO:0035039\_male\_pronucleus\_formation | 1 | 0 |  |  |  |  |  |  |  |  |
| GO:0035066\_positive\_regulation\_of\_histone\_acetylation | 1 | 0 |  |  |  |  |  |  |  |  |
| GO:0035083\_cilium\_axoneme\_assembly | 1 | 0 |  |  |  |  |  |  |  |  |
| GO:0035090\_maintenance\_of\_apical\_basal\_cell\_polarity | 1 | 0 |  |  |  |  |  |  |  |  |
| GO:0035106\_operant\_conditioning | 1 | 0 |  |  |  |  |  |  |  |  |
| GO:0035172\_hemocyte\_proliferation | 1 | 0 |  |  |  |  |  |  |  |  |
| GO:0035227\_regulation\_of\_glutamate-cysteine\_ligase\_activity | 1 | 0 |  |  |  |  |  |  |  |  |
| GO:0035229\_positive\_regulation\_of\_glutamate-cysteine\_ligase\_activity | 1 | 0 |  |  |  |  |  |  |  |  |
| GO:0035260\_internal\_genitalia\_morphogenesis | 1 | 0 |  |  |  |  |  |  |  |  |
| GO:0035262\_gonad\_morphogenesis | 1 | 0 |  |  |  |  |  |  |  |  |
| GO:0035287\_head\_segmentation | 1 | 0 |  |  |  |  |  |  |  |  |
| GO:0035289\_posterior\_head\_segmentation | 1 | 0 |  |  |  |  |  |  |  |  |
| GO:0035303\_regulation\_of\_dephosphorylation | 1 | 0 |  |  |  |  |  |  |  |  |
| GO:0035304\_regulation\_of\_protein\_amino\_acid\_dephosphorylation | 1 | 0 |  |  |  |  |  |  |  |  |
| GO:0035305\_negative\_regulation\_of\_dephosphorylation | 1 | 0 |  |  |  |  |  |  |  |  |
| GO:0035308\_negative\_regulation\_of\_protein\_amino\_acid\_dephosphorylation | 1 | 0 |  |  |  |  |  |  |  |  |
| GO:0035313\_wound\_healing\_\_spreading\_of\_epidermal\_cells | 1 | 0 |  |  |  |  |  |  |  |  |
| GO:0040013\_negative\_regulation\_of\_locomotion | 1 | 0 |  |  |  |  |  |  |  |  |
| GO:0040019\_positive\_regulation\_of\_embryonic\_development | 1 | 0 |  |  |  |  |  |  |  |  |
| GO:0040032\_post-embryonic\_body\_morphogenesis | 1 | 0 |  |  |  |  |  |  |  |  |
| GO:0040038\_polar\_body\_extrusion\_after\_meiotic\_divisions | 1 | 0 |  |  |  |  |  |  |  |  |
| GO:0042026\_protein\_refolding | 1 | 0 |  |  |  |  |  |  |  |  |
| GO:0042048\_olfactory\_behavior | 1 | 0 |  |  |  |  |  |  |  |  |
| GO:0042059\_negative\_regulation\_of\_epidermal\_growth\_factor\_receptor\_signaling\_pathway | 1 | 0 |  |  |  |  |  |  |  |  |
| GO:0042073\_intraflagellar\_transport | 1 | 0 |  |  |  |  |  |  |  |  |
| GO:0042078\_germ-line\_stem\_cell\_division | 1 | 0 |  |  |  |  |  |  |  |  |
| GO:0042091\_interleukin-10\_biosynthetic\_process | 1 | 0 |  |  |  |  |  |  |  |  |
| GO:0042103\_positive\_regulation\_of\_T\_cell\_homeostatic\_proliferation | 1 | 0 |  |  |  |  |  |  |  |  |
| GO:0042136\_neurotransmitter\_biosynthetic\_process | 1 | 0 |  |  |  |  |  |  |  |  |
| GO:0042137\_sequestering\_of\_neurotransmitter | 1 | 0 |  |  |  |  |  |  |  |  |
| GO:0042138\_meiotic\_DNA\_double-strand\_break\_formation | 1 | 0 |  |  |  |  |  |  |  |  |
| GO:0042178\_xenobiotic\_catabolic\_process | 1 | 0 |  |  |  |  |  |  |  |  |
| GO:0042225\_interleukin-5\_biosynthetic\_process | 1 | 0 |  |  |  |  |  |  |  |  |
| GO:0042231\_interleukin-13\_biosynthetic\_process | 1 | 0 |  |  |  |  |  |  |  |  |
| GO:0042255\_ribosome\_assembly | 1 | 0 |  |  |  |  |  |  |  |  |
| GO:0042257\_ribosomal\_subunit\_assembly | 1 | 0 |  |  |  |  |  |  |  |  |
| GO:0042264\_peptidyl-aspartic\_acid\_hydroxylation | 1 | 0 |  |  |  |  |  |  |  |  |
| GO:0042276\_error-prone\_postreplication\_DNA\_repair | 1 | 0 |  |  |  |  |  |  |  |  |
| GO:0042297\_vocal\_learning | 1 | 0 |  |  |  |  |  |  |  |  |
| GO:0042309\_homoiothermy | 1 | 0 |  |  |  |  |  |  |  |  |
| GO:0042320\_regulation\_of\_circadian\_sleep\_wake\_cycle\_\_REM\_sleep | 1 | 0 |  |  |  |  |  |  |  |  |
| GO:0042339\_keratan\_sulfate\_metabolic\_process | 1 | 0 |  |  |  |  |  |  |  |  |
| GO:0042347\_negative\_regulation\_of\_NF-kappaB\_import\_into\_nucleus | 1 | 0 |  |  |  |  |  |  |  |  |
| GO:0042360\_vitamin\_E\_metabolic\_process | 1 | 0 |  |  |  |  |  |  |  |  |
| GO:0042363\_fat-soluble\_vitamin\_catabolic\_process | 1 | 0 |  |  |  |  |  |  |  |  |
| GO:0042369\_vitamin\_D\_catabolic\_process | 1 | 0 |  |  |  |  |  |  |  |  |
| GO:0042373\_vitamin\_K\_metabolic\_process | 1 | 0 |  |  |  |  |  |  |  |  |
| GO:0042404\_thyroid\_hormone\_catabolic\_process | 1 | 0 |  |  |  |  |  |  |  |  |
| GO:0042414\_epinephrine\_metabolic\_process | 1 | 0 |  |  |  |  |  |  |  |  |
| GO:0042436\_indole\_derivative\_catabolic\_process | 1 | 0 |  |  |  |  |  |  |  |  |
| GO:0042489\_negative\_regulation\_of\_odontogenesis\_of\_dentine-containing\_tooth | 1 | 0 |  |  |  |  |  |  |  |  |
| GO:0042508\_tyrosine\_phosphorylation\_of\_Stat1\_protein | 1 | 0 |  |  |  |  |  |  |  |  |
| GO:0042518\_negative\_regulation\_of\_tyrosine\_phosphorylation\_of\_Stat3\_protein | 1 | 0 |  |  |  |  |  |  |  |  |
| GO:0042524\_negative\_regulation\_of\_tyrosine\_phosphorylation\_of\_Stat5\_protein | 1 | 0 |  |  |  |  |  |  |  |  |
| GO:0042536\_negative\_regulation\_of\_tumor\_necrosis\_factor\_biosynthetic\_process | 1 | 0 |  |  |  |  |  |  |  |  |
| GO:0042538\_hyperosmotic\_salinity\_response | 1 | 0 |  |  |  |  |  |  |  |  |
| GO:0042628\_mating\_plug\_formation | 1 | 0 |  |  |  |  |  |  |  |  |
| GO:0042631\_cellular\_response\_to\_water\_deprivation | 1 | 0 |  |  |  |  |  |  |  |  |
| GO:0042637\_catagen | 1 | 0 |  |  |  |  |  |  |  |  |
| GO:0042660\_positive\_regulation\_of\_cell\_fate\_specification | 1 | 0 |  |  |  |  |  |  |  |  |
| GO:0042663\_regulation\_of\_endodermal\_cell\_fate\_specification | 1 | 0 |  |  |  |  |  |  |  |  |
| GO:0042664\_negative\_regulation\_of\_endodermal\_cell\_fate\_specification | 1 | 0 |  |  |  |  |  |  |  |  |
| GO:0042667\_auditory\_receptor\_cell\_fate\_specification | 1 | 0 |  |  |  |  |  |  |  |  |
| GO:0042694\_muscle\_cell\_fate\_specification | 1 | 0 |  |  |  |  |  |  |  |  |
| GO:0042706\_eye\_photoreceptor\_cell\_fate\_commitment | 1 | 0 |  |  |  |  |  |  |  |  |
| GO:0042713\_sperm\_ejaculation | 1 | 0 |  |  |  |  |  |  |  |  |
| GO:0042723\_thiamin\_and\_derivative\_metabolic\_process | 1 | 0 |  |  |  |  |  |  |  |  |
| GO:0042737\_drug\_catabolic\_process | 1 | 0 |  |  |  |  |  |  |  |  |
| GO:0042738\_exogenous\_drug\_catabolic\_process | 1 | 0 |  |  |  |  |  |  |  |  |
| GO:0042747\_circadian\_sleep\_wake\_cycle\_\_REM\_sleep | 1 | 0 |  |  |  |  |  |  |  |  |
| GO:0042748\_circadian\_sleep\_wake\_cycle\_\_non-REM\_sleep | 1 | 0 |  |  |  |  |  |  |  |  |
| GO:0042772\_DNA\_damage\_response\_\_signal\_transduction\_resulting\_in\_transcription | 1 | 0 |  |  |  |  |  |  |  |  |
| GO:0042790\_transcription\_of\_nuclear\_rRNA\_large\_RNA\_polymerase\_I\_transcript | 1 | 0 |  |  |  |  |  |  |  |  |
| GO:0042839\_D-glucuronate\_metabolic\_process | 1 | 0 |  |  |  |  |  |  |  |  |
| GO:0042840\_D-glucuronate\_catabolic\_process | 1 | 0 |  |  |  |  |  |  |  |  |
| GO:0042891\_antibiotic\_transport | 1 | 0 |  |  |  |  |  |  |  |  |
| GO:0042892\_chloramphenicol\_transport | 1 | 0 |  |  |  |  |  |  |  |  |
| GO:0042940\_D-amino\_acid\_transport | 1 | 0 |  |  |  |  |  |  |  |  |
| GO:0042941\_D-alanine\_transport | 1 | 0 |  |  |  |  |  |  |  |  |
| GO:0042942\_D-serine\_transport | 1 | 0 |  |  |  |  |  |  |  |  |
| GO:0042983\_amyloid\_precursor\_protein\_biosynthetic\_process | 1 | 0 |  |  |  |  |  |  |  |  |
| GO:0042984\_regulation\_of\_amyloid\_precursor\_protein\_biosynthetic\_process | 1 | 0 |  |  |  |  |  |  |  |  |
| GO:0042985\_negative\_regulation\_of\_amyloid\_precursor\_protein\_biosynthetic\_process | 1 | 0 |  |  |  |  |  |  |  |  |
| GO:0042989\_sequestering\_of\_actin\_monomers | 1 | 0 |  |  |  |  |  |  |  |  |
| GO:0043044\_ATP-dependent\_chromatin\_remodeling | 1 | 0 |  |  |  |  |  |  |  |  |
| GO:0043056\_forward\_locomotion | 1 | 0 |  |  |  |  |  |  |  |  |
| GO:0043060\_meiotic\_metaphase\_I\_plate\_congression | 1 | 0 |  |  |  |  |  |  |  |  |
| GO:0043091\_L-arginine\_import | 1 | 0 |  |  |  |  |  |  |  |  |
| GO:0043124\_negative\_regulation\_of\_I-kappaB\_kinase\_NF-kappaB\_cascade | 1 | 0 |  |  |  |  |  |  |  |  |
| GO:0043132\_NAD\_transport | 1 | 0 |  |  |  |  |  |  |  |  |
| GO:0043153\_entrainment\_of\_circadian\_clock\_by\_photoperiod | 1 | 0 |  |  |  |  |  |  |  |  |
| GO:0043171\_peptide\_catabolic\_process | 1 | 0 |  |  |  |  |  |  |  |  |
| GO:0043179\_rhythmic\_excitation | 1 | 0 |  |  |  |  |  |  |  |  |
| GO:0043206\_fibril\_organization | 1 | 0 |  |  |  |  |  |  |  |  |
| GO:0043217\_myelin\_maintenance | 1 | 0 |  |  |  |  |  |  |  |  |
| GO:0043313\_regulation\_of\_neutrophil\_degranulation | 1 | 0 |  |  |  |  |  |  |  |  |
| GO:0043316\_cytotoxic\_T\_cell\_degranulation | 1 | 0 |  |  |  |  |  |  |  |  |
| GO:0043369\_CD4-positive\_or\_CD8-positive\_\_alpha-beta\_T\_cell\_lineage\_commitment | 1 | 0 |  |  |  |  |  |  |  |  |
| GO:0043375\_CD8-positive\_\_alpha-beta\_T\_cell\_lineage\_commitment | 1 | 0 |  |  |  |  |  |  |  |  |
| GO:0043379\_memory\_T\_cell\_differentiation | 1 | 0 |  |  |  |  |  |  |  |  |
| GO:0043380\_regulation\_of\_memory\_T\_cell\_differentiation | 1 | 0 |  |  |  |  |  |  |  |  |
| GO:0043400\_cortisol\_secretion | 1 | 0 |  |  |  |  |  |  |  |  |
| GO:0043415\_positive\_regulation\_of\_skeletal\_muscle\_regeneration | 1 | 0 |  |  |  |  |  |  |  |  |
| GO:0043416\_regulation\_of\_skeletal\_muscle\_regeneration | 1 | 0 |  |  |  |  |  |  |  |  |
| GO:0043437\_butanoic\_acid\_metabolic\_process | 1 | 0 |  |  |  |  |  |  |  |  |
| GO:0043438\_acetoacetic\_acid\_metabolic\_process | 1 | 0 |  |  |  |  |  |  |  |  |
| GO:0043480\_pigment\_accumulation\_in\_tissues | 1 | 0 |  |  |  |  |  |  |  |  |
| GO:0043482\_cellular\_pigment\_accumulation | 1 | 0 |  |  |  |  |  |  |  |  |
| GO:0043486\_histone\_exchange | 1 | 0 |  |  |  |  |  |  |  |  |
| GO:0043496\_regulation\_of\_protein\_homodimerization\_activity | 1 | 0 |  |  |  |  |  |  |  |  |
| GO:0043501\_skeletal\_muscle\_adaptation | 1 | 0 |  |  |  |  |  |  |  |  |
| GO:0043508\_negative\_regulation\_of\_JUN\_kinase\_activity | 1 | 0 |  |  |  |  |  |  |  |  |
| GO:0043517\_positive\_regulation\_of\_DNA\_damage\_response\_\_signal\_transduction\_by\_p53\_class\_mediator | 1 | 0 |  |  |  |  |  |  |  |  |
| GO:0043535\_regulation\_of\_blood\_vessel\_endothelial\_cell\_migration | 1 | 0 |  |  |  |  |  |  |  |  |
| GO:0043537\_negative\_regulation\_of\_blood\_vessel\_endothelial\_cell\_migration | 1 | 0 |  |  |  |  |  |  |  |  |
| GO:0043545\_molybdopterin\_cofactor\_metabolic\_process | 1 | 0 |  |  |  |  |  |  |  |  |
| GO:0043587\_tongue\_morphogenesis | 1 | 0 |  |  |  |  |  |  |  |  |
| GO:0043604\_amide\_biosynthetic\_process | 1 | 0 |  |  |  |  |  |  |  |  |
| GO:0043628\_ncRNA\_3'-end\_processing | 1 | 0 |  |  |  |  |  |  |  |  |
| GO:0044254\_multicellular\_organismal\_protein\_catabolic\_process | 1 | 0 |  |  |  |  |  |  |  |  |
| GO:0044256\_protein\_digestion | 1 | 0 |  |  |  |  |  |  |  |  |
| GO:0044266\_multicellular\_organismal\_macromolecule\_catabolic\_process | 1 | 0 |  |  |  |  |  |  |  |  |
| GO:0045004\_DNA\_replication\_proofreading | 1 | 0 |  |  |  |  |  |  |  |  |
| GO:0045019\_negative\_regulation\_of\_nitric\_oxide\_biosynthetic\_process | 1 | 0 |  |  |  |  |  |  |  |  |
| GO:0045020\_error-prone\_DNA\_repair | 1 | 0 |  |  |  |  |  |  |  |  |
| GO:0045022\_early\_endosome\_to\_late\_endosome\_transport | 1 | 0 |  |  |  |  |  |  |  |  |
| GO:0045062\_extrathymic\_T\_cell\_selection | 1 | 0 |  |  |  |  |  |  |  |  |
| GO:0045069\_regulation\_of\_viral\_genome\_replication | 1 | 0 |  |  |  |  |  |  |  |  |
| GO:0045074\_regulation\_of\_interleukin-10\_biosynthetic\_process | 1 | 0 |  |  |  |  |  |  |  |  |
| GO:0045082\_positive\_regulation\_of\_interleukin-10\_biosynthetic\_process | 1 | 0 |  |  |  |  |  |  |  |  |
| GO:0045083\_negative\_regulation\_of\_interleukin-12\_biosynthetic\_process | 1 | 0 |  |  |  |  |  |  |  |  |
| GO:0045112\_integrin\_biosynthetic\_process | 1 | 0 |  |  |  |  |  |  |  |  |
| GO:0045113\_regulation\_of\_integrin\_biosynthetic\_process | 1 | 0 |  |  |  |  |  |  |  |  |
| GO:0045188\_regulation\_of\_circadian\_sleep\_wake\_cycle\_\_non-REM\_sleep | 1 | 0 |  |  |  |  |  |  |  |  |
| GO:0045210\_FasL\_biosynthetic\_process | 1 | 0 |  |  |  |  |  |  |  |  |
| GO:0045297\_post-mating\_behavior | 1 | 0 |  |  |  |  |  |  |  |  |
| GO:0045299\_otolith\_mineralization | 1 | 0 |  |  |  |  |  |  |  |  |
| GO:0045329\_carnitine\_biosynthetic\_process | 1 | 0 |  |  |  |  |  |  |  |  |
| GO:0045341\_MHC\_class\_I\_biosynthetic\_process | 1 | 0 |  |  |  |  |  |  |  |  |
| GO:0045343\_regulation\_of\_MHC\_class\_I\_biosynthetic\_process | 1 | 0 |  |  |  |  |  |  |  |  |
| GO:0045347\_negative\_regulation\_of\_MHC\_class\_II\_biosynthetic\_process | 1 | 0 |  |  |  |  |  |  |  |  |
| GO:0045405\_regulation\_of\_interleukin-5\_biosynthetic\_process | 1 | 0 |  |  |  |  |  |  |  |  |
| GO:0045407\_positive\_regulation\_of\_interleukin-5\_biosynthetic\_process | 1 | 0 |  |  |  |  |  |  |  |  |
| GO:0045426\_quinone\_cofactor\_biosynthetic\_process | 1 | 0 |  |  |  |  |  |  |  |  |
| GO:0045448\_mitotic\_cell\_cycle\_\_embryonic | 1 | 0 |  |  |  |  |  |  |  |  |
| GO:0045454\_cell\_redox\_homeostasis | 1 | 0 |  |  |  |  |  |  |  |  |
| GO:0045583\_regulation\_of\_cytotoxic\_T\_cell\_differentiation | 1 | 0 |  |  |  |  |  |  |  |  |
| GO:0045585\_positive\_regulation\_of\_cytotoxic\_T\_cell\_differentiation | 1 | 0 |  |  |  |  |  |  |  |  |
| GO:0045601\_regulation\_of\_endothelial\_cell\_differentiation | 1 | 0 |  |  |  |  |  |  |  |  |
| GO:0045602\_negative\_regulation\_of\_endothelial\_cell\_differentiation | 1 | 0 |  |  |  |  |  |  |  |  |
| GO:0045605\_negative\_regulation\_of\_epidermal\_cell\_differentiation | 1 | 0 |  |  |  |  |  |  |  |  |
| GO:0045606\_positive\_regulation\_of\_epidermal\_cell\_differentiation | 1 | 0 |  |  |  |  |  |  |  |  |
| GO:0045609\_positive\_regulation\_of\_auditory\_receptor\_cell\_differentiation | 1 | 0 |  |  |  |  |  |  |  |  |
| GO:0045617\_negative\_regulation\_of\_keratinocyte\_differentiation | 1 | 0 |  |  |  |  |  |  |  |  |
| GO:0045618\_positive\_regulation\_of\_keratinocyte\_differentiation | 1 | 0 |  |  |  |  |  |  |  |  |
| GO:0045626\_negative\_regulation\_of\_T-helper\_1\_cell\_differentiation | 1 | 0 |  |  |  |  |  |  |  |  |
| GO:0045633\_positive\_regulation\_of\_mechanoreceptor\_differentiation | 1 | 0 |  |  |  |  |  |  |  |  |
| GO:0045650\_negative\_regulation\_of\_macrophage\_differentiation | 1 | 0 |  |  |  |  |  |  |  |  |
| GO:0045656\_negative\_regulation\_of\_monocyte\_differentiation | 1 | 0 |  |  |  |  |  |  |  |  |
| GO:0045657\_positive\_regulation\_of\_monocyte\_differentiation | 1 | 0 |  |  |  |  |  |  |  |  |
| GO:0045659\_negative\_regulation\_of\_neutrophil\_differentiation | 1 | 0 |  |  |  |  |  |  |  |  |
| GO:0045660\_positive\_regulation\_of\_neutrophil\_differentiation | 1 | 0 |  |  |  |  |  |  |  |  |
| GO:0045721\_negative\_regulation\_of\_gluconeogenesis | 1 | 0 |  |  |  |  |  |  |  |  |
| GO:0045724\_positive\_regulation\_of\_flagellum\_assembly | 1 | 0 |  |  |  |  |  |  |  |  |
| GO:0045725\_positive\_regulation\_of\_glycogen\_biosynthetic\_process | 1 | 0 |  |  |  |  |  |  |  |  |
| GO:0045740\_positive\_regulation\_of\_DNA\_replication | 1 | 0 |  |  |  |  |  |  |  |  |
| GO:0045759\_negative\_regulation\_of\_action\_potential | 1 | 0 |  |  |  |  |  |  |  |  |
| GO:0045768\_positive\_regulation\_of\_anti-apoptosis | 1 | 0 |  |  |  |  |  |  |  |  |
| GO:0045769\_negative\_regulation\_of\_asymmetric\_cell\_division | 1 | 0 |  |  |  |  |  |  |  |  |
| GO:0045794\_negative\_regulation\_of\_cell\_volume | 1 | 0 |  |  |  |  |  |  |  |  |
| GO:0045815\_positive\_regulation\_of\_gene\_expression\_\_epigenetic | 1 | 0 |  |  |  |  |  |  |  |  |
| GO:0045818\_negative\_regulation\_of\_glycogen\_catabolic\_process | 1 | 0 |  |  |  |  |  |  |  |  |
| GO:0045842\_positive\_regulation\_of\_mitotic\_metaphase\_anaphase\_transition | 1 | 0 |  |  |  |  |  |  |  |  |
| GO:0045875\_negative\_regulation\_of\_sister\_chromatid\_cohesion | 1 | 0 |  |  |  |  |  |  |  |  |
| GO:0045898\_regulation\_of\_transcriptional\_preinitiation\_complex\_assembly | 1 | 0 |  |  |  |  |  |  |  |  |
| GO:0045899\_positive\_regulation\_of\_transcriptional\_preinitiation\_complex\_assembly | 1 | 0 |  |  |  |  |  |  |  |  |
| GO:0045906\_negative\_regulation\_of\_vasoconstriction | 1 | 0 |  |  |  |  |  |  |  |  |
| GO:0045908\_negative\_regulation\_of\_vasodilation | 1 | 0 |  |  |  |  |  |  |  |  |
| GO:0045909\_positive\_regulation\_of\_vasodilation | 1 | 0 |  |  |  |  |  |  |  |  |
| GO:0045915\_positive\_regulation\_of\_catecholamine\_metabolic\_process | 1 | 0 |  |  |  |  |  |  |  |  |
| GO:0045920\_negative\_regulation\_of\_exocytosis | 1 | 0 |  |  |  |  |  |  |  |  |
| GO:0045924\_regulation\_of\_female\_receptivity | 1 | 0 |  |  |  |  |  |  |  |  |
| GO:0045947\_negative\_regulation\_of\_translational\_initiation | 1 | 0 |  |  |  |  |  |  |  |  |
| GO:0045955\_negative\_regulation\_of\_calcium\_ion-dependent\_exocytosis | 1 | 0 |  |  |  |  |  |  |  |  |
| GO:0045956\_positive\_regulation\_of\_calcium\_ion-dependent\_exocytosis | 1 | 0 |  |  |  |  |  |  |  |  |
| GO:0045964\_positive\_regulation\_of\_dopamine\_metabolic\_process | 1 | 0 |  |  |  |  |  |  |  |  |
| GO:0045988\_negative\_regulation\_of\_striated\_muscle\_contraction | 1 | 0 |  |  |  |  |  |  |  |  |
| GO:0045989\_positive\_regulation\_of\_striated\_muscle\_contraction | 1 | 0 |  |  |  |  |  |  |  |  |
| GO:0045990\_regulation\_of\_transcription\_by\_carbon\_catabolites | 1 | 0 |  |  |  |  |  |  |  |  |
| GO:0045991\_positive\_regulation\_of\_transcription\_by\_carbon\_catabolites | 1 | 0 |  |  |  |  |  |  |  |  |
| GO:0045994\_positive\_regulation\_of\_translational\_initiation\_by\_iron | 1 | 0 |  |  |  |  |  |  |  |  |
| GO:0046007\_negative\_regulation\_of\_activated\_T\_cell\_proliferation | 1 | 0 |  |  |  |  |  |  |  |  |
| GO:0046014\_negative\_regulation\_of\_T\_cell\_homeostatic\_proliferation | 1 | 0 |  |  |  |  |  |  |  |  |
| GO:0046015\_regulation\_of\_transcription\_by\_glucose | 1 | 0 |  |  |  |  |  |  |  |  |
| GO:0046016\_positive\_regulation\_of\_transcription\_by\_glucose | 1 | 0 |  |  |  |  |  |  |  |  |
| GO:0046031\_ADP\_metabolic\_process | 1 | 0 |  |  |  |  |  |  |  |  |
| GO:0046032\_ADP\_catabolic\_process | 1 | 0 |  |  |  |  |  |  |  |  |
| GO:0046061\_dATP\_catabolic\_process | 1 | 0 |  |  |  |  |  |  |  |  |
| GO:0046075\_dTTP\_metabolic\_process | 1 | 0 |  |  |  |  |  |  |  |  |
| GO:0046078\_dUMP\_metabolic\_process | 1 | 0 |  |  |  |  |  |  |  |  |
| GO:0046079\_dUMP\_catabolic\_process | 1 | 0 |  |  |  |  |  |  |  |  |
| GO:0046086\_adenosine\_biosynthetic\_process | 1 | 0 |  |  |  |  |  |  |  |  |
| GO:0046090\_deoxyadenosine\_metabolic\_process | 1 | 0 |  |  |  |  |  |  |  |  |
| GO:0046098\_guanine\_metabolic\_process | 1 | 0 |  |  |  |  |  |  |  |  |
| GO:0046101\_hypoxanthine\_biosynthetic\_process | 1 | 0 |  |  |  |  |  |  |  |  |
| GO:0046102\_inosine\_metabolic\_process | 1 | 0 |  |  |  |  |  |  |  |  |
| GO:0046103\_inosine\_biosynthetic\_process | 1 | 0 |  |  |  |  |  |  |  |  |
| GO:0046108\_uridine\_metabolic\_process | 1 | 0 |  |  |  |  |  |  |  |  |
| GO:0046110\_xanthine\_metabolic\_process | 1 | 0 |  |  |  |  |  |  |  |  |
| GO:0046111\_xanthine\_biosynthetic\_process | 1 | 0 |  |  |  |  |  |  |  |  |
| GO:0046112\_nucleobase\_biosynthetic\_process | 1 | 0 |  |  |  |  |  |  |  |  |
| GO:0046113\_nucleobase\_catabolic\_process | 1 | 0 |  |  |  |  |  |  |  |  |
| GO:0046121\_deoxyribonucleoside\_catabolic\_process | 1 | 0 |  |  |  |  |  |  |  |  |
| GO:0046122\_purine\_deoxyribonucleoside\_metabolic\_process | 1 | 0 |  |  |  |  |  |  |  |  |
| GO:0046124\_purine\_deoxyribonucleoside\_catabolic\_process | 1 | 0 |  |  |  |  |  |  |  |  |
| GO:0046125\_pyrimidine\_deoxyribonucleoside\_metabolic\_process | 1 | 0 |  |  |  |  |  |  |  |  |
| GO:0046131\_pyrimidine\_ribonucleoside\_metabolic\_process | 1 | 0 |  |  |  |  |  |  |  |  |
| GO:0046160\_heme\_a\_metabolic\_process | 1 | 0 |  |  |  |  |  |  |  |  |
| GO:0046218\_indolalkylamine\_catabolic\_process | 1 | 0 |  |  |  |  |  |  |  |  |
| GO:0046292\_formaldehyde\_metabolic\_process | 1 | 0 |  |  |  |  |  |  |  |  |
| GO:0046294\_formaldehyde\_catabolic\_process | 1 | 0 |  |  |  |  |  |  |  |  |
| GO:0046314\_phosphocreatine\_biosynthetic\_process | 1 | 0 |  |  |  |  |  |  |  |  |
| GO:0046327\_glycerol\_biosynthetic\_process\_from\_pyruvate | 1 | 0 |  |  |  |  |  |  |  |  |
| GO:0046329\_negative\_regulation\_of\_JNK\_cascade | 1 | 0 |  |  |  |  |  |  |  |  |
| GO:0046340\_diacylglycerol\_catabolic\_process | 1 | 0 |  |  |  |  |  |  |  |  |
| GO:0046351\_disaccharide\_biosynthetic\_process | 1 | 0 |  |  |  |  |  |  |  |  |
| GO:0046356\_acetyl-CoA\_catabolic\_process | 1 | 0 |  |  |  |  |  |  |  |  |
| GO:0046358\_butyrate\_biosynthetic\_process | 1 | 0 |  |  |  |  |  |  |  |  |
| GO:0046359\_butyrate\_catabolic\_process | 1 | 0 |  |  |  |  |  |  |  |  |
| GO:0046381\_CMP-N-acetylneuraminate\_metabolic\_process | 1 | 0 |  |  |  |  |  |  |  |  |
| GO:0046415\_urate\_metabolic\_process | 1 | 0 |  |  |  |  |  |  |  |  |
| GO:0046416\_D-amino\_acid\_metabolic\_process | 1 | 0 |  |  |  |  |  |  |  |  |
| GO:0046434\_organophosphate\_catabolic\_process | 1 | 0 |  |  |  |  |  |  |  |  |
| GO:0046437\_D-amino\_acid\_biosynthetic\_process | 1 | 0 |  |  |  |  |  |  |  |  |
| GO:0046440\_L-lysine\_metabolic\_process | 1 | 0 |  |  |  |  |  |  |  |  |
| GO:0046449\_creatinine\_metabolic\_process | 1 | 0 |  |  |  |  |  |  |  |  |
| GO:0046471\_phosphatidylglycerol\_metabolic\_process | 1 | 0 |  |  |  |  |  |  |  |  |
| GO:0046473\_phosphatidic\_acid\_metabolic\_process | 1 | 0 |  |  |  |  |  |  |  |  |
| GO:0046476\_glycosylceramide\_biosynthetic\_process | 1 | 0 |  |  |  |  |  |  |  |  |
| GO:0046477\_glycosylceramide\_catabolic\_process | 1 | 0 |  |  |  |  |  |  |  |  |
| GO:0046485\_ether\_lipid\_metabolic\_process | 1 | 0 |  |  |  |  |  |  |  |  |
| GO:0046487\_glyoxylate\_metabolic\_process | 1 | 0 |  |  |  |  |  |  |  |  |
| GO:0046498\_S-adenosylhomocysteine\_metabolic\_process | 1 | 0 |  |  |  |  |  |  |  |  |
| GO:0046552\_photoreceptor\_cell\_fate\_commitment | 1 | 0 |  |  |  |  |  |  |  |  |
| GO:0046586\_regulation\_of\_calcium-dependent\_cell-cell\_adhesion | 1 | 0 |  |  |  |  |  |  |  |  |
| GO:0046587\_positive\_regulation\_of\_calcium-dependent\_cell-cell\_adhesion | 1 | 0 |  |  |  |  |  |  |  |  |
| GO:0046602\_regulation\_of\_mitotic\_centrosome\_separation | 1 | 0 |  |  |  |  |  |  |  |  |
| GO:0046604\_positive\_regulation\_of\_mitotic\_centrosome\_separation | 1 | 0 |  |  |  |  |  |  |  |  |
| GO:0046607\_positive\_regulation\_of\_centrosome\_cycle | 1 | 0 |  |  |  |  |  |  |  |  |
| GO:0046655\_folic\_acid\_metabolic\_process | 1 | 0 |  |  |  |  |  |  |  |  |
| GO:0046671\_negative\_regulation\_of\_retinal\_cell\_programmed\_cell\_death | 1 | 0 |  |  |  |  |  |  |  |  |
| GO:0046685\_response\_to\_arsenic | 1 | 0 |  |  |  |  |  |  |  |  |
| GO:0046692\_sperm\_competition | 1 | 0 |  |  |  |  |  |  |  |  |
| GO:0046707\_IDP\_metabolic\_process | 1 | 0 |  |  |  |  |  |  |  |  |
| GO:0046709\_IDP\_catabolic\_process | 1 | 0 |  |  |  |  |  |  |  |  |
| GO:0046724\_oxalic\_acid\_secretion | 1 | 0 |  |  |  |  |  |  |  |  |
| GO:0046753\_non-lytic\_viral\_release | 1 | 0 |  |  |  |  |  |  |  |  |
| GO:0046755\_non-lytic\_virus\_budding | 1 | 0 |  |  |  |  |  |  |  |  |
| GO:0046826\_negative\_regulation\_of\_protein\_export\_from\_nucleus | 1 | 0 |  |  |  |  |  |  |  |  |
| GO:0046827\_positive\_regulation\_of\_protein\_export\_from\_nucleus | 1 | 0 |  |  |  |  |  |  |  |  |
| GO:0046831\_regulation\_of\_RNA\_export\_from\_nucleus | 1 | 0 |  |  |  |  |  |  |  |  |
| GO:0046834\_lipid\_phosphorylation | 1 | 0 |  |  |  |  |  |  |  |  |
| GO:0046853\_inositol\_and\_derivative\_phosphorylation | 1 | 0 |  |  |  |  |  |  |  |  |
| GO:0046864\_isoprenoid\_transport | 1 | 0 |  |  |  |  |  |  |  |  |
| GO:0046865\_terpenoid\_transport | 1 | 0 |  |  |  |  |  |  |  |  |
| GO:0046877\_regulation\_of\_saliva\_secretion | 1 | 0 |  |  |  |  |  |  |  |  |
| GO:0046878\_positive\_regulation\_of\_saliva\_secretion | 1 | 0 |  |  |  |  |  |  |  |  |
| GO:0046884\_follicle-stimulating\_hormone\_secretion | 1 | 0 |  |  |  |  |  |  |  |  |
| GO:0046898\_response\_to\_cycloheximide | 1 | 0 |  |  |  |  |  |  |  |  |
| GO:0046929\_negative\_regulation\_of\_neurotransmitter\_secretion | 1 | 0 |  |  |  |  |  |  |  |  |
| GO:0046931\_pore\_complex\_biogenesis | 1 | 0 |  |  |  |  |  |  |  |  |
| GO:0046949\_acyl-CoA\_biosynthetic\_process | 1 | 0 |  |  |  |  |  |  |  |  |
| GO:0046958\_nonassociative\_learning | 1 | 0 |  |  |  |  |  |  |  |  |
| GO:0046960\_sensitization | 1 | 0 |  |  |  |  |  |  |  |  |
| GO:0046986\_negative\_regulation\_of\_hemoglobin\_biosynthetic\_process | 1 | 0 |  |  |  |  |  |  |  |  |
| GO:0047497\_mitochondrion\_transport\_along\_microtubule | 1 | 0 |  |  |  |  |  |  |  |  |
| GO:0048047\_mating\_behavior\_\_sex\_discrimination | 1 | 0 |  |  |  |  |  |  |  |  |
| GO:0048133\_male\_germ-line\_stem\_cell\_division | 1 | 0 |  |  |  |  |  |  |  |  |
| GO:0048137\_spermatocyte\_division | 1 | 0 |  |  |  |  |  |  |  |  |
| GO:0048143\_astrocyte\_activation | 1 | 0 |  |  |  |  |  |  |  |  |
| GO:0048170\_positive\_regulation\_of\_long-term\_neuronal\_synaptic\_plasticity | 1 | 0 |  |  |  |  |  |  |  |  |
| GO:0048199\_vesicle\_targeting\_\_to\_\_from\_or\_within\_Golgi | 1 | 0 |  |  |  |  |  |  |  |  |
| GO:0048241\_epinephrine\_transport | 1 | 0 |  |  |  |  |  |  |  |  |
| GO:0048242\_epinephrine\_secretion | 1 | 0 |  |  |  |  |  |  |  |  |
| GO:0048243\_norepinephrine\_secretion | 1 | 0 |  |  |  |  |  |  |  |  |
| GO:0048247\_lymphocyte\_chemotaxis | 1 | 0 |  |  |  |  |  |  |  |  |
| GO:0048250\_mitochondrial\_iron\_ion\_transport | 1 | 0 |  |  |  |  |  |  |  |  |
| GO:0048259\_regulation\_of\_receptor-mediated\_endocytosis | 1 | 0 |  |  |  |  |  |  |  |  |
| GO:0048260\_positive\_regulation\_of\_receptor-mediated\_endocytosis | 1 | 0 |  |  |  |  |  |  |  |  |
| GO:0048290\_isotype\_switching\_to\_IgA\_isotypes | 1 | 0 |  |  |  |  |  |  |  |  |
| GO:0048296\_regulation\_of\_isotype\_switching\_to\_IgA\_isotypes | 1 | 0 |  |  |  |  |  |  |  |  |
| GO:0048298\_positive\_regulation\_of\_isotype\_switching\_to\_IgA\_isotypes | 1 | 0 |  |  |  |  |  |  |  |  |
| GO:0048319\_axial\_mesoderm\_morphogenesis | 1 | 0 |  |  |  |  |  |  |  |  |
| GO:0048320\_axial\_mesoderm\_formation | 1 | 0 |  |  |  |  |  |  |  |  |
| GO:0048385\_regulation\_of\_retinoic\_acid\_receptor\_signaling\_pathway | 1 | 0 |  |  |  |  |  |  |  |  |
| GO:0048387\_negative\_regulation\_of\_retinoic\_acid\_receptor\_signaling\_pathway | 1 | 0 |  |  |  |  |  |  |  |  |
| GO:0048388\_endosomal\_lumen\_acidification | 1 | 0 |  |  |  |  |  |  |  |  |
| GO:0048389\_intermediate\_mesoderm\_development | 1 | 0 |  |  |  |  |  |  |  |  |
| GO:0048478\_replication\_fork\_protection | 1 | 0 |  |  |  |  |  |  |  |  |
| GO:0048496\_maintenance\_of\_organ\_identity | 1 | 0 |  |  |  |  |  |  |  |  |
| GO:0048525\_negative\_regulation\_of\_viral\_reproduction | 1 | 0 |  |  |  |  |  |  |  |  |
| GO:0048539\_bone\_marrow\_development | 1 | 0 |  |  |  |  |  |  |  |  |
| GO:0048548\_regulation\_of\_pinocytosis | 1 | 0 |  |  |  |  |  |  |  |  |
| GO:0048549\_positive\_regulation\_of\_pinocytosis | 1 | 0 |  |  |  |  |  |  |  |  |
| GO:0048553\_negative\_regulation\_of\_metalloenzyme\_activity | 1 | 0 |  |  |  |  |  |  |  |  |
| GO:0048588\_developmental\_cell\_growth | 1 | 0 |  |  |  |  |  |  |  |  |
| GO:0048601\_oocyte\_morphogenesis | 1 | 0 |  |  |  |  |  |  |  |  |
| GO:0048621\_post-embryonic\_gut\_morphogenesis | 1 | 0 |  |  |  |  |  |  |  |  |
| GO:0048640\_negative\_regulation\_of\_developmental\_growth | 1 | 0 |  |  |  |  |  |  |  |  |
| GO:0048642\_negative\_regulation\_of\_skeletal\_muscle\_tissue\_development | 1 | 0 |  |  |  |  |  |  |  |  |
| GO:0048669\_collateral\_sprouting\_in\_the\_absence\_of\_injury | 1 | 0 |  |  |  |  |  |  |  |  |
| GO:0048680\_positive\_regulation\_of\_axon\_regeneration | 1 | 0 |  |  |  |  |  |  |  |  |
| GO:0048681\_negative\_regulation\_of\_axon\_regeneration | 1 | 0 |  |  |  |  |  |  |  |  |
| GO:0048686\_regulation\_of\_sprouting\_of\_injured\_axon | 1 | 0 |  |  |  |  |  |  |  |  |
| GO:0048687\_positive\_regulation\_of\_sprouting\_of\_injured\_axon | 1 | 0 |  |  |  |  |  |  |  |  |
| GO:0048690\_regulation\_of\_axon\_extension\_involved\_in\_regeneration | 1 | 0 |  |  |  |  |  |  |  |  |
| GO:0048691\_positive\_regulation\_of\_axon\_extension\_involved\_in\_regeneration | 1 | 0 |  |  |  |  |  |  |  |  |
| GO:0048714\_positive\_regulation\_of\_oligodendrocyte\_differentiation | 1 | 0 |  |  |  |  |  |  |  |  |
| GO:0048733\_sebaceous\_gland\_development | 1 | 0 |  |  |  |  |  |  |  |  |
| GO:0048743\_positive\_regulation\_of\_skeletal\_muscle\_fiber\_development | 1 | 0 |  |  |  |  |  |  |  |  |
| GO:0048752\_semicircular\_canal\_morphogenesis | 1 | 0 |  |  |  |  |  |  |  |  |
| GO:0048773\_erythrophore\_differentiation | 1 | 0 |  |  |  |  |  |  |  |  |
| GO:0048790\_maintenance\_of\_presynaptic\_active\_zone\_structure | 1 | 0 |  |  |  |  |  |  |  |  |
| GO:0048791\_calcium\_ion-dependent\_exocytosis\_of\_neurotransmitter | 1 | 0 |  |  |  |  |  |  |  |  |
| GO:0048822\_enucleate\_erythrocyte\_development | 1 | 0 |  |  |  |  |  |  |  |  |
| GO:0048866\_stem\_cell\_fate\_specification | 1 | 0 |  |  |  |  |  |  |  |  |
| GO:0048936\_peripheral\_nervous\_system\_neuron\_axonogenesis | 1 | 0 |  |  |  |  |  |  |  |  |
| GO:0050427\_3'-phosphoadenosine\_5'-phosphosulfate\_metabolic\_process | 1 | 0 |  |  |  |  |  |  |  |  |
| GO:0050428\_3'-phosphoadenosine\_5'-phosphosulfate\_biosynthetic\_process | 1 | 0 |  |  |  |  |  |  |  |  |
| GO:0050482\_arachidonic\_acid\_secretion | 1 | 0 |  |  |  |  |  |  |  |  |
| GO:0050667\_homocysteine\_metabolic\_process | 1 | 0 |  |  |  |  |  |  |  |  |
| GO:0050674\_urothelial\_cell\_proliferation | 1 | 0 |  |  |  |  |  |  |  |  |
| GO:0050675\_regulation\_of\_urothelial\_cell\_proliferation | 1 | 0 |  |  |  |  |  |  |  |  |
| GO:0050677\_positive\_regulation\_of\_urothelial\_cell\_proliferation | 1 | 0 |  |  |  |  |  |  |  |  |
| GO:0050691\_regulation\_of\_defense\_response\_to\_virus\_by\_host | 1 | 0 |  |  |  |  |  |  |  |  |
| GO:0050748\_negative\_regulation\_of\_lipoprotein\_metabolic\_process | 1 | 0 |  |  |  |  |  |  |  |  |
| GO:0050757\_thymidylate\_synthase\_biosynthetic\_process | 1 | 0 |  |  |  |  |  |  |  |  |
| GO:0050758\_regulation\_of\_thymidylate\_synthase\_biosynthetic\_process | 1 | 0 |  |  |  |  |  |  |  |  |
| GO:0050760\_negative\_regulation\_of\_thymidylate\_synthase\_biosynthetic\_process | 1 | 0 |  |  |  |  |  |  |  |  |
| GO:0050812\_regulation\_of\_acyl-CoA\_biosynthetic\_process | 1 | 0 |  |  |  |  |  |  |  |  |
| GO:0050832\_defense\_response\_to\_fungus | 1 | 0 |  |  |  |  |  |  |  |  |
| GO:0050861\_positive\_regulation\_of\_B\_cell\_receptor\_signaling\_pathway | 1 | 0 |  |  |  |  |  |  |  |  |
| GO:0050862\_positive\_regulation\_of\_T\_cell\_receptor\_signaling\_pathway | 1 | 0 |  |  |  |  |  |  |  |  |
| GO:0050916\_sensory\_perception\_of\_sweet\_taste | 1 | 0 |  |  |  |  |  |  |  |  |
| GO:0050975\_sensory\_perception\_of\_touch | 1 | 0 |  |  |  |  |  |  |  |  |
| GO:0050995\_negative\_regulation\_of\_lipid\_catabolic\_process | 1 | 0 |  |  |  |  |  |  |  |  |
| GO:0051001\_negative\_regulation\_of\_nitric-oxide\_synthase\_activity | 1 | 0 |  |  |  |  |  |  |  |  |
| GO:0051005\_negative\_regulation\_of\_lipoprotein\_lipase\_activity | 1 | 0 |  |  |  |  |  |  |  |  |
| GO:0051006\_positive\_regulation\_of\_lipoprotein\_lipase\_activity | 1 | 0 |  |  |  |  |  |  |  |  |
| GO:0051016\_barbed-end\_actin\_filament\_capping | 1 | 0 |  |  |  |  |  |  |  |  |
| GO:0051043\_regulation\_of\_membrane\_protein\_ectodomain\_proteolysis | 1 | 0 |  |  |  |  |  |  |  |  |
| GO:0051044\_positive\_regulation\_of\_membrane\_protein\_ectodomain\_proteolysis | 1 | 0 |  |  |  |  |  |  |  |  |
| GO:0051088\_PMA-inducible\_membrane\_protein\_ectodomain\_proteolysis | 1 | 0 |  |  |  |  |  |  |  |  |
| GO:0051102\_DNA\_ligation\_during\_DNA\_recombination | 1 | 0 |  |  |  |  |  |  |  |  |
| GO:0051103\_DNA\_ligation\_during\_DNA\_repair | 1 | 0 |  |  |  |  |  |  |  |  |
| GO:0051123\_transcriptional\_preinitiation\_complex\_assembly | 1 | 0 |  |  |  |  |  |  |  |  |
| GO:0051125\_regulation\_of\_actin\_nucleation | 1 | 0 |  |  |  |  |  |  |  |  |
| GO:0051127\_positive\_regulation\_of\_actin\_nucleation | 1 | 0 |  |  |  |  |  |  |  |  |
| GO:0051151\_negative\_regulation\_of\_smooth\_muscle\_cell\_differentiation | 1 | 0 |  |  |  |  |  |  |  |  |
| GO:0051154\_negative\_regulation\_of\_striated\_muscle\_cell\_differentiation | 1 | 0 |  |  |  |  |  |  |  |  |
| GO:0051155\_positive\_regulation\_of\_striated\_muscle\_cell\_differentiation | 1 | 0 |  |  |  |  |  |  |  |  |
| GO:0051156\_glucose\_6-phosphate\_metabolic\_process | 1 | 0 |  |  |  |  |  |  |  |  |
| GO:0051187\_cofactor\_catabolic\_process | 1 | 0 |  |  |  |  |  |  |  |  |
| GO:0051189\_prosthetic\_group\_metabolic\_process | 1 | 0 |  |  |  |  |  |  |  |  |
| GO:0051193\_regulation\_of\_cofactor\_metabolic\_process | 1 | 0 |  |  |  |  |  |  |  |  |
| GO:0051196\_regulation\_of\_coenzyme\_metabolic\_process | 1 | 0 |  |  |  |  |  |  |  |  |
| GO:0051255\_spindle\_midzone\_assembly | 1 | 0 |  |  |  |  |  |  |  |  |
| GO:0051257\_spindle\_midzone\_assembly\_involved\_in\_meiosis | 1 | 0 |  |  |  |  |  |  |  |  |
| GO:0051281\_positive\_regulation\_of\_release\_of\_sequestered\_calcium\_ion\_into\_cytosol | 1 | 0 |  |  |  |  |  |  |  |  |
| GO:0051290\_protein\_heterotetramerization | 1 | 0 |  |  |  |  |  |  |  |  |
| GO:0051305\_chromosome\_movement\_towards\_spindle\_pole | 1 | 0 |  |  |  |  |  |  |  |  |
| GO:0051310\_metaphase\_plate\_congression | 1 | 0 |  |  |  |  |  |  |  |  |
| GO:0051311\_meiotic\_metaphase\_plate\_congression | 1 | 0 |  |  |  |  |  |  |  |  |
| GO:0051340\_regulation\_of\_ligase\_activity | 1 | 0 |  |  |  |  |  |  |  |  |
| GO:0051351\_positive\_regulation\_of\_ligase\_activity | 1 | 0 |  |  |  |  |  |  |  |  |
| GO:0051354\_negative\_regulation\_of\_oxidoreductase\_activity | 1 | 0 |  |  |  |  |  |  |  |  |
| GO:0051355\_proprioception\_during\_equilibrioception | 1 | 0 |  |  |  |  |  |  |  |  |
| GO:0051383\_kinetochore\_organization | 1 | 0 |  |  |  |  |  |  |  |  |
| GO:0051386\_regulation\_of\_nerve\_growth\_factor\_receptor\_signaling\_pathway | 1 | 0 |  |  |  |  |  |  |  |  |
| GO:0051409\_response\_to\_nitrosative\_stress | 1 | 0 |  |  |  |  |  |  |  |  |
| GO:0051457\_maintenance\_of\_protein\_location\_in\_nucleus | 1 | 0 |  |  |  |  |  |  |  |  |
| GO:0051462\_regulation\_of\_cortisol\_secretion | 1 | 0 |  |  |  |  |  |  |  |  |
| GO:0051463\_negative\_regulation\_of\_cortisol\_secretion | 1 | 0 |  |  |  |  |  |  |  |  |
| GO:0051481\_reduction\_of\_cytosolic\_calcium\_ion\_concentration | 1 | 0 |  |  |  |  |  |  |  |  |
| GO:0051482\_elevation\_of\_cytosolic\_calcium\_ion\_concentration\_during\_G-protein\_signaling\_\_coupled\_to\_IP3\_second\_messenger\_(phospholipase\_C\_activating) | 1 | 0 |  |  |  |  |  |  |  |  |
| GO:0051542\_elastin\_biosynthetic\_process | 1 | 0 |  |  |  |  |  |  |  |  |
| GO:0051568\_histone\_H3-K4\_methylation | 1 | 0 |  |  |  |  |  |  |  |  |
| GO:0051569\_regulation\_of\_histone\_H3-K4\_methylation | 1 | 0 |  |  |  |  |  |  |  |  |
| GO:0051570\_regulation\_of\_histone\_H3-K9\_methylation | 1 | 0 |  |  |  |  |  |  |  |  |
| GO:0051573\_negative\_regulation\_of\_histone\_H3-K9\_methylation | 1 | 0 |  |  |  |  |  |  |  |  |
| GO:0051580\_regulation\_of\_neurotransmitter\_uptake | 1 | 0 |  |  |  |  |  |  |  |  |
| GO:0051582\_positive\_regulation\_of\_neurotransmitter\_uptake | 1 | 0 |  |  |  |  |  |  |  |  |
| GO:0051584\_regulation\_of\_dopamine\_uptake | 1 | 0 |  |  |  |  |  |  |  |  |
| GO:0051586\_positive\_regulation\_of\_dopamine\_uptake | 1 | 0 |  |  |  |  |  |  |  |  |
| GO:0051589\_negative\_regulation\_of\_neurotransmitter\_transport | 1 | 0 |  |  |  |  |  |  |  |  |
| GO:0051593\_response\_to\_folic\_acid | 1 | 0 |  |  |  |  |  |  |  |  |
| GO:0051615\_histamine\_uptake | 1 | 0 |  |  |  |  |  |  |  |  |
| GO:0051646\_mitochondrion\_localization | 1 | 0 |  |  |  |  |  |  |  |  |
| GO:0051654\_establishment\_of\_mitochondrion\_localization | 1 | 0 |  |  |  |  |  |  |  |  |
| GO:0051661\_maintenance\_of\_centrosome\_location | 1 | 0 |  |  |  |  |  |  |  |  |
| GO:0051665\_membrane\_raft\_localization | 1 | 0 |  |  |  |  |  |  |  |  |
| GO:0051685\_maintenance\_of\_ER\_location | 1 | 0 |  |  |  |  |  |  |  |  |
| GO:0051693\_actin\_filament\_capping | 1 | 0 |  |  |  |  |  |  |  |  |
| GO:0051701\_interaction\_with\_host | 1 | 0 |  |  |  |  |  |  |  |  |
| GO:0051754\_meiotic\_sister\_chromatid\_cohesion\_\_centromeric | 1 | 0 |  |  |  |  |  |  |  |  |
| GO:0051782\_negative\_regulation\_of\_cell\_division | 1 | 0 |  |  |  |  |  |  |  |  |
| GO:0051790\_short-chain\_fatty\_acid\_biosynthetic\_process | 1 | 0 |  |  |  |  |  |  |  |  |
| GO:0051799\_negative\_regulation\_of\_hair\_follicle\_development | 1 | 0 |  |  |  |  |  |  |  |  |
| GO:0051823\_regulation\_of\_synapse\_structural\_plasticity | 1 | 0 |  |  |  |  |  |  |  |  |
| GO:0051865\_protein\_autoubiquitination | 1 | 0 |  |  |  |  |  |  |  |  |
| GO:0051901\_positive\_regulation\_of\_mitochondrial\_depolarization | 1 | 0 |  |  |  |  |  |  |  |  |
| GO:0051917\_regulation\_of\_fibrinolysis | 1 | 0 |  |  |  |  |  |  |  |  |
| GO:0051918\_negative\_regulation\_of\_fibrinolysis | 1 | 0 |  |  |  |  |  |  |  |  |
| GO:0051929\_positive\_regulation\_of\_calcium\_ion\_transport\_via\_voltage-gated\_calcium\_channel\_activity | 1 | 0 |  |  |  |  |  |  |  |  |
| GO:0051933\_amino\_acid\_uptake\_during\_transmission\_of\_nerve\_impulse | 1 | 0 |  |  |  |  |  |  |  |  |
| GO:0051935\_glutamate\_uptake\_during\_transmission\_of\_nerve\_impulse | 1 | 0 |  |  |  |  |  |  |  |  |
| GO:0051940\_regulation\_of\_catecholamine\_uptake\_during\_transmission\_of\_nerve\_impulse | 1 | 0 |  |  |  |  |  |  |  |  |
| GO:0051944\_positive\_regulation\_of\_catecholamine\_uptake\_during\_transmission\_of\_nerve\_impulse | 1 | 0 |  |  |  |  |  |  |  |  |
| GO:0051961\_negative\_regulation\_of\_nervous\_system\_development | 1 | 0 |  |  |  |  |  |  |  |  |
| GO:0051964\_negative\_regulation\_of\_synaptogenesis | 1 | 0 |  |  |  |  |  |  |  |  |
| GO:0051968\_positive\_regulation\_of\_synaptic\_transmission\_\_glutamatergic | 1 | 0 |  |  |  |  |  |  |  |  |
| GO:0051984\_positive\_regulation\_of\_chromosome\_segregation | 1 | 0 |  |  |  |  |  |  |  |  |
| GO:0051987\_positive\_regulation\_of\_attachment\_of\_spindle\_microtubules\_to\_kinetochore | 1 | 0 |  |  |  |  |  |  |  |  |
| GO:0052173\_response\_to\_defenses\_of\_other\_organism\_during\_symbiotic\_interaction | 1 | 0 |  |  |  |  |  |  |  |  |
| GO:0052200\_response\_to\_host\_defenses | 1 | 0 |  |  |  |  |  |  |  |  |
| GO:0052551\_response\_to\_defense-related\_nitric\_oxide\_production\_by\_other\_organism\_during\_symbiotic\_interaction | 1 | 0 |  |  |  |  |  |  |  |  |
| GO:0052564\_response\_to\_immune\_response\_of\_other\_organism\_during\_symbiotic\_interaction | 1 | 0 |  |  |  |  |  |  |  |  |
| GO:0052565\_response\_to\_defense-related\_host\_nitric\_oxide\_production | 1 | 0 |  |  |  |  |  |  |  |  |
| GO:0052572\_response\_to\_host\_immune\_response | 1 | 0 |  |  |  |  |  |  |  |  |
| GO:0055005\_ventricular\_cardiac\_myofibril\_development | 1 | 0 |  |  |  |  |  |  |  |  |
| GO:0055011\_atrial\_cardiac\_muscle\_cell\_differentiation | 1 | 0 |  |  |  |  |  |  |  |  |
| GO:0055014\_atrial\_cardiac\_muscle\_cell\_development | 1 | 0 |  |  |  |  |  |  |  |  |
| GO:0055078\_sodium\_ion\_homeostasis | 1 | 0 |  |  |  |  |  |  |  |  |
| GO:0055089\_fatty\_acid\_homeostasis | 1 | 0 |  |  |  |  |  |  |  |  |
| GO:0055093\_response\_to\_hyperoxia | 1 | 0 |  |  |  |  |  |  |  |  |
| GO:0060003\_copper\_ion\_export | 1 | 0 |  |  |  |  |  |  |  |  |
| GO:0060005\_vestibular\_reflex | 1 | 0 |  |  |  |  |  |  |  |  |
| GO:0060014\_granulosa\_cell\_differentiation | 1 | 0 |  |  |  |  |  |  |  |  |
| GO:0060018\_astrocyte\_fate\_commitment | 1 | 0 |  |  |  |  |  |  |  |  |
| GO:0060020\_Bergmann\_glial\_cell\_differentiation | 1 | 0 |  |  |  |  |  |  |  |  |
| GO:0060022\_hard\_palate\_development | 1 | 0 |  |  |  |  |  |  |  |  |
| GO:0060034\_notochord\_cell\_differentiation | 1 | 0 |  |  |  |  |  |  |  |  |
| GO:0060035\_notochord\_cell\_development | 1 | 0 |  |  |  |  |  |  |  |  |
| GO:0060046\_regulation\_of\_acrosome\_reaction | 1 | 0 |  |  |  |  |  |  |  |  |
| GO:0060054\_positive\_regulation\_of\_epithelial\_cell\_proliferation\_involved\_in\_wound\_healing | 1 | 0 |  |  |  |  |  |  |  |  |
| GO:0060059\_embryonic\_retina\_morphogenesis\_in\_camera-type\_eye | 1 | 0 |  |  |  |  |  |  |  |  |
| GO:0060061\_Spemann\_organizer\_formation | 1 | 0 |  |  |  |  |  |  |  |  |
| GO:0060064\_Spemann\_organizer\_formation\_at\_the\_anterior\_end\_of\_the\_primitive\_streak | 1 | 0 |  |  |  |  |  |  |  |  |
| GO:0060071\_Wnt\_receptor\_signaling\_pathway\_\_planar\_cell\_polarity\_pathway | 1 | 0 |  |  |  |  |  |  |  |  |
| GO:0060075\_regulation\_of\_resting\_membrane\_potential | 1 | 0 |  |  |  |  |  |  |  |  |
| GO:0060082\_eye\_blink\_reflex | 1 | 0 |  |  |  |  |  |  |  |  |
| GO:0060112\_generation\_of\_ovulation\_cycle\_rhythm | 1 | 0 |  |  |  |  |  |  |  |  |
| GO:0060125\_negative\_regulation\_of\_growth\_hormone\_secretion | 1 | 0 |  |  |  |  |  |  |  |  |
| GO:0060161\_positive\_regulation\_of\_dopamine\_receptor\_signaling\_pathway | 1 | 0 |  |  |  |  |  |  |  |  |
| GO:0060163\_subpallium\_neuron\_fate\_commitment | 1 | 0 |  |  |  |  |  |  |  |  |
| GO:0060165\_regulation\_of\_timing\_of\_subpallium\_neuron\_differentiation | 1 | 0 |  |  |  |  |  |  |  |  |
| GO:0060174\_limb\_bud\_formation | 1 | 0 |  |  |  |  |  |  |  |  |
| GO:0060177\_regulation\_of\_angiotensin\_metabolic\_process | 1 | 0 |  |  |  |  |  |  |  |  |
| GO:0060197\_cloacal\_septation | 1 | 0 |  |  |  |  |  |  |  |  |
| GO:0060215\_primitive\_hemopoiesis | 1 | 0 |  |  |  |  |  |  |  |  |
| GO:0060231\_mesenchymal\_to\_epithelial\_transition | 1 | 0 |  |  |  |  |  |  |  |  |
| GO:0060254\_regulation\_of\_N-terminal\_protein\_palmitoylation | 1 | 0 |  |  |  |  |  |  |  |  |
| GO:0060261\_positive\_regulation\_of\_transcription\_initiation\_from\_RNA\_polymerase\_II\_promoter | 1 | 0 |  |  |  |  |  |  |  |  |
| GO:0060262\_negative\_regulation\_of\_N-terminal\_protein\_palmitoylation | 1 | 0 |  |  |  |  |  |  |  |  |
| GO:0060263\_regulation\_of\_respiratory\_burst | 1 | 0 |  |  |  |  |  |  |  |  |
| GO:0060264\_regulation\_of\_respiratory\_burst\_during\_acute\_inflammatory\_response | 1 | 0 |  |  |  |  |  |  |  |  |
| GO:0060265\_positive\_regulation\_of\_respiratory\_burst\_during\_acute\_inflammatory\_response | 1 | 0 |  |  |  |  |  |  |  |  |
| GO:0060267\_positive\_regulation\_of\_respiratory\_burst | 1 | 0 |  |  |  |  |  |  |  |  |
| GO:0060272\_embryonic\_skeletal\_joint\_morphogenesis | 1 | 0 |  |  |  |  |  |  |  |  |
| GO:0060297\_regulation\_of\_sarcomere\_organization | 1 | 0 |  |  |  |  |  |  |  |  |
| GO:0060298\_positive\_regulation\_of\_sarcomere\_organization | 1 | 0 |  |  |  |  |  |  |  |  |
| GO:0060315\_negative\_regulation\_of\_ryanodine-sensitive\_calcium-release\_channel\_activity | 1 | 0 |  |  |  |  |  |  |  |  |
| GO:0060319\_primitive\_erythrocyte\_differentiation | 1 | 0 |  |  |  |  |  |  |  |  |
| GO:0060371\_regulation\_of\_atrial\_cardiomyocyte\_membrane\_depolarization | 1 | 0 |  |  |  |  |  |  |  |  |
| GO:0060374\_mast\_cell\_differentiation | 1 | 0 |  |  |  |  |  |  |  |  |
| GO:0060375\_regulation\_of\_mast\_cell\_differentiation | 1 | 0 |  |  |  |  |  |  |  |  |
| GO:0060376\_positive\_regulation\_of\_mast\_cell\_differentiation | 1 | 0 |  |  |  |  |  |  |  |  |
| GO:0060390\_regulation\_of\_SMAD\_protein\_nuclear\_translocation | 1 | 0 |  |  |  |  |  |  |  |  |
| GO:0060391\_positive\_regulation\_of\_SMAD\_protein\_nuclear\_translocation | 1 | 0 |  |  |  |  |  |  |  |  |
| GO:0060398\_regulation\_of\_growth\_hormone\_receptor\_signaling\_pathway | 1 | 0 |  |  |  |  |  |  |  |  |
| GO:0060399\_positive\_regulation\_of\_growth\_hormone\_receptor\_signaling\_pathway | 1 | 0 |  |  |  |  |  |  |  |  |
| GO:0060405\_regulation\_of\_penile\_erection | 1 | 0 |  |  |  |  |  |  |  |  |
| GO:0060407\_negative\_regulation\_of\_penile\_erection | 1 | 0 |  |  |  |  |  |  |  |  |
| GO:0060413\_atrial\_septum\_morphogenesis | 1 | 0 |  |  |  |  |  |  |  |  |
| GO:0060414\_aorta\_smooth\_muscle\_tissue\_morphogenesis | 1 | 0 |  |  |  |  |  |  |  |  |
| GO:0060419\_heart\_growth | 1 | 0 |  |  |  |  |  |  |  |  |
| GO:0060420\_regulation\_of\_heart\_growth | 1 | 0 |  |  |  |  |  |  |  |  |
| GO:0060421\_positive\_regulation\_of\_heart\_growth | 1 | 0 |  |  |  |  |  |  |  |  |
| GO:0060431\_primary\_lung\_bud\_formation | 1 | 0 |  |  |  |  |  |  |  |  |
| GO:0060436\_bronchiole\_morphogenesis | 1 | 0 |  |  |  |  |  |  |  |  |
| GO:0060440\_trachea\_formation | 1 | 0 |  |  |  |  |  |  |  |  |
| GO:0060449\_bud\_elongation\_involved\_in\_lung\_branching | 1 | 0 |  |  |  |  |  |  |  |  |
| GO:0060456\_positive\_regulation\_of\_digestive\_system\_process | 1 | 0 |  |  |  |  |  |  |  |  |
| GO:0060461\_right\_lung\_morphogenesis | 1 | 0 |  |  |  |  |  |  |  |  |
| GO:0060481\_lobar\_bronchus\_epithelium\_development | 1 | 0 |  |  |  |  |  |  |  |  |
| GO:0060482\_lobar\_bronchus\_development | 1 | 0 |  |  |  |  |  |  |  |  |
| GO:0060484\_lung-associated\_mesenchyme\_development | 1 | 0 |  |  |  |  |  |  |  |  |
| GO:0060486\_Clara\_cell\_differentiation | 1 | 0 |  |  |  |  |  |  |  |  |
| GO:0060510\_Type\_II\_pneumocyte\_differentiation | 1 | 0 |  |  |  |  |  |  |  |  |
| GO:0060514\_prostate\_induction | 1 | 0 |  |  |  |  |  |  |  |  |
| GO:0060515\_prostate\_field\_specification | 1 | 0 |  |  |  |  |  |  |  |  |
| GO:0060517\_epithelial\_cell\_proliferation\_involved\_in\_prostatic\_bud\_elongation | 1 | 0 |  |  |  |  |  |  |  |  |
| GO:0060520\_activation\_of\_prostate\_induction\_by\_androgen\_receptor\_signaling\_pathway | 1 | 0 |  |  |  |  |  |  |  |  |
| GO:0060535\_trachea\_cartilage\_morphogenesis | 1 | 0 |  |  |  |  |  |  |  |  |
| GO:0060536\_cartilage\_morphogenesis | 1 | 0 |  |  |  |  |  |  |  |  |
| GO:0060563\_neuroepithelial\_cell\_differentiation | 1 | 0 |  |  |  |  |  |  |  |  |
| GO:0060577\_pulmonary\_vein\_morphogenesis | 1 | 0 |  |  |  |  |  |  |  |  |
| GO:0060578\_superior\_vena\_cava\_morphogenesis | 1 | 0 |  |  |  |  |  |  |  |  |
| GO:0060584\_regulation\_of\_prostaglandin-endoperoxide\_synthase\_activity | 1 | 0 |  |  |  |  |  |  |  |  |
| GO:0060585\_positive\_regulation\_of\_prostaglandin-endoperoxidase\_synthase\_activity | 1 | 0 |  |  |  |  |  |  |  |  |
| GO:0060598\_dichotomous\_subdivision\_of\_terminal\_units\_involved\_in\_mammary\_gland\_duct\_morphogenesis | 1 | 0 |  |  |  |  |  |  |  |  |
| GO:0060611\_mammary\_gland\_fat\_development | 1 | 0 |  |  |  |  |  |  |  |  |
| GO:0060618\_nipple\_development | 1 | 0 |  |  |  |  |  |  |  |  |
| GO:0060631\_regulation\_of\_meiosis\_I | 1 | 0 |  |  |  |  |  |  |  |  |
| GO:0060649\_mammary\_gland\_bud\_elongation | 1 | 0 |  |  |  |  |  |  |  |  |
| GO:0060658\_nipple\_morphogenesis | 1 | 0 |  |  |  |  |  |  |  |  |
| GO:0060659\_nipple\_sheath\_formation | 1 | 0 |  |  |  |  |  |  |  |  |
| GO:0060668\_regulation\_of\_branching\_involved\_in\_salivary\_gland\_morphogenesis\_by\_extracellular\_matrix-epithelial\_cell\_signaling | 1 | 0 |  |  |  |  |  |  |  |  |
| GO:0060683\_regulation\_of\_branching\_involved\_in\_salivary\_gland\_morphogenesis\_by\_epithelial-mesenchymal\_signaling | 1 | 0 |  |  |  |  |  |  |  |  |
| GO:0060691\_epithelial\_cell\_maturation\_involved\_in\_salivary\_gland\_development | 1 | 0 |  |  |  |  |  |  |  |  |
| GO:0060709\_glycogen\_cell\_development\_involved\_in\_embryonic\_placenta\_development | 1 | 0 |  |  |  |  |  |  |  |  |
| GO:0060732\_positive\_regulation\_of\_inositol\_phosphate\_biosynthetic\_process | 1 | 0 |  |  |  |  |  |  |  |  |
| GO:0060739\_mesenchymal-epithelial\_cell\_signaling\_involved\_in\_prostate\_gland\_development | 1 | 0 |  |  |  |  |  |  |  |  |
| GO:0060781\_mesenchymal\_cell\_proliferation\_involved\_in\_prostate\_gland\_development | 1 | 0 |  |  |  |  |  |  |  |  |
| GO:0060782\_regulation\_of\_mesenchymal\_cell\_proliferation\_involved\_in\_prostate\_gland\_development | 1 | 0 |  |  |  |  |  |  |  |  |
| GO:0060783\_mesenchymal\_smoothened\_signaling\_pathway\_involved\_in\_prostate\_gland\_development | 1 | 0 |  |  |  |  |  |  |  |  |
| GO:0060872\_semicircular\_canal\_development | 1 | 0 |  |  |  |  |  |  |  |  |
| GO:0060896\_neural\_plate\_pattern\_specification | 1 | 0 |  |  |  |  |  |  |  |  |
| GO:0070091\_glucagon\_secretion | 1 | 0 |  |  |  |  |  |  |  |  |
| GO:0070162\_adiponectin\_secretion | 1 | 0 |  |  |  |  |  |  |  |  |
| GO:0070163\_regulation\_of\_adiponectin\_secretion | 1 | 0 |  |  |  |  |  |  |  |  |
| GO:0070164\_negative\_regulation\_of\_adiponectin\_secretion | 1 | 0 |  |  |  |  |  |  |  |  |
| GO:0070178\_D-serine\_metabolic\_process | 1 | 0 |  |  |  |  |  |  |  |  |
| GO:0070179\_D-serine\_biosynthetic\_process | 1 | 0 |  |  |  |  |  |  |  |  |
| GO:0070296\_sarcoplasmic\_reticulum\_calcium\_ion\_transport | 1 | 0 |  |  |  |  |  |  |  |  |
| GO:0070303\_negative\_regulation\_of\_stress-activated\_protein\_kinase\_signaling\_pathway | 1 | 0 |  |  |  |  |  |  |  |  |
| GO:0070328\_triglyceride\_homeostasis | 1 | 0 |  |  |  |  |  |  |  |  |
| GO:0070365\_hepatocyte\_differentiation | 1 | 0 |  |  |  |  |  |  |  |  |
| GO:0070384\_Harderian\_gland\_development | 1 | 0 |  |  |  |  |  |  |  |  |
| GO:0070391\_response\_to\_lipoteichoic\_acid | 1 | 0 |  |  |  |  |  |  |  |  |
| GO:0070424\_regulation\_of\_nucleotide-binding\_oligomerization\_domain\_containing\_signaling\_pathway | 1 | 0 |  |  |  |  |  |  |  |  |
| GO:0070426\_positive\_regulation\_of\_nucleotide-binding\_oligomerization\_domain\_containing\_signaling\_pathway | 1 | 0 |  |  |  |  |  |  |  |  |
| GO:0070428\_regulation\_of\_nucleotide-binding\_oligomerization\_domain\_containing\_1\_signaling\_pathway | 1 | 0 |  |  |  |  |  |  |  |  |
| GO:0070430\_positive\_regulation\_of\_nucleotide-binding\_oligomerization\_domain\_containing\_1\_signaling\_pathway | 1 | 0 |  |  |  |  |  |  |  |  |
| GO:0070432\_regulation\_of\_nucleotide-binding\_oligomerization\_domain\_containing\_2\_signaling\_pathway | 1 | 0 |  |  |  |  |  |  |  |  |
| GO:0070434\_positive\_regulation\_of\_nucleotide-binding\_oligomerization\_domain\_containing\_2\_signaling\_pathway | 1 | 0 |  |  |  |  |  |  |  |  |
| GO:0070493\_thrombin\_receptor\_signaling\_pathway | 1 | 0 |  |  |  |  |  |  |  |  |
| GO:0070508\_cholesterol\_import | 1 | 0 |  |  |  |  |  |  |  |  |
| GO:0070527\_platelet\_aggregation | 1 | 0 |  |  |  |  |  |  |  |  |
| GO:0070528\_protein\_kinase\_C\_signaling\_cascade | 1 | 0 |  |  |  |  |  |  |  |  |
| GO:0070555\_response\_to\_interleukin-1 | 1 | 0 |  |  |  |  |  |  |  |  |
| GO:0070560\_protein\_secretion\_by\_platelet | 1 | 0 |  |  |  |  |  |  |  |  |
| GO:0070561\_vitamin\_D\_receptor\_signaling\_pathway | 1 | 0 |  |  |  |  |  |  |  |  |
| GO:0070562\_regulation\_of\_vitamin\_D\_receptor\_signaling\_pathway | 1 | 0 |  |  |  |  |  |  |  |  |
| GO:0070571\_negative\_regulation\_of\_neuron\_projection\_regeneration | 1 | 0 |  |  |  |  |  |  |  |  |
| GO:0070572\_positive\_regulation\_of\_neuron\_projection\_regeneration | 1 | 0 |  |  |  |  |  |  |  |  |
| GO:0070613\_regulation\_of\_protein\_processing | 1 | 0 |  |  |  |  |  |  |  |  |
| GO:0070627\_ferrous\_iron\_import | 1 | 0 |  |  |  |  |  |  |  |  |
| GO:0070669\_response\_to\_interleukin-2 | 1 | 0 |  |  |  |  |  |  |  |  |
| GO:0070670\_response\_to\_interleukin-4 | 1 | 0 |  |  |  |  |  |  |  |  |
| GO:0070671\_response\_to\_interleukin-12 | 1 | 0 |  |  |  |  |  |  |  |  |
| GO:0070672\_response\_to\_interleukin-15 | 1 | 0 |  |  |  |  |  |  |  |  |
| GO:0070673\_response\_to\_interleukin-18 | 1 | 0 |  |  |  |  |  |  |  |  |
| GO:0070828\_heterochromatin\_organization | 1 | 0 |  |  |  |  |  |  |  |  |
| GO:0070874\_negative\_regulation\_of\_glycogen\_metabolic\_process | 1 | 0 |  |  |  |  |  |  |  |  |
| GO:0075136\_response\_to\_host | 1 | 0 |  |  |  |  |  |  |  |  |
| GO:0080010\_regulation\_of\_oxygen\_and\_reactive\_oxygen\_species\_metabolic\_process | 1 | 0 |  |  |  |  |  |  |  |  |
| GO:0090032\_negative\_regulation\_of\_steroid\_hormone\_biosynthetic\_process | 1 | 0 |  |  |  |  |  |  |  |  |
| GO:0000910\_cytokinesis | 8 | 0 | 0.000000 | -0.000000 | 781 | 764.713997 | 840.64 | 916.566003 | 1.076364 |  |
| GO:0001783\_B\_cell\_apoptosis | 8 | 0 | 0.000000 | -0.000000 | 781 | 764.713997 | 840.64 | 916.566003 | 1.076364 |  |
| GO:0001833\_inner\_cell\_mass\_cell\_proliferation | 8 | 0 | 0.000000 | -0.000000 | 781 | 764.713997 | 840.64 | 916.566003 | 1.076364 |  |
| GO:0001840\_neural\_plate\_development | 8 | 0 | 0.000000 | -0.000000 | 781 | 764.713997 | 840.64 | 916.566003 | 1.076364 |  |
| GO:0001893\_maternal\_placenta\_development | 8 | 0 | 0.000000 | -0.000000 | 781 | 764.713997 | 840.64 | 916.566003 | 1.076364 |  |
| GO:0001911\_negative\_regulation\_of\_leukocyte\_mediated\_cytotoxicity | 8 | 0 | 0.000000 | -0.000000 | 781 | 764.713997 | 840.64 | 916.566003 | 1.076364 |  |
| GO:0001916\_positive\_regulation\_of\_T\_cell\_mediated\_cytotoxicity | 8 | 0 | 0.000000 | -0.000000 | 781 | 764.713997 | 840.64 | 916.566003 | 1.076364 |  |
| GO:0002065\_columnar\_cuboidal\_epithelial\_cell\_differentiation | 8 | 0 | 0.000000 | -0.000000 | 781 | 764.713997 | 840.64 | 916.566003 | 1.076364 |  |
| GO:0002320\_lymphoid\_progenitor\_cell\_differentiation | 8 | 0 | 0.000000 | -0.000000 | 781 | 764.713997 | 840.64 | 916.566003 | 1.076364 |  |
| GO:0002438\_acute\_inflammatory\_response\_to\_antigenic\_stimulus | 8 | 0 | 0.000000 | -0.000000 | 781 | 764.713997 | 840.64 | 916.566003 | 1.076364 |  |
| GO:0002524\_hypersensitivity | 8 | 0 | 0.000000 | -0.000000 | 781 | 764.713997 | 840.64 | 916.566003 | 1.076364 |  |
| GO:0002566\_somatic\_diversification\_of\_immune\_receptors\_via\_somatic\_mutation | 8 | 0 | 0.000000 | -0.000000 | 781 | 764.713997 | 840.64 | 916.566003 | 1.076364 |  |
| GO:0002864\_regulation\_of\_acute\_inflammatory\_response\_to\_antigenic\_stimulus | 8 | 0 | 0.000000 | -0.000000 | 781 | 764.713997 | 840.64 | 916.566003 | 1.076364 |  |
| GO:0002883\_regulation\_of\_hypersensitivity | 8 | 0 | 0.000000 | -0.000000 | 781 | 764.713997 | 840.64 | 916.566003 | 1.076364 |  |
| GO:0003081\_regulation\_of\_systemic\_arterial\_blood\_pressure\_by\_renin-angiotensin | 8 | 0 | 0.000000 | -0.000000 | 781 | 764.713997 | 840.64 | 916.566003 | 1.076364 |  |
| GO:0006020\_inositol\_metabolic\_process | 8 | 0 | 0.000000 | -0.000000 | 781 | 764.713997 | 840.64 | 916.566003 | 1.076364 |  |
| GO:0006195\_purine\_nucleotide\_catabolic\_process | 8 | 0 | 0.000000 | -0.000000 | 781 | 764.713997 | 840.64 | 916.566003 | 1.076364 |  |
| GO:0006284\_base-excision\_repair | 8 | 0 | 0.000000 | -0.000000 | 781 | 764.713997 | 840.64 | 916.566003 | 1.076364 |  |
| GO:0006349\_genetic\_imprinting | 8 | 0 | 0.000000 | -0.000000 | 781 | 764.713997 | 840.64 | 916.566003 | 1.076364 |  |
| GO:0006360\_transcription\_from\_RNA\_polymerase\_I\_promoter | 8 | 0 | 0.000000 | -0.000000 | 781 | 764.713997 | 840.64 | 916.566003 | 1.076364 |  |
| GO:0006399\_tRNA\_metabolic\_process | 8 | 0 | 0.000000 | -0.000000 | 781 | 764.713997 | 840.64 | 916.566003 | 1.076364 |  |
| GO:0006458\_'de\_novo'\_protein\_folding | 8 | 0 | 0.000000 | -0.000000 | 781 | 764.713997 | 840.64 | 916.566003 | 1.076364 |  |
| GO:0006493\_protein\_amino\_acid\_O-linked\_glycosylation | 8 | 0 | 0.000000 | -0.000000 | 781 | 764.713997 | 840.64 | 916.566003 | 1.076364 |  |
| GO:0006582\_melanin\_metabolic\_process | 8 | 0 | 0.000000 | -0.000000 | 781 | 764.713997 | 840.64 | 916.566003 | 1.076364 |  |
| GO:0006733\_oxidoreduction\_coenzyme\_metabolic\_process | 8 | 0 | 0.000000 | -0.000000 | 781 | 764.713997 | 840.64 | 916.566003 | 1.076364 |  |
| GO:0006829\_zinc\_ion\_transport | 8 | 0 | 0.000000 | -0.000000 | 781 | 764.713997 | 840.64 | 916.566003 | 1.076364 |  |
| GO:0007009\_plasma\_membrane\_organization | 8 | 0 | 0.000000 | -0.000000 | 781 | 764.713997 | 840.64 | 916.566003 | 1.076364 |  |
| GO:0007131\_reciprocal\_meiotic\_recombination | 8 | 0 | 0.000000 | -0.000000 | 781 | 764.713997 | 840.64 | 916.566003 | 1.076364 |  |
| GO:0007141\_male\_meiosis\_I | 8 | 0 | 0.000000 | -0.000000 | 781 | 764.713997 | 840.64 | 916.566003 | 1.076364 |  |
| GO:0007625\_grooming\_behavior | 8 | 0 | 0.000000 | -0.000000 | 781 | 764.713997 | 840.64 | 916.566003 | 1.076364 |  |
| GO:0008105\_asymmetric\_protein\_localization | 8 | 0 | 0.000000 | -0.000000 | 781 | 764.713997 | 840.64 | 916.566003 | 1.076364 |  |
| GO:0008593\_regulation\_of\_Notch\_signaling\_pathway | 8 | 0 | 0.000000 | -0.000000 | 781 | 764.713997 | 840.64 | 916.566003 | 1.076364 |  |
| GO:0009072\_aromatic\_amino\_acid\_family\_metabolic\_process | 8 | 0 | 0.000000 | -0.000000 | 781 | 764.713997 | 840.64 | 916.566003 | 1.076364 |  |
| GO:0009144\_purine\_nucleoside\_triphosphate\_metabolic\_process | 8 | 0 | 0.000000 | -0.000000 | 781 | 764.713997 | 840.64 | 916.566003 | 1.076364 |  |
| GO:0009746\_response\_to\_hexose\_stimulus | 8 | 0 | 0.000000 | -0.000000 | 781 | 764.713997 | 840.64 | 916.566003 | 1.076364 |  |
| GO:0009749\_response\_to\_glucose\_stimulus | 8 | 0 | 0.000000 | -0.000000 | 781 | 764.713997 | 840.64 | 916.566003 | 1.076364 |  |
| GO:0014014\_negative\_regulation\_of\_gliogenesis | 8 | 0 | 0.000000 | -0.000000 | 781 | 764.713997 | 840.64 | 916.566003 | 1.076364 |  |
| GO:0014046\_dopamine\_secretion | 8 | 0 | 0.000000 | -0.000000 | 781 | 764.713997 | 840.64 | 916.566003 | 1.076364 |  |
| GO:0014059\_regulation\_of\_dopamine\_secretion | 8 | 0 | 0.000000 | -0.000000 | 781 | 764.713997 | 840.64 | 916.566003 | 1.076364 |  |
| GO:0014065\_phosphoinositide\_3-kinase\_cascade | 8 | 0 | 0.000000 | -0.000000 | 781 | 764.713997 | 840.64 | 916.566003 | 1.076364 |  |
| GO:0015800\_acidic\_amino\_acid\_transport | 8 | 0 | 0.000000 | -0.000000 | 781 | 764.713997 | 840.64 | 916.566003 | 1.076364 |  |
| GO:0015804\_neutral\_amino\_acid\_transport | 8 | 0 | 0.000000 | -0.000000 | 781 | 764.713997 | 840.64 | 916.566003 | 1.076364 |  |
| GO:0016236\_macroautophagy | 8 | 0 | 0.000000 | -0.000000 | 781 | 764.713997 | 840.64 | 916.566003 | 1.076364 |  |
| GO:0016446\_somatic\_hypermutation\_of\_immunoglobulin\_genes | 8 | 0 | 0.000000 | -0.000000 | 781 | 764.713997 | 840.64 | 916.566003 | 1.076364 |  |
| GO:0018107\_peptidyl-threonine\_phosphorylation | 8 | 0 | 0.000000 | -0.000000 | 781 | 764.713997 | 840.64 | 916.566003 | 1.076364 |  |
| GO:0018210\_peptidyl-threonine\_modification | 8 | 0 | 0.000000 | -0.000000 | 781 | 764.713997 | 840.64 | 916.566003 | 1.076364 |  |
| GO:0018345\_protein\_palmitoylation | 8 | 0 | 0.000000 | -0.000000 | 781 | 764.713997 | 840.64 | 916.566003 | 1.076364 |  |
| GO:0019229\_regulation\_of\_vasoconstriction | 8 | 0 | 0.000000 | -0.000000 | 781 | 764.713997 | 840.64 | 916.566003 | 1.076364 |  |
| GO:0019400\_alditol\_metabolic\_process | 8 | 0 | 0.000000 | -0.000000 | 781 | 764.713997 | 840.64 | 916.566003 | 1.076364 |  |
| GO:0021692\_cerebellar\_Purkinje\_cell\_layer\_morphogenesis | 8 | 0 | 0.000000 | -0.000000 | 781 | 764.713997 | 840.64 | 916.566003 | 1.076364 |  |
| GO:0021694\_cerebellar\_Purkinje\_cell\_layer\_formation | 8 | 0 | 0.000000 | -0.000000 | 781 | 764.713997 | 840.64 | 916.566003 | 1.076364 |  |
| GO:0021702\_cerebellar\_Purkinje\_cell\_differentiation | 8 | 0 | 0.000000 | -0.000000 | 781 | 764.713997 | 840.64 | 916.566003 | 1.076364 |  |
| GO:0021781\_glial\_cell\_fate\_commitment | 8 | 0 | 0.000000 | -0.000000 | 781 | 764.713997 | 840.64 | 916.566003 | 1.076364 |  |
| GO:0021799\_cerebral\_cortex\_radially\_oriented\_cell\_migration | 8 | 0 | 0.000000 | -0.000000 | 781 | 764.713997 | 840.64 | 916.566003 | 1.076364 |  |
| GO:0022898\_regulation\_of\_transmembrane\_transporter\_activity | 8 | 0 | 0.000000 | -0.000000 | 781 | 764.713997 | 840.64 | 916.566003 | 1.076364 |  |
| GO:0030035\_microspike\_assembly | 8 | 0 | 0.000000 | -0.000000 | 781 | 764.713997 | 840.64 | 916.566003 | 1.076364 |  |
| GO:0030193\_regulation\_of\_blood\_coagulation | 8 | 0 | 0.000000 | -0.000000 | 781 | 764.713997 | 840.64 | 916.566003 | 1.076364 |  |
| GO:0030204\_chondroitin\_sulfate\_metabolic\_process | 8 | 0 | 0.000000 | -0.000000 | 781 | 764.713997 | 840.64 | 916.566003 | 1.076364 |  |
| GO:0030500\_regulation\_of\_bone\_mineralization | 8 | 0 | 0.000000 | -0.000000 | 781 | 764.713997 | 840.64 | 916.566003 | 1.076364 |  |
| GO:0030511\_positive\_regulation\_of\_transforming\_growth\_factor\_beta\_receptor\_signaling\_pathway | 8 | 0 | 0.000000 | -0.000000 | 781 | 764.713997 | 840.64 | 916.566003 | 1.076364 |  |
| GO:0031102\_neuron\_projection\_regeneration | 8 | 0 | 0.000000 | -0.000000 | 781 | 764.713997 | 840.64 | 916.566003 | 1.076364 |  |
| GO:0031103\_axon\_regeneration | 8 | 0 | 0.000000 | -0.000000 | 781 | 764.713997 | 840.64 | 916.566003 | 1.076364 |  |
| GO:0031111\_negative\_regulation\_of\_microtubule\_polymerization\_or\_depolymerization | 8 | 0 | 0.000000 | -0.000000 | 781 | 764.713997 | 840.64 | 916.566003 | 1.076364 |  |
| GO:0031123\_RNA\_3'-end\_processing | 8 | 0 | 0.000000 | -0.000000 | 781 | 764.713997 | 840.64 | 916.566003 | 1.076364 |  |
| GO:0031294\_lymphocyte\_costimulation | 8 | 0 | 0.000000 | -0.000000 | 781 | 764.713997 | 840.64 | 916.566003 | 1.076364 |  |
| GO:0031295\_T\_cell\_costimulation | 8 | 0 | 0.000000 | -0.000000 | 781 | 764.713997 | 840.64 | 916.566003 | 1.076364 |  |
| GO:0031334\_positive\_regulation\_of\_protein\_complex\_assembly | 8 | 0 | 0.000000 | -0.000000 | 781 | 764.713997 | 840.64 | 916.566003 | 1.076364 |  |
| GO:0031342\_negative\_regulation\_of\_cell\_killing | 8 | 0 | 0.000000 | -0.000000 | 781 | 764.713997 | 840.64 | 916.566003 | 1.076364 |  |
| GO:0031396\_regulation\_of\_protein\_ubiquitination | 8 | 0 | 0.000000 | -0.000000 | 781 | 764.713997 | 840.64 | 916.566003 | 1.076364 |  |
| GO:0032094\_response\_to\_food | 8 | 0 | 0.000000 | -0.000000 | 781 | 764.713997 | 840.64 | 916.566003 | 1.076364 |  |
| GO:0032273\_positive\_regulation\_of\_protein\_polymerization | 8 | 0 | 0.000000 | -0.000000 | 781 | 764.713997 | 840.64 | 916.566003 | 1.076364 |  |
| GO:0032409\_regulation\_of\_transporter\_activity | 8 | 0 | 0.000000 | -0.000000 | 781 | 764.713997 | 840.64 | 916.566003 | 1.076364 |  |
| GO:0032412\_regulation\_of\_ion\_transmembrane\_transporter\_activity | 8 | 0 | 0.000000 | -0.000000 | 781 | 764.713997 | 840.64 | 916.566003 | 1.076364 |  |
| GO:0032613\_interleukin-10\_production | 8 | 0 | 0.000000 | -0.000000 | 781 | 764.713997 | 840.64 | 916.566003 | 1.076364 |  |
| GO:0033198\_response\_to\_ATP | 8 | 0 | 0.000000 | -0.000000 | 781 | 764.713997 | 840.64 | 916.566003 | 1.076364 |  |
| GO:0034284\_response\_to\_monosaccharide\_stimulus | 8 | 0 | 0.000000 | -0.000000 | 781 | 764.713997 | 840.64 | 916.566003 | 1.076364 |  |
| GO:0034728\_nucleosome\_organization | 8 | 0 | 0.000000 | -0.000000 | 781 | 764.713997 | 840.64 | 916.566003 | 1.076364 |  |
| GO:0035023\_regulation\_of\_Rho\_protein\_signal\_transduction | 8 | 0 | 0.000000 | -0.000000 | 781 | 764.713997 | 840.64 | 916.566003 | 1.076364 |  |
| GO:0035112\_genitalia\_morphogenesis | 8 | 0 | 0.000000 | -0.000000 | 781 | 764.713997 | 840.64 | 916.566003 | 1.076364 |  |
| GO:0040017\_positive\_regulation\_of\_locomotion | 8 | 0 | 0.000000 | -0.000000 | 781 | 764.713997 | 840.64 | 916.566003 | 1.076364 |  |
| GO:0040034\_regulation\_of\_development\_\_heterochronic | 8 | 0 | 0.000000 | -0.000000 | 781 | 764.713997 | 840.64 | 916.566003 | 1.076364 |  |
| GO:0042074\_cell\_migration\_involved\_in\_gastrulation | 8 | 0 | 0.000000 | -0.000000 | 781 | 764.713997 | 840.64 | 916.566003 | 1.076364 |  |
| GO:0042090\_interleukin-12\_biosynthetic\_process | 8 | 0 | 0.000000 | -0.000000 | 781 | 764.713997 | 840.64 | 916.566003 | 1.076364 |  |
| GO:0042092\_T-helper\_2\_type\_immune\_response | 8 | 0 | 0.000000 | -0.000000 | 781 | 764.713997 | 840.64 | 916.566003 | 1.076364 |  |
| GO:0042095\_interferon-gamma\_biosynthetic\_process | 8 | 0 | 0.000000 | -0.000000 | 781 | 764.713997 | 840.64 | 916.566003 | 1.076364 |  |
| GO:0042104\_positive\_regulation\_of\_activated\_T\_cell\_proliferation | 8 | 0 | 0.000000 | -0.000000 | 781 | 764.713997 | 840.64 | 916.566003 | 1.076364 |  |
| GO:0042226\_interleukin-6\_biosynthetic\_process | 8 | 0 | 0.000000 | -0.000000 | 781 | 764.713997 | 840.64 | 916.566003 | 1.076364 |  |
| GO:0042304\_regulation\_of\_fatty\_acid\_biosynthetic\_process | 8 | 0 | 0.000000 | -0.000000 | 781 | 764.713997 | 840.64 | 916.566003 | 1.076364 |  |
| GO:0042423\_catecholamine\_biosynthetic\_process | 8 | 0 | 0.000000 | -0.000000 | 781 | 764.713997 | 840.64 | 916.566003 | 1.076364 |  |
| GO:0042771\_DNA\_damage\_response\_\_signal\_transduction\_by\_p53\_class\_mediator\_resulting\_in\_induction\_of\_apoptosis | 8 | 0 | 0.000000 | -0.000000 | 781 | 764.713997 | 840.64 | 916.566003 | 1.076364 |  |
| GO:0042990\_regulation\_of\_transcription\_factor\_import\_into\_nucleus | 8 | 0 | 0.000000 | -0.000000 | 781 | 764.713997 | 840.64 | 916.566003 | 1.076364 |  |
| GO:0042991\_transcription\_factor\_import\_into\_nucleus | 8 | 0 | 0.000000 | -0.000000 | 781 | 764.713997 | 840.64 | 916.566003 | 1.076364 |  |
| GO:0043011\_myeloid\_dendritic\_cell\_differentiation | 8 | 0 | 0.000000 | -0.000000 | 781 | 764.713997 | 840.64 | 916.566003 | 1.076364 |  |
| GO:0043368\_positive\_T\_cell\_selection | 8 | 0 | 0.000000 | -0.000000 | 781 | 764.713997 | 840.64 | 916.566003 | 1.076364 |  |
| GO:0043370\_regulation\_of\_CD4-positive\_\_alpha\_beta\_T\_cell\_differentiation | 8 | 0 | 0.000000 | -0.000000 | 781 | 764.713997 | 840.64 | 916.566003 | 1.076364 |  |
| GO:0043542\_endothelial\_cell\_migration | 8 | 0 | 0.000000 | -0.000000 | 781 | 764.713997 | 840.64 | 916.566003 | 1.076364 |  |
| GO:0043616\_keratinocyte\_proliferation | 8 | 0 | 0.000000 | -0.000000 | 781 | 764.713997 | 840.64 | 916.566003 | 1.076364 |  |
| GO:0045075\_regulation\_of\_interleukin-12\_biosynthetic\_process | 8 | 0 | 0.000000 | -0.000000 | 781 | 764.713997 | 840.64 | 916.566003 | 1.076364 |  |
| GO:0045086\_positive\_regulation\_of\_interleukin-2\_biosynthetic\_process | 8 | 0 | 0.000000 | -0.000000 | 781 | 764.713997 | 840.64 | 916.566003 | 1.076364 |  |
| GO:0045351\_type\_I\_interferon\_biosynthetic\_process | 8 | 0 | 0.000000 | -0.000000 | 781 | 764.713997 | 840.64 | 916.566003 | 1.076364 |  |
| GO:0045408\_regulation\_of\_interleukin-6\_biosynthetic\_process | 8 | 0 | 0.000000 | -0.000000 | 781 | 764.713997 | 840.64 | 916.566003 | 1.076364 |  |
| GO:0045429\_positive\_regulation\_of\_nitric\_oxide\_biosynthetic\_process | 8 | 0 | 0.000000 | -0.000000 | 781 | 764.713997 | 840.64 | 916.566003 | 1.076364 |  |
| GO:0045494\_photoreceptor\_cell\_maintenance | 8 | 0 | 0.000000 | -0.000000 | 781 | 764.713997 | 840.64 | 916.566003 | 1.076364 |  |
| GO:0045686\_negative\_regulation\_of\_glial\_cell\_differentiation | 8 | 0 | 0.000000 | -0.000000 | 781 | 764.713997 | 840.64 | 916.566003 | 1.076364 |  |
| GO:0045910\_negative\_regulation\_of\_DNA\_recombination | 8 | 0 | 0.000000 | -0.000000 | 781 | 764.713997 | 840.64 | 916.566003 | 1.076364 |  |
| GO:0045921\_positive\_regulation\_of\_exocytosis | 8 | 0 | 0.000000 | -0.000000 | 781 | 764.713997 | 840.64 | 916.566003 | 1.076364 |  |
| GO:0045932\_negative\_regulation\_of\_muscle\_contraction | 8 | 0 | 0.000000 | -0.000000 | 781 | 764.713997 | 840.64 | 916.566003 | 1.076364 |  |
| GO:0046470\_phosphatidylcholine\_metabolic\_process | 8 | 0 | 0.000000 | -0.000000 | 781 | 764.713997 | 840.64 | 916.566003 | 1.076364 |  |
| GO:0048266\_behavioral\_response\_to\_pain | 8 | 0 | 0.000000 | -0.000000 | 781 | 764.713997 | 840.64 | 916.566003 | 1.076364 |  |
| GO:0048505\_regulation\_of\_timing\_of\_cell\_differentiation | 8 | 0 | 0.000000 | -0.000000 | 781 | 764.713997 | 840.64 | 916.566003 | 1.076364 |  |
| GO:0048520\_positive\_regulation\_of\_behavior | 8 | 0 | 0.000000 | -0.000000 | 781 | 764.713997 | 840.64 | 916.566003 | 1.076364 |  |
| GO:0048557\_embryonic\_digestive\_tract\_morphogenesis | 8 | 0 | 0.000000 | -0.000000 | 781 | 764.713997 | 840.64 | 916.566003 | 1.076364 |  |
| GO:0048638\_regulation\_of\_developmental\_growth | 8 | 0 | 0.000000 | -0.000000 | 781 | 764.713997 | 840.64 | 916.566003 | 1.076364 |  |
| GO:0048742\_regulation\_of\_skeletal\_muscle\_fiber\_development | 8 | 0 | 0.000000 | -0.000000 | 781 | 764.713997 | 840.64 | 916.566003 | 1.076364 |  |
| GO:0050707\_regulation\_of\_cytokine\_secretion | 8 | 0 | 0.000000 | -0.000000 | 781 | 764.713997 | 840.64 | 916.566003 | 1.076364 |  |
| GO:0050909\_sensory\_perception\_of\_taste | 8 | 0 | 0.000000 | -0.000000 | 781 | 764.713997 | 840.64 | 916.566003 | 1.076364 |  |
| GO:0050920\_regulation\_of\_chemotaxis | 8 | 0 | 0.000000 | -0.000000 | 781 | 764.713997 | 840.64 | 916.566003 | 1.076364 |  |
| GO:0050921\_positive\_regulation\_of\_chemotaxis | 8 | 0 | 0.000000 | -0.000000 | 781 | 764.713997 | 840.64 | 916.566003 | 1.076364 |  |
| GO:0050926\_regulation\_of\_positive\_chemotaxis | 8 | 0 | 0.000000 | -0.000000 | 781 | 764.713997 | 840.64 | 916.566003 | 1.076364 |  |
| GO:0050927\_positive\_regulation\_of\_positive\_chemotaxis | 8 | 0 | 0.000000 | -0.000000 | 781 | 764.713997 | 840.64 | 916.566003 | 1.076364 |  |
| GO:0050930\_induction\_of\_positive\_chemotaxis | 8 | 0 | 0.000000 | -0.000000 | 781 | 764.713997 | 840.64 | 916.566003 | 1.076364 |  |
| GO:0051084\_'de\_novo'\_posttranslational\_protein\_folding | 8 | 0 | 0.000000 | -0.000000 | 781 | 764.713997 | 840.64 | 916.566003 | 1.076364 |  |
| GO:0051181\_cofactor\_transport | 8 | 0 | 0.000000 | -0.000000 | 781 | 764.713997 | 840.64 | 916.566003 | 1.076364 |  |
| GO:0060043\_regulation\_of\_cardiac\_muscle\_cell\_proliferation | 8 | 0 | 0.000000 | -0.000000 | 781 | 764.713997 | 840.64 | 916.566003 | 1.076364 |  |
| GO:0060347\_heart\_trabecula\_formation | 8 | 0 | 0.000000 | -0.000000 | 781 | 764.713997 | 840.64 | 916.566003 | 1.076364 |  |
| GO:0060670\_branching\_involved\_in\_embryonic\_placenta\_morphogenesis | 8 | 0 | 0.000000 | -0.000000 | 781 | 764.713997 | 840.64 | 916.566003 | 1.076364 |  |
| GO:0060712\_spongiotrophoblast\_layer\_development | 8 | 0 | 0.000000 | -0.000000 | 781 | 764.713997 | 840.64 | 916.566003 | 1.076364 |  |
| GO:0070167\_regulation\_of\_biomineral\_formation | 8 | 0 | 0.000000 | -0.000000 | 781 | 764.713997 | 840.64 | 916.566003 | 1.076364 |  |
| GO:0070193\_synaptonemal\_complex\_organization | 8 | 0 | 0.000000 | -0.000000 | 781 | 764.713997 | 840.64 | 916.566003 | 1.076364 |  |
| GO:0070231\_T\_cell\_apoptosis | 8 | 0 | 0.000000 | -0.000000 | 781 | 764.713997 | 840.64 | 916.566003 | 1.076364 |  |
| GO:0070584\_mitochondrion\_morphogenesis | 8 | 0 | 0.000000 | -0.000000 | 781 | 764.713997 | 840.64 | 916.566003 | 1.076364 |  |
| GO:0006753\_nucleoside\_phosphate\_metabolic\_process | 94 | 0 | 0.000000 | -0.000000 | 786 | 769.581127 | 844.8 | 920.018873 | 1.074809 |  |
| GO:0008610\_lipid\_biosynthetic\_process | 94 | 0 | 0.000000 | -0.000000 | 786 | 769.581127 | 844.8 | 920.018873 | 1.074809 |  |
| GO:0009117\_nucleotide\_metabolic\_process | 94 | 0 | 0.000000 | -0.000000 | 786 | 769.581127 | 844.8 | 920.018873 | 1.074809 |  |
| GO:0032943\_mononuclear\_cell\_proliferation | 94 | 0 | 0.000000 | -0.000000 | 786 | 769.581127 | 844.8 | 920.018873 | 1.074809 |  |
| GO:0046651\_lymphocyte\_proliferation | 94 | 0 | 0.000000 | -0.000000 | 786 | 769.581127 | 844.8 | 920.018873 | 1.074809 |  |
| GO:0007281\_germ\_cell\_development | 75 | 0 | 0.000000 | -0.000000 | 790 | 772.845597 | 847.73 | 922.614403 | 1.073076 |  |
| GO:0044265\_cellular\_macromolecule\_catabolic\_process | 75 | 0 | 0.000000 | -0.000000 | 790 | 772.845597 | 847.73 | 922.614403 | 1.073076 |  |
| GO:0048589\_developmental\_growth | 75 | 0 | 0.000000 | -0.000000 | 790 | 772.845597 | 847.73 | 922.614403 | 1.073076 |  |
| GO:0051050\_positive\_regulation\_of\_transport | 75 | 0 | 0.000000 | -0.000000 | 790 | 772.845597 | 847.73 | 922.614403 | 1.073076 |  |
| GO:0002694\_regulation\_of\_leukocyte\_activation | 121 | 0 | 0.000000 | -0.000000 | 791 | 775.866357 | 850.17 | 924.473643 | 1.074804 |  |
| GO:0001503\_ossification | 88 | 0 | 0.000000 | -0.000000 | 794 | 778.367150 | 852.35 | 926.332850 | 1.073489 |  |
| GO:0048754\_branching\_morphogenesis\_of\_a\_tube | 88 | 0 | 0.000000 | -0.000000 | 794 | 778.367150 | 852.35 | 926.332850 | 1.073489 |  |
| GO:0050863\_regulation\_of\_T\_cell\_activation | 88 | 0 | 0.000000 | -0.000000 | 794 | 778.367150 | 852.35 | 926.332850 | 1.073489 |  |
| GO:0000012\_single\_strand\_break\_repair | 2 | 0 |  |  |  |  |  |  |  |  |
| GO:0000019\_regulation\_of\_mitotic\_recombination | 2 | 0 |  |  |  |  |  |  |  |  |
| GO:0000080\_G1\_phase\_of\_mitotic\_cell\_cycle | 2 | 0 |  |  |  |  |  |  |  |  |
| GO:0000083\_regulation\_of\_transcription\_of\_G1\_S-phase\_of\_mitotic\_cell\_cycle | 2 | 0 |  |  |  |  |  |  |  |  |
| GO:0000085\_G2\_phase\_of\_mitotic\_cell\_cycle | 2 | 0 |  |  |  |  |  |  |  |  |
| GO:0000289\_nuclear-transcribed\_mRNA\_poly(A)\_tail\_shortening | 2 | 0 |  |  |  |  |  |  |  |  |
| GO:0000381\_regulation\_of\_alternative\_nuclear\_mRNA\_splicing\_\_via\_spliceosome | 2 | 0 |  |  |  |  |  |  |  |  |
| GO:0000712\_resolution\_of\_meiotic\_joint\_molecules\_as\_recombinants | 2 | 0 |  |  |  |  |  |  |  |  |
| GO:0000720\_pyrimidine\_dimer\_repair\_by\_nucleotide-excision\_repair | 2 | 0 |  |  |  |  |  |  |  |  |
| GO:0001302\_replicative\_cell\_aging | 2 | 0 |  |  |  |  |  |  |  |  |
| GO:0001306\_age-dependent\_response\_to\_oxidative\_stress | 2 | 0 |  |  |  |  |  |  |  |  |
| GO:0001514\_selenocysteine\_incorporation | 2 | 0 |  |  |  |  |  |  |  |  |
| GO:0001522\_pseudouridine\_synthesis | 2 | 0 |  |  |  |  |  |  |  |  |
| GO:0001543\_ovarian\_follicle\_rupture | 2 | 0 |  |  |  |  |  |  |  |  |
| GO:0001675\_acrosome\_assembly | 2 | 0 |  |  |  |  |  |  |  |  |
| GO:0001743\_optic\_placode\_formation | 2 | 0 |  |  |  |  |  |  |  |  |
| GO:0001767\_establishment\_of\_lymphocyte\_polarity | 2 | 0 |  |  |  |  |  |  |  |  |
| GO:0001768\_establishment\_of\_T\_cell\_polarity | 2 | 0 |  |  |  |  |  |  |  |  |
| GO:0001771\_formation\_of\_immunological\_synapse | 2 | 0 |  |  |  |  |  |  |  |  |
| GO:0001774\_microglial\_cell\_activation | 2 | 0 |  |  |  |  |  |  |  |  |
| GO:0001781\_neutrophil\_apoptosis | 2 | 0 |  |  |  |  |  |  |  |  |
| GO:0001787\_natural\_killer\_cell\_proliferation | 2 | 0 |  |  |  |  |  |  |  |  |
| GO:0001788\_antibody-dependent\_cellular\_cytotoxicity | 2 | 0 |  |  |  |  |  |  |  |  |
| GO:0001806\_type\_IV\_hypersensitivity | 2 | 0 |  |  |  |  |  |  |  |  |
| GO:0001807\_regulation\_of\_type\_IV\_hypersensitivity | 2 | 0 |  |  |  |  |  |  |  |  |
| GO:0001808\_negative\_regulation\_of\_type\_IV\_hypersensitivity | 2 | 0 |  |  |  |  |  |  |  |  |
| GO:0001823\_mesonephros\_development | 2 | 0 |  |  |  |  |  |  |  |  |
| GO:0001845\_phagolysosome\_formation | 2 | 0 |  |  |  |  |  |  |  |  |
| GO:0001866\_NK\_T\_cell\_proliferation | 2 | 0 |  |  |  |  |  |  |  |  |
| GO:0001879\_detection\_of\_yeast | 2 | 0 |  |  |  |  |  |  |  |  |
| GO:0001886\_endothelial\_cell\_morphogenesis | 2 | 0 |  |  |  |  |  |  |  |  |
| GO:0001919\_regulation\_of\_receptor\_recycling | 2 | 0 |  |  |  |  |  |  |  |  |
| GO:0001954\_positive\_regulation\_of\_cell-matrix\_adhesion | 2 | 0 |  |  |  |  |  |  |  |  |
| GO:0001977\_renal\_system\_process\_involved\_in\_regulation\_of\_blood\_volume | 2 | 0 |  |  |  |  |  |  |  |  |
| GO:0001982\_baroreceptor\_response\_to\_decreased\_systemic\_arterial\_blood\_pressure | 2 | 0 |  |  |  |  |  |  |  |  |
| GO:0001983\_baroreceptor\_response\_to\_increased\_systemic\_arterial\_blood\_pressure | 2 | 0 |  |  |  |  |  |  |  |  |
| GO:0001992\_regulation\_of\_systemic\_arterial\_blood\_pressure\_by\_vasopressin | 2 | 0 |  |  |  |  |  |  |  |  |
| GO:0001997\_positive\_regulation\_of\_the\_force\_of\_heart\_contraction\_by\_epinephrine-norepinephrine | 2 | 0 |  |  |  |  |  |  |  |  |
| GO:0001998\_angiotensin\_mediated\_vasoconstriction\_involved\_in\_regulation\_of\_systemic\_arterial\_blood\_pressure | 2 | 0 |  |  |  |  |  |  |  |  |
| GO:0001999\_renal\_response\_to\_blood\_flow\_during\_renin-angiotensin\_regulation\_of\_systemic\_arterial\_blood\_pressure | 2 | 0 |  |  |  |  |  |  |  |  |
| GO:0002018\_renin-angiotensin\_regulation\_of\_aldosterone\_production | 2 | 0 |  |  |  |  |  |  |  |  |
| GO:0002019\_regulation\_of\_renal\_output\_by\_angiotensin | 2 | 0 |  |  |  |  |  |  |  |  |
| GO:0002024\_diet\_induced\_thermogenesis | 2 | 0 |  |  |  |  |  |  |  |  |
| GO:0002025\_vasodilation\_by\_norepinephrine-epinephrine\_involved\_in\_regulation\_of\_systemic\_arterial\_blood\_pressure | 2 | 0 |  |  |  |  |  |  |  |  |
| GO:0002029\_desensitization\_of\_G-protein\_coupled\_receptor\_protein\_signaling\_pathway | 2 | 0 |  |  |  |  |  |  |  |  |
| GO:0002033\_vasodilation\_by\_angiotensin\_involved\_in\_regulation\_of\_systemic\_arterial\_blood\_pressure | 2 | 0 |  |  |  |  |  |  |  |  |
| GO:0002066\_columnar\_cuboidal\_epithelial\_cell\_development | 2 | 0 |  |  |  |  |  |  |  |  |
| GO:0002072\_optic\_cup\_morphogenesis\_involved\_in\_camera-type\_eye\_development | 2 | 0 |  |  |  |  |  |  |  |  |
| GO:0002074\_extraocular\_skeletal\_muscle\_development | 2 | 0 |  |  |  |  |  |  |  |  |
| GO:0002138\_retinoic\_acid\_biosynthetic\_process | 2 | 0 |  |  |  |  |  |  |  |  |
| GO:0002223\_stimulatory\_C-type\_lectin\_receptor\_signaling\_pathway | 2 | 0 |  |  |  |  |  |  |  |  |
| GO:0002246\_healing\_during\_inflammatory\_response | 2 | 0 |  |  |  |  |  |  |  |  |
| GO:0002251\_organ\_or\_tissue\_specific\_immune\_response | 2 | 0 |  |  |  |  |  |  |  |  |
| GO:0002266\_follicular\_dendritic\_cell\_activation | 2 | 0 |  |  |  |  |  |  |  |  |
| GO:0002268\_follicular\_dendritic\_cell\_differentiation | 2 | 0 |  |  |  |  |  |  |  |  |
| GO:0002327\_immature\_B\_cell\_differentiation | 2 | 0 |  |  |  |  |  |  |  |  |
| GO:0002329\_pre-B\_cell\_differentiation | 2 | 0 |  |  |  |  |  |  |  |  |
| GO:0002339\_B\_cell\_selection | 2 | 0 |  |  |  |  |  |  |  |  |
| GO:0002352\_B\_cell\_negative\_selection | 2 | 0 |  |  |  |  |  |  |  |  |
| GO:0002358\_B\_cell\_homeostatic\_proliferation | 2 | 0 |  |  |  |  |  |  |  |  |
| GO:0002385\_mucosal\_immune\_response | 2 | 0 |  |  |  |  |  |  |  |  |
| GO:0002514\_B\_cell\_tolerance\_induction | 2 | 0 |  |  |  |  |  |  |  |  |
| GO:0002523\_leukocyte\_migration\_during\_inflammatory\_response | 2 | 0 |  |  |  |  |  |  |  |  |
| GO:0002536\_respiratory\_burst\_during\_acute\_inflammatory\_response | 2 | 0 |  |  |  |  |  |  |  |  |
| GO:0002537\_production\_of\_nitric\_oxide\_during\_acute\_inflammatory\_response | 2 | 0 |  |  |  |  |  |  |  |  |
| GO:0002576\_platelet\_degranulation | 2 | 0 |  |  |  |  |  |  |  |  |
| GO:0002639\_positive\_regulation\_of\_immunoglobulin\_production | 2 | 0 |  |  |  |  |  |  |  |  |
| GO:0002661\_regulation\_of\_B\_cell\_tolerance\_induction | 2 | 0 |  |  |  |  |  |  |  |  |
| GO:0002663\_positive\_regulation\_of\_B\_cell\_tolerance\_induction | 2 | 0 |  |  |  |  |  |  |  |  |
| GO:0002676\_regulation\_of\_chronic\_inflammatory\_response | 2 | 0 |  |  |  |  |  |  |  |  |
| GO:0002679\_respiratory\_burst\_during\_defense\_response | 2 | 0 |  |  |  |  |  |  |  |  |
| GO:0002686\_negative\_regulation\_of\_leukocyte\_migration | 2 | 0 |  |  |  |  |  |  |  |  |
| GO:0002720\_positive\_regulation\_of\_cytokine\_production\_during\_immune\_response | 2 | 0 |  |  |  |  |  |  |  |  |
| GO:0002752\_cell\_surface\_pattern\_recognition\_receptor\_signaling\_pathway | 2 | 0 |  |  |  |  |  |  |  |  |
| GO:0002755\_MyD88-dependent\_toll-like\_receptor\_signaling\_pathway | 2 | 0 |  |  |  |  |  |  |  |  |
| GO:0002765\_immune\_response-inhibiting\_signal\_transduction | 2 | 0 |  |  |  |  |  |  |  |  |
| GO:0002921\_negative\_regulation\_of\_humoral\_immune\_response | 2 | 0 |  |  |  |  |  |  |  |  |
| GO:0002922\_positive\_regulation\_of\_humoral\_immune\_response | 2 | 0 |  |  |  |  |  |  |  |  |
| GO:0002924\_negative\_regulation\_of\_humoral\_immune\_response\_mediated\_by\_circulating\_immunoglobulin | 2 | 0 |  |  |  |  |  |  |  |  |
| GO:0002925\_positive\_regulation\_of\_humoral\_immune\_response\_mediated\_by\_circulating\_immunoglobulin | 2 | 0 |  |  |  |  |  |  |  |  |
| GO:0003057\_regulation\_of\_the\_force\_of\_heart\_contraction\_by\_chemical\_signal | 2 | 0 |  |  |  |  |  |  |  |  |
| GO:0003099\_positive\_regulation\_of\_the\_force\_of\_heart\_contraction\_by\_chemical\_signal | 2 | 0 |  |  |  |  |  |  |  |  |
| GO:0005981\_regulation\_of\_glycogen\_catabolic\_process | 2 | 0 |  |  |  |  |  |  |  |  |
| GO:0006021\_inositol\_biosynthetic\_process | 2 | 0 |  |  |  |  |  |  |  |  |
| GO:0006042\_glucosamine\_biosynthetic\_process | 2 | 0 |  |  |  |  |  |  |  |  |
| GO:0006045\_N-acetylglucosamine\_biosynthetic\_process | 2 | 0 |  |  |  |  |  |  |  |  |
| GO:0006048\_UDP-N-acetylglucosamine\_biosynthetic\_process | 2 | 0 |  |  |  |  |  |  |  |  |
| GO:0006054\_N-acetylneuraminate\_metabolic\_process | 2 | 0 |  |  |  |  |  |  |  |  |
| GO:0006059\_hexitol\_metabolic\_process | 2 | 0 |  |  |  |  |  |  |  |  |
| GO:0006063\_uronic\_acid\_metabolic\_process | 2 | 0 |  |  |  |  |  |  |  |  |
| GO:0006068\_ethanol\_catabolic\_process | 2 | 0 |  |  |  |  |  |  |  |  |
| GO:0006083\_acetate\_metabolic\_process | 2 | 0 |  |  |  |  |  |  |  |  |
| GO:0006089\_lactate\_metabolic\_process | 2 | 0 |  |  |  |  |  |  |  |  |
| GO:0006105\_succinate\_metabolic\_process | 2 | 0 |  |  |  |  |  |  |  |  |
| GO:0006106\_fumarate\_metabolic\_process | 2 | 0 |  |  |  |  |  |  |  |  |
| GO:0006110\_regulation\_of\_glycolysis | 2 | 0 |  |  |  |  |  |  |  |  |
| GO:0006113\_fermentation | 2 | 0 |  |  |  |  |  |  |  |  |
| GO:0006114\_glycerol\_biosynthetic\_process | 2 | 0 |  |  |  |  |  |  |  |  |
| GO:0006122\_mitochondrial\_electron\_transport\_\_ubiquinol\_to\_cytochrome\_c | 2 | 0 |  |  |  |  |  |  |  |  |
| GO:0006152\_purine\_nucleoside\_catabolic\_process | 2 | 0 |  |  |  |  |  |  |  |  |
| GO:0006168\_adenine\_salvage | 2 | 0 |  |  |  |  |  |  |  |  |
| GO:0006200\_ATP\_catabolic\_process | 2 | 0 |  |  |  |  |  |  |  |  |
| GO:0006206\_pyrimidine\_base\_metabolic\_process | 2 | 0 |  |  |  |  |  |  |  |  |
| GO:0006213\_pyrimidine\_nucleoside\_metabolic\_process | 2 | 0 |  |  |  |  |  |  |  |  |
| GO:0006265\_DNA\_topological\_change | 2 | 0 |  |  |  |  |  |  |  |  |
| GO:0006278\_RNA-dependent\_DNA\_replication | 2 | 0 |  |  |  |  |  |  |  |  |
| GO:0006312\_mitotic\_recombination | 2 | 0 |  |  |  |  |  |  |  |  |
| GO:0006398\_histone\_mRNA\_3'-end\_processing | 2 | 0 |  |  |  |  |  |  |  |  |
| GO:0006418\_tRNA\_aminoacylation\_for\_protein\_translation | 2 | 0 |  |  |  |  |  |  |  |  |
| GO:0006451\_translational\_readthrough | 2 | 0 |  |  |  |  |  |  |  |  |
| GO:0006477\_protein\_amino\_acid\_sulfation | 2 | 0 |  |  |  |  |  |  |  |  |
| GO:0006482\_protein\_amino\_acid\_demethylation | 2 | 0 |  |  |  |  |  |  |  |  |
| GO:0006499\_N-terminal\_protein\_myristoylation | 2 | 0 |  |  |  |  |  |  |  |  |
| GO:0006525\_arginine\_metabolic\_process | 2 | 0 |  |  |  |  |  |  |  |  |
| GO:0006527\_arginine\_catabolic\_process | 2 | 0 |  |  |  |  |  |  |  |  |
| GO:0006532\_aspartate\_biosynthetic\_process | 2 | 0 |  |  |  |  |  |  |  |  |
| GO:0006538\_glutamate\_catabolic\_process | 2 | 0 |  |  |  |  |  |  |  |  |
| GO:0006558\_L-phenylalanine\_metabolic\_process | 2 | 0 |  |  |  |  |  |  |  |  |
| GO:0006563\_L-serine\_metabolic\_process | 2 | 0 |  |  |  |  |  |  |  |  |
| GO:0006566\_threonine\_metabolic\_process | 2 | 0 |  |  |  |  |  |  |  |  |
| GO:0006568\_tryptophan\_metabolic\_process | 2 | 0 |  |  |  |  |  |  |  |  |
| GO:0006583\_melanin\_biosynthetic\_process\_from\_tyrosine | 2 | 0 |  |  |  |  |  |  |  |  |
| GO:0006600\_creatine\_metabolic\_process | 2 | 0 |  |  |  |  |  |  |  |  |
| GO:0006603\_phosphocreatine\_metabolic\_process | 2 | 0 |  |  |  |  |  |  |  |  |
| GO:0006610\_ribosomal\_protein\_import\_into\_nucleus | 2 | 0 |  |  |  |  |  |  |  |  |
| GO:0006642\_triglyceride\_mobilization | 2 | 0 |  |  |  |  |  |  |  |  |
| GO:0006649\_phospholipid\_transfer\_to\_membrane | 2 | 0 |  |  |  |  |  |  |  |  |
| GO:0006681\_galactosylceramide\_metabolic\_process | 2 | 0 |  |  |  |  |  |  |  |  |
| GO:0006686\_sphingomyelin\_biosynthetic\_process | 2 | 0 |  |  |  |  |  |  |  |  |
| GO:0006702\_androgen\_biosynthetic\_process | 2 | 0 |  |  |  |  |  |  |  |  |
| GO:0006750\_glutathione\_biosynthetic\_process | 2 | 0 |  |  |  |  |  |  |  |  |
| GO:0006760\_folic\_acid\_and\_derivative\_metabolic\_process | 2 | 0 |  |  |  |  |  |  |  |  |
| GO:0006808\_regulation\_of\_nitrogen\_utilization | 2 | 0 |  |  |  |  |  |  |  |  |
| GO:0006868\_glutamine\_transport | 2 | 0 |  |  |  |  |  |  |  |  |
| GO:0006907\_pinocytosis | 2 | 0 |  |  |  |  |  |  |  |  |
| GO:0006925\_inflammatory\_cell\_apoptosis | 2 | 0 |  |  |  |  |  |  |  |  |
| GO:0006977\_DNA\_damage\_response\_\_signal\_transduction\_by\_p53\_class\_mediator\_resulting\_in\_cell\_cycle\_arrest | 2 | 0 |  |  |  |  |  |  |  |  |
| GO:0006991\_response\_to\_sterol\_depletion | 2 | 0 |  |  |  |  |  |  |  |  |
| GO:0007004\_telomere\_maintenance\_via\_telomerase | 2 | 0 |  |  |  |  |  |  |  |  |
| GO:0007020\_microtubule\_nucleation | 2 | 0 |  |  |  |  |  |  |  |  |
| GO:0007030\_Golgi\_organization | 2 | 0 |  |  |  |  |  |  |  |  |
| GO:0007035\_vacuolar\_acidification | 2 | 0 |  |  |  |  |  |  |  |  |
| GO:0007042\_lysosomal\_lumen\_acidification | 2 | 0 |  |  |  |  |  |  |  |  |
| GO:0007060\_male\_meiosis\_chromosome\_segregation | 2 | 0 |  |  |  |  |  |  |  |  |
| GO:0007089\_traversing\_start\_control\_point\_of\_mitotic\_cell\_cycle | 2 | 0 |  |  |  |  |  |  |  |  |
| GO:0007094\_mitotic\_cell\_cycle\_spindle\_assembly\_checkpoint | 2 | 0 |  |  |  |  |  |  |  |  |
| GO:0007097\_nuclear\_migration | 2 | 0 |  |  |  |  |  |  |  |  |
| GO:0007100\_mitotic\_centrosome\_separation | 2 | 0 |  |  |  |  |  |  |  |  |
| GO:0007132\_meiotic\_metaphase\_I | 2 | 0 |  |  |  |  |  |  |  |  |
| GO:0007171\_activation\_of\_transmembrane\_receptor\_protein\_tyrosine\_kinase\_activity | 2 | 0 |  |  |  |  |  |  |  |  |
| GO:0007182\_common-partner\_SMAD\_protein\_phosphorylation | 2 | 0 |  |  |  |  |  |  |  |  |
| GO:0007185\_transmembrane\_receptor\_protein\_tyrosine\_phosphatase\_signaling\_pathway | 2 | 0 |  |  |  |  |  |  |  |  |
| GO:0007205\_activation\_of\_protein\_kinase\_C\_activity\_by\_G-protein\_coupled\_receptor\_protein\_signaling\_pathway | 2 | 0 |  |  |  |  |  |  |  |  |
| GO:0007210\_serotonin\_receptor\_signaling\_pathway | 2 | 0 |  |  |  |  |  |  |  |  |
| GO:0007220\_Notch\_receptor\_processing | 2 | 0 |  |  |  |  |  |  |  |  |
| GO:0007256\_activation\_of\_JNKK\_activity | 2 | 0 |  |  |  |  |  |  |  |  |
| GO:0007258\_JUN\_phosphorylation | 2 | 0 |  |  |  |  |  |  |  |  |
| GO:0007263\_nitric\_oxide\_mediated\_signal\_transduction | 2 | 0 |  |  |  |  |  |  |  |  |
| GO:0007289\_spermatid\_nucleus\_differentiation | 2 | 0 |  |  |  |  |  |  |  |  |
| GO:0007343\_egg\_activation | 2 | 0 |  |  |  |  |  |  |  |  |
| GO:0007351\_tripartite\_regional\_subdivision | 2 | 0 |  |  |  |  |  |  |  |  |
| GO:0007418\_ventral\_midline\_development | 2 | 0 |  |  |  |  |  |  |  |  |
| GO:0007494\_midgut\_development | 2 | 0 |  |  |  |  |  |  |  |  |
| GO:0007527\_adult\_somatic\_muscle\_development | 2 | 0 |  |  |  |  |  |  |  |  |
| GO:0007549\_dosage\_compensation | 2 | 0 |  |  |  |  |  |  |  |  |
| GO:0007571\_age-dependent\_general\_metabolic\_decline | 2 | 0 |  |  |  |  |  |  |  |  |
| GO:0007603\_phototransduction\_\_visible\_light | 2 | 0 |  |  |  |  |  |  |  |  |
| GO:0007619\_courtship\_behavior | 2 | 0 |  |  |  |  |  |  |  |  |
| GO:0008065\_establishment\_of\_blood-nerve\_barrier | 2 | 0 |  |  |  |  |  |  |  |  |
| GO:0008089\_anterograde\_axon\_cargo\_transport | 2 | 0 |  |  |  |  |  |  |  |  |
| GO:0008210\_estrogen\_metabolic\_process | 2 | 0 |  |  |  |  |  |  |  |  |
| GO:0008212\_mineralocorticoid\_metabolic\_process | 2 | 0 |  |  |  |  |  |  |  |  |
| GO:0008214\_protein\_amino\_acid\_dealkylation | 2 | 0 |  |  |  |  |  |  |  |  |
| GO:0008228\_opsonization | 2 | 0 |  |  |  |  |  |  |  |  |
| GO:0008272\_sulfate\_transport | 2 | 0 |  |  |  |  |  |  |  |  |
| GO:0008291\_acetylcholine\_metabolic\_process | 2 | 0 |  |  |  |  |  |  |  |  |
| GO:0008298\_intracellular\_mRNA\_localization | 2 | 0 |  |  |  |  |  |  |  |  |
| GO:0008334\_histone\_mRNA\_metabolic\_process | 2 | 0 |  |  |  |  |  |  |  |  |
| GO:0008356\_asymmetric\_cell\_division | 2 | 0 |  |  |  |  |  |  |  |  |
| GO:0008582\_regulation\_of\_synaptic\_growth\_at\_neuromuscular\_junction | 2 | 0 |  |  |  |  |  |  |  |  |
| GO:0008594\_photoreceptor\_cell\_morphogenesis | 2 | 0 |  |  |  |  |  |  |  |  |
| GO:0008595\_determination\_of\_anterior\_posterior\_axis\_\_embryo | 2 | 0 |  |  |  |  |  |  |  |  |
| GO:0008608\_attachment\_of\_spindle\_microtubules\_to\_kinetochore | 2 | 0 |  |  |  |  |  |  |  |  |
| GO:0008616\_queuosine\_biosynthetic\_process | 2 | 0 |  |  |  |  |  |  |  |  |
| GO:0008617\_guanosine\_metabolic\_process | 2 | 0 |  |  |  |  |  |  |  |  |
| GO:0008618\_7-methylguanosine\_metabolic\_process | 2 | 0 |  |  |  |  |  |  |  |  |
| GO:0008634\_negative\_regulation\_of\_survival\_gene\_product\_expression | 2 | 0 |  |  |  |  |  |  |  |  |
| GO:0009048\_dosage\_compensation\_\_by\_inactivation\_of\_X\_chromosome | 2 | 0 |  |  |  |  |  |  |  |  |
| GO:0009070\_serine\_family\_amino\_acid\_biosynthetic\_process | 2 | 0 |  |  |  |  |  |  |  |  |
| GO:0009071\_serine\_family\_amino\_acid\_catabolic\_process | 2 | 0 |  |  |  |  |  |  |  |  |
| GO:0009074\_aromatic\_amino\_acid\_family\_catabolic\_process | 2 | 0 |  |  |  |  |  |  |  |  |
| GO:0009083\_branched\_chain\_family\_amino\_acid\_catabolic\_process | 2 | 0 |  |  |  |  |  |  |  |  |
| GO:0009093\_cysteine\_catabolic\_process | 2 | 0 |  |  |  |  |  |  |  |  |
| GO:0009120\_deoxyribonucleoside\_metabolic\_process | 2 | 0 |  |  |  |  |  |  |  |  |
| GO:0009125\_nucleoside\_monophosphate\_catabolic\_process | 2 | 0 |  |  |  |  |  |  |  |  |
| GO:0009126\_purine\_nucleoside\_monophosphate\_metabolic\_process | 2 | 0 |  |  |  |  |  |  |  |  |
| GO:0009142\_nucleoside\_triphosphate\_biosynthetic\_process | 2 | 0 |  |  |  |  |  |  |  |  |
| GO:0009161\_ribonucleoside\_monophosphate\_metabolic\_process | 2 | 0 |  |  |  |  |  |  |  |  |
| GO:0009164\_nucleoside\_catabolic\_process | 2 | 0 |  |  |  |  |  |  |  |  |
| GO:0009167\_purine\_ribonucleoside\_monophosphate\_metabolic\_process | 2 | 0 |  |  |  |  |  |  |  |  |
| GO:0009202\_deoxyribonucleoside\_triphosphate\_biosynthetic\_process | 2 | 0 |  |  |  |  |  |  |  |  |
| GO:0009203\_ribonucleoside\_triphosphate\_catabolic\_process | 2 | 0 |  |  |  |  |  |  |  |  |
| GO:0009207\_purine\_ribonucleoside\_triphosphate\_catabolic\_process | 2 | 0 |  |  |  |  |  |  |  |  |
| GO:0009219\_pyrimidine\_deoxyribonucleotide\_metabolic\_process | 2 | 0 |  |  |  |  |  |  |  |  |
| GO:0009265\_2'-deoxyribonucleotide\_biosynthetic\_process | 2 | 0 |  |  |  |  |  |  |  |  |
| GO:0009268\_response\_to\_pH | 2 | 0 |  |  |  |  |  |  |  |  |
| GO:0009313\_oligosaccharide\_catabolic\_process | 2 | 0 |  |  |  |  |  |  |  |  |
| GO:0009395\_phospholipid\_catabolic\_process | 2 | 0 |  |  |  |  |  |  |  |  |
| GO:0009435\_NAD\_biosynthetic\_process | 2 | 0 |  |  |  |  |  |  |  |  |
| GO:0009608\_response\_to\_symbiont | 2 | 0 |  |  |  |  |  |  |  |  |
| GO:0009609\_response\_to\_symbiotic\_bacterium | 2 | 0 |  |  |  |  |  |  |  |  |
| GO:0009649\_entrainment\_of\_circadian\_clock | 2 | 0 |  |  |  |  |  |  |  |  |
| GO:0009996\_negative\_regulation\_of\_cell\_fate\_specification | 2 | 0 |  |  |  |  |  |  |  |  |
| GO:0010002\_cardioblast\_differentiation | 2 | 0 |  |  |  |  |  |  |  |  |
| GO:0010149\_senescence | 2 | 0 |  |  |  |  |  |  |  |  |
| GO:0010225\_response\_to\_UV-C | 2 | 0 |  |  |  |  |  |  |  |  |
| GO:0010389\_regulation\_of\_G2\_M\_transition\_of\_mitotic\_cell\_cycle | 2 | 0 |  |  |  |  |  |  |  |  |
| GO:0010458\_exit\_from\_mitosis | 2 | 0 |  |  |  |  |  |  |  |  |
| GO:0010459\_negative\_regulation\_of\_heart\_rate | 2 | 0 |  |  |  |  |  |  |  |  |
| GO:0010559\_regulation\_of\_glycoprotein\_biosynthetic\_process | 2 | 0 |  |  |  |  |  |  |  |  |
| GO:0010633\_negative\_regulation\_of\_epithelial\_cell\_migration | 2 | 0 |  |  |  |  |  |  |  |  |
| GO:0010677\_negative\_regulation\_of\_cellular\_carbohydrate\_metabolic\_process | 2 | 0 |  |  |  |  |  |  |  |  |
| GO:0010718\_positive\_regulation\_of\_epithelial\_to\_mesenchymal\_transition | 2 | 0 |  |  |  |  |  |  |  |  |
| GO:0010742\_foam\_cell\_differentiation | 2 | 0 |  |  |  |  |  |  |  |  |
| GO:0010743\_regulation\_of\_foam\_cell\_differentiation | 2 | 0 |  |  |  |  |  |  |  |  |
| GO:0010744\_positive\_regulation\_of\_foam\_cell\_differentiation | 2 | 0 |  |  |  |  |  |  |  |  |
| GO:0010765\_positive\_regulation\_of\_sodium\_ion\_transport | 2 | 0 |  |  |  |  |  |  |  |  |
| GO:0010766\_negative\_regulation\_of\_sodium\_ion\_transport | 2 | 0 |  |  |  |  |  |  |  |  |
| GO:0010770\_positive\_regulation\_of\_cell\_morphogenesis\_involved\_in\_differentiation | 2 | 0 |  |  |  |  |  |  |  |  |
| GO:0010771\_negative\_regulation\_of\_cell\_morphogenesis\_involved\_in\_differentiation | 2 | 0 |  |  |  |  |  |  |  |  |
| GO:0010833\_telomere\_maintenance\_via\_telomere\_lengthening | 2 | 0 |  |  |  |  |  |  |  |  |
| GO:0010862\_positive\_regulation\_of\_pathway-restricted\_SMAD\_protein\_phosphorylation | 2 | 0 |  |  |  |  |  |  |  |  |
| GO:0010872\_regulation\_of\_cholesterol\_esterification | 2 | 0 |  |  |  |  |  |  |  |  |
| GO:0010878\_cholesterol\_storage | 2 | 0 |  |  |  |  |  |  |  |  |
| GO:0010885\_regulation\_of\_cholesterol\_storage | 2 | 0 |  |  |  |  |  |  |  |  |
| GO:0010886\_positive\_regulation\_of\_cholesterol\_storage | 2 | 0 |  |  |  |  |  |  |  |  |
| GO:0010891\_negative\_regulation\_of\_sequestering\_of\_triglyceride | 2 | 0 |  |  |  |  |  |  |  |  |
| GO:0010896\_regulation\_of\_triglyceride\_catabolic\_process | 2 | 0 |  |  |  |  |  |  |  |  |
| GO:0010898\_positive\_regulation\_of\_triglyceride\_catabolic\_process | 2 | 0 |  |  |  |  |  |  |  |  |
| GO:0010907\_positive\_regulation\_of\_glucose\_metabolic\_process | 2 | 0 |  |  |  |  |  |  |  |  |
| GO:0014028\_notochord\_formation | 2 | 0 |  |  |  |  |  |  |  |  |
| GO:0014048\_regulation\_of\_glutamate\_secretion | 2 | 0 |  |  |  |  |  |  |  |  |
| GO:0014052\_regulation\_of\_gamma-aminobutyric\_acid\_secretion | 2 | 0 |  |  |  |  |  |  |  |  |
| GO:0014054\_positive\_regulation\_of\_gamma-aminobutyric\_acid\_secretion | 2 | 0 |  |  |  |  |  |  |  |  |
| GO:0014055\_acetylcholine\_secretion | 2 | 0 |  |  |  |  |  |  |  |  |
| GO:0014056\_regulation\_of\_acetylcholine\_secretion | 2 | 0 |  |  |  |  |  |  |  |  |
| GO:0014067\_negative\_regulation\_of\_phosphoinositide\_3-kinase\_cascade | 2 | 0 |  |  |  |  |  |  |  |  |
| GO:0014745\_negative\_regulation\_of\_muscle\_adaptation | 2 | 0 |  |  |  |  |  |  |  |  |
| GO:0014829\_vascular\_smooth\_muscle\_contraction | 2 | 0 |  |  |  |  |  |  |  |  |
| GO:0014850\_response\_to\_muscle\_activity | 2 | 0 |  |  |  |  |  |  |  |  |
| GO:0014866\_skeletal\_myofibril\_assembly | 2 | 0 |  |  |  |  |  |  |  |  |
| GO:0014888\_striated\_muscle\_adaptation | 2 | 0 |  |  |  |  |  |  |  |  |
| GO:0014916\_regulation\_of\_lung\_blood\_pressure | 2 | 0 |  |  |  |  |  |  |  |  |
| GO:0015671\_oxygen\_transport | 2 | 0 |  |  |  |  |  |  |  |  |
| GO:0015696\_ammonium\_transport | 2 | 0 |  |  |  |  |  |  |  |  |
| GO:0015732\_prostaglandin\_transport | 2 | 0 |  |  |  |  |  |  |  |  |
| GO:0015819\_lysine\_transport | 2 | 0 |  |  |  |  |  |  |  |  |
| GO:0015840\_urea\_transport | 2 | 0 |  |  |  |  |  |  |  |  |
| GO:0015860\_purine\_nucleoside\_transport | 2 | 0 |  |  |  |  |  |  |  |  |
| GO:0015870\_acetylcholine\_transport | 2 | 0 |  |  |  |  |  |  |  |  |
| GO:0015937\_coenzyme\_A\_biosynthetic\_process | 2 | 0 |  |  |  |  |  |  |  |  |
| GO:0016045\_detection\_of\_bacterium | 2 | 0 |  |  |  |  |  |  |  |  |
| GO:0016046\_detection\_of\_fungus | 2 | 0 |  |  |  |  |  |  |  |  |
| GO:0016080\_synaptic\_vesicle\_targeting | 2 | 0 |  |  |  |  |  |  |  |  |
| GO:0016199\_axon\_midline\_choice\_point\_recognition | 2 | 0 |  |  |  |  |  |  |  |  |
| GO:0016226\_iron-sulfur\_cluster\_assembly | 2 | 0 |  |  |  |  |  |  |  |  |
| GO:0016233\_telomere\_capping | 2 | 0 |  |  |  |  |  |  |  |  |
| GO:0016242\_negative\_regulation\_of\_macroautophagy | 2 | 0 |  |  |  |  |  |  |  |  |
| GO:0016441\_posttranscriptional\_gene\_silencing | 2 | 0 |  |  |  |  |  |  |  |  |
| GO:0016540\_protein\_autoprocessing | 2 | 0 |  |  |  |  |  |  |  |  |
| GO:0016572\_histone\_phosphorylation | 2 | 0 |  |  |  |  |  |  |  |  |
| GO:0016577\_histone\_demethylation | 2 | 0 |  |  |  |  |  |  |  |  |
| GO:0016584\_nucleosome\_positioning | 2 | 0 |  |  |  |  |  |  |  |  |
| GO:0016926\_protein\_desumoylation | 2 | 0 |  |  |  |  |  |  |  |  |
| GO:0017014\_protein\_amino\_acid\_nitrosylation | 2 | 0 |  |  |  |  |  |  |  |  |
| GO:0017144\_drug\_metabolic\_process | 2 | 0 |  |  |  |  |  |  |  |  |
| GO:0018094\_protein\_polyglycylation | 2 | 0 |  |  |  |  |  |  |  |  |
| GO:0018119\_peptidyl-cysteine\_S-nitrosylation | 2 | 0 |  |  |  |  |  |  |  |  |
| GO:0018125\_peptidyl-cysteine\_methylation | 2 | 0 |  |  |  |  |  |  |  |  |
| GO:0018205\_peptidyl-lysine\_modification | 2 | 0 |  |  |  |  |  |  |  |  |
| GO:0018319\_protein\_amino\_acid\_myristoylation | 2 | 0 |  |  |  |  |  |  |  |  |
| GO:0018377\_protein\_myristoylation | 2 | 0 |  |  |  |  |  |  |  |  |
| GO:0018401\_peptidyl-proline\_hydroxylation\_to\_4-hydroxy-L-proline | 2 | 0 |  |  |  |  |  |  |  |  |
| GO:0018993\_somatic\_sex\_determination | 2 | 0 |  |  |  |  |  |  |  |  |
| GO:0019067\_viral\_assembly\_\_maturation\_\_egress\_\_and\_release | 2 | 0 |  |  |  |  |  |  |  |  |
| GO:0019322\_pentose\_biosynthetic\_process | 2 | 0 |  |  |  |  |  |  |  |  |
| GO:0019370\_leukotriene\_biosynthetic\_process | 2 | 0 |  |  |  |  |  |  |  |  |
| GO:0019374\_galactolipid\_metabolic\_process | 2 | 0 |  |  |  |  |  |  |  |  |
| GO:0019401\_alditol\_biosynthetic\_process | 2 | 0 |  |  |  |  |  |  |  |  |
| GO:0019448\_L-cysteine\_catabolic\_process | 2 | 0 |  |  |  |  |  |  |  |  |
| GO:0019452\_L-cysteine\_catabolic\_process\_to\_taurine | 2 | 0 |  |  |  |  |  |  |  |  |
| GO:0019471\_4-hydroxyproline\_metabolic\_process | 2 | 0 |  |  |  |  |  |  |  |  |
| GO:0019511\_peptidyl-proline\_hydroxylation | 2 | 0 |  |  |  |  |  |  |  |  |
| GO:0019550\_glutamate\_catabolic\_process\_to\_aspartate | 2 | 0 |  |  |  |  |  |  |  |  |
| GO:0019551\_glutamate\_catabolic\_process\_to\_2-oxoglutarate | 2 | 0 |  |  |  |  |  |  |  |  |
| GO:0019585\_glucuronate\_metabolic\_process | 2 | 0 |  |  |  |  |  |  |  |  |
| GO:0019730\_antimicrobial\_humoral\_response | 2 | 0 |  |  |  |  |  |  |  |  |
| GO:0019740\_nitrogen\_utilization | 2 | 0 |  |  |  |  |  |  |  |  |
| GO:0019853\_L-ascorbic\_acid\_biosynthetic\_process | 2 | 0 |  |  |  |  |  |  |  |  |
| GO:0021506\_anterior\_neuropore\_closure | 2 | 0 |  |  |  |  |  |  |  |  |
| GO:0021524\_visceral\_motor\_neuron\_differentiation | 2 | 0 |  |  |  |  |  |  |  |  |
| GO:0021526\_medial\_motor\_column\_neuron\_differentiation | 2 | 0 |  |  |  |  |  |  |  |  |
| GO:0021557\_oculomotor\_nerve\_development | 2 | 0 |  |  |  |  |  |  |  |  |
| GO:0021558\_trochlear\_nerve\_development | 2 | 0 |  |  |  |  |  |  |  |  |
| GO:0021562\_vestibulocochlear\_nerve\_development | 2 | 0 |  |  |  |  |  |  |  |  |
| GO:0021568\_rhombomere\_2\_development | 2 | 0 |  |  |  |  |  |  |  |  |
| GO:0021578\_hindbrain\_maturation | 2 | 0 |  |  |  |  |  |  |  |  |
| GO:0021593\_rhombomere\_morphogenesis | 2 | 0 |  |  |  |  |  |  |  |  |
| GO:0021626\_central\_nervous\_system\_maturation | 2 | 0 |  |  |  |  |  |  |  |  |
| GO:0021658\_rhombomere\_3\_morphogenesis | 2 | 0 |  |  |  |  |  |  |  |  |
| GO:0021754\_facial\_nucleus\_development | 2 | 0 |  |  |  |  |  |  |  |  |
| GO:0021775\_smoothened\_signaling\_pathway\_involved\_in\_ventral\_spinal\_cord\_interneuron\_specification | 2 | 0 |  |  |  |  |  |  |  |  |
| GO:0021776\_smoothened\_signaling\_pathway\_involved\_in\_spinal\_cord\_motor\_neuron\_cell\_fate\_specification | 2 | 0 |  |  |  |  |  |  |  |  |
| GO:0021796\_cerebral\_cortex\_regionalization | 2 | 0 |  |  |  |  |  |  |  |  |
| GO:0021831\_embryonic\_olfactory\_bulb\_interneuron\_precursor\_migration | 2 | 0 |  |  |  |  |  |  |  |  |
| GO:0021869\_forebrain\_ventricular\_zone\_progenitor\_cell\_division | 2 | 0 |  |  |  |  |  |  |  |  |
| GO:0021873\_forebrain\_neuroblast\_division | 2 | 0 |  |  |  |  |  |  |  |  |
| GO:0021882\_regulation\_of\_transcription\_from\_RNA\_polymerase\_II\_promoter\_involved\_in\_forebrain\_neuron\_fate\_commitment | 2 | 0 |  |  |  |  |  |  |  |  |
| GO:0021893\_cerebral\_cortex\_GABAergic\_interneuron\_fate\_commitment | 2 | 0 |  |  |  |  |  |  |  |  |
| GO:0021898\_commitment\_of\_multipotent\_stem\_cells\_to\_the\_neuronal\_lineage\_in\_the\_forebrain | 2 | 0 |  |  |  |  |  |  |  |  |
| GO:0021932\_hindbrain\_radial\_glia\_guided\_cell\_migration | 2 | 0 |  |  |  |  |  |  |  |  |
| GO:0021965\_spinal\_cord\_ventral\_commissure\_morphogenesis | 2 | 0 |  |  |  |  |  |  |  |  |
| GO:0021985\_neurohypophysis\_development | 2 | 0 |  |  |  |  |  |  |  |  |
| GO:0021990\_neural\_plate\_formation | 2 | 0 |  |  |  |  |  |  |  |  |
| GO:0021995\_neuropore\_closure | 2 | 0 |  |  |  |  |  |  |  |  |
| GO:0022028\_tangential\_migration\_from\_the\_subventricular\_zone\_to\_the\_olfactory\_bulb | 2 | 0 |  |  |  |  |  |  |  |  |
| GO:0022401\_adaptation\_of\_signaling\_pathway | 2 | 0 |  |  |  |  |  |  |  |  |
| GO:0022408\_negative\_regulation\_of\_cell-cell\_adhesion | 2 | 0 |  |  |  |  |  |  |  |  |
| GO:0022410\_circadian\_sleep\_wake\_cycle\_process | 2 | 0 |  |  |  |  |  |  |  |  |
| GO:0030046\_parallel\_actin\_filament\_bundle\_formation | 2 | 0 |  |  |  |  |  |  |  |  |
| GO:0030049\_muscle\_filament\_sliding | 2 | 0 |  |  |  |  |  |  |  |  |
| GO:0030050\_vesicle\_transport\_along\_actin\_filament | 2 | 0 |  |  |  |  |  |  |  |  |
| GO:0030071\_regulation\_of\_mitotic\_metaphase\_anaphase\_transition | 2 | 0 |  |  |  |  |  |  |  |  |
| GO:0030147\_natriuresis | 2 | 0 |  |  |  |  |  |  |  |  |
| GO:0030202\_heparin\_metabolic\_process | 2 | 0 |  |  |  |  |  |  |  |  |
| GO:0030219\_megakaryocyte\_differentiation | 2 | 0 |  |  |  |  |  |  |  |  |
| GO:0030223\_neutrophil\_differentiation | 2 | 0 |  |  |  |  |  |  |  |  |
| GO:0030240\_muscle\_thin\_filament\_assembly | 2 | 0 |  |  |  |  |  |  |  |  |
| GO:0030259\_lipid\_glycosylation | 2 | 0 |  |  |  |  |  |  |  |  |
| GO:0030397\_membrane\_disassembly | 2 | 0 |  |  |  |  |  |  |  |  |
| GO:0030502\_negative\_regulation\_of\_bone\_mineralization | 2 | 0 |  |  |  |  |  |  |  |  |
| GO:0030644\_cellular\_chloride\_ion\_homeostasis | 2 | 0 |  |  |  |  |  |  |  |  |
| GO:0030825\_positive\_regulation\_of\_cGMP\_metabolic\_process | 2 | 0 |  |  |  |  |  |  |  |  |
| GO:0030828\_positive\_regulation\_of\_cGMP\_biosynthetic\_process | 2 | 0 |  |  |  |  |  |  |  |  |
| GO:0030835\_negative\_regulation\_of\_actin\_filament\_depolymerization | 2 | 0 |  |  |  |  |  |  |  |  |
| GO:0030837\_negative\_regulation\_of\_actin\_filament\_polymerization | 2 | 0 |  |  |  |  |  |  |  |  |
| GO:0030852\_regulation\_of\_granulocyte\_differentiation | 2 | 0 |  |  |  |  |  |  |  |  |
| GO:0030885\_regulation\_of\_myeloid\_dendritic\_cell\_activation | 2 | 0 |  |  |  |  |  |  |  |  |
| GO:0030910\_olfactory\_placode\_formation | 2 | 0 |  |  |  |  |  |  |  |  |
| GO:0030948\_negative\_regulation\_of\_vascular\_endothelial\_growth\_factor\_receptor\_signaling\_pathway | 2 | 0 |  |  |  |  |  |  |  |  |
| GO:0030953\_spindle\_astral\_microtubule\_organization | 2 | 0 |  |  |  |  |  |  |  |  |
| GO:0031050\_dsRNA\_fragmentation | 2 | 0 |  |  |  |  |  |  |  |  |
| GO:0031061\_negative\_regulation\_of\_histone\_methylation | 2 | 0 |  |  |  |  |  |  |  |  |
| GO:0031119\_tRNA\_pseudouridine\_synthesis | 2 | 0 |  |  |  |  |  |  |  |  |
| GO:0031163\_metallo-sulfur\_cluster\_assembly | 2 | 0 |  |  |  |  |  |  |  |  |
| GO:0031223\_auditory\_behavior | 2 | 0 |  |  |  |  |  |  |  |  |
| GO:0031296\_B\_cell\_costimulation | 2 | 0 |  |  |  |  |  |  |  |  |
| GO:0031338\_regulation\_of\_vesicle\_fusion | 2 | 0 |  |  |  |  |  |  |  |  |
| GO:0031573\_intra-S\_DNA\_damage\_checkpoint | 2 | 0 |  |  |  |  |  |  |  |  |
| GO:0031577\_spindle\_checkpoint | 2 | 0 |  |  |  |  |  |  |  |  |
| GO:0031629\_synaptic\_vesicle\_fusion\_to\_presynaptic\_membrane | 2 | 0 |  |  |  |  |  |  |  |  |
| GO:0031630\_regulation\_of\_synaptic\_vesicle\_fusion\_to\_presynaptic\_membrane | 2 | 0 |  |  |  |  |  |  |  |  |
| GO:0031664\_regulation\_of\_lipopolysaccharide-mediated\_signaling\_pathway | 2 | 0 |  |  |  |  |  |  |  |  |
| GO:0031670\_cellular\_response\_to\_nutrient | 2 | 0 |  |  |  |  |  |  |  |  |
| GO:0031848\_protection\_from\_non-homologous\_end\_joining\_at\_telomere | 2 | 0 |  |  |  |  |  |  |  |  |
| GO:0031946\_regulation\_of\_glucocorticoid\_biosynthetic\_process | 2 | 0 |  |  |  |  |  |  |  |  |
| GO:0031952\_regulation\_of\_protein\_amino\_acid\_autophosphorylation | 2 | 0 |  |  |  |  |  |  |  |  |
| GO:0031953\_negative\_regulation\_of\_protein\_amino\_acid\_autophosphorylation | 2 | 0 |  |  |  |  |  |  |  |  |
| GO:0031958\_corticosteroid\_receptor\_signaling\_pathway | 2 | 0 |  |  |  |  |  |  |  |  |
| GO:0031987\_locomotion\_involved\_in\_locomotory\_behavior | 2 | 0 |  |  |  |  |  |  |  |  |
| GO:0032096\_negative\_regulation\_of\_response\_to\_food | 2 | 0 |  |  |  |  |  |  |  |  |
| GO:0032099\_negative\_regulation\_of\_appetite | 2 | 0 |  |  |  |  |  |  |  |  |
| GO:0032106\_positive\_regulation\_of\_response\_to\_extracellular\_stimulus | 2 | 0 |  |  |  |  |  |  |  |  |
| GO:0032109\_positive\_regulation\_of\_response\_to\_nutrient\_levels | 2 | 0 |  |  |  |  |  |  |  |  |
| GO:0032226\_positive\_regulation\_of\_synaptic\_transmission\_\_dopaminergic | 2 | 0 |  |  |  |  |  |  |  |  |
| GO:0032230\_positive\_regulation\_of\_synaptic\_transmission\_\_GABAergic | 2 | 0 |  |  |  |  |  |  |  |  |
| GO:0032234\_regulation\_of\_calcium\_ion\_transport\_via\_store-operated\_calcium\_channel\_activity | 2 | 0 |  |  |  |  |  |  |  |  |
| GO:0032236\_positive\_regulation\_of\_calcium\_ion\_transport\_via\_store-operated\_calcium\_channel\_activity | 2 | 0 |  |  |  |  |  |  |  |  |
| GO:0032309\_icosanoid\_secretion | 2 | 0 |  |  |  |  |  |  |  |  |
| GO:0032328\_alanine\_transport | 2 | 0 |  |  |  |  |  |  |  |  |
| GO:0032341\_aldosterone\_metabolic\_process | 2 | 0 |  |  |  |  |  |  |  |  |
| GO:0032351\_negative\_regulation\_of\_hormone\_metabolic\_process | 2 | 0 |  |  |  |  |  |  |  |  |
| GO:0032353\_negative\_regulation\_of\_hormone\_biosynthetic\_process | 2 | 0 |  |  |  |  |  |  |  |  |
| GO:0032435\_negative\_regulation\_of\_proteasomal\_ubiquitin-dependent\_protein\_catabolic\_process | 2 | 0 |  |  |  |  |  |  |  |  |
| GO:0032471\_reduction\_of\_endoplasmic\_reticulum\_calcium\_ion\_concentration | 2 | 0 |  |  |  |  |  |  |  |  |
| GO:0032481\_positive\_regulation\_of\_type\_I\_interferon\_production | 2 | 0 |  |  |  |  |  |  |  |  |
| GO:0032488\_Cdc42\_protein\_signal\_transduction | 2 | 0 |  |  |  |  |  |  |  |  |
| GO:0032489\_regulation\_of\_Cdc42\_protein\_signal\_transduction | 2 | 0 |  |  |  |  |  |  |  |  |
| GO:0032495\_response\_to\_muramyl\_dipeptide | 2 | 0 |  |  |  |  |  |  |  |  |
| GO:0032604\_granulocyte\_macrophage\_colony-stimulating\_factor\_production | 2 | 0 |  |  |  |  |  |  |  |  |
| GO:0032616\_interleukin-13\_production | 2 | 0 |  |  |  |  |  |  |  |  |
| GO:0032645\_regulation\_of\_granulocyte\_macrophage\_colony-stimulating\_factor\_production | 2 | 0 |  |  |  |  |  |  |  |  |
| GO:0032672\_regulation\_of\_interleukin-3\_production | 2 | 0 |  |  |  |  |  |  |  |  |
| GO:0032695\_negative\_regulation\_of\_interleukin-12\_production | 2 | 0 |  |  |  |  |  |  |  |  |
| GO:0032714\_negative\_regulation\_of\_interleukin-5\_production | 2 | 0 |  |  |  |  |  |  |  |  |
| GO:0032722\_positive\_regulation\_of\_chemokine\_production | 2 | 0 |  |  |  |  |  |  |  |  |
| GO:0032743\_positive\_regulation\_of\_interleukin-2\_production | 2 | 0 |  |  |  |  |  |  |  |  |
| GO:0032762\_mast\_cell\_cytokine\_production | 2 | 0 |  |  |  |  |  |  |  |  |
| GO:0032763\_regulation\_of\_mast\_cell\_cytokine\_production | 2 | 0 |  |  |  |  |  |  |  |  |
| GO:0032768\_regulation\_of\_monooxygenase\_activity | 2 | 0 |  |  |  |  |  |  |  |  |
| GO:0032788\_saturated\_monocarboxylic\_acid\_metabolic\_process | 2 | 0 |  |  |  |  |  |  |  |  |
| GO:0032789\_unsaturated\_monocarboxylic\_acid\_metabolic\_process | 2 | 0 |  |  |  |  |  |  |  |  |
| GO:0032796\_uropod\_organization | 2 | 0 |  |  |  |  |  |  |  |  |
| GO:0032800\_receptor\_biosynthetic\_process | 2 | 0 |  |  |  |  |  |  |  |  |
| GO:0032801\_receptor\_catabolic\_process | 2 | 0 |  |  |  |  |  |  |  |  |
| GO:0032829\_regulation\_of\_CD4-positive\_\_CD25-positive\_\_alpha-beta\_regulatory\_T\_cell\_differentiation | 2 | 0 |  |  |  |  |  |  |  |  |
| GO:0032831\_positive\_regulation\_of\_CD4-positive\_\_CD25-positive\_\_alpha-beta\_regulatory\_T\_cell\_differentiation | 2 | 0 |  |  |  |  |  |  |  |  |
| GO:0032892\_positive\_regulation\_of\_organic\_acid\_transport | 2 | 0 |  |  |  |  |  |  |  |  |
| GO:0032905\_transforming\_growth\_factor-beta1\_production | 2 | 0 |  |  |  |  |  |  |  |  |
| GO:0032908\_regulation\_of\_transforming\_growth\_factor-beta1\_production | 2 | 0 |  |  |  |  |  |  |  |  |
| GO:0032914\_positive\_regulation\_of\_transforming\_growth\_factor-beta1\_production | 2 | 0 |  |  |  |  |  |  |  |  |
| GO:0032933\_SREBP-mediated\_signaling\_pathway | 2 | 0 |  |  |  |  |  |  |  |  |
| GO:0032957\_inositol\_trisphosphate\_metabolic\_process | 2 | 0 |  |  |  |  |  |  |  |  |
| GO:0032958\_inositol\_phosphate\_biosynthetic\_process | 2 | 0 |  |  |  |  |  |  |  |  |
| GO:0032959\_inositol\_trisphosphate\_biosynthetic\_process | 2 | 0 |  |  |  |  |  |  |  |  |
| GO:0033092\_positive\_regulation\_of\_immature\_T\_cell\_proliferation\_in\_the\_thymus | 2 | 0 |  |  |  |  |  |  |  |  |
| GO:0033136\_serine\_phosphorylation\_of\_STAT3\_protein | 2 | 0 |  |  |  |  |  |  |  |  |
| GO:0033145\_positive\_regulation\_of\_steroid\_hormone\_receptor\_signaling\_pathway | 2 | 0 |  |  |  |  |  |  |  |  |
| GO:0033147\_negative\_regulation\_of\_estrogen\_receptor\_signaling\_pathway | 2 | 0 |  |  |  |  |  |  |  |  |
| GO:0033148\_positive\_regulation\_of\_estrogen\_receptor\_signaling\_pathway | 2 | 0 |  |  |  |  |  |  |  |  |
| GO:0033194\_response\_to\_hydroperoxide | 2 | 0 |  |  |  |  |  |  |  |  |
| GO:0033275\_actin-myosin\_filament\_sliding | 2 | 0 |  |  |  |  |  |  |  |  |
| GO:0033280\_response\_to\_vitamin\_D | 2 | 0 |  |  |  |  |  |  |  |  |
| GO:0033364\_mast\_cell\_secretory\_granule\_organization | 2 | 0 |  |  |  |  |  |  |  |  |
| GO:0033504\_floor\_plate\_development | 2 | 0 |  |  |  |  |  |  |  |  |
| GO:0033603\_positive\_regulation\_of\_dopamine\_secretion | 2 | 0 |  |  |  |  |  |  |  |  |
| GO:0033605\_positive\_regulation\_of\_catecholamine\_secretion | 2 | 0 |  |  |  |  |  |  |  |  |
| GO:0033622\_integrin\_activation | 2 | 0 |  |  |  |  |  |  |  |  |
| GO:0033623\_regulation\_of\_integrin\_activation | 2 | 0 |  |  |  |  |  |  |  |  |
| GO:0033625\_positive\_regulation\_of\_integrin\_activation | 2 | 0 |  |  |  |  |  |  |  |  |
| GO:0033700\_phospholipid\_efflux | 2 | 0 |  |  |  |  |  |  |  |  |
| GO:0034142\_toll-like\_receptor\_4\_signaling\_pathway | 2 | 0 |  |  |  |  |  |  |  |  |
| GO:0034310\_monohydric\_alcohol\_catabolic\_process | 2 | 0 |  |  |  |  |  |  |  |  |
| GO:0034341\_response\_to\_interferon-gamma | 2 | 0 |  |  |  |  |  |  |  |  |
| GO:0034370\_triglyceride-rich\_lipoprotein\_particle\_remodeling | 2 | 0 |  |  |  |  |  |  |  |  |
| GO:0034374\_low-density\_lipoprotein\_particle\_remodeling | 2 | 0 |  |  |  |  |  |  |  |  |
| GO:0034377\_plasma\_lipoprotein\_particle\_assembly | 2 | 0 |  |  |  |  |  |  |  |  |
| GO:0034384\_high-density\_lipoprotein\_particle\_clearance | 2 | 0 |  |  |  |  |  |  |  |  |
| GO:0034433\_steroid\_esterification | 2 | 0 |  |  |  |  |  |  |  |  |
| GO:0034434\_sterol\_esterification | 2 | 0 |  |  |  |  |  |  |  |  |
| GO:0034435\_cholesterol\_esterification | 2 | 0 |  |  |  |  |  |  |  |  |
| GO:0034453\_microtubule\_anchoring | 2 | 0 |  |  |  |  |  |  |  |  |
| GO:0034644\_cellular\_response\_to\_UV | 2 | 0 |  |  |  |  |  |  |  |  |
| GO:0034755\_iron\_ion\_transmembrane\_transport | 2 | 0 |  |  |  |  |  |  |  |  |
| GO:0034764\_positive\_regulation\_of\_transmembrane\_transport | 2 | 0 |  |  |  |  |  |  |  |  |
| GO:0035021\_negative\_regulation\_of\_Rac\_protein\_signal\_transduction | 2 | 0 |  |  |  |  |  |  |  |  |
| GO:0035054\_embryonic\_heart\_tube\_anterior\_posterior\_pattern\_formation | 2 | 0 |  |  |  |  |  |  |  |  |
| GO:0035092\_sperm\_chromatin\_condensation | 2 | 0 |  |  |  |  |  |  |  |  |
| GO:0035110\_leg\_morphogenesis | 2 | 0 |  |  |  |  |  |  |  |  |
| GO:0035117\_embryonic\_arm\_morphogenesis | 2 | 0 |  |  |  |  |  |  |  |  |
| GO:0035120\_post-embryonic\_appendage\_morphogenesis | 2 | 0 |  |  |  |  |  |  |  |  |
| GO:0035127\_post-embryonic\_limb\_morphogenesis | 2 | 0 |  |  |  |  |  |  |  |  |
| GO:0035129\_post-embryonic\_hindlimb\_morphogenesis | 2 | 0 |  |  |  |  |  |  |  |  |
| GO:0035140\_arm\_morphogenesis | 2 | 0 |  |  |  |  |  |  |  |  |
| GO:0035194\_posttranscriptional\_gene\_silencing\_by\_RNA | 2 | 0 |  |  |  |  |  |  |  |  |
| GO:0035195\_gene\_silencing\_by\_miRNA | 2 | 0 |  |  |  |  |  |  |  |  |
| GO:0035196\_gene\_silencing\_by\_miRNA\_\_production\_of\_miRNAs | 2 | 0 |  |  |  |  |  |  |  |  |
| GO:0035315\_hair\_cell\_differentiation | 2 | 0 |  |  |  |  |  |  |  |  |
| GO:0040009\_regulation\_of\_growth\_rate | 2 | 0 |  |  |  |  |  |  |  |  |
| GO:0040037\_negative\_regulation\_of\_fibroblast\_growth\_factor\_receptor\_signaling\_pathway | 2 | 0 |  |  |  |  |  |  |  |  |
| GO:0042119\_neutrophil\_activation | 2 | 0 |  |  |  |  |  |  |  |  |
| GO:0042147\_retrograde\_transport\_\_endosome\_to\_Golgi | 2 | 0 |  |  |  |  |  |  |  |  |
| GO:0042223\_interleukin-3\_biosynthetic\_process | 2 | 0 |  |  |  |  |  |  |  |  |
| GO:0042249\_establishment\_of\_polarity\_of\_embryonic\_epithelium | 2 | 0 |  |  |  |  |  |  |  |  |
| GO:0042253\_granulocyte\_macrophage\_colony-stimulating\_factor\_biosynthetic\_process | 2 | 0 |  |  |  |  |  |  |  |  |
| GO:0042270\_protection\_from\_natural\_killer\_cell\_mediated\_cytotoxicity | 2 | 0 |  |  |  |  |  |  |  |  |
| GO:0042312\_regulation\_of\_vasodilation | 2 | 0 |  |  |  |  |  |  |  |  |
| GO:0042346\_positive\_regulation\_of\_NF-kappaB\_import\_into\_nucleus | 2 | 0 |  |  |  |  |  |  |  |  |
| GO:0042396\_phosphagen\_biosynthetic\_process | 2 | 0 |  |  |  |  |  |  |  |  |
| GO:0042454\_ribonucleoside\_catabolic\_process | 2 | 0 |  |  |  |  |  |  |  |  |
| GO:0042482\_positive\_regulation\_of\_odontogenesis | 2 | 0 |  |  |  |  |  |  |  |  |
| GO:0042483\_negative\_regulation\_of\_odontogenesis | 2 | 0 |  |  |  |  |  |  |  |  |
| GO:0042488\_positive\_regulation\_of\_odontogenesis\_of\_dentine-containing\_tooth | 2 | 0 |  |  |  |  |  |  |  |  |
| GO:0042501\_serine\_phosphorylation\_of\_STAT\_protein | 2 | 0 |  |  |  |  |  |  |  |  |
| GO:0042517\_positive\_regulation\_of\_tyrosine\_phosphorylation\_of\_Stat3\_protein | 2 | 0 |  |  |  |  |  |  |  |  |
| GO:0042532\_negative\_regulation\_of\_tyrosine\_phosphorylation\_of\_STAT\_protein | 2 | 0 |  |  |  |  |  |  |  |  |
| GO:0042559\_pteridine\_and\_derivative\_biosynthetic\_process | 2 | 0 |  |  |  |  |  |  |  |  |
| GO:0042730\_fibrinolysis | 2 | 0 |  |  |  |  |  |  |  |  |
| GO:0042749\_regulation\_of\_circadian\_sleep\_wake\_cycle | 2 | 0 |  |  |  |  |  |  |  |  |
| GO:0042886\_amide\_transport | 2 | 0 |  |  |  |  |  |  |  |  |
| GO:0042921\_glucocorticoid\_receptor\_signaling\_pathway | 2 | 0 |  |  |  |  |  |  |  |  |
| GO:0042987\_amyloid\_precursor\_protein\_catabolic\_process | 2 | 0 |  |  |  |  |  |  |  |  |
| GO:0042993\_positive\_regulation\_of\_transcription\_factor\_import\_into\_nucleus | 2 | 0 |  |  |  |  |  |  |  |  |
| GO:0042994\_cytoplasmic\_sequestering\_of\_transcription\_factor | 2 | 0 |  |  |  |  |  |  |  |  |
| GO:0043032\_positive\_regulation\_of\_macrophage\_activation | 2 | 0 |  |  |  |  |  |  |  |  |
| GO:0043038\_amino\_acid\_activation | 2 | 0 |  |  |  |  |  |  |  |  |
| GO:0043039\_tRNA\_aminoacylation | 2 | 0 |  |  |  |  |  |  |  |  |
| GO:0043084\_penile\_erection | 2 | 0 |  |  |  |  |  |  |  |  |
| GO:0043088\_regulation\_of\_Cdc42\_GTPase\_activity | 2 | 0 |  |  |  |  |  |  |  |  |
| GO:0043089\_positive\_regulation\_of\_Cdc42\_GTPase\_activity | 2 | 0 |  |  |  |  |  |  |  |  |
| GO:0043096\_purine\_base\_salvage | 2 | 0 |  |  |  |  |  |  |  |  |
| GO:0043247\_telomere\_maintenance\_in\_response\_to\_DNA\_damage | 2 | 0 |  |  |  |  |  |  |  |  |
| GO:0043297\_apical\_junction\_assembly | 2 | 0 |  |  |  |  |  |  |  |  |
| GO:0043312\_neutrophil\_degranulation | 2 | 0 |  |  |  |  |  |  |  |  |
| GO:0043320\_natural\_killer\_cell\_degranulation | 2 | 0 |  |  |  |  |  |  |  |  |
| GO:0043366\_beta\_selection | 2 | 0 |  |  |  |  |  |  |  |  |
| GO:0043450\_alkene\_biosynthetic\_process | 2 | 0 |  |  |  |  |  |  |  |  |
| GO:0043476\_pigment\_accumulation | 2 | 0 |  |  |  |  |  |  |  |  |
| GO:0043490\_malate-aspartate\_shuttle | 2 | 0 |  |  |  |  |  |  |  |  |
| GO:0043502\_regulation\_of\_muscle\_adaptation | 2 | 0 |  |  |  |  |  |  |  |  |
| GO:0043568\_positive\_regulation\_of\_insulin-like\_growth\_factor\_receptor\_signaling\_pathway | 2 | 0 |  |  |  |  |  |  |  |  |
| GO:0043589\_skin\_morphogenesis | 2 | 0 |  |  |  |  |  |  |  |  |
| GO:0043618\_regulation\_of\_transcription\_from\_RNA\_polymerase\_II\_promoter\_in\_response\_to\_stress | 2 | 0 |  |  |  |  |  |  |  |  |
| GO:0043619\_regulation\_of\_transcription\_from\_RNA\_polymerase\_II\_promoter\_in\_response\_to\_oxidative\_stress | 2 | 0 |  |  |  |  |  |  |  |  |
| GO:0043620\_regulation\_of\_transcription\_in\_response\_to\_stress | 2 | 0 |  |  |  |  |  |  |  |  |
| GO:0043647\_inositol\_phosphate\_metabolic\_process | 2 | 0 |  |  |  |  |  |  |  |  |
| GO:0043654\_recognition\_of\_apoptotic\_cell | 2 | 0 |  |  |  |  |  |  |  |  |
| GO:0043966\_histone\_H3\_acetylation | 2 | 0 |  |  |  |  |  |  |  |  |
| GO:0043967\_histone\_H4\_acetylation | 2 | 0 |  |  |  |  |  |  |  |  |
| GO:0044070\_regulation\_of\_anion\_transport | 2 | 0 |  |  |  |  |  |  |  |  |
| GO:0044246\_regulation\_of\_multicellular\_organismal\_metabolic\_process | 2 | 0 |  |  |  |  |  |  |  |  |
| GO:0044253\_positive\_regulation\_of\_multicellular\_organismal\_metabolic\_process | 2 | 0 |  |  |  |  |  |  |  |  |
| GO:0044268\_multicellular\_organismal\_protein\_metabolic\_process | 2 | 0 |  |  |  |  |  |  |  |  |
| GO:0045005\_maintenance\_of\_fidelity\_during\_DNA-dependent\_DNA\_replication | 2 | 0 |  |  |  |  |  |  |  |  |
| GO:0045010\_actin\_nucleation | 2 | 0 |  |  |  |  |  |  |  |  |
| GO:0045065\_cytotoxic\_T\_cell\_differentiation | 2 | 0 |  |  |  |  |  |  |  |  |
| GO:0045077\_negative\_regulation\_of\_interferon-gamma\_biosynthetic\_process | 2 | 0 |  |  |  |  |  |  |  |  |
| GO:0045079\_negative\_regulation\_of\_chemokine\_biosynthetic\_process | 2 | 0 |  |  |  |  |  |  |  |  |
| GO:0045116\_protein\_neddylation | 2 | 0 |  |  |  |  |  |  |  |  |
| GO:0045187\_regulation\_of\_circadian\_sleep\_wake\_cycle\_\_sleep | 2 | 0 |  |  |  |  |  |  |  |  |
| GO:0045212\_neurotransmitter\_receptor\_biosynthetic\_process | 2 | 0 |  |  |  |  |  |  |  |  |
| GO:0045399\_regulation\_of\_interleukin-3\_biosynthetic\_process | 2 | 0 |  |  |  |  |  |  |  |  |
| GO:0045401\_positive\_regulation\_of\_interleukin-3\_biosynthetic\_process | 2 | 0 |  |  |  |  |  |  |  |  |
| GO:0045409\_negative\_regulation\_of\_interleukin-6\_biosynthetic\_process | 2 | 0 |  |  |  |  |  |  |  |  |
| GO:0045423\_regulation\_of\_granulocyte\_macrophage\_colony-stimulating\_factor\_biosynthetic\_process | 2 | 0 |  |  |  |  |  |  |  |  |
| GO:0045425\_positive\_regulation\_of\_granulocyte\_macrophage\_colony-stimulating\_factor\_biosynthetic\_process | 2 | 0 |  |  |  |  |  |  |  |  |
| GO:0045475\_locomotor\_rhythm | 2 | 0 |  |  |  |  |  |  |  |  |
| GO:0045578\_negative\_regulation\_of\_B\_cell\_differentiation | 2 | 0 |  |  |  |  |  |  |  |  |
| GO:0045589\_regulation\_of\_regulatory\_T\_cell\_differentiation | 2 | 0 |  |  |  |  |  |  |  |  |
| GO:0045591\_positive\_regulation\_of\_regulatory\_T\_cell\_differentiation | 2 | 0 |  |  |  |  |  |  |  |  |
| GO:0045608\_negative\_regulation\_of\_auditory\_receptor\_cell\_differentiation | 2 | 0 |  |  |  |  |  |  |  |  |
| GO:0045627\_positive\_regulation\_of\_T-helper\_1\_cell\_differentiation | 2 | 0 |  |  |  |  |  |  |  |  |
| GO:0045629\_negative\_regulation\_of\_T-helper\_2\_cell\_differentiation | 2 | 0 |  |  |  |  |  |  |  |  |
| GO:0045630\_positive\_regulation\_of\_T-helper\_2\_cell\_differentiation | 2 | 0 |  |  |  |  |  |  |  |  |
| GO:0045632\_negative\_regulation\_of\_mechanoreceptor\_differentiation | 2 | 0 |  |  |  |  |  |  |  |  |
| GO:0045636\_positive\_regulation\_of\_melanocyte\_differentiation | 2 | 0 |  |  |  |  |  |  |  |  |
| GO:0045655\_regulation\_of\_monocyte\_differentiation | 2 | 0 |  |  |  |  |  |  |  |  |
| GO:0045658\_regulation\_of\_neutrophil\_differentiation | 2 | 0 |  |  |  |  |  |  |  |  |
| GO:0045662\_negative\_regulation\_of\_myoblast\_differentiation | 2 | 0 |  |  |  |  |  |  |  |  |
| GO:0045663\_positive\_regulation\_of\_myoblast\_differentiation | 2 | 0 |  |  |  |  |  |  |  |  |
| GO:0045683\_negative\_regulation\_of\_epidermis\_development | 2 | 0 |  |  |  |  |  |  |  |  |
| GO:0045737\_positive\_regulation\_of\_cyclin-dependent\_protein\_kinase\_activity | 2 | 0 |  |  |  |  |  |  |  |  |
| GO:0045739\_positive\_regulation\_of\_DNA\_repair | 2 | 0 |  |  |  |  |  |  |  |  |
| GO:0045741\_positive\_regulation\_of\_epidermal\_growth\_factor\_receptor\_activity | 2 | 0 |  |  |  |  |  |  |  |  |
| GO:0045743\_positive\_regulation\_of\_fibroblast\_growth\_factor\_receptor\_signaling\_pathway | 2 | 0 |  |  |  |  |  |  |  |  |
| GO:0045749\_negative\_regulation\_of\_S\_phase\_of\_mitotic\_cell\_cycle | 2 | 0 |  |  |  |  |  |  |  |  |
| GO:0045819\_positive\_regulation\_of\_glycogen\_catabolic\_process | 2 | 0 |  |  |  |  |  |  |  |  |
| GO:0045821\_positive\_regulation\_of\_glycolysis | 2 | 0 |  |  |  |  |  |  |  |  |
| GO:0045835\_negative\_regulation\_of\_meiosis | 2 | 0 |  |  |  |  |  |  |  |  |
| GO:0045836\_positive\_regulation\_of\_meiosis | 2 | 0 |  |  |  |  |  |  |  |  |
| GO:0045839\_negative\_regulation\_of\_mitosis | 2 | 0 |  |  |  |  |  |  |  |  |
| GO:0045841\_negative\_regulation\_of\_mitotic\_metaphase\_anaphase\_transition | 2 | 0 |  |  |  |  |  |  |  |  |
| GO:0045872\_positive\_regulation\_of\_rhodopsin\_gene\_expression | 2 | 0 |  |  |  |  |  |  |  |  |
| GO:0045912\_negative\_regulation\_of\_carbohydrate\_metabolic\_process | 2 | 0 |  |  |  |  |  |  |  |  |
| GO:0045948\_positive\_regulation\_of\_translational\_initiation | 2 | 0 |  |  |  |  |  |  |  |  |
| GO:0045950\_negative\_regulation\_of\_mitotic\_recombination | 2 | 0 |  |  |  |  |  |  |  |  |
| GO:0046033\_AMP\_metabolic\_process | 2 | 0 |  |  |  |  |  |  |  |  |
| GO:0046060\_dATP\_metabolic\_process | 2 | 0 |  |  |  |  |  |  |  |  |
| GO:0046070\_dGTP\_metabolic\_process | 2 | 0 |  |  |  |  |  |  |  |  |
| GO:0046083\_adenine\_metabolic\_process | 2 | 0 |  |  |  |  |  |  |  |  |
| GO:0046085\_adenosine\_metabolic\_process | 2 | 0 |  |  |  |  |  |  |  |  |
| GO:0046100\_hypoxanthine\_metabolic\_process | 2 | 0 |  |  |  |  |  |  |  |  |
| GO:0046114\_guanosine\_biosynthetic\_process | 2 | 0 |  |  |  |  |  |  |  |  |
| GO:0046116\_queuosine\_metabolic\_process | 2 | 0 |  |  |  |  |  |  |  |  |
| GO:0046118\_7-methylguanosine\_biosynthetic\_process | 2 | 0 |  |  |  |  |  |  |  |  |
| GO:0046130\_purine\_ribonucleoside\_catabolic\_process | 2 | 0 |  |  |  |  |  |  |  |  |
| GO:0046146\_tetrahydrobiopterin\_metabolic\_process | 2 | 0 |  |  |  |  |  |  |  |  |
| GO:0046185\_aldehyde\_catabolic\_process | 2 | 0 |  |  |  |  |  |  |  |  |
| GO:0046208\_spermine\_catabolic\_process | 2 | 0 |  |  |  |  |  |  |  |  |
| GO:0046349\_amino\_sugar\_biosynthetic\_process | 2 | 0 |  |  |  |  |  |  |  |  |
| GO:0046439\_L-cysteine\_metabolic\_process | 2 | 0 |  |  |  |  |  |  |  |  |
| GO:0046500\_S-adenosylmethionine\_metabolic\_process | 2 | 0 |  |  |  |  |  |  |  |  |
| GO:0046501\_protoporphyrinogen\_IX\_metabolic\_process | 2 | 0 |  |  |  |  |  |  |  |  |
| GO:0046514\_ceramide\_catabolic\_process | 2 | 0 |  |  |  |  |  |  |  |  |
| GO:0046521\_sphingoid\_catabolic\_process | 2 | 0 |  |  |  |  |  |  |  |  |
| GO:0046532\_regulation\_of\_photoreceptor\_cell\_differentiation | 2 | 0 |  |  |  |  |  |  |  |  |
| GO:0046533\_negative\_regulation\_of\_photoreceptor\_cell\_differentiation | 2 | 0 |  |  |  |  |  |  |  |  |
| GO:0046544\_development\_of\_secondary\_male\_sexual\_characteristics | 2 | 0 |  |  |  |  |  |  |  |  |
| GO:0046619\_optic\_placode\_formation\_involved\_in\_camera-type\_eye | 2 | 0 |  |  |  |  |  |  |  |  |
| GO:0046950\_cellular\_ketone\_body\_metabolic\_process | 2 | 0 |  |  |  |  |  |  |  |  |
| GO:0046984\_regulation\_of\_hemoglobin\_biosynthetic\_process | 2 | 0 |  |  |  |  |  |  |  |  |
| GO:0047484\_regulation\_of\_response\_to\_osmotic\_stress | 2 | 0 |  |  |  |  |  |  |  |  |
| GO:0048134\_germ-line\_cyst\_formation | 2 | 0 |  |  |  |  |  |  |  |  |
| GO:0048136\_male\_germ-line\_cyst\_formation | 2 | 0 |  |  |  |  |  |  |  |  |
| GO:0048172\_regulation\_of\_short-term\_neuronal\_synaptic\_plasticity | 2 | 0 |  |  |  |  |  |  |  |  |
| GO:0048295\_positive\_regulation\_of\_isotype\_switching\_to\_IgE\_isotypes | 2 | 0 |  |  |  |  |  |  |  |  |
| GO:0048342\_paraxial\_mesodermal\_cell\_differentiation | 2 | 0 |  |  |  |  |  |  |  |  |
| GO:0048343\_paraxial\_mesodermal\_cell\_fate\_commitment | 2 | 0 |  |  |  |  |  |  |  |  |
| GO:0048382\_mesendoderm\_development | 2 | 0 |  |  |  |  |  |  |  |  |
| GO:0048552\_regulation\_of\_metalloenzyme\_activity | 2 | 0 |  |  |  |  |  |  |  |  |
| GO:0048554\_positive\_regulation\_of\_metalloenzyme\_activity | 2 | 0 |  |  |  |  |  |  |  |  |
| GO:0048619\_embryonic\_hindgut\_morphogenesis | 2 | 0 |  |  |  |  |  |  |  |  |
| GO:0048625\_myoblast\_cell\_fate\_commitment | 2 | 0 |  |  |  |  |  |  |  |  |
| GO:0048627\_myoblast\_development | 2 | 0 |  |  |  |  |  |  |  |  |
| GO:0048643\_positive\_regulation\_of\_skeletal\_muscle\_tissue\_development | 2 | 0 |  |  |  |  |  |  |  |  |
| GO:0048661\_positive\_regulation\_of\_smooth\_muscle\_cell\_proliferation | 2 | 0 |  |  |  |  |  |  |  |  |
| GO:0048670\_regulation\_of\_collateral\_sprouting | 2 | 0 |  |  |  |  |  |  |  |  |
| GO:0048671\_negative\_regulation\_of\_collateral\_sprouting | 2 | 0 |  |  |  |  |  |  |  |  |
| GO:0048677\_axon\_extension\_involved\_in\_regeneration | 2 | 0 |  |  |  |  |  |  |  |  |
| GO:0048679\_regulation\_of\_axon\_regeneration | 2 | 0 |  |  |  |  |  |  |  |  |
| GO:0048682\_sprouting\_of\_injured\_axon | 2 | 0 |  |  |  |  |  |  |  |  |
| GO:0048702\_embryonic\_neurocranium\_morphogenesis | 2 | 0 |  |  |  |  |  |  |  |  |
| GO:0048711\_positive\_regulation\_of\_astrocyte\_differentiation | 2 | 0 |  |  |  |  |  |  |  |  |
| GO:0048712\_negative\_regulation\_of\_astrocyte\_differentiation | 2 | 0 |  |  |  |  |  |  |  |  |
| GO:0048739\_cardiac\_muscle\_fiber\_development | 2 | 0 |  |  |  |  |  |  |  |  |
| GO:0048807\_female\_genitalia\_morphogenesis | 2 | 0 |  |  |  |  |  |  |  |  |
| GO:0048808\_male\_genitalia\_morphogenesis | 2 | 0 |  |  |  |  |  |  |  |  |
| GO:0048840\_otolith\_development | 2 | 0 |  |  |  |  |  |  |  |  |
| GO:0048850\_hypophysis\_morphogenesis | 2 | 0 |  |  |  |  |  |  |  |  |
| GO:0048867\_stem\_cell\_fate\_determination | 2 | 0 |  |  |  |  |  |  |  |  |
| GO:0050000\_chromosome\_localization | 2 | 0 |  |  |  |  |  |  |  |  |
| GO:0050688\_regulation\_of\_defense\_response\_to\_virus | 2 | 0 |  |  |  |  |  |  |  |  |
| GO:0050746\_regulation\_of\_lipoprotein\_metabolic\_process | 2 | 0 |  |  |  |  |  |  |  |  |
| GO:0050779\_RNA\_destabilization | 2 | 0 |  |  |  |  |  |  |  |  |
| GO:0050792\_regulation\_of\_viral\_reproduction | 2 | 0 |  |  |  |  |  |  |  |  |
| GO:0050802\_circadian\_sleep\_wake\_cycle\_\_sleep | 2 | 0 |  |  |  |  |  |  |  |  |
| GO:0050847\_progesterone\_receptor\_signaling\_pathway | 2 | 0 |  |  |  |  |  |  |  |  |
| GO:0050855\_regulation\_of\_B\_cell\_receptor\_signaling\_pathway | 2 | 0 |  |  |  |  |  |  |  |  |
| GO:0050883\_musculoskeletal\_movement\_\_spinal\_reflex\_action | 2 | 0 |  |  |  |  |  |  |  |  |
| GO:0050901\_leukocyte\_tethering\_or\_rolling | 2 | 0 |  |  |  |  |  |  |  |  |
| GO:0050907\_detection\_of\_chemical\_stimulus\_involved\_in\_sensory\_perception | 2 | 0 |  |  |  |  |  |  |  |  |
| GO:0050917\_sensory\_perception\_of\_umami\_taste | 2 | 0 |  |  |  |  |  |  |  |  |
| GO:0050942\_positive\_regulation\_of\_pigment\_cell\_differentiation | 2 | 0 |  |  |  |  |  |  |  |  |
| GO:0050955\_thermoception | 2 | 0 |  |  |  |  |  |  |  |  |
| GO:0050968\_detection\_of\_chemical\_stimulus\_involved\_in\_sensory\_perception\_of\_pain | 2 | 0 |  |  |  |  |  |  |  |  |
| GO:0050973\_detection\_of\_mechanical\_stimulus\_involved\_in\_equilibrioception | 2 | 0 |  |  |  |  |  |  |  |  |
| GO:0050999\_regulation\_of\_nitric-oxide\_synthase\_activity | 2 | 0 |  |  |  |  |  |  |  |  |
| GO:0051004\_regulation\_of\_lipoprotein\_lipase\_activity | 2 | 0 |  |  |  |  |  |  |  |  |
| GO:0051014\_actin\_filament\_severing | 2 | 0 |  |  |  |  |  |  |  |  |
| GO:0051026\_chiasma\_formation | 2 | 0 |  |  |  |  |  |  |  |  |
| GO:0051081\_nuclear\_envelope\_disassembly | 2 | 0 |  |  |  |  |  |  |  |  |
| GO:0051132\_NK\_T\_cell\_activation | 2 | 0 |  |  |  |  |  |  |  |  |
| GO:0051133\_regulation\_of\_NK\_T\_cell\_activation | 2 | 0 |  |  |  |  |  |  |  |  |
| GO:0051135\_positive\_regulation\_of\_NK\_T\_cell\_activation | 2 | 0 |  |  |  |  |  |  |  |  |
| GO:0051150\_regulation\_of\_smooth\_muscle\_cell\_differentiation | 2 | 0 |  |  |  |  |  |  |  |  |
| GO:0051220\_cytoplasmic\_sequestering\_of\_protein | 2 | 0 |  |  |  |  |  |  |  |  |
| GO:0051279\_regulation\_of\_release\_of\_sequestered\_calcium\_ion\_into\_cytosol | 2 | 0 |  |  |  |  |  |  |  |  |
| GO:0051293\_establishment\_of\_spindle\_localization | 2 | 0 |  |  |  |  |  |  |  |  |
| GO:0051295\_establishment\_of\_meiotic\_spindle\_localization | 2 | 0 |  |  |  |  |  |  |  |  |
| GO:0051299\_centrosome\_separation | 2 | 0 |  |  |  |  |  |  |  |  |
| GO:0051303\_establishment\_of\_chromosome\_localization | 2 | 0 |  |  |  |  |  |  |  |  |
| GO:0051304\_chromosome\_separation | 2 | 0 |  |  |  |  |  |  |  |  |
| GO:0051307\_meiotic\_chromosome\_separation | 2 | 0 |  |  |  |  |  |  |  |  |
| GO:0051313\_attachment\_of\_spindle\_microtubules\_to\_chromosome | 2 | 0 |  |  |  |  |  |  |  |  |
| GO:0051318\_G1\_phase | 2 | 0 |  |  |  |  |  |  |  |  |
| GO:0051319\_G2\_phase | 2 | 0 |  |  |  |  |  |  |  |  |
| GO:0051353\_positive\_regulation\_of\_oxidoreductase\_activity | 2 | 0 |  |  |  |  |  |  |  |  |
| GO:0051451\_myoblast\_migration | 2 | 0 |  |  |  |  |  |  |  |  |
| GO:0051489\_regulation\_of\_filopodium\_assembly | 2 | 0 |  |  |  |  |  |  |  |  |
| GO:0051491\_positive\_regulation\_of\_filopodium\_assembly | 2 | 0 |  |  |  |  |  |  |  |  |
| GO:0051541\_elastin\_metabolic\_process | 2 | 0 |  |  |  |  |  |  |  |  |
| GO:0051546\_keratinocyte\_migration | 2 | 0 |  |  |  |  |  |  |  |  |
| GO:0051563\_smooth\_endoplasmic\_reticulum\_calcium\_ion\_homeostasis | 2 | 0 |  |  |  |  |  |  |  |  |
| GO:0051590\_positive\_regulation\_of\_neurotransmitter\_transport | 2 | 0 |  |  |  |  |  |  |  |  |
| GO:0051602\_response\_to\_electrical\_stimulus | 2 | 0 |  |  |  |  |  |  |  |  |
| GO:0051608\_histamine\_transport | 2 | 0 |  |  |  |  |  |  |  |  |
| GO:0051643\_ER\_localization | 2 | 0 |  |  |  |  |  |  |  |  |
| GO:0051653\_spindle\_localization | 2 | 0 |  |  |  |  |  |  |  |  |
| GO:0051657\_maintenance\_of\_organelle\_location | 2 | 0 |  |  |  |  |  |  |  |  |
| GO:0051702\_interaction\_with\_symbiont | 2 | 0 |  |  |  |  |  |  |  |  |
| GO:0051781\_positive\_regulation\_of\_cell\_division | 2 | 0 |  |  |  |  |  |  |  |  |
| GO:0051784\_negative\_regulation\_of\_nuclear\_division | 2 | 0 |  |  |  |  |  |  |  |  |
| GO:0051890\_regulation\_of\_cardioblast\_differentiation | 2 | 0 |  |  |  |  |  |  |  |  |
| GO:0051891\_positive\_regulation\_of\_cardioblast\_differentiation | 2 | 0 |  |  |  |  |  |  |  |  |
| GO:0051923\_sulfation | 2 | 0 |  |  |  |  |  |  |  |  |
| GO:0051938\_L-glutamate\_import | 2 | 0 |  |  |  |  |  |  |  |  |
| GO:0051957\_positive\_regulation\_of\_amino\_acid\_transport | 2 | 0 |  |  |  |  |  |  |  |  |
| GO:0051988\_regulation\_of\_attachment\_of\_spindle\_microtubules\_to\_kinetochore | 2 | 0 |  |  |  |  |  |  |  |  |
| GO:0055057\_neuroblast\_division | 2 | 0 |  |  |  |  |  |  |  |  |
| GO:0055064\_chloride\_ion\_homeostasis | 2 | 0 |  |  |  |  |  |  |  |  |
| GO:0055075\_potassium\_ion\_homeostasis | 2 | 0 |  |  |  |  |  |  |  |  |
| GO:0055090\_acylglycerol\_homeostasis | 2 | 0 |  |  |  |  |  |  |  |  |
| GO:0055091\_phospholipid\_homeostasis | 2 | 0 |  |  |  |  |  |  |  |  |
| GO:0060012\_synaptic\_transmission\_\_glycinergic | 2 | 0 |  |  |  |  |  |  |  |  |
| GO:0060023\_soft\_palate\_development | 2 | 0 |  |  |  |  |  |  |  |  |
| GO:0060032\_notochord\_regression | 2 | 0 |  |  |  |  |  |  |  |  |
| GO:0060039\_pericardium\_development | 2 | 0 |  |  |  |  |  |  |  |  |
| GO:0060044\_negative\_regulation\_of\_cardiac\_muscle\_cell\_proliferation | 2 | 0 |  |  |  |  |  |  |  |  |
| GO:0060060\_post-embryonic\_retina\_morphogenesis\_in\_camera-type\_eye | 2 | 0 |  |  |  |  |  |  |  |  |
| GO:0060083\_smooth\_muscle\_contraction\_involved\_in\_micturition | 2 | 0 |  |  |  |  |  |  |  |  |
| GO:0060124\_positive\_regulation\_of\_growth\_hormone\_secretion | 2 | 0 |  |  |  |  |  |  |  |  |
| GO:0060133\_somatotropin\_secreting\_cell\_development | 2 | 0 |  |  |  |  |  |  |  |  |
| GO:0060155\_platelet\_dense\_granule\_organization | 2 | 0 |  |  |  |  |  |  |  |  |
| GO:0060159\_regulation\_of\_dopamine\_receptor\_signaling\_pathway | 2 | 0 |  |  |  |  |  |  |  |  |
| GO:0060160\_negative\_regulation\_of\_dopamine\_receptor\_signaling\_pathway | 2 | 0 |  |  |  |  |  |  |  |  |
| GO:0060166\_olfactory\_pit\_development | 2 | 0 |  |  |  |  |  |  |  |  |
| GO:0060179\_male\_mating\_behavior | 2 | 0 |  |  |  |  |  |  |  |  |
| GO:0060180\_female\_mating\_behavior | 2 | 0 |  |  |  |  |  |  |  |  |
| GO:0060214\_endocardium\_formation | 2 | 0 |  |  |  |  |  |  |  |  |
| GO:0060218\_hemopoietic\_stem\_cell\_differentiation | 2 | 0 |  |  |  |  |  |  |  |  |
| GO:0060259\_regulation\_of\_feeding\_behavior | 2 | 0 |  |  |  |  |  |  |  |  |
| GO:0060260\_regulation\_of\_transcription\_initiation\_from\_RNA\_polymerase\_II\_promoter | 2 | 0 |  |  |  |  |  |  |  |  |
| GO:0060292\_long\_term\_synaptic\_depression | 2 | 0 |  |  |  |  |  |  |  |  |
| GO:0060318\_definitive\_erythrocyte\_differentiation | 2 | 0 |  |  |  |  |  |  |  |  |
| GO:0060346\_bone\_trabecula\_formation | 2 | 0 |  |  |  |  |  |  |  |  |
| GO:0060363\_cranial\_suture\_morphogenesis | 2 | 0 |  |  |  |  |  |  |  |  |
| GO:0060393\_regulation\_of\_pathway-restricted\_SMAD\_protein\_phosphorylation | 2 | 0 |  |  |  |  |  |  |  |  |
| GO:0060397\_JAK-STAT\_cascade\_involved\_in\_growth\_hormone\_signaling\_pathway | 2 | 0 |  |  |  |  |  |  |  |  |
| GO:0060426\_lung\_vasculature\_development | 2 | 0 |  |  |  |  |  |  |  |  |
| GO:0060430\_lung\_saccule\_development | 2 | 0 |  |  |  |  |  |  |  |  |
| GO:0060434\_bronchus\_morphogenesis | 2 | 0 |  |  |  |  |  |  |  |  |
| GO:0060439\_trachea\_morphogenesis | 2 | 0 |  |  |  |  |  |  |  |  |
| GO:0060458\_right\_lung\_development | 2 | 0 |  |  |  |  |  |  |  |  |
| GO:0060462\_lung\_lobe\_development | 2 | 0 |  |  |  |  |  |  |  |  |
| GO:0060463\_lung\_lobe\_morphogenesis | 2 | 0 |  |  |  |  |  |  |  |  |
| GO:0060479\_lung\_cell\_differentiation | 2 | 0 |  |  |  |  |  |  |  |  |
| GO:0060487\_lung\_epithelial\_cell\_differentiation | 2 | 0 |  |  |  |  |  |  |  |  |
| GO:0060516\_primary\_prostatic\_bud\_elongation | 2 | 0 |  |  |  |  |  |  |  |  |
| GO:0060529\_squamous\_basal\_epithelial\_stem\_cell\_differentiation\_involved\_in\_prostate\_gland\_acinus\_development | 2 | 0 |  |  |  |  |  |  |  |  |
| GO:0060534\_trachea\_cartilage\_development | 2 | 0 |  |  |  |  |  |  |  |  |
| GO:0060599\_lateral\_sprouting\_involved\_in\_mammary\_gland\_duct\_morphogenesis | 2 | 0 |  |  |  |  |  |  |  |  |
| GO:0060612\_adipose\_tissue\_development | 2 | 0 |  |  |  |  |  |  |  |  |
| GO:0060615\_mammary\_gland\_bud\_formation | 2 | 0 |  |  |  |  |  |  |  |  |
| GO:0060667\_branch\_elongation\_involved\_in\_salivary\_gland\_morphogenesis | 2 | 0 |  |  |  |  |  |  |  |  |
| GO:0060690\_epithelial\_cell\_differentiation\_involved\_in\_salivary\_gland\_development | 2 | 0 |  |  |  |  |  |  |  |  |
| GO:0060738\_epithelial-mesenchymal\_signaling\_involved\_in\_prostate\_gland\_development | 2 | 0 |  |  |  |  |  |  |  |  |
| GO:0060741\_prostate\_gland\_stromal\_morphogenesis | 2 | 0 |  |  |  |  |  |  |  |  |
| GO:0060763\_mammary\_duct\_terminal\_end\_bud\_growth | 2 | 0 |  |  |  |  |  |  |  |  |
| GO:0060765\_regulation\_of\_androgen\_receptor\_signaling\_pathway | 2 | 0 |  |  |  |  |  |  |  |  |
| GO:0060766\_negative\_regulation\_of\_androgen\_receptor\_signaling\_pathway | 2 | 0 |  |  |  |  |  |  |  |  |
| GO:0060769\_positive\_regulation\_of\_epithelial\_cell\_proliferation\_involved\_in\_prostate\_gland\_development | 2 | 0 |  |  |  |  |  |  |  |  |
| GO:0065005\_protein-lipid\_complex\_assembly | 2 | 0 |  |  |  |  |  |  |  |  |
| GO:0070076\_histone\_lysine\_demethylation | 2 | 0 |  |  |  |  |  |  |  |  |
| GO:0070168\_negative\_regulation\_of\_biomineral\_formation | 2 | 0 |  |  |  |  |  |  |  |  |
| GO:0070252\_actin-mediated\_cell\_contraction | 2 | 0 |  |  |  |  |  |  |  |  |
| GO:0070256\_negative\_regulation\_of\_mucus\_secretion | 2 | 0 |  |  |  |  |  |  |  |  |
| GO:0070257\_positive\_regulation\_of\_mucus\_secretion | 2 | 0 |  |  |  |  |  |  |  |  |
| GO:0070570\_regulation\_of\_neuron\_projection\_regeneration | 2 | 0 |  |  |  |  |  |  |  |  |
| GO:0070723\_response\_to\_cholesterol | 2 | 0 |  |  |  |  |  |  |  |  |
| GO:0090030\_regulation\_of\_steroid\_hormone\_biosynthetic\_process | 2 | 0 |  |  |  |  |  |  |  |  |
| GO:0002200\_somatic\_diversification\_of\_immune\_receptors | 34 | 0 | 0.000000 | -0.000000 | 808 | 795.211302 | 868.37 | 941.528698 | 1.074715 |  |
| GO:0002237\_response\_to\_molecule\_of\_bacterial\_origin | 34 | 0 | 0.000000 | -0.000000 | 808 | 795.211302 | 868.37 | 941.528698 | 1.074715 |  |
| GO:0002699\_positive\_regulation\_of\_immune\_effector\_process | 34 | 0 | 0.000000 | -0.000000 | 808 | 795.211302 | 868.37 | 941.528698 | 1.074715 |  |
| GO:0007338\_single\_fertilization | 34 | 0 | 0.000000 | -0.000000 | 808 | 795.211302 | 868.37 | 941.528698 | 1.074715 |  |
| GO:0007568\_aging | 34 | 0 | 0.000000 | -0.000000 | 808 | 795.211302 | 868.37 | 941.528698 | 1.074715 |  |
| GO:0010720\_positive\_regulation\_of\_cell\_development | 34 | 0 | 0.000000 | -0.000000 | 808 | 795.211302 | 868.37 | 941.528698 | 1.074715 |  |
| GO:0010721\_negative\_regulation\_of\_cell\_development | 34 | 0 | 0.000000 | -0.000000 | 808 | 795.211302 | 868.37 | 941.528698 | 1.074715 |  |
| GO:0019882\_antigen\_processing\_and\_presentation | 34 | 0 | 0.000000 | -0.000000 | 808 | 795.211302 | 868.37 | 941.528698 | 1.074715 |  |
| GO:0030509\_BMP\_signaling\_pathway | 34 | 0 | 0.000000 | -0.000000 | 808 | 795.211302 | 868.37 | 941.528698 | 1.074715 |  |
| GO:0045927\_positive\_regulation\_of\_growth | 34 | 0 | 0.000000 | -0.000000 | 808 | 795.211302 | 868.37 | 941.528698 | 1.074715 |  |
| GO:0050730\_regulation\_of\_peptidyl-tyrosine\_phosphorylation | 34 | 0 | 0.000000 | -0.000000 | 808 | 795.211302 | 868.37 | 941.528698 | 1.074715 |  |
| GO:0051047\_positive\_regulation\_of\_secretion | 34 | 0 | 0.000000 | -0.000000 | 808 | 795.211302 | 868.37 | 941.528698 | 1.074715 |  |
| GO:0060443\_mammary\_gland\_morphogenesis | 34 | 0 | 0.000000 | -0.000000 | 808 | 795.211302 | 868.37 | 941.528698 | 1.074715 |  |
| GO:0060711\_labyrinthine\_layer\_development | 34 | 0 | 0.000000 | -0.000000 | 808 | 795.211302 | 868.37 | 941.528698 | 1.074715 |  |
| GO:0001756\_somitogenesis | 35 | 0 | 0.000000 | -0.000000 | 819 | 807.614575 | 880.42 | 953.225425 | 1.074994 |  |
| GO:0001906\_cell\_killing | 35 | 0 | 0.000000 | -0.000000 | 819 | 807.614575 | 880.42 | 953.225425 | 1.074994 |  |
| GO:0001909\_leukocyte\_mediated\_cytotoxicity | 35 | 0 | 0.000000 | -0.000000 | 819 | 807.614575 | 880.42 | 953.225425 | 1.074994 |  |
| GO:0006869\_lipid\_transport | 35 | 0 | 0.000000 | -0.000000 | 819 | 807.614575 | 880.42 | 953.225425 | 1.074994 |  |
| GO:0007292\_female\_gamete\_generation | 35 | 0 | 0.000000 | -0.000000 | 819 | 807.614575 | 880.42 | 953.225425 | 1.074994 |  |
| GO:0010810\_regulation\_of\_cell-substrate\_adhesion | 35 | 0 | 0.000000 | -0.000000 | 819 | 807.614575 | 880.42 | 953.225425 | 1.074994 |  |
| GO:0016051\_carbohydrate\_biosynthetic\_process | 35 | 0 | 0.000000 | -0.000000 | 819 | 807.614575 | 880.42 | 953.225425 | 1.074994 |  |
| GO:0016567\_protein\_ubiquitination | 35 | 0 | 0.000000 | -0.000000 | 819 | 807.614575 | 880.42 | 953.225425 | 1.074994 |  |
| GO:0018904\_organic\_ether\_metabolic\_process | 35 | 0 | 0.000000 | -0.000000 | 819 | 807.614575 | 880.42 | 953.225425 | 1.074994 |  |
| GO:0043406\_positive\_regulation\_of\_MAP\_kinase\_activity | 35 | 0 | 0.000000 | -0.000000 | 819 | 807.614575 | 880.42 | 953.225425 | 1.074994 |  |
| GO:0051051\_negative\_regulation\_of\_transport | 35 | 0 | 0.000000 | -0.000000 | 819 | 807.614575 | 880.42 | 953.225425 | 1.074994 |  |
| GO:0003013\_circulatory\_system\_process | 103 | 0 | 0.000000 | -0.000000 | 822 | 809.898160 | 882.46 | 955.021840 | 1.073552 |  |
| GO:0008015\_blood\_circulation | 103 | 0 | 0.000000 | -0.000000 | 822 | 809.898160 | 882.46 | 955.021840 | 1.073552 |  |
| GO:0009968\_negative\_regulation\_of\_signal\_transduction | 103 | 0 | 0.000000 | -0.000000 | 822 | 809.898160 | 882.46 | 955.021840 | 1.073552 |  |
| GO:0006519\_cellular\_amino\_acid\_and\_derivative\_metabolic\_process | 118 | 0 | 0.000000 | -0.000000 | 824 | 811.287784 | 883.72 | 956.152216 | 1.072476 |  |
| GO:0051960\_regulation\_of\_nervous\_system\_development | 118 | 0 | 0.000000 | -0.000000 | 824 | 811.287784 | 883.72 | 956.152216 | 1.072476 |  |
| GO:0006163\_purine\_nucleotide\_metabolic\_process | 73 | 0 | 0.000000 | -0.000000 | 828 | 816.129331 | 888.03 | 959.930669 | 1.072500 |  |
| GO:0006936\_muscle\_contraction | 73 | 0 | 0.000000 | -0.000000 | 828 | 816.129331 | 888.03 | 959.930669 | 1.072500 |  |
| GO:0048706\_embryonic\_skeletal\_system\_development | 73 | 0 | 0.000000 | -0.000000 | 828 | 816.129331 | 888.03 | 959.930669 | 1.072500 |  |
| GO:0051270\_regulation\_of\_cell\_motion | 73 | 0 | 0.000000 | -0.000000 | 828 | 816.129331 | 888.03 | 959.930669 | 1.072500 |  |
| GO:0001944\_vasculature\_development | 208 | 0 | 0.000000 | -0.000000 | 829 | 817.944556 | 889.62 | 961.295444 | 1.073124 |  |
| GO:0001824\_blastocyst\_development | 40 | 0 | 0.000000 | -0.000000 | 838 | 829.372044 | 900.64 | 971.907956 | 1.074749 |  |
| GO:0007599\_hemostasis | 40 | 0 | 0.000000 | -0.000000 | 838 | 829.372044 | 900.64 | 971.907956 | 1.074749 |  |
| GO:0008203\_cholesterol\_metabolic\_process | 40 | 0 | 0.000000 | -0.000000 | 838 | 829.372044 | 900.64 | 971.907956 | 1.074749 |  |
| GO:0014031\_mesenchymal\_cell\_development | 40 | 0 | 0.000000 | -0.000000 | 838 | 829.372044 | 900.64 | 971.907956 | 1.074749 |  |
| GO:0016485\_protein\_processing | 40 | 0 | 0.000000 | -0.000000 | 838 | 829.372044 | 900.64 | 971.907956 | 1.074749 |  |
| GO:0019935\_cyclic-nucleotide-mediated\_signaling | 40 | 0 | 0.000000 | -0.000000 | 838 | 829.372044 | 900.64 | 971.907956 | 1.074749 |  |
| GO:0035272\_exocrine\_system\_development | 40 | 0 | 0.000000 | -0.000000 | 838 | 829.372044 | 900.64 | 971.907956 | 1.074749 |  |
| GO:0046850\_regulation\_of\_bone\_remodeling | 40 | 0 | 0.000000 | -0.000000 | 838 | 829.372044 | 900.64 | 971.907956 | 1.074749 |  |
| GO:0051129\_negative\_regulation\_of\_cellular\_component\_organization | 40 | 0 | 0.000000 | -0.000000 | 838 | 829.372044 | 900.64 | 971.907956 | 1.074749 |  |
| GO:0060341\_regulation\_of\_cellular\_localization | 97 | 0 | 0.000000 | -0.000000 | 839 | 830.826210 | 901.95 | 973.073790 | 1.075030 |  |
| GO:0000209\_protein\_polyubiquitination | 10 | 0 | 0.000000 | -0.000000 | 957 | 951.393519 | 1021.01 | 1090.626481 | 1.066886 |  |
| GO:0000724\_double-strand\_break\_repair\_via\_homologous\_recombination | 10 | 0 | 0.000000 | -0.000000 | 957 | 951.393519 | 1021.01 | 1090.626481 | 1.066886 |  |
| GO:0000725\_recombinational\_repair | 10 | 0 | 0.000000 | -0.000000 | 957 | 951.393519 | 1021.01 | 1090.626481 | 1.066886 |  |
| GO:0001578\_microtubule\_bundle\_formation | 10 | 0 | 0.000000 | -0.000000 | 957 | 951.393519 | 1021.01 | 1090.626481 | 1.066886 |  |
| GO:0001659\_temperature\_homeostasis | 10 | 0 | 0.000000 | -0.000000 | 957 | 951.393519 | 1021.01 | 1090.626481 | 1.066886 |  |
| GO:0001773\_myeloid\_dendritic\_cell\_activation | 10 | 0 | 0.000000 | -0.000000 | 957 | 951.393519 | 1021.01 | 1090.626481 | 1.066886 |  |
| GO:0001832\_blastocyst\_growth | 10 | 0 | 0.000000 | -0.000000 | 957 | 951.393519 | 1021.01 | 1090.626481 | 1.066886 |  |
| GO:0001914\_regulation\_of\_T\_cell\_mediated\_cytotoxicity | 10 | 0 | 0.000000 | -0.000000 | 957 | 951.393519 | 1021.01 | 1090.626481 | 1.066886 |  |
| GO:0001990\_regulation\_of\_systemic\_arterial\_blood\_pressure\_by\_hormone | 10 | 0 | 0.000000 | -0.000000 | 957 | 951.393519 | 1021.01 | 1090.626481 | 1.066886 |  |
| GO:0002070\_epithelial\_cell\_maturation | 10 | 0 | 0.000000 | -0.000000 | 957 | 951.393519 | 1021.01 | 1090.626481 | 1.066886 |  |
| GO:0002673\_regulation\_of\_acute\_inflammatory\_response | 10 | 0 | 0.000000 | -0.000000 | 957 | 951.393519 | 1021.01 | 1090.626481 | 1.066886 |  |
| GO:0002711\_positive\_regulation\_of\_T\_cell\_mediated\_immunity | 10 | 0 | 0.000000 | -0.000000 | 957 | 951.393519 | 1021.01 | 1090.626481 | 1.066886 |  |
| GO:0002762\_negative\_regulation\_of\_myeloid\_leukocyte\_differentiation | 10 | 0 | 0.000000 | -0.000000 | 957 | 951.393519 | 1021.01 | 1090.626481 | 1.066886 |  |
| GO:0006040\_amino\_sugar\_metabolic\_process | 10 | 0 | 0.000000 | -0.000000 | 957 | 951.393519 | 1021.01 | 1090.626481 | 1.066886 |  |
| GO:0006081\_cellular\_aldehyde\_metabolic\_process | 10 | 0 | 0.000000 | -0.000000 | 957 | 951.393519 | 1021.01 | 1090.626481 | 1.066886 |  |
| GO:0006109\_regulation\_of\_carbohydrate\_metabolic\_process | 10 | 0 | 0.000000 | -0.000000 | 957 | 951.393519 | 1021.01 | 1090.626481 | 1.066886 |  |
| GO:0006289\_nucleotide-excision\_repair | 10 | 0 | 0.000000 | -0.000000 | 957 | 951.393519 | 1021.01 | 1090.626481 | 1.066886 |  |
| GO:0006342\_chromatin\_silencing | 10 | 0 | 0.000000 | -0.000000 | 957 | 951.393519 | 1021.01 | 1090.626481 | 1.066886 |  |
| GO:0006801\_superoxide\_metabolic\_process | 10 | 0 | 0.000000 | -0.000000 | 957 | 951.393519 | 1021.01 | 1090.626481 | 1.066886 |  |
| GO:0006805\_xenobiotic\_metabolic\_process | 10 | 0 | 0.000000 | -0.000000 | 957 | 951.393519 | 1021.01 | 1090.626481 | 1.066886 |  |
| GO:0006826\_iron\_ion\_transport | 10 | 0 | 0.000000 | -0.000000 | 957 | 951.393519 | 1021.01 | 1090.626481 | 1.066886 |  |
| GO:0006921\_cell\_structure\_disassembly\_during\_apoptosis | 10 | 0 | 0.000000 | -0.000000 | 957 | 951.393519 | 1021.01 | 1090.626481 | 1.066886 |  |
| GO:0006968\_cellular\_defense\_response | 10 | 0 | 0.000000 | -0.000000 | 957 | 951.393519 | 1021.01 | 1090.626481 | 1.066886 |  |
| GO:0007006\_mitochondrial\_membrane\_organization | 10 | 0 | 0.000000 | -0.000000 | 957 | 951.393519 | 1021.01 | 1090.626481 | 1.066886 |  |
| GO:0007044\_cell-substrate\_junction\_assembly | 10 | 0 | 0.000000 | -0.000000 | 957 | 951.393519 | 1021.01 | 1090.626481 | 1.066886 |  |
| GO:0007093\_mitotic\_cell\_cycle\_checkpoint | 10 | 0 | 0.000000 | -0.000000 | 957 | 951.393519 | 1021.01 | 1090.626481 | 1.066886 |  |
| GO:0007194\_negative\_regulation\_of\_adenylate\_cyclase\_activity | 10 | 0 | 0.000000 | -0.000000 | 957 | 951.393519 | 1021.01 | 1090.626481 | 1.066886 |  |
| GO:0008088\_axon\_cargo\_transport | 10 | 0 | 0.000000 | -0.000000 | 957 | 951.393519 | 1021.01 | 1090.626481 | 1.066886 |  |
| GO:0008206\_bile\_acid\_metabolic\_process | 10 | 0 | 0.000000 | -0.000000 | 957 | 951.393519 | 1021.01 | 1090.626481 | 1.066886 |  |
| GO:0008211\_glucocorticoid\_metabolic\_process | 10 | 0 | 0.000000 | -0.000000 | 957 | 951.393519 | 1021.01 | 1090.626481 | 1.066886 |  |
| GO:0009066\_aspartate\_family\_amino\_acid\_metabolic\_process | 10 | 0 | 0.000000 | -0.000000 | 957 | 951.393519 | 1021.01 | 1090.626481 | 1.066886 |  |
| GO:0009110\_vitamin\_biosynthetic\_process | 10 | 0 | 0.000000 | -0.000000 | 957 | 951.393519 | 1021.01 | 1090.626481 | 1.066886 |  |
| GO:0009620\_response\_to\_fungus | 10 | 0 | 0.000000 | -0.000000 | 957 | 951.393519 | 1021.01 | 1090.626481 | 1.066886 |  |
| GO:0009743\_response\_to\_carbohydrate\_stimulus | 10 | 0 | 0.000000 | -0.000000 | 957 | 951.393519 | 1021.01 | 1090.626481 | 1.066886 |  |
| GO:0009948\_anterior\_posterior\_axis\_specification | 10 | 0 | 0.000000 | -0.000000 | 957 | 951.393519 | 1021.01 | 1090.626481 | 1.066886 |  |
| GO:0010827\_regulation\_of\_glucose\_transport | 10 | 0 | 0.000000 | -0.000000 | 957 | 951.393519 | 1021.01 | 1090.626481 | 1.066886 |  |
| GO:0015718\_monocarboxylic\_acid\_transport | 10 | 0 | 0.000000 | -0.000000 | 957 | 951.393519 | 1021.01 | 1090.626481 | 1.066886 |  |
| GO:0016197\_endosome\_transport | 10 | 0 | 0.000000 | -0.000000 | 957 | 951.393519 | 1021.01 | 1090.626481 | 1.066886 |  |
| GO:0016486\_peptide\_hormone\_processing | 10 | 0 | 0.000000 | -0.000000 | 957 | 951.393519 | 1021.01 | 1090.626481 | 1.066886 |  |
| GO:0017156\_calcium\_ion-dependent\_exocytosis | 10 | 0 | 0.000000 | -0.000000 | 957 | 951.393519 | 1021.01 | 1090.626481 | 1.066886 |  |
| GO:0018149\_peptide\_cross-linking | 10 | 0 | 0.000000 | -0.000000 | 957 | 951.393519 | 1021.01 | 1090.626481 | 1.066886 |  |
| GO:0019321\_pentose\_metabolic\_process | 10 | 0 | 0.000000 | -0.000000 | 957 | 951.393519 | 1021.01 | 1090.626481 | 1.066886 |  |
| GO:0021534\_cell\_proliferation\_in\_hindbrain | 10 | 0 | 0.000000 | -0.000000 | 957 | 951.393519 | 1021.01 | 1090.626481 | 1.066886 |  |
| GO:0021871\_forebrain\_regionalization | 10 | 0 | 0.000000 | -0.000000 | 957 | 951.393519 | 1021.01 | 1090.626481 | 1.066886 |  |
| GO:0021895\_cerebral\_cortex\_neuron\_differentiation | 10 | 0 | 0.000000 | -0.000000 | 957 | 951.393519 | 1021.01 | 1090.626481 | 1.066886 |  |
| GO:0021924\_cell\_proliferation\_in\_the\_external\_granule\_layer | 10 | 0 | 0.000000 | -0.000000 | 957 | 951.393519 | 1021.01 | 1090.626481 | 1.066886 |  |
| GO:0021930\_granule\_cell\_precursor\_proliferation | 10 | 0 | 0.000000 | -0.000000 | 957 | 951.393519 | 1021.01 | 1090.626481 | 1.066886 |  |
| GO:0021952\_central\_nervous\_system\_projection\_neuron\_axonogenesis | 10 | 0 | 0.000000 | -0.000000 | 957 | 951.393519 | 1021.01 | 1090.626481 | 1.066886 |  |
| GO:0022900\_electron\_transport\_chain | 10 | 0 | 0.000000 | -0.000000 | 957 | 951.393519 | 1021.01 | 1090.626481 | 1.066886 |  |
| GO:0022904\_respiratory\_electron\_transport\_chain | 10 | 0 | 0.000000 | -0.000000 | 957 | 951.393519 | 1021.01 | 1090.626481 | 1.066886 |  |
| GO:0030168\_platelet\_activation | 10 | 0 | 0.000000 | -0.000000 | 957 | 951.393519 | 1021.01 | 1090.626481 | 1.066886 |  |
| GO:0030833\_regulation\_of\_actin\_filament\_polymerization | 10 | 0 | 0.000000 | -0.000000 | 957 | 951.393519 | 1021.01 | 1090.626481 | 1.066886 |  |
| GO:0031018\_endocrine\_pancreas\_development | 10 | 0 | 0.000000 | -0.000000 | 957 | 951.393519 | 1021.01 | 1090.626481 | 1.066886 |  |
| GO:0031280\_negative\_regulation\_of\_cyclase\_activity | 10 | 0 | 0.000000 | -0.000000 | 957 | 951.393519 | 1021.01 | 1090.626481 | 1.066886 |  |
| GO:0031331\_positive\_regulation\_of\_cellular\_catabolic\_process | 10 | 0 | 0.000000 | -0.000000 | 957 | 951.393519 | 1021.01 | 1090.626481 | 1.066886 |  |
| GO:0031645\_negative\_regulation\_of\_neurological\_system\_process | 10 | 0 | 0.000000 | -0.000000 | 957 | 951.393519 | 1021.01 | 1090.626481 | 1.066886 |  |
| GO:0032318\_regulation\_of\_Ras\_GTPase\_activity | 10 | 0 | 0.000000 | -0.000000 | 957 | 951.393519 | 1021.01 | 1090.626481 | 1.066886 |  |
| GO:0032602\_chemokine\_production | 10 | 0 | 0.000000 | -0.000000 | 957 | 951.393519 | 1021.01 | 1090.626481 | 1.066886 |  |
| GO:0032633\_interleukin-4\_production | 10 | 0 | 0.000000 | -0.000000 | 957 | 951.393519 | 1021.01 | 1090.626481 | 1.066886 |  |
| GO:0032642\_regulation\_of\_chemokine\_production | 10 | 0 | 0.000000 | -0.000000 | 957 | 951.393519 | 1021.01 | 1090.626481 | 1.066886 |  |
| GO:0032673\_regulation\_of\_interleukin-4\_production | 10 | 0 | 0.000000 | -0.000000 | 957 | 951.393519 | 1021.01 | 1090.626481 | 1.066886 |  |
| GO:0032760\_positive\_regulation\_of\_tumor\_necrosis\_factor\_production | 10 | 0 | 0.000000 | -0.000000 | 957 | 951.393519 | 1021.01 | 1090.626481 | 1.066886 |  |
| GO:0033081\_regulation\_of\_T\_cell\_differentiation\_in\_the\_thymus | 10 | 0 | 0.000000 | -0.000000 | 957 | 951.393519 | 1021.01 | 1090.626481 | 1.066886 |  |
| GO:0034105\_positive\_regulation\_of\_tissue\_remodeling | 10 | 0 | 0.000000 | -0.000000 | 957 | 951.393519 | 1021.01 | 1090.626481 | 1.066886 |  |
| GO:0034637\_cellular\_carbohydrate\_biosynthetic\_process | 10 | 0 | 0.000000 | -0.000000 | 957 | 951.393519 | 1021.01 | 1090.626481 | 1.066886 |  |
| GO:0040015\_negative\_regulation\_of\_multicellular\_organism\_growth | 10 | 0 | 0.000000 | -0.000000 | 957 | 951.393519 | 1021.01 | 1090.626481 | 1.066886 |  |
| GO:0042088\_T-helper\_1\_type\_immune\_response | 10 | 0 | 0.000000 | -0.000000 | 957 | 951.393519 | 1021.01 | 1090.626481 | 1.066886 |  |
| GO:0042116\_macrophage\_activation | 10 | 0 | 0.000000 | -0.000000 | 957 | 951.393519 | 1021.01 | 1090.626481 | 1.066886 |  |
| GO:0042177\_negative\_regulation\_of\_protein\_catabolic\_process | 10 | 0 | 0.000000 | -0.000000 | 957 | 951.393519 | 1021.01 | 1090.626481 | 1.066886 |  |
| GO:0042755\_eating\_behavior | 10 | 0 | 0.000000 | -0.000000 | 957 | 951.393519 | 1021.01 | 1090.626481 | 1.066886 |  |
| GO:0043330\_response\_to\_exogenous\_dsRNA | 10 | 0 | 0.000000 | -0.000000 | 957 | 951.393519 | 1021.01 | 1090.626481 | 1.066886 |  |
| GO:0043488\_regulation\_of\_mRNA\_stability | 10 | 0 | 0.000000 | -0.000000 | 957 | 951.393519 | 1021.01 | 1090.626481 | 1.066886 |  |
| GO:0043506\_regulation\_of\_JUN\_kinase\_activity | 10 | 0 | 0.000000 | -0.000000 | 957 | 951.393519 | 1021.01 | 1090.626481 | 1.066886 |  |
| GO:0043525\_positive\_regulation\_of\_neuron\_apoptosis | 10 | 0 | 0.000000 | -0.000000 | 957 | 951.393519 | 1021.01 | 1090.626481 | 1.066886 |  |
| GO:0044259\_multicellular\_organismal\_macromolecule\_metabolic\_process | 10 | 0 | 0.000000 | -0.000000 | 957 | 951.393519 | 1021.01 | 1090.626481 | 1.066886 |  |
| GO:0045132\_meiotic\_chromosome\_segregation | 10 | 0 | 0.000000 | -0.000000 | 957 | 951.393519 | 1021.01 | 1090.626481 | 1.066886 |  |
| GO:0045446\_endothelial\_cell\_differentiation | 10 | 0 | 0.000000 | -0.000000 | 957 | 951.393519 | 1021.01 | 1090.626481 | 1.066886 |  |
| GO:0045576\_mast\_cell\_activation | 10 | 0 | 0.000000 | -0.000000 | 957 | 951.393519 | 1021.01 | 1090.626481 | 1.066886 |  |
| GO:0045669\_positive\_regulation\_of\_osteoblast\_differentiation | 10 | 0 | 0.000000 | -0.000000 | 957 | 951.393519 | 1021.01 | 1090.626481 | 1.066886 |  |
| GO:0045776\_negative\_regulation\_of\_blood\_pressure | 10 | 0 | 0.000000 | -0.000000 | 957 | 951.393519 | 1021.01 | 1090.626481 | 1.066886 |  |
| GO:0045777\_positive\_regulation\_of\_blood\_pressure | 10 | 0 | 0.000000 | -0.000000 | 957 | 951.393519 | 1021.01 | 1090.626481 | 1.066886 |  |
| GO:0045814\_negative\_regulation\_of\_gene\_expression\_\_epigenetic | 10 | 0 | 0.000000 | -0.000000 | 957 | 951.393519 | 1021.01 | 1090.626481 | 1.066886 |  |
| GO:0045911\_positive\_regulation\_of\_DNA\_recombination | 10 | 0 | 0.000000 | -0.000000 | 957 | 951.393519 | 1021.01 | 1090.626481 | 1.066886 |  |
| GO:0046887\_positive\_regulation\_of\_hormone\_secretion | 10 | 0 | 0.000000 | -0.000000 | 957 | 951.393519 | 1021.01 | 1090.626481 | 1.066886 |  |
| GO:0048291\_isotype\_switching\_to\_IgG\_isotypes | 10 | 0 | 0.000000 | -0.000000 | 957 | 951.393519 | 1021.01 | 1090.626481 | 1.066886 |  |
| GO:0048302\_regulation\_of\_isotype\_switching\_to\_IgG\_isotypes | 10 | 0 | 0.000000 | -0.000000 | 957 | 951.393519 | 1021.01 | 1090.626481 | 1.066886 |  |
| GO:0048339\_paraxial\_mesoderm\_development | 10 | 0 | 0.000000 | -0.000000 | 957 | 951.393519 | 1021.01 | 1090.626481 | 1.066886 |  |
| GO:0048384\_retinoic\_acid\_receptor\_signaling\_pathway | 10 | 0 | 0.000000 | -0.000000 | 957 | 951.393519 | 1021.01 | 1090.626481 | 1.066886 |  |
| GO:0048596\_embryonic\_camera-type\_eye\_morphogenesis | 10 | 0 | 0.000000 | -0.000000 | 957 | 951.393519 | 1021.01 | 1090.626481 | 1.066886 |  |
| GO:0048641\_regulation\_of\_skeletal\_muscle\_tissue\_development | 10 | 0 | 0.000000 | -0.000000 | 957 | 951.393519 | 1021.01 | 1090.626481 | 1.066886 |  |
| GO:0048738\_cardiac\_muscle\_tissue\_development | 10 | 0 | 0.000000 | -0.000000 | 957 | 951.393519 | 1021.01 | 1090.626481 | 1.066886 |  |
| GO:0050654\_chondroitin\_sulfate\_proteoglycan\_metabolic\_process | 10 | 0 | 0.000000 | -0.000000 | 957 | 951.393519 | 1021.01 | 1090.626481 | 1.066886 |  |
| GO:0050663\_cytokine\_secretion | 10 | 0 | 0.000000 | -0.000000 | 957 | 951.393519 | 1021.01 | 1090.626481 | 1.066886 |  |
| GO:0050714\_positive\_regulation\_of\_protein\_secretion | 10 | 0 | 0.000000 | -0.000000 | 957 | 951.393519 | 1021.01 | 1090.626481 | 1.066886 |  |
| GO:0050879\_multicellular\_organismal\_movement | 10 | 0 | 0.000000 | -0.000000 | 957 | 951.393519 | 1021.01 | 1090.626481 | 1.066886 |  |
| GO:0050881\_musculoskeletal\_movement | 10 | 0 | 0.000000 | -0.000000 | 957 | 951.393519 | 1021.01 | 1090.626481 | 1.066886 |  |
| GO:0050886\_endocrine\_process | 10 | 0 | 0.000000 | -0.000000 | 957 | 951.393519 | 1021.01 | 1090.626481 | 1.066886 |  |
| GO:0050892\_intestinal\_absorption | 10 | 0 | 0.000000 | -0.000000 | 957 | 951.393519 | 1021.01 | 1090.626481 | 1.066886 |  |
| GO:0051147\_regulation\_of\_muscle\_cell\_differentiation | 10 | 0 | 0.000000 | -0.000000 | 957 | 951.393519 | 1021.01 | 1090.626481 | 1.066886 |  |
| GO:0051208\_sequestering\_of\_calcium\_ion | 10 | 0 | 0.000000 | -0.000000 | 957 | 951.393519 | 1021.01 | 1090.626481 | 1.066886 |  |
| GO:0051209\_release\_of\_sequestered\_calcium\_ion\_into\_cytosol | 10 | 0 | 0.000000 | -0.000000 | 957 | 951.393519 | 1021.01 | 1090.626481 | 1.066886 |  |
| GO:0051224\_negative\_regulation\_of\_protein\_transport | 10 | 0 | 0.000000 | -0.000000 | 957 | 951.393519 | 1021.01 | 1090.626481 | 1.066886 |  |
| GO:0051238\_sequestering\_of\_metal\_ion | 10 | 0 | 0.000000 | -0.000000 | 957 | 951.393519 | 1021.01 | 1090.626481 | 1.066886 |  |
| GO:0051262\_protein\_tetramerization | 10 | 0 | 0.000000 | -0.000000 | 957 | 951.393519 | 1021.01 | 1090.626481 | 1.066886 |  |
| GO:0051282\_regulation\_of\_sequestering\_of\_calcium\_ion | 10 | 0 | 0.000000 | -0.000000 | 957 | 951.393519 | 1021.01 | 1090.626481 | 1.066886 |  |
| GO:0051283\_negative\_regulation\_of\_sequestering\_of\_calcium\_ion | 10 | 0 | 0.000000 | -0.000000 | 957 | 951.393519 | 1021.01 | 1090.626481 | 1.066886 |  |
| GO:0051350\_negative\_regulation\_of\_lyase\_activity | 10 | 0 | 0.000000 | -0.000000 | 957 | 951.393519 | 1021.01 | 1090.626481 | 1.066886 |  |
| GO:0051445\_regulation\_of\_meiotic\_cell\_cycle | 10 | 0 | 0.000000 | -0.000000 | 957 | 951.393519 | 1021.01 | 1090.626481 | 1.066886 |  |
| GO:0051650\_establishment\_of\_vesicle\_localization | 10 | 0 | 0.000000 | -0.000000 | 957 | 951.393519 | 1021.01 | 1090.626481 | 1.066886 |  |
| GO:0051651\_maintenance\_of\_location\_in\_cell | 10 | 0 | 0.000000 | -0.000000 | 957 | 951.393519 | 1021.01 | 1090.626481 | 1.066886 |  |
| GO:0060135\_maternal\_process\_involved\_in\_female\_pregnancy | 10 | 0 | 0.000000 | -0.000000 | 957 | 951.393519 | 1021.01 | 1090.626481 | 1.066886 |  |
| GO:0060216\_definitive\_hemopoiesis | 10 | 0 | 0.000000 | -0.000000 | 957 | 951.393519 | 1021.01 | 1090.626481 | 1.066886 |  |
| GO:0060323\_head\_morphogenesis | 10 | 0 | 0.000000 | -0.000000 | 957 | 951.393519 | 1021.01 | 1090.626481 | 1.066886 |  |
| GO:0060343\_trabecula\_formation | 10 | 0 | 0.000000 | -0.000000 | 957 | 951.393519 | 1021.01 | 1090.626481 | 1.066886 |  |
| GO:0060601\_lateral\_sprouting\_from\_an\_epithelium | 10 | 0 | 0.000000 | -0.000000 | 957 | 951.393519 | 1021.01 | 1090.626481 | 1.066886 |  |
| GO:0060669\_embryonic\_placenta\_morphogenesis | 10 | 0 | 0.000000 | -0.000000 | 957 | 951.393519 | 1021.01 | 1090.626481 | 1.066886 |  |
| GO:0060706\_cell\_differentiation\_involved\_in\_embryonic\_placenta\_development | 10 | 0 | 0.000000 | -0.000000 | 957 | 951.393519 | 1021.01 | 1090.626481 | 1.066886 |  |
| GO:0060768\_regulation\_of\_epithelial\_cell\_proliferation\_involved\_in\_prostate\_gland\_development | 10 | 0 | 0.000000 | -0.000000 | 957 | 951.393519 | 1021.01 | 1090.626481 | 1.066886 |  |
| GO:0048872\_homeostasis\_of\_number\_of\_cells | 105 | 0 | 0.000000 | -0.000000 | 958 | 952.606848 | 1022.08 | 1091.553152 | 1.066889 |  |
| GO:0002757\_immune\_response-activating\_signal\_transduction | 47 | 0 | 0.000000 | -0.000000 | 969 | 963.209989 | 1031.96 | 1100.710011 | 1.064974 |  |
| GO:0006140\_regulation\_of\_nucleotide\_metabolic\_process | 47 | 0 | 0.000000 | -0.000000 | 969 | 963.209989 | 1031.96 | 1100.710011 | 1.064974 |  |
| GO:0016570\_histone\_modification | 47 | 0 | 0.000000 | -0.000000 | 969 | 963.209989 | 1031.96 | 1100.710011 | 1.064974 |  |
| GO:0030183\_B\_cell\_differentiation | 47 | 0 | 0.000000 | -0.000000 | 969 | 963.209989 | 1031.96 | 1100.710011 | 1.064974 |  |
| GO:0030799\_regulation\_of\_cyclic\_nucleotide\_metabolic\_process | 47 | 0 | 0.000000 | -0.000000 | 969 | 963.209989 | 1031.96 | 1100.710011 | 1.064974 |  |
| GO:0031667\_response\_to\_nutrient\_levels | 47 | 0 | 0.000000 | -0.000000 | 969 | 963.209989 | 1031.96 | 1100.710011 | 1.064974 |  |
| GO:0034754\_cellular\_hormone\_metabolic\_process | 47 | 0 | 0.000000 | -0.000000 | 969 | 963.209989 | 1031.96 | 1100.710011 | 1.064974 |  |
| GO:0045087\_innate\_immune\_response | 47 | 0 | 0.000000 | -0.000000 | 969 | 963.209989 | 1031.96 | 1100.710011 | 1.064974 |  |
| GO:0045619\_regulation\_of\_lymphocyte\_differentiation | 47 | 0 | 0.000000 | -0.000000 | 969 | 963.209989 | 1031.96 | 1100.710011 | 1.064974 |  |
| GO:0048871\_multicellular\_organismal\_homeostasis | 47 | 0 | 0.000000 | -0.000000 | 969 | 963.209989 | 1031.96 | 1100.710011 | 1.064974 |  |
| GO:0060627\_regulation\_of\_vesicle-mediated\_transport | 47 | 0 | 0.000000 | -0.000000 | 969 | 963.209989 | 1031.96 | 1100.710011 | 1.064974 |  |
| GO:0015674\_di-\_\_tri-valent\_inorganic\_cation\_transport | 79 | 0 | 0.000000 | -0.000000 | 971 | 964.827103 | 1033.36 | 1101.892897 | 1.064222 |  |
| GO:0051046\_regulation\_of\_secretion | 79 | 0 | 0.000000 | -0.000000 | 971 | 964.827103 | 1033.36 | 1101.892897 | 1.064222 |  |
| GO:0048732\_gland\_development | 179 | 0 | 0.000000 | -0.000000 | 972 | 966.386749 | 1034.71 | 1103.033251 | 1.064516 |  |
| GO:0002697\_regulation\_of\_immune\_effector\_process | 68 | 0 | 0.000000 | -0.000000 | 976 | 970.996920 | 1038.7 | 1106.403080 | 1.064242 |  |
| GO:0019932\_second-messenger-mediated\_signaling | 68 | 0 | 0.000000 | -0.000000 | 976 | 970.996920 | 1038.7 | 1106.403080 | 1.064242 |  |
| GO:0034962\_cellular\_biopolymer\_catabolic\_process | 68 | 0 | 0.000000 | -0.000000 | 976 | 970.996920 | 1038.7 | 1106.403080 | 1.064242 |  |
| GO:0042692\_muscle\_cell\_differentiation | 68 | 0 | 0.000000 | -0.000000 | 976 | 970.996920 | 1038.7 | 1106.403080 | 1.064242 |  |
| GO:0030326\_embryonic\_limb\_morphogenesis | 78 | 0 | 0.000000 | -0.000000 | 979 | 976.966134 | 1044.25 | 1111.533866 | 1.066650 |  |
| GO:0035113\_embryonic\_appendage\_morphogenesis | 78 | 0 | 0.000000 | -0.000000 | 979 | 976.966134 | 1044.25 | 1111.533866 | 1.066650 |  |
| GO:0051251\_positive\_regulation\_of\_lymphocyte\_activation | 78 | 0 | 0.000000 | -0.000000 | 979 | 976.966134 | 1044.25 | 1111.533866 | 1.066650 |  |
| GO:0001738\_morphogenesis\_of\_a\_polarized\_epithelium | 12 | 0 | 0.000000 | -0.000000 | 1055 | 1057.325484 | 1123.09 | 1188.854516 | 1.064540 |  |
| GO:0002026\_regulation\_of\_the\_force\_of\_heart\_contraction | 12 | 0 | 0.000000 | -0.000000 | 1055 | 1057.325484 | 1123.09 | 1188.854516 | 1.064540 |  |
| GO:0002089\_lens\_morphogenesis\_in\_camera-type\_eye | 12 | 0 | 0.000000 | -0.000000 | 1055 | 1057.325484 | 1123.09 | 1188.854516 | 1.064540 |  |
| GO:0002218\_activation\_of\_innate\_immune\_response | 12 | 0 | 0.000000 | -0.000000 | 1055 | 1057.325484 | 1123.09 | 1188.854516 | 1.064540 |  |
| GO:0002244\_hemopoietic\_progenitor\_cell\_differentiation | 12 | 0 | 0.000000 | -0.000000 | 1055 | 1057.325484 | 1123.09 | 1188.854516 | 1.064540 |  |
| GO:0002437\_inflammatory\_response\_to\_antigenic\_stimulus | 12 | 0 | 0.000000 | -0.000000 | 1055 | 1057.325484 | 1123.09 | 1188.854516 | 1.064540 |  |
| GO:0002763\_positive\_regulation\_of\_myeloid\_leukocyte\_differentiation | 12 | 0 | 0.000000 | -0.000000 | 1055 | 1057.325484 | 1123.09 | 1188.854516 | 1.064540 |  |
| GO:0002820\_negative\_regulation\_of\_adaptive\_immune\_response | 12 | 0 | 0.000000 | -0.000000 | 1055 | 1057.325484 | 1123.09 | 1188.854516 | 1.064540 |  |
| GO:0002823\_negative\_regulation\_of\_adaptive\_immune\_response\_based\_on\_somatic\_recombination\_of\_immune\_receptors\_built\_from\_immunoglobulin\_superfamily\_domains | 12 | 0 | 0.000000 | -0.000000 | 1055 | 1057.325484 | 1123.09 | 1188.854516 | 1.064540 |  |
| GO:0002861\_regulation\_of\_inflammatory\_response\_to\_antigenic\_stimulus | 12 | 0 | 0.000000 | -0.000000 | 1055 | 1057.325484 | 1123.09 | 1188.854516 | 1.064540 |  |
| GO:0006094\_gluconeogenesis | 12 | 0 | 0.000000 | -0.000000 | 1055 | 1057.325484 | 1123.09 | 1188.854516 | 1.064540 |  |
| GO:0006308\_DNA\_catabolic\_process | 12 | 0 | 0.000000 | -0.000000 | 1055 | 1057.325484 | 1123.09 | 1188.854516 | 1.064540 |  |
| GO:0006413\_translational\_initiation | 12 | 0 | 0.000000 | -0.000000 | 1055 | 1057.325484 | 1123.09 | 1188.854516 | 1.064540 |  |
| GO:0006446\_regulation\_of\_translational\_initiation | 12 | 0 | 0.000000 | -0.000000 | 1055 | 1057.325484 | 1123.09 | 1188.854516 | 1.064540 |  |
| GO:0006839\_mitochondrial\_transport | 12 | 0 | 0.000000 | -0.000000 | 1055 | 1057.325484 | 1123.09 | 1188.854516 | 1.064540 |  |
| GO:0006879\_cellular\_iron\_ion\_homeostasis | 12 | 0 | 0.000000 | -0.000000 | 1055 | 1057.325484 | 1123.09 | 1188.854516 | 1.064540 |  |
| GO:0007143\_female\_meiosis | 12 | 0 | 0.000000 | -0.000000 | 1055 | 1057.325484 | 1123.09 | 1188.854516 | 1.064540 |  |
| GO:0007340\_acrosome\_reaction | 12 | 0 | 0.000000 | -0.000000 | 1055 | 1057.325484 | 1123.09 | 1188.854516 | 1.064540 |  |
| GO:0008038\_neuron\_recognition | 12 | 0 | 0.000000 | -0.000000 | 1055 | 1057.325484 | 1123.09 | 1188.854516 | 1.064540 |  |
| GO:0008045\_motor\_axon\_guidance | 12 | 0 | 0.000000 | -0.000000 | 1055 | 1057.325484 | 1123.09 | 1188.854516 | 1.064540 |  |
| GO:0008277\_regulation\_of\_G-protein\_coupled\_receptor\_protein\_signaling\_pathway | 12 | 0 | 0.000000 | -0.000000 | 1055 | 1057.325484 | 1123.09 | 1188.854516 | 1.064540 |  |
| GO:0009262\_deoxyribonucleotide\_metabolic\_process | 12 | 0 | 0.000000 | -0.000000 | 1055 | 1057.325484 | 1123.09 | 1188.854516 | 1.064540 |  |
| GO:0009886\_post-embryonic\_morphogenesis | 12 | 0 | 0.000000 | -0.000000 | 1055 | 1057.325484 | 1123.09 | 1188.854516 | 1.064540 |  |
| GO:0015872\_dopamine\_transport | 12 | 0 | 0.000000 | -0.000000 | 1055 | 1057.325484 | 1123.09 | 1188.854516 | 1.064540 |  |
| GO:0019319\_hexose\_biosynthetic\_process | 12 | 0 | 0.000000 | -0.000000 | 1055 | 1057.325484 | 1123.09 | 1188.854516 | 1.064540 |  |
| GO:0021513\_spinal\_cord\_dorsal\_ventral\_patterning | 12 | 0 | 0.000000 | -0.000000 | 1055 | 1057.325484 | 1123.09 | 1188.854516 | 1.064540 |  |
| GO:0021680\_cerebellar\_Purkinje\_cell\_layer\_development | 12 | 0 | 0.000000 | -0.000000 | 1055 | 1057.325484 | 1123.09 | 1188.854516 | 1.064540 |  |
| GO:0021697\_cerebellar\_cortex\_formation | 12 | 0 | 0.000000 | -0.000000 | 1055 | 1057.325484 | 1123.09 | 1188.854516 | 1.064540 |  |
| GO:0030010\_establishment\_of\_cell\_polarity | 12 | 0 | 0.000000 | -0.000000 | 1055 | 1057.325484 | 1123.09 | 1188.854516 | 1.064540 |  |
| GO:0030166\_proteoglycan\_biosynthetic\_process | 12 | 0 | 0.000000 | -0.000000 | 1055 | 1057.325484 | 1123.09 | 1188.854516 | 1.064540 |  |
| GO:0030225\_macrophage\_differentiation | 12 | 0 | 0.000000 | -0.000000 | 1055 | 1057.325484 | 1123.09 | 1188.854516 | 1.064540 |  |
| GO:0030239\_myofibril\_assembly | 12 | 0 | 0.000000 | -0.000000 | 1055 | 1057.325484 | 1123.09 | 1188.854516 | 1.064540 |  |
| GO:0030262\_apoptotic\_nuclear\_changes | 12 | 0 | 0.000000 | -0.000000 | 1055 | 1057.325484 | 1123.09 | 1188.854516 | 1.064540 |  |
| GO:0030514\_negative\_regulation\_of\_BMP\_signaling\_pathway | 12 | 0 | 0.000000 | -0.000000 | 1055 | 1057.325484 | 1123.09 | 1188.854516 | 1.064540 |  |
| GO:0031056\_regulation\_of\_histone\_modification | 12 | 0 | 0.000000 | -0.000000 | 1055 | 1057.325484 | 1123.09 | 1188.854516 | 1.064540 |  |
| GO:0031109\_microtubule\_polymerization\_or\_depolymerization | 12 | 0 | 0.000000 | -0.000000 | 1055 | 1057.325484 | 1123.09 | 1188.854516 | 1.064540 |  |
| GO:0032755\_positive\_regulation\_of\_interleukin-6\_production | 12 | 0 | 0.000000 | -0.000000 | 1055 | 1057.325484 | 1123.09 | 1188.854516 | 1.064540 |  |
| GO:0033598\_mammary\_gland\_epithelial\_cell\_proliferation | 12 | 0 | 0.000000 | -0.000000 | 1055 | 1057.325484 | 1123.09 | 1188.854516 | 1.064540 |  |
| GO:0035050\_embryonic\_heart\_tube\_development | 12 | 0 | 0.000000 | -0.000000 | 1055 | 1057.325484 | 1123.09 | 1188.854516 | 1.064540 |  |
| GO:0042278\_purine\_nucleoside\_metabolic\_process | 12 | 0 | 0.000000 | -0.000000 | 1055 | 1057.325484 | 1123.09 | 1188.854516 | 1.064540 |  |
| GO:0042446\_hormone\_biosynthetic\_process | 12 | 0 | 0.000000 | -0.000000 | 1055 | 1057.325484 | 1123.09 | 1188.854516 | 1.064540 |  |
| GO:0042743\_hydrogen\_peroxide\_metabolic\_process | 12 | 0 | 0.000000 | -0.000000 | 1055 | 1057.325484 | 1123.09 | 1188.854516 | 1.064540 |  |
| GO:0043331\_response\_to\_dsRNA | 12 | 0 | 0.000000 | -0.000000 | 1055 | 1057.325484 | 1123.09 | 1188.854516 | 1.064540 |  |
| GO:0043392\_negative\_regulation\_of\_DNA\_binding | 12 | 0 | 0.000000 | -0.000000 | 1055 | 1057.325484 | 1123.09 | 1188.854516 | 1.064540 |  |
| GO:0043487\_regulation\_of\_RNA\_stability | 12 | 0 | 0.000000 | -0.000000 | 1055 | 1057.325484 | 1123.09 | 1188.854516 | 1.064540 |  |
| GO:0043624\_cellular\_protein\_complex\_disassembly | 12 | 0 | 0.000000 | -0.000000 | 1055 | 1057.325484 | 1123.09 | 1188.854516 | 1.064540 |  |
| GO:0045076\_regulation\_of\_interleukin-2\_biosynthetic\_process | 12 | 0 | 0.000000 | -0.000000 | 1055 | 1057.325484 | 1123.09 | 1188.854516 | 1.064540 |  |
| GO:0045445\_myoblast\_differentiation | 12 | 0 | 0.000000 | -0.000000 | 1055 | 1057.325484 | 1123.09 | 1188.854516 | 1.064540 |  |
| GO:0045471\_response\_to\_ethanol | 12 | 0 | 0.000000 | -0.000000 | 1055 | 1057.325484 | 1123.09 | 1188.854516 | 1.064540 |  |
| GO:0045792\_negative\_regulation\_of\_cell\_size | 12 | 0 | 0.000000 | -0.000000 | 1055 | 1057.325484 | 1123.09 | 1188.854516 | 1.064540 |  |
| GO:0045995\_regulation\_of\_embryonic\_development | 12 | 0 | 0.000000 | -0.000000 | 1055 | 1057.325484 | 1123.09 | 1188.854516 | 1.064540 |  |
| GO:0046068\_cGMP\_metabolic\_process | 12 | 0 | 0.000000 | -0.000000 | 1055 | 1057.325484 | 1123.09 | 1188.854516 | 1.064540 |  |
| GO:0046128\_purine\_ribonucleoside\_metabolic\_process | 12 | 0 | 0.000000 | -0.000000 | 1055 | 1057.325484 | 1123.09 | 1188.854516 | 1.064540 |  |
| GO:0046330\_positive\_regulation\_of\_JNK\_cascade | 12 | 0 | 0.000000 | -0.000000 | 1055 | 1057.325484 | 1123.09 | 1188.854516 | 1.064540 |  |
| GO:0048169\_regulation\_of\_long-term\_neuronal\_synaptic\_plasticity | 12 | 0 | 0.000000 | -0.000000 | 1055 | 1057.325484 | 1123.09 | 1188.854516 | 1.064540 |  |
| GO:0048820\_hair\_follicle\_maturation | 12 | 0 | 0.000000 | -0.000000 | 1055 | 1057.325484 | 1123.09 | 1188.854516 | 1.064540 |  |
| GO:0048821\_erythrocyte\_development | 12 | 0 | 0.000000 | -0.000000 | 1055 | 1057.325484 | 1123.09 | 1188.854516 | 1.064540 |  |
| GO:0048854\_brain\_morphogenesis | 12 | 0 | 0.000000 | -0.000000 | 1055 | 1057.325484 | 1123.09 | 1188.854516 | 1.064540 |  |
| GO:0050432\_catecholamine\_secretion | 12 | 0 | 0.000000 | -0.000000 | 1055 | 1057.325484 | 1123.09 | 1188.854516 | 1.064540 |  |
| GO:0050795\_regulation\_of\_behavior | 12 | 0 | 0.000000 | -0.000000 | 1055 | 1057.325484 | 1123.09 | 1188.854516 | 1.064540 |  |
| GO:0050803\_regulation\_of\_synapse\_structure\_and\_activity | 12 | 0 | 0.000000 | -0.000000 | 1055 | 1057.325484 | 1123.09 | 1188.854516 | 1.064540 |  |
| GO:0050848\_regulation\_of\_calcium-mediated\_signaling | 12 | 0 | 0.000000 | -0.000000 | 1055 | 1057.325484 | 1123.09 | 1188.854516 | 1.064540 |  |
| GO:0050850\_positive\_regulation\_of\_calcium-mediated\_signaling | 12 | 0 | 0.000000 | -0.000000 | 1055 | 1057.325484 | 1123.09 | 1188.854516 | 1.064540 |  |
| GO:0050853\_B\_cell\_receptor\_signaling\_pathway | 12 | 0 | 0.000000 | -0.000000 | 1055 | 1057.325484 | 1123.09 | 1188.854516 | 1.064540 |  |
| GO:0050869\_negative\_regulation\_of\_B\_cell\_activation | 12 | 0 | 0.000000 | -0.000000 | 1055 | 1057.325484 | 1123.09 | 1188.854516 | 1.064540 |  |
| GO:0051145\_smooth\_muscle\_cell\_differentiation | 12 | 0 | 0.000000 | -0.000000 | 1055 | 1057.325484 | 1123.09 | 1188.854516 | 1.064540 |  |
| GO:0051261\_protein\_depolymerization | 12 | 0 | 0.000000 | -0.000000 | 1055 | 1057.325484 | 1123.09 | 1188.854516 | 1.064540 |  |
| GO:0051588\_regulation\_of\_neurotransmitter\_transport | 12 | 0 | 0.000000 | -0.000000 | 1055 | 1057.325484 | 1123.09 | 1188.854516 | 1.064540 |  |
| GO:0055002\_striated\_muscle\_cell\_development | 12 | 0 | 0.000000 | -0.000000 | 1055 | 1057.325484 | 1123.09 | 1188.854516 | 1.064540 |  |
| GO:0055114\_oxidation\_reduction | 12 | 0 | 0.000000 | -0.000000 | 1055 | 1057.325484 | 1123.09 | 1188.854516 | 1.064540 |  |
| GO:0060042\_retina\_morphogenesis\_in\_camera-type\_eye | 12 | 0 | 0.000000 | -0.000000 | 1055 | 1057.325484 | 1123.09 | 1188.854516 | 1.064540 |  |
| GO:0060048\_cardiac\_muscle\_contraction | 12 | 0 | 0.000000 | -0.000000 | 1055 | 1057.325484 | 1123.09 | 1188.854516 | 1.064540 |  |
| GO:0060525\_prostate\_glandular\_acinus\_development | 12 | 0 | 0.000000 | -0.000000 | 1055 | 1057.325484 | 1123.09 | 1188.854516 | 1.064540 |  |
| GO:0060572\_morphogenesis\_of\_an\_epithelial\_bud | 12 | 0 | 0.000000 | -0.000000 | 1055 | 1057.325484 | 1123.09 | 1188.854516 | 1.064540 |  |
| GO:0060736\_prostate\_gland\_growth | 12 | 0 | 0.000000 | -0.000000 | 1055 | 1057.325484 | 1123.09 | 1188.854516 | 1.064540 |  |
| GO:0070304\_positive\_regulation\_of\_stress-activated\_protein\_kinase\_signaling\_pathway | 12 | 0 | 0.000000 | -0.000000 | 1055 | 1057.325484 | 1123.09 | 1188.854516 | 1.064540 |  |
| GO:0001656\_metanephros\_development | 50 | 0 | 0.000000 | -0.000000 | 1062 | 1064.436664 | 1129.7 | 1194.963336 | 1.063748 |  |
| GO:0002573\_myeloid\_leukocyte\_differentiation | 50 | 0 | 0.000000 | -0.000000 | 1062 | 1064.436664 | 1129.7 | 1194.963336 | 1.063748 |  |
| GO:0007015\_actin\_filament\_organization | 50 | 0 | 0.000000 | -0.000000 | 1062 | 1064.436664 | 1129.7 | 1194.963336 | 1.063748 |  |
| GO:0009190\_cyclic\_nucleotide\_biosynthetic\_process | 50 | 0 | 0.000000 | -0.000000 | 1062 | 1064.436664 | 1129.7 | 1194.963336 | 1.063748 |  |
| GO:0042129\_regulation\_of\_T\_cell\_proliferation | 50 | 0 | 0.000000 | -0.000000 | 1062 | 1064.436664 | 1129.7 | 1194.963336 | 1.063748 |  |
| GO:0051606\_detection\_of\_stimulus | 50 | 0 | 0.000000 | -0.000000 | 1062 | 1064.436664 | 1129.7 | 1194.963336 | 1.063748 |  |
| GO:0070647\_protein\_modification\_by\_small\_protein\_conjugation\_or\_removal | 50 | 0 | 0.000000 | -0.000000 | 1062 | 1064.436664 | 1129.7 | 1194.963336 | 1.063748 |  |
| GO:0001932\_regulation\_of\_protein\_amino\_acid\_phosphorylation | 69 | 0 | 0.000000 | -0.000000 | 1067 | 1068.852628 | 1133.6 | 1198.347372 | 1.062418 |  |
| GO:0005996\_monosaccharide\_metabolic\_process | 69 | 0 | 0.000000 | -0.000000 | 1067 | 1068.852628 | 1133.6 | 1198.347372 | 1.062418 |  |
| GO:0006816\_calcium\_ion\_transport | 69 | 0 | 0.000000 | -0.000000 | 1067 | 1068.852628 | 1133.6 | 1198.347372 | 1.062418 |  |
| GO:0032101\_regulation\_of\_response\_to\_external\_stimulus | 69 | 0 | 0.000000 | -0.000000 | 1067 | 1068.852628 | 1133.6 | 1198.347372 | 1.062418 |  |
| GO:0055065\_metal\_ion\_homeostasis | 69 | 0 | 0.000000 | -0.000000 | 1067 | 1068.852628 | 1133.6 | 1198.347372 | 1.062418 |  |
| GO:0051246\_regulation\_of\_protein\_metabolic\_process | 170 | 0 | 0.000000 | -0.000000 | 1068 | 1070.051687 | 1134.59 | 1199.128313 | 1.062350 |  |
| GO:0040008\_regulation\_of\_growth | 113 | 0 | 0.000000 | -0.000000 | 1069 | 1071.967925 | 1136.32 | 1200.672075 | 1.062975 |  |
| GO:0007264\_small\_GTPase\_mediated\_signal\_transduction | 72 | 0 | 0.000000 | -0.000000 | 1077 | 1080.595073 | 1144.32 | 1208.044927 | 1.062507 |  |
| GO:0016568\_chromatin\_modification | 72 | 0 | 0.000000 | -0.000000 | 1077 | 1080.595073 | 1144.32 | 1208.044927 | 1.062507 |  |
| GO:0021915\_neural\_tube\_development | 72 | 0 | 0.000000 | -0.000000 | 1077 | 1080.595073 | 1144.32 | 1208.044927 | 1.062507 |  |
| GO:0030879\_mammary\_gland\_development | 72 | 0 | 0.000000 | -0.000000 | 1077 | 1080.595073 | 1144.32 | 1208.044927 | 1.062507 |  |
| GO:0040012\_regulation\_of\_locomotion | 72 | 0 | 0.000000 | -0.000000 | 1077 | 1080.595073 | 1144.32 | 1208.044927 | 1.062507 |  |
| GO:0042098\_T\_cell\_proliferation | 72 | 0 | 0.000000 | -0.000000 | 1077 | 1080.595073 | 1144.32 | 1208.044927 | 1.062507 |  |
| GO:0048839\_inner\_ear\_development | 72 | 0 | 0.000000 | -0.000000 | 1077 | 1080.595073 | 1144.32 | 1208.044927 | 1.062507 |  |
| GO:0050673\_epithelial\_cell\_proliferation | 72 | 0 | 0.000000 | -0.000000 | 1077 | 1080.595073 | 1144.32 | 1208.044927 | 1.062507 |  |
| GO:0035295\_tube\_development | 212 | 0 | 0.000000 | -0.000000 | 1078 | 1081.197940 | 1144.72 | 1208.242060 | 1.061892 |  |
| GO:0016337\_cell-cell\_adhesion | 87 | 0 | 0.000000 | -0.000000 | 1082 | 1086.565550 | 1149.57 | 1212.574450 | 1.062449 |  |
| GO:0022612\_gland\_morphogenesis | 87 | 0 | 0.000000 | -0.000000 | 1082 | 1086.565550 | 1149.57 | 1212.574450 | 1.062449 |  |
| GO:0043583\_ear\_development | 87 | 0 | 0.000000 | -0.000000 | 1082 | 1086.565550 | 1149.57 | 1212.574450 | 1.062449 |  |
| GO:0050778\_positive\_regulation\_of\_immune\_response | 87 | 0 | 0.000000 | -0.000000 | 1082 | 1086.565550 | 1149.57 | 1212.574450 | 1.062449 |  |
| GO:0002250\_adaptive\_immune\_response | 80 | 0 | 0.000000 | -0.000000 | 1085 | 1090.746427 | 1153.13 | 1215.513573 | 1.062793 |  |
| GO:0002460\_adaptive\_immune\_response\_based\_on\_somatic\_recombination\_of\_immune\_receptors\_built\_from\_immunoglobulin\_superfamily\_domains | 80 | 0 | 0.000000 | -0.000000 | 1085 | 1090.746427 | 1153.13 | 1215.513573 | 1.062793 |  |
| GO:0044092\_negative\_regulation\_of\_molecular\_function | 80 | 0 | 0.000000 | -0.000000 | 1085 | 1090.746427 | 1153.13 | 1215.513573 | 1.062793 |  |
| GO:0005976\_polysaccharide\_metabolic\_process | 39 | 0 | 0.000000 | -0.000000 | 1100 | 1103.706585 | 1165.64 | 1227.573415 | 1.059673 |  |
| GO:0006511\_ubiquitin-dependent\_protein\_catabolic\_process | 39 | 0 | 0.000000 | -0.000000 | 1100 | 1103.706585 | 1165.64 | 1227.573415 | 1.059673 |  |
| GO:0006644\_phospholipid\_metabolic\_process | 39 | 0 | 0.000000 | -0.000000 | 1100 | 1103.706585 | 1165.64 | 1227.573415 | 1.059673 |  |
| GO:0006730\_one-carbon\_metabolic\_process | 39 | 0 | 0.000000 | -0.000000 | 1100 | 1103.706585 | 1165.64 | 1227.573415 | 1.059673 |  |
| GO:0007160\_cell-matrix\_adhesion | 39 | 0 | 0.000000 | -0.000000 | 1100 | 1103.706585 | 1165.64 | 1227.573415 | 1.059673 |  |
| GO:0007286\_spermatid\_development | 39 | 0 | 0.000000 | -0.000000 | 1100 | 1103.706585 | 1165.64 | 1227.573415 | 1.059673 |  |
| GO:0008037\_cell\_recognition | 39 | 0 | 0.000000 | -0.000000 | 1100 | 1103.706585 | 1165.64 | 1227.573415 | 1.059673 |  |
| GO:0021953\_central\_nervous\_system\_neuron\_differentiation | 39 | 0 | 0.000000 | -0.000000 | 1100 | 1103.706585 | 1165.64 | 1227.573415 | 1.059673 |  |
| GO:0031279\_regulation\_of\_cyclase\_activity | 39 | 0 | 0.000000 | -0.000000 | 1100 | 1103.706585 | 1165.64 | 1227.573415 | 1.059673 |  |
| GO:0035148\_tube\_lumen\_formation | 39 | 0 | 0.000000 | -0.000000 | 1100 | 1103.706585 | 1165.64 | 1227.573415 | 1.059673 |  |
| GO:0042475\_odontogenesis\_of\_dentine-containing\_tooth | 39 | 0 | 0.000000 | -0.000000 | 1100 | 1103.706585 | 1165.64 | 1227.573415 | 1.059673 |  |
| GO:0043524\_negative\_regulation\_of\_neuron\_apoptosis | 39 | 0 | 0.000000 | -0.000000 | 1100 | 1103.706585 | 1165.64 | 1227.573415 | 1.059673 |  |
| GO:0048663\_neuron\_fate\_commitment | 39 | 0 | 0.000000 | -0.000000 | 1100 | 1103.706585 | 1165.64 | 1227.573415 | 1.059673 |  |
| GO:0051339\_regulation\_of\_lyase\_activity | 39 | 0 | 0.000000 | -0.000000 | 1100 | 1103.706585 | 1165.64 | 1227.573415 | 1.059673 |  |
| GO:0070201\_regulation\_of\_establishment\_of\_protein\_localization | 39 | 0 | 0.000000 | -0.000000 | 1100 | 1103.706585 | 1165.64 | 1227.573415 | 1.059673 |  |
| GO:0007369\_gastrulation | 63 | 0 | 0.000000 | -0.000000 | 1106 | 1108.962879 | 1170.36 | 1231.757121 | 1.058192 |  |
| GO:0009165\_nucleotide\_biosynthetic\_process | 63 | 0 | 0.000000 | -0.000000 | 1106 | 1108.962879 | 1170.36 | 1231.757121 | 1.058192 |  |
| GO:0051186\_cofactor\_metabolic\_process | 63 | 0 | 0.000000 | -0.000000 | 1106 | 1108.962879 | 1170.36 | 1231.757121 | 1.058192 |  |
| GO:0051216\_cartilage\_development | 63 | 0 | 0.000000 | -0.000000 | 1106 | 1108.962879 | 1170.36 | 1231.757121 | 1.058192 |  |
| GO:0070662\_mast\_cell\_proliferation | 63 | 0 | 0.000000 | -0.000000 | 1106 | 1108.962879 | 1170.36 | 1231.757121 | 1.058192 |  |
| GO:0070666\_regulation\_of\_mast\_cell\_proliferation | 63 | 0 | 0.000000 | -0.000000 | 1106 | 1108.962879 | 1170.36 | 1231.757121 | 1.058192 |  |
| GO:0048584\_positive\_regulation\_of\_response\_to\_stimulus | 115 | 0 | 0.000000 | -0.000000 | 1107 | 1110.408410 | 1171.44 | 1232.471590 | 1.058211 |  |
| GO:0055082\_cellular\_chemical\_homeostasis | 181 | 0 | 0.000000 | -0.000000 | 1108 | 1111.877916 | 1172.72 | 1233.562084 | 1.058412 |  |
| GO:0001816\_cytokine\_production | 122 | 0 | 0.000000 | -0.000000 | 1113 | 1116.261738 | 1176.37 | 1236.478262 | 1.056936 |  |
| GO:0002252\_immune\_effector\_process | 122 | 0 | 0.000000 | -0.000000 | 1113 | 1116.261738 | 1176.37 | 1236.478262 | 1.056936 |  |
| GO:0030001\_metal\_ion\_transport | 122 | 0 | 0.000000 | -0.000000 | 1113 | 1116.261738 | 1176.37 | 1236.478262 | 1.056936 |  |
| GO:0050865\_regulation\_of\_cell\_activation | 122 | 0 | 0.000000 | -0.000000 | 1113 | 1116.261738 | 1176.37 | 1236.478262 | 1.056936 |  |
| GO:0060284\_regulation\_of\_cell\_development | 122 | 0 | 0.000000 | -0.000000 | 1113 | 1116.261738 | 1176.37 | 1236.478262 | 1.056936 |  |
| GO:0001508\_regulation\_of\_action\_potential | 43 | 0 | 0.000000 | -0.000000 | 1131 | 1131.972157 | 1191.49 | 1251.007843 | 1.053484 |  |
| GO:0001841\_neural\_tube\_formation | 43 | 0 | 0.000000 | -0.000000 | 1131 | 1131.972157 | 1191.49 | 1251.007843 | 1.053484 |  |
| GO:0001894\_tissue\_homeostasis | 43 | 0 | 0.000000 | -0.000000 | 1131 | 1131.972157 | 1191.49 | 1251.007843 | 1.053484 |  |
| GO:0002819\_regulation\_of\_adaptive\_immune\_response | 43 | 0 | 0.000000 | -0.000000 | 1131 | 1131.972157 | 1191.49 | 1251.007843 | 1.053484 |  |
| GO:0002822\_regulation\_of\_adaptive\_immune\_response\_based\_on\_somatic\_recombination\_of\_immune\_receptors\_built\_from\_immunoglobulin\_superfamily\_domains | 43 | 0 | 0.000000 | -0.000000 | 1131 | 1131.972157 | 1191.49 | 1251.007843 | 1.053484 |  |
| GO:0006766\_vitamin\_metabolic\_process | 43 | 0 | 0.000000 | -0.000000 | 1131 | 1131.972157 | 1191.49 | 1251.007843 | 1.053484 |  |
| GO:0007224\_smoothened\_signaling\_pathway | 43 | 0 | 0.000000 | -0.000000 | 1131 | 1131.972157 | 1191.49 | 1251.007843 | 1.053484 |  |
| GO:0009582\_detection\_of\_abiotic\_stimulus | 43 | 0 | 0.000000 | -0.000000 | 1131 | 1131.972157 | 1191.49 | 1251.007843 | 1.053484 |  |
| GO:0010001\_glial\_cell\_differentiation | 43 | 0 | 0.000000 | -0.000000 | 1131 | 1131.972157 | 1191.49 | 1251.007843 | 1.053484 |  |
| GO:0019637\_organophosphate\_metabolic\_process | 43 | 0 | 0.000000 | -0.000000 | 1131 | 1131.972157 | 1191.49 | 1251.007843 | 1.053484 |  |
| GO:0030814\_regulation\_of\_cAMP\_metabolic\_process | 43 | 0 | 0.000000 | -0.000000 | 1131 | 1131.972157 | 1191.49 | 1251.007843 | 1.053484 |  |
| GO:0031098\_stress-activated\_protein\_kinase\_signaling\_pathway | 43 | 0 | 0.000000 | -0.000000 | 1131 | 1131.972157 | 1191.49 | 1251.007843 | 1.053484 |  |
| GO:0032446\_protein\_modification\_by\_small\_protein\_conjugation | 43 | 0 | 0.000000 | -0.000000 | 1131 | 1131.972157 | 1191.49 | 1251.007843 | 1.053484 |  |
| GO:0032868\_response\_to\_insulin\_stimulus | 43 | 0 | 0.000000 | -0.000000 | 1131 | 1131.972157 | 1191.49 | 1251.007843 | 1.053484 |  |
| GO:0046879\_hormone\_secretion | 43 | 0 | 0.000000 | -0.000000 | 1131 | 1131.972157 | 1191.49 | 1251.007843 | 1.053484 |  |
| GO:0048762\_mesenchymal\_cell\_differentiation | 43 | 0 | 0.000000 | -0.000000 | 1131 | 1131.972157 | 1191.49 | 1251.007843 | 1.053484 |  |
| GO:0051604\_protein\_maturation | 43 | 0 | 0.000000 | -0.000000 | 1131 | 1131.972157 | 1191.49 | 1251.007843 | 1.053484 |  |
| GO:0051789\_response\_to\_protein\_stimulus | 43 | 0 | 0.000000 | -0.000000 | 1131 | 1131.972157 | 1191.49 | 1251.007843 | 1.053484 |  |
| GO:0006576\_biogenic\_amine\_metabolic\_process | 53 | 0 | 0.000000 | -0.000000 | 1137 | 1138.934595 | 1197.96 | 1256.985405 | 1.053615 |  |
| GO:0006935\_chemotaxis | 53 | 0 | 0.000000 | -0.000000 | 1137 | 1138.934595 | 1197.96 | 1256.985405 | 1.053615 |  |
| GO:0030031\_cell\_projection\_assembly | 53 | 0 | 0.000000 | -0.000000 | 1137 | 1138.934595 | 1197.96 | 1256.985405 | 1.053615 |  |
| GO:0042330\_taxis | 53 | 0 | 0.000000 | -0.000000 | 1137 | 1138.934595 | 1197.96 | 1256.985405 | 1.053615 |  |
| GO:0050905\_neuromuscular\_process | 53 | 0 | 0.000000 | -0.000000 | 1137 | 1138.934595 | 1197.96 | 1256.985405 | 1.053615 |  |
| GO:0051248\_negative\_regulation\_of\_protein\_metabolic\_process | 53 | 0 | 0.000000 | -0.000000 | 1137 | 1138.934595 | 1197.96 | 1256.985405 | 1.053615 |  |
| GO:0001568\_blood\_vessel\_development | 203 | 0 | 0.000000 | -0.000000 | 1138 | 1139.476564 | 1198.45 | 1257.423436 | 1.053120 |  |
| GO:0002062\_chondrocyte\_differentiation | 28 | 0 | 0.000000 | -0.000000 | 1156 | 1156.166815 | 1214.56 | 1272.953185 | 1.050657 |  |
| GO:0002088\_lens\_development\_in\_camera-type\_eye | 28 | 0 | 0.000000 | -0.000000 | 1156 | 1156.166815 | 1214.56 | 1272.953185 | 1.050657 |  |
| GO:0002705\_positive\_regulation\_of\_leukocyte\_mediated\_immunity | 28 | 0 | 0.000000 | -0.000000 | 1156 | 1156.166815 | 1214.56 | 1272.953185 | 1.050657 |  |
| GO:0002708\_positive\_regulation\_of\_lymphocyte\_mediated\_immunity | 28 | 0 | 0.000000 | -0.000000 | 1156 | 1156.166815 | 1214.56 | 1272.953185 | 1.050657 |  |
| GO:0006470\_protein\_amino\_acid\_dephosphorylation | 28 | 0 | 0.000000 | -0.000000 | 1156 | 1156.166815 | 1214.56 | 1272.953185 | 1.050657 |  |
| GO:0006997\_nucleus\_organization | 28 | 0 | 0.000000 | -0.000000 | 1156 | 1156.166815 | 1214.56 | 1272.953185 | 1.050657 |  |
| GO:0007127\_meiosis\_I | 28 | 0 | 0.000000 | -0.000000 | 1156 | 1156.166815 | 1214.56 | 1272.953185 | 1.050657 |  |
| GO:0007585\_respiratory\_gaseous\_exchange | 28 | 0 | 0.000000 | -0.000000 | 1156 | 1156.166815 | 1214.56 | 1272.953185 | 1.050657 |  |
| GO:0021549\_cerebellum\_development | 28 | 0 | 0.000000 | -0.000000 | 1156 | 1156.166815 | 1214.56 | 1272.953185 | 1.050657 |  |
| GO:0030073\_insulin\_secretion | 28 | 0 | 0.000000 | -0.000000 | 1156 | 1156.166815 | 1214.56 | 1272.953185 | 1.050657 |  |
| GO:0030111\_regulation\_of\_Wnt\_receptor\_signaling\_pathway | 28 | 0 | 0.000000 | -0.000000 | 1156 | 1156.166815 | 1214.56 | 1272.953185 | 1.050657 |  |
| GO:0042100\_B\_cell\_proliferation | 28 | 0 | 0.000000 | -0.000000 | 1156 | 1156.166815 | 1214.56 | 1272.953185 | 1.050657 |  |
| GO:0043193\_positive\_regulation\_of\_gene-specific\_transcription | 28 | 0 | 0.000000 | -0.000000 | 1156 | 1156.166815 | 1214.56 | 1272.953185 | 1.050657 |  |
| GO:0045926\_negative\_regulation\_of\_growth | 28 | 0 | 0.000000 | -0.000000 | 1156 | 1156.166815 | 1214.56 | 1272.953185 | 1.050657 |  |
| GO:0046328\_regulation\_of\_JNK\_cascade | 28 | 0 | 0.000000 | -0.000000 | 1156 | 1156.166815 | 1214.56 | 1272.953185 | 1.050657 |  |
| GO:0048863\_stem\_cell\_differentiation | 28 | 0 | 0.000000 | -0.000000 | 1156 | 1156.166815 | 1214.56 | 1272.953185 | 1.050657 |  |
| GO:0050871\_positive\_regulation\_of\_B\_cell\_activation | 28 | 0 | 0.000000 | -0.000000 | 1156 | 1156.166815 | 1214.56 | 1272.953185 | 1.050657 |  |
| GO:0051188\_cofactor\_biosynthetic\_process | 28 | 0 | 0.000000 | -0.000000 | 1156 | 1156.166815 | 1214.56 | 1272.953185 | 1.050657 |  |
| GO:0035239\_tube\_morphogenesis | 143 | 0 | 0.000000 | -0.000000 | 1157 | 1157.288005 | 1215.5 | 1273.711995 | 1.050562 |  |
| GO:0009952\_anterior\_posterior\_pattern\_formation | 133 | 0 | 0.000000 | -0.000000 | 1159 | 1159.241830 | 1217.11 | 1274.978170 | 1.050138 |  |
| GO:0044057\_regulation\_of\_system\_process | 133 | 0 | 0.000000 | -0.000000 | 1159 | 1159.241830 | 1217.11 | 1274.978170 | 1.050138 |  |
| GO:0009416\_response\_to\_light\_stimulus | 74 | 0 | 0.000000 | -0.000000 | 1161 | 1162.106679 | 1219.51 | 1276.913321 | 1.050396 |  |
| GO:0048771\_tissue\_remodeling | 74 | 0 | 0.000000 | -0.000000 | 1161 | 1162.106679 | 1219.51 | 1276.913321 | 1.050396 |  |
| GO:0001843\_neural\_tube\_closure | 33 | 0 | 0.000000 | -0.000000 | 1174 | 1176.519348 | 1233.23 | 1289.940652 | 1.050451 |  |
| GO:0002562\_somatic\_diversification\_of\_immune\_receptors\_via\_germline\_recombination\_within\_a\_single\_locus | 33 | 0 | 0.000000 | -0.000000 | 1174 | 1176.519348 | 1233.23 | 1289.940652 | 1.050451 |  |
| GO:0006643\_membrane\_lipid\_metabolic\_process | 33 | 0 | 0.000000 | -0.000000 | 1174 | 1176.519348 | 1233.23 | 1289.940652 | 1.050451 |  |
| GO:0007188\_G-protein\_signaling\_\_coupled\_to\_cAMP\_nucleotide\_second\_messenger | 33 | 0 | 0.000000 | -0.000000 | 1174 | 1176.519348 | 1233.23 | 1289.940652 | 1.050451 |  |
| GO:0007431\_salivary\_gland\_development | 33 | 0 | 0.000000 | -0.000000 | 1174 | 1176.519348 | 1233.23 | 1289.940652 | 1.050451 |  |
| GO:0007565\_female\_pregnancy | 33 | 0 | 0.000000 | -0.000000 | 1174 | 1176.519348 | 1233.23 | 1289.940652 | 1.050451 |  |
| GO:0008584\_male\_gonad\_development | 33 | 0 | 0.000000 | -0.000000 | 1174 | 1176.519348 | 1233.23 | 1289.940652 | 1.050451 |  |
| GO:0008643\_carbohydrate\_transport | 33 | 0 | 0.000000 | -0.000000 | 1174 | 1176.519348 | 1233.23 | 1289.940652 | 1.050451 |  |
| GO:0016444\_somatic\_cell\_DNA\_recombination | 33 | 0 | 0.000000 | -0.000000 | 1174 | 1176.519348 | 1233.23 | 1289.940652 | 1.050451 |  |
| GO:0021536\_diencephalon\_development | 33 | 0 | 0.000000 | -0.000000 | 1174 | 1176.519348 | 1233.23 | 1289.940652 | 1.050451 |  |
| GO:0022037\_metencephalon\_development | 33 | 0 | 0.000000 | -0.000000 | 1174 | 1176.519348 | 1233.23 | 1289.940652 | 1.050451 |  |
| GO:0042108\_positive\_regulation\_of\_cytokine\_biosynthetic\_process | 33 | 0 | 0.000000 | -0.000000 | 1174 | 1176.519348 | 1233.23 | 1289.940652 | 1.050451 |  |
| GO:0060606\_tube\_closure | 33 | 0 | 0.000000 | -0.000000 | 1174 | 1176.519348 | 1233.23 | 1289.940652 | 1.050451 |  |
| GO:0007283\_spermatogenesis | 134 | 0 | 0.000000 | -0.000000 | 1176 | 1177.586904 | 1234.33 | 1291.073096 | 1.049600 |  |
| GO:0048232\_male\_gamete\_generation | 134 | 0 | 0.000000 | -0.000000 | 1176 | 1177.586904 | 1234.33 | 1291.073096 | 1.049600 |  |
| GO:0030036\_actin\_cytoskeleton\_organization | 102 | 0 | 0.000000 | -0.000000 | 1177 | 1178.168380 | 1234.86 | 1291.551620 | 1.049159 |  |
| GO:0050767\_regulation\_of\_neurogenesis | 104 | 0 | 0.000000 | -0.000000 | 1179 | 1179.712473 | 1236.19 | 1292.667527 | 1.048507 |  |
| GO:0055086\_nucleobase\_\_nucleoside\_and\_nucleotide\_metabolic\_process | 104 | 0 | 0.000000 | -0.000000 | 1179 | 1179.712473 | 1236.19 | 1292.667527 | 1.048507 |  |
| GO:0001934\_positive\_regulation\_of\_protein\_amino\_acid\_phosphorylation | 29 | 0 | 0.000000 | -0.000000 | 1197 | 1200.064732 | 1255.42 | 1310.775268 | 1.048805 |  |
| GO:0006417\_regulation\_of\_translation | 29 | 0 | 0.000000 | -0.000000 | 1197 | 1200.064732 | 1255.42 | 1310.775268 | 1.048805 |  |
| GO:0006641\_triglyceride\_metabolic\_process | 29 | 0 | 0.000000 | -0.000000 | 1197 | 1200.064732 | 1255.42 | 1310.775268 | 1.048805 |  |
| GO:0006909\_phagocytosis | 29 | 0 | 0.000000 | -0.000000 | 1197 | 1200.064732 | 1255.42 | 1310.775268 | 1.048805 |  |
| GO:0007190\_activation\_of\_adenylate\_cyclase\_activity | 29 | 0 | 0.000000 | -0.000000 | 1197 | 1200.064732 | 1255.42 | 1310.775268 | 1.048805 |  |
| GO:0016447\_somatic\_recombination\_of\_immunoglobulin\_gene\_segments | 29 | 0 | 0.000000 | -0.000000 | 1197 | 1200.064732 | 1255.42 | 1310.775268 | 1.048805 |  |
| GO:0021761\_limbic\_system\_development | 29 | 0 | 0.000000 | -0.000000 | 1197 | 1200.064732 | 1255.42 | 1310.775268 | 1.048805 |  |
| GO:0042176\_regulation\_of\_protein\_catabolic\_process | 29 | 0 | 0.000000 | -0.000000 | 1197 | 1200.064732 | 1255.42 | 1310.775268 | 1.048805 |  |
| GO:0042490\_mechanoreceptor\_differentiation | 29 | 0 | 0.000000 | -0.000000 | 1197 | 1200.064732 | 1255.42 | 1310.775268 | 1.048805 |  |
| GO:0044087\_regulation\_of\_cellular\_component\_biogenesis | 29 | 0 | 0.000000 | -0.000000 | 1197 | 1200.064732 | 1255.42 | 1310.775268 | 1.048805 |  |
| GO:0044270\_nitrogen\_compound\_catabolic\_process | 29 | 0 | 0.000000 | -0.000000 | 1197 | 1200.064732 | 1255.42 | 1310.775268 | 1.048805 |  |
| GO:0045621\_positive\_regulation\_of\_lymphocyte\_differentiation | 29 | 0 | 0.000000 | -0.000000 | 1197 | 1200.064732 | 1255.42 | 1310.775268 | 1.048805 |  |
| GO:0046634\_regulation\_of\_alpha-beta\_T\_cell\_activation | 29 | 0 | 0.000000 | -0.000000 | 1197 | 1200.064732 | 1255.42 | 1310.775268 | 1.048805 |  |
| GO:0048066\_pigmentation\_during\_development | 29 | 0 | 0.000000 | -0.000000 | 1197 | 1200.064732 | 1255.42 | 1310.775268 | 1.048805 |  |
| GO:0050769\_positive\_regulation\_of\_neurogenesis | 29 | 0 | 0.000000 | -0.000000 | 1197 | 1200.064732 | 1255.42 | 1310.775268 | 1.048805 |  |
| GO:0051301\_cell\_division | 29 | 0 | 0.000000 | -0.000000 | 1197 | 1200.064732 | 1255.42 | 1310.775268 | 1.048805 |  |
| GO:0060041\_retina\_development\_in\_camera-type\_eye | 29 | 0 | 0.000000 | -0.000000 | 1197 | 1200.064732 | 1255.42 | 1310.775268 | 1.048805 |  |
| GO:0070302\_regulation\_of\_stress-activated\_protein\_kinase\_signaling\_pathway | 29 | 0 | 0.000000 | -0.000000 | 1197 | 1200.064732 | 1255.42 | 1310.775268 | 1.048805 |  |
| GO:0000165\_MAPKKK\_cascade | 114 | 0 | 0.000000 | -0.000000 | 1199 | 1201.258291 | 1256.5 | 1311.741709 | 1.047957 |  |
| GO:0009607\_response\_to\_biotic\_stimulus | 114 | 0 | 0.000000 | -0.000000 | 1199 | 1201.258291 | 1256.5 | 1311.741709 | 1.047957 |  |
| GO:0000038\_very-long-chain\_fatty\_acid\_metabolic\_process | 6 | 0 | 0.000000 | -0.000000 | 1384 | 1385.400288 | 1439.13 | 1492.859712 | 1.039834 |  |
| GO:0000245\_spliceosome\_assembly | 6 | 0 | 0.000000 | -0.000000 | 1384 | 1385.400288 | 1439.13 | 1492.859712 | 1.039834 |  |
| GO:0000768\_syncytium\_formation\_by\_plasma\_membrane\_fusion | 6 | 0 | 0.000000 | -0.000000 | 1384 | 1385.400288 | 1439.13 | 1492.859712 | 1.039834 |  |
| GO:0001710\_mesodermal\_cell\_fate\_commitment | 6 | 0 | 0.000000 | -0.000000 | 1384 | 1385.400288 | 1439.13 | 1492.859712 | 1.039834 |  |
| GO:0001779\_natural\_killer\_cell\_differentiation | 6 | 0 | 0.000000 | -0.000000 | 1384 | 1385.400288 | 1439.13 | 1492.859712 | 1.039834 |  |
| GO:0001885\_endothelial\_cell\_development | 6 | 0 | 0.000000 | -0.000000 | 1384 | 1385.400288 | 1439.13 | 1492.859712 | 1.039834 |  |
| GO:0002016\_regulation\_of\_blood\_volume\_by\_renin-angiotensin | 6 | 0 | 0.000000 | -0.000000 | 1384 | 1385.400288 | 1439.13 | 1492.859712 | 1.039834 |  |
| GO:0002335\_mature\_B\_cell\_differentiation | 6 | 0 | 0.000000 | -0.000000 | 1384 | 1385.400288 | 1439.13 | 1492.859712 | 1.039834 |  |
| GO:0002360\_T\_cell\_lineage\_commitment | 6 | 0 | 0.000000 | -0.000000 | 1384 | 1385.400288 | 1439.13 | 1492.859712 | 1.039834 |  |
| GO:0002367\_cytokine\_production\_during\_immune\_response | 6 | 0 | 0.000000 | -0.000000 | 1384 | 1385.400288 | 1439.13 | 1492.859712 | 1.039834 |  |
| GO:0002474\_antigen\_processing\_and\_presentation\_of\_peptide\_antigen\_via\_MHC\_class\_I | 6 | 0 | 0.000000 | -0.000000 | 1384 | 1385.400288 | 1439.13 | 1492.859712 | 1.039834 |  |
| GO:0002475\_antigen\_processing\_and\_presentation\_via\_MHC\_class\_Ib | 6 | 0 | 0.000000 | -0.000000 | 1384 | 1385.400288 | 1439.13 | 1492.859712 | 1.039834 |  |
| GO:0002532\_production\_of\_molecular\_mediator\_of\_acute\_inflammatory\_response | 6 | 0 | 0.000000 | -0.000000 | 1384 | 1385.400288 | 1439.13 | 1492.859712 | 1.039834 |  |
| GO:0002541\_activation\_of\_plasma\_proteins\_involved\_in\_acute\_inflammatory\_response | 6 | 0 | 0.000000 | -0.000000 | 1384 | 1385.400288 | 1439.13 | 1492.859712 | 1.039834 |  |
| GO:0002675\_positive\_regulation\_of\_acute\_inflammatory\_response | 6 | 0 | 0.000000 | -0.000000 | 1384 | 1385.400288 | 1439.13 | 1492.859712 | 1.039834 |  |
| GO:0002685\_regulation\_of\_leukocyte\_migration | 6 | 0 | 0.000000 | -0.000000 | 1384 | 1385.400288 | 1439.13 | 1492.859712 | 1.039834 |  |
| GO:0002831\_regulation\_of\_response\_to\_biotic\_stimulus | 6 | 0 | 0.000000 | -0.000000 | 1384 | 1385.400288 | 1439.13 | 1492.859712 | 1.039834 |  |
| GO:0002920\_regulation\_of\_humoral\_immune\_response | 6 | 0 | 0.000000 | -0.000000 | 1384 | 1385.400288 | 1439.13 | 1492.859712 | 1.039834 |  |
| GO:0006071\_glycerol\_metabolic\_process | 6 | 0 | 0.000000 | -0.000000 | 1384 | 1385.400288 | 1439.13 | 1492.859712 | 1.039834 |  |
| GO:0006084\_acetyl-CoA\_metabolic\_process | 6 | 0 | 0.000000 | -0.000000 | 1384 | 1385.400288 | 1439.13 | 1492.859712 | 1.039834 |  |
| GO:0006264\_mitochondrial\_DNA\_replication | 6 | 0 | 0.000000 | -0.000000 | 1384 | 1385.400288 | 1439.13 | 1492.859712 | 1.039834 |  |
| GO:0006402\_mRNA\_catabolic\_process | 6 | 0 | 0.000000 | -0.000000 | 1384 | 1385.400288 | 1439.13 | 1492.859712 | 1.039834 |  |
| GO:0006471\_protein\_amino\_acid\_ADP-ribosylation | 6 | 0 | 0.000000 | -0.000000 | 1384 | 1385.400288 | 1439.13 | 1492.859712 | 1.039834 |  |
| GO:0006536\_glutamate\_metabolic\_process | 6 | 0 | 0.000000 | -0.000000 | 1384 | 1385.400288 | 1439.13 | 1492.859712 | 1.039834 |  |
| GO:0006656\_phosphatidylcholine\_biosynthetic\_process | 6 | 0 | 0.000000 | -0.000000 | 1384 | 1385.400288 | 1439.13 | 1492.859712 | 1.039834 |  |
| GO:0006692\_prostanoid\_metabolic\_process | 6 | 0 | 0.000000 | -0.000000 | 1384 | 1385.400288 | 1439.13 | 1492.859712 | 1.039834 |  |
| GO:0006693\_prostaglandin\_metabolic\_process | 6 | 0 | 0.000000 | -0.000000 | 1384 | 1385.400288 | 1439.13 | 1492.859712 | 1.039834 |  |
| GO:0006706\_steroid\_catabolic\_process | 6 | 0 | 0.000000 | -0.000000 | 1384 | 1385.400288 | 1439.13 | 1492.859712 | 1.039834 |  |
| GO:0006752\_group\_transfer\_coenzyme\_metabolic\_process | 6 | 0 | 0.000000 | -0.000000 | 1384 | 1385.400288 | 1439.13 | 1492.859712 | 1.039834 |  |
| GO:0006882\_cellular\_zinc\_ion\_homeostasis | 6 | 0 | 0.000000 | -0.000000 | 1384 | 1385.400288 | 1439.13 | 1492.859712 | 1.039834 |  |
| GO:0006942\_regulation\_of\_striated\_muscle\_contraction | 6 | 0 | 0.000000 | -0.000000 | 1384 | 1385.400288 | 1439.13 | 1492.859712 | 1.039834 |  |
| GO:0006956\_complement\_activation | 6 | 0 | 0.000000 | -0.000000 | 1384 | 1385.400288 | 1439.13 | 1492.859712 | 1.039834 |  |
| GO:0006998\_nuclear\_envelope\_organization | 6 | 0 | 0.000000 | -0.000000 | 1384 | 1385.400288 | 1439.13 | 1492.859712 | 1.039834 |  |
| GO:0007032\_endosome\_organization | 6 | 0 | 0.000000 | -0.000000 | 1384 | 1385.400288 | 1439.13 | 1492.859712 | 1.039834 |  |
| GO:0007176\_regulation\_of\_epidermal\_growth\_factor\_receptor\_activity | 6 | 0 | 0.000000 | -0.000000 | 1384 | 1385.400288 | 1439.13 | 1492.859712 | 1.039834 |  |
| GO:0007214\_gamma-aminobutyric\_acid\_signaling\_pathway | 6 | 0 | 0.000000 | -0.000000 | 1384 | 1385.400288 | 1439.13 | 1492.859712 | 1.039834 |  |
| GO:0007257\_activation\_of\_JUN\_kinase\_activity | 6 | 0 | 0.000000 | -0.000000 | 1384 | 1385.400288 | 1439.13 | 1492.859712 | 1.039834 |  |
| GO:0007341\_penetration\_of\_zona\_pellucida | 6 | 0 | 0.000000 | -0.000000 | 1384 | 1385.400288 | 1439.13 | 1492.859712 | 1.039834 |  |
| GO:0007406\_negative\_regulation\_of\_neuroblast\_proliferation | 6 | 0 | 0.000000 | -0.000000 | 1384 | 1385.400288 | 1439.13 | 1492.859712 | 1.039834 |  |
| GO:0007442\_hindgut\_morphogenesis | 6 | 0 | 0.000000 | -0.000000 | 1384 | 1385.400288 | 1439.13 | 1492.859712 | 1.039834 |  |
| GO:0007520\_myoblast\_fusion | 6 | 0 | 0.000000 | -0.000000 | 1384 | 1385.400288 | 1439.13 | 1492.859712 | 1.039834 |  |
| GO:0007620\_copulation | 6 | 0 | 0.000000 | -0.000000 | 1384 | 1385.400288 | 1439.13 | 1492.859712 | 1.039834 |  |
| GO:0008209\_androgen\_metabolic\_process | 6 | 0 | 0.000000 | -0.000000 | 1384 | 1385.400288 | 1439.13 | 1492.859712 | 1.039834 |  |
| GO:0008625\_induction\_of\_apoptosis\_via\_death\_domain\_receptors | 6 | 0 | 0.000000 | -0.000000 | 1384 | 1385.400288 | 1439.13 | 1492.859712 | 1.039834 |  |
| GO:0009067\_aspartate\_family\_amino\_acid\_biosynthetic\_process | 6 | 0 | 0.000000 | -0.000000 | 1384 | 1385.400288 | 1439.13 | 1492.859712 | 1.039834 |  |
| GO:0009069\_serine\_family\_amino\_acid\_metabolic\_process | 6 | 0 | 0.000000 | -0.000000 | 1384 | 1385.400288 | 1439.13 | 1492.859712 | 1.039834 |  |
| GO:0009112\_nucleobase\_metabolic\_process | 6 | 0 | 0.000000 | -0.000000 | 1384 | 1385.400288 | 1439.13 | 1492.859712 | 1.039834 |  |
| GO:0009143\_nucleoside\_triphosphate\_catabolic\_process | 6 | 0 | 0.000000 | -0.000000 | 1384 | 1385.400288 | 1439.13 | 1492.859712 | 1.039834 |  |
| GO:0009247\_glycolipid\_biosynthetic\_process | 6 | 0 | 0.000000 | -0.000000 | 1384 | 1385.400288 | 1439.13 | 1492.859712 | 1.039834 |  |
| GO:0009650\_UV\_protection | 6 | 0 | 0.000000 | -0.000000 | 1384 | 1385.400288 | 1439.13 | 1492.859712 | 1.039834 |  |
| GO:0009651\_response\_to\_salt\_stress | 6 | 0 | 0.000000 | -0.000000 | 1384 | 1385.400288 | 1439.13 | 1492.859712 | 1.039834 |  |
| GO:0010466\_negative\_regulation\_of\_peptidase\_activity | 6 | 0 | 0.000000 | -0.000000 | 1384 | 1385.400288 | 1439.13 | 1492.859712 | 1.039834 |  |
| GO:0010883\_regulation\_of\_lipid\_storage | 6 | 0 | 0.000000 | -0.000000 | 1384 | 1385.400288 | 1439.13 | 1492.859712 | 1.039834 |  |
| GO:0010906\_regulation\_of\_glucose\_metabolic\_process | 6 | 0 | 0.000000 | -0.000000 | 1384 | 1385.400288 | 1439.13 | 1492.859712 | 1.039834 |  |
| GO:0014003\_oligodendrocyte\_development | 6 | 0 | 0.000000 | -0.000000 | 1384 | 1385.400288 | 1439.13 | 1492.859712 | 1.039834 |  |
| GO:0014051\_gamma-aminobutyric\_acid\_secretion | 6 | 0 | 0.000000 | -0.000000 | 1384 | 1385.400288 | 1439.13 | 1492.859712 | 1.039834 |  |
| GO:0014072\_response\_to\_isoquinoline\_alkaloid | 6 | 0 | 0.000000 | -0.000000 | 1384 | 1385.400288 | 1439.13 | 1492.859712 | 1.039834 |  |
| GO:0014812\_muscle\_cell\_migration | 6 | 0 | 0.000000 | -0.000000 | 1384 | 1385.400288 | 1439.13 | 1492.859712 | 1.039834 |  |
| GO:0014823\_response\_to\_activity | 6 | 0 | 0.000000 | -0.000000 | 1384 | 1385.400288 | 1439.13 | 1492.859712 | 1.039834 |  |
| GO:0015012\_heparan\_sulfate\_proteoglycan\_biosynthetic\_process | 6 | 0 | 0.000000 | -0.000000 | 1384 | 1385.400288 | 1439.13 | 1492.859712 | 1.039834 |  |
| GO:0015812\_gamma-aminobutyric\_acid\_transport | 6 | 0 | 0.000000 | -0.000000 | 1384 | 1385.400288 | 1439.13 | 1492.859712 | 1.039834 |  |
| GO:0016032\_viral\_reproduction | 6 | 0 | 0.000000 | -0.000000 | 1384 | 1385.400288 | 1439.13 | 1492.859712 | 1.039834 |  |
| GO:0016574\_histone\_ubiquitination | 6 | 0 | 0.000000 | -0.000000 | 1384 | 1385.400288 | 1439.13 | 1492.859712 | 1.039834 |  |
| GO:0016925\_protein\_sumoylation | 6 | 0 | 0.000000 | -0.000000 | 1384 | 1385.400288 | 1439.13 | 1492.859712 | 1.039834 |  |
| GO:0019433\_triglyceride\_catabolic\_process | 6 | 0 | 0.000000 | -0.000000 | 1384 | 1385.400288 | 1439.13 | 1492.859712 | 1.039834 |  |
| GO:0019835\_cytolysis | 6 | 0 | 0.000000 | -0.000000 | 1384 | 1385.400288 | 1439.13 | 1492.859712 | 1.039834 |  |
| GO:0021548\_pons\_development | 6 | 0 | 0.000000 | -0.000000 | 1384 | 1385.400288 | 1439.13 | 1492.859712 | 1.039834 |  |
| GO:0021783\_preganglionic\_parasympathetic\_nervous\_system\_development | 6 | 0 | 0.000000 | -0.000000 | 1384 | 1385.400288 | 1439.13 | 1492.859712 | 1.039834 |  |
| GO:0021892\_cerebral\_cortex\_GABAergic\_interneuron\_differentiation | 6 | 0 | 0.000000 | -0.000000 | 1384 | 1385.400288 | 1439.13 | 1492.859712 | 1.039834 |  |
| GO:0021937\_Purkinje\_cell-granule\_cell\_precursor\_cell\_signaling\_involved\_in\_regulation\_of\_granule\_cell\_precursor\_cell\_proliferation | 6 | 0 | 0.000000 | -0.000000 | 1384 | 1385.400288 | 1439.13 | 1492.859712 | 1.039834 |  |
| GO:0022409\_positive\_regulation\_of\_cell-cell\_adhesion | 6 | 0 | 0.000000 | -0.000000 | 1384 | 1385.400288 | 1439.13 | 1492.859712 | 1.039834 |  |
| GO:0030002\_cellular\_anion\_homeostasis | 6 | 0 | 0.000000 | -0.000000 | 1384 | 1385.400288 | 1439.13 | 1492.859712 | 1.039834 |  |
| GO:0030149\_sphingolipid\_catabolic\_process | 6 | 0 | 0.000000 | -0.000000 | 1384 | 1385.400288 | 1439.13 | 1492.859712 | 1.039834 |  |
| GO:0030252\_growth\_hormone\_secretion | 6 | 0 | 0.000000 | -0.000000 | 1384 | 1385.400288 | 1439.13 | 1492.859712 | 1.039834 |  |
| GO:0030865\_cortical\_cytoskeleton\_organization | 6 | 0 | 0.000000 | -0.000000 | 1384 | 1385.400288 | 1439.13 | 1492.859712 | 1.039834 |  |
| GO:0030947\_regulation\_of\_vascular\_endothelial\_growth\_factor\_receptor\_signaling\_pathway | 6 | 0 | 0.000000 | -0.000000 | 1384 | 1385.400288 | 1439.13 | 1492.859712 | 1.039834 |  |
| GO:0031077\_post-embryonic\_camera-type\_eye\_development | 6 | 0 | 0.000000 | -0.000000 | 1384 | 1385.400288 | 1439.13 | 1492.859712 | 1.039834 |  |
| GO:0031330\_negative\_regulation\_of\_cellular\_catabolic\_process | 6 | 0 | 0.000000 | -0.000000 | 1384 | 1385.400288 | 1439.13 | 1492.859712 | 1.039834 |  |
| GO:0031575\_G1\_S\_transition\_checkpoint | 6 | 0 | 0.000000 | -0.000000 | 1384 | 1385.400288 | 1439.13 | 1492.859712 | 1.039834 |  |
| GO:0031960\_response\_to\_corticosteroid\_stimulus | 6 | 0 | 0.000000 | -0.000000 | 1384 | 1385.400288 | 1439.13 | 1492.859712 | 1.039834 |  |
| GO:0032042\_mitochondrial\_DNA\_metabolic\_process | 6 | 0 | 0.000000 | -0.000000 | 1384 | 1385.400288 | 1439.13 | 1492.859712 | 1.039834 |  |
| GO:0032331\_negative\_regulation\_of\_chondrocyte\_differentiation | 6 | 0 | 0.000000 | -0.000000 | 1384 | 1385.400288 | 1439.13 | 1492.859712 | 1.039834 |  |
| GO:0032392\_DNA\_geometric\_change | 6 | 0 | 0.000000 | -0.000000 | 1384 | 1385.400288 | 1439.13 | 1492.859712 | 1.039834 |  |
| GO:0032438\_melanosome\_organization | 6 | 0 | 0.000000 | -0.000000 | 1384 | 1385.400288 | 1439.13 | 1492.859712 | 1.039834 |  |
| GO:0032469\_endoplasmic\_reticulum\_calcium\_ion\_homeostasis | 6 | 0 | 0.000000 | -0.000000 | 1384 | 1385.400288 | 1439.13 | 1492.859712 | 1.039834 |  |
| GO:0032653\_regulation\_of\_interleukin-10\_production | 6 | 0 | 0.000000 | -0.000000 | 1384 | 1385.400288 | 1439.13 | 1492.859712 | 1.039834 |  |
| GO:0033238\_regulation\_of\_cellular\_amine\_metabolic\_process | 6 | 0 | 0.000000 | -0.000000 | 1384 | 1385.400288 | 1439.13 | 1492.859712 | 1.039834 |  |
| GO:0034968\_histone\_lysine\_methylation | 6 | 0 | 0.000000 | -0.000000 | 1384 | 1385.400288 | 1439.13 | 1492.859712 | 1.039834 |  |
| GO:0035019\_somatic\_stem\_cell\_maintenance | 6 | 0 | 0.000000 | -0.000000 | 1384 | 1385.400288 | 1439.13 | 1492.859712 | 1.039834 |  |
| GO:0035094\_response\_to\_nicotine | 6 | 0 | 0.000000 | -0.000000 | 1384 | 1385.400288 | 1439.13 | 1492.859712 | 1.039834 |  |
| GO:0035121\_tail\_morphogenesis | 6 | 0 | 0.000000 | -0.000000 | 1384 | 1385.400288 | 1439.13 | 1492.859712 | 1.039834 |  |
| GO:0040016\_embryonic\_cleavage | 6 | 0 | 0.000000 | -0.000000 | 1384 | 1385.400288 | 1439.13 | 1492.859712 | 1.039834 |  |
| GO:0040023\_establishment\_of\_nucleus\_localization | 6 | 0 | 0.000000 | -0.000000 | 1384 | 1385.400288 | 1439.13 | 1492.859712 | 1.039834 |  |
| GO:0040036\_regulation\_of\_fibroblast\_growth\_factor\_receptor\_signaling\_pathway | 6 | 0 | 0.000000 | -0.000000 | 1384 | 1385.400288 | 1439.13 | 1492.859712 | 1.039834 |  |
| GO:0042053\_regulation\_of\_dopamine\_metabolic\_process | 6 | 0 | 0.000000 | -0.000000 | 1384 | 1385.400288 | 1439.13 | 1492.859712 | 1.039834 |  |
| GO:0042069\_regulation\_of\_catecholamine\_metabolic\_process | 6 | 0 | 0.000000 | -0.000000 | 1384 | 1385.400288 | 1439.13 | 1492.859712 | 1.039834 |  |
| GO:0042246\_tissue\_regeneration | 6 | 0 | 0.000000 | -0.000000 | 1384 | 1385.400288 | 1439.13 | 1492.859712 | 1.039834 |  |
| GO:0042307\_positive\_regulation\_of\_protein\_import\_into\_nucleus | 6 | 0 | 0.000000 | -0.000000 | 1384 | 1385.400288 | 1439.13 | 1492.859712 | 1.039834 |  |
| GO:0042308\_negative\_regulation\_of\_protein\_import\_into\_nucleus | 6 | 0 | 0.000000 | -0.000000 | 1384 | 1385.400288 | 1439.13 | 1492.859712 | 1.039834 |  |
| GO:0042403\_thyroid\_hormone\_metabolic\_process | 6 | 0 | 0.000000 | -0.000000 | 1384 | 1385.400288 | 1439.13 | 1492.859712 | 1.039834 |  |
| GO:0042481\_regulation\_of\_odontogenesis | 6 | 0 | 0.000000 | -0.000000 | 1384 | 1385.400288 | 1439.13 | 1492.859712 | 1.039834 |  |
| GO:0042492\_gamma-delta\_T\_cell\_differentiation | 6 | 0 | 0.000000 | -0.000000 | 1384 | 1385.400288 | 1439.13 | 1492.859712 | 1.039834 |  |
| GO:0042953\_lipoprotein\_transport | 6 | 0 | 0.000000 | -0.000000 | 1384 | 1385.400288 | 1439.13 | 1492.859712 | 1.039834 |  |
| GO:0043064\_flagellum\_organization | 6 | 0 | 0.000000 | -0.000000 | 1384 | 1385.400288 | 1439.13 | 1492.859712 | 1.039834 |  |
| GO:0043154\_negative\_regulation\_of\_caspase\_activity | 6 | 0 | 0.000000 | -0.000000 | 1384 | 1385.400288 | 1439.13 | 1492.859712 | 1.039834 |  |
| GO:0043255\_regulation\_of\_carbohydrate\_biosynthetic\_process | 6 | 0 | 0.000000 | -0.000000 | 1384 | 1385.400288 | 1439.13 | 1492.859712 | 1.039834 |  |
| GO:0043271\_negative\_regulation\_of\_ion\_transport | 6 | 0 | 0.000000 | -0.000000 | 1384 | 1385.400288 | 1439.13 | 1492.859712 | 1.039834 |  |
| GO:0043278\_response\_to\_morphine | 6 | 0 | 0.000000 | -0.000000 | 1384 | 1385.400288 | 1439.13 | 1492.859712 | 1.039834 |  |
| GO:0043300\_regulation\_of\_leukocyte\_degranulation | 6 | 0 | 0.000000 | -0.000000 | 1384 | 1385.400288 | 1439.13 | 1492.859712 | 1.039834 |  |
| GO:0043467\_regulation\_of\_generation\_of\_precursor\_metabolites\_and\_energy | 6 | 0 | 0.000000 | -0.000000 | 1384 | 1385.400288 | 1439.13 | 1492.859712 | 1.039834 |  |
| GO:0043547\_positive\_regulation\_of\_GTPase\_activity | 6 | 0 | 0.000000 | -0.000000 | 1384 | 1385.400288 | 1439.13 | 1492.859712 | 1.039834 |  |
| GO:0043627\_response\_to\_estrogen\_stimulus | 6 | 0 | 0.000000 | -0.000000 | 1384 | 1385.400288 | 1439.13 | 1492.859712 | 1.039834 |  |
| GO:0044269\_glycerol\_ether\_catabolic\_process | 6 | 0 | 0.000000 | -0.000000 | 1384 | 1385.400288 | 1439.13 | 1492.859712 | 1.039834 |  |
| GO:0045072\_regulation\_of\_interferon-gamma\_biosynthetic\_process | 6 | 0 | 0.000000 | -0.000000 | 1384 | 1385.400288 | 1439.13 | 1492.859712 | 1.039834 |  |
| GO:0045084\_positive\_regulation\_of\_interleukin-12\_biosynthetic\_process | 6 | 0 | 0.000000 | -0.000000 | 1384 | 1385.400288 | 1439.13 | 1492.859712 | 1.039834 |  |
| GO:0045124\_regulation\_of\_bone\_resorption | 6 | 0 | 0.000000 | -0.000000 | 1384 | 1385.400288 | 1439.13 | 1492.859712 | 1.039834 |  |
| GO:0045176\_apical\_protein\_localization | 6 | 0 | 0.000000 | -0.000000 | 1384 | 1385.400288 | 1439.13 | 1492.859712 | 1.039834 |  |
| GO:0045540\_regulation\_of\_cholesterol\_biosynthetic\_process | 6 | 0 | 0.000000 | -0.000000 | 1384 | 1385.400288 | 1439.13 | 1492.859712 | 1.039834 |  |
| GO:0045579\_positive\_regulation\_of\_B\_cell\_differentiation | 6 | 0 | 0.000000 | -0.000000 | 1384 | 1385.400288 | 1439.13 | 1492.859712 | 1.039834 |  |
| GO:0045649\_regulation\_of\_macrophage\_differentiation | 6 | 0 | 0.000000 | -0.000000 | 1384 | 1385.400288 | 1439.13 | 1492.859712 | 1.039834 |  |
| GO:0045727\_positive\_regulation\_of\_translation | 6 | 0 | 0.000000 | -0.000000 | 1384 | 1385.400288 | 1439.13 | 1492.859712 | 1.039834 |  |
| GO:0045778\_positive\_regulation\_of\_ossification | 6 | 0 | 0.000000 | -0.000000 | 1384 | 1385.400288 | 1439.13 | 1492.859712 | 1.039834 |  |
| GO:0045822\_negative\_regulation\_of\_heart\_contraction | 6 | 0 | 0.000000 | -0.000000 | 1384 | 1385.400288 | 1439.13 | 1492.859712 | 1.039834 |  |
| GO:0045824\_negative\_regulation\_of\_innate\_immune\_response | 6 | 0 | 0.000000 | -0.000000 | 1384 | 1385.400288 | 1439.13 | 1492.859712 | 1.039834 |  |
| GO:0045833\_negative\_regulation\_of\_lipid\_metabolic\_process | 6 | 0 | 0.000000 | -0.000000 | 1384 | 1385.400288 | 1439.13 | 1492.859712 | 1.039834 |  |
| GO:0045843\_negative\_regulation\_of\_striated\_muscle\_development | 6 | 0 | 0.000000 | -0.000000 | 1384 | 1385.400288 | 1439.13 | 1492.859712 | 1.039834 |  |
| GO:0045861\_negative\_regulation\_of\_proteolysis | 6 | 0 | 0.000000 | -0.000000 | 1384 | 1385.400288 | 1439.13 | 1492.859712 | 1.039834 |  |
| GO:0045913\_positive\_regulation\_of\_carbohydrate\_metabolic\_process | 6 | 0 | 0.000000 | -0.000000 | 1384 | 1385.400288 | 1439.13 | 1492.859712 | 1.039834 |  |
| GO:0045931\_positive\_regulation\_of\_mitotic\_cell\_cycle | 6 | 0 | 0.000000 | -0.000000 | 1384 | 1385.400288 | 1439.13 | 1492.859712 | 1.039834 |  |
| GO:0045933\_positive\_regulation\_of\_muscle\_contraction | 6 | 0 | 0.000000 | -0.000000 | 1384 | 1385.400288 | 1439.13 | 1492.859712 | 1.039834 |  |
| GO:0046427\_positive\_regulation\_of\_JAK-STAT\_cascade | 6 | 0 | 0.000000 | -0.000000 | 1384 | 1385.400288 | 1439.13 | 1492.859712 | 1.039834 |  |
| GO:0046460\_neutral\_lipid\_biosynthetic\_process | 6 | 0 | 0.000000 | -0.000000 | 1384 | 1385.400288 | 1439.13 | 1492.859712 | 1.039834 |  |
| GO:0046461\_neutral\_lipid\_catabolic\_process | 6 | 0 | 0.000000 | -0.000000 | 1384 | 1385.400288 | 1439.13 | 1492.859712 | 1.039834 |  |
| GO:0046463\_acylglycerol\_biosynthetic\_process | 6 | 0 | 0.000000 | -0.000000 | 1384 | 1385.400288 | 1439.13 | 1492.859712 | 1.039834 |  |
| GO:0046464\_acylglycerol\_catabolic\_process | 6 | 0 | 0.000000 | -0.000000 | 1384 | 1385.400288 | 1439.13 | 1492.859712 | 1.039834 |  |
| GO:0046466\_membrane\_lipid\_catabolic\_process | 6 | 0 | 0.000000 | -0.000000 | 1384 | 1385.400288 | 1439.13 | 1492.859712 | 1.039834 |  |
| GO:0046503\_glycerolipid\_catabolic\_process | 6 | 0 | 0.000000 | -0.000000 | 1384 | 1385.400288 | 1439.13 | 1492.859712 | 1.039834 |  |
| GO:0046580\_negative\_regulation\_of\_Ras\_protein\_signal\_transduction | 6 | 0 | 0.000000 | -0.000000 | 1384 | 1385.400288 | 1439.13 | 1492.859712 | 1.039834 |  |
| GO:0046627\_negative\_regulation\_of\_insulin\_receptor\_signaling\_pathway | 6 | 0 | 0.000000 | -0.000000 | 1384 | 1385.400288 | 1439.13 | 1492.859712 | 1.039834 |  |
| GO:0046629\_gamma-delta\_T\_cell\_activation | 6 | 0 | 0.000000 | -0.000000 | 1384 | 1385.400288 | 1439.13 | 1492.859712 | 1.039834 |  |
| GO:0046666\_retinal\_cell\_programmed\_cell\_death | 6 | 0 | 0.000000 | -0.000000 | 1384 | 1385.400288 | 1439.13 | 1492.859712 | 1.039834 |  |
| GO:0046852\_positive\_regulation\_of\_bone\_remodeling | 6 | 0 | 0.000000 | -0.000000 | 1384 | 1385.400288 | 1439.13 | 1492.859712 | 1.039834 |  |
| GO:0046889\_positive\_regulation\_of\_lipid\_biosynthetic\_process | 6 | 0 | 0.000000 | -0.000000 | 1384 | 1385.400288 | 1439.13 | 1492.859712 | 1.039834 |  |
| GO:0048041\_focal\_adhesion\_formation | 6 | 0 | 0.000000 | -0.000000 | 1384 | 1385.400288 | 1439.13 | 1492.859712 | 1.039834 |  |
| GO:0048103\_somatic\_stem\_cell\_division | 6 | 0 | 0.000000 | -0.000000 | 1384 | 1385.400288 | 1439.13 | 1492.859712 | 1.039834 |  |
| GO:0048147\_negative\_regulation\_of\_fibroblast\_proliferation | 6 | 0 | 0.000000 | -0.000000 | 1384 | 1385.400288 | 1439.13 | 1492.859712 | 1.039834 |  |
| GO:0048333\_mesodermal\_cell\_differentiation | 6 | 0 | 0.000000 | -0.000000 | 1384 | 1385.400288 | 1439.13 | 1492.859712 | 1.039834 |  |
| GO:0048340\_paraxial\_mesoderm\_morphogenesis | 6 | 0 | 0.000000 | -0.000000 | 1384 | 1385.400288 | 1439.13 | 1492.859712 | 1.039834 |  |
| GO:0048541\_Peyer's\_patch\_development | 6 | 0 | 0.000000 | -0.000000 | 1384 | 1385.400288 | 1439.13 | 1492.859712 | 1.039834 |  |
| GO:0048563\_post-embryonic\_organ\_morphogenesis | 6 | 0 | 0.000000 | -0.000000 | 1384 | 1385.400288 | 1439.13 | 1492.859712 | 1.039834 |  |
| GO:0048617\_embryonic\_foregut\_morphogenesis | 6 | 0 | 0.000000 | -0.000000 | 1384 | 1385.400288 | 1439.13 | 1492.859712 | 1.039834 |  |
| GO:0048635\_negative\_regulation\_of\_muscle\_development | 6 | 0 | 0.000000 | -0.000000 | 1384 | 1385.400288 | 1439.13 | 1492.859712 | 1.039834 |  |
| GO:0048644\_muscle\_organ\_morphogenesis | 6 | 0 | 0.000000 | -0.000000 | 1384 | 1385.400288 | 1439.13 | 1492.859712 | 1.039834 |  |
| GO:0048703\_embryonic\_viscerocranium\_morphogenesis | 6 | 0 | 0.000000 | -0.000000 | 1384 | 1385.400288 | 1439.13 | 1492.859712 | 1.039834 |  |
| GO:0048713\_regulation\_of\_oligodendrocyte\_differentiation | 6 | 0 | 0.000000 | -0.000000 | 1384 | 1385.400288 | 1439.13 | 1492.859712 | 1.039834 |  |
| GO:0048853\_forebrain\_morphogenesis | 6 | 0 | 0.000000 | -0.000000 | 1384 | 1385.400288 | 1439.13 | 1492.859712 | 1.039834 |  |
| GO:0050732\_negative\_regulation\_of\_peptidyl-tyrosine\_phosphorylation | 6 | 0 | 0.000000 | -0.000000 | 1384 | 1385.400288 | 1439.13 | 1492.859712 | 1.039834 |  |
| GO:0050805\_negative\_regulation\_of\_synaptic\_transmission | 6 | 0 | 0.000000 | -0.000000 | 1384 | 1385.400288 | 1439.13 | 1492.859712 | 1.039834 |  |
| GO:0050821\_protein\_stabilization | 6 | 0 | 0.000000 | -0.000000 | 1384 | 1385.400288 | 1439.13 | 1492.859712 | 1.039834 |  |
| GO:0050829\_defense\_response\_to\_Gram-negative\_bacterium | 6 | 0 | 0.000000 | -0.000000 | 1384 | 1385.400288 | 1439.13 | 1492.859712 | 1.039834 |  |
| GO:0050872\_white\_fat\_cell\_differentiation | 6 | 0 | 0.000000 | -0.000000 | 1384 | 1385.400288 | 1439.13 | 1492.859712 | 1.039834 |  |
| GO:0050951\_sensory\_perception\_of\_temperature\_stimulus | 6 | 0 | 0.000000 | -0.000000 | 1384 | 1385.400288 | 1439.13 | 1492.859712 | 1.039834 |  |
| GO:0050966\_detection\_of\_mechanical\_stimulus\_involved\_in\_sensory\_perception\_of\_pain | 6 | 0 | 0.000000 | -0.000000 | 1384 | 1385.400288 | 1439.13 | 1492.859712 | 1.039834 |  |
| GO:0051058\_negative\_regulation\_of\_small\_GTPase\_mediated\_signal\_transduction | 6 | 0 | 0.000000 | -0.000000 | 1384 | 1385.400288 | 1439.13 | 1492.859712 | 1.039834 |  |
| GO:0051085\_chaperone\_mediated\_protein\_folding\_requiring\_cofactor | 6 | 0 | 0.000000 | -0.000000 | 1384 | 1385.400288 | 1439.13 | 1492.859712 | 1.039834 |  |
| GO:0051180\_vitamin\_transport | 6 | 0 | 0.000000 | -0.000000 | 1384 | 1385.400288 | 1439.13 | 1492.859712 | 1.039834 |  |
| GO:0051384\_response\_to\_glucocorticoid\_stimulus | 6 | 0 | 0.000000 | -0.000000 | 1384 | 1385.400288 | 1439.13 | 1492.859712 | 1.039834 |  |
| GO:0051592\_response\_to\_calcium\_ion | 6 | 0 | 0.000000 | -0.000000 | 1384 | 1385.400288 | 1439.13 | 1492.859712 | 1.039834 |  |
| GO:0051875\_pigment\_granule\_localization | 6 | 0 | 0.000000 | -0.000000 | 1384 | 1385.400288 | 1439.13 | 1492.859712 | 1.039834 |  |
| GO:0051881\_regulation\_of\_mitochondrial\_membrane\_potential | 6 | 0 | 0.000000 | -0.000000 | 1384 | 1385.400288 | 1439.13 | 1492.859712 | 1.039834 |  |
| GO:0051970\_negative\_regulation\_of\_transmission\_of\_nerve\_impulse | 6 | 0 | 0.000000 | -0.000000 | 1384 | 1385.400288 | 1439.13 | 1492.859712 | 1.039834 |  |
| GO:0055081\_anion\_homeostasis | 6 | 0 | 0.000000 | -0.000000 | 1384 | 1385.400288 | 1439.13 | 1492.859712 | 1.039834 |  |
| GO:0060013\_righting\_reflex | 6 | 0 | 0.000000 | -0.000000 | 1384 | 1385.400288 | 1439.13 | 1492.859712 | 1.039834 |  |
| GO:0060017\_parathyroid\_gland\_development | 6 | 0 | 0.000000 | -0.000000 | 1384 | 1385.400288 | 1439.13 | 1492.859712 | 1.039834 |  |
| GO:0060056\_mammary\_gland\_involution | 6 | 0 | 0.000000 | -0.000000 | 1384 | 1385.400288 | 1439.13 | 1492.859712 | 1.039834 |  |
| GO:0060068\_vagina\_development | 6 | 0 | 0.000000 | -0.000000 | 1384 | 1385.400288 | 1439.13 | 1492.859712 | 1.039834 |  |
| GO:0060134\_prepulse\_inhibition | 6 | 0 | 0.000000 | -0.000000 | 1384 | 1385.400288 | 1439.13 | 1492.859712 | 1.039834 |  |
| GO:0060136\_embryonic\_process\_involved\_in\_female\_pregnancy | 6 | 0 | 0.000000 | -0.000000 | 1384 | 1385.400288 | 1439.13 | 1492.859712 | 1.039834 |  |
| GO:0060271\_cilium\_morphogenesis | 6 | 0 | 0.000000 | -0.000000 | 1384 | 1385.400288 | 1439.13 | 1492.859712 | 1.039834 |  |
| GO:0060389\_pathway-restricted\_SMAD\_protein\_phosphorylation | 6 | 0 | 0.000000 | -0.000000 | 1384 | 1385.400288 | 1439.13 | 1492.859712 | 1.039834 |  |
| GO:0060411\_heart\_septum\_morphogenesis | 6 | 0 | 0.000000 | -0.000000 | 1384 | 1385.400288 | 1439.13 | 1492.859712 | 1.039834 |  |
| GO:0060638\_mesenchymal-epithelial\_cell\_signaling | 6 | 0 | 0.000000 | -0.000000 | 1384 | 1385.400288 | 1439.13 | 1492.859712 | 1.039834 |  |
| GO:0060685\_regulation\_of\_prostatic\_bud\_formation | 6 | 0 | 0.000000 | -0.000000 | 1384 | 1385.400288 | 1439.13 | 1492.859712 | 1.039834 |  |
| GO:0060710\_chorio-allantoic\_fusion | 6 | 0 | 0.000000 | -0.000000 | 1384 | 1385.400288 | 1439.13 | 1492.859712 | 1.039834 |  |
| GO:0065004\_protein-DNA\_complex\_assembly | 6 | 0 | 0.000000 | -0.000000 | 1384 | 1385.400288 | 1439.13 | 1492.859712 | 1.039834 |  |
| GO:0008406\_gonad\_development | 70 | 0 | 0.000000 | -0.000000 | 1388 | 1389.227110 | 1442.67 | 1496.112890 | 1.039388 |  |
| GO:0009617\_response\_to\_bacterium | 70 | 0 | 0.000000 | -0.000000 | 1388 | 1389.227110 | 1442.67 | 1496.112890 | 1.039388 |  |
| GO:0048592\_eye\_morphogenesis | 70 | 0 | 0.000000 | -0.000000 | 1388 | 1389.227110 | 1442.67 | 1496.112890 | 1.039388 |  |
| GO:0070838\_divalent\_metal\_ion\_transport | 70 | 0 | 0.000000 | -0.000000 | 1388 | 1389.227110 | 1442.67 | 1496.112890 | 1.039388 |  |
| GO:0000302\_response\_to\_reactive\_oxygen\_species | 16 | 0 | 0.000000 | -0.000000 | 1434 | 1435.755643 | 1488.12 | 1540.484357 | 1.037741 |  |
| GO:0001933\_negative\_regulation\_of\_protein\_amino\_acid\_phosphorylation | 16 | 0 | 0.000000 | -0.000000 | 1434 | 1435.755643 | 1488.12 | 1540.484357 | 1.037741 |  |
| GO:0003044\_regulation\_of\_systemic\_arterial\_blood\_pressure\_mediated\_by\_a\_chemical\_signal | 16 | 0 | 0.000000 | -0.000000 | 1434 | 1435.755643 | 1488.12 | 1540.484357 | 1.037741 |  |
| GO:0006664\_glycolipid\_metabolic\_process | 16 | 0 | 0.000000 | -0.000000 | 1434 | 1435.755643 | 1488.12 | 1540.484357 | 1.037741 |  |
| GO:0006821\_chloride\_transport | 16 | 0 | 0.000000 | -0.000000 | 1434 | 1435.755643 | 1488.12 | 1540.484357 | 1.037741 |  |
| GO:0007033\_vacuole\_organization | 16 | 0 | 0.000000 | -0.000000 | 1434 | 1435.755643 | 1488.12 | 1540.484357 | 1.037741 |  |
| GO:0007156\_homophilic\_cell\_adhesion | 16 | 0 | 0.000000 | -0.000000 | 1434 | 1435.755643 | 1488.12 | 1540.484357 | 1.037741 |  |
| GO:0007602\_phototransduction | 16 | 0 | 0.000000 | -0.000000 | 1434 | 1435.755643 | 1488.12 | 1540.484357 | 1.037741 |  |
| GO:0008654\_phospholipid\_biosynthetic\_process | 16 | 0 | 0.000000 | -0.000000 | 1434 | 1435.755643 | 1488.12 | 1540.484357 | 1.037741 |  |
| GO:0009988\_cell-cell\_recognition | 16 | 0 | 0.000000 | -0.000000 | 1434 | 1435.755643 | 1488.12 | 1540.484357 | 1.037741 |  |
| GO:0010038\_response\_to\_metal\_ion | 16 | 0 | 0.000000 | -0.000000 | 1434 | 1435.755643 | 1488.12 | 1540.484357 | 1.037741 |  |
| GO:0010243\_response\_to\_organic\_nitrogen | 16 | 0 | 0.000000 | -0.000000 | 1434 | 1435.755643 | 1488.12 | 1540.484357 | 1.037741 |  |
| GO:0010876\_lipid\_localization | 16 | 0 | 0.000000 | -0.000000 | 1434 | 1435.755643 | 1488.12 | 1540.484357 | 1.037741 |  |
| GO:0014075\_response\_to\_amine\_stimulus | 16 | 0 | 0.000000 | -0.000000 | 1434 | 1435.755643 | 1488.12 | 1540.484357 | 1.037741 |  |
| GO:0016126\_sterol\_biosynthetic\_process | 16 | 0 | 0.000000 | -0.000000 | 1434 | 1435.755643 | 1488.12 | 1540.484357 | 1.037741 |  |
| GO:0019722\_calcium-mediated\_signaling | 16 | 0 | 0.000000 | -0.000000 | 1434 | 1435.755643 | 1488.12 | 1540.484357 | 1.037741 |  |
| GO:0019751\_polyol\_metabolic\_process | 16 | 0 | 0.000000 | -0.000000 | 1434 | 1435.755643 | 1488.12 | 1540.484357 | 1.037741 |  |
| GO:0019915\_lipid\_storage | 16 | 0 | 0.000000 | -0.000000 | 1434 | 1435.755643 | 1488.12 | 1540.484357 | 1.037741 |  |
| GO:0021522\_spinal\_cord\_motor\_neuron\_differentiation | 16 | 0 | 0.000000 | -0.000000 | 1434 | 1435.755643 | 1488.12 | 1540.484357 | 1.037741 |  |
| GO:0021696\_cerebellar\_cortex\_morphogenesis | 16 | 0 | 0.000000 | -0.000000 | 1434 | 1435.755643 | 1488.12 | 1540.484357 | 1.037741 |  |
| GO:0030890\_positive\_regulation\_of\_B\_cell\_proliferation | 16 | 0 | 0.000000 | -0.000000 | 1434 | 1435.755643 | 1488.12 | 1540.484357 | 1.037741 |  |
| GO:0031345\_negative\_regulation\_of\_cell\_projection\_organization | 16 | 0 | 0.000000 | -0.000000 | 1434 | 1435.755643 | 1488.12 | 1540.484357 | 1.037741 |  |
| GO:0031669\_cellular\_response\_to\_nutrient\_levels | 16 | 0 | 0.000000 | -0.000000 | 1434 | 1435.755643 | 1488.12 | 1540.484357 | 1.037741 |  |
| GO:0032663\_regulation\_of\_interleukin-2\_production | 16 | 0 | 0.000000 | -0.000000 | 1434 | 1435.755643 | 1488.12 | 1540.484357 | 1.037741 |  |
| GO:0032956\_regulation\_of\_actin\_cytoskeleton\_organization | 16 | 0 | 0.000000 | -0.000000 | 1434 | 1435.755643 | 1488.12 | 1540.484357 | 1.037741 |  |
| GO:0034976\_response\_to\_endoplasmic\_reticulum\_stress | 16 | 0 | 0.000000 | -0.000000 | 1434 | 1435.755643 | 1488.12 | 1540.484357 | 1.037741 |  |
| GO:0042311\_vasodilation | 16 | 0 | 0.000000 | -0.000000 | 1434 | 1435.755643 | 1488.12 | 1540.484357 | 1.037741 |  |
| GO:0042594\_response\_to\_starvation | 16 | 0 | 0.000000 | -0.000000 | 1434 | 1435.755643 | 1488.12 | 1540.484357 | 1.037741 |  |
| GO:0043087\_regulation\_of\_GTPase\_activity | 16 | 0 | 0.000000 | -0.000000 | 1434 | 1435.755643 | 1488.12 | 1540.484357 | 1.037741 |  |
| GO:0043122\_regulation\_of\_I-kappaB\_kinase\_NF-kappaB\_cascade | 16 | 0 | 0.000000 | -0.000000 | 1434 | 1435.755643 | 1488.12 | 1540.484357 | 1.037741 |  |
| GO:0043367\_CD4-positive\_\_alpha\_beta\_T\_cell\_differentiation | 16 | 0 | 0.000000 | -0.000000 | 1434 | 1435.755643 | 1488.12 | 1540.484357 | 1.037741 |  |
| GO:0045104\_intermediate\_filament\_cytoskeleton\_organization | 16 | 0 | 0.000000 | -0.000000 | 1434 | 1435.755643 | 1488.12 | 1540.484357 | 1.037741 |  |
| GO:0046148\_pigment\_biosynthetic\_process | 16 | 0 | 0.000000 | -0.000000 | 1434 | 1435.755643 | 1488.12 | 1540.484357 | 1.037741 |  |
| GO:0046364\_monosaccharide\_biosynthetic\_process | 16 | 0 | 0.000000 | -0.000000 | 1434 | 1435.755643 | 1488.12 | 1540.484357 | 1.037741 |  |
| GO:0046467\_membrane\_lipid\_biosynthetic\_process | 16 | 0 | 0.000000 | -0.000000 | 1434 | 1435.755643 | 1488.12 | 1540.484357 | 1.037741 |  |
| GO:0046633\_alpha-beta\_T\_cell\_proliferation | 16 | 0 | 0.000000 | -0.000000 | 1434 | 1435.755643 | 1488.12 | 1540.484357 | 1.037741 |  |
| GO:0046700\_heterocycle\_catabolic\_process | 16 | 0 | 0.000000 | -0.000000 | 1434 | 1435.755643 | 1488.12 | 1540.484357 | 1.037741 |  |
| GO:0048015\_phosphoinositide-mediated\_signaling | 16 | 0 | 0.000000 | -0.000000 | 1434 | 1435.755643 | 1488.12 | 1540.484357 | 1.037741 |  |
| GO:0048286\_lung\_alveolus\_development | 16 | 0 | 0.000000 | -0.000000 | 1434 | 1435.755643 | 1488.12 | 1540.484357 | 1.037741 |  |
| GO:0048483\_autonomic\_nervous\_system\_development | 16 | 0 | 0.000000 | -0.000000 | 1434 | 1435.755643 | 1488.12 | 1540.484357 | 1.037741 |  |
| GO:0050974\_detection\_of\_mechanical\_stimulus\_involved\_in\_sensory\_perception | 16 | 0 | 0.000000 | -0.000000 | 1434 | 1435.755643 | 1488.12 | 1540.484357 | 1.037741 |  |
| GO:0051048\_negative\_regulation\_of\_secretion | 16 | 0 | 0.000000 | -0.000000 | 1434 | 1435.755643 | 1488.12 | 1540.484357 | 1.037741 |  |
| GO:0051937\_catecholamine\_transport | 16 | 0 | 0.000000 | -0.000000 | 1434 | 1435.755643 | 1488.12 | 1540.484357 | 1.037741 |  |
| GO:0055007\_cardiac\_muscle\_cell\_differentiation | 16 | 0 | 0.000000 | -0.000000 | 1434 | 1435.755643 | 1488.12 | 1540.484357 | 1.037741 |  |
| GO:0060193\_positive\_regulation\_of\_lipase\_activity | 16 | 0 | 0.000000 | -0.000000 | 1434 | 1435.755643 | 1488.12 | 1540.484357 | 1.037741 |  |
| GO:0060713\_labyrinthine\_layer\_morphogenesis | 16 | 0 | 0.000000 | -0.000000 | 1434 | 1435.755643 | 1488.12 | 1540.484357 | 1.037741 |  |
| GO:0000082\_G1\_S\_transition\_of\_mitotic\_cell\_cycle | 23 | 0 | 0.000000 | -0.000000 | 1455 | 1458.481510 | 1509.94 | 1561.398490 | 1.037759 |  |
| GO:0002204\_somatic\_recombination\_of\_immunoglobulin\_genes\_during\_immune\_response | 23 | 0 | 0.000000 | -0.000000 | 1455 | 1458.481510 | 1509.94 | 1561.398490 | 1.037759 |  |
| GO:0002208\_somatic\_diversification\_of\_immunoglobulins\_during\_immune\_response | 23 | 0 | 0.000000 | -0.000000 | 1455 | 1458.481510 | 1509.94 | 1561.398490 | 1.037759 |  |
| GO:0002228\_natural\_killer\_cell\_mediated\_immunity | 23 | 0 | 0.000000 | -0.000000 | 1455 | 1458.481510 | 1509.94 | 1561.398490 | 1.037759 |  |
| GO:0002821\_positive\_regulation\_of\_adaptive\_immune\_response | 23 | 0 | 0.000000 | -0.000000 | 1455 | 1458.481510 | 1509.94 | 1561.398490 | 1.037759 |  |
| GO:0002824\_positive\_regulation\_of\_adaptive\_immune\_response\_based\_on\_somatic\_recombination\_of\_immune\_receptors\_built\_from\_immunoglobulin\_superfamily\_domains | 23 | 0 | 0.000000 | -0.000000 | 1455 | 1458.481510 | 1509.94 | 1561.398490 | 1.037759 |  |
| GO:0003073\_regulation\_of\_systemic\_arterial\_blood\_pressure | 23 | 0 | 0.000000 | -0.000000 | 1455 | 1458.481510 | 1509.94 | 1561.398490 | 1.037759 |  |
| GO:0007018\_microtubule-based\_movement | 23 | 0 | 0.000000 | -0.000000 | 1455 | 1458.481510 | 1509.94 | 1561.398490 | 1.037759 |  |
| GO:0007163\_establishment\_or\_maintenance\_of\_cell\_polarity | 23 | 0 | 0.000000 | -0.000000 | 1455 | 1458.481510 | 1509.94 | 1561.398490 | 1.037759 |  |
| GO:0007584\_response\_to\_nutrient | 23 | 0 | 0.000000 | -0.000000 | 1455 | 1458.481510 | 1509.94 | 1561.398490 | 1.037759 |  |
| GO:0008542\_visual\_learning | 23 | 0 | 0.000000 | -0.000000 | 1455 | 1458.481510 | 1509.94 | 1561.398490 | 1.037759 |  |
| GO:0009954\_proximal\_distal\_pattern\_formation | 23 | 0 | 0.000000 | -0.000000 | 1455 | 1458.481510 | 1509.94 | 1561.398490 | 1.037759 |  |
| GO:0015698\_inorganic\_anion\_transport | 23 | 0 | 0.000000 | -0.000000 | 1455 | 1458.481510 | 1509.94 | 1561.398490 | 1.037759 |  |
| GO:0030512\_negative\_regulation\_of\_transforming\_growth\_factor\_beta\_receptor\_signaling\_pathway | 23 | 0 | 0.000000 | -0.000000 | 1455 | 1458.481510 | 1509.94 | 1561.398490 | 1.037759 |  |
| GO:0032635\_interleukin-6\_production | 23 | 0 | 0.000000 | -0.000000 | 1455 | 1458.481510 | 1509.94 | 1561.398490 | 1.037759 |  |
| GO:0032675\_regulation\_of\_interleukin-6\_production | 23 | 0 | 0.000000 | -0.000000 | 1455 | 1458.481510 | 1509.94 | 1561.398490 | 1.037759 |  |
| GO:0042267\_natural\_killer\_cell\_mediated\_cytotoxicity | 23 | 0 | 0.000000 | -0.000000 | 1455 | 1458.481510 | 1509.94 | 1561.398490 | 1.037759 |  |
| GO:0043388\_positive\_regulation\_of\_DNA\_binding | 23 | 0 | 0.000000 | -0.000000 | 1455 | 1458.481510 | 1509.94 | 1561.398490 | 1.037759 |  |
| GO:0045190\_isotype\_switching | 23 | 0 | 0.000000 | -0.000000 | 1455 | 1458.481510 | 1509.94 | 1561.398490 | 1.037759 |  |
| GO:0060349\_bone\_morphogenesis | 23 | 0 | 0.000000 | -0.000000 | 1455 | 1458.481510 | 1509.94 | 1561.398490 | 1.037759 |  |
| GO:0060445\_branching\_involved\_in\_salivary\_gland\_morphogenesis | 23 | 0 | 0.000000 | -0.000000 | 1455 | 1458.481510 | 1509.94 | 1561.398490 | 1.037759 |  |
| GO:0032268\_regulation\_of\_cellular\_protein\_metabolic\_process | 152 | 0 | 0.000000 | -0.000000 | 1456 | 1459.197593 | 1510.52 | 1561.842407 | 1.037445 |  |
| GO:0002791\_regulation\_of\_peptide\_secretion | 17 | 0 | 0.000000 | -0.000000 | 1494 | 1503.258804 | 1553.97 | 1604.681196 | 1.040141 |  |
| GO:0006767\_water-soluble\_vitamin\_metabolic\_process | 17 | 0 | 0.000000 | -0.000000 | 1494 | 1503.258804 | 1553.97 | 1604.681196 | 1.040141 |  |
| GO:0006813\_potassium\_ion\_transport | 17 | 0 | 0.000000 | -0.000000 | 1494 | 1503.258804 | 1553.97 | 1604.681196 | 1.040141 |  |
| GO:0006984\_ER-nuclear\_signaling\_pathway | 17 | 0 | 0.000000 | -0.000000 | 1494 | 1503.258804 | 1553.97 | 1604.681196 | 1.040141 |  |
| GO:0009408\_response\_to\_heat | 17 | 0 | 0.000000 | -0.000000 | 1494 | 1503.258804 | 1553.97 | 1604.681196 | 1.040141 |  |
| GO:0010035\_response\_to\_inorganic\_substance | 17 | 0 | 0.000000 | -0.000000 | 1494 | 1503.258804 | 1553.97 | 1604.681196 | 1.040141 |  |
| GO:0010517\_regulation\_of\_phospholipase\_activity | 17 | 0 | 0.000000 | -0.000000 | 1494 | 1503.258804 | 1553.97 | 1604.681196 | 1.040141 |  |
| GO:0010565\_regulation\_of\_cellular\_ketone\_metabolic\_process | 17 | 0 | 0.000000 | -0.000000 | 1494 | 1503.258804 | 1553.97 | 1604.681196 | 1.040141 |  |
| GO:0010741\_negative\_regulation\_of\_protein\_kinase\_cascade | 17 | 0 | 0.000000 | -0.000000 | 1494 | 1503.258804 | 1553.97 | 1604.681196 | 1.040141 |  |
| GO:0015807\_L-amino\_acid\_transport | 17 | 0 | 0.000000 | -0.000000 | 1494 | 1503.258804 | 1553.97 | 1604.681196 | 1.040141 |  |
| GO:0016052\_carbohydrate\_catabolic\_process | 17 | 0 | 0.000000 | -0.000000 | 1494 | 1503.258804 | 1553.97 | 1604.681196 | 1.040141 |  |
| GO:0021545\_cranial\_nerve\_development | 17 | 0 | 0.000000 | -0.000000 | 1494 | 1503.258804 | 1553.97 | 1604.681196 | 1.040141 |  |
| GO:0030101\_natural\_killer\_cell\_activation | 17 | 0 | 0.000000 | -0.000000 | 1494 | 1503.258804 | 1553.97 | 1604.681196 | 1.040141 |  |
| GO:0030317\_sperm\_motility | 17 | 0 | 0.000000 | -0.000000 | 1494 | 1503.258804 | 1553.97 | 1604.681196 | 1.040141 |  |
| GO:0032535\_regulation\_of\_cellular\_component\_size | 17 | 0 | 0.000000 | -0.000000 | 1494 | 1503.258804 | 1553.97 | 1604.681196 | 1.040141 |  |
| GO:0032970\_regulation\_of\_actin\_filament-based\_process | 17 | 0 | 0.000000 | -0.000000 | 1494 | 1503.258804 | 1553.97 | 1604.681196 | 1.040141 |  |
| GO:0034470\_ncRNA\_processing | 17 | 0 | 0.000000 | -0.000000 | 1494 | 1503.258804 | 1553.97 | 1604.681196 | 1.040141 |  |
| GO:0035115\_embryonic\_forelimb\_morphogenesis | 17 | 0 | 0.000000 | -0.000000 | 1494 | 1503.258804 | 1553.97 | 1604.681196 | 1.040141 |  |
| GO:0042384\_cilium\_assembly | 17 | 0 | 0.000000 | -0.000000 | 1494 | 1503.258804 | 1553.97 | 1604.681196 | 1.040141 |  |
| GO:0042398\_cellular\_amino\_acid\_derivative\_biosynthetic\_process | 17 | 0 | 0.000000 | -0.000000 | 1494 | 1503.258804 | 1553.97 | 1604.681196 | 1.040141 |  |
| GO:0042417\_dopamine\_metabolic\_process | 17 | 0 | 0.000000 | -0.000000 | 1494 | 1503.258804 | 1553.97 | 1604.681196 | 1.040141 |  |
| GO:0042440\_pigment\_metabolic\_process | 17 | 0 | 0.000000 | -0.000000 | 1494 | 1503.258804 | 1553.97 | 1604.681196 | 1.040141 |  |
| GO:0043407\_negative\_regulation\_of\_MAP\_kinase\_activity | 17 | 0 | 0.000000 | -0.000000 | 1494 | 1503.258804 | 1553.97 | 1604.681196 | 1.040141 |  |
| GO:0045333\_cellular\_respiration | 17 | 0 | 0.000000 | -0.000000 | 1494 | 1503.258804 | 1553.97 | 1604.681196 | 1.040141 |  |
| GO:0045667\_regulation\_of\_osteoblast\_differentiation | 17 | 0 | 0.000000 | -0.000000 | 1494 | 1503.258804 | 1553.97 | 1604.681196 | 1.040141 |  |
| GO:0045670\_regulation\_of\_osteoclast\_differentiation | 17 | 0 | 0.000000 | -0.000000 | 1494 | 1503.258804 | 1553.97 | 1604.681196 | 1.040141 |  |
| GO:0046637\_regulation\_of\_alpha-beta\_T\_cell\_differentiation | 17 | 0 | 0.000000 | -0.000000 | 1494 | 1503.258804 | 1553.97 | 1604.681196 | 1.040141 |  |
| GO:0048168\_regulation\_of\_neuronal\_synaptic\_plasticity | 17 | 0 | 0.000000 | -0.000000 | 1494 | 1503.258804 | 1553.97 | 1604.681196 | 1.040141 |  |
| GO:0048265\_response\_to\_pain | 17 | 0 | 0.000000 | -0.000000 | 1494 | 1503.258804 | 1553.97 | 1604.681196 | 1.040141 |  |
| GO:0048873\_homeostasis\_of\_number\_of\_cells\_within\_a\_tissue | 17 | 0 | 0.000000 | -0.000000 | 1494 | 1503.258804 | 1553.97 | 1604.681196 | 1.040141 |  |
| GO:0050708\_regulation\_of\_protein\_secretion | 17 | 0 | 0.000000 | -0.000000 | 1494 | 1503.258804 | 1553.97 | 1604.681196 | 1.040141 |  |
| GO:0051091\_positive\_regulation\_of\_transcription\_factor\_activity | 17 | 0 | 0.000000 | -0.000000 | 1494 | 1503.258804 | 1553.97 | 1604.681196 | 1.040141 |  |
| GO:0051605\_protein\_maturation\_by\_peptide\_bond\_cleavage | 17 | 0 | 0.000000 | -0.000000 | 1494 | 1503.258804 | 1553.97 | 1604.681196 | 1.040141 |  |
| GO:0055001\_muscle\_cell\_development | 17 | 0 | 0.000000 | -0.000000 | 1494 | 1503.258804 | 1553.97 | 1604.681196 | 1.040141 |  |
| GO:0055067\_monovalent\_inorganic\_cation\_homeostasis | 17 | 0 | 0.000000 | -0.000000 | 1494 | 1503.258804 | 1553.97 | 1604.681196 | 1.040141 |  |
| GO:0055072\_iron\_ion\_homeostasis | 17 | 0 | 0.000000 | -0.000000 | 1494 | 1503.258804 | 1553.97 | 1604.681196 | 1.040141 |  |
| GO:0060350\_endochondral\_bone\_morphogenesis | 17 | 0 | 0.000000 | -0.000000 | 1494 | 1503.258804 | 1553.97 | 1604.681196 | 1.040141 |  |
| GO:0090047\_positive\_regulation\_of\_transcription\_regulator\_activity | 17 | 0 | 0.000000 | -0.000000 | 1494 | 1503.258804 | 1553.97 | 1604.681196 | 1.040141 |  |
| GO:0002440\_production\_of\_molecular\_mediator\_of\_immune\_response | 49 | 0 | 0.000000 | -0.000000 | 1504 | 1513.671478 | 1563.8 | 1613.928522 | 1.039761 |  |
| GO:0003015\_heart\_process | 49 | 0 | 0.000000 | -0.000000 | 1504 | 1513.671478 | 1563.8 | 1613.928522 | 1.039761 |  |
| GO:0006725\_cellular\_aromatic\_compound\_metabolic\_process | 49 | 0 | 0.000000 | -0.000000 | 1504 | 1513.671478 | 1563.8 | 1613.928522 | 1.039761 |  |
| GO:0007606\_sensory\_perception\_of\_chemical\_stimulus | 49 | 0 | 0.000000 | -0.000000 | 1504 | 1513.671478 | 1563.8 | 1613.928522 | 1.039761 |  |
| GO:0034101\_erythrocyte\_homeostasis | 49 | 0 | 0.000000 | -0.000000 | 1504 | 1513.671478 | 1563.8 | 1613.928522 | 1.039761 |  |
| GO:0042035\_regulation\_of\_cytokine\_biosynthetic\_process | 49 | 0 | 0.000000 | -0.000000 | 1504 | 1513.671478 | 1563.8 | 1613.928522 | 1.039761 |  |
| GO:0043473\_pigmentation | 49 | 0 | 0.000000 | -0.000000 | 1504 | 1513.671478 | 1563.8 | 1613.928522 | 1.039761 |  |
| GO:0046660\_female\_sex\_differentiation | 49 | 0 | 0.000000 | -0.000000 | 1504 | 1513.671478 | 1563.8 | 1613.928522 | 1.039761 |  |
| GO:0046661\_male\_sex\_differentiation | 49 | 0 | 0.000000 | -0.000000 | 1504 | 1513.671478 | 1563.8 | 1613.928522 | 1.039761 |  |
| GO:0060047\_heart\_contraction | 49 | 0 | 0.000000 | -0.000000 | 1504 | 1513.671478 | 1563.8 | 1613.928522 | 1.039761 |  |
| GO:0000087\_M\_phase\_of\_mitotic\_cell\_cycle | 25 | 0 | 0.000000 | -0.000000 | 1529 | 1537.170976 | 1586.63 | 1636.089024 | 1.037691 |  |
| GO:0002526\_acute\_inflammatory\_response | 25 | 0 | 0.000000 | -0.000000 | 1529 | 1537.170976 | 1586.63 | 1636.089024 | 1.037691 |  |
| GO:0002700\_regulation\_of\_production\_of\_molecular\_mediator\_of\_immune\_response | 25 | 0 | 0.000000 | -0.000000 | 1529 | 1537.170976 | 1586.63 | 1636.089024 | 1.037691 |  |
| GO:0006302\_double-strand\_break\_repair | 25 | 0 | 0.000000 | -0.000000 | 1529 | 1537.170976 | 1586.63 | 1636.089024 | 1.037691 |  |
| GO:0006775\_fat-soluble\_vitamin\_metabolic\_process | 25 | 0 | 0.000000 | -0.000000 | 1529 | 1537.170976 | 1586.63 | 1636.089024 | 1.037691 |  |
| GO:0007492\_endoderm\_development | 25 | 0 | 0.000000 | -0.000000 | 1529 | 1537.170976 | 1586.63 | 1636.089024 | 1.037691 |  |
| GO:0007628\_adult\_walking\_behavior | 25 | 0 | 0.000000 | -0.000000 | 1529 | 1537.170976 | 1586.63 | 1636.089024 | 1.037691 |  |
| GO:0010811\_positive\_regulation\_of\_cell-substrate\_adhesion | 25 | 0 | 0.000000 | -0.000000 | 1529 | 1537.170976 | 1586.63 | 1636.089024 | 1.037691 |  |
| GO:0021983\_pituitary\_gland\_development | 25 | 0 | 0.000000 | -0.000000 | 1529 | 1537.170976 | 1586.63 | 1636.089024 | 1.037691 |  |
| GO:0031348\_negative\_regulation\_of\_defense\_response | 25 | 0 | 0.000000 | -0.000000 | 1529 | 1537.170976 | 1586.63 | 1636.089024 | 1.037691 |  |
| GO:0031400\_negative\_regulation\_of\_protein\_modification\_process | 25 | 0 | 0.000000 | -0.000000 | 1529 | 1537.170976 | 1586.63 | 1636.089024 | 1.037691 |  |
| GO:0032103\_positive\_regulation\_of\_response\_to\_external\_stimulus | 25 | 0 | 0.000000 | -0.000000 | 1529 | 1537.170976 | 1586.63 | 1636.089024 | 1.037691 |  |
| GO:0032945\_negative\_regulation\_of\_mononuclear\_cell\_proliferation | 25 | 0 | 0.000000 | -0.000000 | 1529 | 1537.170976 | 1586.63 | 1636.089024 | 1.037691 |  |
| GO:0035137\_hindlimb\_morphogenesis | 25 | 0 | 0.000000 | -0.000000 | 1529 | 1537.170976 | 1586.63 | 1636.089024 | 1.037691 |  |
| GO:0043543\_protein\_amino\_acid\_acylation | 25 | 0 | 0.000000 | -0.000000 | 1529 | 1537.170976 | 1586.63 | 1636.089024 | 1.037691 |  |
| GO:0045089\_positive\_regulation\_of\_innate\_immune\_response | 25 | 0 | 0.000000 | -0.000000 | 1529 | 1537.170976 | 1586.63 | 1636.089024 | 1.037691 |  |
| GO:0048285\_organelle\_fission | 25 | 0 | 0.000000 | -0.000000 | 1529 | 1537.170976 | 1586.63 | 1636.089024 | 1.037691 |  |
| GO:0050672\_negative\_regulation\_of\_lymphocyte\_proliferation | 25 | 0 | 0.000000 | -0.000000 | 1529 | 1537.170976 | 1586.63 | 1636.089024 | 1.037691 |  |
| GO:0050852\_T\_cell\_receptor\_signaling\_pathway | 25 | 0 | 0.000000 | -0.000000 | 1529 | 1537.170976 | 1586.63 | 1636.089024 | 1.037691 |  |
| GO:0051090\_regulation\_of\_transcription\_factor\_activity | 25 | 0 | 0.000000 | -0.000000 | 1529 | 1537.170976 | 1586.63 | 1636.089024 | 1.037691 |  |
| GO:0055123\_digestive\_system\_development | 25 | 0 | 0.000000 | -0.000000 | 1529 | 1537.170976 | 1586.63 | 1636.089024 | 1.037691 |  |
| GO:0060603\_mammary\_gland\_duct\_morphogenesis | 25 | 0 | 0.000000 | -0.000000 | 1529 | 1537.170976 | 1586.63 | 1636.089024 | 1.037691 |  |
| GO:0060688\_regulation\_of\_morphogenesis\_of\_a\_branching\_structure | 25 | 0 | 0.000000 | -0.000000 | 1529 | 1537.170976 | 1586.63 | 1636.089024 | 1.037691 |  |
| GO:0070664\_negative\_regulation\_of\_leukocyte\_proliferation | 25 | 0 | 0.000000 | -0.000000 | 1529 | 1537.170976 | 1586.63 | 1636.089024 | 1.037691 |  |
| GO:0090046\_regulation\_of\_transcription\_regulator\_activity | 25 | 0 | 0.000000 | -0.000000 | 1529 | 1537.170976 | 1586.63 | 1636.089024 | 1.037691 |  |
| GO:0007186\_G-protein\_coupled\_receptor\_protein\_signaling\_pathway | 144 | 0 | 0.000000 | -0.000000 | 1531 | 1538.834667 | 1588.11 | 1637.385333 | 1.037302 |  |
| GO:0045596\_negative\_regulation\_of\_cell\_differentiation | 144 | 0 | 0.000000 | -0.000000 | 1531 | 1538.834667 | 1588.11 | 1637.385333 | 1.037302 |  |
| GO:0001818\_negative\_regulation\_of\_cytokine\_production | 18 | 0 | 0.000000 | -0.000000 | 1581 | 1589.032314 | 1637.46 | 1685.887686 | 1.035712 |  |
| GO:0001825\_blastocyst\_formation | 18 | 0 | 0.000000 | -0.000000 | 1581 | 1589.032314 | 1637.46 | 1685.887686 | 1.035712 |  |
| GO:0001974\_blood\_vessel\_remodeling | 18 | 0 | 0.000000 | -0.000000 | 1581 | 1589.032314 | 1637.46 | 1685.887686 | 1.035712 |  |
| GO:0002064\_epithelial\_cell\_development | 18 | 0 | 0.000000 | -0.000000 | 1581 | 1589.032314 | 1637.46 | 1685.887686 | 1.035712 |  |
| GO:0002285\_lymphocyte\_activation\_during\_immune\_response | 18 | 0 | 0.000000 | -0.000000 | 1581 | 1589.032314 | 1637.46 | 1685.887686 | 1.035712 |  |
| GO:0002715\_regulation\_of\_natural\_killer\_cell\_mediated\_immunity | 18 | 0 | 0.000000 | -0.000000 | 1581 | 1589.032314 | 1637.46 | 1685.887686 | 1.035712 |  |
| GO:0003014\_renal\_system\_process | 18 | 0 | 0.000000 | -0.000000 | 1581 | 1589.032314 | 1637.46 | 1685.887686 | 1.035712 |  |
| GO:0006022\_aminoglycan\_metabolic\_process | 18 | 0 | 0.000000 | -0.000000 | 1581 | 1589.032314 | 1637.46 | 1685.887686 | 1.035712 |  |
| GO:0006457\_protein\_folding | 18 | 0 | 0.000000 | -0.000000 | 1581 | 1589.032314 | 1637.46 | 1685.887686 | 1.035712 |  |
| GO:0006940\_regulation\_of\_smooth\_muscle\_contraction | 18 | 0 | 0.000000 | -0.000000 | 1581 | 1589.032314 | 1637.46 | 1685.887686 | 1.035712 |  |
| GO:0007140\_male\_meiosis | 18 | 0 | 0.000000 | -0.000000 | 1581 | 1589.032314 | 1637.46 | 1685.887686 | 1.035712 |  |
| GO:0007608\_sensory\_perception\_of\_smell | 18 | 0 | 0.000000 | -0.000000 | 1581 | 1589.032314 | 1637.46 | 1685.887686 | 1.035712 |  |
| GO:0008589\_regulation\_of\_smoothened\_signaling\_pathway | 18 | 0 | 0.000000 | -0.000000 | 1581 | 1589.032314 | 1637.46 | 1685.887686 | 1.035712 |  |
| GO:0009063\_cellular\_amino\_acid\_catabolic\_process | 18 | 0 | 0.000000 | -0.000000 | 1581 | 1589.032314 | 1637.46 | 1685.887686 | 1.035712 |  |
| GO:0010498\_proteasomal\_protein\_catabolic\_process | 18 | 0 | 0.000000 | -0.000000 | 1581 | 1589.032314 | 1637.46 | 1685.887686 | 1.035712 |  |
| GO:0010553\_negative\_regulation\_of\_specific\_transcription\_from\_RNA\_polymerase\_II\_promoter | 18 | 0 | 0.000000 | -0.000000 | 1581 | 1589.032314 | 1637.46 | 1685.887686 | 1.035712 |  |
| GO:0015711\_organic\_anion\_transport | 18 | 0 | 0.000000 | -0.000000 | 1581 | 1589.032314 | 1637.46 | 1685.887686 | 1.035712 |  |
| GO:0016458\_gene\_silencing | 18 | 0 | 0.000000 | -0.000000 | 1581 | 1589.032314 | 1637.46 | 1685.887686 | 1.035712 |  |
| GO:0021517\_ventral\_spinal\_cord\_development | 18 | 0 | 0.000000 | -0.000000 | 1581 | 1589.032314 | 1637.46 | 1685.887686 | 1.035712 |  |
| GO:0030178\_negative\_regulation\_of\_Wnt\_receptor\_signaling\_pathway | 18 | 0 | 0.000000 | -0.000000 | 1581 | 1589.032314 | 1637.46 | 1685.887686 | 1.035712 |  |
| GO:0030203\_glycosaminoglycan\_metabolic\_process | 18 | 0 | 0.000000 | -0.000000 | 1581 | 1589.032314 | 1637.46 | 1685.887686 | 1.035712 |  |
| GO:0030282\_bone\_mineralization | 18 | 0 | 0.000000 | -0.000000 | 1581 | 1589.032314 | 1637.46 | 1685.887686 | 1.035712 |  |
| GO:0030318\_melanocyte\_differentiation | 18 | 0 | 0.000000 | -0.000000 | 1581 | 1589.032314 | 1637.46 | 1685.887686 | 1.035712 |  |
| GO:0030336\_negative\_regulation\_of\_cell\_migration | 18 | 0 | 0.000000 | -0.000000 | 1581 | 1589.032314 | 1637.46 | 1685.887686 | 1.035712 |  |
| GO:0030510\_regulation\_of\_BMP\_signaling\_pathway | 18 | 0 | 0.000000 | -0.000000 | 1581 | 1589.032314 | 1637.46 | 1685.887686 | 1.035712 |  |
| GO:0030901\_midbrain\_development | 18 | 0 | 0.000000 | -0.000000 | 1581 | 1589.032314 | 1637.46 | 1685.887686 | 1.035712 |  |
| GO:0032623\_interleukin-2\_production | 18 | 0 | 0.000000 | -0.000000 | 1581 | 1589.032314 | 1637.46 | 1685.887686 | 1.035712 |  |
| GO:0032984\_macromolecular\_complex\_disassembly | 18 | 0 | 0.000000 | -0.000000 | 1581 | 1589.032314 | 1637.46 | 1685.887686 | 1.035712 |  |
| GO:0033157\_regulation\_of\_intracellular\_protein\_transport | 18 | 0 | 0.000000 | -0.000000 | 1581 | 1589.032314 | 1637.46 | 1685.887686 | 1.035712 |  |
| GO:0035051\_cardiac\_cell\_differentiation | 18 | 0 | 0.000000 | -0.000000 | 1581 | 1589.032314 | 1637.46 | 1685.887686 | 1.035712 |  |
| GO:0042269\_regulation\_of\_natural\_killer\_cell\_mediated\_cytotoxicity | 18 | 0 | 0.000000 | -0.000000 | 1581 | 1589.032314 | 1637.46 | 1685.887686 | 1.035712 |  |
| GO:0043029\_T\_cell\_homeostasis | 18 | 0 | 0.000000 | -0.000000 | 1581 | 1589.032314 | 1637.46 | 1685.887686 | 1.035712 |  |
| GO:0043161\_proteasomal\_ubiquitin-dependent\_protein\_catabolic\_process | 18 | 0 | 0.000000 | -0.000000 | 1581 | 1589.032314 | 1637.46 | 1685.887686 | 1.035712 |  |
| GO:0044272\_sulfur\_compound\_biosynthetic\_process | 18 | 0 | 0.000000 | -0.000000 | 1581 | 1589.032314 | 1637.46 | 1685.887686 | 1.035712 |  |
| GO:0045058\_T\_cell\_selection | 18 | 0 | 0.000000 | -0.000000 | 1581 | 1589.032314 | 1637.46 | 1685.887686 | 1.035712 |  |
| GO:0045103\_intermediate\_filament-based\_process | 18 | 0 | 0.000000 | -0.000000 | 1581 | 1589.032314 | 1637.46 | 1685.887686 | 1.035712 |  |
| GO:0045638\_negative\_regulation\_of\_myeloid\_cell\_differentiation | 18 | 0 | 0.000000 | -0.000000 | 1581 | 1589.032314 | 1637.46 | 1685.887686 | 1.035712 |  |
| GO:0045807\_positive\_regulation\_of\_endocytosis | 18 | 0 | 0.000000 | -0.000000 | 1581 | 1589.032314 | 1637.46 | 1685.887686 | 1.035712 |  |
| GO:0046578\_regulation\_of\_Ras\_protein\_signal\_transduction | 18 | 0 | 0.000000 | -0.000000 | 1581 | 1589.032314 | 1637.46 | 1685.887686 | 1.035712 |  |
| GO:0046620\_regulation\_of\_organ\_growth | 18 | 0 | 0.000000 | -0.000000 | 1581 | 1589.032314 | 1637.46 | 1685.887686 | 1.035712 |  |
| GO:0048535\_lymph\_node\_development | 18 | 0 | 0.000000 | -0.000000 | 1581 | 1589.032314 | 1637.46 | 1685.887686 | 1.035712 |  |
| GO:0048730\_epidermis\_morphogenesis | 18 | 0 | 0.000000 | -0.000000 | 1581 | 1589.032314 | 1637.46 | 1685.887686 | 1.035712 |  |
| GO:0050731\_positive\_regulation\_of\_peptidyl-tyrosine\_phosphorylation | 18 | 0 | 0.000000 | -0.000000 | 1581 | 1589.032314 | 1637.46 | 1685.887686 | 1.035712 |  |
| GO:0050982\_detection\_of\_mechanical\_stimulus | 18 | 0 | 0.000000 | -0.000000 | 1581 | 1589.032314 | 1637.46 | 1685.887686 | 1.035712 |  |
| GO:0051222\_positive\_regulation\_of\_protein\_transport | 18 | 0 | 0.000000 | -0.000000 | 1581 | 1589.032314 | 1637.46 | 1685.887686 | 1.035712 |  |
| GO:0051924\_regulation\_of\_calcium\_ion\_transport | 18 | 0 | 0.000000 | -0.000000 | 1581 | 1589.032314 | 1637.46 | 1685.887686 | 1.035712 |  |
| GO:0055008\_cardiac\_muscle\_tissue\_morphogenesis | 18 | 0 | 0.000000 | -0.000000 | 1581 | 1589.032314 | 1637.46 | 1685.887686 | 1.035712 |  |
| GO:0060415\_muscle\_tissue\_morphogenesis | 18 | 0 | 0.000000 | -0.000000 | 1581 | 1589.032314 | 1637.46 | 1685.887686 | 1.035712 |  |
| GO:0060571\_morphogenesis\_of\_an\_epithelial\_fold | 18 | 0 | 0.000000 | -0.000000 | 1581 | 1589.032314 | 1637.46 | 1685.887686 | 1.035712 |  |
| GO:0060674\_placenta\_blood\_vessel\_development | 18 | 0 | 0.000000 | -0.000000 | 1581 | 1589.032314 | 1637.46 | 1685.887686 | 1.035712 |  |
| GO:0000122\_negative\_regulation\_of\_transcription\_from\_RNA\_polymerase\_II\_promoter | 175 | 0 | 0.000000 | -0.000000 | 1582 | 1590.695251 | 1638.93 | 1687.164749 | 1.035986 |  |
| GO:0045165\_cell\_fate\_commitment | 130 | 0 | 0.000000 | -0.000000 | 1584 | 1592.676808 | 1640.69 | 1688.703192 | 1.035789 |  |
| GO:0050776\_regulation\_of\_immune\_response | 130 | 0 | 0.000000 | -0.000000 | 1584 | 1592.676808 | 1640.69 | 1688.703192 | 1.035789 |  |
| GO:0006006\_glucose\_metabolic\_process | 42 | 0 | 0.000000 | -0.000000 | 1600 | 1609.324412 | 1656.39 | 1703.455588 | 1.035244 |  |
| GO:0006171\_cAMP\_biosynthetic\_process | 42 | 0 | 0.000000 | -0.000000 | 1600 | 1609.324412 | 1656.39 | 1703.455588 | 1.035244 |  |
| GO:0010740\_positive\_regulation\_of\_protein\_kinase\_cascade | 42 | 0 | 0.000000 | -0.000000 | 1600 | 1609.324412 | 1656.39 | 1703.455588 | 1.035244 |  |
| GO:0010769\_regulation\_of\_cell\_morphogenesis\_involved\_in\_differentiation | 42 | 0 | 0.000000 | -0.000000 | 1600 | 1609.324412 | 1656.39 | 1703.455588 | 1.035244 |  |
| GO:0015672\_monovalent\_inorganic\_cation\_transport | 42 | 0 | 0.000000 | -0.000000 | 1600 | 1609.324412 | 1656.39 | 1703.455588 | 1.035244 |  |
| GO:0016125\_sterol\_metabolic\_process | 42 | 0 | 0.000000 | -0.000000 | 1600 | 1609.324412 | 1656.39 | 1703.455588 | 1.035244 |  |
| GO:0019221\_cytokine-mediated\_signaling\_pathway | 42 | 0 | 0.000000 | -0.000000 | 1600 | 1609.324412 | 1656.39 | 1703.455588 | 1.035244 |  |
| GO:0019941\_modification-dependent\_protein\_catabolic\_process | 42 | 0 | 0.000000 | -0.000000 | 1600 | 1609.324412 | 1656.39 | 1703.455588 | 1.035244 |  |
| GO:0032946\_positive\_regulation\_of\_mononuclear\_cell\_proliferation | 42 | 0 | 0.000000 | -0.000000 | 1600 | 1609.324412 | 1656.39 | 1703.455588 | 1.035244 |  |
| GO:0042476\_odontogenesis | 42 | 0 | 0.000000 | -0.000000 | 1600 | 1609.324412 | 1656.39 | 1703.455588 | 1.035244 |  |
| GO:0043632\_modification-dependent\_macromolecule\_catabolic\_process | 42 | 0 | 0.000000 | -0.000000 | 1600 | 1609.324412 | 1656.39 | 1703.455588 | 1.035244 |  |
| GO:0045637\_regulation\_of\_myeloid\_cell\_differentiation | 42 | 0 | 0.000000 | -0.000000 | 1600 | 1609.324412 | 1656.39 | 1703.455588 | 1.035244 |  |
| GO:0048515\_spermatid\_differentiation | 42 | 0 | 0.000000 | -0.000000 | 1600 | 1609.324412 | 1656.39 | 1703.455588 | 1.035244 |  |
| GO:0050671\_positive\_regulation\_of\_lymphocyte\_proliferation | 42 | 0 | 0.000000 | -0.000000 | 1600 | 1609.324412 | 1656.39 | 1703.455588 | 1.035244 |  |
| GO:0050817\_coagulation | 42 | 0 | 0.000000 | -0.000000 | 1600 | 1609.324412 | 1656.39 | 1703.455588 | 1.035244 |  |
| GO:0051603\_proteolysis\_involved\_in\_cellular\_protein\_catabolic\_process | 42 | 0 | 0.000000 | -0.000000 | 1600 | 1609.324412 | 1656.39 | 1703.455588 | 1.035244 |  |
| GO:0000060\_protein\_import\_into\_nucleus\_\_translocation | 14 | 0 | 0.000000 | -0.000000 | 1664 | 1671.443480 | 1717.68 | 1763.916520 | 1.032260 |  |
| GO:0000077\_DNA\_damage\_checkpoint | 14 | 0 | 0.000000 | -0.000000 | 1664 | 1671.443480 | 1717.68 | 1763.916520 | 1.032260 |  |
| GO:0001502\_cartilage\_condensation | 14 | 0 | 0.000000 | -0.000000 | 1664 | 1671.443480 | 1717.68 | 1763.916520 | 1.032260 |  |
| GO:0001829\_trophectodermal\_cell\_differentiation | 14 | 0 | 0.000000 | -0.000000 | 1664 | 1671.443480 | 1717.68 | 1763.916520 | 1.032260 |  |
| GO:0002027\_regulation\_of\_heart\_rate | 14 | 0 | 0.000000 | -0.000000 | 1664 | 1671.443480 | 1717.68 | 1763.916520 | 1.032260 |  |
| GO:0002262\_myeloid\_cell\_homeostasis | 14 | 0 | 0.000000 | -0.000000 | 1664 | 1671.443480 | 1717.68 | 1763.916520 | 1.032260 |  |
| GO:0002698\_negative\_regulation\_of\_immune\_effector\_process | 14 | 0 | 0.000000 | -0.000000 | 1664 | 1671.443480 | 1717.68 | 1763.916520 | 1.032260 |  |
| GO:0006304\_DNA\_modification | 14 | 0 | 0.000000 | -0.000000 | 1664 | 1671.443480 | 1717.68 | 1763.916520 | 1.032260 |  |
| GO:0006305\_DNA\_alkylation | 14 | 0 | 0.000000 | -0.000000 | 1664 | 1671.443480 | 1717.68 | 1763.916520 | 1.032260 |  |
| GO:0006306\_DNA\_methylation | 14 | 0 | 0.000000 | -0.000000 | 1664 | 1671.443480 | 1717.68 | 1763.916520 | 1.032260 |  |
| GO:0006695\_cholesterol\_biosynthetic\_process | 14 | 0 | 0.000000 | -0.000000 | 1664 | 1671.443480 | 1717.68 | 1763.916520 | 1.032260 |  |
| GO:0006809\_nitric\_oxide\_biosynthetic\_process | 14 | 0 | 0.000000 | -0.000000 | 1664 | 1671.443480 | 1717.68 | 1763.916520 | 1.032260 |  |
| GO:0006914\_autophagy | 14 | 0 | 0.000000 | -0.000000 | 1664 | 1671.443480 | 1717.68 | 1763.916520 | 1.032260 |  |
| GO:0006970\_response\_to\_osmotic\_stress | 14 | 0 | 0.000000 | -0.000000 | 1664 | 1671.443480 | 1717.68 | 1763.916520 | 1.032260 |  |
| GO:0007157\_heterophilic\_cell\_adhesion | 14 | 0 | 0.000000 | -0.000000 | 1664 | 1671.443480 | 1717.68 | 1763.916520 | 1.032260 |  |
| GO:0007530\_sex\_determination | 14 | 0 | 0.000000 | -0.000000 | 1664 | 1671.443480 | 1717.68 | 1763.916520 | 1.032260 |  |
| GO:0007589\_body\_fluid\_secretion | 14 | 0 | 0.000000 | -0.000000 | 1664 | 1671.443480 | 1717.68 | 1763.916520 | 1.032260 |  |
| GO:0008064\_regulation\_of\_actin\_polymerization\_or\_depolymerization | 14 | 0 | 0.000000 | -0.000000 | 1664 | 1671.443480 | 1717.68 | 1763.916520 | 1.032260 |  |
| GO:0008306\_associative\_learning | 14 | 0 | 0.000000 | -0.000000 | 1664 | 1671.443480 | 1717.68 | 1763.916520 | 1.032260 |  |
| GO:0008630\_DNA\_damage\_response\_\_signal\_transduction\_resulting\_in\_induction\_of\_apoptosis | 14 | 0 | 0.000000 | -0.000000 | 1664 | 1671.443480 | 1717.68 | 1763.916520 | 1.032260 |  |
| GO:0009108\_coenzyme\_biosynthetic\_process | 14 | 0 | 0.000000 | -0.000000 | 1664 | 1671.443480 | 1717.68 | 1763.916520 | 1.032260 |  |
| GO:0009267\_cellular\_response\_to\_starvation | 14 | 0 | 0.000000 | -0.000000 | 1664 | 1671.443480 | 1717.68 | 1763.916520 | 1.032260 |  |
| GO:0009895\_negative\_regulation\_of\_catabolic\_process | 14 | 0 | 0.000000 | -0.000000 | 1664 | 1671.443480 | 1717.68 | 1763.916520 | 1.032260 |  |
| GO:0010332\_response\_to\_gamma\_radiation | 14 | 0 | 0.000000 | -0.000000 | 1664 | 1671.443480 | 1717.68 | 1763.916520 | 1.032260 |  |
| GO:0014855\_striated\_muscle\_cell\_proliferation | 14 | 0 | 0.000000 | -0.000000 | 1664 | 1671.443480 | 1717.68 | 1763.916520 | 1.032260 |  |
| GO:0016573\_histone\_acetylation | 14 | 0 | 0.000000 | -0.000000 | 1664 | 1671.443480 | 1717.68 | 1763.916520 | 1.032260 |  |
| GO:0018130\_heterocycle\_biosynthetic\_process | 14 | 0 | 0.000000 | -0.000000 | 1664 | 1671.443480 | 1717.68 | 1763.916520 | 1.032260 |  |
| GO:0019217\_regulation\_of\_fatty\_acid\_metabolic\_process | 14 | 0 | 0.000000 | -0.000000 | 1664 | 1671.443480 | 1717.68 | 1763.916520 | 1.032260 |  |
| GO:0021782\_glial\_cell\_development | 14 | 0 | 0.000000 | -0.000000 | 1664 | 1671.443480 | 1717.68 | 1763.916520 | 1.032260 |  |
| GO:0021904\_dorsal\_ventral\_neural\_tube\_patterning | 14 | 0 | 0.000000 | -0.000000 | 1664 | 1671.443480 | 1717.68 | 1763.916520 | 1.032260 |  |
| GO:0030032\_lamellipodium\_assembly | 14 | 0 | 0.000000 | -0.000000 | 1664 | 1671.443480 | 1717.68 | 1763.916520 | 1.032260 |  |
| GO:0030148\_sphingolipid\_biosynthetic\_process | 14 | 0 | 0.000000 | -0.000000 | 1664 | 1671.443480 | 1717.68 | 1763.916520 | 1.032260 |  |
| GO:0030162\_regulation\_of\_proteolysis | 14 | 0 | 0.000000 | -0.000000 | 1664 | 1671.443480 | 1717.68 | 1763.916520 | 1.032260 |  |
| GO:0030832\_regulation\_of\_actin\_filament\_length | 14 | 0 | 0.000000 | -0.000000 | 1664 | 1671.443480 | 1717.68 | 1763.916520 | 1.032260 |  |
| GO:0031099\_regeneration | 14 | 0 | 0.000000 | -0.000000 | 1664 | 1671.443480 | 1717.68 | 1763.916520 | 1.032260 |  |
| GO:0031346\_positive\_regulation\_of\_cell\_projection\_organization | 14 | 0 | 0.000000 | -0.000000 | 1664 | 1671.443480 | 1717.68 | 1763.916520 | 1.032260 |  |
| GO:0031663\_lipopolysaccharide-mediated\_signaling\_pathway | 14 | 0 | 0.000000 | -0.000000 | 1664 | 1671.443480 | 1717.68 | 1763.916520 | 1.032260 |  |
| GO:0032271\_regulation\_of\_protein\_polymerization | 14 | 0 | 0.000000 | -0.000000 | 1664 | 1671.443480 | 1717.68 | 1763.916520 | 1.032260 |  |
| GO:0033044\_regulation\_of\_chromosome\_organization | 14 | 0 | 0.000000 | -0.000000 | 1664 | 1671.443480 | 1717.68 | 1763.916520 | 1.032260 |  |
| GO:0034104\_negative\_regulation\_of\_tissue\_remodeling | 14 | 0 | 0.000000 | -0.000000 | 1664 | 1671.443480 | 1717.68 | 1763.916520 | 1.032260 |  |
| GO:0034623\_cellular\_macromolecular\_complex\_disassembly | 14 | 0 | 0.000000 | -0.000000 | 1664 | 1671.443480 | 1717.68 | 1763.916520 | 1.032260 |  |
| GO:0035036\_sperm-egg\_recognition | 14 | 0 | 0.000000 | -0.000000 | 1664 | 1671.443480 | 1717.68 | 1763.916520 | 1.032260 |  |
| GO:0042310\_vasoconstriction | 14 | 0 | 0.000000 | -0.000000 | 1664 | 1671.443480 | 1717.68 | 1763.916520 | 1.032260 |  |
| GO:0042573\_retinoic\_acid\_metabolic\_process | 14 | 0 | 0.000000 | -0.000000 | 1664 | 1671.443480 | 1717.68 | 1763.916520 | 1.032260 |  |
| GO:0043123\_positive\_regulation\_of\_I-kappaB\_kinase\_NF-kappaB\_cascade | 14 | 0 | 0.000000 | -0.000000 | 1664 | 1671.443480 | 1717.68 | 1763.916520 | 1.032260 |  |
| GO:0043254\_regulation\_of\_protein\_complex\_assembly | 14 | 0 | 0.000000 | -0.000000 | 1664 | 1671.443480 | 1717.68 | 1763.916520 | 1.032260 |  |
| GO:0043491\_protein\_kinase\_B\_signaling\_cascade | 14 | 0 | 0.000000 | -0.000000 | 1664 | 1671.443480 | 1717.68 | 1763.916520 | 1.032260 |  |
| GO:0044236\_multicellular\_organismal\_metabolic\_process | 14 | 0 | 0.000000 | -0.000000 | 1664 | 1671.443480 | 1717.68 | 1763.916520 | 1.032260 |  |
| GO:0045061\_thymic\_T\_cell\_selection | 14 | 0 | 0.000000 | -0.000000 | 1664 | 1671.443480 | 1717.68 | 1763.916520 | 1.032260 |  |
| GO:0045453\_bone\_resorption | 14 | 0 | 0.000000 | -0.000000 | 1664 | 1671.443480 | 1717.68 | 1763.916520 | 1.032260 |  |
| GO:0045598\_regulation\_of\_fat\_cell\_differentiation | 14 | 0 | 0.000000 | -0.000000 | 1664 | 1671.443480 | 1717.68 | 1763.916520 | 1.032260 |  |
| GO:0045732\_positive\_regulation\_of\_protein\_catabolic\_process | 14 | 0 | 0.000000 | -0.000000 | 1664 | 1671.443480 | 1717.68 | 1763.916520 | 1.032260 |  |
| GO:0046209\_nitric\_oxide\_metabolic\_process | 14 | 0 | 0.000000 | -0.000000 | 1664 | 1671.443480 | 1717.68 | 1763.916520 | 1.032260 |  |
| GO:0048048\_embryonic\_eye\_morphogenesis | 14 | 0 | 0.000000 | -0.000000 | 1664 | 1671.443480 | 1717.68 | 1763.916520 | 1.032260 |  |
| GO:0048545\_response\_to\_steroid\_hormone\_stimulus | 14 | 0 | 0.000000 | -0.000000 | 1664 | 1671.443480 | 1717.68 | 1763.916520 | 1.032260 |  |
| GO:0048665\_neuron\_fate\_specification | 14 | 0 | 0.000000 | -0.000000 | 1664 | 1671.443480 | 1717.68 | 1763.916520 | 1.032260 |  |
| GO:0048844\_artery\_morphogenesis | 14 | 0 | 0.000000 | -0.000000 | 1664 | 1671.443480 | 1717.68 | 1763.916520 | 1.032260 |  |
| GO:0050810\_regulation\_of\_steroid\_biosynthetic\_process | 14 | 0 | 0.000000 | -0.000000 | 1664 | 1671.443480 | 1717.68 | 1763.916520 | 1.032260 |  |
| GO:0051017\_actin\_filament\_bundle\_formation | 14 | 0 | 0.000000 | -0.000000 | 1664 | 1671.443480 | 1717.68 | 1763.916520 | 1.032260 |  |
| GO:0051054\_positive\_regulation\_of\_DNA\_metabolic\_process | 14 | 0 | 0.000000 | -0.000000 | 1664 | 1671.443480 | 1717.68 | 1763.916520 | 1.032260 |  |
| GO:0051100\_negative\_regulation\_of\_binding | 14 | 0 | 0.000000 | -0.000000 | 1664 | 1671.443480 | 1717.68 | 1763.916520 | 1.032260 |  |
| GO:0051952\_regulation\_of\_amine\_transport | 14 | 0 | 0.000000 | -0.000000 | 1664 | 1671.443480 | 1717.68 | 1763.916520 | 1.032260 |  |
| GO:0060716\_labyrinthine\_layer\_blood\_vessel\_development | 14 | 0 | 0.000000 | -0.000000 | 1664 | 1671.443480 | 1717.68 | 1763.916520 | 1.032260 |  |
| GO:0060840\_artery\_development | 14 | 0 | 0.000000 | -0.000000 | 1664 | 1671.443480 | 1717.68 | 1763.916520 | 1.032260 |  |
| GO:0007276\_gamete\_generation | 188 | 0 | 0.000000 | -0.000000 | 1665 | 1671.896335 | 1718.15 | 1764.403665 | 1.031922 |  |
| GO:0002706\_regulation\_of\_lymphocyte\_mediated\_immunity | 52 | 0 | 0.000000 | -0.000000 | 1670 | 1676.438515 | 1722.26 | 1768.081485 | 1.031293 |  |
| GO:0009124\_nucleoside\_monophosphate\_biosynthetic\_process | 52 | 0 | 0.000000 | -0.000000 | 1670 | 1676.438515 | 1722.26 | 1768.081485 | 1.031293 |  |
| GO:0010608\_posttranscriptional\_regulation\_of\_gene\_expression | 52 | 0 | 0.000000 | -0.000000 | 1670 | 1676.438515 | 1722.26 | 1768.081485 | 1.031293 |  |
| GO:0048585\_negative\_regulation\_of\_response\_to\_stimulus | 52 | 0 | 0.000000 | -0.000000 | 1670 | 1676.438515 | 1722.26 | 1768.081485 | 1.031293 |  |
| GO:0050953\_sensory\_perception\_of\_light\_stimulus | 52 | 0 | 0.000000 | -0.000000 | 1670 | 1676.438515 | 1722.26 | 1768.081485 | 1.031293 |  |
| GO:0046483\_heterocycle\_metabolic\_process | 116 | 0 | 0.000000 | -0.000000 | 1672 | 1678.569055 | 1724.11 | 1769.650945 | 1.031166 |  |
| GO:0048608\_reproductive\_structure\_development | 116 | 0 | 0.000000 | -0.000000 | 1672 | 1678.569055 | 1724.11 | 1769.650945 | 1.031166 |  |
| GO:0006811\_ion\_transport | 186 | 0 | 0.000000 | -0.000000 | 1675 | 1680.080596 | 1725.38 | 1770.679404 | 1.030078 |  |
| GO:0007155\_cell\_adhesion | 186 | 0 | 0.000000 | -0.000000 | 1675 | 1680.080596 | 1725.38 | 1770.679404 | 1.030078 |  |
| GO:0022610\_biological\_adhesion | 186 | 0 | 0.000000 | -0.000000 | 1675 | 1680.080596 | 1725.38 | 1770.679404 | 1.030078 |  |
| GO:0000045\_autophagic\_vacuole\_formation | 5 | 0 | 0.000000 | -0.000000 | 1960 | 1964.662348 | 2007.28 | 2049.897652 | 1.024122 |  |
| GO:0000070\_mitotic\_sister\_chromatid\_segregation | 5 | 0 | 0.000000 | -0.000000 | 1960 | 1964.662348 | 2007.28 | 2049.897652 | 1.024122 |  |
| GO:0000097\_sulfur\_amino\_acid\_biosynthetic\_process | 5 | 0 | 0.000000 | -0.000000 | 1960 | 1964.662348 | 2007.28 | 2049.897652 | 1.024122 |  |
| GO:0000239\_pachytene | 5 | 0 | 0.000000 | -0.000000 | 1960 | 1964.662348 | 2007.28 | 2049.897652 | 1.024122 |  |
| GO:0000272\_polysaccharide\_catabolic\_process | 5 | 0 | 0.000000 | -0.000000 | 1960 | 1964.662348 | 2007.28 | 2049.897652 | 1.024122 |  |
| GO:0000288\_nuclear-transcribed\_mRNA\_catabolic\_process\_\_deadenylation-dependent\_decay | 5 | 0 | 0.000000 | -0.000000 | 1960 | 1964.662348 | 2007.28 | 2049.897652 | 1.024122 |  |
| GO:0000578\_embryonic\_axis\_specification | 5 | 0 | 0.000000 | -0.000000 | 1960 | 1964.662348 | 2007.28 | 2049.897652 | 1.024122 |  |
| GO:0000726\_non-recombinational\_repair | 5 | 0 | 0.000000 | -0.000000 | 1960 | 1964.662348 | 2007.28 | 2049.897652 | 1.024122 |  |
| GO:0000819\_sister\_chromatid\_segregation | 5 | 0 | 0.000000 | -0.000000 | 1960 | 1964.662348 | 2007.28 | 2049.897652 | 1.024122 |  |
| GO:0000956\_nuclear-transcribed\_mRNA\_catabolic\_process | 5 | 0 | 0.000000 | -0.000000 | 1960 | 1964.662348 | 2007.28 | 2049.897652 | 1.024122 |  |
| GO:0001562\_response\_to\_protozoan | 5 | 0 | 0.000000 | -0.000000 | 1960 | 1964.662348 | 2007.28 | 2049.897652 | 1.024122 |  |
| GO:0001706\_endoderm\_formation | 5 | 0 | 0.000000 | -0.000000 | 1960 | 1964.662348 | 2007.28 | 2049.897652 | 1.024122 |  |
| GO:0001865\_NK\_T\_cell\_differentiation | 5 | 0 | 0.000000 | -0.000000 | 1960 | 1964.662348 | 2007.28 | 2049.897652 | 1.024122 |  |
| GO:0001945\_lymph\_vessel\_development | 5 | 0 | 0.000000 | -0.000000 | 1960 | 1964.662348 | 2007.28 | 2049.897652 | 1.024122 |  |
| GO:0001957\_intramembranous\_ossification | 5 | 0 | 0.000000 | -0.000000 | 1960 | 1964.662348 | 2007.28 | 2049.897652 | 1.024122 |  |
| GO:0001959\_regulation\_of\_cytokine-mediated\_signaling\_pathway | 5 | 0 | 0.000000 | -0.000000 | 1960 | 1964.662348 | 2007.28 | 2049.897652 | 1.024122 |  |
| GO:0001991\_regulation\_of\_systemic\_arterial\_blood\_pressure\_by\_circulatory\_renin-angiotensin | 5 | 0 | 0.000000 | -0.000000 | 1960 | 1964.662348 | 2007.28 | 2049.897652 | 1.024122 |  |
| GO:0001993\_regulation\_of\_systemic\_arterial\_blood\_pressure\_by\_norepinephrine-epinephrine | 5 | 0 | 0.000000 | -0.000000 | 1960 | 1964.662348 | 2007.28 | 2049.897652 | 1.024122 |  |
| GO:0002328\_pro-B\_cell\_differentiation | 5 | 0 | 0.000000 | -0.000000 | 1960 | 1964.662348 | 2007.28 | 2049.897652 | 1.024122 |  |
| GO:0002448\_mast\_cell\_mediated\_immunity | 5 | 0 | 0.000000 | -0.000000 | 1960 | 1964.662348 | 2007.28 | 2049.897652 | 1.024122 |  |
| GO:0002517\_T\_cell\_tolerance\_induction | 5 | 0 | 0.000000 | -0.000000 | 1960 | 1964.662348 | 2007.28 | 2049.897652 | 1.024122 |  |
| GO:0002634\_regulation\_of\_germinal\_center\_formation | 5 | 0 | 0.000000 | -0.000000 | 1960 | 1964.662348 | 2007.28 | 2049.897652 | 1.024122 |  |
| GO:0002664\_regulation\_of\_T\_cell\_tolerance\_induction | 5 | 0 | 0.000000 | -0.000000 | 1960 | 1964.662348 | 2007.28 | 2049.897652 | 1.024122 |  |
| GO:0002666\_positive\_regulation\_of\_T\_cell\_tolerance\_induction | 5 | 0 | 0.000000 | -0.000000 | 1960 | 1964.662348 | 2007.28 | 2049.897652 | 1.024122 |  |
| GO:0002710\_negative\_regulation\_of\_T\_cell\_mediated\_immunity | 5 | 0 | 0.000000 | -0.000000 | 1960 | 1964.662348 | 2007.28 | 2049.897652 | 1.024122 |  |
| GO:0002716\_negative\_regulation\_of\_natural\_killer\_cell\_mediated\_immunity | 5 | 0 | 0.000000 | -0.000000 | 1960 | 1964.662348 | 2007.28 | 2049.897652 | 1.024122 |  |
| GO:0002825\_regulation\_of\_T-helper\_1\_type\_immune\_response | 5 | 0 | 0.000000 | -0.000000 | 1960 | 1964.662348 | 2007.28 | 2049.897652 | 1.024122 |  |
| GO:0002862\_negative\_regulation\_of\_inflammatory\_response\_to\_antigenic\_stimulus | 5 | 0 | 0.000000 | -0.000000 | 1960 | 1964.662348 | 2007.28 | 2049.897652 | 1.024122 |  |
| GO:0002866\_positive\_regulation\_of\_acute\_inflammatory\_response\_to\_antigenic\_stimulus | 5 | 0 | 0.000000 | -0.000000 | 1960 | 1964.662348 | 2007.28 | 2049.897652 | 1.024122 |  |
| GO:0002885\_positive\_regulation\_of\_hypersensitivity | 5 | 0 | 0.000000 | -0.000000 | 1960 | 1964.662348 | 2007.28 | 2049.897652 | 1.024122 |  |
| GO:0002902\_regulation\_of\_B\_cell\_apoptosis | 5 | 0 | 0.000000 | -0.000000 | 1960 | 1964.662348 | 2007.28 | 2049.897652 | 1.024122 |  |
| GO:0003071\_renal\_system\_process\_involved\_in\_regulation\_of\_systemic\_arterial\_blood\_pressure | 5 | 0 | 0.000000 | -0.000000 | 1960 | 1964.662348 | 2007.28 | 2049.897652 | 1.024122 |  |
| GO:0005980\_glycogen\_catabolic\_process | 5 | 0 | 0.000000 | -0.000000 | 1960 | 1964.662348 | 2007.28 | 2049.897652 | 1.024122 |  |
| GO:0006023\_aminoglycan\_biosynthetic\_process | 5 | 0 | 0.000000 | -0.000000 | 1960 | 1964.662348 | 2007.28 | 2049.897652 | 1.024122 |  |
| GO:0006024\_glycosaminoglycan\_biosynthetic\_process | 5 | 0 | 0.000000 | -0.000000 | 1960 | 1964.662348 | 2007.28 | 2049.897652 | 1.024122 |  |
| GO:0006268\_DNA\_unwinding\_during\_replication | 5 | 0 | 0.000000 | -0.000000 | 1960 | 1964.662348 | 2007.28 | 2049.897652 | 1.024122 |  |
| GO:0006303\_double-strand\_break\_repair\_via\_nonhomologous\_end\_joining | 5 | 0 | 0.000000 | -0.000000 | 1960 | 1964.662348 | 2007.28 | 2049.897652 | 1.024122 |  |
| GO:0006346\_methylation-dependent\_chromatin\_silencing | 5 | 0 | 0.000000 | -0.000000 | 1960 | 1964.662348 | 2007.28 | 2049.897652 | 1.024122 |  |
| GO:0006376\_mRNA\_splice\_site\_selection | 5 | 0 | 0.000000 | -0.000000 | 1960 | 1964.662348 | 2007.28 | 2049.897652 | 1.024122 |  |
| GO:0006378\_mRNA\_polyadenylation | 5 | 0 | 0.000000 | -0.000000 | 1960 | 1964.662348 | 2007.28 | 2049.897652 | 1.024122 |  |
| GO:0006400\_tRNA\_modification | 5 | 0 | 0.000000 | -0.000000 | 1960 | 1964.662348 | 2007.28 | 2049.897652 | 1.024122 |  |
| GO:0006506\_GPI\_anchor\_biosynthetic\_process | 5 | 0 | 0.000000 | -0.000000 | 1960 | 1964.662348 | 2007.28 | 2049.897652 | 1.024122 |  |
| GO:0006509\_membrane\_protein\_ectodomain\_proteolysis | 5 | 0 | 0.000000 | -0.000000 | 1960 | 1964.662348 | 2007.28 | 2049.897652 | 1.024122 |  |
| GO:0006515\_misfolded\_or\_incompletely\_synthesized\_protein\_catabolic\_process | 5 | 0 | 0.000000 | -0.000000 | 1960 | 1964.662348 | 2007.28 | 2049.897652 | 1.024122 |  |
| GO:0006541\_glutamine\_metabolic\_process | 5 | 0 | 0.000000 | -0.000000 | 1960 | 1964.662348 | 2007.28 | 2049.897652 | 1.024122 |  |
| GO:0006570\_tyrosine\_metabolic\_process | 5 | 0 | 0.000000 | -0.000000 | 1960 | 1964.662348 | 2007.28 | 2049.897652 | 1.024122 |  |
| GO:0006577\_betaine\_metabolic\_process | 5 | 0 | 0.000000 | -0.000000 | 1960 | 1964.662348 | 2007.28 | 2049.897652 | 1.024122 |  |
| GO:0006586\_indolalkylamine\_metabolic\_process | 5 | 0 | 0.000000 | -0.000000 | 1960 | 1964.662348 | 2007.28 | 2049.897652 | 1.024122 |  |
| GO:0006636\_unsaturated\_fatty\_acid\_biosynthetic\_process | 5 | 0 | 0.000000 | -0.000000 | 1960 | 1964.662348 | 2007.28 | 2049.897652 | 1.024122 |  |
| GO:0006691\_leukotriene\_metabolic\_process | 5 | 0 | 0.000000 | -0.000000 | 1960 | 1964.662348 | 2007.28 | 2049.897652 | 1.024122 |  |
| GO:0006704\_glucocorticoid\_biosynthetic\_process | 5 | 0 | 0.000000 | -0.000000 | 1960 | 1964.662348 | 2007.28 | 2049.897652 | 1.024122 |  |
| GO:0006903\_vesicle\_targeting | 5 | 0 | 0.000000 | -0.000000 | 1960 | 1964.662348 | 2007.28 | 2049.897652 | 1.024122 |  |
| GO:0006924\_activation-induced\_cell\_death\_of\_T\_cells | 5 | 0 | 0.000000 | -0.000000 | 1960 | 1964.662348 | 2007.28 | 2049.897652 | 1.024122 |  |
| GO:0006929\_substrate-bound\_cell\_migration | 5 | 0 | 0.000000 | -0.000000 | 1960 | 1964.662348 | 2007.28 | 2049.897652 | 1.024122 |  |
| GO:0006983\_ER\_overload\_response | 5 | 0 | 0.000000 | -0.000000 | 1960 | 1964.662348 | 2007.28 | 2049.897652 | 1.024122 |  |
| GO:0007029\_endoplasmic\_reticulum\_organization | 5 | 0 | 0.000000 | -0.000000 | 1960 | 1964.662348 | 2007.28 | 2049.897652 | 1.024122 |  |
| GO:0007091\_mitotic\_metaphase\_anaphase\_transition | 5 | 0 | 0.000000 | -0.000000 | 1960 | 1964.662348 | 2007.28 | 2049.897652 | 1.024122 |  |
| GO:0007320\_insemination | 5 | 0 | 0.000000 | -0.000000 | 1960 | 1964.662348 | 2007.28 | 2049.897652 | 1.024122 |  |
| GO:0007622\_rhythmic\_behavior | 5 | 0 | 0.000000 | -0.000000 | 1960 | 1964.662348 | 2007.28 | 2049.897652 | 1.024122 |  |
| GO:0007638\_mechanosensory\_behavior | 5 | 0 | 0.000000 | -0.000000 | 1960 | 1964.662348 | 2007.28 | 2049.897652 | 1.024122 |  |
| GO:0008343\_adult\_feeding\_behavior | 5 | 0 | 0.000000 | -0.000000 | 1960 | 1964.662348 | 2007.28 | 2049.897652 | 1.024122 |  |
| GO:0009132\_nucleoside\_diphosphate\_metabolic\_process | 5 | 0 | 0.000000 | -0.000000 | 1960 | 1964.662348 | 2007.28 | 2049.897652 | 1.024122 |  |
| GO:0009146\_purine\_nucleoside\_triphosphate\_catabolic\_process | 5 | 0 | 0.000000 | -0.000000 | 1960 | 1964.662348 | 2007.28 | 2049.897652 | 1.024122 |  |
| GO:0009151\_purine\_deoxyribonucleotide\_metabolic\_process | 5 | 0 | 0.000000 | -0.000000 | 1960 | 1964.662348 | 2007.28 | 2049.897652 | 1.024122 |  |
| GO:0009154\_purine\_ribonucleotide\_catabolic\_process | 5 | 0 | 0.000000 | -0.000000 | 1960 | 1964.662348 | 2007.28 | 2049.897652 | 1.024122 |  |
| GO:0009215\_purine\_deoxyribonucleoside\_triphosphate\_metabolic\_process | 5 | 0 | 0.000000 | -0.000000 | 1960 | 1964.662348 | 2007.28 | 2049.897652 | 1.024122 |  |
| GO:0009251\_glucan\_catabolic\_process | 5 | 0 | 0.000000 | -0.000000 | 1960 | 1964.662348 | 2007.28 | 2049.897652 | 1.024122 |  |
| GO:0009261\_ribonucleotide\_catabolic\_process | 5 | 0 | 0.000000 | -0.000000 | 1960 | 1964.662348 | 2007.28 | 2049.897652 | 1.024122 |  |
| GO:0009263\_deoxyribonucleotide\_biosynthetic\_process | 5 | 0 | 0.000000 | -0.000000 | 1960 | 1964.662348 | 2007.28 | 2049.897652 | 1.024122 |  |
| GO:0009264\_deoxyribonucleotide\_catabolic\_process | 5 | 0 | 0.000000 | -0.000000 | 1960 | 1964.662348 | 2007.28 | 2049.897652 | 1.024122 |  |
| GO:0009296\_flagellum\_assembly | 5 | 0 | 0.000000 | -0.000000 | 1960 | 1964.662348 | 2007.28 | 2049.897652 | 1.024122 |  |
| GO:0009303\_rRNA\_transcription | 5 | 0 | 0.000000 | -0.000000 | 1960 | 1964.662348 | 2007.28 | 2049.897652 | 1.024122 |  |
| GO:0009312\_oligosaccharide\_biosynthetic\_process | 5 | 0 | 0.000000 | -0.000000 | 1960 | 1964.662348 | 2007.28 | 2049.897652 | 1.024122 |  |
| GO:0009437\_carnitine\_metabolic\_process | 5 | 0 | 0.000000 | -0.000000 | 1960 | 1964.662348 | 2007.28 | 2049.897652 | 1.024122 |  |
| GO:0009950\_dorsal\_ventral\_axis\_specification | 5 | 0 | 0.000000 | -0.000000 | 1960 | 1964.662348 | 2007.28 | 2049.897652 | 1.024122 |  |
| GO:0010460\_positive\_regulation\_of\_heart\_rate | 5 | 0 | 0.000000 | -0.000000 | 1960 | 1964.662348 | 2007.28 | 2049.897652 | 1.024122 |  |
| GO:0010522\_regulation\_of\_calcium\_ion\_transport\_into\_cytosol | 5 | 0 | 0.000000 | -0.000000 | 1960 | 1964.662348 | 2007.28 | 2049.897652 | 1.024122 |  |
| GO:0010676\_positive\_regulation\_of\_cellular\_carbohydrate\_metabolic\_process | 5 | 0 | 0.000000 | -0.000000 | 1960 | 1964.662348 | 2007.28 | 2049.897652 | 1.024122 |  |
| GO:0010761\_fibroblast\_migration | 5 | 0 | 0.000000 | -0.000000 | 1960 | 1964.662348 | 2007.28 | 2049.897652 | 1.024122 |  |
| GO:0010828\_positive\_regulation\_of\_glucose\_transport | 5 | 0 | 0.000000 | -0.000000 | 1960 | 1964.662348 | 2007.28 | 2049.897652 | 1.024122 |  |
| GO:0014015\_positive\_regulation\_of\_gliogenesis | 5 | 0 | 0.000000 | -0.000000 | 1960 | 1964.662348 | 2007.28 | 2049.897652 | 1.024122 |  |
| GO:0014044\_Schwann\_cell\_development | 5 | 0 | 0.000000 | -0.000000 | 1960 | 1964.662348 | 2007.28 | 2049.897652 | 1.024122 |  |
| GO:0014068\_positive\_regulation\_of\_phosphoinositide\_3-kinase\_cascade | 5 | 0 | 0.000000 | -0.000000 | 1960 | 1964.662348 | 2007.28 | 2049.897652 | 1.024122 |  |
| GO:0015802\_basic\_amino\_acid\_transport | 5 | 0 | 0.000000 | -0.000000 | 1960 | 1964.662348 | 2007.28 | 2049.897652 | 1.024122 |  |
| GO:0016048\_detection\_of\_temperature\_stimulus | 5 | 0 | 0.000000 | -0.000000 | 1960 | 1964.662348 | 2007.28 | 2049.897652 | 1.024122 |  |
| GO:0016188\_synaptic\_vesicle\_maturation | 5 | 0 | 0.000000 | -0.000000 | 1960 | 1964.662348 | 2007.28 | 2049.897652 | 1.024122 |  |
| GO:0016579\_protein\_deubiquitination | 5 | 0 | 0.000000 | -0.000000 | 1960 | 1964.662348 | 2007.28 | 2049.897652 | 1.024122 |  |
| GO:0017148\_negative\_regulation\_of\_translation | 5 | 0 | 0.000000 | -0.000000 | 1960 | 1964.662348 | 2007.28 | 2049.897652 | 1.024122 |  |
| GO:0017158\_regulation\_of\_calcium\_ion-dependent\_exocytosis | 5 | 0 | 0.000000 | -0.000000 | 1960 | 1964.662348 | 2007.28 | 2049.897652 | 1.024122 |  |
| GO:0019227\_neuronal\_action\_potential\_propagation | 5 | 0 | 0.000000 | -0.000000 | 1960 | 1964.662348 | 2007.28 | 2049.897652 | 1.024122 |  |
| GO:0019430\_removal\_of\_superoxide\_radicals | 5 | 0 | 0.000000 | -0.000000 | 1960 | 1964.662348 | 2007.28 | 2049.897652 | 1.024122 |  |
| GO:0021554\_optic\_nerve\_development | 5 | 0 | 0.000000 | -0.000000 | 1960 | 1964.662348 | 2007.28 | 2049.897652 | 1.024122 |  |
| GO:0021772\_olfactory\_bulb\_development | 5 | 0 | 0.000000 | -0.000000 | 1960 | 1964.662348 | 2007.28 | 2049.897652 | 1.024122 |  |
| GO:0021826\_substrate-independent\_telencephalic\_tangential\_migration | 5 | 0 | 0.000000 | -0.000000 | 1960 | 1964.662348 | 2007.28 | 2049.897652 | 1.024122 |  |
| GO:0021843\_substrate-independent\_telencephalic\_tangential\_interneuron\_migration | 5 | 0 | 0.000000 | -0.000000 | 1960 | 1964.662348 | 2007.28 | 2049.897652 | 1.024122 |  |
| GO:0021854\_hypothalamus\_development | 5 | 0 | 0.000000 | -0.000000 | 1960 | 1964.662348 | 2007.28 | 2049.897652 | 1.024122 |  |
| GO:0021988\_olfactory\_lobe\_development | 5 | 0 | 0.000000 | -0.000000 | 1960 | 1964.662348 | 2007.28 | 2049.897652 | 1.024122 |  |
| GO:0022415\_viral\_reproductive\_process | 5 | 0 | 0.000000 | -0.000000 | 1960 | 1964.662348 | 2007.28 | 2049.897652 | 1.024122 |  |
| GO:0030004\_cellular\_monovalent\_inorganic\_cation\_homeostasis | 5 | 0 | 0.000000 | -0.000000 | 1960 | 1964.662348 | 2007.28 | 2049.897652 | 1.024122 |  |
| GO:0030042\_actin\_filament\_depolymerization | 5 | 0 | 0.000000 | -0.000000 | 1960 | 1964.662348 | 2007.28 | 2049.897652 | 1.024122 |  |
| GO:0030177\_positive\_regulation\_of\_Wnt\_receptor\_signaling\_pathway | 5 | 0 | 0.000000 | -0.000000 | 1960 | 1964.662348 | 2007.28 | 2049.897652 | 1.024122 |  |
| GO:0030261\_chromosome\_condensation | 5 | 0 | 0.000000 | -0.000000 | 1960 | 1964.662348 | 2007.28 | 2049.897652 | 1.024122 |  |
| GO:0030299\_intestinal\_cholesterol\_absorption | 5 | 0 | 0.000000 | -0.000000 | 1960 | 1964.662348 | 2007.28 | 2049.897652 | 1.024122 |  |
| GO:0030431\_sleep | 5 | 0 | 0.000000 | -0.000000 | 1960 | 1964.662348 | 2007.28 | 2049.897652 | 1.024122 |  |
| GO:0030433\_ER-associated\_protein\_catabolic\_process | 5 | 0 | 0.000000 | -0.000000 | 1960 | 1964.662348 | 2007.28 | 2049.897652 | 1.024122 |  |
| GO:0030641\_regulation\_of\_cellular\_pH | 5 | 0 | 0.000000 | -0.000000 | 1960 | 1964.662348 | 2007.28 | 2049.897652 | 1.024122 |  |
| GO:0030801\_positive\_regulation\_of\_cyclic\_nucleotide\_metabolic\_process | 5 | 0 | 0.000000 | -0.000000 | 1960 | 1964.662348 | 2007.28 | 2049.897652 | 1.024122 |  |
| GO:0030804\_positive\_regulation\_of\_cyclic\_nucleotide\_biosynthetic\_process | 5 | 0 | 0.000000 | -0.000000 | 1960 | 1964.662348 | 2007.28 | 2049.897652 | 1.024122 |  |
| GO:0030810\_positive\_regulation\_of\_nucleotide\_biosynthetic\_process | 5 | 0 | 0.000000 | -0.000000 | 1960 | 1964.662348 | 2007.28 | 2049.897652 | 1.024122 |  |
| GO:0030823\_regulation\_of\_cGMP\_metabolic\_process | 5 | 0 | 0.000000 | -0.000000 | 1960 | 1964.662348 | 2007.28 | 2049.897652 | 1.024122 |  |
| GO:0030834\_regulation\_of\_actin\_filament\_depolymerization | 5 | 0 | 0.000000 | -0.000000 | 1960 | 1964.662348 | 2007.28 | 2049.897652 | 1.024122 |  |
| GO:0030838\_positive\_regulation\_of\_actin\_filament\_polymerization | 5 | 0 | 0.000000 | -0.000000 | 1960 | 1964.662348 | 2007.28 | 2049.897652 | 1.024122 |  |
| GO:0030851\_granulocyte\_differentiation | 5 | 0 | 0.000000 | -0.000000 | 1960 | 1964.662348 | 2007.28 | 2049.897652 | 1.024122 |  |
| GO:0030866\_cortical\_actin\_cytoskeleton\_organization | 5 | 0 | 0.000000 | -0.000000 | 1960 | 1964.662348 | 2007.28 | 2049.897652 | 1.024122 |  |
| GO:0030889\_negative\_regulation\_of\_B\_cell\_proliferation | 5 | 0 | 0.000000 | -0.000000 | 1960 | 1964.662348 | 2007.28 | 2049.897652 | 1.024122 |  |
| GO:0030917\_midbrain-hindbrain\_boundary\_development | 5 | 0 | 0.000000 | -0.000000 | 1960 | 1964.662348 | 2007.28 | 2049.897652 | 1.024122 |  |
| GO:0031047\_gene\_silencing\_by\_RNA | 5 | 0 | 0.000000 | -0.000000 | 1960 | 1964.662348 | 2007.28 | 2049.897652 | 1.024122 |  |
| GO:0031057\_negative\_regulation\_of\_histone\_modification | 5 | 0 | 0.000000 | -0.000000 | 1960 | 1964.662348 | 2007.28 | 2049.897652 | 1.024122 |  |
| GO:0031058\_positive\_regulation\_of\_histone\_modification | 5 | 0 | 0.000000 | -0.000000 | 1960 | 1964.662348 | 2007.28 | 2049.897652 | 1.024122 |  |
| GO:0031060\_regulation\_of\_histone\_methylation | 5 | 0 | 0.000000 | -0.000000 | 1960 | 1964.662348 | 2007.28 | 2049.897652 | 1.024122 |  |
| GO:0031122\_cytoplasmic\_microtubule\_organization | 5 | 0 | 0.000000 | -0.000000 | 1960 | 1964.662348 | 2007.28 | 2049.897652 | 1.024122 |  |
| GO:0031532\_actin\_cytoskeleton\_reorganization | 5 | 0 | 0.000000 | -0.000000 | 1960 | 1964.662348 | 2007.28 | 2049.897652 | 1.024122 |  |
| GO:0032024\_positive\_regulation\_of\_insulin\_secretion | 5 | 0 | 0.000000 | -0.000000 | 1960 | 1964.662348 | 2007.28 | 2049.897652 | 1.024122 |  |
| GO:0032320\_positive\_regulation\_of\_Ras\_GTPase\_activity | 5 | 0 | 0.000000 | -0.000000 | 1960 | 1964.662348 | 2007.28 | 2049.897652 | 1.024122 |  |
| GO:0032332\_positive\_regulation\_of\_chondrocyte\_differentiation | 5 | 0 | 0.000000 | -0.000000 | 1960 | 1964.662348 | 2007.28 | 2049.897652 | 1.024122 |  |
| GO:0032350\_regulation\_of\_hormone\_metabolic\_process | 5 | 0 | 0.000000 | -0.000000 | 1960 | 1964.662348 | 2007.28 | 2049.897652 | 1.024122 |  |
| GO:0032368\_regulation\_of\_lipid\_transport | 5 | 0 | 0.000000 | -0.000000 | 1960 | 1964.662348 | 2007.28 | 2049.897652 | 1.024122 |  |
| GO:0032400\_melanosome\_localization | 5 | 0 | 0.000000 | -0.000000 | 1960 | 1964.662348 | 2007.28 | 2049.897652 | 1.024122 |  |
| GO:0032434\_regulation\_of\_proteasomal\_ubiquitin-dependent\_protein\_catabolic\_process | 5 | 0 | 0.000000 | -0.000000 | 1960 | 1964.662348 | 2007.28 | 2049.897652 | 1.024122 |  |
| GO:0032479\_regulation\_of\_type\_I\_interferon\_production | 5 | 0 | 0.000000 | -0.000000 | 1960 | 1964.662348 | 2007.28 | 2049.897652 | 1.024122 |  |
| GO:0032494\_response\_to\_peptidoglycan | 5 | 0 | 0.000000 | -0.000000 | 1960 | 1964.662348 | 2007.28 | 2049.897652 | 1.024122 |  |
| GO:0032508\_DNA\_duplex\_unwinding | 5 | 0 | 0.000000 | -0.000000 | 1960 | 1964.662348 | 2007.28 | 2049.897652 | 1.024122 |  |
| GO:0032720\_negative\_regulation\_of\_tumor\_necrosis\_factor\_production | 5 | 0 | 0.000000 | -0.000000 | 1960 | 1964.662348 | 2007.28 | 2049.897652 | 1.024122 |  |
| GO:0032845\_negative\_regulation\_of\_homeostatic\_process | 5 | 0 | 0.000000 | -0.000000 | 1960 | 1964.662348 | 2007.28 | 2049.897652 | 1.024122 |  |
| GO:0032846\_positive\_regulation\_of\_homeostatic\_process | 5 | 0 | 0.000000 | -0.000000 | 1960 | 1964.662348 | 2007.28 | 2049.897652 | 1.024122 |  |
| GO:0033003\_regulation\_of\_mast\_cell\_activation | 5 | 0 | 0.000000 | -0.000000 | 1960 | 1964.662348 | 2007.28 | 2049.897652 | 1.024122 |  |
| GO:0033005\_positive\_regulation\_of\_mast\_cell\_activation | 5 | 0 | 0.000000 | -0.000000 | 1960 | 1964.662348 | 2007.28 | 2049.897652 | 1.024122 |  |
| GO:0033023\_mast\_cell\_homeostasis | 5 | 0 | 0.000000 | -0.000000 | 1960 | 1964.662348 | 2007.28 | 2049.897652 | 1.024122 |  |
| GO:0033024\_mast\_cell\_apoptosis | 5 | 0 | 0.000000 | -0.000000 | 1960 | 1964.662348 | 2007.28 | 2049.897652 | 1.024122 |  |
| GO:0033025\_regulation\_of\_mast\_cell\_apoptosis | 5 | 0 | 0.000000 | -0.000000 | 1960 | 1964.662348 | 2007.28 | 2049.897652 | 1.024122 |  |
| GO:0033033\_negative\_regulation\_of\_myeloid\_cell\_apoptosis | 5 | 0 | 0.000000 | -0.000000 | 1960 | 1964.662348 | 2007.28 | 2049.897652 | 1.024122 |  |
| GO:0033144\_negative\_regulation\_of\_steroid\_hormone\_receptor\_signaling\_pathway | 5 | 0 | 0.000000 | -0.000000 | 1960 | 1964.662348 | 2007.28 | 2049.897652 | 1.024122 |  |
| GO:0033146\_regulation\_of\_estrogen\_receptor\_signaling\_pathway | 5 | 0 | 0.000000 | -0.000000 | 1960 | 1964.662348 | 2007.28 | 2049.897652 | 1.024122 |  |
| GO:0033152\_immunoglobulin\_V(D)J\_recombination | 5 | 0 | 0.000000 | -0.000000 | 1960 | 1964.662348 | 2007.28 | 2049.897652 | 1.024122 |  |
| GO:0033205\_cytokinesis\_during\_cell\_cycle | 5 | 0 | 0.000000 | -0.000000 | 1960 | 1964.662348 | 2007.28 | 2049.897652 | 1.024122 |  |
| GO:0033619\_membrane\_protein\_proteolysis | 5 | 0 | 0.000000 | -0.000000 | 1960 | 1964.662348 | 2007.28 | 2049.897652 | 1.024122 |  |
| GO:0033627\_cell\_adhesion\_mediated\_by\_integrin | 5 | 0 | 0.000000 | -0.000000 | 1960 | 1964.662348 | 2007.28 | 2049.897652 | 1.024122 |  |
| GO:0033692\_cellular\_polysaccharide\_biosynthetic\_process | 5 | 0 | 0.000000 | -0.000000 | 1960 | 1964.662348 | 2007.28 | 2049.897652 | 1.024122 |  |
| GO:0034109\_homotypic\_cell-cell\_adhesion | 5 | 0 | 0.000000 | -0.000000 | 1960 | 1964.662348 | 2007.28 | 2049.897652 | 1.024122 |  |
| GO:0034367\_macromolecular\_complex\_remodeling | 5 | 0 | 0.000000 | -0.000000 | 1960 | 1964.662348 | 2007.28 | 2049.897652 | 1.024122 |  |
| GO:0034368\_protein-lipid\_complex\_remodeling | 5 | 0 | 0.000000 | -0.000000 | 1960 | 1964.662348 | 2007.28 | 2049.897652 | 1.024122 |  |
| GO:0034369\_plasma\_lipoprotein\_particle\_remodeling | 5 | 0 | 0.000000 | -0.000000 | 1960 | 1964.662348 | 2007.28 | 2049.897652 | 1.024122 |  |
| GO:0035058\_sensory\_cilium\_assembly | 5 | 0 | 0.000000 | -0.000000 | 1960 | 1964.662348 | 2007.28 | 2049.897652 | 1.024122 |  |
| GO:0035065\_regulation\_of\_histone\_acetylation | 5 | 0 | 0.000000 | -0.000000 | 1960 | 1964.662348 | 2007.28 | 2049.897652 | 1.024122 |  |
| GO:0035089\_establishment\_of\_apical\_basal\_cell\_polarity | 5 | 0 | 0.000000 | -0.000000 | 1960 | 1964.662348 | 2007.28 | 2049.897652 | 1.024122 |  |
| GO:0035095\_behavioral\_response\_to\_nicotine | 5 | 0 | 0.000000 | -0.000000 | 1960 | 1964.662348 | 2007.28 | 2049.897652 | 1.024122 |  |
| GO:0035234\_germ\_cell\_programmed\_cell\_death | 5 | 0 | 0.000000 | -0.000000 | 1960 | 1964.662348 | 2007.28 | 2049.897652 | 1.024122 |  |
| GO:0035238\_vitamin\_A\_biosynthetic\_process | 5 | 0 | 0.000000 | -0.000000 | 1960 | 1964.662348 | 2007.28 | 2049.897652 | 1.024122 |  |
| GO:0042159\_lipoprotein\_catabolic\_process | 5 | 0 | 0.000000 | -0.000000 | 1960 | 1964.662348 | 2007.28 | 2049.897652 | 1.024122 |  |
| GO:0042362\_fat-soluble\_vitamin\_biosynthetic\_process | 5 | 0 | 0.000000 | -0.000000 | 1960 | 1964.662348 | 2007.28 | 2049.897652 | 1.024122 |  |
| GO:0042364\_water-soluble\_vitamin\_biosynthetic\_process | 5 | 0 | 0.000000 | -0.000000 | 1960 | 1964.662348 | 2007.28 | 2049.897652 | 1.024122 |  |
| GO:0042416\_dopamine\_biosynthetic\_process | 5 | 0 | 0.000000 | -0.000000 | 1960 | 1964.662348 | 2007.28 | 2049.897652 | 1.024122 |  |
| GO:0042430\_indole\_and\_derivative\_metabolic\_process | 5 | 0 | 0.000000 | -0.000000 | 1960 | 1964.662348 | 2007.28 | 2049.897652 | 1.024122 |  |
| GO:0042434\_indole\_derivative\_metabolic\_process | 5 | 0 | 0.000000 | -0.000000 | 1960 | 1964.662348 | 2007.28 | 2049.897652 | 1.024122 |  |
| GO:0042487\_regulation\_of\_odontogenesis\_of\_dentine-containing\_tooth | 5 | 0 | 0.000000 | -0.000000 | 1960 | 1964.662348 | 2007.28 | 2049.897652 | 1.024122 |  |
| GO:0042506\_tyrosine\_phosphorylation\_of\_Stat5\_protein | 5 | 0 | 0.000000 | -0.000000 | 1960 | 1964.662348 | 2007.28 | 2049.897652 | 1.024122 |  |
| GO:0042516\_regulation\_of\_tyrosine\_phosphorylation\_of\_Stat3\_protein | 5 | 0 | 0.000000 | -0.000000 | 1960 | 1964.662348 | 2007.28 | 2049.897652 | 1.024122 |  |
| GO:0042531\_positive\_regulation\_of\_tyrosine\_phosphorylation\_of\_STAT\_protein | 5 | 0 | 0.000000 | -0.000000 | 1960 | 1964.662348 | 2007.28 | 2049.897652 | 1.024122 |  |
| GO:0042533\_tumor\_necrosis\_factor\_biosynthetic\_process | 5 | 0 | 0.000000 | -0.000000 | 1960 | 1964.662348 | 2007.28 | 2049.897652 | 1.024122 |  |
| GO:0042534\_regulation\_of\_tumor\_necrosis\_factor\_biosynthetic\_process | 5 | 0 | 0.000000 | -0.000000 | 1960 | 1964.662348 | 2007.28 | 2049.897652 | 1.024122 |  |
| GO:0042537\_benzene\_and\_derivative\_metabolic\_process | 5 | 0 | 0.000000 | -0.000000 | 1960 | 1964.662348 | 2007.28 | 2049.897652 | 1.024122 |  |
| GO:0042554\_superoxide\_anion\_generation | 5 | 0 | 0.000000 | -0.000000 | 1960 | 1964.662348 | 2007.28 | 2049.897652 | 1.024122 |  |
| GO:0042574\_retinal\_metabolic\_process | 5 | 0 | 0.000000 | -0.000000 | 1960 | 1964.662348 | 2007.28 | 2049.897652 | 1.024122 |  |
| GO:0042590\_antigen\_processing\_and\_presentation\_of\_exogenous\_peptide\_antigen\_via\_MHC\_class\_I | 5 | 0 | 0.000000 | -0.000000 | 1960 | 1964.662348 | 2007.28 | 2049.897652 | 1.024122 |  |
| GO:0042695\_thelarche | 5 | 0 | 0.000000 | -0.000000 | 1960 | 1964.662348 | 2007.28 | 2049.897652 | 1.024122 |  |
| GO:0042752\_regulation\_of\_circadian\_rhythm | 5 | 0 | 0.000000 | -0.000000 | 1960 | 1964.662348 | 2007.28 | 2049.897652 | 1.024122 |  |
| GO:0042756\_drinking\_behavior | 5 | 0 | 0.000000 | -0.000000 | 1960 | 1964.662348 | 2007.28 | 2049.897652 | 1.024122 |  |
| GO:0042904\_9-cis-retinoic\_acid\_biosynthetic\_process | 5 | 0 | 0.000000 | -0.000000 | 1960 | 1964.662348 | 2007.28 | 2049.897652 | 1.024122 |  |
| GO:0042905\_9-cis-retinoic\_acid\_metabolic\_process | 5 | 0 | 0.000000 | -0.000000 | 1960 | 1964.662348 | 2007.28 | 2049.897652 | 1.024122 |  |
| GO:0043030\_regulation\_of\_macrophage\_activation | 5 | 0 | 0.000000 | -0.000000 | 1960 | 1964.662348 | 2007.28 | 2049.897652 | 1.024122 |  |
| GO:0043046\_DNA\_methylation\_during\_gametogenesis | 5 | 0 | 0.000000 | -0.000000 | 1960 | 1964.662348 | 2007.28 | 2049.897652 | 1.024122 |  |
| GO:0043266\_regulation\_of\_potassium\_ion\_transport | 5 | 0 | 0.000000 | -0.000000 | 1960 | 1964.662348 | 2007.28 | 2049.897652 | 1.024122 |  |
| GO:0043277\_apoptotic\_cell\_clearance | 5 | 0 | 0.000000 | -0.000000 | 1960 | 1964.662348 | 2007.28 | 2049.897652 | 1.024122 |  |
| GO:0043288\_apocarotenoid\_metabolic\_process | 5 | 0 | 0.000000 | -0.000000 | 1960 | 1964.662348 | 2007.28 | 2049.897652 | 1.024122 |  |
| GO:0043302\_positive\_regulation\_of\_leukocyte\_degranulation | 5 | 0 | 0.000000 | -0.000000 | 1960 | 1964.662348 | 2007.28 | 2049.897652 | 1.024122 |  |
| GO:0043303\_mast\_cell\_degranulation | 5 | 0 | 0.000000 | -0.000000 | 1960 | 1964.662348 | 2007.28 | 2049.897652 | 1.024122 |  |
| GO:0043304\_regulation\_of\_mast\_cell\_degranulation | 5 | 0 | 0.000000 | -0.000000 | 1960 | 1964.662348 | 2007.28 | 2049.897652 | 1.024122 |  |
| GO:0043306\_positive\_regulation\_of\_mast\_cell\_degranulation | 5 | 0 | 0.000000 | -0.000000 | 1960 | 1964.662348 | 2007.28 | 2049.897652 | 1.024122 |  |
| GO:0043393\_regulation\_of\_protein\_binding | 5 | 0 | 0.000000 | -0.000000 | 1960 | 1964.662348 | 2007.28 | 2049.897652 | 1.024122 |  |
| GO:0043403\_skeletal\_muscle\_regeneration | 5 | 0 | 0.000000 | -0.000000 | 1960 | 1964.662348 | 2007.28 | 2049.897652 | 1.024122 |  |
| GO:0043489\_RNA\_stabilization | 5 | 0 | 0.000000 | -0.000000 | 1960 | 1964.662348 | 2007.28 | 2049.897652 | 1.024122 |  |
| GO:0043526\_neuroprotection | 5 | 0 | 0.000000 | -0.000000 | 1960 | 1964.662348 | 2007.28 | 2049.897652 | 1.024122 |  |
| GO:0043550\_regulation\_of\_lipid\_kinase\_activity | 5 | 0 | 0.000000 | -0.000000 | 1960 | 1964.662348 | 2007.28 | 2049.897652 | 1.024122 |  |
| GO:0043551\_regulation\_of\_phosphoinositide\_3-kinase\_activity | 5 | 0 | 0.000000 | -0.000000 | 1960 | 1964.662348 | 2007.28 | 2049.897652 | 1.024122 |  |
| GO:0043552\_positive\_regulation\_of\_phosphoinositide\_3-kinase\_activity | 5 | 0 | 0.000000 | -0.000000 | 1960 | 1964.662348 | 2007.28 | 2049.897652 | 1.024122 |  |
| GO:0043555\_regulation\_of\_translation\_in\_response\_to\_stress | 5 | 0 | 0.000000 | -0.000000 | 1960 | 1964.662348 | 2007.28 | 2049.897652 | 1.024122 |  |
| GO:0043558\_regulation\_of\_translational\_initiation\_in\_response\_to\_stress | 5 | 0 | 0.000000 | -0.000000 | 1960 | 1964.662348 | 2007.28 | 2049.897652 | 1.024122 |  |
| GO:0043631\_RNA\_polyadenylation | 5 | 0 | 0.000000 | -0.000000 | 1960 | 1964.662348 | 2007.28 | 2049.897652 | 1.024122 |  |
| GO:0043648\_dicarboxylic\_acid\_metabolic\_process | 5 | 0 | 0.000000 | -0.000000 | 1960 | 1964.662348 | 2007.28 | 2049.897652 | 1.024122 |  |
| GO:0044058\_regulation\_of\_digestive\_system\_process | 5 | 0 | 0.000000 | -0.000000 | 1960 | 1964.662348 | 2007.28 | 2049.897652 | 1.024122 |  |
| GO:0044241\_lipid\_digestion | 5 | 0 | 0.000000 | -0.000000 | 1960 | 1964.662348 | 2007.28 | 2049.897652 | 1.024122 |  |
| GO:0044247\_cellular\_polysaccharide\_catabolic\_process | 5 | 0 | 0.000000 | -0.000000 | 1960 | 1964.662348 | 2007.28 | 2049.897652 | 1.024122 |  |
| GO:0045063\_T-helper\_1\_cell\_differentiation | 5 | 0 | 0.000000 | -0.000000 | 1960 | 1964.662348 | 2007.28 | 2049.897652 | 1.024122 |  |
| GO:0045064\_T-helper\_2\_cell\_differentiation | 5 | 0 | 0.000000 | -0.000000 | 1960 | 1964.662348 | 2007.28 | 2049.897652 | 1.024122 |  |
| GO:0045080\_positive\_regulation\_of\_chemokine\_biosynthetic\_process | 5 | 0 | 0.000000 | -0.000000 | 1960 | 1964.662348 | 2007.28 | 2049.897652 | 1.024122 |  |
| GO:0045123\_cellular\_extravasation | 5 | 0 | 0.000000 | -0.000000 | 1960 | 1964.662348 | 2007.28 | 2049.897652 | 1.024122 |  |
| GO:0045197\_establishment\_or\_maintenance\_of\_epithelial\_cell\_apical\_basal\_polarity | 5 | 0 | 0.000000 | -0.000000 | 1960 | 1964.662348 | 2007.28 | 2049.897652 | 1.024122 |  |
| GO:0045213\_neurotransmitter\_receptor\_metabolic\_process | 5 | 0 | 0.000000 | -0.000000 | 1960 | 1964.662348 | 2007.28 | 2049.897652 | 1.024122 |  |
| GO:0045342\_MHC\_class\_II\_biosynthetic\_process | 5 | 0 | 0.000000 | -0.000000 | 1960 | 1964.662348 | 2007.28 | 2049.897652 | 1.024122 |  |
| GO:0045410\_positive\_regulation\_of\_interleukin-6\_biosynthetic\_process | 5 | 0 | 0.000000 | -0.000000 | 1960 | 1964.662348 | 2007.28 | 2049.897652 | 1.024122 |  |
| GO:0045586\_regulation\_of\_gamma-delta\_T\_cell\_differentiation | 5 | 0 | 0.000000 | -0.000000 | 1960 | 1964.662348 | 2007.28 | 2049.897652 | 1.024122 |  |
| GO:0045588\_positive\_regulation\_of\_gamma-delta\_T\_cell\_differentiation | 5 | 0 | 0.000000 | -0.000000 | 1960 | 1964.662348 | 2007.28 | 2049.897652 | 1.024122 |  |
| GO:0045622\_regulation\_of\_T-helper\_cell\_differentiation | 5 | 0 | 0.000000 | -0.000000 | 1960 | 1964.662348 | 2007.28 | 2049.897652 | 1.024122 |  |
| GO:0045648\_positive\_regulation\_of\_erythrocyte\_differentiation | 5 | 0 | 0.000000 | -0.000000 | 1960 | 1964.662348 | 2007.28 | 2049.897652 | 1.024122 |  |
| GO:0045651\_positive\_regulation\_of\_macrophage\_differentiation | 5 | 0 | 0.000000 | -0.000000 | 1960 | 1964.662348 | 2007.28 | 2049.897652 | 1.024122 |  |
| GO:0045661\_regulation\_of\_myoblast\_differentiation | 5 | 0 | 0.000000 | -0.000000 | 1960 | 1964.662348 | 2007.28 | 2049.897652 | 1.024122 |  |
| GO:0045687\_positive\_regulation\_of\_glial\_cell\_differentiation | 5 | 0 | 0.000000 | -0.000000 | 1960 | 1964.662348 | 2007.28 | 2049.897652 | 1.024122 |  |
| GO:0045730\_respiratory\_burst | 5 | 0 | 0.000000 | -0.000000 | 1960 | 1964.662348 | 2007.28 | 2049.897652 | 1.024122 |  |
| GO:0045744\_negative\_regulation\_of\_G-protein\_coupled\_receptor\_protein\_signaling\_pathway | 5 | 0 | 0.000000 | -0.000000 | 1960 | 1964.662348 | 2007.28 | 2049.897652 | 1.024122 |  |
| GO:0045745\_positive\_regulation\_of\_G-protein\_coupled\_receptor\_protein\_signaling\_pathway | 5 | 0 | 0.000000 | -0.000000 | 1960 | 1964.662348 | 2007.28 | 2049.897652 | 1.024122 |  |
| GO:0045773\_positive\_regulation\_of\_axon\_extension | 5 | 0 | 0.000000 | -0.000000 | 1960 | 1964.662348 | 2007.28 | 2049.897652 | 1.024122 |  |
| GO:0045793\_positive\_regulation\_of\_cell\_size | 5 | 0 | 0.000000 | -0.000000 | 1960 | 1964.662348 | 2007.28 | 2049.897652 | 1.024122 |  |
| GO:0045851\_pH\_reduction | 5 | 0 | 0.000000 | -0.000000 | 1960 | 1964.662348 | 2007.28 | 2049.897652 | 1.024122 |  |
| GO:0045885\_positive\_regulation\_of\_survival\_gene\_product\_expression | 5 | 0 | 0.000000 | -0.000000 | 1960 | 1964.662348 | 2007.28 | 2049.897652 | 1.024122 |  |
| GO:0045953\_negative\_regulation\_of\_natural\_killer\_cell\_mediated\_cytotoxicity | 5 | 0 | 0.000000 | -0.000000 | 1960 | 1964.662348 | 2007.28 | 2049.897652 | 1.024122 |  |
| GO:0045981\_positive\_regulation\_of\_nucleotide\_metabolic\_process | 5 | 0 | 0.000000 | -0.000000 | 1960 | 1964.662348 | 2007.28 | 2049.897652 | 1.024122 |  |
| GO:0045987\_positive\_regulation\_of\_smooth\_muscle\_contraction | 5 | 0 | 0.000000 | -0.000000 | 1960 | 1964.662348 | 2007.28 | 2049.897652 | 1.024122 |  |
| GO:0046326\_positive\_regulation\_of\_glucose\_import | 5 | 0 | 0.000000 | -0.000000 | 1960 | 1964.662348 | 2007.28 | 2049.897652 | 1.024122 |  |
| GO:0046339\_diacylglycerol\_metabolic\_process | 5 | 0 | 0.000000 | -0.000000 | 1960 | 1964.662348 | 2007.28 | 2049.897652 | 1.024122 |  |
| GO:0046456\_icosanoid\_biosynthetic\_process | 5 | 0 | 0.000000 | -0.000000 | 1960 | 1964.662348 | 2007.28 | 2049.897652 | 1.024122 |  |
| GO:0046459\_short-chain\_fatty\_acid\_metabolic\_process | 5 | 0 | 0.000000 | -0.000000 | 1960 | 1964.662348 | 2007.28 | 2049.897652 | 1.024122 |  |
| GO:0046489\_phosphoinositide\_biosynthetic\_process | 5 | 0 | 0.000000 | -0.000000 | 1960 | 1964.662348 | 2007.28 | 2049.897652 | 1.024122 |  |
| GO:0046621\_negative\_regulation\_of\_organ\_growth | 5 | 0 | 0.000000 | -0.000000 | 1960 | 1964.662348 | 2007.28 | 2049.897652 | 1.024122 |  |
| GO:0046643\_regulation\_of\_gamma-delta\_T\_cell\_activation | 5 | 0 | 0.000000 | -0.000000 | 1960 | 1964.662348 | 2007.28 | 2049.897652 | 1.024122 |  |
| GO:0046645\_positive\_regulation\_of\_gamma-delta\_T\_cell\_activation | 5 | 0 | 0.000000 | -0.000000 | 1960 | 1964.662348 | 2007.28 | 2049.897652 | 1.024122 |  |
| GO:0046697\_decidualization | 5 | 0 | 0.000000 | -0.000000 | 1960 | 1964.662348 | 2007.28 | 2049.897652 | 1.024122 |  |
| GO:0046785\_microtubule\_polymerization | 5 | 0 | 0.000000 | -0.000000 | 1960 | 1964.662348 | 2007.28 | 2049.897652 | 1.024122 |  |
| GO:0048013\_ephrin\_receptor\_signaling\_pathway | 5 | 0 | 0.000000 | -0.000000 | 1960 | 1964.662348 | 2007.28 | 2049.897652 | 1.024122 |  |
| GO:0048149\_behavioral\_response\_to\_ethanol | 5 | 0 | 0.000000 | -0.000000 | 1960 | 1964.662348 | 2007.28 | 2049.897652 | 1.024122 |  |
| GO:0048255\_mRNA\_stabilization | 5 | 0 | 0.000000 | -0.000000 | 1960 | 1964.662348 | 2007.28 | 2049.897652 | 1.024122 |  |
| GO:0048289\_isotype\_switching\_to\_IgE\_isotypes | 5 | 0 | 0.000000 | -0.000000 | 1960 | 1964.662348 | 2007.28 | 2049.897652 | 1.024122 |  |
| GO:0048293\_regulation\_of\_isotype\_switching\_to\_IgE\_isotypes | 5 | 0 | 0.000000 | -0.000000 | 1960 | 1964.662348 | 2007.28 | 2049.897652 | 1.024122 |  |
| GO:0048485\_sympathetic\_nervous\_system\_development | 5 | 0 | 0.000000 | -0.000000 | 1960 | 1964.662348 | 2007.28 | 2049.897652 | 1.024122 |  |
| GO:0048532\_anatomical\_structure\_arrangement | 5 | 0 | 0.000000 | -0.000000 | 1960 | 1964.662348 | 2007.28 | 2049.897652 | 1.024122 |  |
| GO:0048570\_notochord\_morphogenesis | 5 | 0 | 0.000000 | -0.000000 | 1960 | 1964.662348 | 2007.28 | 2049.897652 | 1.024122 |  |
| GO:0048659\_smooth\_muscle\_cell\_proliferation | 5 | 0 | 0.000000 | -0.000000 | 1960 | 1964.662348 | 2007.28 | 2049.897652 | 1.024122 |  |
| GO:0048664\_neuron\_fate\_determination | 5 | 0 | 0.000000 | -0.000000 | 1960 | 1964.662348 | 2007.28 | 2049.897652 | 1.024122 |  |
| GO:0048715\_negative\_regulation\_of\_oligodendrocyte\_differentiation | 5 | 0 | 0.000000 | -0.000000 | 1960 | 1964.662348 | 2007.28 | 2049.897652 | 1.024122 |  |
| GO:0048934\_peripheral\_nervous\_system\_neuron\_differentiation | 5 | 0 | 0.000000 | -0.000000 | 1960 | 1964.662348 | 2007.28 | 2049.897652 | 1.024122 |  |
| GO:0050665\_hydrogen\_peroxide\_biosynthetic\_process | 5 | 0 | 0.000000 | -0.000000 | 1960 | 1964.662348 | 2007.28 | 2049.897652 | 1.024122 |  |
| GO:0050701\_interleukin-1\_secretion | 5 | 0 | 0.000000 | -0.000000 | 1960 | 1964.662348 | 2007.28 | 2049.897652 | 1.024122 |  |
| GO:0050715\_positive\_regulation\_of\_cytokine\_secretion | 5 | 0 | 0.000000 | -0.000000 | 1960 | 1964.662348 | 2007.28 | 2049.897652 | 1.024122 |  |
| GO:0050819\_negative\_regulation\_of\_coagulation | 5 | 0 | 0.000000 | -0.000000 | 1960 | 1964.662348 | 2007.28 | 2049.897652 | 1.024122 |  |
| GO:0050858\_negative\_regulation\_of\_antigen\_receptor-mediated\_signaling\_pathway | 5 | 0 | 0.000000 | -0.000000 | 1960 | 1964.662348 | 2007.28 | 2049.897652 | 1.024122 |  |
| GO:0050860\_negative\_regulation\_of\_T\_cell\_receptor\_signaling\_pathway | 5 | 0 | 0.000000 | -0.000000 | 1960 | 1964.662348 | 2007.28 | 2049.897652 | 1.024122 |  |
| GO:0051057\_positive\_regulation\_of\_small\_GTPase\_mediated\_signal\_transduction | 5 | 0 | 0.000000 | -0.000000 | 1960 | 1964.662348 | 2007.28 | 2049.897652 | 1.024122 |  |
| GO:0051136\_regulation\_of\_NK\_T\_cell\_differentiation | 5 | 0 | 0.000000 | -0.000000 | 1960 | 1964.662348 | 2007.28 | 2049.897652 | 1.024122 |  |
| GO:0051138\_positive\_regulation\_of\_NK\_T\_cell\_differentiation | 5 | 0 | 0.000000 | -0.000000 | 1960 | 1964.662348 | 2007.28 | 2049.897652 | 1.024122 |  |
| GO:0051177\_meiotic\_sister\_chromatid\_cohesion | 5 | 0 | 0.000000 | -0.000000 | 1960 | 1964.662348 | 2007.28 | 2049.897652 | 1.024122 |  |
| GO:0051289\_protein\_homotetramerization | 5 | 0 | 0.000000 | -0.000000 | 1960 | 1964.662348 | 2007.28 | 2049.897652 | 1.024122 |  |
| GO:0051323\_metaphase | 5 | 0 | 0.000000 | -0.000000 | 1960 | 1964.662348 | 2007.28 | 2049.897652 | 1.024122 |  |
| GO:0051403\_stress-activated\_MAPK\_cascade | 5 | 0 | 0.000000 | -0.000000 | 1960 | 1964.662348 | 2007.28 | 2049.897652 | 1.024122 |  |
| GO:0051453\_regulation\_of\_intracellular\_pH | 5 | 0 | 0.000000 | -0.000000 | 1960 | 1964.662348 | 2007.28 | 2049.897652 | 1.024122 |  |
| GO:0051668\_localization\_within\_membrane | 5 | 0 | 0.000000 | -0.000000 | 1960 | 1964.662348 | 2007.28 | 2049.897652 | 1.024122 |  |
| GO:0051898\_negative\_regulation\_of\_protein\_kinase\_B\_signaling\_cascade | 5 | 0 | 0.000000 | -0.000000 | 1960 | 1964.662348 | 2007.28 | 2049.897652 | 1.024122 |  |
| GO:0051905\_establishment\_of\_pigment\_granule\_localization | 5 | 0 | 0.000000 | -0.000000 | 1960 | 1964.662348 | 2007.28 | 2049.897652 | 1.024122 |  |
| GO:0051954\_positive\_regulation\_of\_amine\_transport | 5 | 0 | 0.000000 | -0.000000 | 1960 | 1964.662348 | 2007.28 | 2049.897652 | 1.024122 |  |
| GO:0051966\_regulation\_of\_synaptic\_transmission\_\_glutamatergic | 5 | 0 | 0.000000 | -0.000000 | 1960 | 1964.662348 | 2007.28 | 2049.897652 | 1.024122 |  |
| GO:0055003\_cardiac\_myofibril\_assembly | 5 | 0 | 0.000000 | -0.000000 | 1960 | 1964.662348 | 2007.28 | 2049.897652 | 1.024122 |  |
| GO:0055015\_ventricular\_cardiac\_muscle\_cell\_development | 5 | 0 | 0.000000 | -0.000000 | 1960 | 1964.662348 | 2007.28 | 2049.897652 | 1.024122 |  |
| GO:0060045\_positive\_regulation\_of\_cardiac\_muscle\_cell\_proliferation | 5 | 0 | 0.000000 | -0.000000 | 1960 | 1964.662348 | 2007.28 | 2049.897652 | 1.024122 |  |
| GO:0060073\_micturition | 5 | 0 | 0.000000 | -0.000000 | 1960 | 1964.662348 | 2007.28 | 2049.897652 | 1.024122 |  |
| GO:0060351\_cartilage\_development\_involved\_in\_endochondral\_bone\_morphogenesis | 5 | 0 | 0.000000 | -0.000000 | 1960 | 1964.662348 | 2007.28 | 2049.897652 | 1.024122 |  |
| GO:0060438\_trachea\_development | 5 | 0 | 0.000000 | -0.000000 | 1960 | 1964.662348 | 2007.28 | 2049.897652 | 1.024122 |  |
| GO:0060600\_dichotomous\_subdivision\_of\_an\_epithelial\_terminal\_unit | 5 | 0 | 0.000000 | -0.000000 | 1960 | 1964.662348 | 2007.28 | 2049.897652 | 1.024122 |  |
| GO:0060605\_tube\_lumen\_cavitation | 5 | 0 | 0.000000 | -0.000000 | 1960 | 1964.662348 | 2007.28 | 2049.897652 | 1.024122 |  |
| GO:0060662\_salivary\_gland\_cavitation | 5 | 0 | 0.000000 | -0.000000 | 1960 | 1964.662348 | 2007.28 | 2049.897652 | 1.024122 |  |
| GO:0060665\_regulation\_of\_branching\_involved\_in\_salivary\_gland\_morphogenesis\_by\_mesenchymal-epithelial\_signaling | 5 | 0 | 0.000000 | -0.000000 | 1960 | 1964.662348 | 2007.28 | 2049.897652 | 1.024122 |  |
| GO:0060707\_trophoblast\_giant\_cell\_differentiation | 5 | 0 | 0.000000 | -0.000000 | 1960 | 1964.662348 | 2007.28 | 2049.897652 | 1.024122 |  |
| GO:0060744\_mammary\_gland\_branching\_involved\_in\_thelarche | 5 | 0 | 0.000000 | -0.000000 | 1960 | 1964.662348 | 2007.28 | 2049.897652 | 1.024122 |  |
| GO:0060745\_mammary\_gland\_branching\_involved\_in\_pregnancy | 5 | 0 | 0.000000 | -0.000000 | 1960 | 1964.662348 | 2007.28 | 2049.897652 | 1.024122 |  |
| GO:0060762\_regulation\_of\_branching\_involved\_in\_mammary\_gland\_duct\_morphogenesis | 5 | 0 | 0.000000 | -0.000000 | 1960 | 1964.662348 | 2007.28 | 2049.897652 | 1.024122 |  |
| GO:0070371\_ERK1\_and\_ERK2\_cascade | 5 | 0 | 0.000000 | -0.000000 | 1960 | 1964.662348 | 2007.28 | 2049.897652 | 1.024122 |  |
| GO:0070372\_regulation\_of\_ERK1\_and\_ERK2\_cascade | 5 | 0 | 0.000000 | -0.000000 | 1960 | 1964.662348 | 2007.28 | 2049.897652 | 1.024122 |  |
| GO:0070374\_positive\_regulation\_of\_ERK1\_and\_ERK2\_cascade | 5 | 0 | 0.000000 | -0.000000 | 1960 | 1964.662348 | 2007.28 | 2049.897652 | 1.024122 |  |
| GO:0006897\_endocytosis | 86 | 0 | 0.000000 | -0.000000 | 1965 | 1968.950323 | 2011.32 | 2053.689677 | 1.023573 |  |
| GO:0010324\_membrane\_invagination | 86 | 0 | 0.000000 | -0.000000 | 1965 | 1968.950323 | 2011.32 | 2053.689677 | 1.023573 |  |
| GO:0032504\_multicellular\_organism\_reproduction | 86 | 0 | 0.000000 | -0.000000 | 1965 | 1968.950323 | 2011.32 | 2053.689677 | 1.023573 |  |
| GO:0034641\_cellular\_nitrogen\_compound\_metabolic\_process | 86 | 0 | 0.000000 | -0.000000 | 1965 | 1968.950323 | 2011.32 | 2053.689677 | 1.023573 |  |
| GO:0048609\_reproductive\_process\_in\_a\_multicellular\_organism | 86 | 0 | 0.000000 | -0.000000 | 1965 | 1968.950323 | 2011.32 | 2053.689677 | 1.023573 |  |
| GO:0001570\_vasculogenesis | 38 | 0 | 0.000000 | -0.000000 | 1984 | 1987.651536 | 2028.95 | 2070.248464 | 1.022656 |  |
| GO:0001649\_osteoblast\_differentiation | 38 | 0 | 0.000000 | -0.000000 | 1984 | 1987.651536 | 2028.95 | 2070.248464 | 1.022656 |  |
| GO:0001657\_ureteric\_bud\_development | 38 | 0 | 0.000000 | -0.000000 | 1984 | 1987.651536 | 2028.95 | 2070.248464 | 1.022656 |  |
| GO:0002695\_negative\_regulation\_of\_leukocyte\_activation | 38 | 0 | 0.000000 | -0.000000 | 1984 | 1987.651536 | 2028.95 | 2070.248464 | 1.022656 |  |
| GO:0006820\_anion\_transport | 38 | 0 | 0.000000 | -0.000000 | 1984 | 1987.651536 | 2028.95 | 2070.248464 | 1.022656 |  |
| GO:0007596\_blood\_coagulation | 38 | 0 | 0.000000 | -0.000000 | 1984 | 1987.651536 | 2028.95 | 2070.248464 | 1.022656 |  |
| GO:0008016\_regulation\_of\_heart\_contraction | 38 | 0 | 0.000000 | -0.000000 | 1984 | 1987.651536 | 2028.95 | 2070.248464 | 1.022656 |  |
| GO:0010975\_regulation\_of\_neuron\_projection\_development | 38 | 0 | 0.000000 | -0.000000 | 1984 | 1987.651536 | 2028.95 | 2070.248464 | 1.022656 |  |
| GO:0016053\_organic\_acid\_biosynthetic\_process | 38 | 0 | 0.000000 | -0.000000 | 1984 | 1987.651536 | 2028.95 | 2070.248464 | 1.022656 |  |
| GO:0031401\_positive\_regulation\_of\_protein\_modification\_process | 38 | 0 | 0.000000 | -0.000000 | 1984 | 1987.651536 | 2028.95 | 2070.248464 | 1.022656 |  |
| GO:0032259\_methylation | 38 | 0 | 0.000000 | -0.000000 | 1984 | 1987.651536 | 2028.95 | 2070.248464 | 1.022656 |  |
| GO:0042493\_response\_to\_drug | 38 | 0 | 0.000000 | -0.000000 | 1984 | 1987.651536 | 2028.95 | 2070.248464 | 1.022656 |  |
| GO:0043414\_biopolymer\_methylation | 38 | 0 | 0.000000 | -0.000000 | 1984 | 1987.651536 | 2028.95 | 2070.248464 | 1.022656 |  |
| GO:0045580\_regulation\_of\_T\_cell\_differentiation | 38 | 0 | 0.000000 | -0.000000 | 1984 | 1987.651536 | 2028.95 | 2070.248464 | 1.022656 |  |
| GO:0046394\_carboxylic\_acid\_biosynthetic\_process | 38 | 0 | 0.000000 | -0.000000 | 1984 | 1987.651536 | 2028.95 | 2070.248464 | 1.022656 |  |
| GO:0046777\_protein\_amino\_acid\_autophosphorylation | 38 | 0 | 0.000000 | -0.000000 | 1984 | 1987.651536 | 2028.95 | 2070.248464 | 1.022656 |  |
| GO:0050727\_regulation\_of\_inflammatory\_response | 38 | 0 | 0.000000 | -0.000000 | 1984 | 1987.651536 | 2028.95 | 2070.248464 | 1.022656 |  |
| GO:0050866\_negative\_regulation\_of\_cell\_activation | 38 | 0 | 0.000000 | -0.000000 | 1984 | 1987.651536 | 2028.95 | 2070.248464 | 1.022656 |  |
| GO:0051348\_negative\_regulation\_of\_transferase\_activity | 38 | 0 | 0.000000 | -0.000000 | 1984 | 1987.651536 | 2028.95 | 2070.248464 | 1.022656 |  |
| GO:0022604\_regulation\_of\_cell\_morphogenesis | 62 | 0 | 0.000000 | -0.000000 | 1991 | 1995.128950 | 2035.7 | 2076.271050 | 1.022451 |  |
| GO:0030155\_regulation\_of\_cell\_adhesion | 62 | 0 | 0.000000 | -0.000000 | 1991 | 1995.128950 | 2035.7 | 2076.271050 | 1.022451 |  |
| GO:0030855\_epithelial\_cell\_differentiation | 62 | 0 | 0.000000 | -0.000000 | 1991 | 1995.128950 | 2035.7 | 2076.271050 | 1.022451 |  |
| GO:0032944\_regulation\_of\_mononuclear\_cell\_proliferation | 62 | 0 | 0.000000 | -0.000000 | 1991 | 1995.128950 | 2035.7 | 2076.271050 | 1.022451 |  |
| GO:0040014\_regulation\_of\_multicellular\_organism\_growth | 62 | 0 | 0.000000 | -0.000000 | 1991 | 1995.128950 | 2035.7 | 2076.271050 | 1.022451 |  |
| GO:0050670\_regulation\_of\_lymphocyte\_proliferation | 62 | 0 | 0.000000 | -0.000000 | 1991 | 1995.128950 | 2035.7 | 2076.271050 | 1.022451 |  |
| GO:0050954\_sensory\_perception\_of\_mechanical\_stimulus | 62 | 0 | 0.000000 | -0.000000 | 1991 | 1995.128950 | 2035.7 | 2076.271050 | 1.022451 |  |
| GO:0016044\_membrane\_organization | 140 | 0 | 0.000000 | -0.000000 | 1992 | 1996.254189 | 2036.71 | 2077.165811 | 1.022445 |  |
| GO:0001890\_placenta\_development | 77 | 0 | 0.000000 | -0.000000 | 1994 | 1997.878913 | 2038.19 | 2078.501087 | 1.022161 |  |
| GO:0051241\_negative\_regulation\_of\_multicellular\_organismal\_process | 77 | 0 | 0.000000 | -0.000000 | 1994 | 1997.878913 | 2038.19 | 2078.501087 | 1.022161 |  |
| GO:0001817\_regulation\_of\_cytokine\_production | 99 | 0 | 0.000000 | -0.000000 | 1998 | 2001.083672 | 2040.99 | 2080.896328 | 1.021517 |  |
| GO:0007398\_ectoderm\_development | 99 | 0 | 0.000000 | -0.000000 | 1998 | 2001.083672 | 2040.99 | 2080.896328 | 1.021517 |  |
| GO:0060348\_bone\_development | 99 | 0 | 0.000000 | -0.000000 | 1998 | 2001.083672 | 2040.99 | 2080.896328 | 1.021517 |  |
| GO:0060562\_epithelial\_tube\_morphogenesis | 99 | 0 | 0.000000 | -0.000000 | 1998 | 2001.083672 | 2040.99 | 2080.896328 | 1.021517 |  |
| GO:0001658\_branching\_involved\_in\_ureteric\_bud\_morphogenesis | 26 | 0 | 0.000000 | -0.000000 | 2022 | 2023.530557 | 2062.65 | 2101.769443 | 1.020104 |  |
| GO:0001666\_response\_to\_hypoxia | 26 | 0 | 0.000000 | -0.000000 | 2022 | 2023.530557 | 2062.65 | 2101.769443 | 1.020104 |  |
| GO:0006720\_isoprenoid\_metabolic\_process | 26 | 0 | 0.000000 | -0.000000 | 2022 | 2023.530557 | 2062.65 | 2101.769443 | 1.020104 |  |
| GO:0006800\_oxygen\_and\_reactive\_oxygen\_species\_metabolic\_process | 26 | 0 | 0.000000 | -0.000000 | 2022 | 2023.530557 | 2062.65 | 2101.769443 | 1.020104 |  |
| GO:0007405\_neuroblast\_proliferation | 26 | 0 | 0.000000 | -0.000000 | 2022 | 2023.530557 | 2062.65 | 2101.769443 | 1.020104 |  |
| GO:0007613\_memory | 26 | 0 | 0.000000 | -0.000000 | 2022 | 2023.530557 | 2062.65 | 2101.769443 | 1.020104 |  |
| GO:0007623\_circadian\_rhythm | 26 | 0 | 0.000000 | -0.000000 | 2022 | 2023.530557 | 2062.65 | 2101.769443 | 1.020104 |  |
| GO:0009310\_amine\_catabolic\_process | 26 | 0 | 0.000000 | -0.000000 | 2022 | 2023.530557 | 2062.65 | 2101.769443 | 1.020104 |  |
| GO:0009636\_response\_to\_toxin | 26 | 0 | 0.000000 | -0.000000 | 2022 | 2023.530557 | 2062.65 | 2101.769443 | 1.020104 |  |
| GO:0009880\_embryonic\_pattern\_specification | 26 | 0 | 0.000000 | -0.000000 | 2022 | 2023.530557 | 2062.65 | 2101.769443 | 1.020104 |  |
| GO:0010212\_response\_to\_ionizing\_radiation | 26 | 0 | 0.000000 | -0.000000 | 2022 | 2023.530557 | 2062.65 | 2101.769443 | 1.020104 |  |
| GO:0010959\_regulation\_of\_metal\_ion\_transport | 26 | 0 | 0.000000 | -0.000000 | 2022 | 2023.530557 | 2062.65 | 2101.769443 | 1.020104 |  |
| GO:0019233\_sensory\_perception\_of\_pain | 26 | 0 | 0.000000 | -0.000000 | 2022 | 2023.530557 | 2062.65 | 2101.769443 | 1.020104 |  |
| GO:0032609\_interferon-gamma\_production | 26 | 0 | 0.000000 | -0.000000 | 2022 | 2023.530557 | 2062.65 | 2101.769443 | 1.020104 |  |
| GO:0040029\_regulation\_of\_gene\_expression\_\_epigenetic | 26 | 0 | 0.000000 | -0.000000 | 2022 | 2023.530557 | 2062.65 | 2101.769443 | 1.020104 |  |
| GO:0045665\_negative\_regulation\_of\_neuron\_differentiation | 26 | 0 | 0.000000 | -0.000000 | 2022 | 2023.530557 | 2062.65 | 2101.769443 | 1.020104 |  |
| GO:0045765\_regulation\_of\_angiogenesis | 26 | 0 | 0.000000 | -0.000000 | 2022 | 2023.530557 | 2062.65 | 2101.769443 | 1.020104 |  |
| GO:0046530\_photoreceptor\_cell\_differentiation | 26 | 0 | 0.000000 | -0.000000 | 2022 | 2023.530557 | 2062.65 | 2101.769443 | 1.020104 |  |
| GO:0048645\_organ\_formation | 26 | 0 | 0.000000 | -0.000000 | 2022 | 2023.530557 | 2062.65 | 2101.769443 | 1.020104 |  |
| GO:0050680\_negative\_regulation\_of\_epithelial\_cell\_proliferation | 26 | 0 | 0.000000 | -0.000000 | 2022 | 2023.530557 | 2062.65 | 2101.769443 | 1.020104 |  |
| GO:0050873\_brown\_fat\_cell\_differentiation | 26 | 0 | 0.000000 | -0.000000 | 2022 | 2023.530557 | 2062.65 | 2101.769443 | 1.020104 |  |
| GO:0051235\_maintenance\_of\_location | 26 | 0 | 0.000000 | -0.000000 | 2022 | 2023.530557 | 2062.65 | 2101.769443 | 1.020104 |  |
| GO:0051480\_cytosolic\_calcium\_ion\_homeostasis | 26 | 0 | 0.000000 | -0.000000 | 2022 | 2023.530557 | 2062.65 | 2101.769443 | 1.020104 |  |
| GO:0060675\_ureteric\_bud\_morphogenesis | 26 | 0 | 0.000000 | -0.000000 | 2022 | 2023.530557 | 2062.65 | 2101.769443 | 1.020104 |  |
| GO:0000003\_reproduction | 379 | 0 | 0.000000 | 0.000000 | 2059 | 2060.624033 | 2090.14 | 2119.655967 | 1.015124 |  |
| GO:0001501\_skeletal\_system\_development | 236 | 0 | 0.000000 | 0.000000 | 2059 | 2060.624033 | 2090.14 | 2119.655967 | 1.015124 |  |
| GO:0001701\_in\_utero\_embryonic\_development | 221 | 0 | 0.000000 | 0.000000 | 2059 | 2060.624033 | 2090.14 | 2119.655967 | 1.015124 |  |
| GO:0002520\_immune\_system\_development | 295 | 0 | 0.000000 | 0.000000 | 2059 | 2060.624033 | 2090.14 | 2119.655967 | 1.015124 |  |
| GO:0002682\_regulation\_of\_immune\_system\_process | 228 | 0 | 0.000000 | 0.000000 | 2059 | 2060.624033 | 2090.14 | 2119.655967 | 1.015124 |  |
| GO:0006357\_regulation\_of\_transcription\_from\_RNA\_polymerase\_II\_promoter | 435 | 0 | 0.000000 | 0.000000 | 2059 | 2060.624033 | 2090.14 | 2119.655967 | 1.015124 |  |
| GO:0006366\_transcription\_from\_RNA\_polymerase\_II\_promoter | 444 | 0 | 0.000000 | 0.000000 | 2059 | 2060.624033 | 2090.14 | 2119.655967 | 1.015124 |  |
| GO:0006468\_protein\_amino\_acid\_phosphorylation | 237 | 0 | 0.000000 | 0.000000 | 2059 | 2060.624033 | 2090.14 | 2119.655967 | 1.015124 |  |
| GO:0007389\_pattern\_specification\_process | 250 | 0 | 0.000000 | 0.000000 | 2059 | 2060.624033 | 2090.14 | 2119.655967 | 1.015124 |  |
| GO:0007423\_sensory\_organ\_development | 219 | 0 | 0.000000 | 0.000000 | 2059 | 2060.624033 | 2090.14 | 2119.655967 | 1.015124 |  |
| GO:0008150\_biological\_process | 4605 | 20 | 1.000000 | 0.000000 | 2059 | 2060.624033 | 2090.14 | 2119.655967 | 1.015124 |  |
| GO:0009605\_response\_to\_external\_stimulus | 339 | 0 | 0.000000 | 0.000000 | 2059 | 2060.624033 | 2090.14 | 2119.655967 | 1.015124 |  |
| GO:0009790\_embryonic\_development | 567 | 0 | 0.000000 | 0.000000 | 2059 | 2060.624033 | 2090.14 | 2119.655967 | 1.015124 |  |
| GO:0009792\_embryonic\_development\_ending\_in\_birth\_or\_egg\_hatching | 368 | 0 | 0.000000 | 0.000000 | 2059 | 2060.624033 | 2090.14 | 2119.655967 | 1.015124 |  |
| GO:0010557\_positive\_regulation\_of\_macromolecule\_biosynthetic\_process | 371 | 0 | 0.000000 | 0.000000 | 2059 | 2060.624033 | 2090.14 | 2119.655967 | 1.015124 |  |
| GO:0010604\_positive\_regulation\_of\_macromolecule\_metabolic\_process | 433 | 0 | 0.000000 | 0.000000 | 2059 | 2060.624033 | 2090.14 | 2119.655967 | 1.015124 |  |
| GO:0010628\_positive\_regulation\_of\_gene\_expression | 346 | 0 | 0.000000 | 0.000000 | 2059 | 2060.624033 | 2090.14 | 2119.655967 | 1.015124 |  |
| GO:0019953\_sexual\_reproduction | 228 | 0 | 0.000000 | 0.000000 | 2059 | 2060.624033 | 2090.14 | 2119.655967 | 1.015124 |  |
| GO:0022414\_reproductive\_process | 376 | 0 | 0.000000 | 0.000000 | 2059 | 2060.624033 | 2090.14 | 2119.655967 | 1.015124 |  |
| GO:0030097\_hemopoiesis | 253 | 0 | 0.000000 | 0.000000 | 2059 | 2060.624033 | 2090.14 | 2119.655967 | 1.015124 |  |
| GO:0032879\_regulation\_of\_localization | 248 | 0 | 0.000000 | 0.000000 | 2059 | 2060.624033 | 2090.14 | 2119.655967 | 1.015124 |  |
| GO:0042221\_response\_to\_chemical\_stimulus | 409 | 0 | 0.000000 | 0.000000 | 2059 | 2060.624033 | 2090.14 | 2119.655967 | 1.015124 |  |
| GO:0043009\_chordate\_embryonic\_development | 365 | 0 | 0.000000 | 0.000000 | 2059 | 2060.624033 | 2090.14 | 2119.655967 | 1.015124 |  |
| GO:0043687\_post-translational\_protein\_modification | 384 | 0 | 0.000000 | 0.000000 | 2059 | 2060.624033 | 2090.14 | 2119.655967 | 1.015124 |  |
| GO:0045595\_regulation\_of\_cell\_differentiation | 295 | 0 | 0.000000 | 0.000000 | 2059 | 2060.624033 | 2090.14 | 2119.655967 | 1.015124 |  |
| GO:0045893\_positive\_regulation\_of\_transcription\_\_DNA-dependent | 306 | 0 | 0.000000 | 0.000000 | 2059 | 2060.624033 | 2090.14 | 2119.655967 | 1.015124 |  |
| GO:0045935\_positive\_regulation\_of\_nucleobase\_\_nucleoside\_\_nucleotide\_and\_nucleic\_acid\_metabolic\_process | 352 | 0 | 0.000000 | 0.000000 | 2059 | 2060.624033 | 2090.14 | 2119.655967 | 1.015124 |  |
| GO:0045941\_positive\_regulation\_of\_transcription | 338 | 0 | 0.000000 | 0.000000 | 2059 | 2060.624033 | 2090.14 | 2119.655967 | 1.015124 |  |
| GO:0045944\_positive\_regulation\_of\_transcription\_from\_RNA\_polymerase\_II\_promoter | 269 | 0 | 0.000000 | 0.000000 | 2059 | 2060.624033 | 2090.14 | 2119.655967 | 1.015124 |  |
| GO:0048534\_hemopoietic\_or\_lymphoid\_organ\_development | 277 | 0 | 0.000000 | 0.000000 | 2059 | 2060.624033 | 2090.14 | 2119.655967 | 1.015124 |  |
| GO:0048598\_embryonic\_morphogenesis | 299 | 0 | 0.000000 | 0.000000 | 2059 | 2060.624033 | 2090.14 | 2119.655967 | 1.015124 |  |
| GO:0048646\_anatomical\_structure\_formation\_involved\_in\_morphogenesis | 277 | 0 | 0.000000 | 0.000000 | 2059 | 2060.624033 | 2090.14 | 2119.655967 | 1.015124 |  |
| GO:0048729\_tissue\_morphogenesis | 255 | 0 | 0.000000 | 0.000000 | 2059 | 2060.624033 | 2090.14 | 2119.655967 | 1.015124 |  |
| GO:0048878\_chemical\_homeostasis | 254 | 0 | 0.000000 | 0.000000 | 2059 | 2060.624033 | 2090.14 | 2119.655967 | 1.015124 |  |
| GO:0051173\_positive\_regulation\_of\_nitrogen\_compound\_metabolic\_process | 361 | 0 | 0.000000 | 0.000000 | 2059 | 2060.624033 | 2090.14 | 2119.655967 | 1.015124 |  |
| GO:0051239\_regulation\_of\_multicellular\_organismal\_process | 587 | 0 | 0.000000 | 0.000000 | 2059 | 2060.624033 | 2090.14 | 2119.655967 | 1.015124 |  |
| GO:0051254\_positive\_regulation\_of\_RNA\_metabolic\_process | 306 | 0 | 0.000000 | 0.000000 | 2059 | 2060.624033 | 2090.14 | 2119.655967 | 1.015124 |  |
| GO:0010647\_positive\_regulation\_of\_cell\_communication | 110 | 0 | 0.000000 | 0.000000 | 2063 | 2063.461204 | 2092.59 | 2121.718796 | 1.014343 |  |
| GO:0010648\_negative\_regulation\_of\_cell\_communication | 110 | 0 | 0.000000 | 0.000000 | 2063 | 2063.461204 | 2092.59 | 2121.718796 | 1.014343 |  |
| GO:0043010\_camera-type\_eye\_development | 110 | 0 | 0.000000 | 0.000000 | 2063 | 2063.461204 | 2092.59 | 2121.718796 | 1.014343 |  |
| GO:0055080\_cation\_homeostasis | 110 | 0 | 0.000000 | 0.000000 | 2063 | 2063.461204 | 2092.59 | 2121.718796 | 1.014343 |  |
| GO:0003007\_heart\_morphogenesis | 67 | 0 | 0.000000 | 0.000000 | 2068 | 2067.611776 | 2096.47 | 2125.328224 | 1.013767 |  |
| GO:0009791\_post-embryonic\_development | 67 | 0 | 0.000000 | 0.000000 | 2068 | 2067.611776 | 2096.47 | 2125.328224 | 1.013767 |  |
| GO:0031347\_regulation\_of\_defense\_response | 67 | 0 | 0.000000 | 0.000000 | 2068 | 2067.611776 | 2096.47 | 2125.328224 | 1.013767 |  |
| GO:0042445\_hormone\_metabolic\_process | 67 | 0 | 0.000000 | 0.000000 | 2068 | 2067.611776 | 2096.47 | 2125.328224 | 1.013767 |  |
| GO:0051247\_positive\_regulation\_of\_protein\_metabolic\_process | 67 | 0 | 0.000000 | 0.000000 | 2068 | 2067.611776 | 2096.47 | 2125.328224 | 1.013767 |  |
| GO:0006812\_cation\_transport | 146 | 0 | 0.000000 | 0.000000 | 2069 | 2069.538400 | 2098.09 | 2126.641600 | 1.014060 |  |
| GO:0002696\_positive\_regulation\_of\_leukocyte\_activation | 82 | 0 | 0.000000 | 0.000000 | 2074 | 2073.896316 | 2101.92 | 2129.943684 | 1.013462 |  |
| GO:0007411\_axon\_guidance | 82 | 0 | 0.000000 | 0.000000 | 2074 | 2073.896316 | 2101.92 | 2129.943684 | 1.013462 |  |
| GO:0008202\_steroid\_metabolic\_process | 82 | 0 | 0.000000 | 0.000000 | 2074 | 2073.896316 | 2101.92 | 2129.943684 | 1.013462 |  |
| GO:0010627\_regulation\_of\_protein\_kinase\_cascade | 82 | 0 | 0.000000 | 0.000000 | 2074 | 2073.896316 | 2101.92 | 2129.943684 | 1.013462 |  |
| GO:0045664\_regulation\_of\_neuron\_differentiation | 82 | 0 | 0.000000 | 0.000000 | 2074 | 2073.896316 | 2101.92 | 2129.943684 | 1.013462 |  |
| GO:0002009\_morphogenesis\_of\_an\_epithelium | 198 | 0 | 0.000000 | 0.000000 | 2076 | 2074.893338 | 2102.68 | 2130.466662 | 1.012852 |  |
| GO:0060429\_epithelium\_development | 198 | 0 | 0.000000 | 0.000000 | 2076 | 2074.893338 | 2102.68 | 2130.466662 | 1.012852 |  |
| GO:0022603\_regulation\_of\_anatomical\_structure\_morphogenesis | 147 | 0 | 0.000000 | 0.000000 | 2077 | 2075.464458 | 2103.17 | 2130.875542 | 1.012600 |  |
| GO:0002790\_peptide\_secretion | 37 | 0 | 0.000000 | 0.000000 | 2089 | 2086.397204 | 2113.52 | 2140.642796 | 1.011738 |  |
| GO:0009799\_determination\_of\_symmetry | 37 | 0 | 0.000000 | 0.000000 | 2089 | 2086.397204 | 2113.52 | 2140.642796 | 1.011738 |  |
| GO:0009855\_determination\_of\_bilateral\_symmetry | 37 | 0 | 0.000000 | 0.000000 | 2089 | 2086.397204 | 2113.52 | 2140.642796 | 1.011738 |  |
| GO:0019933\_cAMP-mediated\_signaling | 37 | 0 | 0.000000 | 0.000000 | 2089 | 2086.397204 | 2113.52 | 2140.642796 | 1.011738 |  |
| GO:0031349\_positive\_regulation\_of\_defense\_response | 37 | 0 | 0.000000 | 0.000000 | 2089 | 2086.397204 | 2113.52 | 2140.642796 | 1.011738 |  |
| GO:0032869\_cellular\_response\_to\_insulin\_stimulus | 37 | 0 | 0.000000 | 0.000000 | 2089 | 2086.397204 | 2113.52 | 2140.642796 | 1.011738 |  |
| GO:0042698\_ovulation\_cycle | 37 | 0 | 0.000000 | 0.000000 | 2089 | 2086.397204 | 2113.52 | 2140.642796 | 1.011738 |  |
| GO:0045761\_regulation\_of\_adenylate\_cyclase\_activity | 37 | 0 | 0.000000 | 0.000000 | 2089 | 2086.397204 | 2113.52 | 2140.642796 | 1.011738 |  |
| GO:0045785\_positive\_regulation\_of\_cell\_adhesion | 37 | 0 | 0.000000 | 0.000000 | 2089 | 2086.397204 | 2113.52 | 2140.642796 | 1.011738 |  |
| GO:0050906\_detection\_of\_stimulus\_involved\_in\_sensory\_perception | 37 | 0 | 0.000000 | 0.000000 | 2089 | 2086.397204 | 2113.52 | 2140.642796 | 1.011738 |  |
| GO:0051101\_regulation\_of\_DNA\_binding | 37 | 0 | 0.000000 | 0.000000 | 2089 | 2086.397204 | 2113.52 | 2140.642796 | 1.011738 |  |
| GO:0051250\_negative\_regulation\_of\_lymphocyte\_activation | 37 | 0 | 0.000000 | 0.000000 | 2089 | 2086.397204 | 2113.52 | 2140.642796 | 1.011738 |  |
| GO:0016192\_vesicle-mediated\_transport | 184 | 0 | 0.000000 | 0.000000 | 2090 | 2087.303362 | 2114.28 | 2141.256638 | 1.011617 |  |
| GO:0001838\_embryonic\_epithelial\_tube\_formation | 45 | 0 | 0.000000 | 0.000000 | 2095 | 2094.191027 | 2120.55 | 2146.908973 | 1.012196 |  |
| GO:0032870\_cellular\_response\_to\_hormone\_stimulus | 45 | 0 | 0.000000 | 0.000000 | 2095 | 2094.191027 | 2120.55 | 2146.908973 | 1.012196 |  |
| GO:0034103\_regulation\_of\_tissue\_remodeling | 45 | 0 | 0.000000 | 0.000000 | 2095 | 2094.191027 | 2120.55 | 2146.908973 | 1.012196 |  |
| GO:0046058\_cAMP\_metabolic\_process | 45 | 0 | 0.000000 | 0.000000 | 2095 | 2094.191027 | 2120.55 | 2146.908973 | 1.012196 |  |
| GO:0046546\_development\_of\_primary\_male\_sexual\_characteristics | 45 | 0 | 0.000000 | 0.000000 | 2095 | 2094.191027 | 2120.55 | 2146.908973 | 1.012196 |  |
| GO:0000002\_mitochondrial\_genome\_maintenance | 9 | 0 | 0.000000 | 0.000000 | 2212 | 2211.525618 | 2235.81 | 2260.094382 | 1.010764 |  |
| GO:0000186\_activation\_of\_MAPKK\_activity | 9 | 0 | 0.000000 | 0.000000 | 2212 | 2211.525618 | 2235.81 | 2260.094382 | 1.010764 |  |
| GO:0001539\_ciliary\_or\_flagellar\_motility | 9 | 0 | 0.000000 | 0.000000 | 2212 | 2211.525618 | 2235.81 | 2260.094382 | 1.010764 |  |
| GO:0001542\_ovulation\_from\_ovarian\_follicle | 9 | 0 | 0.000000 | 0.000000 | 2212 | 2211.525618 | 2235.81 | 2260.094382 | 1.010764 |  |
| GO:0001667\_ameboidal\_cell\_migration | 9 | 0 | 0.000000 | 0.000000 | 2212 | 2211.525618 | 2235.81 | 2260.094382 | 1.010764 |  |
| GO:0001676\_long-chain\_fatty\_acid\_metabolic\_process | 9 | 0 | 0.000000 | 0.000000 | 2212 | 2211.525618 | 2235.81 | 2260.094382 | 1.010764 |  |
| GO:0001935\_endothelial\_cell\_proliferation | 9 | 0 | 0.000000 | 0.000000 | 2212 | 2211.525618 | 2235.81 | 2260.094382 | 1.010764 |  |
| GO:0002021\_response\_to\_dietary\_excess | 9 | 0 | 0.000000 | 0.000000 | 2212 | 2211.525618 | 2235.81 | 2260.094382 | 1.010764 |  |
| GO:0002028\_regulation\_of\_sodium\_ion\_transport | 9 | 0 | 0.000000 | 0.000000 | 2212 | 2211.525618 | 2235.81 | 2260.094382 | 1.010764 |  |
| GO:0002221\_pattern\_recognition\_receptor\_signaling\_pathway | 9 | 0 | 0.000000 | 0.000000 | 2212 | 2211.525618 | 2235.81 | 2260.094382 | 1.010764 |  |
| GO:0002292\_T\_cell\_differentiation\_during\_immune\_response | 9 | 0 | 0.000000 | 0.000000 | 2212 | 2211.525618 | 2235.81 | 2260.094382 | 1.010764 |  |
| GO:0002293\_alpha-beta\_T\_cell\_differentiation\_during\_immune\_response | 9 | 0 | 0.000000 | 0.000000 | 2212 | 2211.525618 | 2235.81 | 2260.094382 | 1.010764 |  |
| GO:0002294\_CD4-positive\_\_alpha-beta\_T\_cell\_differentiation\_during\_immune\_response | 9 | 0 | 0.000000 | 0.000000 | 2212 | 2211.525618 | 2235.81 | 2260.094382 | 1.010764 |  |
| GO:0002507\_tolerance\_induction | 9 | 0 | 0.000000 | 0.000000 | 2212 | 2211.525618 | 2235.81 | 2260.094382 | 1.010764 |  |
| GO:0002886\_regulation\_of\_myeloid\_leukocyte\_mediated\_immunity | 9 | 0 | 0.000000 | 0.000000 | 2212 | 2211.525618 | 2235.81 | 2260.094382 | 1.010764 |  |
| GO:0006007\_glucose\_catabolic\_process | 9 | 0 | 0.000000 | 0.000000 | 2212 | 2211.525618 | 2235.81 | 2260.094382 | 1.010764 |  |
| GO:0006182\_cGMP\_biosynthetic\_process | 9 | 0 | 0.000000 | 0.000000 | 2212 | 2211.525618 | 2235.81 | 2260.094382 | 1.010764 |  |
| GO:0006309\_DNA\_fragmentation\_involved\_in\_apoptosis | 9 | 0 | 0.000000 | 0.000000 | 2212 | 2211.525618 | 2235.81 | 2260.094382 | 1.010764 |  |
| GO:0006364\_rRNA\_processing | 9 | 0 | 0.000000 | 0.000000 | 2212 | 2211.525618 | 2235.81 | 2260.094382 | 1.010764 |  |
| GO:0006476\_protein\_amino\_acid\_deacetylation | 9 | 0 | 0.000000 | 0.000000 | 2212 | 2211.525618 | 2235.81 | 2260.094382 | 1.010764 |  |
| GO:0006595\_polyamine\_metabolic\_process | 9 | 0 | 0.000000 | 0.000000 | 2212 | 2211.525618 | 2235.81 | 2260.094382 | 1.010764 |  |
| GO:0006910\_phagocytosis\_\_recognition | 9 | 0 | 0.000000 | 0.000000 | 2212 | 2211.525618 | 2235.81 | 2260.094382 | 1.010764 |  |
| GO:0006911\_phagocytosis\_\_engulfment | 9 | 0 | 0.000000 | 0.000000 | 2212 | 2211.525618 | 2235.81 | 2260.094382 | 1.010764 |  |
| GO:0007128\_meiotic\_prophase\_I | 9 | 0 | 0.000000 | 0.000000 | 2212 | 2211.525618 | 2235.81 | 2260.094382 | 1.010764 |  |
| GO:0007193\_inhibition\_of\_adenylate\_cyclase\_activity\_by\_G-protein\_signaling | 9 | 0 | 0.000000 | 0.000000 | 2212 | 2211.525618 | 2235.81 | 2260.094382 | 1.010764 |  |
| GO:0007379\_segment\_specification | 9 | 0 | 0.000000 | 0.000000 | 2212 | 2211.525618 | 2235.81 | 2260.094382 | 1.010764 |  |
| GO:0007617\_mating\_behavior | 9 | 0 | 0.000000 | 0.000000 | 2212 | 2211.525618 | 2235.81 | 2260.094382 | 1.010764 |  |
| GO:0009451\_RNA\_modification | 9 | 0 | 0.000000 | 0.000000 | 2212 | 2211.525618 | 2235.81 | 2260.094382 | 1.010764 |  |
| GO:0010165\_response\_to\_X-ray | 9 | 0 | 0.000000 | 0.000000 | 2212 | 2211.525618 | 2235.81 | 2260.094382 | 1.010764 |  |
| GO:0010675\_regulation\_of\_cellular\_carbohydrate\_metabolic\_process | 9 | 0 | 0.000000 | 0.000000 | 2212 | 2211.525618 | 2235.81 | 2260.094382 | 1.010764 |  |
| GO:0014037\_Schwann\_cell\_differentiation | 9 | 0 | 0.000000 | 0.000000 | 2212 | 2211.525618 | 2235.81 | 2260.094382 | 1.010764 |  |
| GO:0014073\_response\_to\_tropane | 9 | 0 | 0.000000 | 0.000000 | 2212 | 2211.525618 | 2235.81 | 2260.094382 | 1.010764 |  |
| GO:0015695\_organic\_cation\_transport | 9 | 0 | 0.000000 | 0.000000 | 2212 | 2211.525618 | 2235.81 | 2260.094382 | 1.010764 |  |
| GO:0016072\_rRNA\_metabolic\_process | 9 | 0 | 0.000000 | 0.000000 | 2212 | 2211.525618 | 2235.81 | 2260.094382 | 1.010764 |  |
| GO:0016601\_Rac\_protein\_signal\_transduction | 9 | 0 | 0.000000 | 0.000000 | 2212 | 2211.525618 | 2235.81 | 2260.094382 | 1.010764 |  |
| GO:0017145\_stem\_cell\_division | 9 | 0 | 0.000000 | 0.000000 | 2212 | 2211.525618 | 2235.81 | 2260.094382 | 1.010764 |  |
| GO:0019320\_hexose\_catabolic\_process | 9 | 0 | 0.000000 | 0.000000 | 2212 | 2211.525618 | 2235.81 | 2260.094382 | 1.010764 |  |
| GO:0021544\_subpallium\_development | 9 | 0 | 0.000000 | 0.000000 | 2212 | 2211.525618 | 2235.81 | 2260.094382 | 1.010764 |  |
| GO:0021936\_regulation\_of\_granule\_cell\_precursor\_proliferation | 9 | 0 | 0.000000 | 0.000000 | 2212 | 2211.525618 | 2235.81 | 2260.094382 | 1.010764 |  |
| GO:0021940\_positive\_regulation\_of\_granule\_cell\_precursor\_proliferation | 9 | 0 | 0.000000 | 0.000000 | 2212 | 2211.525618 | 2235.81 | 2260.094382 | 1.010764 |  |
| GO:0030048\_actin\_filament-based\_movement | 9 | 0 | 0.000000 | 0.000000 | 2212 | 2211.525618 | 2235.81 | 2260.094382 | 1.010764 |  |
| GO:0030279\_negative\_regulation\_of\_ossification | 9 | 0 | 0.000000 | 0.000000 | 2212 | 2211.525618 | 2235.81 | 2260.094382 | 1.010764 |  |
| GO:0030325\_adrenal\_gland\_development | 9 | 0 | 0.000000 | 0.000000 | 2212 | 2211.525618 | 2235.81 | 2260.094382 | 1.010764 |  |
| GO:0030728\_ovulation | 9 | 0 | 0.000000 | 0.000000 | 2212 | 2211.525618 | 2235.81 | 2260.094382 | 1.010764 |  |
| GO:0032388\_positive\_regulation\_of\_intracellular\_transport | 9 | 0 | 0.000000 | 0.000000 | 2212 | 2211.525618 | 2235.81 | 2260.094382 | 1.010764 |  |
| GO:0032606\_type\_I\_interferon\_production | 9 | 0 | 0.000000 | 0.000000 | 2212 | 2211.525618 | 2235.81 | 2260.094382 | 1.010764 |  |
| GO:0032814\_regulation\_of\_natural\_killer\_cell\_activation | 9 | 0 | 0.000000 | 0.000000 | 2212 | 2211.525618 | 2235.81 | 2260.094382 | 1.010764 |  |
| GO:0032816\_positive\_regulation\_of\_natural\_killer\_cell\_activation | 9 | 0 | 0.000000 | 0.000000 | 2212 | 2211.525618 | 2235.81 | 2260.094382 | 1.010764 |  |
| GO:0032963\_collagen\_metabolic\_process | 9 | 0 | 0.000000 | 0.000000 | 2212 | 2211.525618 | 2235.81 | 2260.094382 | 1.010764 |  |
| GO:0033028\_myeloid\_cell\_apoptosis | 9 | 0 | 0.000000 | 0.000000 | 2212 | 2211.525618 | 2235.81 | 2260.094382 | 1.010764 |  |
| GO:0033143\_regulation\_of\_steroid\_hormone\_receptor\_signaling\_pathway | 9 | 0 | 0.000000 | 0.000000 | 2212 | 2211.525618 | 2235.81 | 2260.094382 | 1.010764 |  |
| GO:0033151\_V(D)J\_recombination | 9 | 0 | 0.000000 | 0.000000 | 2212 | 2211.525618 | 2235.81 | 2260.094382 | 1.010764 |  |
| GO:0033344\_cholesterol\_efflux | 9 | 0 | 0.000000 | 0.000000 | 2212 | 2211.525618 | 2235.81 | 2260.094382 | 1.010764 |  |
| GO:0034605\_cellular\_response\_to\_heat | 9 | 0 | 0.000000 | 0.000000 | 2212 | 2211.525618 | 2235.81 | 2260.094382 | 1.010764 |  |
| GO:0035088\_establishment\_or\_maintenance\_of\_apical\_basal\_cell\_polarity | 9 | 0 | 0.000000 | 0.000000 | 2212 | 2211.525618 | 2235.81 | 2260.094382 | 1.010764 |  |
| GO:0035162\_embryonic\_hemopoiesis | 9 | 0 | 0.000000 | 0.000000 | 2212 | 2211.525618 | 2235.81 | 2260.094382 | 1.010764 |  |
| GO:0040020\_regulation\_of\_meiosis | 9 | 0 | 0.000000 | 0.000000 | 2212 | 2211.525618 | 2235.81 | 2260.094382 | 1.010764 |  |
| GO:0042058\_regulation\_of\_epidermal\_growth\_factor\_receptor\_signaling\_pathway | 9 | 0 | 0.000000 | 0.000000 | 2212 | 2211.525618 | 2235.81 | 2260.094382 | 1.010764 |  |
| GO:0042093\_T-helper\_cell\_differentiation | 9 | 0 | 0.000000 | 0.000000 | 2212 | 2211.525618 | 2235.81 | 2260.094382 | 1.010764 |  |
| GO:0042220\_response\_to\_cocaine | 9 | 0 | 0.000000 | 0.000000 | 2212 | 2211.525618 | 2235.81 | 2260.094382 | 1.010764 |  |
| GO:0042402\_biogenic\_amine\_catabolic\_process | 9 | 0 | 0.000000 | 0.000000 | 2212 | 2211.525618 | 2235.81 | 2260.094382 | 1.010764 |  |
| GO:0042509\_regulation\_of\_tyrosine\_phosphorylation\_of\_STAT\_protein | 9 | 0 | 0.000000 | 0.000000 | 2212 | 2211.525618 | 2235.81 | 2260.094382 | 1.010764 |  |
| GO:0042640\_anagen | 9 | 0 | 0.000000 | 0.000000 | 2212 | 2211.525618 | 2235.81 | 2260.094382 | 1.010764 |  |
| GO:0043242\_negative\_regulation\_of\_protein\_complex\_disassembly | 9 | 0 | 0.000000 | 0.000000 | 2212 | 2211.525618 | 2235.81 | 2260.094382 | 1.010764 |  |
| GO:0043299\_leukocyte\_degranulation | 9 | 0 | 0.000000 | 0.000000 | 2212 | 2211.525618 | 2235.81 | 2260.094382 | 1.010764 |  |
| GO:0043383\_negative\_T\_cell\_selection | 9 | 0 | 0.000000 | 0.000000 | 2212 | 2211.525618 | 2235.81 | 2260.094382 | 1.010764 |  |
| GO:0043409\_negative\_regulation\_of\_MAPKKK\_cascade | 9 | 0 | 0.000000 | 0.000000 | 2212 | 2211.525618 | 2235.81 | 2260.094382 | 1.010764 |  |
| GO:0043433\_negative\_regulation\_of\_transcription\_factor\_activity | 9 | 0 | 0.000000 | 0.000000 | 2212 | 2211.525618 | 2235.81 | 2260.094382 | 1.010764 |  |
| GO:0043603\_cellular\_amide\_metabolic\_process | 9 | 0 | 0.000000 | 0.000000 | 2212 | 2211.525618 | 2235.81 | 2260.094382 | 1.010764 |  |
| GO:0045060\_negative\_thymic\_T\_cell\_selection | 9 | 0 | 0.000000 | 0.000000 | 2212 | 2211.525618 | 2235.81 | 2260.094382 | 1.010764 |  |
| GO:0045109\_intermediate\_filament\_organization | 9 | 0 | 0.000000 | 0.000000 | 2212 | 2211.525618 | 2235.81 | 2260.094382 | 1.010764 |  |
| GO:0045136\_development\_of\_secondary\_sexual\_characteristics | 9 | 0 | 0.000000 | 0.000000 | 2212 | 2211.525618 | 2235.81 | 2260.094382 | 1.010764 |  |
| GO:0045185\_maintenance\_of\_protein\_location | 9 | 0 | 0.000000 | 0.000000 | 2212 | 2211.525618 | 2235.81 | 2260.094382 | 1.010764 |  |
| GO:0045214\_sarcomere\_organization | 9 | 0 | 0.000000 | 0.000000 | 2212 | 2211.525618 | 2235.81 | 2260.094382 | 1.010764 |  |
| GO:0045428\_regulation\_of\_nitric\_oxide\_biosynthetic\_process | 9 | 0 | 0.000000 | 0.000000 | 2212 | 2211.525618 | 2235.81 | 2260.094382 | 1.010764 |  |
| GO:0045620\_negative\_regulation\_of\_lymphocyte\_differentiation | 9 | 0 | 0.000000 | 0.000000 | 2212 | 2211.525618 | 2235.81 | 2260.094382 | 1.010764 |  |
| GO:0045646\_regulation\_of\_erythrocyte\_differentiation | 9 | 0 | 0.000000 | 0.000000 | 2212 | 2211.525618 | 2235.81 | 2260.094382 | 1.010764 |  |
| GO:0045671\_negative\_regulation\_of\_osteoclast\_differentiation | 9 | 0 | 0.000000 | 0.000000 | 2212 | 2211.525618 | 2235.81 | 2260.094382 | 1.010764 |  |
| GO:0045766\_positive\_regulation\_of\_angiogenesis | 9 | 0 | 0.000000 | 0.000000 | 2212 | 2211.525618 | 2235.81 | 2260.094382 | 1.010764 |  |
| GO:0045830\_positive\_regulation\_of\_isotype\_switching | 9 | 0 | 0.000000 | 0.000000 | 2212 | 2211.525618 | 2235.81 | 2260.094382 | 1.010764 |  |
| GO:0045884\_regulation\_of\_survival\_gene\_product\_expression | 9 | 0 | 0.000000 | 0.000000 | 2212 | 2211.525618 | 2235.81 | 2260.094382 | 1.010764 |  |
| GO:0046006\_regulation\_of\_activated\_T\_cell\_proliferation | 9 | 0 | 0.000000 | 0.000000 | 2212 | 2211.525618 | 2235.81 | 2260.094382 | 1.010764 |  |
| GO:0046324\_regulation\_of\_glucose\_import | 9 | 0 | 0.000000 | 0.000000 | 2212 | 2211.525618 | 2235.81 | 2260.094382 | 1.010764 |  |
| GO:0046365\_monosaccharide\_catabolic\_process | 9 | 0 | 0.000000 | 0.000000 | 2212 | 2211.525618 | 2235.81 | 2260.094382 | 1.010764 |  |
| GO:0046636\_negative\_regulation\_of\_alpha-beta\_T\_cell\_activation | 9 | 0 | 0.000000 | 0.000000 | 2212 | 2211.525618 | 2235.81 | 2260.094382 | 1.010764 |  |
| GO:0046641\_positive\_regulation\_of\_alpha-beta\_T\_cell\_proliferation | 9 | 0 | 0.000000 | 0.000000 | 2212 | 2211.525618 | 2235.81 | 2260.094382 | 1.010764 |  |
| GO:0046888\_negative\_regulation\_of\_hormone\_secretion | 9 | 0 | 0.000000 | 0.000000 | 2212 | 2211.525618 | 2235.81 | 2260.094382 | 1.010764 |  |
| GO:0048070\_regulation\_of\_pigmentation\_during\_development | 9 | 0 | 0.000000 | 0.000000 | 2212 | 2211.525618 | 2235.81 | 2260.094382 | 1.010764 |  |
| GO:0048146\_positive\_regulation\_of\_fibroblast\_proliferation | 9 | 0 | 0.000000 | 0.000000 | 2212 | 2211.525618 | 2235.81 | 2260.094382 | 1.010764 |  |
| GO:0048284\_organelle\_fusion | 9 | 0 | 0.000000 | 0.000000 | 2212 | 2211.525618 | 2235.81 | 2260.094382 | 1.010764 |  |
| GO:0048488\_synaptic\_vesicle\_endocytosis | 9 | 0 | 0.000000 | 0.000000 | 2212 | 2211.525618 | 2235.81 | 2260.094382 | 1.010764 |  |
| GO:0048569\_post-embryonic\_organ\_development | 9 | 0 | 0.000000 | 0.000000 | 2212 | 2211.525618 | 2235.81 | 2260.094382 | 1.010764 |  |
| GO:0048708\_astrocyte\_differentiation | 9 | 0 | 0.000000 | 0.000000 | 2212 | 2211.525618 | 2235.81 | 2260.094382 | 1.010764 |  |
| GO:0050433\_regulation\_of\_catecholamine\_secretion | 9 | 0 | 0.000000 | 0.000000 | 2212 | 2211.525618 | 2235.81 | 2260.094382 | 1.010764 |  |
| GO:0050856\_regulation\_of\_T\_cell\_receptor\_signaling\_pathway | 9 | 0 | 0.000000 | 0.000000 | 2212 | 2211.525618 | 2235.81 | 2260.094382 | 1.010764 |  |
| GO:0050884\_neuromuscular\_process\_controlling\_posture | 9 | 0 | 0.000000 | 0.000000 | 2212 | 2211.525618 | 2235.81 | 2260.094382 | 1.010764 |  |
| GO:0050910\_detection\_of\_mechanical\_stimulus\_involved\_in\_sensory\_perception\_of\_sound | 9 | 0 | 0.000000 | 0.000000 | 2212 | 2211.525618 | 2235.81 | 2260.094382 | 1.010764 |  |
| GO:0050918\_positive\_chemotaxis | 9 | 0 | 0.000000 | 0.000000 | 2212 | 2211.525618 | 2235.81 | 2260.094382 | 1.010764 |  |
| GO:0051023\_regulation\_of\_immunoglobulin\_secretion | 9 | 0 | 0.000000 | 0.000000 | 2212 | 2211.525618 | 2235.81 | 2260.094382 | 1.010764 |  |
| GO:0051324\_prophase | 9 | 0 | 0.000000 | 0.000000 | 2212 | 2211.525618 | 2235.81 | 2260.094382 | 1.010764 |  |
| GO:0051607\_defense\_response\_to\_virus | 9 | 0 | 0.000000 | 0.000000 | 2212 | 2211.525618 | 2235.81 | 2260.094382 | 1.010764 |  |
| GO:0051647\_nucleus\_localization | 9 | 0 | 0.000000 | 0.000000 | 2212 | 2211.525618 | 2235.81 | 2260.094382 | 1.010764 |  |
| GO:0051896\_regulation\_of\_protein\_kinase\_B\_signaling\_cascade | 9 | 0 | 0.000000 | 0.000000 | 2212 | 2211.525618 | 2235.81 | 2260.094382 | 1.010764 |  |
| GO:0051932\_synaptic\_transmission\_\_GABAergic | 9 | 0 | 0.000000 | 0.000000 | 2212 | 2211.525618 | 2235.81 | 2260.094382 | 1.010764 |  |
| GO:0051963\_regulation\_of\_synaptogenesis | 9 | 0 | 0.000000 | 0.000000 | 2212 | 2211.525618 | 2235.81 | 2260.094382 | 1.010764 |  |
| GO:0055012\_ventricular\_cardiac\_muscle\_cell\_differentiation | 9 | 0 | 0.000000 | 0.000000 | 2212 | 2211.525618 | 2235.81 | 2260.094382 | 1.010764 |  |
| GO:0055013\_cardiac\_muscle\_cell\_development | 9 | 0 | 0.000000 | 0.000000 | 2212 | 2211.525618 | 2235.81 | 2260.094382 | 1.010764 |  |
| GO:0060052\_neurofilament\_cytoskeleton\_organization | 9 | 0 | 0.000000 | 0.000000 | 2212 | 2211.525618 | 2235.81 | 2260.094382 | 1.010764 |  |
| GO:0060081\_membrane\_hyperpolarization | 9 | 0 | 0.000000 | 0.000000 | 2212 | 2211.525618 | 2235.81 | 2260.094382 | 1.010764 |  |
| GO:0060119\_inner\_ear\_receptor\_cell\_development | 9 | 0 | 0.000000 | 0.000000 | 2212 | 2211.525618 | 2235.81 | 2260.094382 | 1.010764 |  |
| GO:0060122\_inner\_ear\_receptor\_stereocilium\_organization | 9 | 0 | 0.000000 | 0.000000 | 2212 | 2211.525618 | 2235.81 | 2260.094382 | 1.010764 |  |
| GO:0060325\_face\_morphogenesis | 9 | 0 | 0.000000 | 0.000000 | 2212 | 2211.525618 | 2235.81 | 2260.094382 | 1.010764 |  |
| GO:0060513\_prostatic\_bud\_formation | 9 | 0 | 0.000000 | 0.000000 | 2212 | 2211.525618 | 2235.81 | 2260.094382 | 1.010764 |  |
| GO:0060602\_branch\_elongation\_of\_an\_epithelium | 9 | 0 | 0.000000 | 0.000000 | 2212 | 2211.525618 | 2235.81 | 2260.094382 | 1.010764 |  |
| GO:0060693\_regulation\_of\_branching\_involved\_in\_salivary\_gland\_morphogenesis | 9 | 0 | 0.000000 | 0.000000 | 2212 | 2211.525618 | 2235.81 | 2260.094382 | 1.010764 |  |
| GO:0070306\_lens\_fiber\_cell\_differentiation | 9 | 0 | 0.000000 | 0.000000 | 2212 | 2211.525618 | 2235.81 | 2260.094382 | 1.010764 |  |
| GO:0090048\_negative\_regulation\_of\_transcription\_regulator\_activity | 9 | 0 | 0.000000 | 0.000000 | 2212 | 2211.525618 | 2235.81 | 2260.094382 | 1.010764 |  |
| GO:0009057\_macromolecule\_catabolic\_process | 137 | 0 | 0.000000 | 0.000000 | 2213 | 2212.099946 | 2236.34 | 2260.580054 | 1.010547 |  |
| GO:0002253\_activation\_of\_immune\_response | 54 | 0 | 0.000000 | 0.000000 | 2221 | 2219.818006 | 2243.42 | 2267.021994 | 1.010095 |  |
| GO:0006091\_generation\_of\_precursor\_metabolites\_and\_energy | 54 | 0 | 0.000000 | 0.000000 | 2221 | 2219.818006 | 2243.42 | 2267.021994 | 1.010095 |  |
| GO:0006164\_purine\_nucleotide\_biosynthetic\_process | 54 | 0 | 0.000000 | 0.000000 | 2221 | 2219.818006 | 2243.42 | 2267.021994 | 1.010095 |  |
| GO:0006412\_translation | 54 | 0 | 0.000000 | 0.000000 | 2221 | 2219.818006 | 2243.42 | 2267.021994 | 1.010095 |  |
| GO:0007265\_Ras\_protein\_signal\_transduction | 54 | 0 | 0.000000 | 0.000000 | 2221 | 2219.818006 | 2243.42 | 2267.021994 | 1.010095 |  |
| GO:0009566\_fertilization | 54 | 0 | 0.000000 | 0.000000 | 2221 | 2219.818006 | 2243.42 | 2267.021994 | 1.010095 |  |
| GO:0043405\_regulation\_of\_MAP\_kinase\_activity | 54 | 0 | 0.000000 | 0.000000 | 2221 | 2219.818006 | 2243.42 | 2267.021994 | 1.010095 |  |
| GO:0044271\_nitrogen\_compound\_biosynthetic\_process | 54 | 0 | 0.000000 | 0.000000 | 2221 | 2219.818006 | 2243.42 | 2267.021994 | 1.010095 |  |
| GO:0006873\_cellular\_ion\_homeostasis | 176 | 0 | 0.000000 | 0.000000 | 2222 | 2221.350190 | 2244.74 | 2268.129810 | 1.010234 |  |
| GO:0009123\_nucleoside\_monophosphate\_metabolic\_process | 60 | 0 | 0.000000 | 0.000000 | 2223 | 2222.282761 | 2245.52 | 2268.757239 | 1.010130 |  |
| GO:0003006\_reproductive\_developmental\_process | 141 | 0 | 0.000000 | 0.000000 | 2224 | 2223.394325 | 2246.61 | 2269.825675 | 1.010166 |  |
| GO:0006338\_chromatin\_remodeling | 19 | 0 | 0.000000 | 0.000000 | 2256 | 2255.110103 | 2278.1 | 2301.089897 | 1.009796 |  |
| GO:0006497\_protein\_amino\_acid\_lipidation | 19 | 0 | 0.000000 | 0.000000 | 2256 | 2255.110103 | 2278.1 | 2301.089897 | 1.009796 |  |
| GO:0006672\_ceramide\_metabolic\_process | 19 | 0 | 0.000000 | 0.000000 | 2256 | 2255.110103 | 2278.1 | 2301.089897 | 1.009796 |  |
| GO:0006776\_vitamin\_A\_metabolic\_process | 19 | 0 | 0.000000 | 0.000000 | 2256 | 2255.110103 | 2278.1 | 2301.089897 | 1.009796 |  |
| GO:0007569\_cell\_aging | 19 | 0 | 0.000000 | 0.000000 | 2256 | 2255.110103 | 2278.1 | 2301.089897 | 1.009796 |  |
| GO:0007595\_lactation | 19 | 0 | 0.000000 | 0.000000 | 2256 | 2255.110103 | 2278.1 | 2301.089897 | 1.009796 |  |
| GO:0009584\_detection\_of\_visible\_light | 19 | 0 | 0.000000 | 0.000000 | 2256 | 2255.110103 | 2278.1 | 2301.089897 | 1.009796 |  |
| GO:0009798\_axis\_specification | 19 | 0 | 0.000000 | 0.000000 | 2256 | 2255.110103 | 2278.1 | 2301.089897 | 1.009796 |  |
| GO:0010639\_negative\_regulation\_of\_organelle\_organization | 19 | 0 | 0.000000 | 0.000000 | 2256 | 2255.110103 | 2278.1 | 2301.089897 | 1.009796 |  |
| GO:0019218\_regulation\_of\_steroid\_metabolic\_process | 19 | 0 | 0.000000 | 0.000000 | 2256 | 2255.110103 | 2278.1 | 2301.089897 | 1.009796 |  |
| GO:0021587\_cerebellum\_morphogenesis | 19 | 0 | 0.000000 | 0.000000 | 2256 | 2255.110103 | 2278.1 | 2301.089897 | 1.009796 |  |
| GO:0030199\_collagen\_fibril\_organization | 19 | 0 | 0.000000 | 0.000000 | 2256 | 2255.110103 | 2278.1 | 2301.089897 | 1.009796 |  |
| GO:0030518\_steroid\_hormone\_receptor\_signaling\_pathway | 19 | 0 | 0.000000 | 0.000000 | 2256 | 2255.110103 | 2278.1 | 2301.089897 | 1.009796 |  |
| GO:0030595\_leukocyte\_chemotaxis | 19 | 0 | 0.000000 | 0.000000 | 2256 | 2255.110103 | 2278.1 | 2301.089897 | 1.009796 |  |
| GO:0032526\_response\_to\_retinoic\_acid | 19 | 0 | 0.000000 | 0.000000 | 2256 | 2255.110103 | 2278.1 | 2301.089897 | 1.009796 |  |
| GO:0033002\_muscle\_cell\_proliferation | 19 | 0 | 0.000000 | 0.000000 | 2256 | 2255.110103 | 2278.1 | 2301.089897 | 1.009796 |  |
| GO:0033189\_response\_to\_vitamin\_A | 19 | 0 | 0.000000 | 0.000000 | 2256 | 2255.110103 | 2278.1 | 2301.089897 | 1.009796 |  |
| GO:0042462\_eye\_photoreceptor\_cell\_development | 19 | 0 | 0.000000 | 0.000000 | 2256 | 2255.110103 | 2278.1 | 2301.089897 | 1.009796 |  |
| GO:0042491\_auditory\_receptor\_cell\_differentiation | 19 | 0 | 0.000000 | 0.000000 | 2256 | 2255.110103 | 2278.1 | 2301.089897 | 1.009796 |  |
| GO:0046165\_alcohol\_biosynthetic\_process | 19 | 0 | 0.000000 | 0.000000 | 2256 | 2255.110103 | 2278.1 | 2301.089897 | 1.009796 |  |
| GO:0046890\_regulation\_of\_lipid\_biosynthetic\_process | 19 | 0 | 0.000000 | 0.000000 | 2256 | 2255.110103 | 2278.1 | 2301.089897 | 1.009796 |  |
| GO:0048536\_spleen\_development | 19 | 0 | 0.000000 | 0.000000 | 2256 | 2255.110103 | 2278.1 | 2301.089897 | 1.009796 |  |
| GO:0048547\_gut\_morphogenesis | 19 | 0 | 0.000000 | 0.000000 | 2256 | 2255.110103 | 2278.1 | 2301.089897 | 1.009796 |  |
| GO:0048701\_embryonic\_cranial\_skeleton\_morphogenesis | 19 | 0 | 0.000000 | 0.000000 | 2256 | 2255.110103 | 2278.1 | 2301.089897 | 1.009796 |  |
| GO:0050728\_negative\_regulation\_of\_inflammatory\_response | 19 | 0 | 0.000000 | 0.000000 | 2256 | 2255.110103 | 2278.1 | 2301.089897 | 1.009796 |  |
| GO:0050908\_detection\_of\_light\_stimulus\_involved\_in\_visual\_perception | 19 | 0 | 0.000000 | 0.000000 | 2256 | 2255.110103 | 2278.1 | 2301.089897 | 1.009796 |  |
| GO:0050931\_pigment\_cell\_differentiation | 19 | 0 | 0.000000 | 0.000000 | 2256 | 2255.110103 | 2278.1 | 2301.089897 | 1.009796 |  |
| GO:0050962\_detection\_of\_light\_stimulus\_involved\_in\_sensory\_perception | 19 | 0 | 0.000000 | 0.000000 | 2256 | 2255.110103 | 2278.1 | 2301.089897 | 1.009796 |  |
| GO:0051056\_regulation\_of\_small\_GTPase\_mediated\_signal\_transduction | 19 | 0 | 0.000000 | 0.000000 | 2256 | 2255.110103 | 2278.1 | 2301.089897 | 1.009796 |  |
| GO:0060079\_regulation\_of\_excitatory\_postsynaptic\_membrane\_potential | 19 | 0 | 0.000000 | 0.000000 | 2256 | 2255.110103 | 2278.1 | 2301.089897 | 1.009796 |  |
| GO:0060326\_cell\_chemotaxis | 19 | 0 | 0.000000 | 0.000000 | 2256 | 2255.110103 | 2278.1 | 2301.089897 | 1.009796 |  |
| GO:0060444\_branching\_involved\_in\_mammary\_gland\_duct\_morphogenesis | 19 | 0 | 0.000000 | 0.000000 | 2256 | 2255.110103 | 2278.1 | 2301.089897 | 1.009796 |  |
| GO:0031589\_cell-substrate\_adhesion | 66 | 0 | 0.000000 | 0.000000 | 2258 | 2259.799952 | 2282.49 | 2305.180048 | 1.010846 |  |
| GO:0051402\_neuron\_apoptosis | 66 | 0 | 0.000000 | 0.000000 | 2258 | 2259.799952 | 2282.49 | 2305.180048 | 1.010846 |  |
| GO:0006955\_immune\_response | 205 | 0 | 0.000000 | 0.000000 | 2260 | 2260.838614 | 2283.25 | 2305.661386 | 1.010288 |  |
| GO:0007243\_protein\_kinase\_cascade | 205 | 0 | 0.000000 | 0.000000 | 2260 | 2260.838614 | 2283.25 | 2305.661386 | 1.010288 |  |
| GO:0006732\_coenzyme\_metabolic\_process | 46 | 0 | 0.000000 | 0.000000 | 2269 | 2271.411690 | 2292.9 | 2314.388310 | 1.010533 |  |
| GO:0008217\_regulation\_of\_blood\_pressure | 46 | 0 | 0.000000 | 0.000000 | 2269 | 2271.411690 | 2292.9 | 2314.388310 | 1.010533 |  |
| GO:0009581\_detection\_of\_external\_stimulus | 46 | 0 | 0.000000 | 0.000000 | 2269 | 2271.411690 | 2292.9 | 2314.388310 | 1.010533 |  |
| GO:0019724\_B\_cell\_mediated\_immunity | 46 | 0 | 0.000000 | 0.000000 | 2269 | 2271.411690 | 2292.9 | 2314.388310 | 1.010533 |  |
| GO:0030218\_erythrocyte\_differentiation | 46 | 0 | 0.000000 | 0.000000 | 2269 | 2271.411690 | 2292.9 | 2314.388310 | 1.010533 |  |
| GO:0030850\_prostate\_gland\_development | 46 | 0 | 0.000000 | 0.000000 | 2269 | 2271.411690 | 2292.9 | 2314.388310 | 1.010533 |  |
| GO:0042063\_gliogenesis | 46 | 0 | 0.000000 | 0.000000 | 2269 | 2271.411690 | 2292.9 | 2314.388310 | 1.010533 |  |
| GO:0046631\_alpha-beta\_T\_cell\_activation | 46 | 0 | 0.000000 | 0.000000 | 2269 | 2271.411690 | 2292.9 | 2314.388310 | 1.010533 |  |
| GO:0051098\_regulation\_of\_binding | 46 | 0 | 0.000000 | 0.000000 | 2269 | 2271.411690 | 2292.9 | 2314.388310 | 1.010533 |  |
| GO:0006874\_cellular\_calcium\_ion\_homeostasis | 61 | 0 | 0.000000 | 0.000000 | 2273 | 2275.583496 | 2296.71 | 2317.836504 | 1.010431 |  |
| GO:0009991\_response\_to\_extracellular\_stimulus | 61 | 0 | 0.000000 | 0.000000 | 2273 | 2275.583496 | 2296.71 | 2317.836504 | 1.010431 |  |
| GO:0032270\_positive\_regulation\_of\_cellular\_protein\_metabolic\_process | 61 | 0 | 0.000000 | 0.000000 | 2273 | 2275.583496 | 2296.71 | 2317.836504 | 1.010431 |  |
| GO:0051969\_regulation\_of\_transmission\_of\_nerve\_impulse | 61 | 0 | 0.000000 | 0.000000 | 2273 | 2275.583496 | 2296.71 | 2317.836504 | 1.010431 |  |
| GO:0009719\_response\_to\_endogenous\_stimulus | 92 | 0 | 0.000000 | 0.000000 | 2276 | 2278.375526 | 2299.16 | 2319.944474 | 1.010176 |  |
| GO:0030217\_T\_cell\_differentiation | 92 | 0 | 0.000000 | 0.000000 | 2276 | 2278.375526 | 2299.16 | 2319.944474 | 1.010176 |  |
| GO:0030323\_respiratory\_tube\_development | 92 | 0 | 0.000000 | 0.000000 | 2276 | 2278.375526 | 2299.16 | 2319.944474 | 1.010176 |  |
| GO:0042471\_ear\_morphogenesis | 65 | 0 | 0.000000 | 0.000000 | 2279 | 2280.880310 | 2301.46 | 2322.039690 | 1.009855 |  |
| GO:0043086\_negative\_regulation\_of\_catalytic\_activity | 65 | 0 | 0.000000 | 0.000000 | 2279 | 2280.880310 | 2301.46 | 2322.039690 | 1.009855 |  |
| GO:0048511\_rhythmic\_process | 65 | 0 | 0.000000 | 0.000000 | 2279 | 2280.880310 | 2301.46 | 2322.039690 | 1.009855 |  |
| GO:0042391\_regulation\_of\_membrane\_potential | 95 | 0 | 0.000000 | 0.000000 | 2281 | 2282.264886 | 2302.82 | 2323.375114 | 1.009566 |  |
| GO:0051707\_response\_to\_other\_organism | 95 | 0 | 0.000000 | 0.000000 | 2281 | 2282.264886 | 2302.82 | 2323.375114 | 1.009566 |  |
| GO:0001942\_hair\_follicle\_development | 44 | 0 | 0.000000 | 0.000000 | 2301 | 2301.820219 | 2320.67 | 2339.519781 | 1.008548 |  |
| GO:0002377\_immunoglobulin\_production | 44 | 0 | 0.000000 | 0.000000 | 2301 | 2301.820219 | 2320.67 | 2339.519781 | 1.008548 |  |
| GO:0002768\_immune\_response-regulating\_cell\_surface\_receptor\_signaling\_pathway | 44 | 0 | 0.000000 | 0.000000 | 2301 | 2301.820219 | 2320.67 | 2339.519781 | 1.008548 |  |
| GO:0006606\_protein\_import\_into\_nucleus | 44 | 0 | 0.000000 | 0.000000 | 2301 | 2301.820219 | 2320.67 | 2339.519781 | 1.008548 |  |
| GO:0009914\_hormone\_transport | 44 | 0 | 0.000000 | 0.000000 | 2301 | 2301.820219 | 2320.67 | 2339.519781 | 1.008548 |  |
| GO:0016064\_immunoglobulin\_mediated\_immune\_response | 44 | 0 | 0.000000 | 0.000000 | 2301 | 2301.820219 | 2320.67 | 2339.519781 | 1.008548 |  |
| GO:0022404\_molting\_cycle\_process | 44 | 0 | 0.000000 | 0.000000 | 2301 | 2301.820219 | 2320.67 | 2339.519781 | 1.008548 |  |
| GO:0022405\_hair\_cycle\_process | 44 | 0 | 0.000000 | 0.000000 | 2301 | 2301.820219 | 2320.67 | 2339.519781 | 1.008548 |  |
| GO:0030802\_regulation\_of\_cyclic\_nucleotide\_biosynthetic\_process | 44 | 0 | 0.000000 | 0.000000 | 2301 | 2301.820219 | 2320.67 | 2339.519781 | 1.008548 |  |
| GO:0030808\_regulation\_of\_nucleotide\_biosynthetic\_process | 44 | 0 | 0.000000 | 0.000000 | 2301 | 2301.820219 | 2320.67 | 2339.519781 | 1.008548 |  |
| GO:0035282\_segmentation | 44 | 0 | 0.000000 | 0.000000 | 2301 | 2301.820219 | 2320.67 | 2339.519781 | 1.008548 |  |
| GO:0042303\_molting\_cycle | 44 | 0 | 0.000000 | 0.000000 | 2301 | 2301.820219 | 2320.67 | 2339.519781 | 1.008548 |  |
| GO:0042633\_hair\_cycle | 44 | 0 | 0.000000 | 0.000000 | 2301 | 2301.820219 | 2320.67 | 2339.519781 | 1.008548 |  |
| GO:0044257\_cellular\_protein\_catabolic\_process | 44 | 0 | 0.000000 | 0.000000 | 2301 | 2301.820219 | 2320.67 | 2339.519781 | 1.008548 |  |
| GO:0046545\_development\_of\_primary\_female\_sexual\_characteristics | 44 | 0 | 0.000000 | 0.000000 | 2301 | 2301.820219 | 2320.67 | 2339.519781 | 1.008548 |  |
| GO:0048593\_camera-type\_eye\_morphogenesis | 44 | 0 | 0.000000 | 0.000000 | 2301 | 2301.820219 | 2320.67 | 2339.519781 | 1.008548 |  |
| GO:0051170\_nuclear\_import | 44 | 0 | 0.000000 | 0.000000 | 2301 | 2301.820219 | 2320.67 | 2339.519781 | 1.008548 |  |
| GO:0060485\_mesenchyme\_development | 44 | 0 | 0.000000 | 0.000000 | 2301 | 2301.820219 | 2320.67 | 2339.519781 | 1.008548 |  |
| GO:0070665\_positive\_regulation\_of\_leukocyte\_proliferation | 44 | 0 | 0.000000 | 0.000000 | 2301 | 2301.820219 | 2320.67 | 2339.519781 | 1.008548 |  |
| GO:0070668\_positive\_regulation\_of\_mast\_cell\_proliferation | 44 | 0 | 0.000000 | 0.000000 | 2301 | 2301.820219 | 2320.67 | 2339.519781 | 1.008548 |  |
| GO:0048705\_skeletal\_system\_morphogenesis | 111 | 0 | 0.000000 | 0.000000 | 2302 | 2302.486396 | 2321.32 | 2340.153604 | 1.008393 |  |
| GO:0030902\_hindbrain\_development | 58 | 0 | 0.000000 | 0.000000 | 2304 | 2305.774762 | 2324.29 | 2342.805238 | 1.008806 |  |
| GO:0050804\_regulation\_of\_synaptic\_transmission | 58 | 0 | 0.000000 | 0.000000 | 2304 | 2305.774762 | 2324.29 | 2342.805238 | 1.008806 |  |
| GO:0009628\_response\_to\_abiotic\_stimulus | 162 | 0 | 0.000000 | 0.000000 | 2305 | 2306.329889 | 2324.75 | 2343.170111 | 1.008568 |  |
| GO:0000096\_sulfur\_amino\_acid\_metabolic\_process | 11 | 0 | 0.000000 | 0.000000 | 2389 | 2388.327129 | 2405.13 | 2421.932871 | 1.006752 |  |
| GO:0000271\_polysaccharide\_biosynthetic\_process | 11 | 0 | 0.000000 | 0.000000 | 2389 | 2388.327129 | 2405.13 | 2421.932871 | 1.006752 |  |
| GO:0000737\_DNA\_catabolic\_process\_\_endonucleolytic | 11 | 0 | 0.000000 | 0.000000 | 2389 | 2388.327129 | 2405.13 | 2421.932871 | 1.006752 |  |
| GO:0001101\_response\_to\_acid | 11 | 0 | 0.000000 | 0.000000 | 2389 | 2388.327129 | 2405.13 | 2421.932871 | 1.006752 |  |
| GO:0001837\_epithelial\_to\_mesenchymal\_transition | 11 | 0 | 0.000000 | 0.000000 | 2389 | 2388.327129 | 2405.13 | 2421.932871 | 1.006752 |  |
| GO:0001913\_T\_cell\_mediated\_cytotoxicity | 11 | 0 | 0.000000 | 0.000000 | 2389 | 2388.327129 | 2405.13 | 2421.932871 | 1.006752 |  |
| GO:0001952\_regulation\_of\_cell-matrix\_adhesion | 11 | 0 | 0.000000 | 0.000000 | 2389 | 2388.327129 | 2405.13 | 2421.932871 | 1.006752 |  |
| GO:0001963\_synaptic\_transmission\_\_dopaminergic | 11 | 0 | 0.000000 | 0.000000 | 2389 | 2388.327129 | 2405.13 | 2421.932871 | 1.006752 |  |
| GO:0002444\_myeloid\_leukocyte\_mediated\_immunity | 11 | 0 | 0.000000 | 0.000000 | 2389 | 2388.327129 | 2405.13 | 2421.932871 | 1.006752 |  |
| GO:0002467\_germinal\_center\_formation | 11 | 0 | 0.000000 | 0.000000 | 2389 | 2388.327129 | 2405.13 | 2421.932871 | 1.006752 |  |
| GO:0002758\_innate\_immune\_response-activating\_signal\_transduction | 11 | 0 | 0.000000 | 0.000000 | 2389 | 2388.327129 | 2405.13 | 2421.932871 | 1.006752 |  |
| GO:0006333\_chromatin\_assembly\_or\_disassembly | 11 | 0 | 0.000000 | 0.000000 | 2389 | 2388.327129 | 2405.13 | 2421.932871 | 1.006752 |  |
| GO:0006635\_fatty\_acid\_beta-oxidation | 11 | 0 | 0.000000 | 0.000000 | 2389 | 2388.327129 | 2405.13 | 2421.932871 | 1.006752 |  |
| GO:0006637\_acyl-CoA\_metabolic\_process | 11 | 0 | 0.000000 | 0.000000 | 2389 | 2388.327129 | 2405.13 | 2421.932871 | 1.006752 |  |
| GO:0006690\_icosanoid\_metabolic\_process | 11 | 0 | 0.000000 | 0.000000 | 2389 | 2388.327129 | 2405.13 | 2421.932871 | 1.006752 |  |
| GO:0006779\_porphyrin\_biosynthetic\_process | 11 | 0 | 0.000000 | 0.000000 | 2389 | 2388.327129 | 2405.13 | 2421.932871 | 1.006752 |  |
| GO:0007051\_spindle\_organization | 11 | 0 | 0.000000 | 0.000000 | 2389 | 2388.327129 | 2405.13 | 2421.932871 | 1.006752 |  |
| GO:0007088\_regulation\_of\_mitosis | 11 | 0 | 0.000000 | 0.000000 | 2389 | 2388.327129 | 2405.13 | 2421.932871 | 1.006752 |  |
| GO:0007159\_leukocyte\_adhesion | 11 | 0 | 0.000000 | 0.000000 | 2389 | 2388.327129 | 2405.13 | 2421.932871 | 1.006752 |  |
| GO:0007162\_negative\_regulation\_of\_cell\_adhesion | 11 | 0 | 0.000000 | 0.000000 | 2389 | 2388.327129 | 2405.13 | 2421.932871 | 1.006752 |  |
| GO:0007215\_glutamate\_signaling\_pathway | 11 | 0 | 0.000000 | 0.000000 | 2389 | 2388.327129 | 2405.13 | 2421.932871 | 1.006752 |  |
| GO:0007229\_integrin-mediated\_signaling\_pathway | 11 | 0 | 0.000000 | 0.000000 | 2389 | 2388.327129 | 2405.13 | 2421.932871 | 1.006752 |  |
| GO:0007260\_tyrosine\_phosphorylation\_of\_STAT\_protein | 11 | 0 | 0.000000 | 0.000000 | 2389 | 2388.327129 | 2405.13 | 2421.932871 | 1.006752 |  |
| GO:0008354\_germ\_cell\_migration | 11 | 0 | 0.000000 | 0.000000 | 2389 | 2388.327129 | 2405.13 | 2421.932871 | 1.006752 |  |
| GO:0008652\_cellular\_amino\_acid\_biosynthetic\_process | 11 | 0 | 0.000000 | 0.000000 | 2389 | 2388.327129 | 2405.13 | 2421.932871 | 1.006752 |  |
| GO:0009064\_glutamine\_family\_amino\_acid\_metabolic\_process | 11 | 0 | 0.000000 | 0.000000 | 2389 | 2388.327129 | 2405.13 | 2421.932871 | 1.006752 |  |
| GO:0009141\_nucleoside\_triphosphate\_metabolic\_process | 11 | 0 | 0.000000 | 0.000000 | 2389 | 2388.327129 | 2405.13 | 2421.932871 | 1.006752 |  |
| GO:0009166\_nucleotide\_catabolic\_process | 11 | 0 | 0.000000 | 0.000000 | 2389 | 2388.327129 | 2405.13 | 2421.932871 | 1.006752 |  |
| GO:0009409\_response\_to\_cold | 11 | 0 | 0.000000 | 0.000000 | 2389 | 2388.327129 | 2405.13 | 2421.932871 | 1.006752 |  |
| GO:0010259\_multicellular\_organismal\_aging | 11 | 0 | 0.000000 | 0.000000 | 2389 | 2388.327129 | 2405.13 | 2421.932871 | 1.006752 |  |
| GO:0014013\_regulation\_of\_gliogenesis | 11 | 0 | 0.000000 | 0.000000 | 2389 | 2388.327129 | 2405.13 | 2421.932871 | 1.006752 |  |
| GO:0014902\_myotube\_differentiation | 11 | 0 | 0.000000 | 0.000000 | 2389 | 2388.327129 | 2405.13 | 2421.932871 | 1.006752 |  |
| GO:0016079\_synaptic\_vesicle\_exocytosis | 11 | 0 | 0.000000 | 0.000000 | 2389 | 2388.327129 | 2405.13 | 2421.932871 | 1.006752 |  |
| GO:0021602\_cranial\_nerve\_morphogenesis | 11 | 0 | 0.000000 | 0.000000 | 2389 | 2388.327129 | 2405.13 | 2421.932871 | 1.006752 |  |
| GO:0021846\_cell\_proliferation\_in\_forebrain | 11 | 0 | 0.000000 | 0.000000 | 2389 | 2388.327129 | 2405.13 | 2421.932871 | 1.006752 |  |
| GO:0030238\_male\_sex\_determination | 11 | 0 | 0.000000 | 0.000000 | 2389 | 2388.327129 | 2405.13 | 2421.932871 | 1.006752 |  |
| GO:0030308\_negative\_regulation\_of\_cell\_growth | 11 | 0 | 0.000000 | 0.000000 | 2389 | 2388.327129 | 2405.13 | 2421.932871 | 1.006752 |  |
| GO:0030593\_neutrophil\_chemotaxis | 11 | 0 | 0.000000 | 0.000000 | 2389 | 2388.327129 | 2405.13 | 2421.932871 | 1.006752 |  |
| GO:0030856\_regulation\_of\_epithelial\_cell\_differentiation | 11 | 0 | 0.000000 | 0.000000 | 2389 | 2388.327129 | 2405.13 | 2421.932871 | 1.006752 |  |
| GO:0030878\_thyroid\_gland\_development | 11 | 0 | 0.000000 | 0.000000 | 2389 | 2388.327129 | 2405.13 | 2421.932871 | 1.006752 |  |
| GO:0030968\_endoplasmic\_reticulum\_unfolded\_protein\_response | 11 | 0 | 0.000000 | 0.000000 | 2389 | 2388.327129 | 2405.13 | 2421.932871 | 1.006752 |  |
| GO:0031110\_regulation\_of\_microtubule\_polymerization\_or\_depolymerization | 11 | 0 | 0.000000 | 0.000000 | 2389 | 2388.327129 | 2405.13 | 2421.932871 | 1.006752 |  |
| GO:0031646\_positive\_regulation\_of\_neurological\_system\_process | 11 | 0 | 0.000000 | 0.000000 | 2389 | 2388.327129 | 2405.13 | 2421.932871 | 1.006752 |  |
| GO:0031647\_regulation\_of\_protein\_stability | 11 | 0 | 0.000000 | 0.000000 | 2389 | 2388.327129 | 2405.13 | 2421.932871 | 1.006752 |  |
| GO:0032655\_regulation\_of\_interleukin-12\_production | 11 | 0 | 0.000000 | 0.000000 | 2389 | 2388.327129 | 2405.13 | 2421.932871 | 1.006752 |  |
| GO:0033014\_tetrapyrrole\_biosynthetic\_process | 11 | 0 | 0.000000 | 0.000000 | 2389 | 2388.327129 | 2405.13 | 2421.932871 | 1.006752 |  |
| GO:0033059\_cellular\_pigmentation | 11 | 0 | 0.000000 | 0.000000 | 2389 | 2388.327129 | 2405.13 | 2421.932871 | 1.006752 |  |
| GO:0033559\_unsaturated\_fatty\_acid\_metabolic\_process | 11 | 0 | 0.000000 | 0.000000 | 2389 | 2388.327129 | 2405.13 | 2421.932871 | 1.006752 |  |
| GO:0034620\_cellular\_response\_to\_unfolded\_protein | 11 | 0 | 0.000000 | 0.000000 | 2389 | 2388.327129 | 2405.13 | 2421.932871 | 1.006752 |  |
| GO:0034762\_regulation\_of\_transmembrane\_transport | 11 | 0 | 0.000000 | 0.000000 | 2389 | 2388.327129 | 2405.13 | 2421.932871 | 1.006752 |  |
| GO:0035176\_social\_behavior | 11 | 0 | 0.000000 | 0.000000 | 2389 | 2388.327129 | 2405.13 | 2421.932871 | 1.006752 |  |
| GO:0042036\_negative\_regulation\_of\_cytokine\_biosynthetic\_process | 11 | 0 | 0.000000 | 0.000000 | 2389 | 2388.327129 | 2405.13 | 2421.932871 | 1.006752 |  |
| GO:0042219\_cellular\_amino\_acid\_derivative\_catabolic\_process | 11 | 0 | 0.000000 | 0.000000 | 2389 | 2388.327129 | 2405.13 | 2421.932871 | 1.006752 |  |
| GO:0042401\_biogenic\_amine\_biosynthetic\_process | 11 | 0 | 0.000000 | 0.000000 | 2389 | 2388.327129 | 2405.13 | 2421.932871 | 1.006752 |  |
| GO:0042439\_ethanolamine\_and\_derivative\_metabolic\_process | 11 | 0 | 0.000000 | 0.000000 | 2389 | 2388.327129 | 2405.13 | 2421.932871 | 1.006752 |  |
| GO:0042542\_response\_to\_hydrogen\_peroxide | 11 | 0 | 0.000000 | 0.000000 | 2389 | 2388.327129 | 2405.13 | 2421.932871 | 1.006752 |  |
| GO:0042551\_neuron\_maturation | 11 | 0 | 0.000000 | 0.000000 | 2389 | 2388.327129 | 2405.13 | 2421.932871 | 1.006752 |  |
| GO:0043270\_positive\_regulation\_of\_ion\_transport | 11 | 0 | 0.000000 | 0.000000 | 2389 | 2388.327129 | 2405.13 | 2421.932871 | 1.006752 |  |
| GO:0043576\_regulation\_of\_respiratory\_gaseous\_exchange | 11 | 0 | 0.000000 | 0.000000 | 2389 | 2388.327129 | 2405.13 | 2421.932871 | 1.006752 |  |
| GO:0045026\_plasma\_membrane\_fusion | 11 | 0 | 0.000000 | 0.000000 | 2389 | 2388.327129 | 2405.13 | 2421.932871 | 1.006752 |  |
| GO:0045055\_regulated\_secretory\_pathway | 11 | 0 | 0.000000 | 0.000000 | 2389 | 2388.327129 | 2405.13 | 2421.932871 | 1.006752 |  |
| GO:0045216\_cell-cell\_junction\_organization | 11 | 0 | 0.000000 | 0.000000 | 2389 | 2388.327129 | 2405.13 | 2421.932871 | 1.006752 |  |
| GO:0045685\_regulation\_of\_glial\_cell\_differentiation | 11 | 0 | 0.000000 | 0.000000 | 2389 | 2388.327129 | 2405.13 | 2421.932871 | 1.006752 |  |
| GO:0045834\_positive\_regulation\_of\_lipid\_metabolic\_process | 11 | 0 | 0.000000 | 0.000000 | 2389 | 2388.327129 | 2405.13 | 2421.932871 | 1.006752 |  |
| GO:0046323\_glucose\_import | 11 | 0 | 0.000000 | 0.000000 | 2389 | 2388.327129 | 2405.13 | 2421.932871 | 1.006752 |  |
| GO:0046425\_regulation\_of\_JAK-STAT\_cascade | 11 | 0 | 0.000000 | 0.000000 | 2389 | 2388.327129 | 2405.13 | 2421.932871 | 1.006752 |  |
| GO:0046716\_muscle\_maintenance | 11 | 0 | 0.000000 | 0.000000 | 2389 | 2388.327129 | 2405.13 | 2421.932871 | 1.006752 |  |
| GO:0046928\_regulation\_of\_neurotransmitter\_secretion | 11 | 0 | 0.000000 | 0.000000 | 2389 | 2388.327129 | 2405.13 | 2421.932871 | 1.006752 |  |
| GO:0048009\_insulin-like\_growth\_factor\_receptor\_signaling\_pathway | 11 | 0 | 0.000000 | 0.000000 | 2389 | 2388.327129 | 2405.13 | 2421.932871 | 1.006752 |  |
| GO:0048193\_Golgi\_vesicle\_transport | 11 | 0 | 0.000000 | 0.000000 | 2389 | 2388.327129 | 2405.13 | 2421.932871 | 1.006752 |  |
| GO:0048678\_response\_to\_axon\_injury | 11 | 0 | 0.000000 | 0.000000 | 2389 | 2388.327129 | 2405.13 | 2421.932871 | 1.006752 |  |
| GO:0048745\_smooth\_muscle\_tissue\_development | 11 | 0 | 0.000000 | 0.000000 | 2389 | 2388.327129 | 2405.13 | 2421.932871 | 1.006752 |  |
| GO:0050772\_positive\_regulation\_of\_axonogenesis | 11 | 0 | 0.000000 | 0.000000 | 2389 | 2388.327129 | 2405.13 | 2421.932871 | 1.006752 |  |
| GO:0050806\_positive\_regulation\_of\_synaptic\_transmission | 11 | 0 | 0.000000 | 0.000000 | 2389 | 2388.327129 | 2405.13 | 2421.932871 | 1.006752 |  |
| GO:0050807\_regulation\_of\_synapse\_organization | 11 | 0 | 0.000000 | 0.000000 | 2389 | 2388.327129 | 2405.13 | 2421.932871 | 1.006752 |  |
| GO:0050830\_defense\_response\_to\_Gram-positive\_bacterium | 11 | 0 | 0.000000 | 0.000000 | 2389 | 2388.327129 | 2405.13 | 2421.932871 | 1.006752 |  |
| GO:0050854\_regulation\_of\_antigen\_receptor-mediated\_signaling\_pathway | 11 | 0 | 0.000000 | 0.000000 | 2389 | 2388.327129 | 2405.13 | 2421.932871 | 1.006752 |  |
| GO:0051092\_positive\_regulation\_of\_NF-kappaB\_transcription\_factor\_activity | 11 | 0 | 0.000000 | 0.000000 | 2389 | 2388.327129 | 2405.13 | 2421.932871 | 1.006752 |  |
| GO:0051494\_negative\_regulation\_of\_cytoskeleton\_organization | 11 | 0 | 0.000000 | 0.000000 | 2389 | 2388.327129 | 2405.13 | 2421.932871 | 1.006752 |  |
| GO:0051648\_vesicle\_localization | 11 | 0 | 0.000000 | 0.000000 | 2389 | 2388.327129 | 2405.13 | 2421.932871 | 1.006752 |  |
| GO:0051783\_regulation\_of\_nuclear\_division | 11 | 0 | 0.000000 | 0.000000 | 2389 | 2388.327129 | 2405.13 | 2421.932871 | 1.006752 |  |
| GO:0051971\_positive\_regulation\_of\_transmission\_of\_nerve\_impulse | 11 | 0 | 0.000000 | 0.000000 | 2389 | 2388.327129 | 2405.13 | 2421.932871 | 1.006752 |  |
| GO:0060004\_reflex | 11 | 0 | 0.000000 | 0.000000 | 2389 | 2388.327129 | 2405.13 | 2421.932871 | 1.006752 |  |
| GO:0060767\_epithelial\_cell\_proliferation\_involved\_in\_prostate\_gland\_development | 11 | 0 | 0.000000 | 0.000000 | 2389 | 2388.327129 | 2405.13 | 2421.932871 | 1.006752 |  |
| GO:0030099\_myeloid\_cell\_differentiation | 108 | 0 | 0.000000 | 0.000000 | 2391 | 2389.962748 | 2406.53 | 2423.097252 | 1.006495 |  |
| GO:0051240\_positive\_regulation\_of\_multicellular\_organismal\_process | 108 | 0 | 0.000000 | 0.000000 | 2391 | 2389.962748 | 2406.53 | 2423.097252 | 1.006495 |  |
| GO:0000086\_G2\_M\_transition\_of\_mitotic\_cell\_cycle | 4 | 0 |  |  |  |  |  |  |  |  |
| GO:0000305\_response\_to\_oxygen\_radical | 4 | 0 |  |  |  |  |  |  |  |  |
| GO:0001661\_conditioned\_taste\_aversion | 4 | 0 |  |  |  |  |  |  |  |  |
| GO:0001678\_cellular\_glucose\_homeostasis | 4 | 0 |  |  |  |  |  |  |  |  |
| GO:0001777\_T\_cell\_homeostatic\_proliferation | 4 | 0 |  |  |  |  |  |  |  |  |
| GO:0001794\_type\_IIa\_hypersensitivity | 4 | 0 |  |  |  |  |  |  |  |  |
| GO:0001796\_regulation\_of\_type\_IIa\_hypersensitivity | 4 | 0 |  |  |  |  |  |  |  |  |
| GO:0001798\_positive\_regulation\_of\_type\_IIa\_hypersensitivity | 4 | 0 |  |  |  |  |  |  |  |  |
| GO:0001810\_regulation\_of\_type\_I\_hypersensitivity | 4 | 0 |  |  |  |  |  |  |  |  |
| GO:0001820\_serotonin\_secretion | 4 | 0 |  |  |  |  |  |  |  |  |
| GO:0001835\_blastocyst\_hatching | 4 | 0 |  |  |  |  |  |  |  |  |
| GO:0001842\_neural\_fold\_formation | 4 | 0 |  |  |  |  |  |  |  |  |
| GO:0001881\_receptor\_recycling | 4 | 0 |  |  |  |  |  |  |  |  |
| GO:0001938\_positive\_regulation\_of\_endothelial\_cell\_proliferation | 4 | 0 |  |  |  |  |  |  |  |  |
| GO:0001978\_regulation\_of\_systemic\_arterial\_blood\_pressure\_by\_carotid\_sinus\_baroreceptor\_feedback | 4 | 0 |  |  |  |  |  |  |  |  |
| GO:0002035\_brain\_renin-angiotensin\_system | 4 | 0 |  |  |  |  |  |  |  |  |
| GO:0002051\_osteoblast\_fate\_commitment | 4 | 0 |  |  |  |  |  |  |  |  |
| GO:0002220\_innate\_immune\_response\_activating\_cell\_surface\_receptor\_signaling\_pathway | 4 | 0 |  |  |  |  |  |  |  |  |
| GO:0002249\_lymphocyte\_anergy | 4 | 0 |  |  |  |  |  |  |  |  |
| GO:0002312\_B\_cell\_activation\_during\_immune\_response | 4 | 0 |  |  |  |  |  |  |  |  |
| GO:0002313\_mature\_B\_cell\_differentiation\_during\_immune\_response | 4 | 0 |  |  |  |  |  |  |  |  |
| GO:0002318\_myeloid\_progenitor\_cell\_differentiation | 4 | 0 |  |  |  |  |  |  |  |  |
| GO:0002326\_B\_cell\_lineage\_commitment | 4 | 0 |  |  |  |  |  |  |  |  |
| GO:0002347\_response\_to\_tumor\_cell | 4 | 0 |  |  |  |  |  |  |  |  |
| GO:0002418\_immune\_response\_to\_tumor\_cell | 4 | 0 |  |  |  |  |  |  |  |  |
| GO:0002445\_type\_II\_hypersensitivity | 4 | 0 |  |  |  |  |  |  |  |  |
| GO:0002544\_chronic\_inflammatory\_response | 4 | 0 |  |  |  |  |  |  |  |  |
| GO:0002636\_positive\_regulation\_of\_germinal\_center\_formation | 4 | 0 |  |  |  |  |  |  |  |  |
| GO:0002667\_regulation\_of\_T\_cell\_anergy | 4 | 0 |  |  |  |  |  |  |  |  |
| GO:0002669\_positive\_regulation\_of\_T\_cell\_anergy | 4 | 0 |  |  |  |  |  |  |  |  |
| GO:0002687\_positive\_regulation\_of\_leukocyte\_migration | 4 | 0 |  |  |  |  |  |  |  |  |
| GO:0002702\_positive\_regulation\_of\_production\_of\_molecular\_mediator\_of\_immune\_response | 4 | 0 |  |  |  |  |  |  |  |  |
| GO:0002718\_regulation\_of\_cytokine\_production\_during\_immune\_response | 4 | 0 |  |  |  |  |  |  |  |  |
| GO:0002829\_negative\_regulation\_of\_T-helper\_2\_type\_immune\_response | 4 | 0 |  |  |  |  |  |  |  |  |
| GO:0002833\_positive\_regulation\_of\_response\_to\_biotic\_stimulus | 4 | 0 |  |  |  |  |  |  |  |  |
| GO:0002834\_regulation\_of\_response\_to\_tumor\_cell | 4 | 0 |  |  |  |  |  |  |  |  |
| GO:0002836\_positive\_regulation\_of\_response\_to\_tumor\_cell | 4 | 0 |  |  |  |  |  |  |  |  |
| GO:0002837\_regulation\_of\_immune\_response\_to\_tumor\_cell | 4 | 0 |  |  |  |  |  |  |  |  |
| GO:0002839\_positive\_regulation\_of\_immune\_response\_to\_tumor\_cell | 4 | 0 |  |  |  |  |  |  |  |  |
| GO:0002870\_T\_cell\_anergy | 4 | 0 |  |  |  |  |  |  |  |  |
| GO:0002888\_positive\_regulation\_of\_myeloid\_leukocyte\_mediated\_immunity | 4 | 0 |  |  |  |  |  |  |  |  |
| GO:0002892\_regulation\_of\_type\_II\_hypersensitivity | 4 | 0 |  |  |  |  |  |  |  |  |
| GO:0002894\_positive\_regulation\_of\_type\_II\_hypersensitivity | 4 | 0 |  |  |  |  |  |  |  |  |
| GO:0002911\_regulation\_of\_lymphocyte\_anergy | 4 | 0 |  |  |  |  |  |  |  |  |
| GO:0002913\_positive\_regulation\_of\_lymphocyte\_anergy | 4 | 0 |  |  |  |  |  |  |  |  |
| GO:0002923\_regulation\_of\_humoral\_immune\_response\_mediated\_by\_circulating\_immunoglobulin | 4 | 0 |  |  |  |  |  |  |  |  |
| GO:0003025\_regulation\_of\_systemic\_arterial\_blood\_pressure\_by\_baroreceptor\_feedback | 4 | 0 |  |  |  |  |  |  |  |  |
| GO:0003091\_renal\_water\_homeostasis | 4 | 0 |  |  |  |  |  |  |  |  |
| GO:0005978\_glycogen\_biosynthetic\_process | 4 | 0 |  |  |  |  |  |  |  |  |
| GO:0006012\_galactose\_metabolic\_process | 4 | 0 |  |  |  |  |  |  |  |  |
| GO:0006085\_acetyl-CoA\_biosynthetic\_process | 4 | 0 |  |  |  |  |  |  |  |  |
| GO:0006111\_regulation\_of\_gluconeogenesis | 4 | 0 |  |  |  |  |  |  |  |  |
| GO:0006144\_purine\_base\_metabolic\_process | 4 | 0 |  |  |  |  |  |  |  |  |
| GO:0006290\_pyrimidine\_dimer\_repair | 4 | 0 |  |  |  |  |  |  |  |  |
| GO:0006334\_nucleosome\_assembly | 4 | 0 |  |  |  |  |  |  |  |  |
| GO:0006534\_cysteine\_metabolic\_process | 4 | 0 |  |  |  |  |  |  |  |  |
| GO:0006547\_histidine\_metabolic\_process | 4 | 0 |  |  |  |  |  |  |  |  |
| GO:0006548\_histidine\_catabolic\_process | 4 | 0 |  |  |  |  |  |  |  |  |
| GO:0006555\_methionine\_metabolic\_process | 4 | 0 |  |  |  |  |  |  |  |  |
| GO:0006599\_phosphagen\_metabolic\_process | 4 | 0 |  |  |  |  |  |  |  |  |
| GO:0006623\_protein\_targeting\_to\_vacuole | 4 | 0 |  |  |  |  |  |  |  |  |
| GO:0006626\_protein\_targeting\_to\_mitochondrion | 4 | 0 |  |  |  |  |  |  |  |  |
| GO:0006684\_sphingomyelin\_metabolic\_process | 4 | 0 |  |  |  |  |  |  |  |  |
| GO:0006688\_glycosphingolipid\_biosynthetic\_process | 4 | 0 |  |  |  |  |  |  |  |  |
| GO:0006707\_cholesterol\_catabolic\_process | 4 | 0 |  |  |  |  |  |  |  |  |
| GO:0006739\_NADP\_metabolic\_process | 4 | 0 |  |  |  |  |  |  |  |  |
| GO:0006835\_dicarboxylic\_acid\_transport | 4 | 0 |  |  |  |  |  |  |  |  |
| GO:0006837\_serotonin\_transport | 4 | 0 |  |  |  |  |  |  |  |  |
| GO:0006888\_ER\_to\_Golgi\_vesicle-mediated\_transport | 4 | 0 |  |  |  |  |  |  |  |  |
| GO:0006906\_vesicle\_fusion | 4 | 0 |  |  |  |  |  |  |  |  |
| GO:0006927\_transformed\_cell\_apoptosis | 4 | 0 |  |  |  |  |  |  |  |  |
| GO:0006972\_hyperosmotic\_response | 4 | 0 |  |  |  |  |  |  |  |  |
| GO:0007028\_cytoplasm\_organization | 4 | 0 |  |  |  |  |  |  |  |  |
| GO:0007066\_female\_meiosis\_sister\_chromatid\_cohesion | 4 | 0 |  |  |  |  |  |  |  |  |
| GO:0007144\_female\_meiosis\_I | 4 | 0 |  |  |  |  |  |  |  |  |
| GO:0007184\_SMAD\_protein\_nuclear\_translocation | 4 | 0 |  |  |  |  |  |  |  |  |
| GO:0007216\_metabotropic\_glutamate\_receptor\_signaling\_pathway | 4 | 0 |  |  |  |  |  |  |  |  |
| GO:0007342\_fusion\_of\_sperm\_to\_egg\_plasma\_membrane | 4 | 0 |  |  |  |  |  |  |  |  |
| GO:0007386\_compartment\_specification | 4 | 0 |  |  |  |  |  |  |  |  |
| GO:0008053\_mitochondrial\_fusion | 4 | 0 |  |  |  |  |  |  |  |  |
| GO:0008207\_C21-steroid\_hormone\_metabolic\_process | 4 | 0 |  |  |  |  |  |  |  |  |
| GO:0008215\_spermine\_metabolic\_process | 4 | 0 |  |  |  |  |  |  |  |  |
| GO:0009065\_glutamine\_family\_amino\_acid\_catabolic\_process | 4 | 0 |  |  |  |  |  |  |  |  |
| GO:0009075\_histidine\_family\_amino\_acid\_metabolic\_process | 4 | 0 |  |  |  |  |  |  |  |  |
| GO:0009077\_histidine\_family\_amino\_acid\_catabolic\_process | 4 | 0 |  |  |  |  |  |  |  |  |
| GO:0009134\_nucleoside\_diphosphate\_catabolic\_process | 4 | 0 |  |  |  |  |  |  |  |  |
| GO:0009163\_nucleoside\_biosynthetic\_process | 4 | 0 |  |  |  |  |  |  |  |  |
| GO:0009225\_nucleotide-sugar\_metabolic\_process | 4 | 0 |  |  |  |  |  |  |  |  |
| GO:0009250\_glucan\_biosynthetic\_process | 4 | 0 |  |  |  |  |  |  |  |  |
| GO:0009404\_toxin\_metabolic\_process | 4 | 0 |  |  |  |  |  |  |  |  |
| GO:0009593\_detection\_of\_chemical\_stimulus | 4 | 0 |  |  |  |  |  |  |  |  |
| GO:0009595\_detection\_of\_biotic\_stimulus | 4 | 0 |  |  |  |  |  |  |  |  |
| GO:0009755\_hormone-mediated\_signaling | 4 | 0 |  |  |  |  |  |  |  |  |
| GO:0009912\_auditory\_receptor\_cell\_fate\_commitment | 4 | 0 |  |  |  |  |  |  |  |  |
| GO:0010224\_response\_to\_UV-B | 4 | 0 |  |  |  |  |  |  |  |  |
| GO:0010453\_regulation\_of\_cell\_fate\_commitment | 4 | 0 |  |  |  |  |  |  |  |  |
| GO:0010506\_regulation\_of\_autophagy | 4 | 0 |  |  |  |  |  |  |  |  |
| GO:0010631\_epithelial\_cell\_migration | 4 | 0 |  |  |  |  |  |  |  |  |
| GO:0010812\_negative\_regulation\_of\_cell-substrate\_adhesion | 4 | 0 |  |  |  |  |  |  |  |  |
| GO:0010829\_negative\_regulation\_of\_glucose\_transport | 4 | 0 |  |  |  |  |  |  |  |  |
| GO:0014002\_astrocyte\_development | 4 | 0 |  |  |  |  |  |  |  |  |
| GO:0014832\_urinary\_bladder\_smooth\_muscle\_contraction | 4 | 0 |  |  |  |  |  |  |  |  |
| GO:0014848\_urinary\_tract\_smooth\_muscle\_contraction | 4 | 0 |  |  |  |  |  |  |  |  |
| GO:0015701\_bicarbonate\_transport | 4 | 0 |  |  |  |  |  |  |  |  |
| GO:0015809\_arginine\_transport | 4 | 0 |  |  |  |  |  |  |  |  |
| GO:0015850\_organic\_alcohol\_transport | 4 | 0 |  |  |  |  |  |  |  |  |
| GO:0015858\_nucleoside\_transport | 4 | 0 |  |  |  |  |  |  |  |  |
| GO:0016068\_type\_I\_hypersensitivity | 4 | 0 |  |  |  |  |  |  |  |  |
| GO:0016127\_sterol\_catabolic\_process | 4 | 0 |  |  |  |  |  |  |  |  |
| GO:0016198\_axon\_choice\_point\_recognition | 4 | 0 |  |  |  |  |  |  |  |  |
| GO:0016338\_calcium-independent\_cell-cell\_adhesion | 4 | 0 |  |  |  |  |  |  |  |  |
| GO:0018198\_peptidyl-cysteine\_modification | 4 | 0 |  |  |  |  |  |  |  |  |
| GO:0018409\_peptide\_or\_protein\_amino-terminal\_blocking | 4 | 0 |  |  |  |  |  |  |  |  |
| GO:0019377\_glycolipid\_catabolic\_process | 4 | 0 |  |  |  |  |  |  |  |  |
| GO:0019432\_triglyceride\_biosynthetic\_process | 4 | 0 |  |  |  |  |  |  |  |  |
| GO:0019530\_taurine\_metabolic\_process | 4 | 0 |  |  |  |  |  |  |  |  |
| GO:0021523\_somatic\_motor\_neuron\_differentiation | 4 | 0 |  |  |  |  |  |  |  |  |
| GO:0021535\_cell\_migration\_in\_hindbrain | 4 | 0 |  |  |  |  |  |  |  |  |
| GO:0021542\_dentate\_gyrus\_development | 4 | 0 |  |  |  |  |  |  |  |  |
| GO:0021561\_facial\_nerve\_development | 4 | 0 |  |  |  |  |  |  |  |  |
| GO:0021569\_rhombomere\_3\_development | 4 | 0 |  |  |  |  |  |  |  |  |
| GO:0021571\_rhombomere\_5\_development | 4 | 0 |  |  |  |  |  |  |  |  |
| GO:0021604\_cranial\_nerve\_structural\_organization | 4 | 0 |  |  |  |  |  |  |  |  |
| GO:0021610\_facial\_nerve\_morphogenesis | 4 | 0 |  |  |  |  |  |  |  |  |
| GO:0021612\_facial\_nerve\_structural\_organization | 4 | 0 |  |  |  |  |  |  |  |  |
| GO:0021631\_optic\_nerve\_morphogenesis | 4 | 0 |  |  |  |  |  |  |  |  |
| GO:0021681\_cerebellar\_granular\_layer\_development | 4 | 0 |  |  |  |  |  |  |  |  |
| GO:0021683\_cerebellar\_granular\_layer\_morphogenesis | 4 | 0 |  |  |  |  |  |  |  |  |
| GO:0021684\_cerebellar\_granular\_layer\_formation | 4 | 0 |  |  |  |  |  |  |  |  |
| GO:0021707\_cerebellar\_granule\_cell\_differentiation | 4 | 0 |  |  |  |  |  |  |  |  |
| GO:0021778\_oligodendrocyte\_cell\_fate\_specification | 4 | 0 |  |  |  |  |  |  |  |  |
| GO:0021779\_oligodendrocyte\_cell\_fate\_commitment | 4 | 0 |  |  |  |  |  |  |  |  |
| GO:0021780\_glial\_cell\_fate\_specification | 4 | 0 |  |  |  |  |  |  |  |  |
| GO:0021801\_cerebral\_cortex\_radial\_glia\_guided\_migration | 4 | 0 |  |  |  |  |  |  |  |  |
| GO:0021830\_interneuron\_migration\_from\_the\_subpallium\_to\_the\_cortex | 4 | 0 |  |  |  |  |  |  |  |  |
| GO:0021853\_cerebral\_cortex\_GABAergic\_interneuron\_migration | 4 | 0 |  |  |  |  |  |  |  |  |
| GO:0021877\_forebrain\_neuron\_fate\_commitment | 4 | 0 |  |  |  |  |  |  |  |  |
| GO:0021894\_cerebral\_cortex\_GABAergic\_interneuron\_development | 4 | 0 |  |  |  |  |  |  |  |  |
| GO:0021910\_smoothened\_signaling\_pathway\_involved\_in\_ventral\_spinal\_cord\_patterning | 4 | 0 |  |  |  |  |  |  |  |  |
| GO:0021913\_regulation\_of\_transcription\_from\_RNA\_polymerase\_II\_promoter\_involved\_in\_ventral\_spinal\_cord\_interneuron\_specification | 4 | 0 |  |  |  |  |  |  |  |  |
| GO:0021938\_smoothened\_signaling\_pathway\_involved\_in\_regulation\_of\_granule\_cell\_precursor\_cell\_proliferation | 4 | 0 |  |  |  |  |  |  |  |  |
| GO:0021978\_telencephalon\_regionalization | 4 | 0 |  |  |  |  |  |  |  |  |
| GO:0022011\_myelination\_in\_the\_peripheral\_nervous\_system | 4 | 0 |  |  |  |  |  |  |  |  |
| GO:0030146\_diuresis | 4 | 0 |  |  |  |  |  |  |  |  |
| GO:0030300\_regulation\_of\_intestinal\_cholesterol\_absorption | 4 | 0 |  |  |  |  |  |  |  |  |
| GO:0030800\_negative\_regulation\_of\_cyclic\_nucleotide\_metabolic\_process | 4 | 0 |  |  |  |  |  |  |  |  |
| GO:0030803\_negative\_regulation\_of\_cyclic\_nucleotide\_biosynthetic\_process | 4 | 0 |  |  |  |  |  |  |  |  |
| GO:0030809\_negative\_regulation\_of\_nucleotide\_biosynthetic\_process | 4 | 0 |  |  |  |  |  |  |  |  |
| GO:0030815\_negative\_regulation\_of\_cAMP\_metabolic\_process | 4 | 0 |  |  |  |  |  |  |  |  |
| GO:0030816\_positive\_regulation\_of\_cAMP\_metabolic\_process | 4 | 0 |  |  |  |  |  |  |  |  |
| GO:0030818\_negative\_regulation\_of\_cAMP\_biosynthetic\_process | 4 | 0 |  |  |  |  |  |  |  |  |
| GO:0030819\_positive\_regulation\_of\_cAMP\_biosynthetic\_process | 4 | 0 |  |  |  |  |  |  |  |  |
| GO:0030826\_regulation\_of\_cGMP\_biosynthetic\_process | 4 | 0 |  |  |  |  |  |  |  |  |
| GO:0030858\_positive\_regulation\_of\_epithelial\_cell\_differentiation | 4 | 0 |  |  |  |  |  |  |  |  |
| GO:0030859\_polarized\_epithelial\_cell\_differentiation | 4 | 0 |  |  |  |  |  |  |  |  |
| GO:0030949\_positive\_regulation\_of\_vascular\_endothelial\_growth\_factor\_receptor\_signaling\_pathway | 4 | 0 |  |  |  |  |  |  |  |  |
| GO:0031113\_regulation\_of\_microtubule\_polymerization | 4 | 0 |  |  |  |  |  |  |  |  |
| GO:0031365\_N-terminal\_protein\_amino\_acid\_modification | 4 | 0 |  |  |  |  |  |  |  |  |
| GO:0031424\_keratinization | 4 | 0 |  |  |  |  |  |  |  |  |
| GO:0031557\_induction\_of\_programmed\_cell\_death\_in\_response\_to\_chemical\_stimulus | 4 | 0 |  |  |  |  |  |  |  |  |
| GO:0031558\_induction\_of\_apoptosis\_in\_response\_to\_chemical\_stimulus | 4 | 0 |  |  |  |  |  |  |  |  |
| GO:0031623\_receptor\_internalization | 4 | 0 |  |  |  |  |  |  |  |  |
| GO:0032088\_negative\_regulation\_of\_NF-kappaB\_transcription\_factor\_activity | 4 | 0 |  |  |  |  |  |  |  |  |
| GO:0032098\_regulation\_of\_appetite | 4 | 0 |  |  |  |  |  |  |  |  |
| GO:0032105\_negative\_regulation\_of\_response\_to\_extracellular\_stimulus | 4 | 0 |  |  |  |  |  |  |  |  |
| GO:0032108\_negative\_regulation\_of\_response\_to\_nutrient\_levels | 4 | 0 |  |  |  |  |  |  |  |  |
| GO:0032225\_regulation\_of\_synaptic\_transmission\_\_dopaminergic | 4 | 0 |  |  |  |  |  |  |  |  |
| GO:0032292\_ensheathment\_of\_axons\_in\_the\_peripheral\_nervous\_system | 4 | 0 |  |  |  |  |  |  |  |  |
| GO:0032321\_positive\_regulation\_of\_Rho\_GTPase\_activity | 4 | 0 |  |  |  |  |  |  |  |  |
| GO:0032371\_regulation\_of\_sterol\_transport | 4 | 0 |  |  |  |  |  |  |  |  |
| GO:0032374\_regulation\_of\_cholesterol\_transport | 4 | 0 |  |  |  |  |  |  |  |  |
| GO:0032401\_establishment\_of\_melanosome\_localization | 4 | 0 |  |  |  |  |  |  |  |  |
| GO:0032608\_interferon-beta\_production | 4 | 0 |  |  |  |  |  |  |  |  |
| GO:0032611\_interleukin-1\_beta\_production | 4 | 0 |  |  |  |  |  |  |  |  |
| GO:0032612\_interleukin-1\_production | 4 | 0 |  |  |  |  |  |  |  |  |
| GO:0032648\_regulation\_of\_interferon-beta\_production | 4 | 0 |  |  |  |  |  |  |  |  |
| GO:0032651\_regulation\_of\_interleukin-1\_beta\_production | 4 | 0 |  |  |  |  |  |  |  |  |
| GO:0032652\_regulation\_of\_interleukin-1\_production | 4 | 0 |  |  |  |  |  |  |  |  |
| GO:0032689\_negative\_regulation\_of\_interferon-gamma\_production | 4 | 0 |  |  |  |  |  |  |  |  |
| GO:0032713\_negative\_regulation\_of\_interleukin-4\_production | 4 | 0 |  |  |  |  |  |  |  |  |
| GO:0032715\_negative\_regulation\_of\_interleukin-6\_production | 4 | 0 |  |  |  |  |  |  |  |  |
| GO:0032733\_positive\_regulation\_of\_interleukin-10\_production | 4 | 0 |  |  |  |  |  |  |  |  |
| GO:0032808\_lacrimal\_gland\_development | 4 | 0 |  |  |  |  |  |  |  |  |
| GO:0032835\_glomerulus\_development | 4 | 0 |  |  |  |  |  |  |  |  |
| GO:0032872\_regulation\_of\_stress-activated\_MAPK\_cascade | 4 | 0 |  |  |  |  |  |  |  |  |
| GO:0032922\_circadian\_regulation\_of\_gene\_expression | 4 | 0 |  |  |  |  |  |  |  |  |
| GO:0033026\_negative\_regulation\_of\_mast\_cell\_apoptosis | 4 | 0 |  |  |  |  |  |  |  |  |
| GO:0033079\_immature\_T\_cell\_proliferation | 4 | 0 |  |  |  |  |  |  |  |  |
| GO:0033083\_regulation\_of\_immature\_T\_cell\_proliferation | 4 | 0 |  |  |  |  |  |  |  |  |
| GO:0033089\_positive\_regulation\_of\_T\_cell\_differentiation\_in\_the\_thymus | 4 | 0 |  |  |  |  |  |  |  |  |
| GO:0033135\_regulation\_of\_peptidyl-serine\_phosphorylation | 4 | 0 |  |  |  |  |  |  |  |  |
| GO:0033299\_secretion\_of\_lysosomal\_enzymes | 4 | 0 |  |  |  |  |  |  |  |  |
| GO:0033327\_Leydig\_cell\_differentiation | 4 | 0 |  |  |  |  |  |  |  |  |
| GO:0033363\_secretory\_granule\_organization | 4 | 0 |  |  |  |  |  |  |  |  |
| GO:0033599\_regulation\_of\_mammary\_gland\_epithelial\_cell\_proliferation | 4 | 0 |  |  |  |  |  |  |  |  |
| GO:0033865\_nucleoside\_bisphosphate\_metabolic\_process | 4 | 0 |  |  |  |  |  |  |  |  |
| GO:0034204\_lipid\_translocation | 4 | 0 |  |  |  |  |  |  |  |  |
| GO:0034404\_nucleobase\_\_nucleoside\_and\_nucleotide\_biosynthetic\_process | 4 | 0 |  |  |  |  |  |  |  |  |
| GO:0034587\_piRNA\_metabolic\_process | 4 | 0 |  |  |  |  |  |  |  |  |
| GO:0034614\_cellular\_response\_to\_reactive\_oxygen\_species | 4 | 0 |  |  |  |  |  |  |  |  |
| GO:0034654\_nucleobase\_\_nucleoside\_\_nucleotide\_and\_nucleic\_acid\_biosynthetic\_process | 4 | 0 |  |  |  |  |  |  |  |  |
| GO:0035020\_regulation\_of\_Rac\_protein\_signal\_transduction | 4 | 0 |  |  |  |  |  |  |  |  |
| GO:0035082\_axoneme\_assembly | 4 | 0 |  |  |  |  |  |  |  |  |
| GO:0035188\_hatching | 4 | 0 |  |  |  |  |  |  |  |  |
| GO:0035235\_ionotropic\_glutamate\_receptor\_signaling\_pathway | 4 | 0 |  |  |  |  |  |  |  |  |
| GO:0042345\_regulation\_of\_NF-kappaB\_import\_into\_nucleus | 4 | 0 |  |  |  |  |  |  |  |  |
| GO:0042348\_NF-kappaB\_import\_into\_nucleus | 4 | 0 |  |  |  |  |  |  |  |  |
| GO:0042359\_vitamin\_D\_metabolic\_process | 4 | 0 |  |  |  |  |  |  |  |  |
| GO:0042428\_serotonin\_metabolic\_process | 4 | 0 |  |  |  |  |  |  |  |  |
| GO:0042451\_purine\_nucleoside\_biosynthetic\_process | 4 | 0 |  |  |  |  |  |  |  |  |
| GO:0042455\_ribonucleoside\_biosynthetic\_process | 4 | 0 |  |  |  |  |  |  |  |  |
| GO:0042473\_outer\_ear\_morphogenesis | 4 | 0 |  |  |  |  |  |  |  |  |
| GO:0042522\_regulation\_of\_tyrosine\_phosphorylation\_of\_Stat5\_protein | 4 | 0 |  |  |  |  |  |  |  |  |
| GO:0042535\_positive\_regulation\_of\_tumor\_necrosis\_factor\_biosynthetic\_process | 4 | 0 |  |  |  |  |  |  |  |  |
| GO:0042541\_hemoglobin\_biosynthetic\_process | 4 | 0 |  |  |  |  |  |  |  |  |
| GO:0042558\_pteridine\_and\_derivative\_metabolic\_process | 4 | 0 |  |  |  |  |  |  |  |  |
| GO:0042634\_regulation\_of\_hair\_cycle | 4 | 0 |  |  |  |  |  |  |  |  |
| GO:0042744\_hydrogen\_peroxide\_catabolic\_process | 4 | 0 |  |  |  |  |  |  |  |  |
| GO:0042773\_ATP\_synthesis\_coupled\_electron\_transport | 4 | 0 |  |  |  |  |  |  |  |  |
| GO:0042775\_mitochondrial\_ATP\_synthesis\_coupled\_electron\_transport | 4 | 0 |  |  |  |  |  |  |  |  |
| GO:0042832\_defense\_response\_to\_protozoan | 4 | 0 |  |  |  |  |  |  |  |  |
| GO:0042982\_amyloid\_precursor\_protein\_metabolic\_process | 4 | 0 |  |  |  |  |  |  |  |  |
| GO:0042992\_negative\_regulation\_of\_transcription\_factor\_import\_into\_nucleus | 4 | 0 |  |  |  |  |  |  |  |  |
| GO:0043043\_peptide\_biosynthetic\_process | 4 | 0 |  |  |  |  |  |  |  |  |
| GO:0043129\_surfactant\_homeostasis | 4 | 0 |  |  |  |  |  |  |  |  |
| GO:0043374\_CD8-positive\_\_alpha-beta\_T\_cell\_differentiation | 4 | 0 |  |  |  |  |  |  |  |  |
| GO:0043470\_regulation\_of\_carbohydrate\_catabolic\_process | 4 | 0 |  |  |  |  |  |  |  |  |
| GO:0043471\_regulation\_of\_cellular\_carbohydrate\_catabolic\_process | 4 | 0 |  |  |  |  |  |  |  |  |
| GO:0043500\_muscle\_adaptation | 4 | 0 |  |  |  |  |  |  |  |  |
| GO:0043534\_blood\_vessel\_endothelial\_cell\_migration | 4 | 0 |  |  |  |  |  |  |  |  |
| GO:0043691\_reverse\_cholesterol\_transport | 4 | 0 |  |  |  |  |  |  |  |  |
| GO:0044243\_multicellular\_organismal\_catabolic\_process | 4 | 0 |  |  |  |  |  |  |  |  |
| GO:0044403\_symbiosis\_\_encompassing\_mutualism\_through\_parasitism | 4 | 0 |  |  |  |  |  |  |  |  |
| GO:0044419\_interspecies\_interaction\_between\_organisms | 4 | 0 |  |  |  |  |  |  |  |  |
| GO:0045066\_regulatory\_T\_cell\_differentiation | 4 | 0 |  |  |  |  |  |  |  |  |
| GO:0045078\_positive\_regulation\_of\_interferon-gamma\_biosynthetic\_process | 4 | 0 |  |  |  |  |  |  |  |  |
| GO:0045332\_phospholipid\_translocation | 4 | 0 |  |  |  |  |  |  |  |  |
| GO:0045346\_regulation\_of\_MHC\_class\_II\_biosynthetic\_process | 4 | 0 |  |  |  |  |  |  |  |  |
| GO:0045350\_interferon-beta\_biosynthetic\_process | 4 | 0 |  |  |  |  |  |  |  |  |
| GO:0045357\_regulation\_of\_interferon-beta\_biosynthetic\_process | 4 | 0 |  |  |  |  |  |  |  |  |
| GO:0045359\_positive\_regulation\_of\_interferon-beta\_biosynthetic\_process | 4 | 0 |  |  |  |  |  |  |  |  |
| GO:0045600\_positive\_regulation\_of\_fat\_cell\_differentiation | 4 | 0 |  |  |  |  |  |  |  |  |
| GO:0045616\_regulation\_of\_keratinocyte\_differentiation | 4 | 0 |  |  |  |  |  |  |  |  |
| GO:0045624\_positive\_regulation\_of\_T-helper\_cell\_differentiation | 4 | 0 |  |  |  |  |  |  |  |  |
| GO:0045628\_regulation\_of\_T-helper\_2\_cell\_differentiation | 4 | 0 |  |  |  |  |  |  |  |  |
| GO:0045634\_regulation\_of\_melanocyte\_differentiation | 4 | 0 |  |  |  |  |  |  |  |  |
| GO:0045647\_negative\_regulation\_of\_erythrocyte\_differentiation | 4 | 0 |  |  |  |  |  |  |  |  |
| GO:0045672\_positive\_regulation\_of\_osteoclast\_differentiation | 4 | 0 |  |  |  |  |  |  |  |  |
| GO:0045684\_positive\_regulation\_of\_epidermis\_development | 4 | 0 |  |  |  |  |  |  |  |  |
| GO:0045736\_negative\_regulation\_of\_cyclin-dependent\_protein\_kinase\_activity | 4 | 0 |  |  |  |  |  |  |  |  |
| GO:0045742\_positive\_regulation\_of\_epidermal\_growth\_factor\_receptor\_signaling\_pathway | 4 | 0 |  |  |  |  |  |  |  |  |
| GO:0045747\_positive\_regulation\_of\_Notch\_signaling\_pathway | 4 | 0 |  |  |  |  |  |  |  |  |
| GO:0045767\_regulation\_of\_anti-apoptosis | 4 | 0 |  |  |  |  |  |  |  |  |
| GO:0045779\_negative\_regulation\_of\_bone\_resorption | 4 | 0 |  |  |  |  |  |  |  |  |
| GO:0045923\_positive\_regulation\_of\_fatty\_acid\_metabolic\_process | 4 | 0 |  |  |  |  |  |  |  |  |
| GO:0045930\_negative\_regulation\_of\_mitotic\_cell\_cycle | 4 | 0 |  |  |  |  |  |  |  |  |
| GO:0045940\_positive\_regulation\_of\_steroid\_metabolic\_process | 4 | 0 |  |  |  |  |  |  |  |  |
| GO:0045980\_negative\_regulation\_of\_nucleotide\_metabolic\_process | 4 | 0 |  |  |  |  |  |  |  |  |
| GO:0046129\_purine\_ribonucleoside\_biosynthetic\_process | 4 | 0 |  |  |  |  |  |  |  |  |
| GO:0046173\_polyol\_biosynthetic\_process | 4 | 0 |  |  |  |  |  |  |  |  |
| GO:0046541\_saliva\_secretion | 4 | 0 |  |  |  |  |  |  |  |  |
| GO:0046548\_retinal\_rod\_cell\_development | 4 | 0 |  |  |  |  |  |  |  |  |
| GO:0046579\_positive\_regulation\_of\_Ras\_protein\_signal\_transduction | 4 | 0 |  |  |  |  |  |  |  |  |
| GO:0046639\_negative\_regulation\_of\_alpha-beta\_T\_cell\_differentiation | 4 | 0 |  |  |  |  |  |  |  |  |
| GO:0046642\_negative\_regulation\_of\_alpha-beta\_T\_cell\_proliferation | 4 | 0 |  |  |  |  |  |  |  |  |
| GO:0046668\_regulation\_of\_retinal\_cell\_programmed\_cell\_death | 4 | 0 |  |  |  |  |  |  |  |  |
| GO:0046686\_response\_to\_cadmium\_ion | 4 | 0 |  |  |  |  |  |  |  |  |
| GO:0046835\_carbohydrate\_phosphorylation | 4 | 0 |  |  |  |  |  |  |  |  |
| GO:0046902\_regulation\_of\_mitochondrial\_membrane\_permeability | 4 | 0 |  |  |  |  |  |  |  |  |
| GO:0047496\_vesicle\_transport\_along\_microtubule | 4 | 0 |  |  |  |  |  |  |  |  |
| GO:0048011\_nerve\_growth\_factor\_receptor\_signaling\_pathway | 4 | 0 |  |  |  |  |  |  |  |  |
| GO:0048240\_sperm\_capacitation | 4 | 0 |  |  |  |  |  |  |  |  |
| GO:0048341\_paraxial\_mesoderm\_formation | 4 | 0 |  |  |  |  |  |  |  |  |
| GO:0048484\_enteric\_nervous\_system\_development | 4 | 0 |  |  |  |  |  |  |  |  |
| GO:0048512\_circadian\_behavior | 4 | 0 |  |  |  |  |  |  |  |  |
| GO:0048558\_embryonic\_gut\_morphogenesis | 4 | 0 |  |  |  |  |  |  |  |  |
| GO:0048639\_positive\_regulation\_of\_developmental\_growth | 4 | 0 |  |  |  |  |  |  |  |  |
| GO:0048710\_regulation\_of\_astrocyte\_differentiation | 4 | 0 |  |  |  |  |  |  |  |  |
| GO:0048841\_regulation\_of\_axon\_extension\_involved\_in\_axon\_guidance | 4 | 0 |  |  |  |  |  |  |  |  |
| GO:0048843\_negative\_regulation\_of\_axon\_extension\_involved\_in\_axon\_guidance | 4 | 0 |  |  |  |  |  |  |  |  |
| GO:0048846\_axon\_extension\_involved\_in\_axon\_guidance | 4 | 0 |  |  |  |  |  |  |  |  |
| GO:0048875\_chemical\_homeostasis\_within\_a\_tissue | 4 | 0 |  |  |  |  |  |  |  |  |
| GO:0048935\_peripheral\_nervous\_system\_neuron\_development | 4 | 0 |  |  |  |  |  |  |  |  |
| GO:0050702\_interleukin-1\_beta\_secretion | 4 | 0 |  |  |  |  |  |  |  |  |
| GO:0050704\_regulation\_of\_interleukin-1\_secretion | 4 | 0 |  |  |  |  |  |  |  |  |
| GO:0050706\_regulation\_of\_interleukin-1\_beta\_secretion | 4 | 0 |  |  |  |  |  |  |  |  |
| GO:0050716\_positive\_regulation\_of\_interleukin-1\_secretion | 4 | 0 |  |  |  |  |  |  |  |  |
| GO:0050718\_positive\_regulation\_of\_interleukin-1\_beta\_secretion | 4 | 0 |  |  |  |  |  |  |  |  |
| GO:0050820\_positive\_regulation\_of\_coagulation | 4 | 0 |  |  |  |  |  |  |  |  |
| GO:0050891\_multicellular\_organismal\_water\_homeostasis | 4 | 0 |  |  |  |  |  |  |  |  |
| GO:0050919\_negative\_chemotaxis | 4 | 0 |  |  |  |  |  |  |  |  |
| GO:0050932\_regulation\_of\_pigment\_cell\_differentiation | 4 | 0 |  |  |  |  |  |  |  |  |
| GO:0050961\_detection\_of\_temperature\_stimulus\_involved\_in\_sensory\_perception | 4 | 0 |  |  |  |  |  |  |  |  |
| GO:0050965\_detection\_of\_temperature\_stimulus\_involved\_in\_sensory\_perception\_of\_pain | 4 | 0 |  |  |  |  |  |  |  |  |
| GO:0050994\_regulation\_of\_lipid\_catabolic\_process | 4 | 0 |  |  |  |  |  |  |  |  |
| GO:0051024\_positive\_regulation\_of\_immunoglobulin\_secretion | 4 | 0 |  |  |  |  |  |  |  |  |
| GO:0051055\_negative\_regulation\_of\_lipid\_biosynthetic\_process | 4 | 0 |  |  |  |  |  |  |  |  |
| GO:0051124\_synaptic\_growth\_at\_neuromuscular\_junction | 4 | 0 |  |  |  |  |  |  |  |  |
| GO:0051148\_negative\_regulation\_of\_muscle\_cell\_differentiation | 4 | 0 |  |  |  |  |  |  |  |  |
| GO:0051205\_protein\_insertion\_into\_membrane | 4 | 0 |  |  |  |  |  |  |  |  |
| GO:0051225\_spindle\_assembly | 4 | 0 |  |  |  |  |  |  |  |  |
| GO:0051341\_regulation\_of\_oxidoreductase\_activity | 4 | 0 |  |  |  |  |  |  |  |  |
| GO:0051452\_intracellular\_pH\_reduction | 4 | 0 |  |  |  |  |  |  |  |  |
| GO:0051567\_histone\_H3-K9\_methylation | 4 | 0 |  |  |  |  |  |  |  |  |
| GO:0051642\_centrosome\_localization | 4 | 0 |  |  |  |  |  |  |  |  |
| GO:0051797\_regulation\_of\_hair\_follicle\_development | 4 | 0 |  |  |  |  |  |  |  |  |
| GO:0051897\_positive\_regulation\_of\_protein\_kinase\_B\_signaling\_cascade | 4 | 0 |  |  |  |  |  |  |  |  |
| GO:0051904\_pigment\_granule\_transport | 4 | 0 |  |  |  |  |  |  |  |  |
| GO:0055009\_atrial\_cardiac\_muscle\_morphogenesis | 4 | 0 |  |  |  |  |  |  |  |  |
| GO:0060008\_Sertoli\_cell\_differentiation | 4 | 0 |  |  |  |  |  |  |  |  |
| GO:0060011\_Sertoli\_cell\_proliferation | 4 | 0 |  |  |  |  |  |  |  |  |
| GO:0060057\_apoptosis\_involved\_in\_mammary\_gland\_involution | 4 | 0 |  |  |  |  |  |  |  |  |
| GO:0060058\_positive\_regulation\_of\_apoptosis\_involved\_in\_mammary\_gland\_involution | 4 | 0 |  |  |  |  |  |  |  |  |
| GO:0060065\_uterus\_development | 4 | 0 |  |  |  |  |  |  |  |  |
| GO:0060087\_relaxation\_of\_vascular\_smooth\_muscle | 4 | 0 |  |  |  |  |  |  |  |  |
| GO:0060120\_inner\_ear\_receptor\_cell\_fate\_commitment | 4 | 0 |  |  |  |  |  |  |  |  |
| GO:0060157\_urinary\_bladder\_development | 4 | 0 |  |  |  |  |  |  |  |  |
| GO:0060158\_activation\_of\_phospholipase\_C\_activity\_by\_dopamine\_receptor\_signaling\_pathway | 4 | 0 |  |  |  |  |  |  |  |  |
| GO:0060164\_regulation\_of\_timing\_of\_neuron\_differentiation | 4 | 0 |  |  |  |  |  |  |  |  |
| GO:0060235\_lens\_induction\_in\_camera-type\_eye | 4 | 0 |  |  |  |  |  |  |  |  |
| GO:0060291\_long-term\_synaptic\_potentiation | 4 | 0 |  |  |  |  |  |  |  |  |
| GO:0060412\_ventricular\_septum\_morphogenesis | 4 | 0 |  |  |  |  |  |  |  |  |
| GO:0060459\_left\_lung\_development | 4 | 0 |  |  |  |  |  |  |  |  |
| GO:0060528\_secretory\_columnal\_luminar\_epithelial\_cell\_differentiation\_involved\_in\_prostate\_glandular\_acinus\_development | 4 | 0 |  |  |  |  |  |  |  |  |
| GO:0060561\_apoptosis\_involved\_in\_morphogenesis | 4 | 0 |  |  |  |  |  |  |  |  |
| GO:0060592\_mammary\_gland\_formation | 4 | 0 |  |  |  |  |  |  |  |  |
| GO:0060644\_mammary\_gland\_epithelial\_cell\_differentiation | 4 | 0 |  |  |  |  |  |  |  |  |
| GO:0060666\_dichotomous\_subdivision\_of\_terminal\_units\_involved\_in\_salivary\_gland\_branching | 4 | 0 |  |  |  |  |  |  |  |  |
| GO:0060737\_prostate\_gland\_morphogenetic\_growth | 4 | 0 |  |  |  |  |  |  |  |  |
| GO:0060743\_epithelial\_cell\_maturation\_involved\_in\_prostate\_gland\_development | 4 | 0 |  |  |  |  |  |  |  |  |
| GO:0060751\_mammary\_gland\_duct\_branch\_elongation | 4 | 0 |  |  |  |  |  |  |  |  |
| GO:0060900\_embryonic\_camera-type\_eye\_formation | 4 | 0 |  |  |  |  |  |  |  |  |
| GO:0070059\_apoptosis\_in\_response\_to\_endoplasmic\_reticulum\_stress | 4 | 0 |  |  |  |  |  |  |  |  |
| GO:0070254\_mucus\_secretion | 4 | 0 |  |  |  |  |  |  |  |  |
| GO:0070255\_regulation\_of\_mucus\_secretion | 4 | 0 |  |  |  |  |  |  |  |  |
| GO:0070301\_cellular\_response\_to\_hydrogen\_peroxide | 4 | 0 |  |  |  |  |  |  |  |  |
| GO:0070585\_protein\_localization\_in\_mitochondrion | 4 | 0 |  |  |  |  |  |  |  |  |
| GO:0000089\_mitotic\_metaphase | 3 | 0 |  |  |  |  |  |  |  |  |
| GO:0000098\_sulfur\_amino\_acid\_catabolic\_process | 3 | 0 |  |  |  |  |  |  |  |  |
| GO:0000103\_sulfate\_assimilation | 3 | 0 |  |  |  |  |  |  |  |  |
| GO:0000212\_meiotic\_spindle\_organization | 3 | 0 |  |  |  |  |  |  |  |  |
| GO:0000281\_cytokinesis\_after\_mitosis | 3 | 0 |  |  |  |  |  |  |  |  |
| GO:0000303\_response\_to\_superoxide | 3 | 0 |  |  |  |  |  |  |  |  |
| GO:0000320\_re-entry\_into\_mitotic\_cell\_cycle | 3 | 0 |  |  |  |  |  |  |  |  |
| GO:0000380\_alternative\_nuclear\_mRNA\_splicing\_\_via\_spliceosome | 3 | 0 |  |  |  |  |  |  |  |  |
| GO:0001516\_prostaglandin\_biosynthetic\_process | 3 | 0 |  |  |  |  |  |  |  |  |
| GO:0001553\_luteinization | 3 | 0 |  |  |  |  |  |  |  |  |
| GO:0001574\_ganglioside\_biosynthetic\_process | 3 | 0 |  |  |  |  |  |  |  |  |
| GO:0001705\_ectoderm\_formation | 3 | 0 |  |  |  |  |  |  |  |  |
| GO:0001711\_endodermal\_cell\_fate\_commitment | 3 | 0 |  |  |  |  |  |  |  |  |
| GO:0001757\_somite\_specification | 3 | 0 |  |  |  |  |  |  |  |  |
| GO:0001778\_plasma\_membrane\_repair | 3 | 0 |  |  |  |  |  |  |  |  |
| GO:0001780\_neutrophil\_homeostasis | 3 | 0 |  |  |  |  |  |  |  |  |
| GO:0001802\_type\_III\_hypersensitivity | 3 | 0 |  |  |  |  |  |  |  |  |
| GO:0001803\_regulation\_of\_type\_III\_hypersensitivity | 3 | 0 |  |  |  |  |  |  |  |  |
| GO:0001805\_positive\_regulation\_of\_type\_III\_hypersensitivity | 3 | 0 |  |  |  |  |  |  |  |  |
| GO:0001812\_positive\_regulation\_of\_type\_I\_hypersensitivity | 3 | 0 |  |  |  |  |  |  |  |  |
| GO:0001831\_trophectodermal\_cellular\_morphogenesis | 3 | 0 |  |  |  |  |  |  |  |  |
| GO:0001844\_protein\_insertion\_into\_mitochondrial\_membrane\_during\_induction\_of\_apoptosis | 3 | 0 |  |  |  |  |  |  |  |  |
| GO:0001878\_response\_to\_yeast | 3 | 0 |  |  |  |  |  |  |  |  |
| GO:0001895\_retina\_homeostasis | 3 | 0 |  |  |  |  |  |  |  |  |
| GO:0001915\_negative\_regulation\_of\_T\_cell\_mediated\_cytotoxicity | 3 | 0 |  |  |  |  |  |  |  |  |
| GO:0001937\_negative\_regulation\_of\_endothelial\_cell\_proliferation | 3 | 0 |  |  |  |  |  |  |  |  |
| GO:0001953\_negative\_regulation\_of\_cell-matrix\_adhesion | 3 | 0 |  |  |  |  |  |  |  |  |
| GO:0001955\_blood\_vessel\_maturation | 3 | 0 |  |  |  |  |  |  |  |  |
| GO:0001960\_negative\_regulation\_of\_cytokine-mediated\_signaling\_pathway | 3 | 0 |  |  |  |  |  |  |  |  |
| GO:0001973\_adenosine\_receptor\_signaling\_pathway | 3 | 0 |  |  |  |  |  |  |  |  |
| GO:0001996\_positive\_regulation\_of\_heart\_rate\_by\_epinephrine-norepinephrine | 3 | 0 |  |  |  |  |  |  |  |  |
| GO:0002034\_regulation\_of\_blood\_vessel\_size\_by\_renin-angiotensin | 3 | 0 |  |  |  |  |  |  |  |  |
| GO:0002238\_response\_to\_molecule\_of\_fungal\_origin | 3 | 0 |  |  |  |  |  |  |  |  |
| GO:0002275\_myeloid\_cell\_activation\_during\_immune\_response | 3 | 0 |  |  |  |  |  |  |  |  |
| GO:0002281\_macrophage\_activation\_during\_immune\_response | 3 | 0 |  |  |  |  |  |  |  |  |
| GO:0002309\_T\_cell\_proliferation\_during\_immune\_response | 3 | 0 |  |  |  |  |  |  |  |  |
| GO:0002361\_CD4-positive\_\_CD25-positive\_\_alpha-beta\_regulatory\_T\_cell\_differentiation | 3 | 0 |  |  |  |  |  |  |  |  |
| GO:0002369\_T\_cell\_cytokine\_production | 3 | 0 |  |  |  |  |  |  |  |  |
| GO:0002428\_antigen\_processing\_and\_presentation\_of\_peptide\_antigen\_via\_MHC\_class\_Ib | 3 | 0 |  |  |  |  |  |  |  |  |
| GO:0002446\_neutrophil\_mediated\_immunity | 3 | 0 |  |  |  |  |  |  |  |  |
| GO:0002477\_antigen\_processing\_and\_presentation\_of\_exogenous\_peptide\_antigen\_via\_MHC\_class\_Ib | 3 | 0 |  |  |  |  |  |  |  |  |
| GO:0002481\_antigen\_processing\_and\_presentation\_of\_exogenous\_protein\_antigen\_via\_MHC\_class\_Ib\_\_TAP-dependent | 3 | 0 |  |  |  |  |  |  |  |  |
| GO:0002513\_tolerance\_induction\_to\_self\_antigen | 3 | 0 |  |  |  |  |  |  |  |  |
| GO:0002568\_somatic\_diversification\_of\_T\_cell\_receptor\_genes | 3 | 0 |  |  |  |  |  |  |  |  |
| GO:0002674\_negative\_regulation\_of\_acute\_inflammatory\_response | 3 | 0 |  |  |  |  |  |  |  |  |
| GO:0002681\_somatic\_recombination\_of\_T\_cell\_receptor\_gene\_segments | 3 | 0 |  |  |  |  |  |  |  |  |
| GO:0002713\_negative\_regulation\_of\_B\_cell\_mediated\_immunity | 3 | 0 |  |  |  |  |  |  |  |  |
| GO:0002827\_positive\_regulation\_of\_T-helper\_1\_type\_immune\_response | 3 | 0 |  |  |  |  |  |  |  |  |
| GO:0002865\_negative\_regulation\_of\_acute\_inflammatory\_response\_to\_antigenic\_stimulus | 3 | 0 |  |  |  |  |  |  |  |  |
| GO:0002884\_negative\_regulation\_of\_hypersensitivity | 3 | 0 |  |  |  |  |  |  |  |  |
| GO:0002890\_negative\_regulation\_of\_immunoglobulin\_mediated\_immune\_response | 3 | 0 |  |  |  |  |  |  |  |  |
| GO:0002904\_positive\_regulation\_of\_B\_cell\_apoptosis | 3 | 0 |  |  |  |  |  |  |  |  |
| GO:0003009\_skeletal\_muscle\_contraction | 3 | 0 |  |  |  |  |  |  |  |  |
| GO:0003072\_renal\_control\_of\_peripheral\_vascular\_resistance\_involved\_in\_regulation\_of\_systemic\_arterial\_blood\_pressure | 3 | 0 |  |  |  |  |  |  |  |  |
| GO:0006047\_UDP-N-acetylglucosamine\_metabolic\_process | 3 | 0 |  |  |  |  |  |  |  |  |
| GO:0006067\_ethanol\_metabolic\_process | 3 | 0 |  |  |  |  |  |  |  |  |
| GO:0006072\_glycerol-3-phosphate\_metabolic\_process | 3 | 0 |  |  |  |  |  |  |  |  |
| GO:0006103\_2-oxoglutarate\_metabolic\_process | 3 | 0 |  |  |  |  |  |  |  |  |
| GO:0006107\_oxaloacetate\_metabolic\_process | 3 | 0 |  |  |  |  |  |  |  |  |
| GO:0006166\_purine\_ribonucleoside\_salvage | 3 | 0 |  |  |  |  |  |  |  |  |
| GO:0006220\_pyrimidine\_nucleotide\_metabolic\_process | 3 | 0 |  |  |  |  |  |  |  |  |
| GO:0006266\_DNA\_ligation | 3 | 0 |  |  |  |  |  |  |  |  |
| GO:0006282\_regulation\_of\_DNA\_repair | 3 | 0 |  |  |  |  |  |  |  |  |
| GO:0006287\_base-excision\_repair\_\_gap-filling | 3 | 0 |  |  |  |  |  |  |  |  |
| GO:0006301\_postreplication\_repair | 3 | 0 |  |  |  |  |  |  |  |  |
| GO:0006361\_transcription\_initiation\_from\_RNA\_polymerase\_I\_promoter | 3 | 0 |  |  |  |  |  |  |  |  |
| GO:0006367\_transcription\_initiation\_from\_RNA\_polymerase\_II\_promoter | 3 | 0 |  |  |  |  |  |  |  |  |
| GO:0006414\_translational\_elongation | 3 | 0 |  |  |  |  |  |  |  |  |
| GO:0006491\_N-glycan\_processing | 3 | 0 |  |  |  |  |  |  |  |  |
| GO:0006498\_N-terminal\_protein\_lipidation | 3 | 0 |  |  |  |  |  |  |  |  |
| GO:0006531\_aspartate\_metabolic\_process | 3 | 0 |  |  |  |  |  |  |  |  |
| GO:0006598\_polyamine\_catabolic\_process | 3 | 0 |  |  |  |  |  |  |  |  |
| GO:0006620\_posttranslational\_protein\_targeting\_to\_membrane | 3 | 0 |  |  |  |  |  |  |  |  |
| GO:0006651\_diacylglycerol\_biosynthetic\_process | 3 | 0 |  |  |  |  |  |  |  |  |
| GO:0006670\_sphingosine\_metabolic\_process | 3 | 0 |  |  |  |  |  |  |  |  |
| GO:0006677\_glycosylceramide\_metabolic\_process | 3 | 0 |  |  |  |  |  |  |  |  |
| GO:0006689\_ganglioside\_catabolic\_process | 3 | 0 |  |  |  |  |  |  |  |  |
| GO:0006699\_bile\_acid\_biosynthetic\_process | 3 | 0 |  |  |  |  |  |  |  |  |
| GO:0006791\_sulfur\_utilization | 3 | 0 |  |  |  |  |  |  |  |  |
| GO:0006817\_phosphate\_transport | 3 | 0 |  |  |  |  |  |  |  |  |
| GO:0006825\_copper\_ion\_transport | 3 | 0 |  |  |  |  |  |  |  |  |
| GO:0006828\_manganese\_ion\_transport | 3 | 0 |  |  |  |  |  |  |  |  |
| GO:0006857\_oligopeptide\_transport | 3 | 0 |  |  |  |  |  |  |  |  |
| GO:0006892\_post-Golgi\_vesicle-mediated\_transport | 3 | 0 |  |  |  |  |  |  |  |  |
| GO:0006904\_vesicle\_docking\_during\_exocytosis | 3 | 0 |  |  |  |  |  |  |  |  |
| GO:0006926\_virus-infected\_cell\_apoptosis | 3 | 0 |  |  |  |  |  |  |  |  |
| GO:0006953\_acute-phase\_response | 3 | 0 |  |  |  |  |  |  |  |  |
| GO:0007000\_nucleolus\_organization | 3 | 0 |  |  |  |  |  |  |  |  |
| GO:0007041\_lysosomal\_transport | 3 | 0 |  |  |  |  |  |  |  |  |
| GO:0007043\_cell-cell\_junction\_assembly | 3 | 0 |  |  |  |  |  |  |  |  |
| GO:0007195\_inhibition\_of\_adenylate\_cyclase\_activity\_by\_dopamine\_receptor\_signaling\_pathway | 3 | 0 |  |  |  |  |  |  |  |  |
| GO:0007199\_G-protein\_signaling\_\_coupled\_to\_cGMP\_nucleotide\_second\_messenger | 3 | 0 |  |  |  |  |  |  |  |  |
| GO:0007213\_muscarinic\_acetylcholine\_receptor\_signaling\_pathway | 3 | 0 |  |  |  |  |  |  |  |  |
| GO:0007250\_activation\_of\_NF-kappaB-inducing\_kinase\_activity | 3 | 0 |  |  |  |  |  |  |  |  |
| GO:0007252\_I-kappaB\_phosphorylation | 3 | 0 |  |  |  |  |  |  |  |  |
| GO:0007262\_STAT\_protein\_nuclear\_translocation | 3 | 0 |  |  |  |  |  |  |  |  |
| GO:0007288\_sperm\_axoneme\_assembly | 3 | 0 |  |  |  |  |  |  |  |  |
| GO:0007350\_blastoderm\_segmentation | 3 | 0 |  |  |  |  |  |  |  |  |
| GO:0007403\_glial\_cell\_fate\_determination | 3 | 0 |  |  |  |  |  |  |  |  |
| GO:0007412\_axon\_target\_recognition | 3 | 0 |  |  |  |  |  |  |  |  |
| GO:0007468\_regulation\_of\_rhodopsin\_gene\_expression | 3 | 0 |  |  |  |  |  |  |  |  |
| GO:0007525\_somatic\_muscle\_development | 3 | 0 |  |  |  |  |  |  |  |  |
| GO:0007635\_chemosensory\_behavior | 3 | 0 |  |  |  |  |  |  |  |  |
| GO:0008090\_retrograde\_axon\_cargo\_transport | 3 | 0 |  |  |  |  |  |  |  |  |
| GO:0008347\_glial\_cell\_migration | 3 | 0 |  |  |  |  |  |  |  |  |
| GO:0008635\_activation\_of\_caspase\_activity\_by\_cytochrome\_c | 3 | 0 |  |  |  |  |  |  |  |  |
| GO:0009060\_aerobic\_respiration | 3 | 0 |  |  |  |  |  |  |  |  |
| GO:0009081\_branched\_chain\_family\_amino\_acid\_metabolic\_process | 3 | 0 |  |  |  |  |  |  |  |  |
| GO:0009086\_methionine\_biosynthetic\_process | 3 | 0 |  |  |  |  |  |  |  |  |
| GO:0009135\_purine\_nucleoside\_diphosphate\_metabolic\_process | 3 | 0 |  |  |  |  |  |  |  |  |
| GO:0009137\_purine\_nucleoside\_diphosphate\_catabolic\_process | 3 | 0 |  |  |  |  |  |  |  |  |
| GO:0009155\_purine\_deoxyribonucleotide\_catabolic\_process | 3 | 0 |  |  |  |  |  |  |  |  |
| GO:0009179\_purine\_ribonucleoside\_diphosphate\_metabolic\_process | 3 | 0 |  |  |  |  |  |  |  |  |
| GO:0009181\_purine\_ribonucleoside\_diphosphate\_catabolic\_process | 3 | 0 |  |  |  |  |  |  |  |  |
| GO:0009185\_ribonucleoside\_diphosphate\_metabolic\_process | 3 | 0 |  |  |  |  |  |  |  |  |
| GO:0009191\_ribonucleoside\_diphosphate\_catabolic\_process | 3 | 0 |  |  |  |  |  |  |  |  |
| GO:0009199\_ribonucleoside\_triphosphate\_metabolic\_process | 3 | 0 |  |  |  |  |  |  |  |  |
| GO:0009204\_deoxyribonucleoside\_triphosphate\_catabolic\_process | 3 | 0 |  |  |  |  |  |  |  |  |
| GO:0009205\_purine\_ribonucleoside\_triphosphate\_metabolic\_process | 3 | 0 |  |  |  |  |  |  |  |  |
| GO:0009217\_purine\_deoxyribonucleoside\_triphosphate\_catabolic\_process | 3 | 0 |  |  |  |  |  |  |  |  |
| GO:0009448\_gamma-aminobutyric\_acid\_metabolic\_process | 3 | 0 |  |  |  |  |  |  |  |  |
| GO:0010043\_response\_to\_zinc\_ion | 3 | 0 |  |  |  |  |  |  |  |  |
| GO:0010159\_specification\_of\_organ\_position | 3 | 0 |  |  |  |  |  |  |  |  |
| GO:0010172\_embryonic\_body\_morphogenesis | 3 | 0 |  |  |  |  |  |  |  |  |
| GO:0010216\_maintenance\_of\_DNA\_methylation | 3 | 0 |  |  |  |  |  |  |  |  |
| GO:0010273\_detoxification\_of\_copper\_ion | 3 | 0 |  |  |  |  |  |  |  |  |
| GO:0010454\_negative\_regulation\_of\_cell\_fate\_commitment | 3 | 0 |  |  |  |  |  |  |  |  |
| GO:0010507\_negative\_regulation\_of\_autophagy | 3 | 0 |  |  |  |  |  |  |  |  |
| GO:0010524\_positive\_regulation\_of\_calcium\_ion\_transport\_into\_cytosol | 3 | 0 |  |  |  |  |  |  |  |  |
| GO:0010573\_vascular\_endothelial\_growth\_factor\_production | 3 | 0 |  |  |  |  |  |  |  |  |
| GO:0010574\_regulation\_of\_vascular\_endothelial\_growth\_factor\_production | 3 | 0 |  |  |  |  |  |  |  |  |
| GO:0010575\_positive\_regulation\_vascular\_endothelial\_growth\_factor\_production | 3 | 0 |  |  |  |  |  |  |  |  |
| GO:0010632\_regulation\_of\_epithelial\_cell\_migration | 3 | 0 |  |  |  |  |  |  |  |  |
| GO:0010717\_regulation\_of\_epithelial\_to\_mesenchymal\_transition | 3 | 0 |  |  |  |  |  |  |  |  |
| GO:0010884\_positive\_regulation\_of\_lipid\_storage | 3 | 0 |  |  |  |  |  |  |  |  |
| GO:0010888\_negative\_regulation\_of\_lipid\_storage | 3 | 0 |  |  |  |  |  |  |  |  |
| GO:0010889\_regulation\_of\_sequestering\_of\_triglyceride | 3 | 0 |  |  |  |  |  |  |  |  |
| GO:0010893\_positive\_regulation\_of\_steroid\_biosynthetic\_process | 3 | 0 |  |  |  |  |  |  |  |  |
| GO:0010894\_negative\_regulation\_of\_steroid\_biosynthetic\_process | 3 | 0 |  |  |  |  |  |  |  |  |
| GO:0010998\_regulation\_of\_translational\_initiation\_by\_eIF2\_alpha\_phosphorylation | 3 | 0 |  |  |  |  |  |  |  |  |
| GO:0010999\_regulation\_of\_eIF2\_alpha\_phosphorylation\_by\_heme | 3 | 0 |  |  |  |  |  |  |  |  |
| GO:0014074\_response\_to\_purine | 3 | 0 |  |  |  |  |  |  |  |  |
| GO:0014909\_smooth\_muscle\_cell\_migration | 3 | 0 |  |  |  |  |  |  |  |  |
| GO:0015669\_gas\_transport | 3 | 0 |  |  |  |  |  |  |  |  |
| GO:0015760\_glucose-6-phosphate\_transport | 3 | 0 |  |  |  |  |  |  |  |  |
| GO:0015816\_glycine\_transport | 3 | 0 |  |  |  |  |  |  |  |  |
| GO:0015838\_betaine\_transport | 3 | 0 |  |  |  |  |  |  |  |  |
| GO:0015871\_choline\_transport | 3 | 0 |  |  |  |  |  |  |  |  |
| GO:0015879\_carnitine\_transport | 3 | 0 |  |  |  |  |  |  |  |  |
| GO:0015893\_drug\_transport | 3 | 0 |  |  |  |  |  |  |  |  |
| GO:0015909\_long-chain\_fatty\_acid\_transport | 3 | 0 |  |  |  |  |  |  |  |  |
| GO:0015936\_coenzyme\_A\_metabolic\_process | 3 | 0 |  |  |  |  |  |  |  |  |
| GO:0015988\_energy\_coupled\_proton\_transport\_\_against\_electrochemical\_gradient | 3 | 0 |  |  |  |  |  |  |  |  |
| GO:0015991\_ATP\_hydrolysis\_coupled\_proton\_transport | 3 | 0 |  |  |  |  |  |  |  |  |
| GO:0016241\_regulation\_of\_macroautophagy | 3 | 0 |  |  |  |  |  |  |  |  |
| GO:0016322\_neuron\_remodeling | 3 | 0 |  |  |  |  |  |  |  |  |
| GO:0016556\_mRNA\_modification | 3 | 0 |  |  |  |  |  |  |  |  |
| GO:0016973\_poly(A)+\_mRNA\_export\_from\_nucleus | 3 | 0 |  |  |  |  |  |  |  |  |
| GO:0018208\_peptidyl-proline\_modification | 3 | 0 |  |  |  |  |  |  |  |  |
| GO:0018894\_dibenzo-p-dioxin\_metabolic\_process | 3 | 0 |  |  |  |  |  |  |  |  |
| GO:0019058\_viral\_infectious\_cycle | 3 | 0 |  |  |  |  |  |  |  |  |
| GO:0019230\_proprioception | 3 | 0 |  |  |  |  |  |  |  |  |
| GO:0019236\_response\_to\_pheromone | 3 | 0 |  |  |  |  |  |  |  |  |
| GO:0019359\_nicotinamide\_nucleotide\_biosynthetic\_process | 3 | 0 |  |  |  |  |  |  |  |  |
| GO:0019363\_pyridine\_nucleotide\_biosynthetic\_process | 3 | 0 |  |  |  |  |  |  |  |  |
| GO:0019438\_aromatic\_compound\_biosynthetic\_process | 3 | 0 |  |  |  |  |  |  |  |  |
| GO:0019439\_aromatic\_compound\_catabolic\_process | 3 | 0 |  |  |  |  |  |  |  |  |
| GO:0019605\_butyrate\_metabolic\_process | 3 | 0 |  |  |  |  |  |  |  |  |
| GO:0019614\_catechol\_catabolic\_process | 3 | 0 |  |  |  |  |  |  |  |  |
| GO:0019674\_NAD\_metabolic\_process | 3 | 0 |  |  |  |  |  |  |  |  |
| GO:0019852\_L-ascorbic\_acid\_metabolic\_process | 3 | 0 |  |  |  |  |  |  |  |  |
| GO:0019934\_cGMP-mediated\_signaling | 3 | 0 |  |  |  |  |  |  |  |  |
| GO:0019987\_negative\_regulation\_of\_anti-apoptosis | 3 | 0 |  |  |  |  |  |  |  |  |
| GO:0021527\_spinal\_cord\_association\_neuron\_differentiation | 3 | 0 |  |  |  |  |  |  |  |  |
| GO:0021529\_spinal\_cord\_oligodendrocyte\_cell\_differentiation | 3 | 0 |  |  |  |  |  |  |  |  |
| GO:0021530\_spinal\_cord\_oligodendrocyte\_cell\_fate\_specification | 3 | 0 |  |  |  |  |  |  |  |  |
| GO:0021555\_midbrain-hindbrain\_boundary\_morphogenesis | 3 | 0 |  |  |  |  |  |  |  |  |
| GO:0021563\_glossopharyngeal\_nerve\_development | 3 | 0 |  |  |  |  |  |  |  |  |
| GO:0021570\_rhombomere\_4\_development | 3 | 0 |  |  |  |  |  |  |  |  |
| GO:0021591\_ventricular\_system\_development | 3 | 0 |  |  |  |  |  |  |  |  |
| GO:0021615\_glossopharyngeal\_nerve\_morphogenesis | 3 | 0 |  |  |  |  |  |  |  |  |
| GO:0021794\_thalamus\_development | 3 | 0 |  |  |  |  |  |  |  |  |
| GO:0021797\_forebrain\_anterior\_posterior\_pattern\_formation | 3 | 0 |  |  |  |  |  |  |  |  |
| GO:0021798\_forebrain\_dorsal\_ventral\_pattern\_formation | 3 | 0 |  |  |  |  |  |  |  |  |
| GO:0021800\_cerebral\_cortex\_tangential\_migration | 3 | 0 |  |  |  |  |  |  |  |  |
| GO:0021819\_layer\_formation\_in\_the\_cerebral\_cortex | 3 | 0 |  |  |  |  |  |  |  |  |
| GO:0021859\_pyramidal\_neuron\_differentiation | 3 | 0 |  |  |  |  |  |  |  |  |
| GO:0021860\_pyramidal\_neuron\_development | 3 | 0 |  |  |  |  |  |  |  |  |
| GO:0021889\_olfactory\_bulb\_interneuron\_differentiation | 3 | 0 |  |  |  |  |  |  |  |  |
| GO:0021891\_olfactory\_bulb\_interneuron\_development | 3 | 0 |  |  |  |  |  |  |  |  |
| GO:0021912\_regulation\_of\_transcription\_from\_RNA\_polymerase\_II\_promoter\_involved\_in\_spinal\_cord\_motor\_neuron\_fate\_specification | 3 | 0 |  |  |  |  |  |  |  |  |
| GO:0021979\_hypothalamus\_cell\_differentiation | 3 | 0 |  |  |  |  |  |  |  |  |
| GO:0022010\_myelination\_in\_the\_central\_nervous\_system | 3 | 0 |  |  |  |  |  |  |  |  |
| GO:0022027\_interkinetic\_nuclear\_migration | 3 | 0 |  |  |  |  |  |  |  |  |
| GO:0022406\_membrane\_docking | 3 | 0 |  |  |  |  |  |  |  |  |
| GO:0030033\_microvillus\_assembly | 3 | 0 |  |  |  |  |  |  |  |  |
| GO:0030091\_protein\_repair | 3 | 0 |  |  |  |  |  |  |  |  |
| GO:0030195\_negative\_regulation\_of\_blood\_coagulation | 3 | 0 |  |  |  |  |  |  |  |  |
| GO:0030224\_monocyte\_differentiation | 3 | 0 |  |  |  |  |  |  |  |  |
| GO:0030307\_positive\_regulation\_of\_cell\_growth | 3 | 0 |  |  |  |  |  |  |  |  |
| GO:0030319\_cellular\_di-\_\_tri-valent\_inorganic\_anion\_homeostasis | 3 | 0 |  |  |  |  |  |  |  |  |
| GO:0030320\_cellular\_monovalent\_inorganic\_anion\_homeostasis | 3 | 0 |  |  |  |  |  |  |  |  |
| GO:0030321\_transepithelial\_chloride\_transport | 3 | 0 |  |  |  |  |  |  |  |  |
| GO:0030501\_positive\_regulation\_of\_bone\_mineralization | 3 | 0 |  |  |  |  |  |  |  |  |
| GO:0030513\_positive\_regulation\_of\_BMP\_signaling\_pathway | 3 | 0 |  |  |  |  |  |  |  |  |
| GO:0030538\_embryonic\_genitalia\_morphogenesis | 3 | 0 |  |  |  |  |  |  |  |  |
| GO:0030540\_female\_genitalia\_development | 3 | 0 |  |  |  |  |  |  |  |  |
| GO:0030574\_collagen\_catabolic\_process | 3 | 0 |  |  |  |  |  |  |  |  |
| GO:0030643\_cellular\_phosphate\_ion\_homeostasis | 3 | 0 |  |  |  |  |  |  |  |  |
| GO:0030718\_germ-line\_stem\_cell\_maintenance | 3 | 0 |  |  |  |  |  |  |  |  |
| GO:0030730\_sequestering\_of\_triglyceride | 3 | 0 |  |  |  |  |  |  |  |  |
| GO:0030836\_positive\_regulation\_of\_actin\_filament\_depolymerization | 3 | 0 |  |  |  |  |  |  |  |  |
| GO:0030857\_negative\_regulation\_of\_epithelial\_cell\_differentiation | 3 | 0 |  |  |  |  |  |  |  |  |
| GO:0030916\_otic\_vesicle\_formation | 3 | 0 |  |  |  |  |  |  |  |  |
| GO:0031000\_response\_to\_caffeine | 3 | 0 |  |  |  |  |  |  |  |  |
| GO:0031063\_regulation\_of\_histone\_deacetylation | 3 | 0 |  |  |  |  |  |  |  |  |
| GO:0031065\_positive\_regulation\_of\_histone\_deacetylation | 3 | 0 |  |  |  |  |  |  |  |  |
| GO:0031112\_positive\_regulation\_of\_microtubule\_polymerization\_or\_depolymerization | 3 | 0 |  |  |  |  |  |  |  |  |
| GO:0031116\_positive\_regulation\_of\_microtubule\_polymerization | 3 | 0 |  |  |  |  |  |  |  |  |
| GO:0031133\_regulation\_of\_axon\_diameter | 3 | 0 |  |  |  |  |  |  |  |  |
| GO:0031282\_regulation\_of\_guanylate\_cyclase\_activity | 3 | 0 |  |  |  |  |  |  |  |  |
| GO:0031333\_negative\_regulation\_of\_protein\_complex\_assembly | 3 | 0 |  |  |  |  |  |  |  |  |
| GO:0031397\_negative\_regulation\_of\_protein\_ubiquitination | 3 | 0 |  |  |  |  |  |  |  |  |
| GO:0031398\_positive\_regulation\_of\_protein\_ubiquitination | 3 | 0 |  |  |  |  |  |  |  |  |
| GO:0031503\_protein\_complex\_localization | 3 | 0 |  |  |  |  |  |  |  |  |
| GO:0031571\_G1\_DNA\_damage\_checkpoint | 3 | 0 |  |  |  |  |  |  |  |  |
| GO:0031579\_membrane\_raft\_organization | 3 | 0 |  |  |  |  |  |  |  |  |
| GO:0031638\_zymogen\_activation | 3 | 0 |  |  |  |  |  |  |  |  |
| GO:0031641\_regulation\_of\_myelination | 3 | 0 |  |  |  |  |  |  |  |  |
| GO:0031642\_negative\_regulation\_of\_myelination | 3 | 0 |  |  |  |  |  |  |  |  |
| GO:0031649\_heat\_generation | 3 | 0 |  |  |  |  |  |  |  |  |
| GO:0031943\_regulation\_of\_glucocorticoid\_metabolic\_process | 3 | 0 |  |  |  |  |  |  |  |  |
| GO:0032020\_ISG15-protein\_conjugation | 3 | 0 |  |  |  |  |  |  |  |  |
| GO:0032060\_bleb\_formation | 3 | 0 |  |  |  |  |  |  |  |  |
| GO:0032095\_regulation\_of\_response\_to\_food | 3 | 0 |  |  |  |  |  |  |  |  |
| GO:0032272\_negative\_regulation\_of\_protein\_polymerization | 3 | 0 |  |  |  |  |  |  |  |  |
| GO:0032288\_myelin\_assembly | 3 | 0 |  |  |  |  |  |  |  |  |
| GO:0032291\_ensheathment\_of\_axons\_in\_the\_central\_nervous\_system | 3 | 0 |  |  |  |  |  |  |  |  |
| GO:0032355\_response\_to\_estradiol\_stimulus | 3 | 0 |  |  |  |  |  |  |  |  |
| GO:0032402\_melanosome\_transport | 3 | 0 |  |  |  |  |  |  |  |  |
| GO:0032411\_positive\_regulation\_of\_transporter\_activity | 3 | 0 |  |  |  |  |  |  |  |  |
| GO:0032414\_positive\_regulation\_of\_ion\_transmembrane\_transporter\_activity | 3 | 0 |  |  |  |  |  |  |  |  |
| GO:0032436\_positive\_regulation\_of\_proteasomal\_ubiquitin-dependent\_protein\_catabolic\_process | 3 | 0 |  |  |  |  |  |  |  |  |
| GO:0032528\_microvillus\_organization | 3 | 0 |  |  |  |  |  |  |  |  |
| GO:0032536\_regulation\_of\_cell\_projection\_size | 3 | 0 |  |  |  |  |  |  |  |  |
| GO:0032632\_interleukin-3\_production | 3 | 0 |  |  |  |  |  |  |  |  |
| GO:0032634\_interleukin-5\_production | 3 | 0 |  |  |  |  |  |  |  |  |
| GO:0032674\_regulation\_of\_interleukin-5\_production | 3 | 0 |  |  |  |  |  |  |  |  |
| GO:0032703\_negative\_regulation\_of\_interleukin-2\_production | 3 | 0 |  |  |  |  |  |  |  |  |
| GO:0032753\_positive\_regulation\_of\_interleukin-4\_production | 3 | 0 |  |  |  |  |  |  |  |  |
| GO:0032823\_regulation\_of\_natural\_killer\_cell\_differentiation | 3 | 0 |  |  |  |  |  |  |  |  |
| GO:0032825\_positive\_regulation\_of\_natural\_killer\_cell\_differentiation | 3 | 0 |  |  |  |  |  |  |  |  |
| GO:0032856\_activation\_of\_Ras\_GTPase\_activity | 3 | 0 |  |  |  |  |  |  |  |  |
| GO:0032862\_activation\_of\_Rho\_GTPase\_activity | 3 | 0 |  |  |  |  |  |  |  |  |
| GO:0032874\_positive\_regulation\_of\_stress-activated\_MAPK\_cascade | 3 | 0 |  |  |  |  |  |  |  |  |
| GO:0032881\_regulation\_of\_polysaccharide\_metabolic\_process | 3 | 0 |  |  |  |  |  |  |  |  |
| GO:0032890\_regulation\_of\_organic\_acid\_transport | 3 | 0 |  |  |  |  |  |  |  |  |
| GO:0033058\_directional\_locomotion | 3 | 0 |  |  |  |  |  |  |  |  |
| GO:0033080\_immature\_T\_cell\_proliferation\_in\_the\_thymus | 3 | 0 |  |  |  |  |  |  |  |  |
| GO:0033084\_regulation\_of\_immature\_T\_cell\_proliferation\_in\_the\_thymus | 3 | 0 |  |  |  |  |  |  |  |  |
| GO:0033091\_positive\_regulation\_of\_immature\_T\_cell\_proliferation | 3 | 0 |  |  |  |  |  |  |  |  |
| GO:0033137\_negative\_regulation\_of\_peptidyl-serine\_phosphorylation | 3 | 0 |  |  |  |  |  |  |  |  |
| GO:0033153\_T\_cell\_receptor\_V(D)J\_recombination | 3 | 0 |  |  |  |  |  |  |  |  |
| GO:0033209\_tumor\_necrosis\_factor-mediated\_signaling\_pathway | 3 | 0 |  |  |  |  |  |  |  |  |
| GO:0033600\_negative\_regulation\_of\_mammary\_gland\_epithelial\_cell\_proliferation | 3 | 0 |  |  |  |  |  |  |  |  |
| GO:0033631\_cell-cell\_adhesion\_mediated\_by\_integrin | 3 | 0 |  |  |  |  |  |  |  |  |
| GO:0033993\_response\_to\_lipid | 3 | 0 |  |  |  |  |  |  |  |  |
| GO:0034220\_ion\_transmembrane\_transport | 3 | 0 |  |  |  |  |  |  |  |  |
| GO:0034308\_monohydric\_alcohol\_metabolic\_process | 3 | 0 |  |  |  |  |  |  |  |  |
| GO:0034313\_diol\_catabolic\_process | 3 | 0 |  |  |  |  |  |  |  |  |
| GO:0034331\_cell\_junction\_maintenance | 3 | 0 |  |  |  |  |  |  |  |  |
| GO:0034332\_adherens\_junction\_organization | 3 | 0 |  |  |  |  |  |  |  |  |
| GO:0034375\_high-density\_lipoprotein\_particle\_remodeling | 3 | 0 |  |  |  |  |  |  |  |  |
| GO:0034381\_lipoprotein\_particle\_clearance | 3 | 0 |  |  |  |  |  |  |  |  |
| GO:0034612\_response\_to\_tumor\_necrosis\_factor | 3 | 0 |  |  |  |  |  |  |  |  |
| GO:0034655\_nucleobase\_\_nucleoside\_\_nucleotide\_and\_nucleic\_acid\_catabolic\_process | 3 | 0 |  |  |  |  |  |  |  |  |
| GO:0034656\_nucleobase\_\_nucleoside\_and\_nucleotide\_catabolic\_process | 3 | 0 |  |  |  |  |  |  |  |  |
| GO:0035067\_negative\_regulation\_of\_histone\_acetylation | 3 | 0 |  |  |  |  |  |  |  |  |
| GO:0035084\_flagellar\_axoneme\_assembly | 3 | 0 |  |  |  |  |  |  |  |  |
| GO:0035166\_post-embryonic\_hemopoiesis | 3 | 0 |  |  |  |  |  |  |  |  |
| GO:0035283\_central\_nervous\_system\_segmentation | 3 | 0 |  |  |  |  |  |  |  |  |
| GO:0035284\_brain\_segmentation | 3 | 0 |  |  |  |  |  |  |  |  |
| GO:0042097\_interleukin-4\_biosynthetic\_process | 3 | 0 |  |  |  |  |  |  |  |  |
| GO:0042135\_neurotransmitter\_catabolic\_process | 3 | 0 |  |  |  |  |  |  |  |  |
| GO:0042271\_susceptibility\_to\_natural\_killer\_cell\_mediated\_cytotoxicity | 3 | 0 |  |  |  |  |  |  |  |  |
| GO:0042375\_quinone\_cofactor\_metabolic\_process | 3 | 0 |  |  |  |  |  |  |  |  |
| GO:0042420\_dopamine\_catabolic\_process | 3 | 0 |  |  |  |  |  |  |  |  |
| GO:0042421\_norepinephrine\_biosynthetic\_process | 3 | 0 |  |  |  |  |  |  |  |  |
| GO:0042424\_catecholamine\_catabolic\_process | 3 | 0 |  |  |  |  |  |  |  |  |
| GO:0042447\_hormone\_catabolic\_process | 3 | 0 |  |  |  |  |  |  |  |  |
| GO:0042448\_progesterone\_metabolic\_process | 3 | 0 |  |  |  |  |  |  |  |  |
| GO:0042523\_positive\_regulation\_of\_tyrosine\_phosphorylation\_of\_Stat5\_protein | 3 | 0 |  |  |  |  |  |  |  |  |
| GO:0042659\_regulation\_of\_cell\_fate\_specification | 3 | 0 |  |  |  |  |  |  |  |  |
| GO:0042668\_auditory\_receptor\_cell\_fate\_determination | 3 | 0 |  |  |  |  |  |  |  |  |
| GO:0042670\_retinal\_cone\_cell\_differentiation | 3 | 0 |  |  |  |  |  |  |  |  |
| GO:0042693\_muscle\_cell\_fate\_commitment | 3 | 0 |  |  |  |  |  |  |  |  |
| GO:0042711\_maternal\_behavior | 3 | 0 |  |  |  |  |  |  |  |  |
| GO:0042745\_circadian\_sleep\_wake\_cycle | 3 | 0 |  |  |  |  |  |  |  |  |
| GO:0042759\_long-chain\_fatty\_acid\_biosynthetic\_process | 3 | 0 |  |  |  |  |  |  |  |  |
| GO:0042787\_protein\_ubiquitination\_during\_ubiquitin-dependent\_protein\_catabolic\_process | 3 | 0 |  |  |  |  |  |  |  |  |
| GO:0043045\_DNA\_methylation\_during\_embryonic\_development | 3 | 0 |  |  |  |  |  |  |  |  |
| GO:0043090\_amino\_acid\_import | 3 | 0 |  |  |  |  |  |  |  |  |
| GO:0043092\_L-amino\_acid\_import | 3 | 0 |  |  |  |  |  |  |  |  |
| GO:0043094\_cellular\_metabolic\_compound\_salvage | 3 | 0 |  |  |  |  |  |  |  |  |
| GO:0043101\_purine\_salvage | 3 | 0 |  |  |  |  |  |  |  |  |
| GO:0043149\_stress\_fiber\_formation | 3 | 0 |  |  |  |  |  |  |  |  |
| GO:0043174\_nucleoside\_salvage | 3 | 0 |  |  |  |  |  |  |  |  |
| GO:0043200\_response\_to\_amino\_acid\_stimulus | 3 | 0 |  |  |  |  |  |  |  |  |
| GO:0043243\_positive\_regulation\_of\_protein\_complex\_disassembly | 3 | 0 |  |  |  |  |  |  |  |  |
| GO:0043249\_erythrocyte\_maturation | 3 | 0 |  |  |  |  |  |  |  |  |
| GO:0043267\_negative\_regulation\_of\_potassium\_ion\_transport | 3 | 0 |  |  |  |  |  |  |  |  |
| GO:0043371\_negative\_regulation\_of\_CD4-positive\_\_alpha\_beta\_T\_cell\_differentiation | 3 | 0 |  |  |  |  |  |  |  |  |
| GO:0043462\_regulation\_of\_ATPase\_activity | 3 | 0 |  |  |  |  |  |  |  |  |
| GO:0043569\_negative\_regulation\_of\_insulin-like\_growth\_factor\_receptor\_signaling\_pathway | 3 | 0 |  |  |  |  |  |  |  |  |
| GO:0043586\_tongue\_development | 3 | 0 |  |  |  |  |  |  |  |  |
| GO:0043900\_regulation\_of\_multi-organism\_process | 3 | 0 |  |  |  |  |  |  |  |  |
| GO:0043954\_cellular\_component\_maintenance | 3 | 0 |  |  |  |  |  |  |  |  |
| GO:0044030\_regulation\_of\_DNA\_methylation | 3 | 0 |  |  |  |  |  |  |  |  |
| GO:0044089\_positive\_regulation\_of\_cellular\_component\_biogenesis | 3 | 0 |  |  |  |  |  |  |  |  |
| GO:0044273\_sulfur\_compound\_catabolic\_process | 3 | 0 |  |  |  |  |  |  |  |  |
| GO:0045047\_protein\_targeting\_to\_ER | 3 | 0 |  |  |  |  |  |  |  |  |
| GO:0045085\_negative\_regulation\_of\_interleukin-2\_biosynthetic\_process | 3 | 0 |  |  |  |  |  |  |  |  |
| GO:0045110\_intermediate\_filament\_bundle\_assembly | 3 | 0 |  |  |  |  |  |  |  |  |
| GO:0045143\_homologous\_chromosome\_segregation | 3 | 0 |  |  |  |  |  |  |  |  |
| GO:0045198\_establishment\_of\_epithelial\_cell\_apical\_basal\_polarity | 3 | 0 |  |  |  |  |  |  |  |  |
| GO:0045217\_cell-cell\_junction\_maintenance | 3 | 0 |  |  |  |  |  |  |  |  |
| GO:0045348\_positive\_regulation\_of\_MHC\_class\_II\_biosynthetic\_process | 3 | 0 |  |  |  |  |  |  |  |  |
| GO:0045402\_regulation\_of\_interleukin-4\_biosynthetic\_process | 3 | 0 |  |  |  |  |  |  |  |  |
| GO:0045404\_positive\_regulation\_of\_interleukin-4\_biosynthetic\_process | 3 | 0 |  |  |  |  |  |  |  |  |
| GO:0045542\_positive\_regulation\_of\_cholesterol\_biosynthetic\_process | 3 | 0 |  |  |  |  |  |  |  |  |
| GO:0045607\_regulation\_of\_auditory\_receptor\_cell\_differentiation | 3 | 0 |  |  |  |  |  |  |  |  |
| GO:0045623\_negative\_regulation\_of\_T-helper\_cell\_differentiation | 3 | 0 |  |  |  |  |  |  |  |  |
| GO:0045625\_regulation\_of\_T-helper\_1\_cell\_differentiation | 3 | 0 |  |  |  |  |  |  |  |  |
| GO:0045631\_regulation\_of\_mechanoreceptor\_differentiation | 3 | 0 |  |  |  |  |  |  |  |  |
| GO:0045717\_negative\_regulation\_of\_fatty\_acid\_biosynthetic\_process | 3 | 0 |  |  |  |  |  |  |  |  |
| GO:0045723\_positive\_regulation\_of\_fatty\_acid\_biosynthetic\_process | 3 | 0 |  |  |  |  |  |  |  |  |
| GO:0045746\_negative\_regulation\_of\_Notch\_signaling\_pathway | 3 | 0 |  |  |  |  |  |  |  |  |
| GO:0045806\_negative\_regulation\_of\_endocytosis | 3 | 0 |  |  |  |  |  |  |  |  |
| GO:0045829\_negative\_regulation\_of\_isotype\_switching | 3 | 0 |  |  |  |  |  |  |  |  |
| GO:0045844\_positive\_regulation\_of\_striated\_muscle\_development | 3 | 0 |  |  |  |  |  |  |  |  |
| GO:0045907\_positive\_regulation\_of\_vasoconstriction | 3 | 0 |  |  |  |  |  |  |  |  |
| GO:0045922\_negative\_regulation\_of\_fatty\_acid\_metabolic\_process | 3 | 0 |  |  |  |  |  |  |  |  |
| GO:0045939\_negative\_regulation\_of\_steroid\_metabolic\_process | 3 | 0 |  |  |  |  |  |  |  |  |
| GO:0046013\_regulation\_of\_T\_cell\_homeostatic\_proliferation | 3 | 0 |  |  |  |  |  |  |  |  |
| GO:0046034\_ATP\_metabolic\_process | 3 | 0 |  |  |  |  |  |  |  |  |
| GO:0046325\_negative\_regulation\_of\_glucose\_import | 3 | 0 |  |  |  |  |  |  |  |  |
| GO:0046426\_negative\_regulation\_of\_JAK-STAT\_cascade | 3 | 0 |  |  |  |  |  |  |  |  |
| GO:0046457\_prostanoid\_biosynthetic\_process | 3 | 0 |  |  |  |  |  |  |  |  |
| GO:0046479\_glycosphingolipid\_catabolic\_process | 3 | 0 |  |  |  |  |  |  |  |  |
| GO:0046488\_phosphatidylinositol\_metabolic\_process | 3 | 0 |  |  |  |  |  |  |  |  |
| GO:0046549\_retinal\_cone\_cell\_development | 3 | 0 |  |  |  |  |  |  |  |  |
| GO:0046688\_response\_to\_copper\_ion | 3 | 0 |  |  |  |  |  |  |  |  |
| GO:0046717\_acid\_secretion | 3 | 0 |  |  |  |  |  |  |  |  |
| GO:0046825\_regulation\_of\_protein\_export\_from\_nucleus | 3 | 0 |  |  |  |  |  |  |  |  |
| GO:0046885\_regulation\_of\_hormone\_biosynthetic\_process | 3 | 0 |  |  |  |  |  |  |  |  |
| GO:0048003\_antigen\_processing\_and\_presentation\_of\_lipid\_antigen\_via\_MHC\_class\_Ib | 3 | 0 |  |  |  |  |  |  |  |  |
| GO:0048007\_antigen\_processing\_and\_presentation\_\_exogenous\_lipid\_antigen\_via\_MHC\_class\_Ib | 3 | 0 |  |  |  |  |  |  |  |  |
| GO:0048012\_hepatocyte\_growth\_factor\_receptor\_signaling\_pathway | 3 | 0 |  |  |  |  |  |  |  |  |
| GO:0048050\_post-embryonic\_eye\_morphogenesis | 3 | 0 |  |  |  |  |  |  |  |  |
| GO:0048087\_positive\_regulation\_of\_pigmentation\_during\_development | 3 | 0 |  |  |  |  |  |  |  |  |
| GO:0048246\_macrophage\_chemotaxis | 3 | 0 |  |  |  |  |  |  |  |  |
| GO:0048251\_elastic\_fiber\_assembly | 3 | 0 |  |  |  |  |  |  |  |  |
| GO:0048278\_vesicle\_docking | 3 | 0 |  |  |  |  |  |  |  |  |
| GO:0048294\_negative\_regulation\_of\_isotype\_switching\_to\_IgE\_isotypes | 3 | 0 |  |  |  |  |  |  |  |  |
| GO:0048318\_axial\_mesoderm\_development | 3 | 0 |  |  |  |  |  |  |  |  |
| GO:0048597\_post-embryonic\_camera-type\_eye\_morphogenesis | 3 | 0 |  |  |  |  |  |  |  |  |
| GO:0048636\_positive\_regulation\_of\_muscle\_development | 3 | 0 |  |  |  |  |  |  |  |  |
| GO:0048660\_regulation\_of\_smooth\_muscle\_cell\_proliferation | 3 | 0 |  |  |  |  |  |  |  |  |
| GO:0048668\_collateral\_sprouting | 3 | 0 |  |  |  |  |  |  |  |  |
| GO:0048676\_axon\_extension\_involved\_in\_development | 3 | 0 |  |  |  |  |  |  |  |  |
| GO:0048755\_branching\_morphogenesis\_of\_a\_nerve | 3 | 0 |  |  |  |  |  |  |  |  |
| GO:0048845\_venous\_blood\_vessel\_morphogenesis | 3 | 0 |  |  |  |  |  |  |  |  |
| GO:0048852\_diencephalon\_morphogenesis | 3 | 0 |  |  |  |  |  |  |  |  |
| GO:0048859\_formation\_of\_anatomical\_boundary | 3 | 0 |  |  |  |  |  |  |  |  |
| GO:0048865\_stem\_cell\_fate\_commitment | 3 | 0 |  |  |  |  |  |  |  |  |
| GO:0050435\_beta-amyloid\_metabolic\_process | 3 | 0 |  |  |  |  |  |  |  |  |
| GO:0050650\_chondroitin\_sulfate\_proteoglycan\_biosynthetic\_process | 3 | 0 |  |  |  |  |  |  |  |  |
| GO:0050703\_interleukin-1\_alpha\_secretion | 3 | 0 |  |  |  |  |  |  |  |  |
| GO:0050705\_regulation\_of\_interleukin-1\_alpha\_secretion | 3 | 0 |  |  |  |  |  |  |  |  |
| GO:0050709\_negative\_regulation\_of\_protein\_secretion | 3 | 0 |  |  |  |  |  |  |  |  |
| GO:0050710\_negative\_regulation\_of\_cytokine\_secretion | 3 | 0 |  |  |  |  |  |  |  |  |
| GO:0050717\_positive\_regulation\_of\_interleukin-1\_alpha\_secretion | 3 | 0 |  |  |  |  |  |  |  |  |
| GO:0050774\_negative\_regulation\_of\_dendrite\_morphogenesis | 3 | 0 |  |  |  |  |  |  |  |  |
| GO:0050857\_positive\_regulation\_of\_antigen\_receptor-mediated\_signaling\_pathway | 3 | 0 |  |  |  |  |  |  |  |  |
| GO:0050882\_voluntary\_musculoskeletal\_movement | 3 | 0 |  |  |  |  |  |  |  |  |
| GO:0050913\_sensory\_perception\_of\_bitter\_taste | 3 | 0 |  |  |  |  |  |  |  |  |
| GO:0050957\_equilibrioception | 3 | 0 |  |  |  |  |  |  |  |  |
| GO:0050996\_positive\_regulation\_of\_lipid\_catabolic\_process | 3 | 0 |  |  |  |  |  |  |  |  |
| GO:0051149\_positive\_regulation\_of\_muscle\_cell\_differentiation | 3 | 0 |  |  |  |  |  |  |  |  |
| GO:0051153\_regulation\_of\_striated\_muscle\_cell\_differentiation | 3 | 0 |  |  |  |  |  |  |  |  |
| GO:0051204\_protein\_insertion\_into\_mitochondrial\_membrane | 3 | 0 |  |  |  |  |  |  |  |  |
| GO:0051291\_protein\_heterooligomerization | 3 | 0 |  |  |  |  |  |  |  |  |
| GO:0051302\_regulation\_of\_cell\_division | 3 | 0 |  |  |  |  |  |  |  |  |
| GO:0051450\_myoblast\_proliferation | 3 | 0 |  |  |  |  |  |  |  |  |
| GO:0051583\_dopamine\_uptake | 3 | 0 |  |  |  |  |  |  |  |  |
| GO:0051798\_positive\_regulation\_of\_hair\_follicle\_development | 3 | 0 |  |  |  |  |  |  |  |  |
| GO:0051882\_mitochondrial\_depolarization | 3 | 0 |  |  |  |  |  |  |  |  |
| GO:0051900\_regulation\_of\_mitochondrial\_depolarization | 3 | 0 |  |  |  |  |  |  |  |  |
| GO:0051925\_regulation\_of\_calcium\_ion\_transport\_via\_voltage-gated\_calcium\_channel\_activity | 3 | 0 |  |  |  |  |  |  |  |  |
| GO:0051926\_negative\_regulation\_of\_calcium\_ion\_transport | 3 | 0 |  |  |  |  |  |  |  |  |
| GO:0051930\_regulation\_of\_sensory\_perception\_of\_pain | 3 | 0 |  |  |  |  |  |  |  |  |
| GO:0051931\_regulation\_of\_sensory\_perception | 3 | 0 |  |  |  |  |  |  |  |  |
| GO:0051934\_catecholamine\_uptake\_during\_transmission\_of\_nerve\_impulse | 3 | 0 |  |  |  |  |  |  |  |  |
| GO:0051955\_regulation\_of\_amino\_acid\_transport | 3 | 0 |  |  |  |  |  |  |  |  |
| GO:0051962\_positive\_regulation\_of\_nervous\_system\_development | 3 | 0 |  |  |  |  |  |  |  |  |
| GO:0051965\_positive\_regulation\_of\_synaptogenesis | 3 | 0 |  |  |  |  |  |  |  |  |
| GO:0051967\_negative\_regulation\_of\_synaptic\_transmission\_\_glutamatergic | 3 | 0 |  |  |  |  |  |  |  |  |
| GO:0051983\_regulation\_of\_chromosome\_segregation | 3 | 0 |  |  |  |  |  |  |  |  |
| GO:0055061\_di-\_\_tri-valent\_inorganic\_anion\_homeostasis | 3 | 0 |  |  |  |  |  |  |  |  |
| GO:0055062\_phosphate\_ion\_homeostasis | 3 | 0 |  |  |  |  |  |  |  |  |
| GO:0055083\_monovalent\_inorganic\_anion\_homeostasis | 3 | 0 |  |  |  |  |  |  |  |  |
| GO:0055117\_regulation\_of\_cardiac\_muscle\_contraction | 3 | 0 |  |  |  |  |  |  |  |  |
| GO:0060009\_Sertoli\_cell\_development | 3 | 0 |  |  |  |  |  |  |  |  |
| GO:0060024\_rhythmic\_synaptic\_transmission | 3 | 0 |  |  |  |  |  |  |  |  |
| GO:0060033\_anatomical\_structure\_regression | 3 | 0 |  |  |  |  |  |  |  |  |
| GO:0060040\_retinal\_bipolar\_neuron\_differentiation | 3 | 0 |  |  |  |  |  |  |  |  |
| GO:0060055\_angiogenesis\_involved\_in\_wound\_healing | 3 | 0 |  |  |  |  |  |  |  |  |
| GO:0060084\_synaptic\_transmission\_involved\_in\_micturition | 3 | 0 |  |  |  |  |  |  |  |  |
| GO:0060123\_regulation\_of\_growth\_hormone\_secretion | 3 | 0 |  |  |  |  |  |  |  |  |
| GO:0060126\_somatotropin\_secreting\_cell\_differentiation | 3 | 0 |  |  |  |  |  |  |  |  |
| GO:0060192\_negative\_regulation\_of\_lipase\_activity | 3 | 0 |  |  |  |  |  |  |  |  |
| GO:0060219\_camera-type\_eye\_photoreceptor\_cell\_differentiation | 3 | 0 |  |  |  |  |  |  |  |  |
| GO:0060285\_ciliary\_cell\_motility | 3 | 0 |  |  |  |  |  |  |  |  |
| GO:0060294\_cilium\_movement\_involved\_in\_ciliary\_motility | 3 | 0 |  |  |  |  |  |  |  |  |
| GO:0060295\_regulation\_of\_cilium\_movement\_involved\_in\_ciliary\_motility | 3 | 0 |  |  |  |  |  |  |  |  |
| GO:0060296\_regulation\_of\_cilium\_beat\_frequency\_involved\_in\_ciliary\_motility | 3 | 0 |  |  |  |  |  |  |  |  |
| GO:0060314\_regulation\_of\_ryanodine-sensitive\_calcium-release\_channel\_activity | 3 | 0 |  |  |  |  |  |  |  |  |
| GO:0060396\_growth\_hormone\_receptor\_signaling\_pathway | 3 | 0 |  |  |  |  |  |  |  |  |
| GO:0060416\_response\_to\_growth\_hormone\_stimulus | 3 | 0 |  |  |  |  |  |  |  |  |
| GO:0060428\_lung\_epithelium\_development | 3 | 0 |  |  |  |  |  |  |  |  |
| GO:0060433\_bronchus\_development | 3 | 0 |  |  |  |  |  |  |  |  |
| GO:0060435\_bronchiole\_development | 3 | 0 |  |  |  |  |  |  |  |  |
| GO:0060460\_left\_lung\_morphogenesis | 3 | 0 |  |  |  |  |  |  |  |  |
| GO:0060491\_regulation\_of\_cell\_projection\_assembly | 3 | 0 |  |  |  |  |  |  |  |  |
| GO:0060523\_prostate\_epithelial\_cord\_elongation | 3 | 0 |  |  |  |  |  |  |  |  |
| GO:0060586\_multicellular\_organismal\_iron\_ion\_homeostasis | 3 | 0 |  |  |  |  |  |  |  |  |
| GO:0060596\_mammary\_placode\_formation | 3 | 0 |  |  |  |  |  |  |  |  |
| GO:0060632\_regulation\_of\_microtubule-based\_movement | 3 | 0 |  |  |  |  |  |  |  |  |
| GO:0060648\_mammary\_gland\_bud\_morphogenesis | 3 | 0 |  |  |  |  |  |  |  |  |
| GO:0060684\_epithelial-mesenchymal\_cell\_signaling | 3 | 0 |  |  |  |  |  |  |  |  |
| GO:0060686\_negative\_regulation\_of\_prostatic\_bud\_formation | 3 | 0 |  |  |  |  |  |  |  |  |
| GO:0060689\_cell\_differentiation\_involved\_in\_salivary\_gland\_development | 3 | 0 |  |  |  |  |  |  |  |  |
| GO:0060708\_spongiotrophoblast\_differentiation | 3 | 0 |  |  |  |  |  |  |  |  |
| GO:0060746\_parental\_behavior | 3 | 0 |  |  |  |  |  |  |  |  |
| GO:0060748\_tertiary\_branching\_involved\_in\_mammary\_gland\_duct\_morphogenesis | 3 | 0 |  |  |  |  |  |  |  |  |
| GO:0060750\_epithelial\_cell\_proliferation\_involved\_in\_mammary\_gland\_duct\_elongation | 3 | 0 |  |  |  |  |  |  |  |  |
| GO:0060841\_venous\_blood\_vessel\_development | 3 | 0 |  |  |  |  |  |  |  |  |
| GO:0070102\_interleukin-6-mediated\_signaling\_pathway | 3 | 0 |  |  |  |  |  |  |  |  |
| GO:0070169\_positive\_regulation\_of\_biomineral\_formation | 3 | 0 |  |  |  |  |  |  |  |  |
| GO:0070206\_protein\_trimerization | 3 | 0 |  |  |  |  |  |  |  |  |
| GO:0070207\_protein\_homotrimerization | 3 | 0 |  |  |  |  |  |  |  |  |
| GO:0070229\_negative\_regulation\_of\_lymphocyte\_apoptosis | 3 | 0 |  |  |  |  |  |  |  |  |
| GO:0070230\_positive\_regulation\_of\_lymphocyte\_apoptosis | 3 | 0 |  |  |  |  |  |  |  |  |
| GO:0070232\_regulation\_of\_T\_cell\_apoptosis | 3 | 0 |  |  |  |  |  |  |  |  |
| GO:0070233\_negative\_regulation\_of\_T\_cell\_apoptosis | 3 | 0 |  |  |  |  |  |  |  |  |
| GO:0070242\_thymocyte\_apoptosis | 3 | 0 |  |  |  |  |  |  |  |  |
| GO:0070243\_regulation\_of\_thymocyte\_apoptosis | 3 | 0 |  |  |  |  |  |  |  |  |
| GO:0070244\_negative\_regulation\_of\_thymocyte\_apoptosis | 3 | 0 |  |  |  |  |  |  |  |  |
| GO:0070307\_lens\_fiber\_cell\_development | 3 | 0 |  |  |  |  |  |  |  |  |
| GO:0070309\_lens\_fiber\_cell\_morphogenesis | 3 | 0 |  |  |  |  |  |  |  |  |
| GO:0070423\_nucleotide-binding\_oligomerization\_domain\_containing\_signaling\_pathway | 3 | 0 |  |  |  |  |  |  |  |  |
| GO:0070427\_nucleotide-binding\_oligomerization\_domain\_containing\_1\_signaling\_pathway | 3 | 0 |  |  |  |  |  |  |  |  |
| GO:0070431\_nucleotide-binding\_oligomerization\_domain\_containing\_2\_signaling\_pathway | 3 | 0 |  |  |  |  |  |  |  |  |
| GO:0070633\_transepithelial\_transport | 3 | 0 |  |  |  |  |  |  |  |  |
| GO:0070846\_Hsp90\_deacetylation | 3 | 0 |  |  |  |  |  |  |  |  |
| GO:0070873\_regulation\_of\_glycogen\_metabolic\_process | 3 | 0 |  |  |  |  |  |  |  |  |
| GO:0070875\_positive\_regulation\_of\_glycogen\_metabolic\_process | 3 | 0 |  |  |  |  |  |  |  |  |
| GO:0000041\_transition\_metal\_ion\_transport | 22 | 0 | 0.000000 | 0.000000 | 2434 | 2433.099628 | 2448.79 | 2464.480372 | 1.006076 |  |
| GO:0001523\_retinoid\_metabolic\_process | 22 | 0 | 0.000000 | 0.000000 | 2434 | 2433.099628 | 2448.79 | 2464.480372 | 1.006076 |  |
| GO:0001558\_regulation\_of\_cell\_growth | 22 | 0 | 0.000000 | 0.000000 | 2434 | 2433.099628 | 2448.79 | 2464.480372 | 1.006076 |  |
| GO:0001569\_patterning\_of\_blood\_vessels | 22 | 0 | 0.000000 | 0.000000 | 2434 | 2433.099628 | 2448.79 | 2464.480372 | 1.006076 |  |
| GO:0001947\_heart\_looping | 22 | 0 | 0.000000 | 0.000000 | 2434 | 2433.099628 | 2448.79 | 2464.480372 | 1.006076 |  |
| GO:0002478\_antigen\_processing\_and\_presentation\_of\_exogenous\_peptide\_antigen | 22 | 0 | 0.000000 | 0.000000 | 2434 | 2433.099628 | 2448.79 | 2464.480372 | 1.006076 |  |
| GO:0002637\_regulation\_of\_immunoglobulin\_production | 22 | 0 | 0.000000 | 0.000000 | 2434 | 2433.099628 | 2448.79 | 2464.480372 | 1.006076 |  |
| GO:0002712\_regulation\_of\_B\_cell\_mediated\_immunity | 22 | 0 | 0.000000 | 0.000000 | 2434 | 2433.099628 | 2448.79 | 2464.480372 | 1.006076 |  |
| GO:0002889\_regulation\_of\_immunoglobulin\_mediated\_immune\_response | 22 | 0 | 0.000000 | 0.000000 | 2434 | 2433.099628 | 2448.79 | 2464.480372 | 1.006076 |  |
| GO:0006029\_proteoglycan\_metabolic\_process | 22 | 0 | 0.000000 | 0.000000 | 2434 | 2433.099628 | 2448.79 | 2464.480372 | 1.006076 |  |
| GO:0006112\_energy\_reserve\_metabolic\_process | 22 | 0 | 0.000000 | 0.000000 | 2434 | 2433.099628 | 2448.79 | 2464.480372 | 1.006076 |  |
| GO:0006721\_terpenoid\_metabolic\_process | 22 | 0 | 0.000000 | 0.000000 | 2434 | 2433.099628 | 2448.79 | 2464.480372 | 1.006076 |  |
| GO:0007059\_chromosome\_segregation | 22 | 0 | 0.000000 | 0.000000 | 2434 | 2433.099628 | 2448.79 | 2464.480372 | 1.006076 |  |
| GO:0008624\_induction\_of\_apoptosis\_by\_extracellular\_signals | 22 | 0 | 0.000000 | 0.000000 | 2434 | 2433.099628 | 2448.79 | 2464.480372 | 1.006076 |  |
| GO:0009309\_amine\_biosynthetic\_process | 22 | 0 | 0.000000 | 0.000000 | 2434 | 2433.099628 | 2448.79 | 2464.480372 | 1.006076 |  |
| GO:0009896\_positive\_regulation\_of\_catabolic\_process | 22 | 0 | 0.000000 | 0.000000 | 2434 | 2433.099628 | 2448.79 | 2464.480372 | 1.006076 |  |
| GO:0010463\_mesenchymal\_cell\_proliferation | 22 | 0 | 0.000000 | 0.000000 | 2434 | 2433.099628 | 2448.79 | 2464.480372 | 1.006076 |  |
| GO:0010464\_regulation\_of\_mesenchymal\_cell\_proliferation | 22 | 0 | 0.000000 | 0.000000 | 2434 | 2433.099628 | 2448.79 | 2464.480372 | 1.006076 |  |
| GO:0015918\_sterol\_transport | 22 | 0 | 0.000000 | 0.000000 | 2434 | 2433.099628 | 2448.79 | 2464.480372 | 1.006076 |  |
| GO:0016101\_diterpenoid\_metabolic\_process | 22 | 0 | 0.000000 | 0.000000 | 2434 | 2433.099628 | 2448.79 | 2464.480372 | 1.006076 |  |
| GO:0021575\_hindbrain\_morphogenesis | 22 | 0 | 0.000000 | 0.000000 | 2434 | 2433.099628 | 2448.79 | 2464.480372 | 1.006076 |  |
| GO:0021675\_nerve\_development | 22 | 0 | 0.000000 | 0.000000 | 2434 | 2433.099628 | 2448.79 | 2464.480372 | 1.006076 |  |
| GO:0021766\_hippocampus\_development | 22 | 0 | 0.000000 | 0.000000 | 2434 | 2433.099628 | 2448.79 | 2464.480372 | 1.006076 |  |
| GO:0030301\_cholesterol\_transport | 22 | 0 | 0.000000 | 0.000000 | 2434 | 2433.099628 | 2448.79 | 2464.480372 | 1.006076 |  |
| GO:0030316\_osteoclast\_differentiation | 22 | 0 | 0.000000 | 0.000000 | 2434 | 2433.099628 | 2448.79 | 2464.480372 | 1.006076 |  |
| GO:0030335\_positive\_regulation\_of\_cell\_migration | 22 | 0 | 0.000000 | 0.000000 | 2434 | 2433.099628 | 2448.79 | 2464.480372 | 1.006076 |  |
| GO:0030705\_cytoskeleton-dependent\_intracellular\_transport | 22 | 0 | 0.000000 | 0.000000 | 2434 | 2433.099628 | 2448.79 | 2464.480372 | 1.006076 |  |
| GO:0032649\_regulation\_of\_interferon-gamma\_production | 22 | 0 | 0.000000 | 0.000000 | 2434 | 2433.099628 | 2448.79 | 2464.480372 | 1.006076 |  |
| GO:0033273\_response\_to\_vitamin | 22 | 0 | 0.000000 | 0.000000 | 2434 | 2433.099628 | 2448.79 | 2464.480372 | 1.006076 |  |
| GO:0034097\_response\_to\_cytokine\_stimulus | 22 | 0 | 0.000000 | 0.000000 | 2434 | 2433.099628 | 2448.79 | 2464.480372 | 1.006076 |  |
| GO:0034660\_ncRNA\_metabolic\_process | 22 | 0 | 0.000000 | 0.000000 | 2434 | 2433.099628 | 2448.79 | 2464.480372 | 1.006076 |  |
| GO:0040018\_positive\_regulation\_of\_multicellular\_organism\_growth | 22 | 0 | 0.000000 | 0.000000 | 2434 | 2433.099628 | 2448.79 | 2464.480372 | 1.006076 |  |
| GO:0042130\_negative\_regulation\_of\_T\_cell\_proliferation | 22 | 0 | 0.000000 | 0.000000 | 2434 | 2433.099628 | 2448.79 | 2464.480372 | 1.006076 |  |
| GO:0042461\_photoreceptor\_cell\_development | 22 | 0 | 0.000000 | 0.000000 | 2434 | 2433.099628 | 2448.79 | 2464.480372 | 1.006076 |  |
| GO:0042733\_embryonic\_digit\_morphogenesis | 22 | 0 | 0.000000 | 0.000000 | 2434 | 2433.099628 | 2448.79 | 2464.480372 | 1.006076 |  |
| GO:0044264\_cellular\_polysaccharide\_metabolic\_process | 22 | 0 | 0.000000 | 0.000000 | 2434 | 2433.099628 | 2448.79 | 2464.480372 | 1.006076 |  |
| GO:0045787\_positive\_regulation\_of\_cell\_cycle | 22 | 0 | 0.000000 | 0.000000 | 2434 | 2433.099628 | 2448.79 | 2464.480372 | 1.006076 |  |
| GO:0046635\_positive\_regulation\_of\_alpha-beta\_T\_cell\_activation | 22 | 0 | 0.000000 | 0.000000 | 2434 | 2433.099628 | 2448.79 | 2464.480372 | 1.006076 |  |
| GO:0046883\_regulation\_of\_hormone\_secretion | 22 | 0 | 0.000000 | 0.000000 | 2434 | 2433.099628 | 2448.79 | 2464.480372 | 1.006076 |  |
| GO:0048477\_oogenesis | 22 | 0 | 0.000000 | 0.000000 | 2434 | 2433.099628 | 2448.79 | 2464.480372 | 1.006076 |  |
| GO:0048489\_synaptic\_vesicle\_transport | 22 | 0 | 0.000000 | 0.000000 | 2434 | 2433.099628 | 2448.79 | 2464.480372 | 1.006076 |  |
| GO:0048864\_stem\_cell\_development | 22 | 0 | 0.000000 | 0.000000 | 2434 | 2433.099628 | 2448.79 | 2464.480372 | 1.006076 |  |
| GO:0051260\_protein\_homooligomerization | 22 | 0 | 0.000000 | 0.000000 | 2434 | 2433.099628 | 2448.79 | 2464.480372 | 1.006076 |  |
| GO:0002521\_leukocyte\_differentiation | 161 | 0 | 0.000000 | 0.000000 | 2435 | 2433.763258 | 2449.28 | 2464.796742 | 1.005864 |  |
| GO:0030005\_cellular\_di-\_\_tri-valent\_inorganic\_cation\_homeostasis | 84 | 0 | 0.000000 | 0.000000 | 2437 | 2435.172288 | 2450.58 | 2465.987712 | 1.005572 |  |
| GO:0045137\_development\_of\_primary\_sexual\_characteristics | 84 | 0 | 0.000000 | 0.000000 | 2437 | 2435.172288 | 2450.58 | 2465.987712 | 1.005572 |  |
| GO:0006875\_cellular\_metal\_ion\_homeostasis | 64 | 0 | 0.000000 | 0.000000 | 2442 | 2439.393483 | 2454.34 | 2469.286517 | 1.005053 |  |
| GO:0031644\_regulation\_of\_neurological\_system\_process | 64 | 0 | 0.000000 | 0.000000 | 2442 | 2439.393483 | 2454.34 | 2469.286517 | 1.005053 |  |
| GO:0042060\_wound\_healing | 64 | 0 | 0.000000 | 0.000000 | 2442 | 2439.393483 | 2454.34 | 2469.286517 | 1.005053 |  |
| GO:0055074\_calcium\_ion\_homeostasis | 64 | 0 | 0.000000 | 0.000000 | 2442 | 2439.393483 | 2454.34 | 2469.286517 | 1.005053 |  |
| GO:0070663\_regulation\_of\_leukocyte\_proliferation | 64 | 0 | 0.000000 | 0.000000 | 2442 | 2439.393483 | 2454.34 | 2469.286517 | 1.005053 |  |
| GO:0030163\_protein\_catabolic\_process | 101 | 0 | 0.000000 | 0.000000 | 2443 | 2440.037358 | 2454.96 | 2469.882642 | 1.004896 |  |
| GO:0007548\_sex\_differentiation | 98 | 0 | 0.000000 | 0.000000 | 2447 | 2443.583326 | 2458.07 | 2472.556674 | 1.004524 |  |
| GO:0009314\_response\_to\_radiation | 98 | 0 | 0.000000 | 0.000000 | 2447 | 2443.583326 | 2458.07 | 2472.556674 | 1.004524 |  |
| GO:0009967\_positive\_regulation\_of\_signal\_transduction | 98 | 0 | 0.000000 | 0.000000 | 2447 | 2443.583326 | 2458.07 | 2472.556674 | 1.004524 |  |
| GO:0060541\_respiratory\_system\_development | 98 | 0 | 0.000000 | 0.000000 | 2447 | 2443.583326 | 2458.07 | 2472.556674 | 1.004524 |  |
| GO:0000279\_M\_phase | 85 | 0 | 0.000000 | 0.000000 | 2450 | 2445.899889 | 2460.24 | 2474.580111 | 1.004180 |  |
| GO:0002449\_lymphocyte\_mediated\_immunity | 85 | 0 | 0.000000 | 0.000000 | 2450 | 2445.899889 | 2460.24 | 2474.580111 | 1.004180 |  |
| GO:0070887\_cellular\_response\_to\_chemical\_stimulus | 85 | 0 | 0.000000 | 0.000000 | 2450 | 2445.899889 | 2460.24 | 2474.580111 | 1.004180 |  |
| GO:0002684\_positive\_regulation\_of\_immune\_system\_process | 148 | 0 | 0.000000 | 0.000000 | 2451 | 2447.354051 | 2461.27 | 2475.185949 | 1.004190 |  |
| GO:0010817\_regulation\_of\_hormone\_levels | 106 | 0 | 0.000000 | 0.000000 | 2452 | 2448.117049 | 2461.98 | 2475.842951 | 1.004070 |  |
| GO:0050801\_ion\_homeostasis | 197 | 0 | 0.000000 | 0.000000 | 2453 | 2450.173056 | 2463.6 | 2477.026944 | 1.004321 |  |
| GO:0000723\_telomere\_maintenance | 13 | 0 | 0.000000 | 0.000000 | 2528 | 2522.320847 | 2534.91 | 2547.499153 | 1.002733 |  |
| GO:0001836\_release\_of\_cytochrome\_c\_from\_mitochondria | 13 | 0 | 0.000000 | 0.000000 | 2528 | 2522.320847 | 2534.91 | 2547.499153 | 1.002733 |  |
| GO:0001958\_endochondral\_ossification | 13 | 0 | 0.000000 | 0.000000 | 2528 | 2522.320847 | 2534.91 | 2547.499153 | 1.002733 |  |
| GO:0001975\_response\_to\_amphetamine | 13 | 0 | 0.000000 | 0.000000 | 2528 | 2522.320847 | 2534.91 | 2547.499153 | 1.002733 |  |
| GO:0001976\_neurological\_system\_process\_involved\_in\_regulation\_of\_systemic\_arterial\_blood\_pressure | 13 | 0 | 0.000000 | 0.000000 | 2528 | 2522.320847 | 2534.91 | 2547.499153 | 1.002733 |  |
| GO:0002704\_negative\_regulation\_of\_leukocyte\_mediated\_immunity | 13 | 0 | 0.000000 | 0.000000 | 2528 | 2522.320847 | 2534.91 | 2547.499153 | 1.002733 |  |
| GO:0002707\_negative\_regulation\_of\_lymphocyte\_mediated\_immunity | 13 | 0 | 0.000000 | 0.000000 | 2528 | 2522.320847 | 2534.91 | 2547.499153 | 1.002733 |  |
| GO:0002717\_positive\_regulation\_of\_natural\_killer\_cell\_mediated\_immunity | 13 | 0 | 0.000000 | 0.000000 | 2528 | 2522.320847 | 2534.91 | 2547.499153 | 1.002733 |  |
| GO:0003016\_respiratory\_system\_process | 13 | 0 | 0.000000 | 0.000000 | 2528 | 2522.320847 | 2534.91 | 2547.499153 | 1.002733 |  |
| GO:0006090\_pyruvate\_metabolic\_process | 13 | 0 | 0.000000 | 0.000000 | 2528 | 2522.320847 | 2534.91 | 2547.499153 | 1.002733 |  |
| GO:0006687\_glycosphingolipid\_metabolic\_process | 13 | 0 | 0.000000 | 0.000000 | 2528 | 2522.320847 | 2534.91 | 2547.499153 | 1.002733 |  |
| GO:0006778\_porphyrin\_metabolic\_process | 13 | 0 | 0.000000 | 0.000000 | 2528 | 2522.320847 | 2534.91 | 2547.499153 | 1.002733 |  |
| GO:0006833\_water\_transport | 13 | 0 | 0.000000 | 0.000000 | 2528 | 2522.320847 | 2534.91 | 2547.499153 | 1.002733 |  |
| GO:0006898\_receptor-mediated\_endocytosis | 13 | 0 | 0.000000 | 0.000000 | 2528 | 2522.320847 | 2534.91 | 2547.499153 | 1.002733 |  |
| GO:0006986\_response\_to\_unfolded\_protein | 13 | 0 | 0.000000 | 0.000000 | 2528 | 2522.320847 | 2534.91 | 2547.499153 | 1.002733 |  |
| GO:0007129\_synapsis | 13 | 0 | 0.000000 | 0.000000 | 2528 | 2522.320847 | 2534.91 | 2547.499153 | 1.002733 |  |
| GO:0007212\_dopamine\_receptor\_signaling\_pathway | 13 | 0 | 0.000000 | 0.000000 | 2528 | 2522.320847 | 2534.91 | 2547.499153 | 1.002733 |  |
| GO:0007274\_neuromuscular\_synaptic\_transmission | 13 | 0 | 0.000000 | 0.000000 | 2528 | 2522.320847 | 2534.91 | 2547.499153 | 1.002733 |  |
| GO:0007339\_binding\_of\_sperm\_to\_zona\_pellucida | 13 | 0 | 0.000000 | 0.000000 | 2528 | 2522.320847 | 2534.91 | 2547.499153 | 1.002733 |  |
| GO:0007439\_ectodermal\_gut\_development | 13 | 0 | 0.000000 | 0.000000 | 2528 | 2522.320847 | 2534.91 | 2547.499153 | 1.002733 |  |
| GO:0007512\_adult\_heart\_development | 13 | 0 | 0.000000 | 0.000000 | 2528 | 2522.320847 | 2534.91 | 2547.499153 | 1.002733 |  |
| GO:0007566\_embryo\_implantation | 13 | 0 | 0.000000 | 0.000000 | 2528 | 2522.320847 | 2534.91 | 2547.499153 | 1.002733 |  |
| GO:0009119\_ribonucleoside\_metabolic\_process | 13 | 0 | 0.000000 | 0.000000 | 2528 | 2522.320847 | 2534.91 | 2547.499153 | 1.002733 |  |
| GO:0009410\_response\_to\_xenobiotic\_stimulus | 13 | 0 | 0.000000 | 0.000000 | 2528 | 2522.320847 | 2534.91 | 2547.499153 | 1.002733 |  |
| GO:0009994\_oocyte\_differentiation | 13 | 0 | 0.000000 | 0.000000 | 2528 | 2522.320847 | 2534.91 | 2547.499153 | 1.002733 |  |
| GO:0010623\_developmental\_programmed\_cell\_death | 13 | 0 | 0.000000 | 0.000000 | 2528 | 2522.320847 | 2534.91 | 2547.499153 | 1.002733 |  |
| GO:0010970\_microtubule-based\_transport | 13 | 0 | 0.000000 | 0.000000 | 2528 | 2522.320847 | 2534.91 | 2547.499153 | 1.002733 |  |
| GO:0016525\_negative\_regulation\_of\_angiogenesis | 13 | 0 | 0.000000 | 0.000000 | 2528 | 2522.320847 | 2534.91 | 2547.499153 | 1.002733 |  |
| GO:0018105\_peptidyl-serine\_phosphorylation | 13 | 0 | 0.000000 | 0.000000 | 2528 | 2522.320847 | 2534.91 | 2547.499153 | 1.002733 |  |
| GO:0019098\_reproductive\_behavior | 13 | 0 | 0.000000 | 0.000000 | 2528 | 2522.320847 | 2534.91 | 2547.499153 | 1.002733 |  |
| GO:0021511\_spinal\_cord\_patterning | 13 | 0 | 0.000000 | 0.000000 | 2528 | 2522.320847 | 2534.91 | 2547.499153 | 1.002733 |  |
| GO:0021533\_cell\_differentiation\_in\_hindbrain | 13 | 0 | 0.000000 | 0.000000 | 2528 | 2522.320847 | 2534.91 | 2547.499153 | 1.002733 |  |
| GO:0021879\_forebrain\_neuron\_differentiation | 13 | 0 | 0.000000 | 0.000000 | 2528 | 2522.320847 | 2534.91 | 2547.499153 | 1.002733 |  |
| GO:0021955\_central\_nervous\_system\_neuron\_axonogenesis | 13 | 0 | 0.000000 | 0.000000 | 2528 | 2522.320847 | 2534.91 | 2547.499153 | 1.002733 |  |
| GO:0030384\_phosphoinositide\_metabolic\_process | 13 | 0 | 0.000000 | 0.000000 | 2528 | 2522.320847 | 2534.91 | 2547.499153 | 1.002733 |  |
| GO:0030516\_regulation\_of\_axon\_extension | 13 | 0 | 0.000000 | 0.000000 | 2528 | 2522.320847 | 2534.91 | 2547.499153 | 1.002733 |  |
| GO:0030539\_male\_genitalia\_development | 13 | 0 | 0.000000 | 0.000000 | 2528 | 2522.320847 | 2534.91 | 2547.499153 | 1.002733 |  |
| GO:0031032\_actomyosin\_structure\_organization | 13 | 0 | 0.000000 | 0.000000 | 2528 | 2522.320847 | 2534.91 | 2547.499153 | 1.002733 |  |
| GO:0031290\_retinal\_ganglion\_cell\_axon\_guidance | 13 | 0 | 0.000000 | 0.000000 | 2528 | 2522.320847 | 2534.91 | 2547.499153 | 1.002733 |  |
| GO:0032200\_telomere\_organization | 13 | 0 | 0.000000 | 0.000000 | 2528 | 2522.320847 | 2534.91 | 2547.499153 | 1.002733 |  |
| GO:0032330\_regulation\_of\_chondrocyte\_differentiation | 13 | 0 | 0.000000 | 0.000000 | 2528 | 2522.320847 | 2534.91 | 2547.499153 | 1.002733 |  |
| GO:0032615\_interleukin-12\_production | 13 | 0 | 0.000000 | 0.000000 | 2528 | 2522.320847 | 2534.91 | 2547.499153 | 1.002733 |  |
| GO:0032729\_positive\_regulation\_of\_interferon-gamma\_production | 13 | 0 | 0.000000 | 0.000000 | 2528 | 2522.320847 | 2534.91 | 2547.499153 | 1.002733 |  |
| GO:0033013\_tetrapyrrole\_metabolic\_process | 13 | 0 | 0.000000 | 0.000000 | 2528 | 2522.320847 | 2534.91 | 2547.499153 | 1.002733 |  |
| GO:0034329\_cell\_junction\_assembly | 13 | 0 | 0.000000 | 0.000000 | 2528 | 2522.320847 | 2534.91 | 2547.499153 | 1.002733 |  |
| GO:0042044\_fluid\_transport | 13 | 0 | 0.000000 | 0.000000 | 2528 | 2522.320847 | 2534.91 | 2547.499153 | 1.002733 |  |
| GO:0042094\_interleukin-2\_biosynthetic\_process | 13 | 0 | 0.000000 | 0.000000 | 2528 | 2522.320847 | 2534.91 | 2547.499153 | 1.002733 |  |
| GO:0042474\_middle\_ear\_morphogenesis | 13 | 0 | 0.000000 | 0.000000 | 2528 | 2522.320847 | 2534.91 | 2547.499153 | 1.002733 |  |
| GO:0043241\_protein\_complex\_disassembly | 13 | 0 | 0.000000 | 0.000000 | 2528 | 2522.320847 | 2534.91 | 2547.499153 | 1.002733 |  |
| GO:0043244\_regulation\_of\_protein\_complex\_disassembly | 13 | 0 | 0.000000 | 0.000000 | 2528 | 2522.320847 | 2534.91 | 2547.499153 | 1.002733 |  |
| GO:0045191\_regulation\_of\_isotype\_switching | 13 | 0 | 0.000000 | 0.000000 | 2528 | 2522.320847 | 2534.91 | 2547.499153 | 1.002733 |  |
| GO:0045577\_regulation\_of\_B\_cell\_differentiation | 13 | 0 | 0.000000 | 0.000000 | 2528 | 2522.320847 | 2534.91 | 2547.499153 | 1.002733 |  |
| GO:0045682\_regulation\_of\_epidermis\_development | 13 | 0 | 0.000000 | 0.000000 | 2528 | 2522.320847 | 2534.91 | 2547.499153 | 1.002733 |  |
| GO:0045954\_positive\_regulation\_of\_natural\_killer\_cell\_mediated\_cytotoxicity | 13 | 0 | 0.000000 | 0.000000 | 2528 | 2522.320847 | 2534.91 | 2547.499153 | 1.002733 |  |
| GO:0046474\_glycerophospholipid\_biosynthetic\_process | 13 | 0 | 0.000000 | 0.000000 | 2528 | 2522.320847 | 2534.91 | 2547.499153 | 1.002733 |  |
| GO:0046640\_regulation\_of\_alpha-beta\_T\_cell\_proliferation | 13 | 0 | 0.000000 | 0.000000 | 2528 | 2522.320847 | 2534.91 | 2547.499153 | 1.002733 |  |
| GO:0046851\_negative\_regulation\_of\_bone\_remodeling | 13 | 0 | 0.000000 | 0.000000 | 2528 | 2522.320847 | 2534.91 | 2547.499153 | 1.002733 |  |
| GO:0048305\_immunoglobulin\_secretion | 13 | 0 | 0.000000 | 0.000000 | 2528 | 2522.320847 | 2534.91 | 2547.499153 | 1.002733 |  |
| GO:0048566\_embryonic\_gut\_development | 13 | 0 | 0.000000 | 0.000000 | 2528 | 2522.320847 | 2534.91 | 2547.499153 | 1.002733 |  |
| GO:0048567\_ectodermal\_gut\_morphogenesis | 13 | 0 | 0.000000 | 0.000000 | 2528 | 2522.320847 | 2534.91 | 2547.499153 | 1.002733 |  |
| GO:0048599\_oocyte\_development | 13 | 0 | 0.000000 | 0.000000 | 2528 | 2522.320847 | 2534.91 | 2547.499153 | 1.002733 |  |
| GO:0050764\_regulation\_of\_phagocytosis | 13 | 0 | 0.000000 | 0.000000 | 2528 | 2522.320847 | 2534.91 | 2547.499153 | 1.002733 |  |
| GO:0050766\_positive\_regulation\_of\_phagocytosis | 13 | 0 | 0.000000 | 0.000000 | 2528 | 2522.320847 | 2534.91 | 2547.499153 | 1.002733 |  |
| GO:0050771\_negative\_regulation\_of\_axonogenesis | 13 | 0 | 0.000000 | 0.000000 | 2528 | 2522.320847 | 2534.91 | 2547.499153 | 1.002733 |  |
| GO:0050818\_regulation\_of\_coagulation | 13 | 0 | 0.000000 | 0.000000 | 2528 | 2522.320847 | 2534.91 | 2547.499153 | 1.002733 |  |
| GO:0051346\_negative\_regulation\_of\_hydrolase\_activity | 13 | 0 | 0.000000 | 0.000000 | 2528 | 2522.320847 | 2534.91 | 2547.499153 | 1.002733 |  |
| GO:0051495\_positive\_regulation\_of\_cytoskeleton\_organization | 13 | 0 | 0.000000 | 0.000000 | 2528 | 2522.320847 | 2534.91 | 2547.499153 | 1.002733 |  |
| GO:0060038\_cardiac\_muscle\_cell\_proliferation | 13 | 0 | 0.000000 | 0.000000 | 2528 | 2522.320847 | 2534.91 | 2547.499153 | 1.002733 |  |
| GO:0060070\_Wnt\_receptor\_signaling\_pathway\_through\_beta-catenin | 13 | 0 | 0.000000 | 0.000000 | 2528 | 2522.320847 | 2534.91 | 2547.499153 | 1.002733 |  |
| GO:0060324\_face\_development | 13 | 0 | 0.000000 | 0.000000 | 2528 | 2522.320847 | 2534.91 | 2547.499153 | 1.002733 |  |
| GO:0060401\_cytosolic\_calcium\_ion\_transport | 13 | 0 | 0.000000 | 0.000000 | 2528 | 2522.320847 | 2534.91 | 2547.499153 | 1.002733 |  |
| GO:0060402\_calcium\_ion\_transport\_into\_cytosol | 13 | 0 | 0.000000 | 0.000000 | 2528 | 2522.320847 | 2534.91 | 2547.499153 | 1.002733 |  |
| GO:0060560\_developmental\_growth\_involved\_in\_morphogenesis | 13 | 0 | 0.000000 | 0.000000 | 2528 | 2522.320847 | 2534.91 | 2547.499153 | 1.002733 |  |
| GO:0060742\_epithelial\_cell\_differentiation\_involved\_in\_prostate\_gland\_development | 13 | 0 | 0.000000 | 0.000000 | 2528 | 2522.320847 | 2534.91 | 2547.499153 | 1.002733 |  |
| GO:0070192\_chromosome\_organization\_involved\_in\_meiosis | 13 | 0 | 0.000000 | 0.000000 | 2528 | 2522.320847 | 2534.91 | 2547.499153 | 1.002733 |  |
| GO:0006281\_DNA\_repair | 71 | 0 | 0.000000 | 0.000000 | 2530 | 2525.924032 | 2538.37 | 2550.815968 | 1.003308 |  |
| GO:0016331\_morphogenesis\_of\_embryonic\_epithelium | 71 | 0 | 0.000000 | 0.000000 | 2530 | 2525.924032 | 2538.37 | 2550.815968 | 1.003308 |  |
| GO:0030003\_cellular\_cation\_homeostasis | 90 | 0 | 0.000000 | 0.000000 | 2534 | 2530.674976 | 2542.66 | 2554.645024 | 1.003418 |  |
| GO:0030324\_lung\_development | 90 | 0 | 0.000000 | 0.000000 | 2534 | 2530.674976 | 2542.66 | 2554.645024 | 1.003418 |  |
| GO:0035264\_multicellular\_organism\_growth | 90 | 0 | 0.000000 | 0.000000 | 2534 | 2530.674976 | 2542.66 | 2554.645024 | 1.003418 |  |
| GO:0042113\_B\_cell\_activation | 90 | 0 | 0.000000 | 0.000000 | 2534 | 2530.674976 | 2542.66 | 2554.645024 | 1.003418 |  |
| GO:0001704\_formation\_of\_primary\_germ\_layer | 36 | 0 | 0.000000 | 0.000000 | 2550 | 2546.944888 | 2558.25 | 2569.555112 | 1.003235 |  |
| GO:0001819\_positive\_regulation\_of\_cytokine\_production | 36 | 0 | 0.000000 | 0.000000 | 2550 | 2546.944888 | 2558.25 | 2569.555112 | 1.003235 |  |
| GO:0006469\_negative\_regulation\_of\_protein\_kinase\_activity | 36 | 0 | 0.000000 | 0.000000 | 2550 | 2546.944888 | 2558.25 | 2569.555112 | 1.003235 |  |
| GO:0007187\_G-protein\_signaling\_\_coupled\_to\_cyclic\_nucleotide\_second\_messenger | 36 | 0 | 0.000000 | 0.000000 | 2550 | 2546.944888 | 2558.25 | 2569.555112 | 1.003235 |  |
| GO:0007368\_determination\_of\_left\_right\_symmetry | 36 | 0 | 0.000000 | 0.000000 | 2550 | 2546.944888 | 2558.25 | 2569.555112 | 1.003235 |  |
| GO:0014020\_primary\_neural\_tube\_formation | 36 | 0 | 0.000000 | 0.000000 | 2550 | 2546.944888 | 2558.25 | 2569.555112 | 1.003235 |  |
| GO:0019228\_regulation\_of\_action\_potential\_in\_neuron | 36 | 0 | 0.000000 | 0.000000 | 2550 | 2546.944888 | 2558.25 | 2569.555112 | 1.003235 |  |
| GO:0021510\_spinal\_cord\_development | 36 | 0 | 0.000000 | 0.000000 | 2550 | 2546.944888 | 2558.25 | 2569.555112 | 1.003235 |  |
| GO:0022602\_ovulation\_cycle\_process | 36 | 0 | 0.000000 | 0.000000 | 2550 | 2546.944888 | 2558.25 | 2569.555112 | 1.003235 |  |
| GO:0030072\_peptide\_hormone\_secretion | 36 | 0 | 0.000000 | 0.000000 | 2550 | 2546.944888 | 2558.25 | 2569.555112 | 1.003235 |  |
| GO:0030278\_regulation\_of\_ossification | 36 | 0 | 0.000000 | 0.000000 | 2550 | 2546.944888 | 2558.25 | 2569.555112 | 1.003235 |  |
| GO:0033673\_negative\_regulation\_of\_kinase\_activity | 36 | 0 | 0.000000 | 0.000000 | 2550 | 2546.944888 | 2558.25 | 2569.555112 | 1.003235 |  |
| GO:0042742\_defense\_response\_to\_bacterium | 36 | 0 | 0.000000 | 0.000000 | 2550 | 2546.944888 | 2558.25 | 2569.555112 | 1.003235 |  |
| GO:0050851\_antigen\_receptor-mediated\_signaling\_pathway | 36 | 0 | 0.000000 | 0.000000 | 2550 | 2546.944888 | 2558.25 | 2569.555112 | 1.003235 |  |
| GO:0050900\_leukocyte\_migration | 36 | 0 | 0.000000 | 0.000000 | 2550 | 2546.944888 | 2558.25 | 2569.555112 | 1.003235 |  |
| GO:0051223\_regulation\_of\_protein\_transport | 36 | 0 | 0.000000 | 0.000000 | 2550 | 2546.944888 | 2558.25 | 2569.555112 | 1.003235 |  |
| GO:0006325\_chromatin\_organization | 83 | 0 | 0.000000 | 0.000000 | 2555 | 2551.627250 | 2562.44 | 2573.252750 | 1.002912 |  |
| GO:0006575\_cellular\_amino\_acid\_derivative\_metabolic\_process | 83 | 0 | 0.000000 | 0.000000 | 2555 | 2551.627250 | 2562.44 | 2573.252750 | 1.002912 |  |
| GO:0030198\_extracellular\_matrix\_organization | 83 | 0 | 0.000000 | 0.000000 | 2555 | 2551.627250 | 2562.44 | 2573.252750 | 1.002912 |  |
| GO:0030534\_adult\_behavior | 83 | 0 | 0.000000 | 0.000000 | 2555 | 2551.627250 | 2562.44 | 2573.252750 | 1.002912 |  |
| GO:0050867\_positive\_regulation\_of\_cell\_activation | 83 | 0 | 0.000000 | 0.000000 | 2555 | 2551.627250 | 2562.44 | 2573.252750 | 1.002912 |  |
| GO:0051049\_regulation\_of\_transport | 167 | 0 | 0.000000 | 0.000000 | 2556 | 2553.563386 | 2563.91 | 2574.256614 | 1.003095 |  |
| GO:0001707\_mesoderm\_formation | 32 | 0 | 0.000000 | 0.000000 | 2570 | 2567.369289 | 2577.12 | 2586.870711 | 1.002770 |  |
| GO:0002274\_myeloid\_leukocyte\_activation | 32 | 0 | 0.000000 | 0.000000 | 2570 | 2567.369289 | 2577.12 | 2586.870711 | 1.002770 |  |
| GO:0006638\_neutral\_lipid\_metabolic\_process | 32 | 0 | 0.000000 | 0.000000 | 2570 | 2567.369289 | 2577.12 | 2586.870711 | 1.002770 |  |
| GO:0006662\_glycerol\_ether\_metabolic\_process | 32 | 0 | 0.000000 | 0.000000 | 2570 | 2567.369289 | 2577.12 | 2586.870711 | 1.002770 |  |
| GO:0006937\_regulation\_of\_muscle\_contraction | 32 | 0 | 0.000000 | 0.000000 | 2570 | 2567.369289 | 2577.12 | 2586.870711 | 1.002770 |  |
| GO:0007249\_I-kappaB\_kinase\_NF-kappaB\_cascade | 32 | 0 | 0.000000 | 0.000000 | 2570 | 2567.369289 | 2577.12 | 2586.870711 | 1.002770 |  |
| GO:0007272\_ensheathment\_of\_neurons | 32 | 0 | 0.000000 | 0.000000 | 2570 | 2567.369289 | 2577.12 | 2586.870711 | 1.002770 |  |
| GO:0008366\_axon\_ensheathment | 32 | 0 | 0.000000 | 0.000000 | 2570 | 2567.369289 | 2577.12 | 2586.870711 | 1.002770 |  |
| GO:0042102\_positive\_regulation\_of\_T\_cell\_proliferation | 32 | 0 | 0.000000 | 0.000000 | 2570 | 2567.369289 | 2577.12 | 2586.870711 | 1.002770 |  |
| GO:0048332\_mesoderm\_morphogenesis | 32 | 0 | 0.000000 | 0.000000 | 2570 | 2567.369289 | 2577.12 | 2586.870711 | 1.002770 |  |
| GO:0050768\_negative\_regulation\_of\_neurogenesis | 32 | 0 | 0.000000 | 0.000000 | 2570 | 2567.369289 | 2577.12 | 2586.870711 | 1.002770 |  |
| GO:0050770\_regulation\_of\_axonogenesis | 32 | 0 | 0.000000 | 0.000000 | 2570 | 2567.369289 | 2577.12 | 2586.870711 | 1.002770 |  |
| GO:0050885\_neuromuscular\_process\_controlling\_balance | 32 | 0 | 0.000000 | 0.000000 | 2570 | 2567.369289 | 2577.12 | 2586.870711 | 1.002770 |  |
| GO:0051259\_protein\_oligomerization | 32 | 0 | 0.000000 | 0.000000 | 2570 | 2567.369289 | 2577.12 | 2586.870711 | 1.002770 |  |
| GO:0001654\_eye\_development | 136 | 0 | 0.000000 | 0.000000 | 2571 | 2568.005760 | 2577.64 | 2587.274240 | 1.002583 |  |
| GO:0002443\_leukocyte\_mediated\_immunity | 91 | 0 | 0.000000 | 0.000000 | 2574 | 2570.584766 | 2579.81 | 2589.035234 | 1.002257 |  |
| GO:0008544\_epidermis\_development | 91 | 0 | 0.000000 | 0.000000 | 2574 | 2570.584766 | 2579.81 | 2589.035234 | 1.002257 |  |
| GO:0031399\_regulation\_of\_protein\_modification\_process | 91 | 0 | 0.000000 | 0.000000 | 2574 | 2570.584766 | 2579.81 | 2589.035234 | 1.002257 |  |
| GO:0051249\_regulation\_of\_lymphocyte\_activation | 112 | 0 | 0.000000 | 0.000000 | 2575 | 2571.963689 | 2580.88 | 2589.796311 | 1.002283 |  |
| GO:0007498\_mesoderm\_development | 48 | 0 | 0.000000 | 0.000000 | 2580 | 2578.172598 | 2586.83 | 2595.487402 | 1.002647 |  |
| GO:0019318\_hexose\_metabolic\_process | 48 | 0 | 0.000000 | 0.000000 | 2580 | 2578.172598 | 2586.83 | 2595.487402 | 1.002647 |  |
| GO:0032269\_negative\_regulation\_of\_cellular\_protein\_metabolic\_process | 48 | 0 | 0.000000 | 0.000000 | 2580 | 2578.172598 | 2586.83 | 2595.487402 | 1.002647 |  |
| GO:0034504\_protein\_localization\_in\_nucleus | 48 | 0 | 0.000000 | 0.000000 | 2580 | 2578.172598 | 2586.83 | 2595.487402 | 1.002647 |  |
| GO:0046849\_bone\_remodeling | 48 | 0 | 0.000000 | 0.000000 | 2580 | 2578.172598 | 2586.83 | 2595.487402 | 1.002647 |  |
| GO:0007600\_sensory\_perception | 172 | 0 | 0.000000 | 0.000000 | 2582 | 2579.222460 | 2587.7 | 2596.177540 | 1.002208 |  |
| GO:0009611\_response\_to\_wounding | 172 | 0 | 0.000000 | 0.000000 | 2582 | 2579.222460 | 2587.7 | 2596.177540 | 1.002208 |  |
| GO:0001525\_angiogenesis | 100 | 0 | 0.000000 | 0.000000 | 2583 | 2579.857133 | 2588.35 | 2596.842867 | 1.002071 |  |
| GO:0045597\_positive\_regulation\_of\_cell\_differentiation | 128 | 0 | 0.000000 | 0.000000 | 2584 | 2581.916428 | 2590.12 | 2598.323572 | 1.002368 |  |
| GO:0003002\_regionalization | 195 | 0 | 0.000000 | 0.000000 | 2586 | 2583.392569 | 2591.35 | 2599.307431 | 1.002069 |  |
| GO:0007507\_heart\_development | 195 | 0 | 0.000000 | 0.000000 | 2586 | 2583.392569 | 2591.35 | 2599.307431 | 1.002069 |  |
| GO:0001910\_regulation\_of\_leukocyte\_mediated\_cytotoxicity | 27 | 0 | 0.000000 | 0.000000 | 2603 | 2600.065567 | 2607.6 | 2615.134433 | 1.001767 |  |
| GO:0002761\_regulation\_of\_myeloid\_leukocyte\_differentiation | 27 | 0 | 0.000000 | 0.000000 | 2603 | 2600.065567 | 2607.6 | 2615.134433 | 1.001767 |  |
| GO:0006479\_protein\_amino\_acid\_methylation | 27 | 0 | 0.000000 | 0.000000 | 2603 | 2600.065567 | 2607.6 | 2615.134433 | 1.001767 |  |
| GO:0007422\_peripheral\_nervous\_system\_development | 27 | 0 | 0.000000 | 0.000000 | 2603 | 2600.065567 | 2607.6 | 2615.134433 | 1.001767 |  |
| GO:0008213\_protein\_amino\_acid\_alkylation | 27 | 0 | 0.000000 | 0.000000 | 2603 | 2600.065567 | 2607.6 | 2615.134433 | 1.001767 |  |
| GO:0008286\_insulin\_receptor\_signaling\_pathway | 27 | 0 | 0.000000 | 0.000000 | 2603 | 2600.065567 | 2607.6 | 2615.134433 | 1.001767 |  |
| GO:0009411\_response\_to\_UV | 27 | 0 | 0.000000 | 0.000000 | 2603 | 2600.065567 | 2607.6 | 2615.134433 | 1.001767 |  |
| GO:0009913\_epidermal\_cell\_differentiation | 27 | 0 | 0.000000 | 0.000000 | 2603 | 2600.065567 | 2607.6 | 2615.134433 | 1.001767 |  |
| GO:0016050\_vesicle\_organization | 27 | 0 | 0.000000 | 0.000000 | 2603 | 2600.065567 | 2607.6 | 2615.134433 | 1.001767 |  |
| GO:0019884\_antigen\_processing\_and\_presentation\_of\_exogenous\_antigen | 27 | 0 | 0.000000 | 0.000000 | 2603 | 2600.065567 | 2607.6 | 2615.134433 | 1.001767 |  |
| GO:0030100\_regulation\_of\_endocytosis | 27 | 0 | 0.000000 | 0.000000 | 2603 | 2600.065567 | 2607.6 | 2615.134433 | 1.001767 |  |
| GO:0031016\_pancreas\_development | 27 | 0 | 0.000000 | 0.000000 | 2603 | 2600.065567 | 2607.6 | 2615.134433 | 1.001767 |  |
| GO:0031341\_regulation\_of\_cell\_killing | 27 | 0 | 0.000000 | 0.000000 | 2603 | 2600.065567 | 2607.6 | 2615.134433 | 1.001767 |  |
| GO:0032496\_response\_to\_lipopolysaccharide | 27 | 0 | 0.000000 | 0.000000 | 2603 | 2600.065567 | 2607.6 | 2615.134433 | 1.001767 |  |
| GO:0045582\_positive\_regulation\_of\_T\_cell\_differentiation | 27 | 0 | 0.000000 | 0.000000 | 2603 | 2600.065567 | 2607.6 | 2615.134433 | 1.001767 |  |
| GO:0051272\_positive\_regulation\_of\_cell\_motion | 27 | 0 | 0.000000 | 0.000000 | 2603 | 2600.065567 | 2607.6 | 2615.134433 | 1.001767 |  |
| GO:0070482\_response\_to\_oxygen\_levels | 27 | 0 | 0.000000 | 0.000000 | 2603 | 2600.065567 | 2607.6 | 2615.134433 | 1.001767 |  |
| GO:0035107\_appendage\_morphogenesis | 93 | 0 | 0.000000 | 0.000000 | 2606 | 2603.160857 | 2610.39 | 2617.619143 | 1.001685 |  |
| GO:0035108\_limb\_morphogenesis | 93 | 0 | 0.000000 | 0.000000 | 2606 | 2603.160857 | 2610.39 | 2617.619143 | 1.001685 |  |
| GO:0055066\_di-\_\_tri-valent\_inorganic\_cation\_homeostasis | 93 | 0 | 0.000000 | 0.000000 | 2606 | 2603.160857 | 2610.39 | 2617.619143 | 1.001685 |  |
| GO:0006066\_alcohol\_metabolic\_process | 158 | 0 | 0.000000 | 0.000000 | 2609 | 2605.045058 | 2611.99 | 2618.934942 | 1.001146 |  |
| GO:0007409\_axonogenesis | 158 | 0 | 0.000000 | 0.000000 | 2609 | 2605.045058 | 2611.99 | 2618.934942 | 1.001146 |  |
| GO:0048514\_blood\_vessel\_morphogenesis | 158 | 0 | 0.000000 | 0.000000 | 2609 | 2605.045058 | 2611.99 | 2618.934942 | 1.001146 |  |
| GO:0001912\_positive\_regulation\_of\_leukocyte\_mediated\_cytotoxicity | 20 | 0 | 0.000000 | 0.000000 | 2639 | 2634.137897 | 2640.39 | 2646.642103 | 1.000527 |  |
| GO:0005977\_glycogen\_metabolic\_process | 20 | 0 | 0.000000 | 0.000000 | 2639 | 2634.137897 | 2640.39 | 2646.642103 | 1.000527 |  |
| GO:0006073\_cellular\_glucan\_metabolic\_process | 20 | 0 | 0.000000 | 0.000000 | 2639 | 2634.137897 | 2640.39 | 2646.642103 | 1.000527 |  |
| GO:0006518\_peptide\_metabolic\_process | 20 | 0 | 0.000000 | 0.000000 | 2639 | 2634.137897 | 2640.39 | 2646.642103 | 1.000527 |  |
| GO:0006584\_catecholamine\_metabolic\_process | 20 | 0 | 0.000000 | 0.000000 | 2639 | 2634.137897 | 2640.39 | 2646.642103 | 1.000527 |  |
| GO:0007416\_synaptogenesis | 20 | 0 | 0.000000 | 0.000000 | 2639 | 2634.137897 | 2640.39 | 2646.642103 | 1.000527 |  |
| GO:0007586\_digestion | 20 | 0 | 0.000000 | 0.000000 | 2639 | 2634.137897 | 2640.39 | 2646.642103 | 1.000527 |  |
| GO:0008360\_regulation\_of\_cell\_shape | 20 | 0 | 0.000000 | 0.000000 | 2639 | 2634.137897 | 2640.39 | 2646.642103 | 1.000527 |  |
| GO:0009615\_response\_to\_virus | 20 | 0 | 0.000000 | 0.000000 | 2639 | 2634.137897 | 2640.39 | 2646.642103 | 1.000527 |  |
| GO:0009712\_catechol\_metabolic\_process | 20 | 0 | 0.000000 | 0.000000 | 2639 | 2634.137897 | 2640.39 | 2646.642103 | 1.000527 |  |
| GO:0010927\_cellular\_component\_assembly\_involved\_in\_morphogenesis | 20 | 0 | 0.000000 | 0.000000 | 2639 | 2634.137897 | 2640.39 | 2646.642103 | 1.000527 |  |
| GO:0016571\_histone\_methylation | 20 | 0 | 0.000000 | 0.000000 | 2639 | 2634.137897 | 2640.39 | 2646.642103 | 1.000527 |  |
| GO:0018209\_peptidyl-serine\_modification | 20 | 0 | 0.000000 | 0.000000 | 2639 | 2634.137897 | 2640.39 | 2646.642103 | 1.000527 |  |
| GO:0018958\_phenol\_metabolic\_process | 20 | 0 | 0.000000 | 0.000000 | 2639 | 2634.137897 | 2640.39 | 2646.642103 | 1.000527 |  |
| GO:0021695\_cerebellar\_cortex\_development | 20 | 0 | 0.000000 | 0.000000 | 2639 | 2634.137897 | 2640.39 | 2646.642103 | 1.000527 |  |
| GO:0031128\_developmental\_induction | 20 | 0 | 0.000000 | 0.000000 | 2639 | 2634.137897 | 2640.39 | 2646.642103 | 1.000527 |  |
| GO:0031214\_biomineral\_formation | 20 | 0 | 0.000000 | 0.000000 | 2639 | 2634.137897 | 2640.39 | 2646.642103 | 1.000527 |  |
| GO:0031343\_positive\_regulation\_of\_cell\_killing | 20 | 0 | 0.000000 | 0.000000 | 2639 | 2634.137897 | 2640.39 | 2646.642103 | 1.000527 |  |
| GO:0032582\_negative\_regulation\_of\_gene-specific\_transcription | 20 | 0 | 0.000000 | 0.000000 | 2639 | 2634.137897 | 2640.39 | 2646.642103 | 1.000527 |  |
| GO:0032640\_tumor\_necrosis\_factor\_production | 20 | 0 | 0.000000 | 0.000000 | 2639 | 2634.137897 | 2640.39 | 2646.642103 | 1.000527 |  |
| GO:0032680\_regulation\_of\_tumor\_necrosis\_factor\_production | 20 | 0 | 0.000000 | 0.000000 | 2639 | 2634.137897 | 2640.39 | 2646.642103 | 1.000527 |  |
| GO:0034311\_diol\_metabolic\_process | 20 | 0 | 0.000000 | 0.000000 | 2639 | 2634.137897 | 2640.39 | 2646.642103 | 1.000527 |  |
| GO:0042326\_negative\_regulation\_of\_phosphorylation | 20 | 0 | 0.000000 | 0.000000 | 2639 | 2634.137897 | 2640.39 | 2646.642103 | 1.000527 |  |
| GO:0044042\_glucan\_metabolic\_process | 20 | 0 | 0.000000 | 0.000000 | 2639 | 2634.137897 | 2640.39 | 2646.642103 | 1.000527 |  |
| GO:0045017\_glycerolipid\_biosynthetic\_process | 20 | 0 | 0.000000 | 0.000000 | 2639 | 2634.137897 | 2640.39 | 2646.642103 | 1.000527 |  |
| GO:0045168\_cell-cell\_signaling\_involved\_in\_cell\_fate\_specification | 20 | 0 | 0.000000 | 0.000000 | 2639 | 2634.137897 | 2640.39 | 2646.642103 | 1.000527 |  |
| GO:0045639\_positive\_regulation\_of\_myeloid\_cell\_differentiation | 20 | 0 | 0.000000 | 0.000000 | 2639 | 2634.137897 | 2640.39 | 2646.642103 | 1.000527 |  |
| GO:0046822\_regulation\_of\_nucleocytoplasmic\_transport | 20 | 0 | 0.000000 | 0.000000 | 2639 | 2634.137897 | 2640.39 | 2646.642103 | 1.000527 |  |
| GO:0048806\_genitalia\_development | 20 | 0 | 0.000000 | 0.000000 | 2639 | 2634.137897 | 2640.39 | 2646.642103 | 1.000527 |  |
| GO:0060191\_regulation\_of\_lipase\_activity | 20 | 0 | 0.000000 | 0.000000 | 2639 | 2634.137897 | 2640.39 | 2646.642103 | 1.000527 |  |
| GO:0010033\_response\_to\_organic\_substance | 216 | 0 | 0.000000 | 0.000000 | 2640 | 2634.557181 | 2640.75 | 2646.942819 | 1.000284 |  |
| GO:0043285\_biopolymer\_catabolic\_process | 129 | 0 | 0.000000 | 0.000000 | 2642 | 2635.632990 | 2641.81 | 2647.987010 | 0.999928 |  |
| GO:0051276\_chromosome\_organization | 129 | 0 | 0.000000 | 0.000000 | 2642 | 2635.632990 | 2641.81 | 2647.987010 | 0.999928 |  |
| GO:0000018\_regulation\_of\_DNA\_recombination | 21 | 0 | 0.000000 | 0.000000 | 2680 | 2674.643853 | 2679.37 | 2684.096147 | 0.999765 |  |
| GO:0001702\_gastrulation\_with\_mouth\_forming\_second | 21 | 0 | 0.000000 | 0.000000 | 2680 | 2674.643853 | 2679.37 | 2684.096147 | 0.999765 |  |
| GO:0001709\_cell\_fate\_determination | 21 | 0 | 0.000000 | 0.000000 | 2680 | 2674.643853 | 2679.37 | 2684.096147 | 0.999765 |  |
| GO:0001754\_eye\_photoreceptor\_cell\_differentiation | 21 | 0 | 0.000000 | 0.000000 | 2680 | 2674.643853 | 2679.37 | 2684.096147 | 0.999765 |  |
| GO:0001755\_neural\_crest\_cell\_migration | 21 | 0 | 0.000000 | 0.000000 | 2680 | 2674.643853 | 2679.37 | 2684.096147 | 0.999765 |  |
| GO:0002053\_positive\_regulation\_of\_mesenchymal\_cell\_proliferation | 21 | 0 | 0.000000 | 0.000000 | 2680 | 2674.643853 | 2679.37 | 2684.096147 | 0.999765 |  |
| GO:0002263\_cell\_activation\_during\_immune\_response | 21 | 0 | 0.000000 | 0.000000 | 2680 | 2674.643853 | 2679.37 | 2684.096147 | 0.999765 |  |
| GO:0002366\_leukocyte\_activation\_during\_immune\_response | 21 | 0 | 0.000000 | 0.000000 | 2680 | 2674.643853 | 2679.37 | 2684.096147 | 0.999765 |  |
| GO:0002456\_T\_cell\_mediated\_immunity | 21 | 0 | 0.000000 | 0.000000 | 2680 | 2674.643853 | 2679.37 | 2684.096147 | 0.999765 |  |
| GO:0006633\_fatty\_acid\_biosynthetic\_process | 21 | 0 | 0.000000 | 0.000000 | 2680 | 2674.643853 | 2679.37 | 2684.096147 | 0.999765 |  |
| GO:0006814\_sodium\_ion\_transport | 21 | 0 | 0.000000 | 0.000000 | 2680 | 2674.643853 | 2679.37 | 2684.096147 | 0.999765 |  |
| GO:0006944\_membrane\_fusion | 21 | 0 | 0.000000 | 0.000000 | 2680 | 2674.643853 | 2679.37 | 2684.096147 | 0.999765 |  |
| GO:0007189\_activation\_of\_adenylate\_cyclase\_activity\_by\_G-protein\_signaling\_pathway | 21 | 0 | 0.000000 | 0.000000 | 2680 | 2674.643853 | 2679.37 | 2684.096147 | 0.999765 |  |
| GO:0008154\_actin\_polymerization\_or\_depolymerization | 21 | 0 | 0.000000 | 0.000000 | 2680 | 2674.643853 | 2679.37 | 2684.096147 | 0.999765 |  |
| GO:0009583\_detection\_of\_light\_stimulus | 21 | 0 | 0.000000 | 0.000000 | 2680 | 2674.643853 | 2679.37 | 2684.096147 | 0.999765 |  |
| GO:0010552\_positive\_regulation\_of\_specific\_transcription\_from\_RNA\_polymerase\_II\_promoter | 21 | 0 | 0.000000 | 0.000000 | 2680 | 2674.643853 | 2679.37 | 2684.096147 | 0.999765 |  |
| GO:0010563\_negative\_regulation\_of\_phosphorus\_metabolic\_process | 21 | 0 | 0.000000 | 0.000000 | 2680 | 2674.643853 | 2679.37 | 2684.096147 | 0.999765 |  |
| GO:0010578\_regulation\_of\_adenylate\_cyclase\_activity\_involved\_in\_G-protein\_signaling | 21 | 0 | 0.000000 | 0.000000 | 2680 | 2674.643853 | 2679.37 | 2684.096147 | 0.999765 |  |
| GO:0010579\_positive\_regulation\_of\_adenylate\_cyclase\_activity\_by\_G-protein\_signaling\_pathway | 21 | 0 | 0.000000 | 0.000000 | 2680 | 2674.643853 | 2679.37 | 2684.096147 | 0.999765 |  |
| GO:0015844\_monoamine\_transport | 21 | 0 | 0.000000 | 0.000000 | 2680 | 2674.643853 | 2679.37 | 2684.096147 | 0.999765 |  |
| GO:0016202\_regulation\_of\_striated\_muscle\_tissue\_development | 21 | 0 | 0.000000 | 0.000000 | 2680 | 2674.643853 | 2679.37 | 2684.096147 | 0.999765 |  |
| GO:0017157\_regulation\_of\_exocytosis | 21 | 0 | 0.000000 | 0.000000 | 2680 | 2674.643853 | 2679.37 | 2684.096147 | 0.999765 |  |
| GO:0019827\_stem\_cell\_maintenance | 21 | 0 | 0.000000 | 0.000000 | 2680 | 2674.643853 | 2679.37 | 2684.096147 | 0.999765 |  |
| GO:0021532\_neural\_tube\_patterning | 21 | 0 | 0.000000 | 0.000000 | 2680 | 2674.643853 | 2679.37 | 2684.096147 | 0.999765 |  |
| GO:0030216\_keratinocyte\_differentiation | 21 | 0 | 0.000000 | 0.000000 | 2680 | 2674.643853 | 2679.37 | 2684.096147 | 0.999765 |  |
| GO:0030888\_regulation\_of\_B\_cell\_proliferation | 21 | 0 | 0.000000 | 0.000000 | 2680 | 2674.643853 | 2679.37 | 2684.096147 | 0.999765 |  |
| GO:0032147\_activation\_of\_protein\_kinase\_activity | 21 | 0 | 0.000000 | 0.000000 | 2680 | 2674.643853 | 2679.37 | 2684.096147 | 0.999765 |  |
| GO:0034330\_cell\_junction\_organization | 21 | 0 | 0.000000 | 0.000000 | 2680 | 2674.643853 | 2679.37 | 2684.096147 | 0.999765 |  |
| GO:0035136\_forelimb\_morphogenesis | 21 | 0 | 0.000000 | 0.000000 | 2680 | 2674.643853 | 2679.37 | 2684.096147 | 0.999765 |  |
| GO:0043279\_response\_to\_alkaloid | 21 | 0 | 0.000000 | 0.000000 | 2680 | 2674.643853 | 2679.37 | 2684.096147 | 0.999765 |  |
| GO:0045936\_negative\_regulation\_of\_phosphate\_metabolic\_process | 21 | 0 | 0.000000 | 0.000000 | 2680 | 2674.643853 | 2679.37 | 2684.096147 | 0.999765 |  |
| GO:0046519\_sphingoid\_metabolic\_process | 21 | 0 | 0.000000 | 0.000000 | 2680 | 2674.643853 | 2679.37 | 2684.096147 | 0.999765 |  |
| GO:0048538\_thymus\_development | 21 | 0 | 0.000000 | 0.000000 | 2680 | 2674.643853 | 2679.37 | 2684.096147 | 0.999765 |  |
| GO:0048634\_regulation\_of\_muscle\_development | 21 | 0 | 0.000000 | 0.000000 | 2680 | 2674.643853 | 2679.37 | 2684.096147 | 0.999765 |  |
| GO:0048675\_axon\_extension | 21 | 0 | 0.000000 | 0.000000 | 2680 | 2674.643853 | 2679.37 | 2684.096147 | 0.999765 |  |
| GO:0050777\_negative\_regulation\_of\_immune\_response | 21 | 0 | 0.000000 | 0.000000 | 2680 | 2674.643853 | 2679.37 | 2684.096147 | 0.999765 |  |
| GO:0051258\_protein\_polymerization | 21 | 0 | 0.000000 | 0.000000 | 2680 | 2674.643853 | 2679.37 | 2684.096147 | 0.999765 |  |
| GO:0051271\_negative\_regulation\_of\_cell\_motion | 21 | 0 | 0.000000 | 0.000000 | 2680 | 2674.643853 | 2679.37 | 2684.096147 | 0.999765 |  |
| GO:0003018\_vascular\_process\_in\_circulatory\_system | 31 | 0 | 0.000000 | 0.000000 | 2706 | 2704.096221 | 2707.99 | 2711.883779 | 1.000735 |  |
| GO:0006639\_acylglycerol\_metabolic\_process | 31 | 0 | 0.000000 | 0.000000 | 2706 | 2704.096221 | 2707.99 | 2711.883779 | 1.000735 |  |
| GO:0006665\_sphingolipid\_metabolic\_process | 31 | 0 | 0.000000 | 0.000000 | 2706 | 2704.096221 | 2707.99 | 2711.883779 | 1.000735 |  |
| GO:0006694\_steroid\_biosynthetic\_process | 31 | 0 | 0.000000 | 0.000000 | 2706 | 2704.096221 | 2707.99 | 2711.883779 | 1.000735 |  |
| GO:0006939\_smooth\_muscle\_contraction | 31 | 0 | 0.000000 | 0.000000 | 2706 | 2704.096221 | 2707.99 | 2711.883779 | 1.000735 |  |
| GO:0008645\_hexose\_transport | 31 | 0 | 0.000000 | 0.000000 | 2706 | 2704.096221 | 2707.99 | 2711.883779 | 1.000735 |  |
| GO:0009306\_protein\_secretion | 31 | 0 | 0.000000 | 0.000000 | 2706 | 2704.096221 | 2707.99 | 2711.883779 | 1.000735 |  |
| GO:0010562\_positive\_regulation\_of\_phosphorus\_metabolic\_process | 31 | 0 | 0.000000 | 0.000000 | 2706 | 2704.096221 | 2707.99 | 2711.883779 | 1.000735 |  |
| GO:0015749\_monosaccharide\_transport | 31 | 0 | 0.000000 | 0.000000 | 2706 | 2704.096221 | 2707.99 | 2711.883779 | 1.000735 |  |
| GO:0015758\_glucose\_transport | 31 | 0 | 0.000000 | 0.000000 | 2706 | 2704.096221 | 2707.99 | 2711.883779 | 1.000735 |  |
| GO:0016311\_dephosphorylation | 31 | 0 | 0.000000 | 0.000000 | 2706 | 2704.096221 | 2707.99 | 2711.883779 | 1.000735 |  |
| GO:0021954\_central\_nervous\_system\_neuron\_development | 31 | 0 | 0.000000 | 0.000000 | 2706 | 2704.096221 | 2707.99 | 2711.883779 | 1.000735 |  |
| GO:0035150\_regulation\_of\_tube\_size | 31 | 0 | 0.000000 | 0.000000 | 2706 | 2704.096221 | 2707.99 | 2711.883779 | 1.000735 |  |
| GO:0042157\_lipoprotein\_metabolic\_process | 31 | 0 | 0.000000 | 0.000000 | 2706 | 2704.096221 | 2707.99 | 2711.883779 | 1.000735 |  |
| GO:0042327\_positive\_regulation\_of\_phosphorylation | 31 | 0 | 0.000000 | 0.000000 | 2706 | 2704.096221 | 2707.99 | 2711.883779 | 1.000735 |  |
| GO:0043269\_regulation\_of\_ion\_transport | 31 | 0 | 0.000000 | 0.000000 | 2706 | 2704.096221 | 2707.99 | 2711.883779 | 1.000735 |  |
| GO:0045088\_regulation\_of\_innate\_immune\_response | 31 | 0 | 0.000000 | 0.000000 | 2706 | 2704.096221 | 2707.99 | 2711.883779 | 1.000735 |  |
| GO:0045937\_positive\_regulation\_of\_phosphate\_metabolic\_process | 31 | 0 | 0.000000 | 0.000000 | 2706 | 2704.096221 | 2707.99 | 2711.883779 | 1.000735 |  |
| GO:0046632\_alpha-beta\_T\_cell\_differentiation | 31 | 0 | 0.000000 | 0.000000 | 2706 | 2704.096221 | 2707.99 | 2711.883779 | 1.000735 |  |
| GO:0048167\_regulation\_of\_synaptic\_plasticity | 31 | 0 | 0.000000 | 0.000000 | 2706 | 2704.096221 | 2707.99 | 2711.883779 | 1.000735 |  |
| GO:0048562\_embryonic\_organ\_morphogenesis | 31 | 0 | 0.000000 | 0.000000 | 2706 | 2704.096221 | 2707.99 | 2711.883779 | 1.000735 |  |
| GO:0050868\_negative\_regulation\_of\_T\_cell\_activation | 31 | 0 | 0.000000 | 0.000000 | 2706 | 2704.096221 | 2707.99 | 2711.883779 | 1.000735 |  |
| GO:0050880\_regulation\_of\_blood\_vessel\_size | 31 | 0 | 0.000000 | 0.000000 | 2706 | 2704.096221 | 2707.99 | 2711.883779 | 1.000735 |  |
| GO:0051899\_membrane\_depolarization | 31 | 0 | 0.000000 | 0.000000 | 2706 | 2704.096221 | 2707.99 | 2711.883779 | 1.000735 |  |
| GO:0055088\_lipid\_homeostasis | 31 | 0 | 0.000000 | 0.000000 | 2706 | 2704.096221 | 2707.99 | 2711.883779 | 1.000735 |  |
| GO:0060512\_prostate\_gland\_morphogenesis | 31 | 0 | 0.000000 | 0.000000 | 2706 | 2704.096221 | 2707.99 | 2711.883779 | 1.000735 |  |
| GO:0001892\_embryonic\_placenta\_development | 57 | 0 | 0.000000 | 0.000000 | 2716 | 2713.877679 | 2717.5 | 2721.122321 | 1.000552 |  |
| GO:0008344\_adult\_locomotory\_behavior | 57 | 0 | 0.000000 | 0.000000 | 2716 | 2713.877679 | 2717.5 | 2721.122321 | 1.000552 |  |
| GO:0009953\_dorsal\_ventral\_pattern\_formation | 57 | 0 | 0.000000 | 0.000000 | 2716 | 2713.877679 | 2717.5 | 2721.122321 | 1.000552 |  |
| GO:0018108\_peptidyl-tyrosine\_phosphorylation | 57 | 0 | 0.000000 | 0.000000 | 2716 | 2713.877679 | 2717.5 | 2721.122321 | 1.000552 |  |
| GO:0018212\_peptidyl-tyrosine\_modification | 57 | 0 | 0.000000 | 0.000000 | 2716 | 2713.877679 | 2717.5 | 2721.122321 | 1.000552 |  |
| GO:0033365\_protein\_localization\_in\_organelle | 57 | 0 | 0.000000 | 0.000000 | 2716 | 2713.877679 | 2717.5 | 2721.122321 | 1.000552 |  |
| GO:0042472\_inner\_ear\_morphogenesis | 57 | 0 | 0.000000 | 0.000000 | 2716 | 2713.877679 | 2717.5 | 2721.122321 | 1.000552 |  |
| GO:0043523\_regulation\_of\_neuron\_apoptosis | 57 | 0 | 0.000000 | 0.000000 | 2716 | 2713.877679 | 2717.5 | 2721.122321 | 1.000552 |  |
| GO:0045444\_fat\_cell\_differentiation | 57 | 0 | 0.000000 | 0.000000 | 2716 | 2713.877679 | 2717.5 | 2721.122321 | 1.000552 |  |
| GO:0050878\_regulation\_of\_body\_fluid\_levels | 57 | 0 | 0.000000 | 0.000000 | 2716 | 2713.877679 | 2717.5 | 2721.122321 | 1.000552 |  |
| GO:0000187\_activation\_of\_MAPK\_activity | 30 | 0 | 0.000000 | 0.000000 | 2739 | 2736.186168 | 2738.75 | 2741.313832 | 0.999909 |  |
| GO:0002260\_lymphocyte\_homeostasis | 30 | 0 | 0.000000 | 0.000000 | 2739 | 2736.186168 | 2738.75 | 2741.313832 | 0.999909 |  |
| GO:0007219\_Notch\_signaling\_pathway | 30 | 0 | 0.000000 | 0.000000 | 2739 | 2736.186168 | 2738.75 | 2741.313832 | 0.999909 |  |
| GO:0007435\_salivary\_gland\_morphogenesis | 30 | 0 | 0.000000 | 0.000000 | 2739 | 2736.186168 | 2738.75 | 2741.313832 | 0.999909 |  |
| GO:0009266\_response\_to\_temperature\_stimulus | 30 | 0 | 0.000000 | 0.000000 | 2739 | 2736.186168 | 2738.75 | 2741.313832 | 0.999909 |  |
| GO:0014032\_neural\_crest\_cell\_development | 30 | 0 | 0.000000 | 0.000000 | 2739 | 2736.186168 | 2738.75 | 2741.313832 | 0.999909 |  |
| GO:0014033\_neural\_crest\_cell\_differentiation | 30 | 0 | 0.000000 | 0.000000 | 2739 | 2736.186168 | 2738.75 | 2741.313832 | 0.999909 |  |
| GO:0016445\_somatic\_diversification\_of\_immunoglobulins | 30 | 0 | 0.000000 | 0.000000 | 2739 | 2736.186168 | 2738.75 | 2741.313832 | 0.999909 |  |
| GO:0022411\_cellular\_component\_disassembly | 30 | 0 | 0.000000 | 0.000000 | 2739 | 2736.186168 | 2738.75 | 2741.313832 | 0.999909 |  |
| GO:0030522\_intracellular\_receptor-mediated\_signaling\_pathway | 30 | 0 | 0.000000 | 0.000000 | 2739 | 2736.186168 | 2738.75 | 2741.313832 | 0.999909 |  |
| GO:0031281\_positive\_regulation\_of\_cyclase\_activity | 30 | 0 | 0.000000 | 0.000000 | 2739 | 2736.186168 | 2738.75 | 2741.313832 | 0.999909 |  |
| GO:0031668\_cellular\_response\_to\_extracellular\_stimulus | 30 | 0 | 0.000000 | 0.000000 | 2739 | 2736.186168 | 2738.75 | 2741.313832 | 0.999909 |  |
| GO:0032102\_negative\_regulation\_of\_response\_to\_external\_stimulus | 30 | 0 | 0.000000 | 0.000000 | 2739 | 2736.186168 | 2738.75 | 2741.313832 | 0.999909 |  |
| GO:0033500\_carbohydrate\_homeostasis | 30 | 0 | 0.000000 | 0.000000 | 2739 | 2736.186168 | 2738.75 | 2741.313832 | 0.999909 |  |
| GO:0035265\_organ\_growth | 30 | 0 | 0.000000 | 0.000000 | 2739 | 2736.186168 | 2738.75 | 2741.313832 | 0.999909 |  |
| GO:0042552\_myelination | 30 | 0 | 0.000000 | 0.000000 | 2739 | 2736.186168 | 2738.75 | 2741.313832 | 0.999909 |  |
| GO:0042593\_glucose\_homeostasis | 30 | 0 | 0.000000 | 0.000000 | 2739 | 2736.186168 | 2738.75 | 2741.313832 | 0.999909 |  |
| GO:0045762\_positive\_regulation\_of\_adenylate\_cyclase\_activity | 30 | 0 | 0.000000 | 0.000000 | 2739 | 2736.186168 | 2738.75 | 2741.313832 | 0.999909 |  |
| GO:0048565\_gut\_development | 30 | 0 | 0.000000 | 0.000000 | 2739 | 2736.186168 | 2738.75 | 2741.313832 | 0.999909 |  |
| GO:0051146\_striated\_muscle\_cell\_differentiation | 30 | 0 | 0.000000 | 0.000000 | 2739 | 2736.186168 | 2738.75 | 2741.313832 | 0.999909 |  |
| GO:0051349\_positive\_regulation\_of\_lyase\_activity | 30 | 0 | 0.000000 | 0.000000 | 2739 | 2736.186168 | 2738.75 | 2741.313832 | 0.999909 |  |
| GO:0060021\_palate\_development | 30 | 0 | 0.000000 | 0.000000 | 2739 | 2736.186168 | 2738.75 | 2741.313832 | 0.999909 |  |
| GO:0060740\_prostate\_gland\_epithelium\_morphogenesis | 30 | 0 | 0.000000 | 0.000000 | 2739 | 2736.186168 | 2738.75 | 2741.313832 | 0.999909 |  |
| GO:0001763\_morphogenesis\_of\_a\_branching\_structure | 125 | 0 | 0.000000 | 0.000000 | 2740 | 2737.611359 | 2740.05 | 2742.488641 | 1.000018 |  |
| GO:0030334\_regulation\_of\_cell\_migration | 59 | 0 | 0.000000 | 0.000000 | 2743 | 2741.765986 | 2743.97 | 2746.174014 | 1.000354 |  |
| GO:0035270\_endocrine\_system\_development | 59 | 0 | 0.000000 | 0.000000 | 2743 | 2741.765986 | 2743.97 | 2746.174014 | 1.000354 |  |
| GO:0050870\_positive\_regulation\_of\_T\_cell\_activation | 59 | 0 | 0.000000 | 0.000000 | 2743 | 2741.765986 | 2743.97 | 2746.174014 | 1.000354 |  |
| GO:0009308\_amine\_metabolic\_process | 124 | 0 | 0.000000 | 0.000000 | 2745 | 2743.111625 | 2745.13 | 2747.148375 | 1.000047 |  |
| GO:0030098\_lymphocyte\_differentiation | 124 | 0 | 0.000000 | 0.000000 | 2745 | 2743.111625 | 2745.13 | 2747.148375 | 1.000047 |  |
| GO:0003012\_muscle\_system\_process | 76 | 0 | 0.000000 | 0.000000 | 2748 | 2746.859024 | 2748.36 | 2749.860976 | 1.000131 |  |
| GO:0006508\_proteolysis | 76 | 0 | 0.000000 | 0.000000 | 2748 | 2746.859024 | 2748.36 | 2749.860976 | 1.000131 |  |
| GO:0009725\_response\_to\_hormone\_stimulus | 76 | 0 | 0.000000 | 0.000000 | 2748 | 2746.859024 | 2748.36 | 2749.860976 | 1.000131 |  |
| GO:0006310\_DNA\_recombination | 55 | 0 | 0.000000 | 0.000000 | 2755 | 2755.000000 | 2755.0 | 2755.000000 | 1.000000 |  |
| GO:0007126\_meiosis | 55 | 0 | 0.000000 | 0.000000 | 2755 | 2755.000000 | 2755.0 | 2755.000000 | 1.000000 |  |
| GO:0007605\_sensory\_perception\_of\_sound | 55 | 0 | 0.000000 | 0.000000 | 2755 | 2755.000000 | 2755.0 | 2755.000000 | 1.000000 |  |
| GO:0043434\_response\_to\_peptide\_hormone\_stimulus | 55 | 0 | 0.000000 | 0.000000 | 2755 | 2755.000000 | 2755.0 | 2755.000000 | 1.000000 |  |
| GO:0048568\_embryonic\_organ\_development | 55 | 0 | 0.000000 | 0.000000 | 2755 | 2755.000000 | 2755.0 | 2755.000000 | 1.000000 |  |
| GO:0048704\_embryonic\_skeletal\_system\_morphogenesis | 55 | 0 | 0.000000 | 0.000000 | 2755 | 2755.000000 | 2755.0 | 2755.000000 | 1.000000 |  |
| GO:0051327\_M\_phase\_of\_meiotic\_cell\_cycle | 55 | 0 | 0.000000 | 0.000000 | 2755 | 2755.000000 | 2755.0 | 2755.000000 | 1.000000 |  |
