# Supplementary material for: RedundancyMiner: De-replication of redundant GO categories in microarray and proteomics analysis
Source: BMC Bioinformatics. 2011 Feb 10;12:52. doi: 10.1186/1471-2105-12-52 (PMC3223614; doi:10.1186/1471-2105-12-52)
Supplement: Additional file 8 — Retinal development HTGM download. compressed package of the results of running HTGM on the retinal development genes list. [file 1471-2105-12-52-S8.ZIP › SCENARIO_2_MODIFIED/total.txt.total.txt.dir/Exp1_BestClusterMap_LEIGS_KM_24.csv.join.6.txt.dir/Exp1_BestClusterMap_LEIGS_KM_24.csv.join.6.txt.change.gce.html]

Gene Category Report for Exp1\_BestClusterMap\_LEIGS\_KM\_24.csv.join.6.txt

# Gene Category Report for Exp1\_BestClusterMap\_LEIGS\_KM\_24.csv.join.6.txt

| HYPERLINKED GO CATEGORY | HYPERLINKED GENE NAME | TOTAL GENES | CHANGED GENES | ENRICHMENT | LOG10(p) | CUMULATIVE NUMBER OF CATEGORIES | CUMULATIVE RANDOMS MEAN | FALSE DISCOVERY RATE |  |
| --- | --- | --- | --- | --- | --- | --- | --- | --- | --- |
| GO:0006670\_sphingosine\_metabolic\_process | SGPP1 | 3 | 2 |  |  |  |  |  |  |
| GO:0006670\_sphingosine\_metabolic\_process | PPAP2A | 3 | 2 |  |  |  |  |  |  |
| GO:0006665\_sphingolipid\_metabolic\_process | SGPP1 | 31 | 4 | 8.738140 | -2.994186 | 1 | 0.67 | 0.670000 |  |
| GO:0006665\_sphingolipid\_metabolic\_process | HEXB | 31 | 4 | 8.738140 | -2.994186 | 1 | 0.67 | 0.670000 |  |
| GO:0006665\_sphingolipid\_metabolic\_process | SGMS1 | 31 | 4 | 8.738140 | -2.994186 | 1 | 0.67 | 0.670000 |  |
| GO:0006665\_sphingolipid\_metabolic\_process | PPAP2A | 31 | 4 | 8.738140 | -2.994186 | 1 | 0.67 | 0.670000 |  |
| GO:0007399\_nervous\_system\_development | DCC | 621 | 19 | 2.071966 | -2.928808 | 2 | 0.77 | 0.385000 |  |
| GO:0007399\_nervous\_system\_development | IRX5 | 621 | 19 | 2.071966 | -2.928808 | 2 | 0.77 | 0.385000 |  |
| GO:0007399\_nervous\_system\_development | MYO6 | 621 | 19 | 2.071966 | -2.928808 | 2 | 0.77 | 0.385000 |  |
| GO:0007399\_nervous\_system\_development | TBX3 | 621 | 19 | 2.071966 | -2.928808 | 2 | 0.77 | 0.385000 |  |
| GO:0007399\_nervous\_system\_development | PLXNA2 | 621 | 19 | 2.071966 | -2.928808 | 2 | 0.77 | 0.385000 |  |
| GO:0007399\_nervous\_system\_development | HEXB | 621 | 19 | 2.071966 | -2.928808 | 2 | 0.77 | 0.385000 |  |
| GO:0007399\_nervous\_system\_development | SOX5 | 621 | 19 | 2.071966 | -2.928808 | 2 | 0.77 | 0.385000 |  |
| GO:0007399\_nervous\_system\_development | NR2E1 | 621 | 19 | 2.071966 | -2.928808 | 2 | 0.77 | 0.385000 |  |
| GO:0007399\_nervous\_system\_development | TCF7L2 | 621 | 19 | 2.071966 | -2.928808 | 2 | 0.77 | 0.385000 |  |
| GO:0007399\_nervous\_system\_development | NKX6-1 | 621 | 19 | 2.071966 | -2.928808 | 2 | 0.77 | 0.385000 |  |
| GO:0007399\_nervous\_system\_development | ALCAM | 621 | 19 | 2.071966 | -2.928808 | 2 | 0.77 | 0.385000 |  |
| GO:0007399\_nervous\_system\_development | ASCL1 | 621 | 19 | 2.071966 | -2.928808 | 2 | 0.77 | 0.385000 |  |
| GO:0007399\_nervous\_system\_development | MSX1 | 621 | 19 | 2.071966 | -2.928808 | 2 | 0.77 | 0.385000 |  |
| GO:0007399\_nervous\_system\_development | ETV1 | 621 | 19 | 2.071966 | -2.928808 | 2 | 0.77 | 0.385000 |  |
| GO:0007399\_nervous\_system\_development | NEUROD1 | 621 | 19 | 2.071966 | -2.928808 | 2 | 0.77 | 0.385000 |  |
| GO:0007399\_nervous\_system\_development | VPS13A | 621 | 19 | 2.071966 | -2.928808 | 2 | 0.77 | 0.385000 |  |
| GO:0007399\_nervous\_system\_development | B3GNT2 | 621 | 19 | 2.071966 | -2.928808 | 2 | 0.77 | 0.385000 |  |
| GO:0007399\_nervous\_system\_development | HDAC9 | 621 | 19 | 2.071966 | -2.928808 | 2 | 0.77 | 0.385000 |  |
| GO:0007399\_nervous\_system\_development | BOC | 621 | 19 | 2.071966 | -2.928808 | 2 | 0.77 | 0.385000 |  |
| GO:0060164\_regulation\_of\_timing\_of\_neuron\_differentiation | ASCL1 | 4 | 2 |  |  |  |  |  |  |
| GO:0060164\_regulation\_of\_timing\_of\_neuron\_differentiation | SOX5 | 4 | 2 |  |  |  |  |  |  |
| GO:0006643\_membrane\_lipid\_metabolic\_process | SGPP1 | 33 | 4 | 8.208556 | -2.889715 | 3 | 0.93 | 0.310000 |  |
| GO:0006643\_membrane\_lipid\_metabolic\_process | HEXB | 33 | 4 | 8.208556 | -2.889715 | 3 | 0.93 | 0.310000 |  |
| GO:0006643\_membrane\_lipid\_metabolic\_process | SGMS1 | 33 | 4 | 8.208556 | -2.889715 | 3 | 0.93 | 0.310000 |  |
| GO:0006643\_membrane\_lipid\_metabolic\_process | PPAP2A | 33 | 4 | 8.208556 | -2.889715 | 3 | 0.93 | 0.310000 |  |
| GO:0032400\_melanosome\_localization | MLPH | 5 | 2 | 27.088235 | -2.680282 | 5 | 2.11 | 0.422000 |  |
| GO:0032400\_melanosome\_localization | RAB27A | 5 | 2 | 27.088235 | -2.680282 | 5 | 2.11 | 0.422000 |  |
| GO:0045661\_regulation\_of\_myoblast\_differentiation | MBNL3 | 5 | 2 | 27.088235 | -2.680282 | 5 | 2.11 | 0.422000 |  |
| GO:0045661\_regulation\_of\_myoblast\_differentiation | BOC | 5 | 2 | 27.088235 | -2.680282 | 5 | 2.11 | 0.422000 |  |
| GO:0021953\_central\_nervous\_system\_neuron\_differentiation | DCC | 39 | 4 | 6.945701 | -2.615381 | 6 | 2.32 | 0.386667 |  |
| GO:0021953\_central\_nervous\_system\_neuron\_differentiation | ASCL1 | 39 | 4 | 6.945701 | -2.615381 | 6 | 2.32 | 0.386667 |  |
| GO:0021953\_central\_nervous\_system\_neuron\_differentiation | SOX5 | 39 | 4 | 6.945701 | -2.615381 | 6 | 2.32 | 0.386667 |  |
| GO:0021953\_central\_nervous\_system\_neuron\_differentiation | NR2E1 | 39 | 4 | 6.945701 | -2.615381 | 6 | 2.32 | 0.386667 |  |
| GO:0051875\_pigment\_granule\_localization | MLPH | 6 | 2 | 22.573529 | -2.508349 | 7 | 3.52 | 0.502857 |  |
| GO:0051875\_pigment\_granule\_localization | RAB27A | 6 | 2 | 22.573529 | -2.508349 | 7 | 3.52 | 0.502857 |  |
| GO:0016202\_regulation\_of\_striated\_muscle\_tissue\_development | MBNL3 | 21 | 3 | 9.674370 | -2.470286 | 10 | 3.78 | 0.378000 |  |
| GO:0016202\_regulation\_of\_striated\_muscle\_tissue\_development | HDAC9 | 21 | 3 | 9.674370 | -2.470286 | 10 | 3.78 | 0.378000 |  |
| GO:0016202\_regulation\_of\_striated\_muscle\_tissue\_development | BOC | 21 | 3 | 9.674370 | -2.470286 | 10 | 3.78 | 0.378000 |  |
| GO:0046519\_sphingoid\_metabolic\_process | SGPP1 | 21 | 3 | 9.674370 | -2.470286 | 10 | 3.78 | 0.378000 |  |
| GO:0046519\_sphingoid\_metabolic\_process | HEXB | 21 | 3 | 9.674370 | -2.470286 | 10 | 3.78 | 0.378000 |  |
| GO:0046519\_sphingoid\_metabolic\_process | PPAP2A | 21 | 3 | 9.674370 | -2.470286 | 10 | 3.78 | 0.378000 |  |
| GO:0048634\_regulation\_of\_muscle\_development | MBNL3 | 21 | 3 | 9.674370 | -2.470286 | 10 | 3.78 | 0.378000 |  |
| GO:0048634\_regulation\_of\_muscle\_development | HDAC9 | 21 | 3 | 9.674370 | -2.470286 | 10 | 3.78 | 0.378000 |  |
| GO:0048634\_regulation\_of\_muscle\_development | BOC | 21 | 3 | 9.674370 | -2.470286 | 10 | 3.78 | 0.378000 |  |
| GO:0051705\_behavioral\_interaction\_between\_organisms | HEXB | 23 | 3 | 8.833120 | -2.355073 | 11 | 5.43 | 0.493636 |  |
| GO:0051705\_behavioral\_interaction\_between\_organisms | VPS13A | 23 | 3 | 8.833120 | -2.355073 | 11 | 5.43 | 0.493636 |  |
| GO:0051705\_behavioral\_interaction\_between\_organisms | NR2E1 | 23 | 3 | 8.833120 | -2.355073 | 11 | 5.43 | 0.493636 |  |
| GO:0040034\_regulation\_of\_development\_\_heterochronic | ASCL1 | 8 | 2 | 16.930147 | -2.245586 | 14 | 6.88 | 0.491429 |  |
| GO:0040034\_regulation\_of\_development\_\_heterochronic | SOX5 | 8 | 2 | 16.930147 | -2.245586 | 14 | 6.88 | 0.491429 |  |
| GO:0048505\_regulation\_of\_timing\_of\_cell\_differentiation | ASCL1 | 8 | 2 | 16.930147 | -2.245586 | 14 | 6.88 | 0.491429 |  |
| GO:0048505\_regulation\_of\_timing\_of\_cell\_differentiation | SOX5 | 8 | 2 | 16.930147 | -2.245586 | 14 | 6.88 | 0.491429 |  |
| GO:0048742\_regulation\_of\_skeletal\_muscle\_fiber\_development | MBNL3 | 8 | 2 | 16.930147 | -2.245586 | 14 | 6.88 | 0.491429 |  |
| GO:0048742\_regulation\_of\_skeletal\_muscle\_fiber\_development | BOC | 8 | 2 | 16.930147 | -2.245586 | 14 | 6.88 | 0.491429 |  |
| GO:0032502\_developmental\_process | DCC | 2060 | 41 | 1.347837 | -2.171148 | 15 | 7.4 | 0.493333 |  |
| GO:0032502\_developmental\_process | FGF18 | 2060 | 41 | 1.347837 | -2.171148 | 15 | 7.4 | 0.493333 |  |
| GO:0032502\_developmental\_process | IRX5 | 2060 | 41 | 1.347837 | -2.171148 | 15 | 7.4 | 0.493333 |  |
| GO:0032502\_developmental\_process | BBS7 | 2060 | 41 | 1.347837 | -2.171148 | 15 | 7.4 | 0.493333 |  |
| GO:0032502\_developmental\_process | MLPH | 2060 | 41 | 1.347837 | -2.171148 | 15 | 7.4 | 0.493333 |  |
| GO:0032502\_developmental\_process | PLXNA2 | 2060 | 41 | 1.347837 | -2.171148 | 15 | 7.4 | 0.493333 |  |
| GO:0032502\_developmental\_process | TBX20 | 2060 | 41 | 1.347837 | -2.171148 | 15 | 7.4 | 0.493333 |  |
| GO:0032502\_developmental\_process | HEXB | 2060 | 41 | 1.347837 | -2.171148 | 15 | 7.4 | 0.493333 |  |
| GO:0032502\_developmental\_process | SOX5 | 2060 | 41 | 1.347837 | -2.171148 | 15 | 7.4 | 0.493333 |  |
| GO:0032502\_developmental\_process | SGMS1 | 2060 | 41 | 1.347837 | -2.171148 | 15 | 7.4 | 0.493333 |  |
| GO:0032502\_developmental\_process | NR3C1 | 2060 | 41 | 1.347837 | -2.171148 | 15 | 7.4 | 0.493333 |  |
| GO:0032502\_developmental\_process | TCF7L2 | 2060 | 41 | 1.347837 | -2.171148 | 15 | 7.4 | 0.493333 |  |
| GO:0032502\_developmental\_process | NR2E1 | 2060 | 41 | 1.347837 | -2.171148 | 15 | 7.4 | 0.493333 |  |
| GO:0032502\_developmental\_process | SERINC3 | 2060 | 41 | 1.347837 | -2.171148 | 15 | 7.4 | 0.493333 |  |
| GO:0032502\_developmental\_process | ALCAM | 2060 | 41 | 1.347837 | -2.171148 | 15 | 7.4 | 0.493333 |  |
| GO:0032502\_developmental\_process | AQP11 | 2060 | 41 | 1.347837 | -2.171148 | 15 | 7.4 | 0.493333 |  |
| GO:0032502\_developmental\_process | DMD | 2060 | 41 | 1.347837 | -2.171148 | 15 | 7.4 | 0.493333 |  |
| GO:0032502\_developmental\_process | ETV1 | 2060 | 41 | 1.347837 | -2.171148 | 15 | 7.4 | 0.493333 |  |
| GO:0032502\_developmental\_process | VPS13A | 2060 | 41 | 1.347837 | -2.171148 | 15 | 7.4 | 0.493333 |  |
| GO:0032502\_developmental\_process | MBNL3 | 2060 | 41 | 1.347837 | -2.171148 | 15 | 7.4 | 0.493333 |  |
| GO:0032502\_developmental\_process | B3GNT2 | 2060 | 41 | 1.347837 | -2.171148 | 15 | 7.4 | 0.493333 |  |
| GO:0032502\_developmental\_process | THBS1 | 2060 | 41 | 1.347837 | -2.171148 | 15 | 7.4 | 0.493333 |  |
| GO:0032502\_developmental\_process | BOC | 2060 | 41 | 1.347837 | -2.171148 | 15 | 7.4 | 0.493333 |  |
| GO:0032502\_developmental\_process | CD27 | 2060 | 41 | 1.347837 | -2.171148 | 15 | 7.4 | 0.493333 |  |
| GO:0032502\_developmental\_process | RAB27A | 2060 | 41 | 1.347837 | -2.171148 | 15 | 7.4 | 0.493333 |  |
| GO:0032502\_developmental\_process | PTPRC | 2060 | 41 | 1.347837 | -2.171148 | 15 | 7.4 | 0.493333 |  |
| GO:0032502\_developmental\_process | MYO6 | 2060 | 41 | 1.347837 | -2.171148 | 15 | 7.4 | 0.493333 |  |
| GO:0032502\_developmental\_process | TBX3 | 2060 | 41 | 1.347837 | -2.171148 | 15 | 7.4 | 0.493333 |  |
| GO:0032502\_developmental\_process | TCFAP2B | 2060 | 41 | 1.347837 | -2.171148 | 15 | 7.4 | 0.493333 |  |
| GO:0032502\_developmental\_process | ARID5B | 2060 | 41 | 1.347837 | -2.171148 | 15 | 7.4 | 0.493333 |  |
| GO:0032502\_developmental\_process | ZFX | 2060 | 41 | 1.347837 | -2.171148 | 15 | 7.4 | 0.493333 |  |
| GO:0032502\_developmental\_process | PCDH8 | 2060 | 41 | 1.347837 | -2.171148 | 15 | 7.4 | 0.493333 |  |
| GO:0032502\_developmental\_process | ITGA4 | 2060 | 41 | 1.347837 | -2.171148 | 15 | 7.4 | 0.493333 |  |
| GO:0032502\_developmental\_process | NKX6-1 | 2060 | 41 | 1.347837 | -2.171148 | 15 | 7.4 | 0.493333 |  |
| GO:0032502\_developmental\_process | ASCL1 | 2060 | 41 | 1.347837 | -2.171148 | 15 | 7.4 | 0.493333 |  |
| GO:0032502\_developmental\_process | AFP | 2060 | 41 | 1.347837 | -2.171148 | 15 | 7.4 | 0.493333 |  |
| GO:0032502\_developmental\_process | MSX1 | 2060 | 41 | 1.347837 | -2.171148 | 15 | 7.4 | 0.493333 |  |
| GO:0032502\_developmental\_process | NEUROD1 | 2060 | 41 | 1.347837 | -2.171148 | 15 | 7.4 | 0.493333 |  |
| GO:0032502\_developmental\_process | JAK2 | 2060 | 41 | 1.347837 | -2.171148 | 15 | 7.4 | 0.493333 |  |
| GO:0032502\_developmental\_process | HDAC9 | 2060 | 41 | 1.347837 | -2.171148 | 15 | 7.4 | 0.493333 |  |
| GO:0032502\_developmental\_process | CAMK1D | 2060 | 41 | 1.347837 | -2.171148 | 15 | 7.4 | 0.493333 |  |
| GO:0007517\_muscle\_organ\_development | MSX1 | 153 | 7 | 3.098328 | -2.160996 | 16 | 7.52 | 0.470000 |  |
| GO:0007517\_muscle\_organ\_development | DMD | 153 | 7 | 3.098328 | -2.160996 | 16 | 7.52 | 0.470000 |  |
| GO:0007517\_muscle\_organ\_development | ARID5B | 153 | 7 | 3.098328 | -2.160996 | 16 | 7.52 | 0.470000 |  |
| GO:0007517\_muscle\_organ\_development | ETV1 | 153 | 7 | 3.098328 | -2.160996 | 16 | 7.52 | 0.470000 |  |
| GO:0007517\_muscle\_organ\_development | MBNL3 | 153 | 7 | 3.098328 | -2.160996 | 16 | 7.52 | 0.470000 |  |
| GO:0007517\_muscle\_organ\_development | HDAC9 | 153 | 7 | 3.098328 | -2.160996 | 16 | 7.52 | 0.470000 |  |
| GO:0007517\_muscle\_organ\_development | BOC | 153 | 7 | 3.098328 | -2.160996 | 16 | 7.52 | 0.470000 |  |
| GO:0048731\_system\_development | DCC | 1609 | 34 | 1.431013 | -2.144964 | 17 | 7.72 | 0.454118 |  |
| GO:0048731\_system\_development | FGF18 | 1609 | 34 | 1.431013 | -2.144964 | 17 | 7.72 | 0.454118 |  |
| GO:0048731\_system\_development | IRX5 | 1609 | 34 | 1.431013 | -2.144964 | 17 | 7.72 | 0.454118 |  |
| GO:0048731\_system\_development | PLXNA2 | 1609 | 34 | 1.431013 | -2.144964 | 17 | 7.72 | 0.454118 |  |
| GO:0048731\_system\_development | TBX20 | 1609 | 34 | 1.431013 | -2.144964 | 17 | 7.72 | 0.454118 |  |
| GO:0048731\_system\_development | HEXB | 1609 | 34 | 1.431013 | -2.144964 | 17 | 7.72 | 0.454118 |  |
| GO:0048731\_system\_development | SOX5 | 1609 | 34 | 1.431013 | -2.144964 | 17 | 7.72 | 0.454118 |  |
| GO:0048731\_system\_development | NR3C1 | 1609 | 34 | 1.431013 | -2.144964 | 17 | 7.72 | 0.454118 |  |
| GO:0048731\_system\_development | NR2E1 | 1609 | 34 | 1.431013 | -2.144964 | 17 | 7.72 | 0.454118 |  |
| GO:0048731\_system\_development | TCF7L2 | 1609 | 34 | 1.431013 | -2.144964 | 17 | 7.72 | 0.454118 |  |
| GO:0048731\_system\_development | ALCAM | 1609 | 34 | 1.431013 | -2.144964 | 17 | 7.72 | 0.454118 |  |
| GO:0048731\_system\_development | AQP11 | 1609 | 34 | 1.431013 | -2.144964 | 17 | 7.72 | 0.454118 |  |
| GO:0048731\_system\_development | DMD | 1609 | 34 | 1.431013 | -2.144964 | 17 | 7.72 | 0.454118 |  |
| GO:0048731\_system\_development | ETV1 | 1609 | 34 | 1.431013 | -2.144964 | 17 | 7.72 | 0.454118 |  |
| GO:0048731\_system\_development | VPS13A | 1609 | 34 | 1.431013 | -2.144964 | 17 | 7.72 | 0.454118 |  |
| GO:0048731\_system\_development | MBNL3 | 1609 | 34 | 1.431013 | -2.144964 | 17 | 7.72 | 0.454118 |  |
| GO:0048731\_system\_development | B3GNT2 | 1609 | 34 | 1.431013 | -2.144964 | 17 | 7.72 | 0.454118 |  |
| GO:0048731\_system\_development | THBS1 | 1609 | 34 | 1.431013 | -2.144964 | 17 | 7.72 | 0.454118 |  |
| GO:0048731\_system\_development | BOC | 1609 | 34 | 1.431013 | -2.144964 | 17 | 7.72 | 0.454118 |  |
| GO:0048731\_system\_development | PTPRC | 1609 | 34 | 1.431013 | -2.144964 | 17 | 7.72 | 0.454118 |  |
| GO:0048731\_system\_development | MYO6 | 1609 | 34 | 1.431013 | -2.144964 | 17 | 7.72 | 0.454118 |  |
| GO:0048731\_system\_development | TBX3 | 1609 | 34 | 1.431013 | -2.144964 | 17 | 7.72 | 0.454118 |  |
| GO:0048731\_system\_development | TCFAP2B | 1609 | 34 | 1.431013 | -2.144964 | 17 | 7.72 | 0.454118 |  |
| GO:0048731\_system\_development | ARID5B | 1609 | 34 | 1.431013 | -2.144964 | 17 | 7.72 | 0.454118 |  |
| GO:0048731\_system\_development | ZFX | 1609 | 34 | 1.431013 | -2.144964 | 17 | 7.72 | 0.454118 |  |
| GO:0048731\_system\_development | PCDH8 | 1609 | 34 | 1.431013 | -2.144964 | 17 | 7.72 | 0.454118 |  |
| GO:0048731\_system\_development | ITGA4 | 1609 | 34 | 1.431013 | -2.144964 | 17 | 7.72 | 0.454118 |  |
| GO:0048731\_system\_development | NKX6-1 | 1609 | 34 | 1.431013 | -2.144964 | 17 | 7.72 | 0.454118 |  |
| GO:0048731\_system\_development | AFP | 1609 | 34 | 1.431013 | -2.144964 | 17 | 7.72 | 0.454118 |  |
| GO:0048731\_system\_development | ASCL1 | 1609 | 34 | 1.431013 | -2.144964 | 17 | 7.72 | 0.454118 |  |
| GO:0048731\_system\_development | MSX1 | 1609 | 34 | 1.431013 | -2.144964 | 17 | 7.72 | 0.454118 |  |
| GO:0048731\_system\_development | NEUROD1 | 1609 | 34 | 1.431013 | -2.144964 | 17 | 7.72 | 0.454118 |  |
| GO:0048731\_system\_development | JAK2 | 1609 | 34 | 1.431013 | -2.144964 | 17 | 7.72 | 0.454118 |  |
| GO:0048731\_system\_development | HDAC9 | 1609 | 34 | 1.431013 | -2.144964 | 17 | 7.72 | 0.454118 |  |
| GO:0060284\_regulation\_of\_cell\_development | ASCL1 | 122 | 6 | 3.330521 | -2.057512 | 18 | 9.54 | 0.530000 |  |
| GO:0060284\_regulation\_of\_cell\_development | SOX5 | 122 | 6 | 3.330521 | -2.057512 | 18 | 9.54 | 0.530000 |  |
| GO:0060284\_regulation\_of\_cell\_development | MBNL3 | 122 | 6 | 3.330521 | -2.057512 | 18 | 9.54 | 0.530000 |  |
| GO:0060284\_regulation\_of\_cell\_development | NR2E1 | 122 | 6 | 3.330521 | -2.057512 | 18 | 9.54 | 0.530000 |  |
| GO:0060284\_regulation\_of\_cell\_development | BOC | 122 | 6 | 3.330521 | -2.057512 | 18 | 9.54 | 0.530000 |  |
| GO:0060284\_regulation\_of\_cell\_development | NKX6-1 | 122 | 6 | 3.330521 | -2.057512 | 18 | 9.54 | 0.530000 |  |
| GO:0021952\_central\_nervous\_system\_projection\_neuron\_axonogenesis | DCC | 10 | 2 | 13.544118 | -2.047816 | 22 | 10.63 | 0.483182 |  |
| GO:0021952\_central\_nervous\_system\_projection\_neuron\_axonogenesis | NR2E1 | 10 | 2 | 13.544118 | -2.047816 | 22 | 10.63 | 0.483182 |  |
| GO:0043525\_positive\_regulation\_of\_neuron\_apoptosis | ASCL1 | 10 | 2 | 13.544118 | -2.047816 | 22 | 10.63 | 0.483182 |  |
| GO:0043525\_positive\_regulation\_of\_neuron\_apoptosis | NR3C1 | 10 | 2 | 13.544118 | -2.047816 | 22 | 10.63 | 0.483182 |  |
| GO:0048641\_regulation\_of\_skeletal\_muscle\_tissue\_development | MBNL3 | 10 | 2 | 13.544118 | -2.047816 | 22 | 10.63 | 0.483182 |  |
| GO:0048641\_regulation\_of\_skeletal\_muscle\_tissue\_development | BOC | 10 | 2 | 13.544118 | -2.047816 | 22 | 10.63 | 0.483182 |  |
| GO:0051147\_regulation\_of\_muscle\_cell\_differentiation | MBNL3 | 10 | 2 | 13.544118 | -2.047816 | 22 | 10.63 | 0.483182 |  |
| GO:0051147\_regulation\_of\_muscle\_cell\_differentiation | BOC | 10 | 2 | 13.544118 | -2.047816 | 22 | 10.63 | 0.483182 |  |
| GO:0021954\_central\_nervous\_system\_neuron\_development | DCC | 31 | 3 | 6.553605 | -1.987067 | 24 | 11.55 | 0.481250 |  |
| GO:0021954\_central\_nervous\_system\_neuron\_development | ASCL1 | 31 | 3 | 6.553605 | -1.987067 | 24 | 11.55 | 0.481250 |  |
| GO:0021954\_central\_nervous\_system\_neuron\_development | NR2E1 | 31 | 3 | 6.553605 | -1.987067 | 24 | 11.55 | 0.481250 |  |
| GO:0051640\_organelle\_localization | MLPH | 31 | 3 | 6.553605 | -1.987067 | 24 | 11.55 | 0.481250 |  |
| GO:0051640\_organelle\_localization | PLXNA2 | 31 | 3 | 6.553605 | -1.987067 | 24 | 11.55 | 0.481250 |  |
| GO:0051640\_organelle\_localization | RAB27A | 31 | 3 | 6.553605 | -1.987067 | 24 | 11.55 | 0.481250 |  |
| GO:0001913\_T\_cell\_mediated\_cytotoxicity | PTPRC | 11 | 2 | 12.312834 | -1.964802 | 30 | 12.63 | 0.421000 |  |
| GO:0001913\_T\_cell\_mediated\_cytotoxicity | RAB27A | 11 | 2 | 12.312834 | -1.964802 | 30 | 12.63 | 0.421000 |  |
| GO:0007159\_leukocyte\_adhesion | PTPRC | 11 | 2 | 12.312834 | -1.964802 | 30 | 12.63 | 0.421000 |  |
| GO:0007159\_leukocyte\_adhesion | ITGA4 | 11 | 2 | 12.312834 | -1.964802 | 30 | 12.63 | 0.421000 |  |
| GO:0033059\_cellular\_pigmentation | MLPH | 11 | 2 | 12.312834 | -1.964802 | 30 | 12.63 | 0.421000 |  |
| GO:0033059\_cellular\_pigmentation | RAB27A | 11 | 2 | 12.312834 | -1.964802 | 30 | 12.63 | 0.421000 |  |
| GO:0035176\_social\_behavior | VPS13A | 11 | 2 | 12.312834 | -1.964802 | 30 | 12.63 | 0.421000 |  |
| GO:0035176\_social\_behavior | NR2E1 | 11 | 2 | 12.312834 | -1.964802 | 30 | 12.63 | 0.421000 |  |
| GO:0050854\_regulation\_of\_antigen\_receptor-mediated\_signaling\_pathway | PTPRC | 11 | 2 | 12.312834 | -1.964802 | 30 | 12.63 | 0.421000 |  |
| GO:0050854\_regulation\_of\_antigen\_receptor-mediated\_signaling\_pathway | ELF1 | 11 | 2 | 12.312834 | -1.964802 | 30 | 12.63 | 0.421000 |  |
| GO:0051648\_vesicle\_localization | MLPH | 11 | 2 | 12.312834 | -1.964802 | 30 | 12.63 | 0.421000 |  |
| GO:0051648\_vesicle\_localization | RAB27A | 11 | 2 | 12.312834 | -1.964802 | 30 | 12.63 | 0.421000 |  |
| GO:0045445\_myoblast\_differentiation | MBNL3 | 12 | 2 | 11.286765 | -1.889753 | 31 | 14.39 | 0.464194 |  |
| GO:0045445\_myoblast\_differentiation | BOC | 12 | 2 | 11.286765 | -1.889753 | 31 | 14.39 | 0.464194 |  |
| GO:0016265\_death | PTPRC | 450 | 13 | 1.956373 | -1.885645 | 32 | 14.41 | 0.450312 |  |
| GO:0016265\_death | SGPP1 | 450 | 13 | 1.956373 | -1.885645 | 32 | 14.41 | 0.450312 |  |
| GO:0016265\_death | ZFX | 450 | 13 | 1.956373 | -1.885645 | 32 | 14.41 | 0.450312 |  |
| GO:0016265\_death | NR3C1 | 450 | 13 | 1.956373 | -1.885645 | 32 | 14.41 | 0.450312 |  |
| GO:0016265\_death | SGMS1 | 450 | 13 | 1.956373 | -1.885645 | 32 | 14.41 | 0.450312 |  |
| GO:0016265\_death | NR2E1 | 450 | 13 | 1.956373 | -1.885645 | 32 | 14.41 | 0.450312 |  |
| GO:0016265\_death | SERINC3 | 450 | 13 | 1.956373 | -1.885645 | 32 | 14.41 | 0.450312 |  |
| GO:0016265\_death | ASCL1 | 450 | 13 | 1.956373 | -1.885645 | 32 | 14.41 | 0.450312 |  |
| GO:0016265\_death | MSX1 | 450 | 13 | 1.956373 | -1.885645 | 32 | 14.41 | 0.450312 |  |
| GO:0016265\_death | JAK2 | 450 | 13 | 1.956373 | -1.885645 | 32 | 14.41 | 0.450312 |  |
| GO:0016265\_death | CD27 | 450 | 13 | 1.956373 | -1.885645 | 32 | 14.41 | 0.450312 |  |
| GO:0016265\_death | RAB27A | 450 | 13 | 1.956373 | -1.885645 | 32 | 14.41 | 0.450312 |  |
| GO:0016265\_death | CAMK1D | 450 | 13 | 1.956373 | -1.885645 | 32 | 14.41 | 0.450312 |  |
| GO:0048666\_neuron\_development | ALCAM | 262 | 9 | 2.326280 | -1.853982 | 33 | 15.11 | 0.457879 |  |
| GO:0048666\_neuron\_development | DCC | 262 | 9 | 2.326280 | -1.853982 | 33 | 15.11 | 0.457879 |  |
| GO:0048666\_neuron\_development | ASCL1 | 262 | 9 | 2.326280 | -1.853982 | 33 | 15.11 | 0.457879 |  |
| GO:0048666\_neuron\_development | IRX5 | 262 | 9 | 2.326280 | -1.853982 | 33 | 15.11 | 0.457879 |  |
| GO:0048666\_neuron\_development | MYO6 | 262 | 9 | 2.326280 | -1.853982 | 33 | 15.11 | 0.457879 |  |
| GO:0048666\_neuron\_development | ETV1 | 262 | 9 | 2.326280 | -1.853982 | 33 | 15.11 | 0.457879 |  |
| GO:0048666\_neuron\_development | B3GNT2 | 262 | 9 | 2.326280 | -1.853982 | 33 | 15.11 | 0.457879 |  |
| GO:0048666\_neuron\_development | BOC | 262 | 9 | 2.326280 | -1.853982 | 33 | 15.11 | 0.457879 |  |
| GO:0048666\_neuron\_development | NR2E1 | 262 | 9 | 2.326280 | -1.853982 | 33 | 15.11 | 0.457879 |  |
| GO:0030182\_neuron\_differentiation | ALCAM | 356 | 11 | 2.092490 | -1.849845 | 34 | 15.13 | 0.445000 |  |
| GO:0030182\_neuron\_differentiation | DCC | 356 | 11 | 2.092490 | -1.849845 | 34 | 15.13 | 0.445000 |  |
| GO:0030182\_neuron\_differentiation | ASCL1 | 356 | 11 | 2.092490 | -1.849845 | 34 | 15.13 | 0.445000 |  |
| GO:0030182\_neuron\_differentiation | IRX5 | 356 | 11 | 2.092490 | -1.849845 | 34 | 15.13 | 0.445000 |  |
| GO:0030182\_neuron\_differentiation | MYO6 | 356 | 11 | 2.092490 | -1.849845 | 34 | 15.13 | 0.445000 |  |
| GO:0030182\_neuron\_differentiation | SOX5 | 356 | 11 | 2.092490 | -1.849845 | 34 | 15.13 | 0.445000 |  |
| GO:0030182\_neuron\_differentiation | ETV1 | 356 | 11 | 2.092490 | -1.849845 | 34 | 15.13 | 0.445000 |  |
| GO:0030182\_neuron\_differentiation | B3GNT2 | 356 | 11 | 2.092490 | -1.849845 | 34 | 15.13 | 0.445000 |  |
| GO:0030182\_neuron\_differentiation | BOC | 356 | 11 | 2.092490 | -1.849845 | 34 | 15.13 | 0.445000 |  |
| GO:0030182\_neuron\_differentiation | NR2E1 | 356 | 11 | 2.092490 | -1.849845 | 34 | 15.13 | 0.445000 |  |
| GO:0030182\_neuron\_differentiation | NKX6-1 | 356 | 11 | 2.092490 | -1.849845 | 34 | 15.13 | 0.445000 |  |
| GO:0009967\_positive\_regulation\_of\_signal\_transduction | ASCL1 | 98 | 5 | 3.455132 | -1.845870 | 35 | 15.25 | 0.435714 |  |
| GO:0009967\_positive\_regulation\_of\_signal\_transduction | FGF18 | 98 | 5 | 3.455132 | -1.845870 | 35 | 15.25 | 0.435714 |  |
| GO:0009967\_positive\_regulation\_of\_signal\_transduction | PTPRC | 98 | 5 | 3.455132 | -1.845870 | 35 | 15.25 | 0.435714 |  |
| GO:0009967\_positive\_regulation\_of\_signal\_transduction | JAK2 | 98 | 5 | 3.455132 | -1.845870 | 35 | 15.25 | 0.435714 |  |
| GO:0009967\_positive\_regulation\_of\_signal\_transduction | CD27 | 98 | 5 | 3.455132 | -1.845870 | 35 | 15.25 | 0.435714 |  |
| GO:0007292\_female\_gamete\_generation | AFP | 35 | 3 | 5.804622 | -1.842074 | 36 | 15.51 | 0.430833 |  |
| GO:0007292\_female\_gamete\_generation | HEXB | 35 | 3 | 5.804622 | -1.842074 | 36 | 15.51 | 0.430833 |  |
| GO:0007292\_female\_gamete\_generation | ZFX | 35 | 3 | 5.804622 | -1.842074 | 36 | 15.51 | 0.430833 |  |
| GO:0048513\_organ\_development | FGF18 | 1365 | 29 | 1.438752 | -1.832090 | 37 | 15.56 | 0.420541 |  |
| GO:0048513\_organ\_development | IRX5 | 1365 | 29 | 1.438752 | -1.832090 | 37 | 15.56 | 0.420541 |  |
| GO:0048513\_organ\_development | PLXNA2 | 1365 | 29 | 1.438752 | -1.832090 | 37 | 15.56 | 0.420541 |  |
| GO:0048513\_organ\_development | TBX20 | 1365 | 29 | 1.438752 | -1.832090 | 37 | 15.56 | 0.420541 |  |
| GO:0048513\_organ\_development | SOX5 | 1365 | 29 | 1.438752 | -1.832090 | 37 | 15.56 | 0.420541 |  |
| GO:0048513\_organ\_development | NR3C1 | 1365 | 29 | 1.438752 | -1.832090 | 37 | 15.56 | 0.420541 |  |
| GO:0048513\_organ\_development | TCF7L2 | 1365 | 29 | 1.438752 | -1.832090 | 37 | 15.56 | 0.420541 |  |
| GO:0048513\_organ\_development | NR2E1 | 1365 | 29 | 1.438752 | -1.832090 | 37 | 15.56 | 0.420541 |  |
| GO:0048513\_organ\_development | AQP11 | 1365 | 29 | 1.438752 | -1.832090 | 37 | 15.56 | 0.420541 |  |
| GO:0048513\_organ\_development | DMD | 1365 | 29 | 1.438752 | -1.832090 | 37 | 15.56 | 0.420541 |  |
| GO:0048513\_organ\_development | ETV1 | 1365 | 29 | 1.438752 | -1.832090 | 37 | 15.56 | 0.420541 |  |
| GO:0048513\_organ\_development | MBNL3 | 1365 | 29 | 1.438752 | -1.832090 | 37 | 15.56 | 0.420541 |  |
| GO:0048513\_organ\_development | THBS1 | 1365 | 29 | 1.438752 | -1.832090 | 37 | 15.56 | 0.420541 |  |
| GO:0048513\_organ\_development | BOC | 1365 | 29 | 1.438752 | -1.832090 | 37 | 15.56 | 0.420541 |  |
| GO:0048513\_organ\_development | PTPRC | 1365 | 29 | 1.438752 | -1.832090 | 37 | 15.56 | 0.420541 |  |
| GO:0048513\_organ\_development | MYO6 | 1365 | 29 | 1.438752 | -1.832090 | 37 | 15.56 | 0.420541 |  |
| GO:0048513\_organ\_development | TBX3 | 1365 | 29 | 1.438752 | -1.832090 | 37 | 15.56 | 0.420541 |  |
| GO:0048513\_organ\_development | TCFAP2B | 1365 | 29 | 1.438752 | -1.832090 | 37 | 15.56 | 0.420541 |  |
| GO:0048513\_organ\_development | ARID5B | 1365 | 29 | 1.438752 | -1.832090 | 37 | 15.56 | 0.420541 |  |
| GO:0048513\_organ\_development | ZFX | 1365 | 29 | 1.438752 | -1.832090 | 37 | 15.56 | 0.420541 |  |
| GO:0048513\_organ\_development | PCDH8 | 1365 | 29 | 1.438752 | -1.832090 | 37 | 15.56 | 0.420541 |  |
| GO:0048513\_organ\_development | ITGA4 | 1365 | 29 | 1.438752 | -1.832090 | 37 | 15.56 | 0.420541 |  |
| GO:0048513\_organ\_development | NKX6-1 | 1365 | 29 | 1.438752 | -1.832090 | 37 | 15.56 | 0.420541 |  |
| GO:0048513\_organ\_development | AFP | 1365 | 29 | 1.438752 | -1.832090 | 37 | 15.56 | 0.420541 |  |
| GO:0048513\_organ\_development | ASCL1 | 1365 | 29 | 1.438752 | -1.832090 | 37 | 15.56 | 0.420541 |  |
| GO:0048513\_organ\_development | MSX1 | 1365 | 29 | 1.438752 | -1.832090 | 37 | 15.56 | 0.420541 |  |
| GO:0048513\_organ\_development | NEUROD1 | 1365 | 29 | 1.438752 | -1.832090 | 37 | 15.56 | 0.420541 |  |
| GO:0048513\_organ\_development | JAK2 | 1365 | 29 | 1.438752 | -1.832090 | 37 | 15.56 | 0.420541 |  |
| GO:0048513\_organ\_development | HDAC9 | 1365 | 29 | 1.438752 | -1.832090 | 37 | 15.56 | 0.420541 |  |
| GO:0006667\_sphinganine\_metabolic\_process | SGPP1 | 1 | 1 |  |  |  |  |  |  |
| GO:0006668\_sphinganine-1-phosphate\_metabolic\_process | SGPP1 | 1 | 1 |  |  |  |  |  |  |
| GO:0007400\_neuroblast\_fate\_determination | ASCL1 | 1 | 1 |  |  |  |  |  |  |
| GO:0008049\_male\_courtship\_behavior | HEXB | 1 | 1 |  |  |  |  |  |  |
| GO:0014016\_neuroblast\_differentiation | ASCL1 | 1 | 1 |  |  |  |  |  |  |
| GO:0014017\_neuroblast\_fate\_commitment | ASCL1 | 1 | 1 |  |  |  |  |  |  |
| GO:0021934\_hindbrain\_tangential\_cell\_migration | PLXNA2 | 1 | 1 |  |  |  |  |  |  |
| GO:0021935\_granule\_cell\_precursor\_tangential\_migration | PLXNA2 | 1 | 1 |  |  |  |  |  |  |
| GO:0021960\_anterior\_commissure\_morphogenesis | NR2E1 | 1 | 1 |  |  |  |  |  |  |
| GO:0034113\_heterotypic\_cell-cell\_adhesion | PTPRC | 1 | 1 |  |  |  |  |  |  |
| GO:0043316\_cytotoxic\_T\_cell\_degranulation | RAB27A | 1 | 1 |  |  |  |  |  |  |
| GO:0045988\_negative\_regulation\_of\_striated\_muscle\_contraction | ATP1A2 | 1 | 1 |  |  |  |  |  |  |
| GO:0048388\_endosomal\_lumen\_acidification | AQP11 | 1 | 1 |  |  |  |  |  |  |
| GO:0051481\_reduction\_of\_cytosolic\_calcium\_ion\_concentration | ATP1A2 | 1 | 1 |  |  |  |  |  |  |
| GO:0060163\_subpallium\_neuron\_fate\_commitment | ASCL1 | 1 | 1 |  |  |  |  |  |  |
| GO:0060165\_regulation\_of\_timing\_of\_subpallium\_neuron\_differentiation | ASCL1 | 1 | 1 |  |  |  |  |  |  |
| GO:0060398\_regulation\_of\_growth\_hormone\_receptor\_signaling\_pathway | JAK2 | 1 | 1 |  |  |  |  |  |  |
| GO:0060399\_positive\_regulation\_of\_growth\_hormone\_receptor\_signaling\_pathway | JAK2 | 1 | 1 |  |  |  |  |  |  |
| GO:0021955\_central\_nervous\_system\_neuron\_axonogenesis | DCC | 13 | 2 | 10.418552 | -1.821329 | 39 | 16.88 | 0.432821 |  |
| GO:0021955\_central\_nervous\_system\_neuron\_axonogenesis | NR2E1 | 13 | 2 | 10.418552 | -1.821329 | 39 | 16.88 | 0.432821 |  |
| GO:0060324\_face\_development | ARID5B | 13 | 2 | 10.418552 | -1.821329 | 39 | 16.88 | 0.432821 |  |
| GO:0060324\_face\_development | ITGA4 | 13 | 2 | 10.418552 | -1.821329 | 39 | 16.88 | 0.432821 |  |
| GO:0048856\_anatomical\_structure\_development | DCC | 1688 | 34 | 1.364040 | -1.795628 | 40 | 17.35 | 0.433750 |  |
| GO:0048856\_anatomical\_structure\_development | FGF18 | 1688 | 34 | 1.364040 | -1.795628 | 40 | 17.35 | 0.433750 |  |
| GO:0048856\_anatomical\_structure\_development | IRX5 | 1688 | 34 | 1.364040 | -1.795628 | 40 | 17.35 | 0.433750 |  |
| GO:0048856\_anatomical\_structure\_development | PLXNA2 | 1688 | 34 | 1.364040 | -1.795628 | 40 | 17.35 | 0.433750 |  |
| GO:0048856\_anatomical\_structure\_development | HEXB | 1688 | 34 | 1.364040 | -1.795628 | 40 | 17.35 | 0.433750 |  |
| GO:0048856\_anatomical\_structure\_development | TBX20 | 1688 | 34 | 1.364040 | -1.795628 | 40 | 17.35 | 0.433750 |  |
| GO:0048856\_anatomical\_structure\_development | SOX5 | 1688 | 34 | 1.364040 | -1.795628 | 40 | 17.35 | 0.433750 |  |
| GO:0048856\_anatomical\_structure\_development | NR3C1 | 1688 | 34 | 1.364040 | -1.795628 | 40 | 17.35 | 0.433750 |  |
| GO:0048856\_anatomical\_structure\_development | TCF7L2 | 1688 | 34 | 1.364040 | -1.795628 | 40 | 17.35 | 0.433750 |  |
| GO:0048856\_anatomical\_structure\_development | NR2E1 | 1688 | 34 | 1.364040 | -1.795628 | 40 | 17.35 | 0.433750 |  |
| GO:0048856\_anatomical\_structure\_development | ALCAM | 1688 | 34 | 1.364040 | -1.795628 | 40 | 17.35 | 0.433750 |  |
| GO:0048856\_anatomical\_structure\_development | AQP11 | 1688 | 34 | 1.364040 | -1.795628 | 40 | 17.35 | 0.433750 |  |
| GO:0048856\_anatomical\_structure\_development | DMD | 1688 | 34 | 1.364040 | -1.795628 | 40 | 17.35 | 0.433750 |  |
| GO:0048856\_anatomical\_structure\_development | ETV1 | 1688 | 34 | 1.364040 | -1.795628 | 40 | 17.35 | 0.433750 |  |
| GO:0048856\_anatomical\_structure\_development | VPS13A | 1688 | 34 | 1.364040 | -1.795628 | 40 | 17.35 | 0.433750 |  |
| GO:0048856\_anatomical\_structure\_development | B3GNT2 | 1688 | 34 | 1.364040 | -1.795628 | 40 | 17.35 | 0.433750 |  |
| GO:0048856\_anatomical\_structure\_development | MBNL3 | 1688 | 34 | 1.364040 | -1.795628 | 40 | 17.35 | 0.433750 |  |
| GO:0048856\_anatomical\_structure\_development | THBS1 | 1688 | 34 | 1.364040 | -1.795628 | 40 | 17.35 | 0.433750 |  |
| GO:0048856\_anatomical\_structure\_development | BOC | 1688 | 34 | 1.364040 | -1.795628 | 40 | 17.35 | 0.433750 |  |
| GO:0048856\_anatomical\_structure\_development | PTPRC | 1688 | 34 | 1.364040 | -1.795628 | 40 | 17.35 | 0.433750 |  |
| GO:0048856\_anatomical\_structure\_development | MYO6 | 1688 | 34 | 1.364040 | -1.795628 | 40 | 17.35 | 0.433750 |  |
| GO:0048856\_anatomical\_structure\_development | TCFAP2B | 1688 | 34 | 1.364040 | -1.795628 | 40 | 17.35 | 0.433750 |  |
| GO:0048856\_anatomical\_structure\_development | TBX3 | 1688 | 34 | 1.364040 | -1.795628 | 40 | 17.35 | 0.433750 |  |
| GO:0048856\_anatomical\_structure\_development | ARID5B | 1688 | 34 | 1.364040 | -1.795628 | 40 | 17.35 | 0.433750 |  |
| GO:0048856\_anatomical\_structure\_development | ZFX | 1688 | 34 | 1.364040 | -1.795628 | 40 | 17.35 | 0.433750 |  |
| GO:0048856\_anatomical\_structure\_development | PCDH8 | 1688 | 34 | 1.364040 | -1.795628 | 40 | 17.35 | 0.433750 |  |
| GO:0048856\_anatomical\_structure\_development | ITGA4 | 1688 | 34 | 1.364040 | -1.795628 | 40 | 17.35 | 0.433750 |  |
| GO:0048856\_anatomical\_structure\_development | NKX6-1 | 1688 | 34 | 1.364040 | -1.795628 | 40 | 17.35 | 0.433750 |  |
| GO:0048856\_anatomical\_structure\_development | AFP | 1688 | 34 | 1.364040 | -1.795628 | 40 | 17.35 | 0.433750 |  |
| GO:0048856\_anatomical\_structure\_development | ASCL1 | 1688 | 34 | 1.364040 | -1.795628 | 40 | 17.35 | 0.433750 |  |
| GO:0048856\_anatomical\_structure\_development | MSX1 | 1688 | 34 | 1.364040 | -1.795628 | 40 | 17.35 | 0.433750 |  |
| GO:0048856\_anatomical\_structure\_development | NEUROD1 | 1688 | 34 | 1.364040 | -1.795628 | 40 | 17.35 | 0.433750 |  |
| GO:0048856\_anatomical\_structure\_development | JAK2 | 1688 | 34 | 1.364040 | -1.795628 | 40 | 17.35 | 0.433750 |  |
| GO:0048856\_anatomical\_structure\_development | HDAC9 | 1688 | 34 | 1.364040 | -1.795628 | 40 | 17.35 | 0.433750 |  |
| GO:0048665\_neuron\_fate\_specification | ASCL1 | 14 | 2 | 9.674370 | -1.758505 | 41 | 19.0 | 0.463415 |  |
| GO:0048665\_neuron\_fate\_specification | NKX6-1 | 14 | 2 | 9.674370 | -1.758505 | 41 | 19.0 | 0.463415 |  |
| GO:0006644\_phospholipid\_metabolic\_process | SGPP1 | 39 | 3 | 5.209276 | -1.715225 | 42 | 20.08 | 0.478095 |  |
| GO:0006644\_phospholipid\_metabolic\_process | HEXB | 39 | 3 | 5.209276 | -1.715225 | 42 | 20.08 | 0.478095 |  |
| GO:0006644\_phospholipid\_metabolic\_process | SGMS1 | 39 | 3 | 5.209276 | -1.715225 | 42 | 20.08 | 0.478095 |  |
| GO:0006885\_regulation\_of\_pH | AQP11 | 15 | 2 | 9.029412 | -1.700475 | 45 | 21.47 | 0.477111 |  |
| GO:0006885\_regulation\_of\_pH | ATP6V1B1 | 15 | 2 | 9.029412 | -1.700475 | 45 | 21.47 | 0.477111 |  |
| GO:0048709\_oligodendrocyte\_differentiation | ASCL1 | 15 | 2 | 9.029412 | -1.700475 | 45 | 21.47 | 0.477111 |  |
| GO:0048709\_oligodendrocyte\_differentiation | NKX6-1 | 15 | 2 | 9.029412 | -1.700475 | 45 | 21.47 | 0.477111 |  |
| GO:0060322\_head\_development | ARID5B | 15 | 2 | 9.029412 | -1.700475 | 45 | 21.47 | 0.477111 |  |
| GO:0060322\_head\_development | ITGA4 | 15 | 2 | 9.029412 | -1.700475 | 45 | 21.47 | 0.477111 |  |
| GO:0006915\_apoptosis | SERINC3 | 427 | 12 | 1.903155 | -1.681421 | 46 | 22.05 | 0.479348 |  |
| GO:0006915\_apoptosis | ASCL1 | 427 | 12 | 1.903155 | -1.681421 | 46 | 22.05 | 0.479348 |  |
| GO:0006915\_apoptosis | PTPRC | 427 | 12 | 1.903155 | -1.681421 | 46 | 22.05 | 0.479348 |  |
| GO:0006915\_apoptosis | MSX1 | 427 | 12 | 1.903155 | -1.681421 | 46 | 22.05 | 0.479348 |  |
| GO:0006915\_apoptosis | SGPP1 | 427 | 12 | 1.903155 | -1.681421 | 46 | 22.05 | 0.479348 |  |
| GO:0006915\_apoptosis | JAK2 | 427 | 12 | 1.903155 | -1.681421 | 46 | 22.05 | 0.479348 |  |
| GO:0006915\_apoptosis | SGMS1 | 427 | 12 | 1.903155 | -1.681421 | 46 | 22.05 | 0.479348 |  |
| GO:0006915\_apoptosis | NR3C1 | 427 | 12 | 1.903155 | -1.681421 | 46 | 22.05 | 0.479348 |  |
| GO:0006915\_apoptosis | NR2E1 | 427 | 12 | 1.903155 | -1.681421 | 46 | 22.05 | 0.479348 |  |
| GO:0006915\_apoptosis | CD27 | 427 | 12 | 1.903155 | -1.681421 | 46 | 22.05 | 0.479348 |  |
| GO:0006915\_apoptosis | CAMK1D | 427 | 12 | 1.903155 | -1.681421 | 46 | 22.05 | 0.479348 |  |
| GO:0006915\_apoptosis | RAB27A | 427 | 12 | 1.903155 | -1.681421 | 46 | 22.05 | 0.479348 |  |
| GO:0006928\_cell\_motion | DCC | 330 | 10 | 2.052139 | -1.662701 | 48 | 22.37 | 0.466042 |  |
| GO:0006928\_cell\_motion | ALCAM | 330 | 10 | 2.052139 | -1.662701 | 48 | 22.37 | 0.466042 |  |
| GO:0006928\_cell\_motion | ASCL1 | 330 | 10 | 2.052139 | -1.662701 | 48 | 22.37 | 0.466042 |  |
| GO:0006928\_cell\_motion | PLXNA2 | 330 | 10 | 2.052139 | -1.662701 | 48 | 22.37 | 0.466042 |  |
| GO:0006928\_cell\_motion | ARID5B | 330 | 10 | 2.052139 | -1.662701 | 48 | 22.37 | 0.466042 |  |
| GO:0006928\_cell\_motion | ETV1 | 330 | 10 | 2.052139 | -1.662701 | 48 | 22.37 | 0.466042 |  |
| GO:0006928\_cell\_motion | ITGA4 | 330 | 10 | 2.052139 | -1.662701 | 48 | 22.37 | 0.466042 |  |
| GO:0006928\_cell\_motion | B3GNT2 | 330 | 10 | 2.052139 | -1.662701 | 48 | 22.37 | 0.466042 |  |
| GO:0006928\_cell\_motion | NR2E1 | 330 | 10 | 2.052139 | -1.662701 | 48 | 22.37 | 0.466042 |  |
| GO:0006928\_cell\_motion | BOC | 330 | 10 | 2.052139 | -1.662701 | 48 | 22.37 | 0.466042 |  |
| GO:0051674\_localization\_of\_cell | DCC | 330 | 10 | 2.052139 | -1.662701 | 48 | 22.37 | 0.466042 |  |
| GO:0051674\_localization\_of\_cell | ALCAM | 330 | 10 | 2.052139 | -1.662701 | 48 | 22.37 | 0.466042 |  |
| GO:0051674\_localization\_of\_cell | ASCL1 | 330 | 10 | 2.052139 | -1.662701 | 48 | 22.37 | 0.466042 |  |
| GO:0051674\_localization\_of\_cell | PLXNA2 | 330 | 10 | 2.052139 | -1.662701 | 48 | 22.37 | 0.466042 |  |
| GO:0051674\_localization\_of\_cell | ARID5B | 330 | 10 | 2.052139 | -1.662701 | 48 | 22.37 | 0.466042 |  |
| GO:0051674\_localization\_of\_cell | ETV1 | 330 | 10 | 2.052139 | -1.662701 | 48 | 22.37 | 0.466042 |  |
| GO:0051674\_localization\_of\_cell | ITGA4 | 330 | 10 | 2.052139 | -1.662701 | 48 | 22.37 | 0.466042 |  |
| GO:0051674\_localization\_of\_cell | B3GNT2 | 330 | 10 | 2.052139 | -1.662701 | 48 | 22.37 | 0.466042 |  |
| GO:0051674\_localization\_of\_cell | NR2E1 | 330 | 10 | 2.052139 | -1.662701 | 48 | 22.37 | 0.466042 |  |
| GO:0051674\_localization\_of\_cell | BOC | 330 | 10 | 2.052139 | -1.662701 | 48 | 22.37 | 0.466042 |  |
| GO:0051239\_regulation\_of\_multicellular\_organismal\_process | PTPRC | 587 | 15 | 1.730509 | -1.661834 | 49 | 22.38 | 0.456735 |  |
| GO:0051239\_regulation\_of\_multicellular\_organismal\_process | ELF1 | 587 | 15 | 1.730509 | -1.661834 | 49 | 22.38 | 0.456735 |  |
| GO:0051239\_regulation\_of\_multicellular\_organismal\_process | IRX5 | 587 | 15 | 1.730509 | -1.661834 | 49 | 22.38 | 0.456735 |  |
| GO:0051239\_regulation\_of\_multicellular\_organismal\_process | MYO6 | 587 | 15 | 1.730509 | -1.661834 | 49 | 22.38 | 0.456735 |  |
| GO:0051239\_regulation\_of\_multicellular\_organismal\_process | ATRN | 587 | 15 | 1.730509 | -1.661834 | 49 | 22.38 | 0.456735 |  |
| GO:0051239\_regulation\_of\_multicellular\_organismal\_process | SOX5 | 587 | 15 | 1.730509 | -1.661834 | 49 | 22.38 | 0.456735 |  |
| GO:0051239\_regulation\_of\_multicellular\_organismal\_process | ATP1A2 | 587 | 15 | 1.730509 | -1.661834 | 49 | 22.38 | 0.456735 |  |
| GO:0051239\_regulation\_of\_multicellular\_organismal\_process | NR2E1 | 587 | 15 | 1.730509 | -1.661834 | 49 | 22.38 | 0.456735 |  |
| GO:0051239\_regulation\_of\_multicellular\_organismal\_process | NKX6-1 | 587 | 15 | 1.730509 | -1.661834 | 49 | 22.38 | 0.456735 |  |
| GO:0051239\_regulation\_of\_multicellular\_organismal\_process | ASCL1 | 587 | 15 | 1.730509 | -1.661834 | 49 | 22.38 | 0.456735 |  |
| GO:0051239\_regulation\_of\_multicellular\_organismal\_process | ATXN2 | 587 | 15 | 1.730509 | -1.661834 | 49 | 22.38 | 0.456735 |  |
| GO:0051239\_regulation\_of\_multicellular\_organismal\_process | MBNL3 | 587 | 15 | 1.730509 | -1.661834 | 49 | 22.38 | 0.456735 |  |
| GO:0051239\_regulation\_of\_multicellular\_organismal\_process | THBS1 | 587 | 15 | 1.730509 | -1.661834 | 49 | 22.38 | 0.456735 |  |
| GO:0051239\_regulation\_of\_multicellular\_organismal\_process | HDAC9 | 587 | 15 | 1.730509 | -1.661834 | 49 | 22.38 | 0.456735 |  |
| GO:0051239\_regulation\_of\_multicellular\_organismal\_process | BOC | 587 | 15 | 1.730509 | -1.661834 | 49 | 22.38 | 0.456735 |  |
| GO:0010647\_positive\_regulation\_of\_cell\_communication | ASCL1 | 110 | 5 | 3.078209 | -1.648705 | 51 | 22.99 | 0.450784 |  |
| GO:0010647\_positive\_regulation\_of\_cell\_communication | FGF18 | 110 | 5 | 3.078209 | -1.648705 | 51 | 22.99 | 0.450784 |  |
| GO:0010647\_positive\_regulation\_of\_cell\_communication | PTPRC | 110 | 5 | 3.078209 | -1.648705 | 51 | 22.99 | 0.450784 |  |
| GO:0010647\_positive\_regulation\_of\_cell\_communication | JAK2 | 110 | 5 | 3.078209 | -1.648705 | 51 | 22.99 | 0.450784 |  |
| GO:0010647\_positive\_regulation\_of\_cell\_communication | CD27 | 110 | 5 | 3.078209 | -1.648705 | 51 | 22.99 | 0.450784 |  |
| GO:0055080\_cation\_homeostasis | PTPRC | 110 | 5 | 3.078209 | -1.648705 | 51 | 22.99 | 0.450784 |  |
| GO:0055080\_cation\_homeostasis | AQP11 | 110 | 5 | 3.078209 | -1.648705 | 51 | 22.99 | 0.450784 |  |
| GO:0055080\_cation\_homeostasis | HEXB | 110 | 5 | 3.078209 | -1.648705 | 51 | 22.99 | 0.450784 |  |
| GO:0055080\_cation\_homeostasis | ATP1A2 | 110 | 5 | 3.078209 | -1.648705 | 51 | 22.99 | 0.450784 |  |
| GO:0055080\_cation\_homeostasis | ATP6V1B1 | 110 | 5 | 3.078209 | -1.648705 | 51 | 22.99 | 0.450784 |  |
| GO:0008654\_phospholipid\_biosynthetic\_process | HEXB | 16 | 2 | 8.465074 | -1.646596 | 52 | 24.27 | 0.466731 |  |
| GO:0008654\_phospholipid\_biosynthetic\_process | SGMS1 | 16 | 2 | 8.465074 | -1.646596 | 52 | 24.27 | 0.466731 |  |
| GO:0012501\_programmed\_cell\_death | SERINC3 | 433 | 12 | 1.876783 | -1.638476 | 53 | 24.38 | 0.460000 |  |
| GO:0012501\_programmed\_cell\_death | ASCL1 | 433 | 12 | 1.876783 | -1.638476 | 53 | 24.38 | 0.460000 |  |
| GO:0012501\_programmed\_cell\_death | PTPRC | 433 | 12 | 1.876783 | -1.638476 | 53 | 24.38 | 0.460000 |  |
| GO:0012501\_programmed\_cell\_death | MSX1 | 433 | 12 | 1.876783 | -1.638476 | 53 | 24.38 | 0.460000 |  |
| GO:0012501\_programmed\_cell\_death | SGPP1 | 433 | 12 | 1.876783 | -1.638476 | 53 | 24.38 | 0.460000 |  |
| GO:0012501\_programmed\_cell\_death | JAK2 | 433 | 12 | 1.876783 | -1.638476 | 53 | 24.38 | 0.460000 |  |
| GO:0012501\_programmed\_cell\_death | SGMS1 | 433 | 12 | 1.876783 | -1.638476 | 53 | 24.38 | 0.460000 |  |
| GO:0012501\_programmed\_cell\_death | NR3C1 | 433 | 12 | 1.876783 | -1.638476 | 53 | 24.38 | 0.460000 |  |
| GO:0012501\_programmed\_cell\_death | NR2E1 | 433 | 12 | 1.876783 | -1.638476 | 53 | 24.38 | 0.460000 |  |
| GO:0012501\_programmed\_cell\_death | CD27 | 433 | 12 | 1.876783 | -1.638476 | 53 | 24.38 | 0.460000 |  |
| GO:0012501\_programmed\_cell\_death | CAMK1D | 433 | 12 | 1.876783 | -1.638476 | 53 | 24.38 | 0.460000 |  |
| GO:0012501\_programmed\_cell\_death | RAB27A | 433 | 12 | 1.876783 | -1.638476 | 53 | 24.38 | 0.460000 |  |
| GO:0010740\_positive\_regulation\_of\_protein\_kinase\_cascade | PTPRC | 42 | 3 | 4.837185 | -1.629757 | 54 | 24.96 | 0.462222 |  |
| GO:0010740\_positive\_regulation\_of\_protein\_kinase\_cascade | JAK2 | 42 | 3 | 4.837185 | -1.629757 | 54 | 24.96 | 0.462222 |  |
| GO:0010740\_positive\_regulation\_of\_protein\_kinase\_cascade | CD27 | 42 | 3 | 4.837185 | -1.629757 | 54 | 24.96 | 0.462222 |  |
| GO:0050793\_regulation\_of\_developmental\_process | PTPRC | 703 | 17 | 1.637624 | -1.623542 | 55 | 25.04 | 0.455273 |  |
| GO:0050793\_regulation\_of\_developmental\_process | SOX5 | 703 | 17 | 1.637624 | -1.623542 | 55 | 25.04 | 0.455273 |  |
| GO:0050793\_regulation\_of\_developmental\_process | NR3C1 | 703 | 17 | 1.637624 | -1.623542 | 55 | 25.04 | 0.455273 |  |
| GO:0050793\_regulation\_of\_developmental\_process | SGMS1 | 703 | 17 | 1.637624 | -1.623542 | 55 | 25.04 | 0.455273 |  |
| GO:0050793\_regulation\_of\_developmental\_process | TCF7L2 | 703 | 17 | 1.637624 | -1.623542 | 55 | 25.04 | 0.455273 |  |
| GO:0050793\_regulation\_of\_developmental\_process | NR2E1 | 703 | 17 | 1.637624 | -1.623542 | 55 | 25.04 | 0.455273 |  |
| GO:0050793\_regulation\_of\_developmental\_process | NKX6-1 | 703 | 17 | 1.637624 | -1.623542 | 55 | 25.04 | 0.455273 |  |
| GO:0050793\_regulation\_of\_developmental\_process | SERINC3 | 703 | 17 | 1.637624 | -1.623542 | 55 | 25.04 | 0.455273 |  |
| GO:0050793\_regulation\_of\_developmental\_process | ASCL1 | 703 | 17 | 1.637624 | -1.623542 | 55 | 25.04 | 0.455273 |  |
| GO:0050793\_regulation\_of\_developmental\_process | MSX1 | 703 | 17 | 1.637624 | -1.623542 | 55 | 25.04 | 0.455273 |  |
| GO:0050793\_regulation\_of\_developmental\_process | MBNL3 | 703 | 17 | 1.637624 | -1.623542 | 55 | 25.04 | 0.455273 |  |
| GO:0050793\_regulation\_of\_developmental\_process | HDAC9 | 703 | 17 | 1.637624 | -1.623542 | 55 | 25.04 | 0.455273 |  |
| GO:0050793\_regulation\_of\_developmental\_process | THBS1 | 703 | 17 | 1.637624 | -1.623542 | 55 | 25.04 | 0.455273 |  |
| GO:0050793\_regulation\_of\_developmental\_process | BOC | 703 | 17 | 1.637624 | -1.623542 | 55 | 25.04 | 0.455273 |  |
| GO:0050793\_regulation\_of\_developmental\_process | CD27 | 703 | 17 | 1.637624 | -1.623542 | 55 | 25.04 | 0.455273 |  |
| GO:0050793\_regulation\_of\_developmental\_process | RAB27A | 703 | 17 | 1.637624 | -1.623542 | 55 | 25.04 | 0.455273 |  |
| GO:0050793\_regulation\_of\_developmental\_process | CAMK1D | 703 | 17 | 1.637624 | -1.623542 | 55 | 25.04 | 0.455273 |  |
| GO:0019725\_cellular\_homeostasis | PTPRC | 195 | 7 | 2.430995 | -1.621862 | 56 | 25.11 | 0.448393 |  |
| GO:0019725\_cellular\_homeostasis | AQP11 | 195 | 7 | 2.430995 | -1.621862 | 56 | 25.11 | 0.448393 |  |
| GO:0019725\_cellular\_homeostasis | DMD | 195 | 7 | 2.430995 | -1.621862 | 56 | 25.11 | 0.448393 |  |
| GO:0019725\_cellular\_homeostasis | HEXB | 195 | 7 | 2.430995 | -1.621862 | 56 | 25.11 | 0.448393 |  |
| GO:0019725\_cellular\_homeostasis | KCNA5 | 195 | 7 | 2.430995 | -1.621862 | 56 | 25.11 | 0.448393 |  |
| GO:0019725\_cellular\_homeostasis | ATP1A2 | 195 | 7 | 2.430995 | -1.621862 | 56 | 25.11 | 0.448393 |  |
| GO:0019725\_cellular\_homeostasis | SLC9A3R1 | 195 | 7 | 2.430995 | -1.621862 | 56 | 25.11 | 0.448393 |  |
| GO:0007417\_central\_nervous\_system\_development | DCC | 287 | 9 | 2.123642 | -1.619725 | 57 | 25.13 | 0.440877 |  |
| GO:0007417\_central\_nervous\_system\_development | ASCL1 | 287 | 9 | 2.123642 | -1.619725 | 57 | 25.13 | 0.440877 |  |
| GO:0007417\_central\_nervous\_system\_development | MSX1 | 287 | 9 | 2.123642 | -1.619725 | 57 | 25.13 | 0.440877 |  |
| GO:0007417\_central\_nervous\_system\_development | TBX3 | 287 | 9 | 2.123642 | -1.619725 | 57 | 25.13 | 0.440877 |  |
| GO:0007417\_central\_nervous\_system\_development | PLXNA2 | 287 | 9 | 2.123642 | -1.619725 | 57 | 25.13 | 0.440877 |  |
| GO:0007417\_central\_nervous\_system\_development | SOX5 | 287 | 9 | 2.123642 | -1.619725 | 57 | 25.13 | 0.440877 |  |
| GO:0007417\_central\_nervous\_system\_development | NEUROD1 | 287 | 9 | 2.123642 | -1.619725 | 57 | 25.13 | 0.440877 |  |
| GO:0007417\_central\_nervous\_system\_development | NR2E1 | 287 | 9 | 2.123642 | -1.619725 | 57 | 25.13 | 0.440877 |  |
| GO:0007417\_central\_nervous\_system\_development | NKX6-1 | 287 | 9 | 2.123642 | -1.619725 | 57 | 25.13 | 0.440877 |  |
| GO:0019637\_organophosphate\_metabolic\_process | SGPP1 | 43 | 3 | 4.724692 | -1.602868 | 58 | 25.68 | 0.442759 |  |
| GO:0019637\_organophosphate\_metabolic\_process | HEXB | 43 | 3 | 4.724692 | -1.602868 | 58 | 25.68 | 0.442759 |  |
| GO:0019637\_organophosphate\_metabolic\_process | SGMS1 | 43 | 3 | 4.724692 | -1.602868 | 58 | 25.68 | 0.442759 |  |
| GO:0031175\_neuron\_projection\_development | DCC | 197 | 7 | 2.406315 | -1.600399 | 60 | 25.74 | 0.429000 |  |
| GO:0031175\_neuron\_projection\_development | ALCAM | 197 | 7 | 2.406315 | -1.600399 | 60 | 25.74 | 0.429000 |  |
| GO:0031175\_neuron\_projection\_development | MYO6 | 197 | 7 | 2.406315 | -1.600399 | 60 | 25.74 | 0.429000 |  |
| GO:0031175\_neuron\_projection\_development | ETV1 | 197 | 7 | 2.406315 | -1.600399 | 60 | 25.74 | 0.429000 |  |
| GO:0031175\_neuron\_projection\_development | B3GNT2 | 197 | 7 | 2.406315 | -1.600399 | 60 | 25.74 | 0.429000 |  |
| GO:0031175\_neuron\_projection\_development | NR2E1 | 197 | 7 | 2.406315 | -1.600399 | 60 | 25.74 | 0.429000 |  |
| GO:0031175\_neuron\_projection\_development | BOC | 197 | 7 | 2.406315 | -1.600399 | 60 | 25.74 | 0.429000 |  |
| GO:0050801\_ion\_homeostasis | PTPRC | 197 | 7 | 2.406315 | -1.600399 | 60 | 25.74 | 0.429000 |  |
| GO:0050801\_ion\_homeostasis | AQP11 | 197 | 7 | 2.406315 | -1.600399 | 60 | 25.74 | 0.429000 |  |
| GO:0050801\_ion\_homeostasis | HEXB | 197 | 7 | 2.406315 | -1.600399 | 60 | 25.74 | 0.429000 |  |
| GO:0050801\_ion\_homeostasis | KCNA5 | 197 | 7 | 2.406315 | -1.600399 | 60 | 25.74 | 0.429000 |  |
| GO:0050801\_ion\_homeostasis | ATP1A2 | 197 | 7 | 2.406315 | -1.600399 | 60 | 25.74 | 0.429000 |  |
| GO:0050801\_ion\_homeostasis | ATP6V1B1 | 197 | 7 | 2.406315 | -1.600399 | 60 | 25.74 | 0.429000 |  |
| GO:0050801\_ion\_homeostasis | SLC9A3R1 | 197 | 7 | 2.406315 | -1.600399 | 60 | 25.74 | 0.429000 |  |
| GO:0055067\_monovalent\_inorganic\_cation\_homeostasis | AQP11 | 17 | 2 | 7.967128 | -1.596347 | 61 | 27.11 | 0.444426 |  |
| GO:0055067\_monovalent\_inorganic\_cation\_homeostasis | ATP6V1B1 | 17 | 2 | 7.967128 | -1.596347 | 61 | 27.11 | 0.444426 |  |
| GO:0008219\_cell\_death | SERINC3 | 444 | 12 | 1.830286 | -1.562407 | 62 | 28.0 | 0.451613 |  |
| GO:0008219\_cell\_death | ASCL1 | 444 | 12 | 1.830286 | -1.562407 | 62 | 28.0 | 0.451613 |  |
| GO:0008219\_cell\_death | PTPRC | 444 | 12 | 1.830286 | -1.562407 | 62 | 28.0 | 0.451613 |  |
| GO:0008219\_cell\_death | MSX1 | 444 | 12 | 1.830286 | -1.562407 | 62 | 28.0 | 0.451613 |  |
| GO:0008219\_cell\_death | SGPP1 | 444 | 12 | 1.830286 | -1.562407 | 62 | 28.0 | 0.451613 |  |
| GO:0008219\_cell\_death | JAK2 | 444 | 12 | 1.830286 | -1.562407 | 62 | 28.0 | 0.451613 |  |
| GO:0008219\_cell\_death | SGMS1 | 444 | 12 | 1.830286 | -1.562407 | 62 | 28.0 | 0.451613 |  |
| GO:0008219\_cell\_death | NR3C1 | 444 | 12 | 1.830286 | -1.562407 | 62 | 28.0 | 0.451613 |  |
| GO:0008219\_cell\_death | NR2E1 | 444 | 12 | 1.830286 | -1.562407 | 62 | 28.0 | 0.451613 |  |
| GO:0008219\_cell\_death | CD27 | 444 | 12 | 1.830286 | -1.562407 | 62 | 28.0 | 0.451613 |  |
| GO:0008219\_cell\_death | CAMK1D | 444 | 12 | 1.830286 | -1.562407 | 62 | 28.0 | 0.451613 |  |
| GO:0008219\_cell\_death | RAB27A | 444 | 12 | 1.830286 | -1.562407 | 62 | 28.0 | 0.451613 |  |
| GO:0007409\_axonogenesis | DCC | 158 | 6 | 2.571668 | -1.550319 | 63 | 28.41 | 0.450952 |  |
| GO:0007409\_axonogenesis | ALCAM | 158 | 6 | 2.571668 | -1.550319 | 63 | 28.41 | 0.450952 |  |
| GO:0007409\_axonogenesis | ETV1 | 158 | 6 | 2.571668 | -1.550319 | 63 | 28.41 | 0.450952 |  |
| GO:0007409\_axonogenesis | B3GNT2 | 158 | 6 | 2.571668 | -1.550319 | 63 | 28.41 | 0.450952 |  |
| GO:0007409\_axonogenesis | NR2E1 | 158 | 6 | 2.571668 | -1.550319 | 63 | 28.41 | 0.450952 |  |
| GO:0007409\_axonogenesis | BOC | 158 | 6 | 2.571668 | -1.550319 | 63 | 28.41 | 0.450952 |  |
| GO:0006022\_aminoglycan\_metabolic\_process | MAMDC2 | 18 | 2 | 7.524510 | -1.549299 | 66 | 29.77 | 0.451061 |  |
| GO:0006022\_aminoglycan\_metabolic\_process | HEXB | 18 | 2 | 7.524510 | -1.549299 | 66 | 29.77 | 0.451061 |  |
| GO:0030203\_glycosaminoglycan\_metabolic\_process | MAMDC2 | 18 | 2 | 7.524510 | -1.549299 | 66 | 29.77 | 0.451061 |  |
| GO:0030203\_glycosaminoglycan\_metabolic\_process | HEXB | 18 | 2 | 7.524510 | -1.549299 | 66 | 29.77 | 0.451061 |  |
| GO:0030318\_melanocyte\_differentiation | MLPH | 18 | 2 | 7.524510 | -1.549299 | 66 | 29.77 | 0.451061 |  |
| GO:0030318\_melanocyte\_differentiation | RAB27A | 18 | 2 | 7.524510 | -1.549299 | 66 | 29.77 | 0.451061 |  |
| GO:0048699\_generation\_of\_neurons | ALCAM | 396 | 11 | 1.881127 | -1.538863 | 67 | 29.9 | 0.446269 |  |
| GO:0048699\_generation\_of\_neurons | DCC | 396 | 11 | 1.881127 | -1.538863 | 67 | 29.9 | 0.446269 |  |
| GO:0048699\_generation\_of\_neurons | ASCL1 | 396 | 11 | 1.881127 | -1.538863 | 67 | 29.9 | 0.446269 |  |
| GO:0048699\_generation\_of\_neurons | IRX5 | 396 | 11 | 1.881127 | -1.538863 | 67 | 29.9 | 0.446269 |  |
| GO:0048699\_generation\_of\_neurons | MYO6 | 396 | 11 | 1.881127 | -1.538863 | 67 | 29.9 | 0.446269 |  |
| GO:0048699\_generation\_of\_neurons | SOX5 | 396 | 11 | 1.881127 | -1.538863 | 67 | 29.9 | 0.446269 |  |
| GO:0048699\_generation\_of\_neurons | ETV1 | 396 | 11 | 1.881127 | -1.538863 | 67 | 29.9 | 0.446269 |  |
| GO:0048699\_generation\_of\_neurons | B3GNT2 | 396 | 11 | 1.881127 | -1.538863 | 67 | 29.9 | 0.446269 |  |
| GO:0048699\_generation\_of\_neurons | BOC | 396 | 11 | 1.881127 | -1.538863 | 67 | 29.9 | 0.446269 |  |
| GO:0048699\_generation\_of\_neurons | NR2E1 | 396 | 11 | 1.881127 | -1.538863 | 67 | 29.9 | 0.446269 |  |
| GO:0048699\_generation\_of\_neurons | NKX6-1 | 396 | 11 | 1.881127 | -1.538863 | 67 | 29.9 | 0.446269 |  |
| GO:0002922\_positive\_regulation\_of\_humoral\_immune\_response | PTPRC | 2 | 1 |  |  |  |  |  |  |
| GO:0002925\_positive\_regulation\_of\_humoral\_immune\_response\_mediated\_by\_circulating\_immunoglobulin | PTPRC | 2 | 1 |  |  |  |  |  |  |
| GO:0006686\_sphingomyelin\_biosynthetic\_process | SGMS1 | 2 | 1 |  |  |  |  |  |  |
| GO:0007619\_courtship\_behavior | HEXB | 2 | 1 |  |  |  |  |  |  |
| GO:0009313\_oligosaccharide\_catabolic\_process | HEXB | 2 | 1 |  |  |  |  |  |  |
| GO:0021965\_spinal\_cord\_ventral\_commissure\_morphogenesis | DCC | 2 | 1 |  |  |  |  |  |  |
| GO:0031946\_regulation\_of\_glucocorticoid\_biosynthetic\_process | NR3C1 | 2 | 1 |  |  |  |  |  |  |
| GO:0031952\_regulation\_of\_protein\_amino\_acid\_autophosphorylation | PTPRC | 2 | 1 |  |  |  |  |  |  |
| GO:0031953\_negative\_regulation\_of\_protein\_amino\_acid\_autophosphorylation | PTPRC | 2 | 1 |  |  |  |  |  |  |
| GO:0031958\_corticosteroid\_receptor\_signaling\_pathway | NR3C1 | 2 | 1 |  |  |  |  |  |  |
| GO:0033700\_phospholipid\_efflux | ABCA1 | 2 | 1 |  |  |  |  |  |  |
| GO:0042517\_positive\_regulation\_of\_tyrosine\_phosphorylation\_of\_Stat3\_protein | JAK2 | 2 | 1 |  |  |  |  |  |  |
| GO:0042921\_glucocorticoid\_receptor\_signaling\_pathway | NR3C1 | 2 | 1 |  |  |  |  |  |  |
| GO:0043320\_natural\_killer\_cell\_degranulation | RAB27A | 2 | 1 |  |  |  |  |  |  |
| GO:0045662\_negative\_regulation\_of\_myoblast\_differentiation | MBNL3 | 2 | 1 |  |  |  |  |  |  |
| GO:0045663\_positive\_regulation\_of\_myoblast\_differentiation | BOC | 2 | 1 |  |  |  |  |  |  |
| GO:0048619\_embryonic\_hindgut\_morphogenesis | TCF7L2 | 2 | 1 |  |  |  |  |  |  |
| GO:0048867\_stem\_cell\_fate\_determination | ASCL1 | 2 | 1 |  |  |  |  |  |  |
| GO:0050855\_regulation\_of\_B\_cell\_receptor\_signaling\_pathway | PTPRC | 2 | 1 |  |  |  |  |  |  |
| GO:0060166\_olfactory\_pit\_development | ASCL1 | 2 | 1 |  |  |  |  |  |  |
| GO:0060179\_male\_mating\_behavior | HEXB | 2 | 1 |  |  |  |  |  |  |
| GO:0060397\_JAK-STAT\_cascade\_involved\_in\_growth\_hormone\_signaling\_pathway | JAK2 | 2 | 1 |  |  |  |  |  |  |
| GO:0090030\_regulation\_of\_steroid\_hormone\_biosynthetic\_process | NR3C1 | 2 | 1 |  |  |  |  |  |  |
| GO:0042063\_gliogenesis | ASCL1 | 46 | 3 | 4.416560 | -1.526487 | 68 | 30.34 | 0.446176 |  |
| GO:0042063\_gliogenesis | NR2E1 | 46 | 3 | 4.416560 | -1.526487 | 68 | 30.34 | 0.446176 |  |
| GO:0042063\_gliogenesis | NKX6-1 | 46 | 3 | 4.416560 | -1.526487 | 68 | 30.34 | 0.446176 |  |
| GO:0048522\_positive\_regulation\_of\_cellular\_process | PTPRC | 895 | 20 | 1.513309 | -1.515412 | 69 | 30.48 | 0.441739 |  |
| GO:0048522\_positive\_regulation\_of\_cellular\_process | FGF18 | 895 | 20 | 1.513309 | -1.515412 | 69 | 30.48 | 0.441739 |  |
| GO:0048522\_positive\_regulation\_of\_cellular\_process | TBX3 | 895 | 20 | 1.513309 | -1.515412 | 69 | 30.48 | 0.441739 |  |
| GO:0048522\_positive\_regulation\_of\_cellular\_process | TBX20 | 895 | 20 | 1.513309 | -1.515412 | 69 | 30.48 | 0.441739 |  |
| GO:0048522\_positive\_regulation\_of\_cellular\_process | ESRRG | 895 | 20 | 1.513309 | -1.515412 | 69 | 30.48 | 0.441739 |  |
| GO:0048522\_positive\_regulation\_of\_cellular\_process | SOX5 | 895 | 20 | 1.513309 | -1.515412 | 69 | 30.48 | 0.441739 |  |
| GO:0048522\_positive\_regulation\_of\_cellular\_process | NR3C1 | 895 | 20 | 1.513309 | -1.515412 | 69 | 30.48 | 0.441739 |  |
| GO:0048522\_positive\_regulation\_of\_cellular\_process | NR2E1 | 895 | 20 | 1.513309 | -1.515412 | 69 | 30.48 | 0.441739 |  |
| GO:0048522\_positive\_regulation\_of\_cellular\_process | TCF7L2 | 895 | 20 | 1.513309 | -1.515412 | 69 | 30.48 | 0.441739 |  |
| GO:0048522\_positive\_regulation\_of\_cellular\_process | NKX6-1 | 895 | 20 | 1.513309 | -1.515412 | 69 | 30.48 | 0.441739 |  |
| GO:0048522\_positive\_regulation\_of\_cellular\_process | SERINC3 | 895 | 20 | 1.513309 | -1.515412 | 69 | 30.48 | 0.441739 |  |
| GO:0048522\_positive\_regulation\_of\_cellular\_process | ASCL1 | 895 | 20 | 1.513309 | -1.515412 | 69 | 30.48 | 0.441739 |  |
| GO:0048522\_positive\_regulation\_of\_cellular\_process | MSX1 | 895 | 20 | 1.513309 | -1.515412 | 69 | 30.48 | 0.441739 |  |
| GO:0048522\_positive\_regulation\_of\_cellular\_process | ETV1 | 895 | 20 | 1.513309 | -1.515412 | 69 | 30.48 | 0.441739 |  |
| GO:0048522\_positive\_regulation\_of\_cellular\_process | NEUROD1 | 895 | 20 | 1.513309 | -1.515412 | 69 | 30.48 | 0.441739 |  |
| GO:0048522\_positive\_regulation\_of\_cellular\_process | JAK2 | 895 | 20 | 1.513309 | -1.515412 | 69 | 30.48 | 0.441739 |  |
| GO:0048522\_positive\_regulation\_of\_cellular\_process | THBS1 | 895 | 20 | 1.513309 | -1.515412 | 69 | 30.48 | 0.441739 |  |
| GO:0048522\_positive\_regulation\_of\_cellular\_process | BOC | 895 | 20 | 1.513309 | -1.515412 | 69 | 30.48 | 0.441739 |  |
| GO:0048522\_positive\_regulation\_of\_cellular\_process | CD27 | 895 | 20 | 1.513309 | -1.515412 | 69 | 30.48 | 0.441739 |  |
| GO:0048522\_positive\_regulation\_of\_cellular\_process | RAB27A | 895 | 20 | 1.513309 | -1.515412 | 69 | 30.48 | 0.441739 |  |
| GO:0007275\_multicellular\_organismal\_development | DCC | 1760 | 34 | 1.308239 | -1.512578 | 70 | 30.52 | 0.436000 |  |
| GO:0007275\_multicellular\_organismal\_development | FGF18 | 1760 | 34 | 1.308239 | -1.512578 | 70 | 30.52 | 0.436000 |  |
| GO:0007275\_multicellular\_organismal\_development | IRX5 | 1760 | 34 | 1.308239 | -1.512578 | 70 | 30.52 | 0.436000 |  |
| GO:0007275\_multicellular\_organismal\_development | PLXNA2 | 1760 | 34 | 1.308239 | -1.512578 | 70 | 30.52 | 0.436000 |  |
| GO:0007275\_multicellular\_organismal\_development | TBX20 | 1760 | 34 | 1.308239 | -1.512578 | 70 | 30.52 | 0.436000 |  |
| GO:0007275\_multicellular\_organismal\_development | HEXB | 1760 | 34 | 1.308239 | -1.512578 | 70 | 30.52 | 0.436000 |  |
| GO:0007275\_multicellular\_organismal\_development | SOX5 | 1760 | 34 | 1.308239 | -1.512578 | 70 | 30.52 | 0.436000 |  |
| GO:0007275\_multicellular\_organismal\_development | NR3C1 | 1760 | 34 | 1.308239 | -1.512578 | 70 | 30.52 | 0.436000 |  |
| GO:0007275\_multicellular\_organismal\_development | TCF7L2 | 1760 | 34 | 1.308239 | -1.512578 | 70 | 30.52 | 0.436000 |  |
| GO:0007275\_multicellular\_organismal\_development | NR2E1 | 1760 | 34 | 1.308239 | -1.512578 | 70 | 30.52 | 0.436000 |  |
| GO:0007275\_multicellular\_organismal\_development | ALCAM | 1760 | 34 | 1.308239 | -1.512578 | 70 | 30.52 | 0.436000 |  |
| GO:0007275\_multicellular\_organismal\_development | AQP11 | 1760 | 34 | 1.308239 | -1.512578 | 70 | 30.52 | 0.436000 |  |
| GO:0007275\_multicellular\_organismal\_development | DMD | 1760 | 34 | 1.308239 | -1.512578 | 70 | 30.52 | 0.436000 |  |
| GO:0007275\_multicellular\_organismal\_development | ETV1 | 1760 | 34 | 1.308239 | -1.512578 | 70 | 30.52 | 0.436000 |  |
| GO:0007275\_multicellular\_organismal\_development | VPS13A | 1760 | 34 | 1.308239 | -1.512578 | 70 | 30.52 | 0.436000 |  |
| GO:0007275\_multicellular\_organismal\_development | MBNL3 | 1760 | 34 | 1.308239 | -1.512578 | 70 | 30.52 | 0.436000 |  |
| GO:0007275\_multicellular\_organismal\_development | B3GNT2 | 1760 | 34 | 1.308239 | -1.512578 | 70 | 30.52 | 0.436000 |  |
| GO:0007275\_multicellular\_organismal\_development | THBS1 | 1760 | 34 | 1.308239 | -1.512578 | 70 | 30.52 | 0.436000 |  |
| GO:0007275\_multicellular\_organismal\_development | BOC | 1760 | 34 | 1.308239 | -1.512578 | 70 | 30.52 | 0.436000 |  |
| GO:0007275\_multicellular\_organismal\_development | PTPRC | 1760 | 34 | 1.308239 | -1.512578 | 70 | 30.52 | 0.436000 |  |
| GO:0007275\_multicellular\_organismal\_development | MYO6 | 1760 | 34 | 1.308239 | -1.512578 | 70 | 30.52 | 0.436000 |  |
| GO:0007275\_multicellular\_organismal\_development | TBX3 | 1760 | 34 | 1.308239 | -1.512578 | 70 | 30.52 | 0.436000 |  |
| GO:0007275\_multicellular\_organismal\_development | TCFAP2B | 1760 | 34 | 1.308239 | -1.512578 | 70 | 30.52 | 0.436000 |  |
| GO:0007275\_multicellular\_organismal\_development | ARID5B | 1760 | 34 | 1.308239 | -1.512578 | 70 | 30.52 | 0.436000 |  |
| GO:0007275\_multicellular\_organismal\_development | ZFX | 1760 | 34 | 1.308239 | -1.512578 | 70 | 30.52 | 0.436000 |  |
| GO:0007275\_multicellular\_organismal\_development | PCDH8 | 1760 | 34 | 1.308239 | -1.512578 | 70 | 30.52 | 0.436000 |  |
| GO:0007275\_multicellular\_organismal\_development | ITGA4 | 1760 | 34 | 1.308239 | -1.512578 | 70 | 30.52 | 0.436000 |  |
| GO:0007275\_multicellular\_organismal\_development | NKX6-1 | 1760 | 34 | 1.308239 | -1.512578 | 70 | 30.52 | 0.436000 |  |
| GO:0007275\_multicellular\_organismal\_development | ASCL1 | 1760 | 34 | 1.308239 | -1.512578 | 70 | 30.52 | 0.436000 |  |
| GO:0007275\_multicellular\_organismal\_development | AFP | 1760 | 34 | 1.308239 | -1.512578 | 70 | 30.52 | 0.436000 |  |
| GO:0007275\_multicellular\_organismal\_development | MSX1 | 1760 | 34 | 1.308239 | -1.512578 | 70 | 30.52 | 0.436000 |  |
| GO:0007275\_multicellular\_organismal\_development | NEUROD1 | 1760 | 34 | 1.308239 | -1.512578 | 70 | 30.52 | 0.436000 |  |
| GO:0007275\_multicellular\_organismal\_development | JAK2 | 1760 | 34 | 1.308239 | -1.512578 | 70 | 30.52 | 0.436000 |  |
| GO:0007275\_multicellular\_organismal\_development | HDAC9 | 1760 | 34 | 1.308239 | -1.512578 | 70 | 30.52 | 0.436000 |  |
| GO:0006672\_ceramide\_metabolic\_process | HEXB | 19 | 2 | 7.128483 | -1.505094 | 72 | 31.31 | 0.434861 |  |
| GO:0006672\_ceramide\_metabolic\_process | PPAP2A | 19 | 2 | 7.128483 | -1.505094 | 72 | 31.31 | 0.434861 |  |
| GO:0050931\_pigment\_cell\_differentiation | MLPH | 19 | 2 | 7.128483 | -1.505094 | 72 | 31.31 | 0.434861 |  |
| GO:0050931\_pigment\_cell\_differentiation | RAB27A | 19 | 2 | 7.128483 | -1.505094 | 72 | 31.31 | 0.434861 |  |
| GO:0007411\_axon\_guidance | ALCAM | 82 | 4 | 3.303443 | -1.492769 | 74 | 31.99 | 0.432297 |  |
| GO:0007411\_axon\_guidance | ETV1 | 82 | 4 | 3.303443 | -1.492769 | 74 | 31.99 | 0.432297 |  |
| GO:0007411\_axon\_guidance | B3GNT2 | 82 | 4 | 3.303443 | -1.492769 | 74 | 31.99 | 0.432297 |  |
| GO:0007411\_axon\_guidance | BOC | 82 | 4 | 3.303443 | -1.492769 | 74 | 31.99 | 0.432297 |  |
| GO:0045664\_regulation\_of\_neuron\_differentiation | ASCL1 | 82 | 4 | 3.303443 | -1.492769 | 74 | 31.99 | 0.432297 |  |
| GO:0045664\_regulation\_of\_neuron\_differentiation | SOX5 | 82 | 4 | 3.303443 | -1.492769 | 74 | 31.99 | 0.432297 |  |
| GO:0045664\_regulation\_of\_neuron\_differentiation | NR2E1 | 82 | 4 | 3.303443 | -1.492769 | 74 | 31.99 | 0.432297 |  |
| GO:0045664\_regulation\_of\_neuron\_differentiation | NKX6-1 | 82 | 4 | 3.303443 | -1.492769 | 74 | 31.99 | 0.432297 |  |
| GO:0045935\_positive\_regulation\_of\_nucleobase\_\_nucleoside\_\_nucleotide\_and\_nucleic\_acid\_metabolic\_process | PTPRC | 352 | 10 | 1.923880 | -1.490975 | 75 | 32.17 | 0.428933 |  |
| GO:0045935\_positive\_regulation\_of\_nucleobase\_\_nucleoside\_\_nucleotide\_and\_nucleic\_acid\_metabolic\_process | ASCL1 | 352 | 10 | 1.923880 | -1.490975 | 75 | 32.17 | 0.428933 |  |
| GO:0045935\_positive\_regulation\_of\_nucleobase\_\_nucleoside\_\_nucleotide\_and\_nucleic\_acid\_metabolic\_process | MSX1 | 352 | 10 | 1.923880 | -1.490975 | 75 | 32.17 | 0.428933 |  |
| GO:0045935\_positive\_regulation\_of\_nucleobase\_\_nucleoside\_\_nucleotide\_and\_nucleic\_acid\_metabolic\_process | TBX3 | 352 | 10 | 1.923880 | -1.490975 | 75 | 32.17 | 0.428933 |  |
| GO:0045935\_positive\_regulation\_of\_nucleobase\_\_nucleoside\_\_nucleotide\_and\_nucleic\_acid\_metabolic\_process | TBX20 | 352 | 10 | 1.923880 | -1.490975 | 75 | 32.17 | 0.428933 |  |
| GO:0045935\_positive\_regulation\_of\_nucleobase\_\_nucleoside\_\_nucleotide\_and\_nucleic\_acid\_metabolic\_process | ESRRG | 352 | 10 | 1.923880 | -1.490975 | 75 | 32.17 | 0.428933 |  |
| GO:0045935\_positive\_regulation\_of\_nucleobase\_\_nucleoside\_\_nucleotide\_and\_nucleic\_acid\_metabolic\_process | SOX5 | 352 | 10 | 1.923880 | -1.490975 | 75 | 32.17 | 0.428933 |  |
| GO:0045935\_positive\_regulation\_of\_nucleobase\_\_nucleoside\_\_nucleotide\_and\_nucleic\_acid\_metabolic\_process | ETV1 | 352 | 10 | 1.923880 | -1.490975 | 75 | 32.17 | 0.428933 |  |
| GO:0045935\_positive\_regulation\_of\_nucleobase\_\_nucleoside\_\_nucleotide\_and\_nucleic\_acid\_metabolic\_process | NEUROD1 | 352 | 10 | 1.923880 | -1.490975 | 75 | 32.17 | 0.428933 |  |
| GO:0045935\_positive\_regulation\_of\_nucleobase\_\_nucleoside\_\_nucleotide\_and\_nucleic\_acid\_metabolic\_process | TCF7L2 | 352 | 10 | 1.923880 | -1.490975 | 75 | 32.17 | 0.428933 |  |
| GO:0006886\_intracellular\_protein\_transport | MYO6 | 122 | 5 | 2.775434 | -1.478336 | 76 | 32.72 | 0.430526 |  |
| GO:0006886\_intracellular\_protein\_transport | AP2A1 | 122 | 5 | 2.775434 | -1.478336 | 76 | 32.72 | 0.430526 |  |
| GO:0006886\_intracellular\_protein\_transport | MLPH | 122 | 5 | 2.775434 | -1.478336 | 76 | 32.72 | 0.430526 |  |
| GO:0006886\_intracellular\_protein\_transport | JAK2 | 122 | 5 | 2.775434 | -1.478336 | 76 | 32.72 | 0.430526 |  |
| GO:0006886\_intracellular\_protein\_transport | RAB27A | 122 | 5 | 2.775434 | -1.478336 | 76 | 32.72 | 0.430526 |  |
| GO:0043065\_positive\_regulation\_of\_apoptosis | SERINC3 | 166 | 6 | 2.447732 | -1.459219 | 77 | 34.28 | 0.445195 |  |
| GO:0043065\_positive\_regulation\_of\_apoptosis | ASCL1 | 166 | 6 | 2.447732 | -1.459219 | 77 | 34.28 | 0.445195 |  |
| GO:0043065\_positive\_regulation\_of\_apoptosis | PTPRC | 166 | 6 | 2.447732 | -1.459219 | 77 | 34.28 | 0.445195 |  |
| GO:0043065\_positive\_regulation\_of\_apoptosis | NR3C1 | 166 | 6 | 2.447732 | -1.459219 | 77 | 34.28 | 0.445195 |  |
| GO:0043065\_positive\_regulation\_of\_apoptosis | CD27 | 166 | 6 | 2.447732 | -1.459219 | 77 | 34.28 | 0.445195 |  |
| GO:0043065\_positive\_regulation\_of\_apoptosis | RAB27A | 166 | 6 | 2.447732 | -1.459219 | 77 | 34.28 | 0.445195 |  |
| GO:0043473\_pigmentation | MLPH | 49 | 3 | 4.146158 | -1.455888 | 78 | 34.66 | 0.444359 |  |
| GO:0043473\_pigmentation | ATRN | 49 | 3 | 4.146158 | -1.455888 | 78 | 34.66 | 0.444359 |  |
| GO:0043473\_pigmentation | RAB27A | 49 | 3 | 4.146158 | -1.455888 | 78 | 34.66 | 0.444359 |  |
| GO:0010942\_positive\_regulation\_of\_cell\_death | SERINC3 | 167 | 6 | 2.433075 | -1.448279 | 80 | 34.81 | 0.435125 |  |
| GO:0010942\_positive\_regulation\_of\_cell\_death | ASCL1 | 167 | 6 | 2.433075 | -1.448279 | 80 | 34.81 | 0.435125 |  |
| GO:0010942\_positive\_regulation\_of\_cell\_death | PTPRC | 167 | 6 | 2.433075 | -1.448279 | 80 | 34.81 | 0.435125 |  |
| GO:0010942\_positive\_regulation\_of\_cell\_death | NR3C1 | 167 | 6 | 2.433075 | -1.448279 | 80 | 34.81 | 0.435125 |  |
| GO:0010942\_positive\_regulation\_of\_cell\_death | CD27 | 167 | 6 | 2.433075 | -1.448279 | 80 | 34.81 | 0.435125 |  |
| GO:0010942\_positive\_regulation\_of\_cell\_death | RAB27A | 167 | 6 | 2.433075 | -1.448279 | 80 | 34.81 | 0.435125 |  |
| GO:0043068\_positive\_regulation\_of\_programmed\_cell\_death | SERINC3 | 167 | 6 | 2.433075 | -1.448279 | 80 | 34.81 | 0.435125 |  |
| GO:0043068\_positive\_regulation\_of\_programmed\_cell\_death | ASCL1 | 167 | 6 | 2.433075 | -1.448279 | 80 | 34.81 | 0.435125 |  |
| GO:0043068\_positive\_regulation\_of\_programmed\_cell\_death | PTPRC | 167 | 6 | 2.433075 | -1.448279 | 80 | 34.81 | 0.435125 |  |
| GO:0043068\_positive\_regulation\_of\_programmed\_cell\_death | NR3C1 | 167 | 6 | 2.433075 | -1.448279 | 80 | 34.81 | 0.435125 |  |
| GO:0043068\_positive\_regulation\_of\_programmed\_cell\_death | CD27 | 167 | 6 | 2.433075 | -1.448279 | 80 | 34.81 | 0.435125 |  |
| GO:0043068\_positive\_regulation\_of\_programmed\_cell\_death | RAB27A | 167 | 6 | 2.433075 | -1.448279 | 80 | 34.81 | 0.435125 |  |
| GO:0042981\_regulation\_of\_apoptosis | SERINC3 | 360 | 10 | 1.881127 | -1.432959 | 81 | 35.31 | 0.435926 |  |
| GO:0042981\_regulation\_of\_apoptosis | ASCL1 | 360 | 10 | 1.881127 | -1.432959 | 81 | 35.31 | 0.435926 |  |
| GO:0042981\_regulation\_of\_apoptosis | PTPRC | 360 | 10 | 1.881127 | -1.432959 | 81 | 35.31 | 0.435926 |  |
| GO:0042981\_regulation\_of\_apoptosis | MSX1 | 360 | 10 | 1.881127 | -1.432959 | 81 | 35.31 | 0.435926 |  |
| GO:0042981\_regulation\_of\_apoptosis | SGMS1 | 360 | 10 | 1.881127 | -1.432959 | 81 | 35.31 | 0.435926 |  |
| GO:0042981\_regulation\_of\_apoptosis | NR3C1 | 360 | 10 | 1.881127 | -1.432959 | 81 | 35.31 | 0.435926 |  |
| GO:0042981\_regulation\_of\_apoptosis | NR2E1 | 360 | 10 | 1.881127 | -1.432959 | 81 | 35.31 | 0.435926 |  |
| GO:0042981\_regulation\_of\_apoptosis | CD27 | 360 | 10 | 1.881127 | -1.432959 | 81 | 35.31 | 0.435926 |  |
| GO:0042981\_regulation\_of\_apoptosis | CAMK1D | 360 | 10 | 1.881127 | -1.432959 | 81 | 35.31 | 0.435926 |  |
| GO:0042981\_regulation\_of\_apoptosis | RAB27A | 360 | 10 | 1.881127 | -1.432959 | 81 | 35.31 | 0.435926 |  |
| GO:0006605\_protein\_targeting | MYO6 | 86 | 4 | 3.149795 | -1.427405 | 82 | 35.51 | 0.433049 |  |
| GO:0006605\_protein\_targeting | MLPH | 86 | 4 | 3.149795 | -1.427405 | 82 | 35.51 | 0.433049 |  |
| GO:0006605\_protein\_targeting | JAK2 | 86 | 4 | 3.149795 | -1.427405 | 82 | 35.51 | 0.433049 |  |
| GO:0006605\_protein\_targeting | RAB27A | 86 | 4 | 3.149795 | -1.427405 | 82 | 35.51 | 0.433049 |  |
| GO:0051173\_positive\_regulation\_of\_nitrogen\_compound\_metabolic\_process | PTPRC | 361 | 10 | 1.875917 | -1.425864 | 83 | 35.52 | 0.427952 |  |
| GO:0051173\_positive\_regulation\_of\_nitrogen\_compound\_metabolic\_process | ASCL1 | 361 | 10 | 1.875917 | -1.425864 | 83 | 35.52 | 0.427952 |  |
| GO:0051173\_positive\_regulation\_of\_nitrogen\_compound\_metabolic\_process | MSX1 | 361 | 10 | 1.875917 | -1.425864 | 83 | 35.52 | 0.427952 |  |
| GO:0051173\_positive\_regulation\_of\_nitrogen\_compound\_metabolic\_process | TBX3 | 361 | 10 | 1.875917 | -1.425864 | 83 | 35.52 | 0.427952 |  |
| GO:0051173\_positive\_regulation\_of\_nitrogen\_compound\_metabolic\_process | TBX20 | 361 | 10 | 1.875917 | -1.425864 | 83 | 35.52 | 0.427952 |  |
| GO:0051173\_positive\_regulation\_of\_nitrogen\_compound\_metabolic\_process | ESRRG | 361 | 10 | 1.875917 | -1.425864 | 83 | 35.52 | 0.427952 |  |
| GO:0051173\_positive\_regulation\_of\_nitrogen\_compound\_metabolic\_process | SOX5 | 361 | 10 | 1.875917 | -1.425864 | 83 | 35.52 | 0.427952 |  |
| GO:0051173\_positive\_regulation\_of\_nitrogen\_compound\_metabolic\_process | ETV1 | 361 | 10 | 1.875917 | -1.425864 | 83 | 35.52 | 0.427952 |  |
| GO:0051173\_positive\_regulation\_of\_nitrogen\_compound\_metabolic\_process | NEUROD1 | 361 | 10 | 1.875917 | -1.425864 | 83 | 35.52 | 0.427952 |  |
| GO:0051173\_positive\_regulation\_of\_nitrogen\_compound\_metabolic\_process | TCF7L2 | 361 | 10 | 1.875917 | -1.425864 | 83 | 35.52 | 0.427952 |  |
| GO:0002456\_T\_cell\_mediated\_immunity | PTPRC | 21 | 2 | 6.449580 | -1.424057 | 84 | 36.9 | 0.439286 |  |
| GO:0002456\_T\_cell\_mediated\_immunity | RAB27A | 21 | 2 | 6.449580 | -1.424057 | 84 | 36.9 | 0.439286 |  |
| GO:0032501\_multicellular\_organismal\_process | DCC | 2183 | 40 | 1.240872 | -1.423244 | 85 | 36.91 | 0.434235 |  |
| GO:0032501\_multicellular\_organismal\_process | FGF18 | 2183 | 40 | 1.240872 | -1.423244 | 85 | 36.91 | 0.434235 |  |
| GO:0032501\_multicellular\_organismal\_process | ELF1 | 2183 | 40 | 1.240872 | -1.423244 | 85 | 36.91 | 0.434235 |  |
| GO:0032501\_multicellular\_organismal\_process | IRX5 | 2183 | 40 | 1.240872 | -1.423244 | 85 | 36.91 | 0.434235 |  |
| GO:0032501\_multicellular\_organismal\_process | PLXNA2 | 2183 | 40 | 1.240872 | -1.423244 | 85 | 36.91 | 0.434235 |  |
| GO:0032501\_multicellular\_organismal\_process | HEXB | 2183 | 40 | 1.240872 | -1.423244 | 85 | 36.91 | 0.434235 |  |
| GO:0032501\_multicellular\_organismal\_process | TBX20 | 2183 | 40 | 1.240872 | -1.423244 | 85 | 36.91 | 0.434235 |  |
| GO:0032501\_multicellular\_organismal\_process | SOX5 | 2183 | 40 | 1.240872 | -1.423244 | 85 | 36.91 | 0.434235 |  |
| GO:0032501\_multicellular\_organismal\_process | NR3C1 | 2183 | 40 | 1.240872 | -1.423244 | 85 | 36.91 | 0.434235 |  |
| GO:0032501\_multicellular\_organismal\_process | NR2E1 | 2183 | 40 | 1.240872 | -1.423244 | 85 | 36.91 | 0.434235 |  |
| GO:0032501\_multicellular\_organismal\_process | TCF7L2 | 2183 | 40 | 1.240872 | -1.423244 | 85 | 36.91 | 0.434235 |  |
| GO:0032501\_multicellular\_organismal\_process | ALCAM | 2183 | 40 | 1.240872 | -1.423244 | 85 | 36.91 | 0.434235 |  |
| GO:0032501\_multicellular\_organismal\_process | MYOM2 | 2183 | 40 | 1.240872 | -1.423244 | 85 | 36.91 | 0.434235 |  |
| GO:0032501\_multicellular\_organismal\_process | AQP11 | 2183 | 40 | 1.240872 | -1.423244 | 85 | 36.91 | 0.434235 |  |
| GO:0032501\_multicellular\_organismal\_process | DMD | 2183 | 40 | 1.240872 | -1.423244 | 85 | 36.91 | 0.434235 |  |
| GO:0032501\_multicellular\_organismal\_process | ETV1 | 2183 | 40 | 1.240872 | -1.423244 | 85 | 36.91 | 0.434235 |  |
| GO:0032501\_multicellular\_organismal\_process | VPS13A | 2183 | 40 | 1.240872 | -1.423244 | 85 | 36.91 | 0.434235 |  |
| GO:0032501\_multicellular\_organismal\_process | MBNL3 | 2183 | 40 | 1.240872 | -1.423244 | 85 | 36.91 | 0.434235 |  |
| GO:0032501\_multicellular\_organismal\_process | B3GNT2 | 2183 | 40 | 1.240872 | -1.423244 | 85 | 36.91 | 0.434235 |  |
| GO:0032501\_multicellular\_organismal\_process | THBS1 | 2183 | 40 | 1.240872 | -1.423244 | 85 | 36.91 | 0.434235 |  |
| GO:0032501\_multicellular\_organismal\_process | BOC | 2183 | 40 | 1.240872 | -1.423244 | 85 | 36.91 | 0.434235 |  |
| GO:0032501\_multicellular\_organismal\_process | RAB27A | 2183 | 40 | 1.240872 | -1.423244 | 85 | 36.91 | 0.434235 |  |
| GO:0032501\_multicellular\_organismal\_process | PTPRC | 2183 | 40 | 1.240872 | -1.423244 | 85 | 36.91 | 0.434235 |  |
| GO:0032501\_multicellular\_organismal\_process | MYO6 | 2183 | 40 | 1.240872 | -1.423244 | 85 | 36.91 | 0.434235 |  |
| GO:0032501\_multicellular\_organismal\_process | TBX3 | 2183 | 40 | 1.240872 | -1.423244 | 85 | 36.91 | 0.434235 |  |
| GO:0032501\_multicellular\_organismal\_process | TCFAP2B | 2183 | 40 | 1.240872 | -1.423244 | 85 | 36.91 | 0.434235 |  |
| GO:0032501\_multicellular\_organismal\_process | ARID5B | 2183 | 40 | 1.240872 | -1.423244 | 85 | 36.91 | 0.434235 |  |
| GO:0032501\_multicellular\_organismal\_process | ATRN | 2183 | 40 | 1.240872 | -1.423244 | 85 | 36.91 | 0.434235 |  |
| GO:0032501\_multicellular\_organismal\_process | ZFX | 2183 | 40 | 1.240872 | -1.423244 | 85 | 36.91 | 0.434235 |  |
| GO:0032501\_multicellular\_organismal\_process | PCDH8 | 2183 | 40 | 1.240872 | -1.423244 | 85 | 36.91 | 0.434235 |  |
| GO:0032501\_multicellular\_organismal\_process | ITGA4 | 2183 | 40 | 1.240872 | -1.423244 | 85 | 36.91 | 0.434235 |  |
| GO:0032501\_multicellular\_organismal\_process | ATP1A2 | 2183 | 40 | 1.240872 | -1.423244 | 85 | 36.91 | 0.434235 |  |
| GO:0032501\_multicellular\_organismal\_process | NKX6-1 | 2183 | 40 | 1.240872 | -1.423244 | 85 | 36.91 | 0.434235 |  |
| GO:0032501\_multicellular\_organismal\_process | ATXN2 | 2183 | 40 | 1.240872 | -1.423244 | 85 | 36.91 | 0.434235 |  |
| GO:0032501\_multicellular\_organismal\_process | ASCL1 | 2183 | 40 | 1.240872 | -1.423244 | 85 | 36.91 | 0.434235 |  |
| GO:0032501\_multicellular\_organismal\_process | AFP | 2183 | 40 | 1.240872 | -1.423244 | 85 | 36.91 | 0.434235 |  |
| GO:0032501\_multicellular\_organismal\_process | MSX1 | 2183 | 40 | 1.240872 | -1.423244 | 85 | 36.91 | 0.434235 |  |
| GO:0032501\_multicellular\_organismal\_process | NEUROD1 | 2183 | 40 | 1.240872 | -1.423244 | 85 | 36.91 | 0.434235 |  |
| GO:0032501\_multicellular\_organismal\_process | JAK2 | 2183 | 40 | 1.240872 | -1.423244 | 85 | 36.91 | 0.434235 |  |
| GO:0032501\_multicellular\_organismal\_process | HDAC9 | 2183 | 40 | 1.240872 | -1.423244 | 85 | 36.91 | 0.434235 |  |
| GO:0048812\_neuron\_projection\_morphogenesis | DCC | 170 | 6 | 2.390138 | -1.416026 | 86 | 36.96 | 0.429767 |  |
| GO:0048812\_neuron\_projection\_morphogenesis | ALCAM | 170 | 6 | 2.390138 | -1.416026 | 86 | 36.96 | 0.429767 |  |
| GO:0048812\_neuron\_projection\_morphogenesis | ETV1 | 170 | 6 | 2.390138 | -1.416026 | 86 | 36.96 | 0.429767 |  |
| GO:0048812\_neuron\_projection\_morphogenesis | B3GNT2 | 170 | 6 | 2.390138 | -1.416026 | 86 | 36.96 | 0.429767 |  |
| GO:0048812\_neuron\_projection\_morphogenesis | NR2E1 | 170 | 6 | 2.390138 | -1.416026 | 86 | 36.96 | 0.429767 |  |
| GO:0048812\_neuron\_projection\_morphogenesis | BOC | 170 | 6 | 2.390138 | -1.416026 | 86 | 36.96 | 0.429767 |  |
| GO:0030030\_cell\_projection\_organization | DCC | 263 | 8 | 2.059942 | -1.415702 | 87 | 36.97 | 0.424943 |  |
| GO:0030030\_cell\_projection\_organization | ALCAM | 263 | 8 | 2.059942 | -1.415702 | 87 | 36.97 | 0.424943 |  |
| GO:0030030\_cell\_projection\_organization | MYO6 | 263 | 8 | 2.059942 | -1.415702 | 87 | 36.97 | 0.424943 |  |
| GO:0030030\_cell\_projection\_organization | ETV1 | 263 | 8 | 2.059942 | -1.415702 | 87 | 36.97 | 0.424943 |  |
| GO:0030030\_cell\_projection\_organization | B3GNT2 | 263 | 8 | 2.059942 | -1.415702 | 87 | 36.97 | 0.424943 |  |
| GO:0030030\_cell\_projection\_organization | NR2E1 | 263 | 8 | 2.059942 | -1.415702 | 87 | 36.97 | 0.424943 |  |
| GO:0030030\_cell\_projection\_organization | BOC | 263 | 8 | 2.059942 | -1.415702 | 87 | 36.97 | 0.424943 |  |
| GO:0030030\_cell\_projection\_organization | FGD4 | 263 | 8 | 2.059942 | -1.415702 | 87 | 36.97 | 0.424943 |  |
| GO:0016337\_cell-cell\_adhesion | PTPRC | 87 | 4 | 3.113590 | -1.411682 | 88 | 37.16 | 0.422273 |  |
| GO:0016337\_cell-cell\_adhesion | CLDN1 | 87 | 4 | 3.113590 | -1.411682 | 88 | 37.16 | 0.422273 |  |
| GO:0016337\_cell-cell\_adhesion | ITGA4 | 87 | 4 | 3.113590 | -1.411682 | 88 | 37.16 | 0.422273 |  |
| GO:0016337\_cell-cell\_adhesion | BOC | 87 | 4 | 3.113590 | -1.411682 | 88 | 37.16 | 0.422273 |  |
| GO:0010941\_regulation\_of\_cell\_death | SERINC3 | 365 | 10 | 1.855359 | -1.397820 | 90 | 37.92 | 0.421333 |  |
| GO:0010941\_regulation\_of\_cell\_death | ASCL1 | 365 | 10 | 1.855359 | -1.397820 | 90 | 37.92 | 0.421333 |  |
| GO:0010941\_regulation\_of\_cell\_death | PTPRC | 365 | 10 | 1.855359 | -1.397820 | 90 | 37.92 | 0.421333 |  |
| GO:0010941\_regulation\_of\_cell\_death | MSX1 | 365 | 10 | 1.855359 | -1.397820 | 90 | 37.92 | 0.421333 |  |
| GO:0010941\_regulation\_of\_cell\_death | SGMS1 | 365 | 10 | 1.855359 | -1.397820 | 90 | 37.92 | 0.421333 |  |
| GO:0010941\_regulation\_of\_cell\_death | NR3C1 | 365 | 10 | 1.855359 | -1.397820 | 90 | 37.92 | 0.421333 |  |
| GO:0010941\_regulation\_of\_cell\_death | NR2E1 | 365 | 10 | 1.855359 | -1.397820 | 90 | 37.92 | 0.421333 |  |
| GO:0010941\_regulation\_of\_cell\_death | CD27 | 365 | 10 | 1.855359 | -1.397820 | 90 | 37.92 | 0.421333 |  |
| GO:0010941\_regulation\_of\_cell\_death | CAMK1D | 365 | 10 | 1.855359 | -1.397820 | 90 | 37.92 | 0.421333 |  |
| GO:0010941\_regulation\_of\_cell\_death | RAB27A | 365 | 10 | 1.855359 | -1.397820 | 90 | 37.92 | 0.421333 |  |
| GO:0043067\_regulation\_of\_programmed\_cell\_death | SERINC3 | 365 | 10 | 1.855359 | -1.397820 | 90 | 37.92 | 0.421333 |  |
| GO:0043067\_regulation\_of\_programmed\_cell\_death | ASCL1 | 365 | 10 | 1.855359 | -1.397820 | 90 | 37.92 | 0.421333 |  |
| GO:0043067\_regulation\_of\_programmed\_cell\_death | PTPRC | 365 | 10 | 1.855359 | -1.397820 | 90 | 37.92 | 0.421333 |  |
| GO:0043067\_regulation\_of\_programmed\_cell\_death | MSX1 | 365 | 10 | 1.855359 | -1.397820 | 90 | 37.92 | 0.421333 |  |
| GO:0043067\_regulation\_of\_programmed\_cell\_death | SGMS1 | 365 | 10 | 1.855359 | -1.397820 | 90 | 37.92 | 0.421333 |  |
| GO:0043067\_regulation\_of\_programmed\_cell\_death | NR3C1 | 365 | 10 | 1.855359 | -1.397820 | 90 | 37.92 | 0.421333 |  |
| GO:0043067\_regulation\_of\_programmed\_cell\_death | NR2E1 | 365 | 10 | 1.855359 | -1.397820 | 90 | 37.92 | 0.421333 |  |
| GO:0043067\_regulation\_of\_programmed\_cell\_death | CD27 | 365 | 10 | 1.855359 | -1.397820 | 90 | 37.92 | 0.421333 |  |
| GO:0043067\_regulation\_of\_programmed\_cell\_death | CAMK1D | 365 | 10 | 1.855359 | -1.397820 | 90 | 37.92 | 0.421333 |  |
| GO:0043067\_regulation\_of\_programmed\_cell\_death | RAB27A | 365 | 10 | 1.855359 | -1.397820 | 90 | 37.92 | 0.421333 |  |
| GO:0001947\_heart\_looping | TBX3 | 22 | 2 | 6.156417 | -1.386750 | 92 | 40.17 | 0.436630 |  |
| GO:0001947\_heart\_looping | TBX20 | 22 | 2 | 6.156417 | -1.386750 | 92 | 40.17 | 0.436630 |  |
| GO:0048477\_oogenesis | HEXB | 22 | 2 | 6.156417 | -1.386750 | 92 | 40.17 | 0.436630 |  |
| GO:0048477\_oogenesis | ZFX | 22 | 2 | 6.156417 | -1.386750 | 92 | 40.17 | 0.436630 |  |
| GO:0048667\_cell\_morphogenesis\_involved\_in\_neuron\_differentiation | DCC | 173 | 6 | 2.348691 | -1.384600 | 93 | 40.21 | 0.432366 |  |
| GO:0048667\_cell\_morphogenesis\_involved\_in\_neuron\_differentiation | ALCAM | 173 | 6 | 2.348691 | -1.384600 | 93 | 40.21 | 0.432366 |  |
| GO:0048667\_cell\_morphogenesis\_involved\_in\_neuron\_differentiation | ETV1 | 173 | 6 | 2.348691 | -1.384600 | 93 | 40.21 | 0.432366 |  |
| GO:0048667\_cell\_morphogenesis\_involved\_in\_neuron\_differentiation | B3GNT2 | 173 | 6 | 2.348691 | -1.384600 | 93 | 40.21 | 0.432366 |  |
| GO:0048667\_cell\_morphogenesis\_involved\_in\_neuron\_differentiation | NR2E1 | 173 | 6 | 2.348691 | -1.384600 | 93 | 40.21 | 0.432366 |  |
| GO:0048667\_cell\_morphogenesis\_involved\_in\_neuron\_differentiation | BOC | 173 | 6 | 2.348691 | -1.384600 | 93 | 40.21 | 0.432366 |  |
| GO:0042592\_homeostatic\_process | PTPRC | 419 | 11 | 1.777867 | -1.383498 | 94 | 40.23 | 0.427979 |  |
| GO:0042592\_homeostatic\_process | AQP11 | 419 | 11 | 1.777867 | -1.383498 | 94 | 40.23 | 0.427979 |  |
| GO:0042592\_homeostatic\_process | DMD | 419 | 11 | 1.777867 | -1.383498 | 94 | 40.23 | 0.427979 |  |
| GO:0042592\_homeostatic\_process | ZFX | 419 | 11 | 1.777867 | -1.383498 | 94 | 40.23 | 0.427979 |  |
| GO:0042592\_homeostatic\_process | HEXB | 419 | 11 | 1.777867 | -1.383498 | 94 | 40.23 | 0.427979 |  |
| GO:0042592\_homeostatic\_process | HSPA1A | 419 | 11 | 1.777867 | -1.383498 | 94 | 40.23 | 0.427979 |  |
| GO:0042592\_homeostatic\_process | KCNA5 | 419 | 11 | 1.777867 | -1.383498 | 94 | 40.23 | 0.427979 |  |
| GO:0042592\_homeostatic\_process | ATP1A2 | 419 | 11 | 1.777867 | -1.383498 | 94 | 40.23 | 0.427979 |  |
| GO:0042592\_homeostatic\_process | ATP6V1B1 | 419 | 11 | 1.777867 | -1.383498 | 94 | 40.23 | 0.427979 |  |
| GO:0042592\_homeostatic\_process | SLC9A3R1 | 419 | 11 | 1.777867 | -1.383498 | 94 | 40.23 | 0.427979 |  |
| GO:0042592\_homeostatic\_process | TCF7L2 | 419 | 11 | 1.777867 | -1.383498 | 94 | 40.23 | 0.427979 |  |
| GO:0065008\_regulation\_of\_biological\_quality | PTPRC | 693 | 16 | 1.563535 | -1.380626 | 95 | 40.31 | 0.424316 |  |
| GO:0065008\_regulation\_of\_biological\_quality | IRX5 | 693 | 16 | 1.563535 | -1.380626 | 95 | 40.31 | 0.424316 |  |
| GO:0065008\_regulation\_of\_biological\_quality | HEXB | 693 | 16 | 1.563535 | -1.380626 | 95 | 40.31 | 0.424316 |  |
| GO:0065008\_regulation\_of\_biological\_quality | ZFX | 693 | 16 | 1.563535 | -1.380626 | 95 | 40.31 | 0.424316 |  |
| GO:0065008\_regulation\_of\_biological\_quality | HSPA1A | 693 | 16 | 1.563535 | -1.380626 | 95 | 40.31 | 0.424316 |  |
| GO:0065008\_regulation\_of\_biological\_quality | ATP1A2 | 693 | 16 | 1.563535 | -1.380626 | 95 | 40.31 | 0.424316 |  |
| GO:0065008\_regulation\_of\_biological\_quality | SGMS1 | 693 | 16 | 1.563535 | -1.380626 | 95 | 40.31 | 0.424316 |  |
| GO:0065008\_regulation\_of\_biological\_quality | NR3C1 | 693 | 16 | 1.563535 | -1.380626 | 95 | 40.31 | 0.424316 |  |
| GO:0065008\_regulation\_of\_biological\_quality | KCNA5 | 693 | 16 | 1.563535 | -1.380626 | 95 | 40.31 | 0.424316 |  |
| GO:0065008\_regulation\_of\_biological\_quality | SLC9A3R1 | 693 | 16 | 1.563535 | -1.380626 | 95 | 40.31 | 0.424316 |  |
| GO:0065008\_regulation\_of\_biological\_quality | ATP6V1B1 | 693 | 16 | 1.563535 | -1.380626 | 95 | 40.31 | 0.424316 |  |
| GO:0065008\_regulation\_of\_biological\_quality | TCF7L2 | 693 | 16 | 1.563535 | -1.380626 | 95 | 40.31 | 0.424316 |  |
| GO:0065008\_regulation\_of\_biological\_quality | AFP | 693 | 16 | 1.563535 | -1.380626 | 95 | 40.31 | 0.424316 |  |
| GO:0065008\_regulation\_of\_biological\_quality | AQP11 | 693 | 16 | 1.563535 | -1.380626 | 95 | 40.31 | 0.424316 |  |
| GO:0065008\_regulation\_of\_biological\_quality | DMD | 693 | 16 | 1.563535 | -1.380626 | 95 | 40.31 | 0.424316 |  |
| GO:0065008\_regulation\_of\_biological\_quality | RAB27A | 693 | 16 | 1.563535 | -1.380626 | 95 | 40.31 | 0.424316 |  |
| GO:0045165\_cell\_fate\_commitment | ASCL1 | 130 | 5 | 2.604638 | -1.377032 | 96 | 40.45 | 0.421354 |  |
| GO:0045165\_cell\_fate\_commitment | SOX5 | 130 | 5 | 2.604638 | -1.377032 | 96 | 40.45 | 0.421354 |  |
| GO:0045165\_cell\_fate\_commitment | NEUROD1 | 130 | 5 | 2.604638 | -1.377032 | 96 | 40.45 | 0.421354 |  |
| GO:0045165\_cell\_fate\_commitment | NR2E1 | 130 | 5 | 2.604638 | -1.377032 | 96 | 40.45 | 0.421354 |  |
| GO:0045165\_cell\_fate\_commitment | NKX6-1 | 130 | 5 | 2.604638 | -1.377032 | 96 | 40.45 | 0.421354 |  |
| GO:0030003\_cellular\_cation\_homeostasis | PTPRC | 90 | 4 | 3.009804 | -1.365907 | 98 | 41.07 | 0.419082 |  |
| GO:0030003\_cellular\_cation\_homeostasis | AQP11 | 90 | 4 | 3.009804 | -1.365907 | 98 | 41.07 | 0.419082 |  |
| GO:0030003\_cellular\_cation\_homeostasis | HEXB | 90 | 4 | 3.009804 | -1.365907 | 98 | 41.07 | 0.419082 |  |
| GO:0030003\_cellular\_cation\_homeostasis | ATP1A2 | 90 | 4 | 3.009804 | -1.365907 | 98 | 41.07 | 0.419082 |  |
| GO:0035264\_multicellular\_organism\_growth | ATXN2 | 90 | 4 | 3.009804 | -1.365907 | 98 | 41.07 | 0.419082 |  |
| GO:0035264\_multicellular\_organism\_growth | ARID5B | 90 | 4 | 3.009804 | -1.365907 | 98 | 41.07 | 0.419082 |  |
| GO:0035264\_multicellular\_organism\_growth | ZFX | 90 | 4 | 3.009804 | -1.365907 | 98 | 41.07 | 0.419082 |  |
| GO:0035264\_multicellular\_organism\_growth | ATRN | 90 | 4 | 3.009804 | -1.365907 | 98 | 41.07 | 0.419082 |  |
| GO:0001915\_negative\_regulation\_of\_T\_cell\_mediated\_cytotoxicity | PTPRC | 3 | 1 |  |  |  |  |  |  |
| GO:0001960\_negative\_regulation\_of\_cytokine-mediated\_signaling\_pathway | PTPRC | 3 | 1 |  |  |  |  |  |  |
| GO:0006651\_diacylglycerol\_biosynthetic\_process | PPAP2A | 3 | 1 |  |  |  |  |  |  |
| GO:0006689\_ganglioside\_catabolic\_process | HEXB | 3 | 1 |  |  |  |  |  |  |
| GO:0007262\_STAT\_protein\_nuclear\_translocation | JAK2 | 3 | 1 |  |  |  |  |  |  |
| GO:0008347\_glial\_cell\_migration | NR2E1 | 3 | 1 |  |  |  |  |  |  |
| GO:0010159\_specification\_of\_organ\_position | TBX3 | 3 | 1 |  |  |  |  |  |  |
| GO:0021527\_spinal\_cord\_association\_neuron\_differentiation | ASCL1 | 3 | 1 |  |  |  |  |  |  |
| GO:0021529\_spinal\_cord\_oligodendrocyte\_cell\_differentiation | ASCL1 | 3 | 1 |  |  |  |  |  |  |
| GO:0021530\_spinal\_cord\_oligodendrocyte\_cell\_fate\_specification | ASCL1 | 3 | 1 |  |  |  |  |  |  |
| GO:0021912\_regulation\_of\_transcription\_from\_RNA\_polymerase\_II\_promoter\_involved\_in\_spinal\_cord\_motor\_neuron\_fate\_specification | NKX6-1 | 3 | 1 |  |  |  |  |  |  |
| GO:0030319\_cellular\_di-\_\_tri-valent\_inorganic\_anion\_homeostasis | SLC9A3R1 | 3 | 1 |  |  |  |  |  |  |
| GO:0030538\_embryonic\_genitalia\_morphogenesis | TCF7L2 | 3 | 1 |  |  |  |  |  |  |
| GO:0030643\_cellular\_phosphate\_ion\_homeostasis | SLC9A3R1 | 3 | 1 |  |  |  |  |  |  |
| GO:0031943\_regulation\_of\_glucocorticoid\_metabolic\_process | NR3C1 | 3 | 1 |  |  |  |  |  |  |
| GO:0032402\_melanosome\_transport | RAB27A | 3 | 1 |  |  |  |  |  |  |
| GO:0042448\_progesterone\_metabolic\_process | AFP | 3 | 1 |  |  |  |  |  |  |
| GO:0042523\_positive\_regulation\_of\_tyrosine\_phosphorylation\_of\_Stat5\_protein | JAK2 | 3 | 1 |  |  |  |  |  |  |
| GO:0043267\_negative\_regulation\_of\_potassium\_ion\_transport | KCNA5 | 3 | 1 |  |  |  |  |  |  |
| GO:0046479\_glycosphingolipid\_catabolic\_process | HEXB | 3 | 1 |  |  |  |  |  |  |
| GO:0046885\_regulation\_of\_hormone\_biosynthetic\_process | NR3C1 | 3 | 1 |  |  |  |  |  |  |
| GO:0048865\_stem\_cell\_fate\_commitment | ASCL1 | 3 | 1 |  |  |  |  |  |  |
| GO:0050857\_positive\_regulation\_of\_antigen\_receptor-mediated\_signaling\_pathway | PTPRC | 3 | 1 |  |  |  |  |  |  |
| GO:0051149\_positive\_regulation\_of\_muscle\_cell\_differentiation | BOC | 3 | 1 |  |  |  |  |  |  |
| GO:0055061\_di-\_\_tri-valent\_inorganic\_anion\_homeostasis | SLC9A3R1 | 3 | 1 |  |  |  |  |  |  |
| GO:0055062\_phosphate\_ion\_homeostasis | SLC9A3R1 | 3 | 1 |  |  |  |  |  |  |
| GO:0060040\_retinal\_bipolar\_neuron\_differentiation | IRX5 | 3 | 1 |  |  |  |  |  |  |
| GO:0060396\_growth\_hormone\_receptor\_signaling\_pathway | JAK2 | 3 | 1 |  |  |  |  |  |  |
| GO:0060416\_response\_to\_growth\_hormone\_stimulus | JAK2 | 3 | 1 |  |  |  |  |  |  |
| GO:0060596\_mammary\_placode\_formation | TBX3 | 3 | 1 |  |  |  |  |  |  |
| GO:0022008\_neurogenesis | ALCAM | 423 | 11 | 1.761055 | -1.358032 | 99 | 41.24 | 0.416566 |  |
| GO:0022008\_neurogenesis | DCC | 423 | 11 | 1.761055 | -1.358032 | 99 | 41.24 | 0.416566 |  |
| GO:0022008\_neurogenesis | ASCL1 | 423 | 11 | 1.761055 | -1.358032 | 99 | 41.24 | 0.416566 |  |
| GO:0022008\_neurogenesis | IRX5 | 423 | 11 | 1.761055 | -1.358032 | 99 | 41.24 | 0.416566 |  |
| GO:0022008\_neurogenesis | MYO6 | 423 | 11 | 1.761055 | -1.358032 | 99 | 41.24 | 0.416566 |  |
| GO:0022008\_neurogenesis | SOX5 | 423 | 11 | 1.761055 | -1.358032 | 99 | 41.24 | 0.416566 |  |
| GO:0022008\_neurogenesis | ETV1 | 423 | 11 | 1.761055 | -1.358032 | 99 | 41.24 | 0.416566 |  |
| GO:0022008\_neurogenesis | B3GNT2 | 423 | 11 | 1.761055 | -1.358032 | 99 | 41.24 | 0.416566 |  |
| GO:0022008\_neurogenesis | BOC | 423 | 11 | 1.761055 | -1.358032 | 99 | 41.24 | 0.416566 |  |
| GO:0022008\_neurogenesis | NR2E1 | 423 | 11 | 1.761055 | -1.358032 | 99 | 41.24 | 0.416566 |  |
| GO:0022008\_neurogenesis | NKX6-1 | 423 | 11 | 1.761055 | -1.358032 | 99 | 41.24 | 0.416566 |  |
| GO:0006873\_cellular\_ion\_homeostasis | PTPRC | 176 | 6 | 2.308656 | -1.353972 | 101 | 41.39 | 0.409802 |  |
| GO:0006873\_cellular\_ion\_homeostasis | AQP11 | 176 | 6 | 2.308656 | -1.353972 | 101 | 41.39 | 0.409802 |  |
| GO:0006873\_cellular\_ion\_homeostasis | HEXB | 176 | 6 | 2.308656 | -1.353972 | 101 | 41.39 | 0.409802 |  |
| GO:0006873\_cellular\_ion\_homeostasis | KCNA5 | 176 | 6 | 2.308656 | -1.353972 | 101 | 41.39 | 0.409802 |  |
| GO:0006873\_cellular\_ion\_homeostasis | ATP1A2 | 176 | 6 | 2.308656 | -1.353972 | 101 | 41.39 | 0.409802 |  |
| GO:0006873\_cellular\_ion\_homeostasis | SLC9A3R1 | 176 | 6 | 2.308656 | -1.353972 | 101 | 41.39 | 0.409802 |  |
| GO:0048858\_cell\_projection\_morphogenesis | DCC | 176 | 6 | 2.308656 | -1.353972 | 101 | 41.39 | 0.409802 |  |
| GO:0048858\_cell\_projection\_morphogenesis | ALCAM | 176 | 6 | 2.308656 | -1.353972 | 101 | 41.39 | 0.409802 |  |
| GO:0048858\_cell\_projection\_morphogenesis | ETV1 | 176 | 6 | 2.308656 | -1.353972 | 101 | 41.39 | 0.409802 |  |
| GO:0048858\_cell\_projection\_morphogenesis | B3GNT2 | 176 | 6 | 2.308656 | -1.353972 | 101 | 41.39 | 0.409802 |  |
| GO:0048858\_cell\_projection\_morphogenesis | NR2E1 | 176 | 6 | 2.308656 | -1.353972 | 101 | 41.39 | 0.409802 |  |
| GO:0048858\_cell\_projection\_morphogenesis | BOC | 176 | 6 | 2.308656 | -1.353972 | 101 | 41.39 | 0.409802 |  |
| GO:0048518\_positive\_regulation\_of\_biological\_process | FGF18 | 995 | 21 | 1.429279 | -1.333125 | 102 | 43.36 | 0.425098 |  |
| GO:0048518\_positive\_regulation\_of\_biological\_process | PTPRC | 995 | 21 | 1.429279 | -1.333125 | 102 | 43.36 | 0.425098 |  |
| GO:0048518\_positive\_regulation\_of\_biological\_process | ELF1 | 995 | 21 | 1.429279 | -1.333125 | 102 | 43.36 | 0.425098 |  |
| GO:0048518\_positive\_regulation\_of\_biological\_process | TBX3 | 995 | 21 | 1.429279 | -1.333125 | 102 | 43.36 | 0.425098 |  |
| GO:0048518\_positive\_regulation\_of\_biological\_process | TBX20 | 995 | 21 | 1.429279 | -1.333125 | 102 | 43.36 | 0.425098 |  |
| GO:0048518\_positive\_regulation\_of\_biological\_process | SOX5 | 995 | 21 | 1.429279 | -1.333125 | 102 | 43.36 | 0.425098 |  |
| GO:0048518\_positive\_regulation\_of\_biological\_process | ESRRG | 995 | 21 | 1.429279 | -1.333125 | 102 | 43.36 | 0.425098 |  |
| GO:0048518\_positive\_regulation\_of\_biological\_process | NR3C1 | 995 | 21 | 1.429279 | -1.333125 | 102 | 43.36 | 0.425098 |  |
| GO:0048518\_positive\_regulation\_of\_biological\_process | TCF7L2 | 995 | 21 | 1.429279 | -1.333125 | 102 | 43.36 | 0.425098 |  |
| GO:0048518\_positive\_regulation\_of\_biological\_process | NR2E1 | 995 | 21 | 1.429279 | -1.333125 | 102 | 43.36 | 0.425098 |  |
| GO:0048518\_positive\_regulation\_of\_biological\_process | NKX6-1 | 995 | 21 | 1.429279 | -1.333125 | 102 | 43.36 | 0.425098 |  |
| GO:0048518\_positive\_regulation\_of\_biological\_process | SERINC3 | 995 | 21 | 1.429279 | -1.333125 | 102 | 43.36 | 0.425098 |  |
| GO:0048518\_positive\_regulation\_of\_biological\_process | ASCL1 | 995 | 21 | 1.429279 | -1.333125 | 102 | 43.36 | 0.425098 |  |
| GO:0048518\_positive\_regulation\_of\_biological\_process | MSX1 | 995 | 21 | 1.429279 | -1.333125 | 102 | 43.36 | 0.425098 |  |
| GO:0048518\_positive\_regulation\_of\_biological\_process | NEUROD1 | 995 | 21 | 1.429279 | -1.333125 | 102 | 43.36 | 0.425098 |  |
| GO:0048518\_positive\_regulation\_of\_biological\_process | ETV1 | 995 | 21 | 1.429279 | -1.333125 | 102 | 43.36 | 0.425098 |  |
| GO:0048518\_positive\_regulation\_of\_biological\_process | JAK2 | 995 | 21 | 1.429279 | -1.333125 | 102 | 43.36 | 0.425098 |  |
| GO:0048518\_positive\_regulation\_of\_biological\_process | THBS1 | 995 | 21 | 1.429279 | -1.333125 | 102 | 43.36 | 0.425098 |  |
| GO:0048518\_positive\_regulation\_of\_biological\_process | BOC | 995 | 21 | 1.429279 | -1.333125 | 102 | 43.36 | 0.425098 |  |
| GO:0048518\_positive\_regulation\_of\_biological\_process | CD27 | 995 | 21 | 1.429279 | -1.333125 | 102 | 43.36 | 0.425098 |  |
| GO:0048518\_positive\_regulation\_of\_biological\_process | RAB27A | 995 | 21 | 1.429279 | -1.333125 | 102 | 43.36 | 0.425098 |  |
| GO:0021515\_cell\_differentiation\_in\_spinal\_cord | ASCL1 | 24 | 2 | 5.643382 | -1.317611 | 104 | 45.44 | 0.436923 |  |
| GO:0021515\_cell\_differentiation\_in\_spinal\_cord | NKX6-1 | 24 | 2 | 5.643382 | -1.317611 | 104 | 45.44 | 0.436923 |  |
| GO:0043410\_positive\_regulation\_of\_MAPKKK\_cascade | PTPRC | 24 | 2 | 5.643382 | -1.317611 | 104 | 45.44 | 0.436923 |  |
| GO:0043410\_positive\_regulation\_of\_MAPKKK\_cascade | CD27 | 24 | 2 | 5.643382 | -1.317611 | 104 | 45.44 | 0.436923 |  |
| GO:0030154\_cell\_differentiation | DCC | 1060 | 22 | 1.405522 | -1.313199 | 105 | 45.49 | 0.433238 |  |
| GO:0030154\_cell\_differentiation | FGF18 | 1060 | 22 | 1.405522 | -1.313199 | 105 | 45.49 | 0.433238 |  |
| GO:0030154\_cell\_differentiation | PTPRC | 1060 | 22 | 1.405522 | -1.313199 | 105 | 45.49 | 0.433238 |  |
| GO:0030154\_cell\_differentiation | IRX5 | 1060 | 22 | 1.405522 | -1.313199 | 105 | 45.49 | 0.433238 |  |
| GO:0030154\_cell\_differentiation | MYO6 | 1060 | 22 | 1.405522 | -1.313199 | 105 | 45.49 | 0.433238 |  |
| GO:0030154\_cell\_differentiation | BBS7 | 1060 | 22 | 1.405522 | -1.313199 | 105 | 45.49 | 0.433238 |  |
| GO:0030154\_cell\_differentiation | MLPH | 1060 | 22 | 1.405522 | -1.313199 | 105 | 45.49 | 0.433238 |  |
| GO:0030154\_cell\_differentiation | ZFX | 1060 | 22 | 1.405522 | -1.313199 | 105 | 45.49 | 0.433238 |  |
| GO:0030154\_cell\_differentiation | SOX5 | 1060 | 22 | 1.405522 | -1.313199 | 105 | 45.49 | 0.433238 |  |
| GO:0030154\_cell\_differentiation | TCF7L2 | 1060 | 22 | 1.405522 | -1.313199 | 105 | 45.49 | 0.433238 |  |
| GO:0030154\_cell\_differentiation | NR2E1 | 1060 | 22 | 1.405522 | -1.313199 | 105 | 45.49 | 0.433238 |  |
| GO:0030154\_cell\_differentiation | NKX6-1 | 1060 | 22 | 1.405522 | -1.313199 | 105 | 45.49 | 0.433238 |  |
| GO:0030154\_cell\_differentiation | ALCAM | 1060 | 22 | 1.405522 | -1.313199 | 105 | 45.49 | 0.433238 |  |
| GO:0030154\_cell\_differentiation | ASCL1 | 1060 | 22 | 1.405522 | -1.313199 | 105 | 45.49 | 0.433238 |  |
| GO:0030154\_cell\_differentiation | NEUROD1 | 1060 | 22 | 1.405522 | -1.313199 | 105 | 45.49 | 0.433238 |  |
| GO:0030154\_cell\_differentiation | ETV1 | 1060 | 22 | 1.405522 | -1.313199 | 105 | 45.49 | 0.433238 |  |
| GO:0030154\_cell\_differentiation | JAK2 | 1060 | 22 | 1.405522 | -1.313199 | 105 | 45.49 | 0.433238 |  |
| GO:0030154\_cell\_differentiation | MBNL3 | 1060 | 22 | 1.405522 | -1.313199 | 105 | 45.49 | 0.433238 |  |
| GO:0030154\_cell\_differentiation | B3GNT2 | 1060 | 22 | 1.405522 | -1.313199 | 105 | 45.49 | 0.433238 |  |
| GO:0030154\_cell\_differentiation | HDAC9 | 1060 | 22 | 1.405522 | -1.313199 | 105 | 45.49 | 0.433238 |  |
| GO:0030154\_cell\_differentiation | BOC | 1060 | 22 | 1.405522 | -1.313199 | 105 | 45.49 | 0.433238 |  |
| GO:0030154\_cell\_differentiation | RAB27A | 1060 | 22 | 1.405522 | -1.313199 | 105 | 45.49 | 0.433238 |  |
| GO:0008610\_lipid\_biosynthetic\_process | HEXB | 94 | 4 | 2.881727 | -1.307929 | 106 | 46.22 | 0.436038 |  |
| GO:0008610\_lipid\_biosynthetic\_process | SGMS1 | 94 | 4 | 2.881727 | -1.307929 | 106 | 46.22 | 0.436038 |  |
| GO:0008610\_lipid\_biosynthetic\_process | NR3C1 | 94 | 4 | 2.881727 | -1.307929 | 106 | 46.22 | 0.436038 |  |
| GO:0008610\_lipid\_biosynthetic\_process | PPAP2A | 94 | 4 | 2.881727 | -1.307929 | 106 | 46.22 | 0.436038 |  |
| GO:0055082\_cellular\_chemical\_homeostasis | PTPRC | 181 | 6 | 2.244881 | -1.304623 | 107 | 46.57 | 0.435234 |  |
| GO:0055082\_cellular\_chemical\_homeostasis | AQP11 | 181 | 6 | 2.244881 | -1.304623 | 107 | 46.57 | 0.435234 |  |
| GO:0055082\_cellular\_chemical\_homeostasis | HEXB | 181 | 6 | 2.244881 | -1.304623 | 107 | 46.57 | 0.435234 |  |
| GO:0055082\_cellular\_chemical\_homeostasis | KCNA5 | 181 | 6 | 2.244881 | -1.304623 | 107 | 46.57 | 0.435234 |  |
| GO:0055082\_cellular\_chemical\_homeostasis | ATP1A2 | 181 | 6 | 2.244881 | -1.304623 | 107 | 46.57 | 0.435234 |  |
| GO:0055082\_cellular\_chemical\_homeostasis | SLC9A3R1 | 181 | 6 | 2.244881 | -1.304623 | 107 | 46.57 | 0.435234 |  |
| GO:0010604\_positive\_regulation\_of\_macromolecule\_metabolic\_process | PTPRC | 433 | 11 | 1.720384 | -1.296264 | 108 | 46.79 | 0.433241 |  |
| GO:0010604\_positive\_regulation\_of\_macromolecule\_metabolic\_process | ASCL1 | 433 | 11 | 1.720384 | -1.296264 | 108 | 46.79 | 0.433241 |  |
| GO:0010604\_positive\_regulation\_of\_macromolecule\_metabolic\_process | MSX1 | 433 | 11 | 1.720384 | -1.296264 | 108 | 46.79 | 0.433241 |  |
| GO:0010604\_positive\_regulation\_of\_macromolecule\_metabolic\_process | TBX3 | 433 | 11 | 1.720384 | -1.296264 | 108 | 46.79 | 0.433241 |  |
| GO:0010604\_positive\_regulation\_of\_macromolecule\_metabolic\_process | TBX20 | 433 | 11 | 1.720384 | -1.296264 | 108 | 46.79 | 0.433241 |  |
| GO:0010604\_positive\_regulation\_of\_macromolecule\_metabolic\_process | ESRRG | 433 | 11 | 1.720384 | -1.296264 | 108 | 46.79 | 0.433241 |  |
| GO:0010604\_positive\_regulation\_of\_macromolecule\_metabolic\_process | SOX5 | 433 | 11 | 1.720384 | -1.296264 | 108 | 46.79 | 0.433241 |  |
| GO:0010604\_positive\_regulation\_of\_macromolecule\_metabolic\_process | ETV1 | 433 | 11 | 1.720384 | -1.296264 | 108 | 46.79 | 0.433241 |  |
| GO:0010604\_positive\_regulation\_of\_macromolecule\_metabolic\_process | NEUROD1 | 433 | 11 | 1.720384 | -1.296264 | 108 | 46.79 | 0.433241 |  |
| GO:0010604\_positive\_regulation\_of\_macromolecule\_metabolic\_process | JAK2 | 433 | 11 | 1.720384 | -1.296264 | 108 | 46.79 | 0.433241 |  |
| GO:0010604\_positive\_regulation\_of\_macromolecule\_metabolic\_process | TCF7L2 | 433 | 11 | 1.720384 | -1.296264 | 108 | 46.79 | 0.433241 |  |
| GO:0048468\_cell\_development | DCC | 654 | 15 | 1.553225 | -1.292954 | 109 | 46.97 | 0.430917 |  |
| GO:0048468\_cell\_development | FGF18 | 654 | 15 | 1.553225 | -1.292954 | 109 | 46.97 | 0.430917 |  |
| GO:0048468\_cell\_development | MYO6 | 654 | 15 | 1.553225 | -1.292954 | 109 | 46.97 | 0.430917 |  |
| GO:0048468\_cell\_development | IRX5 | 654 | 15 | 1.553225 | -1.292954 | 109 | 46.97 | 0.430917 |  |
| GO:0048468\_cell\_development | ZFX | 654 | 15 | 1.553225 | -1.292954 | 109 | 46.97 | 0.430917 |  |
| GO:0048468\_cell\_development | SOX5 | 654 | 15 | 1.553225 | -1.292954 | 109 | 46.97 | 0.430917 |  |
| GO:0048468\_cell\_development | TCF7L2 | 654 | 15 | 1.553225 | -1.292954 | 109 | 46.97 | 0.430917 |  |
| GO:0048468\_cell\_development | NR2E1 | 654 | 15 | 1.553225 | -1.292954 | 109 | 46.97 | 0.430917 |  |
| GO:0048468\_cell\_development | NKX6-1 | 654 | 15 | 1.553225 | -1.292954 | 109 | 46.97 | 0.430917 |  |
| GO:0048468\_cell\_development | ALCAM | 654 | 15 | 1.553225 | -1.292954 | 109 | 46.97 | 0.430917 |  |
| GO:0048468\_cell\_development | ASCL1 | 654 | 15 | 1.553225 | -1.292954 | 109 | 46.97 | 0.430917 |  |
| GO:0048468\_cell\_development | ETV1 | 654 | 15 | 1.553225 | -1.292954 | 109 | 46.97 | 0.430917 |  |
| GO:0048468\_cell\_development | MBNL3 | 654 | 15 | 1.553225 | -1.292954 | 109 | 46.97 | 0.430917 |  |
| GO:0048468\_cell\_development | B3GNT2 | 654 | 15 | 1.553225 | -1.292954 | 109 | 46.97 | 0.430917 |  |
| GO:0048468\_cell\_development | BOC | 654 | 15 | 1.553225 | -1.292954 | 109 | 46.97 | 0.430917 |  |
| GO:0060603\_mammary\_gland\_duct\_morphogenesis | TBX3 | 25 | 2 | 5.417647 | -1.285471 | 110 | 48.75 | 0.443182 |  |
| GO:0060603\_mammary\_gland\_duct\_morphogenesis | NR3C1 | 25 | 2 | 5.417647 | -1.285471 | 110 | 48.75 | 0.443182 |  |
| GO:0051093\_negative\_regulation\_of\_developmental\_process | MSX1 | 331 | 9 | 1.841345 | -1.278521 | 111 | 49.19 | 0.443153 |  |
| GO:0051093\_negative\_regulation\_of\_developmental\_process | MBNL3 | 331 | 9 | 1.841345 | -1.278521 | 111 | 49.19 | 0.443153 |  |
| GO:0051093\_negative\_regulation\_of\_developmental\_process | SGMS1 | 331 | 9 | 1.841345 | -1.278521 | 111 | 49.19 | 0.443153 |  |
| GO:0051093\_negative\_regulation\_of\_developmental\_process | HDAC9 | 331 | 9 | 1.841345 | -1.278521 | 111 | 49.19 | 0.443153 |  |
| GO:0051093\_negative\_regulation\_of\_developmental\_process | THBS1 | 331 | 9 | 1.841345 | -1.278521 | 111 | 49.19 | 0.443153 |  |
| GO:0051093\_negative\_regulation\_of\_developmental\_process | TCF7L2 | 331 | 9 | 1.841345 | -1.278521 | 111 | 49.19 | 0.443153 |  |
| GO:0051093\_negative\_regulation\_of\_developmental\_process | NR2E1 | 331 | 9 | 1.841345 | -1.278521 | 111 | 49.19 | 0.443153 |  |
| GO:0051093\_negative\_regulation\_of\_developmental\_process | NKX6-1 | 331 | 9 | 1.841345 | -1.278521 | 111 | 49.19 | 0.443153 |  |
| GO:0051093\_negative\_regulation\_of\_developmental\_process | CD27 | 331 | 9 | 1.841345 | -1.278521 | 111 | 49.19 | 0.443153 |  |
| GO:0032990\_cell\_part\_morphogenesis | DCC | 184 | 6 | 2.208280 | -1.275990 | 112 | 49.27 | 0.439911 |  |
| GO:0032990\_cell\_part\_morphogenesis | ALCAM | 184 | 6 | 2.208280 | -1.275990 | 112 | 49.27 | 0.439911 |  |
| GO:0032990\_cell\_part\_morphogenesis | ETV1 | 184 | 6 | 2.208280 | -1.275990 | 112 | 49.27 | 0.439911 |  |
| GO:0032990\_cell\_part\_morphogenesis | B3GNT2 | 184 | 6 | 2.208280 | -1.275990 | 112 | 49.27 | 0.439911 |  |
| GO:0032990\_cell\_part\_morphogenesis | NR2E1 | 184 | 6 | 2.208280 | -1.275990 | 112 | 49.27 | 0.439911 |  |
| GO:0032990\_cell\_part\_morphogenesis | BOC | 184 | 6 | 2.208280 | -1.275990 | 112 | 49.27 | 0.439911 |  |
| GO:0034613\_cellular\_protein\_localization | MYO6 | 139 | 5 | 2.435992 | -1.273064 | 113 | 49.39 | 0.437080 |  |
| GO:0034613\_cellular\_protein\_localization | AP2A1 | 139 | 5 | 2.435992 | -1.273064 | 113 | 49.39 | 0.437080 |  |
| GO:0034613\_cellular\_protein\_localization | MLPH | 139 | 5 | 2.435992 | -1.273064 | 113 | 49.39 | 0.437080 |  |
| GO:0034613\_cellular\_protein\_localization | JAK2 | 139 | 5 | 2.435992 | -1.273064 | 113 | 49.39 | 0.437080 |  |
| GO:0034613\_cellular\_protein\_localization | RAB27A | 139 | 5 | 2.435992 | -1.273064 | 113 | 49.39 | 0.437080 |  |
| GO:0007155\_cell\_adhesion | PTPRC | 186 | 6 | 2.184535 | -1.257293 | 115 | 50.06 | 0.435304 |  |
| GO:0007155\_cell\_adhesion | CLDN1 | 186 | 6 | 2.184535 | -1.257293 | 115 | 50.06 | 0.435304 |  |
| GO:0007155\_cell\_adhesion | NID2 | 186 | 6 | 2.184535 | -1.257293 | 115 | 50.06 | 0.435304 |  |
| GO:0007155\_cell\_adhesion | ITGA4 | 186 | 6 | 2.184535 | -1.257293 | 115 | 50.06 | 0.435304 |  |
| GO:0007155\_cell\_adhesion | THBS1 | 186 | 6 | 2.184535 | -1.257293 | 115 | 50.06 | 0.435304 |  |
| GO:0007155\_cell\_adhesion | BOC | 186 | 6 | 2.184535 | -1.257293 | 115 | 50.06 | 0.435304 |  |
| GO:0022610\_biological\_adhesion | PTPRC | 186 | 6 | 2.184535 | -1.257293 | 115 | 50.06 | 0.435304 |  |
| GO:0022610\_biological\_adhesion | CLDN1 | 186 | 6 | 2.184535 | -1.257293 | 115 | 50.06 | 0.435304 |  |
| GO:0022610\_biological\_adhesion | NID2 | 186 | 6 | 2.184535 | -1.257293 | 115 | 50.06 | 0.435304 |  |
| GO:0022610\_biological\_adhesion | ITGA4 | 186 | 6 | 2.184535 | -1.257293 | 115 | 50.06 | 0.435304 |  |
| GO:0022610\_biological\_adhesion | THBS1 | 186 | 6 | 2.184535 | -1.257293 | 115 | 50.06 | 0.435304 |  |
| GO:0022610\_biological\_adhesion | BOC | 186 | 6 | 2.184535 | -1.257293 | 115 | 50.06 | 0.435304 |  |
| GO:0051480\_cytosolic\_calcium\_ion\_homeostasis | PTPRC | 26 | 2 | 5.209276 | -1.254776 | 116 | 51.19 | 0.441293 |  |
| GO:0051480\_cytosolic\_calcium\_ion\_homeostasis | ATP1A2 | 26 | 2 | 5.209276 | -1.254776 | 116 | 51.19 | 0.441293 |  |
| GO:0070727\_cellular\_macromolecule\_localization | MYO6 | 141 | 5 | 2.401439 | -1.251262 | 117 | 51.76 | 0.442393 |  |
| GO:0070727\_cellular\_macromolecule\_localization | AP2A1 | 141 | 5 | 2.401439 | -1.251262 | 117 | 51.76 | 0.442393 |  |
| GO:0070727\_cellular\_macromolecule\_localization | MLPH | 141 | 5 | 2.401439 | -1.251262 | 117 | 51.76 | 0.442393 |  |
| GO:0070727\_cellular\_macromolecule\_localization | JAK2 | 141 | 5 | 2.401439 | -1.251262 | 117 | 51.76 | 0.442393 |  |
| GO:0070727\_cellular\_macromolecule\_localization | RAB27A | 141 | 5 | 2.401439 | -1.251262 | 117 | 51.76 | 0.442393 |  |
| GO:0031325\_positive\_regulation\_of\_cellular\_metabolic\_process | PTPRC | 442 | 11 | 1.685354 | -1.242903 | 118 | 51.87 | 0.439576 |  |
| GO:0031325\_positive\_regulation\_of\_cellular\_metabolic\_process | ASCL1 | 442 | 11 | 1.685354 | -1.242903 | 118 | 51.87 | 0.439576 |  |
| GO:0031325\_positive\_regulation\_of\_cellular\_metabolic\_process | MSX1 | 442 | 11 | 1.685354 | -1.242903 | 118 | 51.87 | 0.439576 |  |
| GO:0031325\_positive\_regulation\_of\_cellular\_metabolic\_process | TBX3 | 442 | 11 | 1.685354 | -1.242903 | 118 | 51.87 | 0.439576 |  |
| GO:0031325\_positive\_regulation\_of\_cellular\_metabolic\_process | TBX20 | 442 | 11 | 1.685354 | -1.242903 | 118 | 51.87 | 0.439576 |  |
| GO:0031325\_positive\_regulation\_of\_cellular\_metabolic\_process | ESRRG | 442 | 11 | 1.685354 | -1.242903 | 118 | 51.87 | 0.439576 |  |
| GO:0031325\_positive\_regulation\_of\_cellular\_metabolic\_process | SOX5 | 442 | 11 | 1.685354 | -1.242903 | 118 | 51.87 | 0.439576 |  |
| GO:0031325\_positive\_regulation\_of\_cellular\_metabolic\_process | ETV1 | 442 | 11 | 1.685354 | -1.242903 | 118 | 51.87 | 0.439576 |  |
| GO:0031325\_positive\_regulation\_of\_cellular\_metabolic\_process | NEUROD1 | 442 | 11 | 1.685354 | -1.242903 | 118 | 51.87 | 0.439576 |  |
| GO:0031325\_positive\_regulation\_of\_cellular\_metabolic\_process | JAK2 | 442 | 11 | 1.685354 | -1.242903 | 118 | 51.87 | 0.439576 |  |
| GO:0031325\_positive\_regulation\_of\_cellular\_metabolic\_process | TCF7L2 | 442 | 11 | 1.685354 | -1.242903 | 118 | 51.87 | 0.439576 |  |
| GO:0002923\_regulation\_of\_humoral\_immune\_response\_mediated\_by\_circulating\_immunoglobulin | PTPRC | 4 | 1 |  |  |  |  |  |  |
| GO:0006111\_regulation\_of\_gluconeogenesis | NR3C1 | 4 | 1 |  |  |  |  |  |  |
| GO:0006684\_sphingomyelin\_metabolic\_process | SGMS1 | 4 | 1 |  |  |  |  |  |  |
| GO:0008207\_C21-steroid\_hormone\_metabolic\_process | AFP | 4 | 1 |  |  |  |  |  |  |
| GO:0016338\_calcium-independent\_cell-cell\_adhesion | CLDN1 | 4 | 1 |  |  |  |  |  |  |
| GO:0019377\_glycolipid\_catabolic\_process | HEXB | 4 | 1 |  |  |  |  |  |  |
| GO:0021535\_cell\_migration\_in\_hindbrain | PLXNA2 | 4 | 1 |  |  |  |  |  |  |
| GO:0021778\_oligodendrocyte\_cell\_fate\_specification | ASCL1 | 4 | 1 |  |  |  |  |  |  |
| GO:0021779\_oligodendrocyte\_cell\_fate\_commitment | ASCL1 | 4 | 1 |  |  |  |  |  |  |
| GO:0021780\_glial\_cell\_fate\_specification | ASCL1 | 4 | 1 |  |  |  |  |  |  |
| GO:0021913\_regulation\_of\_transcription\_from\_RNA\_polymerase\_II\_promoter\_involved\_in\_ventral\_spinal\_cord\_interneuron\_specification | NKX6-1 | 4 | 1 |  |  |  |  |  |  |
| GO:0030949\_positive\_regulation\_of\_vascular\_endothelial\_growth\_factor\_receptor\_signaling\_pathway | FGF18 | 4 | 1 |  |  |  |  |  |  |
| GO:0032401\_establishment\_of\_melanosome\_localization | RAB27A | 4 | 1 |  |  |  |  |  |  |
| GO:0034204\_lipid\_translocation | ABCA1 | 4 | 1 |  |  |  |  |  |  |
| GO:0042522\_regulation\_of\_tyrosine\_phosphorylation\_of\_Stat5\_protein | JAK2 | 4 | 1 |  |  |  |  |  |  |
| GO:0043691\_reverse\_cholesterol\_transport | ABCA1 | 4 | 1 |  |  |  |  |  |  |
| GO:0045332\_phospholipid\_translocation | ABCA1 | 4 | 1 |  |  |  |  |  |  |
| GO:0045747\_positive\_regulation\_of\_Notch\_signaling\_pathway | ASCL1 | 4 | 1 |  |  |  |  |  |  |
| GO:0051148\_negative\_regulation\_of\_muscle\_cell\_differentiation | MBNL3 | 4 | 1 |  |  |  |  |  |  |
| GO:0051452\_intracellular\_pH\_reduction | AQP11 | 4 | 1 |  |  |  |  |  |  |
| GO:0051642\_centrosome\_localization | PLXNA2 | 4 | 1 |  |  |  |  |  |  |
| GO:0051904\_pigment\_granule\_transport | RAB27A | 4 | 1 |  |  |  |  |  |  |
| GO:0060592\_mammary\_gland\_formation | TBX3 | 4 | 1 |  |  |  |  |  |  |
| GO:0045941\_positive\_regulation\_of\_transcription | ASCL1 | 338 | 9 | 1.803211 | -1.231262 | 119 | 52.34 | 0.439832 |  |
| GO:0045941\_positive\_regulation\_of\_transcription | MSX1 | 338 | 9 | 1.803211 | -1.231262 | 119 | 52.34 | 0.439832 |  |
| GO:0045941\_positive\_regulation\_of\_transcription | TBX3 | 338 | 9 | 1.803211 | -1.231262 | 119 | 52.34 | 0.439832 |  |
| GO:0045941\_positive\_regulation\_of\_transcription | TBX20 | 338 | 9 | 1.803211 | -1.231262 | 119 | 52.34 | 0.439832 |  |
| GO:0045941\_positive\_regulation\_of\_transcription | ESRRG | 338 | 9 | 1.803211 | -1.231262 | 119 | 52.34 | 0.439832 |  |
| GO:0045941\_positive\_regulation\_of\_transcription | SOX5 | 338 | 9 | 1.803211 | -1.231262 | 119 | 52.34 | 0.439832 |  |
| GO:0045941\_positive\_regulation\_of\_transcription | ETV1 | 338 | 9 | 1.803211 | -1.231262 | 119 | 52.34 | 0.439832 |  |
| GO:0045941\_positive\_regulation\_of\_transcription | NEUROD1 | 338 | 9 | 1.803211 | -1.231262 | 119 | 52.34 | 0.439832 |  |
| GO:0045941\_positive\_regulation\_of\_transcription | TCF7L2 | 338 | 9 | 1.803211 | -1.231262 | 119 | 52.34 | 0.439832 |  |
| GO:0006874\_cellular\_calcium\_ion\_homeostasis | PTPRC | 61 | 3 | 3.330521 | -1.218871 | 120 | 53.9 | 0.449167 |  |
| GO:0006874\_cellular\_calcium\_ion\_homeostasis | HEXB | 61 | 3 | 3.330521 | -1.218871 | 120 | 53.9 | 0.449167 |  |
| GO:0006874\_cellular\_calcium\_ion\_homeostasis | ATP1A2 | 61 | 3 | 3.330521 | -1.218871 | 120 | 53.9 | 0.449167 |  |
| GO:0065007\_biological\_regulation | FGF18 | 2593 | 45 | 1.175251 | -1.208457 | 121 | 54.35 | 0.449174 |  |
| GO:0065007\_biological\_regulation | ELF1 | 2593 | 45 | 1.175251 | -1.208457 | 121 | 54.35 | 0.449174 |  |
| GO:0065007\_biological\_regulation | IRX5 | 2593 | 45 | 1.175251 | -1.208457 | 121 | 54.35 | 0.449174 |  |
| GO:0065007\_biological\_regulation | PLXNA2 | 2593 | 45 | 1.175251 | -1.208457 | 121 | 54.35 | 0.449174 |  |
| GO:0065007\_biological\_regulation | TBX20 | 2593 | 45 | 1.175251 | -1.208457 | 121 | 54.35 | 0.449174 |  |
| GO:0065007\_biological\_regulation | HEXB | 2593 | 45 | 1.175251 | -1.208457 | 121 | 54.35 | 0.449174 |  |
| GO:0065007\_biological\_regulation | SOX5 | 2593 | 45 | 1.175251 | -1.208457 | 121 | 54.35 | 0.449174 |  |
| GO:0065007\_biological\_regulation | HSPA1A | 2593 | 45 | 1.175251 | -1.208457 | 121 | 54.35 | 0.449174 |  |
| GO:0065007\_biological\_regulation | SGMS1 | 2593 | 45 | 1.175251 | -1.208457 | 121 | 54.35 | 0.449174 |  |
| GO:0065007\_biological\_regulation | NR3C1 | 2593 | 45 | 1.175251 | -1.208457 | 121 | 54.35 | 0.449174 |  |
| GO:0065007\_biological\_regulation | KCNA5 | 2593 | 45 | 1.175251 | -1.208457 | 121 | 54.35 | 0.449174 |  |
| GO:0065007\_biological\_regulation | ATP6V1B1 | 2593 | 45 | 1.175251 | -1.208457 | 121 | 54.35 | 0.449174 |  |
| GO:0065007\_biological\_regulation | TCF7L2 | 2593 | 45 | 1.175251 | -1.208457 | 121 | 54.35 | 0.449174 |  |
| GO:0065007\_biological\_regulation | NR2E1 | 2593 | 45 | 1.175251 | -1.208457 | 121 | 54.35 | 0.449174 |  |
| GO:0065007\_biological\_regulation | SERINC3 | 2593 | 45 | 1.175251 | -1.208457 | 121 | 54.35 | 0.449174 |  |
| GO:0065007\_biological\_regulation | AQP11 | 2593 | 45 | 1.175251 | -1.208457 | 121 | 54.35 | 0.449174 |  |
| GO:0065007\_biological\_regulation | DMD | 2593 | 45 | 1.175251 | -1.208457 | 121 | 54.35 | 0.449174 |  |
| GO:0065007\_biological\_regulation | ETV1 | 2593 | 45 | 1.175251 | -1.208457 | 121 | 54.35 | 0.449174 |  |
| GO:0065007\_biological\_regulation | MBNL3 | 2593 | 45 | 1.175251 | -1.208457 | 121 | 54.35 | 0.449174 |  |
| GO:0065007\_biological\_regulation | PPAP2A | 2593 | 45 | 1.175251 | -1.208457 | 121 | 54.35 | 0.449174 |  |
| GO:0065007\_biological\_regulation | THBS1 | 2593 | 45 | 1.175251 | -1.208457 | 121 | 54.35 | 0.449174 |  |
| GO:0065007\_biological\_regulation | BOC | 2593 | 45 | 1.175251 | -1.208457 | 121 | 54.35 | 0.449174 |  |
| GO:0065007\_biological\_regulation | CD27 | 2593 | 45 | 1.175251 | -1.208457 | 121 | 54.35 | 0.449174 |  |
| GO:0065007\_biological\_regulation | RAB27A | 2593 | 45 | 1.175251 | -1.208457 | 121 | 54.35 | 0.449174 |  |
| GO:0065007\_biological\_regulation | PTPRC | 2593 | 45 | 1.175251 | -1.208457 | 121 | 54.35 | 0.449174 |  |
| GO:0065007\_biological\_regulation | MYO6 | 2593 | 45 | 1.175251 | -1.208457 | 121 | 54.35 | 0.449174 |  |
| GO:0065007\_biological\_regulation | TBX3 | 2593 | 45 | 1.175251 | -1.208457 | 121 | 54.35 | 0.449174 |  |
| GO:0065007\_biological\_regulation | ARID5B | 2593 | 45 | 1.175251 | -1.208457 | 121 | 54.35 | 0.449174 |  |
| GO:0065007\_biological\_regulation | ZFX | 2593 | 45 | 1.175251 | -1.208457 | 121 | 54.35 | 0.449174 |  |
| GO:0065007\_biological\_regulation | ATRN | 2593 | 45 | 1.175251 | -1.208457 | 121 | 54.35 | 0.449174 |  |
| GO:0065007\_biological\_regulation | ESRRG | 2593 | 45 | 1.175251 | -1.208457 | 121 | 54.35 | 0.449174 |  |
| GO:0065007\_biological\_regulation | ATP1A2 | 2593 | 45 | 1.175251 | -1.208457 | 121 | 54.35 | 0.449174 |  |
| GO:0065007\_biological\_regulation | SLC9A3R1 | 2593 | 45 | 1.175251 | -1.208457 | 121 | 54.35 | 0.449174 |  |
| GO:0065007\_biological\_regulation | NKX6-1 | 2593 | 45 | 1.175251 | -1.208457 | 121 | 54.35 | 0.449174 |  |
| GO:0065007\_biological\_regulation | RGS13 | 2593 | 45 | 1.175251 | -1.208457 | 121 | 54.35 | 0.449174 |  |
| GO:0065007\_biological\_regulation | AFP | 2593 | 45 | 1.175251 | -1.208457 | 121 | 54.35 | 0.449174 |  |
| GO:0065007\_biological\_regulation | ASCL1 | 2593 | 45 | 1.175251 | -1.208457 | 121 | 54.35 | 0.449174 |  |
| GO:0065007\_biological\_regulation | ATXN2 | 2593 | 45 | 1.175251 | -1.208457 | 121 | 54.35 | 0.449174 |  |
| GO:0065007\_biological\_regulation | MSX1 | 2593 | 45 | 1.175251 | -1.208457 | 121 | 54.35 | 0.449174 |  |
| GO:0065007\_biological\_regulation | NEUROD1 | 2593 | 45 | 1.175251 | -1.208457 | 121 | 54.35 | 0.449174 |  |
| GO:0065007\_biological\_regulation | RGS8 | 2593 | 45 | 1.175251 | -1.208457 | 121 | 54.35 | 0.449174 |  |
| GO:0065007\_biological\_regulation | JAK2 | 2593 | 45 | 1.175251 | -1.208457 | 121 | 54.35 | 0.449174 |  |
| GO:0065007\_biological\_regulation | HDAC9 | 2593 | 45 | 1.175251 | -1.208457 | 121 | 54.35 | 0.449174 |  |
| GO:0065007\_biological\_regulation | GADD45B | 2593 | 45 | 1.175251 | -1.208457 | 121 | 54.35 | 0.449174 |  |
| GO:0065007\_biological\_regulation | CAMK1D | 2593 | 45 | 1.175251 | -1.208457 | 121 | 54.35 | 0.449174 |  |
| GO:0005975\_carbohydrate\_metabolic\_process | FUT9 | 146 | 5 | 2.319198 | -1.198671 | 122 | 55.2 | 0.452459 |  |
| GO:0005975\_carbohydrate\_metabolic\_process | MAMDC2 | 146 | 5 | 2.319198 | -1.198671 | 122 | 55.2 | 0.452459 |  |
| GO:0005975\_carbohydrate\_metabolic\_process | HEXB | 146 | 5 | 2.319198 | -1.198671 | 122 | 55.2 | 0.452459 |  |
| GO:0005975\_carbohydrate\_metabolic\_process | B3GNT2 | 146 | 5 | 2.319198 | -1.198671 | 122 | 55.2 | 0.452459 |  |
| GO:0005975\_carbohydrate\_metabolic\_process | NR3C1 | 146 | 5 | 2.319198 | -1.198671 | 122 | 55.2 | 0.452459 |  |
| GO:0006470\_protein\_amino\_acid\_dephosphorylation | PTPRC | 28 | 2 | 4.837185 | -1.197289 | 124 | 56.15 | 0.452823 |  |
| GO:0006470\_protein\_amino\_acid\_dephosphorylation | PPAP2A | 28 | 2 | 4.837185 | -1.197289 | 124 | 56.15 | 0.452823 |  |
| GO:0048863\_stem\_cell\_differentiation | ASCL1 | 28 | 2 | 4.837185 | -1.197289 | 124 | 56.15 | 0.452823 |  |
| GO:0048863\_stem\_cell\_differentiation | TCF7L2 | 28 | 2 | 4.837185 | -1.197289 | 124 | 56.15 | 0.452823 |  |
| GO:0010628\_positive\_regulation\_of\_gene\_expression | ASCL1 | 346 | 9 | 1.761518 | -1.179328 | 125 | 57.0 | 0.456000 |  |
| GO:0010628\_positive\_regulation\_of\_gene\_expression | MSX1 | 346 | 9 | 1.761518 | -1.179328 | 125 | 57.0 | 0.456000 |  |
| GO:0010628\_positive\_regulation\_of\_gene\_expression | TBX3 | 346 | 9 | 1.761518 | -1.179328 | 125 | 57.0 | 0.456000 |  |
| GO:0010628\_positive\_regulation\_of\_gene\_expression | TBX20 | 346 | 9 | 1.761518 | -1.179328 | 125 | 57.0 | 0.456000 |  |
| GO:0010628\_positive\_regulation\_of\_gene\_expression | ESRRG | 346 | 9 | 1.761518 | -1.179328 | 125 | 57.0 | 0.456000 |  |
| GO:0010628\_positive\_regulation\_of\_gene\_expression | SOX5 | 346 | 9 | 1.761518 | -1.179328 | 125 | 57.0 | 0.456000 |  |
| GO:0010628\_positive\_regulation\_of\_gene\_expression | ETV1 | 346 | 9 | 1.761518 | -1.179328 | 125 | 57.0 | 0.456000 |  |
| GO:0010628\_positive\_regulation\_of\_gene\_expression | NEUROD1 | 346 | 9 | 1.761518 | -1.179328 | 125 | 57.0 | 0.456000 |  |
| GO:0010628\_positive\_regulation\_of\_gene\_expression | TCF7L2 | 346 | 9 | 1.761518 | -1.179328 | 125 | 57.0 | 0.456000 |  |
| GO:0050767\_regulation\_of\_neurogenesis | ASCL1 | 104 | 4 | 2.604638 | -1.176498 | 126 | 57.4 | 0.455556 |  |
| GO:0050767\_regulation\_of\_neurogenesis | SOX5 | 104 | 4 | 2.604638 | -1.176498 | 126 | 57.4 | 0.455556 |  |
| GO:0050767\_regulation\_of\_neurogenesis | NR2E1 | 104 | 4 | 2.604638 | -1.176498 | 126 | 57.4 | 0.455556 |  |
| GO:0050767\_regulation\_of\_neurogenesis | NKX6-1 | 104 | 4 | 2.604638 | -1.176498 | 126 | 57.4 | 0.455556 |  |
| GO:0045595\_regulation\_of\_cell\_differentiation | PTPRC | 295 | 8 | 1.836491 | -1.173096 | 127 | 57.56 | 0.453228 |  |
| GO:0045595\_regulation\_of\_cell\_differentiation | ASCL1 | 295 | 8 | 1.836491 | -1.173096 | 127 | 57.56 | 0.453228 |  |
| GO:0045595\_regulation\_of\_cell\_differentiation | SOX5 | 295 | 8 | 1.836491 | -1.173096 | 127 | 57.56 | 0.453228 |  |
| GO:0045595\_regulation\_of\_cell\_differentiation | MBNL3 | 295 | 8 | 1.836491 | -1.173096 | 127 | 57.56 | 0.453228 |  |
| GO:0045595\_regulation\_of\_cell\_differentiation | BOC | 295 | 8 | 1.836491 | -1.173096 | 127 | 57.56 | 0.453228 |  |
| GO:0045595\_regulation\_of\_cell\_differentiation | NR2E1 | 295 | 8 | 1.836491 | -1.173096 | 127 | 57.56 | 0.453228 |  |
| GO:0045595\_regulation\_of\_cell\_differentiation | TCF7L2 | 295 | 8 | 1.836491 | -1.173096 | 127 | 57.56 | 0.453228 |  |
| GO:0045595\_regulation\_of\_cell\_differentiation | NKX6-1 | 295 | 8 | 1.836491 | -1.173096 | 127 | 57.56 | 0.453228 |  |
| GO:0048066\_pigmentation\_during\_development | MLPH | 29 | 2 | 4.670385 | -1.170308 | 130 | 58.93 | 0.453308 |  |
| GO:0048066\_pigmentation\_during\_development | RAB27A | 29 | 2 | 4.670385 | -1.170308 | 130 | 58.93 | 0.453308 |  |
| GO:0050769\_positive\_regulation\_of\_neurogenesis | ASCL1 | 29 | 2 | 4.670385 | -1.170308 | 130 | 58.93 | 0.453308 |  |
| GO:0050769\_positive\_regulation\_of\_neurogenesis | NKX6-1 | 29 | 2 | 4.670385 | -1.170308 | 130 | 58.93 | 0.453308 |  |
| GO:0060041\_retina\_development\_in\_camera-type\_eye | IRX5 | 29 | 2 | 4.670385 | -1.170308 | 130 | 58.93 | 0.453308 |  |
| GO:0060041\_retina\_development\_in\_camera-type\_eye | NR2E1 | 29 | 2 | 4.670385 | -1.170308 | 130 | 58.93 | 0.453308 |  |
| GO:0006875\_cellular\_metal\_ion\_homeostasis | PTPRC | 64 | 3 | 3.174403 | -1.168668 | 132 | 59.34 | 0.449545 |  |
| GO:0006875\_cellular\_metal\_ion\_homeostasis | HEXB | 64 | 3 | 3.174403 | -1.168668 | 132 | 59.34 | 0.449545 |  |
| GO:0006875\_cellular\_metal\_ion\_homeostasis | ATP1A2 | 64 | 3 | 3.174403 | -1.168668 | 132 | 59.34 | 0.449545 |  |
| GO:0055074\_calcium\_ion\_homeostasis | PTPRC | 64 | 3 | 3.174403 | -1.168668 | 132 | 59.34 | 0.449545 |  |
| GO:0055074\_calcium\_ion\_homeostasis | HEXB | 64 | 3 | 3.174403 | -1.168668 | 132 | 59.34 | 0.449545 |  |
| GO:0055074\_calcium\_ion\_homeostasis | ATP1A2 | 64 | 3 | 3.174403 | -1.168668 | 132 | 59.34 | 0.449545 |  |
| GO:0048519\_negative\_regulation\_of\_biological\_process | PTPRC | 859 | 18 | 1.419058 | -1.156416 | 133 | 59.75 | 0.449248 |  |
| GO:0048519\_negative\_regulation\_of\_biological\_process | ELF1 | 859 | 18 | 1.419058 | -1.156416 | 133 | 59.75 | 0.449248 |  |
| GO:0048519\_negative\_regulation\_of\_biological\_process | TBX3 | 859 | 18 | 1.419058 | -1.156416 | 133 | 59.75 | 0.449248 |  |
| GO:0048519\_negative\_regulation\_of\_biological\_process | TBX20 | 859 | 18 | 1.419058 | -1.156416 | 133 | 59.75 | 0.449248 |  |
| GO:0048519\_negative\_regulation\_of\_biological\_process | ATP1A2 | 859 | 18 | 1.419058 | -1.156416 | 133 | 59.75 | 0.449248 |  |
| GO:0048519\_negative\_regulation\_of\_biological\_process | KCNA5 | 859 | 18 | 1.419058 | -1.156416 | 133 | 59.75 | 0.449248 |  |
| GO:0048519\_negative\_regulation\_of\_biological\_process | SGMS1 | 859 | 18 | 1.419058 | -1.156416 | 133 | 59.75 | 0.449248 |  |
| GO:0048519\_negative\_regulation\_of\_biological\_process | NR2E1 | 859 | 18 | 1.419058 | -1.156416 | 133 | 59.75 | 0.449248 |  |
| GO:0048519\_negative\_regulation\_of\_biological\_process | TCF7L2 | 859 | 18 | 1.419058 | -1.156416 | 133 | 59.75 | 0.449248 |  |
| GO:0048519\_negative\_regulation\_of\_biological\_process | NKX6-1 | 859 | 18 | 1.419058 | -1.156416 | 133 | 59.75 | 0.449248 |  |
| GO:0048519\_negative\_regulation\_of\_biological\_process | RGS13 | 859 | 18 | 1.419058 | -1.156416 | 133 | 59.75 | 0.449248 |  |
| GO:0048519\_negative\_regulation\_of\_biological\_process | ATXN2 | 859 | 18 | 1.419058 | -1.156416 | 133 | 59.75 | 0.449248 |  |
| GO:0048519\_negative\_regulation\_of\_biological\_process | MSX1 | 859 | 18 | 1.419058 | -1.156416 | 133 | 59.75 | 0.449248 |  |
| GO:0048519\_negative\_regulation\_of\_biological\_process | JAK2 | 859 | 18 | 1.419058 | -1.156416 | 133 | 59.75 | 0.449248 |  |
| GO:0048519\_negative\_regulation\_of\_biological\_process | MBNL3 | 859 | 18 | 1.419058 | -1.156416 | 133 | 59.75 | 0.449248 |  |
| GO:0048519\_negative\_regulation\_of\_biological\_process | THBS1 | 859 | 18 | 1.419058 | -1.156416 | 133 | 59.75 | 0.449248 |  |
| GO:0048519\_negative\_regulation\_of\_biological\_process | HDAC9 | 859 | 18 | 1.419058 | -1.156416 | 133 | 59.75 | 0.449248 |  |
| GO:0048519\_negative\_regulation\_of\_biological\_process | CD27 | 859 | 18 | 1.419058 | -1.156416 | 133 | 59.75 | 0.449248 |  |
| GO:0009893\_positive\_regulation\_of\_metabolic\_process | PTPRC | 458 | 11 | 1.626477 | -1.152979 | 134 | 59.79 | 0.446194 |  |
| GO:0009893\_positive\_regulation\_of\_metabolic\_process | ASCL1 | 458 | 11 | 1.626477 | -1.152979 | 134 | 59.79 | 0.446194 |  |
| GO:0009893\_positive\_regulation\_of\_metabolic\_process | MSX1 | 458 | 11 | 1.626477 | -1.152979 | 134 | 59.79 | 0.446194 |  |
| GO:0009893\_positive\_regulation\_of\_metabolic\_process | TBX3 | 458 | 11 | 1.626477 | -1.152979 | 134 | 59.79 | 0.446194 |  |
| GO:0009893\_positive\_regulation\_of\_metabolic\_process | TBX20 | 458 | 11 | 1.626477 | -1.152979 | 134 | 59.79 | 0.446194 |  |
| GO:0009893\_positive\_regulation\_of\_metabolic\_process | ESRRG | 458 | 11 | 1.626477 | -1.152979 | 134 | 59.79 | 0.446194 |  |
| GO:0009893\_positive\_regulation\_of\_metabolic\_process | SOX5 | 458 | 11 | 1.626477 | -1.152979 | 134 | 59.79 | 0.446194 |  |
| GO:0009893\_positive\_regulation\_of\_metabolic\_process | ETV1 | 458 | 11 | 1.626477 | -1.152979 | 134 | 59.79 | 0.446194 |  |
| GO:0009893\_positive\_regulation\_of\_metabolic\_process | NEUROD1 | 458 | 11 | 1.626477 | -1.152979 | 134 | 59.79 | 0.446194 |  |
| GO:0009893\_positive\_regulation\_of\_metabolic\_process | JAK2 | 458 | 11 | 1.626477 | -1.152979 | 134 | 59.79 | 0.446194 |  |
| GO:0009893\_positive\_regulation\_of\_metabolic\_process | TCF7L2 | 458 | 11 | 1.626477 | -1.152979 | 134 | 59.79 | 0.446194 |  |
| GO:0001957\_intramembranous\_ossification | FGF18 | 5 | 1 | 13.544118 | -1.144393 | 164 | 80.47 | 0.490671 |  |
| GO:0001959\_regulation\_of\_cytokine-mediated\_signaling\_pathway | PTPRC | 5 | 1 | 13.544118 | -1.144393 | 164 | 80.47 | 0.490671 |  |
| GO:0002710\_negative\_regulation\_of\_T\_cell\_mediated\_immunity | PTPRC | 5 | 1 | 13.544118 | -1.144393 | 164 | 80.47 | 0.490671 |  |
| GO:0006704\_glucocorticoid\_biosynthetic\_process | NR3C1 | 5 | 1 | 13.544118 | -1.144393 | 164 | 80.47 | 0.490671 |  |
| GO:0007638\_mechanosensory\_behavior | ETV1 | 5 | 1 | 13.544118 | -1.144393 | 164 | 80.47 | 0.490671 |  |
| GO:0010761\_fibroblast\_migration | ARID5B | 5 | 1 | 13.544118 | -1.144393 | 164 | 80.47 | 0.490671 |  |
| GO:0014015\_positive\_regulation\_of\_gliogenesis | NKX6-1 | 5 | 1 | 13.544118 | -1.144393 | 164 | 80.47 | 0.490671 |  |
| GO:0014068\_positive\_regulation\_of\_phosphoinositide\_3-kinase\_cascade | JAK2 | 5 | 1 | 13.544118 | -1.144393 | 164 | 80.47 | 0.490671 |  |
| GO:0021772\_olfactory\_bulb\_development | NR2E1 | 5 | 1 | 13.544118 | -1.144393 | 164 | 80.47 | 0.490671 |  |
| GO:0021988\_olfactory\_lobe\_development | NR2E1 | 5 | 1 | 13.544118 | -1.144393 | 164 | 80.47 | 0.490671 |  |
| GO:0030004\_cellular\_monovalent\_inorganic\_cation\_homeostasis | AQP11 | 5 | 1 | 13.544118 | -1.144393 | 164 | 80.47 | 0.490671 |  |
| GO:0030641\_regulation\_of\_cellular\_pH | AQP11 | 5 | 1 | 13.544118 | -1.144393 | 164 | 80.47 | 0.490671 |  |
| GO:0032350\_regulation\_of\_hormone\_metabolic\_process | NR3C1 | 5 | 1 | 13.544118 | -1.144393 | 164 | 80.47 | 0.490671 |  |
| GO:0042506\_tyrosine\_phosphorylation\_of\_Stat5\_protein | JAK2 | 5 | 1 | 13.544118 | -1.144393 | 164 | 80.47 | 0.490671 |  |
| GO:0042516\_regulation\_of\_tyrosine\_phosphorylation\_of\_Stat3\_protein | JAK2 | 5 | 1 | 13.544118 | -1.144393 | 164 | 80.47 | 0.490671 |  |
| GO:0042531\_positive\_regulation\_of\_tyrosine\_phosphorylation\_of\_STAT\_protein | JAK2 | 5 | 1 | 13.544118 | -1.144393 | 164 | 80.47 | 0.490671 |  |
| GO:0043266\_regulation\_of\_potassium\_ion\_transport | KCNA5 | 5 | 1 | 13.544118 | -1.144393 | 164 | 80.47 | 0.490671 |  |
| GO:0045213\_neurotransmitter\_receptor\_metabolic\_process | DMD | 5 | 1 | 13.544118 | -1.144393 | 164 | 80.47 | 0.490671 |  |
| GO:0045586\_regulation\_of\_gamma-delta\_T\_cell\_differentiation | PTPRC | 5 | 1 | 13.544118 | -1.144393 | 164 | 80.47 | 0.490671 |  |
| GO:0045588\_positive\_regulation\_of\_gamma-delta\_T\_cell\_differentiation | PTPRC | 5 | 1 | 13.544118 | -1.144393 | 164 | 80.47 | 0.490671 |  |
| GO:0045687\_positive\_regulation\_of\_glial\_cell\_differentiation | NKX6-1 | 5 | 1 | 13.544118 | -1.144393 | 164 | 80.47 | 0.490671 |  |
| GO:0045744\_negative\_regulation\_of\_G-protein\_coupled\_receptor\_protein\_signaling\_pathway | RGS13 | 5 | 1 | 13.544118 | -1.144393 | 164 | 80.47 | 0.490671 |  |
| GO:0045851\_pH\_reduction | AQP11 | 5 | 1 | 13.544118 | -1.144393 | 164 | 80.47 | 0.490671 |  |
| GO:0046339\_diacylglycerol\_metabolic\_process | PPAP2A | 5 | 1 | 13.544118 | -1.144393 | 164 | 80.47 | 0.490671 |  |
| GO:0046643\_regulation\_of\_gamma-delta\_T\_cell\_activation | PTPRC | 5 | 1 | 13.544118 | -1.144393 | 164 | 80.47 | 0.490671 |  |
| GO:0046645\_positive\_regulation\_of\_gamma-delta\_T\_cell\_activation | PTPRC | 5 | 1 | 13.544118 | -1.144393 | 164 | 80.47 | 0.490671 |  |
| GO:0050858\_negative\_regulation\_of\_antigen\_receptor-mediated\_signaling\_pathway | ELF1 | 5 | 1 | 13.544118 | -1.144393 | 164 | 80.47 | 0.490671 |  |
| GO:0050860\_negative\_regulation\_of\_T\_cell\_receptor\_signaling\_pathway | ELF1 | 5 | 1 | 13.544118 | -1.144393 | 164 | 80.47 | 0.490671 |  |
| GO:0051453\_regulation\_of\_intracellular\_pH | AQP11 | 5 | 1 | 13.544118 | -1.144393 | 164 | 80.47 | 0.490671 |  |
| GO:0051905\_establishment\_of\_pigment\_granule\_localization | RAB27A | 5 | 1 | 13.544118 | -1.144393 | 164 | 80.47 | 0.490671 |  |
| GO:0000904\_cell\_morphogenesis\_involved\_in\_differentiation | DCC | 199 | 6 | 2.041827 | -1.142912 | 165 | 82.35 | 0.499091 |  |
| GO:0000904\_cell\_morphogenesis\_involved\_in\_differentiation | ALCAM | 199 | 6 | 2.041827 | -1.142912 | 165 | 82.35 | 0.499091 |  |
| GO:0000904\_cell\_morphogenesis\_involved\_in\_differentiation | ETV1 | 199 | 6 | 2.041827 | -1.142912 | 165 | 82.35 | 0.499091 |  |
| GO:0000904\_cell\_morphogenesis\_involved\_in\_differentiation | B3GNT2 | 199 | 6 | 2.041827 | -1.142912 | 165 | 82.35 | 0.499091 |  |
| GO:0000904\_cell\_morphogenesis\_involved\_in\_differentiation | NR2E1 | 199 | 6 | 2.041827 | -1.142912 | 165 | 82.35 | 0.499091 |  |
| GO:0000904\_cell\_morphogenesis\_involved\_in\_differentiation | BOC | 199 | 6 | 2.041827 | -1.142912 | 165 | 82.35 | 0.499091 |  |
| GO:0003007\_heart\_morphogenesis | MSX1 | 67 | 3 | 3.032265 | -1.121395 | 166 | 83.63 | 0.503795 |  |
| GO:0003007\_heart\_morphogenesis | TBX3 | 67 | 3 | 3.032265 | -1.121395 | 166 | 83.63 | 0.503795 |  |
| GO:0003007\_heart\_morphogenesis | TBX20 | 67 | 3 | 3.032265 | -1.121395 | 166 | 83.63 | 0.503795 |  |
| GO:0006486\_protein\_amino\_acid\_glycosylation | FUT9 | 31 | 2 | 4.369070 | -1.119473 | 170 | 85.92 | 0.505412 |  |
| GO:0006486\_protein\_amino\_acid\_glycosylation | B3GNT2 | 31 | 2 | 4.369070 | -1.119473 | 170 | 85.92 | 0.505412 |  |
| GO:0016311\_dephosphorylation | PTPRC | 31 | 2 | 4.369070 | -1.119473 | 170 | 85.92 | 0.505412 |  |
| GO:0016311\_dephosphorylation | PPAP2A | 31 | 2 | 4.369070 | -1.119473 | 170 | 85.92 | 0.505412 |  |
| GO:0043413\_biopolymer\_glycosylation | FUT9 | 31 | 2 | 4.369070 | -1.119473 | 170 | 85.92 | 0.505412 |  |
| GO:0043413\_biopolymer\_glycosylation | B3GNT2 | 31 | 2 | 4.369070 | -1.119473 | 170 | 85.92 | 0.505412 |  |
| GO:0070085\_glycosylation | FUT9 | 31 | 2 | 4.369070 | -1.119473 | 170 | 85.92 | 0.505412 |  |
| GO:0070085\_glycosylation | B3GNT2 | 31 | 2 | 4.369070 | -1.119473 | 170 | 85.92 | 0.505412 |  |
| GO:0048869\_cellular\_developmental\_process | DCC | 1113 | 22 | 1.338592 | -1.112328 | 171 | 86.12 | 0.503626 |  |
| GO:0048869\_cellular\_developmental\_process | FGF18 | 1113 | 22 | 1.338592 | -1.112328 | 171 | 86.12 | 0.503626 |  |
| GO:0048869\_cellular\_developmental\_process | PTPRC | 1113 | 22 | 1.338592 | -1.112328 | 171 | 86.12 | 0.503626 |  |
| GO:0048869\_cellular\_developmental\_process | IRX5 | 1113 | 22 | 1.338592 | -1.112328 | 171 | 86.12 | 0.503626 |  |
| GO:0048869\_cellular\_developmental\_process | MYO6 | 1113 | 22 | 1.338592 | -1.112328 | 171 | 86.12 | 0.503626 |  |
| GO:0048869\_cellular\_developmental\_process | BBS7 | 1113 | 22 | 1.338592 | -1.112328 | 171 | 86.12 | 0.503626 |  |
| GO:0048869\_cellular\_developmental\_process | MLPH | 1113 | 22 | 1.338592 | -1.112328 | 171 | 86.12 | 0.503626 |  |
| GO:0048869\_cellular\_developmental\_process | ZFX | 1113 | 22 | 1.338592 | -1.112328 | 171 | 86.12 | 0.503626 |  |
| GO:0048869\_cellular\_developmental\_process | SOX5 | 1113 | 22 | 1.338592 | -1.112328 | 171 | 86.12 | 0.503626 |  |
| GO:0048869\_cellular\_developmental\_process | TCF7L2 | 1113 | 22 | 1.338592 | -1.112328 | 171 | 86.12 | 0.503626 |  |
| GO:0048869\_cellular\_developmental\_process | NR2E1 | 1113 | 22 | 1.338592 | -1.112328 | 171 | 86.12 | 0.503626 |  |
| GO:0048869\_cellular\_developmental\_process | NKX6-1 | 1113 | 22 | 1.338592 | -1.112328 | 171 | 86.12 | 0.503626 |  |
| GO:0048869\_cellular\_developmental\_process | ALCAM | 1113 | 22 | 1.338592 | -1.112328 | 171 | 86.12 | 0.503626 |  |
| GO:0048869\_cellular\_developmental\_process | ASCL1 | 1113 | 22 | 1.338592 | -1.112328 | 171 | 86.12 | 0.503626 |  |
| GO:0048869\_cellular\_developmental\_process | NEUROD1 | 1113 | 22 | 1.338592 | -1.112328 | 171 | 86.12 | 0.503626 |  |
| GO:0048869\_cellular\_developmental\_process | ETV1 | 1113 | 22 | 1.338592 | -1.112328 | 171 | 86.12 | 0.503626 |  |
| GO:0048869\_cellular\_developmental\_process | JAK2 | 1113 | 22 | 1.338592 | -1.112328 | 171 | 86.12 | 0.503626 |  |
| GO:0048869\_cellular\_developmental\_process | MBNL3 | 1113 | 22 | 1.338592 | -1.112328 | 171 | 86.12 | 0.503626 |  |
| GO:0048869\_cellular\_developmental\_process | B3GNT2 | 1113 | 22 | 1.338592 | -1.112328 | 171 | 86.12 | 0.503626 |  |
| GO:0048869\_cellular\_developmental\_process | HDAC9 | 1113 | 22 | 1.338592 | -1.112328 | 171 | 86.12 | 0.503626 |  |
| GO:0048869\_cellular\_developmental\_process | BOC | 1113 | 22 | 1.338592 | -1.112328 | 171 | 86.12 | 0.503626 |  |
| GO:0048869\_cellular\_developmental\_process | RAB27A | 1113 | 22 | 1.338592 | -1.112328 | 171 | 86.12 | 0.503626 |  |
| GO:0048878\_chemical\_homeostasis | PTPRC | 254 | 7 | 1.866315 | -1.102309 | 172 | 87.14 | 0.506628 |  |
| GO:0048878\_chemical\_homeostasis | AQP11 | 254 | 7 | 1.866315 | -1.102309 | 172 | 87.14 | 0.506628 |  |
| GO:0048878\_chemical\_homeostasis | HEXB | 254 | 7 | 1.866315 | -1.102309 | 172 | 87.14 | 0.506628 |  |
| GO:0048878\_chemical\_homeostasis | KCNA5 | 254 | 7 | 1.866315 | -1.102309 | 172 | 87.14 | 0.506628 |  |
| GO:0048878\_chemical\_homeostasis | ATP1A2 | 254 | 7 | 1.866315 | -1.102309 | 172 | 87.14 | 0.506628 |  |
| GO:0048878\_chemical\_homeostasis | ATP6V1B1 | 254 | 7 | 1.866315 | -1.102309 | 172 | 87.14 | 0.506628 |  |
| GO:0048878\_chemical\_homeostasis | SLC9A3R1 | 254 | 7 | 1.866315 | -1.102309 | 172 | 87.14 | 0.506628 |  |
| GO:0045893\_positive\_regulation\_of\_transcription\_\_DNA-dependent | ASCL1 | 306 | 8 | 1.770473 | -1.099752 | 174 | 87.25 | 0.501437 |  |
| GO:0045893\_positive\_regulation\_of\_transcription\_\_DNA-dependent | MSX1 | 306 | 8 | 1.770473 | -1.099752 | 174 | 87.25 | 0.501437 |  |
| GO:0045893\_positive\_regulation\_of\_transcription\_\_DNA-dependent | TBX3 | 306 | 8 | 1.770473 | -1.099752 | 174 | 87.25 | 0.501437 |  |
| GO:0045893\_positive\_regulation\_of\_transcription\_\_DNA-dependent | TBX20 | 306 | 8 | 1.770473 | -1.099752 | 174 | 87.25 | 0.501437 |  |
| GO:0045893\_positive\_regulation\_of\_transcription\_\_DNA-dependent | ESRRG | 306 | 8 | 1.770473 | -1.099752 | 174 | 87.25 | 0.501437 |  |
| GO:0045893\_positive\_regulation\_of\_transcription\_\_DNA-dependent | SOX5 | 306 | 8 | 1.770473 | -1.099752 | 174 | 87.25 | 0.501437 |  |
| GO:0045893\_positive\_regulation\_of\_transcription\_\_DNA-dependent | NEUROD1 | 306 | 8 | 1.770473 | -1.099752 | 174 | 87.25 | 0.501437 |  |
| GO:0045893\_positive\_regulation\_of\_transcription\_\_DNA-dependent | TCF7L2 | 306 | 8 | 1.770473 | -1.099752 | 174 | 87.25 | 0.501437 |  |
| GO:0051254\_positive\_regulation\_of\_RNA\_metabolic\_process | ASCL1 | 306 | 8 | 1.770473 | -1.099752 | 174 | 87.25 | 0.501437 |  |
| GO:0051254\_positive\_regulation\_of\_RNA\_metabolic\_process | MSX1 | 306 | 8 | 1.770473 | -1.099752 | 174 | 87.25 | 0.501437 |  |
| GO:0051254\_positive\_regulation\_of\_RNA\_metabolic\_process | TBX3 | 306 | 8 | 1.770473 | -1.099752 | 174 | 87.25 | 0.501437 |  |
| GO:0051254\_positive\_regulation\_of\_RNA\_metabolic\_process | TBX20 | 306 | 8 | 1.770473 | -1.099752 | 174 | 87.25 | 0.501437 |  |
| GO:0051254\_positive\_regulation\_of\_RNA\_metabolic\_process | ESRRG | 306 | 8 | 1.770473 | -1.099752 | 174 | 87.25 | 0.501437 |  |
| GO:0051254\_positive\_regulation\_of\_RNA\_metabolic\_process | SOX5 | 306 | 8 | 1.770473 | -1.099752 | 174 | 87.25 | 0.501437 |  |
| GO:0051254\_positive\_regulation\_of\_RNA\_metabolic\_process | NEUROD1 | 306 | 8 | 1.770473 | -1.099752 | 174 | 87.25 | 0.501437 |  |
| GO:0051254\_positive\_regulation\_of\_RNA\_metabolic\_process | TCF7L2 | 306 | 8 | 1.770473 | -1.099752 | 174 | 87.25 | 0.501437 |  |
| GO:0055065\_metal\_ion\_homeostasis | PTPRC | 69 | 3 | 2.944373 | -1.091377 | 175 | 89.26 | 0.510057 |  |
| GO:0055065\_metal\_ion\_homeostasis | HEXB | 69 | 3 | 2.944373 | -1.091377 | 175 | 89.26 | 0.510057 |  |
| GO:0055065\_metal\_ion\_homeostasis | ATP1A2 | 69 | 3 | 2.944373 | -1.091377 | 175 | 89.26 | 0.510057 |  |
| GO:0051179\_localization | DCC | 1058 | 21 | 1.344170 | -1.089230 | 176 | 89.37 | 0.507784 |  |
| GO:0051179\_localization | PTPRC | 1058 | 21 | 1.344170 | -1.089230 | 176 | 89.37 | 0.507784 |  |
| GO:0051179\_localization | MYO6 | 1058 | 21 | 1.344170 | -1.089230 | 176 | 89.37 | 0.507784 |  |
| GO:0051179\_localization | MLPH | 1058 | 21 | 1.344170 | -1.089230 | 176 | 89.37 | 0.507784 |  |
| GO:0051179\_localization | PLXNA2 | 1058 | 21 | 1.344170 | -1.089230 | 176 | 89.37 | 0.507784 |  |
| GO:0051179\_localization | ARID5B | 1058 | 21 | 1.344170 | -1.089230 | 176 | 89.37 | 0.507784 |  |
| GO:0051179\_localization | HEXB | 1058 | 21 | 1.344170 | -1.089230 | 176 | 89.37 | 0.507784 |  |
| GO:0051179\_localization | ITGA4 | 1058 | 21 | 1.344170 | -1.089230 | 176 | 89.37 | 0.507784 |  |
| GO:0051179\_localization | KCNA5 | 1058 | 21 | 1.344170 | -1.089230 | 176 | 89.37 | 0.507784 |  |
| GO:0051179\_localization | ABCA1 | 1058 | 21 | 1.344170 | -1.089230 | 176 | 89.37 | 0.507784 |  |
| GO:0051179\_localization | ATP1A2 | 1058 | 21 | 1.344170 | -1.089230 | 176 | 89.37 | 0.507784 |  |
| GO:0051179\_localization | NR2E1 | 1058 | 21 | 1.344170 | -1.089230 | 176 | 89.37 | 0.507784 |  |
| GO:0051179\_localization | ALCAM | 1058 | 21 | 1.344170 | -1.089230 | 176 | 89.37 | 0.507784 |  |
| GO:0051179\_localization | ASCL1 | 1058 | 21 | 1.344170 | -1.089230 | 176 | 89.37 | 0.507784 |  |
| GO:0051179\_localization | AP2A1 | 1058 | 21 | 1.344170 | -1.089230 | 176 | 89.37 | 0.507784 |  |
| GO:0051179\_localization | ETV1 | 1058 | 21 | 1.344170 | -1.089230 | 176 | 89.37 | 0.507784 |  |
| GO:0051179\_localization | JAK2 | 1058 | 21 | 1.344170 | -1.089230 | 176 | 89.37 | 0.507784 |  |
| GO:0051179\_localization | B3GNT2 | 1058 | 21 | 1.344170 | -1.089230 | 176 | 89.37 | 0.507784 |  |
| GO:0051179\_localization | BOC | 1058 | 21 | 1.344170 | -1.089230 | 176 | 89.37 | 0.507784 |  |
| GO:0051179\_localization | SLC40A1 | 1058 | 21 | 1.344170 | -1.089230 | 176 | 89.37 | 0.507784 |  |
| GO:0051179\_localization | RAB27A | 1058 | 21 | 1.344170 | -1.089230 | 176 | 89.37 | 0.507784 |  |
| GO:0009966\_regulation\_of\_signal\_transduction | FGF18 | 256 | 7 | 1.851735 | -1.088136 | 177 | 89.48 | 0.505537 |  |
| GO:0009966\_regulation\_of\_signal\_transduction | ASCL1 | 256 | 7 | 1.851735 | -1.088136 | 177 | 89.48 | 0.505537 |  |
| GO:0009966\_regulation\_of\_signal\_transduction | PTPRC | 256 | 7 | 1.851735 | -1.088136 | 177 | 89.48 | 0.505537 |  |
| GO:0009966\_regulation\_of\_signal\_transduction | ELF1 | 256 | 7 | 1.851735 | -1.088136 | 177 | 89.48 | 0.505537 |  |
| GO:0009966\_regulation\_of\_signal\_transduction | JAK2 | 256 | 7 | 1.851735 | -1.088136 | 177 | 89.48 | 0.505537 |  |
| GO:0009966\_regulation\_of\_signal\_transduction | CD27 | 256 | 7 | 1.851735 | -1.088136 | 177 | 89.48 | 0.505537 |  |
| GO:0009966\_regulation\_of\_signal\_transduction | RGS13 | 256 | 7 | 1.851735 | -1.088136 | 177 | 89.48 | 0.505537 |  |
| GO:0051094\_positive\_regulation\_of\_developmental\_process | SERINC3 | 308 | 8 | 1.758976 | -1.086912 | 178 | 89.54 | 0.503034 |  |
| GO:0051094\_positive\_regulation\_of\_developmental\_process | ASCL1 | 308 | 8 | 1.758976 | -1.086912 | 178 | 89.54 | 0.503034 |  |
| GO:0051094\_positive\_regulation\_of\_developmental\_process | PTPRC | 308 | 8 | 1.758976 | -1.086912 | 178 | 89.54 | 0.503034 |  |
| GO:0051094\_positive\_regulation\_of\_developmental\_process | NR3C1 | 308 | 8 | 1.758976 | -1.086912 | 178 | 89.54 | 0.503034 |  |
| GO:0051094\_positive\_regulation\_of\_developmental\_process | BOC | 308 | 8 | 1.758976 | -1.086912 | 178 | 89.54 | 0.503034 |  |
| GO:0051094\_positive\_regulation\_of\_developmental\_process | NKX6-1 | 308 | 8 | 1.758976 | -1.086912 | 178 | 89.54 | 0.503034 |  |
| GO:0051094\_positive\_regulation\_of\_developmental\_process | CD27 | 308 | 8 | 1.758976 | -1.086912 | 178 | 89.54 | 0.503034 |  |
| GO:0051094\_positive\_regulation\_of\_developmental\_process | RAB27A | 308 | 8 | 1.758976 | -1.086912 | 178 | 89.54 | 0.503034 |  |
| GO:0006350\_transcription | TBX3 | 701 | 15 | 1.449085 | -1.077862 | 179 | 90.13 | 0.503520 |  |
| GO:0006350\_transcription | TBX20 | 701 | 15 | 1.449085 | -1.077862 | 179 | 90.13 | 0.503520 |  |
| GO:0006350\_transcription | SOX5 | 701 | 15 | 1.449085 | -1.077862 | 179 | 90.13 | 0.503520 |  |
| GO:0006350\_transcription | ESRRG | 701 | 15 | 1.449085 | -1.077862 | 179 | 90.13 | 0.503520 |  |
| GO:0006350\_transcription | NR3C1 | 701 | 15 | 1.449085 | -1.077862 | 179 | 90.13 | 0.503520 |  |
| GO:0006350\_transcription | NR2E1 | 701 | 15 | 1.449085 | -1.077862 | 179 | 90.13 | 0.503520 |  |
| GO:0006350\_transcription | TCF7L2 | 701 | 15 | 1.449085 | -1.077862 | 179 | 90.13 | 0.503520 |  |
| GO:0006350\_transcription | NKX6-1 | 701 | 15 | 1.449085 | -1.077862 | 179 | 90.13 | 0.503520 |  |
| GO:0006350\_transcription | ASCL1 | 701 | 15 | 1.449085 | -1.077862 | 179 | 90.13 | 0.503520 |  |
| GO:0006350\_transcription | TCERG1 | 701 | 15 | 1.449085 | -1.077862 | 179 | 90.13 | 0.503520 |  |
| GO:0006350\_transcription | MSX1 | 701 | 15 | 1.449085 | -1.077862 | 179 | 90.13 | 0.503520 |  |
| GO:0006350\_transcription | DMD | 701 | 15 | 1.449085 | -1.077862 | 179 | 90.13 | 0.503520 |  |
| GO:0006350\_transcription | NEUROD1 | 701 | 15 | 1.449085 | -1.077862 | 179 | 90.13 | 0.503520 |  |
| GO:0006350\_transcription | ETV1 | 701 | 15 | 1.449085 | -1.077862 | 179 | 90.13 | 0.503520 |  |
| GO:0006350\_transcription | HDAC9 | 701 | 15 | 1.449085 | -1.077862 | 179 | 90.13 | 0.503520 |  |
| GO:0021987\_cerebral\_cortex\_development | ASCL1 | 33 | 2 | 4.104278 | -1.072366 | 180 | 91.64 | 0.509111 |  |
| GO:0021987\_cerebral\_cortex\_development | NR2E1 | 33 | 2 | 4.104278 | -1.072366 | 180 | 91.64 | 0.509111 |  |
| GO:0002920\_regulation\_of\_humoral\_immune\_response | PTPRC | 6 | 1 | 11.286765 | -1.068354 | 206 | 107.47 | 0.521699 |  |
| GO:0006942\_regulation\_of\_striated\_muscle\_contraction | ATP1A2 | 6 | 1 | 11.286765 | -1.068354 | 206 | 107.47 | 0.521699 |  |
| GO:0007032\_endosome\_organization | AQP11 | 6 | 1 | 11.286765 | -1.068354 | 206 | 107.47 | 0.521699 |  |
| GO:0007341\_penetration\_of\_zona\_pellucida | HEXB | 6 | 1 | 11.286765 | -1.068354 | 206 | 107.47 | 0.521699 |  |
| GO:0007442\_hindgut\_morphogenesis | TCF7L2 | 6 | 1 | 11.286765 | -1.068354 | 206 | 107.47 | 0.521699 |  |
| GO:0010906\_regulation\_of\_glucose\_metabolic\_process | NR3C1 | 6 | 1 | 11.286765 | -1.068354 | 206 | 107.47 | 0.521699 |  |
| GO:0014003\_oligodendrocyte\_development | ASCL1 | 6 | 1 | 11.286765 | -1.068354 | 206 | 107.47 | 0.521699 |  |
| GO:0030002\_cellular\_anion\_homeostasis | SLC9A3R1 | 6 | 1 | 11.286765 | -1.068354 | 206 | 107.47 | 0.521699 |  |
| GO:0030149\_sphingolipid\_catabolic\_process | HEXB | 6 | 1 | 11.286765 | -1.068354 | 206 | 107.47 | 0.521699 |  |
| GO:0030947\_regulation\_of\_vascular\_endothelial\_growth\_factor\_receptor\_signaling\_pathway | FGF18 | 6 | 1 | 11.286765 | -1.068354 | 206 | 107.47 | 0.521699 |  |
| GO:0035019\_somatic\_stem\_cell\_maintenance | TCF7L2 | 6 | 1 | 11.286765 | -1.068354 | 206 | 107.47 | 0.521699 |  |
| GO:0042492\_gamma-delta\_T\_cell\_differentiation | PTPRC | 6 | 1 | 11.286765 | -1.068354 | 206 | 107.47 | 0.521699 |  |
| GO:0043255\_regulation\_of\_carbohydrate\_biosynthetic\_process | NR3C1 | 6 | 1 | 11.286765 | -1.068354 | 206 | 107.47 | 0.521699 |  |
| GO:0043271\_negative\_regulation\_of\_ion\_transport | KCNA5 | 6 | 1 | 11.286765 | -1.068354 | 206 | 107.47 | 0.521699 |  |
| GO:0045822\_negative\_regulation\_of\_heart\_contraction | ATP1A2 | 6 | 1 | 11.286765 | -1.068354 | 206 | 107.47 | 0.521699 |  |
| GO:0045843\_negative\_regulation\_of\_striated\_muscle\_development | HDAC9 | 6 | 1 | 11.286765 | -1.068354 | 206 | 107.47 | 0.521699 |  |
| GO:0046427\_positive\_regulation\_of\_JAK-STAT\_cascade | JAK2 | 6 | 1 | 11.286765 | -1.068354 | 206 | 107.47 | 0.521699 |  |
| GO:0046460\_neutral\_lipid\_biosynthetic\_process | PPAP2A | 6 | 1 | 11.286765 | -1.068354 | 206 | 107.47 | 0.521699 |  |
| GO:0046463\_acylglycerol\_biosynthetic\_process | PPAP2A | 6 | 1 | 11.286765 | -1.068354 | 206 | 107.47 | 0.521699 |  |
| GO:0046466\_membrane\_lipid\_catabolic\_process | HEXB | 6 | 1 | 11.286765 | -1.068354 | 206 | 107.47 | 0.521699 |  |
| GO:0046629\_gamma-delta\_T\_cell\_activation | PTPRC | 6 | 1 | 11.286765 | -1.068354 | 206 | 107.47 | 0.521699 |  |
| GO:0048635\_negative\_regulation\_of\_muscle\_development | HDAC9 | 6 | 1 | 11.286765 | -1.068354 | 206 | 107.47 | 0.521699 |  |
| GO:0048644\_muscle\_organ\_morphogenesis | ARID5B | 6 | 1 | 11.286765 | -1.068354 | 206 | 107.47 | 0.521699 |  |
| GO:0050732\_negative\_regulation\_of\_peptidyl-tyrosine\_phosphorylation | PTPRC | 6 | 1 | 11.286765 | -1.068354 | 206 | 107.47 | 0.521699 |  |
| GO:0055081\_anion\_homeostasis | SLC9A3R1 | 6 | 1 | 11.286765 | -1.068354 | 206 | 107.47 | 0.521699 |  |
| GO:0060710\_chorio-allantoic\_fusion | ITGA4 | 6 | 1 | 11.286765 | -1.068354 | 206 | 107.47 | 0.521699 |  |
| GO:0009100\_glycoprotein\_metabolic\_process | FUT9 | 71 | 3 | 2.861433 | -1.062474 | 207 | 108.07 | 0.522077 |  |
| GO:0009100\_glycoprotein\_metabolic\_process | MAMDC2 | 71 | 3 | 2.861433 | -1.062474 | 207 | 108.07 | 0.522077 |  |
| GO:0009100\_glycoprotein\_metabolic\_process | B3GNT2 | 71 | 3 | 2.861433 | -1.062474 | 207 | 108.07 | 0.522077 |  |
| GO:0048584\_positive\_regulation\_of\_response\_to\_stimulus | PTPRC | 115 | 4 | 2.355499 | -1.050747 | 208 | 108.45 | 0.521394 |  |
| GO:0048584\_positive\_regulation\_of\_response\_to\_stimulus | ELF1 | 115 | 4 | 2.355499 | -1.050747 | 208 | 108.45 | 0.521394 |  |
| GO:0048584\_positive\_regulation\_of\_response\_to\_stimulus | JAK2 | 115 | 4 | 2.355499 | -1.050747 | 208 | 108.45 | 0.521394 |  |
| GO:0048584\_positive\_regulation\_of\_response\_to\_stimulus | CD27 | 115 | 4 | 2.355499 | -1.050747 | 208 | 108.45 | 0.521394 |  |
| GO:0010720\_positive\_regulation\_of\_cell\_development | ASCL1 | 34 | 2 | 3.983564 | -1.050067 | 211 | 109.96 | 0.521137 |  |
| GO:0010720\_positive\_regulation\_of\_cell\_development | NKX6-1 | 34 | 2 | 3.983564 | -1.050067 | 211 | 109.96 | 0.521137 |  |
| GO:0050730\_regulation\_of\_peptidyl-tyrosine\_phosphorylation | PTPRC | 34 | 2 | 3.983564 | -1.050067 | 211 | 109.96 | 0.521137 |  |
| GO:0050730\_regulation\_of\_peptidyl-tyrosine\_phosphorylation | JAK2 | 34 | 2 | 3.983564 | -1.050067 | 211 | 109.96 | 0.521137 |  |
| GO:0060443\_mammary\_gland\_morphogenesis | TBX3 | 34 | 2 | 3.983564 | -1.050067 | 211 | 109.96 | 0.521137 |  |
| GO:0060443\_mammary\_gland\_morphogenesis | NR3C1 | 34 | 2 | 3.983564 | -1.050067 | 211 | 109.96 | 0.521137 |  |
| GO:0044262\_cellular\_carbohydrate\_metabolic\_process | FUT9 | 72 | 3 | 2.821691 | -1.048420 | 212 | 110.55 | 0.521462 |  |
| GO:0044262\_cellular\_carbohydrate\_metabolic\_process | NR3C1 | 72 | 3 | 2.821691 | -1.048420 | 212 | 110.55 | 0.521462 |  |
| GO:0044262\_cellular\_carbohydrate\_metabolic\_process | B3GNT2 | 72 | 3 | 2.821691 | -1.048420 | 212 | 110.55 | 0.521462 |  |
| GO:0006936\_muscle\_contraction | MYOM2 | 73 | 3 | 2.783038 | -1.034622 | 213 | 111.82 | 0.524977 |  |
| GO:0006936\_muscle\_contraction | TBX20 | 73 | 3 | 2.783038 | -1.034622 | 213 | 111.82 | 0.524977 |  |
| GO:0006936\_muscle\_contraction | ATP1A2 | 73 | 3 | 2.783038 | -1.034622 | 213 | 111.82 | 0.524977 |  |
| GO:0044255\_cellular\_lipid\_metabolic\_process | AFP | 264 | 7 | 1.795622 | -1.033275 | 214 | 112.03 | 0.523505 |  |
| GO:0044255\_cellular\_lipid\_metabolic\_process | SGPP1 | 264 | 7 | 1.795622 | -1.033275 | 214 | 112.03 | 0.523505 |  |
| GO:0044255\_cellular\_lipid\_metabolic\_process | HEXB | 264 | 7 | 1.795622 | -1.033275 | 214 | 112.03 | 0.523505 |  |
| GO:0044255\_cellular\_lipid\_metabolic\_process | ABCA1 | 264 | 7 | 1.795622 | -1.033275 | 214 | 112.03 | 0.523505 |  |
| GO:0044255\_cellular\_lipid\_metabolic\_process | NR3C1 | 264 | 7 | 1.795622 | -1.033275 | 214 | 112.03 | 0.523505 |  |
| GO:0044255\_cellular\_lipid\_metabolic\_process | SGMS1 | 264 | 7 | 1.795622 | -1.033275 | 214 | 112.03 | 0.523505 |  |
| GO:0044255\_cellular\_lipid\_metabolic\_process | PPAP2A | 264 | 7 | 1.795622 | -1.033275 | 214 | 112.03 | 0.523505 |  |
| GO:0010557\_positive\_regulation\_of\_macromolecule\_biosynthetic\_process | ASCL1 | 371 | 9 | 1.642818 | -1.030222 | 215 | 112.13 | 0.521535 |  |
| GO:0010557\_positive\_regulation\_of\_macromolecule\_biosynthetic\_process | MSX1 | 371 | 9 | 1.642818 | -1.030222 | 215 | 112.13 | 0.521535 |  |
| GO:0010557\_positive\_regulation\_of\_macromolecule\_biosynthetic\_process | TBX3 | 371 | 9 | 1.642818 | -1.030222 | 215 | 112.13 | 0.521535 |  |
| GO:0010557\_positive\_regulation\_of\_macromolecule\_biosynthetic\_process | TBX20 | 371 | 9 | 1.642818 | -1.030222 | 215 | 112.13 | 0.521535 |  |
| GO:0010557\_positive\_regulation\_of\_macromolecule\_biosynthetic\_process | ESRRG | 371 | 9 | 1.642818 | -1.030222 | 215 | 112.13 | 0.521535 |  |
| GO:0010557\_positive\_regulation\_of\_macromolecule\_biosynthetic\_process | SOX5 | 371 | 9 | 1.642818 | -1.030222 | 215 | 112.13 | 0.521535 |  |
| GO:0010557\_positive\_regulation\_of\_macromolecule\_biosynthetic\_process | ETV1 | 371 | 9 | 1.642818 | -1.030222 | 215 | 112.13 | 0.521535 |  |
| GO:0010557\_positive\_regulation\_of\_macromolecule\_biosynthetic\_process | NEUROD1 | 371 | 9 | 1.642818 | -1.030222 | 215 | 112.13 | 0.521535 |  |
| GO:0010557\_positive\_regulation\_of\_macromolecule\_biosynthetic\_process | TCF7L2 | 371 | 9 | 1.642818 | -1.030222 | 215 | 112.13 | 0.521535 |  |
| GO:0001906\_cell\_killing | PTPRC | 35 | 2 | 3.869748 | -1.028537 | 217 | 113.56 | 0.523318 |  |
| GO:0001906\_cell\_killing | RAB27A | 35 | 2 | 3.869748 | -1.028537 | 217 | 113.56 | 0.523318 |  |
| GO:0001909\_leukocyte\_mediated\_cytotoxicity | PTPRC | 35 | 2 | 3.869748 | -1.028537 | 217 | 113.56 | 0.523318 |  |
| GO:0001909\_leukocyte\_mediated\_cytotoxicity | RAB27A | 35 | 2 | 3.869748 | -1.028537 | 217 | 113.56 | 0.523318 |  |
| GO:0007166\_cell\_surface\_receptor\_linked\_signal\_transduction | PTPRC | 597 | 13 | 1.474653 | -1.024871 | 218 | 113.62 | 0.521193 |  |
| GO:0007166\_cell\_surface\_receptor\_linked\_signal\_transduction | FGF18 | 597 | 13 | 1.474653 | -1.024871 | 218 | 113.62 | 0.521193 |  |
| GO:0007166\_cell\_surface\_receptor\_linked\_signal\_transduction | ELF1 | 597 | 13 | 1.474653 | -1.024871 | 218 | 113.62 | 0.521193 |  |
| GO:0007166\_cell\_surface\_receptor\_linked\_signal\_transduction | PLXNA2 | 597 | 13 | 1.474653 | -1.024871 | 218 | 113.62 | 0.521193 |  |
| GO:0007166\_cell\_surface\_receptor\_linked\_signal\_transduction | ARID5B | 597 | 13 | 1.474653 | -1.024871 | 218 | 113.62 | 0.521193 |  |
| GO:0007166\_cell\_surface\_receptor\_linked\_signal\_transduction | TCF7L2 | 597 | 13 | 1.474653 | -1.024871 | 218 | 113.62 | 0.521193 |  |
| GO:0007166\_cell\_surface\_receptor\_linked\_signal\_transduction | NKX6-1 | 597 | 13 | 1.474653 | -1.024871 | 218 | 113.62 | 0.521193 |  |
| GO:0007166\_cell\_surface\_receptor\_linked\_signal\_transduction | RGS13 | 597 | 13 | 1.474653 | -1.024871 | 218 | 113.62 | 0.521193 |  |
| GO:0007166\_cell\_surface\_receptor\_linked\_signal\_transduction | ASCL1 | 597 | 13 | 1.474653 | -1.024871 | 218 | 113.62 | 0.521193 |  |
| GO:0007166\_cell\_surface\_receptor\_linked\_signal\_transduction | MSX1 | 597 | 13 | 1.474653 | -1.024871 | 218 | 113.62 | 0.521193 |  |
| GO:0007166\_cell\_surface\_receptor\_linked\_signal\_transduction | JAK2 | 597 | 13 | 1.474653 | -1.024871 | 218 | 113.62 | 0.521193 |  |
| GO:0007166\_cell\_surface\_receptor\_linked\_signal\_transduction | RGS8 | 597 | 13 | 1.474653 | -1.024871 | 218 | 113.62 | 0.521193 |  |
| GO:0007166\_cell\_surface\_receptor\_linked\_signal\_transduction | BOC | 597 | 13 | 1.474653 | -1.024871 | 218 | 113.62 | 0.521193 |  |
| GO:0051960\_regulation\_of\_nervous\_system\_development | ASCL1 | 118 | 4 | 2.295613 | -1.019381 | 219 | 114.31 | 0.521963 |  |
| GO:0051960\_regulation\_of\_nervous\_system\_development | SOX5 | 118 | 4 | 2.295613 | -1.019381 | 219 | 114.31 | 0.521963 |  |
| GO:0051960\_regulation\_of\_nervous\_system\_development | NR2E1 | 118 | 4 | 2.295613 | -1.019381 | 219 | 114.31 | 0.521963 |  |
| GO:0051960\_regulation\_of\_nervous\_system\_development | NKX6-1 | 118 | 4 | 2.295613 | -1.019381 | 219 | 114.31 | 0.521963 |  |
| GO:0034960\_cellular\_biopolymer\_metabolic\_process | FUT9 | 1395 | 26 | 1.262176 | -1.008895 | 220 | 114.62 | 0.521000 |  |
| GO:0034960\_cellular\_biopolymer\_metabolic\_process | MAMDC2 | 1395 | 26 | 1.262176 | -1.008895 | 220 | 114.62 | 0.521000 |  |
| GO:0034960\_cellular\_biopolymer\_metabolic\_process | HEXB | 1395 | 26 | 1.262176 | -1.008895 | 220 | 114.62 | 0.521000 |  |
| GO:0034960\_cellular\_biopolymer\_metabolic\_process | TBX20 | 1395 | 26 | 1.262176 | -1.008895 | 220 | 114.62 | 0.521000 |  |
| GO:0034960\_cellular\_biopolymer\_metabolic\_process | SOX5 | 1395 | 26 | 1.262176 | -1.008895 | 220 | 114.62 | 0.521000 |  |
| GO:0034960\_cellular\_biopolymer\_metabolic\_process | HSPA1A | 1395 | 26 | 1.262176 | -1.008895 | 220 | 114.62 | 0.521000 |  |
| GO:0034960\_cellular\_biopolymer\_metabolic\_process | NR3C1 | 1395 | 26 | 1.262176 | -1.008895 | 220 | 114.62 | 0.521000 |  |
| GO:0034960\_cellular\_biopolymer\_metabolic\_process | ABCA1 | 1395 | 26 | 1.262176 | -1.008895 | 220 | 114.62 | 0.521000 |  |
| GO:0034960\_cellular\_biopolymer\_metabolic\_process | TCF7L2 | 1395 | 26 | 1.262176 | -1.008895 | 220 | 114.62 | 0.521000 |  |
| GO:0034960\_cellular\_biopolymer\_metabolic\_process | NR2E1 | 1395 | 26 | 1.262176 | -1.008895 | 220 | 114.62 | 0.521000 |  |
| GO:0034960\_cellular\_biopolymer\_metabolic\_process | TCERG1 | 1395 | 26 | 1.262176 | -1.008895 | 220 | 114.62 | 0.521000 |  |
| GO:0034960\_cellular\_biopolymer\_metabolic\_process | DMD | 1395 | 26 | 1.262176 | -1.008895 | 220 | 114.62 | 0.521000 |  |
| GO:0034960\_cellular\_biopolymer\_metabolic\_process | ETV1 | 1395 | 26 | 1.262176 | -1.008895 | 220 | 114.62 | 0.521000 |  |
| GO:0034960\_cellular\_biopolymer\_metabolic\_process | B3GNT2 | 1395 | 26 | 1.262176 | -1.008895 | 220 | 114.62 | 0.521000 |  |
| GO:0034960\_cellular\_biopolymer\_metabolic\_process | PPAP2A | 1395 | 26 | 1.262176 | -1.008895 | 220 | 114.62 | 0.521000 |  |
| GO:0034960\_cellular\_biopolymer\_metabolic\_process | PTPRC | 1395 | 26 | 1.262176 | -1.008895 | 220 | 114.62 | 0.521000 |  |
| GO:0034960\_cellular\_biopolymer\_metabolic\_process | TBX3 | 1395 | 26 | 1.262176 | -1.008895 | 220 | 114.62 | 0.521000 |  |
| GO:0034960\_cellular\_biopolymer\_metabolic\_process | ESRRG | 1395 | 26 | 1.262176 | -1.008895 | 220 | 114.62 | 0.521000 |  |
| GO:0034960\_cellular\_biopolymer\_metabolic\_process | TRIM63 | 1395 | 26 | 1.262176 | -1.008895 | 220 | 114.62 | 0.521000 |  |
| GO:0034960\_cellular\_biopolymer\_metabolic\_process | NKX6-1 | 1395 | 26 | 1.262176 | -1.008895 | 220 | 114.62 | 0.521000 |  |
| GO:0034960\_cellular\_biopolymer\_metabolic\_process | ASCL1 | 1395 | 26 | 1.262176 | -1.008895 | 220 | 114.62 | 0.521000 |  |
| GO:0034960\_cellular\_biopolymer\_metabolic\_process | MSX1 | 1395 | 26 | 1.262176 | -1.008895 | 220 | 114.62 | 0.521000 |  |
| GO:0034960\_cellular\_biopolymer\_metabolic\_process | NEUROD1 | 1395 | 26 | 1.262176 | -1.008895 | 220 | 114.62 | 0.521000 |  |
| GO:0034960\_cellular\_biopolymer\_metabolic\_process | JAK2 | 1395 | 26 | 1.262176 | -1.008895 | 220 | 114.62 | 0.521000 |  |
| GO:0034960\_cellular\_biopolymer\_metabolic\_process | HDAC9 | 1395 | 26 | 1.262176 | -1.008895 | 220 | 114.62 | 0.521000 |  |
| GO:0034960\_cellular\_biopolymer\_metabolic\_process | GADD45B | 1395 | 26 | 1.262176 | -1.008895 | 220 | 114.62 | 0.521000 |  |
| GO:0006469\_negative\_regulation\_of\_protein\_kinase\_activity | PTPRC | 36 | 2 | 3.762255 | -1.007734 | 225 | 116.39 | 0.517289 |  |
| GO:0006469\_negative\_regulation\_of\_protein\_kinase\_activity | GADD45B | 36 | 2 | 3.762255 | -1.007734 | 225 | 116.39 | 0.517289 |  |
| GO:0021510\_spinal\_cord\_development | ASCL1 | 36 | 2 | 3.762255 | -1.007734 | 225 | 116.39 | 0.517289 |  |
| GO:0021510\_spinal\_cord\_development | NKX6-1 | 36 | 2 | 3.762255 | -1.007734 | 225 | 116.39 | 0.517289 |  |
| GO:0022602\_ovulation\_cycle\_process | AFP | 36 | 2 | 3.762255 | -1.007734 | 225 | 116.39 | 0.517289 |  |
| GO:0022602\_ovulation\_cycle\_process | ZFX | 36 | 2 | 3.762255 | -1.007734 | 225 | 116.39 | 0.517289 |  |
| GO:0033673\_negative\_regulation\_of\_kinase\_activity | PTPRC | 36 | 2 | 3.762255 | -1.007734 | 225 | 116.39 | 0.517289 |  |
| GO:0033673\_negative\_regulation\_of\_kinase\_activity | GADD45B | 36 | 2 | 3.762255 | -1.007734 | 225 | 116.39 | 0.517289 |  |
| GO:0050851\_antigen\_receptor-mediated\_signaling\_pathway | PTPRC | 36 | 2 | 3.762255 | -1.007734 | 225 | 116.39 | 0.517289 |  |
| GO:0050851\_antigen\_receptor-mediated\_signaling\_pathway | ELF1 | 36 | 2 | 3.762255 | -1.007734 | 225 | 116.39 | 0.517289 |  |
| GO:0001504\_neurotransmitter\_uptake | ATP1A2 | 7 | 1 | 9.674370 | -1.004542 | 250 | 134.22 | 0.536880 |  |
| GO:0001573\_ganglioside\_metabolic\_process | HEXB | 7 | 1 | 9.674370 | -1.004542 | 250 | 134.22 | 0.536880 |  |
| GO:0002063\_chondrocyte\_development | FGF18 | 7 | 1 | 9.674370 | -1.004542 | 250 | 134.22 | 0.536880 |  |
| GO:0002087\_regulation\_of\_respiratory\_gaseous\_exchange\_by\_neurological\_system\_process | ATP1A2 | 7 | 1 | 9.674370 | -1.004542 | 250 | 134.22 | 0.536880 |  |
| GO:0002455\_humoral\_immune\_response\_mediated\_by\_circulating\_immunoglobulin | PTPRC | 7 | 1 | 9.674370 | -1.004542 | 250 | 134.22 | 0.536880 |  |
| GO:0002714\_positive\_regulation\_of\_B\_cell\_mediated\_immunity | PTPRC | 7 | 1 | 9.674370 | -1.004542 | 250 | 134.22 | 0.536880 |  |
| GO:0002891\_positive\_regulation\_of\_immunoglobulin\_mediated\_immune\_response | PTPRC | 7 | 1 | 9.674370 | -1.004542 | 250 | 134.22 | 0.536880 |  |
| GO:0009311\_oligosaccharide\_metabolic\_process | HEXB | 7 | 1 | 9.674370 | -1.004542 | 250 | 134.22 | 0.536880 |  |
| GO:0014047\_glutamate\_secretion | MYO6 | 7 | 1 | 9.674370 | -1.004542 | 250 | 134.22 | 0.536880 |  |
| GO:0014066\_regulation\_of\_phosphoinositide\_3-kinase\_cascade | JAK2 | 7 | 1 | 9.674370 | -1.004542 | 250 | 134.22 | 0.536880 |  |
| GO:0015914\_phospholipid\_transport | ABCA1 | 7 | 1 | 9.674370 | -1.004542 | 250 | 134.22 | 0.536880 |  |
| GO:0019800\_peptide\_cross-linking\_via\_chondroitin\_4-sulfate\_glycosaminoglycan | MAMDC2 | 7 | 1 | 9.674370 | -1.004542 | 250 | 134.22 | 0.536880 |  |
| GO:0021514\_ventral\_spinal\_cord\_interneuron\_differentiation | NKX6-1 | 7 | 1 | 9.674370 | -1.004542 | 250 | 134.22 | 0.536880 |  |
| GO:0021516\_dorsal\_spinal\_cord\_development | ASCL1 | 7 | 1 | 9.674370 | -1.004542 | 250 | 134.22 | 0.536880 |  |
| GO:0021520\_spinal\_cord\_motor\_neuron\_cell\_fate\_specification | NKX6-1 | 7 | 1 | 9.674370 | -1.004542 | 250 | 134.22 | 0.536880 |  |
| GO:0021521\_ventral\_spinal\_cord\_interneuron\_specification | NKX6-1 | 7 | 1 | 9.674370 | -1.004542 | 250 | 134.22 | 0.536880 |  |
| GO:0033057\_reproductive\_behavior\_in\_a\_multicellular\_organism | HEXB | 7 | 1 | 9.674370 | -1.004542 | 250 | 134.22 | 0.536880 |  |
| GO:0042503\_tyrosine\_phosphorylation\_of\_Stat3\_protein | JAK2 | 7 | 1 | 9.674370 | -1.004542 | 250 | 134.22 | 0.536880 |  |
| GO:0043584\_nose\_development | ASCL1 | 7 | 1 | 9.674370 | -1.004542 | 250 | 134.22 | 0.536880 |  |
| GO:0044065\_regulation\_of\_respiratory\_system\_process | ATP1A2 | 7 | 1 | 9.674370 | -1.004542 | 250 | 134.22 | 0.536880 |  |
| GO:0045059\_positive\_thymic\_T\_cell\_selection | PTPRC | 7 | 1 | 9.674370 | -1.004542 | 250 | 134.22 | 0.536880 |  |
| GO:0046504\_glycerol\_ether\_biosynthetic\_process | PPAP2A | 7 | 1 | 9.674370 | -1.004542 | 250 | 134.22 | 0.536880 |  |
| GO:0048304\_positive\_regulation\_of\_isotype\_switching\_to\_IgG\_isotypes | PTPRC | 7 | 1 | 9.674370 | -1.004542 | 250 | 134.22 | 0.536880 |  |
| GO:0060579\_ventral\_spinal\_cord\_interneuron\_fate\_commitment | NKX6-1 | 7 | 1 | 9.674370 | -1.004542 | 250 | 134.22 | 0.536880 |  |
| GO:0060788\_ectodermal\_placode\_formation | TBX3 | 7 | 1 | 9.674370 | -1.004542 | 250 | 134.22 | 0.536880 |  |
| GO:0014706\_striated\_muscle\_tissue\_development | DMD | 120 | 4 | 2.257353 | -0.999103 | 251 | 134.67 | 0.536534 |  |
| GO:0014706\_striated\_muscle\_tissue\_development | MBNL3 | 120 | 4 | 2.257353 | -0.999103 | 251 | 134.67 | 0.536534 |  |
| GO:0014706\_striated\_muscle\_tissue\_development | HDAC9 | 120 | 4 | 2.257353 | -0.999103 | 251 | 134.67 | 0.536534 |  |
| GO:0014706\_striated\_muscle\_tissue\_development | BOC | 120 | 4 | 2.257353 | -0.999103 | 251 | 134.67 | 0.536534 |  |
| GO:0006807\_nitrogen\_compound\_metabolic\_process | PTPRC | 1147 | 22 | 1.298913 | -0.996250 | 252 | 134.72 | 0.534603 |  |
| GO:0006807\_nitrogen\_compound\_metabolic\_process | MAMDC2 | 1147 | 22 | 1.298913 | -0.996250 | 252 | 134.72 | 0.534603 |  |
| GO:0006807\_nitrogen\_compound\_metabolic\_process | TBX3 | 1147 | 22 | 1.298913 | -0.996250 | 252 | 134.72 | 0.534603 |  |
| GO:0006807\_nitrogen\_compound\_metabolic\_process | SGPP1 | 1147 | 22 | 1.298913 | -0.996250 | 252 | 134.72 | 0.534603 |  |
| GO:0006807\_nitrogen\_compound\_metabolic\_process | ARID5B | 1147 | 22 | 1.298913 | -0.996250 | 252 | 134.72 | 0.534603 |  |
| GO:0006807\_nitrogen\_compound\_metabolic\_process | HEXB | 1147 | 22 | 1.298913 | -0.996250 | 252 | 134.72 | 0.534603 |  |
| GO:0006807\_nitrogen\_compound\_metabolic\_process | TBX20 | 1147 | 22 | 1.298913 | -0.996250 | 252 | 134.72 | 0.534603 |  |
| GO:0006807\_nitrogen\_compound\_metabolic\_process | SOX5 | 1147 | 22 | 1.298913 | -0.996250 | 252 | 134.72 | 0.534603 |  |
| GO:0006807\_nitrogen\_compound\_metabolic\_process | ESRRG | 1147 | 22 | 1.298913 | -0.996250 | 252 | 134.72 | 0.534603 |  |
| GO:0006807\_nitrogen\_compound\_metabolic\_process | HSPA1A | 1147 | 22 | 1.298913 | -0.996250 | 252 | 134.72 | 0.534603 |  |
| GO:0006807\_nitrogen\_compound\_metabolic\_process | NR3C1 | 1147 | 22 | 1.298913 | -0.996250 | 252 | 134.72 | 0.534603 |  |
| GO:0006807\_nitrogen\_compound\_metabolic\_process | NR2E1 | 1147 | 22 | 1.298913 | -0.996250 | 252 | 134.72 | 0.534603 |  |
| GO:0006807\_nitrogen\_compound\_metabolic\_process | TCF7L2 | 1147 | 22 | 1.298913 | -0.996250 | 252 | 134.72 | 0.534603 |  |
| GO:0006807\_nitrogen\_compound\_metabolic\_process | NKX6-1 | 1147 | 22 | 1.298913 | -0.996250 | 252 | 134.72 | 0.534603 |  |
| GO:0006807\_nitrogen\_compound\_metabolic\_process | ASCL1 | 1147 | 22 | 1.298913 | -0.996250 | 252 | 134.72 | 0.534603 |  |
| GO:0006807\_nitrogen\_compound\_metabolic\_process | TCERG1 | 1147 | 22 | 1.298913 | -0.996250 | 252 | 134.72 | 0.534603 |  |
| GO:0006807\_nitrogen\_compound\_metabolic\_process | MSX1 | 1147 | 22 | 1.298913 | -0.996250 | 252 | 134.72 | 0.534603 |  |
| GO:0006807\_nitrogen\_compound\_metabolic\_process | DMD | 1147 | 22 | 1.298913 | -0.996250 | 252 | 134.72 | 0.534603 |  |
| GO:0006807\_nitrogen\_compound\_metabolic\_process | ETV1 | 1147 | 22 | 1.298913 | -0.996250 | 252 | 134.72 | 0.534603 |  |
| GO:0006807\_nitrogen\_compound\_metabolic\_process | NEUROD1 | 1147 | 22 | 1.298913 | -0.996250 | 252 | 134.72 | 0.534603 |  |
| GO:0006807\_nitrogen\_compound\_metabolic\_process | PPAP2A | 1147 | 22 | 1.298913 | -0.996250 | 252 | 134.72 | 0.534603 |  |
| GO:0006807\_nitrogen\_compound\_metabolic\_process | HDAC9 | 1147 | 22 | 1.298913 | -0.996250 | 252 | 134.72 | 0.534603 |  |
| GO:0034645\_cellular\_macromolecule\_biosynthetic\_process | FUT9 | 901 | 18 | 1.352909 | -0.995757 | 253 | 134.77 | 0.532688 |  |
| GO:0034645\_cellular\_macromolecule\_biosynthetic\_process | TBX3 | 901 | 18 | 1.352909 | -0.995757 | 253 | 134.77 | 0.532688 |  |
| GO:0034645\_cellular\_macromolecule\_biosynthetic\_process | TBX20 | 901 | 18 | 1.352909 | -0.995757 | 253 | 134.77 | 0.532688 |  |
| GO:0034645\_cellular\_macromolecule\_biosynthetic\_process | SOX5 | 901 | 18 | 1.352909 | -0.995757 | 253 | 134.77 | 0.532688 |  |
| GO:0034645\_cellular\_macromolecule\_biosynthetic\_process | ESRRG | 901 | 18 | 1.352909 | -0.995757 | 253 | 134.77 | 0.532688 |  |
| GO:0034645\_cellular\_macromolecule\_biosynthetic\_process | ABCA1 | 901 | 18 | 1.352909 | -0.995757 | 253 | 134.77 | 0.532688 |  |
| GO:0034645\_cellular\_macromolecule\_biosynthetic\_process | NR3C1 | 901 | 18 | 1.352909 | -0.995757 | 253 | 134.77 | 0.532688 |  |
| GO:0034645\_cellular\_macromolecule\_biosynthetic\_process | NR2E1 | 901 | 18 | 1.352909 | -0.995757 | 253 | 134.77 | 0.532688 |  |
| GO:0034645\_cellular\_macromolecule\_biosynthetic\_process | TCF7L2 | 901 | 18 | 1.352909 | -0.995757 | 253 | 134.77 | 0.532688 |  |
| GO:0034645\_cellular\_macromolecule\_biosynthetic\_process | NKX6-1 | 901 | 18 | 1.352909 | -0.995757 | 253 | 134.77 | 0.532688 |  |
| GO:0034645\_cellular\_macromolecule\_biosynthetic\_process | ASCL1 | 901 | 18 | 1.352909 | -0.995757 | 253 | 134.77 | 0.532688 |  |
| GO:0034645\_cellular\_macromolecule\_biosynthetic\_process | TCERG1 | 901 | 18 | 1.352909 | -0.995757 | 253 | 134.77 | 0.532688 |  |
| GO:0034645\_cellular\_macromolecule\_biosynthetic\_process | MSX1 | 901 | 18 | 1.352909 | -0.995757 | 253 | 134.77 | 0.532688 |  |
| GO:0034645\_cellular\_macromolecule\_biosynthetic\_process | DMD | 901 | 18 | 1.352909 | -0.995757 | 253 | 134.77 | 0.532688 |  |
| GO:0034645\_cellular\_macromolecule\_biosynthetic\_process | ETV1 | 901 | 18 | 1.352909 | -0.995757 | 253 | 134.77 | 0.532688 |  |
| GO:0034645\_cellular\_macromolecule\_biosynthetic\_process | NEUROD1 | 901 | 18 | 1.352909 | -0.995757 | 253 | 134.77 | 0.532688 |  |
| GO:0034645\_cellular\_macromolecule\_biosynthetic\_process | B3GNT2 | 901 | 18 | 1.352909 | -0.995757 | 253 | 134.77 | 0.532688 |  |
| GO:0034645\_cellular\_macromolecule\_biosynthetic\_process | HDAC9 | 901 | 18 | 1.352909 | -0.995757 | 253 | 134.77 | 0.532688 |  |
| GO:0003012\_muscle\_system\_process | MYOM2 | 76 | 3 | 2.673181 | -0.994689 | 254 | 135.12 | 0.531969 |  |
| GO:0003012\_muscle\_system\_process | TBX20 | 76 | 3 | 2.673181 | -0.994689 | 254 | 135.12 | 0.531969 |  |
| GO:0003012\_muscle\_system\_process | ATP1A2 | 76 | 3 | 2.673181 | -0.994689 | 254 | 135.12 | 0.531969 |  |
| GO:0006917\_induction\_of\_apoptosis | SERINC3 | 121 | 4 | 2.238697 | -0.989146 | 256 | 135.55 | 0.529492 |  |
| GO:0006917\_induction\_of\_apoptosis | PTPRC | 121 | 4 | 2.238697 | -0.989146 | 256 | 135.55 | 0.529492 |  |
| GO:0006917\_induction\_of\_apoptosis | CD27 | 121 | 4 | 2.238697 | -0.989146 | 256 | 135.55 | 0.529492 |  |
| GO:0006917\_induction\_of\_apoptosis | RAB27A | 121 | 4 | 2.238697 | -0.989146 | 256 | 135.55 | 0.529492 |  |
| GO:0012502\_induction\_of\_programmed\_cell\_death | SERINC3 | 121 | 4 | 2.238697 | -0.989146 | 256 | 135.55 | 0.529492 |  |
| GO:0012502\_induction\_of\_programmed\_cell\_death | PTPRC | 121 | 4 | 2.238697 | -0.989146 | 256 | 135.55 | 0.529492 |  |
| GO:0012502\_induction\_of\_programmed\_cell\_death | CD27 | 121 | 4 | 2.238697 | -0.989146 | 256 | 135.55 | 0.529492 |  |
| GO:0012502\_induction\_of\_programmed\_cell\_death | RAB27A | 121 | 4 | 2.238697 | -0.989146 | 256 | 135.55 | 0.529492 |  |
| GO:0042698\_ovulation\_cycle | AFP | 37 | 2 | 3.660572 | -0.987615 | 257 | 136.65 | 0.531712 |  |
| GO:0042698\_ovulation\_cycle | ZFX | 37 | 2 | 3.660572 | -0.987615 | 257 | 136.65 | 0.531712 |  |
| GO:0044249\_cellular\_biosynthetic\_process | FUT9 | 1150 | 22 | 1.295524 | -0.986462 | 258 | 136.75 | 0.530039 |  |
| GO:0044249\_cellular\_biosynthetic\_process | TBX3 | 1150 | 22 | 1.295524 | -0.986462 | 258 | 136.75 | 0.530039 |  |
| GO:0044249\_cellular\_biosynthetic\_process | HEXB | 1150 | 22 | 1.295524 | -0.986462 | 258 | 136.75 | 0.530039 |  |
| GO:0044249\_cellular\_biosynthetic\_process | TBX20 | 1150 | 22 | 1.295524 | -0.986462 | 258 | 136.75 | 0.530039 |  |
| GO:0044249\_cellular\_biosynthetic\_process | ESRRG | 1150 | 22 | 1.295524 | -0.986462 | 258 | 136.75 | 0.530039 |  |
| GO:0044249\_cellular\_biosynthetic\_process | SOX5 | 1150 | 22 | 1.295524 | -0.986462 | 258 | 136.75 | 0.530039 |  |
| GO:0044249\_cellular\_biosynthetic\_process | NR3C1 | 1150 | 22 | 1.295524 | -0.986462 | 258 | 136.75 | 0.530039 |  |
| GO:0044249\_cellular\_biosynthetic\_process | ABCA1 | 1150 | 22 | 1.295524 | -0.986462 | 258 | 136.75 | 0.530039 |  |
| GO:0044249\_cellular\_biosynthetic\_process | SGMS1 | 1150 | 22 | 1.295524 | -0.986462 | 258 | 136.75 | 0.530039 |  |
| GO:0044249\_cellular\_biosynthetic\_process | NR2E1 | 1150 | 22 | 1.295524 | -0.986462 | 258 | 136.75 | 0.530039 |  |
| GO:0044249\_cellular\_biosynthetic\_process | TCF7L2 | 1150 | 22 | 1.295524 | -0.986462 | 258 | 136.75 | 0.530039 |  |
| GO:0044249\_cellular\_biosynthetic\_process | NKX6-1 | 1150 | 22 | 1.295524 | -0.986462 | 258 | 136.75 | 0.530039 |  |
| GO:0044249\_cellular\_biosynthetic\_process | ASCL1 | 1150 | 22 | 1.295524 | -0.986462 | 258 | 136.75 | 0.530039 |  |
| GO:0044249\_cellular\_biosynthetic\_process | AFP | 1150 | 22 | 1.295524 | -0.986462 | 258 | 136.75 | 0.530039 |  |
| GO:0044249\_cellular\_biosynthetic\_process | TCERG1 | 1150 | 22 | 1.295524 | -0.986462 | 258 | 136.75 | 0.530039 |  |
| GO:0044249\_cellular\_biosynthetic\_process | MSX1 | 1150 | 22 | 1.295524 | -0.986462 | 258 | 136.75 | 0.530039 |  |
| GO:0044249\_cellular\_biosynthetic\_process | DMD | 1150 | 22 | 1.295524 | -0.986462 | 258 | 136.75 | 0.530039 |  |
| GO:0044249\_cellular\_biosynthetic\_process | ETV1 | 1150 | 22 | 1.295524 | -0.986462 | 258 | 136.75 | 0.530039 |  |
| GO:0044249\_cellular\_biosynthetic\_process | NEUROD1 | 1150 | 22 | 1.295524 | -0.986462 | 258 | 136.75 | 0.530039 |  |
| GO:0044249\_cellular\_biosynthetic\_process | B3GNT2 | 1150 | 22 | 1.295524 | -0.986462 | 258 | 136.75 | 0.530039 |  |
| GO:0044249\_cellular\_biosynthetic\_process | PPAP2A | 1150 | 22 | 1.295524 | -0.986462 | 258 | 136.75 | 0.530039 |  |
| GO:0044249\_cellular\_biosynthetic\_process | HDAC9 | 1150 | 22 | 1.295524 | -0.986462 | 258 | 136.75 | 0.530039 |  |
| GO:0007519\_skeletal\_muscle\_tissue\_development | DMD | 78 | 3 | 2.604638 | -0.969221 | 260 | 138.46 | 0.532538 |  |
| GO:0007519\_skeletal\_muscle\_tissue\_development | MBNL3 | 78 | 3 | 2.604638 | -0.969221 | 260 | 138.46 | 0.532538 |  |
| GO:0007519\_skeletal\_muscle\_tissue\_development | BOC | 78 | 3 | 2.604638 | -0.969221 | 260 | 138.46 | 0.532538 |  |
| GO:0060538\_skeletal\_muscle\_organ\_development | DMD | 78 | 3 | 2.604638 | -0.969221 | 260 | 138.46 | 0.532538 |  |
| GO:0060538\_skeletal\_muscle\_organ\_development | MBNL3 | 78 | 3 | 2.604638 | -0.969221 | 260 | 138.46 | 0.532538 |  |
| GO:0060538\_skeletal\_muscle\_organ\_development | BOC | 78 | 3 | 2.604638 | -0.969221 | 260 | 138.46 | 0.532538 |  |
| GO:0008016\_regulation\_of\_heart\_contraction | IRX5 | 38 | 2 | 3.564241 | -0.968145 | 262 | 140.51 | 0.536298 |  |
| GO:0008016\_regulation\_of\_heart\_contraction | ATP1A2 | 38 | 2 | 3.564241 | -0.968145 | 262 | 140.51 | 0.536298 |  |
| GO:0051348\_negative\_regulation\_of\_transferase\_activity | PTPRC | 38 | 2 | 3.564241 | -0.968145 | 262 | 140.51 | 0.536298 |  |
| GO:0051348\_negative\_regulation\_of\_transferase\_activity | GADD45B | 38 | 2 | 3.564241 | -0.968145 | 262 | 140.51 | 0.536298 |  |
| GO:0009059\_macromolecule\_biosynthetic\_process | FUT9 | 910 | 18 | 1.339528 | -0.963740 | 263 | 140.67 | 0.534867 |  |
| GO:0009059\_macromolecule\_biosynthetic\_process | TBX3 | 910 | 18 | 1.339528 | -0.963740 | 263 | 140.67 | 0.534867 |  |
| GO:0009059\_macromolecule\_biosynthetic\_process | TBX20 | 910 | 18 | 1.339528 | -0.963740 | 263 | 140.67 | 0.534867 |  |
| GO:0009059\_macromolecule\_biosynthetic\_process | SOX5 | 910 | 18 | 1.339528 | -0.963740 | 263 | 140.67 | 0.534867 |  |
| GO:0009059\_macromolecule\_biosynthetic\_process | ESRRG | 910 | 18 | 1.339528 | -0.963740 | 263 | 140.67 | 0.534867 |  |
| GO:0009059\_macromolecule\_biosynthetic\_process | ABCA1 | 910 | 18 | 1.339528 | -0.963740 | 263 | 140.67 | 0.534867 |  |
| GO:0009059\_macromolecule\_biosynthetic\_process | NR3C1 | 910 | 18 | 1.339528 | -0.963740 | 263 | 140.67 | 0.534867 |  |
| GO:0009059\_macromolecule\_biosynthetic\_process | TCF7L2 | 910 | 18 | 1.339528 | -0.963740 | 263 | 140.67 | 0.534867 |  |
| GO:0009059\_macromolecule\_biosynthetic\_process | NR2E1 | 910 | 18 | 1.339528 | -0.963740 | 263 | 140.67 | 0.534867 |  |
| GO:0009059\_macromolecule\_biosynthetic\_process | NKX6-1 | 910 | 18 | 1.339528 | -0.963740 | 263 | 140.67 | 0.534867 |  |
| GO:0009059\_macromolecule\_biosynthetic\_process | ASCL1 | 910 | 18 | 1.339528 | -0.963740 | 263 | 140.67 | 0.534867 |  |
| GO:0009059\_macromolecule\_biosynthetic\_process | TCERG1 | 910 | 18 | 1.339528 | -0.963740 | 263 | 140.67 | 0.534867 |  |
| GO:0009059\_macromolecule\_biosynthetic\_process | MSX1 | 910 | 18 | 1.339528 | -0.963740 | 263 | 140.67 | 0.534867 |  |
| GO:0009059\_macromolecule\_biosynthetic\_process | DMD | 910 | 18 | 1.339528 | -0.963740 | 263 | 140.67 | 0.534867 |  |
| GO:0009059\_macromolecule\_biosynthetic\_process | NEUROD1 | 910 | 18 | 1.339528 | -0.963740 | 263 | 140.67 | 0.534867 |  |
| GO:0009059\_macromolecule\_biosynthetic\_process | ETV1 | 910 | 18 | 1.339528 | -0.963740 | 263 | 140.67 | 0.534867 |  |
| GO:0009059\_macromolecule\_biosynthetic\_process | B3GNT2 | 910 | 18 | 1.339528 | -0.963740 | 263 | 140.67 | 0.534867 |  |
| GO:0009059\_macromolecule\_biosynthetic\_process | HDAC9 | 910 | 18 | 1.339528 | -0.963740 | 263 | 140.67 | 0.534867 |  |
| GO:0009308\_amine\_metabolic\_process | MAMDC2 | 124 | 4 | 2.184535 | -0.959986 | 264 | 140.92 | 0.533788 |  |
| GO:0009308\_amine\_metabolic\_process | SGPP1 | 124 | 4 | 2.184535 | -0.959986 | 264 | 140.92 | 0.533788 |  |
| GO:0009308\_amine\_metabolic\_process | HEXB | 124 | 4 | 2.184535 | -0.959986 | 264 | 140.92 | 0.533788 |  |
| GO:0009308\_amine\_metabolic\_process | PPAP2A | 124 | 4 | 2.184535 | -0.959986 | 264 | 140.92 | 0.533788 |  |
| GO:0010646\_regulation\_of\_cell\_communication | FGF18 | 330 | 8 | 1.641711 | -0.954977 | 265 | 141.76 | 0.534943 |  |
| GO:0010646\_regulation\_of\_cell\_communication | ASCL1 | 330 | 8 | 1.641711 | -0.954977 | 265 | 141.76 | 0.534943 |  |
| GO:0010646\_regulation\_of\_cell\_communication | PTPRC | 330 | 8 | 1.641711 | -0.954977 | 265 | 141.76 | 0.534943 |  |
| GO:0010646\_regulation\_of\_cell\_communication | ELF1 | 330 | 8 | 1.641711 | -0.954977 | 265 | 141.76 | 0.534943 |  |
| GO:0010646\_regulation\_of\_cell\_communication | MYO6 | 330 | 8 | 1.641711 | -0.954977 | 265 | 141.76 | 0.534943 |  |
| GO:0010646\_regulation\_of\_cell\_communication | JAK2 | 330 | 8 | 1.641711 | -0.954977 | 265 | 141.76 | 0.534943 |  |
| GO:0010646\_regulation\_of\_cell\_communication | CD27 | 330 | 8 | 1.641711 | -0.954977 | 265 | 141.76 | 0.534943 |  |
| GO:0010646\_regulation\_of\_cell\_communication | RGS13 | 330 | 8 | 1.641711 | -0.954977 | 265 | 141.76 | 0.534943 |  |
| GO:0001911\_negative\_regulation\_of\_leukocyte\_mediated\_cytotoxicity | PTPRC | 8 | 1 | 8.465074 | -0.949678 | 280 | 155.77 | 0.556321 |  |
| GO:0001916\_positive\_regulation\_of\_T\_cell\_mediated\_cytotoxicity | PTPRC | 8 | 1 | 8.465074 | -0.949678 | 280 | 155.77 | 0.556321 |  |
| GO:0008593\_regulation\_of\_Notch\_signaling\_pathway | ASCL1 | 8 | 1 | 8.465074 | -0.949678 | 280 | 155.77 | 0.556321 |  |
| GO:0014014\_negative\_regulation\_of\_gliogenesis | NKX6-1 | 8 | 1 | 8.465074 | -0.949678 | 280 | 155.77 | 0.556321 |  |
| GO:0014065\_phosphoinositide\_3-kinase\_cascade | JAK2 | 8 | 1 | 8.465074 | -0.949678 | 280 | 155.77 | 0.556321 |  |
| GO:0019229\_regulation\_of\_vasoconstriction | ATP1A2 | 8 | 1 | 8.465074 | -0.949678 | 280 | 155.77 | 0.556321 |  |
| GO:0021781\_glial\_cell\_fate\_commitment | ASCL1 | 8 | 1 | 8.465074 | -0.949678 | 280 | 155.77 | 0.556321 |  |
| GO:0030035\_microspike\_assembly | FGD4 | 8 | 1 | 8.465074 | -0.949678 | 280 | 155.77 | 0.556321 |  |
| GO:0030204\_chondroitin\_sulfate\_metabolic\_process | MAMDC2 | 8 | 1 | 8.465074 | -0.949678 | 280 | 155.77 | 0.556321 |  |
| GO:0031342\_negative\_regulation\_of\_cell\_killing | PTPRC | 8 | 1 | 8.465074 | -0.949678 | 280 | 155.77 | 0.556321 |  |
| GO:0035112\_genitalia\_morphogenesis | TCF7L2 | 8 | 1 | 8.465074 | -0.949678 | 280 | 155.77 | 0.556321 |  |
| GO:0043368\_positive\_T\_cell\_selection | PTPRC | 8 | 1 | 8.465074 | -0.949678 | 280 | 155.77 | 0.556321 |  |
| GO:0045686\_negative\_regulation\_of\_glial\_cell\_differentiation | NKX6-1 | 8 | 1 | 8.465074 | -0.949678 | 280 | 155.77 | 0.556321 |  |
| GO:0045932\_negative\_regulation\_of\_muscle\_contraction | ATP1A2 | 8 | 1 | 8.465074 | -0.949678 | 280 | 155.77 | 0.556321 |  |
| GO:0048557\_embryonic\_digestive\_tract\_morphogenesis | TCF7L2 | 8 | 1 | 8.465074 | -0.949678 | 280 | 155.77 | 0.556321 |  |
| GO:0005976\_polysaccharide\_metabolic\_process | MAMDC2 | 39 | 2 | 3.472851 | -0.949288 | 282 | 157.74 | 0.559362 |  |
| GO:0005976\_polysaccharide\_metabolic\_process | HEXB | 39 | 2 | 3.472851 | -0.949288 | 282 | 157.74 | 0.559362 |  |
| GO:0048663\_neuron\_fate\_commitment | ASCL1 | 39 | 2 | 3.472851 | -0.949288 | 282 | 157.74 | 0.559362 |  |
| GO:0048663\_neuron\_fate\_commitment | NKX6-1 | 39 | 2 | 3.472851 | -0.949288 | 282 | 157.74 | 0.559362 |  |
| GO:0044092\_negative\_regulation\_of\_molecular\_function | PTPRC | 80 | 3 | 2.539522 | -0.944618 | 283 | 158.23 | 0.559117 |  |
| GO:0044092\_negative\_regulation\_of\_molecular\_function | JAK2 | 80 | 3 | 2.539522 | -0.944618 | 283 | 158.23 | 0.559117 |  |
| GO:0044092\_negative\_regulation\_of\_molecular\_function | GADD45B | 80 | 3 | 2.539522 | -0.944618 | 283 | 158.23 | 0.559117 |  |
| GO:0031328\_positive\_regulation\_of\_cellular\_biosynthetic\_process | ASCL1 | 387 | 9 | 1.574897 | -0.944330 | 284 | 158.27 | 0.557289 |  |
| GO:0031328\_positive\_regulation\_of\_cellular\_biosynthetic\_process | MSX1 | 387 | 9 | 1.574897 | -0.944330 | 284 | 158.27 | 0.557289 |  |
| GO:0031328\_positive\_regulation\_of\_cellular\_biosynthetic\_process | TBX3 | 387 | 9 | 1.574897 | -0.944330 | 284 | 158.27 | 0.557289 |  |
| GO:0031328\_positive\_regulation\_of\_cellular\_biosynthetic\_process | TBX20 | 387 | 9 | 1.574897 | -0.944330 | 284 | 158.27 | 0.557289 |  |
| GO:0031328\_positive\_regulation\_of\_cellular\_biosynthetic\_process | ESRRG | 387 | 9 | 1.574897 | -0.944330 | 284 | 158.27 | 0.557289 |  |
| GO:0031328\_positive\_regulation\_of\_cellular\_biosynthetic\_process | SOX5 | 387 | 9 | 1.574897 | -0.944330 | 284 | 158.27 | 0.557289 |  |
| GO:0031328\_positive\_regulation\_of\_cellular\_biosynthetic\_process | ETV1 | 387 | 9 | 1.574897 | -0.944330 | 284 | 158.27 | 0.557289 |  |
| GO:0031328\_positive\_regulation\_of\_cellular\_biosynthetic\_process | NEUROD1 | 387 | 9 | 1.574897 | -0.944330 | 284 | 158.27 | 0.557289 |  |
| GO:0031328\_positive\_regulation\_of\_cellular\_biosynthetic\_process | TCF7L2 | 387 | 9 | 1.574897 | -0.944330 | 284 | 158.27 | 0.557289 |  |
| GO:0045449\_regulation\_of\_transcription | TBX3 | 676 | 14 | 1.402497 | -0.939595 | 285 | 158.61 | 0.556526 |  |
| GO:0045449\_regulation\_of\_transcription | TBX20 | 676 | 14 | 1.402497 | -0.939595 | 285 | 158.61 | 0.556526 |  |
| GO:0045449\_regulation\_of\_transcription | SOX5 | 676 | 14 | 1.402497 | -0.939595 | 285 | 158.61 | 0.556526 |  |
| GO:0045449\_regulation\_of\_transcription | ESRRG | 676 | 14 | 1.402497 | -0.939595 | 285 | 158.61 | 0.556526 |  |
| GO:0045449\_regulation\_of\_transcription | NR3C1 | 676 | 14 | 1.402497 | -0.939595 | 285 | 158.61 | 0.556526 |  |
| GO:0045449\_regulation\_of\_transcription | NR2E1 | 676 | 14 | 1.402497 | -0.939595 | 285 | 158.61 | 0.556526 |  |
| GO:0045449\_regulation\_of\_transcription | TCF7L2 | 676 | 14 | 1.402497 | -0.939595 | 285 | 158.61 | 0.556526 |  |
| GO:0045449\_regulation\_of\_transcription | NKX6-1 | 676 | 14 | 1.402497 | -0.939595 | 285 | 158.61 | 0.556526 |  |
| GO:0045449\_regulation\_of\_transcription | ASCL1 | 676 | 14 | 1.402497 | -0.939595 | 285 | 158.61 | 0.556526 |  |
| GO:0045449\_regulation\_of\_transcription | MSX1 | 676 | 14 | 1.402497 | -0.939595 | 285 | 158.61 | 0.556526 |  |
| GO:0045449\_regulation\_of\_transcription | DMD | 676 | 14 | 1.402497 | -0.939595 | 285 | 158.61 | 0.556526 |  |
| GO:0045449\_regulation\_of\_transcription | NEUROD1 | 676 | 14 | 1.402497 | -0.939595 | 285 | 158.61 | 0.556526 |  |
| GO:0045449\_regulation\_of\_transcription | ETV1 | 676 | 14 | 1.402497 | -0.939595 | 285 | 158.61 | 0.556526 |  |
| GO:0045449\_regulation\_of\_transcription | HDAC9 | 676 | 14 | 1.402497 | -0.939595 | 285 | 158.61 | 0.556526 |  |
| GO:0015031\_protein\_transport | MYO6 | 175 | 5 | 1.934874 | -0.939507 | 286 | 158.8 | 0.555245 |  |
| GO:0015031\_protein\_transport | AP2A1 | 175 | 5 | 1.934874 | -0.939507 | 286 | 158.8 | 0.555245 |  |
| GO:0015031\_protein\_transport | MLPH | 175 | 5 | 1.934874 | -0.939507 | 286 | 158.8 | 0.555245 |  |
| GO:0015031\_protein\_transport | JAK2 | 175 | 5 | 1.934874 | -0.939507 | 286 | 158.8 | 0.555245 |  |
| GO:0015031\_protein\_transport | RAB27A | 175 | 5 | 1.934874 | -0.939507 | 286 | 158.8 | 0.555245 |  |
| GO:0009891\_positive\_regulation\_of\_biosynthetic\_process | ASCL1 | 388 | 9 | 1.570838 | -0.939190 | 287 | 158.84 | 0.553449 |  |
| GO:0009891\_positive\_regulation\_of\_biosynthetic\_process | MSX1 | 388 | 9 | 1.570838 | -0.939190 | 287 | 158.84 | 0.553449 |  |
| GO:0009891\_positive\_regulation\_of\_biosynthetic\_process | TBX3 | 388 | 9 | 1.570838 | -0.939190 | 287 | 158.84 | 0.553449 |  |
| GO:0009891\_positive\_regulation\_of\_biosynthetic\_process | TBX20 | 388 | 9 | 1.570838 | -0.939190 | 287 | 158.84 | 0.553449 |  |
| GO:0009891\_positive\_regulation\_of\_biosynthetic\_process | ESRRG | 388 | 9 | 1.570838 | -0.939190 | 287 | 158.84 | 0.553449 |  |
| GO:0009891\_positive\_regulation\_of\_biosynthetic\_process | SOX5 | 388 | 9 | 1.570838 | -0.939190 | 287 | 158.84 | 0.553449 |  |
| GO:0009891\_positive\_regulation\_of\_biosynthetic\_process | ETV1 | 388 | 9 | 1.570838 | -0.939190 | 287 | 158.84 | 0.553449 |  |
| GO:0009891\_positive\_regulation\_of\_biosynthetic\_process | NEUROD1 | 388 | 9 | 1.570838 | -0.939190 | 287 | 158.84 | 0.553449 |  |
| GO:0009891\_positive\_regulation\_of\_biosynthetic\_process | TCF7L2 | 388 | 9 | 1.570838 | -0.939190 | 287 | 158.84 | 0.553449 |  |
| GO:0007610\_behavior | MYO6 | 279 | 7 | 1.699083 | -0.937818 | 288 | 158.98 | 0.552014 |  |
| GO:0007610\_behavior | ZFX | 279 | 7 | 1.699083 | -0.937818 | 288 | 158.98 | 0.552014 |  |
| GO:0007610\_behavior | HEXB | 279 | 7 | 1.699083 | -0.937818 | 288 | 158.98 | 0.552014 |  |
| GO:0007610\_behavior | ETV1 | 279 | 7 | 1.699083 | -0.937818 | 288 | 158.98 | 0.552014 |  |
| GO:0007610\_behavior | VPS13A | 279 | 7 | 1.699083 | -0.937818 | 288 | 158.98 | 0.552014 |  |
| GO:0007610\_behavior | ATP1A2 | 279 | 7 | 1.699083 | -0.937818 | 288 | 158.98 | 0.552014 |  |
| GO:0007610\_behavior | NR2E1 | 279 | 7 | 1.699083 | -0.937818 | 288 | 158.98 | 0.552014 |  |
| GO:0045597\_positive\_regulation\_of\_cell\_differentiation | PTPRC | 128 | 4 | 2.116268 | -0.922685 | 290 | 161.3 | 0.556207 |  |
| GO:0045597\_positive\_regulation\_of\_cell\_differentiation | ASCL1 | 128 | 4 | 2.116268 | -0.922685 | 290 | 161.3 | 0.556207 |  |
| GO:0045597\_positive\_regulation\_of\_cell\_differentiation | BOC | 128 | 4 | 2.116268 | -0.922685 | 290 | 161.3 | 0.556207 |  |
| GO:0045597\_positive\_regulation\_of\_cell\_differentiation | NKX6-1 | 128 | 4 | 2.116268 | -0.922685 | 290 | 161.3 | 0.556207 |  |
| GO:0060537\_muscle\_tissue\_development | DMD | 128 | 4 | 2.116268 | -0.922685 | 290 | 161.3 | 0.556207 |  |
| GO:0060537\_muscle\_tissue\_development | MBNL3 | 128 | 4 | 2.116268 | -0.922685 | 290 | 161.3 | 0.556207 |  |
| GO:0060537\_muscle\_tissue\_development | HDAC9 | 128 | 4 | 2.116268 | -0.922685 | 290 | 161.3 | 0.556207 |  |
| GO:0060537\_muscle\_tissue\_development | BOC | 128 | 4 | 2.116268 | -0.922685 | 290 | 161.3 | 0.556207 |  |
| GO:0008202\_steroid\_metabolic\_process | AFP | 82 | 3 | 2.477582 | -0.920838 | 292 | 161.78 | 0.554041 |  |
| GO:0008202\_steroid\_metabolic\_process | ABCA1 | 82 | 3 | 2.477582 | -0.920838 | 292 | 161.78 | 0.554041 |  |
| GO:0008202\_steroid\_metabolic\_process | NR3C1 | 82 | 3 | 2.477582 | -0.920838 | 292 | 161.78 | 0.554041 |  |
| GO:0010627\_regulation\_of\_protein\_kinase\_cascade | PTPRC | 82 | 3 | 2.477582 | -0.920838 | 292 | 161.78 | 0.554041 |  |
| GO:0010627\_regulation\_of\_protein\_kinase\_cascade | JAK2 | 82 | 3 | 2.477582 | -0.920838 | 292 | 161.78 | 0.554041 |  |
| GO:0010627\_regulation\_of\_protein\_kinase\_cascade | CD27 | 82 | 3 | 2.477582 | -0.920838 | 292 | 161.78 | 0.554041 |  |
| GO:0002429\_immune\_response-activating\_cell\_surface\_receptor\_signaling\_pathway | PTPRC | 41 | 2 | 3.303443 | -0.913289 | 294 | 164.42 | 0.559252 |  |
| GO:0002429\_immune\_response-activating\_cell\_surface\_receptor\_signaling\_pathway | ELF1 | 41 | 2 | 3.303443 | -0.913289 | 294 | 164.42 | 0.559252 |  |
| GO:0008585\_female\_gonad\_development | AFP | 41 | 2 | 3.303443 | -0.913289 | 294 | 164.42 | 0.559252 |  |
| GO:0008585\_female\_gonad\_development | ZFX | 41 | 2 | 3.303443 | -0.913289 | 294 | 164.42 | 0.559252 |  |
| GO:0009058\_biosynthetic\_process | FUT9 | 1175 | 22 | 1.267960 | -0.907657 | 295 | 165.55 | 0.561186 |  |
| GO:0009058\_biosynthetic\_process | TBX3 | 1175 | 22 | 1.267960 | -0.907657 | 295 | 165.55 | 0.561186 |  |
| GO:0009058\_biosynthetic\_process | HEXB | 1175 | 22 | 1.267960 | -0.907657 | 295 | 165.55 | 0.561186 |  |
| GO:0009058\_biosynthetic\_process | TBX20 | 1175 | 22 | 1.267960 | -0.907657 | 295 | 165.55 | 0.561186 |  |
| GO:0009058\_biosynthetic\_process | SOX5 | 1175 | 22 | 1.267960 | -0.907657 | 295 | 165.55 | 0.561186 |  |
| GO:0009058\_biosynthetic\_process | ESRRG | 1175 | 22 | 1.267960 | -0.907657 | 295 | 165.55 | 0.561186 |  |
| GO:0009058\_biosynthetic\_process | ABCA1 | 1175 | 22 | 1.267960 | -0.907657 | 295 | 165.55 | 0.561186 |  |
| GO:0009058\_biosynthetic\_process | NR3C1 | 1175 | 22 | 1.267960 | -0.907657 | 295 | 165.55 | 0.561186 |  |
| GO:0009058\_biosynthetic\_process | SGMS1 | 1175 | 22 | 1.267960 | -0.907657 | 295 | 165.55 | 0.561186 |  |
| GO:0009058\_biosynthetic\_process | NR2E1 | 1175 | 22 | 1.267960 | -0.907657 | 295 | 165.55 | 0.561186 |  |
| GO:0009058\_biosynthetic\_process | TCF7L2 | 1175 | 22 | 1.267960 | -0.907657 | 295 | 165.55 | 0.561186 |  |
| GO:0009058\_biosynthetic\_process | NKX6-1 | 1175 | 22 | 1.267960 | -0.907657 | 295 | 165.55 | 0.561186 |  |
| GO:0009058\_biosynthetic\_process | AFP | 1175 | 22 | 1.267960 | -0.907657 | 295 | 165.55 | 0.561186 |  |
| GO:0009058\_biosynthetic\_process | ASCL1 | 1175 | 22 | 1.267960 | -0.907657 | 295 | 165.55 | 0.561186 |  |
| GO:0009058\_biosynthetic\_process | TCERG1 | 1175 | 22 | 1.267960 | -0.907657 | 295 | 165.55 | 0.561186 |  |
| GO:0009058\_biosynthetic\_process | MSX1 | 1175 | 22 | 1.267960 | -0.907657 | 295 | 165.55 | 0.561186 |  |
| GO:0009058\_biosynthetic\_process | DMD | 1175 | 22 | 1.267960 | -0.907657 | 295 | 165.55 | 0.561186 |  |
| GO:0009058\_biosynthetic\_process | NEUROD1 | 1175 | 22 | 1.267960 | -0.907657 | 295 | 165.55 | 0.561186 |  |
| GO:0009058\_biosynthetic\_process | ETV1 | 1175 | 22 | 1.267960 | -0.907657 | 295 | 165.55 | 0.561186 |  |
| GO:0009058\_biosynthetic\_process | B3GNT2 | 1175 | 22 | 1.267960 | -0.907657 | 295 | 165.55 | 0.561186 |  |
| GO:0009058\_biosynthetic\_process | HDAC9 | 1175 | 22 | 1.267960 | -0.907657 | 295 | 165.55 | 0.561186 |  |
| GO:0009058\_biosynthetic\_process | PPAP2A | 1175 | 22 | 1.267960 | -0.907657 | 295 | 165.55 | 0.561186 |  |
| GO:0007420\_brain\_development | ASCL1 | 231 | 6 | 1.758976 | -0.906382 | 296 | 165.62 | 0.559527 |  |
| GO:0007420\_brain\_development | MSX1 | 231 | 6 | 1.758976 | -0.906382 | 296 | 165.62 | 0.559527 |  |
| GO:0007420\_brain\_development | TBX3 | 231 | 6 | 1.758976 | -0.906382 | 296 | 165.62 | 0.559527 |  |
| GO:0007420\_brain\_development | PLXNA2 | 231 | 6 | 1.758976 | -0.906382 | 296 | 165.62 | 0.559527 |  |
| GO:0007420\_brain\_development | NEUROD1 | 231 | 6 | 1.758976 | -0.906382 | 296 | 165.62 | 0.559527 |  |
| GO:0007420\_brain\_development | NR2E1 | 231 | 6 | 1.758976 | -0.906382 | 296 | 165.62 | 0.559527 |  |
| GO:0006629\_lipid\_metabolic\_process | AFP | 285 | 7 | 1.663313 | -0.902143 | 297 | 166.27 | 0.559832 |  |
| GO:0006629\_lipid\_metabolic\_process | SGPP1 | 285 | 7 | 1.663313 | -0.902143 | 297 | 166.27 | 0.559832 |  |
| GO:0006629\_lipid\_metabolic\_process | HEXB | 285 | 7 | 1.663313 | -0.902143 | 297 | 166.27 | 0.559832 |  |
| GO:0006629\_lipid\_metabolic\_process | ABCA1 | 285 | 7 | 1.663313 | -0.902143 | 297 | 166.27 | 0.559832 |  |
| GO:0006629\_lipid\_metabolic\_process | SGMS1 | 285 | 7 | 1.663313 | -0.902143 | 297 | 166.27 | 0.559832 |  |
| GO:0006629\_lipid\_metabolic\_process | NR3C1 | 285 | 7 | 1.663313 | -0.902143 | 297 | 166.27 | 0.559832 |  |
| GO:0006629\_lipid\_metabolic\_process | PPAP2A | 285 | 7 | 1.663313 | -0.902143 | 297 | 166.27 | 0.559832 |  |
| GO:0000186\_activation\_of\_MAPKK\_activity | GADD45B | 9 | 1 | 7.524510 | -0.901646 | 316 | 180.18 | 0.570190 |  |
| GO:0001542\_ovulation\_from\_ovarian\_follicle | AFP | 9 | 1 | 7.524510 | -0.901646 | 316 | 180.18 | 0.570190 |  |
| GO:0001667\_ameboidal\_cell\_migration | ARID5B | 9 | 1 | 7.524510 | -0.901646 | 316 | 180.18 | 0.570190 |  |
| GO:0006911\_phagocytosis\_\_engulfment | ABCA1 | 9 | 1 | 7.524510 | -0.901646 | 316 | 180.18 | 0.570190 |  |
| GO:0007617\_mating\_behavior | HEXB | 9 | 1 | 7.524510 | -0.901646 | 316 | 180.18 | 0.570190 |  |
| GO:0010675\_regulation\_of\_cellular\_carbohydrate\_metabolic\_process | NR3C1 | 9 | 1 | 7.524510 | -0.901646 | 316 | 180.18 | 0.570190 |  |
| GO:0021544\_subpallium\_development | ASCL1 | 9 | 1 | 7.524510 | -0.901646 | 316 | 180.18 | 0.570190 |  |
| GO:0030325\_adrenal\_gland\_development | NR3C1 | 9 | 1 | 7.524510 | -0.901646 | 316 | 180.18 | 0.570190 |  |
| GO:0030728\_ovulation | AFP | 9 | 1 | 7.524510 | -0.901646 | 316 | 180.18 | 0.570190 |  |
| GO:0033344\_cholesterol\_efflux | ABCA1 | 9 | 1 | 7.524510 | -0.901646 | 316 | 180.18 | 0.570190 |  |
| GO:0042509\_regulation\_of\_tyrosine\_phosphorylation\_of\_STAT\_protein | JAK2 | 9 | 1 | 7.524510 | -0.901646 | 316 | 180.18 | 0.570190 |  |
| GO:0043299\_leukocyte\_degranulation | RAB27A | 9 | 1 | 7.524510 | -0.901646 | 316 | 180.18 | 0.570190 |  |
| GO:0043383\_negative\_T\_cell\_selection | PTPRC | 9 | 1 | 7.524510 | -0.901646 | 316 | 180.18 | 0.570190 |  |
| GO:0045060\_negative\_thymic\_T\_cell\_selection | PTPRC | 9 | 1 | 7.524510 | -0.901646 | 316 | 180.18 | 0.570190 |  |
| GO:0045830\_positive\_regulation\_of\_isotype\_switching | PTPRC | 9 | 1 | 7.524510 | -0.901646 | 316 | 180.18 | 0.570190 |  |
| GO:0046641\_positive\_regulation\_of\_alpha-beta\_T\_cell\_proliferation | PTPRC | 9 | 1 | 7.524510 | -0.901646 | 316 | 180.18 | 0.570190 |  |
| GO:0050856\_regulation\_of\_T\_cell\_receptor\_signaling\_pathway | ELF1 | 9 | 1 | 7.524510 | -0.901646 | 316 | 180.18 | 0.570190 |  |
| GO:0051607\_defense\_response\_to\_virus | PTPRC | 9 | 1 | 7.524510 | -0.901646 | 316 | 180.18 | 0.570190 |  |
| GO:0060325\_face\_morphogenesis | ARID5B | 9 | 1 | 7.524510 | -0.901646 | 316 | 180.18 | 0.570190 |  |
| GO:0045184\_establishment\_of\_protein\_localization | MYO6 | 180 | 5 | 1.881127 | -0.901501 | 317 | 180.3 | 0.568770 |  |
| GO:0045184\_establishment\_of\_protein\_localization | AP2A1 | 180 | 5 | 1.881127 | -0.901501 | 317 | 180.3 | 0.568770 |  |
| GO:0045184\_establishment\_of\_protein\_localization | MLPH | 180 | 5 | 1.881127 | -0.901501 | 317 | 180.3 | 0.568770 |  |
| GO:0045184\_establishment\_of\_protein\_localization | JAK2 | 180 | 5 | 1.881127 | -0.901501 | 317 | 180.3 | 0.568770 |  |
| GO:0045184\_establishment\_of\_protein\_localization | RAB27A | 180 | 5 | 1.881127 | -0.901501 | 317 | 180.3 | 0.568770 |  |
| GO:0030005\_cellular\_di-\_\_tri-valent\_inorganic\_cation\_homeostasis | PTPRC | 84 | 3 | 2.418592 | -0.897840 | 318 | 180.71 | 0.568270 |  |
| GO:0030005\_cellular\_di-\_\_tri-valent\_inorganic\_cation\_homeostasis | HEXB | 84 | 3 | 2.418592 | -0.897840 | 318 | 180.71 | 0.568270 |  |
| GO:0030005\_cellular\_di-\_\_tri-valent\_inorganic\_cation\_homeostasis | ATP1A2 | 84 | 3 | 2.418592 | -0.897840 | 318 | 180.71 | 0.568270 |  |
| GO:0019221\_cytokine-mediated\_signaling\_pathway | PTPRC | 42 | 2 | 3.224790 | -0.896090 | 319 | 182.8 | 0.573041 |  |
| GO:0019221\_cytokine-mediated\_signaling\_pathway | JAK2 | 42 | 2 | 3.224790 | -0.896090 | 319 | 182.8 | 0.573041 |  |
| GO:0016477\_cell\_migration | DCC | 234 | 6 | 1.736425 | -0.887006 | 320 | 183.54 | 0.573562 |  |
| GO:0016477\_cell\_migration | ASCL1 | 234 | 6 | 1.736425 | -0.887006 | 320 | 183.54 | 0.573562 |  |
| GO:0016477\_cell\_migration | PLXNA2 | 234 | 6 | 1.736425 | -0.887006 | 320 | 183.54 | 0.573562 |  |
| GO:0016477\_cell\_migration | ARID5B | 234 | 6 | 1.736425 | -0.887006 | 320 | 183.54 | 0.573562 |  |
| GO:0016477\_cell\_migration | ITGA4 | 234 | 6 | 1.736425 | -0.887006 | 320 | 183.54 | 0.573562 |  |
| GO:0016477\_cell\_migration | NR2E1 | 234 | 6 | 1.736425 | -0.887006 | 320 | 183.54 | 0.573562 |  |
| GO:0007224\_smoothened\_signaling\_pathway | BOC | 43 | 2 | 3.149795 | -0.879392 | 323 | 186.41 | 0.577121 |  |
| GO:0007224\_smoothened\_signaling\_pathway | NKX6-1 | 43 | 2 | 3.149795 | -0.879392 | 323 | 186.41 | 0.577121 |  |
| GO:0010001\_glial\_cell\_differentiation | ASCL1 | 43 | 2 | 3.149795 | -0.879392 | 323 | 186.41 | 0.577121 |  |
| GO:0010001\_glial\_cell\_differentiation | NKX6-1 | 43 | 2 | 3.149795 | -0.879392 | 323 | 186.41 | 0.577121 |  |
| GO:0051789\_response\_to\_protein\_stimulus | MSX1 | 43 | 2 | 3.149795 | -0.879392 | 323 | 186.41 | 0.577121 |  |
| GO:0051789\_response\_to\_protein\_stimulus | BOC | 43 | 2 | 3.149795 | -0.879392 | 323 | 186.41 | 0.577121 |  |
| GO:0032504\_multicellular\_organism\_reproduction | AFP | 86 | 3 | 2.362346 | -0.875585 | 325 | 187.32 | 0.576369 |  |
| GO:0032504\_multicellular\_organism\_reproduction | ZFX | 86 | 3 | 2.362346 | -0.875585 | 325 | 187.32 | 0.576369 |  |
| GO:0032504\_multicellular\_organism\_reproduction | HEXB | 86 | 3 | 2.362346 | -0.875585 | 325 | 187.32 | 0.576369 |  |
| GO:0048609\_reproductive\_process\_in\_a\_multicellular\_organism | AFP | 86 | 3 | 2.362346 | -0.875585 | 325 | 187.32 | 0.576369 |  |
| GO:0048609\_reproductive\_process\_in\_a\_multicellular\_organism | ZFX | 86 | 3 | 2.362346 | -0.875585 | 325 | 187.32 | 0.576369 |  |
| GO:0048609\_reproductive\_process\_in\_a\_multicellular\_organism | HEXB | 86 | 3 | 2.362346 | -0.875585 | 325 | 187.32 | 0.576369 |  |
| GO:0006355\_regulation\_of\_transcription\_\_DNA-dependent | ASCL1 | 575 | 12 | 1.413299 | -0.871769 | 326 | 187.86 | 0.576258 |  |
| GO:0006355\_regulation\_of\_transcription\_\_DNA-dependent | MSX1 | 575 | 12 | 1.413299 | -0.871769 | 326 | 187.86 | 0.576258 |  |
| GO:0006355\_regulation\_of\_transcription\_\_DNA-dependent | TBX3 | 575 | 12 | 1.413299 | -0.871769 | 326 | 187.86 | 0.576258 |  |
| GO:0006355\_regulation\_of\_transcription\_\_DNA-dependent | TBX20 | 575 | 12 | 1.413299 | -0.871769 | 326 | 187.86 | 0.576258 |  |
| GO:0006355\_regulation\_of\_transcription\_\_DNA-dependent | ESRRG | 575 | 12 | 1.413299 | -0.871769 | 326 | 187.86 | 0.576258 |  |
| GO:0006355\_regulation\_of\_transcription\_\_DNA-dependent | SOX5 | 575 | 12 | 1.413299 | -0.871769 | 326 | 187.86 | 0.576258 |  |
| GO:0006355\_regulation\_of\_transcription\_\_DNA-dependent | NEUROD1 | 575 | 12 | 1.413299 | -0.871769 | 326 | 187.86 | 0.576258 |  |
| GO:0006355\_regulation\_of\_transcription\_\_DNA-dependent | NR3C1 | 575 | 12 | 1.413299 | -0.871769 | 326 | 187.86 | 0.576258 |  |
| GO:0006355\_regulation\_of\_transcription\_\_DNA-dependent | HDAC9 | 575 | 12 | 1.413299 | -0.871769 | 326 | 187.86 | 0.576258 |  |
| GO:0006355\_regulation\_of\_transcription\_\_DNA-dependent | TCF7L2 | 575 | 12 | 1.413299 | -0.871769 | 326 | 187.86 | 0.576258 |  |
| GO:0006355\_regulation\_of\_transcription\_\_DNA-dependent | NR2E1 | 575 | 12 | 1.413299 | -0.871769 | 326 | 187.86 | 0.576258 |  |
| GO:0006355\_regulation\_of\_transcription\_\_DNA-dependent | NKX6-1 | 575 | 12 | 1.413299 | -0.871769 | 326 | 187.86 | 0.576258 |  |
| GO:0001822\_kidney\_development | TCFAP2B | 87 | 3 | 2.335193 | -0.864725 | 327 | 189.05 | 0.578135 |  |
| GO:0001822\_kidney\_development | AQP11 | 87 | 3 | 2.335193 | -0.864725 | 327 | 189.05 | 0.578135 |  |
| GO:0001822\_kidney\_development | ARID5B | 87 | 3 | 2.335193 | -0.864725 | 327 | 189.05 | 0.578135 |  |
| GO:0002768\_immune\_response-regulating\_cell\_surface\_receptor\_signaling\_pathway | PTPRC | 44 | 2 | 3.078209 | -0.863170 | 329 | 191.45 | 0.581915 |  |
| GO:0002768\_immune\_response-regulating\_cell\_surface\_receptor\_signaling\_pathway | ELF1 | 44 | 2 | 3.078209 | -0.863170 | 329 | 191.45 | 0.581915 |  |
| GO:0046545\_development\_of\_primary\_female\_sexual\_characteristics | AFP | 44 | 2 | 3.078209 | -0.863170 | 329 | 191.45 | 0.581915 |  |
| GO:0046545\_development\_of\_primary\_female\_sexual\_characteristics | ZFX | 44 | 2 | 3.078209 | -0.863170 | 329 | 191.45 | 0.581915 |  |
| GO:0019219\_regulation\_of\_nucleobase\_\_nucleoside\_\_nucleotide\_and\_nucleic\_acid\_metabolic\_process | PTPRC | 757 | 15 | 1.341887 | -0.861055 | 330 | 191.61 | 0.580636 |  |
| GO:0019219\_regulation\_of\_nucleobase\_\_nucleoside\_\_nucleotide\_and\_nucleic\_acid\_metabolic\_process | TBX3 | 757 | 15 | 1.341887 | -0.861055 | 330 | 191.61 | 0.580636 |  |
| GO:0019219\_regulation\_of\_nucleobase\_\_nucleoside\_\_nucleotide\_and\_nucleic\_acid\_metabolic\_process | TBX20 | 757 | 15 | 1.341887 | -0.861055 | 330 | 191.61 | 0.580636 |  |
| GO:0019219\_regulation\_of\_nucleobase\_\_nucleoside\_\_nucleotide\_and\_nucleic\_acid\_metabolic\_process | SOX5 | 757 | 15 | 1.341887 | -0.861055 | 330 | 191.61 | 0.580636 |  |
| GO:0019219\_regulation\_of\_nucleobase\_\_nucleoside\_\_nucleotide\_and\_nucleic\_acid\_metabolic\_process | ESRRG | 757 | 15 | 1.341887 | -0.861055 | 330 | 191.61 | 0.580636 |  |
| GO:0019219\_regulation\_of\_nucleobase\_\_nucleoside\_\_nucleotide\_and\_nucleic\_acid\_metabolic\_process | NR3C1 | 757 | 15 | 1.341887 | -0.861055 | 330 | 191.61 | 0.580636 |  |
| GO:0019219\_regulation\_of\_nucleobase\_\_nucleoside\_\_nucleotide\_and\_nucleic\_acid\_metabolic\_process | TCF7L2 | 757 | 15 | 1.341887 | -0.861055 | 330 | 191.61 | 0.580636 |  |
| GO:0019219\_regulation\_of\_nucleobase\_\_nucleoside\_\_nucleotide\_and\_nucleic\_acid\_metabolic\_process | NR2E1 | 757 | 15 | 1.341887 | -0.861055 | 330 | 191.61 | 0.580636 |  |
| GO:0019219\_regulation\_of\_nucleobase\_\_nucleoside\_\_nucleotide\_and\_nucleic\_acid\_metabolic\_process | NKX6-1 | 757 | 15 | 1.341887 | -0.861055 | 330 | 191.61 | 0.580636 |  |
| GO:0019219\_regulation\_of\_nucleobase\_\_nucleoside\_\_nucleotide\_and\_nucleic\_acid\_metabolic\_process | ASCL1 | 757 | 15 | 1.341887 | -0.861055 | 330 | 191.61 | 0.580636 |  |
| GO:0019219\_regulation\_of\_nucleobase\_\_nucleoside\_\_nucleotide\_and\_nucleic\_acid\_metabolic\_process | MSX1 | 757 | 15 | 1.341887 | -0.861055 | 330 | 191.61 | 0.580636 |  |
| GO:0019219\_regulation\_of\_nucleobase\_\_nucleoside\_\_nucleotide\_and\_nucleic\_acid\_metabolic\_process | DMD | 757 | 15 | 1.341887 | -0.861055 | 330 | 191.61 | 0.580636 |  |
| GO:0019219\_regulation\_of\_nucleobase\_\_nucleoside\_\_nucleotide\_and\_nucleic\_acid\_metabolic\_process | NEUROD1 | 757 | 15 | 1.341887 | -0.861055 | 330 | 191.61 | 0.580636 |  |
| GO:0019219\_regulation\_of\_nucleobase\_\_nucleoside\_\_nucleotide\_and\_nucleic\_acid\_metabolic\_process | ETV1 | 757 | 15 | 1.341887 | -0.861055 | 330 | 191.61 | 0.580636 |  |
| GO:0019219\_regulation\_of\_nucleobase\_\_nucleoside\_\_nucleotide\_and\_nucleic\_acid\_metabolic\_process | HDAC9 | 757 | 15 | 1.341887 | -0.861055 | 330 | 191.61 | 0.580636 |  |
| GO:0001914\_regulation\_of\_T\_cell\_mediated\_cytotoxicity | PTPRC | 10 | 1 | 6.772059 | -0.859001 | 350 | 208.01 | 0.594314 |  |
| GO:0002711\_positive\_regulation\_of\_T\_cell\_mediated\_immunity | PTPRC | 10 | 1 | 6.772059 | -0.859001 | 350 | 208.01 | 0.594314 |  |
| GO:0006109\_regulation\_of\_carbohydrate\_metabolic\_process | NR3C1 | 10 | 1 | 6.772059 | -0.859001 | 350 | 208.01 | 0.594314 |  |
| GO:0006826\_iron\_ion\_transport | SLC40A1 | 10 | 1 | 6.772059 | -0.859001 | 350 | 208.01 | 0.594314 |  |
| GO:0008211\_glucocorticoid\_metabolic\_process | NR3C1 | 10 | 1 | 6.772059 | -0.859001 | 350 | 208.01 | 0.594314 |  |
| GO:0018149\_peptide\_cross-linking | MAMDC2 | 10 | 1 | 6.772059 | -0.859001 | 350 | 208.01 | 0.594314 |  |
| GO:0031018\_endocrine\_pancreas\_development | NKX6-1 | 10 | 1 | 6.772059 | -0.859001 | 350 | 208.01 | 0.594314 |  |
| GO:0040015\_negative\_regulation\_of\_multicellular\_organism\_growth | ATXN2 | 10 | 1 | 6.772059 | -0.859001 | 350 | 208.01 | 0.594314 |  |
| GO:0045576\_mast\_cell\_activation | NDRG1 | 10 | 1 | 6.772059 | -0.859001 | 350 | 208.01 | 0.594314 |  |
| GO:0045911\_positive\_regulation\_of\_DNA\_recombination | PTPRC | 10 | 1 | 6.772059 | -0.859001 | 350 | 208.01 | 0.594314 |  |
| GO:0048291\_isotype\_switching\_to\_IgG\_isotypes | PTPRC | 10 | 1 | 6.772059 | -0.859001 | 350 | 208.01 | 0.594314 |  |
| GO:0048302\_regulation\_of\_isotype\_switching\_to\_IgG\_isotypes | PTPRC | 10 | 1 | 6.772059 | -0.859001 | 350 | 208.01 | 0.594314 |  |
| GO:0050654\_chondroitin\_sulfate\_proteoglycan\_metabolic\_process | MAMDC2 | 10 | 1 | 6.772059 | -0.859001 | 350 | 208.01 | 0.594314 |  |
| GO:0051208\_sequestering\_of\_calcium\_ion | PTPRC | 10 | 1 | 6.772059 | -0.859001 | 350 | 208.01 | 0.594314 |  |
| GO:0051209\_release\_of\_sequestered\_calcium\_ion\_into\_cytosol | PTPRC | 10 | 1 | 6.772059 | -0.859001 | 350 | 208.01 | 0.594314 |  |
| GO:0051238\_sequestering\_of\_metal\_ion | PTPRC | 10 | 1 | 6.772059 | -0.859001 | 350 | 208.01 | 0.594314 |  |
| GO:0051282\_regulation\_of\_sequestering\_of\_calcium\_ion | PTPRC | 10 | 1 | 6.772059 | -0.859001 | 350 | 208.01 | 0.594314 |  |
| GO:0051283\_negative\_regulation\_of\_sequestering\_of\_calcium\_ion | PTPRC | 10 | 1 | 6.772059 | -0.859001 | 350 | 208.01 | 0.594314 |  |
| GO:0051650\_establishment\_of\_vesicle\_localization | RAB27A | 10 | 1 | 6.772059 | -0.859001 | 350 | 208.01 | 0.594314 |  |
| GO:0060323\_head\_morphogenesis | ARID5B | 10 | 1 | 6.772059 | -0.859001 | 350 | 208.01 | 0.594314 |  |
| GO:0044260\_cellular\_macromolecule\_metabolic\_process | FUT9 | 1447 | 26 | 1.216818 | -0.857484 | 351 | 208.63 | 0.594387 |  |
| GO:0044260\_cellular\_macromolecule\_metabolic\_process | MAMDC2 | 1447 | 26 | 1.216818 | -0.857484 | 351 | 208.63 | 0.594387 |  |
| GO:0044260\_cellular\_macromolecule\_metabolic\_process | HEXB | 1447 | 26 | 1.216818 | -0.857484 | 351 | 208.63 | 0.594387 |  |
| GO:0044260\_cellular\_macromolecule\_metabolic\_process | TBX20 | 1447 | 26 | 1.216818 | -0.857484 | 351 | 208.63 | 0.594387 |  |
| GO:0044260\_cellular\_macromolecule\_metabolic\_process | SOX5 | 1447 | 26 | 1.216818 | -0.857484 | 351 | 208.63 | 0.594387 |  |
| GO:0044260\_cellular\_macromolecule\_metabolic\_process | HSPA1A | 1447 | 26 | 1.216818 | -0.857484 | 351 | 208.63 | 0.594387 |  |
| GO:0044260\_cellular\_macromolecule\_metabolic\_process | NR3C1 | 1447 | 26 | 1.216818 | -0.857484 | 351 | 208.63 | 0.594387 |  |
| GO:0044260\_cellular\_macromolecule\_metabolic\_process | ABCA1 | 1447 | 26 | 1.216818 | -0.857484 | 351 | 208.63 | 0.594387 |  |
| GO:0044260\_cellular\_macromolecule\_metabolic\_process | TCF7L2 | 1447 | 26 | 1.216818 | -0.857484 | 351 | 208.63 | 0.594387 |  |
| GO:0044260\_cellular\_macromolecule\_metabolic\_process | NR2E1 | 1447 | 26 | 1.216818 | -0.857484 | 351 | 208.63 | 0.594387 |  |
| GO:0044260\_cellular\_macromolecule\_metabolic\_process | TCERG1 | 1447 | 26 | 1.216818 | -0.857484 | 351 | 208.63 | 0.594387 |  |
| GO:0044260\_cellular\_macromolecule\_metabolic\_process | DMD | 1447 | 26 | 1.216818 | -0.857484 | 351 | 208.63 | 0.594387 |  |
| GO:0044260\_cellular\_macromolecule\_metabolic\_process | ETV1 | 1447 | 26 | 1.216818 | -0.857484 | 351 | 208.63 | 0.594387 |  |
| GO:0044260\_cellular\_macromolecule\_metabolic\_process | B3GNT2 | 1447 | 26 | 1.216818 | -0.857484 | 351 | 208.63 | 0.594387 |  |
| GO:0044260\_cellular\_macromolecule\_metabolic\_process | PPAP2A | 1447 | 26 | 1.216818 | -0.857484 | 351 | 208.63 | 0.594387 |  |
| GO:0044260\_cellular\_macromolecule\_metabolic\_process | PTPRC | 1447 | 26 | 1.216818 | -0.857484 | 351 | 208.63 | 0.594387 |  |
| GO:0044260\_cellular\_macromolecule\_metabolic\_process | TBX3 | 1447 | 26 | 1.216818 | -0.857484 | 351 | 208.63 | 0.594387 |  |
| GO:0044260\_cellular\_macromolecule\_metabolic\_process | ESRRG | 1447 | 26 | 1.216818 | -0.857484 | 351 | 208.63 | 0.594387 |  |
| GO:0044260\_cellular\_macromolecule\_metabolic\_process | TRIM63 | 1447 | 26 | 1.216818 | -0.857484 | 351 | 208.63 | 0.594387 |  |
| GO:0044260\_cellular\_macromolecule\_metabolic\_process | NKX6-1 | 1447 | 26 | 1.216818 | -0.857484 | 351 | 208.63 | 0.594387 |  |
| GO:0044260\_cellular\_macromolecule\_metabolic\_process | ASCL1 | 1447 | 26 | 1.216818 | -0.857484 | 351 | 208.63 | 0.594387 |  |
| GO:0044260\_cellular\_macromolecule\_metabolic\_process | MSX1 | 1447 | 26 | 1.216818 | -0.857484 | 351 | 208.63 | 0.594387 |  |
| GO:0044260\_cellular\_macromolecule\_metabolic\_process | NEUROD1 | 1447 | 26 | 1.216818 | -0.857484 | 351 | 208.63 | 0.594387 |  |
| GO:0044260\_cellular\_macromolecule\_metabolic\_process | JAK2 | 1447 | 26 | 1.216818 | -0.857484 | 351 | 208.63 | 0.594387 |  |
| GO:0044260\_cellular\_macromolecule\_metabolic\_process | HDAC9 | 1447 | 26 | 1.216818 | -0.857484 | 351 | 208.63 | 0.594387 |  |
| GO:0044260\_cellular\_macromolecule\_metabolic\_process | GADD45B | 1447 | 26 | 1.216818 | -0.857484 | 351 | 208.63 | 0.594387 |  |
| GO:0040011\_locomotion | DCC | 295 | 7 | 1.606929 | -0.845631 | 352 | 210.15 | 0.597017 |  |
| GO:0040011\_locomotion | ASCL1 | 295 | 7 | 1.606929 | -0.845631 | 352 | 210.15 | 0.597017 |  |
| GO:0040011\_locomotion | PLXNA2 | 295 | 7 | 1.606929 | -0.845631 | 352 | 210.15 | 0.597017 |  |
| GO:0040011\_locomotion | ARID5B | 295 | 7 | 1.606929 | -0.845631 | 352 | 210.15 | 0.597017 |  |
| GO:0040011\_locomotion | ATP1A2 | 295 | 7 | 1.606929 | -0.845631 | 352 | 210.15 | 0.597017 |  |
| GO:0040011\_locomotion | ITGA4 | 295 | 7 | 1.606929 | -0.845631 | 352 | 210.15 | 0.597017 |  |
| GO:0040011\_locomotion | NR2E1 | 295 | 7 | 1.606929 | -0.845631 | 352 | 210.15 | 0.597017 |  |
| GO:0007260\_tyrosine\_phosphorylation\_of\_STAT\_protein | JAK2 | 11 | 1 | 6.156417 | -0.820714 | 360 | 225.32 | 0.625889 |  |
| GO:0014013\_regulation\_of\_gliogenesis | NKX6-1 | 11 | 1 | 6.156417 | -0.820714 | 360 | 225.32 | 0.625889 |  |
| GO:0042551\_neuron\_maturation | IRX5 | 11 | 1 | 6.156417 | -0.820714 | 360 | 225.32 | 0.625889 |  |
| GO:0043576\_regulation\_of\_respiratory\_gaseous\_exchange | ATP1A2 | 11 | 1 | 6.156417 | -0.820714 | 360 | 225.32 | 0.625889 |  |
| GO:0045055\_regulated\_secretory\_pathway | RAB27A | 11 | 1 | 6.156417 | -0.820714 | 360 | 225.32 | 0.625889 |  |
| GO:0045685\_regulation\_of\_glial\_cell\_differentiation | NKX6-1 | 11 | 1 | 6.156417 | -0.820714 | 360 | 225.32 | 0.625889 |  |
| GO:0046425\_regulation\_of\_JAK-STAT\_cascade | JAK2 | 11 | 1 | 6.156417 | -0.820714 | 360 | 225.32 | 0.625889 |  |
| GO:0046716\_muscle\_maintenance | DMD | 11 | 1 | 6.156417 | -0.820714 | 360 | 225.32 | 0.625889 |  |
| GO:0002757\_immune\_response-activating\_signal\_transduction | PTPRC | 47 | 2 | 2.881727 | -0.817150 | 363 | 227.12 | 0.625675 |  |
| GO:0002757\_immune\_response-activating\_signal\_transduction | ELF1 | 47 | 2 | 2.881727 | -0.817150 | 363 | 227.12 | 0.625675 |  |
| GO:0030183\_B\_cell\_differentiation | PTPRC | 47 | 2 | 2.881727 | -0.817150 | 363 | 227.12 | 0.625675 |  |
| GO:0030183\_B\_cell\_differentiation | HDAC9 | 47 | 2 | 2.881727 | -0.817150 | 363 | 227.12 | 0.625675 |  |
| GO:0034754\_cellular\_hormone\_metabolic\_process | AFP | 47 | 2 | 2.881727 | -0.817150 | 363 | 227.12 | 0.625675 |  |
| GO:0034754\_cellular\_hormone\_metabolic\_process | NR3C1 | 47 | 2 | 2.881727 | -0.817150 | 363 | 227.12 | 0.625675 |  |
| GO:0051252\_regulation\_of\_RNA\_metabolic\_process | ASCL1 | 590 | 12 | 1.377368 | -0.813126 | 364 | 227.18 | 0.624121 |  |
| GO:0051252\_regulation\_of\_RNA\_metabolic\_process | MSX1 | 590 | 12 | 1.377368 | -0.813126 | 364 | 227.18 | 0.624121 |  |
| GO:0051252\_regulation\_of\_RNA\_metabolic\_process | TBX3 | 590 | 12 | 1.377368 | -0.813126 | 364 | 227.18 | 0.624121 |  |
| GO:0051252\_regulation\_of\_RNA\_metabolic\_process | TBX20 | 590 | 12 | 1.377368 | -0.813126 | 364 | 227.18 | 0.624121 |  |
| GO:0051252\_regulation\_of\_RNA\_metabolic\_process | ESRRG | 590 | 12 | 1.377368 | -0.813126 | 364 | 227.18 | 0.624121 |  |
| GO:0051252\_regulation\_of\_RNA\_metabolic\_process | SOX5 | 590 | 12 | 1.377368 | -0.813126 | 364 | 227.18 | 0.624121 |  |
| GO:0051252\_regulation\_of\_RNA\_metabolic\_process | NEUROD1 | 590 | 12 | 1.377368 | -0.813126 | 364 | 227.18 | 0.624121 |  |
| GO:0051252\_regulation\_of\_RNA\_metabolic\_process | NR3C1 | 590 | 12 | 1.377368 | -0.813126 | 364 | 227.18 | 0.624121 |  |
| GO:0051252\_regulation\_of\_RNA\_metabolic\_process | HDAC9 | 590 | 12 | 1.377368 | -0.813126 | 364 | 227.18 | 0.624121 |  |
| GO:0051252\_regulation\_of\_RNA\_metabolic\_process | TCF7L2 | 590 | 12 | 1.377368 | -0.813126 | 364 | 227.18 | 0.624121 |  |
| GO:0051252\_regulation\_of\_RNA\_metabolic\_process | NR2E1 | 590 | 12 | 1.377368 | -0.813126 | 364 | 227.18 | 0.624121 |  |
| GO:0051252\_regulation\_of\_RNA\_metabolic\_process | NKX6-1 | 590 | 12 | 1.377368 | -0.813126 | 364 | 227.18 | 0.624121 |  |
| GO:0051171\_regulation\_of\_nitrogen\_compound\_metabolic\_process | PTPRC | 771 | 15 | 1.317521 | -0.812829 | 365 | 227.61 | 0.623589 |  |
| GO:0051171\_regulation\_of\_nitrogen\_compound\_metabolic\_process | TBX3 | 771 | 15 | 1.317521 | -0.812829 | 365 | 227.61 | 0.623589 |  |
| GO:0051171\_regulation\_of\_nitrogen\_compound\_metabolic\_process | TBX20 | 771 | 15 | 1.317521 | -0.812829 | 365 | 227.61 | 0.623589 |  |
| GO:0051171\_regulation\_of\_nitrogen\_compound\_metabolic\_process | SOX5 | 771 | 15 | 1.317521 | -0.812829 | 365 | 227.61 | 0.623589 |  |
| GO:0051171\_regulation\_of\_nitrogen\_compound\_metabolic\_process | ESRRG | 771 | 15 | 1.317521 | -0.812829 | 365 | 227.61 | 0.623589 |  |
| GO:0051171\_regulation\_of\_nitrogen\_compound\_metabolic\_process | NR3C1 | 771 | 15 | 1.317521 | -0.812829 | 365 | 227.61 | 0.623589 |  |
| GO:0051171\_regulation\_of\_nitrogen\_compound\_metabolic\_process | TCF7L2 | 771 | 15 | 1.317521 | -0.812829 | 365 | 227.61 | 0.623589 |  |
| GO:0051171\_regulation\_of\_nitrogen\_compound\_metabolic\_process | NR2E1 | 771 | 15 | 1.317521 | -0.812829 | 365 | 227.61 | 0.623589 |  |
| GO:0051171\_regulation\_of\_nitrogen\_compound\_metabolic\_process | NKX6-1 | 771 | 15 | 1.317521 | -0.812829 | 365 | 227.61 | 0.623589 |  |
| GO:0051171\_regulation\_of\_nitrogen\_compound\_metabolic\_process | ASCL1 | 771 | 15 | 1.317521 | -0.812829 | 365 | 227.61 | 0.623589 |  |
| GO:0051171\_regulation\_of\_nitrogen\_compound\_metabolic\_process | MSX1 | 771 | 15 | 1.317521 | -0.812829 | 365 | 227.61 | 0.623589 |  |
| GO:0051171\_regulation\_of\_nitrogen\_compound\_metabolic\_process | DMD | 771 | 15 | 1.317521 | -0.812829 | 365 | 227.61 | 0.623589 |  |
| GO:0051171\_regulation\_of\_nitrogen\_compound\_metabolic\_process | NEUROD1 | 771 | 15 | 1.317521 | -0.812829 | 365 | 227.61 | 0.623589 |  |
| GO:0051171\_regulation\_of\_nitrogen\_compound\_metabolic\_process | ETV1 | 771 | 15 | 1.317521 | -0.812829 | 365 | 227.61 | 0.623589 |  |
| GO:0051171\_regulation\_of\_nitrogen\_compound\_metabolic\_process | HDAC9 | 771 | 15 | 1.317521 | -0.812829 | 365 | 227.61 | 0.623589 |  |
| GO:0009653\_anatomical\_structure\_morphogenesis | DCC | 958 | 18 | 1.272412 | -0.806292 | 366 | 227.96 | 0.622842 |  |
| GO:0009653\_anatomical\_structure\_morphogenesis | FGF18 | 958 | 18 | 1.272412 | -0.806292 | 366 | 227.96 | 0.622842 |  |
| GO:0009653\_anatomical\_structure\_morphogenesis | MYO6 | 958 | 18 | 1.272412 | -0.806292 | 366 | 227.96 | 0.622842 |  |
| GO:0009653\_anatomical\_structure\_morphogenesis | IRX5 | 958 | 18 | 1.272412 | -0.806292 | 366 | 227.96 | 0.622842 |  |
| GO:0009653\_anatomical\_structure\_morphogenesis | TBX3 | 958 | 18 | 1.272412 | -0.806292 | 366 | 227.96 | 0.622842 |  |
| GO:0009653\_anatomical\_structure\_morphogenesis | ARID5B | 958 | 18 | 1.272412 | -0.806292 | 366 | 227.96 | 0.622842 |  |
| GO:0009653\_anatomical\_structure\_morphogenesis | TBX20 | 958 | 18 | 1.272412 | -0.806292 | 366 | 227.96 | 0.622842 |  |
| GO:0009653\_anatomical\_structure\_morphogenesis | PCDH8 | 958 | 18 | 1.272412 | -0.806292 | 366 | 227.96 | 0.622842 |  |
| GO:0009653\_anatomical\_structure\_morphogenesis | ITGA4 | 958 | 18 | 1.272412 | -0.806292 | 366 | 227.96 | 0.622842 |  |
| GO:0009653\_anatomical\_structure\_morphogenesis | NR3C1 | 958 | 18 | 1.272412 | -0.806292 | 366 | 227.96 | 0.622842 |  |
| GO:0009653\_anatomical\_structure\_morphogenesis | NR2E1 | 958 | 18 | 1.272412 | -0.806292 | 366 | 227.96 | 0.622842 |  |
| GO:0009653\_anatomical\_structure\_morphogenesis | TCF7L2 | 958 | 18 | 1.272412 | -0.806292 | 366 | 227.96 | 0.622842 |  |
| GO:0009653\_anatomical\_structure\_morphogenesis | ALCAM | 958 | 18 | 1.272412 | -0.806292 | 366 | 227.96 | 0.622842 |  |
| GO:0009653\_anatomical\_structure\_morphogenesis | MSX1 | 958 | 18 | 1.272412 | -0.806292 | 366 | 227.96 | 0.622842 |  |
| GO:0009653\_anatomical\_structure\_morphogenesis | ETV1 | 958 | 18 | 1.272412 | -0.806292 | 366 | 227.96 | 0.622842 |  |
| GO:0009653\_anatomical\_structure\_morphogenesis | B3GNT2 | 958 | 18 | 1.272412 | -0.806292 | 366 | 227.96 | 0.622842 |  |
| GO:0009653\_anatomical\_structure\_morphogenesis | THBS1 | 958 | 18 | 1.272412 | -0.806292 | 366 | 227.96 | 0.622842 |  |
| GO:0009653\_anatomical\_structure\_morphogenesis | BOC | 958 | 18 | 1.272412 | -0.806292 | 366 | 227.96 | 0.622842 |  |
| GO:0046907\_intracellular\_transport | MYO6 | 194 | 5 | 1.745376 | -0.803666 | 367 | 228.07 | 0.621444 |  |
| GO:0046907\_intracellular\_transport | AP2A1 | 194 | 5 | 1.745376 | -0.803666 | 367 | 228.07 | 0.621444 |  |
| GO:0046907\_intracellular\_transport | MLPH | 194 | 5 | 1.745376 | -0.803666 | 367 | 228.07 | 0.621444 |  |
| GO:0046907\_intracellular\_transport | JAK2 | 194 | 5 | 1.745376 | -0.803666 | 367 | 228.07 | 0.621444 |  |
| GO:0046907\_intracellular\_transport | RAB27A | 194 | 5 | 1.745376 | -0.803666 | 367 | 228.07 | 0.621444 |  |
| GO:0055066\_di-\_\_tri-valent\_inorganic\_cation\_homeostasis | PTPRC | 93 | 3 | 2.184535 | -0.803057 | 368 | 228.4 | 0.620652 |  |
| GO:0055066\_di-\_\_tri-valent\_inorganic\_cation\_homeostasis | HEXB | 93 | 3 | 2.184535 | -0.803057 | 368 | 228.4 | 0.620652 |  |
| GO:0055066\_di-\_\_tri-valent\_inorganic\_cation\_homeostasis | ATP1A2 | 93 | 3 | 2.184535 | -0.803057 | 368 | 228.4 | 0.620652 |  |
| GO:0009101\_glycoprotein\_biosynthetic\_process | FUT9 | 48 | 2 | 2.821691 | -0.802628 | 369 | 229.22 | 0.621192 |  |
| GO:0009101\_glycoprotein\_biosynthetic\_process | B3GNT2 | 48 | 2 | 2.821691 | -0.802628 | 369 | 229.22 | 0.621192 |  |
| GO:0006351\_transcription\_\_DNA-dependent | ASCL1 | 594 | 12 | 1.368093 | -0.798067 | 370 | 229.52 | 0.620324 |  |
| GO:0006351\_transcription\_\_DNA-dependent | MSX1 | 594 | 12 | 1.368093 | -0.798067 | 370 | 229.52 | 0.620324 |  |
| GO:0006351\_transcription\_\_DNA-dependent | TBX3 | 594 | 12 | 1.368093 | -0.798067 | 370 | 229.52 | 0.620324 |  |
| GO:0006351\_transcription\_\_DNA-dependent | TBX20 | 594 | 12 | 1.368093 | -0.798067 | 370 | 229.52 | 0.620324 |  |
| GO:0006351\_transcription\_\_DNA-dependent | ESRRG | 594 | 12 | 1.368093 | -0.798067 | 370 | 229.52 | 0.620324 |  |
| GO:0006351\_transcription\_\_DNA-dependent | SOX5 | 594 | 12 | 1.368093 | -0.798067 | 370 | 229.52 | 0.620324 |  |
| GO:0006351\_transcription\_\_DNA-dependent | NEUROD1 | 594 | 12 | 1.368093 | -0.798067 | 370 | 229.52 | 0.620324 |  |
| GO:0006351\_transcription\_\_DNA-dependent | NR3C1 | 594 | 12 | 1.368093 | -0.798067 | 370 | 229.52 | 0.620324 |  |
| GO:0006351\_transcription\_\_DNA-dependent | HDAC9 | 594 | 12 | 1.368093 | -0.798067 | 370 | 229.52 | 0.620324 |  |
| GO:0006351\_transcription\_\_DNA-dependent | TCF7L2 | 594 | 12 | 1.368093 | -0.798067 | 370 | 229.52 | 0.620324 |  |
| GO:0006351\_transcription\_\_DNA-dependent | NR2E1 | 594 | 12 | 1.368093 | -0.798067 | 370 | 229.52 | 0.620324 |  |
| GO:0006351\_transcription\_\_DNA-dependent | NKX6-1 | 594 | 12 | 1.368093 | -0.798067 | 370 | 229.52 | 0.620324 |  |
| GO:0003002\_regionalization | TBX3 | 195 | 5 | 1.736425 | -0.797127 | 372 | 229.83 | 0.617823 |  |
| GO:0003002\_regionalization | TBX20 | 195 | 5 | 1.736425 | -0.797127 | 372 | 229.83 | 0.617823 |  |
| GO:0003002\_regionalization | NEUROD1 | 195 | 5 | 1.736425 | -0.797127 | 372 | 229.83 | 0.617823 |  |
| GO:0003002\_regionalization | PCDH8 | 195 | 5 | 1.736425 | -0.797127 | 372 | 229.83 | 0.617823 |  |
| GO:0003002\_regionalization | NKX6-1 | 195 | 5 | 1.736425 | -0.797127 | 372 | 229.83 | 0.617823 |  |
| GO:0007507\_heart\_development | MSX1 | 195 | 5 | 1.736425 | -0.797127 | 372 | 229.83 | 0.617823 |  |
| GO:0007507\_heart\_development | TBX3 | 195 | 5 | 1.736425 | -0.797127 | 372 | 229.83 | 0.617823 |  |
| GO:0007507\_heart\_development | TBX20 | 195 | 5 | 1.736425 | -0.797127 | 372 | 229.83 | 0.617823 |  |
| GO:0007507\_heart\_development | ITGA4 | 195 | 5 | 1.736425 | -0.797127 | 372 | 229.83 | 0.617823 |  |
| GO:0007507\_heart\_development | HDAC9 | 195 | 5 | 1.736425 | -0.797127 | 372 | 229.83 | 0.617823 |  |
| GO:0032774\_RNA\_biosynthetic\_process | ASCL1 | 595 | 12 | 1.365793 | -0.794339 | 373 | 230.25 | 0.617292 |  |
| GO:0032774\_RNA\_biosynthetic\_process | MSX1 | 595 | 12 | 1.365793 | -0.794339 | 373 | 230.25 | 0.617292 |  |
| GO:0032774\_RNA\_biosynthetic\_process | TBX3 | 595 | 12 | 1.365793 | -0.794339 | 373 | 230.25 | 0.617292 |  |
| GO:0032774\_RNA\_biosynthetic\_process | TBX20 | 595 | 12 | 1.365793 | -0.794339 | 373 | 230.25 | 0.617292 |  |
| GO:0032774\_RNA\_biosynthetic\_process | ESRRG | 595 | 12 | 1.365793 | -0.794339 | 373 | 230.25 | 0.617292 |  |
| GO:0032774\_RNA\_biosynthetic\_process | SOX5 | 595 | 12 | 1.365793 | -0.794339 | 373 | 230.25 | 0.617292 |  |
| GO:0032774\_RNA\_biosynthetic\_process | NEUROD1 | 595 | 12 | 1.365793 | -0.794339 | 373 | 230.25 | 0.617292 |  |
| GO:0032774\_RNA\_biosynthetic\_process | NR3C1 | 595 | 12 | 1.365793 | -0.794339 | 373 | 230.25 | 0.617292 |  |
| GO:0032774\_RNA\_biosynthetic\_process | HDAC9 | 595 | 12 | 1.365793 | -0.794339 | 373 | 230.25 | 0.617292 |  |
| GO:0032774\_RNA\_biosynthetic\_process | TCF7L2 | 595 | 12 | 1.365793 | -0.794339 | 373 | 230.25 | 0.617292 |  |
| GO:0032774\_RNA\_biosynthetic\_process | NR2E1 | 595 | 12 | 1.365793 | -0.794339 | 373 | 230.25 | 0.617292 |  |
| GO:0032774\_RNA\_biosynthetic\_process | NKX6-1 | 595 | 12 | 1.365793 | -0.794339 | 373 | 230.25 | 0.617292 |  |
| GO:0007389\_pattern\_specification\_process | ASCL1 | 250 | 6 | 1.625294 | -0.790581 | 374 | 231.27 | 0.618369 |  |
| GO:0007389\_pattern\_specification\_process | TBX3 | 250 | 6 | 1.625294 | -0.790581 | 374 | 231.27 | 0.618369 |  |
| GO:0007389\_pattern\_specification\_process | TBX20 | 250 | 6 | 1.625294 | -0.790581 | 374 | 231.27 | 0.618369 |  |
| GO:0007389\_pattern\_specification\_process | NEUROD1 | 250 | 6 | 1.625294 | -0.790581 | 374 | 231.27 | 0.618369 |  |
| GO:0007389\_pattern\_specification\_process | PCDH8 | 250 | 6 | 1.625294 | -0.790581 | 374 | 231.27 | 0.618369 |  |
| GO:0007389\_pattern\_specification\_process | NKX6-1 | 250 | 6 | 1.625294 | -0.790581 | 374 | 231.27 | 0.618369 |  |
| GO:0010468\_regulation\_of\_gene\_expression | IRX5 | 778 | 15 | 1.305667 | -0.789549 | 375 | 231.43 | 0.617147 |  |
| GO:0010468\_regulation\_of\_gene\_expression | TBX3 | 778 | 15 | 1.305667 | -0.789549 | 375 | 231.43 | 0.617147 |  |
| GO:0010468\_regulation\_of\_gene\_expression | TBX20 | 778 | 15 | 1.305667 | -0.789549 | 375 | 231.43 | 0.617147 |  |
| GO:0010468\_regulation\_of\_gene\_expression | SOX5 | 778 | 15 | 1.305667 | -0.789549 | 375 | 231.43 | 0.617147 |  |
| GO:0010468\_regulation\_of\_gene\_expression | ESRRG | 778 | 15 | 1.305667 | -0.789549 | 375 | 231.43 | 0.617147 |  |
| GO:0010468\_regulation\_of\_gene\_expression | NR3C1 | 778 | 15 | 1.305667 | -0.789549 | 375 | 231.43 | 0.617147 |  |
| GO:0010468\_regulation\_of\_gene\_expression | TCF7L2 | 778 | 15 | 1.305667 | -0.789549 | 375 | 231.43 | 0.617147 |  |
| GO:0010468\_regulation\_of\_gene\_expression | NR2E1 | 778 | 15 | 1.305667 | -0.789549 | 375 | 231.43 | 0.617147 |  |
| GO:0010468\_regulation\_of\_gene\_expression | NKX6-1 | 778 | 15 | 1.305667 | -0.789549 | 375 | 231.43 | 0.617147 |  |
| GO:0010468\_regulation\_of\_gene\_expression | ASCL1 | 778 | 15 | 1.305667 | -0.789549 | 375 | 231.43 | 0.617147 |  |
| GO:0010468\_regulation\_of\_gene\_expression | MSX1 | 778 | 15 | 1.305667 | -0.789549 | 375 | 231.43 | 0.617147 |  |
| GO:0010468\_regulation\_of\_gene\_expression | DMD | 778 | 15 | 1.305667 | -0.789549 | 375 | 231.43 | 0.617147 |  |
| GO:0010468\_regulation\_of\_gene\_expression | NEUROD1 | 778 | 15 | 1.305667 | -0.789549 | 375 | 231.43 | 0.617147 |  |
| GO:0010468\_regulation\_of\_gene\_expression | ETV1 | 778 | 15 | 1.305667 | -0.789549 | 375 | 231.43 | 0.617147 |  |
| GO:0010468\_regulation\_of\_gene\_expression | HDAC9 | 778 | 15 | 1.305667 | -0.789549 | 375 | 231.43 | 0.617147 |  |
| GO:0045596\_negative\_regulation\_of\_cell\_differentiation | MBNL3 | 144 | 4 | 1.881127 | -0.789462 | 376 | 231.61 | 0.615984 |  |
| GO:0045596\_negative\_regulation\_of\_cell\_differentiation | NR2E1 | 144 | 4 | 1.881127 | -0.789462 | 376 | 231.61 | 0.615984 |  |
| GO:0045596\_negative\_regulation\_of\_cell\_differentiation | TCF7L2 | 144 | 4 | 1.881127 | -0.789462 | 376 | 231.61 | 0.615984 |  |
| GO:0045596\_negative\_regulation\_of\_cell\_differentiation | NKX6-1 | 144 | 4 | 1.881127 | -0.789462 | 376 | 231.61 | 0.615984 |  |
| GO:0003015\_heart\_process | IRX5 | 49 | 2 | 2.764106 | -0.788487 | 381 | 233.23 | 0.612152 |  |
| GO:0003015\_heart\_process | ATP1A2 | 49 | 2 | 2.764106 | -0.788487 | 381 | 233.23 | 0.612152 |  |
| GO:0021543\_pallium\_development | ASCL1 | 49 | 2 | 2.764106 | -0.788487 | 381 | 233.23 | 0.612152 |  |
| GO:0021543\_pallium\_development | NR2E1 | 49 | 2 | 2.764106 | -0.788487 | 381 | 233.23 | 0.612152 |  |
| GO:0046660\_female\_sex\_differentiation | AFP | 49 | 2 | 2.764106 | -0.788487 | 381 | 233.23 | 0.612152 |  |
| GO:0046660\_female\_sex\_differentiation | ZFX | 49 | 2 | 2.764106 | -0.788487 | 381 | 233.23 | 0.612152 |  |
| GO:0048741\_skeletal\_muscle\_fiber\_development | MBNL3 | 49 | 2 | 2.764106 | -0.788487 | 381 | 233.23 | 0.612152 |  |
| GO:0048741\_skeletal\_muscle\_fiber\_development | BOC | 49 | 2 | 2.764106 | -0.788487 | 381 | 233.23 | 0.612152 |  |
| GO:0060047\_heart\_contraction | IRX5 | 49 | 2 | 2.764106 | -0.788487 | 381 | 233.23 | 0.612152 |  |
| GO:0060047\_heart\_contraction | ATP1A2 | 49 | 2 | 2.764106 | -0.788487 | 381 | 233.23 | 0.612152 |  |
| GO:0002026\_regulation\_of\_the\_force\_of\_heart\_contraction | ATP1A2 | 12 | 1 | 5.643382 | -0.786024 | 396 | 246.49 | 0.622449 |  |
| GO:0002820\_negative\_regulation\_of\_adaptive\_immune\_response | PTPRC | 12 | 1 | 5.643382 | -0.786024 | 396 | 246.49 | 0.622449 |  |
| GO:0002823\_negative\_regulation\_of\_adaptive\_immune\_response\_based\_on\_somatic\_recombination\_of\_immune\_receptors\_built\_from\_immunoglobulin\_superfamily\_domains | PTPRC | 12 | 1 | 5.643382 | -0.786024 | 396 | 246.49 | 0.622449 |  |
| GO:0006094\_gluconeogenesis | NR3C1 | 12 | 1 | 5.643382 | -0.786024 | 396 | 246.49 | 0.622449 |  |
| GO:0008045\_motor\_axon\_guidance | ALCAM | 12 | 1 | 5.643382 | -0.786024 | 396 | 246.49 | 0.622449 |  |
| GO:0008277\_regulation\_of\_G-protein\_coupled\_receptor\_protein\_signaling\_pathway | RGS13 | 12 | 1 | 5.643382 | -0.786024 | 396 | 246.49 | 0.622449 |  |
| GO:0019319\_hexose\_biosynthetic\_process | NR3C1 | 12 | 1 | 5.643382 | -0.786024 | 396 | 246.49 | 0.622449 |  |
| GO:0021513\_spinal\_cord\_dorsal\_ventral\_patterning | NKX6-1 | 12 | 1 | 5.643382 | -0.786024 | 396 | 246.49 | 0.622449 |  |
| GO:0035050\_embryonic\_heart\_tube\_development | TBX20 | 12 | 1 | 5.643382 | -0.786024 | 396 | 246.49 | 0.622449 |  |
| GO:0042446\_hormone\_biosynthetic\_process | NR3C1 | 12 | 1 | 5.643382 | -0.786024 | 396 | 246.49 | 0.622449 |  |
| GO:0043392\_negative\_regulation\_of\_DNA\_binding | JAK2 | 12 | 1 | 5.643382 | -0.786024 | 396 | 246.49 | 0.622449 |  |
| GO:0046330\_positive\_regulation\_of\_JNK\_cascade | CD27 | 12 | 1 | 5.643382 | -0.786024 | 396 | 246.49 | 0.622449 |  |
| GO:0050853\_B\_cell\_receptor\_signaling\_pathway | PTPRC | 12 | 1 | 5.643382 | -0.786024 | 396 | 246.49 | 0.622449 |  |
| GO:0060042\_retina\_morphogenesis\_in\_camera-type\_eye | IRX5 | 12 | 1 | 5.643382 | -0.786024 | 396 | 246.49 | 0.622449 |  |
| GO:0070304\_positive\_regulation\_of\_stress-activated\_protein\_kinase\_signaling\_pathway | CD27 | 12 | 1 | 5.643382 | -0.786024 | 396 | 246.49 | 0.622449 |  |
| GO:0030900\_forebrain\_development | ASCL1 | 146 | 4 | 1.855359 | -0.774414 | 397 | 249.24 | 0.627809 |  |
| GO:0030900\_forebrain\_development | MSX1 | 146 | 4 | 1.855359 | -0.774414 | 397 | 249.24 | 0.627809 |  |
| GO:0030900\_forebrain\_development | TBX3 | 146 | 4 | 1.855359 | -0.774414 | 397 | 249.24 | 0.627809 |  |
| GO:0030900\_forebrain\_development | NR2E1 | 146 | 4 | 1.855359 | -0.774414 | 397 | 249.24 | 0.627809 |  |
| GO:0060249\_anatomical\_structure\_homeostasis | DMD | 96 | 3 | 2.116268 | -0.774304 | 398 | 249.66 | 0.627286 |  |
| GO:0060249\_anatomical\_structure\_homeostasis | HSPA1A | 96 | 3 | 2.116268 | -0.774304 | 398 | 249.66 | 0.627286 |  |
| GO:0060249\_anatomical\_structure\_homeostasis | TCF7L2 | 96 | 3 | 2.116268 | -0.774304 | 398 | 249.66 | 0.627286 |  |
| GO:0007154\_cell\_communication | PTPRC | 1096 | 20 | 1.235777 | -0.769595 | 399 | 250.06 | 0.626717 |  |
| GO:0007154\_cell\_communication | FGF18 | 1096 | 20 | 1.235777 | -0.769595 | 399 | 250.06 | 0.626717 |  |
| GO:0007154\_cell\_communication | ELF1 | 1096 | 20 | 1.235777 | -0.769595 | 399 | 250.06 | 0.626717 |  |
| GO:0007154\_cell\_communication | MYO6 | 1096 | 20 | 1.235777 | -0.769595 | 399 | 250.06 | 0.626717 |  |
| GO:0007154\_cell\_communication | PLXNA2 | 1096 | 20 | 1.235777 | -0.769595 | 399 | 250.06 | 0.626717 |  |
| GO:0007154\_cell\_communication | ARID5B | 1096 | 20 | 1.235777 | -0.769595 | 399 | 250.06 | 0.626717 |  |
| GO:0007154\_cell\_communication | ATP1A2 | 1096 | 20 | 1.235777 | -0.769595 | 399 | 250.06 | 0.626717 |  |
| GO:0007154\_cell\_communication | NR3C1 | 1096 | 20 | 1.235777 | -0.769595 | 399 | 250.06 | 0.626717 |  |
| GO:0007154\_cell\_communication | TCF7L2 | 1096 | 20 | 1.235777 | -0.769595 | 399 | 250.06 | 0.626717 |  |
| GO:0007154\_cell\_communication | NKX6-1 | 1096 | 20 | 1.235777 | -0.769595 | 399 | 250.06 | 0.626717 |  |
| GO:0007154\_cell\_communication | RGS13 | 1096 | 20 | 1.235777 | -0.769595 | 399 | 250.06 | 0.626717 |  |
| GO:0007154\_cell\_communication | ASCL1 | 1096 | 20 | 1.235777 | -0.769595 | 399 | 250.06 | 0.626717 |  |
| GO:0007154\_cell\_communication | MSX1 | 1096 | 20 | 1.235777 | -0.769595 | 399 | 250.06 | 0.626717 |  |
| GO:0007154\_cell\_communication | DMD | 1096 | 20 | 1.235777 | -0.769595 | 399 | 250.06 | 0.626717 |  |
| GO:0007154\_cell\_communication | JAK2 | 1096 | 20 | 1.235777 | -0.769595 | 399 | 250.06 | 0.626717 |  |
| GO:0007154\_cell\_communication | RGS8 | 1096 | 20 | 1.235777 | -0.769595 | 399 | 250.06 | 0.626717 |  |
| GO:0007154\_cell\_communication | PPAP2A | 1096 | 20 | 1.235777 | -0.769595 | 399 | 250.06 | 0.626717 |  |
| GO:0007154\_cell\_communication | GADD45B | 1096 | 20 | 1.235777 | -0.769595 | 399 | 250.06 | 0.626717 |  |
| GO:0007154\_cell\_communication | BOC | 1096 | 20 | 1.235777 | -0.769595 | 399 | 250.06 | 0.626717 |  |
| GO:0007154\_cell\_communication | CD27 | 1096 | 20 | 1.235777 | -0.769595 | 399 | 250.06 | 0.626717 |  |
| GO:0018193\_peptidyl-amino\_acid\_modification | PTPRC | 97 | 3 | 2.094451 | -0.765006 | 400 | 250.45 | 0.626125 |  |
| GO:0018193\_peptidyl-amino\_acid\_modification | MAMDC2 | 97 | 3 | 2.094451 | -0.765006 | 400 | 250.45 | 0.626125 |  |
| GO:0018193\_peptidyl-amino\_acid\_modification | JAK2 | 97 | 3 | 2.094451 | -0.765006 | 400 | 250.45 | 0.626125 |  |
| GO:0002764\_immune\_response-regulating\_signal\_transduction | PTPRC | 51 | 2 | 2.655709 | -0.761282 | 403 | 251.9 | 0.625062 |  |
| GO:0002764\_immune\_response-regulating\_signal\_transduction | ELF1 | 51 | 2 | 2.655709 | -0.761282 | 403 | 251.9 | 0.625062 |  |
| GO:0043408\_regulation\_of\_MAPKKK\_cascade | PTPRC | 51 | 2 | 2.655709 | -0.761282 | 403 | 251.9 | 0.625062 |  |
| GO:0043408\_regulation\_of\_MAPKKK\_cascade | CD27 | 51 | 2 | 2.655709 | -0.761282 | 403 | 251.9 | 0.625062 |  |
| GO:0048747\_muscle\_fiber\_development | MBNL3 | 51 | 2 | 2.655709 | -0.761282 | 403 | 251.9 | 0.625062 |  |
| GO:0048747\_muscle\_fiber\_development | BOC | 51 | 2 | 2.655709 | -0.761282 | 403 | 251.9 | 0.625062 |  |
| GO:0007548\_sex\_differentiation | AFP | 98 | 3 | 2.073079 | -0.755845 | 404 | 252.7 | 0.625495 |  |
| GO:0007548\_sex\_differentiation | ZFX | 98 | 3 | 2.073079 | -0.755845 | 404 | 252.7 | 0.625495 |  |
| GO:0007548\_sex\_differentiation | TCF7L2 | 98 | 3 | 2.073079 | -0.755845 | 404 | 252.7 | 0.625495 |  |
| GO:0000723\_telomere\_maintenance | HSPA1A | 13 | 1 | 5.209276 | -0.754353 | 424 | 264.61 | 0.624080 |  |
| GO:0001958\_endochondral\_ossification | FGF18 | 13 | 1 | 5.209276 | -0.754353 | 424 | 264.61 | 0.624080 |  |
| GO:0002704\_negative\_regulation\_of\_leukocyte\_mediated\_immunity | PTPRC | 13 | 1 | 5.209276 | -0.754353 | 424 | 264.61 | 0.624080 |  |
| GO:0002707\_negative\_regulation\_of\_lymphocyte\_mediated\_immunity | PTPRC | 13 | 1 | 5.209276 | -0.754353 | 424 | 264.61 | 0.624080 |  |
| GO:0003016\_respiratory\_system\_process | ATP1A2 | 13 | 1 | 5.209276 | -0.754353 | 424 | 264.61 | 0.624080 |  |
| GO:0006090\_pyruvate\_metabolic\_process | NR3C1 | 13 | 1 | 5.209276 | -0.754353 | 424 | 264.61 | 0.624080 |  |
| GO:0006687\_glycosphingolipid\_metabolic\_process | HEXB | 13 | 1 | 5.209276 | -0.754353 | 424 | 264.61 | 0.624080 |  |
| GO:0007439\_ectodermal\_gut\_development | TCF7L2 | 13 | 1 | 5.209276 | -0.754353 | 424 | 264.61 | 0.624080 |  |
| GO:0009994\_oocyte\_differentiation | ZFX | 13 | 1 | 5.209276 | -0.754353 | 424 | 264.61 | 0.624080 |  |
| GO:0016525\_negative\_regulation\_of\_angiogenesis | THBS1 | 13 | 1 | 5.209276 | -0.754353 | 424 | 264.61 | 0.624080 |  |
| GO:0019098\_reproductive\_behavior | HEXB | 13 | 1 | 5.209276 | -0.754353 | 424 | 264.61 | 0.624080 |  |
| GO:0021511\_spinal\_cord\_patterning | NKX6-1 | 13 | 1 | 5.209276 | -0.754353 | 424 | 264.61 | 0.624080 |  |
| GO:0032200\_telomere\_organization | HSPA1A | 13 | 1 | 5.209276 | -0.754353 | 424 | 264.61 | 0.624080 |  |
| GO:0045191\_regulation\_of\_isotype\_switching | PTPRC | 13 | 1 | 5.209276 | -0.754353 | 424 | 264.61 | 0.624080 |  |
| GO:0045577\_regulation\_of\_B\_cell\_differentiation | PTPRC | 13 | 1 | 5.209276 | -0.754353 | 424 | 264.61 | 0.624080 |  |
| GO:0046640\_regulation\_of\_alpha-beta\_T\_cell\_proliferation | PTPRC | 13 | 1 | 5.209276 | -0.754353 | 424 | 264.61 | 0.624080 |  |
| GO:0048567\_ectodermal\_gut\_morphogenesis | TCF7L2 | 13 | 1 | 5.209276 | -0.754353 | 424 | 264.61 | 0.624080 |  |
| GO:0048599\_oocyte\_development | ZFX | 13 | 1 | 5.209276 | -0.754353 | 424 | 264.61 | 0.624080 |  |
| GO:0060401\_cytosolic\_calcium\_ion\_transport | PTPRC | 13 | 1 | 5.209276 | -0.754353 | 424 | 264.61 | 0.624080 |  |
| GO:0060402\_calcium\_ion\_transport\_into\_cytosol | PTPRC | 13 | 1 | 5.209276 | -0.754353 | 424 | 264.61 | 0.624080 |  |
| GO:0048870\_cell\_motility | DCC | 257 | 6 | 1.581025 | -0.751788 | 425 | 264.84 | 0.623153 |  |
| GO:0048870\_cell\_motility | ASCL1 | 257 | 6 | 1.581025 | -0.751788 | 425 | 264.84 | 0.623153 |  |
| GO:0048870\_cell\_motility | PLXNA2 | 257 | 6 | 1.581025 | -0.751788 | 425 | 264.84 | 0.623153 |  |
| GO:0048870\_cell\_motility | ARID5B | 257 | 6 | 1.581025 | -0.751788 | 425 | 264.84 | 0.623153 |  |
| GO:0048870\_cell\_motility | ITGA4 | 257 | 6 | 1.581025 | -0.751788 | 425 | 264.84 | 0.623153 |  |
| GO:0048870\_cell\_motility | NR2E1 | 257 | 6 | 1.581025 | -0.751788 | 425 | 264.84 | 0.623153 |  |
| GO:0007165\_signal\_transduction | PTPRC | 915 | 17 | 1.258197 | -0.748120 | 426 | 265.73 | 0.623779 |  |
| GO:0007165\_signal\_transduction | FGF18 | 915 | 17 | 1.258197 | -0.748120 | 426 | 265.73 | 0.623779 |  |
| GO:0007165\_signal\_transduction | ELF1 | 915 | 17 | 1.258197 | -0.748120 | 426 | 265.73 | 0.623779 |  |
| GO:0007165\_signal\_transduction | PLXNA2 | 915 | 17 | 1.258197 | -0.748120 | 426 | 265.73 | 0.623779 |  |
| GO:0007165\_signal\_transduction | ARID5B | 915 | 17 | 1.258197 | -0.748120 | 426 | 265.73 | 0.623779 |  |
| GO:0007165\_signal\_transduction | NR3C1 | 915 | 17 | 1.258197 | -0.748120 | 426 | 265.73 | 0.623779 |  |
| GO:0007165\_signal\_transduction | TCF7L2 | 915 | 17 | 1.258197 | -0.748120 | 426 | 265.73 | 0.623779 |  |
| GO:0007165\_signal\_transduction | NKX6-1 | 915 | 17 | 1.258197 | -0.748120 | 426 | 265.73 | 0.623779 |  |
| GO:0007165\_signal\_transduction | RGS13 | 915 | 17 | 1.258197 | -0.748120 | 426 | 265.73 | 0.623779 |  |
| GO:0007165\_signal\_transduction | ASCL1 | 915 | 17 | 1.258197 | -0.748120 | 426 | 265.73 | 0.623779 |  |
| GO:0007165\_signal\_transduction | MSX1 | 915 | 17 | 1.258197 | -0.748120 | 426 | 265.73 | 0.623779 |  |
| GO:0007165\_signal\_transduction | JAK2 | 915 | 17 | 1.258197 | -0.748120 | 426 | 265.73 | 0.623779 |  |
| GO:0007165\_signal\_transduction | RGS8 | 915 | 17 | 1.258197 | -0.748120 | 426 | 265.73 | 0.623779 |  |
| GO:0007165\_signal\_transduction | PPAP2A | 915 | 17 | 1.258197 | -0.748120 | 426 | 265.73 | 0.623779 |  |
| GO:0007165\_signal\_transduction | GADD45B | 915 | 17 | 1.258197 | -0.748120 | 426 | 265.73 | 0.623779 |  |
| GO:0007165\_signal\_transduction | BOC | 915 | 17 | 1.258197 | -0.748120 | 426 | 265.73 | 0.623779 |  |
| GO:0007165\_signal\_transduction | CD27 | 915 | 17 | 1.258197 | -0.748120 | 426 | 265.73 | 0.623779 |  |
| GO:0001568\_blood\_vessel\_development | FGF18 | 203 | 5 | 1.667995 | -0.746790 | 427 | 266.3 | 0.623653 |  |
| GO:0001568\_blood\_vessel\_development | TBX3 | 203 | 5 | 1.667995 | -0.746790 | 427 | 266.3 | 0.623653 |  |
| GO:0001568\_blood\_vessel\_development | TBX20 | 203 | 5 | 1.667995 | -0.746790 | 427 | 266.3 | 0.623653 |  |
| GO:0001568\_blood\_vessel\_development | ITGA4 | 203 | 5 | 1.667995 | -0.746790 | 427 | 266.3 | 0.623653 |  |
| GO:0001568\_blood\_vessel\_development | THBS1 | 203 | 5 | 1.667995 | -0.746790 | 427 | 266.3 | 0.623653 |  |
| GO:0043283\_biopolymer\_metabolic\_process | FUT9 | 1490 | 26 | 1.181702 | -0.745126 | 428 | 266.4 | 0.622430 |  |
| GO:0043283\_biopolymer\_metabolic\_process | MAMDC2 | 1490 | 26 | 1.181702 | -0.745126 | 428 | 266.4 | 0.622430 |  |
| GO:0043283\_biopolymer\_metabolic\_process | HEXB | 1490 | 26 | 1.181702 | -0.745126 | 428 | 266.4 | 0.622430 |  |
| GO:0043283\_biopolymer\_metabolic\_process | TBX20 | 1490 | 26 | 1.181702 | -0.745126 | 428 | 266.4 | 0.622430 |  |
| GO:0043283\_biopolymer\_metabolic\_process | SOX5 | 1490 | 26 | 1.181702 | -0.745126 | 428 | 266.4 | 0.622430 |  |
| GO:0043283\_biopolymer\_metabolic\_process | HSPA1A | 1490 | 26 | 1.181702 | -0.745126 | 428 | 266.4 | 0.622430 |  |
| GO:0043283\_biopolymer\_metabolic\_process | ABCA1 | 1490 | 26 | 1.181702 | -0.745126 | 428 | 266.4 | 0.622430 |  |
| GO:0043283\_biopolymer\_metabolic\_process | NR3C1 | 1490 | 26 | 1.181702 | -0.745126 | 428 | 266.4 | 0.622430 |  |
| GO:0043283\_biopolymer\_metabolic\_process | NR2E1 | 1490 | 26 | 1.181702 | -0.745126 | 428 | 266.4 | 0.622430 |  |
| GO:0043283\_biopolymer\_metabolic\_process | TCF7L2 | 1490 | 26 | 1.181702 | -0.745126 | 428 | 266.4 | 0.622430 |  |
| GO:0043283\_biopolymer\_metabolic\_process | TCERG1 | 1490 | 26 | 1.181702 | -0.745126 | 428 | 266.4 | 0.622430 |  |
| GO:0043283\_biopolymer\_metabolic\_process | DMD | 1490 | 26 | 1.181702 | -0.745126 | 428 | 266.4 | 0.622430 |  |
| GO:0043283\_biopolymer\_metabolic\_process | ETV1 | 1490 | 26 | 1.181702 | -0.745126 | 428 | 266.4 | 0.622430 |  |
| GO:0043283\_biopolymer\_metabolic\_process | B3GNT2 | 1490 | 26 | 1.181702 | -0.745126 | 428 | 266.4 | 0.622430 |  |
| GO:0043283\_biopolymer\_metabolic\_process | PPAP2A | 1490 | 26 | 1.181702 | -0.745126 | 428 | 266.4 | 0.622430 |  |
| GO:0043283\_biopolymer\_metabolic\_process | PTPRC | 1490 | 26 | 1.181702 | -0.745126 | 428 | 266.4 | 0.622430 |  |
| GO:0043283\_biopolymer\_metabolic\_process | TBX3 | 1490 | 26 | 1.181702 | -0.745126 | 428 | 266.4 | 0.622430 |  |
| GO:0043283\_biopolymer\_metabolic\_process | ESRRG | 1490 | 26 | 1.181702 | -0.745126 | 428 | 266.4 | 0.622430 |  |
| GO:0043283\_biopolymer\_metabolic\_process | TRIM63 | 1490 | 26 | 1.181702 | -0.745126 | 428 | 266.4 | 0.622430 |  |
| GO:0043283\_biopolymer\_metabolic\_process | NKX6-1 | 1490 | 26 | 1.181702 | -0.745126 | 428 | 266.4 | 0.622430 |  |
| GO:0043283\_biopolymer\_metabolic\_process | ASCL1 | 1490 | 26 | 1.181702 | -0.745126 | 428 | 266.4 | 0.622430 |  |
| GO:0043283\_biopolymer\_metabolic\_process | MSX1 | 1490 | 26 | 1.181702 | -0.745126 | 428 | 266.4 | 0.622430 |  |
| GO:0043283\_biopolymer\_metabolic\_process | NEUROD1 | 1490 | 26 | 1.181702 | -0.745126 | 428 | 266.4 | 0.622430 |  |
| GO:0043283\_biopolymer\_metabolic\_process | JAK2 | 1490 | 26 | 1.181702 | -0.745126 | 428 | 266.4 | 0.622430 |  |
| GO:0043283\_biopolymer\_metabolic\_process | HDAC9 | 1490 | 26 | 1.181702 | -0.745126 | 428 | 266.4 | 0.622430 |  |
| GO:0043283\_biopolymer\_metabolic\_process | GADD45B | 1490 | 26 | 1.181702 | -0.745126 | 428 | 266.4 | 0.622430 |  |
| GO:0001525\_angiogenesis | FGF18 | 100 | 3 | 2.031618 | -0.737926 | 429 | 266.8 | 0.621911 |  |
| GO:0001525\_angiogenesis | TBX20 | 100 | 3 | 2.031618 | -0.737926 | 429 | 266.8 | 0.621911 |  |
| GO:0001525\_angiogenesis | THBS1 | 100 | 3 | 2.031618 | -0.737926 | 429 | 266.8 | 0.621911 |  |
| GO:0050789\_regulation\_of\_biological\_process | FGF18 | 2357 | 39 | 1.120536 | -0.736781 | 430 | 266.87 | 0.620628 |  |
| GO:0050789\_regulation\_of\_biological\_process | ELF1 | 2357 | 39 | 1.120536 | -0.736781 | 430 | 266.87 | 0.620628 |  |
| GO:0050789\_regulation\_of\_biological\_process | IRX5 | 2357 | 39 | 1.120536 | -0.736781 | 430 | 266.87 | 0.620628 |  |
| GO:0050789\_regulation\_of\_biological\_process | PLXNA2 | 2357 | 39 | 1.120536 | -0.736781 | 430 | 266.87 | 0.620628 |  |
| GO:0050789\_regulation\_of\_biological\_process | TBX20 | 2357 | 39 | 1.120536 | -0.736781 | 430 | 266.87 | 0.620628 |  |
| GO:0050789\_regulation\_of\_biological\_process | HEXB | 2357 | 39 | 1.120536 | -0.736781 | 430 | 266.87 | 0.620628 |  |
| GO:0050789\_regulation\_of\_biological\_process | SOX5 | 2357 | 39 | 1.120536 | -0.736781 | 430 | 266.87 | 0.620628 |  |
| GO:0050789\_regulation\_of\_biological\_process | SGMS1 | 2357 | 39 | 1.120536 | -0.736781 | 430 | 266.87 | 0.620628 |  |
| GO:0050789\_regulation\_of\_biological\_process | NR3C1 | 2357 | 39 | 1.120536 | -0.736781 | 430 | 266.87 | 0.620628 |  |
| GO:0050789\_regulation\_of\_biological\_process | KCNA5 | 2357 | 39 | 1.120536 | -0.736781 | 430 | 266.87 | 0.620628 |  |
| GO:0050789\_regulation\_of\_biological\_process | TCF7L2 | 2357 | 39 | 1.120536 | -0.736781 | 430 | 266.87 | 0.620628 |  |
| GO:0050789\_regulation\_of\_biological\_process | NR2E1 | 2357 | 39 | 1.120536 | -0.736781 | 430 | 266.87 | 0.620628 |  |
| GO:0050789\_regulation\_of\_biological\_process | SERINC3 | 2357 | 39 | 1.120536 | -0.736781 | 430 | 266.87 | 0.620628 |  |
| GO:0050789\_regulation\_of\_biological\_process | DMD | 2357 | 39 | 1.120536 | -0.736781 | 430 | 266.87 | 0.620628 |  |
| GO:0050789\_regulation\_of\_biological\_process | ETV1 | 2357 | 39 | 1.120536 | -0.736781 | 430 | 266.87 | 0.620628 |  |
| GO:0050789\_regulation\_of\_biological\_process | MBNL3 | 2357 | 39 | 1.120536 | -0.736781 | 430 | 266.87 | 0.620628 |  |
| GO:0050789\_regulation\_of\_biological\_process | PPAP2A | 2357 | 39 | 1.120536 | -0.736781 | 430 | 266.87 | 0.620628 |  |
| GO:0050789\_regulation\_of\_biological\_process | THBS1 | 2357 | 39 | 1.120536 | -0.736781 | 430 | 266.87 | 0.620628 |  |
| GO:0050789\_regulation\_of\_biological\_process | BOC | 2357 | 39 | 1.120536 | -0.736781 | 430 | 266.87 | 0.620628 |  |
| GO:0050789\_regulation\_of\_biological\_process | CD27 | 2357 | 39 | 1.120536 | -0.736781 | 430 | 266.87 | 0.620628 |  |
| GO:0050789\_regulation\_of\_biological\_process | RAB27A | 2357 | 39 | 1.120536 | -0.736781 | 430 | 266.87 | 0.620628 |  |
| GO:0050789\_regulation\_of\_biological\_process | PTPRC | 2357 | 39 | 1.120536 | -0.736781 | 430 | 266.87 | 0.620628 |  |
| GO:0050789\_regulation\_of\_biological\_process | MYO6 | 2357 | 39 | 1.120536 | -0.736781 | 430 | 266.87 | 0.620628 |  |
| GO:0050789\_regulation\_of\_biological\_process | TBX3 | 2357 | 39 | 1.120536 | -0.736781 | 430 | 266.87 | 0.620628 |  |
| GO:0050789\_regulation\_of\_biological\_process | ARID5B | 2357 | 39 | 1.120536 | -0.736781 | 430 | 266.87 | 0.620628 |  |
| GO:0050789\_regulation\_of\_biological\_process | ATRN | 2357 | 39 | 1.120536 | -0.736781 | 430 | 266.87 | 0.620628 |  |
| GO:0050789\_regulation\_of\_biological\_process | ESRRG | 2357 | 39 | 1.120536 | -0.736781 | 430 | 266.87 | 0.620628 |  |
| GO:0050789\_regulation\_of\_biological\_process | ATP1A2 | 2357 | 39 | 1.120536 | -0.736781 | 430 | 266.87 | 0.620628 |  |
| GO:0050789\_regulation\_of\_biological\_process | NKX6-1 | 2357 | 39 | 1.120536 | -0.736781 | 430 | 266.87 | 0.620628 |  |
| GO:0050789\_regulation\_of\_biological\_process | RGS13 | 2357 | 39 | 1.120536 | -0.736781 | 430 | 266.87 | 0.620628 |  |
| GO:0050789\_regulation\_of\_biological\_process | ASCL1 | 2357 | 39 | 1.120536 | -0.736781 | 430 | 266.87 | 0.620628 |  |
| GO:0050789\_regulation\_of\_biological\_process | ATXN2 | 2357 | 39 | 1.120536 | -0.736781 | 430 | 266.87 | 0.620628 |  |
| GO:0050789\_regulation\_of\_biological\_process | MSX1 | 2357 | 39 | 1.120536 | -0.736781 | 430 | 266.87 | 0.620628 |  |
| GO:0050789\_regulation\_of\_biological\_process | NEUROD1 | 2357 | 39 | 1.120536 | -0.736781 | 430 | 266.87 | 0.620628 |  |
| GO:0050789\_regulation\_of\_biological\_process | RGS8 | 2357 | 39 | 1.120536 | -0.736781 | 430 | 266.87 | 0.620628 |  |
| GO:0050789\_regulation\_of\_biological\_process | JAK2 | 2357 | 39 | 1.120536 | -0.736781 | 430 | 266.87 | 0.620628 |  |
| GO:0050789\_regulation\_of\_biological\_process | HDAC9 | 2357 | 39 | 1.120536 | -0.736781 | 430 | 266.87 | 0.620628 |  |
| GO:0050789\_regulation\_of\_biological\_process | GADD45B | 2357 | 39 | 1.120536 | -0.736781 | 430 | 266.87 | 0.620628 |  |
| GO:0050789\_regulation\_of\_biological\_process | CAMK1D | 2357 | 39 | 1.120536 | -0.736781 | 430 | 266.87 | 0.620628 |  |
| GO:0000060\_protein\_import\_into\_nucleus\_\_translocation | JAK2 | 14 | 1 | 4.837185 | -0.725251 | 442 | 280.03 | 0.633552 |  |
| GO:0002027\_regulation\_of\_heart\_rate | IRX5 | 14 | 1 | 4.837185 | -0.725251 | 442 | 280.03 | 0.633552 |  |
| GO:0002698\_negative\_regulation\_of\_immune\_effector\_process | PTPRC | 14 | 1 | 4.837185 | -0.725251 | 442 | 280.03 | 0.633552 |  |
| GO:0007157\_heterophilic\_cell\_adhesion | ITGA4 | 14 | 1 | 4.837185 | -0.725251 | 442 | 280.03 | 0.633552 |  |
| GO:0021782\_glial\_cell\_development | ASCL1 | 14 | 1 | 4.837185 | -0.725251 | 442 | 280.03 | 0.633552 |  |
| GO:0030032\_lamellipodium\_assembly | FGD4 | 14 | 1 | 4.837185 | -0.725251 | 442 | 280.03 | 0.633552 |  |
| GO:0030148\_sphingolipid\_biosynthetic\_process | SGMS1 | 14 | 1 | 4.837185 | -0.725251 | 442 | 280.03 | 0.633552 |  |
| GO:0042310\_vasoconstriction | ATP1A2 | 14 | 1 | 4.837185 | -0.725251 | 442 | 280.03 | 0.633552 |  |
| GO:0045061\_thymic\_T\_cell\_selection | PTPRC | 14 | 1 | 4.837185 | -0.725251 | 442 | 280.03 | 0.633552 |  |
| GO:0050810\_regulation\_of\_steroid\_biosynthetic\_process | NR3C1 | 14 | 1 | 4.837185 | -0.725251 | 442 | 280.03 | 0.633552 |  |
| GO:0051054\_positive\_regulation\_of\_DNA\_metabolic\_process | PTPRC | 14 | 1 | 4.837185 | -0.725251 | 442 | 280.03 | 0.633552 |  |
| GO:0051100\_negative\_regulation\_of\_binding | JAK2 | 14 | 1 | 4.837185 | -0.725251 | 442 | 280.03 | 0.633552 |  |
| GO:0002253\_activation\_of\_immune\_response | PTPRC | 54 | 2 | 2.508170 | -0.722963 | 444 | 281.7 | 0.634459 |  |
| GO:0002253\_activation\_of\_immune\_response | ELF1 | 54 | 2 | 2.508170 | -0.722963 | 444 | 281.7 | 0.634459 |  |
| GO:0009566\_fertilization | ZFX | 54 | 2 | 2.508170 | -0.722963 | 444 | 281.7 | 0.634459 |  |
| GO:0009566\_fertilization | HEXB | 54 | 2 | 2.508170 | -0.722963 | 444 | 281.7 | 0.634459 |  |
| GO:0001944\_vasculature\_development | FGF18 | 208 | 5 | 1.627899 | -0.717030 | 445 | 282.38 | 0.634562 |  |
| GO:0001944\_vasculature\_development | TBX3 | 208 | 5 | 1.627899 | -0.717030 | 445 | 282.38 | 0.634562 |  |
| GO:0001944\_vasculature\_development | TBX20 | 208 | 5 | 1.627899 | -0.717030 | 445 | 282.38 | 0.634562 |  |
| GO:0001944\_vasculature\_development | ITGA4 | 208 | 5 | 1.627899 | -0.717030 | 445 | 282.38 | 0.634562 |  |
| GO:0001944\_vasculature\_development | THBS1 | 208 | 5 | 1.627899 | -0.717030 | 445 | 282.38 | 0.634562 |  |
| GO:0003013\_circulatory\_system\_process | IRX5 | 103 | 3 | 1.972444 | -0.712012 | 448 | 283.11 | 0.631942 |  |
| GO:0003013\_circulatory\_system\_process | TBX20 | 103 | 3 | 1.972444 | -0.712012 | 448 | 283.11 | 0.631942 |  |
| GO:0003013\_circulatory\_system\_process | ATP1A2 | 103 | 3 | 1.972444 | -0.712012 | 448 | 283.11 | 0.631942 |  |
| GO:0008015\_blood\_circulation | IRX5 | 103 | 3 | 1.972444 | -0.712012 | 448 | 283.11 | 0.631942 |  |
| GO:0008015\_blood\_circulation | TBX20 | 103 | 3 | 1.972444 | -0.712012 | 448 | 283.11 | 0.631942 |  |
| GO:0008015\_blood\_circulation | ATP1A2 | 103 | 3 | 1.972444 | -0.712012 | 448 | 283.11 | 0.631942 |  |
| GO:0009968\_negative\_regulation\_of\_signal\_transduction | PTPRC | 103 | 3 | 1.972444 | -0.712012 | 448 | 283.11 | 0.631942 |  |
| GO:0009968\_negative\_regulation\_of\_signal\_transduction | ELF1 | 103 | 3 | 1.972444 | -0.712012 | 448 | 283.11 | 0.631942 |  |
| GO:0009968\_negative\_regulation\_of\_signal\_transduction | RGS13 | 103 | 3 | 1.972444 | -0.712012 | 448 | 283.11 | 0.631942 |  |
| GO:0007605\_sensory\_perception\_of\_sound | MYO6 | 55 | 2 | 2.462567 | -0.710803 | 449 | 284.09 | 0.632717 |  |
| GO:0007605\_sensory\_perception\_of\_sound | HEXB | 55 | 2 | 2.462567 | -0.710803 | 449 | 284.09 | 0.632717 |  |
| GO:0034961\_cellular\_biopolymer\_biosynthetic\_process | TBX3 | 804 | 15 | 1.263444 | -0.707728 | 450 | 284.45 | 0.632111 |  |
| GO:0034961\_cellular\_biopolymer\_biosynthetic\_process | TBX20 | 804 | 15 | 1.263444 | -0.707728 | 450 | 284.45 | 0.632111 |  |
| GO:0034961\_cellular\_biopolymer\_biosynthetic\_process | SOX5 | 804 | 15 | 1.263444 | -0.707728 | 450 | 284.45 | 0.632111 |  |
| GO:0034961\_cellular\_biopolymer\_biosynthetic\_process | ESRRG | 804 | 15 | 1.263444 | -0.707728 | 450 | 284.45 | 0.632111 |  |
| GO:0034961\_cellular\_biopolymer\_biosynthetic\_process | NR3C1 | 804 | 15 | 1.263444 | -0.707728 | 450 | 284.45 | 0.632111 |  |
| GO:0034961\_cellular\_biopolymer\_biosynthetic\_process | NR2E1 | 804 | 15 | 1.263444 | -0.707728 | 450 | 284.45 | 0.632111 |  |
| GO:0034961\_cellular\_biopolymer\_biosynthetic\_process | TCF7L2 | 804 | 15 | 1.263444 | -0.707728 | 450 | 284.45 | 0.632111 |  |
| GO:0034961\_cellular\_biopolymer\_biosynthetic\_process | NKX6-1 | 804 | 15 | 1.263444 | -0.707728 | 450 | 284.45 | 0.632111 |  |
| GO:0034961\_cellular\_biopolymer\_biosynthetic\_process | ASCL1 | 804 | 15 | 1.263444 | -0.707728 | 450 | 284.45 | 0.632111 |  |
| GO:0034961\_cellular\_biopolymer\_biosynthetic\_process | TCERG1 | 804 | 15 | 1.263444 | -0.707728 | 450 | 284.45 | 0.632111 |  |
| GO:0034961\_cellular\_biopolymer\_biosynthetic\_process | MSX1 | 804 | 15 | 1.263444 | -0.707728 | 450 | 284.45 | 0.632111 |  |
| GO:0034961\_cellular\_biopolymer\_biosynthetic\_process | DMD | 804 | 15 | 1.263444 | -0.707728 | 450 | 284.45 | 0.632111 |  |
| GO:0034961\_cellular\_biopolymer\_biosynthetic\_process | NEUROD1 | 804 | 15 | 1.263444 | -0.707728 | 450 | 284.45 | 0.632111 |  |
| GO:0034961\_cellular\_biopolymer\_biosynthetic\_process | ETV1 | 804 | 15 | 1.263444 | -0.707728 | 450 | 284.45 | 0.632111 |  |
| GO:0034961\_cellular\_biopolymer\_biosynthetic\_process | HDAC9 | 804 | 15 | 1.263444 | -0.707728 | 450 | 284.45 | 0.632111 |  |
| GO:0001708\_cell\_fate\_specification | ASCL1 | 56 | 2 | 2.418592 | -0.698931 | 452 | 286.34 | 0.633496 |  |
| GO:0001708\_cell\_fate\_specification | NKX6-1 | 56 | 2 | 2.418592 | -0.698931 | 452 | 286.34 | 0.633496 |  |
| GO:0002683\_negative\_regulation\_of\_immune\_system\_process | PTPRC | 56 | 2 | 2.418592 | -0.698931 | 452 | 286.34 | 0.633496 |  |
| GO:0002683\_negative\_regulation\_of\_immune\_system\_process | ELF1 | 56 | 2 | 2.418592 | -0.698931 | 452 | 286.34 | 0.633496 |  |
| GO:0043284\_biopolymer\_biosynthetic\_process | TBX3 | 807 | 15 | 1.258747 | -0.698743 | 453 | 286.39 | 0.632208 |  |
| GO:0043284\_biopolymer\_biosynthetic\_process | TBX20 | 807 | 15 | 1.258747 | -0.698743 | 453 | 286.39 | 0.632208 |  |
| GO:0043284\_biopolymer\_biosynthetic\_process | SOX5 | 807 | 15 | 1.258747 | -0.698743 | 453 | 286.39 | 0.632208 |  |
| GO:0043284\_biopolymer\_biosynthetic\_process | ESRRG | 807 | 15 | 1.258747 | -0.698743 | 453 | 286.39 | 0.632208 |  |
| GO:0043284\_biopolymer\_biosynthetic\_process | NR3C1 | 807 | 15 | 1.258747 | -0.698743 | 453 | 286.39 | 0.632208 |  |
| GO:0043284\_biopolymer\_biosynthetic\_process | NR2E1 | 807 | 15 | 1.258747 | -0.698743 | 453 | 286.39 | 0.632208 |  |
| GO:0043284\_biopolymer\_biosynthetic\_process | TCF7L2 | 807 | 15 | 1.258747 | -0.698743 | 453 | 286.39 | 0.632208 |  |
| GO:0043284\_biopolymer\_biosynthetic\_process | NKX6-1 | 807 | 15 | 1.258747 | -0.698743 | 453 | 286.39 | 0.632208 |  |
| GO:0043284\_biopolymer\_biosynthetic\_process | ASCL1 | 807 | 15 | 1.258747 | -0.698743 | 453 | 286.39 | 0.632208 |  |
| GO:0043284\_biopolymer\_biosynthetic\_process | TCERG1 | 807 | 15 | 1.258747 | -0.698743 | 453 | 286.39 | 0.632208 |  |
| GO:0043284\_biopolymer\_biosynthetic\_process | MSX1 | 807 | 15 | 1.258747 | -0.698743 | 453 | 286.39 | 0.632208 |  |
| GO:0043284\_biopolymer\_biosynthetic\_process | DMD | 807 | 15 | 1.258747 | -0.698743 | 453 | 286.39 | 0.632208 |  |
| GO:0043284\_biopolymer\_biosynthetic\_process | NEUROD1 | 807 | 15 | 1.258747 | -0.698743 | 453 | 286.39 | 0.632208 |  |
| GO:0043284\_biopolymer\_biosynthetic\_process | ETV1 | 807 | 15 | 1.258747 | -0.698743 | 453 | 286.39 | 0.632208 |  |
| GO:0043284\_biopolymer\_biosynthetic\_process | HDAC9 | 807 | 15 | 1.258747 | -0.698743 | 453 | 286.39 | 0.632208 |  |
| GO:0010556\_regulation\_of\_macromolecule\_biosynthetic\_process | TBX3 | 745 | 14 | 1.272602 | -0.698646 | 454 | 286.51 | 0.631079 |  |
| GO:0010556\_regulation\_of\_macromolecule\_biosynthetic\_process | TBX20 | 745 | 14 | 1.272602 | -0.698646 | 454 | 286.51 | 0.631079 |  |
| GO:0010556\_regulation\_of\_macromolecule\_biosynthetic\_process | SOX5 | 745 | 14 | 1.272602 | -0.698646 | 454 | 286.51 | 0.631079 |  |
| GO:0010556\_regulation\_of\_macromolecule\_biosynthetic\_process | ESRRG | 745 | 14 | 1.272602 | -0.698646 | 454 | 286.51 | 0.631079 |  |
| GO:0010556\_regulation\_of\_macromolecule\_biosynthetic\_process | NR3C1 | 745 | 14 | 1.272602 | -0.698646 | 454 | 286.51 | 0.631079 |  |
| GO:0010556\_regulation\_of\_macromolecule\_biosynthetic\_process | TCF7L2 | 745 | 14 | 1.272602 | -0.698646 | 454 | 286.51 | 0.631079 |  |
| GO:0010556\_regulation\_of\_macromolecule\_biosynthetic\_process | NR2E1 | 745 | 14 | 1.272602 | -0.698646 | 454 | 286.51 | 0.631079 |  |
| GO:0010556\_regulation\_of\_macromolecule\_biosynthetic\_process | NKX6-1 | 745 | 14 | 1.272602 | -0.698646 | 454 | 286.51 | 0.631079 |  |
| GO:0010556\_regulation\_of\_macromolecule\_biosynthetic\_process | ASCL1 | 745 | 14 | 1.272602 | -0.698646 | 454 | 286.51 | 0.631079 |  |
| GO:0010556\_regulation\_of\_macromolecule\_biosynthetic\_process | MSX1 | 745 | 14 | 1.272602 | -0.698646 | 454 | 286.51 | 0.631079 |  |
| GO:0010556\_regulation\_of\_macromolecule\_biosynthetic\_process | DMD | 745 | 14 | 1.272602 | -0.698646 | 454 | 286.51 | 0.631079 |  |
| GO:0010556\_regulation\_of\_macromolecule\_biosynthetic\_process | NEUROD1 | 745 | 14 | 1.272602 | -0.698646 | 454 | 286.51 | 0.631079 |  |
| GO:0010556\_regulation\_of\_macromolecule\_biosynthetic\_process | ETV1 | 745 | 14 | 1.272602 | -0.698646 | 454 | 286.51 | 0.631079 |  |
| GO:0010556\_regulation\_of\_macromolecule\_biosynthetic\_process | HDAC9 | 745 | 14 | 1.272602 | -0.698646 | 454 | 286.51 | 0.631079 |  |
| GO:0002709\_regulation\_of\_T\_cell\_mediated\_immunity | PTPRC | 15 | 1 | 4.514706 | -0.698364 | 461 | 296.3 | 0.642733 |  |
| GO:0007040\_lysosome\_organization | HEXB | 15 | 1 | 4.514706 | -0.698364 | 461 | 296.3 | 0.642733 |  |
| GO:0007618\_mating | HEXB | 15 | 1 | 4.514706 | -0.698364 | 461 | 296.3 | 0.642733 |  |
| GO:0010171\_body\_morphogenesis | ARID5B | 15 | 1 | 4.514706 | -0.698364 | 461 | 296.3 | 0.642733 |  |
| GO:0045666\_positive\_regulation\_of\_neuron\_differentiation | NKX6-1 | 15 | 1 | 4.514706 | -0.698364 | 461 | 296.3 | 0.642733 |  |
| GO:0048008\_platelet-derived\_growth\_factor\_receptor\_signaling\_pathway | ARID5B | 15 | 1 | 4.514706 | -0.698364 | 461 | 296.3 | 0.642733 |  |
| GO:0048010\_vascular\_endothelial\_growth\_factor\_receptor\_signaling\_pathway | FGF18 | 15 | 1 | 4.514706 | -0.698364 | 461 | 296.3 | 0.642733 |  |
| GO:0051704\_multi-organism\_process | PTPRC | 157 | 4 | 1.725365 | -0.697180 | 462 | 296.42 | 0.641602 |  |
| GO:0051704\_multi-organism\_process | HEXB | 157 | 4 | 1.725365 | -0.697180 | 462 | 296.42 | 0.641602 |  |
| GO:0051704\_multi-organism\_process | VPS13A | 157 | 4 | 1.725365 | -0.697180 | 462 | 296.42 | 0.641602 |  |
| GO:0051704\_multi-organism\_process | NR2E1 | 157 | 4 | 1.725365 | -0.697180 | 462 | 296.42 | 0.641602 |  |
| GO:0043170\_macromolecule\_metabolic\_process | FUT9 | 1576 | 27 | 1.160188 | -0.695012 | 463 | 296.75 | 0.640929 |  |
| GO:0043170\_macromolecule\_metabolic\_process | IRX5 | 1576 | 27 | 1.160188 | -0.695012 | 463 | 296.75 | 0.640929 |  |
| GO:0043170\_macromolecule\_metabolic\_process | MAMDC2 | 1576 | 27 | 1.160188 | -0.695012 | 463 | 296.75 | 0.640929 |  |
| GO:0043170\_macromolecule\_metabolic\_process | TBX20 | 1576 | 27 | 1.160188 | -0.695012 | 463 | 296.75 | 0.640929 |  |
| GO:0043170\_macromolecule\_metabolic\_process | HEXB | 1576 | 27 | 1.160188 | -0.695012 | 463 | 296.75 | 0.640929 |  |
| GO:0043170\_macromolecule\_metabolic\_process | SOX5 | 1576 | 27 | 1.160188 | -0.695012 | 463 | 296.75 | 0.640929 |  |
| GO:0043170\_macromolecule\_metabolic\_process | HSPA1A | 1576 | 27 | 1.160188 | -0.695012 | 463 | 296.75 | 0.640929 |  |
| GO:0043170\_macromolecule\_metabolic\_process | NR3C1 | 1576 | 27 | 1.160188 | -0.695012 | 463 | 296.75 | 0.640929 |  |
| GO:0043170\_macromolecule\_metabolic\_process | ABCA1 | 1576 | 27 | 1.160188 | -0.695012 | 463 | 296.75 | 0.640929 |  |
| GO:0043170\_macromolecule\_metabolic\_process | NR2E1 | 1576 | 27 | 1.160188 | -0.695012 | 463 | 296.75 | 0.640929 |  |
| GO:0043170\_macromolecule\_metabolic\_process | TCF7L2 | 1576 | 27 | 1.160188 | -0.695012 | 463 | 296.75 | 0.640929 |  |
| GO:0043170\_macromolecule\_metabolic\_process | TCERG1 | 1576 | 27 | 1.160188 | -0.695012 | 463 | 296.75 | 0.640929 |  |
| GO:0043170\_macromolecule\_metabolic\_process | DMD | 1576 | 27 | 1.160188 | -0.695012 | 463 | 296.75 | 0.640929 |  |
| GO:0043170\_macromolecule\_metabolic\_process | ETV1 | 1576 | 27 | 1.160188 | -0.695012 | 463 | 296.75 | 0.640929 |  |
| GO:0043170\_macromolecule\_metabolic\_process | B3GNT2 | 1576 | 27 | 1.160188 | -0.695012 | 463 | 296.75 | 0.640929 |  |
| GO:0043170\_macromolecule\_metabolic\_process | PPAP2A | 1576 | 27 | 1.160188 | -0.695012 | 463 | 296.75 | 0.640929 |  |
| GO:0043170\_macromolecule\_metabolic\_process | PTPRC | 1576 | 27 | 1.160188 | -0.695012 | 463 | 296.75 | 0.640929 |  |
| GO:0043170\_macromolecule\_metabolic\_process | TBX3 | 1576 | 27 | 1.160188 | -0.695012 | 463 | 296.75 | 0.640929 |  |
| GO:0043170\_macromolecule\_metabolic\_process | ESRRG | 1576 | 27 | 1.160188 | -0.695012 | 463 | 296.75 | 0.640929 |  |
| GO:0043170\_macromolecule\_metabolic\_process | TRIM63 | 1576 | 27 | 1.160188 | -0.695012 | 463 | 296.75 | 0.640929 |  |
| GO:0043170\_macromolecule\_metabolic\_process | NKX6-1 | 1576 | 27 | 1.160188 | -0.695012 | 463 | 296.75 | 0.640929 |  |
| GO:0043170\_macromolecule\_metabolic\_process | ASCL1 | 1576 | 27 | 1.160188 | -0.695012 | 463 | 296.75 | 0.640929 |  |
| GO:0043170\_macromolecule\_metabolic\_process | MSX1 | 1576 | 27 | 1.160188 | -0.695012 | 463 | 296.75 | 0.640929 |  |
| GO:0043170\_macromolecule\_metabolic\_process | NEUROD1 | 1576 | 27 | 1.160188 | -0.695012 | 463 | 296.75 | 0.640929 |  |
| GO:0043170\_macromolecule\_metabolic\_process | JAK2 | 1576 | 27 | 1.160188 | -0.695012 | 463 | 296.75 | 0.640929 |  |
| GO:0043170\_macromolecule\_metabolic\_process | HDAC9 | 1576 | 27 | 1.160188 | -0.695012 | 463 | 296.75 | 0.640929 |  |
| GO:0043170\_macromolecule\_metabolic\_process | GADD45B | 1576 | 27 | 1.160188 | -0.695012 | 463 | 296.75 | 0.640929 |  |
| GO:0006066\_alcohol\_metabolic\_process | SGPP1 | 158 | 4 | 1.714445 | -0.690594 | 465 | 297.65 | 0.640108 |  |
| GO:0006066\_alcohol\_metabolic\_process | ABCA1 | 158 | 4 | 1.714445 | -0.690594 | 465 | 297.65 | 0.640108 |  |
| GO:0006066\_alcohol\_metabolic\_process | NR3C1 | 158 | 4 | 1.714445 | -0.690594 | 465 | 297.65 | 0.640108 |  |
| GO:0006066\_alcohol\_metabolic\_process | PPAP2A | 158 | 4 | 1.714445 | -0.690594 | 465 | 297.65 | 0.640108 |  |
| GO:0048514\_blood\_vessel\_morphogenesis | FGF18 | 158 | 4 | 1.714445 | -0.690594 | 465 | 297.65 | 0.640108 |  |
| GO:0048514\_blood\_vessel\_morphogenesis | TBX20 | 158 | 4 | 1.714445 | -0.690594 | 465 | 297.65 | 0.640108 |  |
| GO:0048514\_blood\_vessel\_morphogenesis | ITGA4 | 158 | 4 | 1.714445 | -0.690594 | 465 | 297.65 | 0.640108 |  |
| GO:0048514\_blood\_vessel\_morphogenesis | THBS1 | 158 | 4 | 1.714445 | -0.690594 | 465 | 297.65 | 0.640108 |  |
| GO:0060255\_regulation\_of\_macromolecule\_metabolic\_process | PTPRC | 936 | 17 | 1.229968 | -0.687908 | 466 | 297.96 | 0.639399 |  |
| GO:0060255\_regulation\_of\_macromolecule\_metabolic\_process | IRX5 | 936 | 17 | 1.229968 | -0.687908 | 466 | 297.96 | 0.639399 |  |
| GO:0060255\_regulation\_of\_macromolecule\_metabolic\_process | TBX3 | 936 | 17 | 1.229968 | -0.687908 | 466 | 297.96 | 0.639399 |  |
| GO:0060255\_regulation\_of\_macromolecule\_metabolic\_process | TBX20 | 936 | 17 | 1.229968 | -0.687908 | 466 | 297.96 | 0.639399 |  |
| GO:0060255\_regulation\_of\_macromolecule\_metabolic\_process | SOX5 | 936 | 17 | 1.229968 | -0.687908 | 466 | 297.96 | 0.639399 |  |
| GO:0060255\_regulation\_of\_macromolecule\_metabolic\_process | ESRRG | 936 | 17 | 1.229968 | -0.687908 | 466 | 297.96 | 0.639399 |  |
| GO:0060255\_regulation\_of\_macromolecule\_metabolic\_process | NR3C1 | 936 | 17 | 1.229968 | -0.687908 | 466 | 297.96 | 0.639399 |  |
| GO:0060255\_regulation\_of\_macromolecule\_metabolic\_process | TCF7L2 | 936 | 17 | 1.229968 | -0.687908 | 466 | 297.96 | 0.639399 |  |
| GO:0060255\_regulation\_of\_macromolecule\_metabolic\_process | NR2E1 | 936 | 17 | 1.229968 | -0.687908 | 466 | 297.96 | 0.639399 |  |
| GO:0060255\_regulation\_of\_macromolecule\_metabolic\_process | NKX6-1 | 936 | 17 | 1.229968 | -0.687908 | 466 | 297.96 | 0.639399 |  |
| GO:0060255\_regulation\_of\_macromolecule\_metabolic\_process | ASCL1 | 936 | 17 | 1.229968 | -0.687908 | 466 | 297.96 | 0.639399 |  |
| GO:0060255\_regulation\_of\_macromolecule\_metabolic\_process | MSX1 | 936 | 17 | 1.229968 | -0.687908 | 466 | 297.96 | 0.639399 |  |
| GO:0060255\_regulation\_of\_macromolecule\_metabolic\_process | DMD | 936 | 17 | 1.229968 | -0.687908 | 466 | 297.96 | 0.639399 |  |
| GO:0060255\_regulation\_of\_macromolecule\_metabolic\_process | NEUROD1 | 936 | 17 | 1.229968 | -0.687908 | 466 | 297.96 | 0.639399 |  |
| GO:0060255\_regulation\_of\_macromolecule\_metabolic\_process | ETV1 | 936 | 17 | 1.229968 | -0.687908 | 466 | 297.96 | 0.639399 |  |
| GO:0060255\_regulation\_of\_macromolecule\_metabolic\_process | JAK2 | 936 | 17 | 1.229968 | -0.687908 | 466 | 297.96 | 0.639399 |  |
| GO:0060255\_regulation\_of\_macromolecule\_metabolic\_process | HDAC9 | 936 | 17 | 1.229968 | -0.687908 | 466 | 297.96 | 0.639399 |  |
| GO:0001764\_neuron\_migration | DCC | 57 | 2 | 2.376161 | -0.687336 | 471 | 299.81 | 0.636539 |  |
| GO:0001764\_neuron\_migration | ASCL1 | 57 | 2 | 2.376161 | -0.687336 | 471 | 299.81 | 0.636539 |  |
| GO:0009953\_dorsal\_ventral\_pattern\_formation | TBX20 | 57 | 2 | 2.376161 | -0.687336 | 471 | 299.81 | 0.636539 |  |
| GO:0009953\_dorsal\_ventral\_pattern\_formation | NKX6-1 | 57 | 2 | 2.376161 | -0.687336 | 471 | 299.81 | 0.636539 |  |
| GO:0018108\_peptidyl-tyrosine\_phosphorylation | PTPRC | 57 | 2 | 2.376161 | -0.687336 | 471 | 299.81 | 0.636539 |  |
| GO:0018108\_peptidyl-tyrosine\_phosphorylation | JAK2 | 57 | 2 | 2.376161 | -0.687336 | 471 | 299.81 | 0.636539 |  |
| GO:0018212\_peptidyl-tyrosine\_modification | PTPRC | 57 | 2 | 2.376161 | -0.687336 | 471 | 299.81 | 0.636539 |  |
| GO:0018212\_peptidyl-tyrosine\_modification | JAK2 | 57 | 2 | 2.376161 | -0.687336 | 471 | 299.81 | 0.636539 |  |
| GO:0043523\_regulation\_of\_neuron\_apoptosis | ASCL1 | 57 | 2 | 2.376161 | -0.687336 | 471 | 299.81 | 0.636539 |  |
| GO:0043523\_regulation\_of\_neuron\_apoptosis | NR3C1 | 57 | 2 | 2.376161 | -0.687336 | 471 | 299.81 | 0.636539 |  |
| GO:0030902\_hindbrain\_development | PLXNA2 | 58 | 2 | 2.335193 | -0.676007 | 472 | 301.62 | 0.639025 |  |
| GO:0030902\_hindbrain\_development | NEUROD1 | 58 | 2 | 2.335193 | -0.676007 | 472 | 301.62 | 0.639025 |  |
| GO:0001933\_negative\_regulation\_of\_protein\_amino\_acid\_phosphorylation | PTPRC | 16 | 1 | 4.232537 | -0.673404 | 483 | 311.36 | 0.644638 |  |
| GO:0006664\_glycolipid\_metabolic\_process | HEXB | 16 | 1 | 4.232537 | -0.673404 | 483 | 311.36 | 0.644638 |  |
| GO:0007033\_vacuole\_organization | HEXB | 16 | 1 | 4.232537 | -0.673404 | 483 | 311.36 | 0.644638 |  |
| GO:0010876\_lipid\_localization | HEXB | 16 | 1 | 4.232537 | -0.673404 | 483 | 311.36 | 0.644638 |  |
| GO:0019915\_lipid\_storage | HEXB | 16 | 1 | 4.232537 | -0.673404 | 483 | 311.36 | 0.644638 |  |
| GO:0021522\_spinal\_cord\_motor\_neuron\_differentiation | NKX6-1 | 16 | 1 | 4.232537 | -0.673404 | 483 | 311.36 | 0.644638 |  |
| GO:0030890\_positive\_regulation\_of\_B\_cell\_proliferation | PTPRC | 16 | 1 | 4.232537 | -0.673404 | 483 | 311.36 | 0.644638 |  |
| GO:0046364\_monosaccharide\_biosynthetic\_process | NR3C1 | 16 | 1 | 4.232537 | -0.673404 | 483 | 311.36 | 0.644638 |  |
| GO:0046467\_membrane\_lipid\_biosynthetic\_process | SGMS1 | 16 | 1 | 4.232537 | -0.673404 | 483 | 311.36 | 0.644638 |  |
| GO:0046633\_alpha-beta\_T\_cell\_proliferation | PTPRC | 16 | 1 | 4.232537 | -0.673404 | 483 | 311.36 | 0.644638 |  |
| GO:0060713\_labyrinthine\_layer\_morphogenesis | ITGA4 | 16 | 1 | 4.232537 | -0.673404 | 483 | 311.36 | 0.644638 |  |
| GO:0040007\_growth | ATXN2 | 217 | 5 | 1.560382 | -0.666516 | 484 | 312.75 | 0.646178 |  |
| GO:0040007\_growth | ARID5B | 217 | 5 | 1.560382 | -0.666516 | 484 | 312.75 | 0.646178 |  |
| GO:0040007\_growth | ZFX | 217 | 5 | 1.560382 | -0.666516 | 484 | 312.75 | 0.646178 |  |
| GO:0040007\_growth | ATRN | 217 | 5 | 1.560382 | -0.666516 | 484 | 312.75 | 0.646178 |  |
| GO:0040007\_growth | SGMS1 | 217 | 5 | 1.560382 | -0.666516 | 484 | 312.75 | 0.646178 |  |
| GO:0033036\_macromolecule\_localization | MYO6 | 274 | 6 | 1.482933 | -0.665262 | 485 | 312.96 | 0.645278 |  |
| GO:0033036\_macromolecule\_localization | AP2A1 | 274 | 6 | 1.482933 | -0.665262 | 485 | 312.96 | 0.645278 |  |
| GO:0033036\_macromolecule\_localization | MLPH | 274 | 6 | 1.482933 | -0.665262 | 485 | 312.96 | 0.645278 |  |
| GO:0033036\_macromolecule\_localization | HEXB | 274 | 6 | 1.482933 | -0.665262 | 485 | 312.96 | 0.645278 |  |
| GO:0033036\_macromolecule\_localization | JAK2 | 274 | 6 | 1.482933 | -0.665262 | 485 | 312.96 | 0.645278 |  |
| GO:0033036\_macromolecule\_localization | RAB27A | 274 | 6 | 1.482933 | -0.665262 | 485 | 312.96 | 0.645278 |  |
| GO:0035270\_endocrine\_system\_development | NR3C1 | 59 | 2 | 2.295613 | -0.664937 | 486 | 313.63 | 0.645329 |  |
| GO:0035270\_endocrine\_system\_development | NKX6-1 | 59 | 2 | 2.295613 | -0.664937 | 486 | 313.63 | 0.645329 |  |
| GO:0009987\_cellular\_process | FGF18 | 3868 | 60 | 1.050474 | -0.662283 | 487 | 314.24 | 0.645257 |  |
| GO:0009987\_cellular\_process | ELF1 | 3868 | 60 | 1.050474 | -0.662283 | 487 | 314.24 | 0.645257 |  |
| GO:0009987\_cellular\_process | PLXNA2 | 3868 | 60 | 1.050474 | -0.662283 | 487 | 314.24 | 0.645257 |  |
| GO:0009987\_cellular\_process | SGPP1 | 3868 | 60 | 1.050474 | -0.662283 | 487 | 314.24 | 0.645257 |  |
| GO:0009987\_cellular\_process | MLPH | 3868 | 60 | 1.050474 | -0.662283 | 487 | 314.24 | 0.645257 |  |
| GO:0009987\_cellular\_process | HEXB | 3868 | 60 | 1.050474 | -0.662283 | 487 | 314.24 | 0.645257 |  |
| GO:0009987\_cellular\_process | TBX20 | 3868 | 60 | 1.050474 | -0.662283 | 487 | 314.24 | 0.645257 |  |
| GO:0009987\_cellular\_process | SGMS1 | 3868 | 60 | 1.050474 | -0.662283 | 487 | 314.24 | 0.645257 |  |
| GO:0009987\_cellular\_process | NR2E1 | 3868 | 60 | 1.050474 | -0.662283 | 487 | 314.24 | 0.645257 |  |
| GO:0009987\_cellular\_process | LATS2 | 3868 | 60 | 1.050474 | -0.662283 | 487 | 314.24 | 0.645257 |  |
| GO:0009987\_cellular\_process | AQP11 | 3868 | 60 | 1.050474 | -0.662283 | 487 | 314.24 | 0.645257 |  |
| GO:0009987\_cellular\_process | BOC | 3868 | 60 | 1.050474 | -0.662283 | 487 | 314.24 | 0.645257 |  |
| GO:0009987\_cellular\_process | RAB27A | 3868 | 60 | 1.050474 | -0.662283 | 487 | 314.24 | 0.645257 |  |
| GO:0009987\_cellular\_process | MYO6 | 3868 | 60 | 1.050474 | -0.662283 | 487 | 314.24 | 0.645257 |  |
| GO:0009987\_cellular\_process | ARID5B | 3868 | 60 | 1.050474 | -0.662283 | 487 | 314.24 | 0.645257 |  |
| GO:0009987\_cellular\_process | ZFX | 3868 | 60 | 1.050474 | -0.662283 | 487 | 314.24 | 0.645257 |  |
| GO:0009987\_cellular\_process | SLC9A3R1 | 3868 | 60 | 1.050474 | -0.662283 | 487 | 314.24 | 0.645257 |  |
| GO:0009987\_cellular\_process | ASCL1 | 3868 | 60 | 1.050474 | -0.662283 | 487 | 314.24 | 0.645257 |  |
| GO:0009987\_cellular\_process | MSX1 | 3868 | 60 | 1.050474 | -0.662283 | 487 | 314.24 | 0.645257 |  |
| GO:0009987\_cellular\_process | CLDN1 | 3868 | 60 | 1.050474 | -0.662283 | 487 | 314.24 | 0.645257 |  |
| GO:0009987\_cellular\_process | GADD45B | 3868 | 60 | 1.050474 | -0.662283 | 487 | 314.24 | 0.645257 |  |
| GO:0009987\_cellular\_process | CAMK1D | 3868 | 60 | 1.050474 | -0.662283 | 487 | 314.24 | 0.645257 |  |
| GO:0009987\_cellular\_process | DCC | 3868 | 60 | 1.050474 | -0.662283 | 487 | 314.24 | 0.645257 |  |
| GO:0009987\_cellular\_process | IRX5 | 3868 | 60 | 1.050474 | -0.662283 | 487 | 314.24 | 0.645257 |  |
| GO:0009987\_cellular\_process | FUT9 | 3868 | 60 | 1.050474 | -0.662283 | 487 | 314.24 | 0.645257 |  |
| GO:0009987\_cellular\_process | BBS7 | 3868 | 60 | 1.050474 | -0.662283 | 487 | 314.24 | 0.645257 |  |
| GO:0009987\_cellular\_process | MAMDC2 | 3868 | 60 | 1.050474 | -0.662283 | 487 | 314.24 | 0.645257 |  |
| GO:0009987\_cellular\_process | SOX5 | 3868 | 60 | 1.050474 | -0.662283 | 487 | 314.24 | 0.645257 |  |
| GO:0009987\_cellular\_process | HSPA1A | 3868 | 60 | 1.050474 | -0.662283 | 487 | 314.24 | 0.645257 |  |
| GO:0009987\_cellular\_process | ABCA1 | 3868 | 60 | 1.050474 | -0.662283 | 487 | 314.24 | 0.645257 |  |
| GO:0009987\_cellular\_process | NR3C1 | 3868 | 60 | 1.050474 | -0.662283 | 487 | 314.24 | 0.645257 |  |
| GO:0009987\_cellular\_process | KCNA5 | 3868 | 60 | 1.050474 | -0.662283 | 487 | 314.24 | 0.645257 |  |
| GO:0009987\_cellular\_process | TCF7L2 | 3868 | 60 | 1.050474 | -0.662283 | 487 | 314.24 | 0.645257 |  |
| GO:0009987\_cellular\_process | ALCAM | 3868 | 60 | 1.050474 | -0.662283 | 487 | 314.24 | 0.645257 |  |
| GO:0009987\_cellular\_process | SERINC3 | 3868 | 60 | 1.050474 | -0.662283 | 487 | 314.24 | 0.645257 |  |
| GO:0009987\_cellular\_process | TCERG1 | 3868 | 60 | 1.050474 | -0.662283 | 487 | 314.24 | 0.645257 |  |
| GO:0009987\_cellular\_process | DMD | 3868 | 60 | 1.050474 | -0.662283 | 487 | 314.24 | 0.645257 |  |
| GO:0009987\_cellular\_process | ETV1 | 3868 | 60 | 1.050474 | -0.662283 | 487 | 314.24 | 0.645257 |  |
| GO:0009987\_cellular\_process | NDRG1 | 3868 | 60 | 1.050474 | -0.662283 | 487 | 314.24 | 0.645257 |  |
| GO:0009987\_cellular\_process | MBNL3 | 3868 | 60 | 1.050474 | -0.662283 | 487 | 314.24 | 0.645257 |  |
| GO:0009987\_cellular\_process | B3GNT2 | 3868 | 60 | 1.050474 | -0.662283 | 487 | 314.24 | 0.645257 |  |
| GO:0009987\_cellular\_process | THBS1 | 3868 | 60 | 1.050474 | -0.662283 | 487 | 314.24 | 0.645257 |  |
| GO:0009987\_cellular\_process | PPAP2A | 3868 | 60 | 1.050474 | -0.662283 | 487 | 314.24 | 0.645257 |  |
| GO:0009987\_cellular\_process | CD27 | 3868 | 60 | 1.050474 | -0.662283 | 487 | 314.24 | 0.645257 |  |
| GO:0009987\_cellular\_process | FGD4 | 3868 | 60 | 1.050474 | -0.662283 | 487 | 314.24 | 0.645257 |  |
| GO:0009987\_cellular\_process | PTPRC | 3868 | 60 | 1.050474 | -0.662283 | 487 | 314.24 | 0.645257 |  |
| GO:0009987\_cellular\_process | TBX3 | 3868 | 60 | 1.050474 | -0.662283 | 487 | 314.24 | 0.645257 |  |
| GO:0009987\_cellular\_process | ESRRG | 3868 | 60 | 1.050474 | -0.662283 | 487 | 314.24 | 0.645257 |  |
| GO:0009987\_cellular\_process | ITGA4 | 3868 | 60 | 1.050474 | -0.662283 | 487 | 314.24 | 0.645257 |  |
| GO:0009987\_cellular\_process | NID2 | 3868 | 60 | 1.050474 | -0.662283 | 487 | 314.24 | 0.645257 |  |
| GO:0009987\_cellular\_process | ATP1A2 | 3868 | 60 | 1.050474 | -0.662283 | 487 | 314.24 | 0.645257 |  |
| GO:0009987\_cellular\_process | TRIM63 | 3868 | 60 | 1.050474 | -0.662283 | 487 | 314.24 | 0.645257 |  |
| GO:0009987\_cellular\_process | NKX6-1 | 3868 | 60 | 1.050474 | -0.662283 | 487 | 314.24 | 0.645257 |  |
| GO:0009987\_cellular\_process | RGS13 | 3868 | 60 | 1.050474 | -0.662283 | 487 | 314.24 | 0.645257 |  |
| GO:0009987\_cellular\_process | AFP | 3868 | 60 | 1.050474 | -0.662283 | 487 | 314.24 | 0.645257 |  |
| GO:0009987\_cellular\_process | AP2A1 | 3868 | 60 | 1.050474 | -0.662283 | 487 | 314.24 | 0.645257 |  |
| GO:0009987\_cellular\_process | NEUROD1 | 3868 | 60 | 1.050474 | -0.662283 | 487 | 314.24 | 0.645257 |  |
| GO:0009987\_cellular\_process | JAK2 | 3868 | 60 | 1.050474 | -0.662283 | 487 | 314.24 | 0.645257 |  |
| GO:0009987\_cellular\_process | RGS8 | 3868 | 60 | 1.050474 | -0.662283 | 487 | 314.24 | 0.645257 |  |
| GO:0009987\_cellular\_process | HDAC9 | 3868 | 60 | 1.050474 | -0.662283 | 487 | 314.24 | 0.645257 |  |
| GO:0045892\_negative\_regulation\_of\_transcription\_\_DNA-dependent | MSX1 | 218 | 5 | 1.553225 | -0.661134 | 488 | 314.4 | 0.644262 |  |
| GO:0045892\_negative\_regulation\_of\_transcription\_\_DNA-dependent | TBX3 | 218 | 5 | 1.553225 | -0.661134 | 488 | 314.4 | 0.644262 |  |
| GO:0045892\_negative\_regulation\_of\_transcription\_\_DNA-dependent | TBX20 | 218 | 5 | 1.553225 | -0.661134 | 488 | 314.4 | 0.644262 |  |
| GO:0045892\_negative\_regulation\_of\_transcription\_\_DNA-dependent | HDAC9 | 218 | 5 | 1.553225 | -0.661134 | 488 | 314.4 | 0.644262 |  |
| GO:0045892\_negative\_regulation\_of\_transcription\_\_DNA-dependent | NR2E1 | 218 | 5 | 1.553225 | -0.661134 | 488 | 314.4 | 0.644262 |  |
| GO:0007626\_locomotory\_behavior | MYO6 | 163 | 4 | 1.661855 | -0.658666 | 489 | 314.81 | 0.643783 |  |
| GO:0007626\_locomotory\_behavior | HEXB | 163 | 4 | 1.661855 | -0.658666 | 489 | 314.81 | 0.643783 |  |
| GO:0007626\_locomotory\_behavior | VPS13A | 163 | 4 | 1.661855 | -0.658666 | 489 | 314.81 | 0.643783 |  |
| GO:0007626\_locomotory\_behavior | ATP1A2 | 163 | 4 | 1.661855 | -0.658666 | 489 | 314.81 | 0.643783 |  |
| GO:0007423\_sensory\_organ\_development | ASCL1 | 219 | 5 | 1.546132 | -0.655796 | 490 | 315.04 | 0.642939 |  |
| GO:0007423\_sensory\_organ\_development | MYO6 | 219 | 5 | 1.546132 | -0.655796 | 490 | 315.04 | 0.642939 |  |
| GO:0007423\_sensory\_organ\_development | IRX5 | 219 | 5 | 1.546132 | -0.655796 | 490 | 315.04 | 0.642939 |  |
| GO:0007423\_sensory\_organ\_development | NEUROD1 | 219 | 5 | 1.546132 | -0.655796 | 490 | 315.04 | 0.642939 |  |
| GO:0007423\_sensory\_organ\_development | NR2E1 | 219 | 5 | 1.546132 | -0.655796 | 490 | 315.04 | 0.642939 |  |
| GO:0010648\_negative\_regulation\_of\_cell\_communication | PTPRC | 110 | 3 | 1.846925 | -0.655696 | 492 | 315.59 | 0.641443 |  |
| GO:0010648\_negative\_regulation\_of\_cell\_communication | ELF1 | 110 | 3 | 1.846925 | -0.655696 | 492 | 315.59 | 0.641443 |  |
| GO:0010648\_negative\_regulation\_of\_cell\_communication | RGS13 | 110 | 3 | 1.846925 | -0.655696 | 492 | 315.59 | 0.641443 |  |
| GO:0043010\_camera-type\_eye\_development | IRX5 | 110 | 3 | 1.846925 | -0.655696 | 492 | 315.59 | 0.641443 |  |
| GO:0043010\_camera-type\_eye\_development | NEUROD1 | 110 | 3 | 1.846925 | -0.655696 | 492 | 315.59 | 0.641443 |  |
| GO:0043010\_camera-type\_eye\_development | NR2E1 | 110 | 3 | 1.846925 | -0.655696 | 492 | 315.59 | 0.641443 |  |
| GO:0051253\_negative\_regulation\_of\_RNA\_metabolic\_process | MSX1 | 220 | 5 | 1.539104 | -0.650502 | 493 | 316.39 | 0.641765 |  |
| GO:0051253\_negative\_regulation\_of\_RNA\_metabolic\_process | TBX3 | 220 | 5 | 1.539104 | -0.650502 | 493 | 316.39 | 0.641765 |  |
| GO:0051253\_negative\_regulation\_of\_RNA\_metabolic\_process | TBX20 | 220 | 5 | 1.539104 | -0.650502 | 493 | 316.39 | 0.641765 |  |
| GO:0051253\_negative\_regulation\_of\_RNA\_metabolic\_process | HDAC9 | 220 | 5 | 1.539104 | -0.650502 | 493 | 316.39 | 0.641765 |  |
| GO:0051253\_negative\_regulation\_of\_RNA\_metabolic\_process | NR2E1 | 220 | 5 | 1.539104 | -0.650502 | 493 | 316.39 | 0.641765 |  |
| GO:0006813\_potassium\_ion\_transport | KCNA5 | 17 | 1 | 3.983564 | -0.650137 | 499 | 325.52 | 0.652345 |  |
| GO:0009408\_response\_to\_heat | HSPA1A | 17 | 1 | 3.983564 | -0.650137 | 499 | 325.52 | 0.652345 |  |
| GO:0010565\_regulation\_of\_cellular\_ketone\_metabolic\_process | NR3C1 | 17 | 1 | 3.983564 | -0.650137 | 499 | 325.52 | 0.652345 |  |
| GO:0016052\_carbohydrate\_catabolic\_process | HEXB | 17 | 1 | 3.983564 | -0.650137 | 499 | 325.52 | 0.652345 |  |
| GO:0030101\_natural\_killer\_cell\_activation | RAB27A | 17 | 1 | 3.983564 | -0.650137 | 499 | 325.52 | 0.652345 |  |
| GO:0060350\_endochondral\_bone\_morphogenesis | FGF18 | 17 | 1 | 3.983564 | -0.650137 | 499 | 325.52 | 0.652345 |  |
| GO:0031323\_regulation\_of\_cellular\_metabolic\_process | PTPRC | 1015 | 18 | 1.200956 | -0.646051 | 500 | 326.24 | 0.652480 |  |
| GO:0031323\_regulation\_of\_cellular\_metabolic\_process | TBX3 | 1015 | 18 | 1.200956 | -0.646051 | 500 | 326.24 | 0.652480 |  |
| GO:0031323\_regulation\_of\_cellular\_metabolic\_process | HEXB | 1015 | 18 | 1.200956 | -0.646051 | 500 | 326.24 | 0.652480 |  |
| GO:0031323\_regulation\_of\_cellular\_metabolic\_process | TBX20 | 1015 | 18 | 1.200956 | -0.646051 | 500 | 326.24 | 0.652480 |  |
| GO:0031323\_regulation\_of\_cellular\_metabolic\_process | SOX5 | 1015 | 18 | 1.200956 | -0.646051 | 500 | 326.24 | 0.652480 |  |
| GO:0031323\_regulation\_of\_cellular\_metabolic\_process | ESRRG | 1015 | 18 | 1.200956 | -0.646051 | 500 | 326.24 | 0.652480 |  |
| GO:0031323\_regulation\_of\_cellular\_metabolic\_process | NR3C1 | 1015 | 18 | 1.200956 | -0.646051 | 500 | 326.24 | 0.652480 |  |
| GO:0031323\_regulation\_of\_cellular\_metabolic\_process | TCF7L2 | 1015 | 18 | 1.200956 | -0.646051 | 500 | 326.24 | 0.652480 |  |
| GO:0031323\_regulation\_of\_cellular\_metabolic\_process | NR2E1 | 1015 | 18 | 1.200956 | -0.646051 | 500 | 326.24 | 0.652480 |  |
| GO:0031323\_regulation\_of\_cellular\_metabolic\_process | NKX6-1 | 1015 | 18 | 1.200956 | -0.646051 | 500 | 326.24 | 0.652480 |  |
| GO:0031323\_regulation\_of\_cellular\_metabolic\_process | ASCL1 | 1015 | 18 | 1.200956 | -0.646051 | 500 | 326.24 | 0.652480 |  |
| GO:0031323\_regulation\_of\_cellular\_metabolic\_process | MSX1 | 1015 | 18 | 1.200956 | -0.646051 | 500 | 326.24 | 0.652480 |  |
| GO:0031323\_regulation\_of\_cellular\_metabolic\_process | DMD | 1015 | 18 | 1.200956 | -0.646051 | 500 | 326.24 | 0.652480 |  |
| GO:0031323\_regulation\_of\_cellular\_metabolic\_process | NEUROD1 | 1015 | 18 | 1.200956 | -0.646051 | 500 | 326.24 | 0.652480 |  |
| GO:0031323\_regulation\_of\_cellular\_metabolic\_process | ETV1 | 1015 | 18 | 1.200956 | -0.646051 | 500 | 326.24 | 0.652480 |  |
| GO:0031323\_regulation\_of\_cellular\_metabolic\_process | JAK2 | 1015 | 18 | 1.200956 | -0.646051 | 500 | 326.24 | 0.652480 |  |
| GO:0031323\_regulation\_of\_cellular\_metabolic\_process | GADD45B | 1015 | 18 | 1.200956 | -0.646051 | 500 | 326.24 | 0.652480 |  |
| GO:0031323\_regulation\_of\_cellular\_metabolic\_process | HDAC9 | 1015 | 18 | 1.200956 | -0.646051 | 500 | 326.24 | 0.652480 |  |
| GO:0009887\_organ\_morphogenesis | FGF18 | 642 | 12 | 1.265805 | -0.634911 | 501 | 329.09 | 0.656866 |  |
| GO:0009887\_organ\_morphogenesis | IRX5 | 642 | 12 | 1.265805 | -0.634911 | 501 | 329.09 | 0.656866 |  |
| GO:0009887\_organ\_morphogenesis | MYO6 | 642 | 12 | 1.265805 | -0.634911 | 501 | 329.09 | 0.656866 |  |
| GO:0009887\_organ\_morphogenesis | MSX1 | 642 | 12 | 1.265805 | -0.634911 | 501 | 329.09 | 0.656866 |  |
| GO:0009887\_organ\_morphogenesis | TBX3 | 642 | 12 | 1.265805 | -0.634911 | 501 | 329.09 | 0.656866 |  |
| GO:0009887\_organ\_morphogenesis | ARID5B | 642 | 12 | 1.265805 | -0.634911 | 501 | 329.09 | 0.656866 |  |
| GO:0009887\_organ\_morphogenesis | TBX20 | 642 | 12 | 1.265805 | -0.634911 | 501 | 329.09 | 0.656866 |  |
| GO:0009887\_organ\_morphogenesis | PCDH8 | 642 | 12 | 1.265805 | -0.634911 | 501 | 329.09 | 0.656866 |  |
| GO:0009887\_organ\_morphogenesis | NR3C1 | 642 | 12 | 1.265805 | -0.634911 | 501 | 329.09 | 0.656866 |  |
| GO:0009887\_organ\_morphogenesis | ITGA4 | 642 | 12 | 1.265805 | -0.634911 | 501 | 329.09 | 0.656866 |  |
| GO:0009887\_organ\_morphogenesis | THBS1 | 642 | 12 | 1.265805 | -0.634911 | 501 | 329.09 | 0.656866 |  |
| GO:0009887\_organ\_morphogenesis | TCF7L2 | 642 | 12 | 1.265805 | -0.634911 | 501 | 329.09 | 0.656866 |  |
| GO:0021537\_telencephalon\_development | ASCL1 | 62 | 2 | 2.184535 | -0.633185 | 504 | 331.55 | 0.657837 |  |
| GO:0021537\_telencephalon\_development | NR2E1 | 62 | 2 | 2.184535 | -0.633185 | 504 | 331.55 | 0.657837 |  |
| GO:0040014\_regulation\_of\_multicellular\_organism\_growth | ATXN2 | 62 | 2 | 2.184535 | -0.633185 | 504 | 331.55 | 0.657837 |  |
| GO:0040014\_regulation\_of\_multicellular\_organism\_growth | ATRN | 62 | 2 | 2.184535 | -0.633185 | 504 | 331.55 | 0.657837 |  |
| GO:0050954\_sensory\_perception\_of\_mechanical\_stimulus | MYO6 | 62 | 2 | 2.184535 | -0.633185 | 504 | 331.55 | 0.657837 |  |
| GO:0050954\_sensory\_perception\_of\_mechanical\_stimulus | HEXB | 62 | 2 | 2.184535 | -0.633185 | 504 | 331.55 | 0.657837 |  |
| GO:0001974\_blood\_vessel\_remodeling | ITGA4 | 18 | 1 | 3.762255 | -0.628367 | 513 | 342.76 | 0.668148 |  |
| GO:0006940\_regulation\_of\_smooth\_muscle\_contraction | ATP1A2 | 18 | 1 | 3.762255 | -0.628367 | 513 | 342.76 | 0.668148 |  |
| GO:0007608\_sensory\_perception\_of\_smell | B3GNT2 | 18 | 1 | 3.762255 | -0.628367 | 513 | 342.76 | 0.668148 |  |
| GO:0010498\_proteasomal\_protein\_catabolic\_process | TRIM63 | 18 | 1 | 3.762255 | -0.628367 | 513 | 342.76 | 0.668148 |  |
| GO:0021517\_ventral\_spinal\_cord\_development | NKX6-1 | 18 | 1 | 3.762255 | -0.628367 | 513 | 342.76 | 0.668148 |  |
| GO:0030901\_midbrain\_development | MSX1 | 18 | 1 | 3.762255 | -0.628367 | 513 | 342.76 | 0.668148 |  |
| GO:0043161\_proteasomal\_ubiquitin-dependent\_protein\_catabolic\_process | TRIM63 | 18 | 1 | 3.762255 | -0.628367 | 513 | 342.76 | 0.668148 |  |
| GO:0045058\_T\_cell\_selection | PTPRC | 18 | 1 | 3.762255 | -0.628367 | 513 | 342.76 | 0.668148 |  |
| GO:0050731\_positive\_regulation\_of\_peptidyl-tyrosine\_phosphorylation | JAK2 | 18 | 1 | 3.762255 | -0.628367 | 513 | 342.76 | 0.668148 |  |
| GO:0000165\_MAPKKK\_cascade | PTPRC | 114 | 3 | 1.782121 | -0.625897 | 514 | 343.11 | 0.667529 |  |
| GO:0000165\_MAPKKK\_cascade | GADD45B | 114 | 3 | 1.782121 | -0.625897 | 514 | 343.11 | 0.667529 |  |
| GO:0000165\_MAPKKK\_cascade | CD27 | 114 | 3 | 1.782121 | -0.625897 | 514 | 343.11 | 0.667529 |  |
| GO:0000902\_cell\_morphogenesis | DCC | 283 | 6 | 1.435772 | -0.623484 | 515 | 343.29 | 0.666583 |  |
| GO:0000902\_cell\_morphogenesis | ALCAM | 283 | 6 | 1.435772 | -0.623484 | 515 | 343.29 | 0.666583 |  |
| GO:0000902\_cell\_morphogenesis | ETV1 | 283 | 6 | 1.435772 | -0.623484 | 515 | 343.29 | 0.666583 |  |
| GO:0000902\_cell\_morphogenesis | B3GNT2 | 283 | 6 | 1.435772 | -0.623484 | 515 | 343.29 | 0.666583 |  |
| GO:0000902\_cell\_morphogenesis | NR2E1 | 283 | 6 | 1.435772 | -0.623484 | 515 | 343.29 | 0.666583 |  |
| GO:0000902\_cell\_morphogenesis | BOC | 283 | 6 | 1.435772 | -0.623484 | 515 | 343.29 | 0.666583 |  |
| GO:0019222\_regulation\_of\_metabolic\_process | PTPRC | 1088 | 19 | 1.182621 | -0.623217 | 516 | 343.39 | 0.665484 |  |
| GO:0019222\_regulation\_of\_metabolic\_process | IRX5 | 1088 | 19 | 1.182621 | -0.623217 | 516 | 343.39 | 0.665484 |  |
| GO:0019222\_regulation\_of\_metabolic\_process | TBX3 | 1088 | 19 | 1.182621 | -0.623217 | 516 | 343.39 | 0.665484 |  |
| GO:0019222\_regulation\_of\_metabolic\_process | HEXB | 1088 | 19 | 1.182621 | -0.623217 | 516 | 343.39 | 0.665484 |  |
| GO:0019222\_regulation\_of\_metabolic\_process | TBX20 | 1088 | 19 | 1.182621 | -0.623217 | 516 | 343.39 | 0.665484 |  |
| GO:0019222\_regulation\_of\_metabolic\_process | SOX5 | 1088 | 19 | 1.182621 | -0.623217 | 516 | 343.39 | 0.665484 |  |
| GO:0019222\_regulation\_of\_metabolic\_process | ESRRG | 1088 | 19 | 1.182621 | -0.623217 | 516 | 343.39 | 0.665484 |  |
| GO:0019222\_regulation\_of\_metabolic\_process | NR3C1 | 1088 | 19 | 1.182621 | -0.623217 | 516 | 343.39 | 0.665484 |  |
| GO:0019222\_regulation\_of\_metabolic\_process | NR2E1 | 1088 | 19 | 1.182621 | -0.623217 | 516 | 343.39 | 0.665484 |  |
| GO:0019222\_regulation\_of\_metabolic\_process | TCF7L2 | 1088 | 19 | 1.182621 | -0.623217 | 516 | 343.39 | 0.665484 |  |
| GO:0019222\_regulation\_of\_metabolic\_process | NKX6-1 | 1088 | 19 | 1.182621 | -0.623217 | 516 | 343.39 | 0.665484 |  |
| GO:0019222\_regulation\_of\_metabolic\_process | ASCL1 | 1088 | 19 | 1.182621 | -0.623217 | 516 | 343.39 | 0.665484 |  |
| GO:0019222\_regulation\_of\_metabolic\_process | MSX1 | 1088 | 19 | 1.182621 | -0.623217 | 516 | 343.39 | 0.665484 |  |
| GO:0019222\_regulation\_of\_metabolic\_process | DMD | 1088 | 19 | 1.182621 | -0.623217 | 516 | 343.39 | 0.665484 |  |
| GO:0019222\_regulation\_of\_metabolic\_process | NEUROD1 | 1088 | 19 | 1.182621 | -0.623217 | 516 | 343.39 | 0.665484 |  |
| GO:0019222\_regulation\_of\_metabolic\_process | ETV1 | 1088 | 19 | 1.182621 | -0.623217 | 516 | 343.39 | 0.665484 |  |
| GO:0019222\_regulation\_of\_metabolic\_process | JAK2 | 1088 | 19 | 1.182621 | -0.623217 | 516 | 343.39 | 0.665484 |  |
| GO:0019222\_regulation\_of\_metabolic\_process | GADD45B | 1088 | 19 | 1.182621 | -0.623217 | 516 | 343.39 | 0.665484 |  |
| GO:0019222\_regulation\_of\_metabolic\_process | HDAC9 | 1088 | 19 | 1.182621 | -0.623217 | 516 | 343.39 | 0.665484 |  |
| GO:0051216\_cartilage\_development | FGF18 | 63 | 2 | 2.149860 | -0.623060 | 517 | 344.48 | 0.666306 |  |
| GO:0051216\_cartilage\_development | SOX5 | 63 | 2 | 2.149860 | -0.623060 | 517 | 344.48 | 0.666306 |  |
| GO:0048523\_negative\_regulation\_of\_cellular\_process | PTPRC | 774 | 14 | 1.224920 | -0.613756 | 518 | 345.94 | 0.667838 |  |
| GO:0048523\_negative\_regulation\_of\_cellular\_process | ELF1 | 774 | 14 | 1.224920 | -0.613756 | 518 | 345.94 | 0.667838 |  |
| GO:0048523\_negative\_regulation\_of\_cellular\_process | TBX3 | 774 | 14 | 1.224920 | -0.613756 | 518 | 345.94 | 0.667838 |  |
| GO:0048523\_negative\_regulation\_of\_cellular\_process | TBX20 | 774 | 14 | 1.224920 | -0.613756 | 518 | 345.94 | 0.667838 |  |
| GO:0048523\_negative\_regulation\_of\_cellular\_process | SGMS1 | 774 | 14 | 1.224920 | -0.613756 | 518 | 345.94 | 0.667838 |  |
| GO:0048523\_negative\_regulation\_of\_cellular\_process | NR2E1 | 774 | 14 | 1.224920 | -0.613756 | 518 | 345.94 | 0.667838 |  |
| GO:0048523\_negative\_regulation\_of\_cellular\_process | TCF7L2 | 774 | 14 | 1.224920 | -0.613756 | 518 | 345.94 | 0.667838 |  |
| GO:0048523\_negative\_regulation\_of\_cellular\_process | NKX6-1 | 774 | 14 | 1.224920 | -0.613756 | 518 | 345.94 | 0.667838 |  |
| GO:0048523\_negative\_regulation\_of\_cellular\_process | RGS13 | 774 | 14 | 1.224920 | -0.613756 | 518 | 345.94 | 0.667838 |  |
| GO:0048523\_negative\_regulation\_of\_cellular\_process | MSX1 | 774 | 14 | 1.224920 | -0.613756 | 518 | 345.94 | 0.667838 |  |
| GO:0048523\_negative\_regulation\_of\_cellular\_process | JAK2 | 774 | 14 | 1.224920 | -0.613756 | 518 | 345.94 | 0.667838 |  |
| GO:0048523\_negative\_regulation\_of\_cellular\_process | MBNL3 | 774 | 14 | 1.224920 | -0.613756 | 518 | 345.94 | 0.667838 |  |
| GO:0048523\_negative\_regulation\_of\_cellular\_process | HDAC9 | 774 | 14 | 1.224920 | -0.613756 | 518 | 345.94 | 0.667838 |  |
| GO:0048523\_negative\_regulation\_of\_cellular\_process | CD27 | 774 | 14 | 1.224920 | -0.613756 | 518 | 345.94 | 0.667838 |  |
| GO:0006497\_protein\_amino\_acid\_lipidation | ABCA1 | 19 | 1 | 3.564241 | -0.607933 | 527 | 355.62 | 0.674801 |  |
| GO:0007569\_cell\_aging | TBX3 | 19 | 1 | 3.564241 | -0.607933 | 527 | 355.62 | 0.674801 |  |
| GO:0019218\_regulation\_of\_steroid\_metabolic\_process | NR3C1 | 19 | 1 | 3.564241 | -0.607933 | 527 | 355.62 | 0.674801 |  |
| GO:0030518\_steroid\_hormone\_receptor\_signaling\_pathway | NR3C1 | 19 | 1 | 3.564241 | -0.607933 | 527 | 355.62 | 0.674801 |  |
| GO:0042491\_auditory\_receptor\_cell\_differentiation | MYO6 | 19 | 1 | 3.564241 | -0.607933 | 527 | 355.62 | 0.674801 |  |
| GO:0046165\_alcohol\_biosynthetic\_process | NR3C1 | 19 | 1 | 3.564241 | -0.607933 | 527 | 355.62 | 0.674801 |  |
| GO:0046890\_regulation\_of\_lipid\_biosynthetic\_process | NR3C1 | 19 | 1 | 3.564241 | -0.607933 | 527 | 355.62 | 0.674801 |  |
| GO:0048547\_gut\_morphogenesis | TCF7L2 | 19 | 1 | 3.564241 | -0.607933 | 527 | 355.62 | 0.674801 |  |
| GO:0060444\_branching\_involved\_in\_mammary\_gland\_duct\_morphogenesis | TBX3 | 19 | 1 | 3.564241 | -0.607933 | 527 | 355.62 | 0.674801 |  |
| GO:0007600\_sensory\_perception | MYO6 | 172 | 4 | 1.574897 | -0.605155 | 528 | 356.2 | 0.674621 |  |
| GO:0007600\_sensory\_perception | HEXB | 172 | 4 | 1.574897 | -0.605155 | 528 | 356.2 | 0.674621 |  |
| GO:0007600\_sensory\_perception | B3GNT2 | 172 | 4 | 1.574897 | -0.605155 | 528 | 356.2 | 0.674621 |  |
| GO:0007600\_sensory\_perception | NR2E1 | 172 | 4 | 1.574897 | -0.605155 | 528 | 356.2 | 0.674621 |  |
| GO:0043086\_negative\_regulation\_of\_catalytic\_activity | PTPRC | 65 | 2 | 2.083710 | -0.603457 | 530 | 357.26 | 0.674075 |  |
| GO:0043086\_negative\_regulation\_of\_catalytic\_activity | GADD45B | 65 | 2 | 2.083710 | -0.603457 | 530 | 357.26 | 0.674075 |  |
| GO:0048511\_rhythmic\_process | AFP | 65 | 2 | 2.083710 | -0.603457 | 530 | 357.26 | 0.674075 |  |
| GO:0048511\_rhythmic\_process | ZFX | 65 | 2 | 2.083710 | -0.603457 | 530 | 357.26 | 0.674075 |  |
| GO:0010467\_gene\_expression | IRX5 | 905 | 16 | 1.197270 | -0.601683 | 531 | 357.47 | 0.673202 |  |
| GO:0010467\_gene\_expression | TBX3 | 905 | 16 | 1.197270 | -0.601683 | 531 | 357.47 | 0.673202 |  |
| GO:0010467\_gene\_expression | TBX20 | 905 | 16 | 1.197270 | -0.601683 | 531 | 357.47 | 0.673202 |  |
| GO:0010467\_gene\_expression | SOX5 | 905 | 16 | 1.197270 | -0.601683 | 531 | 357.47 | 0.673202 |  |
| GO:0010467\_gene\_expression | ESRRG | 905 | 16 | 1.197270 | -0.601683 | 531 | 357.47 | 0.673202 |  |
| GO:0010467\_gene\_expression | NR3C1 | 905 | 16 | 1.197270 | -0.601683 | 531 | 357.47 | 0.673202 |  |
| GO:0010467\_gene\_expression | NR2E1 | 905 | 16 | 1.197270 | -0.601683 | 531 | 357.47 | 0.673202 |  |
| GO:0010467\_gene\_expression | TCF7L2 | 905 | 16 | 1.197270 | -0.601683 | 531 | 357.47 | 0.673202 |  |
| GO:0010467\_gene\_expression | NKX6-1 | 905 | 16 | 1.197270 | -0.601683 | 531 | 357.47 | 0.673202 |  |
| GO:0010467\_gene\_expression | ASCL1 | 905 | 16 | 1.197270 | -0.601683 | 531 | 357.47 | 0.673202 |  |
| GO:0010467\_gene\_expression | TCERG1 | 905 | 16 | 1.197270 | -0.601683 | 531 | 357.47 | 0.673202 |  |
| GO:0010467\_gene\_expression | MSX1 | 905 | 16 | 1.197270 | -0.601683 | 531 | 357.47 | 0.673202 |  |
| GO:0010467\_gene\_expression | DMD | 905 | 16 | 1.197270 | -0.601683 | 531 | 357.47 | 0.673202 |  |
| GO:0010467\_gene\_expression | NEUROD1 | 905 | 16 | 1.197270 | -0.601683 | 531 | 357.47 | 0.673202 |  |
| GO:0010467\_gene\_expression | ETV1 | 905 | 16 | 1.197270 | -0.601683 | 531 | 357.47 | 0.673202 |  |
| GO:0010467\_gene\_expression | HDAC9 | 905 | 16 | 1.197270 | -0.601683 | 531 | 357.47 | 0.673202 |  |
| GO:0031589\_cell-substrate\_adhesion | NID2 | 66 | 2 | 2.052139 | -0.593963 | 534 | 360.2 | 0.674532 |  |
| GO:0031589\_cell-substrate\_adhesion | THBS1 | 66 | 2 | 2.052139 | -0.593963 | 534 | 360.2 | 0.674532 |  |
| GO:0045860\_positive\_regulation\_of\_protein\_kinase\_activity | PTPRC | 66 | 2 | 2.052139 | -0.593963 | 534 | 360.2 | 0.674532 |  |
| GO:0045860\_positive\_regulation\_of\_protein\_kinase\_activity | GADD45B | 66 | 2 | 2.052139 | -0.593963 | 534 | 360.2 | 0.674532 |  |
| GO:0051402\_neuron\_apoptosis | ASCL1 | 66 | 2 | 2.052139 | -0.593963 | 534 | 360.2 | 0.674532 |  |
| GO:0051402\_neuron\_apoptosis | NR3C1 | 66 | 2 | 2.052139 | -0.593963 | 534 | 360.2 | 0.674532 |  |
| GO:0001912\_positive\_regulation\_of\_leukocyte\_mediated\_cytotoxicity | PTPRC | 20 | 1 | 3.386029 | -0.588696 | 542 | 367.91 | 0.678801 |  |
| GO:0007416\_synaptogenesis | MYO6 | 20 | 1 | 3.386029 | -0.588696 | 542 | 367.91 | 0.678801 |  |
| GO:0009615\_response\_to\_virus | PTPRC | 20 | 1 | 3.386029 | -0.588696 | 542 | 367.91 | 0.678801 |  |
| GO:0018209\_peptidyl-serine\_modification | MAMDC2 | 20 | 1 | 3.386029 | -0.588696 | 542 | 367.91 | 0.678801 |  |
| GO:0031343\_positive\_regulation\_of\_cell\_killing | PTPRC | 20 | 1 | 3.386029 | -0.588696 | 542 | 367.91 | 0.678801 |  |
| GO:0042326\_negative\_regulation\_of\_phosphorylation | PTPRC | 20 | 1 | 3.386029 | -0.588696 | 542 | 367.91 | 0.678801 |  |
| GO:0045017\_glycerolipid\_biosynthetic\_process | PPAP2A | 20 | 1 | 3.386029 | -0.588696 | 542 | 367.91 | 0.678801 |  |
| GO:0048806\_genitalia\_development | TCF7L2 | 20 | 1 | 3.386029 | -0.588696 | 542 | 367.91 | 0.678801 |  |
| GO:0016070\_RNA\_metabolic\_process | ASCL1 | 658 | 12 | 1.235026 | -0.587166 | 543 | 368.45 | 0.678545 |  |
| GO:0016070\_RNA\_metabolic\_process | MSX1 | 658 | 12 | 1.235026 | -0.587166 | 543 | 368.45 | 0.678545 |  |
| GO:0016070\_RNA\_metabolic\_process | TBX3 | 658 | 12 | 1.235026 | -0.587166 | 543 | 368.45 | 0.678545 |  |
| GO:0016070\_RNA\_metabolic\_process | TBX20 | 658 | 12 | 1.235026 | -0.587166 | 543 | 368.45 | 0.678545 |  |
| GO:0016070\_RNA\_metabolic\_process | ESRRG | 658 | 12 | 1.235026 | -0.587166 | 543 | 368.45 | 0.678545 |  |
| GO:0016070\_RNA\_metabolic\_process | SOX5 | 658 | 12 | 1.235026 | -0.587166 | 543 | 368.45 | 0.678545 |  |
| GO:0016070\_RNA\_metabolic\_process | NEUROD1 | 658 | 12 | 1.235026 | -0.587166 | 543 | 368.45 | 0.678545 |  |
| GO:0016070\_RNA\_metabolic\_process | NR3C1 | 658 | 12 | 1.235026 | -0.587166 | 543 | 368.45 | 0.678545 |  |
| GO:0016070\_RNA\_metabolic\_process | HDAC9 | 658 | 12 | 1.235026 | -0.587166 | 543 | 368.45 | 0.678545 |  |
| GO:0016070\_RNA\_metabolic\_process | TCF7L2 | 658 | 12 | 1.235026 | -0.587166 | 543 | 368.45 | 0.678545 |  |
| GO:0016070\_RNA\_metabolic\_process | NR2E1 | 658 | 12 | 1.235026 | -0.587166 | 543 | 368.45 | 0.678545 |  |
| GO:0016070\_RNA\_metabolic\_process | NKX6-1 | 658 | 12 | 1.235026 | -0.587166 | 543 | 368.45 | 0.678545 |  |
| GO:0050890\_cognition | MYO6 | 233 | 5 | 1.453232 | -0.585498 | 544 | 368.76 | 0.677868 |  |
| GO:0050890\_cognition | HEXB | 233 | 5 | 1.453232 | -0.585498 | 544 | 368.76 | 0.677868 |  |
| GO:0050890\_cognition | ATP1A2 | 233 | 5 | 1.453232 | -0.585498 | 544 | 368.76 | 0.677868 |  |
| GO:0050890\_cognition | B3GNT2 | 233 | 5 | 1.453232 | -0.585498 | 544 | 368.76 | 0.677868 |  |
| GO:0050890\_cognition | NR2E1 | 233 | 5 | 1.453232 | -0.585498 | 544 | 368.76 | 0.677868 |  |
| GO:0009791\_post-embryonic\_development | ARID5B | 67 | 2 | 2.021510 | -0.584668 | 546 | 369.8 | 0.677289 |  |
| GO:0009791\_post-embryonic\_development | ZFX | 67 | 2 | 2.021510 | -0.584668 | 546 | 369.8 | 0.677289 |  |
| GO:0042445\_hormone\_metabolic\_process | AFP | 67 | 2 | 2.021510 | -0.584668 | 546 | 369.8 | 0.677289 |  |
| GO:0042445\_hormone\_metabolic\_process | NR3C1 | 67 | 2 | 2.021510 | -0.584668 | 546 | 369.8 | 0.677289 |  |
| GO:0043066\_negative\_regulation\_of\_apoptosis | MSX1 | 176 | 4 | 1.539104 | -0.582885 | 547 | 370.67 | 0.677642 |  |
| GO:0043066\_negative\_regulation\_of\_apoptosis | SGMS1 | 176 | 4 | 1.539104 | -0.582885 | 547 | 370.67 | 0.677642 |  |
| GO:0043066\_negative\_regulation\_of\_apoptosis | NR2E1 | 176 | 4 | 1.539104 | -0.582885 | 547 | 370.67 | 0.677642 |  |
| GO:0043066\_negative\_regulation\_of\_apoptosis | CD27 | 176 | 4 | 1.539104 | -0.582885 | 547 | 370.67 | 0.677642 |  |
| GO:0042692\_muscle\_cell\_differentiation | MBNL3 | 68 | 2 | 1.991782 | -0.575564 | 548 | 372.69 | 0.680091 |  |
| GO:0042692\_muscle\_cell\_differentiation | BOC | 68 | 2 | 1.991782 | -0.575564 | 548 | 372.69 | 0.680091 |  |
| GO:0030001\_metal\_ion\_transport | PTPRC | 122 | 3 | 1.665260 | -0.570911 | 549 | 374.2 | 0.681603 |  |
| GO:0030001\_metal\_ion\_transport | KCNA5 | 122 | 3 | 1.665260 | -0.570911 | 549 | 374.2 | 0.681603 |  |
| GO:0030001\_metal\_ion\_transport | SLC40A1 | 122 | 3 | 1.665260 | -0.570911 | 549 | 374.2 | 0.681603 |  |
| GO:0000018\_regulation\_of\_DNA\_recombination | PTPRC | 21 | 1 | 3.224790 | -0.570538 | 558 | 383.38 | 0.687061 |  |
| GO:0001709\_cell\_fate\_determination | ASCL1 | 21 | 1 | 3.224790 | -0.570538 | 558 | 383.38 | 0.687061 |  |
| GO:0010563\_negative\_regulation\_of\_phosphorus\_metabolic\_process | PTPRC | 21 | 1 | 3.224790 | -0.570538 | 558 | 383.38 | 0.687061 |  |
| GO:0019827\_stem\_cell\_maintenance | TCF7L2 | 21 | 1 | 3.224790 | -0.570538 | 558 | 383.38 | 0.687061 |  |
| GO:0030888\_regulation\_of\_B\_cell\_proliferation | PTPRC | 21 | 1 | 3.224790 | -0.570538 | 558 | 383.38 | 0.687061 |  |
| GO:0032147\_activation\_of\_protein\_kinase\_activity | GADD45B | 21 | 1 | 3.224790 | -0.570538 | 558 | 383.38 | 0.687061 |  |
| GO:0045936\_negative\_regulation\_of\_phosphate\_metabolic\_process | PTPRC | 21 | 1 | 3.224790 | -0.570538 | 558 | 383.38 | 0.687061 |  |
| GO:0050777\_negative\_regulation\_of\_immune\_response | PTPRC | 21 | 1 | 3.224790 | -0.570538 | 558 | 383.38 | 0.687061 |  |
| GO:0051656\_establishment\_of\_organelle\_localization | RAB27A | 21 | 1 | 3.224790 | -0.570538 | 558 | 383.38 | 0.687061 |  |
| GO:0043069\_negative\_regulation\_of\_programmed\_cell\_death | MSX1 | 179 | 4 | 1.513309 | -0.566754 | 560 | 384.25 | 0.686161 |  |
| GO:0043069\_negative\_regulation\_of\_programmed\_cell\_death | SGMS1 | 179 | 4 | 1.513309 | -0.566754 | 560 | 384.25 | 0.686161 |  |
| GO:0043069\_negative\_regulation\_of\_programmed\_cell\_death | NR2E1 | 179 | 4 | 1.513309 | -0.566754 | 560 | 384.25 | 0.686161 |  |
| GO:0043069\_negative\_regulation\_of\_programmed\_cell\_death | CD27 | 179 | 4 | 1.513309 | -0.566754 | 560 | 384.25 | 0.686161 |  |
| GO:0060548\_negative\_regulation\_of\_cell\_death | MSX1 | 179 | 4 | 1.513309 | -0.566754 | 560 | 384.25 | 0.686161 |  |
| GO:0060548\_negative\_regulation\_of\_cell\_death | SGMS1 | 179 | 4 | 1.513309 | -0.566754 | 560 | 384.25 | 0.686161 |  |
| GO:0060548\_negative\_regulation\_of\_cell\_death | NR2E1 | 179 | 4 | 1.513309 | -0.566754 | 560 | 384.25 | 0.686161 |  |
| GO:0060548\_negative\_regulation\_of\_cell\_death | CD27 | 179 | 4 | 1.513309 | -0.566754 | 560 | 384.25 | 0.686161 |  |
| GO:0001932\_regulation\_of\_protein\_amino\_acid\_phosphorylation | PTPRC | 69 | 2 | 1.962916 | -0.566645 | 561 | 385.21 | 0.686649 |  |
| GO:0001932\_regulation\_of\_protein\_amino\_acid\_phosphorylation | JAK2 | 69 | 2 | 1.962916 | -0.566645 | 561 | 385.21 | 0.686649 |  |
| GO:0008406\_gonad\_development | AFP | 70 | 2 | 1.934874 | -0.557906 | 562 | 387.12 | 0.688826 |  |
| GO:0008406\_gonad\_development | ZFX | 70 | 2 | 1.934874 | -0.557906 | 562 | 387.12 | 0.688826 |  |
| GO:0000041\_transition\_metal\_ion\_transport | SLC40A1 | 22 | 1 | 3.078209 | -0.553360 | 573 | 399.13 | 0.696562 |  |
| GO:0001569\_patterning\_of\_blood\_vessels | TBX20 | 22 | 1 | 3.078209 | -0.553360 | 573 | 399.13 | 0.696562 |  |
| GO:0002637\_regulation\_of\_immunoglobulin\_production | PTPRC | 22 | 1 | 3.078209 | -0.553360 | 573 | 399.13 | 0.696562 |  |
| GO:0002712\_regulation\_of\_B\_cell\_mediated\_immunity | PTPRC | 22 | 1 | 3.078209 | -0.553360 | 573 | 399.13 | 0.696562 |  |
| GO:0002889\_regulation\_of\_immunoglobulin\_mediated\_immune\_response | PTPRC | 22 | 1 | 3.078209 | -0.553360 | 573 | 399.13 | 0.696562 |  |
| GO:0006029\_proteoglycan\_metabolic\_process | MAMDC2 | 22 | 1 | 3.078209 | -0.553360 | 573 | 399.13 | 0.696562 |  |
| GO:0015918\_sterol\_transport | ABCA1 | 22 | 1 | 3.078209 | -0.553360 | 573 | 399.13 | 0.696562 |  |
| GO:0030301\_cholesterol\_transport | ABCA1 | 22 | 1 | 3.078209 | -0.553360 | 573 | 399.13 | 0.696562 |  |
| GO:0043112\_receptor\_metabolic\_process | DMD | 22 | 1 | 3.078209 | -0.553360 | 573 | 399.13 | 0.696562 |  |
| GO:0046635\_positive\_regulation\_of\_alpha-beta\_T\_cell\_activation | PTPRC | 22 | 1 | 3.078209 | -0.553360 | 573 | 399.13 | 0.696562 |  |
| GO:0048864\_stem\_cell\_development | TCF7L2 | 22 | 1 | 3.078209 | -0.553360 | 573 | 399.13 | 0.696562 |  |
| GO:0080090\_regulation\_of\_primary\_metabolic\_process | PTPRC | 926 | 16 | 1.170118 | -0.549626 | 574 | 400.03 | 0.696916 |  |
| GO:0080090\_regulation\_of\_primary\_metabolic\_process | TBX3 | 926 | 16 | 1.170118 | -0.549626 | 574 | 400.03 | 0.696916 |  |
| GO:0080090\_regulation\_of\_primary\_metabolic\_process | TBX20 | 926 | 16 | 1.170118 | -0.549626 | 574 | 400.03 | 0.696916 |  |
| GO:0080090\_regulation\_of\_primary\_metabolic\_process | SOX5 | 926 | 16 | 1.170118 | -0.549626 | 574 | 400.03 | 0.696916 |  |
| GO:0080090\_regulation\_of\_primary\_metabolic\_process | ESRRG | 926 | 16 | 1.170118 | -0.549626 | 574 | 400.03 | 0.696916 |  |
| GO:0080090\_regulation\_of\_primary\_metabolic\_process | NR3C1 | 926 | 16 | 1.170118 | -0.549626 | 574 | 400.03 | 0.696916 |  |
| GO:0080090\_regulation\_of\_primary\_metabolic\_process | TCF7L2 | 926 | 16 | 1.170118 | -0.549626 | 574 | 400.03 | 0.696916 |  |
| GO:0080090\_regulation\_of\_primary\_metabolic\_process | NR2E1 | 926 | 16 | 1.170118 | -0.549626 | 574 | 400.03 | 0.696916 |  |
| GO:0080090\_regulation\_of\_primary\_metabolic\_process | NKX6-1 | 926 | 16 | 1.170118 | -0.549626 | 574 | 400.03 | 0.696916 |  |
| GO:0080090\_regulation\_of\_primary\_metabolic\_process | ASCL1 | 926 | 16 | 1.170118 | -0.549626 | 574 | 400.03 | 0.696916 |  |
| GO:0080090\_regulation\_of\_primary\_metabolic\_process | MSX1 | 926 | 16 | 1.170118 | -0.549626 | 574 | 400.03 | 0.696916 |  |
| GO:0080090\_regulation\_of\_primary\_metabolic\_process | DMD | 926 | 16 | 1.170118 | -0.549626 | 574 | 400.03 | 0.696916 |  |
| GO:0080090\_regulation\_of\_primary\_metabolic\_process | NEUROD1 | 926 | 16 | 1.170118 | -0.549626 | 574 | 400.03 | 0.696916 |  |
| GO:0080090\_regulation\_of\_primary\_metabolic\_process | ETV1 | 926 | 16 | 1.170118 | -0.549626 | 574 | 400.03 | 0.696916 |  |
| GO:0080090\_regulation\_of\_primary\_metabolic\_process | JAK2 | 926 | 16 | 1.170118 | -0.549626 | 574 | 400.03 | 0.696916 |  |
| GO:0080090\_regulation\_of\_primary\_metabolic\_process | HDAC9 | 926 | 16 | 1.170118 | -0.549626 | 574 | 400.03 | 0.696916 |  |
| GO:0033674\_positive\_regulation\_of\_kinase\_activity | PTPRC | 71 | 2 | 1.907622 | -0.549342 | 575 | 401.09 | 0.697548 |  |
| GO:0033674\_positive\_regulation\_of\_kinase\_activity | GADD45B | 71 | 2 | 1.907622 | -0.549342 | 575 | 401.09 | 0.697548 |  |
| GO:0030879\_mammary\_gland\_development | TBX3 | 72 | 2 | 1.881127 | -0.540947 | 577 | 403.99 | 0.700156 |  |
| GO:0030879\_mammary\_gland\_development | NR3C1 | 72 | 2 | 1.881127 | -0.540947 | 577 | 403.99 | 0.700156 |  |
| GO:0051347\_positive\_regulation\_of\_transferase\_activity | PTPRC | 72 | 2 | 1.881127 | -0.540947 | 577 | 403.99 | 0.700156 |  |
| GO:0051347\_positive\_regulation\_of\_transferase\_activity | GADD45B | 72 | 2 | 1.881127 | -0.540947 | 577 | 403.99 | 0.700156 |  |
| GO:0016192\_vesicle-mediated\_transport | MYO6 | 184 | 4 | 1.472187 | -0.540906 | 578 | 404.34 | 0.699550 |  |
| GO:0016192\_vesicle-mediated\_transport | AP2A1 | 184 | 4 | 1.472187 | -0.540906 | 578 | 404.34 | 0.699550 |  |
| GO:0016192\_vesicle-mediated\_transport | ABCA1 | 184 | 4 | 1.472187 | -0.540906 | 578 | 404.34 | 0.699550 |  |
| GO:0016192\_vesicle-mediated\_transport | RAB27A | 184 | 4 | 1.472187 | -0.540906 | 578 | 404.34 | 0.699550 |  |
| GO:0000082\_G1\_S\_transition\_of\_mitotic\_cell\_cycle | LATS2 | 23 | 1 | 2.944373 | -0.537072 | 588 | 410.7 | 0.698469 |  |
| GO:0002204\_somatic\_recombination\_of\_immunoglobulin\_genes\_during\_immune\_response | PTPRC | 23 | 1 | 2.944373 | -0.537072 | 588 | 410.7 | 0.698469 |  |
| GO:0002208\_somatic\_diversification\_of\_immunoglobulins\_during\_immune\_response | PTPRC | 23 | 1 | 2.944373 | -0.537072 | 588 | 410.7 | 0.698469 |  |
| GO:0002228\_natural\_killer\_cell\_mediated\_immunity | RAB27A | 23 | 1 | 2.944373 | -0.537072 | 588 | 410.7 | 0.698469 |  |
| GO:0002821\_positive\_regulation\_of\_adaptive\_immune\_response | PTPRC | 23 | 1 | 2.944373 | -0.537072 | 588 | 410.7 | 0.698469 |  |
| GO:0002824\_positive\_regulation\_of\_adaptive\_immune\_response\_based\_on\_somatic\_recombination\_of\_immune\_receptors\_built\_from\_immunoglobulin\_superfamily\_domains | PTPRC | 23 | 1 | 2.944373 | -0.537072 | 588 | 410.7 | 0.698469 |  |
| GO:0008542\_visual\_learning | ATP1A2 | 23 | 1 | 2.944373 | -0.537072 | 588 | 410.7 | 0.698469 |  |
| GO:0042267\_natural\_killer\_cell\_mediated\_cytotoxicity | RAB27A | 23 | 1 | 2.944373 | -0.537072 | 588 | 410.7 | 0.698469 |  |
| GO:0045190\_isotype\_switching | PTPRC | 23 | 1 | 2.944373 | -0.537072 | 588 | 410.7 | 0.698469 |  |
| GO:0060349\_bone\_morphogenesis | FGF18 | 23 | 1 | 2.944373 | -0.537072 | 588 | 410.7 | 0.698469 |  |
| GO:0001655\_urogenital\_system\_development | TCFAP2B | 128 | 3 | 1.587201 | -0.533300 | 589 | 411.92 | 0.699355 |  |
| GO:0001655\_urogenital\_system\_development | AQP11 | 128 | 3 | 1.587201 | -0.533300 | 589 | 411.92 | 0.699355 |  |
| GO:0001655\_urogenital\_system\_development | ARID5B | 128 | 3 | 1.587201 | -0.533300 | 589 | 411.92 | 0.699355 |  |
| GO:0006952\_defense\_response | PTPRC | 187 | 4 | 1.448569 | -0.525992 | 590 | 414.4 | 0.702373 |  |
| GO:0006952\_defense\_response | THBS1 | 187 | 4 | 1.448569 | -0.525992 | 590 | 414.4 | 0.702373 |  |
| GO:0006952\_defense\_response | HDAC9 | 187 | 4 | 1.448569 | -0.525992 | 590 | 414.4 | 0.702373 |  |
| GO:0006952\_defense\_response | RAB27A | 187 | 4 | 1.448569 | -0.525992 | 590 | 414.4 | 0.702373 |  |
| GO:0032989\_cellular\_component\_morphogenesis | DCC | 307 | 6 | 1.323529 | -0.524059 | 591 | 415.09 | 0.702352 |  |
| GO:0032989\_cellular\_component\_morphogenesis | ALCAM | 307 | 6 | 1.323529 | -0.524059 | 591 | 415.09 | 0.702352 |  |
| GO:0032989\_cellular\_component\_morphogenesis | ETV1 | 307 | 6 | 1.323529 | -0.524059 | 591 | 415.09 | 0.702352 |  |
| GO:0032989\_cellular\_component\_morphogenesis | B3GNT2 | 307 | 6 | 1.323529 | -0.524059 | 591 | 415.09 | 0.702352 |  |
| GO:0032989\_cellular\_component\_morphogenesis | NR2E1 | 307 | 6 | 1.323529 | -0.524059 | 591 | 415.09 | 0.702352 |  |
| GO:0032989\_cellular\_component\_morphogenesis | BOC | 307 | 6 | 1.323529 | -0.524059 | 591 | 415.09 | 0.702352 |  |
| GO:0006139\_nucleobase\_\_nucleoside\_\_nucleotide\_and\_nucleic\_acid\_metabolic\_process | PTPRC | 1002 | 17 | 1.148952 | -0.522848 | 592 | 415.2 | 0.701351 |  |
| GO:0006139\_nucleobase\_\_nucleoside\_\_nucleotide\_and\_nucleic\_acid\_metabolic\_process | TBX3 | 1002 | 17 | 1.148952 | -0.522848 | 592 | 415.2 | 0.701351 |  |
| GO:0006139\_nucleobase\_\_nucleoside\_\_nucleotide\_and\_nucleic\_acid\_metabolic\_process | TBX20 | 1002 | 17 | 1.148952 | -0.522848 | 592 | 415.2 | 0.701351 |  |
| GO:0006139\_nucleobase\_\_nucleoside\_\_nucleotide\_and\_nucleic\_acid\_metabolic\_process | SOX5 | 1002 | 17 | 1.148952 | -0.522848 | 592 | 415.2 | 0.701351 |  |
| GO:0006139\_nucleobase\_\_nucleoside\_\_nucleotide\_and\_nucleic\_acid\_metabolic\_process | ESRRG | 1002 | 17 | 1.148952 | -0.522848 | 592 | 415.2 | 0.701351 |  |
| GO:0006139\_nucleobase\_\_nucleoside\_\_nucleotide\_and\_nucleic\_acid\_metabolic\_process | HSPA1A | 1002 | 17 | 1.148952 | -0.522848 | 592 | 415.2 | 0.701351 |  |
| GO:0006139\_nucleobase\_\_nucleoside\_\_nucleotide\_and\_nucleic\_acid\_metabolic\_process | NR3C1 | 1002 | 17 | 1.148952 | -0.522848 | 592 | 415.2 | 0.701351 |  |
| GO:0006139\_nucleobase\_\_nucleoside\_\_nucleotide\_and\_nucleic\_acid\_metabolic\_process | NR2E1 | 1002 | 17 | 1.148952 | -0.522848 | 592 | 415.2 | 0.701351 |  |
| GO:0006139\_nucleobase\_\_nucleoside\_\_nucleotide\_and\_nucleic\_acid\_metabolic\_process | TCF7L2 | 1002 | 17 | 1.148952 | -0.522848 | 592 | 415.2 | 0.701351 |  |
| GO:0006139\_nucleobase\_\_nucleoside\_\_nucleotide\_and\_nucleic\_acid\_metabolic\_process | NKX6-1 | 1002 | 17 | 1.148952 | -0.522848 | 592 | 415.2 | 0.701351 |  |
| GO:0006139\_nucleobase\_\_nucleoside\_\_nucleotide\_and\_nucleic\_acid\_metabolic\_process | ASCL1 | 1002 | 17 | 1.148952 | -0.522848 | 592 | 415.2 | 0.701351 |  |
| GO:0006139\_nucleobase\_\_nucleoside\_\_nucleotide\_and\_nucleic\_acid\_metabolic\_process | TCERG1 | 1002 | 17 | 1.148952 | -0.522848 | 592 | 415.2 | 0.701351 |  |
| GO:0006139\_nucleobase\_\_nucleoside\_\_nucleotide\_and\_nucleic\_acid\_metabolic\_process | MSX1 | 1002 | 17 | 1.148952 | -0.522848 | 592 | 415.2 | 0.701351 |  |
| GO:0006139\_nucleobase\_\_nucleoside\_\_nucleotide\_and\_nucleic\_acid\_metabolic\_process | DMD | 1002 | 17 | 1.148952 | -0.522848 | 592 | 415.2 | 0.701351 |  |
| GO:0006139\_nucleobase\_\_nucleoside\_\_nucleotide\_and\_nucleic\_acid\_metabolic\_process | ETV1 | 1002 | 17 | 1.148952 | -0.522848 | 592 | 415.2 | 0.701351 |  |
| GO:0006139\_nucleobase\_\_nucleoside\_\_nucleotide\_and\_nucleic\_acid\_metabolic\_process | NEUROD1 | 1002 | 17 | 1.148952 | -0.522848 | 592 | 415.2 | 0.701351 |  |
| GO:0006139\_nucleobase\_\_nucleoside\_\_nucleotide\_and\_nucleic\_acid\_metabolic\_process | HDAC9 | 1002 | 17 | 1.148952 | -0.522848 | 592 | 415.2 | 0.701351 |  |
| GO:0001541\_ovarian\_follicle\_development | ZFX | 24 | 1 | 2.821691 | -0.521598 | 603 | 422.62 | 0.700862 |  |
| GO:0002381\_immunoglobulin\_production\_during\_immune\_response | PTPRC | 24 | 1 | 2.821691 | -0.521598 | 603 | 422.62 | 0.700862 |  |
| GO:0006941\_striated\_muscle\_contraction | ATP1A2 | 24 | 1 | 2.821691 | -0.521598 | 603 | 422.62 | 0.700862 |  |
| GO:0006959\_humoral\_immune\_response | PTPRC | 24 | 1 | 2.821691 | -0.521598 | 603 | 422.62 | 0.700862 |  |
| GO:0007204\_elevation\_of\_cytosolic\_calcium\_ion\_concentration | PTPRC | 24 | 1 | 2.821691 | -0.521598 | 603 | 422.62 | 0.700862 |  |
| GO:0007259\_JAK-STAT\_cascade | JAK2 | 24 | 1 | 2.821691 | -0.521598 | 603 | 422.62 | 0.700862 |  |
| GO:0007632\_visual\_behavior | ATP1A2 | 24 | 1 | 2.821691 | -0.521598 | 603 | 422.62 | 0.700862 |  |
| GO:0009612\_response\_to\_mechanical\_stimulus | ETV1 | 24 | 1 | 2.821691 | -0.521598 | 603 | 422.62 | 0.700862 |  |
| GO:0042158\_lipoprotein\_biosynthetic\_process | ABCA1 | 24 | 1 | 2.821691 | -0.521598 | 603 | 422.62 | 0.700862 |  |
| GO:0048546\_digestive\_tract\_morphogenesis | TCF7L2 | 24 | 1 | 2.821691 | -0.521598 | 603 | 422.62 | 0.700862 |  |
| GO:0060113\_inner\_ear\_receptor\_cell\_differentiation | MYO6 | 24 | 1 | 2.821691 | -0.521598 | 603 | 422.62 | 0.700862 |  |
| GO:0019226\_transmission\_of\_nerve\_impulse | MYO6 | 189 | 4 | 1.433240 | -0.516288 | 604 | 425.28 | 0.704106 |  |
| GO:0019226\_transmission\_of\_nerve\_impulse | DMD | 189 | 4 | 1.433240 | -0.516288 | 604 | 425.28 | 0.704106 |  |
| GO:0019226\_transmission\_of\_nerve\_impulse | HEXB | 189 | 4 | 1.433240 | -0.516288 | 604 | 425.28 | 0.704106 |  |
| GO:0019226\_transmission\_of\_nerve\_impulse | ATP1A2 | 189 | 4 | 1.433240 | -0.516288 | 604 | 425.28 | 0.704106 |  |
| GO:0031326\_regulation\_of\_cellular\_biosynthetic\_process | TBX3 | 812 | 14 | 1.167596 | -0.515315 | 605 | 425.43 | 0.703190 |  |
| GO:0031326\_regulation\_of\_cellular\_biosynthetic\_process | TBX20 | 812 | 14 | 1.167596 | -0.515315 | 605 | 425.43 | 0.703190 |  |
| GO:0031326\_regulation\_of\_cellular\_biosynthetic\_process | SOX5 | 812 | 14 | 1.167596 | -0.515315 | 605 | 425.43 | 0.703190 |  |
| GO:0031326\_regulation\_of\_cellular\_biosynthetic\_process | ESRRG | 812 | 14 | 1.167596 | -0.515315 | 605 | 425.43 | 0.703190 |  |
| GO:0031326\_regulation\_of\_cellular\_biosynthetic\_process | NR3C1 | 812 | 14 | 1.167596 | -0.515315 | 605 | 425.43 | 0.703190 |  |
| GO:0031326\_regulation\_of\_cellular\_biosynthetic\_process | NR2E1 | 812 | 14 | 1.167596 | -0.515315 | 605 | 425.43 | 0.703190 |  |
| GO:0031326\_regulation\_of\_cellular\_biosynthetic\_process | TCF7L2 | 812 | 14 | 1.167596 | -0.515315 | 605 | 425.43 | 0.703190 |  |
| GO:0031326\_regulation\_of\_cellular\_biosynthetic\_process | NKX6-1 | 812 | 14 | 1.167596 | -0.515315 | 605 | 425.43 | 0.703190 |  |
| GO:0031326\_regulation\_of\_cellular\_biosynthetic\_process | ASCL1 | 812 | 14 | 1.167596 | -0.515315 | 605 | 425.43 | 0.703190 |  |
| GO:0031326\_regulation\_of\_cellular\_biosynthetic\_process | MSX1 | 812 | 14 | 1.167596 | -0.515315 | 605 | 425.43 | 0.703190 |  |
| GO:0031326\_regulation\_of\_cellular\_biosynthetic\_process | DMD | 812 | 14 | 1.167596 | -0.515315 | 605 | 425.43 | 0.703190 |  |
| GO:0031326\_regulation\_of\_cellular\_biosynthetic\_process | NEUROD1 | 812 | 14 | 1.167596 | -0.515315 | 605 | 425.43 | 0.703190 |  |
| GO:0031326\_regulation\_of\_cellular\_biosynthetic\_process | ETV1 | 812 | 14 | 1.167596 | -0.515315 | 605 | 425.43 | 0.703190 |  |
| GO:0031326\_regulation\_of\_cellular\_biosynthetic\_process | HDAC9 | 812 | 14 | 1.167596 | -0.515315 | 605 | 425.43 | 0.703190 |  |
| GO:0044267\_cellular\_protein\_metabolic\_process | PTPRC | 559 | 10 | 1.211460 | -0.511450 | 606 | 426.07 | 0.703086 |  |
| GO:0044267\_cellular\_protein\_metabolic\_process | FUT9 | 559 | 10 | 1.211460 | -0.511450 | 606 | 426.07 | 0.703086 |  |
| GO:0044267\_cellular\_protein\_metabolic\_process | MAMDC2 | 559 | 10 | 1.211460 | -0.511450 | 606 | 426.07 | 0.703086 |  |
| GO:0044267\_cellular\_protein\_metabolic\_process | HEXB | 559 | 10 | 1.211460 | -0.511450 | 606 | 426.07 | 0.703086 |  |
| GO:0044267\_cellular\_protein\_metabolic\_process | JAK2 | 559 | 10 | 1.211460 | -0.511450 | 606 | 426.07 | 0.703086 |  |
| GO:0044267\_cellular\_protein\_metabolic\_process | ABCA1 | 559 | 10 | 1.211460 | -0.511450 | 606 | 426.07 | 0.703086 |  |
| GO:0044267\_cellular\_protein\_metabolic\_process | B3GNT2 | 559 | 10 | 1.211460 | -0.511450 | 606 | 426.07 | 0.703086 |  |
| GO:0044267\_cellular\_protein\_metabolic\_process | TRIM63 | 559 | 10 | 1.211460 | -0.511450 | 606 | 426.07 | 0.703086 |  |
| GO:0044267\_cellular\_protein\_metabolic\_process | PPAP2A | 559 | 10 | 1.211460 | -0.511450 | 606 | 426.07 | 0.703086 |  |
| GO:0044267\_cellular\_protein\_metabolic\_process | GADD45B | 559 | 10 | 1.211460 | -0.511450 | 606 | 426.07 | 0.703086 |  |
| GO:0009889\_regulation\_of\_biosynthetic\_process | TBX3 | 815 | 14 | 1.163298 | -0.508119 | 607 | 427.2 | 0.703789 |  |
| GO:0009889\_regulation\_of\_biosynthetic\_process | TBX20 | 815 | 14 | 1.163298 | -0.508119 | 607 | 427.2 | 0.703789 |  |
| GO:0009889\_regulation\_of\_biosynthetic\_process | SOX5 | 815 | 14 | 1.163298 | -0.508119 | 607 | 427.2 | 0.703789 |  |
| GO:0009889\_regulation\_of\_biosynthetic\_process | ESRRG | 815 | 14 | 1.163298 | -0.508119 | 607 | 427.2 | 0.703789 |  |
| GO:0009889\_regulation\_of\_biosynthetic\_process | NR3C1 | 815 | 14 | 1.163298 | -0.508119 | 607 | 427.2 | 0.703789 |  |
| GO:0009889\_regulation\_of\_biosynthetic\_process | NR2E1 | 815 | 14 | 1.163298 | -0.508119 | 607 | 427.2 | 0.703789 |  |
| GO:0009889\_regulation\_of\_biosynthetic\_process | TCF7L2 | 815 | 14 | 1.163298 | -0.508119 | 607 | 427.2 | 0.703789 |  |
| GO:0009889\_regulation\_of\_biosynthetic\_process | NKX6-1 | 815 | 14 | 1.163298 | -0.508119 | 607 | 427.2 | 0.703789 |  |
| GO:0009889\_regulation\_of\_biosynthetic\_process | ASCL1 | 815 | 14 | 1.163298 | -0.508119 | 607 | 427.2 | 0.703789 |  |
| GO:0009889\_regulation\_of\_biosynthetic\_process | MSX1 | 815 | 14 | 1.163298 | -0.508119 | 607 | 427.2 | 0.703789 |  |
| GO:0009889\_regulation\_of\_biosynthetic\_process | DMD | 815 | 14 | 1.163298 | -0.508119 | 607 | 427.2 | 0.703789 |  |
| GO:0009889\_regulation\_of\_biosynthetic\_process | NEUROD1 | 815 | 14 | 1.163298 | -0.508119 | 607 | 427.2 | 0.703789 |  |
| GO:0009889\_regulation\_of\_biosynthetic\_process | ETV1 | 815 | 14 | 1.163298 | -0.508119 | 607 | 427.2 | 0.703789 |  |
| GO:0009889\_regulation\_of\_biosynthetic\_process | HDAC9 | 815 | 14 | 1.163298 | -0.508119 | 607 | 427.2 | 0.703789 |  |
| GO:0006357\_regulation\_of\_transcription\_from\_RNA\_polymerase\_II\_promoter | ASCL1 | 435 | 8 | 1.245436 | -0.507334 | 608 | 427.35 | 0.702878 |  |
| GO:0006357\_regulation\_of\_transcription\_from\_RNA\_polymerase\_II\_promoter | MSX1 | 435 | 8 | 1.245436 | -0.507334 | 608 | 427.35 | 0.702878 |  |
| GO:0006357\_regulation\_of\_transcription\_from\_RNA\_polymerase\_II\_promoter | SOX5 | 435 | 8 | 1.245436 | -0.507334 | 608 | 427.35 | 0.702878 |  |
| GO:0006357\_regulation\_of\_transcription\_from\_RNA\_polymerase\_II\_promoter | NEUROD1 | 435 | 8 | 1.245436 | -0.507334 | 608 | 427.35 | 0.702878 |  |
| GO:0006357\_regulation\_of\_transcription\_from\_RNA\_polymerase\_II\_promoter | HDAC9 | 435 | 8 | 1.245436 | -0.507334 | 608 | 427.35 | 0.702878 |  |
| GO:0006357\_regulation\_of\_transcription\_from\_RNA\_polymerase\_II\_promoter | TCF7L2 | 435 | 8 | 1.245436 | -0.507334 | 608 | 427.35 | 0.702878 |  |
| GO:0006357\_regulation\_of\_transcription\_from\_RNA\_polymerase\_II\_promoter | NR2E1 | 435 | 8 | 1.245436 | -0.507334 | 608 | 427.35 | 0.702878 |  |
| GO:0006357\_regulation\_of\_transcription\_from\_RNA\_polymerase\_II\_promoter | NKX6-1 | 435 | 8 | 1.245436 | -0.507334 | 608 | 427.35 | 0.702878 |  |
| GO:0002700\_regulation\_of\_production\_of\_molecular\_mediator\_of\_immune\_response | PTPRC | 25 | 1 | 2.708824 | -0.506872 | 613 | 434.1 | 0.708157 |  |
| GO:0010811\_positive\_regulation\_of\_cell-substrate\_adhesion | THBS1 | 25 | 1 | 2.708824 | -0.506872 | 613 | 434.1 | 0.708157 |  |
| GO:0031400\_negative\_regulation\_of\_protein\_modification\_process | PTPRC | 25 | 1 | 2.708824 | -0.506872 | 613 | 434.1 | 0.708157 |  |
| GO:0050852\_T\_cell\_receptor\_signaling\_pathway | ELF1 | 25 | 1 | 2.708824 | -0.506872 | 613 | 434.1 | 0.708157 |  |
| GO:0055123\_digestive\_system\_development | TCF7L2 | 25 | 1 | 2.708824 | -0.506872 | 613 | 434.1 | 0.708157 |  |
| GO:0008104\_protein\_localization | MYO6 | 251 | 5 | 1.349016 | -0.506078 | 614 | 434.26 | 0.707264 |  |
| GO:0008104\_protein\_localization | AP2A1 | 251 | 5 | 1.349016 | -0.506078 | 614 | 434.26 | 0.707264 |  |
| GO:0008104\_protein\_localization | MLPH | 251 | 5 | 1.349016 | -0.506078 | 614 | 434.26 | 0.707264 |  |
| GO:0008104\_protein\_localization | JAK2 | 251 | 5 | 1.349016 | -0.506078 | 614 | 434.26 | 0.707264 |  |
| GO:0008104\_protein\_localization | RAB27A | 251 | 5 | 1.349016 | -0.506078 | 614 | 434.26 | 0.707264 |  |
| GO:0044057\_regulation\_of\_system\_process | MYO6 | 133 | 3 | 1.527532 | -0.504087 | 615 | 434.6 | 0.706667 |  |
| GO:0044057\_regulation\_of\_system\_process | IRX5 | 133 | 3 | 1.527532 | -0.504087 | 615 | 434.6 | 0.706667 |  |
| GO:0044057\_regulation\_of\_system\_process | ATP1A2 | 133 | 3 | 1.527532 | -0.504087 | 615 | 434.6 | 0.706667 |  |
| GO:0051241\_negative\_regulation\_of\_multicellular\_organismal\_process | ATXN2 | 77 | 2 | 1.758976 | -0.501355 | 616 | 435.2 | 0.706494 |  |
| GO:0051241\_negative\_regulation\_of\_multicellular\_organismal\_process | ATP1A2 | 77 | 2 | 1.758976 | -0.501355 | 616 | 435.2 | 0.706494 |  |
| GO:0016481\_negative\_regulation\_of\_transcription | MSX1 | 253 | 5 | 1.338352 | -0.497939 | 617 | 436.02 | 0.706677 |  |
| GO:0016481\_negative\_regulation\_of\_transcription | TBX3 | 253 | 5 | 1.338352 | -0.497939 | 617 | 436.02 | 0.706677 |  |
| GO:0016481\_negative\_regulation\_of\_transcription | TBX20 | 253 | 5 | 1.338352 | -0.497939 | 617 | 436.02 | 0.706677 |  |
| GO:0016481\_negative\_regulation\_of\_transcription | HDAC9 | 253 | 5 | 1.338352 | -0.497939 | 617 | 436.02 | 0.706677 |  |
| GO:0016481\_negative\_regulation\_of\_transcription | NR2E1 | 253 | 5 | 1.338352 | -0.497939 | 617 | 436.02 | 0.706677 |  |
| GO:0006464\_protein\_modification\_process | PTPRC | 439 | 8 | 1.234088 | -0.494884 | 618 | 436.33 | 0.706036 |  |
| GO:0006464\_protein\_modification\_process | FUT9 | 439 | 8 | 1.234088 | -0.494884 | 618 | 436.33 | 0.706036 |  |
| GO:0006464\_protein\_modification\_process | MAMDC2 | 439 | 8 | 1.234088 | -0.494884 | 618 | 436.33 | 0.706036 |  |
| GO:0006464\_protein\_modification\_process | JAK2 | 439 | 8 | 1.234088 | -0.494884 | 618 | 436.33 | 0.706036 |  |
| GO:0006464\_protein\_modification\_process | ABCA1 | 439 | 8 | 1.234088 | -0.494884 | 618 | 436.33 | 0.706036 |  |
| GO:0006464\_protein\_modification\_process | B3GNT2 | 439 | 8 | 1.234088 | -0.494884 | 618 | 436.33 | 0.706036 |  |
| GO:0006464\_protein\_modification\_process | PPAP2A | 439 | 8 | 1.234088 | -0.494884 | 618 | 436.33 | 0.706036 |  |
| GO:0006464\_protein\_modification\_process | GADD45B | 439 | 8 | 1.234088 | -0.494884 | 618 | 436.33 | 0.706036 |  |
| GO:0007405\_neuroblast\_proliferation | ASCL1 | 26 | 1 | 2.604638 | -0.492833 | 623 | 444.59 | 0.713628 |  |
| GO:0010959\_regulation\_of\_metal\_ion\_transport | KCNA5 | 26 | 1 | 2.604638 | -0.492833 | 623 | 444.59 | 0.713628 |  |
| GO:0045665\_negative\_regulation\_of\_neuron\_differentiation | NR2E1 | 26 | 1 | 2.604638 | -0.492833 | 623 | 444.59 | 0.713628 |  |
| GO:0045765\_regulation\_of\_angiogenesis | THBS1 | 26 | 1 | 2.604638 | -0.492833 | 623 | 444.59 | 0.713628 |  |
| GO:0051235\_maintenance\_of\_location | PTPRC | 26 | 1 | 2.604638 | -0.492833 | 623 | 444.59 | 0.713628 |  |
| GO:0001654\_eye\_development | IRX5 | 136 | 3 | 1.493837 | -0.487419 | 624 | 445.95 | 0.714663 |  |
| GO:0001654\_eye\_development | NEUROD1 | 136 | 3 | 1.493837 | -0.487419 | 624 | 445.95 | 0.714663 |  |
| GO:0001654\_eye\_development | NR2E1 | 136 | 3 | 1.493837 | -0.487419 | 624 | 445.95 | 0.714663 |  |
| GO:0015674\_di-\_\_tri-valent\_inorganic\_cation\_transport | PTPRC | 79 | 2 | 1.714445 | -0.486549 | 625 | 446.64 | 0.714624 |  |
| GO:0015674\_di-\_\_tri-valent\_inorganic\_cation\_transport | SLC40A1 | 79 | 2 | 1.714445 | -0.486549 | 625 | 446.64 | 0.714624 |  |
| GO:0006366\_transcription\_from\_RNA\_polymerase\_II\_promoter | ASCL1 | 444 | 8 | 1.220191 | -0.479704 | 626 | 448.0 | 0.715655 |  |
| GO:0006366\_transcription\_from\_RNA\_polymerase\_II\_promoter | MSX1 | 444 | 8 | 1.220191 | -0.479704 | 626 | 448.0 | 0.715655 |  |
| GO:0006366\_transcription\_from\_RNA\_polymerase\_II\_promoter | SOX5 | 444 | 8 | 1.220191 | -0.479704 | 626 | 448.0 | 0.715655 |  |
| GO:0006366\_transcription\_from\_RNA\_polymerase\_II\_promoter | NEUROD1 | 444 | 8 | 1.220191 | -0.479704 | 626 | 448.0 | 0.715655 |  |
| GO:0006366\_transcription\_from\_RNA\_polymerase\_II\_promoter | HDAC9 | 444 | 8 | 1.220191 | -0.479704 | 626 | 448.0 | 0.715655 |  |
| GO:0006366\_transcription\_from\_RNA\_polymerase\_II\_promoter | TCF7L2 | 444 | 8 | 1.220191 | -0.479704 | 626 | 448.0 | 0.715655 |  |
| GO:0006366\_transcription\_from\_RNA\_polymerase\_II\_promoter | NR2E1 | 444 | 8 | 1.220191 | -0.479704 | 626 | 448.0 | 0.715655 |  |
| GO:0006366\_transcription\_from\_RNA\_polymerase\_II\_promoter | NKX6-1 | 444 | 8 | 1.220191 | -0.479704 | 626 | 448.0 | 0.715655 |  |
| GO:0001910\_regulation\_of\_leukocyte\_mediated\_cytotoxicity | PTPRC | 27 | 1 | 2.508170 | -0.479431 | 630 | 453.07 | 0.719159 |  |
| GO:0031016\_pancreas\_development | NKX6-1 | 27 | 1 | 2.508170 | -0.479431 | 630 | 453.07 | 0.719159 |  |
| GO:0031341\_regulation\_of\_cell\_killing | PTPRC | 27 | 1 | 2.508170 | -0.479431 | 630 | 453.07 | 0.719159 |  |
| GO:0045582\_positive\_regulation\_of\_T\_cell\_differentiation | PTPRC | 27 | 1 | 2.508170 | -0.479431 | 630 | 453.07 | 0.719159 |  |
| GO:0002250\_adaptive\_immune\_response | PTPRC | 80 | 2 | 1.693015 | -0.479352 | 632 | 454.31 | 0.718845 |  |
| GO:0002250\_adaptive\_immune\_response | RAB27A | 80 | 2 | 1.693015 | -0.479352 | 632 | 454.31 | 0.718845 |  |
| GO:0002460\_adaptive\_immune\_response\_based\_on\_somatic\_recombination\_of\_immune\_receptors\_built\_from\_immunoglobulin\_superfamily\_domains | PTPRC | 80 | 2 | 1.693015 | -0.479352 | 632 | 454.31 | 0.718845 |  |
| GO:0002460\_adaptive\_immune\_response\_based\_on\_somatic\_recombination\_of\_immune\_receptors\_built\_from\_immunoglobulin\_superfamily\_domains | RAB27A | 80 | 2 | 1.693015 | -0.479352 | 632 | 454.31 | 0.718845 |  |
| GO:0007169\_transmembrane\_receptor\_protein\_tyrosine\_kinase\_signaling\_pathway | FGF18 | 139 | 3 | 1.461595 | -0.471360 | 633 | 455.98 | 0.720348 |  |
| GO:0007169\_transmembrane\_receptor\_protein\_tyrosine\_kinase\_signaling\_pathway | ARID5B | 139 | 3 | 1.461595 | -0.471360 | 633 | 455.98 | 0.720348 |  |
| GO:0007169\_transmembrane\_receptor\_protein\_tyrosine\_kinase\_signaling\_pathway | JAK2 | 139 | 3 | 1.461595 | -0.471360 | 633 | 455.98 | 0.720348 |  |
| GO:0002062\_chondrocyte\_differentiation | FGF18 | 28 | 1 | 2.418592 | -0.466617 | 641 | 461.36 | 0.719750 |  |
| GO:0002705\_positive\_regulation\_of\_leukocyte\_mediated\_immunity | PTPRC | 28 | 1 | 2.418592 | -0.466617 | 641 | 461.36 | 0.719750 |  |
| GO:0002708\_positive\_regulation\_of\_lymphocyte\_mediated\_immunity | PTPRC | 28 | 1 | 2.418592 | -0.466617 | 641 | 461.36 | 0.719750 |  |
| GO:0007585\_respiratory\_gaseous\_exchange | ATP1A2 | 28 | 1 | 2.418592 | -0.466617 | 641 | 461.36 | 0.719750 |  |
| GO:0042100\_B\_cell\_proliferation | PTPRC | 28 | 1 | 2.418592 | -0.466617 | 641 | 461.36 | 0.719750 |  |
| GO:0045926\_negative\_regulation\_of\_growth | ATXN2 | 28 | 1 | 2.418592 | -0.466617 | 641 | 461.36 | 0.719750 |  |
| GO:0046328\_regulation\_of\_JNK\_cascade | CD27 | 28 | 1 | 2.418592 | -0.466617 | 641 | 461.36 | 0.719750 |  |
| GO:0050871\_positive\_regulation\_of\_B\_cell\_activation | PTPRC | 28 | 1 | 2.418592 | -0.466617 | 641 | 461.36 | 0.719750 |  |
| GO:0010629\_negative\_regulation\_of\_gene\_expression | MSX1 | 262 | 5 | 1.292378 | -0.462868 | 642 | 463.57 | 0.722072 |  |
| GO:0010629\_negative\_regulation\_of\_gene\_expression | TBX3 | 262 | 5 | 1.292378 | -0.462868 | 642 | 463.57 | 0.722072 |  |
| GO:0010629\_negative\_regulation\_of\_gene\_expression | TBX20 | 262 | 5 | 1.292378 | -0.462868 | 642 | 463.57 | 0.722072 |  |
| GO:0010629\_negative\_regulation\_of\_gene\_expression | HDAC9 | 262 | 5 | 1.292378 | -0.462868 | 642 | 463.57 | 0.722072 |  |
| GO:0010629\_negative\_regulation\_of\_gene\_expression | NR2E1 | 262 | 5 | 1.292378 | -0.462868 | 642 | 463.57 | 0.722072 |  |
| GO:0003006\_reproductive\_developmental\_process | AFP | 141 | 3 | 1.440864 | -0.460978 | 643 | 464.19 | 0.721913 |  |
| GO:0003006\_reproductive\_developmental\_process | ZFX | 141 | 3 | 1.440864 | -0.460978 | 643 | 464.19 | 0.721913 |  |
| GO:0003006\_reproductive\_developmental\_process | TCF7L2 | 141 | 3 | 1.440864 | -0.460978 | 643 | 464.19 | 0.721913 |  |
| GO:0003008\_system\_process | MYO6 | 516 | 9 | 1.181173 | -0.455215 | 644 | 466.6 | 0.724534 |  |
| GO:0003008\_system\_process | IRX5 | 516 | 9 | 1.181173 | -0.455215 | 644 | 466.6 | 0.724534 |  |
| GO:0003008\_system\_process | MYOM2 | 516 | 9 | 1.181173 | -0.455215 | 644 | 466.6 | 0.724534 |  |
| GO:0003008\_system\_process | DMD | 516 | 9 | 1.181173 | -0.455215 | 644 | 466.6 | 0.724534 |  |
| GO:0003008\_system\_process | HEXB | 516 | 9 | 1.181173 | -0.455215 | 644 | 466.6 | 0.724534 |  |
| GO:0003008\_system\_process | TBX20 | 516 | 9 | 1.181173 | -0.455215 | 644 | 466.6 | 0.724534 |  |
| GO:0003008\_system\_process | B3GNT2 | 516 | 9 | 1.181173 | -0.455215 | 644 | 466.6 | 0.724534 |  |
| GO:0003008\_system\_process | ATP1A2 | 516 | 9 | 1.181173 | -0.455215 | 644 | 466.6 | 0.724534 |  |
| GO:0003008\_system\_process | NR2E1 | 516 | 9 | 1.181173 | -0.455215 | 644 | 466.6 | 0.724534 |  |
| GO:0001934\_positive\_regulation\_of\_protein\_amino\_acid\_phosphorylation | JAK2 | 29 | 1 | 2.335193 | -0.454350 | 652 | 472.42 | 0.724571 |  |
| GO:0006909\_phagocytosis | ABCA1 | 29 | 1 | 2.335193 | -0.454350 | 652 | 472.42 | 0.724571 |  |
| GO:0016447\_somatic\_recombination\_of\_immunoglobulin\_gene\_segments | PTPRC | 29 | 1 | 2.335193 | -0.454350 | 652 | 472.42 | 0.724571 |  |
| GO:0021761\_limbic\_system\_development | TBX3 | 29 | 1 | 2.335193 | -0.454350 | 652 | 472.42 | 0.724571 |  |
| GO:0042490\_mechanoreceptor\_differentiation | MYO6 | 29 | 1 | 2.335193 | -0.454350 | 652 | 472.42 | 0.724571 |  |
| GO:0045621\_positive\_regulation\_of\_lymphocyte\_differentiation | PTPRC | 29 | 1 | 2.335193 | -0.454350 | 652 | 472.42 | 0.724571 |  |
| GO:0046634\_regulation\_of\_alpha-beta\_T\_cell\_activation | PTPRC | 29 | 1 | 2.335193 | -0.454350 | 652 | 472.42 | 0.724571 |  |
| GO:0070302\_regulation\_of\_stress-activated\_protein\_kinase\_signaling\_pathway | CD27 | 29 | 1 | 2.335193 | -0.454350 | 652 | 472.42 | 0.724571 |  |
| GO:0045137\_development\_of\_primary\_sexual\_characteristics | AFP | 84 | 2 | 1.612395 | -0.451851 | 653 | 473.22 | 0.724686 |  |
| GO:0045137\_development\_of\_primary\_sexual\_characteristics | ZFX | 84 | 2 | 1.612395 | -0.451851 | 653 | 473.22 | 0.724686 |  |
| GO:0035239\_tube\_morphogenesis | TBX3 | 143 | 3 | 1.420712 | -0.450846 | 654 | 473.56 | 0.724098 |  |
| GO:0035239\_tube\_morphogenesis | TBX20 | 143 | 3 | 1.420712 | -0.450846 | 654 | 473.56 | 0.724098 |  |
| GO:0035239\_tube\_morphogenesis | NR3C1 | 143 | 3 | 1.420712 | -0.450846 | 654 | 473.56 | 0.724098 |  |
| GO:0002449\_lymphocyte\_mediated\_immunity | PTPRC | 85 | 2 | 1.593426 | -0.445281 | 655 | 474.93 | 0.725084 |  |
| GO:0002449\_lymphocyte\_mediated\_immunity | RAB27A | 85 | 2 | 1.593426 | -0.445281 | 655 | 474.93 | 0.725084 |  |
| GO:0007243\_protein\_kinase\_cascade | PTPRC | 205 | 4 | 1.321377 | -0.445005 | 656 | 475.28 | 0.724512 |  |
| GO:0007243\_protein\_kinase\_cascade | JAK2 | 205 | 4 | 1.321377 | -0.445005 | 656 | 475.28 | 0.724512 |  |
| GO:0007243\_protein\_kinase\_cascade | GADD45B | 205 | 4 | 1.321377 | -0.445005 | 656 | 475.28 | 0.724512 |  |
| GO:0007243\_protein\_kinase\_cascade | CD27 | 205 | 4 | 1.321377 | -0.445005 | 656 | 475.28 | 0.724512 |  |
| GO:0000187\_activation\_of\_MAPK\_activity | PTPRC | 30 | 1 | 2.257353 | -0.442592 | 664 | 482.74 | 0.727018 |  |
| GO:0007219\_Notch\_signaling\_pathway | ASCL1 | 30 | 1 | 2.257353 | -0.442592 | 664 | 482.74 | 0.727018 |  |
| GO:0009266\_response\_to\_temperature\_stimulus | HSPA1A | 30 | 1 | 2.257353 | -0.442592 | 664 | 482.74 | 0.727018 |  |
| GO:0016445\_somatic\_diversification\_of\_immunoglobulins | PTPRC | 30 | 1 | 2.257353 | -0.442592 | 664 | 482.74 | 0.727018 |  |
| GO:0030522\_intracellular\_receptor-mediated\_signaling\_pathway | NR3C1 | 30 | 1 | 2.257353 | -0.442592 | 664 | 482.74 | 0.727018 |  |
| GO:0042552\_myelination | HEXB | 30 | 1 | 2.257353 | -0.442592 | 664 | 482.74 | 0.727018 |  |
| GO:0048565\_gut\_development | TCF7L2 | 30 | 1 | 2.257353 | -0.442592 | 664 | 482.74 | 0.727018 |  |
| GO:0060021\_palate\_development | ARID5B | 30 | 1 | 2.257353 | -0.442592 | 664 | 482.74 | 0.727018 |  |
| GO:0010605\_negative\_regulation\_of\_macromolecule\_metabolic\_process | PTPRC | 331 | 6 | 1.227564 | -0.439777 | 665 | 483.26 | 0.726707 |  |
| GO:0010605\_negative\_regulation\_of\_macromolecule\_metabolic\_process | MSX1 | 331 | 6 | 1.227564 | -0.439777 | 665 | 483.26 | 0.726707 |  |
| GO:0010605\_negative\_regulation\_of\_macromolecule\_metabolic\_process | TBX3 | 331 | 6 | 1.227564 | -0.439777 | 665 | 483.26 | 0.726707 |  |
| GO:0010605\_negative\_regulation\_of\_macromolecule\_metabolic\_process | TBX20 | 331 | 6 | 1.227564 | -0.439777 | 665 | 483.26 | 0.726707 |  |
| GO:0010605\_negative\_regulation\_of\_macromolecule\_metabolic\_process | HDAC9 | 331 | 6 | 1.227564 | -0.439777 | 665 | 483.26 | 0.726707 |  |
| GO:0010605\_negative\_regulation\_of\_macromolecule\_metabolic\_process | NR2E1 | 331 | 6 | 1.227564 | -0.439777 | 665 | 483.26 | 0.726707 |  |
| GO:0043412\_biopolymer\_modification | PTPRC | 458 | 8 | 1.182892 | -0.439367 | 666 | 483.58 | 0.726096 |  |
| GO:0043412\_biopolymer\_modification | FUT9 | 458 | 8 | 1.182892 | -0.439367 | 666 | 483.58 | 0.726096 |  |
| GO:0043412\_biopolymer\_modification | MAMDC2 | 458 | 8 | 1.182892 | -0.439367 | 666 | 483.58 | 0.726096 |  |
| GO:0043412\_biopolymer\_modification | JAK2 | 458 | 8 | 1.182892 | -0.439367 | 666 | 483.58 | 0.726096 |  |
| GO:0043412\_biopolymer\_modification | ABCA1 | 458 | 8 | 1.182892 | -0.439367 | 666 | 483.58 | 0.726096 |  |
| GO:0043412\_biopolymer\_modification | B3GNT2 | 458 | 8 | 1.182892 | -0.439367 | 666 | 483.58 | 0.726096 |  |
| GO:0043412\_biopolymer\_modification | PPAP2A | 458 | 8 | 1.182892 | -0.439367 | 666 | 483.58 | 0.726096 |  |
| GO:0043412\_biopolymer\_modification | GADD45B | 458 | 8 | 1.182892 | -0.439367 | 666 | 483.58 | 0.726096 |  |
| GO:0006897\_endocytosis | MYO6 | 86 | 2 | 1.574897 | -0.438828 | 668 | 484.91 | 0.725913 |  |
| GO:0006897\_endocytosis | ABCA1 | 86 | 2 | 1.574897 | -0.438828 | 668 | 484.91 | 0.725913 |  |
| GO:0010324\_membrane\_invagination | MYO6 | 86 | 2 | 1.574897 | -0.438828 | 668 | 484.91 | 0.725913 |  |
| GO:0010324\_membrane\_invagination | ABCA1 | 86 | 2 | 1.574897 | -0.438828 | 668 | 484.91 | 0.725913 |  |
| GO:0045944\_positive\_regulation\_of\_transcription\_from\_RNA\_polymerase\_II\_promoter | ASCL1 | 269 | 5 | 1.258747 | -0.437262 | 669 | 485.28 | 0.725381 |  |
| GO:0045944\_positive\_regulation\_of\_transcription\_from\_RNA\_polymerase\_II\_promoter | MSX1 | 269 | 5 | 1.258747 | -0.437262 | 669 | 485.28 | 0.725381 |  |
| GO:0045944\_positive\_regulation\_of\_transcription\_from\_RNA\_polymerase\_II\_promoter | SOX5 | 269 | 5 | 1.258747 | -0.437262 | 669 | 485.28 | 0.725381 |  |
| GO:0045944\_positive\_regulation\_of\_transcription\_from\_RNA\_polymerase\_II\_promoter | NEUROD1 | 269 | 5 | 1.258747 | -0.437262 | 669 | 485.28 | 0.725381 |  |
| GO:0045944\_positive\_regulation\_of\_transcription\_from\_RNA\_polymerase\_II\_promoter | TCF7L2 | 269 | 5 | 1.258747 | -0.437262 | 669 | 485.28 | 0.725381 |  |
| GO:0031324\_negative\_regulation\_of\_cellular\_metabolic\_process | PTPRC | 332 | 6 | 1.223866 | -0.436557 | 670 | 485.47 | 0.724582 |  |
| GO:0031324\_negative\_regulation\_of\_cellular\_metabolic\_process | MSX1 | 332 | 6 | 1.223866 | -0.436557 | 670 | 485.47 | 0.724582 |  |
| GO:0031324\_negative\_regulation\_of\_cellular\_metabolic\_process | TBX3 | 332 | 6 | 1.223866 | -0.436557 | 670 | 485.47 | 0.724582 |  |
| GO:0031324\_negative\_regulation\_of\_cellular\_metabolic\_process | TBX20 | 332 | 6 | 1.223866 | -0.436557 | 670 | 485.47 | 0.724582 |  |
| GO:0031324\_negative\_regulation\_of\_cellular\_metabolic\_process | HDAC9 | 332 | 6 | 1.223866 | -0.436557 | 670 | 485.47 | 0.724582 |  |
| GO:0031324\_negative\_regulation\_of\_cellular\_metabolic\_process | NR2E1 | 332 | 6 | 1.223866 | -0.436557 | 670 | 485.47 | 0.724582 |  |
| GO:0006812\_cation\_transport | PTPRC | 146 | 3 | 1.391519 | -0.436101 | 671 | 486.11 | 0.724456 |  |
| GO:0006812\_cation\_transport | KCNA5 | 146 | 3 | 1.391519 | -0.436101 | 671 | 486.11 | 0.724456 |  |
| GO:0006812\_cation\_transport | SLC40A1 | 146 | 3 | 1.391519 | -0.436101 | 671 | 486.11 | 0.724456 |  |
| GO:0045934\_negative\_regulation\_of\_nucleobase\_\_nucleoside\_\_nucleotide\_and\_nucleic\_acid\_metabolic\_process | MSX1 | 270 | 5 | 1.254085 | -0.433717 | 672 | 486.58 | 0.724077 |  |
| GO:0045934\_negative\_regulation\_of\_nucleobase\_\_nucleoside\_\_nucleotide\_and\_nucleic\_acid\_metabolic\_process | TBX3 | 270 | 5 | 1.254085 | -0.433717 | 672 | 486.58 | 0.724077 |  |
| GO:0045934\_negative\_regulation\_of\_nucleobase\_\_nucleoside\_\_nucleotide\_and\_nucleic\_acid\_metabolic\_process | TBX20 | 270 | 5 | 1.254085 | -0.433717 | 672 | 486.58 | 0.724077 |  |
| GO:0045934\_negative\_regulation\_of\_nucleobase\_\_nucleoside\_\_nucleotide\_and\_nucleic\_acid\_metabolic\_process | HDAC9 | 270 | 5 | 1.254085 | -0.433717 | 672 | 486.58 | 0.724077 |  |
| GO:0045934\_negative\_regulation\_of\_nucleobase\_\_nucleoside\_\_nucleotide\_and\_nucleic\_acid\_metabolic\_process | NR2E1 | 270 | 5 | 1.254085 | -0.433717 | 672 | 486.58 | 0.724077 |  |
| GO:0022612\_gland\_morphogenesis | TBX3 | 87 | 2 | 1.556795 | -0.432489 | 674 | 488.53 | 0.724822 |  |
| GO:0022612\_gland\_morphogenesis | NR3C1 | 87 | 2 | 1.556795 | -0.432489 | 674 | 488.53 | 0.724822 |  |
| GO:0050778\_positive\_regulation\_of\_immune\_response | PTPRC | 87 | 2 | 1.556795 | -0.432489 | 674 | 488.53 | 0.724822 |  |
| GO:0050778\_positive\_regulation\_of\_immune\_response | ELF1 | 87 | 2 | 1.556795 | -0.432489 | 674 | 488.53 | 0.724822 |  |
| GO:0009888\_tissue\_development | FGF18 | 525 | 9 | 1.160924 | -0.431542 | 675 | 488.83 | 0.724193 |  |
| GO:0009888\_tissue\_development | TBX3 | 525 | 9 | 1.160924 | -0.431542 | 675 | 488.83 | 0.724193 |  |
| GO:0009888\_tissue\_development | DMD | 525 | 9 | 1.160924 | -0.431542 | 675 | 488.83 | 0.724193 |  |
| GO:0009888\_tissue\_development | SOX5 | 525 | 9 | 1.160924 | -0.431542 | 675 | 488.83 | 0.724193 |  |
| GO:0009888\_tissue\_development | PCDH8 | 525 | 9 | 1.160924 | -0.431542 | 675 | 488.83 | 0.724193 |  |
| GO:0009888\_tissue\_development | MBNL3 | 525 | 9 | 1.160924 | -0.431542 | 675 | 488.83 | 0.724193 |  |
| GO:0009888\_tissue\_development | NR3C1 | 525 | 9 | 1.160924 | -0.431542 | 675 | 488.83 | 0.724193 |  |
| GO:0009888\_tissue\_development | HDAC9 | 525 | 9 | 1.160924 | -0.431542 | 675 | 488.83 | 0.724193 |  |
| GO:0009888\_tissue\_development | BOC | 525 | 9 | 1.160924 | -0.431542 | 675 | 488.83 | 0.724193 |  |
| GO:0003018\_vascular\_process\_in\_circulatory\_system | ATP1A2 | 31 | 1 | 2.184535 | -0.431310 | 689 | 498.07 | 0.722888 |  |
| GO:0006639\_acylglycerol\_metabolic\_process | PPAP2A | 31 | 1 | 2.184535 | -0.431310 | 689 | 498.07 | 0.722888 |  |
| GO:0006694\_steroid\_biosynthetic\_process | NR3C1 | 31 | 1 | 2.184535 | -0.431310 | 689 | 498.07 | 0.722888 |  |
| GO:0006939\_smooth\_muscle\_contraction | ATP1A2 | 31 | 1 | 2.184535 | -0.431310 | 689 | 498.07 | 0.722888 |  |
| GO:0010562\_positive\_regulation\_of\_phosphorus\_metabolic\_process | JAK2 | 31 | 1 | 2.184535 | -0.431310 | 689 | 498.07 | 0.722888 |  |
| GO:0016049\_cell\_growth | SGMS1 | 31 | 1 | 2.184535 | -0.431310 | 689 | 498.07 | 0.722888 |  |
| GO:0035150\_regulation\_of\_tube\_size | ATP1A2 | 31 | 1 | 2.184535 | -0.431310 | 689 | 498.07 | 0.722888 |  |
| GO:0042157\_lipoprotein\_metabolic\_process | ABCA1 | 31 | 1 | 2.184535 | -0.431310 | 689 | 498.07 | 0.722888 |  |
| GO:0042327\_positive\_regulation\_of\_phosphorylation | JAK2 | 31 | 1 | 2.184535 | -0.431310 | 689 | 498.07 | 0.722888 |  |
| GO:0043269\_regulation\_of\_ion\_transport | KCNA5 | 31 | 1 | 2.184535 | -0.431310 | 689 | 498.07 | 0.722888 |  |
| GO:0045937\_positive\_regulation\_of\_phosphate\_metabolic\_process | JAK2 | 31 | 1 | 2.184535 | -0.431310 | 689 | 498.07 | 0.722888 |  |
| GO:0048167\_regulation\_of\_synaptic\_plasticity | MYO6 | 31 | 1 | 2.184535 | -0.431310 | 689 | 498.07 | 0.722888 |  |
| GO:0048562\_embryonic\_organ\_morphogenesis | TCF7L2 | 31 | 1 | 2.184535 | -0.431310 | 689 | 498.07 | 0.722888 |  |
| GO:0050880\_regulation\_of\_blood\_vessel\_size | ATP1A2 | 31 | 1 | 2.184535 | -0.431310 | 689 | 498.07 | 0.722888 |  |
| GO:0051172\_negative\_regulation\_of\_nitrogen\_compound\_metabolic\_process | MSX1 | 271 | 5 | 1.249457 | -0.430200 | 690 | 498.6 | 0.722609 |  |
| GO:0051172\_negative\_regulation\_of\_nitrogen\_compound\_metabolic\_process | TBX3 | 271 | 5 | 1.249457 | -0.430200 | 690 | 498.6 | 0.722609 |  |
| GO:0051172\_negative\_regulation\_of\_nitrogen\_compound\_metabolic\_process | TBX20 | 271 | 5 | 1.249457 | -0.430200 | 690 | 498.6 | 0.722609 |  |
| GO:0051172\_negative\_regulation\_of\_nitrogen\_compound\_metabolic\_process | HDAC9 | 271 | 5 | 1.249457 | -0.430200 | 690 | 498.6 | 0.722609 |  |
| GO:0051172\_negative\_regulation\_of\_nitrogen\_compound\_metabolic\_process | NR2E1 | 271 | 5 | 1.249457 | -0.430200 | 690 | 498.6 | 0.722609 |  |
| GO:0048754\_branching\_morphogenesis\_of\_a\_tube | TBX3 | 88 | 2 | 1.539104 | -0.426260 | 691 | 500.34 | 0.724081 |  |
| GO:0048754\_branching\_morphogenesis\_of\_a\_tube | TBX20 | 88 | 2 | 1.539104 | -0.426260 | 691 | 500.34 | 0.724081 |  |
| GO:0002274\_myeloid\_leukocyte\_activation | NDRG1 | 32 | 1 | 2.116268 | -0.420472 | 700 | 506.01 | 0.722871 |  |
| GO:0006638\_neutral\_lipid\_metabolic\_process | PPAP2A | 32 | 1 | 2.116268 | -0.420472 | 700 | 506.01 | 0.722871 |  |
| GO:0006662\_glycerol\_ether\_metabolic\_process | PPAP2A | 32 | 1 | 2.116268 | -0.420472 | 700 | 506.01 | 0.722871 |  |
| GO:0006937\_regulation\_of\_muscle\_contraction | ATP1A2 | 32 | 1 | 2.116268 | -0.420472 | 700 | 506.01 | 0.722871 |  |
| GO:0007272\_ensheathment\_of\_neurons | HEXB | 32 | 1 | 2.116268 | -0.420472 | 700 | 506.01 | 0.722871 |  |
| GO:0008366\_axon\_ensheathment | HEXB | 32 | 1 | 2.116268 | -0.420472 | 700 | 506.01 | 0.722871 |  |
| GO:0042102\_positive\_regulation\_of\_T\_cell\_proliferation | PTPRC | 32 | 1 | 2.116268 | -0.420472 | 700 | 506.01 | 0.722871 |  |
| GO:0050768\_negative\_regulation\_of\_neurogenesis | NKX6-1 | 32 | 1 | 2.116268 | -0.420472 | 700 | 506.01 | 0.722871 |  |
| GO:0050885\_neuromuscular\_process\_controlling\_balance | HEXB | 32 | 1 | 2.116268 | -0.420472 | 700 | 506.01 | 0.722871 |  |
| GO:0010558\_negative\_regulation\_of\_macromolecule\_biosynthetic\_process | MSX1 | 274 | 5 | 1.235777 | -0.419814 | 701 | 506.42 | 0.722425 |  |
| GO:0010558\_negative\_regulation\_of\_macromolecule\_biosynthetic\_process | TBX3 | 274 | 5 | 1.235777 | -0.419814 | 701 | 506.42 | 0.722425 |  |
| GO:0010558\_negative\_regulation\_of\_macromolecule\_biosynthetic\_process | TBX20 | 274 | 5 | 1.235777 | -0.419814 | 701 | 506.42 | 0.722425 |  |
| GO:0010558\_negative\_regulation\_of\_macromolecule\_biosynthetic\_process | HDAC9 | 274 | 5 | 1.235777 | -0.419814 | 701 | 506.42 | 0.722425 |  |
| GO:0010558\_negative\_regulation\_of\_macromolecule\_biosynthetic\_process | NR2E1 | 274 | 5 | 1.235777 | -0.419814 | 701 | 506.42 | 0.722425 |  |
| GO:0035295\_tube\_development | FGF18 | 212 | 4 | 1.277747 | -0.417054 | 702 | 506.85 | 0.722009 |  |
| GO:0035295\_tube\_development | TBX3 | 212 | 4 | 1.277747 | -0.417054 | 702 | 506.85 | 0.722009 |  |
| GO:0035295\_tube\_development | TBX20 | 212 | 4 | 1.277747 | -0.417054 | 702 | 506.85 | 0.722009 |  |
| GO:0035295\_tube\_development | NR3C1 | 212 | 4 | 1.277747 | -0.417054 | 702 | 506.85 | 0.722009 |  |
| GO:0042113\_B\_cell\_activation | PTPRC | 90 | 2 | 1.504902 | -0.414124 | 703 | 508.11 | 0.722774 |  |
| GO:0042113\_B\_cell\_activation | HDAC9 | 90 | 2 | 1.504902 | -0.414124 | 703 | 508.11 | 0.722774 |  |
| GO:0050794\_regulation\_of\_cellular\_process | FGF18 | 2190 | 34 | 1.051370 | -0.411606 | 704 | 508.29 | 0.722003 |  |
| GO:0050794\_regulation\_of\_cellular\_process | ELF1 | 2190 | 34 | 1.051370 | -0.411606 | 704 | 508.29 | 0.722003 |  |
| GO:0050794\_regulation\_of\_cellular\_process | PLXNA2 | 2190 | 34 | 1.051370 | -0.411606 | 704 | 508.29 | 0.722003 |  |
| GO:0050794\_regulation\_of\_cellular\_process | HEXB | 2190 | 34 | 1.051370 | -0.411606 | 704 | 508.29 | 0.722003 |  |
| GO:0050794\_regulation\_of\_cellular\_process | TBX20 | 2190 | 34 | 1.051370 | -0.411606 | 704 | 508.29 | 0.722003 |  |
| GO:0050794\_regulation\_of\_cellular\_process | SOX5 | 2190 | 34 | 1.051370 | -0.411606 | 704 | 508.29 | 0.722003 |  |
| GO:0050794\_regulation\_of\_cellular\_process | NR3C1 | 2190 | 34 | 1.051370 | -0.411606 | 704 | 508.29 | 0.722003 |  |
| GO:0050794\_regulation\_of\_cellular\_process | SGMS1 | 2190 | 34 | 1.051370 | -0.411606 | 704 | 508.29 | 0.722003 |  |
| GO:0050794\_regulation\_of\_cellular\_process | TCF7L2 | 2190 | 34 | 1.051370 | -0.411606 | 704 | 508.29 | 0.722003 |  |
| GO:0050794\_regulation\_of\_cellular\_process | NR2E1 | 2190 | 34 | 1.051370 | -0.411606 | 704 | 508.29 | 0.722003 |  |
| GO:0050794\_regulation\_of\_cellular\_process | SERINC3 | 2190 | 34 | 1.051370 | -0.411606 | 704 | 508.29 | 0.722003 |  |
| GO:0050794\_regulation\_of\_cellular\_process | DMD | 2190 | 34 | 1.051370 | -0.411606 | 704 | 508.29 | 0.722003 |  |
| GO:0050794\_regulation\_of\_cellular\_process | ETV1 | 2190 | 34 | 1.051370 | -0.411606 | 704 | 508.29 | 0.722003 |  |
| GO:0050794\_regulation\_of\_cellular\_process | MBNL3 | 2190 | 34 | 1.051370 | -0.411606 | 704 | 508.29 | 0.722003 |  |
| GO:0050794\_regulation\_of\_cellular\_process | PPAP2A | 2190 | 34 | 1.051370 | -0.411606 | 704 | 508.29 | 0.722003 |  |
| GO:0050794\_regulation\_of\_cellular\_process | THBS1 | 2190 | 34 | 1.051370 | -0.411606 | 704 | 508.29 | 0.722003 |  |
| GO:0050794\_regulation\_of\_cellular\_process | BOC | 2190 | 34 | 1.051370 | -0.411606 | 704 | 508.29 | 0.722003 |  |
| GO:0050794\_regulation\_of\_cellular\_process | CD27 | 2190 | 34 | 1.051370 | -0.411606 | 704 | 508.29 | 0.722003 |  |
| GO:0050794\_regulation\_of\_cellular\_process | RAB27A | 2190 | 34 | 1.051370 | -0.411606 | 704 | 508.29 | 0.722003 |  |
| GO:0050794\_regulation\_of\_cellular\_process | PTPRC | 2190 | 34 | 1.051370 | -0.411606 | 704 | 508.29 | 0.722003 |  |
| GO:0050794\_regulation\_of\_cellular\_process | MYO6 | 2190 | 34 | 1.051370 | -0.411606 | 704 | 508.29 | 0.722003 |  |
| GO:0050794\_regulation\_of\_cellular\_process | TBX3 | 2190 | 34 | 1.051370 | -0.411606 | 704 | 508.29 | 0.722003 |  |
| GO:0050794\_regulation\_of\_cellular\_process | ARID5B | 2190 | 34 | 1.051370 | -0.411606 | 704 | 508.29 | 0.722003 |  |
| GO:0050794\_regulation\_of\_cellular\_process | ESRRG | 2190 | 34 | 1.051370 | -0.411606 | 704 | 508.29 | 0.722003 |  |
| GO:0050794\_regulation\_of\_cellular\_process | NKX6-1 | 2190 | 34 | 1.051370 | -0.411606 | 704 | 508.29 | 0.722003 |  |
| GO:0050794\_regulation\_of\_cellular\_process | RGS13 | 2190 | 34 | 1.051370 | -0.411606 | 704 | 508.29 | 0.722003 |  |
| GO:0050794\_regulation\_of\_cellular\_process | ASCL1 | 2190 | 34 | 1.051370 | -0.411606 | 704 | 508.29 | 0.722003 |  |
| GO:0050794\_regulation\_of\_cellular\_process | MSX1 | 2190 | 34 | 1.051370 | -0.411606 | 704 | 508.29 | 0.722003 |  |
| GO:0050794\_regulation\_of\_cellular\_process | NEUROD1 | 2190 | 34 | 1.051370 | -0.411606 | 704 | 508.29 | 0.722003 |  |
| GO:0050794\_regulation\_of\_cellular\_process | RGS8 | 2190 | 34 | 1.051370 | -0.411606 | 704 | 508.29 | 0.722003 |  |
| GO:0050794\_regulation\_of\_cellular\_process | JAK2 | 2190 | 34 | 1.051370 | -0.411606 | 704 | 508.29 | 0.722003 |  |
| GO:0050794\_regulation\_of\_cellular\_process | HDAC9 | 2190 | 34 | 1.051370 | -0.411606 | 704 | 508.29 | 0.722003 |  |
| GO:0050794\_regulation\_of\_cellular\_process | GADD45B | 2190 | 34 | 1.051370 | -0.411606 | 704 | 508.29 | 0.722003 |  |
| GO:0050794\_regulation\_of\_cellular\_process | CAMK1D | 2190 | 34 | 1.051370 | -0.411606 | 704 | 508.29 | 0.722003 |  |
| GO:0002562\_somatic\_diversification\_of\_immune\_receptors\_via\_germline\_recombination\_within\_a\_single\_locus | PTPRC | 33 | 1 | 2.052139 | -0.410051 | 706 | 513.34 | 0.727110 |  |
| GO:0016444\_somatic\_cell\_DNA\_recombination | PTPRC | 33 | 1 | 2.052139 | -0.410051 | 706 | 513.34 | 0.727110 |  |
| GO:0002443\_leukocyte\_mediated\_immunity | PTPRC | 91 | 2 | 1.488365 | -0.408212 | 708 | 514.62 | 0.726864 |  |
| GO:0002443\_leukocyte\_mediated\_immunity | RAB27A | 91 | 2 | 1.488365 | -0.408212 | 708 | 514.62 | 0.726864 |  |
| GO:0031399\_regulation\_of\_protein\_modification\_process | PTPRC | 91 | 2 | 1.488365 | -0.408212 | 708 | 514.62 | 0.726864 |  |
| GO:0031399\_regulation\_of\_protein\_modification\_process | JAK2 | 91 | 2 | 1.488365 | -0.408212 | 708 | 514.62 | 0.726864 |  |
| GO:0002200\_somatic\_diversification\_of\_immune\_receptors | PTPRC | 34 | 1 | 1.991782 | -0.400023 | 716 | 522.05 | 0.729120 |  |
| GO:0002699\_positive\_regulation\_of\_immune\_effector\_process | PTPRC | 34 | 1 | 1.991782 | -0.400023 | 716 | 522.05 | 0.729120 |  |
| GO:0007338\_single\_fertilization | HEXB | 34 | 1 | 1.991782 | -0.400023 | 716 | 522.05 | 0.729120 |  |
| GO:0007568\_aging | TBX3 | 34 | 1 | 1.991782 | -0.400023 | 716 | 522.05 | 0.729120 |  |
| GO:0010721\_negative\_regulation\_of\_cell\_development | NKX6-1 | 34 | 1 | 1.991782 | -0.400023 | 716 | 522.05 | 0.729120 |  |
| GO:0030509\_BMP\_signaling\_pathway | MSX1 | 34 | 1 | 1.991782 | -0.400023 | 716 | 522.05 | 0.729120 |  |
| GO:0051052\_regulation\_of\_DNA\_metabolic\_process | PTPRC | 34 | 1 | 1.991782 | -0.400023 | 716 | 522.05 | 0.729120 |  |
| GO:0060711\_labyrinthine\_layer\_development | ITGA4 | 34 | 1 | 1.991782 | -0.400023 | 716 | 522.05 | 0.729120 |  |
| GO:0007268\_synaptic\_transmission | MYO6 | 154 | 3 | 1.319232 | -0.399269 | 717 | 522.28 | 0.728424 |  |
| GO:0007268\_synaptic\_transmission | DMD | 154 | 3 | 1.319232 | -0.399269 | 717 | 522.28 | 0.728424 |  |
| GO:0007268\_synaptic\_transmission | ATP1A2 | 154 | 3 | 1.319232 | -0.399269 | 717 | 522.28 | 0.728424 |  |
| GO:0048583\_regulation\_of\_response\_to\_stimulus | PTPRC | 217 | 4 | 1.248306 | -0.398177 | 718 | 522.75 | 0.728064 |  |
| GO:0048583\_regulation\_of\_response\_to\_stimulus | ELF1 | 217 | 4 | 1.248306 | -0.398177 | 718 | 522.75 | 0.728064 |  |
| GO:0048583\_regulation\_of\_response\_to\_stimulus | JAK2 | 217 | 4 | 1.248306 | -0.398177 | 718 | 522.75 | 0.728064 |  |
| GO:0048583\_regulation\_of\_response\_to\_stimulus | CD27 | 217 | 4 | 1.248306 | -0.398177 | 718 | 522.75 | 0.728064 |  |
| GO:0035107\_appendage\_morphogenesis | MSX1 | 93 | 2 | 1.456357 | -0.396688 | 720 | 524.02 | 0.727806 |  |
| GO:0035107\_appendage\_morphogenesis | TBX3 | 93 | 2 | 1.456357 | -0.396688 | 720 | 524.02 | 0.727806 |  |
| GO:0035108\_limb\_morphogenesis | MSX1 | 93 | 2 | 1.456357 | -0.396688 | 720 | 524.02 | 0.727806 |  |
| GO:0035108\_limb\_morphogenesis | TBX3 | 93 | 2 | 1.456357 | -0.396688 | 720 | 524.02 | 0.727806 |  |
| GO:0031327\_negative\_regulation\_of\_cellular\_biosynthetic\_process | MSX1 | 282 | 5 | 1.200720 | -0.393279 | 721 | 524.96 | 0.728100 |  |
| GO:0031327\_negative\_regulation\_of\_cellular\_biosynthetic\_process | TBX3 | 282 | 5 | 1.200720 | -0.393279 | 721 | 524.96 | 0.728100 |  |
| GO:0031327\_negative\_regulation\_of\_cellular\_biosynthetic\_process | TBX20 | 282 | 5 | 1.200720 | -0.393279 | 721 | 524.96 | 0.728100 |  |
| GO:0031327\_negative\_regulation\_of\_cellular\_biosynthetic\_process | HDAC9 | 282 | 5 | 1.200720 | -0.393279 | 721 | 524.96 | 0.728100 |  |
| GO:0031327\_negative\_regulation\_of\_cellular\_biosynthetic\_process | NR2E1 | 282 | 5 | 1.200720 | -0.393279 | 721 | 524.96 | 0.728100 |  |
| GO:0001756\_somitogenesis | PCDH8 | 35 | 1 | 1.934874 | -0.390362 | 731 | 531.18 | 0.726648 |  |
| GO:0006869\_lipid\_transport | ABCA1 | 35 | 1 | 1.934874 | -0.390362 | 731 | 531.18 | 0.726648 |  |
| GO:0010810\_regulation\_of\_cell-substrate\_adhesion | THBS1 | 35 | 1 | 1.934874 | -0.390362 | 731 | 531.18 | 0.726648 |  |
| GO:0016051\_carbohydrate\_biosynthetic\_process | NR3C1 | 35 | 1 | 1.934874 | -0.390362 | 731 | 531.18 | 0.726648 |  |
| GO:0018904\_organic\_ether\_metabolic\_process | PPAP2A | 35 | 1 | 1.934874 | -0.390362 | 731 | 531.18 | 0.726648 |  |
| GO:0043406\_positive\_regulation\_of\_MAP\_kinase\_activity | PTPRC | 35 | 1 | 1.934874 | -0.390362 | 731 | 531.18 | 0.726648 |  |
| GO:0044242\_cellular\_lipid\_catabolic\_process | HEXB | 35 | 1 | 1.934874 | -0.390362 | 731 | 531.18 | 0.726648 |  |
| GO:0051051\_negative\_regulation\_of\_transport | KCNA5 | 35 | 1 | 1.934874 | -0.390362 | 731 | 531.18 | 0.726648 |  |
| GO:0051325\_interphase | LATS2 | 35 | 1 | 1.934874 | -0.390362 | 731 | 531.18 | 0.726648 |  |
| GO:0051329\_interphase\_of\_mitotic\_cell\_cycle | LATS2 | 35 | 1 | 1.934874 | -0.390362 | 731 | 531.18 | 0.726648 |  |
| GO:0009892\_negative\_regulation\_of\_metabolic\_process | PTPRC | 348 | 6 | 1.167596 | -0.387914 | 732 | 531.91 | 0.726653 |  |
| GO:0009892\_negative\_regulation\_of\_metabolic\_process | MSX1 | 348 | 6 | 1.167596 | -0.387914 | 732 | 531.91 | 0.726653 |  |
| GO:0009892\_negative\_regulation\_of\_metabolic\_process | TBX3 | 348 | 6 | 1.167596 | -0.387914 | 732 | 531.91 | 0.726653 |  |
| GO:0009892\_negative\_regulation\_of\_metabolic\_process | TBX20 | 348 | 6 | 1.167596 | -0.387914 | 732 | 531.91 | 0.726653 |  |
| GO:0009892\_negative\_regulation\_of\_metabolic\_process | HDAC9 | 348 | 6 | 1.167596 | -0.387914 | 732 | 531.91 | 0.726653 |  |
| GO:0009892\_negative\_regulation\_of\_metabolic\_process | NR2E1 | 348 | 6 | 1.167596 | -0.387914 | 732 | 531.91 | 0.726653 |  |
| GO:0009890\_negative\_regulation\_of\_biosynthetic\_process | MSX1 | 284 | 5 | 1.192264 | -0.386900 | 733 | 532.24 | 0.726112 |  |
| GO:0009890\_negative\_regulation\_of\_biosynthetic\_process | TBX3 | 284 | 5 | 1.192264 | -0.386900 | 733 | 532.24 | 0.726112 |  |
| GO:0009890\_negative\_regulation\_of\_biosynthetic\_process | TBX20 | 284 | 5 | 1.192264 | -0.386900 | 733 | 532.24 | 0.726112 |  |
| GO:0009890\_negative\_regulation\_of\_biosynthetic\_process | HDAC9 | 284 | 5 | 1.192264 | -0.386900 | 733 | 532.24 | 0.726112 |  |
| GO:0009890\_negative\_regulation\_of\_biosynthetic\_process | NR2E1 | 284 | 5 | 1.192264 | -0.386900 | 733 | 532.24 | 0.726112 |  |
| GO:0042391\_regulation\_of\_membrane\_potential | HEXB | 95 | 2 | 1.425697 | -0.385548 | 734 | 532.99 | 0.726144 |  |
| GO:0042391\_regulation\_of\_membrane\_potential | KCNA5 | 95 | 2 | 1.425697 | -0.385548 | 734 | 532.99 | 0.726144 |  |
| GO:0019228\_regulation\_of\_action\_potential\_in\_neuron | HEXB | 36 | 1 | 1.881127 | -0.381049 | 735 | 540.38 | 0.735211 |  |
| GO:0006954\_inflammatory\_response | THBS1 | 96 | 2 | 1.410846 | -0.380117 | 738 | 541.86 | 0.734228 |  |
| GO:0006954\_inflammatory\_response | HDAC9 | 96 | 2 | 1.410846 | -0.380117 | 738 | 541.86 | 0.734228 |  |
| GO:0048736\_appendage\_development | MSX1 | 96 | 2 | 1.410846 | -0.380117 | 738 | 541.86 | 0.734228 |  |
| GO:0048736\_appendage\_development | TBX3 | 96 | 2 | 1.410846 | -0.380117 | 738 | 541.86 | 0.734228 |  |
| GO:0060173\_limb\_development | MSX1 | 96 | 2 | 1.410846 | -0.380117 | 738 | 541.86 | 0.734228 |  |
| GO:0060173\_limb\_development | TBX3 | 96 | 2 | 1.410846 | -0.380117 | 738 | 541.86 | 0.734228 |  |
| GO:0002790\_peptide\_secretion | ABCA1 | 37 | 1 | 1.830286 | -0.372063 | 741 | 547.42 | 0.738758 |  |
| GO:0045785\_positive\_regulation\_of\_cell\_adhesion | THBS1 | 37 | 1 | 1.830286 | -0.372063 | 741 | 547.42 | 0.738758 |  |
| GO:0051101\_regulation\_of\_DNA\_binding | JAK2 | 37 | 1 | 1.830286 | -0.372063 | 741 | 547.42 | 0.738758 |  |
| GO:0060541\_respiratory\_system\_development | ASCL1 | 98 | 2 | 1.382053 | -0.369522 | 742 | 548.67 | 0.739447 |  |
| GO:0060541\_respiratory\_system\_development | FGF18 | 98 | 2 | 1.382053 | -0.369522 | 742 | 548.67 | 0.739447 |  |
| GO:0009628\_response\_to\_abiotic\_stimulus | ETV1 | 162 | 3 | 1.254085 | -0.365742 | 743 | 549.19 | 0.739152 |  |
| GO:0009628\_response\_to\_abiotic\_stimulus | HSPA1A | 162 | 3 | 1.254085 | -0.365742 | 743 | 549.19 | 0.739152 |  |
| GO:0009628\_response\_to\_abiotic\_stimulus | ATP1A2 | 162 | 3 | 1.254085 | -0.365742 | 743 | 549.19 | 0.739152 |  |
| GO:0060562\_epithelial\_tube\_morphogenesis | TBX3 | 99 | 2 | 1.368093 | -0.364354 | 744 | 550.32 | 0.739677 |  |
| GO:0060562\_epithelial\_tube\_morphogenesis | NR3C1 | 99 | 2 | 1.368093 | -0.364354 | 744 | 550.32 | 0.739677 |  |
| GO:0007596\_blood\_coagulation | RAB27A | 38 | 1 | 1.782121 | -0.363388 | 749 | 557.13 | 0.743832 |  |
| GO:0016042\_lipid\_catabolic\_process | HEXB | 38 | 1 | 1.782121 | -0.363388 | 749 | 557.13 | 0.743832 |  |
| GO:0031401\_positive\_regulation\_of\_protein\_modification\_process | JAK2 | 38 | 1 | 1.782121 | -0.363388 | 749 | 557.13 | 0.743832 |  |
| GO:0045580\_regulation\_of\_T\_cell\_differentiation | PTPRC | 38 | 1 | 1.782121 | -0.363388 | 749 | 557.13 | 0.743832 |  |
| GO:0046777\_protein\_amino\_acid\_autophosphorylation | PTPRC | 38 | 1 | 1.782121 | -0.363388 | 749 | 557.13 | 0.743832 |  |
| GO:0042325\_regulation\_of\_phosphorylation | PTPRC | 164 | 3 | 1.238791 | -0.357834 | 750 | 559.13 | 0.745507 |  |
| GO:0042325\_regulation\_of\_phosphorylation | JAK2 | 164 | 3 | 1.238791 | -0.357834 | 750 | 559.13 | 0.745507 |  |
| GO:0042325\_regulation\_of\_phosphorylation | GADD45B | 164 | 3 | 1.238791 | -0.357834 | 750 | 559.13 | 0.745507 |  |
| GO:0007167\_enzyme\_linked\_receptor\_protein\_signaling\_pathway | FGF18 | 229 | 4 | 1.182892 | -0.356278 | 751 | 559.33 | 0.744780 |  |
| GO:0007167\_enzyme\_linked\_receptor\_protein\_signaling\_pathway | MSX1 | 229 | 4 | 1.182892 | -0.356278 | 751 | 559.33 | 0.744780 |  |
| GO:0007167\_enzyme\_linked\_receptor\_protein\_signaling\_pathway | ARID5B | 229 | 4 | 1.182892 | -0.356278 | 751 | 559.33 | 0.744780 |  |
| GO:0007167\_enzyme\_linked\_receptor\_protein\_signaling\_pathway | JAK2 | 229 | 4 | 1.182892 | -0.356278 | 751 | 559.33 | 0.744780 |  |
| GO:0006511\_ubiquitin-dependent\_protein\_catabolic\_process | TRIM63 | 39 | 1 | 1.736425 | -0.355006 | 753 | 564.23 | 0.749309 |  |
| GO:0007160\_cell-matrix\_adhesion | NID2 | 39 | 1 | 1.736425 | -0.355006 | 753 | 564.23 | 0.749309 |  |
| GO:0019220\_regulation\_of\_phosphate\_metabolic\_process | PTPRC | 165 | 3 | 1.231283 | -0.353948 | 755 | 565.49 | 0.748993 |  |
| GO:0019220\_regulation\_of\_phosphate\_metabolic\_process | JAK2 | 165 | 3 | 1.231283 | -0.353948 | 755 | 565.49 | 0.748993 |  |
| GO:0019220\_regulation\_of\_phosphate\_metabolic\_process | GADD45B | 165 | 3 | 1.231283 | -0.353948 | 755 | 565.49 | 0.748993 |  |
| GO:0051174\_regulation\_of\_phosphorus\_metabolic\_process | PTPRC | 165 | 3 | 1.231283 | -0.353948 | 755 | 565.49 | 0.748993 |  |
| GO:0051174\_regulation\_of\_phosphorus\_metabolic\_process | JAK2 | 165 | 3 | 1.231283 | -0.353948 | 755 | 565.49 | 0.748993 |  |
| GO:0051174\_regulation\_of\_phosphorus\_metabolic\_process | GADD45B | 165 | 3 | 1.231283 | -0.353948 | 755 | 565.49 | 0.748993 |  |
| GO:0007599\_hemostasis | RAB27A | 40 | 1 | 1.693015 | -0.346903 | 758 | 572.39 | 0.755132 |  |
| GO:0008203\_cholesterol\_metabolic\_process | ABCA1 | 40 | 1 | 1.693015 | -0.346903 | 758 | 572.39 | 0.755132 |  |
| GO:0016358\_dendrite\_development | MYO6 | 40 | 1 | 1.693015 | -0.346903 | 758 | 572.39 | 0.755132 |  |
| GO:0006836\_neurotransmitter\_transport | ATP1A2 | 41 | 1 | 1.651722 | -0.339063 | 767 | 582.55 | 0.759518 |  |
| GO:0007254\_JNK\_cascade | CD27 | 41 | 1 | 1.651722 | -0.339063 | 767 | 582.55 | 0.759518 |  |
| GO:0010551\_regulation\_of\_specific\_transcription\_from\_RNA\_polymerase\_II\_promoter | NKX6-1 | 41 | 1 | 1.651722 | -0.339063 | 767 | 582.55 | 0.759518 |  |
| GO:0015833\_peptide\_transport | ABCA1 | 41 | 1 | 1.651722 | -0.339063 | 767 | 582.55 | 0.759518 |  |
| GO:0019216\_regulation\_of\_lipid\_metabolic\_process | NR3C1 | 41 | 1 | 1.651722 | -0.339063 | 767 | 582.55 | 0.759518 |  |
| GO:0032569\_specific\_transcription\_from\_RNA\_polymerase\_II\_promoter | NKX6-1 | 41 | 1 | 1.651722 | -0.339063 | 767 | 582.55 | 0.759518 |  |
| GO:0032844\_regulation\_of\_homeostatic\_process | PTPRC | 41 | 1 | 1.651722 | -0.339063 | 767 | 582.55 | 0.759518 |  |
| GO:0033077\_T\_cell\_differentiation\_in\_the\_thymus | PTPRC | 41 | 1 | 1.651722 | -0.339063 | 767 | 582.55 | 0.759518 |  |
| GO:0050864\_regulation\_of\_B\_cell\_activation | PTPRC | 41 | 1 | 1.651722 | -0.339063 | 767 | 582.55 | 0.759518 |  |
| GO:0044238\_primary\_metabolic\_process | FUT9 | 1905 | 29 | 1.030917 | -0.336523 | 768 | 582.96 | 0.759063 |  |
| GO:0044238\_primary\_metabolic\_process | MAMDC2 | 1905 | 29 | 1.030917 | -0.336523 | 768 | 582.96 | 0.759063 |  |
| GO:0044238\_primary\_metabolic\_process | SGPP1 | 1905 | 29 | 1.030917 | -0.336523 | 768 | 582.96 | 0.759063 |  |
| GO:0044238\_primary\_metabolic\_process | HEXB | 1905 | 29 | 1.030917 | -0.336523 | 768 | 582.96 | 0.759063 |  |
| GO:0044238\_primary\_metabolic\_process | TBX20 | 1905 | 29 | 1.030917 | -0.336523 | 768 | 582.96 | 0.759063 |  |
| GO:0044238\_primary\_metabolic\_process | SOX5 | 1905 | 29 | 1.030917 | -0.336523 | 768 | 582.96 | 0.759063 |  |
| GO:0044238\_primary\_metabolic\_process | HSPA1A | 1905 | 29 | 1.030917 | -0.336523 | 768 | 582.96 | 0.759063 |  |
| GO:0044238\_primary\_metabolic\_process | ABCA1 | 1905 | 29 | 1.030917 | -0.336523 | 768 | 582.96 | 0.759063 |  |
| GO:0044238\_primary\_metabolic\_process | NR3C1 | 1905 | 29 | 1.030917 | -0.336523 | 768 | 582.96 | 0.759063 |  |
| GO:0044238\_primary\_metabolic\_process | SGMS1 | 1905 | 29 | 1.030917 | -0.336523 | 768 | 582.96 | 0.759063 |  |
| GO:0044238\_primary\_metabolic\_process | TCF7L2 | 1905 | 29 | 1.030917 | -0.336523 | 768 | 582.96 | 0.759063 |  |
| GO:0044238\_primary\_metabolic\_process | NR2E1 | 1905 | 29 | 1.030917 | -0.336523 | 768 | 582.96 | 0.759063 |  |
| GO:0044238\_primary\_metabolic\_process | TCERG1 | 1905 | 29 | 1.030917 | -0.336523 | 768 | 582.96 | 0.759063 |  |
| GO:0044238\_primary\_metabolic\_process | DMD | 1905 | 29 | 1.030917 | -0.336523 | 768 | 582.96 | 0.759063 |  |
| GO:0044238\_primary\_metabolic\_process | ETV1 | 1905 | 29 | 1.030917 | -0.336523 | 768 | 582.96 | 0.759063 |  |
| GO:0044238\_primary\_metabolic\_process | B3GNT2 | 1905 | 29 | 1.030917 | -0.336523 | 768 | 582.96 | 0.759063 |  |
| GO:0044238\_primary\_metabolic\_process | PPAP2A | 1905 | 29 | 1.030917 | -0.336523 | 768 | 582.96 | 0.759063 |  |
| GO:0044238\_primary\_metabolic\_process | PTPRC | 1905 | 29 | 1.030917 | -0.336523 | 768 | 582.96 | 0.759063 |  |
| GO:0044238\_primary\_metabolic\_process | TBX3 | 1905 | 29 | 1.030917 | -0.336523 | 768 | 582.96 | 0.759063 |  |
| GO:0044238\_primary\_metabolic\_process | ESRRG | 1905 | 29 | 1.030917 | -0.336523 | 768 | 582.96 | 0.759063 |  |
| GO:0044238\_primary\_metabolic\_process | TRIM63 | 1905 | 29 | 1.030917 | -0.336523 | 768 | 582.96 | 0.759063 |  |
| GO:0044238\_primary\_metabolic\_process | NKX6-1 | 1905 | 29 | 1.030917 | -0.336523 | 768 | 582.96 | 0.759063 |  |
| GO:0044238\_primary\_metabolic\_process | ASCL1 | 1905 | 29 | 1.030917 | -0.336523 | 768 | 582.96 | 0.759063 |  |
| GO:0044238\_primary\_metabolic\_process | AFP | 1905 | 29 | 1.030917 | -0.336523 | 768 | 582.96 | 0.759063 |  |
| GO:0044238\_primary\_metabolic\_process | MSX1 | 1905 | 29 | 1.030917 | -0.336523 | 768 | 582.96 | 0.759063 |  |
| GO:0044238\_primary\_metabolic\_process | NEUROD1 | 1905 | 29 | 1.030917 | -0.336523 | 768 | 582.96 | 0.759063 |  |
| GO:0044238\_primary\_metabolic\_process | JAK2 | 1905 | 29 | 1.030917 | -0.336523 | 768 | 582.96 | 0.759063 |  |
| GO:0044238\_primary\_metabolic\_process | GADD45B | 1905 | 29 | 1.030917 | -0.336523 | 768 | 582.96 | 0.759063 |  |
| GO:0044238\_primary\_metabolic\_process | HDAC9 | 1905 | 29 | 1.030917 | -0.336523 | 768 | 582.96 | 0.759063 |  |
| GO:0001501\_skeletal\_system\_development | FGF18 | 236 | 4 | 1.147807 | -0.333883 | 769 | 584.41 | 0.759961 |  |
| GO:0001501\_skeletal\_system\_development | ARID5B | 236 | 4 | 1.147807 | -0.333883 | 769 | 584.41 | 0.759961 |  |
| GO:0001501\_skeletal\_system\_development | HEXB | 236 | 4 | 1.147807 | -0.333883 | 769 | 584.41 | 0.759961 |  |
| GO:0001501\_skeletal\_system\_development | SOX5 | 236 | 4 | 1.147807 | -0.333883 | 769 | 584.41 | 0.759961 |  |
| GO:0006006\_glucose\_metabolic\_process | NR3C1 | 42 | 1 | 1.612395 | -0.331475 | 780 | 591.35 | 0.758141 |  |
| GO:0008361\_regulation\_of\_cell\_size | SGMS1 | 42 | 1 | 1.612395 | -0.331475 | 780 | 591.35 | 0.758141 |  |
| GO:0015672\_monovalent\_inorganic\_cation\_transport | KCNA5 | 42 | 1 | 1.612395 | -0.331475 | 780 | 591.35 | 0.758141 |  |
| GO:0016125\_sterol\_metabolic\_process | ABCA1 | 42 | 1 | 1.612395 | -0.331475 | 780 | 591.35 | 0.758141 |  |
| GO:0019941\_modification-dependent\_protein\_catabolic\_process | TRIM63 | 42 | 1 | 1.612395 | -0.331475 | 780 | 591.35 | 0.758141 |  |
| GO:0032946\_positive\_regulation\_of\_mononuclear\_cell\_proliferation | PTPRC | 42 | 1 | 1.612395 | -0.331475 | 780 | 591.35 | 0.758141 |  |
| GO:0043632\_modification-dependent\_macromolecule\_catabolic\_process | TRIM63 | 42 | 1 | 1.612395 | -0.331475 | 780 | 591.35 | 0.758141 |  |
| GO:0050671\_positive\_regulation\_of\_lymphocyte\_proliferation | PTPRC | 42 | 1 | 1.612395 | -0.331475 | 780 | 591.35 | 0.758141 |  |
| GO:0050817\_coagulation | RAB27A | 42 | 1 | 1.612395 | -0.331475 | 780 | 591.35 | 0.758141 |  |
| GO:0051603\_proteolysis\_involved\_in\_cellular\_protein\_catabolic\_process | TRIM63 | 42 | 1 | 1.612395 | -0.331475 | 780 | 591.35 | 0.758141 |  |
| GO:0080135\_regulation\_of\_cellular\_response\_to\_stress | CD27 | 42 | 1 | 1.612395 | -0.331475 | 780 | 591.35 | 0.758141 |  |
| GO:0010817\_regulation\_of\_hormone\_levels | AFP | 106 | 2 | 1.277747 | -0.330439 | 781 | 592.01 | 0.758015 |  |
| GO:0010817\_regulation\_of\_hormone\_levels | NR3C1 | 106 | 2 | 1.277747 | -0.330439 | 781 | 592.01 | 0.758015 |  |
| GO:0051641\_cellular\_localization | MYO6 | 370 | 6 | 1.098172 | -0.329141 | 782 | 592.29 | 0.757404 |  |
| GO:0051641\_cellular\_localization | AP2A1 | 370 | 6 | 1.098172 | -0.329141 | 782 | 592.29 | 0.757404 |  |
| GO:0051641\_cellular\_localization | MLPH | 370 | 6 | 1.098172 | -0.329141 | 782 | 592.29 | 0.757404 |  |
| GO:0051641\_cellular\_localization | PLXNA2 | 370 | 6 | 1.098172 | -0.329141 | 782 | 592.29 | 0.757404 |  |
| GO:0051641\_cellular\_localization | JAK2 | 370 | 6 | 1.098172 | -0.329141 | 782 | 592.29 | 0.757404 |  |
| GO:0051641\_cellular\_localization | RAB27A | 370 | 6 | 1.098172 | -0.329141 | 782 | 592.29 | 0.757404 |  |
| GO:0009611\_response\_to\_wounding | THBS1 | 172 | 3 | 1.181173 | -0.327950 | 783 | 592.63 | 0.756871 |  |
| GO:0009611\_response\_to\_wounding | HDAC9 | 172 | 3 | 1.181173 | -0.327950 | 783 | 592.63 | 0.756871 |  |
| GO:0009611\_response\_to\_wounding | RAB27A | 172 | 3 | 1.181173 | -0.327950 | 783 | 592.63 | 0.756871 |  |
| GO:0045859\_regulation\_of\_protein\_kinase\_activity | PTPRC | 107 | 2 | 1.265805 | -0.325897 | 784 | 593.57 | 0.757105 |  |
| GO:0045859\_regulation\_of\_protein\_kinase\_activity | GADD45B | 107 | 2 | 1.265805 | -0.325897 | 784 | 593.57 | 0.757105 |  |
| GO:0050896\_response\_to\_stimulus | PTPRC | 1107 | 17 | 1.039973 | -0.325225 | 785 | 593.68 | 0.756280 |  |
| GO:0050896\_response\_to\_stimulus | ELF1 | 1107 | 17 | 1.039973 | -0.325225 | 785 | 593.68 | 0.756280 |  |
| GO:0050896\_response\_to\_stimulus | MYO6 | 1107 | 17 | 1.039973 | -0.325225 | 785 | 593.68 | 0.756280 |  |
| GO:0050896\_response\_to\_stimulus | ZFX | 1107 | 17 | 1.039973 | -0.325225 | 785 | 593.68 | 0.756280 |  |
| GO:0050896\_response\_to\_stimulus | HEXB | 1107 | 17 | 1.039973 | -0.325225 | 785 | 593.68 | 0.756280 |  |
| GO:0050896\_response\_to\_stimulus | HSPA1A | 1107 | 17 | 1.039973 | -0.325225 | 785 | 593.68 | 0.756280 |  |
| GO:0050896\_response\_to\_stimulus | ATP1A2 | 1107 | 17 | 1.039973 | -0.325225 | 785 | 593.68 | 0.756280 |  |
| GO:0050896\_response\_to\_stimulus | NR2E1 | 1107 | 17 | 1.039973 | -0.325225 | 785 | 593.68 | 0.756280 |  |
| GO:0050896\_response\_to\_stimulus | MSX1 | 1107 | 17 | 1.039973 | -0.325225 | 785 | 593.68 | 0.756280 |  |
| GO:0050896\_response\_to\_stimulus | ETV1 | 1107 | 17 | 1.039973 | -0.325225 | 785 | 593.68 | 0.756280 |  |
| GO:0050896\_response\_to\_stimulus | JAK2 | 1107 | 17 | 1.039973 | -0.325225 | 785 | 593.68 | 0.756280 |  |
| GO:0050896\_response\_to\_stimulus | VPS13A | 1107 | 17 | 1.039973 | -0.325225 | 785 | 593.68 | 0.756280 |  |
| GO:0050896\_response\_to\_stimulus | HDAC9 | 1107 | 17 | 1.039973 | -0.325225 | 785 | 593.68 | 0.756280 |  |
| GO:0050896\_response\_to\_stimulus | THBS1 | 1107 | 17 | 1.039973 | -0.325225 | 785 | 593.68 | 0.756280 |  |
| GO:0050896\_response\_to\_stimulus | BOC | 1107 | 17 | 1.039973 | -0.325225 | 785 | 593.68 | 0.756280 |  |
| GO:0050896\_response\_to\_stimulus | CD27 | 1107 | 17 | 1.039973 | -0.325225 | 785 | 593.68 | 0.756280 |  |
| GO:0050896\_response\_to\_stimulus | RAB27A | 1107 | 17 | 1.039973 | -0.325225 | 785 | 593.68 | 0.756280 |  |
| GO:0001508\_regulation\_of\_action\_potential | HEXB | 43 | 1 | 1.574897 | -0.324125 | 789 | 600.16 | 0.760659 |  |
| GO:0002819\_regulation\_of\_adaptive\_immune\_response | PTPRC | 43 | 1 | 1.574897 | -0.324125 | 789 | 600.16 | 0.760659 |  |
| GO:0002822\_regulation\_of\_adaptive\_immune\_response\_based\_on\_somatic\_recombination\_of\_immune\_receptors\_built\_from\_immunoglobulin\_superfamily\_domains | PTPRC | 43 | 1 | 1.574897 | -0.324125 | 789 | 600.16 | 0.760659 |  |
| GO:0031098\_stress-activated\_protein\_kinase\_signaling\_pathway | CD27 | 43 | 1 | 1.574897 | -0.324125 | 789 | 600.16 | 0.760659 |  |
| GO:0000122\_negative\_regulation\_of\_transcription\_from\_RNA\_polymerase\_II\_promoter | MSX1 | 175 | 3 | 1.160924 | -0.317424 | 791 | 602.95 | 0.762263 |  |
| GO:0000122\_negative\_regulation\_of\_transcription\_from\_RNA\_polymerase\_II\_promoter | HDAC9 | 175 | 3 | 1.160924 | -0.317424 | 791 | 602.95 | 0.762263 |  |
| GO:0000122\_negative\_regulation\_of\_transcription\_from\_RNA\_polymerase\_II\_promoter | NR2E1 | 175 | 3 | 1.160924 | -0.317424 | 791 | 602.95 | 0.762263 |  |
| GO:0046903\_secretion | MYO6 | 175 | 3 | 1.160924 | -0.317424 | 791 | 602.95 | 0.762263 |  |
| GO:0046903\_secretion | ABCA1 | 175 | 3 | 1.160924 | -0.317424 | 791 | 602.95 | 0.762263 |  |
| GO:0046903\_secretion | RAB27A | 175 | 3 | 1.160924 | -0.317424 | 791 | 602.95 | 0.762263 |  |
| GO:0002377\_immunoglobulin\_production | PTPRC | 44 | 1 | 1.539104 | -0.317003 | 801 | 610.07 | 0.761635 |  |
| GO:0006606\_protein\_import\_into\_nucleus | JAK2 | 44 | 1 | 1.539104 | -0.317003 | 801 | 610.07 | 0.761635 |  |
| GO:0016064\_immunoglobulin\_mediated\_immune\_response | PTPRC | 44 | 1 | 1.539104 | -0.317003 | 801 | 610.07 | 0.761635 |  |
| GO:0035282\_segmentation | PCDH8 | 44 | 1 | 1.539104 | -0.317003 | 801 | 610.07 | 0.761635 |  |
| GO:0044257\_cellular\_protein\_catabolic\_process | TRIM63 | 44 | 1 | 1.539104 | -0.317003 | 801 | 610.07 | 0.761635 |  |
| GO:0048593\_camera-type\_eye\_morphogenesis | IRX5 | 44 | 1 | 1.539104 | -0.317003 | 801 | 610.07 | 0.761635 |  |
| GO:0050808\_synapse\_organization | MYO6 | 44 | 1 | 1.539104 | -0.317003 | 801 | 610.07 | 0.761635 |  |
| GO:0051170\_nuclear\_import | JAK2 | 44 | 1 | 1.539104 | -0.317003 | 801 | 610.07 | 0.761635 |  |
| GO:0070665\_positive\_regulation\_of\_leukocyte\_proliferation | PTPRC | 44 | 1 | 1.539104 | -0.317003 | 801 | 610.07 | 0.761635 |  |
| GO:0070668\_positive\_regulation\_of\_mast\_cell\_proliferation | PTPRC | 44 | 1 | 1.539104 | -0.317003 | 801 | 610.07 | 0.761635 |  |
| GO:0032870\_cellular\_response\_to\_hormone\_stimulus | JAK2 | 45 | 1 | 1.504902 | -0.310098 | 802 | 615.55 | 0.767519 |  |
| GO:0048705\_skeletal\_system\_morphogenesis | FGF18 | 111 | 2 | 1.220191 | -0.308424 | 803 | 615.97 | 0.767086 |  |
| GO:0048705\_skeletal\_system\_morphogenesis | ARID5B | 111 | 2 | 1.220191 | -0.308424 | 803 | 615.97 | 0.767086 |  |
| GO:0043549\_regulation\_of\_kinase\_activity | PTPRC | 112 | 2 | 1.209296 | -0.304223 | 804 | 616.72 | 0.767065 |  |
| GO:0043549\_regulation\_of\_kinase\_activity | GADD45B | 112 | 2 | 1.209296 | -0.304223 | 804 | 616.72 | 0.767065 |  |
| GO:0007612\_learning | ATP1A2 | 46 | 1 | 1.472187 | -0.303401 | 809 | 621.06 | 0.767689 |  |
| GO:0008217\_regulation\_of\_blood\_pressure | ATP1A2 | 46 | 1 | 1.472187 | -0.303401 | 809 | 621.06 | 0.767689 |  |
| GO:0019724\_B\_cell\_mediated\_immunity | PTPRC | 46 | 1 | 1.472187 | -0.303401 | 809 | 621.06 | 0.767689 |  |
| GO:0046631\_alpha-beta\_T\_cell\_activation | PTPRC | 46 | 1 | 1.472187 | -0.303401 | 809 | 621.06 | 0.767689 |  |
| GO:0051098\_regulation\_of\_binding | JAK2 | 46 | 1 | 1.472187 | -0.303401 | 809 | 621.06 | 0.767689 |  |
| GO:0040008\_regulation\_of\_growth | ATXN2 | 113 | 2 | 1.198594 | -0.300086 | 810 | 622.35 | 0.768333 |  |
| GO:0040008\_regulation\_of\_growth | ATRN | 113 | 2 | 1.198594 | -0.300086 | 810 | 622.35 | 0.768333 |  |
| GO:0045321\_leukocyte\_activation | PTPRC | 248 | 4 | 1.092268 | -0.298655 | 811 | 623.02 | 0.768212 |  |
| GO:0045321\_leukocyte\_activation | NDRG1 | 248 | 4 | 1.092268 | -0.298655 | 811 | 623.02 | 0.768212 |  |
| GO:0045321\_leukocyte\_activation | HDAC9 | 248 | 4 | 1.092268 | -0.298655 | 811 | 623.02 | 0.768212 |  |
| GO:0045321\_leukocyte\_activation | RAB27A | 248 | 4 | 1.092268 | -0.298655 | 811 | 623.02 | 0.768212 |  |
| GO:0045087\_innate\_immune\_response | RAB27A | 47 | 1 | 1.440864 | -0.296901 | 813 | 628.09 | 0.772558 |  |
| GO:0045619\_regulation\_of\_lymphocyte\_differentiation | PTPRC | 47 | 1 | 1.440864 | -0.296901 | 813 | 628.09 | 0.772558 |  |
| GO:0019538\_protein\_metabolic\_process | PTPRC | 655 | 10 | 1.033902 | -0.294457 | 814 | 629.3 | 0.773096 |  |
| GO:0019538\_protein\_metabolic\_process | FUT9 | 655 | 10 | 1.033902 | -0.294457 | 814 | 629.3 | 0.773096 |  |
| GO:0019538\_protein\_metabolic\_process | MAMDC2 | 655 | 10 | 1.033902 | -0.294457 | 814 | 629.3 | 0.773096 |  |
| GO:0019538\_protein\_metabolic\_process | HEXB | 655 | 10 | 1.033902 | -0.294457 | 814 | 629.3 | 0.773096 |  |
| GO:0019538\_protein\_metabolic\_process | JAK2 | 655 | 10 | 1.033902 | -0.294457 | 814 | 629.3 | 0.773096 |  |
| GO:0019538\_protein\_metabolic\_process | ABCA1 | 655 | 10 | 1.033902 | -0.294457 | 814 | 629.3 | 0.773096 |  |
| GO:0019538\_protein\_metabolic\_process | B3GNT2 | 655 | 10 | 1.033902 | -0.294457 | 814 | 629.3 | 0.773096 |  |
| GO:0019538\_protein\_metabolic\_process | PPAP2A | 655 | 10 | 1.033902 | -0.294457 | 814 | 629.3 | 0.773096 |  |
| GO:0019538\_protein\_metabolic\_process | GADD45B | 655 | 10 | 1.033902 | -0.294457 | 814 | 629.3 | 0.773096 |  |
| GO:0019538\_protein\_metabolic\_process | TRIM63 | 655 | 10 | 1.033902 | -0.294457 | 814 | 629.3 | 0.773096 |  |
| GO:0051338\_regulation\_of\_transferase\_activity | PTPRC | 115 | 2 | 1.177749 | -0.292001 | 815 | 630.48 | 0.773595 |  |
| GO:0051338\_regulation\_of\_transferase\_activity | GADD45B | 115 | 2 | 1.177749 | -0.292001 | 815 | 630.48 | 0.773595 |  |
| GO:0001505\_regulation\_of\_neurotransmitter\_levels | ATP1A2 | 48 | 1 | 1.410846 | -0.290591 | 819 | 633.25 | 0.773199 |  |
| GO:0019318\_hexose\_metabolic\_process | NR3C1 | 48 | 1 | 1.410846 | -0.290591 | 819 | 633.25 | 0.773199 |  |
| GO:0032269\_negative\_regulation\_of\_cellular\_protein\_metabolic\_process | PTPRC | 48 | 1 | 1.410846 | -0.290591 | 819 | 633.25 | 0.773199 |  |
| GO:0034504\_protein\_localization\_in\_nucleus | JAK2 | 48 | 1 | 1.410846 | -0.290591 | 819 | 633.25 | 0.773199 |  |
| GO:0048608\_reproductive\_structure\_development | AFP | 116 | 2 | 1.167596 | -0.288050 | 820 | 634.78 | 0.774122 |  |
| GO:0048608\_reproductive\_structure\_development | ZFX | 116 | 2 | 1.167596 | -0.288050 | 820 | 634.78 | 0.774122 |  |
| GO:0002440\_production\_of\_molecular\_mediator\_of\_immune\_response | PTPRC | 49 | 1 | 1.382053 | -0.284461 | 822 | 640.9 | 0.779684 |  |
| GO:0007606\_sensory\_perception\_of\_chemical\_stimulus | B3GNT2 | 49 | 1 | 1.382053 | -0.284461 | 822 | 640.9 | 0.779684 |  |
| GO:0050877\_neurological\_system\_process | MYO6 | 390 | 6 | 1.041855 | -0.282871 | 823 | 641.5 | 0.779465 |  |
| GO:0050877\_neurological\_system\_process | DMD | 390 | 6 | 1.041855 | -0.282871 | 823 | 641.5 | 0.779465 |  |
| GO:0050877\_neurological\_system\_process | HEXB | 390 | 6 | 1.041855 | -0.282871 | 823 | 641.5 | 0.779465 |  |
| GO:0050877\_neurological\_system\_process | B3GNT2 | 390 | 6 | 1.041855 | -0.282871 | 823 | 641.5 | 0.779465 |  |
| GO:0050877\_neurological\_system\_process | ATP1A2 | 390 | 6 | 1.041855 | -0.282871 | 823 | 641.5 | 0.779465 |  |
| GO:0050877\_neurological\_system\_process | NR2E1 | 390 | 6 | 1.041855 | -0.282871 | 823 | 641.5 | 0.779465 |  |
| GO:0006811\_ion\_transport | PTPRC | 186 | 3 | 1.092268 | -0.281713 | 824 | 643.04 | 0.780388 |  |
| GO:0006811\_ion\_transport | KCNA5 | 186 | 3 | 1.092268 | -0.281713 | 824 | 643.04 | 0.780388 |  |
| GO:0006811\_ion\_transport | SLC40A1 | 186 | 3 | 1.092268 | -0.281713 | 824 | 643.04 | 0.780388 |  |
| GO:0017038\_protein\_import | JAK2 | 50 | 1 | 1.354412 | -0.278506 | 826 | 647.45 | 0.783838 |  |
| GO:0042129\_regulation\_of\_T\_cell\_proliferation | PTPRC | 50 | 1 | 1.354412 | -0.278506 | 826 | 647.45 | 0.783838 |  |
| GO:0042127\_regulation\_of\_cell\_proliferation | FGF18 | 393 | 6 | 1.033902 | -0.276463 | 827 | 648.37 | 0.784002 |  |
| GO:0042127\_regulation\_of\_cell\_proliferation | PTPRC | 393 | 6 | 1.033902 | -0.276463 | 827 | 648.37 | 0.784002 |  |
| GO:0042127\_regulation\_of\_cell\_proliferation | MSX1 | 393 | 6 | 1.033902 | -0.276463 | 827 | 648.37 | 0.784002 |  |
| GO:0042127\_regulation\_of\_cell\_proliferation | TBX3 | 393 | 6 | 1.033902 | -0.276463 | 827 | 648.37 | 0.784002 |  |
| GO:0042127\_regulation\_of\_cell\_proliferation | JAK2 | 393 | 6 | 1.033902 | -0.276463 | 827 | 648.37 | 0.784002 |  |
| GO:0042127\_regulation\_of\_cell\_proliferation | NR2E1 | 393 | 6 | 1.033902 | -0.276463 | 827 | 648.37 | 0.784002 |  |
| GO:0007276\_gamete\_generation | AFP | 188 | 3 | 1.080648 | -0.275675 | 828 | 648.71 | 0.783466 |  |
| GO:0007276\_gamete\_generation | HEXB | 188 | 3 | 1.080648 | -0.275675 | 828 | 648.71 | 0.783466 |  |
| GO:0007276\_gamete\_generation | ZFX | 188 | 3 | 1.080648 | -0.275675 | 828 | 648.71 | 0.783466 |  |
| GO:0006887\_exocytosis | RAB27A | 51 | 1 | 1.327855 | -0.272716 | 831 | 653.06 | 0.785872 |  |
| GO:0007601\_visual\_perception | NR2E1 | 51 | 1 | 1.327855 | -0.272716 | 831 | 653.06 | 0.785872 |  |
| GO:0032583\_regulation\_of\_gene-specific\_transcription | NKX6-1 | 51 | 1 | 1.327855 | -0.272716 | 831 | 653.06 | 0.785872 |  |
| GO:0051726\_regulation\_of\_cell\_cycle | PTPRC | 121 | 2 | 1.119349 | -0.269166 | 832 | 654.6 | 0.786779 |  |
| GO:0051726\_regulation\_of\_cell\_cycle | GADD45B | 121 | 2 | 1.119349 | -0.269166 | 832 | 654.6 | 0.786779 |  |
| GO:0002706\_regulation\_of\_lymphocyte\_mediated\_immunity | PTPRC | 52 | 1 | 1.302319 | -0.267087 | 835 | 656.84 | 0.786635 |  |
| GO:0048585\_negative\_regulation\_of\_response\_to\_stimulus | PTPRC | 52 | 1 | 1.302319 | -0.267087 | 835 | 656.84 | 0.786635 |  |
| GO:0050953\_sensory\_perception\_of\_light\_stimulus | NR2E1 | 52 | 1 | 1.302319 | -0.267087 | 835 | 656.84 | 0.786635 |  |
| GO:0002252\_immune\_effector\_process | PTPRC | 122 | 2 | 1.110174 | -0.265556 | 836 | 658.41 | 0.787572 |  |
| GO:0002252\_immune\_effector\_process | RAB27A | 122 | 2 | 1.110174 | -0.265556 | 836 | 658.41 | 0.787572 |  |
| GO:0001775\_cell\_activation | PTPRC | 262 | 4 | 1.033902 | -0.262100 | 837 | 660.6 | 0.789247 |  |
| GO:0001775\_cell\_activation | NDRG1 | 262 | 4 | 1.033902 | -0.262100 | 837 | 660.6 | 0.789247 |  |
| GO:0001775\_cell\_activation | HDAC9 | 262 | 4 | 1.033902 | -0.262100 | 837 | 660.6 | 0.789247 |  |
| GO:0001775\_cell\_activation | RAB27A | 262 | 4 | 1.033902 | -0.262100 | 837 | 660.6 | 0.789247 |  |
| GO:0030031\_cell\_projection\_assembly | FGD4 | 53 | 1 | 1.277747 | -0.261610 | 840 | 663.69 | 0.790107 |  |
| GO:0050905\_neuromuscular\_process | HEXB | 53 | 1 | 1.277747 | -0.261610 | 840 | 663.69 | 0.790107 |  |
| GO:0051248\_negative\_regulation\_of\_protein\_metabolic\_process | PTPRC | 53 | 1 | 1.277747 | -0.261610 | 840 | 663.69 | 0.790107 |  |
| GO:0030098\_lymphocyte\_differentiation | PTPRC | 124 | 2 | 1.092268 | -0.258495 | 841 | 664.89 | 0.790595 |  |
| GO:0030098\_lymphocyte\_differentiation | HDAC9 | 124 | 2 | 1.092268 | -0.258495 | 841 | 664.89 | 0.790595 |  |
| GO:0043405\_regulation\_of\_MAP\_kinase\_activity | PTPRC | 54 | 1 | 1.254085 | -0.256281 | 842 | 668.91 | 0.794430 |  |
| GO:0001763\_morphogenesis\_of\_a\_branching\_structure | TBX3 | 125 | 2 | 1.083529 | -0.255042 | 843 | 670.39 | 0.795243 |  |
| GO:0001763\_morphogenesis\_of\_a\_branching\_structure | TBX20 | 125 | 2 | 1.083529 | -0.255042 | 843 | 670.39 | 0.795243 |  |
| GO:0006310\_DNA\_recombination | PTPRC | 55 | 1 | 1.231283 | -0.251094 | 846 | 673.02 | 0.795532 |  |
| GO:0043434\_response\_to\_peptide\_hormone\_stimulus | JAK2 | 55 | 1 | 1.231283 | -0.251094 | 846 | 673.02 | 0.795532 |  |
| GO:0048568\_embryonic\_organ\_development | TCF7L2 | 55 | 1 | 1.231283 | -0.251094 | 846 | 673.02 | 0.795532 |  |
| GO:0044237\_cellular\_metabolic\_process | FUT9 | 1974 | 29 | 0.994882 | -0.250739 | 847 | 673.1 | 0.794687 |  |
| GO:0044237\_cellular\_metabolic\_process | MAMDC2 | 1974 | 29 | 0.994882 | -0.250739 | 847 | 673.1 | 0.794687 |  |
| GO:0044237\_cellular\_metabolic\_process | SGPP1 | 1974 | 29 | 0.994882 | -0.250739 | 847 | 673.1 | 0.794687 |  |
| GO:0044237\_cellular\_metabolic\_process | TBX20 | 1974 | 29 | 0.994882 | -0.250739 | 847 | 673.1 | 0.794687 |  |
| GO:0044237\_cellular\_metabolic\_process | HEXB | 1974 | 29 | 0.994882 | -0.250739 | 847 | 673.1 | 0.794687 |  |
| GO:0044237\_cellular\_metabolic\_process | SOX5 | 1974 | 29 | 0.994882 | -0.250739 | 847 | 673.1 | 0.794687 |  |
| GO:0044237\_cellular\_metabolic\_process | HSPA1A | 1974 | 29 | 0.994882 | -0.250739 | 847 | 673.1 | 0.794687 |  |
| GO:0044237\_cellular\_metabolic\_process | SGMS1 | 1974 | 29 | 0.994882 | -0.250739 | 847 | 673.1 | 0.794687 |  |
| GO:0044237\_cellular\_metabolic\_process | NR3C1 | 1974 | 29 | 0.994882 | -0.250739 | 847 | 673.1 | 0.794687 |  |
| GO:0044237\_cellular\_metabolic\_process | ABCA1 | 1974 | 29 | 0.994882 | -0.250739 | 847 | 673.1 | 0.794687 |  |
| GO:0044237\_cellular\_metabolic\_process | TCF7L2 | 1974 | 29 | 0.994882 | -0.250739 | 847 | 673.1 | 0.794687 |  |
| GO:0044237\_cellular\_metabolic\_process | NR2E1 | 1974 | 29 | 0.994882 | -0.250739 | 847 | 673.1 | 0.794687 |  |
| GO:0044237\_cellular\_metabolic\_process | TCERG1 | 1974 | 29 | 0.994882 | -0.250739 | 847 | 673.1 | 0.794687 |  |
| GO:0044237\_cellular\_metabolic\_process | DMD | 1974 | 29 | 0.994882 | -0.250739 | 847 | 673.1 | 0.794687 |  |
| GO:0044237\_cellular\_metabolic\_process | ETV1 | 1974 | 29 | 0.994882 | -0.250739 | 847 | 673.1 | 0.794687 |  |
| GO:0044237\_cellular\_metabolic\_process | B3GNT2 | 1974 | 29 | 0.994882 | -0.250739 | 847 | 673.1 | 0.794687 |  |
| GO:0044237\_cellular\_metabolic\_process | PPAP2A | 1974 | 29 | 0.994882 | -0.250739 | 847 | 673.1 | 0.794687 |  |
| GO:0044237\_cellular\_metabolic\_process | PTPRC | 1974 | 29 | 0.994882 | -0.250739 | 847 | 673.1 | 0.794687 |  |
| GO:0044237\_cellular\_metabolic\_process | TBX3 | 1974 | 29 | 0.994882 | -0.250739 | 847 | 673.1 | 0.794687 |  |
| GO:0044237\_cellular\_metabolic\_process | ESRRG | 1974 | 29 | 0.994882 | -0.250739 | 847 | 673.1 | 0.794687 |  |
| GO:0044237\_cellular\_metabolic\_process | TRIM63 | 1974 | 29 | 0.994882 | -0.250739 | 847 | 673.1 | 0.794687 |  |
| GO:0044237\_cellular\_metabolic\_process | NKX6-1 | 1974 | 29 | 0.994882 | -0.250739 | 847 | 673.1 | 0.794687 |  |
| GO:0044237\_cellular\_metabolic\_process | ASCL1 | 1974 | 29 | 0.994882 | -0.250739 | 847 | 673.1 | 0.794687 |  |
| GO:0044237\_cellular\_metabolic\_process | AFP | 1974 | 29 | 0.994882 | -0.250739 | 847 | 673.1 | 0.794687 |  |
| GO:0044237\_cellular\_metabolic\_process | MSX1 | 1974 | 29 | 0.994882 | -0.250739 | 847 | 673.1 | 0.794687 |  |
| GO:0044237\_cellular\_metabolic\_process | NEUROD1 | 1974 | 29 | 0.994882 | -0.250739 | 847 | 673.1 | 0.794687 |  |
| GO:0044237\_cellular\_metabolic\_process | JAK2 | 1974 | 29 | 0.994882 | -0.250739 | 847 | 673.1 | 0.794687 |  |
| GO:0044237\_cellular\_metabolic\_process | HDAC9 | 1974 | 29 | 0.994882 | -0.250739 | 847 | 673.1 | 0.794687 |  |
| GO:0044237\_cellular\_metabolic\_process | GADD45B | 1974 | 29 | 0.994882 | -0.250739 | 847 | 673.1 | 0.794687 |  |
| GO:0008283\_cell\_proliferation | FGF18 | 544 | 8 | 0.995891 | -0.250457 | 848 | 673.17 | 0.793833 |  |
| GO:0008283\_cell\_proliferation | PTPRC | 544 | 8 | 0.995891 | -0.250457 | 848 | 673.17 | 0.793833 |  |
| GO:0008283\_cell\_proliferation | ASCL1 | 544 | 8 | 0.995891 | -0.250457 | 848 | 673.17 | 0.793833 |  |
| GO:0008283\_cell\_proliferation | MSX1 | 544 | 8 | 0.995891 | -0.250457 | 848 | 673.17 | 0.793833 |  |
| GO:0008283\_cell\_proliferation | TBX3 | 544 | 8 | 0.995891 | -0.250457 | 848 | 673.17 | 0.793833 |  |
| GO:0008283\_cell\_proliferation | TBX20 | 544 | 8 | 0.995891 | -0.250457 | 848 | 673.17 | 0.793833 |  |
| GO:0008283\_cell\_proliferation | JAK2 | 544 | 8 | 0.995891 | -0.250457 | 848 | 673.17 | 0.793833 |  |
| GO:0008283\_cell\_proliferation | NR2E1 | 544 | 8 | 0.995891 | -0.250457 | 848 | 673.17 | 0.793833 |  |
| GO:0002009\_morphogenesis\_of\_an\_epithelium | TBX3 | 198 | 3 | 1.026070 | -0.247394 | 850 | 674.29 | 0.793282 |  |
| GO:0002009\_morphogenesis\_of\_an\_epithelium | PCDH8 | 198 | 3 | 1.026070 | -0.247394 | 850 | 674.29 | 0.793282 |  |
| GO:0002009\_morphogenesis\_of\_an\_epithelium | NR3C1 | 198 | 3 | 1.026070 | -0.247394 | 850 | 674.29 | 0.793282 |  |
| GO:0060429\_epithelium\_development | TBX3 | 198 | 3 | 1.026070 | -0.247394 | 850 | 674.29 | 0.793282 |  |
| GO:0060429\_epithelium\_development | PCDH8 | 198 | 3 | 1.026070 | -0.247394 | 850 | 674.29 | 0.793282 |  |
| GO:0060429\_epithelium\_development | NR3C1 | 198 | 3 | 1.026070 | -0.247394 | 850 | 674.29 | 0.793282 |  |
| GO:0002703\_regulation\_of\_leukocyte\_mediated\_immunity | PTPRC | 56 | 1 | 1.209296 | -0.246043 | 853 | 678.47 | 0.795393 |  |
| GO:0006790\_sulfur\_metabolic\_process | MAMDC2 | 56 | 1 | 1.209296 | -0.246043 | 853 | 678.47 | 0.795393 |  |
| GO:0046486\_glycerolipid\_metabolic\_process | PPAP2A | 56 | 1 | 1.209296 | -0.246043 | 853 | 678.47 | 0.795393 |  |
| GO:0051276\_chromosome\_organization | HSPA1A | 129 | 2 | 1.049932 | -0.241724 | 854 | 681.69 | 0.798232 |  |
| GO:0051276\_chromosome\_organization | HDAC9 | 129 | 2 | 1.049932 | -0.241724 | 854 | 681.69 | 0.798232 |  |
| GO:0001892\_embryonic\_placenta\_development | ITGA4 | 57 | 1 | 1.188080 | -0.241123 | 860 | 686.24 | 0.797953 |  |
| GO:0008344\_adult\_locomotory\_behavior | ATP1A2 | 57 | 1 | 1.188080 | -0.241123 | 860 | 686.24 | 0.797953 |  |
| GO:0033365\_protein\_localization\_in\_organelle | JAK2 | 57 | 1 | 1.188080 | -0.241123 | 860 | 686.24 | 0.797953 |  |
| GO:0042472\_inner\_ear\_morphogenesis | MYO6 | 57 | 1 | 1.188080 | -0.241123 | 860 | 686.24 | 0.797953 |  |
| GO:0045444\_fat\_cell\_differentiation | BBS7 | 57 | 1 | 1.188080 | -0.241123 | 860 | 686.24 | 0.797953 |  |
| GO:0050878\_regulation\_of\_body\_fluid\_levels | RAB27A | 57 | 1 | 1.188080 | -0.241123 | 860 | 686.24 | 0.797953 |  |
| GO:0051649\_establishment\_of\_localization\_in\_cell | MYO6 | 342 | 5 | 0.990067 | -0.239094 | 861 | 687.19 | 0.798130 |  |
| GO:0051649\_establishment\_of\_localization\_in\_cell | AP2A1 | 342 | 5 | 0.990067 | -0.239094 | 861 | 687.19 | 0.798130 |  |
| GO:0051649\_establishment\_of\_localization\_in\_cell | MLPH | 342 | 5 | 0.990067 | -0.239094 | 861 | 687.19 | 0.798130 |  |
| GO:0051649\_establishment\_of\_localization\_in\_cell | JAK2 | 342 | 5 | 0.990067 | -0.239094 | 861 | 687.19 | 0.798130 |  |
| GO:0051649\_establishment\_of\_localization\_in\_cell | RAB27A | 342 | 5 | 0.990067 | -0.239094 | 861 | 687.19 | 0.798130 |  |
| GO:0050776\_regulation\_of\_immune\_response | PTPRC | 130 | 2 | 1.041855 | -0.238514 | 862 | 687.99 | 0.798132 |  |
| GO:0050776\_regulation\_of\_immune\_response | ELF1 | 130 | 2 | 1.041855 | -0.238514 | 862 | 687.99 | 0.798132 |  |
| GO:0050804\_regulation\_of\_synaptic\_transmission | MYO6 | 58 | 1 | 1.167596 | -0.236329 | 863 | 689.9 | 0.799421 |  |
| GO:0016055\_Wnt\_receptor\_signaling\_pathway | TCF7L2 | 59 | 1 | 1.147807 | -0.231658 | 866 | 693.26 | 0.800531 |  |
| GO:0048469\_cell\_maturation | IRX5 | 59 | 1 | 1.147807 | -0.231658 | 866 | 693.26 | 0.800531 |  |
| GO:0050870\_positive\_regulation\_of\_T\_cell\_activation | PTPRC | 59 | 1 | 1.147807 | -0.231658 | 866 | 693.26 | 0.800531 |  |
| GO:0006955\_immune\_response | PTPRC | 205 | 3 | 0.991033 | -0.229352 | 867 | 694.06 | 0.800531 |  |
| GO:0006955\_immune\_response | ELF1 | 205 | 3 | 0.991033 | -0.229352 | 867 | 694.06 | 0.800531 |  |
| GO:0006955\_immune\_response | RAB27A | 205 | 3 | 0.991033 | -0.229352 | 867 | 694.06 | 0.800531 |  |
| GO:0009952\_anterior\_posterior\_pattern\_formation | NEUROD1 | 133 | 2 | 1.018355 | -0.229157 | 868 | 694.61 | 0.800242 |  |
| GO:0009952\_anterior\_posterior\_pattern\_formation | PCDH8 | 133 | 2 | 1.018355 | -0.229157 | 868 | 694.61 | 0.800242 |  |
| GO:0048646\_anatomical\_structure\_formation\_involved\_in\_morphogenesis | FGF18 | 277 | 4 | 0.977915 | -0.227709 | 869 | 694.96 | 0.799724 |  |
| GO:0048646\_anatomical\_structure\_formation\_involved\_in\_morphogenesis | TBX3 | 277 | 4 | 0.977915 | -0.227709 | 869 | 694.96 | 0.799724 |  |
| GO:0048646\_anatomical\_structure\_formation\_involved\_in\_morphogenesis | TBX20 | 277 | 4 | 0.977915 | -0.227709 | 869 | 694.96 | 0.799724 |  |
| GO:0048646\_anatomical\_structure\_formation\_involved\_in\_morphogenesis | THBS1 | 277 | 4 | 0.977915 | -0.227709 | 869 | 694.96 | 0.799724 |  |
| GO:0008152\_metabolic\_process | IRX5 | 2133 | 31 | 0.984219 | -0.225251 | 870 | 696.52 | 0.800598 |  |
| GO:0008152\_metabolic\_process | FUT9 | 2133 | 31 | 0.984219 | -0.225251 | 870 | 696.52 | 0.800598 |  |
| GO:0008152\_metabolic\_process | MAMDC2 | 2133 | 31 | 0.984219 | -0.225251 | 870 | 696.52 | 0.800598 |  |
| GO:0008152\_metabolic\_process | SGPP1 | 2133 | 31 | 0.984219 | -0.225251 | 870 | 696.52 | 0.800598 |  |
| GO:0008152\_metabolic\_process | TBX20 | 2133 | 31 | 0.984219 | -0.225251 | 870 | 696.52 | 0.800598 |  |
| GO:0008152\_metabolic\_process | HEXB | 2133 | 31 | 0.984219 | -0.225251 | 870 | 696.52 | 0.800598 |  |
| GO:0008152\_metabolic\_process | SOX5 | 2133 | 31 | 0.984219 | -0.225251 | 870 | 696.52 | 0.800598 |  |
| GO:0008152\_metabolic\_process | HSPA1A | 2133 | 31 | 0.984219 | -0.225251 | 870 | 696.52 | 0.800598 |  |
| GO:0008152\_metabolic\_process | ABCA1 | 2133 | 31 | 0.984219 | -0.225251 | 870 | 696.52 | 0.800598 |  |
| GO:0008152\_metabolic\_process | NR3C1 | 2133 | 31 | 0.984219 | -0.225251 | 870 | 696.52 | 0.800598 |  |
| GO:0008152\_metabolic\_process | SGMS1 | 2133 | 31 | 0.984219 | -0.225251 | 870 | 696.52 | 0.800598 |  |
| GO:0008152\_metabolic\_process | TCF7L2 | 2133 | 31 | 0.984219 | -0.225251 | 870 | 696.52 | 0.800598 |  |
| GO:0008152\_metabolic\_process | NR2E1 | 2133 | 31 | 0.984219 | -0.225251 | 870 | 696.52 | 0.800598 |  |
| GO:0008152\_metabolic\_process | TCERG1 | 2133 | 31 | 0.984219 | -0.225251 | 870 | 696.52 | 0.800598 |  |
| GO:0008152\_metabolic\_process | DMD | 2133 | 31 | 0.984219 | -0.225251 | 870 | 696.52 | 0.800598 |  |
| GO:0008152\_metabolic\_process | ETV1 | 2133 | 31 | 0.984219 | -0.225251 | 870 | 696.52 | 0.800598 |  |
| GO:0008152\_metabolic\_process | B3GNT2 | 2133 | 31 | 0.984219 | -0.225251 | 870 | 696.52 | 0.800598 |  |
| GO:0008152\_metabolic\_process | PPAP2A | 2133 | 31 | 0.984219 | -0.225251 | 870 | 696.52 | 0.800598 |  |
| GO:0008152\_metabolic\_process | PTPRC | 2133 | 31 | 0.984219 | -0.225251 | 870 | 696.52 | 0.800598 |  |
| GO:0008152\_metabolic\_process | TBX3 | 2133 | 31 | 0.984219 | -0.225251 | 870 | 696.52 | 0.800598 |  |
| GO:0008152\_metabolic\_process | ARID5B | 2133 | 31 | 0.984219 | -0.225251 | 870 | 696.52 | 0.800598 |  |
| GO:0008152\_metabolic\_process | ESRRG | 2133 | 31 | 0.984219 | -0.225251 | 870 | 696.52 | 0.800598 |  |
| GO:0008152\_metabolic\_process | TRIM63 | 2133 | 31 | 0.984219 | -0.225251 | 870 | 696.52 | 0.800598 |  |
| GO:0008152\_metabolic\_process | NKX6-1 | 2133 | 31 | 0.984219 | -0.225251 | 870 | 696.52 | 0.800598 |  |
| GO:0008152\_metabolic\_process | AFP | 2133 | 31 | 0.984219 | -0.225251 | 870 | 696.52 | 0.800598 |  |
| GO:0008152\_metabolic\_process | ASCL1 | 2133 | 31 | 0.984219 | -0.225251 | 870 | 696.52 | 0.800598 |  |
| GO:0008152\_metabolic\_process | MSX1 | 2133 | 31 | 0.984219 | -0.225251 | 870 | 696.52 | 0.800598 |  |
| GO:0008152\_metabolic\_process | NEUROD1 | 2133 | 31 | 0.984219 | -0.225251 | 870 | 696.52 | 0.800598 |  |
| GO:0008152\_metabolic\_process | JAK2 | 2133 | 31 | 0.984219 | -0.225251 | 870 | 696.52 | 0.800598 |  |
| GO:0008152\_metabolic\_process | GADD45B | 2133 | 31 | 0.984219 | -0.225251 | 870 | 696.52 | 0.800598 |  |
| GO:0008152\_metabolic\_process | HDAC9 | 2133 | 31 | 0.984219 | -0.225251 | 870 | 696.52 | 0.800598 |  |
| GO:0032270\_positive\_regulation\_of\_cellular\_protein\_metabolic\_process | JAK2 | 61 | 1 | 1.110174 | -0.222663 | 872 | 698.96 | 0.801560 |  |
| GO:0051969\_regulation\_of\_transmission\_of\_nerve\_impulse | MYO6 | 61 | 1 | 1.110174 | -0.222663 | 872 | 698.96 | 0.801560 |  |
| GO:0008284\_positive\_regulation\_of\_cell\_proliferation | FGF18 | 208 | 3 | 0.976739 | -0.222028 | 873 | 699.41 | 0.801157 |  |
| GO:0008284\_positive\_regulation\_of\_cell\_proliferation | PTPRC | 208 | 3 | 0.976739 | -0.222028 | 873 | 699.41 | 0.801157 |  |
| GO:0008284\_positive\_regulation\_of\_cell\_proliferation | NR2E1 | 208 | 3 | 0.976739 | -0.222028 | 873 | 699.41 | 0.801157 |  |
| GO:0006916\_anti-apoptosis | CD27 | 62 | 1 | 1.092268 | -0.218331 | 877 | 703.83 | 0.802543 |  |
| GO:0030155\_regulation\_of\_cell\_adhesion | THBS1 | 62 | 1 | 1.092268 | -0.218331 | 877 | 703.83 | 0.802543 |  |
| GO:0032944\_regulation\_of\_mononuclear\_cell\_proliferation | PTPRC | 62 | 1 | 1.092268 | -0.218331 | 877 | 703.83 | 0.802543 |  |
| GO:0050670\_regulation\_of\_lymphocyte\_proliferation | PTPRC | 62 | 1 | 1.092268 | -0.218331 | 877 | 703.83 | 0.802543 |  |
| GO:0070662\_mast\_cell\_proliferation | PTPRC | 63 | 1 | 1.074930 | -0.214106 | 879 | 707.3 | 0.804664 |  |
| GO:0070666\_regulation\_of\_mast\_cell\_proliferation | PTPRC | 63 | 1 | 1.074930 | -0.214106 | 879 | 707.3 | 0.804664 |  |
| GO:0009790\_embryonic\_development | MYO6 | 567 | 8 | 0.955493 | -0.213888 | 880 | 707.56 | 0.804045 |  |
| GO:0009790\_embryonic\_development | MSX1 | 567 | 8 | 0.955493 | -0.213888 | 880 | 707.56 | 0.804045 |  |
| GO:0009790\_embryonic\_development | TBX3 | 567 | 8 | 0.955493 | -0.213888 | 880 | 707.56 | 0.804045 |  |
| GO:0009790\_embryonic\_development | ZFX | 567 | 8 | 0.955493 | -0.213888 | 880 | 707.56 | 0.804045 |  |
| GO:0009790\_embryonic\_development | TBX20 | 567 | 8 | 0.955493 | -0.213888 | 880 | 707.56 | 0.804045 |  |
| GO:0009790\_embryonic\_development | PCDH8 | 567 | 8 | 0.955493 | -0.213888 | 880 | 707.56 | 0.804045 |  |
| GO:0009790\_embryonic\_development | ITGA4 | 567 | 8 | 0.955493 | -0.213888 | 880 | 707.56 | 0.804045 |  |
| GO:0009790\_embryonic\_development | TCF7L2 | 567 | 8 | 0.955493 | -0.213888 | 880 | 707.56 | 0.804045 |  |
| GO:0031644\_regulation\_of\_neurological\_system\_process | MYO6 | 64 | 1 | 1.058134 | -0.209982 | 883 | 711.22 | 0.805459 |  |
| GO:0042060\_wound\_healing | RAB27A | 64 | 1 | 1.058134 | -0.209982 | 883 | 711.22 | 0.805459 |  |
| GO:0070663\_regulation\_of\_leukocyte\_proliferation | PTPRC | 64 | 1 | 1.058134 | -0.209982 | 883 | 711.22 | 0.805459 |  |
| GO:0016044\_membrane\_organization | MYO6 | 140 | 2 | 0.967437 | -0.208825 | 884 | 711.47 | 0.804830 |  |
| GO:0016044\_membrane\_organization | ABCA1 | 140 | 2 | 0.967437 | -0.208825 | 884 | 711.47 | 0.804830 |  |
| GO:0042471\_ear\_morphogenesis | MYO6 | 65 | 1 | 1.041855 | -0.205957 | 885 | 713.94 | 0.806712 |  |
| GO:0010033\_response\_to\_organic\_substance | MSX1 | 216 | 3 | 0.940564 | -0.203612 | 886 | 714.49 | 0.806422 |  |
| GO:0010033\_response\_to\_organic\_substance | JAK2 | 216 | 3 | 0.940564 | -0.203612 | 886 | 714.49 | 0.806422 |  |
| GO:0010033\_response\_to\_organic\_substance | BOC | 216 | 3 | 0.940564 | -0.203612 | 886 | 714.49 | 0.806422 |  |
| GO:0006810\_transport | PTPRC | 718 | 10 | 0.943184 | -0.199166 | 887 | 718.86 | 0.810440 |  |
| GO:0006810\_transport | MYO6 | 718 | 10 | 0.943184 | -0.199166 | 887 | 718.86 | 0.810440 |  |
| GO:0006810\_transport | MLPH | 718 | 10 | 0.943184 | -0.199166 | 887 | 718.86 | 0.810440 |  |
| GO:0006810\_transport | AP2A1 | 718 | 10 | 0.943184 | -0.199166 | 887 | 718.86 | 0.810440 |  |
| GO:0006810\_transport | JAK2 | 718 | 10 | 0.943184 | -0.199166 | 887 | 718.86 | 0.810440 |  |
| GO:0006810\_transport | ATP1A2 | 718 | 10 | 0.943184 | -0.199166 | 887 | 718.86 | 0.810440 |  |
| GO:0006810\_transport | KCNA5 | 718 | 10 | 0.943184 | -0.199166 | 887 | 718.86 | 0.810440 |  |
| GO:0006810\_transport | ABCA1 | 718 | 10 | 0.943184 | -0.199166 | 887 | 718.86 | 0.810440 |  |
| GO:0006810\_transport | SLC40A1 | 718 | 10 | 0.943184 | -0.199166 | 887 | 718.86 | 0.810440 |  |
| GO:0006810\_transport | RAB27A | 718 | 10 | 0.943184 | -0.199166 | 887 | 718.86 | 0.810440 |  |
| GO:0051247\_positive\_regulation\_of\_protein\_metabolic\_process | JAK2 | 67 | 1 | 1.010755 | -0.198191 | 888 | 720.86 | 0.811779 |  |
| GO:0007186\_G-protein\_coupled\_receptor\_protein\_signaling\_pathway | RGS8 | 144 | 2 | 0.940564 | -0.198078 | 889 | 721.42 | 0.811496 |  |
| GO:0007186\_G-protein\_coupled\_receptor\_protein\_signaling\_pathway | RGS13 | 144 | 2 | 0.940564 | -0.198078 | 889 | 721.42 | 0.811496 |  |
| GO:0043009\_chordate\_embryonic\_development | MSX1 | 365 | 5 | 0.927679 | -0.196595 | 890 | 722.41 | 0.811697 |  |
| GO:0043009\_chordate\_embryonic\_development | TBX3 | 365 | 5 | 0.927679 | -0.196595 | 890 | 722.41 | 0.811697 |  |
| GO:0043009\_chordate\_embryonic\_development | PCDH8 | 365 | 5 | 0.927679 | -0.196595 | 890 | 722.41 | 0.811697 |  |
| GO:0043009\_chordate\_embryonic\_development | ITGA4 | 365 | 5 | 0.927679 | -0.196595 | 890 | 722.41 | 0.811697 |  |
| GO:0043009\_chordate\_embryonic\_development | TCF7L2 | 365 | 5 | 0.927679 | -0.196595 | 890 | 722.41 | 0.811697 |  |
| GO:0002697\_regulation\_of\_immune\_effector\_process | PTPRC | 68 | 1 | 0.995891 | -0.194444 | 892 | 724.31 | 0.812007 |  |
| GO:0034962\_cellular\_biopolymer\_catabolic\_process | TRIM63 | 68 | 1 | 0.995891 | -0.194444 | 892 | 724.31 | 0.812007 |  |
| GO:0001701\_in\_utero\_embryonic\_development | MSX1 | 221 | 3 | 0.919284 | -0.192878 | 893 | 725.65 | 0.812598 |  |
| GO:0001701\_in\_utero\_embryonic\_development | TBX3 | 221 | 3 | 0.919284 | -0.192878 | 893 | 725.65 | 0.812598 |  |
| GO:0001701\_in\_utero\_embryonic\_development | ITGA4 | 221 | 3 | 0.919284 | -0.192878 | 893 | 725.65 | 0.812598 |  |
| GO:0009792\_embryonic\_development\_ending\_in\_birth\_or\_egg\_hatching | MSX1 | 368 | 5 | 0.920117 | -0.191594 | 894 | 726.44 | 0.812573 |  |
| GO:0009792\_embryonic\_development\_ending\_in\_birth\_or\_egg\_hatching | TBX3 | 368 | 5 | 0.920117 | -0.191594 | 894 | 726.44 | 0.812573 |  |
| GO:0009792\_embryonic\_development\_ending\_in\_birth\_or\_egg\_hatching | PCDH8 | 368 | 5 | 0.920117 | -0.191594 | 894 | 726.44 | 0.812573 |  |
| GO:0009792\_embryonic\_development\_ending\_in\_birth\_or\_egg\_hatching | ITGA4 | 368 | 5 | 0.920117 | -0.191594 | 894 | 726.44 | 0.812573 |  |
| GO:0009792\_embryonic\_development\_ending\_in\_birth\_or\_egg\_hatching | TCF7L2 | 368 | 5 | 0.920117 | -0.191594 | 894 | 726.44 | 0.812573 |  |
| GO:0005996\_monosaccharide\_metabolic\_process | NR3C1 | 69 | 1 | 0.981458 | -0.190784 | 896 | 728.32 | 0.812857 |  |
| GO:0006816\_calcium\_ion\_transport | PTPRC | 69 | 1 | 0.981458 | -0.190784 | 896 | 728.32 | 0.812857 |  |
| GO:0002684\_positive\_regulation\_of\_immune\_system\_process | PTPRC | 148 | 2 | 0.915143 | -0.187915 | 898 | 729.75 | 0.812639 |  |
| GO:0002684\_positive\_regulation\_of\_immune\_system\_process | ELF1 | 148 | 2 | 0.915143 | -0.187915 | 898 | 729.75 | 0.812639 |  |
| GO:0043085\_positive\_regulation\_of\_catalytic\_activity | PTPRC | 148 | 2 | 0.915143 | -0.187915 | 898 | 729.75 | 0.812639 |  |
| GO:0043085\_positive\_regulation\_of\_catalytic\_activity | GADD45B | 148 | 2 | 0.915143 | -0.187915 | 898 | 729.75 | 0.812639 |  |
| GO:0007611\_learning\_or\_memory | ATP1A2 | 70 | 1 | 0.967437 | -0.187207 | 901 | 731.68 | 0.812075 |  |
| GO:0048592\_eye\_morphogenesis | IRX5 | 70 | 1 | 0.967437 | -0.187207 | 901 | 731.68 | 0.812075 |  |
| GO:0070838\_divalent\_metal\_ion\_transport | PTPRC | 70 | 1 | 0.967437 | -0.187207 | 901 | 731.68 | 0.812075 |  |
| GO:0051234\_establishment\_of\_localization | PTPRC | 729 | 10 | 0.928952 | -0.185532 | 902 | 732.65 | 0.812251 |  |
| GO:0051234\_establishment\_of\_localization | MYO6 | 729 | 10 | 0.928952 | -0.185532 | 902 | 732.65 | 0.812251 |  |
| GO:0051234\_establishment\_of\_localization | MLPH | 729 | 10 | 0.928952 | -0.185532 | 902 | 732.65 | 0.812251 |  |
| GO:0051234\_establishment\_of\_localization | AP2A1 | 729 | 10 | 0.928952 | -0.185532 | 902 | 732.65 | 0.812251 |  |
| GO:0051234\_establishment\_of\_localization | JAK2 | 729 | 10 | 0.928952 | -0.185532 | 902 | 732.65 | 0.812251 |  |
| GO:0051234\_establishment\_of\_localization | ATP1A2 | 729 | 10 | 0.928952 | -0.185532 | 902 | 732.65 | 0.812251 |  |
| GO:0051234\_establishment\_of\_localization | KCNA5 | 729 | 10 | 0.928952 | -0.185532 | 902 | 732.65 | 0.812251 |  |
| GO:0051234\_establishment\_of\_localization | ABCA1 | 729 | 10 | 0.928952 | -0.185532 | 902 | 732.65 | 0.812251 |  |
| GO:0051234\_establishment\_of\_localization | SLC40A1 | 729 | 10 | 0.928952 | -0.185532 | 902 | 732.65 | 0.812251 |  |
| GO:0051234\_establishment\_of\_localization | RAB27A | 729 | 10 | 0.928952 | -0.185532 | 902 | 732.65 | 0.812251 |  |
| GO:0032940\_secretion\_by\_cell | MYO6 | 149 | 2 | 0.909001 | -0.185461 | 903 | 732.92 | 0.811650 |  |
| GO:0032940\_secretion\_by\_cell | RAB27A | 149 | 2 | 0.909001 | -0.185461 | 903 | 732.92 | 0.811650 |  |
| GO:0048598\_embryonic\_morphogenesis | MYO6 | 299 | 4 | 0.905961 | -0.184923 | 904 | 733.48 | 0.811372 |  |
| GO:0048598\_embryonic\_morphogenesis | MSX1 | 299 | 4 | 0.905961 | -0.184923 | 904 | 733.48 | 0.811372 |  |
| GO:0048598\_embryonic\_morphogenesis | PCDH8 | 299 | 4 | 0.905961 | -0.184923 | 904 | 733.48 | 0.811372 |  |
| GO:0048598\_embryonic\_morphogenesis | TCF7L2 | 299 | 4 | 0.905961 | -0.184923 | 904 | 733.48 | 0.811372 |  |
| GO:0006281\_DNA\_repair | HSPA1A | 71 | 1 | 0.953811 | -0.183712 | 907 | 735.42 | 0.810827 |  |
| GO:0006913\_nucleocytoplasmic\_transport | JAK2 | 71 | 1 | 0.953811 | -0.183712 | 907 | 735.42 | 0.810827 |  |
| GO:0016331\_morphogenesis\_of\_embryonic\_epithelium | PCDH8 | 71 | 1 | 0.953811 | -0.183712 | 907 | 735.42 | 0.810827 |  |
| GO:0016568\_chromatin\_modification | HDAC9 | 72 | 1 | 0.940564 | -0.180296 | 912 | 739.75 | 0.811129 |  |
| GO:0021915\_neural\_tube\_development | TCF7L2 | 72 | 1 | 0.940564 | -0.180296 | 912 | 739.75 | 0.811129 |  |
| GO:0042098\_T\_cell\_proliferation | PTPRC | 72 | 1 | 0.940564 | -0.180296 | 912 | 739.75 | 0.811129 |  |
| GO:0048839\_inner\_ear\_development | MYO6 | 72 | 1 | 0.940564 | -0.180296 | 912 | 739.75 | 0.811129 |  |
| GO:0051169\_nuclear\_transport | JAK2 | 72 | 1 | 0.940564 | -0.180296 | 912 | 739.75 | 0.811129 |  |
| GO:0019953\_sexual\_reproduction | AFP | 228 | 3 | 0.891060 | -0.178780 | 914 | 740.8 | 0.810503 |  |
| GO:0019953\_sexual\_reproduction | HEXB | 228 | 3 | 0.891060 | -0.178780 | 914 | 740.8 | 0.810503 |  |
| GO:0019953\_sexual\_reproduction | ZFX | 228 | 3 | 0.891060 | -0.178780 | 914 | 740.8 | 0.810503 |  |
| GO:0046649\_lymphocyte\_activation | PTPRC | 228 | 3 | 0.891060 | -0.178780 | 914 | 740.8 | 0.810503 |  |
| GO:0046649\_lymphocyte\_activation | HDAC9 | 228 | 3 | 0.891060 | -0.178780 | 914 | 740.8 | 0.810503 |  |
| GO:0046649\_lymphocyte\_activation | RAB27A | 228 | 3 | 0.891060 | -0.178780 | 914 | 740.8 | 0.810503 |  |
| GO:0032268\_regulation\_of\_cellular\_protein\_metabolic\_process | PTPRC | 152 | 2 | 0.891060 | -0.178298 | 915 | 741.22 | 0.810077 |  |
| GO:0032268\_regulation\_of\_cellular\_protein\_metabolic\_process | JAK2 | 152 | 2 | 0.891060 | -0.178298 | 915 | 741.22 | 0.810077 |  |
| GO:0009416\_response\_to\_light\_stimulus | ATP1A2 | 74 | 1 | 0.915143 | -0.173691 | 917 | 745.52 | 0.812999 |  |
| GO:0048771\_tissue\_remodeling | ITGA4 | 74 | 1 | 0.915143 | -0.173691 | 917 | 745.52 | 0.812999 |  |
| GO:0008285\_negative\_regulation\_of\_cell\_proliferation | MSX1 | 155 | 2 | 0.873814 | -0.171424 | 918 | 747.58 | 0.814357 |  |
| GO:0008285\_negative\_regulation\_of\_cell\_proliferation | JAK2 | 155 | 2 | 0.873814 | -0.171424 | 918 | 747.58 | 0.814357 |  |
| GO:0007281\_germ\_cell\_development | ZFX | 75 | 1 | 0.902941 | -0.170497 | 920 | 749.32 | 0.814478 |  |
| GO:0044265\_cellular\_macromolecule\_catabolic\_process | TRIM63 | 75 | 1 | 0.902941 | -0.170497 | 920 | 749.32 | 0.814478 |  |
| GO:0006508\_proteolysis | TRIM63 | 76 | 1 | 0.891060 | -0.167374 | 922 | 752.08 | 0.815705 |  |
| GO:0009725\_response\_to\_hormone\_stimulus | JAK2 | 76 | 1 | 0.891060 | -0.167374 | 922 | 752.08 | 0.815705 |  |
| GO:0043687\_post-translational\_protein\_modification | PTPRC | 384 | 5 | 0.881778 | -0.166825 | 923 | 752.8 | 0.815601 |  |
| GO:0043687\_post-translational\_protein\_modification | MAMDC2 | 384 | 5 | 0.881778 | -0.166825 | 923 | 752.8 | 0.815601 |  |
| GO:0043687\_post-translational\_protein\_modification | JAK2 | 384 | 5 | 0.881778 | -0.166825 | 923 | 752.8 | 0.815601 |  |
| GO:0043687\_post-translational\_protein\_modification | PPAP2A | 384 | 5 | 0.881778 | -0.166825 | 923 | 752.8 | 0.815601 |  |
| GO:0043687\_post-translational\_protein\_modification | GADD45B | 384 | 5 | 0.881778 | -0.166825 | 923 | 752.8 | 0.815601 |  |
| GO:0001890\_placenta\_development | ITGA4 | 77 | 1 | 0.879488 | -0.164319 | 924 | 754.99 | 0.817089 |  |
| GO:0006468\_protein\_amino\_acid\_phosphorylation | PTPRC | 237 | 3 | 0.857223 | -0.162127 | 925 | 755.84 | 0.817124 |  |
| GO:0006468\_protein\_amino\_acid\_phosphorylation | JAK2 | 237 | 3 | 0.857223 | -0.162127 | 925 | 755.84 | 0.817124 |  |
| GO:0006468\_protein\_amino\_acid\_phosphorylation | GADD45B | 237 | 3 | 0.857223 | -0.162127 | 925 | 755.84 | 0.817124 |  |
| GO:0030326\_embryonic\_limb\_morphogenesis | MSX1 | 78 | 1 | 0.868213 | -0.161330 | 928 | 758.47 | 0.817317 |  |
| GO:0035113\_embryonic\_appendage\_morphogenesis | MSX1 | 78 | 1 | 0.868213 | -0.161330 | 928 | 758.47 | 0.817317 |  |
| GO:0051251\_positive\_regulation\_of\_lymphocyte\_activation | PTPRC | 78 | 1 | 0.868213 | -0.161330 | 928 | 758.47 | 0.817317 |  |
| GO:0007049\_cell\_cycle | PTPRC | 238 | 3 | 0.853621 | -0.160373 | 929 | 759.04 | 0.817051 |  |
| GO:0007049\_cell\_cycle | GADD45B | 238 | 3 | 0.853621 | -0.160373 | 929 | 759.04 | 0.817051 |  |
| GO:0007049\_cell\_cycle | LATS2 | 238 | 3 | 0.853621 | -0.160373 | 929 | 759.04 | 0.817051 |  |
| GO:0016043\_cellular\_component\_organization | DCC | 964 | 13 | 0.913244 | -0.160348 | 930 | 759.2 | 0.816344 |  |
| GO:0016043\_cellular\_component\_organization | MYO6 | 964 | 13 | 0.913244 | -0.160348 | 930 | 759.2 | 0.816344 |  |
| GO:0016043\_cellular\_component\_organization | HEXB | 964 | 13 | 0.913244 | -0.160348 | 930 | 759.2 | 0.816344 |  |
| GO:0016043\_cellular\_component\_organization | HSPA1A | 964 | 13 | 0.913244 | -0.160348 | 930 | 759.2 | 0.816344 |  |
| GO:0016043\_cellular\_component\_organization | ABCA1 | 964 | 13 | 0.913244 | -0.160348 | 930 | 759.2 | 0.816344 |  |
| GO:0016043\_cellular\_component\_organization | NR2E1 | 964 | 13 | 0.913244 | -0.160348 | 930 | 759.2 | 0.816344 |  |
| GO:0016043\_cellular\_component\_organization | ALCAM | 964 | 13 | 0.913244 | -0.160348 | 930 | 759.2 | 0.816344 |  |
| GO:0016043\_cellular\_component\_organization | AQP11 | 964 | 13 | 0.913244 | -0.160348 | 930 | 759.2 | 0.816344 |  |
| GO:0016043\_cellular\_component\_organization | ETV1 | 964 | 13 | 0.913244 | -0.160348 | 930 | 759.2 | 0.816344 |  |
| GO:0016043\_cellular\_component\_organization | B3GNT2 | 964 | 13 | 0.913244 | -0.160348 | 930 | 759.2 | 0.816344 |  |
| GO:0016043\_cellular\_component\_organization | HDAC9 | 964 | 13 | 0.913244 | -0.160348 | 930 | 759.2 | 0.816344 |  |
| GO:0016043\_cellular\_component\_organization | BOC | 964 | 13 | 0.913244 | -0.160348 | 930 | 759.2 | 0.816344 |  |
| GO:0016043\_cellular\_component\_organization | FGD4 | 964 | 13 | 0.913244 | -0.160348 | 930 | 759.2 | 0.816344 |  |
| GO:0002521\_leukocyte\_differentiation | PTPRC | 161 | 2 | 0.841250 | -0.158491 | 931 | 759.77 | 0.816079 |  |
| GO:0002521\_leukocyte\_differentiation | HDAC9 | 161 | 2 | 0.841250 | -0.158491 | 931 | 759.77 | 0.816079 |  |
| GO:0000278\_mitotic\_cell\_cycle | LATS2 | 80 | 1 | 0.846507 | -0.155543 | 932 | 762.73 | 0.818380 |  |
| GO:0042110\_T\_cell\_activation | PTPRC | 163 | 2 | 0.830927 | -0.154408 | 933 | 763.21 | 0.818017 |  |
| GO:0042110\_T\_cell\_activation | RAB27A | 163 | 2 | 0.830927 | -0.154408 | 933 | 763.21 | 0.818017 |  |
| GO:0021700\_developmental\_maturation | IRX5 | 81 | 1 | 0.836057 | -0.152741 | 934 | 764.06 | 0.818051 |  |
| GO:0006259\_DNA\_metabolic\_process | PTPRC | 165 | 2 | 0.820856 | -0.150434 | 935 | 765.21 | 0.818406 |  |
| GO:0006259\_DNA\_metabolic\_process | HSPA1A | 165 | 2 | 0.820856 | -0.150434 | 935 | 765.21 | 0.818406 |  |
| GO:0002696\_positive\_regulation\_of\_leukocyte\_activation | PTPRC | 82 | 1 | 0.825861 | -0.149998 | 936 | 767.35 | 0.819818 |  |
| GO:0006325\_chromatin\_organization | HDAC9 | 83 | 1 | 0.815911 | -0.147313 | 939 | 770.06 | 0.820085 |  |
| GO:0030534\_adult\_behavior | ATP1A2 | 83 | 1 | 0.815911 | -0.147313 | 939 | 770.06 | 0.820085 |  |
| GO:0050867\_positive\_regulation\_of\_cell\_activation | PTPRC | 83 | 1 | 0.815911 | -0.147313 | 939 | 770.06 | 0.820085 |  |
| GO:0070887\_cellular\_response\_to\_chemical\_stimulus | JAK2 | 85 | 1 | 0.796713 | -0.142109 | 940 | 773.89 | 0.823287 |  |
| GO:0051246\_regulation\_of\_protein\_metabolic\_process | PTPRC | 170 | 2 | 0.796713 | -0.140951 | 941 | 774.36 | 0.822912 |  |
| GO:0051246\_regulation\_of\_protein\_metabolic\_process | JAK2 | 170 | 2 | 0.796713 | -0.140951 | 941 | 774.36 | 0.822912 |  |
| GO:0007267\_cell-cell\_signaling | MYO6 | 252 | 3 | 0.806197 | -0.137663 | 942 | 777.39 | 0.825255 |  |
| GO:0007267\_cell-cell\_signaling | DMD | 252 | 3 | 0.806197 | -0.137663 | 942 | 777.39 | 0.825255 |  |
| GO:0007267\_cell-cell\_signaling | ATP1A2 | 252 | 3 | 0.806197 | -0.137663 | 942 | 777.39 | 0.825255 |  |
| GO:0003001\_generation\_of\_a\_signal\_involved\_in\_cell-cell\_signaling | MYO6 | 87 | 1 | 0.778398 | -0.137117 | 945 | 781.38 | 0.826857 |  |
| GO:0007178\_transmembrane\_receptor\_protein\_serine\_threonine\_kinase\_signaling\_pathway | MSX1 | 87 | 1 | 0.778398 | -0.137117 | 945 | 781.38 | 0.826857 |  |
| GO:0043583\_ear\_development | MYO6 | 87 | 1 | 0.778398 | -0.137117 | 945 | 781.38 | 0.826857 |  |
| GO:0030097\_hemopoiesis | PTPRC | 253 | 3 | 0.803011 | -0.136165 | 946 | 781.95 | 0.826586 |  |
| GO:0030097\_hemopoiesis | JAK2 | 253 | 3 | 0.803011 | -0.136165 | 946 | 781.95 | 0.826586 |  |
| GO:0030097\_hemopoiesis | HDAC9 | 253 | 3 | 0.803011 | -0.136165 | 946 | 781.95 | 0.826586 |  |
| GO:0044093\_positive\_regulation\_of\_molecular\_function | PTPRC | 173 | 2 | 0.782897 | -0.135557 | 948 | 783.07 | 0.826023 |  |
| GO:0044093\_positive\_regulation\_of\_molecular\_function | GADD45B | 173 | 2 | 0.782897 | -0.135557 | 948 | 783.07 | 0.826023 |  |
| GO:0044248\_cellular\_catabolic\_process | HEXB | 173 | 2 | 0.782897 | -0.135557 | 948 | 783.07 | 0.826023 |  |
| GO:0044248\_cellular\_catabolic\_process | TRIM63 | 173 | 2 | 0.782897 | -0.135557 | 948 | 783.07 | 0.826023 |  |
| GO:0001503\_ossification | FGF18 | 88 | 1 | 0.769552 | -0.134697 | 950 | 784.32 | 0.825600 |  |
| GO:0050863\_regulation\_of\_T\_cell\_activation | PTPRC | 88 | 1 | 0.769552 | -0.134697 | 950 | 784.32 | 0.825600 |  |
| GO:0048729\_tissue\_morphogenesis | TBX3 | 255 | 3 | 0.796713 | -0.133218 | 951 | 785.27 | 0.825731 |  |
| GO:0048729\_tissue\_morphogenesis | PCDH8 | 255 | 3 | 0.796713 | -0.133218 | 951 | 785.27 | 0.825731 |  |
| GO:0048729\_tissue\_morphogenesis | NR3C1 | 255 | 3 | 0.796713 | -0.133218 | 951 | 785.27 | 0.825731 |  |
| GO:0007242\_intracellular\_signaling\_cascade | PTPRC | 411 | 5 | 0.823851 | -0.131533 | 952 | 786.38 | 0.826029 |  |
| GO:0007242\_intracellular\_signaling\_cascade | JAK2 | 411 | 5 | 0.823851 | -0.131533 | 952 | 786.38 | 0.826029 |  |
| GO:0007242\_intracellular\_signaling\_cascade | NR3C1 | 411 | 5 | 0.823851 | -0.131533 | 952 | 786.38 | 0.826029 |  |
| GO:0007242\_intracellular\_signaling\_cascade | GADD45B | 411 | 5 | 0.823851 | -0.131533 | 952 | 786.38 | 0.826029 |  |
| GO:0007242\_intracellular\_signaling\_cascade | CD27 | 411 | 5 | 0.823851 | -0.131533 | 952 | 786.38 | 0.826029 |  |
| GO:0030324\_lung\_development | FGF18 | 90 | 1 | 0.752451 | -0.130003 | 953 | 788.83 | 0.827733 |  |
| GO:0009605\_response\_to\_external\_stimulus | ETV1 | 339 | 4 | 0.799063 | -0.125772 | 954 | 790.66 | 0.828784 |  |
| GO:0009605\_response\_to\_external\_stimulus | THBS1 | 339 | 4 | 0.799063 | -0.125772 | 954 | 790.66 | 0.828784 |  |
| GO:0009605\_response\_to\_external\_stimulus | HDAC9 | 339 | 4 | 0.799063 | -0.125772 | 954 | 790.66 | 0.828784 |  |
| GO:0009605\_response\_to\_external\_stimulus | RAB27A | 339 | 4 | 0.799063 | -0.125772 | 954 | 790.66 | 0.828784 |  |
| GO:0009719\_response\_to\_endogenous\_stimulus | JAK2 | 92 | 1 | 0.736093 | -0.125495 | 957 | 791.64 | 0.827210 |  |
| GO:0030217\_T\_cell\_differentiation | PTPRC | 92 | 1 | 0.736093 | -0.125495 | 957 | 791.64 | 0.827210 |  |
| GO:0030323\_respiratory\_tube\_development | FGF18 | 92 | 1 | 0.736093 | -0.125495 | 957 | 791.64 | 0.827210 |  |
| GO:0048732\_gland\_development | TBX3 | 179 | 2 | 0.756655 | -0.125392 | 958 | 792.53 | 0.827276 |  |
| GO:0048732\_gland\_development | NR3C1 | 179 | 2 | 0.756655 | -0.125392 | 958 | 792.53 | 0.827276 |  |
| GO:0006793\_phosphorus\_metabolic\_process | PTPRC | 340 | 4 | 0.796713 | -0.124549 | 960 | 793.24 | 0.826292 |  |
| GO:0006793\_phosphorus\_metabolic\_process | JAK2 | 340 | 4 | 0.796713 | -0.124549 | 960 | 793.24 | 0.826292 |  |
| GO:0006793\_phosphorus\_metabolic\_process | PPAP2A | 340 | 4 | 0.796713 | -0.124549 | 960 | 793.24 | 0.826292 |  |
| GO:0006793\_phosphorus\_metabolic\_process | GADD45B | 340 | 4 | 0.796713 | -0.124549 | 960 | 793.24 | 0.826292 |  |
| GO:0006796\_phosphate\_metabolic\_process | PTPRC | 340 | 4 | 0.796713 | -0.124549 | 960 | 793.24 | 0.826292 |  |
| GO:0006796\_phosphate\_metabolic\_process | JAK2 | 340 | 4 | 0.796713 | -0.124549 | 960 | 793.24 | 0.826292 |  |
| GO:0006796\_phosphate\_metabolic\_process | PPAP2A | 340 | 4 | 0.796713 | -0.124549 | 960 | 793.24 | 0.826292 |  |
| GO:0006796\_phosphate\_metabolic\_process | GADD45B | 340 | 4 | 0.796713 | -0.124549 | 960 | 793.24 | 0.826292 |  |
| GO:0032943\_mononuclear\_cell\_proliferation | PTPRC | 94 | 1 | 0.720432 | -0.121164 | 963 | 799.16 | 0.829865 |  |
| GO:0034984\_cellular\_response\_to\_DNA\_damage\_stimulus | HSPA1A | 94 | 1 | 0.720432 | -0.121164 | 963 | 799.16 | 0.829865 |  |
| GO:0046651\_lymphocyte\_proliferation | PTPRC | 94 | 1 | 0.720432 | -0.121164 | 963 | 799.16 | 0.829865 |  |
| GO:0051707\_response\_to\_other\_organism | PTPRC | 95 | 1 | 0.712848 | -0.119063 | 964 | 800.49 | 0.830384 |  |
| GO:0070661\_leukocyte\_proliferation | PTPRC | 96 | 1 | 0.705423 | -0.117002 | 965 | 803.42 | 0.832560 |  |
| GO:0009314\_response\_to\_radiation | ATP1A2 | 98 | 1 | 0.691026 | -0.113001 | 966 | 807.74 | 0.836170 |  |
| GO:0002376\_immune\_system\_process | PTPRC | 505 | 6 | 0.804601 | -0.112603 | 967 | 808.26 | 0.835843 |  |
| GO:0002376\_immune\_system\_process | ELF1 | 505 | 6 | 0.804601 | -0.112603 | 967 | 808.26 | 0.835843 |  |
| GO:0002376\_immune\_system\_process | NDRG1 | 505 | 6 | 0.804601 | -0.112603 | 967 | 808.26 | 0.835843 |  |
| GO:0002376\_immune\_system\_process | JAK2 | 505 | 6 | 0.804601 | -0.112603 | 967 | 808.26 | 0.835843 |  |
| GO:0002376\_immune\_system\_process | HDAC9 | 505 | 6 | 0.804601 | -0.112603 | 967 | 808.26 | 0.835843 |  |
| GO:0002376\_immune\_system\_process | RAB27A | 505 | 6 | 0.804601 | -0.112603 | 967 | 808.26 | 0.835843 |  |
| GO:0001817\_regulation\_of\_cytokine\_production | ELF1 | 99 | 1 | 0.684046 | -0.111058 | 969 | 810.39 | 0.836316 |  |
| GO:0060348\_bone\_development | FGF18 | 99 | 1 | 0.684046 | -0.111058 | 969 | 810.39 | 0.836316 |  |
| GO:0051716\_cellular\_response\_to\_stimulus | JAK2 | 273 | 3 | 0.744182 | -0.109306 | 970 | 811.36 | 0.836454 |  |
| GO:0051716\_cellular\_response\_to\_stimulus | HSPA1A | 273 | 3 | 0.744182 | -0.109306 | 970 | 811.36 | 0.836454 |  |
| GO:0051716\_cellular\_response\_to\_stimulus | CD27 | 273 | 3 | 0.744182 | -0.109306 | 970 | 811.36 | 0.836454 |  |
| GO:0030163\_protein\_catabolic\_process | TRIM63 | 101 | 1 | 0.670501 | -0.107283 | 971 | 812.77 | 0.837044 |  |
| GO:0048534\_hemopoietic\_or\_lymphoid\_organ\_development | PTPRC | 277 | 3 | 0.733436 | -0.104580 | 972 | 814.38 | 0.837840 |  |
| GO:0048534\_hemopoietic\_or\_lymphoid\_organ\_development | JAK2 | 277 | 3 | 0.733436 | -0.104580 | 972 | 814.38 | 0.837840 |  |
| GO:0048534\_hemopoietic\_or\_lymphoid\_organ\_development | HDAC9 | 277 | 3 | 0.733436 | -0.104580 | 972 | 814.38 | 0.837840 |  |
| GO:0065009\_regulation\_of\_molecular\_function | PTPRC | 279 | 3 | 0.728178 | -0.102291 | 973 | 816.26 | 0.838911 |  |
| GO:0065009\_regulation\_of\_molecular\_function | JAK2 | 279 | 3 | 0.728178 | -0.102291 | 973 | 816.26 | 0.838911 |  |
| GO:0065009\_regulation\_of\_molecular\_function | GADD45B | 279 | 3 | 0.728178 | -0.102291 | 973 | 816.26 | 0.838911 |  |
| GO:0033554\_cellular\_response\_to\_stress | HSPA1A | 196 | 2 | 0.691026 | -0.100581 | 974 | 818.59 | 0.840441 |  |
| GO:0033554\_cellular\_response\_to\_stress | CD27 | 196 | 2 | 0.691026 | -0.100581 | 974 | 818.59 | 0.840441 |  |
| GO:0048872\_homeostasis\_of\_number\_of\_cells | ZFX | 105 | 1 | 0.644958 | -0.100155 | 975 | 819.2 | 0.840205 |  |
| GO:0030099\_myeloid\_cell\_differentiation | JAK2 | 108 | 1 | 0.627042 | -0.095153 | 976 | 823.68 | 0.843934 |  |
| GO:0010926\_anatomical\_structure\_formation | FGF18 | 447 | 5 | 0.757501 | -0.094983 | 977 | 823.91 | 0.843306 |  |
| GO:0010926\_anatomical\_structure\_formation | TBX3 | 447 | 5 | 0.757501 | -0.094983 | 977 | 823.91 | 0.843306 |  |
| GO:0010926\_anatomical\_structure\_formation | TBX20 | 447 | 5 | 0.757501 | -0.094983 | 977 | 823.91 | 0.843306 |  |
| GO:0010926\_anatomical\_structure\_formation | THBS1 | 447 | 5 | 0.757501 | -0.094983 | 977 | 823.91 | 0.843306 |  |
| GO:0010926\_anatomical\_structure\_formation | FGD4 | 447 | 5 | 0.757501 | -0.094983 | 977 | 823.91 | 0.843306 |  |
| GO:0051249\_regulation\_of\_lymphocyte\_activation | PTPRC | 112 | 1 | 0.604648 | -0.088906 | 978 | 829.18 | 0.847832 |  |
| GO:0006974\_response\_to\_DNA\_damage\_stimulus | HSPA1A | 113 | 1 | 0.599297 | -0.087415 | 979 | 829.96 | 0.847763 |  |
| GO:0022414\_reproductive\_process | AFP | 376 | 4 | 0.720432 | -0.087191 | 980 | 830.08 | 0.847020 |  |
| GO:0022414\_reproductive\_process | HEXB | 376 | 4 | 0.720432 | -0.087191 | 980 | 830.08 | 0.847020 |  |
| GO:0022414\_reproductive\_process | ZFX | 376 | 4 | 0.720432 | -0.087191 | 980 | 830.08 | 0.847020 |  |
| GO:0022414\_reproductive\_process | TCF7L2 | 376 | 4 | 0.720432 | -0.087191 | 980 | 830.08 | 0.847020 |  |
| GO:0009607\_response\_to\_biotic\_stimulus | PTPRC | 114 | 1 | 0.594040 | -0.085952 | 981 | 831.66 | 0.847768 |  |
| GO:0002520\_immune\_system\_development | PTPRC | 295 | 3 | 0.688684 | -0.085621 | 982 | 832.63 | 0.847892 |  |
| GO:0002520\_immune\_system\_development | JAK2 | 295 | 3 | 0.688684 | -0.085621 | 982 | 832.63 | 0.847892 |  |
| GO:0002520\_immune\_system\_development | HDAC9 | 295 | 3 | 0.688684 | -0.085621 | 982 | 832.63 | 0.847892 |  |
| GO:0000003\_reproduction | AFP | 379 | 4 | 0.714729 | -0.084601 | 983 | 832.74 | 0.847141 |  |
| GO:0000003\_reproduction | HEXB | 379 | 4 | 0.714729 | -0.084601 | 983 | 832.74 | 0.847141 |  |
| GO:0000003\_reproduction | ZFX | 379 | 4 | 0.714729 | -0.084601 | 983 | 832.74 | 0.847141 |  |
| GO:0000003\_reproduction | TCF7L2 | 379 | 4 | 0.714729 | -0.084601 | 983 | 832.74 | 0.847141 |  |
| GO:0080134\_regulation\_of\_response\_to\_stress | CD27 | 116 | 1 | 0.583798 | -0.083105 | 984 | 834.48 | 0.848049 |  |
| GO:0022403\_cell\_cycle\_phase | LATS2 | 119 | 1 | 0.569081 | -0.079023 | 985 | 836.98 | 0.849726 |  |
| GO:0006950\_response\_to\_stress | PTPRC | 549 | 6 | 0.740116 | -0.077063 | 986 | 838.88 | 0.850791 |  |
| GO:0006950\_response\_to\_stress | HSPA1A | 549 | 6 | 0.740116 | -0.077063 | 986 | 838.88 | 0.850791 |  |
| GO:0006950\_response\_to\_stress | THBS1 | 549 | 6 | 0.740116 | -0.077063 | 986 | 838.88 | 0.850791 |  |
| GO:0006950\_response\_to\_stress | HDAC9 | 549 | 6 | 0.740116 | -0.077063 | 986 | 838.88 | 0.850791 |  |
| GO:0006950\_response\_to\_stress | CD27 | 549 | 6 | 0.740116 | -0.077063 | 986 | 838.88 | 0.850791 |  |
| GO:0006950\_response\_to\_stress | RAB27A | 549 | 6 | 0.740116 | -0.077063 | 986 | 838.88 | 0.850791 |  |
| GO:0002694\_regulation\_of\_leukocyte\_activation | PTPRC | 121 | 1 | 0.559674 | -0.076423 | 987 | 840.41 | 0.851479 |  |
| GO:0001816\_cytokine\_production | ELF1 | 122 | 1 | 0.555087 | -0.075158 | 989 | 842.99 | 0.852366 |  |
| GO:0050865\_regulation\_of\_cell\_activation | PTPRC | 122 | 1 | 0.555087 | -0.075158 | 989 | 842.99 | 0.852366 |  |
| GO:0016310\_phosphorylation | PTPRC | 309 | 3 | 0.657481 | -0.073181 | 990 | 844.24 | 0.852768 |  |
| GO:0016310\_phosphorylation | JAK2 | 309 | 3 | 0.657481 | -0.073181 | 990 | 844.24 | 0.852768 |  |
| GO:0016310\_phosphorylation | GADD45B | 309 | 3 | 0.657481 | -0.073181 | 990 | 844.24 | 0.852768 |  |
| GO:0043062\_extracellular\_structure\_organization | MYO6 | 125 | 1 | 0.541765 | -0.071495 | 991 | 845.74 | 0.853421 |  |
| GO:0043285\_biopolymer\_catabolic\_process | TRIM63 | 129 | 1 | 0.524966 | -0.066906 | 992 | 847.97 | 0.854808 |  |
| GO:0002682\_regulation\_of\_immune\_system\_process | PTPRC | 228 | 2 | 0.594040 | -0.066396 | 993 | 848.52 | 0.854502 |  |
| GO:0002682\_regulation\_of\_immune\_system\_process | ELF1 | 228 | 2 | 0.594040 | -0.066396 | 993 | 848.52 | 0.854502 |  |
| GO:0032787\_monocarboxylic\_acid\_metabolic\_process | NR3C1 | 130 | 1 | 0.520928 | -0.065808 | 994 | 849.33 | 0.854457 |  |
| GO:0050790\_regulation\_of\_catalytic\_activity | PTPRC | 233 | 2 | 0.581293 | -0.062211 | 995 | 851.6 | 0.855879 |  |
| GO:0050790\_regulation\_of\_catalytic\_activity | GADD45B | 233 | 2 | 0.581293 | -0.062211 | 995 | 851.6 | 0.855879 |  |
| GO:0007283\_spermatogenesis | ZFX | 134 | 1 | 0.505378 | -0.061606 | 997 | 852.14 | 0.854704 |  |
| GO:0048232\_male\_gamete\_generation | ZFX | 134 | 1 | 0.505378 | -0.061606 | 997 | 852.14 | 0.854704 |  |
| GO:0044085\_cellular\_component\_biogenesis | MYO6 | 237 | 2 | 0.571482 | -0.059051 | 998 | 854.29 | 0.856002 |  |
| GO:0044085\_cellular\_component\_biogenesis | FGD4 | 237 | 2 | 0.571482 | -0.059051 | 998 | 854.29 | 0.856002 |  |
| GO:0009057\_macromolecule\_catabolic\_process | TRIM63 | 137 | 1 | 0.494311 | -0.058641 | 999 | 854.61 | 0.855465 |  |
| GO:0009056\_catabolic\_process | HEXB | 243 | 2 | 0.557371 | -0.054603 | 1000 | 857.9 | 0.857900 |  |
| GO:0009056\_catabolic\_process | TRIM63 | 243 | 2 | 0.557371 | -0.054603 | 1000 | 857.9 | 0.857900 |  |
| GO:0032879\_regulation\_of\_localization | PTPRC | 248 | 2 | 0.546134 | -0.051148 | 1001 | 860.46 | 0.859600 |  |
| GO:0032879\_regulation\_of\_localization | KCNA5 | 248 | 2 | 0.546134 | -0.051148 | 1001 | 860.46 | 0.859600 |  |
| GO:0022603\_regulation\_of\_anatomical\_structure\_morphogenesis | THBS1 | 147 | 1 | 0.460684 | -0.049796 | 1002 | 862.24 | 0.860519 |  |
| GO:0022402\_cell\_cycle\_process | LATS2 | 155 | 1 | 0.436907 | -0.043729 | 1003 | 867.5 | 0.864905 |  |
| GO:0006996\_organelle\_organization | AQP11 | 449 | 4 | 0.603301 | -0.040966 | 1004 | 869.92 | 0.866454 |  |
| GO:0006996\_organelle\_organization | HEXB | 449 | 4 | 0.603301 | -0.040966 | 1004 | 869.92 | 0.866454 |  |
| GO:0006996\_organelle\_organization | HSPA1A | 449 | 4 | 0.603301 | -0.040966 | 1004 | 869.92 | 0.866454 |  |
| GO:0006996\_organelle\_organization | HDAC9 | 449 | 4 | 0.603301 | -0.040966 | 1004 | 869.92 | 0.866454 |  |
| GO:0051049\_regulation\_of\_transport | KCNA5 | 167 | 1 | 0.405513 | -0.036027 | 1005 | 875.49 | 0.871134 |  |
| GO:0019752\_carboxylic\_acid\_metabolic\_process | NR3C1 | 181 | 1 | 0.374147 | -0.028776 | 1007 | 882.48 | 0.876346 |  |
| GO:0043436\_oxoacid\_metabolic\_process | NR3C1 | 181 | 1 | 0.374147 | -0.028776 | 1007 | 882.48 | 0.876346 |  |
| GO:0006082\_organic\_acid\_metabolic\_process | NR3C1 | 182 | 1 | 0.372091 | -0.028319 | 1008 | 882.87 | 0.875863 |  |
| GO:0042180\_cellular\_ketone\_metabolic\_process | NR3C1 | 183 | 1 | 0.370058 | -0.027869 | 1009 | 883.1 | 0.875223 |  |
| GO:0042221\_response\_to\_chemical\_stimulus | MSX1 | 409 | 3 | 0.496728 | -0.022841 | 1010 | 887.16 | 0.878376 |  |
| GO:0042221\_response\_to\_chemical\_stimulus | JAK2 | 409 | 3 | 0.496728 | -0.022841 | 1010 | 887.16 | 0.878376 |  |
| GO:0042221\_response\_to\_chemical\_stimulus | BOC | 409 | 3 | 0.496728 | -0.022841 | 1010 | 887.16 | 0.878376 |  |
| GO:0022607\_cellular\_component\_assembly | FGD4 | 204 | 1 | 0.331964 | -0.019931 | 1011 | 889.29 | 0.879614 |  |
| GO:0008150\_biological\_process | FGF18 | 4605 | 68 | 1.000000 | 0.000000 | 2421 | 2410.41 | 0.995626 |  |
| GO:0008150\_biological\_process | ELF1 | 4605 | 68 | 1.000000 | 0.000000 | 2421 | 2410.41 | 0.995626 |  |
| GO:0008150\_biological\_process | MLPH | 4605 | 68 | 1.000000 | 0.000000 | 2421 | 2410.41 | 0.995626 |  |
| GO:0008150\_biological\_process | SGPP1 | 4605 | 68 | 1.000000 | 0.000000 | 2421 | 2410.41 | 0.995626 |  |
| GO:0008150\_biological\_process | PLXNA2 | 4605 | 68 | 1.000000 | 0.000000 | 2421 | 2410.41 | 0.995626 |  |
| GO:0008150\_biological\_process | TBX20 | 4605 | 68 | 1.000000 | 0.000000 | 2421 | 2410.41 | 0.995626 |  |
| GO:0008150\_biological\_process | HEXB | 4605 | 68 | 1.000000 | 0.000000 | 2421 | 2410.41 | 0.995626 |  |
| GO:0008150\_biological\_process | SGMS1 | 4605 | 68 | 1.000000 | 0.000000 | 2421 | 2410.41 | 0.995626 |  |
| GO:0008150\_biological\_process | NR2E1 | 4605 | 68 | 1.000000 | 0.000000 | 2421 | 2410.41 | 0.995626 |  |
| GO:0008150\_biological\_process | LATS2 | 4605 | 68 | 1.000000 | 0.000000 | 2421 | 2410.41 | 0.995626 |  |
| GO:0008150\_biological\_process | AQP11 | 4605 | 68 | 1.000000 | 0.000000 | 2421 | 2410.41 | 0.995626 |  |
| GO:0008150\_biological\_process | VPS13A | 4605 | 68 | 1.000000 | 0.000000 | 2421 | 2410.41 | 0.995626 |  |
| GO:0008150\_biological\_process | BOC | 4605 | 68 | 1.000000 | 0.000000 | 2421 | 2410.41 | 0.995626 |  |
| GO:0008150\_biological\_process | RAB27A | 4605 | 68 | 1.000000 | 0.000000 | 2421 | 2410.41 | 0.995626 |  |
| GO:0008150\_biological\_process | MYO6 | 4605 | 68 | 1.000000 | 0.000000 | 2421 | 2410.41 | 0.995626 |  |
| GO:0008150\_biological\_process | TCFAP2B | 4605 | 68 | 1.000000 | 0.000000 | 2421 | 2410.41 | 0.995626 |  |
| GO:0008150\_biological\_process | ARID5B | 4605 | 68 | 1.000000 | 0.000000 | 2421 | 2410.41 | 0.995626 |  |
| GO:0008150\_biological\_process | ZFX | 4605 | 68 | 1.000000 | 0.000000 | 2421 | 2410.41 | 0.995626 |  |
| GO:0008150\_biological\_process | PCDH8 | 4605 | 68 | 1.000000 | 0.000000 | 2421 | 2410.41 | 0.995626 |  |
| GO:0008150\_biological\_process | SLC9A3R1 | 4605 | 68 | 1.000000 | 0.000000 | 2421 | 2410.41 | 0.995626 |  |
| GO:0008150\_biological\_process | ASCL1 | 4605 | 68 | 1.000000 | 0.000000 | 2421 | 2410.41 | 0.995626 |  |
| GO:0008150\_biological\_process | MSX1 | 4605 | 68 | 1.000000 | 0.000000 | 2421 | 2410.41 | 0.995626 |  |
| GO:0008150\_biological\_process | CLDN1 | 4605 | 68 | 1.000000 | 0.000000 | 2421 | 2410.41 | 0.995626 |  |
| GO:0008150\_biological\_process | GADD45B | 4605 | 68 | 1.000000 | 0.000000 | 2421 | 2410.41 | 0.995626 |  |
| GO:0008150\_biological\_process | SLC40A1 | 4605 | 68 | 1.000000 | 0.000000 | 2421 | 2410.41 | 0.995626 |  |
| GO:0008150\_biological\_process | CAMK1D | 4605 | 68 | 1.000000 | 0.000000 | 2421 | 2410.41 | 0.995626 |  |
| GO:0008150\_biological\_process | DCC | 4605 | 68 | 1.000000 | 0.000000 | 2421 | 2410.41 | 0.995626 |  |
| GO:0008150\_biological\_process | FUT9 | 4605 | 68 | 1.000000 | 0.000000 | 2421 | 2410.41 | 0.995626 |  |
| GO:0008150\_biological\_process | IRX5 | 4605 | 68 | 1.000000 | 0.000000 | 2421 | 2410.41 | 0.995626 |  |
| GO:0008150\_biological\_process | BBS7 | 4605 | 68 | 1.000000 | 0.000000 | 2421 | 2410.41 | 0.995626 |  |
| GO:0008150\_biological\_process | MAMDC2 | 4605 | 68 | 1.000000 | 0.000000 | 2421 | 2410.41 | 0.995626 |  |
| GO:0008150\_biological\_process | SOX5 | 4605 | 68 | 1.000000 | 0.000000 | 2421 | 2410.41 | 0.995626 |  |
| GO:0008150\_biological\_process | HSPA1A | 4605 | 68 | 1.000000 | 0.000000 | 2421 | 2410.41 | 0.995626 |  |
| GO:0008150\_biological\_process | KCNA5 | 4605 | 68 | 1.000000 | 0.000000 | 2421 | 2410.41 | 0.995626 |  |
| GO:0008150\_biological\_process | NR3C1 | 4605 | 68 | 1.000000 | 0.000000 | 2421 | 2410.41 | 0.995626 |  |
| GO:0008150\_biological\_process | ABCA1 | 4605 | 68 | 1.000000 | 0.000000 | 2421 | 2410.41 | 0.995626 |  |
| GO:0008150\_biological\_process | ATP6V1B1 | 4605 | 68 | 1.000000 | 0.000000 | 2421 | 2410.41 | 0.995626 |  |
| GO:0008150\_biological\_process | TCF7L2 | 4605 | 68 | 1.000000 | 0.000000 | 2421 | 2410.41 | 0.995626 |  |
| GO:0008150\_biological\_process | SERINC3 | 4605 | 68 | 1.000000 | 0.000000 | 2421 | 2410.41 | 0.995626 |  |
| GO:0008150\_biological\_process | ALCAM | 4605 | 68 | 1.000000 | 0.000000 | 2421 | 2410.41 | 0.995626 |  |
| GO:0008150\_biological\_process | TCERG1 | 4605 | 68 | 1.000000 | 0.000000 | 2421 | 2410.41 | 0.995626 |  |
| GO:0008150\_biological\_process | MYOM2 | 4605 | 68 | 1.000000 | 0.000000 | 2421 | 2410.41 | 0.995626 |  |
| GO:0008150\_biological\_process | DMD | 4605 | 68 | 1.000000 | 0.000000 | 2421 | 2410.41 | 0.995626 |  |
| GO:0008150\_biological\_process | ETV1 | 4605 | 68 | 1.000000 | 0.000000 | 2421 | 2410.41 | 0.995626 |  |
| GO:0008150\_biological\_process | NDRG1 | 4605 | 68 | 1.000000 | 0.000000 | 2421 | 2410.41 | 0.995626 |  |
| GO:0008150\_biological\_process | MBNL3 | 4605 | 68 | 1.000000 | 0.000000 | 2421 | 2410.41 | 0.995626 |  |
| GO:0008150\_biological\_process | B3GNT2 | 4605 | 68 | 1.000000 | 0.000000 | 2421 | 2410.41 | 0.995626 |  |
| GO:0008150\_biological\_process | PPAP2A | 4605 | 68 | 1.000000 | 0.000000 | 2421 | 2410.41 | 0.995626 |  |
| GO:0008150\_biological\_process | THBS1 | 4605 | 68 | 1.000000 | 0.000000 | 2421 | 2410.41 | 0.995626 |  |
| GO:0008150\_biological\_process | CD27 | 4605 | 68 | 1.000000 | 0.000000 | 2421 | 2410.41 | 0.995626 |  |
| GO:0008150\_biological\_process | FGD4 | 4605 | 68 | 1.000000 | 0.000000 | 2421 | 2410.41 | 0.995626 |  |
| GO:0008150\_biological\_process | PTPRC | 4605 | 68 | 1.000000 | 0.000000 | 2421 | 2410.41 | 0.995626 |  |
| GO:0008150\_biological\_process | TBX3 | 4605 | 68 | 1.000000 | 0.000000 | 2421 | 2410.41 | 0.995626 |  |
| GO:0008150\_biological\_process | ATRN | 4605 | 68 | 1.000000 | 0.000000 | 2421 | 2410.41 | 0.995626 |  |
| GO:0008150\_biological\_process | ESRRG | 4605 | 68 | 1.000000 | 0.000000 | 2421 | 2410.41 | 0.995626 |  |
| GO:0008150\_biological\_process | ATP1A2 | 4605 | 68 | 1.000000 | 0.000000 | 2421 | 2410.41 | 0.995626 |  |
| GO:0008150\_biological\_process | ITGA4 | 4605 | 68 | 1.000000 | 0.000000 | 2421 | 2410.41 | 0.995626 |  |
| GO:0008150\_biological\_process | NID2 | 4605 | 68 | 1.000000 | 0.000000 | 2421 | 2410.41 | 0.995626 |  |
| GO:0008150\_biological\_process | TRIM63 | 4605 | 68 | 1.000000 | 0.000000 | 2421 | 2410.41 | 0.995626 |  |
| GO:0008150\_biological\_process | NKX6-1 | 4605 | 68 | 1.000000 | 0.000000 | 2421 | 2410.41 | 0.995626 |  |
| GO:0008150\_biological\_process | RGS13 | 4605 | 68 | 1.000000 | 0.000000 | 2421 | 2410.41 | 0.995626 |  |
| GO:0008150\_biological\_process | AFP | 4605 | 68 | 1.000000 | 0.000000 | 2421 | 2410.41 | 0.995626 |  |
| GO:0008150\_biological\_process | ATXN2 | 4605 | 68 | 1.000000 | 0.000000 | 2421 | 2410.41 | 0.995626 |  |
| GO:0008150\_biological\_process | AP2A1 | 4605 | 68 | 1.000000 | 0.000000 | 2421 | 2410.41 | 0.995626 |  |
| GO:0008150\_biological\_process | NEUROD1 | 4605 | 68 | 1.000000 | 0.000000 | 2421 | 2410.41 | 0.995626 |  |
| GO:0008150\_biological\_process | RGS8 | 4605 | 68 | 1.000000 | 0.000000 | 2421 | 2410.41 | 0.995626 |  |
| GO:0008150\_biological\_process | JAK2 | 4605 | 68 | 1.000000 | 0.000000 | 2421 | 2410.41 | 0.995626 |  |
| GO:0008150\_biological\_process | HDAC9 | 4605 | 68 | 1.000000 | 0.000000 | 2421 | 2410.41 | 0.995626 |  |
