# Supplementary material for: RedundancyMiner: De-replication of redundant GO categories in microarray and proteomics analysis
Source: BMC Bioinformatics. 2011 Feb 10;12:52. doi: 10.1186/1471-2105-12-52 (PMC3223614; doi:10.1186/1471-2105-12-52)
Supplement: Additional file 8 — Retinal development HTGM download. compressed package of the results of running HTGM on the retinal development genes list. [file 1471-2105-12-52-S8.ZIP › SCENARIO_2_MODIFIED/total.txt.total.txt.dir/Exp1_BestClusterMap_LEIGS_KM_24.csv.join.6.txt.dir/Exp1_BestClusterMap_LEIGS_KM_24.csv.join.6.txt.change.html]

Category Summary Report for Exp1\_BestClusterMap\_LEIGS\_KM\_24.csv.join.6.txt

# Category Summary Report for Exp1\_BestClusterMap\_LEIGS\_KM\_24.csv.join.6.txt

| HYPERLINKED GO CATEGORY | TOTAL GENES | CHANGED GENES | ENRICHMENT | LOG10(p) | CUMULATIVE NUMBER OF CATEGORIES | CUMULATIVE RANDOMS LOWER BOUND | CUMULATIVE RANDOMS MEAN | CUMULATIVE RANDOMS UPPER BOUND | FALSE DISCOVERY RATE |  |
| --- | --- | --- | --- | --- | --- | --- | --- | --- | --- | --- |
| GO:0006670\_sphingosine\_metabolic\_process | 3 | 2 |  |  |  |  |  |  |  |  |
| GO:0006665\_sphingolipid\_metabolic\_process | 31 | 4 | 8.738140 | -2.994186 | 1 | -0.701094 | 0.67 | 2.041094 | 0.670000 |  |
| GO:0007399\_nervous\_system\_development | 621 | 19 | 2.071966 | -2.928808 | 2 | -0.657401 | 0.77 | 2.197401 | 0.385000 |  |
| GO:0060164\_regulation\_of\_timing\_of\_neuron\_differentiation | 4 | 2 |  |  |  |  |  |  |  |  |
| GO:0006643\_membrane\_lipid\_metabolic\_process | 33 | 4 | 8.208556 | -2.889715 | 3 | -0.788909 | 0.93 | 2.648909 | 0.310000 |  |
| GO:0032400\_melanosome\_localization | 5 | 2 | 27.088235 | -2.680282 | 5 | -0.514265 | 2.11 | 4.734265 | 0.422000 |  |
| GO:0045661\_regulation\_of\_myoblast\_differentiation | 5 | 2 | 27.088235 | -2.680282 | 5 | -0.514265 | 2.11 | 4.734265 | 0.422000 |  |
| GO:0021953\_central\_nervous\_system\_neuron\_differentiation | 39 | 4 | 6.945701 | -2.615381 | 6 | -0.592460 | 2.32 | 5.232460 | 0.386667 |  |
| GO:0051875\_pigment\_granule\_localization | 6 | 2 | 22.573529 | -2.508349 | 7 | -0.249843 | 3.52 | 7.289843 | 0.502857 |  |
| GO:0016202\_regulation\_of\_striated\_muscle\_tissue\_development | 21 | 3 | 9.674370 | -2.470286 | 10 | -0.286567 | 3.78 | 7.846567 | 0.378000 |  |
| GO:0046519\_sphingoid\_metabolic\_process | 21 | 3 | 9.674370 | -2.470286 | 10 | -0.286567 | 3.78 | 7.846567 | 0.378000 |  |
| GO:0048634\_regulation\_of\_muscle\_development | 21 | 3 | 9.674370 | -2.470286 | 10 | -0.286567 | 3.78 | 7.846567 | 0.378000 |  |
| GO:0051705\_behavioral\_interaction\_between\_organisms | 23 | 3 | 8.833120 | -2.355073 | 11 | 0.344360 | 5.43 | 10.515640 | 0.493636 |  |
| GO:0040034\_regulation\_of\_development\_\_heterochronic | 8 | 2 | 16.930147 | -2.245586 | 14 | 0.954060 | 6.88 | 12.805940 | 0.491429 |  |
| GO:0048505\_regulation\_of\_timing\_of\_cell\_differentiation | 8 | 2 | 16.930147 | -2.245586 | 14 | 0.954060 | 6.88 | 12.805940 | 0.491429 |  |
| GO:0048742\_regulation\_of\_skeletal\_muscle\_fiber\_development | 8 | 2 | 16.930147 | -2.245586 | 14 | 0.954060 | 6.88 | 12.805940 | 0.491429 |  |
| GO:0032502\_developmental\_process | 2060 | 41 | 1.347837 | -2.171148 | 15 | 1.230672 | 7.4 | 13.569328 | 0.493333 |  |
| GO:0007517\_muscle\_organ\_development | 153 | 7 | 3.098328 | -2.160996 | 16 | 1.299344 | 7.52 | 13.740656 | 0.470000 |  |
| GO:0048731\_system\_development | 1609 | 34 | 1.431013 | -2.144964 | 17 | 1.262556 | 7.72 | 14.177444 | 0.454118 |  |
| GO:0060284\_regulation\_of\_cell\_development | 122 | 6 | 3.330521 | -2.057512 | 18 | 2.058818 | 9.54 | 17.021182 | 0.530000 |  |
| GO:0021952\_central\_nervous\_system\_projection\_neuron\_axonogenesis | 10 | 2 | 13.544118 | -2.047816 | 22 | 2.459277 | 10.63 | 18.800723 | 0.483182 |  |
| GO:0043525\_positive\_regulation\_of\_neuron\_apoptosis | 10 | 2 | 13.544118 | -2.047816 | 22 | 2.459277 | 10.63 | 18.800723 | 0.483182 |  |
| GO:0048641\_regulation\_of\_skeletal\_muscle\_tissue\_development | 10 | 2 | 13.544118 | -2.047816 | 22 | 2.459277 | 10.63 | 18.800723 | 0.483182 |  |
| GO:0051147\_regulation\_of\_muscle\_cell\_differentiation | 10 | 2 | 13.544118 | -2.047816 | 22 | 2.459277 | 10.63 | 18.800723 | 0.483182 |  |
| GO:0021954\_central\_nervous\_system\_neuron\_development | 31 | 3 | 6.553605 | -1.987067 | 24 | 2.746448 | 11.55 | 20.353552 | 0.481250 |  |
| GO:0051640\_organelle\_localization | 31 | 3 | 6.553605 | -1.987067 | 24 | 2.746448 | 11.55 | 20.353552 | 0.481250 |  |
| GO:0001913\_T\_cell\_mediated\_cytotoxicity | 11 | 2 | 12.312834 | -1.964802 | 30 | 3.400431 | 12.63 | 21.859569 | 0.421000 |  |
| GO:0007159\_leukocyte\_adhesion | 11 | 2 | 12.312834 | -1.964802 | 30 | 3.400431 | 12.63 | 21.859569 | 0.421000 |  |
| GO:0033059\_cellular\_pigmentation | 11 | 2 | 12.312834 | -1.964802 | 30 | 3.400431 | 12.63 | 21.859569 | 0.421000 |  |
| GO:0035176\_social\_behavior | 11 | 2 | 12.312834 | -1.964802 | 30 | 3.400431 | 12.63 | 21.859569 | 0.421000 |  |
| GO:0050854\_regulation\_of\_antigen\_receptor-mediated\_signaling\_pathway | 11 | 2 | 12.312834 | -1.964802 | 30 | 3.400431 | 12.63 | 21.859569 | 0.421000 |  |
| GO:0051648\_vesicle\_localization | 11 | 2 | 12.312834 | -1.964802 | 30 | 3.400431 | 12.63 | 21.859569 | 0.421000 |  |
| GO:0045445\_myoblast\_differentiation | 12 | 2 | 11.286765 | -1.889753 | 31 | 4.389096 | 14.39 | 24.390904 | 0.464194 |  |
| GO:0016265\_death | 450 | 13 | 1.956373 | -1.885645 | 32 | 4.383676 | 14.41 | 24.436324 | 0.450312 |  |
| GO:0048666\_neuron\_development | 262 | 9 | 2.326280 | -1.853982 | 33 | 4.524232 | 15.11 | 25.695768 | 0.457879 |  |
| GO:0030182\_neuron\_differentiation | 356 | 11 | 2.092490 | -1.849845 | 34 | 4.538737 | 15.13 | 25.721263 | 0.445000 |  |
| GO:0009967\_positive\_regulation\_of\_signal\_transduction | 98 | 5 | 3.455132 | -1.845870 | 35 | 4.601934 | 15.25 | 25.898066 | 0.435714 |  |
| GO:0007292\_female\_gamete\_generation | 35 | 3 | 5.804622 | -1.842074 | 36 | 4.727958 | 15.51 | 26.292042 | 0.430833 |  |
| GO:0048513\_organ\_development | 1365 | 29 | 1.438752 | -1.832090 | 37 | 4.752857 | 15.56 | 26.367143 | 0.420541 |  |
| GO:0006667\_sphinganine\_metabolic\_process | 1 | 1 |  |  |  |  |  |  |  |  |
| GO:0006668\_sphinganine-1-phosphate\_metabolic\_process | 1 | 1 |  |  |  |  |  |  |  |  |
| GO:0007400\_neuroblast\_fate\_determination | 1 | 1 |  |  |  |  |  |  |  |  |
| GO:0008049\_male\_courtship\_behavior | 1 | 1 |  |  |  |  |  |  |  |  |
| GO:0014016\_neuroblast\_differentiation | 1 | 1 |  |  |  |  |  |  |  |  |
| GO:0014017\_neuroblast\_fate\_commitment | 1 | 1 |  |  |  |  |  |  |  |  |
| GO:0021934\_hindbrain\_tangential\_cell\_migration | 1 | 1 |  |  |  |  |  |  |  |  |
| GO:0021935\_granule\_cell\_precursor\_tangential\_migration | 1 | 1 |  |  |  |  |  |  |  |  |
| GO:0021960\_anterior\_commissure\_morphogenesis | 1 | 1 |  |  |  |  |  |  |  |  |
| GO:0034113\_heterotypic\_cell-cell\_adhesion | 1 | 1 |  |  |  |  |  |  |  |  |
| GO:0043316\_cytotoxic\_T\_cell\_degranulation | 1 | 1 |  |  |  |  |  |  |  |  |
| GO:0045988\_negative\_regulation\_of\_striated\_muscle\_contraction | 1 | 1 |  |  |  |  |  |  |  |  |
| GO:0048388\_endosomal\_lumen\_acidification | 1 | 1 |  |  |  |  |  |  |  |  |
| GO:0051481\_reduction\_of\_cytosolic\_calcium\_ion\_concentration | 1 | 1 |  |  |  |  |  |  |  |  |
| GO:0060163\_subpallium\_neuron\_fate\_commitment | 1 | 1 |  |  |  |  |  |  |  |  |
| GO:0060165\_regulation\_of\_timing\_of\_subpallium\_neuron\_differentiation | 1 | 1 |  |  |  |  |  |  |  |  |
| GO:0060398\_regulation\_of\_growth\_hormone\_receptor\_signaling\_pathway | 1 | 1 |  |  |  |  |  |  |  |  |
| GO:0060399\_positive\_regulation\_of\_growth\_hormone\_receptor\_signaling\_pathway | 1 | 1 |  |  |  |  |  |  |  |  |
| GO:0021955\_central\_nervous\_system\_neuron\_axonogenesis | 13 | 2 | 10.418552 | -1.821329 | 39 | 5.248208 | 16.88 | 28.511792 | 0.432821 |  |
| GO:0060324\_face\_development | 13 | 2 | 10.418552 | -1.821329 | 39 | 5.248208 | 16.88 | 28.511792 | 0.432821 |  |
| GO:0048856\_anatomical\_structure\_development | 1688 | 34 | 1.364040 | -1.795628 | 40 | 5.391000 | 17.35 | 29.309000 | 0.433750 |  |
| GO:0048665\_neuron\_fate\_specification | 14 | 2 | 9.674370 | -1.758505 | 41 | 6.210326 | 19.0 | 31.789674 | 0.463415 |  |
| GO:0006644\_phospholipid\_metabolic\_process | 39 | 3 | 5.209276 | -1.715225 | 42 | 6.715128 | 20.08 | 33.444872 | 0.478095 |  |
| GO:0006885\_regulation\_of\_pH | 15 | 2 | 9.029412 | -1.700475 | 45 | 7.579043 | 21.47 | 35.360957 | 0.477111 |  |
| GO:0048709\_oligodendrocyte\_differentiation | 15 | 2 | 9.029412 | -1.700475 | 45 | 7.579043 | 21.47 | 35.360957 | 0.477111 |  |
| GO:0060322\_head\_development | 15 | 2 | 9.029412 | -1.700475 | 45 | 7.579043 | 21.47 | 35.360957 | 0.477111 |  |
| GO:0006915\_apoptosis | 427 | 12 | 1.903155 | -1.681421 | 46 | 7.874067 | 22.05 | 36.225933 | 0.479348 |  |
| GO:0006928\_cell\_motion | 330 | 10 | 2.052139 | -1.662701 | 48 | 8.002771 | 22.37 | 36.737229 | 0.466042 |  |
| GO:0051674\_localization\_of\_cell | 330 | 10 | 2.052139 | -1.662701 | 48 | 8.002771 | 22.37 | 36.737229 | 0.466042 |  |
| GO:0051239\_regulation\_of\_multicellular\_organismal\_process | 587 | 15 | 1.730509 | -1.661834 | 49 | 7.995820 | 22.38 | 36.764180 | 0.456735 |  |
| GO:0010647\_positive\_regulation\_of\_cell\_communication | 110 | 5 | 3.078209 | -1.648705 | 51 | 8.373353 | 22.99 | 37.606647 | 0.450784 |  |
| GO:0055080\_cation\_homeostasis | 110 | 5 | 3.078209 | -1.648705 | 51 | 8.373353 | 22.99 | 37.606647 | 0.450784 |  |
| GO:0008654\_phospholipid\_biosynthetic\_process | 16 | 2 | 8.465074 | -1.646596 | 52 | 8.970396 | 24.27 | 39.569604 | 0.466731 |  |
| GO:0012501\_programmed\_cell\_death | 433 | 12 | 1.876783 | -1.638476 | 53 | 8.943732 | 24.38 | 39.816268 | 0.460000 |  |
| GO:0010740\_positive\_regulation\_of\_protein\_kinase\_cascade | 42 | 3 | 4.837185 | -1.629757 | 54 | 9.174877 | 24.96 | 40.745123 | 0.462222 |  |
| GO:0050793\_regulation\_of\_developmental\_process | 703 | 17 | 1.637624 | -1.623542 | 55 | 9.211423 | 25.04 | 40.868577 | 0.455273 |  |
| GO:0019725\_cellular\_homeostasis | 195 | 7 | 2.430995 | -1.621862 | 56 | 9.255297 | 25.11 | 40.964703 | 0.448393 |  |
| GO:0007417\_central\_nervous\_system\_development | 287 | 9 | 2.123642 | -1.619725 | 57 | 9.259530 | 25.13 | 41.000470 | 0.440877 |  |
| GO:0019637\_organophosphate\_metabolic\_process | 43 | 3 | 4.724692 | -1.602868 | 58 | 9.582519 | 25.68 | 41.777481 | 0.442759 |  |
| GO:0031175\_neuron\_projection\_development | 197 | 7 | 2.406315 | -1.600399 | 60 | 9.618229 | 25.74 | 41.861771 | 0.429000 |  |
| GO:0050801\_ion\_homeostasis | 197 | 7 | 2.406315 | -1.600399 | 60 | 9.618229 | 25.74 | 41.861771 | 0.429000 |  |
| GO:0055067\_monovalent\_inorganic\_cation\_homeostasis | 17 | 2 | 7.967128 | -1.596347 | 61 | 10.197931 | 27.11 | 44.022069 | 0.444426 |  |
| GO:0008219\_cell\_death | 444 | 12 | 1.830286 | -1.562407 | 62 | 10.713934 | 28.0 | 45.286066 | 0.451613 |  |
| GO:0007409\_axonogenesis | 158 | 6 | 2.571668 | -1.550319 | 63 | 10.996958 | 28.41 | 45.823042 | 0.450952 |  |
| GO:0006022\_aminoglycan\_metabolic\_process | 18 | 2 | 7.524510 | -1.549299 | 66 | 11.786924 | 29.77 | 47.753076 | 0.451061 |  |
| GO:0030203\_glycosaminoglycan\_metabolic\_process | 18 | 2 | 7.524510 | -1.549299 | 66 | 11.786924 | 29.77 | 47.753076 | 0.451061 |  |
| GO:0030318\_melanocyte\_differentiation | 18 | 2 | 7.524510 | -1.549299 | 66 | 11.786924 | 29.77 | 47.753076 | 0.451061 |  |
| GO:0048699\_generation\_of\_neurons | 396 | 11 | 1.881127 | -1.538863 | 67 | 11.813507 | 29.9 | 47.986493 | 0.446269 |  |
| GO:0002922\_positive\_regulation\_of\_humoral\_immune\_response | 2 | 1 |  |  |  |  |  |  |  |  |
| GO:0002925\_positive\_regulation\_of\_humoral\_immune\_response\_mediated\_by\_circulating\_immunoglobulin | 2 | 1 |  |  |  |  |  |  |  |  |
| GO:0006686\_sphingomyelin\_biosynthetic\_process | 2 | 1 |  |  |  |  |  |  |  |  |
| GO:0007619\_courtship\_behavior | 2 | 1 |  |  |  |  |  |  |  |  |
| GO:0009313\_oligosaccharide\_catabolic\_process | 2 | 1 |  |  |  |  |  |  |  |  |
| GO:0021965\_spinal\_cord\_ventral\_commissure\_morphogenesis | 2 | 1 |  |  |  |  |  |  |  |  |
| GO:0031946\_regulation\_of\_glucocorticoid\_biosynthetic\_process | 2 | 1 |  |  |  |  |  |  |  |  |
| GO:0031952\_regulation\_of\_protein\_amino\_acid\_autophosphorylation | 2 | 1 |  |  |  |  |  |  |  |  |
| GO:0031953\_negative\_regulation\_of\_protein\_amino\_acid\_autophosphorylation | 2 | 1 |  |  |  |  |  |  |  |  |
| GO:0031958\_corticosteroid\_receptor\_signaling\_pathway | 2 | 1 |  |  |  |  |  |  |  |  |
| GO:0033700\_phospholipid\_efflux | 2 | 1 |  |  |  |  |  |  |  |  |
| GO:0042517\_positive\_regulation\_of\_tyrosine\_phosphorylation\_of\_Stat3\_protein | 2 | 1 |  |  |  |  |  |  |  |  |
| GO:0042921\_glucocorticoid\_receptor\_signaling\_pathway | 2 | 1 |  |  |  |  |  |  |  |  |
| GO:0043320\_natural\_killer\_cell\_degranulation | 2 | 1 |  |  |  |  |  |  |  |  |
| GO:0045662\_negative\_regulation\_of\_myoblast\_differentiation | 2 | 1 |  |  |  |  |  |  |  |  |
| GO:0045663\_positive\_regulation\_of\_myoblast\_differentiation | 2 | 1 |  |  |  |  |  |  |  |  |
| GO:0048619\_embryonic\_hindgut\_morphogenesis | 2 | 1 |  |  |  |  |  |  |  |  |
| GO:0048867\_stem\_cell\_fate\_determination | 2 | 1 |  |  |  |  |  |  |  |  |
| GO:0050855\_regulation\_of\_B\_cell\_receptor\_signaling\_pathway | 2 | 1 |  |  |  |  |  |  |  |  |
| GO:0060166\_olfactory\_pit\_development | 2 | 1 |  |  |  |  |  |  |  |  |
| GO:0060179\_male\_mating\_behavior | 2 | 1 |  |  |  |  |  |  |  |  |
| GO:0060397\_JAK-STAT\_cascade\_involved\_in\_growth\_hormone\_signaling\_pathway | 2 | 1 |  |  |  |  |  |  |  |  |
| GO:0090030\_regulation\_of\_steroid\_hormone\_biosynthetic\_process | 2 | 1 |  |  |  |  |  |  |  |  |
| GO:0042063\_gliogenesis | 46 | 3 | 4.416560 | -1.526487 | 68 | 12.016190 | 30.34 | 48.663810 | 0.446176 |  |
| GO:0048522\_positive\_regulation\_of\_cellular\_process | 895 | 20 | 1.513309 | -1.515412 | 69 | 12.080132 | 30.48 | 48.879868 | 0.441739 |  |
| GO:0007275\_multicellular\_organismal\_development | 1760 | 34 | 1.308239 | -1.512578 | 70 | 12.089963 | 30.52 | 48.950037 | 0.436000 |  |
| GO:0006672\_ceramide\_metabolic\_process | 19 | 2 | 7.128483 | -1.505094 | 72 | 12.692264 | 31.31 | 49.927736 | 0.434861 |  |
| GO:0050931\_pigment\_cell\_differentiation | 19 | 2 | 7.128483 | -1.505094 | 72 | 12.692264 | 31.31 | 49.927736 | 0.434861 |  |
| GO:0007411\_axon\_guidance | 82 | 4 | 3.303443 | -1.492769 | 74 | 13.270651 | 31.99 | 50.709349 | 0.432297 |  |
| GO:0045664\_regulation\_of\_neuron\_differentiation | 82 | 4 | 3.303443 | -1.492769 | 74 | 13.270651 | 31.99 | 50.709349 | 0.432297 |  |
| GO:0045935\_positive\_regulation\_of\_nucleobase\_\_nucleoside\_\_nucleotide\_and\_nucleic\_acid\_metabolic\_process | 352 | 10 | 1.923880 | -1.490975 | 75 | 13.426621 | 32.17 | 50.913379 | 0.428933 |  |
| GO:0006886\_intracellular\_protein\_transport | 122 | 5 | 2.775434 | -1.478336 | 76 | 13.575109 | 32.72 | 51.864891 | 0.430526 |  |
| GO:0043065\_positive\_regulation\_of\_apoptosis | 166 | 6 | 2.447732 | -1.459219 | 77 | 14.261786 | 34.28 | 54.298214 | 0.445195 |  |
| GO:0043473\_pigmentation | 49 | 3 | 4.146158 | -1.455888 | 78 | 14.407942 | 34.66 | 54.912058 | 0.444359 |  |
| GO:0010942\_positive\_regulation\_of\_cell\_death | 167 | 6 | 2.433075 | -1.448279 | 80 | 14.509383 | 34.81 | 55.110617 | 0.435125 |  |
| GO:0043068\_positive\_regulation\_of\_programmed\_cell\_death | 167 | 6 | 2.433075 | -1.448279 | 80 | 14.509383 | 34.81 | 55.110617 | 0.435125 |  |
| GO:0042981\_regulation\_of\_apoptosis | 360 | 10 | 1.881127 | -1.432959 | 81 | 14.825616 | 35.31 | 55.794384 | 0.435926 |  |
| GO:0006605\_protein\_targeting | 86 | 4 | 3.149795 | -1.427405 | 82 | 14.965151 | 35.51 | 56.054849 | 0.433049 |  |
| GO:0051173\_positive\_regulation\_of\_nitrogen\_compound\_metabolic\_process | 361 | 10 | 1.875917 | -1.425864 | 83 | 14.976634 | 35.52 | 56.063366 | 0.427952 |  |
| GO:0002456\_T\_cell\_mediated\_immunity | 21 | 2 | 6.449580 | -1.424057 | 84 | 15.906735 | 36.9 | 57.893265 | 0.439286 |  |
| GO:0032501\_multicellular\_organismal\_process | 2183 | 40 | 1.240872 | -1.423244 | 85 | 15.921261 | 36.91 | 57.898739 | 0.434235 |  |
| GO:0048812\_neuron\_projection\_morphogenesis | 170 | 6 | 2.390138 | -1.416026 | 86 | 15.933840 | 36.96 | 57.986160 | 0.429767 |  |
| GO:0030030\_cell\_projection\_organization | 263 | 8 | 2.059942 | -1.415702 | 87 | 15.951752 | 36.97 | 57.988248 | 0.424943 |  |
| GO:0016337\_cell-cell\_adhesion | 87 | 4 | 3.113590 | -1.411682 | 88 | 16.111369 | 37.16 | 58.208631 | 0.422273 |  |
| GO:0010941\_regulation\_of\_cell\_death | 365 | 10 | 1.855359 | -1.397820 | 90 | 16.667939 | 37.92 | 59.172061 | 0.421333 |  |
| GO:0043067\_regulation\_of\_programmed\_cell\_death | 365 | 10 | 1.855359 | -1.397820 | 90 | 16.667939 | 37.92 | 59.172061 | 0.421333 |  |
| GO:0001947\_heart\_looping | 22 | 2 | 6.156417 | -1.386750 | 92 | 18.171352 | 40.17 | 62.168648 | 0.436630 |  |
| GO:0048477\_oogenesis | 22 | 2 | 6.156417 | -1.386750 | 92 | 18.171352 | 40.17 | 62.168648 | 0.436630 |  |
| GO:0048667\_cell\_morphogenesis\_involved\_in\_neuron\_differentiation | 173 | 6 | 2.348691 | -1.384600 | 93 | 18.198848 | 40.21 | 62.221152 | 0.432366 |  |
| GO:0042592\_homeostatic\_process | 419 | 11 | 1.777867 | -1.383498 | 94 | 18.237873 | 40.23 | 62.222127 | 0.427979 |  |
| GO:0065008\_regulation\_of\_biological\_quality | 693 | 16 | 1.563535 | -1.380626 | 95 | 18.294076 | 40.31 | 62.325924 | 0.424316 |  |
| GO:0045165\_cell\_fate\_commitment | 130 | 5 | 2.604638 | -1.377032 | 96 | 18.363222 | 40.45 | 62.536778 | 0.421354 |  |
| GO:0030003\_cellular\_cation\_homeostasis | 90 | 4 | 3.009804 | -1.365907 | 98 | 18.892454 | 41.07 | 63.247546 | 0.419082 |  |
| GO:0035264\_multicellular\_organism\_growth | 90 | 4 | 3.009804 | -1.365907 | 98 | 18.892454 | 41.07 | 63.247546 | 0.419082 |  |
| GO:0001915\_negative\_regulation\_of\_T\_cell\_mediated\_cytotoxicity | 3 | 1 |  |  |  |  |  |  |  |  |
| GO:0001960\_negative\_regulation\_of\_cytokine-mediated\_signaling\_pathway | 3 | 1 |  |  |  |  |  |  |  |  |
| GO:0006651\_diacylglycerol\_biosynthetic\_process | 3 | 1 |  |  |  |  |  |  |  |  |
| GO:0006689\_ganglioside\_catabolic\_process | 3 | 1 |  |  |  |  |  |  |  |  |
| GO:0007262\_STAT\_protein\_nuclear\_translocation | 3 | 1 |  |  |  |  |  |  |  |  |
| GO:0008347\_glial\_cell\_migration | 3 | 1 |  |  |  |  |  |  |  |  |
| GO:0010159\_specification\_of\_organ\_position | 3 | 1 |  |  |  |  |  |  |  |  |
| GO:0021527\_spinal\_cord\_association\_neuron\_differentiation | 3 | 1 |  |  |  |  |  |  |  |  |
| GO:0021529\_spinal\_cord\_oligodendrocyte\_cell\_differentiation | 3 | 1 |  |  |  |  |  |  |  |  |
| GO:0021530\_spinal\_cord\_oligodendrocyte\_cell\_fate\_specification | 3 | 1 |  |  |  |  |  |  |  |  |
| GO:0021912\_regulation\_of\_transcription\_from\_RNA\_polymerase\_II\_promoter\_involved\_in\_spinal\_cord\_motor\_neuron\_fate\_specification | 3 | 1 |  |  |  |  |  |  |  |  |
| GO:0030319\_cellular\_di-\_\_tri-valent\_inorganic\_anion\_homeostasis | 3 | 1 |  |  |  |  |  |  |  |  |
| GO:0030538\_embryonic\_genitalia\_morphogenesis | 3 | 1 |  |  |  |  |  |  |  |  |
| GO:0030643\_cellular\_phosphate\_ion\_homeostasis | 3 | 1 |  |  |  |  |  |  |  |  |
| GO:0031943\_regulation\_of\_glucocorticoid\_metabolic\_process | 3 | 1 |  |  |  |  |  |  |  |  |
| GO:0032402\_melanosome\_transport | 3 | 1 |  |  |  |  |  |  |  |  |
| GO:0042448\_progesterone\_metabolic\_process | 3 | 1 |  |  |  |  |  |  |  |  |
| GO:0042523\_positive\_regulation\_of\_tyrosine\_phosphorylation\_of\_Stat5\_protein | 3 | 1 |  |  |  |  |  |  |  |  |
| GO:0043267\_negative\_regulation\_of\_potassium\_ion\_transport | 3 | 1 |  |  |  |  |  |  |  |  |
| GO:0046479\_glycosphingolipid\_catabolic\_process | 3 | 1 |  |  |  |  |  |  |  |  |
| GO:0046885\_regulation\_of\_hormone\_biosynthetic\_process | 3 | 1 |  |  |  |  |  |  |  |  |
| GO:0048865\_stem\_cell\_fate\_commitment | 3 | 1 |  |  |  |  |  |  |  |  |
| GO:0050857\_positive\_regulation\_of\_antigen\_receptor-mediated\_signaling\_pathway | 3 | 1 |  |  |  |  |  |  |  |  |
| GO:0051149\_positive\_regulation\_of\_muscle\_cell\_differentiation | 3 | 1 |  |  |  |  |  |  |  |  |
| GO:0055061\_di-\_\_tri-valent\_inorganic\_anion\_homeostasis | 3 | 1 |  |  |  |  |  |  |  |  |
| GO:0055062\_phosphate\_ion\_homeostasis | 3 | 1 |  |  |  |  |  |  |  |  |
| GO:0060040\_retinal\_bipolar\_neuron\_differentiation | 3 | 1 |  |  |  |  |  |  |  |  |
| GO:0060396\_growth\_hormone\_receptor\_signaling\_pathway | 3 | 1 |  |  |  |  |  |  |  |  |
| GO:0060416\_response\_to\_growth\_hormone\_stimulus | 3 | 1 |  |  |  |  |  |  |  |  |
| GO:0060596\_mammary\_placode\_formation | 3 | 1 |  |  |  |  |  |  |  |  |
| GO:0022008\_neurogenesis | 423 | 11 | 1.761055 | -1.358032 | 99 | 19.066615 | 41.24 | 63.413385 | 0.416566 |  |
| GO:0006873\_cellular\_ion\_homeostasis | 176 | 6 | 2.308656 | -1.353972 | 101 | 19.168028 | 41.39 | 63.611972 | 0.409802 |  |
| GO:0048858\_cell\_projection\_morphogenesis | 176 | 6 | 2.308656 | -1.353972 | 101 | 19.168028 | 41.39 | 63.611972 | 0.409802 |  |
| GO:0048518\_positive\_regulation\_of\_biological\_process | 995 | 21 | 1.429279 | -1.333125 | 102 | 20.424200 | 43.36 | 66.295800 | 0.425098 |  |
| GO:0021515\_cell\_differentiation\_in\_spinal\_cord | 24 | 2 | 5.643382 | -1.317611 | 104 | 21.933147 | 45.44 | 68.946853 | 0.436923 |  |
| GO:0043410\_positive\_regulation\_of\_MAPKKK\_cascade | 24 | 2 | 5.643382 | -1.317611 | 104 | 21.933147 | 45.44 | 68.946853 | 0.436923 |  |
| GO:0030154\_cell\_differentiation | 1060 | 22 | 1.405522 | -1.313199 | 105 | 21.956016 | 45.49 | 69.023984 | 0.433238 |  |
| GO:0008610\_lipid\_biosynthetic\_process | 94 | 4 | 2.881727 | -1.307929 | 106 | 22.411174 | 46.22 | 70.028826 | 0.436038 |  |
| GO:0055082\_cellular\_chemical\_homeostasis | 181 | 6 | 2.244881 | -1.304623 | 107 | 22.577049 | 46.57 | 70.562951 | 0.435234 |  |
| GO:0010604\_positive\_regulation\_of\_macromolecule\_metabolic\_process | 433 | 11 | 1.720384 | -1.296264 | 108 | 22.671900 | 46.79 | 70.908100 | 0.433241 |  |
| GO:0048468\_cell\_development | 654 | 15 | 1.553225 | -1.292954 | 109 | 22.819188 | 46.97 | 71.120812 | 0.430917 |  |
| GO:0060603\_mammary\_gland\_duct\_morphogenesis | 25 | 2 | 5.417647 | -1.285471 | 110 | 24.119652 | 48.75 | 73.380348 | 0.443182 |  |
| GO:0051093\_negative\_regulation\_of\_developmental\_process | 331 | 9 | 1.841345 | -1.278521 | 111 | 24.387876 | 49.19 | 73.992124 | 0.443153 |  |
| GO:0032990\_cell\_part\_morphogenesis | 184 | 6 | 2.208280 | -1.275990 | 112 | 24.478809 | 49.27 | 74.061191 | 0.439911 |  |
| GO:0034613\_cellular\_protein\_localization | 139 | 5 | 2.435992 | -1.273064 | 113 | 24.573544 | 49.39 | 74.206456 | 0.437080 |  |
| GO:0007155\_cell\_adhesion | 186 | 6 | 2.184535 | -1.257293 | 115 | 24.951822 | 50.06 | 75.168178 | 0.435304 |  |
| GO:0022610\_biological\_adhesion | 186 | 6 | 2.184535 | -1.257293 | 115 | 24.951822 | 50.06 | 75.168178 | 0.435304 |  |
| GO:0051480\_cytosolic\_calcium\_ion\_homeostasis | 26 | 2 | 5.209276 | -1.254776 | 116 | 25.634477 | 51.19 | 76.745523 | 0.441293 |  |
| GO:0070727\_cellular\_macromolecule\_localization | 141 | 5 | 2.401439 | -1.251262 | 117 | 25.941238 | 51.76 | 77.578762 | 0.442393 |  |
| GO:0031325\_positive\_regulation\_of\_cellular\_metabolic\_process | 442 | 11 | 1.685354 | -1.242903 | 118 | 26.033820 | 51.87 | 77.706180 | 0.439576 |  |
| GO:0002923\_regulation\_of\_humoral\_immune\_response\_mediated\_by\_circulating\_immunoglobulin | 4 | 1 |  |  |  |  |  |  |  |  |
| GO:0006111\_regulation\_of\_gluconeogenesis | 4 | 1 |  |  |  |  |  |  |  |  |
| GO:0006684\_sphingomyelin\_metabolic\_process | 4 | 1 |  |  |  |  |  |  |  |  |
| GO:0008207\_C21-steroid\_hormone\_metabolic\_process | 4 | 1 |  |  |  |  |  |  |  |  |
| GO:0016338\_calcium-independent\_cell-cell\_adhesion | 4 | 1 |  |  |  |  |  |  |  |  |
| GO:0019377\_glycolipid\_catabolic\_process | 4 | 1 |  |  |  |  |  |  |  |  |
| GO:0021535\_cell\_migration\_in\_hindbrain | 4 | 1 |  |  |  |  |  |  |  |  |
| GO:0021778\_oligodendrocyte\_cell\_fate\_specification | 4 | 1 |  |  |  |  |  |  |  |  |
| GO:0021779\_oligodendrocyte\_cell\_fate\_commitment | 4 | 1 |  |  |  |  |  |  |  |  |
| GO:0021780\_glial\_cell\_fate\_specification | 4 | 1 |  |  |  |  |  |  |  |  |
| GO:0021913\_regulation\_of\_transcription\_from\_RNA\_polymerase\_II\_promoter\_involved\_in\_ventral\_spinal\_cord\_interneuron\_specification | 4 | 1 |  |  |  |  |  |  |  |  |
| GO:0030949\_positive\_regulation\_of\_vascular\_endothelial\_growth\_factor\_receptor\_signaling\_pathway | 4 | 1 |  |  |  |  |  |  |  |  |
| GO:0032401\_establishment\_of\_melanosome\_localization | 4 | 1 |  |  |  |  |  |  |  |  |
| GO:0034204\_lipid\_translocation | 4 | 1 |  |  |  |  |  |  |  |  |
| GO:0042522\_regulation\_of\_tyrosine\_phosphorylation\_of\_Stat5\_protein | 4 | 1 |  |  |  |  |  |  |  |  |
| GO:0043691\_reverse\_cholesterol\_transport | 4 | 1 |  |  |  |  |  |  |  |  |
| GO:0045332\_phospholipid\_translocation | 4 | 1 |  |  |  |  |  |  |  |  |
| GO:0045747\_positive\_regulation\_of\_Notch\_signaling\_pathway | 4 | 1 |  |  |  |  |  |  |  |  |
| GO:0051148\_negative\_regulation\_of\_muscle\_cell\_differentiation | 4 | 1 |  |  |  |  |  |  |  |  |
| GO:0051452\_intracellular\_pH\_reduction | 4 | 1 |  |  |  |  |  |  |  |  |
| GO:0051642\_centrosome\_localization | 4 | 1 |  |  |  |  |  |  |  |  |
| GO:0051904\_pigment\_granule\_transport | 4 | 1 |  |  |  |  |  |  |  |  |
| GO:0060592\_mammary\_gland\_formation | 4 | 1 |  |  |  |  |  |  |  |  |
| GO:0045941\_positive\_regulation\_of\_transcription | 338 | 9 | 1.803211 | -1.231262 | 119 | 26.384630 | 52.34 | 78.295370 | 0.439832 |  |
| GO:0006874\_cellular\_calcium\_ion\_homeostasis | 61 | 3 | 3.330521 | -1.218871 | 120 | 27.333331 | 53.9 | 80.466669 | 0.449167 |  |
| GO:0065007\_biological\_regulation | 2593 | 45 | 1.175251 | -1.208457 | 121 | 27.599988 | 54.35 | 81.100012 | 0.449174 |  |
| GO:0005975\_carbohydrate\_metabolic\_process | 146 | 5 | 2.319198 | -1.198671 | 122 | 28.077383 | 55.2 | 82.322617 | 0.452459 |  |
| GO:0006470\_protein\_amino\_acid\_dephosphorylation | 28 | 2 | 4.837185 | -1.197289 | 124 | 28.770374 | 56.15 | 83.529626 | 0.452823 |  |
| GO:0048863\_stem\_cell\_differentiation | 28 | 2 | 4.837185 | -1.197289 | 124 | 28.770374 | 56.15 | 83.529626 | 0.452823 |  |
| GO:0010628\_positive\_regulation\_of\_gene\_expression | 346 | 9 | 1.761518 | -1.179328 | 125 | 29.368222 | 57.0 | 84.631778 | 0.456000 |  |
| GO:0050767\_regulation\_of\_neurogenesis | 104 | 4 | 2.604638 | -1.176498 | 126 | 29.525461 | 57.4 | 85.274539 | 0.455556 |  |
| GO:0045595\_regulation\_of\_cell\_differentiation | 295 | 8 | 1.836491 | -1.173096 | 127 | 29.593816 | 57.56 | 85.526184 | 0.453228 |  |
| GO:0048066\_pigmentation\_during\_development | 29 | 2 | 4.670385 | -1.170308 | 130 | 30.486807 | 58.93 | 87.373193 | 0.453308 |  |
| GO:0050769\_positive\_regulation\_of\_neurogenesis | 29 | 2 | 4.670385 | -1.170308 | 130 | 30.486807 | 58.93 | 87.373193 | 0.453308 |  |
| GO:0060041\_retina\_development\_in\_camera-type\_eye | 29 | 2 | 4.670385 | -1.170308 | 130 | 30.486807 | 58.93 | 87.373193 | 0.453308 |  |
| GO:0006875\_cellular\_metal\_ion\_homeostasis | 64 | 3 | 3.174403 | -1.168668 | 132 | 30.703575 | 59.34 | 87.976425 | 0.449545 |  |
| GO:0055074\_calcium\_ion\_homeostasis | 64 | 3 | 3.174403 | -1.168668 | 132 | 30.703575 | 59.34 | 87.976425 | 0.449545 |  |
| GO:0048519\_negative\_regulation\_of\_biological\_process | 859 | 18 | 1.419058 | -1.156416 | 133 | 30.983656 | 59.75 | 88.516344 | 0.449248 |  |
| GO:0009893\_positive\_regulation\_of\_metabolic\_process | 458 | 11 | 1.626477 | -1.152979 | 134 | 30.985436 | 59.79 | 88.594564 | 0.446194 |  |
| GO:0001957\_intramembranous\_ossification | 5 | 1 | 13.544118 | -1.144393 | 164 | 47.188084 | 80.47 | 113.751916 | 0.490671 |  |
| GO:0001959\_regulation\_of\_cytokine-mediated\_signaling\_pathway | 5 | 1 | 13.544118 | -1.144393 | 164 | 47.188084 | 80.47 | 113.751916 | 0.490671 |  |
| GO:0002710\_negative\_regulation\_of\_T\_cell\_mediated\_immunity | 5 | 1 | 13.544118 | -1.144393 | 164 | 47.188084 | 80.47 | 113.751916 | 0.490671 |  |
| GO:0006704\_glucocorticoid\_biosynthetic\_process | 5 | 1 | 13.544118 | -1.144393 | 164 | 47.188084 | 80.47 | 113.751916 | 0.490671 |  |
| GO:0007638\_mechanosensory\_behavior | 5 | 1 | 13.544118 | -1.144393 | 164 | 47.188084 | 80.47 | 113.751916 | 0.490671 |  |
| GO:0010761\_fibroblast\_migration | 5 | 1 | 13.544118 | -1.144393 | 164 | 47.188084 | 80.47 | 113.751916 | 0.490671 |  |
| GO:0014015\_positive\_regulation\_of\_gliogenesis | 5 | 1 | 13.544118 | -1.144393 | 164 | 47.188084 | 80.47 | 113.751916 | 0.490671 |  |
| GO:0014068\_positive\_regulation\_of\_phosphoinositide\_3-kinase\_cascade | 5 | 1 | 13.544118 | -1.144393 | 164 | 47.188084 | 80.47 | 113.751916 | 0.490671 |  |
| GO:0021772\_olfactory\_bulb\_development | 5 | 1 | 13.544118 | -1.144393 | 164 | 47.188084 | 80.47 | 113.751916 | 0.490671 |  |
| GO:0021988\_olfactory\_lobe\_development | 5 | 1 | 13.544118 | -1.144393 | 164 | 47.188084 | 80.47 | 113.751916 | 0.490671 |  |
| GO:0030004\_cellular\_monovalent\_inorganic\_cation\_homeostasis | 5 | 1 | 13.544118 | -1.144393 | 164 | 47.188084 | 80.47 | 113.751916 | 0.490671 |  |
| GO:0030641\_regulation\_of\_cellular\_pH | 5 | 1 | 13.544118 | -1.144393 | 164 | 47.188084 | 80.47 | 113.751916 | 0.490671 |  |
| GO:0032350\_regulation\_of\_hormone\_metabolic\_process | 5 | 1 | 13.544118 | -1.144393 | 164 | 47.188084 | 80.47 | 113.751916 | 0.490671 |  |
| GO:0042506\_tyrosine\_phosphorylation\_of\_Stat5\_protein | 5 | 1 | 13.544118 | -1.144393 | 164 | 47.188084 | 80.47 | 113.751916 | 0.490671 |  |
| GO:0042516\_regulation\_of\_tyrosine\_phosphorylation\_of\_Stat3\_protein | 5 | 1 | 13.544118 | -1.144393 | 164 | 47.188084 | 80.47 | 113.751916 | 0.490671 |  |
| GO:0042531\_positive\_regulation\_of\_tyrosine\_phosphorylation\_of\_STAT\_protein | 5 | 1 | 13.544118 | -1.144393 | 164 | 47.188084 | 80.47 | 113.751916 | 0.490671 |  |
| GO:0043266\_regulation\_of\_potassium\_ion\_transport | 5 | 1 | 13.544118 | -1.144393 | 164 | 47.188084 | 80.47 | 113.751916 | 0.490671 |  |
| GO:0045213\_neurotransmitter\_receptor\_metabolic\_process | 5 | 1 | 13.544118 | -1.144393 | 164 | 47.188084 | 80.47 | 113.751916 | 0.490671 |  |
| GO:0045586\_regulation\_of\_gamma-delta\_T\_cell\_differentiation | 5 | 1 | 13.544118 | -1.144393 | 164 | 47.188084 | 80.47 | 113.751916 | 0.490671 |  |
| GO:0045588\_positive\_regulation\_of\_gamma-delta\_T\_cell\_differentiation | 5 | 1 | 13.544118 | -1.144393 | 164 | 47.188084 | 80.47 | 113.751916 | 0.490671 |  |
| GO:0045687\_positive\_regulation\_of\_glial\_cell\_differentiation | 5 | 1 | 13.544118 | -1.144393 | 164 | 47.188084 | 80.47 | 113.751916 | 0.490671 |  |
| GO:0045744\_negative\_regulation\_of\_G-protein\_coupled\_receptor\_protein\_signaling\_pathway | 5 | 1 | 13.544118 | -1.144393 | 164 | 47.188084 | 80.47 | 113.751916 | 0.490671 |  |
| GO:0045851\_pH\_reduction | 5 | 1 | 13.544118 | -1.144393 | 164 | 47.188084 | 80.47 | 113.751916 | 0.490671 |  |
| GO:0046339\_diacylglycerol\_metabolic\_process | 5 | 1 | 13.544118 | -1.144393 | 164 | 47.188084 | 80.47 | 113.751916 | 0.490671 |  |
| GO:0046643\_regulation\_of\_gamma-delta\_T\_cell\_activation | 5 | 1 | 13.544118 | -1.144393 | 164 | 47.188084 | 80.47 | 113.751916 | 0.490671 |  |
| GO:0046645\_positive\_regulation\_of\_gamma-delta\_T\_cell\_activation | 5 | 1 | 13.544118 | -1.144393 | 164 | 47.188084 | 80.47 | 113.751916 | 0.490671 |  |
| GO:0050858\_negative\_regulation\_of\_antigen\_receptor-mediated\_signaling\_pathway | 5 | 1 | 13.544118 | -1.144393 | 164 | 47.188084 | 80.47 | 113.751916 | 0.490671 |  |
| GO:0050860\_negative\_regulation\_of\_T\_cell\_receptor\_signaling\_pathway | 5 | 1 | 13.544118 | -1.144393 | 164 | 47.188084 | 80.47 | 113.751916 | 0.490671 |  |
| GO:0051453\_regulation\_of\_intracellular\_pH | 5 | 1 | 13.544118 | -1.144393 | 164 | 47.188084 | 80.47 | 113.751916 | 0.490671 |  |
| GO:0051905\_establishment\_of\_pigment\_granule\_localization | 5 | 1 | 13.544118 | -1.144393 | 164 | 47.188084 | 80.47 | 113.751916 | 0.490671 |  |
| GO:0000904\_cell\_morphogenesis\_involved\_in\_differentiation | 199 | 6 | 2.041827 | -1.142912 | 165 | 48.151124 | 82.35 | 116.548876 | 0.499091 |  |
| GO:0003007\_heart\_morphogenesis | 67 | 3 | 3.032265 | -1.121395 | 166 | 48.693505 | 83.63 | 118.566495 | 0.503795 |  |
| GO:0006486\_protein\_amino\_acid\_glycosylation | 31 | 2 | 4.369070 | -1.119473 | 170 | 49.984120 | 85.92 | 121.855880 | 0.505412 |  |
| GO:0016311\_dephosphorylation | 31 | 2 | 4.369070 | -1.119473 | 170 | 49.984120 | 85.92 | 121.855880 | 0.505412 |  |
| GO:0043413\_biopolymer\_glycosylation | 31 | 2 | 4.369070 | -1.119473 | 170 | 49.984120 | 85.92 | 121.855880 | 0.505412 |  |
| GO:0070085\_glycosylation | 31 | 2 | 4.369070 | -1.119473 | 170 | 49.984120 | 85.92 | 121.855880 | 0.505412 |  |
| GO:0048869\_cellular\_developmental\_process | 1113 | 22 | 1.338592 | -1.112328 | 171 | 50.152484 | 86.12 | 122.087516 | 0.503626 |  |
| GO:0048878\_chemical\_homeostasis | 254 | 7 | 1.866315 | -1.102309 | 172 | 50.861888 | 87.14 | 123.418112 | 0.506628 |  |
| GO:0045893\_positive\_regulation\_of\_transcription\_\_DNA-dependent | 306 | 8 | 1.770473 | -1.099752 | 174 | 50.990449 | 87.25 | 123.509551 | 0.501437 |  |
| GO:0051254\_positive\_regulation\_of\_RNA\_metabolic\_process | 306 | 8 | 1.770473 | -1.099752 | 174 | 50.990449 | 87.25 | 123.509551 | 0.501437 |  |
| GO:0055065\_metal\_ion\_homeostasis | 69 | 3 | 2.944373 | -1.091377 | 175 | 52.277170 | 89.26 | 126.242830 | 0.510057 |  |
| GO:0051179\_localization | 1058 | 21 | 1.344170 | -1.089230 | 176 | 52.307493 | 89.37 | 126.432507 | 0.507784 |  |
| GO:0009966\_regulation\_of\_signal\_transduction | 256 | 7 | 1.851735 | -1.088136 | 177 | 52.360624 | 89.48 | 126.599376 | 0.505537 |  |
| GO:0051094\_positive\_regulation\_of\_developmental\_process | 308 | 8 | 1.758976 | -1.086912 | 178 | 52.395613 | 89.54 | 126.684387 | 0.503034 |  |
| GO:0006350\_transcription | 701 | 15 | 1.449085 | -1.077862 | 179 | 52.735393 | 90.13 | 127.524607 | 0.503520 |  |
| GO:0021987\_cerebral\_cortex\_development | 33 | 2 | 4.104278 | -1.072366 | 180 | 53.444194 | 91.64 | 129.835806 | 0.509111 |  |
| GO:0002920\_regulation\_of\_humoral\_immune\_response | 6 | 1 | 11.286765 | -1.068354 | 206 | 65.854042 | 107.47 | 149.085958 | 0.521699 |  |
| GO:0006942\_regulation\_of\_striated\_muscle\_contraction | 6 | 1 | 11.286765 | -1.068354 | 206 | 65.854042 | 107.47 | 149.085958 | 0.521699 |  |
| GO:0007032\_endosome\_organization | 6 | 1 | 11.286765 | -1.068354 | 206 | 65.854042 | 107.47 | 149.085958 | 0.521699 |  |
| GO:0007341\_penetration\_of\_zona\_pellucida | 6 | 1 | 11.286765 | -1.068354 | 206 | 65.854042 | 107.47 | 149.085958 | 0.521699 |  |
| GO:0007442\_hindgut\_morphogenesis | 6 | 1 | 11.286765 | -1.068354 | 206 | 65.854042 | 107.47 | 149.085958 | 0.521699 |  |
| GO:0010906\_regulation\_of\_glucose\_metabolic\_process | 6 | 1 | 11.286765 | -1.068354 | 206 | 65.854042 | 107.47 | 149.085958 | 0.521699 |  |
| GO:0014003\_oligodendrocyte\_development | 6 | 1 | 11.286765 | -1.068354 | 206 | 65.854042 | 107.47 | 149.085958 | 0.521699 |  |
| GO:0030002\_cellular\_anion\_homeostasis | 6 | 1 | 11.286765 | -1.068354 | 206 | 65.854042 | 107.47 | 149.085958 | 0.521699 |  |
| GO:0030149\_sphingolipid\_catabolic\_process | 6 | 1 | 11.286765 | -1.068354 | 206 | 65.854042 | 107.47 | 149.085958 | 0.521699 |  |
| GO:0030947\_regulation\_of\_vascular\_endothelial\_growth\_factor\_receptor\_signaling\_pathway | 6 | 1 | 11.286765 | -1.068354 | 206 | 65.854042 | 107.47 | 149.085958 | 0.521699 |  |
| GO:0035019\_somatic\_stem\_cell\_maintenance | 6 | 1 | 11.286765 | -1.068354 | 206 | 65.854042 | 107.47 | 149.085958 | 0.521699 |  |
| GO:0042492\_gamma-delta\_T\_cell\_differentiation | 6 | 1 | 11.286765 | -1.068354 | 206 | 65.854042 | 107.47 | 149.085958 | 0.521699 |  |
| GO:0043255\_regulation\_of\_carbohydrate\_biosynthetic\_process | 6 | 1 | 11.286765 | -1.068354 | 206 | 65.854042 | 107.47 | 149.085958 | 0.521699 |  |
| GO:0043271\_negative\_regulation\_of\_ion\_transport | 6 | 1 | 11.286765 | -1.068354 | 206 | 65.854042 | 107.47 | 149.085958 | 0.521699 |  |
| GO:0045822\_negative\_regulation\_of\_heart\_contraction | 6 | 1 | 11.286765 | -1.068354 | 206 | 65.854042 | 107.47 | 149.085958 | 0.521699 |  |
| GO:0045843\_negative\_regulation\_of\_striated\_muscle\_development | 6 | 1 | 11.286765 | -1.068354 | 206 | 65.854042 | 107.47 | 149.085958 | 0.521699 |  |
| GO:0046427\_positive\_regulation\_of\_JAK-STAT\_cascade | 6 | 1 | 11.286765 | -1.068354 | 206 | 65.854042 | 107.47 | 149.085958 | 0.521699 |  |
| GO:0046460\_neutral\_lipid\_biosynthetic\_process | 6 | 1 | 11.286765 | -1.068354 | 206 | 65.854042 | 107.47 | 149.085958 | 0.521699 |  |
| GO:0046463\_acylglycerol\_biosynthetic\_process | 6 | 1 | 11.286765 | -1.068354 | 206 | 65.854042 | 107.47 | 149.085958 | 0.521699 |  |
| GO:0046466\_membrane\_lipid\_catabolic\_process | 6 | 1 | 11.286765 | -1.068354 | 206 | 65.854042 | 107.47 | 149.085958 | 0.521699 |  |
| GO:0046629\_gamma-delta\_T\_cell\_activation | 6 | 1 | 11.286765 | -1.068354 | 206 | 65.854042 | 107.47 | 149.085958 | 0.521699 |  |
| GO:0048635\_negative\_regulation\_of\_muscle\_development | 6 | 1 | 11.286765 | -1.068354 | 206 | 65.854042 | 107.47 | 149.085958 | 0.521699 |  |
| GO:0048644\_muscle\_organ\_morphogenesis | 6 | 1 | 11.286765 | -1.068354 | 206 | 65.854042 | 107.47 | 149.085958 | 0.521699 |  |
| GO:0050732\_negative\_regulation\_of\_peptidyl-tyrosine\_phosphorylation | 6 | 1 | 11.286765 | -1.068354 | 206 | 65.854042 | 107.47 | 149.085958 | 0.521699 |  |
| GO:0055081\_anion\_homeostasis | 6 | 1 | 11.286765 | -1.068354 | 206 | 65.854042 | 107.47 | 149.085958 | 0.521699 |  |
| GO:0060710\_chorio-allantoic\_fusion | 6 | 1 | 11.286765 | -1.068354 | 206 | 65.854042 | 107.47 | 149.085958 | 0.521699 |  |
| GO:0009100\_glycoprotein\_metabolic\_process | 71 | 3 | 2.861433 | -1.062474 | 207 | 66.266164 | 108.07 | 149.873836 | 0.522077 |  |
| GO:0048584\_positive\_regulation\_of\_response\_to\_stimulus | 115 | 4 | 2.355499 | -1.050747 | 208 | 66.496352 | 108.45 | 150.403648 | 0.521394 |  |
| GO:0010720\_positive\_regulation\_of\_cell\_development | 34 | 2 | 3.983564 | -1.050067 | 211 | 67.459633 | 109.96 | 152.460367 | 0.521137 |  |
| GO:0050730\_regulation\_of\_peptidyl-tyrosine\_phosphorylation | 34 | 2 | 3.983564 | -1.050067 | 211 | 67.459633 | 109.96 | 152.460367 | 0.521137 |  |
| GO:0060443\_mammary\_gland\_morphogenesis | 34 | 2 | 3.983564 | -1.050067 | 211 | 67.459633 | 109.96 | 152.460367 | 0.521137 |  |
| GO:0044262\_cellular\_carbohydrate\_metabolic\_process | 72 | 3 | 2.821691 | -1.048420 | 212 | 67.813699 | 110.55 | 153.286301 | 0.521462 |  |
| GO:0006936\_muscle\_contraction | 73 | 3 | 2.783038 | -1.034622 | 213 | 68.643739 | 111.82 | 154.996261 | 0.524977 |  |
| GO:0044255\_cellular\_lipid\_metabolic\_process | 264 | 7 | 1.795622 | -1.033275 | 214 | 68.891170 | 112.03 | 155.168830 | 0.523505 |  |
| GO:0010557\_positive\_regulation\_of\_macromolecule\_biosynthetic\_process | 371 | 9 | 1.642818 | -1.030222 | 215 | 68.928416 | 112.13 | 155.331584 | 0.521535 |  |
| GO:0001906\_cell\_killing | 35 | 2 | 3.869748 | -1.028537 | 217 | 69.986929 | 113.56 | 157.133071 | 0.523318 |  |
| GO:0001909\_leukocyte\_mediated\_cytotoxicity | 35 | 2 | 3.869748 | -1.028537 | 217 | 69.986929 | 113.56 | 157.133071 | 0.523318 |  |
| GO:0007166\_cell\_surface\_receptor\_linked\_signal\_transduction | 597 | 13 | 1.474653 | -1.024871 | 218 | 70.017160 | 113.62 | 157.222840 | 0.521193 |  |
| GO:0051960\_regulation\_of\_nervous\_system\_development | 118 | 4 | 2.295613 | -1.019381 | 219 | 70.381984 | 114.31 | 158.238016 | 0.521963 |  |
| GO:0034960\_cellular\_biopolymer\_metabolic\_process | 1395 | 26 | 1.262176 | -1.008895 | 220 | 70.451411 | 114.62 | 158.788589 | 0.521000 |  |
| GO:0006469\_negative\_regulation\_of\_protein\_kinase\_activity | 36 | 2 | 3.762255 | -1.007734 | 225 | 71.650824 | 116.39 | 161.129176 | 0.517289 |  |
| GO:0021510\_spinal\_cord\_development | 36 | 2 | 3.762255 | -1.007734 | 225 | 71.650824 | 116.39 | 161.129176 | 0.517289 |  |
| GO:0022602\_ovulation\_cycle\_process | 36 | 2 | 3.762255 | -1.007734 | 225 | 71.650824 | 116.39 | 161.129176 | 0.517289 |  |
| GO:0033673\_negative\_regulation\_of\_kinase\_activity | 36 | 2 | 3.762255 | -1.007734 | 225 | 71.650824 | 116.39 | 161.129176 | 0.517289 |  |
| GO:0050851\_antigen\_receptor-mediated\_signaling\_pathway | 36 | 2 | 3.762255 | -1.007734 | 225 | 71.650824 | 116.39 | 161.129176 | 0.517289 |  |
| GO:0001504\_neurotransmitter\_uptake | 7 | 1 | 9.674370 | -1.004542 | 250 | 87.081995 | 134.22 | 181.358005 | 0.536880 |  |
| GO:0001573\_ganglioside\_metabolic\_process | 7 | 1 | 9.674370 | -1.004542 | 250 | 87.081995 | 134.22 | 181.358005 | 0.536880 |  |
| GO:0002063\_chondrocyte\_development | 7 | 1 | 9.674370 | -1.004542 | 250 | 87.081995 | 134.22 | 181.358005 | 0.536880 |  |
| GO:0002087\_regulation\_of\_respiratory\_gaseous\_exchange\_by\_neurological\_system\_process | 7 | 1 | 9.674370 | -1.004542 | 250 | 87.081995 | 134.22 | 181.358005 | 0.536880 |  |
| GO:0002455\_humoral\_immune\_response\_mediated\_by\_circulating\_immunoglobulin | 7 | 1 | 9.674370 | -1.004542 | 250 | 87.081995 | 134.22 | 181.358005 | 0.536880 |  |
| GO:0002714\_positive\_regulation\_of\_B\_cell\_mediated\_immunity | 7 | 1 | 9.674370 | -1.004542 | 250 | 87.081995 | 134.22 | 181.358005 | 0.536880 |  |
| GO:0002891\_positive\_regulation\_of\_immunoglobulin\_mediated\_immune\_response | 7 | 1 | 9.674370 | -1.004542 | 250 | 87.081995 | 134.22 | 181.358005 | 0.536880 |  |
| GO:0009311\_oligosaccharide\_metabolic\_process | 7 | 1 | 9.674370 | -1.004542 | 250 | 87.081995 | 134.22 | 181.358005 | 0.536880 |  |
| GO:0014047\_glutamate\_secretion | 7 | 1 | 9.674370 | -1.004542 | 250 | 87.081995 | 134.22 | 181.358005 | 0.536880 |  |
| GO:0014066\_regulation\_of\_phosphoinositide\_3-kinase\_cascade | 7 | 1 | 9.674370 | -1.004542 | 250 | 87.081995 | 134.22 | 181.358005 | 0.536880 |  |
| GO:0015914\_phospholipid\_transport | 7 | 1 | 9.674370 | -1.004542 | 250 | 87.081995 | 134.22 | 181.358005 | 0.536880 |  |
| GO:0019800\_peptide\_cross-linking\_via\_chondroitin\_4-sulfate\_glycosaminoglycan | 7 | 1 | 9.674370 | -1.004542 | 250 | 87.081995 | 134.22 | 181.358005 | 0.536880 |  |
| GO:0021514\_ventral\_spinal\_cord\_interneuron\_differentiation | 7 | 1 | 9.674370 | -1.004542 | 250 | 87.081995 | 134.22 | 181.358005 | 0.536880 |  |
| GO:0021516\_dorsal\_spinal\_cord\_development | 7 | 1 | 9.674370 | -1.004542 | 250 | 87.081995 | 134.22 | 181.358005 | 0.536880 |  |
| GO:0021520\_spinal\_cord\_motor\_neuron\_cell\_fate\_specification | 7 | 1 | 9.674370 | -1.004542 | 250 | 87.081995 | 134.22 | 181.358005 | 0.536880 |  |
| GO:0021521\_ventral\_spinal\_cord\_interneuron\_specification | 7 | 1 | 9.674370 | -1.004542 | 250 | 87.081995 | 134.22 | 181.358005 | 0.536880 |  |
| GO:0033057\_reproductive\_behavior\_in\_a\_multicellular\_organism | 7 | 1 | 9.674370 | -1.004542 | 250 | 87.081995 | 134.22 | 181.358005 | 0.536880 |  |
| GO:0042503\_tyrosine\_phosphorylation\_of\_Stat3\_protein | 7 | 1 | 9.674370 | -1.004542 | 250 | 87.081995 | 134.22 | 181.358005 | 0.536880 |  |
| GO:0043584\_nose\_development | 7 | 1 | 9.674370 | -1.004542 | 250 | 87.081995 | 134.22 | 181.358005 | 0.536880 |  |
| GO:0044065\_regulation\_of\_respiratory\_system\_process | 7 | 1 | 9.674370 | -1.004542 | 250 | 87.081995 | 134.22 | 181.358005 | 0.536880 |  |
| GO:0045059\_positive\_thymic\_T\_cell\_selection | 7 | 1 | 9.674370 | -1.004542 | 250 | 87.081995 | 134.22 | 181.358005 | 0.536880 |  |
| GO:0046504\_glycerol\_ether\_biosynthetic\_process | 7 | 1 | 9.674370 | -1.004542 | 250 | 87.081995 | 134.22 | 181.358005 | 0.536880 |  |
| GO:0048304\_positive\_regulation\_of\_isotype\_switching\_to\_IgG\_isotypes | 7 | 1 | 9.674370 | -1.004542 | 250 | 87.081995 | 134.22 | 181.358005 | 0.536880 |  |
| GO:0060579\_ventral\_spinal\_cord\_interneuron\_fate\_commitment | 7 | 1 | 9.674370 | -1.004542 | 250 | 87.081995 | 134.22 | 181.358005 | 0.536880 |  |
| GO:0060788\_ectodermal\_placode\_formation | 7 | 1 | 9.674370 | -1.004542 | 250 | 87.081995 | 134.22 | 181.358005 | 0.536880 |  |
| GO:0014706\_striated\_muscle\_tissue\_development | 120 | 4 | 2.257353 | -0.999103 | 251 | 87.220706 | 134.67 | 182.119294 | 0.536534 |  |
| GO:0006807\_nitrogen\_compound\_metabolic\_process | 1147 | 22 | 1.298913 | -0.996250 | 252 | 87.261335 | 134.72 | 182.178665 | 0.534603 |  |
| GO:0034645\_cellular\_macromolecule\_biosynthetic\_process | 901 | 18 | 1.352909 | -0.995757 | 253 | 87.328839 | 134.77 | 182.211161 | 0.532688 |  |
| GO:0003012\_muscle\_system\_process | 76 | 3 | 2.673181 | -0.994689 | 254 | 87.612365 | 135.12 | 182.627635 | 0.531969 |  |
| GO:0006917\_induction\_of\_apoptosis | 121 | 4 | 2.238697 | -0.989146 | 256 | 87.799941 | 135.55 | 183.300059 | 0.529492 |  |
| GO:0012502\_induction\_of\_programmed\_cell\_death | 121 | 4 | 2.238697 | -0.989146 | 256 | 87.799941 | 135.55 | 183.300059 | 0.529492 |  |
| GO:0042698\_ovulation\_cycle | 37 | 2 | 3.660572 | -0.987615 | 257 | 88.550487 | 136.65 | 184.749513 | 0.531712 |  |
| GO:0044249\_cellular\_biosynthetic\_process | 1150 | 22 | 1.295524 | -0.986462 | 258 | 88.604519 | 136.75 | 184.895481 | 0.530039 |  |
| GO:0007519\_skeletal\_muscle\_tissue\_development | 78 | 3 | 2.604638 | -0.969221 | 260 | 89.592953 | 138.46 | 187.327047 | 0.532538 |  |
| GO:0060538\_skeletal\_muscle\_organ\_development | 78 | 3 | 2.604638 | -0.969221 | 260 | 89.592953 | 138.46 | 187.327047 | 0.532538 |  |
| GO:0008016\_regulation\_of\_heart\_contraction | 38 | 2 | 3.564241 | -0.968145 | 262 | 90.879752 | 140.51 | 190.140248 | 0.536298 |  |
| GO:0051348\_negative\_regulation\_of\_transferase\_activity | 38 | 2 | 3.564241 | -0.968145 | 262 | 90.879752 | 140.51 | 190.140248 | 0.536298 |  |
| GO:0009059\_macromolecule\_biosynthetic\_process | 910 | 18 | 1.339528 | -0.963740 | 263 | 91.000170 | 140.67 | 190.339830 | 0.534867 |  |
| GO:0009308\_amine\_metabolic\_process | 124 | 4 | 2.184535 | -0.959986 | 264 | 91.116613 | 140.92 | 190.723387 | 0.533788 |  |
| GO:0010646\_regulation\_of\_cell\_communication | 330 | 8 | 1.641711 | -0.954977 | 265 | 91.622186 | 141.76 | 191.897814 | 0.534943 |  |
| GO:0001911\_negative\_regulation\_of\_leukocyte\_mediated\_cytotoxicity | 8 | 1 | 8.465074 | -0.949678 | 280 | 103.198768 | 155.77 | 208.341232 | 0.556321 |  |
| GO:0001916\_positive\_regulation\_of\_T\_cell\_mediated\_cytotoxicity | 8 | 1 | 8.465074 | -0.949678 | 280 | 103.198768 | 155.77 | 208.341232 | 0.556321 |  |
| GO:0008593\_regulation\_of\_Notch\_signaling\_pathway | 8 | 1 | 8.465074 | -0.949678 | 280 | 103.198768 | 155.77 | 208.341232 | 0.556321 |  |
| GO:0014014\_negative\_regulation\_of\_gliogenesis | 8 | 1 | 8.465074 | -0.949678 | 280 | 103.198768 | 155.77 | 208.341232 | 0.556321 |  |
| GO:0014065\_phosphoinositide\_3-kinase\_cascade | 8 | 1 | 8.465074 | -0.949678 | 280 | 103.198768 | 155.77 | 208.341232 | 0.556321 |  |
| GO:0019229\_regulation\_of\_vasoconstriction | 8 | 1 | 8.465074 | -0.949678 | 280 | 103.198768 | 155.77 | 208.341232 | 0.556321 |  |
| GO:0021781\_glial\_cell\_fate\_commitment | 8 | 1 | 8.465074 | -0.949678 | 280 | 103.198768 | 155.77 | 208.341232 | 0.556321 |  |
| GO:0030035\_microspike\_assembly | 8 | 1 | 8.465074 | -0.949678 | 280 | 103.198768 | 155.77 | 208.341232 | 0.556321 |  |
| GO:0030204\_chondroitin\_sulfate\_metabolic\_process | 8 | 1 | 8.465074 | -0.949678 | 280 | 103.198768 | 155.77 | 208.341232 | 0.556321 |  |
| GO:0031342\_negative\_regulation\_of\_cell\_killing | 8 | 1 | 8.465074 | -0.949678 | 280 | 103.198768 | 155.77 | 208.341232 | 0.556321 |  |
| GO:0035112\_genitalia\_morphogenesis | 8 | 1 | 8.465074 | -0.949678 | 280 | 103.198768 | 155.77 | 208.341232 | 0.556321 |  |
| GO:0043368\_positive\_T\_cell\_selection | 8 | 1 | 8.465074 | -0.949678 | 280 | 103.198768 | 155.77 | 208.341232 | 0.556321 |  |
| GO:0045686\_negative\_regulation\_of\_glial\_cell\_differentiation | 8 | 1 | 8.465074 | -0.949678 | 280 | 103.198768 | 155.77 | 208.341232 | 0.556321 |  |
| GO:0045932\_negative\_regulation\_of\_muscle\_contraction | 8 | 1 | 8.465074 | -0.949678 | 280 | 103.198768 | 155.77 | 208.341232 | 0.556321 |  |
| GO:0048557\_embryonic\_digestive\_tract\_morphogenesis | 8 | 1 | 8.465074 | -0.949678 | 280 | 103.198768 | 155.77 | 208.341232 | 0.556321 |  |
| GO:0005976\_polysaccharide\_metabolic\_process | 39 | 2 | 3.472851 | -0.949288 | 282 | 104.335385 | 157.74 | 211.144615 | 0.559362 |  |
| GO:0048663\_neuron\_fate\_commitment | 39 | 2 | 3.472851 | -0.949288 | 282 | 104.335385 | 157.74 | 211.144615 | 0.559362 |  |
| GO:0044092\_negative\_regulation\_of\_molecular\_function | 80 | 3 | 2.539522 | -0.944618 | 283 | 104.602807 | 158.23 | 211.857193 | 0.559117 |  |
| GO:0031328\_positive\_regulation\_of\_cellular\_biosynthetic\_process | 387 | 9 | 1.574897 | -0.944330 | 284 | 104.640170 | 158.27 | 211.899830 | 0.557289 |  |
| GO:0045449\_regulation\_of\_transcription | 676 | 14 | 1.402497 | -0.939595 | 285 | 104.779008 | 158.61 | 212.440992 | 0.556526 |  |
| GO:0015031\_protein\_transport | 175 | 5 | 1.934874 | -0.939507 | 286 | 104.916662 | 158.8 | 212.683338 | 0.555245 |  |
| GO:0009891\_positive\_regulation\_of\_biosynthetic\_process | 388 | 9 | 1.570838 | -0.939190 | 287 | 104.953527 | 158.84 | 212.726473 | 0.553449 |  |
| GO:0007610\_behavior | 279 | 7 | 1.699083 | -0.937818 | 288 | 105.067055 | 158.98 | 212.892945 | 0.552014 |  |
| GO:0045597\_positive\_regulation\_of\_cell\_differentiation | 128 | 4 | 2.116268 | -0.922685 | 290 | 106.600730 | 161.3 | 215.999270 | 0.556207 |  |
| GO:0060537\_muscle\_tissue\_development | 128 | 4 | 2.116268 | -0.922685 | 290 | 106.600730 | 161.3 | 215.999270 | 0.556207 |  |
| GO:0008202\_steroid\_metabolic\_process | 82 | 3 | 2.477582 | -0.920838 | 292 | 106.910351 | 161.78 | 216.649649 | 0.554041 |  |
| GO:0010627\_regulation\_of\_protein\_kinase\_cascade | 82 | 3 | 2.477582 | -0.920838 | 292 | 106.910351 | 161.78 | 216.649649 | 0.554041 |  |
| GO:0002429\_immune\_response-activating\_cell\_surface\_receptor\_signaling\_pathway | 41 | 2 | 3.303443 | -0.913289 | 294 | 108.597377 | 164.42 | 220.242623 | 0.559252 |  |
| GO:0008585\_female\_gonad\_development | 41 | 2 | 3.303443 | -0.913289 | 294 | 108.597377 | 164.42 | 220.242623 | 0.559252 |  |
| GO:0009058\_biosynthetic\_process | 1175 | 22 | 1.267960 | -0.907657 | 295 | 109.117619 | 165.55 | 221.982381 | 0.561186 |  |
| GO:0007420\_brain\_development | 231 | 6 | 1.758976 | -0.906382 | 296 | 109.155158 | 165.62 | 222.084842 | 0.559527 |  |
| GO:0006629\_lipid\_metabolic\_process | 285 | 7 | 1.663313 | -0.902143 | 297 | 109.592665 | 166.27 | 222.947335 | 0.559832 |  |
| GO:0000186\_activation\_of\_MAPKK\_activity | 9 | 1 | 7.524510 | -0.901646 | 316 | 121.544957 | 180.18 | 238.815043 | 0.570190 |  |
| GO:0001542\_ovulation\_from\_ovarian\_follicle | 9 | 1 | 7.524510 | -0.901646 | 316 | 121.544957 | 180.18 | 238.815043 | 0.570190 |  |
| GO:0001667\_ameboidal\_cell\_migration | 9 | 1 | 7.524510 | -0.901646 | 316 | 121.544957 | 180.18 | 238.815043 | 0.570190 |  |
| GO:0006911\_phagocytosis\_\_engulfment | 9 | 1 | 7.524510 | -0.901646 | 316 | 121.544957 | 180.18 | 238.815043 | 0.570190 |  |
| GO:0007617\_mating\_behavior | 9 | 1 | 7.524510 | -0.901646 | 316 | 121.544957 | 180.18 | 238.815043 | 0.570190 |  |
| GO:0010675\_regulation\_of\_cellular\_carbohydrate\_metabolic\_process | 9 | 1 | 7.524510 | -0.901646 | 316 | 121.544957 | 180.18 | 238.815043 | 0.570190 |  |
| GO:0021544\_subpallium\_development | 9 | 1 | 7.524510 | -0.901646 | 316 | 121.544957 | 180.18 | 238.815043 | 0.570190 |  |
| GO:0030325\_adrenal\_gland\_development | 9 | 1 | 7.524510 | -0.901646 | 316 | 121.544957 | 180.18 | 238.815043 | 0.570190 |  |
| GO:0030728\_ovulation | 9 | 1 | 7.524510 | -0.901646 | 316 | 121.544957 | 180.18 | 238.815043 | 0.570190 |  |
| GO:0033344\_cholesterol\_efflux | 9 | 1 | 7.524510 | -0.901646 | 316 | 121.544957 | 180.18 | 238.815043 | 0.570190 |  |
| GO:0042509\_regulation\_of\_tyrosine\_phosphorylation\_of\_STAT\_protein | 9 | 1 | 7.524510 | -0.901646 | 316 | 121.544957 | 180.18 | 238.815043 | 0.570190 |  |
| GO:0043299\_leukocyte\_degranulation | 9 | 1 | 7.524510 | -0.901646 | 316 | 121.544957 | 180.18 | 238.815043 | 0.570190 |  |
| GO:0043383\_negative\_T\_cell\_selection | 9 | 1 | 7.524510 | -0.901646 | 316 | 121.544957 | 180.18 | 238.815043 | 0.570190 |  |
| GO:0045060\_negative\_thymic\_T\_cell\_selection | 9 | 1 | 7.524510 | -0.901646 | 316 | 121.544957 | 180.18 | 238.815043 | 0.570190 |  |
| GO:0045830\_positive\_regulation\_of\_isotype\_switching | 9 | 1 | 7.524510 | -0.901646 | 316 | 121.544957 | 180.18 | 238.815043 | 0.570190 |  |
| GO:0046641\_positive\_regulation\_of\_alpha-beta\_T\_cell\_proliferation | 9 | 1 | 7.524510 | -0.901646 | 316 | 121.544957 | 180.18 | 238.815043 | 0.570190 |  |
| GO:0050856\_regulation\_of\_T\_cell\_receptor\_signaling\_pathway | 9 | 1 | 7.524510 | -0.901646 | 316 | 121.544957 | 180.18 | 238.815043 | 0.570190 |  |
| GO:0051607\_defense\_response\_to\_virus | 9 | 1 | 7.524510 | -0.901646 | 316 | 121.544957 | 180.18 | 238.815043 | 0.570190 |  |
| GO:0060325\_face\_morphogenesis | 9 | 1 | 7.524510 | -0.901646 | 316 | 121.544957 | 180.18 | 238.815043 | 0.570190 |  |
| GO:0045184\_establishment\_of\_protein\_localization | 180 | 5 | 1.881127 | -0.901501 | 317 | 121.604845 | 180.3 | 238.995155 | 0.568770 |  |
| GO:0030005\_cellular\_di-\_\_tri-valent\_inorganic\_cation\_homeostasis | 84 | 3 | 2.418592 | -0.897840 | 318 | 121.854022 | 180.71 | 239.565978 | 0.568270 |  |
| GO:0019221\_cytokine-mediated\_signaling\_pathway | 42 | 2 | 3.224790 | -0.896090 | 319 | 123.165423 | 182.8 | 242.434577 | 0.573041 |  |
| GO:0016477\_cell\_migration | 234 | 6 | 1.736425 | -0.887006 | 320 | 123.476831 | 183.54 | 243.603169 | 0.573562 |  |
| GO:0007224\_smoothened\_signaling\_pathway | 43 | 2 | 3.149795 | -0.879392 | 323 | 125.325509 | 186.41 | 247.494491 | 0.577121 |  |
| GO:0010001\_glial\_cell\_differentiation | 43 | 2 | 3.149795 | -0.879392 | 323 | 125.325509 | 186.41 | 247.494491 | 0.577121 |  |
| GO:0051789\_response\_to\_protein\_stimulus | 43 | 2 | 3.149795 | -0.879392 | 323 | 125.325509 | 186.41 | 247.494491 | 0.577121 |  |
| GO:0032504\_multicellular\_organism\_reproduction | 86 | 3 | 2.362346 | -0.875585 | 325 | 125.955403 | 187.32 | 248.684597 | 0.576369 |  |
| GO:0048609\_reproductive\_process\_in\_a\_multicellular\_organism | 86 | 3 | 2.362346 | -0.875585 | 325 | 125.955403 | 187.32 | 248.684597 | 0.576369 |  |
| GO:0006355\_regulation\_of\_transcription\_\_DNA-dependent | 575 | 12 | 1.413299 | -0.871769 | 326 | 126.416913 | 187.86 | 249.303087 | 0.576258 |  |
| GO:0001822\_kidney\_development | 87 | 3 | 2.335193 | -0.864725 | 327 | 127.241502 | 189.05 | 250.858498 | 0.578135 |  |
| GO:0002768\_immune\_response-regulating\_cell\_surface\_receptor\_signaling\_pathway | 44 | 2 | 3.078209 | -0.863170 | 329 | 128.431104 | 191.45 | 254.468896 | 0.581915 |  |
| GO:0046545\_development\_of\_primary\_female\_sexual\_characteristics | 44 | 2 | 3.078209 | -0.863170 | 329 | 128.431104 | 191.45 | 254.468896 | 0.581915 |  |
| GO:0019219\_regulation\_of\_nucleobase\_\_nucleoside\_\_nucleotide\_and\_nucleic\_acid\_metabolic\_process | 757 | 15 | 1.341887 | -0.861055 | 330 | 128.594386 | 191.61 | 254.625614 | 0.580636 |  |
| GO:0001914\_regulation\_of\_T\_cell\_mediated\_cytotoxicity | 10 | 1 | 6.772059 | -0.859001 | 350 | 142.166062 | 208.01 | 273.853938 | 0.594314 |  |
| GO:0002711\_positive\_regulation\_of\_T\_cell\_mediated\_immunity | 10 | 1 | 6.772059 | -0.859001 | 350 | 142.166062 | 208.01 | 273.853938 | 0.594314 |  |
| GO:0006109\_regulation\_of\_carbohydrate\_metabolic\_process | 10 | 1 | 6.772059 | -0.859001 | 350 | 142.166062 | 208.01 | 273.853938 | 0.594314 |  |
| GO:0006826\_iron\_ion\_transport | 10 | 1 | 6.772059 | -0.859001 | 350 | 142.166062 | 208.01 | 273.853938 | 0.594314 |  |
| GO:0008211\_glucocorticoid\_metabolic\_process | 10 | 1 | 6.772059 | -0.859001 | 350 | 142.166062 | 208.01 | 273.853938 | 0.594314 |  |
| GO:0018149\_peptide\_cross-linking | 10 | 1 | 6.772059 | -0.859001 | 350 | 142.166062 | 208.01 | 273.853938 | 0.594314 |  |
| GO:0031018\_endocrine\_pancreas\_development | 10 | 1 | 6.772059 | -0.859001 | 350 | 142.166062 | 208.01 | 273.853938 | 0.594314 |  |
| GO:0040015\_negative\_regulation\_of\_multicellular\_organism\_growth | 10 | 1 | 6.772059 | -0.859001 | 350 | 142.166062 | 208.01 | 273.853938 | 0.594314 |  |
| GO:0045576\_mast\_cell\_activation | 10 | 1 | 6.772059 | -0.859001 | 350 | 142.166062 | 208.01 | 273.853938 | 0.594314 |  |
| GO:0045911\_positive\_regulation\_of\_DNA\_recombination | 10 | 1 | 6.772059 | -0.859001 | 350 | 142.166062 | 208.01 | 273.853938 | 0.594314 |  |
| GO:0048291\_isotype\_switching\_to\_IgG\_isotypes | 10 | 1 | 6.772059 | -0.859001 | 350 | 142.166062 | 208.01 | 273.853938 | 0.594314 |  |
| GO:0048302\_regulation\_of\_isotype\_switching\_to\_IgG\_isotypes | 10 | 1 | 6.772059 | -0.859001 | 350 | 142.166062 | 208.01 | 273.853938 | 0.594314 |  |
| GO:0050654\_chondroitin\_sulfate\_proteoglycan\_metabolic\_process | 10 | 1 | 6.772059 | -0.859001 | 350 | 142.166062 | 208.01 | 273.853938 | 0.594314 |  |
| GO:0051208\_sequestering\_of\_calcium\_ion | 10 | 1 | 6.772059 | -0.859001 | 350 | 142.166062 | 208.01 | 273.853938 | 0.594314 |  |
| GO:0051209\_release\_of\_sequestered\_calcium\_ion\_into\_cytosol | 10 | 1 | 6.772059 | -0.859001 | 350 | 142.166062 | 208.01 | 273.853938 | 0.594314 |  |
| GO:0051238\_sequestering\_of\_metal\_ion | 10 | 1 | 6.772059 | -0.859001 | 350 | 142.166062 | 208.01 | 273.853938 | 0.594314 |  |
| GO:0051282\_regulation\_of\_sequestering\_of\_calcium\_ion | 10 | 1 | 6.772059 | -0.859001 | 350 | 142.166062 | 208.01 | 273.853938 | 0.594314 |  |
| GO:0051283\_negative\_regulation\_of\_sequestering\_of\_calcium\_ion | 10 | 1 | 6.772059 | -0.859001 | 350 | 142.166062 | 208.01 | 273.853938 | 0.594314 |  |
| GO:0051650\_establishment\_of\_vesicle\_localization | 10 | 1 | 6.772059 | -0.859001 | 350 | 142.166062 | 208.01 | 273.853938 | 0.594314 |  |
| GO:0060323\_head\_morphogenesis | 10 | 1 | 6.772059 | -0.859001 | 350 | 142.166062 | 208.01 | 273.853938 | 0.594314 |  |
| GO:0044260\_cellular\_macromolecule\_metabolic\_process | 1447 | 26 | 1.216818 | -0.857484 | 351 | 142.603122 | 208.63 | 274.656878 | 0.594387 |  |
| GO:0040011\_locomotion | 295 | 7 | 1.606929 | -0.845631 | 352 | 143.325131 | 210.15 | 276.974869 | 0.597017 |  |
| GO:0007260\_tyrosine\_phosphorylation\_of\_STAT\_protein | 11 | 1 | 6.156417 | -0.820714 | 360 | 155.133829 | 225.32 | 295.506171 | 0.625889 |  |
| GO:0014013\_regulation\_of\_gliogenesis | 11 | 1 | 6.156417 | -0.820714 | 360 | 155.133829 | 225.32 | 295.506171 | 0.625889 |  |
| GO:0042551\_neuron\_maturation | 11 | 1 | 6.156417 | -0.820714 | 360 | 155.133829 | 225.32 | 295.506171 | 0.625889 |  |
| GO:0043576\_regulation\_of\_respiratory\_gaseous\_exchange | 11 | 1 | 6.156417 | -0.820714 | 360 | 155.133829 | 225.32 | 295.506171 | 0.625889 |  |
| GO:0045055\_regulated\_secretory\_pathway | 11 | 1 | 6.156417 | -0.820714 | 360 | 155.133829 | 225.32 | 295.506171 | 0.625889 |  |
| GO:0045685\_regulation\_of\_glial\_cell\_differentiation | 11 | 1 | 6.156417 | -0.820714 | 360 | 155.133829 | 225.32 | 295.506171 | 0.625889 |  |
| GO:0046425\_regulation\_of\_JAK-STAT\_cascade | 11 | 1 | 6.156417 | -0.820714 | 360 | 155.133829 | 225.32 | 295.506171 | 0.625889 |  |
| GO:0046716\_muscle\_maintenance | 11 | 1 | 6.156417 | -0.820714 | 360 | 155.133829 | 225.32 | 295.506171 | 0.625889 |  |
| GO:0002757\_immune\_response-activating\_signal\_transduction | 47 | 2 | 2.881727 | -0.817150 | 363 | 156.359588 | 227.12 | 297.880412 | 0.625675 |  |
| GO:0030183\_B\_cell\_differentiation | 47 | 2 | 2.881727 | -0.817150 | 363 | 156.359588 | 227.12 | 297.880412 | 0.625675 |  |
| GO:0034754\_cellular\_hormone\_metabolic\_process | 47 | 2 | 2.881727 | -0.817150 | 363 | 156.359588 | 227.12 | 297.880412 | 0.625675 |  |
| GO:0051252\_regulation\_of\_RNA\_metabolic\_process | 590 | 12 | 1.377368 | -0.813126 | 364 | 156.453270 | 227.18 | 297.906730 | 0.624121 |  |
| GO:0051171\_regulation\_of\_nitrogen\_compound\_metabolic\_process | 771 | 15 | 1.317521 | -0.812829 | 365 | 156.762761 | 227.61 | 298.457239 | 0.623589 |  |
| GO:0009653\_anatomical\_structure\_morphogenesis | 958 | 18 | 1.272412 | -0.806292 | 366 | 156.918126 | 227.96 | 299.001874 | 0.622842 |  |
| GO:0046907\_intracellular\_transport | 194 | 5 | 1.745376 | -0.803666 | 367 | 156.986999 | 228.07 | 299.153001 | 0.621444 |  |
| GO:0055066\_di-\_\_tri-valent\_inorganic\_cation\_homeostasis | 93 | 3 | 2.184535 | -0.803057 | 368 | 157.206282 | 228.4 | 299.593718 | 0.620652 |  |
| GO:0009101\_glycoprotein\_biosynthetic\_process | 48 | 2 | 2.821691 | -0.802628 | 369 | 157.836758 | 229.22 | 300.603242 | 0.621192 |  |
| GO:0006351\_transcription\_\_DNA-dependent | 594 | 12 | 1.368093 | -0.798067 | 370 | 157.993148 | 229.52 | 301.046852 | 0.620324 |  |
| GO:0003002\_regionalization | 195 | 5 | 1.736425 | -0.797127 | 372 | 158.260855 | 229.83 | 301.399145 | 0.617823 |  |
| GO:0007507\_heart\_development | 195 | 5 | 1.736425 | -0.797127 | 372 | 158.260855 | 229.83 | 301.399145 | 0.617823 |  |
| GO:0032774\_RNA\_biosynthetic\_process | 595 | 12 | 1.365793 | -0.794339 | 373 | 158.569681 | 230.25 | 301.930319 | 0.617292 |  |
| GO:0007389\_pattern\_specification\_process | 250 | 6 | 1.625294 | -0.790581 | 374 | 159.318297 | 231.27 | 303.221703 | 0.618369 |  |
| GO:0010468\_regulation\_of\_gene\_expression | 778 | 15 | 1.305667 | -0.789549 | 375 | 159.350323 | 231.43 | 303.509677 | 0.617147 |  |
| GO:0045596\_negative\_regulation\_of\_cell\_differentiation | 144 | 4 | 1.881127 | -0.789462 | 376 | 159.413733 | 231.61 | 303.806267 | 0.615984 |  |
| GO:0003015\_heart\_process | 49 | 2 | 2.764106 | -0.788487 | 381 | 160.555848 | 233.23 | 305.904152 | 0.612152 |  |
| GO:0021543\_pallium\_development | 49 | 2 | 2.764106 | -0.788487 | 381 | 160.555848 | 233.23 | 305.904152 | 0.612152 |  |
| GO:0046660\_female\_sex\_differentiation | 49 | 2 | 2.764106 | -0.788487 | 381 | 160.555848 | 233.23 | 305.904152 | 0.612152 |  |
| GO:0048741\_skeletal\_muscle\_fiber\_development | 49 | 2 | 2.764106 | -0.788487 | 381 | 160.555848 | 233.23 | 305.904152 | 0.612152 |  |
| GO:0060047\_heart\_contraction | 49 | 2 | 2.764106 | -0.788487 | 381 | 160.555848 | 233.23 | 305.904152 | 0.612152 |  |
| GO:0002026\_regulation\_of\_the\_force\_of\_heart\_contraction | 12 | 1 | 5.643382 | -0.786024 | 396 | 171.514247 | 246.49 | 321.465753 | 0.622449 |  |
| GO:0002820\_negative\_regulation\_of\_adaptive\_immune\_response | 12 | 1 | 5.643382 | -0.786024 | 396 | 171.514247 | 246.49 | 321.465753 | 0.622449 |  |
| GO:0002823\_negative\_regulation\_of\_adaptive\_immune\_response\_based\_on\_somatic\_recombination\_of\_immune\_receptors\_built\_from\_immunoglobulin\_superfamily\_domains | 12 | 1 | 5.643382 | -0.786024 | 396 | 171.514247 | 246.49 | 321.465753 | 0.622449 |  |
| GO:0006094\_gluconeogenesis | 12 | 1 | 5.643382 | -0.786024 | 396 | 171.514247 | 246.49 | 321.465753 | 0.622449 |  |
| GO:0008045\_motor\_axon\_guidance | 12 | 1 | 5.643382 | -0.786024 | 396 | 171.514247 | 246.49 | 321.465753 | 0.622449 |  |
| GO:0008277\_regulation\_of\_G-protein\_coupled\_receptor\_protein\_signaling\_pathway | 12 | 1 | 5.643382 | -0.786024 | 396 | 171.514247 | 246.49 | 321.465753 | 0.622449 |  |
| GO:0019319\_hexose\_biosynthetic\_process | 12 | 1 | 5.643382 | -0.786024 | 396 | 171.514247 | 246.49 | 321.465753 | 0.622449 |  |
| GO:0021513\_spinal\_cord\_dorsal\_ventral\_patterning | 12 | 1 | 5.643382 | -0.786024 | 396 | 171.514247 | 246.49 | 321.465753 | 0.622449 |  |
| GO:0035050\_embryonic\_heart\_tube\_development | 12 | 1 | 5.643382 | -0.786024 | 396 | 171.514247 | 246.49 | 321.465753 | 0.622449 |  |
| GO:0042446\_hormone\_biosynthetic\_process | 12 | 1 | 5.643382 | -0.786024 | 396 | 171.514247 | 246.49 | 321.465753 | 0.622449 |  |
| GO:0043392\_negative\_regulation\_of\_DNA\_binding | 12 | 1 | 5.643382 | -0.786024 | 396 | 171.514247 | 246.49 | 321.465753 | 0.622449 |  |
| GO:0046330\_positive\_regulation\_of\_JNK\_cascade | 12 | 1 | 5.643382 | -0.786024 | 396 | 171.514247 | 246.49 | 321.465753 | 0.622449 |  |
| GO:0050853\_B\_cell\_receptor\_signaling\_pathway | 12 | 1 | 5.643382 | -0.786024 | 396 | 171.514247 | 246.49 | 321.465753 | 0.622449 |  |
| GO:0060042\_retina\_morphogenesis\_in\_camera-type\_eye | 12 | 1 | 5.643382 | -0.786024 | 396 | 171.514247 | 246.49 | 321.465753 | 0.622449 |  |
| GO:0070304\_positive\_regulation\_of\_stress-activated\_protein\_kinase\_signaling\_pathway | 12 | 1 | 5.643382 | -0.786024 | 396 | 171.514247 | 246.49 | 321.465753 | 0.622449 |  |
| GO:0030900\_forebrain\_development | 146 | 4 | 1.855359 | -0.774414 | 397 | 173.295028 | 249.24 | 325.184972 | 0.627809 |  |
| GO:0060249\_anatomical\_structure\_homeostasis | 96 | 3 | 2.116268 | -0.774304 | 398 | 173.444301 | 249.66 | 325.875699 | 0.627286 |  |
| GO:0007154\_cell\_communication | 1096 | 20 | 1.235777 | -0.769595 | 399 | 173.612775 | 250.06 | 326.507225 | 0.626717 |  |
| GO:0018193\_peptidyl-amino\_acid\_modification | 97 | 3 | 2.094451 | -0.765006 | 400 | 173.974431 | 250.45 | 326.925569 | 0.626125 |  |
| GO:0002764\_immune\_response-regulating\_signal\_transduction | 51 | 2 | 2.655709 | -0.761282 | 403 | 174.886096 | 251.9 | 328.913904 | 0.625062 |  |
| GO:0043408\_regulation\_of\_MAPKKK\_cascade | 51 | 2 | 2.655709 | -0.761282 | 403 | 174.886096 | 251.9 | 328.913904 | 0.625062 |  |
| GO:0048747\_muscle\_fiber\_development | 51 | 2 | 2.655709 | -0.761282 | 403 | 174.886096 | 251.9 | 328.913904 | 0.625062 |  |
| GO:0007548\_sex\_differentiation | 98 | 3 | 2.073079 | -0.755845 | 404 | 175.347925 | 252.7 | 330.052075 | 0.625495 |  |
| GO:0000723\_telomere\_maintenance | 13 | 1 | 5.209276 | -0.754353 | 424 | 185.644863 | 264.61 | 343.575137 | 0.624080 |  |
| GO:0001958\_endochondral\_ossification | 13 | 1 | 5.209276 | -0.754353 | 424 | 185.644863 | 264.61 | 343.575137 | 0.624080 |  |
| GO:0002704\_negative\_regulation\_of\_leukocyte\_mediated\_immunity | 13 | 1 | 5.209276 | -0.754353 | 424 | 185.644863 | 264.61 | 343.575137 | 0.624080 |  |
| GO:0002707\_negative\_regulation\_of\_lymphocyte\_mediated\_immunity | 13 | 1 | 5.209276 | -0.754353 | 424 | 185.644863 | 264.61 | 343.575137 | 0.624080 |  |
| GO:0003016\_respiratory\_system\_process | 13 | 1 | 5.209276 | -0.754353 | 424 | 185.644863 | 264.61 | 343.575137 | 0.624080 |  |
| GO:0006090\_pyruvate\_metabolic\_process | 13 | 1 | 5.209276 | -0.754353 | 424 | 185.644863 | 264.61 | 343.575137 | 0.624080 |  |
| GO:0006687\_glycosphingolipid\_metabolic\_process | 13 | 1 | 5.209276 | -0.754353 | 424 | 185.644863 | 264.61 | 343.575137 | 0.624080 |  |
| GO:0007439\_ectodermal\_gut\_development | 13 | 1 | 5.209276 | -0.754353 | 424 | 185.644863 | 264.61 | 343.575137 | 0.624080 |  |
| GO:0009994\_oocyte\_differentiation | 13 | 1 | 5.209276 | -0.754353 | 424 | 185.644863 | 264.61 | 343.575137 | 0.624080 |  |
| GO:0016525\_negative\_regulation\_of\_angiogenesis | 13 | 1 | 5.209276 | -0.754353 | 424 | 185.644863 | 264.61 | 343.575137 | 0.624080 |  |
| GO:0019098\_reproductive\_behavior | 13 | 1 | 5.209276 | -0.754353 | 424 | 185.644863 | 264.61 | 343.575137 | 0.624080 |  |
| GO:0021511\_spinal\_cord\_patterning | 13 | 1 | 5.209276 | -0.754353 | 424 | 185.644863 | 264.61 | 343.575137 | 0.624080 |  |
| GO:0032200\_telomere\_organization | 13 | 1 | 5.209276 | -0.754353 | 424 | 185.644863 | 264.61 | 343.575137 | 0.624080 |  |
| GO:0045191\_regulation\_of\_isotype\_switching | 13 | 1 | 5.209276 | -0.754353 | 424 | 185.644863 | 264.61 | 343.575137 | 0.624080 |  |
| GO:0045577\_regulation\_of\_B\_cell\_differentiation | 13 | 1 | 5.209276 | -0.754353 | 424 | 185.644863 | 264.61 | 343.575137 | 0.624080 |  |
| GO:0046640\_regulation\_of\_alpha-beta\_T\_cell\_proliferation | 13 | 1 | 5.209276 | -0.754353 | 424 | 185.644863 | 264.61 | 343.575137 | 0.624080 |  |
| GO:0048567\_ectodermal\_gut\_morphogenesis | 13 | 1 | 5.209276 | -0.754353 | 424 | 185.644863 | 264.61 | 343.575137 | 0.624080 |  |
| GO:0048599\_oocyte\_development | 13 | 1 | 5.209276 | -0.754353 | 424 | 185.644863 | 264.61 | 343.575137 | 0.624080 |  |
| GO:0060401\_cytosolic\_calcium\_ion\_transport | 13 | 1 | 5.209276 | -0.754353 | 424 | 185.644863 | 264.61 | 343.575137 | 0.624080 |  |
| GO:0060402\_calcium\_ion\_transport\_into\_cytosol | 13 | 1 | 5.209276 | -0.754353 | 424 | 185.644863 | 264.61 | 343.575137 | 0.624080 |  |
| GO:0048870\_cell\_motility | 257 | 6 | 1.581025 | -0.751788 | 425 | 185.741963 | 264.84 | 343.938037 | 0.623153 |  |
| GO:0007165\_signal\_transduction | 915 | 17 | 1.258197 | -0.748120 | 426 | 186.200721 | 265.73 | 345.259279 | 0.623779 |  |
| GO:0001568\_blood\_vessel\_development | 203 | 5 | 1.667995 | -0.746790 | 427 | 186.580986 | 266.3 | 346.019014 | 0.623653 |  |
| GO:0043283\_biopolymer\_metabolic\_process | 1490 | 26 | 1.181702 | -0.745126 | 428 | 186.627090 | 266.4 | 346.172910 | 0.622430 |  |
| GO:0001525\_angiogenesis | 100 | 3 | 2.031618 | -0.737926 | 429 | 186.973673 | 266.8 | 346.626327 | 0.621911 |  |
| GO:0050789\_regulation\_of\_biological\_process | 2357 | 39 | 1.120536 | -0.736781 | 430 | 187.030432 | 266.87 | 346.709568 | 0.620628 |  |
| GO:0000060\_protein\_import\_into\_nucleus\_\_translocation | 14 | 1 | 4.837185 | -0.725251 | 442 | 198.230608 | 280.03 | 361.829392 | 0.633552 |  |
| GO:0002027\_regulation\_of\_heart\_rate | 14 | 1 | 4.837185 | -0.725251 | 442 | 198.230608 | 280.03 | 361.829392 | 0.633552 |  |
| GO:0002698\_negative\_regulation\_of\_immune\_effector\_process | 14 | 1 | 4.837185 | -0.725251 | 442 | 198.230608 | 280.03 | 361.829392 | 0.633552 |  |
| GO:0007157\_heterophilic\_cell\_adhesion | 14 | 1 | 4.837185 | -0.725251 | 442 | 198.230608 | 280.03 | 361.829392 | 0.633552 |  |
| GO:0021782\_glial\_cell\_development | 14 | 1 | 4.837185 | -0.725251 | 442 | 198.230608 | 280.03 | 361.829392 | 0.633552 |  |
| GO:0030032\_lamellipodium\_assembly | 14 | 1 | 4.837185 | -0.725251 | 442 | 198.230608 | 280.03 | 361.829392 | 0.633552 |  |
| GO:0030148\_sphingolipid\_biosynthetic\_process | 14 | 1 | 4.837185 | -0.725251 | 442 | 198.230608 | 280.03 | 361.829392 | 0.633552 |  |
| GO:0042310\_vasoconstriction | 14 | 1 | 4.837185 | -0.725251 | 442 | 198.230608 | 280.03 | 361.829392 | 0.633552 |  |
| GO:0045061\_thymic\_T\_cell\_selection | 14 | 1 | 4.837185 | -0.725251 | 442 | 198.230608 | 280.03 | 361.829392 | 0.633552 |  |
| GO:0050810\_regulation\_of\_steroid\_biosynthetic\_process | 14 | 1 | 4.837185 | -0.725251 | 442 | 198.230608 | 280.03 | 361.829392 | 0.633552 |  |
| GO:0051054\_positive\_regulation\_of\_DNA\_metabolic\_process | 14 | 1 | 4.837185 | -0.725251 | 442 | 198.230608 | 280.03 | 361.829392 | 0.633552 |  |
| GO:0051100\_negative\_regulation\_of\_binding | 14 | 1 | 4.837185 | -0.725251 | 442 | 198.230608 | 280.03 | 361.829392 | 0.633552 |  |
| GO:0002253\_activation\_of\_immune\_response | 54 | 2 | 2.508170 | -0.722963 | 444 | 199.477402 | 281.7 | 363.922598 | 0.634459 |  |
| GO:0009566\_fertilization | 54 | 2 | 2.508170 | -0.722963 | 444 | 199.477402 | 281.7 | 363.922598 | 0.634459 |  |
| GO:0001944\_vasculature\_development | 208 | 5 | 1.627899 | -0.717030 | 445 | 200.039026 | 282.38 | 364.720974 | 0.634562 |  |
| GO:0003013\_circulatory\_system\_process | 103 | 3 | 1.972444 | -0.712012 | 448 | 200.540299 | 283.11 | 365.679701 | 0.631942 |  |
| GO:0008015\_blood\_circulation | 103 | 3 | 1.972444 | -0.712012 | 448 | 200.540299 | 283.11 | 365.679701 | 0.631942 |  |
| GO:0009968\_negative\_regulation\_of\_signal\_transduction | 103 | 3 | 1.972444 | -0.712012 | 448 | 200.540299 | 283.11 | 365.679701 | 0.631942 |  |
| GO:0007605\_sensory\_perception\_of\_sound | 55 | 2 | 2.462567 | -0.710803 | 449 | 201.247556 | 284.09 | 366.932444 | 0.632717 |  |
| GO:0034961\_cellular\_biopolymer\_biosynthetic\_process | 804 | 15 | 1.263444 | -0.707728 | 450 | 201.418136 | 284.45 | 367.481864 | 0.632111 |  |
| GO:0001708\_cell\_fate\_specification | 56 | 2 | 2.418592 | -0.698931 | 452 | 202.626899 | 286.34 | 370.053101 | 0.633496 |  |
| GO:0002683\_negative\_regulation\_of\_immune\_system\_process | 56 | 2 | 2.418592 | -0.698931 | 452 | 202.626899 | 286.34 | 370.053101 | 0.633496 |  |
| GO:0043284\_biopolymer\_biosynthetic\_process | 807 | 15 | 1.258747 | -0.698743 | 453 | 202.671026 | 286.39 | 370.108974 | 0.632208 |  |
| GO:0010556\_regulation\_of\_macromolecule\_biosynthetic\_process | 745 | 14 | 1.272602 | -0.698646 | 454 | 202.756213 | 286.51 | 370.263787 | 0.631079 |  |
| GO:0002709\_regulation\_of\_T\_cell\_mediated\_immunity | 15 | 1 | 4.514706 | -0.698364 | 461 | 211.030433 | 296.3 | 381.569567 | 0.642733 |  |
| GO:0007040\_lysosome\_organization | 15 | 1 | 4.514706 | -0.698364 | 461 | 211.030433 | 296.3 | 381.569567 | 0.642733 |  |
| GO:0007618\_mating | 15 | 1 | 4.514706 | -0.698364 | 461 | 211.030433 | 296.3 | 381.569567 | 0.642733 |  |
| GO:0010171\_body\_morphogenesis | 15 | 1 | 4.514706 | -0.698364 | 461 | 211.030433 | 296.3 | 381.569567 | 0.642733 |  |
| GO:0045666\_positive\_regulation\_of\_neuron\_differentiation | 15 | 1 | 4.514706 | -0.698364 | 461 | 211.030433 | 296.3 | 381.569567 | 0.642733 |  |
| GO:0048008\_platelet-derived\_growth\_factor\_receptor\_signaling\_pathway | 15 | 1 | 4.514706 | -0.698364 | 461 | 211.030433 | 296.3 | 381.569567 | 0.642733 |  |
| GO:0048010\_vascular\_endothelial\_growth\_factor\_receptor\_signaling\_pathway | 15 | 1 | 4.514706 | -0.698364 | 461 | 211.030433 | 296.3 | 381.569567 | 0.642733 |  |
| GO:0051704\_multi-organism\_process | 157 | 4 | 1.725365 | -0.697180 | 462 | 211.149997 | 296.42 | 381.690003 | 0.641602 |  |
| GO:0043170\_macromolecule\_metabolic\_process | 1576 | 27 | 1.160188 | -0.695012 | 463 | 211.344683 | 296.75 | 382.155317 | 0.640929 |  |
| GO:0006066\_alcohol\_metabolic\_process | 158 | 4 | 1.714445 | -0.690594 | 465 | 212.038312 | 297.65 | 383.261688 | 0.640108 |  |
| GO:0048514\_blood\_vessel\_morphogenesis | 158 | 4 | 1.714445 | -0.690594 | 465 | 212.038312 | 297.65 | 383.261688 | 0.640108 |  |
| GO:0060255\_regulation\_of\_macromolecule\_metabolic\_process | 936 | 17 | 1.229968 | -0.687908 | 466 | 212.134017 | 297.96 | 383.785983 | 0.639399 |  |
| GO:0001764\_neuron\_migration | 57 | 2 | 2.376161 | -0.687336 | 471 | 213.473602 | 299.81 | 386.146398 | 0.636539 |  |
| GO:0009953\_dorsal\_ventral\_pattern\_formation | 57 | 2 | 2.376161 | -0.687336 | 471 | 213.473602 | 299.81 | 386.146398 | 0.636539 |  |
| GO:0018108\_peptidyl-tyrosine\_phosphorylation | 57 | 2 | 2.376161 | -0.687336 | 471 | 213.473602 | 299.81 | 386.146398 | 0.636539 |  |
| GO:0018212\_peptidyl-tyrosine\_modification | 57 | 2 | 2.376161 | -0.687336 | 471 | 213.473602 | 299.81 | 386.146398 | 0.636539 |  |
| GO:0043523\_regulation\_of\_neuron\_apoptosis | 57 | 2 | 2.376161 | -0.687336 | 471 | 213.473602 | 299.81 | 386.146398 | 0.636539 |  |
| GO:0030902\_hindbrain\_development | 58 | 2 | 2.335193 | -0.676007 | 472 | 214.811750 | 301.62 | 388.428250 | 0.639025 |  |
| GO:0001933\_negative\_regulation\_of\_protein\_amino\_acid\_phosphorylation | 16 | 1 | 4.232537 | -0.673404 | 483 | 223.168001 | 311.36 | 399.551999 | 0.644638 |  |
| GO:0006664\_glycolipid\_metabolic\_process | 16 | 1 | 4.232537 | -0.673404 | 483 | 223.168001 | 311.36 | 399.551999 | 0.644638 |  |
| GO:0007033\_vacuole\_organization | 16 | 1 | 4.232537 | -0.673404 | 483 | 223.168001 | 311.36 | 399.551999 | 0.644638 |  |
| GO:0010876\_lipid\_localization | 16 | 1 | 4.232537 | -0.673404 | 483 | 223.168001 | 311.36 | 399.551999 | 0.644638 |  |
| GO:0019915\_lipid\_storage | 16 | 1 | 4.232537 | -0.673404 | 483 | 223.168001 | 311.36 | 399.551999 | 0.644638 |  |
| GO:0021522\_spinal\_cord\_motor\_neuron\_differentiation | 16 | 1 | 4.232537 | -0.673404 | 483 | 223.168001 | 311.36 | 399.551999 | 0.644638 |  |
| GO:0030890\_positive\_regulation\_of\_B\_cell\_proliferation | 16 | 1 | 4.232537 | -0.673404 | 483 | 223.168001 | 311.36 | 399.551999 | 0.644638 |  |
| GO:0046364\_monosaccharide\_biosynthetic\_process | 16 | 1 | 4.232537 | -0.673404 | 483 | 223.168001 | 311.36 | 399.551999 | 0.644638 |  |
| GO:0046467\_membrane\_lipid\_biosynthetic\_process | 16 | 1 | 4.232537 | -0.673404 | 483 | 223.168001 | 311.36 | 399.551999 | 0.644638 |  |
| GO:0046633\_alpha-beta\_T\_cell\_proliferation | 16 | 1 | 4.232537 | -0.673404 | 483 | 223.168001 | 311.36 | 399.551999 | 0.644638 |  |
| GO:0060713\_labyrinthine\_layer\_morphogenesis | 16 | 1 | 4.232537 | -0.673404 | 483 | 223.168001 | 311.36 | 399.551999 | 0.644638 |  |
| GO:0040007\_growth | 217 | 5 | 1.560382 | -0.666516 | 484 | 224.061079 | 312.75 | 401.438921 | 0.646178 |  |
| GO:0033036\_macromolecule\_localization | 274 | 6 | 1.482933 | -0.665262 | 485 | 224.167207 | 312.96 | 401.752793 | 0.645278 |  |
| GO:0035270\_endocrine\_system\_development | 59 | 2 | 2.295613 | -0.664937 | 486 | 224.709350 | 313.63 | 402.550650 | 0.645329 |  |
| GO:0009987\_cellular\_process | 3868 | 60 | 1.050474 | -0.662283 | 487 | 225.318502 | 314.24 | 403.161498 | 0.645257 |  |
| GO:0045892\_negative\_regulation\_of\_transcription\_\_DNA-dependent | 218 | 5 | 1.553225 | -0.661134 | 488 | 225.408569 | 314.4 | 403.391431 | 0.644262 |  |
| GO:0007626\_locomotory\_behavior | 163 | 4 | 1.661855 | -0.658666 | 489 | 225.669129 | 314.81 | 403.950871 | 0.643783 |  |
| GO:0007423\_sensory\_organ\_development | 219 | 5 | 1.546132 | -0.655796 | 490 | 225.798989 | 315.04 | 404.281011 | 0.642939 |  |
| GO:0010648\_negative\_regulation\_of\_cell\_communication | 110 | 3 | 1.846925 | -0.655696 | 492 | 226.261292 | 315.59 | 404.918708 | 0.641443 |  |
| GO:0043010\_camera-type\_eye\_development | 110 | 3 | 1.846925 | -0.655696 | 492 | 226.261292 | 315.59 | 404.918708 | 0.641443 |  |
| GO:0051253\_negative\_regulation\_of\_RNA\_metabolic\_process | 220 | 5 | 1.539104 | -0.650502 | 493 | 226.809846 | 316.39 | 405.970154 | 0.641765 |  |
| GO:0006813\_potassium\_ion\_transport | 17 | 1 | 3.983564 | -0.650137 | 499 | 234.724695 | 325.52 | 416.315305 | 0.652345 |  |
| GO:0009408\_response\_to\_heat | 17 | 1 | 3.983564 | -0.650137 | 499 | 234.724695 | 325.52 | 416.315305 | 0.652345 |  |
| GO:0010565\_regulation\_of\_cellular\_ketone\_metabolic\_process | 17 | 1 | 3.983564 | -0.650137 | 499 | 234.724695 | 325.52 | 416.315305 | 0.652345 |  |
| GO:0016052\_carbohydrate\_catabolic\_process | 17 | 1 | 3.983564 | -0.650137 | 499 | 234.724695 | 325.52 | 416.315305 | 0.652345 |  |
| GO:0030101\_natural\_killer\_cell\_activation | 17 | 1 | 3.983564 | -0.650137 | 499 | 234.724695 | 325.52 | 416.315305 | 0.652345 |  |
| GO:0060350\_endochondral\_bone\_morphogenesis | 17 | 1 | 3.983564 | -0.650137 | 499 | 234.724695 | 325.52 | 416.315305 | 0.652345 |  |
| GO:0031323\_regulation\_of\_cellular\_metabolic\_process | 1015 | 18 | 1.200956 | -0.646051 | 500 | 235.224171 | 326.24 | 417.255829 | 0.652480 |  |
| GO:0009887\_organ\_morphogenesis | 642 | 12 | 1.265805 | -0.634911 | 501 | 237.286620 | 329.09 | 420.893380 | 0.656866 |  |
| GO:0021537\_telencephalon\_development | 62 | 2 | 2.184535 | -0.633185 | 504 | 239.012115 | 331.55 | 424.087885 | 0.657837 |  |
| GO:0040014\_regulation\_of\_multicellular\_organism\_growth | 62 | 2 | 2.184535 | -0.633185 | 504 | 239.012115 | 331.55 | 424.087885 | 0.657837 |  |
| GO:0050954\_sensory\_perception\_of\_mechanical\_stimulus | 62 | 2 | 2.184535 | -0.633185 | 504 | 239.012115 | 331.55 | 424.087885 | 0.657837 |  |
| GO:0001974\_blood\_vessel\_remodeling | 18 | 1 | 3.762255 | -0.628367 | 513 | 248.678140 | 342.76 | 436.841860 | 0.668148 |  |
| GO:0006940\_regulation\_of\_smooth\_muscle\_contraction | 18 | 1 | 3.762255 | -0.628367 | 513 | 248.678140 | 342.76 | 436.841860 | 0.668148 |  |
| GO:0007608\_sensory\_perception\_of\_smell | 18 | 1 | 3.762255 | -0.628367 | 513 | 248.678140 | 342.76 | 436.841860 | 0.668148 |  |
| GO:0010498\_proteasomal\_protein\_catabolic\_process | 18 | 1 | 3.762255 | -0.628367 | 513 | 248.678140 | 342.76 | 436.841860 | 0.668148 |  |
| GO:0021517\_ventral\_spinal\_cord\_development | 18 | 1 | 3.762255 | -0.628367 | 513 | 248.678140 | 342.76 | 436.841860 | 0.668148 |  |
| GO:0030901\_midbrain\_development | 18 | 1 | 3.762255 | -0.628367 | 513 | 248.678140 | 342.76 | 436.841860 | 0.668148 |  |
| GO:0043161\_proteasomal\_ubiquitin-dependent\_protein\_catabolic\_process | 18 | 1 | 3.762255 | -0.628367 | 513 | 248.678140 | 342.76 | 436.841860 | 0.668148 |  |
| GO:0045058\_T\_cell\_selection | 18 | 1 | 3.762255 | -0.628367 | 513 | 248.678140 | 342.76 | 436.841860 | 0.668148 |  |
| GO:0050731\_positive\_regulation\_of\_peptidyl-tyrosine\_phosphorylation | 18 | 1 | 3.762255 | -0.628367 | 513 | 248.678140 | 342.76 | 436.841860 | 0.668148 |  |
| GO:0000165\_MAPKKK\_cascade | 114 | 3 | 1.782121 | -0.625897 | 514 | 248.970849 | 343.11 | 437.249151 | 0.667529 |  |
| GO:0000902\_cell\_morphogenesis | 283 | 6 | 1.435772 | -0.623484 | 515 | 249.161859 | 343.29 | 437.418141 | 0.666583 |  |
| GO:0019222\_regulation\_of\_metabolic\_process | 1088 | 19 | 1.182621 | -0.623217 | 516 | 249.212015 | 343.39 | 437.567985 | 0.665484 |  |
| GO:0051216\_cartilage\_development | 63 | 2 | 2.149860 | -0.623060 | 517 | 249.845084 | 344.48 | 439.114916 | 0.666306 |  |
| GO:0048523\_negative\_regulation\_of\_cellular\_process | 774 | 14 | 1.224920 | -0.613756 | 518 | 250.800568 | 345.94 | 441.079432 | 0.667838 |  |
| GO:0006497\_protein\_amino\_acid\_lipidation | 19 | 1 | 3.564241 | -0.607933 | 527 | 259.066693 | 355.62 | 452.173307 | 0.674801 |  |
| GO:0007569\_cell\_aging | 19 | 1 | 3.564241 | -0.607933 | 527 | 259.066693 | 355.62 | 452.173307 | 0.674801 |  |
| GO:0019218\_regulation\_of\_steroid\_metabolic\_process | 19 | 1 | 3.564241 | -0.607933 | 527 | 259.066693 | 355.62 | 452.173307 | 0.674801 |  |
| GO:0030518\_steroid\_hormone\_receptor\_signaling\_pathway | 19 | 1 | 3.564241 | -0.607933 | 527 | 259.066693 | 355.62 | 452.173307 | 0.674801 |  |
| GO:0042491\_auditory\_receptor\_cell\_differentiation | 19 | 1 | 3.564241 | -0.607933 | 527 | 259.066693 | 355.62 | 452.173307 | 0.674801 |  |
| GO:0046165\_alcohol\_biosynthetic\_process | 19 | 1 | 3.564241 | -0.607933 | 527 | 259.066693 | 355.62 | 452.173307 | 0.674801 |  |
| GO:0046890\_regulation\_of\_lipid\_biosynthetic\_process | 19 | 1 | 3.564241 | -0.607933 | 527 | 259.066693 | 355.62 | 452.173307 | 0.674801 |  |
| GO:0048547\_gut\_morphogenesis | 19 | 1 | 3.564241 | -0.607933 | 527 | 259.066693 | 355.62 | 452.173307 | 0.674801 |  |
| GO:0060444\_branching\_involved\_in\_mammary\_gland\_duct\_morphogenesis | 19 | 1 | 3.564241 | -0.607933 | 527 | 259.066693 | 355.62 | 452.173307 | 0.674801 |  |
| GO:0007600\_sensory\_perception | 172 | 4 | 1.574897 | -0.605155 | 528 | 259.576185 | 356.2 | 452.823815 | 0.674621 |  |
| GO:0043086\_negative\_regulation\_of\_catalytic\_activity | 65 | 2 | 2.083710 | -0.603457 | 530 | 260.477873 | 357.26 | 454.042127 | 0.674075 |  |
| GO:0048511\_rhythmic\_process | 65 | 2 | 2.083710 | -0.603457 | 530 | 260.477873 | 357.26 | 454.042127 | 0.674075 |  |
| GO:0010467\_gene\_expression | 905 | 16 | 1.197270 | -0.601683 | 531 | 260.699058 | 357.47 | 454.240942 | 0.673202 |  |
| GO:0031589\_cell-substrate\_adhesion | 66 | 2 | 2.052139 | -0.593963 | 534 | 262.909225 | 360.2 | 457.490775 | 0.674532 |  |
| GO:0045860\_positive\_regulation\_of\_protein\_kinase\_activity | 66 | 2 | 2.052139 | -0.593963 | 534 | 262.909225 | 360.2 | 457.490775 | 0.674532 |  |
| GO:0051402\_neuron\_apoptosis | 66 | 2 | 2.052139 | -0.593963 | 534 | 262.909225 | 360.2 | 457.490775 | 0.674532 |  |
| GO:0001912\_positive\_regulation\_of\_leukocyte\_mediated\_cytotoxicity | 20 | 1 | 3.386029 | -0.588696 | 542 | 269.016823 | 367.91 | 466.803177 | 0.678801 |  |
| GO:0007416\_synaptogenesis | 20 | 1 | 3.386029 | -0.588696 | 542 | 269.016823 | 367.91 | 466.803177 | 0.678801 |  |
| GO:0009615\_response\_to\_virus | 20 | 1 | 3.386029 | -0.588696 | 542 | 269.016823 | 367.91 | 466.803177 | 0.678801 |  |
| GO:0018209\_peptidyl-serine\_modification | 20 | 1 | 3.386029 | -0.588696 | 542 | 269.016823 | 367.91 | 466.803177 | 0.678801 |  |
| GO:0031343\_positive\_regulation\_of\_cell\_killing | 20 | 1 | 3.386029 | -0.588696 | 542 | 269.016823 | 367.91 | 466.803177 | 0.678801 |  |
| GO:0042326\_negative\_regulation\_of\_phosphorylation | 20 | 1 | 3.386029 | -0.588696 | 542 | 269.016823 | 367.91 | 466.803177 | 0.678801 |  |
| GO:0045017\_glycerolipid\_biosynthetic\_process | 20 | 1 | 3.386029 | -0.588696 | 542 | 269.016823 | 367.91 | 466.803177 | 0.678801 |  |
| GO:0048806\_genitalia\_development | 20 | 1 | 3.386029 | -0.588696 | 542 | 269.016823 | 367.91 | 466.803177 | 0.678801 |  |
| GO:0016070\_RNA\_metabolic\_process | 658 | 12 | 1.235026 | -0.587166 | 543 | 269.389833 | 368.45 | 467.510167 | 0.678545 |  |
| GO:0050890\_cognition | 233 | 5 | 1.453232 | -0.585498 | 544 | 269.682322 | 368.76 | 467.837678 | 0.677868 |  |
| GO:0009791\_post-embryonic\_development | 67 | 2 | 2.021510 | -0.584668 | 546 | 270.622270 | 369.8 | 468.977730 | 0.677289 |  |
| GO:0042445\_hormone\_metabolic\_process | 67 | 2 | 2.021510 | -0.584668 | 546 | 270.622270 | 369.8 | 468.977730 | 0.677289 |  |
| GO:0043066\_negative\_regulation\_of\_apoptosis | 176 | 4 | 1.539104 | -0.582885 | 547 | 271.345203 | 370.67 | 469.994797 | 0.677642 |  |
| GO:0042692\_muscle\_cell\_differentiation | 68 | 2 | 1.991782 | -0.575564 | 548 | 272.689425 | 372.69 | 472.690575 | 0.680091 |  |
| GO:0030001\_metal\_ion\_transport | 122 | 3 | 1.665260 | -0.570911 | 549 | 273.914851 | 374.2 | 474.485149 | 0.681603 |  |
| GO:0000018\_regulation\_of\_DNA\_recombination | 21 | 1 | 3.224790 | -0.570538 | 558 | 281.605266 | 383.38 | 485.154734 | 0.687061 |  |
| GO:0001709\_cell\_fate\_determination | 21 | 1 | 3.224790 | -0.570538 | 558 | 281.605266 | 383.38 | 485.154734 | 0.687061 |  |
| GO:0010563\_negative\_regulation\_of\_phosphorus\_metabolic\_process | 21 | 1 | 3.224790 | -0.570538 | 558 | 281.605266 | 383.38 | 485.154734 | 0.687061 |  |
| GO:0019827\_stem\_cell\_maintenance | 21 | 1 | 3.224790 | -0.570538 | 558 | 281.605266 | 383.38 | 485.154734 | 0.687061 |  |
| GO:0030888\_regulation\_of\_B\_cell\_proliferation | 21 | 1 | 3.224790 | -0.570538 | 558 | 281.605266 | 383.38 | 485.154734 | 0.687061 |  |
| GO:0032147\_activation\_of\_protein\_kinase\_activity | 21 | 1 | 3.224790 | -0.570538 | 558 | 281.605266 | 383.38 | 485.154734 | 0.687061 |  |
| GO:0045936\_negative\_regulation\_of\_phosphate\_metabolic\_process | 21 | 1 | 3.224790 | -0.570538 | 558 | 281.605266 | 383.38 | 485.154734 | 0.687061 |  |
| GO:0050777\_negative\_regulation\_of\_immune\_response | 21 | 1 | 3.224790 | -0.570538 | 558 | 281.605266 | 383.38 | 485.154734 | 0.687061 |  |
| GO:0051656\_establishment\_of\_organelle\_localization | 21 | 1 | 3.224790 | -0.570538 | 558 | 281.605266 | 383.38 | 485.154734 | 0.687061 |  |
| GO:0043069\_negative\_regulation\_of\_programmed\_cell\_death | 179 | 4 | 1.513309 | -0.566754 | 560 | 282.263333 | 384.25 | 486.236667 | 0.686161 |  |
| GO:0060548\_negative\_regulation\_of\_cell\_death | 179 | 4 | 1.513309 | -0.566754 | 560 | 282.263333 | 384.25 | 486.236667 | 0.686161 |  |
| GO:0001932\_regulation\_of\_protein\_amino\_acid\_phosphorylation | 69 | 2 | 1.962916 | -0.566645 | 561 | 283.073303 | 385.21 | 487.346697 | 0.686649 |  |
| GO:0008406\_gonad\_development | 70 | 2 | 1.934874 | -0.557906 | 562 | 284.823216 | 387.12 | 489.416784 | 0.688826 |  |
| GO:0000041\_transition\_metal\_ion\_transport | 22 | 1 | 3.078209 | -0.553360 | 573 | 295.053818 | 399.13 | 503.206182 | 0.696562 |  |
| GO:0001569\_patterning\_of\_blood\_vessels | 22 | 1 | 3.078209 | -0.553360 | 573 | 295.053818 | 399.13 | 503.206182 | 0.696562 |  |
| GO:0002637\_regulation\_of\_immunoglobulin\_production | 22 | 1 | 3.078209 | -0.553360 | 573 | 295.053818 | 399.13 | 503.206182 | 0.696562 |  |
| GO:0002712\_regulation\_of\_B\_cell\_mediated\_immunity | 22 | 1 | 3.078209 | -0.553360 | 573 | 295.053818 | 399.13 | 503.206182 | 0.696562 |  |
| GO:0002889\_regulation\_of\_immunoglobulin\_mediated\_immune\_response | 22 | 1 | 3.078209 | -0.553360 | 573 | 295.053818 | 399.13 | 503.206182 | 0.696562 |  |
| GO:0006029\_proteoglycan\_metabolic\_process | 22 | 1 | 3.078209 | -0.553360 | 573 | 295.053818 | 399.13 | 503.206182 | 0.696562 |  |
| GO:0015918\_sterol\_transport | 22 | 1 | 3.078209 | -0.553360 | 573 | 295.053818 | 399.13 | 503.206182 | 0.696562 |  |
| GO:0030301\_cholesterol\_transport | 22 | 1 | 3.078209 | -0.553360 | 573 | 295.053818 | 399.13 | 503.206182 | 0.696562 |  |
| GO:0043112\_receptor\_metabolic\_process | 22 | 1 | 3.078209 | -0.553360 | 573 | 295.053818 | 399.13 | 503.206182 | 0.696562 |  |
| GO:0046635\_positive\_regulation\_of\_alpha-beta\_T\_cell\_activation | 22 | 1 | 3.078209 | -0.553360 | 573 | 295.053818 | 399.13 | 503.206182 | 0.696562 |  |
| GO:0048864\_stem\_cell\_development | 22 | 1 | 3.078209 | -0.553360 | 573 | 295.053818 | 399.13 | 503.206182 | 0.696562 |  |
| GO:0080090\_regulation\_of\_primary\_metabolic\_process | 926 | 16 | 1.170118 | -0.549626 | 574 | 295.663566 | 400.03 | 504.396434 | 0.696916 |  |
| GO:0033674\_positive\_regulation\_of\_kinase\_activity | 71 | 2 | 1.907622 | -0.549342 | 575 | 296.516108 | 401.09 | 505.663892 | 0.697548 |  |
| GO:0030879\_mammary\_gland\_development | 72 | 2 | 1.881127 | -0.540947 | 577 | 298.905090 | 403.99 | 509.074910 | 0.700156 |  |
| GO:0051347\_positive\_regulation\_of\_transferase\_activity | 72 | 2 | 1.881127 | -0.540947 | 577 | 298.905090 | 403.99 | 509.074910 | 0.700156 |  |
| GO:0016192\_vesicle-mediated\_transport | 184 | 4 | 1.472187 | -0.540906 | 578 | 299.146458 | 404.34 | 509.533542 | 0.699550 |  |
| GO:0000082\_G1\_S\_transition\_of\_mitotic\_cell\_cycle | 23 | 1 | 2.944373 | -0.537072 | 588 | 304.271241 | 410.7 | 517.128759 | 0.698469 |  |
| GO:0002204\_somatic\_recombination\_of\_immunoglobulin\_genes\_during\_immune\_response | 23 | 1 | 2.944373 | -0.537072 | 588 | 304.271241 | 410.7 | 517.128759 | 0.698469 |  |
| GO:0002208\_somatic\_diversification\_of\_immunoglobulins\_during\_immune\_response | 23 | 1 | 2.944373 | -0.537072 | 588 | 304.271241 | 410.7 | 517.128759 | 0.698469 |  |
| GO:0002228\_natural\_killer\_cell\_mediated\_immunity | 23 | 1 | 2.944373 | -0.537072 | 588 | 304.271241 | 410.7 | 517.128759 | 0.698469 |  |
| GO:0002821\_positive\_regulation\_of\_adaptive\_immune\_response | 23 | 1 | 2.944373 | -0.537072 | 588 | 304.271241 | 410.7 | 517.128759 | 0.698469 |  |
| GO:0002824\_positive\_regulation\_of\_adaptive\_immune\_response\_based\_on\_somatic\_recombination\_of\_immune\_receptors\_built\_from\_immunoglobulin\_superfamily\_domains | 23 | 1 | 2.944373 | -0.537072 | 588 | 304.271241 | 410.7 | 517.128759 | 0.698469 |  |
| GO:0008542\_visual\_learning | 23 | 1 | 2.944373 | -0.537072 | 588 | 304.271241 | 410.7 | 517.128759 | 0.698469 |  |
| GO:0042267\_natural\_killer\_cell\_mediated\_cytotoxicity | 23 | 1 | 2.944373 | -0.537072 | 588 | 304.271241 | 410.7 | 517.128759 | 0.698469 |  |
| GO:0045190\_isotype\_switching | 23 | 1 | 2.944373 | -0.537072 | 588 | 304.271241 | 410.7 | 517.128759 | 0.698469 |  |
| GO:0060349\_bone\_morphogenesis | 23 | 1 | 2.944373 | -0.537072 | 588 | 304.271241 | 410.7 | 517.128759 | 0.698469 |  |
| GO:0001655\_urogenital\_system\_development | 128 | 3 | 1.587201 | -0.533300 | 589 | 305.284239 | 411.92 | 518.555761 | 0.699355 |  |
| GO:0006952\_defense\_response | 187 | 4 | 1.448569 | -0.525992 | 590 | 307.352290 | 414.4 | 521.447710 | 0.702373 |  |
| GO:0032989\_cellular\_component\_morphogenesis | 307 | 6 | 1.323529 | -0.524059 | 591 | 307.743483 | 415.09 | 522.436517 | 0.702352 |  |
| GO:0006139\_nucleobase\_\_nucleoside\_\_nucleotide\_and\_nucleic\_acid\_metabolic\_process | 1002 | 17 | 1.148952 | -0.522848 | 592 | 307.879184 | 415.2 | 522.520816 | 0.701351 |  |
| GO:0001541\_ovarian\_follicle\_development | 24 | 1 | 2.821691 | -0.521598 | 603 | 314.357899 | 422.62 | 530.882101 | 0.700862 |  |
| GO:0002381\_immunoglobulin\_production\_during\_immune\_response | 24 | 1 | 2.821691 | -0.521598 | 603 | 314.357899 | 422.62 | 530.882101 | 0.700862 |  |
| GO:0006941\_striated\_muscle\_contraction | 24 | 1 | 2.821691 | -0.521598 | 603 | 314.357899 | 422.62 | 530.882101 | 0.700862 |  |
| GO:0006959\_humoral\_immune\_response | 24 | 1 | 2.821691 | -0.521598 | 603 | 314.357899 | 422.62 | 530.882101 | 0.700862 |  |
| GO:0007204\_elevation\_of\_cytosolic\_calcium\_ion\_concentration | 24 | 1 | 2.821691 | -0.521598 | 603 | 314.357899 | 422.62 | 530.882101 | 0.700862 |  |
| GO:0007259\_JAK-STAT\_cascade | 24 | 1 | 2.821691 | -0.521598 | 603 | 314.357899 | 422.62 | 530.882101 | 0.700862 |  |
| GO:0007632\_visual\_behavior | 24 | 1 | 2.821691 | -0.521598 | 603 | 314.357899 | 422.62 | 530.882101 | 0.700862 |  |
| GO:0009612\_response\_to\_mechanical\_stimulus | 24 | 1 | 2.821691 | -0.521598 | 603 | 314.357899 | 422.62 | 530.882101 | 0.700862 |  |
| GO:0042158\_lipoprotein\_biosynthetic\_process | 24 | 1 | 2.821691 | -0.521598 | 603 | 314.357899 | 422.62 | 530.882101 | 0.700862 |  |
| GO:0048546\_digestive\_tract\_morphogenesis | 24 | 1 | 2.821691 | -0.521598 | 603 | 314.357899 | 422.62 | 530.882101 | 0.700862 |  |
| GO:0060113\_inner\_ear\_receptor\_cell\_differentiation | 24 | 1 | 2.821691 | -0.521598 | 603 | 314.357899 | 422.62 | 530.882101 | 0.700862 |  |
| GO:0019226\_transmission\_of\_nerve\_impulse | 189 | 4 | 1.433240 | -0.516288 | 604 | 316.318040 | 425.28 | 534.241960 | 0.704106 |  |
| GO:0031326\_regulation\_of\_cellular\_biosynthetic\_process | 812 | 14 | 1.167596 | -0.515315 | 605 | 316.455232 | 425.43 | 534.404768 | 0.703190 |  |
| GO:0044267\_cellular\_protein\_metabolic\_process | 559 | 10 | 1.211460 | -0.511450 | 606 | 316.824354 | 426.07 | 535.315646 | 0.703086 |  |
| GO:0009889\_regulation\_of\_biosynthetic\_process | 815 | 14 | 1.163298 | -0.508119 | 607 | 317.754381 | 427.2 | 536.645619 | 0.703789 |  |
| GO:0006357\_regulation\_of\_transcription\_from\_RNA\_polymerase\_II\_promoter | 435 | 8 | 1.245436 | -0.507334 | 608 | 317.916438 | 427.35 | 536.783562 | 0.702878 |  |
| GO:0002700\_regulation\_of\_production\_of\_molecular\_mediator\_of\_immune\_response | 25 | 1 | 2.708824 | -0.506872 | 613 | 323.414761 | 434.1 | 544.785239 | 0.708157 |  |
| GO:0010811\_positive\_regulation\_of\_cell-substrate\_adhesion | 25 | 1 | 2.708824 | -0.506872 | 613 | 323.414761 | 434.1 | 544.785239 | 0.708157 |  |
| GO:0031400\_negative\_regulation\_of\_protein\_modification\_process | 25 | 1 | 2.708824 | -0.506872 | 613 | 323.414761 | 434.1 | 544.785239 | 0.708157 |  |
| GO:0050852\_T\_cell\_receptor\_signaling\_pathway | 25 | 1 | 2.708824 | -0.506872 | 613 | 323.414761 | 434.1 | 544.785239 | 0.708157 |  |
| GO:0055123\_digestive\_system\_development | 25 | 1 | 2.708824 | -0.506872 | 613 | 323.414761 | 434.1 | 544.785239 | 0.708157 |  |
| GO:0008104\_protein\_localization | 251 | 5 | 1.349016 | -0.506078 | 614 | 323.545368 | 434.26 | 544.974632 | 0.707264 |  |
| GO:0044057\_regulation\_of\_system\_process | 133 | 3 | 1.527532 | -0.504087 | 615 | 323.913894 | 434.6 | 545.286106 | 0.706667 |  |
| GO:0051241\_negative\_regulation\_of\_multicellular\_organismal\_process | 77 | 2 | 1.758976 | -0.501355 | 616 | 324.349022 | 435.2 | 546.050978 | 0.706494 |  |
| GO:0016481\_negative\_regulation\_of\_transcription | 253 | 5 | 1.338352 | -0.497939 | 617 | 324.970009 | 436.02 | 547.069991 | 0.706677 |  |
| GO:0006464\_protein\_modification\_process | 439 | 8 | 1.234088 | -0.494884 | 618 | 325.208713 | 436.33 | 547.451287 | 0.706036 |  |
| GO:0007405\_neuroblast\_proliferation | 26 | 1 | 2.604638 | -0.492833 | 623 | 332.343599 | 444.59 | 556.836401 | 0.713628 |  |
| GO:0010959\_regulation\_of\_metal\_ion\_transport | 26 | 1 | 2.604638 | -0.492833 | 623 | 332.343599 | 444.59 | 556.836401 | 0.713628 |  |
| GO:0045665\_negative\_regulation\_of\_neuron\_differentiation | 26 | 1 | 2.604638 | -0.492833 | 623 | 332.343599 | 444.59 | 556.836401 | 0.713628 |  |
| GO:0045765\_regulation\_of\_angiogenesis | 26 | 1 | 2.604638 | -0.492833 | 623 | 332.343599 | 444.59 | 556.836401 | 0.713628 |  |
| GO:0051235\_maintenance\_of\_location | 26 | 1 | 2.604638 | -0.492833 | 623 | 332.343599 | 444.59 | 556.836401 | 0.713628 |  |
| GO:0001654\_eye\_development | 136 | 3 | 1.493837 | -0.487419 | 624 | 333.544226 | 445.95 | 558.355774 | 0.714663 |  |
| GO:0015674\_di-\_\_tri-valent\_inorganic\_cation\_transport | 79 | 2 | 1.714445 | -0.486549 | 625 | 334.116645 | 446.64 | 559.163355 | 0.714624 |  |
| GO:0006366\_transcription\_from\_RNA\_polymerase\_II\_promoter | 444 | 8 | 1.220191 | -0.479704 | 626 | 335.249717 | 448.0 | 560.750283 | 0.715655 |  |
| GO:0001910\_regulation\_of\_leukocyte\_mediated\_cytotoxicity | 27 | 1 | 2.508170 | -0.479431 | 630 | 339.541180 | 453.07 | 566.598820 | 0.719159 |  |
| GO:0031016\_pancreas\_development | 27 | 1 | 2.508170 | -0.479431 | 630 | 339.541180 | 453.07 | 566.598820 | 0.719159 |  |
| GO:0031341\_regulation\_of\_cell\_killing | 27 | 1 | 2.508170 | -0.479431 | 630 | 339.541180 | 453.07 | 566.598820 | 0.719159 |  |
| GO:0045582\_positive\_regulation\_of\_T\_cell\_differentiation | 27 | 1 | 2.508170 | -0.479431 | 630 | 339.541180 | 453.07 | 566.598820 | 0.719159 |  |
| GO:0002250\_adaptive\_immune\_response | 80 | 2 | 1.693015 | -0.479352 | 632 | 340.610174 | 454.31 | 568.009826 | 0.718845 |  |
| GO:0002460\_adaptive\_immune\_response\_based\_on\_somatic\_recombination\_of\_immune\_receptors\_built\_from\_immunoglobulin\_superfamily\_domains | 80 | 2 | 1.693015 | -0.479352 | 632 | 340.610174 | 454.31 | 568.009826 | 0.718845 |  |
| GO:0007169\_transmembrane\_receptor\_protein\_tyrosine\_kinase\_signaling\_pathway | 139 | 3 | 1.461595 | -0.471360 | 633 | 342.208034 | 455.98 | 569.751966 | 0.720348 |  |
| GO:0002062\_chondrocyte\_differentiation | 28 | 1 | 2.418592 | -0.466617 | 641 | 346.968282 | 461.36 | 575.751718 | 0.719750 |  |
| GO:0002705\_positive\_regulation\_of\_leukocyte\_mediated\_immunity | 28 | 1 | 2.418592 | -0.466617 | 641 | 346.968282 | 461.36 | 575.751718 | 0.719750 |  |
| GO:0002708\_positive\_regulation\_of\_lymphocyte\_mediated\_immunity | 28 | 1 | 2.418592 | -0.466617 | 641 | 346.968282 | 461.36 | 575.751718 | 0.719750 |  |
| GO:0007585\_respiratory\_gaseous\_exchange | 28 | 1 | 2.418592 | -0.466617 | 641 | 346.968282 | 461.36 | 575.751718 | 0.719750 |  |
| GO:0042100\_B\_cell\_proliferation | 28 | 1 | 2.418592 | -0.466617 | 641 | 346.968282 | 461.36 | 575.751718 | 0.719750 |  |
| GO:0045926\_negative\_regulation\_of\_growth | 28 | 1 | 2.418592 | -0.466617 | 641 | 346.968282 | 461.36 | 575.751718 | 0.719750 |  |
| GO:0046328\_regulation\_of\_JNK\_cascade | 28 | 1 | 2.418592 | -0.466617 | 641 | 346.968282 | 461.36 | 575.751718 | 0.719750 |  |
| GO:0050871\_positive\_regulation\_of\_B\_cell\_activation | 28 | 1 | 2.418592 | -0.466617 | 641 | 346.968282 | 461.36 | 575.751718 | 0.719750 |  |
| GO:0010629\_negative\_regulation\_of\_gene\_expression | 262 | 5 | 1.292378 | -0.462868 | 642 | 348.825113 | 463.57 | 578.314887 | 0.722072 |  |
| GO:0003006\_reproductive\_developmental\_process | 141 | 3 | 1.440864 | -0.460978 | 643 | 349.423069 | 464.19 | 578.956931 | 0.721913 |  |
| GO:0003008\_system\_process | 516 | 9 | 1.181173 | -0.455215 | 644 | 351.359978 | 466.6 | 581.840022 | 0.724534 |  |
| GO:0001934\_positive\_regulation\_of\_protein\_amino\_acid\_phosphorylation | 29 | 1 | 2.335193 | -0.454350 | 652 | 356.204406 | 472.42 | 588.635594 | 0.724571 |  |
| GO:0006909\_phagocytosis | 29 | 1 | 2.335193 | -0.454350 | 652 | 356.204406 | 472.42 | 588.635594 | 0.724571 |  |
| GO:0016447\_somatic\_recombination\_of\_immunoglobulin\_gene\_segments | 29 | 1 | 2.335193 | -0.454350 | 652 | 356.204406 | 472.42 | 588.635594 | 0.724571 |  |
| GO:0021761\_limbic\_system\_development | 29 | 1 | 2.335193 | -0.454350 | 652 | 356.204406 | 472.42 | 588.635594 | 0.724571 |  |
| GO:0042490\_mechanoreceptor\_differentiation | 29 | 1 | 2.335193 | -0.454350 | 652 | 356.204406 | 472.42 | 588.635594 | 0.724571 |  |
| GO:0045621\_positive\_regulation\_of\_lymphocyte\_differentiation | 29 | 1 | 2.335193 | -0.454350 | 652 | 356.204406 | 472.42 | 588.635594 | 0.724571 |  |
| GO:0046634\_regulation\_of\_alpha-beta\_T\_cell\_activation | 29 | 1 | 2.335193 | -0.454350 | 652 | 356.204406 | 472.42 | 588.635594 | 0.724571 |  |
| GO:0070302\_regulation\_of\_stress-activated\_protein\_kinase\_signaling\_pathway | 29 | 1 | 2.335193 | -0.454350 | 652 | 356.204406 | 472.42 | 588.635594 | 0.724571 |  |
| GO:0045137\_development\_of\_primary\_sexual\_characteristics | 84 | 2 | 1.612395 | -0.451851 | 653 | 357.070968 | 473.22 | 589.369032 | 0.724686 |  |
| GO:0035239\_tube\_morphogenesis | 143 | 3 | 1.420712 | -0.450846 | 654 | 357.307461 | 473.56 | 589.812539 | 0.724098 |  |
| GO:0002449\_lymphocyte\_mediated\_immunity | 85 | 2 | 1.593426 | -0.445281 | 655 | 358.370633 | 474.93 | 591.489367 | 0.725084 |  |
| GO:0007243\_protein\_kinase\_cascade | 205 | 4 | 1.321377 | -0.445005 | 656 | 358.732261 | 475.28 | 591.827739 | 0.724512 |  |
| GO:0000187\_activation\_of\_MAPK\_activity | 30 | 1 | 2.257353 | -0.442592 | 664 | 365.227600 | 482.74 | 600.252400 | 0.727018 |  |
| GO:0007219\_Notch\_signaling\_pathway | 30 | 1 | 2.257353 | -0.442592 | 664 | 365.227600 | 482.74 | 600.252400 | 0.727018 |  |
| GO:0009266\_response\_to\_temperature\_stimulus | 30 | 1 | 2.257353 | -0.442592 | 664 | 365.227600 | 482.74 | 600.252400 | 0.727018 |  |
| GO:0016445\_somatic\_diversification\_of\_immunoglobulins | 30 | 1 | 2.257353 | -0.442592 | 664 | 365.227600 | 482.74 | 600.252400 | 0.727018 |  |
| GO:0030522\_intracellular\_receptor-mediated\_signaling\_pathway | 30 | 1 | 2.257353 | -0.442592 | 664 | 365.227600 | 482.74 | 600.252400 | 0.727018 |  |
| GO:0042552\_myelination | 30 | 1 | 2.257353 | -0.442592 | 664 | 365.227600 | 482.74 | 600.252400 | 0.727018 |  |
| GO:0048565\_gut\_development | 30 | 1 | 2.257353 | -0.442592 | 664 | 365.227600 | 482.74 | 600.252400 | 0.727018 |  |
| GO:0060021\_palate\_development | 30 | 1 | 2.257353 | -0.442592 | 664 | 365.227600 | 482.74 | 600.252400 | 0.727018 |  |
| GO:0010605\_negative\_regulation\_of\_macromolecule\_metabolic\_process | 331 | 6 | 1.227564 | -0.439777 | 665 | 365.611867 | 483.26 | 600.908133 | 0.726707 |  |
| GO:0043412\_biopolymer\_modification | 458 | 8 | 1.182892 | -0.439367 | 666 | 365.830550 | 483.58 | 601.329450 | 0.726096 |  |
| GO:0006897\_endocytosis | 86 | 2 | 1.574897 | -0.438828 | 668 | 367.076605 | 484.91 | 602.743395 | 0.725913 |  |
| GO:0010324\_membrane\_invagination | 86 | 2 | 1.574897 | -0.438828 | 668 | 367.076605 | 484.91 | 602.743395 | 0.725913 |  |
| GO:0045944\_positive\_regulation\_of\_transcription\_from\_RNA\_polymerase\_II\_promoter | 269 | 5 | 1.258747 | -0.437262 | 669 | 367.411979 | 485.28 | 603.148021 | 0.725381 |  |
| GO:0031324\_negative\_regulation\_of\_cellular\_metabolic\_process | 332 | 6 | 1.223866 | -0.436557 | 670 | 367.585666 | 485.47 | 603.354334 | 0.724582 |  |
| GO:0006812\_cation\_transport | 146 | 3 | 1.391519 | -0.436101 | 671 | 368.174057 | 486.11 | 604.045943 | 0.724456 |  |
| GO:0045934\_negative\_regulation\_of\_nucleobase\_\_nucleoside\_\_nucleotide\_and\_nucleic\_acid\_metabolic\_process | 270 | 5 | 1.254085 | -0.433717 | 672 | 368.564577 | 486.58 | 604.595423 | 0.724077 |  |
| GO:0022612\_gland\_morphogenesis | 87 | 2 | 1.556795 | -0.432489 | 674 | 370.355633 | 488.53 | 606.704367 | 0.724822 |  |
| GO:0050778\_positive\_regulation\_of\_immune\_response | 87 | 2 | 1.556795 | -0.432489 | 674 | 370.355633 | 488.53 | 606.704367 | 0.724822 |  |
| GO:0009888\_tissue\_development | 525 | 9 | 1.160924 | -0.431542 | 675 | 370.601830 | 488.83 | 607.058170 | 0.724193 |  |
| GO:0003018\_vascular\_process\_in\_circulatory\_system | 31 | 1 | 2.184535 | -0.431310 | 689 | 379.177870 | 498.07 | 616.962130 | 0.722888 |  |
| GO:0006639\_acylglycerol\_metabolic\_process | 31 | 1 | 2.184535 | -0.431310 | 689 | 379.177870 | 498.07 | 616.962130 | 0.722888 |  |
| GO:0006694\_steroid\_biosynthetic\_process | 31 | 1 | 2.184535 | -0.431310 | 689 | 379.177870 | 498.07 | 616.962130 | 0.722888 |  |
| GO:0006939\_smooth\_muscle\_contraction | 31 | 1 | 2.184535 | -0.431310 | 689 | 379.177870 | 498.07 | 616.962130 | 0.722888 |  |
| GO:0010562\_positive\_regulation\_of\_phosphorus\_metabolic\_process | 31 | 1 | 2.184535 | -0.431310 | 689 | 379.177870 | 498.07 | 616.962130 | 0.722888 |  |
| GO:0016049\_cell\_growth | 31 | 1 | 2.184535 | -0.431310 | 689 | 379.177870 | 498.07 | 616.962130 | 0.722888 |  |
| GO:0035150\_regulation\_of\_tube\_size | 31 | 1 | 2.184535 | -0.431310 | 689 | 379.177870 | 498.07 | 616.962130 | 0.722888 |  |
| GO:0042157\_lipoprotein\_metabolic\_process | 31 | 1 | 2.184535 | -0.431310 | 689 | 379.177870 | 498.07 | 616.962130 | 0.722888 |  |
| GO:0042327\_positive\_regulation\_of\_phosphorylation | 31 | 1 | 2.184535 | -0.431310 | 689 | 379.177870 | 498.07 | 616.962130 | 0.722888 |  |
| GO:0043269\_regulation\_of\_ion\_transport | 31 | 1 | 2.184535 | -0.431310 | 689 | 379.177870 | 498.07 | 616.962130 | 0.722888 |  |
| GO:0045937\_positive\_regulation\_of\_phosphate\_metabolic\_process | 31 | 1 | 2.184535 | -0.431310 | 689 | 379.177870 | 498.07 | 616.962130 | 0.722888 |  |
| GO:0048167\_regulation\_of\_synaptic\_plasticity | 31 | 1 | 2.184535 | -0.431310 | 689 | 379.177870 | 498.07 | 616.962130 | 0.722888 |  |
| GO:0048562\_embryonic\_organ\_morphogenesis | 31 | 1 | 2.184535 | -0.431310 | 689 | 379.177870 | 498.07 | 616.962130 | 0.722888 |  |
| GO:0050880\_regulation\_of\_blood\_vessel\_size | 31 | 1 | 2.184535 | -0.431310 | 689 | 379.177870 | 498.07 | 616.962130 | 0.722888 |  |
| GO:0051172\_negative\_regulation\_of\_nitrogen\_compound\_metabolic\_process | 271 | 5 | 1.249457 | -0.430200 | 690 | 379.562191 | 498.6 | 617.637809 | 0.722609 |  |
| GO:0048754\_branching\_morphogenesis\_of\_a\_tube | 88 | 2 | 1.539104 | -0.426260 | 691 | 380.955702 | 500.34 | 619.724298 | 0.724081 |  |
| GO:0002274\_myeloid\_leukocyte\_activation | 32 | 1 | 2.116268 | -0.420472 | 700 | 385.970655 | 506.01 | 626.049345 | 0.722871 |  |
| GO:0006638\_neutral\_lipid\_metabolic\_process | 32 | 1 | 2.116268 | -0.420472 | 700 | 385.970655 | 506.01 | 626.049345 | 0.722871 |  |
| GO:0006662\_glycerol\_ether\_metabolic\_process | 32 | 1 | 2.116268 | -0.420472 | 700 | 385.970655 | 506.01 | 626.049345 | 0.722871 |  |
| GO:0006937\_regulation\_of\_muscle\_contraction | 32 | 1 | 2.116268 | -0.420472 | 700 | 385.970655 | 506.01 | 626.049345 | 0.722871 |  |
| GO:0007272\_ensheathment\_of\_neurons | 32 | 1 | 2.116268 | -0.420472 | 700 | 385.970655 | 506.01 | 626.049345 | 0.722871 |  |
| GO:0008366\_axon\_ensheathment | 32 | 1 | 2.116268 | -0.420472 | 700 | 385.970655 | 506.01 | 626.049345 | 0.722871 |  |
| GO:0042102\_positive\_regulation\_of\_T\_cell\_proliferation | 32 | 1 | 2.116268 | -0.420472 | 700 | 385.970655 | 506.01 | 626.049345 | 0.722871 |  |
| GO:0050768\_negative\_regulation\_of\_neurogenesis | 32 | 1 | 2.116268 | -0.420472 | 700 | 385.970655 | 506.01 | 626.049345 | 0.722871 |  |
| GO:0050885\_neuromuscular\_process\_controlling\_balance | 32 | 1 | 2.116268 | -0.420472 | 700 | 385.970655 | 506.01 | 626.049345 | 0.722871 |  |
| GO:0010558\_negative\_regulation\_of\_macromolecule\_biosynthetic\_process | 274 | 5 | 1.235777 | -0.419814 | 701 | 386.331634 | 506.42 | 626.508366 | 0.722425 |  |
| GO:0035295\_tube\_development | 212 | 4 | 1.277747 | -0.417054 | 702 | 386.665598 | 506.85 | 627.034402 | 0.722009 |  |
| GO:0042113\_B\_cell\_activation | 90 | 2 | 1.504902 | -0.414124 | 703 | 387.865141 | 508.11 | 628.354859 | 0.722774 |  |
| GO:0050794\_regulation\_of\_cellular\_process | 2190 | 34 | 1.051370 | -0.411606 | 704 | 388.027468 | 508.29 | 628.552532 | 0.722003 |  |
| GO:0002562\_somatic\_diversification\_of\_immune\_receptors\_via\_germline\_recombination\_within\_a\_single\_locus | 33 | 1 | 2.052139 | -0.410051 | 706 | 392.441995 | 513.34 | 634.238005 | 0.727110 |  |
| GO:0016444\_somatic\_cell\_DNA\_recombination | 33 | 1 | 2.052139 | -0.410051 | 706 | 392.441995 | 513.34 | 634.238005 | 0.727110 |  |
| GO:0002443\_leukocyte\_mediated\_immunity | 91 | 2 | 1.488365 | -0.408212 | 708 | 393.463304 | 514.62 | 635.776696 | 0.726864 |  |
| GO:0031399\_regulation\_of\_protein\_modification\_process | 91 | 2 | 1.488365 | -0.408212 | 708 | 393.463304 | 514.62 | 635.776696 | 0.726864 |  |
| GO:0002200\_somatic\_diversification\_of\_immune\_receptors | 34 | 1 | 1.991782 | -0.400023 | 716 | 400.264348 | 522.05 | 643.835652 | 0.729120 |  |
| GO:0002699\_positive\_regulation\_of\_immune\_effector\_process | 34 | 1 | 1.991782 | -0.400023 | 716 | 400.264348 | 522.05 | 643.835652 | 0.729120 |  |
| GO:0007338\_single\_fertilization | 34 | 1 | 1.991782 | -0.400023 | 716 | 400.264348 | 522.05 | 643.835652 | 0.729120 |  |
| GO:0007568\_aging | 34 | 1 | 1.991782 | -0.400023 | 716 | 400.264348 | 522.05 | 643.835652 | 0.729120 |  |
| GO:0010721\_negative\_regulation\_of\_cell\_development | 34 | 1 | 1.991782 | -0.400023 | 716 | 400.264348 | 522.05 | 643.835652 | 0.729120 |  |
| GO:0030509\_BMP\_signaling\_pathway | 34 | 1 | 1.991782 | -0.400023 | 716 | 400.264348 | 522.05 | 643.835652 | 0.729120 |  |
| GO:0051052\_regulation\_of\_DNA\_metabolic\_process | 34 | 1 | 1.991782 | -0.400023 | 716 | 400.264348 | 522.05 | 643.835652 | 0.729120 |  |
| GO:0060711\_labyrinthine\_layer\_development | 34 | 1 | 1.991782 | -0.400023 | 716 | 400.264348 | 522.05 | 643.835652 | 0.729120 |  |
| GO:0007268\_synaptic\_transmission | 154 | 3 | 1.319232 | -0.399269 | 717 | 400.471649 | 522.28 | 644.088351 | 0.728424 |  |
| GO:0048583\_regulation\_of\_response\_to\_stimulus | 217 | 4 | 1.248306 | -0.398177 | 718 | 400.939800 | 522.75 | 644.560200 | 0.728064 |  |
| GO:0035107\_appendage\_morphogenesis | 93 | 2 | 1.456357 | -0.396688 | 720 | 402.215555 | 524.02 | 645.824445 | 0.727806 |  |
| GO:0035108\_limb\_morphogenesis | 93 | 2 | 1.456357 | -0.396688 | 720 | 402.215555 | 524.02 | 645.824445 | 0.727806 |  |
| GO:0031327\_negative\_regulation\_of\_cellular\_biosynthetic\_process | 282 | 5 | 1.200720 | -0.393279 | 721 | 403.033385 | 524.96 | 646.886615 | 0.728100 |  |
| GO:0001756\_somitogenesis | 35 | 1 | 1.934874 | -0.390362 | 731 | 408.866495 | 531.18 | 653.493505 | 0.726648 |  |
| GO:0006869\_lipid\_transport | 35 | 1 | 1.934874 | -0.390362 | 731 | 408.866495 | 531.18 | 653.493505 | 0.726648 |  |
| GO:0010810\_regulation\_of\_cell-substrate\_adhesion | 35 | 1 | 1.934874 | -0.390362 | 731 | 408.866495 | 531.18 | 653.493505 | 0.726648 |  |
| GO:0016051\_carbohydrate\_biosynthetic\_process | 35 | 1 | 1.934874 | -0.390362 | 731 | 408.866495 | 531.18 | 653.493505 | 0.726648 |  |
| GO:0018904\_organic\_ether\_metabolic\_process | 35 | 1 | 1.934874 | -0.390362 | 731 | 408.866495 | 531.18 | 653.493505 | 0.726648 |  |
| GO:0043406\_positive\_regulation\_of\_MAP\_kinase\_activity | 35 | 1 | 1.934874 | -0.390362 | 731 | 408.866495 | 531.18 | 653.493505 | 0.726648 |  |
| GO:0044242\_cellular\_lipid\_catabolic\_process | 35 | 1 | 1.934874 | -0.390362 | 731 | 408.866495 | 531.18 | 653.493505 | 0.726648 |  |
| GO:0051051\_negative\_regulation\_of\_transport | 35 | 1 | 1.934874 | -0.390362 | 731 | 408.866495 | 531.18 | 653.493505 | 0.726648 |  |
| GO:0051325\_interphase | 35 | 1 | 1.934874 | -0.390362 | 731 | 408.866495 | 531.18 | 653.493505 | 0.726648 |  |
| GO:0051329\_interphase\_of\_mitotic\_cell\_cycle | 35 | 1 | 1.934874 | -0.390362 | 731 | 408.866495 | 531.18 | 653.493505 | 0.726648 |  |
| GO:0009892\_negative\_regulation\_of\_metabolic\_process | 348 | 6 | 1.167596 | -0.387914 | 732 | 409.470314 | 531.91 | 654.349686 | 0.726653 |  |
| GO:0009890\_negative\_regulation\_of\_biosynthetic\_process | 284 | 5 | 1.192264 | -0.386900 | 733 | 409.758905 | 532.24 | 654.721095 | 0.726112 |  |
| GO:0042391\_regulation\_of\_membrane\_potential | 95 | 2 | 1.425697 | -0.385548 | 734 | 410.453055 | 532.99 | 655.526945 | 0.726144 |  |
| GO:0019228\_regulation\_of\_action\_potential\_in\_neuron | 36 | 1 | 1.881127 | -0.381049 | 735 | 416.703101 | 540.38 | 664.056899 | 0.735211 |  |
| GO:0006954\_inflammatory\_response | 96 | 2 | 1.410846 | -0.380117 | 738 | 417.990729 | 541.86 | 665.729271 | 0.734228 |  |
| GO:0048736\_appendage\_development | 96 | 2 | 1.410846 | -0.380117 | 738 | 417.990729 | 541.86 | 665.729271 | 0.734228 |  |
| GO:0060173\_limb\_development | 96 | 2 | 1.410846 | -0.380117 | 738 | 417.990729 | 541.86 | 665.729271 | 0.734228 |  |
| GO:0002790\_peptide\_secretion | 37 | 1 | 1.830286 | -0.372063 | 741 | 422.988024 | 547.42 | 671.851976 | 0.738758 |  |
| GO:0045785\_positive\_regulation\_of\_cell\_adhesion | 37 | 1 | 1.830286 | -0.372063 | 741 | 422.988024 | 547.42 | 671.851976 | 0.738758 |  |
| GO:0051101\_regulation\_of\_DNA\_binding | 37 | 1 | 1.830286 | -0.372063 | 741 | 422.988024 | 547.42 | 671.851976 | 0.738758 |  |
| GO:0060541\_respiratory\_system\_development | 98 | 2 | 1.382053 | -0.369522 | 742 | 424.116977 | 548.67 | 673.223023 | 0.739447 |  |
| GO:0009628\_response\_to\_abiotic\_stimulus | 162 | 3 | 1.254085 | -0.365742 | 743 | 424.623869 | 549.19 | 673.756131 | 0.739152 |  |
| GO:0060562\_epithelial\_tube\_morphogenesis | 99 | 2 | 1.368093 | -0.364354 | 744 | 425.542227 | 550.32 | 675.097773 | 0.739677 |  |
| GO:0007596\_blood\_coagulation | 38 | 1 | 1.782121 | -0.363388 | 749 | 431.693417 | 557.13 | 682.566583 | 0.743832 |  |
| GO:0016042\_lipid\_catabolic\_process | 38 | 1 | 1.782121 | -0.363388 | 749 | 431.693417 | 557.13 | 682.566583 | 0.743832 |  |
| GO:0031401\_positive\_regulation\_of\_protein\_modification\_process | 38 | 1 | 1.782121 | -0.363388 | 749 | 431.693417 | 557.13 | 682.566583 | 0.743832 |  |
| GO:0045580\_regulation\_of\_T\_cell\_differentiation | 38 | 1 | 1.782121 | -0.363388 | 749 | 431.693417 | 557.13 | 682.566583 | 0.743832 |  |
| GO:0046777\_protein\_amino\_acid\_autophosphorylation | 38 | 1 | 1.782121 | -0.363388 | 749 | 431.693417 | 557.13 | 682.566583 | 0.743832 |  |
| GO:0042325\_regulation\_of\_phosphorylation | 164 | 3 | 1.238791 | -0.357834 | 750 | 433.547667 | 559.13 | 684.712333 | 0.745507 |  |
| GO:0007167\_enzyme\_linked\_receptor\_protein\_signaling\_pathway | 229 | 4 | 1.182892 | -0.356278 | 751 | 433.774020 | 559.33 | 684.885980 | 0.744780 |  |
| GO:0006511\_ubiquitin-dependent\_protein\_catabolic\_process | 39 | 1 | 1.736425 | -0.355006 | 753 | 438.540640 | 564.23 | 689.919360 | 0.749309 |  |
| GO:0007160\_cell-matrix\_adhesion | 39 | 1 | 1.736425 | -0.355006 | 753 | 438.540640 | 564.23 | 689.919360 | 0.749309 |  |
| GO:0019220\_regulation\_of\_phosphate\_metabolic\_process | 165 | 3 | 1.231283 | -0.353948 | 755 | 439.663802 | 565.49 | 691.316198 | 0.748993 |  |
| GO:0051174\_regulation\_of\_phosphorus\_metabolic\_process | 165 | 3 | 1.231283 | -0.353948 | 755 | 439.663802 | 565.49 | 691.316198 | 0.748993 |  |
| GO:0007599\_hemostasis | 40 | 1 | 1.693015 | -0.346903 | 758 | 446.059434 | 572.39 | 698.720566 | 0.755132 |  |
| GO:0008203\_cholesterol\_metabolic\_process | 40 | 1 | 1.693015 | -0.346903 | 758 | 446.059434 | 572.39 | 698.720566 | 0.755132 |  |
| GO:0016358\_dendrite\_development | 40 | 1 | 1.693015 | -0.346903 | 758 | 446.059434 | 572.39 | 698.720566 | 0.755132 |  |
| GO:0006836\_neurotransmitter\_transport | 41 | 1 | 1.651722 | -0.339063 | 767 | 454.923463 | 582.55 | 710.176537 | 0.759518 |  |
| GO:0007254\_JNK\_cascade | 41 | 1 | 1.651722 | -0.339063 | 767 | 454.923463 | 582.55 | 710.176537 | 0.759518 |  |
| GO:0010551\_regulation\_of\_specific\_transcription\_from\_RNA\_polymerase\_II\_promoter | 41 | 1 | 1.651722 | -0.339063 | 767 | 454.923463 | 582.55 | 710.176537 | 0.759518 |  |
| GO:0015833\_peptide\_transport | 41 | 1 | 1.651722 | -0.339063 | 767 | 454.923463 | 582.55 | 710.176537 | 0.759518 |  |
| GO:0019216\_regulation\_of\_lipid\_metabolic\_process | 41 | 1 | 1.651722 | -0.339063 | 767 | 454.923463 | 582.55 | 710.176537 | 0.759518 |  |
| GO:0032569\_specific\_transcription\_from\_RNA\_polymerase\_II\_promoter | 41 | 1 | 1.651722 | -0.339063 | 767 | 454.923463 | 582.55 | 710.176537 | 0.759518 |  |
| GO:0032844\_regulation\_of\_homeostatic\_process | 41 | 1 | 1.651722 | -0.339063 | 767 | 454.923463 | 582.55 | 710.176537 | 0.759518 |  |
| GO:0033077\_T\_cell\_differentiation\_in\_the\_thymus | 41 | 1 | 1.651722 | -0.339063 | 767 | 454.923463 | 582.55 | 710.176537 | 0.759518 |  |
| GO:0050864\_regulation\_of\_B\_cell\_activation | 41 | 1 | 1.651722 | -0.339063 | 767 | 454.923463 | 582.55 | 710.176537 | 0.759518 |  |
| GO:0044238\_primary\_metabolic\_process | 1905 | 29 | 1.030917 | -0.336523 | 768 | 455.349804 | 582.96 | 710.570196 | 0.759063 |  |
| GO:0001501\_skeletal\_system\_development | 236 | 4 | 1.147807 | -0.333883 | 769 | 456.742099 | 584.41 | 712.077901 | 0.759961 |  |
| GO:0006006\_glucose\_metabolic\_process | 42 | 1 | 1.612395 | -0.331475 | 780 | 463.559659 | 591.35 | 719.140341 | 0.758141 |  |
| GO:0008361\_regulation\_of\_cell\_size | 42 | 1 | 1.612395 | -0.331475 | 780 | 463.559659 | 591.35 | 719.140341 | 0.758141 |  |
| GO:0015672\_monovalent\_inorganic\_cation\_transport | 42 | 1 | 1.612395 | -0.331475 | 780 | 463.559659 | 591.35 | 719.140341 | 0.758141 |  |
| GO:0016125\_sterol\_metabolic\_process | 42 | 1 | 1.612395 | -0.331475 | 780 | 463.559659 | 591.35 | 719.140341 | 0.758141 |  |
| GO:0019941\_modification-dependent\_protein\_catabolic\_process | 42 | 1 | 1.612395 | -0.331475 | 780 | 463.559659 | 591.35 | 719.140341 | 0.758141 |  |
| GO:0032946\_positive\_regulation\_of\_mononuclear\_cell\_proliferation | 42 | 1 | 1.612395 | -0.331475 | 780 | 463.559659 | 591.35 | 719.140341 | 0.758141 |  |
| GO:0043632\_modification-dependent\_macromolecule\_catabolic\_process | 42 | 1 | 1.612395 | -0.331475 | 780 | 463.559659 | 591.35 | 719.140341 | 0.758141 |  |
| GO:0050671\_positive\_regulation\_of\_lymphocyte\_proliferation | 42 | 1 | 1.612395 | -0.331475 | 780 | 463.559659 | 591.35 | 719.140341 | 0.758141 |  |
| GO:0050817\_coagulation | 42 | 1 | 1.612395 | -0.331475 | 780 | 463.559659 | 591.35 | 719.140341 | 0.758141 |  |
| GO:0051603\_proteolysis\_involved\_in\_cellular\_protein\_catabolic\_process | 42 | 1 | 1.612395 | -0.331475 | 780 | 463.559659 | 591.35 | 719.140341 | 0.758141 |  |
| GO:0080135\_regulation\_of\_cellular\_response\_to\_stress | 42 | 1 | 1.612395 | -0.331475 | 780 | 463.559659 | 591.35 | 719.140341 | 0.758141 |  |
| GO:0010817\_regulation\_of\_hormone\_levels | 106 | 2 | 1.277747 | -0.330439 | 781 | 464.137155 | 592.01 | 719.882845 | 0.758015 |  |
| GO:0051641\_cellular\_localization | 370 | 6 | 1.098172 | -0.329141 | 782 | 464.336387 | 592.29 | 720.243613 | 0.757404 |  |
| GO:0009611\_response\_to\_wounding | 172 | 3 | 1.181173 | -0.327950 | 783 | 464.640918 | 592.63 | 720.619082 | 0.756871 |  |
| GO:0045859\_regulation\_of\_protein\_kinase\_activity | 107 | 2 | 1.265805 | -0.325897 | 784 | 465.522561 | 593.57 | 721.617439 | 0.757105 |  |
| GO:0050896\_response\_to\_stimulus | 1107 | 17 | 1.039973 | -0.325225 | 785 | 465.671724 | 593.68 | 721.688276 | 0.756280 |  |
| GO:0001508\_regulation\_of\_action\_potential | 43 | 1 | 1.574897 | -0.324125 | 789 | 471.870734 | 600.16 | 728.449266 | 0.760659 |  |
| GO:0002819\_regulation\_of\_adaptive\_immune\_response | 43 | 1 | 1.574897 | -0.324125 | 789 | 471.870734 | 600.16 | 728.449266 | 0.760659 |  |
| GO:0002822\_regulation\_of\_adaptive\_immune\_response\_based\_on\_somatic\_recombination\_of\_immune\_receptors\_built\_from\_immunoglobulin\_superfamily\_domains | 43 | 1 | 1.574897 | -0.324125 | 789 | 471.870734 | 600.16 | 728.449266 | 0.760659 |  |
| GO:0031098\_stress-activated\_protein\_kinase\_signaling\_pathway | 43 | 1 | 1.574897 | -0.324125 | 789 | 471.870734 | 600.16 | 728.449266 | 0.760659 |  |
| GO:0000122\_negative\_regulation\_of\_transcription\_from\_RNA\_polymerase\_II\_promoter | 175 | 3 | 1.160924 | -0.317424 | 791 | 474.702341 | 602.95 | 731.197659 | 0.762263 |  |
| GO:0046903\_secretion | 175 | 3 | 1.160924 | -0.317424 | 791 | 474.702341 | 602.95 | 731.197659 | 0.762263 |  |
| GO:0002377\_immunoglobulin\_production | 44 | 1 | 1.539104 | -0.317003 | 801 | 480.603632 | 610.07 | 739.536368 | 0.761635 |  |
| GO:0006606\_protein\_import\_into\_nucleus | 44 | 1 | 1.539104 | -0.317003 | 801 | 480.603632 | 610.07 | 739.536368 | 0.761635 |  |
| GO:0016064\_immunoglobulin\_mediated\_immune\_response | 44 | 1 | 1.539104 | -0.317003 | 801 | 480.603632 | 610.07 | 739.536368 | 0.761635 |  |
| GO:0035282\_segmentation | 44 | 1 | 1.539104 | -0.317003 | 801 | 480.603632 | 610.07 | 739.536368 | 0.761635 |  |
| GO:0044257\_cellular\_protein\_catabolic\_process | 44 | 1 | 1.539104 | -0.317003 | 801 | 480.603632 | 610.07 | 739.536368 | 0.761635 |  |
| GO:0048593\_camera-type\_eye\_morphogenesis | 44 | 1 | 1.539104 | -0.317003 | 801 | 480.603632 | 610.07 | 739.536368 | 0.761635 |  |
| GO:0050808\_synapse\_organization | 44 | 1 | 1.539104 | -0.317003 | 801 | 480.603632 | 610.07 | 739.536368 | 0.761635 |  |
| GO:0051170\_nuclear\_import | 44 | 1 | 1.539104 | -0.317003 | 801 | 480.603632 | 610.07 | 739.536368 | 0.761635 |  |
| GO:0070665\_positive\_regulation\_of\_leukocyte\_proliferation | 44 | 1 | 1.539104 | -0.317003 | 801 | 480.603632 | 610.07 | 739.536368 | 0.761635 |  |
| GO:0070668\_positive\_regulation\_of\_mast\_cell\_proliferation | 44 | 1 | 1.539104 | -0.317003 | 801 | 480.603632 | 610.07 | 739.536368 | 0.761635 |  |
| GO:0032870\_cellular\_response\_to\_hormone\_stimulus | 45 | 1 | 1.504902 | -0.310098 | 802 | 486.046256 | 615.55 | 745.053744 | 0.767519 |  |
| GO:0048705\_skeletal\_system\_morphogenesis | 111 | 2 | 1.220191 | -0.308424 | 803 | 486.479978 | 615.97 | 745.460022 | 0.767086 |  |
| GO:0043549\_regulation\_of\_kinase\_activity | 112 | 2 | 1.209296 | -0.304223 | 804 | 487.155455 | 616.72 | 746.284545 | 0.767065 |  |
| GO:0007612\_learning | 46 | 1 | 1.472187 | -0.303401 | 809 | 491.264915 | 621.06 | 750.855085 | 0.767689 |  |
| GO:0008217\_regulation\_of\_blood\_pressure | 46 | 1 | 1.472187 | -0.303401 | 809 | 491.264915 | 621.06 | 750.855085 | 0.767689 |  |
| GO:0019724\_B\_cell\_mediated\_immunity | 46 | 1 | 1.472187 | -0.303401 | 809 | 491.264915 | 621.06 | 750.855085 | 0.767689 |  |
| GO:0046631\_alpha-beta\_T\_cell\_activation | 46 | 1 | 1.472187 | -0.303401 | 809 | 491.264915 | 621.06 | 750.855085 | 0.767689 |  |
| GO:0051098\_regulation\_of\_binding | 46 | 1 | 1.472187 | -0.303401 | 809 | 491.264915 | 621.06 | 750.855085 | 0.767689 |  |
| GO:0040008\_regulation\_of\_growth | 113 | 2 | 1.198594 | -0.300086 | 810 | 492.580400 | 622.35 | 752.119600 | 0.768333 |  |
| GO:0045321\_leukocyte\_activation | 248 | 4 | 1.092268 | -0.298655 | 811 | 493.237744 | 623.02 | 752.802256 | 0.768212 |  |
| GO:0045087\_innate\_immune\_response | 47 | 1 | 1.440864 | -0.296901 | 813 | 497.796230 | 628.09 | 758.383770 | 0.772558 |  |
| GO:0045619\_regulation\_of\_lymphocyte\_differentiation | 47 | 1 | 1.440864 | -0.296901 | 813 | 497.796230 | 628.09 | 758.383770 | 0.772558 |  |
| GO:0019538\_protein\_metabolic\_process | 655 | 10 | 1.033902 | -0.294457 | 814 | 499.064898 | 629.3 | 759.535102 | 0.773096 |  |
| GO:0051338\_regulation\_of\_transferase\_activity | 115 | 2 | 1.177749 | -0.292001 | 815 | 500.118461 | 630.48 | 760.841539 | 0.773595 |  |
| GO:0001505\_regulation\_of\_neurotransmitter\_levels | 48 | 1 | 1.410846 | -0.290591 | 819 | 502.705192 | 633.25 | 763.794808 | 0.773199 |  |
| GO:0019318\_hexose\_metabolic\_process | 48 | 1 | 1.410846 | -0.290591 | 819 | 502.705192 | 633.25 | 763.794808 | 0.773199 |  |
| GO:0032269\_negative\_regulation\_of\_cellular\_protein\_metabolic\_process | 48 | 1 | 1.410846 | -0.290591 | 819 | 502.705192 | 633.25 | 763.794808 | 0.773199 |  |
| GO:0034504\_protein\_localization\_in\_nucleus | 48 | 1 | 1.410846 | -0.290591 | 819 | 502.705192 | 633.25 | 763.794808 | 0.773199 |  |
| GO:0048608\_reproductive\_structure\_development | 116 | 2 | 1.167596 | -0.288050 | 820 | 504.265124 | 634.78 | 765.294876 | 0.774122 |  |
| GO:0002440\_production\_of\_molecular\_mediator\_of\_immune\_response | 49 | 1 | 1.382053 | -0.284461 | 822 | 510.095458 | 640.9 | 771.704542 | 0.779684 |  |
| GO:0007606\_sensory\_perception\_of\_chemical\_stimulus | 49 | 1 | 1.382053 | -0.284461 | 822 | 510.095458 | 640.9 | 771.704542 | 0.779684 |  |
| GO:0050877\_neurological\_system\_process | 390 | 6 | 1.041855 | -0.282871 | 823 | 510.715074 | 641.5 | 772.284926 | 0.779465 |  |
| GO:0006811\_ion\_transport | 186 | 3 | 1.092268 | -0.281713 | 824 | 512.408904 | 643.04 | 773.671096 | 0.780388 |  |
| GO:0017038\_protein\_import | 50 | 1 | 1.354412 | -0.278506 | 826 | 516.458183 | 647.45 | 778.441817 | 0.783838 |  |
| GO:0042129\_regulation\_of\_T\_cell\_proliferation | 50 | 1 | 1.354412 | -0.278506 | 826 | 516.458183 | 647.45 | 778.441817 | 0.783838 |  |
| GO:0042127\_regulation\_of\_cell\_proliferation | 393 | 6 | 1.033902 | -0.276463 | 827 | 517.345625 | 648.37 | 779.394375 | 0.784002 |  |
| GO:0007276\_gamete\_generation | 188 | 3 | 1.080648 | -0.275675 | 828 | 517.714334 | 648.71 | 779.705666 | 0.783466 |  |
| GO:0006887\_exocytosis | 51 | 1 | 1.327855 | -0.272716 | 831 | 521.981658 | 653.06 | 784.138342 | 0.785872 |  |
| GO:0007601\_visual\_perception | 51 | 1 | 1.327855 | -0.272716 | 831 | 521.981658 | 653.06 | 784.138342 | 0.785872 |  |
| GO:0032583\_regulation\_of\_gene-specific\_transcription | 51 | 1 | 1.327855 | -0.272716 | 831 | 521.981658 | 653.06 | 784.138342 | 0.785872 |  |
| GO:0051726\_regulation\_of\_cell\_cycle | 121 | 2 | 1.119349 | -0.269166 | 832 | 523.393632 | 654.6 | 785.806368 | 0.786779 |  |
| GO:0002706\_regulation\_of\_lymphocyte\_mediated\_immunity | 52 | 1 | 1.302319 | -0.267087 | 835 | 525.796039 | 656.84 | 787.883961 | 0.786635 |  |
| GO:0048585\_negative\_regulation\_of\_response\_to\_stimulus | 52 | 1 | 1.302319 | -0.267087 | 835 | 525.796039 | 656.84 | 787.883961 | 0.786635 |  |
| GO:0050953\_sensory\_perception\_of\_light\_stimulus | 52 | 1 | 1.302319 | -0.267087 | 835 | 525.796039 | 656.84 | 787.883961 | 0.786635 |  |
| GO:0002252\_immune\_effector\_process | 122 | 2 | 1.110174 | -0.265556 | 836 | 527.127815 | 658.41 | 789.692185 | 0.787572 |  |
| GO:0001775\_cell\_activation | 262 | 4 | 1.033902 | -0.262100 | 837 | 529.222450 | 660.6 | 791.977550 | 0.789247 |  |
| GO:0030031\_cell\_projection\_assembly | 53 | 1 | 1.277747 | -0.261610 | 840 | 532.175612 | 663.69 | 795.204388 | 0.790107 |  |
| GO:0050905\_neuromuscular\_process | 53 | 1 | 1.277747 | -0.261610 | 840 | 532.175612 | 663.69 | 795.204388 | 0.790107 |  |
| GO:0051248\_negative\_regulation\_of\_protein\_metabolic\_process | 53 | 1 | 1.277747 | -0.261610 | 840 | 532.175612 | 663.69 | 795.204388 | 0.790107 |  |
| GO:0030098\_lymphocyte\_differentiation | 124 | 2 | 1.092268 | -0.258495 | 841 | 533.301270 | 664.89 | 796.478730 | 0.790595 |  |
| GO:0043405\_regulation\_of\_MAP\_kinase\_activity | 54 | 1 | 1.254085 | -0.256281 | 842 | 537.263849 | 668.91 | 800.556151 | 0.794430 |  |
| GO:0001763\_morphogenesis\_of\_a\_branching\_structure | 125 | 2 | 1.083529 | -0.255042 | 843 | 538.808639 | 670.39 | 801.971361 | 0.795243 |  |
| GO:0006310\_DNA\_recombination | 55 | 1 | 1.231283 | -0.251094 | 846 | 541.555293 | 673.02 | 804.484707 | 0.795532 |  |
| GO:0043434\_response\_to\_peptide\_hormone\_stimulus | 55 | 1 | 1.231283 | -0.251094 | 846 | 541.555293 | 673.02 | 804.484707 | 0.795532 |  |
| GO:0048568\_embryonic\_organ\_development | 55 | 1 | 1.231283 | -0.251094 | 846 | 541.555293 | 673.02 | 804.484707 | 0.795532 |  |
| GO:0044237\_cellular\_metabolic\_process | 1974 | 29 | 0.994882 | -0.250739 | 847 | 541.675366 | 673.1 | 804.524634 | 0.794687 |  |
| GO:0008283\_cell\_proliferation | 544 | 8 | 0.995891 | -0.250457 | 848 | 541.744786 | 673.17 | 804.595214 | 0.793833 |  |
| GO:0002009\_morphogenesis\_of\_an\_epithelium | 198 | 3 | 1.026070 | -0.247394 | 850 | 542.692182 | 674.29 | 805.887818 | 0.793282 |  |
| GO:0060429\_epithelium\_development | 198 | 3 | 1.026070 | -0.247394 | 850 | 542.692182 | 674.29 | 805.887818 | 0.793282 |  |
| GO:0002703\_regulation\_of\_leukocyte\_mediated\_immunity | 56 | 1 | 1.209296 | -0.246043 | 853 | 546.516729 | 678.47 | 810.423271 | 0.795393 |  |
| GO:0006790\_sulfur\_metabolic\_process | 56 | 1 | 1.209296 | -0.246043 | 853 | 546.516729 | 678.47 | 810.423271 | 0.795393 |  |
| GO:0046486\_glycerolipid\_metabolic\_process | 56 | 1 | 1.209296 | -0.246043 | 853 | 546.516729 | 678.47 | 810.423271 | 0.795393 |  |
| GO:0051276\_chromosome\_organization | 129 | 2 | 1.049932 | -0.241724 | 854 | 549.591958 | 681.69 | 813.788042 | 0.798232 |  |
| GO:0001892\_embryonic\_placenta\_development | 57 | 1 | 1.188080 | -0.241123 | 860 | 554.176416 | 686.24 | 818.303584 | 0.797953 |  |
| GO:0008344\_adult\_locomotory\_behavior | 57 | 1 | 1.188080 | -0.241123 | 860 | 554.176416 | 686.24 | 818.303584 | 0.797953 |  |
| GO:0033365\_protein\_localization\_in\_organelle | 57 | 1 | 1.188080 | -0.241123 | 860 | 554.176416 | 686.24 | 818.303584 | 0.797953 |  |
| GO:0042472\_inner\_ear\_morphogenesis | 57 | 1 | 1.188080 | -0.241123 | 860 | 554.176416 | 686.24 | 818.303584 | 0.797953 |  |
| GO:0045444\_fat\_cell\_differentiation | 57 | 1 | 1.188080 | -0.241123 | 860 | 554.176416 | 686.24 | 818.303584 | 0.797953 |  |
| GO:0050878\_regulation\_of\_body\_fluid\_levels | 57 | 1 | 1.188080 | -0.241123 | 860 | 554.176416 | 686.24 | 818.303584 | 0.797953 |  |
| GO:0051649\_establishment\_of\_localization\_in\_cell | 342 | 5 | 0.990067 | -0.239094 | 861 | 555.212600 | 687.19 | 819.167400 | 0.798130 |  |
| GO:0050776\_regulation\_of\_immune\_response | 130 | 2 | 1.041855 | -0.238514 | 862 | 555.877522 | 687.99 | 820.102478 | 0.798132 |  |
| GO:0050804\_regulation\_of\_synaptic\_transmission | 58 | 1 | 1.167596 | -0.236329 | 863 | 557.984929 | 689.9 | 821.815071 | 0.799421 |  |
| GO:0016055\_Wnt\_receptor\_signaling\_pathway | 59 | 1 | 1.147807 | -0.231658 | 866 | 561.340249 | 693.26 | 825.179751 | 0.800531 |  |
| GO:0048469\_cell\_maturation | 59 | 1 | 1.147807 | -0.231658 | 866 | 561.340249 | 693.26 | 825.179751 | 0.800531 |  |
| GO:0050870\_positive\_regulation\_of\_T\_cell\_activation | 59 | 1 | 1.147807 | -0.231658 | 866 | 561.340249 | 693.26 | 825.179751 | 0.800531 |  |
| GO:0006955\_immune\_response | 205 | 3 | 0.991033 | -0.229352 | 867 | 562.097362 | 694.06 | 826.022638 | 0.800531 |  |
| GO:0009952\_anterior\_posterior\_pattern\_formation | 133 | 2 | 1.018355 | -0.229157 | 868 | 562.620875 | 694.61 | 826.599125 | 0.800242 |  |
| GO:0048646\_anatomical\_structure\_formation\_involved\_in\_morphogenesis | 277 | 4 | 0.977915 | -0.227709 | 869 | 562.961384 | 694.96 | 826.958616 | 0.799724 |  |
| GO:0008152\_metabolic\_process | 2133 | 31 | 0.984219 | -0.225251 | 870 | 564.601026 | 696.52 | 828.438974 | 0.800598 |  |
| GO:0032270\_positive\_regulation\_of\_cellular\_protein\_metabolic\_process | 61 | 1 | 1.110174 | -0.222663 | 872 | 566.988323 | 698.96 | 830.931677 | 0.801560 |  |
| GO:0051969\_regulation\_of\_transmission\_of\_nerve\_impulse | 61 | 1 | 1.110174 | -0.222663 | 872 | 566.988323 | 698.96 | 830.931677 | 0.801560 |  |
| GO:0008284\_positive\_regulation\_of\_cell\_proliferation | 208 | 3 | 0.976739 | -0.222028 | 873 | 567.473139 | 699.41 | 831.346861 | 0.801157 |  |
| GO:0006916\_anti-apoptosis | 62 | 1 | 1.092268 | -0.218331 | 877 | 571.760455 | 703.83 | 835.899545 | 0.802543 |  |
| GO:0030155\_regulation\_of\_cell\_adhesion | 62 | 1 | 1.092268 | -0.218331 | 877 | 571.760455 | 703.83 | 835.899545 | 0.802543 |  |
| GO:0032944\_regulation\_of\_mononuclear\_cell\_proliferation | 62 | 1 | 1.092268 | -0.218331 | 877 | 571.760455 | 703.83 | 835.899545 | 0.802543 |  |
| GO:0050670\_regulation\_of\_lymphocyte\_proliferation | 62 | 1 | 1.092268 | -0.218331 | 877 | 571.760455 | 703.83 | 835.899545 | 0.802543 |  |
| GO:0070662\_mast\_cell\_proliferation | 63 | 1 | 1.074930 | -0.214106 | 879 | 575.279226 | 707.3 | 839.320774 | 0.804664 |  |
| GO:0070666\_regulation\_of\_mast\_cell\_proliferation | 63 | 1 | 1.074930 | -0.214106 | 879 | 575.279226 | 707.3 | 839.320774 | 0.804664 |  |
| GO:0009790\_embryonic\_development | 567 | 8 | 0.955493 | -0.213888 | 880 | 575.508103 | 707.56 | 839.611897 | 0.804045 |  |
| GO:0031644\_regulation\_of\_neurological\_system\_process | 64 | 1 | 1.058134 | -0.209982 | 883 | 579.344747 | 711.22 | 843.095253 | 0.805459 |  |
| GO:0042060\_wound\_healing | 64 | 1 | 1.058134 | -0.209982 | 883 | 579.344747 | 711.22 | 843.095253 | 0.805459 |  |
| GO:0070663\_regulation\_of\_leukocyte\_proliferation | 64 | 1 | 1.058134 | -0.209982 | 883 | 579.344747 | 711.22 | 843.095253 | 0.805459 |  |
| GO:0016044\_membrane\_organization | 140 | 2 | 0.967437 | -0.208825 | 884 | 579.600578 | 711.47 | 843.339422 | 0.804830 |  |
| GO:0042471\_ear\_morphogenesis | 65 | 1 | 1.041855 | -0.205957 | 885 | 582.075069 | 713.94 | 845.804931 | 0.806712 |  |
| GO:0010033\_response\_to\_organic\_substance | 216 | 3 | 0.940564 | -0.203612 | 886 | 582.705397 | 714.49 | 846.274603 | 0.806422 |  |
| GO:0006810\_transport | 718 | 10 | 0.943184 | -0.199166 | 887 | 587.114146 | 718.86 | 850.605854 | 0.810440 |  |
| GO:0051247\_positive\_regulation\_of\_protein\_metabolic\_process | 67 | 1 | 1.010755 | -0.198191 | 888 | 589.124113 | 720.86 | 852.595887 | 0.811779 |  |
| GO:0007186\_G-protein\_coupled\_receptor\_protein\_signaling\_pathway | 144 | 2 | 0.940564 | -0.198078 | 889 | 589.790571 | 721.42 | 853.049429 | 0.811496 |  |
| GO:0043009\_chordate\_embryonic\_development | 365 | 5 | 0.927679 | -0.196595 | 890 | 590.810201 | 722.41 | 854.009799 | 0.811697 |  |
| GO:0002697\_regulation\_of\_immune\_effector\_process | 68 | 1 | 0.995891 | -0.194444 | 892 | 592.768349 | 724.31 | 855.851651 | 0.812007 |  |
| GO:0034962\_cellular\_biopolymer\_catabolic\_process | 68 | 1 | 0.995891 | -0.194444 | 892 | 592.768349 | 724.31 | 855.851651 | 0.812007 |  |
| GO:0001701\_in\_utero\_embryonic\_development | 221 | 3 | 0.919284 | -0.192878 | 893 | 593.968154 | 725.65 | 857.331846 | 0.812598 |  |
| GO:0009792\_embryonic\_development\_ending\_in\_birth\_or\_egg\_hatching | 368 | 5 | 0.920117 | -0.191594 | 894 | 594.697880 | 726.44 | 858.182120 | 0.812573 |  |
| GO:0005996\_monosaccharide\_metabolic\_process | 69 | 1 | 0.981458 | -0.190784 | 896 | 596.598540 | 728.32 | 860.041460 | 0.812857 |  |
| GO:0006816\_calcium\_ion\_transport | 69 | 1 | 0.981458 | -0.190784 | 896 | 596.598540 | 728.32 | 860.041460 | 0.812857 |  |
| GO:0002684\_positive\_regulation\_of\_immune\_system\_process | 148 | 2 | 0.915143 | -0.187915 | 898 | 598.048442 | 729.75 | 861.451558 | 0.812639 |  |
| GO:0043085\_positive\_regulation\_of\_catalytic\_activity | 148 | 2 | 0.915143 | -0.187915 | 898 | 598.048442 | 729.75 | 861.451558 | 0.812639 |  |
| GO:0007611\_learning\_or\_memory | 70 | 1 | 0.967437 | -0.187207 | 901 | 599.950872 | 731.68 | 863.409128 | 0.812075 |  |
| GO:0048592\_eye\_morphogenesis | 70 | 1 | 0.967437 | -0.187207 | 901 | 599.950872 | 731.68 | 863.409128 | 0.812075 |  |
| GO:0070838\_divalent\_metal\_ion\_transport | 70 | 1 | 0.967437 | -0.187207 | 901 | 599.950872 | 731.68 | 863.409128 | 0.812075 |  |
| GO:0051234\_establishment\_of\_localization | 729 | 10 | 0.928952 | -0.185532 | 902 | 600.764959 | 732.65 | 864.535041 | 0.812251 |  |
| GO:0032940\_secretion\_by\_cell | 149 | 2 | 0.909001 | -0.185461 | 903 | 601.078445 | 732.92 | 864.761555 | 0.811650 |  |
| GO:0048598\_embryonic\_morphogenesis | 299 | 4 | 0.905961 | -0.184923 | 904 | 601.707585 | 733.48 | 865.252415 | 0.811372 |  |
| GO:0006281\_DNA\_repair | 71 | 1 | 0.953811 | -0.183712 | 907 | 603.583464 | 735.42 | 867.256536 | 0.810827 |  |
| GO:0006913\_nucleocytoplasmic\_transport | 71 | 1 | 0.953811 | -0.183712 | 907 | 603.583464 | 735.42 | 867.256536 | 0.810827 |  |
| GO:0016331\_morphogenesis\_of\_embryonic\_epithelium | 71 | 1 | 0.953811 | -0.183712 | 907 | 603.583464 | 735.42 | 867.256536 | 0.810827 |  |
| GO:0016568\_chromatin\_modification | 72 | 1 | 0.940564 | -0.180296 | 912 | 608.003275 | 739.75 | 871.496725 | 0.811129 |  |
| GO:0021915\_neural\_tube\_development | 72 | 1 | 0.940564 | -0.180296 | 912 | 608.003275 | 739.75 | 871.496725 | 0.811129 |  |
| GO:0042098\_T\_cell\_proliferation | 72 | 1 | 0.940564 | -0.180296 | 912 | 608.003275 | 739.75 | 871.496725 | 0.811129 |  |
| GO:0048839\_inner\_ear\_development | 72 | 1 | 0.940564 | -0.180296 | 912 | 608.003275 | 739.75 | 871.496725 | 0.811129 |  |
| GO:0051169\_nuclear\_transport | 72 | 1 | 0.940564 | -0.180296 | 912 | 608.003275 | 739.75 | 871.496725 | 0.811129 |  |
| GO:0019953\_sexual\_reproduction | 228 | 3 | 0.891060 | -0.178780 | 914 | 609.143959 | 740.8 | 872.456041 | 0.810503 |  |
| GO:0046649\_lymphocyte\_activation | 228 | 3 | 0.891060 | -0.178780 | 914 | 609.143959 | 740.8 | 872.456041 | 0.810503 |  |
| GO:0032268\_regulation\_of\_cellular\_protein\_metabolic\_process | 152 | 2 | 0.891060 | -0.178298 | 915 | 609.619320 | 741.22 | 872.820680 | 0.810077 |  |
| GO:0009416\_response\_to\_light\_stimulus | 74 | 1 | 0.915143 | -0.173691 | 917 | 614.445846 | 745.52 | 876.594154 | 0.812999 |  |
| GO:0048771\_tissue\_remodeling | 74 | 1 | 0.915143 | -0.173691 | 917 | 614.445846 | 745.52 | 876.594154 | 0.812999 |  |
| GO:0008285\_negative\_regulation\_of\_cell\_proliferation | 155 | 2 | 0.873814 | -0.171424 | 918 | 616.386552 | 747.58 | 878.773448 | 0.814357 |  |
| GO:0007281\_germ\_cell\_development | 75 | 1 | 0.902941 | -0.170497 | 920 | 618.133043 | 749.32 | 880.506957 | 0.814478 |  |
| GO:0044265\_cellular\_macromolecule\_catabolic\_process | 75 | 1 | 0.902941 | -0.170497 | 920 | 618.133043 | 749.32 | 880.506957 | 0.814478 |  |
| GO:0006508\_proteolysis | 76 | 1 | 0.891060 | -0.167374 | 922 | 621.150172 | 752.08 | 883.009828 | 0.815705 |  |
| GO:0009725\_response\_to\_hormone\_stimulus | 76 | 1 | 0.891060 | -0.167374 | 922 | 621.150172 | 752.08 | 883.009828 | 0.815705 |  |
| GO:0043687\_post-translational\_protein\_modification | 384 | 5 | 0.881778 | -0.166825 | 923 | 621.800193 | 752.8 | 883.799807 | 0.815601 |  |
| GO:0001890\_placenta\_development | 77 | 1 | 0.879488 | -0.164319 | 924 | 624.119604 | 754.99 | 885.860396 | 0.817089 |  |
| GO:0006468\_protein\_amino\_acid\_phosphorylation | 237 | 3 | 0.857223 | -0.162127 | 925 | 625.009651 | 755.84 | 886.670349 | 0.817124 |  |
| GO:0030326\_embryonic\_limb\_morphogenesis | 78 | 1 | 0.868213 | -0.161330 | 928 | 627.632260 | 758.47 | 889.307740 | 0.817317 |  |
| GO:0035113\_embryonic\_appendage\_morphogenesis | 78 | 1 | 0.868213 | -0.161330 | 928 | 627.632260 | 758.47 | 889.307740 | 0.817317 |  |
| GO:0051251\_positive\_regulation\_of\_lymphocyte\_activation | 78 | 1 | 0.868213 | -0.161330 | 928 | 627.632260 | 758.47 | 889.307740 | 0.817317 |  |
| GO:0007049\_cell\_cycle | 238 | 3 | 0.853621 | -0.160373 | 929 | 628.226545 | 759.04 | 889.853455 | 0.817051 |  |
| GO:0016043\_cellular\_component\_organization | 964 | 13 | 0.913244 | -0.160348 | 930 | 628.374494 | 759.2 | 890.025506 | 0.816344 |  |
| GO:0002521\_leukocyte\_differentiation | 161 | 2 | 0.841250 | -0.158491 | 931 | 628.987209 | 759.77 | 890.552791 | 0.816079 |  |
| GO:0000278\_mitotic\_cell\_cycle | 80 | 1 | 0.846507 | -0.155543 | 932 | 632.083754 | 762.73 | 893.376246 | 0.818380 |  |
| GO:0042110\_T\_cell\_activation | 163 | 2 | 0.830927 | -0.154408 | 933 | 632.521049 | 763.21 | 893.898951 | 0.818017 |  |
| GO:0021700\_developmental\_maturation | 81 | 1 | 0.836057 | -0.152741 | 934 | 633.435562 | 764.06 | 894.684438 | 0.818051 |  |
| GO:0006259\_DNA\_metabolic\_process | 165 | 2 | 0.820856 | -0.150434 | 935 | 634.904353 | 765.21 | 895.515647 | 0.818406 |  |
| GO:0002696\_positive\_regulation\_of\_leukocyte\_activation | 82 | 1 | 0.825861 | -0.149998 | 936 | 637.407171 | 767.35 | 897.292829 | 0.819818 |  |
| GO:0006325\_chromatin\_organization | 83 | 1 | 0.815911 | -0.147313 | 939 | 640.357635 | 770.06 | 899.762365 | 0.820085 |  |
| GO:0030534\_adult\_behavior | 83 | 1 | 0.815911 | -0.147313 | 939 | 640.357635 | 770.06 | 899.762365 | 0.820085 |  |
| GO:0050867\_positive\_regulation\_of\_cell\_activation | 83 | 1 | 0.815911 | -0.147313 | 939 | 640.357635 | 770.06 | 899.762365 | 0.820085 |  |
| GO:0070887\_cellular\_response\_to\_chemical\_stimulus | 85 | 1 | 0.796713 | -0.142109 | 940 | 644.463222 | 773.89 | 903.316778 | 0.823287 |  |
| GO:0051246\_regulation\_of\_protein\_metabolic\_process | 170 | 2 | 0.796713 | -0.140951 | 941 | 644.982663 | 774.36 | 903.737337 | 0.822912 |  |
| GO:0007267\_cell-cell\_signaling | 252 | 3 | 0.806197 | -0.137663 | 942 | 648.183494 | 777.39 | 906.596506 | 0.825255 |  |
| GO:0003001\_generation\_of\_a\_signal\_involved\_in\_cell-cell\_signaling | 87 | 1 | 0.778398 | -0.137117 | 945 | 652.186325 | 781.38 | 910.573675 | 0.826857 |  |
| GO:0007178\_transmembrane\_receptor\_protein\_serine\_threonine\_kinase\_signaling\_pathway | 87 | 1 | 0.778398 | -0.137117 | 945 | 652.186325 | 781.38 | 910.573675 | 0.826857 |  |
| GO:0043583\_ear\_development | 87 | 1 | 0.778398 | -0.137117 | 945 | 652.186325 | 781.38 | 910.573675 | 0.826857 |  |
| GO:0030097\_hemopoiesis | 253 | 3 | 0.803011 | -0.136165 | 946 | 652.791467 | 781.95 | 911.108533 | 0.826586 |  |
| GO:0044093\_positive\_regulation\_of\_molecular\_function | 173 | 2 | 0.782897 | -0.135557 | 948 | 654.074952 | 783.07 | 912.065048 | 0.826023 |  |
| GO:0044248\_cellular\_catabolic\_process | 173 | 2 | 0.782897 | -0.135557 | 948 | 654.074952 | 783.07 | 912.065048 | 0.826023 |  |
| GO:0001503\_ossification | 88 | 1 | 0.769552 | -0.134697 | 950 | 655.247483 | 784.32 | 913.392517 | 0.825600 |  |
| GO:0050863\_regulation\_of\_T\_cell\_activation | 88 | 1 | 0.769552 | -0.134697 | 950 | 655.247483 | 784.32 | 913.392517 | 0.825600 |  |
| GO:0048729\_tissue\_morphogenesis | 255 | 3 | 0.796713 | -0.133218 | 951 | 656.348142 | 785.27 | 914.191858 | 0.825731 |  |
| GO:0007242\_intracellular\_signaling\_cascade | 411 | 5 | 0.823851 | -0.131533 | 952 | 657.641146 | 786.38 | 915.118854 | 0.826029 |  |
| GO:0030324\_lung\_development | 90 | 1 | 0.752451 | -0.130003 | 953 | 660.109564 | 788.83 | 917.550436 | 0.827733 |  |
| GO:0009605\_response\_to\_external\_stimulus | 339 | 4 | 0.799063 | -0.125772 | 954 | 662.131481 | 790.66 | 919.188519 | 0.828784 |  |
| GO:0009719\_response\_to\_endogenous\_stimulus | 92 | 1 | 0.736093 | -0.125495 | 957 | 663.121124 | 791.64 | 920.158876 | 0.827210 |  |
| GO:0030217\_T\_cell\_differentiation | 92 | 1 | 0.736093 | -0.125495 | 957 | 663.121124 | 791.64 | 920.158876 | 0.827210 |  |
| GO:0030323\_respiratory\_tube\_development | 92 | 1 | 0.736093 | -0.125495 | 957 | 663.121124 | 791.64 | 920.158876 | 0.827210 |  |
| GO:0048732\_gland\_development | 179 | 2 | 0.756655 | -0.125392 | 958 | 664.016081 | 792.53 | 921.043919 | 0.827276 |  |
| GO:0006793\_phosphorus\_metabolic\_process | 340 | 4 | 0.796713 | -0.124549 | 960 | 664.768322 | 793.24 | 921.711678 | 0.826292 |  |
| GO:0006796\_phosphate\_metabolic\_process | 340 | 4 | 0.796713 | -0.124549 | 960 | 664.768322 | 793.24 | 921.711678 | 0.826292 |  |
| GO:0032943\_mononuclear\_cell\_proliferation | 94 | 1 | 0.720432 | -0.121164 | 963 | 671.099115 | 799.16 | 927.220885 | 0.829865 |  |
| GO:0034984\_cellular\_response\_to\_DNA\_damage\_stimulus | 94 | 1 | 0.720432 | -0.121164 | 963 | 671.099115 | 799.16 | 927.220885 | 0.829865 |  |
| GO:0046651\_lymphocyte\_proliferation | 94 | 1 | 0.720432 | -0.121164 | 963 | 671.099115 | 799.16 | 927.220885 | 0.829865 |  |
| GO:0051707\_response\_to\_other\_organism | 95 | 1 | 0.712848 | -0.119063 | 964 | 672.401213 | 800.49 | 928.578787 | 0.830384 |  |
| GO:0070661\_leukocyte\_proliferation | 96 | 1 | 0.705423 | -0.117002 | 965 | 675.812792 | 803.42 | 931.027208 | 0.832560 |  |
| GO:0009314\_response\_to\_radiation | 98 | 1 | 0.691026 | -0.113001 | 966 | 680.500814 | 807.74 | 934.979186 | 0.836170 |  |
| GO:0002376\_immune\_system\_process | 505 | 6 | 0.804601 | -0.112603 | 967 | 681.052175 | 808.26 | 935.467825 | 0.835843 |  |
| GO:0001817\_regulation\_of\_cytokine\_production | 99 | 1 | 0.684046 | -0.111058 | 969 | 683.259438 | 810.39 | 937.520562 | 0.836316 |  |
| GO:0060348\_bone\_development | 99 | 1 | 0.684046 | -0.111058 | 969 | 683.259438 | 810.39 | 937.520562 | 0.836316 |  |
| GO:0051716\_cellular\_response\_to\_stimulus | 273 | 3 | 0.744182 | -0.109306 | 970 | 684.320078 | 811.36 | 938.399922 | 0.836454 |  |
| GO:0030163\_protein\_catabolic\_process | 101 | 1 | 0.670501 | -0.107283 | 971 | 685.799244 | 812.77 | 939.740756 | 0.837044 |  |
| GO:0048534\_hemopoietic\_or\_lymphoid\_organ\_development | 277 | 3 | 0.733436 | -0.104580 | 972 | 687.681856 | 814.38 | 941.078144 | 0.837840 |  |
| GO:0065009\_regulation\_of\_molecular\_function | 279 | 3 | 0.728178 | -0.102291 | 973 | 689.967841 | 816.26 | 942.552159 | 0.838911 |  |
| GO:0033554\_cellular\_response\_to\_stress | 196 | 2 | 0.691026 | -0.100581 | 974 | 692.572197 | 818.59 | 944.607803 | 0.840441 |  |
| GO:0048872\_homeostasis\_of\_number\_of\_cells | 105 | 1 | 0.644958 | -0.100155 | 975 | 693.225897 | 819.2 | 945.174103 | 0.840205 |  |
| GO:0030099\_myeloid\_cell\_differentiation | 108 | 1 | 0.627042 | -0.095153 | 976 | 697.992486 | 823.68 | 949.367514 | 0.843934 |  |
| GO:0010926\_anatomical\_structure\_formation | 447 | 5 | 0.757501 | -0.094983 | 977 | 698.257594 | 823.91 | 949.562406 | 0.843306 |  |
| GO:0051249\_regulation\_of\_lymphocyte\_activation | 112 | 1 | 0.604648 | -0.088906 | 978 | 703.882828 | 829.18 | 954.477172 | 0.847832 |  |
| GO:0006974\_response\_to\_DNA\_damage\_stimulus | 113 | 1 | 0.599297 | -0.087415 | 979 | 704.711889 | 829.96 | 955.208111 | 0.847763 |  |
| GO:0022414\_reproductive\_process | 376 | 4 | 0.720432 | -0.087191 | 980 | 704.854089 | 830.08 | 955.305911 | 0.847020 |  |
| GO:0009607\_response\_to\_biotic\_stimulus | 114 | 1 | 0.594040 | -0.085952 | 981 | 706.441144 | 831.66 | 956.878856 | 0.847768 |  |
| GO:0002520\_immune\_system\_development | 295 | 3 | 0.688684 | -0.085621 | 982 | 707.630634 | 832.63 | 957.629366 | 0.847892 |  |
| GO:0000003\_reproduction | 379 | 4 | 0.714729 | -0.084601 | 983 | 707.768761 | 832.74 | 957.711239 | 0.847141 |  |
| GO:0080134\_regulation\_of\_response\_to\_stress | 116 | 1 | 0.583798 | -0.083105 | 984 | 709.561725 | 834.48 | 959.398275 | 0.848049 |  |
| GO:0022403\_cell\_cycle\_phase | 119 | 1 | 0.569081 | -0.079023 | 985 | 712.678722 | 836.98 | 961.281278 | 0.849726 |  |
| GO:0006950\_response\_to\_stress | 549 | 6 | 0.740116 | -0.077063 | 986 | 715.179541 | 838.88 | 962.580459 | 0.850791 |  |
| GO:0002694\_regulation\_of\_leukocyte\_activation | 121 | 1 | 0.559674 | -0.076423 | 987 | 717.076905 | 840.41 | 963.743095 | 0.851479 |  |
| GO:0001816\_cytokine\_production | 122 | 1 | 0.555087 | -0.075158 | 989 | 719.938028 | 842.99 | 966.041972 | 0.852366 |  |
| GO:0050865\_regulation\_of\_cell\_activation | 122 | 1 | 0.555087 | -0.075158 | 989 | 719.938028 | 842.99 | 966.041972 | 0.852366 |  |
| GO:0016310\_phosphorylation | 309 | 3 | 0.657481 | -0.073181 | 990 | 721.483847 | 844.24 | 966.996153 | 0.852768 |  |
| GO:0043062\_extracellular\_structure\_organization | 125 | 1 | 0.541765 | -0.071495 | 991 | 723.337153 | 845.74 | 968.142847 | 0.853421 |  |
| GO:0043285\_biopolymer\_catabolic\_process | 129 | 1 | 0.524966 | -0.066906 | 992 | 725.965160 | 847.97 | 969.974840 | 0.854808 |  |
| GO:0002682\_regulation\_of\_immune\_system\_process | 228 | 2 | 0.594040 | -0.066396 | 993 | 726.611152 | 848.52 | 970.428848 | 0.854502 |  |
| GO:0032787\_monocarboxylic\_acid\_metabolic\_process | 130 | 1 | 0.520928 | -0.065808 | 994 | 727.647431 | 849.33 | 971.012569 | 0.854457 |  |
| GO:0050790\_regulation\_of\_catalytic\_activity | 233 | 2 | 0.581293 | -0.062211 | 995 | 730.490733 | 851.6 | 972.709267 | 0.855879 |  |
| GO:0007283\_spermatogenesis | 134 | 1 | 0.505378 | -0.061606 | 997 | 731.166005 | 852.14 | 973.113995 | 0.854704 |  |
| GO:0048232\_male\_gamete\_generation | 134 | 1 | 0.505378 | -0.061606 | 997 | 731.166005 | 852.14 | 973.113995 | 0.854704 |  |
| GO:0044085\_cellular\_component\_biogenesis | 237 | 2 | 0.571482 | -0.059051 | 998 | 733.700263 | 854.29 | 974.879737 | 0.856002 |  |
| GO:0009057\_macromolecule\_catabolic\_process | 137 | 1 | 0.494311 | -0.058641 | 999 | 734.093109 | 854.61 | 975.126891 | 0.855465 |  |
| GO:0009056\_catabolic\_process | 243 | 2 | 0.557371 | -0.054603 | 1000 | 738.105015 | 857.9 | 977.694985 | 0.857900 |  |
| GO:0032879\_regulation\_of\_localization | 248 | 2 | 0.546134 | -0.051148 | 1001 | 741.376257 | 860.46 | 979.543743 | 0.859600 |  |
| GO:0022603\_regulation\_of\_anatomical\_structure\_morphogenesis | 147 | 1 | 0.460684 | -0.049796 | 1002 | 743.439163 | 862.24 | 981.040837 | 0.860519 |  |
| GO:0022402\_cell\_cycle\_process | 155 | 1 | 0.436907 | -0.043729 | 1003 | 749.935618 | 867.5 | 985.064382 | 0.864905 |  |
| GO:0006996\_organelle\_organization | 449 | 4 | 0.603301 | -0.040966 | 1004 | 752.893224 | 869.92 | 986.946776 | 0.866454 |  |
| GO:0051049\_regulation\_of\_transport | 167 | 1 | 0.405513 | -0.036027 | 1005 | 760.720094 | 875.49 | 990.259906 | 0.871134 |  |
| GO:0019752\_carboxylic\_acid\_metabolic\_process | 181 | 1 | 0.374147 | -0.028776 | 1007 | 770.110706 | 882.48 | 994.849294 | 0.876346 |  |
| GO:0043436\_oxoacid\_metabolic\_process | 181 | 1 | 0.374147 | -0.028776 | 1007 | 770.110706 | 882.48 | 994.849294 | 0.876346 |  |
| GO:0006082\_organic\_acid\_metabolic\_process | 182 | 1 | 0.372091 | -0.028319 | 1008 | 770.629219 | 882.87 | 995.110781 | 0.875863 |  |
| GO:0042180\_cellular\_ketone\_metabolic\_process | 183 | 1 | 0.370058 | -0.027869 | 1009 | 770.915763 | 883.1 | 995.284237 | 0.875223 |  |
| GO:0042221\_response\_to\_chemical\_stimulus | 409 | 3 | 0.496728 | -0.022841 | 1010 | 775.843750 | 887.16 | 998.476250 | 0.878376 |  |
| GO:0022607\_cellular\_component\_assembly | 204 | 1 | 0.331964 | -0.019931 | 1011 | 778.960347 | 889.29 | 999.619653 | 0.879614 |  |
| GO:0009187\_cyclic\_nucleotide\_metabolic\_process | 56 | 0 | 0.000000 | -0.000000 | 1016 | 803.925076 | 906.89 | 1009.854924 | 0.892608 |  |
| GO:0042089\_cytokine\_biosynthetic\_process | 56 | 0 | 0.000000 | -0.000000 | 1016 | 803.925076 | 906.89 | 1009.854924 | 0.892608 |  |
| GO:0042107\_cytokine\_metabolic\_process | 56 | 0 | 0.000000 | -0.000000 | 1016 | 803.925076 | 906.89 | 1009.854924 | 0.892608 |  |
| GO:0050678\_regulation\_of\_epithelial\_cell\_proliferation | 56 | 0 | 0.000000 | -0.000000 | 1016 | 803.925076 | 906.89 | 1009.854924 | 0.892608 |  |
| GO:0051321\_meiotic\_cell\_cycle | 56 | 0 | 0.000000 | -0.000000 | 1016 | 803.925076 | 906.89 | 1009.854924 | 0.892608 |  |
| GO:0001843\_neural\_tube\_closure | 33 | 0 | 0.000000 | -0.000000 | 1027 | 814.194630 | 915.78 | 1017.365370 | 0.891704 |  |
| GO:0007188\_G-protein\_signaling\_\_coupled\_to\_cAMP\_nucleotide\_second\_messenger | 33 | 0 | 0.000000 | -0.000000 | 1027 | 814.194630 | 915.78 | 1017.365370 | 0.891704 |  |
| GO:0007270\_nerve-nerve\_synaptic\_transmission | 33 | 0 | 0.000000 | -0.000000 | 1027 | 814.194630 | 915.78 | 1017.365370 | 0.891704 |  |
| GO:0007431\_salivary\_gland\_development | 33 | 0 | 0.000000 | -0.000000 | 1027 | 814.194630 | 915.78 | 1017.365370 | 0.891704 |  |
| GO:0007565\_female\_pregnancy | 33 | 0 | 0.000000 | -0.000000 | 1027 | 814.194630 | 915.78 | 1017.365370 | 0.891704 |  |
| GO:0008584\_male\_gonad\_development | 33 | 0 | 0.000000 | -0.000000 | 1027 | 814.194630 | 915.78 | 1017.365370 | 0.891704 |  |
| GO:0008643\_carbohydrate\_transport | 33 | 0 | 0.000000 | -0.000000 | 1027 | 814.194630 | 915.78 | 1017.365370 | 0.891704 |  |
| GO:0021536\_diencephalon\_development | 33 | 0 | 0.000000 | -0.000000 | 1027 | 814.194630 | 915.78 | 1017.365370 | 0.891704 |  |
| GO:0022037\_metencephalon\_development | 33 | 0 | 0.000000 | -0.000000 | 1027 | 814.194630 | 915.78 | 1017.365370 | 0.891704 |  |
| GO:0042108\_positive\_regulation\_of\_cytokine\_biosynthetic\_process | 33 | 0 | 0.000000 | -0.000000 | 1027 | 814.194630 | 915.78 | 1017.365370 | 0.891704 |  |
| GO:0060606\_tube\_closure | 33 | 0 | 0.000000 | -0.000000 | 1027 | 814.194630 | 915.78 | 1017.365370 | 0.891704 |  |
| GO:0000910\_cytokinesis | 8 | 0 | 0.000000 | -0.000000 | 1141 | 934.530567 | 1033.35 | 1132.169433 | 0.905653 |  |
| GO:0001783\_B\_cell\_apoptosis | 8 | 0 | 0.000000 | -0.000000 | 1141 | 934.530567 | 1033.35 | 1132.169433 | 0.905653 |  |
| GO:0001833\_inner\_cell\_mass\_cell\_proliferation | 8 | 0 | 0.000000 | -0.000000 | 1141 | 934.530567 | 1033.35 | 1132.169433 | 0.905653 |  |
| GO:0001840\_neural\_plate\_development | 8 | 0 | 0.000000 | -0.000000 | 1141 | 934.530567 | 1033.35 | 1132.169433 | 0.905653 |  |
| GO:0001893\_maternal\_placenta\_development | 8 | 0 | 0.000000 | -0.000000 | 1141 | 934.530567 | 1033.35 | 1132.169433 | 0.905653 |  |
| GO:0002065\_columnar\_cuboidal\_epithelial\_cell\_differentiation | 8 | 0 | 0.000000 | -0.000000 | 1141 | 934.530567 | 1033.35 | 1132.169433 | 0.905653 |  |
| GO:0002320\_lymphoid\_progenitor\_cell\_differentiation | 8 | 0 | 0.000000 | -0.000000 | 1141 | 934.530567 | 1033.35 | 1132.169433 | 0.905653 |  |
| GO:0002438\_acute\_inflammatory\_response\_to\_antigenic\_stimulus | 8 | 0 | 0.000000 | -0.000000 | 1141 | 934.530567 | 1033.35 | 1132.169433 | 0.905653 |  |
| GO:0002524\_hypersensitivity | 8 | 0 | 0.000000 | -0.000000 | 1141 | 934.530567 | 1033.35 | 1132.169433 | 0.905653 |  |
| GO:0002566\_somatic\_diversification\_of\_immune\_receptors\_via\_somatic\_mutation | 8 | 0 | 0.000000 | -0.000000 | 1141 | 934.530567 | 1033.35 | 1132.169433 | 0.905653 |  |
| GO:0002864\_regulation\_of\_acute\_inflammatory\_response\_to\_antigenic\_stimulus | 8 | 0 | 0.000000 | -0.000000 | 1141 | 934.530567 | 1033.35 | 1132.169433 | 0.905653 |  |
| GO:0002883\_regulation\_of\_hypersensitivity | 8 | 0 | 0.000000 | -0.000000 | 1141 | 934.530567 | 1033.35 | 1132.169433 | 0.905653 |  |
| GO:0003081\_regulation\_of\_systemic\_arterial\_blood\_pressure\_by\_renin-angiotensin | 8 | 0 | 0.000000 | -0.000000 | 1141 | 934.530567 | 1033.35 | 1132.169433 | 0.905653 |  |
| GO:0006020\_inositol\_metabolic\_process | 8 | 0 | 0.000000 | -0.000000 | 1141 | 934.530567 | 1033.35 | 1132.169433 | 0.905653 |  |
| GO:0006195\_purine\_nucleotide\_catabolic\_process | 8 | 0 | 0.000000 | -0.000000 | 1141 | 934.530567 | 1033.35 | 1132.169433 | 0.905653 |  |
| GO:0006284\_base-excision\_repair | 8 | 0 | 0.000000 | -0.000000 | 1141 | 934.530567 | 1033.35 | 1132.169433 | 0.905653 |  |
| GO:0006349\_genetic\_imprinting | 8 | 0 | 0.000000 | -0.000000 | 1141 | 934.530567 | 1033.35 | 1132.169433 | 0.905653 |  |
| GO:0006360\_transcription\_from\_RNA\_polymerase\_I\_promoter | 8 | 0 | 0.000000 | -0.000000 | 1141 | 934.530567 | 1033.35 | 1132.169433 | 0.905653 |  |
| GO:0006399\_tRNA\_metabolic\_process | 8 | 0 | 0.000000 | -0.000000 | 1141 | 934.530567 | 1033.35 | 1132.169433 | 0.905653 |  |
| GO:0006458\_'de\_novo'\_protein\_folding | 8 | 0 | 0.000000 | -0.000000 | 1141 | 934.530567 | 1033.35 | 1132.169433 | 0.905653 |  |
| GO:0006493\_protein\_amino\_acid\_O-linked\_glycosylation | 8 | 0 | 0.000000 | -0.000000 | 1141 | 934.530567 | 1033.35 | 1132.169433 | 0.905653 |  |
| GO:0006582\_melanin\_metabolic\_process | 8 | 0 | 0.000000 | -0.000000 | 1141 | 934.530567 | 1033.35 | 1132.169433 | 0.905653 |  |
| GO:0006733\_oxidoreduction\_coenzyme\_metabolic\_process | 8 | 0 | 0.000000 | -0.000000 | 1141 | 934.530567 | 1033.35 | 1132.169433 | 0.905653 |  |
| GO:0006829\_zinc\_ion\_transport | 8 | 0 | 0.000000 | -0.000000 | 1141 | 934.530567 | 1033.35 | 1132.169433 | 0.905653 |  |
| GO:0007009\_plasma\_membrane\_organization | 8 | 0 | 0.000000 | -0.000000 | 1141 | 934.530567 | 1033.35 | 1132.169433 | 0.905653 |  |
| GO:0007098\_centrosome\_cycle | 8 | 0 | 0.000000 | -0.000000 | 1141 | 934.530567 | 1033.35 | 1132.169433 | 0.905653 |  |
| GO:0007131\_reciprocal\_meiotic\_recombination | 8 | 0 | 0.000000 | -0.000000 | 1141 | 934.530567 | 1033.35 | 1132.169433 | 0.905653 |  |
| GO:0007141\_male\_meiosis\_I | 8 | 0 | 0.000000 | -0.000000 | 1141 | 934.530567 | 1033.35 | 1132.169433 | 0.905653 |  |
| GO:0007625\_grooming\_behavior | 8 | 0 | 0.000000 | -0.000000 | 1141 | 934.530567 | 1033.35 | 1132.169433 | 0.905653 |  |
| GO:0008105\_asymmetric\_protein\_localization | 8 | 0 | 0.000000 | -0.000000 | 1141 | 934.530567 | 1033.35 | 1132.169433 | 0.905653 |  |
| GO:0009072\_aromatic\_amino\_acid\_family\_metabolic\_process | 8 | 0 | 0.000000 | -0.000000 | 1141 | 934.530567 | 1033.35 | 1132.169433 | 0.905653 |  |
| GO:0009144\_purine\_nucleoside\_triphosphate\_metabolic\_process | 8 | 0 | 0.000000 | -0.000000 | 1141 | 934.530567 | 1033.35 | 1132.169433 | 0.905653 |  |
| GO:0009746\_response\_to\_hexose\_stimulus | 8 | 0 | 0.000000 | -0.000000 | 1141 | 934.530567 | 1033.35 | 1132.169433 | 0.905653 |  |
| GO:0009749\_response\_to\_glucose\_stimulus | 8 | 0 | 0.000000 | -0.000000 | 1141 | 934.530567 | 1033.35 | 1132.169433 | 0.905653 |  |
| GO:0014046\_dopamine\_secretion | 8 | 0 | 0.000000 | -0.000000 | 1141 | 934.530567 | 1033.35 | 1132.169433 | 0.905653 |  |
| GO:0014059\_regulation\_of\_dopamine\_secretion | 8 | 0 | 0.000000 | -0.000000 | 1141 | 934.530567 | 1033.35 | 1132.169433 | 0.905653 |  |
| GO:0015800\_acidic\_amino\_acid\_transport | 8 | 0 | 0.000000 | -0.000000 | 1141 | 934.530567 | 1033.35 | 1132.169433 | 0.905653 |  |
| GO:0015804\_neutral\_amino\_acid\_transport | 8 | 0 | 0.000000 | -0.000000 | 1141 | 934.530567 | 1033.35 | 1132.169433 | 0.905653 |  |
| GO:0016236\_macroautophagy | 8 | 0 | 0.000000 | -0.000000 | 1141 | 934.530567 | 1033.35 | 1132.169433 | 0.905653 |  |
| GO:0016446\_somatic\_hypermutation\_of\_immunoglobulin\_genes | 8 | 0 | 0.000000 | -0.000000 | 1141 | 934.530567 | 1033.35 | 1132.169433 | 0.905653 |  |
| GO:0018107\_peptidyl-threonine\_phosphorylation | 8 | 0 | 0.000000 | -0.000000 | 1141 | 934.530567 | 1033.35 | 1132.169433 | 0.905653 |  |
| GO:0018210\_peptidyl-threonine\_modification | 8 | 0 | 0.000000 | -0.000000 | 1141 | 934.530567 | 1033.35 | 1132.169433 | 0.905653 |  |
| GO:0018345\_protein\_palmitoylation | 8 | 0 | 0.000000 | -0.000000 | 1141 | 934.530567 | 1033.35 | 1132.169433 | 0.905653 |  |
| GO:0019400\_alditol\_metabolic\_process | 8 | 0 | 0.000000 | -0.000000 | 1141 | 934.530567 | 1033.35 | 1132.169433 | 0.905653 |  |
| GO:0021692\_cerebellar\_Purkinje\_cell\_layer\_morphogenesis | 8 | 0 | 0.000000 | -0.000000 | 1141 | 934.530567 | 1033.35 | 1132.169433 | 0.905653 |  |
| GO:0021694\_cerebellar\_Purkinje\_cell\_layer\_formation | 8 | 0 | 0.000000 | -0.000000 | 1141 | 934.530567 | 1033.35 | 1132.169433 | 0.905653 |  |
| GO:0021702\_cerebellar\_Purkinje\_cell\_differentiation | 8 | 0 | 0.000000 | -0.000000 | 1141 | 934.530567 | 1033.35 | 1132.169433 | 0.905653 |  |
| GO:0021799\_cerebral\_cortex\_radially\_oriented\_cell\_migration | 8 | 0 | 0.000000 | -0.000000 | 1141 | 934.530567 | 1033.35 | 1132.169433 | 0.905653 |  |
| GO:0022898\_regulation\_of\_transmembrane\_transporter\_activity | 8 | 0 | 0.000000 | -0.000000 | 1141 | 934.530567 | 1033.35 | 1132.169433 | 0.905653 |  |
| GO:0030193\_regulation\_of\_blood\_coagulation | 8 | 0 | 0.000000 | -0.000000 | 1141 | 934.530567 | 1033.35 | 1132.169433 | 0.905653 |  |
| GO:0030500\_regulation\_of\_bone\_mineralization | 8 | 0 | 0.000000 | -0.000000 | 1141 | 934.530567 | 1033.35 | 1132.169433 | 0.905653 |  |
| GO:0030511\_positive\_regulation\_of\_transforming\_growth\_factor\_beta\_receptor\_signaling\_pathway | 8 | 0 | 0.000000 | -0.000000 | 1141 | 934.530567 | 1033.35 | 1132.169433 | 0.905653 |  |
| GO:0031102\_neuron\_projection\_regeneration | 8 | 0 | 0.000000 | -0.000000 | 1141 | 934.530567 | 1033.35 | 1132.169433 | 0.905653 |  |
| GO:0031103\_axon\_regeneration | 8 | 0 | 0.000000 | -0.000000 | 1141 | 934.530567 | 1033.35 | 1132.169433 | 0.905653 |  |
| GO:0031111\_negative\_regulation\_of\_microtubule\_polymerization\_or\_depolymerization | 8 | 0 | 0.000000 | -0.000000 | 1141 | 934.530567 | 1033.35 | 1132.169433 | 0.905653 |  |
| GO:0031123\_RNA\_3'-end\_processing | 8 | 0 | 0.000000 | -0.000000 | 1141 | 934.530567 | 1033.35 | 1132.169433 | 0.905653 |  |
| GO:0031294\_lymphocyte\_costimulation | 8 | 0 | 0.000000 | -0.000000 | 1141 | 934.530567 | 1033.35 | 1132.169433 | 0.905653 |  |
| GO:0031295\_T\_cell\_costimulation | 8 | 0 | 0.000000 | -0.000000 | 1141 | 934.530567 | 1033.35 | 1132.169433 | 0.905653 |  |
| GO:0031334\_positive\_regulation\_of\_protein\_complex\_assembly | 8 | 0 | 0.000000 | -0.000000 | 1141 | 934.530567 | 1033.35 | 1132.169433 | 0.905653 |  |
| GO:0031396\_regulation\_of\_protein\_ubiquitination | 8 | 0 | 0.000000 | -0.000000 | 1141 | 934.530567 | 1033.35 | 1132.169433 | 0.905653 |  |
| GO:0032094\_response\_to\_food | 8 | 0 | 0.000000 | -0.000000 | 1141 | 934.530567 | 1033.35 | 1132.169433 | 0.905653 |  |
| GO:0032273\_positive\_regulation\_of\_protein\_polymerization | 8 | 0 | 0.000000 | -0.000000 | 1141 | 934.530567 | 1033.35 | 1132.169433 | 0.905653 |  |
| GO:0032409\_regulation\_of\_transporter\_activity | 8 | 0 | 0.000000 | -0.000000 | 1141 | 934.530567 | 1033.35 | 1132.169433 | 0.905653 |  |
| GO:0032412\_regulation\_of\_ion\_transmembrane\_transporter\_activity | 8 | 0 | 0.000000 | -0.000000 | 1141 | 934.530567 | 1033.35 | 1132.169433 | 0.905653 |  |
| GO:0032613\_interleukin-10\_production | 8 | 0 | 0.000000 | -0.000000 | 1141 | 934.530567 | 1033.35 | 1132.169433 | 0.905653 |  |
| GO:0033198\_response\_to\_ATP | 8 | 0 | 0.000000 | -0.000000 | 1141 | 934.530567 | 1033.35 | 1132.169433 | 0.905653 |  |
| GO:0034284\_response\_to\_monosaccharide\_stimulus | 8 | 0 | 0.000000 | -0.000000 | 1141 | 934.530567 | 1033.35 | 1132.169433 | 0.905653 |  |
| GO:0034728\_nucleosome\_organization | 8 | 0 | 0.000000 | -0.000000 | 1141 | 934.530567 | 1033.35 | 1132.169433 | 0.905653 |  |
| GO:0035023\_regulation\_of\_Rho\_protein\_signal\_transduction | 8 | 0 | 0.000000 | -0.000000 | 1141 | 934.530567 | 1033.35 | 1132.169433 | 0.905653 |  |
| GO:0040017\_positive\_regulation\_of\_locomotion | 8 | 0 | 0.000000 | -0.000000 | 1141 | 934.530567 | 1033.35 | 1132.169433 | 0.905653 |  |
| GO:0042074\_cell\_migration\_involved\_in\_gastrulation | 8 | 0 | 0.000000 | -0.000000 | 1141 | 934.530567 | 1033.35 | 1132.169433 | 0.905653 |  |
| GO:0042090\_interleukin-12\_biosynthetic\_process | 8 | 0 | 0.000000 | -0.000000 | 1141 | 934.530567 | 1033.35 | 1132.169433 | 0.905653 |  |
| GO:0042092\_T-helper\_2\_type\_immune\_response | 8 | 0 | 0.000000 | -0.000000 | 1141 | 934.530567 | 1033.35 | 1132.169433 | 0.905653 |  |
| GO:0042095\_interferon-gamma\_biosynthetic\_process | 8 | 0 | 0.000000 | -0.000000 | 1141 | 934.530567 | 1033.35 | 1132.169433 | 0.905653 |  |
| GO:0042104\_positive\_regulation\_of\_activated\_T\_cell\_proliferation | 8 | 0 | 0.000000 | -0.000000 | 1141 | 934.530567 | 1033.35 | 1132.169433 | 0.905653 |  |
| GO:0042226\_interleukin-6\_biosynthetic\_process | 8 | 0 | 0.000000 | -0.000000 | 1141 | 934.530567 | 1033.35 | 1132.169433 | 0.905653 |  |
| GO:0042304\_regulation\_of\_fatty\_acid\_biosynthetic\_process | 8 | 0 | 0.000000 | -0.000000 | 1141 | 934.530567 | 1033.35 | 1132.169433 | 0.905653 |  |
| GO:0042423\_catecholamine\_biosynthetic\_process | 8 | 0 | 0.000000 | -0.000000 | 1141 | 934.530567 | 1033.35 | 1132.169433 | 0.905653 |  |
| GO:0042771\_DNA\_damage\_response\_\_signal\_transduction\_by\_p53\_class\_mediator\_resulting\_in\_induction\_of\_apoptosis | 8 | 0 | 0.000000 | -0.000000 | 1141 | 934.530567 | 1033.35 | 1132.169433 | 0.905653 |  |
| GO:0042990\_regulation\_of\_transcription\_factor\_import\_into\_nucleus | 8 | 0 | 0.000000 | -0.000000 | 1141 | 934.530567 | 1033.35 | 1132.169433 | 0.905653 |  |
| GO:0042991\_transcription\_factor\_import\_into\_nucleus | 8 | 0 | 0.000000 | -0.000000 | 1141 | 934.530567 | 1033.35 | 1132.169433 | 0.905653 |  |
| GO:0043011\_myeloid\_dendritic\_cell\_differentiation | 8 | 0 | 0.000000 | -0.000000 | 1141 | 934.530567 | 1033.35 | 1132.169433 | 0.905653 |  |
| GO:0043370\_regulation\_of\_CD4-positive\_\_alpha\_beta\_T\_cell\_differentiation | 8 | 0 | 0.000000 | -0.000000 | 1141 | 934.530567 | 1033.35 | 1132.169433 | 0.905653 |  |
| GO:0043542\_endothelial\_cell\_migration | 8 | 0 | 0.000000 | -0.000000 | 1141 | 934.530567 | 1033.35 | 1132.169433 | 0.905653 |  |
| GO:0043616\_keratinocyte\_proliferation | 8 | 0 | 0.000000 | -0.000000 | 1141 | 934.530567 | 1033.35 | 1132.169433 | 0.905653 |  |
| GO:0045075\_regulation\_of\_interleukin-12\_biosynthetic\_process | 8 | 0 | 0.000000 | -0.000000 | 1141 | 934.530567 | 1033.35 | 1132.169433 | 0.905653 |  |
| GO:0045086\_positive\_regulation\_of\_interleukin-2\_biosynthetic\_process | 8 | 0 | 0.000000 | -0.000000 | 1141 | 934.530567 | 1033.35 | 1132.169433 | 0.905653 |  |
| GO:0045351\_type\_I\_interferon\_biosynthetic\_process | 8 | 0 | 0.000000 | -0.000000 | 1141 | 934.530567 | 1033.35 | 1132.169433 | 0.905653 |  |
| GO:0045408\_regulation\_of\_interleukin-6\_biosynthetic\_process | 8 | 0 | 0.000000 | -0.000000 | 1141 | 934.530567 | 1033.35 | 1132.169433 | 0.905653 |  |
| GO:0045429\_positive\_regulation\_of\_nitric\_oxide\_biosynthetic\_process | 8 | 0 | 0.000000 | -0.000000 | 1141 | 934.530567 | 1033.35 | 1132.169433 | 0.905653 |  |
| GO:0045494\_photoreceptor\_cell\_maintenance | 8 | 0 | 0.000000 | -0.000000 | 1141 | 934.530567 | 1033.35 | 1132.169433 | 0.905653 |  |
| GO:0045910\_negative\_regulation\_of\_DNA\_recombination | 8 | 0 | 0.000000 | -0.000000 | 1141 | 934.530567 | 1033.35 | 1132.169433 | 0.905653 |  |
| GO:0045921\_positive\_regulation\_of\_exocytosis | 8 | 0 | 0.000000 | -0.000000 | 1141 | 934.530567 | 1033.35 | 1132.169433 | 0.905653 |  |
| GO:0046470\_phosphatidylcholine\_metabolic\_process | 8 | 0 | 0.000000 | -0.000000 | 1141 | 934.530567 | 1033.35 | 1132.169433 | 0.905653 |  |
| GO:0048266\_behavioral\_response\_to\_pain | 8 | 0 | 0.000000 | -0.000000 | 1141 | 934.530567 | 1033.35 | 1132.169433 | 0.905653 |  |
| GO:0048520\_positive\_regulation\_of\_behavior | 8 | 0 | 0.000000 | -0.000000 | 1141 | 934.530567 | 1033.35 | 1132.169433 | 0.905653 |  |
| GO:0048638\_regulation\_of\_developmental\_growth | 8 | 0 | 0.000000 | -0.000000 | 1141 | 934.530567 | 1033.35 | 1132.169433 | 0.905653 |  |
| GO:0050707\_regulation\_of\_cytokine\_secretion | 8 | 0 | 0.000000 | -0.000000 | 1141 | 934.530567 | 1033.35 | 1132.169433 | 0.905653 |  |
| GO:0050909\_sensory\_perception\_of\_taste | 8 | 0 | 0.000000 | -0.000000 | 1141 | 934.530567 | 1033.35 | 1132.169433 | 0.905653 |  |
| GO:0050920\_regulation\_of\_chemotaxis | 8 | 0 | 0.000000 | -0.000000 | 1141 | 934.530567 | 1033.35 | 1132.169433 | 0.905653 |  |
| GO:0050921\_positive\_regulation\_of\_chemotaxis | 8 | 0 | 0.000000 | -0.000000 | 1141 | 934.530567 | 1033.35 | 1132.169433 | 0.905653 |  |
| GO:0050926\_regulation\_of\_positive\_chemotaxis | 8 | 0 | 0.000000 | -0.000000 | 1141 | 934.530567 | 1033.35 | 1132.169433 | 0.905653 |  |
| GO:0050927\_positive\_regulation\_of\_positive\_chemotaxis | 8 | 0 | 0.000000 | -0.000000 | 1141 | 934.530567 | 1033.35 | 1132.169433 | 0.905653 |  |
| GO:0050930\_induction\_of\_positive\_chemotaxis | 8 | 0 | 0.000000 | -0.000000 | 1141 | 934.530567 | 1033.35 | 1132.169433 | 0.905653 |  |
| GO:0051084\_'de\_novo'\_posttranslational\_protein\_folding | 8 | 0 | 0.000000 | -0.000000 | 1141 | 934.530567 | 1033.35 | 1132.169433 | 0.905653 |  |
| GO:0051181\_cofactor\_transport | 8 | 0 | 0.000000 | -0.000000 | 1141 | 934.530567 | 1033.35 | 1132.169433 | 0.905653 |  |
| GO:0060043\_regulation\_of\_cardiac\_muscle\_cell\_proliferation | 8 | 0 | 0.000000 | -0.000000 | 1141 | 934.530567 | 1033.35 | 1132.169433 | 0.905653 |  |
| GO:0060347\_heart\_trabecula\_formation | 8 | 0 | 0.000000 | -0.000000 | 1141 | 934.530567 | 1033.35 | 1132.169433 | 0.905653 |  |
| GO:0060670\_branching\_involved\_in\_embryonic\_placenta\_morphogenesis | 8 | 0 | 0.000000 | -0.000000 | 1141 | 934.530567 | 1033.35 | 1132.169433 | 0.905653 |  |
| GO:0060712\_spongiotrophoblast\_layer\_development | 8 | 0 | 0.000000 | -0.000000 | 1141 | 934.530567 | 1033.35 | 1132.169433 | 0.905653 |  |
| GO:0070167\_regulation\_of\_biomineral\_formation | 8 | 0 | 0.000000 | -0.000000 | 1141 | 934.530567 | 1033.35 | 1132.169433 | 0.905653 |  |
| GO:0070193\_synaptonemal\_complex\_organization | 8 | 0 | 0.000000 | -0.000000 | 1141 | 934.530567 | 1033.35 | 1132.169433 | 0.905653 |  |
| GO:0070231\_T\_cell\_apoptosis | 8 | 0 | 0.000000 | -0.000000 | 1141 | 934.530567 | 1033.35 | 1132.169433 | 0.905653 |  |
| GO:0070584\_mitochondrion\_morphogenesis | 8 | 0 | 0.000000 | -0.000000 | 1141 | 934.530567 | 1033.35 | 1132.169433 | 0.905653 |  |
| GO:0000280\_nuclear\_division | 24 | 0 | 0.000000 | -0.000000 | 1157 | 956.347302 | 1053.68 | 1151.012698 | 0.910700 |  |
| GO:0006650\_glycerophospholipid\_metabolic\_process | 24 | 0 | 0.000000 | -0.000000 | 1157 | 956.347302 | 1053.68 | 1151.012698 | 0.910700 |  |
| GO:0007050\_cell\_cycle\_arrest | 24 | 0 | 0.000000 | -0.000000 | 1157 | 956.347302 | 1053.68 | 1151.012698 | 0.910700 |  |
| GO:0007067\_mitosis | 24 | 0 | 0.000000 | -0.000000 | 1157 | 956.347302 | 1053.68 | 1151.012698 | 0.910700 |  |
| GO:0007266\_Rho\_protein\_signal\_transduction | 24 | 0 | 0.000000 | -0.000000 | 1157 | 956.347302 | 1053.68 | 1151.012698 | 0.910700 |  |
| GO:0008629\_induction\_of\_apoptosis\_by\_intracellular\_signals | 24 | 0 | 0.000000 | -0.000000 | 1157 | 956.347302 | 1053.68 | 1151.012698 | 0.910700 |  |
| GO:0014070\_response\_to\_organic\_cyclic\_substance | 24 | 0 | 0.000000 | -0.000000 | 1157 | 956.347302 | 1053.68 | 1151.012698 | 0.910700 |  |
| GO:0032386\_regulation\_of\_intracellular\_transport | 24 | 0 | 0.000000 | -0.000000 | 1157 | 956.347302 | 1053.68 | 1151.012698 | 0.910700 |  |
| GO:0042632\_cholesterol\_homeostasis | 24 | 0 | 0.000000 | -0.000000 | 1157 | 956.347302 | 1053.68 | 1151.012698 | 0.910700 |  |
| GO:0043588\_skin\_development | 24 | 0 | 0.000000 | -0.000000 | 1157 | 956.347302 | 1053.68 | 1151.012698 | 0.910700 |  |
| GO:0048002\_antigen\_processing\_and\_presentation\_of\_peptide\_antigen | 24 | 0 | 0.000000 | -0.000000 | 1157 | 956.347302 | 1053.68 | 1151.012698 | 0.910700 |  |
| GO:0050679\_positive\_regulation\_of\_epithelial\_cell\_proliferation | 24 | 0 | 0.000000 | -0.000000 | 1157 | 956.347302 | 1053.68 | 1151.012698 | 0.910700 |  |
| GO:0051099\_positive\_regulation\_of\_binding | 24 | 0 | 0.000000 | -0.000000 | 1157 | 956.347302 | 1053.68 | 1151.012698 | 0.910700 |  |
| GO:0055092\_sterol\_homeostasis | 24 | 0 | 0.000000 | -0.000000 | 1157 | 956.347302 | 1053.68 | 1151.012698 | 0.910700 |  |
| GO:0060078\_regulation\_of\_postsynaptic\_membrane\_potential | 24 | 0 | 0.000000 | -0.000000 | 1157 | 956.347302 | 1053.68 | 1151.012698 | 0.910700 |  |
| GO:0070667\_negative\_regulation\_of\_mast\_cell\_proliferation | 24 | 0 | 0.000000 | -0.000000 | 1157 | 956.347302 | 1053.68 | 1151.012698 | 0.910700 |  |
| GO:0001824\_blastocyst\_development | 40 | 0 | 0.000000 | -0.000000 | 1167 | 963.880201 | 1060.43 | 1156.979799 | 0.908680 |  |
| GO:0007346\_regulation\_of\_mitotic\_cell\_cycle | 40 | 0 | 0.000000 | -0.000000 | 1167 | 963.880201 | 1060.43 | 1156.979799 | 0.908680 |  |
| GO:0014031\_mesenchymal\_cell\_development | 40 | 0 | 0.000000 | -0.000000 | 1167 | 963.880201 | 1060.43 | 1156.979799 | 0.908680 |  |
| GO:0016071\_mRNA\_metabolic\_process | 40 | 0 | 0.000000 | -0.000000 | 1167 | 963.880201 | 1060.43 | 1156.979799 | 0.908680 |  |
| GO:0016485\_protein\_processing | 40 | 0 | 0.000000 | -0.000000 | 1167 | 963.880201 | 1060.43 | 1156.979799 | 0.908680 |  |
| GO:0017015\_regulation\_of\_transforming\_growth\_factor\_beta\_receptor\_signaling\_pathway | 40 | 0 | 0.000000 | -0.000000 | 1167 | 963.880201 | 1060.43 | 1156.979799 | 0.908680 |  |
| GO:0019935\_cyclic-nucleotide-mediated\_signaling | 40 | 0 | 0.000000 | -0.000000 | 1167 | 963.880201 | 1060.43 | 1156.979799 | 0.908680 |  |
| GO:0035272\_exocrine\_system\_development | 40 | 0 | 0.000000 | -0.000000 | 1167 | 963.880201 | 1060.43 | 1156.979799 | 0.908680 |  |
| GO:0046850\_regulation\_of\_bone\_remodeling | 40 | 0 | 0.000000 | -0.000000 | 1167 | 963.880201 | 1060.43 | 1156.979799 | 0.908680 |  |
| GO:0051129\_negative\_regulation\_of\_cellular\_component\_organization | 40 | 0 | 0.000000 | -0.000000 | 1167 | 963.880201 | 1060.43 | 1156.979799 | 0.908680 |  |
| GO:0000077\_DNA\_damage\_checkpoint | 14 | 0 | 0.000000 | -0.000000 | 1219 | 1018.168059 | 1113.26 | 1208.351941 | 0.913257 |  |
| GO:0001502\_cartilage\_condensation | 14 | 0 | 0.000000 | -0.000000 | 1219 | 1018.168059 | 1113.26 | 1208.351941 | 0.913257 |  |
| GO:0001829\_trophectodermal\_cell\_differentiation | 14 | 0 | 0.000000 | -0.000000 | 1219 | 1018.168059 | 1113.26 | 1208.351941 | 0.913257 |  |
| GO:0002262\_myeloid\_cell\_homeostasis | 14 | 0 | 0.000000 | -0.000000 | 1219 | 1018.168059 | 1113.26 | 1208.351941 | 0.913257 |  |
| GO:0006304\_DNA\_modification | 14 | 0 | 0.000000 | -0.000000 | 1219 | 1018.168059 | 1113.26 | 1208.351941 | 0.913257 |  |
| GO:0006305\_DNA\_alkylation | 14 | 0 | 0.000000 | -0.000000 | 1219 | 1018.168059 | 1113.26 | 1208.351941 | 0.913257 |  |
| GO:0006306\_DNA\_methylation | 14 | 0 | 0.000000 | -0.000000 | 1219 | 1018.168059 | 1113.26 | 1208.351941 | 0.913257 |  |
| GO:0006695\_cholesterol\_biosynthetic\_process | 14 | 0 | 0.000000 | -0.000000 | 1219 | 1018.168059 | 1113.26 | 1208.351941 | 0.913257 |  |
| GO:0006809\_nitric\_oxide\_biosynthetic\_process | 14 | 0 | 0.000000 | -0.000000 | 1219 | 1018.168059 | 1113.26 | 1208.351941 | 0.913257 |  |
| GO:0006914\_autophagy | 14 | 0 | 0.000000 | -0.000000 | 1219 | 1018.168059 | 1113.26 | 1208.351941 | 0.913257 |  |
| GO:0006970\_response\_to\_osmotic\_stress | 14 | 0 | 0.000000 | -0.000000 | 1219 | 1018.168059 | 1113.26 | 1208.351941 | 0.913257 |  |
| GO:0007530\_sex\_determination | 14 | 0 | 0.000000 | -0.000000 | 1219 | 1018.168059 | 1113.26 | 1208.351941 | 0.913257 |  |
| GO:0007589\_body\_fluid\_secretion | 14 | 0 | 0.000000 | -0.000000 | 1219 | 1018.168059 | 1113.26 | 1208.351941 | 0.913257 |  |
| GO:0008064\_regulation\_of\_actin\_polymerization\_or\_depolymerization | 14 | 0 | 0.000000 | -0.000000 | 1219 | 1018.168059 | 1113.26 | 1208.351941 | 0.913257 |  |
| GO:0008306\_associative\_learning | 14 | 0 | 0.000000 | -0.000000 | 1219 | 1018.168059 | 1113.26 | 1208.351941 | 0.913257 |  |
| GO:0008630\_DNA\_damage\_response\_\_signal\_transduction\_resulting\_in\_induction\_of\_apoptosis | 14 | 0 | 0.000000 | -0.000000 | 1219 | 1018.168059 | 1113.26 | 1208.351941 | 0.913257 |  |
| GO:0009108\_coenzyme\_biosynthetic\_process | 14 | 0 | 0.000000 | -0.000000 | 1219 | 1018.168059 | 1113.26 | 1208.351941 | 0.913257 |  |
| GO:0009267\_cellular\_response\_to\_starvation | 14 | 0 | 0.000000 | -0.000000 | 1219 | 1018.168059 | 1113.26 | 1208.351941 | 0.913257 |  |
| GO:0009895\_negative\_regulation\_of\_catabolic\_process | 14 | 0 | 0.000000 | -0.000000 | 1219 | 1018.168059 | 1113.26 | 1208.351941 | 0.913257 |  |
| GO:0010332\_response\_to\_gamma\_radiation | 14 | 0 | 0.000000 | -0.000000 | 1219 | 1018.168059 | 1113.26 | 1208.351941 | 0.913257 |  |
| GO:0014855\_striated\_muscle\_cell\_proliferation | 14 | 0 | 0.000000 | -0.000000 | 1219 | 1018.168059 | 1113.26 | 1208.351941 | 0.913257 |  |
| GO:0016573\_histone\_acetylation | 14 | 0 | 0.000000 | -0.000000 | 1219 | 1018.168059 | 1113.26 | 1208.351941 | 0.913257 |  |
| GO:0018130\_heterocycle\_biosynthetic\_process | 14 | 0 | 0.000000 | -0.000000 | 1219 | 1018.168059 | 1113.26 | 1208.351941 | 0.913257 |  |
| GO:0019217\_regulation\_of\_fatty\_acid\_metabolic\_process | 14 | 0 | 0.000000 | -0.000000 | 1219 | 1018.168059 | 1113.26 | 1208.351941 | 0.913257 |  |
| GO:0021904\_dorsal\_ventral\_neural\_tube\_patterning | 14 | 0 | 0.000000 | -0.000000 | 1219 | 1018.168059 | 1113.26 | 1208.351941 | 0.913257 |  |
| GO:0030162\_regulation\_of\_proteolysis | 14 | 0 | 0.000000 | -0.000000 | 1219 | 1018.168059 | 1113.26 | 1208.351941 | 0.913257 |  |
| GO:0030832\_regulation\_of\_actin\_filament\_length | 14 | 0 | 0.000000 | -0.000000 | 1219 | 1018.168059 | 1113.26 | 1208.351941 | 0.913257 |  |
| GO:0031099\_regeneration | 14 | 0 | 0.000000 | -0.000000 | 1219 | 1018.168059 | 1113.26 | 1208.351941 | 0.913257 |  |
| GO:0031346\_positive\_regulation\_of\_cell\_projection\_organization | 14 | 0 | 0.000000 | -0.000000 | 1219 | 1018.168059 | 1113.26 | 1208.351941 | 0.913257 |  |
| GO:0031663\_lipopolysaccharide-mediated\_signaling\_pathway | 14 | 0 | 0.000000 | -0.000000 | 1219 | 1018.168059 | 1113.26 | 1208.351941 | 0.913257 |  |
| GO:0032271\_regulation\_of\_protein\_polymerization | 14 | 0 | 0.000000 | -0.000000 | 1219 | 1018.168059 | 1113.26 | 1208.351941 | 0.913257 |  |
| GO:0033044\_regulation\_of\_chromosome\_organization | 14 | 0 | 0.000000 | -0.000000 | 1219 | 1018.168059 | 1113.26 | 1208.351941 | 0.913257 |  |
| GO:0034104\_negative\_regulation\_of\_tissue\_remodeling | 14 | 0 | 0.000000 | -0.000000 | 1219 | 1018.168059 | 1113.26 | 1208.351941 | 0.913257 |  |
| GO:0034623\_cellular\_macromolecular\_complex\_disassembly | 14 | 0 | 0.000000 | -0.000000 | 1219 | 1018.168059 | 1113.26 | 1208.351941 | 0.913257 |  |
| GO:0035036\_sperm-egg\_recognition | 14 | 0 | 0.000000 | -0.000000 | 1219 | 1018.168059 | 1113.26 | 1208.351941 | 0.913257 |  |
| GO:0042573\_retinoic\_acid\_metabolic\_process | 14 | 0 | 0.000000 | -0.000000 | 1219 | 1018.168059 | 1113.26 | 1208.351941 | 0.913257 |  |
| GO:0043123\_positive\_regulation\_of\_I-kappaB\_kinase\_NF-kappaB\_cascade | 14 | 0 | 0.000000 | -0.000000 | 1219 | 1018.168059 | 1113.26 | 1208.351941 | 0.913257 |  |
| GO:0043254\_regulation\_of\_protein\_complex\_assembly | 14 | 0 | 0.000000 | -0.000000 | 1219 | 1018.168059 | 1113.26 | 1208.351941 | 0.913257 |  |
| GO:0043491\_protein\_kinase\_B\_signaling\_cascade | 14 | 0 | 0.000000 | -0.000000 | 1219 | 1018.168059 | 1113.26 | 1208.351941 | 0.913257 |  |
| GO:0044236\_multicellular\_organismal\_metabolic\_process | 14 | 0 | 0.000000 | -0.000000 | 1219 | 1018.168059 | 1113.26 | 1208.351941 | 0.913257 |  |
| GO:0045453\_bone\_resorption | 14 | 0 | 0.000000 | -0.000000 | 1219 | 1018.168059 | 1113.26 | 1208.351941 | 0.913257 |  |
| GO:0045598\_regulation\_of\_fat\_cell\_differentiation | 14 | 0 | 0.000000 | -0.000000 | 1219 | 1018.168059 | 1113.26 | 1208.351941 | 0.913257 |  |
| GO:0045732\_positive\_regulation\_of\_protein\_catabolic\_process | 14 | 0 | 0.000000 | -0.000000 | 1219 | 1018.168059 | 1113.26 | 1208.351941 | 0.913257 |  |
| GO:0046209\_nitric\_oxide\_metabolic\_process | 14 | 0 | 0.000000 | -0.000000 | 1219 | 1018.168059 | 1113.26 | 1208.351941 | 0.913257 |  |
| GO:0048048\_embryonic\_eye\_morphogenesis | 14 | 0 | 0.000000 | -0.000000 | 1219 | 1018.168059 | 1113.26 | 1208.351941 | 0.913257 |  |
| GO:0048545\_response\_to\_steroid\_hormone\_stimulus | 14 | 0 | 0.000000 | -0.000000 | 1219 | 1018.168059 | 1113.26 | 1208.351941 | 0.913257 |  |
| GO:0048844\_artery\_morphogenesis | 14 | 0 | 0.000000 | -0.000000 | 1219 | 1018.168059 | 1113.26 | 1208.351941 | 0.913257 |  |
| GO:0051017\_actin\_filament\_bundle\_formation | 14 | 0 | 0.000000 | -0.000000 | 1219 | 1018.168059 | 1113.26 | 1208.351941 | 0.913257 |  |
| GO:0051053\_negative\_regulation\_of\_DNA\_metabolic\_process | 14 | 0 | 0.000000 | -0.000000 | 1219 | 1018.168059 | 1113.26 | 1208.351941 | 0.913257 |  |
| GO:0051952\_regulation\_of\_amine\_transport | 14 | 0 | 0.000000 | -0.000000 | 1219 | 1018.168059 | 1113.26 | 1208.351941 | 0.913257 |  |
| GO:0060716\_labyrinthine\_layer\_blood\_vessel\_development | 14 | 0 | 0.000000 | -0.000000 | 1219 | 1018.168059 | 1113.26 | 1208.351941 | 0.913257 |  |
| GO:0060840\_artery\_development | 14 | 0 | 0.000000 | -0.000000 | 1219 | 1018.168059 | 1113.26 | 1208.351941 | 0.913257 |  |
| GO:0001759\_induction\_of\_an\_organ | 15 | 0 | 0.000000 | -0.000000 | 1265 | 1065.163749 | 1158.06 | 1250.956251 | 0.915462 |  |
| GO:0001782\_B\_cell\_homeostasis | 15 | 0 | 0.000000 | -0.000000 | 1265 | 1065.163749 | 1158.06 | 1250.956251 | 0.915462 |  |
| GO:0001964\_startle\_response | 15 | 0 | 0.000000 | -0.000000 | 1265 | 1065.163749 | 1158.06 | 1250.956251 | 0.915462 |  |
| GO:0002286\_T\_cell\_activation\_during\_immune\_response | 15 | 0 | 0.000000 | -0.000000 | 1265 | 1065.163749 | 1158.06 | 1250.956251 | 0.915462 |  |
| GO:0002495\_antigen\_processing\_and\_presentation\_of\_peptide\_antigen\_via\_MHC\_class\_II | 15 | 0 | 0.000000 | -0.000000 | 1265 | 1065.163749 | 1158.06 | 1250.956251 | 0.915462 |  |
| GO:0002504\_antigen\_processing\_and\_presentation\_of\_peptide\_or\_polysaccharide\_antigen\_via\_MHC\_class\_II | 15 | 0 | 0.000000 | -0.000000 | 1265 | 1065.163749 | 1158.06 | 1250.956251 | 0.915462 |  |
| GO:0006473\_protein\_amino\_acid\_acetylation | 15 | 0 | 0.000000 | -0.000000 | 1265 | 1065.163749 | 1158.06 | 1250.956251 | 0.915462 |  |
| GO:0006487\_protein\_amino\_acid\_N-linked\_glycosylation | 15 | 0 | 0.000000 | -0.000000 | 1265 | 1065.163749 | 1158.06 | 1250.956251 | 0.915462 |  |
| GO:0006749\_glutathione\_metabolic\_process | 15 | 0 | 0.000000 | -0.000000 | 1265 | 1065.163749 | 1158.06 | 1250.956251 | 0.915462 |  |
| GO:0007173\_epidermal\_growth\_factor\_receptor\_signaling\_pathway | 15 | 0 | 0.000000 | -0.000000 | 1265 | 1065.163749 | 1158.06 | 1250.956251 | 0.915462 |  |
| GO:0007200\_activation\_of\_phospholipase\_C\_activity\_by\_G-protein\_coupled\_receptor\_protein\_signaling\_pathway\_coupled\_to\_IP3\_second\_messenger | 15 | 0 | 0.000000 | -0.000000 | 1265 | 1065.163749 | 1158.06 | 1250.956251 | 0.915462 |  |
| GO:0007202\_activation\_of\_phospholipase\_C\_activity | 15 | 0 | 0.000000 | -0.000000 | 1265 | 1065.163749 | 1158.06 | 1250.956251 | 0.915462 |  |
| GO:0007218\_neuropeptide\_signaling\_pathway | 15 | 0 | 0.000000 | -0.000000 | 1265 | 1065.163749 | 1158.06 | 1250.956251 | 0.915462 |  |
| GO:0007588\_excretion | 15 | 0 | 0.000000 | -0.000000 | 1265 | 1065.163749 | 1158.06 | 1250.956251 | 0.915462 |  |
| GO:0008543\_fibroblast\_growth\_factor\_receptor\_signaling\_pathway | 15 | 0 | 0.000000 | -0.000000 | 1265 | 1065.163749 | 1158.06 | 1250.956251 | 0.915462 |  |
| GO:0009062\_fatty\_acid\_catabolic\_process | 15 | 0 | 0.000000 | -0.000000 | 1265 | 1065.163749 | 1158.06 | 1250.956251 | 0.915462 |  |
| GO:0009116\_nucleoside\_metabolic\_process | 15 | 0 | 0.000000 | -0.000000 | 1265 | 1065.163749 | 1158.06 | 1250.956251 | 0.915462 |  |
| GO:0010092\_specification\_of\_organ\_identity | 15 | 0 | 0.000000 | -0.000000 | 1265 | 1065.163749 | 1158.06 | 1250.956251 | 0.915462 |  |
| GO:0010518\_positive\_regulation\_of\_phospholipase\_activity | 15 | 0 | 0.000000 | -0.000000 | 1265 | 1065.163749 | 1158.06 | 1250.956251 | 0.915462 |  |
| GO:0010863\_positive\_regulation\_of\_phospholipase\_C\_activity | 15 | 0 | 0.000000 | -0.000000 | 1265 | 1065.163749 | 1158.06 | 1250.956251 | 0.915462 |  |
| GO:0015931\_nucleobase\_\_nucleoside\_\_nucleotide\_and\_nucleic\_acid\_transport | 15 | 0 | 0.000000 | -0.000000 | 1265 | 1065.163749 | 1158.06 | 1250.956251 | 0.915462 |  |
| GO:0019886\_antigen\_processing\_and\_presentation\_of\_exogenous\_peptide\_antigen\_via\_MHC\_class\_II | 15 | 0 | 0.000000 | -0.000000 | 1265 | 1065.163749 | 1158.06 | 1250.956251 | 0.915462 |  |
| GO:0021795\_cerebral\_cortex\_cell\_migration | 15 | 0 | 0.000000 | -0.000000 | 1265 | 1065.163749 | 1158.06 | 1250.956251 | 0.915462 |  |
| GO:0021872\_generation\_of\_neurons\_in\_the\_forebrain | 15 | 0 | 0.000000 | -0.000000 | 1265 | 1065.163749 | 1158.06 | 1250.956251 | 0.915462 |  |
| GO:0022600\_digestive\_system\_process | 15 | 0 | 0.000000 | -0.000000 | 1265 | 1065.163749 | 1158.06 | 1250.956251 | 0.915462 |  |
| GO:0030041\_actin\_filament\_polymerization | 15 | 0 | 0.000000 | -0.000000 | 1265 | 1065.163749 | 1158.06 | 1250.956251 | 0.915462 |  |
| GO:0031069\_hair\_follicle\_morphogenesis | 15 | 0 | 0.000000 | -0.000000 | 1265 | 1065.163749 | 1158.06 | 1250.956251 | 0.915462 |  |
| GO:0031076\_embryonic\_camera-type\_eye\_development | 15 | 0 | 0.000000 | -0.000000 | 1265 | 1065.163749 | 1158.06 | 1250.956251 | 0.915462 |  |
| GO:0031329\_regulation\_of\_cellular\_catabolic\_process | 15 | 0 | 0.000000 | -0.000000 | 1265 | 1065.163749 | 1158.06 | 1250.956251 | 0.915462 |  |
| GO:0035116\_embryonic\_hindlimb\_morphogenesis | 15 | 0 | 0.000000 | -0.000000 | 1265 | 1065.163749 | 1158.06 | 1250.956251 | 0.915462 |  |
| GO:0035249\_synaptic\_transmission\_\_glutamatergic | 15 | 0 | 0.000000 | -0.000000 | 1265 | 1065.163749 | 1158.06 | 1250.956251 | 0.915462 |  |
| GO:0042306\_regulation\_of\_protein\_import\_into\_nucleus | 15 | 0 | 0.000000 | -0.000000 | 1265 | 1065.163749 | 1158.06 | 1250.956251 | 0.915462 |  |
| GO:0046164\_alcohol\_catabolic\_process | 15 | 0 | 0.000000 | -0.000000 | 1265 | 1065.163749 | 1158.06 | 1250.956251 | 0.915462 |  |
| GO:0046638\_positive\_regulation\_of\_alpha-beta\_T\_cell\_differentiation | 15 | 0 | 0.000000 | -0.000000 | 1265 | 1065.163749 | 1158.06 | 1250.956251 | 0.915462 |  |
| GO:0048144\_fibroblast\_proliferation | 15 | 0 | 0.000000 | -0.000000 | 1265 | 1065.163749 | 1158.06 | 1250.956251 | 0.915462 |  |
| GO:0048145\_regulation\_of\_fibroblast\_proliferation | 15 | 0 | 0.000000 | -0.000000 | 1265 | 1065.163749 | 1158.06 | 1250.956251 | 0.915462 |  |
| GO:0048610\_reproductive\_cellular\_process | 15 | 0 | 0.000000 | -0.000000 | 1265 | 1065.163749 | 1158.06 | 1250.956251 | 0.915462 |  |
| GO:0050729\_positive\_regulation\_of\_inflammatory\_response | 15 | 0 | 0.000000 | -0.000000 | 1265 | 1065.163749 | 1158.06 | 1250.956251 | 0.915462 |  |
| GO:0050796\_regulation\_of\_insulin\_secretion | 15 | 0 | 0.000000 | -0.000000 | 1265 | 1065.163749 | 1158.06 | 1250.956251 | 0.915462 |  |
| GO:0050798\_activated\_T\_cell\_proliferation | 15 | 0 | 0.000000 | -0.000000 | 1265 | 1065.163749 | 1158.06 | 1250.956251 | 0.915462 |  |
| GO:0055010\_ventricular\_cardiac\_muscle\_morphogenesis | 15 | 0 | 0.000000 | -0.000000 | 1265 | 1065.163749 | 1158.06 | 1250.956251 | 0.915462 |  |
| GO:0060425\_lung\_morphogenesis | 15 | 0 | 0.000000 | -0.000000 | 1265 | 1065.163749 | 1158.06 | 1250.956251 | 0.915462 |  |
| GO:0060442\_branching\_involved\_in\_prostate\_gland\_morphogenesis | 15 | 0 | 0.000000 | -0.000000 | 1265 | 1065.163749 | 1158.06 | 1250.956251 | 0.915462 |  |
| GO:0060749\_mammary\_gland\_alveolus\_development | 15 | 0 | 0.000000 | -0.000000 | 1265 | 1065.163749 | 1158.06 | 1250.956251 | 0.915462 |  |
| GO:0070227\_lymphocyte\_apoptosis | 15 | 0 | 0.000000 | -0.000000 | 1265 | 1065.163749 | 1158.06 | 1250.956251 | 0.915462 |  |
| GO:0070507\_regulation\_of\_microtubule\_cytoskeleton\_organization | 15 | 0 | 0.000000 | -0.000000 | 1265 | 1065.163749 | 1158.06 | 1250.956251 | 0.915462 |  |
| GO:0002237\_response\_to\_molecule\_of\_bacterial\_origin | 34 | 0 | 0.000000 | -0.000000 | 1272 | 1077.470950 | 1169.03 | 1260.589050 | 0.919049 |  |
| GO:0007269\_neurotransmitter\_secretion | 34 | 0 | 0.000000 | -0.000000 | 1272 | 1077.470950 | 1169.03 | 1260.589050 | 0.919049 |  |
| GO:0016054\_organic\_acid\_catabolic\_process | 34 | 0 | 0.000000 | -0.000000 | 1272 | 1077.470950 | 1169.03 | 1260.589050 | 0.919049 |  |
| GO:0019882\_antigen\_processing\_and\_presentation | 34 | 0 | 0.000000 | -0.000000 | 1272 | 1077.470950 | 1169.03 | 1260.589050 | 0.919049 |  |
| GO:0045927\_positive\_regulation\_of\_growth | 34 | 0 | 0.000000 | -0.000000 | 1272 | 1077.470950 | 1169.03 | 1260.589050 | 0.919049 |  |
| GO:0046395\_carboxylic\_acid\_catabolic\_process | 34 | 0 | 0.000000 | -0.000000 | 1272 | 1077.470950 | 1169.03 | 1260.589050 | 0.919049 |  |
| GO:0051047\_positive\_regulation\_of\_secretion | 34 | 0 | 0.000000 | -0.000000 | 1272 | 1077.470950 | 1169.03 | 1260.589050 | 0.919049 |  |
| GO:0000084\_S\_phase\_of\_mitotic\_cell\_cycle | 3 | 0 |  |  |  |  |  |  |  |  |
| GO:0000089\_mitotic\_metaphase | 3 | 0 |  |  |  |  |  |  |  |  |
| GO:0000098\_sulfur\_amino\_acid\_catabolic\_process | 3 | 0 |  |  |  |  |  |  |  |  |
| GO:0000103\_sulfate\_assimilation | 3 | 0 |  |  |  |  |  |  |  |  |
| GO:0000212\_meiotic\_spindle\_organization | 3 | 0 |  |  |  |  |  |  |  |  |
| GO:0000281\_cytokinesis\_after\_mitosis | 3 | 0 |  |  |  |  |  |  |  |  |
| GO:0000303\_response\_to\_superoxide | 3 | 0 |  |  |  |  |  |  |  |  |
| GO:0000320\_re-entry\_into\_mitotic\_cell\_cycle | 3 | 0 |  |  |  |  |  |  |  |  |
| GO:0000380\_alternative\_nuclear\_mRNA\_splicing\_\_via\_spliceosome | 3 | 0 |  |  |  |  |  |  |  |  |
| GO:0001516\_prostaglandin\_biosynthetic\_process | 3 | 0 |  |  |  |  |  |  |  |  |
| GO:0001553\_luteinization | 3 | 0 |  |  |  |  |  |  |  |  |
| GO:0001574\_ganglioside\_biosynthetic\_process | 3 | 0 |  |  |  |  |  |  |  |  |
| GO:0001705\_ectoderm\_formation | 3 | 0 |  |  |  |  |  |  |  |  |
| GO:0001711\_endodermal\_cell\_fate\_commitment | 3 | 0 |  |  |  |  |  |  |  |  |
| GO:0001757\_somite\_specification | 3 | 0 |  |  |  |  |  |  |  |  |
| GO:0001778\_plasma\_membrane\_repair | 3 | 0 |  |  |  |  |  |  |  |  |
| GO:0001780\_neutrophil\_homeostasis | 3 | 0 |  |  |  |  |  |  |  |  |
| GO:0001802\_type\_III\_hypersensitivity | 3 | 0 |  |  |  |  |  |  |  |  |
| GO:0001803\_regulation\_of\_type\_III\_hypersensitivity | 3 | 0 |  |  |  |  |  |  |  |  |
| GO:0001805\_positive\_regulation\_of\_type\_III\_hypersensitivity | 3 | 0 |  |  |  |  |  |  |  |  |
| GO:0001812\_positive\_regulation\_of\_type\_I\_hypersensitivity | 3 | 0 |  |  |  |  |  |  |  |  |
| GO:0001831\_trophectodermal\_cellular\_morphogenesis | 3 | 0 |  |  |  |  |  |  |  |  |
| GO:0001844\_protein\_insertion\_into\_mitochondrial\_membrane\_during\_induction\_of\_apoptosis | 3 | 0 |  |  |  |  |  |  |  |  |
| GO:0001878\_response\_to\_yeast | 3 | 0 |  |  |  |  |  |  |  |  |
| GO:0001895\_retina\_homeostasis | 3 | 0 |  |  |  |  |  |  |  |  |
| GO:0001937\_negative\_regulation\_of\_endothelial\_cell\_proliferation | 3 | 0 |  |  |  |  |  |  |  |  |
| GO:0001953\_negative\_regulation\_of\_cell-matrix\_adhesion | 3 | 0 |  |  |  |  |  |  |  |  |
| GO:0001955\_blood\_vessel\_maturation | 3 | 0 |  |  |  |  |  |  |  |  |
| GO:0001973\_adenosine\_receptor\_signaling\_pathway | 3 | 0 |  |  |  |  |  |  |  |  |
| GO:0001996\_positive\_regulation\_of\_heart\_rate\_by\_epinephrine-norepinephrine | 3 | 0 |  |  |  |  |  |  |  |  |
| GO:0002034\_regulation\_of\_blood\_vessel\_size\_by\_renin-angiotensin | 3 | 0 |  |  |  |  |  |  |  |  |
| GO:0002238\_response\_to\_molecule\_of\_fungal\_origin | 3 | 0 |  |  |  |  |  |  |  |  |
| GO:0002275\_myeloid\_cell\_activation\_during\_immune\_response | 3 | 0 |  |  |  |  |  |  |  |  |
| GO:0002281\_macrophage\_activation\_during\_immune\_response | 3 | 0 |  |  |  |  |  |  |  |  |
| GO:0002309\_T\_cell\_proliferation\_during\_immune\_response | 3 | 0 |  |  |  |  |  |  |  |  |
| GO:0002361\_CD4-positive\_\_CD25-positive\_\_alpha-beta\_regulatory\_T\_cell\_differentiation | 3 | 0 |  |  |  |  |  |  |  |  |
| GO:0002369\_T\_cell\_cytokine\_production | 3 | 0 |  |  |  |  |  |  |  |  |
| GO:0002428\_antigen\_processing\_and\_presentation\_of\_peptide\_antigen\_via\_MHC\_class\_Ib | 3 | 0 |  |  |  |  |  |  |  |  |
| GO:0002446\_neutrophil\_mediated\_immunity | 3 | 0 |  |  |  |  |  |  |  |  |
| GO:0002477\_antigen\_processing\_and\_presentation\_of\_exogenous\_peptide\_antigen\_via\_MHC\_class\_Ib | 3 | 0 |  |  |  |  |  |  |  |  |
| GO:0002481\_antigen\_processing\_and\_presentation\_of\_exogenous\_protein\_antigen\_via\_MHC\_class\_Ib\_\_TAP-dependent | 3 | 0 |  |  |  |  |  |  |  |  |
| GO:0002513\_tolerance\_induction\_to\_self\_antigen | 3 | 0 |  |  |  |  |  |  |  |  |
| GO:0002568\_somatic\_diversification\_of\_T\_cell\_receptor\_genes | 3 | 0 |  |  |  |  |  |  |  |  |
| GO:0002674\_negative\_regulation\_of\_acute\_inflammatory\_response | 3 | 0 |  |  |  |  |  |  |  |  |
| GO:0002681\_somatic\_recombination\_of\_T\_cell\_receptor\_gene\_segments | 3 | 0 |  |  |  |  |  |  |  |  |
| GO:0002713\_negative\_regulation\_of\_B\_cell\_mediated\_immunity | 3 | 0 |  |  |  |  |  |  |  |  |
| GO:0002827\_positive\_regulation\_of\_T-helper\_1\_type\_immune\_response | 3 | 0 |  |  |  |  |  |  |  |  |
| GO:0002865\_negative\_regulation\_of\_acute\_inflammatory\_response\_to\_antigenic\_stimulus | 3 | 0 |  |  |  |  |  |  |  |  |
| GO:0002884\_negative\_regulation\_of\_hypersensitivity | 3 | 0 |  |  |  |  |  |  |  |  |
| GO:0002890\_negative\_regulation\_of\_immunoglobulin\_mediated\_immune\_response | 3 | 0 |  |  |  |  |  |  |  |  |
| GO:0002904\_positive\_regulation\_of\_B\_cell\_apoptosis | 3 | 0 |  |  |  |  |  |  |  |  |
| GO:0003009\_skeletal\_muscle\_contraction | 3 | 0 |  |  |  |  |  |  |  |  |
| GO:0003072\_renal\_control\_of\_peripheral\_vascular\_resistance\_involved\_in\_regulation\_of\_systemic\_arterial\_blood\_pressure | 3 | 0 |  |  |  |  |  |  |  |  |
| GO:0006047\_UDP-N-acetylglucosamine\_metabolic\_process | 3 | 0 |  |  |  |  |  |  |  |  |
| GO:0006067\_ethanol\_metabolic\_process | 3 | 0 |  |  |  |  |  |  |  |  |
| GO:0006072\_glycerol-3-phosphate\_metabolic\_process | 3 | 0 |  |  |  |  |  |  |  |  |
| GO:0006103\_2-oxoglutarate\_metabolic\_process | 3 | 0 |  |  |  |  |  |  |  |  |
| GO:0006107\_oxaloacetate\_metabolic\_process | 3 | 0 |  |  |  |  |  |  |  |  |
| GO:0006166\_purine\_ribonucleoside\_salvage | 3 | 0 |  |  |  |  |  |  |  |  |
| GO:0006220\_pyrimidine\_nucleotide\_metabolic\_process | 3 | 0 |  |  |  |  |  |  |  |  |
| GO:0006266\_DNA\_ligation | 3 | 0 |  |  |  |  |  |  |  |  |
| GO:0006282\_regulation\_of\_DNA\_repair | 3 | 0 |  |  |  |  |  |  |  |  |
| GO:0006287\_base-excision\_repair\_\_gap-filling | 3 | 0 |  |  |  |  |  |  |  |  |
| GO:0006301\_postreplication\_repair | 3 | 0 |  |  |  |  |  |  |  |  |
| GO:0006361\_transcription\_initiation\_from\_RNA\_polymerase\_I\_promoter | 3 | 0 |  |  |  |  |  |  |  |  |
| GO:0006367\_transcription\_initiation\_from\_RNA\_polymerase\_II\_promoter | 3 | 0 |  |  |  |  |  |  |  |  |
| GO:0006414\_translational\_elongation | 3 | 0 |  |  |  |  |  |  |  |  |
| GO:0006491\_N-glycan\_processing | 3 | 0 |  |  |  |  |  |  |  |  |
| GO:0006498\_N-terminal\_protein\_lipidation | 3 | 0 |  |  |  |  |  |  |  |  |
| GO:0006531\_aspartate\_metabolic\_process | 3 | 0 |  |  |  |  |  |  |  |  |
| GO:0006598\_polyamine\_catabolic\_process | 3 | 0 |  |  |  |  |  |  |  |  |
| GO:0006620\_posttranslational\_protein\_targeting\_to\_membrane | 3 | 0 |  |  |  |  |  |  |  |  |
| GO:0006625\_protein\_targeting\_to\_peroxisome | 3 | 0 |  |  |  |  |  |  |  |  |
| GO:0006677\_glycosylceramide\_metabolic\_process | 3 | 0 |  |  |  |  |  |  |  |  |
| GO:0006699\_bile\_acid\_biosynthetic\_process | 3 | 0 |  |  |  |  |  |  |  |  |
| GO:0006791\_sulfur\_utilization | 3 | 0 |  |  |  |  |  |  |  |  |
| GO:0006817\_phosphate\_transport | 3 | 0 |  |  |  |  |  |  |  |  |
| GO:0006825\_copper\_ion\_transport | 3 | 0 |  |  |  |  |  |  |  |  |
| GO:0006828\_manganese\_ion\_transport | 3 | 0 |  |  |  |  |  |  |  |  |
| GO:0006857\_oligopeptide\_transport | 3 | 0 |  |  |  |  |  |  |  |  |
| GO:0006892\_post-Golgi\_vesicle-mediated\_transport | 3 | 0 |  |  |  |  |  |  |  |  |
| GO:0006904\_vesicle\_docking\_during\_exocytosis | 3 | 0 |  |  |  |  |  |  |  |  |
| GO:0006926\_virus-infected\_cell\_apoptosis | 3 | 0 |  |  |  |  |  |  |  |  |
| GO:0006953\_acute-phase\_response | 3 | 0 |  |  |  |  |  |  |  |  |
| GO:0007000\_nucleolus\_organization | 3 | 0 |  |  |  |  |  |  |  |  |
| GO:0007041\_lysosomal\_transport | 3 | 0 |  |  |  |  |  |  |  |  |
| GO:0007043\_cell-cell\_junction\_assembly | 3 | 0 |  |  |  |  |  |  |  |  |
| GO:0007090\_regulation\_of\_S\_phase\_of\_mitotic\_cell\_cycle | 3 | 0 |  |  |  |  |  |  |  |  |
| GO:0007195\_inhibition\_of\_adenylate\_cyclase\_activity\_by\_dopamine\_receptor\_signaling\_pathway | 3 | 0 |  |  |  |  |  |  |  |  |
| GO:0007199\_G-protein\_signaling\_\_coupled\_to\_cGMP\_nucleotide\_second\_messenger | 3 | 0 |  |  |  |  |  |  |  |  |
| GO:0007213\_muscarinic\_acetylcholine\_receptor\_signaling\_pathway | 3 | 0 |  |  |  |  |  |  |  |  |
| GO:0007250\_activation\_of\_NF-kappaB-inducing\_kinase\_activity | 3 | 0 |  |  |  |  |  |  |  |  |
| GO:0007252\_I-kappaB\_phosphorylation | 3 | 0 |  |  |  |  |  |  |  |  |
| GO:0007288\_sperm\_axoneme\_assembly | 3 | 0 |  |  |  |  |  |  |  |  |
| GO:0007350\_blastoderm\_segmentation | 3 | 0 |  |  |  |  |  |  |  |  |
| GO:0007403\_glial\_cell\_fate\_determination | 3 | 0 |  |  |  |  |  |  |  |  |
| GO:0007412\_axon\_target\_recognition | 3 | 0 |  |  |  |  |  |  |  |  |
| GO:0007468\_regulation\_of\_rhodopsin\_gene\_expression | 3 | 0 |  |  |  |  |  |  |  |  |
| GO:0007525\_somatic\_muscle\_development | 3 | 0 |  |  |  |  |  |  |  |  |
| GO:0007635\_chemosensory\_behavior | 3 | 0 |  |  |  |  |  |  |  |  |
| GO:0008090\_retrograde\_axon\_cargo\_transport | 3 | 0 |  |  |  |  |  |  |  |  |
| GO:0008635\_activation\_of\_caspase\_activity\_by\_cytochrome\_c | 3 | 0 |  |  |  |  |  |  |  |  |
| GO:0009060\_aerobic\_respiration | 3 | 0 |  |  |  |  |  |  |  |  |
| GO:0009081\_branched\_chain\_family\_amino\_acid\_metabolic\_process | 3 | 0 |  |  |  |  |  |  |  |  |
| GO:0009086\_methionine\_biosynthetic\_process | 3 | 0 |  |  |  |  |  |  |  |  |
| GO:0009135\_purine\_nucleoside\_diphosphate\_metabolic\_process | 3 | 0 |  |  |  |  |  |  |  |  |
| GO:0009137\_purine\_nucleoside\_diphosphate\_catabolic\_process | 3 | 0 |  |  |  |  |  |  |  |  |
| GO:0009155\_purine\_deoxyribonucleotide\_catabolic\_process | 3 | 0 |  |  |  |  |  |  |  |  |
| GO:0009179\_purine\_ribonucleoside\_diphosphate\_metabolic\_process | 3 | 0 |  |  |  |  |  |  |  |  |
| GO:0009181\_purine\_ribonucleoside\_diphosphate\_catabolic\_process | 3 | 0 |  |  |  |  |  |  |  |  |
| GO:0009185\_ribonucleoside\_diphosphate\_metabolic\_process | 3 | 0 |  |  |  |  |  |  |  |  |
| GO:0009191\_ribonucleoside\_diphosphate\_catabolic\_process | 3 | 0 |  |  |  |  |  |  |  |  |
| GO:0009199\_ribonucleoside\_triphosphate\_metabolic\_process | 3 | 0 |  |  |  |  |  |  |  |  |
| GO:0009204\_deoxyribonucleoside\_triphosphate\_catabolic\_process | 3 | 0 |  |  |  |  |  |  |  |  |
| GO:0009205\_purine\_ribonucleoside\_triphosphate\_metabolic\_process | 3 | 0 |  |  |  |  |  |  |  |  |
| GO:0009217\_purine\_deoxyribonucleoside\_triphosphate\_catabolic\_process | 3 | 0 |  |  |  |  |  |  |  |  |
| GO:0009448\_gamma-aminobutyric\_acid\_metabolic\_process | 3 | 0 |  |  |  |  |  |  |  |  |
| GO:0010043\_response\_to\_zinc\_ion | 3 | 0 |  |  |  |  |  |  |  |  |
| GO:0010172\_embryonic\_body\_morphogenesis | 3 | 0 |  |  |  |  |  |  |  |  |
| GO:0010216\_maintenance\_of\_DNA\_methylation | 3 | 0 |  |  |  |  |  |  |  |  |
| GO:0010273\_detoxification\_of\_copper\_ion | 3 | 0 |  |  |  |  |  |  |  |  |
| GO:0010454\_negative\_regulation\_of\_cell\_fate\_commitment | 3 | 0 |  |  |  |  |  |  |  |  |
| GO:0010507\_negative\_regulation\_of\_autophagy | 3 | 0 |  |  |  |  |  |  |  |  |
| GO:0010524\_positive\_regulation\_of\_calcium\_ion\_transport\_into\_cytosol | 3 | 0 |  |  |  |  |  |  |  |  |
| GO:0010573\_vascular\_endothelial\_growth\_factor\_production | 3 | 0 |  |  |  |  |  |  |  |  |
| GO:0010574\_regulation\_of\_vascular\_endothelial\_growth\_factor\_production | 3 | 0 |  |  |  |  |  |  |  |  |
| GO:0010575\_positive\_regulation\_vascular\_endothelial\_growth\_factor\_production | 3 | 0 |  |  |  |  |  |  |  |  |
| GO:0010632\_regulation\_of\_epithelial\_cell\_migration | 3 | 0 |  |  |  |  |  |  |  |  |
| GO:0010717\_regulation\_of\_epithelial\_to\_mesenchymal\_transition | 3 | 0 |  |  |  |  |  |  |  |  |
| GO:0010884\_positive\_regulation\_of\_lipid\_storage | 3 | 0 |  |  |  |  |  |  |  |  |
| GO:0010888\_negative\_regulation\_of\_lipid\_storage | 3 | 0 |  |  |  |  |  |  |  |  |
| GO:0010889\_regulation\_of\_sequestering\_of\_triglyceride | 3 | 0 |  |  |  |  |  |  |  |  |
| GO:0010893\_positive\_regulation\_of\_steroid\_biosynthetic\_process | 3 | 0 |  |  |  |  |  |  |  |  |
| GO:0010894\_negative\_regulation\_of\_steroid\_biosynthetic\_process | 3 | 0 |  |  |  |  |  |  |  |  |
| GO:0010998\_regulation\_of\_translational\_initiation\_by\_eIF2\_alpha\_phosphorylation | 3 | 0 |  |  |  |  |  |  |  |  |
| GO:0010999\_regulation\_of\_eIF2\_alpha\_phosphorylation\_by\_heme | 3 | 0 |  |  |  |  |  |  |  |  |
| GO:0014074\_response\_to\_purine | 3 | 0 |  |  |  |  |  |  |  |  |
| GO:0014909\_smooth\_muscle\_cell\_migration | 3 | 0 |  |  |  |  |  |  |  |  |
| GO:0015669\_gas\_transport | 3 | 0 |  |  |  |  |  |  |  |  |
| GO:0015760\_glucose-6-phosphate\_transport | 3 | 0 |  |  |  |  |  |  |  |  |
| GO:0015816\_glycine\_transport | 3 | 0 |  |  |  |  |  |  |  |  |
| GO:0015838\_betaine\_transport | 3 | 0 |  |  |  |  |  |  |  |  |
| GO:0015871\_choline\_transport | 3 | 0 |  |  |  |  |  |  |  |  |
| GO:0015879\_carnitine\_transport | 3 | 0 |  |  |  |  |  |  |  |  |
| GO:0015893\_drug\_transport | 3 | 0 |  |  |  |  |  |  |  |  |
| GO:0015909\_long-chain\_fatty\_acid\_transport | 3 | 0 |  |  |  |  |  |  |  |  |
| GO:0015936\_coenzyme\_A\_metabolic\_process | 3 | 0 |  |  |  |  |  |  |  |  |
| GO:0015988\_energy\_coupled\_proton\_transport\_\_against\_electrochemical\_gradient | 3 | 0 |  |  |  |  |  |  |  |  |
| GO:0015991\_ATP\_hydrolysis\_coupled\_proton\_transport | 3 | 0 |  |  |  |  |  |  |  |  |
| GO:0016241\_regulation\_of\_macroautophagy | 3 | 0 |  |  |  |  |  |  |  |  |
| GO:0016322\_neuron\_remodeling | 3 | 0 |  |  |  |  |  |  |  |  |
| GO:0016556\_mRNA\_modification | 3 | 0 |  |  |  |  |  |  |  |  |
| GO:0016973\_poly(A)+\_mRNA\_export\_from\_nucleus | 3 | 0 |  |  |  |  |  |  |  |  |
| GO:0018196\_peptidyl-asparagine\_modification | 3 | 0 |  |  |  |  |  |  |  |  |
| GO:0018208\_peptidyl-proline\_modification | 3 | 0 |  |  |  |  |  |  |  |  |
| GO:0018279\_protein\_amino\_acid\_N-linked\_glycosylation\_via\_asparagine | 3 | 0 |  |  |  |  |  |  |  |  |
| GO:0018894\_dibenzo-p-dioxin\_metabolic\_process | 3 | 0 |  |  |  |  |  |  |  |  |
| GO:0019058\_viral\_infectious\_cycle | 3 | 0 |  |  |  |  |  |  |  |  |
| GO:0019230\_proprioception | 3 | 0 |  |  |  |  |  |  |  |  |
| GO:0019236\_response\_to\_pheromone | 3 | 0 |  |  |  |  |  |  |  |  |
| GO:0019359\_nicotinamide\_nucleotide\_biosynthetic\_process | 3 | 0 |  |  |  |  |  |  |  |  |
| GO:0019363\_pyridine\_nucleotide\_biosynthetic\_process | 3 | 0 |  |  |  |  |  |  |  |  |
| GO:0019438\_aromatic\_compound\_biosynthetic\_process | 3 | 0 |  |  |  |  |  |  |  |  |
| GO:0019439\_aromatic\_compound\_catabolic\_process | 3 | 0 |  |  |  |  |  |  |  |  |
| GO:0019605\_butyrate\_metabolic\_process | 3 | 0 |  |  |  |  |  |  |  |  |
| GO:0019614\_catechol\_catabolic\_process | 3 | 0 |  |  |  |  |  |  |  |  |
| GO:0019674\_NAD\_metabolic\_process | 3 | 0 |  |  |  |  |  |  |  |  |
| GO:0019852\_L-ascorbic\_acid\_metabolic\_process | 3 | 0 |  |  |  |  |  |  |  |  |
| GO:0019934\_cGMP-mediated\_signaling | 3 | 0 |  |  |  |  |  |  |  |  |
| GO:0019987\_negative\_regulation\_of\_anti-apoptosis | 3 | 0 |  |  |  |  |  |  |  |  |
| GO:0021555\_midbrain-hindbrain\_boundary\_morphogenesis | 3 | 0 |  |  |  |  |  |  |  |  |
| GO:0021563\_glossopharyngeal\_nerve\_development | 3 | 0 |  |  |  |  |  |  |  |  |
| GO:0021570\_rhombomere\_4\_development | 3 | 0 |  |  |  |  |  |  |  |  |
| GO:0021591\_ventricular\_system\_development | 3 | 0 |  |  |  |  |  |  |  |  |
| GO:0021615\_glossopharyngeal\_nerve\_morphogenesis | 3 | 0 |  |  |  |  |  |  |  |  |
| GO:0021794\_thalamus\_development | 3 | 0 |  |  |  |  |  |  |  |  |
| GO:0021797\_forebrain\_anterior\_posterior\_pattern\_formation | 3 | 0 |  |  |  |  |  |  |  |  |
| GO:0021798\_forebrain\_dorsal\_ventral\_pattern\_formation | 3 | 0 |  |  |  |  |  |  |  |  |
| GO:0021800\_cerebral\_cortex\_tangential\_migration | 3 | 0 |  |  |  |  |  |  |  |  |
| GO:0021819\_layer\_formation\_in\_the\_cerebral\_cortex | 3 | 0 |  |  |  |  |  |  |  |  |
| GO:0021859\_pyramidal\_neuron\_differentiation | 3 | 0 |  |  |  |  |  |  |  |  |
| GO:0021860\_pyramidal\_neuron\_development | 3 | 0 |  |  |  |  |  |  |  |  |
| GO:0021889\_olfactory\_bulb\_interneuron\_differentiation | 3 | 0 |  |  |  |  |  |  |  |  |
| GO:0021891\_olfactory\_bulb\_interneuron\_development | 3 | 0 |  |  |  |  |  |  |  |  |
| GO:0021979\_hypothalamus\_cell\_differentiation | 3 | 0 |  |  |  |  |  |  |  |  |
| GO:0022010\_myelination\_in\_the\_central\_nervous\_system | 3 | 0 |  |  |  |  |  |  |  |  |
| GO:0022027\_interkinetic\_nuclear\_migration | 3 | 0 |  |  |  |  |  |  |  |  |
| GO:0022406\_membrane\_docking | 3 | 0 |  |  |  |  |  |  |  |  |
| GO:0030033\_microvillus\_assembly | 3 | 0 |  |  |  |  |  |  |  |  |
| GO:0030091\_protein\_repair | 3 | 0 |  |  |  |  |  |  |  |  |
| GO:0030195\_negative\_regulation\_of\_blood\_coagulation | 3 | 0 |  |  |  |  |  |  |  |  |
| GO:0030224\_monocyte\_differentiation | 3 | 0 |  |  |  |  |  |  |  |  |
| GO:0030307\_positive\_regulation\_of\_cell\_growth | 3 | 0 |  |  |  |  |  |  |  |  |
| GO:0030320\_cellular\_monovalent\_inorganic\_anion\_homeostasis | 3 | 0 |  |  |  |  |  |  |  |  |
| GO:0030321\_transepithelial\_chloride\_transport | 3 | 0 |  |  |  |  |  |  |  |  |
| GO:0030501\_positive\_regulation\_of\_bone\_mineralization | 3 | 0 |  |  |  |  |  |  |  |  |
| GO:0030513\_positive\_regulation\_of\_BMP\_signaling\_pathway | 3 | 0 |  |  |  |  |  |  |  |  |
| GO:0030540\_female\_genitalia\_development | 3 | 0 |  |  |  |  |  |  |  |  |
| GO:0030574\_collagen\_catabolic\_process | 3 | 0 |  |  |  |  |  |  |  |  |
| GO:0030718\_germ-line\_stem\_cell\_maintenance | 3 | 0 |  |  |  |  |  |  |  |  |
| GO:0030730\_sequestering\_of\_triglyceride | 3 | 0 |  |  |  |  |  |  |  |  |
| GO:0030836\_positive\_regulation\_of\_actin\_filament\_depolymerization | 3 | 0 |  |  |  |  |  |  |  |  |
| GO:0030857\_negative\_regulation\_of\_epithelial\_cell\_differentiation | 3 | 0 |  |  |  |  |  |  |  |  |
| GO:0030916\_otic\_vesicle\_formation | 3 | 0 |  |  |  |  |  |  |  |  |
| GO:0031000\_response\_to\_caffeine | 3 | 0 |  |  |  |  |  |  |  |  |
| GO:0031063\_regulation\_of\_histone\_deacetylation | 3 | 0 |  |  |  |  |  |  |  |  |
| GO:0031065\_positive\_regulation\_of\_histone\_deacetylation | 3 | 0 |  |  |  |  |  |  |  |  |
| GO:0031112\_positive\_regulation\_of\_microtubule\_polymerization\_or\_depolymerization | 3 | 0 |  |  |  |  |  |  |  |  |
| GO:0031116\_positive\_regulation\_of\_microtubule\_polymerization | 3 | 0 |  |  |  |  |  |  |  |  |
| GO:0031133\_regulation\_of\_axon\_diameter | 3 | 0 |  |  |  |  |  |  |  |  |
| GO:0031282\_regulation\_of\_guanylate\_cyclase\_activity | 3 | 0 |  |  |  |  |  |  |  |  |
| GO:0031333\_negative\_regulation\_of\_protein\_complex\_assembly | 3 | 0 |  |  |  |  |  |  |  |  |
| GO:0031397\_negative\_regulation\_of\_protein\_ubiquitination | 3 | 0 |  |  |  |  |  |  |  |  |
| GO:0031398\_positive\_regulation\_of\_protein\_ubiquitination | 3 | 0 |  |  |  |  |  |  |  |  |
| GO:0031503\_protein\_complex\_localization | 3 | 0 |  |  |  |  |  |  |  |  |
| GO:0031571\_G1\_DNA\_damage\_checkpoint | 3 | 0 |  |  |  |  |  |  |  |  |
| GO:0031579\_membrane\_raft\_organization | 3 | 0 |  |  |  |  |  |  |  |  |
| GO:0031638\_zymogen\_activation | 3 | 0 |  |  |  |  |  |  |  |  |
| GO:0031641\_regulation\_of\_myelination | 3 | 0 |  |  |  |  |  |  |  |  |
| GO:0031642\_negative\_regulation\_of\_myelination | 3 | 0 |  |  |  |  |  |  |  |  |
| GO:0031649\_heat\_generation | 3 | 0 |  |  |  |  |  |  |  |  |
| GO:0032020\_ISG15-protein\_conjugation | 3 | 0 |  |  |  |  |  |  |  |  |
| GO:0032060\_bleb\_formation | 3 | 0 |  |  |  |  |  |  |  |  |
| GO:0032095\_regulation\_of\_response\_to\_food | 3 | 0 |  |  |  |  |  |  |  |  |
| GO:0032272\_negative\_regulation\_of\_protein\_polymerization | 3 | 0 |  |  |  |  |  |  |  |  |
| GO:0032288\_myelin\_assembly | 3 | 0 |  |  |  |  |  |  |  |  |
| GO:0032291\_ensheathment\_of\_axons\_in\_the\_central\_nervous\_system | 3 | 0 |  |  |  |  |  |  |  |  |
| GO:0032355\_response\_to\_estradiol\_stimulus | 3 | 0 |  |  |  |  |  |  |  |  |
| GO:0032411\_positive\_regulation\_of\_transporter\_activity | 3 | 0 |  |  |  |  |  |  |  |  |
| GO:0032414\_positive\_regulation\_of\_ion\_transmembrane\_transporter\_activity | 3 | 0 |  |  |  |  |  |  |  |  |
| GO:0032436\_positive\_regulation\_of\_proteasomal\_ubiquitin-dependent\_protein\_catabolic\_process | 3 | 0 |  |  |  |  |  |  |  |  |
| GO:0032528\_microvillus\_organization | 3 | 0 |  |  |  |  |  |  |  |  |
| GO:0032536\_regulation\_of\_cell\_projection\_size | 3 | 0 |  |  |  |  |  |  |  |  |
| GO:0032632\_interleukin-3\_production | 3 | 0 |  |  |  |  |  |  |  |  |
| GO:0032634\_interleukin-5\_production | 3 | 0 |  |  |  |  |  |  |  |  |
| GO:0032674\_regulation\_of\_interleukin-5\_production | 3 | 0 |  |  |  |  |  |  |  |  |
| GO:0032703\_negative\_regulation\_of\_interleukin-2\_production | 3 | 0 |  |  |  |  |  |  |  |  |
| GO:0032753\_positive\_regulation\_of\_interleukin-4\_production | 3 | 0 |  |  |  |  |  |  |  |  |
| GO:0032823\_regulation\_of\_natural\_killer\_cell\_differentiation | 3 | 0 |  |  |  |  |  |  |  |  |
| GO:0032825\_positive\_regulation\_of\_natural\_killer\_cell\_differentiation | 3 | 0 |  |  |  |  |  |  |  |  |
| GO:0032856\_activation\_of\_Ras\_GTPase\_activity | 3 | 0 |  |  |  |  |  |  |  |  |
| GO:0032862\_activation\_of\_Rho\_GTPase\_activity | 3 | 0 |  |  |  |  |  |  |  |  |
| GO:0032874\_positive\_regulation\_of\_stress-activated\_MAPK\_cascade | 3 | 0 |  |  |  |  |  |  |  |  |
| GO:0032881\_regulation\_of\_polysaccharide\_metabolic\_process | 3 | 0 |  |  |  |  |  |  |  |  |
| GO:0032890\_regulation\_of\_organic\_acid\_transport | 3 | 0 |  |  |  |  |  |  |  |  |
| GO:0033058\_directional\_locomotion | 3 | 0 |  |  |  |  |  |  |  |  |
| GO:0033080\_immature\_T\_cell\_proliferation\_in\_the\_thymus | 3 | 0 |  |  |  |  |  |  |  |  |
| GO:0033084\_regulation\_of\_immature\_T\_cell\_proliferation\_in\_the\_thymus | 3 | 0 |  |  |  |  |  |  |  |  |
| GO:0033091\_positive\_regulation\_of\_immature\_T\_cell\_proliferation | 3 | 0 |  |  |  |  |  |  |  |  |
| GO:0033137\_negative\_regulation\_of\_peptidyl-serine\_phosphorylation | 3 | 0 |  |  |  |  |  |  |  |  |
| GO:0033153\_T\_cell\_receptor\_V(D)J\_recombination | 3 | 0 |  |  |  |  |  |  |  |  |
| GO:0033209\_tumor\_necrosis\_factor-mediated\_signaling\_pathway | 3 | 0 |  |  |  |  |  |  |  |  |
| GO:0033261\_regulation\_of\_S\_phase | 3 | 0 |  |  |  |  |  |  |  |  |
| GO:0033600\_negative\_regulation\_of\_mammary\_gland\_epithelial\_cell\_proliferation | 3 | 0 |  |  |  |  |  |  |  |  |
| GO:0033631\_cell-cell\_adhesion\_mediated\_by\_integrin | 3 | 0 |  |  |  |  |  |  |  |  |
| GO:0033993\_response\_to\_lipid | 3 | 0 |  |  |  |  |  |  |  |  |
| GO:0034220\_ion\_transmembrane\_transport | 3 | 0 |  |  |  |  |  |  |  |  |
| GO:0034308\_monohydric\_alcohol\_metabolic\_process | 3 | 0 |  |  |  |  |  |  |  |  |
| GO:0034313\_diol\_catabolic\_process | 3 | 0 |  |  |  |  |  |  |  |  |
| GO:0034331\_cell\_junction\_maintenance | 3 | 0 |  |  |  |  |  |  |  |  |
| GO:0034332\_adherens\_junction\_organization | 3 | 0 |  |  |  |  |  |  |  |  |
| GO:0034375\_high-density\_lipoprotein\_particle\_remodeling | 3 | 0 |  |  |  |  |  |  |  |  |
| GO:0034381\_lipoprotein\_particle\_clearance | 3 | 0 |  |  |  |  |  |  |  |  |
| GO:0034612\_response\_to\_tumor\_necrosis\_factor | 3 | 0 |  |  |  |  |  |  |  |  |
| GO:0034655\_nucleobase\_\_nucleoside\_\_nucleotide\_and\_nucleic\_acid\_catabolic\_process | 3 | 0 |  |  |  |  |  |  |  |  |
| GO:0034656\_nucleobase\_\_nucleoside\_and\_nucleotide\_catabolic\_process | 3 | 0 |  |  |  |  |  |  |  |  |
| GO:0035067\_negative\_regulation\_of\_histone\_acetylation | 3 | 0 |  |  |  |  |  |  |  |  |
| GO:0035084\_flagellar\_axoneme\_assembly | 3 | 0 |  |  |  |  |  |  |  |  |
| GO:0035166\_post-embryonic\_hemopoiesis | 3 | 0 |  |  |  |  |  |  |  |  |
| GO:0035283\_central\_nervous\_system\_segmentation | 3 | 0 |  |  |  |  |  |  |  |  |
| GO:0035284\_brain\_segmentation | 3 | 0 |  |  |  |  |  |  |  |  |
| GO:0042097\_interleukin-4\_biosynthetic\_process | 3 | 0 |  |  |  |  |  |  |  |  |
| GO:0042135\_neurotransmitter\_catabolic\_process | 3 | 0 |  |  |  |  |  |  |  |  |
| GO:0042271\_susceptibility\_to\_natural\_killer\_cell\_mediated\_cytotoxicity | 3 | 0 |  |  |  |  |  |  |  |  |
| GO:0042273\_ribosomal\_large\_subunit\_biogenesis | 3 | 0 |  |  |  |  |  |  |  |  |
| GO:0042375\_quinone\_cofactor\_metabolic\_process | 3 | 0 |  |  |  |  |  |  |  |  |
| GO:0042420\_dopamine\_catabolic\_process | 3 | 0 |  |  |  |  |  |  |  |  |
| GO:0042421\_norepinephrine\_biosynthetic\_process | 3 | 0 |  |  |  |  |  |  |  |  |
| GO:0042424\_catecholamine\_catabolic\_process | 3 | 0 |  |  |  |  |  |  |  |  |
| GO:0042447\_hormone\_catabolic\_process | 3 | 0 |  |  |  |  |  |  |  |  |
| GO:0042659\_regulation\_of\_cell\_fate\_specification | 3 | 0 |  |  |  |  |  |  |  |  |
| GO:0042668\_auditory\_receptor\_cell\_fate\_determination | 3 | 0 |  |  |  |  |  |  |  |  |
| GO:0042670\_retinal\_cone\_cell\_differentiation | 3 | 0 |  |  |  |  |  |  |  |  |
| GO:0042693\_muscle\_cell\_fate\_commitment | 3 | 0 |  |  |  |  |  |  |  |  |
| GO:0042711\_maternal\_behavior | 3 | 0 |  |  |  |  |  |  |  |  |
| GO:0042745\_circadian\_sleep\_wake\_cycle | 3 | 0 |  |  |  |  |  |  |  |  |
| GO:0042759\_long-chain\_fatty\_acid\_biosynthetic\_process | 3 | 0 |  |  |  |  |  |  |  |  |
| GO:0042787\_protein\_ubiquitination\_during\_ubiquitin-dependent\_protein\_catabolic\_process | 3 | 0 |  |  |  |  |  |  |  |  |
| GO:0043045\_DNA\_methylation\_during\_embryonic\_development | 3 | 0 |  |  |  |  |  |  |  |  |
| GO:0043090\_amino\_acid\_import | 3 | 0 |  |  |  |  |  |  |  |  |
| GO:0043092\_L-amino\_acid\_import | 3 | 0 |  |  |  |  |  |  |  |  |
| GO:0043094\_cellular\_metabolic\_compound\_salvage | 3 | 0 |  |  |  |  |  |  |  |  |
| GO:0043101\_purine\_salvage | 3 | 0 |  |  |  |  |  |  |  |  |
| GO:0043149\_stress\_fiber\_formation | 3 | 0 |  |  |  |  |  |  |  |  |
| GO:0043174\_nucleoside\_salvage | 3 | 0 |  |  |  |  |  |  |  |  |
| GO:0043200\_response\_to\_amino\_acid\_stimulus | 3 | 0 |  |  |  |  |  |  |  |  |
| GO:0043243\_positive\_regulation\_of\_protein\_complex\_disassembly | 3 | 0 |  |  |  |  |  |  |  |  |
| GO:0043249\_erythrocyte\_maturation | 3 | 0 |  |  |  |  |  |  |  |  |
| GO:0043371\_negative\_regulation\_of\_CD4-positive\_\_alpha\_beta\_T\_cell\_differentiation | 3 | 0 |  |  |  |  |  |  |  |  |
| GO:0043462\_regulation\_of\_ATPase\_activity | 3 | 0 |  |  |  |  |  |  |  |  |
| GO:0043569\_negative\_regulation\_of\_insulin-like\_growth\_factor\_receptor\_signaling\_pathway | 3 | 0 |  |  |  |  |  |  |  |  |
| GO:0043574\_peroxisomal\_transport | 3 | 0 |  |  |  |  |  |  |  |  |
| GO:0043586\_tongue\_development | 3 | 0 |  |  |  |  |  |  |  |  |
| GO:0043900\_regulation\_of\_multi-organism\_process | 3 | 0 |  |  |  |  |  |  |  |  |
| GO:0043954\_cellular\_component\_maintenance | 3 | 0 |  |  |  |  |  |  |  |  |
| GO:0044030\_regulation\_of\_DNA\_methylation | 3 | 0 |  |  |  |  |  |  |  |  |
| GO:0044089\_positive\_regulation\_of\_cellular\_component\_biogenesis | 3 | 0 |  |  |  |  |  |  |  |  |
| GO:0044273\_sulfur\_compound\_catabolic\_process | 3 | 0 |  |  |  |  |  |  |  |  |
| GO:0045047\_protein\_targeting\_to\_ER | 3 | 0 |  |  |  |  |  |  |  |  |
| GO:0045085\_negative\_regulation\_of\_interleukin-2\_biosynthetic\_process | 3 | 0 |  |  |  |  |  |  |  |  |
| GO:0045110\_intermediate\_filament\_bundle\_assembly | 3 | 0 |  |  |  |  |  |  |  |  |
| GO:0045143\_homologous\_chromosome\_segregation | 3 | 0 |  |  |  |  |  |  |  |  |
| GO:0045198\_establishment\_of\_epithelial\_cell\_apical\_basal\_polarity | 3 | 0 |  |  |  |  |  |  |  |  |
| GO:0045217\_cell-cell\_junction\_maintenance | 3 | 0 |  |  |  |  |  |  |  |  |
| GO:0045348\_positive\_regulation\_of\_MHC\_class\_II\_biosynthetic\_process | 3 | 0 |  |  |  |  |  |  |  |  |
| GO:0045402\_regulation\_of\_interleukin-4\_biosynthetic\_process | 3 | 0 |  |  |  |  |  |  |  |  |
| GO:0045404\_positive\_regulation\_of\_interleukin-4\_biosynthetic\_process | 3 | 0 |  |  |  |  |  |  |  |  |
| GO:0045542\_positive\_regulation\_of\_cholesterol\_biosynthetic\_process | 3 | 0 |  |  |  |  |  |  |  |  |
| GO:0045607\_regulation\_of\_auditory\_receptor\_cell\_differentiation | 3 | 0 |  |  |  |  |  |  |  |  |
| GO:0045623\_negative\_regulation\_of\_T-helper\_cell\_differentiation | 3 | 0 |  |  |  |  |  |  |  |  |
| GO:0045625\_regulation\_of\_T-helper\_1\_cell\_differentiation | 3 | 0 |  |  |  |  |  |  |  |  |
| GO:0045631\_regulation\_of\_mechanoreceptor\_differentiation | 3 | 0 |  |  |  |  |  |  |  |  |
| GO:0045717\_negative\_regulation\_of\_fatty\_acid\_biosynthetic\_process | 3 | 0 |  |  |  |  |  |  |  |  |
| GO:0045723\_positive\_regulation\_of\_fatty\_acid\_biosynthetic\_process | 3 | 0 |  |  |  |  |  |  |  |  |
| GO:0045746\_negative\_regulation\_of\_Notch\_signaling\_pathway | 3 | 0 |  |  |  |  |  |  |  |  |
| GO:0045806\_negative\_regulation\_of\_endocytosis | 3 | 0 |  |  |  |  |  |  |  |  |
| GO:0045829\_negative\_regulation\_of\_isotype\_switching | 3 | 0 |  |  |  |  |  |  |  |  |
| GO:0045844\_positive\_regulation\_of\_striated\_muscle\_development | 3 | 0 |  |  |  |  |  |  |  |  |
| GO:0045907\_positive\_regulation\_of\_vasoconstriction | 3 | 0 |  |  |  |  |  |  |  |  |
| GO:0045922\_negative\_regulation\_of\_fatty\_acid\_metabolic\_process | 3 | 0 |  |  |  |  |  |  |  |  |
| GO:0045939\_negative\_regulation\_of\_steroid\_metabolic\_process | 3 | 0 |  |  |  |  |  |  |  |  |
| GO:0046013\_regulation\_of\_T\_cell\_homeostatic\_proliferation | 3 | 0 |  |  |  |  |  |  |  |  |
| GO:0046034\_ATP\_metabolic\_process | 3 | 0 |  |  |  |  |  |  |  |  |
| GO:0046325\_negative\_regulation\_of\_glucose\_import | 3 | 0 |  |  |  |  |  |  |  |  |
| GO:0046426\_negative\_regulation\_of\_JAK-STAT\_cascade | 3 | 0 |  |  |  |  |  |  |  |  |
| GO:0046457\_prostanoid\_biosynthetic\_process | 3 | 0 |  |  |  |  |  |  |  |  |
| GO:0046488\_phosphatidylinositol\_metabolic\_process | 3 | 0 |  |  |  |  |  |  |  |  |
| GO:0046549\_retinal\_cone\_cell\_development | 3 | 0 |  |  |  |  |  |  |  |  |
| GO:0046605\_regulation\_of\_centrosome\_cycle | 3 | 0 |  |  |  |  |  |  |  |  |
| GO:0046688\_response\_to\_copper\_ion | 3 | 0 |  |  |  |  |  |  |  |  |
| GO:0046717\_acid\_secretion | 3 | 0 |  |  |  |  |  |  |  |  |
| GO:0046825\_regulation\_of\_protein\_export\_from\_nucleus | 3 | 0 |  |  |  |  |  |  |  |  |
| GO:0048003\_antigen\_processing\_and\_presentation\_of\_lipid\_antigen\_via\_MHC\_class\_Ib | 3 | 0 |  |  |  |  |  |  |  |  |
| GO:0048007\_antigen\_processing\_and\_presentation\_\_exogenous\_lipid\_antigen\_via\_MHC\_class\_Ib | 3 | 0 |  |  |  |  |  |  |  |  |
| GO:0048012\_hepatocyte\_growth\_factor\_receptor\_signaling\_pathway | 3 | 0 |  |  |  |  |  |  |  |  |
| GO:0048050\_post-embryonic\_eye\_morphogenesis | 3 | 0 |  |  |  |  |  |  |  |  |
| GO:0048087\_positive\_regulation\_of\_pigmentation\_during\_development | 3 | 0 |  |  |  |  |  |  |  |  |
| GO:0048246\_macrophage\_chemotaxis | 3 | 0 |  |  |  |  |  |  |  |  |
| GO:0048251\_elastic\_fiber\_assembly | 3 | 0 |  |  |  |  |  |  |  |  |
| GO:0048278\_vesicle\_docking | 3 | 0 |  |  |  |  |  |  |  |  |
| GO:0048294\_negative\_regulation\_of\_isotype\_switching\_to\_IgE\_isotypes | 3 | 0 |  |  |  |  |  |  |  |  |
| GO:0048318\_axial\_mesoderm\_development | 3 | 0 |  |  |  |  |  |  |  |  |
| GO:0048597\_post-embryonic\_camera-type\_eye\_morphogenesis | 3 | 0 |  |  |  |  |  |  |  |  |
| GO:0048636\_positive\_regulation\_of\_muscle\_development | 3 | 0 |  |  |  |  |  |  |  |  |
| GO:0048660\_regulation\_of\_smooth\_muscle\_cell\_proliferation | 3 | 0 |  |  |  |  |  |  |  |  |
| GO:0048668\_collateral\_sprouting | 3 | 0 |  |  |  |  |  |  |  |  |
| GO:0048676\_axon\_extension\_involved\_in\_development | 3 | 0 |  |  |  |  |  |  |  |  |
| GO:0048755\_branching\_morphogenesis\_of\_a\_nerve | 3 | 0 |  |  |  |  |  |  |  |  |
| GO:0048845\_venous\_blood\_vessel\_morphogenesis | 3 | 0 |  |  |  |  |  |  |  |  |
| GO:0048852\_diencephalon\_morphogenesis | 3 | 0 |  |  |  |  |  |  |  |  |
| GO:0048859\_formation\_of\_anatomical\_boundary | 3 | 0 |  |  |  |  |  |  |  |  |
| GO:0050435\_beta-amyloid\_metabolic\_process | 3 | 0 |  |  |  |  |  |  |  |  |
| GO:0050650\_chondroitin\_sulfate\_proteoglycan\_biosynthetic\_process | 3 | 0 |  |  |  |  |  |  |  |  |
| GO:0050703\_interleukin-1\_alpha\_secretion | 3 | 0 |  |  |  |  |  |  |  |  |
| GO:0050705\_regulation\_of\_interleukin-1\_alpha\_secretion | 3 | 0 |  |  |  |  |  |  |  |  |
| GO:0050709\_negative\_regulation\_of\_protein\_secretion | 3 | 0 |  |  |  |  |  |  |  |  |
| GO:0050710\_negative\_regulation\_of\_cytokine\_secretion | 3 | 0 |  |  |  |  |  |  |  |  |
| GO:0050717\_positive\_regulation\_of\_interleukin-1\_alpha\_secretion | 3 | 0 |  |  |  |  |  |  |  |  |
| GO:0050774\_negative\_regulation\_of\_dendrite\_morphogenesis | 3 | 0 |  |  |  |  |  |  |  |  |
| GO:0050882\_voluntary\_musculoskeletal\_movement | 3 | 0 |  |  |  |  |  |  |  |  |
| GO:0050913\_sensory\_perception\_of\_bitter\_taste | 3 | 0 |  |  |  |  |  |  |  |  |
| GO:0050957\_equilibrioception | 3 | 0 |  |  |  |  |  |  |  |  |
| GO:0050996\_positive\_regulation\_of\_lipid\_catabolic\_process | 3 | 0 |  |  |  |  |  |  |  |  |
| GO:0051153\_regulation\_of\_striated\_muscle\_cell\_differentiation | 3 | 0 |  |  |  |  |  |  |  |  |
| GO:0051204\_protein\_insertion\_into\_mitochondrial\_membrane | 3 | 0 |  |  |  |  |  |  |  |  |
| GO:0051291\_protein\_heterooligomerization | 3 | 0 |  |  |  |  |  |  |  |  |
| GO:0051302\_regulation\_of\_cell\_division | 3 | 0 |  |  |  |  |  |  |  |  |
| GO:0051320\_S\_phase | 3 | 0 |  |  |  |  |  |  |  |  |
| GO:0051450\_myoblast\_proliferation | 3 | 0 |  |  |  |  |  |  |  |  |
| GO:0051583\_dopamine\_uptake | 3 | 0 |  |  |  |  |  |  |  |  |
| GO:0051798\_positive\_regulation\_of\_hair\_follicle\_development | 3 | 0 |  |  |  |  |  |  |  |  |
| GO:0051882\_mitochondrial\_depolarization | 3 | 0 |  |  |  |  |  |  |  |  |
| GO:0051900\_regulation\_of\_mitochondrial\_depolarization | 3 | 0 |  |  |  |  |  |  |  |  |
| GO:0051925\_regulation\_of\_calcium\_ion\_transport\_via\_voltage-gated\_calcium\_channel\_activity | 3 | 0 |  |  |  |  |  |  |  |  |
| GO:0051926\_negative\_regulation\_of\_calcium\_ion\_transport | 3 | 0 |  |  |  |  |  |  |  |  |
| GO:0051930\_regulation\_of\_sensory\_perception\_of\_pain | 3 | 0 |  |  |  |  |  |  |  |  |
| GO:0051931\_regulation\_of\_sensory\_perception | 3 | 0 |  |  |  |  |  |  |  |  |
| GO:0051934\_catecholamine\_uptake\_during\_transmission\_of\_nerve\_impulse | 3 | 0 |  |  |  |  |  |  |  |  |
| GO:0051955\_regulation\_of\_amino\_acid\_transport | 3 | 0 |  |  |  |  |  |  |  |  |
| GO:0051962\_positive\_regulation\_of\_nervous\_system\_development | 3 | 0 |  |  |  |  |  |  |  |  |
| GO:0051965\_positive\_regulation\_of\_synaptogenesis | 3 | 0 |  |  |  |  |  |  |  |  |
| GO:0051967\_negative\_regulation\_of\_synaptic\_transmission\_\_glutamatergic | 3 | 0 |  |  |  |  |  |  |  |  |
| GO:0051983\_regulation\_of\_chromosome\_segregation | 3 | 0 |  |  |  |  |  |  |  |  |
| GO:0055083\_monovalent\_inorganic\_anion\_homeostasis | 3 | 0 |  |  |  |  |  |  |  |  |
| GO:0055117\_regulation\_of\_cardiac\_muscle\_contraction | 3 | 0 |  |  |  |  |  |  |  |  |
| GO:0060009\_Sertoli\_cell\_development | 3 | 0 |  |  |  |  |  |  |  |  |
| GO:0060024\_rhythmic\_synaptic\_transmission | 3 | 0 |  |  |  |  |  |  |  |  |
| GO:0060033\_anatomical\_structure\_regression | 3 | 0 |  |  |  |  |  |  |  |  |
| GO:0060055\_angiogenesis\_involved\_in\_wound\_healing | 3 | 0 |  |  |  |  |  |  |  |  |
| GO:0060084\_synaptic\_transmission\_involved\_in\_micturition | 3 | 0 |  |  |  |  |  |  |  |  |
| GO:0060123\_regulation\_of\_growth\_hormone\_secretion | 3 | 0 |  |  |  |  |  |  |  |  |
| GO:0060126\_somatotropin\_secreting\_cell\_differentiation | 3 | 0 |  |  |  |  |  |  |  |  |
| GO:0060192\_negative\_regulation\_of\_lipase\_activity | 3 | 0 |  |  |  |  |  |  |  |  |
| GO:0060219\_camera-type\_eye\_photoreceptor\_cell\_differentiation | 3 | 0 |  |  |  |  |  |  |  |  |
| GO:0060285\_ciliary\_cell\_motility | 3 | 0 |  |  |  |  |  |  |  |  |
| GO:0060294\_cilium\_movement\_involved\_in\_ciliary\_motility | 3 | 0 |  |  |  |  |  |  |  |  |
| GO:0060295\_regulation\_of\_cilium\_movement\_involved\_in\_ciliary\_motility | 3 | 0 |  |  |  |  |  |  |  |  |
| GO:0060296\_regulation\_of\_cilium\_beat\_frequency\_involved\_in\_ciliary\_motility | 3 | 0 |  |  |  |  |  |  |  |  |
| GO:0060314\_regulation\_of\_ryanodine-sensitive\_calcium-release\_channel\_activity | 3 | 0 |  |  |  |  |  |  |  |  |
| GO:0060428\_lung\_epithelium\_development | 3 | 0 |  |  |  |  |  |  |  |  |
| GO:0060433\_bronchus\_development | 3 | 0 |  |  |  |  |  |  |  |  |
| GO:0060435\_bronchiole\_development | 3 | 0 |  |  |  |  |  |  |  |  |
| GO:0060460\_left\_lung\_morphogenesis | 3 | 0 |  |  |  |  |  |  |  |  |
| GO:0060491\_regulation\_of\_cell\_projection\_assembly | 3 | 0 |  |  |  |  |  |  |  |  |
| GO:0060523\_prostate\_epithelial\_cord\_elongation | 3 | 0 |  |  |  |  |  |  |  |  |
| GO:0060586\_multicellular\_organismal\_iron\_ion\_homeostasis | 3 | 0 |  |  |  |  |  |  |  |  |
| GO:0060632\_regulation\_of\_microtubule-based\_movement | 3 | 0 |  |  |  |  |  |  |  |  |
| GO:0060648\_mammary\_gland\_bud\_morphogenesis | 3 | 0 |  |  |  |  |  |  |  |  |
| GO:0060684\_epithelial-mesenchymal\_cell\_signaling | 3 | 0 |  |  |  |  |  |  |  |  |
| GO:0060686\_negative\_regulation\_of\_prostatic\_bud\_formation | 3 | 0 |  |  |  |  |  |  |  |  |
| GO:0060689\_cell\_differentiation\_involved\_in\_salivary\_gland\_development | 3 | 0 |  |  |  |  |  |  |  |  |
| GO:0060708\_spongiotrophoblast\_differentiation | 3 | 0 |  |  |  |  |  |  |  |  |
| GO:0060746\_parental\_behavior | 3 | 0 |  |  |  |  |  |  |  |  |
| GO:0060748\_tertiary\_branching\_involved\_in\_mammary\_gland\_duct\_morphogenesis | 3 | 0 |  |  |  |  |  |  |  |  |
| GO:0060750\_epithelial\_cell\_proliferation\_involved\_in\_mammary\_gland\_duct\_elongation | 3 | 0 |  |  |  |  |  |  |  |  |
| GO:0060841\_venous\_blood\_vessel\_development | 3 | 0 |  |  |  |  |  |  |  |  |
| GO:0070102\_interleukin-6-mediated\_signaling\_pathway | 3 | 0 |  |  |  |  |  |  |  |  |
| GO:0070169\_positive\_regulation\_of\_biomineral\_formation | 3 | 0 |  |  |  |  |  |  |  |  |
| GO:0070206\_protein\_trimerization | 3 | 0 |  |  |  |  |  |  |  |  |
| GO:0070207\_protein\_homotrimerization | 3 | 0 |  |  |  |  |  |  |  |  |
| GO:0070229\_negative\_regulation\_of\_lymphocyte\_apoptosis | 3 | 0 |  |  |  |  |  |  |  |  |
| GO:0070230\_positive\_regulation\_of\_lymphocyte\_apoptosis | 3 | 0 |  |  |  |  |  |  |  |  |
| GO:0070232\_regulation\_of\_T\_cell\_apoptosis | 3 | 0 |  |  |  |  |  |  |  |  |
| GO:0070233\_negative\_regulation\_of\_T\_cell\_apoptosis | 3 | 0 |  |  |  |  |  |  |  |  |
| GO:0070242\_thymocyte\_apoptosis | 3 | 0 |  |  |  |  |  |  |  |  |
| GO:0070243\_regulation\_of\_thymocyte\_apoptosis | 3 | 0 |  |  |  |  |  |  |  |  |
| GO:0070244\_negative\_regulation\_of\_thymocyte\_apoptosis | 3 | 0 |  |  |  |  |  |  |  |  |
| GO:0070307\_lens\_fiber\_cell\_development | 3 | 0 |  |  |  |  |  |  |  |  |
| GO:0070309\_lens\_fiber\_cell\_morphogenesis | 3 | 0 |  |  |  |  |  |  |  |  |
| GO:0070423\_nucleotide-binding\_oligomerization\_domain\_containing\_signaling\_pathway | 3 | 0 |  |  |  |  |  |  |  |  |
| GO:0070427\_nucleotide-binding\_oligomerization\_domain\_containing\_1\_signaling\_pathway | 3 | 0 |  |  |  |  |  |  |  |  |
| GO:0070431\_nucleotide-binding\_oligomerization\_domain\_containing\_2\_signaling\_pathway | 3 | 0 |  |  |  |  |  |  |  |  |
| GO:0070633\_transepithelial\_transport | 3 | 0 |  |  |  |  |  |  |  |  |
| GO:0070846\_Hsp90\_deacetylation | 3 | 0 |  |  |  |  |  |  |  |  |
| GO:0070873\_regulation\_of\_glycogen\_metabolic\_process | 3 | 0 |  |  |  |  |  |  |  |  |
| GO:0070875\_positive\_regulation\_of\_glycogen\_metabolic\_process | 3 | 0 |  |  |  |  |  |  |  |  |
| GO:0001656\_metanephros\_development | 50 | 0 | 0.000000 | -0.000000 | 1278 | 1081.526357 | 1172.54 | 1263.553643 | 0.917480 |  |
| GO:0002573\_myeloid\_leukocyte\_differentiation | 50 | 0 | 0.000000 | -0.000000 | 1278 | 1081.526357 | 1172.54 | 1263.553643 | 0.917480 |  |
| GO:0007015\_actin\_filament\_organization | 50 | 0 | 0.000000 | -0.000000 | 1278 | 1081.526357 | 1172.54 | 1263.553643 | 0.917480 |  |
| GO:0009190\_cyclic\_nucleotide\_biosynthetic\_process | 50 | 0 | 0.000000 | -0.000000 | 1278 | 1081.526357 | 1172.54 | 1263.553643 | 0.917480 |  |
| GO:0051606\_detection\_of\_stimulus | 50 | 0 | 0.000000 | -0.000000 | 1278 | 1081.526357 | 1172.54 | 1263.553643 | 0.917480 |  |
| GO:0070647\_protein\_modification\_by\_small\_protein\_conjugation\_or\_removal | 50 | 0 | 0.000000 | -0.000000 | 1278 | 1081.526357 | 1172.54 | 1263.553643 | 0.917480 |  |
| GO:0006520\_cellular\_amino\_acid\_metabolic\_process | 51 | 0 | 0.000000 | -0.000000 | 1282 | 1086.513531 | 1177.06 | 1267.606469 | 0.918144 |  |
| GO:0016569\_covalent\_chromatin\_modification | 51 | 0 | 0.000000 | -0.000000 | 1282 | 1086.513531 | 1177.06 | 1267.606469 | 0.918144 |  |
| GO:0032880\_regulation\_of\_protein\_localization | 51 | 0 | 0.000000 | -0.000000 | 1282 | 1086.513531 | 1177.06 | 1267.606469 | 0.918144 |  |
| GO:0044106\_cellular\_amine\_metabolic\_process | 51 | 0 | 0.000000 | -0.000000 | 1282 | 1086.513531 | 1177.06 | 1267.606469 | 0.918144 |  |
| GO:0001776\_leukocyte\_homeostasis | 41 | 0 | 0.000000 | -0.000000 | 1291 | 1099.329581 | 1188.06 | 1276.790419 | 0.920263 |  |
| GO:0006260\_DNA\_replication | 41 | 0 | 0.000000 | -0.000000 | 1291 | 1099.329581 | 1188.06 | 1276.790419 | 0.920263 |  |
| GO:0006865\_amino\_acid\_transport | 41 | 0 | 0.000000 | -0.000000 | 1291 | 1099.329581 | 1188.06 | 1276.790419 | 0.920263 |  |
| GO:0006979\_response\_to\_oxidative\_stress | 41 | 0 | 0.000000 | -0.000000 | 1291 | 1099.329581 | 1188.06 | 1276.790419 | 0.920263 |  |
| GO:0009894\_regulation\_of\_catabolic\_process | 41 | 0 | 0.000000 | -0.000000 | 1291 | 1099.329581 | 1188.06 | 1276.790419 | 0.920263 |  |
| GO:0015980\_energy\_derivation\_by\_oxidation\_of\_organic\_compounds | 41 | 0 | 0.000000 | -0.000000 | 1291 | 1099.329581 | 1188.06 | 1276.790419 | 0.920263 |  |
| GO:0019748\_secondary\_metabolic\_process | 41 | 0 | 0.000000 | -0.000000 | 1291 | 1099.329581 | 1188.06 | 1276.790419 | 0.920263 |  |
| GO:0030817\_regulation\_of\_cAMP\_biosynthetic\_process | 41 | 0 | 0.000000 | -0.000000 | 1291 | 1099.329581 | 1188.06 | 1276.790419 | 0.920263 |  |
| GO:0031344\_regulation\_of\_cell\_projection\_organization | 41 | 0 | 0.000000 | -0.000000 | 1291 | 1099.329581 | 1188.06 | 1276.790419 | 0.920263 |  |
| GO:0003073\_regulation\_of\_systemic\_arterial\_blood\_pressure | 23 | 0 | 0.000000 | -0.000000 | 1304 | 1117.827594 | 1205.07 | 1292.312406 | 0.924133 |  |
| GO:0006397\_mRNA\_processing | 23 | 0 | 0.000000 | -0.000000 | 1304 | 1117.827594 | 1205.07 | 1292.312406 | 0.924133 |  |
| GO:0007018\_microtubule-based\_movement | 23 | 0 | 0.000000 | -0.000000 | 1304 | 1117.827594 | 1205.07 | 1292.312406 | 0.924133 |  |
| GO:0007163\_establishment\_or\_maintenance\_of\_cell\_polarity | 23 | 0 | 0.000000 | -0.000000 | 1304 | 1117.827594 | 1205.07 | 1292.312406 | 0.924133 |  |
| GO:0007584\_response\_to\_nutrient | 23 | 0 | 0.000000 | -0.000000 | 1304 | 1117.827594 | 1205.07 | 1292.312406 | 0.924133 |  |
| GO:0009954\_proximal\_distal\_pattern\_formation | 23 | 0 | 0.000000 | -0.000000 | 1304 | 1117.827594 | 1205.07 | 1292.312406 | 0.924133 |  |
| GO:0015698\_inorganic\_anion\_transport | 23 | 0 | 0.000000 | -0.000000 | 1304 | 1117.827594 | 1205.07 | 1292.312406 | 0.924133 |  |
| GO:0022613\_ribonucleoprotein\_complex\_biogenesis | 23 | 0 | 0.000000 | -0.000000 | 1304 | 1117.827594 | 1205.07 | 1292.312406 | 0.924133 |  |
| GO:0030512\_negative\_regulation\_of\_transforming\_growth\_factor\_beta\_receptor\_signaling\_pathway | 23 | 0 | 0.000000 | -0.000000 | 1304 | 1117.827594 | 1205.07 | 1292.312406 | 0.924133 |  |
| GO:0032635\_interleukin-6\_production | 23 | 0 | 0.000000 | -0.000000 | 1304 | 1117.827594 | 1205.07 | 1292.312406 | 0.924133 |  |
| GO:0032675\_regulation\_of\_interleukin-6\_production | 23 | 0 | 0.000000 | -0.000000 | 1304 | 1117.827594 | 1205.07 | 1292.312406 | 0.924133 |  |
| GO:0043388\_positive\_regulation\_of\_DNA\_binding | 23 | 0 | 0.000000 | -0.000000 | 1304 | 1117.827594 | 1205.07 | 1292.312406 | 0.924133 |  |
| GO:0060445\_branching\_involved\_in\_salivary\_gland\_morphogenesis | 23 | 0 | 0.000000 | -0.000000 | 1304 | 1117.827594 | 1205.07 | 1292.312406 | 0.924133 |  |
| GO:0006725\_cellular\_aromatic\_compound\_metabolic\_process | 49 | 0 | 0.000000 | -0.000000 | 1308 | 1124.193860 | 1210.43 | 1296.666140 | 0.925405 |  |
| GO:0034101\_erythrocyte\_homeostasis | 49 | 0 | 0.000000 | -0.000000 | 1308 | 1124.193860 | 1210.43 | 1296.666140 | 0.925405 |  |
| GO:0042035\_regulation\_of\_cytokine\_biosynthetic\_process | 49 | 0 | 0.000000 | -0.000000 | 1308 | 1124.193860 | 1210.43 | 1296.666140 | 0.925405 |  |
| GO:0046661\_male\_sex\_differentiation | 49 | 0 | 0.000000 | -0.000000 | 1308 | 1124.193860 | 1210.43 | 1296.666140 | 0.925405 |  |
| GO:0006140\_regulation\_of\_nucleotide\_metabolic\_process | 47 | 0 | 0.000000 | -0.000000 | 1315 | 1131.134860 | 1216.3 | 1301.465140 | 0.924943 |  |
| GO:0006396\_RNA\_processing | 47 | 0 | 0.000000 | -0.000000 | 1315 | 1131.134860 | 1216.3 | 1301.465140 | 0.924943 |  |
| GO:0016570\_histone\_modification | 47 | 0 | 0.000000 | -0.000000 | 1315 | 1131.134860 | 1216.3 | 1301.465140 | 0.924943 |  |
| GO:0030799\_regulation\_of\_cyclic\_nucleotide\_metabolic\_process | 47 | 0 | 0.000000 | -0.000000 | 1315 | 1131.134860 | 1216.3 | 1301.465140 | 0.924943 |  |
| GO:0031667\_response\_to\_nutrient\_levels | 47 | 0 | 0.000000 | -0.000000 | 1315 | 1131.134860 | 1216.3 | 1301.465140 | 0.924943 |  |
| GO:0048871\_multicellular\_organismal\_homeostasis | 47 | 0 | 0.000000 | -0.000000 | 1315 | 1131.134860 | 1216.3 | 1301.465140 | 0.924943 |  |
| GO:0060627\_regulation\_of\_vesicle-mediated\_transport | 47 | 0 | 0.000000 | -0.000000 | 1315 | 1131.134860 | 1216.3 | 1301.465140 | 0.924943 |  |
| GO:0001841\_neural\_tube\_formation | 43 | 0 | 0.000000 | -0.000000 | 1325 | 1142.024733 | 1226.01 | 1309.995267 | 0.925291 |  |
| GO:0001894\_tissue\_homeostasis | 43 | 0 | 0.000000 | -0.000000 | 1325 | 1142.024733 | 1226.01 | 1309.995267 | 0.925291 |  |
| GO:0006766\_vitamin\_metabolic\_process | 43 | 0 | 0.000000 | -0.000000 | 1325 | 1142.024733 | 1226.01 | 1309.995267 | 0.925291 |  |
| GO:0009582\_detection\_of\_abiotic\_stimulus | 43 | 0 | 0.000000 | -0.000000 | 1325 | 1142.024733 | 1226.01 | 1309.995267 | 0.925291 |  |
| GO:0030814\_regulation\_of\_cAMP\_metabolic\_process | 43 | 0 | 0.000000 | -0.000000 | 1325 | 1142.024733 | 1226.01 | 1309.995267 | 0.925291 |  |
| GO:0032446\_protein\_modification\_by\_small\_protein\_conjugation | 43 | 0 | 0.000000 | -0.000000 | 1325 | 1142.024733 | 1226.01 | 1309.995267 | 0.925291 |  |
| GO:0032868\_response\_to\_insulin\_stimulus | 43 | 0 | 0.000000 | -0.000000 | 1325 | 1142.024733 | 1226.01 | 1309.995267 | 0.925291 |  |
| GO:0046879\_hormone\_secretion | 43 | 0 | 0.000000 | -0.000000 | 1325 | 1142.024733 | 1226.01 | 1309.995267 | 0.925291 |  |
| GO:0048762\_mesenchymal\_cell\_differentiation | 43 | 0 | 0.000000 | -0.000000 | 1325 | 1142.024733 | 1226.01 | 1309.995267 | 0.925291 |  |
| GO:0051604\_protein\_maturation | 43 | 0 | 0.000000 | -0.000000 | 1325 | 1142.024733 | 1226.01 | 1309.995267 | 0.925291 |  |
| GO:0000086\_G2\_M\_transition\_of\_mitotic\_cell\_cycle | 4 | 0 |  |  |  |  |  |  |  |  |
| GO:0000305\_response\_to\_oxygen\_radical | 4 | 0 |  |  |  |  |  |  |  |  |
| GO:0001661\_conditioned\_taste\_aversion | 4 | 0 |  |  |  |  |  |  |  |  |
| GO:0001678\_cellular\_glucose\_homeostasis | 4 | 0 |  |  |  |  |  |  |  |  |
| GO:0001777\_T\_cell\_homeostatic\_proliferation | 4 | 0 |  |  |  |  |  |  |  |  |
| GO:0001794\_type\_IIa\_hypersensitivity | 4 | 0 |  |  |  |  |  |  |  |  |
| GO:0001796\_regulation\_of\_type\_IIa\_hypersensitivity | 4 | 0 |  |  |  |  |  |  |  |  |
| GO:0001798\_positive\_regulation\_of\_type\_IIa\_hypersensitivity | 4 | 0 |  |  |  |  |  |  |  |  |
| GO:0001810\_regulation\_of\_type\_I\_hypersensitivity | 4 | 0 |  |  |  |  |  |  |  |  |
| GO:0001820\_serotonin\_secretion | 4 | 0 |  |  |  |  |  |  |  |  |
| GO:0001835\_blastocyst\_hatching | 4 | 0 |  |  |  |  |  |  |  |  |
| GO:0001842\_neural\_fold\_formation | 4 | 0 |  |  |  |  |  |  |  |  |
| GO:0001881\_receptor\_recycling | 4 | 0 |  |  |  |  |  |  |  |  |
| GO:0001938\_positive\_regulation\_of\_endothelial\_cell\_proliferation | 4 | 0 |  |  |  |  |  |  |  |  |
| GO:0001978\_regulation\_of\_systemic\_arterial\_blood\_pressure\_by\_carotid\_sinus\_baroreceptor\_feedback | 4 | 0 |  |  |  |  |  |  |  |  |
| GO:0002035\_brain\_renin-angiotensin\_system | 4 | 0 |  |  |  |  |  |  |  |  |
| GO:0002051\_osteoblast\_fate\_commitment | 4 | 0 |  |  |  |  |  |  |  |  |
| GO:0002220\_innate\_immune\_response\_activating\_cell\_surface\_receptor\_signaling\_pathway | 4 | 0 |  |  |  |  |  |  |  |  |
| GO:0002249\_lymphocyte\_anergy | 4 | 0 |  |  |  |  |  |  |  |  |
| GO:0002312\_B\_cell\_activation\_during\_immune\_response | 4 | 0 |  |  |  |  |  |  |  |  |
| GO:0002313\_mature\_B\_cell\_differentiation\_during\_immune\_response | 4 | 0 |  |  |  |  |  |  |  |  |
| GO:0002318\_myeloid\_progenitor\_cell\_differentiation | 4 | 0 |  |  |  |  |  |  |  |  |
| GO:0002326\_B\_cell\_lineage\_commitment | 4 | 0 |  |  |  |  |  |  |  |  |
| GO:0002347\_response\_to\_tumor\_cell | 4 | 0 |  |  |  |  |  |  |  |  |
| GO:0002418\_immune\_response\_to\_tumor\_cell | 4 | 0 |  |  |  |  |  |  |  |  |
| GO:0002445\_type\_II\_hypersensitivity | 4 | 0 |  |  |  |  |  |  |  |  |
| GO:0002544\_chronic\_inflammatory\_response | 4 | 0 |  |  |  |  |  |  |  |  |
| GO:0002636\_positive\_regulation\_of\_germinal\_center\_formation | 4 | 0 |  |  |  |  |  |  |  |  |
| GO:0002667\_regulation\_of\_T\_cell\_anergy | 4 | 0 |  |  |  |  |  |  |  |  |
| GO:0002669\_positive\_regulation\_of\_T\_cell\_anergy | 4 | 0 |  |  |  |  |  |  |  |  |
| GO:0002687\_positive\_regulation\_of\_leukocyte\_migration | 4 | 0 |  |  |  |  |  |  |  |  |
| GO:0002702\_positive\_regulation\_of\_production\_of\_molecular\_mediator\_of\_immune\_response | 4 | 0 |  |  |  |  |  |  |  |  |
| GO:0002718\_regulation\_of\_cytokine\_production\_during\_immune\_response | 4 | 0 |  |  |  |  |  |  |  |  |
| GO:0002829\_negative\_regulation\_of\_T-helper\_2\_type\_immune\_response | 4 | 0 |  |  |  |  |  |  |  |  |
| GO:0002833\_positive\_regulation\_of\_response\_to\_biotic\_stimulus | 4 | 0 |  |  |  |  |  |  |  |  |
| GO:0002834\_regulation\_of\_response\_to\_tumor\_cell | 4 | 0 |  |  |  |  |  |  |  |  |
| GO:0002836\_positive\_regulation\_of\_response\_to\_tumor\_cell | 4 | 0 |  |  |  |  |  |  |  |  |
| GO:0002837\_regulation\_of\_immune\_response\_to\_tumor\_cell | 4 | 0 |  |  |  |  |  |  |  |  |
| GO:0002839\_positive\_regulation\_of\_immune\_response\_to\_tumor\_cell | 4 | 0 |  |  |  |  |  |  |  |  |
| GO:0002870\_T\_cell\_anergy | 4 | 0 |  |  |  |  |  |  |  |  |
| GO:0002888\_positive\_regulation\_of\_myeloid\_leukocyte\_mediated\_immunity | 4 | 0 |  |  |  |  |  |  |  |  |
| GO:0002892\_regulation\_of\_type\_II\_hypersensitivity | 4 | 0 |  |  |  |  |  |  |  |  |
| GO:0002894\_positive\_regulation\_of\_type\_II\_hypersensitivity | 4 | 0 |  |  |  |  |  |  |  |  |
| GO:0002911\_regulation\_of\_lymphocyte\_anergy | 4 | 0 |  |  |  |  |  |  |  |  |
| GO:0002913\_positive\_regulation\_of\_lymphocyte\_anergy | 4 | 0 |  |  |  |  |  |  |  |  |
| GO:0003025\_regulation\_of\_systemic\_arterial\_blood\_pressure\_by\_baroreceptor\_feedback | 4 | 0 |  |  |  |  |  |  |  |  |
| GO:0003091\_renal\_water\_homeostasis | 4 | 0 |  |  |  |  |  |  |  |  |
| GO:0005978\_glycogen\_biosynthetic\_process | 4 | 0 |  |  |  |  |  |  |  |  |
| GO:0006012\_galactose\_metabolic\_process | 4 | 0 |  |  |  |  |  |  |  |  |
| GO:0006085\_acetyl-CoA\_biosynthetic\_process | 4 | 0 |  |  |  |  |  |  |  |  |
| GO:0006144\_purine\_base\_metabolic\_process | 4 | 0 |  |  |  |  |  |  |  |  |
| GO:0006290\_pyrimidine\_dimer\_repair | 4 | 0 |  |  |  |  |  |  |  |  |
| GO:0006334\_nucleosome\_assembly | 4 | 0 |  |  |  |  |  |  |  |  |
| GO:0006534\_cysteine\_metabolic\_process | 4 | 0 |  |  |  |  |  |  |  |  |
| GO:0006547\_histidine\_metabolic\_process | 4 | 0 |  |  |  |  |  |  |  |  |
| GO:0006548\_histidine\_catabolic\_process | 4 | 0 |  |  |  |  |  |  |  |  |
| GO:0006555\_methionine\_metabolic\_process | 4 | 0 |  |  |  |  |  |  |  |  |
| GO:0006599\_phosphagen\_metabolic\_process | 4 | 0 |  |  |  |  |  |  |  |  |
| GO:0006623\_protein\_targeting\_to\_vacuole | 4 | 0 |  |  |  |  |  |  |  |  |
| GO:0006626\_protein\_targeting\_to\_mitochondrion | 4 | 0 |  |  |  |  |  |  |  |  |
| GO:0006688\_glycosphingolipid\_biosynthetic\_process | 4 | 0 |  |  |  |  |  |  |  |  |
| GO:0006707\_cholesterol\_catabolic\_process | 4 | 0 |  |  |  |  |  |  |  |  |
| GO:0006739\_NADP\_metabolic\_process | 4 | 0 |  |  |  |  |  |  |  |  |
| GO:0006835\_dicarboxylic\_acid\_transport | 4 | 0 |  |  |  |  |  |  |  |  |
| GO:0006837\_serotonin\_transport | 4 | 0 |  |  |  |  |  |  |  |  |
| GO:0006888\_ER\_to\_Golgi\_vesicle-mediated\_transport | 4 | 0 |  |  |  |  |  |  |  |  |
| GO:0006906\_vesicle\_fusion | 4 | 0 |  |  |  |  |  |  |  |  |
| GO:0006927\_transformed\_cell\_apoptosis | 4 | 0 |  |  |  |  |  |  |  |  |
| GO:0006972\_hyperosmotic\_response | 4 | 0 |  |  |  |  |  |  |  |  |
| GO:0007028\_cytoplasm\_organization | 4 | 0 |  |  |  |  |  |  |  |  |
| GO:0007031\_peroxisome\_organization | 4 | 0 |  |  |  |  |  |  |  |  |
| GO:0007066\_female\_meiosis\_sister\_chromatid\_cohesion | 4 | 0 |  |  |  |  |  |  |  |  |
| GO:0007144\_female\_meiosis\_I | 4 | 0 |  |  |  |  |  |  |  |  |
| GO:0007184\_SMAD\_protein\_nuclear\_translocation | 4 | 0 |  |  |  |  |  |  |  |  |
| GO:0007216\_metabotropic\_glutamate\_receptor\_signaling\_pathway | 4 | 0 |  |  |  |  |  |  |  |  |
| GO:0007342\_fusion\_of\_sperm\_to\_egg\_plasma\_membrane | 4 | 0 |  |  |  |  |  |  |  |  |
| GO:0007386\_compartment\_specification | 4 | 0 |  |  |  |  |  |  |  |  |
| GO:0008053\_mitochondrial\_fusion | 4 | 0 |  |  |  |  |  |  |  |  |
| GO:0008215\_spermine\_metabolic\_process | 4 | 0 |  |  |  |  |  |  |  |  |
| GO:0009065\_glutamine\_family\_amino\_acid\_catabolic\_process | 4 | 0 |  |  |  |  |  |  |  |  |
| GO:0009075\_histidine\_family\_amino\_acid\_metabolic\_process | 4 | 0 |  |  |  |  |  |  |  |  |
| GO:0009077\_histidine\_family\_amino\_acid\_catabolic\_process | 4 | 0 |  |  |  |  |  |  |  |  |
| GO:0009134\_nucleoside\_diphosphate\_catabolic\_process | 4 | 0 |  |  |  |  |  |  |  |  |
| GO:0009163\_nucleoside\_biosynthetic\_process | 4 | 0 |  |  |  |  |  |  |  |  |
| GO:0009225\_nucleotide-sugar\_metabolic\_process | 4 | 0 |  |  |  |  |  |  |  |  |
| GO:0009250\_glucan\_biosynthetic\_process | 4 | 0 |  |  |  |  |  |  |  |  |
| GO:0009404\_toxin\_metabolic\_process | 4 | 0 |  |  |  |  |  |  |  |  |
| GO:0009593\_detection\_of\_chemical\_stimulus | 4 | 0 |  |  |  |  |  |  |  |  |
| GO:0009595\_detection\_of\_biotic\_stimulus | 4 | 0 |  |  |  |  |  |  |  |  |
| GO:0009755\_hormone-mediated\_signaling | 4 | 0 |  |  |  |  |  |  |  |  |
| GO:0009912\_auditory\_receptor\_cell\_fate\_commitment | 4 | 0 |  |  |  |  |  |  |  |  |
| GO:0010224\_response\_to\_UV-B | 4 | 0 |  |  |  |  |  |  |  |  |
| GO:0010453\_regulation\_of\_cell\_fate\_commitment | 4 | 0 |  |  |  |  |  |  |  |  |
| GO:0010506\_regulation\_of\_autophagy | 4 | 0 |  |  |  |  |  |  |  |  |
| GO:0010631\_epithelial\_cell\_migration | 4 | 0 |  |  |  |  |  |  |  |  |
| GO:0010812\_negative\_regulation\_of\_cell-substrate\_adhesion | 4 | 0 |  |  |  |  |  |  |  |  |
| GO:0010829\_negative\_regulation\_of\_glucose\_transport | 4 | 0 |  |  |  |  |  |  |  |  |
| GO:0014002\_astrocyte\_development | 4 | 0 |  |  |  |  |  |  |  |  |
| GO:0014832\_urinary\_bladder\_smooth\_muscle\_contraction | 4 | 0 |  |  |  |  |  |  |  |  |
| GO:0014848\_urinary\_tract\_smooth\_muscle\_contraction | 4 | 0 |  |  |  |  |  |  |  |  |
| GO:0015701\_bicarbonate\_transport | 4 | 0 |  |  |  |  |  |  |  |  |
| GO:0015809\_arginine\_transport | 4 | 0 |  |  |  |  |  |  |  |  |
| GO:0015850\_organic\_alcohol\_transport | 4 | 0 |  |  |  |  |  |  |  |  |
| GO:0015858\_nucleoside\_transport | 4 | 0 |  |  |  |  |  |  |  |  |
| GO:0016068\_type\_I\_hypersensitivity | 4 | 0 |  |  |  |  |  |  |  |  |
| GO:0016127\_sterol\_catabolic\_process | 4 | 0 |  |  |  |  |  |  |  |  |
| GO:0016198\_axon\_choice\_point\_recognition | 4 | 0 |  |  |  |  |  |  |  |  |
| GO:0018198\_peptidyl-cysteine\_modification | 4 | 0 |  |  |  |  |  |  |  |  |
| GO:0018409\_peptide\_or\_protein\_amino-terminal\_blocking | 4 | 0 |  |  |  |  |  |  |  |  |
| GO:0019432\_triglyceride\_biosynthetic\_process | 4 | 0 |  |  |  |  |  |  |  |  |
| GO:0019530\_taurine\_metabolic\_process | 4 | 0 |  |  |  |  |  |  |  |  |
| GO:0021523\_somatic\_motor\_neuron\_differentiation | 4 | 0 |  |  |  |  |  |  |  |  |
| GO:0021542\_dentate\_gyrus\_development | 4 | 0 |  |  |  |  |  |  |  |  |
| GO:0021561\_facial\_nerve\_development | 4 | 0 |  |  |  |  |  |  |  |  |
| GO:0021569\_rhombomere\_3\_development | 4 | 0 |  |  |  |  |  |  |  |  |
| GO:0021571\_rhombomere\_5\_development | 4 | 0 |  |  |  |  |  |  |  |  |
| GO:0021604\_cranial\_nerve\_structural\_organization | 4 | 0 |  |  |  |  |  |  |  |  |
| GO:0021610\_facial\_nerve\_morphogenesis | 4 | 0 |  |  |  |  |  |  |  |  |
| GO:0021612\_facial\_nerve\_structural\_organization | 4 | 0 |  |  |  |  |  |  |  |  |
| GO:0021631\_optic\_nerve\_morphogenesis | 4 | 0 |  |  |  |  |  |  |  |  |
| GO:0021681\_cerebellar\_granular\_layer\_development | 4 | 0 |  |  |  |  |  |  |  |  |
| GO:0021683\_cerebellar\_granular\_layer\_morphogenesis | 4 | 0 |  |  |  |  |  |  |  |  |
| GO:0021684\_cerebellar\_granular\_layer\_formation | 4 | 0 |  |  |  |  |  |  |  |  |
| GO:0021707\_cerebellar\_granule\_cell\_differentiation | 4 | 0 |  |  |  |  |  |  |  |  |
| GO:0021801\_cerebral\_cortex\_radial\_glia\_guided\_migration | 4 | 0 |  |  |  |  |  |  |  |  |
| GO:0021830\_interneuron\_migration\_from\_the\_subpallium\_to\_the\_cortex | 4 | 0 |  |  |  |  |  |  |  |  |
| GO:0021853\_cerebral\_cortex\_GABAergic\_interneuron\_migration | 4 | 0 |  |  |  |  |  |  |  |  |
| GO:0021877\_forebrain\_neuron\_fate\_commitment | 4 | 0 |  |  |  |  |  |  |  |  |
| GO:0021894\_cerebral\_cortex\_GABAergic\_interneuron\_development | 4 | 0 |  |  |  |  |  |  |  |  |
| GO:0021910\_smoothened\_signaling\_pathway\_involved\_in\_ventral\_spinal\_cord\_patterning | 4 | 0 |  |  |  |  |  |  |  |  |
| GO:0021938\_smoothened\_signaling\_pathway\_involved\_in\_regulation\_of\_granule\_cell\_precursor\_cell\_proliferation | 4 | 0 |  |  |  |  |  |  |  |  |
| GO:0021978\_telencephalon\_regionalization | 4 | 0 |  |  |  |  |  |  |  |  |
| GO:0022011\_myelination\_in\_the\_peripheral\_nervous\_system | 4 | 0 |  |  |  |  |  |  |  |  |
| GO:0030146\_diuresis | 4 | 0 |  |  |  |  |  |  |  |  |
| GO:0030300\_regulation\_of\_intestinal\_cholesterol\_absorption | 4 | 0 |  |  |  |  |  |  |  |  |
| GO:0030800\_negative\_regulation\_of\_cyclic\_nucleotide\_metabolic\_process | 4 | 0 |  |  |  |  |  |  |  |  |
| GO:0030803\_negative\_regulation\_of\_cyclic\_nucleotide\_biosynthetic\_process | 4 | 0 |  |  |  |  |  |  |  |  |
| GO:0030809\_negative\_regulation\_of\_nucleotide\_biosynthetic\_process | 4 | 0 |  |  |  |  |  |  |  |  |
| GO:0030815\_negative\_regulation\_of\_cAMP\_metabolic\_process | 4 | 0 |  |  |  |  |  |  |  |  |
| GO:0030816\_positive\_regulation\_of\_cAMP\_metabolic\_process | 4 | 0 |  |  |  |  |  |  |  |  |
| GO:0030818\_negative\_regulation\_of\_cAMP\_biosynthetic\_process | 4 | 0 |  |  |  |  |  |  |  |  |
| GO:0030819\_positive\_regulation\_of\_cAMP\_biosynthetic\_process | 4 | 0 |  |  |  |  |  |  |  |  |
| GO:0030826\_regulation\_of\_cGMP\_biosynthetic\_process | 4 | 0 |  |  |  |  |  |  |  |  |
| GO:0030858\_positive\_regulation\_of\_epithelial\_cell\_differentiation | 4 | 0 |  |  |  |  |  |  |  |  |
| GO:0030859\_polarized\_epithelial\_cell\_differentiation | 4 | 0 |  |  |  |  |  |  |  |  |
| GO:0031113\_regulation\_of\_microtubule\_polymerization | 4 | 0 |  |  |  |  |  |  |  |  |
| GO:0031365\_N-terminal\_protein\_amino\_acid\_modification | 4 | 0 |  |  |  |  |  |  |  |  |
| GO:0031424\_keratinization | 4 | 0 |  |  |  |  |  |  |  |  |
| GO:0031557\_induction\_of\_programmed\_cell\_death\_in\_response\_to\_chemical\_stimulus | 4 | 0 |  |  |  |  |  |  |  |  |
| GO:0031558\_induction\_of\_apoptosis\_in\_response\_to\_chemical\_stimulus | 4 | 0 |  |  |  |  |  |  |  |  |
| GO:0031623\_receptor\_internalization | 4 | 0 |  |  |  |  |  |  |  |  |
| GO:0032088\_negative\_regulation\_of\_NF-kappaB\_transcription\_factor\_activity | 4 | 0 |  |  |  |  |  |  |  |  |
| GO:0032098\_regulation\_of\_appetite | 4 | 0 |  |  |  |  |  |  |  |  |
| GO:0032105\_negative\_regulation\_of\_response\_to\_extracellular\_stimulus | 4 | 0 |  |  |  |  |  |  |  |  |
| GO:0032108\_negative\_regulation\_of\_response\_to\_nutrient\_levels | 4 | 0 |  |  |  |  |  |  |  |  |
| GO:0032225\_regulation\_of\_synaptic\_transmission\_\_dopaminergic | 4 | 0 |  |  |  |  |  |  |  |  |
| GO:0032292\_ensheathment\_of\_axons\_in\_the\_peripheral\_nervous\_system | 4 | 0 |  |  |  |  |  |  |  |  |
| GO:0032321\_positive\_regulation\_of\_Rho\_GTPase\_activity | 4 | 0 |  |  |  |  |  |  |  |  |
| GO:0032371\_regulation\_of\_sterol\_transport | 4 | 0 |  |  |  |  |  |  |  |  |
| GO:0032374\_regulation\_of\_cholesterol\_transport | 4 | 0 |  |  |  |  |  |  |  |  |
| GO:0032608\_interferon-beta\_production | 4 | 0 |  |  |  |  |  |  |  |  |
| GO:0032611\_interleukin-1\_beta\_production | 4 | 0 |  |  |  |  |  |  |  |  |
| GO:0032612\_interleukin-1\_production | 4 | 0 |  |  |  |  |  |  |  |  |
| GO:0032648\_regulation\_of\_interferon-beta\_production | 4 | 0 |  |  |  |  |  |  |  |  |
| GO:0032651\_regulation\_of\_interleukin-1\_beta\_production | 4 | 0 |  |  |  |  |  |  |  |  |
| GO:0032652\_regulation\_of\_interleukin-1\_production | 4 | 0 |  |  |  |  |  |  |  |  |
| GO:0032689\_negative\_regulation\_of\_interferon-gamma\_production | 4 | 0 |  |  |  |  |  |  |  |  |
| GO:0032713\_negative\_regulation\_of\_interleukin-4\_production | 4 | 0 |  |  |  |  |  |  |  |  |
| GO:0032715\_negative\_regulation\_of\_interleukin-6\_production | 4 | 0 |  |  |  |  |  |  |  |  |
| GO:0032733\_positive\_regulation\_of\_interleukin-10\_production | 4 | 0 |  |  |  |  |  |  |  |  |
| GO:0032808\_lacrimal\_gland\_development | 4 | 0 |  |  |  |  |  |  |  |  |
| GO:0032835\_glomerulus\_development | 4 | 0 |  |  |  |  |  |  |  |  |
| GO:0032872\_regulation\_of\_stress-activated\_MAPK\_cascade | 4 | 0 |  |  |  |  |  |  |  |  |
| GO:0032922\_circadian\_regulation\_of\_gene\_expression | 4 | 0 |  |  |  |  |  |  |  |  |
| GO:0033026\_negative\_regulation\_of\_mast\_cell\_apoptosis | 4 | 0 |  |  |  |  |  |  |  |  |
| GO:0033079\_immature\_T\_cell\_proliferation | 4 | 0 |  |  |  |  |  |  |  |  |
| GO:0033083\_regulation\_of\_immature\_T\_cell\_proliferation | 4 | 0 |  |  |  |  |  |  |  |  |
| GO:0033089\_positive\_regulation\_of\_T\_cell\_differentiation\_in\_the\_thymus | 4 | 0 |  |  |  |  |  |  |  |  |
| GO:0033135\_regulation\_of\_peptidyl-serine\_phosphorylation | 4 | 0 |  |  |  |  |  |  |  |  |
| GO:0033299\_secretion\_of\_lysosomal\_enzymes | 4 | 0 |  |  |  |  |  |  |  |  |
| GO:0033327\_Leydig\_cell\_differentiation | 4 | 0 |  |  |  |  |  |  |  |  |
| GO:0033363\_secretory\_granule\_organization | 4 | 0 |  |  |  |  |  |  |  |  |
| GO:0033599\_regulation\_of\_mammary\_gland\_epithelial\_cell\_proliferation | 4 | 0 |  |  |  |  |  |  |  |  |
| GO:0033865\_nucleoside\_bisphosphate\_metabolic\_process | 4 | 0 |  |  |  |  |  |  |  |  |
| GO:0034404\_nucleobase\_\_nucleoside\_and\_nucleotide\_biosynthetic\_process | 4 | 0 |  |  |  |  |  |  |  |  |
| GO:0034587\_piRNA\_metabolic\_process | 4 | 0 |  |  |  |  |  |  |  |  |
| GO:0034614\_cellular\_response\_to\_reactive\_oxygen\_species | 4 | 0 |  |  |  |  |  |  |  |  |
| GO:0034654\_nucleobase\_\_nucleoside\_\_nucleotide\_and\_nucleic\_acid\_biosynthetic\_process | 4 | 0 |  |  |  |  |  |  |  |  |
| GO:0035020\_regulation\_of\_Rac\_protein\_signal\_transduction | 4 | 0 |  |  |  |  |  |  |  |  |
| GO:0035082\_axoneme\_assembly | 4 | 0 |  |  |  |  |  |  |  |  |
| GO:0035188\_hatching | 4 | 0 |  |  |  |  |  |  |  |  |
| GO:0035235\_ionotropic\_glutamate\_receptor\_signaling\_pathway | 4 | 0 |  |  |  |  |  |  |  |  |
| GO:0042345\_regulation\_of\_NF-kappaB\_import\_into\_nucleus | 4 | 0 |  |  |  |  |  |  |  |  |
| GO:0042348\_NF-kappaB\_import\_into\_nucleus | 4 | 0 |  |  |  |  |  |  |  |  |
| GO:0042359\_vitamin\_D\_metabolic\_process | 4 | 0 |  |  |  |  |  |  |  |  |
| GO:0042428\_serotonin\_metabolic\_process | 4 | 0 |  |  |  |  |  |  |  |  |
| GO:0042451\_purine\_nucleoside\_biosynthetic\_process | 4 | 0 |  |  |  |  |  |  |  |  |
| GO:0042455\_ribonucleoside\_biosynthetic\_process | 4 | 0 |  |  |  |  |  |  |  |  |
| GO:0042473\_outer\_ear\_morphogenesis | 4 | 0 |  |  |  |  |  |  |  |  |
| GO:0042535\_positive\_regulation\_of\_tumor\_necrosis\_factor\_biosynthetic\_process | 4 | 0 |  |  |  |  |  |  |  |  |
| GO:0042541\_hemoglobin\_biosynthetic\_process | 4 | 0 |  |  |  |  |  |  |  |  |
| GO:0042558\_pteridine\_and\_derivative\_metabolic\_process | 4 | 0 |  |  |  |  |  |  |  |  |
| GO:0042634\_regulation\_of\_hair\_cycle | 4 | 0 |  |  |  |  |  |  |  |  |
| GO:0042744\_hydrogen\_peroxide\_catabolic\_process | 4 | 0 |  |  |  |  |  |  |  |  |
| GO:0042773\_ATP\_synthesis\_coupled\_electron\_transport | 4 | 0 |  |  |  |  |  |  |  |  |
| GO:0042775\_mitochondrial\_ATP\_synthesis\_coupled\_electron\_transport | 4 | 0 |  |  |  |  |  |  |  |  |
| GO:0042832\_defense\_response\_to\_protozoan | 4 | 0 |  |  |  |  |  |  |  |  |
| GO:0042982\_amyloid\_precursor\_protein\_metabolic\_process | 4 | 0 |  |  |  |  |  |  |  |  |
| GO:0042992\_negative\_regulation\_of\_transcription\_factor\_import\_into\_nucleus | 4 | 0 |  |  |  |  |  |  |  |  |
| GO:0043043\_peptide\_biosynthetic\_process | 4 | 0 |  |  |  |  |  |  |  |  |
| GO:0043129\_surfactant\_homeostasis | 4 | 0 |  |  |  |  |  |  |  |  |
| GO:0043374\_CD8-positive\_\_alpha-beta\_T\_cell\_differentiation | 4 | 0 |  |  |  |  |  |  |  |  |
| GO:0043470\_regulation\_of\_carbohydrate\_catabolic\_process | 4 | 0 |  |  |  |  |  |  |  |  |
| GO:0043471\_regulation\_of\_cellular\_carbohydrate\_catabolic\_process | 4 | 0 |  |  |  |  |  |  |  |  |
| GO:0043484\_regulation\_of\_RNA\_splicing | 4 | 0 |  |  |  |  |  |  |  |  |
| GO:0043500\_muscle\_adaptation | 4 | 0 |  |  |  |  |  |  |  |  |
| GO:0043534\_blood\_vessel\_endothelial\_cell\_migration | 4 | 0 |  |  |  |  |  |  |  |  |
| GO:0044243\_multicellular\_organismal\_catabolic\_process | 4 | 0 |  |  |  |  |  |  |  |  |
| GO:0044403\_symbiosis\_\_encompassing\_mutualism\_through\_parasitism | 4 | 0 |  |  |  |  |  |  |  |  |
| GO:0044419\_interspecies\_interaction\_between\_organisms | 4 | 0 |  |  |  |  |  |  |  |  |
| GO:0045066\_regulatory\_T\_cell\_differentiation | 4 | 0 |  |  |  |  |  |  |  |  |
| GO:0045078\_positive\_regulation\_of\_interferon-gamma\_biosynthetic\_process | 4 | 0 |  |  |  |  |  |  |  |  |
| GO:0045346\_regulation\_of\_MHC\_class\_II\_biosynthetic\_process | 4 | 0 |  |  |  |  |  |  |  |  |
| GO:0045350\_interferon-beta\_biosynthetic\_process | 4 | 0 |  |  |  |  |  |  |  |  |
| GO:0045357\_regulation\_of\_interferon-beta\_biosynthetic\_process | 4 | 0 |  |  |  |  |  |  |  |  |
| GO:0045359\_positive\_regulation\_of\_interferon-beta\_biosynthetic\_process | 4 | 0 |  |  |  |  |  |  |  |  |
| GO:0045600\_positive\_regulation\_of\_fat\_cell\_differentiation | 4 | 0 |  |  |  |  |  |  |  |  |
| GO:0045616\_regulation\_of\_keratinocyte\_differentiation | 4 | 0 |  |  |  |  |  |  |  |  |
| GO:0045624\_positive\_regulation\_of\_T-helper\_cell\_differentiation | 4 | 0 |  |  |  |  |  |  |  |  |
| GO:0045628\_regulation\_of\_T-helper\_2\_cell\_differentiation | 4 | 0 |  |  |  |  |  |  |  |  |
| GO:0045634\_regulation\_of\_melanocyte\_differentiation | 4 | 0 |  |  |  |  |  |  |  |  |
| GO:0045647\_negative\_regulation\_of\_erythrocyte\_differentiation | 4 | 0 |  |  |  |  |  |  |  |  |
| GO:0045672\_positive\_regulation\_of\_osteoclast\_differentiation | 4 | 0 |  |  |  |  |  |  |  |  |
| GO:0045684\_positive\_regulation\_of\_epidermis\_development | 4 | 0 |  |  |  |  |  |  |  |  |
| GO:0045736\_negative\_regulation\_of\_cyclin-dependent\_protein\_kinase\_activity | 4 | 0 |  |  |  |  |  |  |  |  |
| GO:0045742\_positive\_regulation\_of\_epidermal\_growth\_factor\_receptor\_signaling\_pathway | 4 | 0 |  |  |  |  |  |  |  |  |
| GO:0045767\_regulation\_of\_anti-apoptosis | 4 | 0 |  |  |  |  |  |  |  |  |
| GO:0045779\_negative\_regulation\_of\_bone\_resorption | 4 | 0 |  |  |  |  |  |  |  |  |
| GO:0045923\_positive\_regulation\_of\_fatty\_acid\_metabolic\_process | 4 | 0 |  |  |  |  |  |  |  |  |
| GO:0045930\_negative\_regulation\_of\_mitotic\_cell\_cycle | 4 | 0 |  |  |  |  |  |  |  |  |
| GO:0045940\_positive\_regulation\_of\_steroid\_metabolic\_process | 4 | 0 |  |  |  |  |  |  |  |  |
| GO:0045980\_negative\_regulation\_of\_nucleotide\_metabolic\_process | 4 | 0 |  |  |  |  |  |  |  |  |
| GO:0046129\_purine\_ribonucleoside\_biosynthetic\_process | 4 | 0 |  |  |  |  |  |  |  |  |
| GO:0046173\_polyol\_biosynthetic\_process | 4 | 0 |  |  |  |  |  |  |  |  |
| GO:0046541\_saliva\_secretion | 4 | 0 |  |  |  |  |  |  |  |  |
| GO:0046548\_retinal\_rod\_cell\_development | 4 | 0 |  |  |  |  |  |  |  |  |
| GO:0046579\_positive\_regulation\_of\_Ras\_protein\_signal\_transduction | 4 | 0 |  |  |  |  |  |  |  |  |
| GO:0046639\_negative\_regulation\_of\_alpha-beta\_T\_cell\_differentiation | 4 | 0 |  |  |  |  |  |  |  |  |
| GO:0046642\_negative\_regulation\_of\_alpha-beta\_T\_cell\_proliferation | 4 | 0 |  |  |  |  |  |  |  |  |
| GO:0046668\_regulation\_of\_retinal\_cell\_programmed\_cell\_death | 4 | 0 |  |  |  |  |  |  |  |  |
| GO:0046686\_response\_to\_cadmium\_ion | 4 | 0 |  |  |  |  |  |  |  |  |
| GO:0046835\_carbohydrate\_phosphorylation | 4 | 0 |  |  |  |  |  |  |  |  |
| GO:0046902\_regulation\_of\_mitochondrial\_membrane\_permeability | 4 | 0 |  |  |  |  |  |  |  |  |
| GO:0047496\_vesicle\_transport\_along\_microtubule | 4 | 0 |  |  |  |  |  |  |  |  |
| GO:0048011\_nerve\_growth\_factor\_receptor\_signaling\_pathway | 4 | 0 |  |  |  |  |  |  |  |  |
| GO:0048024\_regulation\_of\_nuclear\_mRNA\_splicing\_\_via\_spliceosome | 4 | 0 |  |  |  |  |  |  |  |  |
| GO:0048240\_sperm\_capacitation | 4 | 0 |  |  |  |  |  |  |  |  |
| GO:0048341\_paraxial\_mesoderm\_formation | 4 | 0 |  |  |  |  |  |  |  |  |
| GO:0048484\_enteric\_nervous\_system\_development | 4 | 0 |  |  |  |  |  |  |  |  |
| GO:0048512\_circadian\_behavior | 4 | 0 |  |  |  |  |  |  |  |  |
| GO:0048558\_embryonic\_gut\_morphogenesis | 4 | 0 |  |  |  |  |  |  |  |  |
| GO:0048639\_positive\_regulation\_of\_developmental\_growth | 4 | 0 |  |  |  |  |  |  |  |  |
| GO:0048710\_regulation\_of\_astrocyte\_differentiation | 4 | 0 |  |  |  |  |  |  |  |  |
| GO:0048841\_regulation\_of\_axon\_extension\_involved\_in\_axon\_guidance | 4 | 0 |  |  |  |  |  |  |  |  |
| GO:0048843\_negative\_regulation\_of\_axon\_extension\_involved\_in\_axon\_guidance | 4 | 0 |  |  |  |  |  |  |  |  |
| GO:0048846\_axon\_extension\_involved\_in\_axon\_guidance | 4 | 0 |  |  |  |  |  |  |  |  |
| GO:0048875\_chemical\_homeostasis\_within\_a\_tissue | 4 | 0 |  |  |  |  |  |  |  |  |
| GO:0048935\_peripheral\_nervous\_system\_neuron\_development | 4 | 0 |  |  |  |  |  |  |  |  |
| GO:0050702\_interleukin-1\_beta\_secretion | 4 | 0 |  |  |  |  |  |  |  |  |
| GO:0050704\_regulation\_of\_interleukin-1\_secretion | 4 | 0 |  |  |  |  |  |  |  |  |
| GO:0050706\_regulation\_of\_interleukin-1\_beta\_secretion | 4 | 0 |  |  |  |  |  |  |  |  |
| GO:0050716\_positive\_regulation\_of\_interleukin-1\_secretion | 4 | 0 |  |  |  |  |  |  |  |  |
| GO:0050718\_positive\_regulation\_of\_interleukin-1\_beta\_secretion | 4 | 0 |  |  |  |  |  |  |  |  |
| GO:0050820\_positive\_regulation\_of\_coagulation | 4 | 0 |  |  |  |  |  |  |  |  |
| GO:0050891\_multicellular\_organismal\_water\_homeostasis | 4 | 0 |  |  |  |  |  |  |  |  |
| GO:0050919\_negative\_chemotaxis | 4 | 0 |  |  |  |  |  |  |  |  |
| GO:0050932\_regulation\_of\_pigment\_cell\_differentiation | 4 | 0 |  |  |  |  |  |  |  |  |
| GO:0050961\_detection\_of\_temperature\_stimulus\_involved\_in\_sensory\_perception | 4 | 0 |  |  |  |  |  |  |  |  |
| GO:0050965\_detection\_of\_temperature\_stimulus\_involved\_in\_sensory\_perception\_of\_pain | 4 | 0 |  |  |  |  |  |  |  |  |
| GO:0050994\_regulation\_of\_lipid\_catabolic\_process | 4 | 0 |  |  |  |  |  |  |  |  |
| GO:0051024\_positive\_regulation\_of\_immunoglobulin\_secretion | 4 | 0 |  |  |  |  |  |  |  |  |
| GO:0051055\_negative\_regulation\_of\_lipid\_biosynthetic\_process | 4 | 0 |  |  |  |  |  |  |  |  |
| GO:0051124\_synaptic\_growth\_at\_neuromuscular\_junction | 4 | 0 |  |  |  |  |  |  |  |  |
| GO:0051205\_protein\_insertion\_into\_membrane | 4 | 0 |  |  |  |  |  |  |  |  |
| GO:0051225\_spindle\_assembly | 4 | 0 |  |  |  |  |  |  |  |  |
| GO:0051341\_regulation\_of\_oxidoreductase\_activity | 4 | 0 |  |  |  |  |  |  |  |  |
| GO:0051567\_histone\_H3-K9\_methylation | 4 | 0 |  |  |  |  |  |  |  |  |
| GO:0051797\_regulation\_of\_hair\_follicle\_development | 4 | 0 |  |  |  |  |  |  |  |  |
| GO:0051897\_positive\_regulation\_of\_protein\_kinase\_B\_signaling\_cascade | 4 | 0 |  |  |  |  |  |  |  |  |
| GO:0055009\_atrial\_cardiac\_muscle\_morphogenesis | 4 | 0 |  |  |  |  |  |  |  |  |
| GO:0060008\_Sertoli\_cell\_differentiation | 4 | 0 |  |  |  |  |  |  |  |  |
| GO:0060011\_Sertoli\_cell\_proliferation | 4 | 0 |  |  |  |  |  |  |  |  |
| GO:0060057\_apoptosis\_involved\_in\_mammary\_gland\_involution | 4 | 0 |  |  |  |  |  |  |  |  |
| GO:0060058\_positive\_regulation\_of\_apoptosis\_involved\_in\_mammary\_gland\_involution | 4 | 0 |  |  |  |  |  |  |  |  |
| GO:0060065\_uterus\_development | 4 | 0 |  |  |  |  |  |  |  |  |
| GO:0060087\_relaxation\_of\_vascular\_smooth\_muscle | 4 | 0 |  |  |  |  |  |  |  |  |
| GO:0060120\_inner\_ear\_receptor\_cell\_fate\_commitment | 4 | 0 |  |  |  |  |  |  |  |  |
| GO:0060157\_urinary\_bladder\_development | 4 | 0 |  |  |  |  |  |  |  |  |
| GO:0060158\_activation\_of\_phospholipase\_C\_activity\_by\_dopamine\_receptor\_signaling\_pathway | 4 | 0 |  |  |  |  |  |  |  |  |
| GO:0060235\_lens\_induction\_in\_camera-type\_eye | 4 | 0 |  |  |  |  |  |  |  |  |
| GO:0060291\_long-term\_synaptic\_potentiation | 4 | 0 |  |  |  |  |  |  |  |  |
| GO:0060412\_ventricular\_septum\_morphogenesis | 4 | 0 |  |  |  |  |  |  |  |  |
| GO:0060459\_left\_lung\_development | 4 | 0 |  |  |  |  |  |  |  |  |
| GO:0060528\_secretory\_columnal\_luminar\_epithelial\_cell\_differentiation\_involved\_in\_prostate\_glandular\_acinus\_development | 4 | 0 |  |  |  |  |  |  |  |  |
| GO:0060561\_apoptosis\_involved\_in\_morphogenesis | 4 | 0 |  |  |  |  |  |  |  |  |
| GO:0060644\_mammary\_gland\_epithelial\_cell\_differentiation | 4 | 0 |  |  |  |  |  |  |  |  |
| GO:0060666\_dichotomous\_subdivision\_of\_terminal\_units\_involved\_in\_salivary\_gland\_branching | 4 | 0 |  |  |  |  |  |  |  |  |
| GO:0060737\_prostate\_gland\_morphogenetic\_growth | 4 | 0 |  |  |  |  |  |  |  |  |
| GO:0060743\_epithelial\_cell\_maturation\_involved\_in\_prostate\_gland\_development | 4 | 0 |  |  |  |  |  |  |  |  |
| GO:0060751\_mammary\_gland\_duct\_branch\_elongation | 4 | 0 |  |  |  |  |  |  |  |  |
| GO:0060900\_embryonic\_camera-type\_eye\_formation | 4 | 0 |  |  |  |  |  |  |  |  |
| GO:0070059\_apoptosis\_in\_response\_to\_endoplasmic\_reticulum\_stress | 4 | 0 |  |  |  |  |  |  |  |  |
| GO:0070254\_mucus\_secretion | 4 | 0 |  |  |  |  |  |  |  |  |
| GO:0070255\_regulation\_of\_mucus\_secretion | 4 | 0 |  |  |  |  |  |  |  |  |
| GO:0070301\_cellular\_response\_to\_hydrogen\_peroxide | 4 | 0 |  |  |  |  |  |  |  |  |
| GO:0070585\_protein\_localization\_in\_mitochondrion | 4 | 0 |  |  |  |  |  |  |  |  |
| GO:0000302\_response\_to\_reactive\_oxygen\_species | 16 | 0 | 0.000000 | -0.000000 | 1361 | 1181.236407 | 1263.31 | 1345.383593 | 0.928222 |  |
| GO:0003044\_regulation\_of\_systemic\_arterial\_blood\_pressure\_mediated\_by\_a\_chemical\_signal | 16 | 0 | 0.000000 | -0.000000 | 1361 | 1181.236407 | 1263.31 | 1345.383593 | 0.928222 |  |
| GO:0006821\_chloride\_transport | 16 | 0 | 0.000000 | -0.000000 | 1361 | 1181.236407 | 1263.31 | 1345.383593 | 0.928222 |  |
| GO:0007156\_homophilic\_cell\_adhesion | 16 | 0 | 0.000000 | -0.000000 | 1361 | 1181.236407 | 1263.31 | 1345.383593 | 0.928222 |  |
| GO:0007602\_phototransduction | 16 | 0 | 0.000000 | -0.000000 | 1361 | 1181.236407 | 1263.31 | 1345.383593 | 0.928222 |  |
| GO:0009988\_cell-cell\_recognition | 16 | 0 | 0.000000 | -0.000000 | 1361 | 1181.236407 | 1263.31 | 1345.383593 | 0.928222 |  |
| GO:0010038\_response\_to\_metal\_ion | 16 | 0 | 0.000000 | -0.000000 | 1361 | 1181.236407 | 1263.31 | 1345.383593 | 0.928222 |  |
| GO:0010243\_response\_to\_organic\_nitrogen | 16 | 0 | 0.000000 | -0.000000 | 1361 | 1181.236407 | 1263.31 | 1345.383593 | 0.928222 |  |
| GO:0014075\_response\_to\_amine\_stimulus | 16 | 0 | 0.000000 | -0.000000 | 1361 | 1181.236407 | 1263.31 | 1345.383593 | 0.928222 |  |
| GO:0016126\_sterol\_biosynthetic\_process | 16 | 0 | 0.000000 | -0.000000 | 1361 | 1181.236407 | 1263.31 | 1345.383593 | 0.928222 |  |
| GO:0019722\_calcium-mediated\_signaling | 16 | 0 | 0.000000 | -0.000000 | 1361 | 1181.236407 | 1263.31 | 1345.383593 | 0.928222 |  |
| GO:0019751\_polyol\_metabolic\_process | 16 | 0 | 0.000000 | -0.000000 | 1361 | 1181.236407 | 1263.31 | 1345.383593 | 0.928222 |  |
| GO:0021696\_cerebellar\_cortex\_morphogenesis | 16 | 0 | 0.000000 | -0.000000 | 1361 | 1181.236407 | 1263.31 | 1345.383593 | 0.928222 |  |
| GO:0031345\_negative\_regulation\_of\_cell\_projection\_organization | 16 | 0 | 0.000000 | -0.000000 | 1361 | 1181.236407 | 1263.31 | 1345.383593 | 0.928222 |  |
| GO:0031570\_DNA\_integrity\_checkpoint | 16 | 0 | 0.000000 | -0.000000 | 1361 | 1181.236407 | 1263.31 | 1345.383593 | 0.928222 |  |
| GO:0031669\_cellular\_response\_to\_nutrient\_levels | 16 | 0 | 0.000000 | -0.000000 | 1361 | 1181.236407 | 1263.31 | 1345.383593 | 0.928222 |  |
| GO:0032663\_regulation\_of\_interleukin-2\_production | 16 | 0 | 0.000000 | -0.000000 | 1361 | 1181.236407 | 1263.31 | 1345.383593 | 0.928222 |  |
| GO:0032956\_regulation\_of\_actin\_cytoskeleton\_organization | 16 | 0 | 0.000000 | -0.000000 | 1361 | 1181.236407 | 1263.31 | 1345.383593 | 0.928222 |  |
| GO:0034976\_response\_to\_endoplasmic\_reticulum\_stress | 16 | 0 | 0.000000 | -0.000000 | 1361 | 1181.236407 | 1263.31 | 1345.383593 | 0.928222 |  |
| GO:0042311\_vasodilation | 16 | 0 | 0.000000 | -0.000000 | 1361 | 1181.236407 | 1263.31 | 1345.383593 | 0.928222 |  |
| GO:0042594\_response\_to\_starvation | 16 | 0 | 0.000000 | -0.000000 | 1361 | 1181.236407 | 1263.31 | 1345.383593 | 0.928222 |  |
| GO:0042596\_fear\_response | 16 | 0 | 0.000000 | -0.000000 | 1361 | 1181.236407 | 1263.31 | 1345.383593 | 0.928222 |  |
| GO:0043087\_regulation\_of\_GTPase\_activity | 16 | 0 | 0.000000 | -0.000000 | 1361 | 1181.236407 | 1263.31 | 1345.383593 | 0.928222 |  |
| GO:0043122\_regulation\_of\_I-kappaB\_kinase\_NF-kappaB\_cascade | 16 | 0 | 0.000000 | -0.000000 | 1361 | 1181.236407 | 1263.31 | 1345.383593 | 0.928222 |  |
| GO:0043367\_CD4-positive\_\_alpha\_beta\_T\_cell\_differentiation | 16 | 0 | 0.000000 | -0.000000 | 1361 | 1181.236407 | 1263.31 | 1345.383593 | 0.928222 |  |
| GO:0045104\_intermediate\_filament\_cytoskeleton\_organization | 16 | 0 | 0.000000 | -0.000000 | 1361 | 1181.236407 | 1263.31 | 1345.383593 | 0.928222 |  |
| GO:0046148\_pigment\_biosynthetic\_process | 16 | 0 | 0.000000 | -0.000000 | 1361 | 1181.236407 | 1263.31 | 1345.383593 | 0.928222 |  |
| GO:0046700\_heterocycle\_catabolic\_process | 16 | 0 | 0.000000 | -0.000000 | 1361 | 1181.236407 | 1263.31 | 1345.383593 | 0.928222 |  |
| GO:0048015\_phosphoinositide-mediated\_signaling | 16 | 0 | 0.000000 | -0.000000 | 1361 | 1181.236407 | 1263.31 | 1345.383593 | 0.928222 |  |
| GO:0048286\_lung\_alveolus\_development | 16 | 0 | 0.000000 | -0.000000 | 1361 | 1181.236407 | 1263.31 | 1345.383593 | 0.928222 |  |
| GO:0048483\_autonomic\_nervous\_system\_development | 16 | 0 | 0.000000 | -0.000000 | 1361 | 1181.236407 | 1263.31 | 1345.383593 | 0.928222 |  |
| GO:0050974\_detection\_of\_mechanical\_stimulus\_involved\_in\_sensory\_perception | 16 | 0 | 0.000000 | -0.000000 | 1361 | 1181.236407 | 1263.31 | 1345.383593 | 0.928222 |  |
| GO:0051048\_negative\_regulation\_of\_secretion | 16 | 0 | 0.000000 | -0.000000 | 1361 | 1181.236407 | 1263.31 | 1345.383593 | 0.928222 |  |
| GO:0051937\_catecholamine\_transport | 16 | 0 | 0.000000 | -0.000000 | 1361 | 1181.236407 | 1263.31 | 1345.383593 | 0.928222 |  |
| GO:0055007\_cardiac\_muscle\_cell\_differentiation | 16 | 0 | 0.000000 | -0.000000 | 1361 | 1181.236407 | 1263.31 | 1345.383593 | 0.928222 |  |
| GO:0060193\_positive\_regulation\_of\_lipase\_activity | 16 | 0 | 0.000000 | -0.000000 | 1361 | 1181.236407 | 1263.31 | 1345.383593 | 0.928222 |  |
| GO:0000002\_mitochondrial\_genome\_maintenance | 9 | 0 | 0.000000 | -0.000000 | 1462 | 1289.553102 | 1368.51 | 1447.466898 | 0.936053 |  |
| GO:0001539\_ciliary\_or\_flagellar\_motility | 9 | 0 | 0.000000 | -0.000000 | 1462 | 1289.553102 | 1368.51 | 1447.466898 | 0.936053 |  |
| GO:0001676\_long-chain\_fatty\_acid\_metabolic\_process | 9 | 0 | 0.000000 | -0.000000 | 1462 | 1289.553102 | 1368.51 | 1447.466898 | 0.936053 |  |
| GO:0001935\_endothelial\_cell\_proliferation | 9 | 0 | 0.000000 | -0.000000 | 1462 | 1289.553102 | 1368.51 | 1447.466898 | 0.936053 |  |
| GO:0002021\_response\_to\_dietary\_excess | 9 | 0 | 0.000000 | -0.000000 | 1462 | 1289.553102 | 1368.51 | 1447.466898 | 0.936053 |  |
| GO:0002028\_regulation\_of\_sodium\_ion\_transport | 9 | 0 | 0.000000 | -0.000000 | 1462 | 1289.553102 | 1368.51 | 1447.466898 | 0.936053 |  |
| GO:0002221\_pattern\_recognition\_receptor\_signaling\_pathway | 9 | 0 | 0.000000 | -0.000000 | 1462 | 1289.553102 | 1368.51 | 1447.466898 | 0.936053 |  |
| GO:0002292\_T\_cell\_differentiation\_during\_immune\_response | 9 | 0 | 0.000000 | -0.000000 | 1462 | 1289.553102 | 1368.51 | 1447.466898 | 0.936053 |  |
| GO:0002293\_alpha-beta\_T\_cell\_differentiation\_during\_immune\_response | 9 | 0 | 0.000000 | -0.000000 | 1462 | 1289.553102 | 1368.51 | 1447.466898 | 0.936053 |  |
| GO:0002294\_CD4-positive\_\_alpha-beta\_T\_cell\_differentiation\_during\_immune\_response | 9 | 0 | 0.000000 | -0.000000 | 1462 | 1289.553102 | 1368.51 | 1447.466898 | 0.936053 |  |
| GO:0002507\_tolerance\_induction | 9 | 0 | 0.000000 | -0.000000 | 1462 | 1289.553102 | 1368.51 | 1447.466898 | 0.936053 |  |
| GO:0002886\_regulation\_of\_myeloid\_leukocyte\_mediated\_immunity | 9 | 0 | 0.000000 | -0.000000 | 1462 | 1289.553102 | 1368.51 | 1447.466898 | 0.936053 |  |
| GO:0006007\_glucose\_catabolic\_process | 9 | 0 | 0.000000 | -0.000000 | 1462 | 1289.553102 | 1368.51 | 1447.466898 | 0.936053 |  |
| GO:0006182\_cGMP\_biosynthetic\_process | 9 | 0 | 0.000000 | -0.000000 | 1462 | 1289.553102 | 1368.51 | 1447.466898 | 0.936053 |  |
| GO:0006309\_DNA\_fragmentation\_involved\_in\_apoptosis | 9 | 0 | 0.000000 | -0.000000 | 1462 | 1289.553102 | 1368.51 | 1447.466898 | 0.936053 |  |
| GO:0006364\_rRNA\_processing | 9 | 0 | 0.000000 | -0.000000 | 1462 | 1289.553102 | 1368.51 | 1447.466898 | 0.936053 |  |
| GO:0006476\_protein\_amino\_acid\_deacetylation | 9 | 0 | 0.000000 | -0.000000 | 1462 | 1289.553102 | 1368.51 | 1447.466898 | 0.936053 |  |
| GO:0006595\_polyamine\_metabolic\_process | 9 | 0 | 0.000000 | -0.000000 | 1462 | 1289.553102 | 1368.51 | 1447.466898 | 0.936053 |  |
| GO:0006611\_protein\_export\_from\_nucleus | 9 | 0 | 0.000000 | -0.000000 | 1462 | 1289.553102 | 1368.51 | 1447.466898 | 0.936053 |  |
| GO:0006910\_phagocytosis\_\_recognition | 9 | 0 | 0.000000 | -0.000000 | 1462 | 1289.553102 | 1368.51 | 1447.466898 | 0.936053 |  |
| GO:0007128\_meiotic\_prophase\_I | 9 | 0 | 0.000000 | -0.000000 | 1462 | 1289.553102 | 1368.51 | 1447.466898 | 0.936053 |  |
| GO:0007193\_inhibition\_of\_adenylate\_cyclase\_activity\_by\_G-protein\_signaling | 9 | 0 | 0.000000 | -0.000000 | 1462 | 1289.553102 | 1368.51 | 1447.466898 | 0.936053 |  |
| GO:0007379\_segment\_specification | 9 | 0 | 0.000000 | -0.000000 | 1462 | 1289.553102 | 1368.51 | 1447.466898 | 0.936053 |  |
| GO:0009451\_RNA\_modification | 9 | 0 | 0.000000 | -0.000000 | 1462 | 1289.553102 | 1368.51 | 1447.466898 | 0.936053 |  |
| GO:0010165\_response\_to\_X-ray | 9 | 0 | 0.000000 | -0.000000 | 1462 | 1289.553102 | 1368.51 | 1447.466898 | 0.936053 |  |
| GO:0014037\_Schwann\_cell\_differentiation | 9 | 0 | 0.000000 | -0.000000 | 1462 | 1289.553102 | 1368.51 | 1447.466898 | 0.936053 |  |
| GO:0014073\_response\_to\_tropane | 9 | 0 | 0.000000 | -0.000000 | 1462 | 1289.553102 | 1368.51 | 1447.466898 | 0.936053 |  |
| GO:0015695\_organic\_cation\_transport | 9 | 0 | 0.000000 | -0.000000 | 1462 | 1289.553102 | 1368.51 | 1447.466898 | 0.936053 |  |
| GO:0016072\_rRNA\_metabolic\_process | 9 | 0 | 0.000000 | -0.000000 | 1462 | 1289.553102 | 1368.51 | 1447.466898 | 0.936053 |  |
| GO:0016601\_Rac\_protein\_signal\_transduction | 9 | 0 | 0.000000 | -0.000000 | 1462 | 1289.553102 | 1368.51 | 1447.466898 | 0.936053 |  |
| GO:0017145\_stem\_cell\_division | 9 | 0 | 0.000000 | -0.000000 | 1462 | 1289.553102 | 1368.51 | 1447.466898 | 0.936053 |  |
| GO:0019320\_hexose\_catabolic\_process | 9 | 0 | 0.000000 | -0.000000 | 1462 | 1289.553102 | 1368.51 | 1447.466898 | 0.936053 |  |
| GO:0021936\_regulation\_of\_granule\_cell\_precursor\_proliferation | 9 | 0 | 0.000000 | -0.000000 | 1462 | 1289.553102 | 1368.51 | 1447.466898 | 0.936053 |  |
| GO:0021940\_positive\_regulation\_of\_granule\_cell\_precursor\_proliferation | 9 | 0 | 0.000000 | -0.000000 | 1462 | 1289.553102 | 1368.51 | 1447.466898 | 0.936053 |  |
| GO:0030048\_actin\_filament-based\_movement | 9 | 0 | 0.000000 | -0.000000 | 1462 | 1289.553102 | 1368.51 | 1447.466898 | 0.936053 |  |
| GO:0030279\_negative\_regulation\_of\_ossification | 9 | 0 | 0.000000 | -0.000000 | 1462 | 1289.553102 | 1368.51 | 1447.466898 | 0.936053 |  |
| GO:0031023\_microtubule\_organizing\_center\_organization | 9 | 0 | 0.000000 | -0.000000 | 1462 | 1289.553102 | 1368.51 | 1447.466898 | 0.936053 |  |
| GO:0032388\_positive\_regulation\_of\_intracellular\_transport | 9 | 0 | 0.000000 | -0.000000 | 1462 | 1289.553102 | 1368.51 | 1447.466898 | 0.936053 |  |
| GO:0032606\_type\_I\_interferon\_production | 9 | 0 | 0.000000 | -0.000000 | 1462 | 1289.553102 | 1368.51 | 1447.466898 | 0.936053 |  |
| GO:0032814\_regulation\_of\_natural\_killer\_cell\_activation | 9 | 0 | 0.000000 | -0.000000 | 1462 | 1289.553102 | 1368.51 | 1447.466898 | 0.936053 |  |
| GO:0032816\_positive\_regulation\_of\_natural\_killer\_cell\_activation | 9 | 0 | 0.000000 | -0.000000 | 1462 | 1289.553102 | 1368.51 | 1447.466898 | 0.936053 |  |
| GO:0032963\_collagen\_metabolic\_process | 9 | 0 | 0.000000 | -0.000000 | 1462 | 1289.553102 | 1368.51 | 1447.466898 | 0.936053 |  |
| GO:0033028\_myeloid\_cell\_apoptosis | 9 | 0 | 0.000000 | -0.000000 | 1462 | 1289.553102 | 1368.51 | 1447.466898 | 0.936053 |  |
| GO:0033143\_regulation\_of\_steroid\_hormone\_receptor\_signaling\_pathway | 9 | 0 | 0.000000 | -0.000000 | 1462 | 1289.553102 | 1368.51 | 1447.466898 | 0.936053 |  |
| GO:0033151\_V(D)J\_recombination | 9 | 0 | 0.000000 | -0.000000 | 1462 | 1289.553102 | 1368.51 | 1447.466898 | 0.936053 |  |
| GO:0034605\_cellular\_response\_to\_heat | 9 | 0 | 0.000000 | -0.000000 | 1462 | 1289.553102 | 1368.51 | 1447.466898 | 0.936053 |  |
| GO:0035088\_establishment\_or\_maintenance\_of\_apical\_basal\_cell\_polarity | 9 | 0 | 0.000000 | -0.000000 | 1462 | 1289.553102 | 1368.51 | 1447.466898 | 0.936053 |  |
| GO:0035162\_embryonic\_hemopoiesis | 9 | 0 | 0.000000 | -0.000000 | 1462 | 1289.553102 | 1368.51 | 1447.466898 | 0.936053 |  |
| GO:0040020\_regulation\_of\_meiosis | 9 | 0 | 0.000000 | -0.000000 | 1462 | 1289.553102 | 1368.51 | 1447.466898 | 0.936053 |  |
| GO:0042058\_regulation\_of\_epidermal\_growth\_factor\_receptor\_signaling\_pathway | 9 | 0 | 0.000000 | -0.000000 | 1462 | 1289.553102 | 1368.51 | 1447.466898 | 0.936053 |  |
| GO:0042093\_T-helper\_cell\_differentiation | 9 | 0 | 0.000000 | -0.000000 | 1462 | 1289.553102 | 1368.51 | 1447.466898 | 0.936053 |  |
| GO:0042220\_response\_to\_cocaine | 9 | 0 | 0.000000 | -0.000000 | 1462 | 1289.553102 | 1368.51 | 1447.466898 | 0.936053 |  |
| GO:0042402\_biogenic\_amine\_catabolic\_process | 9 | 0 | 0.000000 | -0.000000 | 1462 | 1289.553102 | 1368.51 | 1447.466898 | 0.936053 |  |
| GO:0042640\_anagen | 9 | 0 | 0.000000 | -0.000000 | 1462 | 1289.553102 | 1368.51 | 1447.466898 | 0.936053 |  |
| GO:0043242\_negative\_regulation\_of\_protein\_complex\_disassembly | 9 | 0 | 0.000000 | -0.000000 | 1462 | 1289.553102 | 1368.51 | 1447.466898 | 0.936053 |  |
| GO:0043409\_negative\_regulation\_of\_MAPKKK\_cascade | 9 | 0 | 0.000000 | -0.000000 | 1462 | 1289.553102 | 1368.51 | 1447.466898 | 0.936053 |  |
| GO:0043433\_negative\_regulation\_of\_transcription\_factor\_activity | 9 | 0 | 0.000000 | -0.000000 | 1462 | 1289.553102 | 1368.51 | 1447.466898 | 0.936053 |  |
| GO:0043603\_cellular\_amide\_metabolic\_process | 9 | 0 | 0.000000 | -0.000000 | 1462 | 1289.553102 | 1368.51 | 1447.466898 | 0.936053 |  |
| GO:0045109\_intermediate\_filament\_organization | 9 | 0 | 0.000000 | -0.000000 | 1462 | 1289.553102 | 1368.51 | 1447.466898 | 0.936053 |  |
| GO:0045136\_development\_of\_secondary\_sexual\_characteristics | 9 | 0 | 0.000000 | -0.000000 | 1462 | 1289.553102 | 1368.51 | 1447.466898 | 0.936053 |  |
| GO:0045185\_maintenance\_of\_protein\_location | 9 | 0 | 0.000000 | -0.000000 | 1462 | 1289.553102 | 1368.51 | 1447.466898 | 0.936053 |  |
| GO:0045214\_sarcomere\_organization | 9 | 0 | 0.000000 | -0.000000 | 1462 | 1289.553102 | 1368.51 | 1447.466898 | 0.936053 |  |
| GO:0045428\_regulation\_of\_nitric\_oxide\_biosynthetic\_process | 9 | 0 | 0.000000 | -0.000000 | 1462 | 1289.553102 | 1368.51 | 1447.466898 | 0.936053 |  |
| GO:0045620\_negative\_regulation\_of\_lymphocyte\_differentiation | 9 | 0 | 0.000000 | -0.000000 | 1462 | 1289.553102 | 1368.51 | 1447.466898 | 0.936053 |  |
| GO:0045646\_regulation\_of\_erythrocyte\_differentiation | 9 | 0 | 0.000000 | -0.000000 | 1462 | 1289.553102 | 1368.51 | 1447.466898 | 0.936053 |  |
| GO:0045671\_negative\_regulation\_of\_osteoclast\_differentiation | 9 | 0 | 0.000000 | -0.000000 | 1462 | 1289.553102 | 1368.51 | 1447.466898 | 0.936053 |  |
| GO:0045766\_positive\_regulation\_of\_angiogenesis | 9 | 0 | 0.000000 | -0.000000 | 1462 | 1289.553102 | 1368.51 | 1447.466898 | 0.936053 |  |
| GO:0045884\_regulation\_of\_survival\_gene\_product\_expression | 9 | 0 | 0.000000 | -0.000000 | 1462 | 1289.553102 | 1368.51 | 1447.466898 | 0.936053 |  |
| GO:0046006\_regulation\_of\_activated\_T\_cell\_proliferation | 9 | 0 | 0.000000 | -0.000000 | 1462 | 1289.553102 | 1368.51 | 1447.466898 | 0.936053 |  |
| GO:0046324\_regulation\_of\_glucose\_import | 9 | 0 | 0.000000 | -0.000000 | 1462 | 1289.553102 | 1368.51 | 1447.466898 | 0.936053 |  |
| GO:0046365\_monosaccharide\_catabolic\_process | 9 | 0 | 0.000000 | -0.000000 | 1462 | 1289.553102 | 1368.51 | 1447.466898 | 0.936053 |  |
| GO:0046636\_negative\_regulation\_of\_alpha-beta\_T\_cell\_activation | 9 | 0 | 0.000000 | -0.000000 | 1462 | 1289.553102 | 1368.51 | 1447.466898 | 0.936053 |  |
| GO:0046888\_negative\_regulation\_of\_hormone\_secretion | 9 | 0 | 0.000000 | -0.000000 | 1462 | 1289.553102 | 1368.51 | 1447.466898 | 0.936053 |  |
| GO:0048070\_regulation\_of\_pigmentation\_during\_development | 9 | 0 | 0.000000 | -0.000000 | 1462 | 1289.553102 | 1368.51 | 1447.466898 | 0.936053 |  |
| GO:0048146\_positive\_regulation\_of\_fibroblast\_proliferation | 9 | 0 | 0.000000 | -0.000000 | 1462 | 1289.553102 | 1368.51 | 1447.466898 | 0.936053 |  |
| GO:0048284\_organelle\_fusion | 9 | 0 | 0.000000 | -0.000000 | 1462 | 1289.553102 | 1368.51 | 1447.466898 | 0.936053 |  |
| GO:0048488\_synaptic\_vesicle\_endocytosis | 9 | 0 | 0.000000 | -0.000000 | 1462 | 1289.553102 | 1368.51 | 1447.466898 | 0.936053 |  |
| GO:0048569\_post-embryonic\_organ\_development | 9 | 0 | 0.000000 | -0.000000 | 1462 | 1289.553102 | 1368.51 | 1447.466898 | 0.936053 |  |
| GO:0048708\_astrocyte\_differentiation | 9 | 0 | 0.000000 | -0.000000 | 1462 | 1289.553102 | 1368.51 | 1447.466898 | 0.936053 |  |
| GO:0050433\_regulation\_of\_catecholamine\_secretion | 9 | 0 | 0.000000 | -0.000000 | 1462 | 1289.553102 | 1368.51 | 1447.466898 | 0.936053 |  |
| GO:0050884\_neuromuscular\_process\_controlling\_posture | 9 | 0 | 0.000000 | -0.000000 | 1462 | 1289.553102 | 1368.51 | 1447.466898 | 0.936053 |  |
| GO:0050910\_detection\_of\_mechanical\_stimulus\_involved\_in\_sensory\_perception\_of\_sound | 9 | 0 | 0.000000 | -0.000000 | 1462 | 1289.553102 | 1368.51 | 1447.466898 | 0.936053 |  |
| GO:0050918\_positive\_chemotaxis | 9 | 0 | 0.000000 | -0.000000 | 1462 | 1289.553102 | 1368.51 | 1447.466898 | 0.936053 |  |
| GO:0051023\_regulation\_of\_immunoglobulin\_secretion | 9 | 0 | 0.000000 | -0.000000 | 1462 | 1289.553102 | 1368.51 | 1447.466898 | 0.936053 |  |
| GO:0051297\_centrosome\_organization | 9 | 0 | 0.000000 | -0.000000 | 1462 | 1289.553102 | 1368.51 | 1447.466898 | 0.936053 |  |
| GO:0051324\_prophase | 9 | 0 | 0.000000 | -0.000000 | 1462 | 1289.553102 | 1368.51 | 1447.466898 | 0.936053 |  |
| GO:0051647\_nucleus\_localization | 9 | 0 | 0.000000 | -0.000000 | 1462 | 1289.553102 | 1368.51 | 1447.466898 | 0.936053 |  |
| GO:0051896\_regulation\_of\_protein\_kinase\_B\_signaling\_cascade | 9 | 0 | 0.000000 | -0.000000 | 1462 | 1289.553102 | 1368.51 | 1447.466898 | 0.936053 |  |
| GO:0051932\_synaptic\_transmission\_\_GABAergic | 9 | 0 | 0.000000 | -0.000000 | 1462 | 1289.553102 | 1368.51 | 1447.466898 | 0.936053 |  |
| GO:0051963\_regulation\_of\_synaptogenesis | 9 | 0 | 0.000000 | -0.000000 | 1462 | 1289.553102 | 1368.51 | 1447.466898 | 0.936053 |  |
| GO:0055012\_ventricular\_cardiac\_muscle\_cell\_differentiation | 9 | 0 | 0.000000 | -0.000000 | 1462 | 1289.553102 | 1368.51 | 1447.466898 | 0.936053 |  |
| GO:0055013\_cardiac\_muscle\_cell\_development | 9 | 0 | 0.000000 | -0.000000 | 1462 | 1289.553102 | 1368.51 | 1447.466898 | 0.936053 |  |
| GO:0060052\_neurofilament\_cytoskeleton\_organization | 9 | 0 | 0.000000 | -0.000000 | 1462 | 1289.553102 | 1368.51 | 1447.466898 | 0.936053 |  |
| GO:0060081\_membrane\_hyperpolarization | 9 | 0 | 0.000000 | -0.000000 | 1462 | 1289.553102 | 1368.51 | 1447.466898 | 0.936053 |  |
| GO:0060119\_inner\_ear\_receptor\_cell\_development | 9 | 0 | 0.000000 | -0.000000 | 1462 | 1289.553102 | 1368.51 | 1447.466898 | 0.936053 |  |
| GO:0060122\_inner\_ear\_receptor\_stereocilium\_organization | 9 | 0 | 0.000000 | -0.000000 | 1462 | 1289.553102 | 1368.51 | 1447.466898 | 0.936053 |  |
| GO:0060513\_prostatic\_bud\_formation | 9 | 0 | 0.000000 | -0.000000 | 1462 | 1289.553102 | 1368.51 | 1447.466898 | 0.936053 |  |
| GO:0060602\_branch\_elongation\_of\_an\_epithelium | 9 | 0 | 0.000000 | -0.000000 | 1462 | 1289.553102 | 1368.51 | 1447.466898 | 0.936053 |  |
| GO:0060693\_regulation\_of\_branching\_involved\_in\_salivary\_gland\_morphogenesis | 9 | 0 | 0.000000 | -0.000000 | 1462 | 1289.553102 | 1368.51 | 1447.466898 | 0.936053 |  |
| GO:0070306\_lens\_fiber\_cell\_differentiation | 9 | 0 | 0.000000 | -0.000000 | 1462 | 1289.553102 | 1368.51 | 1447.466898 | 0.936053 |  |
| GO:0090048\_negative\_regulation\_of\_transcription\_regulator\_activity | 9 | 0 | 0.000000 | -0.000000 | 1462 | 1289.553102 | 1368.51 | 1447.466898 | 0.936053 |  |
| GO:0006576\_biogenic\_amine\_metabolic\_process | 53 | 0 | 0.000000 | -0.000000 | 1467 | 1293.473499 | 1371.91 | 1450.346501 | 0.935181 |  |
| GO:0006935\_chemotaxis | 53 | 0 | 0.000000 | -0.000000 | 1467 | 1293.473499 | 1371.91 | 1450.346501 | 0.935181 |  |
| GO:0042330\_taxis | 53 | 0 | 0.000000 | -0.000000 | 1467 | 1293.473499 | 1371.91 | 1450.346501 | 0.935181 |  |
| GO:0046942\_carboxylic\_acid\_transport | 53 | 0 | 0.000000 | -0.000000 | 1467 | 1293.473499 | 1371.91 | 1450.346501 | 0.935181 |  |
| GO:0055085\_transmembrane\_transport | 53 | 0 | 0.000000 | -0.000000 | 1467 | 1293.473499 | 1371.91 | 1450.346501 | 0.935181 |  |
| GO:0006417\_regulation\_of\_translation | 29 | 0 | 0.000000 | -0.000000 | 1478 | 1309.730854 | 1386.54 | 1463.349146 | 0.938119 |  |
| GO:0006641\_triglyceride\_metabolic\_process | 29 | 0 | 0.000000 | -0.000000 | 1478 | 1309.730854 | 1386.54 | 1463.349146 | 0.938119 |  |
| GO:0007190\_activation\_of\_adenylate\_cyclase\_activity | 29 | 0 | 0.000000 | -0.000000 | 1478 | 1309.730854 | 1386.54 | 1463.349146 | 0.938119 |  |
| GO:0010564\_regulation\_of\_cell\_cycle\_process | 29 | 0 | 0.000000 | -0.000000 | 1478 | 1309.730854 | 1386.54 | 1463.349146 | 0.938119 |  |
| GO:0042176\_regulation\_of\_protein\_catabolic\_process | 29 | 0 | 0.000000 | -0.000000 | 1478 | 1309.730854 | 1386.54 | 1463.349146 | 0.938119 |  |
| GO:0042770\_DNA\_damage\_response\_\_signal\_transduction | 29 | 0 | 0.000000 | -0.000000 | 1478 | 1309.730854 | 1386.54 | 1463.349146 | 0.938119 |  |
| GO:0043281\_regulation\_of\_caspase\_activity | 29 | 0 | 0.000000 | -0.000000 | 1478 | 1309.730854 | 1386.54 | 1463.349146 | 0.938119 |  |
| GO:0044087\_regulation\_of\_cellular\_component\_biogenesis | 29 | 0 | 0.000000 | -0.000000 | 1478 | 1309.730854 | 1386.54 | 1463.349146 | 0.938119 |  |
| GO:0044270\_nitrogen\_compound\_catabolic\_process | 29 | 0 | 0.000000 | -0.000000 | 1478 | 1309.730854 | 1386.54 | 1463.349146 | 0.938119 |  |
| GO:0051301\_cell\_division | 29 | 0 | 0.000000 | -0.000000 | 1478 | 1309.730854 | 1386.54 | 1463.349146 | 0.938119 |  |
| GO:0052548\_regulation\_of\_endopeptidase\_activity | 29 | 0 | 0.000000 | -0.000000 | 1478 | 1309.730854 | 1386.54 | 1463.349146 | 0.938119 |  |
| GO:0006730\_one-carbon\_metabolic\_process | 39 | 0 | 0.000000 | -0.000000 | 1487 | 1318.837260 | 1394.71 | 1470.582740 | 0.937935 |  |
| GO:0007286\_spermatid\_development | 39 | 0 | 0.000000 | -0.000000 | 1487 | 1318.837260 | 1394.71 | 1470.582740 | 0.937935 |  |
| GO:0008037\_cell\_recognition | 39 | 0 | 0.000000 | -0.000000 | 1487 | 1318.837260 | 1394.71 | 1470.582740 | 0.937935 |  |
| GO:0031279\_regulation\_of\_cyclase\_activity | 39 | 0 | 0.000000 | -0.000000 | 1487 | 1318.837260 | 1394.71 | 1470.582740 | 0.937935 |  |
| GO:0035148\_tube\_lumen\_formation | 39 | 0 | 0.000000 | -0.000000 | 1487 | 1318.837260 | 1394.71 | 1470.582740 | 0.937935 |  |
| GO:0042475\_odontogenesis\_of\_dentine-containing\_tooth | 39 | 0 | 0.000000 | -0.000000 | 1487 | 1318.837260 | 1394.71 | 1470.582740 | 0.937935 |  |
| GO:0043524\_negative\_regulation\_of\_neuron\_apoptosis | 39 | 0 | 0.000000 | -0.000000 | 1487 | 1318.837260 | 1394.71 | 1470.582740 | 0.937935 |  |
| GO:0051339\_regulation\_of\_lyase\_activity | 39 | 0 | 0.000000 | -0.000000 | 1487 | 1318.837260 | 1394.71 | 1470.582740 | 0.937935 |  |
| GO:0070201\_regulation\_of\_establishment\_of\_protein\_localization | 39 | 0 | 0.000000 | -0.000000 | 1487 | 1318.837260 | 1394.71 | 1470.582740 | 0.937935 |  |
| GO:0006091\_generation\_of\_precursor\_metabolites\_and\_energy | 54 | 0 | 0.000000 | -0.000000 | 1493 | 1323.148842 | 1398.54 | 1473.931158 | 0.936731 |  |
| GO:0006164\_purine\_nucleotide\_biosynthetic\_process | 54 | 0 | 0.000000 | -0.000000 | 1493 | 1323.148842 | 1398.54 | 1473.931158 | 0.936731 |  |
| GO:0006412\_translation | 54 | 0 | 0.000000 | -0.000000 | 1493 | 1323.148842 | 1398.54 | 1473.931158 | 0.936731 |  |
| GO:0007265\_Ras\_protein\_signal\_transduction | 54 | 0 | 0.000000 | -0.000000 | 1493 | 1323.148842 | 1398.54 | 1473.931158 | 0.936731 |  |
| GO:0015849\_organic\_acid\_transport | 54 | 0 | 0.000000 | -0.000000 | 1493 | 1323.148842 | 1398.54 | 1473.931158 | 0.936731 |  |
| GO:0044271\_nitrogen\_compound\_biosynthetic\_process | 54 | 0 | 0.000000 | -0.000000 | 1493 | 1323.148842 | 1398.54 | 1473.931158 | 0.936731 |  |
| GO:0009123\_nucleoside\_monophosphate\_metabolic\_process | 60 | 0 | 0.000000 | -0.000000 | 1494 | 1323.602417 | 1398.89 | 1474.177583 | 0.936339 |  |
| GO:0007498\_mesoderm\_development | 48 | 0 | 0.000000 | -0.000000 | 1496 | 1327.551602 | 1402.41 | 1477.268398 | 0.937440 |  |
| GO:0046849\_bone\_remodeling | 48 | 0 | 0.000000 | -0.000000 | 1496 | 1327.551602 | 1402.41 | 1477.268398 | 0.937440 |  |
| GO:0007005\_mitochondrion\_organization | 61 | 0 | 0.000000 | -0.000000 | 1498 | 1329.778273 | 1404.26 | 1478.741727 | 0.937423 |  |
| GO:0009991\_response\_to\_extracellular\_stimulus | 61 | 0 | 0.000000 | -0.000000 | 1498 | 1329.778273 | 1404.26 | 1478.741727 | 0.937423 |  |
| GO:0001836\_release\_of\_cytochrome\_c\_from\_mitochondria | 13 | 0 | 0.000000 | -0.000000 | 1551 | 1394.031535 | 1466.07 | 1538.108465 | 0.945242 |  |
| GO:0001975\_response\_to\_amphetamine | 13 | 0 | 0.000000 | -0.000000 | 1551 | 1394.031535 | 1466.07 | 1538.108465 | 0.945242 |  |
| GO:0001976\_neurological\_system\_process\_involved\_in\_regulation\_of\_systemic\_arterial\_blood\_pressure | 13 | 0 | 0.000000 | -0.000000 | 1551 | 1394.031535 | 1466.07 | 1538.108465 | 0.945242 |  |
| GO:0002717\_positive\_regulation\_of\_natural\_killer\_cell\_mediated\_immunity | 13 | 0 | 0.000000 | -0.000000 | 1551 | 1394.031535 | 1466.07 | 1538.108465 | 0.945242 |  |
| GO:0006778\_porphyrin\_metabolic\_process | 13 | 0 | 0.000000 | -0.000000 | 1551 | 1394.031535 | 1466.07 | 1538.108465 | 0.945242 |  |
| GO:0006833\_water\_transport | 13 | 0 | 0.000000 | -0.000000 | 1551 | 1394.031535 | 1466.07 | 1538.108465 | 0.945242 |  |
| GO:0006898\_receptor-mediated\_endocytosis | 13 | 0 | 0.000000 | -0.000000 | 1551 | 1394.031535 | 1466.07 | 1538.108465 | 0.945242 |  |
| GO:0006986\_response\_to\_unfolded\_protein | 13 | 0 | 0.000000 | -0.000000 | 1551 | 1394.031535 | 1466.07 | 1538.108465 | 0.945242 |  |
| GO:0007129\_synapsis | 13 | 0 | 0.000000 | -0.000000 | 1551 | 1394.031535 | 1466.07 | 1538.108465 | 0.945242 |  |
| GO:0007212\_dopamine\_receptor\_signaling\_pathway | 13 | 0 | 0.000000 | -0.000000 | 1551 | 1394.031535 | 1466.07 | 1538.108465 | 0.945242 |  |
| GO:0007274\_neuromuscular\_synaptic\_transmission | 13 | 0 | 0.000000 | -0.000000 | 1551 | 1394.031535 | 1466.07 | 1538.108465 | 0.945242 |  |
| GO:0007339\_binding\_of\_sperm\_to\_zona\_pellucida | 13 | 0 | 0.000000 | -0.000000 | 1551 | 1394.031535 | 1466.07 | 1538.108465 | 0.945242 |  |
| GO:0007512\_adult\_heart\_development | 13 | 0 | 0.000000 | -0.000000 | 1551 | 1394.031535 | 1466.07 | 1538.108465 | 0.945242 |  |
| GO:0007566\_embryo\_implantation | 13 | 0 | 0.000000 | -0.000000 | 1551 | 1394.031535 | 1466.07 | 1538.108465 | 0.945242 |  |
| GO:0009119\_ribonucleoside\_metabolic\_process | 13 | 0 | 0.000000 | -0.000000 | 1551 | 1394.031535 | 1466.07 | 1538.108465 | 0.945242 |  |
| GO:0009410\_response\_to\_xenobiotic\_stimulus | 13 | 0 | 0.000000 | -0.000000 | 1551 | 1394.031535 | 1466.07 | 1538.108465 | 0.945242 |  |
| GO:0010623\_developmental\_programmed\_cell\_death | 13 | 0 | 0.000000 | -0.000000 | 1551 | 1394.031535 | 1466.07 | 1538.108465 | 0.945242 |  |
| GO:0010970\_microtubule-based\_transport | 13 | 0 | 0.000000 | -0.000000 | 1551 | 1394.031535 | 1466.07 | 1538.108465 | 0.945242 |  |
| GO:0018105\_peptidyl-serine\_phosphorylation | 13 | 0 | 0.000000 | -0.000000 | 1551 | 1394.031535 | 1466.07 | 1538.108465 | 0.945242 |  |
| GO:0021533\_cell\_differentiation\_in\_hindbrain | 13 | 0 | 0.000000 | -0.000000 | 1551 | 1394.031535 | 1466.07 | 1538.108465 | 0.945242 |  |
| GO:0021879\_forebrain\_neuron\_differentiation | 13 | 0 | 0.000000 | -0.000000 | 1551 | 1394.031535 | 1466.07 | 1538.108465 | 0.945242 |  |
| GO:0030384\_phosphoinositide\_metabolic\_process | 13 | 0 | 0.000000 | -0.000000 | 1551 | 1394.031535 | 1466.07 | 1538.108465 | 0.945242 |  |
| GO:0030516\_regulation\_of\_axon\_extension | 13 | 0 | 0.000000 | -0.000000 | 1551 | 1394.031535 | 1466.07 | 1538.108465 | 0.945242 |  |
| GO:0030539\_male\_genitalia\_development | 13 | 0 | 0.000000 | -0.000000 | 1551 | 1394.031535 | 1466.07 | 1538.108465 | 0.945242 |  |
| GO:0031032\_actomyosin\_structure\_organization | 13 | 0 | 0.000000 | -0.000000 | 1551 | 1394.031535 | 1466.07 | 1538.108465 | 0.945242 |  |
| GO:0031290\_retinal\_ganglion\_cell\_axon\_guidance | 13 | 0 | 0.000000 | -0.000000 | 1551 | 1394.031535 | 1466.07 | 1538.108465 | 0.945242 |  |
| GO:0032330\_regulation\_of\_chondrocyte\_differentiation | 13 | 0 | 0.000000 | -0.000000 | 1551 | 1394.031535 | 1466.07 | 1538.108465 | 0.945242 |  |
| GO:0032615\_interleukin-12\_production | 13 | 0 | 0.000000 | -0.000000 | 1551 | 1394.031535 | 1466.07 | 1538.108465 | 0.945242 |  |
| GO:0032729\_positive\_regulation\_of\_interferon-gamma\_production | 13 | 0 | 0.000000 | -0.000000 | 1551 | 1394.031535 | 1466.07 | 1538.108465 | 0.945242 |  |
| GO:0033013\_tetrapyrrole\_metabolic\_process | 13 | 0 | 0.000000 | -0.000000 | 1551 | 1394.031535 | 1466.07 | 1538.108465 | 0.945242 |  |
| GO:0034329\_cell\_junction\_assembly | 13 | 0 | 0.000000 | -0.000000 | 1551 | 1394.031535 | 1466.07 | 1538.108465 | 0.945242 |  |
| GO:0042044\_fluid\_transport | 13 | 0 | 0.000000 | -0.000000 | 1551 | 1394.031535 | 1466.07 | 1538.108465 | 0.945242 |  |
| GO:0042094\_interleukin-2\_biosynthetic\_process | 13 | 0 | 0.000000 | -0.000000 | 1551 | 1394.031535 | 1466.07 | 1538.108465 | 0.945242 |  |
| GO:0042474\_middle\_ear\_morphogenesis | 13 | 0 | 0.000000 | -0.000000 | 1551 | 1394.031535 | 1466.07 | 1538.108465 | 0.945242 |  |
| GO:0043241\_protein\_complex\_disassembly | 13 | 0 | 0.000000 | -0.000000 | 1551 | 1394.031535 | 1466.07 | 1538.108465 | 0.945242 |  |
| GO:0043244\_regulation\_of\_protein\_complex\_disassembly | 13 | 0 | 0.000000 | -0.000000 | 1551 | 1394.031535 | 1466.07 | 1538.108465 | 0.945242 |  |
| GO:0045682\_regulation\_of\_epidermis\_development | 13 | 0 | 0.000000 | -0.000000 | 1551 | 1394.031535 | 1466.07 | 1538.108465 | 0.945242 |  |
| GO:0045954\_positive\_regulation\_of\_natural\_killer\_cell\_mediated\_cytotoxicity | 13 | 0 | 0.000000 | -0.000000 | 1551 | 1394.031535 | 1466.07 | 1538.108465 | 0.945242 |  |
| GO:0046474\_glycerophospholipid\_biosynthetic\_process | 13 | 0 | 0.000000 | -0.000000 | 1551 | 1394.031535 | 1466.07 | 1538.108465 | 0.945242 |  |
| GO:0046851\_negative\_regulation\_of\_bone\_remodeling | 13 | 0 | 0.000000 | -0.000000 | 1551 | 1394.031535 | 1466.07 | 1538.108465 | 0.945242 |  |
| GO:0048305\_immunoglobulin\_secretion | 13 | 0 | 0.000000 | -0.000000 | 1551 | 1394.031535 | 1466.07 | 1538.108465 | 0.945242 |  |
| GO:0048566\_embryonic\_gut\_development | 13 | 0 | 0.000000 | -0.000000 | 1551 | 1394.031535 | 1466.07 | 1538.108465 | 0.945242 |  |
| GO:0050764\_regulation\_of\_phagocytosis | 13 | 0 | 0.000000 | -0.000000 | 1551 | 1394.031535 | 1466.07 | 1538.108465 | 0.945242 |  |
| GO:0050766\_positive\_regulation\_of\_phagocytosis | 13 | 0 | 0.000000 | -0.000000 | 1551 | 1394.031535 | 1466.07 | 1538.108465 | 0.945242 |  |
| GO:0050771\_negative\_regulation\_of\_axonogenesis | 13 | 0 | 0.000000 | -0.000000 | 1551 | 1394.031535 | 1466.07 | 1538.108465 | 0.945242 |  |
| GO:0050818\_regulation\_of\_coagulation | 13 | 0 | 0.000000 | -0.000000 | 1551 | 1394.031535 | 1466.07 | 1538.108465 | 0.945242 |  |
| GO:0051346\_negative\_regulation\_of\_hydrolase\_activity | 13 | 0 | 0.000000 | -0.000000 | 1551 | 1394.031535 | 1466.07 | 1538.108465 | 0.945242 |  |
| GO:0051495\_positive\_regulation\_of\_cytoskeleton\_organization | 13 | 0 | 0.000000 | -0.000000 | 1551 | 1394.031535 | 1466.07 | 1538.108465 | 0.945242 |  |
| GO:0060038\_cardiac\_muscle\_cell\_proliferation | 13 | 0 | 0.000000 | -0.000000 | 1551 | 1394.031535 | 1466.07 | 1538.108465 | 0.945242 |  |
| GO:0060070\_Wnt\_receptor\_signaling\_pathway\_through\_beta-catenin | 13 | 0 | 0.000000 | -0.000000 | 1551 | 1394.031535 | 1466.07 | 1538.108465 | 0.945242 |  |
| GO:0060560\_developmental\_growth\_involved\_in\_morphogenesis | 13 | 0 | 0.000000 | -0.000000 | 1551 | 1394.031535 | 1466.07 | 1538.108465 | 0.945242 |  |
| GO:0060742\_epithelial\_cell\_differentiation\_involved\_in\_prostate\_gland\_development | 13 | 0 | 0.000000 | -0.000000 | 1551 | 1394.031535 | 1466.07 | 1538.108465 | 0.945242 |  |
| GO:0070192\_chromosome\_organization\_involved\_in\_meiosis | 13 | 0 | 0.000000 | -0.000000 | 1551 | 1394.031535 | 1466.07 | 1538.108465 | 0.945242 |  |
| GO:0001818\_negative\_regulation\_of\_cytokine\_production | 18 | 0 | 0.000000 | -0.000000 | 1592 | 1436.912428 | 1506.81 | 1576.707572 | 0.946489 |  |
| GO:0001825\_blastocyst\_formation | 18 | 0 | 0.000000 | -0.000000 | 1592 | 1436.912428 | 1506.81 | 1576.707572 | 0.946489 |  |
| GO:0002064\_epithelial\_cell\_development | 18 | 0 | 0.000000 | -0.000000 | 1592 | 1436.912428 | 1506.81 | 1576.707572 | 0.946489 |  |
| GO:0002285\_lymphocyte\_activation\_during\_immune\_response | 18 | 0 | 0.000000 | -0.000000 | 1592 | 1436.912428 | 1506.81 | 1576.707572 | 0.946489 |  |
| GO:0002715\_regulation\_of\_natural\_killer\_cell\_mediated\_immunity | 18 | 0 | 0.000000 | -0.000000 | 1592 | 1436.912428 | 1506.81 | 1576.707572 | 0.946489 |  |
| GO:0003014\_renal\_system\_process | 18 | 0 | 0.000000 | -0.000000 | 1592 | 1436.912428 | 1506.81 | 1576.707572 | 0.946489 |  |
| GO:0006457\_protein\_folding | 18 | 0 | 0.000000 | -0.000000 | 1592 | 1436.912428 | 1506.81 | 1576.707572 | 0.946489 |  |
| GO:0007140\_male\_meiosis | 18 | 0 | 0.000000 | -0.000000 | 1592 | 1436.912428 | 1506.81 | 1576.707572 | 0.946489 |  |
| GO:0008589\_regulation\_of\_smoothened\_signaling\_pathway | 18 | 0 | 0.000000 | -0.000000 | 1592 | 1436.912428 | 1506.81 | 1576.707572 | 0.946489 |  |
| GO:0009063\_cellular\_amino\_acid\_catabolic\_process | 18 | 0 | 0.000000 | -0.000000 | 1592 | 1436.912428 | 1506.81 | 1576.707572 | 0.946489 |  |
| GO:0010553\_negative\_regulation\_of\_specific\_transcription\_from\_RNA\_polymerase\_II\_promoter | 18 | 0 | 0.000000 | -0.000000 | 1592 | 1436.912428 | 1506.81 | 1576.707572 | 0.946489 |  |
| GO:0015711\_organic\_anion\_transport | 18 | 0 | 0.000000 | -0.000000 | 1592 | 1436.912428 | 1506.81 | 1576.707572 | 0.946489 |  |
| GO:0016458\_gene\_silencing | 18 | 0 | 0.000000 | -0.000000 | 1592 | 1436.912428 | 1506.81 | 1576.707572 | 0.946489 |  |
| GO:0021885\_forebrain\_cell\_migration | 18 | 0 | 0.000000 | -0.000000 | 1592 | 1436.912428 | 1506.81 | 1576.707572 | 0.946489 |  |
| GO:0030178\_negative\_regulation\_of\_Wnt\_receptor\_signaling\_pathway | 18 | 0 | 0.000000 | -0.000000 | 1592 | 1436.912428 | 1506.81 | 1576.707572 | 0.946489 |  |
| GO:0030282\_bone\_mineralization | 18 | 0 | 0.000000 | -0.000000 | 1592 | 1436.912428 | 1506.81 | 1576.707572 | 0.946489 |  |
| GO:0030336\_negative\_regulation\_of\_cell\_migration | 18 | 0 | 0.000000 | -0.000000 | 1592 | 1436.912428 | 1506.81 | 1576.707572 | 0.946489 |  |
| GO:0030510\_regulation\_of\_BMP\_signaling\_pathway | 18 | 0 | 0.000000 | -0.000000 | 1592 | 1436.912428 | 1506.81 | 1576.707572 | 0.946489 |  |
| GO:0032623\_interleukin-2\_production | 18 | 0 | 0.000000 | -0.000000 | 1592 | 1436.912428 | 1506.81 | 1576.707572 | 0.946489 |  |
| GO:0032984\_macromolecular\_complex\_disassembly | 18 | 0 | 0.000000 | -0.000000 | 1592 | 1436.912428 | 1506.81 | 1576.707572 | 0.946489 |  |
| GO:0033157\_regulation\_of\_intracellular\_protein\_transport | 18 | 0 | 0.000000 | -0.000000 | 1592 | 1436.912428 | 1506.81 | 1576.707572 | 0.946489 |  |
| GO:0035051\_cardiac\_cell\_differentiation | 18 | 0 | 0.000000 | -0.000000 | 1592 | 1436.912428 | 1506.81 | 1576.707572 | 0.946489 |  |
| GO:0042269\_regulation\_of\_natural\_killer\_cell\_mediated\_cytotoxicity | 18 | 0 | 0.000000 | -0.000000 | 1592 | 1436.912428 | 1506.81 | 1576.707572 | 0.946489 |  |
| GO:0043029\_T\_cell\_homeostasis | 18 | 0 | 0.000000 | -0.000000 | 1592 | 1436.912428 | 1506.81 | 1576.707572 | 0.946489 |  |
| GO:0044272\_sulfur\_compound\_biosynthetic\_process | 18 | 0 | 0.000000 | -0.000000 | 1592 | 1436.912428 | 1506.81 | 1576.707572 | 0.946489 |  |
| GO:0045103\_intermediate\_filament-based\_process | 18 | 0 | 0.000000 | -0.000000 | 1592 | 1436.912428 | 1506.81 | 1576.707572 | 0.946489 |  |
| GO:0045638\_negative\_regulation\_of\_myeloid\_cell\_differentiation | 18 | 0 | 0.000000 | -0.000000 | 1592 | 1436.912428 | 1506.81 | 1576.707572 | 0.946489 |  |
| GO:0045807\_positive\_regulation\_of\_endocytosis | 18 | 0 | 0.000000 | -0.000000 | 1592 | 1436.912428 | 1506.81 | 1576.707572 | 0.946489 |  |
| GO:0046578\_regulation\_of\_Ras\_protein\_signal\_transduction | 18 | 0 | 0.000000 | -0.000000 | 1592 | 1436.912428 | 1506.81 | 1576.707572 | 0.946489 |  |
| GO:0046620\_regulation\_of\_organ\_growth | 18 | 0 | 0.000000 | -0.000000 | 1592 | 1436.912428 | 1506.81 | 1576.707572 | 0.946489 |  |
| GO:0048535\_lymph\_node\_development | 18 | 0 | 0.000000 | -0.000000 | 1592 | 1436.912428 | 1506.81 | 1576.707572 | 0.946489 |  |
| GO:0048730\_epidermis\_morphogenesis | 18 | 0 | 0.000000 | -0.000000 | 1592 | 1436.912428 | 1506.81 | 1576.707572 | 0.946489 |  |
| GO:0048813\_dendrite\_morphogenesis | 18 | 0 | 0.000000 | -0.000000 | 1592 | 1436.912428 | 1506.81 | 1576.707572 | 0.946489 |  |
| GO:0050982\_detection\_of\_mechanical\_stimulus | 18 | 0 | 0.000000 | -0.000000 | 1592 | 1436.912428 | 1506.81 | 1576.707572 | 0.946489 |  |
| GO:0051168\_nuclear\_export | 18 | 0 | 0.000000 | -0.000000 | 1592 | 1436.912428 | 1506.81 | 1576.707572 | 0.946489 |  |
| GO:0051222\_positive\_regulation\_of\_protein\_transport | 18 | 0 | 0.000000 | -0.000000 | 1592 | 1436.912428 | 1506.81 | 1576.707572 | 0.946489 |  |
| GO:0051924\_regulation\_of\_calcium\_ion\_transport | 18 | 0 | 0.000000 | -0.000000 | 1592 | 1436.912428 | 1506.81 | 1576.707572 | 0.946489 |  |
| GO:0055008\_cardiac\_muscle\_tissue\_morphogenesis | 18 | 0 | 0.000000 | -0.000000 | 1592 | 1436.912428 | 1506.81 | 1576.707572 | 0.946489 |  |
| GO:0060415\_muscle\_tissue\_morphogenesis | 18 | 0 | 0.000000 | -0.000000 | 1592 | 1436.912428 | 1506.81 | 1576.707572 | 0.946489 |  |
| GO:0060571\_morphogenesis\_of\_an\_epithelial\_fold | 18 | 0 | 0.000000 | -0.000000 | 1592 | 1436.912428 | 1506.81 | 1576.707572 | 0.946489 |  |
| GO:0060674\_placenta\_blood\_vessel\_development | 18 | 0 | 0.000000 | -0.000000 | 1592 | 1436.912428 | 1506.81 | 1576.707572 | 0.946489 |  |
| GO:0007369\_gastrulation | 63 | 0 | 0.000000 | -0.000000 | 1595 | 1439.535991 | 1508.88 | 1578.224009 | 0.946006 |  |
| GO:0009165\_nucleotide\_biosynthetic\_process | 63 | 0 | 0.000000 | -0.000000 | 1595 | 1439.535991 | 1508.88 | 1578.224009 | 0.946006 |  |
| GO:0051186\_cofactor\_metabolic\_process | 63 | 0 | 0.000000 | -0.000000 | 1595 | 1439.535991 | 1508.88 | 1578.224009 | 0.946006 |  |
| GO:0001704\_formation\_of\_primary\_germ\_layer | 36 | 0 | 0.000000 | -0.000000 | 1607 | 1451.325845 | 1519.17 | 1587.014155 | 0.945345 |  |
| GO:0001819\_positive\_regulation\_of\_cytokine\_production | 36 | 0 | 0.000000 | -0.000000 | 1607 | 1451.325845 | 1519.17 | 1587.014155 | 0.945345 |  |
| GO:0001889\_liver\_development | 36 | 0 | 0.000000 | -0.000000 | 1607 | 1451.325845 | 1519.17 | 1587.014155 | 0.945345 |  |
| GO:0007187\_G-protein\_signaling\_\_coupled\_to\_cyclic\_nucleotide\_second\_messenger | 36 | 0 | 0.000000 | -0.000000 | 1607 | 1451.325845 | 1519.17 | 1587.014155 | 0.945345 |  |
| GO:0007368\_determination\_of\_left\_right\_symmetry | 36 | 0 | 0.000000 | -0.000000 | 1607 | 1451.325845 | 1519.17 | 1587.014155 | 0.945345 |  |
| GO:0007631\_feeding\_behavior | 36 | 0 | 0.000000 | -0.000000 | 1607 | 1451.325845 | 1519.17 | 1587.014155 | 0.945345 |  |
| GO:0014020\_primary\_neural\_tube\_formation | 36 | 0 | 0.000000 | -0.000000 | 1607 | 1451.325845 | 1519.17 | 1587.014155 | 0.945345 |  |
| GO:0030072\_peptide\_hormone\_secretion | 36 | 0 | 0.000000 | -0.000000 | 1607 | 1451.325845 | 1519.17 | 1587.014155 | 0.945345 |  |
| GO:0030278\_regulation\_of\_ossification | 36 | 0 | 0.000000 | -0.000000 | 1607 | 1451.325845 | 1519.17 | 1587.014155 | 0.945345 |  |
| GO:0042742\_defense\_response\_to\_bacterium | 36 | 0 | 0.000000 | -0.000000 | 1607 | 1451.325845 | 1519.17 | 1587.014155 | 0.945345 |  |
| GO:0050900\_leukocyte\_migration | 36 | 0 | 0.000000 | -0.000000 | 1607 | 1451.325845 | 1519.17 | 1587.014155 | 0.945345 |  |
| GO:0051223\_regulation\_of\_protein\_transport | 36 | 0 | 0.000000 | -0.000000 | 1607 | 1451.325845 | 1519.17 | 1587.014155 | 0.945345 |  |
| GO:0000012\_single\_strand\_break\_repair | 2 | 0 |  |  |  |  |  |  |  |  |
| GO:0000019\_regulation\_of\_mitotic\_recombination | 2 | 0 |  |  |  |  |  |  |  |  |
| GO:0000076\_DNA\_replication\_checkpoint | 2 | 0 |  |  |  |  |  |  |  |  |
| GO:0000080\_G1\_phase\_of\_mitotic\_cell\_cycle | 2 | 0 |  |  |  |  |  |  |  |  |
| GO:0000083\_regulation\_of\_transcription\_of\_G1\_S-phase\_of\_mitotic\_cell\_cycle | 2 | 0 |  |  |  |  |  |  |  |  |
| GO:0000085\_G2\_phase\_of\_mitotic\_cell\_cycle | 2 | 0 |  |  |  |  |  |  |  |  |
| GO:0000289\_nuclear-transcribed\_mRNA\_poly(A)\_tail\_shortening | 2 | 0 |  |  |  |  |  |  |  |  |
| GO:0000381\_regulation\_of\_alternative\_nuclear\_mRNA\_splicing\_\_via\_spliceosome | 2 | 0 |  |  |  |  |  |  |  |  |
| GO:0000712\_resolution\_of\_meiotic\_joint\_molecules\_as\_recombinants | 2 | 0 |  |  |  |  |  |  |  |  |
| GO:0000720\_pyrimidine\_dimer\_repair\_by\_nucleotide-excision\_repair | 2 | 0 |  |  |  |  |  |  |  |  |
| GO:0001302\_replicative\_cell\_aging | 2 | 0 |  |  |  |  |  |  |  |  |
| GO:0001306\_age-dependent\_response\_to\_oxidative\_stress | 2 | 0 |  |  |  |  |  |  |  |  |
| GO:0001514\_selenocysteine\_incorporation | 2 | 0 |  |  |  |  |  |  |  |  |
| GO:0001522\_pseudouridine\_synthesis | 2 | 0 |  |  |  |  |  |  |  |  |
| GO:0001543\_ovarian\_follicle\_rupture | 2 | 0 |  |  |  |  |  |  |  |  |
| GO:0001561\_fatty\_acid\_alpha-oxidation | 2 | 0 |  |  |  |  |  |  |  |  |
| GO:0001675\_acrosome\_assembly | 2 | 0 |  |  |  |  |  |  |  |  |
| GO:0001743\_optic\_placode\_formation | 2 | 0 |  |  |  |  |  |  |  |  |
| GO:0001767\_establishment\_of\_lymphocyte\_polarity | 2 | 0 |  |  |  |  |  |  |  |  |
| GO:0001768\_establishment\_of\_T\_cell\_polarity | 2 | 0 |  |  |  |  |  |  |  |  |
| GO:0001771\_formation\_of\_immunological\_synapse | 2 | 0 |  |  |  |  |  |  |  |  |
| GO:0001774\_microglial\_cell\_activation | 2 | 0 |  |  |  |  |  |  |  |  |
| GO:0001781\_neutrophil\_apoptosis | 2 | 0 |  |  |  |  |  |  |  |  |
| GO:0001787\_natural\_killer\_cell\_proliferation | 2 | 0 |  |  |  |  |  |  |  |  |
| GO:0001788\_antibody-dependent\_cellular\_cytotoxicity | 2 | 0 |  |  |  |  |  |  |  |  |
| GO:0001806\_type\_IV\_hypersensitivity | 2 | 0 |  |  |  |  |  |  |  |  |
| GO:0001807\_regulation\_of\_type\_IV\_hypersensitivity | 2 | 0 |  |  |  |  |  |  |  |  |
| GO:0001808\_negative\_regulation\_of\_type\_IV\_hypersensitivity | 2 | 0 |  |  |  |  |  |  |  |  |
| GO:0001823\_mesonephros\_development | 2 | 0 |  |  |  |  |  |  |  |  |
| GO:0001845\_phagolysosome\_formation | 2 | 0 |  |  |  |  |  |  |  |  |
| GO:0001866\_NK\_T\_cell\_proliferation | 2 | 0 |  |  |  |  |  |  |  |  |
| GO:0001879\_detection\_of\_yeast | 2 | 0 |  |  |  |  |  |  |  |  |
| GO:0001886\_endothelial\_cell\_morphogenesis | 2 | 0 |  |  |  |  |  |  |  |  |
| GO:0001919\_regulation\_of\_receptor\_recycling | 2 | 0 |  |  |  |  |  |  |  |  |
| GO:0001954\_positive\_regulation\_of\_cell-matrix\_adhesion | 2 | 0 |  |  |  |  |  |  |  |  |
| GO:0001977\_renal\_system\_process\_involved\_in\_regulation\_of\_blood\_volume | 2 | 0 |  |  |  |  |  |  |  |  |
| GO:0001982\_baroreceptor\_response\_to\_decreased\_systemic\_arterial\_blood\_pressure | 2 | 0 |  |  |  |  |  |  |  |  |
| GO:0001983\_baroreceptor\_response\_to\_increased\_systemic\_arterial\_blood\_pressure | 2 | 0 |  |  |  |  |  |  |  |  |
| GO:0001992\_regulation\_of\_systemic\_arterial\_blood\_pressure\_by\_vasopressin | 2 | 0 |  |  |  |  |  |  |  |  |
| GO:0001997\_positive\_regulation\_of\_the\_force\_of\_heart\_contraction\_by\_epinephrine-norepinephrine | 2 | 0 |  |  |  |  |  |  |  |  |
| GO:0001998\_angiotensin\_mediated\_vasoconstriction\_involved\_in\_regulation\_of\_systemic\_arterial\_blood\_pressure | 2 | 0 |  |  |  |  |  |  |  |  |
| GO:0001999\_renal\_response\_to\_blood\_flow\_during\_renin-angiotensin\_regulation\_of\_systemic\_arterial\_blood\_pressure | 2 | 0 |  |  |  |  |  |  |  |  |
| GO:0002018\_renin-angiotensin\_regulation\_of\_aldosterone\_production | 2 | 0 |  |  |  |  |  |  |  |  |
| GO:0002019\_regulation\_of\_renal\_output\_by\_angiotensin | 2 | 0 |  |  |  |  |  |  |  |  |
| GO:0002024\_diet\_induced\_thermogenesis | 2 | 0 |  |  |  |  |  |  |  |  |
| GO:0002025\_vasodilation\_by\_norepinephrine-epinephrine\_involved\_in\_regulation\_of\_systemic\_arterial\_blood\_pressure | 2 | 0 |  |  |  |  |  |  |  |  |
| GO:0002029\_desensitization\_of\_G-protein\_coupled\_receptor\_protein\_signaling\_pathway | 2 | 0 |  |  |  |  |  |  |  |  |
| GO:0002033\_vasodilation\_by\_angiotensin\_involved\_in\_regulation\_of\_systemic\_arterial\_blood\_pressure | 2 | 0 |  |  |  |  |  |  |  |  |
| GO:0002066\_columnar\_cuboidal\_epithelial\_cell\_development | 2 | 0 |  |  |  |  |  |  |  |  |
| GO:0002072\_optic\_cup\_morphogenesis\_involved\_in\_camera-type\_eye\_development | 2 | 0 |  |  |  |  |  |  |  |  |
| GO:0002074\_extraocular\_skeletal\_muscle\_development | 2 | 0 |  |  |  |  |  |  |  |  |
| GO:0002138\_retinoic\_acid\_biosynthetic\_process | 2 | 0 |  |  |  |  |  |  |  |  |
| GO:0002223\_stimulatory\_C-type\_lectin\_receptor\_signaling\_pathway | 2 | 0 |  |  |  |  |  |  |  |  |
| GO:0002246\_healing\_during\_inflammatory\_response | 2 | 0 |  |  |  |  |  |  |  |  |
| GO:0002251\_organ\_or\_tissue\_specific\_immune\_response | 2 | 0 |  |  |  |  |  |  |  |  |
| GO:0002266\_follicular\_dendritic\_cell\_activation | 2 | 0 |  |  |  |  |  |  |  |  |
| GO:0002268\_follicular\_dendritic\_cell\_differentiation | 2 | 0 |  |  |  |  |  |  |  |  |
| GO:0002327\_immature\_B\_cell\_differentiation | 2 | 0 |  |  |  |  |  |  |  |  |
| GO:0002329\_pre-B\_cell\_differentiation | 2 | 0 |  |  |  |  |  |  |  |  |
| GO:0002339\_B\_cell\_selection | 2 | 0 |  |  |  |  |  |  |  |  |
| GO:0002352\_B\_cell\_negative\_selection | 2 | 0 |  |  |  |  |  |  |  |  |
| GO:0002358\_B\_cell\_homeostatic\_proliferation | 2 | 0 |  |  |  |  |  |  |  |  |
| GO:0002385\_mucosal\_immune\_response | 2 | 0 |  |  |  |  |  |  |  |  |
| GO:0002514\_B\_cell\_tolerance\_induction | 2 | 0 |  |  |  |  |  |  |  |  |
| GO:0002523\_leukocyte\_migration\_during\_inflammatory\_response | 2 | 0 |  |  |  |  |  |  |  |  |
| GO:0002536\_respiratory\_burst\_during\_acute\_inflammatory\_response | 2 | 0 |  |  |  |  |  |  |  |  |
| GO:0002537\_production\_of\_nitric\_oxide\_during\_acute\_inflammatory\_response | 2 | 0 |  |  |  |  |  |  |  |  |
| GO:0002576\_platelet\_degranulation | 2 | 0 |  |  |  |  |  |  |  |  |
| GO:0002639\_positive\_regulation\_of\_immunoglobulin\_production | 2 | 0 |  |  |  |  |  |  |  |  |
| GO:0002661\_regulation\_of\_B\_cell\_tolerance\_induction | 2 | 0 |  |  |  |  |  |  |  |  |
| GO:0002663\_positive\_regulation\_of\_B\_cell\_tolerance\_induction | 2 | 0 |  |  |  |  |  |  |  |  |
| GO:0002676\_regulation\_of\_chronic\_inflammatory\_response | 2 | 0 |  |  |  |  |  |  |  |  |
| GO:0002679\_respiratory\_burst\_during\_defense\_response | 2 | 0 |  |  |  |  |  |  |  |  |
| GO:0002686\_negative\_regulation\_of\_leukocyte\_migration | 2 | 0 |  |  |  |  |  |  |  |  |
| GO:0002720\_positive\_regulation\_of\_cytokine\_production\_during\_immune\_response | 2 | 0 |  |  |  |  |  |  |  |  |
| GO:0002752\_cell\_surface\_pattern\_recognition\_receptor\_signaling\_pathway | 2 | 0 |  |  |  |  |  |  |  |  |
| GO:0002755\_MyD88-dependent\_toll-like\_receptor\_signaling\_pathway | 2 | 0 |  |  |  |  |  |  |  |  |
| GO:0002765\_immune\_response-inhibiting\_signal\_transduction | 2 | 0 |  |  |  |  |  |  |  |  |
| GO:0002921\_negative\_regulation\_of\_humoral\_immune\_response | 2 | 0 |  |  |  |  |  |  |  |  |
| GO:0002924\_negative\_regulation\_of\_humoral\_immune\_response\_mediated\_by\_circulating\_immunoglobulin | 2 | 0 |  |  |  |  |  |  |  |  |
| GO:0003057\_regulation\_of\_the\_force\_of\_heart\_contraction\_by\_chemical\_signal | 2 | 0 |  |  |  |  |  |  |  |  |
| GO:0003099\_positive\_regulation\_of\_the\_force\_of\_heart\_contraction\_by\_chemical\_signal | 2 | 0 |  |  |  |  |  |  |  |  |
| GO:0005981\_regulation\_of\_glycogen\_catabolic\_process | 2 | 0 |  |  |  |  |  |  |  |  |
| GO:0006021\_inositol\_biosynthetic\_process | 2 | 0 |  |  |  |  |  |  |  |  |
| GO:0006042\_glucosamine\_biosynthetic\_process | 2 | 0 |  |  |  |  |  |  |  |  |
| GO:0006045\_N-acetylglucosamine\_biosynthetic\_process | 2 | 0 |  |  |  |  |  |  |  |  |
| GO:0006048\_UDP-N-acetylglucosamine\_biosynthetic\_process | 2 | 0 |  |  |  |  |  |  |  |  |
| GO:0006054\_N-acetylneuraminate\_metabolic\_process | 2 | 0 |  |  |  |  |  |  |  |  |
| GO:0006059\_hexitol\_metabolic\_process | 2 | 0 |  |  |  |  |  |  |  |  |
| GO:0006063\_uronic\_acid\_metabolic\_process | 2 | 0 |  |  |  |  |  |  |  |  |
| GO:0006068\_ethanol\_catabolic\_process | 2 | 0 |  |  |  |  |  |  |  |  |
| GO:0006083\_acetate\_metabolic\_process | 2 | 0 |  |  |  |  |  |  |  |  |
| GO:0006089\_lactate\_metabolic\_process | 2 | 0 |  |  |  |  |  |  |  |  |
| GO:0006105\_succinate\_metabolic\_process | 2 | 0 |  |  |  |  |  |  |  |  |
| GO:0006106\_fumarate\_metabolic\_process | 2 | 0 |  |  |  |  |  |  |  |  |
| GO:0006110\_regulation\_of\_glycolysis | 2 | 0 |  |  |  |  |  |  |  |  |
| GO:0006113\_fermentation | 2 | 0 |  |  |  |  |  |  |  |  |
| GO:0006114\_glycerol\_biosynthetic\_process | 2 | 0 |  |  |  |  |  |  |  |  |
| GO:0006122\_mitochondrial\_electron\_transport\_\_ubiquinol\_to\_cytochrome\_c | 2 | 0 |  |  |  |  |  |  |  |  |
| GO:0006152\_purine\_nucleoside\_catabolic\_process | 2 | 0 |  |  |  |  |  |  |  |  |
| GO:0006168\_adenine\_salvage | 2 | 0 |  |  |  |  |  |  |  |  |
| GO:0006200\_ATP\_catabolic\_process | 2 | 0 |  |  |  |  |  |  |  |  |
| GO:0006206\_pyrimidine\_base\_metabolic\_process | 2 | 0 |  |  |  |  |  |  |  |  |
| GO:0006213\_pyrimidine\_nucleoside\_metabolic\_process | 2 | 0 |  |  |  |  |  |  |  |  |
| GO:0006265\_DNA\_topological\_change | 2 | 0 |  |  |  |  |  |  |  |  |
| GO:0006278\_RNA-dependent\_DNA\_replication | 2 | 0 |  |  |  |  |  |  |  |  |
| GO:0006312\_mitotic\_recombination | 2 | 0 |  |  |  |  |  |  |  |  |
| GO:0006398\_histone\_mRNA\_3'-end\_processing | 2 | 0 |  |  |  |  |  |  |  |  |
| GO:0006418\_tRNA\_aminoacylation\_for\_protein\_translation | 2 | 0 |  |  |  |  |  |  |  |  |
| GO:0006451\_translational\_readthrough | 2 | 0 |  |  |  |  |  |  |  |  |
| GO:0006477\_protein\_amino\_acid\_sulfation | 2 | 0 |  |  |  |  |  |  |  |  |
| GO:0006482\_protein\_amino\_acid\_demethylation | 2 | 0 |  |  |  |  |  |  |  |  |
| GO:0006499\_N-terminal\_protein\_myristoylation | 2 | 0 |  |  |  |  |  |  |  |  |
| GO:0006525\_arginine\_metabolic\_process | 2 | 0 |  |  |  |  |  |  |  |  |
| GO:0006527\_arginine\_catabolic\_process | 2 | 0 |  |  |  |  |  |  |  |  |
| GO:0006532\_aspartate\_biosynthetic\_process | 2 | 0 |  |  |  |  |  |  |  |  |
| GO:0006538\_glutamate\_catabolic\_process | 2 | 0 |  |  |  |  |  |  |  |  |
| GO:0006558\_L-phenylalanine\_metabolic\_process | 2 | 0 |  |  |  |  |  |  |  |  |
| GO:0006563\_L-serine\_metabolic\_process | 2 | 0 |  |  |  |  |  |  |  |  |
| GO:0006566\_threonine\_metabolic\_process | 2 | 0 |  |  |  |  |  |  |  |  |
| GO:0006568\_tryptophan\_metabolic\_process | 2 | 0 |  |  |  |  |  |  |  |  |
| GO:0006583\_melanin\_biosynthetic\_process\_from\_tyrosine | 2 | 0 |  |  |  |  |  |  |  |  |
| GO:0006600\_creatine\_metabolic\_process | 2 | 0 |  |  |  |  |  |  |  |  |
| GO:0006603\_phosphocreatine\_metabolic\_process | 2 | 0 |  |  |  |  |  |  |  |  |
| GO:0006610\_ribosomal\_protein\_import\_into\_nucleus | 2 | 0 |  |  |  |  |  |  |  |  |
| GO:0006642\_triglyceride\_mobilization | 2 | 0 |  |  |  |  |  |  |  |  |
| GO:0006649\_phospholipid\_transfer\_to\_membrane | 2 | 0 |  |  |  |  |  |  |  |  |
| GO:0006681\_galactosylceramide\_metabolic\_process | 2 | 0 |  |  |  |  |  |  |  |  |
| GO:0006702\_androgen\_biosynthetic\_process | 2 | 0 |  |  |  |  |  |  |  |  |
| GO:0006750\_glutathione\_biosynthetic\_process | 2 | 0 |  |  |  |  |  |  |  |  |
| GO:0006760\_folic\_acid\_and\_derivative\_metabolic\_process | 2 | 0 |  |  |  |  |  |  |  |  |
| GO:0006808\_regulation\_of\_nitrogen\_utilization | 2 | 0 |  |  |  |  |  |  |  |  |
| GO:0006868\_glutamine\_transport | 2 | 0 |  |  |  |  |  |  |  |  |
| GO:0006907\_pinocytosis | 2 | 0 |  |  |  |  |  |  |  |  |
| GO:0006925\_inflammatory\_cell\_apoptosis | 2 | 0 |  |  |  |  |  |  |  |  |
| GO:0006977\_DNA\_damage\_response\_\_signal\_transduction\_by\_p53\_class\_mediator\_resulting\_in\_cell\_cycle\_arrest | 2 | 0 |  |  |  |  |  |  |  |  |
| GO:0006991\_response\_to\_sterol\_depletion | 2 | 0 |  |  |  |  |  |  |  |  |
| GO:0007004\_telomere\_maintenance\_via\_telomerase | 2 | 0 |  |  |  |  |  |  |  |  |
| GO:0007020\_microtubule\_nucleation | 2 | 0 |  |  |  |  |  |  |  |  |
| GO:0007030\_Golgi\_organization | 2 | 0 |  |  |  |  |  |  |  |  |
| GO:0007035\_vacuolar\_acidification | 2 | 0 |  |  |  |  |  |  |  |  |
| GO:0007042\_lysosomal\_lumen\_acidification | 2 | 0 |  |  |  |  |  |  |  |  |
| GO:0007060\_male\_meiosis\_chromosome\_segregation | 2 | 0 |  |  |  |  |  |  |  |  |
| GO:0007089\_traversing\_start\_control\_point\_of\_mitotic\_cell\_cycle | 2 | 0 |  |  |  |  |  |  |  |  |
| GO:0007094\_mitotic\_cell\_cycle\_spindle\_assembly\_checkpoint | 2 | 0 |  |  |  |  |  |  |  |  |
| GO:0007097\_nuclear\_migration | 2 | 0 |  |  |  |  |  |  |  |  |
| GO:0007100\_mitotic\_centrosome\_separation | 2 | 0 |  |  |  |  |  |  |  |  |
| GO:0007132\_meiotic\_metaphase\_I | 2 | 0 |  |  |  |  |  |  |  |  |
| GO:0007171\_activation\_of\_transmembrane\_receptor\_protein\_tyrosine\_kinase\_activity | 2 | 0 |  |  |  |  |  |  |  |  |
| GO:0007182\_common-partner\_SMAD\_protein\_phosphorylation | 2 | 0 |  |  |  |  |  |  |  |  |
| GO:0007185\_transmembrane\_receptor\_protein\_tyrosine\_phosphatase\_signaling\_pathway | 2 | 0 |  |  |  |  |  |  |  |  |
| GO:0007205\_activation\_of\_protein\_kinase\_C\_activity\_by\_G-protein\_coupled\_receptor\_protein\_signaling\_pathway | 2 | 0 |  |  |  |  |  |  |  |  |
| GO:0007210\_serotonin\_receptor\_signaling\_pathway | 2 | 0 |  |  |  |  |  |  |  |  |
| GO:0007220\_Notch\_receptor\_processing | 2 | 0 |  |  |  |  |  |  |  |  |
| GO:0007256\_activation\_of\_JNKK\_activity | 2 | 0 |  |  |  |  |  |  |  |  |
| GO:0007258\_JUN\_phosphorylation | 2 | 0 |  |  |  |  |  |  |  |  |
| GO:0007263\_nitric\_oxide\_mediated\_signal\_transduction | 2 | 0 |  |  |  |  |  |  |  |  |
| GO:0007289\_spermatid\_nucleus\_differentiation | 2 | 0 |  |  |  |  |  |  |  |  |
| GO:0007343\_egg\_activation | 2 | 0 |  |  |  |  |  |  |  |  |
| GO:0007351\_tripartite\_regional\_subdivision | 2 | 0 |  |  |  |  |  |  |  |  |
| GO:0007418\_ventral\_midline\_development | 2 | 0 |  |  |  |  |  |  |  |  |
| GO:0007494\_midgut\_development | 2 | 0 |  |  |  |  |  |  |  |  |
| GO:0007527\_adult\_somatic\_muscle\_development | 2 | 0 |  |  |  |  |  |  |  |  |
| GO:0007549\_dosage\_compensation | 2 | 0 |  |  |  |  |  |  |  |  |
| GO:0007571\_age-dependent\_general\_metabolic\_decline | 2 | 0 |  |  |  |  |  |  |  |  |
| GO:0007603\_phototransduction\_\_visible\_light | 2 | 0 |  |  |  |  |  |  |  |  |
| GO:0008065\_establishment\_of\_blood-nerve\_barrier | 2 | 0 |  |  |  |  |  |  |  |  |
| GO:0008089\_anterograde\_axon\_cargo\_transport | 2 | 0 |  |  |  |  |  |  |  |  |
| GO:0008210\_estrogen\_metabolic\_process | 2 | 0 |  |  |  |  |  |  |  |  |
| GO:0008212\_mineralocorticoid\_metabolic\_process | 2 | 0 |  |  |  |  |  |  |  |  |
| GO:0008214\_protein\_amino\_acid\_dealkylation | 2 | 0 |  |  |  |  |  |  |  |  |
| GO:0008228\_opsonization | 2 | 0 |  |  |  |  |  |  |  |  |
| GO:0008272\_sulfate\_transport | 2 | 0 |  |  |  |  |  |  |  |  |
| GO:0008291\_acetylcholine\_metabolic\_process | 2 | 0 |  |  |  |  |  |  |  |  |
| GO:0008298\_intracellular\_mRNA\_localization | 2 | 0 |  |  |  |  |  |  |  |  |
| GO:0008334\_histone\_mRNA\_metabolic\_process | 2 | 0 |  |  |  |  |  |  |  |  |
| GO:0008356\_asymmetric\_cell\_division | 2 | 0 |  |  |  |  |  |  |  |  |
| GO:0008582\_regulation\_of\_synaptic\_growth\_at\_neuromuscular\_junction | 2 | 0 |  |  |  |  |  |  |  |  |
| GO:0008594\_photoreceptor\_cell\_morphogenesis | 2 | 0 |  |  |  |  |  |  |  |  |
| GO:0008595\_determination\_of\_anterior\_posterior\_axis\_\_embryo | 2 | 0 |  |  |  |  |  |  |  |  |
| GO:0008608\_attachment\_of\_spindle\_microtubules\_to\_kinetochore | 2 | 0 |  |  |  |  |  |  |  |  |
| GO:0008616\_queuosine\_biosynthetic\_process | 2 | 0 |  |  |  |  |  |  |  |  |
| GO:0008617\_guanosine\_metabolic\_process | 2 | 0 |  |  |  |  |  |  |  |  |
| GO:0008618\_7-methylguanosine\_metabolic\_process | 2 | 0 |  |  |  |  |  |  |  |  |
| GO:0008634\_negative\_regulation\_of\_survival\_gene\_product\_expression | 2 | 0 |  |  |  |  |  |  |  |  |
| GO:0009048\_dosage\_compensation\_\_by\_inactivation\_of\_X\_chromosome | 2 | 0 |  |  |  |  |  |  |  |  |
| GO:0009070\_serine\_family\_amino\_acid\_biosynthetic\_process | 2 | 0 |  |  |  |  |  |  |  |  |
| GO:0009071\_serine\_family\_amino\_acid\_catabolic\_process | 2 | 0 |  |  |  |  |  |  |  |  |
| GO:0009074\_aromatic\_amino\_acid\_family\_catabolic\_process | 2 | 0 |  |  |  |  |  |  |  |  |
| GO:0009083\_branched\_chain\_family\_amino\_acid\_catabolic\_process | 2 | 0 |  |  |  |  |  |  |  |  |
| GO:0009093\_cysteine\_catabolic\_process | 2 | 0 |  |  |  |  |  |  |  |  |
| GO:0009120\_deoxyribonucleoside\_metabolic\_process | 2 | 0 |  |  |  |  |  |  |  |  |
| GO:0009125\_nucleoside\_monophosphate\_catabolic\_process | 2 | 0 |  |  |  |  |  |  |  |  |
| GO:0009126\_purine\_nucleoside\_monophosphate\_metabolic\_process | 2 | 0 |  |  |  |  |  |  |  |  |
| GO:0009142\_nucleoside\_triphosphate\_biosynthetic\_process | 2 | 0 |  |  |  |  |  |  |  |  |
| GO:0009161\_ribonucleoside\_monophosphate\_metabolic\_process | 2 | 0 |  |  |  |  |  |  |  |  |
| GO:0009164\_nucleoside\_catabolic\_process | 2 | 0 |  |  |  |  |  |  |  |  |
| GO:0009167\_purine\_ribonucleoside\_monophosphate\_metabolic\_process | 2 | 0 |  |  |  |  |  |  |  |  |
| GO:0009202\_deoxyribonucleoside\_triphosphate\_biosynthetic\_process | 2 | 0 |  |  |  |  |  |  |  |  |
| GO:0009203\_ribonucleoside\_triphosphate\_catabolic\_process | 2 | 0 |  |  |  |  |  |  |  |  |
| GO:0009207\_purine\_ribonucleoside\_triphosphate\_catabolic\_process | 2 | 0 |  |  |  |  |  |  |  |  |
| GO:0009219\_pyrimidine\_deoxyribonucleotide\_metabolic\_process | 2 | 0 |  |  |  |  |  |  |  |  |
| GO:0009265\_2'-deoxyribonucleotide\_biosynthetic\_process | 2 | 0 |  |  |  |  |  |  |  |  |
| GO:0009268\_response\_to\_pH | 2 | 0 |  |  |  |  |  |  |  |  |
| GO:0009395\_phospholipid\_catabolic\_process | 2 | 0 |  |  |  |  |  |  |  |  |
| GO:0009435\_NAD\_biosynthetic\_process | 2 | 0 |  |  |  |  |  |  |  |  |
| GO:0009608\_response\_to\_symbiont | 2 | 0 |  |  |  |  |  |  |  |  |
| GO:0009609\_response\_to\_symbiotic\_bacterium | 2 | 0 |  |  |  |  |  |  |  |  |
| GO:0009649\_entrainment\_of\_circadian\_clock | 2 | 0 |  |  |  |  |  |  |  |  |
| GO:0009996\_negative\_regulation\_of\_cell\_fate\_specification | 2 | 0 |  |  |  |  |  |  |  |  |
| GO:0010002\_cardioblast\_differentiation | 2 | 0 |  |  |  |  |  |  |  |  |
| GO:0010149\_senescence | 2 | 0 |  |  |  |  |  |  |  |  |
| GO:0010225\_response\_to\_UV-C | 2 | 0 |  |  |  |  |  |  |  |  |
| GO:0010389\_regulation\_of\_G2\_M\_transition\_of\_mitotic\_cell\_cycle | 2 | 0 |  |  |  |  |  |  |  |  |
| GO:0010458\_exit\_from\_mitosis | 2 | 0 |  |  |  |  |  |  |  |  |
| GO:0010459\_negative\_regulation\_of\_heart\_rate | 2 | 0 |  |  |  |  |  |  |  |  |
| GO:0010559\_regulation\_of\_glycoprotein\_biosynthetic\_process | 2 | 0 |  |  |  |  |  |  |  |  |
| GO:0010633\_negative\_regulation\_of\_epithelial\_cell\_migration | 2 | 0 |  |  |  |  |  |  |  |  |
| GO:0010677\_negative\_regulation\_of\_cellular\_carbohydrate\_metabolic\_process | 2 | 0 |  |  |  |  |  |  |  |  |
| GO:0010718\_positive\_regulation\_of\_epithelial\_to\_mesenchymal\_transition | 2 | 0 |  |  |  |  |  |  |  |  |
| GO:0010742\_foam\_cell\_differentiation | 2 | 0 |  |  |  |  |  |  |  |  |
| GO:0010743\_regulation\_of\_foam\_cell\_differentiation | 2 | 0 |  |  |  |  |  |  |  |  |
| GO:0010744\_positive\_regulation\_of\_foam\_cell\_differentiation | 2 | 0 |  |  |  |  |  |  |  |  |
| GO:0010765\_positive\_regulation\_of\_sodium\_ion\_transport | 2 | 0 |  |  |  |  |  |  |  |  |
| GO:0010766\_negative\_regulation\_of\_sodium\_ion\_transport | 2 | 0 |  |  |  |  |  |  |  |  |
| GO:0010770\_positive\_regulation\_of\_cell\_morphogenesis\_involved\_in\_differentiation | 2 | 0 |  |  |  |  |  |  |  |  |
| GO:0010771\_negative\_regulation\_of\_cell\_morphogenesis\_involved\_in\_differentiation | 2 | 0 |  |  |  |  |  |  |  |  |
| GO:0010824\_regulation\_of\_centrosome\_duplication | 2 | 0 |  |  |  |  |  |  |  |  |
| GO:0010833\_telomere\_maintenance\_via\_telomere\_lengthening | 2 | 0 |  |  |  |  |  |  |  |  |
| GO:0010862\_positive\_regulation\_of\_pathway-restricted\_SMAD\_protein\_phosphorylation | 2 | 0 |  |  |  |  |  |  |  |  |
| GO:0010872\_regulation\_of\_cholesterol\_esterification | 2 | 0 |  |  |  |  |  |  |  |  |
| GO:0010878\_cholesterol\_storage | 2 | 0 |  |  |  |  |  |  |  |  |
| GO:0010885\_regulation\_of\_cholesterol\_storage | 2 | 0 |  |  |  |  |  |  |  |  |
| GO:0010886\_positive\_regulation\_of\_cholesterol\_storage | 2 | 0 |  |  |  |  |  |  |  |  |
| GO:0010891\_negative\_regulation\_of\_sequestering\_of\_triglyceride | 2 | 0 |  |  |  |  |  |  |  |  |
| GO:0010896\_regulation\_of\_triglyceride\_catabolic\_process | 2 | 0 |  |  |  |  |  |  |  |  |
| GO:0010898\_positive\_regulation\_of\_triglyceride\_catabolic\_process | 2 | 0 |  |  |  |  |  |  |  |  |
| GO:0010907\_positive\_regulation\_of\_glucose\_metabolic\_process | 2 | 0 |  |  |  |  |  |  |  |  |
| GO:0014028\_notochord\_formation | 2 | 0 |  |  |  |  |  |  |  |  |
| GO:0014048\_regulation\_of\_glutamate\_secretion | 2 | 0 |  |  |  |  |  |  |  |  |
| GO:0014052\_regulation\_of\_gamma-aminobutyric\_acid\_secretion | 2 | 0 |  |  |  |  |  |  |  |  |
| GO:0014054\_positive\_regulation\_of\_gamma-aminobutyric\_acid\_secretion | 2 | 0 |  |  |  |  |  |  |  |  |
| GO:0014055\_acetylcholine\_secretion | 2 | 0 |  |  |  |  |  |  |  |  |
| GO:0014056\_regulation\_of\_acetylcholine\_secretion | 2 | 0 |  |  |  |  |  |  |  |  |
| GO:0014067\_negative\_regulation\_of\_phosphoinositide\_3-kinase\_cascade | 2 | 0 |  |  |  |  |  |  |  |  |
| GO:0014745\_negative\_regulation\_of\_muscle\_adaptation | 2 | 0 |  |  |  |  |  |  |  |  |
| GO:0014829\_vascular\_smooth\_muscle\_contraction | 2 | 0 |  |  |  |  |  |  |  |  |
| GO:0014850\_response\_to\_muscle\_activity | 2 | 0 |  |  |  |  |  |  |  |  |
| GO:0014866\_skeletal\_myofibril\_assembly | 2 | 0 |  |  |  |  |  |  |  |  |
| GO:0014888\_striated\_muscle\_adaptation | 2 | 0 |  |  |  |  |  |  |  |  |
| GO:0014916\_regulation\_of\_lung\_blood\_pressure | 2 | 0 |  |  |  |  |  |  |  |  |
| GO:0015671\_oxygen\_transport | 2 | 0 |  |  |  |  |  |  |  |  |
| GO:0015696\_ammonium\_transport | 2 | 0 |  |  |  |  |  |  |  |  |
| GO:0015732\_prostaglandin\_transport | 2 | 0 |  |  |  |  |  |  |  |  |
| GO:0015819\_lysine\_transport | 2 | 0 |  |  |  |  |  |  |  |  |
| GO:0015840\_urea\_transport | 2 | 0 |  |  |  |  |  |  |  |  |
| GO:0015860\_purine\_nucleoside\_transport | 2 | 0 |  |  |  |  |  |  |  |  |
| GO:0015870\_acetylcholine\_transport | 2 | 0 |  |  |  |  |  |  |  |  |
| GO:0015937\_coenzyme\_A\_biosynthetic\_process | 2 | 0 |  |  |  |  |  |  |  |  |
| GO:0016045\_detection\_of\_bacterium | 2 | 0 |  |  |  |  |  |  |  |  |
| GO:0016046\_detection\_of\_fungus | 2 | 0 |  |  |  |  |  |  |  |  |
| GO:0016080\_synaptic\_vesicle\_targeting | 2 | 0 |  |  |  |  |  |  |  |  |
| GO:0016199\_axon\_midline\_choice\_point\_recognition | 2 | 0 |  |  |  |  |  |  |  |  |
| GO:0016226\_iron-sulfur\_cluster\_assembly | 2 | 0 |  |  |  |  |  |  |  |  |
| GO:0016233\_telomere\_capping | 2 | 0 |  |  |  |  |  |  |  |  |
| GO:0016242\_negative\_regulation\_of\_macroautophagy | 2 | 0 |  |  |  |  |  |  |  |  |
| GO:0016441\_posttranscriptional\_gene\_silencing | 2 | 0 |  |  |  |  |  |  |  |  |
| GO:0016540\_protein\_autoprocessing | 2 | 0 |  |  |  |  |  |  |  |  |
| GO:0016558\_protein\_import\_into\_peroxisome\_matrix | 2 | 0 |  |  |  |  |  |  |  |  |
| GO:0016572\_histone\_phosphorylation | 2 | 0 |  |  |  |  |  |  |  |  |
| GO:0016577\_histone\_demethylation | 2 | 0 |  |  |  |  |  |  |  |  |
| GO:0016584\_nucleosome\_positioning | 2 | 0 |  |  |  |  |  |  |  |  |
| GO:0016926\_protein\_desumoylation | 2 | 0 |  |  |  |  |  |  |  |  |
| GO:0017014\_protein\_amino\_acid\_nitrosylation | 2 | 0 |  |  |  |  |  |  |  |  |
| GO:0017144\_drug\_metabolic\_process | 2 | 0 |  |  |  |  |  |  |  |  |
| GO:0018094\_protein\_polyglycylation | 2 | 0 |  |  |  |  |  |  |  |  |
| GO:0018119\_peptidyl-cysteine\_S-nitrosylation | 2 | 0 |  |  |  |  |  |  |  |  |
| GO:0018125\_peptidyl-cysteine\_methylation | 2 | 0 |  |  |  |  |  |  |  |  |
| GO:0018205\_peptidyl-lysine\_modification | 2 | 0 |  |  |  |  |  |  |  |  |
| GO:0018319\_protein\_amino\_acid\_myristoylation | 2 | 0 |  |  |  |  |  |  |  |  |
| GO:0018377\_protein\_myristoylation | 2 | 0 |  |  |  |  |  |  |  |  |
| GO:0018401\_peptidyl-proline\_hydroxylation\_to\_4-hydroxy-L-proline | 2 | 0 |  |  |  |  |  |  |  |  |
| GO:0018993\_somatic\_sex\_determination | 2 | 0 |  |  |  |  |  |  |  |  |
| GO:0019067\_viral\_assembly\_\_maturation\_\_egress\_\_and\_release | 2 | 0 |  |  |  |  |  |  |  |  |
| GO:0019322\_pentose\_biosynthetic\_process | 2 | 0 |  |  |  |  |  |  |  |  |
| GO:0019370\_leukotriene\_biosynthetic\_process | 2 | 0 |  |  |  |  |  |  |  |  |
| GO:0019374\_galactolipid\_metabolic\_process | 2 | 0 |  |  |  |  |  |  |  |  |
| GO:0019401\_alditol\_biosynthetic\_process | 2 | 0 |  |  |  |  |  |  |  |  |
| GO:0019448\_L-cysteine\_catabolic\_process | 2 | 0 |  |  |  |  |  |  |  |  |
| GO:0019452\_L-cysteine\_catabolic\_process\_to\_taurine | 2 | 0 |  |  |  |  |  |  |  |  |
| GO:0019471\_4-hydroxyproline\_metabolic\_process | 2 | 0 |  |  |  |  |  |  |  |  |
| GO:0019511\_peptidyl-proline\_hydroxylation | 2 | 0 |  |  |  |  |  |  |  |  |
| GO:0019550\_glutamate\_catabolic\_process\_to\_aspartate | 2 | 0 |  |  |  |  |  |  |  |  |
| GO:0019551\_glutamate\_catabolic\_process\_to\_2-oxoglutarate | 2 | 0 |  |  |  |  |  |  |  |  |
| GO:0019585\_glucuronate\_metabolic\_process | 2 | 0 |  |  |  |  |  |  |  |  |
| GO:0019730\_antimicrobial\_humoral\_response | 2 | 0 |  |  |  |  |  |  |  |  |
| GO:0019740\_nitrogen\_utilization | 2 | 0 |  |  |  |  |  |  |  |  |
| GO:0019853\_L-ascorbic\_acid\_biosynthetic\_process | 2 | 0 |  |  |  |  |  |  |  |  |
| GO:0021506\_anterior\_neuropore\_closure | 2 | 0 |  |  |  |  |  |  |  |  |
| GO:0021524\_visceral\_motor\_neuron\_differentiation | 2 | 0 |  |  |  |  |  |  |  |  |
| GO:0021526\_medial\_motor\_column\_neuron\_differentiation | 2 | 0 |  |  |  |  |  |  |  |  |
| GO:0021557\_oculomotor\_nerve\_development | 2 | 0 |  |  |  |  |  |  |  |  |
| GO:0021558\_trochlear\_nerve\_development | 2 | 0 |  |  |  |  |  |  |  |  |
| GO:0021562\_vestibulocochlear\_nerve\_development | 2 | 0 |  |  |  |  |  |  |  |  |
| GO:0021568\_rhombomere\_2\_development | 2 | 0 |  |  |  |  |  |  |  |  |
| GO:0021578\_hindbrain\_maturation | 2 | 0 |  |  |  |  |  |  |  |  |
| GO:0021593\_rhombomere\_morphogenesis | 2 | 0 |  |  |  |  |  |  |  |  |
| GO:0021626\_central\_nervous\_system\_maturation | 2 | 0 |  |  |  |  |  |  |  |  |
| GO:0021658\_rhombomere\_3\_morphogenesis | 2 | 0 |  |  |  |  |  |  |  |  |
| GO:0021754\_facial\_nucleus\_development | 2 | 0 |  |  |  |  |  |  |  |  |
| GO:0021775\_smoothened\_signaling\_pathway\_involved\_in\_ventral\_spinal\_cord\_interneuron\_specification | 2 | 0 |  |  |  |  |  |  |  |  |
| GO:0021776\_smoothened\_signaling\_pathway\_involved\_in\_spinal\_cord\_motor\_neuron\_cell\_fate\_specification | 2 | 0 |  |  |  |  |  |  |  |  |
| GO:0021796\_cerebral\_cortex\_regionalization | 2 | 0 |  |  |  |  |  |  |  |  |
| GO:0021831\_embryonic\_olfactory\_bulb\_interneuron\_precursor\_migration | 2 | 0 |  |  |  |  |  |  |  |  |
| GO:0021869\_forebrain\_ventricular\_zone\_progenitor\_cell\_division | 2 | 0 |  |  |  |  |  |  |  |  |
| GO:0021873\_forebrain\_neuroblast\_division | 2 | 0 |  |  |  |  |  |  |  |  |
| GO:0021882\_regulation\_of\_transcription\_from\_RNA\_polymerase\_II\_promoter\_involved\_in\_forebrain\_neuron\_fate\_commitment | 2 | 0 |  |  |  |  |  |  |  |  |
| GO:0021893\_cerebral\_cortex\_GABAergic\_interneuron\_fate\_commitment | 2 | 0 |  |  |  |  |  |  |  |  |
| GO:0021898\_commitment\_of\_multipotent\_stem\_cells\_to\_the\_neuronal\_lineage\_in\_the\_forebrain | 2 | 0 |  |  |  |  |  |  |  |  |
| GO:0021932\_hindbrain\_radial\_glia\_guided\_cell\_migration | 2 | 0 |  |  |  |  |  |  |  |  |
| GO:0021985\_neurohypophysis\_development | 2 | 0 |  |  |  |  |  |  |  |  |
| GO:0021990\_neural\_plate\_formation | 2 | 0 |  |  |  |  |  |  |  |  |
| GO:0021995\_neuropore\_closure | 2 | 0 |  |  |  |  |  |  |  |  |
| GO:0022028\_tangential\_migration\_from\_the\_subventricular\_zone\_to\_the\_olfactory\_bulb | 2 | 0 |  |  |  |  |  |  |  |  |
| GO:0022401\_adaptation\_of\_signaling\_pathway | 2 | 0 |  |  |  |  |  |  |  |  |
| GO:0022408\_negative\_regulation\_of\_cell-cell\_adhesion | 2 | 0 |  |  |  |  |  |  |  |  |
| GO:0022410\_circadian\_sleep\_wake\_cycle\_process | 2 | 0 |  |  |  |  |  |  |  |  |
| GO:0030046\_parallel\_actin\_filament\_bundle\_formation | 2 | 0 |  |  |  |  |  |  |  |  |
| GO:0030049\_muscle\_filament\_sliding | 2 | 0 |  |  |  |  |  |  |  |  |
| GO:0030050\_vesicle\_transport\_along\_actin\_filament | 2 | 0 |  |  |  |  |  |  |  |  |
| GO:0030071\_regulation\_of\_mitotic\_metaphase\_anaphase\_transition | 2 | 0 |  |  |  |  |  |  |  |  |
| GO:0030147\_natriuresis | 2 | 0 |  |  |  |  |  |  |  |  |
| GO:0030174\_regulation\_of\_DNA\_replication\_initiation | 2 | 0 |  |  |  |  |  |  |  |  |
| GO:0030202\_heparin\_metabolic\_process | 2 | 0 |  |  |  |  |  |  |  |  |
| GO:0030219\_megakaryocyte\_differentiation | 2 | 0 |  |  |  |  |  |  |  |  |
| GO:0030223\_neutrophil\_differentiation | 2 | 0 |  |  |  |  |  |  |  |  |
| GO:0030240\_muscle\_thin\_filament\_assembly | 2 | 0 |  |  |  |  |  |  |  |  |
| GO:0030259\_lipid\_glycosylation | 2 | 0 |  |  |  |  |  |  |  |  |
| GO:0030397\_membrane\_disassembly | 2 | 0 |  |  |  |  |  |  |  |  |
| GO:0030502\_negative\_regulation\_of\_bone\_mineralization | 2 | 0 |  |  |  |  |  |  |  |  |
| GO:0030644\_cellular\_chloride\_ion\_homeostasis | 2 | 0 |  |  |  |  |  |  |  |  |
| GO:0030825\_positive\_regulation\_of\_cGMP\_metabolic\_process | 2 | 0 |  |  |  |  |  |  |  |  |
| GO:0030828\_positive\_regulation\_of\_cGMP\_biosynthetic\_process | 2 | 0 |  |  |  |  |  |  |  |  |
| GO:0030835\_negative\_regulation\_of\_actin\_filament\_depolymerization | 2 | 0 |  |  |  |  |  |  |  |  |
| GO:0030837\_negative\_regulation\_of\_actin\_filament\_polymerization | 2 | 0 |  |  |  |  |  |  |  |  |
| GO:0030852\_regulation\_of\_granulocyte\_differentiation | 2 | 0 |  |  |  |  |  |  |  |  |
| GO:0030885\_regulation\_of\_myeloid\_dendritic\_cell\_activation | 2 | 0 |  |  |  |  |  |  |  |  |
| GO:0030910\_olfactory\_placode\_formation | 2 | 0 |  |  |  |  |  |  |  |  |
| GO:0030948\_negative\_regulation\_of\_vascular\_endothelial\_growth\_factor\_receptor\_signaling\_pathway | 2 | 0 |  |  |  |  |  |  |  |  |
| GO:0030953\_spindle\_astral\_microtubule\_organization | 2 | 0 |  |  |  |  |  |  |  |  |
| GO:0031050\_dsRNA\_fragmentation | 2 | 0 |  |  |  |  |  |  |  |  |
| GO:0031061\_negative\_regulation\_of\_histone\_methylation | 2 | 0 |  |  |  |  |  |  |  |  |
| GO:0031119\_tRNA\_pseudouridine\_synthesis | 2 | 0 |  |  |  |  |  |  |  |  |
| GO:0031163\_metallo-sulfur\_cluster\_assembly | 2 | 0 |  |  |  |  |  |  |  |  |
| GO:0031223\_auditory\_behavior | 2 | 0 |  |  |  |  |  |  |  |  |
| GO:0031296\_B\_cell\_costimulation | 2 | 0 |  |  |  |  |  |  |  |  |
| GO:0031338\_regulation\_of\_vesicle\_fusion | 2 | 0 |  |  |  |  |  |  |  |  |
| GO:0031573\_intra-S\_DNA\_damage\_checkpoint | 2 | 0 |  |  |  |  |  |  |  |  |
| GO:0031577\_spindle\_checkpoint | 2 | 0 |  |  |  |  |  |  |  |  |
| GO:0031629\_synaptic\_vesicle\_fusion\_to\_presynaptic\_membrane | 2 | 0 |  |  |  |  |  |  |  |  |
| GO:0031630\_regulation\_of\_synaptic\_vesicle\_fusion\_to\_presynaptic\_membrane | 2 | 0 |  |  |  |  |  |  |  |  |
| GO:0031664\_regulation\_of\_lipopolysaccharide-mediated\_signaling\_pathway | 2 | 0 |  |  |  |  |  |  |  |  |
| GO:0031670\_cellular\_response\_to\_nutrient | 2 | 0 |  |  |  |  |  |  |  |  |
| GO:0031848\_protection\_from\_non-homologous\_end\_joining\_at\_telomere | 2 | 0 |  |  |  |  |  |  |  |  |
| GO:0031987\_locomotion\_involved\_in\_locomotory\_behavior | 2 | 0 |  |  |  |  |  |  |  |  |
| GO:0032096\_negative\_regulation\_of\_response\_to\_food | 2 | 0 |  |  |  |  |  |  |  |  |
| GO:0032099\_negative\_regulation\_of\_appetite | 2 | 0 |  |  |  |  |  |  |  |  |
| GO:0032106\_positive\_regulation\_of\_response\_to\_extracellular\_stimulus | 2 | 0 |  |  |  |  |  |  |  |  |
| GO:0032109\_positive\_regulation\_of\_response\_to\_nutrient\_levels | 2 | 0 |  |  |  |  |  |  |  |  |
| GO:0032226\_positive\_regulation\_of\_synaptic\_transmission\_\_dopaminergic | 2 | 0 |  |  |  |  |  |  |  |  |
| GO:0032230\_positive\_regulation\_of\_synaptic\_transmission\_\_GABAergic | 2 | 0 |  |  |  |  |  |  |  |  |
| GO:0032234\_regulation\_of\_calcium\_ion\_transport\_via\_store-operated\_calcium\_channel\_activity | 2 | 0 |  |  |  |  |  |  |  |  |
| GO:0032236\_positive\_regulation\_of\_calcium\_ion\_transport\_via\_store-operated\_calcium\_channel\_activity | 2 | 0 |  |  |  |  |  |  |  |  |
| GO:0032297\_negative\_regulation\_of\_DNA\_replication\_initiation | 2 | 0 |  |  |  |  |  |  |  |  |
| GO:0032309\_icosanoid\_secretion | 2 | 0 |  |  |  |  |  |  |  |  |
| GO:0032328\_alanine\_transport | 2 | 0 |  |  |  |  |  |  |  |  |
| GO:0032341\_aldosterone\_metabolic\_process | 2 | 0 |  |  |  |  |  |  |  |  |
| GO:0032351\_negative\_regulation\_of\_hormone\_metabolic\_process | 2 | 0 |  |  |  |  |  |  |  |  |
| GO:0032353\_negative\_regulation\_of\_hormone\_biosynthetic\_process | 2 | 0 |  |  |  |  |  |  |  |  |
| GO:0032435\_negative\_regulation\_of\_proteasomal\_ubiquitin-dependent\_protein\_catabolic\_process | 2 | 0 |  |  |  |  |  |  |  |  |
| GO:0032471\_reduction\_of\_endoplasmic\_reticulum\_calcium\_ion\_concentration | 2 | 0 |  |  |  |  |  |  |  |  |
| GO:0032481\_positive\_regulation\_of\_type\_I\_interferon\_production | 2 | 0 |  |  |  |  |  |  |  |  |
| GO:0032488\_Cdc42\_protein\_signal\_transduction | 2 | 0 |  |  |  |  |  |  |  |  |
| GO:0032489\_regulation\_of\_Cdc42\_protein\_signal\_transduction | 2 | 0 |  |  |  |  |  |  |  |  |
| GO:0032495\_response\_to\_muramyl\_dipeptide | 2 | 0 |  |  |  |  |  |  |  |  |
| GO:0032604\_granulocyte\_macrophage\_colony-stimulating\_factor\_production | 2 | 0 |  |  |  |  |  |  |  |  |
| GO:0032616\_interleukin-13\_production | 2 | 0 |  |  |  |  |  |  |  |  |
| GO:0032645\_regulation\_of\_granulocyte\_macrophage\_colony-stimulating\_factor\_production | 2 | 0 |  |  |  |  |  |  |  |  |
| GO:0032672\_regulation\_of\_interleukin-3\_production | 2 | 0 |  |  |  |  |  |  |  |  |
| GO:0032695\_negative\_regulation\_of\_interleukin-12\_production | 2 | 0 |  |  |  |  |  |  |  |  |
| GO:0032714\_negative\_regulation\_of\_interleukin-5\_production | 2 | 0 |  |  |  |  |  |  |  |  |
| GO:0032722\_positive\_regulation\_of\_chemokine\_production | 2 | 0 |  |  |  |  |  |  |  |  |
| GO:0032743\_positive\_regulation\_of\_interleukin-2\_production | 2 | 0 |  |  |  |  |  |  |  |  |
| GO:0032762\_mast\_cell\_cytokine\_production | 2 | 0 |  |  |  |  |  |  |  |  |
| GO:0032763\_regulation\_of\_mast\_cell\_cytokine\_production | 2 | 0 |  |  |  |  |  |  |  |  |
| GO:0032768\_regulation\_of\_monooxygenase\_activity | 2 | 0 |  |  |  |  |  |  |  |  |
| GO:0032788\_saturated\_monocarboxylic\_acid\_metabolic\_process | 2 | 0 |  |  |  |  |  |  |  |  |
| GO:0032789\_unsaturated\_monocarboxylic\_acid\_metabolic\_process | 2 | 0 |  |  |  |  |  |  |  |  |
| GO:0032796\_uropod\_organization | 2 | 0 |  |  |  |  |  |  |  |  |
| GO:0032800\_receptor\_biosynthetic\_process | 2 | 0 |  |  |  |  |  |  |  |  |
| GO:0032801\_receptor\_catabolic\_process | 2 | 0 |  |  |  |  |  |  |  |  |
| GO:0032829\_regulation\_of\_CD4-positive\_\_CD25-positive\_\_alpha-beta\_regulatory\_T\_cell\_differentiation | 2 | 0 |  |  |  |  |  |  |  |  |
| GO:0032831\_positive\_regulation\_of\_CD4-positive\_\_CD25-positive\_\_alpha-beta\_regulatory\_T\_cell\_differentiation | 2 | 0 |  |  |  |  |  |  |  |  |
| GO:0032892\_positive\_regulation\_of\_organic\_acid\_transport | 2 | 0 |  |  |  |  |  |  |  |  |
| GO:0032905\_transforming\_growth\_factor-beta1\_production | 2 | 0 |  |  |  |  |  |  |  |  |
| GO:0032908\_regulation\_of\_transforming\_growth\_factor-beta1\_production | 2 | 0 |  |  |  |  |  |  |  |  |
| GO:0032914\_positive\_regulation\_of\_transforming\_growth\_factor-beta1\_production | 2 | 0 |  |  |  |  |  |  |  |  |
| GO:0032933\_SREBP-mediated\_signaling\_pathway | 2 | 0 |  |  |  |  |  |  |  |  |
| GO:0032957\_inositol\_trisphosphate\_metabolic\_process | 2 | 0 |  |  |  |  |  |  |  |  |
| GO:0032958\_inositol\_phosphate\_biosynthetic\_process | 2 | 0 |  |  |  |  |  |  |  |  |
| GO:0032959\_inositol\_trisphosphate\_biosynthetic\_process | 2 | 0 |  |  |  |  |  |  |  |  |
| GO:0033092\_positive\_regulation\_of\_immature\_T\_cell\_proliferation\_in\_the\_thymus | 2 | 0 |  |  |  |  |  |  |  |  |
| GO:0033119\_negative\_regulation\_of\_RNA\_splicing | 2 | 0 |  |  |  |  |  |  |  |  |
| GO:0033136\_serine\_phosphorylation\_of\_STAT3\_protein | 2 | 0 |  |  |  |  |  |  |  |  |
| GO:0033145\_positive\_regulation\_of\_steroid\_hormone\_receptor\_signaling\_pathway | 2 | 0 |  |  |  |  |  |  |  |  |
| GO:0033147\_negative\_regulation\_of\_estrogen\_receptor\_signaling\_pathway | 2 | 0 |  |  |  |  |  |  |  |  |
| GO:0033148\_positive\_regulation\_of\_estrogen\_receptor\_signaling\_pathway | 2 | 0 |  |  |  |  |  |  |  |  |
| GO:0033194\_response\_to\_hydroperoxide | 2 | 0 |  |  |  |  |  |  |  |  |
| GO:0033275\_actin-myosin\_filament\_sliding | 2 | 0 |  |  |  |  |  |  |  |  |
| GO:0033280\_response\_to\_vitamin\_D | 2 | 0 |  |  |  |  |  |  |  |  |
| GO:0033364\_mast\_cell\_secretory\_granule\_organization | 2 | 0 |  |  |  |  |  |  |  |  |
| GO:0033504\_floor\_plate\_development | 2 | 0 |  |  |  |  |  |  |  |  |
| GO:0033603\_positive\_regulation\_of\_dopamine\_secretion | 2 | 0 |  |  |  |  |  |  |  |  |
| GO:0033605\_positive\_regulation\_of\_catecholamine\_secretion | 2 | 0 |  |  |  |  |  |  |  |  |
| GO:0033622\_integrin\_activation | 2 | 0 |  |  |  |  |  |  |  |  |
| GO:0033623\_regulation\_of\_integrin\_activation | 2 | 0 |  |  |  |  |  |  |  |  |
| GO:0033625\_positive\_regulation\_of\_integrin\_activation | 2 | 0 |  |  |  |  |  |  |  |  |
| GO:0034142\_toll-like\_receptor\_4\_signaling\_pathway | 2 | 0 |  |  |  |  |  |  |  |  |
| GO:0034310\_monohydric\_alcohol\_catabolic\_process | 2 | 0 |  |  |  |  |  |  |  |  |
| GO:0034341\_response\_to\_interferon-gamma | 2 | 0 |  |  |  |  |  |  |  |  |
| GO:0034370\_triglyceride-rich\_lipoprotein\_particle\_remodeling | 2 | 0 |  |  |  |  |  |  |  |  |
| GO:0034374\_low-density\_lipoprotein\_particle\_remodeling | 2 | 0 |  |  |  |  |  |  |  |  |
| GO:0034377\_plasma\_lipoprotein\_particle\_assembly | 2 | 0 |  |  |  |  |  |  |  |  |
| GO:0034384\_high-density\_lipoprotein\_particle\_clearance | 2 | 0 |  |  |  |  |  |  |  |  |
| GO:0034433\_steroid\_esterification | 2 | 0 |  |  |  |  |  |  |  |  |
| GO:0034434\_sterol\_esterification | 2 | 0 |  |  |  |  |  |  |  |  |
| GO:0034435\_cholesterol\_esterification | 2 | 0 |  |  |  |  |  |  |  |  |
| GO:0034453\_microtubule\_anchoring | 2 | 0 |  |  |  |  |  |  |  |  |
| GO:0034644\_cellular\_response\_to\_UV | 2 | 0 |  |  |  |  |  |  |  |  |
| GO:0034755\_iron\_ion\_transmembrane\_transport | 2 | 0 |  |  |  |  |  |  |  |  |
| GO:0034764\_positive\_regulation\_of\_transmembrane\_transport | 2 | 0 |  |  |  |  |  |  |  |  |
| GO:0035021\_negative\_regulation\_of\_Rac\_protein\_signal\_transduction | 2 | 0 |  |  |  |  |  |  |  |  |
| GO:0035054\_embryonic\_heart\_tube\_anterior\_posterior\_pattern\_formation | 2 | 0 |  |  |  |  |  |  |  |  |
| GO:0035092\_sperm\_chromatin\_condensation | 2 | 0 |  |  |  |  |  |  |  |  |
| GO:0035110\_leg\_morphogenesis | 2 | 0 |  |  |  |  |  |  |  |  |
| GO:0035117\_embryonic\_arm\_morphogenesis | 2 | 0 |  |  |  |  |  |  |  |  |
| GO:0035120\_post-embryonic\_appendage\_morphogenesis | 2 | 0 |  |  |  |  |  |  |  |  |
| GO:0035127\_post-embryonic\_limb\_morphogenesis | 2 | 0 |  |  |  |  |  |  |  |  |
| GO:0035129\_post-embryonic\_hindlimb\_morphogenesis | 2 | 0 |  |  |  |  |  |  |  |  |
| GO:0035140\_arm\_morphogenesis | 2 | 0 |  |  |  |  |  |  |  |  |
| GO:0035194\_posttranscriptional\_gene\_silencing\_by\_RNA | 2 | 0 |  |  |  |  |  |  |  |  |
| GO:0035195\_gene\_silencing\_by\_miRNA | 2 | 0 |  |  |  |  |  |  |  |  |
| GO:0035196\_gene\_silencing\_by\_miRNA\_\_production\_of\_miRNAs | 2 | 0 |  |  |  |  |  |  |  |  |
| GO:0035315\_hair\_cell\_differentiation | 2 | 0 |  |  |  |  |  |  |  |  |
| GO:0040009\_regulation\_of\_growth\_rate | 2 | 0 |  |  |  |  |  |  |  |  |
| GO:0040037\_negative\_regulation\_of\_fibroblast\_growth\_factor\_receptor\_signaling\_pathway | 2 | 0 |  |  |  |  |  |  |  |  |
| GO:0042119\_neutrophil\_activation | 2 | 0 |  |  |  |  |  |  |  |  |
| GO:0042147\_retrograde\_transport\_\_endosome\_to\_Golgi | 2 | 0 |  |  |  |  |  |  |  |  |
| GO:0042223\_interleukin-3\_biosynthetic\_process | 2 | 0 |  |  |  |  |  |  |  |  |
| GO:0042249\_establishment\_of\_polarity\_of\_embryonic\_epithelium | 2 | 0 |  |  |  |  |  |  |  |  |
| GO:0042253\_granulocyte\_macrophage\_colony-stimulating\_factor\_biosynthetic\_process | 2 | 0 |  |  |  |  |  |  |  |  |
| GO:0042270\_protection\_from\_natural\_killer\_cell\_mediated\_cytotoxicity | 2 | 0 |  |  |  |  |  |  |  |  |
| GO:0042274\_ribosomal\_small\_subunit\_biogenesis | 2 | 0 |  |  |  |  |  |  |  |  |
| GO:0042312\_regulation\_of\_vasodilation | 2 | 0 |  |  |  |  |  |  |  |  |
| GO:0042346\_positive\_regulation\_of\_NF-kappaB\_import\_into\_nucleus | 2 | 0 |  |  |  |  |  |  |  |  |
| GO:0042396\_phosphagen\_biosynthetic\_process | 2 | 0 |  |  |  |  |  |  |  |  |
| GO:0042454\_ribonucleoside\_catabolic\_process | 2 | 0 |  |  |  |  |  |  |  |  |
| GO:0042482\_positive\_regulation\_of\_odontogenesis | 2 | 0 |  |  |  |  |  |  |  |  |
| GO:0042483\_negative\_regulation\_of\_odontogenesis | 2 | 0 |  |  |  |  |  |  |  |  |
| GO:0042488\_positive\_regulation\_of\_odontogenesis\_of\_dentine-containing\_tooth | 2 | 0 |  |  |  |  |  |  |  |  |
| GO:0042501\_serine\_phosphorylation\_of\_STAT\_protein | 2 | 0 |  |  |  |  |  |  |  |  |
| GO:0042532\_negative\_regulation\_of\_tyrosine\_phosphorylation\_of\_STAT\_protein | 2 | 0 |  |  |  |  |  |  |  |  |
| GO:0042559\_pteridine\_and\_derivative\_biosynthetic\_process | 2 | 0 |  |  |  |  |  |  |  |  |
| GO:0042730\_fibrinolysis | 2 | 0 |  |  |  |  |  |  |  |  |
| GO:0042749\_regulation\_of\_circadian\_sleep\_wake\_cycle | 2 | 0 |  |  |  |  |  |  |  |  |
| GO:0042886\_amide\_transport | 2 | 0 |  |  |  |  |  |  |  |  |
| GO:0042987\_amyloid\_precursor\_protein\_catabolic\_process | 2 | 0 |  |  |  |  |  |  |  |  |
| GO:0042993\_positive\_regulation\_of\_transcription\_factor\_import\_into\_nucleus | 2 | 0 |  |  |  |  |  |  |  |  |
| GO:0042994\_cytoplasmic\_sequestering\_of\_transcription\_factor | 2 | 0 |  |  |  |  |  |  |  |  |
| GO:0043032\_positive\_regulation\_of\_macrophage\_activation | 2 | 0 |  |  |  |  |  |  |  |  |
| GO:0043038\_amino\_acid\_activation | 2 | 0 |  |  |  |  |  |  |  |  |
| GO:0043039\_tRNA\_aminoacylation | 2 | 0 |  |  |  |  |  |  |  |  |
| GO:0043084\_penile\_erection | 2 | 0 |  |  |  |  |  |  |  |  |
| GO:0043088\_regulation\_of\_Cdc42\_GTPase\_activity | 2 | 0 |  |  |  |  |  |  |  |  |
| GO:0043089\_positive\_regulation\_of\_Cdc42\_GTPase\_activity | 2 | 0 |  |  |  |  |  |  |  |  |
| GO:0043096\_purine\_base\_salvage | 2 | 0 |  |  |  |  |  |  |  |  |
| GO:0043247\_telomere\_maintenance\_in\_response\_to\_DNA\_damage | 2 | 0 |  |  |  |  |  |  |  |  |
| GO:0043297\_apical\_junction\_assembly | 2 | 0 |  |  |  |  |  |  |  |  |
| GO:0043312\_neutrophil\_degranulation | 2 | 0 |  |  |  |  |  |  |  |  |
| GO:0043366\_beta\_selection | 2 | 0 |  |  |  |  |  |  |  |  |
| GO:0043450\_alkene\_biosynthetic\_process | 2 | 0 |  |  |  |  |  |  |  |  |
| GO:0043476\_pigment\_accumulation | 2 | 0 |  |  |  |  |  |  |  |  |
| GO:0043490\_malate-aspartate\_shuttle | 2 | 0 |  |  |  |  |  |  |  |  |
| GO:0043502\_regulation\_of\_muscle\_adaptation | 2 | 0 |  |  |  |  |  |  |  |  |
| GO:0043516\_regulation\_of\_DNA\_damage\_response\_\_signal\_transduction\_by\_p53\_class\_mediator | 2 | 0 |  |  |  |  |  |  |  |  |
| GO:0043568\_positive\_regulation\_of\_insulin-like\_growth\_factor\_receptor\_signaling\_pathway | 2 | 0 |  |  |  |  |  |  |  |  |
| GO:0043589\_skin\_morphogenesis | 2 | 0 |  |  |  |  |  |  |  |  |
| GO:0043618\_regulation\_of\_transcription\_from\_RNA\_polymerase\_II\_promoter\_in\_response\_to\_stress | 2 | 0 |  |  |  |  |  |  |  |  |
| GO:0043619\_regulation\_of\_transcription\_from\_RNA\_polymerase\_II\_promoter\_in\_response\_to\_oxidative\_stress | 2 | 0 |  |  |  |  |  |  |  |  |
| GO:0043620\_regulation\_of\_transcription\_in\_response\_to\_stress | 2 | 0 |  |  |  |  |  |  |  |  |
| GO:0043647\_inositol\_phosphate\_metabolic\_process | 2 | 0 |  |  |  |  |  |  |  |  |
| GO:0043654\_recognition\_of\_apoptotic\_cell | 2 | 0 |  |  |  |  |  |  |  |  |
| GO:0043966\_histone\_H3\_acetylation | 2 | 0 |  |  |  |  |  |  |  |  |
| GO:0043967\_histone\_H4\_acetylation | 2 | 0 |  |  |  |  |  |  |  |  |
| GO:0044070\_regulation\_of\_anion\_transport | 2 | 0 |  |  |  |  |  |  |  |  |
| GO:0044246\_regulation\_of\_multicellular\_organismal\_metabolic\_process | 2 | 0 |  |  |  |  |  |  |  |  |
| GO:0044253\_positive\_regulation\_of\_multicellular\_organismal\_metabolic\_process | 2 | 0 |  |  |  |  |  |  |  |  |
| GO:0044268\_multicellular\_organismal\_protein\_metabolic\_process | 2 | 0 |  |  |  |  |  |  |  |  |
| GO:0045005\_maintenance\_of\_fidelity\_during\_DNA-dependent\_DNA\_replication | 2 | 0 |  |  |  |  |  |  |  |  |
| GO:0045010\_actin\_nucleation | 2 | 0 |  |  |  |  |  |  |  |  |
| GO:0045065\_cytotoxic\_T\_cell\_differentiation | 2 | 0 |  |  |  |  |  |  |  |  |
| GO:0045077\_negative\_regulation\_of\_interferon-gamma\_biosynthetic\_process | 2 | 0 |  |  |  |  |  |  |  |  |
| GO:0045079\_negative\_regulation\_of\_chemokine\_biosynthetic\_process | 2 | 0 |  |  |  |  |  |  |  |  |
| GO:0045116\_protein\_neddylation | 2 | 0 |  |  |  |  |  |  |  |  |
| GO:0045187\_regulation\_of\_circadian\_sleep\_wake\_cycle\_\_sleep | 2 | 0 |  |  |  |  |  |  |  |  |
| GO:0045212\_neurotransmitter\_receptor\_biosynthetic\_process | 2 | 0 |  |  |  |  |  |  |  |  |
| GO:0045399\_regulation\_of\_interleukin-3\_biosynthetic\_process | 2 | 0 |  |  |  |  |  |  |  |  |
| GO:0045401\_positive\_regulation\_of\_interleukin-3\_biosynthetic\_process | 2 | 0 |  |  |  |  |  |  |  |  |
| GO:0045409\_negative\_regulation\_of\_interleukin-6\_biosynthetic\_process | 2 | 0 |  |  |  |  |  |  |  |  |
| GO:0045423\_regulation\_of\_granulocyte\_macrophage\_colony-stimulating\_factor\_biosynthetic\_process | 2 | 0 |  |  |  |  |  |  |  |  |
| GO:0045425\_positive\_regulation\_of\_granulocyte\_macrophage\_colony-stimulating\_factor\_biosynthetic\_process | 2 | 0 |  |  |  |  |  |  |  |  |
| GO:0045475\_locomotor\_rhythm | 2 | 0 |  |  |  |  |  |  |  |  |
| GO:0045578\_negative\_regulation\_of\_B\_cell\_differentiation | 2 | 0 |  |  |  |  |  |  |  |  |
| GO:0045589\_regulation\_of\_regulatory\_T\_cell\_differentiation | 2 | 0 |  |  |  |  |  |  |  |  |
| GO:0045591\_positive\_regulation\_of\_regulatory\_T\_cell\_differentiation | 2 | 0 |  |  |  |  |  |  |  |  |
| GO:0045608\_negative\_regulation\_of\_auditory\_receptor\_cell\_differentiation | 2 | 0 |  |  |  |  |  |  |  |  |
| GO:0045627\_positive\_regulation\_of\_T-helper\_1\_cell\_differentiation | 2 | 0 |  |  |  |  |  |  |  |  |
| GO:0045629\_negative\_regulation\_of\_T-helper\_2\_cell\_differentiation | 2 | 0 |  |  |  |  |  |  |  |  |
| GO:0045630\_positive\_regulation\_of\_T-helper\_2\_cell\_differentiation | 2 | 0 |  |  |  |  |  |  |  |  |
| GO:0045632\_negative\_regulation\_of\_mechanoreceptor\_differentiation | 2 | 0 |  |  |  |  |  |  |  |  |
| GO:0045636\_positive\_regulation\_of\_melanocyte\_differentiation | 2 | 0 |  |  |  |  |  |  |  |  |
| GO:0045655\_regulation\_of\_monocyte\_differentiation | 2 | 0 |  |  |  |  |  |  |  |  |
| GO:0045658\_regulation\_of\_neutrophil\_differentiation | 2 | 0 |  |  |  |  |  |  |  |  |
| GO:0045683\_negative\_regulation\_of\_epidermis\_development | 2 | 0 |  |  |  |  |  |  |  |  |
| GO:0045737\_positive\_regulation\_of\_cyclin-dependent\_protein\_kinase\_activity | 2 | 0 |  |  |  |  |  |  |  |  |
| GO:0045739\_positive\_regulation\_of\_DNA\_repair | 2 | 0 |  |  |  |  |  |  |  |  |
| GO:0045741\_positive\_regulation\_of\_epidermal\_growth\_factor\_receptor\_activity | 2 | 0 |  |  |  |  |  |  |  |  |
| GO:0045743\_positive\_regulation\_of\_fibroblast\_growth\_factor\_receptor\_signaling\_pathway | 2 | 0 |  |  |  |  |  |  |  |  |
| GO:0045749\_negative\_regulation\_of\_S\_phase\_of\_mitotic\_cell\_cycle | 2 | 0 |  |  |  |  |  |  |  |  |
| GO:0045819\_positive\_regulation\_of\_glycogen\_catabolic\_process | 2 | 0 |  |  |  |  |  |  |  |  |
| GO:0045821\_positive\_regulation\_of\_glycolysis | 2 | 0 |  |  |  |  |  |  |  |  |
| GO:0045835\_negative\_regulation\_of\_meiosis | 2 | 0 |  |  |  |  |  |  |  |  |
| GO:0045836\_positive\_regulation\_of\_meiosis | 2 | 0 |  |  |  |  |  |  |  |  |
| GO:0045839\_negative\_regulation\_of\_mitosis | 2 | 0 |  |  |  |  |  |  |  |  |
| GO:0045841\_negative\_regulation\_of\_mitotic\_metaphase\_anaphase\_transition | 2 | 0 |  |  |  |  |  |  |  |  |
| GO:0045872\_positive\_regulation\_of\_rhodopsin\_gene\_expression | 2 | 0 |  |  |  |  |  |  |  |  |
| GO:0045912\_negative\_regulation\_of\_carbohydrate\_metabolic\_process | 2 | 0 |  |  |  |  |  |  |  |  |
| GO:0045948\_positive\_regulation\_of\_translational\_initiation | 2 | 0 |  |  |  |  |  |  |  |  |
| GO:0045950\_negative\_regulation\_of\_mitotic\_recombination | 2 | 0 |  |  |  |  |  |  |  |  |
| GO:0046033\_AMP\_metabolic\_process | 2 | 0 |  |  |  |  |  |  |  |  |
| GO:0046060\_dATP\_metabolic\_process | 2 | 0 |  |  |  |  |  |  |  |  |
| GO:0046070\_dGTP\_metabolic\_process | 2 | 0 |  |  |  |  |  |  |  |  |
| GO:0046083\_adenine\_metabolic\_process | 2 | 0 |  |  |  |  |  |  |  |  |
| GO:0046085\_adenosine\_metabolic\_process | 2 | 0 |  |  |  |  |  |  |  |  |
| GO:0046100\_hypoxanthine\_metabolic\_process | 2 | 0 |  |  |  |  |  |  |  |  |
| GO:0046114\_guanosine\_biosynthetic\_process | 2 | 0 |  |  |  |  |  |  |  |  |
| GO:0046116\_queuosine\_metabolic\_process | 2 | 0 |  |  |  |  |  |  |  |  |
| GO:0046118\_7-methylguanosine\_biosynthetic\_process | 2 | 0 |  |  |  |  |  |  |  |  |
| GO:0046130\_purine\_ribonucleoside\_catabolic\_process | 2 | 0 |  |  |  |  |  |  |  |  |
| GO:0046146\_tetrahydrobiopterin\_metabolic\_process | 2 | 0 |  |  |  |  |  |  |  |  |
| GO:0046185\_aldehyde\_catabolic\_process | 2 | 0 |  |  |  |  |  |  |  |  |
| GO:0046208\_spermine\_catabolic\_process | 2 | 0 |  |  |  |  |  |  |  |  |
| GO:0046349\_amino\_sugar\_biosynthetic\_process | 2 | 0 |  |  |  |  |  |  |  |  |
| GO:0046439\_L-cysteine\_metabolic\_process | 2 | 0 |  |  |  |  |  |  |  |  |
| GO:0046500\_S-adenosylmethionine\_metabolic\_process | 2 | 0 |  |  |  |  |  |  |  |  |
| GO:0046501\_protoporphyrinogen\_IX\_metabolic\_process | 2 | 0 |  |  |  |  |  |  |  |  |
| GO:0046514\_ceramide\_catabolic\_process | 2 | 0 |  |  |  |  |  |  |  |  |
| GO:0046521\_sphingoid\_catabolic\_process | 2 | 0 |  |  |  |  |  |  |  |  |
| GO:0046532\_regulation\_of\_photoreceptor\_cell\_differentiation | 2 | 0 |  |  |  |  |  |  |  |  |
| GO:0046533\_negative\_regulation\_of\_photoreceptor\_cell\_differentiation | 2 | 0 |  |  |  |  |  |  |  |  |
| GO:0046544\_development\_of\_secondary\_male\_sexual\_characteristics | 2 | 0 |  |  |  |  |  |  |  |  |
| GO:0046619\_optic\_placode\_formation\_involved\_in\_camera-type\_eye | 2 | 0 |  |  |  |  |  |  |  |  |
| GO:0046950\_cellular\_ketone\_body\_metabolic\_process | 2 | 0 |  |  |  |  |  |  |  |  |
| GO:0046984\_regulation\_of\_hemoglobin\_biosynthetic\_process | 2 | 0 |  |  |  |  |  |  |  |  |
| GO:0047484\_regulation\_of\_response\_to\_osmotic\_stress | 2 | 0 |  |  |  |  |  |  |  |  |
| GO:0048025\_negative\_regulation\_of\_nuclear\_mRNA\_splicing\_\_via\_spliceosome | 2 | 0 |  |  |  |  |  |  |  |  |
| GO:0048134\_germ-line\_cyst\_formation | 2 | 0 |  |  |  |  |  |  |  |  |
| GO:0048136\_male\_germ-line\_cyst\_formation | 2 | 0 |  |  |  |  |  |  |  |  |
| GO:0048172\_regulation\_of\_short-term\_neuronal\_synaptic\_plasticity | 2 | 0 |  |  |  |  |  |  |  |  |
| GO:0048295\_positive\_regulation\_of\_isotype\_switching\_to\_IgE\_isotypes | 2 | 0 |  |  |  |  |  |  |  |  |
| GO:0048342\_paraxial\_mesodermal\_cell\_differentiation | 2 | 0 |  |  |  |  |  |  |  |  |
| GO:0048343\_paraxial\_mesodermal\_cell\_fate\_commitment | 2 | 0 |  |  |  |  |  |  |  |  |
| GO:0048382\_mesendoderm\_development | 2 | 0 |  |  |  |  |  |  |  |  |
| GO:0048552\_regulation\_of\_metalloenzyme\_activity | 2 | 0 |  |  |  |  |  |  |  |  |
| GO:0048554\_positive\_regulation\_of\_metalloenzyme\_activity | 2 | 0 |  |  |  |  |  |  |  |  |
| GO:0048625\_myoblast\_cell\_fate\_commitment | 2 | 0 |  |  |  |  |  |  |  |  |
| GO:0048627\_myoblast\_development | 2 | 0 |  |  |  |  |  |  |  |  |
| GO:0048643\_positive\_regulation\_of\_skeletal\_muscle\_tissue\_development | 2 | 0 |  |  |  |  |  |  |  |  |
| GO:0048661\_positive\_regulation\_of\_smooth\_muscle\_cell\_proliferation | 2 | 0 |  |  |  |  |  |  |  |  |
| GO:0048670\_regulation\_of\_collateral\_sprouting | 2 | 0 |  |  |  |  |  |  |  |  |
| GO:0048671\_negative\_regulation\_of\_collateral\_sprouting | 2 | 0 |  |  |  |  |  |  |  |  |
| GO:0048677\_axon\_extension\_involved\_in\_regeneration | 2 | 0 |  |  |  |  |  |  |  |  |
| GO:0048679\_regulation\_of\_axon\_regeneration | 2 | 0 |  |  |  |  |  |  |  |  |
| GO:0048682\_sprouting\_of\_injured\_axon | 2 | 0 |  |  |  |  |  |  |  |  |
| GO:0048702\_embryonic\_neurocranium\_morphogenesis | 2 | 0 |  |  |  |  |  |  |  |  |
| GO:0048711\_positive\_regulation\_of\_astrocyte\_differentiation | 2 | 0 |  |  |  |  |  |  |  |  |
| GO:0048712\_negative\_regulation\_of\_astrocyte\_differentiation | 2 | 0 |  |  |  |  |  |  |  |  |
| GO:0048739\_cardiac\_muscle\_fiber\_development | 2 | 0 |  |  |  |  |  |  |  |  |
| GO:0048807\_female\_genitalia\_morphogenesis | 2 | 0 |  |  |  |  |  |  |  |  |
| GO:0048808\_male\_genitalia\_morphogenesis | 2 | 0 |  |  |  |  |  |  |  |  |
| GO:0048840\_otolith\_development | 2 | 0 |  |  |  |  |  |  |  |  |
| GO:0048850\_hypophysis\_morphogenesis | 2 | 0 |  |  |  |  |  |  |  |  |
| GO:0050000\_chromosome\_localization | 2 | 0 |  |  |  |  |  |  |  |  |
| GO:0050686\_negative\_regulation\_of\_mRNA\_processing | 2 | 0 |  |  |  |  |  |  |  |  |
| GO:0050688\_regulation\_of\_defense\_response\_to\_virus | 2 | 0 |  |  |  |  |  |  |  |  |
| GO:0050746\_regulation\_of\_lipoprotein\_metabolic\_process | 2 | 0 |  |  |  |  |  |  |  |  |
| GO:0050779\_RNA\_destabilization | 2 | 0 |  |  |  |  |  |  |  |  |
| GO:0050792\_regulation\_of\_viral\_reproduction | 2 | 0 |  |  |  |  |  |  |  |  |
| GO:0050802\_circadian\_sleep\_wake\_cycle\_\_sleep | 2 | 0 |  |  |  |  |  |  |  |  |
| GO:0050847\_progesterone\_receptor\_signaling\_pathway | 2 | 0 |  |  |  |  |  |  |  |  |
| GO:0050883\_musculoskeletal\_movement\_\_spinal\_reflex\_action | 2 | 0 |  |  |  |  |  |  |  |  |
| GO:0050901\_leukocyte\_tethering\_or\_rolling | 2 | 0 |  |  |  |  |  |  |  |  |
| GO:0050907\_detection\_of\_chemical\_stimulus\_involved\_in\_sensory\_perception | 2 | 0 |  |  |  |  |  |  |  |  |
| GO:0050917\_sensory\_perception\_of\_umami\_taste | 2 | 0 |  |  |  |  |  |  |  |  |
| GO:0050942\_positive\_regulation\_of\_pigment\_cell\_differentiation | 2 | 0 |  |  |  |  |  |  |  |  |
| GO:0050955\_thermoception | 2 | 0 |  |  |  |  |  |  |  |  |
| GO:0050968\_detection\_of\_chemical\_stimulus\_involved\_in\_sensory\_perception\_of\_pain | 2 | 0 |  |  |  |  |  |  |  |  |
| GO:0050973\_detection\_of\_mechanical\_stimulus\_involved\_in\_equilibrioception | 2 | 0 |  |  |  |  |  |  |  |  |
| GO:0050999\_regulation\_of\_nitric-oxide\_synthase\_activity | 2 | 0 |  |  |  |  |  |  |  |  |
| GO:0051004\_regulation\_of\_lipoprotein\_lipase\_activity | 2 | 0 |  |  |  |  |  |  |  |  |
| GO:0051014\_actin\_filament\_severing | 2 | 0 |  |  |  |  |  |  |  |  |
| GO:0051026\_chiasma\_formation | 2 | 0 |  |  |  |  |  |  |  |  |
| GO:0051081\_nuclear\_envelope\_disassembly | 2 | 0 |  |  |  |  |  |  |  |  |
| GO:0051132\_NK\_T\_cell\_activation | 2 | 0 |  |  |  |  |  |  |  |  |
| GO:0051133\_regulation\_of\_NK\_T\_cell\_activation | 2 | 0 |  |  |  |  |  |  |  |  |
| GO:0051135\_positive\_regulation\_of\_NK\_T\_cell\_activation | 2 | 0 |  |  |  |  |  |  |  |  |
| GO:0051150\_regulation\_of\_smooth\_muscle\_cell\_differentiation | 2 | 0 |  |  |  |  |  |  |  |  |
| GO:0051220\_cytoplasmic\_sequestering\_of\_protein | 2 | 0 |  |  |  |  |  |  |  |  |
| GO:0051279\_regulation\_of\_release\_of\_sequestered\_calcium\_ion\_into\_cytosol | 2 | 0 |  |  |  |  |  |  |  |  |
| GO:0051293\_establishment\_of\_spindle\_localization | 2 | 0 |  |  |  |  |  |  |  |  |
| GO:0051295\_establishment\_of\_meiotic\_spindle\_localization | 2 | 0 |  |  |  |  |  |  |  |  |
| GO:0051299\_centrosome\_separation | 2 | 0 |  |  |  |  |  |  |  |  |
| GO:0051303\_establishment\_of\_chromosome\_localization | 2 | 0 |  |  |  |  |  |  |  |  |
| GO:0051304\_chromosome\_separation | 2 | 0 |  |  |  |  |  |  |  |  |
| GO:0051307\_meiotic\_chromosome\_separation | 2 | 0 |  |  |  |  |  |  |  |  |
| GO:0051313\_attachment\_of\_spindle\_microtubules\_to\_chromosome | 2 | 0 |  |  |  |  |  |  |  |  |
| GO:0051318\_G1\_phase | 2 | 0 |  |  |  |  |  |  |  |  |
| GO:0051319\_G2\_phase | 2 | 0 |  |  |  |  |  |  |  |  |
| GO:0051353\_positive\_regulation\_of\_oxidoreductase\_activity | 2 | 0 |  |  |  |  |  |  |  |  |
| GO:0051451\_myoblast\_migration | 2 | 0 |  |  |  |  |  |  |  |  |
| GO:0051489\_regulation\_of\_filopodium\_assembly | 2 | 0 |  |  |  |  |  |  |  |  |
| GO:0051491\_positive\_regulation\_of\_filopodium\_assembly | 2 | 0 |  |  |  |  |  |  |  |  |
| GO:0051541\_elastin\_metabolic\_process | 2 | 0 |  |  |  |  |  |  |  |  |
| GO:0051546\_keratinocyte\_migration | 2 | 0 |  |  |  |  |  |  |  |  |
| GO:0051563\_smooth\_endoplasmic\_reticulum\_calcium\_ion\_homeostasis | 2 | 0 |  |  |  |  |  |  |  |  |
| GO:0051590\_positive\_regulation\_of\_neurotransmitter\_transport | 2 | 0 |  |  |  |  |  |  |  |  |
| GO:0051602\_response\_to\_electrical\_stimulus | 2 | 0 |  |  |  |  |  |  |  |  |
| GO:0051608\_histamine\_transport | 2 | 0 |  |  |  |  |  |  |  |  |
| GO:0051643\_ER\_localization | 2 | 0 |  |  |  |  |  |  |  |  |
| GO:0051653\_spindle\_localization | 2 | 0 |  |  |  |  |  |  |  |  |
| GO:0051657\_maintenance\_of\_organelle\_location | 2 | 0 |  |  |  |  |  |  |  |  |
| GO:0051702\_interaction\_with\_symbiont | 2 | 0 |  |  |  |  |  |  |  |  |
| GO:0051781\_positive\_regulation\_of\_cell\_division | 2 | 0 |  |  |  |  |  |  |  |  |
| GO:0051784\_negative\_regulation\_of\_nuclear\_division | 2 | 0 |  |  |  |  |  |  |  |  |
| GO:0051890\_regulation\_of\_cardioblast\_differentiation | 2 | 0 |  |  |  |  |  |  |  |  |
| GO:0051891\_positive\_regulation\_of\_cardioblast\_differentiation | 2 | 0 |  |  |  |  |  |  |  |  |
| GO:0051923\_sulfation | 2 | 0 |  |  |  |  |  |  |  |  |
| GO:0051938\_L-glutamate\_import | 2 | 0 |  |  |  |  |  |  |  |  |
| GO:0051957\_positive\_regulation\_of\_amino\_acid\_transport | 2 | 0 |  |  |  |  |  |  |  |  |
| GO:0051988\_regulation\_of\_attachment\_of\_spindle\_microtubules\_to\_kinetochore | 2 | 0 |  |  |  |  |  |  |  |  |
| GO:0055057\_neuroblast\_division | 2 | 0 |  |  |  |  |  |  |  |  |
| GO:0055064\_chloride\_ion\_homeostasis | 2 | 0 |  |  |  |  |  |  |  |  |
| GO:0055075\_potassium\_ion\_homeostasis | 2 | 0 |  |  |  |  |  |  |  |  |
| GO:0055090\_acylglycerol\_homeostasis | 2 | 0 |  |  |  |  |  |  |  |  |
| GO:0055091\_phospholipid\_homeostasis | 2 | 0 |  |  |  |  |  |  |  |  |
| GO:0060012\_synaptic\_transmission\_\_glycinergic | 2 | 0 |  |  |  |  |  |  |  |  |
| GO:0060023\_soft\_palate\_development | 2 | 0 |  |  |  |  |  |  |  |  |
| GO:0060032\_notochord\_regression | 2 | 0 |  |  |  |  |  |  |  |  |
| GO:0060039\_pericardium\_development | 2 | 0 |  |  |  |  |  |  |  |  |
| GO:0060044\_negative\_regulation\_of\_cardiac\_muscle\_cell\_proliferation | 2 | 0 |  |  |  |  |  |  |  |  |
| GO:0060060\_post-embryonic\_retina\_morphogenesis\_in\_camera-type\_eye | 2 | 0 |  |  |  |  |  |  |  |  |
| GO:0060083\_smooth\_muscle\_contraction\_involved\_in\_micturition | 2 | 0 |  |  |  |  |  |  |  |  |
| GO:0060124\_positive\_regulation\_of\_growth\_hormone\_secretion | 2 | 0 |  |  |  |  |  |  |  |  |
| GO:0060133\_somatotropin\_secreting\_cell\_development | 2 | 0 |  |  |  |  |  |  |  |  |
| GO:0060155\_platelet\_dense\_granule\_organization | 2 | 0 |  |  |  |  |  |  |  |  |
| GO:0060159\_regulation\_of\_dopamine\_receptor\_signaling\_pathway | 2 | 0 |  |  |  |  |  |  |  |  |
| GO:0060160\_negative\_regulation\_of\_dopamine\_receptor\_signaling\_pathway | 2 | 0 |  |  |  |  |  |  |  |  |
| GO:0060180\_female\_mating\_behavior | 2 | 0 |  |  |  |  |  |  |  |  |
| GO:0060214\_endocardium\_formation | 2 | 0 |  |  |  |  |  |  |  |  |
| GO:0060218\_hemopoietic\_stem\_cell\_differentiation | 2 | 0 |  |  |  |  |  |  |  |  |
| GO:0060259\_regulation\_of\_feeding\_behavior | 2 | 0 |  |  |  |  |  |  |  |  |
| GO:0060260\_regulation\_of\_transcription\_initiation\_from\_RNA\_polymerase\_II\_promoter | 2 | 0 |  |  |  |  |  |  |  |  |
| GO:0060292\_long\_term\_synaptic\_depression | 2 | 0 |  |  |  |  |  |  |  |  |
| GO:0060318\_definitive\_erythrocyte\_differentiation | 2 | 0 |  |  |  |  |  |  |  |  |
| GO:0060346\_bone\_trabecula\_formation | 2 | 0 |  |  |  |  |  |  |  |  |
| GO:0060363\_cranial\_suture\_morphogenesis | 2 | 0 |  |  |  |  |  |  |  |  |
| GO:0060393\_regulation\_of\_pathway-restricted\_SMAD\_protein\_phosphorylation | 2 | 0 |  |  |  |  |  |  |  |  |
| GO:0060426\_lung\_vasculature\_development | 2 | 0 |  |  |  |  |  |  |  |  |
| GO:0060430\_lung\_saccule\_development | 2 | 0 |  |  |  |  |  |  |  |  |
| GO:0060434\_bronchus\_morphogenesis | 2 | 0 |  |  |  |  |  |  |  |  |
| GO:0060439\_trachea\_morphogenesis | 2 | 0 |  |  |  |  |  |  |  |  |
| GO:0060458\_right\_lung\_development | 2 | 0 |  |  |  |  |  |  |  |  |
| GO:0060462\_lung\_lobe\_development | 2 | 0 |  |  |  |  |  |  |  |  |
| GO:0060463\_lung\_lobe\_morphogenesis | 2 | 0 |  |  |  |  |  |  |  |  |
| GO:0060479\_lung\_cell\_differentiation | 2 | 0 |  |  |  |  |  |  |  |  |
| GO:0060487\_lung\_epithelial\_cell\_differentiation | 2 | 0 |  |  |  |  |  |  |  |  |
| GO:0060516\_primary\_prostatic\_bud\_elongation | 2 | 0 |  |  |  |  |  |  |  |  |
| GO:0060529\_squamous\_basal\_epithelial\_stem\_cell\_differentiation\_involved\_in\_prostate\_gland\_acinus\_development | 2 | 0 |  |  |  |  |  |  |  |  |
| GO:0060534\_trachea\_cartilage\_development | 2 | 0 |  |  |  |  |  |  |  |  |
| GO:0060599\_lateral\_sprouting\_involved\_in\_mammary\_gland\_duct\_morphogenesis | 2 | 0 |  |  |  |  |  |  |  |  |
| GO:0060612\_adipose\_tissue\_development | 2 | 0 |  |  |  |  |  |  |  |  |
| GO:0060615\_mammary\_gland\_bud\_formation | 2 | 0 |  |  |  |  |  |  |  |  |
| GO:0060667\_branch\_elongation\_involved\_in\_salivary\_gland\_morphogenesis | 2 | 0 |  |  |  |  |  |  |  |  |
| GO:0060690\_epithelial\_cell\_differentiation\_involved\_in\_salivary\_gland\_development | 2 | 0 |  |  |  |  |  |  |  |  |
| GO:0060738\_epithelial-mesenchymal\_signaling\_involved\_in\_prostate\_gland\_development | 2 | 0 |  |  |  |  |  |  |  |  |
| GO:0060741\_prostate\_gland\_stromal\_morphogenesis | 2 | 0 |  |  |  |  |  |  |  |  |
| GO:0060763\_mammary\_duct\_terminal\_end\_bud\_growth | 2 | 0 |  |  |  |  |  |  |  |  |
| GO:0060765\_regulation\_of\_androgen\_receptor\_signaling\_pathway | 2 | 0 |  |  |  |  |  |  |  |  |
| GO:0060766\_negative\_regulation\_of\_androgen\_receptor\_signaling\_pathway | 2 | 0 |  |  |  |  |  |  |  |  |
| GO:0060769\_positive\_regulation\_of\_epithelial\_cell\_proliferation\_involved\_in\_prostate\_gland\_development | 2 | 0 |  |  |  |  |  |  |  |  |
| GO:0065005\_protein-lipid\_complex\_assembly | 2 | 0 |  |  |  |  |  |  |  |  |
| GO:0070076\_histone\_lysine\_demethylation | 2 | 0 |  |  |  |  |  |  |  |  |
| GO:0070168\_negative\_regulation\_of\_biomineral\_formation | 2 | 0 |  |  |  |  |  |  |  |  |
| GO:0070252\_actin-mediated\_cell\_contraction | 2 | 0 |  |  |  |  |  |  |  |  |
| GO:0070256\_negative\_regulation\_of\_mucus\_secretion | 2 | 0 |  |  |  |  |  |  |  |  |
| GO:0070257\_positive\_regulation\_of\_mucus\_secretion | 2 | 0 |  |  |  |  |  |  |  |  |
| GO:0070570\_regulation\_of\_neuron\_projection\_regeneration | 2 | 0 |  |  |  |  |  |  |  |  |
| GO:0070723\_response\_to\_cholesterol | 2 | 0 |  |  |  |  |  |  |  |  |
| GO:0033043\_regulation\_of\_organelle\_organization | 58 | 0 | 0.000000 | -0.000000 | 1609 | 1453.169227 | 1520.86 | 1588.550773 | 0.945221 |  |
| GO:0034622\_cellular\_macromolecular\_complex\_assembly | 58 | 0 | 0.000000 | -0.000000 | 1609 | 1453.169227 | 1520.86 | 1588.550773 | 0.945221 |  |
| GO:0000027\_ribosomal\_large\_subunit\_assembly | 1 | 0 |  |  |  |  |  |  |  |  |
| GO:0000042\_protein\_targeting\_to\_Golgi | 1 | 0 |  |  |  |  |  |  |  |  |
| GO:0000046\_autophagic\_vacuole\_fusion | 1 | 0 |  |  |  |  |  |  |  |  |
| GO:0000050\_urea\_cycle | 1 | 0 |  |  |  |  |  |  |  |  |
| GO:0000054\_ribosome\_export\_from\_nucleus | 1 | 0 |  |  |  |  |  |  |  |  |
| GO:0000055\_ribosomal\_large\_subunit\_export\_from\_nucleus | 1 | 0 |  |  |  |  |  |  |  |  |
| GO:0000056\_ribosomal\_small\_subunit\_export\_from\_nucleus | 1 | 0 |  |  |  |  |  |  |  |  |
| GO:0000072\_M\_phase\_specific\_microtubule\_process | 1 | 0 |  |  |  |  |  |  |  |  |
| GO:0000101\_sulfur\_amino\_acid\_transport | 1 | 0 |  |  |  |  |  |  |  |  |
| GO:0000147\_actin\_cortical\_patch\_assembly | 1 | 0 |  |  |  |  |  |  |  |  |
| GO:0000154\_rRNA\_modification | 1 | 0 |  |  |  |  |  |  |  |  |
| GO:0000183\_chromatin\_silencing\_at\_rDNA | 1 | 0 |  |  |  |  |  |  |  |  |
| GO:0000185\_activation\_of\_MAPKKK\_activity | 1 | 0 |  |  |  |  |  |  |  |  |
| GO:0000238\_zygotene | 1 | 0 |  |  |  |  |  |  |  |  |
| GO:0000255\_allantoin\_metabolic\_process | 1 | 0 |  |  |  |  |  |  |  |  |
| GO:0000266\_mitochondrial\_fission | 1 | 0 |  |  |  |  |  |  |  |  |
| GO:0000273\_lipoic\_acid\_metabolic\_process | 1 | 0 |  |  |  |  |  |  |  |  |
| GO:0000301\_retrograde\_transport\_\_vesicle\_recycling\_within\_Golgi | 1 | 0 |  |  |  |  |  |  |  |  |
| GO:0000394\_RNA\_splicing\_\_via\_endonucleolytic\_cleavage\_and\_ligation | 1 | 0 |  |  |  |  |  |  |  |  |
| GO:0000429\_regulation\_of\_transcription\_from\_RNA\_polymerase\_II\_promoter\_by\_carbon\_catabolites | 1 | 0 |  |  |  |  |  |  |  |  |
| GO:0000430\_regulation\_of\_transcription\_from\_RNA\_polymerase\_II\_promoter\_by\_glucose | 1 | 0 |  |  |  |  |  |  |  |  |
| GO:0000432\_positive\_regulation\_of\_transcription\_from\_RNA\_polymerase\_II\_promoter\_by\_glucose | 1 | 0 |  |  |  |  |  |  |  |  |
| GO:0000436\_positive\_regulation\_of\_transcription\_from\_RNA\_polymerase\_II\_promoter\_by\_carbon\_catabolites | 1 | 0 |  |  |  |  |  |  |  |  |
| GO:0000448\_cleavage\_in\_ITS2\_between\_5.8S\_rRNA\_and\_LSU-rRNA\_of\_tricistronic\_rRNA\_transcript\_(SSU-rRNA\_\_5.8S\_rRNA\_\_LSU-rRNA) | 1 | 0 |  |  |  |  |  |  |  |  |
| GO:0000460\_maturation\_of\_5.8S\_rRNA | 1 | 0 |  |  |  |  |  |  |  |  |
| GO:0000463\_maturation\_of\_LSU-rRNA\_from\_tricistronic\_rRNA\_transcript\_(SSU-rRNA\_\_5.8S\_rRNA\_\_LSU-rRNA) | 1 | 0 |  |  |  |  |  |  |  |  |
| GO:0000466\_maturation\_of\_5.8S\_rRNA\_from\_tricistronic\_rRNA\_transcript\_(SSU-rRNA\_\_5.8S\_rRNA\_\_LSU-rRNA) | 1 | 0 |  |  |  |  |  |  |  |  |
| GO:0000469\_cleavages\_during\_rRNA\_processing | 1 | 0 |  |  |  |  |  |  |  |  |
| GO:0000470\_maturation\_of\_LSU-rRNA | 1 | 0 |  |  |  |  |  |  |  |  |
| GO:0000478\_endonucleolytic\_cleavages\_during\_rRNA\_processing | 1 | 0 |  |  |  |  |  |  |  |  |
| GO:0000479\_endonucleolytic\_cleavage\_of\_tricistronic\_rRNA\_transcript\_(SSU-rRNA\_\_5.8S\_rRNA\_\_LSU-rRNA) | 1 | 0 |  |  |  |  |  |  |  |  |
| GO:0000705\_achiasmate\_meiosis\_I | 1 | 0 |  |  |  |  |  |  |  |  |
| GO:0000966\_RNA\_5'-end\_processing | 1 | 0 |  |  |  |  |  |  |  |  |
| GO:0001300\_chronological\_cell\_aging | 1 | 0 |  |  |  |  |  |  |  |  |
| GO:0001547\_antral\_ovarian\_follicle\_growth | 1 | 0 |  |  |  |  |  |  |  |  |
| GO:0001555\_oocyte\_growth | 1 | 0 |  |  |  |  |  |  |  |  |
| GO:0001560\_regulation\_of\_cell\_growth\_by\_extracellular\_stimulus | 1 | 0 |  |  |  |  |  |  |  |  |
| GO:0001660\_fever | 1 | 0 |  |  |  |  |  |  |  |  |
| GO:0001696\_gastric\_acid\_secretion | 1 | 0 |  |  |  |  |  |  |  |  |
| GO:0001712\_ectodermal\_cell\_fate\_commitment | 1 | 0 |  |  |  |  |  |  |  |  |
| GO:0001714\_endodermal\_cell\_fate\_specification | 1 | 0 |  |  |  |  |  |  |  |  |
| GO:0001762\_beta-alanine\_transport | 1 | 0 |  |  |  |  |  |  |  |  |
| GO:0001766\_membrane\_raft\_polarization | 1 | 0 |  |  |  |  |  |  |  |  |
| GO:0001811\_negative\_regulation\_of\_type\_I\_hypersensitivity | 1 | 0 |  |  |  |  |  |  |  |  |
| GO:0001821\_histamine\_secretion | 1 | 0 |  |  |  |  |  |  |  |  |
| GO:0001826\_inner\_cell\_mass\_cell\_differentiation | 1 | 0 |  |  |  |  |  |  |  |  |
| GO:0001830\_trophectodermal\_cell\_fate\_commitment | 1 | 0 |  |  |  |  |  |  |  |  |
| GO:0001834\_trophectodermal\_cell\_proliferation | 1 | 0 |  |  |  |  |  |  |  |  |
| GO:0001867\_complement\_activation\_\_lectin\_pathway | 1 | 0 |  |  |  |  |  |  |  |  |
| GO:0001880\_Mullerian\_duct\_regression | 1 | 0 |  |  |  |  |  |  |  |  |
| GO:0001887\_selenium\_metabolic\_process | 1 | 0 |  |  |  |  |  |  |  |  |
| GO:0001922\_B-1\_B\_cell\_homeostasis | 1 | 0 |  |  |  |  |  |  |  |  |
| GO:0001923\_B-1\_B\_cell\_differentiation | 1 | 0 |  |  |  |  |  |  |  |  |
| GO:0001941\_postsynaptic\_membrane\_organization | 1 | 0 |  |  |  |  |  |  |  |  |
| GO:0001946\_lymphangiogenesis | 1 | 0 |  |  |  |  |  |  |  |  |
| GO:0001956\_positive\_regulation\_of\_neurotransmitter\_secretion | 1 | 0 |  |  |  |  |  |  |  |  |
| GO:0001961\_positive\_regulation\_of\_cytokine-mediated\_signaling\_pathway | 1 | 0 |  |  |  |  |  |  |  |  |
| GO:0001979\_regulation\_of\_systemic\_arterial\_blood\_pressure\_by\_chemoreceptor\_signaling | 1 | 0 |  |  |  |  |  |  |  |  |
| GO:0001980\_regulation\_of\_systemic\_arterial\_blood\_pressure\_by\_ischemic\_conditions | 1 | 0 |  |  |  |  |  |  |  |  |
| GO:0001984\_vasodilation\_of\_artery\_during\_baroreceptor\_response\_to\_increased\_systemic\_arterial\_blood\_pressure | 1 | 0 |  |  |  |  |  |  |  |  |
| GO:0001985\_negative\_regulation\_of\_heart\_rate\_in\_baroreceptor\_response\_to\_increased\_systemic\_arterial\_blood\_pressure | 1 | 0 |  |  |  |  |  |  |  |  |
| GO:0001987\_vasoconstriction\_of\_artery\_involved\_in\_baroreceptor\_response\_to\_lowering\_of\_systemic\_arterial\_blood\_pressure | 1 | 0 |  |  |  |  |  |  |  |  |
| GO:0001988\_positive\_regulation\_of\_heart\_rate\_in\_baroreceptor\_response\_to\_decreased\_systemic\_arterial\_blood\_pressure | 1 | 0 |  |  |  |  |  |  |  |  |
| GO:0001994\_norepinephrine-epinephrine\_vasoconstriction\_involved\_in\_regulation\_of\_systemic\_arterial\_blood\_pressure | 1 | 0 |  |  |  |  |  |  |  |  |
| GO:0002001\_renin\_secretion\_into\_blood\_stream | 1 | 0 |  |  |  |  |  |  |  |  |
| GO:0002002\_regulation\_of\_angiotensin\_levels\_in\_blood | 1 | 0 |  |  |  |  |  |  |  |  |
| GO:0002003\_angiotensin\_maturation | 1 | 0 |  |  |  |  |  |  |  |  |
| GO:0002007\_detection\_of\_hypoxic\_conditions\_in\_blood\_by\_chemoreceptor\_signaling | 1 | 0 |  |  |  |  |  |  |  |  |
| GO:0002017\_regulation\_of\_blood\_volume\_by\_renal\_aldosterone | 1 | 0 |  |  |  |  |  |  |  |  |
| GO:0002023\_reduction\_of\_food\_intake\_in\_response\_to\_dietary\_excess | 1 | 0 |  |  |  |  |  |  |  |  |
| GO:0002031\_G-protein\_coupled\_receptor\_internalization | 1 | 0 |  |  |  |  |  |  |  |  |
| GO:0002036\_regulation\_of\_L-glutamate\_transport | 1 | 0 |  |  |  |  |  |  |  |  |
| GO:0002040\_sprouting\_angiogenesis | 1 | 0 |  |  |  |  |  |  |  |  |
| GO:0002041\_intussusceptive\_angiogenesis | 1 | 0 |  |  |  |  |  |  |  |  |
| GO:0002068\_glandular\_epithelial\_cell\_development | 1 | 0 |  |  |  |  |  |  |  |  |
| GO:0002069\_columnar\_cuboidal\_epithelial\_cell\_maturation | 1 | 0 |  |  |  |  |  |  |  |  |
| GO:0002071\_glandular\_epithelial\_cell\_maturation | 1 | 0 |  |  |  |  |  |  |  |  |
| GO:0002082\_regulation\_of\_oxidative\_phosphorylation | 1 | 0 |  |  |  |  |  |  |  |  |
| GO:0002084\_protein\_depalmitoylation | 1 | 0 |  |  |  |  |  |  |  |  |
| GO:0002085\_inhibition\_of\_neuroepithelial\_cell\_differentiation | 1 | 0 |  |  |  |  |  |  |  |  |
| GO:0002086\_diaphragm\_contraction | 1 | 0 |  |  |  |  |  |  |  |  |
| GO:0002118\_aggressive\_behavior | 1 | 0 |  |  |  |  |  |  |  |  |
| GO:0002121\_inter-male\_aggressive\_behavior | 1 | 0 |  |  |  |  |  |  |  |  |
| GO:0002124\_territorial\_aggressive\_behavior | 1 | 0 |  |  |  |  |  |  |  |  |
| GO:0002227\_innate\_immune\_response\_in\_mucosa | 1 | 0 |  |  |  |  |  |  |  |  |
| GO:0002232\_leukocyte\_chemotaxis\_during\_inflammatory\_response | 1 | 0 |  |  |  |  |  |  |  |  |
| GO:0002248\_connective\_tissue\_replacement\_during\_inflammatory\_response | 1 | 0 |  |  |  |  |  |  |  |  |
| GO:0002282\_microglial\_cell\_activation\_during\_immune\_response | 1 | 0 |  |  |  |  |  |  |  |  |
| GO:0002287\_alpha-beta\_T\_cell\_activation\_during\_immune\_response | 1 | 0 |  |  |  |  |  |  |  |  |
| GO:0002314\_germinal\_center\_B\_cell\_differentiation | 1 | 0 |  |  |  |  |  |  |  |  |
| GO:0002315\_marginal\_zone\_B\_cell\_differentiation | 1 | 0 |  |  |  |  |  |  |  |  |
| GO:0002316\_follicular\_B\_cell\_differentiation | 1 | 0 |  |  |  |  |  |  |  |  |
| GO:0002317\_plasma\_cell\_differentiation | 1 | 0 |  |  |  |  |  |  |  |  |
| GO:0002349\_histamine\_production\_during\_acute\_inflammatory\_response | 1 | 0 |  |  |  |  |  |  |  |  |
| GO:0002351\_serotonin\_production\_during\_acute\_inflammatory\_response | 1 | 0 |  |  |  |  |  |  |  |  |
| GO:0002355\_detection\_of\_tumor\_cell | 1 | 0 |  |  |  |  |  |  |  |  |
| GO:0002370\_natural\_killer\_cell\_cytokine\_production | 1 | 0 |  |  |  |  |  |  |  |  |
| GO:0002371\_dendritic\_cell\_cytokine\_production | 1 | 0 |  |  |  |  |  |  |  |  |
| GO:0002380\_immunoglobulin\_secretion\_during\_immune\_response | 1 | 0 |  |  |  |  |  |  |  |  |
| GO:0002396\_MHC\_protein\_complex\_assembly | 1 | 0 |  |  |  |  |  |  |  |  |
| GO:0002397\_MHC\_class\_I\_protein\_complex\_assembly | 1 | 0 |  |  |  |  |  |  |  |  |
| GO:0002420\_natural\_killer\_cell\_mediated\_cytotoxicity\_directed\_against\_tumor\_cell\_target | 1 | 0 |  |  |  |  |  |  |  |  |
| GO:0002423\_natural\_killer\_cell\_mediated\_immune\_response\_to\_tumor\_cell | 1 | 0 |  |  |  |  |  |  |  |  |
| GO:0002424\_T\_cell\_mediated\_immune\_response\_to\_tumor\_cell | 1 | 0 |  |  |  |  |  |  |  |  |
| GO:0002426\_immunoglobulin\_production\_in\_mucosal\_tissue | 1 | 0 |  |  |  |  |  |  |  |  |
| GO:0002431\_Fc\_receptor\_mediated\_stimulatory\_signaling\_pathway | 1 | 0 |  |  |  |  |  |  |  |  |
| GO:0002432\_granuloma\_formation | 1 | 0 |  |  |  |  |  |  |  |  |
| GO:0002441\_histamine\_secretion\_during\_acute\_inflammatory\_response | 1 | 0 |  |  |  |  |  |  |  |  |
| GO:0002442\_serotonin\_secretion\_during\_acute\_inflammatory\_response | 1 | 0 |  |  |  |  |  |  |  |  |
| GO:0002457\_T\_cell\_antigen\_processing\_and\_presentation | 1 | 0 |  |  |  |  |  |  |  |  |
| GO:0002458\_peripheral\_T\_cell\_tolerance\_induction | 1 | 0 |  |  |  |  |  |  |  |  |
| GO:0002461\_tolerance\_induction\_dependent\_upon\_immune\_response | 1 | 0 |  |  |  |  |  |  |  |  |
| GO:0002465\_peripheral\_tolerance\_induction | 1 | 0 |  |  |  |  |  |  |  |  |
| GO:0002468\_dendritic\_cell\_antigen\_processing\_and\_presentation | 1 | 0 |  |  |  |  |  |  |  |  |
| GO:0002476\_antigen\_processing\_and\_presentation\_of\_endogenous\_peptide\_antigen\_via\_MHC\_class\_Ib | 1 | 0 |  |  |  |  |  |  |  |  |
| GO:0002479\_antigen\_processing\_and\_presentation\_of\_exogenous\_peptide\_antigen\_via\_MHC\_class\_I\_\_TAP-dependent | 1 | 0 |  |  |  |  |  |  |  |  |
| GO:0002483\_antigen\_processing\_and\_presentation\_of\_endogenous\_peptide\_antigen | 1 | 0 |  |  |  |  |  |  |  |  |
| GO:0002501\_peptide\_antigen\_assembly\_with\_MHC\_protein\_complex | 1 | 0 |  |  |  |  |  |  |  |  |
| GO:0002502\_peptide\_antigen\_assembly\_with\_MHC\_class\_I\_protein\_complex | 1 | 0 |  |  |  |  |  |  |  |  |
| GO:0002508\_central\_tolerance\_induction | 1 | 0 |  |  |  |  |  |  |  |  |
| GO:0002510\_central\_B\_cell\_tolerance\_induction | 1 | 0 |  |  |  |  |  |  |  |  |
| GO:0002545\_chronic\_inflammatory\_response\_to\_non-antigenic\_stimulus | 1 | 0 |  |  |  |  |  |  |  |  |
| GO:0002553\_histamine\_secretion\_by\_mast\_cell | 1 | 0 |  |  |  |  |  |  |  |  |
| GO:0002554\_serotonin\_secretion\_by\_platelet | 1 | 0 |  |  |  |  |  |  |  |  |
| GO:0002572\_pro-T\_cell\_differentiation | 1 | 0 |  |  |  |  |  |  |  |  |
| GO:0002577\_regulation\_of\_antigen\_processing\_and\_presentation | 1 | 0 |  |  |  |  |  |  |  |  |
| GO:0002579\_positive\_regulation\_of\_antigen\_processing\_and\_presentation | 1 | 0 |  |  |  |  |  |  |  |  |
| GO:0002604\_regulation\_of\_dendritic\_cell\_antigen\_processing\_and\_presentation | 1 | 0 |  |  |  |  |  |  |  |  |
| GO:0002606\_positive\_regulation\_of\_dendritic\_cell\_antigen\_processing\_and\_presentation | 1 | 0 |  |  |  |  |  |  |  |  |
| GO:0002635\_negative\_regulation\_of\_germinal\_center\_formation | 1 | 0 |  |  |  |  |  |  |  |  |
| GO:0002646\_regulation\_of\_central\_tolerance\_induction | 1 | 0 |  |  |  |  |  |  |  |  |
| GO:0002648\_positive\_regulation\_of\_central\_tolerance\_induction | 1 | 0 |  |  |  |  |  |  |  |  |
| GO:0002649\_regulation\_of\_tolerance\_induction\_to\_self\_antigen | 1 | 0 |  |  |  |  |  |  |  |  |
| GO:0002651\_positive\_regulation\_of\_tolerance\_induction\_to\_self\_antigen | 1 | 0 |  |  |  |  |  |  |  |  |
| GO:0002652\_regulation\_of\_tolerance\_induction\_dependent\_upon\_immune\_response | 1 | 0 |  |  |  |  |  |  |  |  |
| GO:0002654\_positive\_regulation\_of\_tolerance\_induction\_dependent\_upon\_immune\_response | 1 | 0 |  |  |  |  |  |  |  |  |
| GO:0002658\_regulation\_of\_peripheral\_tolerance\_induction | 1 | 0 |  |  |  |  |  |  |  |  |
| GO:0002660\_positive\_regulation\_of\_peripheral\_tolerance\_induction | 1 | 0 |  |  |  |  |  |  |  |  |
| GO:0002677\_negative\_regulation\_of\_chronic\_inflammatory\_response | 1 | 0 |  |  |  |  |  |  |  |  |
| GO:0002678\_positive\_regulation\_of\_chronic\_inflammatory\_response | 1 | 0 |  |  |  |  |  |  |  |  |
| GO:0002701\_negative\_regulation\_of\_production\_of\_molecular\_mediator\_of\_immune\_response | 1 | 0 |  |  |  |  |  |  |  |  |
| GO:0002719\_negative\_regulation\_of\_cytokine\_production\_during\_immune\_response | 1 | 0 |  |  |  |  |  |  |  |  |
| GO:0002724\_regulation\_of\_T\_cell\_cytokine\_production | 1 | 0 |  |  |  |  |  |  |  |  |
| GO:0002727\_regulation\_of\_natural\_killer\_cell\_cytokine\_production | 1 | 0 |  |  |  |  |  |  |  |  |
| GO:0002729\_positive\_regulation\_of\_natural\_killer\_cell\_cytokine\_production | 1 | 0 |  |  |  |  |  |  |  |  |
| GO:0002730\_regulation\_of\_dendritic\_cell\_cytokine\_production | 1 | 0 |  |  |  |  |  |  |  |  |
| GO:0002756\_MyD88-independent\_toll-like\_receptor\_signaling\_pathway | 1 | 0 |  |  |  |  |  |  |  |  |
| GO:0002767\_immune\_response-inhibiting\_cell\_surface\_receptor\_signaling\_pathway | 1 | 0 |  |  |  |  |  |  |  |  |
| GO:0002769\_natural\_killer\_cell\_inhibitory\_signaling\_pathway | 1 | 0 |  |  |  |  |  |  |  |  |
| GO:0002840\_regulation\_of\_T\_cell\_mediated\_immune\_response\_to\_tumor\_cell | 1 | 0 |  |  |  |  |  |  |  |  |
| GO:0002842\_positive\_regulation\_of\_T\_cell\_mediated\_immune\_response\_to\_tumor\_cell | 1 | 0 |  |  |  |  |  |  |  |  |
| GO:0002849\_regulation\_of\_peripheral\_T\_cell\_tolerance\_induction | 1 | 0 |  |  |  |  |  |  |  |  |
| GO:0002851\_positive\_regulation\_of\_peripheral\_T\_cell\_tolerance\_induction | 1 | 0 |  |  |  |  |  |  |  |  |
| GO:0002855\_regulation\_of\_natural\_killer\_cell\_mediated\_immune\_response\_to\_tumor\_cell | 1 | 0 |  |  |  |  |  |  |  |  |
| GO:0002857\_positive\_regulation\_of\_natural\_killer\_cell\_mediated\_immune\_response\_to\_tumor\_cell | 1 | 0 |  |  |  |  |  |  |  |  |
| GO:0002858\_regulation\_of\_natural\_killer\_cell\_mediated\_cytotoxicity\_directed\_against\_tumor\_cell\_target | 1 | 0 |  |  |  |  |  |  |  |  |
| GO:0002860\_positive\_regulation\_of\_natural\_killer\_cell\_mediated\_cytotoxicity\_directed\_against\_tumor\_cell\_target | 1 | 0 |  |  |  |  |  |  |  |  |
| GO:0002880\_regulation\_of\_chronic\_inflammatory\_response\_to\_non-antigenic\_stimulus | 1 | 0 |  |  |  |  |  |  |  |  |
| GO:0002882\_positive\_regulation\_of\_chronic\_inflammatory\_response\_to\_non-antigenic\_stimulus | 1 | 0 |  |  |  |  |  |  |  |  |
| GO:0002895\_regulation\_of\_central\_B\_cell\_tolerance\_induction | 1 | 0 |  |  |  |  |  |  |  |  |
| GO:0002897\_positive\_regulation\_of\_central\_B\_cell\_tolerance\_induction | 1 | 0 |  |  |  |  |  |  |  |  |
| GO:0002901\_mature\_B\_cell\_apoptosis | 1 | 0 |  |  |  |  |  |  |  |  |
| GO:0002903\_negative\_regulation\_of\_B\_cell\_apoptosis | 1 | 0 |  |  |  |  |  |  |  |  |
| GO:0002905\_regulation\_of\_mature\_B\_cell\_apoptosis | 1 | 0 |  |  |  |  |  |  |  |  |
| GO:0002906\_negative\_regulation\_of\_mature\_B\_cell\_apoptosis | 1 | 0 |  |  |  |  |  |  |  |  |
| GO:0003011\_involuntary\_skeletal\_muscle\_contraction | 1 | 0 |  |  |  |  |  |  |  |  |
| GO:0003027\_regulation\_of\_systemic\_arterial\_blood\_pressure\_by\_carotid\_body\_chemoreceptor\_signaling | 1 | 0 |  |  |  |  |  |  |  |  |
| GO:0003029\_detection\_of\_hypoxic\_conditions\_in\_blood\_by\_carotid\_body\_chemoreceptor\_signaling | 1 | 0 |  |  |  |  |  |  |  |  |
| GO:0003032\_detection\_of\_oxygen | 1 | 0 |  |  |  |  |  |  |  |  |
| GO:0003056\_regulation\_of\_vascular\_smooth\_muscle\_contraction | 1 | 0 |  |  |  |  |  |  |  |  |
| GO:0003062\_regulation\_of\_heart\_rate\_by\_chemical\_signal | 1 | 0 |  |  |  |  |  |  |  |  |
| GO:0003065\_positive\_regulation\_of\_heart\_rate\_by\_epinephrine | 1 | 0 |  |  |  |  |  |  |  |  |
| GO:0003068\_regulation\_of\_systemic\_arterial\_blood\_pressure\_by\_acetylcholine | 1 | 0 |  |  |  |  |  |  |  |  |
| GO:0003069\_vasodilation\_by\_acetylcholine\_involved\_in\_regulation\_of\_systemic\_arterial\_blood\_pressure | 1 | 0 |  |  |  |  |  |  |  |  |
| GO:0003070\_regulation\_of\_systemic\_arterial\_blood\_pressure\_by\_neurotransmitter | 1 | 0 |  |  |  |  |  |  |  |  |
| GO:0003097\_renal\_water\_transport | 1 | 0 |  |  |  |  |  |  |  |  |
| GO:0005979\_regulation\_of\_glycogen\_biosynthetic\_process | 1 | 0 |  |  |  |  |  |  |  |  |
| GO:0005984\_disaccharide\_metabolic\_process | 1 | 0 |  |  |  |  |  |  |  |  |
| GO:0005988\_lactose\_metabolic\_process | 1 | 0 |  |  |  |  |  |  |  |  |
| GO:0005989\_lactose\_biosynthetic\_process | 1 | 0 |  |  |  |  |  |  |  |  |
| GO:0005997\_xylulose\_metabolic\_process | 1 | 0 |  |  |  |  |  |  |  |  |
| GO:0006000\_fructose\_metabolic\_process | 1 | 0 |  |  |  |  |  |  |  |  |
| GO:0006002\_fructose\_6-phosphate\_metabolic\_process | 1 | 0 |  |  |  |  |  |  |  |  |
| GO:0006004\_fucose\_metabolic\_process | 1 | 0 |  |  |  |  |  |  |  |  |
| GO:0006013\_mannose\_metabolic\_process | 1 | 0 |  |  |  |  |  |  |  |  |
| GO:0006060\_sorbitol\_metabolic\_process | 1 | 0 |  |  |  |  |  |  |  |  |
| GO:0006064\_glucuronate\_catabolic\_process | 1 | 0 |  |  |  |  |  |  |  |  |
| GO:0006086\_acetyl-CoA\_biosynthetic\_process\_from\_pyruvate | 1 | 0 |  |  |  |  |  |  |  |  |
| GO:0006098\_pentose-phosphate\_shunt | 1 | 0 |  |  |  |  |  |  |  |  |
| GO:0006101\_citrate\_metabolic\_process | 1 | 0 |  |  |  |  |  |  |  |  |
| GO:0006104\_succinyl-CoA\_metabolic\_process | 1 | 0 |  |  |  |  |  |  |  |  |
| GO:0006116\_NADH\_oxidation | 1 | 0 |  |  |  |  |  |  |  |  |
| GO:0006120\_mitochondrial\_electron\_transport\_\_NADH\_to\_ubiquinone | 1 | 0 |  |  |  |  |  |  |  |  |
| GO:0006154\_adenosine\_catabolic\_process | 1 | 0 |  |  |  |  |  |  |  |  |
| GO:0006157\_deoxyadenosine\_catabolic\_process | 1 | 0 |  |  |  |  |  |  |  |  |
| GO:0006167\_AMP\_biosynthetic\_process | 1 | 0 |  |  |  |  |  |  |  |  |
| GO:0006175\_dATP\_biosynthetic\_process | 1 | 0 |  |  |  |  |  |  |  |  |
| GO:0006178\_guanine\_salvage | 1 | 0 |  |  |  |  |  |  |  |  |
| GO:0006196\_AMP\_catabolic\_process | 1 | 0 |  |  |  |  |  |  |  |  |
| GO:0006203\_dGTP\_catabolic\_process | 1 | 0 |  |  |  |  |  |  |  |  |
| GO:0006208\_pyrimidine\_base\_catabolic\_process | 1 | 0 |  |  |  |  |  |  |  |  |
| GO:0006221\_pyrimidine\_nucleotide\_biosynthetic\_process | 1 | 0 |  |  |  |  |  |  |  |  |
| GO:0006235\_dTTP\_biosynthetic\_process | 1 | 0 |  |  |  |  |  |  |  |  |
| GO:0006244\_pyrimidine\_nucleotide\_catabolic\_process | 1 | 0 |  |  |  |  |  |  |  |  |
| GO:0006269\_DNA\_replication\_\_synthesis\_of\_RNA\_primer | 1 | 0 |  |  |  |  |  |  |  |  |
| GO:0006283\_transcription-coupled\_nucleotide-excision\_repair | 1 | 0 |  |  |  |  |  |  |  |  |
| GO:0006296\_nucleotide-excision\_repair\_\_DNA\_incision\_\_5'-to\_lesion | 1 | 0 |  |  |  |  |  |  |  |  |
| GO:0006307\_DNA\_dealkylation | 1 | 0 |  |  |  |  |  |  |  |  |
| GO:0006337\_nucleosome\_disassembly | 1 | 0 |  |  |  |  |  |  |  |  |
| GO:0006344\_maintenance\_of\_chromatin\_silencing | 1 | 0 |  |  |  |  |  |  |  |  |
| GO:0006356\_regulation\_of\_transcription\_from\_RNA\_polymerase\_I\_promoter | 1 | 0 |  |  |  |  |  |  |  |  |
| GO:0006388\_tRNA\_splicing\_\_via\_endonucleolytic\_cleavage\_and\_ligation | 1 | 0 |  |  |  |  |  |  |  |  |
| GO:0006407\_rRNA\_export\_from\_nucleus | 1 | 0 |  |  |  |  |  |  |  |  |
| GO:0006419\_alanyl-tRNA\_aminoacylation | 1 | 0 |  |  |  |  |  |  |  |  |
| GO:0006434\_seryl-tRNA\_aminoacylation | 1 | 0 |  |  |  |  |  |  |  |  |
| GO:0006447\_regulation\_of\_translational\_initiation\_by\_iron | 1 | 0 |  |  |  |  |  |  |  |  |
| GO:0006463\_steroid\_hormone\_receptor\_complex\_assembly | 1 | 0 |  |  |  |  |  |  |  |  |
| GO:0006467\_protein\_thiol-disulfide\_exchange | 1 | 0 |  |  |  |  |  |  |  |  |
| GO:0006474\_N-terminal\_protein\_amino\_acid\_acetylation | 1 | 0 |  |  |  |  |  |  |  |  |
| GO:0006481\_C-terminal\_protein\_amino\_acid\_methylation | 1 | 0 |  |  |  |  |  |  |  |  |
| GO:0006488\_dolichol-linked\_oligosaccharide\_biosynthetic\_process | 1 | 0 |  |  |  |  |  |  |  |  |
| GO:0006494\_protein\_amino\_acid\_terminal\_glycosylation | 1 | 0 |  |  |  |  |  |  |  |  |
| GO:0006496\_protein\_amino\_acid\_terminal\_N-glycosylation | 1 | 0 |  |  |  |  |  |  |  |  |
| GO:0006500\_N-terminal\_protein\_palmitoylation | 1 | 0 |  |  |  |  |  |  |  |  |
| GO:0006507\_GPI\_anchor\_release | 1 | 0 |  |  |  |  |  |  |  |  |
| GO:0006537\_glutamate\_biosynthetic\_process | 1 | 0 |  |  |  |  |  |  |  |  |
| GO:0006544\_glycine\_metabolic\_process | 1 | 0 |  |  |  |  |  |  |  |  |
| GO:0006549\_isoleucine\_metabolic\_process | 1 | 0 |  |  |  |  |  |  |  |  |
| GO:0006553\_lysine\_metabolic\_process | 1 | 0 |  |  |  |  |  |  |  |  |
| GO:0006554\_lysine\_catabolic\_process | 1 | 0 |  |  |  |  |  |  |  |  |
| GO:0006556\_S-adenosylmethionine\_biosynthetic\_process | 1 | 0 |  |  |  |  |  |  |  |  |
| GO:0006559\_L-phenylalanine\_catabolic\_process | 1 | 0 |  |  |  |  |  |  |  |  |
| GO:0006569\_tryptophan\_catabolic\_process | 1 | 0 |  |  |  |  |  |  |  |  |
| GO:0006572\_tyrosine\_catabolic\_process | 1 | 0 |  |  |  |  |  |  |  |  |
| GO:0006573\_valine\_metabolic\_process | 1 | 0 |  |  |  |  |  |  |  |  |
| GO:0006581\_acetylcholine\_catabolic\_process | 1 | 0 |  |  |  |  |  |  |  |  |
| GO:0006585\_dopamine\_biosynthetic\_process\_from\_tyrosine | 1 | 0 |  |  |  |  |  |  |  |  |
| GO:0006590\_thyroid\_hormone\_generation | 1 | 0 |  |  |  |  |  |  |  |  |
| GO:0006591\_ornithine\_metabolic\_process | 1 | 0 |  |  |  |  |  |  |  |  |
| GO:0006596\_polyamine\_biosynthetic\_process | 1 | 0 |  |  |  |  |  |  |  |  |
| GO:0006597\_spermine\_biosynthetic\_process | 1 | 0 |  |  |  |  |  |  |  |  |
| GO:0006601\_creatine\_biosynthetic\_process | 1 | 0 |  |  |  |  |  |  |  |  |
| GO:0006613\_cotranslational\_protein\_targeting\_to\_membrane | 1 | 0 |  |  |  |  |  |  |  |  |
| GO:0006622\_protein\_targeting\_to\_lysosome | 1 | 0 |  |  |  |  |  |  |  |  |
| GO:0006627\_mitochondrial\_protein\_processing\_during\_import | 1 | 0 |  |  |  |  |  |  |  |  |
| GO:0006653\_lecithin\_metabolic\_process | 1 | 0 |  |  |  |  |  |  |  |  |
| GO:0006654\_phosphatidic\_acid\_biosynthetic\_process | 1 | 0 |  |  |  |  |  |  |  |  |
| GO:0006658\_phosphatidylserine\_metabolic\_process | 1 | 0 |  |  |  |  |  |  |  |  |
| GO:0006659\_phosphatidylserine\_biosynthetic\_process | 1 | 0 |  |  |  |  |  |  |  |  |
| GO:0006678\_glucosylceramide\_metabolic\_process | 1 | 0 |  |  |  |  |  |  |  |  |
| GO:0006682\_galactosylceramide\_biosynthetic\_process | 1 | 0 |  |  |  |  |  |  |  |  |
| GO:0006685\_sphingomyelin\_catabolic\_process | 1 | 0 |  |  |  |  |  |  |  |  |
| GO:0006700\_C21-steroid\_hormone\_biosynthetic\_process | 1 | 0 |  |  |  |  |  |  |  |  |
| GO:0006705\_mineralocorticoid\_biosynthetic\_process | 1 | 0 |  |  |  |  |  |  |  |  |
| GO:0006709\_progesterone\_catabolic\_process | 1 | 0 |  |  |  |  |  |  |  |  |
| GO:0006729\_tetrahydrobiopterin\_biosynthetic\_process | 1 | 0 |  |  |  |  |  |  |  |  |
| GO:0006734\_NADH\_metabolic\_process | 1 | 0 |  |  |  |  |  |  |  |  |
| GO:0006740\_NADPH\_regeneration | 1 | 0 |  |  |  |  |  |  |  |  |
| GO:0006741\_NADP\_biosynthetic\_process | 1 | 0 |  |  |  |  |  |  |  |  |
| GO:0006743\_ubiquinone\_metabolic\_process | 1 | 0 |  |  |  |  |  |  |  |  |
| GO:0006744\_ubiquinone\_biosynthetic\_process | 1 | 0 |  |  |  |  |  |  |  |  |
| GO:0006772\_thiamin\_metabolic\_process | 1 | 0 |  |  |  |  |  |  |  |  |
| GO:0006784\_heme\_a\_biosynthetic\_process | 1 | 0 |  |  |  |  |  |  |  |  |
| GO:0006797\_polyphosphate\_metabolic\_process | 1 | 0 |  |  |  |  |  |  |  |  |
| GO:0006798\_polyphosphate\_catabolic\_process | 1 | 0 |  |  |  |  |  |  |  |  |
| GO:0006824\_cobalt\_ion\_transport | 1 | 0 |  |  |  |  |  |  |  |  |
| GO:0006842\_tricarboxylic\_acid\_transport | 1 | 0 |  |  |  |  |  |  |  |  |
| GO:0006844\_acyl\_carnitine\_transport | 1 | 0 |  |  |  |  |  |  |  |  |
| GO:0006855\_multidrug\_transport | 1 | 0 |  |  |  |  |  |  |  |  |
| GO:0006863\_purine\_transport | 1 | 0 |  |  |  |  |  |  |  |  |
| GO:0006890\_retrograde\_vesicle-mediated\_transport\_\_Golgi\_to\_ER | 1 | 0 |  |  |  |  |  |  |  |  |
| GO:0006891\_intra-Golgi\_vesicle-mediated\_transport | 1 | 0 |  |  |  |  |  |  |  |  |
| GO:0006893\_Golgi\_to\_plasma\_membrane\_transport | 1 | 0 |  |  |  |  |  |  |  |  |
| GO:0006895\_Golgi\_to\_endosome\_transport | 1 | 0 |  |  |  |  |  |  |  |  |
| GO:0006896\_Golgi\_to\_vacuole\_transport | 1 | 0 |  |  |  |  |  |  |  |  |
| GO:0006900\_membrane\_budding | 1 | 0 |  |  |  |  |  |  |  |  |
| GO:0006930\_substrate-bound\_cell\_migration\_\_cell\_extension | 1 | 0 |  |  |  |  |  |  |  |  |
| GO:0006931\_substrate-bound\_cell\_migration\_\_cell\_attachment\_to\_substrate | 1 | 0 |  |  |  |  |  |  |  |  |
| GO:0006933\_negative\_regulation\_of\_cell\_adhesion\_involved\_in\_substrate-bound\_cell\_migration | 1 | 0 |  |  |  |  |  |  |  |  |
| GO:0006957\_complement\_activation\_\_alternative\_pathway | 1 | 0 |  |  |  |  |  |  |  |  |
| GO:0006958\_complement\_activation\_\_classical\_pathway | 1 | 0 |  |  |  |  |  |  |  |  |
| GO:0006978\_DNA\_damage\_response\_\_signal\_transduction\_by\_p53\_class\_mediator\_resulting\_in\_transcription\_of\_p21\_class\_mediator | 1 | 0 |  |  |  |  |  |  |  |  |
| GO:0007016\_cytoskeletal\_anchoring\_at\_plasma\_membrane | 1 | 0 |  |  |  |  |  |  |  |  |
| GO:0007021\_tubulin\_complex\_assembly | 1 | 0 |  |  |  |  |  |  |  |  |
| GO:0007052\_mitotic\_spindle\_organization | 1 | 0 |  |  |  |  |  |  |  |  |
| GO:0007056\_spindle\_assembly\_involved\_in\_female\_meiosis | 1 | 0 |  |  |  |  |  |  |  |  |
| GO:0007057\_spindle\_assembly\_involved\_in\_female\_meiosis\_I | 1 | 0 |  |  |  |  |  |  |  |  |
| GO:0007063\_regulation\_of\_sister\_chromatid\_cohesion | 1 | 0 |  |  |  |  |  |  |  |  |
| GO:0007065\_male\_meiosis\_sister\_chromatid\_cohesion | 1 | 0 |  |  |  |  |  |  |  |  |
| GO:0007076\_mitotic\_chromosome\_condensation | 1 | 0 |  |  |  |  |  |  |  |  |
| GO:0007095\_mitotic\_cell\_cycle\_G2\_M\_transition\_DNA\_damage\_checkpoint | 1 | 0 |  |  |  |  |  |  |  |  |
| GO:0007096\_regulation\_of\_exit\_from\_mitosis | 1 | 0 |  |  |  |  |  |  |  |  |
| GO:0007158\_neuron\_adhesion | 1 | 0 |  |  |  |  |  |  |  |  |
| GO:0007168\_receptor\_guanylyl\_cyclase\_signaling\_pathway | 1 | 0 |  |  |  |  |  |  |  |  |
| GO:0007197\_inhibition\_of\_adenylate\_cyclase\_activity\_by\_muscarinic\_acetylcholine\_receptor\_signaling\_pathway | 1 | 0 |  |  |  |  |  |  |  |  |
| GO:0007207\_activation\_of\_phospholipase\_C\_activity\_by\_muscarinic\_acetylcholine\_receptor\_signaling\_pathway | 1 | 0 |  |  |  |  |  |  |  |  |
| GO:0007208\_activation\_of\_phospholipase\_C\_activity\_by\_serotonin\_receptor\_signaling\_pathway | 1 | 0 |  |  |  |  |  |  |  |  |
| GO:0007217\_tachykinin\_receptor\_signaling\_pathway | 1 | 0 |  |  |  |  |  |  |  |  |
| GO:0007221\_positive\_regulation\_of\_transcription\_of\_Notch\_receptor\_target | 1 | 0 |  |  |  |  |  |  |  |  |
| GO:0007223\_Wnt\_receptor\_signaling\_pathway\_\_calcium\_modulating\_pathway | 1 | 0 |  |  |  |  |  |  |  |  |
| GO:0007225\_patched\_ligand\_processing | 1 | 0 |  |  |  |  |  |  |  |  |
| GO:0007227\_signal\_transduction\_downstream\_of\_smoothened | 1 | 0 |  |  |  |  |  |  |  |  |
| GO:0007228\_positive\_regulation\_of\_hh\_target\_transcription\_factor\_activity | 1 | 0 |  |  |  |  |  |  |  |  |
| GO:0007231\_osmosensory\_signaling\_pathway | 1 | 0 |  |  |  |  |  |  |  |  |
| GO:0007284\_spermatogonial\_cell\_division | 1 | 0 |  |  |  |  |  |  |  |  |
| GO:0007290\_spermatid\_nucleus\_elongation | 1 | 0 |  |  |  |  |  |  |  |  |
| GO:0007296\_vitellogenesis | 1 | 0 |  |  |  |  |  |  |  |  |
| GO:0007321\_sperm\_displacement | 1 | 0 |  |  |  |  |  |  |  |  |
| GO:0007380\_specification\_of\_segmental\_identity\_\_head | 1 | 0 |  |  |  |  |  |  |  |  |
| GO:0007382\_specification\_of\_segmental\_identity\_\_maxillary\_segment | 1 | 0 |  |  |  |  |  |  |  |  |
| GO:0007402\_ganglion\_mother\_cell\_fate\_determination | 1 | 0 |  |  |  |  |  |  |  |  |
| GO:0007495\_visceral\_mesoderm-endoderm\_interaction\_involved\_in\_midgut\_development | 1 | 0 |  |  |  |  |  |  |  |  |
| GO:0007497\_posterior\_midgut\_development | 1 | 0 |  |  |  |  |  |  |  |  |
| GO:0007499\_ectoderm\_and\_mesoderm\_interaction | 1 | 0 |  |  |  |  |  |  |  |  |
| GO:0007500\_mesodermal\_cell\_fate\_determination | 1 | 0 |  |  |  |  |  |  |  |  |
| GO:0007509\_mesoderm\_migration | 1 | 0 |  |  |  |  |  |  |  |  |
| GO:0007518\_myoblast\_cell\_fate\_determination | 1 | 0 |  |  |  |  |  |  |  |  |
| GO:0007521\_muscle\_cell\_fate\_determination | 1 | 0 |  |  |  |  |  |  |  |  |
| GO:0007522\_visceral\_muscle\_development | 1 | 0 |  |  |  |  |  |  |  |  |
| GO:0007529\_establishment\_of\_synaptic\_specificity\_at\_neuromuscular\_junction | 1 | 0 |  |  |  |  |  |  |  |  |
| GO:0007538\_primary\_sex\_determination | 1 | 0 |  |  |  |  |  |  |  |  |
| GO:0007542\_primary\_sex\_determination\_\_germ-line | 1 | 0 |  |  |  |  |  |  |  |  |
| GO:0007567\_parturition | 1 | 0 |  |  |  |  |  |  |  |  |
| GO:0007614\_short-term\_memory | 1 | 0 |  |  |  |  |  |  |  |  |
| GO:0007621\_negative\_regulation\_of\_female\_receptivity | 1 | 0 |  |  |  |  |  |  |  |  |
| GO:0008050\_female\_courtship\_behavior | 1 | 0 |  |  |  |  |  |  |  |  |
| GO:0008052\_sensory\_organ\_boundary\_specification | 1 | 0 |  |  |  |  |  |  |  |  |
| GO:0008054\_cyclin\_catabolic\_process | 1 | 0 |  |  |  |  |  |  |  |  |
| GO:0008057\_eye\_pigment\_granule\_organization | 1 | 0 |  |  |  |  |  |  |  |  |
| GO:0008078\_mesodermal\_cell\_migration | 1 | 0 |  |  |  |  |  |  |  |  |
| GO:0008208\_C21-steroid\_hormone\_catabolic\_process | 1 | 0 |  |  |  |  |  |  |  |  |
| GO:0008216\_spermidine\_metabolic\_process | 1 | 0 |  |  |  |  |  |  |  |  |
| GO:0008292\_acetylcholine\_biosynthetic\_process | 1 | 0 |  |  |  |  |  |  |  |  |
| GO:0008295\_spermidine\_biosynthetic\_process | 1 | 0 |  |  |  |  |  |  |  |  |
| GO:0008300\_isoprenoid\_catabolic\_process | 1 | 0 |  |  |  |  |  |  |  |  |
| GO:0008333\_endosome\_to\_lysosome\_transport | 1 | 0 |  |  |  |  |  |  |  |  |
| GO:0008355\_olfactory\_learning | 1 | 0 |  |  |  |  |  |  |  |  |
| GO:0008611\_ether\_lipid\_biosynthetic\_process | 1 | 0 |  |  |  |  |  |  |  |  |
| GO:0008626\_induction\_of\_apoptosis\_by\_granzyme | 1 | 0 |  |  |  |  |  |  |  |  |
| GO:0008633\_activation\_of\_pro-apoptotic\_gene\_products | 1 | 0 |  |  |  |  |  |  |  |  |
| GO:0008653\_lipopolysaccharide\_metabolic\_process | 1 | 0 |  |  |  |  |  |  |  |  |
| GO:0009068\_aspartate\_family\_amino\_acid\_catabolic\_process | 1 | 0 |  |  |  |  |  |  |  |  |
| GO:0009084\_glutamine\_family\_amino\_acid\_biosynthetic\_process | 1 | 0 |  |  |  |  |  |  |  |  |
| GO:0009088\_threonine\_biosynthetic\_process | 1 | 0 |  |  |  |  |  |  |  |  |
| GO:0009105\_lipoic\_acid\_biosynthetic\_process | 1 | 0 |  |  |  |  |  |  |  |  |
| GO:0009109\_coenzyme\_catabolic\_process | 1 | 0 |  |  |  |  |  |  |  |  |
| GO:0009111\_vitamin\_catabolic\_process | 1 | 0 |  |  |  |  |  |  |  |  |
| GO:0009113\_purine\_base\_biosynthetic\_process | 1 | 0 |  |  |  |  |  |  |  |  |
| GO:0009127\_purine\_nucleoside\_monophosphate\_biosynthetic\_process | 1 | 0 |  |  |  |  |  |  |  |  |
| GO:0009128\_purine\_nucleoside\_monophosphate\_catabolic\_process | 1 | 0 |  |  |  |  |  |  |  |  |
| GO:0009129\_pyrimidine\_nucleoside\_monophosphate\_metabolic\_process | 1 | 0 |  |  |  |  |  |  |  |  |
| GO:0009131\_pyrimidine\_nucleoside\_monophosphate\_catabolic\_process | 1 | 0 |  |  |  |  |  |  |  |  |
| GO:0009133\_nucleoside\_diphosphate\_biosynthetic\_process | 1 | 0 |  |  |  |  |  |  |  |  |
| GO:0009145\_purine\_nucleoside\_triphosphate\_biosynthetic\_process | 1 | 0 |  |  |  |  |  |  |  |  |
| GO:0009147\_pyrimidine\_nucleoside\_triphosphate\_metabolic\_process | 1 | 0 |  |  |  |  |  |  |  |  |
| GO:0009148\_pyrimidine\_nucleoside\_triphosphate\_biosynthetic\_process | 1 | 0 |  |  |  |  |  |  |  |  |
| GO:0009152\_purine\_ribonucleotide\_biosynthetic\_process | 1 | 0 |  |  |  |  |  |  |  |  |
| GO:0009153\_purine\_deoxyribonucleotide\_biosynthetic\_process | 1 | 0 |  |  |  |  |  |  |  |  |
| GO:0009156\_ribonucleoside\_monophosphate\_biosynthetic\_process | 1 | 0 |  |  |  |  |  |  |  |  |
| GO:0009158\_ribonucleoside\_monophosphate\_catabolic\_process | 1 | 0 |  |  |  |  |  |  |  |  |
| GO:0009159\_deoxyribonucleoside\_monophosphate\_catabolic\_process | 1 | 0 |  |  |  |  |  |  |  |  |
| GO:0009162\_deoxyribonucleoside\_monophosphate\_metabolic\_process | 1 | 0 |  |  |  |  |  |  |  |  |
| GO:0009168\_purine\_ribonucleoside\_monophosphate\_biosynthetic\_process | 1 | 0 |  |  |  |  |  |  |  |  |
| GO:0009169\_purine\_ribonucleoside\_monophosphate\_catabolic\_process | 1 | 0 |  |  |  |  |  |  |  |  |
| GO:0009176\_pyrimidine\_deoxyribonucleoside\_monophosphate\_metabolic\_process | 1 | 0 |  |  |  |  |  |  |  |  |
| GO:0009178\_pyrimidine\_deoxyribonucleoside\_monophosphate\_catabolic\_process | 1 | 0 |  |  |  |  |  |  |  |  |
| GO:0009211\_pyrimidine\_deoxyribonucleoside\_triphosphate\_metabolic\_process | 1 | 0 |  |  |  |  |  |  |  |  |
| GO:0009212\_pyrimidine\_deoxyribonucleoside\_triphosphate\_biosynthetic\_process | 1 | 0 |  |  |  |  |  |  |  |  |
| GO:0009216\_purine\_deoxyribonucleoside\_triphosphate\_biosynthetic\_process | 1 | 0 |  |  |  |  |  |  |  |  |
| GO:0009221\_pyrimidine\_deoxyribonucleotide\_biosynthetic\_process | 1 | 0 |  |  |  |  |  |  |  |  |
| GO:0009223\_pyrimidine\_deoxyribonucleotide\_catabolic\_process | 1 | 0 |  |  |  |  |  |  |  |  |
| GO:0009260\_ribonucleotide\_biosynthetic\_process | 1 | 0 |  |  |  |  |  |  |  |  |
| GO:0009405\_pathogenesis | 1 | 0 |  |  |  |  |  |  |  |  |
| GO:0009414\_response\_to\_water\_deprivation | 1 | 0 |  |  |  |  |  |  |  |  |
| GO:0009415\_response\_to\_water | 1 | 0 |  |  |  |  |  |  |  |  |
| GO:0009449\_gamma-aminobutyric\_acid\_biosynthetic\_process | 1 | 0 |  |  |  |  |  |  |  |  |
| GO:0009450\_gamma-aminobutyric\_acid\_catabolic\_process | 1 | 0 |  |  |  |  |  |  |  |  |
| GO:0009589\_detection\_of\_UV | 1 | 0 |  |  |  |  |  |  |  |  |
| GO:0009590\_detection\_of\_gravity | 1 | 0 |  |  |  |  |  |  |  |  |
| GO:0009624\_response\_to\_nematode | 1 | 0 |  |  |  |  |  |  |  |  |
| GO:0009629\_response\_to\_gravity | 1 | 0 |  |  |  |  |  |  |  |  |
| GO:0009648\_photoperiodism | 1 | 0 |  |  |  |  |  |  |  |  |
| GO:0009690\_cytokinin\_metabolic\_process | 1 | 0 |  |  |  |  |  |  |  |  |
| GO:0009691\_cytokinin\_biosynthetic\_process | 1 | 0 |  |  |  |  |  |  |  |  |
| GO:0009786\_regulation\_of\_asymmetric\_cell\_division | 1 | 0 |  |  |  |  |  |  |  |  |
| GO:0009794\_regulation\_of\_mitotic\_cell\_cycle\_\_embryonic | 1 | 0 |  |  |  |  |  |  |  |  |
| GO:0009956\_radial\_pattern\_formation | 1 | 0 |  |  |  |  |  |  |  |  |
| GO:0009957\_epidermal\_cell\_fate\_specification | 1 | 0 |  |  |  |  |  |  |  |  |
| GO:0009992\_cellular\_water\_homeostasis | 1 | 0 |  |  |  |  |  |  |  |  |
| GO:0010032\_meiotic\_chromosome\_condensation | 1 | 0 |  |  |  |  |  |  |  |  |
| GO:0010039\_response\_to\_iron\_ion | 1 | 0 |  |  |  |  |  |  |  |  |
| GO:0010042\_response\_to\_manganese\_ion | 1 | 0 |  |  |  |  |  |  |  |  |
| GO:0010045\_response\_to\_nickel\_ion | 1 | 0 |  |  |  |  |  |  |  |  |
| GO:0010046\_response\_to\_mycotoxin | 1 | 0 |  |  |  |  |  |  |  |  |
| GO:0010107\_potassium\_ion\_import | 1 | 0 |  |  |  |  |  |  |  |  |
| GO:0010155\_regulation\_of\_proton\_transport | 1 | 0 |  |  |  |  |  |  |  |  |
| GO:0010160\_formation\_of\_organ\_boundary | 1 | 0 |  |  |  |  |  |  |  |  |
| GO:0010260\_organ\_senescence | 1 | 0 |  |  |  |  |  |  |  |  |
| GO:0010310\_regulation\_of\_hydrogen\_peroxide\_metabolic\_process | 1 | 0 |  |  |  |  |  |  |  |  |
| GO:0010447\_response\_to\_acidity | 1 | 0 |  |  |  |  |  |  |  |  |
| GO:0010452\_histone\_H3-K36\_methylation | 1 | 0 |  |  |  |  |  |  |  |  |
| GO:0010455\_positive\_regulation\_of\_cell\_fate\_commitment | 1 | 0 |  |  |  |  |  |  |  |  |
| GO:0010470\_regulation\_of\_gastrulation | 1 | 0 |  |  |  |  |  |  |  |  |
| GO:0010508\_positive\_regulation\_of\_autophagy | 1 | 0 |  |  |  |  |  |  |  |  |
| GO:0010519\_negative\_regulation\_of\_phospholipase\_activity | 1 | 0 |  |  |  |  |  |  |  |  |
| GO:0010520\_regulation\_of\_reciprocal\_meiotic\_recombination | 1 | 0 |  |  |  |  |  |  |  |  |
| GO:0010523\_negative\_regulation\_of\_calcium\_ion\_transport\_into\_cytosol | 1 | 0 |  |  |  |  |  |  |  |  |
| GO:0010543\_regulation\_of\_platelet\_activation | 1 | 0 |  |  |  |  |  |  |  |  |
| GO:0010561\_negative\_regulation\_of\_glycoprotein\_biosynthetic\_process | 1 | 0 |  |  |  |  |  |  |  |  |
| GO:0010569\_regulation\_of\_double-strand\_break\_repair\_via\_homologous\_recombination | 1 | 0 |  |  |  |  |  |  |  |  |
| GO:0010572\_positive\_regulation\_of\_platelet\_activation | 1 | 0 |  |  |  |  |  |  |  |  |
| GO:0010594\_regulation\_of\_endothelial\_cell\_migration | 1 | 0 |  |  |  |  |  |  |  |  |
| GO:0010596\_negative\_regulation\_of\_endothelial\_cell\_migration | 1 | 0 |  |  |  |  |  |  |  |  |
| GO:0010611\_regulation\_of\_cardiac\_muscle\_hypertrophy | 1 | 0 |  |  |  |  |  |  |  |  |
| GO:0010612\_regulation\_of\_cardiac\_muscle\_adaptation | 1 | 0 |  |  |  |  |  |  |  |  |
| GO:0010614\_negative\_regulation\_of\_cardiac\_muscle\_hypertrophy | 1 | 0 |  |  |  |  |  |  |  |  |
| GO:0010616\_negative\_regulation\_of\_cardiac\_muscle\_adaptation | 1 | 0 |  |  |  |  |  |  |  |  |
| GO:0010634\_positive\_regulation\_of\_epithelial\_cell\_migration | 1 | 0 |  |  |  |  |  |  |  |  |
| GO:0010656\_negative\_regulation\_of\_muscle\_cell\_apoptosis | 1 | 0 |  |  |  |  |  |  |  |  |
| GO:0010657\_muscle\_cell\_apoptosis | 1 | 0 |  |  |  |  |  |  |  |  |
| GO:0010658\_striated\_muscle\_cell\_apoptosis | 1 | 0 |  |  |  |  |  |  |  |  |
| GO:0010659\_cardiac\_muscle\_cell\_apoptosis | 1 | 0 |  |  |  |  |  |  |  |  |
| GO:0010660\_regulation\_of\_muscle\_cell\_apoptosis | 1 | 0 |  |  |  |  |  |  |  |  |
| GO:0010662\_regulation\_of\_striated\_muscle\_cell\_apoptosis | 1 | 0 |  |  |  |  |  |  |  |  |
| GO:0010664\_negative\_regulation\_of\_striated\_muscle\_cell\_apoptosis | 1 | 0 |  |  |  |  |  |  |  |  |
| GO:0010665\_regulation\_of\_cardiac\_muscle\_cell\_apoptosis | 1 | 0 |  |  |  |  |  |  |  |  |
| GO:0010667\_negative\_regulation\_of\_cardiac\_muscle\_cell\_apoptosis | 1 | 0 |  |  |  |  |  |  |  |  |
| GO:0010668\_ectodermal\_cell\_differentiation | 1 | 0 |  |  |  |  |  |  |  |  |
| GO:0010671\_negative\_regulation\_of\_oxygen\_and\_reactive\_oxygen\_species\_metabolic\_process | 1 | 0 |  |  |  |  |  |  |  |  |
| GO:0010719\_negative\_regulation\_of\_epithelial\_to\_mesenchymal\_transition | 1 | 0 |  |  |  |  |  |  |  |  |
| GO:0010735\_positive\_regulation\_of\_transcription\_via\_serum\_response\_element\_binding | 1 | 0 |  |  |  |  |  |  |  |  |
| GO:0010825\_positive\_regulation\_of\_centrosome\_duplication | 1 | 0 |  |  |  |  |  |  |  |  |
| GO:0010845\_positive\_regulation\_of\_reciprocal\_meiotic\_recombination | 1 | 0 |  |  |  |  |  |  |  |  |
| GO:0010850\_chemoreceptor\_signaling\_pathway\_involved\_in\_regulation\_of\_blood\_pressure | 1 | 0 |  |  |  |  |  |  |  |  |
| GO:0010873\_positive\_regulation\_of\_cholesterol\_esterification | 1 | 0 |  |  |  |  |  |  |  |  |
| GO:0010880\_regulation\_of\_release\_of\_sequestered\_calcium\_ion\_into\_cytosol\_by\_sarcoplasmic\_reticulum | 1 | 0 |  |  |  |  |  |  |  |  |
| GO:0010881\_regulation\_of\_cardiac\_muscle\_contraction\_by\_regulation\_of\_the\_release\_of\_sequestered\_calcium\_ion | 1 | 0 |  |  |  |  |  |  |  |  |
| GO:0010882\_regulation\_of\_cardiac\_muscle\_contraction\_by\_calcium\_ion\_signaling | 1 | 0 |  |  |  |  |  |  |  |  |
| GO:0010890\_positive\_regulation\_of\_sequestering\_of\_triglyceride | 1 | 0 |  |  |  |  |  |  |  |  |
| GO:0010919\_regulation\_of\_inositol\_phosphate\_biosynthetic\_process | 1 | 0 |  |  |  |  |  |  |  |  |
| GO:0010931\_macrophage\_tolerance\_induction | 1 | 0 |  |  |  |  |  |  |  |  |
| GO:0010932\_regulation\_of\_macrophage\_tolerance\_induction | 1 | 0 |  |  |  |  |  |  |  |  |
| GO:0010933\_positive\_regulation\_of\_macrophage\_tolerance\_induction | 1 | 0 |  |  |  |  |  |  |  |  |
| GO:0010934\_macrophage\_cytokine\_production | 1 | 0 |  |  |  |  |  |  |  |  |
| GO:0010935\_regulation\_of\_macrophage\_cytokine\_production | 1 | 0 |  |  |  |  |  |  |  |  |
| GO:0010936\_negative\_regulation\_of\_macrophage\_cytokine\_production | 1 | 0 |  |  |  |  |  |  |  |  |
| GO:0010953\_regulation\_of\_protein\_maturation\_by\_peptide\_bond\_cleavage | 1 | 0 |  |  |  |  |  |  |  |  |
| GO:0010962\_regulation\_of\_glucan\_biosynthetic\_process | 1 | 0 |  |  |  |  |  |  |  |  |
| GO:0010966\_regulation\_of\_phosphate\_transport | 1 | 0 |  |  |  |  |  |  |  |  |
| GO:0014012\_axon\_regeneration\_in\_the\_peripheral\_nervous\_system | 1 | 0 |  |  |  |  |  |  |  |  |
| GO:0014041\_regulation\_of\_neuron\_maturation | 1 | 0 |  |  |  |  |  |  |  |  |
| GO:0014042\_positive\_regulation\_of\_neuron\_maturation | 1 | 0 |  |  |  |  |  |  |  |  |
| GO:0014049\_positive\_regulation\_of\_glutamate\_secretion | 1 | 0 |  |  |  |  |  |  |  |  |
| GO:0014061\_regulation\_of\_norepinephrine\_secretion | 1 | 0 |  |  |  |  |  |  |  |  |
| GO:0014071\_response\_to\_cycloalkane | 1 | 0 |  |  |  |  |  |  |  |  |
| GO:0014707\_branchiomeric\_skeletal\_muscle\_development | 1 | 0 |  |  |  |  |  |  |  |  |
| GO:0014738\_regulation\_of\_muscle\_hyperplasia | 1 | 0 |  |  |  |  |  |  |  |  |
| GO:0014740\_negative\_regulation\_of\_muscle\_hyperplasia | 1 | 0 |  |  |  |  |  |  |  |  |
| GO:0014741\_negative\_regulation\_of\_muscle\_hypertrophy | 1 | 0 |  |  |  |  |  |  |  |  |
| GO:0014743\_regulation\_of\_muscle\_hypertrophy | 1 | 0 |  |  |  |  |  |  |  |  |
| GO:0014805\_smooth\_muscle\_adaptation | 1 | 0 |  |  |  |  |  |  |  |  |
| GO:0014806\_smooth\_muscle\_hyperplasia | 1 | 0 |  |  |  |  |  |  |  |  |
| GO:0014807\_regulation\_of\_somitogenesis | 1 | 0 |  |  |  |  |  |  |  |  |
| GO:0014808\_release\_of\_sequestered\_calcium\_ion\_into\_cytosol\_by\_sarcoplasmic\_reticulum | 1 | 0 |  |  |  |  |  |  |  |  |
| GO:0014813\_satellite\_cell\_commitment | 1 | 0 |  |  |  |  |  |  |  |  |
| GO:0014816\_satellite\_cell\_differentiation | 1 | 0 |  |  |  |  |  |  |  |  |
| GO:0014819\_regulation\_of\_skeletal\_muscle\_contraction | 1 | 0 |  |  |  |  |  |  |  |  |
| GO:0014852\_regulation\_of\_skeletal\_muscle\_contraction\_by\_neural\_stimulation\_via\_neuromuscular\_junction | 1 | 0 |  |  |  |  |  |  |  |  |
| GO:0014853\_regulation\_of\_excitatory\_postsynaptic\_membrane\_potential\_involved\_in\_skeletal\_muscle\_contraction | 1 | 0 |  |  |  |  |  |  |  |  |
| GO:0014856\_skeletal\_muscle\_cell\_proliferation | 1 | 0 |  |  |  |  |  |  |  |  |
| GO:0014857\_regulation\_of\_skeletal\_muscle\_cell\_proliferation | 1 | 0 |  |  |  |  |  |  |  |  |
| GO:0014858\_positive\_regulation\_of\_skeletal\_muscle\_cell\_proliferation | 1 | 0 |  |  |  |  |  |  |  |  |
| GO:0014887\_cardiac\_muscle\_adaptation | 1 | 0 |  |  |  |  |  |  |  |  |
| GO:0014889\_muscle\_atrophy | 1 | 0 |  |  |  |  |  |  |  |  |
| GO:0014896\_muscle\_hypertrophy | 1 | 0 |  |  |  |  |  |  |  |  |
| GO:0014897\_striated\_muscle\_hypertrophy | 1 | 0 |  |  |  |  |  |  |  |  |
| GO:0014898\_cardiac\_muscle\_hypertrophy | 1 | 0 |  |  |  |  |  |  |  |  |
| GO:0014900\_muscle\_hyperplasia | 1 | 0 |  |  |  |  |  |  |  |  |
| GO:0014910\_regulation\_of\_smooth\_muscle\_cell\_migration | 1 | 0 |  |  |  |  |  |  |  |  |
| GO:0014911\_positive\_regulation\_of\_smooth\_muscle\_cell\_migration | 1 | 0 |  |  |  |  |  |  |  |  |
| GO:0015014\_heparan\_sulfate\_proteoglycan\_biosynthetic\_process\_\_polysaccharide\_chain\_biosynthetic\_process | 1 | 0 |  |  |  |  |  |  |  |  |
| GO:0015074\_DNA\_integration | 1 | 0 |  |  |  |  |  |  |  |  |
| GO:0015670\_carbon\_dioxide\_transport | 1 | 0 |  |  |  |  |  |  |  |  |
| GO:0015677\_copper\_ion\_import | 1 | 0 |  |  |  |  |  |  |  |  |
| GO:0015680\_intracellular\_copper\_ion\_transport | 1 | 0 |  |  |  |  |  |  |  |  |
| GO:0015684\_ferrous\_iron\_transport | 1 | 0 |  |  |  |  |  |  |  |  |
| GO:0015707\_nitrite\_transport | 1 | 0 |  |  |  |  |  |  |  |  |
| GO:0015724\_formate\_transport | 1 | 0 |  |  |  |  |  |  |  |  |
| GO:0015734\_taurine\_transport | 1 | 0 |  |  |  |  |  |  |  |  |
| GO:0015740\_C4-dicarboxylate\_transport | 1 | 0 |  |  |  |  |  |  |  |  |
| GO:0015744\_succinate\_transport | 1 | 0 |  |  |  |  |  |  |  |  |
| GO:0015746\_citrate\_transport | 1 | 0 |  |  |  |  |  |  |  |  |
| GO:0015747\_urate\_transport | 1 | 0 |  |  |  |  |  |  |  |  |
| GO:0015791\_polyol\_transport | 1 | 0 |  |  |  |  |  |  |  |  |
| GO:0015798\_myo-inositol\_transport | 1 | 0 |  |  |  |  |  |  |  |  |
| GO:0015808\_L-alanine\_transport | 1 | 0 |  |  |  |  |  |  |  |  |
| GO:0015810\_aspartate\_transport | 1 | 0 |  |  |  |  |  |  |  |  |
| GO:0015811\_L-cystine\_transport | 1 | 0 |  |  |  |  |  |  |  |  |
| GO:0015817\_histidine\_transport | 1 | 0 |  |  |  |  |  |  |  |  |
| GO:0015822\_ornithine\_transport | 1 | 0 |  |  |  |  |  |  |  |  |
| GO:0015824\_proline\_transport | 1 | 0 |  |  |  |  |  |  |  |  |
| GO:0015851\_nucleobase\_transport | 1 | 0 |  |  |  |  |  |  |  |  |
| GO:0015864\_pyrimidine\_nucleoside\_transport | 1 | 0 |  |  |  |  |  |  |  |  |
| GO:0015874\_norepinephrine\_transport | 1 | 0 |  |  |  |  |  |  |  |  |
| GO:0015881\_creatine\_transport | 1 | 0 |  |  |  |  |  |  |  |  |
| GO:0015884\_folic\_acid\_transport | 1 | 0 |  |  |  |  |  |  |  |  |
| GO:0015886\_heme\_transport | 1 | 0 |  |  |  |  |  |  |  |  |
| GO:0015888\_thiamin\_transport | 1 | 0 |  |  |  |  |  |  |  |  |
| GO:0015938\_coenzyme\_A\_catabolic\_process | 1 | 0 |  |  |  |  |  |  |  |  |
| GO:0015939\_pantothenate\_metabolic\_process | 1 | 0 |  |  |  |  |  |  |  |  |
| GO:0016073\_snRNA\_metabolic\_process | 1 | 0 |  |  |  |  |  |  |  |  |
| GO:0016074\_snoRNA\_metabolic\_process | 1 | 0 |  |  |  |  |  |  |  |  |
| GO:0016082\_synaptic\_vesicle\_priming | 1 | 0 |  |  |  |  |  |  |  |  |
| GO:0016090\_prenol\_metabolic\_process | 1 | 0 |  |  |  |  |  |  |  |  |
| GO:0016093\_polyprenol\_metabolic\_process | 1 | 0 |  |  |  |  |  |  |  |  |
| GO:0016180\_snRNA\_processing | 1 | 0 |  |  |  |  |  |  |  |  |
| GO:0016239\_positive\_regulation\_of\_macroautophagy | 1 | 0 |  |  |  |  |  |  |  |  |
| GO:0016246\_RNA\_interference | 1 | 0 |  |  |  |  |  |  |  |  |
| GO:0016255\_attachment\_of\_GPI\_anchor\_to\_protein | 1 | 0 |  |  |  |  |  |  |  |  |
| GO:0016333\_morphogenesis\_of\_follicular\_epithelium | 1 | 0 |  |  |  |  |  |  |  |  |
| GO:0016340\_calcium-dependent\_cell-matrix\_adhesion | 1 | 0 |  |  |  |  |  |  |  |  |
| GO:0016344\_meiotic\_chromosome\_movement\_towards\_spindle\_pole | 1 | 0 |  |  |  |  |  |  |  |  |
| GO:0016482\_cytoplasmic\_transport | 1 | 0 |  |  |  |  |  |  |  |  |
| GO:0016553\_base\_conversion\_or\_substitution\_editing | 1 | 0 |  |  |  |  |  |  |  |  |
| GO:0016554\_cytidine\_to\_uridine\_editing | 1 | 0 |  |  |  |  |  |  |  |  |
| GO:0016560\_protein\_import\_into\_peroxisome\_matrix\_\_docking | 1 | 0 |  |  |  |  |  |  |  |  |
| GO:0016578\_histone\_deubiquitination | 1 | 0 |  |  |  |  |  |  |  |  |
| GO:0016598\_protein\_arginylation | 1 | 0 |  |  |  |  |  |  |  |  |
| GO:0017004\_cytochrome\_complex\_assembly | 1 | 0 |  |  |  |  |  |  |  |  |
| GO:0018022\_peptidyl-lysine\_methylation | 1 | 0 |  |  |  |  |  |  |  |  |
| GO:0018023\_peptidyl-lysine\_trimethylation | 1 | 0 |  |  |  |  |  |  |  |  |
| GO:0018120\_peptidyl-arginine\_ADP-ribosylation | 1 | 0 |  |  |  |  |  |  |  |  |
| GO:0018126\_protein\_amino\_acid\_hydroxylation | 1 | 0 |  |  |  |  |  |  |  |  |
| GO:0018146\_keratan\_sulfate\_biosynthetic\_process | 1 | 0 |  |  |  |  |  |  |  |  |
| GO:0018158\_protein\_amino\_acid\_oxidation | 1 | 0 |  |  |  |  |  |  |  |  |
| GO:0018195\_peptidyl-arginine\_modification | 1 | 0 |  |  |  |  |  |  |  |  |
| GO:0018197\_peptidyl-aspartic\_acid\_modification | 1 | 0 |  |  |  |  |  |  |  |  |
| GO:0018282\_metal\_incorporation\_into\_metallo-sulfur\_cluster | 1 | 0 |  |  |  |  |  |  |  |  |
| GO:0018283\_iron\_incorporation\_into\_metallo-sulfur\_cluster | 1 | 0 |  |  |  |  |  |  |  |  |
| GO:0018318\_protein\_amino\_acid\_palmitoylation | 1 | 0 |  |  |  |  |  |  |  |  |
| GO:0018342\_protein\_prenylation | 1 | 0 |  |  |  |  |  |  |  |  |
| GO:0018344\_protein\_geranylgeranylation | 1 | 0 |  |  |  |  |  |  |  |  |
| GO:0018410\_peptide\_or\_protein\_carboxyl-terminal\_blocking | 1 | 0 |  |  |  |  |  |  |  |  |
| GO:0018916\_nitrobenzene\_metabolic\_process | 1 | 0 |  |  |  |  |  |  |  |  |
| GO:0018931\_naphthalene\_metabolic\_process | 1 | 0 |  |  |  |  |  |  |  |  |
| GO:0018992\_germ-line\_sex\_determination | 1 | 0 |  |  |  |  |  |  |  |  |
| GO:0019042\_latent\_virus\_infection | 1 | 0 |  |  |  |  |  |  |  |  |
| GO:0019046\_reactivation\_of\_latent\_virus | 1 | 0 |  |  |  |  |  |  |  |  |
| GO:0019047\_provirus\_integration | 1 | 0 |  |  |  |  |  |  |  |  |
| GO:0019076\_release\_of\_virus\_from\_host | 1 | 0 |  |  |  |  |  |  |  |  |
| GO:0019079\_viral\_genome\_replication | 1 | 0 |  |  |  |  |  |  |  |  |
| GO:0019100\_male\_germ-line\_sex\_determination | 1 | 0 |  |  |  |  |  |  |  |  |
| GO:0019101\_female\_somatic\_sex\_determination | 1 | 0 |  |  |  |  |  |  |  |  |
| GO:0019102\_male\_somatic\_sex\_determination | 1 | 0 |  |  |  |  |  |  |  |  |
| GO:0019255\_glucose\_1-phosphate\_metabolic\_process | 1 | 0 |  |  |  |  |  |  |  |  |
| GO:0019276\_UDP-N-acetylgalactosamine\_metabolic\_process | 1 | 0 |  |  |  |  |  |  |  |  |
| GO:0019344\_cysteine\_biosynthetic\_process | 1 | 0 |  |  |  |  |  |  |  |  |
| GO:0019348\_dolichol\_metabolic\_process | 1 | 0 |  |  |  |  |  |  |  |  |
| GO:0019375\_galactolipid\_biosynthetic\_process | 1 | 0 |  |  |  |  |  |  |  |  |
| GO:0019402\_galactitol\_metabolic\_process | 1 | 0 |  |  |  |  |  |  |  |  |
| GO:0019441\_tryptophan\_catabolic\_process\_to\_kynurenine | 1 | 0 |  |  |  |  |  |  |  |  |
| GO:0019477\_L-lysine\_catabolic\_process | 1 | 0 |  |  |  |  |  |  |  |  |
| GO:0019510\_S-adenosylhomocysteine\_catabolic\_process | 1 | 0 |  |  |  |  |  |  |  |  |
| GO:0019532\_oxalate\_transport | 1 | 0 |  |  |  |  |  |  |  |  |
| GO:0019626\_short-chain\_fatty\_acid\_catabolic\_process | 1 | 0 |  |  |  |  |  |  |  |  |
| GO:0019627\_urea\_metabolic\_process | 1 | 0 |  |  |  |  |  |  |  |  |
| GO:0019676\_ammonia\_assimilation\_cycle | 1 | 0 |  |  |  |  |  |  |  |  |
| GO:0019682\_glyceraldehyde-3-phosphate\_metabolic\_process | 1 | 0 |  |  |  |  |  |  |  |  |
| GO:0019695\_choline\_metabolic\_process | 1 | 0 |  |  |  |  |  |  |  |  |
| GO:0019731\_antibacterial\_humoral\_response | 1 | 0 |  |  |  |  |  |  |  |  |
| GO:0019794\_nonprotein\_amino\_acid\_metabolic\_process | 1 | 0 |  |  |  |  |  |  |  |  |
| GO:0019858\_cytosine\_metabolic\_process | 1 | 0 |  |  |  |  |  |  |  |  |
| GO:0019883\_antigen\_processing\_and\_presentation\_of\_endogenous\_antigen | 1 | 0 |  |  |  |  |  |  |  |  |
| GO:0019889\_pteridine\_metabolic\_process | 1 | 0 |  |  |  |  |  |  |  |  |
| GO:0019896\_axon\_transport\_of\_mitochondrion | 1 | 0 |  |  |  |  |  |  |  |  |
| GO:0021508\_floor\_plate\_formation | 1 | 0 |  |  |  |  |  |  |  |  |
| GO:0021528\_commissural\_neuron\_differentiation\_in\_the\_spinal\_cord | 1 | 0 |  |  |  |  |  |  |  |  |
| GO:0021572\_rhombomere\_6\_development | 1 | 0 |  |  |  |  |  |  |  |  |
| GO:0021577\_hindbrain\_structural\_organization | 1 | 0 |  |  |  |  |  |  |  |  |
| GO:0021586\_pons\_maturation | 1 | 0 |  |  |  |  |  |  |  |  |
| GO:0021589\_cerebellum\_structural\_organization | 1 | 0 |  |  |  |  |  |  |  |  |
| GO:0021590\_cerebellum\_maturation | 1 | 0 |  |  |  |  |  |  |  |  |
| GO:0021592\_fourth\_ventricle\_development | 1 | 0 |  |  |  |  |  |  |  |  |
| GO:0021594\_rhombomere\_formation | 1 | 0 |  |  |  |  |  |  |  |  |
| GO:0021660\_rhombomere\_3\_formation | 1 | 0 |  |  |  |  |  |  |  |  |
| GO:0021664\_rhombomere\_5\_morphogenesis | 1 | 0 |  |  |  |  |  |  |  |  |
| GO:0021666\_rhombomere\_5\_formation | 1 | 0 |  |  |  |  |  |  |  |  |
| GO:0021670\_lateral\_ventricle\_development | 1 | 0 |  |  |  |  |  |  |  |  |
| GO:0021678\_third\_ventricle\_development | 1 | 0 |  |  |  |  |  |  |  |  |
| GO:0021679\_cerebellar\_molecular\_layer\_development | 1 | 0 |  |  |  |  |  |  |  |  |
| GO:0021703\_locus\_ceruleus\_development | 1 | 0 |  |  |  |  |  |  |  |  |
| GO:0021732\_midbrain-hindbrain\_boundary\_maturation | 1 | 0 |  |  |  |  |  |  |  |  |
| GO:0021747\_cochlear\_nucleus\_development | 1 | 0 |  |  |  |  |  |  |  |  |
| GO:0021750\_vestibular\_nucleus\_development | 1 | 0 |  |  |  |  |  |  |  |  |
| GO:0021759\_globus\_pallidus\_development | 1 | 0 |  |  |  |  |  |  |  |  |
| GO:0021768\_nucleus\_accumbens\_development | 1 | 0 |  |  |  |  |  |  |  |  |
| GO:0021771\_lateral\_geniculate\_nucleus\_development | 1 | 0 |  |  |  |  |  |  |  |  |
| GO:0021812\_neuronal-glial\_interaction\_involved\_in\_cerebral\_cortex\_radial\_glia\_guided\_migration | 1 | 0 |  |  |  |  |  |  |  |  |
| GO:0021813\_cell-cell\_adhesion\_involved\_in\_neuronal-glial\_interactions\_involved\_in\_cerebral\_cortex\_radial\_glia\_guided\_migration | 1 | 0 |  |  |  |  |  |  |  |  |
| GO:0021870\_Cajal-Retzius\_cell\_differentiation | 1 | 0 |  |  |  |  |  |  |  |  |
| GO:0021874\_Wnt\_receptor\_signaling\_pathway\_in\_forebrain\_neuroblast\_division | 1 | 0 |  |  |  |  |  |  |  |  |
| GO:0021896\_forebrain\_astrocyte\_differentiation | 1 | 0 |  |  |  |  |  |  |  |  |
| GO:0021897\_forebrain\_astrocyte\_development | 1 | 0 |  |  |  |  |  |  |  |  |
| GO:0021902\_commitment\_of\_a\_neuronal\_cell\_to\_a\_specific\_type\_of\_neuron\_in\_the\_forebrain | 1 | 0 |  |  |  |  |  |  |  |  |
| GO:0021905\_forebrain-midbrain\_boundary\_formation | 1 | 0 |  |  |  |  |  |  |  |  |
| GO:0021914\_negative\_regulation\_of\_smoothened\_signaling\_pathway\_involved\_in\_ventral\_spinal\_cord\_patterning | 1 | 0 |  |  |  |  |  |  |  |  |
| GO:0021917\_somatic\_motor\_neuron\_fate\_commitment | 1 | 0 |  |  |  |  |  |  |  |  |
| GO:0021918\_regulation\_of\_transcription\_from\_RNA\_polymerase\_II\_promoter\_involved\_in\_somatic\_motor\_neuron\_fate\_commitment | 1 | 0 |  |  |  |  |  |  |  |  |
| GO:0021933\_radial\_glia\_guided\_migration\_of\_granule\_cell | 1 | 0 |  |  |  |  |  |  |  |  |
| GO:0021942\_radial\_glia\_guided\_migration\_of\_Purkinje\_cell | 1 | 0 |  |  |  |  |  |  |  |  |
| GO:0021997\_neural\_plate\_axis\_specification | 1 | 0 |  |  |  |  |  |  |  |  |
| GO:0021999\_neural\_plate\_anterior\_posterior\_pattern\_formation | 1 | 0 |  |  |  |  |  |  |  |  |
| GO:0022004\_midbrain-hindbrain\_boundary\_maturation\_during\_brain\_development | 1 | 0 |  |  |  |  |  |  |  |  |
| GO:0022038\_corpus\_callosum\_development | 1 | 0 |  |  |  |  |  |  |  |  |
| GO:0022605\_oogenesis\_stage | 1 | 0 |  |  |  |  |  |  |  |  |
| GO:0030011\_maintenance\_of\_cell\_polarity | 1 | 0 |  |  |  |  |  |  |  |  |
| GO:0030069\_lysogeny | 1 | 0 |  |  |  |  |  |  |  |  |
| GO:0030070\_insulin\_processing | 1 | 0 |  |  |  |  |  |  |  |  |
| GO:0030092\_regulation\_of\_flagellum\_assembly | 1 | 0 |  |  |  |  |  |  |  |  |
| GO:0030103\_vasopressin\_secretion | 1 | 0 |  |  |  |  |  |  |  |  |
| GO:0030194\_positive\_regulation\_of\_blood\_coagulation | 1 | 0 |  |  |  |  |  |  |  |  |
| GO:0030206\_chondroitin\_sulfate\_biosynthetic\_process | 1 | 0 |  |  |  |  |  |  |  |  |
| GO:0030210\_heparin\_biosynthetic\_process | 1 | 0 |  |  |  |  |  |  |  |  |
| GO:0030220\_platelet\_formation | 1 | 0 |  |  |  |  |  |  |  |  |
| GO:0030222\_eosinophil\_differentiation | 1 | 0 |  |  |  |  |  |  |  |  |
| GO:0030237\_female\_sex\_determination | 1 | 0 |  |  |  |  |  |  |  |  |
| GO:0030264\_nuclear\_fragmentation\_during\_apoptosis | 1 | 0 |  |  |  |  |  |  |  |  |
| GO:0030322\_stabilization\_of\_membrane\_potential | 1 | 0 |  |  |  |  |  |  |  |  |
| GO:0030327\_prenylated\_protein\_catabolic\_process | 1 | 0 |  |  |  |  |  |  |  |  |
| GO:0030328\_prenylcysteine\_catabolic\_process | 1 | 0 |  |  |  |  |  |  |  |  |
| GO:0030329\_prenylcysteine\_metabolic\_process | 1 | 0 |  |  |  |  |  |  |  |  |
| GO:0030382\_sperm\_mitochondrion\_organization | 1 | 0 |  |  |  |  |  |  |  |  |
| GO:0030389\_fructosamine\_metabolic\_process | 1 | 0 |  |  |  |  |  |  |  |  |
| GO:0030422\_RNA\_interference\_\_production\_of\_siRNA | 1 | 0 |  |  |  |  |  |  |  |  |
| GO:0030449\_regulation\_of\_complement\_activation | 1 | 0 |  |  |  |  |  |  |  |  |
| GO:0030497\_fatty\_acid\_elongation | 1 | 0 |  |  |  |  |  |  |  |  |
| GO:0030575\_nuclear\_body\_organization | 1 | 0 |  |  |  |  |  |  |  |  |
| GO:0030578\_PML\_body\_organization | 1 | 0 |  |  |  |  |  |  |  |  |
| GO:0030853\_negative\_regulation\_of\_granulocyte\_differentiation | 1 | 0 |  |  |  |  |  |  |  |  |
| GO:0030854\_positive\_regulation\_of\_granulocyte\_differentiation | 1 | 0 |  |  |  |  |  |  |  |  |
| GO:0030886\_negative\_regulation\_of\_myeloid\_dendritic\_cell\_activation | 1 | 0 |  |  |  |  |  |  |  |  |
| GO:0030913\_paranodal\_junction\_assembly | 1 | 0 |  |  |  |  |  |  |  |  |
| GO:0031033\_myosin\_filament\_assembly\_or\_disassembly | 1 | 0 |  |  |  |  |  |  |  |  |
| GO:0031034\_myosin\_filament\_assembly | 1 | 0 |  |  |  |  |  |  |  |  |
| GO:0031055\_chromatin\_remodeling\_at\_centromere | 1 | 0 |  |  |  |  |  |  |  |  |
| GO:0031062\_positive\_regulation\_of\_histone\_methylation | 1 | 0 |  |  |  |  |  |  |  |  |
| GO:0031115\_negative\_regulation\_of\_microtubule\_polymerization | 1 | 0 |  |  |  |  |  |  |  |  |
| GO:0031129\_inductive\_cell-cell\_signaling | 1 | 0 |  |  |  |  |  |  |  |  |
| GO:0031284\_positive\_regulation\_of\_guanylate\_cyclase\_activity | 1 | 0 |  |  |  |  |  |  |  |  |
| GO:0031498\_chromatin\_disassembly | 1 | 0 |  |  |  |  |  |  |  |  |
| GO:0031507\_heterochromatin\_formation | 1 | 0 |  |  |  |  |  |  |  |  |
| GO:0031508\_centromeric\_heterochromatin\_formation | 1 | 0 |  |  |  |  |  |  |  |  |
| GO:0031529\_ruffle\_organization | 1 | 0 |  |  |  |  |  |  |  |  |
| GO:0031536\_positive\_regulation\_of\_exit\_from\_mitosis | 1 | 0 |  |  |  |  |  |  |  |  |
| GO:0031572\_G2\_M\_transition\_DNA\_damage\_checkpoint | 1 | 0 |  |  |  |  |  |  |  |  |
| GO:0031576\_G2\_M\_transition\_checkpoint | 1 | 0 |  |  |  |  |  |  |  |  |
| GO:0031580\_membrane\_raft\_distribution | 1 | 0 |  |  |  |  |  |  |  |  |
| GO:0031583\_activation\_of\_phospholipase\_D\_activity\_by\_G-protein\_coupled\_receptor\_protein\_signaling\_pathway | 1 | 0 |  |  |  |  |  |  |  |  |
| GO:0031584\_activation\_of\_phospholipase\_D\_activity | 1 | 0 |  |  |  |  |  |  |  |  |
| GO:0031585\_regulation\_of\_inositol-1\_4\_5-triphosphate\_receptor\_activity | 1 | 0 |  |  |  |  |  |  |  |  |
| GO:0031639\_plasminogen\_activation | 1 | 0 |  |  |  |  |  |  |  |  |
| GO:0031648\_protein\_destabilization | 1 | 0 |  |  |  |  |  |  |  |  |
| GO:0031665\_negative\_regulation\_of\_lipopolysaccharide-mediated\_signaling\_pathway | 1 | 0 |  |  |  |  |  |  |  |  |
| GO:0031914\_negative\_regulation\_of\_synaptic\_plasticity | 1 | 0 |  |  |  |  |  |  |  |  |
| GO:0031944\_negative\_regulation\_of\_glucocorticoid\_metabolic\_process | 1 | 0 |  |  |  |  |  |  |  |  |
| GO:0031947\_negative\_regulation\_of\_glucocorticoid\_biosynthetic\_process | 1 | 0 |  |  |  |  |  |  |  |  |
| GO:0032025\_response\_to\_cobalt\_ion | 1 | 0 |  |  |  |  |  |  |  |  |
| GO:0032026\_response\_to\_magnesium\_ion | 1 | 0 |  |  |  |  |  |  |  |  |
| GO:0032048\_cardiolipin\_metabolic\_process | 1 | 0 |  |  |  |  |  |  |  |  |
| GO:0032066\_nucleolus\_to\_nucleoplasm\_transport | 1 | 0 |  |  |  |  |  |  |  |  |
| GO:0032091\_negative\_regulation\_of\_protein\_binding | 1 | 0 |  |  |  |  |  |  |  |  |
| GO:0032092\_positive\_regulation\_of\_protein\_binding | 1 | 0 |  |  |  |  |  |  |  |  |
| GO:0032097\_positive\_regulation\_of\_response\_to\_food | 1 | 0 |  |  |  |  |  |  |  |  |
| GO:0032100\_positive\_regulation\_of\_appetite | 1 | 0 |  |  |  |  |  |  |  |  |
| GO:0032204\_regulation\_of\_telomere\_maintenance | 1 | 0 |  |  |  |  |  |  |  |  |
| GO:0032206\_positive\_regulation\_of\_telomere\_maintenance | 1 | 0 |  |  |  |  |  |  |  |  |
| GO:0032222\_regulation\_of\_synaptic\_transmission\_\_cholinergic | 1 | 0 |  |  |  |  |  |  |  |  |
| GO:0032224\_positive\_regulation\_of\_synaptic\_transmission\_\_cholinergic | 1 | 0 |  |  |  |  |  |  |  |  |
| GO:0032229\_negative\_regulation\_of\_synaptic\_transmission\_\_GABAergic | 1 | 0 |  |  |  |  |  |  |  |  |
| GO:0032237\_activation\_of\_store-operated\_calcium\_channel\_activity | 1 | 0 |  |  |  |  |  |  |  |  |
| GO:0032239\_regulation\_of\_nucleobase\_\_nucleoside\_\_nucleotide\_and\_nucleic\_acid\_transport | 1 | 0 |  |  |  |  |  |  |  |  |
| GO:0032252\_secretory\_granule\_localization | 1 | 0 |  |  |  |  |  |  |  |  |
| GO:0032274\_gonadotropin\_secretion | 1 | 0 |  |  |  |  |  |  |  |  |
| GO:0032275\_luteinizing\_hormone\_secretion | 1 | 0 |  |  |  |  |  |  |  |  |
| GO:0032287\_myelin\_maintenance\_in\_the\_peripheral\_nervous\_system | 1 | 0 |  |  |  |  |  |  |  |  |
| GO:0032289\_myelin\_formation\_in\_the\_central\_nervous\_system | 1 | 0 |  |  |  |  |  |  |  |  |
| GO:0032303\_regulation\_of\_icosanoid\_secretion | 1 | 0 |  |  |  |  |  |  |  |  |
| GO:0032305\_positive\_regulation\_of\_icosanoid\_secretion | 1 | 0 |  |  |  |  |  |  |  |  |
| GO:0032306\_regulation\_of\_prostaglandin\_secretion | 1 | 0 |  |  |  |  |  |  |  |  |
| GO:0032308\_positive\_regulation\_of\_prostaglandin\_secretion | 1 | 0 |  |  |  |  |  |  |  |  |
| GO:0032310\_prostaglandin\_secretion | 1 | 0 |  |  |  |  |  |  |  |  |
| GO:0032313\_regulation\_of\_Rab\_GTPase\_activity | 1 | 0 |  |  |  |  |  |  |  |  |
| GO:0032314\_regulation\_of\_Rac\_GTPase\_activity | 1 | 0 |  |  |  |  |  |  |  |  |
| GO:0032317\_regulation\_of\_Rap\_GTPase\_activity | 1 | 0 |  |  |  |  |  |  |  |  |
| GO:0032324\_molybdopterin\_cofactor\_biosynthetic\_process | 1 | 0 |  |  |  |  |  |  |  |  |
| GO:0032329\_serine\_transport | 1 | 0 |  |  |  |  |  |  |  |  |
| GO:0032342\_aldosterone\_biosynthetic\_process | 1 | 0 |  |  |  |  |  |  |  |  |
| GO:0032344\_regulation\_of\_aldosterone\_metabolic\_process | 1 | 0 |  |  |  |  |  |  |  |  |
| GO:0032365\_intracellular\_lipid\_transport | 1 | 0 |  |  |  |  |  |  |  |  |
| GO:0032366\_intracellular\_sterol\_transport | 1 | 0 |  |  |  |  |  |  |  |  |
| GO:0032367\_intracellular\_cholesterol\_transport | 1 | 0 |  |  |  |  |  |  |  |  |
| GO:0032370\_positive\_regulation\_of\_lipid\_transport | 1 | 0 |  |  |  |  |  |  |  |  |
| GO:0032410\_negative\_regulation\_of\_transporter\_activity | 1 | 0 |  |  |  |  |  |  |  |  |
| GO:0032413\_negative\_regulation\_of\_ion\_transmembrane\_transporter\_activity | 1 | 0 |  |  |  |  |  |  |  |  |
| GO:0032429\_regulation\_of\_phospholipase\_A2\_activity | 1 | 0 |  |  |  |  |  |  |  |  |
| GO:0032474\_otolith\_morphogenesis | 1 | 0 |  |  |  |  |  |  |  |  |
| GO:0032482\_Rab\_protein\_signal\_transduction | 1 | 0 |  |  |  |  |  |  |  |  |
| GO:0032483\_regulation\_of\_Rab\_protein\_signal\_transduction | 1 | 0 |  |  |  |  |  |  |  |  |
| GO:0032486\_Rap\_protein\_signal\_transduction | 1 | 0 |  |  |  |  |  |  |  |  |
| GO:0032487\_regulation\_of\_Rap\_protein\_signal\_transduction | 1 | 0 |  |  |  |  |  |  |  |  |
| GO:0032594\_protein\_transport\_within\_lipid\_bilayer | 1 | 0 |  |  |  |  |  |  |  |  |
| GO:0032599\_protein\_transport\_out\_of\_membrane\_raft | 1 | 0 |  |  |  |  |  |  |  |  |
| GO:0032600\_chemokine\_receptor\_transport\_out\_of\_membrane\_raft | 1 | 0 |  |  |  |  |  |  |  |  |
| GO:0032607\_interferon-alpha\_production | 1 | 0 |  |  |  |  |  |  |  |  |
| GO:0032621\_interleukin-18\_production | 1 | 0 |  |  |  |  |  |  |  |  |
| GO:0032647\_regulation\_of\_interferon-alpha\_production | 1 | 0 |  |  |  |  |  |  |  |  |
| GO:0032656\_regulation\_of\_interleukin-13\_production | 1 | 0 |  |  |  |  |  |  |  |  |
| GO:0032682\_negative\_regulation\_of\_chemokine\_production | 1 | 0 |  |  |  |  |  |  |  |  |
| GO:0032691\_negative\_regulation\_of\_interleukin-1\_beta\_production | 1 | 0 |  |  |  |  |  |  |  |  |
| GO:0032692\_negative\_regulation\_of\_interleukin-1\_production | 1 | 0 |  |  |  |  |  |  |  |  |
| GO:0032693\_negative\_regulation\_of\_interleukin-10\_production | 1 | 0 |  |  |  |  |  |  |  |  |
| GO:0032696\_negative\_regulation\_of\_interleukin-13\_production | 1 | 0 |  |  |  |  |  |  |  |  |
| GO:0032727\_positive\_regulation\_of\_interferon-alpha\_production | 1 | 0 |  |  |  |  |  |  |  |  |
| GO:0032731\_positive\_regulation\_of\_interleukin-1\_beta\_production | 1 | 0 |  |  |  |  |  |  |  |  |
| GO:0032732\_positive\_regulation\_of\_interleukin-1\_production | 1 | 0 |  |  |  |  |  |  |  |  |
| GO:0032735\_positive\_regulation\_of\_interleukin-12\_production | 1 | 0 |  |  |  |  |  |  |  |  |
| GO:0032764\_negative\_regulation\_of\_mast\_cell\_cytokine\_production | 1 | 0 |  |  |  |  |  |  |  |  |
| GO:0032765\_positive\_regulation\_of\_mast\_cell\_cytokine\_production | 1 | 0 |  |  |  |  |  |  |  |  |
| GO:0032769\_negative\_regulation\_of\_monooxygenase\_activity | 1 | 0 |  |  |  |  |  |  |  |  |
| GO:0032781\_positive\_regulation\_of\_ATPase\_activity | 1 | 0 |  |  |  |  |  |  |  |  |
| GO:0032790\_ribosome\_disassembly | 1 | 0 |  |  |  |  |  |  |  |  |
| GO:0032799\_low-density\_lipoprotein\_receptor\_metabolic\_process | 1 | 0 |  |  |  |  |  |  |  |  |
| GO:0032802\_low-density\_lipoprotein\_receptor\_catabolic\_process | 1 | 0 |  |  |  |  |  |  |  |  |
| GO:0032803\_regulation\_of\_low-density\_lipoprotein\_receptor\_catabolic\_process | 1 | 0 |  |  |  |  |  |  |  |  |
| GO:0032817\_regulation\_of\_natural\_killer\_cell\_proliferation | 1 | 0 |  |  |  |  |  |  |  |  |
| GO:0032819\_positive\_regulation\_of\_natural\_killer\_cell\_proliferation | 1 | 0 |  |  |  |  |  |  |  |  |
| GO:0032836\_glomerular\_basement\_membrane\_development | 1 | 0 |  |  |  |  |  |  |  |  |
| GO:0032855\_positive\_regulation\_of\_Rac\_GTPase\_activity | 1 | 0 |  |  |  |  |  |  |  |  |
| GO:0032863\_activation\_of\_Rac\_GTPase\_activity | 1 | 0 |  |  |  |  |  |  |  |  |
| GO:0032864\_activation\_of\_Cdc42\_GTPase\_activity | 1 | 0 |  |  |  |  |  |  |  |  |
| GO:0032885\_regulation\_of\_polysaccharide\_biosynthetic\_process | 1 | 0 |  |  |  |  |  |  |  |  |
| GO:0032907\_transforming\_growth\_factor-beta3\_production | 1 | 0 |  |  |  |  |  |  |  |  |
| GO:0032910\_regulation\_of\_transforming\_growth\_factor-beta3\_production | 1 | 0 |  |  |  |  |  |  |  |  |
| GO:0032913\_negative\_regulation\_of\_transforming\_growth\_factor-beta3\_production | 1 | 0 |  |  |  |  |  |  |  |  |
| GO:0032924\_activin\_receptor\_signaling\_pathway | 1 | 0 |  |  |  |  |  |  |  |  |
| GO:0032925\_regulation\_of\_activin\_receptor\_signaling\_pathway | 1 | 0 |  |  |  |  |  |  |  |  |
| GO:0032960\_regulation\_of\_inositol\_trisphosphate\_biosynthetic\_process | 1 | 0 |  |  |  |  |  |  |  |  |
| GO:0032962\_positive\_regulation\_of\_inositol\_trisphosphate\_biosynthetic\_process | 1 | 0 |  |  |  |  |  |  |  |  |
| GO:0032964\_collagen\_biosynthetic\_process | 1 | 0 |  |  |  |  |  |  |  |  |
| GO:0032971\_regulation\_of\_muscle\_filament\_sliding | 1 | 0 |  |  |  |  |  |  |  |  |
| GO:0032972\_regulation\_of\_muscle\_filament\_sliding\_speed | 1 | 0 |  |  |  |  |  |  |  |  |
| GO:0032986\_protein-DNA\_complex\_disassembly | 1 | 0 |  |  |  |  |  |  |  |  |
| GO:0032988\_ribonucleoprotein\_complex\_disassembly | 1 | 0 |  |  |  |  |  |  |  |  |
| GO:0033037\_polysaccharide\_localization | 1 | 0 |  |  |  |  |  |  |  |  |
| GO:0033078\_extrathymic\_T\_cell\_differentiation | 1 | 0 |  |  |  |  |  |  |  |  |
| GO:0033085\_negative\_regulation\_of\_T\_cell\_differentiation\_in\_the\_thymus | 1 | 0 |  |  |  |  |  |  |  |  |
| GO:0033087\_negative\_regulation\_of\_immature\_T\_cell\_proliferation | 1 | 0 |  |  |  |  |  |  |  |  |
| GO:0033088\_negative\_regulation\_of\_immature\_T\_cell\_proliferation\_in\_the\_thymus | 1 | 0 |  |  |  |  |  |  |  |  |
| GO:0033108\_mitochondrial\_respiratory\_chain\_complex\_assembly | 1 | 0 |  |  |  |  |  |  |  |  |
| GO:0033127\_regulation\_of\_histone\_phosphorylation | 1 | 0 |  |  |  |  |  |  |  |  |
| GO:0033128\_negative\_regulation\_of\_histone\_phosphorylation | 1 | 0 |  |  |  |  |  |  |  |  |
| GO:0033138\_positive\_regulation\_of\_peptidyl-serine\_phosphorylation | 1 | 0 |  |  |  |  |  |  |  |  |
| GO:0033158\_regulation\_of\_protein\_import\_into\_nucleus\_\_translocation | 1 | 0 |  |  |  |  |  |  |  |  |
| GO:0033160\_positive\_regulation\_of\_protein\_import\_into\_nucleus\_\_translocation | 1 | 0 |  |  |  |  |  |  |  |  |
| GO:0033169\_histone\_H3-K9\_demethylation | 1 | 0 |  |  |  |  |  |  |  |  |
| GO:0033206\_cytokinesis\_after\_meiosis | 1 | 0 |  |  |  |  |  |  |  |  |
| GO:0033240\_positive\_regulation\_of\_cellular\_amine\_metabolic\_process | 1 | 0 |  |  |  |  |  |  |  |  |
| GO:0033313\_meiotic\_cell\_cycle\_checkpoint | 1 | 0 |  |  |  |  |  |  |  |  |
| GO:0033315\_meiotic\_cell\_cycle\_DNA\_replication\_checkpoint | 1 | 0 |  |  |  |  |  |  |  |  |
| GO:0033326\_cerebrospinal\_fluid\_secretion | 1 | 0 |  |  |  |  |  |  |  |  |
| GO:0033366\_protein\_localization\_in\_secretory\_granule | 1 | 0 |  |  |  |  |  |  |  |  |
| GO:0033367\_protein\_localization\_in\_mast\_cell\_secretory\_granule | 1 | 0 |  |  |  |  |  |  |  |  |
| GO:0033368\_protease\_localization\_in\_mast\_cell\_secretory\_granule | 1 | 0 |  |  |  |  |  |  |  |  |
| GO:0033370\_maintenance\_of\_protein\_location\_in\_mast\_cell\_secretory\_granule | 1 | 0 |  |  |  |  |  |  |  |  |
| GO:0033371\_T\_cell\_secretory\_granule\_organization | 1 | 0 |  |  |  |  |  |  |  |  |
| GO:0033373\_maintenance\_of\_protease\_location\_in\_mast\_cell\_secretory\_granule | 1 | 0 |  |  |  |  |  |  |  |  |
| GO:0033374\_protein\_localization\_in\_T\_cell\_secretory\_granule | 1 | 0 |  |  |  |  |  |  |  |  |
| GO:0033375\_protease\_localization\_in\_T\_cell\_secretory\_granule | 1 | 0 |  |  |  |  |  |  |  |  |
| GO:0033377\_maintenance\_of\_protein\_location\_in\_T\_cell\_secretory\_granule | 1 | 0 |  |  |  |  |  |  |  |  |
| GO:0033379\_maintenance\_of\_protease\_location\_in\_T\_cell\_secretory\_granule | 1 | 0 |  |  |  |  |  |  |  |  |
| GO:0033380\_granzyme\_B\_localization\_in\_T\_cell\_secretory\_granule | 1 | 0 |  |  |  |  |  |  |  |  |
| GO:0033382\_maintenance\_of\_granzyme\_B\_location\_in\_T\_cell\_secretory\_granule | 1 | 0 |  |  |  |  |  |  |  |  |
| GO:0033483\_gas\_homeostasis | 1 | 0 |  |  |  |  |  |  |  |  |
| GO:0033484\_nitric\_oxide\_homeostasis | 1 | 0 |  |  |  |  |  |  |  |  |
| GO:0033505\_floor\_plate\_morphogenesis | 1 | 0 |  |  |  |  |  |  |  |  |
| GO:0033522\_histone\_H2A\_ubiquitination | 1 | 0 |  |  |  |  |  |  |  |  |
| GO:0033523\_histone\_H2B\_ubiquitination | 1 | 0 |  |  |  |  |  |  |  |  |
| GO:0033574\_response\_to\_testosterone\_stimulus | 1 | 0 |  |  |  |  |  |  |  |  |
| GO:0033606\_chemokine\_receptor\_transport\_within\_lipid\_bilayer | 1 | 0 |  |  |  |  |  |  |  |  |
| GO:0033628\_regulation\_of\_cell\_adhesion\_mediated\_by\_integrin | 1 | 0 |  |  |  |  |  |  |  |  |
| GO:0033630\_positive\_regulation\_of\_cell\_adhesion\_mediated\_by\_integrin | 1 | 0 |  |  |  |  |  |  |  |  |
| GO:0033632\_regulation\_of\_cell-cell\_adhesion\_mediated\_by\_integrin | 1 | 0 |  |  |  |  |  |  |  |  |
| GO:0033634\_positive\_regulation\_of\_cell-cell\_adhesion\_mediated\_by\_integrin | 1 | 0 |  |  |  |  |  |  |  |  |
| GO:0033683\_nucleotide-excision\_repair\_\_DNA\_incision | 1 | 0 |  |  |  |  |  |  |  |  |
| GO:0033687\_osteoblast\_proliferation | 1 | 0 |  |  |  |  |  |  |  |  |
| GO:0033688\_regulation\_of\_osteoblast\_proliferation | 1 | 0 |  |  |  |  |  |  |  |  |
| GO:0033689\_negative\_regulation\_of\_osteoblast\_proliferation | 1 | 0 |  |  |  |  |  |  |  |  |
| GO:0033750\_ribosome\_localization | 1 | 0 |  |  |  |  |  |  |  |  |
| GO:0033753\_establishment\_of\_ribosome\_localization | 1 | 0 |  |  |  |  |  |  |  |  |
| GO:0033866\_nucleoside\_bisphosphate\_biosynthetic\_process | 1 | 0 |  |  |  |  |  |  |  |  |
| GO:0033875\_ribonucleoside\_bisphosphate\_metabolic\_process | 1 | 0 |  |  |  |  |  |  |  |  |
| GO:0034030\_ribonucleoside\_bisphosphate\_biosynthetic\_process | 1 | 0 |  |  |  |  |  |  |  |  |
| GO:0034032\_purine\_nucleoside\_bisphosphate\_metabolic\_process | 1 | 0 |  |  |  |  |  |  |  |  |
| GO:0034033\_purine\_nucleoside\_bisphosphate\_biosynthetic\_process | 1 | 0 |  |  |  |  |  |  |  |  |
| GO:0034035\_purine\_ribonucleoside\_bisphosphate\_metabolic\_process | 1 | 0 |  |  |  |  |  |  |  |  |
| GO:0034036\_purine\_ribonucleoside\_bisphosphate\_biosynthetic\_process | 1 | 0 |  |  |  |  |  |  |  |  |
| GO:0034067\_protein\_localization\_in\_Golgi\_apparatus | 1 | 0 |  |  |  |  |  |  |  |  |
| GO:0034102\_erythrocyte\_clearance | 1 | 0 |  |  |  |  |  |  |  |  |
| GO:0034106\_regulation\_of\_erythrocyte\_clearance | 1 | 0 |  |  |  |  |  |  |  |  |
| GO:0034107\_negative\_regulation\_of\_erythrocyte\_clearance | 1 | 0 |  |  |  |  |  |  |  |  |
| GO:0034110\_regulation\_of\_homotypic\_cell-cell\_adhesion | 1 | 0 |  |  |  |  |  |  |  |  |
| GO:0034111\_negative\_regulation\_of\_homotypic\_cell-cell\_adhesion | 1 | 0 |  |  |  |  |  |  |  |  |
| GO:0034117\_erythrocyte\_aggregation | 1 | 0 |  |  |  |  |  |  |  |  |
| GO:0034118\_regulation\_of\_erythrocyte\_aggregation | 1 | 0 |  |  |  |  |  |  |  |  |
| GO:0034119\_negative\_regulation\_of\_erythrocyte\_aggregation | 1 | 0 |  |  |  |  |  |  |  |  |
| GO:0034121\_regulation\_of\_toll-like\_receptor\_signaling\_pathway | 1 | 0 |  |  |  |  |  |  |  |  |
| GO:0034122\_negative\_regulation\_of\_toll-like\_receptor\_signaling\_pathway | 1 | 0 |  |  |  |  |  |  |  |  |
| GO:0034230\_enkephalin\_processing | 1 | 0 |  |  |  |  |  |  |  |  |
| GO:0034372\_very-low-density\_lipoprotein\_particle\_remodeling | 1 | 0 |  |  |  |  |  |  |  |  |
| GO:0034379\_very-low-density\_lipoprotein\_particle\_assembly | 1 | 0 |  |  |  |  |  |  |  |  |
| GO:0034380\_high-density\_lipoprotein\_particle\_assembly | 1 | 0 |  |  |  |  |  |  |  |  |
| GO:0034394\_protein\_localization\_at\_cell\_surface | 1 | 0 |  |  |  |  |  |  |  |  |
| GO:0034405\_response\_to\_fluid\_shear\_stress | 1 | 0 |  |  |  |  |  |  |  |  |
| GO:0034472\_snRNA\_3'-end\_processing | 1 | 0 |  |  |  |  |  |  |  |  |
| GO:0034474\_U2\_snRNA\_3'-end\_processing | 1 | 0 |  |  |  |  |  |  |  |  |
| GO:0034502\_protein\_localization\_to\_chromosome | 1 | 0 |  |  |  |  |  |  |  |  |
| GO:0034505\_tooth\_mineralization | 1 | 0 |  |  |  |  |  |  |  |  |
| GO:0034508\_centromere\_complex\_assembly | 1 | 0 |  |  |  |  |  |  |  |  |
| GO:0034633\_retinol\_transport | 1 | 0 |  |  |  |  |  |  |  |  |
| GO:0034643\_mitochondrion\_localization\_\_microtubule-mediated | 1 | 0 |  |  |  |  |  |  |  |  |
| GO:0034969\_histone\_arginine\_methylation | 1 | 0 |  |  |  |  |  |  |  |  |
| GO:0034982\_mitochondrial\_protein\_processing | 1 | 0 |  |  |  |  |  |  |  |  |
| GO:0035022\_positive\_regulation\_of\_Rac\_protein\_signal\_transduction | 1 | 0 |  |  |  |  |  |  |  |  |
| GO:0035024\_negative\_regulation\_of\_Rho\_protein\_signal\_transduction | 1 | 0 |  |  |  |  |  |  |  |  |
| GO:0035026\_leading\_edge\_cell\_differentiation | 1 | 0 |  |  |  |  |  |  |  |  |
| GO:0035037\_sperm\_entry | 1 | 0 |  |  |  |  |  |  |  |  |
| GO:0035039\_male\_pronucleus\_formation | 1 | 0 |  |  |  |  |  |  |  |  |
| GO:0035066\_positive\_regulation\_of\_histone\_acetylation | 1 | 0 |  |  |  |  |  |  |  |  |
| GO:0035083\_cilium\_axoneme\_assembly | 1 | 0 |  |  |  |  |  |  |  |  |
| GO:0035090\_maintenance\_of\_apical\_basal\_cell\_polarity | 1 | 0 |  |  |  |  |  |  |  |  |
| GO:0035106\_operant\_conditioning | 1 | 0 |  |  |  |  |  |  |  |  |
| GO:0035172\_hemocyte\_proliferation | 1 | 0 |  |  |  |  |  |  |  |  |
| GO:0035227\_regulation\_of\_glutamate-cysteine\_ligase\_activity | 1 | 0 |  |  |  |  |  |  |  |  |
| GO:0035229\_positive\_regulation\_of\_glutamate-cysteine\_ligase\_activity | 1 | 0 |  |  |  |  |  |  |  |  |
| GO:0035260\_internal\_genitalia\_morphogenesis | 1 | 0 |  |  |  |  |  |  |  |  |
| GO:0035262\_gonad\_morphogenesis | 1 | 0 |  |  |  |  |  |  |  |  |
| GO:0035287\_head\_segmentation | 1 | 0 |  |  |  |  |  |  |  |  |
| GO:0035289\_posterior\_head\_segmentation | 1 | 0 |  |  |  |  |  |  |  |  |
| GO:0035303\_regulation\_of\_dephosphorylation | 1 | 0 |  |  |  |  |  |  |  |  |
| GO:0035304\_regulation\_of\_protein\_amino\_acid\_dephosphorylation | 1 | 0 |  |  |  |  |  |  |  |  |
| GO:0035305\_negative\_regulation\_of\_dephosphorylation | 1 | 0 |  |  |  |  |  |  |  |  |
| GO:0035308\_negative\_regulation\_of\_protein\_amino\_acid\_dephosphorylation | 1 | 0 |  |  |  |  |  |  |  |  |
| GO:0035313\_wound\_healing\_\_spreading\_of\_epidermal\_cells | 1 | 0 |  |  |  |  |  |  |  |  |
| GO:0040013\_negative\_regulation\_of\_locomotion | 1 | 0 |  |  |  |  |  |  |  |  |
| GO:0040019\_positive\_regulation\_of\_embryonic\_development | 1 | 0 |  |  |  |  |  |  |  |  |
| GO:0040032\_post-embryonic\_body\_morphogenesis | 1 | 0 |  |  |  |  |  |  |  |  |
| GO:0040038\_polar\_body\_extrusion\_after\_meiotic\_divisions | 1 | 0 |  |  |  |  |  |  |  |  |
| GO:0042026\_protein\_refolding | 1 | 0 |  |  |  |  |  |  |  |  |
| GO:0042048\_olfactory\_behavior | 1 | 0 |  |  |  |  |  |  |  |  |
| GO:0042059\_negative\_regulation\_of\_epidermal\_growth\_factor\_receptor\_signaling\_pathway | 1 | 0 |  |  |  |  |  |  |  |  |
| GO:0042073\_intraflagellar\_transport | 1 | 0 |  |  |  |  |  |  |  |  |
| GO:0042078\_germ-line\_stem\_cell\_division | 1 | 0 |  |  |  |  |  |  |  |  |
| GO:0042091\_interleukin-10\_biosynthetic\_process | 1 | 0 |  |  |  |  |  |  |  |  |
| GO:0042103\_positive\_regulation\_of\_T\_cell\_homeostatic\_proliferation | 1 | 0 |  |  |  |  |  |  |  |  |
| GO:0042136\_neurotransmitter\_biosynthetic\_process | 1 | 0 |  |  |  |  |  |  |  |  |
| GO:0042137\_sequestering\_of\_neurotransmitter | 1 | 0 |  |  |  |  |  |  |  |  |
| GO:0042138\_meiotic\_DNA\_double-strand\_break\_formation | 1 | 0 |  |  |  |  |  |  |  |  |
| GO:0042178\_xenobiotic\_catabolic\_process | 1 | 0 |  |  |  |  |  |  |  |  |
| GO:0042225\_interleukin-5\_biosynthetic\_process | 1 | 0 |  |  |  |  |  |  |  |  |
| GO:0042231\_interleukin-13\_biosynthetic\_process | 1 | 0 |  |  |  |  |  |  |  |  |
| GO:0042255\_ribosome\_assembly | 1 | 0 |  |  |  |  |  |  |  |  |
| GO:0042257\_ribosomal\_subunit\_assembly | 1 | 0 |  |  |  |  |  |  |  |  |
| GO:0042264\_peptidyl-aspartic\_acid\_hydroxylation | 1 | 0 |  |  |  |  |  |  |  |  |
| GO:0042276\_error-prone\_postreplication\_DNA\_repair | 1 | 0 |  |  |  |  |  |  |  |  |
| GO:0042297\_vocal\_learning | 1 | 0 |  |  |  |  |  |  |  |  |
| GO:0042309\_homoiothermy | 1 | 0 |  |  |  |  |  |  |  |  |
| GO:0042320\_regulation\_of\_circadian\_sleep\_wake\_cycle\_\_REM\_sleep | 1 | 0 |  |  |  |  |  |  |  |  |
| GO:0042339\_keratan\_sulfate\_metabolic\_process | 1 | 0 |  |  |  |  |  |  |  |  |
| GO:0042347\_negative\_regulation\_of\_NF-kappaB\_import\_into\_nucleus | 1 | 0 |  |  |  |  |  |  |  |  |
| GO:0042360\_vitamin\_E\_metabolic\_process | 1 | 0 |  |  |  |  |  |  |  |  |
| GO:0042363\_fat-soluble\_vitamin\_catabolic\_process | 1 | 0 |  |  |  |  |  |  |  |  |
| GO:0042369\_vitamin\_D\_catabolic\_process | 1 | 0 |  |  |  |  |  |  |  |  |
| GO:0042373\_vitamin\_K\_metabolic\_process | 1 | 0 |  |  |  |  |  |  |  |  |
| GO:0042404\_thyroid\_hormone\_catabolic\_process | 1 | 0 |  |  |  |  |  |  |  |  |
| GO:0042414\_epinephrine\_metabolic\_process | 1 | 0 |  |  |  |  |  |  |  |  |
| GO:0042436\_indole\_derivative\_catabolic\_process | 1 | 0 |  |  |  |  |  |  |  |  |
| GO:0042489\_negative\_regulation\_of\_odontogenesis\_of\_dentine-containing\_tooth | 1 | 0 |  |  |  |  |  |  |  |  |
| GO:0042508\_tyrosine\_phosphorylation\_of\_Stat1\_protein | 1 | 0 |  |  |  |  |  |  |  |  |
| GO:0042518\_negative\_regulation\_of\_tyrosine\_phosphorylation\_of\_Stat3\_protein | 1 | 0 |  |  |  |  |  |  |  |  |
| GO:0042524\_negative\_regulation\_of\_tyrosine\_phosphorylation\_of\_Stat5\_protein | 1 | 0 |  |  |  |  |  |  |  |  |
| GO:0042536\_negative\_regulation\_of\_tumor\_necrosis\_factor\_biosynthetic\_process | 1 | 0 |  |  |  |  |  |  |  |  |
| GO:0042538\_hyperosmotic\_salinity\_response | 1 | 0 |  |  |  |  |  |  |  |  |
| GO:0042628\_mating\_plug\_formation | 1 | 0 |  |  |  |  |  |  |  |  |
| GO:0042631\_cellular\_response\_to\_water\_deprivation | 1 | 0 |  |  |  |  |  |  |  |  |
| GO:0042637\_catagen | 1 | 0 |  |  |  |  |  |  |  |  |
| GO:0042660\_positive\_regulation\_of\_cell\_fate\_specification | 1 | 0 |  |  |  |  |  |  |  |  |
| GO:0042663\_regulation\_of\_endodermal\_cell\_fate\_specification | 1 | 0 |  |  |  |  |  |  |  |  |
| GO:0042664\_negative\_regulation\_of\_endodermal\_cell\_fate\_specification | 1 | 0 |  |  |  |  |  |  |  |  |
| GO:0042667\_auditory\_receptor\_cell\_fate\_specification | 1 | 0 |  |  |  |  |  |  |  |  |
| GO:0042694\_muscle\_cell\_fate\_specification | 1 | 0 |  |  |  |  |  |  |  |  |
| GO:0042706\_eye\_photoreceptor\_cell\_fate\_commitment | 1 | 0 |  |  |  |  |  |  |  |  |
| GO:0042713\_sperm\_ejaculation | 1 | 0 |  |  |  |  |  |  |  |  |
| GO:0042723\_thiamin\_and\_derivative\_metabolic\_process | 1 | 0 |  |  |  |  |  |  |  |  |
| GO:0042737\_drug\_catabolic\_process | 1 | 0 |  |  |  |  |  |  |  |  |
| GO:0042738\_exogenous\_drug\_catabolic\_process | 1 | 0 |  |  |  |  |  |  |  |  |
| GO:0042747\_circadian\_sleep\_wake\_cycle\_\_REM\_sleep | 1 | 0 |  |  |  |  |  |  |  |  |
| GO:0042748\_circadian\_sleep\_wake\_cycle\_\_non-REM\_sleep | 1 | 0 |  |  |  |  |  |  |  |  |
| GO:0042772\_DNA\_damage\_response\_\_signal\_transduction\_resulting\_in\_transcription | 1 | 0 |  |  |  |  |  |  |  |  |
| GO:0042790\_transcription\_of\_nuclear\_rRNA\_large\_RNA\_polymerase\_I\_transcript | 1 | 0 |  |  |  |  |  |  |  |  |
| GO:0042839\_D-glucuronate\_metabolic\_process | 1 | 0 |  |  |  |  |  |  |  |  |
| GO:0042840\_D-glucuronate\_catabolic\_process | 1 | 0 |  |  |  |  |  |  |  |  |
| GO:0042891\_antibiotic\_transport | 1 | 0 |  |  |  |  |  |  |  |  |
| GO:0042892\_chloramphenicol\_transport | 1 | 0 |  |  |  |  |  |  |  |  |
| GO:0042940\_D-amino\_acid\_transport | 1 | 0 |  |  |  |  |  |  |  |  |
| GO:0042941\_D-alanine\_transport | 1 | 0 |  |  |  |  |  |  |  |  |
| GO:0042942\_D-serine\_transport | 1 | 0 |  |  |  |  |  |  |  |  |
| GO:0042983\_amyloid\_precursor\_protein\_biosynthetic\_process | 1 | 0 |  |  |  |  |  |  |  |  |
| GO:0042984\_regulation\_of\_amyloid\_precursor\_protein\_biosynthetic\_process | 1 | 0 |  |  |  |  |  |  |  |  |
| GO:0042985\_negative\_regulation\_of\_amyloid\_precursor\_protein\_biosynthetic\_process | 1 | 0 |  |  |  |  |  |  |  |  |
| GO:0042989\_sequestering\_of\_actin\_monomers | 1 | 0 |  |  |  |  |  |  |  |  |
| GO:0043044\_ATP-dependent\_chromatin\_remodeling | 1 | 0 |  |  |  |  |  |  |  |  |
| GO:0043056\_forward\_locomotion | 1 | 0 |  |  |  |  |  |  |  |  |
| GO:0043060\_meiotic\_metaphase\_I\_plate\_congression | 1 | 0 |  |  |  |  |  |  |  |  |
| GO:0043091\_L-arginine\_import | 1 | 0 |  |  |  |  |  |  |  |  |
| GO:0043124\_negative\_regulation\_of\_I-kappaB\_kinase\_NF-kappaB\_cascade | 1 | 0 |  |  |  |  |  |  |  |  |
| GO:0043132\_NAD\_transport | 1 | 0 |  |  |  |  |  |  |  |  |
| GO:0043153\_entrainment\_of\_circadian\_clock\_by\_photoperiod | 1 | 0 |  |  |  |  |  |  |  |  |
| GO:0043171\_peptide\_catabolic\_process | 1 | 0 |  |  |  |  |  |  |  |  |
| GO:0043179\_rhythmic\_excitation | 1 | 0 |  |  |  |  |  |  |  |  |
| GO:0043206\_fibril\_organization | 1 | 0 |  |  |  |  |  |  |  |  |
| GO:0043217\_myelin\_maintenance | 1 | 0 |  |  |  |  |  |  |  |  |
| GO:0043313\_regulation\_of\_neutrophil\_degranulation | 1 | 0 |  |  |  |  |  |  |  |  |
| GO:0043369\_CD4-positive\_or\_CD8-positive\_\_alpha-beta\_T\_cell\_lineage\_commitment | 1 | 0 |  |  |  |  |  |  |  |  |
| GO:0043375\_CD8-positive\_\_alpha-beta\_T\_cell\_lineage\_commitment | 1 | 0 |  |  |  |  |  |  |  |  |
| GO:0043379\_memory\_T\_cell\_differentiation | 1 | 0 |  |  |  |  |  |  |  |  |
| GO:0043380\_regulation\_of\_memory\_T\_cell\_differentiation | 1 | 0 |  |  |  |  |  |  |  |  |
| GO:0043400\_cortisol\_secretion | 1 | 0 |  |  |  |  |  |  |  |  |
| GO:0043415\_positive\_regulation\_of\_skeletal\_muscle\_regeneration | 1 | 0 |  |  |  |  |  |  |  |  |
| GO:0043416\_regulation\_of\_skeletal\_muscle\_regeneration | 1 | 0 |  |  |  |  |  |  |  |  |
| GO:0043437\_butanoic\_acid\_metabolic\_process | 1 | 0 |  |  |  |  |  |  |  |  |
| GO:0043438\_acetoacetic\_acid\_metabolic\_process | 1 | 0 |  |  |  |  |  |  |  |  |
| GO:0043480\_pigment\_accumulation\_in\_tissues | 1 | 0 |  |  |  |  |  |  |  |  |
| GO:0043482\_cellular\_pigment\_accumulation | 1 | 0 |  |  |  |  |  |  |  |  |
| GO:0043486\_histone\_exchange | 1 | 0 |  |  |  |  |  |  |  |  |
| GO:0043496\_regulation\_of\_protein\_homodimerization\_activity | 1 | 0 |  |  |  |  |  |  |  |  |
| GO:0043501\_skeletal\_muscle\_adaptation | 1 | 0 |  |  |  |  |  |  |  |  |
| GO:0043508\_negative\_regulation\_of\_JUN\_kinase\_activity | 1 | 0 |  |  |  |  |  |  |  |  |
| GO:0043517\_positive\_regulation\_of\_DNA\_damage\_response\_\_signal\_transduction\_by\_p53\_class\_mediator | 1 | 0 |  |  |  |  |  |  |  |  |
| GO:0043535\_regulation\_of\_blood\_vessel\_endothelial\_cell\_migration | 1 | 0 |  |  |  |  |  |  |  |  |
| GO:0043537\_negative\_regulation\_of\_blood\_vessel\_endothelial\_cell\_migration | 1 | 0 |  |  |  |  |  |  |  |  |
| GO:0043545\_molybdopterin\_cofactor\_metabolic\_process | 1 | 0 |  |  |  |  |  |  |  |  |
| GO:0043587\_tongue\_morphogenesis | 1 | 0 |  |  |  |  |  |  |  |  |
| GO:0043604\_amide\_biosynthetic\_process | 1 | 0 |  |  |  |  |  |  |  |  |
| GO:0043628\_ncRNA\_3'-end\_processing | 1 | 0 |  |  |  |  |  |  |  |  |
| GO:0044254\_multicellular\_organismal\_protein\_catabolic\_process | 1 | 0 |  |  |  |  |  |  |  |  |
| GO:0044256\_protein\_digestion | 1 | 0 |  |  |  |  |  |  |  |  |
| GO:0044266\_multicellular\_organismal\_macromolecule\_catabolic\_process | 1 | 0 |  |  |  |  |  |  |  |  |
| GO:0045004\_DNA\_replication\_proofreading | 1 | 0 |  |  |  |  |  |  |  |  |
| GO:0045019\_negative\_regulation\_of\_nitric\_oxide\_biosynthetic\_process | 1 | 0 |  |  |  |  |  |  |  |  |
| GO:0045020\_error-prone\_DNA\_repair | 1 | 0 |  |  |  |  |  |  |  |  |
| GO:0045022\_early\_endosome\_to\_late\_endosome\_transport | 1 | 0 |  |  |  |  |  |  |  |  |
| GO:0045062\_extrathymic\_T\_cell\_selection | 1 | 0 |  |  |  |  |  |  |  |  |
| GO:0045069\_regulation\_of\_viral\_genome\_replication | 1 | 0 |  |  |  |  |  |  |  |  |
| GO:0045074\_regulation\_of\_interleukin-10\_biosynthetic\_process | 1 | 0 |  |  |  |  |  |  |  |  |
| GO:0045082\_positive\_regulation\_of\_interleukin-10\_biosynthetic\_process | 1 | 0 |  |  |  |  |  |  |  |  |
| GO:0045083\_negative\_regulation\_of\_interleukin-12\_biosynthetic\_process | 1 | 0 |  |  |  |  |  |  |  |  |
| GO:0045112\_integrin\_biosynthetic\_process | 1 | 0 |  |  |  |  |  |  |  |  |
| GO:0045113\_regulation\_of\_integrin\_biosynthetic\_process | 1 | 0 |  |  |  |  |  |  |  |  |
| GO:0045188\_regulation\_of\_circadian\_sleep\_wake\_cycle\_\_non-REM\_sleep | 1 | 0 |  |  |  |  |  |  |  |  |
| GO:0045210\_FasL\_biosynthetic\_process | 1 | 0 |  |  |  |  |  |  |  |  |
| GO:0045297\_post-mating\_behavior | 1 | 0 |  |  |  |  |  |  |  |  |
| GO:0045299\_otolith\_mineralization | 1 | 0 |  |  |  |  |  |  |  |  |
| GO:0045329\_carnitine\_biosynthetic\_process | 1 | 0 |  |  |  |  |  |  |  |  |
| GO:0045341\_MHC\_class\_I\_biosynthetic\_process | 1 | 0 |  |  |  |  |  |  |  |  |
| GO:0045343\_regulation\_of\_MHC\_class\_I\_biosynthetic\_process | 1 | 0 |  |  |  |  |  |  |  |  |
| GO:0045347\_negative\_regulation\_of\_MHC\_class\_II\_biosynthetic\_process | 1 | 0 |  |  |  |  |  |  |  |  |
| GO:0045405\_regulation\_of\_interleukin-5\_biosynthetic\_process | 1 | 0 |  |  |  |  |  |  |  |  |
| GO:0045407\_positive\_regulation\_of\_interleukin-5\_biosynthetic\_process | 1 | 0 |  |  |  |  |  |  |  |  |
| GO:0045426\_quinone\_cofactor\_biosynthetic\_process | 1 | 0 |  |  |  |  |  |  |  |  |
| GO:0045448\_mitotic\_cell\_cycle\_\_embryonic | 1 | 0 |  |  |  |  |  |  |  |  |
| GO:0045454\_cell\_redox\_homeostasis | 1 | 0 |  |  |  |  |  |  |  |  |
| GO:0045583\_regulation\_of\_cytotoxic\_T\_cell\_differentiation | 1 | 0 |  |  |  |  |  |  |  |  |
| GO:0045585\_positive\_regulation\_of\_cytotoxic\_T\_cell\_differentiation | 1 | 0 |  |  |  |  |  |  |  |  |
| GO:0045601\_regulation\_of\_endothelial\_cell\_differentiation | 1 | 0 |  |  |  |  |  |  |  |  |
| GO:0045602\_negative\_regulation\_of\_endothelial\_cell\_differentiation | 1 | 0 |  |  |  |  |  |  |  |  |
| GO:0045605\_negative\_regulation\_of\_epidermal\_cell\_differentiation | 1 | 0 |  |  |  |  |  |  |  |  |
| GO:0045606\_positive\_regulation\_of\_epidermal\_cell\_differentiation | 1 | 0 |  |  |  |  |  |  |  |  |
| GO:0045609\_positive\_regulation\_of\_auditory\_receptor\_cell\_differentiation | 1 | 0 |  |  |  |  |  |  |  |  |
| GO:0045617\_negative\_regulation\_of\_keratinocyte\_differentiation | 1 | 0 |  |  |  |  |  |  |  |  |
| GO:0045618\_positive\_regulation\_of\_keratinocyte\_differentiation | 1 | 0 |  |  |  |  |  |  |  |  |
| GO:0045626\_negative\_regulation\_of\_T-helper\_1\_cell\_differentiation | 1 | 0 |  |  |  |  |  |  |  |  |
| GO:0045633\_positive\_regulation\_of\_mechanoreceptor\_differentiation | 1 | 0 |  |  |  |  |  |  |  |  |
| GO:0045650\_negative\_regulation\_of\_macrophage\_differentiation | 1 | 0 |  |  |  |  |  |  |  |  |
| GO:0045656\_negative\_regulation\_of\_monocyte\_differentiation | 1 | 0 |  |  |  |  |  |  |  |  |
| GO:0045657\_positive\_regulation\_of\_monocyte\_differentiation | 1 | 0 |  |  |  |  |  |  |  |  |
| GO:0045659\_negative\_regulation\_of\_neutrophil\_differentiation | 1 | 0 |  |  |  |  |  |  |  |  |
| GO:0045660\_positive\_regulation\_of\_neutrophil\_differentiation | 1 | 0 |  |  |  |  |  |  |  |  |
| GO:0045721\_negative\_regulation\_of\_gluconeogenesis | 1 | 0 |  |  |  |  |  |  |  |  |
| GO:0045724\_positive\_regulation\_of\_flagellum\_assembly | 1 | 0 |  |  |  |  |  |  |  |  |
| GO:0045725\_positive\_regulation\_of\_glycogen\_biosynthetic\_process | 1 | 0 |  |  |  |  |  |  |  |  |
| GO:0045740\_positive\_regulation\_of\_DNA\_replication | 1 | 0 |  |  |  |  |  |  |  |  |
| GO:0045759\_negative\_regulation\_of\_action\_potential | 1 | 0 |  |  |  |  |  |  |  |  |
| GO:0045768\_positive\_regulation\_of\_anti-apoptosis | 1 | 0 |  |  |  |  |  |  |  |  |
| GO:0045769\_negative\_regulation\_of\_asymmetric\_cell\_division | 1 | 0 |  |  |  |  |  |  |  |  |
| GO:0045794\_negative\_regulation\_of\_cell\_volume | 1 | 0 |  |  |  |  |  |  |  |  |
| GO:0045815\_positive\_regulation\_of\_gene\_expression\_\_epigenetic | 1 | 0 |  |  |  |  |  |  |  |  |
| GO:0045818\_negative\_regulation\_of\_glycogen\_catabolic\_process | 1 | 0 |  |  |  |  |  |  |  |  |
| GO:0045842\_positive\_regulation\_of\_mitotic\_metaphase\_anaphase\_transition | 1 | 0 |  |  |  |  |  |  |  |  |
| GO:0045875\_negative\_regulation\_of\_sister\_chromatid\_cohesion | 1 | 0 |  |  |  |  |  |  |  |  |
| GO:0045898\_regulation\_of\_transcriptional\_preinitiation\_complex\_assembly | 1 | 0 |  |  |  |  |  |  |  |  |
| GO:0045899\_positive\_regulation\_of\_transcriptional\_preinitiation\_complex\_assembly | 1 | 0 |  |  |  |  |  |  |  |  |
| GO:0045906\_negative\_regulation\_of\_vasoconstriction | 1 | 0 |  |  |  |  |  |  |  |  |
| GO:0045908\_negative\_regulation\_of\_vasodilation | 1 | 0 |  |  |  |  |  |  |  |  |
| GO:0045909\_positive\_regulation\_of\_vasodilation | 1 | 0 |  |  |  |  |  |  |  |  |
| GO:0045915\_positive\_regulation\_of\_catecholamine\_metabolic\_process | 1 | 0 |  |  |  |  |  |  |  |  |
| GO:0045920\_negative\_regulation\_of\_exocytosis | 1 | 0 |  |  |  |  |  |  |  |  |
| GO:0045924\_regulation\_of\_female\_receptivity | 1 | 0 |  |  |  |  |  |  |  |  |
| GO:0045947\_negative\_regulation\_of\_translational\_initiation | 1 | 0 |  |  |  |  |  |  |  |  |
| GO:0045955\_negative\_regulation\_of\_calcium\_ion-dependent\_exocytosis | 1 | 0 |  |  |  |  |  |  |  |  |
| GO:0045956\_positive\_regulation\_of\_calcium\_ion-dependent\_exocytosis | 1 | 0 |  |  |  |  |  |  |  |  |
| GO:0045964\_positive\_regulation\_of\_dopamine\_metabolic\_process | 1 | 0 |  |  |  |  |  |  |  |  |
| GO:0045989\_positive\_regulation\_of\_striated\_muscle\_contraction | 1 | 0 |  |  |  |  |  |  |  |  |
| GO:0045990\_regulation\_of\_transcription\_by\_carbon\_catabolites | 1 | 0 |  |  |  |  |  |  |  |  |
| GO:0045991\_positive\_regulation\_of\_transcription\_by\_carbon\_catabolites | 1 | 0 |  |  |  |  |  |  |  |  |
| GO:0045994\_positive\_regulation\_of\_translational\_initiation\_by\_iron | 1 | 0 |  |  |  |  |  |  |  |  |
| GO:0046007\_negative\_regulation\_of\_activated\_T\_cell\_proliferation | 1 | 0 |  |  |  |  |  |  |  |  |
| GO:0046014\_negative\_regulation\_of\_T\_cell\_homeostatic\_proliferation | 1 | 0 |  |  |  |  |  |  |  |  |
| GO:0046015\_regulation\_of\_transcription\_by\_glucose | 1 | 0 |  |  |  |  |  |  |  |  |
| GO:0046016\_positive\_regulation\_of\_transcription\_by\_glucose | 1 | 0 |  |  |  |  |  |  |  |  |
| GO:0046031\_ADP\_metabolic\_process | 1 | 0 |  |  |  |  |  |  |  |  |
| GO:0046032\_ADP\_catabolic\_process | 1 | 0 |  |  |  |  |  |  |  |  |
| GO:0046061\_dATP\_catabolic\_process | 1 | 0 |  |  |  |  |  |  |  |  |
| GO:0046075\_dTTP\_metabolic\_process | 1 | 0 |  |  |  |  |  |  |  |  |
| GO:0046078\_dUMP\_metabolic\_process | 1 | 0 |  |  |  |  |  |  |  |  |
| GO:0046079\_dUMP\_catabolic\_process | 1 | 0 |  |  |  |  |  |  |  |  |
| GO:0046086\_adenosine\_biosynthetic\_process | 1 | 0 |  |  |  |  |  |  |  |  |
| GO:0046090\_deoxyadenosine\_metabolic\_process | 1 | 0 |  |  |  |  |  |  |  |  |
| GO:0046098\_guanine\_metabolic\_process | 1 | 0 |  |  |  |  |  |  |  |  |
| GO:0046101\_hypoxanthine\_biosynthetic\_process | 1 | 0 |  |  |  |  |  |  |  |  |
| GO:0046102\_inosine\_metabolic\_process | 1 | 0 |  |  |  |  |  |  |  |  |
| GO:0046103\_inosine\_biosynthetic\_process | 1 | 0 |  |  |  |  |  |  |  |  |
| GO:0046108\_uridine\_metabolic\_process | 1 | 0 |  |  |  |  |  |  |  |  |
| GO:0046110\_xanthine\_metabolic\_process | 1 | 0 |  |  |  |  |  |  |  |  |
| GO:0046111\_xanthine\_biosynthetic\_process | 1 | 0 |  |  |  |  |  |  |  |  |
| GO:0046112\_nucleobase\_biosynthetic\_process | 1 | 0 |  |  |  |  |  |  |  |  |
| GO:0046113\_nucleobase\_catabolic\_process | 1 | 0 |  |  |  |  |  |  |  |  |
| GO:0046121\_deoxyribonucleoside\_catabolic\_process | 1 | 0 |  |  |  |  |  |  |  |  |
| GO:0046122\_purine\_deoxyribonucleoside\_metabolic\_process | 1 | 0 |  |  |  |  |  |  |  |  |
| GO:0046124\_purine\_deoxyribonucleoside\_catabolic\_process | 1 | 0 |  |  |  |  |  |  |  |  |
| GO:0046125\_pyrimidine\_deoxyribonucleoside\_metabolic\_process | 1 | 0 |  |  |  |  |  |  |  |  |
| GO:0046131\_pyrimidine\_ribonucleoside\_metabolic\_process | 1 | 0 |  |  |  |  |  |  |  |  |
| GO:0046160\_heme\_a\_metabolic\_process | 1 | 0 |  |  |  |  |  |  |  |  |
| GO:0046218\_indolalkylamine\_catabolic\_process | 1 | 0 |  |  |  |  |  |  |  |  |
| GO:0046292\_formaldehyde\_metabolic\_process | 1 | 0 |  |  |  |  |  |  |  |  |
| GO:0046294\_formaldehyde\_catabolic\_process | 1 | 0 |  |  |  |  |  |  |  |  |
| GO:0046314\_phosphocreatine\_biosynthetic\_process | 1 | 0 |  |  |  |  |  |  |  |  |
| GO:0046327\_glycerol\_biosynthetic\_process\_from\_pyruvate | 1 | 0 |  |  |  |  |  |  |  |  |
| GO:0046329\_negative\_regulation\_of\_JNK\_cascade | 1 | 0 |  |  |  |  |  |  |  |  |
| GO:0046340\_diacylglycerol\_catabolic\_process | 1 | 0 |  |  |  |  |  |  |  |  |
| GO:0046351\_disaccharide\_biosynthetic\_process | 1 | 0 |  |  |  |  |  |  |  |  |
| GO:0046356\_acetyl-CoA\_catabolic\_process | 1 | 0 |  |  |  |  |  |  |  |  |
| GO:0046358\_butyrate\_biosynthetic\_process | 1 | 0 |  |  |  |  |  |  |  |  |
| GO:0046359\_butyrate\_catabolic\_process | 1 | 0 |  |  |  |  |  |  |  |  |
| GO:0046381\_CMP-N-acetylneuraminate\_metabolic\_process | 1 | 0 |  |  |  |  |  |  |  |  |
| GO:0046415\_urate\_metabolic\_process | 1 | 0 |  |  |  |  |  |  |  |  |
| GO:0046416\_D-amino\_acid\_metabolic\_process | 1 | 0 |  |  |  |  |  |  |  |  |
| GO:0046434\_organophosphate\_catabolic\_process | 1 | 0 |  |  |  |  |  |  |  |  |
| GO:0046437\_D-amino\_acid\_biosynthetic\_process | 1 | 0 |  |  |  |  |  |  |  |  |
| GO:0046440\_L-lysine\_metabolic\_process | 1 | 0 |  |  |  |  |  |  |  |  |
| GO:0046449\_creatinine\_metabolic\_process | 1 | 0 |  |  |  |  |  |  |  |  |
| GO:0046471\_phosphatidylglycerol\_metabolic\_process | 1 | 0 |  |  |  |  |  |  |  |  |
| GO:0046473\_phosphatidic\_acid\_metabolic\_process | 1 | 0 |  |  |  |  |  |  |  |  |
| GO:0046476\_glycosylceramide\_biosynthetic\_process | 1 | 0 |  |  |  |  |  |  |  |  |
| GO:0046477\_glycosylceramide\_catabolic\_process | 1 | 0 |  |  |  |  |  |  |  |  |
| GO:0046485\_ether\_lipid\_metabolic\_process | 1 | 0 |  |  |  |  |  |  |  |  |
| GO:0046487\_glyoxylate\_metabolic\_process | 1 | 0 |  |  |  |  |  |  |  |  |
| GO:0046498\_S-adenosylhomocysteine\_metabolic\_process | 1 | 0 |  |  |  |  |  |  |  |  |
| GO:0046552\_photoreceptor\_cell\_fate\_commitment | 1 | 0 |  |  |  |  |  |  |  |  |
| GO:0046586\_regulation\_of\_calcium-dependent\_cell-cell\_adhesion | 1 | 0 |  |  |  |  |  |  |  |  |
| GO:0046587\_positive\_regulation\_of\_calcium-dependent\_cell-cell\_adhesion | 1 | 0 |  |  |  |  |  |  |  |  |
| GO:0046602\_regulation\_of\_mitotic\_centrosome\_separation | 1 | 0 |  |  |  |  |  |  |  |  |
| GO:0046604\_positive\_regulation\_of\_mitotic\_centrosome\_separation | 1 | 0 |  |  |  |  |  |  |  |  |
| GO:0046607\_positive\_regulation\_of\_centrosome\_cycle | 1 | 0 |  |  |  |  |  |  |  |  |
| GO:0046655\_folic\_acid\_metabolic\_process | 1 | 0 |  |  |  |  |  |  |  |  |
| GO:0046671\_negative\_regulation\_of\_retinal\_cell\_programmed\_cell\_death | 1 | 0 |  |  |  |  |  |  |  |  |
| GO:0046685\_response\_to\_arsenic | 1 | 0 |  |  |  |  |  |  |  |  |
| GO:0046692\_sperm\_competition | 1 | 0 |  |  |  |  |  |  |  |  |
| GO:0046707\_IDP\_metabolic\_process | 1 | 0 |  |  |  |  |  |  |  |  |
| GO:0046709\_IDP\_catabolic\_process | 1 | 0 |  |  |  |  |  |  |  |  |
| GO:0046724\_oxalic\_acid\_secretion | 1 | 0 |  |  |  |  |  |  |  |  |
| GO:0046753\_non-lytic\_viral\_release | 1 | 0 |  |  |  |  |  |  |  |  |
| GO:0046755\_non-lytic\_virus\_budding | 1 | 0 |  |  |  |  |  |  |  |  |
| GO:0046826\_negative\_regulation\_of\_protein\_export\_from\_nucleus | 1 | 0 |  |  |  |  |  |  |  |  |
| GO:0046827\_positive\_regulation\_of\_protein\_export\_from\_nucleus | 1 | 0 |  |  |  |  |  |  |  |  |
| GO:0046831\_regulation\_of\_RNA\_export\_from\_nucleus | 1 | 0 |  |  |  |  |  |  |  |  |
| GO:0046834\_lipid\_phosphorylation | 1 | 0 |  |  |  |  |  |  |  |  |
| GO:0046853\_inositol\_and\_derivative\_phosphorylation | 1 | 0 |  |  |  |  |  |  |  |  |
| GO:0046864\_isoprenoid\_transport | 1 | 0 |  |  |  |  |  |  |  |  |
| GO:0046865\_terpenoid\_transport | 1 | 0 |  |  |  |  |  |  |  |  |
| GO:0046877\_regulation\_of\_saliva\_secretion | 1 | 0 |  |  |  |  |  |  |  |  |
| GO:0046878\_positive\_regulation\_of\_saliva\_secretion | 1 | 0 |  |  |  |  |  |  |  |  |
| GO:0046884\_follicle-stimulating\_hormone\_secretion | 1 | 0 |  |  |  |  |  |  |  |  |
| GO:0046898\_response\_to\_cycloheximide | 1 | 0 |  |  |  |  |  |  |  |  |
| GO:0046929\_negative\_regulation\_of\_neurotransmitter\_secretion | 1 | 0 |  |  |  |  |  |  |  |  |
| GO:0046931\_pore\_complex\_biogenesis | 1 | 0 |  |  |  |  |  |  |  |  |
| GO:0046949\_acyl-CoA\_biosynthetic\_process | 1 | 0 |  |  |  |  |  |  |  |  |
| GO:0046958\_nonassociative\_learning | 1 | 0 |  |  |  |  |  |  |  |  |
| GO:0046960\_sensitization | 1 | 0 |  |  |  |  |  |  |  |  |
| GO:0046986\_negative\_regulation\_of\_hemoglobin\_biosynthetic\_process | 1 | 0 |  |  |  |  |  |  |  |  |
| GO:0047497\_mitochondrion\_transport\_along\_microtubule | 1 | 0 |  |  |  |  |  |  |  |  |
| GO:0048047\_mating\_behavior\_\_sex\_discrimination | 1 | 0 |  |  |  |  |  |  |  |  |
| GO:0048133\_male\_germ-line\_stem\_cell\_division | 1 | 0 |  |  |  |  |  |  |  |  |
| GO:0048137\_spermatocyte\_division | 1 | 0 |  |  |  |  |  |  |  |  |
| GO:0048143\_astrocyte\_activation | 1 | 0 |  |  |  |  |  |  |  |  |
| GO:0048170\_positive\_regulation\_of\_long-term\_neuronal\_synaptic\_plasticity | 1 | 0 |  |  |  |  |  |  |  |  |
| GO:0048199\_vesicle\_targeting\_\_to\_\_from\_or\_within\_Golgi | 1 | 0 |  |  |  |  |  |  |  |  |
| GO:0048241\_epinephrine\_transport | 1 | 0 |  |  |  |  |  |  |  |  |
| GO:0048242\_epinephrine\_secretion | 1 | 0 |  |  |  |  |  |  |  |  |
| GO:0048243\_norepinephrine\_secretion | 1 | 0 |  |  |  |  |  |  |  |  |
| GO:0048247\_lymphocyte\_chemotaxis | 1 | 0 |  |  |  |  |  |  |  |  |
| GO:0048250\_mitochondrial\_iron\_ion\_transport | 1 | 0 |  |  |  |  |  |  |  |  |
| GO:0048259\_regulation\_of\_receptor-mediated\_endocytosis | 1 | 0 |  |  |  |  |  |  |  |  |
| GO:0048260\_positive\_regulation\_of\_receptor-mediated\_endocytosis | 1 | 0 |  |  |  |  |  |  |  |  |
| GO:0048290\_isotype\_switching\_to\_IgA\_isotypes | 1 | 0 |  |  |  |  |  |  |  |  |
| GO:0048296\_regulation\_of\_isotype\_switching\_to\_IgA\_isotypes | 1 | 0 |  |  |  |  |  |  |  |  |
| GO:0048298\_positive\_regulation\_of\_isotype\_switching\_to\_IgA\_isotypes | 1 | 0 |  |  |  |  |  |  |  |  |
| GO:0048319\_axial\_mesoderm\_morphogenesis | 1 | 0 |  |  |  |  |  |  |  |  |
| GO:0048320\_axial\_mesoderm\_formation | 1 | 0 |  |  |  |  |  |  |  |  |
| GO:0048385\_regulation\_of\_retinoic\_acid\_receptor\_signaling\_pathway | 1 | 0 |  |  |  |  |  |  |  |  |
| GO:0048387\_negative\_regulation\_of\_retinoic\_acid\_receptor\_signaling\_pathway | 1 | 0 |  |  |  |  |  |  |  |  |
| GO:0048389\_intermediate\_mesoderm\_development | 1 | 0 |  |  |  |  |  |  |  |  |
| GO:0048478\_replication\_fork\_protection | 1 | 0 |  |  |  |  |  |  |  |  |
| GO:0048496\_maintenance\_of\_organ\_identity | 1 | 0 |  |  |  |  |  |  |  |  |
| GO:0048525\_negative\_regulation\_of\_viral\_reproduction | 1 | 0 |  |  |  |  |  |  |  |  |
| GO:0048539\_bone\_marrow\_development | 1 | 0 |  |  |  |  |  |  |  |  |
| GO:0048548\_regulation\_of\_pinocytosis | 1 | 0 |  |  |  |  |  |  |  |  |
| GO:0048549\_positive\_regulation\_of\_pinocytosis | 1 | 0 |  |  |  |  |  |  |  |  |
| GO:0048553\_negative\_regulation\_of\_metalloenzyme\_activity | 1 | 0 |  |  |  |  |  |  |  |  |
| GO:0048588\_developmental\_cell\_growth | 1 | 0 |  |  |  |  |  |  |  |  |
| GO:0048601\_oocyte\_morphogenesis | 1 | 0 |  |  |  |  |  |  |  |  |
| GO:0048621\_post-embryonic\_gut\_morphogenesis | 1 | 0 |  |  |  |  |  |  |  |  |
| GO:0048640\_negative\_regulation\_of\_developmental\_growth | 1 | 0 |  |  |  |  |  |  |  |  |
| GO:0048642\_negative\_regulation\_of\_skeletal\_muscle\_tissue\_development | 1 | 0 |  |  |  |  |  |  |  |  |
| GO:0048669\_collateral\_sprouting\_in\_the\_absence\_of\_injury | 1 | 0 |  |  |  |  |  |  |  |  |
| GO:0048680\_positive\_regulation\_of\_axon\_regeneration | 1 | 0 |  |  |  |  |  |  |  |  |
| GO:0048681\_negative\_regulation\_of\_axon\_regeneration | 1 | 0 |  |  |  |  |  |  |  |  |
| GO:0048686\_regulation\_of\_sprouting\_of\_injured\_axon | 1 | 0 |  |  |  |  |  |  |  |  |
| GO:0048687\_positive\_regulation\_of\_sprouting\_of\_injured\_axon | 1 | 0 |  |  |  |  |  |  |  |  |
| GO:0048690\_regulation\_of\_axon\_extension\_involved\_in\_regeneration | 1 | 0 |  |  |  |  |  |  |  |  |
| GO:0048691\_positive\_regulation\_of\_axon\_extension\_involved\_in\_regeneration | 1 | 0 |  |  |  |  |  |  |  |  |
| GO:0048714\_positive\_regulation\_of\_oligodendrocyte\_differentiation | 1 | 0 |  |  |  |  |  |  |  |  |
| GO:0048733\_sebaceous\_gland\_development | 1 | 0 |  |  |  |  |  |  |  |  |
| GO:0048743\_positive\_regulation\_of\_skeletal\_muscle\_fiber\_development | 1 | 0 |  |  |  |  |  |  |  |  |
| GO:0048752\_semicircular\_canal\_morphogenesis | 1 | 0 |  |  |  |  |  |  |  |  |
| GO:0048773\_erythrophore\_differentiation | 1 | 0 |  |  |  |  |  |  |  |  |
| GO:0048790\_maintenance\_of\_presynaptic\_active\_zone\_structure | 1 | 0 |  |  |  |  |  |  |  |  |
| GO:0048791\_calcium\_ion-dependent\_exocytosis\_of\_neurotransmitter | 1 | 0 |  |  |  |  |  |  |  |  |
| GO:0048822\_enucleate\_erythrocyte\_development | 1 | 0 |  |  |  |  |  |  |  |  |
| GO:0048866\_stem\_cell\_fate\_specification | 1 | 0 |  |  |  |  |  |  |  |  |
| GO:0048936\_peripheral\_nervous\_system\_neuron\_axonogenesis | 1 | 0 |  |  |  |  |  |  |  |  |
| GO:0050427\_3'-phosphoadenosine\_5'-phosphosulfate\_metabolic\_process | 1 | 0 |  |  |  |  |  |  |  |  |
| GO:0050428\_3'-phosphoadenosine\_5'-phosphosulfate\_biosynthetic\_process | 1 | 0 |  |  |  |  |  |  |  |  |
| GO:0050482\_arachidonic\_acid\_secretion | 1 | 0 |  |  |  |  |  |  |  |  |
| GO:0050667\_homocysteine\_metabolic\_process | 1 | 0 |  |  |  |  |  |  |  |  |
| GO:0050674\_urothelial\_cell\_proliferation | 1 | 0 |  |  |  |  |  |  |  |  |
| GO:0050675\_regulation\_of\_urothelial\_cell\_proliferation | 1 | 0 |  |  |  |  |  |  |  |  |
| GO:0050677\_positive\_regulation\_of\_urothelial\_cell\_proliferation | 1 | 0 |  |  |  |  |  |  |  |  |
| GO:0050691\_regulation\_of\_defense\_response\_to\_virus\_by\_host | 1 | 0 |  |  |  |  |  |  |  |  |
| GO:0050748\_negative\_regulation\_of\_lipoprotein\_metabolic\_process | 1 | 0 |  |  |  |  |  |  |  |  |
| GO:0050757\_thymidylate\_synthase\_biosynthetic\_process | 1 | 0 |  |  |  |  |  |  |  |  |
| GO:0050758\_regulation\_of\_thymidylate\_synthase\_biosynthetic\_process | 1 | 0 |  |  |  |  |  |  |  |  |
| GO:0050760\_negative\_regulation\_of\_thymidylate\_synthase\_biosynthetic\_process | 1 | 0 |  |  |  |  |  |  |  |  |
| GO:0050812\_regulation\_of\_acyl-CoA\_biosynthetic\_process | 1 | 0 |  |  |  |  |  |  |  |  |
| GO:0050832\_defense\_response\_to\_fungus | 1 | 0 |  |  |  |  |  |  |  |  |
| GO:0050861\_positive\_regulation\_of\_B\_cell\_receptor\_signaling\_pathway | 1 | 0 |  |  |  |  |  |  |  |  |
| GO:0050862\_positive\_regulation\_of\_T\_cell\_receptor\_signaling\_pathway | 1 | 0 |  |  |  |  |  |  |  |  |
| GO:0050916\_sensory\_perception\_of\_sweet\_taste | 1 | 0 |  |  |  |  |  |  |  |  |
| GO:0050975\_sensory\_perception\_of\_touch | 1 | 0 |  |  |  |  |  |  |  |  |
| GO:0050995\_negative\_regulation\_of\_lipid\_catabolic\_process | 1 | 0 |  |  |  |  |  |  |  |  |
| GO:0051001\_negative\_regulation\_of\_nitric-oxide\_synthase\_activity | 1 | 0 |  |  |  |  |  |  |  |  |
| GO:0051005\_negative\_regulation\_of\_lipoprotein\_lipase\_activity | 1 | 0 |  |  |  |  |  |  |  |  |
| GO:0051006\_positive\_regulation\_of\_lipoprotein\_lipase\_activity | 1 | 0 |  |  |  |  |  |  |  |  |
| GO:0051016\_barbed-end\_actin\_filament\_capping | 1 | 0 |  |  |  |  |  |  |  |  |
| GO:0051029\_rRNA\_transport | 1 | 0 |  |  |  |  |  |  |  |  |
| GO:0051043\_regulation\_of\_membrane\_protein\_ectodomain\_proteolysis | 1 | 0 |  |  |  |  |  |  |  |  |
| GO:0051044\_positive\_regulation\_of\_membrane\_protein\_ectodomain\_proteolysis | 1 | 0 |  |  |  |  |  |  |  |  |
| GO:0051088\_PMA-inducible\_membrane\_protein\_ectodomain\_proteolysis | 1 | 0 |  |  |  |  |  |  |  |  |
| GO:0051102\_DNA\_ligation\_during\_DNA\_recombination | 1 | 0 |  |  |  |  |  |  |  |  |
| GO:0051103\_DNA\_ligation\_during\_DNA\_repair | 1 | 0 |  |  |  |  |  |  |  |  |
| GO:0051123\_transcriptional\_preinitiation\_complex\_assembly | 1 | 0 |  |  |  |  |  |  |  |  |
| GO:0051125\_regulation\_of\_actin\_nucleation | 1 | 0 |  |  |  |  |  |  |  |  |
| GO:0051127\_positive\_regulation\_of\_actin\_nucleation | 1 | 0 |  |  |  |  |  |  |  |  |
| GO:0051151\_negative\_regulation\_of\_smooth\_muscle\_cell\_differentiation | 1 | 0 |  |  |  |  |  |  |  |  |
| GO:0051154\_negative\_regulation\_of\_striated\_muscle\_cell\_differentiation | 1 | 0 |  |  |  |  |  |  |  |  |
| GO:0051155\_positive\_regulation\_of\_striated\_muscle\_cell\_differentiation | 1 | 0 |  |  |  |  |  |  |  |  |
| GO:0051156\_glucose\_6-phosphate\_metabolic\_process | 1 | 0 |  |  |  |  |  |  |  |  |
| GO:0051187\_cofactor\_catabolic\_process | 1 | 0 |  |  |  |  |  |  |  |  |
| GO:0051189\_prosthetic\_group\_metabolic\_process | 1 | 0 |  |  |  |  |  |  |  |  |
| GO:0051193\_regulation\_of\_cofactor\_metabolic\_process | 1 | 0 |  |  |  |  |  |  |  |  |
| GO:0051196\_regulation\_of\_coenzyme\_metabolic\_process | 1 | 0 |  |  |  |  |  |  |  |  |
| GO:0051255\_spindle\_midzone\_assembly | 1 | 0 |  |  |  |  |  |  |  |  |
| GO:0051257\_spindle\_midzone\_assembly\_involved\_in\_meiosis | 1 | 0 |  |  |  |  |  |  |  |  |
| GO:0051281\_positive\_regulation\_of\_release\_of\_sequestered\_calcium\_ion\_into\_cytosol | 1 | 0 |  |  |  |  |  |  |  |  |
| GO:0051290\_protein\_heterotetramerization | 1 | 0 |  |  |  |  |  |  |  |  |
| GO:0051305\_chromosome\_movement\_towards\_spindle\_pole | 1 | 0 |  |  |  |  |  |  |  |  |
| GO:0051310\_metaphase\_plate\_congression | 1 | 0 |  |  |  |  |  |  |  |  |
| GO:0051311\_meiotic\_metaphase\_plate\_congression | 1 | 0 |  |  |  |  |  |  |  |  |
| GO:0051340\_regulation\_of\_ligase\_activity | 1 | 0 |  |  |  |  |  |  |  |  |
| GO:0051351\_positive\_regulation\_of\_ligase\_activity | 1 | 0 |  |  |  |  |  |  |  |  |
| GO:0051354\_negative\_regulation\_of\_oxidoreductase\_activity | 1 | 0 |  |  |  |  |  |  |  |  |
| GO:0051355\_proprioception\_during\_equilibrioception | 1 | 0 |  |  |  |  |  |  |  |  |
| GO:0051383\_kinetochore\_organization | 1 | 0 |  |  |  |  |  |  |  |  |
| GO:0051386\_regulation\_of\_nerve\_growth\_factor\_receptor\_signaling\_pathway | 1 | 0 |  |  |  |  |  |  |  |  |
| GO:0051409\_response\_to\_nitrosative\_stress | 1 | 0 |  |  |  |  |  |  |  |  |
| GO:0051457\_maintenance\_of\_protein\_location\_in\_nucleus | 1 | 0 |  |  |  |  |  |  |  |  |
| GO:0051462\_regulation\_of\_cortisol\_secretion | 1 | 0 |  |  |  |  |  |  |  |  |
| GO:0051463\_negative\_regulation\_of\_cortisol\_secretion | 1 | 0 |  |  |  |  |  |  |  |  |
| GO:0051482\_elevation\_of\_cytosolic\_calcium\_ion\_concentration\_during\_G-protein\_signaling\_\_coupled\_to\_IP3\_second\_messenger\_(phospholipase\_C\_activating) | 1 | 0 |  |  |  |  |  |  |  |  |
| GO:0051542\_elastin\_biosynthetic\_process | 1 | 0 |  |  |  |  |  |  |  |  |
| GO:0051568\_histone\_H3-K4\_methylation | 1 | 0 |  |  |  |  |  |  |  |  |
| GO:0051569\_regulation\_of\_histone\_H3-K4\_methylation | 1 | 0 |  |  |  |  |  |  |  |  |
| GO:0051570\_regulation\_of\_histone\_H3-K9\_methylation | 1 | 0 |  |  |  |  |  |  |  |  |
| GO:0051573\_negative\_regulation\_of\_histone\_H3-K9\_methylation | 1 | 0 |  |  |  |  |  |  |  |  |
| GO:0051580\_regulation\_of\_neurotransmitter\_uptake | 1 | 0 |  |  |  |  |  |  |  |  |
| GO:0051582\_positive\_regulation\_of\_neurotransmitter\_uptake | 1 | 0 |  |  |  |  |  |  |  |  |
| GO:0051584\_regulation\_of\_dopamine\_uptake | 1 | 0 |  |  |  |  |  |  |  |  |
| GO:0051586\_positive\_regulation\_of\_dopamine\_uptake | 1 | 0 |  |  |  |  |  |  |  |  |
| GO:0051589\_negative\_regulation\_of\_neurotransmitter\_transport | 1 | 0 |  |  |  |  |  |  |  |  |
| GO:0051593\_response\_to\_folic\_acid | 1 | 0 |  |  |  |  |  |  |  |  |
| GO:0051615\_histamine\_uptake | 1 | 0 |  |  |  |  |  |  |  |  |
| GO:0051646\_mitochondrion\_localization | 1 | 0 |  |  |  |  |  |  |  |  |
| GO:0051654\_establishment\_of\_mitochondrion\_localization | 1 | 0 |  |  |  |  |  |  |  |  |
| GO:0051661\_maintenance\_of\_centrosome\_location | 1 | 0 |  |  |  |  |  |  |  |  |
| GO:0051665\_membrane\_raft\_localization | 1 | 0 |  |  |  |  |  |  |  |  |
| GO:0051685\_maintenance\_of\_ER\_location | 1 | 0 |  |  |  |  |  |  |  |  |
| GO:0051693\_actin\_filament\_capping | 1 | 0 |  |  |  |  |  |  |  |  |
| GO:0051701\_interaction\_with\_host | 1 | 0 |  |  |  |  |  |  |  |  |
| GO:0051754\_meiotic\_sister\_chromatid\_cohesion\_\_centromeric | 1 | 0 |  |  |  |  |  |  |  |  |
| GO:0051782\_negative\_regulation\_of\_cell\_division | 1 | 0 |  |  |  |  |  |  |  |  |
| GO:0051790\_short-chain\_fatty\_acid\_biosynthetic\_process | 1 | 0 |  |  |  |  |  |  |  |  |
| GO:0051799\_negative\_regulation\_of\_hair\_follicle\_development | 1 | 0 |  |  |  |  |  |  |  |  |
| GO:0051823\_regulation\_of\_synapse\_structural\_plasticity | 1 | 0 |  |  |  |  |  |  |  |  |
| GO:0051865\_protein\_autoubiquitination | 1 | 0 |  |  |  |  |  |  |  |  |
| GO:0051901\_positive\_regulation\_of\_mitochondrial\_depolarization | 1 | 0 |  |  |  |  |  |  |  |  |
| GO:0051917\_regulation\_of\_fibrinolysis | 1 | 0 |  |  |  |  |  |  |  |  |
| GO:0051918\_negative\_regulation\_of\_fibrinolysis | 1 | 0 |  |  |  |  |  |  |  |  |
| GO:0051929\_positive\_regulation\_of\_calcium\_ion\_transport\_via\_voltage-gated\_calcium\_channel\_activity | 1 | 0 |  |  |  |  |  |  |  |  |
| GO:0051933\_amino\_acid\_uptake\_during\_transmission\_of\_nerve\_impulse | 1 | 0 |  |  |  |  |  |  |  |  |
| GO:0051935\_glutamate\_uptake\_during\_transmission\_of\_nerve\_impulse | 1 | 0 |  |  |  |  |  |  |  |  |
| GO:0051940\_regulation\_of\_catecholamine\_uptake\_during\_transmission\_of\_nerve\_impulse | 1 | 0 |  |  |  |  |  |  |  |  |
| GO:0051944\_positive\_regulation\_of\_catecholamine\_uptake\_during\_transmission\_of\_nerve\_impulse | 1 | 0 |  |  |  |  |  |  |  |  |
| GO:0051961\_negative\_regulation\_of\_nervous\_system\_development | 1 | 0 |  |  |  |  |  |  |  |  |
| GO:0051964\_negative\_regulation\_of\_synaptogenesis | 1 | 0 |  |  |  |  |  |  |  |  |
| GO:0051968\_positive\_regulation\_of\_synaptic\_transmission\_\_glutamatergic | 1 | 0 |  |  |  |  |  |  |  |  |
| GO:0051984\_positive\_regulation\_of\_chromosome\_segregation | 1 | 0 |  |  |  |  |  |  |  |  |
| GO:0051987\_positive\_regulation\_of\_attachment\_of\_spindle\_microtubules\_to\_kinetochore | 1 | 0 |  |  |  |  |  |  |  |  |
| GO:0052173\_response\_to\_defenses\_of\_other\_organism\_during\_symbiotic\_interaction | 1 | 0 |  |  |  |  |  |  |  |  |
| GO:0052200\_response\_to\_host\_defenses | 1 | 0 |  |  |  |  |  |  |  |  |
| GO:0052551\_response\_to\_defense-related\_nitric\_oxide\_production\_by\_other\_organism\_during\_symbiotic\_interaction | 1 | 0 |  |  |  |  |  |  |  |  |
| GO:0052564\_response\_to\_immune\_response\_of\_other\_organism\_during\_symbiotic\_interaction | 1 | 0 |  |  |  |  |  |  |  |  |
| GO:0052565\_response\_to\_defense-related\_host\_nitric\_oxide\_production | 1 | 0 |  |  |  |  |  |  |  |  |
| GO:0052572\_response\_to\_host\_immune\_response | 1 | 0 |  |  |  |  |  |  |  |  |
| GO:0055005\_ventricular\_cardiac\_myofibril\_development | 1 | 0 |  |  |  |  |  |  |  |  |
| GO:0055011\_atrial\_cardiac\_muscle\_cell\_differentiation | 1 | 0 |  |  |  |  |  |  |  |  |
| GO:0055014\_atrial\_cardiac\_muscle\_cell\_development | 1 | 0 |  |  |  |  |  |  |  |  |
| GO:0055078\_sodium\_ion\_homeostasis | 1 | 0 |  |  |  |  |  |  |  |  |
| GO:0055089\_fatty\_acid\_homeostasis | 1 | 0 |  |  |  |  |  |  |  |  |
| GO:0055093\_response\_to\_hyperoxia | 1 | 0 |  |  |  |  |  |  |  |  |
| GO:0060003\_copper\_ion\_export | 1 | 0 |  |  |  |  |  |  |  |  |
| GO:0060005\_vestibular\_reflex | 1 | 0 |  |  |  |  |  |  |  |  |
| GO:0060014\_granulosa\_cell\_differentiation | 1 | 0 |  |  |  |  |  |  |  |  |
| GO:0060018\_astrocyte\_fate\_commitment | 1 | 0 |  |  |  |  |  |  |  |  |
| GO:0060020\_Bergmann\_glial\_cell\_differentiation | 1 | 0 |  |  |  |  |  |  |  |  |
| GO:0060022\_hard\_palate\_development | 1 | 0 |  |  |  |  |  |  |  |  |
| GO:0060034\_notochord\_cell\_differentiation | 1 | 0 |  |  |  |  |  |  |  |  |
| GO:0060035\_notochord\_cell\_development | 1 | 0 |  |  |  |  |  |  |  |  |
| GO:0060046\_regulation\_of\_acrosome\_reaction | 1 | 0 |  |  |  |  |  |  |  |  |
| GO:0060054\_positive\_regulation\_of\_epithelial\_cell\_proliferation\_involved\_in\_wound\_healing | 1 | 0 |  |  |  |  |  |  |  |  |
| GO:0060059\_embryonic\_retina\_morphogenesis\_in\_camera-type\_eye | 1 | 0 |  |  |  |  |  |  |  |  |
| GO:0060061\_Spemann\_organizer\_formation | 1 | 0 |  |  |  |  |  |  |  |  |
| GO:0060064\_Spemann\_organizer\_formation\_at\_the\_anterior\_end\_of\_the\_primitive\_streak | 1 | 0 |  |  |  |  |  |  |  |  |
| GO:0060071\_Wnt\_receptor\_signaling\_pathway\_\_planar\_cell\_polarity\_pathway | 1 | 0 |  |  |  |  |  |  |  |  |
| GO:0060075\_regulation\_of\_resting\_membrane\_potential | 1 | 0 |  |  |  |  |  |  |  |  |
| GO:0060082\_eye\_blink\_reflex | 1 | 0 |  |  |  |  |  |  |  |  |
| GO:0060112\_generation\_of\_ovulation\_cycle\_rhythm | 1 | 0 |  |  |  |  |  |  |  |  |
| GO:0060125\_negative\_regulation\_of\_growth\_hormone\_secretion | 1 | 0 |  |  |  |  |  |  |  |  |
| GO:0060151\_peroxisome\_localization | 1 | 0 |  |  |  |  |  |  |  |  |
| GO:0060152\_microtubule-based\_peroxisome\_localization | 1 | 0 |  |  |  |  |  |  |  |  |
| GO:0060161\_positive\_regulation\_of\_dopamine\_receptor\_signaling\_pathway | 1 | 0 |  |  |  |  |  |  |  |  |
| GO:0060174\_limb\_bud\_formation | 1 | 0 |  |  |  |  |  |  |  |  |
| GO:0060177\_regulation\_of\_angiotensin\_metabolic\_process | 1 | 0 |  |  |  |  |  |  |  |  |
| GO:0060197\_cloacal\_septation | 1 | 0 |  |  |  |  |  |  |  |  |
| GO:0060215\_primitive\_hemopoiesis | 1 | 0 |  |  |  |  |  |  |  |  |
| GO:0060231\_mesenchymal\_to\_epithelial\_transition | 1 | 0 |  |  |  |  |  |  |  |  |
| GO:0060254\_regulation\_of\_N-terminal\_protein\_palmitoylation | 1 | 0 |  |  |  |  |  |  |  |  |
| GO:0060261\_positive\_regulation\_of\_transcription\_initiation\_from\_RNA\_polymerase\_II\_promoter | 1 | 0 |  |  |  |  |  |  |  |  |
| GO:0060262\_negative\_regulation\_of\_N-terminal\_protein\_palmitoylation | 1 | 0 |  |  |  |  |  |  |  |  |
| GO:0060263\_regulation\_of\_respiratory\_burst | 1 | 0 |  |  |  |  |  |  |  |  |
| GO:0060264\_regulation\_of\_respiratory\_burst\_during\_acute\_inflammatory\_response | 1 | 0 |  |  |  |  |  |  |  |  |
| GO:0060265\_positive\_regulation\_of\_respiratory\_burst\_during\_acute\_inflammatory\_response | 1 | 0 |  |  |  |  |  |  |  |  |
| GO:0060267\_positive\_regulation\_of\_respiratory\_burst | 1 | 0 |  |  |  |  |  |  |  |  |
| GO:0060272\_embryonic\_skeletal\_joint\_morphogenesis | 1 | 0 |  |  |  |  |  |  |  |  |
| GO:0060297\_regulation\_of\_sarcomere\_organization | 1 | 0 |  |  |  |  |  |  |  |  |
| GO:0060298\_positive\_regulation\_of\_sarcomere\_organization | 1 | 0 |  |  |  |  |  |  |  |  |
| GO:0060315\_negative\_regulation\_of\_ryanodine-sensitive\_calcium-release\_channel\_activity | 1 | 0 |  |  |  |  |  |  |  |  |
| GO:0060319\_primitive\_erythrocyte\_differentiation | 1 | 0 |  |  |  |  |  |  |  |  |
| GO:0060371\_regulation\_of\_atrial\_cardiomyocyte\_membrane\_depolarization | 1 | 0 |  |  |  |  |  |  |  |  |
| GO:0060374\_mast\_cell\_differentiation | 1 | 0 |  |  |  |  |  |  |  |  |
| GO:0060375\_regulation\_of\_mast\_cell\_differentiation | 1 | 0 |  |  |  |  |  |  |  |  |
| GO:0060376\_positive\_regulation\_of\_mast\_cell\_differentiation | 1 | 0 |  |  |  |  |  |  |  |  |
| GO:0060390\_regulation\_of\_SMAD\_protein\_nuclear\_translocation | 1 | 0 |  |  |  |  |  |  |  |  |
| GO:0060391\_positive\_regulation\_of\_SMAD\_protein\_nuclear\_translocation | 1 | 0 |  |  |  |  |  |  |  |  |
| GO:0060405\_regulation\_of\_penile\_erection | 1 | 0 |  |  |  |  |  |  |  |  |
| GO:0060407\_negative\_regulation\_of\_penile\_erection | 1 | 0 |  |  |  |  |  |  |  |  |
| GO:0060413\_atrial\_septum\_morphogenesis | 1 | 0 |  |  |  |  |  |  |  |  |
| GO:0060414\_aorta\_smooth\_muscle\_tissue\_morphogenesis | 1 | 0 |  |  |  |  |  |  |  |  |
| GO:0060419\_heart\_growth | 1 | 0 |  |  |  |  |  |  |  |  |
| GO:0060420\_regulation\_of\_heart\_growth | 1 | 0 |  |  |  |  |  |  |  |  |
| GO:0060421\_positive\_regulation\_of\_heart\_growth | 1 | 0 |  |  |  |  |  |  |  |  |
| GO:0060431\_primary\_lung\_bud\_formation | 1 | 0 |  |  |  |  |  |  |  |  |
| GO:0060436\_bronchiole\_morphogenesis | 1 | 0 |  |  |  |  |  |  |  |  |
| GO:0060440\_trachea\_formation | 1 | 0 |  |  |  |  |  |  |  |  |
| GO:0060449\_bud\_elongation\_involved\_in\_lung\_branching | 1 | 0 |  |  |  |  |  |  |  |  |
| GO:0060456\_positive\_regulation\_of\_digestive\_system\_process | 1 | 0 |  |  |  |  |  |  |  |  |
| GO:0060461\_right\_lung\_morphogenesis | 1 | 0 |  |  |  |  |  |  |  |  |
| GO:0060481\_lobar\_bronchus\_epithelium\_development | 1 | 0 |  |  |  |  |  |  |  |  |
| GO:0060482\_lobar\_bronchus\_development | 1 | 0 |  |  |  |  |  |  |  |  |
| GO:0060484\_lung-associated\_mesenchyme\_development | 1 | 0 |  |  |  |  |  |  |  |  |
| GO:0060486\_Clara\_cell\_differentiation | 1 | 0 |  |  |  |  |  |  |  |  |
| GO:0060510\_Type\_II\_pneumocyte\_differentiation | 1 | 0 |  |  |  |  |  |  |  |  |
| GO:0060514\_prostate\_induction | 1 | 0 |  |  |  |  |  |  |  |  |
| GO:0060515\_prostate\_field\_specification | 1 | 0 |  |  |  |  |  |  |  |  |
| GO:0060517\_epithelial\_cell\_proliferation\_involved\_in\_prostatic\_bud\_elongation | 1 | 0 |  |  |  |  |  |  |  |  |
| GO:0060520\_activation\_of\_prostate\_induction\_by\_androgen\_receptor\_signaling\_pathway | 1 | 0 |  |  |  |  |  |  |  |  |
| GO:0060535\_trachea\_cartilage\_morphogenesis | 1 | 0 |  |  |  |  |  |  |  |  |
| GO:0060536\_cartilage\_morphogenesis | 1 | 0 |  |  |  |  |  |  |  |  |
| GO:0060563\_neuroepithelial\_cell\_differentiation | 1 | 0 |  |  |  |  |  |  |  |  |
| GO:0060577\_pulmonary\_vein\_morphogenesis | 1 | 0 |  |  |  |  |  |  |  |  |
| GO:0060578\_superior\_vena\_cava\_morphogenesis | 1 | 0 |  |  |  |  |  |  |  |  |
| GO:0060584\_regulation\_of\_prostaglandin-endoperoxide\_synthase\_activity | 1 | 0 |  |  |  |  |  |  |  |  |
| GO:0060585\_positive\_regulation\_of\_prostaglandin-endoperoxidase\_synthase\_activity | 1 | 0 |  |  |  |  |  |  |  |  |
| GO:0060598\_dichotomous\_subdivision\_of\_terminal\_units\_involved\_in\_mammary\_gland\_duct\_morphogenesis | 1 | 0 |  |  |  |  |  |  |  |  |
| GO:0060611\_mammary\_gland\_fat\_development | 1 | 0 |  |  |  |  |  |  |  |  |
| GO:0060618\_nipple\_development | 1 | 0 |  |  |  |  |  |  |  |  |
| GO:0060631\_regulation\_of\_meiosis\_I | 1 | 0 |  |  |  |  |  |  |  |  |
| GO:0060649\_mammary\_gland\_bud\_elongation | 1 | 0 |  |  |  |  |  |  |  |  |
| GO:0060658\_nipple\_morphogenesis | 1 | 0 |  |  |  |  |  |  |  |  |
| GO:0060659\_nipple\_sheath\_formation | 1 | 0 |  |  |  |  |  |  |  |  |
| GO:0060668\_regulation\_of\_branching\_involved\_in\_salivary\_gland\_morphogenesis\_by\_extracellular\_matrix-epithelial\_cell\_signaling | 1 | 0 |  |  |  |  |  |  |  |  |
| GO:0060683\_regulation\_of\_branching\_involved\_in\_salivary\_gland\_morphogenesis\_by\_epithelial-mesenchymal\_signaling | 1 | 0 |  |  |  |  |  |  |  |  |
| GO:0060691\_epithelial\_cell\_maturation\_involved\_in\_salivary\_gland\_development | 1 | 0 |  |  |  |  |  |  |  |  |
| GO:0060709\_glycogen\_cell\_development\_involved\_in\_embryonic\_placenta\_development | 1 | 0 |  |  |  |  |  |  |  |  |
| GO:0060732\_positive\_regulation\_of\_inositol\_phosphate\_biosynthetic\_process | 1 | 0 |  |  |  |  |  |  |  |  |
| GO:0060739\_mesenchymal-epithelial\_cell\_signaling\_involved\_in\_prostate\_gland\_development | 1 | 0 |  |  |  |  |  |  |  |  |
| GO:0060781\_mesenchymal\_cell\_proliferation\_involved\_in\_prostate\_gland\_development | 1 | 0 |  |  |  |  |  |  |  |  |
| GO:0060782\_regulation\_of\_mesenchymal\_cell\_proliferation\_involved\_in\_prostate\_gland\_development | 1 | 0 |  |  |  |  |  |  |  |  |
| GO:0060783\_mesenchymal\_smoothened\_signaling\_pathway\_involved\_in\_prostate\_gland\_development | 1 | 0 |  |  |  |  |  |  |  |  |
| GO:0060872\_semicircular\_canal\_development | 1 | 0 |  |  |  |  |  |  |  |  |
| GO:0060896\_neural\_plate\_pattern\_specification | 1 | 0 |  |  |  |  |  |  |  |  |
| GO:0070091\_glucagon\_secretion | 1 | 0 |  |  |  |  |  |  |  |  |
| GO:0070162\_adiponectin\_secretion | 1 | 0 |  |  |  |  |  |  |  |  |
| GO:0070163\_regulation\_of\_adiponectin\_secretion | 1 | 0 |  |  |  |  |  |  |  |  |
| GO:0070164\_negative\_regulation\_of\_adiponectin\_secretion | 1 | 0 |  |  |  |  |  |  |  |  |
| GO:0070178\_D-serine\_metabolic\_process | 1 | 0 |  |  |  |  |  |  |  |  |
| GO:0070179\_D-serine\_biosynthetic\_process | 1 | 0 |  |  |  |  |  |  |  |  |
| GO:0070296\_sarcoplasmic\_reticulum\_calcium\_ion\_transport | 1 | 0 |  |  |  |  |  |  |  |  |
| GO:0070303\_negative\_regulation\_of\_stress-activated\_protein\_kinase\_signaling\_pathway | 1 | 0 |  |  |  |  |  |  |  |  |
| GO:0070328\_triglyceride\_homeostasis | 1 | 0 |  |  |  |  |  |  |  |  |
| GO:0070365\_hepatocyte\_differentiation | 1 | 0 |  |  |  |  |  |  |  |  |
| GO:0070384\_Harderian\_gland\_development | 1 | 0 |  |  |  |  |  |  |  |  |
| GO:0070391\_response\_to\_lipoteichoic\_acid | 1 | 0 |  |  |  |  |  |  |  |  |
| GO:0070424\_regulation\_of\_nucleotide-binding\_oligomerization\_domain\_containing\_signaling\_pathway | 1 | 0 |  |  |  |  |  |  |  |  |
| GO:0070426\_positive\_regulation\_of\_nucleotide-binding\_oligomerization\_domain\_containing\_signaling\_pathway | 1 | 0 |  |  |  |  |  |  |  |  |
| GO:0070428\_regulation\_of\_nucleotide-binding\_oligomerization\_domain\_containing\_1\_signaling\_pathway | 1 | 0 |  |  |  |  |  |  |  |  |
| GO:0070430\_positive\_regulation\_of\_nucleotide-binding\_oligomerization\_domain\_containing\_1\_signaling\_pathway | 1 | 0 |  |  |  |  |  |  |  |  |
| GO:0070432\_regulation\_of\_nucleotide-binding\_oligomerization\_domain\_containing\_2\_signaling\_pathway | 1 | 0 |  |  |  |  |  |  |  |  |
| GO:0070434\_positive\_regulation\_of\_nucleotide-binding\_oligomerization\_domain\_containing\_2\_signaling\_pathway | 1 | 0 |  |  |  |  |  |  |  |  |
| GO:0070493\_thrombin\_receptor\_signaling\_pathway | 1 | 0 |  |  |  |  |  |  |  |  |
| GO:0070508\_cholesterol\_import | 1 | 0 |  |  |  |  |  |  |  |  |
| GO:0070527\_platelet\_aggregation | 1 | 0 |  |  |  |  |  |  |  |  |
| GO:0070528\_protein\_kinase\_C\_signaling\_cascade | 1 | 0 |  |  |  |  |  |  |  |  |
| GO:0070555\_response\_to\_interleukin-1 | 1 | 0 |  |  |  |  |  |  |  |  |
| GO:0070560\_protein\_secretion\_by\_platelet | 1 | 0 |  |  |  |  |  |  |  |  |
| GO:0070561\_vitamin\_D\_receptor\_signaling\_pathway | 1 | 0 |  |  |  |  |  |  |  |  |
| GO:0070562\_regulation\_of\_vitamin\_D\_receptor\_signaling\_pathway | 1 | 0 |  |  |  |  |  |  |  |  |
| GO:0070571\_negative\_regulation\_of\_neuron\_projection\_regeneration | 1 | 0 |  |  |  |  |  |  |  |  |
| GO:0070572\_positive\_regulation\_of\_neuron\_projection\_regeneration | 1 | 0 |  |  |  |  |  |  |  |  |
| GO:0070613\_regulation\_of\_protein\_processing | 1 | 0 |  |  |  |  |  |  |  |  |
| GO:0070627\_ferrous\_iron\_import | 1 | 0 |  |  |  |  |  |  |  |  |
| GO:0070669\_response\_to\_interleukin-2 | 1 | 0 |  |  |  |  |  |  |  |  |
| GO:0070670\_response\_to\_interleukin-4 | 1 | 0 |  |  |  |  |  |  |  |  |
| GO:0070671\_response\_to\_interleukin-12 | 1 | 0 |  |  |  |  |  |  |  |  |
| GO:0070672\_response\_to\_interleukin-15 | 1 | 0 |  |  |  |  |  |  |  |  |
| GO:0070673\_response\_to\_interleukin-18 | 1 | 0 |  |  |  |  |  |  |  |  |
| GO:0070828\_heterochromatin\_organization | 1 | 0 |  |  |  |  |  |  |  |  |
| GO:0070874\_negative\_regulation\_of\_glycogen\_metabolic\_process | 1 | 0 |  |  |  |  |  |  |  |  |
| GO:0075136\_response\_to\_host | 1 | 0 |  |  |  |  |  |  |  |  |
| GO:0080010\_regulation\_of\_oxygen\_and\_reactive\_oxygen\_species\_metabolic\_process | 1 | 0 |  |  |  |  |  |  |  |  |
| GO:0090032\_negative\_regulation\_of\_steroid\_hormone\_biosynthetic\_process | 1 | 0 |  |  |  |  |  |  |  |  |
| GO:0008645\_hexose\_transport | 31 | 0 | 0.000000 | -0.000000 | 1620 | 1475.226192 | 1541.16 | 1607.093808 | 0.951333 |  |
| GO:0009306\_protein\_secretion | 31 | 0 | 0.000000 | -0.000000 | 1620 | 1475.226192 | 1541.16 | 1607.093808 | 0.951333 |  |
| GO:0015749\_monosaccharide\_transport | 31 | 0 | 0.000000 | -0.000000 | 1620 | 1475.226192 | 1541.16 | 1607.093808 | 0.951333 |  |
| GO:0015758\_glucose\_transport | 31 | 0 | 0.000000 | -0.000000 | 1620 | 1475.226192 | 1541.16 | 1607.093808 | 0.951333 |  |
| GO:0033555\_multicellular\_organismal\_response\_to\_stress | 31 | 0 | 0.000000 | -0.000000 | 1620 | 1475.226192 | 1541.16 | 1607.093808 | 0.951333 |  |
| GO:0045088\_regulation\_of\_innate\_immune\_response | 31 | 0 | 0.000000 | -0.000000 | 1620 | 1475.226192 | 1541.16 | 1607.093808 | 0.951333 |  |
| GO:0046632\_alpha-beta\_T\_cell\_differentiation | 31 | 0 | 0.000000 | -0.000000 | 1620 | 1475.226192 | 1541.16 | 1607.093808 | 0.951333 |  |
| GO:0050868\_negative\_regulation\_of\_T\_cell\_activation | 31 | 0 | 0.000000 | -0.000000 | 1620 | 1475.226192 | 1541.16 | 1607.093808 | 0.951333 |  |
| GO:0051899\_membrane\_depolarization | 31 | 0 | 0.000000 | -0.000000 | 1620 | 1475.226192 | 1541.16 | 1607.093808 | 0.951333 |  |
| GO:0055088\_lipid\_homeostasis | 31 | 0 | 0.000000 | -0.000000 | 1620 | 1475.226192 | 1541.16 | 1607.093808 | 0.951333 |  |
| GO:0060512\_prostate\_gland\_morphogenesis | 31 | 0 | 0.000000 | -0.000000 | 1620 | 1475.226192 | 1541.16 | 1607.093808 | 0.951333 |  |
| GO:0002260\_lymphocyte\_homeostasis | 30 | 0 | 0.000000 | -0.000000 | 1636 | 1492.381760 | 1556.32 | 1620.258240 | 0.951296 |  |
| GO:0007435\_salivary\_gland\_morphogenesis | 30 | 0 | 0.000000 | -0.000000 | 1636 | 1492.381760 | 1556.32 | 1620.258240 | 0.951296 |  |
| GO:0014032\_neural\_crest\_cell\_development | 30 | 0 | 0.000000 | -0.000000 | 1636 | 1492.381760 | 1556.32 | 1620.258240 | 0.951296 |  |
| GO:0014033\_neural\_crest\_cell\_differentiation | 30 | 0 | 0.000000 | -0.000000 | 1636 | 1492.381760 | 1556.32 | 1620.258240 | 0.951296 |  |
| GO:0022411\_cellular\_component\_disassembly | 30 | 0 | 0.000000 | -0.000000 | 1636 | 1492.381760 | 1556.32 | 1620.258240 | 0.951296 |  |
| GO:0031281\_positive\_regulation\_of\_cyclase\_activity | 30 | 0 | 0.000000 | -0.000000 | 1636 | 1492.381760 | 1556.32 | 1620.258240 | 0.951296 |  |
| GO:0031668\_cellular\_response\_to\_extracellular\_stimulus | 30 | 0 | 0.000000 | -0.000000 | 1636 | 1492.381760 | 1556.32 | 1620.258240 | 0.951296 |  |
| GO:0032102\_negative\_regulation\_of\_response\_to\_external\_stimulus | 30 | 0 | 0.000000 | -0.000000 | 1636 | 1492.381760 | 1556.32 | 1620.258240 | 0.951296 |  |
| GO:0033500\_carbohydrate\_homeostasis | 30 | 0 | 0.000000 | -0.000000 | 1636 | 1492.381760 | 1556.32 | 1620.258240 | 0.951296 |  |
| GO:0035265\_organ\_growth | 30 | 0 | 0.000000 | -0.000000 | 1636 | 1492.381760 | 1556.32 | 1620.258240 | 0.951296 |  |
| GO:0042593\_glucose\_homeostasis | 30 | 0 | 0.000000 | -0.000000 | 1636 | 1492.381760 | 1556.32 | 1620.258240 | 0.951296 |  |
| GO:0045762\_positive\_regulation\_of\_adenylate\_cyclase\_activity | 30 | 0 | 0.000000 | -0.000000 | 1636 | 1492.381760 | 1556.32 | 1620.258240 | 0.951296 |  |
| GO:0051146\_striated\_muscle\_cell\_differentiation | 30 | 0 | 0.000000 | -0.000000 | 1636 | 1492.381760 | 1556.32 | 1620.258240 | 0.951296 |  |
| GO:0051349\_positive\_regulation\_of\_lyase\_activity | 30 | 0 | 0.000000 | -0.000000 | 1636 | 1492.381760 | 1556.32 | 1620.258240 | 0.951296 |  |
| GO:0052547\_regulation\_of\_peptidase\_activity | 30 | 0 | 0.000000 | -0.000000 | 1636 | 1492.381760 | 1556.32 | 1620.258240 | 0.951296 |  |
| GO:0060740\_prostate\_gland\_epithelium\_morphogenesis | 30 | 0 | 0.000000 | -0.000000 | 1636 | 1492.381760 | 1556.32 | 1620.258240 | 0.951296 |  |
| GO:0000079\_regulation\_of\_cyclin-dependent\_protein\_kinase\_activity | 7 | 0 | 0.000000 | -0.000000 | 1793 | 1658.829499 | 1719.9 | 1780.970501 | 0.959230 |  |
| GO:0000188\_inactivation\_of\_MAPK\_activity | 7 | 0 | 0.000000 | -0.000000 | 1793 | 1658.829499 | 1719.9 | 1780.970501 | 0.959230 |  |
| GO:0001556\_oocyte\_maturation | 7 | 0 | 0.000000 | -0.000000 | 1793 | 1658.829499 | 1719.9 | 1780.970501 | 0.959230 |  |
| GO:0001736\_establishment\_of\_planar\_polarity | 7 | 0 | 0.000000 | -0.000000 | 1793 | 1658.829499 | 1719.9 | 1780.970501 | 0.959230 |  |
| GO:0001839\_neural\_plate\_morphogenesis | 7 | 0 | 0.000000 | -0.000000 | 1793 | 1658.829499 | 1719.9 | 1780.970501 | 0.959230 |  |
| GO:0001936\_regulation\_of\_endothelial\_cell\_proliferation | 7 | 0 | 0.000000 | -0.000000 | 1793 | 1658.829499 | 1719.9 | 1780.970501 | 0.959230 |  |
| GO:0001967\_suckling\_behavior | 7 | 0 | 0.000000 | -0.000000 | 1793 | 1658.829499 | 1719.9 | 1780.970501 | 0.959230 |  |
| GO:0002011\_morphogenesis\_of\_an\_epithelial\_sheet | 7 | 0 | 0.000000 | -0.000000 | 1793 | 1658.829499 | 1719.9 | 1780.970501 | 0.959230 |  |
| GO:0002052\_positive\_regulation\_of\_neuroblast\_proliferation | 7 | 0 | 0.000000 | -0.000000 | 1793 | 1658.829499 | 1719.9 | 1780.970501 | 0.959230 |  |
| GO:0002067\_glandular\_epithelial\_cell\_differentiation | 7 | 0 | 0.000000 | -0.000000 | 1793 | 1658.829499 | 1719.9 | 1780.970501 | 0.959230 |  |
| GO:0002076\_osteoblast\_development | 7 | 0 | 0.000000 | -0.000000 | 1793 | 1658.829499 | 1719.9 | 1780.970501 | 0.959230 |  |
| GO:0002093\_auditory\_receptor\_cell\_morphogenesis | 7 | 0 | 0.000000 | -0.000000 | 1793 | 1658.829499 | 1719.9 | 1780.970501 | 0.959230 |  |
| GO:0002224\_toll-like\_receptor\_signaling\_pathway | 7 | 0 | 0.000000 | -0.000000 | 1793 | 1658.829499 | 1719.9 | 1780.970501 | 0.959230 |  |
| GO:0002643\_regulation\_of\_tolerance\_induction | 7 | 0 | 0.000000 | -0.000000 | 1793 | 1658.829499 | 1719.9 | 1780.970501 | 0.959230 |  |
| GO:0002645\_positive\_regulation\_of\_tolerance\_induction | 7 | 0 | 0.000000 | -0.000000 | 1793 | 1658.829499 | 1719.9 | 1780.970501 | 0.959230 |  |
| GO:0002792\_negative\_regulation\_of\_peptide\_secretion | 7 | 0 | 0.000000 | -0.000000 | 1793 | 1658.829499 | 1719.9 | 1780.970501 | 0.959230 |  |
| GO:0002793\_positive\_regulation\_of\_peptide\_secretion | 7 | 0 | 0.000000 | -0.000000 | 1793 | 1658.829499 | 1719.9 | 1780.970501 | 0.959230 |  |
| GO:0002828\_regulation\_of\_T-helper\_2\_type\_immune\_response | 7 | 0 | 0.000000 | -0.000000 | 1793 | 1658.829499 | 1719.9 | 1780.970501 | 0.959230 |  |
| GO:0002863\_positive\_regulation\_of\_inflammatory\_response\_to\_antigenic\_stimulus | 7 | 0 | 0.000000 | -0.000000 | 1793 | 1658.829499 | 1719.9 | 1780.970501 | 0.959230 |  |
| GO:0003084\_positive\_regulation\_of\_systemic\_arterial\_blood\_pressure | 7 | 0 | 0.000000 | -0.000000 | 1793 | 1658.829499 | 1719.9 | 1780.970501 | 0.959230 |  |
| GO:0003085\_negative\_regulation\_of\_systemic\_arterial\_blood\_pressure | 7 | 0 | 0.000000 | -0.000000 | 1793 | 1658.829499 | 1719.9 | 1780.970501 | 0.959230 |  |
| GO:0006014\_D-ribose\_metabolic\_process | 7 | 0 | 0.000000 | -0.000000 | 1793 | 1658.829499 | 1719.9 | 1780.970501 | 0.959230 |  |
| GO:0006041\_glucosamine\_metabolic\_process | 7 | 0 | 0.000000 | -0.000000 | 1793 | 1658.829499 | 1719.9 | 1780.970501 | 0.959230 |  |
| GO:0006044\_N-acetylglucosamine\_metabolic\_process | 7 | 0 | 0.000000 | -0.000000 | 1793 | 1658.829499 | 1719.9 | 1780.970501 | 0.959230 |  |
| GO:0006096\_glycolysis | 7 | 0 | 0.000000 | -0.000000 | 1793 | 1658.829499 | 1719.9 | 1780.970501 | 0.959230 |  |
| GO:0006119\_oxidative\_phosphorylation | 7 | 0 | 0.000000 | -0.000000 | 1793 | 1658.829499 | 1719.9 | 1780.970501 | 0.959230 |  |
| GO:0006275\_regulation\_of\_DNA\_replication | 7 | 0 | 0.000000 | -0.000000 | 1793 | 1658.829499 | 1719.9 | 1780.970501 | 0.959230 |  |
| GO:0006298\_mismatch\_repair | 7 | 0 | 0.000000 | -0.000000 | 1793 | 1658.829499 | 1719.9 | 1780.970501 | 0.959230 |  |
| GO:0006352\_transcription\_initiation | 7 | 0 | 0.000000 | -0.000000 | 1793 | 1658.829499 | 1719.9 | 1780.970501 | 0.959230 |  |
| GO:0006401\_RNA\_catabolic\_process | 7 | 0 | 0.000000 | -0.000000 | 1793 | 1658.829499 | 1719.9 | 1780.970501 | 0.959230 |  |
| GO:0006406\_mRNA\_export\_from\_nucleus | 7 | 0 | 0.000000 | -0.000000 | 1793 | 1658.829499 | 1719.9 | 1780.970501 | 0.959230 |  |
| GO:0006505\_GPI\_anchor\_metabolic\_process | 7 | 0 | 0.000000 | -0.000000 | 1793 | 1658.829499 | 1719.9 | 1780.970501 | 0.959230 |  |
| GO:0006516\_glycoprotein\_catabolic\_process | 7 | 0 | 0.000000 | -0.000000 | 1793 | 1658.829499 | 1719.9 | 1780.970501 | 0.959230 |  |
| GO:0006612\_protein\_targeting\_to\_membrane | 7 | 0 | 0.000000 | -0.000000 | 1793 | 1658.829499 | 1719.9 | 1780.970501 | 0.959230 |  |
| GO:0006769\_nicotinamide\_metabolic\_process | 7 | 0 | 0.000000 | -0.000000 | 1793 | 1658.829499 | 1719.9 | 1780.970501 | 0.959230 |  |
| GO:0006783\_heme\_biosynthetic\_process | 7 | 0 | 0.000000 | -0.000000 | 1793 | 1658.829499 | 1719.9 | 1780.970501 | 0.959230 |  |
| GO:0006818\_hydrogen\_transport | 7 | 0 | 0.000000 | -0.000000 | 1793 | 1658.829499 | 1719.9 | 1780.970501 | 0.959230 |  |
| GO:0006878\_cellular\_copper\_ion\_homeostasis | 7 | 0 | 0.000000 | -0.000000 | 1793 | 1658.829499 | 1719.9 | 1780.970501 | 0.959230 |  |
| GO:0006884\_cell\_volume\_homeostasis | 7 | 0 | 0.000000 | -0.000000 | 1793 | 1658.829499 | 1719.9 | 1780.970501 | 0.959230 |  |
| GO:0006949\_syncytium\_formation | 7 | 0 | 0.000000 | -0.000000 | 1793 | 1658.829499 | 1719.9 | 1780.970501 | 0.959230 |  |
| GO:0007019\_microtubule\_depolymerization | 7 | 0 | 0.000000 | -0.000000 | 1793 | 1658.829499 | 1719.9 | 1780.970501 | 0.959230 |  |
| GO:0007026\_negative\_regulation\_of\_microtubule\_depolymerization | 7 | 0 | 0.000000 | -0.000000 | 1793 | 1658.829499 | 1719.9 | 1780.970501 | 0.959230 |  |
| GO:0007034\_vacuolar\_transport | 7 | 0 | 0.000000 | -0.000000 | 1793 | 1658.829499 | 1719.9 | 1780.970501 | 0.959230 |  |
| GO:0007062\_sister\_chromatid\_cohesion | 7 | 0 | 0.000000 | -0.000000 | 1793 | 1658.829499 | 1719.9 | 1780.970501 | 0.959230 |  |
| GO:0007130\_synaptonemal\_complex\_assembly | 7 | 0 | 0.000000 | -0.000000 | 1793 | 1658.829499 | 1719.9 | 1780.970501 | 0.959230 |  |
| GO:0007164\_establishment\_of\_tissue\_polarity | 7 | 0 | 0.000000 | -0.000000 | 1793 | 1658.829499 | 1719.9 | 1780.970501 | 0.959230 |  |
| GO:0007191\_activation\_of\_adenylate\_cyclase\_activity\_by\_dopamine\_receptor\_signaling\_pathway | 7 | 0 | 0.000000 | -0.000000 | 1793 | 1658.829499 | 1719.9 | 1780.970501 | 0.959230 |  |
| GO:0007271\_synaptic\_transmission\_\_cholinergic | 7 | 0 | 0.000000 | -0.000000 | 1793 | 1658.829499 | 1719.9 | 1780.970501 | 0.959230 |  |
| GO:0007413\_axonal\_fasciculation | 7 | 0 | 0.000000 | -0.000000 | 1793 | 1658.829499 | 1719.9 | 1780.970501 | 0.959230 |  |
| GO:0007440\_foregut\_morphogenesis | 7 | 0 | 0.000000 | -0.000000 | 1793 | 1658.829499 | 1719.9 | 1780.970501 | 0.959230 |  |
| GO:0007616\_long-term\_memory | 7 | 0 | 0.000000 | -0.000000 | 1793 | 1658.829499 | 1719.9 | 1780.970501 | 0.959230 |  |
| GO:0008033\_tRNA\_processing | 7 | 0 | 0.000000 | -0.000000 | 1793 | 1658.829499 | 1719.9 | 1780.970501 | 0.959230 |  |
| GO:0008299\_isoprenoid\_biosynthetic\_process | 7 | 0 | 0.000000 | -0.000000 | 1793 | 1658.829499 | 1719.9 | 1780.970501 | 0.959230 |  |
| GO:0008340\_determination\_of\_adult\_lifespan | 7 | 0 | 0.000000 | -0.000000 | 1793 | 1658.829499 | 1719.9 | 1780.970501 | 0.959230 |  |
| GO:0009150\_purine\_ribonucleotide\_metabolic\_process | 7 | 0 | 0.000000 | -0.000000 | 1793 | 1658.829499 | 1719.9 | 1780.970501 | 0.959230 |  |
| GO:0009200\_deoxyribonucleoside\_triphosphate\_metabolic\_process | 7 | 0 | 0.000000 | -0.000000 | 1793 | 1658.829499 | 1719.9 | 1780.970501 | 0.959230 |  |
| GO:0009259\_ribonucleotide\_metabolic\_process | 7 | 0 | 0.000000 | -0.000000 | 1793 | 1658.829499 | 1719.9 | 1780.970501 | 0.959230 |  |
| GO:0009394\_2'-deoxyribonucleotide\_metabolic\_process | 7 | 0 | 0.000000 | -0.000000 | 1793 | 1658.829499 | 1719.9 | 1780.970501 | 0.959230 |  |
| GO:0009820\_alkaloid\_metabolic\_process | 7 | 0 | 0.000000 | -0.000000 | 1793 | 1658.829499 | 1719.9 | 1780.970501 | 0.959230 |  |
| GO:0010469\_regulation\_of\_receptor\_activity | 7 | 0 | 0.000000 | -0.000000 | 1793 | 1658.829499 | 1719.9 | 1780.970501 | 0.959230 |  |
| GO:0010948\_negative\_regulation\_of\_cell\_cycle\_process | 7 | 0 | 0.000000 | -0.000000 | 1793 | 1658.829499 | 1719.9 | 1780.970501 | 0.959230 |  |
| GO:0014821\_phasic\_smooth\_muscle\_contraction | 7 | 0 | 0.000000 | -0.000000 | 1793 | 1658.829499 | 1719.9 | 1780.970501 | 0.959230 |  |
| GO:0015697\_quaternary\_ammonium\_group\_transport | 7 | 0 | 0.000000 | -0.000000 | 1793 | 1658.829499 | 1719.9 | 1780.970501 | 0.959230 |  |
| GO:0015813\_L-glutamate\_transport | 7 | 0 | 0.000000 | -0.000000 | 1793 | 1658.829499 | 1719.9 | 1780.970501 | 0.959230 |  |
| GO:0015908\_fatty\_acid\_transport | 7 | 0 | 0.000000 | -0.000000 | 1793 | 1658.829499 | 1719.9 | 1780.970501 | 0.959230 |  |
| GO:0015992\_proton\_transport | 7 | 0 | 0.000000 | -0.000000 | 1793 | 1658.829499 | 1719.9 | 1780.970501 | 0.959230 |  |
| GO:0016339\_calcium-dependent\_cell-cell\_adhesion | 7 | 0 | 0.000000 | -0.000000 | 1793 | 1658.829499 | 1719.9 | 1780.970501 | 0.959230 |  |
| GO:0016575\_histone\_deacetylation | 7 | 0 | 0.000000 | -0.000000 | 1793 | 1658.829499 | 1719.9 | 1780.970501 | 0.959230 |  |
| GO:0019362\_pyridine\_nucleotide\_metabolic\_process | 7 | 0 | 0.000000 | -0.000000 | 1793 | 1658.829499 | 1719.9 | 1780.970501 | 0.959230 |  |
| GO:0019692\_deoxyribose\_phosphate\_metabolic\_process | 7 | 0 | 0.000000 | -0.000000 | 1793 | 1658.829499 | 1719.9 | 1780.970501 | 0.959230 |  |
| GO:0020027\_hemoglobin\_metabolic\_process | 7 | 0 | 0.000000 | -0.000000 | 1793 | 1658.829499 | 1719.9 | 1780.970501 | 0.959230 |  |
| GO:0021546\_rhombomere\_development | 7 | 0 | 0.000000 | -0.000000 | 1793 | 1658.829499 | 1719.9 | 1780.970501 | 0.959230 |  |
| GO:0021756\_striatum\_development | 7 | 0 | 0.000000 | -0.000000 | 1793 | 1658.829499 | 1719.9 | 1780.970501 | 0.959230 |  |
| GO:0021884\_forebrain\_neuron\_development | 7 | 0 | 0.000000 | -0.000000 | 1793 | 1658.829499 | 1719.9 | 1780.970501 | 0.959230 |  |
| GO:0021903\_rostrocaudal\_neural\_tube\_patterning | 7 | 0 | 0.000000 | -0.000000 | 1793 | 1658.829499 | 1719.9 | 1780.970501 | 0.959230 |  |
| GO:0021984\_adenohypophysis\_development | 7 | 0 | 0.000000 | -0.000000 | 1793 | 1658.829499 | 1719.9 | 1780.970501 | 0.959230 |  |
| GO:0022407\_regulation\_of\_cell-cell\_adhesion | 7 | 0 | 0.000000 | -0.000000 | 1793 | 1658.829499 | 1719.9 | 1780.970501 | 0.959230 |  |
| GO:0022618\_ribonucleoprotein\_complex\_assembly | 7 | 0 | 0.000000 | -0.000000 | 1793 | 1658.829499 | 1719.9 | 1780.970501 | 0.959230 |  |
| GO:0030104\_water\_homeostasis | 7 | 0 | 0.000000 | -0.000000 | 1793 | 1658.829499 | 1719.9 | 1780.970501 | 0.959230 |  |
| GO:0030201\_heparan\_sulfate\_proteoglycan\_metabolic\_process | 7 | 0 | 0.000000 | -0.000000 | 1793 | 1658.829499 | 1719.9 | 1780.970501 | 0.959230 |  |
| GO:0030432\_peristalsis | 7 | 0 | 0.000000 | -0.000000 | 1793 | 1658.829499 | 1719.9 | 1780.970501 | 0.959230 |  |
| GO:0030517\_negative\_regulation\_of\_axon\_extension | 7 | 0 | 0.000000 | -0.000000 | 1793 | 1658.829499 | 1719.9 | 1780.970501 | 0.959230 |  |
| GO:0030520\_estrogen\_receptor\_signaling\_pathway | 7 | 0 | 0.000000 | -0.000000 | 1793 | 1658.829499 | 1719.9 | 1780.970501 | 0.959230 |  |
| GO:0030521\_androgen\_receptor\_signaling\_pathway | 7 | 0 | 0.000000 | -0.000000 | 1793 | 1658.829499 | 1719.9 | 1780.970501 | 0.959230 |  |
| GO:0030903\_notochord\_development | 7 | 0 | 0.000000 | -0.000000 | 1793 | 1658.829499 | 1719.9 | 1780.970501 | 0.959230 |  |
| GO:0031017\_exocrine\_pancreas\_development | 7 | 0 | 0.000000 | -0.000000 | 1793 | 1658.829499 | 1719.9 | 1780.970501 | 0.959230 |  |
| GO:0031114\_regulation\_of\_microtubule\_depolymerization | 7 | 0 | 0.000000 | -0.000000 | 1793 | 1658.829499 | 1719.9 | 1780.970501 | 0.959230 |  |
| GO:0031124\_mRNA\_3'-end\_processing | 7 | 0 | 0.000000 | -0.000000 | 1793 | 1658.829499 | 1719.9 | 1780.970501 | 0.959230 |  |
| GO:0031497\_chromatin\_assembly | 7 | 0 | 0.000000 | -0.000000 | 1793 | 1658.829499 | 1719.9 | 1780.970501 | 0.959230 |  |
| GO:0032104\_regulation\_of\_response\_to\_extracellular\_stimulus | 7 | 0 | 0.000000 | -0.000000 | 1793 | 1658.829499 | 1719.9 | 1780.970501 | 0.959230 |  |
| GO:0032107\_regulation\_of\_response\_to\_nutrient\_levels | 7 | 0 | 0.000000 | -0.000000 | 1793 | 1658.829499 | 1719.9 | 1780.970501 | 0.959230 |  |
| GO:0032228\_regulation\_of\_synaptic\_transmission\_\_GABAergic | 7 | 0 | 0.000000 | -0.000000 | 1793 | 1658.829499 | 1719.9 | 1780.970501 | 0.959230 |  |
| GO:0032319\_regulation\_of\_Rho\_GTPase\_activity | 7 | 0 | 0.000000 | -0.000000 | 1793 | 1658.829499 | 1719.9 | 1780.970501 | 0.959230 |  |
| GO:0032387\_negative\_regulation\_of\_intracellular\_transport | 7 | 0 | 0.000000 | -0.000000 | 1793 | 1658.829499 | 1719.9 | 1780.970501 | 0.959230 |  |
| GO:0032507\_maintenance\_of\_protein\_location\_in\_cell | 7 | 0 | 0.000000 | -0.000000 | 1793 | 1658.829499 | 1719.9 | 1780.970501 | 0.959230 |  |
| GO:0033032\_regulation\_of\_myeloid\_cell\_apoptosis | 7 | 0 | 0.000000 | -0.000000 | 1793 | 1658.829499 | 1719.9 | 1780.970501 | 0.959230 |  |
| GO:0034599\_cellular\_response\_to\_oxidative\_stress | 7 | 0 | 0.000000 | -0.000000 | 1793 | 1658.829499 | 1719.9 | 1780.970501 | 0.959230 |  |
| GO:0042033\_chemokine\_biosynthetic\_process | 7 | 0 | 0.000000 | -0.000000 | 1793 | 1658.829499 | 1719.9 | 1780.970501 | 0.959230 |  |
| GO:0042133\_neurotransmitter\_metabolic\_process | 7 | 0 | 0.000000 | -0.000000 | 1793 | 1658.829499 | 1719.9 | 1780.970501 | 0.959230 |  |
| GO:0042168\_heme\_metabolic\_process | 7 | 0 | 0.000000 | -0.000000 | 1793 | 1658.829499 | 1719.9 | 1780.970501 | 0.959230 |  |
| GO:0042415\_norepinephrine\_metabolic\_process | 7 | 0 | 0.000000 | -0.000000 | 1793 | 1658.829499 | 1719.9 | 1780.970501 | 0.959230 |  |
| GO:0042438\_melanin\_biosynthetic\_process | 7 | 0 | 0.000000 | -0.000000 | 1793 | 1658.829499 | 1719.9 | 1780.970501 | 0.959230 |  |
| GO:0042572\_retinol\_metabolic\_process | 7 | 0 | 0.000000 | -0.000000 | 1793 | 1658.829499 | 1719.9 | 1780.970501 | 0.959230 |  |
| GO:0043353\_enucleate\_erythrocyte\_differentiation | 7 | 0 | 0.000000 | -0.000000 | 1793 | 1658.829499 | 1719.9 | 1780.970501 | 0.959230 |  |
| GO:0043372\_positive\_regulation\_of\_CD4-positive\_\_alpha\_beta\_T\_cell\_differentiation | 7 | 0 | 0.000000 | -0.000000 | 1793 | 1658.829499 | 1719.9 | 1780.970501 | 0.959230 |  |
| GO:0043449\_cellular\_alkene\_metabolic\_process | 7 | 0 | 0.000000 | -0.000000 | 1793 | 1658.829499 | 1719.9 | 1780.970501 | 0.959230 |  |
| GO:0043507\_positive\_regulation\_of\_JUN\_kinase\_activity | 7 | 0 | 0.000000 | -0.000000 | 1793 | 1658.829499 | 1719.9 | 1780.970501 | 0.959230 |  |
| GO:0043567\_regulation\_of\_insulin-like\_growth\_factor\_receptor\_signaling\_pathway | 7 | 0 | 0.000000 | -0.000000 | 1793 | 1658.829499 | 1719.9 | 1780.970501 | 0.959230 |  |
| GO:0044275\_cellular\_carbohydrate\_catabolic\_process | 7 | 0 | 0.000000 | -0.000000 | 1793 | 1658.829499 | 1719.9 | 1780.970501 | 0.959230 |  |
| GO:0045073\_regulation\_of\_chemokine\_biosynthetic\_process | 7 | 0 | 0.000000 | -0.000000 | 1793 | 1658.829499 | 1719.9 | 1780.970501 | 0.959230 |  |
| GO:0045581\_negative\_regulation\_of\_T\_cell\_differentiation | 7 | 0 | 0.000000 | -0.000000 | 1793 | 1658.829499 | 1719.9 | 1780.970501 | 0.959230 |  |
| GO:0045599\_negative\_regulation\_of\_fat\_cell\_differentiation | 7 | 0 | 0.000000 | -0.000000 | 1793 | 1658.829499 | 1719.9 | 1780.970501 | 0.959230 |  |
| GO:0045604\_regulation\_of\_epidermal\_cell\_differentiation | 7 | 0 | 0.000000 | -0.000000 | 1793 | 1658.829499 | 1719.9 | 1780.970501 | 0.959230 |  |
| GO:0045668\_negative\_regulation\_of\_osteoblast\_differentiation | 7 | 0 | 0.000000 | -0.000000 | 1793 | 1658.829499 | 1719.9 | 1780.970501 | 0.959230 |  |
| GO:0045823\_positive\_regulation\_of\_heart\_contraction | 7 | 0 | 0.000000 | -0.000000 | 1793 | 1658.829499 | 1719.9 | 1780.970501 | 0.959230 |  |
| GO:0045840\_positive\_regulation\_of\_mitosis | 7 | 0 | 0.000000 | -0.000000 | 1793 | 1658.829499 | 1719.9 | 1780.970501 | 0.959230 |  |
| GO:0045862\_positive\_regulation\_of\_proteolysis | 7 | 0 | 0.000000 | -0.000000 | 1793 | 1658.829499 | 1719.9 | 1780.970501 | 0.959230 |  |
| GO:0045879\_negative\_regulation\_of\_smoothened\_signaling\_pathway | 7 | 0 | 0.000000 | -0.000000 | 1793 | 1658.829499 | 1719.9 | 1780.970501 | 0.959230 |  |
| GO:0045880\_positive\_regulation\_of\_smoothened\_signaling\_pathway | 7 | 0 | 0.000000 | -0.000000 | 1793 | 1658.829499 | 1719.9 | 1780.970501 | 0.959230 |  |
| GO:0045986\_negative\_regulation\_of\_smooth\_muscle\_contraction | 7 | 0 | 0.000000 | -0.000000 | 1793 | 1658.829499 | 1719.9 | 1780.970501 | 0.959230 |  |
| GO:0046496\_nicotinamide\_nucleotide\_metabolic\_process | 7 | 0 | 0.000000 | -0.000000 | 1793 | 1658.829499 | 1719.9 | 1780.970501 | 0.959230 |  |
| GO:0046513\_ceramide\_biosynthetic\_process | 7 | 0 | 0.000000 | -0.000000 | 1793 | 1658.829499 | 1719.9 | 1780.970501 | 0.959230 |  |
| GO:0046520\_sphingoid\_biosynthetic\_process | 7 | 0 | 0.000000 | -0.000000 | 1793 | 1658.829499 | 1719.9 | 1780.970501 | 0.959230 |  |
| GO:0046543\_development\_of\_secondary\_female\_sexual\_characteristics | 7 | 0 | 0.000000 | -0.000000 | 1793 | 1658.829499 | 1719.9 | 1780.970501 | 0.959230 |  |
| GO:0046622\_positive\_regulation\_of\_organ\_growth | 7 | 0 | 0.000000 | -0.000000 | 1793 | 1658.829499 | 1719.9 | 1780.970501 | 0.959230 |  |
| GO:0046626\_regulation\_of\_insulin\_receptor\_signaling\_pathway | 7 | 0 | 0.000000 | -0.000000 | 1793 | 1658.829499 | 1719.9 | 1780.970501 | 0.959230 |  |
| GO:0046676\_negative\_regulation\_of\_insulin\_secretion | 7 | 0 | 0.000000 | -0.000000 | 1793 | 1658.829499 | 1719.9 | 1780.970501 | 0.959230 |  |
| GO:0046677\_response\_to\_antibiotic | 7 | 0 | 0.000000 | -0.000000 | 1793 | 1658.829499 | 1719.9 | 1780.970501 | 0.959230 |  |
| GO:0046823\_negative\_regulation\_of\_nucleocytoplasmic\_transport | 7 | 0 | 0.000000 | -0.000000 | 1793 | 1658.829499 | 1719.9 | 1780.970501 | 0.959230 |  |
| GO:0046824\_positive\_regulation\_of\_nucleocytoplasmic\_transport | 7 | 0 | 0.000000 | -0.000000 | 1793 | 1658.829499 | 1719.9 | 1780.970501 | 0.959230 |  |
| GO:0046847\_filopodium\_assembly | 7 | 0 | 0.000000 | -0.000000 | 1793 | 1658.829499 | 1719.9 | 1780.970501 | 0.959230 |  |
| GO:0048148\_behavioral\_response\_to\_cocaine | 7 | 0 | 0.000000 | -0.000000 | 1793 | 1658.829499 | 1719.9 | 1780.970501 | 0.959230 |  |
| GO:0048486\_parasympathetic\_nervous\_system\_development | 7 | 0 | 0.000000 | -0.000000 | 1793 | 1658.829499 | 1719.9 | 1780.970501 | 0.959230 |  |
| GO:0048537\_mucosal-associated\_lymphoid\_tissue\_development | 7 | 0 | 0.000000 | -0.000000 | 1793 | 1658.829499 | 1719.9 | 1780.970501 | 0.959230 |  |
| GO:0048753\_pigment\_granule\_organization | 7 | 0 | 0.000000 | -0.000000 | 1793 | 1658.829499 | 1719.9 | 1780.970501 | 0.959230 |  |
| GO:0048814\_regulation\_of\_dendrite\_morphogenesis | 7 | 0 | 0.000000 | -0.000000 | 1793 | 1658.829499 | 1719.9 | 1780.970501 | 0.959230 |  |
| GO:0048857\_neural\_nucleus\_development | 7 | 0 | 0.000000 | -0.000000 | 1793 | 1658.829499 | 1719.9 | 1780.970501 | 0.959230 |  |
| GO:0050755\_chemokine\_metabolic\_process | 7 | 0 | 0.000000 | -0.000000 | 1793 | 1658.829499 | 1719.9 | 1780.970501 | 0.959230 |  |
| GO:0050773\_regulation\_of\_dendrite\_development | 7 | 0 | 0.000000 | -0.000000 | 1793 | 1658.829499 | 1719.9 | 1780.970501 | 0.959230 |  |
| GO:0051028\_mRNA\_transport | 7 | 0 | 0.000000 | -0.000000 | 1793 | 1658.829499 | 1719.9 | 1780.970501 | 0.959230 |  |
| GO:0051785\_positive\_regulation\_of\_nuclear\_division | 7 | 0 | 0.000000 | -0.000000 | 1793 | 1658.829499 | 1719.9 | 1780.970501 | 0.959230 |  |
| GO:0051928\_positive\_regulation\_of\_calcium\_ion\_transport | 7 | 0 | 0.000000 | -0.000000 | 1793 | 1658.829499 | 1719.9 | 1780.970501 | 0.959230 |  |
| GO:0055069\_zinc\_ion\_homeostasis | 7 | 0 | 0.000000 | -0.000000 | 1793 | 1658.829499 | 1719.9 | 1780.970501 | 0.959230 |  |
| GO:0055070\_copper\_ion\_homeostasis | 7 | 0 | 0.000000 | -0.000000 | 1793 | 1658.829499 | 1719.9 | 1780.970501 | 0.959230 |  |
| GO:0060037\_pharyngeal\_system\_development | 7 | 0 | 0.000000 | -0.000000 | 1793 | 1658.829499 | 1719.9 | 1780.970501 | 0.959230 |  |
| GO:0060080\_regulation\_of\_inhibitory\_postsynaptic\_membrane\_potential | 7 | 0 | 0.000000 | -0.000000 | 1793 | 1658.829499 | 1719.9 | 1780.970501 | 0.959230 |  |
| GO:0060088\_auditory\_receptor\_cell\_stereocilium\_organization | 7 | 0 | 0.000000 | -0.000000 | 1793 | 1658.829499 | 1719.9 | 1780.970501 | 0.959230 |  |
| GO:0060117\_auditory\_receptor\_cell\_development | 7 | 0 | 0.000000 | -0.000000 | 1793 | 1658.829499 | 1719.9 | 1780.970501 | 0.959230 |  |
| GO:0060441\_branching\_involved\_in\_lung\_morphogenesis | 7 | 0 | 0.000000 | -0.000000 | 1793 | 1658.829499 | 1719.9 | 1780.970501 | 0.959230 |  |
| GO:0060526\_prostate\_glandular\_acinus\_morphogenesis | 7 | 0 | 0.000000 | -0.000000 | 1793 | 1658.829499 | 1719.9 | 1780.970501 | 0.959230 |  |
| GO:0060527\_prostate\_epithelial\_cord\_arborization\_involved\_in\_prostate\_glandular\_acinus\_morphogenesis | 7 | 0 | 0.000000 | -0.000000 | 1793 | 1658.829499 | 1719.9 | 1780.970501 | 0.959230 |  |
| GO:0060664\_epithelial\_cell\_proliferation\_involved\_in\_salivary\_gland\_morphogenesis | 7 | 0 | 0.000000 | -0.000000 | 1793 | 1658.829499 | 1719.9 | 1780.970501 | 0.959230 |  |
| GO:0060687\_regulation\_of\_branching\_involved\_in\_prostate\_gland\_morphogenesis | 7 | 0 | 0.000000 | -0.000000 | 1793 | 1658.829499 | 1719.9 | 1780.970501 | 0.959230 |  |
| GO:0060770\_negative\_regulation\_of\_epithelial\_cell\_proliferation\_involved\_in\_prostate\_gland\_development | 7 | 0 | 0.000000 | -0.000000 | 1793 | 1658.829499 | 1719.9 | 1780.970501 | 0.959230 |  |
| GO:0060795\_cell\_fate\_commitment\_involved\_in\_the\_formation\_of\_primary\_germ\_layers | 7 | 0 | 0.000000 | -0.000000 | 1793 | 1658.829499 | 1719.9 | 1780.970501 | 0.959230 |  |
| GO:0070228\_regulation\_of\_lymphocyte\_apoptosis | 7 | 0 | 0.000000 | -0.000000 | 1793 | 1658.829499 | 1719.9 | 1780.970501 | 0.959230 |  |
| GO:0070646\_protein\_modification\_by\_small\_protein\_removal | 7 | 0 | 0.000000 | -0.000000 | 1793 | 1658.829499 | 1719.9 | 1780.970501 | 0.959230 |  |
| GO:0000087\_M\_phase\_of\_mitotic\_cell\_cycle | 25 | 0 | 0.000000 | -0.000000 | 1812 | 1677.721069 | 1736.96 | 1796.198931 | 0.958587 |  |
| GO:0002526\_acute\_inflammatory\_response | 25 | 0 | 0.000000 | -0.000000 | 1812 | 1677.721069 | 1736.96 | 1796.198931 | 0.958587 |  |
| GO:0006302\_double-strand\_break\_repair | 25 | 0 | 0.000000 | -0.000000 | 1812 | 1677.721069 | 1736.96 | 1796.198931 | 0.958587 |  |
| GO:0006775\_fat-soluble\_vitamin\_metabolic\_process | 25 | 0 | 0.000000 | -0.000000 | 1812 | 1677.721069 | 1736.96 | 1796.198931 | 0.958587 |  |
| GO:0007492\_endoderm\_development | 25 | 0 | 0.000000 | -0.000000 | 1812 | 1677.721069 | 1736.96 | 1796.198931 | 0.958587 |  |
| GO:0007628\_adult\_walking\_behavior | 25 | 0 | 0.000000 | -0.000000 | 1812 | 1677.721069 | 1736.96 | 1796.198931 | 0.958587 |  |
| GO:0021983\_pituitary\_gland\_development | 25 | 0 | 0.000000 | -0.000000 | 1812 | 1677.721069 | 1736.96 | 1796.198931 | 0.958587 |  |
| GO:0031348\_negative\_regulation\_of\_defense\_response | 25 | 0 | 0.000000 | -0.000000 | 1812 | 1677.721069 | 1736.96 | 1796.198931 | 0.958587 |  |
| GO:0032103\_positive\_regulation\_of\_response\_to\_external\_stimulus | 25 | 0 | 0.000000 | -0.000000 | 1812 | 1677.721069 | 1736.96 | 1796.198931 | 0.958587 |  |
| GO:0032945\_negative\_regulation\_of\_mononuclear\_cell\_proliferation | 25 | 0 | 0.000000 | -0.000000 | 1812 | 1677.721069 | 1736.96 | 1796.198931 | 0.958587 |  |
| GO:0035137\_hindlimb\_morphogenesis | 25 | 0 | 0.000000 | -0.000000 | 1812 | 1677.721069 | 1736.96 | 1796.198931 | 0.958587 |  |
| GO:0043543\_protein\_amino\_acid\_acylation | 25 | 0 | 0.000000 | -0.000000 | 1812 | 1677.721069 | 1736.96 | 1796.198931 | 0.958587 |  |
| GO:0045089\_positive\_regulation\_of\_innate\_immune\_response | 25 | 0 | 0.000000 | -0.000000 | 1812 | 1677.721069 | 1736.96 | 1796.198931 | 0.958587 |  |
| GO:0048285\_organelle\_fission | 25 | 0 | 0.000000 | -0.000000 | 1812 | 1677.721069 | 1736.96 | 1796.198931 | 0.958587 |  |
| GO:0050672\_negative\_regulation\_of\_lymphocyte\_proliferation | 25 | 0 | 0.000000 | -0.000000 | 1812 | 1677.721069 | 1736.96 | 1796.198931 | 0.958587 |  |
| GO:0051090\_regulation\_of\_transcription\_factor\_activity | 25 | 0 | 0.000000 | -0.000000 | 1812 | 1677.721069 | 1736.96 | 1796.198931 | 0.958587 |  |
| GO:0060688\_regulation\_of\_morphogenesis\_of\_a\_branching\_structure | 25 | 0 | 0.000000 | -0.000000 | 1812 | 1677.721069 | 1736.96 | 1796.198931 | 0.958587 |  |
| GO:0070664\_negative\_regulation\_of\_leukocyte\_proliferation | 25 | 0 | 0.000000 | -0.000000 | 1812 | 1677.721069 | 1736.96 | 1796.198931 | 0.958587 |  |
| GO:0090046\_regulation\_of\_transcription\_regulator\_activity | 25 | 0 | 0.000000 | -0.000000 | 1812 | 1677.721069 | 1736.96 | 1796.198931 | 0.958587 |  |
| GO:0000209\_protein\_polyubiquitination | 10 | 0 | 0.000000 | -0.000000 | 1912 | 1787.764685 | 1843.46 | 1899.155315 | 0.964153 |  |
| GO:0000724\_double-strand\_break\_repair\_via\_homologous\_recombination | 10 | 0 | 0.000000 | -0.000000 | 1912 | 1787.764685 | 1843.46 | 1899.155315 | 0.964153 |  |
| GO:0000725\_recombinational\_repair | 10 | 0 | 0.000000 | -0.000000 | 1912 | 1787.764685 | 1843.46 | 1899.155315 | 0.964153 |  |
| GO:0001578\_microtubule\_bundle\_formation | 10 | 0 | 0.000000 | -0.000000 | 1912 | 1787.764685 | 1843.46 | 1899.155315 | 0.964153 |  |
| GO:0001659\_temperature\_homeostasis | 10 | 0 | 0.000000 | -0.000000 | 1912 | 1787.764685 | 1843.46 | 1899.155315 | 0.964153 |  |
| GO:0001773\_myeloid\_dendritic\_cell\_activation | 10 | 0 | 0.000000 | -0.000000 | 1912 | 1787.764685 | 1843.46 | 1899.155315 | 0.964153 |  |
| GO:0001832\_blastocyst\_growth | 10 | 0 | 0.000000 | -0.000000 | 1912 | 1787.764685 | 1843.46 | 1899.155315 | 0.964153 |  |
| GO:0001990\_regulation\_of\_systemic\_arterial\_blood\_pressure\_by\_hormone | 10 | 0 | 0.000000 | -0.000000 | 1912 | 1787.764685 | 1843.46 | 1899.155315 | 0.964153 |  |
| GO:0002070\_epithelial\_cell\_maturation | 10 | 0 | 0.000000 | -0.000000 | 1912 | 1787.764685 | 1843.46 | 1899.155315 | 0.964153 |  |
| GO:0002673\_regulation\_of\_acute\_inflammatory\_response | 10 | 0 | 0.000000 | -0.000000 | 1912 | 1787.764685 | 1843.46 | 1899.155315 | 0.964153 |  |
| GO:0002762\_negative\_regulation\_of\_myeloid\_leukocyte\_differentiation | 10 | 0 | 0.000000 | -0.000000 | 1912 | 1787.764685 | 1843.46 | 1899.155315 | 0.964153 |  |
| GO:0006040\_amino\_sugar\_metabolic\_process | 10 | 0 | 0.000000 | -0.000000 | 1912 | 1787.764685 | 1843.46 | 1899.155315 | 0.964153 |  |
| GO:0006081\_cellular\_aldehyde\_metabolic\_process | 10 | 0 | 0.000000 | -0.000000 | 1912 | 1787.764685 | 1843.46 | 1899.155315 | 0.964153 |  |
| GO:0006289\_nucleotide-excision\_repair | 10 | 0 | 0.000000 | -0.000000 | 1912 | 1787.764685 | 1843.46 | 1899.155315 | 0.964153 |  |
| GO:0006342\_chromatin\_silencing | 10 | 0 | 0.000000 | -0.000000 | 1912 | 1787.764685 | 1843.46 | 1899.155315 | 0.964153 |  |
| GO:0006405\_RNA\_export\_from\_nucleus | 10 | 0 | 0.000000 | -0.000000 | 1912 | 1787.764685 | 1843.46 | 1899.155315 | 0.964153 |  |
| GO:0006801\_superoxide\_metabolic\_process | 10 | 0 | 0.000000 | -0.000000 | 1912 | 1787.764685 | 1843.46 | 1899.155315 | 0.964153 |  |
| GO:0006805\_xenobiotic\_metabolic\_process | 10 | 0 | 0.000000 | -0.000000 | 1912 | 1787.764685 | 1843.46 | 1899.155315 | 0.964153 |  |
| GO:0006921\_cell\_structure\_disassembly\_during\_apoptosis | 10 | 0 | 0.000000 | -0.000000 | 1912 | 1787.764685 | 1843.46 | 1899.155315 | 0.964153 |  |
| GO:0006968\_cellular\_defense\_response | 10 | 0 | 0.000000 | -0.000000 | 1912 | 1787.764685 | 1843.46 | 1899.155315 | 0.964153 |  |
| GO:0007006\_mitochondrial\_membrane\_organization | 10 | 0 | 0.000000 | -0.000000 | 1912 | 1787.764685 | 1843.46 | 1899.155315 | 0.964153 |  |
| GO:0007044\_cell-substrate\_junction\_assembly | 10 | 0 | 0.000000 | -0.000000 | 1912 | 1787.764685 | 1843.46 | 1899.155315 | 0.964153 |  |
| GO:0007093\_mitotic\_cell\_cycle\_checkpoint | 10 | 0 | 0.000000 | -0.000000 | 1912 | 1787.764685 | 1843.46 | 1899.155315 | 0.964153 |  |
| GO:0007172\_signal\_complex\_assembly | 10 | 0 | 0.000000 | -0.000000 | 1912 | 1787.764685 | 1843.46 | 1899.155315 | 0.964153 |  |
| GO:0007194\_negative\_regulation\_of\_adenylate\_cyclase\_activity | 10 | 0 | 0.000000 | -0.000000 | 1912 | 1787.764685 | 1843.46 | 1899.155315 | 0.964153 |  |
| GO:0008088\_axon\_cargo\_transport | 10 | 0 | 0.000000 | -0.000000 | 1912 | 1787.764685 | 1843.46 | 1899.155315 | 0.964153 |  |
| GO:0008206\_bile\_acid\_metabolic\_process | 10 | 0 | 0.000000 | -0.000000 | 1912 | 1787.764685 | 1843.46 | 1899.155315 | 0.964153 |  |
| GO:0009066\_aspartate\_family\_amino\_acid\_metabolic\_process | 10 | 0 | 0.000000 | -0.000000 | 1912 | 1787.764685 | 1843.46 | 1899.155315 | 0.964153 |  |
| GO:0009110\_vitamin\_biosynthetic\_process | 10 | 0 | 0.000000 | -0.000000 | 1912 | 1787.764685 | 1843.46 | 1899.155315 | 0.964153 |  |
| GO:0009620\_response\_to\_fungus | 10 | 0 | 0.000000 | -0.000000 | 1912 | 1787.764685 | 1843.46 | 1899.155315 | 0.964153 |  |
| GO:0009743\_response\_to\_carbohydrate\_stimulus | 10 | 0 | 0.000000 | -0.000000 | 1912 | 1787.764685 | 1843.46 | 1899.155315 | 0.964153 |  |
| GO:0009948\_anterior\_posterior\_axis\_specification | 10 | 0 | 0.000000 | -0.000000 | 1912 | 1787.764685 | 1843.46 | 1899.155315 | 0.964153 |  |
| GO:0010827\_regulation\_of\_glucose\_transport | 10 | 0 | 0.000000 | -0.000000 | 1912 | 1787.764685 | 1843.46 | 1899.155315 | 0.964153 |  |
| GO:0015718\_monocarboxylic\_acid\_transport | 10 | 0 | 0.000000 | -0.000000 | 1912 | 1787.764685 | 1843.46 | 1899.155315 | 0.964153 |  |
| GO:0016197\_endosome\_transport | 10 | 0 | 0.000000 | -0.000000 | 1912 | 1787.764685 | 1843.46 | 1899.155315 | 0.964153 |  |
| GO:0016486\_peptide\_hormone\_processing | 10 | 0 | 0.000000 | -0.000000 | 1912 | 1787.764685 | 1843.46 | 1899.155315 | 0.964153 |  |
| GO:0017156\_calcium\_ion-dependent\_exocytosis | 10 | 0 | 0.000000 | -0.000000 | 1912 | 1787.764685 | 1843.46 | 1899.155315 | 0.964153 |  |
| GO:0019321\_pentose\_metabolic\_process | 10 | 0 | 0.000000 | -0.000000 | 1912 | 1787.764685 | 1843.46 | 1899.155315 | 0.964153 |  |
| GO:0021534\_cell\_proliferation\_in\_hindbrain | 10 | 0 | 0.000000 | -0.000000 | 1912 | 1787.764685 | 1843.46 | 1899.155315 | 0.964153 |  |
| GO:0021871\_forebrain\_regionalization | 10 | 0 | 0.000000 | -0.000000 | 1912 | 1787.764685 | 1843.46 | 1899.155315 | 0.964153 |  |
| GO:0021895\_cerebral\_cortex\_neuron\_differentiation | 10 | 0 | 0.000000 | -0.000000 | 1912 | 1787.764685 | 1843.46 | 1899.155315 | 0.964153 |  |
| GO:0021924\_cell\_proliferation\_in\_the\_external\_granule\_layer | 10 | 0 | 0.000000 | -0.000000 | 1912 | 1787.764685 | 1843.46 | 1899.155315 | 0.964153 |  |
| GO:0021930\_granule\_cell\_precursor\_proliferation | 10 | 0 | 0.000000 | -0.000000 | 1912 | 1787.764685 | 1843.46 | 1899.155315 | 0.964153 |  |
| GO:0022900\_electron\_transport\_chain | 10 | 0 | 0.000000 | -0.000000 | 1912 | 1787.764685 | 1843.46 | 1899.155315 | 0.964153 |  |
| GO:0022904\_respiratory\_electron\_transport\_chain | 10 | 0 | 0.000000 | -0.000000 | 1912 | 1787.764685 | 1843.46 | 1899.155315 | 0.964153 |  |
| GO:0030168\_platelet\_activation | 10 | 0 | 0.000000 | -0.000000 | 1912 | 1787.764685 | 1843.46 | 1899.155315 | 0.964153 |  |
| GO:0030833\_regulation\_of\_actin\_filament\_polymerization | 10 | 0 | 0.000000 | -0.000000 | 1912 | 1787.764685 | 1843.46 | 1899.155315 | 0.964153 |  |
| GO:0031280\_negative\_regulation\_of\_cyclase\_activity | 10 | 0 | 0.000000 | -0.000000 | 1912 | 1787.764685 | 1843.46 | 1899.155315 | 0.964153 |  |
| GO:0031331\_positive\_regulation\_of\_cellular\_catabolic\_process | 10 | 0 | 0.000000 | -0.000000 | 1912 | 1787.764685 | 1843.46 | 1899.155315 | 0.964153 |  |
| GO:0031645\_negative\_regulation\_of\_neurological\_system\_process | 10 | 0 | 0.000000 | -0.000000 | 1912 | 1787.764685 | 1843.46 | 1899.155315 | 0.964153 |  |
| GO:0032318\_regulation\_of\_Ras\_GTPase\_activity | 10 | 0 | 0.000000 | -0.000000 | 1912 | 1787.764685 | 1843.46 | 1899.155315 | 0.964153 |  |
| GO:0032602\_chemokine\_production | 10 | 0 | 0.000000 | -0.000000 | 1912 | 1787.764685 | 1843.46 | 1899.155315 | 0.964153 |  |
| GO:0032633\_interleukin-4\_production | 10 | 0 | 0.000000 | -0.000000 | 1912 | 1787.764685 | 1843.46 | 1899.155315 | 0.964153 |  |
| GO:0032642\_regulation\_of\_chemokine\_production | 10 | 0 | 0.000000 | -0.000000 | 1912 | 1787.764685 | 1843.46 | 1899.155315 | 0.964153 |  |
| GO:0032673\_regulation\_of\_interleukin-4\_production | 10 | 0 | 0.000000 | -0.000000 | 1912 | 1787.764685 | 1843.46 | 1899.155315 | 0.964153 |  |
| GO:0032760\_positive\_regulation\_of\_tumor\_necrosis\_factor\_production | 10 | 0 | 0.000000 | -0.000000 | 1912 | 1787.764685 | 1843.46 | 1899.155315 | 0.964153 |  |
| GO:0033081\_regulation\_of\_T\_cell\_differentiation\_in\_the\_thymus | 10 | 0 | 0.000000 | -0.000000 | 1912 | 1787.764685 | 1843.46 | 1899.155315 | 0.964153 |  |
| GO:0034105\_positive\_regulation\_of\_tissue\_remodeling | 10 | 0 | 0.000000 | -0.000000 | 1912 | 1787.764685 | 1843.46 | 1899.155315 | 0.964153 |  |
| GO:0034637\_cellular\_carbohydrate\_biosynthetic\_process | 10 | 0 | 0.000000 | -0.000000 | 1912 | 1787.764685 | 1843.46 | 1899.155315 | 0.964153 |  |
| GO:0042088\_T-helper\_1\_type\_immune\_response | 10 | 0 | 0.000000 | -0.000000 | 1912 | 1787.764685 | 1843.46 | 1899.155315 | 0.964153 |  |
| GO:0042116\_macrophage\_activation | 10 | 0 | 0.000000 | -0.000000 | 1912 | 1787.764685 | 1843.46 | 1899.155315 | 0.964153 |  |
| GO:0042177\_negative\_regulation\_of\_protein\_catabolic\_process | 10 | 0 | 0.000000 | -0.000000 | 1912 | 1787.764685 | 1843.46 | 1899.155315 | 0.964153 |  |
| GO:0042755\_eating\_behavior | 10 | 0 | 0.000000 | -0.000000 | 1912 | 1787.764685 | 1843.46 | 1899.155315 | 0.964153 |  |
| GO:0043113\_receptor\_clustering | 10 | 0 | 0.000000 | -0.000000 | 1912 | 1787.764685 | 1843.46 | 1899.155315 | 0.964153 |  |
| GO:0043330\_response\_to\_exogenous\_dsRNA | 10 | 0 | 0.000000 | -0.000000 | 1912 | 1787.764685 | 1843.46 | 1899.155315 | 0.964153 |  |
| GO:0043488\_regulation\_of\_mRNA\_stability | 10 | 0 | 0.000000 | -0.000000 | 1912 | 1787.764685 | 1843.46 | 1899.155315 | 0.964153 |  |
| GO:0043506\_regulation\_of\_JUN\_kinase\_activity | 10 | 0 | 0.000000 | -0.000000 | 1912 | 1787.764685 | 1843.46 | 1899.155315 | 0.964153 |  |
| GO:0044259\_multicellular\_organismal\_macromolecule\_metabolic\_process | 10 | 0 | 0.000000 | -0.000000 | 1912 | 1787.764685 | 1843.46 | 1899.155315 | 0.964153 |  |
| GO:0045132\_meiotic\_chromosome\_segregation | 10 | 0 | 0.000000 | -0.000000 | 1912 | 1787.764685 | 1843.46 | 1899.155315 | 0.964153 |  |
| GO:0045446\_endothelial\_cell\_differentiation | 10 | 0 | 0.000000 | -0.000000 | 1912 | 1787.764685 | 1843.46 | 1899.155315 | 0.964153 |  |
| GO:0045669\_positive\_regulation\_of\_osteoblast\_differentiation | 10 | 0 | 0.000000 | -0.000000 | 1912 | 1787.764685 | 1843.46 | 1899.155315 | 0.964153 |  |
| GO:0045776\_negative\_regulation\_of\_blood\_pressure | 10 | 0 | 0.000000 | -0.000000 | 1912 | 1787.764685 | 1843.46 | 1899.155315 | 0.964153 |  |
| GO:0045777\_positive\_regulation\_of\_blood\_pressure | 10 | 0 | 0.000000 | -0.000000 | 1912 | 1787.764685 | 1843.46 | 1899.155315 | 0.964153 |  |
| GO:0045814\_negative\_regulation\_of\_gene\_expression\_\_epigenetic | 10 | 0 | 0.000000 | -0.000000 | 1912 | 1787.764685 | 1843.46 | 1899.155315 | 0.964153 |  |
| GO:0046887\_positive\_regulation\_of\_hormone\_secretion | 10 | 0 | 0.000000 | -0.000000 | 1912 | 1787.764685 | 1843.46 | 1899.155315 | 0.964153 |  |
| GO:0048339\_paraxial\_mesoderm\_development | 10 | 0 | 0.000000 | -0.000000 | 1912 | 1787.764685 | 1843.46 | 1899.155315 | 0.964153 |  |
| GO:0048384\_retinoic\_acid\_receptor\_signaling\_pathway | 10 | 0 | 0.000000 | -0.000000 | 1912 | 1787.764685 | 1843.46 | 1899.155315 | 0.964153 |  |
| GO:0048596\_embryonic\_camera-type\_eye\_morphogenesis | 10 | 0 | 0.000000 | -0.000000 | 1912 | 1787.764685 | 1843.46 | 1899.155315 | 0.964153 |  |
| GO:0048738\_cardiac\_muscle\_tissue\_development | 10 | 0 | 0.000000 | -0.000000 | 1912 | 1787.764685 | 1843.46 | 1899.155315 | 0.964153 |  |
| GO:0050657\_nucleic\_acid\_transport | 10 | 0 | 0.000000 | -0.000000 | 1912 | 1787.764685 | 1843.46 | 1899.155315 | 0.964153 |  |
| GO:0050658\_RNA\_transport | 10 | 0 | 0.000000 | -0.000000 | 1912 | 1787.764685 | 1843.46 | 1899.155315 | 0.964153 |  |
| GO:0050663\_cytokine\_secretion | 10 | 0 | 0.000000 | -0.000000 | 1912 | 1787.764685 | 1843.46 | 1899.155315 | 0.964153 |  |
| GO:0050714\_positive\_regulation\_of\_protein\_secretion | 10 | 0 | 0.000000 | -0.000000 | 1912 | 1787.764685 | 1843.46 | 1899.155315 | 0.964153 |  |
| GO:0050879\_multicellular\_organismal\_movement | 10 | 0 | 0.000000 | -0.000000 | 1912 | 1787.764685 | 1843.46 | 1899.155315 | 0.964153 |  |
| GO:0050881\_musculoskeletal\_movement | 10 | 0 | 0.000000 | -0.000000 | 1912 | 1787.764685 | 1843.46 | 1899.155315 | 0.964153 |  |
| GO:0050886\_endocrine\_process | 10 | 0 | 0.000000 | -0.000000 | 1912 | 1787.764685 | 1843.46 | 1899.155315 | 0.964153 |  |
| GO:0050892\_intestinal\_absorption | 10 | 0 | 0.000000 | -0.000000 | 1912 | 1787.764685 | 1843.46 | 1899.155315 | 0.964153 |  |
| GO:0051224\_negative\_regulation\_of\_protein\_transport | 10 | 0 | 0.000000 | -0.000000 | 1912 | 1787.764685 | 1843.46 | 1899.155315 | 0.964153 |  |
| GO:0051236\_establishment\_of\_RNA\_localization | 10 | 0 | 0.000000 | -0.000000 | 1912 | 1787.764685 | 1843.46 | 1899.155315 | 0.964153 |  |
| GO:0051262\_protein\_tetramerization | 10 | 0 | 0.000000 | -0.000000 | 1912 | 1787.764685 | 1843.46 | 1899.155315 | 0.964153 |  |
| GO:0051350\_negative\_regulation\_of\_lyase\_activity | 10 | 0 | 0.000000 | -0.000000 | 1912 | 1787.764685 | 1843.46 | 1899.155315 | 0.964153 |  |
| GO:0051445\_regulation\_of\_meiotic\_cell\_cycle | 10 | 0 | 0.000000 | -0.000000 | 1912 | 1787.764685 | 1843.46 | 1899.155315 | 0.964153 |  |
| GO:0051651\_maintenance\_of\_location\_in\_cell | 10 | 0 | 0.000000 | -0.000000 | 1912 | 1787.764685 | 1843.46 | 1899.155315 | 0.964153 |  |
| GO:0060135\_maternal\_process\_involved\_in\_female\_pregnancy | 10 | 0 | 0.000000 | -0.000000 | 1912 | 1787.764685 | 1843.46 | 1899.155315 | 0.964153 |  |
| GO:0060216\_definitive\_hemopoiesis | 10 | 0 | 0.000000 | -0.000000 | 1912 | 1787.764685 | 1843.46 | 1899.155315 | 0.964153 |  |
| GO:0060343\_trabecula\_formation | 10 | 0 | 0.000000 | -0.000000 | 1912 | 1787.764685 | 1843.46 | 1899.155315 | 0.964153 |  |
| GO:0060601\_lateral\_sprouting\_from\_an\_epithelium | 10 | 0 | 0.000000 | -0.000000 | 1912 | 1787.764685 | 1843.46 | 1899.155315 | 0.964153 |  |
| GO:0060669\_embryonic\_placenta\_morphogenesis | 10 | 0 | 0.000000 | -0.000000 | 1912 | 1787.764685 | 1843.46 | 1899.155315 | 0.964153 |  |
| GO:0060706\_cell\_differentiation\_involved\_in\_embryonic\_placenta\_development | 10 | 0 | 0.000000 | -0.000000 | 1912 | 1787.764685 | 1843.46 | 1899.155315 | 0.964153 |  |
| GO:0060768\_regulation\_of\_epithelial\_cell\_proliferation\_involved\_in\_prostate\_gland\_development | 10 | 0 | 0.000000 | -0.000000 | 1912 | 1787.764685 | 1843.46 | 1899.155315 | 0.964153 |  |
| GO:0006732\_coenzyme\_metabolic\_process | 46 | 0 | 0.000000 | -0.000000 | 1916 | 1794.515790 | 1849.56 | 1904.604210 | 0.965324 |  |
| GO:0009581\_detection\_of\_external\_stimulus | 46 | 0 | 0.000000 | -0.000000 | 1916 | 1794.515790 | 1849.56 | 1904.604210 | 0.965324 |  |
| GO:0030218\_erythrocyte\_differentiation | 46 | 0 | 0.000000 | -0.000000 | 1916 | 1794.515790 | 1849.56 | 1904.604210 | 0.965324 |  |
| GO:0030850\_prostate\_gland\_development | 46 | 0 | 0.000000 | -0.000000 | 1916 | 1794.515790 | 1849.56 | 1904.604210 | 0.965324 |  |
| GO:0006171\_cAMP\_biosynthetic\_process | 42 | 0 | 0.000000 | -0.000000 | 1922 | 1805.770169 | 1859.55 | 1913.329831 | 0.967508 |  |
| GO:0010769\_regulation\_of\_cell\_morphogenesis\_involved\_in\_differentiation | 42 | 0 | 0.000000 | -0.000000 | 1922 | 1805.770169 | 1859.55 | 1913.329831 | 0.967508 |  |
| GO:0042476\_odontogenesis | 42 | 0 | 0.000000 | -0.000000 | 1922 | 1805.770169 | 1859.55 | 1913.329831 | 0.967508 |  |
| GO:0045637\_regulation\_of\_myeloid\_cell\_differentiation | 42 | 0 | 0.000000 | -0.000000 | 1922 | 1805.770169 | 1859.55 | 1913.329831 | 0.967508 |  |
| GO:0048515\_spermatid\_differentiation | 42 | 0 | 0.000000 | -0.000000 | 1922 | 1805.770169 | 1859.55 | 1913.329831 | 0.967508 |  |
| GO:0051345\_positive\_regulation\_of\_hydrolase\_activity | 42 | 0 | 0.000000 | -0.000000 | 1922 | 1805.770169 | 1859.55 | 1913.329831 | 0.967508 |  |
| GO:0005977\_glycogen\_metabolic\_process | 20 | 0 | 0.000000 | -0.000000 | 1945 | 1830.492337 | 1882.24 | 1933.987663 | 0.967733 |  |
| GO:0006073\_cellular\_glucan\_metabolic\_process | 20 | 0 | 0.000000 | -0.000000 | 1945 | 1830.492337 | 1882.24 | 1933.987663 | 0.967733 |  |
| GO:0006518\_peptide\_metabolic\_process | 20 | 0 | 0.000000 | -0.000000 | 1945 | 1830.492337 | 1882.24 | 1933.987663 | 0.967733 |  |
| GO:0006584\_catecholamine\_metabolic\_process | 20 | 0 | 0.000000 | -0.000000 | 1945 | 1830.492337 | 1882.24 | 1933.987663 | 0.967733 |  |
| GO:0007528\_neuromuscular\_junction\_development | 20 | 0 | 0.000000 | -0.000000 | 1945 | 1830.492337 | 1882.24 | 1933.987663 | 0.967733 |  |
| GO:0007586\_digestion | 20 | 0 | 0.000000 | -0.000000 | 1945 | 1830.492337 | 1882.24 | 1933.987663 | 0.967733 |  |
| GO:0008360\_regulation\_of\_cell\_shape | 20 | 0 | 0.000000 | -0.000000 | 1945 | 1830.492337 | 1882.24 | 1933.987663 | 0.967733 |  |
| GO:0009712\_catechol\_metabolic\_process | 20 | 0 | 0.000000 | -0.000000 | 1945 | 1830.492337 | 1882.24 | 1933.987663 | 0.967733 |  |
| GO:0010927\_cellular\_component\_assembly\_involved\_in\_morphogenesis | 20 | 0 | 0.000000 | -0.000000 | 1945 | 1830.492337 | 1882.24 | 1933.987663 | 0.967733 |  |
| GO:0016571\_histone\_methylation | 20 | 0 | 0.000000 | -0.000000 | 1945 | 1830.492337 | 1882.24 | 1933.987663 | 0.967733 |  |
| GO:0018958\_phenol\_metabolic\_process | 20 | 0 | 0.000000 | -0.000000 | 1945 | 1830.492337 | 1882.24 | 1933.987663 | 0.967733 |  |
| GO:0021695\_cerebellar\_cortex\_development | 20 | 0 | 0.000000 | -0.000000 | 1945 | 1830.492337 | 1882.24 | 1933.987663 | 0.967733 |  |
| GO:0031128\_developmental\_induction | 20 | 0 | 0.000000 | -0.000000 | 1945 | 1830.492337 | 1882.24 | 1933.987663 | 0.967733 |  |
| GO:0031214\_biomineral\_formation | 20 | 0 | 0.000000 | -0.000000 | 1945 | 1830.492337 | 1882.24 | 1933.987663 | 0.967733 |  |
| GO:0032582\_negative\_regulation\_of\_gene-specific\_transcription | 20 | 0 | 0.000000 | -0.000000 | 1945 | 1830.492337 | 1882.24 | 1933.987663 | 0.967733 |  |
| GO:0032640\_tumor\_necrosis\_factor\_production | 20 | 0 | 0.000000 | -0.000000 | 1945 | 1830.492337 | 1882.24 | 1933.987663 | 0.967733 |  |
| GO:0032680\_regulation\_of\_tumor\_necrosis\_factor\_production | 20 | 0 | 0.000000 | -0.000000 | 1945 | 1830.492337 | 1882.24 | 1933.987663 | 0.967733 |  |
| GO:0034311\_diol\_metabolic\_process | 20 | 0 | 0.000000 | -0.000000 | 1945 | 1830.492337 | 1882.24 | 1933.987663 | 0.967733 |  |
| GO:0044042\_glucan\_metabolic\_process | 20 | 0 | 0.000000 | -0.000000 | 1945 | 1830.492337 | 1882.24 | 1933.987663 | 0.967733 |  |
| GO:0045168\_cell-cell\_signaling\_involved\_in\_cell\_fate\_specification | 20 | 0 | 0.000000 | -0.000000 | 1945 | 1830.492337 | 1882.24 | 1933.987663 | 0.967733 |  |
| GO:0045639\_positive\_regulation\_of\_myeloid\_cell\_differentiation | 20 | 0 | 0.000000 | -0.000000 | 1945 | 1830.492337 | 1882.24 | 1933.987663 | 0.967733 |  |
| GO:0046822\_regulation\_of\_nucleocytoplasmic\_transport | 20 | 0 | 0.000000 | -0.000000 | 1945 | 1830.492337 | 1882.24 | 1933.987663 | 0.967733 |  |
| GO:0060191\_regulation\_of\_lipase\_activity | 20 | 0 | 0.000000 | -0.000000 | 1945 | 1830.492337 | 1882.24 | 1933.987663 | 0.967733 |  |
| GO:0001570\_vasculogenesis | 38 | 0 | 0.000000 | -0.000000 | 1958 | 1842.853062 | 1893.29 | 1943.726938 | 0.966951 |  |
| GO:0001649\_osteoblast\_differentiation | 38 | 0 | 0.000000 | -0.000000 | 1958 | 1842.853062 | 1893.29 | 1943.726938 | 0.966951 |  |
| GO:0001657\_ureteric\_bud\_development | 38 | 0 | 0.000000 | -0.000000 | 1958 | 1842.853062 | 1893.29 | 1943.726938 | 0.966951 |  |
| GO:0002695\_negative\_regulation\_of\_leukocyte\_activation | 38 | 0 | 0.000000 | -0.000000 | 1958 | 1842.853062 | 1893.29 | 1943.726938 | 0.966951 |  |
| GO:0006820\_anion\_transport | 38 | 0 | 0.000000 | -0.000000 | 1958 | 1842.853062 | 1893.29 | 1943.726938 | 0.966951 |  |
| GO:0010975\_regulation\_of\_neuron\_projection\_development | 38 | 0 | 0.000000 | -0.000000 | 1958 | 1842.853062 | 1893.29 | 1943.726938 | 0.966951 |  |
| GO:0016053\_organic\_acid\_biosynthetic\_process | 38 | 0 | 0.000000 | -0.000000 | 1958 | 1842.853062 | 1893.29 | 1943.726938 | 0.966951 |  |
| GO:0032259\_methylation | 38 | 0 | 0.000000 | -0.000000 | 1958 | 1842.853062 | 1893.29 | 1943.726938 | 0.966951 |  |
| GO:0042493\_response\_to\_drug | 38 | 0 | 0.000000 | -0.000000 | 1958 | 1842.853062 | 1893.29 | 1943.726938 | 0.966951 |  |
| GO:0043414\_biopolymer\_methylation | 38 | 0 | 0.000000 | -0.000000 | 1958 | 1842.853062 | 1893.29 | 1943.726938 | 0.966951 |  |
| GO:0046394\_carboxylic\_acid\_biosynthetic\_process | 38 | 0 | 0.000000 | -0.000000 | 1958 | 1842.853062 | 1893.29 | 1943.726938 | 0.966951 |  |
| GO:0050727\_regulation\_of\_inflammatory\_response | 38 | 0 | 0.000000 | -0.000000 | 1958 | 1842.853062 | 1893.29 | 1943.726938 | 0.966951 |  |
| GO:0050866\_negative\_regulation\_of\_cell\_activation | 38 | 0 | 0.000000 | -0.000000 | 1958 | 1842.853062 | 1893.29 | 1943.726938 | 0.966951 |  |
| GO:0001707\_mesoderm\_formation | 32 | 0 | 0.000000 | -0.000000 | 1964 | 1852.739710 | 1902.27 | 1951.800290 | 0.968569 |  |
| GO:0007249\_I-kappaB\_kinase\_NF-kappaB\_cascade | 32 | 0 | 0.000000 | -0.000000 | 1964 | 1852.739710 | 1902.27 | 1951.800290 | 0.968569 |  |
| GO:0048332\_mesoderm\_morphogenesis | 32 | 0 | 0.000000 | -0.000000 | 1964 | 1852.739710 | 1902.27 | 1951.800290 | 0.968569 |  |
| GO:0050770\_regulation\_of\_axonogenesis | 32 | 0 | 0.000000 | -0.000000 | 1964 | 1852.739710 | 1902.27 | 1951.800290 | 0.968569 |  |
| GO:0051259\_protein\_oligomerization | 32 | 0 | 0.000000 | -0.000000 | 1964 | 1852.739710 | 1902.27 | 1951.800290 | 0.968569 |  |
| GO:0051493\_regulation\_of\_cytoskeleton\_organization | 32 | 0 | 0.000000 | -0.000000 | 1964 | 1852.739710 | 1902.27 | 1951.800290 | 0.968569 |  |
| GO:0000375\_RNA\_splicing\_\_via\_transesterification\_reactions | 12 | 0 | 0.000000 | -0.000000 | 2031 | 1924.251154 | 1971.43 | 2018.608846 | 0.970670 |  |
| GO:0000377\_RNA\_splicing\_\_via\_transesterification\_reactions\_with\_bulged\_adenosine\_as\_nucleophile | 12 | 0 | 0.000000 | -0.000000 | 2031 | 1924.251154 | 1971.43 | 2018.608846 | 0.970670 |  |
| GO:0000398\_nuclear\_mRNA\_splicing\_\_via\_spliceosome | 12 | 0 | 0.000000 | -0.000000 | 2031 | 1924.251154 | 1971.43 | 2018.608846 | 0.970670 |  |
| GO:0001662\_behavioral\_fear\_response | 12 | 0 | 0.000000 | -0.000000 | 2031 | 1924.251154 | 1971.43 | 2018.608846 | 0.970670 |  |
| GO:0001738\_morphogenesis\_of\_a\_polarized\_epithelium | 12 | 0 | 0.000000 | -0.000000 | 2031 | 1924.251154 | 1971.43 | 2018.608846 | 0.970670 |  |
| GO:0002089\_lens\_morphogenesis\_in\_camera-type\_eye | 12 | 0 | 0.000000 | -0.000000 | 2031 | 1924.251154 | 1971.43 | 2018.608846 | 0.970670 |  |
| GO:0002209\_behavioral\_defense\_response | 12 | 0 | 0.000000 | -0.000000 | 2031 | 1924.251154 | 1971.43 | 2018.608846 | 0.970670 |  |
| GO:0002218\_activation\_of\_innate\_immune\_response | 12 | 0 | 0.000000 | -0.000000 | 2031 | 1924.251154 | 1971.43 | 2018.608846 | 0.970670 |  |
| GO:0002244\_hemopoietic\_progenitor\_cell\_differentiation | 12 | 0 | 0.000000 | -0.000000 | 2031 | 1924.251154 | 1971.43 | 2018.608846 | 0.970670 |  |
| GO:0002437\_inflammatory\_response\_to\_antigenic\_stimulus | 12 | 0 | 0.000000 | -0.000000 | 2031 | 1924.251154 | 1971.43 | 2018.608846 | 0.970670 |  |
| GO:0002763\_positive\_regulation\_of\_myeloid\_leukocyte\_differentiation | 12 | 0 | 0.000000 | -0.000000 | 2031 | 1924.251154 | 1971.43 | 2018.608846 | 0.970670 |  |
| GO:0002861\_regulation\_of\_inflammatory\_response\_to\_antigenic\_stimulus | 12 | 0 | 0.000000 | -0.000000 | 2031 | 1924.251154 | 1971.43 | 2018.608846 | 0.970670 |  |
| GO:0006308\_DNA\_catabolic\_process | 12 | 0 | 0.000000 | -0.000000 | 2031 | 1924.251154 | 1971.43 | 2018.608846 | 0.970670 |  |
| GO:0006403\_RNA\_localization | 12 | 0 | 0.000000 | -0.000000 | 2031 | 1924.251154 | 1971.43 | 2018.608846 | 0.970670 |  |
| GO:0006413\_translational\_initiation | 12 | 0 | 0.000000 | -0.000000 | 2031 | 1924.251154 | 1971.43 | 2018.608846 | 0.970670 |  |
| GO:0006446\_regulation\_of\_translational\_initiation | 12 | 0 | 0.000000 | -0.000000 | 2031 | 1924.251154 | 1971.43 | 2018.608846 | 0.970670 |  |
| GO:0006839\_mitochondrial\_transport | 12 | 0 | 0.000000 | -0.000000 | 2031 | 1924.251154 | 1971.43 | 2018.608846 | 0.970670 |  |
| GO:0006879\_cellular\_iron\_ion\_homeostasis | 12 | 0 | 0.000000 | -0.000000 | 2031 | 1924.251154 | 1971.43 | 2018.608846 | 0.970670 |  |
| GO:0007143\_female\_meiosis | 12 | 0 | 0.000000 | -0.000000 | 2031 | 1924.251154 | 1971.43 | 2018.608846 | 0.970670 |  |
| GO:0007340\_acrosome\_reaction | 12 | 0 | 0.000000 | -0.000000 | 2031 | 1924.251154 | 1971.43 | 2018.608846 | 0.970670 |  |
| GO:0008038\_neuron\_recognition | 12 | 0 | 0.000000 | -0.000000 | 2031 | 1924.251154 | 1971.43 | 2018.608846 | 0.970670 |  |
| GO:0009262\_deoxyribonucleotide\_metabolic\_process | 12 | 0 | 0.000000 | -0.000000 | 2031 | 1924.251154 | 1971.43 | 2018.608846 | 0.970670 |  |
| GO:0009886\_post-embryonic\_morphogenesis | 12 | 0 | 0.000000 | -0.000000 | 2031 | 1924.251154 | 1971.43 | 2018.608846 | 0.970670 |  |
| GO:0015872\_dopamine\_transport | 12 | 0 | 0.000000 | -0.000000 | 2031 | 1924.251154 | 1971.43 | 2018.608846 | 0.970670 |  |
| GO:0021680\_cerebellar\_Purkinje\_cell\_layer\_development | 12 | 0 | 0.000000 | -0.000000 | 2031 | 1924.251154 | 1971.43 | 2018.608846 | 0.970670 |  |
| GO:0021697\_cerebellar\_cortex\_formation | 12 | 0 | 0.000000 | -0.000000 | 2031 | 1924.251154 | 1971.43 | 2018.608846 | 0.970670 |  |
| GO:0030010\_establishment\_of\_cell\_polarity | 12 | 0 | 0.000000 | -0.000000 | 2031 | 1924.251154 | 1971.43 | 2018.608846 | 0.970670 |  |
| GO:0030166\_proteoglycan\_biosynthetic\_process | 12 | 0 | 0.000000 | -0.000000 | 2031 | 1924.251154 | 1971.43 | 2018.608846 | 0.970670 |  |
| GO:0030225\_macrophage\_differentiation | 12 | 0 | 0.000000 | -0.000000 | 2031 | 1924.251154 | 1971.43 | 2018.608846 | 0.970670 |  |
| GO:0030239\_myofibril\_assembly | 12 | 0 | 0.000000 | -0.000000 | 2031 | 1924.251154 | 1971.43 | 2018.608846 | 0.970670 |  |
| GO:0030262\_apoptotic\_nuclear\_changes | 12 | 0 | 0.000000 | -0.000000 | 2031 | 1924.251154 | 1971.43 | 2018.608846 | 0.970670 |  |
| GO:0030330\_DNA\_damage\_response\_\_signal\_transduction\_by\_p53\_class\_mediator | 12 | 0 | 0.000000 | -0.000000 | 2031 | 1924.251154 | 1971.43 | 2018.608846 | 0.970670 |  |
| GO:0030514\_negative\_regulation\_of\_BMP\_signaling\_pathway | 12 | 0 | 0.000000 | -0.000000 | 2031 | 1924.251154 | 1971.43 | 2018.608846 | 0.970670 |  |
| GO:0031056\_regulation\_of\_histone\_modification | 12 | 0 | 0.000000 | -0.000000 | 2031 | 1924.251154 | 1971.43 | 2018.608846 | 0.970670 |  |
| GO:0031109\_microtubule\_polymerization\_or\_depolymerization | 12 | 0 | 0.000000 | -0.000000 | 2031 | 1924.251154 | 1971.43 | 2018.608846 | 0.970670 |  |
| GO:0032755\_positive\_regulation\_of\_interleukin-6\_production | 12 | 0 | 0.000000 | -0.000000 | 2031 | 1924.251154 | 1971.43 | 2018.608846 | 0.970670 |  |
| GO:0033598\_mammary\_gland\_epithelial\_cell\_proliferation | 12 | 0 | 0.000000 | -0.000000 | 2031 | 1924.251154 | 1971.43 | 2018.608846 | 0.970670 |  |
| GO:0042278\_purine\_nucleoside\_metabolic\_process | 12 | 0 | 0.000000 | -0.000000 | 2031 | 1924.251154 | 1971.43 | 2018.608846 | 0.970670 |  |
| GO:0042743\_hydrogen\_peroxide\_metabolic\_process | 12 | 0 | 0.000000 | -0.000000 | 2031 | 1924.251154 | 1971.43 | 2018.608846 | 0.970670 |  |
| GO:0043331\_response\_to\_dsRNA | 12 | 0 | 0.000000 | -0.000000 | 2031 | 1924.251154 | 1971.43 | 2018.608846 | 0.970670 |  |
| GO:0043487\_regulation\_of\_RNA\_stability | 12 | 0 | 0.000000 | -0.000000 | 2031 | 1924.251154 | 1971.43 | 2018.608846 | 0.970670 |  |
| GO:0043624\_cellular\_protein\_complex\_disassembly | 12 | 0 | 0.000000 | -0.000000 | 2031 | 1924.251154 | 1971.43 | 2018.608846 | 0.970670 |  |
| GO:0045076\_regulation\_of\_interleukin-2\_biosynthetic\_process | 12 | 0 | 0.000000 | -0.000000 | 2031 | 1924.251154 | 1971.43 | 2018.608846 | 0.970670 |  |
| GO:0045471\_response\_to\_ethanol | 12 | 0 | 0.000000 | -0.000000 | 2031 | 1924.251154 | 1971.43 | 2018.608846 | 0.970670 |  |
| GO:0045792\_negative\_regulation\_of\_cell\_size | 12 | 0 | 0.000000 | -0.000000 | 2031 | 1924.251154 | 1971.43 | 2018.608846 | 0.970670 |  |
| GO:0045995\_regulation\_of\_embryonic\_development | 12 | 0 | 0.000000 | -0.000000 | 2031 | 1924.251154 | 1971.43 | 2018.608846 | 0.970670 |  |
| GO:0046068\_cGMP\_metabolic\_process | 12 | 0 | 0.000000 | -0.000000 | 2031 | 1924.251154 | 1971.43 | 2018.608846 | 0.970670 |  |
| GO:0046128\_purine\_ribonucleoside\_metabolic\_process | 12 | 0 | 0.000000 | -0.000000 | 2031 | 1924.251154 | 1971.43 | 2018.608846 | 0.970670 |  |
| GO:0048169\_regulation\_of\_long-term\_neuronal\_synaptic\_plasticity | 12 | 0 | 0.000000 | -0.000000 | 2031 | 1924.251154 | 1971.43 | 2018.608846 | 0.970670 |  |
| GO:0048820\_hair\_follicle\_maturation | 12 | 0 | 0.000000 | -0.000000 | 2031 | 1924.251154 | 1971.43 | 2018.608846 | 0.970670 |  |
| GO:0048821\_erythrocyte\_development | 12 | 0 | 0.000000 | -0.000000 | 2031 | 1924.251154 | 1971.43 | 2018.608846 | 0.970670 |  |
| GO:0048854\_brain\_morphogenesis | 12 | 0 | 0.000000 | -0.000000 | 2031 | 1924.251154 | 1971.43 | 2018.608846 | 0.970670 |  |
| GO:0050432\_catecholamine\_secretion | 12 | 0 | 0.000000 | -0.000000 | 2031 | 1924.251154 | 1971.43 | 2018.608846 | 0.970670 |  |
| GO:0050795\_regulation\_of\_behavior | 12 | 0 | 0.000000 | -0.000000 | 2031 | 1924.251154 | 1971.43 | 2018.608846 | 0.970670 |  |
| GO:0050803\_regulation\_of\_synapse\_structure\_and\_activity | 12 | 0 | 0.000000 | -0.000000 | 2031 | 1924.251154 | 1971.43 | 2018.608846 | 0.970670 |  |
| GO:0050848\_regulation\_of\_calcium-mediated\_signaling | 12 | 0 | 0.000000 | -0.000000 | 2031 | 1924.251154 | 1971.43 | 2018.608846 | 0.970670 |  |
| GO:0050850\_positive\_regulation\_of\_calcium-mediated\_signaling | 12 | 0 | 0.000000 | -0.000000 | 2031 | 1924.251154 | 1971.43 | 2018.608846 | 0.970670 |  |
| GO:0050869\_negative\_regulation\_of\_B\_cell\_activation | 12 | 0 | 0.000000 | -0.000000 | 2031 | 1924.251154 | 1971.43 | 2018.608846 | 0.970670 |  |
| GO:0051145\_smooth\_muscle\_cell\_differentiation | 12 | 0 | 0.000000 | -0.000000 | 2031 | 1924.251154 | 1971.43 | 2018.608846 | 0.970670 |  |
| GO:0051261\_protein\_depolymerization | 12 | 0 | 0.000000 | -0.000000 | 2031 | 1924.251154 | 1971.43 | 2018.608846 | 0.970670 |  |
| GO:0051588\_regulation\_of\_neurotransmitter\_transport | 12 | 0 | 0.000000 | -0.000000 | 2031 | 1924.251154 | 1971.43 | 2018.608846 | 0.970670 |  |
| GO:0055002\_striated\_muscle\_cell\_development | 12 | 0 | 0.000000 | -0.000000 | 2031 | 1924.251154 | 1971.43 | 2018.608846 | 0.970670 |  |
| GO:0055114\_oxidation\_reduction | 12 | 0 | 0.000000 | -0.000000 | 2031 | 1924.251154 | 1971.43 | 2018.608846 | 0.970670 |  |
| GO:0060048\_cardiac\_muscle\_contraction | 12 | 0 | 0.000000 | -0.000000 | 2031 | 1924.251154 | 1971.43 | 2018.608846 | 0.970670 |  |
| GO:0060525\_prostate\_glandular\_acinus\_development | 12 | 0 | 0.000000 | -0.000000 | 2031 | 1924.251154 | 1971.43 | 2018.608846 | 0.970670 |  |
| GO:0060572\_morphogenesis\_of\_an\_epithelial\_bud | 12 | 0 | 0.000000 | -0.000000 | 2031 | 1924.251154 | 1971.43 | 2018.608846 | 0.970670 |  |
| GO:0060736\_prostate\_gland\_growth | 12 | 0 | 0.000000 | -0.000000 | 2031 | 1924.251154 | 1971.43 | 2018.608846 | 0.970670 |  |
| GO:0006338\_chromatin\_remodeling | 19 | 0 | 0.000000 | -0.000000 | 2054 | 1950.710249 | 1996.83 | 2042.949751 | 0.972167 |  |
| GO:0006776\_vitamin\_A\_metabolic\_process | 19 | 0 | 0.000000 | -0.000000 | 2054 | 1950.710249 | 1996.83 | 2042.949751 | 0.972167 |  |
| GO:0007595\_lactation | 19 | 0 | 0.000000 | -0.000000 | 2054 | 1950.710249 | 1996.83 | 2042.949751 | 0.972167 |  |
| GO:0009584\_detection\_of\_visible\_light | 19 | 0 | 0.000000 | -0.000000 | 2054 | 1950.710249 | 1996.83 | 2042.949751 | 0.972167 |  |
| GO:0009798\_axis\_specification | 19 | 0 | 0.000000 | -0.000000 | 2054 | 1950.710249 | 1996.83 | 2042.949751 | 0.972167 |  |
| GO:0010639\_negative\_regulation\_of\_organelle\_organization | 19 | 0 | 0.000000 | -0.000000 | 2054 | 1950.710249 | 1996.83 | 2042.949751 | 0.972167 |  |
| GO:0010952\_positive\_regulation\_of\_peptidase\_activity | 19 | 0 | 0.000000 | -0.000000 | 2054 | 1950.710249 | 1996.83 | 2042.949751 | 0.972167 |  |
| GO:0021587\_cerebellum\_morphogenesis | 19 | 0 | 0.000000 | -0.000000 | 2054 | 1950.710249 | 1996.83 | 2042.949751 | 0.972167 |  |
| GO:0030199\_collagen\_fibril\_organization | 19 | 0 | 0.000000 | -0.000000 | 2054 | 1950.710249 | 1996.83 | 2042.949751 | 0.972167 |  |
| GO:0030595\_leukocyte\_chemotaxis | 19 | 0 | 0.000000 | -0.000000 | 2054 | 1950.710249 | 1996.83 | 2042.949751 | 0.972167 |  |
| GO:0032526\_response\_to\_retinoic\_acid | 19 | 0 | 0.000000 | -0.000000 | 2054 | 1950.710249 | 1996.83 | 2042.949751 | 0.972167 |  |
| GO:0033002\_muscle\_cell\_proliferation | 19 | 0 | 0.000000 | -0.000000 | 2054 | 1950.710249 | 1996.83 | 2042.949751 | 0.972167 |  |
| GO:0033189\_response\_to\_vitamin\_A | 19 | 0 | 0.000000 | -0.000000 | 2054 | 1950.710249 | 1996.83 | 2042.949751 | 0.972167 |  |
| GO:0042462\_eye\_photoreceptor\_cell\_development | 19 | 0 | 0.000000 | -0.000000 | 2054 | 1950.710249 | 1996.83 | 2042.949751 | 0.972167 |  |
| GO:0043280\_positive\_regulation\_of\_caspase\_activity | 19 | 0 | 0.000000 | -0.000000 | 2054 | 1950.710249 | 1996.83 | 2042.949751 | 0.972167 |  |
| GO:0048536\_spleen\_development | 19 | 0 | 0.000000 | -0.000000 | 2054 | 1950.710249 | 1996.83 | 2042.949751 | 0.972167 |  |
| GO:0048701\_embryonic\_cranial\_skeleton\_morphogenesis | 19 | 0 | 0.000000 | -0.000000 | 2054 | 1950.710249 | 1996.83 | 2042.949751 | 0.972167 |  |
| GO:0050728\_negative\_regulation\_of\_inflammatory\_response | 19 | 0 | 0.000000 | -0.000000 | 2054 | 1950.710249 | 1996.83 | 2042.949751 | 0.972167 |  |
| GO:0050908\_detection\_of\_light\_stimulus\_involved\_in\_visual\_perception | 19 | 0 | 0.000000 | -0.000000 | 2054 | 1950.710249 | 1996.83 | 2042.949751 | 0.972167 |  |
| GO:0050962\_detection\_of\_light\_stimulus\_involved\_in\_sensory\_perception | 19 | 0 | 0.000000 | -0.000000 | 2054 | 1950.710249 | 1996.83 | 2042.949751 | 0.972167 |  |
| GO:0051056\_regulation\_of\_small\_GTPase\_mediated\_signal\_transduction | 19 | 0 | 0.000000 | -0.000000 | 2054 | 1950.710249 | 1996.83 | 2042.949751 | 0.972167 |  |
| GO:0060079\_regulation\_of\_excitatory\_postsynaptic\_membrane\_potential | 19 | 0 | 0.000000 | -0.000000 | 2054 | 1950.710249 | 1996.83 | 2042.949751 | 0.972167 |  |
| GO:0060326\_cell\_chemotaxis | 19 | 0 | 0.000000 | -0.000000 | 2054 | 1950.710249 | 1996.83 | 2042.949751 | 0.972167 |  |
| GO:0001942\_hair\_follicle\_development | 44 | 0 | 0.000000 | -0.000000 | 2063 | 1964.517881 | 2008.14 | 2051.762119 | 0.973408 |  |
| GO:0009914\_hormone\_transport | 44 | 0 | 0.000000 | -0.000000 | 2063 | 1964.517881 | 2008.14 | 2051.762119 | 0.973408 |  |
| GO:0022404\_molting\_cycle\_process | 44 | 0 | 0.000000 | -0.000000 | 2063 | 1964.517881 | 2008.14 | 2051.762119 | 0.973408 |  |
| GO:0022405\_hair\_cycle\_process | 44 | 0 | 0.000000 | -0.000000 | 2063 | 1964.517881 | 2008.14 | 2051.762119 | 0.973408 |  |
| GO:0030802\_regulation\_of\_cyclic\_nucleotide\_biosynthetic\_process | 44 | 0 | 0.000000 | -0.000000 | 2063 | 1964.517881 | 2008.14 | 2051.762119 | 0.973408 |  |
| GO:0030808\_regulation\_of\_nucleotide\_biosynthetic\_process | 44 | 0 | 0.000000 | -0.000000 | 2063 | 1964.517881 | 2008.14 | 2051.762119 | 0.973408 |  |
| GO:0042303\_molting\_cycle | 44 | 0 | 0.000000 | -0.000000 | 2063 | 1964.517881 | 2008.14 | 2051.762119 | 0.973408 |  |
| GO:0042633\_hair\_cycle | 44 | 0 | 0.000000 | -0.000000 | 2063 | 1964.517881 | 2008.14 | 2051.762119 | 0.973408 |  |
| GO:0060485\_mesenchyme\_development | 44 | 0 | 0.000000 | -0.000000 | 2063 | 1964.517881 | 2008.14 | 2051.762119 | 0.973408 |  |
| GO:0002761\_regulation\_of\_myeloid\_leukocyte\_differentiation | 27 | 0 | 0.000000 | -0.000000 | 2077 | 1977.596009 | 2020.09 | 2062.583991 | 0.972600 |  |
| GO:0006479\_protein\_amino\_acid\_methylation | 27 | 0 | 0.000000 | -0.000000 | 2077 | 1977.596009 | 2020.09 | 2062.583991 | 0.972600 |  |
| GO:0007422\_peripheral\_nervous\_system\_development | 27 | 0 | 0.000000 | -0.000000 | 2077 | 1977.596009 | 2020.09 | 2062.583991 | 0.972600 |  |
| GO:0008213\_protein\_amino\_acid\_alkylation | 27 | 0 | 0.000000 | -0.000000 | 2077 | 1977.596009 | 2020.09 | 2062.583991 | 0.972600 |  |
| GO:0008286\_insulin\_receptor\_signaling\_pathway | 27 | 0 | 0.000000 | -0.000000 | 2077 | 1977.596009 | 2020.09 | 2062.583991 | 0.972600 |  |
| GO:0009411\_response\_to\_UV | 27 | 0 | 0.000000 | -0.000000 | 2077 | 1977.596009 | 2020.09 | 2062.583991 | 0.972600 |  |
| GO:0009913\_epidermal\_cell\_differentiation | 27 | 0 | 0.000000 | -0.000000 | 2077 | 1977.596009 | 2020.09 | 2062.583991 | 0.972600 |  |
| GO:0010638\_positive\_regulation\_of\_organelle\_organization | 27 | 0 | 0.000000 | -0.000000 | 2077 | 1977.596009 | 2020.09 | 2062.583991 | 0.972600 |  |
| GO:0016050\_vesicle\_organization | 27 | 0 | 0.000000 | -0.000000 | 2077 | 1977.596009 | 2020.09 | 2062.583991 | 0.972600 |  |
| GO:0019884\_antigen\_processing\_and\_presentation\_of\_exogenous\_antigen | 27 | 0 | 0.000000 | -0.000000 | 2077 | 1977.596009 | 2020.09 | 2062.583991 | 0.972600 |  |
| GO:0030100\_regulation\_of\_endocytosis | 27 | 0 | 0.000000 | -0.000000 | 2077 | 1977.596009 | 2020.09 | 2062.583991 | 0.972600 |  |
| GO:0032496\_response\_to\_lipopolysaccharide | 27 | 0 | 0.000000 | -0.000000 | 2077 | 1977.596009 | 2020.09 | 2062.583991 | 0.972600 |  |
| GO:0051272\_positive\_regulation\_of\_cell\_motion | 27 | 0 | 0.000000 | -0.000000 | 2077 | 1977.596009 | 2020.09 | 2062.583991 | 0.972600 |  |
| GO:0070482\_response\_to\_oxygen\_levels | 27 | 0 | 0.000000 | -0.000000 | 2077 | 1977.596009 | 2020.09 | 2062.583991 | 0.972600 |  |
| GO:0030334\_regulation\_of\_cell\_migration | 59 | 0 | 0.000000 | -0.000000 | 2078 | 1980.138733 | 2022.39 | 2064.641267 | 0.973239 |  |
| GO:0000075\_cell\_cycle\_checkpoint | 21 | 0 | 0.000000 | -0.000000 | 2106 | 2012.459833 | 2052.76 | 2093.060167 | 0.974720 |  |
| GO:0001702\_gastrulation\_with\_mouth\_forming\_second | 21 | 0 | 0.000000 | -0.000000 | 2106 | 2012.459833 | 2052.76 | 2093.060167 | 0.974720 |  |
| GO:0001754\_eye\_photoreceptor\_cell\_differentiation | 21 | 0 | 0.000000 | -0.000000 | 2106 | 2012.459833 | 2052.76 | 2093.060167 | 0.974720 |  |
| GO:0001755\_neural\_crest\_cell\_migration | 21 | 0 | 0.000000 | -0.000000 | 2106 | 2012.459833 | 2052.76 | 2093.060167 | 0.974720 |  |
| GO:0002053\_positive\_regulation\_of\_mesenchymal\_cell\_proliferation | 21 | 0 | 0.000000 | -0.000000 | 2106 | 2012.459833 | 2052.76 | 2093.060167 | 0.974720 |  |
| GO:0002263\_cell\_activation\_during\_immune\_response | 21 | 0 | 0.000000 | -0.000000 | 2106 | 2012.459833 | 2052.76 | 2093.060167 | 0.974720 |  |
| GO:0002366\_leukocyte\_activation\_during\_immune\_response | 21 | 0 | 0.000000 | -0.000000 | 2106 | 2012.459833 | 2052.76 | 2093.060167 | 0.974720 |  |
| GO:0006633\_fatty\_acid\_biosynthetic\_process | 21 | 0 | 0.000000 | -0.000000 | 2106 | 2012.459833 | 2052.76 | 2093.060167 | 0.974720 |  |
| GO:0006814\_sodium\_ion\_transport | 21 | 0 | 0.000000 | -0.000000 | 2106 | 2012.459833 | 2052.76 | 2093.060167 | 0.974720 |  |
| GO:0006944\_membrane\_fusion | 21 | 0 | 0.000000 | -0.000000 | 2106 | 2012.459833 | 2052.76 | 2093.060167 | 0.974720 |  |
| GO:0007189\_activation\_of\_adenylate\_cyclase\_activity\_by\_G-protein\_signaling\_pathway | 21 | 0 | 0.000000 | -0.000000 | 2106 | 2012.459833 | 2052.76 | 2093.060167 | 0.974720 |  |
| GO:0008154\_actin\_polymerization\_or\_depolymerization | 21 | 0 | 0.000000 | -0.000000 | 2106 | 2012.459833 | 2052.76 | 2093.060167 | 0.974720 |  |
| GO:0008637\_apoptotic\_mitochondrial\_changes | 21 | 0 | 0.000000 | -0.000000 | 2106 | 2012.459833 | 2052.76 | 2093.060167 | 0.974720 |  |
| GO:0009583\_detection\_of\_light\_stimulus | 21 | 0 | 0.000000 | -0.000000 | 2106 | 2012.459833 | 2052.76 | 2093.060167 | 0.974720 |  |
| GO:0010552\_positive\_regulation\_of\_specific\_transcription\_from\_RNA\_polymerase\_II\_promoter | 21 | 0 | 0.000000 | -0.000000 | 2106 | 2012.459833 | 2052.76 | 2093.060167 | 0.974720 |  |
| GO:0010578\_regulation\_of\_adenylate\_cyclase\_activity\_involved\_in\_G-protein\_signaling | 21 | 0 | 0.000000 | -0.000000 | 2106 | 2012.459833 | 2052.76 | 2093.060167 | 0.974720 |  |
| GO:0010579\_positive\_regulation\_of\_adenylate\_cyclase\_activity\_by\_G-protein\_signaling\_pathway | 21 | 0 | 0.000000 | -0.000000 | 2106 | 2012.459833 | 2052.76 | 2093.060167 | 0.974720 |  |
| GO:0015844\_monoamine\_transport | 21 | 0 | 0.000000 | -0.000000 | 2106 | 2012.459833 | 2052.76 | 2093.060167 | 0.974720 |  |
| GO:0017157\_regulation\_of\_exocytosis | 21 | 0 | 0.000000 | -0.000000 | 2106 | 2012.459833 | 2052.76 | 2093.060167 | 0.974720 |  |
| GO:0021532\_neural\_tube\_patterning | 21 | 0 | 0.000000 | -0.000000 | 2106 | 2012.459833 | 2052.76 | 2093.060167 | 0.974720 |  |
| GO:0030216\_keratinocyte\_differentiation | 21 | 0 | 0.000000 | -0.000000 | 2106 | 2012.459833 | 2052.76 | 2093.060167 | 0.974720 |  |
| GO:0034330\_cell\_junction\_organization | 21 | 0 | 0.000000 | -0.000000 | 2106 | 2012.459833 | 2052.76 | 2093.060167 | 0.974720 |  |
| GO:0035136\_forelimb\_morphogenesis | 21 | 0 | 0.000000 | -0.000000 | 2106 | 2012.459833 | 2052.76 | 2093.060167 | 0.974720 |  |
| GO:0043279\_response\_to\_alkaloid | 21 | 0 | 0.000000 | -0.000000 | 2106 | 2012.459833 | 2052.76 | 2093.060167 | 0.974720 |  |
| GO:0048538\_thymus\_development | 21 | 0 | 0.000000 | -0.000000 | 2106 | 2012.459833 | 2052.76 | 2093.060167 | 0.974720 |  |
| GO:0048675\_axon\_extension | 21 | 0 | 0.000000 | -0.000000 | 2106 | 2012.459833 | 2052.76 | 2093.060167 | 0.974720 |  |
| GO:0051258\_protein\_polymerization | 21 | 0 | 0.000000 | -0.000000 | 2106 | 2012.459833 | 2052.76 | 2093.060167 | 0.974720 |  |
| GO:0051271\_negative\_regulation\_of\_cell\_motion | 21 | 0 | 0.000000 | -0.000000 | 2106 | 2012.459833 | 2052.76 | 2093.060167 | 0.974720 |  |
| GO:0002088\_lens\_development\_in\_camera-type\_eye | 28 | 0 | 0.000000 | -0.000000 | 2114 | 2025.573099 | 2065.07 | 2104.566901 | 0.976854 |  |
| GO:0006997\_nucleus\_organization | 28 | 0 | 0.000000 | -0.000000 | 2114 | 2025.573099 | 2065.07 | 2104.566901 | 0.976854 |  |
| GO:0007127\_meiosis\_I | 28 | 0 | 0.000000 | -0.000000 | 2114 | 2025.573099 | 2065.07 | 2104.566901 | 0.976854 |  |
| GO:0021549\_cerebellum\_development | 28 | 0 | 0.000000 | -0.000000 | 2114 | 2025.573099 | 2065.07 | 2104.566901 | 0.976854 |  |
| GO:0030073\_insulin\_secretion | 28 | 0 | 0.000000 | -0.000000 | 2114 | 2025.573099 | 2065.07 | 2104.566901 | 0.976854 |  |
| GO:0030111\_regulation\_of\_Wnt\_receptor\_signaling\_pathway | 28 | 0 | 0.000000 | -0.000000 | 2114 | 2025.573099 | 2065.07 | 2104.566901 | 0.976854 |  |
| GO:0043193\_positive\_regulation\_of\_gene-specific\_transcription | 28 | 0 | 0.000000 | -0.000000 | 2114 | 2025.573099 | 2065.07 | 2104.566901 | 0.976854 |  |
| GO:0051188\_cofactor\_biosynthetic\_process | 28 | 0 | 0.000000 | -0.000000 | 2114 | 2025.573099 | 2065.07 | 2104.566901 | 0.976854 |  |
| GO:0002791\_regulation\_of\_peptide\_secretion | 17 | 0 | 0.000000 | -0.000000 | 2154 | 2063.730107 | 2101.67 | 2139.609893 | 0.975706 |  |
| GO:0006261\_DNA-dependent\_DNA\_replication | 17 | 0 | 0.000000 | -0.000000 | 2154 | 2063.730107 | 2101.67 | 2139.609893 | 0.975706 |  |
| GO:0006323\_DNA\_packaging | 17 | 0 | 0.000000 | -0.000000 | 2154 | 2063.730107 | 2101.67 | 2139.609893 | 0.975706 |  |
| GO:0006767\_water-soluble\_vitamin\_metabolic\_process | 17 | 0 | 0.000000 | -0.000000 | 2154 | 2063.730107 | 2101.67 | 2139.609893 | 0.975706 |  |
| GO:0006919\_activation\_of\_caspase\_activity | 17 | 0 | 0.000000 | -0.000000 | 2154 | 2063.730107 | 2101.67 | 2139.609893 | 0.975706 |  |
| GO:0006984\_ER-nuclear\_signaling\_pathway | 17 | 0 | 0.000000 | -0.000000 | 2154 | 2063.730107 | 2101.67 | 2139.609893 | 0.975706 |  |
| GO:0008380\_RNA\_splicing | 17 | 0 | 0.000000 | -0.000000 | 2154 | 2063.730107 | 2101.67 | 2139.609893 | 0.975706 |  |
| GO:0010035\_response\_to\_inorganic\_substance | 17 | 0 | 0.000000 | -0.000000 | 2154 | 2063.730107 | 2101.67 | 2139.609893 | 0.975706 |  |
| GO:0010517\_regulation\_of\_phospholipase\_activity | 17 | 0 | 0.000000 | -0.000000 | 2154 | 2063.730107 | 2101.67 | 2139.609893 | 0.975706 |  |
| GO:0010741\_negative\_regulation\_of\_protein\_kinase\_cascade | 17 | 0 | 0.000000 | -0.000000 | 2154 | 2063.730107 | 2101.67 | 2139.609893 | 0.975706 |  |
| GO:0015807\_L-amino\_acid\_transport | 17 | 0 | 0.000000 | -0.000000 | 2154 | 2063.730107 | 2101.67 | 2139.609893 | 0.975706 |  |
| GO:0019395\_fatty\_acid\_oxidation | 17 | 0 | 0.000000 | -0.000000 | 2154 | 2063.730107 | 2101.67 | 2139.609893 | 0.975706 |  |
| GO:0021545\_cranial\_nerve\_development | 17 | 0 | 0.000000 | -0.000000 | 2154 | 2063.730107 | 2101.67 | 2139.609893 | 0.975706 |  |
| GO:0022029\_telencephalon\_cell\_migration | 17 | 0 | 0.000000 | -0.000000 | 2154 | 2063.730107 | 2101.67 | 2139.609893 | 0.975706 |  |
| GO:0030317\_sperm\_motility | 17 | 0 | 0.000000 | -0.000000 | 2154 | 2063.730107 | 2101.67 | 2139.609893 | 0.975706 |  |
| GO:0032535\_regulation\_of\_cellular\_component\_size | 17 | 0 | 0.000000 | -0.000000 | 2154 | 2063.730107 | 2101.67 | 2139.609893 | 0.975706 |  |
| GO:0032970\_regulation\_of\_actin\_filament-based\_process | 17 | 0 | 0.000000 | -0.000000 | 2154 | 2063.730107 | 2101.67 | 2139.609893 | 0.975706 |  |
| GO:0034440\_lipid\_oxidation | 17 | 0 | 0.000000 | -0.000000 | 2154 | 2063.730107 | 2101.67 | 2139.609893 | 0.975706 |  |
| GO:0034470\_ncRNA\_processing | 17 | 0 | 0.000000 | -0.000000 | 2154 | 2063.730107 | 2101.67 | 2139.609893 | 0.975706 |  |
| GO:0035115\_embryonic\_forelimb\_morphogenesis | 17 | 0 | 0.000000 | -0.000000 | 2154 | 2063.730107 | 2101.67 | 2139.609893 | 0.975706 |  |
| GO:0042254\_ribosome\_biogenesis | 17 | 0 | 0.000000 | -0.000000 | 2154 | 2063.730107 | 2101.67 | 2139.609893 | 0.975706 |  |
| GO:0042384\_cilium\_assembly | 17 | 0 | 0.000000 | -0.000000 | 2154 | 2063.730107 | 2101.67 | 2139.609893 | 0.975706 |  |
| GO:0042398\_cellular\_amino\_acid\_derivative\_biosynthetic\_process | 17 | 0 | 0.000000 | -0.000000 | 2154 | 2063.730107 | 2101.67 | 2139.609893 | 0.975706 |  |
| GO:0042417\_dopamine\_metabolic\_process | 17 | 0 | 0.000000 | -0.000000 | 2154 | 2063.730107 | 2101.67 | 2139.609893 | 0.975706 |  |
| GO:0042440\_pigment\_metabolic\_process | 17 | 0 | 0.000000 | -0.000000 | 2154 | 2063.730107 | 2101.67 | 2139.609893 | 0.975706 |  |
| GO:0043407\_negative\_regulation\_of\_MAP\_kinase\_activity | 17 | 0 | 0.000000 | -0.000000 | 2154 | 2063.730107 | 2101.67 | 2139.609893 | 0.975706 |  |
| GO:0045333\_cellular\_respiration | 17 | 0 | 0.000000 | -0.000000 | 2154 | 2063.730107 | 2101.67 | 2139.609893 | 0.975706 |  |
| GO:0045667\_regulation\_of\_osteoblast\_differentiation | 17 | 0 | 0.000000 | -0.000000 | 2154 | 2063.730107 | 2101.67 | 2139.609893 | 0.975706 |  |
| GO:0045670\_regulation\_of\_osteoclast\_differentiation | 17 | 0 | 0.000000 | -0.000000 | 2154 | 2063.730107 | 2101.67 | 2139.609893 | 0.975706 |  |
| GO:0045786\_negative\_regulation\_of\_cell\_cycle | 17 | 0 | 0.000000 | -0.000000 | 2154 | 2063.730107 | 2101.67 | 2139.609893 | 0.975706 |  |
| GO:0046637\_regulation\_of\_alpha-beta\_T\_cell\_differentiation | 17 | 0 | 0.000000 | -0.000000 | 2154 | 2063.730107 | 2101.67 | 2139.609893 | 0.975706 |  |
| GO:0048168\_regulation\_of\_neuronal\_synaptic\_plasticity | 17 | 0 | 0.000000 | -0.000000 | 2154 | 2063.730107 | 2101.67 | 2139.609893 | 0.975706 |  |
| GO:0048265\_response\_to\_pain | 17 | 0 | 0.000000 | -0.000000 | 2154 | 2063.730107 | 2101.67 | 2139.609893 | 0.975706 |  |
| GO:0048873\_homeostasis\_of\_number\_of\_cells\_within\_a\_tissue | 17 | 0 | 0.000000 | -0.000000 | 2154 | 2063.730107 | 2101.67 | 2139.609893 | 0.975706 |  |
| GO:0050708\_regulation\_of\_protein\_secretion | 17 | 0 | 0.000000 | -0.000000 | 2154 | 2063.730107 | 2101.67 | 2139.609893 | 0.975706 |  |
| GO:0051091\_positive\_regulation\_of\_transcription\_factor\_activity | 17 | 0 | 0.000000 | -0.000000 | 2154 | 2063.730107 | 2101.67 | 2139.609893 | 0.975706 |  |
| GO:0051605\_protein\_maturation\_by\_peptide\_bond\_cleavage | 17 | 0 | 0.000000 | -0.000000 | 2154 | 2063.730107 | 2101.67 | 2139.609893 | 0.975706 |  |
| GO:0055001\_muscle\_cell\_development | 17 | 0 | 0.000000 | -0.000000 | 2154 | 2063.730107 | 2101.67 | 2139.609893 | 0.975706 |  |
| GO:0055072\_iron\_ion\_homeostasis | 17 | 0 | 0.000000 | -0.000000 | 2154 | 2063.730107 | 2101.67 | 2139.609893 | 0.975706 |  |
| GO:0090047\_positive\_regulation\_of\_transcription\_regulator\_activity | 17 | 0 | 0.000000 | -0.000000 | 2154 | 2063.730107 | 2101.67 | 2139.609893 | 0.975706 |  |
| GO:0001523\_retinoid\_metabolic\_process | 22 | 0 | 0.000000 | -0.000000 | 2187 | 2098.976177 | 2134.56 | 2170.143823 | 0.976022 |  |
| GO:0001558\_regulation\_of\_cell\_growth | 22 | 0 | 0.000000 | -0.000000 | 2187 | 2098.976177 | 2134.56 | 2170.143823 | 0.976022 |  |
| GO:0002478\_antigen\_processing\_and\_presentation\_of\_exogenous\_peptide\_antigen | 22 | 0 | 0.000000 | -0.000000 | 2187 | 2098.976177 | 2134.56 | 2170.143823 | 0.976022 |  |
| GO:0006112\_energy\_reserve\_metabolic\_process | 22 | 0 | 0.000000 | -0.000000 | 2187 | 2098.976177 | 2134.56 | 2170.143823 | 0.976022 |  |
| GO:0006721\_terpenoid\_metabolic\_process | 22 | 0 | 0.000000 | -0.000000 | 2187 | 2098.976177 | 2134.56 | 2170.143823 | 0.976022 |  |
| GO:0007059\_chromosome\_segregation | 22 | 0 | 0.000000 | -0.000000 | 2187 | 2098.976177 | 2134.56 | 2170.143823 | 0.976022 |  |
| GO:0008624\_induction\_of\_apoptosis\_by\_extracellular\_signals | 22 | 0 | 0.000000 | -0.000000 | 2187 | 2098.976177 | 2134.56 | 2170.143823 | 0.976022 |  |
| GO:0009309\_amine\_biosynthetic\_process | 22 | 0 | 0.000000 | -0.000000 | 2187 | 2098.976177 | 2134.56 | 2170.143823 | 0.976022 |  |
| GO:0009896\_positive\_regulation\_of\_catabolic\_process | 22 | 0 | 0.000000 | -0.000000 | 2187 | 2098.976177 | 2134.56 | 2170.143823 | 0.976022 |  |
| GO:0010463\_mesenchymal\_cell\_proliferation | 22 | 0 | 0.000000 | -0.000000 | 2187 | 2098.976177 | 2134.56 | 2170.143823 | 0.976022 |  |
| GO:0010464\_regulation\_of\_mesenchymal\_cell\_proliferation | 22 | 0 | 0.000000 | -0.000000 | 2187 | 2098.976177 | 2134.56 | 2170.143823 | 0.976022 |  |
| GO:0016101\_diterpenoid\_metabolic\_process | 22 | 0 | 0.000000 | -0.000000 | 2187 | 2098.976177 | 2134.56 | 2170.143823 | 0.976022 |  |
| GO:0021575\_hindbrain\_morphogenesis | 22 | 0 | 0.000000 | -0.000000 | 2187 | 2098.976177 | 2134.56 | 2170.143823 | 0.976022 |  |
| GO:0021675\_nerve\_development | 22 | 0 | 0.000000 | -0.000000 | 2187 | 2098.976177 | 2134.56 | 2170.143823 | 0.976022 |  |
| GO:0021766\_hippocampus\_development | 22 | 0 | 0.000000 | -0.000000 | 2187 | 2098.976177 | 2134.56 | 2170.143823 | 0.976022 |  |
| GO:0030258\_lipid\_modification | 22 | 0 | 0.000000 | -0.000000 | 2187 | 2098.976177 | 2134.56 | 2170.143823 | 0.976022 |  |
| GO:0030316\_osteoclast\_differentiation | 22 | 0 | 0.000000 | -0.000000 | 2187 | 2098.976177 | 2134.56 | 2170.143823 | 0.976022 |  |
| GO:0030335\_positive\_regulation\_of\_cell\_migration | 22 | 0 | 0.000000 | -0.000000 | 2187 | 2098.976177 | 2134.56 | 2170.143823 | 0.976022 |  |
| GO:0030705\_cytoskeleton-dependent\_intracellular\_transport | 22 | 0 | 0.000000 | -0.000000 | 2187 | 2098.976177 | 2134.56 | 2170.143823 | 0.976022 |  |
| GO:0032649\_regulation\_of\_interferon-gamma\_production | 22 | 0 | 0.000000 | -0.000000 | 2187 | 2098.976177 | 2134.56 | 2170.143823 | 0.976022 |  |
| GO:0032886\_regulation\_of\_microtubule-based\_process | 22 | 0 | 0.000000 | -0.000000 | 2187 | 2098.976177 | 2134.56 | 2170.143823 | 0.976022 |  |
| GO:0033273\_response\_to\_vitamin | 22 | 0 | 0.000000 | -0.000000 | 2187 | 2098.976177 | 2134.56 | 2170.143823 | 0.976022 |  |
| GO:0034097\_response\_to\_cytokine\_stimulus | 22 | 0 | 0.000000 | -0.000000 | 2187 | 2098.976177 | 2134.56 | 2170.143823 | 0.976022 |  |
| GO:0034660\_ncRNA\_metabolic\_process | 22 | 0 | 0.000000 | -0.000000 | 2187 | 2098.976177 | 2134.56 | 2170.143823 | 0.976022 |  |
| GO:0040018\_positive\_regulation\_of\_multicellular\_organism\_growth | 22 | 0 | 0.000000 | -0.000000 | 2187 | 2098.976177 | 2134.56 | 2170.143823 | 0.976022 |  |
| GO:0042130\_negative\_regulation\_of\_T\_cell\_proliferation | 22 | 0 | 0.000000 | -0.000000 | 2187 | 2098.976177 | 2134.56 | 2170.143823 | 0.976022 |  |
| GO:0042461\_photoreceptor\_cell\_development | 22 | 0 | 0.000000 | -0.000000 | 2187 | 2098.976177 | 2134.56 | 2170.143823 | 0.976022 |  |
| GO:0042733\_embryonic\_digit\_morphogenesis | 22 | 0 | 0.000000 | -0.000000 | 2187 | 2098.976177 | 2134.56 | 2170.143823 | 0.976022 |  |
| GO:0044264\_cellular\_polysaccharide\_metabolic\_process | 22 | 0 | 0.000000 | -0.000000 | 2187 | 2098.976177 | 2134.56 | 2170.143823 | 0.976022 |  |
| GO:0045787\_positive\_regulation\_of\_cell\_cycle | 22 | 0 | 0.000000 | -0.000000 | 2187 | 2098.976177 | 2134.56 | 2170.143823 | 0.976022 |  |
| GO:0046883\_regulation\_of\_hormone\_secretion | 22 | 0 | 0.000000 | -0.000000 | 2187 | 2098.976177 | 2134.56 | 2170.143823 | 0.976022 |  |
| GO:0048489\_synaptic\_vesicle\_transport | 22 | 0 | 0.000000 | -0.000000 | 2187 | 2098.976177 | 2134.56 | 2170.143823 | 0.976022 |  |
| GO:0051260\_protein\_homooligomerization | 22 | 0 | 0.000000 | -0.000000 | 2187 | 2098.976177 | 2134.56 | 2170.143823 | 0.976022 |  |
| GO:0000038\_very-long-chain\_fatty\_acid\_metabolic\_process | 6 | 0 | 0.000000 | -0.000000 | 2347 | 2272.880208 | 2305.46 | 2338.039792 | 0.982301 |  |
| GO:0000245\_spliceosome\_assembly | 6 | 0 | 0.000000 | -0.000000 | 2347 | 2272.880208 | 2305.46 | 2338.039792 | 0.982301 |  |
| GO:0000768\_syncytium\_formation\_by\_plasma\_membrane\_fusion | 6 | 0 | 0.000000 | -0.000000 | 2347 | 2272.880208 | 2305.46 | 2338.039792 | 0.982301 |  |
| GO:0001710\_mesodermal\_cell\_fate\_commitment | 6 | 0 | 0.000000 | -0.000000 | 2347 | 2272.880208 | 2305.46 | 2338.039792 | 0.982301 |  |
| GO:0001779\_natural\_killer\_cell\_differentiation | 6 | 0 | 0.000000 | -0.000000 | 2347 | 2272.880208 | 2305.46 | 2338.039792 | 0.982301 |  |
| GO:0001885\_endothelial\_cell\_development | 6 | 0 | 0.000000 | -0.000000 | 2347 | 2272.880208 | 2305.46 | 2338.039792 | 0.982301 |  |
| GO:0002016\_regulation\_of\_blood\_volume\_by\_renin-angiotensin | 6 | 0 | 0.000000 | -0.000000 | 2347 | 2272.880208 | 2305.46 | 2338.039792 | 0.982301 |  |
| GO:0002335\_mature\_B\_cell\_differentiation | 6 | 0 | 0.000000 | -0.000000 | 2347 | 2272.880208 | 2305.46 | 2338.039792 | 0.982301 |  |
| GO:0002360\_T\_cell\_lineage\_commitment | 6 | 0 | 0.000000 | -0.000000 | 2347 | 2272.880208 | 2305.46 | 2338.039792 | 0.982301 |  |
| GO:0002367\_cytokine\_production\_during\_immune\_response | 6 | 0 | 0.000000 | -0.000000 | 2347 | 2272.880208 | 2305.46 | 2338.039792 | 0.982301 |  |
| GO:0002474\_antigen\_processing\_and\_presentation\_of\_peptide\_antigen\_via\_MHC\_class\_I | 6 | 0 | 0.000000 | -0.000000 | 2347 | 2272.880208 | 2305.46 | 2338.039792 | 0.982301 |  |
| GO:0002475\_antigen\_processing\_and\_presentation\_via\_MHC\_class\_Ib | 6 | 0 | 0.000000 | -0.000000 | 2347 | 2272.880208 | 2305.46 | 2338.039792 | 0.982301 |  |
| GO:0002532\_production\_of\_molecular\_mediator\_of\_acute\_inflammatory\_response | 6 | 0 | 0.000000 | -0.000000 | 2347 | 2272.880208 | 2305.46 | 2338.039792 | 0.982301 |  |
| GO:0002541\_activation\_of\_plasma\_proteins\_involved\_in\_acute\_inflammatory\_response | 6 | 0 | 0.000000 | -0.000000 | 2347 | 2272.880208 | 2305.46 | 2338.039792 | 0.982301 |  |
| GO:0002675\_positive\_regulation\_of\_acute\_inflammatory\_response | 6 | 0 | 0.000000 | -0.000000 | 2347 | 2272.880208 | 2305.46 | 2338.039792 | 0.982301 |  |
| GO:0002685\_regulation\_of\_leukocyte\_migration | 6 | 0 | 0.000000 | -0.000000 | 2347 | 2272.880208 | 2305.46 | 2338.039792 | 0.982301 |  |
| GO:0002831\_regulation\_of\_response\_to\_biotic\_stimulus | 6 | 0 | 0.000000 | -0.000000 | 2347 | 2272.880208 | 2305.46 | 2338.039792 | 0.982301 |  |
| GO:0006071\_glycerol\_metabolic\_process | 6 | 0 | 0.000000 | -0.000000 | 2347 | 2272.880208 | 2305.46 | 2338.039792 | 0.982301 |  |
| GO:0006084\_acetyl-CoA\_metabolic\_process | 6 | 0 | 0.000000 | -0.000000 | 2347 | 2272.880208 | 2305.46 | 2338.039792 | 0.982301 |  |
| GO:0006264\_mitochondrial\_DNA\_replication | 6 | 0 | 0.000000 | -0.000000 | 2347 | 2272.880208 | 2305.46 | 2338.039792 | 0.982301 |  |
| GO:0006402\_mRNA\_catabolic\_process | 6 | 0 | 0.000000 | -0.000000 | 2347 | 2272.880208 | 2305.46 | 2338.039792 | 0.982301 |  |
| GO:0006471\_protein\_amino\_acid\_ADP-ribosylation | 6 | 0 | 0.000000 | -0.000000 | 2347 | 2272.880208 | 2305.46 | 2338.039792 | 0.982301 |  |
| GO:0006536\_glutamate\_metabolic\_process | 6 | 0 | 0.000000 | -0.000000 | 2347 | 2272.880208 | 2305.46 | 2338.039792 | 0.982301 |  |
| GO:0006656\_phosphatidylcholine\_biosynthetic\_process | 6 | 0 | 0.000000 | -0.000000 | 2347 | 2272.880208 | 2305.46 | 2338.039792 | 0.982301 |  |
| GO:0006692\_prostanoid\_metabolic\_process | 6 | 0 | 0.000000 | -0.000000 | 2347 | 2272.880208 | 2305.46 | 2338.039792 | 0.982301 |  |
| GO:0006693\_prostaglandin\_metabolic\_process | 6 | 0 | 0.000000 | -0.000000 | 2347 | 2272.880208 | 2305.46 | 2338.039792 | 0.982301 |  |
| GO:0006706\_steroid\_catabolic\_process | 6 | 0 | 0.000000 | -0.000000 | 2347 | 2272.880208 | 2305.46 | 2338.039792 | 0.982301 |  |
| GO:0006752\_group\_transfer\_coenzyme\_metabolic\_process | 6 | 0 | 0.000000 | -0.000000 | 2347 | 2272.880208 | 2305.46 | 2338.039792 | 0.982301 |  |
| GO:0006882\_cellular\_zinc\_ion\_homeostasis | 6 | 0 | 0.000000 | -0.000000 | 2347 | 2272.880208 | 2305.46 | 2338.039792 | 0.982301 |  |
| GO:0006956\_complement\_activation | 6 | 0 | 0.000000 | -0.000000 | 2347 | 2272.880208 | 2305.46 | 2338.039792 | 0.982301 |  |
| GO:0006998\_nuclear\_envelope\_organization | 6 | 0 | 0.000000 | -0.000000 | 2347 | 2272.880208 | 2305.46 | 2338.039792 | 0.982301 |  |
| GO:0007176\_regulation\_of\_epidermal\_growth\_factor\_receptor\_activity | 6 | 0 | 0.000000 | -0.000000 | 2347 | 2272.880208 | 2305.46 | 2338.039792 | 0.982301 |  |
| GO:0007214\_gamma-aminobutyric\_acid\_signaling\_pathway | 6 | 0 | 0.000000 | -0.000000 | 2347 | 2272.880208 | 2305.46 | 2338.039792 | 0.982301 |  |
| GO:0007257\_activation\_of\_JUN\_kinase\_activity | 6 | 0 | 0.000000 | -0.000000 | 2347 | 2272.880208 | 2305.46 | 2338.039792 | 0.982301 |  |
| GO:0007406\_negative\_regulation\_of\_neuroblast\_proliferation | 6 | 0 | 0.000000 | -0.000000 | 2347 | 2272.880208 | 2305.46 | 2338.039792 | 0.982301 |  |
| GO:0007520\_myoblast\_fusion | 6 | 0 | 0.000000 | -0.000000 | 2347 | 2272.880208 | 2305.46 | 2338.039792 | 0.982301 |  |
| GO:0007620\_copulation | 6 | 0 | 0.000000 | -0.000000 | 2347 | 2272.880208 | 2305.46 | 2338.039792 | 0.982301 |  |
| GO:0008156\_negative\_regulation\_of\_DNA\_replication | 6 | 0 | 0.000000 | -0.000000 | 2347 | 2272.880208 | 2305.46 | 2338.039792 | 0.982301 |  |
| GO:0008209\_androgen\_metabolic\_process | 6 | 0 | 0.000000 | -0.000000 | 2347 | 2272.880208 | 2305.46 | 2338.039792 | 0.982301 |  |
| GO:0008625\_induction\_of\_apoptosis\_via\_death\_domain\_receptors | 6 | 0 | 0.000000 | -0.000000 | 2347 | 2272.880208 | 2305.46 | 2338.039792 | 0.982301 |  |
| GO:0009067\_aspartate\_family\_amino\_acid\_biosynthetic\_process | 6 | 0 | 0.000000 | -0.000000 | 2347 | 2272.880208 | 2305.46 | 2338.039792 | 0.982301 |  |
| GO:0009069\_serine\_family\_amino\_acid\_metabolic\_process | 6 | 0 | 0.000000 | -0.000000 | 2347 | 2272.880208 | 2305.46 | 2338.039792 | 0.982301 |  |
| GO:0009112\_nucleobase\_metabolic\_process | 6 | 0 | 0.000000 | -0.000000 | 2347 | 2272.880208 | 2305.46 | 2338.039792 | 0.982301 |  |
| GO:0009143\_nucleoside\_triphosphate\_catabolic\_process | 6 | 0 | 0.000000 | -0.000000 | 2347 | 2272.880208 | 2305.46 | 2338.039792 | 0.982301 |  |
| GO:0009247\_glycolipid\_biosynthetic\_process | 6 | 0 | 0.000000 | -0.000000 | 2347 | 2272.880208 | 2305.46 | 2338.039792 | 0.982301 |  |
| GO:0009650\_UV\_protection | 6 | 0 | 0.000000 | -0.000000 | 2347 | 2272.880208 | 2305.46 | 2338.039792 | 0.982301 |  |
| GO:0009651\_response\_to\_salt\_stress | 6 | 0 | 0.000000 | -0.000000 | 2347 | 2272.880208 | 2305.46 | 2338.039792 | 0.982301 |  |
| GO:0010466\_negative\_regulation\_of\_peptidase\_activity | 6 | 0 | 0.000000 | -0.000000 | 2347 | 2272.880208 | 2305.46 | 2338.039792 | 0.982301 |  |
| GO:0010883\_regulation\_of\_lipid\_storage | 6 | 0 | 0.000000 | -0.000000 | 2347 | 2272.880208 | 2305.46 | 2338.039792 | 0.982301 |  |
| GO:0014051\_gamma-aminobutyric\_acid\_secretion | 6 | 0 | 0.000000 | -0.000000 | 2347 | 2272.880208 | 2305.46 | 2338.039792 | 0.982301 |  |
| GO:0014072\_response\_to\_isoquinoline\_alkaloid | 6 | 0 | 0.000000 | -0.000000 | 2347 | 2272.880208 | 2305.46 | 2338.039792 | 0.982301 |  |
| GO:0014812\_muscle\_cell\_migration | 6 | 0 | 0.000000 | -0.000000 | 2347 | 2272.880208 | 2305.46 | 2338.039792 | 0.982301 |  |
| GO:0014823\_response\_to\_activity | 6 | 0 | 0.000000 | -0.000000 | 2347 | 2272.880208 | 2305.46 | 2338.039792 | 0.982301 |  |
| GO:0015012\_heparan\_sulfate\_proteoglycan\_biosynthetic\_process | 6 | 0 | 0.000000 | -0.000000 | 2347 | 2272.880208 | 2305.46 | 2338.039792 | 0.982301 |  |
| GO:0015812\_gamma-aminobutyric\_acid\_transport | 6 | 0 | 0.000000 | -0.000000 | 2347 | 2272.880208 | 2305.46 | 2338.039792 | 0.982301 |  |
| GO:0016032\_viral\_reproduction | 6 | 0 | 0.000000 | -0.000000 | 2347 | 2272.880208 | 2305.46 | 2338.039792 | 0.982301 |  |
| GO:0016574\_histone\_ubiquitination | 6 | 0 | 0.000000 | -0.000000 | 2347 | 2272.880208 | 2305.46 | 2338.039792 | 0.982301 |  |
| GO:0016925\_protein\_sumoylation | 6 | 0 | 0.000000 | -0.000000 | 2347 | 2272.880208 | 2305.46 | 2338.039792 | 0.982301 |  |
| GO:0019433\_triglyceride\_catabolic\_process | 6 | 0 | 0.000000 | -0.000000 | 2347 | 2272.880208 | 2305.46 | 2338.039792 | 0.982301 |  |
| GO:0019835\_cytolysis | 6 | 0 | 0.000000 | -0.000000 | 2347 | 2272.880208 | 2305.46 | 2338.039792 | 0.982301 |  |
| GO:0021548\_pons\_development | 6 | 0 | 0.000000 | -0.000000 | 2347 | 2272.880208 | 2305.46 | 2338.039792 | 0.982301 |  |
| GO:0021783\_preganglionic\_parasympathetic\_nervous\_system\_development | 6 | 0 | 0.000000 | -0.000000 | 2347 | 2272.880208 | 2305.46 | 2338.039792 | 0.982301 |  |
| GO:0021892\_cerebral\_cortex\_GABAergic\_interneuron\_differentiation | 6 | 0 | 0.000000 | -0.000000 | 2347 | 2272.880208 | 2305.46 | 2338.039792 | 0.982301 |  |
| GO:0021937\_Purkinje\_cell-granule\_cell\_precursor\_cell\_signaling\_involved\_in\_regulation\_of\_granule\_cell\_precursor\_cell\_proliferation | 6 | 0 | 0.000000 | -0.000000 | 2347 | 2272.880208 | 2305.46 | 2338.039792 | 0.982301 |  |
| GO:0022409\_positive\_regulation\_of\_cell-cell\_adhesion | 6 | 0 | 0.000000 | -0.000000 | 2347 | 2272.880208 | 2305.46 | 2338.039792 | 0.982301 |  |
| GO:0030252\_growth\_hormone\_secretion | 6 | 0 | 0.000000 | -0.000000 | 2347 | 2272.880208 | 2305.46 | 2338.039792 | 0.982301 |  |
| GO:0030865\_cortical\_cytoskeleton\_organization | 6 | 0 | 0.000000 | -0.000000 | 2347 | 2272.880208 | 2305.46 | 2338.039792 | 0.982301 |  |
| GO:0031077\_post-embryonic\_camera-type\_eye\_development | 6 | 0 | 0.000000 | -0.000000 | 2347 | 2272.880208 | 2305.46 | 2338.039792 | 0.982301 |  |
| GO:0031330\_negative\_regulation\_of\_cellular\_catabolic\_process | 6 | 0 | 0.000000 | -0.000000 | 2347 | 2272.880208 | 2305.46 | 2338.039792 | 0.982301 |  |
| GO:0031575\_G1\_S\_transition\_checkpoint | 6 | 0 | 0.000000 | -0.000000 | 2347 | 2272.880208 | 2305.46 | 2338.039792 | 0.982301 |  |
| GO:0031960\_response\_to\_corticosteroid\_stimulus | 6 | 0 | 0.000000 | -0.000000 | 2347 | 2272.880208 | 2305.46 | 2338.039792 | 0.982301 |  |
| GO:0032042\_mitochondrial\_DNA\_metabolic\_process | 6 | 0 | 0.000000 | -0.000000 | 2347 | 2272.880208 | 2305.46 | 2338.039792 | 0.982301 |  |
| GO:0032331\_negative\_regulation\_of\_chondrocyte\_differentiation | 6 | 0 | 0.000000 | -0.000000 | 2347 | 2272.880208 | 2305.46 | 2338.039792 | 0.982301 |  |
| GO:0032392\_DNA\_geometric\_change | 6 | 0 | 0.000000 | -0.000000 | 2347 | 2272.880208 | 2305.46 | 2338.039792 | 0.982301 |  |
| GO:0032438\_melanosome\_organization | 6 | 0 | 0.000000 | -0.000000 | 2347 | 2272.880208 | 2305.46 | 2338.039792 | 0.982301 |  |
| GO:0032469\_endoplasmic\_reticulum\_calcium\_ion\_homeostasis | 6 | 0 | 0.000000 | -0.000000 | 2347 | 2272.880208 | 2305.46 | 2338.039792 | 0.982301 |  |
| GO:0032653\_regulation\_of\_interleukin-10\_production | 6 | 0 | 0.000000 | -0.000000 | 2347 | 2272.880208 | 2305.46 | 2338.039792 | 0.982301 |  |
| GO:0033238\_regulation\_of\_cellular\_amine\_metabolic\_process | 6 | 0 | 0.000000 | -0.000000 | 2347 | 2272.880208 | 2305.46 | 2338.039792 | 0.982301 |  |
| GO:0034968\_histone\_lysine\_methylation | 6 | 0 | 0.000000 | -0.000000 | 2347 | 2272.880208 | 2305.46 | 2338.039792 | 0.982301 |  |
| GO:0035094\_response\_to\_nicotine | 6 | 0 | 0.000000 | -0.000000 | 2347 | 2272.880208 | 2305.46 | 2338.039792 | 0.982301 |  |
| GO:0035121\_tail\_morphogenesis | 6 | 0 | 0.000000 | -0.000000 | 2347 | 2272.880208 | 2305.46 | 2338.039792 | 0.982301 |  |
| GO:0040016\_embryonic\_cleavage | 6 | 0 | 0.000000 | -0.000000 | 2347 | 2272.880208 | 2305.46 | 2338.039792 | 0.982301 |  |
| GO:0040023\_establishment\_of\_nucleus\_localization | 6 | 0 | 0.000000 | -0.000000 | 2347 | 2272.880208 | 2305.46 | 2338.039792 | 0.982301 |  |
| GO:0040036\_regulation\_of\_fibroblast\_growth\_factor\_receptor\_signaling\_pathway | 6 | 0 | 0.000000 | -0.000000 | 2347 | 2272.880208 | 2305.46 | 2338.039792 | 0.982301 |  |
| GO:0042053\_regulation\_of\_dopamine\_metabolic\_process | 6 | 0 | 0.000000 | -0.000000 | 2347 | 2272.880208 | 2305.46 | 2338.039792 | 0.982301 |  |
| GO:0042069\_regulation\_of\_catecholamine\_metabolic\_process | 6 | 0 | 0.000000 | -0.000000 | 2347 | 2272.880208 | 2305.46 | 2338.039792 | 0.982301 |  |
| GO:0042246\_tissue\_regeneration | 6 | 0 | 0.000000 | -0.000000 | 2347 | 2272.880208 | 2305.46 | 2338.039792 | 0.982301 |  |
| GO:0042307\_positive\_regulation\_of\_protein\_import\_into\_nucleus | 6 | 0 | 0.000000 | -0.000000 | 2347 | 2272.880208 | 2305.46 | 2338.039792 | 0.982301 |  |
| GO:0042308\_negative\_regulation\_of\_protein\_import\_into\_nucleus | 6 | 0 | 0.000000 | -0.000000 | 2347 | 2272.880208 | 2305.46 | 2338.039792 | 0.982301 |  |
| GO:0042403\_thyroid\_hormone\_metabolic\_process | 6 | 0 | 0.000000 | -0.000000 | 2347 | 2272.880208 | 2305.46 | 2338.039792 | 0.982301 |  |
| GO:0042481\_regulation\_of\_odontogenesis | 6 | 0 | 0.000000 | -0.000000 | 2347 | 2272.880208 | 2305.46 | 2338.039792 | 0.982301 |  |
| GO:0042953\_lipoprotein\_transport | 6 | 0 | 0.000000 | -0.000000 | 2347 | 2272.880208 | 2305.46 | 2338.039792 | 0.982301 |  |
| GO:0043064\_flagellum\_organization | 6 | 0 | 0.000000 | -0.000000 | 2347 | 2272.880208 | 2305.46 | 2338.039792 | 0.982301 |  |
| GO:0043154\_negative\_regulation\_of\_caspase\_activity | 6 | 0 | 0.000000 | -0.000000 | 2347 | 2272.880208 | 2305.46 | 2338.039792 | 0.982301 |  |
| GO:0043278\_response\_to\_morphine | 6 | 0 | 0.000000 | -0.000000 | 2347 | 2272.880208 | 2305.46 | 2338.039792 | 0.982301 |  |
| GO:0043300\_regulation\_of\_leukocyte\_degranulation | 6 | 0 | 0.000000 | -0.000000 | 2347 | 2272.880208 | 2305.46 | 2338.039792 | 0.982301 |  |
| GO:0043467\_regulation\_of\_generation\_of\_precursor\_metabolites\_and\_energy | 6 | 0 | 0.000000 | -0.000000 | 2347 | 2272.880208 | 2305.46 | 2338.039792 | 0.982301 |  |
| GO:0043547\_positive\_regulation\_of\_GTPase\_activity | 6 | 0 | 0.000000 | -0.000000 | 2347 | 2272.880208 | 2305.46 | 2338.039792 | 0.982301 |  |
| GO:0043627\_response\_to\_estrogen\_stimulus | 6 | 0 | 0.000000 | -0.000000 | 2347 | 2272.880208 | 2305.46 | 2338.039792 | 0.982301 |  |
| GO:0044269\_glycerol\_ether\_catabolic\_process | 6 | 0 | 0.000000 | -0.000000 | 2347 | 2272.880208 | 2305.46 | 2338.039792 | 0.982301 |  |
| GO:0045072\_regulation\_of\_interferon-gamma\_biosynthetic\_process | 6 | 0 | 0.000000 | -0.000000 | 2347 | 2272.880208 | 2305.46 | 2338.039792 | 0.982301 |  |
| GO:0045084\_positive\_regulation\_of\_interleukin-12\_biosynthetic\_process | 6 | 0 | 0.000000 | -0.000000 | 2347 | 2272.880208 | 2305.46 | 2338.039792 | 0.982301 |  |
| GO:0045124\_regulation\_of\_bone\_resorption | 6 | 0 | 0.000000 | -0.000000 | 2347 | 2272.880208 | 2305.46 | 2338.039792 | 0.982301 |  |
| GO:0045176\_apical\_protein\_localization | 6 | 0 | 0.000000 | -0.000000 | 2347 | 2272.880208 | 2305.46 | 2338.039792 | 0.982301 |  |
| GO:0045540\_regulation\_of\_cholesterol\_biosynthetic\_process | 6 | 0 | 0.000000 | -0.000000 | 2347 | 2272.880208 | 2305.46 | 2338.039792 | 0.982301 |  |
| GO:0045579\_positive\_regulation\_of\_B\_cell\_differentiation | 6 | 0 | 0.000000 | -0.000000 | 2347 | 2272.880208 | 2305.46 | 2338.039792 | 0.982301 |  |
| GO:0045649\_regulation\_of\_macrophage\_differentiation | 6 | 0 | 0.000000 | -0.000000 | 2347 | 2272.880208 | 2305.46 | 2338.039792 | 0.982301 |  |
| GO:0045727\_positive\_regulation\_of\_translation | 6 | 0 | 0.000000 | -0.000000 | 2347 | 2272.880208 | 2305.46 | 2338.039792 | 0.982301 |  |
| GO:0045778\_positive\_regulation\_of\_ossification | 6 | 0 | 0.000000 | -0.000000 | 2347 | 2272.880208 | 2305.46 | 2338.039792 | 0.982301 |  |
| GO:0045824\_negative\_regulation\_of\_innate\_immune\_response | 6 | 0 | 0.000000 | -0.000000 | 2347 | 2272.880208 | 2305.46 | 2338.039792 | 0.982301 |  |
| GO:0045833\_negative\_regulation\_of\_lipid\_metabolic\_process | 6 | 0 | 0.000000 | -0.000000 | 2347 | 2272.880208 | 2305.46 | 2338.039792 | 0.982301 |  |
| GO:0045861\_negative\_regulation\_of\_proteolysis | 6 | 0 | 0.000000 | -0.000000 | 2347 | 2272.880208 | 2305.46 | 2338.039792 | 0.982301 |  |
| GO:0045913\_positive\_regulation\_of\_carbohydrate\_metabolic\_process | 6 | 0 | 0.000000 | -0.000000 | 2347 | 2272.880208 | 2305.46 | 2338.039792 | 0.982301 |  |
| GO:0045931\_positive\_regulation\_of\_mitotic\_cell\_cycle | 6 | 0 | 0.000000 | -0.000000 | 2347 | 2272.880208 | 2305.46 | 2338.039792 | 0.982301 |  |
| GO:0045933\_positive\_regulation\_of\_muscle\_contraction | 6 | 0 | 0.000000 | -0.000000 | 2347 | 2272.880208 | 2305.46 | 2338.039792 | 0.982301 |  |
| GO:0046461\_neutral\_lipid\_catabolic\_process | 6 | 0 | 0.000000 | -0.000000 | 2347 | 2272.880208 | 2305.46 | 2338.039792 | 0.982301 |  |
| GO:0046464\_acylglycerol\_catabolic\_process | 6 | 0 | 0.000000 | -0.000000 | 2347 | 2272.880208 | 2305.46 | 2338.039792 | 0.982301 |  |
| GO:0046503\_glycerolipid\_catabolic\_process | 6 | 0 | 0.000000 | -0.000000 | 2347 | 2272.880208 | 2305.46 | 2338.039792 | 0.982301 |  |
| GO:0046580\_negative\_regulation\_of\_Ras\_protein\_signal\_transduction | 6 | 0 | 0.000000 | -0.000000 | 2347 | 2272.880208 | 2305.46 | 2338.039792 | 0.982301 |  |
| GO:0046627\_negative\_regulation\_of\_insulin\_receptor\_signaling\_pathway | 6 | 0 | 0.000000 | -0.000000 | 2347 | 2272.880208 | 2305.46 | 2338.039792 | 0.982301 |  |
| GO:0046666\_retinal\_cell\_programmed\_cell\_death | 6 | 0 | 0.000000 | -0.000000 | 2347 | 2272.880208 | 2305.46 | 2338.039792 | 0.982301 |  |
| GO:0046852\_positive\_regulation\_of\_bone\_remodeling | 6 | 0 | 0.000000 | -0.000000 | 2347 | 2272.880208 | 2305.46 | 2338.039792 | 0.982301 |  |
| GO:0046889\_positive\_regulation\_of\_lipid\_biosynthetic\_process | 6 | 0 | 0.000000 | -0.000000 | 2347 | 2272.880208 | 2305.46 | 2338.039792 | 0.982301 |  |
| GO:0048041\_focal\_adhesion\_formation | 6 | 0 | 0.000000 | -0.000000 | 2347 | 2272.880208 | 2305.46 | 2338.039792 | 0.982301 |  |
| GO:0048103\_somatic\_stem\_cell\_division | 6 | 0 | 0.000000 | -0.000000 | 2347 | 2272.880208 | 2305.46 | 2338.039792 | 0.982301 |  |
| GO:0048147\_negative\_regulation\_of\_fibroblast\_proliferation | 6 | 0 | 0.000000 | -0.000000 | 2347 | 2272.880208 | 2305.46 | 2338.039792 | 0.982301 |  |
| GO:0048333\_mesodermal\_cell\_differentiation | 6 | 0 | 0.000000 | -0.000000 | 2347 | 2272.880208 | 2305.46 | 2338.039792 | 0.982301 |  |
| GO:0048340\_paraxial\_mesoderm\_morphogenesis | 6 | 0 | 0.000000 | -0.000000 | 2347 | 2272.880208 | 2305.46 | 2338.039792 | 0.982301 |  |
| GO:0048541\_Peyer's\_patch\_development | 6 | 0 | 0.000000 | -0.000000 | 2347 | 2272.880208 | 2305.46 | 2338.039792 | 0.982301 |  |
| GO:0048563\_post-embryonic\_organ\_morphogenesis | 6 | 0 | 0.000000 | -0.000000 | 2347 | 2272.880208 | 2305.46 | 2338.039792 | 0.982301 |  |
| GO:0048617\_embryonic\_foregut\_morphogenesis | 6 | 0 | 0.000000 | -0.000000 | 2347 | 2272.880208 | 2305.46 | 2338.039792 | 0.982301 |  |
| GO:0048703\_embryonic\_viscerocranium\_morphogenesis | 6 | 0 | 0.000000 | -0.000000 | 2347 | 2272.880208 | 2305.46 | 2338.039792 | 0.982301 |  |
| GO:0048713\_regulation\_of\_oligodendrocyte\_differentiation | 6 | 0 | 0.000000 | -0.000000 | 2347 | 2272.880208 | 2305.46 | 2338.039792 | 0.982301 |  |
| GO:0048853\_forebrain\_morphogenesis | 6 | 0 | 0.000000 | -0.000000 | 2347 | 2272.880208 | 2305.46 | 2338.039792 | 0.982301 |  |
| GO:0050684\_regulation\_of\_mRNA\_processing | 6 | 0 | 0.000000 | -0.000000 | 2347 | 2272.880208 | 2305.46 | 2338.039792 | 0.982301 |  |
| GO:0050805\_negative\_regulation\_of\_synaptic\_transmission | 6 | 0 | 0.000000 | -0.000000 | 2347 | 2272.880208 | 2305.46 | 2338.039792 | 0.982301 |  |
| GO:0050821\_protein\_stabilization | 6 | 0 | 0.000000 | -0.000000 | 2347 | 2272.880208 | 2305.46 | 2338.039792 | 0.982301 |  |
| GO:0050829\_defense\_response\_to\_Gram-negative\_bacterium | 6 | 0 | 0.000000 | -0.000000 | 2347 | 2272.880208 | 2305.46 | 2338.039792 | 0.982301 |  |
| GO:0050872\_white\_fat\_cell\_differentiation | 6 | 0 | 0.000000 | -0.000000 | 2347 | 2272.880208 | 2305.46 | 2338.039792 | 0.982301 |  |
| GO:0050951\_sensory\_perception\_of\_temperature\_stimulus | 6 | 0 | 0.000000 | -0.000000 | 2347 | 2272.880208 | 2305.46 | 2338.039792 | 0.982301 |  |
| GO:0050966\_detection\_of\_mechanical\_stimulus\_involved\_in\_sensory\_perception\_of\_pain | 6 | 0 | 0.000000 | -0.000000 | 2347 | 2272.880208 | 2305.46 | 2338.039792 | 0.982301 |  |
| GO:0051058\_negative\_regulation\_of\_small\_GTPase\_mediated\_signal\_transduction | 6 | 0 | 0.000000 | -0.000000 | 2347 | 2272.880208 | 2305.46 | 2338.039792 | 0.982301 |  |
| GO:0051085\_chaperone\_mediated\_protein\_folding\_requiring\_cofactor | 6 | 0 | 0.000000 | -0.000000 | 2347 | 2272.880208 | 2305.46 | 2338.039792 | 0.982301 |  |
| GO:0051180\_vitamin\_transport | 6 | 0 | 0.000000 | -0.000000 | 2347 | 2272.880208 | 2305.46 | 2338.039792 | 0.982301 |  |
| GO:0051384\_response\_to\_glucocorticoid\_stimulus | 6 | 0 | 0.000000 | -0.000000 | 2347 | 2272.880208 | 2305.46 | 2338.039792 | 0.982301 |  |
| GO:0051592\_response\_to\_calcium\_ion | 6 | 0 | 0.000000 | -0.000000 | 2347 | 2272.880208 | 2305.46 | 2338.039792 | 0.982301 |  |
| GO:0051881\_regulation\_of\_mitochondrial\_membrane\_potential | 6 | 0 | 0.000000 | -0.000000 | 2347 | 2272.880208 | 2305.46 | 2338.039792 | 0.982301 |  |
| GO:0051970\_negative\_regulation\_of\_transmission\_of\_nerve\_impulse | 6 | 0 | 0.000000 | -0.000000 | 2347 | 2272.880208 | 2305.46 | 2338.039792 | 0.982301 |  |
| GO:0060013\_righting\_reflex | 6 | 0 | 0.000000 | -0.000000 | 2347 | 2272.880208 | 2305.46 | 2338.039792 | 0.982301 |  |
| GO:0060017\_parathyroid\_gland\_development | 6 | 0 | 0.000000 | -0.000000 | 2347 | 2272.880208 | 2305.46 | 2338.039792 | 0.982301 |  |
| GO:0060056\_mammary\_gland\_involution | 6 | 0 | 0.000000 | -0.000000 | 2347 | 2272.880208 | 2305.46 | 2338.039792 | 0.982301 |  |
| GO:0060068\_vagina\_development | 6 | 0 | 0.000000 | -0.000000 | 2347 | 2272.880208 | 2305.46 | 2338.039792 | 0.982301 |  |
| GO:0060134\_prepulse\_inhibition | 6 | 0 | 0.000000 | -0.000000 | 2347 | 2272.880208 | 2305.46 | 2338.039792 | 0.982301 |  |
| GO:0060136\_embryonic\_process\_involved\_in\_female\_pregnancy | 6 | 0 | 0.000000 | -0.000000 | 2347 | 2272.880208 | 2305.46 | 2338.039792 | 0.982301 |  |
| GO:0060271\_cilium\_morphogenesis | 6 | 0 | 0.000000 | -0.000000 | 2347 | 2272.880208 | 2305.46 | 2338.039792 | 0.982301 |  |
| GO:0060389\_pathway-restricted\_SMAD\_protein\_phosphorylation | 6 | 0 | 0.000000 | -0.000000 | 2347 | 2272.880208 | 2305.46 | 2338.039792 | 0.982301 |  |
| GO:0060411\_heart\_septum\_morphogenesis | 6 | 0 | 0.000000 | -0.000000 | 2347 | 2272.880208 | 2305.46 | 2338.039792 | 0.982301 |  |
| GO:0060638\_mesenchymal-epithelial\_cell\_signaling | 6 | 0 | 0.000000 | -0.000000 | 2347 | 2272.880208 | 2305.46 | 2338.039792 | 0.982301 |  |
| GO:0060685\_regulation\_of\_prostatic\_bud\_formation | 6 | 0 | 0.000000 | -0.000000 | 2347 | 2272.880208 | 2305.46 | 2338.039792 | 0.982301 |  |
| GO:0065004\_protein-DNA\_complex\_assembly | 6 | 0 | 0.000000 | -0.000000 | 2347 | 2272.880208 | 2305.46 | 2338.039792 | 0.982301 |  |
| GO:0022604\_regulation\_of\_cell\_morphogenesis | 62 | 0 | 0.000000 | -0.000000 | 2349 | 2277.253595 | 2308.92 | 2340.586405 | 0.982937 |  |
| GO:0030855\_epithelial\_cell\_differentiation | 62 | 0 | 0.000000 | -0.000000 | 2349 | 2277.253595 | 2308.92 | 2340.586405 | 0.982937 |  |
| GO:0000226\_microtubule\_cytoskeleton\_organization | 57 | 0 | 0.000000 | -0.000000 | 2350 | 2283.031265 | 2313.94 | 2344.848735 | 0.984655 |  |
| GO:0001658\_branching\_involved\_in\_ureteric\_bud\_morphogenesis | 26 | 0 | 0.000000 | -0.000000 | 2368 | 2300.606134 | 2330.59 | 2360.573866 | 0.984202 |  |
| GO:0001666\_response\_to\_hypoxia | 26 | 0 | 0.000000 | -0.000000 | 2368 | 2300.606134 | 2330.59 | 2360.573866 | 0.984202 |  |
| GO:0006720\_isoprenoid\_metabolic\_process | 26 | 0 | 0.000000 | -0.000000 | 2368 | 2300.606134 | 2330.59 | 2360.573866 | 0.984202 |  |
| GO:0006800\_oxygen\_and\_reactive\_oxygen\_species\_metabolic\_process | 26 | 0 | 0.000000 | -0.000000 | 2368 | 2300.606134 | 2330.59 | 2360.573866 | 0.984202 |  |
| GO:0007613\_memory | 26 | 0 | 0.000000 | -0.000000 | 2368 | 2300.606134 | 2330.59 | 2360.573866 | 0.984202 |  |
| GO:0007623\_circadian\_rhythm | 26 | 0 | 0.000000 | -0.000000 | 2368 | 2300.606134 | 2330.59 | 2360.573866 | 0.984202 |  |
| GO:0009310\_amine\_catabolic\_process | 26 | 0 | 0.000000 | -0.000000 | 2368 | 2300.606134 | 2330.59 | 2360.573866 | 0.984202 |  |
| GO:0009636\_response\_to\_toxin | 26 | 0 | 0.000000 | -0.000000 | 2368 | 2300.606134 | 2330.59 | 2360.573866 | 0.984202 |  |
| GO:0009880\_embryonic\_pattern\_specification | 26 | 0 | 0.000000 | -0.000000 | 2368 | 2300.606134 | 2330.59 | 2360.573866 | 0.984202 |  |
| GO:0010212\_response\_to\_ionizing\_radiation | 26 | 0 | 0.000000 | -0.000000 | 2368 | 2300.606134 | 2330.59 | 2360.573866 | 0.984202 |  |
| GO:0019233\_sensory\_perception\_of\_pain | 26 | 0 | 0.000000 | -0.000000 | 2368 | 2300.606134 | 2330.59 | 2360.573866 | 0.984202 |  |
| GO:0032609\_interferon-gamma\_production | 26 | 0 | 0.000000 | -0.000000 | 2368 | 2300.606134 | 2330.59 | 2360.573866 | 0.984202 |  |
| GO:0040029\_regulation\_of\_gene\_expression\_\_epigenetic | 26 | 0 | 0.000000 | -0.000000 | 2368 | 2300.606134 | 2330.59 | 2360.573866 | 0.984202 |  |
| GO:0046530\_photoreceptor\_cell\_differentiation | 26 | 0 | 0.000000 | -0.000000 | 2368 | 2300.606134 | 2330.59 | 2360.573866 | 0.984202 |  |
| GO:0048645\_organ\_formation | 26 | 0 | 0.000000 | -0.000000 | 2368 | 2300.606134 | 2330.59 | 2360.573866 | 0.984202 |  |
| GO:0050680\_negative\_regulation\_of\_epithelial\_cell\_proliferation | 26 | 0 | 0.000000 | -0.000000 | 2368 | 2300.606134 | 2330.59 | 2360.573866 | 0.984202 |  |
| GO:0050873\_brown\_fat\_cell\_differentiation | 26 | 0 | 0.000000 | -0.000000 | 2368 | 2300.606134 | 2330.59 | 2360.573866 | 0.984202 |  |
| GO:0060675\_ureteric\_bud\_morphogenesis | 26 | 0 | 0.000000 | -0.000000 | 2368 | 2300.606134 | 2330.59 | 2360.573866 | 0.984202 |  |
| GO:0016567\_protein\_ubiquitination | 35 | 0 | 0.000000 | -0.000000 | 2369 | 2309.232504 | 2338.7 | 2368.167496 | 0.987210 |  |
| GO:0009799\_determination\_of\_symmetry | 37 | 0 | 0.000000 | -0.000000 | 2377 | 2316.909250 | 2345.35 | 2373.790750 | 0.986685 |  |
| GO:0009855\_determination\_of\_bilateral\_symmetry | 37 | 0 | 0.000000 | -0.000000 | 2377 | 2316.909250 | 2345.35 | 2373.790750 | 0.986685 |  |
| GO:0019933\_cAMP-mediated\_signaling | 37 | 0 | 0.000000 | -0.000000 | 2377 | 2316.909250 | 2345.35 | 2373.790750 | 0.986685 |  |
| GO:0031349\_positive\_regulation\_of\_defense\_response | 37 | 0 | 0.000000 | -0.000000 | 2377 | 2316.909250 | 2345.35 | 2373.790750 | 0.986685 |  |
| GO:0032869\_cellular\_response\_to\_insulin\_stimulus | 37 | 0 | 0.000000 | -0.000000 | 2377 | 2316.909250 | 2345.35 | 2373.790750 | 0.986685 |  |
| GO:0045761\_regulation\_of\_adenylate\_cyclase\_activity | 37 | 0 | 0.000000 | -0.000000 | 2377 | 2316.909250 | 2345.35 | 2373.790750 | 0.986685 |  |
| GO:0050906\_detection\_of\_stimulus\_involved\_in\_sensory\_perception | 37 | 0 | 0.000000 | -0.000000 | 2377 | 2316.909250 | 2345.35 | 2373.790750 | 0.986685 |  |
| GO:0051250\_negative\_regulation\_of\_lymphocyte\_activation | 37 | 0 | 0.000000 | -0.000000 | 2377 | 2316.909250 | 2345.35 | 2373.790750 | 0.986685 |  |
| GO:0009124\_nucleoside\_monophosphate\_biosynthetic\_process | 52 | 0 | 0.000000 | -0.000000 | 2379 | 2321.271652 | 2349.09 | 2376.908348 | 0.987427 |  |
| GO:0010608\_posttranscriptional\_regulation\_of\_gene\_expression | 52 | 0 | 0.000000 | -0.000000 | 2379 | 2321.271652 | 2349.09 | 2376.908348 | 0.987427 |  |
| GO:0000279\_M\_phase | 85 | 0 | 0.000000 | 0.000000 | 2421 | 2399.378278 | 2410.41 | 2421.441722 | 0.995626 |  |
| GO:0006163\_purine\_nucleotide\_metabolic\_process | 73 | 0 | 0.000000 | 0.000000 | 2421 | 2399.378278 | 2410.41 | 2421.441722 | 0.995626 |  |
| GO:0006461\_protein\_complex\_assembly | 78 | 0 | 0.000000 | 0.000000 | 2421 | 2399.378278 | 2410.41 | 2421.441722 | 0.995626 |  |
| GO:0006519\_cellular\_amino\_acid\_and\_derivative\_metabolic\_process | 118 | 0 | 0.000000 | 0.000000 | 2421 | 2399.378278 | 2410.41 | 2421.441722 | 0.995626 |  |
| GO:0006575\_cellular\_amino\_acid\_derivative\_metabolic\_process | 83 | 0 | 0.000000 | 0.000000 | 2421 | 2399.378278 | 2410.41 | 2421.441722 | 0.995626 |  |
| GO:0006631\_fatty\_acid\_metabolic\_process | 80 | 0 | 0.000000 | 0.000000 | 2421 | 2399.378278 | 2410.41 | 2421.441722 | 0.995626 |  |
| GO:0006753\_nucleoside\_phosphate\_metabolic\_process | 94 | 0 | 0.000000 | 0.000000 | 2421 | 2399.378278 | 2410.41 | 2421.441722 | 0.995626 |  |
| GO:0007010\_cytoskeleton\_organization | 185 | 0 | 0.000000 | 0.000000 | 2421 | 2399.378278 | 2410.41 | 2421.441722 | 0.995626 |  |
| GO:0007017\_microtubule-based\_process | 83 | 0 | 0.000000 | 0.000000 | 2421 | 2399.378278 | 2410.41 | 2421.441722 | 0.995626 |  |
| GO:0007179\_transforming\_growth\_factor\_beta\_receptor\_signaling\_pathway | 66 | 0 | 0.000000 | 0.000000 | 2421 | 2399.378278 | 2410.41 | 2421.441722 | 0.995626 |  |
| GO:0007264\_small\_GTPase\_mediated\_signal\_transduction | 72 | 0 | 0.000000 | 0.000000 | 2421 | 2399.378278 | 2410.41 | 2421.441722 | 0.995626 |  |
| GO:0007398\_ectoderm\_development | 99 | 0 | 0.000000 | 0.000000 | 2421 | 2399.378278 | 2410.41 | 2421.441722 | 0.995626 |  |
| GO:0008150\_biological\_process | 4605 | 68 | 1.000000 | 0.000000 | 2421 | 2399.378278 | 2410.41 | 2421.441722 | 0.995626 |  |
| GO:0008544\_epidermis\_development | 91 | 0 | 0.000000 | 0.000000 | 2421 | 2399.378278 | 2410.41 | 2421.441722 | 0.995626 |  |
| GO:0009117\_nucleotide\_metabolic\_process | 94 | 0 | 0.000000 | 0.000000 | 2421 | 2399.378278 | 2410.41 | 2421.441722 | 0.995626 |  |
| GO:0009617\_response\_to\_bacterium | 70 | 0 | 0.000000 | 0.000000 | 2421 | 2399.378278 | 2410.41 | 2421.441722 | 0.995626 |  |
| GO:0015837\_amine\_transport | 66 | 0 | 0.000000 | 0.000000 | 2421 | 2399.378278 | 2410.41 | 2421.441722 | 0.995626 |  |
| GO:0019932\_second-messenger-mediated\_signaling | 68 | 0 | 0.000000 | 0.000000 | 2421 | 2399.378278 | 2410.41 | 2421.441722 | 0.995626 |  |
| GO:0030029\_actin\_filament-based\_process | 109 | 0 | 0.000000 | 0.000000 | 2421 | 2399.378278 | 2410.41 | 2421.441722 | 0.995626 |  |
| GO:0030036\_actin\_cytoskeleton\_organization | 102 | 0 | 0.000000 | 0.000000 | 2421 | 2399.378278 | 2410.41 | 2421.441722 | 0.995626 |  |
| GO:0030198\_extracellular\_matrix\_organization | 83 | 0 | 0.000000 | 0.000000 | 2421 | 2399.378278 | 2410.41 | 2421.441722 | 0.995626 |  |
| GO:0031347\_regulation\_of\_defense\_response | 67 | 0 | 0.000000 | 0.000000 | 2421 | 2399.378278 | 2410.41 | 2421.441722 | 0.995626 |  |
| GO:0032101\_regulation\_of\_response\_to\_external\_stimulus | 69 | 0 | 0.000000 | 0.000000 | 2421 | 2399.378278 | 2410.41 | 2421.441722 | 0.995626 |  |
| GO:0034621\_cellular\_macromolecular\_complex\_subunit\_organization | 76 | 0 | 0.000000 | 0.000000 | 2421 | 2399.378278 | 2410.41 | 2421.441722 | 0.995626 |  |
| GO:0034641\_cellular\_nitrogen\_compound\_metabolic\_process | 86 | 0 | 0.000000 | 0.000000 | 2421 | 2399.378278 | 2410.41 | 2421.441722 | 0.995626 |  |
| GO:0040012\_regulation\_of\_locomotion | 72 | 0 | 0.000000 | 0.000000 | 2421 | 2399.378278 | 2410.41 | 2421.441722 | 0.995626 |  |
| GO:0043933\_macromolecular\_complex\_subunit\_organization | 117 | 0 | 0.000000 | 0.000000 | 2421 | 2399.378278 | 2410.41 | 2421.441722 | 0.995626 |  |
| GO:0046483\_heterocycle\_metabolic\_process | 116 | 0 | 0.000000 | 0.000000 | 2421 | 2399.378278 | 2410.41 | 2421.441722 | 0.995626 |  |
| GO:0048589\_developmental\_growth | 75 | 0 | 0.000000 | 0.000000 | 2421 | 2399.378278 | 2410.41 | 2421.441722 | 0.995626 |  |
| GO:0048706\_embryonic\_skeletal\_system\_development | 73 | 0 | 0.000000 | 0.000000 | 2421 | 2399.378278 | 2410.41 | 2421.441722 | 0.995626 |  |
| GO:0050673\_epithelial\_cell\_proliferation | 72 | 0 | 0.000000 | 0.000000 | 2421 | 2399.378278 | 2410.41 | 2421.441722 | 0.995626 |  |
| GO:0051046\_regulation\_of\_secretion | 79 | 0 | 0.000000 | 0.000000 | 2421 | 2399.378278 | 2410.41 | 2421.441722 | 0.995626 |  |
| GO:0051050\_positive\_regulation\_of\_transport | 75 | 0 | 0.000000 | 0.000000 | 2421 | 2399.378278 | 2410.41 | 2421.441722 | 0.995626 |  |
| GO:0051128\_regulation\_of\_cellular\_component\_organization | 160 | 0 | 0.000000 | 0.000000 | 2421 | 2399.378278 | 2410.41 | 2421.441722 | 0.995626 |  |
| GO:0051130\_positive\_regulation\_of\_cellular\_component\_organization | 66 | 0 | 0.000000 | 0.000000 | 2421 | 2399.378278 | 2410.41 | 2421.441722 | 0.995626 |  |
| GO:0051240\_positive\_regulation\_of\_multicellular\_organismal\_process | 108 | 0 | 0.000000 | 0.000000 | 2421 | 2399.378278 | 2410.41 | 2421.441722 | 0.995626 |  |
| GO:0051270\_regulation\_of\_cell\_motion | 73 | 0 | 0.000000 | 0.000000 | 2421 | 2399.378278 | 2410.41 | 2421.441722 | 0.995626 |  |
| GO:0051336\_regulation\_of\_hydrolase\_activity | 73 | 0 | 0.000000 | 0.000000 | 2421 | 2399.378278 | 2410.41 | 2421.441722 | 0.995626 |  |
| GO:0055086\_nucleobase\_\_nucleoside\_and\_nucleotide\_metabolic\_process | 104 | 0 | 0.000000 | 0.000000 | 2421 | 2399.378278 | 2410.41 | 2421.441722 | 0.995626 |  |
| GO:0060341\_regulation\_of\_cellular\_localization | 97 | 0 | 0.000000 | 0.000000 | 2421 | 2399.378278 | 2410.41 | 2421.441722 | 0.995626 |  |
| GO:0065003\_macromolecular\_complex\_assembly | 93 | 0 | 0.000000 | 0.000000 | 2421 | 2399.378278 | 2410.41 | 2421.441722 | 0.995626 |  |
| GO:0070271\_protein\_complex\_biogenesis | 78 | 0 | 0.000000 | 0.000000 | 2421 | 2399.378278 | 2410.41 | 2421.441722 | 0.995626 |  |
| GO:0000045\_autophagic\_vacuole\_formation | 5 | 0 | 0.000000 | 0.000000 | 2677 | 2672.502146 | 2677.49 | 2682.477854 | 1.000183 |  |
| GO:0000070\_mitotic\_sister\_chromatid\_segregation | 5 | 0 | 0.000000 | 0.000000 | 2677 | 2672.502146 | 2677.49 | 2682.477854 | 1.000183 |  |
| GO:0000097\_sulfur\_amino\_acid\_biosynthetic\_process | 5 | 0 | 0.000000 | 0.000000 | 2677 | 2672.502146 | 2677.49 | 2682.477854 | 1.000183 |  |
| GO:0000239\_pachytene | 5 | 0 | 0.000000 | 0.000000 | 2677 | 2672.502146 | 2677.49 | 2682.477854 | 1.000183 |  |
| GO:0000272\_polysaccharide\_catabolic\_process | 5 | 0 | 0.000000 | 0.000000 | 2677 | 2672.502146 | 2677.49 | 2682.477854 | 1.000183 |  |
| GO:0000288\_nuclear-transcribed\_mRNA\_catabolic\_process\_\_deadenylation-dependent\_decay | 5 | 0 | 0.000000 | 0.000000 | 2677 | 2672.502146 | 2677.49 | 2682.477854 | 1.000183 |  |
| GO:0000578\_embryonic\_axis\_specification | 5 | 0 | 0.000000 | 0.000000 | 2677 | 2672.502146 | 2677.49 | 2682.477854 | 1.000183 |  |
| GO:0000726\_non-recombinational\_repair | 5 | 0 | 0.000000 | 0.000000 | 2677 | 2672.502146 | 2677.49 | 2682.477854 | 1.000183 |  |
| GO:0000819\_sister\_chromatid\_segregation | 5 | 0 | 0.000000 | 0.000000 | 2677 | 2672.502146 | 2677.49 | 2682.477854 | 1.000183 |  |
| GO:0000956\_nuclear-transcribed\_mRNA\_catabolic\_process | 5 | 0 | 0.000000 | 0.000000 | 2677 | 2672.502146 | 2677.49 | 2682.477854 | 1.000183 |  |
| GO:0001562\_response\_to\_protozoan | 5 | 0 | 0.000000 | 0.000000 | 2677 | 2672.502146 | 2677.49 | 2682.477854 | 1.000183 |  |
| GO:0001706\_endoderm\_formation | 5 | 0 | 0.000000 | 0.000000 | 2677 | 2672.502146 | 2677.49 | 2682.477854 | 1.000183 |  |
| GO:0001865\_NK\_T\_cell\_differentiation | 5 | 0 | 0.000000 | 0.000000 | 2677 | 2672.502146 | 2677.49 | 2682.477854 | 1.000183 |  |
| GO:0001945\_lymph\_vessel\_development | 5 | 0 | 0.000000 | 0.000000 | 2677 | 2672.502146 | 2677.49 | 2682.477854 | 1.000183 |  |
| GO:0001991\_regulation\_of\_systemic\_arterial\_blood\_pressure\_by\_circulatory\_renin-angiotensin | 5 | 0 | 0.000000 | 0.000000 | 2677 | 2672.502146 | 2677.49 | 2682.477854 | 1.000183 |  |
| GO:0001993\_regulation\_of\_systemic\_arterial\_blood\_pressure\_by\_norepinephrine-epinephrine | 5 | 0 | 0.000000 | 0.000000 | 2677 | 2672.502146 | 2677.49 | 2682.477854 | 1.000183 |  |
| GO:0002328\_pro-B\_cell\_differentiation | 5 | 0 | 0.000000 | 0.000000 | 2677 | 2672.502146 | 2677.49 | 2682.477854 | 1.000183 |  |
| GO:0002448\_mast\_cell\_mediated\_immunity | 5 | 0 | 0.000000 | 0.000000 | 2677 | 2672.502146 | 2677.49 | 2682.477854 | 1.000183 |  |
| GO:0002517\_T\_cell\_tolerance\_induction | 5 | 0 | 0.000000 | 0.000000 | 2677 | 2672.502146 | 2677.49 | 2682.477854 | 1.000183 |  |
| GO:0002634\_regulation\_of\_germinal\_center\_formation | 5 | 0 | 0.000000 | 0.000000 | 2677 | 2672.502146 | 2677.49 | 2682.477854 | 1.000183 |  |
| GO:0002664\_regulation\_of\_T\_cell\_tolerance\_induction | 5 | 0 | 0.000000 | 0.000000 | 2677 | 2672.502146 | 2677.49 | 2682.477854 | 1.000183 |  |
| GO:0002666\_positive\_regulation\_of\_T\_cell\_tolerance\_induction | 5 | 0 | 0.000000 | 0.000000 | 2677 | 2672.502146 | 2677.49 | 2682.477854 | 1.000183 |  |
| GO:0002716\_negative\_regulation\_of\_natural\_killer\_cell\_mediated\_immunity | 5 | 0 | 0.000000 | 0.000000 | 2677 | 2672.502146 | 2677.49 | 2682.477854 | 1.000183 |  |
| GO:0002825\_regulation\_of\_T-helper\_1\_type\_immune\_response | 5 | 0 | 0.000000 | 0.000000 | 2677 | 2672.502146 | 2677.49 | 2682.477854 | 1.000183 |  |
| GO:0002862\_negative\_regulation\_of\_inflammatory\_response\_to\_antigenic\_stimulus | 5 | 0 | 0.000000 | 0.000000 | 2677 | 2672.502146 | 2677.49 | 2682.477854 | 1.000183 |  |
| GO:0002866\_positive\_regulation\_of\_acute\_inflammatory\_response\_to\_antigenic\_stimulus | 5 | 0 | 0.000000 | 0.000000 | 2677 | 2672.502146 | 2677.49 | 2682.477854 | 1.000183 |  |
| GO:0002885\_positive\_regulation\_of\_hypersensitivity | 5 | 0 | 0.000000 | 0.000000 | 2677 | 2672.502146 | 2677.49 | 2682.477854 | 1.000183 |  |
| GO:0002902\_regulation\_of\_B\_cell\_apoptosis | 5 | 0 | 0.000000 | 0.000000 | 2677 | 2672.502146 | 2677.49 | 2682.477854 | 1.000183 |  |
| GO:0003071\_renal\_system\_process\_involved\_in\_regulation\_of\_systemic\_arterial\_blood\_pressure | 5 | 0 | 0.000000 | 0.000000 | 2677 | 2672.502146 | 2677.49 | 2682.477854 | 1.000183 |  |
| GO:0005980\_glycogen\_catabolic\_process | 5 | 0 | 0.000000 | 0.000000 | 2677 | 2672.502146 | 2677.49 | 2682.477854 | 1.000183 |  |
| GO:0006023\_aminoglycan\_biosynthetic\_process | 5 | 0 | 0.000000 | 0.000000 | 2677 | 2672.502146 | 2677.49 | 2682.477854 | 1.000183 |  |
| GO:0006024\_glycosaminoglycan\_biosynthetic\_process | 5 | 0 | 0.000000 | 0.000000 | 2677 | 2672.502146 | 2677.49 | 2682.477854 | 1.000183 |  |
| GO:0006268\_DNA\_unwinding\_during\_replication | 5 | 0 | 0.000000 | 0.000000 | 2677 | 2672.502146 | 2677.49 | 2682.477854 | 1.000183 |  |
| GO:0006270\_DNA\_replication\_initiation | 5 | 0 | 0.000000 | 0.000000 | 2677 | 2672.502146 | 2677.49 | 2682.477854 | 1.000183 |  |
| GO:0006303\_double-strand\_break\_repair\_via\_nonhomologous\_end\_joining | 5 | 0 | 0.000000 | 0.000000 | 2677 | 2672.502146 | 2677.49 | 2682.477854 | 1.000183 |  |
| GO:0006346\_methylation-dependent\_chromatin\_silencing | 5 | 0 | 0.000000 | 0.000000 | 2677 | 2672.502146 | 2677.49 | 2682.477854 | 1.000183 |  |
| GO:0006376\_mRNA\_splice\_site\_selection | 5 | 0 | 0.000000 | 0.000000 | 2677 | 2672.502146 | 2677.49 | 2682.477854 | 1.000183 |  |
| GO:0006378\_mRNA\_polyadenylation | 5 | 0 | 0.000000 | 0.000000 | 2677 | 2672.502146 | 2677.49 | 2682.477854 | 1.000183 |  |
| GO:0006400\_tRNA\_modification | 5 | 0 | 0.000000 | 0.000000 | 2677 | 2672.502146 | 2677.49 | 2682.477854 | 1.000183 |  |
| GO:0006506\_GPI\_anchor\_biosynthetic\_process | 5 | 0 | 0.000000 | 0.000000 | 2677 | 2672.502146 | 2677.49 | 2682.477854 | 1.000183 |  |
| GO:0006509\_membrane\_protein\_ectodomain\_proteolysis | 5 | 0 | 0.000000 | 0.000000 | 2677 | 2672.502146 | 2677.49 | 2682.477854 | 1.000183 |  |
| GO:0006515\_misfolded\_or\_incompletely\_synthesized\_protein\_catabolic\_process | 5 | 0 | 0.000000 | 0.000000 | 2677 | 2672.502146 | 2677.49 | 2682.477854 | 1.000183 |  |
| GO:0006541\_glutamine\_metabolic\_process | 5 | 0 | 0.000000 | 0.000000 | 2677 | 2672.502146 | 2677.49 | 2682.477854 | 1.000183 |  |
| GO:0006570\_tyrosine\_metabolic\_process | 5 | 0 | 0.000000 | 0.000000 | 2677 | 2672.502146 | 2677.49 | 2682.477854 | 1.000183 |  |
| GO:0006577\_betaine\_metabolic\_process | 5 | 0 | 0.000000 | 0.000000 | 2677 | 2672.502146 | 2677.49 | 2682.477854 | 1.000183 |  |
| GO:0006586\_indolalkylamine\_metabolic\_process | 5 | 0 | 0.000000 | 0.000000 | 2677 | 2672.502146 | 2677.49 | 2682.477854 | 1.000183 |  |
| GO:0006636\_unsaturated\_fatty\_acid\_biosynthetic\_process | 5 | 0 | 0.000000 | 0.000000 | 2677 | 2672.502146 | 2677.49 | 2682.477854 | 1.000183 |  |
| GO:0006691\_leukotriene\_metabolic\_process | 5 | 0 | 0.000000 | 0.000000 | 2677 | 2672.502146 | 2677.49 | 2682.477854 | 1.000183 |  |
| GO:0006903\_vesicle\_targeting | 5 | 0 | 0.000000 | 0.000000 | 2677 | 2672.502146 | 2677.49 | 2682.477854 | 1.000183 |  |
| GO:0006924\_activation-induced\_cell\_death\_of\_T\_cells | 5 | 0 | 0.000000 | 0.000000 | 2677 | 2672.502146 | 2677.49 | 2682.477854 | 1.000183 |  |
| GO:0006929\_substrate-bound\_cell\_migration | 5 | 0 | 0.000000 | 0.000000 | 2677 | 2672.502146 | 2677.49 | 2682.477854 | 1.000183 |  |
| GO:0006983\_ER\_overload\_response | 5 | 0 | 0.000000 | 0.000000 | 2677 | 2672.502146 | 2677.49 | 2682.477854 | 1.000183 |  |
| GO:0007029\_endoplasmic\_reticulum\_organization | 5 | 0 | 0.000000 | 0.000000 | 2677 | 2672.502146 | 2677.49 | 2682.477854 | 1.000183 |  |
| GO:0007091\_mitotic\_metaphase\_anaphase\_transition | 5 | 0 | 0.000000 | 0.000000 | 2677 | 2672.502146 | 2677.49 | 2682.477854 | 1.000183 |  |
| GO:0007320\_insemination | 5 | 0 | 0.000000 | 0.000000 | 2677 | 2672.502146 | 2677.49 | 2682.477854 | 1.000183 |  |
| GO:0007622\_rhythmic\_behavior | 5 | 0 | 0.000000 | 0.000000 | 2677 | 2672.502146 | 2677.49 | 2682.477854 | 1.000183 |  |
| GO:0008343\_adult\_feeding\_behavior | 5 | 0 | 0.000000 | 0.000000 | 2677 | 2672.502146 | 2677.49 | 2682.477854 | 1.000183 |  |
| GO:0008631\_induction\_of\_apoptosis\_by\_oxidative\_stress | 5 | 0 | 0.000000 | 0.000000 | 2677 | 2672.502146 | 2677.49 | 2682.477854 | 1.000183 |  |
| GO:0009132\_nucleoside\_diphosphate\_metabolic\_process | 5 | 0 | 0.000000 | 0.000000 | 2677 | 2672.502146 | 2677.49 | 2682.477854 | 1.000183 |  |
| GO:0009146\_purine\_nucleoside\_triphosphate\_catabolic\_process | 5 | 0 | 0.000000 | 0.000000 | 2677 | 2672.502146 | 2677.49 | 2682.477854 | 1.000183 |  |
| GO:0009151\_purine\_deoxyribonucleotide\_metabolic\_process | 5 | 0 | 0.000000 | 0.000000 | 2677 | 2672.502146 | 2677.49 | 2682.477854 | 1.000183 |  |
| GO:0009154\_purine\_ribonucleotide\_catabolic\_process | 5 | 0 | 0.000000 | 0.000000 | 2677 | 2672.502146 | 2677.49 | 2682.477854 | 1.000183 |  |
| GO:0009215\_purine\_deoxyribonucleoside\_triphosphate\_metabolic\_process | 5 | 0 | 0.000000 | 0.000000 | 2677 | 2672.502146 | 2677.49 | 2682.477854 | 1.000183 |  |
| GO:0009251\_glucan\_catabolic\_process | 5 | 0 | 0.000000 | 0.000000 | 2677 | 2672.502146 | 2677.49 | 2682.477854 | 1.000183 |  |
| GO:0009261\_ribonucleotide\_catabolic\_process | 5 | 0 | 0.000000 | 0.000000 | 2677 | 2672.502146 | 2677.49 | 2682.477854 | 1.000183 |  |
| GO:0009263\_deoxyribonucleotide\_biosynthetic\_process | 5 | 0 | 0.000000 | 0.000000 | 2677 | 2672.502146 | 2677.49 | 2682.477854 | 1.000183 |  |
| GO:0009264\_deoxyribonucleotide\_catabolic\_process | 5 | 0 | 0.000000 | 0.000000 | 2677 | 2672.502146 | 2677.49 | 2682.477854 | 1.000183 |  |
| GO:0009296\_flagellum\_assembly | 5 | 0 | 0.000000 | 0.000000 | 2677 | 2672.502146 | 2677.49 | 2682.477854 | 1.000183 |  |
| GO:0009303\_rRNA\_transcription | 5 | 0 | 0.000000 | 0.000000 | 2677 | 2672.502146 | 2677.49 | 2682.477854 | 1.000183 |  |
| GO:0009312\_oligosaccharide\_biosynthetic\_process | 5 | 0 | 0.000000 | 0.000000 | 2677 | 2672.502146 | 2677.49 | 2682.477854 | 1.000183 |  |
| GO:0009437\_carnitine\_metabolic\_process | 5 | 0 | 0.000000 | 0.000000 | 2677 | 2672.502146 | 2677.49 | 2682.477854 | 1.000183 |  |
| GO:0009950\_dorsal\_ventral\_axis\_specification | 5 | 0 | 0.000000 | 0.000000 | 2677 | 2672.502146 | 2677.49 | 2682.477854 | 1.000183 |  |
| GO:0010460\_positive\_regulation\_of\_heart\_rate | 5 | 0 | 0.000000 | 0.000000 | 2677 | 2672.502146 | 2677.49 | 2682.477854 | 1.000183 |  |
| GO:0010522\_regulation\_of\_calcium\_ion\_transport\_into\_cytosol | 5 | 0 | 0.000000 | 0.000000 | 2677 | 2672.502146 | 2677.49 | 2682.477854 | 1.000183 |  |
| GO:0010676\_positive\_regulation\_of\_cellular\_carbohydrate\_metabolic\_process | 5 | 0 | 0.000000 | 0.000000 | 2677 | 2672.502146 | 2677.49 | 2682.477854 | 1.000183 |  |
| GO:0010828\_positive\_regulation\_of\_glucose\_transport | 5 | 0 | 0.000000 | 0.000000 | 2677 | 2672.502146 | 2677.49 | 2682.477854 | 1.000183 |  |
| GO:0014044\_Schwann\_cell\_development | 5 | 0 | 0.000000 | 0.000000 | 2677 | 2672.502146 | 2677.49 | 2682.477854 | 1.000183 |  |
| GO:0015802\_basic\_amino\_acid\_transport | 5 | 0 | 0.000000 | 0.000000 | 2677 | 2672.502146 | 2677.49 | 2682.477854 | 1.000183 |  |
| GO:0016048\_detection\_of\_temperature\_stimulus | 5 | 0 | 0.000000 | 0.000000 | 2677 | 2672.502146 | 2677.49 | 2682.477854 | 1.000183 |  |
| GO:0016188\_synaptic\_vesicle\_maturation | 5 | 0 | 0.000000 | 0.000000 | 2677 | 2672.502146 | 2677.49 | 2682.477854 | 1.000183 |  |
| GO:0016579\_protein\_deubiquitination | 5 | 0 | 0.000000 | 0.000000 | 2677 | 2672.502146 | 2677.49 | 2682.477854 | 1.000183 |  |
| GO:0017148\_negative\_regulation\_of\_translation | 5 | 0 | 0.000000 | 0.000000 | 2677 | 2672.502146 | 2677.49 | 2682.477854 | 1.000183 |  |
| GO:0017158\_regulation\_of\_calcium\_ion-dependent\_exocytosis | 5 | 0 | 0.000000 | 0.000000 | 2677 | 2672.502146 | 2677.49 | 2682.477854 | 1.000183 |  |
| GO:0019227\_neuronal\_action\_potential\_propagation | 5 | 0 | 0.000000 | 0.000000 | 2677 | 2672.502146 | 2677.49 | 2682.477854 | 1.000183 |  |
| GO:0019430\_removal\_of\_superoxide\_radicals | 5 | 0 | 0.000000 | 0.000000 | 2677 | 2672.502146 | 2677.49 | 2682.477854 | 1.000183 |  |
| GO:0021554\_optic\_nerve\_development | 5 | 0 | 0.000000 | 0.000000 | 2677 | 2672.502146 | 2677.49 | 2682.477854 | 1.000183 |  |
| GO:0021826\_substrate-independent\_telencephalic\_tangential\_migration | 5 | 0 | 0.000000 | 0.000000 | 2677 | 2672.502146 | 2677.49 | 2682.477854 | 1.000183 |  |
| GO:0021843\_substrate-independent\_telencephalic\_tangential\_interneuron\_migration | 5 | 0 | 0.000000 | 0.000000 | 2677 | 2672.502146 | 2677.49 | 2682.477854 | 1.000183 |  |
| GO:0021854\_hypothalamus\_development | 5 | 0 | 0.000000 | 0.000000 | 2677 | 2672.502146 | 2677.49 | 2682.477854 | 1.000183 |  |
| GO:0022415\_viral\_reproductive\_process | 5 | 0 | 0.000000 | 0.000000 | 2677 | 2672.502146 | 2677.49 | 2682.477854 | 1.000183 |  |
| GO:0030042\_actin\_filament\_depolymerization | 5 | 0 | 0.000000 | 0.000000 | 2677 | 2672.502146 | 2677.49 | 2682.477854 | 1.000183 |  |
| GO:0030177\_positive\_regulation\_of\_Wnt\_receptor\_signaling\_pathway | 5 | 0 | 0.000000 | 0.000000 | 2677 | 2672.502146 | 2677.49 | 2682.477854 | 1.000183 |  |
| GO:0030261\_chromosome\_condensation | 5 | 0 | 0.000000 | 0.000000 | 2677 | 2672.502146 | 2677.49 | 2682.477854 | 1.000183 |  |
| GO:0030299\_intestinal\_cholesterol\_absorption | 5 | 0 | 0.000000 | 0.000000 | 2677 | 2672.502146 | 2677.49 | 2682.477854 | 1.000183 |  |
| GO:0030431\_sleep | 5 | 0 | 0.000000 | 0.000000 | 2677 | 2672.502146 | 2677.49 | 2682.477854 | 1.000183 |  |
| GO:0030433\_ER-associated\_protein\_catabolic\_process | 5 | 0 | 0.000000 | 0.000000 | 2677 | 2672.502146 | 2677.49 | 2682.477854 | 1.000183 |  |
| GO:0030801\_positive\_regulation\_of\_cyclic\_nucleotide\_metabolic\_process | 5 | 0 | 0.000000 | 0.000000 | 2677 | 2672.502146 | 2677.49 | 2682.477854 | 1.000183 |  |
| GO:0030804\_positive\_regulation\_of\_cyclic\_nucleotide\_biosynthetic\_process | 5 | 0 | 0.000000 | 0.000000 | 2677 | 2672.502146 | 2677.49 | 2682.477854 | 1.000183 |  |
| GO:0030810\_positive\_regulation\_of\_nucleotide\_biosynthetic\_process | 5 | 0 | 0.000000 | 0.000000 | 2677 | 2672.502146 | 2677.49 | 2682.477854 | 1.000183 |  |
| GO:0030823\_regulation\_of\_cGMP\_metabolic\_process | 5 | 0 | 0.000000 | 0.000000 | 2677 | 2672.502146 | 2677.49 | 2682.477854 | 1.000183 |  |
| GO:0030834\_regulation\_of\_actin\_filament\_depolymerization | 5 | 0 | 0.000000 | 0.000000 | 2677 | 2672.502146 | 2677.49 | 2682.477854 | 1.000183 |  |
| GO:0030838\_positive\_regulation\_of\_actin\_filament\_polymerization | 5 | 0 | 0.000000 | 0.000000 | 2677 | 2672.502146 | 2677.49 | 2682.477854 | 1.000183 |  |
| GO:0030851\_granulocyte\_differentiation | 5 | 0 | 0.000000 | 0.000000 | 2677 | 2672.502146 | 2677.49 | 2682.477854 | 1.000183 |  |
| GO:0030866\_cortical\_actin\_cytoskeleton\_organization | 5 | 0 | 0.000000 | 0.000000 | 2677 | 2672.502146 | 2677.49 | 2682.477854 | 1.000183 |  |
| GO:0030889\_negative\_regulation\_of\_B\_cell\_proliferation | 5 | 0 | 0.000000 | 0.000000 | 2677 | 2672.502146 | 2677.49 | 2682.477854 | 1.000183 |  |
| GO:0030917\_midbrain-hindbrain\_boundary\_development | 5 | 0 | 0.000000 | 0.000000 | 2677 | 2672.502146 | 2677.49 | 2682.477854 | 1.000183 |  |
| GO:0031047\_gene\_silencing\_by\_RNA | 5 | 0 | 0.000000 | 0.000000 | 2677 | 2672.502146 | 2677.49 | 2682.477854 | 1.000183 |  |
| GO:0031057\_negative\_regulation\_of\_histone\_modification | 5 | 0 | 0.000000 | 0.000000 | 2677 | 2672.502146 | 2677.49 | 2682.477854 | 1.000183 |  |
| GO:0031058\_positive\_regulation\_of\_histone\_modification | 5 | 0 | 0.000000 | 0.000000 | 2677 | 2672.502146 | 2677.49 | 2682.477854 | 1.000183 |  |
| GO:0031060\_regulation\_of\_histone\_methylation | 5 | 0 | 0.000000 | 0.000000 | 2677 | 2672.502146 | 2677.49 | 2682.477854 | 1.000183 |  |
| GO:0031122\_cytoplasmic\_microtubule\_organization | 5 | 0 | 0.000000 | 0.000000 | 2677 | 2672.502146 | 2677.49 | 2682.477854 | 1.000183 |  |
| GO:0031532\_actin\_cytoskeleton\_reorganization | 5 | 0 | 0.000000 | 0.000000 | 2677 | 2672.502146 | 2677.49 | 2682.477854 | 1.000183 |  |
| GO:0032024\_positive\_regulation\_of\_insulin\_secretion | 5 | 0 | 0.000000 | 0.000000 | 2677 | 2672.502146 | 2677.49 | 2682.477854 | 1.000183 |  |
| GO:0032320\_positive\_regulation\_of\_Ras\_GTPase\_activity | 5 | 0 | 0.000000 | 0.000000 | 2677 | 2672.502146 | 2677.49 | 2682.477854 | 1.000183 |  |
| GO:0032332\_positive\_regulation\_of\_chondrocyte\_differentiation | 5 | 0 | 0.000000 | 0.000000 | 2677 | 2672.502146 | 2677.49 | 2682.477854 | 1.000183 |  |
| GO:0032368\_regulation\_of\_lipid\_transport | 5 | 0 | 0.000000 | 0.000000 | 2677 | 2672.502146 | 2677.49 | 2682.477854 | 1.000183 |  |
| GO:0032434\_regulation\_of\_proteasomal\_ubiquitin-dependent\_protein\_catabolic\_process | 5 | 0 | 0.000000 | 0.000000 | 2677 | 2672.502146 | 2677.49 | 2682.477854 | 1.000183 |  |
| GO:0032479\_regulation\_of\_type\_I\_interferon\_production | 5 | 0 | 0.000000 | 0.000000 | 2677 | 2672.502146 | 2677.49 | 2682.477854 | 1.000183 |  |
| GO:0032494\_response\_to\_peptidoglycan | 5 | 0 | 0.000000 | 0.000000 | 2677 | 2672.502146 | 2677.49 | 2682.477854 | 1.000183 |  |
| GO:0032508\_DNA\_duplex\_unwinding | 5 | 0 | 0.000000 | 0.000000 | 2677 | 2672.502146 | 2677.49 | 2682.477854 | 1.000183 |  |
| GO:0032720\_negative\_regulation\_of\_tumor\_necrosis\_factor\_production | 5 | 0 | 0.000000 | 0.000000 | 2677 | 2672.502146 | 2677.49 | 2682.477854 | 1.000183 |  |
| GO:0032845\_negative\_regulation\_of\_homeostatic\_process | 5 | 0 | 0.000000 | 0.000000 | 2677 | 2672.502146 | 2677.49 | 2682.477854 | 1.000183 |  |
| GO:0032846\_positive\_regulation\_of\_homeostatic\_process | 5 | 0 | 0.000000 | 0.000000 | 2677 | 2672.502146 | 2677.49 | 2682.477854 | 1.000183 |  |
| GO:0033003\_regulation\_of\_mast\_cell\_activation | 5 | 0 | 0.000000 | 0.000000 | 2677 | 2672.502146 | 2677.49 | 2682.477854 | 1.000183 |  |
| GO:0033005\_positive\_regulation\_of\_mast\_cell\_activation | 5 | 0 | 0.000000 | 0.000000 | 2677 | 2672.502146 | 2677.49 | 2682.477854 | 1.000183 |  |
| GO:0033023\_mast\_cell\_homeostasis | 5 | 0 | 0.000000 | 0.000000 | 2677 | 2672.502146 | 2677.49 | 2682.477854 | 1.000183 |  |
| GO:0033024\_mast\_cell\_apoptosis | 5 | 0 | 0.000000 | 0.000000 | 2677 | 2672.502146 | 2677.49 | 2682.477854 | 1.000183 |  |
| GO:0033025\_regulation\_of\_mast\_cell\_apoptosis | 5 | 0 | 0.000000 | 0.000000 | 2677 | 2672.502146 | 2677.49 | 2682.477854 | 1.000183 |  |
| GO:0033033\_negative\_regulation\_of\_myeloid\_cell\_apoptosis | 5 | 0 | 0.000000 | 0.000000 | 2677 | 2672.502146 | 2677.49 | 2682.477854 | 1.000183 |  |
| GO:0033144\_negative\_regulation\_of\_steroid\_hormone\_receptor\_signaling\_pathway | 5 | 0 | 0.000000 | 0.000000 | 2677 | 2672.502146 | 2677.49 | 2682.477854 | 1.000183 |  |
| GO:0033146\_regulation\_of\_estrogen\_receptor\_signaling\_pathway | 5 | 0 | 0.000000 | 0.000000 | 2677 | 2672.502146 | 2677.49 | 2682.477854 | 1.000183 |  |
| GO:0033152\_immunoglobulin\_V(D)J\_recombination | 5 | 0 | 0.000000 | 0.000000 | 2677 | 2672.502146 | 2677.49 | 2682.477854 | 1.000183 |  |
| GO:0033205\_cytokinesis\_during\_cell\_cycle | 5 | 0 | 0.000000 | 0.000000 | 2677 | 2672.502146 | 2677.49 | 2682.477854 | 1.000183 |  |
| GO:0033619\_membrane\_protein\_proteolysis | 5 | 0 | 0.000000 | 0.000000 | 2677 | 2672.502146 | 2677.49 | 2682.477854 | 1.000183 |  |
| GO:0033627\_cell\_adhesion\_mediated\_by\_integrin | 5 | 0 | 0.000000 | 0.000000 | 2677 | 2672.502146 | 2677.49 | 2682.477854 | 1.000183 |  |
| GO:0033692\_cellular\_polysaccharide\_biosynthetic\_process | 5 | 0 | 0.000000 | 0.000000 | 2677 | 2672.502146 | 2677.49 | 2682.477854 | 1.000183 |  |
| GO:0034109\_homotypic\_cell-cell\_adhesion | 5 | 0 | 0.000000 | 0.000000 | 2677 | 2672.502146 | 2677.49 | 2682.477854 | 1.000183 |  |
| GO:0034367\_macromolecular\_complex\_remodeling | 5 | 0 | 0.000000 | 0.000000 | 2677 | 2672.502146 | 2677.49 | 2682.477854 | 1.000183 |  |
| GO:0034368\_protein-lipid\_complex\_remodeling | 5 | 0 | 0.000000 | 0.000000 | 2677 | 2672.502146 | 2677.49 | 2682.477854 | 1.000183 |  |
| GO:0034369\_plasma\_lipoprotein\_particle\_remodeling | 5 | 0 | 0.000000 | 0.000000 | 2677 | 2672.502146 | 2677.49 | 2682.477854 | 1.000183 |  |
| GO:0035058\_sensory\_cilium\_assembly | 5 | 0 | 0.000000 | 0.000000 | 2677 | 2672.502146 | 2677.49 | 2682.477854 | 1.000183 |  |
| GO:0035065\_regulation\_of\_histone\_acetylation | 5 | 0 | 0.000000 | 0.000000 | 2677 | 2672.502146 | 2677.49 | 2682.477854 | 1.000183 |  |
| GO:0035089\_establishment\_of\_apical\_basal\_cell\_polarity | 5 | 0 | 0.000000 | 0.000000 | 2677 | 2672.502146 | 2677.49 | 2682.477854 | 1.000183 |  |
| GO:0035095\_behavioral\_response\_to\_nicotine | 5 | 0 | 0.000000 | 0.000000 | 2677 | 2672.502146 | 2677.49 | 2682.477854 | 1.000183 |  |
| GO:0035234\_germ\_cell\_programmed\_cell\_death | 5 | 0 | 0.000000 | 0.000000 | 2677 | 2672.502146 | 2677.49 | 2682.477854 | 1.000183 |  |
| GO:0035238\_vitamin\_A\_biosynthetic\_process | 5 | 0 | 0.000000 | 0.000000 | 2677 | 2672.502146 | 2677.49 | 2682.477854 | 1.000183 |  |
| GO:0042159\_lipoprotein\_catabolic\_process | 5 | 0 | 0.000000 | 0.000000 | 2677 | 2672.502146 | 2677.49 | 2682.477854 | 1.000183 |  |
| GO:0042362\_fat-soluble\_vitamin\_biosynthetic\_process | 5 | 0 | 0.000000 | 0.000000 | 2677 | 2672.502146 | 2677.49 | 2682.477854 | 1.000183 |  |
| GO:0042364\_water-soluble\_vitamin\_biosynthetic\_process | 5 | 0 | 0.000000 | 0.000000 | 2677 | 2672.502146 | 2677.49 | 2682.477854 | 1.000183 |  |
| GO:0042416\_dopamine\_biosynthetic\_process | 5 | 0 | 0.000000 | 0.000000 | 2677 | 2672.502146 | 2677.49 | 2682.477854 | 1.000183 |  |
| GO:0042430\_indole\_and\_derivative\_metabolic\_process | 5 | 0 | 0.000000 | 0.000000 | 2677 | 2672.502146 | 2677.49 | 2682.477854 | 1.000183 |  |
| GO:0042434\_indole\_derivative\_metabolic\_process | 5 | 0 | 0.000000 | 0.000000 | 2677 | 2672.502146 | 2677.49 | 2682.477854 | 1.000183 |  |
| GO:0042487\_regulation\_of\_odontogenesis\_of\_dentine-containing\_tooth | 5 | 0 | 0.000000 | 0.000000 | 2677 | 2672.502146 | 2677.49 | 2682.477854 | 1.000183 |  |
| GO:0042533\_tumor\_necrosis\_factor\_biosynthetic\_process | 5 | 0 | 0.000000 | 0.000000 | 2677 | 2672.502146 | 2677.49 | 2682.477854 | 1.000183 |  |
| GO:0042534\_regulation\_of\_tumor\_necrosis\_factor\_biosynthetic\_process | 5 | 0 | 0.000000 | 0.000000 | 2677 | 2672.502146 | 2677.49 | 2682.477854 | 1.000183 |  |
| GO:0042537\_benzene\_and\_derivative\_metabolic\_process | 5 | 0 | 0.000000 | 0.000000 | 2677 | 2672.502146 | 2677.49 | 2682.477854 | 1.000183 |  |
| GO:0042554\_superoxide\_anion\_generation | 5 | 0 | 0.000000 | 0.000000 | 2677 | 2672.502146 | 2677.49 | 2682.477854 | 1.000183 |  |
| GO:0042574\_retinal\_metabolic\_process | 5 | 0 | 0.000000 | 0.000000 | 2677 | 2672.502146 | 2677.49 | 2682.477854 | 1.000183 |  |
| GO:0042590\_antigen\_processing\_and\_presentation\_of\_exogenous\_peptide\_antigen\_via\_MHC\_class\_I | 5 | 0 | 0.000000 | 0.000000 | 2677 | 2672.502146 | 2677.49 | 2682.477854 | 1.000183 |  |
| GO:0042695\_thelarche | 5 | 0 | 0.000000 | 0.000000 | 2677 | 2672.502146 | 2677.49 | 2682.477854 | 1.000183 |  |
| GO:0042752\_regulation\_of\_circadian\_rhythm | 5 | 0 | 0.000000 | 0.000000 | 2677 | 2672.502146 | 2677.49 | 2682.477854 | 1.000183 |  |
| GO:0042756\_drinking\_behavior | 5 | 0 | 0.000000 | 0.000000 | 2677 | 2672.502146 | 2677.49 | 2682.477854 | 1.000183 |  |
| GO:0042904\_9-cis-retinoic\_acid\_biosynthetic\_process | 5 | 0 | 0.000000 | 0.000000 | 2677 | 2672.502146 | 2677.49 | 2682.477854 | 1.000183 |  |
| GO:0042905\_9-cis-retinoic\_acid\_metabolic\_process | 5 | 0 | 0.000000 | 0.000000 | 2677 | 2672.502146 | 2677.49 | 2682.477854 | 1.000183 |  |
| GO:0043030\_regulation\_of\_macrophage\_activation | 5 | 0 | 0.000000 | 0.000000 | 2677 | 2672.502146 | 2677.49 | 2682.477854 | 1.000183 |  |
| GO:0043046\_DNA\_methylation\_during\_gametogenesis | 5 | 0 | 0.000000 | 0.000000 | 2677 | 2672.502146 | 2677.49 | 2682.477854 | 1.000183 |  |
| GO:0043277\_apoptotic\_cell\_clearance | 5 | 0 | 0.000000 | 0.000000 | 2677 | 2672.502146 | 2677.49 | 2682.477854 | 1.000183 |  |
| GO:0043288\_apocarotenoid\_metabolic\_process | 5 | 0 | 0.000000 | 0.000000 | 2677 | 2672.502146 | 2677.49 | 2682.477854 | 1.000183 |  |
| GO:0043302\_positive\_regulation\_of\_leukocyte\_degranulation | 5 | 0 | 0.000000 | 0.000000 | 2677 | 2672.502146 | 2677.49 | 2682.477854 | 1.000183 |  |
| GO:0043303\_mast\_cell\_degranulation | 5 | 0 | 0.000000 | 0.000000 | 2677 | 2672.502146 | 2677.49 | 2682.477854 | 1.000183 |  |
| GO:0043304\_regulation\_of\_mast\_cell\_degranulation | 5 | 0 | 0.000000 | 0.000000 | 2677 | 2672.502146 | 2677.49 | 2682.477854 | 1.000183 |  |
| GO:0043306\_positive\_regulation\_of\_mast\_cell\_degranulation | 5 | 0 | 0.000000 | 0.000000 | 2677 | 2672.502146 | 2677.49 | 2682.477854 | 1.000183 |  |
| GO:0043393\_regulation\_of\_protein\_binding | 5 | 0 | 0.000000 | 0.000000 | 2677 | 2672.502146 | 2677.49 | 2682.477854 | 1.000183 |  |
| GO:0043403\_skeletal\_muscle\_regeneration | 5 | 0 | 0.000000 | 0.000000 | 2677 | 2672.502146 | 2677.49 | 2682.477854 | 1.000183 |  |
| GO:0043489\_RNA\_stabilization | 5 | 0 | 0.000000 | 0.000000 | 2677 | 2672.502146 | 2677.49 | 2682.477854 | 1.000183 |  |
| GO:0043526\_neuroprotection | 5 | 0 | 0.000000 | 0.000000 | 2677 | 2672.502146 | 2677.49 | 2682.477854 | 1.000183 |  |
| GO:0043550\_regulation\_of\_lipid\_kinase\_activity | 5 | 0 | 0.000000 | 0.000000 | 2677 | 2672.502146 | 2677.49 | 2682.477854 | 1.000183 |  |
| GO:0043551\_regulation\_of\_phosphoinositide\_3-kinase\_activity | 5 | 0 | 0.000000 | 0.000000 | 2677 | 2672.502146 | 2677.49 | 2682.477854 | 1.000183 |  |
| GO:0043552\_positive\_regulation\_of\_phosphoinositide\_3-kinase\_activity | 5 | 0 | 0.000000 | 0.000000 | 2677 | 2672.502146 | 2677.49 | 2682.477854 | 1.000183 |  |
| GO:0043555\_regulation\_of\_translation\_in\_response\_to\_stress | 5 | 0 | 0.000000 | 0.000000 | 2677 | 2672.502146 | 2677.49 | 2682.477854 | 1.000183 |  |
| GO:0043558\_regulation\_of\_translational\_initiation\_in\_response\_to\_stress | 5 | 0 | 0.000000 | 0.000000 | 2677 | 2672.502146 | 2677.49 | 2682.477854 | 1.000183 |  |
| GO:0043631\_RNA\_polyadenylation | 5 | 0 | 0.000000 | 0.000000 | 2677 | 2672.502146 | 2677.49 | 2682.477854 | 1.000183 |  |
| GO:0043648\_dicarboxylic\_acid\_metabolic\_process | 5 | 0 | 0.000000 | 0.000000 | 2677 | 2672.502146 | 2677.49 | 2682.477854 | 1.000183 |  |
| GO:0044058\_regulation\_of\_digestive\_system\_process | 5 | 0 | 0.000000 | 0.000000 | 2677 | 2672.502146 | 2677.49 | 2682.477854 | 1.000183 |  |
| GO:0044241\_lipid\_digestion | 5 | 0 | 0.000000 | 0.000000 | 2677 | 2672.502146 | 2677.49 | 2682.477854 | 1.000183 |  |
| GO:0044247\_cellular\_polysaccharide\_catabolic\_process | 5 | 0 | 0.000000 | 0.000000 | 2677 | 2672.502146 | 2677.49 | 2682.477854 | 1.000183 |  |
| GO:0045063\_T-helper\_1\_cell\_differentiation | 5 | 0 | 0.000000 | 0.000000 | 2677 | 2672.502146 | 2677.49 | 2682.477854 | 1.000183 |  |
| GO:0045064\_T-helper\_2\_cell\_differentiation | 5 | 0 | 0.000000 | 0.000000 | 2677 | 2672.502146 | 2677.49 | 2682.477854 | 1.000183 |  |
| GO:0045080\_positive\_regulation\_of\_chemokine\_biosynthetic\_process | 5 | 0 | 0.000000 | 0.000000 | 2677 | 2672.502146 | 2677.49 | 2682.477854 | 1.000183 |  |
| GO:0045123\_cellular\_extravasation | 5 | 0 | 0.000000 | 0.000000 | 2677 | 2672.502146 | 2677.49 | 2682.477854 | 1.000183 |  |
| GO:0045197\_establishment\_or\_maintenance\_of\_epithelial\_cell\_apical\_basal\_polarity | 5 | 0 | 0.000000 | 0.000000 | 2677 | 2672.502146 | 2677.49 | 2682.477854 | 1.000183 |  |
| GO:0045342\_MHC\_class\_II\_biosynthetic\_process | 5 | 0 | 0.000000 | 0.000000 | 2677 | 2672.502146 | 2677.49 | 2682.477854 | 1.000183 |  |
| GO:0045410\_positive\_regulation\_of\_interleukin-6\_biosynthetic\_process | 5 | 0 | 0.000000 | 0.000000 | 2677 | 2672.502146 | 2677.49 | 2682.477854 | 1.000183 |  |
| GO:0045622\_regulation\_of\_T-helper\_cell\_differentiation | 5 | 0 | 0.000000 | 0.000000 | 2677 | 2672.502146 | 2677.49 | 2682.477854 | 1.000183 |  |
| GO:0045648\_positive\_regulation\_of\_erythrocyte\_differentiation | 5 | 0 | 0.000000 | 0.000000 | 2677 | 2672.502146 | 2677.49 | 2682.477854 | 1.000183 |  |
| GO:0045651\_positive\_regulation\_of\_macrophage\_differentiation | 5 | 0 | 0.000000 | 0.000000 | 2677 | 2672.502146 | 2677.49 | 2682.477854 | 1.000183 |  |
| GO:0045730\_respiratory\_burst | 5 | 0 | 0.000000 | 0.000000 | 2677 | 2672.502146 | 2677.49 | 2682.477854 | 1.000183 |  |
| GO:0045745\_positive\_regulation\_of\_G-protein\_coupled\_receptor\_protein\_signaling\_pathway | 5 | 0 | 0.000000 | 0.000000 | 2677 | 2672.502146 | 2677.49 | 2682.477854 | 1.000183 |  |
| GO:0045773\_positive\_regulation\_of\_axon\_extension | 5 | 0 | 0.000000 | 0.000000 | 2677 | 2672.502146 | 2677.49 | 2682.477854 | 1.000183 |  |
| GO:0045793\_positive\_regulation\_of\_cell\_size | 5 | 0 | 0.000000 | 0.000000 | 2677 | 2672.502146 | 2677.49 | 2682.477854 | 1.000183 |  |
| GO:0045885\_positive\_regulation\_of\_survival\_gene\_product\_expression | 5 | 0 | 0.000000 | 0.000000 | 2677 | 2672.502146 | 2677.49 | 2682.477854 | 1.000183 |  |
| GO:0045953\_negative\_regulation\_of\_natural\_killer\_cell\_mediated\_cytotoxicity | 5 | 0 | 0.000000 | 0.000000 | 2677 | 2672.502146 | 2677.49 | 2682.477854 | 1.000183 |  |
| GO:0045981\_positive\_regulation\_of\_nucleotide\_metabolic\_process | 5 | 0 | 0.000000 | 0.000000 | 2677 | 2672.502146 | 2677.49 | 2682.477854 | 1.000183 |  |
| GO:0045987\_positive\_regulation\_of\_smooth\_muscle\_contraction | 5 | 0 | 0.000000 | 0.000000 | 2677 | 2672.502146 | 2677.49 | 2682.477854 | 1.000183 |  |
| GO:0046326\_positive\_regulation\_of\_glucose\_import | 5 | 0 | 0.000000 | 0.000000 | 2677 | 2672.502146 | 2677.49 | 2682.477854 | 1.000183 |  |
| GO:0046456\_icosanoid\_biosynthetic\_process | 5 | 0 | 0.000000 | 0.000000 | 2677 | 2672.502146 | 2677.49 | 2682.477854 | 1.000183 |  |
| GO:0046459\_short-chain\_fatty\_acid\_metabolic\_process | 5 | 0 | 0.000000 | 0.000000 | 2677 | 2672.502146 | 2677.49 | 2682.477854 | 1.000183 |  |
| GO:0046489\_phosphoinositide\_biosynthetic\_process | 5 | 0 | 0.000000 | 0.000000 | 2677 | 2672.502146 | 2677.49 | 2682.477854 | 1.000183 |  |
| GO:0046621\_negative\_regulation\_of\_organ\_growth | 5 | 0 | 0.000000 | 0.000000 | 2677 | 2672.502146 | 2677.49 | 2682.477854 | 1.000183 |  |
| GO:0046697\_decidualization | 5 | 0 | 0.000000 | 0.000000 | 2677 | 2672.502146 | 2677.49 | 2682.477854 | 1.000183 |  |
| GO:0046785\_microtubule\_polymerization | 5 | 0 | 0.000000 | 0.000000 | 2677 | 2672.502146 | 2677.49 | 2682.477854 | 1.000183 |  |
| GO:0048013\_ephrin\_receptor\_signaling\_pathway | 5 | 0 | 0.000000 | 0.000000 | 2677 | 2672.502146 | 2677.49 | 2682.477854 | 1.000183 |  |
| GO:0048149\_behavioral\_response\_to\_ethanol | 5 | 0 | 0.000000 | 0.000000 | 2677 | 2672.502146 | 2677.49 | 2682.477854 | 1.000183 |  |
| GO:0048255\_mRNA\_stabilization | 5 | 0 | 0.000000 | 0.000000 | 2677 | 2672.502146 | 2677.49 | 2682.477854 | 1.000183 |  |
| GO:0048289\_isotype\_switching\_to\_IgE\_isotypes | 5 | 0 | 0.000000 | 0.000000 | 2677 | 2672.502146 | 2677.49 | 2682.477854 | 1.000183 |  |
| GO:0048293\_regulation\_of\_isotype\_switching\_to\_IgE\_isotypes | 5 | 0 | 0.000000 | 0.000000 | 2677 | 2672.502146 | 2677.49 | 2682.477854 | 1.000183 |  |
| GO:0048485\_sympathetic\_nervous\_system\_development | 5 | 0 | 0.000000 | 0.000000 | 2677 | 2672.502146 | 2677.49 | 2682.477854 | 1.000183 |  |
| GO:0048532\_anatomical\_structure\_arrangement | 5 | 0 | 0.000000 | 0.000000 | 2677 | 2672.502146 | 2677.49 | 2682.477854 | 1.000183 |  |
| GO:0048570\_notochord\_morphogenesis | 5 | 0 | 0.000000 | 0.000000 | 2677 | 2672.502146 | 2677.49 | 2682.477854 | 1.000183 |  |
| GO:0048659\_smooth\_muscle\_cell\_proliferation | 5 | 0 | 0.000000 | 0.000000 | 2677 | 2672.502146 | 2677.49 | 2682.477854 | 1.000183 |  |
| GO:0048664\_neuron\_fate\_determination | 5 | 0 | 0.000000 | 0.000000 | 2677 | 2672.502146 | 2677.49 | 2682.477854 | 1.000183 |  |
| GO:0048715\_negative\_regulation\_of\_oligodendrocyte\_differentiation | 5 | 0 | 0.000000 | 0.000000 | 2677 | 2672.502146 | 2677.49 | 2682.477854 | 1.000183 |  |
| GO:0048934\_peripheral\_nervous\_system\_neuron\_differentiation | 5 | 0 | 0.000000 | 0.000000 | 2677 | 2672.502146 | 2677.49 | 2682.477854 | 1.000183 |  |
| GO:0050665\_hydrogen\_peroxide\_biosynthetic\_process | 5 | 0 | 0.000000 | 0.000000 | 2677 | 2672.502146 | 2677.49 | 2682.477854 | 1.000183 |  |
| GO:0050701\_interleukin-1\_secretion | 5 | 0 | 0.000000 | 0.000000 | 2677 | 2672.502146 | 2677.49 | 2682.477854 | 1.000183 |  |
| GO:0050715\_positive\_regulation\_of\_cytokine\_secretion | 5 | 0 | 0.000000 | 0.000000 | 2677 | 2672.502146 | 2677.49 | 2682.477854 | 1.000183 |  |
| GO:0050819\_negative\_regulation\_of\_coagulation | 5 | 0 | 0.000000 | 0.000000 | 2677 | 2672.502146 | 2677.49 | 2682.477854 | 1.000183 |  |
| GO:0051057\_positive\_regulation\_of\_small\_GTPase\_mediated\_signal\_transduction | 5 | 0 | 0.000000 | 0.000000 | 2677 | 2672.502146 | 2677.49 | 2682.477854 | 1.000183 |  |
| GO:0051136\_regulation\_of\_NK\_T\_cell\_differentiation | 5 | 0 | 0.000000 | 0.000000 | 2677 | 2672.502146 | 2677.49 | 2682.477854 | 1.000183 |  |
| GO:0051138\_positive\_regulation\_of\_NK\_T\_cell\_differentiation | 5 | 0 | 0.000000 | 0.000000 | 2677 | 2672.502146 | 2677.49 | 2682.477854 | 1.000183 |  |
| GO:0051177\_meiotic\_sister\_chromatid\_cohesion | 5 | 0 | 0.000000 | 0.000000 | 2677 | 2672.502146 | 2677.49 | 2682.477854 | 1.000183 |  |
| GO:0051289\_protein\_homotetramerization | 5 | 0 | 0.000000 | 0.000000 | 2677 | 2672.502146 | 2677.49 | 2682.477854 | 1.000183 |  |
| GO:0051298\_centrosome\_duplication | 5 | 0 | 0.000000 | 0.000000 | 2677 | 2672.502146 | 2677.49 | 2682.477854 | 1.000183 |  |
| GO:0051323\_metaphase | 5 | 0 | 0.000000 | 0.000000 | 2677 | 2672.502146 | 2677.49 | 2682.477854 | 1.000183 |  |
| GO:0051403\_stress-activated\_MAPK\_cascade | 5 | 0 | 0.000000 | 0.000000 | 2677 | 2672.502146 | 2677.49 | 2682.477854 | 1.000183 |  |
| GO:0051668\_localization\_within\_membrane | 5 | 0 | 0.000000 | 0.000000 | 2677 | 2672.502146 | 2677.49 | 2682.477854 | 1.000183 |  |
| GO:0051898\_negative\_regulation\_of\_protein\_kinase\_B\_signaling\_cascade | 5 | 0 | 0.000000 | 0.000000 | 2677 | 2672.502146 | 2677.49 | 2682.477854 | 1.000183 |  |
| GO:0051954\_positive\_regulation\_of\_amine\_transport | 5 | 0 | 0.000000 | 0.000000 | 2677 | 2672.502146 | 2677.49 | 2682.477854 | 1.000183 |  |
| GO:0051966\_regulation\_of\_synaptic\_transmission\_\_glutamatergic | 5 | 0 | 0.000000 | 0.000000 | 2677 | 2672.502146 | 2677.49 | 2682.477854 | 1.000183 |  |
| GO:0055003\_cardiac\_myofibril\_assembly | 5 | 0 | 0.000000 | 0.000000 | 2677 | 2672.502146 | 2677.49 | 2682.477854 | 1.000183 |  |
| GO:0055015\_ventricular\_cardiac\_muscle\_cell\_development | 5 | 0 | 0.000000 | 0.000000 | 2677 | 2672.502146 | 2677.49 | 2682.477854 | 1.000183 |  |
| GO:0060045\_positive\_regulation\_of\_cardiac\_muscle\_cell\_proliferation | 5 | 0 | 0.000000 | 0.000000 | 2677 | 2672.502146 | 2677.49 | 2682.477854 | 1.000183 |  |
| GO:0060073\_micturition | 5 | 0 | 0.000000 | 0.000000 | 2677 | 2672.502146 | 2677.49 | 2682.477854 | 1.000183 |  |
| GO:0060351\_cartilage\_development\_involved\_in\_endochondral\_bone\_morphogenesis | 5 | 0 | 0.000000 | 0.000000 | 2677 | 2672.502146 | 2677.49 | 2682.477854 | 1.000183 |  |
| GO:0060438\_trachea\_development | 5 | 0 | 0.000000 | 0.000000 | 2677 | 2672.502146 | 2677.49 | 2682.477854 | 1.000183 |  |
| GO:0060600\_dichotomous\_subdivision\_of\_an\_epithelial\_terminal\_unit | 5 | 0 | 0.000000 | 0.000000 | 2677 | 2672.502146 | 2677.49 | 2682.477854 | 1.000183 |  |
| GO:0060605\_tube\_lumen\_cavitation | 5 | 0 | 0.000000 | 0.000000 | 2677 | 2672.502146 | 2677.49 | 2682.477854 | 1.000183 |  |
| GO:0060662\_salivary\_gland\_cavitation | 5 | 0 | 0.000000 | 0.000000 | 2677 | 2672.502146 | 2677.49 | 2682.477854 | 1.000183 |  |
| GO:0060665\_regulation\_of\_branching\_involved\_in\_salivary\_gland\_morphogenesis\_by\_mesenchymal-epithelial\_signaling | 5 | 0 | 0.000000 | 0.000000 | 2677 | 2672.502146 | 2677.49 | 2682.477854 | 1.000183 |  |
| GO:0060707\_trophoblast\_giant\_cell\_differentiation | 5 | 0 | 0.000000 | 0.000000 | 2677 | 2672.502146 | 2677.49 | 2682.477854 | 1.000183 |  |
| GO:0060744\_mammary\_gland\_branching\_involved\_in\_thelarche | 5 | 0 | 0.000000 | 0.000000 | 2677 | 2672.502146 | 2677.49 | 2682.477854 | 1.000183 |  |
| GO:0060745\_mammary\_gland\_branching\_involved\_in\_pregnancy | 5 | 0 | 0.000000 | 0.000000 | 2677 | 2672.502146 | 2677.49 | 2682.477854 | 1.000183 |  |
| GO:0060762\_regulation\_of\_branching\_involved\_in\_mammary\_gland\_duct\_morphogenesis | 5 | 0 | 0.000000 | 0.000000 | 2677 | 2672.502146 | 2677.49 | 2682.477854 | 1.000183 |  |
| GO:0070371\_ERK1\_and\_ERK2\_cascade | 5 | 0 | 0.000000 | 0.000000 | 2677 | 2672.502146 | 2677.49 | 2682.477854 | 1.000183 |  |
| GO:0070372\_regulation\_of\_ERK1\_and\_ERK2\_cascade | 5 | 0 | 0.000000 | 0.000000 | 2677 | 2672.502146 | 2677.49 | 2682.477854 | 1.000183 |  |
| GO:0070374\_positive\_regulation\_of\_ERK1\_and\_ERK2\_cascade | 5 | 0 | 0.000000 | 0.000000 | 2677 | 2672.502146 | 2677.49 | 2682.477854 | 1.000183 |  |
| GO:0000096\_sulfur\_amino\_acid\_metabolic\_process | 11 | 0 | 0.000000 | 0.000000 | 2747 | 2746.562663 | 2748.84 | 2751.117337 | 1.000670 |  |
| GO:0000271\_polysaccharide\_biosynthetic\_process | 11 | 0 | 0.000000 | 0.000000 | 2747 | 2746.562663 | 2748.84 | 2751.117337 | 1.000670 |  |
| GO:0000737\_DNA\_catabolic\_process\_\_endonucleolytic | 11 | 0 | 0.000000 | 0.000000 | 2747 | 2746.562663 | 2748.84 | 2751.117337 | 1.000670 |  |
| GO:0001101\_response\_to\_acid | 11 | 0 | 0.000000 | 0.000000 | 2747 | 2746.562663 | 2748.84 | 2751.117337 | 1.000670 |  |
| GO:0001837\_epithelial\_to\_mesenchymal\_transition | 11 | 0 | 0.000000 | 0.000000 | 2747 | 2746.562663 | 2748.84 | 2751.117337 | 1.000670 |  |
| GO:0001952\_regulation\_of\_cell-matrix\_adhesion | 11 | 0 | 0.000000 | 0.000000 | 2747 | 2746.562663 | 2748.84 | 2751.117337 | 1.000670 |  |
| GO:0001963\_synaptic\_transmission\_\_dopaminergic | 11 | 0 | 0.000000 | 0.000000 | 2747 | 2746.562663 | 2748.84 | 2751.117337 | 1.000670 |  |
| GO:0002444\_myeloid\_leukocyte\_mediated\_immunity | 11 | 0 | 0.000000 | 0.000000 | 2747 | 2746.562663 | 2748.84 | 2751.117337 | 1.000670 |  |
| GO:0002467\_germinal\_center\_formation | 11 | 0 | 0.000000 | 0.000000 | 2747 | 2746.562663 | 2748.84 | 2751.117337 | 1.000670 |  |
| GO:0002758\_innate\_immune\_response-activating\_signal\_transduction | 11 | 0 | 0.000000 | 0.000000 | 2747 | 2746.562663 | 2748.84 | 2751.117337 | 1.000670 |  |
| GO:0006333\_chromatin\_assembly\_or\_disassembly | 11 | 0 | 0.000000 | 0.000000 | 2747 | 2746.562663 | 2748.84 | 2751.117337 | 1.000670 |  |
| GO:0006635\_fatty\_acid\_beta-oxidation | 11 | 0 | 0.000000 | 0.000000 | 2747 | 2746.562663 | 2748.84 | 2751.117337 | 1.000670 |  |
| GO:0006637\_acyl-CoA\_metabolic\_process | 11 | 0 | 0.000000 | 0.000000 | 2747 | 2746.562663 | 2748.84 | 2751.117337 | 1.000670 |  |
| GO:0006690\_icosanoid\_metabolic\_process | 11 | 0 | 0.000000 | 0.000000 | 2747 | 2746.562663 | 2748.84 | 2751.117337 | 1.000670 |  |
| GO:0006779\_porphyrin\_biosynthetic\_process | 11 | 0 | 0.000000 | 0.000000 | 2747 | 2746.562663 | 2748.84 | 2751.117337 | 1.000670 |  |
| GO:0007051\_spindle\_organization | 11 | 0 | 0.000000 | 0.000000 | 2747 | 2746.562663 | 2748.84 | 2751.117337 | 1.000670 |  |
| GO:0007088\_regulation\_of\_mitosis | 11 | 0 | 0.000000 | 0.000000 | 2747 | 2746.562663 | 2748.84 | 2751.117337 | 1.000670 |  |
| GO:0007162\_negative\_regulation\_of\_cell\_adhesion | 11 | 0 | 0.000000 | 0.000000 | 2747 | 2746.562663 | 2748.84 | 2751.117337 | 1.000670 |  |
| GO:0007215\_glutamate\_signaling\_pathway | 11 | 0 | 0.000000 | 0.000000 | 2747 | 2746.562663 | 2748.84 | 2751.117337 | 1.000670 |  |
| GO:0007229\_integrin-mediated\_signaling\_pathway | 11 | 0 | 0.000000 | 0.000000 | 2747 | 2746.562663 | 2748.84 | 2751.117337 | 1.000670 |  |
| GO:0008354\_germ\_cell\_migration | 11 | 0 | 0.000000 | 0.000000 | 2747 | 2746.562663 | 2748.84 | 2751.117337 | 1.000670 |  |
| GO:0008652\_cellular\_amino\_acid\_biosynthetic\_process | 11 | 0 | 0.000000 | 0.000000 | 2747 | 2746.562663 | 2748.84 | 2751.117337 | 1.000670 |  |
| GO:0009064\_glutamine\_family\_amino\_acid\_metabolic\_process | 11 | 0 | 0.000000 | 0.000000 | 2747 | 2746.562663 | 2748.84 | 2751.117337 | 1.000670 |  |
| GO:0009141\_nucleoside\_triphosphate\_metabolic\_process | 11 | 0 | 0.000000 | 0.000000 | 2747 | 2746.562663 | 2748.84 | 2751.117337 | 1.000670 |  |
| GO:0009166\_nucleotide\_catabolic\_process | 11 | 0 | 0.000000 | 0.000000 | 2747 | 2746.562663 | 2748.84 | 2751.117337 | 1.000670 |  |
| GO:0009409\_response\_to\_cold | 11 | 0 | 0.000000 | 0.000000 | 2747 | 2746.562663 | 2748.84 | 2751.117337 | 1.000670 |  |
| GO:0010259\_multicellular\_organismal\_aging | 11 | 0 | 0.000000 | 0.000000 | 2747 | 2746.562663 | 2748.84 | 2751.117337 | 1.000670 |  |
| GO:0014902\_myotube\_differentiation | 11 | 0 | 0.000000 | 0.000000 | 2747 | 2746.562663 | 2748.84 | 2751.117337 | 1.000670 |  |
| GO:0016079\_synaptic\_vesicle\_exocytosis | 11 | 0 | 0.000000 | 0.000000 | 2747 | 2746.562663 | 2748.84 | 2751.117337 | 1.000670 |  |
| GO:0021602\_cranial\_nerve\_morphogenesis | 11 | 0 | 0.000000 | 0.000000 | 2747 | 2746.562663 | 2748.84 | 2751.117337 | 1.000670 |  |
| GO:0021846\_cell\_proliferation\_in\_forebrain | 11 | 0 | 0.000000 | 0.000000 | 2747 | 2746.562663 | 2748.84 | 2751.117337 | 1.000670 |  |
| GO:0030238\_male\_sex\_determination | 11 | 0 | 0.000000 | 0.000000 | 2747 | 2746.562663 | 2748.84 | 2751.117337 | 1.000670 |  |
| GO:0030308\_negative\_regulation\_of\_cell\_growth | 11 | 0 | 0.000000 | 0.000000 | 2747 | 2746.562663 | 2748.84 | 2751.117337 | 1.000670 |  |
| GO:0030593\_neutrophil\_chemotaxis | 11 | 0 | 0.000000 | 0.000000 | 2747 | 2746.562663 | 2748.84 | 2751.117337 | 1.000670 |  |
| GO:0030856\_regulation\_of\_epithelial\_cell\_differentiation | 11 | 0 | 0.000000 | 0.000000 | 2747 | 2746.562663 | 2748.84 | 2751.117337 | 1.000670 |  |
| GO:0030878\_thyroid\_gland\_development | 11 | 0 | 0.000000 | 0.000000 | 2747 | 2746.562663 | 2748.84 | 2751.117337 | 1.000670 |  |
| GO:0030968\_endoplasmic\_reticulum\_unfolded\_protein\_response | 11 | 0 | 0.000000 | 0.000000 | 2747 | 2746.562663 | 2748.84 | 2751.117337 | 1.000670 |  |
| GO:0031110\_regulation\_of\_microtubule\_polymerization\_or\_depolymerization | 11 | 0 | 0.000000 | 0.000000 | 2747 | 2746.562663 | 2748.84 | 2751.117337 | 1.000670 |  |
| GO:0031646\_positive\_regulation\_of\_neurological\_system\_process | 11 | 0 | 0.000000 | 0.000000 | 2747 | 2746.562663 | 2748.84 | 2751.117337 | 1.000670 |  |
| GO:0031647\_regulation\_of\_protein\_stability | 11 | 0 | 0.000000 | 0.000000 | 2747 | 2746.562663 | 2748.84 | 2751.117337 | 1.000670 |  |
| GO:0032655\_regulation\_of\_interleukin-12\_production | 11 | 0 | 0.000000 | 0.000000 | 2747 | 2746.562663 | 2748.84 | 2751.117337 | 1.000670 |  |
| GO:0033014\_tetrapyrrole\_biosynthetic\_process | 11 | 0 | 0.000000 | 0.000000 | 2747 | 2746.562663 | 2748.84 | 2751.117337 | 1.000670 |  |
| GO:0033559\_unsaturated\_fatty\_acid\_metabolic\_process | 11 | 0 | 0.000000 | 0.000000 | 2747 | 2746.562663 | 2748.84 | 2751.117337 | 1.000670 |  |
| GO:0034620\_cellular\_response\_to\_unfolded\_protein | 11 | 0 | 0.000000 | 0.000000 | 2747 | 2746.562663 | 2748.84 | 2751.117337 | 1.000670 |  |
| GO:0034762\_regulation\_of\_transmembrane\_transport | 11 | 0 | 0.000000 | 0.000000 | 2747 | 2746.562663 | 2748.84 | 2751.117337 | 1.000670 |  |
| GO:0042036\_negative\_regulation\_of\_cytokine\_biosynthetic\_process | 11 | 0 | 0.000000 | 0.000000 | 2747 | 2746.562663 | 2748.84 | 2751.117337 | 1.000670 |  |
| GO:0042219\_cellular\_amino\_acid\_derivative\_catabolic\_process | 11 | 0 | 0.000000 | 0.000000 | 2747 | 2746.562663 | 2748.84 | 2751.117337 | 1.000670 |  |
| GO:0042401\_biogenic\_amine\_biosynthetic\_process | 11 | 0 | 0.000000 | 0.000000 | 2747 | 2746.562663 | 2748.84 | 2751.117337 | 1.000670 |  |
| GO:0042439\_ethanolamine\_and\_derivative\_metabolic\_process | 11 | 0 | 0.000000 | 0.000000 | 2747 | 2746.562663 | 2748.84 | 2751.117337 | 1.000670 |  |
| GO:0042542\_response\_to\_hydrogen\_peroxide | 11 | 0 | 0.000000 | 0.000000 | 2747 | 2746.562663 | 2748.84 | 2751.117337 | 1.000670 |  |
| GO:0043270\_positive\_regulation\_of\_ion\_transport | 11 | 0 | 0.000000 | 0.000000 | 2747 | 2746.562663 | 2748.84 | 2751.117337 | 1.000670 |  |
| GO:0045026\_plasma\_membrane\_fusion | 11 | 0 | 0.000000 | 0.000000 | 2747 | 2746.562663 | 2748.84 | 2751.117337 | 1.000670 |  |
| GO:0045216\_cell-cell\_junction\_organization | 11 | 0 | 0.000000 | 0.000000 | 2747 | 2746.562663 | 2748.84 | 2751.117337 | 1.000670 |  |
| GO:0045834\_positive\_regulation\_of\_lipid\_metabolic\_process | 11 | 0 | 0.000000 | 0.000000 | 2747 | 2746.562663 | 2748.84 | 2751.117337 | 1.000670 |  |
| GO:0046323\_glucose\_import | 11 | 0 | 0.000000 | 0.000000 | 2747 | 2746.562663 | 2748.84 | 2751.117337 | 1.000670 |  |
| GO:0046928\_regulation\_of\_neurotransmitter\_secretion | 11 | 0 | 0.000000 | 0.000000 | 2747 | 2746.562663 | 2748.84 | 2751.117337 | 1.000670 |  |
| GO:0048009\_insulin-like\_growth\_factor\_receptor\_signaling\_pathway | 11 | 0 | 0.000000 | 0.000000 | 2747 | 2746.562663 | 2748.84 | 2751.117337 | 1.000670 |  |
| GO:0048193\_Golgi\_vesicle\_transport | 11 | 0 | 0.000000 | 0.000000 | 2747 | 2746.562663 | 2748.84 | 2751.117337 | 1.000670 |  |
| GO:0048678\_response\_to\_axon\_injury | 11 | 0 | 0.000000 | 0.000000 | 2747 | 2746.562663 | 2748.84 | 2751.117337 | 1.000670 |  |
| GO:0048745\_smooth\_muscle\_tissue\_development | 11 | 0 | 0.000000 | 0.000000 | 2747 | 2746.562663 | 2748.84 | 2751.117337 | 1.000670 |  |
| GO:0050772\_positive\_regulation\_of\_axonogenesis | 11 | 0 | 0.000000 | 0.000000 | 2747 | 2746.562663 | 2748.84 | 2751.117337 | 1.000670 |  |
| GO:0050806\_positive\_regulation\_of\_synaptic\_transmission | 11 | 0 | 0.000000 | 0.000000 | 2747 | 2746.562663 | 2748.84 | 2751.117337 | 1.000670 |  |
| GO:0050807\_regulation\_of\_synapse\_organization | 11 | 0 | 0.000000 | 0.000000 | 2747 | 2746.562663 | 2748.84 | 2751.117337 | 1.000670 |  |
| GO:0050830\_defense\_response\_to\_Gram-positive\_bacterium | 11 | 0 | 0.000000 | 0.000000 | 2747 | 2746.562663 | 2748.84 | 2751.117337 | 1.000670 |  |
| GO:0051092\_positive\_regulation\_of\_NF-kappaB\_transcription\_factor\_activity | 11 | 0 | 0.000000 | 0.000000 | 2747 | 2746.562663 | 2748.84 | 2751.117337 | 1.000670 |  |
| GO:0051494\_negative\_regulation\_of\_cytoskeleton\_organization | 11 | 0 | 0.000000 | 0.000000 | 2747 | 2746.562663 | 2748.84 | 2751.117337 | 1.000670 |  |
| GO:0051783\_regulation\_of\_nuclear\_division | 11 | 0 | 0.000000 | 0.000000 | 2747 | 2746.562663 | 2748.84 | 2751.117337 | 1.000670 |  |
| GO:0051971\_positive\_regulation\_of\_transmission\_of\_nerve\_impulse | 11 | 0 | 0.000000 | 0.000000 | 2747 | 2746.562663 | 2748.84 | 2751.117337 | 1.000670 |  |
| GO:0060004\_reflex | 11 | 0 | 0.000000 | 0.000000 | 2747 | 2746.562663 | 2748.84 | 2751.117337 | 1.000670 |  |
| GO:0060767\_epithelial\_cell\_proliferation\_involved\_in\_prostate\_gland\_development | 11 | 0 | 0.000000 | 0.000000 | 2747 | 2746.562663 | 2748.84 | 2751.117337 | 1.000670 |  |
| GO:0001838\_embryonic\_epithelial\_tube\_formation | 45 | 0 | 0.000000 | 0.000000 | 2752 | 2750.210539 | 2751.71 | 2753.209461 | 0.999895 |  |
| GO:0034103\_regulation\_of\_tissue\_remodeling | 45 | 0 | 0.000000 | 0.000000 | 2752 | 2750.210539 | 2751.71 | 2753.209461 | 0.999895 |  |
| GO:0043623\_cellular\_protein\_complex\_assembly | 45 | 0 | 0.000000 | 0.000000 | 2752 | 2750.210539 | 2751.71 | 2753.209461 | 0.999895 |  |
| GO:0046058\_cAMP\_metabolic\_process | 45 | 0 | 0.000000 | 0.000000 | 2752 | 2750.210539 | 2751.71 | 2753.209461 | 0.999895 |  |
| GO:0046546\_development\_of\_primary\_male\_sexual\_characteristics | 45 | 0 | 0.000000 | 0.000000 | 2752 | 2750.210539 | 2751.71 | 2753.209461 | 0.999895 |  |
| GO:0007126\_meiosis | 55 | 0 | 0.000000 | 0.000000 | 2755 | 2755.000000 | 2755.0 | 2755.000000 | 1.000000 |  |
| GO:0048704\_embryonic\_skeletal\_system\_morphogenesis | 55 | 0 | 0.000000 | 0.000000 | 2755 | 2755.000000 | 2755.0 | 2755.000000 | 1.000000 |  |
| GO:0051327\_M\_phase\_of\_meiotic\_cell\_cycle | 55 | 0 | 0.000000 | 0.000000 | 2755 | 2755.000000 | 2755.0 | 2755.000000 | 1.000000 |  |
