# Supplementary material for: RedundancyMiner: De-replication of redundant GO categories in microarray and proteomics analysis
Source: BMC Bioinformatics. 2011 Feb 10;12:52. doi: 10.1186/1471-2105-12-52 (PMC3223614; doi:10.1186/1471-2105-12-52)
Supplement: Additional file 8 — Retinal development HTGM download. compressed package of the results of running HTGM on the retinal development genes list. [file 1471-2105-12-52-S8.ZIP › SCENARIO_2_MODIFIED/total.txt.total.txt.dir/Exp1_BestClusterMap_LEIGS_KM_24.csv.join.7.txt.dir/Exp1_BestClusterMap_LEIGS_KM_24.csv.join.7.txt.change.gce.html]

Gene Category Report for Exp1\_BestClusterMap\_LEIGS\_KM\_24.csv.join.7.txt

# Gene Category Report for Exp1\_BestClusterMap\_LEIGS\_KM\_24.csv.join.7.txt

| HYPERLINKED GO CATEGORY | HYPERLINKED GENE NAME | TOTAL GENES | CHANGED GENES | ENRICHMENT | LOG10(p) | CUMULATIVE NUMBER OF CATEGORIES | CUMULATIVE RANDOMS MEAN | FALSE DISCOVERY RATE |  |
| --- | --- | --- | --- | --- | --- | --- | --- | --- | --- |
| GO:0010508\_positive\_regulation\_of\_autophagy | BECN1 | 1 | 1 |  |  |  |  |  |  |
| GO:0016239\_positive\_regulation\_of\_macroautophagy | BECN1 | 1 | 1 |  |  |  |  |  |  |
| GO:0030206\_chondroitin\_sulfate\_biosynthetic\_process | CHST11 | 1 | 1 |  |  |  |  |  |  |
| GO:0032410\_negative\_regulation\_of\_transporter\_activity | PKD2 | 1 | 1 |  |  |  |  |  |  |
| GO:0032413\_negative\_regulation\_of\_ion\_transmembrane\_transporter\_activity | PKD2 | 1 | 1 |  |  |  |  |  |  |
| GO:0033037\_polysaccharide\_localization | CHST11 | 1 | 1 |  |  |  |  |  |  |
| GO:0060315\_negative\_regulation\_of\_ryanodine-sensitive\_calcium-release\_channel\_activity | PKD2 | 1 | 1 |  |  |  |  |  |  |
| GO:0010389\_regulation\_of\_G2\_M\_transition\_of\_mitotic\_cell\_cycle | PKIA | 2 | 1 |  |  |  |  |  |  |
| GO:0032106\_positive\_regulation\_of\_response\_to\_extracellular\_stimulus | BECN1 | 2 | 1 |  |  |  |  |  |  |
| GO:0032109\_positive\_regulation\_of\_response\_to\_nutrient\_levels | BECN1 | 2 | 1 |  |  |  |  |  |  |
| GO:0051132\_NK\_T\_cell\_activation | CD1D1 | 2 | 1 |  |  |  |  |  |  |
| GO:0051133\_regulation\_of\_NK\_T\_cell\_activation | CD1D1 | 2 | 1 |  |  |  |  |  |  |
| GO:0051135\_positive\_regulation\_of\_NK\_T\_cell\_activation | CD1D1 | 2 | 1 |  |  |  |  |  |  |
| GO:0007049\_cell\_cycle | PSMD13 | 238 | 5 | 3.224790 | -1.758370 | 1 | 9.78 | 9.780000 |  |
| GO:0007049\_cell\_cycle | HUS1 | 238 | 5 | 3.224790 | -1.758370 | 1 | 9.78 | 9.780000 |  |
| GO:0007049\_cell\_cycle | PKD2 | 238 | 5 | 3.224790 | -1.758370 | 1 | 9.78 | 9.780000 |  |
| GO:0007049\_cell\_cycle | PTEN | 238 | 5 | 3.224790 | -1.758370 | 1 | 9.78 | 9.780000 |  |
| GO:0007049\_cell\_cycle | PKIA | 238 | 5 | 3.224790 | -1.758370 | 1 | 9.78 | 9.780000 |  |
| GO:0001955\_blood\_vessel\_maturation | RECK | 3 | 1 |  |  |  |  |  |  |
| GO:0016241\_regulation\_of\_macroautophagy | BECN1 | 3 | 1 |  |  |  |  |  |  |
| GO:0042097\_interleukin-4\_biosynthetic\_process | CD1D1 | 3 | 1 |  |  |  |  |  |  |
| GO:0043045\_DNA\_methylation\_during\_embryonic\_development | DNMT3A | 3 | 1 |  |  |  |  |  |  |
| GO:0045402\_regulation\_of\_interleukin-4\_biosynthetic\_process | CD1D1 | 3 | 1 |  |  |  |  |  |  |
| GO:0045404\_positive\_regulation\_of\_interleukin-4\_biosynthetic\_process | CD1D1 | 3 | 1 |  |  |  |  |  |  |
| GO:0048003\_antigen\_processing\_and\_presentation\_of\_lipid\_antigen\_via\_MHC\_class\_Ib | CD1D1 | 3 | 1 |  |  |  |  |  |  |
| GO:0048007\_antigen\_processing\_and\_presentation\_\_exogenous\_lipid\_antigen\_via\_MHC\_class\_Ib | CD1D1 | 3 | 1 |  |  |  |  |  |  |
| GO:0050650\_chondroitin\_sulfate\_proteoglycan\_biosynthetic\_process | CHST11 | 3 | 1 |  |  |  |  |  |  |
| GO:0060314\_regulation\_of\_ryanodine-sensitive\_calcium-release\_channel\_activity | PKD2 | 3 | 1 |  |  |  |  |  |  |
| GO:0016051\_carbohydrate\_biosynthetic\_process | B3GALNT1 | 35 | 2 | 8.771429 | -1.670236 | 3 | 11.52 | 3.840000 |  |
| GO:0016051\_carbohydrate\_biosynthetic\_process | CHST11 | 35 | 2 | 8.771429 | -1.670236 | 3 | 11.52 | 3.840000 |  |
| GO:0051051\_negative\_regulation\_of\_transport | PKD2 | 35 | 2 | 8.771429 | -1.670236 | 3 | 11.52 | 3.840000 |  |
| GO:0051051\_negative\_regulation\_of\_transport | PKIA | 35 | 2 | 8.771429 | -1.670236 | 3 | 11.52 | 3.840000 |  |
| GO:0000086\_G2\_M\_transition\_of\_mitotic\_cell\_cycle | PKIA | 4 | 1 |  |  |  |  |  |  |
| GO:0010506\_regulation\_of\_autophagy | BECN1 | 4 | 1 |  |  |  |  |  |  |
| GO:0045078\_positive\_regulation\_of\_interferon-gamma\_biosynthetic\_process | CD1D1 | 4 | 1 |  |  |  |  |  |  |
| GO:0007346\_regulation\_of\_mitotic\_cell\_cycle | HUS1 | 40 | 2 | 7.675000 | -1.561371 | 4 | 14.36 | 3.590000 |  |
| GO:0007346\_regulation\_of\_mitotic\_cell\_cycle | PKIA | 40 | 2 | 7.675000 | -1.561371 | 4 | 14.36 | 3.590000 |  |
| GO:0045934\_negative\_regulation\_of\_nucleobase\_\_nucleoside\_\_nucleotide\_and\_nucleic\_acid\_metabolic\_process | TBL1XR1 | 270 | 5 | 2.842593 | -1.545809 | 5 | 14.65 | 2.930000 |  |
| GO:0045934\_negative\_regulation\_of\_nucleobase\_\_nucleoside\_\_nucleotide\_and\_nucleic\_acid\_metabolic\_process | DNMT3A | 270 | 5 | 2.842593 | -1.545809 | 5 | 14.65 | 2.930000 |  |
| GO:0045934\_negative\_regulation\_of\_nucleobase\_\_nucleoside\_\_nucleotide\_and\_nucleic\_acid\_metabolic\_process | HUS1 | 270 | 5 | 2.842593 | -1.545809 | 5 | 14.65 | 2.930000 |  |
| GO:0045934\_negative\_regulation\_of\_nucleobase\_\_nucleoside\_\_nucleotide\_and\_nucleic\_acid\_metabolic\_process | ZFP238 | 270 | 5 | 2.842593 | -1.545809 | 5 | 14.65 | 2.930000 |  |
| GO:0045934\_negative\_regulation\_of\_nucleobase\_\_nucleoside\_\_nucleotide\_and\_nucleic\_acid\_metabolic\_process | PKIA | 270 | 5 | 2.842593 | -1.545809 | 5 | 14.65 | 2.930000 |  |
| GO:0051172\_negative\_regulation\_of\_nitrogen\_compound\_metabolic\_process | TBL1XR1 | 271 | 5 | 2.832103 | -1.539707 | 6 | 15.27 | 2.545000 |  |
| GO:0051172\_negative\_regulation\_of\_nitrogen\_compound\_metabolic\_process | DNMT3A | 271 | 5 | 2.832103 | -1.539707 | 6 | 15.27 | 2.545000 |  |
| GO:0051172\_negative\_regulation\_of\_nitrogen\_compound\_metabolic\_process | HUS1 | 271 | 5 | 2.832103 | -1.539707 | 6 | 15.27 | 2.545000 |  |
| GO:0051172\_negative\_regulation\_of\_nitrogen\_compound\_metabolic\_process | ZFP238 | 271 | 5 | 2.832103 | -1.539707 | 6 | 15.27 | 2.545000 |  |
| GO:0051172\_negative\_regulation\_of\_nitrogen\_compound\_metabolic\_process | PKIA | 271 | 5 | 2.832103 | -1.539707 | 6 | 15.27 | 2.545000 |  |
| GO:0010558\_negative\_regulation\_of\_macromolecule\_biosynthetic\_process | TBL1XR1 | 274 | 5 | 2.801095 | -1.521582 | 7 | 16.12 | 2.302857 |  |
| GO:0010558\_negative\_regulation\_of\_macromolecule\_biosynthetic\_process | DNMT3A | 274 | 5 | 2.801095 | -1.521582 | 7 | 16.12 | 2.302857 |  |
| GO:0010558\_negative\_regulation\_of\_macromolecule\_biosynthetic\_process | HUS1 | 274 | 5 | 2.801095 | -1.521582 | 7 | 16.12 | 2.302857 |  |
| GO:0010558\_negative\_regulation\_of\_macromolecule\_biosynthetic\_process | ZFP238 | 274 | 5 | 2.801095 | -1.521582 | 7 | 16.12 | 2.302857 |  |
| GO:0010558\_negative\_regulation\_of\_macromolecule\_biosynthetic\_process | PKIA | 274 | 5 | 2.801095 | -1.521582 | 7 | 16.12 | 2.302857 |  |
| GO:0001865\_NK\_T\_cell\_differentiation | CD1D1 | 5 | 1 | 30.700000 | -1.492611 | 17 | 26.17 | 1.539412 |  |
| GO:0002902\_regulation\_of\_B\_cell\_apoptosis | PTEN | 5 | 1 | 30.700000 | -1.492611 | 17 | 26.17 | 1.539412 |  |
| GO:0006023\_aminoglycan\_biosynthetic\_process | CHST11 | 5 | 1 | 30.700000 | -1.492611 | 17 | 26.17 | 1.539412 |  |
| GO:0006024\_glycosaminoglycan\_biosynthetic\_process | CHST11 | 5 | 1 | 30.700000 | -1.492611 | 17 | 26.17 | 1.539412 |  |
| GO:0006346\_methylation-dependent\_chromatin\_silencing | DNMT3A | 5 | 1 | 30.700000 | -1.492611 | 17 | 26.17 | 1.539412 |  |
| GO:0009312\_oligosaccharide\_biosynthetic\_process | B3GALNT1 | 5 | 1 | 30.700000 | -1.492611 | 17 | 26.17 | 1.539412 |  |
| GO:0043046\_DNA\_methylation\_during\_gametogenesis | DNMT3A | 5 | 1 | 30.700000 | -1.492611 | 17 | 26.17 | 1.539412 |  |
| GO:0051136\_regulation\_of\_NK\_T\_cell\_differentiation | CD1D1 | 5 | 1 | 30.700000 | -1.492611 | 17 | 26.17 | 1.539412 |  |
| GO:0051138\_positive\_regulation\_of\_NK\_T\_cell\_differentiation | CD1D1 | 5 | 1 | 30.700000 | -1.492611 | 17 | 26.17 | 1.539412 |  |
| GO:0051898\_negative\_regulation\_of\_protein\_kinase\_B\_signaling\_cascade | PTEN | 5 | 1 | 30.700000 | -1.492611 | 17 | 26.17 | 1.539412 |  |
| GO:0031327\_negative\_regulation\_of\_cellular\_biosynthetic\_process | TBL1XR1 | 282 | 5 | 2.721631 | -1.474524 | 18 | 26.87 | 1.492778 |  |
| GO:0031327\_negative\_regulation\_of\_cellular\_biosynthetic\_process | DNMT3A | 282 | 5 | 2.721631 | -1.474524 | 18 | 26.87 | 1.492778 |  |
| GO:0031327\_negative\_regulation\_of\_cellular\_biosynthetic\_process | HUS1 | 282 | 5 | 2.721631 | -1.474524 | 18 | 26.87 | 1.492778 |  |
| GO:0031327\_negative\_regulation\_of\_cellular\_biosynthetic\_process | ZFP238 | 282 | 5 | 2.721631 | -1.474524 | 18 | 26.87 | 1.492778 |  |
| GO:0031327\_negative\_regulation\_of\_cellular\_biosynthetic\_process | PKIA | 282 | 5 | 2.721631 | -1.474524 | 18 | 26.87 | 1.492778 |  |
| GO:0009890\_negative\_regulation\_of\_biosynthetic\_process | TBL1XR1 | 284 | 5 | 2.702465 | -1.463040 | 19 | 27.14 | 1.428421 |  |
| GO:0009890\_negative\_regulation\_of\_biosynthetic\_process | DNMT3A | 284 | 5 | 2.702465 | -1.463040 | 19 | 27.14 | 1.428421 |  |
| GO:0009890\_negative\_regulation\_of\_biosynthetic\_process | HUS1 | 284 | 5 | 2.702465 | -1.463040 | 19 | 27.14 | 1.428421 |  |
| GO:0009890\_negative\_regulation\_of\_biosynthetic\_process | ZFP238 | 284 | 5 | 2.702465 | -1.463040 | 19 | 27.14 | 1.428421 |  |
| GO:0009890\_negative\_regulation\_of\_biosynthetic\_process | PKIA | 284 | 5 | 2.702465 | -1.463040 | 19 | 27.14 | 1.428421 |  |
| GO:0007507\_heart\_development | FGF15 | 195 | 4 | 3.148718 | -1.442662 | 20 | 27.98 | 1.399000 |  |
| GO:0007507\_heart\_development | TRP53BP2 | 195 | 4 | 3.148718 | -1.442662 | 20 | 27.98 | 1.399000 |  |
| GO:0007507\_heart\_development | PKD2 | 195 | 4 | 3.148718 | -1.442662 | 20 | 27.98 | 1.399000 |  |
| GO:0007507\_heart\_development | PTEN | 195 | 4 | 3.148718 | -1.442662 | 20 | 27.98 | 1.399000 |  |
| GO:0002475\_antigen\_processing\_and\_presentation\_via\_MHC\_class\_Ib | CD1D1 | 6 | 1 | 25.583333 | -1.414794 | 28 | 35.74 | 1.276429 |  |
| GO:0006471\_protein\_amino\_acid\_ADP-ribosylation | ARL6 | 6 | 1 | 25.583333 | -1.414794 | 28 | 35.74 | 1.276429 |  |
| GO:0008156\_negative\_regulation\_of\_DNA\_replication | HUS1 | 6 | 1 | 25.583333 | -1.414794 | 28 | 35.74 | 1.276429 |  |
| GO:0031575\_G1\_S\_transition\_checkpoint | HUS1 | 6 | 1 | 25.583333 | -1.414794 | 28 | 35.74 | 1.276429 |  |
| GO:0035121\_tail\_morphogenesis | CHST11 | 6 | 1 | 25.583333 | -1.414794 | 28 | 35.74 | 1.276429 |  |
| GO:0042308\_negative\_regulation\_of\_protein\_import\_into\_nucleus | PKIA | 6 | 1 | 25.583333 | -1.414794 | 28 | 35.74 | 1.276429 |  |
| GO:0045072\_regulation\_of\_interferon-gamma\_biosynthetic\_process | CD1D1 | 6 | 1 | 25.583333 | -1.414794 | 28 | 35.74 | 1.276429 |  |
| GO:0048703\_embryonic\_viscerocranium\_morphogenesis | CHST11 | 6 | 1 | 25.583333 | -1.414794 | 28 | 35.74 | 1.276429 |  |
| GO:0048519\_negative\_regulation\_of\_biological\_process | TBL1XR1 | 859 | 10 | 1.786962 | -1.400202 | 29 | 36.02 | 1.242069 |  |
| GO:0048519\_negative\_regulation\_of\_biological\_process | DNMT3A | 859 | 10 | 1.786962 | -1.400202 | 29 | 36.02 | 1.242069 |  |
| GO:0048519\_negative\_regulation\_of\_biological\_process | BECN1 | 859 | 10 | 1.786962 | -1.400202 | 29 | 36.02 | 1.242069 |  |
| GO:0048519\_negative\_regulation\_of\_biological\_process | CHST11 | 859 | 10 | 1.786962 | -1.400202 | 29 | 36.02 | 1.242069 |  |
| GO:0048519\_negative\_regulation\_of\_biological\_process | HUS1 | 859 | 10 | 1.786962 | -1.400202 | 29 | 36.02 | 1.242069 |  |
| GO:0048519\_negative\_regulation\_of\_biological\_process | PKD2 | 859 | 10 | 1.786962 | -1.400202 | 29 | 36.02 | 1.242069 |  |
| GO:0048519\_negative\_regulation\_of\_biological\_process | ZFP238 | 859 | 10 | 1.786962 | -1.400202 | 29 | 36.02 | 1.242069 |  |
| GO:0048519\_negative\_regulation\_of\_biological\_process | PTEN | 859 | 10 | 1.786962 | -1.400202 | 29 | 36.02 | 1.242069 |  |
| GO:0048519\_negative\_regulation\_of\_biological\_process | PKIA | 859 | 10 | 1.786962 | -1.400202 | 29 | 36.02 | 1.242069 |  |
| GO:0048519\_negative\_regulation\_of\_biological\_process | VASH1 | 859 | 10 | 1.786962 | -1.400202 | 29 | 36.02 | 1.242069 |  |
| GO:0006917\_induction\_of\_apoptosis | CD1D1 | 121 | 3 | 3.805785 | -1.367503 | 32 | 37.26 | 1.164375 |  |
| GO:0006917\_induction\_of\_apoptosis | PTEN | 121 | 3 | 3.805785 | -1.367503 | 32 | 37.26 | 1.164375 |  |
| GO:0006917\_induction\_of\_apoptosis | CASP2 | 121 | 3 | 3.805785 | -1.367503 | 32 | 37.26 | 1.164375 |  |
| GO:0012502\_induction\_of\_programmed\_cell\_death | CD1D1 | 121 | 3 | 3.805785 | -1.367503 | 32 | 37.26 | 1.164375 |  |
| GO:0012502\_induction\_of\_programmed\_cell\_death | PTEN | 121 | 3 | 3.805785 | -1.367503 | 32 | 37.26 | 1.164375 |  |
| GO:0012502\_induction\_of\_programmed\_cell\_death | CASP2 | 121 | 3 | 3.805785 | -1.367503 | 32 | 37.26 | 1.164375 |  |
| GO:0051726\_regulation\_of\_cell\_cycle | HUS1 | 121 | 3 | 3.805785 | -1.367503 | 32 | 37.26 | 1.164375 |  |
| GO:0051726\_regulation\_of\_cell\_cycle | PTEN | 121 | 3 | 3.805785 | -1.367503 | 32 | 37.26 | 1.164375 |  |
| GO:0051726\_regulation\_of\_cell\_cycle | PKIA | 121 | 3 | 3.805785 | -1.367503 | 32 | 37.26 | 1.164375 |  |
| GO:0016569\_covalent\_chromatin\_modification | DNMT3A | 51 | 2 | 6.019608 | -1.367065 | 33 | 37.64 | 1.140606 |  |
| GO:0016569\_covalent\_chromatin\_modification | HDAC2 | 51 | 2 | 6.019608 | -1.367065 | 33 | 37.64 | 1.140606 |  |
| GO:0002063\_chondrocyte\_development | CHST11 | 7 | 1 | 21.928571 | -1.349211 | 45 | 46.45 | 1.032222 |  |
| GO:0006275\_regulation\_of\_DNA\_replication | HUS1 | 7 | 1 | 21.928571 | -1.349211 | 45 | 46.45 | 1.032222 |  |
| GO:0009311\_oligosaccharide\_metabolic\_process | B3GALNT1 | 7 | 1 | 21.928571 | -1.349211 | 45 | 46.45 | 1.032222 |  |
| GO:0016575\_histone\_deacetylation | HDAC2 | 7 | 1 | 21.928571 | -1.349211 | 45 | 46.45 | 1.032222 |  |
| GO:0032104\_regulation\_of\_response\_to\_extracellular\_stimulus | BECN1 | 7 | 1 | 21.928571 | -1.349211 | 45 | 46.45 | 1.032222 |  |
| GO:0032107\_regulation\_of\_response\_to\_nutrient\_levels | BECN1 | 7 | 1 | 21.928571 | -1.349211 | 45 | 46.45 | 1.032222 |  |
| GO:0032387\_negative\_regulation\_of\_intracellular\_transport | PKIA | 7 | 1 | 21.928571 | -1.349211 | 45 | 46.45 | 1.032222 |  |
| GO:0033032\_regulation\_of\_myeloid\_cell\_apoptosis | PTEN | 7 | 1 | 21.928571 | -1.349211 | 45 | 46.45 | 1.032222 |  |
| GO:0042572\_retinol\_metabolic\_process | RBP1 | 7 | 1 | 21.928571 | -1.349211 | 45 | 46.45 | 1.032222 |  |
| GO:0045059\_positive\_thymic\_T\_cell\_selection | CD1D1 | 7 | 1 | 21.928571 | -1.349211 | 45 | 46.45 | 1.032222 |  |
| GO:0046823\_negative\_regulation\_of\_nucleocytoplasmic\_transport | PKIA | 7 | 1 | 21.928571 | -1.349211 | 45 | 46.45 | 1.032222 |  |
| GO:0070228\_regulation\_of\_lymphocyte\_apoptosis | PTEN | 7 | 1 | 21.928571 | -1.349211 | 45 | 46.45 | 1.032222 |  |
| GO:0001783\_B\_cell\_apoptosis | PTEN | 8 | 1 | 19.187500 | -1.292582 | 57 | 55.38 | 0.971579 |  |
| GO:0001916\_positive\_regulation\_of\_T\_cell\_mediated\_cytotoxicity | CD1D1 | 8 | 1 | 19.187500 | -1.292582 | 57 | 55.38 | 0.971579 |  |
| GO:0006349\_genetic\_imprinting | DNMT3A | 8 | 1 | 19.187500 | -1.292582 | 57 | 55.38 | 0.971579 |  |
| GO:0016236\_macroautophagy | BECN1 | 8 | 1 | 19.187500 | -1.292582 | 57 | 55.38 | 0.971579 |  |
| GO:0022898\_regulation\_of\_transmembrane\_transporter\_activity | PKD2 | 8 | 1 | 19.187500 | -1.292582 | 57 | 55.38 | 0.971579 |  |
| GO:0030204\_chondroitin\_sulfate\_metabolic\_process | CHST11 | 8 | 1 | 19.187500 | -1.292582 | 57 | 55.38 | 0.971579 |  |
| GO:0032409\_regulation\_of\_transporter\_activity | PKD2 | 8 | 1 | 19.187500 | -1.292582 | 57 | 55.38 | 0.971579 |  |
| GO:0032412\_regulation\_of\_ion\_transmembrane\_transporter\_activity | PKD2 | 8 | 1 | 19.187500 | -1.292582 | 57 | 55.38 | 0.971579 |  |
| GO:0042095\_interferon-gamma\_biosynthetic\_process | CD1D1 | 8 | 1 | 19.187500 | -1.292582 | 57 | 55.38 | 0.971579 |  |
| GO:0043368\_positive\_T\_cell\_selection | CD1D1 | 8 | 1 | 19.187500 | -1.292582 | 57 | 55.38 | 0.971579 |  |
| GO:0043542\_endothelial\_cell\_migration | PTEN | 8 | 1 | 19.187500 | -1.292582 | 57 | 55.38 | 0.971579 |  |
| GO:0045086\_positive\_regulation\_of\_interleukin-2\_biosynthetic\_process | CD1D1 | 8 | 1 | 19.187500 | -1.292582 | 57 | 55.38 | 0.971579 |  |
| GO:0045892\_negative\_regulation\_of\_transcription\_\_DNA-dependent | TBL1XR1 | 218 | 4 | 2.816514 | -1.292428 | 58 | 55.39 | 0.955000 |  |
| GO:0045892\_negative\_regulation\_of\_transcription\_\_DNA-dependent | DNMT3A | 218 | 4 | 2.816514 | -1.292428 | 58 | 55.39 | 0.955000 |  |
| GO:0045892\_negative\_regulation\_of\_transcription\_\_DNA-dependent | ZFP238 | 218 | 4 | 2.816514 | -1.292428 | 58 | 55.39 | 0.955000 |  |
| GO:0045892\_negative\_regulation\_of\_transcription\_\_DNA-dependent | PKIA | 218 | 4 | 2.816514 | -1.292428 | 58 | 55.39 | 0.955000 |  |
| GO:0051253\_negative\_regulation\_of\_RNA\_metabolic\_process | TBL1XR1 | 220 | 4 | 2.790909 | -1.280352 | 59 | 55.78 | 0.945424 |  |
| GO:0051253\_negative\_regulation\_of\_RNA\_metabolic\_process | DNMT3A | 220 | 4 | 2.790909 | -1.280352 | 59 | 55.78 | 0.945424 |  |
| GO:0051253\_negative\_regulation\_of\_RNA\_metabolic\_process | ZFP238 | 220 | 4 | 2.790909 | -1.280352 | 59 | 55.78 | 0.945424 |  |
| GO:0051253\_negative\_regulation\_of\_RNA\_metabolic\_process | PKIA | 220 | 4 | 2.790909 | -1.280352 | 59 | 55.78 | 0.945424 |  |
| GO:0006476\_protein\_amino\_acid\_deacetylation | HDAC2 | 9 | 1 | 17.055556 | -1.242790 | 64 | 63.46 | 0.991562 |  |
| GO:0031023\_microtubule\_organizing\_center\_organization | UXT | 9 | 1 | 17.055556 | -1.242790 | 64 | 63.46 | 0.991562 |  |
| GO:0033028\_myeloid\_cell\_apoptosis | PTEN | 9 | 1 | 17.055556 | -1.242790 | 64 | 63.46 | 0.991562 |  |
| GO:0051297\_centrosome\_organization | UXT | 9 | 1 | 17.055556 | -1.242790 | 64 | 63.46 | 0.991562 |  |
| GO:0051896\_regulation\_of\_protein\_kinase\_B\_signaling\_cascade | PTEN | 9 | 1 | 17.055556 | -1.242790 | 64 | 63.46 | 0.991562 |  |
| GO:0010605\_negative\_regulation\_of\_macromolecule\_metabolic\_process | TBL1XR1 | 331 | 5 | 2.318731 | -1.221614 | 65 | 64.21 | 0.987846 |  |
| GO:0010605\_negative\_regulation\_of\_macromolecule\_metabolic\_process | DNMT3A | 331 | 5 | 2.318731 | -1.221614 | 65 | 64.21 | 0.987846 |  |
| GO:0010605\_negative\_regulation\_of\_macromolecule\_metabolic\_process | HUS1 | 331 | 5 | 2.318731 | -1.221614 | 65 | 64.21 | 0.987846 |  |
| GO:0010605\_negative\_regulation\_of\_macromolecule\_metabolic\_process | ZFP238 | 331 | 5 | 2.318731 | -1.221614 | 65 | 64.21 | 0.987846 |  |
| GO:0010605\_negative\_regulation\_of\_macromolecule\_metabolic\_process | PKIA | 331 | 5 | 2.318731 | -1.221614 | 65 | 64.21 | 0.987846 |  |
| GO:0031324\_negative\_regulation\_of\_cellular\_metabolic\_process | TBL1XR1 | 332 | 5 | 2.311747 | -1.217008 | 66 | 64.34 | 0.974848 |  |
| GO:0031324\_negative\_regulation\_of\_cellular\_metabolic\_process | DNMT3A | 332 | 5 | 2.311747 | -1.217008 | 66 | 64.34 | 0.974848 |  |
| GO:0031324\_negative\_regulation\_of\_cellular\_metabolic\_process | HUS1 | 332 | 5 | 2.311747 | -1.217008 | 66 | 64.34 | 0.974848 |  |
| GO:0031324\_negative\_regulation\_of\_cellular\_metabolic\_process | ZFP238 | 332 | 5 | 2.311747 | -1.217008 | 66 | 64.34 | 0.974848 |  |
| GO:0031324\_negative\_regulation\_of\_cellular\_metabolic\_process | PKIA | 332 | 5 | 2.311747 | -1.217008 | 66 | 64.34 | 0.974848 |  |
| GO:0000724\_double-strand\_break\_repair\_via\_homologous\_recombination | HUS1 | 10 | 1 | 15.350000 | -1.198393 | 84 | 73.21 | 0.871548 |  |
| GO:0000725\_recombinational\_repair | HUS1 | 10 | 1 | 15.350000 | -1.198393 | 84 | 73.21 | 0.871548 |  |
| GO:0001914\_regulation\_of\_T\_cell\_mediated\_cytotoxicity | CD1D1 | 10 | 1 | 15.350000 | -1.198393 | 84 | 73.21 | 0.871548 |  |
| GO:0002711\_positive\_regulation\_of\_T\_cell\_mediated\_immunity | CD1D1 | 10 | 1 | 15.350000 | -1.198393 | 84 | 73.21 | 0.871548 |  |
| GO:0006342\_chromatin\_silencing | DNMT3A | 10 | 1 | 15.350000 | -1.198393 | 84 | 73.21 | 0.871548 |  |
| GO:0007093\_mitotic\_cell\_cycle\_checkpoint | HUS1 | 10 | 1 | 15.350000 | -1.198393 | 84 | 73.21 | 0.871548 |  |
| GO:0031331\_positive\_regulation\_of\_cellular\_catabolic\_process | BECN1 | 10 | 1 | 15.350000 | -1.198393 | 84 | 73.21 | 0.871548 |  |
| GO:0032633\_interleukin-4\_production | CD1D1 | 10 | 1 | 15.350000 | -1.198393 | 84 | 73.21 | 0.871548 |  |
| GO:0032673\_regulation\_of\_interleukin-4\_production | CD1D1 | 10 | 1 | 15.350000 | -1.198393 | 84 | 73.21 | 0.871548 |  |
| GO:0045814\_negative\_regulation\_of\_gene\_expression\_\_epigenetic | DNMT3A | 10 | 1 | 15.350000 | -1.198393 | 84 | 73.21 | 0.871548 |  |
| GO:0048738\_cardiac\_muscle\_tissue\_development | PTEN | 10 | 1 | 15.350000 | -1.198393 | 84 | 73.21 | 0.871548 |  |
| GO:0050654\_chondroitin\_sulfate\_proteoglycan\_metabolic\_process | CHST11 | 10 | 1 | 15.350000 | -1.198393 | 84 | 73.21 | 0.871548 |  |
| GO:0051208\_sequestering\_of\_calcium\_ion | PKD2 | 10 | 1 | 15.350000 | -1.198393 | 84 | 73.21 | 0.871548 |  |
| GO:0051209\_release\_of\_sequestered\_calcium\_ion\_into\_cytosol | PKD2 | 10 | 1 | 15.350000 | -1.198393 | 84 | 73.21 | 0.871548 |  |
| GO:0051224\_negative\_regulation\_of\_protein\_transport | PKIA | 10 | 1 | 15.350000 | -1.198393 | 84 | 73.21 | 0.871548 |  |
| GO:0051238\_sequestering\_of\_metal\_ion | PKD2 | 10 | 1 | 15.350000 | -1.198393 | 84 | 73.21 | 0.871548 |  |
| GO:0051282\_regulation\_of\_sequestering\_of\_calcium\_ion | PKD2 | 10 | 1 | 15.350000 | -1.198393 | 84 | 73.21 | 0.871548 |  |
| GO:0051283\_negative\_regulation\_of\_sequestering\_of\_calcium\_ion | PKD2 | 10 | 1 | 15.350000 | -1.198393 | 84 | 73.21 | 0.871548 |  |
| GO:0009790\_embryonic\_development | DNMT3A | 567 | 7 | 1.895062 | -1.172212 | 85 | 74.43 | 0.875647 |  |
| GO:0009790\_embryonic\_development | PRMT1 | 567 | 7 | 1.895062 | -1.172212 | 85 | 74.43 | 0.875647 |  |
| GO:0009790\_embryonic\_development | TRP53BP2 | 567 | 7 | 1.895062 | -1.172212 | 85 | 74.43 | 0.875647 |  |
| GO:0009790\_embryonic\_development | HUS1 | 567 | 7 | 1.895062 | -1.172212 | 85 | 74.43 | 0.875647 |  |
| GO:0009790\_embryonic\_development | CHST11 | 567 | 7 | 1.895062 | -1.172212 | 85 | 74.43 | 0.875647 |  |
| GO:0009790\_embryonic\_development | PKD2 | 567 | 7 | 1.895062 | -1.172212 | 85 | 74.43 | 0.875647 |  |
| GO:0009790\_embryonic\_development | FBN2 | 567 | 7 | 1.895062 | -1.172212 | 85 | 74.43 | 0.875647 |  |
| GO:0000271\_polysaccharide\_biosynthetic\_process | CHST11 | 11 | 1 | 13.954545 | -1.158359 | 88 | 80.89 | 0.919205 |  |
| GO:0001913\_T\_cell\_mediated\_cytotoxicity | CD1D1 | 11 | 1 | 13.954545 | -1.158359 | 88 | 80.89 | 0.919205 |  |
| GO:0034762\_regulation\_of\_transmembrane\_transport | PKD2 | 11 | 1 | 13.954545 | -1.158359 | 88 | 80.89 | 0.919205 |  |
| GO:0009892\_negative\_regulation\_of\_metabolic\_process | TBL1XR1 | 348 | 5 | 2.205460 | -1.145941 | 89 | 81.61 | 0.916966 |  |
| GO:0009892\_negative\_regulation\_of\_metabolic\_process | DNMT3A | 348 | 5 | 2.205460 | -1.145941 | 89 | 81.61 | 0.916966 |  |
| GO:0009892\_negative\_regulation\_of\_metabolic\_process | HUS1 | 348 | 5 | 2.205460 | -1.145941 | 89 | 81.61 | 0.916966 |  |
| GO:0009892\_negative\_regulation\_of\_metabolic\_process | ZFP238 | 348 | 5 | 2.205460 | -1.145941 | 89 | 81.61 | 0.916966 |  |
| GO:0009892\_negative\_regulation\_of\_metabolic\_process | PKIA | 348 | 5 | 2.205460 | -1.145941 | 89 | 81.61 | 0.916966 |  |
| GO:0030166\_proteoglycan\_biosynthetic\_process | CHST11 | 12 | 1 | 12.791667 | -1.121928 | 92 | 88.77 | 0.964891 |  |
| GO:0045076\_regulation\_of\_interleukin-2\_biosynthetic\_process | CD1D1 | 12 | 1 | 12.791667 | -1.121928 | 92 | 88.77 | 0.964891 |  |
| GO:0060736\_prostate\_gland\_growth | PTEN | 12 | 1 | 12.791667 | -1.121928 | 92 | 88.77 | 0.964891 |  |
| GO:0022402\_cell\_cycle\_process | PSMD13 | 155 | 3 | 2.970968 | -1.106229 | 93 | 89.38 | 0.961075 |  |
| GO:0022402\_cell\_cycle\_process | PKD2 | 155 | 3 | 2.970968 | -1.106229 | 93 | 89.38 | 0.961075 |  |
| GO:0022402\_cell\_cycle\_process | PKIA | 155 | 3 | 2.970968 | -1.106229 | 93 | 89.38 | 0.961075 |  |
| GO:0016568\_chromatin\_modification | DNMT3A | 72 | 2 | 4.263889 | -1.101268 | 95 | 90.06 | 0.948000 |  |
| GO:0016568\_chromatin\_modification | HDAC2 | 72 | 2 | 4.263889 | -1.101268 | 95 | 90.06 | 0.948000 |  |
| GO:0050673\_epithelial\_cell\_proliferation | PTEN | 72 | 2 | 4.263889 | -1.101268 | 95 | 90.06 | 0.948000 |  |
| GO:0050673\_epithelial\_cell\_proliferation | LGR4 | 72 | 2 | 4.263889 | -1.101268 | 95 | 90.06 | 0.948000 |  |
| GO:0016481\_negative\_regulation\_of\_transcription | TBL1XR1 | 253 | 4 | 2.426877 | -1.100227 | 96 | 90.15 | 0.939062 |  |
| GO:0016481\_negative\_regulation\_of\_transcription | DNMT3A | 253 | 4 | 2.426877 | -1.100227 | 96 | 90.15 | 0.939062 |  |
| GO:0016481\_negative\_regulation\_of\_transcription | ZFP238 | 253 | 4 | 2.426877 | -1.100227 | 96 | 90.15 | 0.939062 |  |
| GO:0016481\_negative\_regulation\_of\_transcription | PKIA | 253 | 4 | 2.426877 | -1.100227 | 96 | 90.15 | 0.939062 |  |
| GO:0016525\_negative\_regulation\_of\_angiogenesis | VASH1 | 13 | 1 | 11.807692 | -1.088522 | 101 | 96.45 | 0.954950 |  |
| GO:0030539\_male\_genitalia\_development | LGR4 | 13 | 1 | 11.807692 | -1.088522 | 101 | 96.45 | 0.954950 |  |
| GO:0042094\_interleukin-2\_biosynthetic\_process | CD1D1 | 13 | 1 | 11.807692 | -1.088522 | 101 | 96.45 | 0.954950 |  |
| GO:0060401\_cytosolic\_calcium\_ion\_transport | PKD2 | 13 | 1 | 11.807692 | -1.088522 | 101 | 96.45 | 0.954950 |  |
| GO:0060402\_calcium\_ion\_transport\_into\_cytosol | PKD2 | 13 | 1 | 11.807692 | -1.088522 | 101 | 96.45 | 0.954950 |  |
| GO:0048589\_developmental\_growth | CHST11 | 75 | 2 | 4.093333 | -1.070720 | 102 | 97.57 | 0.956569 |  |
| GO:0048589\_developmental\_growth | PTEN | 75 | 2 | 4.093333 | -1.070720 | 102 | 97.57 | 0.956569 |  |
| GO:0009628\_response\_to\_abiotic\_stimulus | TRP53BP2 | 162 | 3 | 2.842593 | -1.061384 | 103 | 97.79 | 0.949417 |  |
| GO:0009628\_response\_to\_abiotic\_stimulus | HUS1 | 162 | 3 | 2.842593 | -1.061384 | 103 | 97.79 | 0.949417 |  |
| GO:0009628\_response\_to\_abiotic\_stimulus | PKD2 | 162 | 3 | 2.842593 | -1.061384 | 103 | 97.79 | 0.949417 |  |
| GO:0000077\_DNA\_damage\_checkpoint | HUS1 | 14 | 1 | 10.964286 | -1.057692 | 113 | 103.33 | 0.914425 |  |
| GO:0006304\_DNA\_modification | DNMT3A | 14 | 1 | 10.964286 | -1.057692 | 113 | 103.33 | 0.914425 |  |
| GO:0006305\_DNA\_alkylation | DNMT3A | 14 | 1 | 10.964286 | -1.057692 | 113 | 103.33 | 0.914425 |  |
| GO:0006306\_DNA\_methylation | DNMT3A | 14 | 1 | 10.964286 | -1.057692 | 113 | 103.33 | 0.914425 |  |
| GO:0006914\_autophagy | BECN1 | 14 | 1 | 10.964286 | -1.057692 | 113 | 103.33 | 0.914425 |  |
| GO:0009267\_cellular\_response\_to\_starvation | BECN1 | 14 | 1 | 10.964286 | -1.057692 | 113 | 103.33 | 0.914425 |  |
| GO:0042573\_retinoic\_acid\_metabolic\_process | RBP1 | 14 | 1 | 10.964286 | -1.057692 | 113 | 103.33 | 0.914425 |  |
| GO:0043491\_protein\_kinase\_B\_signaling\_cascade | PTEN | 14 | 1 | 10.964286 | -1.057692 | 113 | 103.33 | 0.914425 |  |
| GO:0045061\_thymic\_T\_cell\_selection | CD1D1 | 14 | 1 | 10.964286 | -1.057692 | 113 | 103.33 | 0.914425 |  |
| GO:0051053\_negative\_regulation\_of\_DNA\_metabolic\_process | HUS1 | 14 | 1 | 10.964286 | -1.057692 | 113 | 103.33 | 0.914425 |  |
| GO:0010629\_negative\_regulation\_of\_gene\_expression | TBL1XR1 | 262 | 4 | 2.343511 | -1.056628 | 114 | 103.49 | 0.907807 |  |
| GO:0010629\_negative\_regulation\_of\_gene\_expression | DNMT3A | 262 | 4 | 2.343511 | -1.056628 | 114 | 103.49 | 0.907807 |  |
| GO:0010629\_negative\_regulation\_of\_gene\_expression | ZFP238 | 262 | 4 | 2.343511 | -1.056628 | 114 | 103.49 | 0.907807 |  |
| GO:0010629\_negative\_regulation\_of\_gene\_expression | PKIA | 262 | 4 | 2.343511 | -1.056628 | 114 | 103.49 | 0.907807 |  |
| GO:0030326\_embryonic\_limb\_morphogenesis | CHST11 | 78 | 2 | 3.935897 | -1.041575 | 116 | 105.0 | 0.905172 |  |
| GO:0030326\_embryonic\_limb\_morphogenesis | FBN2 | 78 | 2 | 3.935897 | -1.041575 | 116 | 105.0 | 0.905172 |  |
| GO:0035113\_embryonic\_appendage\_morphogenesis | CHST11 | 78 | 2 | 3.935897 | -1.041575 | 116 | 105.0 | 0.905172 |  |
| GO:0035113\_embryonic\_appendage\_morphogenesis | FBN2 | 78 | 2 | 3.935897 | -1.041575 | 116 | 105.0 | 0.905172 |  |
| GO:0043065\_positive\_regulation\_of\_apoptosis | CD1D1 | 166 | 3 | 2.774096 | -1.036870 | 117 | 105.07 | 0.898034 |  |
| GO:0043065\_positive\_regulation\_of\_apoptosis | PTEN | 166 | 3 | 2.774096 | -1.036870 | 117 | 105.07 | 0.898034 |  |
| GO:0043065\_positive\_regulation\_of\_apoptosis | CASP2 | 166 | 3 | 2.774096 | -1.036870 | 117 | 105.07 | 0.898034 |  |
| GO:0010942\_positive\_regulation\_of\_cell\_death | CD1D1 | 167 | 3 | 2.757485 | -1.030862 | 119 | 105.6 | 0.887395 |  |
| GO:0010942\_positive\_regulation\_of\_cell\_death | PTEN | 167 | 3 | 2.757485 | -1.030862 | 119 | 105.6 | 0.887395 |  |
| GO:0010942\_positive\_regulation\_of\_cell\_death | CASP2 | 167 | 3 | 2.757485 | -1.030862 | 119 | 105.6 | 0.887395 |  |
| GO:0043068\_positive\_regulation\_of\_programmed\_cell\_death | CD1D1 | 167 | 3 | 2.757485 | -1.030862 | 119 | 105.6 | 0.887395 |  |
| GO:0043068\_positive\_regulation\_of\_programmed\_cell\_death | PTEN | 167 | 3 | 2.757485 | -1.030862 | 119 | 105.6 | 0.887395 |  |
| GO:0043068\_positive\_regulation\_of\_programmed\_cell\_death | CASP2 | 167 | 3 | 2.757485 | -1.030862 | 119 | 105.6 | 0.887395 |  |
| GO:0002709\_regulation\_of\_T\_cell\_mediated\_immunity | CD1D1 | 15 | 1 | 10.233333 | -1.029083 | 125 | 110.5 | 0.884000 |  |
| GO:0031329\_regulation\_of\_cellular\_catabolic\_process | BECN1 | 15 | 1 | 10.233333 | -1.029083 | 125 | 110.5 | 0.884000 |  |
| GO:0042306\_regulation\_of\_protein\_import\_into\_nucleus | PKIA | 15 | 1 | 10.233333 | -1.029083 | 125 | 110.5 | 0.884000 |  |
| GO:0046638\_positive\_regulation\_of\_alpha-beta\_T\_cell\_differentiation | CD1D1 | 15 | 1 | 10.233333 | -1.029083 | 125 | 110.5 | 0.884000 |  |
| GO:0048008\_platelet-derived\_growth\_factor\_receptor\_signaling\_pathway | TXNIP | 15 | 1 | 10.233333 | -1.029083 | 125 | 110.5 | 0.884000 |  |
| GO:0070227\_lymphocyte\_apoptosis | PTEN | 15 | 1 | 10.233333 | -1.029083 | 125 | 110.5 | 0.884000 |  |
| GO:0000278\_mitotic\_cell\_cycle | HUS1 | 80 | 2 | 3.837500 | -1.022871 | 127 | 111.02 | 0.874173 |  |
| GO:0000278\_mitotic\_cell\_cycle | PKIA | 80 | 2 | 3.837500 | -1.022871 | 127 | 111.02 | 0.874173 |  |
| GO:0044092\_negative\_regulation\_of\_molecular\_function | PKD2 | 80 | 2 | 3.837500 | -1.022871 | 127 | 111.02 | 0.874173 |  |
| GO:0044092\_negative\_regulation\_of\_molecular\_function | PKIA | 80 | 2 | 3.837500 | -1.022871 | 127 | 111.02 | 0.874173 |  |
| GO:0031570\_DNA\_integrity\_checkpoint | HUS1 | 16 | 1 | 9.593750 | -1.002406 | 131 | 116.4 | 0.888550 |  |
| GO:0031669\_cellular\_response\_to\_nutrient\_levels | BECN1 | 16 | 1 | 9.593750 | -1.002406 | 131 | 116.4 | 0.888550 |  |
| GO:0032663\_regulation\_of\_interleukin-2\_production | CD1D1 | 16 | 1 | 9.593750 | -1.002406 | 131 | 116.4 | 0.888550 |  |
| GO:0042594\_response\_to\_starvation | BECN1 | 16 | 1 | 9.593750 | -1.002406 | 131 | 116.4 | 0.888550 |  |
| GO:0006325\_chromatin\_organization | DNMT3A | 83 | 2 | 3.698795 | -0.995833 | 132 | 117.24 | 0.888182 |  |
| GO:0006325\_chromatin\_organization | HDAC2 | 83 | 2 | 3.698795 | -0.995833 | 132 | 117.24 | 0.888182 |  |
| GO:0000122\_negative\_regulation\_of\_transcription\_from\_RNA\_polymerase\_II\_promoter | DNMT3A | 175 | 3 | 2.631429 | -0.984438 | 133 | 118.28 | 0.889323 |  |
| GO:0000122\_negative\_regulation\_of\_transcription\_from\_RNA\_polymerase\_II\_promoter | ZFP238 | 175 | 3 | 2.631429 | -0.984438 | 133 | 118.28 | 0.889323 |  |
| GO:0000122\_negative\_regulation\_of\_transcription\_from\_RNA\_polymerase\_II\_promoter | PKIA | 175 | 3 | 2.631429 | -0.984438 | 133 | 118.28 | 0.889323 |  |
| GO:0010741\_negative\_regulation\_of\_protein\_kinase\_cascade | PTEN | 17 | 1 | 9.029412 | -0.977428 | 135 | 123.99 | 0.918444 |  |
| GO:0046637\_regulation\_of\_alpha-beta\_T\_cell\_differentiation | CD1D1 | 17 | 1 | 9.029412 | -0.977428 | 135 | 123.99 | 0.918444 |  |
| GO:0006022\_aminoglycan\_metabolic\_process | CHST11 | 18 | 1 | 8.527778 | -0.953955 | 146 | 131.49 | 0.900616 |  |
| GO:0010498\_proteasomal\_protein\_catabolic\_process | TBL1XR1 | 18 | 1 | 8.527778 | -0.953955 | 146 | 131.49 | 0.900616 |  |
| GO:0010553\_negative\_regulation\_of\_specific\_transcription\_from\_RNA\_polymerase\_II\_promoter | ZFP238 | 18 | 1 | 8.527778 | -0.953955 | 146 | 131.49 | 0.900616 |  |
| GO:0016458\_gene\_silencing | DNMT3A | 18 | 1 | 8.527778 | -0.953955 | 146 | 131.49 | 0.900616 |  |
| GO:0030203\_glycosaminoglycan\_metabolic\_process | CHST11 | 18 | 1 | 8.527778 | -0.953955 | 146 | 131.49 | 0.900616 |  |
| GO:0032623\_interleukin-2\_production | CD1D1 | 18 | 1 | 8.527778 | -0.953955 | 146 | 131.49 | 0.900616 |  |
| GO:0033157\_regulation\_of\_intracellular\_protein\_transport | PKIA | 18 | 1 | 8.527778 | -0.953955 | 146 | 131.49 | 0.900616 |  |
| GO:0043161\_proteasomal\_ubiquitin-dependent\_protein\_catabolic\_process | TBL1XR1 | 18 | 1 | 8.527778 | -0.953955 | 146 | 131.49 | 0.900616 |  |
| GO:0044272\_sulfur\_compound\_biosynthetic\_process | CHST11 | 18 | 1 | 8.527778 | -0.953955 | 146 | 131.49 | 0.900616 |  |
| GO:0045058\_T\_cell\_selection | CD1D1 | 18 | 1 | 8.527778 | -0.953955 | 146 | 131.49 | 0.900616 |  |
| GO:0050982\_detection\_of\_mechanical\_stimulus | PKD2 | 18 | 1 | 8.527778 | -0.953955 | 146 | 131.49 | 0.900616 |  |
| GO:0006776\_vitamin\_A\_metabolic\_process | RBP1 | 19 | 1 | 8.078947 | -0.931822 | 148 | 136.96 | 0.925405 |  |
| GO:0048701\_embryonic\_cranial\_skeleton\_morphogenesis | CHST11 | 19 | 1 | 8.078947 | -0.931822 | 148 | 136.96 | 0.925405 |  |
| GO:0048523\_negative\_regulation\_of\_cellular\_process | TBL1XR1 | 774 | 8 | 1.586563 | -0.930727 | 149 | 137.03 | 0.919664 |  |
| GO:0048523\_negative\_regulation\_of\_cellular\_process | DNMT3A | 774 | 8 | 1.586563 | -0.930727 | 149 | 137.03 | 0.919664 |  |
| GO:0048523\_negative\_regulation\_of\_cellular\_process | BECN1 | 774 | 8 | 1.586563 | -0.930727 | 149 | 137.03 | 0.919664 |  |
| GO:0048523\_negative\_regulation\_of\_cellular\_process | HUS1 | 774 | 8 | 1.586563 | -0.930727 | 149 | 137.03 | 0.919664 |  |
| GO:0048523\_negative\_regulation\_of\_cellular\_process | CHST11 | 774 | 8 | 1.586563 | -0.930727 | 149 | 137.03 | 0.919664 |  |
| GO:0048523\_negative\_regulation\_of\_cellular\_process | ZFP238 | 774 | 8 | 1.586563 | -0.930727 | 149 | 137.03 | 0.919664 |  |
| GO:0048523\_negative\_regulation\_of\_cellular\_process | PKIA | 774 | 8 | 1.586563 | -0.930727 | 149 | 137.03 | 0.919664 |  |
| GO:0048523\_negative\_regulation\_of\_cellular\_process | PTEN | 774 | 8 | 1.586563 | -0.930727 | 149 | 137.03 | 0.919664 |  |
| GO:0035107\_appendage\_morphogenesis | CHST11 | 93 | 2 | 3.301075 | -0.913531 | 151 | 139.22 | 0.921987 |  |
| GO:0035107\_appendage\_morphogenesis | FBN2 | 93 | 2 | 3.301075 | -0.913531 | 151 | 139.22 | 0.921987 |  |
| GO:0035108\_limb\_morphogenesis | CHST11 | 93 | 2 | 3.301075 | -0.913531 | 151 | 139.22 | 0.921987 |  |
| GO:0035108\_limb\_morphogenesis | FBN2 | 93 | 2 | 3.301075 | -0.913531 | 151 | 139.22 | 0.921987 |  |
| GO:0001912\_positive\_regulation\_of\_leukocyte\_mediated\_cytotoxicity | CD1D1 | 20 | 1 | 7.675000 | -0.910893 | 156 | 142.94 | 0.916282 |  |
| GO:0031343\_positive\_regulation\_of\_cell\_killing | CD1D1 | 20 | 1 | 7.675000 | -0.910893 | 156 | 142.94 | 0.916282 |  |
| GO:0032582\_negative\_regulation\_of\_gene-specific\_transcription | ZFP238 | 20 | 1 | 7.675000 | -0.910893 | 156 | 142.94 | 0.916282 |  |
| GO:0046822\_regulation\_of\_nucleocytoplasmic\_transport | PKIA | 20 | 1 | 7.675000 | -0.910893 | 156 | 142.94 | 0.916282 |  |
| GO:0048806\_genitalia\_development | LGR4 | 20 | 1 | 7.675000 | -0.910893 | 156 | 142.94 | 0.916282 |  |
| GO:0007275\_multicellular\_organismal\_development | RECK | 1760 | 15 | 1.308239 | -0.896139 | 157 | 144.09 | 0.917771 |  |
| GO:0007275\_multicellular\_organismal\_development | DNMT3A | 1760 | 15 | 1.308239 | -0.896139 | 157 | 144.09 | 0.917771 |  |
| GO:0007275\_multicellular\_organismal\_development | FGF15 | 1760 | 15 | 1.308239 | -0.896139 | 157 | 144.09 | 0.917771 |  |
| GO:0007275\_multicellular\_organismal\_development | HUS1 | 1760 | 15 | 1.308239 | -0.896139 | 157 | 144.09 | 0.917771 |  |
| GO:0007275\_multicellular\_organismal\_development | CD1D1 | 1760 | 15 | 1.308239 | -0.896139 | 157 | 144.09 | 0.917771 |  |
| GO:0007275\_multicellular\_organismal\_development | PTEN | 1760 | 15 | 1.308239 | -0.896139 | 157 | 144.09 | 0.917771 |  |
| GO:0007275\_multicellular\_organismal\_development | LGR4 | 1760 | 15 | 1.308239 | -0.896139 | 157 | 144.09 | 0.917771 |  |
| GO:0007275\_multicellular\_organismal\_development | VASH1 | 1760 | 15 | 1.308239 | -0.896139 | 157 | 144.09 | 0.917771 |  |
| GO:0007275\_multicellular\_organismal\_development | ANKRD17 | 1760 | 15 | 1.308239 | -0.896139 | 157 | 144.09 | 0.917771 |  |
| GO:0007275\_multicellular\_organismal\_development | PRMT1 | 1760 | 15 | 1.308239 | -0.896139 | 157 | 144.09 | 0.917771 |  |
| GO:0007275\_multicellular\_organismal\_development | HDAC2 | 1760 | 15 | 1.308239 | -0.896139 | 157 | 144.09 | 0.917771 |  |
| GO:0007275\_multicellular\_organismal\_development | TRP53BP2 | 1760 | 15 | 1.308239 | -0.896139 | 157 | 144.09 | 0.917771 |  |
| GO:0007275\_multicellular\_organismal\_development | CHST11 | 1760 | 15 | 1.308239 | -0.896139 | 157 | 144.09 | 0.917771 |  |
| GO:0007275\_multicellular\_organismal\_development | PKD2 | 1760 | 15 | 1.308239 | -0.896139 | 157 | 144.09 | 0.917771 |  |
| GO:0007275\_multicellular\_organismal\_development | FBN2 | 1760 | 15 | 1.308239 | -0.896139 | 157 | 144.09 | 0.917771 |  |
| GO:0032502\_developmental\_process | RECK | 2060 | 17 | 1.266748 | -0.891167 | 158 | 144.12 | 0.912152 |  |
| GO:0032502\_developmental\_process | DNMT3A | 2060 | 17 | 1.266748 | -0.891167 | 158 | 144.12 | 0.912152 |  |
| GO:0032502\_developmental\_process | FGF15 | 2060 | 17 | 1.266748 | -0.891167 | 158 | 144.12 | 0.912152 |  |
| GO:0032502\_developmental\_process | HUS1 | 2060 | 17 | 1.266748 | -0.891167 | 158 | 144.12 | 0.912152 |  |
| GO:0032502\_developmental\_process | ARL6 | 2060 | 17 | 1.266748 | -0.891167 | 158 | 144.12 | 0.912152 |  |
| GO:0032502\_developmental\_process | CD1D1 | 2060 | 17 | 1.266748 | -0.891167 | 158 | 144.12 | 0.912152 |  |
| GO:0032502\_developmental\_process | PTEN | 2060 | 17 | 1.266748 | -0.891167 | 158 | 144.12 | 0.912152 |  |
| GO:0032502\_developmental\_process | LGR4 | 2060 | 17 | 1.266748 | -0.891167 | 158 | 144.12 | 0.912152 |  |
| GO:0032502\_developmental\_process | VASH1 | 2060 | 17 | 1.266748 | -0.891167 | 158 | 144.12 | 0.912152 |  |
| GO:0032502\_developmental\_process | ANKRD17 | 2060 | 17 | 1.266748 | -0.891167 | 158 | 144.12 | 0.912152 |  |
| GO:0032502\_developmental\_process | PRMT1 | 2060 | 17 | 1.266748 | -0.891167 | 158 | 144.12 | 0.912152 |  |
| GO:0032502\_developmental\_process | HDAC2 | 2060 | 17 | 1.266748 | -0.891167 | 158 | 144.12 | 0.912152 |  |
| GO:0032502\_developmental\_process | TRP53BP2 | 2060 | 17 | 1.266748 | -0.891167 | 158 | 144.12 | 0.912152 |  |
| GO:0032502\_developmental\_process | CHST11 | 2060 | 17 | 1.266748 | -0.891167 | 158 | 144.12 | 0.912152 |  |
| GO:0032502\_developmental\_process | PKD2 | 2060 | 17 | 1.266748 | -0.891167 | 158 | 144.12 | 0.912152 |  |
| GO:0032502\_developmental\_process | FBN2 | 2060 | 17 | 1.266748 | -0.891167 | 158 | 144.12 | 0.912152 |  |
| GO:0032502\_developmental\_process | CASP2 | 2060 | 17 | 1.266748 | -0.891167 | 158 | 144.12 | 0.912152 |  |
| GO:0000075\_cell\_cycle\_checkpoint | HUS1 | 21 | 1 | 7.309524 | -0.891050 | 161 | 148.94 | 0.925093 |  |
| GO:0001755\_neural\_crest\_cell\_migration | FGF15 | 21 | 1 | 7.309524 | -0.891050 | 161 | 148.94 | 0.925093 |  |
| GO:0002456\_T\_cell\_mediated\_immunity | CD1D1 | 21 | 1 | 7.309524 | -0.891050 | 161 | 148.94 | 0.925093 |  |
| GO:0048736\_appendage\_development | CHST11 | 96 | 2 | 3.197917 | -0.890915 | 163 | 149.38 | 0.916442 |  |
| GO:0048736\_appendage\_development | FBN2 | 96 | 2 | 3.197917 | -0.890915 | 163 | 149.38 | 0.916442 |  |
| GO:0060173\_limb\_development | CHST11 | 96 | 2 | 3.197917 | -0.890915 | 163 | 149.38 | 0.916442 |  |
| GO:0060173\_limb\_development | FBN2 | 96 | 2 | 3.197917 | -0.890915 | 163 | 149.38 | 0.916442 |  |
| GO:0009314\_response\_to\_radiation | TRP53BP2 | 98 | 2 | 3.132653 | -0.876314 | 164 | 150.68 | 0.918780 |  |
| GO:0009314\_response\_to\_radiation | HUS1 | 98 | 2 | 3.132653 | -0.876314 | 164 | 150.68 | 0.918780 |  |
| GO:0001523\_retinoid\_metabolic\_process | RBP1 | 22 | 1 | 6.977273 | -0.872191 | 173 | 156.91 | 0.906994 |  |
| GO:0006029\_proteoglycan\_metabolic\_process | CHST11 | 22 | 1 | 6.977273 | -0.872191 | 173 | 156.91 | 0.906994 |  |
| GO:0006721\_terpenoid\_metabolic\_process | RBP1 | 22 | 1 | 6.977273 | -0.872191 | 173 | 156.91 | 0.906994 |  |
| GO:0009896\_positive\_regulation\_of\_catabolic\_process | BECN1 | 22 | 1 | 6.977273 | -0.872191 | 173 | 156.91 | 0.906994 |  |
| GO:0016101\_diterpenoid\_metabolic\_process | RBP1 | 22 | 1 | 6.977273 | -0.872191 | 173 | 156.91 | 0.906994 |  |
| GO:0021766\_hippocampus\_development | HDAC2 | 22 | 1 | 6.977273 | -0.872191 | 173 | 156.91 | 0.906994 |  |
| GO:0032649\_regulation\_of\_interferon-gamma\_production | CD1D1 | 22 | 1 | 6.977273 | -0.872191 | 173 | 156.91 | 0.906994 |  |
| GO:0042733\_embryonic\_digit\_morphogenesis | CHST11 | 22 | 1 | 6.977273 | -0.872191 | 173 | 156.91 | 0.906994 |  |
| GO:0046635\_positive\_regulation\_of\_alpha-beta\_T\_cell\_activation | CD1D1 | 22 | 1 | 6.977273 | -0.872191 | 173 | 156.91 | 0.906994 |  |
| GO:0001525\_angiogenesis | PTEN | 100 | 2 | 3.070000 | -0.862076 | 174 | 158.51 | 0.910977 |  |
| GO:0001525\_angiogenesis | VASH1 | 100 | 2 | 3.070000 | -0.862076 | 174 | 158.51 | 0.910977 |  |
| GO:0002821\_positive\_regulation\_of\_adaptive\_immune\_response | CD1D1 | 23 | 1 | 6.673913 | -0.854230 | 177 | 162.57 | 0.918475 |  |
| GO:0002824\_positive\_regulation\_of\_adaptive\_immune\_response\_based\_on\_somatic\_recombination\_of\_immune\_receptors\_built\_from\_immunoglobulin\_superfamily\_domains | CD1D1 | 23 | 1 | 6.673913 | -0.854230 | 177 | 162.57 | 0.918475 |  |
| GO:0030512\_negative\_regulation\_of\_transforming\_growth\_factor\_beta\_receptor\_signaling\_pathway | CHST11 | 23 | 1 | 6.673913 | -0.854230 | 177 | 162.57 | 0.918475 |  |
| GO:0001568\_blood\_vessel\_development | RECK | 203 | 3 | 2.268473 | -0.841970 | 178 | 162.8 | 0.914607 |  |
| GO:0001568\_blood\_vessel\_development | PTEN | 203 | 3 | 2.268473 | -0.841970 | 178 | 162.8 | 0.914607 |  |
| GO:0001568\_blood\_vessel\_development | VASH1 | 203 | 3 | 2.268473 | -0.841970 | 178 | 162.8 | 0.914607 |  |
| GO:0009968\_negative\_regulation\_of\_signal\_transduction | CHST11 | 103 | 2 | 2.980583 | -0.841367 | 179 | 163.12 | 0.911285 |  |
| GO:0009968\_negative\_regulation\_of\_signal\_transduction | PTEN | 103 | 2 | 2.980583 | -0.841367 | 179 | 163.12 | 0.911285 |  |
| GO:0007050\_cell\_cycle\_arrest | PKD2 | 24 | 1 | 6.395833 | -0.837089 | 184 | 167.32 | 0.909348 |  |
| GO:0007204\_elevation\_of\_cytosolic\_calcium\_ion\_concentration | PKD2 | 24 | 1 | 6.395833 | -0.837089 | 184 | 167.32 | 0.909348 |  |
| GO:0007259\_JAK-STAT\_cascade | PKD2 | 24 | 1 | 6.395833 | -0.837089 | 184 | 167.32 | 0.909348 |  |
| GO:0009612\_response\_to\_mechanical\_stimulus | PKD2 | 24 | 1 | 6.395833 | -0.837089 | 184 | 167.32 | 0.909348 |  |
| GO:0032386\_regulation\_of\_intracellular\_transport | PKIA | 24 | 1 | 6.395833 | -0.837089 | 184 | 167.32 | 0.909348 |  |
| GO:0006302\_double-strand\_break\_repair | HUS1 | 25 | 1 | 6.140000 | -0.820701 | 188 | 171.69 | 0.913245 |  |
| GO:0006775\_fat-soluble\_vitamin\_metabolic\_process | RBP1 | 25 | 1 | 6.140000 | -0.820701 | 188 | 171.69 | 0.913245 |  |
| GO:0007492\_endoderm\_development | ANKRD17 | 25 | 1 | 6.140000 | -0.820701 | 188 | 171.69 | 0.913245 |  |
| GO:0032103\_positive\_regulation\_of\_response\_to\_external\_stimulus | BECN1 | 25 | 1 | 6.140000 | -0.820701 | 188 | 171.69 | 0.913245 |  |
| GO:0001944\_vasculature\_development | RECK | 208 | 3 | 2.213942 | -0.819353 | 189 | 171.9 | 0.909524 |  |
| GO:0001944\_vasculature\_development | PTEN | 208 | 3 | 2.213942 | -0.819353 | 189 | 171.9 | 0.909524 |  |
| GO:0001944\_vasculature\_development | VASH1 | 208 | 3 | 2.213942 | -0.819353 | 189 | 171.9 | 0.909524 |  |
| GO:0006720\_isoprenoid\_metabolic\_process | RBP1 | 26 | 1 | 5.903846 | -0.805008 | 197 | 176.58 | 0.896345 |  |
| GO:0010212\_response\_to\_ionizing\_radiation | TRP53BP2 | 26 | 1 | 5.903846 | -0.805008 | 197 | 176.58 | 0.896345 |  |
| GO:0032609\_interferon-gamma\_production | CD1D1 | 26 | 1 | 5.903846 | -0.805008 | 197 | 176.58 | 0.896345 |  |
| GO:0040029\_regulation\_of\_gene\_expression\_\_epigenetic | DNMT3A | 26 | 1 | 5.903846 | -0.805008 | 197 | 176.58 | 0.896345 |  |
| GO:0045765\_regulation\_of\_angiogenesis | VASH1 | 26 | 1 | 5.903846 | -0.805008 | 197 | 176.58 | 0.896345 |  |
| GO:0050680\_negative\_regulation\_of\_epithelial\_cell\_proliferation | PTEN | 26 | 1 | 5.903846 | -0.805008 | 197 | 176.58 | 0.896345 |  |
| GO:0051235\_maintenance\_of\_location | PKD2 | 26 | 1 | 5.903846 | -0.805008 | 197 | 176.58 | 0.896345 |  |
| GO:0051480\_cytosolic\_calcium\_ion\_homeostasis | PKD2 | 26 | 1 | 5.903846 | -0.805008 | 197 | 176.58 | 0.896345 |  |
| GO:0010648\_negative\_regulation\_of\_cell\_communication | CHST11 | 110 | 2 | 2.790909 | -0.795843 | 198 | 177.59 | 0.896919 |  |
| GO:0010648\_negative\_regulation\_of\_cell\_communication | PTEN | 110 | 2 | 2.790909 | -0.795843 | 198 | 177.59 | 0.896919 |  |
| GO:0001910\_regulation\_of\_leukocyte\_mediated\_cytotoxicity | CD1D1 | 27 | 1 | 5.685185 | -0.789956 | 203 | 180.7 | 0.890148 |  |
| GO:0009411\_response\_to\_UV | HUS1 | 27 | 1 | 5.685185 | -0.789956 | 203 | 180.7 | 0.890148 |  |
| GO:0019884\_antigen\_processing\_and\_presentation\_of\_exogenous\_antigen | CD1D1 | 27 | 1 | 5.685185 | -0.789956 | 203 | 180.7 | 0.890148 |  |
| GO:0031341\_regulation\_of\_cell\_killing | CD1D1 | 27 | 1 | 5.685185 | -0.789956 | 203 | 180.7 | 0.890148 |  |
| GO:0045582\_positive\_regulation\_of\_T\_cell\_differentiation | CD1D1 | 27 | 1 | 5.685185 | -0.789956 | 203 | 180.7 | 0.890148 |  |
| GO:0006974\_response\_to\_DNA\_damage\_stimulus | HUS1 | 113 | 2 | 2.716814 | -0.777436 | 204 | 182.92 | 0.896667 |  |
| GO:0006974\_response\_to\_DNA\_damage\_stimulus | RAD17 | 113 | 2 | 2.716814 | -0.777436 | 204 | 182.92 | 0.896667 |  |
| GO:0002062\_chondrocyte\_differentiation | CHST11 | 28 | 1 | 5.482143 | -0.775499 | 209 | 185.95 | 0.889713 |  |
| GO:0002705\_positive\_regulation\_of\_leukocyte\_mediated\_immunity | CD1D1 | 28 | 1 | 5.482143 | -0.775499 | 209 | 185.95 | 0.889713 |  |
| GO:0002708\_positive\_regulation\_of\_lymphocyte\_mediated\_immunity | CD1D1 | 28 | 1 | 5.482143 | -0.775499 | 209 | 185.95 | 0.889713 |  |
| GO:0007127\_meiosis\_I | PSMD13 | 28 | 1 | 5.482143 | -0.775499 | 209 | 185.95 | 0.889713 |  |
| GO:0007585\_respiratory\_gaseous\_exchange | CHST11 | 28 | 1 | 5.482143 | -0.775499 | 209 | 185.95 | 0.889713 |  |
| GO:0048584\_positive\_regulation\_of\_response\_to\_stimulus | BECN1 | 115 | 2 | 2.669565 | -0.765507 | 210 | 186.87 | 0.889857 |  |
| GO:0048584\_positive\_regulation\_of\_response\_to\_stimulus | CD1D1 | 115 | 2 | 2.669565 | -0.765507 | 210 | 186.87 | 0.889857 |  |
| GO:0010564\_regulation\_of\_cell\_cycle\_process | PKIA | 29 | 1 | 5.293103 | -0.761595 | 215 | 190.48 | 0.885953 |  |
| GO:0021761\_limbic\_system\_development | HDAC2 | 29 | 1 | 5.293103 | -0.761595 | 215 | 190.48 | 0.885953 |  |
| GO:0042770\_DNA\_damage\_response\_\_signal\_transduction | HUS1 | 29 | 1 | 5.293103 | -0.761595 | 215 | 190.48 | 0.885953 |  |
| GO:0045621\_positive\_regulation\_of\_lymphocyte\_differentiation | CD1D1 | 29 | 1 | 5.293103 | -0.761595 | 215 | 190.48 | 0.885953 |  |
| GO:0046634\_regulation\_of\_alpha-beta\_T\_cell\_activation | CD1D1 | 29 | 1 | 5.293103 | -0.761595 | 215 | 190.48 | 0.885953 |  |
| GO:0014032\_neural\_crest\_cell\_development | FGF15 | 30 | 1 | 5.116667 | -0.748206 | 218 | 195.34 | 0.896055 |  |
| GO:0014033\_neural\_crest\_cell\_differentiation | FGF15 | 30 | 1 | 5.116667 | -0.748206 | 218 | 195.34 | 0.896055 |  |
| GO:0031668\_cellular\_response\_to\_extracellular\_stimulus | BECN1 | 30 | 1 | 5.116667 | -0.748206 | 218 | 195.34 | 0.896055 |  |
| GO:0022403\_cell\_cycle\_phase | PSMD13 | 119 | 2 | 2.579832 | -0.742425 | 219 | 195.72 | 0.893699 |  |
| GO:0022403\_cell\_cycle\_phase | PKIA | 119 | 2 | 2.579832 | -0.742425 | 219 | 195.72 | 0.893699 |  |
| GO:0046632\_alpha-beta\_T\_cell\_differentiation | CD1D1 | 31 | 1 | 4.951613 | -0.735299 | 220 | 202.18 | 0.919000 |  |
| GO:0042108\_positive\_regulation\_of\_cytokine\_biosynthetic\_process | CD1D1 | 33 | 1 | 4.651515 | -0.710810 | 221 | 210.5 | 0.952489 |  |
| GO:0031323\_regulation\_of\_cellular\_metabolic\_process | TBL1XR1 | 1015 | 9 | 1.361084 | -0.702351 | 222 | 211.31 | 0.951847 |  |
| GO:0031323\_regulation\_of\_cellular\_metabolic\_process | DNMT3A | 1015 | 9 | 1.361084 | -0.702351 | 222 | 211.31 | 0.951847 |  |
| GO:0031323\_regulation\_of\_cellular\_metabolic\_process | BECN1 | 1015 | 9 | 1.361084 | -0.702351 | 222 | 211.31 | 0.951847 |  |
| GO:0031323\_regulation\_of\_cellular\_metabolic\_process | HUS1 | 1015 | 9 | 1.361084 | -0.702351 | 222 | 211.31 | 0.951847 |  |
| GO:0031323\_regulation\_of\_cellular\_metabolic\_process | TFDP2 | 1015 | 9 | 1.361084 | -0.702351 | 222 | 211.31 | 0.951847 |  |
| GO:0031323\_regulation\_of\_cellular\_metabolic\_process | ZFP238 | 1015 | 9 | 1.361084 | -0.702351 | 222 | 211.31 | 0.951847 |  |
| GO:0031323\_regulation\_of\_cellular\_metabolic\_process | PKD2 | 1015 | 9 | 1.361084 | -0.702351 | 222 | 211.31 | 0.951847 |  |
| GO:0031323\_regulation\_of\_cellular\_metabolic\_process | CD1D1 | 1015 | 9 | 1.361084 | -0.702351 | 222 | 211.31 | 0.951847 |  |
| GO:0031323\_regulation\_of\_cellular\_metabolic\_process | PKIA | 1015 | 9 | 1.361084 | -0.702351 | 222 | 211.31 | 0.951847 |  |
| GO:0002699\_positive\_regulation\_of\_immune\_effector\_process | CD1D1 | 34 | 1 | 4.514706 | -0.699175 | 225 | 214.74 | 0.954400 |  |
| GO:0019882\_antigen\_processing\_and\_presentation | CD1D1 | 34 | 1 | 4.514706 | -0.699175 | 225 | 214.74 | 0.954400 |  |
| GO:0051052\_regulation\_of\_DNA\_metabolic\_process | HUS1 | 34 | 1 | 4.514706 | -0.699175 | 225 | 214.74 | 0.954400 |  |
| GO:0010556\_regulation\_of\_macromolecule\_biosynthetic\_process | TBL1XR1 | 745 | 7 | 1.442282 | -0.697272 | 226 | 214.93 | 0.951018 |  |
| GO:0010556\_regulation\_of\_macromolecule\_biosynthetic\_process | DNMT3A | 745 | 7 | 1.442282 | -0.697272 | 226 | 214.93 | 0.951018 |  |
| GO:0010556\_regulation\_of\_macromolecule\_biosynthetic\_process | HUS1 | 745 | 7 | 1.442282 | -0.697272 | 226 | 214.93 | 0.951018 |  |
| GO:0010556\_regulation\_of\_macromolecule\_biosynthetic\_process | TFDP2 | 745 | 7 | 1.442282 | -0.697272 | 226 | 214.93 | 0.951018 |  |
| GO:0010556\_regulation\_of\_macromolecule\_biosynthetic\_process | ZFP238 | 745 | 7 | 1.442282 | -0.697272 | 226 | 214.93 | 0.951018 |  |
| GO:0010556\_regulation\_of\_macromolecule\_biosynthetic\_process | CD1D1 | 745 | 7 | 1.442282 | -0.697272 | 226 | 214.93 | 0.951018 |  |
| GO:0010556\_regulation\_of\_macromolecule\_biosynthetic\_process | PKIA | 745 | 7 | 1.442282 | -0.697272 | 226 | 214.93 | 0.951018 |  |
| GO:0001655\_urogenital\_system\_development | PKD2 | 128 | 2 | 2.398438 | -0.693969 | 227 | 215.4 | 0.948899 |  |
| GO:0001655\_urogenital\_system\_development | PTEN | 128 | 2 | 2.398438 | -0.693969 | 227 | 215.4 | 0.948899 |  |
| GO:0042981\_regulation\_of\_apoptosis | CHST11 | 360 | 4 | 1.705556 | -0.691060 | 228 | 215.46 | 0.945000 |  |
| GO:0042981\_regulation\_of\_apoptosis | CD1D1 | 360 | 4 | 1.705556 | -0.691060 | 228 | 215.46 | 0.945000 |  |
| GO:0042981\_regulation\_of\_apoptosis | CASP2 | 360 | 4 | 1.705556 | -0.691060 | 228 | 215.46 | 0.945000 |  |
| GO:0042981\_regulation\_of\_apoptosis | PTEN | 360 | 4 | 1.705556 | -0.691060 | 228 | 215.46 | 0.945000 |  |
| GO:0051276\_chromosome\_organization | DNMT3A | 129 | 2 | 2.379845 | -0.688860 | 229 | 215.98 | 0.943144 |  |
| GO:0051276\_chromosome\_organization | HDAC2 | 129 | 2 | 2.379845 | -0.688860 | 229 | 215.98 | 0.943144 |  |
| GO:0001906\_cell\_killing | CD1D1 | 35 | 1 | 4.385714 | -0.687914 | 233 | 218.97 | 0.939785 |  |
| GO:0001909\_leukocyte\_mediated\_cytotoxicity | CD1D1 | 35 | 1 | 4.385714 | -0.687914 | 233 | 218.97 | 0.939785 |  |
| GO:0051325\_interphase | PKIA | 35 | 1 | 4.385714 | -0.687914 | 233 | 218.97 | 0.939785 |  |
| GO:0051329\_interphase\_of\_mitotic\_cell\_cycle | PKIA | 35 | 1 | 4.385714 | -0.687914 | 233 | 218.97 | 0.939785 |  |
| GO:0006469\_negative\_regulation\_of\_protein\_kinase\_activity | PKIA | 36 | 1 | 4.263889 | -0.677007 | 237 | 223.18 | 0.941688 |  |
| GO:0007368\_determination\_of\_left\_right\_symmetry | PKD2 | 36 | 1 | 4.263889 | -0.677007 | 237 | 223.18 | 0.941688 |  |
| GO:0033673\_negative\_regulation\_of\_kinase\_activity | PKIA | 36 | 1 | 4.263889 | -0.677007 | 237 | 223.18 | 0.941688 |  |
| GO:0051223\_regulation\_of\_protein\_transport | PKIA | 36 | 1 | 4.263889 | -0.677007 | 237 | 223.18 | 0.941688 |  |
| GO:0010941\_regulation\_of\_cell\_death | CHST11 | 365 | 4 | 1.682192 | -0.676583 | 239 | 223.44 | 0.934895 |  |
| GO:0010941\_regulation\_of\_cell\_death | CD1D1 | 365 | 4 | 1.682192 | -0.676583 | 239 | 223.44 | 0.934895 |  |
| GO:0010941\_regulation\_of\_cell\_death | PTEN | 365 | 4 | 1.682192 | -0.676583 | 239 | 223.44 | 0.934895 |  |
| GO:0010941\_regulation\_of\_cell\_death | CASP2 | 365 | 4 | 1.682192 | -0.676583 | 239 | 223.44 | 0.934895 |  |
| GO:0043067\_regulation\_of\_programmed\_cell\_death | CHST11 | 365 | 4 | 1.682192 | -0.676583 | 239 | 223.44 | 0.934895 |  |
| GO:0043067\_regulation\_of\_programmed\_cell\_death | CD1D1 | 365 | 4 | 1.682192 | -0.676583 | 239 | 223.44 | 0.934895 |  |
| GO:0043067\_regulation\_of\_programmed\_cell\_death | CASP2 | 365 | 4 | 1.682192 | -0.676583 | 239 | 223.44 | 0.934895 |  |
| GO:0043067\_regulation\_of\_programmed\_cell\_death | PTEN | 365 | 4 | 1.682192 | -0.676583 | 239 | 223.44 | 0.934895 |  |
| GO:0019219\_regulation\_of\_nucleobase\_\_nucleoside\_\_nucleotide\_and\_nucleic\_acid\_metabolic\_process | TBL1XR1 | 757 | 7 | 1.419419 | -0.672755 | 240 | 223.55 | 0.931458 |  |
| GO:0019219\_regulation\_of\_nucleobase\_\_nucleoside\_\_nucleotide\_and\_nucleic\_acid\_metabolic\_process | DNMT3A | 757 | 7 | 1.419419 | -0.672755 | 240 | 223.55 | 0.931458 |  |
| GO:0019219\_regulation\_of\_nucleobase\_\_nucleoside\_\_nucleotide\_and\_nucleic\_acid\_metabolic\_process | HUS1 | 757 | 7 | 1.419419 | -0.672755 | 240 | 223.55 | 0.931458 |  |
| GO:0019219\_regulation\_of\_nucleobase\_\_nucleoside\_\_nucleotide\_and\_nucleic\_acid\_metabolic\_process | TFDP2 | 757 | 7 | 1.419419 | -0.672755 | 240 | 223.55 | 0.931458 |  |
| GO:0019219\_regulation\_of\_nucleobase\_\_nucleoside\_\_nucleotide\_and\_nucleic\_acid\_metabolic\_process | ZFP238 | 757 | 7 | 1.419419 | -0.672755 | 240 | 223.55 | 0.931458 |  |
| GO:0019219\_regulation\_of\_nucleobase\_\_nucleoside\_\_nucleotide\_and\_nucleic\_acid\_metabolic\_process | PKD2 | 757 | 7 | 1.419419 | -0.672755 | 240 | 223.55 | 0.931458 |  |
| GO:0019219\_regulation\_of\_nucleobase\_\_nucleoside\_\_nucleotide\_and\_nucleic\_acid\_metabolic\_process | PKIA | 757 | 7 | 1.419419 | -0.672755 | 240 | 223.55 | 0.931458 |  |
| GO:0009792\_embryonic\_development\_ending\_in\_birth\_or\_egg\_hatching | PRMT1 | 368 | 4 | 1.668478 | -0.668052 | 241 | 224.22 | 0.930373 |  |
| GO:0009792\_embryonic\_development\_ending\_in\_birth\_or\_egg\_hatching | TRP53BP2 | 368 | 4 | 1.668478 | -0.668052 | 241 | 224.22 | 0.930373 |  |
| GO:0009792\_embryonic\_development\_ending\_in\_birth\_or\_egg\_hatching | CHST11 | 368 | 4 | 1.668478 | -0.668052 | 241 | 224.22 | 0.930373 |  |
| GO:0009792\_embryonic\_development\_ending\_in\_birth\_or\_egg\_hatching | PKD2 | 368 | 4 | 1.668478 | -0.668052 | 241 | 224.22 | 0.930373 |  |
| GO:0009799\_determination\_of\_symmetry | PKD2 | 37 | 1 | 4.148649 | -0.666433 | 243 | 226.58 | 0.932428 |  |
| GO:0009855\_determination\_of\_bilateral\_symmetry | PKD2 | 37 | 1 | 4.148649 | -0.666433 | 243 | 226.58 | 0.932428 |  |
| GO:0034645\_cellular\_macromolecule\_biosynthetic\_process | TBL1XR1 | 901 | 8 | 1.362930 | -0.658157 | 244 | 227.94 | 0.934180 |  |
| GO:0034645\_cellular\_macromolecule\_biosynthetic\_process | DNMT3A | 901 | 8 | 1.362930 | -0.658157 | 244 | 227.94 | 0.934180 |  |
| GO:0034645\_cellular\_macromolecule\_biosynthetic\_process | HUS1 | 901 | 8 | 1.362930 | -0.658157 | 244 | 227.94 | 0.934180 |  |
| GO:0034645\_cellular\_macromolecule\_biosynthetic\_process | CHST11 | 901 | 8 | 1.362930 | -0.658157 | 244 | 227.94 | 0.934180 |  |
| GO:0034645\_cellular\_macromolecule\_biosynthetic\_process | TFDP2 | 901 | 8 | 1.362930 | -0.658157 | 244 | 227.94 | 0.934180 |  |
| GO:0034645\_cellular\_macromolecule\_biosynthetic\_process | ZFP238 | 901 | 8 | 1.362930 | -0.658157 | 244 | 227.94 | 0.934180 |  |
| GO:0034645\_cellular\_macromolecule\_biosynthetic\_process | CD1D1 | 901 | 8 | 1.362930 | -0.658157 | 244 | 227.94 | 0.934180 |  |
| GO:0034645\_cellular\_macromolecule\_biosynthetic\_process | PKIA | 901 | 8 | 1.362930 | -0.658157 | 244 | 227.94 | 0.934180 |  |
| GO:0032259\_methylation | DNMT3A | 38 | 1 | 4.039474 | -0.656176 | 248 | 231.99 | 0.935444 |  |
| GO:0043414\_biopolymer\_methylation | DNMT3A | 38 | 1 | 4.039474 | -0.656176 | 248 | 231.99 | 0.935444 |  |
| GO:0045580\_regulation\_of\_T\_cell\_differentiation | CD1D1 | 38 | 1 | 4.039474 | -0.656176 | 248 | 231.99 | 0.935444 |  |
| GO:0051348\_negative\_regulation\_of\_transferase\_activity | PKIA | 38 | 1 | 4.039474 | -0.656176 | 248 | 231.99 | 0.935444 |  |
| GO:0005976\_polysaccharide\_metabolic\_process | CHST11 | 39 | 1 | 3.935897 | -0.646218 | 252 | 236.05 | 0.936706 |  |
| GO:0006511\_ubiquitin-dependent\_protein\_catabolic\_process | TBL1XR1 | 39 | 1 | 3.935897 | -0.646218 | 252 | 236.05 | 0.936706 |  |
| GO:0006730\_one-carbon\_metabolic\_process | DNMT3A | 39 | 1 | 3.935897 | -0.646218 | 252 | 236.05 | 0.936706 |  |
| GO:0070201\_regulation\_of\_establishment\_of\_protein\_localization | PKIA | 39 | 1 | 3.935897 | -0.646218 | 252 | 236.05 | 0.936706 |  |
| GO:0051171\_regulation\_of\_nitrogen\_compound\_metabolic\_process | TBL1XR1 | 771 | 7 | 1.393645 | -0.645126 | 253 | 236.57 | 0.935059 |  |
| GO:0051171\_regulation\_of\_nitrogen\_compound\_metabolic\_process | DNMT3A | 771 | 7 | 1.393645 | -0.645126 | 253 | 236.57 | 0.935059 |  |
| GO:0051171\_regulation\_of\_nitrogen\_compound\_metabolic\_process | HUS1 | 771 | 7 | 1.393645 | -0.645126 | 253 | 236.57 | 0.935059 |  |
| GO:0051171\_regulation\_of\_nitrogen\_compound\_metabolic\_process | TFDP2 | 771 | 7 | 1.393645 | -0.645126 | 253 | 236.57 | 0.935059 |  |
| GO:0051171\_regulation\_of\_nitrogen\_compound\_metabolic\_process | ZFP238 | 771 | 7 | 1.393645 | -0.645126 | 253 | 236.57 | 0.935059 |  |
| GO:0051171\_regulation\_of\_nitrogen\_compound\_metabolic\_process | PKD2 | 771 | 7 | 1.393645 | -0.645126 | 253 | 236.57 | 0.935059 |  |
| GO:0051171\_regulation\_of\_nitrogen\_compound\_metabolic\_process | PKIA | 771 | 7 | 1.393645 | -0.645126 | 253 | 236.57 | 0.935059 |  |
| GO:0009059\_macromolecule\_biosynthetic\_process | TBL1XR1 | 910 | 8 | 1.349451 | -0.641797 | 254 | 237.06 | 0.933307 |  |
| GO:0009059\_macromolecule\_biosynthetic\_process | DNMT3A | 910 | 8 | 1.349451 | -0.641797 | 254 | 237.06 | 0.933307 |  |
| GO:0009059\_macromolecule\_biosynthetic\_process | CHST11 | 910 | 8 | 1.349451 | -0.641797 | 254 | 237.06 | 0.933307 |  |
| GO:0009059\_macromolecule\_biosynthetic\_process | HUS1 | 910 | 8 | 1.349451 | -0.641797 | 254 | 237.06 | 0.933307 |  |
| GO:0009059\_macromolecule\_biosynthetic\_process | TFDP2 | 910 | 8 | 1.349451 | -0.641797 | 254 | 237.06 | 0.933307 |  |
| GO:0009059\_macromolecule\_biosynthetic\_process | ZFP238 | 910 | 8 | 1.349451 | -0.641797 | 254 | 237.06 | 0.933307 |  |
| GO:0009059\_macromolecule\_biosynthetic\_process | CD1D1 | 910 | 8 | 1.349451 | -0.641797 | 254 | 237.06 | 0.933307 |  |
| GO:0009059\_macromolecule\_biosynthetic\_process | PKIA | 910 | 8 | 1.349451 | -0.641797 | 254 | 237.06 | 0.933307 |  |
| GO:0014031\_mesenchymal\_cell\_development | FGF15 | 40 | 1 | 3.837500 | -0.636545 | 256 | 240.29 | 0.938633 |  |
| GO:0017015\_regulation\_of\_transforming\_growth\_factor\_beta\_receptor\_signaling\_pathway | CHST11 | 40 | 1 | 3.837500 | -0.636545 | 256 | 240.29 | 0.938633 |  |
| GO:0003006\_reproductive\_developmental\_process | PTEN | 141 | 2 | 2.177305 | -0.631378 | 257 | 241.34 | 0.939066 |  |
| GO:0003006\_reproductive\_developmental\_process | LGR4 | 141 | 2 | 2.177305 | -0.631378 | 257 | 241.34 | 0.939066 |  |
| GO:0006260\_DNA\_replication | HUS1 | 41 | 1 | 3.743902 | -0.627142 | 265 | 245.72 | 0.927245 |  |
| GO:0006979\_response\_to\_oxidative\_stress | TXNIP | 41 | 1 | 3.743902 | -0.627142 | 265 | 245.72 | 0.927245 |  |
| GO:0009894\_regulation\_of\_catabolic\_process | BECN1 | 41 | 1 | 3.743902 | -0.627142 | 265 | 245.72 | 0.927245 |  |
| GO:0010551\_regulation\_of\_specific\_transcription\_from\_RNA\_polymerase\_II\_promoter | ZFP238 | 41 | 1 | 3.743902 | -0.627142 | 265 | 245.72 | 0.927245 |  |
| GO:0019748\_secondary\_metabolic\_process | RBP1 | 41 | 1 | 3.743902 | -0.627142 | 265 | 245.72 | 0.927245 |  |
| GO:0032569\_specific\_transcription\_from\_RNA\_polymerase\_II\_promoter | ZFP238 | 41 | 1 | 3.743902 | -0.627142 | 265 | 245.72 | 0.927245 |  |
| GO:0032844\_regulation\_of\_homeostatic\_process | PKD2 | 41 | 1 | 3.743902 | -0.627142 | 265 | 245.72 | 0.927245 |  |
| GO:0033077\_T\_cell\_differentiation\_in\_the\_thymus | CD1D1 | 41 | 1 | 3.743902 | -0.627142 | 265 | 245.72 | 0.927245 |  |
| GO:0019221\_cytokine-mediated\_signaling\_pathway | ZC3H15 | 42 | 1 | 3.654762 | -0.617996 | 270 | 250.63 | 0.928259 |  |
| GO:0019941\_modification-dependent\_protein\_catabolic\_process | TBL1XR1 | 42 | 1 | 3.654762 | -0.617996 | 270 | 250.63 | 0.928259 |  |
| GO:0043632\_modification-dependent\_macromolecule\_catabolic\_process | TBL1XR1 | 42 | 1 | 3.654762 | -0.617996 | 270 | 250.63 | 0.928259 |  |
| GO:0051603\_proteolysis\_involved\_in\_cellular\_protein\_catabolic\_process | TBL1XR1 | 42 | 1 | 3.654762 | -0.617996 | 270 | 250.63 | 0.928259 |  |
| GO:0080135\_regulation\_of\_cellular\_response\_to\_stress | BECN1 | 42 | 1 | 3.654762 | -0.617996 | 270 | 250.63 | 0.928259 |  |
| GO:0005975\_carbohydrate\_metabolic\_process | B3GALNT1 | 146 | 2 | 2.102740 | -0.609331 | 271 | 252.39 | 0.931328 |  |
| GO:0005975\_carbohydrate\_metabolic\_process | CHST11 | 146 | 2 | 2.102740 | -0.609331 | 271 | 252.39 | 0.931328 |  |
| GO:0002819\_regulation\_of\_adaptive\_immune\_response | CD1D1 | 43 | 1 | 3.569767 | -0.609095 | 278 | 256.45 | 0.922482 |  |
| GO:0002822\_regulation\_of\_adaptive\_immune\_response\_based\_on\_somatic\_recombination\_of\_immune\_receptors\_built\_from\_immunoglobulin\_superfamily\_domains | CD1D1 | 43 | 1 | 3.569767 | -0.609095 | 278 | 256.45 | 0.922482 |  |
| GO:0006766\_vitamin\_metabolic\_process | RBP1 | 43 | 1 | 3.569767 | -0.609095 | 278 | 256.45 | 0.922482 |  |
| GO:0009582\_detection\_of\_abiotic\_stimulus | PKD2 | 43 | 1 | 3.569767 | -0.609095 | 278 | 256.45 | 0.922482 |  |
| GO:0030814\_regulation\_of\_cAMP\_metabolic\_process | PKD2 | 43 | 1 | 3.569767 | -0.609095 | 278 | 256.45 | 0.922482 |  |
| GO:0048762\_mesenchymal\_cell\_differentiation | FGF15 | 43 | 1 | 3.569767 | -0.609095 | 278 | 256.45 | 0.922482 |  |
| GO:0051789\_response\_to\_protein\_stimulus | VASH1 | 43 | 1 | 3.569767 | -0.609095 | 278 | 256.45 | 0.922482 |  |
| GO:0006606\_protein\_import\_into\_nucleus | PKIA | 44 | 1 | 3.488636 | -0.600427 | 282 | 262.24 | 0.929929 |  |
| GO:0044257\_cellular\_protein\_catabolic\_process | TBL1XR1 | 44 | 1 | 3.488636 | -0.600427 | 282 | 262.24 | 0.929929 |  |
| GO:0051170\_nuclear\_import | PKIA | 44 | 1 | 3.488636 | -0.600427 | 282 | 262.24 | 0.929929 |  |
| GO:0060485\_mesenchyme\_development | FGF15 | 44 | 1 | 3.488636 | -0.600427 | 282 | 262.24 | 0.929929 |  |
| GO:0048513\_organ\_development | RECK | 1365 | 11 | 1.236996 | -0.593977 | 283 | 262.97 | 0.929223 |  |
| GO:0048513\_organ\_development | ANKRD17 | 1365 | 11 | 1.236996 | -0.593977 | 283 | 262.97 | 0.929223 |  |
| GO:0048513\_organ\_development | HDAC2 | 1365 | 11 | 1.236996 | -0.593977 | 283 | 262.97 | 0.929223 |  |
| GO:0048513\_organ\_development | FGF15 | 1365 | 11 | 1.236996 | -0.593977 | 283 | 262.97 | 0.929223 |  |
| GO:0048513\_organ\_development | TRP53BP2 | 1365 | 11 | 1.236996 | -0.593977 | 283 | 262.97 | 0.929223 |  |
| GO:0048513\_organ\_development | CHST11 | 1365 | 11 | 1.236996 | -0.593977 | 283 | 262.97 | 0.929223 |  |
| GO:0048513\_organ\_development | PKD2 | 1365 | 11 | 1.236996 | -0.593977 | 283 | 262.97 | 0.929223 |  |
| GO:0048513\_organ\_development | CD1D1 | 1365 | 11 | 1.236996 | -0.593977 | 283 | 262.97 | 0.929223 |  |
| GO:0048513\_organ\_development | PTEN | 1365 | 11 | 1.236996 | -0.593977 | 283 | 262.97 | 0.929223 |  |
| GO:0048513\_organ\_development | LGR4 | 1365 | 11 | 1.236996 | -0.593977 | 283 | 262.97 | 0.929223 |  |
| GO:0048513\_organ\_development | VASH1 | 1365 | 11 | 1.236996 | -0.593977 | 283 | 262.97 | 0.929223 |  |
| GO:0046058\_cAMP\_metabolic\_process | PKD2 | 45 | 1 | 3.411111 | -0.591983 | 285 | 264.39 | 0.927684 |  |
| GO:0046546\_development\_of\_primary\_male\_sexual\_characteristics | LGR4 | 45 | 1 | 3.411111 | -0.591983 | 285 | 264.39 | 0.927684 |  |
| GO:0034961\_cellular\_biopolymer\_biosynthetic\_process | TBL1XR1 | 804 | 7 | 1.336443 | -0.583937 | 286 | 265.01 | 0.926608 |  |
| GO:0034961\_cellular\_biopolymer\_biosynthetic\_process | DNMT3A | 804 | 7 | 1.336443 | -0.583937 | 286 | 265.01 | 0.926608 |  |
| GO:0034961\_cellular\_biopolymer\_biosynthetic\_process | CHST11 | 804 | 7 | 1.336443 | -0.583937 | 286 | 265.01 | 0.926608 |  |
| GO:0034961\_cellular\_biopolymer\_biosynthetic\_process | HUS1 | 804 | 7 | 1.336443 | -0.583937 | 286 | 265.01 | 0.926608 |  |
| GO:0034961\_cellular\_biopolymer\_biosynthetic\_process | TFDP2 | 804 | 7 | 1.336443 | -0.583937 | 286 | 265.01 | 0.926608 |  |
| GO:0034961\_cellular\_biopolymer\_biosynthetic\_process | ZFP238 | 804 | 7 | 1.336443 | -0.583937 | 286 | 265.01 | 0.926608 |  |
| GO:0034961\_cellular\_biopolymer\_biosynthetic\_process | PKIA | 804 | 7 | 1.336443 | -0.583937 | 286 | 265.01 | 0.926608 |  |
| GO:0009581\_detection\_of\_external\_stimulus | PKD2 | 46 | 1 | 3.336957 | -0.583752 | 289 | 267.58 | 0.925882 |  |
| GO:0030850\_prostate\_gland\_development | PTEN | 46 | 1 | 3.336957 | -0.583752 | 289 | 267.58 | 0.925882 |  |
| GO:0046631\_alpha-beta\_T\_cell\_activation | CD1D1 | 46 | 1 | 3.336957 | -0.583752 | 289 | 267.58 | 0.925882 |  |
| GO:0019222\_regulation\_of\_metabolic\_process | TBL1XR1 | 1088 | 9 | 1.269761 | -0.578846 | 290 | 268.31 | 0.925207 |  |
| GO:0019222\_regulation\_of\_metabolic\_process | DNMT3A | 1088 | 9 | 1.269761 | -0.578846 | 290 | 268.31 | 0.925207 |  |
| GO:0019222\_regulation\_of\_metabolic\_process | BECN1 | 1088 | 9 | 1.269761 | -0.578846 | 290 | 268.31 | 0.925207 |  |
| GO:0019222\_regulation\_of\_metabolic\_process | HUS1 | 1088 | 9 | 1.269761 | -0.578846 | 290 | 268.31 | 0.925207 |  |
| GO:0019222\_regulation\_of\_metabolic\_process | PKD2 | 1088 | 9 | 1.269761 | -0.578846 | 290 | 268.31 | 0.925207 |  |
| GO:0019222\_regulation\_of\_metabolic\_process | TFDP2 | 1088 | 9 | 1.269761 | -0.578846 | 290 | 268.31 | 0.925207 |  |
| GO:0019222\_regulation\_of\_metabolic\_process | ZFP238 | 1088 | 9 | 1.269761 | -0.578846 | 290 | 268.31 | 0.925207 |  |
| GO:0019222\_regulation\_of\_metabolic\_process | CD1D1 | 1088 | 9 | 1.269761 | -0.578846 | 290 | 268.31 | 0.925207 |  |
| GO:0019222\_regulation\_of\_metabolic\_process | PKIA | 1088 | 9 | 1.269761 | -0.578846 | 290 | 268.31 | 0.925207 |  |
| GO:0043284\_biopolymer\_biosynthetic\_process | TBL1XR1 | 807 | 7 | 1.331475 | -0.578637 | 291 | 268.47 | 0.922577 |  |
| GO:0043284\_biopolymer\_biosynthetic\_process | DNMT3A | 807 | 7 | 1.331475 | -0.578637 | 291 | 268.47 | 0.922577 |  |
| GO:0043284\_biopolymer\_biosynthetic\_process | HUS1 | 807 | 7 | 1.331475 | -0.578637 | 291 | 268.47 | 0.922577 |  |
| GO:0043284\_biopolymer\_biosynthetic\_process | CHST11 | 807 | 7 | 1.331475 | -0.578637 | 291 | 268.47 | 0.922577 |  |
| GO:0043284\_biopolymer\_biosynthetic\_process | TFDP2 | 807 | 7 | 1.331475 | -0.578637 | 291 | 268.47 | 0.922577 |  |
| GO:0043284\_biopolymer\_biosynthetic\_process | ZFP238 | 807 | 7 | 1.331475 | -0.578637 | 291 | 268.47 | 0.922577 |  |
| GO:0043284\_biopolymer\_biosynthetic\_process | PKIA | 807 | 7 | 1.331475 | -0.578637 | 291 | 268.47 | 0.922577 |  |
| GO:0006140\_regulation\_of\_nucleotide\_metabolic\_process | PKD2 | 47 | 1 | 3.265957 | -0.575725 | 297 | 271.25 | 0.913300 |  |
| GO:0016570\_histone\_modification | HDAC2 | 47 | 1 | 3.265957 | -0.575725 | 297 | 271.25 | 0.913300 |  |
| GO:0030799\_regulation\_of\_cyclic\_nucleotide\_metabolic\_process | PKD2 | 47 | 1 | 3.265957 | -0.575725 | 297 | 271.25 | 0.913300 |  |
| GO:0031667\_response\_to\_nutrient\_levels | BECN1 | 47 | 1 | 3.265957 | -0.575725 | 297 | 271.25 | 0.913300 |  |
| GO:0034754\_cellular\_hormone\_metabolic\_process | RBP1 | 47 | 1 | 3.265957 | -0.575725 | 297 | 271.25 | 0.913300 |  |
| GO:0045619\_regulation\_of\_lymphocyte\_differentiation | CD1D1 | 47 | 1 | 3.265957 | -0.575725 | 297 | 271.25 | 0.913300 |  |
| GO:0008285\_negative\_regulation\_of\_cell\_proliferation | BECN1 | 155 | 2 | 1.980645 | -0.572138 | 298 | 271.55 | 0.911242 |  |
| GO:0008285\_negative\_regulation\_of\_cell\_proliferation | PTEN | 155 | 2 | 1.980645 | -0.572138 | 298 | 271.55 | 0.911242 |  |
| GO:0031326\_regulation\_of\_cellular\_biosynthetic\_process | TBL1XR1 | 812 | 7 | 1.323276 | -0.569896 | 299 | 271.99 | 0.909666 |  |
| GO:0031326\_regulation\_of\_cellular\_biosynthetic\_process | DNMT3A | 812 | 7 | 1.323276 | -0.569896 | 299 | 271.99 | 0.909666 |  |
| GO:0031326\_regulation\_of\_cellular\_biosynthetic\_process | HUS1 | 812 | 7 | 1.323276 | -0.569896 | 299 | 271.99 | 0.909666 |  |
| GO:0031326\_regulation\_of\_cellular\_biosynthetic\_process | TFDP2 | 812 | 7 | 1.323276 | -0.569896 | 299 | 271.99 | 0.909666 |  |
| GO:0031326\_regulation\_of\_cellular\_biosynthetic\_process | ZFP238 | 812 | 7 | 1.323276 | -0.569896 | 299 | 271.99 | 0.909666 |  |
| GO:0031326\_regulation\_of\_cellular\_biosynthetic\_process | CD1D1 | 812 | 7 | 1.323276 | -0.569896 | 299 | 271.99 | 0.909666 |  |
| GO:0031326\_regulation\_of\_cellular\_biosynthetic\_process | PKIA | 812 | 7 | 1.323276 | -0.569896 | 299 | 271.99 | 0.909666 |  |
| GO:0009101\_glycoprotein\_biosynthetic\_process | CHST11 | 48 | 1 | 3.197917 | -0.567894 | 301 | 273.89 | 0.909934 |  |
| GO:0034504\_protein\_localization\_in\_nucleus | PKIA | 48 | 1 | 3.197917 | -0.567894 | 301 | 273.89 | 0.909934 |  |
| GO:0009889\_regulation\_of\_biosynthetic\_process | TBL1XR1 | 815 | 7 | 1.318405 | -0.564707 | 302 | 274.63 | 0.909371 |  |
| GO:0009889\_regulation\_of\_biosynthetic\_process | DNMT3A | 815 | 7 | 1.318405 | -0.564707 | 302 | 274.63 | 0.909371 |  |
| GO:0009889\_regulation\_of\_biosynthetic\_process | HUS1 | 815 | 7 | 1.318405 | -0.564707 | 302 | 274.63 | 0.909371 |  |
| GO:0009889\_regulation\_of\_biosynthetic\_process | TFDP2 | 815 | 7 | 1.318405 | -0.564707 | 302 | 274.63 | 0.909371 |  |
| GO:0009889\_regulation\_of\_biosynthetic\_process | ZFP238 | 815 | 7 | 1.318405 | -0.564707 | 302 | 274.63 | 0.909371 |  |
| GO:0009889\_regulation\_of\_biosynthetic\_process | CD1D1 | 815 | 7 | 1.318405 | -0.564707 | 302 | 274.63 | 0.909371 |  |
| GO:0009889\_regulation\_of\_biosynthetic\_process | PKIA | 815 | 7 | 1.318405 | -0.564707 | 302 | 274.63 | 0.909371 |  |
| GO:0048514\_blood\_vessel\_morphogenesis | PTEN | 158 | 2 | 1.943038 | -0.560398 | 303 | 275.69 | 0.909868 |  |
| GO:0048514\_blood\_vessel\_morphogenesis | VASH1 | 158 | 2 | 1.943038 | -0.560398 | 303 | 275.69 | 0.909868 |  |
| GO:0021543\_pallium\_development | HDAC2 | 49 | 1 | 3.132653 | -0.560249 | 306 | 278.59 | 0.910425 |  |
| GO:0042035\_regulation\_of\_cytokine\_biosynthetic\_process | CD1D1 | 49 | 1 | 3.132653 | -0.560249 | 306 | 278.59 | 0.910425 |  |
| GO:0046661\_male\_sex\_differentiation | LGR4 | 49 | 1 | 3.132653 | -0.560249 | 306 | 278.59 | 0.910425 |  |
| GO:0007242\_intracellular\_signaling\_cascade | HUS1 | 411 | 4 | 1.493917 | -0.557400 | 307 | 278.86 | 0.908339 |  |
| GO:0007242\_intracellular\_signaling\_cascade | PKD2 | 411 | 4 | 1.493917 | -0.557400 | 307 | 278.86 | 0.908339 |  |
| GO:0007242\_intracellular\_signaling\_cascade | ARL6 | 411 | 4 | 1.493917 | -0.557400 | 307 | 278.86 | 0.908339 |  |
| GO:0007242\_intracellular\_signaling\_cascade | PTEN | 411 | 4 | 1.493917 | -0.557400 | 307 | 278.86 | 0.908339 |  |
| GO:0017038\_protein\_import | PKIA | 50 | 1 | 3.070000 | -0.552784 | 309 | 281.27 | 0.910259 |  |
| GO:0051606\_detection\_of\_stimulus | PKD2 | 50 | 1 | 3.070000 | -0.552784 | 309 | 281.27 | 0.910259 |  |
| GO:0032583\_regulation\_of\_gene-specific\_transcription | ZFP238 | 51 | 1 | 3.009804 | -0.545492 | 311 | 284.85 | 0.915916 |  |
| GO:0032880\_regulation\_of\_protein\_localization | PKIA | 51 | 1 | 3.009804 | -0.545492 | 311 | 284.85 | 0.915916 |  |
| GO:0007417\_central\_nervous\_system\_development | HDAC2 | 287 | 3 | 1.604530 | -0.542595 | 312 | 285.18 | 0.914038 |  |
| GO:0007417\_central\_nervous\_system\_development | TRP53BP2 | 287 | 3 | 1.604530 | -0.542595 | 312 | 285.18 | 0.914038 |  |
| GO:0007417\_central\_nervous\_system\_development | PTEN | 287 | 3 | 1.604530 | -0.542595 | 312 | 285.18 | 0.914038 |  |
| GO:0002706\_regulation\_of\_lymphocyte\_mediated\_immunity | CD1D1 | 52 | 1 | 2.951923 | -0.538366 | 313 | 287.18 | 0.917508 |  |
| GO:0006259\_DNA\_metabolic\_process | DNMT3A | 165 | 2 | 1.860606 | -0.534183 | 314 | 288.26 | 0.918025 |  |
| GO:0006259\_DNA\_metabolic\_process | HUS1 | 165 | 2 | 1.860606 | -0.534183 | 314 | 288.26 | 0.918025 |  |
| GO:0055085\_transmembrane\_transport | PKD2 | 53 | 1 | 2.896226 | -0.531399 | 315 | 290.41 | 0.921937 |  |
| GO:0051049\_regulation\_of\_transport | PKD2 | 167 | 2 | 1.838323 | -0.526980 | 316 | 291.54 | 0.922595 |  |
| GO:0051049\_regulation\_of\_transport | PKIA | 167 | 2 | 1.838323 | -0.526980 | 316 | 291.54 | 0.922595 |  |
| GO:0007265\_Ras\_protein\_signal\_transduction | ARL6 | 54 | 1 | 2.842593 | -0.524585 | 317 | 293.79 | 0.926782 |  |
| GO:0006915\_apoptosis | CHST11 | 427 | 4 | 1.437939 | -0.521200 | 318 | 293.89 | 0.924182 |  |
| GO:0006915\_apoptosis | CD1D1 | 427 | 4 | 1.437939 | -0.521200 | 318 | 293.89 | 0.924182 |  |
| GO:0006915\_apoptosis | CASP2 | 427 | 4 | 1.437939 | -0.521200 | 318 | 293.89 | 0.924182 |  |
| GO:0006915\_apoptosis | PTEN | 427 | 4 | 1.437939 | -0.521200 | 318 | 293.89 | 0.924182 |  |
| GO:0006310\_DNA\_recombination | HUS1 | 55 | 1 | 2.790909 | -0.517919 | 322 | 296.12 | 0.919627 |  |
| GO:0007126\_meiosis | PSMD13 | 55 | 1 | 2.790909 | -0.517919 | 322 | 296.12 | 0.919627 |  |
| GO:0048704\_embryonic\_skeletal\_system\_morphogenesis | CHST11 | 55 | 1 | 2.790909 | -0.517919 | 322 | 296.12 | 0.919627 |  |
| GO:0051327\_M\_phase\_of\_meiotic\_cell\_cycle | PSMD13 | 55 | 1 | 2.790909 | -0.517919 | 322 | 296.12 | 0.919627 |  |
| GO:0002703\_regulation\_of\_leukocyte\_mediated\_immunity | CD1D1 | 56 | 1 | 2.741071 | -0.511395 | 329 | 299.2 | 0.909422 |  |
| GO:0006790\_sulfur\_metabolic\_process | CHST11 | 56 | 1 | 2.741071 | -0.511395 | 329 | 299.2 | 0.909422 |  |
| GO:0009187\_cyclic\_nucleotide\_metabolic\_process | PKD2 | 56 | 1 | 2.741071 | -0.511395 | 329 | 299.2 | 0.909422 |  |
| GO:0042089\_cytokine\_biosynthetic\_process | CD1D1 | 56 | 1 | 2.741071 | -0.511395 | 329 | 299.2 | 0.909422 |  |
| GO:0042107\_cytokine\_metabolic\_process | CD1D1 | 56 | 1 | 2.741071 | -0.511395 | 329 | 299.2 | 0.909422 |  |
| GO:0050678\_regulation\_of\_epithelial\_cell\_proliferation | PTEN | 56 | 1 | 2.741071 | -0.511395 | 329 | 299.2 | 0.909422 |  |
| GO:0051321\_meiotic\_cell\_cycle | PSMD13 | 56 | 1 | 2.741071 | -0.511395 | 329 | 299.2 | 0.909422 |  |
| GO:0012501\_programmed\_cell\_death | CHST11 | 433 | 4 | 1.418014 | -0.508245 | 330 | 300.06 | 0.909273 |  |
| GO:0012501\_programmed\_cell\_death | CD1D1 | 433 | 4 | 1.418014 | -0.508245 | 330 | 300.06 | 0.909273 |  |
| GO:0012501\_programmed\_cell\_death | CASP2 | 433 | 4 | 1.418014 | -0.508245 | 330 | 300.06 | 0.909273 |  |
| GO:0012501\_programmed\_cell\_death | PTEN | 433 | 4 | 1.418014 | -0.508245 | 330 | 300.06 | 0.909273 |  |
| GO:0044248\_cellular\_catabolic\_process | TBL1XR1 | 173 | 2 | 1.774566 | -0.506094 | 331 | 300.78 | 0.908701 |  |
| GO:0044248\_cellular\_catabolic\_process | BECN1 | 173 | 2 | 1.774566 | -0.506094 | 331 | 300.78 | 0.908701 |  |
| GO:0000226\_microtubule\_cytoskeleton\_organization | UXT | 57 | 1 | 2.692982 | -0.505008 | 335 | 304.08 | 0.907701 |  |
| GO:0001892\_embryonic\_placenta\_development | PKD2 | 57 | 1 | 2.692982 | -0.505008 | 335 | 304.08 | 0.907701 |  |
| GO:0033365\_protein\_localization\_in\_organelle | PKIA | 57 | 1 | 2.692982 | -0.505008 | 335 | 304.08 | 0.907701 |  |
| GO:0045444\_fat\_cell\_differentiation | ARL6 | 57 | 1 | 2.692982 | -0.505008 | 335 | 304.08 | 0.907701 |  |
| GO:0006355\_regulation\_of\_transcription\_\_DNA-dependent | TBL1XR1 | 575 | 5 | 1.334783 | -0.497976 | 336 | 306.35 | 0.911756 |  |
| GO:0006355\_regulation\_of\_transcription\_\_DNA-dependent | DNMT3A | 575 | 5 | 1.334783 | -0.497976 | 336 | 306.35 | 0.911756 |  |
| GO:0006355\_regulation\_of\_transcription\_\_DNA-dependent | TFDP2 | 575 | 5 | 1.334783 | -0.497976 | 336 | 306.35 | 0.911756 |  |
| GO:0006355\_regulation\_of\_transcription\_\_DNA-dependent | ZFP238 | 575 | 5 | 1.334783 | -0.497976 | 336 | 306.35 | 0.911756 |  |
| GO:0006355\_regulation\_of\_transcription\_\_DNA-dependent | PKIA | 575 | 5 | 1.334783 | -0.497976 | 336 | 306.35 | 0.911756 |  |
| GO:0043066\_negative\_regulation\_of\_apoptosis | CHST11 | 176 | 2 | 1.744318 | -0.496038 | 337 | 307.16 | 0.911454 |  |
| GO:0043066\_negative\_regulation\_of\_apoptosis | PTEN | 176 | 2 | 1.744318 | -0.496038 | 337 | 307.16 | 0.911454 |  |
| GO:0050870\_positive\_regulation\_of\_T\_cell\_activation | CD1D1 | 59 | 1 | 2.601695 | -0.492628 | 338 | 309.42 | 0.915444 |  |
| GO:0051094\_positive\_regulation\_of\_developmental\_process | CD1D1 | 308 | 3 | 1.495130 | -0.488137 | 339 | 310.24 | 0.915162 |  |
| GO:0051094\_positive\_regulation\_of\_developmental\_process | PTEN | 308 | 3 | 1.495130 | -0.488137 | 339 | 310.24 | 0.915162 |  |
| GO:0051094\_positive\_regulation\_of\_developmental\_process | CASP2 | 308 | 3 | 1.495130 | -0.488137 | 339 | 310.24 | 0.915162 |  |
| GO:0009123\_nucleoside\_monophosphate\_metabolic\_process | PKD2 | 60 | 1 | 2.558333 | -0.486625 | 340 | 310.5 | 0.913235 |  |
| GO:0043069\_negative\_regulation\_of\_programmed\_cell\_death | CHST11 | 179 | 2 | 1.715084 | -0.486228 | 342 | 311.11 | 0.909678 |  |
| GO:0043069\_negative\_regulation\_of\_programmed\_cell\_death | PTEN | 179 | 2 | 1.715084 | -0.486228 | 342 | 311.11 | 0.909678 |  |
| GO:0060548\_negative\_regulation\_of\_cell\_death | CHST11 | 179 | 2 | 1.715084 | -0.486228 | 342 | 311.11 | 0.909678 |  |
| GO:0060548\_negative\_regulation\_of\_cell\_death | PTEN | 179 | 2 | 1.715084 | -0.486228 | 342 | 311.11 | 0.909678 |  |
| GO:0008219\_cell\_death | CHST11 | 444 | 4 | 1.382883 | -0.485326 | 343 | 311.67 | 0.908659 |  |
| GO:0008219\_cell\_death | CD1D1 | 444 | 4 | 1.382883 | -0.485326 | 343 | 311.67 | 0.908659 |  |
| GO:0008219\_cell\_death | CASP2 | 444 | 4 | 1.382883 | -0.485326 | 343 | 311.67 | 0.908659 |  |
| GO:0008219\_cell\_death | PTEN | 444 | 4 | 1.382883 | -0.485326 | 343 | 311.67 | 0.908659 |  |
| GO:0006874\_cellular\_calcium\_ion\_homeostasis | PKD2 | 61 | 1 | 2.516393 | -0.480741 | 345 | 313.31 | 0.908145 |  |
| GO:0009991\_response\_to\_extracellular\_stimulus | BECN1 | 61 | 1 | 2.516393 | -0.480741 | 345 | 313.31 | 0.908145 |  |
| GO:0021537\_telencephalon\_development | HDAC2 | 62 | 1 | 2.475806 | -0.474973 | 346 | 316.97 | 0.916098 |  |
| GO:0016265\_death | CHST11 | 450 | 4 | 1.364444 | -0.473263 | 347 | 317.63 | 0.915360 |  |
| GO:0016265\_death | CD1D1 | 450 | 4 | 1.364444 | -0.473263 | 347 | 317.63 | 0.915360 |  |
| GO:0016265\_death | CASP2 | 450 | 4 | 1.364444 | -0.473263 | 347 | 317.63 | 0.915360 |  |
| GO:0016265\_death | PTEN | 450 | 4 | 1.364444 | -0.473263 | 347 | 317.63 | 0.915360 |  |
| GO:0051252\_regulation\_of\_RNA\_metabolic\_process | TBL1XR1 | 590 | 5 | 1.300847 | -0.471115 | 348 | 317.79 | 0.913190 |  |
| GO:0051252\_regulation\_of\_RNA\_metabolic\_process | DNMT3A | 590 | 5 | 1.300847 | -0.471115 | 348 | 317.79 | 0.913190 |  |
| GO:0051252\_regulation\_of\_RNA\_metabolic\_process | TFDP2 | 590 | 5 | 1.300847 | -0.471115 | 348 | 317.79 | 0.913190 |  |
| GO:0051252\_regulation\_of\_RNA\_metabolic\_process | ZFP238 | 590 | 5 | 1.300847 | -0.471115 | 348 | 317.79 | 0.913190 |  |
| GO:0051252\_regulation\_of\_RNA\_metabolic\_process | PKIA | 590 | 5 | 1.300847 | -0.471115 | 348 | 317.79 | 0.913190 |  |
| GO:0051216\_cartilage\_development | CHST11 | 63 | 1 | 2.436508 | -0.469317 | 349 | 320.17 | 0.917393 |  |
| GO:0006351\_transcription\_\_DNA-dependent | TBL1XR1 | 594 | 5 | 1.292088 | -0.464185 | 350 | 321.51 | 0.918600 |  |
| GO:0006351\_transcription\_\_DNA-dependent | DNMT3A | 594 | 5 | 1.292088 | -0.464185 | 350 | 321.51 | 0.918600 |  |
| GO:0006351\_transcription\_\_DNA-dependent | TFDP2 | 594 | 5 | 1.292088 | -0.464185 | 350 | 321.51 | 0.918600 |  |
| GO:0006351\_transcription\_\_DNA-dependent | ZFP238 | 594 | 5 | 1.292088 | -0.464185 | 350 | 321.51 | 0.918600 |  |
| GO:0006351\_transcription\_\_DNA-dependent | PKIA | 594 | 5 | 1.292088 | -0.464185 | 350 | 321.51 | 0.918600 |  |
| GO:0006875\_cellular\_metal\_ion\_homeostasis | PKD2 | 64 | 1 | 2.398438 | -0.463769 | 352 | 323.13 | 0.917983 |  |
| GO:0055074\_calcium\_ion\_homeostasis | PKD2 | 64 | 1 | 2.398438 | -0.463769 | 352 | 323.13 | 0.917983 |  |
| GO:0032774\_RNA\_biosynthetic\_process | TBL1XR1 | 595 | 5 | 1.289916 | -0.462467 | 353 | 323.5 | 0.916431 |  |
| GO:0032774\_RNA\_biosynthetic\_process | DNMT3A | 595 | 5 | 1.289916 | -0.462467 | 353 | 323.5 | 0.916431 |  |
| GO:0032774\_RNA\_biosynthetic\_process | TFDP2 | 595 | 5 | 1.289916 | -0.462467 | 353 | 323.5 | 0.916431 |  |
| GO:0032774\_RNA\_biosynthetic\_process | ZFP238 | 595 | 5 | 1.289916 | -0.462467 | 353 | 323.5 | 0.916431 |  |
| GO:0032774\_RNA\_biosynthetic\_process | PKIA | 595 | 5 | 1.289916 | -0.462467 | 353 | 323.5 | 0.916431 |  |
| GO:0043086\_negative\_regulation\_of\_catalytic\_activity | PKIA | 65 | 1 | 2.361538 | -0.458325 | 354 | 324.7 | 0.917232 |  |
| GO:0043412\_biopolymer\_modification | DNMT3A | 458 | 4 | 1.340611 | -0.457639 | 355 | 325.37 | 0.916535 |  |
| GO:0043412\_biopolymer\_modification | HDAC2 | 458 | 4 | 1.340611 | -0.457639 | 355 | 325.37 | 0.916535 |  |
| GO:0043412\_biopolymer\_modification | HUS1 | 458 | 4 | 1.340611 | -0.457639 | 355 | 325.37 | 0.916535 |  |
| GO:0043412\_biopolymer\_modification | ARL6 | 458 | 4 | 1.340611 | -0.457639 | 355 | 325.37 | 0.916535 |  |
| GO:0009058\_biosynthetic\_process | TBL1XR1 | 1175 | 9 | 1.175745 | -0.455408 | 356 | 325.46 | 0.914213 |  |
| GO:0009058\_biosynthetic\_process | DNMT3A | 1175 | 9 | 1.175745 | -0.455408 | 356 | 325.46 | 0.914213 |  |
| GO:0009058\_biosynthetic\_process | B3GALNT1 | 1175 | 9 | 1.175745 | -0.455408 | 356 | 325.46 | 0.914213 |  |
| GO:0009058\_biosynthetic\_process | CHST11 | 1175 | 9 | 1.175745 | -0.455408 | 356 | 325.46 | 0.914213 |  |
| GO:0009058\_biosynthetic\_process | HUS1 | 1175 | 9 | 1.175745 | -0.455408 | 356 | 325.46 | 0.914213 |  |
| GO:0009058\_biosynthetic\_process | TFDP2 | 1175 | 9 | 1.175745 | -0.455408 | 356 | 325.46 | 0.914213 |  |
| GO:0009058\_biosynthetic\_process | ZFP238 | 1175 | 9 | 1.175745 | -0.455408 | 356 | 325.46 | 0.914213 |  |
| GO:0009058\_biosynthetic\_process | CD1D1 | 1175 | 9 | 1.175745 | -0.455408 | 356 | 325.46 | 0.914213 |  |
| GO:0009058\_biosynthetic\_process | PKIA | 1175 | 9 | 1.175745 | -0.455408 | 356 | 325.46 | 0.914213 |  |
| GO:0007179\_transforming\_growth\_factor\_beta\_receptor\_signaling\_pathway | CHST11 | 66 | 1 | 2.325758 | -0.452983 | 357 | 327.48 | 0.917311 |  |
| GO:0009791\_post-embryonic\_development | CHST11 | 67 | 1 | 2.291045 | -0.447740 | 359 | 329.35 | 0.917409 |  |
| GO:0042445\_hormone\_metabolic\_process | RBP1 | 67 | 1 | 2.291045 | -0.447740 | 359 | 329.35 | 0.917409 |  |
| GO:0002697\_regulation\_of\_immune\_effector\_process | CD1D1 | 68 | 1 | 2.257353 | -0.442592 | 361 | 330.44 | 0.915346 |  |
| GO:0034962\_cellular\_biopolymer\_catabolic\_process | TBL1XR1 | 68 | 1 | 2.257353 | -0.442592 | 361 | 330.44 | 0.915346 |  |
| GO:0006816\_calcium\_ion\_transport | PKD2 | 69 | 1 | 2.224638 | -0.437536 | 364 | 333.22 | 0.915440 |  |
| GO:0032101\_regulation\_of\_response\_to\_external\_stimulus | BECN1 | 69 | 1 | 2.224638 | -0.437536 | 364 | 333.22 | 0.915440 |  |
| GO:0055065\_metal\_ion\_homeostasis | PKD2 | 69 | 1 | 2.224638 | -0.437536 | 364 | 333.22 | 0.915440 |  |
| GO:0010646\_regulation\_of\_cell\_communication | BECN1 | 330 | 3 | 1.395455 | -0.437362 | 365 | 333.69 | 0.914219 |  |
| GO:0010646\_regulation\_of\_cell\_communication | CHST11 | 330 | 3 | 1.395455 | -0.437362 | 365 | 333.69 | 0.914219 |  |
| GO:0010646\_regulation\_of\_cell\_communication | PTEN | 330 | 3 | 1.395455 | -0.437362 | 365 | 333.69 | 0.914219 |  |
| GO:0051093\_negative\_regulation\_of\_developmental\_process | CHST11 | 331 | 3 | 1.391239 | -0.435192 | 366 | 334.05 | 0.912705 |  |
| GO:0051093\_negative\_regulation\_of\_developmental\_process | PTEN | 331 | 3 | 1.391239 | -0.435192 | 366 | 334.05 | 0.912705 |  |
| GO:0051093\_negative\_regulation\_of\_developmental\_process | VASH1 | 331 | 3 | 1.391239 | -0.435192 | 366 | 334.05 | 0.912705 |  |
| GO:0033554\_cellular\_response\_to\_stress | BECN1 | 196 | 2 | 1.566327 | -0.434906 | 367 | 334.3 | 0.910899 |  |
| GO:0033554\_cellular\_response\_to\_stress | HUS1 | 196 | 2 | 1.566327 | -0.434906 | 367 | 334.3 | 0.910899 |  |
| GO:0070838\_divalent\_metal\_ion\_transport | PKD2 | 70 | 1 | 2.192857 | -0.432571 | 368 | 336.13 | 0.913397 |  |
| GO:0006281\_DNA\_repair | HUS1 | 71 | 1 | 2.161972 | -0.427693 | 371 | 339.11 | 0.914043 |  |
| GO:0006913\_nucleocytoplasmic\_transport | PKIA | 71 | 1 | 2.161972 | -0.427693 | 371 | 339.11 | 0.914043 |  |
| GO:0009100\_glycoprotein\_metabolic\_process | CHST11 | 71 | 1 | 2.161972 | -0.427693 | 371 | 339.11 | 0.914043 |  |
| GO:0007264\_small\_GTPase\_mediated\_signal\_transduction | ARL6 | 72 | 1 | 2.131944 | -0.422900 | 373 | 342.84 | 0.919142 |  |
| GO:0051169\_nuclear\_transport | PKIA | 72 | 1 | 2.131944 | -0.422900 | 373 | 342.84 | 0.919142 |  |
| GO:0006163\_purine\_nucleotide\_metabolic\_process | PKD2 | 73 | 1 | 2.102740 | -0.418189 | 375 | 344.93 | 0.919813 |  |
| GO:0048706\_embryonic\_skeletal\_system\_development | CHST11 | 73 | 1 | 2.102740 | -0.418189 | 375 | 344.93 | 0.919813 |  |
| GO:0009416\_response\_to\_light\_stimulus | HUS1 | 74 | 1 | 2.074324 | -0.413558 | 376 | 346.48 | 0.921489 |  |
| GO:0007165\_signal\_transduction | TXNIP | 915 | 7 | 1.174317 | -0.413546 | 377 | 346.64 | 0.919469 |  |
| GO:0007165\_signal\_transduction | ZC3H15 | 915 | 7 | 1.174317 | -0.413546 | 377 | 346.64 | 0.919469 |  |
| GO:0007165\_signal\_transduction | HUS1 | 915 | 7 | 1.174317 | -0.413546 | 377 | 346.64 | 0.919469 |  |
| GO:0007165\_signal\_transduction | CHST11 | 915 | 7 | 1.174317 | -0.413546 | 377 | 346.64 | 0.919469 |  |
| GO:0007165\_signal\_transduction | PKD2 | 915 | 7 | 1.174317 | -0.413546 | 377 | 346.64 | 0.919469 |  |
| GO:0007165\_signal\_transduction | ARL6 | 915 | 7 | 1.174317 | -0.413546 | 377 | 346.64 | 0.919469 |  |
| GO:0007165\_signal\_transduction | PTEN | 915 | 7 | 1.174317 | -0.413546 | 377 | 346.64 | 0.919469 |  |
| GO:0007243\_protein\_kinase\_cascade | PKD2 | 205 | 2 | 1.497561 | -0.410375 | 378 | 347.72 | 0.919894 |  |
| GO:0007243\_protein\_kinase\_cascade | PTEN | 205 | 2 | 1.497561 | -0.410375 | 378 | 347.72 | 0.919894 |  |
| GO:0044265\_cellular\_macromolecule\_catabolic\_process | TBL1XR1 | 75 | 1 | 2.046667 | -0.409006 | 379 | 348.9 | 0.920580 |  |
| GO:0006508\_proteolysis | TBL1XR1 | 76 | 1 | 2.019737 | -0.404529 | 380 | 350.39 | 0.922079 |  |
| GO:0001890\_placenta\_development | PKD2 | 77 | 1 | 1.993506 | -0.400127 | 381 | 352.11 | 0.924173 |  |
| GO:0080090\_regulation\_of\_primary\_metabolic\_process | TBL1XR1 | 926 | 7 | 1.160367 | -0.399267 | 382 | 352.44 | 0.922618 |  |
| GO:0080090\_regulation\_of\_primary\_metabolic\_process | DNMT3A | 926 | 7 | 1.160367 | -0.399267 | 382 | 352.44 | 0.922618 |  |
| GO:0080090\_regulation\_of\_primary\_metabolic\_process | HUS1 | 926 | 7 | 1.160367 | -0.399267 | 382 | 352.44 | 0.922618 |  |
| GO:0080090\_regulation\_of\_primary\_metabolic\_process | TFDP2 | 926 | 7 | 1.160367 | -0.399267 | 382 | 352.44 | 0.922618 |  |
| GO:0080090\_regulation\_of\_primary\_metabolic\_process | ZFP238 | 926 | 7 | 1.160367 | -0.399267 | 382 | 352.44 | 0.922618 |  |
| GO:0080090\_regulation\_of\_primary\_metabolic\_process | PKD2 | 926 | 7 | 1.160367 | -0.399267 | 382 | 352.44 | 0.922618 |  |
| GO:0080090\_regulation\_of\_primary\_metabolic\_process | PKIA | 926 | 7 | 1.160367 | -0.399267 | 382 | 352.44 | 0.922618 |  |
| GO:0051251\_positive\_regulation\_of\_lymphocyte\_activation | CD1D1 | 78 | 1 | 1.967949 | -0.395796 | 383 | 354.41 | 0.925352 |  |
| GO:0015674\_di-\_\_tri-valent\_inorganic\_cation\_transport | PKD2 | 79 | 1 | 1.943038 | -0.391535 | 384 | 355.78 | 0.926510 |  |
| GO:0002250\_adaptive\_immune\_response | CD1D1 | 80 | 1 | 1.918750 | -0.387343 | 386 | 357.34 | 0.925751 |  |
| GO:0002460\_adaptive\_immune\_response\_based\_on\_somatic\_recombination\_of\_immune\_receptors\_built\_from\_immunoglobulin\_superfamily\_domains | CD1D1 | 80 | 1 | 1.918750 | -0.387343 | 386 | 357.34 | 0.925751 |  |
| GO:0060255\_regulation\_of\_macromolecule\_metabolic\_process | TBL1XR1 | 936 | 7 | 1.147970 | -0.386651 | 387 | 357.47 | 0.923695 |  |
| GO:0060255\_regulation\_of\_macromolecule\_metabolic\_process | DNMT3A | 936 | 7 | 1.147970 | -0.386651 | 387 | 357.47 | 0.923695 |  |
| GO:0060255\_regulation\_of\_macromolecule\_metabolic\_process | HUS1 | 936 | 7 | 1.147970 | -0.386651 | 387 | 357.47 | 0.923695 |  |
| GO:0060255\_regulation\_of\_macromolecule\_metabolic\_process | TFDP2 | 936 | 7 | 1.147970 | -0.386651 | 387 | 357.47 | 0.923695 |  |
| GO:0060255\_regulation\_of\_macromolecule\_metabolic\_process | ZFP238 | 936 | 7 | 1.147970 | -0.386651 | 387 | 357.47 | 0.923695 |  |
| GO:0060255\_regulation\_of\_macromolecule\_metabolic\_process | CD1D1 | 936 | 7 | 1.147970 | -0.386651 | 387 | 357.47 | 0.923695 |  |
| GO:0060255\_regulation\_of\_macromolecule\_metabolic\_process | PKIA | 936 | 7 | 1.147970 | -0.386651 | 387 | 357.47 | 0.923695 |  |
| GO:0021700\_developmental\_maturation | RECK | 81 | 1 | 1.895062 | -0.383217 | 388 | 358.15 | 0.923067 |  |
| GO:0040007\_growth | CHST11 | 217 | 2 | 1.414747 | -0.380152 | 390 | 359.07 | 0.920692 |  |
| GO:0040007\_growth | PTEN | 217 | 2 | 1.414747 | -0.380152 | 390 | 359.07 | 0.920692 |  |
| GO:0048583\_regulation\_of\_response\_to\_stimulus | BECN1 | 217 | 2 | 1.414747 | -0.380152 | 390 | 359.07 | 0.920692 |  |
| GO:0048583\_regulation\_of\_response\_to\_stimulus | CD1D1 | 217 | 2 | 1.414747 | -0.380152 | 390 | 359.07 | 0.920692 |  |
| GO:0002696\_positive\_regulation\_of\_leukocyte\_activation | CD1D1 | 82 | 1 | 1.871951 | -0.379156 | 392 | 360.74 | 0.920255 |  |
| GO:0010627\_regulation\_of\_protein\_kinase\_cascade | PTEN | 82 | 1 | 1.871951 | -0.379156 | 392 | 360.74 | 0.920255 |  |
| GO:0048856\_anatomical\_structure\_development | RECK | 1688 | 12 | 1.091232 | -0.378845 | 393 | 360.94 | 0.918422 |  |
| GO:0048856\_anatomical\_structure\_development | ANKRD17 | 1688 | 12 | 1.091232 | -0.378845 | 393 | 360.94 | 0.918422 |  |
| GO:0048856\_anatomical\_structure\_development | HDAC2 | 1688 | 12 | 1.091232 | -0.378845 | 393 | 360.94 | 0.918422 |  |
| GO:0048856\_anatomical\_structure\_development | FGF15 | 1688 | 12 | 1.091232 | -0.378845 | 393 | 360.94 | 0.918422 |  |
| GO:0048856\_anatomical\_structure\_development | TRP53BP2 | 1688 | 12 | 1.091232 | -0.378845 | 393 | 360.94 | 0.918422 |  |
| GO:0048856\_anatomical\_structure\_development | CHST11 | 1688 | 12 | 1.091232 | -0.378845 | 393 | 360.94 | 0.918422 |  |
| GO:0048856\_anatomical\_structure\_development | PKD2 | 1688 | 12 | 1.091232 | -0.378845 | 393 | 360.94 | 0.918422 |  |
| GO:0048856\_anatomical\_structure\_development | FBN2 | 1688 | 12 | 1.091232 | -0.378845 | 393 | 360.94 | 0.918422 |  |
| GO:0048856\_anatomical\_structure\_development | CD1D1 | 1688 | 12 | 1.091232 | -0.378845 | 393 | 360.94 | 0.918422 |  |
| GO:0048856\_anatomical\_structure\_development | PTEN | 1688 | 12 | 1.091232 | -0.378845 | 393 | 360.94 | 0.918422 |  |
| GO:0048856\_anatomical\_structure\_development | LGR4 | 1688 | 12 | 1.091232 | -0.378845 | 393 | 360.94 | 0.918422 |  |
| GO:0048856\_anatomical\_structure\_development | VASH1 | 1688 | 12 | 1.091232 | -0.378845 | 393 | 360.94 | 0.918422 |  |
| GO:0007017\_microtubule-based\_process | UXT | 83 | 1 | 1.849398 | -0.375159 | 396 | 363.87 | 0.918864 |  |
| GO:0030198\_extracellular\_matrix\_organization | RECK | 83 | 1 | 1.849398 | -0.375159 | 396 | 363.87 | 0.918864 |  |
| GO:0050867\_positive\_regulation\_of\_cell\_activation | CD1D1 | 83 | 1 | 1.849398 | -0.375159 | 396 | 363.87 | 0.918864 |  |
| GO:0007154\_cell\_communication | TXNIP | 1096 | 8 | 1.120438 | -0.372402 | 397 | 364.5 | 0.918136 |  |
| GO:0007154\_cell\_communication | ZC3H15 | 1096 | 8 | 1.120438 | -0.372402 | 397 | 364.5 | 0.918136 |  |
| GO:0007154\_cell\_communication | BECN1 | 1096 | 8 | 1.120438 | -0.372402 | 397 | 364.5 | 0.918136 |  |
| GO:0007154\_cell\_communication | HUS1 | 1096 | 8 | 1.120438 | -0.372402 | 397 | 364.5 | 0.918136 |  |
| GO:0007154\_cell\_communication | CHST11 | 1096 | 8 | 1.120438 | -0.372402 | 397 | 364.5 | 0.918136 |  |
| GO:0007154\_cell\_communication | PKD2 | 1096 | 8 | 1.120438 | -0.372402 | 397 | 364.5 | 0.918136 |  |
| GO:0007154\_cell\_communication | ARL6 | 1096 | 8 | 1.120438 | -0.372402 | 397 | 364.5 | 0.918136 |  |
| GO:0007154\_cell\_communication | PTEN | 1096 | 8 | 1.120438 | -0.372402 | 397 | 364.5 | 0.918136 |  |
| GO:0030005\_cellular\_di-\_\_tri-valent\_inorganic\_cation\_homeostasis | PKD2 | 84 | 1 | 1.827381 | -0.371223 | 399 | 365.3 | 0.915539 |  |
| GO:0045137\_development\_of\_primary\_sexual\_characteristics | LGR4 | 84 | 1 | 1.827381 | -0.371223 | 399 | 365.3 | 0.915539 |  |
| GO:0001701\_in\_utero\_embryonic\_development | PRMT1 | 221 | 2 | 1.389140 | -0.370658 | 400 | 365.65 | 0.914125 |  |
| GO:0001701\_in\_utero\_embryonic\_development | PKD2 | 221 | 2 | 1.389140 | -0.370658 | 400 | 365.65 | 0.914125 |  |
| GO:0043009\_chordate\_embryonic\_development | PRMT1 | 365 | 3 | 1.261644 | -0.367742 | 401 | 366.48 | 0.913915 |  |
| GO:0043009\_chordate\_embryonic\_development | CHST11 | 365 | 3 | 1.261644 | -0.367742 | 401 | 366.48 | 0.913915 |  |
| GO:0043009\_chordate\_embryonic\_development | PKD2 | 365 | 3 | 1.261644 | -0.367742 | 401 | 366.48 | 0.913915 |  |
| GO:0000279\_M\_phase | PSMD13 | 85 | 1 | 1.805882 | -0.367347 | 403 | 367.43 | 0.911737 |  |
| GO:0002449\_lymphocyte\_mediated\_immunity | CD1D1 | 85 | 1 | 1.805882 | -0.367347 | 403 | 367.43 | 0.911737 |  |
| GO:0016070\_RNA\_metabolic\_process | TBL1XR1 | 658 | 5 | 1.166413 | -0.365363 | 404 | 367.82 | 0.910446 |  |
| GO:0016070\_RNA\_metabolic\_process | DNMT3A | 658 | 5 | 1.166413 | -0.365363 | 404 | 367.82 | 0.910446 |  |
| GO:0016070\_RNA\_metabolic\_process | TFDP2 | 658 | 5 | 1.166413 | -0.365363 | 404 | 367.82 | 0.910446 |  |
| GO:0016070\_RNA\_metabolic\_process | ZFP238 | 658 | 5 | 1.166413 | -0.365363 | 404 | 367.82 | 0.910446 |  |
| GO:0016070\_RNA\_metabolic\_process | PKIA | 658 | 5 | 1.166413 | -0.365363 | 404 | 367.82 | 0.910446 |  |
| GO:0006605\_protein\_targeting | PKIA | 86 | 1 | 1.784884 | -0.363530 | 405 | 369.34 | 0.911951 |  |
| GO:0050896\_response\_to\_stimulus | TXNIP | 1107 | 8 | 1.109304 | -0.360007 | 406 | 370.03 | 0.911404 |  |
| GO:0050896\_response\_to\_stimulus | TRP53BP2 | 1107 | 8 | 1.109304 | -0.360007 | 406 | 370.03 | 0.911404 |  |
| GO:0050896\_response\_to\_stimulus | BECN1 | 1107 | 8 | 1.109304 | -0.360007 | 406 | 370.03 | 0.911404 |  |
| GO:0050896\_response\_to\_stimulus | HUS1 | 1107 | 8 | 1.109304 | -0.360007 | 406 | 370.03 | 0.911404 |  |
| GO:0050896\_response\_to\_stimulus | PKD2 | 1107 | 8 | 1.109304 | -0.360007 | 406 | 370.03 | 0.911404 |  |
| GO:0050896\_response\_to\_stimulus | CD1D1 | 1107 | 8 | 1.109304 | -0.360007 | 406 | 370.03 | 0.911404 |  |
| GO:0050896\_response\_to\_stimulus | VASH1 | 1107 | 8 | 1.109304 | -0.360007 | 406 | 370.03 | 0.911404 |  |
| GO:0050896\_response\_to\_stimulus | RAD17 | 1107 | 8 | 1.109304 | -0.360007 | 406 | 370.03 | 0.911404 |  |
| GO:0001822\_kidney\_development | PKD2 | 87 | 1 | 1.764368 | -0.359771 | 409 | 372.43 | 0.910587 |  |
| GO:0007178\_transmembrane\_receptor\_protein\_serine\_threonine\_kinase\_signaling\_pathway | CHST11 | 87 | 1 | 1.764368 | -0.359771 | 409 | 372.43 | 0.910587 |  |
| GO:0050778\_positive\_regulation\_of\_immune\_response | CD1D1 | 87 | 1 | 1.764368 | -0.359771 | 409 | 372.43 | 0.910587 |  |
| GO:0050863\_regulation\_of\_T\_cell\_activation | CD1D1 | 88 | 1 | 1.744318 | -0.356068 | 410 | 374.11 | 0.912463 |  |
| GO:0007167\_enzyme\_linked\_receptor\_protein\_signaling\_pathway | TXNIP | 229 | 2 | 1.340611 | -0.352475 | 411 | 375.73 | 0.914185 |  |
| GO:0007167\_enzyme\_linked\_receptor\_protein\_signaling\_pathway | CHST11 | 229 | 2 | 1.340611 | -0.352475 | 411 | 375.73 | 0.914185 |  |
| GO:0030003\_cellular\_cation\_homeostasis | PKD2 | 90 | 1 | 1.705556 | -0.348825 | 412 | 377.08 | 0.915243 |  |
| GO:0022414\_reproductive\_process | DNMT3A | 376 | 3 | 1.224734 | -0.348314 | 413 | 377.22 | 0.913366 |  |
| GO:0022414\_reproductive\_process | PTEN | 376 | 3 | 1.224734 | -0.348314 | 413 | 377.22 | 0.913366 |  |
| GO:0022414\_reproductive\_process | LGR4 | 376 | 3 | 1.224734 | -0.348314 | 413 | 377.22 | 0.913366 |  |
| GO:0002443\_leukocyte\_mediated\_immunity | CD1D1 | 91 | 1 | 1.686813 | -0.345282 | 414 | 378.52 | 0.914300 |  |
| GO:0009888\_tissue\_development | ANKRD17 | 525 | 4 | 1.169524 | -0.345097 | 415 | 378.7 | 0.912530 |  |
| GO:0009888\_tissue\_development | FGF15 | 525 | 4 | 1.169524 | -0.345097 | 415 | 378.7 | 0.912530 |  |
| GO:0009888\_tissue\_development | CHST11 | 525 | 4 | 1.169524 | -0.345097 | 415 | 378.7 | 0.912530 |  |
| GO:0009888\_tissue\_development | PTEN | 525 | 4 | 1.169524 | -0.345097 | 415 | 378.7 | 0.912530 |  |
| GO:0000003\_reproduction | DNMT3A | 379 | 3 | 1.215040 | -0.343200 | 416 | 379.3 | 0.911779 |  |
| GO:0000003\_reproduction | PTEN | 379 | 3 | 1.215040 | -0.343200 | 416 | 379.3 | 0.911779 |  |
| GO:0000003\_reproduction | LGR4 | 379 | 3 | 1.215040 | -0.343200 | 416 | 379.3 | 0.911779 |  |
| GO:0030217\_T\_cell\_differentiation | CD1D1 | 92 | 1 | 1.668478 | -0.341790 | 417 | 380.36 | 0.912134 |  |
| GO:0016477\_cell\_migration | FGF15 | 234 | 2 | 1.311966 | -0.341627 | 418 | 380.6 | 0.910526 |  |
| GO:0016477\_cell\_migration | PTEN | 234 | 2 | 1.311966 | -0.341627 | 418 | 380.6 | 0.910526 |  |
| GO:0045449\_regulation\_of\_transcription | TBL1XR1 | 676 | 5 | 1.135355 | -0.341268 | 419 | 380.77 | 0.908759 |  |
| GO:0045449\_regulation\_of\_transcription | DNMT3A | 676 | 5 | 1.135355 | -0.341268 | 419 | 380.77 | 0.908759 |  |
| GO:0045449\_regulation\_of\_transcription | TFDP2 | 676 | 5 | 1.135355 | -0.341268 | 419 | 380.77 | 0.908759 |  |
| GO:0045449\_regulation\_of\_transcription | ZFP238 | 676 | 5 | 1.135355 | -0.341268 | 419 | 380.77 | 0.908759 |  |
| GO:0045449\_regulation\_of\_transcription | PKIA | 676 | 5 | 1.135355 | -0.341268 | 419 | 380.77 | 0.908759 |  |
| GO:0032501\_multicellular\_organismal\_process | RECK | 2183 | 15 | 1.054741 | -0.338964 | 420 | 381.04 | 0.907238 |  |
| GO:0032501\_multicellular\_organismal\_process | DNMT3A | 2183 | 15 | 1.054741 | -0.338964 | 420 | 381.04 | 0.907238 |  |
| GO:0032501\_multicellular\_organismal\_process | FGF15 | 2183 | 15 | 1.054741 | -0.338964 | 420 | 381.04 | 0.907238 |  |
| GO:0032501\_multicellular\_organismal\_process | HUS1 | 2183 | 15 | 1.054741 | -0.338964 | 420 | 381.04 | 0.907238 |  |
| GO:0032501\_multicellular\_organismal\_process | CD1D1 | 2183 | 15 | 1.054741 | -0.338964 | 420 | 381.04 | 0.907238 |  |
| GO:0032501\_multicellular\_organismal\_process | PTEN | 2183 | 15 | 1.054741 | -0.338964 | 420 | 381.04 | 0.907238 |  |
| GO:0032501\_multicellular\_organismal\_process | LGR4 | 2183 | 15 | 1.054741 | -0.338964 | 420 | 381.04 | 0.907238 |  |
| GO:0032501\_multicellular\_organismal\_process | VASH1 | 2183 | 15 | 1.054741 | -0.338964 | 420 | 381.04 | 0.907238 |  |
| GO:0032501\_multicellular\_organismal\_process | ANKRD17 | 2183 | 15 | 1.054741 | -0.338964 | 420 | 381.04 | 0.907238 |  |
| GO:0032501\_multicellular\_organismal\_process | PRMT1 | 2183 | 15 | 1.054741 | -0.338964 | 420 | 381.04 | 0.907238 |  |
| GO:0032501\_multicellular\_organismal\_process | HDAC2 | 2183 | 15 | 1.054741 | -0.338964 | 420 | 381.04 | 0.907238 |  |
| GO:0032501\_multicellular\_organismal\_process | TRP53BP2 | 2183 | 15 | 1.054741 | -0.338964 | 420 | 381.04 | 0.907238 |  |
| GO:0032501\_multicellular\_organismal\_process | CHST11 | 2183 | 15 | 1.054741 | -0.338964 | 420 | 381.04 | 0.907238 |  |
| GO:0032501\_multicellular\_organismal\_process | PKD2 | 2183 | 15 | 1.054741 | -0.338964 | 420 | 381.04 | 0.907238 |  |
| GO:0032501\_multicellular\_organismal\_process | FBN2 | 2183 | 15 | 1.054741 | -0.338964 | 420 | 381.04 | 0.907238 |  |
| GO:0055066\_di-\_\_tri-valent\_inorganic\_cation\_homeostasis | PKD2 | 93 | 1 | 1.650538 | -0.338349 | 421 | 382.35 | 0.908195 |  |
| GO:0006753\_nucleoside\_phosphate\_metabolic\_process | PKD2 | 94 | 1 | 1.632979 | -0.334957 | 424 | 385.36 | 0.908868 |  |
| GO:0009117\_nucleotide\_metabolic\_process | PKD2 | 94 | 1 | 1.632979 | -0.334957 | 424 | 385.36 | 0.908868 |  |
| GO:0034984\_cellular\_response\_to\_DNA\_damage\_stimulus | HUS1 | 94 | 1 | 1.632979 | -0.334957 | 424 | 385.36 | 0.908868 |  |
| GO:0043687\_post-translational\_protein\_modification | HDAC2 | 384 | 3 | 1.199219 | -0.334845 | 425 | 385.61 | 0.907318 |  |
| GO:0043687\_post-translational\_protein\_modification | HUS1 | 384 | 3 | 1.199219 | -0.334845 | 425 | 385.61 | 0.907318 |  |
| GO:0043687\_post-translational\_protein\_modification | ARL6 | 384 | 3 | 1.199219 | -0.334845 | 425 | 385.61 | 0.907318 |  |
| GO:0050794\_regulation\_of\_cellular\_process | TXNIP | 2190 | 15 | 1.051370 | -0.332660 | 426 | 386.08 | 0.906291 |  |
| GO:0050794\_regulation\_of\_cellular\_process | TBL1XR1 | 2190 | 15 | 1.051370 | -0.332660 | 426 | 386.08 | 0.906291 |  |
| GO:0050794\_regulation\_of\_cellular\_process | DNMT3A | 2190 | 15 | 1.051370 | -0.332660 | 426 | 386.08 | 0.906291 |  |
| GO:0050794\_regulation\_of\_cellular\_process | ZC3H15 | 2190 | 15 | 1.051370 | -0.332660 | 426 | 386.08 | 0.906291 |  |
| GO:0050794\_regulation\_of\_cellular\_process | BECN1 | 2190 | 15 | 1.051370 | -0.332660 | 426 | 386.08 | 0.906291 |  |
| GO:0050794\_regulation\_of\_cellular\_process | HUS1 | 2190 | 15 | 1.051370 | -0.332660 | 426 | 386.08 | 0.906291 |  |
| GO:0050794\_regulation\_of\_cellular\_process | ARL6 | 2190 | 15 | 1.051370 | -0.332660 | 426 | 386.08 | 0.906291 |  |
| GO:0050794\_regulation\_of\_cellular\_process | CD1D1 | 2190 | 15 | 1.051370 | -0.332660 | 426 | 386.08 | 0.906291 |  |
| GO:0050794\_regulation\_of\_cellular\_process | PKIA | 2190 | 15 | 1.051370 | -0.332660 | 426 | 386.08 | 0.906291 |  |
| GO:0050794\_regulation\_of\_cellular\_process | PTEN | 2190 | 15 | 1.051370 | -0.332660 | 426 | 386.08 | 0.906291 |  |
| GO:0050794\_regulation\_of\_cellular\_process | CHST11 | 2190 | 15 | 1.051370 | -0.332660 | 426 | 386.08 | 0.906291 |  |
| GO:0050794\_regulation\_of\_cellular\_process | TFDP2 | 2190 | 15 | 1.051370 | -0.332660 | 426 | 386.08 | 0.906291 |  |
| GO:0050794\_regulation\_of\_cellular\_process | PKD2 | 2190 | 15 | 1.051370 | -0.332660 | 426 | 386.08 | 0.906291 |  |
| GO:0050794\_regulation\_of\_cellular\_process | ZFP238 | 2190 | 15 | 1.051370 | -0.332660 | 426 | 386.08 | 0.906291 |  |
| GO:0050794\_regulation\_of\_cellular\_process | CASP2 | 2190 | 15 | 1.051370 | -0.332660 | 426 | 386.08 | 0.906291 |  |
| GO:0051707\_response\_to\_other\_organism | BECN1 | 95 | 1 | 1.615789 | -0.331612 | 427 | 386.84 | 0.905948 |  |
| GO:0060341\_regulation\_of\_cellular\_localization | PKIA | 97 | 1 | 1.582474 | -0.325062 | 428 | 390.09 | 0.911425 |  |
| GO:0009056\_catabolic\_process | TBL1XR1 | 243 | 2 | 1.263374 | -0.323036 | 429 | 390.34 | 0.909883 |  |
| GO:0009056\_catabolic\_process | BECN1 | 243 | 2 | 1.263374 | -0.323036 | 429 | 390.34 | 0.909883 |  |
| GO:0007548\_sex\_differentiation | LGR4 | 98 | 1 | 1.566327 | -0.321855 | 430 | 391.76 | 0.911070 |  |
| GO:0042127\_regulation\_of\_cell\_proliferation | BECN1 | 393 | 3 | 1.171756 | -0.320321 | 431 | 391.93 | 0.909350 |  |
| GO:0042127\_regulation\_of\_cell\_proliferation | CHST11 | 393 | 3 | 1.171756 | -0.320321 | 431 | 391.93 | 0.909350 |  |
| GO:0042127\_regulation\_of\_cell\_proliferation | PTEN | 393 | 3 | 1.171756 | -0.320321 | 431 | 391.93 | 0.909350 |  |
| GO:0044260\_cellular\_macromolecule\_metabolic\_process | TBL1XR1 | 1447 | 10 | 1.060815 | -0.319904 | 432 | 392.11 | 0.907662 |  |
| GO:0044260\_cellular\_macromolecule\_metabolic\_process | DNMT3A | 1447 | 10 | 1.060815 | -0.319904 | 432 | 392.11 | 0.907662 |  |
| GO:0044260\_cellular\_macromolecule\_metabolic\_process | HDAC2 | 1447 | 10 | 1.060815 | -0.319904 | 432 | 392.11 | 0.907662 |  |
| GO:0044260\_cellular\_macromolecule\_metabolic\_process | HUS1 | 1447 | 10 | 1.060815 | -0.319904 | 432 | 392.11 | 0.907662 |  |
| GO:0044260\_cellular\_macromolecule\_metabolic\_process | CHST11 | 1447 | 10 | 1.060815 | -0.319904 | 432 | 392.11 | 0.907662 |  |
| GO:0044260\_cellular\_macromolecule\_metabolic\_process | TFDP2 | 1447 | 10 | 1.060815 | -0.319904 | 432 | 392.11 | 0.907662 |  |
| GO:0044260\_cellular\_macromolecule\_metabolic\_process | ZFP238 | 1447 | 10 | 1.060815 | -0.319904 | 432 | 392.11 | 0.907662 |  |
| GO:0044260\_cellular\_macromolecule\_metabolic\_process | ARL6 | 1447 | 10 | 1.060815 | -0.319904 | 432 | 392.11 | 0.907662 |  |
| GO:0044260\_cellular\_macromolecule\_metabolic\_process | CD1D1 | 1447 | 10 | 1.060815 | -0.319904 | 432 | 392.11 | 0.907662 |  |
| GO:0044260\_cellular\_macromolecule\_metabolic\_process | PKIA | 1447 | 10 | 1.060815 | -0.319904 | 432 | 392.11 | 0.907662 |  |
| GO:0050789\_regulation\_of\_biological\_process | TXNIP | 2357 | 16 | 1.042003 | -0.319252 | 433 | 392.47 | 0.906397 |  |
| GO:0050789\_regulation\_of\_biological\_process | DNMT3A | 2357 | 16 | 1.042003 | -0.319252 | 433 | 392.47 | 0.906397 |  |
| GO:0050789\_regulation\_of\_biological\_process | TBL1XR1 | 2357 | 16 | 1.042003 | -0.319252 | 433 | 392.47 | 0.906397 |  |
| GO:0050789\_regulation\_of\_biological\_process | ZC3H15 | 2357 | 16 | 1.042003 | -0.319252 | 433 | 392.47 | 0.906397 |  |
| GO:0050789\_regulation\_of\_biological\_process | BECN1 | 2357 | 16 | 1.042003 | -0.319252 | 433 | 392.47 | 0.906397 |  |
| GO:0050789\_regulation\_of\_biological\_process | HUS1 | 2357 | 16 | 1.042003 | -0.319252 | 433 | 392.47 | 0.906397 |  |
| GO:0050789\_regulation\_of\_biological\_process | ARL6 | 2357 | 16 | 1.042003 | -0.319252 | 433 | 392.47 | 0.906397 |  |
| GO:0050789\_regulation\_of\_biological\_process | CD1D1 | 2357 | 16 | 1.042003 | -0.319252 | 433 | 392.47 | 0.906397 |  |
| GO:0050789\_regulation\_of\_biological\_process | PKIA | 2357 | 16 | 1.042003 | -0.319252 | 433 | 392.47 | 0.906397 |  |
| GO:0050789\_regulation\_of\_biological\_process | PTEN | 2357 | 16 | 1.042003 | -0.319252 | 433 | 392.47 | 0.906397 |  |
| GO:0050789\_regulation\_of\_biological\_process | VASH1 | 2357 | 16 | 1.042003 | -0.319252 | 433 | 392.47 | 0.906397 |  |
| GO:0050789\_regulation\_of\_biological\_process | CHST11 | 2357 | 16 | 1.042003 | -0.319252 | 433 | 392.47 | 0.906397 |  |
| GO:0050789\_regulation\_of\_biological\_process | ZFP238 | 2357 | 16 | 1.042003 | -0.319252 | 433 | 392.47 | 0.906397 |  |
| GO:0050789\_regulation\_of\_biological\_process | TFDP2 | 2357 | 16 | 1.042003 | -0.319252 | 433 | 392.47 | 0.906397 |  |
| GO:0050789\_regulation\_of\_biological\_process | PKD2 | 2357 | 16 | 1.042003 | -0.319252 | 433 | 392.47 | 0.906397 |  |
| GO:0050789\_regulation\_of\_biological\_process | CASP2 | 2357 | 16 | 1.042003 | -0.319252 | 433 | 392.47 | 0.906397 |  |
| GO:0001817\_regulation\_of\_cytokine\_production | CD1D1 | 99 | 1 | 1.550505 | -0.318692 | 434 | 394.07 | 0.907995 |  |
| GO:0008283\_cell\_proliferation | BECN1 | 544 | 4 | 1.128676 | -0.318360 | 435 | 394.32 | 0.906483 |  |
| GO:0008283\_cell\_proliferation | CHST11 | 544 | 4 | 1.128676 | -0.318360 | 435 | 394.32 | 0.906483 |  |
| GO:0008283\_cell\_proliferation | PTEN | 544 | 4 | 1.128676 | -0.318360 | 435 | 394.32 | 0.906483 |  |
| GO:0008283\_cell\_proliferation | LGR4 | 544 | 4 | 1.128676 | -0.318360 | 435 | 394.32 | 0.906483 |  |
| GO:0006807\_nitrogen\_compound\_metabolic\_process | TBL1XR1 | 1147 | 8 | 1.070619 | -0.317741 | 436 | 394.52 | 0.904862 |  |
| GO:0006807\_nitrogen\_compound\_metabolic\_process | DNMT3A | 1147 | 8 | 1.070619 | -0.317741 | 436 | 394.52 | 0.904862 |  |
| GO:0006807\_nitrogen\_compound\_metabolic\_process | CHST11 | 1147 | 8 | 1.070619 | -0.317741 | 436 | 394.52 | 0.904862 |  |
| GO:0006807\_nitrogen\_compound\_metabolic\_process | HUS1 | 1147 | 8 | 1.070619 | -0.317741 | 436 | 394.52 | 0.904862 |  |
| GO:0006807\_nitrogen\_compound\_metabolic\_process | PKD2 | 1147 | 8 | 1.070619 | -0.317741 | 436 | 394.52 | 0.904862 |  |
| GO:0006807\_nitrogen\_compound\_metabolic\_process | TFDP2 | 1147 | 8 | 1.070619 | -0.317741 | 436 | 394.52 | 0.904862 |  |
| GO:0006807\_nitrogen\_compound\_metabolic\_process | ZFP238 | 1147 | 8 | 1.070619 | -0.317741 | 436 | 394.52 | 0.904862 |  |
| GO:0006807\_nitrogen\_compound\_metabolic\_process | PKIA | 1147 | 8 | 1.070619 | -0.317741 | 436 | 394.52 | 0.904862 |  |
| GO:0044249\_cellular\_biosynthetic\_process | TBL1XR1 | 1150 | 8 | 1.067826 | -0.314742 | 437 | 395.35 | 0.904691 |  |
| GO:0044249\_cellular\_biosynthetic\_process | DNMT3A | 1150 | 8 | 1.067826 | -0.314742 | 437 | 395.35 | 0.904691 |  |
| GO:0044249\_cellular\_biosynthetic\_process | CHST11 | 1150 | 8 | 1.067826 | -0.314742 | 437 | 395.35 | 0.904691 |  |
| GO:0044249\_cellular\_biosynthetic\_process | HUS1 | 1150 | 8 | 1.067826 | -0.314742 | 437 | 395.35 | 0.904691 |  |
| GO:0044249\_cellular\_biosynthetic\_process | TFDP2 | 1150 | 8 | 1.067826 | -0.314742 | 437 | 395.35 | 0.904691 |  |
| GO:0044249\_cellular\_biosynthetic\_process | ZFP238 | 1150 | 8 | 1.067826 | -0.314742 | 437 | 395.35 | 0.904691 |  |
| GO:0044249\_cellular\_biosynthetic\_process | CD1D1 | 1150 | 8 | 1.067826 | -0.314742 | 437 | 395.35 | 0.904691 |  |
| GO:0044249\_cellular\_biosynthetic\_process | PKIA | 1150 | 8 | 1.067826 | -0.314742 | 437 | 395.35 | 0.904691 |  |
| GO:0032879\_regulation\_of\_localization | PKD2 | 248 | 2 | 1.237903 | -0.313199 | 438 | 395.95 | 0.903995 |  |
| GO:0032879\_regulation\_of\_localization | PKIA | 248 | 2 | 1.237903 | -0.313199 | 438 | 395.95 | 0.903995 |  |
| GO:0030163\_protein\_catabolic\_process | TBL1XR1 | 101 | 1 | 1.519802 | -0.312493 | 439 | 396.3 | 0.902733 |  |
| GO:0006950\_response\_to\_stress | TXNIP | 549 | 4 | 1.118397 | -0.311656 | 440 | 396.45 | 0.901023 |  |
| GO:0006950\_response\_to\_stress | BECN1 | 549 | 4 | 1.118397 | -0.311656 | 440 | 396.45 | 0.901023 |  |
| GO:0006950\_response\_to\_stress | HUS1 | 549 | 4 | 1.118397 | -0.311656 | 440 | 396.45 | 0.901023 |  |
| GO:0006950\_response\_to\_stress | RAD17 | 549 | 4 | 1.118397 | -0.311656 | 440 | 396.45 | 0.901023 |  |
| GO:0006139\_nucleobase\_\_nucleoside\_\_nucleotide\_and\_nucleic\_acid\_metabolic\_process | TBL1XR1 | 1002 | 7 | 1.072355 | -0.311517 | 441 | 396.64 | 0.899410 |  |
| GO:0006139\_nucleobase\_\_nucleoside\_\_nucleotide\_and\_nucleic\_acid\_metabolic\_process | DNMT3A | 1002 | 7 | 1.072355 | -0.311517 | 441 | 396.64 | 0.899410 |  |
| GO:0006139\_nucleobase\_\_nucleoside\_\_nucleotide\_and\_nucleic\_acid\_metabolic\_process | HUS1 | 1002 | 7 | 1.072355 | -0.311517 | 441 | 396.64 | 0.899410 |  |
| GO:0006139\_nucleobase\_\_nucleoside\_\_nucleotide\_and\_nucleic\_acid\_metabolic\_process | PKD2 | 1002 | 7 | 1.072355 | -0.311517 | 441 | 396.64 | 0.899410 |  |
| GO:0006139\_nucleobase\_\_nucleoside\_\_nucleotide\_and\_nucleic\_acid\_metabolic\_process | TFDP2 | 1002 | 7 | 1.072355 | -0.311517 | 441 | 396.64 | 0.899410 |  |
| GO:0006139\_nucleobase\_\_nucleoside\_\_nucleotide\_and\_nucleic\_acid\_metabolic\_process | ZFP238 | 1002 | 7 | 1.072355 | -0.311517 | 441 | 396.64 | 0.899410 |  |
| GO:0006139\_nucleobase\_\_nucleoside\_\_nucleotide\_and\_nucleic\_acid\_metabolic\_process | PKIA | 1002 | 7 | 1.072355 | -0.311517 | 441 | 396.64 | 0.899410 |  |
| GO:0048731\_system\_development | RECK | 1609 | 11 | 1.049410 | -0.310205 | 442 | 396.96 | 0.898100 |  |
| GO:0048731\_system\_development | ANKRD17 | 1609 | 11 | 1.049410 | -0.310205 | 442 | 396.96 | 0.898100 |  |
| GO:0048731\_system\_development | HDAC2 | 1609 | 11 | 1.049410 | -0.310205 | 442 | 396.96 | 0.898100 |  |
| GO:0048731\_system\_development | FGF15 | 1609 | 11 | 1.049410 | -0.310205 | 442 | 396.96 | 0.898100 |  |
| GO:0048731\_system\_development | TRP53BP2 | 1609 | 11 | 1.049410 | -0.310205 | 442 | 396.96 | 0.898100 |  |
| GO:0048731\_system\_development | CHST11 | 1609 | 11 | 1.049410 | -0.310205 | 442 | 396.96 | 0.898100 |  |
| GO:0048731\_system\_development | PKD2 | 1609 | 11 | 1.049410 | -0.310205 | 442 | 396.96 | 0.898100 |  |
| GO:0048731\_system\_development | CD1D1 | 1609 | 11 | 1.049410 | -0.310205 | 442 | 396.96 | 0.898100 |  |
| GO:0048731\_system\_development | PTEN | 1609 | 11 | 1.049410 | -0.310205 | 442 | 396.96 | 0.898100 |  |
| GO:0048731\_system\_development | LGR4 | 1609 | 11 | 1.049410 | -0.310205 | 442 | 396.96 | 0.898100 |  |
| GO:0048731\_system\_development | VASH1 | 1609 | 11 | 1.049410 | -0.310205 | 442 | 396.96 | 0.898100 |  |
| GO:0006350\_transcription | TBL1XR1 | 701 | 5 | 1.094864 | -0.310182 | 443 | 397.14 | 0.896479 |  |
| GO:0006350\_transcription | DNMT3A | 701 | 5 | 1.094864 | -0.310182 | 443 | 397.14 | 0.896479 |  |
| GO:0006350\_transcription | TFDP2 | 701 | 5 | 1.094864 | -0.310182 | 443 | 397.14 | 0.896479 |  |
| GO:0006350\_transcription | ZFP238 | 701 | 5 | 1.094864 | -0.310182 | 443 | 397.14 | 0.896479 |  |
| GO:0006350\_transcription | PKIA | 701 | 5 | 1.094864 | -0.310182 | 443 | 397.14 | 0.896479 |  |
| GO:0050793\_regulation\_of\_developmental\_process | CHST11 | 703 | 5 | 1.091750 | -0.307809 | 444 | 397.82 | 0.895991 |  |
| GO:0050793\_regulation\_of\_developmental\_process | CD1D1 | 703 | 5 | 1.091750 | -0.307809 | 444 | 397.82 | 0.895991 |  |
| GO:0050793\_regulation\_of\_developmental\_process | PTEN | 703 | 5 | 1.091750 | -0.307809 | 444 | 397.82 | 0.895991 |  |
| GO:0050793\_regulation\_of\_developmental\_process | CASP2 | 703 | 5 | 1.091750 | -0.307809 | 444 | 397.82 | 0.895991 |  |
| GO:0050793\_regulation\_of\_developmental\_process | VASH1 | 703 | 5 | 1.091750 | -0.307809 | 444 | 397.82 | 0.895991 |  |
| GO:0055086\_nucleobase\_\_nucleoside\_and\_nucleotide\_metabolic\_process | PKD2 | 104 | 1 | 1.475962 | -0.303503 | 445 | 400.84 | 0.900764 |  |
| GO:0044267\_cellular\_protein\_metabolic\_process | TBL1XR1 | 559 | 4 | 1.098390 | -0.298645 | 446 | 402.22 | 0.901839 |  |
| GO:0044267\_cellular\_protein\_metabolic\_process | HDAC2 | 559 | 4 | 1.098390 | -0.298645 | 446 | 402.22 | 0.901839 |  |
| GO:0044267\_cellular\_protein\_metabolic\_process | HUS1 | 559 | 4 | 1.098390 | -0.298645 | 446 | 402.22 | 0.901839 |  |
| GO:0044267\_cellular\_protein\_metabolic\_process | ARL6 | 559 | 4 | 1.098390 | -0.298645 | 446 | 402.22 | 0.901839 |  |
| GO:0009966\_regulation\_of\_signal\_transduction | CHST11 | 256 | 2 | 1.199219 | -0.298144 | 447 | 402.75 | 0.901007 |  |
| GO:0009966\_regulation\_of\_signal\_transduction | PTEN | 256 | 2 | 1.199219 | -0.298144 | 447 | 402.75 | 0.901007 |  |
| GO:0010817\_regulation\_of\_hormone\_levels | RBP1 | 106 | 1 | 1.448113 | -0.297706 | 448 | 403.24 | 0.900089 |  |
| GO:0048870\_cell\_motility | FGF15 | 257 | 2 | 1.194553 | -0.296319 | 449 | 403.46 | 0.898575 |  |
| GO:0048870\_cell\_motility | PTEN | 257 | 2 | 1.194553 | -0.296319 | 449 | 403.46 | 0.898575 |  |
| GO:0045859\_regulation\_of\_protein\_kinase\_activity | PKIA | 107 | 1 | 1.434579 | -0.294863 | 450 | 404.11 | 0.898022 |  |
| GO:0010647\_positive\_regulation\_of\_cell\_communication | BECN1 | 110 | 1 | 1.395455 | -0.286553 | 452 | 408.44 | 0.903628 |  |
| GO:0055080\_cation\_homeostasis | PKD2 | 110 | 1 | 1.395455 | -0.286553 | 452 | 408.44 | 0.903628 |  |
| GO:0048705\_skeletal\_system\_morphogenesis | CHST11 | 111 | 1 | 1.382883 | -0.283853 | 453 | 409.29 | 0.903510 |  |
| GO:0043549\_regulation\_of\_kinase\_activity | PKIA | 112 | 1 | 1.370536 | -0.281187 | 455 | 410.56 | 0.902330 |  |
| GO:0051249\_regulation\_of\_lymphocyte\_activation | CD1D1 | 112 | 1 | 1.370536 | -0.281187 | 455 | 410.56 | 0.902330 |  |
| GO:0009607\_response\_to\_biotic\_stimulus | BECN1 | 114 | 1 | 1.346491 | -0.275954 | 456 | 412.78 | 0.905219 |  |
| GO:0051338\_regulation\_of\_transferase\_activity | PKIA | 115 | 1 | 1.334783 | -0.273386 | 457 | 414.08 | 0.906083 |  |
| GO:0046483\_heterocycle\_metabolic\_process | PKD2 | 116 | 1 | 1.323276 | -0.270849 | 460 | 415.84 | 0.904000 |  |
| GO:0048608\_reproductive\_structure\_development | PTEN | 116 | 1 | 1.323276 | -0.270849 | 460 | 415.84 | 0.904000 |  |
| GO:0080134\_regulation\_of\_response\_to\_stress | BECN1 | 116 | 1 | 1.323276 | -0.270849 | 460 | 415.84 | 0.904000 |  |
| GO:0051716\_cellular\_response\_to\_stimulus | BECN1 | 273 | 2 | 1.124542 | -0.268721 | 461 | 416.08 | 0.902560 |  |
| GO:0051716\_cellular\_response\_to\_stimulus | HUS1 | 273 | 2 | 1.124542 | -0.268721 | 461 | 416.08 | 0.902560 |  |
| GO:0033036\_macromolecule\_localization | CHST11 | 274 | 2 | 1.120438 | -0.267092 | 462 | 416.92 | 0.902424 |  |
| GO:0033036\_macromolecule\_localization | PKIA | 274 | 2 | 1.120438 | -0.267092 | 462 | 416.92 | 0.902424 |  |
| GO:0048646\_anatomical\_structure\_formation\_involved\_in\_morphogenesis | PTEN | 277 | 2 | 1.108303 | -0.262266 | 463 | 419.55 | 0.906156 |  |
| GO:0048646\_anatomical\_structure\_formation\_involved\_in\_morphogenesis | VASH1 | 277 | 2 | 1.108303 | -0.262266 | 463 | 419.55 | 0.906156 |  |
| GO:0014706\_striated\_muscle\_tissue\_development | PTEN | 120 | 1 | 1.279167 | -0.261008 | 464 | 420.27 | 0.905754 |  |
| GO:0006357\_regulation\_of\_transcription\_from\_RNA\_polymerase\_II\_promoter | DNMT3A | 435 | 3 | 1.058621 | -0.260449 | 465 | 420.5 | 0.904301 |  |
| GO:0006357\_regulation\_of\_transcription\_from\_RNA\_polymerase\_II\_promoter | ZFP238 | 435 | 3 | 1.058621 | -0.260449 | 465 | 420.5 | 0.904301 |  |
| GO:0006357\_regulation\_of\_transcription\_from\_RNA\_polymerase\_II\_promoter | PKIA | 435 | 3 | 1.058621 | -0.260449 | 465 | 420.5 | 0.904301 |  |
| GO:0044237\_cellular\_metabolic\_process | DNMT3A | 1974 | 13 | 1.010892 | -0.260103 | 466 | 420.79 | 0.902983 |  |
| GO:0044237\_cellular\_metabolic\_process | TBL1XR1 | 1974 | 13 | 1.010892 | -0.260103 | 466 | 420.79 | 0.902983 |  |
| GO:0044237\_cellular\_metabolic\_process | RBP1 | 1974 | 13 | 1.010892 | -0.260103 | 466 | 420.79 | 0.902983 |  |
| GO:0044237\_cellular\_metabolic\_process | BECN1 | 1974 | 13 | 1.010892 | -0.260103 | 466 | 420.79 | 0.902983 |  |
| GO:0044237\_cellular\_metabolic\_process | HUS1 | 1974 | 13 | 1.010892 | -0.260103 | 466 | 420.79 | 0.902983 |  |
| GO:0044237\_cellular\_metabolic\_process | CD1D1 | 1974 | 13 | 1.010892 | -0.260103 | 466 | 420.79 | 0.902983 |  |
| GO:0044237\_cellular\_metabolic\_process | ARL6 | 1974 | 13 | 1.010892 | -0.260103 | 466 | 420.79 | 0.902983 |  |
| GO:0044237\_cellular\_metabolic\_process | PKIA | 1974 | 13 | 1.010892 | -0.260103 | 466 | 420.79 | 0.902983 |  |
| GO:0044237\_cellular\_metabolic\_process | HDAC2 | 1974 | 13 | 1.010892 | -0.260103 | 466 | 420.79 | 0.902983 |  |
| GO:0044237\_cellular\_metabolic\_process | CHST11 | 1974 | 13 | 1.010892 | -0.260103 | 466 | 420.79 | 0.902983 |  |
| GO:0044237\_cellular\_metabolic\_process | ZFP238 | 1974 | 13 | 1.010892 | -0.260103 | 466 | 420.79 | 0.902983 |  |
| GO:0044237\_cellular\_metabolic\_process | TFDP2 | 1974 | 13 | 1.010892 | -0.260103 | 466 | 420.79 | 0.902983 |  |
| GO:0044237\_cellular\_metabolic\_process | PKD2 | 1974 | 13 | 1.010892 | -0.260103 | 466 | 420.79 | 0.902983 |  |
| GO:0065009\_regulation\_of\_molecular\_function | PKD2 | 279 | 2 | 1.100358 | -0.259102 | 467 | 421.33 | 0.902206 |  |
| GO:0065009\_regulation\_of\_molecular\_function | PKIA | 279 | 2 | 1.100358 | -0.259102 | 467 | 421.33 | 0.902206 |  |
| GO:0002694\_regulation\_of\_leukocyte\_activation | CD1D1 | 121 | 1 | 1.268595 | -0.258621 | 468 | 422.74 | 0.903291 |  |
| GO:0001816\_cytokine\_production | CD1D1 | 122 | 1 | 1.258197 | -0.256263 | 473 | 425.36 | 0.899281 |  |
| GO:0002252\_immune\_effector\_process | CD1D1 | 122 | 1 | 1.258197 | -0.256263 | 473 | 425.36 | 0.899281 |  |
| GO:0006886\_intracellular\_protein\_transport | PKIA | 122 | 1 | 1.258197 | -0.256263 | 473 | 425.36 | 0.899281 |  |
| GO:0030001\_metal\_ion\_transport | PKD2 | 122 | 1 | 1.258197 | -0.256263 | 473 | 425.36 | 0.899281 |  |
| GO:0050865\_regulation\_of\_cell\_activation | CD1D1 | 122 | 1 | 1.258197 | -0.256263 | 473 | 425.36 | 0.899281 |  |
| GO:0006464\_protein\_modification\_process | HDAC2 | 439 | 3 | 1.048975 | -0.255360 | 474 | 425.95 | 0.898629 |  |
| GO:0006464\_protein\_modification\_process | HUS1 | 439 | 3 | 1.048975 | -0.255360 | 474 | 425.95 | 0.898629 |  |
| GO:0006464\_protein\_modification\_process | ARL6 | 439 | 3 | 1.048975 | -0.255360 | 474 | 425.95 | 0.898629 |  |
| GO:0008152\_metabolic\_process | DNMT3A | 2133 | 14 | 1.007501 | -0.255014 | 475 | 426.05 | 0.896947 |  |
| GO:0008152\_metabolic\_process | TBL1XR1 | 2133 | 14 | 1.007501 | -0.255014 | 475 | 426.05 | 0.896947 |  |
| GO:0008152\_metabolic\_process | RBP1 | 2133 | 14 | 1.007501 | -0.255014 | 475 | 426.05 | 0.896947 |  |
| GO:0008152\_metabolic\_process | BECN1 | 2133 | 14 | 1.007501 | -0.255014 | 475 | 426.05 | 0.896947 |  |
| GO:0008152\_metabolic\_process | HUS1 | 2133 | 14 | 1.007501 | -0.255014 | 475 | 426.05 | 0.896947 |  |
| GO:0008152\_metabolic\_process | CD1D1 | 2133 | 14 | 1.007501 | -0.255014 | 475 | 426.05 | 0.896947 |  |
| GO:0008152\_metabolic\_process | ARL6 | 2133 | 14 | 1.007501 | -0.255014 | 475 | 426.05 | 0.896947 |  |
| GO:0008152\_metabolic\_process | PKIA | 2133 | 14 | 1.007501 | -0.255014 | 475 | 426.05 | 0.896947 |  |
| GO:0008152\_metabolic\_process | HDAC2 | 2133 | 14 | 1.007501 | -0.255014 | 475 | 426.05 | 0.896947 |  |
| GO:0008152\_metabolic\_process | B3GALNT1 | 2133 | 14 | 1.007501 | -0.255014 | 475 | 426.05 | 0.896947 |  |
| GO:0008152\_metabolic\_process | CHST11 | 2133 | 14 | 1.007501 | -0.255014 | 475 | 426.05 | 0.896947 |  |
| GO:0008152\_metabolic\_process | ZFP238 | 2133 | 14 | 1.007501 | -0.255014 | 475 | 426.05 | 0.896947 |  |
| GO:0008152\_metabolic\_process | TFDP2 | 2133 | 14 | 1.007501 | -0.255014 | 475 | 426.05 | 0.896947 |  |
| GO:0008152\_metabolic\_process | PKD2 | 2133 | 14 | 1.007501 | -0.255014 | 475 | 426.05 | 0.896947 |  |
| GO:0009308\_amine\_metabolic\_process | CHST11 | 124 | 1 | 1.237903 | -0.251629 | 477 | 427.76 | 0.896771 |  |
| GO:0030098\_lymphocyte\_differentiation | CD1D1 | 124 | 1 | 1.237903 | -0.251629 | 477 | 427.76 | 0.896771 |  |
| GO:0065007\_biological\_regulation | TXNIP | 2593 | 17 | 1.006363 | -0.251450 | 478 | 428.1 | 0.895607 |  |
| GO:0065007\_biological\_regulation | DNMT3A | 2593 | 17 | 1.006363 | -0.251450 | 478 | 428.1 | 0.895607 |  |
| GO:0065007\_biological\_regulation | TBL1XR1 | 2593 | 17 | 1.006363 | -0.251450 | 478 | 428.1 | 0.895607 |  |
| GO:0065007\_biological\_regulation | ZC3H15 | 2593 | 17 | 1.006363 | -0.251450 | 478 | 428.1 | 0.895607 |  |
| GO:0065007\_biological\_regulation | RBP1 | 2593 | 17 | 1.006363 | -0.251450 | 478 | 428.1 | 0.895607 |  |
| GO:0065007\_biological\_regulation | BECN1 | 2593 | 17 | 1.006363 | -0.251450 | 478 | 428.1 | 0.895607 |  |
| GO:0065007\_biological\_regulation | HUS1 | 2593 | 17 | 1.006363 | -0.251450 | 478 | 428.1 | 0.895607 |  |
| GO:0065007\_biological\_regulation | ARL6 | 2593 | 17 | 1.006363 | -0.251450 | 478 | 428.1 | 0.895607 |  |
| GO:0065007\_biological\_regulation | CD1D1 | 2593 | 17 | 1.006363 | -0.251450 | 478 | 428.1 | 0.895607 |  |
| GO:0065007\_biological\_regulation | PKIA | 2593 | 17 | 1.006363 | -0.251450 | 478 | 428.1 | 0.895607 |  |
| GO:0065007\_biological\_regulation | PTEN | 2593 | 17 | 1.006363 | -0.251450 | 478 | 428.1 | 0.895607 |  |
| GO:0065007\_biological\_regulation | VASH1 | 2593 | 17 | 1.006363 | -0.251450 | 478 | 428.1 | 0.895607 |  |
| GO:0065007\_biological\_regulation | CHST11 | 2593 | 17 | 1.006363 | -0.251450 | 478 | 428.1 | 0.895607 |  |
| GO:0065007\_biological\_regulation | ZFP238 | 2593 | 17 | 1.006363 | -0.251450 | 478 | 428.1 | 0.895607 |  |
| GO:0065007\_biological\_regulation | TFDP2 | 2593 | 17 | 1.006363 | -0.251450 | 478 | 428.1 | 0.895607 |  |
| GO:0065007\_biological\_regulation | PKD2 | 2593 | 17 | 1.006363 | -0.251450 | 478 | 428.1 | 0.895607 |  |
| GO:0065007\_biological\_regulation | CASP2 | 2593 | 17 | 1.006363 | -0.251450 | 478 | 428.1 | 0.895607 |  |
| GO:0043062\_extracellular\_structure\_organization | RECK | 125 | 1 | 1.228000 | -0.249352 | 479 | 429.48 | 0.896618 |  |
| GO:0006366\_transcription\_from\_RNA\_polymerase\_II\_promoter | DNMT3A | 444 | 3 | 1.037162 | -0.249137 | 480 | 429.9 | 0.895625 |  |
| GO:0006366\_transcription\_from\_RNA\_polymerase\_II\_promoter | ZFP238 | 444 | 3 | 1.037162 | -0.249137 | 480 | 429.9 | 0.895625 |  |
| GO:0006366\_transcription\_from\_RNA\_polymerase\_II\_promoter | PKIA | 444 | 3 | 1.037162 | -0.249137 | 480 | 429.9 | 0.895625 |  |
| GO:0006996\_organelle\_organization | UXT | 449 | 3 | 1.025612 | -0.243062 | 481 | 430.86 | 0.895759 |  |
| GO:0006996\_organelle\_organization | DNMT3A | 449 | 3 | 1.025612 | -0.243062 | 481 | 430.86 | 0.895759 |  |
| GO:0006996\_organelle\_organization | HDAC2 | 449 | 3 | 1.025612 | -0.243062 | 481 | 430.86 | 0.895759 |  |
| GO:0045597\_positive\_regulation\_of\_cell\_differentiation | CD1D1 | 128 | 1 | 1.199219 | -0.242680 | 483 | 431.96 | 0.894327 |  |
| GO:0060537\_muscle\_tissue\_development | PTEN | 128 | 1 | 1.199219 | -0.242680 | 483 | 431.96 | 0.894327 |  |
| GO:0043285\_biopolymer\_catabolic\_process | TBL1XR1 | 129 | 1 | 1.189922 | -0.240507 | 484 | 432.78 | 0.894174 |  |
| GO:0032787\_monocarboxylic\_acid\_metabolic\_process | RBP1 | 130 | 1 | 1.180769 | -0.238359 | 486 | 433.79 | 0.892572 |  |
| GO:0050776\_regulation\_of\_immune\_response | CD1D1 | 130 | 1 | 1.180769 | -0.238359 | 486 | 433.79 | 0.892572 |  |
| GO:0040011\_locomotion | FGF15 | 295 | 2 | 1.040678 | -0.235210 | 487 | 435.11 | 0.893450 |  |
| GO:0040011\_locomotion | PTEN | 295 | 2 | 1.040678 | -0.235210 | 487 | 435.11 | 0.893450 |  |
| GO:0034960\_cellular\_biopolymer\_metabolic\_process | TBL1XR1 | 1395 | 9 | 0.990323 | -0.234596 | 488 | 435.25 | 0.891906 |  |
| GO:0034960\_cellular\_biopolymer\_metabolic\_process | DNMT3A | 1395 | 9 | 0.990323 | -0.234596 | 488 | 435.25 | 0.891906 |  |
| GO:0034960\_cellular\_biopolymer\_metabolic\_process | HDAC2 | 1395 | 9 | 0.990323 | -0.234596 | 488 | 435.25 | 0.891906 |  |
| GO:0034960\_cellular\_biopolymer\_metabolic\_process | CHST11 | 1395 | 9 | 0.990323 | -0.234596 | 488 | 435.25 | 0.891906 |  |
| GO:0034960\_cellular\_biopolymer\_metabolic\_process | HUS1 | 1395 | 9 | 0.990323 | -0.234596 | 488 | 435.25 | 0.891906 |  |
| GO:0034960\_cellular\_biopolymer\_metabolic\_process | TFDP2 | 1395 | 9 | 0.990323 | -0.234596 | 488 | 435.25 | 0.891906 |  |
| GO:0034960\_cellular\_biopolymer\_metabolic\_process | ZFP238 | 1395 | 9 | 0.990323 | -0.234596 | 488 | 435.25 | 0.891906 |  |
| GO:0034960\_cellular\_biopolymer\_metabolic\_process | ARL6 | 1395 | 9 | 0.990323 | -0.234596 | 488 | 435.25 | 0.891906 |  |
| GO:0034960\_cellular\_biopolymer\_metabolic\_process | PKIA | 1395 | 9 | 0.990323 | -0.234596 | 488 | 435.25 | 0.891906 |  |
| GO:0007283\_spermatogenesis | DNMT3A | 134 | 1 | 1.145522 | -0.230006 | 490 | 437.46 | 0.892776 |  |
| GO:0048232\_male\_gamete\_generation | DNMT3A | 134 | 1 | 1.145522 | -0.230006 | 490 | 437.46 | 0.892776 |  |
| GO:0010468\_regulation\_of\_gene\_expression | TBL1XR1 | 778 | 5 | 0.986504 | -0.229687 | 491 | 437.66 | 0.891365 |  |
| GO:0010468\_regulation\_of\_gene\_expression | DNMT3A | 778 | 5 | 0.986504 | -0.229687 | 491 | 437.66 | 0.891365 |  |
| GO:0010468\_regulation\_of\_gene\_expression | TFDP2 | 778 | 5 | 0.986504 | -0.229687 | 491 | 437.66 | 0.891365 |  |
| GO:0010468\_regulation\_of\_gene\_expression | ZFP238 | 778 | 5 | 0.986504 | -0.229687 | 491 | 437.66 | 0.891365 |  |
| GO:0010468\_regulation\_of\_gene\_expression | PKIA | 778 | 5 | 0.986504 | -0.229687 | 491 | 437.66 | 0.891365 |  |
| GO:0048598\_embryonic\_morphogenesis | CHST11 | 299 | 2 | 1.026756 | -0.229611 | 492 | 437.9 | 0.890041 |  |
| GO:0048598\_embryonic\_morphogenesis | FBN2 | 299 | 2 | 1.026756 | -0.229611 | 492 | 437.9 | 0.890041 |  |
| GO:0009057\_macromolecule\_catabolic\_process | TBL1XR1 | 137 | 1 | 1.120438 | -0.223983 | 493 | 439.04 | 0.890548 |  |
| GO:0007169\_transmembrane\_receptor\_protein\_tyrosine\_kinase\_signaling\_pathway | TXNIP | 139 | 1 | 1.104317 | -0.220078 | 495 | 440.21 | 0.889313 |  |
| GO:0034613\_cellular\_protein\_localization | PKIA | 139 | 1 | 1.104317 | -0.220078 | 495 | 440.21 | 0.889313 |  |
| GO:0070727\_cellular\_macromolecule\_localization | PKIA | 141 | 1 | 1.088652 | -0.216257 | 496 | 441.98 | 0.891089 |  |
| GO:0016310\_phosphorylation | HUS1 | 309 | 2 | 0.993528 | -0.216218 | 497 | 442.28 | 0.889899 |  |
| GO:0016310\_phosphorylation | PKIA | 309 | 2 | 0.993528 | -0.216218 | 497 | 442.28 | 0.889899 |  |
| GO:0043170\_macromolecule\_metabolic\_process | DNMT3A | 1576 | 10 | 0.973985 | -0.215406 | 498 | 442.4 | 0.888353 |  |
| GO:0043170\_macromolecule\_metabolic\_process | TBL1XR1 | 1576 | 10 | 0.973985 | -0.215406 | 498 | 442.4 | 0.888353 |  |
| GO:0043170\_macromolecule\_metabolic\_process | HDAC2 | 1576 | 10 | 0.973985 | -0.215406 | 498 | 442.4 | 0.888353 |  |
| GO:0043170\_macromolecule\_metabolic\_process | CHST11 | 1576 | 10 | 0.973985 | -0.215406 | 498 | 442.4 | 0.888353 |  |
| GO:0043170\_macromolecule\_metabolic\_process | HUS1 | 1576 | 10 | 0.973985 | -0.215406 | 498 | 442.4 | 0.888353 |  |
| GO:0043170\_macromolecule\_metabolic\_process | TFDP2 | 1576 | 10 | 0.973985 | -0.215406 | 498 | 442.4 | 0.888353 |  |
| GO:0043170\_macromolecule\_metabolic\_process | ZFP238 | 1576 | 10 | 0.973985 | -0.215406 | 498 | 442.4 | 0.888353 |  |
| GO:0043170\_macromolecule\_metabolic\_process | ARL6 | 1576 | 10 | 0.973985 | -0.215406 | 498 | 442.4 | 0.888353 |  |
| GO:0043170\_macromolecule\_metabolic\_process | CD1D1 | 1576 | 10 | 0.973985 | -0.215406 | 498 | 442.4 | 0.888353 |  |
| GO:0043170\_macromolecule\_metabolic\_process | PKIA | 1576 | 10 | 0.973985 | -0.215406 | 498 | 442.4 | 0.888353 |  |
| GO:0035239\_tube\_morphogenesis | LGR4 | 143 | 1 | 1.073427 | -0.212518 | 499 | 442.99 | 0.887756 |  |
| GO:0006812\_cation\_transport | PKD2 | 146 | 1 | 1.051370 | -0.207059 | 501 | 445.24 | 0.888703 |  |
| GO:0030900\_forebrain\_development | HDAC2 | 146 | 1 | 1.051370 | -0.207059 | 501 | 445.24 | 0.888703 |  |
| GO:0022603\_regulation\_of\_anatomical\_structure\_morphogenesis | VASH1 | 147 | 1 | 1.044218 | -0.205278 | 502 | 446.24 | 0.888924 |  |
| GO:0002684\_positive\_regulation\_of\_immune\_system\_process | CD1D1 | 148 | 1 | 1.037162 | -0.203515 | 503 | 447.22 | 0.889105 |  |
| GO:0044238\_primary\_metabolic\_process | TBL1XR1 | 1905 | 12 | 0.966929 | -0.201615 | 504 | 447.77 | 0.888433 |  |
| GO:0044238\_primary\_metabolic\_process | DNMT3A | 1905 | 12 | 0.966929 | -0.201615 | 504 | 447.77 | 0.888433 |  |
| GO:0044238\_primary\_metabolic\_process | HDAC2 | 1905 | 12 | 0.966929 | -0.201615 | 504 | 447.77 | 0.888433 |  |
| GO:0044238\_primary\_metabolic\_process | RBP1 | 1905 | 12 | 0.966929 | -0.201615 | 504 | 447.77 | 0.888433 |  |
| GO:0044238\_primary\_metabolic\_process | B3GALNT1 | 1905 | 12 | 0.966929 | -0.201615 | 504 | 447.77 | 0.888433 |  |
| GO:0044238\_primary\_metabolic\_process | HUS1 | 1905 | 12 | 0.966929 | -0.201615 | 504 | 447.77 | 0.888433 |  |
| GO:0044238\_primary\_metabolic\_process | CHST11 | 1905 | 12 | 0.966929 | -0.201615 | 504 | 447.77 | 0.888433 |  |
| GO:0044238\_primary\_metabolic\_process | PKD2 | 1905 | 12 | 0.966929 | -0.201615 | 504 | 447.77 | 0.888433 |  |
| GO:0044238\_primary\_metabolic\_process | TFDP2 | 1905 | 12 | 0.966929 | -0.201615 | 504 | 447.77 | 0.888433 |  |
| GO:0044238\_primary\_metabolic\_process | ZFP238 | 1905 | 12 | 0.966929 | -0.201615 | 504 | 447.77 | 0.888433 |  |
| GO:0044238\_primary\_metabolic\_process | ARL6 | 1905 | 12 | 0.966929 | -0.201615 | 504 | 447.77 | 0.888433 |  |
| GO:0044238\_primary\_metabolic\_process | PKIA | 1905 | 12 | 0.966929 | -0.201615 | 504 | 447.77 | 0.888433 |  |
| GO:0019538\_protein\_metabolic\_process | TBL1XR1 | 655 | 4 | 0.937405 | -0.196971 | 505 | 448.87 | 0.888851 |  |
| GO:0019538\_protein\_metabolic\_process | HDAC2 | 655 | 4 | 0.937405 | -0.196971 | 505 | 448.87 | 0.888851 |  |
| GO:0019538\_protein\_metabolic\_process | HUS1 | 655 | 4 | 0.937405 | -0.196971 | 505 | 448.87 | 0.888851 |  |
| GO:0019538\_protein\_metabolic\_process | ARL6 | 655 | 4 | 0.937405 | -0.196971 | 505 | 448.87 | 0.888851 |  |
| GO:0007517\_muscle\_organ\_development | PTEN | 153 | 1 | 1.003268 | -0.194976 | 506 | 449.8 | 0.888933 |  |
| GO:0006928\_cell\_motion | FGF15 | 330 | 2 | 0.930303 | -0.190682 | 508 | 452.01 | 0.889783 |  |
| GO:0006928\_cell\_motion | PTEN | 330 | 2 | 0.930303 | -0.190682 | 508 | 452.01 | 0.889783 |  |
| GO:0051674\_localization\_of\_cell | FGF15 | 330 | 2 | 0.930303 | -0.190682 | 508 | 452.01 | 0.889783 |  |
| GO:0051674\_localization\_of\_cell | PTEN | 330 | 2 | 0.930303 | -0.190682 | 508 | 452.01 | 0.889783 |  |
| GO:0051704\_multi-organism\_process | BECN1 | 157 | 1 | 0.977707 | -0.188456 | 509 | 453.11 | 0.890196 |  |
| GO:0002521\_leukocyte\_differentiation | CD1D1 | 161 | 1 | 0.953416 | -0.182198 | 510 | 456.33 | 0.894765 |  |
| GO:0009605\_response\_to\_external\_stimulus | BECN1 | 339 | 2 | 0.905605 | -0.180713 | 511 | 456.84 | 0.894012 |  |
| GO:0009605\_response\_to\_external\_stimulus | PKD2 | 339 | 2 | 0.905605 | -0.180713 | 511 | 456.84 | 0.894012 |  |
| GO:0006793\_phosphorus\_metabolic\_process | HUS1 | 340 | 2 | 0.902941 | -0.179639 | 513 | 457.83 | 0.892456 |  |
| GO:0006793\_phosphorus\_metabolic\_process | PKIA | 340 | 2 | 0.902941 | -0.179639 | 513 | 457.83 | 0.892456 |  |
| GO:0006796\_phosphate\_metabolic\_process | HUS1 | 340 | 2 | 0.902941 | -0.179639 | 513 | 457.83 | 0.892456 |  |
| GO:0006796\_phosphate\_metabolic\_process | PKIA | 340 | 2 | 0.902941 | -0.179639 | 513 | 457.83 | 0.892456 |  |
| GO:0042110\_T\_cell\_activation | CD1D1 | 163 | 1 | 0.941718 | -0.179162 | 514 | 458.91 | 0.892821 |  |
| GO:0042325\_regulation\_of\_phosphorylation | PKIA | 164 | 1 | 0.935976 | -0.177667 | 515 | 459.5 | 0.892233 |  |
| GO:0019220\_regulation\_of\_phosphate\_metabolic\_process | PKIA | 165 | 1 | 0.930303 | -0.176186 | 517 | 460.82 | 0.891335 |  |
| GO:0051174\_regulation\_of\_phosphorus\_metabolic\_process | PKIA | 165 | 1 | 0.930303 | -0.176186 | 517 | 460.82 | 0.891335 |  |
| GO:0043283\_biopolymer\_metabolic\_process | DNMT3A | 1490 | 9 | 0.927181 | -0.170823 | 518 | 463.23 | 0.894266 |  |
| GO:0043283\_biopolymer\_metabolic\_process | TBL1XR1 | 1490 | 9 | 0.927181 | -0.170823 | 518 | 463.23 | 0.894266 |  |
| GO:0043283\_biopolymer\_metabolic\_process | HDAC2 | 1490 | 9 | 0.927181 | -0.170823 | 518 | 463.23 | 0.894266 |  |
| GO:0043283\_biopolymer\_metabolic\_process | HUS1 | 1490 | 9 | 0.927181 | -0.170823 | 518 | 463.23 | 0.894266 |  |
| GO:0043283\_biopolymer\_metabolic\_process | CHST11 | 1490 | 9 | 0.927181 | -0.170823 | 518 | 463.23 | 0.894266 |  |
| GO:0043283\_biopolymer\_metabolic\_process | TFDP2 | 1490 | 9 | 0.927181 | -0.170823 | 518 | 463.23 | 0.894266 |  |
| GO:0043283\_biopolymer\_metabolic\_process | ZFP238 | 1490 | 9 | 0.927181 | -0.170823 | 518 | 463.23 | 0.894266 |  |
| GO:0043283\_biopolymer\_metabolic\_process | ARL6 | 1490 | 9 | 0.927181 | -0.170823 | 518 | 463.23 | 0.894266 |  |
| GO:0043283\_biopolymer\_metabolic\_process | PKIA | 1490 | 9 | 0.927181 | -0.170823 | 518 | 463.23 | 0.894266 |  |
| GO:0015031\_protein\_transport | PKIA | 175 | 1 | 0.877143 | -0.162158 | 519 | 468.3 | 0.902312 |  |
| GO:0006873\_cellular\_ion\_homeostasis | PKD2 | 176 | 1 | 0.872159 | -0.160829 | 520 | 469.5 | 0.902885 |  |
| GO:0048732\_gland\_development | PTEN | 179 | 1 | 0.857542 | -0.156917 | 521 | 471.55 | 0.905086 |  |
| GO:0045184\_establishment\_of\_protein\_localization | PKIA | 180 | 1 | 0.852778 | -0.155638 | 522 | 472.0 | 0.904215 |  |
| GO:0019752\_carboxylic\_acid\_metabolic\_process | RBP1 | 181 | 1 | 0.848066 | -0.154371 | 525 | 473.88 | 0.902629 |  |
| GO:0043436\_oxoacid\_metabolic\_process | RBP1 | 181 | 1 | 0.848066 | -0.154371 | 525 | 473.88 | 0.902629 |  |
| GO:0055082\_cellular\_chemical\_homeostasis | PKD2 | 181 | 1 | 0.848066 | -0.154371 | 525 | 473.88 | 0.902629 |  |
| GO:0006082\_organic\_acid\_metabolic\_process | RBP1 | 182 | 1 | 0.843407 | -0.153115 | 526 | 474.23 | 0.901578 |  |
| GO:0042180\_cellular\_ketone\_metabolic\_process | RBP1 | 183 | 1 | 0.838798 | -0.151872 | 527 | 474.98 | 0.901290 |  |
| GO:0007010\_cytoskeleton\_organization | UXT | 185 | 1 | 0.829730 | -0.149420 | 528 | 476.41 | 0.902292 |  |
| GO:0006811\_ion\_transport | PKD2 | 186 | 1 | 0.825269 | -0.148211 | 531 | 477.87 | 0.899944 |  |
| GO:0007155\_cell\_adhesion | BYSL | 186 | 1 | 0.825269 | -0.148211 | 531 | 477.87 | 0.899944 |  |
| GO:0022610\_biological\_adhesion | BYSL | 186 | 1 | 0.825269 | -0.148211 | 531 | 477.87 | 0.899944 |  |
| GO:0007276\_gamete\_generation | DNMT3A | 188 | 1 | 0.816489 | -0.145827 | 532 | 478.88 | 0.900150 |  |
| GO:0046907\_intracellular\_transport | PKIA | 194 | 1 | 0.791237 | -0.138935 | 533 | 481.38 | 0.903152 |  |
| GO:0019725\_cellular\_homeostasis | PKD2 | 195 | 1 | 0.787179 | -0.137823 | 534 | 482.73 | 0.903989 |  |
| GO:0010467\_gene\_expression | TBL1XR1 | 905 | 5 | 0.848066 | -0.136476 | 535 | 483.28 | 0.903327 |  |
| GO:0010467\_gene\_expression | DNMT3A | 905 | 5 | 0.848066 | -0.136476 | 535 | 483.28 | 0.903327 |  |
| GO:0010467\_gene\_expression | TFDP2 | 905 | 5 | 0.848066 | -0.136476 | 535 | 483.28 | 0.903327 |  |
| GO:0010467\_gene\_expression | ZFP238 | 905 | 5 | 0.848066 | -0.136476 | 535 | 483.28 | 0.903327 |  |
| GO:0010467\_gene\_expression | PKIA | 905 | 5 | 0.848066 | -0.136476 | 535 | 483.28 | 0.903327 |  |
| GO:0050801\_ion\_homeostasis | PKD2 | 197 | 1 | 0.779188 | -0.135629 | 536 | 484.37 | 0.903675 |  |
| GO:0006955\_immune\_response | CD1D1 | 205 | 1 | 0.748780 | -0.127240 | 537 | 489.4 | 0.911359 |  |
| GO:0035295\_tube\_development | LGR4 | 212 | 1 | 0.724057 | -0.120377 | 538 | 491.61 | 0.913773 |  |
| GO:0042221\_response\_to\_chemical\_stimulus | TXNIP | 409 | 2 | 0.750611 | -0.119169 | 539 | 492.48 | 0.913692 |  |
| GO:0042221\_response\_to\_chemical\_stimulus | VASH1 | 409 | 2 | 0.750611 | -0.119169 | 539 | 492.48 | 0.913692 |  |
| GO:0010033\_response\_to\_organic\_substance | VASH1 | 216 | 1 | 0.710648 | -0.116641 | 540 | 493.75 | 0.914352 |  |
| GO:0007166\_cell\_surface\_receptor\_linked\_signal\_transduction | TXNIP | 597 | 3 | 0.771357 | -0.115637 | 541 | 494.9 | 0.914787 |  |
| GO:0007166\_cell\_surface\_receptor\_linked\_signal\_transduction | ZC3H15 | 597 | 3 | 0.771357 | -0.115637 | 541 | 494.9 | 0.914787 |  |
| GO:0007166\_cell\_surface\_receptor\_linked\_signal\_transduction | CHST11 | 597 | 3 | 0.771357 | -0.115637 | 541 | 494.9 | 0.914787 |  |
| GO:0009653\_anatomical\_structure\_morphogenesis | CHST11 | 958 | 5 | 0.801148 | -0.108644 | 542 | 497.77 | 0.918395 |  |
| GO:0009653\_anatomical\_structure\_morphogenesis | FBN2 | 958 | 5 | 0.801148 | -0.108644 | 542 | 497.77 | 0.918395 |  |
| GO:0009653\_anatomical\_structure\_morphogenesis | PTEN | 958 | 5 | 0.801148 | -0.108644 | 542 | 497.77 | 0.918395 |  |
| GO:0009653\_anatomical\_structure\_morphogenesis | LGR4 | 958 | 5 | 0.801148 | -0.108644 | 542 | 497.77 | 0.918395 |  |
| GO:0009653\_anatomical\_structure\_morphogenesis | VASH1 | 958 | 5 | 0.801148 | -0.108644 | 542 | 497.77 | 0.918395 |  |
| GO:0002682\_regulation\_of\_immune\_system\_process | CD1D1 | 228 | 1 | 0.673246 | -0.106183 | 545 | 498.97 | 0.915541 |  |
| GO:0019953\_sexual\_reproduction | DNMT3A | 228 | 1 | 0.673246 | -0.106183 | 545 | 498.97 | 0.915541 |  |
| GO:0046649\_lymphocyte\_activation | CD1D1 | 228 | 1 | 0.673246 | -0.106183 | 545 | 498.97 | 0.915541 |  |
| GO:0007420\_brain\_development | HDAC2 | 231 | 1 | 0.664502 | -0.103733 | 546 | 499.81 | 0.915403 |  |
| GO:0007399\_nervous\_system\_development | HDAC2 | 621 | 3 | 0.741546 | -0.102184 | 547 | 500.44 | 0.914881 |  |
| GO:0007399\_nervous\_system\_development | TRP53BP2 | 621 | 3 | 0.741546 | -0.102184 | 547 | 500.44 | 0.914881 |  |
| GO:0007399\_nervous\_system\_development | PTEN | 621 | 3 | 0.741546 | -0.102184 | 547 | 500.44 | 0.914881 |  |
| GO:0050790\_regulation\_of\_catalytic\_activity | PKIA | 233 | 1 | 0.658798 | -0.102134 | 548 | 501.23 | 0.914653 |  |
| GO:0001501\_skeletal\_system\_development | CHST11 | 236 | 1 | 0.650424 | -0.099786 | 549 | 502.31 | 0.914954 |  |
| GO:0006468\_protein\_amino\_acid\_phosphorylation | HUS1 | 237 | 1 | 0.647679 | -0.099016 | 550 | 503.37 | 0.915218 |  |
| GO:0031325\_positive\_regulation\_of\_cellular\_metabolic\_process | BECN1 | 442 | 2 | 0.694570 | -0.097897 | 551 | 504.22 | 0.915100 |  |
| GO:0031325\_positive\_regulation\_of\_cellular\_metabolic\_process | CD1D1 | 442 | 2 | 0.694570 | -0.097897 | 551 | 504.22 | 0.915100 |  |
| GO:0010926\_anatomical\_structure\_formation | PTEN | 447 | 2 | 0.686801 | -0.095017 | 552 | 505.07 | 0.914982 |  |
| GO:0010926\_anatomical\_structure\_formation | VASH1 | 447 | 2 | 0.686801 | -0.095017 | 552 | 505.07 | 0.914982 |  |
| GO:0009887\_organ\_morphogenesis | CHST11 | 642 | 3 | 0.717290 | -0.091618 | 553 | 506.67 | 0.916221 |  |
| GO:0009887\_organ\_morphogenesis | PTEN | 642 | 3 | 0.717290 | -0.091618 | 553 | 506.67 | 0.916221 |  |
| GO:0009887\_organ\_morphogenesis | VASH1 | 642 | 3 | 0.717290 | -0.091618 | 553 | 506.67 | 0.916221 |  |
| GO:0045321\_leukocyte\_activation | CD1D1 | 248 | 1 | 0.618952 | -0.090963 | 554 | 507.46 | 0.915993 |  |
| GO:0007389\_pattern\_specification\_process | PKD2 | 250 | 1 | 0.614000 | -0.089576 | 555 | 507.89 | 0.915117 |  |
| GO:0009893\_positive\_regulation\_of\_metabolic\_process | BECN1 | 458 | 2 | 0.670306 | -0.088969 | 556 | 508.45 | 0.914478 |  |
| GO:0009893\_positive\_regulation\_of\_metabolic\_process | CD1D1 | 458 | 2 | 0.670306 | -0.088969 | 556 | 508.45 | 0.914478 |  |
| GO:0008104\_protein\_localization | PKIA | 251 | 1 | 0.611554 | -0.088892 | 557 | 508.72 | 0.913321 |  |
| GO:0030097\_hemopoiesis | CD1D1 | 253 | 1 | 0.606719 | -0.087539 | 558 | 509.93 | 0.913853 |  |
| GO:0048878\_chemical\_homeostasis | PKD2 | 254 | 1 | 0.604331 | -0.086871 | 559 | 510.17 | 0.912648 |  |
| GO:0048468\_cell\_development | HDAC2 | 654 | 3 | 0.704128 | -0.086042 | 560 | 510.76 | 0.912071 |  |
| GO:0048468\_cell\_development | FGF15 | 654 | 3 | 0.704128 | -0.086042 | 560 | 510.76 | 0.912071 |  |
| GO:0048468\_cell\_development | CHST11 | 654 | 3 | 0.704128 | -0.086042 | 560 | 510.76 | 0.912071 |  |
| GO:0001775\_cell\_activation | CD1D1 | 262 | 1 | 0.585878 | -0.081719 | 561 | 512.94 | 0.914332 |  |
| GO:0044255\_cellular\_lipid\_metabolic\_process | RBP1 | 264 | 1 | 0.581439 | -0.080483 | 562 | 513.6 | 0.913879 |  |
| GO:0048534\_hemopoietic\_or\_lymphoid\_organ\_development | CD1D1 | 277 | 1 | 0.554152 | -0.072916 | 563 | 516.75 | 0.917851 |  |
| GO:0051179\_localization | FGF15 | 1058 | 5 | 0.725425 | -0.069266 | 564 | 518.67 | 0.919628 |  |
| GO:0051179\_localization | CHST11 | 1058 | 5 | 0.725425 | -0.069266 | 564 | 518.67 | 0.919628 |  |
| GO:0051179\_localization | PKD2 | 1058 | 5 | 0.725425 | -0.069266 | 564 | 518.67 | 0.919628 |  |
| GO:0051179\_localization | PTEN | 1058 | 5 | 0.725425 | -0.069266 | 564 | 518.67 | 0.919628 |  |
| GO:0051179\_localization | PKIA | 1058 | 5 | 0.725425 | -0.069266 | 564 | 518.67 | 0.919628 |  |
| GO:0006629\_lipid\_metabolic\_process | RBP1 | 285 | 1 | 0.538596 | -0.068637 | 565 | 519.37 | 0.919239 |  |
| GO:0030154\_cell\_differentiation | HDAC2 | 1060 | 5 | 0.724057 | -0.068626 | 566 | 519.53 | 0.917898 |  |
| GO:0030154\_cell\_differentiation | FGF15 | 1060 | 5 | 0.724057 | -0.068626 | 566 | 519.53 | 0.917898 |  |
| GO:0030154\_cell\_differentiation | CHST11 | 1060 | 5 | 0.724057 | -0.068626 | 566 | 519.53 | 0.917898 |  |
| GO:0030154\_cell\_differentiation | ARL6 | 1060 | 5 | 0.724057 | -0.068626 | 566 | 519.53 | 0.917898 |  |
| GO:0030154\_cell\_differentiation | CD1D1 | 1060 | 5 | 0.724057 | -0.068626 | 566 | 519.53 | 0.917898 |  |
| GO:0048522\_positive\_regulation\_of\_cellular\_process | BECN1 | 895 | 4 | 0.686034 | -0.064287 | 567 | 520.83 | 0.918571 |  |
| GO:0048522\_positive\_regulation\_of\_cellular\_process | CD1D1 | 895 | 4 | 0.686034 | -0.064287 | 567 | 520.83 | 0.918571 |  |
| GO:0048522\_positive\_regulation\_of\_cellular\_process | PTEN | 895 | 4 | 0.686034 | -0.064287 | 567 | 520.83 | 0.918571 |  |
| GO:0048522\_positive\_regulation\_of\_cellular\_process | CASP2 | 895 | 4 | 0.686034 | -0.064287 | 567 | 520.83 | 0.918571 |  |
| GO:0002520\_immune\_system\_development | CD1D1 | 295 | 1 | 0.520339 | -0.063655 | 569 | 521.71 | 0.916889 |  |
| GO:0045595\_regulation\_of\_cell\_differentiation | CD1D1 | 295 | 1 | 0.520339 | -0.063655 | 569 | 521.71 | 0.916889 |  |
| GO:0048869\_cellular\_developmental\_process | HDAC2 | 1113 | 5 | 0.689578 | -0.053434 | 570 | 525.83 | 0.922509 |  |
| GO:0048869\_cellular\_developmental\_process | FGF15 | 1113 | 5 | 0.689578 | -0.053434 | 570 | 525.83 | 0.922509 |  |
| GO:0048869\_cellular\_developmental\_process | CHST11 | 1113 | 5 | 0.689578 | -0.053434 | 570 | 525.83 | 0.922509 |  |
| GO:0048869\_cellular\_developmental\_process | ARL6 | 1113 | 5 | 0.689578 | -0.053434 | 570 | 525.83 | 0.922509 |  |
| GO:0048869\_cellular\_developmental\_process | CD1D1 | 1113 | 5 | 0.689578 | -0.053434 | 570 | 525.83 | 0.922509 |  |
| GO:0016043\_cellular\_component\_organization | RECK | 964 | 4 | 0.636929 | -0.045355 | 571 | 530.32 | 0.928757 |  |
| GO:0016043\_cellular\_component\_organization | UXT | 964 | 4 | 0.636929 | -0.045355 | 571 | 530.32 | 0.928757 |  |
| GO:0016043\_cellular\_component\_organization | DNMT3A | 964 | 4 | 0.636929 | -0.045355 | 571 | 530.32 | 0.928757 |  |
| GO:0016043\_cellular\_component\_organization | HDAC2 | 964 | 4 | 0.636929 | -0.045355 | 571 | 530.32 | 0.928757 |  |
| GO:0051649\_establishment\_of\_localization\_in\_cell | PKIA | 342 | 1 | 0.448830 | -0.044798 | 572 | 530.77 | 0.927920 |  |
| GO:0009987\_cellular\_process | RECK | 3868 | 23 | 0.912746 | -0.042854 | 573 | 531.47 | 0.927522 |  |
| GO:0009987\_cellular\_process | TXNIP | 3868 | 23 | 0.912746 | -0.042854 | 573 | 531.47 | 0.927522 |  |
| GO:0009987\_cellular\_process | TBL1XR1 | 3868 | 23 | 0.912746 | -0.042854 | 573 | 531.47 | 0.927522 |  |
| GO:0009987\_cellular\_process | DNMT3A | 3868 | 23 | 0.912746 | -0.042854 | 573 | 531.47 | 0.927522 |  |
| GO:0009987\_cellular\_process | ZC3H15 | 3868 | 23 | 0.912746 | -0.042854 | 573 | 531.47 | 0.927522 |  |
| GO:0009987\_cellular\_process | RBP1 | 3868 | 23 | 0.912746 | -0.042854 | 573 | 531.47 | 0.927522 |  |
| GO:0009987\_cellular\_process | FGF15 | 3868 | 23 | 0.912746 | -0.042854 | 573 | 531.47 | 0.927522 |  |
| GO:0009987\_cellular\_process | BECN1 | 3868 | 23 | 0.912746 | -0.042854 | 573 | 531.47 | 0.927522 |  |
| GO:0009987\_cellular\_process | BYSL | 3868 | 23 | 0.912746 | -0.042854 | 573 | 531.47 | 0.927522 |  |
| GO:0009987\_cellular\_process | HUS1 | 3868 | 23 | 0.912746 | -0.042854 | 573 | 531.47 | 0.927522 |  |
| GO:0009987\_cellular\_process | ARL6 | 3868 | 23 | 0.912746 | -0.042854 | 573 | 531.47 | 0.927522 |  |
| GO:0009987\_cellular\_process | CD1D1 | 3868 | 23 | 0.912746 | -0.042854 | 573 | 531.47 | 0.927522 |  |
| GO:0009987\_cellular\_process | PTEN | 3868 | 23 | 0.912746 | -0.042854 | 573 | 531.47 | 0.927522 |  |
| GO:0009987\_cellular\_process | PKIA | 3868 | 23 | 0.912746 | -0.042854 | 573 | 531.47 | 0.927522 |  |
| GO:0009987\_cellular\_process | LGR4 | 3868 | 23 | 0.912746 | -0.042854 | 573 | 531.47 | 0.927522 |  |
| GO:0009987\_cellular\_process | UXT | 3868 | 23 | 0.912746 | -0.042854 | 573 | 531.47 | 0.927522 |  |
| GO:0009987\_cellular\_process | HDAC2 | 3868 | 23 | 0.912746 | -0.042854 | 573 | 531.47 | 0.927522 |  |
| GO:0009987\_cellular\_process | PSMD13 | 3868 | 23 | 0.912746 | -0.042854 | 573 | 531.47 | 0.927522 |  |
| GO:0009987\_cellular\_process | CHST11 | 3868 | 23 | 0.912746 | -0.042854 | 573 | 531.47 | 0.927522 |  |
| GO:0009987\_cellular\_process | TFDP2 | 3868 | 23 | 0.912746 | -0.042854 | 573 | 531.47 | 0.927522 |  |
| GO:0009987\_cellular\_process | ZFP238 | 3868 | 23 | 0.912746 | -0.042854 | 573 | 531.47 | 0.927522 |  |
| GO:0009987\_cellular\_process | PKD2 | 3868 | 23 | 0.912746 | -0.042854 | 573 | 531.47 | 0.927522 |  |
| GO:0009987\_cellular\_process | CASP2 | 3868 | 23 | 0.912746 | -0.042854 | 573 | 531.47 | 0.927522 |  |
| GO:0051239\_regulation\_of\_multicellular\_organismal\_process | CD1D1 | 587 | 2 | 0.522998 | -0.040635 | 574 | 532.1 | 0.927003 |  |
| GO:0051239\_regulation\_of\_multicellular\_organismal\_process | VASH1 | 587 | 2 | 0.522998 | -0.040635 | 574 | 532.1 | 0.927003 |  |
| GO:0030182\_neuron\_differentiation | HDAC2 | 356 | 1 | 0.431180 | -0.040371 | 575 | 532.45 | 0.926000 |  |
| GO:0048518\_positive\_regulation\_of\_biological\_process | BECN1 | 995 | 4 | 0.617085 | -0.038603 | 576 | 533.42 | 0.926076 |  |
| GO:0048518\_positive\_regulation\_of\_biological\_process | CD1D1 | 995 | 4 | 0.617085 | -0.038603 | 576 | 533.42 | 0.926076 |  |
| GO:0048518\_positive\_regulation\_of\_biological\_process | PTEN | 995 | 4 | 0.617085 | -0.038603 | 576 | 533.42 | 0.926076 |  |
| GO:0048518\_positive\_regulation\_of\_biological\_process | CASP2 | 995 | 4 | 0.617085 | -0.038603 | 576 | 533.42 | 0.926076 |  |
| GO:0051641\_cellular\_localization | PKIA | 370 | 1 | 0.414865 | -0.036389 | 577 | 535.37 | 0.927851 |  |
| GO:0010557\_positive\_regulation\_of\_macromolecule\_biosynthetic\_process | CD1D1 | 371 | 1 | 0.413747 | -0.036120 | 578 | 535.55 | 0.926557 |  |
| GO:0031328\_positive\_regulation\_of\_cellular\_biosynthetic\_process | CD1D1 | 387 | 1 | 0.396641 | -0.032081 | 579 | 536.94 | 0.927358 |  |
| GO:0009891\_positive\_regulation\_of\_biosynthetic\_process | CD1D1 | 388 | 1 | 0.395619 | -0.031845 | 580 | 537.1 | 0.926034 |  |
| GO:0048699\_generation\_of\_neurons | HDAC2 | 396 | 1 | 0.387626 | -0.030013 | 581 | 537.9 | 0.925818 |  |
| GO:0042592\_homeostatic\_process | PKD2 | 419 | 1 | 0.366348 | -0.025313 | 582 | 539.18 | 0.926426 |  |
| GO:0022008\_neurogenesis | HDAC2 | 423 | 1 | 0.362884 | -0.024574 | 583 | 539.42 | 0.925249 |  |
| GO:0010604\_positive\_regulation\_of\_macromolecule\_metabolic\_process | CD1D1 | 433 | 1 | 0.354503 | -0.022820 | 584 | 540.36 | 0.925274 |  |
| GO:0065008\_regulation\_of\_biological\_quality | RBP1 | 693 | 2 | 0.443001 | -0.020845 | 585 | 541.86 | 0.926256 |  |
| GO:0065008\_regulation\_of\_biological\_quality | PKD2 | 693 | 2 | 0.443001 | -0.020845 | 585 | 541.86 | 0.926256 |  |
| GO:0006810\_transport | PKD2 | 718 | 2 | 0.427577 | -0.017742 | 586 | 543.41 | 0.927321 |  |
| GO:0006810\_transport | PKIA | 718 | 2 | 0.427577 | -0.017742 | 586 | 543.41 | 0.927321 |  |
| GO:0051234\_establishment\_of\_localization | PKD2 | 729 | 2 | 0.421125 | -0.016519 | 587 | 543.95 | 0.926661 |  |
| GO:0051234\_establishment\_of\_localization | PKIA | 729 | 2 | 0.421125 | -0.016519 | 587 | 543.95 | 0.926661 |  |
| GO:0002376\_immune\_system\_process | CD1D1 | 505 | 1 | 0.303960 | -0.013366 | 588 | 544.65 | 0.926276 |  |
| GO:0008150\_biological\_process | RBP1 | 4605 | 30 | 1.000000 | 0.000000 | 1458 | 1439.9 | 0.987586 |  |
| GO:0008150\_biological\_process | FGF15 | 4605 | 30 | 1.000000 | 0.000000 | 1458 | 1439.9 | 0.987586 |  |
| GO:0008150\_biological\_process | HUS1 | 4605 | 30 | 1.000000 | 0.000000 | 1458 | 1439.9 | 0.987586 |  |
| GO:0008150\_biological\_process | CD1D1 | 4605 | 30 | 1.000000 | 0.000000 | 1458 | 1439.9 | 0.987586 |  |
| GO:0008150\_biological\_process | PTEN | 4605 | 30 | 1.000000 | 0.000000 | 1458 | 1439.9 | 0.987586 |  |
| GO:0008150\_biological\_process | LGR4 | 4605 | 30 | 1.000000 | 0.000000 | 1458 | 1439.9 | 0.987586 |  |
| GO:0008150\_biological\_process | ANKRD17 | 4605 | 30 | 1.000000 | 0.000000 | 1458 | 1439.9 | 0.987586 |  |
| GO:0008150\_biological\_process | PRMT1 | 4605 | 30 | 1.000000 | 0.000000 | 1458 | 1439.9 | 0.987586 |  |
| GO:0008150\_biological\_process | TRP53BP2 | 4605 | 30 | 1.000000 | 0.000000 | 1458 | 1439.9 | 0.987586 |  |
| GO:0008150\_biological\_process | B3GALNT1 | 4605 | 30 | 1.000000 | 0.000000 | 1458 | 1439.9 | 0.987586 |  |
| GO:0008150\_biological\_process | CHST11 | 4605 | 30 | 1.000000 | 0.000000 | 1458 | 1439.9 | 0.987586 |  |
| GO:0008150\_biological\_process | PKD2 | 4605 | 30 | 1.000000 | 0.000000 | 1458 | 1439.9 | 0.987586 |  |
| GO:0008150\_biological\_process | TFDP2 | 4605 | 30 | 1.000000 | 0.000000 | 1458 | 1439.9 | 0.987586 |  |
| GO:0008150\_biological\_process | ZFP238 | 4605 | 30 | 1.000000 | 0.000000 | 1458 | 1439.9 | 0.987586 |  |
| GO:0008150\_biological\_process | FBN2 | 4605 | 30 | 1.000000 | 0.000000 | 1458 | 1439.9 | 0.987586 |  |
| GO:0008150\_biological\_process | CASP2 | 4605 | 30 | 1.000000 | 0.000000 | 1458 | 1439.9 | 0.987586 |  |
| GO:0008150\_biological\_process | TXNIP | 4605 | 30 | 1.000000 | 0.000000 | 1458 | 1439.9 | 0.987586 |  |
| GO:0008150\_biological\_process | RECK | 4605 | 30 | 1.000000 | 0.000000 | 1458 | 1439.9 | 0.987586 |  |
| GO:0008150\_biological\_process | TBL1XR1 | 4605 | 30 | 1.000000 | 0.000000 | 1458 | 1439.9 | 0.987586 |  |
| GO:0008150\_biological\_process | DNMT3A | 4605 | 30 | 1.000000 | 0.000000 | 1458 | 1439.9 | 0.987586 |  |
| GO:0008150\_biological\_process | ZC3H15 | 4605 | 30 | 1.000000 | 0.000000 | 1458 | 1439.9 | 0.987586 |  |
| GO:0008150\_biological\_process | BECN1 | 4605 | 30 | 1.000000 | 0.000000 | 1458 | 1439.9 | 0.987586 |  |
| GO:0008150\_biological\_process | BYSL | 4605 | 30 | 1.000000 | 0.000000 | 1458 | 1439.9 | 0.987586 |  |
| GO:0008150\_biological\_process | ARL6 | 4605 | 30 | 1.000000 | 0.000000 | 1458 | 1439.9 | 0.987586 |  |
| GO:0008150\_biological\_process | PKIA | 4605 | 30 | 1.000000 | 0.000000 | 1458 | 1439.9 | 0.987586 |  |
| GO:0008150\_biological\_process | VASH1 | 4605 | 30 | 1.000000 | 0.000000 | 1458 | 1439.9 | 0.987586 |  |
| GO:0008150\_biological\_process | UXT | 4605 | 30 | 1.000000 | 0.000000 | 1458 | 1439.9 | 0.987586 |  |
| GO:0008150\_biological\_process | HDAC2 | 4605 | 30 | 1.000000 | 0.000000 | 1458 | 1439.9 | 0.987586 |  |
| GO:0008150\_biological\_process | PSMD13 | 4605 | 30 | 1.000000 | 0.000000 | 1458 | 1439.9 | 0.987586 |  |
| GO:0008150\_biological\_process | RAD17 | 4605 | 30 | 1.000000 | 0.000000 | 1458 | 1439.9 | 0.987586 |  |
