# Supplementary material for: RedundancyMiner: De-replication of redundant GO categories in microarray and proteomics analysis
Source: BMC Bioinformatics. 2011 Feb 10;12:52. doi: 10.1186/1471-2105-12-52 (PMC3223614; doi:10.1186/1471-2105-12-52)
Supplement: Additional file 8 — Retinal development HTGM download. compressed package of the results of running HTGM on the retinal development genes list. [file 1471-2105-12-52-S8.ZIP › SCENARIO_2_MODIFIED/total.txt.total.txt.dir/Exp1_BestClusterMap_LEIGS_KM_24.csv.join.8.txt.dir/Exp1_BestClusterMap_LEIGS_KM_24.csv.join.8.txt.change.gce.CIM.dir/cgi_user_info.html]

Input file: **Exp1\_BestClusterMap\_LEIGS\_KM\_24.csv.join.8.txt.change.gce.CIM**
